# Comparative Proteomics and Metabonomics Analysis of Different Diapause Stages Revealed a New Regulation Mechanism of Diapause in *Loxostege sticticalis* (Lepidoptera: Pyralidae)

**DOI:** 10.3390/molecules29153472

**Published:** 2024-07-25

**Authors:** Lijun Shao, Fangzheng Yue, Jinfu Fan, Qin Su, Hairui Liu, Quanyi Zhang, Linbo Xu

**Affiliations:** 1 Institute of Grassland Research, Chinese Academy of Agricultural Sciences, Hohhot 010010, China; s17861507827@163.com (L.S.);; 2 Center for Biological Disaster Prevention and Control, National Forestry and Grassland Administration, Shenyang 110034, China; 3 Erdos Forestry and Grassland Bureau, Erdos 017000, Chinacyzsuqin@126.com (Q.S.); 4 Jiaxiang County Natural Resources and Planning Bureau, Jining 272000, China

**Keywords:** histone deacetylase, ribosomal subunits, proteomics, metabolomics, diapause

## Abstract

Histone acetylation is an important epigenetic mechanism that has been shown to play a role in diapause regulation. To explore the physiological and molecular mechanisms of histone deacetylase in the diapause process, LC-MS/MS analysis was used to perform TMT proteomic and metabolomic analysis on non-diapause (ND), pre-diapause (PreD), diapause (D), cold treatment (CT), and post-diapause (RD) stages of the meadow moth. A total of 5367 proteins were identified by proteomics, including 1179 differentially expressed proteins. We found 975 (602 up-regulated and 373 down-regulated), 997 (608 up-regulated and 389 down-regulated), 1119 (726 up-regulated and 393 down-regulated), 1179 (630 up-regulated and 549 down-regulated), 94 (51 up-regulated and 43 down-regulated), 111 (63 up-regulated and 48 down-regulated), 533 (243 up-regulated and 290 down-regulated), 58 (31 up-regulated and 27 down-regulated), and 516 (228 up-regulated and 288 down-regulated) proteins in ND and PreD, ND and D, ND and CT, ND and RD, PreD and D, PreD and CT, PreD and RD, D and CT, D and RD, and CT and RD stages, respectively. A total of 1255 differentially expressed metabolites were annotated by metabolomics. Through KEGG analysis and time series analysis of differentially expressed metabolites, we found that phospholipids were annotated in significantly different modules, demonstrating their important role in the diapause process of the meadow moth. Using phospholipids as an indicator for weighted gene co-expression network analysis, we analyzed the most relevant differentially expressed proteins in the module and found that ribosomal 40s and 60s subunits were the most relevant proteins for diapause. Because there have been studies that have shown that histone deacetylase is associated with the diapause of meadow moths, we believe that histone deacetylase regulates the 40s and 60s subunits of ribosomes, which in turn affects the diapause of meadow moths. This finding expands our understanding of the regulation of meadow moth diapause and provides new insights into its control mechanism.

## 1. Introduction

The meadow moth is a major migratory pest in China and even the whole northern hemisphere. Diapause is a defensive measure evolved by the meadow moth to adapt to the cold climate in northern China during its migration [1]. It is of great significance to study the diapause mechanism of the meadow moth for scientific prevention and control of the occurrence and damage of meadow moth. Previous studies have shown that the entry of insects into diapause is not only the closure of gene expression [2], but also involves the complex regulation of metabolites and proteins in insect cells. The rapid and comprehensive progress of proteomics and metabonomics sequencing technology has been widely used to study various biological functions and processes of insects. In this study, we sequenced the non-diapause period, pre-diapause period, diapause period, cold treatment period, and post-diapause period of meadow moth by proteomics and metabonomics, in order to further understand the regulation mechanism of meadow moth diapause.

Diapause is an adaptive strategy employed by insects to enhance survival capabilities in response to adverse environmental conditions and seasonal changes. The meadow moth (*Loxostege sticticalis*) over-winters through diapause, with larvae entering diapause under short-day conditions in autumn. They survive the winter at temperatures as low as −20 °C and terminate diapause in the following spring, with the diapause period lasting up to six months. The meadow moth is a long-day developmental-type insect, where under long-day conditions (≥16 h), larvae almost completely avoid diapause; however, under short-day conditions of 15 h, some larvae enter diapause, and the number of diapausing larvae increases as the photoperiod shortens [3]. Since the diapause stage of the meadow moth is spent encased in a cocoon and buried in the soil, it is widely believed that the photoperiod experienced during the late developmental stages is the primary light factor influencing diapause in this species [4].

Histone acetylation is a type of histone modification that can function independently of genomic changes at any stage of insect development, and may therefore play a role in regulating changes in gene expression necessary for diapause initiation, maintenance, and termination [5]. In the cell, the helical chromatin structure is formed by packaging DNA into nucleosomes composed of histones H2a, H2b, H3, and H4. Histones H3 and H4 possess a tail that can be covalently modified to increase or decrease interactions between the DNA chain and nucleosomes, thereby altering transcriptional activity at specific DNA loci [6]. Increasing the interaction between DNA and histones typically results in a compact chromatin structure, while reducing the interaction between histones and DNA creates regions of relaxed chromatin with helicase activity. Evidence that histone acetylation affects diapause transcription comes from studies on the southern ground cricket Allonemobius socius [7], the northern house mosquito Culex pipiens [8], and the flesh fly Sarcophaga bullata [9]. Cui et al. conducted transcriptome analysis of diapause-regulating genes in the fall armyworm, finding that histone deacetylases play a significant role in regulating diapause [10].

Ribosomes, fundamental sites of protein synthesis in eukaryotes, comprise 60s and 40s subunits, which are assembled in the nucleus and independently transported out of the nucleus. The intricate biogenesis of ribosomes plays a critical role in regulating biological development at multiple levels, including translational regulation in multicellular organisms. Xu discovered that histone deacetylases in rice are involved in the deacetylation of ribosomal proteins, potentially affecting ribosomal stability and translational efficiency [11]. Urwanisch found that histone deacetylases can down-regulate ribosomal proteins [12].

Previous studies have investigated the metabolomics of diapause in various insects: in diapausing wheat blossom midge (*Sitodiplosis mosellana*), significant increases were observed in trehalose and glycerol compared to non-diapausing individuals [13]. In diapausing Asian tiger mosquito Aedes albopictus, 84.7% of diapause-specific metabolites were categorized as “lipids and lipid-like molecules” [14]. These metabolites, showing significant differences between diapause and non-diapause stages in insects, likely play roles in energy provision and cold resistance during diapause.

This study utilized proteomics and metabolomics techniques to identify and investigate changes in protein and metabolite levels during different diapause stages of the oriental armyworm, providing foundational research data on proteins and metabolites associated with diapause. This aims to better our understanding of the molecular regulatory mechanisms underlying diapause formation in the oriental armyworm.

## 2. Results

### 2.1. Original Data Analysis, Protein and Metabolite Identification of Meadow Moth with Different Diapause Treatments

In order to explore the key proteins in the diapause process of meadow moths, TMT quantitative proteomics and LC-MS technology were used to study and compare the differences in protein abundance in five stages of diapause of meadow moth larvae. The molecular weight of most identified proteins was less than 101 kda (Supplementary Figure S1). Most peptides are 6–44 amino acids in length (Supplementary Figure S2). Of the 13,111 proteins identified, 5367 were qualified, and the details are in the attached File S3.

In order to accurately measure the metabolic changes in meadow moth larvae at different diapause stages, six biologically replicated meadow moth larvae at different diapause stages were determined by LC-MS. A total of 2020 metabolites were identified and determined. Its details are shown in the attached File S4.

**Figure 1:**
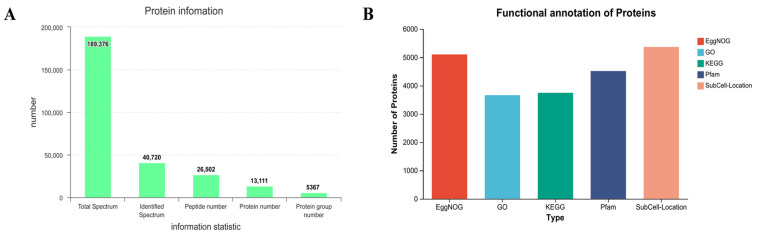
Proteomic sample information of meadow moth in different experimental groups. (**A**) Information on the number of peptides and proteins obtained by proteome. (**B**) The number of proteins annotated in the five databases.

Proteins were quantified based on data correlation acquisition (DDA) proteomics. A total of 26,502 peptides were detected (Figure 1A). The 26,502 peptides were annotated in five databases (EggNOG, GO, KEGG, Pfam, SubCell-Location) [15]. In total, 5367 proteins were annotated in the SubCell-Location database. The minimum number of genes annotated in the GO database was 3664 (Figure 1B).

### 2.2. DEP Analysis of Meadow Moth in Different Diapause Treatments

**Figure 2:**
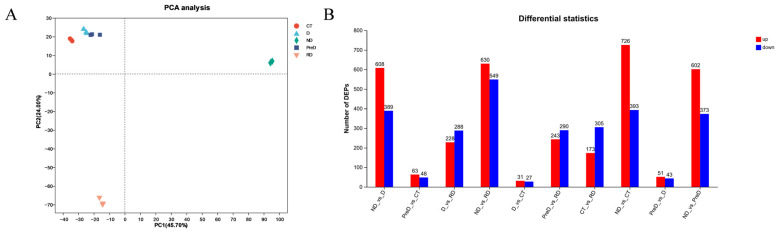
Processing and proteomic sample information of meadow moth in different experimental groups. (**A**) PCA principal component scores of the proteome of meadow moth at different diapause stages. (**B**) The number of genes annotated in the five databases.

Principal component analysis (PCA) of the omics dataset showed that PC 1 (45.70%) and PC 2 (24.00%) were the two main components (Figure 2A), and biological replication had good clustering. In these five different diapause stages, the DEPs whose proteome expression was up-regulated or down-regulated ≥ 2 times were identified, and the proteome was compared every two stages to identify DEPs. A total of 1179 non-redundant DEPs were identified (Figure 2B).

**Figure 3:**
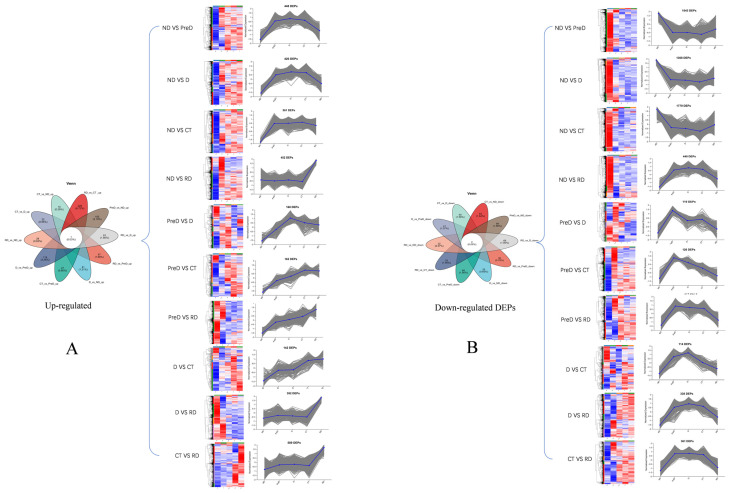
Venn and thermogram analysis of differentially expressed proteins in meadow moth larvae at different diapause stages. (**A**) The Venn diagram of unique and common DEPs in up-regulated proteins in 10 different comparison groups, as well as the heat maps and cluster analysis of DEPs in different comparison groups. (**B**) The Venn diagram of unique and common DEPs in down-regulated proteins in 10 different comparison groups, as well as the heat maps and cluster analysis of DEPs in different comparison groups. The blue part in the picture represents upregulated proteins, while the red part represents downregulated proteins.

Venn diagrams show the unique and common DEPs in 10 different control groups, including DEPs of up-regulated and down regulated genes (ND vs. PreD, ND vs. D, ND vs. CT, ND vs. RD, PreD vs. D, PreD vs. CT, PreD vs. RD, D vs. CT, D vs. RD, CT vs. RD). The heat map and cluster analysis of DEPs in different comparison groups are shown in Figure 3, indicating that there are significant differences in the proteins of meadow moth larvae at different diapause stages.

### 2.3. Functional Classification of DEPs in Different Diapause Stages

**Figure 4:**
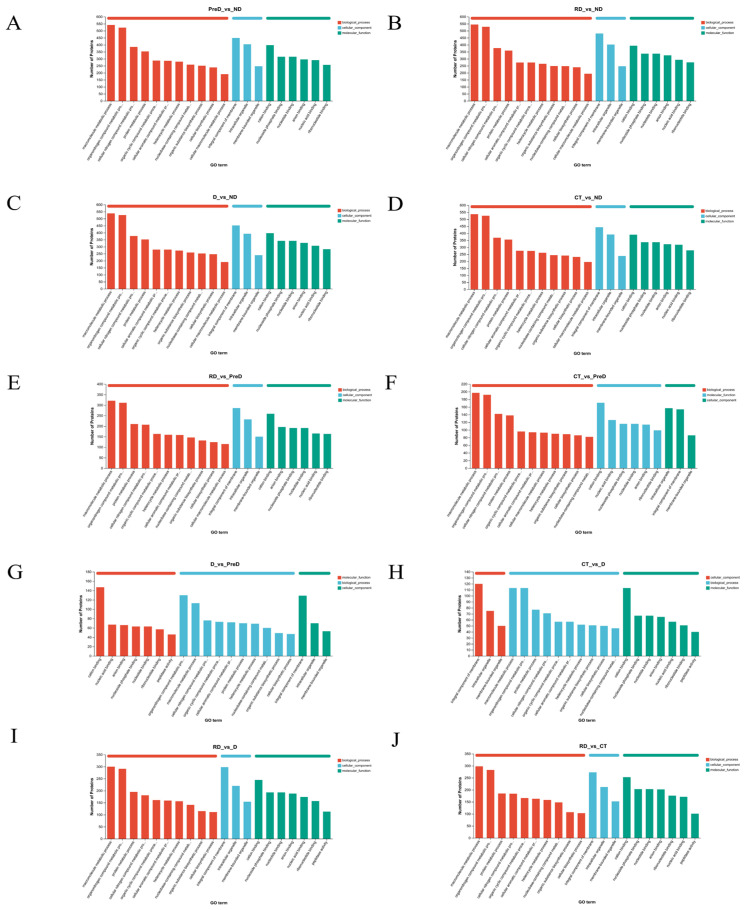
The number of differentially expressed proteins of meadow moth larvae at different diapause stages is related to the molecular function and cellular components during diapause. (**A**–**J**) are the GO annotation diagram of ND vs. PreD, ND vs. D, ND vs. CT, ND vs. RD, PreD vs. D, PreD vs. CT, PreD vs. RD, D vs. CT, D vs. RD, and CT vs. RD.

We conducted a Gene Ontology (GO) functional analysis of differentially expressed proteins (DEPs) across various comparison groups to identify enriched biological processes, cellular components, and molecular functions (Figure 4). Across the groups ND vs. PreD, ND vs. D, ND vs. CT, ND vs. RD, and PreD vs. CT, the top three categories of proteins annotated in biological process, cellular component, and molecular function were consistent. Specifically, the biological processes included the macromolecule metabolic process, the organonitrogen compound metabolic process, and the cellular nitrogen compound metabolic process. The cellular components comprised integral components of membranes, intracellular organelles, and membrane-bounded organelles. In molecular function, the categories were cation binding, nucleoside phosphate binding, and nucleotide binding.

Additionally, in the groups D vs. CT, D vs. RD, and CT vs. RD, the top three annotated proteins in all three processes were the same, with the only difference from the previous five groups being in biological process, where the third most annotated protein was involved in the protein metabolic process. In terms of cellular components, the highest enrichment across all ten comparison groups was seen in integral membrane components, followed by intracellular organelles, and then membrane-bound organelles.

In the ND vs. PreD group, the most highly enriched biological processes were macromolecule metabolic processes (15.49%), followed by organonitrogen compound metabolic processes (14.95%) and cellular nitrogen compound metabolic processes (11.01%). For molecular functions, the category with the highest enrichment was cation binding proteins (21.26%). In the PreD vs. D group, the most highly enriched biological processes were the organonitrogen compound metabolic process (17.13%), the macromolecule metabolic process (14.89%), and the cellular nitrogen compound metabolic process (10.01%). For molecular functions, the most enriched categories were cation binding (28.88%), followed by nucleic acid binding (13.16%) and then anion binding (12.97%).

### 2.4. Metabolic Characteristics of Meadow Moth in Different Diapause Periods

**Figure 5:**
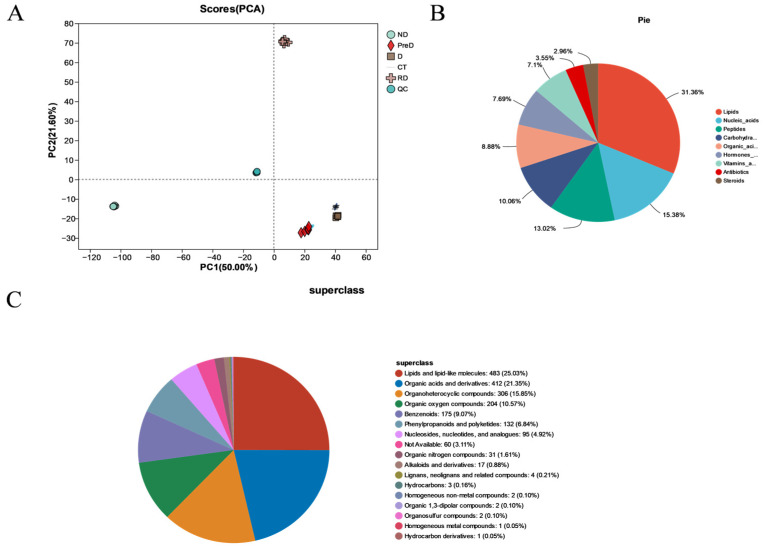
Metabonomic sample information of meadow moth in different diapause treatment groups. (**A**) PCA of different samples. The scores of the first two PCs and the explained variance. (**B**) Classification diagram of KEGG compounds in the metabolome. (**C**) HMDB compound classification diagram.

Using extensive targeted metabonomics technology, 2049 metabolites were successfully detected in different treatment groups of meadow moth. Principal component analysis (PCA) showed that PC 1 (50.0%) and PC 2 (21.6%) were the two main components (Figure 5A). The metabolites detected were various and could be divided into nine categories, namely lipid (31.36%), nucleic acid (15.38%), peptide (13.02%), carbohydrate (10.06%), organic acid (8.88%), hormones and transmitters (7.69%), vitamins and cofactors (7.1%), antibiotics (3.55%), and steroids (2.96%) (Figure 5B). The 9 kinds of metabolites detected were subdivided into 17 kinds of compounds, which were lipids and lipid molecules (483; 25.03%), organic acids and derivatives (412; 21.35%), organic heterocyclic compounds (306; 15.85%), organic oxygen compounds (204; 10.57%), benzene-like compounds (175; 9.07%), phenylpropane and polyketones (132; 6.84%), nucleosides, nucleotides, and analogs (95; 4.92%), organic nitrogen compounds (31; 1.61%), alkaloids and derivatives (17; 0.88%), lignin, new signal elements, and related compounds (4; 0.21%), hydrocarbons (3; 0.16%), homogeneous nonmetallic compounds (2; 0.10%), organic 1,3-dipolar compounds (2) homogeneous metal compounds (1; 0.05%), and others (60; 3.11%) (Figure 5C).

**Figure 6:**
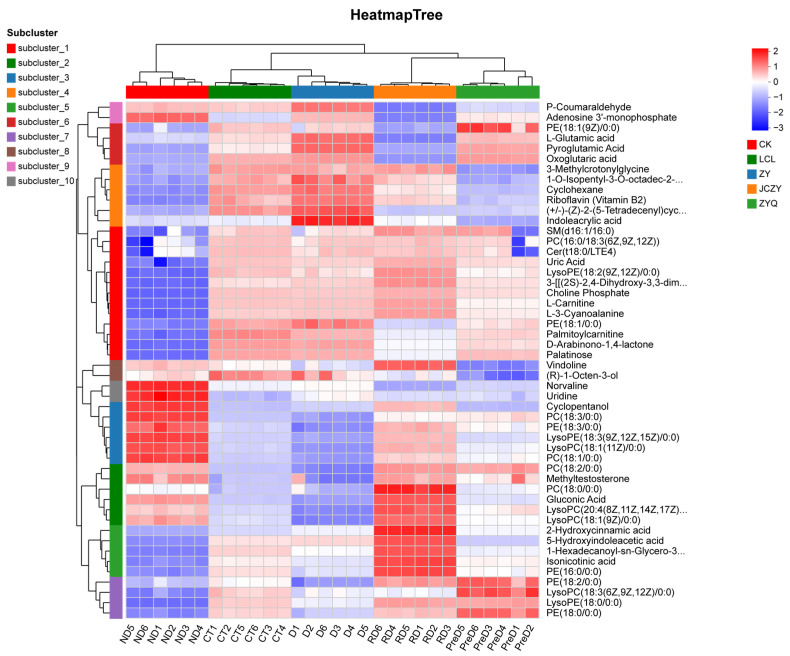
Thermogram of metabolites of meadow moth at different diapause stages. Each sample has 6 biological replicates.

The heatmap and cluster analysis of metabolites in the meadow moths at different diapause stages are shown in Figure 6, and indicate significant differences between the metabolites identified in the five treatment groups studied. CT and D have the highest similarity. Secondly, the metabolic profiles of CT and D have higher similarity than those of RD and PreD (Figure 6).

**Figure 7:**
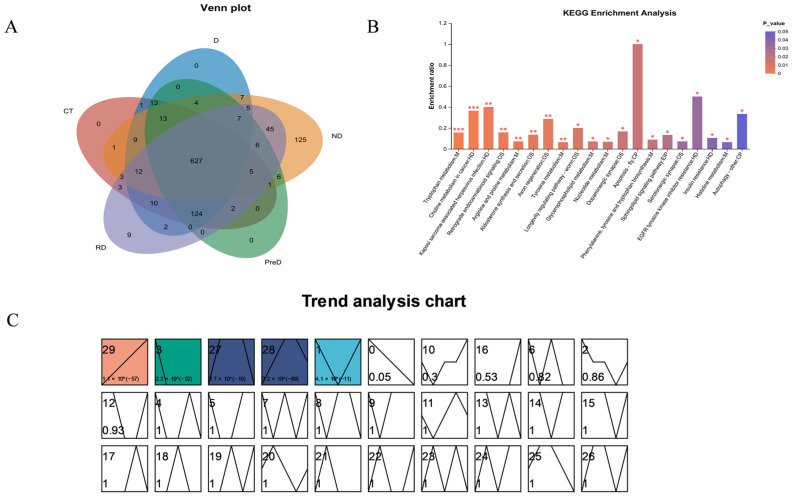
Screening of differentially expressed metabolites in different diapause treatment groups. (**A**) Venn diagram of screening different metabolites among different experimental groups of meadow moth. (**B**) KEGG enrichment analysis of significant correlation groups, * *p* < 0.05, ** *p* < 0.01, and *** *p* < 0.001. (**C**). Time series analysis of meadow moth.

Among the five samples, except for 1255 co-expressed metabolites, the remaining metabolites were not expressed in all samples; 240 metabolites were expressed separately in the ND sample, while 18 metabolites in the RD sample were not expressed in other samples. The remaining samples were expressed in two or more samples (Figure 7A). KEGG enrichment analysis was performed on 1255 co-expressed differential metabolites selected, and the most significant 20 metabolic pathways were identified, including amino acid metabolism, lipid metabolism, nucleotide metabolism, etc. (Figure 7B).

Time series analysis can analyze the dynamic expression and clustering of genes [16]. We performed basic clustering analysis on differentially accumulated metabolites (DAMs) using STEM software (Version 1.3.13; https://www.cs.cmu.edu/~jernst/stem/ accessed on 18 May 2024) and selected clusters with higher correlation. A total of 1255 non-redundant differential accumulation metabolites were divided into 30 files according to their expression trends. Among the 30 files (0–29), 5 files showed significant trends of 29, 3, 27, 28, 1. These five files were divided into four clusters based on similar accumulation trends (Figure 7C).

**Figure 8:**
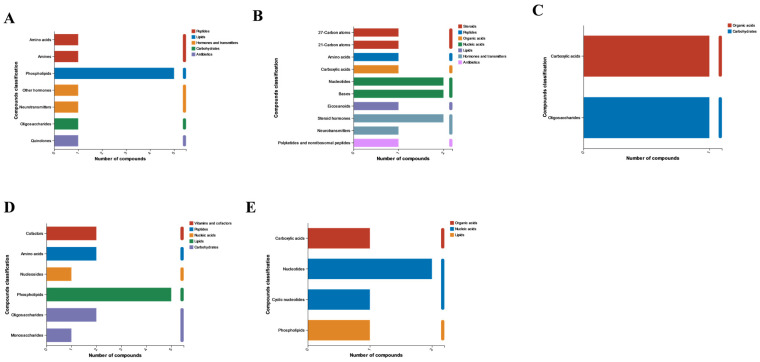
KEGG compound analysis was performed on the five significant modules of time series analysis, with (**A**–**E**) representing time series analysis clusters 1 to 5.

We performed KEGG metabolite annotation analysis on each cluster to identify enriched metabolites. Figure 8A–E show the first to fifth clusters, and we found that phospholipids have been annotated in 145, including substances such as phospholipids and sphingolipids. This indicates that phospholipids play an important role in the diapause process of meadow moths.

### 2.5. WGCNA Analysis of Proteomics of Meadow Moth in Different Diapause Treatment Groups

**Figure 9:**
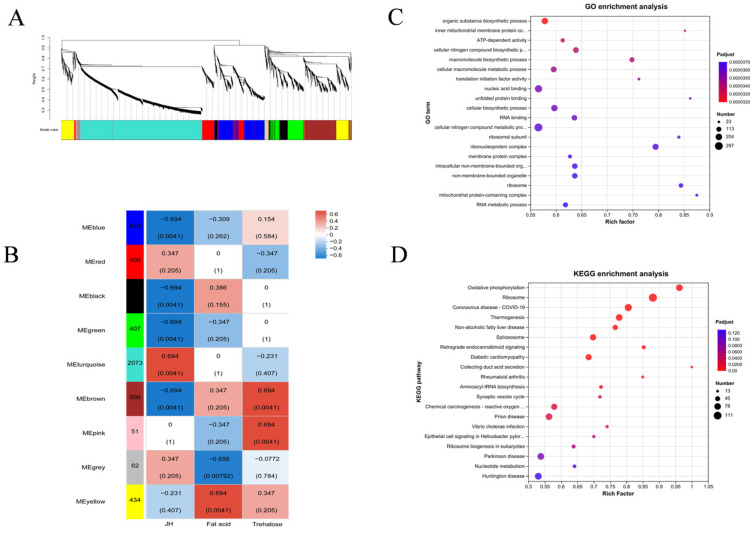
Screening and annotation of JH-related proteins. (**A**,**B**) WGCNA analysis of five experimental treatment groups. Note: genes are divided into modules according to the expression trend. One branch represents one gene, and each color represents one module. Red indicates high abundance, blue indicates low relative expression, and gray indicates genes that are not classified into a specific module. (**C**) GO enrichment analysis of related proteins. (**D**) KEGG enrichment analysis of related proteins.

After data preprocessing of differentially expressed proteins of meadow moth in different diapause treatment groups, 4897 non-redundant proteins were obtained, and 9 WGCNA modules were identified. The correlation between the light blue module and JH was the highest (r = 0.694, *p* = 4.1 × 10^−3^). Therefore, 2073 proteins in the light blue module play an important role in changing the JH content of the meadow moth, thus participating in the diapause of the meadow moth (Figure 9A,B).

The results of KEGG enrichment analysis and GO analysis of 2073 proteins showed that most proteins were related to metabolism. In the secondary metabolic pathway, RNA metabolism-related proteins, ribosomal complex-related proteins, energy metabolism-related proteins, and oxidative phosphorylation-related proteins account for the majority (Figure 9C,D).

**Figure 10:**
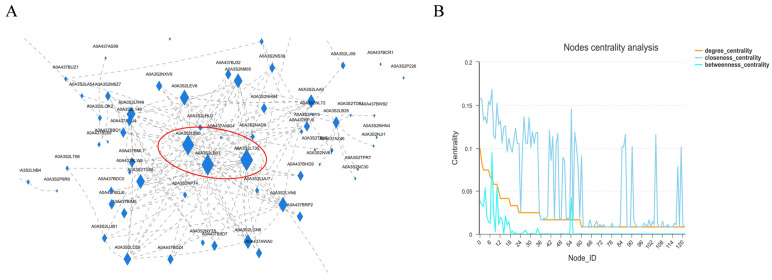
Analysis of diapause-related protein interaction network of meadow moth. (**A**) Protein–protein interaction network. Note: nodes represent proteins and edges represent interactions between two proteins. The size of the node is directly proportional to the connectivity (degree) of the node; that is, the more edges connected to the node, the greater the connectivity (degree) and the larger the node, indicating that the node gene is more important in the network. (**B**) Protein network center coefficient distribution map.

*Chilo suppressalis* of Lepidoptera Pyralidae was selected as the reference species of the turquoise module group of meadow moth, and the top 10% connectivity proteins were used as the hub proteins in the important module to construct the interaction network. The results showed that the centrality of three proteins in the interaction network was large. Therefore, the nodes of these three proteins play the most important role in ensuring the tight connection of the whole network. The compact centrality values of these three nodes are also high (Figure 10A and Table S3). However, the three proteins with the highest centrality were annotated. In addition to A0A3S2LT35, the other two proteins with serial numbers xp9313648.1 and xp9307912.1 in NCBI were annotated with 60s ribosomal subunit and 40s ribosomal subunit, respectively. Studies have shown that histone deacetylase, which regulates lysine acetylation in rice, can regulate ribosomal subunits, thereby affecting plant photosynthesis [17]. Therefore, we believe that 40s and 60s ribosomal subunits are regulated by histone acetylase and deacetylase to compress chromatin structure, which makes it difficult for RNA polymerase to enter, thus reducing protein-related expression to affect diapause.

### 2.6. GSEA of Proteomics

**Figure 11:**
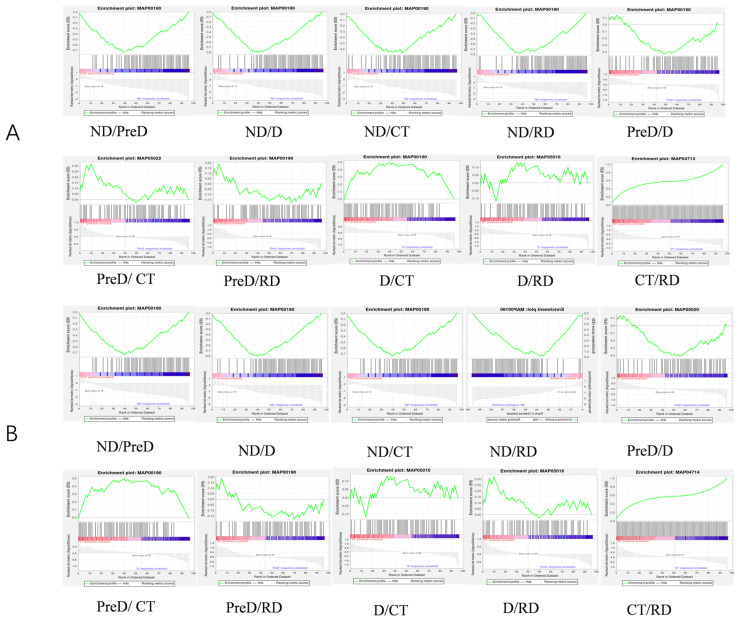
GSEA of proteomics of meadow moth larvae with different diapause treatments. (**A**) Light transduction-related proteins and circadian rhythm proteins play a role in different stages of meadow moth. (**B**) Thermogenic proteins play a role in different stages of meadow moth. The top curve represents the dynamic enrichment score (ES) value, and the highest point represents the ES value of the gene set.

Since there are only changes in light conditions and temperature during the whole diapause process of meadow moths, we analyzed the proteins related to the light transduction pathway (map04744, map04745), the circadian rhythm pathway (map04710, 04711, 04713) and the thermogenesis pathway (map04714) annotated for meadow moth by GSEA. GSEA can reveal whether some genes are enriched in the concentration of circadian gene-related protein/thermogenic gene-related protein, and whether the genes in this gene concentration are randomly distributed or enriched at the top or bottom of the corresponding gene concentration. The results showed that except the PreD/CT and D/CT groups, the other groups were not significant (Figure 11A). In contrast, in the PreD/CT and D/RD groups, the thermogenic pathway-related protein (map04714) showed a significant effect (Figure 11B). From ND to PreD/D, the temperature did not change, but the proteins related to thermogenic pathway had a significant effect on it. In the case of no change in environmental factors, D and CT groups were at the stage of diapause maintenance, and the proteins related to the thermogenic pathway had a significant impact on the difference between D and CT. In this study, RSEM software with default parameters was used for analysis. The five stages of meadow moth larvae were divided into 10 groups, and GSEA was carried out for each group pairing.

### 2.7. Combined Proteomics and Metabonomics Analysis

**Figure 12:**
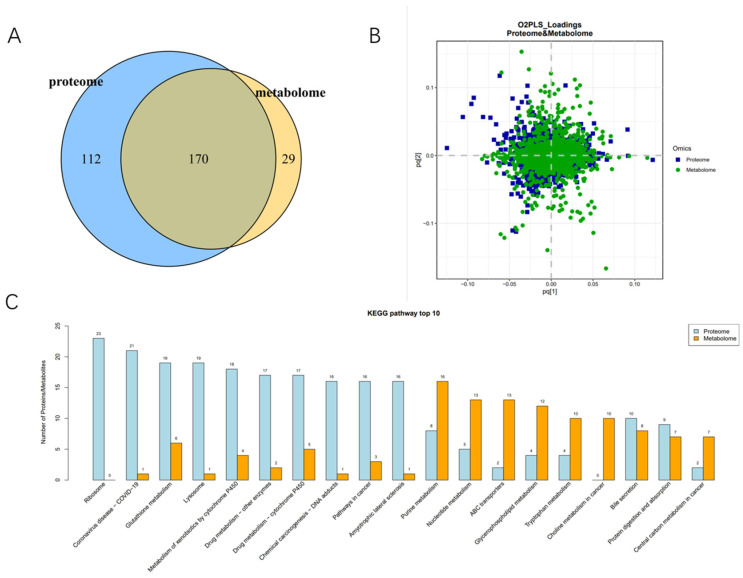
Proteomics and metabolite group joint analysis between D and CT. (**A**) Proteomic and metabonomic annotations of the metabolic pathways. (**B**) Partial least squares discriminant analysis (PLS-DA) score maps of different strains of samples. (**C**) KEGG enriched the top 10 most closely related metabolites and proteins.

In the D/CT group, because it is in the diapause maintenance stage and has a significant impact on the differences in proteins related to the light transduction pathway and thermogenic pathway, the D/CT group was analyzed by proteomics and metabonomics. The common annotation of proteomics and metabolomics detected 170 pathways (Figure 12A and Table S4). Two-way orthogonal partial least squares combined with discriminant analysis (O2PLS) was used to integrate the metabonomic and proteomic analysis (Figure 12B). It showed that the most closely related metabolites and proteins included ribosome, glutathione metabolism, lysosome, nucleotide metabolism, lipid metabolism, tryptophan metabolism, protein digestion and absorption (Figure 12C).

## 3. Discussion

As an important agricultural pest in China, studying its diapause is of great significance for scientific prevention and control of meadow moth outbreaks. During diapause, various changes occur in neuroendocrine, molecular, cellular, enzymatic, metabolic, endocrine, and behavioral aspects [18]. Diapause typically occurs at specific developmental stages of insects, namely embryo, larva/nymph, pupa, and adult [19]. It is divided into three phases: pre-diapause, diapause, and post-diapause. The pre-diapause phase involves the induction and preparation for diapause, encompassing the initiation, maintenance, and termination stages, while the post-diapause phase is characterized by a state of quiescence and rapid development [20,21]. Typically, since diapause occurs at life cycle stages that resist unfavorable developmental environments, the intensity of diapause varies significantly among different insects; the duration of diapause can serve as a measure of its intensity. During diapause, most insects do not feed or, in some cases of larvae and adults, feed minimally. This indicates that insects must have sufficient food reserves before diapause to meet their metabolic needs during diapause, and still have enough reserves to complete development and resume activities post-diapause [21,22].

In this study, a total of 5367 DEP and 2020 DAM species were identified, which may include the proteins and metabolites most closely related to diapause of meadow moth. These proteins and metabolites include not only DEP and DAM in the prophase of diapause, diapause, and diapause termination, but also DEP and DAM, which play a key role in the diapause process of meadow moth. We further analyzed the differentially expressed proteins in proteomics and screened 1179 differentially accumulated proteins at different diapause stages. Through comparative analysis, cluster analysis, and weighted gene co-expression network analysis, the important proteins related to carbohydrate metabolism, stress and defense response, lipid metabolism, and insect development were screened out. Based on the metabonomic time series and KEGG enrichment analysis of five groups of diapause larvae of meadow moth, 1255 different metabolites were identified, including amino acid metabolism, lipid metabolism, nucleotide metabolism, etc. 

KEGG metabolite analysis shows that among 1255 metabolites, phospholipids are closely related to diapause. Most of the secondary metabolic pathways are important regulators in the diapause process of meadow moth. Phospholipids can promote lipid metabolism within insects and are integral components of cell membranes, capable of binding with membrane proteins to regulate cellular metabolism, energy conversion, cell repair, and signal transduction [23]. In this study, certain phospholipids such as PI (Phosphatidyl inositol), PC (Phosphatidyl choline, also known as lecithin), PE (Phosphatidyl ethanolamine, also known as cephalin), PS (Phosphatidyl serine), and PA (Phosphatidic acid) were significantly higher in the diapause-affected Ostrinia furnacalis compared to the non-diapause group. Research on Bombyx mori has also shown that phospholipid levels positively correlate with cold resistance [24]. Increased phospholipid content may influence lipid metabolism, transformation, and intercellular energy transfer during diapause in Ostrinia furnacalis, thereby enhancing cold tolerance. Lysophospholipids, derivatives of phospholipid hydrolysis, are more polar than phospholipids and can enhance membrane fluidity upon extensive contact with cell membranes [25].

To investigate whether circadian rhythm gene-related proteins and thermogenesis gene-related proteins play a role in protein variations during different diapause stages of the fall armyworm, we conducted Gene Set Enrichment Analysis (GSEA) on various treatment groups. The results showed that the role of circadian rhythm gene-related proteins in diapause and diapause release of meadow moth was not obvious. Heat-generating genes are the main reason for the differences in each stage. This may be because the regulation network of circadian rhythm signals is extremely complex, and the analysis result is not significant [26].

WGCNA analysis of differentially expressed proteins revealed that 2073 proteins were highly correlated with JH (r = 0.694, *p* = 4.1 × 10^−3^). We believe that the 2073 proteins most related to JH in the light blue module are the key protein set for regulating diapause of meadow moth. We analyzed the protein interaction network of the light blue module and found that the 40s and 60s subunits of the ribosome played an important role in the whole network. Previous studies have shown that HDAC may play an important role in the diapause of meadow moth [10]. At the same time, it was reported that lysine acetylation may increase the combination diversity of ribosomal activity, and then affect the histone modification in plant cells [11]. Therefore, we speculate that the 40s and 60s subunits of ribosomes are regulated by histone deacetylase to compress the chromatin structure during diapause of meadow moth, which makes it difficult for RNA polymerase to enter, thus reducing protein-related expression. That is to say, there may be an interaction between HDAC and 40s and 60s subunits of ribosomes. These results expand the relevant regulatory network of meadow moth diapause and provide new insights.

However, the regulation of thermogenic pathways directly involved in energy metabolism is more direct than that of circadian rhythm genes. In the next step, we will further verify the functions of ribosomal subunits and HDAC. At the same time, we studied the influence mechanism of histone acetylase-related genes on diapause in meadow moth, and then determined the molecular mechanism of acetylation and endocrine regulation coupling. In addition, in order to mediate acetylation regulation, we will study the cellular signal transduction and co-regulation of acetylation in insect diapause. Acetylation may be a molecular marker for diapause regulation or developmental decision in insects. It is of great significance to analyze the specific role of epigenetic code in diapause regulation, and then to understand the diapause mechanism of insects from a broader perspective.

## 4. Materials and Methods

### 4.1. Insect Collection

The caterpillars used in this experiment were collected from the agricultural fields of Dalad Banner and Otog Front Banner in Ordos City, Inner Mongolia Autonomous Region. To ensure that the experimental insects maintained consistent vitality, we reared the collected over-wintering generation of caterpillars in an insect-controlled climate chamber at 21 °C, with a humidity of 60–70% and an L:D ratio of 16:8 for three generations, after which they underwent diapause treatment.

### 4.2. Acquisition of Diapause and Diapause-Lifting Larvae

**Figure 13:**
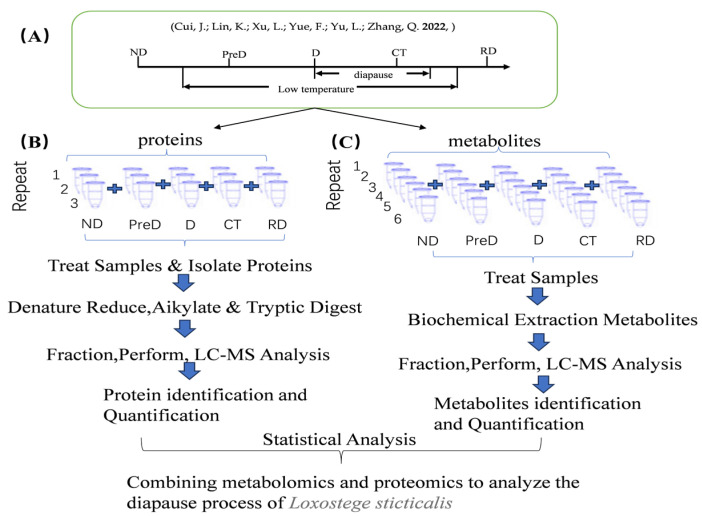
Experimental design of proteomics and metabonomics analysis of meadow moth at different diapause stages using tandem mass spectrometry standard TMT and LC−MS methods. (**A**) Sampling diagram of different experimental groups of meadow moth. Note: as shown in the figure, the lighting conditions are L:D = 12:12, and ND is selected for the first day. The stage of low-temperature treatment was in diapause. RD is the sample after diapause is released. The exact time of diapause start and end is uncertain. The diapause start and end marked on the figure is an estimate, not an accurate time [10]. (**B**,**C**) Proteomics and metabonomics analysis process of meadow moth larvae at different diapause stages.

The diapause of the meadow moth is induced under the conditions of 21 °C and a 12:12 L:D cycle. In this laboratory, the diapause induction training of the meadow moth was conducted according to previous methods [4]. The 1st to 4th instar larvae of the meadow moth were reared under 21 °C and a 16:8 L:D cycle, while the 5th instar larvae were induced under 21 °C and a 12:12 L:D cycle (ND). The feeding conditions for the control group and experimental group were identical, but the control group maintained a 16L:8D light condition after entering the 5th instar. After the control group’s meadow moths burrowed into the soil to make cocoons and emerged, we collected the non-burrowed portion of the diapause-treated group as pre-diapause (PreD), while collecting the cocoons from the control group, cutting off one end of its cocoon, and observing its growth. After the control group pupated for over a week, the diapause-treated group remained in a larval state, and was named the diapause group (D). The meadow moths after two weeks of diapause were transferred to diapause-release conditions (0–5 °C, 0L:24D) for 20 days, serving as the cold treatment group (CT). After being placed under normal cultivation conditions (21 ± 1 °C, 16L:8D) for 7–10 days, some of the moths in the bottle began to pupate, and those that remained in larval form were considered diapause-released larvae (RD). All tested meadow moths were placed in a rearing box measuring 28 × 18 × 13.5 cm, with 50 individuals per box. Five states of meadow moths (ND, PreD, D, CT, RD) were subjected to proteomic sequencing (Figure 13A). Twenty samples were collected from each treatment and replicated three times, with each sample collected near the end of the photoperiod.

### 4.3. Sample Preparation for Proteomics Analysis

An appropriate amount of sample in the frozen state is taken out and transferred to the vibrating tube. An appropriate amount of protein lysate (8 M urea + 1% SDS, containing protease inhibitor) is added. A high-flux tissue grinder was used to vibrate the sample 3 times, for 40 s each time. Pyrolysis was performed on ice for 30 min, and vortex mixing was carried out for 5–10 s every 5 min. The supernatant was collected after centrifugation at 4 °C for 30 min at 12,000 G. Subsequently, the protein content was determined by the BCA method, and the operation was carried out in strict accordance with the instructions of the BCA reagent [26].

The final concentration of teab was made to be 100 mm by adding teab (triethylammonium bicarbonate buffer) to 100 µg of protein; TCEP (tris (2-carboxyethyl) phosphine) was added so that the final concentration of TCEP was 10 mm, and the reaction was carried out at 37 °C for 60 min; IAM (iodoacetamide) was added to make the final concentration of IAM 40 mm, and the mixture was kept in the dark for 40 min at room temperature. Each tube was added with pre-cooled acetone (acetone/sample *v*/*v* = 6:1) and was precipitated at −20 °C for 4 h. Centrifugation was performed at 10,000× *g* for 20 min, and the sediment was collected. The sample was fully dissolved with 100 µL of 100 mm teab. Trypsin was added according to an enzyme/protein ratio (m/m) of 1:50, and enzymolysis was performed overnight at 37 °C. The TMT reagent was taken out at 20 °C and allowed to return to room temperature; acetonitrile was added, vortexed, and centrifuged. Each tube was added with 100 μg of polypeptide into a TMT reagent and incubated at room temperature for 2 h. Hydroxylamine was added, reacted at room temperature for 30 min, mixed with the same amount of labeled product in a tube, and drained with a vacuum concentrator [27,28].

The peptide samples were redissolved with UPLC loading buffer (2% acetonitrile (adjusted to pH 10 with ammonia water)), and high-pH liquid-phase separation was performed on an acquity UPLC beh C18 column of 1.7 µm, 2.1 mm × 150 mm (Waters, Milford, MA, USA). Phase A involved 2% acetonitrile (adjusted to pH 10 with ammonia); phase B involved 80% acetonitrile (adjusted to pH 10 with ammonia), 0–1.9 min, 0–0% B; 9~2 min, 0~5% B; 2–17 min, 5–5% B; 17~18 min, 5~10% B; 5 min, 10−30% B; 5~38 min, 30~36% B; 38~39 min, 36~42% B; 39~40 min, 42~100% B; 40–44 min, 100% B; 44~45 min, 100~0% B; 45~48 min, 0~0% B. The UV detection wavelength was 214 nm, and the volume flow rate was 200 μL/min. The elution time was 48 min. According to the peak shape and time, 28 fractions were collected and combined into 14 fractions, which were concentrated by vacuum centrifugation.

### 4.4. Sample Preparation for Metabonomic Analysis

The solid sample was taken into a 2 mL centrifuge tube, and a grinding ball with a diameter of 6 mm was added; 400 μL of extract (methanol/water = 4:1 (*v*:*v*)) containing 0.02 mg/mL of internal standard (l-2-chlorophenylalanine) was used for the extraction of metabolites. The sample solution was ground for 6 min (−10 °C, 50 Hz) with a frozen tissue grinder, and then subjected to low-temperature ultrasound for 30 min (5 °C, 40 kHz). The sample was then placed at −20 °C for 30 min and centrifuged for 15 min (4 °C, 13,000× *g*), and the supernatant was transferred to the injection vial with an internal cannula for analysis [29].

### 4.5. LC-MS/MS Analysis

Proteomics in the second dimension was analyzed by liquid chromatography tandem mass spectrometry (evosep one combined with obitrap exploris 480 mass spectrometer) (Shanghai Meiji Biomedical Technology Co., Ltd, Shanghai, China). The peptide was dissolved in a mass spectrometry loading buffer and then loaded on a C18 column (150 mm). The volume flow rate was 300 NL/min; 1% formic acid aqueous solution was in phase a, with 100% acetonitrile plus 0. 1% formic acid in phase B, 0~2 min, 5~5% B; 2–30 min, 5–38% B; 30–40 min, 38–90% B; 40–44 min, 90–90% B. The MS scanning range (M/z) was 350–1500, the acquisition mode was DDA, and the fragmentation mode was HCD. The resolution of primary mass spectrometry was 60,000, the resolution of secondary mass spectrometry was 15,000, and the dynamic exclusion time was 30 s. Turbotmt intelligent acquisition improves the resolution of reported ion isotopes.

The instrument platform for metabonomic LC-MS analysis is the uhplc-q exactive system (Shanghai Meiji Biomedical Technology Co., Ltd.) of semefier. Chromatographic conditions: a 3 μL sample was separated by an HSS T3 chromatographic column (100 mm × 2.1 mm I.D., 1.8 µm) and detected by mass spectrometry. Mass spectrum conditions: the sample mass spectrum signal was collected in the positive and negative ion scanning mode, and the mass scanning range was *m*/*z*: 70–1050. The ion spray voltage and positive ion voltage were 3500 V, the negative ion voltage was 2800 V, the sheath gas was 40 psi, the auxiliary heating gas was 10 psi, and the ion source heating temperature was 400 °C, with 20–40–60 V cyclic collision energy, an MS1 resolution of 70,000, and an MS2 resolution of 17,500.

### 4.6. Bioinformatics Analysis

The original files of mass spectrometry off the machine were analyzed by proteomediscoverertm software 2.4, and the database used for searching the database was the Meiji biological self-developed database. The false discovery rate (FDR) of peptide identification was set to FDR ≤ 0.01, the mass tolerance of peptides was set to 20 ppm, and the fragment mass tolerance was set to 0.02 da, containing at least one specific peptide [30].

The original data of LC-MS were imported into the metabonomics processing software progenesis Qi (Waters Corporation, Milford, MA, USA) for baseline filtering, peak identification, integration, retention time correction, and peak alignment. Finally, a data matrix of retention time, mass charge ratio, and peak intensity was obtained. At the same time, MS and MSMS mass spectrometry information was linked to the metabolism public databases HMDB (http://www.hmdb.ca/, accessed on 12 December 2023) and metlin (https://metlin.scripps.edu/, accessed on 17 December 2023) and meggith built its own database to match and obtain the metabolite information. We uploaded the database-searched data matrix to the meggitt cloud platform (cloud.majorbio.com, accessed on 17 December 2023) for analysis. Firstly, the data matrix was preprocessed as follows: the data matrix used an 80% rule to remove missing values, that is, to retain at least 80% of non-zero variables in a group of samples, and then fill the gap value (the minimum value in the original matrix fills the gap value). In order to reduce the error caused by sample preparation and instrument instability, the total normalization method was used to normalize the response intensity of the sample mass spectrum peak to obtain the normalized data matrix. At the same time, the variables with a relative standard deviation (RSD) > 30% of QC samples were deleted, and log10 logarithm processing was performed to obtain the data matrix for subsequent analysis. Secondly, the ropls package (version1.6.2) in R language was used to perform principal component analysis (PCA) and orthogonal least partial binary discriminant analysis (OPLS-DA) on the preprocessed data matrix, and the stability of the model was evaluated by seven cycles of interactive validation. The selection of significantly different metabolites was determined based on the variable weight value (VIP) obtained by the opls-da model and the *p* value of Student’s *t* test. The metabolites with vip > 1 and *p* < 0.05 were significantly different metabolites.

Differential metabolites were annotated by the KEGG database (https://www.kegg.jp/kegg/pathway.html, accessed on 17 December 2023) to obtain the pathways involved by differential metabolites. Python software (Version 3.10) package scipy.stats was used for pathway enrichment analysis, and a Fisher exact test was used to obtain the biological pathway that was most relevant to the experimental treatment.

## 5. Conclusions

In summary, through TMT proteomic and metabolomic analysis on non-diapause (ND), pre-diapause (PreD), diapause (D), cold treatment (CT), and post-diapause (RD) stages of the meadow moth, we found significant differences in protein and metabolic profiles between diapause and non diapause states. Then, we screened for differential proteins and metabolites among the different treatment groups of grassland moths mentioned above, and annotated a total of 1179 differential proteins and 1255 co expressed differential metabolites. We conducted KEGG analysis and time series analysis on the co expressed metabolites, and found that phospholipids were annotated in significantly different modules, indicating their important role in the diapause process of grassland moths. Using phospholipids as a weighted gene co expression network analysis indicator to analyze differentially expressed proteins, it was found that ribosomal 40s and 60s subunits were the most relevant proteins for diapause. Based on previous research in our laboratory, it has been found that the histone deacetylase of the meadow moth is related to its diapause. The results demonstrate that histone deacetylase regulates the 40s and 60s subunits of ribosomes, thereby affecting the diapause of the meadow moth.

## Data Availability

The datasets analyzed during the current study can be found in the iprox repository: https://proteomecentral.proteomexchange.org/cgi/GetDataset?ID=PXD048065,PXD048065 (accessed on 4 March 2024).

## Supplementary Materials

The following supporting information can be downloaded at https://www.mdpi.com/article/10.3390/molecules29153472/s1, Figure S1: protein-molecular-weight; Figure S2: peptide-length-distribut; Table S1: Proteomics Data; Table S2: Metabolomics Data; Table S3: Protein interaction network analysis; Table S4: Pathways co annotated by proteomics and metabonomics.

## References

[1] Frolov A.N. (2015). The beet webworm *Loxostege sticticalis* L. (*Lepidoptera*, *Crambidae*) in the focus of agricultural entomology objectives: I. The periodicity of pest outbreaks. Entomol. Rev..

[2] Santos P.K.F., de Souza Araujo N., Françoso E., Zuntini A.R., Arias M.C. (2018). Diapause in a tropical oil-collecting bee: Molecular basis unveiled by RNA-Seq. BMC Genom..

[3] Huang S.H., Jiang X.F., Luo L.Z. (2009). The effects of photoperiod and temperature on the induction of diapause in grassland moths. J. Entomol..

[4] Jiang X.F., Huang S.H., Luo L.Z., Liu Y., Zhang L. (2010). Diapause termination, post-diapause development and reproduction in the beet webworm, *Loxostege sticticalis* (Lepidoptera: Pyralidae). J. Insect Physiol..

[5] Lennartsson A., Ekwall K. (2009). Histone modification patterns and epigenetic codes. Biochim. Et Biophys. Acta General. Subj..

[6] Huang H., Sabari B.R., Garcia B.A., Allis C.D., Zhao Y. (2014). SnapShot: Histone Modifications. Cell.

[7] Reynolds J.A., Hand S.C. (2009). Decoupling development and energy flow during embryonic diapause in the cricket, *Allonemobius socius*. J. Exp. Biol..

[8] Hickner P.V., Mori A., Zeng E., Tan J.C., Severson D.W. (2015). Whole transcriptome responses among females of the filariasis and arbovirus vector mosquito Culex pipiens implicate TGF-β signaling and chromatin modification as key drivers of diapause induction. Funct. Integr. Genom..

[9] Reynolds J.A., Bautista-Jimenez R., Denlinger D.L. (2016). Changes in histone acetylation as potential mediators of pupal diapause in the flesh fly, *Sarcophaga bullata*. Insect Biochem. Mol. Biol..

[10] Cui J., Lin K., Xu L., Yue F., Yu L., Zhang Q. (2022). Transcriptome Analysis of Beet Webworm Shows That Histone Deacetylase May Affect Diapause by Regulating Juvenile Hormone. Insects.

[11] Xu Q., Liu Q., Chen Z., Yue Y., Liu Y., Zhao Y., Zhou D.-X. (2021). Histone deacetylases control lysine acetylation of ribosomal proteins in rice. Nucleic Acids Res..

[12] Urwanisch L., Unger M.S., Sieberer H., Dang H.-H., Neuper T., Regl C., Horejs-Hoeck J. (2023). The Class IIA Histone Deacetylase (HDAC) Inhibitor TMP269 Downregulates Ribosomal Proteins and Has Anti-Proliferative and Pro-Apoptotic Effects on AML Cells. Cancers.

[13] Huang Q., Ma Q., Li F., Zhu-Salzman K., Cheng W. (2022). Metabolomics Reveals Changes in Metabolite Profiles among Pre-Diapause, Diapause and Post-Diapause Larvae of *Sitodiplosis mosellana* (Diptera: *Cecidomyiidae*). Insects.

[14] Batz Z.A., Armbruster P.A. (2018). Diapause-associated changes in the lipid and metabolite profiles of the Asian tiger mosquito, Aedes albopictus. J. Exp. Biol..

[15] Ju Z., Liang L., Zheng Y., Shi H., Zhao W., Sun W., Pang Y. (2024). Full-Length Transcriptome Sequencing and RNA-Seq Analysis Offer Insights into Terpenoid Biosynthesis in *Blumea balsamifera* (L.) DC. Genes.

[16] Van Delft J.H.M., Mathijs K., Staal Y.C.M., Van Herwijnen M.H.M., Brauers K.J.J., Boorsma A., Kleinjans J.C.S. (2010). Time Series Analysis of Benzo[A]Pyrene-Induced Transcriptome Changes Suggests That a Network of Transcription Factors Regulates the Effects on Functional Gene Sets. Toxicol. Sci..

[17] George S., Gaddelapati S.C., Palli S.R. (2019). Histone deacetylase 1 suppresses Krüppel homolog 1 gene expression and influences juvenile hormone action in *Tribolium castaneum*. Proc. Natl. Acad. Sci. USA.

[18] Meng L.H. (2021). TheEffects of Photoperiod on theGrowth, Development, Flight, Reproduction, and Stress Resistance of theArmyworm. Master’s Thesis.

[19] Scott S.M., Dingle H. (1987). *Insect* Dormancy: An Ecological Perspective. H. V. Danks. Q. Rev. Biol..

[20] Hahn D.A., Denlinger D.L. (2011). Energetics of Insect Diapause. Annu. Rev. Entomol..

[21] Saunders D.S. (2020). Dormancy, Diapause, and the Role of the Circadian System in Insect Photoperiodism. Annu. Rev. Entomol..

[22] Nagao K., Yanagida T. (2002). Physiological functions of phospholipids. Oleoscience J. Jpn. Oil Chem. Soc..

[23] Wu D.Y. (1989). The impact of different temperature for cold storage on the phosphglycerides content of the silkworm eggs. J. Southwest Agric. Univ..

[24] Birgbauer E., Chun J. (2006). New developments in the biological functions of lysophospholipids. Cell. Mol. Life Sci. CMLS.

[25] Liu M., Pile L.A. (2017). The Transcriptional Corepressor SIN3 Directly Regulates Genes Involved in Methionine Catabolism and Affects Histone Methylation, Linking Epigenetics and Metabolism. J. Biol. Chem..

[26] Wen J., Niu X., Chen S., Chen Z., Wu S., Wang X., Ju X. (2022). Chitosan oligosaccharide improves the mucosal immunity of small intestine through activating SIgA production in mice: Proteomic analysis. Int. Immunopharmacol..

[27] El-Sharkawy I., Liang D., Xu K. (2015). Transcriptome analysis of an apple (*Malus* × *domestica*) yellow fruit somatic mutation identifies a gene network module highly associated with anthocyanin and epigenetic regulation. J. Exp. Bot..

[28] Hu S., Hu C., Luo L., Zhang H., Zhao S., Liu Z., Zeng L. (2022). Pu-erh tea increases the metabolite Cinnabarinic acid to improve circadian rhythm disorder-induced obesity. Food Chem..

[29] Bian J., Liao Y., Liu R., An X., Hu C., Liu H., Qu J. (2022). Synergy of cyano groups and cobalt single atoms in graphitic carbon nitride for enhanced bio-denitrification. Water Res..

[30] Ren Y., Yu G., Shi C., Liu L., Guo Q., Han C., Huang H. (2022). Majorbio Cloud: A one-stop, comprehensive bioinformatic platform for multiomics analyses. iMeta.

