# Supplementary material for: Comparative Proteomics and Metabonomics Analysis of Different Diapause Stages Revealed a New Regulation Mechanism of Diapause in Loxostege sticticalis (Lepidoptera: Pyralidae)
Source: Molecules. 2024 Jul 25;29(15):3472. doi: 10.3390/molecules29153472 (PMC11314584; doi:10.3390/molecules29153472)
Supplement: Supplementary file 1 [file molecules-29-03472-s001.zip › analysis process/All analysis results.pdf]

Results

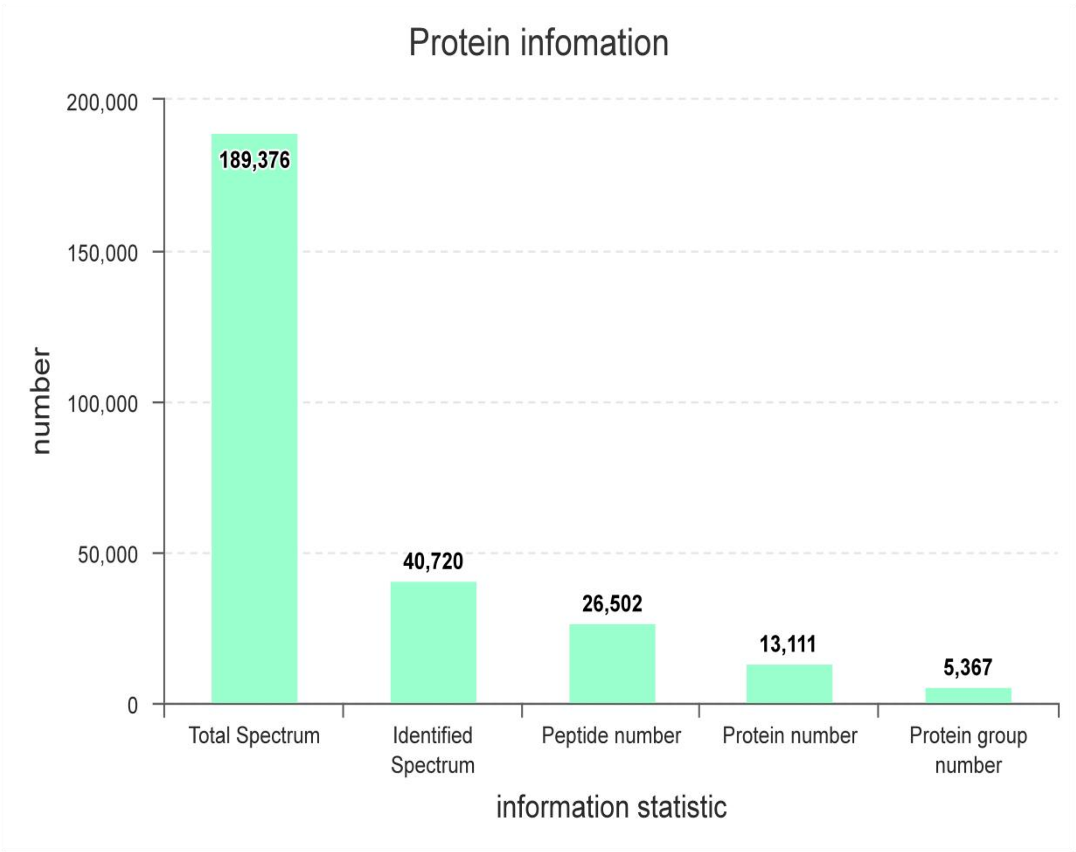
Protein infomation
200,000
150,000
100,000
50,000
0
number
189,376
40,720
26,502
13,111
5,367
Total Spectrum
Identified Spectrum
Peptide number
Protein number
Protein group number
information statistic

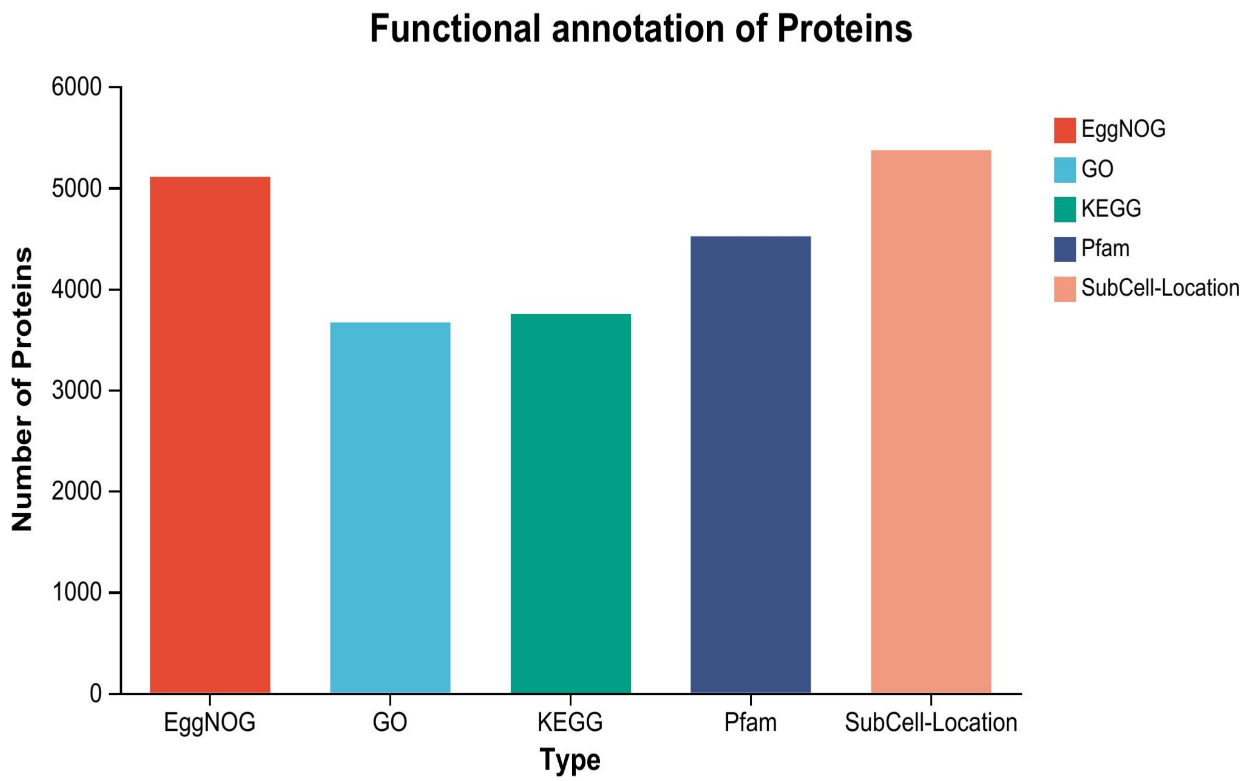
Functional annotation of Proteins
6000
5000
4000
3000
2000
1000
0
Number of Proteins
EggNOG
GO
KEGG
Pfam
SubCell-Location
Type

Results

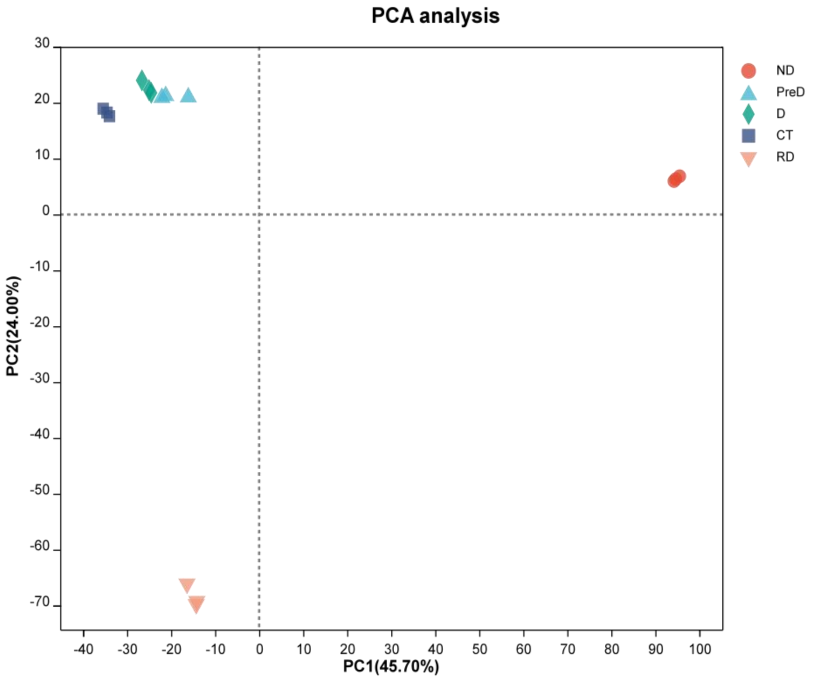
PCA analysis
ND
PreD
D
CT
RD
PC2(24.00%)
PC1(45.70%)
30
20
10
0
-10
-20
-30
-40
-50
-60
-70
-40 -30 -20 -10 0 10 20 30 40 50 60 70 80 90 100

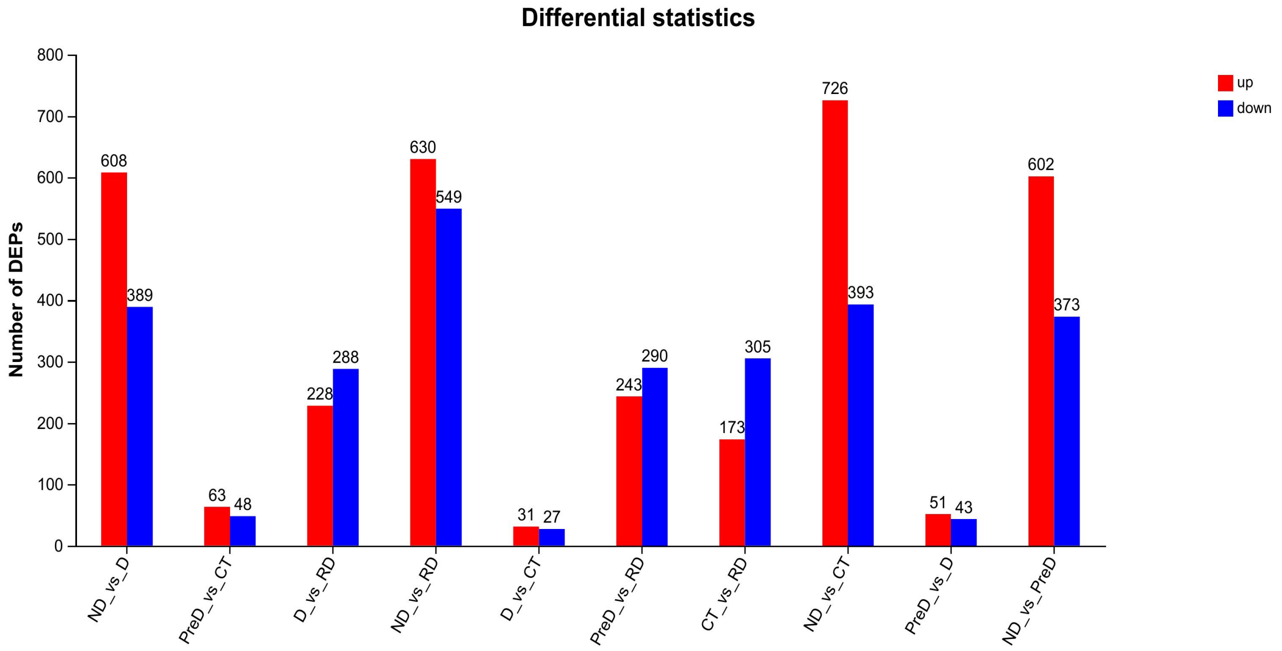
Differential statistics
up
down
Number of DEPs
800
700
600
500
400
300
200
100
0
608
389
63
48
228
288
630
549
31
27
243
290
173
305
726
393
51
43
602
373
ND_vs_D
PreD_vs_CT
D_vs_RD
ND_vs_RD
D_vs_CT
PreD_vs_RD
CT_vs_RD
ND_vs_CT
PreD_vs_D
ND_vs_PreD

Results

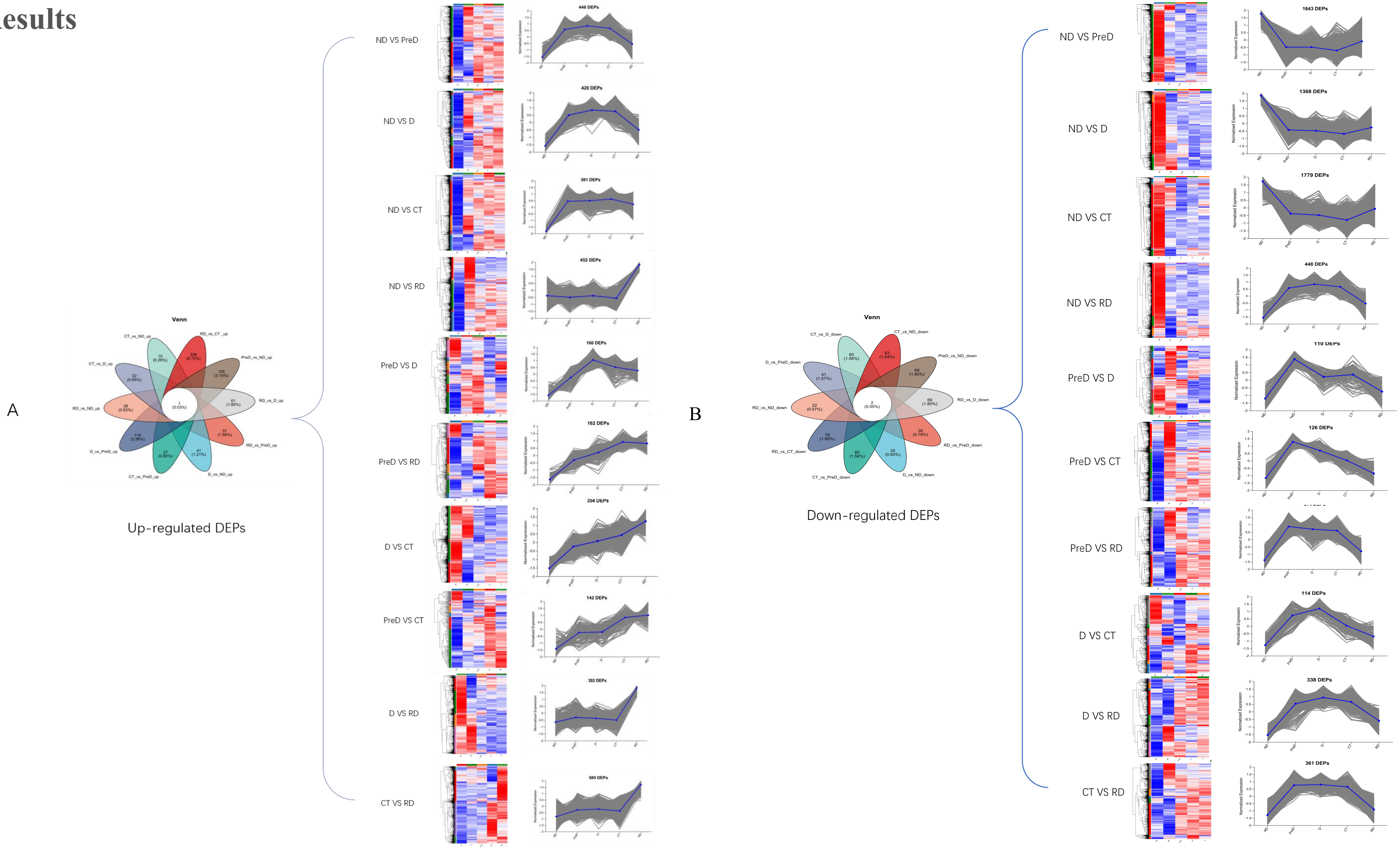
A
Venn
Up-regulated DEPs
ND VS PreD
ND VS D
ND VS CT
ND VS RD
PreD VS D
PreD VS RD
D VS CT
PreD VS CT
D VS RD
CT VS RD
448 DEPs
420 DEPs
561 DEPs
452 DEPs
160 DEPs
162 DEPs
142 DEPs
392 DEPs
589 DEPs
B
Venn
Down-regulated DEPs
ND VS PreD
ND VS D
ND VS CT
ND VS RD
PreD VS D
PreD VS CT
PreD VS RD
D VS CT
D VS RD
CT VS RD
1643 DEPs
1368 DEPs
1779 DEPs
448 DEPs
126 DEPs
114 DEPs
338 DEPs
361 DEPs

Results

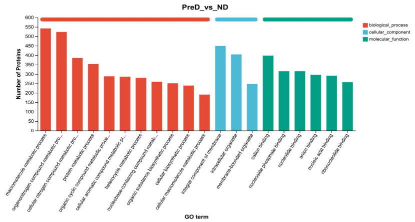
PreD_vs_ND
Number of Proteins
GO term
biological_process
cellular_component
molecular_function

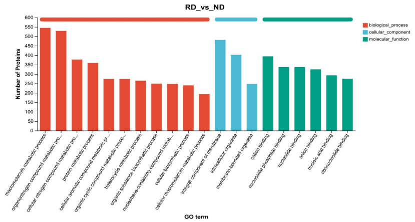
RD_vs_ND
Number of Proteins
GO term
biological_process
cellular_component
molecular_function

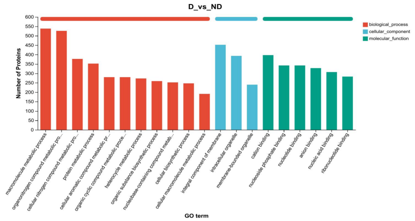
D_vs_ND
Number of Proteins
GO term
biological_process
cellular_component
molecular_function

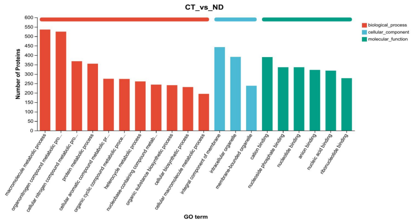
CT_vs_ND
Number of Proteins
GO term
biological_process
cellular_component
molecular_function

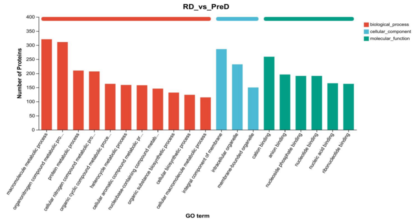
RD_vs_PreD
Number of Proteins
GO term
biological_process
cellular_component
molecular_function

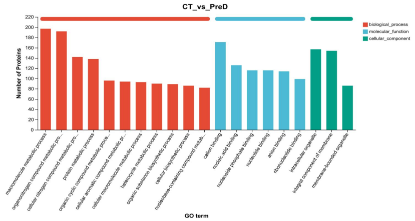
CT_vs_PreD
Number of Proteins
GO term
biological_process
molecular_function
cellular_component

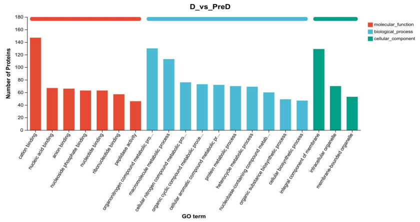
D_vs_PreD
Number of Proteins
GO term
molecular_function
biological_process
cellular_component

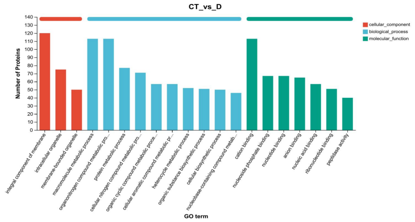
CT_vs_D
Number of Proteins
GO term
cellular_component
biological_process
molecular_function

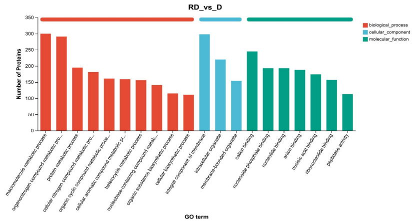
RD_vs_D
Number of Proteins
GO term
biological_process
cellular_component
molecular_function

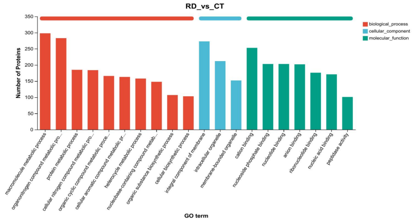
RD_vs_CT
Number of Proteins
GO term
biological_process
cellular_component
molecular_function

Results

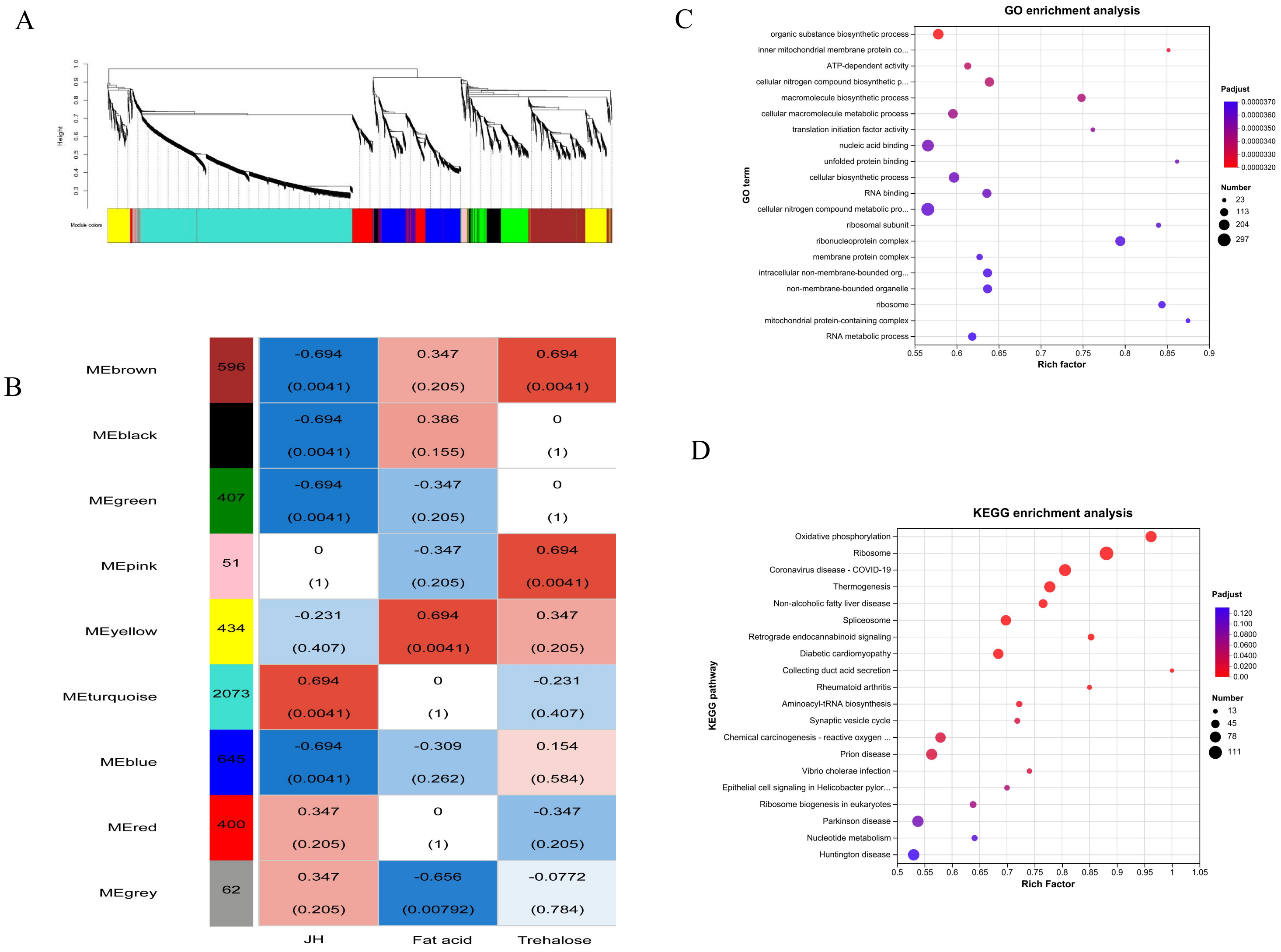
A
Height
Module colors
B
MEbrown
596
-0.694
(0.0041)
0.347
(0.205)
0.694
(0.0041)
MEblack
-0.694
(0.0041)
0.386
(0.155)
0
(1)
MEgreen
407
-0.694
(0.0041)
-0.347
(0.205)
0
(1)
MEpink
51
0
(1)
-0.347
(0.205)
0.694
(0.0041)
MEyellow
434
-0.231
(0.407)
0.694
(0.0041)
0.347
(0.205)
MEturquoise
2073
0.694
(0.0041)
0
(1)
-0.231
(0.407)
MEblue
645
-0.694
(0.0041)
-0.309
(0.262)
0.154
(0.584)
MEred
400
0.347
(0.205)
0
(1)
-0.347
(0.205)
MEgrey
62
0.347
(0.205)
-0.656
(0.00792)
-0.0772
(0.784)
JH
Fat acid
Trehalose
C
GO enrichment analysis
GO term
organic substance biosynthetic process
inner mitochondrial membrane protein co...
ATP-dependent activity
cellular nitrogen compound biosynthetic p...
macromolecule biosynthetic process
cellular macromolecule metabolic process
translation initiation factor activity
nucleic acid binding
unfolded protein binding
cellular biosynthetic process
RNA binding
cellular nitrogen compound metabolic pro...
ribosomal subunit
ribonucleoprotein complex
membrane protein complex
intracellular non-membrane-bounded org...
non-membrane-bounded organelle
ribosome
mitochondrial protein-containing complex
RNA metabolic process
Rich factor
Padjust
0.0000370
0.0000360
0.0000350
0.0000340
0.0000330
0.0000320
Number
23
113
204
297
D
KEGG enrichment analysis
KEGG pathway
Oxidative phosphorylation
Ribosome
Coronavirus disease - COVID-19
Thermogenesis
Non-alcoholic fatty liver disease
Spliceosome
Retrograde endocannabinoid signaling
Diabetic cardiomyopathy
Collecting duct acid secretion
Rheumatoid arthritis
Aminoacyl-tRNA biosynthesis
Synaptic vesicle cycle
Chemical carcinogenesis - reactive oxygen ...
Prion disease
Vibrio cholerae infection
Epithelial cell signaling in Helicobacter pylor...
Ribosome biogenesis in eukaryotes
Parkinson disease
Nucleotide metabolism
Huntington disease
Rich Factor
Padjust
0.120
0.100
0.0800
0.0600
0.0400
0.0200
0.00
Number
13
45
78
111

Results

A

B

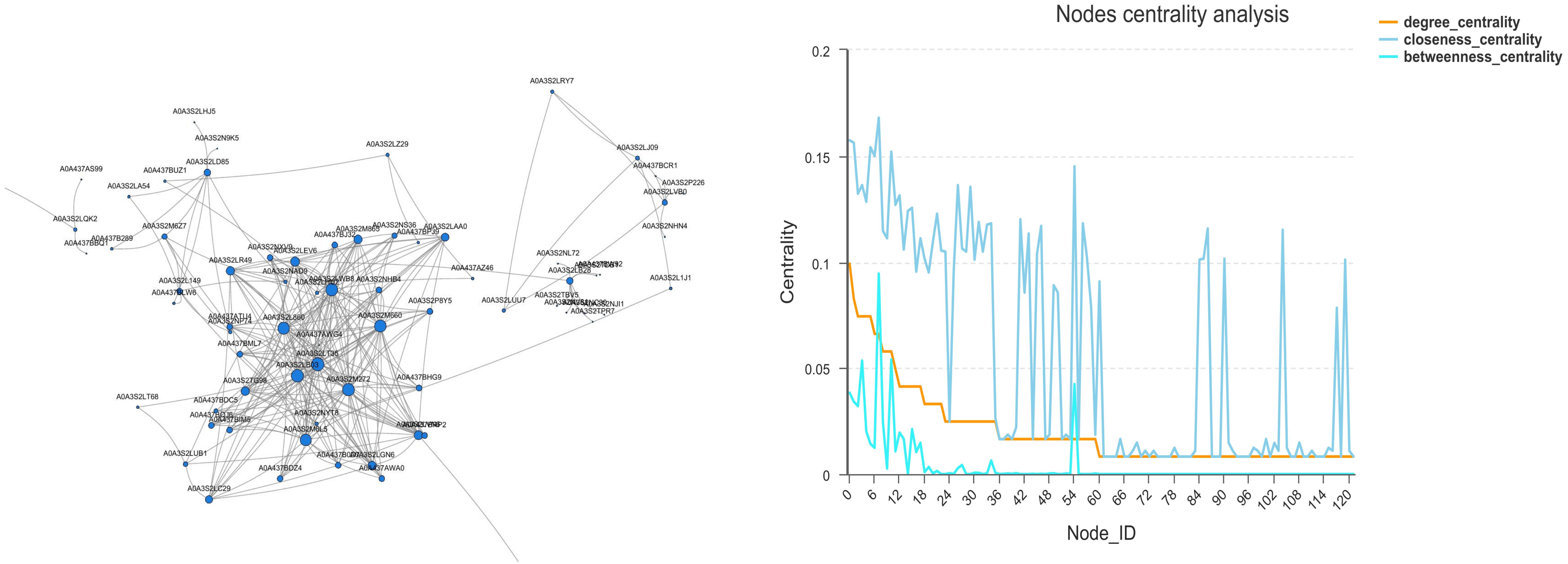

Nodes centrality analysis
degree_centrality
closeness_centrality
betweenness_centrality
Centrality
Node_ID
0.2
0.15
0.1
0.05
0
0
6
12
18
24
30
36
42
48
54
60
66
72
78
84
90
96
102
108
114
120

Results

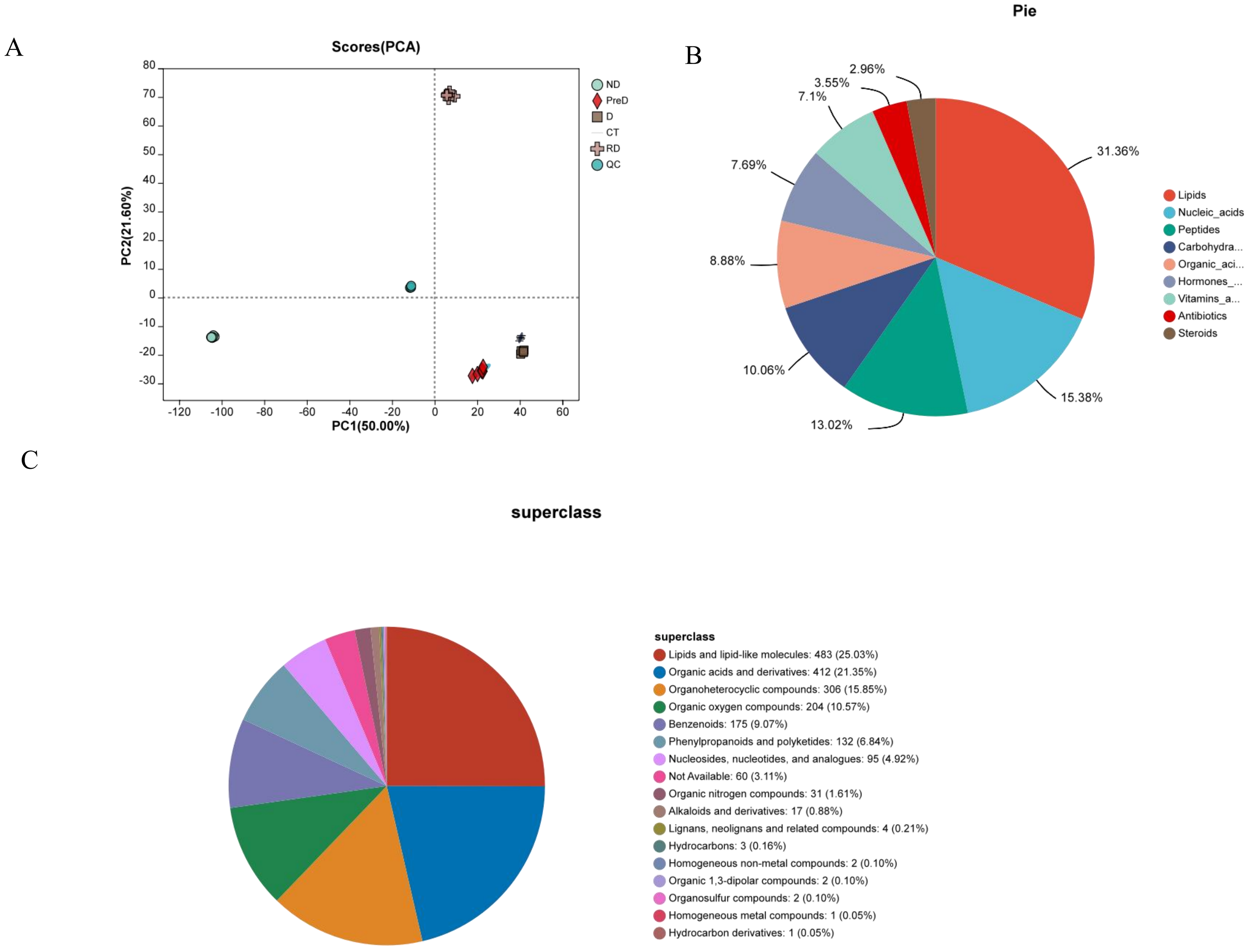
A
Scores(PCA)
ND
PreD
D
CT
RD
QC
PC2(21.60%)
PC1(50.00%)
80
70
60
50
40
30
20
10
0
-10
-20
-30
-120
-100
-80
-60
-40
-20
0
20
40
60
B
Pie
31.36%
15.38%
13.02%
10.06%
8.88%
7.69%
7.1%
3.55%
2.96%
Lipids
Nucleic_acids
Peptides
Carbohydra...
Organic_aci...
Hormones_...
Vitamins_a...
Antibiotics
Steroids
C
superclass
superclass
Lipids and lipid-like molecules: 483 (25.03%)
Organic acids and derivatives: 412 (21.35%)
Organoheterocyclic compounds: 306 (15.85%)
Organic oxygen compounds: 204 (10.57%)
Benzenoids: 175 (9.07%)
Phenylpropanoids and polyketides: 132 (6.84%)
Nucleosides, nucleotides, and analogues: 95 (4.92%)
Not Available: 60 (3.11%)
Organic nitrogen compounds: 31 (1.61%)
Alkaloids and derivatives: 17 (0.88%)
Lignans, neolignans and related compounds: 4 (0.21%)
Hydrocarbons: 3 (0.16%)
Homogeneous non-metal compounds: 2 (0.10%)
Organic 1,3-dipolar compounds: 2 (0.10%)
Organosulfur compounds: 2 (0.10%)
Homogeneous metal compounds: 1 (0.05%)
Hydrocarbon derivatives: 1 (0.05%)

Results

A

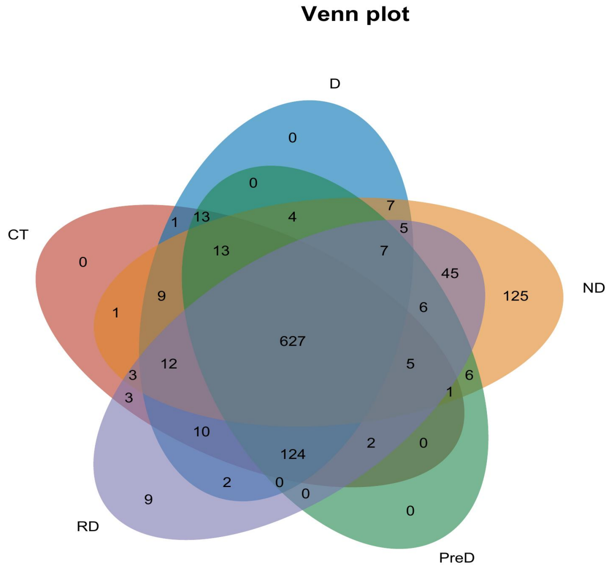
Venn plot
D
CT
ND
RD
PreD

C

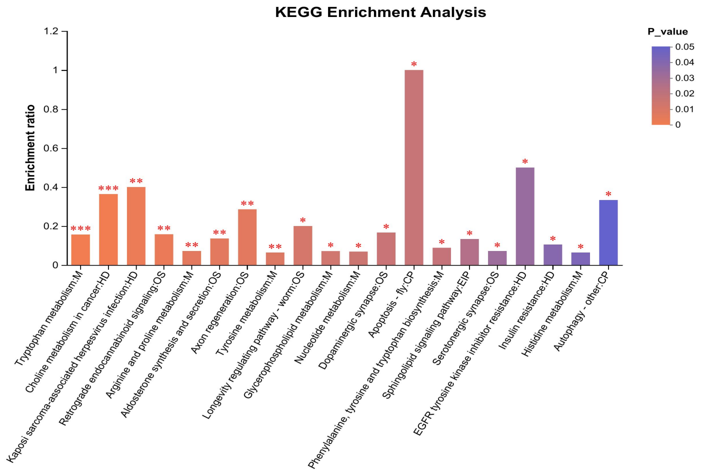
KEGG Enrichment Analysis
Enrichment ratio
P_value
Tryptophan metabolism:M
Choline metabolism in cancer:HD
Kaposi sarcoma-associated herpesvirus infection:HD
Retrograde endocannabinoid signaling:OS
Arginine and proline metabolism:M
Aldosterone synthesis and secretion:OS
Axon regeneration:OS
Tyrosine metabolism:M
Longevity regulating pathway - worm:OS
Glycerophospholipid metabolism:M
Nucleotide metabolism:M
Dopaminergic synapse:OS
Apoptosis - fly:CP
Phenylalanine, tyrosine and tryptophan biosynthesis:M
Sphingolipid signaling pathway:EIP
Serotonergic synapse:OS
EGFR tyrosine kinase inhibitor resistance:HD
Insulin resistance:HD
Histidine metabolism:M
Autophagy - other:CP

B

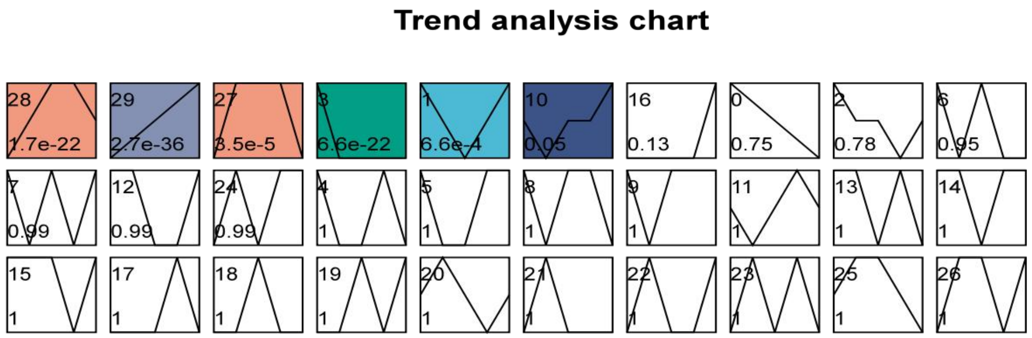
Trend analysis chart

Results

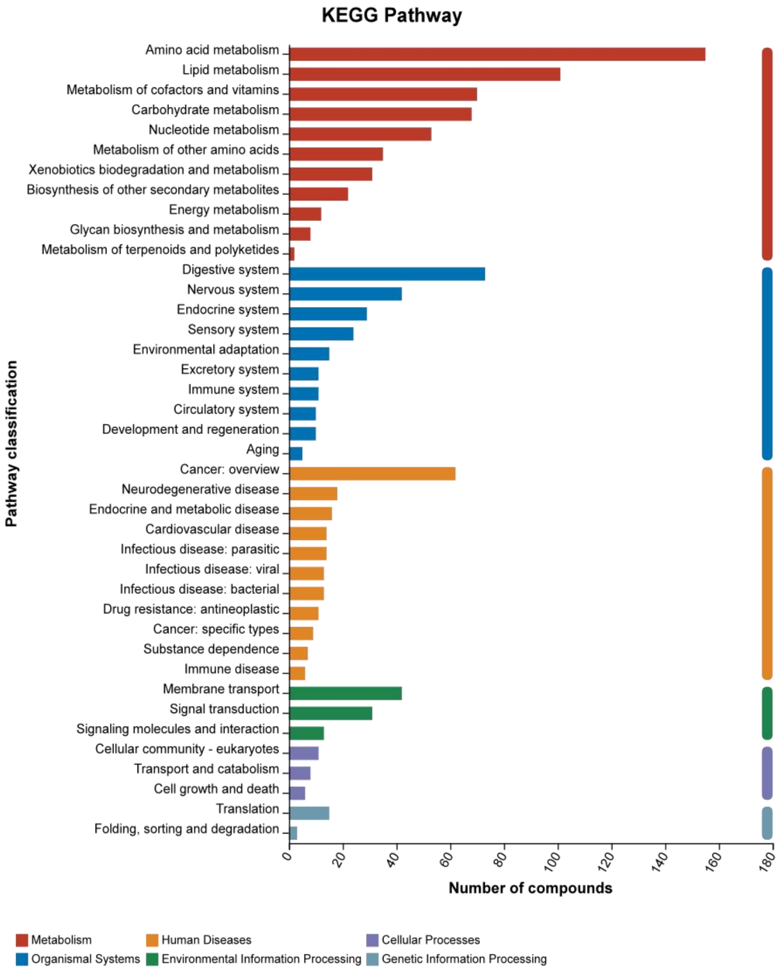
KEGG Pathway
Pathway classification
Amino acid metabolism
Lipid metabolism
Metabolism of cofactors and vitamins
Carbohydrate metabolism
Nucleotide metabolism
Metabolism of other amino acids
Xenobiotics biodegradation and metabolism
Biosynthesis of other secondary metabolites
Energy metabolism
Glycan biosynthesis and metabolism
Metabolism of terpenoids and polyketides
Digestive system
Nervous system
Endocrine system
Sensory system
Environmental adaptation
Excretory system
Immune system
Circulatory system
Development and regeneration
Aging
Cancer: overview
Neurodegenerative disease
Endocrine and metabolic disease
Cardiovascular disease
Infectious disease: parasitic
Infectious disease: viral
Infectious disease: bacterial
Drug resistance: antineoplastic
Cancer: specific types
Substance dependence
Immune disease
Membrane transport
Signal transduction
Signaling molecules and interaction
Cellular community - eukaryotes
Transport and catabolism
Cell growth and death
Translation
Folding, sorting and degradation
0 20 40 60 80 100 120 140 160 180
Number of compounds
Metabolism
Human Diseases
Cellular Processes
Organismal Systems
Environmental Information Processing
Genetic Information Processing

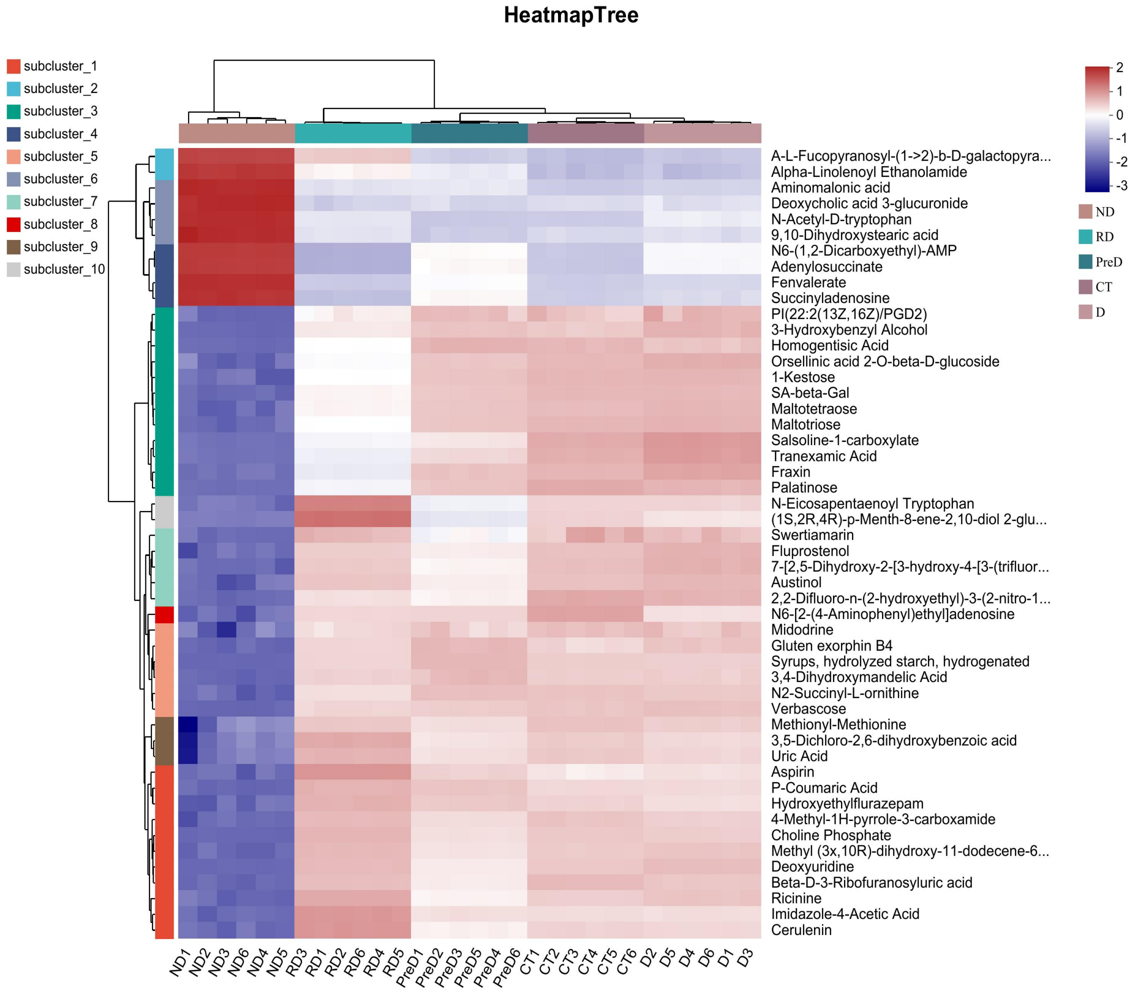
HeatmapTree
subcluster_1
subcluster_2
subcluster_3
subcluster_4
subcluster_5
subcluster_6
subcluster_7
subcluster_8
subcluster_9
subcluster_10
2
1
0
-1
-2
-3
ND
RD
PreD
CT
D
A-L-Fucopyranosyl-(1->2)-b-D-galactopyra...
Alpha-Linolenoyl Ethanolamide
Aminomalonic acid
Deoxycholic acid 3-glucuronide
N-Acetyl-D-tryptophan
9,10-Dihydroxystearic acid
N6-(1,2-Dicarboxyethyl)-AMP
Adenylosuccinate
Fenvalerate
Succinyladenosine
PI(22:2(13Z,16Z)/PGD2)
3-Hydroxybenzyl Alcohol
Homogentisic Acid
Orsellinic acid 2-O-beta-D-glucoside
1-Kestose
SA-beta-Gal
Maltotetraose
Maltotriose
Salsoline-1-carboxylate
Tranexamic Acid
Fraxin
Palatinose
N-Eicosapentaenoyl Tryptophan
(1S,2R,4R)-p-Menth-8-ene-2,10-diol 2-glu...
Swertiamarin
Fluprostenol
7-[2,5-Dihydroxy-2-[3-hydroxy-4-[3-(trifluor...
Austinol
2,2-Difluoro-n-(2-hydroxyethyl)-3-(2-nitro-1...
N6-[2-(4-Aminophenyl)ethyl]adenosine
Midodrine
Gluten exorphin B4
Syrups, hydrolyzed starch, hydrogenated
3,4-Dihydroxymandelic Acid
N2-Succinyl-L-ornithine
Verbascose
Methionyl-Methionine
3,5-Dichloro-2,6-dihydroxybenzoic acid
Uric Acid
Aspirin
P-Coumaric Acid
Hydroxyethylflurazepam
4-Methyl-1H-pyrrole-3-carboxamide
Choline Phosphate
Methyl (3x,10R)-dihydroxy-11-dodecene-6...
Deoxyuridine
Beta-D-3-Ribofuranosyluric acid
Ricinine
Imidazole-4-Acetic Acid
Cerulenin
ND1 ND2 ND3 ND6 ND4 ND5 RD3 RD1 RD2 RD6 RD4 RD5 PreD1 PreD2 PreD3 PreD5 PreD4 PreD6 CT1 CT2 CT3 CT4 CT5 CT6 D2 D5 D4 D6 D1 D3

Results

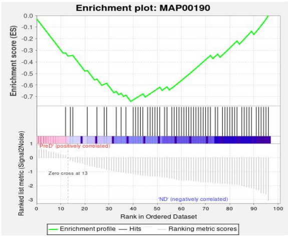

ND/PreD

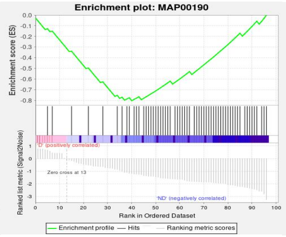

ND/D

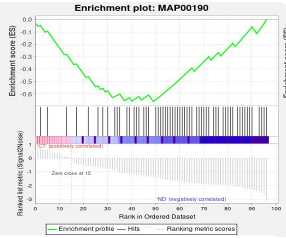

ND/CT

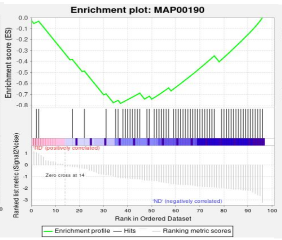

ND/RD

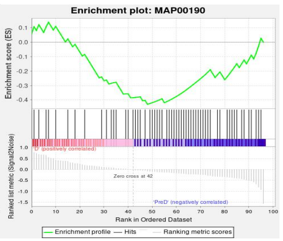

PreD/D

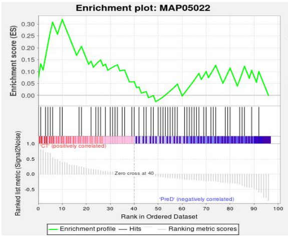

PreD/ CT

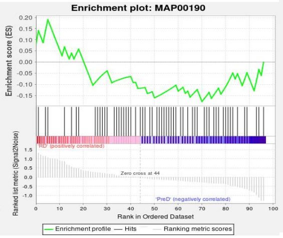

PreD/RD

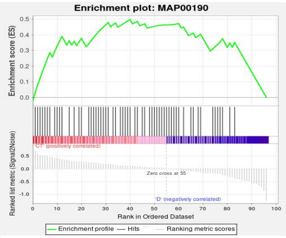

D/CT

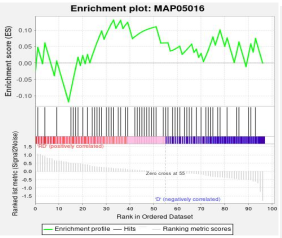

D/RD

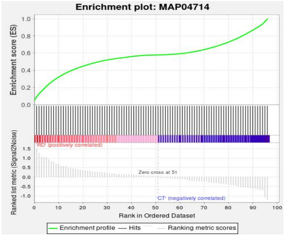

CT/RD

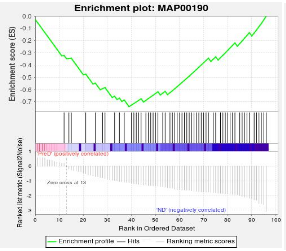

ND/PreD

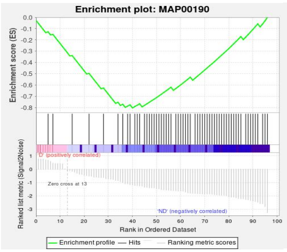

ND/D

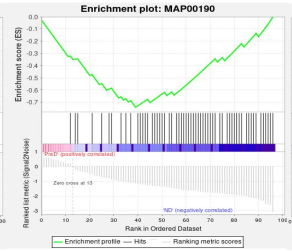

ND/CT

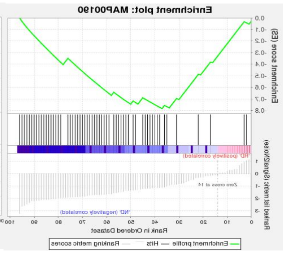
ND/RD

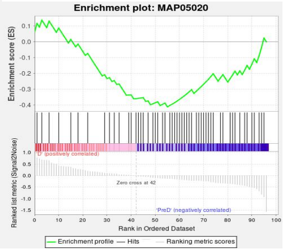

PreD/D

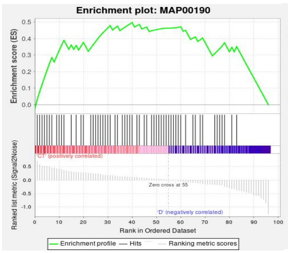

PreD/ CT

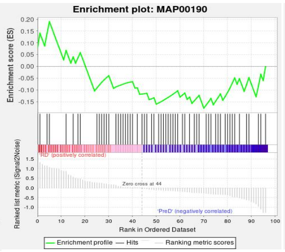

PreD/RD

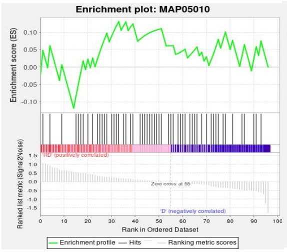

D/CT

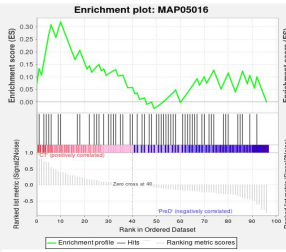

D/RD

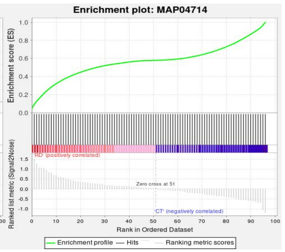

CT/RD

Results

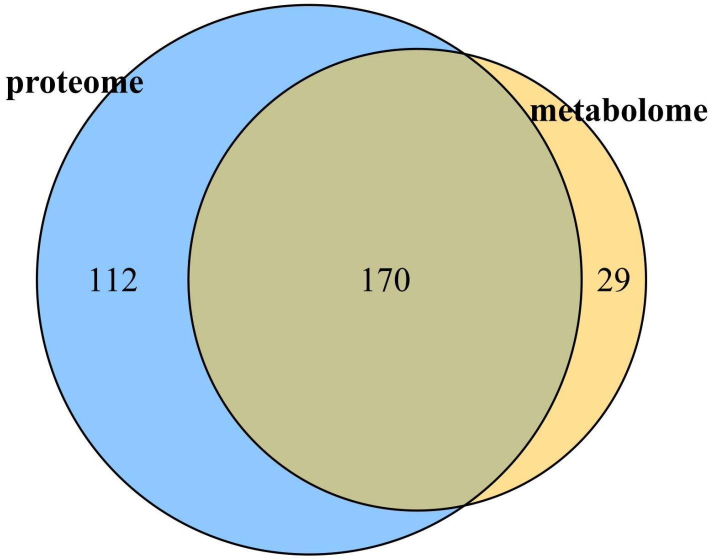
proteome
metabolome
112
170
29

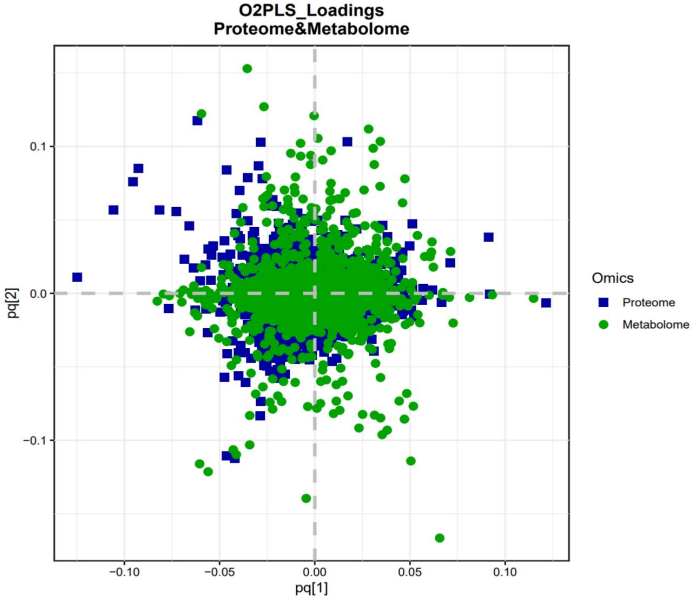
O2PLS_Loadings
Proteome&Metabolome
pq[2]
pq[1]
Omics
Proteome
Metabolome

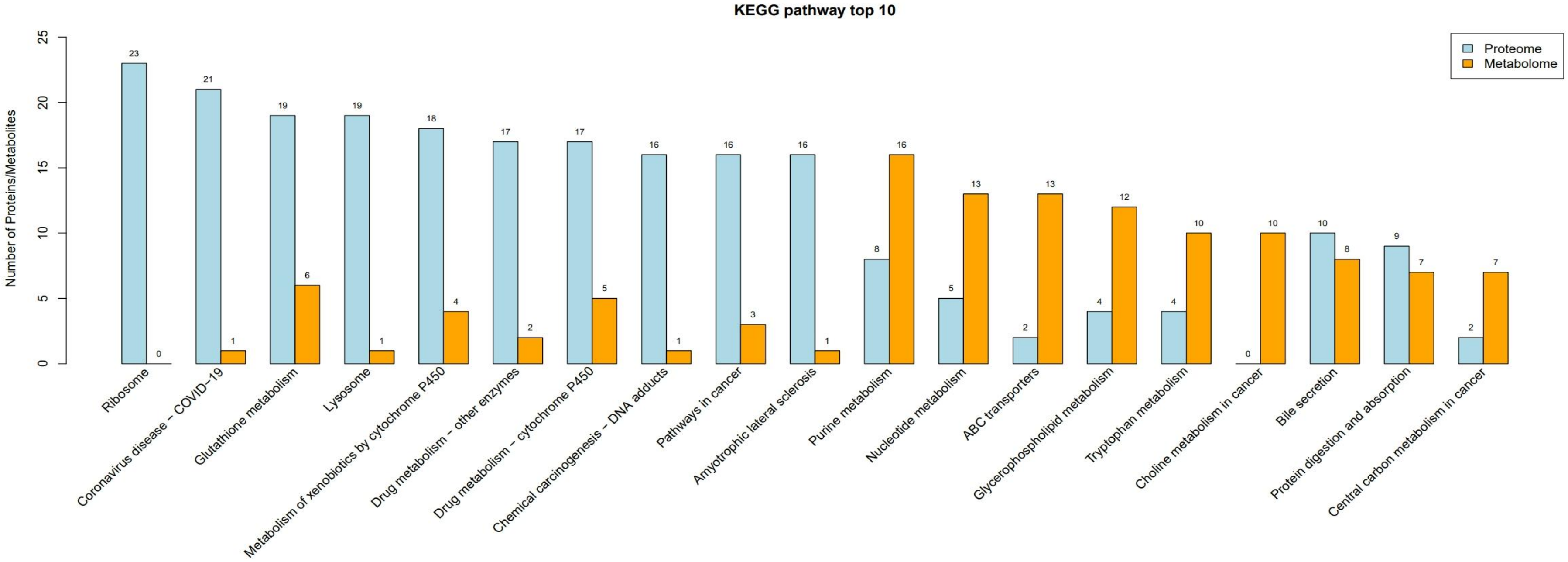
KEGG pathway top 10
Number of Proteins/Metabolites
Proteome
Metabolome
Ribosome
Coronavirus disease – COVID-19
Glutathione metabolism
Lysosome
Metabolism of xenobiotics by cytochrome P450
Drug metabolism – other enzymes
Drug metabolism – cytochrome P450
Chemical carcinogenesis – DNA adducts
Pathways in cancer
Amyotrophic lateral sclerosis
Purine metabolism
Nucleotide metabolism
ABC transporters
Glycerophospholipid metabolism
Tryptophan metabolism
Choline metabolism in cancer
Bile secretion
Protein digestion and absorption
Central carbon metabolism in cancer
