# Supplementary material for: Comparative Proteomics and Metabonomics Analysis of Different Diapause Stages Revealed a New Regulation Mechanism of Diapause in Loxostege sticticalis (Lepidoptera: Pyralidae)
Source: Molecules. 2024 Jul 25;29(15):3472. doi: 10.3390/molecules29153472 (PMC11314584; doi:10.3390/molecules29153472)
Supplement: Supplementary file 1 [file molecules-29-03472-s001.zip › analysis process/Joint analysis of proteomics and metabolomics/CTvsD.O2PLS_Loadings.pdf]

Object	pq[1]	pq[2]	Omics
TRINITY_DN19662_c0_g2_i1_orf1	-0.038935105	-0.035644613	Proteome
TRINITY_DN5080_c0_g1_i5_orf1	-0.046810254	-0.006186747	Proteome
TRINITY_DN11817_c0_g1_i4_orf1	-0.019523037	-0.011383044	Proteome
TRINITY_DN10430_c0_g1_i4_orf1	0.019687471	0.004062209	Proteome
TRINITY_DN841_c0_g1_i4_orf1	0.001544545	-0.008317867	Proteome
TRINITY_DN741_c0_g1_i10_orf1	0.012053465	0.010270144	Proteome
TRINITY_DN1211_c0_g1_i10_orf1	0.007654645	-0.001094722	Proteome
TRINITY_DN8258_c0_g1_i5_orf1	0.010458189	0.012803556	Proteome
TRINITY_DN1173_c0_g1_i11_orf1	0.003465803	0.009015382	Proteome
TRINITY_DN1665_c1_g1_i2_orf1	0.010826178	-0.001212964	Proteome
TRINITY_DN1889_c0_g1_i1_orf1	0.010471081	0.010730017	Proteome
TRINITY_DN41_c0_g1_i3_orf1	0.023546593	0.012096756	Proteome
TRINITY_DN6587_c0_g1_i3_orf1	-0.014159738	0.012097332	Proteome
TRINITY_DN1216_c0_g1_i4_orf1	0.013229043	0.01038316	Proteome
TRINITY_DN2300_c0_g1_i1_orf1	-0.002732934	0.001613523	Proteome
TRINITY_DN41842_c0_g1_i2_orf1	-0.002997623	0.000508534	Proteome
TRINITY_DN53238_c1_g1_i5_orf1	0.008865956	-0.006968002	Proteome
TRINITY_DN7394_c0_g2_i1_orf1	0.003683753	0.01376751	Proteome
TRINITY_DN1103_c0_g1_i18_orf1	-0.001845774	-0.022851449	Proteome
TRINITY_DN1103_c0_g1_i12_orf1	0.010384836	0.009537801	Proteome
TRINITY_DN46173_c0_g3_i1_orf1	0.00652487	0.013692119	Proteome
TRINITY_DN5628_c0_g1_i5_orf1	0.008414499	0.024526509	Proteome
TRINITY_DN39813_c0_g1_i1_orf1	-0.012312512	-0.012669824	Proteome
TRINITY_DN1068_c0_g1_i3_orf1	-0.006735814	0.001880811	Proteome
TRINITY_DN12387_c0_g1_i1_orf1	-0.003783139	0.004256766	Proteome
TRINITY_DN10222_c0_g1_i2_orf1	-0.019192652	-0.032055221	Proteome
TRINITY_DN16187_c0_g1_i1_orf1	0.012192516	0.003108415	Proteome
TRINITY_DN2146_c0_g2_i1_orf1	-0.027883549	0.03058153	Proteome
TRINITY_DN85412_c0_g1_i1_orf1	-0.036956973	0.016499945	Proteome
TRINITY_DN45530_c0_g1_i1_orf1	-0.014246731	0.00395066	Proteome
TRINITY_DN21533_c0_g1_i4_orf1	0.001107789	0.002240855	Proteome
TRINITY_DN1563_c0_g1_i4_orf1	-0.011495343	0.009229052	Proteome
TRINITY_DN67716_c0_g1_i1_orf1	-0.00378367	0.01441077	Proteome
TRINITY_DN120439_c1_g1_i1_orf1	0.00797264	0.013330877	Proteome
TRINITY_DN1196_c0_g1_i4_orf1	0.007609641	0.011883787	Proteome
TRINITY_DN19079_c0_g1_i5_orf1	0.00159712	0.007382189	Proteome
TRINITY_DN19260_c0_g1_i5_orf1	0.007376697	0.004003914	Proteome
TRINITY_DN26688_c0_g1_i2_orf1	0.003412924	-0.002587353	Proteome
TRINITY_DN20279_c0_g1_i1_orf1	-0.004769611	-0.005821334	Proteome
TRINITY_DN6325_c0_g1_i9_orf1	-0.011323802	-0.039613897	Proteome
TRINITY_DN1587_c0_g1_i4_orf1	0.008225303	0.004148543	Proteome
TRINITY_DN2919_c0_g1_i5_orf1	0.005380046	0.003204364	Proteome
TRINITY_DN29604_c0_g2_i2_orf1	0.0036374	0.02214293	Proteome
TRINITY_DN5765_c0_g2_i3_orf1	0.004961771	0.010575567	Proteome
TRINITY_DN91_c0_g1_i9_orf1	0.010378133	0.007597621	Proteome
TRINITY_DN1206_c0_g1_i6_orf1	-0.009729316	0.006925063	Proteome
TRINITY_DN1790_c1_g1_i3_orf1	-0.004454115	0.002721927	Proteome
TRINITY_DN1437_c0_g1_i6_orf1	0.006647481	0.007228323	Proteome
TRINITY_DN42269_c2_g1_i1_orf1	-0.004458618	0.005297232	Proteome
TRINITY_DN9_c0_g1_i11_orf1	-0.005865342	-0.012469567	Proteome
TRINITY_DN20294_c0_g2_i1_orf1	-0.003139057	0.005476595	Proteome
TRINITY_DN1173_c1_g1_i10_orf1	0.002575256	0.021440099	Proteome
TRINITY_DN30178_c0_g1_i3_orf1	0.011752902	-0.004079856	Proteome
TRINITY_DN2173_c0_g1_i1_orf1	-0.004009258	0.007146296	Proteome
TRINITY_DN2515_c0_g1_i6_orf1	0.012496703	0.006442912	Proteome
TRINITY_DN2186_c0_g1_i13_orf1	0.000948322	0.00533058	Proteome
TRINITY_DN1125_c0_g1_i4_orf1	0.014769178	0.004626313	Proteome

TRINITY_DN78492_c0_g1_i1_orf1	0.015200326	0.002172164	Proteome
TRINITY_DN57900_c0_g1_i2_orf1	0.030219561	0.008206854	Proteome
TRINITY_DN8406_c0_g1_i2_orf1	0.005515092	0.017919716	Proteome
TRINITY_DN7579_c1_g3_i1_orf1	-0.012243275	0.011219258	Proteome
TRINITY_DN32487_c0_g1_i1_orf1	0.00115597	-0.008598995	Proteome
TRINITY_DN4561_c0_g1_i3_orf1	0.006670178	0.002682268	Proteome
TRINITY_DN1091_c0_g3_i1_orf1	0.02243194	0.025446583	Proteome
TRINITY_DN1814_c0_g2_i1_orf1	0.012351971	0.011248024	Proteome
TRINITY_DN7991_c0_g1_i9_orf1	0.00881648	0.004131393	Proteome
TRINITY_DN129869_c0_g4_i1_orf1	0.00845935	0.005922216	Proteome
TRINITY_DN4998_c0_g1_i21_orf1	-0.01587828	0.012414269	Proteome
TRINITY_DN668_c0_g1_i4_orf1	0.002254208	-0.010447444	Proteome
TRINITY_DN4779_c0_g1_i5_orf1	0.006827974	-0.003827786	Proteome
TRINITY_DN19662_c4_g1_i1_orf1	-0.036508443	-0.014380339	Proteome
TRINITY_DN19651_c0_g1_i1_orf1	-0.014450216	0.007702146	Proteome
TRINITY_DN812_c2_g1_i1_orf1	-0.007007623	-0.005828265	Proteome
TRINITY_DN1175_c1_g1_i2_orf1	-0.02117443	0.015243851	Proteome
TRINITY_DN6580_c0_g1_i4_orf1	0.018907321	0.006055124	Proteome
TRINITY_DN20984_c0_g1_i4_orf1	0.014582505	0.002233203	Proteome
TRINITY_DN8405_c0_g1_i4_orf1	0.010965988	-0.003693858	Proteome
TRINITY_DN7570_c0_g1_i18_orf1	0.011469998	-0.00020609	Proteome
TRINITY_DN2338_c0_g1_i5_orf1	-0.017771749	0.003977115	Proteome
TRINITY_DN5129_c0_g3_i3_orf1	-0.002653517	-0.011817283	Proteome
TRINITY_DN1084_c0_g1_i2_orf1	-0.013489256	1.72E-05	Proteome
TRINITY_DN5564_c0_g1_i1_orf1	-0.00091415	-0.007075189	Proteome
TRINITY_DN248_c0_g1_i12_orf1	-0.010449307	0.011352803	Proteome
TRINITY_DN1725_c0_g1_i7_orf1	0.009904508	0.002012006	Proteome
TRINITY_DN40126_c0_g1_i1_orf1	0.010149486	-0.024451261	Proteome
TRINITY_DN2338_c0_g2_i2_orf1	-0.009542067	0.012537504	Proteome
TRINITY_DN5768_c0_g1_i2_orf1	-0.011355123	0.002901226	Proteome
TRINITY_DN3784_c0_g1_i1_orf1	-0.002779731	-0.002349228	Proteome
TRINITY_DN11274_c0_g1_i4_orf1	-0.014154414	0.003995009	Proteome
TRINITY_DN1831_c0_g1_i3_orf1	-0.007873583	-0.001586972	Proteome
TRINITY_DN69_c0_g1_i1_orf1	0.027565728	-0.012087259	Proteome
TRINITY_DN22747_c0_g1_i5_orf1	0.011933114	-0.001136155	Proteome
TRINITY_DN9286_c0_g1_i2_orf1	0.011773672	0.002419388	Proteome
TRINITY_DN2251_c0_g1_i4_orf1	-0.013948184	0.019985784	Proteome
TRINITY_DN1175_c1_g1_i1_orf1	0.00735822	0.000798741	Proteome
TRINITY_DN4279_c0_g1_i4_orf1	0.001877883	-0.01933944	Proteome
TRINITY_DN56155_c0_g1_i1_orf1	0.008073627	-0.011201967	Proteome
TRINITY_DN4572_c0_g1_i2_orf1	0.006187175	0.007181084	Proteome
TRINITY_DN7854_c0_g1_i4_orf1	0.003952209	0.013635147	Proteome
TRINITY_DN825_c8_g1_i5_orf1	0.005597264	-0.002208166	Proteome
TRINITY_DN1233_c0_g2_i1_orf1	0.002323834	0.007833002	Proteome
TRINITY_DN931_c0_g1_i4_orf1	-0.005016545	0.005890259	Proteome
TRINITY_DN1180_c0_g1_i4_orf1	0.012656369	0.019760591	Proteome
TRINITY_DN14670_c0_g1_i1_orf1	0.002629592	0.004816663	Proteome
TRINITY_DN73923_c0_g1_i1_orf1	0.00132881	0.009931634	Proteome
TRINITY_DN181_c0_g1_i3_orf1	0.008624859	0.002000812	Proteome
TRINITY_DN9101_c0_g2_i1_orf1	0.004423904	0.011272851	Proteome
TRINITY_DN2069_c1_g1_i8_orf1	0.009992094	-0.015762322	Proteome
TRINITY_DN2807_c0_g1_i4_orf1	0.005002231	0.000890124	Proteome
TRINITY_DN3393_c0_g2_i1_orf1	0.006927458	0.003749588	Proteome
TRINITY_DN1982_c0_g1_i24_orf1	0.003542063	-0.013910911	Proteome
TRINITY_DN18482_c0_g1_i3_orf1	-0.013542278	0.027449104	Proteome
TRINITY_DN1177_c0_g1_i4_orf1	0.012400288	0.005121183	Proteome
TRINITY_DN8535_c0_g1_i9_orf1	-0.003784789	0.010600102	Proteome
TRINITY_DN1775_c0_g1_i3_orf1	-0.001748052	0.005855005	Proteome

TRINITY_DN4125_c1_g1_i5_orf1	-0.000543166	-0.007764732	Proteome
TRINITY_DN23801_c0_g1_i2_orf1	0.011135904	-0.001482714	Proteome
TRINITY_DN2425_c1_g1_i4_orf1	0.008746349	-0.007519835	Proteome
TRINITY_DN55026_c0_g1_i1_orf1	-0.005025688	-0.002315926	Proteome
TRINITY_DN1265_c0_g1_i9_orf1	-0.005687167	-0.026116822	Proteome
TRINITY_DN7770_c0_g1_i4_orf1	0.000979104	-0.004870167	Proteome
TRINITY_DN1669_c0_g1_i6_orf1	0.001589801	-0.015157211	Proteome
TRINITY_DN1444_c1_g1_i5_orf1	0.00973127	-0.000130169	Proteome
TRINITY_DN5439_c0_g1_i2_orf1	0.045005855	-0.005179803	Proteome
TRINITY_DN7251_c0_g1_i3_orf1	0.002281606	0.007638032	Proteome
TRINITY_DN14286_c0_g1_i5_orf1	0.007378961	-0.005835518	Proteome
TRINITY_DN48638_c0_g1_i5_orf1	-0.008719403	-0.003818813	Proteome
TRINITY_DN1772_c1_g2_i1_orf1	0.027136231	-0.019022886	Proteome
TRINITY_DN15812_c0_g1_i2_orf1	-0.010994522	0.022605896	Proteome
TRINITY_DN80560_c0_g1_i1_orf1	-0.001825896	-0.002522031	Proteome
TRINITY_DN5512_c0_g1_i8_orf1	0.008837802	-0.000631074	Proteome
TRINITY_DN1132_c0_g1_i5_orf1	-0.001553536	0.009556312	Proteome
TRINITY_DN13660_c0_g1_i1_orf1	-0.024993156	0.009264553	Proteome
TRINITY_DN664_c0_g1_i18_orf1	0.007501732	0.02064428	Proteome
TRINITY_DN1201_c0_g1_i4_orf1	0.004225331	0.014562255	Proteome
TRINITY_DN1348_c0_g1_i1_orf1	0.004807592	0.003787361	Proteome
TRINITY_DN42753_c0_g1_i2_orf1	-0.003706642	-0.010073058	Proteome
TRINITY_DN3433_c0_g1_i15_orf1	0.024436063	-0.001834693	Proteome
TRINITY_DN32681_c0_g1_i3_orf1	0.009351101	-0.006415652	Proteome
TRINITY_DN4861_c0_g1_i7_orf1	-0.00094272	-0.007785684	Proteome
TRINITY_DN116_c1_g1_i8_orf1	0.004723023	-0.004438381	Proteome
TRINITY_DN86090_c0_g1_i1_orf1	0.000644494	-0.001111059	Proteome
TRINITY_DN1084_c0_g2_i2_orf1	-0.003704373	0.010293782	Proteome
TRINITY_DN565_c0_g1_i1_orf1	-0.004070814	-0.006037635	Proteome
TRINITY_DN21533_c0_g1_i6_orf1	-0.008692833	-0.016091303	Proteome
TRINITY_DN6313_c0_g1_i4_orf1	-0.005858227	-0.002168614	Proteome
TRINITY_DN1334_c0_g1_i2_orf1	-0.009963337	0.001613417	Proteome
TRINITY_DN34455_c0_g1_i1_orf1	-0.000824676	0.011005555	Proteome
TRINITY_DN21722_c0_g1_i3_orf1	0.003542645	-0.001528621	Proteome
TRINITY_DN63536_c0_g1_i1_orf1	-0.010582525	0.018782289	Proteome
TRINITY_DN4592_c0_g1_i1_orf1	0.008752575	-0.010072083	Proteome
TRINITY_DN1265_c0_g1_i4_orf1	0.009384388	0.002120409	Proteome
TRINITY_DN6291_c0_g1_i4_orf1	0.009690584	0.004792363	Proteome
TRINITY_DN98538_c0_g1_i1_orf1	-0.002519625	-0.008719078	Proteome
TRINITY_DN11177_c0_g1_i4_orf1	0.002919052	-0.000125673	Proteome
TRINITY_DN44288_c0_g1_i2_orf1	0.000241587	-0.006003513	Proteome
TRINITY_DN9592_c0_g1_i2_orf1	0.014242145	0.000293565	Proteome
TRINITY_DN841_c0_g1_i8_orf1	0.011638272	0.010861136	Proteome
TRINITY_DN1306_c0_g1_i8_orf1	0.00920836	0.008215791	Proteome
TRINITY_DN2745_c0_g1_i4_orf1	-0.008236181	0.008018782	Proteome
TRINITY_DN48548_c0_g1_i1_orf1	-0.018357793	-0.044393329	Proteome
TRINITY_DN16125_c0_g1_i3_orf1	-0.007349999	0.009694441	Proteome
TRINITY_DN5266_c0_g1_i1_orf1	0.010870226	0.003684029	Proteome
TRINITY_DN9965_c0_g1_i1_orf1	0.002578268	0.001987427	Proteome
TRINITY_DN726_c0_g1_i8_orf1	-0.004762658	0.008392722	Proteome
TRINITY_DN3175_c0_g1_i7_orf1	0.008389858	0.008704391	Proteome
TRINITY_DN2648_c0_g1_i8_orf1	-0.003118219	-0.010903149	Proteome
TRINITY_DN28299_c0_g1_i1_orf1	-0.010154607	-0.011309363	Proteome
TRINITY_DN11152_c0_g1_i8_orf1	0.007777517	-0.003626208	Proteome
TRINITY_DN6822_c0_g2_i4_orf1	-0.000313639	0.019020776	Proteome
TRINITY_DN3442_c0_g1_i4_orf1	0.009473163	-0.005981005	Proteome
TRINITY_DN9239_c0_g1_i1_orf1	-0.03889811	-0.013126124	Proteome
TRINITY_DN29873_c0_g1_i1_orf1	-0.016334768	0.007758131	Proteome

TRINITY_DN1880_c0_g1_i4_orf1	-0.031355828	0.030650415	Proteome
TRINITY_DN121_c0_g1_i9_orf1	-0.002263577	-0.004648857	Proteome
TRINITY_DN5099_c0_g1_i3_orf1	0.020884741	0.003031833	Proteome
TRINITY_DN1827_c0_g1_i4_orf1	-0.012232142	0.021687347	Proteome
TRINITY_DN1533_c0_g2_i1_orf1	0.004561812	0.014354068	Proteome
TRINITY_DN2430_c0_g1_i1_orf1	0.011451608	-0.00159522	Proteome
TRINITY_DN53684_c0_g1_i1_orf1	0.00251567	-9.56E-06	Proteome
TRINITY_DN140_c0_g1_i1_orf1	-0.023134466	0.010890517	Proteome
TRINITY_DN1423_c0_g1_i4_orf1	-0.018489428	0.018371973	Proteome
TRINITY_DN13118_c0_g1_i6_orf1	0.009662701	-0.006405926	Proteome
TRINITY_DN1829_c0_g1_i1_orf1	-0.004256233	0.005396905	Proteome
TRINITY_DN348_c0_g2_i3_orf1	0.000149345	0.000992017	Proteome
TRINITY_DN12301_c0_g1_i1_orf1	-0.017637974	-0.005024365	Proteome
TRINITY_DN6415_c0_g1_i1_orf1	0.020730284	-0.005637857	Proteome
TRINITY_DN9853_c0_g3_i1_orf1	0.006126911	-0.002744961	Proteome
TRINITY_DN7183_c0_g1_i2_orf1	-0.009686763	-0.025433086	Proteome
TRINITY_DN7131_c0_g1_i2_orf1	0.00956778	0.006781844	Proteome
TRINITY_DN741_c0_g1_i1_orf1	-0.002748519	-0.021681091	Proteome
TRINITY_DN3991_c0_g1_i6_orf1	0.019412809	0.00150697	Proteome
TRINITY_DN235_c0_g1_i2_orf1	0.01189107	0.001971556	Proteome
TRINITY_DN2813_c0_g1_i7_orf1	0.045502689	0.015376094	Proteome
TRINITY_DN592_c0_g1_i6_orf1	0.018173485	0.002187895	Proteome
TRINITY_DN3158_c0_g1_i5_orf1	0.009520573	0.009916743	Proteome
TRINITY_DN7464_c1_g1_i1_orf1	0.005602729	0.003357764	Proteome
TRINITY_DN11467_c0_g1_i5_orf1	-0.023571228	0.023709885	Proteome
TRINITY_DN2049_c1_g1_i2_orf1	-0.007863251	-0.024512719	Proteome
TRINITY_DN20776_c0_g1_i3_orf1	0.006918534	-0.001501918	Proteome
TRINITY_DN13368_c0_g1_i1_orf1	0.000949397	-0.006334392	Proteome
TRINITY_DN9234_c0_g1_i5_orf1	-0.010106111	-0.007998433	Proteome
TRINITY_DN2019_c0_g1_i4_orf1	0.004599902	-0.009726518	Proteome
TRINITY_DN6358_c0_g1_i5_orf1	0.021012373	0.005350207	Proteome
TRINITY_DN970_c0_g1_i4_orf1	0.011332572	0.011685254	Proteome
TRINITY_DN19251_c0_g1_i8_orf1	0.002343201	0.01084746	Proteome
TRINITY_DN97138_c0_g1_i2_orf1	0.001592437	-0.020962763	Proteome
TRINITY_DN1423_c0_g1_i8_orf1	-0.027629241	0.002283019	Proteome
TRINITY_DN19727_c0_g1_i7_orf1	-0.001431908	0.00500256	Proteome
TRINITY_DN26209_c0_g1_i6_orf1	0.030584011	0.009242195	Proteome
TRINITY_DN620_c0_g1_i4_orf1	0.009980413	0.002442526	Proteome
TRINITY_DN8258_c0_g1_i6_orf1	0.008450747	0.010712127	Proteome
TRINITY_DN779_c0_g1_i3_orf1	0.001134761	0.022284166	Proteome
TRINITY_DN993_c0_g1_i7_orf1	-0.038014738	-0.001656234	Proteome
TRINITY_DN6483_c0_g1_i6_orf1	0.013648292	0.010625003	Proteome
TRINITY_DN2103_c0_g1_i1_orf1	-0.003218388	0.00581982	Proteome
TRINITY_DN17759_c0_g1_i5_orf1	0.00058061	0.016013395	Proteome
TRINITY_DN1209_c0_g1_i9_orf1	-0.037103494	0.014722907	Proteome
TRINITY_DN11178_c0_g1_i1_orf1	0.010705069	0.005693059	Proteome
TRINITY_DN37372_c0_g1_i1_orf1	0.006376953	0.013010168	Proteome
TRINITY_DN14436_c0_g1_i7_orf1	0.00466322	-0.003951703	Proteome
TRINITY_DN1103_c0_g1_i19_orf1	-0.004878326	0.005366592	Proteome
TRINITY_DN21251_c1_g1_i1_orf1	0.011485814	0.004586185	Proteome
TRINITY_DN5432_c1_g1_i3_orf1	0.000515312	-0.000218944	Proteome
TRINITY_DN18539_c0_g1_i1_orf1	0.001743973	-0.011522433	Proteome
TRINITY_DN30638_c0_g1_i1_orf1	0.00727422	0.002982604	Proteome
TRINITY_DN25633_c0_g1_i1_orf1	0.006409911	-0.007507282	Proteome
TRINITY_DN12767_c0_g1_i2_orf1	0.009909033	0.002278706	Proteome
TRINITY_DN5628_c0_g1_i3_orf1	0.01337285	0.011871815	Proteome
TRINITY_DN760_c1_g2_i6_orf1	-0.005995751	-0.013481737	Proteome
TRINITY_DN3959_c1_g2_i1_orf1	-0.001496122	-0.006360715	Proteome

TRINITY_DN1044_c0_g1_i2_orf1	0.002065126	-0.006949869	Proteome
TRINITY_DN5510_c0_g1_i9_orf1	0.013619149	0.013154603	Proteome
TRINITY_DN5218_c0_g1_i4_orf1	0.00143806	-0.008591663	Proteome
TRINITY_DN21533_c0_g1_i7_orf1	0.002542742	0.021173548	Proteome
TRINITY_DN42646_c0_g2_i1_orf1	0.011174739	-0.000989375	Proteome
TRINITY_DN18323_c0_g1_i5_orf1	-0.010737114	0.032753299	Proteome
TRINITY_DN33967_c0_g1_i1_orf1	0.008891342	-0.00578587	Proteome
TRINITY_DN1329_c0_g1_i5_orf1	0.022600444	0.015149265	Proteome
TRINITY_DN2999_c1_g2_i1_orf1	0.007055036	-0.002597391	Proteome
TRINITY_DN1750_c1_g1_i5_orf1	-0.015661344	-0.004412536	Proteome
TRINITY_DN94625_c0_g1_i1_orf1	0.002610754	0.008020763	Proteome
TRINITY_DN4451_c0_g2_i4_orf1	-0.010835004	0.006297113	Proteome
TRINITY_DN32822_c0_g1_i1_orf1	0.006702195	-0.00909942	Proteome
TRINITY_DN41166_c0_g1_i1_orf1	0.006730256	0.008081434	Proteome
TRINITY_DN25341_c0_g1_i1_orf1	0.00458654	0.008529996	Proteome
TRINITY_DN32011_c0_g1_i1_orf1	0.005280173	0.008060188	Proteome
TRINITY_DN2803_c4_g1_i1_orf1	-0.022190073	-0.001718534	Proteome
TRINITY_DN3805_c0_g1_i2_orf1	0.011479501	0.008129789	Proteome
TRINITY_DN343_c0_g1_i5_orf1	-0.005329926	0.003351925	Proteome
TRINITY_DN2745_c0_g1_i2_orf1	-0.010913437	-0.015424356	Proteome
TRINITY_DN1455_c0_g1_i8_orf1	0.00829993	0.016712798	Proteome
TRINITY_DN1161_c0_g1_i2_orf1	-0.020015337	0.001581579	Proteome
TRINITY_DN4424_c0_g1_i1_orf1	0.01205276	-0.000709802	Proteome
TRINITY_DN585_c0_g1_i5_orf1	-0.011548956	0.016529654	Proteome
TRINITY_DN3534_c0_g1_i2_orf1	0.010959175	9.17E-05	Proteome
TRINITY_DN1756_c0_g1_i3_orf1	0.014481447	-0.005784532	Proteome
TRINITY_DN1008_c0_g1_i2_orf1	0.020045984	-7.35E-05	Proteome
TRINITY_DN655_c0_g1_i3_orf1	0.00569471	0.000371431	Proteome
TRINITY_DN948_c0_g1_i1_orf1	0.003343777	0.006729237	Proteome
TRINITY_DN17913_c0_g1_i8_orf1	-0.01221062	0.000387527	Proteome
TRINITY_DN4920_c0_g1_i5_orf1	0.007483597	0.006912426	Proteome
TRINITY_DN801_c0_g1_i2_orf1	0.00210313	0.024164994	Proteome
TRINITY_DN15160_c0_g1_i1_orf1	-0.017680647	0.003497101	Proteome
TRINITY_DN2956_c0_g1_i6_orf1	0.016472821	-0.003137975	Proteome
TRINITY_DN1572_c0_g1_i6_orf1	0.002564041	0.003229414	Proteome
TRINITY_DN631_c0_g1_i6_orf1	0.010579871	0.002998965	Proteome
TRINITY_DN12106_c0_g1_i4_orf1	0.012953372	0.005115868	Proteome
TRINITY_DN9492_c0_g1_i7_orf1	0.0130492	0.008308661	Proteome
TRINITY_DN53807_c0_g2_i1_orf1	-0.011795904	0.009414895	Proteome
TRINITY_DN4822_c0_g1_i9_orf1	0.020738482	-0.003461858	Proteome
TRINITY_DN20_c0_g1_i1_orf1	-0.013232101	-0.017229303	Proteome
TRINITY_DN5064_c0_g1_i4_orf1	0.014888079	-0.011835016	Proteome
TRINITY_DN2097_c1_g2_i2_orf1	-0.008981672	-0.013520005	Proteome
TRINITY_DN80328_c0_g1_i5_orf1	-0.0334272	-0.010645354	Proteome
TRINITY_DN15420_c0_g3_i2_orf1	0.001373499	-0.008627206	Proteome
TRINITY_DN5497_c0_g1_i6_orf1	0.016739597	-0.004258986	Proteome
TRINITY_DN10521_c0_g1_i7_orf1	0.000738346	-0.004085042	Proteome
TRINITY_DN511_c0_g2_i1_orf1	0.001618019	0.016523816	Proteome
TRINITY_DN244_c1_g1_i5_orf1	0.01512688	0.00684317	Proteome
TRINITY_DN1173_c0_g1_i12_orf1	0.00869235	0.014226116	Proteome
TRINITY_DN21506_c0_g1_i4_orf1	-0.000651303	0.004134327	Proteome
TRINITY_DN19659_c1_g1_i1_orf1	0.007811598	0.008869405	Proteome
TRINITY_DN20_c0_g1_i11_orf1	0.002279069	0.003833868	Proteome
TRINITY_DN9_c0_g1_i7_orf1	0.013981014	-0.010049219	Proteome
TRINITY_DN46367_c0_g1_i2_orf1	-0.003159608	0.004421505	Proteome
TRINITY_DN6415_c0_g2_i1_orf1	0.040938143	-0.006219495	Proteome
TRINITY_DN4410_c0_g1_i1_orf1	-0.007325718	0.002742838	Proteome
TRINITY_DN89711_c0_g1_i1_orf1	0.004335606	0.007099588	Proteome

TRINITY_DN11013_c0_g1_i3_orf1	0.005792111	-0.009957419	Proteome
TRINITY_DN799_c0_g1_i7_orf1	0.010820882	0.004863859	Proteome
TRINITY_DN241_c0_g2_i3_orf1	0.002768958	0.007330272	Proteome
TRINITY_DN2338_c0_g2_i1_orf1	-0.024643607	0.004824138	Proteome
TRINITY_DN2688_c0_g2_i1_orf1	0.014172567	0.008200012	Proteome
TRINITY_DN33_c0_g1_i14_orf1	0.015387054	0.008631064	Proteome
TRINITY_DN33_c0_g1_i1_orf1	0.011979368	0.006481742	Proteome
TRINITY_DN8258_c0_g1_i3_orf1	0.013611764	-0.003782713	Proteome
TRINITY_DN1196_c0_g1_i5_orf1	0.011349106	0.001806434	Proteome
TRINITY_DN981_c0_g1_i1_orf1	0.001287833	-0.004101919	Proteome
TRINITY_DN2186_c0_g1_i17_orf1	0.012023946	0.005642973	Proteome
TRINITY_DN609_c0_g1_i1_orf1	-0.021624939	0.036721942	Proteome
TRINITY_DN5337_c0_g1_i6_orf1	0.009676268	-0.022109007	Proteome
TRINITY_DN27848_c0_g1_i2_orf1	0.000389953	0.00152997	Proteome
TRINITY_DN119797_c0_g1_i1_orf1	0.008996822	-0.002296297	Proteome
TRINITY_DN4794_c1_g1_i9_orf1	-0.020342018	0.006933657	Proteome
TRINITY_DN4152_c0_g1_i1_orf1	-0.002352448	-0.01028769	Proteome
TRINITY_DN11612_c0_g3_i1_orf1	0.009530458	0.009110293	Proteome
TRINITY_DN416_c0_g1_i1_orf1	0.010967934	0.00750645	Proteome
TRINITY_DN7336_c0_g1_i13_orf1	0.00579955	-0.005446782	Proteome
TRINITY_DN68401_c1_g1_i1_orf1	0.008276606	0.00881637	Proteome
TRINITY_DN6325_c0_g1_i8_orf1	-0.003826407	0.010906581	Proteome
TRINITY_DN357_c0_g1_i8_orf1	-0.004777485	0.0086267	Proteome
TRINITY_DN1422_c0_g1_i4_orf1	-0.002156777	0.005971907	Proteome
TRINITY_DN7560_c0_g1_i4_orf1	0.000807737	-0.004475322	Proteome
TRINITY_DN280_c0_g1_i8_orf1	0.000189408	-0.005540637	Proteome
TRINITY_DN7335_c0_g1_i1_orf1	-0.008427803	0.002415898	Proteome
TRINITY_DN24699_c0_g1_i3_orf1	-0.004993672	-0.004673364	Proteome
TRINITY_DN703_c13_g1_i1_orf1	-0.047538107	-0.057130946	Proteome
TRINITY_DN4080_c0_g1_i8_orf1	0.027543613	0.003978619	Proteome
TRINITY_DN12767_c0_g1_i1_orf1	0.010828435	-0.002996583	Proteome
TRINITY_DN5704_c0_g1_i6_orf1	-0.004951473	0.003266608	Proteome
TRINITY_DN1455_c0_g1_i4_orf1	0.004890374	0.020400144	Proteome
TRINITY_DN211_c1_g1_i10_orf1	0.009346026	-0.001720496	Proteome
TRINITY_DN5508_c0_g1_i1_orf1	0.008091117	0.0051218	Proteome
TRINITY_DN3985_c0_g2_i1_orf1	0.004448879	0.004788062	Proteome
TRINITY_DN41645_c0_g1_i1_orf1	0.027472635	0.003202603	Proteome
TRINITY_DN60946_c0_g2_i3_orf1	0.022989386	0.009285529	Proteome
TRINITY_DN13419_c0_g1_i5_orf1	0.02050659	0.006543543	Proteome
TRINITY_DN2577_c0_g1_i1_orf1	0.014793676	0.000167557	Proteome
TRINITY_DN8076_c0_g1_i6_orf1	0.007026604	0.005065753	Proteome
TRINITY_DN9862_c0_g2_i1_orf1	0.007213734	0.007596617	Proteome
TRINITY_DN3257_c0_g1_i4_orf1	0.010172096	0.003373106	Proteome
TRINITY_DN26961_c0_g1_i1_orf1	-0.001751136	0.026322018	Proteome
TRINITY_DN48410_c0_g1_i1_orf1	-0.000312025	-0.003437408	Proteome
TRINITY_DN34479_c0_g1_i2_orf1	0.008280675	0.005929331	Proteome
TRINITY_DN5029_c0_g1_i1_orf1	-0.011683337	0.005494323	Proteome
TRINITY_DN11448_c0_g1_i11_orf1	0.014073942	0.014061538	Proteome
TRINITY_DN54612_c0_g1_i3_orf1	0.020398894	0.000900848	Proteome
TRINITY_DN79000_c1_g1_i1_orf1	0.003036082	0.013261271	Proteome
TRINITY_DN7247_c0_g1_i7_orf1	0.011882863	-0.007106444	Proteome
TRINITY_DN13385_c0_g1_i4_orf1	0.014353549	0.005086563	Proteome
TRINITY_DN9733_c0_g1_i2_orf1	-0.054628358	0.008830772	Proteome
TRINITY_DN11448_c0_g1_i15_orf1	0.008053516	0.014628534	Proteome
TRINITY_DN136906_c0_g1_i1_orf1	0.003169981	0.000447824	Proteome
TRINITY_DN248_c0_g1_i1_orf1	0.008892801	0.00791797	Proteome
TRINITY_DN501_c0_g1_i5_orf1	0.005358022	0.009930095	Proteome
TRINITY_DN3800_c0_g1_i7_orf1	0.011474309	0.002107482	Proteome

TRINITY_DN5080_c0_g1_i1_orf1	-0.043225288	-0.0322567	Proteome
TRINITY_DN2254_c0_g1_i4_orf1	0.016870852	0.00341996	Proteome
TRINITY_DN8944_c0_g1_i1_orf1	0.002775507	-0.000299914	Proteome
TRINITY_DN235_c0_g3_i1_orf1	0.009377925	0.003149032	Proteome
TRINITY_DN45598_c0_g1_i2_orf1	0.006846833	-0.000896724	Proteome
TRINITY_DN2378_c0_g1_i5_orf1	0.017652259	0.008127665	Proteome
TRINITY_DN1366_c0_g1_i5_orf1	-0.000103912	-0.00487677	Proteome
TRINITY_DN36612_c0_g1_i1_orf1	0.011389622	0.009379773	Proteome
TRINITY_DN33619_c0_g1_i1_orf1	0.004030258	0.001645867	Proteome
TRINITY_DN315_c0_g1_i1_orf1	-0.018966995	0.047800351	Proteome
TRINITY_DN5149_c0_g1_i1_orf1	0.005251402	0.013726802	Proteome
TRINITY_DN1868_c0_g1_i1_orf1	-0.009371134	-0.005170319	Proteome
TRINITY_DN2442_c0_g1_i2_orf1	0.00615259	-0.003105392	Proteome
TRINITY_DN276_c0_g1_i1_orf1	-0.017504307	0.034267119	Proteome
TRINITY_DN4279_c0_g1_i6_orf1	-0.014992236	-0.026094865	Proteome
TRINITY_DN4051_c0_g1_i1_orf1	-0.014571719	0.009632627	Proteome
TRINITY_DN4944_c1_g1_i4_orf1	0.007159378	0.005095463	Proteome
TRINITY_DN3433_c0_g1_i6_orf1	0.003578527	-0.008375268	Proteome
TRINITY_DN3073_c0_g1_i7_orf1	0.007769851	0.008171275	Proteome
TRINITY_DN6698_c0_g2_i2_orf1	0.020737485	0.010384345	Proteome
TRINITY_DN618_c0_g1_i3_orf1	0.013973852	0.010275984	Proteome
TRINITY_DN12175_c0_g1_i5_orf1	0.000878519	0.008195724	Proteome
TRINITY_DN2764_c0_g1_i11_orf1	0.008781765	0.003055218	Proteome
TRINITY_DN1982_c0_g1_i17_orf1	0.012198667	-0.00237085	Proteome
TRINITY_DN232_c0_g1_i9_orf1	0.00066074	0.001307168	Proteome
TRINITY_DN628_c0_g1_i1_orf1	-0.011686138	-0.014004586	Proteome
TRINITY_DN19942_c0_g1_i2_orf1	0.000213617	0.010553129	Proteome
TRINITY_DN4822_c0_g1_i6_orf1	-0.032997438	4.91E-05	Proteome
TRINITY_DN4813_c0_g1_i5_orf1	0.020387012	-0.002154934	Proteome
TRINITY_DN703_c0_g1_i2_orf1	-0.063747217	0.017132181	Proteome
TRINITY_DN4141_c0_g1_i9_orf1	0.008452604	-0.003072729	Proteome
TRINITY_DN11153_c0_g1_i1_orf1	0.009498925	-0.00498183	Proteome
TRINITY_DN33801_c0_g1_i1_orf1	0.009331617	0.005947083	Proteome
TRINITY_DN4744_c0_g1_i7_orf1	0.0150006	0.004917913	Proteome
TRINITY_DN934_c2_g1_i7_orf1	0.004928394	-0.001146625	Proteome
TRINITY_DN9340_c0_g1_i4_orf1	-0.010062033	0.010926911	Proteome
TRINITY_DN8915_c0_g1_i3_orf1	0.00892323	0.01028979	Proteome
TRINITY_DN2433_c0_g1_i3_orf1	-0.011754643	0.007216383	Proteome
TRINITY_DN63662_c0_g4_i1_orf1	0.006668287	-0.002009914	Proteome
TRINITY_DN21715_c0_g1_i1_orf1	0.009807999	0.014446065	Proteome
TRINITY_DN2993_c0_g1_i4_orf1	0.000522411	-0.006341063	Proteome
TRINITY_DN848_c0_g1_i1_orf1	0.009435259	0.00294573	Proteome
TRINITY_DN1262_c0_g1_i2_orf1	0.010852015	-0.010499217	Proteome
TRINITY_DN5177_c0_g1_i2_orf1	0.04210077	0.018841976	Proteome
TRINITY_DN10889_c0_g1_i8_orf1	0.017215857	0.011039711	Proteome
TRINITY_DN21123_c0_g1_i1_orf1	0.008501502	0.001283335	Proteome
TRINITY_DN31342_c2_g2_i1_orf1	0.001489315	-0.003019291	Proteome
TRINITY_DN5721_c0_g1_i5_orf1	-0.008674236	-0.006497479	Proteome
TRINITY_DN1081_c0_g1_i7_orf1	0.00888585	0.004946378	Proteome
TRINITY_DN4596_c0_g1_i14_orf1	0.019116942	-0.008722943	Proteome
TRINITY_DN335_c1_g1_i5_orf1	0.029383205	0.013697122	Proteome
TRINITY_DN18230_c1_g1_i1_orf1	0.002331587	0.013341696	Proteome
TRINITY_DN23429_c0_g2_i1_orf1	0.005928989	0.010983122	Proteome
TRINITY_DN3758_c0_g1_i2_orf1	-0.012869229	0.011765861	Proteome
TRINITY_DN810_c0_g1_i4_orf1	0.020604955	-0.001055662	Proteome
TRINITY_DN6669_c0_g1_i3_orf1	0.012304896	0.015612203	Proteome
TRINITY_DN55147_c0_g1_i1_orfp1	0.002716041	0.028122977	Proteome
TRINITY_DN24024_c0_g1_i1_orf1	0.001144331	0.014725351	Proteome

TRINITY_DN1607_c0_g1_i16_orf1	-0.006020632	0.012690133	Proteome
TRINITY_DN48851_c0_g1_i2_orf1	0.007822666	-0.003267637	Proteome
TRINITY_DN1447_c0_g1_i5_orf1	0.010982503	-0.001286875	Proteome
TRINITY_DN2584_c0_g1_i7_orf1	-0.00738028	0.004319235	Proteome
TRINITY_DN21555_c0_g1_i4_orf1	0.01165478	-0.004937659	Proteome
TRINITY_DN24971_c0_g1_i3_orf1	0.01001087	0.012818832	Proteome
TRINITY_DN1294_c0_g1_i3_orf1	0.004647508	-0.001377418	Proteome
TRINITY_DN3840_c0_g1_i1_orf1	0.00100507	-0.006197774	Proteome
TRINITY_DN4724_c0_g1_i4_orf1	0.006038014	0.006238122	Proteome
TRINITY_DN100327_c0_g1_i1_orf1	-0.040131716	-0.040198928	Proteome
TRINITY_DN1287_c0_g1_i5_orf1	-0.014252339	0.011529329	Proteome
TRINITY_DN4434_c0_g1_i7_orf1	-0.00229402	-0.004536381	Proteome
TRINITY_DN2813_c0_g1_i10_orf1	0.017208767	0.022968601	Proteome
TRINITY_DN14701_c0_g1_i2_orf1	0.009698322	0.004401332	Proteome
TRINITY_DN280_c0_g1_i12_orf1	0.001486038	0.010340564	Proteome
TRINITY_DN2719_c1_g1_i6_orf1	0.014563834	0.002135627	Proteome
TRINITY_DN906_c0_g1_i4_orf1	0.000703285	-0.014348282	Proteome
TRINITY_DN1404_c0_g1_i6_orf1	0.004185606	-0.01134768	Proteome
TRINITY_DN3159_c0_g1_i4_orf1	-0.001854136	0.005041231	Proteome
TRINITY_DN47389_c0_g1_i2_orf1	0.011664744	-0.003408619	Proteome
TRINITY_DN7247_c0_g1_i6_orf1	0.024669778	-0.00114814	Proteome
TRINITY_DN136358_c0_g1_i1_orf1	-0.046141017	-0.041091111	Proteome
TRINITY_DN107708_c0_g1_i1_orf1	0.009500371	0.002802612	Proteome
TRINITY_DN4866_c0_g1_i2_orf1	-0.005897325	-5.39E-07	Proteome
TRINITY_DN2047_c0_g1_i1_orf1	-0.0044759	0.00926323	Proteome
TRINITY_DN12367_c0_g1_i4_orf1	0.000276626	0.012524751	Proteome
TRINITY_DN63719_c0_g1_i5_orf1	0.009611065	-0.000500972	Proteome
TRINITY_DN9715_c0_g1_i1_orf1	0.001585387	-0.002270484	Proteome
TRINITY_DN49409_c0_g1_i2_orf1	0.009631118	0.000244368	Proteome
TRINITY_DN4540_c0_g1_i9_orf1	0.009713707	0.007054326	Proteome
TRINITY_DN8076_c0_g1_i5_orf1	0.001230802	-0.012923145	Proteome
TRINITY_DN13576_c0_g1_i1_orf1	0.014114875	-0.00212925	Proteome
TRINITY_DN2468_c0_g1_i7_orf1	0.008593963	0.01444952	Proteome
TRINITY_DN3822_c0_g1_i7_orf1	-0.016586629	0.013992059	Proteome
TRINITY_DN549_c0_g1_i14_orf1	0.014138038	0.003861915	Proteome
TRINITY_DN28654_c0_g1_i2_orf1	-0.040024712	-0.056034413	Proteome
TRINITY_DN3219_c0_g1_i6_orf1	0.021757669	0.000808346	Proteome
TRINITY_DN32509_c0_g1_i3_orf1	0.006692163	0.010688723	Proteome
TRINITY_DN5753_c0_g1_i10_orf1	0.010465494	-0.002890965	Proteome
TRINITY_DN4301_c2_g2_i4_orf1	0.009865173	0.008321903	Proteome
TRINITY_DN2049_c1_g1_i3_orf1	-0.05722614	-0.010588308	Proteome
TRINITY_DN585_c0_g1_i12_orf1	0.005733479	-0.013364523	Proteome
TRINITY_DN12865_c0_g1_i1_orf1	-0.015320813	0.003289674	Proteome
TRINITY_DN36788_c0_g1_i2_orf1	0.008521791	0.00094981	Proteome
TRINITY_DN58751_c0_g1_i2_orf1	0.01763318	0.01457253	Proteome
TRINITY_DN129_c0_g1_i6_orf1	-0.012333588	0.009154273	Proteome
TRINITY_DN2559_c0_g1_i4_orf1	0.009934789	0.000552465	Proteome
TRINITY_DN5243_c1_g1_i1_orf1	0.011685231	0.002781498	Proteome
TRINITY_DN5262_c0_g1_i7_orf1	0.003509059	0.002826131	Proteome
TRINITY_DN6698_c0_g2_i1_orf1	0.012124306	0.014237957	Proteome
TRINITY_DN29698_c0_g1_i3_orf1	-0.000179103	-0.006824191	Proteome
TRINITY_DN6916_c0_g1_i4_orf1	-0.001225645	0.004005193	Proteome
TRINITY_DN83005_c0_g1_i1_orf1	0.001188954	0.000825042	Proteome
TRINITY_DN16816_c0_g1_i1_orf1	0.009729189	0.006649274	Proteome
TRINITY_DN3471_c0_g1_i1_orf1	-0.003191394	0.009211244	Proteome
TRINITY_DN17896_c0_g1_i1_orf1	0.01261078	0.006805165	Proteome
TRINITY_DN391_c0_g1_i4_orf1	-0.006689106	0.019861309	Proteome
TRINITY_DN5976_c0_g1_i1_orf1	0.00399475	0.004285995	Proteome

TRINITY_DN170_c1_g1_i5_orf1	0.004606148	0.007218911	Proteome
TRINITY_DN31118_c0_g1_i1_orf1	0.001805627	0.022782975	Proteome
TRINITY_DN5190_c0_g3_i1_orf1	0.013373227	0.007170299	Proteome
TRINITY_DN125140_c0_g1_i1_orf1	-0.018998172	0.010402507	Proteome
TRINITY_DN71840_c0_g1_i1_orf1	0.009805197	1.80E-05	Proteome
TRINITY_DN174_c1_g1_i1_orf1	0.008969554	-0.00131716	Proteome
TRINITY_DN2936_c0_g1_i1_orf1	0.006633686	-0.00312314	Proteome
TRINITY_DN15382_c0_g1_i3_orf1	-0.016838631	0.003727025	Proteome
TRINITY_DN2873_c0_g1_i7_orf1	0.011645388	0.005856473	Proteome
TRINITY_DN4381_c0_g2_i1_orf1	0.00624498	-0.000460502	Proteome
TRINITY_DN9498_c0_g1_i3_orf1	0.008252404	-0.000840718	Proteome
TRINITY_DN11409_c0_g1_i4_orf1	0.005780843	0.001279181	Proteome
TRINITY_DN11948_c0_g1_i8_orf1	-0.018720958	0.004892898	Proteome
TRINITY_DN688_c0_g1_i8_orf1	-0.01076761	0.002284008	Proteome
TRINITY_DN21000_c0_g1_i1_orf1	0.001153519	0.000678828	Proteome
TRINITY_DN8659_c0_g1_i1_orf1	0.000989313	-0.00411841	Proteome
TRINITY_DN15136_c0_g1_i2_orf1	0.002660876	0.008185972	Proteome
TRINITY_DN2927_c0_g1_i2_orf1	0.007046475	-0.002241106	Proteome
TRINITY_DN1173_c1_g1_i9_orf1	0.008600389	0.005278377	Proteome
TRINITY_DN1103_c0_g1_i15_orf1	-0.005503046	-0.006438381	Proteome
TRINITY_DN4237_c1_g1_i5_orf1	0.007602678	0.00619291	Proteome
TRINITY_DN5756_c0_g1_i4_orf1	0.007751953	-0.00278944	Proteome
TRINITY_DN842_c0_g1_i9_orf1	0.012027831	0.001469004	Proteome
TRINITY_DN15967_c0_g1_i4_orf1	-0.001930669	0.011855863	Proteome
TRINITY_DN32538_c0_g1_i2_orf1	0.004436353	0.007211952	Proteome
TRINITY_DN49038_c0_g4_i1_orf1	-0.002494032	0.005438817	Proteome
TRINITY_DN23194_c0_g1_i4_orf1	0.010339848	0.014920353	Proteome
TRINITY_DN7228_c0_g1_i6_orf1	0.007534776	-0.002581035	Proteome
TRINITY_DN14215_c0_g1_i7_orf1	0.003724522	-0.006544824	Proteome
TRINITY_DN12367_c0_g1_i8_orf1	-0.002709703	-0.013250455	Proteome
TRINITY_DN1732_c0_g1_i15_orf1	-0.022318193	-0.007338425	Proteome
TRINITY_DN5748_c0_g1_i6_orf1	0.005823714	0.028610283	Proteome
TRINITY_DN7960_c0_g1_i2_orf1	-0.018917994	0.007184394	Proteome
TRINITY_DN1718_c6_g1_i4_orf1	0.012930223	0.004881394	Proteome
TRINITY_DN106156_c1_g1_i1_orf1	-0.056182359	-0.026614371	Proteome
TRINITY_DN1023_c1_g1_i1_orf1	0.002446046	-0.018555028	Proteome
TRINITY_DN917_c0_g1_i6_orf1	0.005921414	-0.008359217	Proteome
TRINITY_DN11194_c0_g1_i4_orf1	0.010463801	0.005481543	Proteome
TRINITY_DN4292_c1_g1_i3_orf1	0.013096112	0.001286619	Proteome
TRINITY_DN1443_c0_g1_i4_orf1	0.011073719	-0.001461247	Proteome
TRINITY_DN98334_c0_g1_i1_orf1	-0.000217651	-0.01524964	Proteome
TRINITY_DN28989_c0_g1_i7_orf1	-0.000221161	-0.013375417	Proteome
TRINITY_DN60787_c0_g1_i5_orf1	-0.005780167	-0.001471076	Proteome
TRINITY_DN8466_c0_g1_i1_orf1	-0.002075473	-0.017052913	Proteome
TRINITY_DN14235_c0_g1_i1_orf1	0.003465104	0.009032118	Proteome
TRINITY_DN35301_c0_g1_i3_orf1	0.00268111	0.000649846	Proteome
TRINITY_DN3913_c0_g1_i6_orf1	0.014635398	0.017597162	Proteome
TRINITY_DN45000_c0_g1_i5_orf1	-0.006689239	0.00834938	Proteome
TRINITY_DN9608_c0_g1_i3_orf1	0.049750667	-0.004278663	Proteome
TRINITY_DN975_c0_g1_i1_orf1	-0.001564509	0.048957694	Proteome
TRINITY_DN4065_c0_g1_i5_orf1	-0.005815644	0.004319451	Proteome
TRINITY_DN6991_c0_g1_i24_orf1	-0.007271058	0.013306579	Proteome
TRINITY_DN374_c0_g1_i4_orf1	-0.002690512	0.002549175	Proteome
TRINITY_DN5661_c0_g1_i5_orf1	0.021700795	0.002606783	Proteome
TRINITY_DN1123_c2_g1_i4_orf1	0.010277245	0.010191712	Proteome
TRINITY_DN50517_c0_g1_i5_orf1	0.004686471	0.019773106	Proteome
TRINITY_DN5829_c0_g1_i1_orf1	-0.004619455	-0.019198994	Proteome
TRINITY_DN5081_c0_g1_i5_orf1	0.00786842	0.00745114	Proteome

TRINITY_DN5458_c0_g1_i3_orf1	0.007998095	-0.008178963	Proteome
TRINITY_DN2442_c0_g1_i6_orf1	0.025392439	-0.005565339	Proteome
TRINITY_DN3732_c1_g1_i5_orf1	0.038190282	-0.001846293	Proteome
TRINITY_DN18869_c0_g1_i1_orf1	0.004208288	-0.012595652	Proteome
TRINITY_DN2682_c0_g1_i4_orf1	0.009804706	0.006488894	Proteome
TRINITY_DN3251_c0_g1_i6_orf1	-0.007187381	-0.0142995	Proteome
TRINITY_DN13973_c0_g1_i6_orf1	-0.018028464	-0.00078952	Proteome
TRINITY_DN21357_c0_g1_i5_orf1	0.011496439	0.006198196	Proteome
TRINITY_DN4451_c0_g1_i1_orf1	-0.01221061	-0.013435872	Proteome
TRINITY_DN13718_c0_g1_i7_orf1	-0.007507013	0.001644841	Proteome
TRINITY_DN2591_c0_g1_i4_orf1	-0.008202506	0.001326462	Proteome
TRINITY_DN91989_c0_g1_i1_orf1	-0.005418584	-0.010124115	Proteome
TRINITY_DN7161_c0_g1_i7_orf1	0.007461513	-0.002352629	Proteome
TRINITY_DN276_c0_g1_i2_orf1	-0.044201075	0.026607195	Proteome
TRINITY_DN5122_c0_g1_i3_orf1	0.003962989	0.013302669	Proteome
TRINITY_DN4464_c0_g2_i1_orf1	-0.006820744	0.007933802	Proteome
TRINITY_DN359_c0_g1_i5_orf1	0.014047819	0.003745172	Proteome
TRINITY_DN5914_c1_g1_i9_orf1	0.019654037	0.005709127	Proteome
TRINITY_DN467_c3_g1_i5_orf1	0.034709238	0.019737205	Proteome
TRINITY_DN7128_c0_g1_i7_orf1	0.005969316	0.017257638	Proteome
TRINITY_DN19116_c0_g1_i3_orf1	0.01741504	-0.000886517	Proteome
TRINITY_DN3374_c0_g1_i7_orf1	0.012998756	0.010715028	Proteome
TRINITY_DN3292_c2_g2_i1_orf1	0.010067078	0.013693294	Proteome
TRINITY_DN4469_c0_g1_i2_orf1	0.011057797	-5.68E-05	Proteome
TRINITY_DN3916_c0_g1_i6_orf1	0.015858695	-0.004833935	Proteome
TRINITY_DN8771_c0_g1_i5_orf1	0.016262429	0.010023799	Proteome
TRINITY_DN5310_c2_g1_i2_orf1	0.039473376	-0.006143671	Proteome
TRINITY_DN3126_c0_g1_i4_orf1	-0.01741217	0.010741663	Proteome
TRINITY_DN28711_c0_g1_i1_orf1	-0.03869833	-0.008626224	Proteome
TRINITY_DN4497_c2_g1_i3_orf1	0.016205522	0.001611974	Proteome
TRINITY_DN19990_c0_g1_i1_orf1	-0.011623082	-0.00570209	Proteome
TRINITY_DN10090_c0_g1_i1_orf1	-0.005143461	0.009616793	Proteome
TRINITY_DN95530_c0_g1_i1_orf1	0.010528985	0.010338725	Proteome
TRINITY_DN8367_c0_g1_i1_orf1	0.001591866	0.008577047	Proteome
TRINITY_DN6235_c0_g1_i5_orf1	0.003414305	-0.001259008	Proteome
TRINITY_DN2688_c0_g1_i3_orf1	-0.012409756	0.017639092	Proteome
TRINITY_DN8133_c0_g1_i4_orf1	0.010503258	0.006164663	Proteome
TRINITY_DN27771_c0_g1_i1_orf1	0.0084353	-0.00702637	Proteome
TRINITY_DN17406_c0_g1_i1_orf1	-0.015345709	0.006140882	Proteome
TRINITY_DN14934_c0_g1_i17_orf1	0.007274755	-0.005068918	Proteome
TRINITY_DN2274_c0_g1_i6_orf1	-0.001502974	-0.007828314	Proteome
TRINITY_DN2464_c0_g1_i12_orf1	0.005609165	0.038594696	Proteome
TRINITY_DN33249_c0_g1_i1_orf1	0.0030168	-0.009641778	Proteome
TRINITY_DN44407_c0_g4_i2_orf1	0.001334275	0.017289243	Proteome
TRINITY_DN8595_c0_g1_i3_orf1	0.034495072	-0.010590708	Proteome
TRINITY_DN1355_c0_g1_i7_orf1	-0.01256234	-0.005491429	Proteome
TRINITY_DN2365_c0_g1_i6_orf1	-7.22E-05	0.009884321	Proteome
TRINITY_DN5740_c0_g1_i4_orf1	-0.00192078	-0.005977776	Proteome
TRINITY_DN11065_c0_g2_i1_orf1	0.011378705	-0.006810302	Proteome
TRINITY_DN6317_c1_g2_i3_orf1	0.010219405	0.002370543	Proteome
TRINITY_DN9135_c0_g1_i4_orf1	-0.001805036	0.016775543	Proteome
TRINITY_DN7686_c0_g1_i4_orf1	0.000637184	-0.007263269	Proteome
TRINITY_DN4010_c0_g2_i1_orf1	0.011605465	0.006687975	Proteome
TRINITY_DN12387_c1_g2_i1_orf1	-0.003528736	-0.013559785	Proteome
TRINITY_DN18637_c0_g1_i4_orf1	0.003520268	0.005686996	Proteome
TRINITY_DN5982_c0_g1_i3_orf1	0.007893435	-0.001549915	Proteome
TRINITY_DN2062_c0_g1_i9_orf1	0.011966314	-0.003478774	Proteome
TRINITY_DN2946_c0_g1_i1_orf1	0.011837161	0.000119233	Proteome

TRINITY_DN391_c5_g1_i1_orf1	0.001278615	-0.00429435	Proteome
TRINITY_DN43792_c0_g1_i1_orf1	0.011713132	-0.001622709	Proteome
TRINITY_DN3029_c4_g1_i1_orf1	0.016646674	-0.002670209	Proteome
TRINITY_DN13114_c0_g1_i1_orf1	0.00153516	-0.005857203	Proteome
TRINITY_DN12960_c0_g1_i1_orf1	0.014960948	-0.006031677	Proteome
TRINITY_DN4731_c0_g1_i1_orf1	0.004776997	-0.002750598	Proteome
TRINITY_DN6595_c1_g1_i6_orf1	0.0174451	0.008153628	Proteome
TRINITY_DN2314_c0_g1_i7_orf1	-0.017045248	0.017611553	Proteome
TRINITY_DN11415_c0_g1_i4_orf1	0.003872578	-0.013630999	Proteome
TRINITY_DN35147_c0_g1_i1_orf1	0.023989817	0.008530306	Proteome
TRINITY_DN817_c0_g1_i3_orf1	0.009731555	-0.001316883	Proteome
TRINITY_DN29555_c0_g1_i8_orf1	0.022625592	0.000825932	Proteome
TRINITY_DN2507_c0_g1_i7_orf1	0.007221858	-0.005041772	Proteome
TRINITY_DN493_c0_g1_i4_orf1	0.006428607	0.005289879	Proteome
TRINITY_DN8471_c0_g1_i6_orf1	0.000710495	0.003573023	Proteome
TRINITY_DN2835_c0_g1_i6_orf1	-0.016732557	0.00856923	Proteome
TRINITY_DN3532_c0_g1_i12_orf1	0.009642649	0.005554093	Proteome
TRINITY_DN2425_c0_g1_i3_orf1	0.012399862	0.011138527	Proteome
TRINITY_DN37830_c0_g1_i1_orf1	0.005614867	-0.007036782	Proteome
TRINITY_DN33959_c0_g1_i1_orf1	-0.000765219	-0.001838214	Proteome
TRINITY_DN1172_c0_g1_i1_orf1	0.008675407	0.003687206	Proteome
TRINITY_DN28875_c0_g1_i1_orf1	0.007119359	-0.014802672	Proteome
TRINITY_DN3209_c0_g1_i1_orf1	0.014351826	-0.0045781	Proteome
TRINITY_DN2302_c0_g2_i1_orf1	-0.009298787	-0.007239324	Proteome
TRINITY_DN20582_c0_g1_i1_orf1	-0.00378996	-0.002134791	Proteome
TRINITY_DN9164_c0_g1_i3_orf1	0.006174034	-0.012288374	Proteome
TRINITY_DN31609_c0_g1_i3_orf1	0.021712424	0.00063389	Proteome
TRINITY_DN35694_c0_g1_i1_orf1	0.009386315	0.011445259	Proteome
TRINITY_DN15327_c2_g1_i2_orf1	-0.015552234	0.025433638	Proteome
TRINITY_DN115_c0_g1_i6_orf1	0.005135021	0.008438778	Proteome
TRINITY_DN2594_c0_g2_i4_orf1	-0.006352897	-0.001740426	Proteome
TRINITY_DN9770_c0_g1_i1_orf1	0.011318325	-0.001633188	Proteome
TRINITY_DN6612_c0_g1_i4_orf1	0.014228933	0.001233293	Proteome
TRINITY_DN1593_c0_g1_i1_orf1	-0.003522651	0.030199962	Proteome
TRINITY_DN843_c0_g1_i2_orf1	-0.010886916	0.014960076	Proteome
TRINITY_DN7776_c0_g1_i5_orf1	0.014002837	0.008910812	Proteome
TRINITY_DN5354_c0_g1_i4_orf1	-0.011267102	-0.007635889	Proteome
TRINITY_DN3732_c0_g1_i2_orf1	0.020744338	0.001335436	Proteome
TRINITY_DN7291_c0_g1_i5_orf1	0.02410856	-0.00107296	Proteome
TRINITY_DN5952_c0_g1_i6_orf1	0.006178976	-0.00576161	Proteome
TRINITY_DN17574_c0_g1_i2_orf1	-0.028718624	0.018151504	Proteome
TRINITY_DN2802_c0_g1_i1_orf1	0.006141198	0.003976688	Proteome
TRINITY_DN19375_c0_g1_i2_orf1	0.00429091	0.017160605	Proteome
TRINITY_DN1820_c0_g1_i6_orf1	0.005342537	0.006789271	Proteome
TRINITY_DN4309_c0_g1_i1_orf1	0.011294533	0.007955797	Proteome
TRINITY_DN4341_c0_g1_i4_orf1	0.019677497	-0.004821731	Proteome
TRINITY_DN2205_c0_g1_i3_orf1	0.015930275	-0.000690611	Proteome
TRINITY_DN1014_c0_g2_i8_orf1	0.014192865	-0.015477834	Proteome
TRINITY_DN9239_c0_g2_i2_orf1	-0.031265095	0.029301259	Proteome
TRINITY_DN56308_c0_g1_i2_orf1	-0.035916898	0.001055332	Proteome
TRINITY_DN31253_c0_g1_i2_orf1	0.006687396	-0.007791397	Proteome
TRINITY_DN247_c0_g1_i1_orf1	-0.006666956	-0.017440271	Proteome
TRINITY_DN364_c5_g1_i3_orf1	0.01029284	0.003031158	Proteome
TRINITY_DN26130_c0_g1_i1_orf1	-0.007976097	-0.003222075	Proteome
TRINITY_DN15882_c0_g1_i1_orf1	0.004253002	0.004091944	Proteome
TRINITY_DN41952_c0_g1_i1_orf1	-0.008034253	0.003569192	Proteome
TRINITY_DN9189_c0_g1_i1_orf1	0.002899946	0.002518726	Proteome
TRINITY_DN5874_c0_g1_i2_orf1	0.003913674	0.005829279	Proteome

TRINITY_DN3609_c0_g1_i6_orf1	0.033428479	-0.000637372	Proteome
TRINITY_DN870_c0_g1_i3_orf1	0.013341628	0.011913322	Proteome
TRINITY_DN125427_c0_g1_i1_orf1	-0.008425456	0.014638328	Proteome
TRINITY_DN1400_c0_g1_i21_orf1	0.002265241	-0.0069966	Proteome
TRINITY_DN4628_c0_g1_i1_orf1	0.016103412	-0.002367203	Proteome
TRINITY_DN1622_c0_g1_i6_orf1	0.021566377	-0.004054781	Proteome
TRINITY_DN2516_c0_g2_i10_orf1	0.006669285	-0.008221697	Proteome
TRINITY_DN52944_c0_g1_i1_orf1	-0.032608739	-0.004082268	Proteome
TRINITY_DN8367_c0_g2_i2_orf1	0.014376666	0.01660833	Proteome
TRINITY_DN9717_c0_g2_i1_orf1	-0.003796412	-0.013904227	Proteome
TRINITY_DN892_c7_g1_i2_orf1	0.026908592	0.01401912	Proteome
TRINITY_DN285_c0_g1_i4_orf1	0.018685707	0.006099827	Proteome
TRINITY_DN891_c5_g1_i5_orf1	0.009929915	0.006740814	Proteome
TRINITY_DN240_c2_g1_i1_orf1	0.003018907	-0.018067449	Proteome
TRINITY_DN1540_c0_g1_i14_orf1	0.01763432	0.011132676	Proteome
TRINITY_DN18681_c0_g1_i7_orf1	0.004355417	0.003171988	Proteome
TRINITY_DN1540_c0_g1_i7_orf1	-0.016649096	0.019889211	Proteome
TRINITY_DN15545_c0_g1_i1_orf1	0.022023944	0.019875195	Proteome
TRINITY_DN334_c0_g1_i4_orf1	0.00662475	-0.002436512	Proteome
TRINITY_DN3831_c0_g1_i7_orf1	0.004419633	0.018955052	Proteome
TRINITY_DN82801_c0_g1_i1_orf1	0.000110443	-0.002441076	Proteome
TRINITY_DN2722_c0_g1_i1_orf1	0.010188514	0.012847856	Proteome
TRINITY_DN2267_c0_g1_i1_orf1	-0.003604825	-0.007006784	Proteome
TRINITY_DN50787_c0_g1_i1_orf1	-0.00457773	-0.002384993	Proteome
TRINITY_DN2075_c0_g1_i4_orf1	0.009270576	-0.010540915	Proteome
TRINITY_DN13972_c0_g1_i5_orf1	0.002344487	0.000755036	Proteome
TRINITY_DN23183_c0_g1_i2_orf1	0.008111907	0.006913762	Proteome
TRINITY_DN4125_c0_g1_i6_orf1	0.019081092	-0.010894928	Proteome
TRINITY_DN11172_c0_g1_i4_orf1	0.02405986	-0.005089744	Proteome
TRINITY_DN41736_c0_g2_i1_orf1	-0.002812104	-0.013973253	Proteome
TRINITY_DN4944_c0_g1_i5_orf1	-0.007099272	0.009283384	Proteome
TRINITY_DN14532_c0_g1_i1_orf1	0.010564229	0.003885205	Proteome
TRINITY_DN1402_c1_g1_i6_orf1	-0.015651558	0.011920486	Proteome
TRINITY_DN126127_c0_g1_i1_orf1	0.015964427	0.004038356	Proteome
TRINITY_DN12944_c0_g1_i1_orf1	0.008018691	0.000121246	Proteome
TRINITY_DN107_c0_g1_i1_orf1	0.000447079	0.009363456	Proteome
TRINITY_DN23978_c0_g1_i2_orf1	-0.025294432	0.005353108	Proteome
TRINITY_DN3312_c0_g1_i10_orf1	-0.009936323	-5.88E-05	Proteome
TRINITY_DN4314_c0_g1_i9_orf1	-0.013104488	0.005550509	Proteome
TRINITY_DN26818_c0_g1_i1_orf1	-0.000325433	-0.010898865	Proteome
TRINITY_DN2255_c0_g1_i1_orf1	0.005531544	-0.01899901	Proteome
TRINITY_DN19361_c0_g1_i7_orf1	-0.003344641	0.004246323	Proteome
TRINITY_DN5234_c0_g1_i2_orf1	-0.012557735	-0.002849016	Proteome
TRINITY_DN13496_c0_g1_i7_orf1	0.004308677	-0.005797615	Proteome
TRINITY_DN8980_c0_g1_i2_orf1	-0.006226188	-0.001708595	Proteome
TRINITY_DN12231_c0_g1_i1_orf1	0.004786376	-0.005755784	Proteome
TRINITY_DN14996_c0_g1_i2_orf1	0.008945991	-0.007571021	Proteome
TRINITY_DN7573_c0_g2_i1_orf1	-0.002306456	-0.001985398	Proteome
TRINITY_DN5087_c0_g1_i6_orf1	-0.00150724	-0.000532318	Proteome
TRINITY_DN12474_c0_g1_i6_orf1	0.0004099	-0.007999615	Proteome
TRINITY_DN17738_c0_g1_i2_orf1	0.005985762	0.00046163	Proteome
TRINITY_DN1445_c0_g1_i1_orf1	-0.000619798	-0.002259172	Proteome
TRINITY_DN141_c0_g1_i1_orf1	-0.00032375	0.010821572	Proteome
TRINITY_DN145227_c0_g1_i1_orf1	0.003084997	-0.005566693	Proteome
TRINITY_DN32997_c0_g1_i8_orf1	0.001724435	0.002545362	Proteome
TRINITY_DN36701_c0_g1_i4_orf1	0.005687502	0.007454613	Proteome
TRINITY_DN58207_c0_g1_i1_orf1	0.009906919	0.003817951	Proteome
TRINITY_DN5384_c0_g1_i21_orf1	0.007328435	-0.010728885	Proteome

TRINITY_DN10192_c0_g1_i6_orf1	0.012068299	-0.007730541	Proteome
TRINITY_DN10174_c0_g1_i4_orf1	-0.006214982	-0.01565353	Proteome
TRINITY_DN5893_c0_g1_i7_orf1	-0.002322766	0.020864224	Proteome
TRINITY_DN2146_c0_g1_i1_orf1	0.007357938	0.007569246	Proteome
TRINITY_DN90321_c0_g2_i1_orf1	0.009267014	-0.007304725	Proteome
TRINITY_DN4524_c0_g1_i2_orf1	0.008996464	-0.004071115	Proteome
TRINITY_DN12242_c0_g1_i5_orf1	-0.001297892	-0.000515402	Proteome
TRINITY_DN448_c0_g1_i20_orf1	0.024171789	-0.00880666	Proteome
TRINITY_DN2089_c0_g1_i5_orf1	0.005933928	0.010781027	Proteome
TRINITY_DN24281_c0_g1_i1_orf1	-0.003029382	-0.00615852	Proteome
TRINITY_DN4676_c0_g1_i16_orf1	-0.011690721	0.003374224	Proteome
TRINITY_DN53167_c0_g1_i3_orf1	0.00164587	-0.009295963	Proteome
TRINITY_DN7241_c0_g2_i2_orf1	0.005703157	0.012186095	Proteome
TRINITY_DN5417_c0_g1_i1_orf1	0.000643649	0.003341055	Proteome
TRINITY_DN6522_c0_g1_i6_orf1	-0.000377183	-0.015172768	Proteome
TRINITY_DN9242_c0_g1_i1_orf1	0.002602757	0.004892726	Proteome
TRINITY_DN20676_c0_g1_i6_orf1	0.015820546	0.008719918	Proteome
TRINITY_DN8343_c0_g1_i2_orf1	-0.001012267	-0.005572917	Proteome
TRINITY_DN56459_c0_g1_i2_orf1	-0.009062859	0.054485229	Proteome
TRINITY_DN14298_c0_g1_i1_orf1	0.007492407	0.005140706	Proteome
TRINITY_DN1024_c0_g4_i1_orf1	0.002476644	0.023976427	Proteome
TRINITY_DN1445_c0_g2_i3_orf1	0.001016438	0.003181016	Proteome
TRINITY_DN1540_c0_g1_i9_orf1	0.008889571	-0.033889675	Proteome
TRINITY_DN4501_c0_g2_i1_orf1	0.002915717	0.008889932	Proteome
TRINITY_DN60903_c0_g1_i1_orf1	0.00647184	0.000177619	Proteome
TRINITY_DN63914_c0_g1_i1_orf1	0.000229914	0.007601378	Proteome
TRINITY_DN28516_c0_g1_i2_orf1	-0.004392629	-0.019556273	Proteome
TRINITY_DN35666_c0_g1_i1_orf1	0.006229544	-0.003398135	Proteome
TRINITY_DN3354_c0_g1_i1_orf1	0.000270999	0.007734049	Proteome
TRINITY_DN15362_c0_g1_i1_orf1	0.008933194	0.005865948	Proteome
TRINITY_DN4343_c0_g1_i2_orf1	0.027809167	0.003419079	Proteome
TRINITY_DN100208_c0_g1_i1_orf1	-0.027004328	0.015719462	Proteome
TRINITY_DN30950_c0_g1_i13_orf1	0.003648034	-0.001849508	Proteome
TRINITY_DN9694_c0_g1_i1_orf1	0.015452421	0.007620462	Proteome
TRINITY_DN987_c0_g1_i3_orf1	0.010619156	0.015616536	Proteome
TRINITY_DN932_c0_g1_i4_orf1	0.002861626	-0.000404523	Proteome
TRINITY_DN10476_c0_g1_i1_orf1	0.00625328	0.00263818	Proteome
TRINITY_DN467_c0_g3_i1_orf1	0.006672161	0.010990234	Proteome
TRINITY_DN4245_c0_g1_i5_orf1	0.004802223	0.011730053	Proteome
TRINITY_DN2172_c0_g2_i5_orf1	0.012500139	-0.004334417	Proteome
TRINITY_DN38720_c0_g1_i3_orf1	0.008814033	0.007376639	Proteome
TRINITY_DN3190_c0_g1_i1_orf1	0.008365934	-0.007949235	Proteome
TRINITY_DN26254_c0_g1_i1_orf1	0.003728778	0.021105209	Proteome
TRINITY_DN24310_c0_g1_i2_orf1	-0.012664618	0.001774091	Proteome
TRINITY_DN19058_c1_g1_i1_orf1	0.007333845	0.00043927	Proteome
TRINITY_DN1090_c0_g1_i1_orf1	0.004767934	-0.009929604	Proteome
TRINITY_DN3909_c0_g2_i2_orf1	0.0124009	0.005709628	Proteome
TRINITY_DN6169_c0_g1_i15_orf1	0.006073652	-0.009715636	Proteome
TRINITY_DN9874_c0_g1_i7_orf1	0.007159421	-0.004891763	Proteome
TRINITY_DN3511_c0_g2_i1_orf1	0.009200285	0.000119077	Proteome
TRINITY_DN443_c0_g1_i2_orf1	-0.013193007	-0.000396586	Proteome
TRINITY_DN3428_c0_g1_i1_orf1	0.004361618	0.008803916	Proteome
TRINITY_DN140_c0_g1_i5_orf1	0.04694133	-0.004298151	Proteome
TRINITY_DN2344_c1_g1_i4_orf1	-0.00712371	-0.006096163	Proteome
TRINITY_DN2922_c0_g1_i1_orf1	0.009935618	0.010584731	Proteome
TRINITY_DN642_c0_g1_i6_orf1	0.008501565	-0.009435105	Proteome
TRINITY_DN130575_c0_g1_i1_orfp1	0.023749336	0.010619116	Proteome
TRINITY_DN1445_c0_g2_i4_orf1	-0.00316175	-0.004930867	Proteome

TRINITY_DN19639_c0_g2_i1_orf1	0.029780892	-0.031226439	Proteome
TRINITY_DN50085_c0_g1_i1_orf1	0.004582929	-0.004112632	Proteome
TRINITY_DN5405_c1_g1_i13_orf1	0.013198446	-0.000564899	Proteome
TRINITY_DN64810_c0_g1_i1_orf1	-0.001602549	-0.004629793	Proteome
TRINITY_DN34289_c0_g2_i2_orf1	-0.006474686	0.006061288	Proteome
TRINITY_DN41952_c0_g1_i4_orf1	0.01345782	0.006280688	Proteome
TRINITY_DN96080_c0_g2_i1_orf1	-0.005352324	0.007346838	Proteome
TRINITY_DN44083_c0_g1_i2_orf1	0.013426085	0.010023528	Proteome
TRINITY_DN114834_c0_g1_i1_orf1	0.003516768	0.001713822	Proteome
TRINITY_DN16077_c0_g1_i13_orf1	-0.003274127	-0.012948155	Proteome
TRINITY_DN33146_c0_g1_i1_orf1	-0.004237212	0.017039529	Proteome
TRINITY_DN2813_c0_g1_i3_orf1	-0.006520508	-0.007968497	Proteome
TRINITY_DN9980_c0_g1_i1_orf1	0.009254005	-0.005068546	Proteome
TRINITY_DN2407_c0_g1_i6_orf1	0.000351627	0.008556506	Proteome
TRINITY_DN4869_c0_g1_i10_orf1	0.001292516	-0.00454938	Proteome
TRINITY_DN6147_c0_g1_i2_orf1	-0.00862525	-0.001173962	Proteome
TRINITY_DN24043_c0_g1_i1_orf1	-0.007241294	0.002724688	Proteome
TRINITY_DN610_c1_g1_i1_orf1	0.001766655	0.005948459	Proteome
TRINITY_DN4711_c0_g1_i2_orf1	0.011085631	-0.005408219	Proteome
TRINITY_DN20658_c0_g1_i1_orf1	0.017805432	0.014396534	Proteome
TRINITY_DN4320_c0_g1_i1_orf1	-0.003684147	0.001357277	Proteome
TRINITY_DN565_c0_g2_i1_orf1	0.022600668	0.002142275	Proteome
TRINITY_DN76815_c0_g1_i3_orf1	0.037542325	-0.001229477	Proteome
TRINITY_DN117_c0_g1_i4_orf1	0.001786262	0.001777465	Proteome
TRINITY_DN16539_c0_g1_i7_orf1	0.010275731	-0.006105826	Proteome
TRINITY_DN38506_c0_g1_i4_orf1	-0.011823455	0.036301316	Proteome
TRINITY_DN21469_c0_g1_i4_orf1	-0.002348904	-0.008799592	Proteome
TRINITY_DN80328_c0_g1_i9_orf1	-0.031390134	-0.015793607	Proteome
TRINITY_DN10502_c0_g1_i4_orf1	0.005178009	-0.005609375	Proteome
TRINITY_DN91533_c0_g1_i1_orf1	0.004413047	0.002846299	Proteome
TRINITY_DN791_c0_g1_i2_orf1	0.004790477	-0.005764683	Proteome
TRINITY_DN31611_c0_g1_i2_orf1	-0.018259306	0.013928369	Proteome
TRINITY_DN6108_c0_g1_i5_orf1	-0.03090049	0.002808322	Proteome
TRINITY_DN1093_c0_g1_i1_orf1	0.006687388	-0.007995621	Proteome
TRINITY_DN51050_c0_g1_i3_orf1	0.009310108	0.009303691	Proteome
TRINITY_DN8724_c0_g1_i2_orf1	-0.003472953	-0.002034647	Proteome
TRINITY_DN376_c0_g1_i1_orf1	-0.008101237	-0.002550553	Proteome
TRINITY_DN1465_c0_g2_i1_orf1	-0.00311323	-0.013623234	Proteome
TRINITY_DN20339_c0_g1_i3_orf1	0.005201887	-0.005660519	Proteome
TRINITY_DN8637_c0_g1_i1_orf1	0.005986873	0.012948208	Proteome
TRINITY_DN26168_c0_g1_i1_orf1	0.008089528	0.001189467	Proteome
TRINITY_DN2072_c0_g1_i1_orf1	0.01654231	0.000418142	Proteome
TRINITY_DN4199_c0_g1_i1_orf1	-0.004493869	0.001197431	Proteome
TRINITY_DN1316_c0_g1_i1_orf1	-0.00732154	0.00304263	Proteome
TRINITY_DN14572_c0_g1_i1_orf1	0.008233853	-0.003533416	Proteome
TRINITY_DN696_c1_g1_i10_orf1	0.008197153	0.002946002	Proteome
TRINITY_DN5564_c0_g1_i5_orf1	-0.018259478	-0.01308832	Proteome
TRINITY_DN1718_c1_g1_i5_orf1	0.008357228	0.016092847	Proteome
TRINITY_DN4793_c0_g1_i7_orf1	0.020359348	0.000959605	Proteome
TRINITY_DN1914_c0_g1_i4_orf1	0.028534347	-0.00030691	Proteome
TRINITY_DN3053_c0_g1_i2_orf1	0.007606689	0.000462408	Proteome
TRINITY_DN52316_c0_g1_i1_orf1	-0.033502308	-0.044204755	Proteome
TRINITY_DN54205_c0_g1_i1_orf1	0.007487909	0.008109659	Proteome
TRINITY_DN13322_c0_g1_i6_orf1	0.001462906	0.028504533	Proteome
TRINITY_DN16349_c0_g1_i10_orf1	0.008328293	0.006327913	Proteome
TRINITY_DN1515_c0_g1_i2_orf1	0.010367709	-0.011650904	Proteome
TRINITY_DN4394_c0_g1_i4_orf1	-0.002637778	0.027138352	Proteome
TRINITY_DN37307_c0_g1_i4_orf1	0.004495953	0.0268453	Proteome

TRINITY_DN22213_c0_g1_i3_orf1	0.007075514	0.015518363	Proteome
TRINITY_DN14443_c0_g1_i1_orf1	0.005856658	0.003898122	Proteome
TRINITY_DN6308_c0_g1_i6_orf1	0.004590428	0.00145121	Proteome
TRINITY_DN4816_c0_g2_i3_orf1	-0.015238757	0.005549046	Proteome
TRINITY_DN4532_c0_g1_i1_orf1	0.007446704	0.000539051	Proteome
TRINITY_DN2894_c0_g3_i1_orf1	-0.002679594	-0.006174248	Proteome
TRINITY_DN120500_c0_g1_i1_orf1	0.0312211	0.000371842	Proteome
TRINITY_DN334_c0_g1_i1_orf1	0.000964257	-0.00156179	Proteome
TRINITY_DN2627_c0_g2_i1_orf1	0.012792856	0.009291301	Proteome
TRINITY_DN717_c0_g1_i2_orfp1	-0.002488115	0.008772462	Proteome
TRINITY_DN2097_c1_g1_i1_orf1	-0.001285618	-0.002099617	Proteome
TRINITY_DN31584_c0_g2_i2_orf1	-0.019783257	0.01110093	Proteome
TRINITY_DN14328_c0_g1_i12_orf1	0.034091826	0.022601427	Proteome
TRINITY_DN3321_c0_g1_i3_orf1	0.008401799	0.000669752	Proteome
TRINITY_DN2706_c0_g1_i3_orf1	0.001134227	-0.012901601	Proteome
TRINITY_DN38685_c0_g1_i4_orf1	0.0112829	0.010780879	Proteome
TRINITY_DN24917_c0_g2_i1_orf1	-0.011425517	-0.017989928	Proteome
TRINITY_DN9820_c0_g1_i1_orf1	0.019804687	0.011015478	Proteome
TRINITY_DN338_c1_g1_i9_orf1	-0.019292457	0.006299352	Proteome
TRINITY_DN2038_c0_g1_i2_orf1	-0.010556766	-0.012600594	Proteome
TRINITY_DN11231_c1_g1_i1_orfp1	0.02324371	0.003812981	Proteome
TRINITY_DN43505_c0_g1_i1_orf1	-0.005850645	-0.016365869	Proteome
TRINITY_DN19923_c0_g1_i1_orf1	-0.001351489	0.0122614	Proteome
TRINITY_DN21218_c0_g1_i4_orf1	-0.008218969	-0.008904029	Proteome
TRINITY_DN101_c0_g2_i2_orf1	-0.00184632	-0.022933582	Proteome
TRINITY_DN1732_c0_g1_i17_orf1	-0.013639346	0.012883791	Proteome
TRINITY_DN26503_c0_g1_i1_orf1	0.00664446	-0.00208645	Proteome
TRINITY_DN8724_c0_g1_i5_orf1	0.013379044	0.004898633	Proteome
TRINITY_DN2133_c0_g2_i1_orf1	-0.017167088	0.010532667	Proteome
TRINITY_DN4533_c0_g1_i1_orf1	0.011054791	0.006559944	Proteome
TRINITY_DN2338_c0_g1_i3_orf1	-0.026368846	0.005550447	Proteome
TRINITY_DN10747_c0_g1_i5_orf1	-0.004345212	-0.015189813	Proteome
TRINITY_DN6143_c0_g2_i1_orf1	0.003895661	0.009015277	Proteome
TRINITY_DN5488_c0_g1_i5_orf1	-0.016321187	-0.003560838	Proteome
TRINITY_DN10429_c0_g1_i2_orf1	0.002389569	0.00024499	Proteome
TRINITY_DN7776_c0_g1_i9_orf1	-0.002236711	0.010462747	Proteome
TRINITY_DN27592_c0_g1_i1_orf1	-0.007794597	5.58E-05	Proteome
TRINITY_DN2999_c1_g1_i4_orf1	0.006948131	-0.003438654	Proteome
TRINITY_DN214_c0_g1_i3_orf1	-0.016953551	-0.016187339	Proteome
TRINITY_DN3483_c0_g1_i5_orf1	-0.003207618	0.018984172	Proteome
TRINITY_DN22664_c0_g1_i1_orf1	0.012257451	0.000652496	Proteome
TRINITY_DN11297_c0_g1_i1_orf1	0.008916638	-0.010972997	Proteome
TRINITY_DN7112_c0_g1_i1_orf1	0.007675742	0.003496676	Proteome
TRINITY_DN47842_c0_g1_i1_orf1	0.029869605	0.004498008	Proteome
TRINITY_DN1578_c0_g3_i1_orf1	-0.007244246	-0.005401554	Proteome
TRINITY_DN1280_c0_g1_i1_orf1	0.000820852	0.011046925	Proteome
TRINITY_DN2215_c0_g2_i1_orf1	0.013999443	0.020018814	Proteome
TRINITY_DN81488_c0_g1_i1_orf1	-0.034703915	-0.007091839	Proteome
TRINITY_DN21124_c0_g1_i4_orf1	0.002793133	-0.007868834	Proteome
TRINITY_DN15858_c0_g1_i2_orf1	-0.015154798	-0.014662833	Proteome
TRINITY_DN8116_c0_g1_i2_orf1	0.006300453	0.003859895	Proteome
TRINITY_DN8702_c0_g1_i1_orf1	0.014013516	-0.002620439	Proteome
TRINITY_DN14684_c0_g2_i1_orf1	-0.004960883	-0.000240783	Proteome
TRINITY_DN2627_c0_g1_i2_orf1	-0.002251176	-0.008843703	Proteome
TRINITY_DN230_c2_g1_i5_orf1	0.02008574	0.008083826	Proteome
TRINITY_DN2652_c0_g2_i1_orf1	-0.010614185	0.003843175	Proteome
TRINITY_DN28039_c0_g1_i1_orf1	0.004273493	0.010486855	Proteome
TRINITY_DN465_c0_g1_i3_orf1	-0.000637591	0.001377792	Proteome

TRINITY_DN471_c0_g1_i6_orf1	-0.010187506	0.000813383	Proteome
TRINITY_DN62_c0_g1_i7_orf1	0.009806651	0.005247762	Proteome
TRINITY_DN768_c0_g1_i7_orf1	-0.025730537	0.010246053	Proteome
TRINITY_DN17326_c0_g1_i5_orf1	0.014110524	0.001043764	Proteome
TRINITY_DN13500_c0_g1_i1_orf1	-0.020402696	-0.008626655	Proteome
TRINITY_DN380_c0_g2_i2_orf1	-0.025969415	0.030486813	Proteome
TRINITY_DN7630_c0_g2_i1_orf1	0.015010513	-0.000431887	Proteome
TRINITY_DN5118_c0_g1_i1_orf1	0.009479836	-0.009131013	Proteome
TRINITY_DN6684_c0_g1_i4_orf1	0.012111358	-0.008537723	Proteome
TRINITY_DN4766_c0_g1_i4_orf1	0.006424509	-0.005600088	Proteome
TRINITY_DN12401_c0_g2_i4_orf1	-0.0090729	0.009783481	Proteome
TRINITY_DN11259_c0_g1_i1_orf1	0.003966195	-0.007027956	Proteome
TRINITY_DN5215_c0_g1_i1_orf1	-0.011840666	-0.002636667	Proteome
TRINITY_DN1509_c0_g1_i1_orf1	0.009534771	-0.002623921	Proteome
TRINITY_DN27751_c0_g2_i1_orf1	0.006687027	0.002370817	Proteome
TRINITY_DN3411_c0_g1_i2_orf1	0.002122744	0.003705377	Proteome
TRINITY_DN2198_c0_g1_i1_orf1	0.011031303	-0.002817656	Proteome
TRINITY_DN714_c0_g1_i3_orf1	0.013091831	0.007036358	Proteome
TRINITY_DN15959_c0_g1_i1_orf1	0.002287173	0.001658341	Proteome
TRINITY_DN42542_c0_g1_i1_orf1	-0.000757921	-0.015100236	Proteome
TRINITY_DN2855_c0_g1_i6_orf1	0.013958938	0.00195647	Proteome
TRINITY_DN5244_c0_g1_i1_orf1	0.007160161	0.005240869	Proteome
TRINITY_DN7040_c0_g2_i1_orf1	-0.021637485	-0.023482767	Proteome
TRINITY_DN2416_c0_g1_i5_orf1	0.021385721	0.004464155	Proteome
TRINITY_DN4817_c0_g1_i4_orf1	0.016560889	0.003706891	Proteome
TRINITY_DN14865_c0_g1_i2_orf1	0.007957811	0.008912736	Proteome
TRINITY_DN10264_c1_g1_i5_orf1	-0.007576407	-0.012541706	Proteome
TRINITY_DN15834_c0_g1_i2_orf1	0.004376703	0.011500926	Proteome
TRINITY_DN2823_c0_g1_i6_orf1	0.009904402	-0.012911129	Proteome
TRINITY_DN3588_c0_g1_i1_orf1	0.002844925	-0.007500241	Proteome
TRINITY_DN110231_c0_g1_i1_orf1	-0.000359757	0.006007861	Proteome
TRINITY_DN62_c0_g1_i18_orf1	0.000817336	0.003741111	Proteome
TRINITY_DN16673_c0_g1_i1_orf1	0.005259711	4.58E-05	Proteome
TRINITY_DN36538_c0_g1_i2_orf1	3.14E-05	-0.009562949	Proteome
TRINITY_DN19628_c1_g1_i1_orf1	0.000233124	0.012607099	Proteome
TRINITY_DN31637_c0_g1_i3_orf1	0.005850224	0.005478889	Proteome
TRINITY_DN7808_c0_g1_i1_orf1	0.003167735	0.009875765	Proteome
TRINITY_DN1926_c0_g1_i5_orf1	0.010495858	0.01019156	Proteome
TRINITY_DN3476_c0_g1_i5_orf1	-0.004220344	-0.006424496	Proteome
TRINITY_DN33763_c0_g1_i1_orf1	0.008774279	0.000573691	Proteome
TRINITY_DN1421_c0_g1_i1_orf1	0.008441794	0.02120057	Proteome
TRINITY_DN740_c0_g1_i1_orf1	0.005790259	0.001636309	Proteome
TRINITY_DN7836_c0_g1_i2_orf1	0.004635336	-0.006032915	Proteome
TRINITY_DN25901_c0_g1_i2_orf1	0.014359681	0.002398965	Proteome
TRINITY_DN2704_c0_g1_i5_orf1	-0.002099688	0.010115594	Proteome
TRINITY_DN2953_c1_g1_i11_orf1	-0.006660396	-0.002374817	Proteome
TRINITY_DN24_c0_g1_i1_orf1	0.034969617	-0.001493281	Proteome
TRINITY_DN1465_c2_g1_i2_orf1	0.022490781	0.001527711	Proteome
TRINITY_DN4944_c0_g1_i2_orf1	0.002960037	0.004866255	Proteome
TRINITY_DN18222_c0_g1_i4_orf1	-0.018465882	0.028262162	Proteome
TRINITY_DN863_c0_g1_i6_orf1	0.010348641	-0.010920182	Proteome
TRINITY_DN5382_c0_g2_i1_orf1	-0.012412624	0.007308605	Proteome
TRINITY_DN4572_c0_g3_i1_orf1	0.002523466	0.012732979	Proteome
TRINITY_DN8291_c0_g1_i3_orf1	-0.001783672	-0.004780431	Proteome
TRINITY_DN20658_c0_g2_i3_orf1	0.029589085	-0.019583797	Proteome
TRINITY_DN472_c0_g1_i6_orf1	-0.006412008	-0.013357205	Proteome
TRINITY_DN10512_c0_g1_i1_orf1	0.014347677	0.008461719	Proteome
TRINITY_DN1520_c0_g1_i9_orf1	0.014994854	0.002022545	Proteome

TRINITY_DN351_c14_g1_i2_orf1	0.011521317	0.005905896	Proteome
TRINITY_DN48610_c0_g1_i2_orf1	0.010912387	0.011011632	Proteome
TRINITY_DN31232_c1_g1_i9_orf1	-0.008551953	0.001213223	Proteome
TRINITY_DN32687_c0_g1_i1_orf1	0.012762222	-0.018523332	Proteome
TRINITY_DN625_c2_g2_i2_orf1	-0.020808245	-0.012467541	Proteome
TRINITY_DN5852_c0_g1_i6_orf1	-0.021777602	0.009455952	Proteome
TRINITY_DN5748_c0_g1_i5_orf1	0.011032215	-0.003425908	Proteome
TRINITY_DN50676_c0_g1_i1_orf1	0.016106056	0.00331921	Proteome
TRINITY_DN18222_c0_g1_i5_orf1	-0.011834994	0.001004471	Proteome
TRINITY_DN2930_c0_g1_i8_orf1	-0.003158875	-0.007103525	Proteome
TRINITY_DN441_c0_g2_i1_orf1	0.011917219	0.002072764	Proteome
TRINITY_DN566_c0_g1_i13_orf1	0.021961937	-0.003888789	Proteome
TRINITY_DN8584_c0_g1_i6_orf1	0.005197332	0.010994054	Proteome
TRINITY_DN7464_c0_g1_i14_orf1	0.009582351	-0.002074862	Proteome
TRINITY_DN33_c0_g1_i8_orf1	-0.008433704	0.017005428	Proteome
TRINITY_DN22166_c0_g1_i3_orf1	0.009501147	-0.001796586	Proteome
TRINITY_DN28661_c0_g1_i1_orf1	0.007977092	0.012406886	Proteome
TRINITY_DN8716_c0_g1_i3_orf1	0.015270731	-0.00594315	Proteome
TRINITY_DN257_c0_g1_i7_orf1	0.008821109	0.005499346	Proteome
TRINITY_DN18249_c0_g1_i1_orf1	0.010085783	0.009916138	Proteome
TRINITY_DN31286_c0_g1_i6_orfp1	0.031417063	0.011722773	Proteome
TRINITY_DN334_c0_g1_i3_orf1	-0.001804962	0.008981382	Proteome
TRINITY_DN7626_c0_g1_i1_orf1	-0.000603887	0.006185875	Proteome
TRINITY_DN1212_c0_g1_i8_orf1	0.0145327	0.020336166	Proteome
TRINITY_DN30306_c0_g2_i1_orf1	0.028379826	0.027459395	Proteome
TRINITY_DN5562_c1_g1_i3_orf1	-0.001068932	-0.001055139	Proteome
TRINITY_DN12964_c0_g1_i1_orf1	0.009144664	-0.002902323	Proteome
TRINITY_DN4125_c0_g1_i14_orf1	0.009175853	-0.001942903	Proteome
TRINITY_DN10138_c0_g1_i1_orf1	-0.043308015	0.003826231	Proteome
TRINITY_DN1552_c0_g1_i3_orf1	-0.002358144	0.000112181	Proteome
TRINITY_DN1952_c0_g1_i2_orf1	0.008328601	-0.001440063	Proteome
TRINITY_DN3637_c0_g1_i2_orf1	-0.002455825	-0.011222432	Proteome
TRINITY_DN3588_c0_g1_i4_orf1	0.009934882	-0.006504536	Proteome
TRINITY_DN5653_c0_g1_i4_orf1	-0.003361221	-0.005253727	Proteome
TRINITY_DN81031_c0_g1_i1_orf1	-0.017075625	-0.001200582	Proteome
TRINITY_DN8030_c0_g1_i2_orf1	0.005086965	-0.012734023	Proteome
TRINITY_DN85319_c0_g1_i1_orf1	0.008512117	-0.000466657	Proteome
TRINITY_DN24970_c0_g1_i4_orf1	0.006321026	0.012895222	Proteome
TRINITY_DN8224_c0_g1_i7_orf1	0.010057342	0.003543059	Proteome
TRINITY_DN8729_c0_g1_i7_orf1	-0.007636842	-0.000150689	Proteome
TRINITY_DN2457_c0_g1_i8_orf1	0.011883358	-0.018271395	Proteome
TRINITY_DN11446_c0_g1_i21_orf1	-0.006143298	0.008762283	Proteome
TRINITY_DN348_c0_g2_i1_orf1	0.003969316	0.007406497	Proteome
TRINITY_DN554_c0_g1_i1_orf1	0.012514206	-0.007185554	Proteome
TRINITY_DN6308_c0_g1_i3_orf1	0.006649485	-0.004819478	Proteome
TRINITY_DN8949_c0_g1_i2_orf1	0.011722183	0.007488003	Proteome
TRINITY_DN14274_c0_g1_i3_orf1	-0.015643369	0.006008733	Proteome
TRINITY_DN14587_c0_g1_i7_orf1	0.005921673	0.009766978	Proteome
TRINITY_DN11799_c0_g1_i4_orf1	-0.007460713	-0.000846201	Proteome
TRINITY_DN1592_c0_g1_i1_orf1	0.01975789	0.008271259	Proteome
TRINITY_DN96801_c0_g1_i1_orf1	0.021272013	-0.010295162	Proteome
TRINITY_DN43881_c0_g1_i2_orf1	0.012860192	-0.002097419	Proteome
TRINITY_DN53760_c0_g1_i1_orf1	0.012070725	0.009462584	Proteome
TRINITY_DN43667_c0_g1_i1_orf1	0.021674808	-0.008936809	Proteome
TRINITY_DN7226_c0_g1_i2_orf1	-0.02604001	-0.047712123	Proteome
TRINITY_DN71698_c0_g1_i1_orfp1	-0.031503687	0.034973143	Proteome
TRINITY_DN2794_c1_g1_i8_orf1	0.01293613	-0.004461331	Proteome
TRINITY_DN1888_c0_g2_i1_orf1	0.004684723	-0.011065922	Proteome

TRINITY_DN2876_c0_g1_i1_orf1	0.012734796	-0.023958537	Proteome
TRINITY_DN268_c3_g1_i2_orf1	0.000384039	0.01804859	Proteome
TRINITY_DN28711_c1_g1_i1_orf1	-0.031156568	0.000576498	Proteome
TRINITY_DN3835_c0_g1_i3_orf1	0.008408458	-0.002860603	Proteome
TRINITY_DN10792_c0_g2_i5_orf1	-0.001612683	-0.004607799	Proteome
TRINITY_DN843_c0_g1_i5_orf1	0.013567874	0.005636678	Proteome
TRINITY_DN1914_c0_g1_i6_orf1	0.004518619	-0.001805364	Proteome
TRINITY_DN8241_c0_g1_i3_orf1	0.009212834	0.014672296	Proteome
TRINITY_DN34536_c0_g1_i6_orf1	0.005860426	0.004751697	Proteome
TRINITY_DN53294_c0_g1_i1_orf1	-0.022014696	0.002819223	Proteome
TRINITY_DN2894_c0_g1_i2_orf1	0.000798024	-0.001334731	Proteome
TRINITY_DN19460_c0_g1_i1_orf1	0.007625849	0.013193471	Proteome
TRINITY_DN2043_c0_g1_i11_orf1	-0.008211456	0.016829616	Proteome
TRINITY_DN38667_c0_g1_i9_orf1	-0.003896072	-0.011173452	Proteome
TRINITY_DN143497_c0_g1_i1_orf1	0.009132442	-0.000898324	Proteome
TRINITY_DN2489_c0_g1_i1_orf1	-0.00730906	-0.014572149	Proteome
TRINITY_DN6103_c0_g1_i6_orf1	0.007902789	0.001153971	Proteome
TRINITY_DN14313_c0_g1_i1_orf1	0.00420644	-0.002772251	Proteome
TRINITY_DN152_c0_g1_i4_orf1	0.01055986	0.008176403	Proteome
TRINITY_DN12545_c0_g1_i7_orf1	0.014375545	-0.008810604	Proteome
TRINITY_DN2323_c0_g1_i4_orf1	-0.004364778	0.005710316	Proteome
TRINITY_DN51342_c0_g1_i7_orf1	-0.014496813	0.013581961	Proteome
TRINITY_DN45973_c0_g1_i4_orf1	0.008369597	0.006173594	Proteome
TRINITY_DN2783_c1_g1_i2_orf1	-0.001401662	-0.00230457	Proteome
TRINITY_DN40126_c0_g2_i1_orf1	0.01384506	0.002662471	Proteome
TRINITY_DN114890_c0_g1_i4_orf1	-0.056049003	0.03018828	Proteome
TRINITY_DN17510_c0_g1_i1_orf1	0.00983293	-0.010889191	Proteome
TRINITY_DN7152_c0_g1_i1_orf1	-0.000909096	0.002461666	Proteome
TRINITY_DN3708_c0_g1_i3_orf1	-0.007729162	-0.009447133	Proteome
TRINITY_DN3366_c0_g1_i6_orf1	0.006174019	0.00303437	Proteome
TRINITY_DN1080_c0_g1_i1_orf1	-0.006778415	0.006940756	Proteome
TRINITY_DN1384_c0_g1_i5_orf1	0.002158807	-0.00737314	Proteome
TRINITY_DN7512_c0_g1_i1_orf1	0.002215723	-0.006343741	Proteome
TRINITY_DN679_c0_g1_i2_orf1	-0.002491758	-0.005088785	Proteome
TRINITY_DN1355_c0_g1_i5_orf1	0.007147396	0.01483393	Proteome
TRINITY_DN5873_c0_g4_i1_orf1	0.010264376	0.006029195	Proteome
TRINITY_DN53115_c0_g1_i1_orf1	-0.000314067	-0.010497348	Proteome
TRINITY_DN1816_c0_g1_i5_orf1	0.002060346	0.009498663	Proteome
TRINITY_DN95713_c0_g1_i1_orf1	-0.009262674	0.001464122	Proteome
TRINITY_DN3749_c0_g1_i1_orf1	-0.005317603	0.010010009	Proteome
TRINITY_DN9457_c0_g1_i9_orf1	0.018857955	-0.005470114	Proteome
TRINITY_DN6881_c0_g1_i1_orf1	-0.007156356	0.01718456	Proteome
TRINITY_DN1722_c0_g1_i2_orf1	0.00237337	0.006742001	Proteome
TRINITY_DN7613_c1_g2_i1_orf1	0.009601503	0.004883426	Proteome
TRINITY_DN23167_c0_g1_i4_orf1	0.007793127	0.010105246	Proteome
TRINITY_DN32687_c0_g1_i2_orf1	-0.004334656	-0.007133611	Proteome
TRINITY_DN10637_c0_g1_i4_orf1	-0.001028934	-0.009149307	Proteome
TRINITY_DN9100_c0_g1_i5_orf1	0.004001175	0.003234953	Proteome
TRINITY_DN26790_c0_g1_i3_orf1	-0.002865601	-0.015170978	Proteome
TRINITY_DN10769_c0_g1_i1_orf1	0.008436546	-0.00641909	Proteome
TRINITY_DN122867_c1_g1_i1_orf1	0.008926172	-0.000180043	Proteome
TRINITY_DN582_c0_g1_i5_orf1	0.01056513	0.01441979	Proteome
TRINITY_DN21559_c0_g2_i1_orf1	0.0004185	0.004917583	Proteome
TRINITY_DN43942_c0_g1_i1_orf1	-0.006092517	-0.016615248	Proteome
TRINITY_DN4456_c0_g1_i1_orf1	0.016794299	-0.006955524	Proteome
TRINITY_DN23204_c0_g1_i1_orf1	0.000753315	0.011518327	Proteome
TRINITY_DN50225_c0_g1_i1_orf1	-0.002544542	-0.0158163	Proteome
TRINITY_DN364_c0_g1_i2_orf1	0.004382739	0.013448821	Proteome

TRINITY_DN4394_c0_g2_i1_orf1	-0.028064794	-0.018255237	Proteome
TRINITY_DN10548_c0_g2_i1_orf1	0.009833296	-0.001932504	Proteome
TRINITY_DN19939_c0_g1_i4_orf1	-0.005025957	-0.01274863	Proteome
TRINITY_DN18136_c0_g1_i1_orf1	-0.000459096	-0.010260839	Proteome
TRINITY_DN20560_c0_g1_i6_orf1	0.029452861	0.014071667	Proteome
TRINITY_DN3551_c0_g1_i4_orf1	-0.002911046	-0.008223667	Proteome
TRINITY_DN1759_c0_g1_i4_orf1	0.031261965	0.005762244	Proteome
TRINITY_DN6199_c2_g1_i3_orf1	-0.010943628	0.01397356	Proteome
TRINITY_DN36460_c0_g1_i2_orf1	0.003418321	-0.016947265	Proteome
TRINITY_DN8854_c0_g1_i2_orf1	-0.011731251	0.001062074	Proteome
TRINITY_DN10900_c0_g1_i7_orf1	0.001517845	-0.002954681	Proteome
TRINITY_DN6908_c0_g1_i3_orf1	0.026879798	-0.008135429	Proteome
TRINITY_DN49265_c0_g3_i2_orf1	-0.007986242	0.003065764	Proteome
TRINITY_DN2407_c0_g1_i2_orf1	-0.044458221	-0.000328204	Proteome
TRINITY_DN41259_c0_g1_i6_orf1	0.003590705	-0.003601685	Proteome
TRINITY_DN15858_c0_g1_i1_orf1	-0.009264632	0.007107402	Proteome
TRINITY_DN364_c0_g2_i1_orf1	-0.001174488	0.019384052	Proteome
TRINITY_DN138481_c0_g1_i5_orf1	-0.013562089	0.014275332	Proteome
TRINITY_DN31118_c0_g2_i1_orf1	0.001250854	0.026667025	Proteome
TRINITY_DN2684_c0_g2_i3_orf1	-0.011572825	-0.010828171	Proteome
TRINITY_DN38274_c0_g1_i1_orf1	0.010198203	-0.005330172	Proteome
TRINITY_DN28806_c0_g1_i1_orf1	0.008472075	-0.005126841	Proteome
TRINITY_DN21909_c0_g1_i1_orf1	0.001710009	0.001519311	Proteome
TRINITY_DN2040_c0_g1_i6_orf1	0.001373668	0.010392742	Proteome
TRINITY_DN23740_c0_g1_i3_orf1	0.006470455	-0.002291781	Proteome
TRINITY_DN701_c0_g1_i1_orf1	0.02387147	-0.006332897	Proteome
TRINITY_DN39975_c0_g1_i4_orf1	0.006687541	0.003046698	Proteome
TRINITY_DN1661_c0_g1_i1_orf1	0.007492751	-0.00304302	Proteome
TRINITY_DN2844_c0_g1_i2_orf1	0.018542129	0.001298056	Proteome
TRINITY_DN51096_c0_g1_i1_orf1	0.00428429	-0.010613056	Proteome
TRINITY_DN34432_c0_g1_i1_orf1	0.002083871	-0.011792088	Proteome
TRINITY_DN2600_c0_g1_i7_orf1	0.009196787	0.002970612	Proteome
TRINITY_DN15706_c0_g2_i5_orf1	0.00670824	0.009112313	Proteome
TRINITY_DN17299_c0_g1_i4_orf1	0.004618755	-0.001637565	Proteome
TRINITY_DN11693_c0_g1_i6_orf1	0.013470134	0.002421769	Proteome
TRINITY_DN1771_c0_g2_i1_orf1	-0.009142755	-0.009221722	Proteome
TRINITY_DN25423_c0_g1_i1_orf1	0.011838314	0.005382672	Proteome
TRINITY_DN4041_c0_g1_i6_orf1	-0.001972323	0.019312684	Proteome
TRINITY_DN47661_c0_g1_i8_orf1	-0.009392199	-0.004885857	Proteome
TRINITY_DN3378_c0_g1_i4_orf1	-0.00708413	0.002357062	Proteome
TRINITY_DN2986_c1_g1_i1_orf1	0.01031981	0.001600686	Proteome
TRINITY_DN11113_c0_g1_i1_orf1	0.010036831	-0.002968019	Proteome
TRINITY_DN19043_c0_g2_i1_orf1	-0.000439562	0.015242081	Proteome
TRINITY_DN19092_c0_g1_i2_orf1	0.006342664	-0.014995367	Proteome
TRINITY_DN214_c0_g1_i4_orf1	-0.020808107	0.005866402	Proteome
TRINITY_DN1264_c0_g1_i2_orf1	0.011563206	-0.011345509	Proteome
TRINITY_DN1191_c0_g1_i4_orf1	0.006088536	0.006090834	Proteome
TRINITY_DN542_c0_g2_i1_orf1	0.001849318	-0.008341238	Proteome
TRINITY_DN6423_c0_g1_i6_orf1	0.001878406	0.030434759	Proteome
TRINITY_DN5950_c0_g1_i4_orf1	0.010729939	-0.001248028	Proteome
TRINITY_DN4272_c0_g1_i1_orf1	0.011296686	0.00582357	Proteome
TRINITY_DN4069_c0_g1_i5_orf1	0.026505675	0.000999352	Proteome
TRINITY_DN38301_c0_g1_i2_orf1	0.000652862	0.001486886	Proteome
TRINITY_DN1386_c0_g1_i6_orf1	0.008104014	-0.007972475	Proteome
TRINITY_DN1947_c0_g1_i6_orf1	0.008246644	-0.001786718	Proteome
TRINITY_DN18593_c0_g1_i1_orf1	0.004715925	0.014546682	Proteome
TRINITY_DN13093_c0_g1_i2_orf1	0.01315773	0.004949391	Proteome
TRINITY_DN64230_c0_g1_i1_orf1	0.009945996	0.002326892	Proteome

TRINITY_DN57536_c0_g1_i14_orf1	0.006830492	-0.005418897	Proteome
TRINITY_DN1491_c0_g1_i4_orf1	-0.027466961	0.013661538	Proteome
TRINITY_DN82_c0_g1_i1_orf1	0.008568791	-0.006995214	Proteome
TRINITY_DN27136_c0_g3_i1_orf1	0.006006339	-0.011674644	Proteome
TRINITY_DN2876_c0_g1_i5_orf1	-0.009729643	0.011845742	Proteome
TRINITY_DN1814_c0_g1_i11_orf1	0.013508333	0.012457309	Proteome
TRINITY_DN18273_c0_g1_i4_orf1	-0.019884587	-0.012686796	Proteome
TRINITY_DN364_c1_g1_i2_orf1	-0.009871648	0.012261979	Proteome
TRINITY_DN35099_c0_g1_i1_orf1	0.010016295	-0.003008208	Proteome
TRINITY_DN3492_c0_g1_i1_orf1	0.002291883	-0.003888051	Proteome
TRINITY_DN413_c0_g1_i11_orf1	-0.003173045	-0.008583873	Proteome
TRINITY_DN104663_c1_g1_i2_orf1	-0.023869994	-0.023730937	Proteome
TRINITY_DN25136_c0_g1_i1_orf1	-0.008653046	-0.008453033	Proteome
TRINITY_DN2171_c0_g1_i1_orf1	0.012706216	-0.00111331	Proteome
TRINITY_DN5563_c1_g2_i2_orf1	0.004598728	0.014912964	Proteome
TRINITY_DN38431_c0_g1_i1_orf1	0.005543145	-0.012495609	Proteome
TRINITY_DN574_c0_g1_i4_orf1	0.010172944	0.009556043	Proteome
TRINITY_DN46173_c0_g3_i2_orf1	0.004282414	-0.000584357	Proteome
TRINITY_DN12508_c0_g1_i1_orf1	0.01457137	-0.007520695	Proteome
TRINITY_DN12534_c0_g1_i4_orf1	-0.018456843	0.042282355	Proteome
TRINITY_DN429_c0_g1_i12_orf1	0.004767432	-0.003019572	Proteome
TRINITY_DN7674_c0_g1_i2_orf1	0.005783243	-0.004679021	Proteome
TRINITY_DN13088_c0_g1_i5_orf1	-0.011897979	-0.013243107	Proteome
TRINITY_DN71699_c0_g1_i1_orf1	-0.032785829	0.006428978	Proteome
TRINITY_DN6671_c0_g1_i6_orf1	0.009884323	0.001140471	Proteome
TRINITY_DN20957_c0_g1_i1_orf1	-0.002258781	0.006723138	Proteome
TRINITY_DN1282_c1_g1_i4_orf1	-0.007806745	-0.002797619	Proteome
TRINITY_DN102260_c0_g1_i1_orf1	0.00175903	0.016458934	Proteome
TRINITY_DN2177_c0_g1_i1_orf1	-0.008933474	-0.002098633	Proteome
TRINITY_DN829_c0_g1_i8_orf1	0.021119661	-0.002119853	Proteome
TRINITY_DN9435_c0_g1_i7_orf1	-0.027237458	0.020122345	Proteome
TRINITY_DN21035_c0_g1_i14_orf1	0.02116082	-0.001823568	Proteome
TRINITY_DN8317_c1_g2_i8_orf1	0.01118304	0.004847133	Proteome
TRINITY_DN29100_c0_g1_i2_orf1	0.0114232	0.010715643	Proteome
TRINITY_DN50517_c0_g1_i3_orf1	-0.007020882	-0.016789989	Proteome
TRINITY_DN1154_c0_g1_i1_orf1	-0.004111256	-0.004581872	Proteome
TRINITY_DN3300_c0_g2_i1_orf1	0.006426404	0.01160888	Proteome
TRINITY_DN17326_c0_g1_i8_orf1	0.010927537	0.015807427	Proteome
TRINITY_DN99020_c0_g1_i1_orf1	0.012438685	0.007215966	Proteome
TRINITY_DN344_c1_g1_i1_orf1	-0.021752522	0.006091258	Proteome
TRINITY_DN9132_c0_g1_i5_orf1	-0.013697618	-0.029875149	Proteome
TRINITY_DN50724_c0_g2_i1_orf1	0.003977204	-0.011010117	Proteome
TRINITY_DN40650_c0_g1_i1_orf1	0.009976659	0.004772346	Proteome
TRINITY_DN23732_c0_g1_i1_orf1	-0.01469194	0.001661038	Proteome
TRINITY_DN21451_c0_g1_i3_orf1	0.004559329	-0.002480115	Proteome
TRINITY_DN4835_c0_g1_i2_orf1	0.006870331	-0.00455654	Proteome
TRINITY_DN94248_c0_g2_i3_orf1	-0.003690013	0.001488122	Proteome
TRINITY_DN1960_c5_g1_i3_orf1	-0.003880709	-0.002315898	Proteome
TRINITY_DN14398_c0_g1_i4_orf1	0.0037268	-0.016730325	Proteome
TRINITY_DN15202_c0_g1_i6_orf1	0.041830809	0.014372675	Proteome
TRINITY_DN1921_c1_g1_i5_orf1	-0.001010516	0.002698927	Proteome
TRINITY_DN2258_c0_g2_i1_orf1	-0.003482578	-0.002151081	Proteome
TRINITY_DN2521_c1_g1_i2_orf1	0.007022079	-0.009140644	Proteome
TRINITY_DN4640_c0_g1_i7_orf1	0.003080119	0.01157408	Proteome
TRINITY_DN12228_c0_g1_i2_orf1	0.008790976	0.002075395	Proteome
TRINITY_DN2947_c0_g1_i4_orf1	0.008005328	-0.012220024	Proteome
TRINITY_DN629_c0_g1_i6_orf1	-0.008546772	0.002782587	Proteome
TRINITY_DN4956_c0_g1_i6_orf1	-0.004300147	-0.001995415	Proteome

TRINITY_DN56795_c1_g1_i1_orf1	0.009529931	0.011422923	Proteome
TRINITY_DN120593_c0_g1_i1_orf1	0.007036843	-0.001305075	Proteome
TRINITY_DN3616_c0_g1_i4_orf1	-0.047535074	0.035677582	Proteome
TRINITY_DN757_c3_g1_i2_orf1	0.011356401	-0.001236083	Proteome
TRINITY_DN120089_c0_g1_i1_orf1	0.00745612	-0.000137994	Proteome
TRINITY_DN1935_c0_g1_i1_orf1	-0.001363755	-0.009719052	Proteome
TRINITY_DN1741_c0_g1_i5_orf1	0.003389464	0.00807021	Proteome
TRINITY_DN1956_c1_g1_i5_orf1	0.007600077	-0.007141626	Proteome
TRINITY_DN53281_c0_g1_i11_orf1	0.021313433	0.014501583	Proteome
TRINITY_DN1766_c0_g1_i6_orf1	0.006322623	0.004402566	Proteome
TRINITY_DN1328_c0_g1_i6_orf1	-0.011816989	0.016262049	Proteome
TRINITY_DN2942_c0_g1_i6_orf1	0.009071523	0.010519847	Proteome
TRINITY_DN2488_c0_g1_i4_orf1	0.0018839	-0.014865524	Proteome
TRINITY_DN74538_c0_g1_i1_orf1	-0.004170884	-0.015820916	Proteome
TRINITY_DN5111_c0_g1_i2_orf1	-0.008992819	-0.005843936	Proteome
TRINITY_DN4891_c0_g1_i4_orf1	-0.001088183	-0.002668328	Proteome
TRINITY_DN46_c0_g1_i2_orf1	-0.008008969	-0.002494541	Proteome
TRINITY_DN2302_c0_g1_i1_orf1	0.011374597	-0.006042107	Proteome
TRINITY_DN5182_c0_g1_i5_orf1	-0.001145946	0.003183464	Proteome
TRINITY_DN1004_c0_g1_i6_orf1	0.009462038	-0.00037112	Proteome
TRINITY_DN72999_c0_g1_i1_orf1	0.007212888	0.003309778	Proteome
TRINITY_DN4494_c0_g1_i1_orf1	-0.00381308	-0.003225939	Proteome
TRINITY_DN1268_c0_g1_i1_orf1	0.005989199	-0.012069418	Proteome
TRINITY_DN5684_c0_g1_i4_orf1	0.004529906	-0.004295435	Proteome
TRINITY_DN6994_c0_g1_i3_orf1	-0.007765744	-0.001090869	Proteome
TRINITY_DN30311_c0_g1_i4_orf1	0.007341805	-0.004640202	Proteome
TRINITY_DN144_c0_g1_i4_orf1	-0.000883239	-0.004571058	Proteome
TRINITY_DN5666_c0_g1_i2_orf1	0.009682972	0.008982343	Proteome
TRINITY_DN51008_c0_g1_i1_orf1	-0.003990709	-0.012767572	Proteome
TRINITY_DN288_c0_g1_i9_orf1	0.008659746	0.005691448	Proteome
TRINITY_DN10831_c1_g1_i1_orf1	0.01050626	-0.003255887	Proteome
TRINITY_DN17861_c0_g1_i5_orf1	0.005229264	-0.002588791	Proteome
TRINITY_DN4748_c0_g1_i5_orf1	-0.017093589	0.018015701	Proteome
TRINITY_DN2570_c0_g1_i1_orf1	0.00050833	0.02890802	Proteome
TRINITY_DN10716_c1_g1_i1_orf1	0.002091153	-0.013681963	Proteome
TRINITY_DN805_c0_g1_i5_orf1	-0.007085218	0.01808901	Proteome
TRINITY_DN13312_c0_g2_i1_orf1	-0.00595672	-0.010066021	Proteome
TRINITY_DN32_c0_g1_i4_orf1	0.017652516	-0.004972094	Proteome
TRINITY_DN1073_c0_g1_i4_orf1	0.006268188	-0.005757416	Proteome
TRINITY_DN4408_c6_g1_i1_orf1	0.025735905	0.001334034	Proteome
TRINITY_DN2438_c0_g1_i1_orf1	0.016295139	-0.008349046	Proteome
TRINITY_DN101658_c0_g1_i1_orf1	-0.000812513	-0.019154743	Proteome
TRINITY_DN10994_c0_g1_i4_orf1	-0.005246442	0.008977807	Proteome
TRINITY_DN9085_c0_g1_i1_orf1	0.007938019	0.001565385	Proteome
TRINITY_DN1666_c0_g1_i2_orf1	0.010817402	0.006517763	Proteome
TRINITY_DN4030_c0_g2_i1_orf1	-0.008533655	-0.006456678	Proteome
TRINITY_DN8655_c0_g4_i1_orf1	0.006829597	0.014472625	Proteome
TRINITY_DN650_c0_g1_i3_orf1	0.010756512	-0.008305124	Proteome
TRINITY_DN4068_c1_g2_i1_orf1	-0.004422587	0.014602817	Proteome
TRINITY_DN2847_c0_g1_i20_orf1	0.019929446	4.13E-05	Proteome
TRINITY_DN2668_c0_g1_i7_orf1	0.004065185	0.006198816	Proteome
TRINITY_DN2200_c0_g1_i4_orf1	-0.012171926	0.009600215	Proteome
TRINITY_DN12320_c0_g1_i1_orf1	0.004990874	0.004874999	Proteome
TRINITY_DN2749_c0_g2_i3_orf1	0.02444159	0.002311168	Proteome
TRINITY_DN8116_c0_g1_i1_orf1	0.00255467	0.000739498	Proteome
TRINITY_DN6241_c0_g1_i1_orf1	-0.00279696	0.019828003	Proteome
TRINITY_DN14952_c0_g3_i1_orf1	0.008526377	0.012032475	Proteome
TRINITY_DN26963_c0_g1_i1_orf1	0.009052379	0.003728725	Proteome

TRINITY_DN2876_c0_g1_i3_orf1	-0.007829176	-0.016290831	Proteome
TRINITY_DN7022_c0_g1_i7_orf1	-0.015039004	0.005391228	Proteome
TRINITY_DN8458_c0_g2_i1_orf1	-0.006579183	-0.003262777	Proteome
TRINITY_DN7539_c0_g1_i2_orf1	0.004336079	-0.026343071	Proteome
TRINITY_DN54410_c0_g2_i1_orf1	-0.012604313	0.004438031	Proteome
TRINITY_DN2392_c0_g2_i1_orf1	0.022146529	0.01285511	Proteome
TRINITY_DN59422_c0_g1_i2_orf1	0.017685085	0.022926385	Proteome
TRINITY_DN42903_c0_g1_i4_orf1	0.004001728	0.006780624	Proteome
TRINITY_DN3836_c0_g1_i4_orf1	0.014237755	0.001866048	Proteome
TRINITY_DN19034_c0_g1_i1_orf1	0.010972403	0.0018439	Proteome
TRINITY_DN307_c1_g1_i1_orf1	0.031769038	0.016038041	Proteome
TRINITY_DN5495_c0_g1_i5_orf1	0.009141359	0.013461048	Proteome
TRINITY_DN35245_c0_g1_i1_orf1	-0.006542398	0.003413859	Proteome
TRINITY_DN8511_c0_g1_i1_orf1	-0.000164552	0.007645739	Proteome
TRINITY_DN9079_c0_g1_i5_orf1	-0.013182956	-0.022991305	Proteome
TRINITY_DN3343_c0_g1_i4_orf1	0.002434548	-0.004524795	Proteome
TRINITY_DN3464_c0_g1_i1_orf1	-0.029451117	0.001680437	Proteome
TRINITY_DN23042_c0_g1_i1_orf1	0.010466134	-0.004220893	Proteome
TRINITY_DN1363_c0_g1_i11_orf1	0.013876168	0.014112936	Proteome
TRINITY_DN2914_c0_g1_i1_orf1	0.00612428	0.004559769	Proteome
TRINITY_DN1609_c0_g1_i3_orf1	0.009826367	-0.007151542	Proteome
TRINITY_DN5578_c0_g1_i4_orf1	0.008787477	0.004126036	Proteome
TRINITY_DN59291_c0_g1_i1_orf1	0.011425991	0.002938688	Proteome
TRINITY_DN3332_c0_g1_i2_orf1	0.000742752	-0.008092814	Proteome
TRINITY_DN19678_c0_g1_i1_orf1	0.008795528	-0.004898087	Proteome
TRINITY_DN9558_c0_g1_i2_orf1	-0.007561234	-0.018169906	Proteome
TRINITY_DN8046_c0_g1_i4_orf1	-0.011592694	-0.017251999	Proteome
TRINITY_DN31163_c1_g1_i4_orf1	-0.019886152	-0.005052763	Proteome
TRINITY_DN3582_c0_g1_i2_orf1	0.00483802	0.005158778	Proteome
TRINITY_DN7291_c0_g1_i3_orf1	-0.011666306	-0.01063791	Proteome
TRINITY_DN3601_c0_g1_i5_orf1	0.005263816	-0.00623395	Proteome
TRINITY_DN2320_c0_g1_i4_orf1	-0.00131017	0.010656241	Proteome
TRINITY_DN11402_c0_g1_i1_orf1	-0.000479902	-0.010117592	Proteome
TRINITY_DN10336_c0_g1_i9_orf1	0.009579755	0.002635217	Proteome
TRINITY_DN1330_c0_g1_i1_orf1	-0.007372328	-0.000327687	Proteome
TRINITY_DN452_c9_g1_i1_orf1	0.013188714	-0.011609832	Proteome
TRINITY_DN128_c0_g1_i5_orf1	-0.000475933	-0.009925745	Proteome
TRINITY_DN23586_c0_g1_i3_orf1	-0.000606383	-0.000635414	Proteome
TRINITY_DN117_c0_g1_i5_orf1	0.004916606	0.00507788	Proteome
TRINITY_DN1369_c0_g2_i3_orf1	0.008719422	0.001162752	Proteome
TRINITY_DN31058_c0_g1_i6_orf1	-0.000867766	0.005762916	Proteome
TRINITY_DN1993_c0_g1_i1_orf1	0.004638705	0.005592352	Proteome
TRINITY_DN6015_c1_g1_i3_orf1	-0.008777284	-0.012163817	Proteome
TRINITY_DN1285_c0_g1_i6_orf1	0.008022898	-0.004714862	Proteome
TRINITY_DN23444_c0_g1_i11_orf1	0.006850315	-0.021178329	Proteome
TRINITY_DN15318_c0_g1_i1_orf1	0.014475982	0.003771058	Proteome
TRINITY_DN2574_c0_g1_i5_orf1	0.005487709	0.008382616	Proteome
TRINITY_DN57074_c0_g2_i1_orf1	0.004690179	0.001664783	Proteome
TRINITY_DN104_c0_g1_i4_orf1	0.017543731	0.001740168	Proteome
TRINITY_DN4247_c0_g1_i4_orf1	0.009628359	-0.006959862	Proteome
TRINITY_DN2782_c0_g1_i7_orf1	0.013982009	0.008522576	Proteome
TRINITY_DN3209_c0_g2_i6_orf1	0.015028944	-0.009677859	Proteome
TRINITY_DN726_c0_g1_i2_orf1	-0.009152487	-0.00124626	Proteome
TRINITY_DN3403_c0_g1_i6_orf1	0.007628978	-0.002429622	Proteome
TRINITY_DN225_c0_g1_i6_orf1	0.022987159	-0.001600405	Proteome
TRINITY_DN17351_c0_g1_i3_orf1	0.004214151	0.004235433	Proteome
TRINITY_DN2394_c0_g1_i4_orf1	0.002024645	0.004306585	Proteome
TRINITY_DN87648_c0_g1_i1_orfp1	0.01316932	0.009387634	Proteome

TRINITY_DN3256_c0_g1_i3_orf1	0.006861667	-0.015080675	Proteome
TRINITY_DN2461_c0_g1_i5_orf1	0.014421231	-0.005972693	Proteome
TRINITY_DN2026_c0_g1_i4_orf1	-0.000795357	-0.012076001	Proteome
TRINITY_DN51830_c0_g1_i4_orf1	0.010117158	-0.008961245	Proteome
TRINITY_DN12_c0_g1_i5_orf1	0.012580462	0.00436752	Proteome
TRINITY_DN364_c2_g1_i2_orf1	-0.00190046	0.010221225	Proteome
TRINITY_DN64769_c0_g1_i3_orf1	0.012539353	0.001290255	Proteome
TRINITY_DN7957_c0_g1_i5_orf1	-0.007742231	0.004783168	Proteome
TRINITY_DN3859_c0_g1_i5_orf1	-0.016697641	0.002981708	Proteome
TRINITY_DN397_c0_g1_i1_orf1	0.007326533	0.002281898	Proteome
TRINITY_DN3229_c0_g1_i1_orf1	0.005888069	0.002199763	Proteome
TRINITY_DN3348_c0_g1_i1_orf1	0.005656126	0.009849403	Proteome
TRINITY_DN9464_c0_g1_i1_orf1	0.008566219	0.001751631	Proteome
TRINITY_DN8245_c0_g1_i4_orf1	-0.011910461	-0.005809602	Proteome
TRINITY_DN142376_c0_g1_i1_orf1	-0.014027747	0.025144979	Proteome
TRINITY_DN2694_c0_g1_i3_orf1	-0.003748228	-0.005148586	Proteome
TRINITY_DN381_c0_g1_i1_orf1	0.013345472	0.002462125	Proteome
TRINITY_DN14987_c0_g1_i3_orf1	0.007467282	-0.002515633	Proteome
TRINITY_DN1957_c0_g1_i4_orf1	-0.015890081	-0.004677536	Proteome
TRINITY_DN874_c2_g1_i1_orf1	0.017689655	-0.007394718	Proteome
TRINITY_DN3769_c0_g1_i1_orf1	0.012653907	0.001645303	Proteome
TRINITY_DN8651_c0_g1_i18_orf1	0.021111147	-0.001171101	Proteome
TRINITY_DN2170_c1_g1_i3_orf1	-0.010169078	-0.014876354	Proteome
TRINITY_DN64015_c0_g1_i1_orf1	-0.001973721	0.005845009	Proteome
TRINITY_DN29018_c0_g1_i4_orf1	-0.008666499	-0.00282723	Proteome
TRINITY_DN13186_c0_g1_i1_orf1	-0.013112654	-0.00011776	Proteome
TRINITY_DN13799_c0_g1_i1_orf1	-0.031017083	0.024171478	Proteome
TRINITY_DN2783_c0_g1_i22_orf1	0.012152859	-0.00561024	Proteome
TRINITY_DN1503_c0_g1_i6_orf1	-0.013887228	0.016244852	Proteome
TRINITY_DN3260_c0_g1_i6_orf1	0.008694215	0.000872051	Proteome
TRINITY_DN2318_c1_g1_i1_orf1	0.000848953	0.011722306	Proteome
TRINITY_DN12661_c0_g1_i3_orf1	0.0057156	0.014774616	Proteome
TRINITY_DN8046_c0_g1_i5_orf1	0.006323388	-0.008509858	Proteome
TRINITY_DN8659_c0_g2_i1_orf1	-0.014366873	-0.00320802	Proteome
TRINITY_DN86149_c0_g1_i1_orf1	-0.010579858	-0.000676042	Proteome
TRINITY_DN1134_c0_g1_i4_orf1	0.013504294	6.30E-05	Proteome
TRINITY_DN11108_c0_g1_i4_orf1	0.019373057	-0.001081441	Proteome
TRINITY_DN29009_c0_g2_i3_orf1	-0.004893388	-0.017204805	Proteome
TRINITY_DN11176_c0_g1_i1_orf1	0.007150756	-0.005963379	Proteome
TRINITY_DN19098_c0_g1_i4_orf1	0.021619772	-0.001671735	Proteome
TRINITY_DN19998_c0_g1_i1_orf1	-0.002218385	0.017532128	Proteome
TRINITY_DN16490_c0_g2_i1_orf1	-0.00137094	0.009282392	Proteome
TRINITY_DN972_c0_g1_i6_orf1	0.001825304	-0.01387472	Proteome
TRINITY_DN361_c0_g1_i5_orf1	-0.015921585	-0.006208901	Proteome
TRINITY_DN21420_c0_g1_i2_orf1	-0.032128292	0.014690355	Proteome
TRINITY_DN2490_c0_g2_i1_orfp1	0.004308844	-0.002184696	Proteome
TRINITY_DN32586_c0_g2_i1_orf1	0.014881165	0.015481098	Proteome
TRINITY_DN23564_c0_g1_i7_orf1	0.020195621	-0.000519165	Proteome
TRINITY_DN42824_c0_g1_i5_orf1	-0.003147279	0.002198476	Proteome
TRINITY_DN6693_c0_g1_i1_orf1	-0.002000896	-0.004113293	Proteome
TRINITY_DN28759_c0_g1_i1_orf1	0.007648326	-0.007379282	Proteome
TRINITY_DN21315_c0_g1_i1_orf1	0.01062879	-0.009803572	Proteome
TRINITY_DN712_c0_g2_i1_orf1	0.007425527	-0.011890701	Proteome
TRINITY_DN18773_c0_g1_i3_orf1	0.005572969	0.011667455	Proteome
TRINITY_DN5019_c0_g1_i2_orf1	0.006411164	0.002066201	Proteome
TRINITY_DN3896_c0_g1_i1_orf1	0.001249719	-0.001269618	Proteome
TRINITY_DN2175_c0_g1_i4_orf1	-0.009356253	0.025175809	Proteome
TRINITY_DN3235_c0_g1_i1_orf1	0.007419187	-0.002785406	Proteome

TRINITY_DN8245_c0_g1_i3_orf1	-0.015135409	0.009294173	Proteome
TRINITY_DN13615_c0_g1_i3_orf1	-0.00115294	0.0145246	Proteome
TRINITY_DN36893_c0_g1_i1_orf1	0.006675289	-0.001732019	Proteome
TRINITY_DN35635_c0_g1_i1_orf1	-0.008676829	0.005451952	Proteome
TRINITY_DN12193_c0_g1_i2_orf1	0.003228657	0.008297092	Proteome
TRINITY_DN2266_c0_g1_i6_orf1	-0.017656883	0.006467318	Proteome
TRINITY_DN5092_c0_g1_i2_orf1	0.007887162	-0.001684084	Proteome
TRINITY_DN18502_c0_g1_i1_orf1	0.006447108	-0.001994076	Proteome
TRINITY_DN206_c0_g1_i11_orf1	0.004611594	-0.006847177	Proteome
TRINITY_DN20344_c0_g1_i5_orf1	0.031782156	0.019937486	Proteome
TRINITY_DN3732_c0_g1_i6_orf1	0.009087833	-0.007768032	Proteome
TRINITY_DN3906_c0_g1_i5_orf1	0.016356791	0.00787467	Proteome
TRINITY_DN109540_c0_g1_i3_orf1	5.64E-05	-0.007728285	Proteome
TRINITY_DN21170_c0_g1_i5_orf1	0.008019332	0.016809881	Proteome
TRINITY_DN935_c0_g1_i3_orf1	0.0052286	-0.00858536	Proteome
TRINITY_DN126648_c0_g1_i1_orf1	0.016337334	0.002523626	Proteome
TRINITY_DN9079_c1_g1_i1_orf1	0.016168893	-0.018535757	Proteome
TRINITY_DN3010_c0_g1_i4_orf1	0.015516214	-0.002451967	Proteome
TRINITY_DN15234_c0_g1_i3_orf1	0.004714033	-0.011569359	Proteome
TRINITY_DN280_c4_g1_i5_orf1	-0.013595753	0.003572741	Proteome
TRINITY_DN6908_c0_g1_i1_orf1	0.011337322	-0.044268133	Proteome
TRINITY_DN3878_c0_g1_i4_orf1	-0.002869519	-0.003353359	Proteome
TRINITY_DN1093_c0_g1_i4_orf1	0.002384773	-0.01739592	Proteome
TRINITY_DN2927_c0_g1_i6_orf1	0.000534152	-0.007121969	Proteome
TRINITY_DN12065_c0_g1_i4_orf1	-0.000575435	0.018078593	Proteome
TRINITY_DN60949_c0_g1_i4_orf1	0.01188823	0.001304245	Proteome
TRINITY_DN5867_c0_g1_i1_orf1	0.001787669	0.008655926	Proteome
TRINITY_DN2953_c1_g1_i10_orf1	0.008858181	0.023320237	Proteome
TRINITY_DN14487_c0_g1_i4_orf1	-0.008308954	-0.000838281	Proteome
TRINITY_DN1391_c1_g2_i2_orf1	0.010822234	0.014865597	Proteome
TRINITY_DN11637_c0_g1_i1_orf1	0.015255452	-0.010143285	Proteome
TRINITY_DN1277_c4_g1_i5_orf1	0.00789264	0.000587278	Proteome
TRINITY_DN972_c0_g2_i1_orf1	0.006950858	-0.0041735	Proteome
TRINITY_DN37141_c0_g1_i2_orf1	0.005802167	0.011004287	Proteome
TRINITY_DN3332_c0_g1_i10_orf1	-0.008202638	0.005144301	Proteome
TRINITY_DN53358_c0_g1_i3_orf1	-0.028556061	-0.083383988	Proteome
TRINITY_DN79210_c0_g1_i1_orf1	-0.003162204	-0.010880956	Proteome
TRINITY_DN79734_c0_g2_i3_orf1	0.01261127	0.001113542	Proteome
TRINITY_DN31676_c0_g1_i4_orf1	-0.009818652	-0.015017317	Proteome
TRINITY_DN14597_c0_g1_i5_orf1	0.013348672	-0.010078281	Proteome
TRINITY_DN106476_c0_g1_i3_orf1	0.009084687	-0.000844233	Proteome
TRINITY_DN40669_c0_g2_i1_orf1	0.013359305	-0.004326568	Proteome
TRINITY_DN10774_c0_g2_i3_orf1	0.016651535	-0.008029386	Proteome
TRINITY_DN13098_c2_g1_i2_orf1	-0.013702861	0.010818343	Proteome
TRINITY_DN35763_c0_g1_i2_orf1	0.011604461	-0.004183277	Proteome
TRINITY_DN8654_c0_g1_i1_orf1	-0.013140053	-0.003719888	Proteome
TRINITY_DN5475_c0_g1_i3_orf1	0.013539054	0.002225314	Proteome
TRINITY_DN1673_c0_g1_i2_orf1	0.00368945	-0.007239651	Proteome
TRINITY_DN2795_c0_g1_i1_orf1	0.008433013	-0.003617598	Proteome
TRINITY_DN4281_c0_g1_i1_orf1	0.000509463	0.008052571	Proteome
TRINITY_DN107617_c3_g1_i1_orf1	0.003559399	-0.003081735	Proteome
TRINITY_DN143_c0_g3_i1_orf1	0.015063031	0.006870922	Proteome
TRINITY_DN3889_c0_g1_i7_orfp1	2.82E-05	-0.00171649	Proteome
TRINITY_DN58261_c0_g1_i1_orf1	0.010973092	-0.00617664	Proteome
TRINITY_DN2772_c0_g1_i3_orf1	-0.01763255	-0.013583211	Proteome
TRINITY_DN124654_c0_g1_i1_orf1	0.013344961	0.023733974	Proteome
TRINITY_DN512_c0_g1_i10_orf1	0.014455526	0.003862653	Proteome
TRINITY_DN220_c0_g1_i3_orf1	0.006592539	0.002312504	Proteome

TRINITY_DN30208_c0_g1_i3_orf1	0.0127908	0.017053922	Proteome
TRINITY_DN667_c0_g1_i13_orf1	0.00807618	0.010047399	Proteome
TRINITY_DN97378_c0_g1_i2_orf1	0.000402378	-0.017860731	Proteome
TRINITY_DN1470_c0_g1_i2_orf1	-0.001861303	0.007862691	Proteome
TRINITY_DN22678_c0_g1_i4_orf1	0.011012841	-0.002315438	Proteome
TRINITY_DN20347_c0_g1_i6_orf1	-0.024073229	0.013268984	Proteome
TRINITY_DN1491_c0_g1_i8_orf1	0.002532049	0.000579192	Proteome
TRINITY_DN2283_c0_g2_i1_orf1	0.008788932	0.004797741	Proteome
TRINITY_DN9302_c0_g1_i1_orf1	0.006871533	0.002872793	Proteome
TRINITY_DN66671_c0_g1_i1_orf1	-0.008315689	0.008037387	Proteome
TRINITY_DN1149_c0_g1_i4_orf1	-0.028748599	0.017678706	Proteome
TRINITY_DN8544_c12_g1_i2_orf1	0.007469248	-0.016104051	Proteome
TRINITY_DN7405_c0_g1_i3_orf1	-0.000390249	-0.01490518	Proteome
TRINITY_DN1093_c0_g1_i6_orf1	-0.008168053	-0.019068753	Proteome
TRINITY_DN79657_c0_g1_i1_orf1	0.01631856	-0.006181458	Proteome
TRINITY_DN147676_c0_g1_i1_orf1	0.010936375	-0.004913851	Proteome
TRINITY_DN38075_c0_g1_i1_orf1	0.006723637	0.001125459	Proteome
TRINITY_DN135_c0_g1_i1_orf1	0.001614173	-0.005349206	Proteome
TRINITY_DN23349_c0_g1_i9_orf1	0.004544246	-0.011440027	Proteome
TRINITY_DN36144_c0_g1_i3_orf1	-0.029248531	0.002675502	Proteome
TRINITY_DN37048_c0_g1_i9_orf1	0.007840255	-0.007602753	Proteome
TRINITY_DN14094_c0_g1_i1_orfp1	-0.014455079	0.020420946	Proteome
TRINITY_DN9468_c1_g1_i4_orf1	-0.00105884	0.007841063	Proteome
TRINITY_DN7919_c0_g1_i4_orf1	0.007184611	-0.016704934	Proteome
TRINITY_DN17446_c0_g1_i1_orf1	0.00038192	-0.009651503	Proteome
TRINITY_DN994_c0_g1_i2_orf1	0.001171143	-0.014678371	Proteome
TRINITY_DN7294_c0_g2_i4_orf1	0.005661082	-0.002876109	Proteome
TRINITY_DN875_c0_g1_i3_orf1	-0.019338309	0.014049525	Proteome
TRINITY_DN166_c0_g1_i4_orf1	-0.006572717	-0.014021303	Proteome
TRINITY_DN24789_c0_g1_i9_orfp1	-0.006465582	0.028693226	Proteome
TRINITY_DN64892_c0_g1_i1_orf1	-0.01901821	-0.032587057	Proteome
TRINITY_DN31314_c0_g1_i4_orf1	-0.002478903	-0.010679763	Proteome
TRINITY_DN5559_c0_g1_i1_orf1	-0.000866141	-0.004054114	Proteome
TRINITY_DN4710_c0_g1_i1_orf1	0.000841309	0.009567776	Proteome
TRINITY_DN1266_c6_g1_i1_orf1	0.008083112	-0.003696524	Proteome
TRINITY_DN2861_c0_g2_i1_orf1	-0.00455535	-0.00789449	Proteome
TRINITY_DN1391_c0_g1_i29_orfp1	0.003395957	0.042690942	Proteome
TRINITY_DN11973_c0_g1_i1_orf1	0.006337242	0.005416329	Proteome
TRINITY_DN8306_c0_g1_i4_orf1	0.015493377	-0.003136994	Proteome
TRINITY_DN246_c1_g1_i5_orf1	-0.004174468	-0.001935303	Proteome
TRINITY_DN22046_c1_g1_i5_orf1	0.022340928	0.00270495	Proteome
TRINITY_DN3600_c0_g1_i1_orf1	-0.011884559	0.011517374	Proteome
TRINITY_DN644_c0_g1_i1_orf1	0.013507834	-0.004282383	Proteome
TRINITY_DN3970_c0_g1_i1_orf1	0.009124185	-0.001977026	Proteome
TRINITY_DN34423_c0_g1_i3_orf1	-0.022248761	0.063826452	Proteome
TRINITY_DN517_c0_g1_i5_orf1	0.005015068	-0.007022591	Proteome
TRINITY_DN43420_c0_g2_i1_orf1	0.001582826	-0.000667444	Proteome
TRINITY_DN66822_c0_g1_i1_orf1	0.006649126	-0.010079151	Proteome
TRINITY_DN4808_c0_g1_i3_orf1	0.002723427	0.012162556	Proteome
TRINITY_DN779_c0_g1_i12_orf1	0.016114964	0.006081592	Proteome
TRINITY_DN7024_c0_g1_i1_orf1	9.05E-05	0.001623889	Proteome
TRINITY_DN30027_c0_g1_i1_orf1	0.011283066	0.008091539	Proteome
TRINITY_DN294_c0_g1_i2_orf1	-0.003814308	-0.01619059	Proteome
TRINITY_DN40345_c0_g1_i6_orf1	0.005493561	0.00544849	Proteome
TRINITY_DN12250_c0_g1_i4_orf1	0.003207985	-0.007448194	Proteome
TRINITY_DN10385_c0_g1_i5_orf1	0.009932869	-0.009733765	Proteome
TRINITY_DN327_c1_g1_i4_orf1	0.006434617	-0.002627719	Proteome
TRINITY_DN9718_c0_g1_i7_orf1	-0.002949416	-0.007216847	Proteome

TRINITY_DN27641_c0_g1_i1_orf1	-0.003799845	-0.005188807	Proteome
TRINITY_DN146524_c0_g1_i1_orf1	0.004741156	0.014780102	Proteome
TRINITY_DN29009_c0_g2_i2_orf1	-0.009823254	0.032895103	Proteome
TRINITY_DN26994_c1_g1_i6_orf1	0.011711981	-0.00499143	Proteome
TRINITY_DN25779_c0_g1_i6_orf1	-0.004692598	-0.004815826	Proteome
TRINITY_DN3332_c0_g1_i11_orf1	-0.005177302	-0.01378408	Proteome
TRINITY_DN95665_c0_g1_i1_orf1	-0.008578762	0.007057326	Proteome
TRINITY_DN2193_c0_g1_i7_orf1	0.018105005	-0.002966609	Proteome
TRINITY_DN27670_c0_g1_i4_orf1	0.00553261	0.006027624	Proteome
TRINITY_DN11464_c0_g1_i3_orf1	0.001553457	0.012618116	Proteome
TRINITY_DN59852_c0_g1_i1_orf1	0.009427037	0.015644151	Proteome
TRINITY_DN1194_c0_g1_i5_orf1	-0.000200495	0.020899135	Proteome
TRINITY_DN26649_c0_g1_i2_orf1	-0.004781396	0.000593367	Proteome
TRINITY_DN6063_c1_g2_i1_orf1	-0.002382473	0.008946322	Proteome
TRINITY_DN1097_c0_g1_i1_orf1	-0.00200045	0.010686905	Proteome
TRINITY_DN14458_c0_g1_i2_orf1	-0.010905948	0.006390442	Proteome
TRINITY_DN2896_c0_g1_i2_orf1	0.009842139	0.010071652	Proteome
TRINITY_DN2126_c0_g1_i2_orf1	0.006431836	-0.010169706	Proteome
TRINITY_DN4429_c0_g1_i5_orf1	-0.005964287	-0.006060414	Proteome
TRINITY_DN49530_c0_g1_i1_orf1	-0.003981041	-0.031135889	Proteome
TRINITY_DN12526_c0_g1_i5_orf1	0.041340069	0.020632807	Proteome
TRINITY_DN195_c0_g3_i6_orf1	0.010469409	-0.004959473	Proteome
TRINITY_DN14855_c0_g1_i1_orf1	0.012712004	-0.008333857	Proteome
TRINITY_DN2818_c0_g1_i2_orf1	0.004441823	0.006221786	Proteome
TRINITY_DN4235_c0_g1_i2_orf1	-0.020611899	-0.008806675	Proteome
TRINITY_DN45924_c0_g1_i14_orf1	0.007388369	0.000505602	Proteome
TRINITY_DN13718_c0_g1_i4_orf1	0.019437148	-0.010478359	Proteome
TRINITY_DN15896_c0_g1_i4_orf1	0.00410484	0.002791296	Proteome
TRINITY_DN39404_c0_g1_i7_orf1	0.00596836	-0.001647815	Proteome
TRINITY_DN658_c1_g1_i6_orf1	0.000853666	-0.001958528	Proteome
TRINITY_DN6162_c1_g1_i1_orf1	0.013714914	0.00370199	Proteome
TRINITY_DN36230_c0_g1_i1_orf1	0.012922297	-0.004735478	Proteome
TRINITY_DN1034_c0_g1_i4_orf1	-0.012247406	-0.010533148	Proteome
TRINITY_DN27_c0_g1_i1_orf1	0.007181426	0.004317878	Proteome
TRINITY_DN2110_c0_g1_i3_orf1	-0.005109522	-0.015278837	Proteome
TRINITY_DN695_c0_g1_i5_orf1	-0.003572109	0.019418203	Proteome
TRINITY_DN120979_c0_g1_i1_orf1	-0.001274704	0.010797002	Proteome
TRINITY_DN11374_c0_g1_i4_orf1	0.011634922	-0.012767096	Proteome
TRINITY_DN20710_c0_g1_i2_orf1	-0.001288082	0.00942551	Proteome
TRINITY_DN13746_c0_g1_i5_orf1	0.001314839	-0.002747073	Proteome
TRINITY_DN1376_c0_g1_i4_orf1	-0.0001853	-0.011614768	Proteome
TRINITY_DN14073_c0_g1_i1_orf1	-0.011523307	-0.005514472	Proteome
TRINITY_DN48410_c0_g2_i1_orf1	-0.001429838	-0.001259248	Proteome
TRINITY_DN18374_c0_g1_i1_orf1	-0.008407048	-0.021902359	Proteome
TRINITY_DN83150_c0_g1_i1_orf1	0.017467916	0.0010917	Proteome
TRINITY_DN6248_c0_g1_i1_orf1	0.000454392	-0.011344327	Proteome
TRINITY_DN3411_c0_g2_i1_orf1	0.014092863	-0.003514558	Proteome
TRINITY_DN960_c1_g1_i6_orf1	0.008517738	0.018238055	Proteome
TRINITY_DN4757_c0_g1_i3_orf1	0.003948736	0.007884663	Proteome
TRINITY_DN3132_c0_g1_i10_orf1	0.011857468	-0.002055076	Proteome
TRINITY_DN117_c0_g1_i6_orf1	-0.004352872	-0.004737728	Proteome
TRINITY_DN4189_c0_g2_i1_orf1	0.008218047	0.013810997	Proteome
TRINITY_DN31503_c0_g1_i4_orf1	-0.003586455	-0.009194885	Proteome
TRINITY_DN83948_c0_g1_i3_orf1	-0.006969529	-0.011783205	Proteome
TRINITY_DN38835_c0_g3_i1_orf1	0.009189606	-0.017519786	Proteome
TRINITY_DN11639_c0_g1_i1_orf1	-0.003263396	0.002400576	Proteome
TRINITY_DN1406_c0_g2_i2_orf1	0.007116493	0.01298032	Proteome
TRINITY_DN34784_c0_g1_i1_orf1	0.000809629	0.013843287	Proteome

TRINITY_DN6715_c0_g1_i3_orf1	-0.008380514	-0.013486629	Proteome
TRINITY_DN16931_c0_g1_i1_orf1	0.008979326	-0.002646696	Proteome
TRINITY_DN2170_c4_g1_i2_orf1	-0.004976499	0.019490914	Proteome
TRINITY_DN6044_c0_g1_i4_orf1	-0.003935674	0.003169711	Proteome
TRINITY_DN10530_c0_g1_i1_orf1	-0.004180391	0.000914897	Proteome
TRINITY_DN40028_c0_g1_i1_orf1	0.009591136	-0.0051654	Proteome
TRINITY_DN8095_c0_g1_i3_orf1	-0.007867398	-0.018498582	Proteome
TRINITY_DN10373_c0_g1_i1_orf1	0.020321465	0.010250617	Proteome
TRINITY_DN45220_c0_g1_i1_orf1	-0.015669678	0.015703831	Proteome
TRINITY_DN5238_c0_g1_i2_orf1	0.00845287	-0.002159149	Proteome
TRINITY_DN132_c0_g2_i2_orf1	0.005516421	0.001178048	Proteome
TRINITY_DN901_c0_g1_i8_orf1	0.007956368	0.000669158	Proteome
TRINITY_DN28729_c0_g1_i7_orf1	-0.002728941	-0.000243683	Proteome
TRINITY_DN146217_c0_g1_i1_orf1	0.00160629	0.010022142	Proteome
TRINITY_DN130778_c0_g1_i1_orf1	0.010891485	0.005235861	Proteome
TRINITY_DN1054_c0_g1_i8_orf1	0.014883816	0.008069728	Proteome
TRINITY_DN399_c3_g2_i6_orf1	-0.015710053	0.026524829	Proteome
TRINITY_DN4183_c0_g1_i8_orf1	0.003523125	0.002819768	Proteome
TRINITY_DN142485_c0_g1_i1_orf1	0.001754536	0.011017232	Proteome
TRINITY_DN85476_c0_g1_i1_orf1	0.017417581	0.004728411	Proteome
TRINITY_DN11886_c0_g1_i1_orf1	0.000990503	0.000495117	Proteome
TRINITY_DN20680_c0_g1_i5_orf1	0.020499556	-0.010890164	Proteome
TRINITY_DN11230_c0_g1_i4_orf1	-0.000195417	0.018997853	Proteome
TRINITY_DN52395_c0_g2_i2_orf1	0.000313952	0.00216484	Proteome
TRINITY_DN64141_c0_g1_i4_orf1	-0.021989529	-0.023276016	Proteome
TRINITY_DN2638_c0_g1_i7_orf1	0.001481328	0.012042111	Proteome
TRINITY_DN21984_c0_g1_i6_orf1	-0.002133257	-0.014087279	Proteome
TRINITY_DN135188_c0_g1_i2_orf1	-0.000178218	-0.016402947	Proteome
TRINITY_DN184_c0_g1_i10_orf1	-0.004782146	-0.027029912	Proteome
TRINITY_DN64627_c0_g1_i1_orf1	-0.008182338	0.004326482	Proteome
TRINITY_DN3616_c0_g2_i1_orf1	0.008442438	0.012577538	Proteome
TRINITY_DN4070_c0_g1_i4_orf1	0.006267576	-0.008421364	Proteome
TRINITY_DN8703_c0_g1_i2_orf1	-0.024507568	-0.004727372	Proteome
TRINITY_DN136031_c0_g1_i7_orf1	0.056276582	-0.004602892	Proteome
TRINITY_DN47114_c0_g1_i5_orf1	0.008885251	-0.001156469	Proteome
TRINITY_DN30510_c0_g1_i6_orf1	0.046238123	0.028062114	Proteome
TRINITY_DN22242_c0_g1_i1_orf1	0.020921855	-0.000837521	Proteome
TRINITY_DN15458_c0_g1_i3_orf1	0.008922981	0.001575139	Proteome
TRINITY_DN3310_c0_g1_i1_orf1	0.000204788	0.003505906	Proteome
TRINITY_DN3113_c1_g2_i1_orf1	0.004832536	-0.010519323	Proteome
TRINITY_DN1481_c0_g1_i4_orf1	-0.006805188	-0.022641177	Proteome
TRINITY_DN2836_c0_g1_i4_orf1	-0.003885097	0.022712664	Proteome
TRINITY_DN2650_c0_g1_i1_orf1	0.03099773	-0.002819752	Proteome
TRINITY_DN7451_c0_g1_i10_orf1	0.008332968	0.00015552	Proteome
TRINITY_DN32572_c0_g1_i1_orf1	0.008160955	-0.010707293	Proteome
TRINITY_DN4245_c0_g2_i1_orf1	0.018314776	-0.003495533	Proteome
TRINITY_DN1383_c0_g1_i2_orf1	-0.00637663	0.015168657	Proteome
TRINITY_DN76633_c0_g1_i1_orfp1	-0.027690067	0.024679019	Proteome
TRINITY_DN5907_c0_g1_i4_orf1	-0.002880707	-0.005254957	Proteome
TRINITY_DN98313_c0_g1_i1_orf1	-0.0163971	0.005868997	Proteome
TRINITY_DN2054_c0_g1_i1_orf1	-0.004712264	0.011767396	Proteome
TRINITY_DN1407_c0_g1_i5_orf1	-0.01797616	0.013723275	Proteome
TRINITY_DN2100_c0_g1_i2_orf1	-0.003235992	-0.013612654	Proteome
TRINITY_DN428_c0_g1_i8_orf1	-0.015689057	0.015798806	Proteome
TRINITY_DN11616_c0_g1_i3_orf1	0.007979226	0.002421115	Proteome
TRINITY_DN114344_c0_g1_i4_orf1	0.010444498	0.00445282	Proteome
TRINITY_DN2848_c0_g1_i1_orf1	-0.026235991	-0.001760528	Proteome
TRINITY_DN7580_c0_g1_i1_orf1	0.022372819	-0.002539643	Proteome

TRINITY_DN1707_c0_g1_i1_orf1	-0.00625523	-0.004537323	Proteome
TRINITY_DN5132_c0_g1_i4_orf1	0.004260332	-0.02723559	Proteome
TRINITY_DN745_c7_g1_i1_orf1	-0.024643463	-0.011716909	Proteome
TRINITY_DN5032_c0_g1_i1_orf1	0.009930388	0.009628797	Proteome
TRINITY_DN1298_c0_g1_i3_orf1	0.011306066	0.007617201	Proteome
TRINITY_DN48096_c0_g2_i2_orf1	0.011207719	0.00462977	Proteome
TRINITY_DN3325_c0_g1_i1_orf1	0.01136014	0.014320339	Proteome
TRINITY_DN20363_c1_g1_i1_orf1	-0.008621277	-0.000629771	Proteome
TRINITY_DN82008_c0_g1_i1_orf1	0.009551722	-0.004637064	Proteome
TRINITY_DN1772_c7_g1_i7_orf1	0.002991443	-0.012555417	Proteome
TRINITY_DN36682_c0_g1_i1_orf1	0.008230279	0.005406783	Proteome
TRINITY_DN12671_c0_g1_i4_orf1	-0.001176437	-0.008671314	Proteome
TRINITY_DN5149_c0_g1_i12_orfp1	0.00251647	0.015971669	Proteome
TRINITY_DN139438_c0_g1_i1_orf1	0.009108105	0.007271039	Proteome
TRINITY_DN5383_c0_g1_i4_orf1	0.011048674	-0.001645765	Proteome
TRINITY_DN21782_c0_g1_i8_orf1	0.001197486	-0.005723867	Proteome
TRINITY_DN10644_c0_g1_i2_orf1	-0.002421196	-0.010332046	Proteome
TRINITY_DN3976_c0_g1_i6_orf1	-0.003784551	-0.001953094	Proteome
TRINITY_DN51776_c0_g2_i1_orf1	0.012760895	0.018118906	Proteome
TRINITY_DN69713_c0_g1_i1_orf1	0.003379067	-0.006855236	Proteome
TRINITY_DN9003_c0_g1_i20_orf1	0.006700798	0.004267128	Proteome
TRINITY_DN542_c0_g1_i4_orf1	-0.002616333	-0.000771479	Proteome
TRINITY_DN47219_c0_g1_i3_orf1	-0.009978139	5.55E-05	Proteome
TRINITY_DN2207_c0_g1_i6_orf1	-0.013869331	0.007441864	Proteome
TRINITY_DN482_c0_g1_i1_orf1	-0.000528437	-0.009549161	Proteome
TRINITY_DN20878_c0_g4_i2_orf1	-0.008547221	-0.005409876	Proteome
TRINITY_DN2472_c0_g1_i6_orf1	-0.027377174	0.007173299	Proteome
TRINITY_DN21150_c0_g1_i4_orf1	0.008207876	0.004712957	Proteome
TRINITY_DN37900_c0_g1_i1_orf1	-0.007195784	-0.01063099	Proteome
TRINITY_DN22242_c0_g2_i1_orf1	0.019979759	-0.01522762	Proteome
TRINITY_DN1965_c0_g1_i7_orf1	-0.007999685	-0.003278153	Proteome
TRINITY_DN896_c0_g1_i2_orf1	0.018754531	-0.001280504	Proteome
TRINITY_DN9593_c0_g1_i2_orf1	-0.030331076	0.007015984	Proteome
TRINITY_DN1123_c2_g1_i3_orf1	0.010955232	0.0154971	Proteome
TRINITY_DN125967_c0_g1_i1_orf1	0.007648781	-0.008953437	Proteome
TRINITY_DN2848_c0_g1_i2_orf1	-0.018902878	0.007396617	Proteome
TRINITY_DN4036_c0_g2_i1_orf1	0.017976501	0.001435393	Proteome
TRINITY_DN17772_c0_g1_i4_orf1	0.003587222	-0.014466829	Proteome
TRINITY_DN147427_c0_g1_i1_orf1	0.001213178	0.018115687	Proteome
TRINITY_DN115082_c0_g1_i5_orf1	-0.008866201	0.00205042	Proteome
TRINITY_DN5112_c0_g1_i1_orf1	0.014175949	-0.001732943	Proteome
TRINITY_DN8783_c0_g1_i9_orf1	0.013621436	-0.001612209	Proteome
TRINITY_DN61711_c0_g1_i1_orf1	0.006280918	0.00281239	Proteome
TRINITY_DN32769_c1_g1_i5_orf1	0.00884185	-0.002490774	Proteome
TRINITY_DN17215_c0_g1_i4_orf1	-0.003755084	-0.008411758	Proteome
TRINITY_DN23167_c0_g2_i1_orf1	0.002153097	0.026212402	Proteome
TRINITY_DN69707_c0_g1_i1_orf1	0.005542319	0.013056886	Proteome
TRINITY_DN2101_c0_g1_i6_orf1	0.010719325	0.001142348	Proteome
TRINITY_DN2350_c0_g1_i6_orf1	0.020378157	0.00435229	Proteome
TRINITY_DN4977_c0_g1_i2_orf1	0.000664111	-0.00524282	Proteome
TRINITY_DN13303_c0_g1_i6_orf1	-0.003633108	-0.014513362	Proteome
TRINITY_DN5628_c0_g2_i1_orf1	0.003709813	-0.010230228	Proteome
TRINITY_DN3263_c0_g1_i2_orf1	-0.002039171	-0.041627107	Proteome
TRINITY_DN63030_c0_g1_i5_orf1	0.00813052	0.002508603	Proteome
TRINITY_DN35277_c0_g1_i1_orf1	0.00706921	-0.002659986	Proteome
TRINITY_DN556_c0_g2_i1_orf1	-0.015015393	0.009702332	Proteome
TRINITY_DN3015_c0_g1_i7_orf1	0.003361858	0.005403796	Proteome
TRINITY_DN21943_c1_g1_i1_orf1	0.001437389	0.000430033	Proteome

TRINITY_DN3223_c0_g1_i4_orf1	-0.003672745	-0.009067885	Proteome
TRINITY_DN26040_c0_g1_i11_orf1	0.008780498	-0.004080196	Proteome
TRINITY_DN59429_c0_g1_i6_orf1	-0.039925627	0.002579407	Proteome
TRINITY_DN98016_c0_g1_i1_orf1	0.018330337	0.000304918	Proteome
TRINITY_DN24163_c0_g1_i1_orf1	0.007976447	-0.005858685	Proteome
TRINITY_DN537_c0_g1_i1_orf1	0.004963923	0.00318296	Proteome
TRINITY_DN2331_c0_g1_i1_orf1	-0.010710237	0.012946817	Proteome
TRINITY_DN31851_c0_g1_i2_orf1	-0.009197214	-0.023665824	Proteome
TRINITY_DN2082_c0_g1_i2_orf1	0.008098029	0.00146083	Proteome
TRINITY_DN21852_c0_g1_i1_orf1	0.002348442	0.002003079	Proteome
TRINITY_DN22928_c0_g1_i6_orf1	0.014629492	0.005360051	Proteome
TRINITY_DN7123_c0_g1_i1_orf1	0.004154941	0.00956539	Proteome
TRINITY_DN1047_c0_g1_i6_orf1	0.007311909	0.001466236	Proteome
TRINITY_DN12969_c0_g1_i3_orf1	0.024134691	0.001910467	Proteome
TRINITY_DN112120_c0_g1_i1_orf1	-0.006244691	-0.011022081	Proteome
TRINITY_DN17906_c0_g1_i1_orf1	-0.003108677	-0.017700911	Proteome
TRINITY_DN1063_c0_g1_i16_orf1	0.011109363	0.009649128	Proteome
TRINITY_DN2921_c1_g1_i4_orf1	0.011798616	0.00443353	Proteome
TRINITY_DN13648_c0_g1_i6_orf1	0.012139261	0.000233606	Proteome
TRINITY_DN479_c6_g1_i2_orf1	-0.002246909	-0.000614379	Proteome
TRINITY_DN1543_c0_g2_i2_orf1	0.027287847	-0.010604498	Proteome
TRINITY_DN15755_c0_g1_i1_orf1	0.038791766	-0.007268756	Proteome
TRINITY_DN28729_c0_g1_i9_orf1	-0.004618714	-0.016475866	Proteome
TRINITY_DN50517_c0_g1_i1_orf1	0.001763641	0.026040867	Proteome
TRINITY_DN27833_c0_g2_i1_orf1	0.023839854	0.001002658	Proteome
TRINITY_DN940_c0_g1_i4_orf1	-0.009872248	-0.019283384	Proteome
TRINITY_DN636_c1_g1_i9_orf1	-0.062864629	-0.011352508	Proteome
TRINITY_DN354_c0_g1_i2_orf1	-0.008625745	-0.01408182	Proteome
TRINITY_DN16122_c0_g1_i4_orf1	0.002456182	-0.009914182	Proteome
TRINITY_DN2749_c4_g1_i2_orf1	-0.025806157	0.012534169	Proteome
TRINITY_DN2438_c0_g1_i4_orf1	0.010341901	0.002620159	Proteome
TRINITY_DN646_c0_g1_i5_orf1	0.006716414	-0.003099712	Proteome
TRINITY_DN17172_c0_g1_i5_orf1	0.013754262	-0.004666158	Proteome
TRINITY_DN117412_c0_g1_i1_orf1	-0.005148425	-0.003380634	Proteome
TRINITY_DN68725_c0_g1_i1_orf1	0.000610649	-0.008845136	Proteome
TRINITY_DN6621_c0_g1_i1_orf1	0.007847864	0.004210653	Proteome
TRINITY_DN4403_c0_g1_i3_orf1	-0.002022491	-0.009886543	Proteome
TRINITY_DN4081_c0_g1_i1_orf1	0.005958667	0.013070486	Proteome
TRINITY_DN7246_c0_g1_i7_orf1	0.00209125	-0.003431329	Proteome
TRINITY_DN25975_c0_g3_i2_orf1	0.004451079	-0.000367645	Proteome
TRINITY_DN2542_c0_g2_i1_orf1	-0.004174012	0.009402457	Proteome
TRINITY_DN12193_c0_g1_i6_orf1	-0.01797843	-0.006939455	Proteome
TRINITY_DN11050_c0_g1_i8_orf1	0.010539664	-0.002951862	Proteome
TRINITY_DN3335_c0_g1_i1_orf1	0.009384508	0.013419751	Proteome
TRINITY_DN2624_c0_g1_i6_orf1	0.012061838	-0.009724807	Proteome
TRINITY_DN1326_c0_g1_i1_orf1	-0.013548487	0.008499761	Proteome
TRINITY_DN1436_c0_g1_i5_orf1	-0.010381863	-0.019590437	Proteome
TRINITY_DN185_c0_g1_i6_orf1	0.00366223	0.014350204	Proteome
TRINITY_DN2304_c0_g1_i4_orf1	-0.002674527	-0.022527347	Proteome
TRINITY_DN8783_c0_g1_i5_orf1	0.014531154	0.023995341	Proteome
TRINITY_DN25582_c0_g1_i3_orf1	0.00221836	-0.007581608	Proteome
TRINITY_DN14831_c0_g1_i9_orf1	0.000557719	0.009482961	Proteome
TRINITY_DN13063_c0_g1_i1_orf1	-0.013934748	0.003874786	Proteome
TRINITY_DN11383_c0_g2_i4_orf1	0.006426095	-0.015039759	Proteome
TRINITY_DN295_c3_g1_i1_orfp1	-0.029445648	0.086907689	Proteome
TRINITY_DN6696_c0_g1_i4_orf1	-0.003658607	-0.009148033	Proteome
TRINITY_DN46022_c0_g1_i1_orf1	0.006151042	0.006929774	Proteome
TRINITY_DN30713_c0_g1_i3_orf1	-0.023594775	-0.011015021	Proteome

TRINITY_DN5852_c0_g1_i13_orf1	-0.014157504	-0.000910258	Proteome
TRINITY_DN3952_c0_g1_i3_orf1	-0.018728522	0.016010594	Proteome
TRINITY_DN9920_c0_g1_i1_orf1	0.008307006	-0.007922764	Proteome
TRINITY_DN304_c2_g1_i1_orf1	0.013041228	-0.004472123	Proteome
TRINITY_DN4289_c0_g1_i5_orf1	-0.001099431	-0.017321208	Proteome
TRINITY_DN17049_c0_g1_i6_orf1	0.004740836	0.004342983	Proteome
TRINITY_DN1601_c0_g1_i4_orf1	-0.001937652	0.011699652	Proteome
TRINITY_DN56998_c0_g1_i2_orf1	0.018842172	-0.005374625	Proteome
TRINITY_DN2663_c0_g1_i12_orf1	-0.016580929	-0.007848324	Proteome
TRINITY_DN29969_c0_g1_i5_orf1	-0.006316149	0.008576424	Proteome
TRINITY_DN82324_c0_g1_i4_orf1	0.007894521	0.001915479	Proteome
TRINITY_DN1768_c0_g1_i2_orf1	0.010419193	-0.004319322	Proteome
TRINITY_DN2022_c0_g1_i7_orf1	0.006125046	0.008252291	Proteome
TRINITY_DN7341_c0_g1_i8_orf1	-0.002384947	-0.012172186	Proteome
TRINITY_DN21218_c0_g2_i3_orf1	-0.00224769	0.014057787	Proteome
TRINITY_DN1326_c0_g1_i2_orf1	0.020561456	-0.008426112	Proteome
TRINITY_DN21743_c0_g1_i2_orf1	0.003791303	0.006431613	Proteome
TRINITY_DN24322_c0_g1_i4_orf1	-0.006362931	0.018789642	Proteome
TRINITY_DN20323_c0_g1_i1_orf1	0.011495643	0.013123699	Proteome
TRINITY_DN131603_c0_g1_i4_orfp1	0.121504574	-0.006437961	Proteome
TRINITY_DN3797_c0_g2_i3_orf1	0.002889262	-0.003302696	Proteome
TRINITY_DN116972_c0_g1_i1_orf1	0.000465016	0.011832538	Proteome
TRINITY_DN6994_c0_g1_i4_orf1	0.014850212	0.004669133	Proteome
TRINITY_DN13598_c1_g1_i1_orf1	0.008451047	0.002005094	Proteome
TRINITY_DN10110_c1_g2_i1_orf1	0.011968731	0.002951807	Proteome
TRINITY_DN2340_c0_g1_i4_orf1	0.002625501	-0.007047837	Proteome
TRINITY_DN5312_c4_g1_i2_orf1	-0.000832192	-0.003670768	Proteome
TRINITY_DN1785_c0_g1_i5_orf1	-0.019432342	0.002565721	Proteome
TRINITY_DN24689_c0_g1_i1_orf1	-0.011155946	-0.003381681	Proteome
TRINITY_DN18242_c0_g1_i3_orf1	0.002499257	0.013162615	Proteome
TRINITY_DN3597_c0_g1_i10_orf1	0.009857599	0.012582425	Proteome
TRINITY_DN3616_c0_g2_i2_orf1	0.024734077	0.001341317	Proteome
TRINITY_DN17071_c0_g1_i6_orf1	0.007060069	0.001631335	Proteome
TRINITY_DN15339_c0_g1_i6_orf1	0.014941175	-0.000519547	Proteome
TRINITY_DN22422_c0_g1_i4_orf1	-0.006660895	-0.018507	Proteome
TRINITY_DN12331_c0_g1_i5_orf1	0.001347641	0.012986177	Proteome
TRINITY_DN147517_c0_g1_i1_orf1	0.009100342	0.008668427	Proteome
TRINITY_DN23640_c0_g1_i5_orf1	0.001277412	0.006123788	Proteome
TRINITY_DN6510_c1_g1_i1_orf1	0.000710758	-0.021621976	Proteome
TRINITY_DN3454_c0_g1_i1_orf1	-0.007871672	-0.012079026	Proteome
TRINITY_DN3980_c0_g2_i1_orf1	-0.000996804	-0.012763168	Proteome
TRINITY_DN14774_c0_g1_i4_orf1	0.001687031	0.009274726	Proteome
TRINITY_DN7655_c0_g1_i3_orf1	0.006303349	0.004594113	Proteome
TRINITY_DN2140_c0_g1_i1_orf1	-0.005140226	0.001243822	Proteome
TRINITY_DN12584_c0_g1_i1_orf1	0.009181934	0.00292575	Proteome
TRINITY_DN1318_c0_g1_i5_orf1	-0.001304061	0.001853908	Proteome
TRINITY_DN21743_c0_g1_i1_orf1	-0.023808358	0.004562375	Proteome
TRINITY_DN1352_c0_g1_i5_orf1	-0.012903631	0.033354441	Proteome
TRINITY_DN15624_c0_g1_i1_orf1	-0.009788838	0.007550509	Proteome
TRINITY_DN26488_c0_g1_i6_orf1	0.006500967	0.011141299	Proteome
TRINITY_DN13055_c0_g1_i5_orf1	0.006439299	0.003795162	Proteome
TRINITY_DN661_c1_g2_i1_orf1	0.012270708	0.001167635	Proteome
TRINITY_DN5943_c0_g1_i6_orf1	-0.006046873	-0.00379294	Proteome
TRINITY_DN14298_c0_g1_i3_orf1	0.002753314	0.013867232	Proteome
TRINITY_DN9647_c0_g1_i1_orf1	0.006373027	0.002119044	Proteome
TRINITY_DN7785_c0_g1_i1_orf1	0.002049656	-0.016125924	Proteome
TRINITY_DN2478_c0_g1_i1_orf1	0.005977381	-0.003656883	Proteome
TRINITY_DN2816_c1_g1_i3_orf1	0.009365006	-0.001552035	Proteome

TRINITY_DN77480_c0_g1_i2_orf1	0.013422592	0.01441666	Proteome
TRINITY_DN5383_c0_g1_i1_orf1	0.005057865	0.000580145	Proteome
TRINITY_DN14396_c0_g1_i4_orf1	0.000586371	-0.014179535	Proteome
TRINITY_DN6842_c0_g1_i1_orf1	0.007751865	-0.003986263	Proteome
TRINITY_DN6087_c0_g1_i7_orf1	-0.000354716	0.00027804	Proteome
TRINITY_DN5344_c0_g1_i7_orf1	0.011937993	-0.010656957	Proteome
TRINITY_DN7839_c0_g1_i4_orf1	0.00783432	-0.003462463	Proteome
TRINITY_DN76333_c0_g1_i2_orf1	0.009647894	0.018363965	Proteome
TRINITY_DN23360_c0_g1_i3_orf1	0.003740075	0.002533848	Proteome
TRINITY_DN3539_c0_g1_i7_orf1	0.003595789	0.005949518	Proteome
TRINITY_DN134810_c0_g1_i1_orf1	-0.005622423	-0.015710826	Proteome
TRINITY_DN4631_c0_g1_i7_orf1	-0.001812595	-0.010296241	Proteome
TRINITY_DN33926_c0_g1_i1_orf1	0.00179166	0.000542549	Proteome
TRINITY_DN1470_c0_g1_i8_orf1	0.003219818	0.018094714	Proteome
TRINITY_DN97589_c0_g1_i3_orf1	0.013151784	0.00463612	Proteome
TRINITY_DN29579_c0_g1_i1_orf1	0.000994196	-0.006043866	Proteome
TRINITY_DN1656_c2_g1_i5_orf1	0.018558987	-0.003744003	Proteome
TRINITY_DN17559_c0_g1_i4_orf1	-0.004681984	-0.009901176	Proteome
TRINITY_DN350_c0_g1_i4_orf1	0.012941774	0.004143766	Proteome
TRINITY_DN9316_c1_g1_i1_orf1	0.003054405	0.006756994	Proteome
TRINITY_DN27852_c0_g1_i1_orf1	-0.011197029	-0.000235439	Proteome
TRINITY_DN1310_c0_g1_i4_orf1	-0.002548196	-0.009035337	Proteome
TRINITY_DN4954_c0_g1_i5_orf1	-0.012779	0.002572048	Proteome
TRINITY_DN17031_c0_g1_i1_orf1	0.00586687	-0.004237219	Proteome
TRINITY_DN31399_c0_g1_i3_orf1	0.006207323	0.006232066	Proteome
TRINITY_DN5453_c0_g1_i2_orf1	-0.003961537	-0.007381146	Proteome
TRINITY_DN37599_c0_g1_i1_orf1	0.004018011	0.010633962	Proteome
TRINITY_DN3066_c0_g1_i5_orf1	-0.005270011	-0.020434736	Proteome
TRINITY_DN18388_c0_g1_i6_orf1	-0.023293698	0.021872091	Proteome
TRINITY_DN15786_c0_g1_i1_orf1	0.009033977	0.000442024	Proteome
TRINITY_DN5556_c0_g1_i3_orf1	0.000500438	0.002729396	Proteome
TRINITY_DN18036_c0_g1_i7_orf1	0.003689656	0.003673355	Proteome
TRINITY_DN3472_c0_g1_i6_orf1	-0.009457803	0.011420808	Proteome
TRINITY_DN103475_c0_g1_i4_orf1	0.012795087	0.003247662	Proteome
TRINITY_DN15720_c0_g1_i2_orf1	0.00588799	-0.002795529	Proteome
TRINITY_DN4950_c0_g1_i2_orf1	0.009155164	-0.006664012	Proteome
TRINITY_DN77425_c0_g1_i2_orf1	0.008895892	0.007761075	Proteome
TRINITY_DN62_c1_g1_i3_orf1	-0.006963988	0.00146104	Proteome
TRINITY_DN11670_c0_g1_i1_orf1	0.014495607	0.004181845	Proteome
TRINITY_DN8979_c0_g1_i5_orf1	0.016336258	-0.003526991	Proteome
TRINITY_DN662_c0_g1_i1_orf1	-0.007834296	0.012172954	Proteome
TRINITY_DN1226_c0_g1_i11_orfp1	0.034758072	0.007076638	Proteome
TRINITY_DN4159_c1_g1_i1_orf1	0.000688512	0.016059374	Proteome
TRINITY_DN46715_c0_g1_i1_orf1	-0.000283671	-0.004297724	Proteome
TRINITY_DN16886_c0_g1_i4_orf1	0.001461401	0.00961577	Proteome
TRINITY_DN1436_c0_g1_i3_orf1	0.007168469	-0.007758091	Proteome
TRINITY_DN10415_c0_g1_i5_orf1	0.017216792	0.001190337	Proteome
TRINITY_DN6239_c0_g1_i1_orf1	-0.007846251	0.009272737	Proteome
TRINITY_DN1493_c0_g1_i5_orf1	0.021396578	0.013461912	Proteome
TRINITY_DN2277_c0_g1_i11_orf1	-0.006022326	0.009758503	Proteome
TRINITY_DN1783_c0_g1_i2_orf1	-0.014343082	0.001296635	Proteome
TRINITY_DN4008_c0_g1_i7_orf1	0.007185485	0.007669593	Proteome
TRINITY_DN7633_c0_g1_i1_orf1	0.014979402	0.001292154	Proteome
TRINITY_DN23398_c0_g1_i1_orf1	0.012515321	-0.021799364	Proteome
TRINITY_DN1824_c0_g2_i2_orf1	0.008683808	-0.006811209	Proteome
TRINITY_DN135077_c0_g1_i1_orf1	0.017932668	0.009880874	Proteome
TRINITY_DN43076_c0_g1_i6_orf1	0.004434599	-0.01027572	Proteome
TRINITY_DN9732_c0_g1_i7_orf1	0.006950667	-0.000839374	Proteome

TRINITY_DN4502_c0_g1_i3_orf1	0.010108354	-0.016953506	Proteome
TRINITY_DN7908_c0_g1_i5_orf1	-0.010526544	-0.000988847	Proteome
TRINITY_DN130069_c0_g6_i1_orf1	0.010216286	-0.017490837	Proteome
TRINITY_DN8079_c0_g1_i2_orf1	-0.00916562	-0.016174306	Proteome
TRINITY_DN51523_c0_g1_i4_orf1	-0.007533505	-0.023452718	Proteome
TRINITY_DN29034_c0_g1_i1_orf1	-0.001069125	-0.003716269	Proteome
TRINITY_DN4068_c0_g2_i4_orf1	0.029427185	0.018477571	Proteome
TRINITY_DN20796_c0_g1_i4_orf1	0.008947509	0.003016142	Proteome
TRINITY_DN72056_c0_g1_i1_orf1	0.00464105	-0.00597404	Proteome
TRINITY_DN1497_c0_g2_i6_orf1	-0.011211531	0.001217594	Proteome
TRINITY_DN4012_c0_g4_i2_orf1	0.003219939	-0.011698883	Proteome
TRINITY_DN6545_c0_g1_i6_orf1	-0.013141819	-0.001205547	Proteome
TRINITY_DN22422_c0_g1_i3_orf1	0.004307514	-0.013534738	Proteome
TRINITY_DN745_c5_g1_i2_orf1	0.005188593	-0.001440024	Proteome
TRINITY_DN21411_c0_g1_i1_orf1	0.001739549	0.007901051	Proteome
TRINITY_DN9325_c0_g1_i1_orf1	0.012084074	-0.004631684	Proteome
TRINITY_DN3245_c2_g1_i4_orf1	0.003820919	0.008470531	Proteome
TRINITY_DN6871_c0_g1_i3_orf1	-0.007589827	-0.025423002	Proteome
TRINITY_DN18300_c0_g1_i17_orf1	0.00038354	-0.007418573	Proteome
TRINITY_DN132043_c0_g1_i1_orf1	0.003760129	0.001960916	Proteome
TRINITY_DN8846_c0_g1_i1_orf1	0.017176923	0.000420874	Proteome
TRINITY_DN16525_c0_g1_i4_orf1	-0.00495955	-0.001350053	Proteome
TRINITY_DN47930_c0_g1_i4_orf1	0.004939597	0.01431668	Proteome
TRINITY_DN24121_c1_g1_i6_orf1	0.041553355	0.003469584	Proteome
TRINITY_DN1424_c0_g1_i5_orf1	0.009509497	0.004065207	Proteome
TRINITY_DN12497_c0_g1_i1_orf1	0.005430004	-0.006814675	Proteome
TRINITY_DN231_c1_g2_i1_orf1	0.006195604	0.012981223	Proteome
TRINITY_DN12997_c0_g2_i1_orf1	0.005279654	-0.006185551	Proteome
TRINITY_DN11746_c0_g2_i1_orf1	0.012990021	-0.008499018	Proteome
TRINITY_DN11370_c0_g1_i6_orf1	0.005914645	-0.004805419	Proteome
TRINITY_DN5558_c0_g1_i4_orf1	0.013876117	0.005049444	Proteome
TRINITY_DN59388_c0_g1_i1_orf1	-0.020675914	0.014232812	Proteome
TRINITY_DN23783_c0_g2_i1_orf1	0.013143105	0.004465206	Proteome
TRINITY_DN1767_c0_g2_i15_orf1	-0.006344655	-0.008898786	Proteome
TRINITY_DN36699_c0_g1_i3_orf1	-0.00391762	-0.010943727	Proteome
TRINITY_DN8454_c0_g1_i4_orf1	0.009582548	0.012339632	Proteome
TRINITY_DN3619_c0_g2_i1_orf1	0.000979658	-0.012787975	Proteome
TRINITY_DN98091_c0_g1_i3_orf1	-0.006903191	-0.004580565	Proteome
TRINITY_DN1494_c0_g1_i3_orf1	-9.41E-05	-0.003175162	Proteome
TRINITY_DN8261_c0_g1_i1_orf1	0.000734745	-0.003349969	Proteome
TRINITY_DN49570_c0_g1_i1_orf1	0.006468442	0.006267393	Proteome
TRINITY_DN1255_c0_g1_i17_orf1	0.005901394	-0.014597936	Proteome
TRINITY_DN2593_c0_g1_i1_orf1	0.000775643	-0.014835463	Proteome
TRINITY_DN26301_c0_g1_i1_orf1	0.010344679	-0.006730543	Proteome
TRINITY_DN35377_c0_g1_i3_orf1	-0.007281353	-0.006859667	Proteome
TRINITY_DN4898_c0_g1_i7_orf1	0.003061567	-0.002666984	Proteome
TRINITY_DN1498_c0_g1_i2_orf1	-0.000307782	-0.013316176	Proteome
TRINITY_DN21971_c0_g1_i4_orf1	0.006429509	0.00343658	Proteome
TRINITY_DN3203_c0_g2_i1_orf1	-0.003730641	-0.014336327	Proteome
TRINITY_DN23946_c0_g1_i1_orf1	-0.001077202	0.011884412	Proteome
TRINITY_DN452_c0_g1_i4_orf1	0.023513137	-0.004923022	Proteome
TRINITY_DN7002_c0_g1_i4_orf1	0.010336252	-0.006051458	Proteome
TRINITY_DN138481_c0_g1_i2_orf1	-0.005363297	0.002229109	Proteome
TRINITY_DN3924_c0_g1_i5_orf1	0.007590023	0.010289573	Proteome
TRINITY_DN53866_c0_g1_i1_orf1	0.011930912	0.010654864	Proteome
TRINITY_DN164_c0_g1_i11_orf1	-0.004003256	0.006610746	Proteome
TRINITY_DN35051_c0_g1_i1_orf1	0.00562196	-0.000487381	Proteome
TRINITY_DN8019_c0_g1_i4_orf1	0.002863435	0.006112694	Proteome

TRINITY_DN10007_c0_g1_i1_orf1	-0.004655308	-0.014840859	Proteome
TRINITY_DN41321_c1_g1_i3_orf1	0.008619776	0.002027692	Proteome
TRINITY_DN13221_c0_g1_i3_orf1	-0.027834024	0.003034623	Proteome
TRINITY_DN22956_c0_g1_i1_orf1	0.009704379	-0.0015473	Proteome
TRINITY_DN5731_c0_g1_i13_orf1	-0.001082169	-0.005856198	Proteome
TRINITY_DN11808_c0_g1_i8_orf1	-0.014724731	0.000627281	Proteome
TRINITY_DN11826_c0_g1_i4_orf1	-0.016087778	-0.03419152	Proteome
TRINITY_DN31001_c0_g1_i1_orf1	0.009365958	0.013971096	Proteome
TRINITY_DN10257_c0_g1_i2_orf1	-0.002526651	-0.003713824	Proteome
TRINITY_DN13997_c0_g1_i5_orf1	-0.015526609	0.00565789	Proteome
TRINITY_DN18027_c0_g2_i1_orf1	-0.005982738	-0.001972567	Proteome
TRINITY_DN19000_c0_g1_i4_orf1	0.005022999	0.00130744	Proteome
TRINITY_DN48250_c0_g1_i1_orf1	-0.013868788	0.001810792	Proteome
TRINITY_DN4304_c0_g1_i3_orf1	0.009249473	-0.004439433	Proteome
TRINITY_DN8596_c0_g1_i2_orf1	0.002663027	0.005017875	Proteome
TRINITY_DN7754_c0_g1_i2_orf1	-0.000682092	0.003213708	Proteome
TRINITY_DN5962_c0_g1_i1_orf1	0.028321119	-0.001433349	Proteome
TRINITY_DN661_c0_g3_i5_orf1	0.011076612	-0.010027782	Proteome
TRINITY_DN332_c0_g1_i6_orf1	0.012034337	-0.006401042	Proteome
TRINITY_DN496_c0_g1_i7_orf1	0.010484272	0.004177434	Proteome
TRINITY_DN5462_c0_g2_i1_orf1	-0.015831843	0.008893937	Proteome
TRINITY_DN15513_c0_g1_i6_orf1	0.012859354	-0.003073528	Proteome
TRINITY_DN5562_c1_g2_i1_orf1	-0.003643372	-0.016575299	Proteome
TRINITY_DN3231_c0_g1_i12_orf1	0.017608808	0.001465613	Proteome
TRINITY_DN9794_c0_g2_i8_orf1	0.006425127	0.008519726	Proteome
TRINITY_DN3355_c0_g2_i4_orf1	-0.001255375	-0.0007174	Proteome
TRINITY_DN130075_c1_g2_i1_orf1	-0.008477256	-0.015159614	Proteome
TRINITY_DN14298_c0_g3_i1_orf1	0.010589138	0.011750565	Proteome
TRINITY_DN11044_c0_g1_i4_orf1	-0.00578287	-0.015964428	Proteome
TRINITY_DN65681_c0_g1_i1_orf1	0.008175141	-0.010008691	Proteome
TRINITY_DN10558_c0_g1_i4_orf1	0.00069955	0.021546289	Proteome
TRINITY_DN32448_c0_g1_i1_orf1	-0.019130113	-0.014392285	Proteome
TRINITY_DN12757_c0_g1_i1_orf1	0.007141258	-0.001306795	Proteome
TRINITY_DN3472_c1_g1_i4_orf1	0.011294048	-0.007263454	Proteome
TRINITY_DN578_c0_g1_i3_orf1	0.018768326	0.000940012	Proteome
TRINITY_DN14501_c0_g1_i1_orf1	-0.006652282	0.003066959	Proteome
TRINITY_DN399_c9_g1_i1_orf1	0.00746117	-0.003619538	Proteome
TRINITY_DN24476_c0_g1_i1_orf1	-0.0047379	0.000375093	Proteome
TRINITY_DN2735_c0_g1_i4_orf1	0.00759683	-0.011826643	Proteome
TRINITY_DN27728_c0_g1_i1_orf1	0.001324802	-0.008173955	Proteome
TRINITY_DN8651_c0_g1_i16_orf1	0.02365575	0.011809553	Proteome
TRINITY_DN42856_c0_g1_i1_orf1	0.013526224	0.016307531	Proteome
TRINITY_DN6855_c1_g1_i3_orf1	-0.00461965	-0.018983438	Proteome
TRINITY_DN28638_c0_g1_i1_orf1	0.008723254	-0.003924794	Proteome
TRINITY_DN36199_c0_g1_i1_orf1	-0.00355445	-0.00201538	Proteome
TRINITY_DN23444_c0_g1_i10_orf1	0.003648608	0.010236344	Proteome
TRINITY_DN23364_c0_g1_i1_orf1	-0.009031713	0.017052786	Proteome
TRINITY_DN52553_c0_g1_i1_orf1	0.011694859	-0.006913685	Proteome
TRINITY_DN1370_c0_g1_i2_orf1	-0.029344667	-0.033319114	Proteome
TRINITY_DN14565_c0_g1_i11_orf1	0.00816238	-0.001824935	Proteome
TRINITY_DN4688_c0_g1_i2_orf1	0.014024166	0.001907027	Proteome
TRINITY_DN10745_c0_g1_i14_orf1	0.004426744	0.005124134	Proteome
TRINITY_DN1466_c0_g1_i4_orf1	-0.030445969	0.008152038	Proteome
TRINITY_DN22018_c0_g1_i3_orf1	-0.005355067	0.002502453	Proteome
TRINITY_DN12336_c0_g1_i1_orfp1	0.034349957	0.001731325	Proteome
TRINITY_DN1459_c1_g1_i1_orf1	-0.007810883	-0.002275874	Proteome
TRINITY_DN3332_c0_g1_i9_orf1	0.018950466	-0.017416998	Proteome
TRINITY_DN4380_c0_g1_i9_orf1	0.003223166	0.008753656	Proteome

TRINITY_DN206_c0_g1_i8_orf1	0.016132772	0.007434929	Proteome
TRINITY_DN109503_c0_g1_i4_orf1	-0.027777069	0.013887418	Proteome
TRINITY_DN4228_c0_g1_i5_orf1	0.010675939	0.003348122	Proteome
TRINITY_DN14498_c0_g1_i1_orf1	0.006070259	0.005538359	Proteome
TRINITY_DN7776_c0_g1_i1_orf1	-0.015493196	0.012275148	Proteome
TRINITY_DN535_c1_g1_i2_orf1	3.66E-05	0.019178365	Proteome
TRINITY_DN350_c0_g1_i10_orf1	0.013707055	0.007555024	Proteome
TRINITY_DN5562_c0_g1_i3_orf1	0.001416116	0.002814049	Proteome
TRINITY_DN11655_c0_g1_i1_orf1	-0.003722883	-0.003022304	Proteome
TRINITY_DN18569_c0_g2_i1_orf1	0.007684451	0.00073952	Proteome
TRINITY_DN136028_c0_g2_i1_orf1	0.005624135	-0.005976506	Proteome
TRINITY_DN6992_c0_g1_i6_orf1	0.011010186	-0.006590702	Proteome
TRINITY_DN6144_c0_g1_i2_orf1	-0.002035577	-0.001451355	Proteome
TRINITY_DN452_c1_g1_i3_orf1	0.007853773	-0.011782939	Proteome
TRINITY_DN19411_c0_g1_i1_orf1	-0.006407808	0.007703938	Proteome
TRINITY_DN15737_c0_g1_i7_orf1	0.013620941	-0.003583288	Proteome
TRINITY_DN10680_c0_g1_i5_orf1	-0.01239258	0.012358785	Proteome
TRINITY_DN22842_c0_g1_i4_orf1	-0.002355564	-0.008128581	Proteome
TRINITY_DN27035_c0_g1_i1_orf1	-0.014588895	0.043059019	Proteome
TRINITY_DN17133_c0_g1_i1_orf1	-0.009968055	-6.02E-05	Proteome
TRINITY_DN2264_c0_g1_i1_orf1	0.009403159	0.003002268	Proteome
TRINITY_DN661_c0_g1_i1_orf1	0.017931941	0.0142478	Proteome
TRINITY_DN120_c0_g1_i2_orf1	0.009412727	-0.011754527	Proteome
TRINITY_DN14935_c0_g1_i1_orf1	0.013531299	-0.005038277	Proteome
TRINITY_DN12690_c0_g1_i1_orf1	0.015138497	3.83E-05	Proteome
TRINITY_DN1305_c0_g1_i6_orf1	-0.001411073	-0.026170545	Proteome
TRINITY_DN97042_c0_g1_i6_orf1	-0.035426922	-0.001877632	Proteome
TRINITY_DN1507_c0_g1_i5_orf1	0.005980162	0.01007133	Proteome
TRINITY_DN11121_c0_g1_i5_orf1	0.006024371	0.003549123	Proteome
TRINITY_DN3194_c0_g1_i6_orf1	0.00654983	0.001927496	Proteome
TRINITY_DN3521_c0_g2_i1_orf1	0.01356183	0.009115415	Proteome
TRINITY_DN124171_c0_g1_i4_orf1	0.005789853	-0.004784049	Proteome
TRINITY_DN6588_c0_g1_i4_orf1	0.018004693	0.010152149	Proteome
TRINITY_DN13018_c0_g1_i1_orf1	-0.004645911	0.012472964	Proteome
TRINITY_DN2172_c0_g2_i8_orf1	0.010792439	-0.004934202	Proteome
TRINITY_DN8143_c0_g1_i6_orf1	-0.007854514	0.006295057	Proteome
TRINITY_DN2793_c0_g2_i1_orf1	-0.010001347	-0.021635216	Proteome
TRINITY_DN21872_c0_g1_i2_orf1	-0.005443003	0.014450188	Proteome
TRINITY_DN18291_c0_g1_i1_orf1	0.003468887	0.019217517	Proteome
TRINITY_DN1752_c0_g1_i18_orf1	0.007160523	0.00201217	Proteome
TRINITY_DN11117_c0_g1_i1_orf1	0.003639115	-0.008955382	Proteome
TRINITY_DN22589_c0_g1_i6_orfp1	0.015030629	0.006079526	Proteome
TRINITY_DN35809_c0_g1_i1_orf1	-0.04132074	0.054361379	Proteome
TRINITY_DN7233_c0_g2_i1_orf1	0.006580143	-0.013180095	Proteome
TRINITY_DN34830_c0_g1_i1_orf1	0.007738445	0.002237826	Proteome
TRINITY_DN56690_c0_g1_i4_orf1	-0.022847069	0.00272419	Proteome
TRINITY_DN40945_c0_g1_i1_orf1	0.02331476	-0.000986859	Proteome
TRINITY_DN20215_c0_g2_i1_orf1	-0.007859922	0.000662232	Proteome
TRINITY_DN2716_c0_g2_i1_orf1	0.008586913	0.010168533	Proteome
TRINITY_DN24707_c0_g1_i2_orf1	-0.000651586	-0.017734826	Proteome
TRINITY_DN2714_c0_g1_i3_orf1	-0.001810622	-0.023202756	Proteome
TRINITY_DN2343_c1_g1_i2_orf1	0.04316984	0.002089861	Proteome
TRINITY_DN2035_c0_g1_i1_orf1	-0.005647305	0.014030739	Proteome
TRINITY_DN3434_c0_g1_i1_orf1	0.00710797	-0.011854511	Proteome
TRINITY_DN13347_c0_g1_i1_orf1	0.003838074	0.011002789	Proteome
TRINITY_DN51045_c0_g1_i1_orf1	0.001270309	0.002440868	Proteome
TRINITY_DN6247_c0_g1_i2_orf1	0.008198706	0.002186046	Proteome
TRINITY_DN57137_c0_g1_i1_orfp1	0.051213263	0.047205441	Proteome

TRINITY_DN108051_c0_g1_i2_orf1	-0.001059212	-0.00688274	Proteome
TRINITY_DN9109_c0_g1_i1_orf1	0.017905638	0.002967576	Proteome
TRINITY_DN463_c0_g1_i3_orf1	-0.001975288	0.014201677	Proteome
TRINITY_DN9742_c0_g1_i5_orf1	0.002752791	-0.025344508	Proteome
TRINITY_DN101358_c0_g2_i1_orf1	-0.003449661	0.0115306	Proteome
TRINITY_DN295_c5_g1_i2_orf1	-0.039424967	0.013248557	Proteome
TRINITY_DN8037_c0_g2_i1_orf1	-0.015965004	0.009059455	Proteome
TRINITY_DN2065_c1_g2_i1_orf1	0.000164234	0.002933887	Proteome
TRINITY_DN14389_c0_g1_i4_orf1	-0.005878786	-0.004241462	Proteome
TRINITY_DN3076_c0_g1_i1_orf1	0.011219624	-0.012317012	Proteome
TRINITY_DN139335_c0_g2_i1_orf1	0.015259866	0.013970943	Proteome
TRINITY_DN12064_c0_g2_i1_orf1	-0.009925399	0.004794822	Proteome
TRINITY_DN48413_c1_g1_i2_orf1	-0.00248301	-0.000418798	Proteome
TRINITY_DN1870_c0_g1_i6_orf1	-0.008663374	0.02810866	Proteome
TRINITY_DN144956_c0_g1_i1_orf1	0.009824926	0.010385637	Proteome
TRINITY_DN2472_c2_g1_i7_orf1	0.002110013	0.007286008	Proteome
TRINITY_DN140212_c0_g1_i1_orf1	0.019203409	0.016108828	Proteome
TRINITY_DN11620_c0_g1_i2_orf1	-0.013468857	-0.028629711	Proteome
TRINITY_DN13119_c0_g1_i4_orf1	0.020404823	0.008877676	Proteome
TRINITY_DN9059_c0_g1_i1_orf1	-0.003240025	-0.008714145	Proteome
TRINITY_DN2062_c0_g1_i11_orf1	0.011705805	0.002717297	Proteome
TRINITY_DN8226_c0_g1_i1_orf1	0.011247792	0.010336718	Proteome
TRINITY_DN13901_c0_g1_i4_orf1	0.006467859	0.000612665	Proteome
TRINITY_DN64446_c0_g1_i1_orf1	0.008623388	-0.00415843	Proteome
TRINITY_DN1124_c0_g1_i7_orf1	0.018899452	-0.003470183	Proteome
TRINITY_DN20321_c0_g1_i5_orf1	-0.001447957	0.008813265	Proteome
TRINITY_DN4131_c0_g2_i1_orf1	0.003645077	0.003234893	Proteome
TRINITY_DN27960_c0_g1_i1_orf1	-0.000380111	-0.002083482	Proteome
TRINITY_DN3584_c0_g1_i3_orf1	0.007900628	0.007189074	Proteome
TRINITY_DN1083_c0_g1_i4_orf1	-0.012035054	0.008331994	Proteome
TRINITY_DN19155_c0_g1_i1_orf1	-0.003789286	-0.015697716	Proteome
TRINITY_DN1694_c0_g1_i1_orf1	-0.014414156	-0.000179471	Proteome
TRINITY_DN1473_c0_g1_i8_orf1	0.010428101	-0.005801383	Proteome
TRINITY_DN25534_c0_g1_i1_orf1	-0.008193621	-0.011118182	Proteome
TRINITY_DN9562_c0_g1_i3_orf1	-0.015609062	-0.031123533	Proteome
TRINITY_DN1866_c0_g1_i4_orf1	0.000951543	0.015249875	Proteome
TRINITY_DN6161_c0_g1_i1_orf1	0.006004839	-0.005155381	Proteome
TRINITY_DN30687_c0_g1_i1_orf1	-0.012797271	-0.011279716	Proteome
TRINITY_DN23069_c0_g2_i3_orf1	0.006828063	-0.006860632	Proteome
TRINITY_DN27958_c0_g1_i1_orf1	0.000624366	0.013235664	Proteome
TRINITY_DN20369_c0_g1_i2_orf1	0.004379108	0.00239804	Proteome
TRINITY_DN2083_c0_g1_i4_orf1	-0.006477334	-0.013091321	Proteome
TRINITY_DN10810_c0_g1_i4_orf1	0.000427978	-0.017135881	Proteome
TRINITY_DN3712_c0_g1_i1_orf1	0.013904904	0.007279918	Proteome
TRINITY_DN17376_c0_g1_i2_orf1	0.011106052	0.002453948	Proteome
TRINITY_DN15000_c0_g1_i4_orf1	0.034893031	-0.008287135	Proteome
TRINITY_DN135679_c0_g1_i2_orfp1	-0.02228274	0.009080596	Proteome
TRINITY_DN2212_c0_g1_i1_orf1	-0.008241762	-0.013672661	Proteome
TRINITY_DN1589_c0_g1_i7_orf1	0.011027172	-0.011248456	Proteome
TRINITY_DN72707_c0_g1_i1_orf1	-0.006924583	0.004422693	Proteome
TRINITY_DN22597_c0_g1_i4_orf1	0.010401053	0.021058185	Proteome
TRINITY_DN3856_c0_g1_i7_orf1	0.009147847	-0.000471354	Proteome
TRINITY_DN49785_c1_g1_i3_orf1	0.016284647	0.00402339	Proteome
TRINITY_DN35633_c0_g2_i1_orf1	-0.003307287	-0.007777731	Proteome
TRINITY_DN19746_c0_g1_i5_orf1	0.010080168	0.009358998	Proteome
TRINITY_DN1012_c0_g1_i2_orf1	0.033253907	0.009122613	Proteome
TRINITY_DN920_c0_g1_i6_orf1	0.028785192	0.000754512	Proteome
TRINITY_DN14754_c0_g1_i6_orf1	0.033132957	0.013385091	Proteome

TRINITY_DN26149_c0_g1_i5_orf1	0.006582971	-0.005960176	Proteome
TRINITY_DN8406_c0_g1_i4_orf1	0.001159388	0.016568387	Proteome
TRINITY_DN323_c0_g2_i5_orf1	0.007509571	-0.017728872	Proteome
TRINITY_DN10484_c0_g1_i8_orf1	0.014441561	0.007716458	Proteome
TRINITY_DN2983_c0_g1_i6_orf1	0.002970799	-0.014753589	Proteome
TRINITY_DN54524_c0_g1_i6_orf1	0.007410796	-0.004146124	Proteome
TRINITY_DN6231_c0_g1_i6_orf1	0.009829453	0.003501138	Proteome
TRINITY_DN1716_c0_g1_i14_orf1	-0.008066555	0.010659876	Proteome
TRINITY_DN77005_c0_g2_i1_orf1	-0.012118561	-0.013566077	Proteome
TRINITY_DN61112_c0_g1_i4_orfp1	-0.002103319	-0.004687428	Proteome
TRINITY_DN12009_c0_g1_i1_orf1	0.050689309	-0.011258999	Proteome
TRINITY_DN36581_c0_g1_i5_orf1	-0.014030775	0.003636476	Proteome
TRINITY_DN146119_c0_g1_i1_orf1	0.01416346	0.00153973	Proteome
TRINITY_DN2374_c0_g1_i1_orf1	0.003252273	0.00892959	Proteome
TRINITY_DN119557_c0_g1_i3_orf1	0.008555832	-0.012886506	Proteome
TRINITY_DN619_c0_g1_i1_orf1	0.008108582	-0.003466902	Proteome
TRINITY_DN33365_c0_g1_i1_orf1	0.008257435	-0.008747906	Proteome
TRINITY_DN142588_c0_g1_i1_orf1	0.003777599	0.004355125	Proteome
TRINITY_DN7574_c0_g1_i10_orf1	0.008459345	0.003211007	Proteome
TRINITY_DN12003_c0_g2_i1_orf1	-0.004952837	-0.000664931	Proteome
TRINITY_DN10639_c0_g1_i6_orf1	0.002194545	-0.010705306	Proteome
TRINITY_DN5162_c0_g1_i3_orf1	0.00391845	0.001095328	Proteome
TRINITY_DN60680_c0_g1_i2_orf1	0.006094115	0.004940229	Proteome
TRINITY_DN2473_c0_g1_i2_orf1	0.013165092	-0.010559079	Proteome
TRINITY_DN50151_c0_g1_i1_orf1	0.007520572	-0.002677552	Proteome
TRINITY_DN4770_c0_g1_i4_orf1	0.007207464	-0.00468952	Proteome
TRINITY_DN8908_c0_g1_i1_orf1	-0.000628125	-0.014532947	Proteome
TRINITY_DN3512_c0_g1_i2_orf1	0.000934192	-0.008010575	Proteome
TRINITY_DN5433_c0_g1_i5_orf1	-0.019616287	0.007694384	Proteome
TRINITY_DN661_c0_g2_i2_orf1	-0.002017546	-0.012297081	Proteome
TRINITY_DN2182_c0_g1_i4_orf1	-0.0113787	0.017042257	Proteome
TRINITY_DN14920_c0_g1_i1_orf1	-0.003567343	0.008637832	Proteome
TRINITY_DN16091_c0_g1_i1_orfp1	-0.065970301	0.046019578	Proteome
TRINITY_DN9541_c0_g1_i5_orf1	0.008641264	-0.000222837	Proteome
TRINITY_DN12973_c0_g1_i1_orf1	-0.001307824	-0.012309419	Proteome
TRINITY_DN21545_c0_g1_i2_orf1	0.006639585	0.013560573	Proteome
TRINITY_DN121047_c0_g1_i3_orf1	-0.006612993	0.012293456	Proteome
TRINITY_DN490_c0_g1_i1_orf1	0.002964948	-0.017969429	Proteome
TRINITY_DN65518_c0_g1_i1_orf1	-0.003377112	0.009089762	Proteome
TRINITY_DN31815_c0_g1_i4_orf1	0.003855828	-0.009776396	Proteome
TRINITY_DN7325_c0_g1_i1_orf1	-0.002375538	-0.016242967	Proteome
TRINITY_DN42337_c0_g1_i6_orf1	0.010389953	0.018198887	Proteome
TRINITY_DN955_c0_g1_i2_orf1	0.04650072	-0.002116827	Proteome
TRINITY_DN3488_c0_g1_i2_orf1	-0.006324466	0.002623048	Proteome
TRINITY_DN33893_c0_g1_i1_orf1	0.013614254	0.007843748	Proteome
TRINITY_DN47257_c0_g1_i4_orf1	0.009904338	0.001771568	Proteome
TRINITY_DN6876_c0_g2_i1_orf1	-0.004008542	-0.010477996	Proteome
TRINITY_DN2114_c0_g1_i5_orf1	0.003453676	0.004865165	Proteome
TRINITY_DN4695_c0_g1_i4_orf1	0.011438919	-0.01943798	Proteome
TRINITY_DN3647_c1_g1_i5_orf1	0.013590978	0.011385861	Proteome
TRINITY_DN13384_c0_g1_i1_orf1	0.011833976	-0.005462345	Proteome
TRINITY_DN2070_c1_g1_i1_orf1	0.001813966	-0.01369879	Proteome
TRINITY_DN512_c1_g1_i4_orf1	0.007583167	-0.008157494	Proteome
TRINITY_DN1285_c0_g2_i1_orf1	0.003929717	-0.003497162	Proteome
TRINITY_DN117844_c0_g1_i1_orf1	0.000263343	0.018848077	Proteome
TRINITY_DN64222_c0_g1_i1_orf1	-0.002678142	-0.005719331	Proteome
TRINITY_DN74020_c0_g1_i2_orf1	0.017150627	0.000143639	Proteome
TRINITY_DN461_c0_g1_i5_orf1	-0.002982835	-0.013623986	Proteome

TRINITY_DN1641_c0_g1_i6_orf1	0.006846353	0.004606091	Proteome
TRINITY_DN74654_c0_g1_i4_orf1	-0.011238183	0.000678851	Proteome
TRINITY_DN1232_c0_g1_i1_orf1	0.010495297	-0.007964691	Proteome
TRINITY_DN7226_c0_g1_i5_orf1	-0.024083874	0.003376137	Proteome
TRINITY_DN4276_c0_g1_i11_orf1	-0.007510082	-0.001508183	Proteome
TRINITY_DN556_c0_g2_i2_orf1	-0.011414269	-0.000844561	Proteome
TRINITY_DN8766_c0_g1_i1_orf1	0.02037011	0.01316967	Proteome
TRINITY_DN36434_c0_g2_i3_orf1	0.028806135	-0.009135955	Proteome
TRINITY_DN8407_c0_g1_i2_orf1	-0.00618871	-0.019864685	Proteome
TRINITY_DN17935_c0_g1_i1_orf1	0.006959973	-0.002238865	Proteome
TRINITY_DN14743_c0_g1_i4_orf1	0.010122213	0.000425808	Proteome
TRINITY_DN18148_c0_g2_i1_orf1	-0.014608255	-0.002545604	Proteome
TRINITY_DN9156_c0_g1_i1_orf1	0.002586206	-0.011995962	Proteome
TRINITY_DN3307_c1_g1_i2_orf1	-0.011750898	4.55E-05	Proteome
TRINITY_DN2881_c0_g1_i7_orf1	0.014731339	-0.011904282	Proteome
TRINITY_DN13259_c0_g1_i2_orf1	0.00941943	0.002118225	Proteome
TRINITY_DN2977_c0_g1_i3_orf1	0.004386594	-0.00773527	Proteome
TRINITY_DN29743_c0_g1_i9_orf1	0.004940534	0.002244785	Proteome
TRINITY_DN2043_c0_g1_i3_orf1	0.010887343	-0.004983883	Proteome
TRINITY_DN10520_c0_g1_i2_orf1	-0.002568398	-0.011944205	Proteome
TRINITY_DN42337_c0_g1_i5_orf1	0.007885967	0.022971775	Proteome
TRINITY_DN2207_c0_g1_i4_orf1	0.030724255	0.004991837	Proteome
TRINITY_DN4767_c0_g1_i4_orf1	-0.0417929	0.041711302	Proteome
TRINITY_DN2953_c1_g1_i2_orf1	0.001594108	-0.000640122	Proteome
TRINITY_DN48460_c0_g1_i1_orf1	0.009836015	0.007567544	Proteome
TRINITY_DN8824_c0_g2_i1_orf1	0.013104385	0.001423958	Proteome
TRINITY_DN6004_c0_g1_i1_orf1	0.006828358	-0.005568009	Proteome
TRINITY_DN146841_c0_g1_i1_orf1	-0.003438818	0.016774354	Proteome
TRINITY_DN6497_c0_g1_i1_orf1	-0.026959848	0.017496814	Proteome
TRINITY_DN1853_c0_g1_i3_orf1	0.009247413	0.011655284	Proteome
TRINITY_DN27885_c0_g1_i3_orf1	-0.005103768	-0.006640383	Proteome
TRINITY_DN3835_c0_g1_i4_orf1	-0.002171135	0.005138059	Proteome
TRINITY_DN391_c1_g2_i1_orf1	-0.003923389	-0.010583413	Proteome
TRINITY_DN27979_c0_g1_i2_orf1	0.003109313	-0.006177053	Proteome
TRINITY_DN18648_c0_g1_i1_orf1	-0.00177794	0.004619555	Proteome
TRINITY_DN12567_c0_g1_i1_orf1	0.009566528	-0.007803671	Proteome
TRINITY_DN3929_c0_g1_i1_orf1	0.002090072	-0.010020155	Proteome
TRINITY_DN4108_c0_g1_i6_orf1	0.01641935	0.003090723	Proteome
TRINITY_DN569_c0_g3_i12_orf1	0.002781773	0.003250117	Proteome
TRINITY_DN2227_c0_g1_i5_orf1	0.018703492	-0.004876797	Proteome
TRINITY_DN7388_c0_g1_i7_orf1	0.019550787	-0.004145019	Proteome
TRINITY_DN6586_c0_g1_i1_orf1	0.001481751	0.004516348	Proteome
TRINITY_DN32359_c0_g2_i1_orf1	0.007109663	0.002244041	Proteome
TRINITY_DN5682_c0_g1_i6_orf1	0.002616783	0.026485395	Proteome
TRINITY_DN15046_c0_g1_i8_orf1	-0.007436128	-0.0070895	Proteome
TRINITY_DN8406_c0_g1_i3_orfp1	0.011205874	0.0101822	Proteome
TRINITY_DN3177_c0_g1_i1_orf1	0.015662919	0.001674058	Proteome
TRINITY_DN17726_c0_g1_i1_orf1	0.001597318	-0.01097844	Proteome
TRINITY_DN59829_c0_g1_i1_orf1	0.000701541	-0.000888777	Proteome
TRINITY_DN483_c0_g1_i6_orf1	0.026772854	0.00202018	Proteome
TRINITY_DN18391_c0_g2_i8_orf1	-0.005875704	0.005234677	Proteome
TRINITY_DN3292_c2_g1_i4_orf1	0.003293205	-0.00269463	Proteome
TRINITY_DN11159_c0_g1_i5_orf1	-0.009443475	0.008609451	Proteome
TRINITY_DN71863_c0_g1_i2_orf1	-0.020597417	-0.019026364	Proteome
TRINITY_DN43431_c0_g1_i1_orf1	-0.001282157	0.018804622	Proteome
TRINITY_DN25916_c0_g1_i1_orf1	0.009799645	0.004174529	Proteome
TRINITY_DN19810_c1_g1_i7_orf1	0.010794555	-0.004821467	Proteome
TRINITY_DN4025_c0_g1_i1_orf1	0.009880885	-0.003210179	Proteome

TRINITY_DN41506_c0_g1_i4_orf1	-0.007878548	-0.012842968	Proteome
TRINITY_DN2348_c0_g1_i1_orfp1	-0.009899728	0.022563772	Proteome
TRINITY_DN2345_c0_g1_i4_orf1	0.005013607	0.004245333	Proteome
TRINITY_DN1710_c0_g2_i2_orf1	0.008912516	0.009086599	Proteome
TRINITY_DN12003_c0_g1_i1_orf1	-0.007832931	0.017959616	Proteome
TRINITY_DN16052_c0_g2_i1_orf1	-0.006529821	-0.021972154	Proteome
TRINITY_DN21531_c0_g1_i1_orf1	-0.007912016	0.009301541	Proteome
TRINITY_DN27771_c0_g2_i1_orf1	-0.003216602	-0.008910848	Proteome
TRINITY_DN12885_c0_g1_i1_orf1	-0.011681681	0.013560521	Proteome
TRINITY_DN45859_c0_g1_i1_orf1	0.000390238	-0.004440106	Proteome
TRINITY_DN3377_c0_g1_i1_orf1	-0.015418405	0.020122668	Proteome
TRINITY_DN2514_c1_g1_i13_orf1	0.011571854	-0.023435936	Proteome
TRINITY_DN15400_c0_g1_i1_orf1	0.057036242	0.003373956	Proteome
TRINITY_DN18863_c0_g1_i3_orf1	0.000750655	0.000178152	Proteome
TRINITY_DN26439_c0_g1_i2_orf1	0.009132863	0.02958025	Proteome
TRINITY_DN7682_c0_g1_i2_orf1	0.01161229	0.000353487	Proteome
TRINITY_DN85290_c0_g2_i1_orf1	-0.060089543	0.008668231	Proteome
TRINITY_DN1020_c0_g1_i3_orf1	0.001833043	-0.018698594	Proteome
TRINITY_DN578_c0_g1_i5_orf1	-0.023159711	0.01647099	Proteome
TRINITY_DN100479_c0_g1_i3_orf1	0.00408064	0.003140825	Proteome
TRINITY_DN84883_c0_g1_i1_orf1	-0.002461502	-0.002547192	Proteome
TRINITY_DN11124_c0_g1_i4_orf1	-0.002713122	-0.011476875	Proteome
TRINITY_DN9311_c0_g1_i1_orf1	0.007251264	-0.003255409	Proteome
TRINITY_DN2743_c0_g1_i5_orf1	0.036727263	-0.013434901	Proteome
TRINITY_DN7740_c0_g1_i2_orf1	0.024641507	0.013909303	Proteome
TRINITY_DN12256_c0_g1_i1_orf1	0.016780973	-0.006497681	Proteome
TRINITY_DN24932_c0_g1_i1_orf1	0.007502546	0.009027551	Proteome
TRINITY_DN3664_c0_g1_i8_orf1	0.002723566	0.010680076	Proteome
TRINITY_DN11798_c0_g2_i1_orf1	-0.017864244	0.008810161	Proteome
TRINITY_DN3058_c0_g1_i1_orf1	-0.01574612	0.018279912	Proteome
TRINITY_DN3461_c0_g1_i1_orf1	0.007083516	-0.005466252	Proteome
TRINITY_DN62707_c0_g1_i1_orf1	-0.01105076	-0.015099289	Proteome
TRINITY_DN10290_c0_g1_i7_orf1	-0.013888204	-0.007467214	Proteome
TRINITY_DN140423_c0_g1_i2_orf1	0.011027646	0.002123551	Proteome
TRINITY_DN3861_c0_g3_i2_orf1	0.012872757	-0.004763326	Proteome
TRINITY_DN1073_c0_g1_i3_orf1	0.009857103	0.020722521	Proteome
TRINITY_DN10646_c0_g1_i2_orf1	-0.007564466	0.001300574	Proteome
TRINITY_DN49786_c0_g1_i1_orf1	0.011243478	-0.011777526	Proteome
TRINITY_DN11204_c0_g1_i3_orf1	-0.008480062	0.003481589	Proteome
TRINITY_DN26013_c0_g1_i1_orf1	-0.010125801	0.0077949	Proteome
TRINITY_DN7787_c0_g1_i1_orf1	0.010214716	-0.002360576	Proteome
TRINITY_DN108_c0_g1_i1_orf1	0.006983919	-0.017101651	Proteome
TRINITY_DN6482_c0_g1_i1_orf1	0.010004093	0.018422604	Proteome
TRINITY_DN13941_c0_g1_i6_orf1	-0.008811274	0.000108455	Proteome
TRINITY_DN7170_c0_g1_i11_orf1	0.011095702	0.011733438	Proteome
TRINITY_DN20793_c0_g2_i1_orf1	-0.004048344	-0.012056885	Proteome
TRINITY_DN2238_c0_g2_i1_orf1	-0.006957348	-0.009198832	Proteome
TRINITY_DN17828_c0_g1_i1_orf1	-0.01053724	-0.029608609	Proteome
TRINITY_DN3562_c0_g1_i4_orf1	-0.009891302	0.000322774	Proteome
TRINITY_DN1494_c0_g2_i1_orf1	-0.002791291	-0.000833346	Proteome
TRINITY_DN9938_c0_g2_i1_orf1	0.009788767	0.001865454	Proteome
TRINITY_DN61222_c0_g1_i1_orf1	0.007958571	0.004104408	Proteome
TRINITY_DN1393_c0_g1_i2_orf1	0.01288504	0.006619198	Proteome
TRINITY_DN25210_c0_g1_i1_orf1	0.00555518	0.008792566	Proteome
TRINITY_DN47304_c0_g1_i3_orf1	-0.00832411	-0.008749833	Proteome
TRINITY_DN38482_c0_g1_i4_orf1	-0.009486181	-0.015989578	Proteome
TRINITY_DN1427_c0_g1_i7_orf1	0.012819203	0.002641753	Proteome
TRINITY_DN14477_c0_g1_i12_orf1	-4.53E-05	-0.016079046	Proteome

TRINITY_DN5525_c0_g1_i4_orf1	-0.006979763	0.003208691	Proteome
TRINITY_DN22443_c0_g2_i3_orf1	-0.00468225	-0.017424548	Proteome
TRINITY_DN20007_c0_g1_i1_orf1	0.002499986	-0.01051269	Proteome
TRINITY_DN106038_c0_g1_i1_orf1	0.002719275	0.006907042	Proteome
TRINITY_DN10441_c0_g1_i3_orf1	0.001228731	0.020542878	Proteome
TRINITY_DN15265_c0_g1_i1_orf1	0.004131377	0.003973147	Proteome
TRINITY_DN9000_c0_g2_i1_orf1	0.005275391	-0.009452755	Proteome
TRINITY_DN105506_c0_g1_i8_orf1	0.012614496	-0.000472187	Proteome
TRINITY_DN3673_c0_g1_i10_orf1	-0.009802085	0.001443455	Proteome
TRINITY_DN698_c0_g1_i5_orf1	0.003470142	-0.010091951	Proteome
TRINITY_DN43350_c0_g3_i1_orf1	0.020471809	0.017466646	Proteome
TRINITY_DN103107_c0_g1_i2_orf1	0.001476386	-0.005725631	Proteome
TRINITY_DN1123_c2_g1_i5_orf1	0.001037371	-0.00110608	Proteome
TRINITY_DN21719_c0_g2_i4_orf1	0.004511878	-0.019749184	Proteome
TRINITY_DN2941_c0_g1_i1_orf1	0.009900158	0.008866892	Proteome
TRINITY_DN50787_c0_g2_i2_orf1	0.012929656	0.001862633	Proteome
TRINITY_DN267_c0_g1_i1_orf1	0.010286592	-0.000972893	Proteome
TRINITY_DN14460_c0_g1_i6_orf1	0.007586622	0.014242028	Proteome
TRINITY_DN28509_c0_g1_i1_orf1	-0.007734741	-0.010263802	Proteome
TRINITY_DN2064_c1_g1_i1_orf1	-0.000687211	-0.01664067	Proteome
TRINITY_DN6426_c0_g1_i2_orf1	0.00694058	-0.004686413	Proteome
TRINITY_DN69871_c0_g1_i1_orf1	0.00251741	-0.015979596	Proteome
TRINITY_DN840_c5_g1_i11_orf1	-0.01331795	-0.032831259	Proteome
TRINITY_DN2290_c0_g1_i2_orfp1	-0.003357614	0.010868529	Proteome
TRINITY_DN7942_c0_g1_i1_orf1	-0.000555195	0.003144696	Proteome
TRINITY_DN18538_c0_g3_i1_orf1	0.010204717	0.020881124	Proteome
TRINITY_DN12514_c0_g2_i1_orf1	-0.017219015	-0.025589412	Proteome
TRINITY_DN97472_c0_g1_i5_orf1	0.001486119	0.016775776	Proteome
TRINITY_DN7938_c0_g1_i3_orf1	0.010507789	0.006978287	Proteome
TRINITY_DN14944_c0_g1_i9_orf1	0.012607222	-0.0251247	Proteome
TRINITY_DN418_c1_g1_i3_orf1	-0.05420459	0.026106668	Proteome
TRINITY_DN19115_c0_g1_i1_orf1	-0.007882364	-0.001937097	Proteome
TRINITY_DN14922_c0_g3_i2_orf1	0.002636716	-0.005611084	Proteome
TRINITY_DN23432_c0_g1_i1_orf1	0.003440169	-0.015226846	Proteome
TRINITY_DN6309_c0_g1_i7_orf1	0.000856158	-0.029947152	Proteome
TRINITY_DN50471_c0_g1_i4_orf1	-0.010074743	0.010909841	Proteome
TRINITY_DN27488_c0_g1_i9_orf1	0.009380078	0.001021803	Proteome
TRINITY_DN54366_c0_g1_i1_orf1	0.006567386	-0.000513348	Proteome
TRINITY_DN57111_c0_g1_i1_orf1	-0.003643066	0.025779763	Proteome
TRINITY_DN14306_c0_g1_i1_orf1	-0.00200901	0.023338334	Proteome
TRINITY_DN21758_c0_g1_i1_orf1	-0.004299948	-0.004805241	Proteome
TRINITY_DN4681_c0_g2_i2_orf1	0.008882467	-0.004945331	Proteome
TRINITY_DN11159_c0_g2_i1_orf1	-0.003323333	-0.006737709	Proteome
TRINITY_DN11981_c0_g1_i7_orf1	-0.020397833	0.011603886	Proteome
TRINITY_DN5149_c0_g1_i14_orfp1	0.018710295	0.004186389	Proteome
TRINITY_DN69535_c0_g1_i2_orf1	-0.006892911	-0.003417844	Proteome
TRINITY_DN146126_c0_g1_i1_orf1	0.003287181	0.015695928	Proteome
TRINITY_DN44335_c0_g1_i7_orf1	0.007081929	-0.004709405	Proteome
TRINITY_DN827_c1_g1_i1_orf1	0.01113115	-0.008336744	Proteome
TRINITY_DN41_c0_g1_i5_orf1	-0.004465463	-0.022646862	Proteome
TRINITY_DN1557_c0_g1_i9_orf1	0.004278002	-0.007826427	Proteome
TRINITY_DN7867_c0_g1_i1_orf1	0.004620974	0.004625457	Proteome
TRINITY_DN15865_c0_g1_i1_orf1	0.011240815	-0.006692073	Proteome
TRINITY_DN1617_c0_g1_i5_orf1	0.006445788	0.000395223	Proteome
TRINITY_DN4233_c0_g2_i2_orf1	0.006401316	-0.006258418	Proteome
TRINITY_DN9376_c1_g1_i3_orf1	0.01591132	0.002650256	Proteome
TRINITY_DN4324_c0_g1_i1_orf1	0.010066545	-0.005380104	Proteome
TRINITY_DN2107_c0_g2_i3_orf1	-0.000857997	0.000952058	Proteome

TRINITY_DN11970_c0_g1_i4_orf1	-0.001804184	-0.012638928	Proteome
TRINITY_DN2859_c0_g1_i7_orf1	-0.000147309	0.01059437	Proteome
TRINITY_DN26947_c0_g1_i1_orf1	-0.009639628	-0.008310364	Proteome
TRINITY_DN21792_c0_g1_i1_orf1	-0.00371143	-0.0093403	Proteome
TRINITY_DN66287_c0_g1_i1_orfp1	-0.004677996	0.009866886	Proteome
TRINITY_DN1477_c0_g1_i5_orf1	0.001374964	0.010743833	Proteome
TRINITY_DN2732_c0_g1_i4_orf1	0.011189134	-0.003939514	Proteome
TRINITY_DN28626_c0_g1_i5_orf1	-0.016112453	0.017514635	Proteome
TRINITY_DN5442_c0_g1_i4_orf1	-0.00253642	0.016151834	Proteome
TRINITY_DN4785_c0_g2_i1_orf1	0.012269176	0.006042591	Proteome
TRINITY_DN3179_c0_g1_i1_orf1	0.003449528	-0.005985805	Proteome
TRINITY_DN52788_c0_g1_i1_orf1	-0.00212209	-0.009101225	Proteome
TRINITY_DN33488_c0_g1_i2_orf1	-0.006133657	-0.014860064	Proteome
TRINITY_DN24318_c0_g1_i1_orf1	0.013157118	0.005231889	Proteome
TRINITY_DN2286_c2_g1_i1_orf1	-0.0156285	-0.055021022	Proteome
TRINITY_DN9555_c0_g1_i1_orf1	-0.00062969	-0.013222494	Proteome
TRINITY_DN33967_c2_g2_i1_orf1	-0.010486176	0.005677994	Proteome
TRINITY_DN5346_c0_g1_i5_orf1	9.12E-05	0.010063586	Proteome
TRINITY_DN3406_c0_g1_i17_orf1	0.011475233	0.000432787	Proteome
TRINITY_DN13324_c0_g1_i3_orf1	0.010861685	-0.006902947	Proteome
TRINITY_DN5126_c0_g1_i3_orf1	-0.009098982	-0.010114023	Proteome
TRINITY_DN19702_c0_g1_i4_orf1	0.005458	-0.010367751	Proteome
TRINITY_DN7828_c0_g1_i2_orf1	0.005524581	-0.000558717	Proteome
TRINITY_DN4014_c0_g1_i1_orf1	-0.010036759	-0.011871549	Proteome
TRINITY_DN7688_c0_g1_i2_orf1	0.000762007	0.004900281	Proteome
TRINITY_DN78_c0_g1_i5_orf1	0.002925338	0.000901894	Proteome
TRINITY_DN3439_c0_g2_i2_orf1	0.049446818	0.003876956	Proteome
TRINITY_DN14584_c0_g1_i1_orf1	0.010408067	-0.010186529	Proteome
TRINITY_DN7062_c0_g1_i1_orf1	0.015721306	0.015239406	Proteome
TRINITY_DN7711_c1_g1_i3_orf1	0.009693725	-0.004397756	Proteome
TRINITY_DN649_c1_g1_i13_orf1	0.002805375	0.008010031	Proteome
TRINITY_DN3324_c0_g1_i3_orf1	0.003386792	-0.013456592	Proteome
TRINITY_DN1197_c0_g1_i6_orf1	0.006344504	0.011295435	Proteome
TRINITY_DN15811_c0_g1_i7_orf1	-0.004461848	0.014908147	Proteome
TRINITY_DN4301_c0_g1_i5_orf1	-0.001192005	0.003325554	Proteome
TRINITY_DN25856_c0_g1_i1_orf1	0.003042397	-0.004624423	Proteome
TRINITY_DN1048_c0_g1_i6_orf1	-0.038044994	0.026021823	Proteome
TRINITY_DN9146_c0_g1_i1_orf1	0.002823497	0.005884413	Proteome
TRINITY_DN4401_c0_g2_i1_orf1	0.014046658	-0.008293611	Proteome
TRINITY_DN272_c0_g1_i1_orf1	-0.015187685	0.017725308	Proteome
TRINITY_DN53747_c0_g3_i1_orf1	-0.001611628	0.009223716	Proteome
TRINITY_DN10079_c0_g1_i1_orf1	-0.00069374	0.008697272	Proteome
TRINITY_DN695_c0_g1_i12_orf1	0.010264308	0.015381045	Proteome
TRINITY_DN31417_c0_g1_i3_orf1	-0.00248182	-0.012067146	Proteome
TRINITY_DN10455_c0_g2_i1_orf1	-0.001481552	-0.017012058	Proteome
TRINITY_DN13923_c0_g2_i1_orf1	-0.01548734	-0.00914006	Proteome
TRINITY_DN3029_c1_g2_i1_orf1	-0.010251985	0.002477805	Proteome
TRINITY_DN29879_c0_g1_i3_orf1	0.021939465	0.007933166	Proteome
TRINITY_DN114960_c0_g1_i4_orf1	0.003541074	0.012011578	Proteome
TRINITY_DN69236_c0_g1_i1_orf1	0.014218951	0.00125995	Proteome
TRINITY_DN13287_c0_g1_i5_orf1	0.01169816	0.007306686	Proteome
TRINITY_DN20527_c0_g1_i1_orf1	0.014410782	-0.006091951	Proteome
TRINITY_DN41108_c0_g1_i1_orf1	0.008012611	-0.012230895	Proteome
TRINITY_DN15175_c0_g1_i1_orf1	-0.020345305	0.009350858	Proteome
TRINITY_DN40015_c0_g1_i2_orf1	0.007754862	0.004482148	Proteome
TRINITY_DN4790_c0_g1_i6_orf1	0.005223693	-0.0015351	Proteome
TRINITY_DN18592_c0_g2_i1_orf1	-0.010435214	-0.019563056	Proteome
TRINITY_DN6153_c0_g1_i6_orf1	0.009589697	0.000885159	Proteome

TRINITY_DN98249_c0_g1_i1_orf1	0.003229065	0.015095768	Proteome
TRINITY_DN20130_c0_g1_i1_orf1	0.009696349	0.006552159	Proteome
TRINITY_DN1322_c0_g1_i4_orf1	-0.014914928	-0.029606101	Proteome
TRINITY_DN1585_c0_g1_i1_orf1	0.014917658	0.007233291	Proteome
TRINITY_DN31118_c1_g1_i1_orf1	0.009918058	0.017486921	Proteome
TRINITY_DN28695_c0_g1_i1_orf1	4.87E-05	-0.013438218	Proteome
TRINITY_DN795_c0_g1_i6_orf1	0.006716875	0.008776857	Proteome
TRINITY_DN5133_c0_g1_i7_orf1	0.009418881	0.005266687	Proteome
TRINITY_DN3984_c0_g1_i4_orf1	-0.007113934	0.004633626	Proteome
TRINITY_DN3430_c0_g1_i1_orf1	-0.003363641	-0.000524659	Proteome
TRINITY_DN11060_c0_g1_i6_orf1	0.003232764	0.015261001	Proteome
TRINITY_DN139326_c0_g1_i1_orf1	0.006600088	0.013915672	Proteome
TRINITY_DN2224_c0_g1_i1_orf1	0.004962976	0.00559421	Proteome
TRINITY_DN8717_c0_g1_i5_orf1	0.010584214	0.003256952	Proteome
TRINITY_DN97097_c0_g1_i4_orf1	-0.008694879	0.021783222	Proteome
TRINITY_DN48590_c0_g1_i1_orf1	0.006880074	-0.000756027	Proteome
TRINITY_DN4725_c0_g1_i4_orf1	-0.001635666	-0.009951548	Proteome
TRINITY_DN5296_c0_g2_i1_orf1	0.003508384	0.008497908	Proteome
TRINITY_DN2668_c0_g1_i6_orf1	-0.002400572	-0.006445424	Proteome
TRINITY_DN42759_c0_g2_i1_orf1	0.009037867	-0.003832673	Proteome
TRINITY_DN3062_c0_g1_i1_orf1	0.00808744	0.000990276	Proteome
TRINITY_DN400_c0_g1_i1_orf1	-0.006083104	0.003481174	Proteome
TRINITY_DN3598_c0_g1_i1_orf1	0.000273008	-0.007504106	Proteome
TRINITY_DN21215_c0_g1_i7_orf1	0.017419605	9.05E-05	Proteome
TRINITY_DN61786_c0_g1_i1_orf1	0.008726183	-0.01784779	Proteome
TRINITY_DN15685_c0_g1_i5_orf1	0.010899056	0.00661818	Proteome
TRINITY_DN2967_c0_g1_i4_orf1	0.001219655	0.004300955	Proteome
TRINITY_DN4621_c0_g1_i4_orf1	-0.001138196	-0.02199216	Proteome
TRINITY_DN63561_c1_g1_i2_orf1	0.005489456	-0.004253345	Proteome
TRINITY_DN6365_c0_g1_i4_orf1	0.013861345	0.001274074	Proteome
TRINITY_DN18230_c1_g2_i1_orf1	-0.005179476	-0.001022769	Proteome
TRINITY_DN45949_c0_g1_i1_orf1	-0.018379451	0.014569314	Proteome
TRINITY_DN2401_c0_g2_i1_orf1	0.000247708	0.000962386	Proteome
TRINITY_DN625_c9_g1_i7_orf1	0.00566397	-0.022052086	Proteome
TRINITY_DN389_c0_g1_i2_orf1	-0.002008125	-0.002241086	Proteome
TRINITY_DN1166_c0_g3_i4_orf1	-0.013164644	-0.005016413	Proteome
TRINITY_DN38180_c0_g1_i3_orf1	0.00793923	0.01190458	Proteome
TRINITY_DN28503_c0_g1_i6_orf1	0.009315026	0.015882158	Proteome
TRINITY_DN6988_c0_g1_i3_orf1	-0.022341748	0.010632944	Proteome
TRINITY_DN9979_c0_g1_i1_orf1	0.012877452	-0.001798854	Proteome
TRINITY_DN5037_c0_g1_i3_orf1	-0.003669044	-0.017110287	Proteome
TRINITY_DN5675_c0_g1_i6_orf1	0.008139005	-0.012475213	Proteome
TRINITY_DN50593_c0_g1_i1_orf1	0.007723679	-0.006667994	Proteome
TRINITY_DN5074_c0_g1_i7_orf1	0.0153381	-0.005309608	Proteome
TRINITY_DN184_c0_g1_i1_orf1	-0.027940883	0.001824805	Proteome
TRINITY_DN41065_c0_g1_i5_orf1	-0.007814463	0.012665892	Proteome
TRINITY_DN18172_c0_g1_i6_orf1	0.002325906	-0.007800957	Proteome
TRINITY_DN6307_c0_g1_i5_orf1	-0.006600712	-0.014613866	Proteome
TRINITY_DN446_c0_g1_i20_orf1	-0.009567768	-0.014722705	Proteome
TRINITY_DN4257_c0_g1_i2_orf1	0.015856433	-0.005588801	Proteome
TRINITY_DN75746_c0_g1_i1_orfp1	0.003027293	0.001012215	Proteome
TRINITY_DN6014_c1_g1_i2_orf1	0.008470658	-0.006543356	Proteome
TRINITY_DN40586_c0_g1_i4_orf1	0.002486049	0.008931808	Proteome
TRINITY_DN13233_c0_g1_i3_orf1	0.004900042	0.012105471	Proteome
TRINITY_DN4501_c0_g1_i3_orf1	0.009788195	-0.002810968	Proteome
TRINITY_DN8691_c0_g1_i3_orf1	-0.002682453	0.002770661	Proteome
TRINITY_DN15607_c0_g1_i6_orf1	0.00527435	-0.000238746	Proteome
TRINITY_DN20499_c0_g1_i1_orf1	0.001052406	-0.009530956	Proteome

TRINITY_DN2529_c0_g1_i3_orf1	0.013029146	0.00749169	Proteome
TRINITY_DN19092_c2_g1_i1_orf1	-0.000939536	-0.017627697	Proteome
TRINITY_DN103118_c0_g1_i4_orf1	0.016824743	-0.003064134	Proteome
TRINITY_DN9637_c0_g1_i14_orf1	7.83E-05	0.008934705	Proteome
TRINITY_DN2201_c0_g1_i1_orf1	0.01850669	-0.004338312	Proteome
TRINITY_DN54554_c0_g1_i1_orf1	-0.005775775	-0.004098318	Proteome
TRINITY_DN108573_c0_g1_i1_orf1	-0.006396692	0.00332032	Proteome
TRINITY_DN11133_c0_g1_i5_orf1	0.00963449	-0.010795059	Proteome
TRINITY_DN350_c0_g1_i5_orf1	-0.007450092	0.024035799	Proteome
TRINITY_DN1814_c0_g2_i4_orfp1	0.013114518	0.013083694	Proteome
TRINITY_DN9090_c0_g1_i9_orf1	-0.019211068	0.027642517	Proteome
TRINITY_DN5170_c0_g1_i5_orf1	0.033366435	-0.000307052	Proteome
TRINITY_DN35800_c0_g1_i6_orf1	-0.010716341	0.00647425	Proteome
TRINITY_DN394_c0_g1_i2_orf1	0.05112629	0.002786828	Proteome
TRINITY_DN38562_c0_g1_i3_orf1	-0.003794027	0.018717779	Proteome
TRINITY_DN41761_c0_g1_i4_orf1	-0.006261662	-0.021388224	Proteome
TRINITY_DN19830_c0_g1_i1_orf1	-0.005331662	0.014122088	Proteome
TRINITY_DN108200_c0_g1_i1_orf1	0.005004038	0.004626162	Proteome
TRINITY_DN26824_c0_g1_i1_orf1	0.019308068	0.007445939	Proteome
TRINITY_DN3037_c0_g1_i1_orf1	-0.014020534	0.002615979	Proteome
TRINITY_DN10824_c0_g1_i3_orf1	0.004784164	0.000818125	Proteome
TRINITY_DN286_c0_g1_i2_orf1	0.004275932	-0.007807843	Proteome
TRINITY_DN6535_c0_g2_i1_orf1	-0.002958002	0.006975162	Proteome
TRINITY_DN610_c0_g1_i1_orf1	-0.016403732	-0.008017823	Proteome
TRINITY_DN17845_c0_g1_i3_orf1	0.003042853	0.019198907	Proteome
TRINITY_DN2343_c1_g1_i8_orf1	0.006697572	0.000676879	Proteome
TRINITY_DN52761_c0_g1_i2_orf1	0.031647124	-0.011604174	Proteome
TRINITY_DN14301_c0_g1_i1_orf1	0.004945887	-0.011310309	Proteome
TRINITY_DN16451_c0_g1_i7_orf1	0.011328296	0.006526536	Proteome
TRINITY_DN28622_c0_g1_i1_orf1	0.006730987	-0.007855615	Proteome
TRINITY_DN7861_c0_g1_i5_orf1	0.016483521	0.004465184	Proteome
TRINITY_DN98147_c0_g2_i1_orf1	0.023624067	0.003899235	Proteome
TRINITY_DN38435_c0_g1_i1_orf1	-0.006035288	0.005281706	Proteome
TRINITY_DN34534_c0_g2_i1_orf1	0.003850315	0.001918825	Proteome
TRINITY_DN1034_c0_g2_i1_orf1	-0.003048702	-0.001335983	Proteome
TRINITY_DN2202_c0_g1_i9_orf1	-0.001568151	-0.010261394	Proteome
TRINITY_DN4135_c0_g1_i5_orf1	0.009128435	0.004998819	Proteome
TRINITY_DN101991_c0_g1_i5_orf1	0.005810166	0.006478834	Proteome
TRINITY_DN430_c0_g1_i5_orf1	0.011780968	0.003402732	Proteome
TRINITY_DN15993_c0_g1_i1_orf1	0.009335361	-0.001480891	Proteome
TRINITY_DN2271_c0_g1_i12_orf1	-0.019982587	0.009637571	Proteome
TRINITY_DN80660_c0_g1_i1_orf1	-0.024572178	0.013324747	Proteome
TRINITY_DN6642_c0_g2_i1_orf1	0.000572956	-0.006389154	Proteome
TRINITY_DN30_c0_g1_i6_orf1	0.009147715	-0.008996637	Proteome
TRINITY_DN38650_c0_g1_i2_orf1	0.002407482	-0.005783162	Proteome
TRINITY_DN14262_c0_g1_i5_orf1	-0.023771944	0.008342549	Proteome
TRINITY_DN4497_c0_g1_i4_orf1	0.016746987	0.006725543	Proteome
TRINITY_DN42461_c0_g1_i4_orf1	0.012886891	0.007110457	Proteome
TRINITY_DN3008_c0_g1_i12_orf1	-0.005285394	-0.012655814	Proteome
TRINITY_DN6535_c0_g1_i3_orf1	0.005032761	0.010589366	Proteome
TRINITY_DN5174_c0_g3_i1_orf1	0.019526368	-0.007644388	Proteome
TRINITY_DN14030_c0_g1_i1_orf1	-0.002036613	-0.015564842	Proteome
TRINITY_DN34423_c0_g1_i2_orf1	-0.018192107	0.000451778	Proteome
TRINITY_DN13371_c0_g1_i4_orf1	0.000413591	0.014685668	Proteome
TRINITY_DN16343_c0_g1_i6_orf1	-0.000898783	0.002959458	Proteome
TRINITY_DN886_c0_g1_i1_orf1	-0.003971446	0.007625437	Proteome
TRINITY_DN58261_c0_g1_i2_orf1	0.010250739	-0.008969417	Proteome
TRINITY_DN12476_c0_g1_i4_orf1	-0.003944955	-0.015829148	Proteome

TRINITY_DN50725_c0_g1_i6_orf1	-0.012854523	-0.002964892	Proteome
TRINITY_DN492_c0_g1_i4_orf1	-0.000838349	-0.013821987	Proteome
TRINITY_DN54477_c0_g1_i1_orf1	0.012713325	0.003456246	Proteome
TRINITY_DN57918_c0_g1_i1_orf1	0.004858777	-0.006996931	Proteome
TRINITY_DN84938_c0_g1_i4_orf1	0.029968229	-0.018907304	Proteome
TRINITY_DN2758_c0_g1_i7_orf1	0.0189129	0.009554518	Proteome
TRINITY_DN48554_c0_g1_i1_orf1	-0.005321154	0.001067818	Proteome
TRINITY_DN38835_c0_g2_i1_orf1	-0.001498007	-0.014526781	Proteome
TRINITY_DN3017_c0_g1_i6_orf1	-0.007778936	0.037046764	Proteome
TRINITY_DN753_c0_g1_i4_orf1	0.00969898	0.001761133	Proteome
TRINITY_DN14922_c0_g1_i4_orf1	-0.009126805	0.016482862	Proteome
TRINITY_DN18933_c0_g1_i3_orf1	0.011787964	0.003038877	Proteome
TRINITY_DN19731_c0_g1_i1_orf1	-0.004899441	0.03587657	Proteome
TRINITY_DN48536_c0_g1_i3_orf1	-0.007029599	-0.004552837	Proteome
TRINITY_DN28577_c0_g1_i6_orf1	0.005117759	0.000504289	Proteome
TRINITY_DN1789_c0_g1_i5_orf1	-0.017090892	0.006946497	Proteome
TRINITY_DN11347_c0_g1_i1_orf1	-0.005384851	-0.001814848	Proteome
TRINITY_DN18918_c0_g1_i3_orf1	-0.017606899	-0.000491814	Proteome
TRINITY_DN4938_c0_g1_i13_orf1	-0.002536084	0.00921173	Proteome
TRINITY_DN45953_c0_g1_i1_orf1	0.008596658	0.000404309	Proteome
TRINITY_DN2751_c0_g1_i3_orf1	-0.011507825	0.004344875	Proteome
TRINITY_DN3860_c0_g1_i5_orf1	0.00064232	0.014523251	Proteome
TRINITY_DN582_c0_g1_i2_orf1	-0.012738345	-0.009324338	Proteome
TRINITY_DN25492_c0_g1_i1_orf1	-0.002503426	0.010972912	Proteome
TRINITY_DN5675_c0_g1_i1_orf1	-0.013158601	-0.001266483	Proteome
TRINITY_DN7516_c0_g2_i1_orf1	0.006081115	0.008013902	Proteome
TRINITY_DN14755_c0_g1_i4_orf1	-0.003332154	-0.011031539	Proteome
TRINITY_DN38783_c0_g1_i1_orf1	0.00915164	-0.046449611	Proteome
TRINITY_DN1408_c0_g1_i10_orf1	-0.013097411	0.003962336	Proteome
TRINITY_DN8359_c0_g1_i5_orf1	0.007380167	-0.004391699	Proteome
TRINITY_DN2997_c0_g1_i6_orf1	-0.001833396	0.013570045	Proteome
TRINITY_DN1252_c0_g1_i3_orf1	-0.015528891	0.001663039	Proteome
TRINITY_DN27321_c0_g1_i1_orf1	0.011006545	0.01103347	Proteome
TRINITY_DN3715_c0_g1_i2_orf1	-0.019662602	-0.002729648	Proteome
TRINITY_DN2372_c0_g1_i5_orf1	-0.002436201	-0.004803524	Proteome
TRINITY_DN2456_c0_g1_i2_orf1	-0.000560784	-0.000339201	Proteome
TRINITY_DN3893_c0_g2_i3_orf1	0.008531973	0.007579201	Proteome
TRINITY_DN135780_c0_g1_i1_orf1	0.030674514	0.016779901	Proteome
TRINITY_DN30131_c0_g1_i1_orf1	0.010654566	-0.008788221	Proteome
TRINITY_DN8158_c0_g1_i2_orf1	-0.046239512	-0.110517554	Proteome
TRINITY_DN3649_c0_g1_i6_orf1	0.017696427	0.000225078	Proteome
TRINITY_DN73_c0_g1_i6_orf1	0.003957995	-0.012133319	Proteome
TRINITY_DN14475_c0_g1_i1_orf1	0.004324002	-0.006488579	Proteome
TRINITY_DN2943_c2_g3_i1_orf1	0.00908549	-0.007537082	Proteome
TRINITY_DN31047_c0_g1_i4_orf1	0.005470006	-0.003303243	Proteome
TRINITY_DN8625_c0_g1_i1_orf1	-0.019496379	-0.009473543	Proteome
TRINITY_DN16234_c0_g2_i3_orf1	-0.011730405	0.046987678	Proteome
TRINITY_DN500_c0_g1_i1_orf1	-0.013619094	-0.006843685	Proteome
TRINITY_DN4040_c0_g1_i10_orf1	-0.002165174	-0.001575858	Proteome
TRINITY_DN24399_c0_g1_i1_orf1	-0.007540403	0.005697498	Proteome
TRINITY_DN2013_c0_g1_i15_orf1	-0.005853329	-0.007603569	Proteome
TRINITY_DN19122_c0_g1_i7_orf1	0.002050607	-0.008033777	Proteome
TRINITY_DN7688_c0_g1_i10_orf1	0.003738712	0.001413541	Proteome
TRINITY_DN20185_c0_g1_i6_orf1	-0.000279921	0.00182804	Proteome
TRINITY_DN2376_c0_g1_i6_orf1	0.005206959	-0.005897765	Proteome
TRINITY_DN26195_c0_g1_i6_orf1	-0.004988184	-0.011539322	Proteome
TRINITY_DN1986_c0_g1_i1_orf1	0.003163635	0.010826081	Proteome
TRINITY_DN2343_c1_g1_i12_orf1	0.013602125	0.001178716	Proteome

TRINITY_DN34857_c0_g1_i6_orf1	-0.011009881	0.012901492	Proteome
TRINITY_DN36718_c0_g1_i1_orf1	-0.001107804	-0.008727235	Proteome
TRINITY_DN16147_c0_g1_i4_orf1	-0.016887026	0.010770244	Proteome
TRINITY_DN1450_c0_g2_i1_orf1	0.012014238	0.040026333	Proteome
TRINITY_DN79083_c0_g1_i2_orf1	-6.39E-05	0.006358197	Proteome
TRINITY_DN1249_c0_g1_i10_orf1	-0.002340154	-0.003140549	Proteome
TRINITY_DN640_c0_g1_i5_orf1	0.003264093	-0.015837032	Proteome
TRINITY_DN3478_c0_g1_i10_orf1	-0.01355053	0.000416409	Proteome
TRINITY_DN4476_c0_g1_i5_orf1	0.007529852	0.006234974	Proteome
TRINITY_DN3826_c0_g1_i1_orf1	-0.026712226	-0.000780158	Proteome
TRINITY_DN16258_c0_g1_i2_orf1	-0.000374756	0.014925847	Proteome
TRINITY_DN42205_c0_g1_i4_orf1	0.004137791	-0.008289728	Proteome
TRINITY_DN2880_c0_g1_i2_orf1	0.008476273	0.025685665	Proteome
TRINITY_DN3975_c0_g1_i10_orf1	0.00734231	0.011864479	Proteome
TRINITY_DN9724_c0_g1_i4_orf1	0.009600919	-0.001775628	Proteome
TRINITY_DN9765_c0_g1_i6_orf1	0.004591265	-0.009221615	Proteome
TRINITY_DN2673_c0_g3_i1_orf1	0.011167055	-0.016250358	Proteome
TRINITY_DN2257_c0_g1_i4_orf1	-0.012474594	-0.018383192	Proteome
TRINITY_DN17657_c0_g1_i1_orf1	-0.018926496	-0.006188758	Proteome
TRINITY_DN2450_c0_g1_i6_orf1	0.0015202	-0.007419465	Proteome
TRINITY_DN18563_c2_g1_i1_orf1	-0.007121788	-0.015187494	Proteome
TRINITY_DN4134_c2_g1_i2_orf1	0.006085767	-0.002303143	Proteome
TRINITY_DN1884_c0_g2_i2_orf1	0.001954448	0.001695321	Proteome
TRINITY_DN10871_c0_g1_i3_orf1	0.001440014	-0.013232853	Proteome
TRINITY_DN143852_c0_g1_i1_orf1	0.009430587	0.007835559	Proteome
TRINITY_DN3022_c0_g1_i1_orf1	0.003753951	-0.003087927	Proteome
TRINITY_DN2024_c0_g1_i12_orfp1	-0.021407541	0.024316821	Proteome
TRINITY_DN3134_c0_g1_i1_orf1	-0.004125295	-0.000603573	Proteome
TRINITY_DN22824_c0_g1_i4_orf1	0.019471046	0.003569161	Proteome
TRINITY_DN84669_c0_g1_i1_orf1	0.013167023	0.009607353	Proteome
TRINITY_DN63152_c0_g1_i7_orf1	0.012606158	-0.001330009	Proteome
TRINITY_DN57636_c0_g1_i4_orf1	0.000882883	0.005659334	Proteome
TRINITY_DN5603_c0_g1_i1_orf1	0.004082351	-0.017038542	Proteome
TRINITY_DN7918_c0_g1_i1_orf1	-0.005925465	-0.003493649	Proteome
TRINITY_DN9868_c0_g1_i6_orf1	-0.00230452	-0.007413699	Proteome
TRINITY_DN18329_c0_g1_i2_orf1	0.00981603	0.000484346	Proteome
TRINITY_DN53166_c0_g1_i1_orf1	0.001288838	-0.011981316	Proteome
TRINITY_DN2187_c0_g1_i1_orf1	0.020002803	0.017877504	Proteome
TRINITY_DN109943_c0_g1_i1_orf1	0.007004872	0.003309958	Proteome
TRINITY_DN4767_c0_g1_i6_orf1	-0.040389213	0.039206846	Proteome
TRINITY_DN25680_c0_g1_i1_orf1	0.010454026	0.006849758	Proteome
TRINITY_DN6185_c0_g1_i12_orf1	-0.003860525	0.006775004	Proteome
TRINITY_DN13139_c0_g1_i1_orf1	0.00313443	-0.012331868	Proteome
TRINITY_DN26789_c0_g1_i2_orf1	-0.020780826	0.000607157	Proteome
TRINITY_DN6423_c0_g1_i5_orf1	0.001649771	-0.009239459	Proteome
TRINITY_DN32896_c0_g3_i1_orf1	0.009772628	-0.002847653	Proteome
TRINITY_DN27045_c0_g1_i1_orf1	0.017839045	-0.007127597	Proteome
TRINITY_DN14856_c0_g1_i1_orf1	0.013579323	0.005483876	Proteome
TRINITY_DN2959_c0_g1_i2_orf1	0.01551264	-0.015176288	Proteome
TRINITY_DN659_c0_g2_i1_orf1	0.007856299	-0.001706106	Proteome
TRINITY_DN4707_c0_g1_i1_orf1	0.005581736	-0.001444493	Proteome
TRINITY_DN2040_c0_g1_i15_orfp1	0.001660371	0.002617207	Proteome
TRINITY_DN13602_c0_g1_i4_orf1	-0.011150696	-0.01509081	Proteome
TRINITY_DN1245_c0_g1_i4_orf1	0.014702465	-0.01662861	Proteome
TRINITY_DN25251_c0_g2_i1_orf1	0.002760044	-0.005182671	Proteome
TRINITY_DN8580_c0_g1_i12_orf1	-0.000219725	-0.009482597	Proteome
TRINITY_DN3005_c0_g1_i7_orf1	-0.009723217	0.012117553	Proteome
TRINITY_DN125521_c0_g2_i1_orf1	0.012654647	0.032825725	Proteome

TRINITY_DN2265_c0_g1_i5_orf1	-0.001273863	-0.018296664	Proteome
TRINITY_DN5478_c0_g2_i2_orf1	0.01669021	0.01003512	Proteome
TRINITY_DN2798_c0_g1_i5_orf1	-0.022384599	0.010094836	Proteome
TRINITY_DN2826_c0_g1_i7_orf1	0.008161653	0.010412968	Proteome
TRINITY_DN9437_c0_g1_i1_orf1	0.002576608	-0.01590532	Proteome
TRINITY_DN9544_c0_g1_i1_orf1	0.013974305	0.003691827	Proteome
TRINITY_DN129808_c0_g1_i1_orf1	0.01057775	-0.001342265	Proteome
TRINITY_DN9280_c0_g1_i1_orf1	-0.008741985	0.010776858	Proteome
TRINITY_DN69307_c0_g1_i6_orf1	-0.00830592	0.023306941	Proteome
TRINITY_DN7868_c0_g1_i8_orf1	0.012482654	-0.008581699	Proteome
TRINITY_DN8958_c0_g1_i1_orf1	0.013650192	-0.005577807	Proteome
TRINITY_DN133474_c0_g2_i2_orf1	-0.004969435	0.014927529	Proteome
TRINITY_DN58413_c0_g1_i4_orf1	0.003052897	-0.005059501	Proteome
TRINITY_DN14009_c0_g1_i1_orf1	0.007869145	-0.006290383	Proteome
TRINITY_DN19917_c0_g1_i1_orf1	0.002680533	-0.000728172	Proteome
TRINITY_DN60821_c0_g1_i1_orf1	-0.010945038	-0.013933563	Proteome
TRINITY_DN4270_c0_g1_i1_orf1	-0.00973841	0.014346695	Proteome
TRINITY_DN83374_c0_g1_i1_orf1	-0.002231451	0.004528364	Proteome
TRINITY_DN2483_c0_g1_i1_orf1	-0.006698845	-0.019241619	Proteome
TRINITY_DN5375_c0_g1_i1_orf1	-0.001773694	-0.010196406	Proteome
TRINITY_DN8685_c0_g1_i5_orf1	-0.019051783	0.020989374	Proteome
TRINITY_DN72859_c0_g1_i1_orf1	0.009624726	0.001102486	Proteome
TRINITY_DN19_c0_g1_i8_orf1	0.004359482	-0.011844222	Proteome
TRINITY_DN3301_c0_g1_i2_orf1	-0.033864833	-0.000589382	Proteome
TRINITY_DN11514_c0_g1_i1_orf1	0.017613733	-0.001981494	Proteome
TRINITY_DN9316_c0_g3_i1_orf1	0.009415174	0.006295588	Proteome
TRINITY_DN3433_c2_g1_i2_orf1	0.013276823	-0.007301643	Proteome
TRINITY_DN18338_c0_g1_i6_orf1	0.027897329	0.005495	Proteome
TRINITY_DN35725_c0_g1_i1_orf1	0.001283374	0.008965725	Proteome
TRINITY_DN6016_c0_g1_i8_orf1	-0.011769936	0.001868683	Proteome
TRINITY_DN10581_c0_g1_i5_orf1	0.012415214	0.004135165	Proteome
TRINITY_DN5274_c0_g2_i2_orf1	-0.01432694	0.005515735	Proteome
TRINITY_DN145224_c0_g1_i1_orf1	0.000143676	0.008335837	Proteome
TRINITY_DN172_c1_g1_i3_orf1	0.004675992	6.77E-05	Proteome
TRINITY_DN13167_c0_g1_i1_orf1	0.018045005	0.003845478	Proteome
TRINITY_DN18728_c0_g1_i2_orf1	0.006463504	-0.000677568	Proteome
TRINITY_DN3383_c0_g1_i5_orf1	-0.028591123	0.011962929	Proteome
TRINITY_DN18620_c0_g1_i5_orf1	0.010348251	0.01650336	Proteome
TRINITY_DN33008_c0_g1_i1_orf1	0.002462476	-0.013321056	Proteome
TRINITY_DN2958_c0_g1_i2_orf1	0.003857322	0.010603342	Proteome
TRINITY_DN61048_c0_g1_i2_orf1	0.017639576	0.004475345	Proteome
TRINITY_DN39490_c0_g1_i1_orf1	0.006239581	0.009316065	Proteome
TRINITY_DN486_c0_g1_i5_orf1	0.012119765	-0.002656423	Proteome
TRINITY_DN1841_c0_g1_i2_orf1	-0.013880953	0.011852349	Proteome
TRINITY_DN10364_c0_g1_i5_orf1	0.006052033	-0.00639011	Proteome
TRINITY_DN12586_c0_g1_i4_orf1	-0.034054621	0.025342033	Proteome
TRINITY_DN147458_c0_g1_i1_orf1	0.013879028	0.010325742	Proteome
TRINITY_DN39725_c0_g1_i4_orf1	0.019466198	0.014901809	Proteome
TRINITY_DN21214_c0_g2_i1_orf1	-0.003641509	0.016210556	Proteome
TRINITY_DN259_c0_g1_i8_orf1	0.007261971	-0.01035064	Proteome
TRINITY_DN4276_c0_g1_i6_orf1	-0.012210981	0.025535281	Proteome
TRINITY_DN8366_c0_g1_i4_orf1	0.006157766	0.002519744	Proteome
TRINITY_DN3566_c0_g1_i4_orf1	0.004221567	-0.022896017	Proteome
TRINITY_DN42705_c0_g1_i3_orf1	0.00053525	0.002477245	Proteome
TRINITY_DN10662_c0_g1_i4_orf1	0.010255264	0.002318356	Proteome
TRINITY_DN5956_c1_g1_i5_orf1	0.006611346	0.006039493	Proteome
TRINITY_DN18918_c0_g1_i2_orf1	-0.011712665	-0.025451081	Proteome
TRINITY_DN4538_c0_g1_i4_orf1	0.003080999	-0.011510076	Proteome

TRINITY_DN10940_c0_g1_i10_orfp1	-0.000488147	0.003889686	Proteome
TRINITY_DN9740_c0_g1_i4_orf1	0.011086043	0.024492499	Proteome
TRINITY_DN40_c0_g1_i3_orf1	-0.002156416	-0.008213013	Proteome
TRINITY_DN4602_c0_g1_i4_orf1	0.009255745	-0.007323938	Proteome
TRINITY_DN558_c0_g1_i4_orf1	0.011307742	-0.013222063	Proteome
TRINITY_DN15157_c0_g1_i1_orf1	0.022507263	-0.007635101	Proteome
TRINITY_DN2778_c0_g1_i5_orf1	0.003803429	-0.006092005	Proteome
TRINITY_DN5880_c0_g2_i2_orf1	0.03160165	0.004394512	Proteome
TRINITY_DN321_c0_g1_i1_orf1	0.011610697	-0.00325166	Proteome
TRINITY_DN37538_c0_g2_i1_orf1	-0.001889356	-0.009683813	Proteome
TRINITY_DN27556_c0_g1_i1_orf1	-0.004443697	-0.001086889	Proteome
TRINITY_DN69334_c0_g1_i1_orf1	0.001422409	-0.013086657	Proteome
TRINITY_DN28366_c0_g1_i1_orf1	0.010038864	0.008825717	Proteome
TRINITY_DN17137_c0_g1_i2_orf1	0.012574514	0.009834135	Proteome
TRINITY_DN2780_c0_g1_i5_orf1	-0.003872468	-0.000157663	Proteome
TRINITY_DN4121_c0_g1_i1_orf1	0.003177558	-0.003176594	Proteome
TRINITY_DN755_c0_g1_i3_orf1	0.001065279	-0.006937393	Proteome
TRINITY_DN37532_c0_g1_i1_orf1	0.008218844	-0.001375056	Proteome
TRINITY_DN1337_c0_g2_i1_orf1	0.010578661	-0.004917967	Proteome
TRINITY_DN10095_c0_g1_i5_orf1	0.013342185	0.004383425	Proteome
TRINITY_DN51480_c0_g1_i1_orf1	0.002505754	-0.020516272	Proteome
TRINITY_DN33248_c0_g1_i1_orf1	0.008947128	-0.000187415	Proteome
TRINITY_DN28741_c0_g1_i3_orf1	-0.007311408	-0.006852289	Proteome
TRINITY_DN17409_c0_g1_i5_orf1	0.018060439	0.003440204	Proteome
TRINITY_DN905_c0_g1_i4_orf1	-0.003829294	0.002162939	Proteome
TRINITY_DN12579_c0_g1_i1_orf1	0.009481789	-0.005742052	Proteome
TRINITY_DN28311_c0_g1_i2_orf1	-0.003962845	0.015920908	Proteome
TRINITY_DN3627_c0_g1_i7_orf1	0.003844549	-0.002118342	Proteome
TRINITY_DN57454_c0_g1_i4_orf1	-0.005349025	0.008273833	Proteome
TRINITY_DN3370_c0_g1_i5_orf1	0.000734766	0.005033329	Proteome
TRINITY_DN10455_c0_g1_i2_orf1	0.001527156	-0.010493432	Proteome
TRINITY_DN21609_c0_g1_i1_orf1	0.008326027	0.000127563	Proteome
TRINITY_DN103_c0_g1_i1_orf1	0.011723169	-0.005001954	Proteome
TRINITY_DN518_c0_g1_i1_orf1	0.004295293	-0.009585158	Proteome
TRINITY_DN13350_c0_g1_i4_orf1	-0.007701132	-0.003126861	Proteome
TRINITY_DN4242_c0_g1_i6_orf1	-0.01012468	0.007829462	Proteome
TRINITY_DN3298_c0_g2_i4_orf1	0.021090249	0.007318537	Proteome
TRINITY_DN11215_c0_g1_i1_orf1	-0.003272155	-0.005172401	Proteome
TRINITY_DN4420_c0_g1_i1_orf1	0.006381256	0.009579085	Proteome
TRINITY_DN12134_c0_g1_i4_orf1	0.015096254	0.002361461	Proteome
TRINITY_DN9489_c0_g1_i5_orf1	-0.006408648	-0.008492687	Proteome
TRINITY_DN3929_c0_g3_i3_orf1	0.015698152	0.000566674	Proteome
TRINITY_DN70236_c0_g1_i1_orf1	0.017281884	0.002982996	Proteome
TRINITY_DN9542_c0_g1_i4_orf1	0.007098653	-0.008901099	Proteome
TRINITY_DN472_c1_g1_i3_orf1	0.009038669	-0.005682925	Proteome
TRINITY_DN5595_c0_g1_i1_orf1	0.012494688	-0.008700774	Proteome
TRINITY_DN29717_c0_g1_i2_orf1	0.011619098	-0.002873387	Proteome
TRINITY_DN70_c6_g1_i1_orf1	-0.019788295	0.004822128	Proteome
TRINITY_DN14874_c0_g1_i6_orf1	-0.01891788	-0.011046456	Proteome
TRINITY_DN49047_c0_g1_i2_orf1	0.011563639	-0.008795192	Proteome
TRINITY_DN55154_c0_g2_i1_orf1	-0.017921574	0.005558504	Proteome
TRINITY_DN44777_c0_g1_i2_orf1	-0.000285548	0.009569382	Proteome
TRINITY_DN2967_c0_g1_i7_orf1	0.003244501	-0.007245137	Proteome
TRINITY_DN6275_c0_g1_i3_orf1	0.010872349	-0.013224225	Proteome
TRINITY_DN81926_c0_g1_i1_orf1	0.012220198	-0.005748952	Proteome
TRINITY_DN15591_c0_g1_i3_orf1	0.009563562	0.004772102	Proteome
TRINITY_DN1144_c0_g1_i10_orf1	-0.021255474	0.022321926	Proteome
TRINITY_DN2012_c0_g1_i3_orf1	0.008733607	0.009340777	Proteome

TRINITY_DN7169_c0_g2_i6_orf1	0.002489292	-0.006857948	Proteome
TRINITY_DN5176_c0_g1_i2_orf1	0.010355229	-0.005707489	Proteome
TRINITY_DN12777_c0_g1_i5_orf1	0.011377282	0.012064188	Proteome
TRINITY_DN10796_c0_g2_i1_orf1	0.004000823	0.007966426	Proteome
TRINITY_DN64126_c0_g1_i1_orf1	0.010470943	-0.011804548	Proteome
TRINITY_DN107288_c0_g1_i2_orf1	-0.015332886	0.014528819	Proteome
TRINITY_DN16911_c0_g1_i3_orf1	0.001489409	-0.009235041	Proteome
TRINITY_DN8473_c0_g1_i6_orf1	-0.002327281	-0.033318719	Proteome
TRINITY_DN4952_c0_g1_i1_orf1	0.007023384	0.012096472	Proteome
TRINITY_DN9406_c0_g1_i5_orf1	-6.59E-06	-0.028407382	Proteome
TRINITY_DN38412_c0_g1_i1_orf1	-0.013273562	0.006457576	Proteome
TRINITY_DN41086_c0_g1_i4_orf1	0.005279017	-0.002404198	Proteome
TRINITY_DN46202_c0_g1_i1_orf1	0.005509627	-0.003937431	Proteome
TRINITY_DN143792_c0_g1_i1_orf1	-0.011372834	-0.014827157	Proteome
TRINITY_DN47784_c0_g2_i1_orfp1	0.040686787	0.019955596	Proteome
TRINITY_DN2975_c0_g1_i4_orf1	0.009041527	0.006571082	Proteome
TRINITY_DN6262_c0_g2_i1_orf1	0.008533313	-0.00997086	Proteome
TRINITY_DN3747_c1_g2_i1_orf1	0.002594744	-0.005933106	Proteome
TRINITY_DN37165_c0_g1_i4_orf1	0.012671181	-0.001571681	Proteome
TRINITY_DN9759_c0_g1_i1_orf1	0.011932737	0.004636148	Proteome
TRINITY_DN4619_c0_g1_i1_orf1	-0.001371821	-0.006016306	Proteome
TRINITY_DN107261_c0_g1_i1_orf1	-0.008528387	-0.003360631	Proteome
TRINITY_DN5568_c0_g2_i2_orf1	0.008749017	-0.013211027	Proteome
TRINITY_DN2825_c0_g1_i3_orf1	-0.003718442	0.002505816	Proteome
TRINITY_DN12495_c0_g1_i2_orf1	0.004227694	-0.004635801	Proteome
TRINITY_DN12823_c0_g1_i1_orf1	0.000228773	0.008786261	Proteome
TRINITY_DN4449_c0_g2_i1_orf1	0.0043721	0.008130564	Proteome
TRINITY_DN8714_c0_g1_i6_orf1	0.003632429	-0.007994694	Proteome
TRINITY_DN6532_c2_g1_i1_orf1	-0.008171465	-0.010487545	Proteome
TRINITY_DN10785_c0_g1_i4_orf1	0.006933644	-0.009676824	Proteome
TRINITY_DN38341_c0_g2_i2_orf1	-0.005122346	-0.01149381	Proteome
TRINITY_DN41311_c0_g2_i3_orf1	0.006755582	0.003012536	Proteome
TRINITY_DN6967_c0_g1_i3_orf1	-0.009926677	0.001286999	Proteome
TRINITY_DN43611_c0_g1_i1_orf1	0.004780849	0.001403237	Proteome
TRINITY_DN4752_c0_g1_i3_orf1	0.006558641	-0.00337877	Proteome
TRINITY_DN6310_c0_g2_i10_orf1	-0.013290053	-0.011649118	Proteome
TRINITY_DN7267_c1_g1_i4_orf1	0.010116958	0.000240717	Proteome
TRINITY_DN11657_c0_g1_i2_orf1	-0.008055423	-0.003423426	Proteome
TRINITY_DN34726_c0_g2_i1_orf1	0.009375528	0.004022143	Proteome
TRINITY_DN36476_c1_g1_i1_orfp1	0.049919031	0.032133354	Proteome
TRINITY_DN892_c0_g1_i9_orf1	0.012217815	0.003994424	Proteome
TRINITY_DN7920_c0_g1_i2_orf1	0.003160414	-0.007920302	Proteome
TRINITY_DN2109_c0_g1_i4_orf1	-0.024416861	0.011341446	Proteome
TRINITY_DN8012_c0_g1_i3_orf1	0.002584566	-0.020898992	Proteome
TRINITY_DN3063_c0_g1_i5_orf1	0.007701567	0.005980007	Proteome
TRINITY_DN1738_c0_g1_i5_orf1	0.000443142	-0.011410291	Proteome
TRINITY_DN6074_c0_g1_i1_orf1	-0.006206553	-0.002114004	Proteome
TRINITY_DN7037_c0_g1_i4_orf1	-0.000598483	0.015756516	Proteome
TRINITY_DN1354_c0_g1_i6_orf1	0.007048197	-0.008399716	Proteome
TRINITY_DN20499_c0_g3_i1_orf1	0.004728026	-0.00335915	Proteome
TRINITY_DN1597_c0_g1_i5_orfp1	0.021846941	0.043878057	Proteome
TRINITY_DN1616_c0_g1_i3_orf1	-0.00644422	-9.28E-05	Proteome
TRINITY_DN2615_c0_g1_i1_orf1	0.005196325	0.013202716	Proteome
TRINITY_DN3456_c0_g2_i1_orf1	-0.016698802	0.005407435	Proteome
TRINITY_DN1027_c0_g1_i8_orf1	0.003678071	-0.009211034	Proteome
TRINITY_DN3753_c0_g1_i7_orf1	-0.008158846	-0.005225719	Proteome
TRINITY_DN4217_c0_g1_i2_orf1	0.004840816	-0.01123163	Proteome
TRINITY_DN5444_c0_g2_i1_orf1	0.012932308	0.008310454	Proteome

TRINITY_DN16084_c0_g1_i4_orf1	-0.012277344	0.005639244	Proteome
TRINITY_DN24164_c0_g1_i1_orf1	0.008632976	-0.016260205	Proteome
TRINITY_DN27984_c0_g2_i1_orf1	-0.004495284	-0.019467904	Proteome
TRINITY_DN49143_c0_g1_i1_orf1	-0.001954883	-0.014527679	Proteome
TRINITY_DN7434_c0_g1_i1_orf1	-0.008485611	-0.019032794	Proteome
TRINITY_DN22674_c0_g1_i2_orf1	-0.013756846	-0.024552465	Proteome
TRINITY_DN971_c0_g1_i10_orfp1	0.091238748	0.038216394	Proteome
TRINITY_DN7106_c0_g1_i5_orf1	-0.009792701	-0.02960125	Proteome
TRINITY_DN40508_c0_g1_i1_orf1	-0.002025622	-0.020013225	Proteome
TRINITY_DN11680_c0_g1_i1_orf1	0.016166001	-0.016646236	Proteome
TRINITY_DN36928_c0_g1_i2_orf1	0.009861424	0.006668892	Proteome
TRINITY_DN817_c0_g2_i4_orf1	-0.002123094	-0.015273926	Proteome
TRINITY_DN25896_c0_g1_i6_orf1	-0.006345392	-0.011147329	Proteome
TRINITY_DN3667_c0_g1_i4_orf1	-0.006781342	0.009384019	Proteome
TRINITY_DN48020_c0_g1_i1_orf1	0.008951632	-0.004910331	Proteome
TRINITY_DN2701_c1_g1_i6_orf1	-0.003440796	0.009623309	Proteome
TRINITY_DN2497_c0_g1_i1_orf1	0.006811741	0.000891953	Proteome
TRINITY_DN133760_c0_g1_i1_orf1	-0.001904768	-0.001178463	Proteome
TRINITY_DN63568_c0_g1_i1_orf1	-0.000334355	-0.001855036	Proteome
TRINITY_DN6933_c0_g1_i2_orf1	-0.002324542	0.005375443	Proteome
TRINITY_DN29156_c0_g1_i1_orf1	0.004104424	0.001545178	Proteome
TRINITY_DN1641_c0_g1_i8_orf1	-0.006578031	0.007807687	Proteome
TRINITY_DN16011_c0_g1_i3_orf1	0.007609592	-0.005018997	Proteome
TRINITY_DN34689_c0_g1_i4_orf1	0.002182049	0.008222088	Proteome
TRINITY_DN100_c0_g1_i13_orf1	0.00194392	-0.004337479	Proteome
TRINITY_DN143496_c0_g1_i1_orf1	0.016692691	-0.002759147	Proteome
TRINITY_DN11825_c0_g1_i4_orf1	0.006725225	-0.001518102	Proteome
TRINITY_DN1749_c0_g2_i2_orf1	0.014997015	0.007388374	Proteome
TRINITY_DN51441_c0_g1_i5_orf1	0.005622541	-0.006655533	Proteome
TRINITY_DN94337_c0_g1_i1_orf1	0.002292918	-0.006058463	Proteome
TRINITY_DN1249_c0_g1_i6_orf1	0.002333805	-0.011997578	Proteome
TRINITY_DN1749_c0_g1_i1_orf1	0.013106944	0.009714269	Proteome
TRINITY_DN10701_c0_g2_i2_orf1	0.001604632	0.015421445	Proteome
TRINITY_DN23502_c0_g1_i1_orf1	0.009998407	0.001555649	Proteome
TRINITY_DN34426_c0_g1_i1_orf1	-0.016393475	0.001733528	Proteome
TRINITY_DN2076_c0_g2_i1_orf1	-0.012544868	-0.01060048	Proteome
TRINITY_DN21278_c0_g2_i2_orf1	0.014515367	0.000789567	Proteome
TRINITY_DN52553_c0_g2_i1_orf1	0.014181917	-0.011452788	Proteome
TRINITY_DN12820_c0_g1_i1_orf1	0.010626987	0.00148359	Proteome
TRINITY_DN2830_c0_g1_i9_orf1	0.00089836	-0.008504437	Proteome
TRINITY_DN5841_c0_g1_i2_orf1	0.012120075	-0.002691377	Proteome
TRINITY_DN106_c0_g1_i3_orf1	0.004024943	0.004532761	Proteome
TRINITY_DN37654_c0_g1_i5_orf1	0.000936564	-0.035680873	Proteome
TRINITY_DN6933_c1_g1_i1_orf1	-0.004822706	0.004692889	Proteome
TRINITY_DN5933_c0_g1_i1_orf1	0.006333926	-0.013331363	Proteome
TRINITY_DN9575_c0_g1_i1_orf1	0.012416293	-0.012068424	Proteome
TRINITY_DN18091_c0_g1_i5_orf1	0.002069472	-0.002474019	Proteome
TRINITY_DN19261_c0_g1_i3_orf1	0.01014484	-0.017677412	Proteome
TRINITY_DN1763_c0_g3_i2_orf1	0.017005084	-0.008322241	Proteome
TRINITY_DN10694_c1_g2_i1_orf1	-0.005160664	-0.014943236	Proteome
TRINITY_DN59042_c1_g1_i1_orf1	0.009771745	-0.000873652	Proteome
TRINITY_DN12576_c0_g1_i2_orf1	0.004694197	0.004208086	Proteome
TRINITY_DN19537_c0_g1_i1_orf1	0.013939846	-0.001249366	Proteome
TRINITY_DN24323_c0_g1_i3_orf1	0.0092265	-0.00399341	Proteome
TRINITY_DN4255_c0_g1_i10_orf1	0.020181814	0.00610914	Proteome
TRINITY_DN25273_c0_g1_i1_orf1	-0.002016303	0.009468085	Proteome
TRINITY_DN1865_c1_g1_i3_orf1	0.003914002	-0.018067647	Proteome
TRINITY_DN5648_c0_g1_i5_orf1	0.002475734	0.012395843	Proteome

TRINITY_DN18922_c0_g1_i1_orf1	0.006959085	0.001075244	Proteome
TRINITY_DN33953_c0_g1_i4_orf1	0.011362134	-0.008587417	Proteome
TRINITY_DN1074_c0_g1_i7_orf1	0.010270118	-0.001140066	Proteome
TRINITY_DN9062_c0_g2_i3_orf1	0.004033706	0.011470373	Proteome
TRINITY_DN17003_c1_g1_i1_orf1	0.006491963	0.00839067	Proteome
TRINITY_DN44633_c0_g1_i4_orf1	0.014573389	0.00727822	Proteome
TRINITY_DN3056_c0_g1_i1_orf1	0.012772975	0.006393043	Proteome
TRINITY_DN6470_c0_g3_i2_orf1	0.002448218	0.001559983	Proteome
TRINITY_DN41546_c0_g1_i15_orf1	0.006642739	-0.005376163	Proteome
TRINITY_DN38230_c0_g1_i4_orf1	0.017205835	-0.01115653	Proteome
TRINITY_DN6685_c0_g1_i8_orf1	0.004545111	5.06E-05	Proteome
TRINITY_DN104596_c0_g1_i1_orf1	-0.013581404	-0.015780723	Proteome
TRINITY_DN1116_c0_g1_i6_orf1	0.031338759	-0.001324322	Proteome
TRINITY_DN125071_c0_g1_i1_orf1	0.000777159	0.017870081	Proteome
TRINITY_DN19186_c0_g1_i1_orf1	0.006315727	-0.007014523	Proteome
TRINITY_DN12594_c0_g1_i1_orf1	-0.013288146	0.016638717	Proteome
TRINITY_DN987_c0_g1_i11_orf1	-0.018710102	0.005346305	Proteome
TRINITY_DN30037_c0_g1_i5_orf1	0.015233587	-0.00897768	Proteome
TRINITY_DN19521_c0_g1_i1_orf1	0.00938728	-0.009912135	Proteome
TRINITY_DN135449_c0_g1_i5_orf1	-0.01698545	-0.016676038	Proteome
TRINITY_DN4859_c0_g1_i5_orf1	0.011602505	0.00112936	Proteome
TRINITY_DN21619_c0_g1_i1_orf1	0.013788685	0.002137423	Proteome
TRINITY_DN52887_c0_g1_i1_orf1	-0.019823539	-0.012798185	Proteome
TRINITY_DN4367_c0_g1_i1_orf1	-0.003875926	-0.005370957	Proteome
TRINITY_DN30070_c0_g1_i6_orf1	-0.002232744	-0.009540251	Proteome
TRINITY_DN9324_c1_g2_i2_orf1	-0.010086195	0.005628158	Proteome
TRINITY_DN50237_c0_g1_i8_orf1	-0.022569513	-0.014267607	Proteome
TRINITY_DN5140_c0_g1_i1_orf1	0.005331445	-0.003171744	Proteome
TRINITY_DN2618_c0_g1_i3_orf1	0.012916817	-0.003880173	Proteome
TRINITY_DN55160_c0_g1_i1_orf1	0.002329281	-0.008944393	Proteome
TRINITY_DN19690_c0_g1_i1_orf1	0.010877815	-0.015246532	Proteome
TRINITY_DN29563_c0_g1_i5_orf1	-0.005020184	0.006198256	Proteome
TRINITY_DN95850_c0_g1_i1_orf1	0.026153505	0.002299801	Proteome
TRINITY_DN607_c0_g1_i16_orf1	0.006922431	0.009058285	Proteome
TRINITY_DN2887_c0_g1_i1_orf1	0.015530836	0.002627032	Proteome
TRINITY_DN4810_c0_g1_i3_orf1	0.006129234	0.003367382	Proteome
TRINITY_DN52649_c0_g1_i6_orf1	0.003318487	0.019644064	Proteome
TRINITY_DN39_c0_g1_i1_orf1	0.003202391	-0.012270812	Proteome
TRINITY_DN52859_c0_g1_i4_orf1	-0.009479918	-0.036950011	Proteome
TRINITY_DN1405_c0_g1_i1_orf1	0.004066737	0.001631067	Proteome
TRINITY_DN3838_c0_g1_i8_orf1	0.00801767	-0.001424036	Proteome
TRINITY_DN23824_c0_g1_i1_orf1	-0.004000823	0.007217676	Proteome
TRINITY_DN82017_c0_g1_i5_orf1	-0.007682699	-0.012248091	Proteome
TRINITY_DN2852_c0_g1_i9_orf1	0.00600012	-0.000403573	Proteome
TRINITY_DN821_c0_g1_i8_orf1	-0.002727311	-0.015000645	Proteome
TRINITY_DN5487_c0_g1_i2_orf1	-0.010869276	-0.004984992	Proteome
TRINITY_DN17130_c0_g2_i1_orf1	0.007348549	-0.005192268	Proteome
TRINITY_DN1215_c0_g1_i2_orf1	0.007103	0.014917557	Proteome
TRINITY_DN3456_c0_g1_i1_orf1	-0.006858311	-0.00653175	Proteome
TRINITY_DN4708_c0_g1_i5_orf1	-0.007776082	-0.030497495	Proteome
TRINITY_DN135679_c0_g1_i1_orfp1	0.024105635	-0.00417212	Proteome
TRINITY_DN2718_c0_g1_i6_orf1	0.005739214	0.000772854	Proteome
TRINITY_DN104244_c0_g1_i1_orf1	-0.003101462	0.009337073	Proteome
TRINITY_DN8676_c0_g1_i1_orf1	0.001106091	0.010569104	Proteome
TRINITY_DN45633_c0_g1_i1_orf1	0.008489473	-0.009619969	Proteome
TRINITY_DN2579_c0_g1_i7_orf1	0.000662627	0.00613395	Proteome
TRINITY_DN11376_c0_g2_i1_orf1	-0.001536078	-0.007554279	Proteome
TRINITY_DN21341_c0_g1_i4_orf1	0.002901151	-0.001535008	Proteome

TRINITY_DN2709_c0_g1_i4_orf1	-0.00633744	0.003860598	Proteome
TRINITY_DN338_c2_g1_i2_orf1	-0.006767676	-0.00027105	Proteome
TRINITY_DN17189_c0_g1_i2_orf1	-0.000669967	0.008373002	Proteome
TRINITY_DN2232_c1_g1_i3_orf1	0.00173819	0.005105096	Proteome
TRINITY_DN1826_c0_g2_i4_orf1	-0.002532154	-0.01624262	Proteome
TRINITY_DN4002_c0_g1_i1_orf1	0.004059484	-0.006422397	Proteome
TRINITY_DN6203_c0_g1_i1_orfp1	-0.032400146	0.001995142	Proteome
TRINITY_DN3953_c0_g1_i2_orf1	-0.006478245	-0.001043116	Proteome
TRINITY_DN67649_c0_g1_i1_orf1	-0.002171206	-0.006431362	Proteome
TRINITY_DN1005_c0_g2_i1_orf1	0.005255858	-0.008746358	Proteome
TRINITY_DN277_c1_g1_i1_orf1	0.016520384	0.005778583	Proteome
TRINITY_DN45293_c0_g1_i1_orf1	0.009264916	-0.01298107	Proteome
TRINITY_DN42964_c0_g1_i1_orf1	0.032065521	-0.000619161	Proteome
TRINITY_DN2840_c0_g1_i5_orf1	0.004431523	0.007992973	Proteome
TRINITY_DN5680_c0_g1_i3_orf1	-0.006371875	0.013599636	Proteome
TRINITY_DN5507_c0_g1_i1_orf1	0.003249431	-0.024529419	Proteome
TRINITY_DN101325_c0_g1_i4_orf1	-0.01077416	0.003371455	Proteome
TRINITY_DN1612_c0_g1_i3_orf1	-0.005726148	0.003558857	Proteome
TRINITY_DN57765_c0_g1_i1_orf1	0.004362142	-0.003715078	Proteome
TRINITY_DN12873_c0_g1_i3_orf1	-0.007546813	-0.029331671	Proteome
TRINITY_DN31390_c0_g1_i2_orf1	-0.017306606	-0.016142324	Proteome
TRINITY_DN4262_c0_g1_i16_orf1	0.011198833	-0.002521662	Proteome
TRINITY_DN67623_c0_g1_i1_orf1	0.003368442	0.004309955	Proteome
TRINITY_DN27721_c1_g1_i2_orf1	0.00530101	0.010603303	Proteome
TRINITY_DN16972_c0_g1_i1_orf1	-0.00144182	-0.028104333	Proteome
TRINITY_DN84478_c0_g1_i8_orf1	-0.105788059	0.056609936	Proteome
TRINITY_DN13236_c0_g1_i4_orf1	0.008380581	0.03005591	Proteome
TRINITY_DN547_c0_g1_i1_orf1	0.003315723	0.004728585	Proteome
TRINITY_DN24317_c0_g1_i7_orf1	0.00849738	0.000759566	Proteome
TRINITY_DN5472_c0_g1_i1_orf1	0.005732993	0.001870569	Proteome
TRINITY_DN52893_c0_g1_i1_orf1	0.005722271	0.004692553	Proteome
TRINITY_DN20682_c0_g1_i2_orf1	0.007499217	-0.002920434	Proteome
TRINITY_DN43391_c0_g1_i5_orf1	-0.001852702	-0.006358218	Proteome
TRINITY_DN22983_c0_g1_i2_orfp1	0.004050073	0.003623945	Proteome
TRINITY_DN28501_c0_g1_i2_orfp1	0.018702242	0.016556793	Proteome
TRINITY_DN4026_c0_g1_i4_orf1	-0.000230169	0.009876102	Proteome
TRINITY_DN4425_c0_g1_i4_orf1	0.009878668	0.005628269	Proteome
TRINITY_DN825_c23_g1_i5_orf1	0.014263875	-0.004224259	Proteome
TRINITY_DN886_c0_g2_i4_orf1	0.005832072	0.006582864	Proteome
TRINITY_DN1213_c0_g1_i5_orf1	-0.000953545	-0.023308869	Proteome
TRINITY_DN2352_c0_g1_i15_orf1	0.003023165	-0.006457628	Proteome
TRINITY_DN57998_c1_g3_i1_orf1	-0.005052179	-0.008200911	Proteome
TRINITY_DN6556_c0_g1_i7_orf1	0.014378277	-0.001690362	Proteome
TRINITY_DN48023_c1_g1_i1_orf1	0.006077724	-0.010156991	Proteome
TRINITY_DN85161_c0_g1_i2_orf1	-0.006481491	0.010704513	Proteome
TRINITY_DN44119_c0_g1_i1_orf1	-0.001748682	-0.015606659	Proteome
TRINITY_DN33883_c0_g1_i1_orf1	-0.003832474	-0.008712026	Proteome
TRINITY_DN2338_c3_g2_i3_orf1	0.004975964	-0.008009853	Proteome
TRINITY_DN10304_c0_g2_i1_orf1	-0.010374416	-0.016771274	Proteome
TRINITY_DN2973_c1_g1_i9_orf1	-0.002227464	-0.012792539	Proteome
TRINITY_DN10403_c0_g1_i3_orf1	-0.01282192	0.013185417	Proteome
TRINITY_DN8985_c0_g1_i4_orf1	-0.015128715	0.010894009	Proteome
TRINITY_DN4959_c0_g1_i1_orf1	0.001775545	-0.007396861	Proteome
TRINITY_DN13395_c0_g1_i1_orf1	-0.002534063	0.012651196	Proteome
TRINITY_DN13018_c0_g1_i2_orf1	-0.007933004	-0.016616936	Proteome
TRINITY_DN13411_c0_g1_i4_orf1	0.012133979	0.007602793	Proteome
TRINITY_DN6391_c0_g1_i1_orf1	0.003671806	-0.000268615	Proteome
TRINITY_DN10636_c0_g1_i1_orf1	0.000734449	-0.016649344	Proteome

TRINITY_DN1924_c0_g1_i3_orf1	-0.003519479	-0.017423673	Proteome
TRINITY_DN21341_c0_g1_i1_orf1	-0.034646419	-0.004814274	Proteome
TRINITY_DN3733_c0_g1_i1_orf1	0.014545318	0.007291957	Proteome
TRINITY_DN59335_c0_g1_i2_orf1	0.000652251	-0.000743228	Proteome
TRINITY_DN48619_c0_g1_i1_orf1	0.010909128	-0.002120699	Proteome
TRINITY_DN23774_c0_g1_i1_orf1	0.006682997	-0.00946008	Proteome
TRINITY_DN5235_c0_g1_i7_orf1	0.007807248	-0.00688868	Proteome
TRINITY_DN129863_c0_g1_i1_orf1	-0.013510148	0.044765699	Proteome
TRINITY_DN76216_c0_g2_i3_orf1	-0.016457092	-0.043544021	Proteome
TRINITY_DN2224_c0_g2_i1_orf1	0.012843599	0.000946794	Proteome
TRINITY_DN3127_c0_g1_i9_orf1	-0.006344849	0.000643063	Proteome
TRINITY_DN11612_c0_g2_i1_orf1	0.007511491	0.01391343	Proteome
TRINITY_DN3135_c0_g1_i6_orf1	0.007324133	0.012625855	Proteome
TRINITY_DN9072_c0_g1_i1_orf1	-0.008527007	-0.018484911	Proteome
TRINITY_DN942_c0_g1_i1_orf1	0.006694385	-0.00428687	Proteome
TRINITY_DN95414_c0_g1_i1_orf1	0.011774063	0.002260135	Proteome
TRINITY_DN7603_c0_g1_i5_orf1	0.004231982	-0.005602776	Proteome
TRINITY_DN291_c0_g1_i2_orf1	-0.006971878	0.01379274	Proteome
TRINITY_DN51429_c1_g1_i1_orf1	-0.015172696	-0.009821197	Proteome
TRINITY_DN2563_c0_g1_i4_orf1	-0.006269615	-0.013423997	Proteome
TRINITY_DN5004_c0_g1_i2_orf1	0.002287938	0.012768502	Proteome
TRINITY_DN17947_c0_g1_i4_orf1	0.018983331	-0.00747072	Proteome
TRINITY_DN3027_c0_g1_i4_orf1	0.00019539	0.001987964	Proteome
TRINITY_DN2642_c0_g1_i5_orf1	0.005233206	0.013686804	Proteome
TRINITY_DN23164_c0_g1_i4_orf1	0.004296912	-0.022347923	Proteome
TRINITY_DN6668_c0_g1_i4_orf1	-0.010211671	0.012594402	Proteome
TRINITY_DN3699_c1_g1_i1_orf1	-0.002138875	-0.017711686	Proteome
TRINITY_DN26853_c0_g1_i1_orf1	0.002575956	-0.009029425	Proteome
TRINITY_DN143532_c0_g1_i1_orf1	-0.046155437	-0.005544837	Proteome
TRINITY_DN6747_c0_g1_i7_orf1	0.006163749	0.003434148	Proteome
TRINITY_DN57462_c0_g1_i1_orf1	0.000997486	-0.023646786	Proteome
TRINITY_DN38366_c0_g1_i4_orfp1	0.01892145	0.008873823	Proteome
TRINITY_DN5022_c0_g1_i4_orf1	0.019691845	0.008619861	Proteome
TRINITY_DN6098_c1_g1_i5_orf1	-0.002532764	0.036967414	Proteome
TRINITY_DN2708_c0_g1_i6_orf1	-0.001959914	-0.011915838	Proteome
TRINITY_DN7040_c0_g1_i4_orf1	-0.00449409	0.034732631	Proteome
TRINITY_DN92153_c0_g2_i2_orf1	0.006024881	-0.009021602	Proteome
TRINITY_DN2170_c0_g1_i2_orf1	-0.014644623	0.002049237	Proteome
TRINITY_DN51776_c0_g1_i1_orf1	0.012834355	0.011998357	Proteome
TRINITY_DN37856_c0_g1_i5_orf1	0.00639029	-0.001666845	Proteome
TRINITY_DN17045_c0_g2_i3_orf1	0.006008626	-0.008995034	Proteome
TRINITY_DN29190_c0_g1_i4_orf1	0.050152819	-0.004186812	Proteome
TRINITY_DN38211_c0_g1_i1_orf1	0.007247092	-0.008055557	Proteome
TRINITY_DN34727_c0_g1_i3_orf1	0.010183602	0.001771119	Proteome
TRINITY_DN29369_c0_g1_i1_orf1	0.000783759	0.011262709	Proteome
TRINITY_DN3174_c0_g1_i2_orf1	0.004801914	0.013527911	Proteome
TRINITY_DN1079_c0_g1_i4_orf1	0.005632757	0.007689221	Proteome
TRINITY_DN1860_c0_g1_i2_orf1	0.001890528	-0.019172769	Proteome
TRINITY_DN13511_c0_g1_i4_orf1	-0.014047309	0.044098055	Proteome
TRINITY_DN9872_c0_g1_i2_orf1	-0.004352398	-0.002763646	Proteome
TRINITY_DN1110_c1_g1_i9_orf1	-0.023519934	0.00762274	Proteome
TRINITY_DN22604_c0_g1_i3_orf1	0.00870187	0.009887396	Proteome
TRINITY_DN325_c0_g1_i15_orf1	0.014092776	0.009130247	Proteome
TRINITY_DN4720_c0_g2_i1_orf1	-0.00397437	0.005037684	Proteome
TRINITY_DN53311_c0_g2_i1_orf1	0.008768774	0.00124253	Proteome
TRINITY_DN17271_c0_g1_i1_orf1	0.00700372	-0.001101836	Proteome
TRINITY_DN5954_c0_g1_i2_orf1	-0.003190506	0.002443251	Proteome
TRINITY_DN45477_c0_g1_i1_orf1	0.008188336	-0.000990205	Proteome

TRINITY_DN7277_c0_g1_i1_orf1	-0.005391374	0.013915369	Proteome
TRINITY_DN1833_c0_g1_i5_orf1	-0.024576184	-0.011795513	Proteome
TRINITY_DN4742_c0_g1_i1_orf1	0.01277986	-0.001941925	Proteome
TRINITY_DN756_c0_g1_i11_orf1	0.006559636	-0.002018589	Proteome
TRINITY_DN3809_c0_g1_i7_orf1	0.007303706	-0.017499948	Proteome
TRINITY_DN3244_c0_g1_i4_orf1	0.001010924	0.005953014	Proteome
TRINITY_DN46452_c0_g1_i1_orf1	-0.006299787	0.016645212	Proteome
TRINITY_DN14904_c0_g1_i1_orf1	0.091855465	-0.000654402	Proteome
TRINITY_DN10650_c0_g1_i1_orf1	0.012314189	0.010472002	Proteome
TRINITY_DN3653_c0_g1_i4_orf1	-0.003847713	-0.015718077	Proteome
TRINITY_DN25373_c0_g1_i1_orf1	0.003203899	0.004626942	Proteome
TRINITY_DN1425_c0_g1_i4_orf1	0.0129393	-0.001003153	Proteome
TRINITY_DN36928_c0_g1_i5_orf1	-0.002456344	0.000665704	Proteome
TRINITY_DN76283_c0_g6_i1_orf1	0.003161118	-0.001414446	Proteome
TRINITY_DN2497_c0_g1_i2_orf1	-0.010282896	0.010644851	Proteome
TRINITY_DN783_c0_g1_i7_orf1	0.006377684	0.001159712	Proteome
TRINITY_DN71610_c0_g1_i1_orf1	0.012079313	-0.000336759	Proteome
TRINITY_DN14843_c0_g1_i1_orf1	0.003465583	0.017621173	Proteome
TRINITY_DN590_c0_g1_i4_orf1	0.000938263	0.00875944	Proteome
TRINITY_DN19821_c0_g2_i4_orf1	-0.018910345	-0.043763837	Proteome
TRINITY_DN9661_c0_g1_i1_orf1	0.014938331	-0.014417325	Proteome
TRINITY_DN10766_c0_g1_i1_orf1	0.016040574	-0.002991076	Proteome
TRINITY_DN616_c1_g1_i6_orf1	-0.004891292	-0.013683531	Proteome
TRINITY_DN146957_c0_g1_i1_orf1	-0.007855678	0.013378446	Proteome
TRINITY_DN35983_c0_g1_i2_orf1	0.00142474	0.013609513	Proteome
TRINITY_DN2432_c0_g1_i1_orf1	0.004706194	-0.007210647	Proteome
TRINITY_DN8310_c0_g2_i1_orf1	-0.013051909	0.0022952	Proteome
TRINITY_DN21609_c0_g2_i1_orf1	0.010867299	0.005596945	Proteome
TRINITY_DN53136_c0_g1_i1_orf1	-0.005797935	-0.008584882	Proteome
TRINITY_DN37336_c1_g1_i1_orf1	-0.005332875	-0.01077248	Proteome
TRINITY_DN13856_c0_g1_i1_orf1	-0.01684074	-0.011495989	Proteome
TRINITY_DN920_c0_g1_i4_orf1	0.021800506	-0.004467017	Proteome
TRINITY_DN3283_c0_g2_i1_orf1	0.008477219	0.000748353	Proteome
TRINITY_DN4565_c0_g2_i1_orf1	-0.008021907	-0.015850767	Proteome
TRINITY_DN17312_c0_g1_i1_orf1	-0.000127407	-0.006128886	Proteome
TRINITY_DN14220_c0_g1_i1_orf1	-0.002309983	-0.015808947	Proteome
TRINITY_DN5001_c0_g1_i4_orf1	0.006366582	-0.020093834	Proteome
TRINITY_DN2596_c0_g1_i6_orf1	0.004874599	-0.025181652	Proteome
TRINITY_DN46369_c0_g1_i3_orf1	0.011166737	0.001877848	Proteome
TRINITY_DN22299_c0_g1_i1_orf1	-0.009496513	-0.037647767	Proteome
TRINITY_DN15448_c0_g1_i1_orf1	-0.00581806	0.009821998	Proteome
TRINITY_DN33452_c0_g1_i1_orf1	0.014015526	0.002735254	Proteome
TRINITY_DN99063_c0_g1_i1_orf1	0.006355478	0.007154226	Proteome
TRINITY_DN5031_c0_g1_i1_orf1	0.00991649	0.004223998	Proteome
TRINITY_DN3687_c0_g1_i1_orf1	0.014807707	0.000462457	Proteome
TRINITY_DN18794_c0_g1_i5_orf1	-0.003744072	0.008707486	Proteome
TRINITY_DN2769_c0_g1_i1_orf1	0.005123371	0.009145386	Proteome
TRINITY_DN10630_c0_g1_i2_orf1	0.021652858	0.006331304	Proteome
TRINITY_DN36632_c0_g1_i1_orf1	0.022378756	-0.002807268	Proteome
TRINITY_DN57348_c0_g1_i4_orf1	0.011930608	-0.00242699	Proteome
TRINITY_DN1469_c0_g1_i1_orf1	0.00599241	-0.012304378	Proteome
TRINITY_DN549_c0_g1_i7_orf1	0.005565429	0.022209956	Proteome
TRINITY_DN13887_c0_g1_i5_orf1	0.001016182	0.006389587	Proteome
TRINITY_DN4321_c0_g1_i1_orf1	0.022727638	0.002726775	Proteome
TRINITY_DN4156_c0_g1_i2_orf1	0.013761558	-0.007449813	Proteome
TRINITY_DN14285_c0_g1_i6_orf1	-0.005498428	0.005045074	Proteome
TRINITY_DN65299_c0_g4_i1_orf1	0.003817892	0.017427188	Proteome
TRINITY_DN22_c0_g1_i3_orf1	0.020794374	-0.009291971	Proteome

TRINITY_DN8536_c0_g1_i2_orf1	0.010403502	0.005324808	Proteome
TRINITY_DN76036_c0_g1_i1_orf1	-0.008670263	0.003066854	Proteome
TRINITY_DN51968_c0_g1_i1_orf1	0.005579519	0.004590985	Proteome
TRINITY_DN8394_c1_g1_i9_orf1	0.009036262	0.010547614	Proteome
TRINITY_DN4836_c0_g1_i4_orf1	0.001729678	-0.011967082	Proteome
TRINITY_DN51737_c0_g1_i3_orf1	0.002504807	0.010622226	Proteome
TRINITY_DN938_c0_g1_i7_orf1	0.00474059	-0.009140404	Proteome
TRINITY_DN57856_c0_g2_i1_orf1	0.026155206	-0.002815591	Proteome
TRINITY_DN22609_c0_g2_i3_orf1	-0.004411026	0.004689109	Proteome
TRINITY_DN23416_c1_g1_i2_orf1	0.01240011	0.00597306	Proteome
TRINITY_DN12826_c0_g1_i1_orf1	0.010260536	0.01082952	Proteome
TRINITY_DN82944_c0_g1_i4_orf1	0.001987009	-0.001334328	Proteome
TRINITY_DN6945_c0_g1_i5_orf1	0.007509026	-9.04E-05	Proteome
TRINITY_DN1368_c0_g1_i6_orf1	0.009419523	0.010443755	Proteome
TRINITY_DN22175_c0_g1_i1_orf1	0.012152586	0.00721683	Proteome
TRINITY_DN774_c0_g1_i9_orf1	-0.006166453	-0.002311998	Proteome
TRINITY_DN3545_c0_g1_i6_orf1	0.0040938	-0.019802862	Proteome
TRINITY_DN34056_c0_g1_i4_orf1	-0.014160745	-0.028561525	Proteome
TRINITY_DN29035_c0_g1_i5_orf1	-0.002886657	-0.011111675	Proteome
TRINITY_DN61_c0_g2_i3_orf1	0.007441187	-0.012604508	Proteome
TRINITY_DN28428_c0_g1_i2_orf1	0.010334554	0.00274426	Proteome
TRINITY_DN131471_c0_g1_i1_orf1	0.012278185	-0.012187365	Proteome
TRINITY_DN22515_c0_g1_i10_orf1	-0.004158066	0.04998168	Proteome
TRINITY_DN14937_c0_g1_i7_orf1	0.011412682	-0.002601958	Proteome
TRINITY_DN4908_c1_g1_i5_orf1	0.003600054	-0.005724773	Proteome
TRINITY_DN41280_c0_g1_i2_orf1	0.025420152	0.013700319	Proteome
TRINITY_DN10131_c0_g1_i7_orf1	0.005224462	0.01212412	Proteome
TRINITY_DN51995_c0_g3_i1_orf1	-0.124853546	0.01097942	Proteome
TRINITY_DN6771_c0_g2_i1_orf1	0.010919479	-0.022889234	Proteome
TRINITY_DN2796_c0_g1_i28_orf1	0.00868475	-0.003717677	Proteome
TRINITY_DN18909_c0_g1_i8_orf1	0.008372276	-0.003555917	Proteome
TRINITY_DN8694_c1_g1_i4_orf1	0.023730156	-0.005884071	Proteome
TRINITY_DN27300_c0_g1_i1_orfp1	-0.012269529	0.008828345	Proteome
TRINITY_DN70409_c0_g1_i3_orf1	0.007656384	0.011318151	Proteome
TRINITY_DN33867_c0_g1_i8_orf1	-0.016922073	0.011058858	Proteome
TRINITY_DN4385_c0_g2_i1_orf1	0.009431585	0.001756444	Proteome
TRINITY_DN6914_c0_g1_i2_orf1	-0.002204998	-0.021233062	Proteome
TRINITY_DN2566_c0_g1_i5_orf1	-0.02165078	0.019086986	Proteome
TRINITY_DN5191_c0_g2_i1_orf1	-0.000824039	-0.021019935	Proteome
TRINITY_DN3869_c0_g1_i4_orf1	-0.000118651	-0.026702049	Proteome
TRINITY_DN57904_c0_g2_i1_orf1	0.002773944	0.009202346	Proteome
TRINITY_DN14372_c0_g2_i1_orf1	0.003422809	0.004516043	Proteome
TRINITY_DN5686_c0_g1_i4_orf1	0.012641037	0.001577052	Proteome
TRINITY_DN3747_c1_g1_i3_orf1	0.011645216	0.005499923	Proteome
TRINITY_DN47123_c0_g1_i1_orf1	0.003431111	-0.001091954	Proteome
TRINITY_DN8887_c0_g1_i1_orf1	0.011764165	0.001699863	Proteome
TRINITY_DN2894_c0_g2_i3_orf1	0.009096292	0.002799369	Proteome
TRINITY_DN2192_c1_g1_i1_orf1	-0.007769465	-0.00934389	Proteome
TRINITY_DN36592_c0_g1_i1_orf1	-0.000200263	0.011411156	Proteome
TRINITY_DN73224_c0_g4_i2_orf1	0.006109486	-0.004594144	Proteome
TRINITY_DN42759_c0_g3_i1_orf1	-0.004793136	0.001612528	Proteome
TRINITY_DN4916_c0_g2_i1_orf1	0.007170248	0.003355052	Proteome
TRINITY_DN12683_c0_g1_i3_orf1	0.004452309	0.004696495	Proteome
TRINITY_DN4798_c0_g1_i3_orf1	-0.009788147	0.003655498	Proteome
TRINITY_DN8411_c1_g1_i1_orf1	-0.004374005	-0.02227495	Proteome
TRINITY_DN8780_c0_g1_i3_orf1	-0.020297536	0.020192674	Proteome
TRINITY_DN36006_c0_g1_i5_orf1	0.01513175	0.005442829	Proteome
TRINITY_DN84322_c0_g2_i1_orf1	0.009891532	0.00040129	Proteome

TRINITY_DN10399_c0_g1_i2_orf1	0.007153439	0.0027264	Proteome
TRINITY_DN45948_c1_g1_i1_orf1	0.025850209	0.009845595	Proteome
TRINITY_DN3057_c0_g2_i1_orf1	0.002819232	0.009916108	Proteome
TRINITY_DN18756_c0_g1_i6_orf1	0.006633076	0.0014338	Proteome
TRINITY_DN1237_c0_g1_i4_orf1	-0.008282514	-0.014953741	Proteome
TRINITY_DN1091_c0_g2_i10_orf1	-0.019954563	0.005541505	Proteome
TRINITY_DN21981_c0_g1_i8_orf1	0.0045044	-0.013847009	Proteome
TRINITY_DN1108_c1_g2_i1_orfp1	0.001413059	0.02199127	Proteome
TRINITY_DN72816_c0_g1_i2_orf1	0.012811042	-0.004551622	Proteome
TRINITY_DN64788_c0_g1_i1_orf1	0.005038496	0.01419816	Proteome
TRINITY_DN1362_c0_g1_i4_orf1	-0.009258221	-0.003416142	Proteome
TRINITY_DN2924_c0_g1_i2_orf1	0.018210728	0.006135441	Proteome
TRINITY_DN47605_c0_g2_i1_orf1	0.004623972	-0.007240069	Proteome
TRINITY_DN10045_c0_g1_i1_orf1	0.00272215	-0.020460552	Proteome
TRINITY_DN8369_c0_g1_i1_orf1	-0.000155362	-0.003082075	Proteome
TRINITY_DN24539_c0_g1_i4_orf1	-0.005926032	0.014136585	Proteome
TRINITY_DN4550_c1_g1_i19_orf1	-0.022964858	0.006168099	Proteome
TRINITY_DN4654_c0_g1_i6_orf1	-0.014709763	0.005111746	Proteome
TRINITY_DN10066_c0_g2_i2_orf1	-0.010050471	-0.004685054	Proteome
TRINITY_DN18624_c0_g1_i5_orf1	0.005369968	-0.014308121	Proteome
TRINITY_DN2356_c2_g1_i6_orf1	0.000721234	-0.011810749	Proteome
TRINITY_DN1902_c0_g1_i4_orf1	-0.004587108	-0.018743584	Proteome
TRINITY_DN4144_c0_g1_i7_orf1	-0.006473361	-0.010640373	Proteome
TRINITY_DN17838_c0_g1_i4_orf1	-0.013311211	-0.020580221	Proteome
TRINITY_DN1005_c0_g1_i5_orf1	0.017078426	0.013491943	Proteome
TRINITY_DN4025_c0_g1_i13_orf1	0.014110192	0.001940095	Proteome
TRINITY_DN31225_c0_g1_i1_orf1	0.028430537	0.008445144	Proteome
TRINITY_DN5467_c0_g1_i5_orf1	0.013309493	0.010951113	Proteome
TRINITY_DN16912_c0_g1_i1_orf1	-0.003270457	-0.022370418	Proteome
TRINITY_DN45449_c0_g1_i1_orf1	0.014092061	-0.004752429	Proteome
TRINITY_DN122423_c0_g4_i1_orf1	0.013557317	0.000775113	Proteome
TRINITY_DN34676_c1_g1_i3_orf1	-0.016474543	-0.022261566	Proteome
TRINITY_DN29448_c0_g1_i1_orf1	0.003854727	0.003182022	Proteome
TRINITY_DN40176_c0_g1_i1_orf1	0.010055062	0.018049569	Proteome
TRINITY_DN142442_c0_g1_i1_orf1	-0.020333006	0.038941834	Proteome
TRINITY_DN33867_c0_g1_i9_orf1	-0.011956135	0.000356689	Proteome
TRINITY_DN37986_c0_g1_i2_orf1	0.009843772	0.005719853	Proteome
TRINITY_DN230_c1_g1_i3_orf1	0.00056644	0.000292723	Proteome
TRINITY_DN3418_c0_g1_i3_orf1	-0.001413499	-0.013924333	Proteome
TRINITY_DN761_c0_g1_i3_orf1	0.00855141	-0.019234285	Proteome
TRINITY_DN71308_c0_g1_i4_orf1	-0.004005474	-0.038587955	Proteome
TRINITY_DN19293_c0_g1_i4_orf1	0.015685727	-0.025173364	Proteome
TRINITY_DN63533_c0_g1_i2_orf1	0.013018323	-0.016980573	Proteome
TRINITY_DN63492_c0_g1_i1_orf1	0.012333508	-0.003166343	Proteome
TRINITY_DN31598_c0_g1_i1_orf1	-0.011633608	-0.022693628	Proteome
TRINITY_DN41922_c0_g3_i1_orf1	0.001268821	0.003274934	Proteome
TRINITY_DN30185_c0_g1_i3_orf1	-0.020175726	-0.008126303	Proteome
TRINITY_DN108818_c0_g1_i5_orf1	0.002794669	0.016964515	Proteome
TRINITY_DN14250_c0_g1_i1_orf1	-0.033441626	0.019208634	Proteome
TRINITY_DN2879_c0_g1_i4_orf1	0.008525881	0.011135725	Proteome
TRINITY_DN6163_c0_g1_i4_orf1	0.009052325	-0.00557976	Proteome
TRINITY_DN24350_c0_g1_i1_orf1	0.00549883	0.012677105	Proteome
TRINITY_DN123184_c0_g1_i1_orf1	0.006084611	0.013167218	Proteome
TRINITY_DN35351_c0_g1_i3_orf1	0.009935212	-0.022157356	Proteome
TRINITY_DN14217_c0_g1_i1_orf1	-0.020023662	0.059074347	Proteome
TRINITY_DN18279_c0_g1_i1_orf1	-0.007353566	-0.004991405	Proteome
TRINITY_DN21435_c0_g1_i2_orf1	0.001198082	0.016586965	Proteome
TRINITY_DN442_c0_g1_i10_orf1	0.012179424	0.002775784	Proteome

TRINITY_DN5421_c0_g1_i1_orf1	-0.023567965	0.027386502	Proteome
TRINITY_DN110376_c0_g1_i1_orf1	0.008684822	-0.013656592	Proteome
TRINITY_DN27968_c0_g2_i2_orf1	0.000746437	0.006617165	Proteome
TRINITY_DN20346_c0_g1_i1_orf1	0.004767644	-0.004107596	Proteome
TRINITY_DN111985_c0_g1_i1_orf1	-0.027635568	0.078191933	Proteome
TRINITY_DN86844_c0_g2_i1_orf1	-0.002103533	-0.001048206	Proteome
TRINITY_DN11962_c0_g1_i2_orf1	-0.00613962	-0.00055024	Proteome
TRINITY_DN137_c0_g1_i1_orf1	0.006753276	0.00653675	Proteome
TRINITY_DN36324_c0_g1_i12_orf1	0.015202875	-0.002149992	Proteome
TRINITY_DN1760_c0_g1_i4_orf1	-0.016612258	0.008781351	Proteome
TRINITY_DN122423_c0_g5_i1_orf1	0.011800421	0.001466304	Proteome
TRINITY_DN9931_c0_g1_i1_orf1	0.011654602	0.000735901	Proteome
TRINITY_DN73230_c0_g1_i1_orf1	-0.004304086	-0.013907768	Proteome
TRINITY_DN1480_c0_g1_i5_orf1	0.010272499	0.012169992	Proteome
TRINITY_DN17061_c0_g1_i1_orf1	0.008840109	0.007035638	Proteome
TRINITY_DN10403_c0_g1_i1_orf1	0.013805898	0.015487223	Proteome
TRINITY_DN5458_c1_g1_i9_orf1	0.008226099	-0.006932239	Proteome
TRINITY_DN334_c0_g1_i2_orf1	0.002148341	-0.007002117	Proteome
TRINITY_DN86621_c0_g1_i2_orf1	0.012158014	0.013635408	Proteome
TRINITY_DN3949_c0_g1_i1_orf1	-0.010167289	0.001332563	Proteome
TRINITY_DN14524_c0_g1_i1_orf1	0.006063249	-0.019648719	Proteome
TRINITY_DN101995_c0_g1_i1_orf1	0.017717194	0.008288801	Proteome
TRINITY_DN44094_c0_g1_i1_orf1	0.008132172	0.007568317	Proteome
TRINITY_DN579_c1_g1_i1_orf1	0.006143923	0.014219842	Proteome
TRINITY_DN371_c0_g1_i6_orf1	-0.004305678	-0.001220623	Proteome
TRINITY_DN31663_c0_g1_i2_orf1	0.008685609	-0.002108731	Proteome
TRINITY_DN83622_c0_g1_i2_orf1	-0.015342328	0.032470647	Proteome
TRINITY_DN146006_c0_g1_i1_orf1	-0.005100551	0.016011309	Proteome
TRINITY_DN913_c0_g1_i6_orf1	-0.009105374	0.01727719	Proteome
TRINITY_DN10266_c0_g1_i5_orf1	0.00850139	-0.001698176	Proteome
TRINITY_DN154_c0_g1_i4_orf1	0.004191696	-0.01440656	Proteome
TRINITY_DN1720_c0_g1_i3_orf1	0.005646296	0.013531152	Proteome
TRINITY_DN5578_c0_g1_i10_orf1	-0.007503575	0.001206729	Proteome
TRINITY_DN121802_c0_g1_i6_orfp1	-0.027402905	-0.039402249	Proteome
TRINITY_DN9569_c1_g1_i7_orf1	-0.004795275	0.009081117	Proteome
TRINITY_DN1664_c0_g1_i4_orf1	-0.003579894	0.020910174	Proteome
TRINITY_DN130_c0_g1_i7_orf1	0.009491128	0.002863059	Proteome
TRINITY_DN26993_c1_g1_i8_orf1	0.010666025	-0.005781942	Proteome
TRINITY_DN4439_c0_g2_i1_orf1	0.007303711	0.01149369	Proteome
TRINITY_DN95850_c0_g4_i3_orf1	-0.000418087	-0.028084207	Proteome
TRINITY_DN4213_c0_g1_i4_orf1	-0.001274573	-0.001515213	Proteome
TRINITY_DN1534_c0_g1_i3_orf1	-0.041063993	0.011959964	Proteome
TRINITY_DN44070_c0_g2_i2_orf1	-0.006948395	-0.032716802	Proteome
TRINITY_DN12555_c0_g1_i1_orf1	-0.009987132	-0.025695064	Proteome
TRINITY_DN5581_c0_g1_i4_orf1	0.000255331	-0.012736549	Proteome
TRINITY_DN4820_c0_g2_i2_orf1	-0.008768053	0.004124175	Proteome
TRINITY_DN8646_c0_g1_i2_orf1	-0.007115236	-0.009163008	Proteome
TRINITY_DN11448_c0_g1_i4_orf1	0.006044206	0.006265051	Proteome
TRINITY_DN9904_c0_g1_i1_orf1	-0.015905963	-0.023882205	Proteome
TRINITY_DN16824_c0_g1_i7_orf1	-0.000727099	0.00739388	Proteome
TRINITY_DN141381_c0_g1_i1_orf1	0.001962379	-0.02248014	Proteome
TRINITY_DN2593_c0_g2_i1_orf1	-0.000966159	-0.003961088	Proteome
TRINITY_DN119265_c0_g2_i1_orf1	-0.000728723	0.002303794	Proteome
TRINITY_DN8833_c0_g1_i1_orf1	0.009071959	-0.006193234	Proteome
TRINITY_DN18118_c0_g2_i10_orf1	-0.00416312	0.006951238	Proteome
TRINITY_DN2748_c0_g1_i6_orf1	-0.01054498	0.002793355	Proteome
TRINITY_DN46140_c0_g1_i1_orf1	-0.004405661	0.005862375	Proteome
TRINITY_DN12932_c0_g1_i1_orf1	0.00847098	-0.011986428	Proteome

TRINITY_DN9465_c0_g1_i4_orf1	-0.006222406	-0.026168967	Proteome
TRINITY_DN4182_c0_g1_i6_orf1	0.00347574	0.006076584	Proteome
TRINITY_DN2808_c0_g1_i8_orf1	0.011940231	-0.000542362	Proteome
TRINITY_DN88640_c0_g1_i1_orf1	0.007310741	-0.01623458	Proteome
TRINITY_DN5055_c0_g1_i12_orf1	0.018580647	0.003864745	Proteome
TRINITY_DN1030_c0_g1_i6_orf1	0.017176485	-0.005654588	Proteome
TRINITY_DN3513_c0_g1_i5_orf1	-0.012325487	0.010133274	Proteome
TRINITY_DN11263_c0_g1_i5_orf1	-0.000248523	-0.022261762	Proteome
TRINITY_DN146364_c0_g1_i1_orf1	-0.012763166	0.000899201	Proteome
TRINITY_DN128231_c0_g1_i5_orf1	-0.04789372	0.012665163	Proteome
TRINITY_DN13174_c0_g1_i4_orf1	-0.006971474	-0.004298491	Proteome
TRINITY_DN24325_c0_g1_i12_orf1	-0.002903892	-0.004948243	Proteome
TRINITY_DN30012_c1_g1_i1_orf1	0.00987323	0.02022351	Proteome
TRINITY_DN7976_c0_g1_i4_orf1	0.009777868	0.008380696	Proteome
TRINITY_DN747_c0_g1_i1_orf1	-0.019211578	0.012456362	Proteome
TRINITY_DN18558_c0_g1_i7_orf1	0.002138072	0.018750562	Proteome
TRINITY_DN51568_c0_g1_i1_orf1	0.007510248	-0.00038524	Proteome
TRINITY_DN2299_c0_g1_i3_orf1	0.027220567	-0.004854144	Proteome
TRINITY_DN27300_c0_g1_i7_orfp1	-0.019483224	0.025105775	Proteome
TRINITY_DN394_c0_g1_i4_orf1	0.005452106	-0.003028485	Proteome
TRINITY_DN5585_c0_g1_i4_orf1	0.015812045	0.008072142	Proteome
TRINITY_DN2943_c2_g2_i1_orf1	0.001658764	0.026110852	Proteome
TRINITY_DN31585_c0_g1_i1_orf1	0.002544483	0.009190246	Proteome
TRINITY_DN52244_c1_g1_i1_orf1	-0.022904582	0.002908084	Proteome
TRINITY_DN15059_c0_g1_i9_orf1	0.013485461	0.001977202	Proteome
TRINITY_DN6653_c0_g1_i1_orf1	0.006702997	0.000233005	Proteome
TRINITY_DN2184_c0_g1_i4_orf1	0.00739734	-0.004196715	Proteome
TRINITY_DN12806_c0_g2_i1_orf1	0.010027884	4.06E-05	Proteome
TRINITY_DN64719_c0_g1_i2_orfp1	0.013088898	0.021301708	Proteome
TRINITY_DN3111_c0_g1_i5_orf1	-0.005117832	-0.015634207	Proteome
TRINITY_DN2084_c0_g1_i1_orf1	0.005216707	-0.002377054	Proteome
TRINITY_DN12293_c0_g1_i1_orf1	0.018688961	-0.004454471	Proteome
TRINITY_DN40058_c0_g2_i1_orf1	-0.006688077	-0.009153606	Proteome
TRINITY_DN64118_c0_g1_i4_orf1	-0.001269097	0.010784263	Proteome
TRINITY_DN14301_c0_g2_i1_orf1	0.005818262	0.003333271	Proteome
TRINITY_DN18804_c0_g1_i5_orf1	-0.020988756	0.020880034	Proteome
TRINITY_DN467_c4_g1_i2_orf1	0.007695821	0.003619858	Proteome
TRINITY_DN43355_c0_g1_i1_orf1	0.004582278	-0.013536602	Proteome
TRINITY_DN19669_c0_g1_i1_orf1	0.006568746	0.019776354	Proteome
TRINITY_DN9926_c1_g1_i1_orf1	0.026185545	0.003855399	Proteome
TRINITY_DN53400_c0_g1_i1_orf1	0.001131323	-0.011244269	Proteome
TRINITY_DN103511_c0_g1_i4_orf1	-0.010125147	-0.044802726	Proteome
TRINITY_DN68770_c0_g1_i1_orf1	-0.030008722	-0.00439309	Proteome
TRINITY_DN1772_c1_g3_i1_orf1	0.005771418	-0.00419819	Proteome
TRINITY_DN700_c0_g1_i3_orf1	-0.003542993	-0.005750268	Proteome
TRINITY_DN18592_c0_g1_i4_orf1	0.001980309	-0.03243603	Proteome
TRINITY_DN667_c0_g1_i5_orf1	0.016219456	-0.001344393	Proteome
TRINITY_DN31303_c0_g1_i4_orf1	-0.002676558	-0.011612807	Proteome
TRINITY_DN4076_c1_g2_i2_orf1	-0.007126843	-0.007738369	Proteome
TRINITY_DN10396_c0_g1_i1_orf1	-0.004162017	0.0073795	Proteome
TRINITY_DN2571_c0_g2_i1_orf1	-0.00662707	0.002444448	Proteome
TRINITY_DN838_c0_g1_i18_orf1	0.006855984	0.009145161	Proteome
TRINITY_DN34786_c0_g1_i1_orf1	-0.002783718	-0.005250284	Proteome
TRINITY_DN22941_c0_g1_i1_orf1	0.000411266	0.000357025	Proteome
TRINITY_DN98723_c1_g1_i1_orf1	0.008589802	0.01196169	Proteome
TRINITY_DN72285_c1_g1_i1_orf1	0.014422342	-0.006625873	Proteome
TRINITY_DN15667_c0_g1_i2_orf1	-0.00469459	-0.002764833	Proteome
TRINITY_DN107840_c1_g1_i1_orf1	0.001523392	-0.013150174	Proteome

TRINITY_DN2749_c0_g1_i4_orf1	-0.00695506	0.012187655	Proteome
TRINITY_DN7488_c0_g1_i1_orf1	0.004839488	-0.00861552	Proteome
TRINITY_DN855_c0_g1_i5_orf1	0.013049314	0.013951151	Proteome
TRINITY_DN43841_c0_g1_i1_orf1	-0.000220317	-0.006114672	Proteome
TRINITY_DN35662_c0_g1_i5_orf1	-0.001413117	-0.002283627	Proteome
TRINITY_DN7534_c0_g1_i15_orf1	0.024052515	0.007653372	Proteome
TRINITY_DN7064_c0_g1_i20_orfp1	-0.01752046	-0.031809537	Proteome
TRINITY_DN36817_c0_g1_i1_orf1	-0.000204944	-0.005629509	Proteome
TRINITY_DN44557_c0_g1_i4_orf1	0.007759549	-0.00568382	Proteome
TRINITY_DN1133_c0_g1_i6_orf1	0.007419167	-0.013704051	Proteome
TRINITY_DN6680_c0_g1_i1_orf1	-0.007034104	0.014864989	Proteome
TRINITY_DN5664_c0_g1_i1_orf1	0.009461008	-0.012524426	Proteome
TRINITY_DN7964_c0_g1_i6_orfp1	-0.008096403	0.005523372	Proteome
TRINITY_DN27398_c0_g1_i3_orf1	-0.001489905	0.015306785	Proteome
TRINITY_DN48983_c0_g1_i2_orf1	0.014320287	-0.012627702	Proteome
TRINITY_DN4133_c0_g1_i2_orfp2	0.023873693	0.003078311	Proteome
TRINITY_DN8027_c0_g1_i3_orf1	0.001977327	-0.017505545	Proteome
TRINITY_DN536_c0_g1_i7_orf1	-0.002621427	0.001191995	Proteome
TRINITY_DN3675_c0_g1_i1_orf1	-0.002680409	-0.018919592	Proteome
TRINITY_DN195_c4_g1_i1_orf1	0.000666512	-0.000577839	Proteome
TRINITY_DN15904_c0_g1_i1_orf1	0.012170481	0.010955733	Proteome
TRINITY_DN27264_c0_g1_i1_orf1	-0.03530541	0.012077826	Proteome
TRINITY_DN3133_c0_g1_i6_orf1	-0.008122102	-0.017567871	Proteome
TRINITY_DN72934_c0_g1_i1_orf1	-0.003221509	-0.011842656	Proteome
TRINITY_DN62192_c0_g1_i2_orf1	-0.010663643	-0.022415811	Proteome
TRINITY_DN10398_c0_g1_i12_orf1	-0.012082195	0.006541722	Proteome
TRINITY_DN16924_c0_g1_i1_orf1	0.009669094	0.005261212	Proteome
TRINITY_DN7778_c0_g1_i1_orf1	0.008534461	0.012670822	Proteome
TRINITY_DN3832_c0_g1_i1_orf1	-0.004693976	0.011679196	Proteome
TRINITY_DN3055_c0_g1_i9_orf1	0.013307835	0.011357778	Proteome
TRINITY_DN42333_c0_g1_i5_orf1	-0.003169305	-0.006319583	Proteome
TRINITY_DN5925_c0_g1_i5_orf1	-0.001185493	0.011120047	Proteome
TRINITY_DN25285_c0_g1_i1_orf1	0.006120452	-0.008690046	Proteome
TRINITY_DN9506_c0_g1_i2_orf1	-0.011855591	-0.022450772	Proteome
TRINITY_DN1293_c0_g1_i4_orf1	0.004861243	0.015781921	Proteome
TRINITY_DN129207_c0_g1_i1_orf1	-0.005324506	-0.004749814	Proteome
TRINITY_DN37055_c0_g1_i1_orf1	-0.00153167	-0.017041801	Proteome
TRINITY_DN11125_c0_g1_i1_orf1	0.012453271	0.008080822	Proteome
TRINITY_DN30663_c0_g1_i1_orf1	-0.009350666	0.00632763	Proteome
TRINITY_DN27111_c0_g1_i1_orf1	-0.021147317	0.006313299	Proteome
TRINITY_DN3019_c0_g1_i1_orf1	0.005677117	-0.007454194	Proteome
TRINITY_DN122321_c0_g1_i1_orf1	0.012617636	-0.023509819	Proteome
TRINITY_DN19295_c0_g1_i1_orf1	0.00445562	-0.010693285	Proteome
TRINITY_DN4795_c0_g1_i2_orf1	0.013690297	-0.012807644	Proteome
TRINITY_DN13285_c0_g1_i9_orf1	0.001132733	0.009259206	Proteome
TRINITY_DN14185_c0_g1_i1_orf1	-0.024580223	0.021342621	Proteome
TRINITY_DN45271_c0_g1_i1_orf1	-0.000498934	0.019071643	Proteome
TRINITY_DN65974_c0_g1_i2_orf1	0.017165307	0.009229756	Proteome
TRINITY_DN2962_c0_g1_i7_orf1	0.000861464	-0.006680116	Proteome
TRINITY_DN5256_c0_g1_i1_orf1	-0.009815754	0.015888098	Proteome
TRINITY_DN45097_c0_g1_i5_orf1	-0.003339619	0.021774031	Proteome
TRINITY_DN28018_c0_g6_i1_orf1	-0.004839196	-0.005256186	Proteome
TRINITY_DN25865_c0_g1_i2_orf1	-0.007576862	-0.004572576	Proteome
TRINITY_DN39200_c0_g1_i5_orf1	-0.002201722	-0.003671306	Proteome
TRINITY_DN4085_c0_g1_i1_orf1	0.001783178	-0.014772348	Proteome
TRINITY_DN31119_c0_g1_i1_orf1	0.013401302	0.002741525	Proteome
TRINITY_DN8553_c0_g1_i4_orf1	0.003819041	0.006998206	Proteome
TRINITY_DN14046_c0_g1_i1_orf1	-0.002770335	-0.014252474	Proteome

TRINITY_DN9282_c0_g1_i2_orf1	0.011855903	0.000421952	Proteome
TRINITY_DN11375_c0_g1_i4_orf1	-0.001161194	-0.00520712	Proteome
TRINITY_DN96738_c0_g2_i1_orf1	0.010540613	0.004012347	Proteome
TRINITY_DN6788_c0_g1_i1_orf1	-0.002048782	-0.010910769	Proteome
TRINITY_DN114982_c0_g1_i1_orf1	-0.023780499	0.021704597	Proteome
TRINITY_DN4384_c0_g1_i5_orf1	-0.014357496	0.023596751	Proteome
TRINITY_DN605_c0_g1_i4_orf1	-0.001737929	-0.010017114	Proteome
TRINITY_DN7075_c0_g2_i1_orf1	0.00805309	-0.003597303	Proteome
TRINITY_DN22815_c0_g1_i2_orf1	0.000155684	0.00863962	Proteome
TRINITY_DN140_c1_g1_i2_orf1	-0.000317165	0.004515197	Proteome
TRINITY_DN3670_c0_g1_i2_orf1	-4.23E-05	0.011279444	Proteome
TRINITY_DN2160_c0_g1_i13_orf1	0.005950089	-0.00246287	Proteome
TRINITY_DN41848_c0_g1_i4_orf1	-0.011117544	-0.017843565	Proteome
TRINITY_DN10118_c0_g1_i4_orf1	-0.005917901	0.003008402	Proteome
TRINITY_DN6563_c0_g1_i1_orf1	0.00290193	-0.007812603	Proteome
TRINITY_DN10619_c0_g5_i7_orf1	0.013536063	0.005616565	Proteome
TRINITY_DN104297_c0_g1_i1_orf1	0.01681567	-0.009315373	Proteome
TRINITY_DN1860_c0_g1_i1_orf1	-0.004184851	-0.000807778	Proteome
TRINITY_DN64772_c0_g1_i1_orf1	-0.011407932	0.015624035	Proteome
TRINITY_DN16894_c0_g1_i5_orf1	0.008561658	0.01330395	Proteome
TRINITY_DN5840_c0_g1_i6_orf1	-0.002974068	0.0053738	Proteome
TRINITY_DN4679_c0_g2_i13_orf1	0.015388	0.005931277	Proteome
TRINITY_DN81791_c0_g2_i2_orf1	0.001082796	0.011829704	Proteome
TRINITY_DN3431_c0_g1_i1_orf1	-0.011224777	-0.003235995	Proteome
TRINITY_DN1252_c0_g1_i1_orf1	-0.006523728	0.028379659	Proteome
TRINITY_DN4255_c0_g1_i11_orf1	0.0212435	0.023522019	Proteome
TRINITY_DN85490_c0_g2_i1_orf1	-0.001724505	-0.016171555	Proteome
TRINITY_DN1066_c0_g1_i4_orf1	0.003817715	0.002298569	Proteome
TRINITY_DN8754_c0_g1_i2_orf1	0.001077164	-0.0017184	Proteome
TRINITY_DN1803_c0_g1_i3_orf1	9.08E-05	-0.013753725	Proteome
TRINITY_DN527_c0_g1_i5_orf1	-0.004224264	-5.27E-05	Proteome
TRINITY_DN5919_c0_g1_i4_orf1	0.009312217	-0.003843488	Proteome
TRINITY_DN14507_c0_g1_i5_orf1	-0.003654076	-0.010985165	Proteome
TRINITY_DN21494_c0_g1_i2_orf1	0.00256539	-0.010659606	Proteome
TRINITY_DN4589_c0_g1_i1_orf1	0.005460347	-0.014198944	Proteome
TRINITY_DN2770_c0_g2_i4_orf1	0.015303194	-0.000793134	Proteome
TRINITY_DN5692_c0_g1_i4_orf1	0.008862021	-0.002904602	Proteome
TRINITY_DN13686_c0_g2_i1_orf1	0.0126139	-0.004910268	Proteome
TRINITY_DN113327_c0_g1_i2_orf1	-0.016591534	-0.0038343	Proteome
TRINITY_DN2593_c0_g3_i1_orf1	0.003763622	0.006135748	Proteome
TRINITY_DN26337_c0_g1_i3_orf1	0.014923952	-0.001754848	Proteome
TRINITY_DN26882_c0_g1_i1_orf1	0.00057219	-0.004972869	Proteome
TRINITY_DN28802_c0_g1_i1_orf1	0.017066991	-0.005420211	Proteome
TRINITY_DN46409_c0_g1_i1_orf1	-0.010053918	-0.000246012	Proteome
TRINITY_DN38644_c0_g1_i1_orf1	0.008125846	-0.004043252	Proteome
TRINITY_DN72369_c0_g1_i1_orf1	0.001509333	-0.009243242	Proteome
TRINITY_DN3791_c0_g1_i2_orf1	0.004729582	0.007829179	Proteome
TRINITY_DN5857_c0_g1_i13_orf1	0.009864785	0.004135557	Proteome
TRINITY_DN90289_c0_g1_i5_orf1	-0.005958639	0.000723806	Proteome
TRINITY_DN268_c1_g1_i7_orf1	-0.004907365	-0.001052295	Proteome
TRINITY_DN27723_c0_g1_i1_orf1	0.01283556	-0.006314832	Proteome
TRINITY_DN23941_c0_g1_i5_orf1	0.009702343	-0.001157303	Proteome
TRINITY_DN11396_c0_g1_i1_orf1	-0.000972149	-0.013145939	Proteome
TRINITY_DN4571_c0_g1_i4_orf1	0.019349754	0.006004353	Proteome
TRINITY_DN5349_c0_g1_i1_orf1	0.001654745	-0.007175302	Proteome
TRINITY_DN27332_c0_g2_i1_orf1	0.0025467	0.015074959	Proteome
TRINITY_DN54586_c1_g1_i1_orf1	0.00251463	0.020517388	Proteome
TRINITY_DN96170_c0_g1_i1_orf1	0.005508916	0.011915425	Proteome

TRINITY_DN141352_c0_g1_i1_orf1	0.005252261	-0.011682124	Proteome
TRINITY_DN4886_c0_g1_i6_orf1	-0.00738551	0.017778284	Proteome
TRINITY_DN4747_c0_g1_i4_orf1	0.007511192	0.005640088	Proteome
TRINITY_DN20710_c0_g2_i2_orf1	-0.00206314	0.032068577	Proteome
TRINITY_DN38225_c0_g2_i1_orf1	0.010038218	-0.005237693	Proteome
TRINITY_DN56121_c0_g1_i4_orf1	-0.009603108	-0.002468727	Proteome
TRINITY_DN3847_c1_g1_i1_orf1	-0.003463103	0.001484937	Proteome
TRINITY_DN1407_c0_g1_i12_orf1	-0.029189954	-0.025079647	Proteome
TRINITY_DN16400_c0_g2_i1_orf1	0.001566316	0.020250511	Proteome
TRINITY_DN29402_c0_g1_i1_orf1	0.008605595	-0.009990387	Proteome
TRINITY_DN25997_c1_g2_i4_orf1	0.013184225	-0.002290067	Proteome
TRINITY_DN13866_c0_g1_i4_orf1	-0.007618745	0.00655549	Proteome
TRINITY_DN251_c0_g1_i2_orf1	-0.023946484	0.015860278	Proteome
TRINITY_DN18937_c0_g1_i1_orf1	-0.013191496	-0.006512005	Proteome
TRINITY_DN54269_c0_g1_i3_orf1	0.001887264	-0.008937633	Proteome
TRINITY_DN3028_c0_g1_i1_orf1	0.008633489	-0.005333611	Proteome
TRINITY_DN14183_c0_g1_i3_orf1	0.002986072	-0.003996106	Proteome
TRINITY_DN20009_c0_g1_i1_orf1	-0.035294527	0.049473097	Proteome
TRINITY_DN21719_c0_g1_i2_orf1	-0.010404362	0.007394889	Proteome
TRINITY_DN13330_c0_g1_i4_orf1	0.020907491	0.017192844	Proteome
TRINITY_DN90327_c0_g1_i1_orf1	0.004668198	0.002331741	Proteome
TRINITY_DN15291_c0_g1_i11_orf1	-0.023994466	-0.012185585	Proteome
TRINITY_DN24751_c0_g1_i1_orf1	0.008882743	0.000495	Proteome
TRINITY_DN18909_c0_g1_i6_orf1	0.007864262	-0.022334919	Proteome
TRINITY_DN1882_c0_g1_i4_orf1	0.016589545	0.008853117	Proteome
TRINITY_DN648_c0_g1_i5_orf1	0.000660548	-3.45E-05	Proteome
TRINITY_DN45446_c0_g1_i2_orf1	-0.006313629	0.006044887	Proteome
TRINITY_DN53427_c0_g1_i2_orf1	0.000226414	0.002454749	Proteome
TRINITY_DN17208_c0_g1_i2_orf1	0.006358682	0.001186316	Proteome
TRINITY_DN23740_c1_g1_i1_orf1	-0.014241006	0.014741303	Proteome
TRINITY_DN3865_c0_g1_i5_orf1	0.002017007	-0.001492583	Proteome
TRINITY_DN51424_c0_g2_i1_orf1	-0.009292126	0.013358813	Proteome
TRINITY_DN26985_c0_g1_i5_orf1	-0.01129343	0.01799196	Proteome
TRINITY_DN8136_c0_g1_i1_orf1	-0.009666119	0.01016062	Proteome
TRINITY_DN3647_c2_g1_i3_orf1	0.010944708	0.008014265	Proteome
TRINITY_DN2406_c0_g1_i6_orf1	-0.017268995	0.015577153	Proteome
TRINITY_DN6058_c0_g1_i3_orf1	0.005327913	-0.000724085	Proteome
TRINITY_DN5834_c0_g1_i2_orf1	0.003960755	0.001166953	Proteome
TRINITY_DN89483_c0_g1_i1_orf1	0.025074549	-0.003321244	Proteome
TRINITY_DN15870_c0_g1_i3_orf1	0.000307164	-0.004321922	Proteome
TRINITY_DN21924_c0_g1_i3_orf1	-0.007225087	-0.021868666	Proteome
TRINITY_DN12608_c0_g1_i1_orf1	0.008493267	-0.01266074	Proteome
TRINITY_DN8940_c0_g1_i4_orf1	0.005176872	-0.016205864	Proteome
TRINITY_DN33178_c0_g1_i1_orf1	0.006251605	-0.006776089	Proteome
TRINITY_DN76283_c0_g2_i1_orf1	0.006713239	-0.012432229	Proteome
TRINITY_DN9916_c0_g1_i1_orf1	0.011074535	-0.007695787	Proteome
TRINITY_DN883_c0_g1_i8_orf1	0.013799497	-0.00221066	Proteome
TRINITY_DN4018_c0_g1_i4_orf1	-0.00526337	-0.002903398	Proteome
TRINITY_DN66453_c0_g1_i4_orfp1	0.034946399	0.005457337	Proteome
TRINITY_DN6875_c0_g1_i2_orf1	0.003140223	-0.02015658	Proteome
TRINITY_DN11823_c1_g1_i2_orf1	-0.001582913	-0.01380503	Proteome
TRINITY_DN6688_c0_g1_i5_orf1	0.007236232	-0.002992316	Proteome
TRINITY_DN5169_c0_g1_i5_orf1	0.001924977	0.009951052	Proteome
TRINITY_DN1304_c0_g1_i6_orf1	-0.003949647	0.010384599	Proteome
TRINITY_DN69170_c0_g2_i1_orf1	0.005214922	0.000642515	Proteome
TRINITY_DN7414_c0_g1_i1_orf1	0.010452564	0.008649153	Proteome
TRINITY_DN8953_c0_g1_i4_orf1	0.017044805	0.020112874	Proteome
TRINITY_DN44219_c0_g1_i1_orf1	0.000509212	0.012040143	Proteome

TRINITY_DN15743_c0_g1_i5_orf1	-0.003232612	-0.004398945	Proteome
TRINITY_DN49872_c0_g2_i1_orf1	0.016347939	0.000845534	Proteome
TRINITY_DN139212_c0_g1_i4_orf1	0.002006867	0.003799869	Proteome
TRINITY_DN9510_c0_g2_i1_orf1	0.002703467	0.003904191	Proteome
TRINITY_DN117362_c0_g1_i5_orf1	-0.001396222	-0.022689696	Proteome
TRINITY_DN7316_c0_g2_i1_orf1	0.005313066	-0.002627004	Proteome
TRINITY_DN4695_c0_g1_i3_orf1	-0.012704309	0.021645501	Proteome
TRINITY_DN1109_c0_g1_i6_orf1	-0.005647856	-0.017516619	Proteome
TRINITY_DN1039_c0_g1_i5_orf1	0.015569662	-0.001135736	Proteome
TRINITY_DN295_c2_g1_i2_orf1	-0.04147242	0.011689853	Proteome
TRINITY_DN478_c0_g1_i16_orf1	-0.010024224	-0.000542638	Proteome
TRINITY_DN705_c0_g1_i1_orf1	-0.000542587	0.009275241	Proteome
TRINITY_DN67231_c0_g1_i1_orf1	0.015370439	0.013831536	Proteome
TRINITY_DN38540_c0_g1_i1_orf1	0.00887123	-8.42E-05	Proteome
TRINITY_DN20426_c0_g2_i1_orf1	0.0101429	-0.002036488	Proteome
TRINITY_DN1901_c0_g1_i6_orf1	-0.001435993	0.002180445	Proteome
TRINITY_DN93764_c0_g1_i1_orf1	-0.008671487	0.014010049	Proteome
TRINITY_DN4929_c1_g2_i5_orf1	0.008879369	-0.001201527	Proteome
TRINITY_DN782_c0_g1_i5_orf1	-0.003774553	-0.014528607	Proteome
TRINITY_DN14953_c0_g1_i5_orf1	-0.004331154	2.06E-05	Proteome
TRINITY_DN38693_c0_g1_i4_orf1	0.005859397	-0.016093188	Proteome
TRINITY_DN702_c0_g1_i15_orf1	0.000890505	-0.027146389	Proteome
TRINITY_DN47575_c0_g1_i1_orf1	0.007285468	0.003618378	Proteome
TRINITY_DN2423_c0_g2_i2_orf1	-0.000426091	-0.022804855	Proteome
TRINITY_DN34399_c0_g1_i1_orf1	0.008430075	0.002885353	Proteome
TRINITY_DN117707_c0_g1_i3_orf1	0.001059996	-0.010668297	Proteome
TRINITY_DN3166_c1_g1_i6_orf1	0.071015846	0.020778428	Proteome
TRINITY_DN17212_c0_g1_i6_orf1	0.002493876	-0.001104767	Proteome
TRINITY_DN61135_c0_g1_i1_orf1	-0.008109444	-0.015245612	Proteome
TRINITY_DN22044_c0_g2_i1_orf1	0.014662091	0.013305645	Proteome
TRINITY_DN39904_c0_g1_i1_orf1	-0.009161738	-0.034674873	Proteome
TRINITY_DN3450_c0_g1_i3_orf1	0.013721826	0.000829543	Proteome
TRINITY_DN436_c0_g2_i5_orfp1	0.022468937	0.001744377	Proteome
TRINITY_DN6122_c0_g1_i6_orf1	-0.030995982	-0.004202689	Proteome
TRINITY_DN11327_c0_g1_i1_orf1	0.012957682	-0.014212669	Proteome
TRINITY_DN101544_c0_g1_i1_orf1	0.002560385	-0.003755526	Proteome
TRINITY_DN4300_c0_g1_i5_orf1	0.002006156	-0.003948681	Proteome
TRINITY_DN802_c0_g1_i2_orf1	-0.003441756	0.003781978	Proteome
TRINITY_DN64297_c0_g1_i1_orf1	-0.076756685	-0.010166529	Proteome
TRINITY_DN1353_c0_g1_i1_orf1	-0.000141157	0.001906158	Proteome
TRINITY_DN2630_c0_g3_i3_orf1	-0.013126828	0.00936087	Proteome
TRINITY_DN1978_c0_g1_i4_orf1	0.024227752	0.003575018	Proteome
TRINITY_DN4207_c0_g1_i1_orf1	0.009088644	-0.011735395	Proteome
TRINITY_DN1004_c0_g2_i1_orf1	-0.014349458	0.00238436	Proteome
TRINITY_DN18031_c0_g1_i1_orf1	-0.028338506	0.102783963	Proteome
TRINITY_DN146758_c0_g1_i1_orf1	-0.002442699	0.021288419	Proteome
TRINITY_DN116951_c0_g3_i2_orf1	0.011270665	0.012562828	Proteome
TRINITY_DN93566_c0_g2_i1_orf1	0.006237957	-0.002168578	Proteome
TRINITY_DN1038_c0_g1_i4_orf1	0.003385048	-0.017763837	Proteome
TRINITY_DN105901_c0_g1_i2_orfp1	-0.009519057	-0.009631958	Proteome
TRINITY_DN15682_c0_g1_i4_orf1	-0.049625572	-0.012824512	Proteome
TRINITY_DN535_c3_g2_i1_orf1	-0.005375754	0.015602907	Proteome
TRINITY_DN2802_c1_g1_i1_orf1	-0.0032595	0.013460464	Proteome
TRINITY_DN100821_c0_g1_i1_orf1	0.009965195	0.001518255	Proteome
TRINITY_DN1506_c0_g1_i6_orfp1	0.017185323	0.103250825	Proteome
TRINITY_DN37699_c0_g1_i4_orfp1	0.011895855	-0.004346694	Proteome
TRINITY_DN26251_c0_g1_i1_orf1	0.008886358	0.004738422	Proteome
TRINITY_DN26411_c0_g1_i2_orfp1	0.0213858	-0.002404852	Proteome

TRINITY_DN124300_c0_g1_i2_orf1	0.003845559	0.006155881	Proteome
TRINITY_DN1231_c0_g1_i4_orf1	0.010713753	-0.001976941	Proteome
TRINITY_DN54543_c0_g5_i2_orf1	0.002039192	0.003454136	Proteome
TRINITY_DN56877_c0_g1_i4_orf1	0.011285991	-0.015632879	Proteome
TRINITY_DN66302_c0_g1_i1_orf1	0.006609255	-0.010348536	Proteome
TRINITY_DN9560_c0_g1_i5_orf1	7.59E-05	0.027548743	Proteome
TRINITY_DN36410_c0_g1_i4_orf1	0.002146891	-0.008015135	Proteome
TRINITY_DN112921_c0_g1_i1_orf1	-0.001363824	-0.016226844	Proteome
TRINITY_DN49221_c0_g1_i1_orf1	-0.006690101	-0.015796276	Proteome
TRINITY_DN9991_c0_g1_i4_orf1	0.009220646	0.025421135	Proteome
TRINITY_DN51197_c0_g1_i3_orf1	0.017445079	0.008375181	Proteome
TRINITY_DN9412_c0_g1_i1_orf1	-0.019150447	0.000263643	Proteome
TRINITY_DN26089_c0_g1_i1_orf1	0.021411664	0.009674939	Proteome
TRINITY_DN15478_c0_g1_i1_orf1	-0.00598653	-0.011930569	Proteome
TRINITY_DN6023_c0_g1_i7_orf1	0.010705826	0.00393994	Proteome
TRINITY_DN210_c0_g1_i9_orf1	0.013687278	-0.001627465	Proteome
TRINITY_DN115498_c0_g1_i1_orf1	0.005631423	-0.001782033	Proteome
TRINITY_DN18035_c0_g1_i7_orf1	-0.004136721	-0.018693077	Proteome
TRINITY_DN3059_c0_g1_i1_orf1	-0.005755753	-0.018949159	Proteome
TRINITY_DN38608_c0_g1_i1_orf1	0.0091482	-0.004364098	Proteome
TRINITY_DN1781_c0_g1_i8_orf1	-0.005458458	-0.009193039	Proteome
TRINITY_DN14018_c0_g1_i4_orf1	-0.004003286	-0.002422938	Proteome
TRINITY_DN27723_c0_g1_i2_orf1	0.001656764	-0.004732451	Proteome
TRINITY_DN2968_c0_g1_i3_orf1	0.009671638	0.000810583	Proteome
TRINITY_DN19262_c0_g1_i1_orf1	0.007941722	0.014315548	Proteome
TRINITY_DN40439_c0_g1_i5_orf1	-0.022967578	-0.035883314	Proteome
TRINITY_DN1099_c1_g1_i4_orf1	0.002848805	0.002304961	Proteome
TRINITY_DN3459_c0_g1_i4_orf1	-0.010853631	-0.005006221	Proteome
TRINITY_DN14967_c0_g2_i1_orf1	-0.00226668	-0.00771739	Proteome
TRINITY_DN1920_c0_g1_i4_orf1	-0.000406621	-0.018868913	Proteome
TRINITY_DN12671_c0_g1_i6_orf1	-0.024841821	0.006500901	Proteome
TRINITY_DN338_c0_g1_i1_orf1	-0.001524116	0.001183358	Proteome
TRINITY_DN22049_c0_g1_i1_orf1	0.007007545	-0.015602209	Proteome
TRINITY_DN8641_c0_g1_i1_orf1	0.00022496	-0.007294911	Proteome
TRINITY_DN5105_c0_g1_i10_orf1	0.001191528	-0.023595024	Proteome
TRINITY_DN2784_c0_g1_i3_orf1	-0.006430631	-0.028623007	Proteome
TRINITY_DN36987_c0_g1_i1_orf1	0.020213149	0.007359965	Proteome
TRINITY_DN64510_c0_g1_i1_orf1	0.005181016	-0.009615006	Proteome
TRINITY_DN15244_c0_g1_i5_orf1	0.001384206	0.007844943	Proteome
TRINITY_DN43412_c0_g1_i2_orf1	0.007076775	0.009814008	Proteome
TRINITY_DN701_c1_g1_i4_orf1	0.024223811	0.009602588	Proteome
TRINITY_DN3355_c0_g1_i1_orf1	-0.008767547	-0.013651752	Proteome
TRINITY_DN15836_c0_g1_i1_orf1	-0.003589119	-0.012745983	Proteome
TRINITY_DN62184_c1_g1_i1_orf1	-0.006090405	0.00763287	Proteome
TRINITY_DN3821_c1_g1_i7_orf1	-0.009396587	0.03416219	Proteome
TRINITY_DN112409_c1_g1_i1_orf1	-0.002486526	0.006820568	Proteome
TRINITY_DN53167_c0_g1_i2_orf1	-0.004103381	-0.008803773	Proteome
TRINITY_DN111110_c0_g1_i1_orf1	0.016788468	-0.004401617	Proteome
TRINITY_DN23020_c0_g1_i1_orf1	0.011105465	0.007572568	Proteome
TRINITY_DN110888_c0_g1_i2_orf1	-0.012192165	-0.015351238	Proteome
TRINITY_DN15597_c0_g1_i1_orf1	0.0252016	-0.017470601	Proteome
TRINITY_DN22951_c0_g1_i1_orf1	0.014904072	-0.003169932	Proteome
TRINITY_DN32583_c0_g1_i4_orf1	-0.012519012	-0.027308069	Proteome
TRINITY_DN14973_c0_g1_i1_orf1	-0.000579672	-0.004193975	Proteome
TRINITY_DN76529_c0_g1_i1_orfp1	0.014495229	0.008680397	Proteome
TRINITY_DN83574_c0_g1_i1_orf1	-0.005458194	-0.001659041	Proteome
TRINITY_DN10297_c0_g1_i1_orf1	-0.00931587	-0.005230251	Proteome
TRINITY_DN16487_c0_g1_i1_orf1	-0.003537991	0.0156194	Proteome

TRINITY_DN42337_c0_g1_i4_orf1	-0.01213724	0.01417722	Proteome
TRINITY_DN7618_c0_g1_i4_orf1	0.011372638	0.018431572	Proteome
TRINITY_DN79673_c0_g1_i1_orf1	0.007308293	0.005175153	Proteome
TRINITY_DN10812_c0_g1_i3_orf1	-0.00082458	0.013065411	Proteome
TRINITY_DN105157_c0_g1_i1_orf1	0.008781787	-0.008391821	Proteome
TRINITY_DN22797_c0_g1_i5_orf1	-0.005704846	0.012483827	Proteome
TRINITY_DN46372_c0_g1_i1_orf1	0.003799979	0.016933567	Proteome
TRINITY_DN56250_c0_g1_i7_orf1	0.006409636	-0.015915208	Proteome
TRINITY_DN14391_c1_g1_i2_orf1	-0.019989019	0.00655918	Proteome
TRINITY_DN39266_c0_g1_i1_orf1	-0.01839274	0.003592448	Proteome
TRINITY_DN40211_c0_g1_i1_orf1	-0.005599832	-0.009991512	Proteome
TRINITY_DN119893_c0_g2_i3_orf1	0.009686479	0.001903062	Proteome
TRINITY_DN49936_c0_g2_i1_orf1	0.000224828	-0.013661331	Proteome
TRINITY_DN125150_c0_g1_i1_orf1	0.022109214	0.006416364	Proteome
TRINITY_DN16868_c0_g2_i1_orf1	-0.001637606	-0.001379666	Proteome
TRINITY_DN19493_c0_g1_i5_orf1	0.011568786	0.004305204	Proteome
TRINITY_DN1091_c0_g1_i1_orf1	0.002746403	-0.01810092	Proteome
TRINITY_DN5828_c0_g1_i5_orf1	-0.010864569	-0.008711212	Proteome
TRINITY_DN92_c1_g2_i1_orf1	0.006315184	0.022345352	Proteome
TRINITY_DN2167_c0_g1_i6_orf1	0.00012937	0.005309009	Proteome
TRINITY_DN62729_c0_g1_i13_orf1	0.015732031	-0.005098616	Proteome
TRINITY_DN12424_c0_g1_i2_orf1	0.011881026	0.006459948	Proteome
TRINITY_DN41697_c0_g1_i1_orf1	-0.019868901	0.007660475	Proteome
TRINITY_DN5829_c0_g2_i1_orf1	0.015888534	-0.000712587	Proteome
TRINITY_DN36045_c0_g1_i2_orf1	-0.002099976	0.000540823	Proteome
TRINITY_DN32780_c0_g1_i2_orf1	-0.019004472	-0.01160429	Proteome
TRINITY_DN2710_c0_g1_i4_orf1	-0.000230481	0.021924047	Proteome
TRINITY_DN31433_c0_g1_i1_orf1	0.007960821	-0.000581699	Proteome
TRINITY_DN43656_c0_g1_i1_orf1	-0.002083241	0.009832786	Proteome
TRINITY_DN13783_c0_g4_i2_orf1	0.008863914	0.001882816	Proteome
TRINITY_DN29229_c0_g1_i5_orfp1	0.01089145	0.000766963	Proteome
TRINITY_DN19951_c0_g1_i5_orf1	-0.003732142	-0.004132387	Proteome
TRINITY_DN131264_c0_g1_i2_orf1	-0.010877085	-0.010358199	Proteome
TRINITY_DN42159_c0_g1_i6_orf1	-0.026687301	-0.049077666	Proteome
TRINITY_DN15114_c0_g2_i1_orf1	0.004673018	0.000902378	Proteome
TRINITY_DN36648_c0_g1_i1_orf1	0.004204381	0.011671401	Proteome
TRINITY_DN9383_c0_g1_i3_orf1	-0.0082264	0.030156778	Proteome
TRINITY_DN1956_c7_g1_i1_orf1	0.008171262	0.009590937	Proteome
TRINITY_DN4905_c0_g1_i6_orf1	-0.004820449	-0.016327221	Proteome
TRINITY_DN62091_c0_g1_i1_orf1	0.008142231	-0.026036138	Proteome
TRINITY_DN5161_c0_g1_i5_orf1	0.017286725	-0.00744768	Proteome
TRINITY_DN103457_c0_g1_i1_orf1	-0.003425044	0.00445176	Proteome
TRINITY_DN15411_c0_g1_i4_orf1	-0.011555171	-0.003805711	Proteome
TRINITY_DN29291_c0_g1_i1_orf1	-0.008252222	-0.012355284	Proteome
TRINITY_DN8390_c0_g1_i2_orf1	-0.001344706	-0.003256838	Proteome
TRINITY_DN11894_c1_g1_i5_orf1	0.011142238	0.002509007	Proteome
TRINITY_DN311_c0_g1_i8_orfp1	-0.045220006	0.017620895	Proteome
TRINITY_DN4694_c0_g2_i1_orf1	-0.006505894	-0.009827736	Proteome
TRINITY_DN16749_c0_g1_i1_orf1	-0.000756905	0.00741698	Proteome
TRINITY_DN2265_c0_g2_i1_orf1	0.00162564	-0.007399003	Proteome
TRINITY_DN3276_c0_g1_i4_orf1	0.017894443	0.014626866	Proteome
TRINITY_DN261_c0_g1_i5_orfp1	-0.016563724	0.021755822	Proteome
TRINITY_DN4299_c0_g1_i6_orf1	-0.025814604	-0.023084595	Proteome
TRINITY_DN2120_c0_g1_i2_orf1	0.00950142	0.002891141	Proteome
TRINITY_DN15865_c0_g2_i2_orf1	0.004021157	0.004352023	Proteome
TRINITY_DN86127_c0_g1_i6_orf1	-0.009604124	0.024653787	Proteome
TRINITY_DN16145_c0_g1_i12_orf1	-0.001544794	-0.026537432	Proteome
TRINITY_DN13747_c1_g2_i8_orf1	-0.005948157	-0.006533137	Proteome

TRINITY_DN29166_c0_g1_i1_orf1	-0.002018763	0.012571951	Proteome
TRINITY_DN657_c0_g1_i2_orf1	0.010325801	-0.004735861	Proteome
TRINITY_DN2676_c0_g1_i2_orf1	-0.01792827	0.007575426	Proteome
TRINITY_DN12442_c0_g1_i4_orf1	0.013264374	-0.005352994	Proteome
TRINITY_DN5531_c0_g3_i3_orf1	0.003378813	0.011660669	Proteome
TRINITY_DN7493_c0_g1_i1_orf1	-0.024508974	0.059664623	Proteome
TRINITY_DN13025_c0_g1_i8_orf1	-0.00059584	-0.01853892	Proteome
TRINITY_DN69049_c0_g1_i2_orf1	0.005905835	0.000478395	Proteome
TRINITY_DN30273_c1_g1_i1_orf1	0.009624876	0.013688496	Proteome
TRINITY_DN12181_c0_g2_i1_orf1	0.005446506	-0.014636429	Proteome
TRINITY_DN3971_c0_g1_i1_orf1	-0.002982541	-0.020700367	Proteome
TRINITY_DN11188_c0_g1_i2_orf1	0.006993866	-0.001089021	Proteome
TRINITY_DN106730_c0_g1_i1_orf1	0.007273661	0.00567383	Proteome
TRINITY_DN29026_c0_g1_i4_orf1	0.045704368	-0.014480782	Proteome
TRINITY_DN28844_c0_g1_i10_orf1	-0.00162045	0.019652387	Proteome
TRINITY_DN35002_c0_g2_i2_orf1	-0.000674233	0.0134208	Proteome
TRINITY_DN44110_c0_g1_i4_orf1	0.013685239	-0.008479033	Proteome
TRINITY_DN56708_c0_g3_i1_orfp1	0.005653347	0.013770675	Proteome
TRINITY_DN51836_c0_g3_i1_orf1	-0.020032483	-0.021415282	Proteome
TRINITY_DN63389_c0_g1_i4_orf1	0.011568314	0.004587056	Proteome
TRINITY_DN7047_c0_g1_i1_orf1	0.005656669	-0.000311509	Proteome
TRINITY_DN58125_c0_g1_i1_orf1	0.009099896	0.003067315	Proteome
TRINITY_DN78686_c0_g1_i1_orf1	0.013131494	0.00705983	Proteome
TRINITY_DN13898_c0_g1_i2_orf1	-0.004045406	0.00761438	Proteome
TRINITY_DN8502_c0_g1_i2_orf1	-0.00625141	-0.018555482	Proteome
TRINITY_DN34751_c0_g1_i1_orf1	-0.001794687	0.012069624	Proteome
TRINITY_DN6901_c0_g1_i4_orf1	0.009834129	0.007303237	Proteome
TRINITY_DN40281_c0_g1_i1_orf1	-0.013031223	-0.033720326	Proteome
TRINITY_DN15965_c0_g1_i1_orf1	0.006005673	-0.007236071	Proteome
TRINITY_DN37923_c0_g1_i1_orf1	0.00365753	0.012968731	Proteome
TRINITY_DN1333_c0_g1_i6_orf1	0.008368951	-0.01379578	Proteome
TRINITY_DN7803_c0_g1_i2_orf1	0.013535832	0.002716923	Proteome
TRINITY_DN19748_c0_g1_i4_orf1	0.005676053	-0.0178315	Proteome
TRINITY_DN54134_c0_g1_i1_orf1	-0.006454568	0.014118799	Proteome
TRINITY_DN33183_c0_g1_i4_orf1	0.00428051	-0.006316894	Proteome
TRINITY_DN74086_c0_g1_i1_orf1	0.015222282	0.015446864	Proteome
TRINITY_DN9517_c0_g1_i7_orf1	0.002769046	-0.005445281	Proteome
TRINITY_DN66_c10_g1_i1_orf1	-0.004477824	-0.019882358	Proteome
TRINITY_DN8660_c0_g1_i1_orf1	0.018923233	0.013598525	Proteome
TRINITY_DN2168_c0_g1_i2_orf1	0.012735723	-0.001353765	Proteome
TRINITY_DN17437_c0_g1_i1_orf1	0.011838992	-0.001473023	Proteome
TRINITY_DN172_c8_g2_i1_orf1	-0.000549885	0.012359858	Proteome
TRINITY_DN1375_c0_g1_i5_orf1	0.00160899	-0.002937814	Proteome
TRINITY_DN119291_c0_g1_i1_orf1	0.008929361	-0.004012418	Proteome
TRINITY_DN131662_c0_g1_i4_orf1	0.001793695	0.00145525	Proteome
TRINITY_DN749_c0_g1_i1_orf1	0.006267068	-0.007071714	Proteome
TRINITY_DN919_c0_g1_i7_orf1	0.001898965	0.01623867	Proteome
TRINITY_DN1099_c1_g1_i2_orf1	0.01329448	0.014860435	Proteome
TRINITY_DN928_c0_g2_i1_orf1	0.006876904	-0.000183609	Proteome
TRINITY_DN3275_c0_g1_i4_orf1	-0.015954677	-0.005617439	Proteome
TRINITY_DN18860_c0_g1_i1_orf1	-0.003406456	-0.003826566	Proteome
TRINITY_DN3116_c0_g1_i2_orf1	0.008112829	-0.006146488	Proteome
TRINITY_DN8692_c0_g1_i2_orf1	0.011278287	-0.000208572	Proteome
TRINITY_DN37418_c0_g1_i4_orf1	-0.011069375	0.014556044	Proteome
TRINITY_DN5811_c0_g1_i4_orf1	-0.000783963	0.015514625	Proteome
TRINITY_DN8922_c0_g1_i3_orf1	-0.024471668	-0.029458018	Proteome
TRINITY_DN8926_c0_g1_i4_orf1	0.00343408	-0.0065348	Proteome
TRINITY_DN1630_c0_g1_i6_orf1	-0.009259468	-0.014323213	Proteome

TRINITY_DN18782_c0_g1_i4_orf1	0.007856439	-0.015993433	Proteome
TRINITY_DN12527_c0_g1_i4_orf1	0.007369819	0.010504577	Proteome
TRINITY_DN1505_c0_g1_i1_orf1	0.000649082	0.012240175	Proteome
TRINITY_DN33430_c0_g1_i5_orf1	-0.006039831	-0.028457196	Proteome
TRINITY_DN68397_c0_g1_i2_orf1	-0.013230189	-0.008098251	Proteome
TRINITY_DN8555_c0_g1_i1_orf1	-0.003920467	0.00329772	Proteome
TRINITY_DN33452_c0_g1_i3_orf1	-0.013392428	0.002609486	Proteome
TRINITY_DN8473_c0_g1_i5_orf1	-0.018669031	-0.003565971	Proteome
TRINITY_DN28592_c0_g1_i2_orf1	0.008443503	0.005311672	Proteome
TRINITY_DN1492_c0_g1_i4_orf1	0.019731336	0.003756254	Proteome
TRINITY_DN3193_c0_g1_i3_orf1	-0.001167952	-0.015377303	Proteome
TRINITY_DN82426_c0_g1_i6_orfp1	0.013973485	0.005216123	Proteome
TRINITY_DN61777_c0_g1_i4_orf1	-0.009361249	-0.01845142	Proteome
TRINITY_DN18568_c0_g1_i2_orfp1	-0.025348913	-0.049583276	Proteome
TRINITY_DN5065_c0_g1_i1_orf1	0.003505729	-0.013162159	Proteome
TRINITY_DN959_c0_g1_i7_orf1	-0.006953791	-0.002062238	Proteome
TRINITY_DN4732_c0_g1_i2_orf1	0.012278762	-0.014370675	Proteome
TRINITY_DN59028_c0_g1_i1_orf1	-0.002879166	0.018762865	Proteome
TRINITY_DN4360_c0_g1_i4_orf1	-0.03260928	0.036251988	Proteome
TRINITY_DN344_c0_g1_i1_orf1	0.003001603	-0.004709024	Proteome
TRINITY_DN22944_c0_g3_i1_orf1	0.007139798	-0.010356837	Proteome
TRINITY_DN19187_c0_g1_i1_orf1	0.0122748	0.000446273	Proteome
TRINITY_DN47949_c0_g1_i4_orf1	0.003996913	0.004943534	Proteome
TRINITY_DN33009_c0_g1_i2_orfp1	-0.015205298	0.019279909	Proteome
TRINITY_DN61536_c0_g3_i1_orf1	-0.008093621	0.004041181	Proteome
TRINITY_DN22272_c0_g1_i1_orf1	-0.001603843	-0.012297618	Proteome
TRINITY_DN1532_c0_g1_i6_orf1	-0.013490482	-0.016634561	Proteome
TRINITY_DN3457_c0_g1_i4_orf1	0.008562396	0.009628516	Proteome
TRINITY_DN15154_c0_g1_i5_orf1	0.000801624	0.016947602	Proteome
TRINITY_DN26569_c0_g1_i4_orf1	-0.018278066	-0.047616639	Proteome
TRINITY_DN29017_c0_g1_i4_orf1	-0.010515512	-0.010616211	Proteome
TRINITY_DN8973_c0_g1_i3_orf1	-0.002437895	0.004471525	Proteome
TRINITY_DN31327_c0_g2_i1_orf1	0.012351639	0.004875897	Proteome
TRINITY_DN31967_c0_g1_i5_orf1	0.000317561	-0.008189762	Proteome
TRINITY_DN67026_c0_g1_i6_orf1	-0.015104367	0.005948959	Proteome
TRINITY_DN31980_c0_g1_i1_orf1	-0.011662672	-0.003510315	Proteome
TRINITY_DN754_c1_g1_i8_orf1	0.003965123	-0.010331367	Proteome
TRINITY_DN754_c1_g1_i6_orf1	0.009914916	0.014740085	Proteome
TRINITY_DN37366_c0_g1_i7_orf1	0.008500415	-0.003452714	Proteome
TRINITY_DN213_c0_g1_i3_orf1	-0.004808113	-0.016246685	Proteome
TRINITY_DN3833_c0_g1_i4_orf1	-0.001626786	0.017776991	Proteome
TRINITY_DN6567_c0_g1_i5_orf1	0.011269382	0.00331443	Proteome
TRINITY_DN73900_c0_g1_i1_orf1	-0.022951826	0.027792417	Proteome
TRINITY_DN22772_c0_g1_i5_orf1	-0.005374378	0.002500754	Proteome
TRINITY_DN122393_c0_g1_i1_orf1	0.008703446	0.01441644	Proteome
TRINITY_DN2822_c0_g1_i4_orf1	0.011437519	-0.009475928	Proteome
TRINITY_DN1114_c0_g1_i4_orf1	-0.009073174	0.004291636	Proteome
TRINITY_DN8561_c0_g4_i1_orf1	0.010116522	-0.004807492	Proteome
TRINITY_DN121439_c0_g1_i1_orf1	0.000313064	-0.011437497	Proteome
TRINITY_DN81248_c0_g1_i1_orf1	-0.007031359	-0.021064543	Proteome
TRINITY_DN9002_c0_g1_i1_orf1	0.006301923	-0.000735418	Proteome
TRINITY_DN38424_c0_g1_i1_orf1	0.014591729	-0.01319649	Proteome
TRINITY_DN5813_c0_g1_i9_orf1	-0.001910891	0.010304089	Proteome
TRINITY_DN14677_c0_g2_i3_orf1	0.009185658	0.0013998	Proteome
TRINITY_DN48713_c0_g1_i1_orf1	-0.008920281	-0.004465518	Proteome
TRINITY_DN96739_c0_g1_i1_orf1	0.02144787	0.004777598	Proteome
TRINITY_DN14112_c0_g1_i3_orf1	0.012250317	-0.000628303	Proteome
TRINITY_DN23746_c0_g1_i2_orf1	-0.023655404	-0.00023862	Proteome

TRINITY_DN11820_c0_g1_i1_orf1	0.014038975	0.006510896	Proteome
TRINITY_DN11566_c0_g1_i6_orf1	0.017571404	0.002773812	Proteome
TRINITY_DN3801_c0_g1_i9_orf1	0.001926181	0.003573516	Proteome
TRINITY_DN747_c0_g1_i4_orf1	0.010562423	-0.000808233	Proteome
TRINITY_DN44261_c0_g1_i1_orf1	0.003646837	0.012609697	Proteome
TRINITY_DN32420_c0_g1_i2_orf1	0.012553156	-0.00143575	Proteome
TRINITY_DN54711_c0_g1_i1_orf1	0.007094957	-0.00406454	Proteome
TRINITY_DN7583_c0_g1_i1_orf1	-0.005507341	0.010055102	Proteome
TRINITY_DN2621_c0_g1_i1_orf1	0.005113551	0.004786005	Proteome
TRINITY_DN5891_c0_g2_i4_orf1	0.000771734	-0.007541982	Proteome
TRINITY_DN57150_c0_g2_i1_orf1	0.008909527	0.005424739	Proteome
TRINITY_DN20749_c0_g1_i3_orf1	0.006502071	-0.00343714	Proteome
TRINITY_DN5503_c0_g1_i5_orf1	0.005431995	0.008282376	Proteome
TRINITY_DN30673_c0_g1_i5_orf1	-0.006173859	-0.002179838	Proteome
TRINITY_DN79868_c0_g1_i1_orf1	0.009770478	-0.000577086	Proteome
TRINITY_DN3665_c0_g1_i2_orf1	0.002492097	0.007555409	Proteome
TRINITY_DN132857_c0_g1_i1_orf1	0.007271905	-0.00550968	Proteome
TRINITY_DN15247_c0_g1_i2_orf1	0.035039144	-0.013631188	Proteome
TRINITY_DN1672_c0_g1_i6_orf1	0.029353939	0.002573516	Proteome
TRINITY_DN11069_c0_g2_i1_orf1	0.009872696	0.004645631	Proteome
TRINITY_DN125565_c1_g1_i1_orf1	-0.019986955	-0.038971263	Proteome
TRINITY_DN9492_c1_g1_i1_orf1	0.012821195	-0.015953174	Proteome
TRINITY_DN43942_c0_g2_i1_orf1	0.008221742	-0.003728332	Proteome
TRINITY_DN5086_c0_g1_i1_orf1	0.002255054	0.018303514	Proteome
TRINITY_DN11118_c0_g2_i1_orf1	0.001181541	0.011533901	Proteome
TRINITY_DN43293_c0_g1_i2_orf1	0.018942128	-0.003133795	Proteome
TRINITY_DN36496_c0_g1_i1_orf1	0.021708498	-0.005025266	Proteome
TRINITY_DN62557_c0_g1_i1_orf1	-0.011295564	-0.001486565	Proteome
TRINITY_DN41609_c0_g1_i1_orf1	0.002412088	-0.000804382	Proteome
TRINITY_DN1554_c0_g1_i9_orf1	-0.004754626	-0.002044751	Proteome
TRINITY_DN7750_c0_g1_i1_orf1	0.00198659	-0.012767309	Proteome
TRINITY_DN2117_c0_g1_i1_orf1	0.004697507	-0.004468208	Proteome
TRINITY_DN3109_c0_g1_i5_orf1	0.001537584	-0.018700535	Proteome
TRINITY_DN90108_c0_g1_i1_orf1	-0.016658698	-0.010204233	Proteome
TRINITY_DN6972_c0_g1_i3_orf1	-0.009777482	-0.004904182	Proteome
TRINITY_DN10195_c0_g1_i8_orf1	-0.011418633	0.019175712	Proteome
TRINITY_DN43228_c0_g1_i1_orf1	0.012512292	-0.012163032	Proteome
TRINITY_DN44658_c0_g1_i2_orf1	0.010480518	-0.010206943	Proteome
TRINITY_DN16316_c0_g1_i7_orf1	-0.018362622	-0.010516665	Proteome
TRINITY_DN81803_c0_g2_i1_orf1	0.002104251	-0.001016313	Proteome
TRINITY_DN14107_c0_g1_i4_orf1	-0.003951498	-0.011050619	Proteome
TRINITY_DN15370_c0_g1_i4_orf1	0.000913557	-0.008951105	Proteome
TRINITY_DN39509_c0_g1_i1_orf1	-0.019872986	-0.035004252	Proteome
TRINITY_DN1260_c0_g2_i1_orf1	0.019280552	0.003772728	Proteome
TRINITY_DN1448_c0_g1_i3_orf1	-0.008865406	-0.010792075	Proteome
TRINITY_DN198_c2_g1_i2_orf1	0.015398748	-0.0120507	Proteome
TRINITY_DN10325_c0_g1_i4_orf1	0.002966081	0.020917227	Proteome
TRINITY_DN12087_c0_g1_i2_orf1	-0.000386124	-0.001347669	Proteome
TRINITY_DN49527_c0_g1_i1_orf1	0.00125957	-0.004800291	Proteome
TRINITY_DN23175_c0_g1_i6_orf1	0.000832738	-0.005905138	Proteome
TRINITY_DN13157_c0_g1_i1_orf1	0.00889258	-0.016869521	Proteome
TRINITY_DN7234_c0_g1_i10_orf1	0.000893896	0.012514862	Proteome
TRINITY_DN12432_c0_g1_i2_orf1	-0.01255508	-0.010594542	Proteome
TRINITY_DN6418_c0_g1_i28_orf1	-0.003496575	0.00468735	Proteome
TRINITY_DN5593_c0_g1_i1_orf1	-0.011194755	0.002969347	Proteome
TRINITY_DN7549_c0_g1_i1_orf1	0.010925293	-0.005366635	Proteome
TRINITY_DN16933_c0_g1_i10_orf1	0.009608812	-0.007494472	Proteome
TRINITY_DN29440_c1_g1_i4_orf1	-0.005420823	0.007464636	Proteome

TRINITY_DN26408_c0_g1_i7_orf1	0.001782702	-0.015092325	Proteome
TRINITY_DN34166_c0_g1_i1_orf1	0.003375161	0.012281553	Proteome
TRINITY_DN19980_c0_g1_i4_orf1	0.012095339	-0.007394548	Proteome
TRINITY_DN27500_c0_g1_i4_orf1	0.020577776	0.008405471	Proteome
TRINITY_DN53847_c0_g1_i7_orf1	2.03E-05	0.007520019	Proteome
TRINITY_DN10652_c0_g1_i4_orf1	-0.002612176	-0.022481411	Proteome
TRINITY_DN8543_c0_g1_i1_orf1	0.005517551	0.005963099	Proteome
TRINITY_DN2596_c0_g1_i2_orf1	0.006840638	-0.006925716	Proteome
TRINITY_DN27704_c0_g1_i1_orf1	0.007715133	0.002115161	Proteome
TRINITY_DN51658_c0_g1_i1_orf1	0.000959071	-0.000965345	Proteome
TRINITY_DN1710_c0_g1_i1_orf1	-0.015946456	-0.006513277	Proteome
TRINITY_DN1267_c0_g2_i10_orf1	-0.0125722	0.017649162	Proteome
TRINITY_DN7966_c0_g1_i4_orf1	0.003641423	-0.003008397	Proteome
TRINITY_DN2170_c0_g2_i1_orf1	-0.022271767	0.001954783	Proteome
TRINITY_DN2058_c0_g1_i2_orf1	-0.018355552	0.026321175	Proteome
TRINITY_DN94475_c0_g1_i1_orf1	0.0032156	-0.018198351	Proteome
TRINITY_DN6660_c0_g1_i5_orf1	-0.006375736	-0.02731426	Proteome
TRINITY_DN1576_c0_g1_i4_orf1	-0.009959515	-0.030886354	Proteome
TRINITY_DN3254_c0_g1_i1_orf1	-0.005960886	-0.000567136	Proteome
TRINITY_DN7749_c1_g1_i2_orf1	0.002089265	0.004478485	Proteome
TRINITY_DN61744_c0_g1_i1_orf1	-0.008491551	0.012265071	Proteome
TRINITY_DN19866_c0_g1_i4_orf1	-0.023125249	-0.011793782	Proteome
TRINITY_DN29351_c0_g1_i1_orfp1	-0.008333172	-0.021647897	Proteome
TRINITY_DN1968_c0_g1_i3_orf1	-0.013472845	-0.011995791	Proteome
TRINITY_DN123396_c0_g1_i1_orf1	-0.003938785	0.004002816	Proteome
TRINITY_DN51498_c0_g1_i1_orf1	0.021259471	-0.005261076	Proteome
TRINITY_DN2508_c0_g1_i2_orf1	0.006886842	0.000226468	Proteome
TRINITY_DN32514_c0_g2_i1_orf1	0.00292907	-0.025985593	Proteome
TRINITY_DN146236_c0_g1_i1_orf1	0.01013473	-0.001490508	Proteome
TRINITY_DN25234_c0_g1_i1_orf1	-0.027812376	-0.014217229	Proteome
TRINITY_DN28981_c0_g1_i1_orf1	0.002113397	0.00879277	Proteome
TRINITY_DN12673_c3_g1_i2_orf1	-0.004213139	0.013693922	Proteome
TRINITY_DN48602_c0_g1_i6_orf1	-0.018031084	-0.009690624	Proteome
TRINITY_DN227_c0_g1_i1_orf1	-0.013129434	0.011563816	Proteome
TRINITY_DN5678_c0_g2_i3_orf1	0.007615544	0.005639235	Proteome
TRINITY_DN4514_c0_g1_i1_orf1	-0.012284777	0.011283495	Proteome
TRINITY_DN41349_c0_g3_i1_orf1	-0.010695726	0.002389402	Proteome
TRINITY_DN825_c2_g1_i5_orf1	-0.00863165	0.005610895	Proteome
TRINITY_DN68477_c0_g1_i1_orf1	-0.009127934	-0.014587646	Proteome
TRINITY_DN57475_c0_g1_i1_orf1	-0.001863365	-0.018393717	Proteome
TRINITY_DN32601_c0_g1_i2_orf1	0.007152978	0.013202051	Proteome
TRINITY_DN2402_c1_g1_i8_orf1	-0.006242252	0.012933977	Proteome
TRINITY_DN4356_c0_g1_i6_orf1	-0.010801768	0.00527489	Proteome
TRINITY_DN5363_c0_g1_i1_orf1	-0.003880821	-0.01062915	Proteome
TRINITY_DN1575_c0_g1_i10_orf1	-0.047061426	0.022040775	Proteome
TRINITY_DN24909_c0_g1_i5_orf1	-0.00369385	0.005493651	Proteome
TRINITY_DN5513_c0_g1_i1_orf1	0.012024967	-0.002351322	Proteome
TRINITY_DN34816_c0_g1_i4_orf1	0.007500644	0.011763194	Proteome
TRINITY_DN17772_c0_g2_i3_orf1	-0.01614003	-0.019340934	Proteome
TRINITY_DN121893_c0_g1_i1_orf1	0.014629736	0.002662762	Proteome
TRINITY_DN19250_c0_g2_i2_orf1	0.011456918	-0.010344103	Proteome
TRINITY_DN8107_c0_g1_i1_orf1	0.01280268	0.006829943	Proteome
TRINITY_DN3759_c0_g1_i1_orf1	0.010139638	0.003058192	Proteome
TRINITY_DN18338_c0_g1_i7_orf1	0.006029729	-0.015741139	Proteome
TRINITY_DN2623_c0_g1_i3_orf1	-0.000609606	-0.00508107	Proteome
TRINITY_DN437_c0_g1_i1_orf1	-0.002065521	0.011157768	Proteome
TRINITY_DN5630_c4_g1_i2_orf1	-0.004394818	0.011525895	Proteome
TRINITY_DN19995_c0_g1_i2_orf1	-0.005442642	0.007052947	Proteome

TRINITY_DN3401_c0_g1_i1_orf1	0.004598009	-0.01074614	Proteome
TRINITY_DN72541_c0_g1_i2_orf1	-0.003417601	-0.001072252	Proteome
TRINITY_DN56430_c0_g1_i1_orf1	0.008254583	0.018971935	Proteome
TRINITY_DN1639_c0_g2_i2_orf1	0.005666586	-0.008288534	Proteome
TRINITY_DN1528_c0_g1_i4_orf1	0.007810705	-0.016284933	Proteome
TRINITY_DN27087_c0_g1_i1_orf1	0.012241136	-0.003876631	Proteome
TRINITY_DN87522_c0_g2_i1_orf1	0.011372244	0.001285736	Proteome
TRINITY_DN7329_c0_g1_i6_orf1	0.013546528	-0.005796748	Proteome
TRINITY_DN14611_c0_g1_i5_orf1	0.007103052	0.009108315	Proteome
TRINITY_DN1254_c0_g1_i1_orf1	0.00757933	0.018871549	Proteome
TRINITY_DN18396_c0_g1_i1_orf1	0.009237492	-0.00425033	Proteome
TRINITY_DN16939_c0_g1_i4_orf1	0.005018255	0.006651875	Proteome
TRINITY_DN3591_c0_g1_i1_orf1	-0.000367489	-0.007095797	Proteome
TRINITY_DN143509_c0_g1_i1_orf1	0.012920448	-0.010425452	Proteome
TRINITY_DN5300_c0_g1_i3_orf1	0.012478881	-0.000812911	Proteome
TRINITY_DN24469_c0_g2_i2_orf1	0.0074532	0.011525502	Proteome
TRINITY_DN6136_c0_g1_i1_orf1	-0.014061849	-0.039590248	Proteome
TRINITY_DN34703_c0_g1_i4_orf1	0.015712594	0.018578072	Proteome
TRINITY_DN1701_c0_g1_i4_orf1	0.005041897	0.005074023	Proteome
TRINITY_DN15762_c0_g1_i2_orf1	0.001733404	-0.014884737	Proteome
TRINITY_DN95558_c0_g3_i1_orf1	0.023480415	0.005573224	Proteome
TRINITY_DN30300_c0_g2_i1_orf1	-0.008881638	-0.017316853	Proteome
TRINITY_DN85004_c0_g1_i1_orf1	-0.008629221	0.00510705	Proteome
TRINITY_DN44517_c0_g1_i4_orf1	0.00654862	-0.033779025	Proteome
TRINITY_DN42120_c0_g1_i2_orf1	-0.004474585	-0.006444472	Proteome
TRINITY_DN8771_c0_g2_i1_orf1	-0.043168179	0.015154347	Proteome
TRINITY_DN8838_c0_g1_i1_orf1	0.009767126	-0.013439545	Proteome
TRINITY_DN969_c0_g1_i3_orf1	0.005206768	0.008317411	Proteome
TRINITY_DN22053_c0_g1_i13_orf1	-0.009345749	-0.013367018	Proteome
TRINITY_DN3766_c0_g1_i10_orf1	0.012215721	0.016642419	Proteome
TRINITY_DN1628_c0_g2_i3_orf1	-0.024356778	-0.005953905	Proteome
TRINITY_DN66040_c0_g1_i2_orf1	-0.068586775	0.023097667	Proteome
TRINITY_DN27994_c0_g1_i1_orf1	-0.00501309	-0.004661612	Proteome
TRINITY_DN44557_c0_g2_i1_orf1	0.008365294	0.008992569	Proteome
TRINITY_DN79319_c0_g1_i8_orfp1	-0.000568225	-0.009278122	Proteome
TRINITY_DN25976_c0_g1_i4_orf1	-0.001968275	0.001492017	Proteome
TRINITY_DN66442_c0_g2_i3_orf1	0.007613251	-0.010174493	Proteome
TRINITY_DN4132_c0_g1_i14_orf1	0.017203619	0.000320588	Proteome
TRINITY_DN71465_c0_g1_i1_orf1	0.007544582	0.002086836	Proteome
TRINITY_DN35162_c0_g1_i4_orf1	0.010321589	-0.022357669	Proteome
TRINITY_DN19829_c0_g1_i1_orf1	0.008959919	0.002386402	Proteome
TRINITY_DN28922_c0_g1_i2_orf1	-0.015955069	-0.027044998	Proteome
TRINITY_DN3343_c0_g2_i1_orf1	0.011703208	0.001936846	Proteome
TRINITY_DN4895_c0_g1_i2_orf1	0.003055915	-0.010409477	Proteome
TRINITY_DN2996_c0_g1_i1_orf1	0.001589149	0.021193031	Proteome
TRINITY_DN6503_c0_g1_i8_orf1	-0.004826584	0.010474845	Proteome
TRINITY_DN5491_c0_g1_i4_orf1	-0.014022227	0.003558671	Proteome
TRINITY_DN2738_c1_g1_i3_orf1	0.002201823	-0.014121057	Proteome
TRINITY_DN1029_c0_g1_i1_orfp1	4.59E-05	0.028130506	Proteome
TRINITY_DN108819_c0_g1_i1_orf1	0.006589796	-0.002560886	Proteome
TRINITY_DN95_c0_g1_i5_orf1	-0.013634467	-0.013571386	Proteome
TRINITY_DN36494_c0_g1_i1_orf1	0.006264253	0.00052518	Proteome
TRINITY_DN5581_c0_g1_i1_orf1	0.014434515	0.003179804	Proteome
TRINITY_DN37729_c0_g1_i8_orf1	0.00542463	-0.000320463	Proteome
TRINITY_DN24631_c0_g2_i1_orf1	0.008127313	-0.003619968	Proteome
TRINITY_DN7473_c0_g1_i1_orf1	-0.005907818	0.010899854	Proteome
TRINITY_DN4003_c0_g1_i2_orf1	0.010940298	0.012777376	Proteome
TRINITY_DN31348_c0_g1_i1_orf1	-0.045985162	0.021078614	Proteome

TRINITY_DN104139_c0_g1_i1_orf1	-0.006087715	-0.007335411	Proteome
TRINITY_DN25686_c0_g1_i4_orf1	0.00159788	-0.01276847	Proteome
TRINITY_DN28439_c0_g1_i7_orf1	0.005514532	0.004623296	Proteome
TRINITY_DN18366_c0_g1_i1_orf1	-0.00116318	0.009414914	Proteome
TRINITY_DN10231_c0_g2_i1_orf1	0.033300622	-0.01059087	Proteome
TRINITY_DN27021_c0_g1_i1_orf1	-0.020393908	-0.005589825	Proteome
TRINITY_DN96566_c0_g1_i1_orf1	-0.002640104	-0.009609059	Proteome
TRINITY_DN23474_c1_g1_i1_orf1	0.01640136	0.001372761	Proteome
TRINITY_DN1753_c1_g1_i8_orf1	0.013519623	-0.00179213	Proteome
TRINITY_DN113778_c0_g2_i1_orf1	0.010901232	-0.009576124	Proteome
TRINITY_DN4814_c0_g1_i6_orf1	0.005996419	0.008859469	Proteome
TRINITY_DN5749_c0_g1_i4_orf1	0.011262101	-0.00234343	Proteome
TRINITY_DN20133_c0_g1_i1_orf1	-0.019977349	-0.006736756	Proteome
TRINITY_DN552_c0_g1_i3_orf1	0.00448709	-0.002592796	Proteome
TRINITY_DN825_c0_g1_i18_orfp1	-0.001723502	0.005371005	Proteome
TRINITY_DN2303_c0_g1_i1_orf1	0.00095918	-0.018462609	Proteome
TRINITY_DN14209_c0_g1_i1_orf1	0.011359284	0.016016465	Proteome
TRINITY_DN11584_c0_g1_i2_orf1	-0.009148159	-0.001776586	Proteome
TRINITY_DN91946_c0_g1_i1_orf1	0.024059309	-0.002889473	Proteome
TRINITY_DN143628_c0_g1_i1_orf1	-0.005490872	0.002667324	Proteome
TRINITY_DN84016_c0_g1_i1_orf1	0.002054687	-0.011171766	Proteome
TRINITY_DN7556_c0_g1_i3_orf1	-0.01855293	-0.004600076	Proteome
TRINITY_DN17394_c0_g1_i1_orf1	0.00326652	0.007295207	Proteome
TRINITY_DN8044_c0_g1_i2_orf1	0.008735975	0.01005837	Proteome
TRINITY_DN15930_c0_g1_i5_orf1	0.012955457	0.009168974	Proteome
TRINITY_DN14019_c0_g1_i5_orf1	0.009695987	-0.012001049	Proteome
TRINITY_DN57496_c0_g1_i1_orf1	0.013630379	0.002924974	Proteome
TRINITY_DN77005_c0_g3_i1_orf1	-0.005624	0.011924081	Proteome
TRINITY_DN8812_c0_g1_i1_orf1	-0.009188623	-0.009010809	Proteome
TRINITY_DN5208_c0_g1_i7_orf1	-0.00709722	-0.014928971	Proteome
TRINITY_DN8361_c0_g1_i4_orf1	0.010320144	0.019198555	Proteome
TRINITY_DN4004_c0_g1_i1_orf1	0.009348323	0.0111075	Proteome
TRINITY_DN39673_c0_g1_i1_orf1	0.00896733	0.002971878	Proteome
TRINITY_DN26805_c0_g2_i3_orf1	-0.000142818	-0.011709475	Proteome
TRINITY_DN7122_c0_g1_i1_orf1	0.012060282	0.002560014	Proteome
TRINITY_DN7212_c0_g1_i4_orf1	-0.012893835	-0.023363238	Proteome
TRINITY_DN42719_c0_g1_i1_orf1	-0.024477737	-0.001149451	Proteome
TRINITY_DN2474_c0_g1_i5_orf1	-0.012385144	-0.00360725	Proteome
TRINITY_DN3504_c0_g1_i4_orfp1	0.007810143	0.004316761	Proteome
TRINITY_DN2827_c3_g1_i3_orf1	0.00698084	-0.021682638	Proteome
TRINITY_DN120144_c0_g1_i1_orf1	-0.000274772	0.008566319	Proteome
TRINITY_DN5757_c0_g1_i1_orf1	-0.00755612	-0.013683483	Proteome
TRINITY_DN3499_c0_g1_i8_orf1	-0.010581889	0.012261013	Proteome
TRINITY_DN5460_c0_g1_i5_orf1	-0.004075415	0.003973616	Proteome
TRINITY_DN19885_c0_g1_i1_orf1	0.00931417	-0.001110607	Proteome
TRINITY_DN146181_c0_g1_i1_orf1	0.008326535	-0.022368779	Proteome
TRINITY_DN1073_c0_g1_i1_orf1	-0.007407895	-0.026446264	Proteome
TRINITY_DN3949_c1_g1_i1_orf1	0.01924546	-0.001379221	Proteome
TRINITY_DN12222_c0_g1_i1_orf1	0.014889968	-0.012881119	Proteome
TRINITY_DN11942_c0_g1_i1_orf1	0.020081107	0.006753225	Proteome
TRINITY_DN22875_c0_g1_i6_orf1	0.016606179	0.004585308	Proteome
TRINITY_DN6262_c0_g1_i2_orf1	0.003117347	0.010655516	Proteome
TRINITY_DN37699_c0_g1_i3_orfp1	0.001821889	-0.00480745	Proteome
TRINITY_DN9044_c0_g1_i1_orf1	0.007652924	-0.011669075	Proteome
TRINITY_DN81312_c0_g1_i1_orf1	0.013429216	0.003660136	Proteome
TRINITY_DN32306_c0_g1_i3_orf1	0.010836853	0.007910396	Proteome
TRINITY_DN2821_c0_g1_i1_orf1	-0.00015219	-0.027324308	Proteome
TRINITY_DN6436_c0_g1_i1_orf1	-0.006117856	0.009864416	Proteome

TRINITY_DN112234_c0_g1_i6_orf1	0.001833272	0.008745336	Proteome
TRINITY_DN460_c0_g1_i3_orf1	-0.008919638	-0.023374477	Proteome
TRINITY_DN2812_c0_g1_i5_orf1	-0.00479776	-0.010714126	Proteome
TRINITY_DN1292_c0_g1_i3_orf1	0.020665888	0.012024184	Proteome
TRINITY_DN32532_c0_g1_i1_orf1	-0.01496901	0.00410537	Proteome
TRINITY_DN10722_c0_g3_i1_orf1	0.006186406	0.015737948	Proteome
TRINITY_DN11665_c0_g1_i4_orf1	0.007648378	0.001184936	Proteome
TRINITY_DN25345_c0_g1_i1_orf1	-0.018174057	0.021086376	Proteome
TRINITY_DN16123_c0_g1_i1_orf1	-0.023839422	-0.008667279	Proteome
TRINITY_DN3119_c0_g1_i7_orf1	0.005513779	0.016669049	Proteome
TRINITY_DN2885_c1_g1_i2_orf1	0.006099599	-5.72E-05	Proteome
TRINITY_DN34465_c0_g1_i1_orf1	0.028119694	0.003248735	Proteome
TRINITY_DN11772_c0_g1_i1_orf1	0.010028381	0.005073338	Proteome
TRINITY_DN39170_c0_g1_i4_orf1	-0.011841433	0.019784133	Proteome
TRINITY_DN21539_c0_g1_i1_orf1	0.006781734	0.001024651	Proteome
TRINITY_DN51252_c0_g2_i1_orf1	0.006725401	0.000298487	Proteome
TRINITY_DN2971_c0_g1_i1_orf1	-0.009039084	0.006322237	Proteome
TRINITY_DN1703_c0_g1_i6_orf1	-0.001110455	0.02442925	Proteome
TRINITY_DN3131_c0_g1_i5_orf1	-0.015099162	0.002901937	Proteome
TRINITY_DN49942_c0_g1_i2_orf1	-0.001143521	-0.019421372	Proteome
TRINITY_DN41179_c0_g1_i1_orf1	0.004170723	0.009150666	Proteome
TRINITY_DN5408_c0_g1_i5_orf1	0.048008629	0.003884101	Proteome
TRINITY_DN638_c0_g2_i9_orf1	0.008092998	0.021593893	Proteome
TRINITY_DN9044_c0_g1_i2_orf1	-0.033282229	0.014164254	Proteome
TRINITY_DN14168_c0_g1_i1_orf1	0.003998407	-0.016262556	Proteome
TRINITY_DN4820_c0_g1_i1_orf1	0.00800578	0.007747234	Proteome
TRINITY_DN9836_c0_g1_i2_orf1	0.008813854	-0.005332852	Proteome
TRINITY_DN6330_c0_g1_i1_orfp1	0.043224768	0.002235256	Proteome
TRINITY_DN28938_c0_g1_i1_orf1	0.00780489	0.001389474	Proteome
TRINITY_DN14239_c0_g1_i5_orf1	0.024418094	-0.008758118	Proteome
TRINITY_DN25360_c0_g1_i2_orf1	0.0017138	-0.016710746	Proteome
TRINITY_DN53531_c0_g1_i1_orf1	0.006246802	-0.014348455	Proteome
TRINITY_DN4064_c0_g2_i1_orf1	0.018550391	-0.015666812	Proteome
TRINITY_DN2614_c0_g2_i3_orf1	-0.015300026	-0.026211911	Proteome
TRINITY_DN37218_c0_g1_i12_orf1	0.005296315	0.002595205	Proteome
TRINITY_DN50743_c0_g1_i1_orf1	0.004168671	-0.007674222	Proteome
TRINITY_DN14365_c0_g1_i2_orf1	0.008143445	0.000576254	Proteome
TRINITY_DN43637_c0_g1_i1_orf1	0.007134011	0.009668985	Proteome
TRINITY_DN77572_c0_g1_i1_orf1	0.014709389	-0.002367776	Proteome
TRINITY_DN10030_c0_g1_i2_orf1	0.002178737	-0.01180502	Proteome
TRINITY_DN19377_c0_g1_i4_orf1	0.002129936	-0.002261095	Proteome
TRINITY_DN16390_c0_g1_i4_orf1	0.008197658	0.004934509	Proteome
TRINITY_DN14673_c0_g1_i3_orf1	0.008973388	0.007251229	Proteome
TRINITY_DN4189_c0_g1_i4_orf1	-0.011755013	-0.014471992	Proteome
TRINITY_DN811_c0_g1_i15_orf1	-0.015181084	-0.00692787	Proteome
TRINITY_DN8652_c0_g1_i8_orf1	0.001822718	0.019630336	Proteome
TRINITY_DN1475_c0_g1_i6_orf1	0.002026829	0.007867246	Proteome
TRINITY_DN6312_c0_g1_i1_orf1	0.010935417	-0.014447044	Proteome
TRINITY_DN1757_c0_g1_i4_orf1	0.0053883	-0.017478939	Proteome
TRINITY_DN7739_c0_g1_i2_orf1	0.006873024	0.00317472	Proteome
TRINITY_DN18696_c0_g1_i1_orf1	-0.003737562	0.006348314	Proteome
TRINITY_DN868_c0_g1_i4_orf1	0.019469263	-0.000366193	Proteome
TRINITY_DN17995_c0_g4_i1_orf1	-0.0121988	-0.013601584	Proteome
TRINITY_DN84_c0_g1_i4_orf1	0.009585431	-0.012035353	Proteome
TRINITY_DN4280_c0_g1_i8_orf1	-0.005521492	-0.010975879	Proteome
TRINITY_DN27276_c0_g1_i5_orf1	0.020176006	-0.008986383	Proteome
TRINITY_DN9475_c0_g1_i6_orf1	0.009638445	0.016116277	Proteome
TRINITY_DN45708_c1_g1_i1_orf1	-0.00580382	0.001531157	Proteome

TRINITY_DN23089_c0_g1_i1_orf1	0.001556517	-0.016335341	Proteome
TRINITY_DN23343_c0_g1_i9_orf1	0.009691326	0.01057902	Proteome
TRINITY_DN3047_c0_g2_i6_orf1	0.010237537	-0.006782702	Proteome
TRINITY_DN12286_c1_g1_i2_orf1	-0.018759871	0.003439741	Proteome
TRINITY_DN10379_c0_g1_i3_orf1	-0.002531587	-0.019773035	Proteome
TRINITY_DN19244_c0_g1_i7_orf1	0.008547919	0.005346986	Proteome
TRINITY_DN18128_c0_g1_i4_orf1	-0.026983966	0.008176232	Proteome
TRINITY_DN9198_c0_g1_i4_orf1	0.014705919	0.006900024	Proteome
TRINITY_DN15373_c0_g1_i2_orf1	-0.002976108	-0.018597581	Proteome
TRINITY_DN23714_c0_g1_i4_orf1	-0.007587279	-0.014872815	Proteome
TRINITY_DN81084_c0_g3_i1_orf1	0.004131949	-0.008167405	Proteome
TRINITY_DN12775_c0_g1_i10_orfp1	0.006054428	-0.015062093	Proteome
TRINITY_DN14154_c0_g1_i1_orf1	-0.009570466	-0.016935324	Proteome
TRINITY_DN11368_c0_g1_i1_orf1	0.004219177	0.012413701	Proteome
TRINITY_DN3975_c0_g1_i7_orf1	-0.006433211	0.017324906	Proteome
TRINITY_DN3593_c0_g1_i3_orfp1	0.046410339	-0.012020759	Proteome
TRINITY_DN20118_c0_g1_i4_orfp1	0.007651995	0.002040411	Proteome
TRINITY_DN1895_c0_g1_i2_orf1	0.01417146	0.000126045	Proteome
TRINITY_DN245_c0_g1_i4_orf1	-0.001739754	-0.033282501	Proteome
TRINITY_DN5848_c0_g1_i6_orf1	0.00771005	-0.006482397	Proteome
TRINITY_DN1691_c0_g1_i3_orf1	0.001012234	0.019292935	Proteome
TRINITY_DN2712_c0_g1_i2_orf1	-0.012623872	-0.033869478	Proteome
TRINITY_DN41113_c0_g1_i7_orf1	0.005721944	0.016276419	Proteome
TRINITY_DN10371_c0_g1_i2_orf1	0.019793082	-0.014497808	Proteome
TRINITY_DN52051_c0_g1_i3_orf1	0.010997501	-0.0055691	Proteome
TRINITY_DN21367_c0_g1_i1_orf1	-0.032514394	0.039121494	Proteome
TRINITY_DN86355_c0_g1_i1_orf1	-0.004759552	0.008121651	Proteome
TRINITY_DN42240_c0_g2_i1_orf1	0.009242846	0.019120183	Proteome
TRINITY_DN2803_c2_g1_i8_orf1	0.008183263	0.005210924	Proteome
TRINITY_DN91198_c0_g2_i1_orf1	0.007525311	0.004594864	Proteome
TRINITY_DN23570_c0_g1_i2_orf1	-0.000236884	0.005907201	Proteome
TRINITY_DN16516_c0_g1_i1_orf1	0.00034549	-0.019987792	Proteome
TRINITY_DN17905_c0_g3_i1_orf1	-0.001869662	0.007679378	Proteome
TRINITY_DN14826_c0_g1_i1_orf1	0.004172543	0.004238973	Proteome
TRINITY_DN124950_c0_g2_i1_orf1	-0.004626234	0.011881818	Proteome
TRINITY_DN7881_c1_g1_i5_orf1	-0.003258053	-0.000352618	Proteome
TRINITY_DN95056_c0_g2_i2_orf1	0.01317478	0.000732553	Proteome
TRINITY_DN2044_c0_g1_i5_orfp1	-0.046193963	0.083873069	Proteome
TRINITY_DN5422_c0_g1_i1_orf1	0.025222419	-0.00154447	Proteome
TRINITY_DN5987_c1_g1_i1_orf1	-0.0158128	-0.040316922	Proteome
TRINITY_DN57749_c0_g1_i4_orf1	0.022514448	-0.00975664	Proteome
TRINITY_DN108354_c0_g1_i1_orf1	0.012653279	0.003182697	Proteome
TRINITY_DN17329_c0_g2_i3_orf1	-0.020008361	0.013799276	Proteome
TRINITY_DN1273_c0_g1_i4_orf1	0.002045941	-0.004321124	Proteome
TRINITY_DN24668_c0_g1_i8_orf1	-0.006285093	-0.004002997	Proteome
TRINITY_DN7992_c0_g1_i4_orf1	0.008017481	0.003481408	Proteome
TRINITY_DN1380_c0_g1_i5_orf1	-0.020193652	0.011026781	Proteome
TRINITY_DN65988_c0_g1_i4_orf1	0.008055455	0.008011424	Proteome
TRINITY_DN17671_c1_g2_i3_orf1	0.005427648	-0.011816582	Proteome
TRINITY_DN12858_c0_g1_i5_orf1	0.00474745	-0.006454134	Proteome
TRINITY_DN6439_c0_g1_i1_orf1	0.006300026	-0.006119509	Proteome
TRINITY_DN34413_c0_g1_i1_orf1	0.011372516	0.014662606	Proteome
TRINITY_DN56110_c0_g1_i1_orf1	0.006923616	-0.009924667	Proteome
TRINITY_DN29541_c0_g1_i1_orf1	0.031127774	-0.03938293	Proteome
TRINITY_DN129226_c0_g4_i1_orf1	-0.005164491	-0.007052644	Proteome
TRINITY_DN862_c0_g1_i4_orf1	-0.012495338	0.003417068	Proteome
TRINITY_DN4390_c0_g1_i4_orf1	-0.017725598	-0.017400902	Proteome
TRINITY_DN46372_c0_g2_i1_orf1	0.007282493	0.010095057	Proteome

TRINITY_DN141462_c0_g1_i1_orf1	0.002529656	0.001566786	Proteome
TRINITY_DN27456_c0_g2_i1_orf1	0.018665142	-0.005998003	Proteome
TRINITY_DN6205_c0_g1_i4_orfp1	0.002402835	0.013945808	Proteome
TRINITY_DN3355_c0_g3_i1_orf1	-0.009862806	-0.001198112	Proteome
TRINITY_DN7647_c0_g1_i4_orf1	0.002870248	0.011540902	Proteome
TRINITY_DN2611_c0_g1_i10_orf1	0.006146032	0.014314322	Proteome
TRINITY_DN31645_c0_g1_i3_orf1	0.013000731	0.015078594	Proteome
TRINITY_DN17615_c0_g1_i3_orf1	0.027761793	0.02888819	Proteome
TRINITY_DN30704_c0_g1_i1_orf1	0.007567953	-0.009146348	Proteome
TRINITY_DN38471_c0_g2_i1_orf1	-0.009581381	-0.032063786	Proteome
TRINITY_DN105055_c0_g1_i1_orfp1	3.57E-05	-0.01241426	Proteome
TRINITY_DN30271_c0_g1_i4_orf1	-0.009747711	0.018268874	Proteome
TRINITY_DN2606_c0_g1_i5_orf1	-0.003823739	-0.004625778	Proteome
TRINITY_DN15736_c0_g1_i2_orf1	0.009944499	0.012273345	Proteome
TRINITY_DN207_c0_g2_i3_orf1	-0.000340482	0.001114078	Proteome
TRINITY_DN6084_c0_g1_i4_orf1	0.006997588	-0.00364561	Proteome
TRINITY_DN13944_c0_g1_i1_orf1	0.001106887	-0.018543127	Proteome
TRINITY_DN1652_c0_g1_i12_orf1	0.013829145	0.006720836	Proteome
TRINITY_DN11698_c0_g1_i1_orf1	0.013201051	0.000921228	Proteome
TRINITY_DN1571_c0_g1_i9_orf1	0.00998542	-0.001656052	Proteome
TRINITY_DN39530_c0_g1_i1_orf1	0.002517745	0.010253539	Proteome
TRINITY_DN16643_c0_g2_i4_orf1	0.008884503	0.01106977	Proteome
TRINITY_DN56379_c0_g1_i1_orf1	0.002013119	-0.001427374	Proteome
TRINITY_DN778_c0_g1_i1_orf1	0.016160343	0.010486133	Proteome
TRINITY_DN12920_c0_g3_i1_orf1	0.014384525	-0.002942328	Proteome
TRINITY_DN4731_c0_g2_i1_orf1	-0.000307768	-0.017452209	Proteome
TRINITY_DN4463_c0_g1_i2_orf1	0.013442165	-0.012258639	Proteome
TRINITY_DN14734_c0_g1_i2_orf1	-0.010147684	0.00816589	Proteome
TRINITY_DN1230_c1_g1_i5_orf1	0.023666655	0.001641496	Proteome
TRINITY_DN8440_c0_g1_i9_orf1	0.012311284	-0.020026667	Proteome
TRINITY_DN19807_c0_g1_i1_orf1	-0.001712761	-0.005957652	Proteome
TRINITY_DN109931_c0_g1_i1_orf1	0.013690982	0.023412141	Proteome
TRINITY_DN57202_c0_g1_i1_orf1	0.005645092	-0.003554346	Proteome
TRINITY_DN54150_c0_g1_i1_orf1	-0.003897065	-0.001934923	Proteome
TRINITY_DN12372_c0_g1_i4_orf1	0.010532736	-0.003632011	Proteome
TRINITY_DN20067_c0_g1_i6_orf1	0.005144953	-0.004104244	Proteome
TRINITY_DN5045_c0_g1_i6_orf1	-0.002210126	-0.018505874	Proteome
TRINITY_DN109144_c0_g1_i5_orf1	0.005963215	-6.78E-05	Proteome
TRINITY_DN401_c0_g1_i15_orf1	0.001755978	0.014475659	Proteome
TRINITY_DN33837_c0_g1_i6_orf1	0.001610671	-0.003933302	Proteome
TRINITY_DN27033_c1_g1_i3_orfp1	-0.001345829	-0.042289117	Proteome
TRINITY_DN440_c0_g1_i11_orf1	-0.011078104	-0.010521977	Proteome
TRINITY_DN86580_c0_g1_i1_orf1	0.009416537	-0.002737475	Proteome
TRINITY_DN48970_c0_g1_i1_orf1	0.006776602	0.005146206	Proteome
TRINITY_DN7590_c0_g1_i4_orf1	0.007509157	-0.005197112	Proteome
TRINITY_DN1012_c0_g2_i1_orf1	0.013729404	-0.018999262	Proteome
TRINITY_DN64171_c0_g1_i1_orf1	-0.005903538	-0.018912445	Proteome
TRINITY_DN39933_c0_g1_i2_orf1	-0.005805183	-0.019995535	Proteome
TRINITY_DN5560_c0_g1_i5_orf1	-0.010078363	-0.017438158	Proteome
TRINITY_DN9248_c0_g1_i10_orf1	0.004239761	0.009677521	Proteome
TRINITY_DN9458_c0_g1_i4_orf1	-0.036905048	0.013658186	Proteome
TRINITY_DN37585_c0_g2_i1_orf1	0.013989188	-0.009589416	Proteome
TRINITY_DN6243_c0_g1_i5_orf1	0.005805184	-0.01371171	Proteome
TRINITY_DN53233_c0_g1_i1_orf1	-0.007833996	-0.011052045	Proteome
TRINITY_DN16905_c0_g1_i1_orf1	-0.011206353	-0.004144805	Proteome
TRINITY_DN116467_c0_g1_i1_orf1	-0.007047917	-0.015316059	Proteome
TRINITY_DN71832_c0_g1_i1_orf1	0.030848623	-0.000796494	Proteome
TRINITY_DN7735_c0_g1_i4_orf1	0.006396243	-0.012080803	Proteome

TRINITY_DN2695_c0_g1_i8_orfp1	-0.024636597	0.012494161	Proteome
TRINITY_DN3536_c0_g1_i1_orf1	-0.000350387	0.013431388	Proteome
TRINITY_DN29956_c1_g1_i1_orf1	0.001061997	-0.02887484	Proteome
TRINITY_DN48497_c0_g1_i1_orf1	0.032273742	0.007807585	Proteome
TRINITY_DN6059_c0_g1_i1_orf1	0.001058804	-0.00039315	Proteome
TRINITY_DN33418_c0_g1_i1_orf1	0.011079908	0.008759419	Proteome
TRINITY_DN445_c0_g1_i2_orf1	-0.002143961	0.00130981	Proteome
TRINITY_DN17417_c0_g1_i11_orf1	0.005844292	0.006356865	Proteome
TRINITY_DN38392_c0_g1_i1_orf1	0.028763827	0.008356312	Proteome
TRINITY_DN7064_c0_g1_i19_orf1	0.035609257	0.046355952	Proteome
TRINITY_DN25681_c0_g1_i5_orf1	-0.01116298	-0.004725023	Proteome
TRINITY_DN5767_c0_g1_i4_orf1	0.001062382	-0.014213654	Proteome
TRINITY_DN4248_c0_g1_i4_orf1	0.005179378	-0.025449313	Proteome
TRINITY_DN105574_c0_g1_i1_orf1	-0.003612434	0.027090545	Proteome
TRINITY_DN12014_c0_g1_i2_orf1	0.004625927	0.000901163	Proteome
TRINITY_DN4243_c0_g1_i6_orf1	0.000926946	0.017065587	Proteome
TRINITY_DN14601_c0_g1_i2_orf1	0.00818331	-0.012893014	Proteome
TRINITY_DN3529_c0_g1_i7_orf1	0.017647422	0.008939293	Proteome
TRINITY_DN1170_c0_g1_i8_orf1	0.001237147	-0.010352538	Proteome
TRINITY_DN7565_c0_g1_i3_orf1	0.007798252	0.002642763	Proteome
TRINITY_DN3755_c0_g1_i3_orf1	-0.00597694	-0.012253468	Proteome
TRINITY_DN18839_c0_g1_i4_orf1	0.012661868	-0.006086576	Proteome
TRINITY_DN4194_c0_g1_i1_orf1	0.018091898	0.00068305	Proteome
TRINITY_DN240_c0_g1_i4_orf1	0.009696999	-0.017690152	Proteome
TRINITY_DN12771_c0_g1_i1_orf1	-0.013849807	0.003300554	Proteome
TRINITY_DN9094_c0_g1_i1_orf1	-0.003513191	-0.001111479	Proteome
TRINITY_DN20614_c0_g1_i1_orf1	-0.002854083	-0.045330018	Proteome
TRINITY_DN5569_c0_g1_i1_orf1	0.005303709	-0.010523426	Proteome
TRINITY_DN1313_c0_g1_i2_orf1	-0.013803541	-0.000104047	Proteome
TRINITY_DN32022_c0_g1_i1_orf1	0.006479859	0.008127865	Proteome
TRINITY_DN40_c0_g2_i1_orf1	0.004746033	0.000153517	Proteome
TRINITY_DN356_c2_g1_i3_orf1	0.000775547	-0.024042578	Proteome
TRINITY_DN7238_c0_g1_i7_orf1	0.014847166	0.011834462	Proteome
TRINITY_DN7909_c0_g2_i1_orf1	0.013532081	0.00512307	Proteome
TRINITY_DN3255_c0_g1_i1_orf1	0.008642019	0.002084131	Proteome
TRINITY_DN11552_c0_g1_i4_orf1	0.005653034	0.013522761	Proteome
TRINITY_DN27725_c0_g1_i2_orf1	-0.003308172	-0.00255055	Proteome
TRINITY_DN81181_c0_g1_i6_orfp1	-0.0159495	-0.020329548	Proteome
TRINITY_DN17255_c0_g1_i9_orf1	0.000177567	-0.012000656	Proteome
TRINITY_DN4145_c0_g1_i1_orf1	0.002481499	0.005749458	Proteome
TRINITY_DN7622_c0_g2_i2_orf1	-0.021340616	-0.010975466	Proteome
TRINITY_DN24266_c0_g2_i2_orf1	0.005684817	0.006311179	Proteome
TRINITY_DN3322_c0_g1_i2_orf1	-0.014128389	-0.008313097	Proteome
TRINITY_DN3176_c0_g1_i2_orf1	0.001093615	0.013130772	Proteome
TRINITY_DN16978_c0_g1_i1_orf1	-0.016701113	-0.015886266	Proteome
TRINITY_DN40999_c0_g1_i1_orf1	0.004585232	0.00695629	Proteome
TRINITY_DN19043_c0_g3_i2_orf1	0.010374331	-0.011257391	Proteome
TRINITY_DN5070_c0_g1_i1_orf1	-0.018149138	0.01725132	Proteome
TRINITY_DN19303_c0_g1_i5_orf1	0.013461775	-0.003588532	Proteome
TRINITY_DN44935_c0_g1_i1_orf1	-0.00907295	-0.0213245	Proteome
TRINITY_DN46132_c0_g2_i2_orf1	0.004305989	-0.004086557	Proteome
TRINITY_DN26663_c0_g1_i4_orf1	0.009634808	-0.004357701	Proteome
TRINITY_DN15578_c0_g2_i1_orfp1	-0.032545374	0.005052703	Proteome
TRINITY_DN16830_c0_g1_i5_orf1	0.007321611	0.003090496	Proteome
TRINITY_DN1038_c1_g1_i3_orf1	0.01039065	-0.000943653	Proteome
TRINITY_DN2655_c0_g2_i1_orf1	-0.001954875	0.006608956	Proteome
TRINITY_DN10933_c0_g2_i1_orf1	-0.014529789	-0.019849203	Proteome
TRINITY_DN3651_c0_g1_i5_orf1	0.010675277	-0.005285976	Proteome

TRINITY_DN8621_c0_g1_i4_orf1	0.018750633	0.003781072	Proteome
TRINITY_DN3087_c0_g1_i1_orf1	0.003741462	-0.030038728	Proteome
TRINITY_DN28152_c0_g1_i1_orf1	0.003178475	-0.006807614	Proteome
TRINITY_DN14721_c0_g1_i2_orf1	-0.004790405	0.009298536	Proteome
TRINITY_DN80245_c0_g1_i1_orf1	0.007531992	-0.005047527	Proteome
TRINITY_DN1153_c1_g1_i1_orf1	0.017799769	0.009175895	Proteome
TRINITY_DN19413_c0_g1_i2_orf1	0.007069154	-0.010146877	Proteome
TRINITY_DN55160_c0_g2_i1_orf1	-0.011110255	0.000778134	Proteome
TRINITY_DN5538_c0_g1_i1_orf1	-0.010510954	-0.031394424	Proteome
TRINITY_DN10183_c0_g2_i3_orf1	0.016958225	0.003163321	Proteome
TRINITY_DN14063_c0_g1_i7_orf1	0.010068514	-0.001095841	Proteome
TRINITY_DN26010_c0_g1_i2_orf1	-0.003613978	0.006692391	Proteome
TRINITY_DN82810_c0_g1_i1_orf1	0.009238222	-0.018022323	Proteome
TRINITY_DN133414_c0_g1_i1_orf1	0.008601537	0.007986478	Proteome
TRINITY_DN7868_c0_g1_i2_orf1	-0.019433274	-0.017527098	Proteome
TRINITY_DN11322_c0_g1_i2_orf1	-0.007482208	-0.007960711	Proteome
TRINITY_DN35757_c0_g1_i1_orf1	0.018788911	0.004297619	Proteome
TRINITY_DN48054_c0_g1_i7_orf1	0.008455602	0.011085175	Proteome
TRINITY_DN10538_c0_g1_i7_orf1	-0.002704108	0.030771153	Proteome
TRINITY_DN9444_c0_g1_i3_orf1	0.006208765	0.015884293	Proteome
TRINITY_DN3773_c0_g1_i4_orf1	0.009212806	0.013458353	Proteome
TRINITY_DN3227_c0_g1_i5_orf1	-0.002095145	-0.00963982	Proteome
TRINITY_DN31751_c0_g1_i5_orf1	-0.003234209	-0.003337407	Proteome
TRINITY_DN7964_c0_g1_i1_orfp1	0.003608328	0.00375502	Proteome
TRINITY_DN2311_c0_g3_i1_orf1	0.027841596	-0.003503276	Proteome
TRINITY_DN1675_c0_g1_i1_orf1	-0.00119688	0.016472643	Proteome
TRINITY_DN1567_c0_g1_i15_orf1	-0.003688023	0.009701514	Proteome
TRINITY_DN110519_c0_g1_i1_orf1	0.007268724	0.002508877	Proteome
TRINITY_DN5046_c0_g3_i1_orf1	0.008408999	-0.007933543	Proteome
TRINITY_DN41573_c0_g1_i1_orf1	0.001508793	0.049604016	Proteome
TRINITY_DN2907_c0_g2_i4_orf1	0.011336819	0.009494276	Proteome
TRINITY_DN699_c0_g2_i1_orf1	-0.033491455	0.012702847	Proteome
TRINITY_DN98814_c0_g1_i2_orf1	-0.008993445	-0.011614294	Proteome
TRINITY_DN9536_c0_g1_i4_orf1	-0.009141489	0.005220127	Proteome
TRINITY_DN533_c3_g1_i2_orf1	0.003304537	0.010922989	Proteome
TRINITY_DN87803_c0_g1_i2_orf1	0.000294718	-0.001050851	Proteome
TRINITY_DN834_c0_g1_i1_orf1	-0.013539312	-0.016750382	Proteome
TRINITY_DN122423_c0_g1_i1_orf1	0.005320907	-0.002363517	Proteome
TRINITY_DN16179_c0_g1_i1_orf1	0.005708019	-0.014192788	Proteome
TRINITY_DN14464_c0_g1_i1_orf1	0.005178987	0.005222813	Proteome
TRINITY_DN1989_c0_g1_i1_orf1	-0.009023563	-0.020478513	Proteome
TRINITY_DN86127_c1_g1_i2_orfp1	-0.001610324	0.004465819	Proteome
TRINITY_DN26375_c0_g1_i1_orf1	0.005643986	0.009020439	Proteome
TRINITY_DN11986_c0_g1_i1_orf1	0.012652242	-0.02651394	Proteome
TRINITY_DN16408_c0_g1_i1_orf1	0.00954476	0.002849389	Proteome
TRINITY_DN7391_c0_g1_i2_orf1	0.004923728	-0.013153861	Proteome
TRINITY_DN81719_c0_g1_i1_orf1	-0.004234774	-0.016119213	Proteome
TRINITY_DN46447_c0_g1_i5_orf1	0.006655788	0.00102831	Proteome
TRINITY_DN8569_c1_g2_i7_orf1	0.011575542	0.003669477	Proteome
TRINITY_DN2897_c0_g2_i1_orf1	-0.018227964	0.006654435	Proteome
TRINITY_DN2535_c0_g1_i4_orf1	-0.007817414	0.000570009	Proteome
TRINITY_DN468_c0_g1_i3_orf1	0.005387209	-0.003750307	Proteome
TRINITY_DN25870_c0_g2_i6_orf1	0.006701745	0.010484212	Proteome
TRINITY_DN2196_c0_g1_i2_orf1	-0.006171135	0.005659283	Proteome
TRINITY_DN97883_c0_g1_i2_orf1	-0.021913969	0.016779148	Proteome
TRINITY_DN2954_c0_g1_i1_orf1	0.010682434	0.010167151	Proteome
TRINITY_DN1155_c0_g1_i9_orf1	0.003345629	-0.022462716	Proteome
TRINITY_DN135781_c0_g1_i1_orf1	0.011544979	0.0115846	Proteome

TRINITY_DN8640_c0_g1_i4_orf1	-0.002906539	-0.006081033	Proteome
TRINITY_DN16965_c0_g2_i1_orf1	0.011430415	-0.001130671	Proteome
TRINITY_DN6656_c0_g1_i1_orf1	0.010525835	-0.001729602	Proteome
TRINITY_DN34821_c0_g1_i4_orf1	-0.001959853	0.000487747	Proteome
TRINITY_DN4622_c0_g1_i1_orf1	-0.00240252	0.010421867	Proteome
TRINITY_DN15900_c0_g1_i6_orf1	0.009204858	7.58E-05	Proteome
TRINITY_DN3241_c0_g1_i1_orf1	0.014054544	0.00218183	Proteome
TRINITY_DN218_c0_g1_i1_orf1	0.006273987	0.005498108	Proteome
TRINITY_DN1427_c0_g1_i9_orf1	0.005713163	0.003066732	Proteome
TRINITY_DN12101_c0_g1_i2_orf1	-0.010530347	0.00168485	Proteome
TRINITY_DN3299_c0_g1_i2_orf1	-0.007013701	0.015941668	Proteome
TRINITY_DN5198_c0_g1_i5_orfp1	0.01260493	0.005078849	Proteome
TRINITY_DN4567_c0_g1_i5_orf1	0.005925874	-0.015944084	Proteome
TRINITY_DN24528_c0_g1_i1_orf1	0.024938624	-0.00275202	Proteome
TRINITY_DN858_c0_g1_i3_orf1	0.032986328	0.001492629	Proteome
TRINITY_DN15961_c0_g1_i1_orf1	-0.003145715	0.013176637	Proteome
TRINITY_DN14035_c0_g1_i1_orf1	0.004159273	-0.000871572	Proteome
TRINITY_DN3474_c1_g2_i7_orf1	0.009228618	-0.010824965	Proteome
TRINITY_DN41997_c0_g1_i2_orf1	0.001159261	-0.002018961	Proteome
TRINITY_DN56993_c0_g1_i4_orf1	0.010071735	-0.006129086	Proteome
TRINITY_DN925_c0_g1_i5_orf1	0.004668243	0.002853698	Proteome
TRINITY_DN7064_c0_g1_i6_orf1	0.006954933	0.009368257	Proteome
TRINITY_DN35669_c0_g1_i1_orf1	0.000781995	-0.003249224	Proteome
TRINITY_DN17373_c0_g1_i1_orf1	0.002943497	-0.024907788	Proteome
TRINITY_DN2400_c0_g1_i1_orf1	0.006495384	-0.012051784	Proteome
TRINITY_DN12392_c0_g1_i3_orf1	-0.000258425	-0.014457832	Proteome
TRINITY_DN115210_c0_g4_i1_orf1	0.020214695	0.003114064	Proteome
TRINITY_DN37538_c0_g4_i1_orf1	0.003027842	0.010950541	Proteome
TRINITY_DN7258_c0_g1_i2_orf1	0.006014608	-0.014033748	Proteome
TRINITY_DN11388_c0_g1_i4_orf1	-0.0135573	0.025534583	Proteome
TRINITY_DN44792_c0_g1_i1_orf1	-0.010216921	-0.013905345	Proteome
TRINITY_DN16840_c1_g1_i1_orf1	0.037763793	0.00925943	Proteome
TRINITY_DN4021_c0_g1_i1_orf1	0.018078378	-0.002925661	Proteome
TRINITY_DN7289_c0_g1_i1_orf1	-0.000740697	-0.014916031	Proteome
TRINITY_DN1108_c3_g1_i1_orfp1	-0.081647467	0.056708336	Proteome
TRINITY_DN35865_c0_g1_i1_orf1	0.013782267	-0.001522842	Proteome
TRINITY_DN8701_c0_g1_i3_orf1	0.015029835	0.002509393	Proteome
TRINITY_DN26293_c0_g1_i4_orf1	-0.006549397	0.014148443	Proteome
TRINITY_DN1569_c0_g1_i6_orf1	-0.027573079	0.006317754	Proteome
TRINITY_DN4056_c0_g1_i8_orf1	0.00872984	0.014998816	Proteome
TRINITY_DN8700_c9_g1_i1_orf1	-0.008274244	0.012169828	Proteome
TRINITY_DN15376_c0_g1_i1_orf1	-0.001865379	0.003499075	Proteome
TRINITY_DN29414_c1_g2_i1_orf1	-0.023599642	-0.052847594	Proteome
TRINITY_DN1706_c0_g1_i7_orf1	0.009440222	0.000745713	Proteome
TRINITY_DN6380_c0_g1_i1_orf1	0.001963455	-0.007995929	Proteome
TRINITY_DN9455_c0_g1_i6_orf1	0.000801988	0.020353729	Proteome
TRINITY_DN11928_c0_g1_i3_orf1	-0.002453786	-0.000719347	Proteome
TRINITY_DN19687_c0_g1_i1_orf1	-0.00560557	-0.013535661	Proteome
TRINITY_DN26429_c0_g1_i4_orf1	0.003635999	0.010909832	Proteome
TRINITY_DN33728_c0_g2_i1_orf1	-0.014494439	0.013135077	Proteome
TRINITY_DN5406_c0_g2_i1_orf1	-0.010991281	-0.006897542	Proteome
TRINITY_DN640_c0_g1_i2_orf1	0.00457644	-0.010994048	Proteome
TRINITY_DN14730_c0_g1_i7_orf1	0.002721601	0.000956631	Proteome
TRINITY_DN31_c0_g1_i3_orfp1	0.016084287	0.0016736	Proteome
TRINITY_DN22375_c0_g1_i4_orf1	-0.007689961	0.006375208	Proteome
TRINITY_DN34745_c0_g2_i1_orf1	0.005991104	0.015431331	Proteome
TRINITY_DN466_c0_g1_i5_orf1	0.000202911	-0.014402137	Proteome
TRINITY_DN4686_c0_g2_i1_orf1	0.005849598	-0.015324513	Proteome

TRINITY_DN2890_c0_g1_i2_orf1	0.011702616	-0.003145603	Proteome
TRINITY_DN112706_c0_g1_i2_orf1	0.00828374	0.012150185	Proteome
TRINITY_DN23264_c0_g1_i1_orf1	0.007569314	0.007752067	Proteome
TRINITY_DN5281_c0_g2_i3_orf1	0.001653479	-0.011878655	Proteome
TRINITY_DN17423_c0_g1_i2_orf1	-0.007906411	0.016167799	Proteome
TRINITY_DN8644_c0_g1_i3_orf1	0.005880501	0.028764539	Proteome
TRINITY_DN133228_c0_g1_i3_orf1	0.006735567	-0.006108518	Proteome
TRINITY_DN113272_c0_g1_i1_orf1	0.010237554	0.00883565	Proteome
TRINITY_DN18216_c0_g1_i4_orf1	0.011657244	-0.008506552	Proteome
TRINITY_DN12769_c0_g1_i5_orf1	0.007835883	0.011560021	Proteome
TRINITY_DN44073_c0_g1_i3_orf1	-0.020506871	0.014917896	Proteome
TRINITY_DN116874_c0_g1_i1_orfp1	-0.024762947	-0.01784035	Proteome
TRINITY_DN4911_c0_g1_i6_orf1	0.013530044	-0.009127512	Proteome
TRINITY_DN13361_c0_g1_i1_orf1	0.008870593	0.007813267	Proteome
TRINITY_DN29229_c0_g1_i4_orf1	0.005017867	0.010735918	Proteome
TRINITY_DN25017_c0_g1_i1_orf1	-0.002931158	-0.015722708	Proteome
TRINITY_DN6870_c0_g1_i5_orf1	0.016213219	0.009896564	Proteome
TRINITY_DN169_c0_g1_i3_orf1	0.002601008	0.008486781	Proteome
TRINITY_DN3887_c0_g1_i1_orf1	0.004523343	0.011376943	Proteome
TRINITY_DN70485_c0_g1_i2_orf1	-0.011240806	0.007287871	Proteome
TRINITY_DN10071_c0_g1_i2_orf1	-0.012431941	-0.007616071	Proteome
TRINITY_DN10058_c0_g1_i1_orf1	0.005412379	-0.016365139	Proteome
TRINITY_DN13094_c0_g1_i1_orf1	-0.006731695	-0.01805338	Proteome
TRINITY_DN13515_c0_g1_i1_orf1	-0.002941767	-0.01190346	Proteome
TRINITY_DN37585_c0_g1_i1_orf1	-0.011503087	-0.02300375	Proteome
TRINITY_DN8083_c0_g1_i1_orf1	0.00722765	0.014742836	Proteome
TRINITY_DN34015_c0_g1_i7_orf1	-0.004398152	0.01390638	Proteome
TRINITY_DN6424_c0_g1_i2_orf1	-0.021822345	-0.04395873	Proteome
TRINITY_DN2135_c0_g1_i2_orf1	0.008827884	-0.001978623	Proteome
TRINITY_DN129226_c0_g1_i2_orf1	0.013383272	-0.005913564	Proteome
TRINITY_DN136187_c0_g1_i2_orf1	0.002783207	-0.026087247	Proteome
TRINITY_DN6189_c0_g1_i1_orf1	0.000577023	-0.00246597	Proteome
TRINITY_DN747_c0_g2_i1_orf1	-0.005750296	-0.022135094	Proteome
TRINITY_DN277_c0_g1_i5_orf1	-0.004021065	-0.014716349	Proteome
TRINITY_DN16900_c0_g2_i1_orf1	0.002888153	-0.003239428	Proteome
TRINITY_DN34406_c0_g2_i9_orfp1	0.052192341	-0.015822965	Proteome
TRINITY_DN24218_c0_g1_i1_orf1	0.010409054	-0.015622169	Proteome
TRINITY_DN16899_c0_g2_i1_orf1	0.011517095	0.001149075	Proteome
TRINITY_DN96170_c0_g2_i1_orf1	0.007272885	-0.002217352	Proteome
TRINITY_DN21930_c0_g1_i1_orf1	0.005144577	-0.013897369	Proteome
TRINITY_DN33885_c0_g1_i1_orf1	-0.007707976	0.029209306	Proteome
TRINITY_DN9554_c0_g1_i1_orf1	0.016767535	-0.009305121	Proteome
TRINITY_DN28221_c0_g2_i1_orf1	-0.020938572	0.031016315	Proteome
TRINITY_DN29120_c0_g1_i6_orf1	0.004541889	0.004726957	Proteome
TRINITY_DN130439_c0_g1_i1_orf1	0.005552528	0.033838637	Proteome
TRINITY_DN72_c0_g1_i16_orf1	0.003049547	0.010341921	Proteome
TRINITY_DN4204_c0_g1_i1_orf1	-0.048708802	0.024763895	Proteome
TRINITY_DN1293_c1_g1_i4_orf1	-0.004893268	-0.015333741	Proteome
TRINITY_DN17368_c0_g1_i6_orf1	-0.018488427	-0.031225515	Proteome
TRINITY_DN25987_c0_g1_i5_orf1	0.005617373	-0.027352586	Proteome
TRINITY_DN22654_c0_g2_i4_orf1	0.003926807	0.00777139	Proteome
TRINITY_DN41708_c0_g1_i1_orf1	0.017924168	-0.00996126	Proteome
TRINITY_DN5300_c0_g1_i2_orf1	0.00125397	0.006296837	Proteome
TRINITY_DN1354_c5_g1_i1_orf1	-0.009726135	0.004412919	Proteome
TRINITY_DN13435_c0_g1_i1_orf1	-0.016909773	-0.005822889	Proteome
TRINITY_DN29034_c0_g1_i2_orf1	0.00415096	0.001308406	Proteome
TRINITY_DN20763_c0_g1_i2_orf1	0.003399557	-0.008068434	Proteome
TRINITY_DN8682_c0_g1_i4_orf1	-0.004793708	-0.018526238	Proteome

TRINITY_DN34040_c0_g2_i1_orf1	0.005411404	0.00469307	Proteome
TRINITY_DN98995_c0_g1_i2_orf1	0.021973097	-0.006650706	Proteome
TRINITY_DN27247_c0_g2_i1_orfp1	0.026189182	-0.021439205	Proteome
TRINITY_DN47_c0_g1_i2_orf1	9.39E-05	0.0066199	Proteome
TRINITY_DN4439_c0_g1_i2_orf1	-0.003557925	0.003809123	Proteome
TRINITY_DN21570_c0_g1_i1_orf1	-0.001671743	-0.014202133	Proteome
TRINITY_DN57998_c1_g1_i1_orf1	-0.019179884	0.020543	Proteome
TRINITY_DN125441_c0_g1_i5_orf1	-0.00441099	0.001367733	Proteome
TRINITY_DN119919_c0_g2_i1_orf1	-0.008872892	-0.026462643	Proteome
TRINITY_DN19814_c0_g1_i4_orf1	0.01312639	-0.003879728	Proteome
TRINITY_DN3702_c0_g1_i1_orf1	0.0170551	0.001398757	Proteome
TRINITY_DN10458_c0_g1_i1_orf1	0.005245479	-0.006237257	Proteome
TRINITY_DN34153_c0_g2_i2_orf1	0.015027901	-0.003139007	Proteome
TRINITY_DN29934_c0_g1_i6_orf1	0.001361702	0.003800641	Proteome
TRINITY_DN2700_c2_g1_i1_orf1	-0.003097727	0.024880005	Proteome
TRINITY_DN48765_c0_g1_i7_orf1	-0.015158504	0.000579372	Proteome
TRINITY_DN125_c0_g1_i2_orf1	-0.016595319	-0.019550968	Proteome
TRINITY_DN7635_c0_g1_i1_orf1	0.012975143	-0.006269662	Proteome
TRINITY_DN35991_c0_g1_i2_orf1	-0.004357247	-0.013647556	Proteome
TRINITY_DN6202_c0_g1_i2_orf1	-0.005773469	-0.001930184	Proteome
TRINITY_DN17655_c0_g1_i1_orf1	-0.008121583	-0.003402609	Proteome
TRINITY_DN51766_c0_g1_i2_orf1	0.004061701	0.003839915	Proteome
TRINITY_DN127056_c0_g1_i1_orf1	0.0099891	-0.01170906	Proteome
TRINITY_DN19584_c0_g1_i2_orf1	0.014007911	-0.007917478	Proteome
TRINITY_DN10939_c0_g1_i5_orf1	-0.005497927	-0.007768787	Proteome
TRINITY_DN23790_c0_g1_i1_orf1	0.008928509	-0.006289674	Proteome
TRINITY_DN9871_c0_g1_i11_orf1	0.00769416	0.018005747	Proteome
TRINITY_DN5200_c0_g1_i2_orf1	0.008934829	0.016642568	Proteome
TRINITY_DN27214_c0_g1_i4_orf1	0.00539026	0.011294777	Proteome
TRINITY_DN670_c0_g1_i15_orf1	0.012951378	0.015904426	Proteome
TRINITY_DN1381_c0_g1_i5_orf1	0.014996018	-0.025101648	Proteome
TRINITY_DN3503_c0_g1_i1_orfp1	0.006624487	-0.000158451	Proteome
TRINITY_DN198_c0_g1_i2_orf1	0.000332691	0.015789076	Proteome
TRINITY_DN40197_c0_g1_i1_orf1	0.003185524	-0.007113294	Proteome
TRINITY_DN18164_c0_g1_i7_orf1	-0.002771959	0.003321967	Proteome
TRINITY_DN867_c0_g1_i1_orf1	-0.014652065	0.007100045	Proteome
TRINITY_DN895_c0_g2_i1_orf1	0.003003493	-0.021663346	Proteome
TRINITY_DN142217_c0_g1_i1_orf1	0.013969391	-0.028559314	Proteome
TRINITY_DN14242_c0_g1_i2_orfp1	-0.095595857	0.076044059	Proteome
TRINITY_DN47914_c0_g2_i1_orf1	-0.003069576	0.001287052	Proteome
TRINITY_DN18196_c0_g1_i4_orf1	-0.003465034	-0.027874724	Proteome
TRINITY_DN1391_c1_g2_i4_orf1	0.010584872	0.000522533	Proteome
TRINITY_DN8432_c0_g2_i1_orf1	-0.006432804	-0.021779611	Proteome
TRINITY_DN279_c0_g1_i10_orf1	0.010668621	-0.002315582	Proteome
TRINITY_DN31431_c0_g1_i1_orf1	0.012907005	-0.000299614	Proteome
TRINITY_DN971_c0_g1_i5_orfp1	-0.092522069	0.085118929	Proteome
TRINITY_DN2589_c0_g1_i1_orf1	0.007495166	0.013586402	Proteome
TRINITY_DN1227_c0_g1_i1_orf1	-0.004446179	-0.016609181	Proteome
TRINITY_DN8252_c0_g1_i6_orf1	-0.008916675	-0.042097818	Proteome
TRINITY_DN409_c0_g1_i5_orf1	-0.008366287	-0.002765461	Proteome
TRINITY_DN18218_c0_g1_i7_orf1	-0.039521262	0.069991016	Proteome
TRINITY_DN2371_c0_g1_i10_orf1	0.009462852	0.002352998	Proteome
TRINITY_DN22572_c0_g1_i1_orf1	0.014849589	0.003667316	Proteome
TRINITY_DN7073_c0_g1_i1_orf1	0.006792746	0.008432169	Proteome
TRINITY_DN44491_c0_g1_i12_orf1	0.016223197	-0.006414306	Proteome
TRINITY_DN21596_c0_g1_i1_orf1	0.010946846	0.00378479	Proteome
TRINITY_DN6716_c0_g1_i1_orf1	0.005110497	-0.018510392	Proteome
TRINITY_DN581_c3_g2_i1_orf1	0.035951222	0.000832608	Proteome

TRINITY_DN46124_c0_g1_i1_orf1	0.00417491	0.004811842	Proteome
TRINITY_DN96884_c0_g1_i1_orf1	0.002404944	-0.015047488	Proteome
TRINITY_DN2031_c11_g1_i2_orfp1	0.019973704	0.005495865	Proteome
TRINITY_DN5233_c0_g1_i1_orf1	0.007673574	-0.00397582	Proteome
TRINITY_DN2396_c0_g1_i9_orfp1	-0.061730881	0.117664972	Proteome
TRINITY_DN55148_c0_g1_i1_orf1	0.012800164	0.026829404	Proteome
TRINITY_DN346_c0_g1_i7_orf1	-0.019642043	0.019252387	Proteome
TRINITY_DN6244_c0_g1_i4_orf1	0.001937497	0.020530079	Proteome
TRINITY_DN65247_c1_g1_i1_orf1	-0.010869505	-0.003208973	Proteome
TRINITY_DN11823_c3_g2_i1_orf1	0.010230667	-0.003271992	Proteome
TRINITY_DN5997_c0_g1_i4_orf1	-0.000317875	0.009720064	Proteome
TRINITY_DN21474_c0_g1_i1_orf1	-0.007681244	-0.027556188	Proteome
TRINITY_DN23229_c0_g1_i2_orf1	0.011754034	-0.01014519	Proteome
TRINITY_DN24391_c1_g1_i1_orf1	-0.007402148	0.010890056	Proteome
TRINITY_DN14057_c0_g1_i3_orf1	-0.006826087	-0.000112665	Proteome
TRINITY_DN26355_c0_g1_i4_orf1	0.001429679	0.012517082	Proteome
TRINITY_DN42373_c0_g4_i1_orf1	0.010989184	0.012973061	Proteome
TRINITY_DN31520_c1_g1_i1_orf1	0.007618724	0.008461797	Proteome
TRINITY_DN16128_c0_g1_i5_orf1	0.009463294	0.008998433	Proteome
TRINITY_DN37538_c0_g1_i1_orf1	-0.001853686	0.004374001	Proteome
TRINITY_DN9062_c0_g2_i1_orf1	0.009249153	0.011099111	Proteome
TRINITY_DN5008_c0_g1_i1_orf1	0.009592803	0.006820146	Proteome
TRINITY_DN4558_c0_g2_i1_orf1	-0.005535498	-0.014640468	Proteome
TRINITY_DN49147_c0_g2_i1_orf1	-0.006524869	-0.029855699	Proteome
TRINITY_DN5420_c0_g1_i2_orf1	-0.012652914	-0.010455248	Proteome
TRINITY_DN127151_c0_g1_i1_orf1	-0.003806955	-0.003163393	Proteome
TRINITY_DN98692_c0_g3_i1_orf1	-0.001184116	0.029179562	Proteome
TRINITY_DN1651_c0_g2_i1_orf1	-0.013049864	-0.003094729	Proteome
TRINITY_DN108122_c0_g1_i9_orf1	0.008456801	0.007938256	Proteome
TRINITY_DN34134_c0_g2_i1_orf1	0.021237949	-0.002075495	Proteome
TRINITY_DN4689_c0_g1_i5_orf1	-0.012226205	0.033688765	Proteome
TRINITY_DN22871_c0_g2_i1_orf1	-0.007265515	-0.012752173	Proteome
TRINITY_DN1380_c0_g1_i6_orf1	-0.00420667	0.005810414	Proteome
TRINITY_DN3486_c0_g1_i5_orf1	0.028066095	0.007614355	Proteome
TRINITY_DN131924_c0_g1_i2_orf1	-0.006828077	0.043332117	Proteome
TRINITY_DN30476_c0_g1_i1_orf1	0.012399668	0.017401078	Proteome
TRINITY_DN54387_c0_g1_i1_orf1	0.011444536	0.014695092	Proteome
TRINITY_DN40281_c0_g2_i1_orf1	0.010785292	0.002363167	Proteome
TRINITY_DN143603_c0_g1_i1_orf1	0.007638768	0.004586055	Proteome
TRINITY_DN46090_c0_g2_i1_orf1	0.008644442	0.00442384	Proteome
TRINITY_DN13563_c0_g1_i1_orf1	0.022209819	0.00038385	Proteome
TRINITY_DN42506_c0_g1_i1_orf1	0.005979476	-0.007016632	Proteome
TRINITY_DN67243_c0_g1_i1_orf1	0.001123584	-0.01446767	Proteome
TRINITY_DN4802_c0_g1_i4_orf1	0.061654355	0.002220526	Proteome
TRINITY_DN52761_c0_g2_i1_orf1	0.028240643	-0.002276552	Proteome
TRINITY_DN2441_c0_g1_i1_orf1	0.014264391	-0.00834977	Proteome
TRINITY_DN146544_c0_g1_i1_orf1	-0.001010216	0.011736191	Proteome
TRINITY_DN659_c0_g1_i3_orf1	0.008482314	0.006175695	Proteome
TRINITY_DN61674_c0_g1_i2_orf1	-0.000340963	0.00546891	Proteome
TRINITY_DN52768_c0_g1_i1_orf1	0.003306836	0.008831739	Proteome
TRINITY_DN6974_c0_g2_i1_orf1	6.39E-05	-0.011099051	Proteome
TRINITY_DN1999_c0_g1_i9_orf1	0.021807154	-0.000444544	Proteome
TRINITY_DN7134_c0_g1_i1_orf1	-0.002399287	0.010749483	Proteome
TRINITY_DN4053_c0_g1_i5_orf1	0.017427726	0.006601105	Proteome
TRINITY_DN23968_c0_g1_i2_orf1	-0.011778956	-0.015196416	Proteome
TRINITY_DN2395_c0_g1_i7_orf1	-0.00757434	-0.011695481	Proteome
TRINITY_DN11569_c0_g1_i1_orf1	-0.028457143	-0.073674435	Proteome
TRINITY_DN2270_c0_g2_i1_orf1	-0.001079516	0.001732575	Proteome

TRINITY_DN40028_c0_g2_i3_orf1	-0.000206802	-0.016389145	Proteome
TRINITY_DN9615_c0_g1_i1_orf1	-0.007314635	0.023669232	Proteome
TRINITY_DN313_c0_g1_i5_orf1	0.011194815	0.006468005	Proteome
TRINITY_DN19110_c0_g1_i2_orf1	-0.025342965	0.007029896	Proteome
TRINITY_DN146718_c0_g1_i1_orf1	0.015058707	0.00154656	Proteome
TRINITY_DN5042_c0_g1_i4_orf1	0.009947646	-0.016630693	Proteome
TRINITY_DN639_c0_g1_i10_orf1	0.004860704	-0.015235153	Proteome
TRINITY_DN5597_c0_g1_i2_orf1	-0.000706819	-0.008510414	Proteome
TRINITY_DN23838_c0_g1_i4_orf1	-0.011677951	0.039295029	Proteome
TRINITY_DN798_c1_g1_i3_orf1	0.002540179	0.022357105	Proteome
TRINITY_DN3845_c1_g1_i4_orf1	-0.001848319	-0.031314297	Proteome
TRINITY_DN138086_c0_g1_i1_orf1	0.006663412	0.017888911	Proteome
TRINITY_DN9410_c0_g1_i4_orf1	-0.001485697	-0.010064712	Proteome
TRINITY_DN1875_c0_g1_i1_orf1	0.016870207	0.003067028	Proteome
TRINITY_DN6351_c0_g1_i4_orf1	-0.000768549	0.01360307	Proteome
TRINITY_DN6388_c0_g1_i1_orf1	-0.006862708	-0.010997988	Proteome
TRINITY_DN43328_c0_g1_i1_orf1	0.006489263	0.005554656	Proteome
TRINITY_DN2691_c0_g1_i1_orf1	0.00137307	-0.007458695	Proteome
TRINITY_DN65604_c0_g1_i2_orf1	-0.002797506	0.004638321	Proteome
TRINITY_DN63943_c0_g1_i5_orf1	0.008005604	-0.014055815	Proteome
TRINITY_DN628_c0_g1_i7_orf1	-0.00875877	-0.00231301	Proteome
TRINITY_DN11596_c0_g1_i1_orf1	-0.011348444	0.002229341	Proteome
TRINITY_DN40823_c0_g1_i1_orf1	0.002949017	-0.011647072	Proteome
TRINITY_DN9207_c0_g1_i1_orf1	-0.001285266	-0.023875874	Proteome
TRINITY_DN5153_c1_g1_i1_orf1	0.009328583	-0.01032458	Proteome
TRINITY_DN699_c0_g1_i5_orf1	-0.018413235	-0.025811883	Proteome
TRINITY_DN101682_c0_g1_i1_orf1	0.015325851	0.015083855	Proteome
TRINITY_DN12024_c0_g1_i4_orf1	-0.008769771	0.011802709	Proteome
TRINITY_DN15845_c0_g1_i1_orf1	0.006180376	0.012873957	Proteome
TRINITY_DN1848_c0_g1_i2_orf1	0.006923647	0.000188569	Proteome
TRINITY_DN8598_c0_g1_i2_orf1	-0.000843805	-0.011086771	Proteome
TRINITY_DN4937_c0_g1_i2_orf1	0.011166191	-0.000536002	Proteome
TRINITY_DN28660_c0_g1_i4_orf1	0.004931738	-0.009912237	Proteome
TRINITY_DN42177_c0_g1_i4_orf1	0.008100451	0.006581841	Proteome
TRINITY_DN114198_c0_g1_i1_orf1	0.014296222	-0.004256583	Proteome
TRINITY_DN24693_c1_g1_i1_orf1	0.003931555	-0.009707705	Proteome
TRINITY_DN2475_c0_g2_i1_orf1	-0.002598436	-0.002153709	Proteome
TRINITY_DN48973_c0_g1_i5_orf1	-0.013640666	2.33E-05	Proteome
TRINITY_DN23119_c0_g1_i3_orf1	-0.01170478	0.002208916	Proteome
TRINITY_DN89829_c0_g1_i1_orf1	0.004657933	-0.00805255	Proteome
TRINITY_DN27110_c0_g1_i4_orf1	0.012637417	-0.016800939	Proteome
TRINITY_DN39532_c0_g1_i1_orf1	0.010653229	-0.00232388	Proteome
TRINITY_DN50571_c1_g1_i1_orf1	0.002043764	-0.011822328	Proteome
TRINITY_DN2874_c0_g1_i4_orf1	0.002084301	0.003084454	Proteome
TRINITY_DN7794_c0_g1_i1_orf1	0.013307181	-0.009057163	Proteome
TRINITY_DN4979_c0_g2_i9_orf1	-0.000510517	0.014905305	Proteome
TRINITY_DN2181_c1_g1_i8_orf1	0.007741493	0.008619424	Proteome
TRINITY_DN17907_c0_g1_i13_orf1	0.005262345	0.003195943	Proteome
TRINITY_DN27114_c0_g1_i1_orf1	-0.008817439	-0.016309267	Proteome
TRINITY_DN95971_c0_g5_i1_orf1	0.004803338	0.009929804	Proteome
TRINITY_DN8155_c0_g1_i1_orf1	-0.007685241	0.02102609	Proteome
TRINITY_DN96_c0_g1_i1_orf1	-0.001127971	0.00031436	Proteome
TRINITY_DN48237_c0_g1_i5_orf1	0.007335886	0.002013733	Proteome
TRINITY_DN36061_c0_g4_i2_orf1	-0.00137902	0.013268308	Proteome
TRINITY_DN11069_c0_g1_i6_orf1	0.009191219	-0.00448461	Proteome
TRINITY_DN11076_c0_g2_i1_orf1	0.01048552	0.020910684	Proteome
TRINITY_DN40704_c0_g1_i2_orf1	0.007637254	-0.003735179	Proteome
TRINITY_DN44857_c0_g1_i4_orf1	0.014370497	-0.005455466	Proteome

TRINITY_DN13626_c0_g2_i1_orf1	-0.00260509	0.007521903	Proteome
TRINITY_DN110460_c0_g2_i1_orf1	-0.001432075	0.020046162	Proteome
TRINITY_DN60792_c0_g1_i2_orf1	0.010078989	0.003029687	Proteome
TRINITY_DN12397_c0_g1_i1_orf1	-0.008278762	-0.012100541	Proteome
TRINITY_DN10742_c0_g1_i4_orf1	0.013009953	0.005958488	Proteome
TRINITY_DN38568_c0_g1_i1_orf1	0.015424103	0.009185423	Proteome
TRINITY_DN5637_c0_g1_i3_orf1	-0.016137503	-0.017148498	Proteome
TRINITY_DN384_c0_g1_i8_orf1	0.007907428	-0.015670427	Proteome
TRINITY_DN38307_c0_g1_i1_orfp1	0.041283276	0.021717632	Proteome
TRINITY_DN336_c0_g1_i6_orfp1	-0.009616351	0.004569491	Proteome
TRINITY_DN124303_c0_g1_i4_orf1	-0.00410407	-0.037937705	Proteome
TRINITY_DN6808_c0_g1_i2_orf1	0.002403736	-0.021677548	Proteome
TRINITY_DN10479_c0_g1_i6_orf1	0.010875659	0.001225503	Proteome
TRINITY_DN1650_c0_g1_i5_orf1	0.030352019	-0.01938624	Proteome
TRINITY_DN12787_c1_g1_i1_orf1	-0.001099904	0.001202312	Proteome
TRINITY_DN806_c0_g2_i1_orf1	0.007694015	-0.012721821	Proteome
TRINITY_DN311_c0_g1_i4_orfp1	-0.002020926	0.010499649	Proteome
TRINITY_DN58872_c0_g1_i1_orfp1	-0.003474328	-0.022301577	Proteome
TRINITY_DN1637_c0_g1_i5_orf1	0.005856376	-0.020198471	Proteome
TRINITY_DN1199_c0_g1_i1_orf1	0.005624309	-0.001836104	Proteome
TRINITY_DN1628_c0_g1_i1_orf1	0.013203466	0.011848526	Proteome
TRINITY_DN1272_c1_g1_i4_orf1	0.016665129	0.01054535	Proteome
TRINITY_DN2904_c0_g1_i4_orf1	0.01651108	-0.003940974	Proteome
TRINITY_DN5126_c0_g2_i1_orf1	0.043593545	-0.002209946	Proteome
TRINITY_DN107035_c0_g1_i1_orf1	0.005851959	0.009114345	Proteome
TRINITY_DN7477_c0_g1_i1_orf1	0.012676123	-0.014061118	Proteome
TRINITY_DN3515_c0_g1_i3_orf1	0.013050948	-0.021831894	Proteome
TRINITY_DN34347_c0_g1_i1_orf1	0.006165691	0.012127355	Proteome
TRINITY_DN77830_c0_g2_i2_orf1	-0.007553402	0.005191365	Proteome
TRINITY_DN13651_c0_g1_i2_orf1	0.006923968	0.00093592	Proteome
TRINITY_DN5012_c0_g1_i6_orf1	0.001194587	-0.005935564	Proteome
TRINITY_DN42082_c0_g2_i2_orfp1	0.011199365	-0.003242963	Proteome
TRINITY_DN23616_c0_g1_i4_orf1	-0.009143776	-0.017896497	Proteome
TRINITY_DN11457_c0_g1_i5_orf1	0.002951634	0.012711049	Proteome
TRINITY_DN24873_c0_g1_i4_orf1	0.010061931	0.003019527	Proteome
TRINITY_DN79804_c0_g1_i1_orf1	0.005452494	-0.002421006	Proteome
TRINITY_DN2544_c1_g1_i2_orf1	0.010683951	-0.000869135	Proteome
TRINITY_DN18159_c0_g1_i6_orf1	0.009472786	0.00065372	Proteome
TRINITY_DN14336_c0_g3_i2_orf1	-0.005696817	0.014868931	Proteome
TRINITY_DN6638_c0_g1_i1_orf1	-0.012850412	-0.004941422	Proteome
TRINITY_DN12133_c0_g2_i1_orf1	-0.004674292	-0.009521315	Proteome
TRINITY_DN61536_c0_g7_i1_orf1	-0.001937197	-0.014956564	Proteome
TRINITY_DN64403_c0_g2_i1_orf1	0.011038901	-0.002325275	Proteome
TRINITY_DN36856_c0_g1_i1_orf1	-0.010228582	0.011785366	Proteome
TRINITY_DN5554_c0_g1_i2_orf1	0.001195034	0.002803737	Proteome
TRINITY_DN115373_c0_g1_i1_orf1	0.006396978	0.018209155	Proteome
TRINITY_DN121650_c0_g1_i1_orf1	0.015123968	-0.004496171	Proteome
TRINITY_DN25783_c0_g1_i2_orf1	0.01184378	0.003772699	Proteome
TRINITY_DN5696_c0_g1_i4_orf1	0.034620347	0.003153318	Proteome
TRINITY_DN4062_c0_g2_i1_orf1	0.010389078	0.020428523	Proteome
TRINITY_DN88539_c0_g2_i1_orf1	-0.000816122	-0.010099596	Proteome
TRINITY_DN30932_c0_g1_i2_orf1	-0.000809841	0.000612097	Proteome
TRINITY_DN69557_c0_g1_i1_orf1	0.007428205	-0.006093042	Proteome
TRINITY_DN80424_c0_g1_i1_orf1	0.005854278	-0.008659324	Proteome
TRINITY_DN22257_c0_g1_i7_orf1	-0.008215519	-0.024035096	Proteome
TRINITY_DN21380_c0_g1_i1_orf1	-0.016624014	-0.003921117	Proteome
TRINITY_DN75188_c0_g1_i1_orf1	0.006848586	0.000448086	Proteome
TRINITY_DN4550_c1_g1_i5_orfp2	0.005750502	0.011270658	Proteome

TRINITY_DN1459_c0_g1_i2_orf1	-0.004552058	0.017056539	Proteome
TRINITY_DN106479_c1_g1_i1_orf1	-0.015126323	-0.029732812	Proteome
TRINITY_DN10672_c0_g1_i3_orf1	0.013792677	0.007444326	Proteome
TRINITY_DN50820_c0_g1_i2_orf1	-0.002390516	0.0212954	Proteome
TRINITY_DN18912_c1_g1_i1_orf1	0.004904973	0.004169736	Proteome
TRINITY_DN83621_c0_g1_i1_orf1	-0.000382273	0.026538136	Proteome
TRINITY_DN3760_c0_g1_i1_orf1	-0.00744354	-0.010238523	Proteome
TRINITY_DN8621_c0_g1_i5_orf1	-0.00492507	-0.002464728	Proteome
TRINITY_DN31943_c0_g1_i1_orf1	-0.002999869	0.002977137	Proteome
TRINITY_DN34509_c0_g1_i1_orf1	-0.002257509	-0.013991433	Proteome
TRINITY_DN4142_c0_g1_i5_orf1	0.020935096	-0.01279008	Proteome
TRINITY_DN45037_c0_g1_i1_orf1	-0.04815606	-0.004990846	Proteome
TRINITY_DN1274_c0_g1_i4_orf1	-0.001833803	-0.00584673	Proteome
TRINITY_DN10629_c0_g1_i1_orf1	0.011176103	0.007137562	Proteome
TRINITY_DN766_c0_g1_i1_orf1	0.000799806	-0.007738995	Proteome
TRINITY_DN5211_c0_g1_i1_orf1	0.00341028	-0.00065906	Proteome
TRINITY_DN40562_c0_g2_i1_orf1	0.005919639	-0.012082252	Proteome
TRINITY_DN7986_c1_g1_i4_orf1	-0.000661962	0.012099024	Proteome
TRINITY_DN16605_c0_g1_i3_orf1	0.00688287	0.003999132	Proteome
TRINITY_DN12013_c0_g1_i6_orf1	-0.003526943	0.007847919	Proteome
TRINITY_DN102051_c0_g1_i1_orf1	0.001308392	0.017597483	Proteome
TRINITY_DN3978_c0_g2_i1_orf1	-0.002498869	-0.032190647	Proteome
TRINITY_DN42738_c0_g1_i1_orf1	0.010855888	-0.001250671	Proteome
TRINITY_DN67193_c0_g1_i1_orf1	-0.026574062	-0.03157038	Proteome
TRINITY_DN3520_c0_g1_i4_orf1	0.009180257	-0.01044349	Proteome
TRINITY_DN51813_c0_g1_i1_orf1	-0.053503259	0.032309526	Proteome
TRINITY_DN22314_c0_g1_i2_orf1	0.01150037	-0.01926122	Proteome
TRINITY_DN12951_c1_g2_i2_orf1	0.003281479	-0.004908791	Proteome
TRINITY_DN237_c1_g1_i1_orf1	-0.00614029	-0.018839254	Proteome
TRINITY_DN320_c0_g1_i2_orf1	-0.008263465	-0.033094775	Proteome
TRINITY_DN69691_c0_g2_i1_orf1	-0.003944472	0.033338377	Proteome
TRINITY_DN42273_c0_g1_i1_orf1	-0.015436185	-0.009157391	Proteome
TRINITY_DN928_c0_g1_i3_orf1	-0.008017283	0.009119002	Proteome
TRINITY_DN20558_c0_g1_i2_orf1	0.041321138	-0.008661975	Proteome
TRINITY_DN22009_c0_g1_i1_orf1	0.015717412	0.005952693	Proteome
TRINITY_DN10658_c0_g1_i1_orf1	0.007436219	-0.002230336	Proteome
TRINITY_DN48838_c0_g1_i6_orf1	0.005289429	0.005080341	Proteome
TRINITY_DN6406_c0_g1_i1_orf1	0.006209046	-0.008820343	Proteome
TRINITY_DN83295_c0_g1_i3_orf1	-0.003811083	0.00624775	Proteome
TRINITY_DN13760_c1_g1_i1_orf1	-0.036234187	0.004930057	Proteome
TRINITY_DN18650_c0_g1_i1_orf1	-0.012113663	-0.011491001	Proteome
TRINITY_DN109733_c0_g1_i1_orf1	0.010356787	3.70E-05	Proteome
TRINITY_DN33995_c0_g1_i5_orf1	0.026133828	-0.011426322	Proteome
TRINITY_DN2178_c0_g1_i1_orf1	-0.00299851	-0.014289271	Proteome
TRINITY_DN54336_c0_g1_i1_orf1	-0.007606781	-0.013046532	Proteome
TRINITY_DN27736_c0_g1_i4_orf1	0.001293894	-0.004800745	Proteome
TRINITY_DN10796_c0_g1_i2_orf1	0.00145146	-0.010995873	Proteome
TRINITY_DN8986_c0_g1_i1_orf1	-0.003642149	0.013611543	Proteome
TRINITY_DN21331_c0_g1_i6_orf1	-0.010198047	-0.025848431	Proteome
TRINITY_DN5207_c0_g2_i3_orf1	-0.004766342	0.017644513	Proteome
TRINITY_DN2342_c0_g1_i1_orf1	0.003228305	-0.006576665	Proteome
TRINITY_DN20322_c0_g1_i1_orf1	0.008268234	0.017500873	Proteome
TRINITY_DN33705_c0_g1_i1_orf1	0.011276197	-0.003959293	Proteome
TRINITY_DN3000_c0_g1_i6_orf1	-0.0036684	-0.031778173	Proteome
TRINITY_DN30097_c0_g1_i2_orf1	0.013066246	-0.007203167	Proteome
TRINITY_DN81715_c0_g1_i1_orf1	0.005984432	-0.012153856	Proteome
TRINITY_DN816_c0_g1_i3_orf1	0.004680791	-0.000680114	Proteome
TRINITY_DN54925_c0_g1_i1_orf1	-0.000696772	-0.003135773	Proteome

TRINITY_DN14347_c0_g1_i1_orf1	0.01467056	0.001254236	Proteome
TRINITY_DN48641_c0_g1_i4_orf1	-0.003287224	0.006246404	Proteome
TRINITY_DN8430_c0_g1_i1_orf1	-0.012113294	-0.009572001	Proteome
TRINITY_DN5009_c0_g1_i2_orf1	0.010782284	0.010108848	Proteome
TRINITY_DN19920_c1_g1_i2_orf1	-0.005811248	-0.002680068	Proteome
TRINITY_DN8487_c0_g1_i4_orf1	0.005695814	0.001347493	Proteome
TRINITY_DN18009_c0_g1_i1_orf1	0.009935297	0.012559292	Proteome
TRINITY_DN4955_c0_g1_i2_orf1	0.005471929	-0.032804738	Proteome
TRINITY_DN2649_c0_g1_i3_orf1	0.009795811	0.004785726	Proteome
TRINITY_DN4762_c0_g1_i2_orf1	0.007534277	0.004840531	Proteome
TRINITY_DN4465_c0_g1_i9_orf1	0.000882164	0.015597429	Proteome
TRINITY_DN6071_c0_g1_i1_orf1	-0.003374712	0.011330177	Proteome
TRINITY_DN40519_c0_g1_i4_orf1	-0.010083037	0.006691169	Proteome
TRINITY_DN53810_c0_g1_i1_orf1	-0.001546795	0.018846369	Proteome
TRINITY_DN1351_c0_g1_i1_orf1	0.015549739	0.009118774	Proteome
TRINITY_DN213_c0_g1_i5_orf1	0.016445735	0.00631324	Proteome
TRINITY_DN879_c0_g1_i2_orf1	-0.004094044	-0.014477891	Proteome
TRINITY_DN1659_c0_g1_i3_orf1	0.004675129	-0.008051826	Proteome
TRINITY_DN3698_c0_g1_i4_orf1	0.010630224	0.019712198	Proteome
TRINITY_DN57_c0_g2_i3_orf1	0.010214293	-3.81E-05	Proteome
TRINITY_DN14705_c0_g2_i1_orf1	-0.014408508	-0.009122591	Proteome
TRINITY_DN90497_c0_g1_i1_orf1	0.005783683	0.00045087	Proteome
TRINITY_DN31310_c0_g1_i1_orf1	-0.004250082	0.011209222	Proteome
TRINITY_DN51829_c0_g1_i1_orf1	0.014685389	0.008068657	Proteome
TRINITY_DN6642_c0_g1_i2_orf1	0.023529017	-0.010151304	Proteome
TRINITY_DN12024_c0_g2_i2_orf1	-0.011321244	-0.013342891	Proteome
TRINITY_DN131371_c0_g1_i1_orf1	0.015990857	-0.000120978	Proteome
TRINITY_DN3273_c0_g1_i4_orf1	-0.002812692	-0.005630512	Proteome
TRINITY_DN31619_c0_g1_i2_orf1	0.003542393	-0.001354211	Proteome
TRINITY_DN21567_c0_g1_i7_orf1	-0.012376262	-0.008863517	Proteome
TRINITY_DN44285_c0_g1_i1_orf1	-0.012523425	-0.013558401	Proteome
TRINITY_DN3110_c0_g1_i4_orf1	0.01264089	-0.013835001	Proteome
TRINITY_DN6221_c0_g1_i5_orf1	-0.003986118	-0.013119778	Proteome
TRINITY_DN4116_c0_g1_i3_orf1	0.01027765	-0.007908873	Proteome
TRINITY_DN64759_c0_g1_i1_orf1	0.003379307	-0.017955494	Proteome
TRINITY_DN136467_c0_g1_i1_orf1	0.003681127	0.026946245	Proteome
TRINITY_DN6472_c0_g1_i5_orf1	0.011816772	0.014417759	Proteome
TRINITY_DN6589_c0_g1_i2_orf1	0.007103163	0.00565567	Proteome
TRINITY_DN37538_c0_g3_i1_orf1	-0.003413959	-0.012446659	Proteome
TRINITY_DN42854_c0_g3_i2_orf1	-0.020942719	0.007075262	Proteome
TRINITY_DN47591_c0_g1_i2_orf1	0.004574226	-0.030611711	Proteome
TRINITY_DN2252_c0_g1_i4_orfp1	0.007624905	0.00281284	Proteome
TRINITY_DN57798_c0_g1_i1_orf1	0.005259187	0.003028234	Proteome
TRINITY_DN23534_c0_g2_i2_orf1	-0.006754461	-0.010641481	Proteome
TRINITY_DN86699_c0_g4_i1_orf1	-0.012395853	0.007541121	Proteome
TRINITY_DN19135_c0_g1_i1_orf1	0.009577885	0.002667293	Proteome
TRINITY_DN111_c0_g2_i2_orf1	0.020723112	-0.003620728	Proteome
TRINITY_DN5998_c0_g2_i1_orf1	0.000899754	0.00229271	Proteome
TRINITY_DN60048_c0_g2_i1_orf1	0.006837903	0.003395855	Proteome
TRINITY_DN47260_c0_g1_i2_orf1	-0.002499928	-0.001856871	Proteome
TRINITY_DN467_c9_g1_i2_orf1	-0.00507931	0.004203691	Proteome
TRINITY_DN3469_c0_g1_i4_orf1	-0.005784835	-0.02287986	Proteome
TRINITY_DN64472_c0_g2_i1_orf1	0.014128197	0.00307718	Proteome
TRINITY_DN10287_c0_g1_i1_orf1	0.002077587	0.004019846	Proteome
TRINITY_DN21126_c0_g1_i1_orf1	0.003906193	0.007388753	Proteome
TRINITY_DN2004_c0_g1_i20_orf1	0.005690298	-0.019931914	Proteome
TRINITY_DN5457_c0_g1_i4_orf1	0.005148017	-0.008839621	Proteome
TRINITY_DN376_c1_g1_i1_orf1	-0.014198897	0.005645438	Proteome

TRINITY_DN64_c0_g1_i4_orf1	0.011397611	-0.020454714	Proteome
TRINITY_DN11587_c0_g1_i7_orf1	0.002883289	-0.00177723	Proteome
TRINITY_DN3638_c0_g1_i1_orf1	-0.002743904	-0.001835686	Proteome
TRINITY_DN82320_c0_g1_i2_orf1	-0.008034052	0.006402909	Proteome
TRINITY_DN142652_c0_g1_i1_orf1	0.012773495	0.00266211	Proteome
TRINITY_DN52296_c0_g1_i6_orf1	0.005629102	0.00199802	Proteome
TRINITY_DN1697_c0_g1_i1_orf1	0.008638731	0.005273787	Proteome
TRINITY_DN6140_c0_g3_i3_orf1	0.009983941	0.004764019	Proteome
TRINITY_DN142657_c0_g1_i1_orf1	-0.046037653	-0.012476693	Proteome
TRINITY_DN2828_c0_g1_i4_orf1	0.002830205	-0.023788975	Proteome
TRINITY_DN157_c0_g1_i4_orf1	0.007711876	0.003091543	Proteome
TRINITY_DN8024_c0_g1_i6_orf1	0.009419338	0.003390136	Proteome
TRINITY_DN11876_c0_g1_i2_orf1	-0.003078851	-0.002498683	Proteome
TRINITY_DN99694_c0_g1_i1_orf1	-0.005288517	-0.030356316	Proteome
TRINITY_DN4256_c0_g1_i1_orf1	-0.01699833	-0.014473501	Proteome
TRINITY_DN15222_c0_g1_i4_orf1	0.029099448	-0.001117555	Proteome
TRINITY_DN59885_c0_g1_i3_orf1	-0.004003955	0.050849903	Proteome
TRINITY_DN2499_c0_g1_i4_orf1	-0.013675634	0.009176391	Proteome
TRINITY_DN86309_c0_g1_i4_orf1	0.001563622	0.000319061	Proteome
TRINITY_DN3738_c0_g1_i5_orf1	-0.017179418	-0.013454792	Proteome
TRINITY_DN22526_c0_g1_i3_orf1	0.015215122	-0.017022019	Proteome
TRINITY_DN15388_c0_g1_i5_orf1	0.007920421	-0.007141419	Proteome
TRINITY_DN29144_c0_g3_i1_orf1	0.036541056	-0.003401338	Proteome
TRINITY_DN33272_c0_g1_i1_orf1	-0.012711399	0.00329305	Proteome
TRINITY_DN21961_c0_g2_i5_orf1	0.011779953	-0.003702146	Proteome
TRINITY_DN47723_c0_g1_i1_orf1	-0.005109246	0.007288433	Proteome
TRINITY_DN30679_c0_g1_i5_orf1	-0.011961621	0.01730024	Proteome
TRINITY_DN110523_c0_g2_i1_orf1	-0.012239275	-0.011197772	Proteome
TRINITY_DN113353_c0_g1_i1_orf1	-0.019609772	-0.003953013	Proteome
TRINITY_DN9400_c0_g1_i8_orf1	-0.021261113	-0.037466517	Proteome
TRINITY_DN8674_c0_g2_i1_orf1	0.014638033	-0.00572172	Proteome
TRINITY_DN9019_c0_g1_i5_orf1	0.000124767	-0.031686554	Proteome
TRINITY_DN35582_c0_g1_i1_orf1	-0.000780389	0.005544574	Proteome
TRINITY_DN865_c0_g1_i4_orf1	-0.001622476	-0.002460917	Proteome
TRINITY_DN14944_c0_g1_i7_orf1	-0.004357943	-0.003270492	Proteome
TRINITY_DN5125_c0_g1_i6_orf1	-0.004555742	-0.005832524	Proteome
TRINITY_DN33038_c0_g1_i1_orf1	-0.005398385	-0.017607246	Proteome
TRINITY_DN171_c0_g1_i1_orf1	0.007203206	-0.03186277	Proteome
TRINITY_DN106534_c0_g1_i1_orf1	0.013392009	0.00829485	Proteome
TRINITY_DN144286_c0_g1_i1_orf1	0.009512604	-0.009293841	Proteome
TRINITY_DN8726_c0_g2_i3_orf1	0.00022406	0.006730242	Proteome
TRINITY_DN7378_c0_g1_i5_orf1	0.000931755	0.004261033	Proteome
TRINITY_DN12464_c0_g1_i3_orf1	-0.006504071	0.011441162	Proteome
TRINITY_DN21134_c0_g1_i6_orf1	-0.002088322	-0.024088813	Proteome
TRINITY_DN33272_c0_g1_i5_orf1	0.01657029	0.039058479	Proteome
TRINITY_DN29106_c1_g1_i5_orf1	-0.01191162	-0.03034704	Proteome
TRINITY_DN9741_c0_g1_i3_orf1	0.010190028	0.001442748	Proteome
TRINITY_DN1181_c0_g1_i1_orf1	-0.010299632	-0.017642483	Proteome
TRINITY_DN43253_c0_g1_i5_orf1	0.012737586	0.016924349	Proteome
TRINITY_DN2815_c0_g1_i3_orf1	0.005332348	-0.000210355	Proteome
TRINITY_DN100_c0_g1_i9_orf1	0.010003698	0.004593769	Proteome
TRINITY_DN4741_c0_g1_i4_orf1	0.009080819	-0.006955707	Proteome
TRINITY_DN5910_c1_g1_i6_orf1	0.004970588	0.004482902	Proteome
TRINITY_DN1064_c0_g1_i3_orf1	0.002022907	0.000885701	Proteome
TRINITY_DN2913_c0_g1_i5_orf1	0.008913224	-0.004245376	Proteome
TRINITY_DN37658_c0_g1_i1_orf1	-0.002232081	-0.001241475	Proteome
TRINITY_DN9831_c0_g1_i1_orf1	0.007929676	-0.005681198	Proteome
TRINITY_DN3407_c0_g1_i9_orf1	-0.01406384	0.004194495	Proteome

TRINITY_DN45400_c0_g1_i1_orf1	-0.004013378	0.021756306	Proteome
TRINITY_DN2695_c0_g1_i14_orfp1	0.016214948	0.001146857	Proteome
TRINITY_DN51938_c0_g3_i1_orf1	0.025345356	0.011153317	Proteome
TRINITY_DN8610_c0_g1_i4_orf1	0.001520813	-0.017494584	Proteome
TRINITY_DN5772_c0_g1_i6_orf1	0.00125181	0.014995212	Proteome
TRINITY_DN57092_c0_g1_i1_orf1	-0.010252629	-0.009021753	Proteome
TRINITY_DN14679_c0_g1_i1_orf1	-0.000313365	0.008001723	Proteome
TRINITY_DN49508_c0_g2_i8_orf1	0.004665528	-0.01398294	Proteome
TRINITY_DN4991_c0_g1_i1_orf1	0.008356795	-0.015739556	Proteome
TRINITY_DN124711_c0_g1_i1_orf1	0.030462049	-0.010355422	Proteome
TRINITY_DN4273_c1_g1_i5_orf1	-0.005114682	0.016889383	Proteome
TRINITY_DN147475_c0_g1_i1_orf1	0.004644611	0.011078041	Proteome
TRINITY_DN4143_c0_g1_i1_orf1	-0.006939578	-0.002618465	Proteome
TRINITY_DN47609_c0_g1_i3_orfp1	-0.036375661	-0.060653127	Proteome
TRINITY_DN4636_c0_g3_i1_orf1	0.011077332	0.005391329	Proteome
TRINITY_DN9274_c0_g1_i7_orf1	-0.010763475	-0.001725951	Proteome
TRINITY_DN501_c1_g1_i1_orf1	0.007593959	0.008381157	Proteome
TRINITY_DN1344_c0_g1_i1_orf1	-0.009220988	-0.012296042	Proteome
TRINITY_DN43206_c0_g1_i6_orf1	-0.000440088	-0.013970635	Proteome
TRINITY_DN6572_c0_g1_i2_orf1	0.006029337	0.006859149	Proteome
TRINITY_DN383_c0_g1_i1_orf1	0.00739952	0.01735331	Proteome
TRINITY_DN4291_c1_g2_i1_orf1	-0.012818573	-0.036952681	Proteome
TRINITY_DN2102_c0_g1_i11_orf1	0.009791286	-0.005723433	Proteome
TRINITY_DN48404_c0_g1_i2_orf1	-0.010916463	-0.01980871	Proteome
TRINITY_DN6105_c0_g1_i1_orf1	-0.004820477	-0.004863523	Proteome
TRINITY_DN6381_c0_g1_i2_orf1	0.003000335	-0.015264254	Proteome
TRINITY_DN6813_c1_g1_i1_orf1	-0.008316509	-0.028776454	Proteome
TRINITY_DN723_c0_g1_i2_orf1	0.011565681	0.011408511	Proteome
TRINITY_DN2918_c0_g1_i1_orf1	-0.002431615	-0.003758109	Proteome
TRINITY_DN70382_c0_g1_i10_orf1	0.010761525	0.014103981	Proteome
TRINITY_DN2326_c0_g1_i1_orf1	0.013918194	-0.002700018	Proteome
TRINITY_DN104597_c0_g1_i2_orf1	-0.011013604	-0.007937742	Proteome
TRINITY_DN71549_c0_g1_i1_orf1	-0.010235202	0.00203378	Proteome
TRINITY_DN5437_c0_g1_i1_orf1	0.010995076	0.012383067	Proteome
TRINITY_DN75086_c0_g1_i5_orf1	0.032154503	-0.019409453	Proteome
TRINITY_DN976_c0_g1_i5_orf1	0.01204345	-0.028975513	Proteome
TRINITY_DN21623_c0_g2_i1_orf1	0.011421194	0.01336487	Proteome
TRINITY_DN19080_c0_g1_i4_orf1	0.002082947	0.010063529	Proteome
TRINITY_DN13067_c0_g1_i6_orf1	0.003663809	-0.017699614	Proteome
TRINITY_DN14429_c0_g1_i2_orf1	-0.000809811	-0.007453918	Proteome
TRINITY_DN1309_c0_g2_i1_orf1	0.010127128	-0.026122893	Proteome
TRINITY_DN1575_c0_g1_i7_orf1	0.012816528	0.009288769	Proteome
TRINITY_DN139537_c0_g1_i1_orf1	-0.014016762	-0.030902779	Proteome
TRINITY_DN86772_c0_g1_i3_orfp1	0.044929726	0.02460758	Proteome
TRINITY_DN29288_c0_g1_i1_orf1	-0.002778226	0.0157983	Proteome
TRINITY_DN30224_c0_g1_i1_orf1	0.011422217	0.011278583	Proteome
TRINITY_DN30169_c0_g1_i1_orfp1	-0.019551756	-0.052042803	Proteome
TRINITY_DN12582_c0_g1_i5_orf1	-0.036732875	-0.040484982	Proteome
TRINITY_DN1239_c0_g1_i3_orf1	0.011380716	-0.003599646	Proteome
TRINITY_DN3706_c0_g1_i6_orf1	0.00127055	-0.00206571	Proteome
TRINITY_DN4013_c0_g1_i4_orf1	-0.001175105	0.010003322	Proteome
TRINITY_DN47609_c0_g1_i1_orfp1	-0.016832243	0.012038235	Proteome
TRINITY_DN7275_c0_g1_i14_orf1	-0.003380423	0.008367867	Proteome
TRINITY_DN15256_c0_g1_i8_orf1	0.011844029	0.016162842	Proteome
TRINITY_DN72017_c0_g1_i1_orf1	-0.006193673	-0.017264042	Proteome
TRINITY_DN4929_c0_g1_i1_orf1	0.009403133	0.001896792	Proteome
TRINITY_DN113626_c0_g1_i3_orfp1	0.019636461	-0.006716169	Proteome
TRINITY_DN20356_c0_g1_i5_orf1	-0.021223929	0.007567124	Proteome

TRINITY_DN42364_c0_g1_i4_orf1	0.006549829	0.001793337	Proteome
TRINITY_DN20244_c0_g1_i1_orfp1	-0.011369646	0.009907545	Proteome
TRINITY_DN978_c9_g2_i1_orf1	0.006029805	0.013149032	Proteome
TRINITY_DN86956_c0_g5_i1_orf1	0.001384802	0.006997447	Proteome
TRINITY_DN19829_c0_g2_i1_orf1	-0.007122893	-0.006676592	Proteome
TRINITY_DN121156_c0_g2_i1_orf1	0.01129458	-0.009828711	Proteome
TRINITY_DN6205_c0_g1_i8_orf1	-0.00050567	-0.020497611	Proteome
TRINITY_DN962_c5_g1_i1_orf1	0.028913728	-0.009821686	Proteome
TRINITY_DN34159_c0_g2_i1_orf1	-0.012375566	0.004554256	Proteome
TRINITY_DN1098_c1_g1_i4_orf1	0.011699225	-0.010431032	Proteome
TRINITY_DN28543_c0_g1_i2_orf1	0.00633325	0.015241142	Proteome
TRINITY_DN4589_c0_g2_i1_orf1	-0.021267059	-0.044169826	Proteome
TRINITY_DN4535_c0_g1_i2_orf1	0.007063372	-0.022191954	Proteome
TRINITY_DN8087_c0_g1_i9_orf1	-0.009088272	-0.007097538	Proteome
TRINITY_DN9117_c0_g1_i1_orf1	-0.001602542	-0.003099317	Proteome
TRINITY_DN20717_c0_g1_i1_orf1	0.008356623	0.020410596	Proteome
TRINITY_DN21125_c0_g1_i1_orf1	0.017350894	-0.005415897	Proteome
TRINITY_DN4246_c0_g2_i3_orf1	0.013438639	-0.020816649	Proteome
TRINITY_DN32700_c0_g1_i2_orf1	-0.003166867	-0.018583929	Proteome
TRINITY_DN11649_c0_g1_i4_orf1	0.02044478	-0.002034857	Proteome
TRINITY_DN22836_c0_g1_i5_orf1	0.011762522	0.009369162	Proteome
TRINITY_DN36262_c0_g1_i1_orf1	0.004217118	0.010841849	Proteome
TRINITY_DN64196_c0_g1_i2_orf1	0.007509403	0.018734738	Proteome
TRINITY_DN130051_c0_g1_i1_orf1	0.010321373	0.007587175	Proteome
TRINITY_DN2061_c0_g1_i3_orf1	0.008877127	0.017120429	Proteome
TRINITY_DN2675_c0_g1_i1_orf1	0.004063637	-0.029756739	Proteome
TRINITY_DN921_c3_g3_i1_orf1	0.013385984	0.002061667	Proteome
TRINITY_DN42171_c0_g1_i1_orf1	0.003673911	0.003543875	Proteome
TRINITY_DN11492_c0_g1_i8_orf1	0.00812534	-0.012119448	Proteome
TRINITY_DN131642_c0_g1_i2_orf1	0.011055644	0.009175216	Proteome
TRINITY_DN78873_c0_g1_i4_orf1	0.009611778	0.000667037	Proteome
TRINITY_DN556_c0_g1_i4_orf1	-0.002833034	-0.025181265	Proteome
TRINITY_DN2141_c0_g1_i1_orf1	-0.011569246	-0.014638828	Proteome
TRINITY_DN5553_c0_g1_i4_orf1	-0.020423819	0.015286275	Proteome
TRINITY_DN2098_c0_g1_i1_orf1	0.005425082	-0.013564287	Proteome
TRINITY_DN1104_c0_g1_i1_orfp1	0.018502702	0.008932249	Proteome
TRINITY_DN21181_c0_g1_i6_orf1	0.002402277	-0.013194133	Proteome
TRINITY_DN8971_c1_g1_i4_orf1	0.014131035	-0.000107752	Proteome
TRINITY_DN6118_c0_g1_i13_orf1	-0.009451141	-0.026561822	Proteome
TRINITY_DN49742_c0_g1_i4_orf1	0.004934221	0.014518058	Proteome
TRINITY_DN52_c0_g1_i4_orf1	0.007692238	-0.005857433	Proteome
TRINITY_DN17003_c0_g1_i1_orf1	0.000916325	0.007349308	Proteome
TRINITY_DN9029_c0_g1_i4_orf1	-0.002712739	-0.001157313	Proteome
TRINITY_DN10057_c0_g2_i1_orf1	0.002629587	0.00264366	Proteome
TRINITY_DN710_c0_g1_i11_orfp1	0.043828989	0.0104454	Proteome
TRINITY_DN84357_c0_g1_i1_orf1	-0.014917867	0.002346685	Proteome
TRINITY_DN4842_c0_g1_i5_orf1	-0.005286284	0.008402313	Proteome
TRINITY_DN713_c0_g1_i4_orf1	0.004751212	0.007286196	Proteome
TRINITY_DN32956_c0_g1_i4_orf1	0.006169061	0.006935731	Proteome
TRINITY_DN3614_c0_g2_i1_orf1	0.005735458	0.010866662	Proteome
TRINITY_DN16482_c0_g1_i6_orf1	0.00502245	0.011089774	Proteome
TRINITY_DN14904_c1_g2_i2_orf1	0.007669513	0.014865187	Proteome
TRINITY_DN14134_c0_g2_i3_orf1	0.007144682	-0.013743076	Proteome
TRINITY_DN15903_c0_g1_i2_orf1	0.011112835	-0.004417788	Proteome
TRINITY_DN105749_c0_g1_i1_orf1	0.016179636	-0.029605455	Proteome
TRINITY_DN544_c0_g1_i4_orf1	0.009008023	0.024205988	Proteome
TRINITY_DN45227_c0_g1_i3_orf1	0.00256395	0.009786243	Proteome
TRINITY_DN29633_c0_g1_i8_orf1	0.003399378	-0.004474835	Proteome

TRINITY_DN47591_c1_g1_i1_orf1	0.001751994	-0.013260775	Proteome
TRINITY_DN6785_c0_g1_i1_orf1	0.007800668	-0.00261252	Proteome
TRINITY_DN88876_c0_g1_i1_orf1	0.001013236	0.001680697	Proteome
TRINITY_DN4443_c0_g1_i4_orf1	-0.009559502	0.024934657	Proteome
TRINITY_DN42719_c0_g2_i1_orf1	-0.042876912	-0.000418018	Proteome
TRINITY_DN44877_c0_g1_i2_orf1	0.004890674	0.00805028	Proteome
TRINITY_DN809_c0_g1_i9_orf1	0.005220718	0.022092001	Proteome
TRINITY_DN43369_c0_g2_i1_orf1	0.016561455	-0.00874491	Proteome
TRINITY_DN41129_c0_g1_i1_orf1	-0.014182244	-0.002263065	Proteome
TRINITY_DN1955_c0_g1_i5_orf1	-0.003649869	-0.025202916	Proteome
TRINITY_DN3814_c1_g1_i1_orf1	0.008590726	0.004055505	Proteome
TRINITY_DN18404_c0_g1_i5_orf1	-0.002789377	0.004724978	Proteome
TRINITY_DN32161_c0_g1_i1_orf1	-0.00532331	0.013231647	Proteome
TRINITY_DN2541_c0_g1_i11_orf1	0.003448723	-0.003929522	Proteome
TRINITY_DN5121_c0_g1_i1_orf1	-0.001396834	-0.016845314	Proteome
TRINITY_DN34087_c0_g1_i4_orf1	-0.010007628	0.012786251	Proteome
TRINITY_DN526_c0_g1_i1_orf1	-0.017630942	0.000674663	Proteome
TRINITY_DN3105_c0_g1_i4_orf1	0.002198271	-0.010687211	Proteome
TRINITY_DN9518_c0_g1_i6_orf1	0.003407757	-7.50E-05	Proteome
TRINITY_DN9119_c0_g1_i3_orf1	-0.007413457	0.001476081	Proteome
TRINITY_DN11084_c1_g1_i2_orf1	0.011337508	0.007656126	Proteome
TRINITY_DN94755_c0_g1_i5_orfp1	0.002424368	0.003009996	Proteome
TRINITY_DN2475_c0_g1_i1_orf1	-0.009941105	-0.029327372	Proteome
TRINITY_DN6396_c0_g1_i1_orf1	0.009163418	-0.013152326	Proteome
TRINITY_DN11735_c0_g1_i5_orf1	-0.00471272	-0.002337904	Proteome
TRINITY_DN17693_c0_g1_i10_orf1	-0.03922456	0.016999909	Proteome
TRINITY_DN8527_c0_g2_i1_orfp1	0.006737214	0.004716984	Proteome
TRINITY_DN5667_c0_g1_i4_orf1	-0.018440924	0.027374821	Proteome
TRINITY_DN17651_c0_g1_i2_orf1	-0.001663317	-0.004790798	Proteome
TRINITY_DN23183_c1_g1_i2_orf1	0.016213202	0.002943862	Proteome
TRINITY_DN12700_c0_g1_i7_orf1	0.012777954	-0.00210507	Proteome
TRINITY_DN48846_c0_g1_i1_orf1	-0.000597179	0.005686847	Proteome
TRINITY_DN117042_c0_g1_i2_orf1	0.002998595	-0.005141662	Proteome
TRINITY_DN11015_c0_g1_i8_orf1	0.008540821	-0.019983815	Proteome
TRINITY_DN29521_c0_g1_i1_orf1	0.012829003	0.009977118	Proteome
TRINITY_DN104586_c0_g1_i1_orf1	0.000536409	-0.004558305	Proteome
TRINITY_DN13375_c0_g1_i6_orf1	0.012739728	0.003724973	Proteome
TRINITY_DN6299_c0_g1_i1_orf1	0.010106154	-0.002042586	Proteome
TRINITY_DN129259_c0_g2_i1_orf1	0.009359957	-0.004447022	Proteome
TRINITY_DN36281_c0_g1_i2_orf1	0.00670343	0.007858121	Proteome
TRINITY_DN17825_c1_g1_i1_orf1	0.006208617	0.011173372	Proteome
TRINITY_DN41664_c0_g1_i4_orf1	0.006593679	-0.001088316	Proteome
TRINITY_DN141353_c0_g1_i1_orf1	-0.005039126	-0.029022666	Proteome
TRINITY_DN28018_c0_g5_i1_orf1	0.006526032	0.005907647	Proteome
TRINITY_DN67495_c0_g1_i1_orf1	-0.011908347	0.003002398	Proteome
TRINITY_DN42310_c0_g1_i1_orf1	0.005008429	0.003173485	Proteome
TRINITY_DN21285_c0_g1_i3_orf1	0.003990264	0.007477414	Proteome
TRINITY_DN3918_c0_g1_i1_orf1	-0.008106793	-0.028863692	Proteome
TRINITY_DN94355_c0_g1_i2_orf1	-0.00215199	-0.002942775	Proteome
TRINITY_DN24142_c0_g1_i1_orf1	0.013053526	-0.006949862	Proteome
TRINITY_DN15774_c0_g1_i3_orf1	-0.010619869	0.014145376	Proteome
TRINITY_DN82104_c0_g1_i5_orf1	0.004950241	-0.022289087	Proteome
TRINITY_DN5028_c0_g1_i11_orf1	-0.015899183	-0.00619386	Proteome
TRINITY_DN143833_c0_g1_i1_orf1	0.010010633	-0.000323362	Proteome
TRINITY_DN20960_c0_g1_i1_orf1	0.017333573	-0.009596653	Proteome
TRINITY_DN40911_c0_g1_i1_orf1	-0.008898098	0.005087469	Proteome
TRINITY_DN24132_c0_g1_i2_orf1	0.005665461	0.015977666	Proteome
TRINITY_DN32479_c0_g1_i8_orf1	0.011878182	0.004563767	Proteome

TRINITY_DN31377_c0_g2_i1_orf1	-0.024501609	-0.013810279	Proteome
TRINITY_DN104507_c0_g1_i2_orf1	0.00907766	0.007828723	Proteome
TRINITY_DN11172_c1_g1_i1_orf1	-0.014841269	-0.043420152	Proteome
TRINITY_DN27300_c0_g1_i6_orfp1	0.009755283	0.004822746	Proteome
TRINITY_DN1856_c0_g1_i3_orf1	0.006285264	0.009149568	Proteome
TRINITY_DN4900_c0_g1_i6_orf1	-0.01022183	-0.003803526	Proteome
TRINITY_DN3039_c0_g2_i1_orf1	-0.005883562	0.032640496	Proteome
TRINITY_DN30509_c0_g1_i9_orf1	0.000765817	-0.001075001	Proteome
TRINITY_DN2505_c0_g1_i1_orf1	-0.003894831	0.014609554	Proteome
TRINITY_DN33408_c0_g1_i1_orf1	-0.031021564	0.024095472	Proteome
TRINITY_DN27491_c0_g1_i1_orf1	0.010739724	0.013225953	Proteome
TRINITY_DN5275_c0_g1_i1_orf1	0.00806007	0.006045562	Proteome
TRINITY_DN51239_c0_g1_i5_orf1	0.012453329	-0.006862501	Proteome
TRINITY_DN10234_c0_g1_i1_orf1	-0.005707927	0.00444326	Proteome
TRINITY_DN8173_c0_g1_i3_orf1	0.007709479	-0.005020896	Proteome
TRINITY_DN5298_c0_g1_i3_orf1	-0.013015317	-0.012456939	Proteome
TRINITY_DN20238_c0_g1_i7_orf1	-0.002204914	0.019051627	Proteome
TRINITY_DN937_c0_g1_i2_orf1	0.004658468	0.005130716	Proteome
TRINITY_DN61536_c0_g2_i1_orf1	0.009525885	-0.015262824	Proteome
TRINITY_DN11245_c0_g1_i2_orf1	-0.00247295	0.021736626	Proteome
TRINITY_DN11666_c0_g1_i6_orf1	0.01983839	-0.006935009	Proteome
TRINITY_DN22962_c0_g1_i1_orf1	0.012611733	0.004624575	Proteome
TRINITY_DN4659_c0_g1_i2_orf1	0.00044935	0.005282361	Proteome
TRINITY_DN13160_c0_g1_i1_orf1	-0.001094503	-0.001664692	Proteome
TRINITY_DN5124_c0_g1_i2_orf1	-0.027936298	-0.034979531	Proteome
TRINITY_DN1045_c0_g1_i6_orf1	0.00757972	-0.004071535	Proteome
TRINITY_DN1421_c0_g1_i2_orf1	-0.002353435	0.000962209	Proteome
TRINITY_DN6205_c0_g1_i1_orf1	-0.028532282	-0.003597255	Proteome
TRINITY_DN16226_c0_g1_i1_orf1	-0.002307342	-0.025555064	Proteome
TRINITY_DN49872_c0_g1_i2_orf1	0.012082186	0.007584772	Proteome
TRINITY_DN957_c0_g1_i18_orf1	0.010430369	-0.006679083	Proteome
TRINITY_DN6985_c0_g1_i5_orf1	0.011763745	-0.008926498	Proteome
TRINITY_DN110402_c0_g2_i1_orf1	-0.010129699	-0.008998664	Proteome
TRINITY_DN1066_c0_g1_i8_orf1	0.002413783	0.004611828	Proteome
TRINITY_DN9591_c0_g1_i1_orf1	0.00483664	-0.009251266	Proteome
TRINITY_DN58314_c0_g1_i1_orf1	0.004190045	0.004536889	Proteome
TRINITY_DN143895_c0_g1_i1_orf1	-0.057525123	0.019440744	Proteome
TRINITY_DN15417_c0_g1_i6_orf1	0.005691743	-0.01296238	Proteome
TRINITY_DN47666_c0_g1_i4_orf1	0.004205515	0.003976601	Proteome
TRINITY_DN25625_c0_g2_i1_orf1	-0.017056703	-0.037924723	Proteome
TRINITY_DN50875_c0_g1_i3_orf1	0.00719425	0.003346133	Proteome
TRINITY_DN98242_c0_g1_i1_orf1	0.006253057	-0.000788913	Proteome
TRINITY_DN22156_c0_g1_i1_orf1	0.011324757	-0.002360819	Proteome
TRINITY_DN10360_c0_g1_i16_orf1	0.010608782	-0.018388037	Proteome
TRINITY_DN2908_c0_g1_i1_orf1	0.010905653	-0.016267383	Proteome
TRINITY_DN77318_c0_g2_i1_orf1	0.024433328	0.008024319	Proteome
TRINITY_DN4151_c1_g1_i4_orf1	0.00663034	0.016326169	Proteome
TRINITY_DN15380_c0_g1_i1_orf1	-0.000279944	-0.001634789	Proteome
TRINITY_DN10229_c0_g1_i6_orf1	0.002642026	0.014590805	Proteome
TRINITY_DN2312_c0_g1_i4_orf1	0.001405142	0.018960167	Proteome
TRINITY_DN24723_c2_g1_i1_orf1	-0.021807246	0.007406795	Proteome
TRINITY_DN99900_c0_g1_i3_orf1	0.005996672	0.001483968	Proteome
TRINITY_DN54275_c0_g1_i4_orf1	-0.010795046	-0.020154786	Proteome
TRINITY_DN73945_c0_g5_i3_orf1	0.01594126	-0.002931183	Proteome
TRINITY_DN64181_c0_g1_i1_orf1	-0.014361058	0.040882145	Proteome
TRINITY_DN3962_c0_g1_i6_orf1	0.019758092	0.014450704	Proteome
TRINITY_DN10871_c0_g2_i1_orf1	-0.003829919	0.004089822	Proteome
TRINITY_DN32362_c0_g1_i1_orf1	0.004105664	0.014230036	Proteome

TRINITY_DN7533_c0_g1_i1_orf1	-0.005711944	-0.024089082	Proteome
TRINITY_DN91877_c0_g1_i1_orf1	-0.016856754	-0.017291805	Proteome
TRINITY_DN53246_c0_g7_i1_orf1	0.008633087	-0.019113115	Proteome
TRINITY_DN33485_c0_g1_i4_orf1	-0.010050504	-0.008468466	Proteome
TRINITY_DN6362_c0_g1_i4_orf1	0.017118846	-0.003630502	Proteome
TRINITY_DN43576_c0_g1_i3_orf1	0.010190453	-0.005107831	Proteome
TRINITY_DN45836_c0_g1_i1_orf1	0.015927062	0.000789126	Proteome
TRINITY_DN59804_c0_g1_i1_orf1	-0.009465423	0.000497866	Proteome
TRINITY_DN71917_c0_g3_i1_orf1	0.017582165	-0.014153126	Proteome
TRINITY_DN25997_c1_g1_i1_orf1	-0.006303633	0.008876535	Proteome
TRINITY_DN96557_c0_g1_i1_orf1	0.00656508	0.012551995	Proteome
TRINITY_DN9420_c0_g1_i2_orf1	-0.009263272	0.019500893	Proteome
TRINITY_DN105359_c0_g2_i5_orf1	0.014365678	0.011633374	Proteome
TRINITY_DN74069_c0_g1_i1_orf1	0.007081081	0.001658273	Proteome
TRINITY_DN21559_c0_g1_i2_orf1	0.010864418	-0.002317686	Proteome
TRINITY_DN108433_c0_g1_i1_orf1	-0.009031309	-0.009878589	Proteome
TRINITY_DN46625_c0_g1_i1_orf1	-0.032118528	-0.028684408	Proteome
TRINITY_DN110132_c0_g1_i1_orf1	0.01192138	0.015850091	Proteome
TRINITY_DN8352_c0_g1_i3_orf1	-0.004593075	-0.004342291	Proteome
TRINITY_DN1604_c0_g1_i4_orf1	0.005235518	0.013814053	Proteome
TRINITY_DN298_c0_g1_i4_orf1	-0.002438895	0.005841431	Proteome
TRINITY_DN3106_c0_g1_i6_orf1	0.006925316	-0.004268973	Proteome
TRINITY_DN4345_c0_g1_i9_orf1	0.009679254	0.001275343	Proteome
TRINITY_DN22577_c0_g1_i2_orf1	0.003684715	0.006284506	Proteome
TRINITY_DN33024_c0_g1_i1_orf1	0.023305558	-0.001482705	Proteome
TRINITY_DN36899_c0_g1_i1_orf1	0.013887075	0.021257407	Proteome
TRINITY_DN2532_c0_g3_i1_orf1	-0.008895415	-0.015283835	Proteome
TRINITY_DN4635_c0_g1_i4_orf1	-0.00429245	-0.003092317	Proteome
TRINITY_DN2403_c0_g1_i3_orf1	0.002041567	-0.020053787	Proteome
TRINITY_DN25960_c0_g1_i1_orf1	0.011820082	-0.003887599	Proteome
TRINITY_DN2673_c2_g1_i2_orf1	-0.01999314	0.006990268	Proteome
TRINITY_DN146264_c0_g1_i1_orf1	0.007957217	-0.002422264	Proteome
TRINITY_DN74889_c0_g1_i1_orf1	0.005099941	0.00558098	Proteome
TRINITY_DN111488_c0_g1_i1_orf1	0.002185999	-0.009771059	Proteome
TRINITY_DN7556_c0_g1_i1_orf1	0.000242558	-0.000523713	Proteome
TRINITY_DN16354_c0_g1_i2_orf1	0.013155314	-0.003920542	Proteome
TRINITY_DN70_c2_g1_i1_orf1	-0.011450731	-0.025471139	Proteome
TRINITY_DN47731_c0_g1_i2_orf1	-0.001416927	0.016981518	Proteome
TRINITY_DN3463_c0_g1_i2_orf1	0.008060529	0.006266842	Proteome
TRINITY_DN7670_c0_g1_i1_orf1	0.002772754	-0.005270729	Proteome
TRINITY_DN8964_c0_g1_i4_orf1	0.016813898	0.006386197	Proteome
TRINITY_DN5670_c0_g1_i2_orf1	0.020654983	0.0005064	Proteome
TRINITY_DN122786_c0_g2_i1_orf1	-0.001635366	-0.009201944	Proteome
TRINITY_DN66596_c0_g1_i1_orf1	0.00877514	0.007203836	Proteome
TRINITY_DN26243_c0_g1_i2_orf1	0.011052685	0.003435394	Proteome
TRINITY_DN5107_c0_g1_i4_orf1	-0.000715522	0.005320335	Proteome
TRINITY_DN4565_c0_g1_i3_orf1	-0.002282563	-0.014298025	Proteome
TRINITY_DN146138_c0_g1_i1_orf1	-0.013185766	-0.008504814	Proteome
TRINITY_DN97680_c0_g1_i1_orf1	-0.006824512	-0.006723965	Proteome
TRINITY_DN3482_c0_g2_i1_orf1	0.007848547	-0.000643367	Proteome
TRINITY_DN6130_c0_g1_i6_orf1	0.005890744	0.004433909	Proteome
TRINITY_DN10070_c0_g1_i1_orf1	-0.010785097	0.039369249	Proteome
TRINITY_DN5210_c0_g1_i3_orf1	0.017559093	0.001162186	Proteome
TRINITY_DN90582_c0_g1_i4_orf1	0.011478233	-0.010559979	Proteome
TRINITY_DN804_c0_g1_i7_orf1	0.02177424	0.002258856	Proteome
TRINITY_DN8482_c0_g1_i4_orf1	0.039455693	0.002123451	Proteome
TRINITY_DN99673_c0_g1_i1_orf1	0.02188312	-0.016006871	Proteome
TRINITY_DN4294_c0_g1_i6_orf1	-0.008449342	0.005792211	Proteome

TRINITY_DN144258_c0_g1_i1_orf1	0.005426229	0.012661736	Proteome
TRINITY_DN11392_c0_g1_i4_orf1	0.003291734	-0.005356826	Proteome
TRINITY_DN9998_c0_g1_i2_orf1	0.004283064	0.0170953	Proteome
TRINITY_DN145666_c0_g1_i1_orf1	0.002977377	-0.002195719	Proteome
TRINITY_DN51934_c0_g2_i1_orf1	0.012657479	0.008716911	Proteome
TRINITY_DN8747_c0_g1_i2_orf1	-0.002318112	0.01894098	Proteome
TRINITY_DN6462_c0_g1_i5_orf1	-0.023890583	0.020568618	Proteome
TRINITY_DN61042_c0_g2_i2_orfp1	-0.032032056	0.008360676	Proteome
TRINITY_DN3196_c0_g1_i1_orf1	0.016111982	-0.010115089	Proteome
TRINITY_DN4460_c0_g1_i1_orf1	0.017876942	-0.003407691	Proteome
TRINITY_DN5770_c0_g1_i4_orf1	-0.023856912	-0.008876483	Proteome
TRINITY_DN5991_c0_g1_i6_orf1	0.02682905	0.00175942	Proteome
TRINITY_DN144342_c0_g1_i1_orfp1	0.019212756	-0.011219231	Proteome
TRINITY_DN24410_c0_g2_i1_orf1	-0.004764413	-0.029239852	Proteome
TRINITY_DN830_c0_g1_i4_orf1	0.003467312	-0.026527308	Proteome
TRINITY_DN147596_c0_g1_i1_orf1	0.014295235	-0.011334435	Proteome
TRINITY_DN45963_c0_g1_i1_orf1	0.006820265	-0.028533184	Proteome
TRINITY_DN7213_c0_g1_i2_orf1	0.003224142	0.006052485	Proteome
TRINITY_DN38371_c0_g1_i7_orf1	0.003961694	0.000493119	Proteome
TRINITY_DN9354_c0_g1_i7_orf1	-0.011938424	0.000115999	Proteome
TRINITY_DN39837_c0_g1_i1_orf1	-0.005893048	0.02092937	Proteome
TRINITY_DN82311_c0_g1_i1_orf1	0.007438573	-0.010024305	Proteome
TRINITY_DN106856_c0_g1_i1_orf1	-0.00654946	0.012216458	Proteome
TRINITY_DN47151_c0_g1_i1_orf1	0.022774452	-0.002875787	Proteome
TRINITY_DN58013_c0_g1_i6_orf1	0.009362322	0.007633348	Proteome
TRINITY_DN40434_c0_g1_i2_orf1	0.018814667	0.002700593	Proteome
TRINITY_DN1791_c0_g1_i3_orf1	0.012925898	0.010790037	Proteome
TRINITY_DN7407_c0_g1_i9_orf1	0.000364981	0.007514903	Proteome
TRINITY_DN3082_c1_g1_i7_orf1	0.002456525	0.004710912	Proteome
TRINITY_DN46778_c0_g1_i2_orf1	-0.004678576	0.002884689	Proteome
TRINITY_DN145448_c0_g1_i1_orf1	-0.003304919	0.016181886	Proteome
TRINITY_DN41602_c0_g3_i1_orf1	-0.0088369	-0.021098722	Proteome
TRINITY_DN52864_c0_g1_i1_orf1	-0.009257154	-0.00584726	Proteome
TRINITY_DN16385_c0_g1_i4_orf1	0.011900518	-0.006637277	Proteome
TRINITY_DN59965_c0_g4_i1_orf1	-0.035205533	0.078734865	Proteome
TRINITY_DN38106_c0_g1_i6_orf1	0.01468638	-0.0095468	Proteome
TRINITY_DN2002_c0_g1_i5_orfp1	0.020958809	-0.006049027	Proteome
TRINITY_DN974_c0_g2_i1_orfp1	0.002891189	-0.002239119	Proteome
TRINITY_DN252_c0_g1_i3_orf1	-0.001480895	-0.001872732	Proteome
TRINITY_DN9028_c0_g1_i5_orf1	0.01093096	-0.007662728	Proteome
TRINITY_DN15338_c0_g1_i7_orf1	-0.013318848	-0.033892254	Proteome
TRINITY_DN8603_c0_g1_i1_orf1	0.005736825	-0.018294358	Proteome
TRINITY_DN4123_c0_g1_i1_orf1	0.027093607	0.004328752	Proteome
TRINITY_DN12227_c0_g2_i3_orf1	0.011126977	0.015850924	Proteome
TRINITY_DN5935_c0_g1_i2_orf1	-0.012904975	-0.026950783	Proteome
TRINITY_DN30177_c0_g2_i1_orf1	0.066445478	-0.005994489	Proteome
TRINITY_DN23004_c0_g1_i1_orf1	0.013091704	-0.000797754	Proteome
TRINITY_DN74116_c0_g1_i2_orf1	-0.011168094	-0.021047393	Proteome
TRINITY_DN16493_c0_g1_i2_orf1	-0.005725426	-0.010168391	Proteome
TRINITY_DN140613_c0_g1_i1_orf1	0.01436647	0.015854021	Proteome
TRINITY_DN140669_c0_g1_i1_orf1	0.00246539	-0.005172948	Proteome
TRINITY_DN86833_c0_g3_i1_orf1	-0.020771175	0.016736049	Proteome
TRINITY_DN9243_c0_g1_i4_orf1	0.010281318	0.010470531	Proteome
TRINITY_DN40440_c0_g1_i1_orf1	0.006476318	-0.000560355	Proteome
TRINITY_DN84631_c0_g1_i1_orf1	0.020099731	-0.005785201	Proteome
TRINITY_DN1198_c0_g1_i1_orf1	0.006392616	0.004275186	Proteome
TRINITY_DN110534_c0_g1_i3_orf1	-0.008513528	-0.012572032	Proteome
TRINITY_DN79803_c0_g1_i7_orf1	0.014274187	-0.004802004	Proteome

TRINITY_DN22513_c0_g1_i4_orf1	0.010312998	0.02298773	Proteome
TRINITY_DN3862_c0_g1_i7_orf1	0.006182199	0.004039223	Proteome
TRINITY_DN3618_c0_g1_i4_orf1	0.008362778	0.0014257	Proteome
TRINITY_DN8139_c0_g1_i2_orf1	0.01786638	-0.007146898	Proteome
TRINITY_DN3504_c0_g1_i3_orfp2	0.012367987	0.009596668	Proteome
TRINITY_DN647_c4_g1_i1_orf1	0.006865031	0.005624732	Proteome
TRINITY_DN11868_c0_g1_i2_orf1	0.016321887	0.014103569	Proteome
TRINITY_DN14905_c0_g2_i2_orf1	-0.004605273	0.016543861	Proteome
TRINITY_DN17247_c0_g1_i14_orf1	0.033121657	-0.016509492	Proteome
TRINITY_DN141396_c0_g1_i1_orf1	0.016174578	0.003059919	Proteome
TRINITY_DN144850_c0_g1_i1_orf1	0.011849527	0.004635467	Proteome
TRINITY_DN9309_c0_g1_i5_orf1	0.011693656	-0.002187923	Proteome
TRINITY_DN49204_c0_g1_i1_orf1	0.013174628	0.004741293	Proteome
TRINITY_DN44256_c0_g1_i1_orf1	0.009608862	0.00553339	Proteome
TRINITY_DN2471_c0_g1_i3_orf1	0.012154415	-0.016679152	Proteome
TRINITY_DN58636_c0_g1_i1_orf1	0.003189372	0.009196941	Proteome
TRINITY_DN37393_c0_g1_i1_orf1	0.005971639	-0.00600936	Proteome
TRINITY_DN4782_c0_g1_i1_orf1	0.007039157	0.005444686	Proteome
TRINITY_DN8384_c0_g1_i1_orf1	-0.027374068	-0.041252399	Proteome
TRINITY_DN1504_c0_g1_i1_orf1	0.010887275	0.009781577	Proteome
TRINITY_DN1013_c0_g1_i3_orf1	-0.011093995	0.009569042	Proteome
TRINITY_DN34115_c0_g1_i1_orf1	-0.010347152	-0.020283718	Proteome
TRINITY_DN6025_c0_g2_i1_orfp1	0.015981743	0.049632664	Proteome
TRINITY_DN4694_c0_g1_i6_orf1	0.009125599	-0.004297624	Proteome
TRINITY_DN130159_c0_g2_i1_orf1	0.008781168	0.000794383	Proteome
TRINITY_DN83542_c0_g1_i1_orf1	-0.005271062	-0.012871351	Proteome
TRINITY_DN30498_c0_g1_i3_orf1	-0.025900265	-0.023131367	Proteome
TRINITY_DN3707_c0_g1_i1_orf1	-0.022202696	0.021121589	Proteome
TRINITY_DN23926_c0_g1_i4_orf1	0.014732364	-0.00094201	Proteome
TRINITY_DN5444_c0_g1_i1_orfp1	-0.019226882	0.004137118	Proteome
TRINITY_DN80547_c0_g1_i5_orf1	0.011534514	0.007476345	Proteome
TRINITY_DN26650_c0_g1_i1_orfp1	-0.001101681	-0.01087676	Proteome
TRINITY_DN11566_c2_g1_i2_orf1	0.010169689	-0.007431993	Proteome
TRINITY_DN30150_c0_g1_i7_orf1	-0.007717437	-0.017660952	Proteome
TRINITY_DN29707_c0_g1_i2_orf1	0.002874005	-0.016691858	Proteome
TRINITY_DN4546_c0_g1_i3_orf1	-0.009764956	0.006654738	Proteome
TRINITY_DN5011_c0_g1_i1_orf1	0.009684203	0.013627486	Proteome
TRINITY_DN69697_c0_g1_i1_orf1	-0.011713428	-0.012217445	Proteome
TRINITY_DN1308_c0_g1_i4_orf1	0.00694349	-0.009453318	Proteome
TRINITY_DN2647_c0_g1_i3_orf1	0.001589902	0.015926221	Proteome
TRINITY_DN71494_c0_g1_i2_orf1	0.006894414	-0.002054532	Proteome
TRINITY_DN25542_c0_g1_i1_orf1	0.008145332	-0.001488841	Proteome
TRINITY_DN3628_c0_g1_i5_orf1	-0.000478898	-0.004093458	Proteome
TRINITY_DN44709_c0_g1_i1_orf1	-0.016189408	0.023202081	Proteome
TRINITY_DN12323_c0_g2_i2_orf1	0.011848944	0.006084051	Proteome
TRINITY_DN21787_c0_g1_i1_orf1	-0.011839881	-0.025069493	Proteome
TRINITY_DN101_c0_g1_i4_orf1	-0.025126913	0.006047737	Proteome
TRINITY_DN13999_c0_g1_i4_orf1	-0.013495386	0.001171269	Proteome
TRINITY_DN26879_c0_g1_i1_orf1	0.00767897	0.011968629	Proteome
TRINITY_DN95544_c0_g2_i1_orf1	-0.010032281	-0.02783954	Proteome
TRINITY_DN53462_c0_g1_i1_orf1	0.008915113	0.000923786	Proteome
TRINITY_DN17791_c0_g1_i1_orf1	0.002999873	0.000539217	Proteome
TRINITY_DN62141_c0_g1_i5_orf1	0.004751847	0.014024898	Proteome
TRINITY_DN23962_c0_g1_i3_orf1	0.011187308	-0.001662281	Proteome
TRINITY_DN33346_c0_g1_i1_orf1	0.014821428	0.020865445	Proteome
TRINITY_DN61335_c0_g2_i1_orf1	0.001898964	0.019985971	Proteome
TRINITY_DN2912_c0_g1_i1_orf1	-0.012910915	-0.018761886	Proteome
TRINITY_DN1897_c0_g2_i4_orf1	0.045267692	0.015717807	Proteome

TRINITY_DN27903_c0_g1_i1_orf1	0.016379018	-0.016208518	Proteome
TRINITY_DN1954_c0_g1_i4_orf1	0.011830494	0.004928325	Proteome
TRINITY_DN1266_c2_g1_i1_orf1	0.014461728	-0.002234286	Proteome
TRINITY_DN5775_c0_g1_i1_orf1	0.010483591	0.002189154	Proteome
TRINITY_DN7841_c1_g1_i9_orf1	0.010786035	-0.017243168	Proteome
TRINITY_DN195_c8_g1_i1_orf1	0.003430903	-0.001324348	Proteome
TRINITY_DN20966_c0_g1_i6_orf1	-0.006139289	-0.01494526	Proteome
TRINITY_DN36883_c0_g1_i1_orf1	0.007494126	0.009129696	Proteome
TRINITY_DN9356_c0_g1_i1_orf1	-0.000351245	-0.018491988	Proteome
TRINITY_DN42185_c0_g1_i7_orf1	0.016121691	0.015998296	Proteome
TRINITY_DN64616_c0_g1_i1_orf1	-0.002765722	0.015801377	Proteome
TRINITY_DN78546_c0_g5_i1_orf1	0.011181168	-0.007090816	Proteome
TRINITY_DN5697_c0_g1_i1_orf1	-0.000268615	0.006696546	Proteome
TRINITY_DN3275_c0_g2_i3_orf1	0.03828068	0.03867972	Proteome
TRINITY_DN48694_c0_g1_i1_orfp1	-0.04097396	0.012017571	Proteome
TRINITY_DN8290_c0_g1_i3_orf1	0.006138769	0.01355622	Proteome
TRINITY_DN31216_c0_g1_i2_orf1	0.010201523	0.007035553	Proteome
TRINITY_DN8089_c0_g1_i3_orf1	0.007796041	0.018787613	Proteome
TRINITY_DN50074_c0_g1_i1_orf1	-0.014905312	-0.004232827	Proteome
TRINITY_DN56910_c0_g2_i1_orf1	0.000754925	0.004143422	Proteome
TRINITY_DN1786_c0_g1_i11_orf1	-0.006798419	-0.025310685	Proteome
TRINITY_DN7735_c1_g1_i1_orf1	0.012970876	0.003899406	Proteome
TRINITY_DN1633_c0_g1_i1_orf1	0.001074519	0.003175663	Proteome
TRINITY_DN2455_c0_g1_i12_orf1	-0.01179846	0.01723376	Proteome
TRINITY_DN23354_c0_g1_i7_orf1	-0.031825732	0.042016544	Proteome
TRINITY_DN6125_c0_g1_i2_orf1	-0.00598545	0.023106626	Proteome
TRINITY_DN23582_c0_g1_i1_orf1	-0.020536483	0.005079194	Proteome
TRINITY_DN141738_c0_g1_i1_orf1	0.00419912	-0.00501702	Proteome
TRINITY_DN20682_c0_g2_i1_orf1	0.005403513	-0.00225748	Proteome
TRINITY_DN23798_c0_g1_i1_orf1	-0.011186376	0.011506874	Proteome
TRINITY_DN3550_c0_g1_i4_orf1	0.002247592	0.002207416	Proteome
TRINITY_DN29_c0_g1_i4_orf1	-0.017778975	0.020022586	Proteome
TRINITY_DN2893_c0_g1_i4_orf1	-0.006057039	0.003295221	Proteome
TRINITY_DN46090_c0_g3_i1_orf1	-0.029113374	0.005368293	Proteome
TRINITY_DN271_c0_g2_i6_orf1	0.000771708	-0.015665649	Proteome
TRINITY_DN19286_c0_g1_i1_orf1	-0.004672389	0.001207989	Proteome
TRINITY_DN101922_c0_g1_i1_orf1	-0.004903445	0.005148537	Proteome
TRINITY_DN25733_c0_g1_i3_orf1	0.009290613	0.002202213	Proteome
TRINITY_DN12666_c0_g1_i2_orf1	-0.015955763	-0.007615387	Proteome
TRINITY_DN111621_c0_g3_i1_orf1	-0.012143875	0.026671818	Proteome
TRINITY_DN99_c0_g1_i3_orf1	-0.016483679	0.011405667	Proteome
TRINITY_DN82628_c0_g1_i2_orf1	0.00848675	0.01572227	Proteome
TRINITY_DN45962_c1_g1_i2_orf1	-0.012502434	0.004826704	Proteome
TRINITY_DN29038_c0_g2_i1_orf1	0.008573594	-0.007485199	Proteome
TRINITY_DN15916_c0_g1_i1_orf1	0.008698777	-0.002613403	Proteome
TRINITY_DN66561_c0_g1_i1_orf1	-0.035236591	-0.02128207	Proteome
TRINITY_DN2560_c0_g1_i1_orf1	-0.000634982	-0.004671319	Proteome
TRINITY_DN5902_c0_g1_i4_orf1	0.004873242	-0.040118534	Proteome
TRINITY_DN3089_c0_g1_i1_orf1	0.0114685	-0.007089377	Proteome
TRINITY_DN21492_c0_g1_i1_orf1	0.000503535	-0.014126952	Proteome
TRINITY_DN5908_c0_g1_i2_orf1	-0.008415725	0.006384149	Proteome
TRINITY_DN102712_c0_g1_i1_orf1	-0.024410875	0.001529191	Proteome
TRINITY_DN56164_c0_g1_i1_orf1	-0.002448818	0.03463752	Proteome
TRINITY_DN4612_c0_g1_i1_orf1	-0.002780534	0.003627063	Proteome
TRINITY_DN10877_c0_g1_i1_orf1	-0.072604318	0.055551892	Proteome
TRINITY_DN6841_c0_g2_i1_orf1	-0.0005625	-0.01368726	Proteome
TRINITY_DN15988_c0_g1_i1_orf1	0.010614122	-0.014297725	Proteome
TRINITY_DN4323_c0_g1_i1_orf1	0.00393318	-0.005577292	Proteome

TRINITY_DN8853_c0_g1_i4_orf1	0.04116984	0.003184853	Proteome
TRINITY_DN14409_c0_g1_i1_orf1	0.014141683	0.009908579	Proteome
TRINITY_DN31486_c0_g1_i1_orf1	0.009104218	-0.000844602	Proteome
TRINITY_DN781_c0_g1_i7_orf1	-0.012824173	-0.018259625	Proteome
TRINITY_DN44089_c0_g5_i1_orf1	0.010824796	-0.015461315	Proteome
TRINITY_DN13711_c0_g1_i1_orf1	-0.009189527	0.014169479	Proteome
TRINITY_DN6466_c0_g1_i5_orf1	-0.006202156	0.002488323	Proteome
TRINITY_DN2984_c0_g1_i3_orf1	0.003185227	0.002127269	Proteome
TRINITY_DN21856_c0_g1_i1_orf1	0.003784787	0.005690911	Proteome
TRINITY_DN3092_c0_g1_i2_orf1	-0.026272618	-0.001598025	Proteome
TRINITY_DN144190_c0_g1_i1_orf1	-0.0027517	0.002477889	Proteome
TRINITY_DN10886_c0_g2_i4_orf1	0.011704414	-0.022313887	Proteome
TRINITY_DN65057_c0_g2_i1_orf1	-0.004756461	-0.028343253	Proteome
TRINITY_DN77642_c0_g1_i1_orf1	-0.003376393	0.001284086	Proteome
TRINITY_DN48097_c0_g1_i1_orf1	0.011666443	-0.013214451	Proteome
TRINITY_DN46216_c0_g3_i1_orf1	0.029177154	0.001039002	Proteome
TRINITY_DN23734_c0_g1_i1_orf1	0.010672159	-0.002096662	Proteome
TRINITY_DN16174_c0_g1_i2_orf1	0.007830213	-0.002373028	Proteome
TRINITY_DN15040_c0_g4_i1_orf1	0.009798276	-0.00622847	Proteome
TRINITY_DN6639_c0_g1_i4_orf1	0.009819551	0.010117732	Proteome
TRINITY_DN8008_c0_g1_i6_orf1	-0.034649494	-0.000560081	Proteome
TRINITY_DN147691_c0_g1_i1_orf1	0.01107438	0.010503727	Proteome
TRINITY_DN107962_c0_g1_i1_orf1	0.015868439	-0.000296482	Proteome
TRINITY_DN19328_c0_g1_i1_orf1	0.018339018	0.005172217	Proteome
TRINITY_DN89083_c0_g1_i1_orf1	-0.004477369	0.022806874	Proteome
TRINITY_DN12873_c0_g2_i1_orf1	-0.00241388	-0.024008797	Proteome
TRINITY_DN5818_c1_g1_i2_orf1	-0.000894174	0.009430909	Proteome
TRINITY_DN6027_c0_g1_i13_orf1	-0.00750307	0.00592063	Proteome
TRINITY_DN162_c0_g1_i4_orf1	-0.000216322	0.012401379	Proteome
TRINITY_DN2367_c1_g1_i20_orf1	0.017786694	0.019432448	Proteome
TRINITY_DN22430_c0_g3_i1_orf1	0.001613703	0.000494275	Proteome
TRINITY_DN9486_c1_g1_i7_orfp1	0.013761418	-0.003322904	Proteome
TRINITY_DN13216_c0_g1_i5_orf1	-0.001932368	0.009695219	Proteome
TRINITY_DN140538_c0_g2_i1_orf1	0.013161595	0.005475748	Proteome
TRINITY_DN11736_c0_g1_i1_orf1	0.003356295	-0.00917374	Proteome
TRINITY_DN22441_c0_g1_i1_orf1	0.002773163	-0.012819884	Proteome
TRINITY_DN57105_c0_g1_i2_orf1	-0.003492663	-0.000710097	Proteome
TRINITY_DN7102_c0_g1_i5_orf1	-0.004773921	0.019151143	Proteome
TRINITY_DN52861_c0_g1_i1_orf1	0.002687107	0.000866812	Proteome
TRINITY_DN19160_c0_g1_i1_orf1	-0.002830726	0.00811552	Proteome
TRINITY_DN28376_c0_g1_i15_orfp1	0.015652734	0.008639358	Proteome
TRINITY_DN14219_c0_g1_i7_orf1	0.008692425	-0.016000008	Proteome
TRINITY_DN4567_c0_g3_i1_orf1	-0.004399901	-0.019336799	Proteome
TRINITY_DN87603_c0_g2_i1_orf1	0.015522085	0.012524507	Proteome
TRINITY_DN135846_c0_g1_i1_orf1	0.003679217	-0.033803743	Proteome
TRINITY_DN58531_c0_g1_i1_orf1	0.004797235	0.002335862	Proteome
TRINITY_DN83327_c0_g1_i1_orf1	-0.008685552	0.0132833	Proteome
TRINITY_DN640_c0_g1_i6_orf1	-0.005980174	-0.043241332	Proteome
TRINITY_DN80134_c0_g1_i1_orf1	-0.012673201	-0.006270145	Proteome
TRINITY_DN110400_c0_g1_i1_orf1	0.01396088	-0.00716385	Proteome
TRINITY_DN8386_c0_g1_i6_orf1	-0.014848469	0.00481427	Proteome
TRINITY_DN8480_c0_g1_i1_orf1	0.012520083	-0.026406609	Proteome
TRINITY_DN10106_c0_g2_i1_orf1	-0.003743875	-0.001708025	Proteome
TRINITY_DN48878_c0_g2_i1_orf1	-0.017709148	-0.01321935	Proteome
TRINITY_DN41334_c0_g1_i1_orf1	-0.001758207	0.00154852	Proteome
TRINITY_DN27745_c0_g1_i6_orf1	0.006368392	0.026964607	Proteome
TRINITY_DN15753_c0_g1_i1_orf1	-0.004020135	0.005219029	Proteome
TRINITY_DN21226_c0_g1_i2_orf1	-0.006270389	0.01079144	Proteome

TRINITY_DN9538_c1_g3_i1_orf1	-0.024677036	0.020868927	Proteome
TRINITY_DN2374_c0_g2_i2_orf1	0.00476836	-0.015461647	Proteome
TRINITY_DN12503_c0_g2_i1_orf1	0.001842508	-0.008745196	Proteome
TRINITY_DN3964_c1_g1_i2_orf1	0.018612677	-0.001134901	Proteome
TRINITY_DN4016_c0_g1_i1_orf1	0.007558865	0.007796153	Proteome
TRINITY_DN23266_c0_g2_i1_orf1	0.000639885	0.008462618	Proteome
TRINITY_DN123139_c0_g1_i1_orfp1	0.008604257	0.000271183	Proteome
TRINITY_DN5459_c0_g1_i1_orf1	-0.001897464	0.010813792	Proteome
TRINITY_DN20442_c0_g2_i1_orf1	0.051879165	-0.004352603	Proteome
TRINITY_DN146493_c0_g1_i1_orf1	0.00506927	0.014948869	Proteome
TRINITY_DN2425_c0_g1_i1_orf1	0.016182466	0.003572786	Proteome
TRINITY_DN129835_c0_g1_i2_orf1	-0.005688065	-0.012351001	Proteome
TRINITY_DN89613_c0_g1_i13_orf1	0.01418598	0.008602428	Proteome
TRINITY_DN434_c0_g1_i4_orf1	0.007441117	0.011518614	Proteome
TRINITY_DN6602_c0_g1_i4_orf1	0.012755557	0.009950283	Proteome
TRINITY_DN16127_c0_g1_i2_orf1	-0.004607388	-0.013118442	Proteome
TRINITY_DN3212_c0_g1_i7_orfp1	-0.011032914	-0.031188509	Proteome
TRINITY_DN9400_c0_g1_i1_orf1	0.022949472	0.011625606	Proteome
TRINITY_DN37696_c0_g1_i3_orf1	-0.01044913	-0.012623994	Proteome
TRINITY_DN122170_c0_g1_i2_orfp1	0.003078227	0.006295197	Proteome
TRINITY_DN77559_c0_g1_i1_orf1	0.011544836	0.00883072	Proteome
TRINITY_DN10332_c0_g1_i2_orfp1	0.00814294	0.0045672	Proteome
TRINITY_DN115658_c0_g1_i1_orf1	0.008801257	0.016311262	Proteome
TRINITY_DN9711_c0_g1_i10_orf1	0.002022625	0.003879565	Proteome
TRINITY_DN20767_c0_g2_i1_orf1	0.005478267	-0.012424137	Proteome
TRINITY_DN30154_c0_g1_i1_orf1	0.014368614	-0.00841963	Proteome
TRINITY_DN6768_c0_g1_i1_orf1	0.010194739	-0.013615456	Proteome
TRINITY_DN24490_c0_g1_i6_orf1	0.007719418	-0.014621498	Proteome
TRINITY_DN143637_c0_g1_i1_orf1	-0.020424206	-0.008195728	Proteome
TRINITY_DN4923_c0_g1_i4_orf1	0.007595471	-8.93E-05	Proteome
TRINITY_DN12113_c0_g1_i1_orf1	-0.00926332	0.003502997	Proteome
TRINITY_DN2243_c0_g1_i4_orf1	0.006362531	0.000110512	Proteome
TRINITY_DN3014_c0_g1_i4_orf1	0.004484437	0.003072968	Proteome
TRINITY_DN74037_c0_g5_i1_orf1	0.014270998	0.015249395	Proteome
TRINITY_DN47677_c0_g1_i1_orf1	0.016105465	0.010006164	Proteome
TRINITY_DN76377_c0_g1_i1_orf1	0.015640696	0.002900347	Proteome
TRINITY_DN6710_c0_g1_i6_orf1	-0.00238869	-0.006596005	Proteome
TRINITY_DN40191_c2_g1_i1_orf1	-0.020977665	-0.006591306	Proteome
TRINITY_DN144807_c0_g1_i1_orf1	0.000984856	-0.003147755	Proteome
TRINITY_DN41296_c0_g1_i1_orf1	0.001203916	-0.004662606	Proteome
TRINITY_DN12951_c1_g1_i5_orf1	0.011626	-0.009492767	Proteome
TRINITY_DN145647_c0_g1_i1_orf1	0.003363093	-0.006791609	Proteome
TRINITY_DN5655_c0_g1_i2_orf1	0.016406131	0.014901005	Proteome
TRINITY_DN1416_c0_g2_i1_orf1	0.019990656	-0.002373568	Proteome
TRINITY_DN3045_c0_g1_i7_orf1	0.003822854	0.003263055	Proteome
TRINITY_DN787_c0_g1_i7_orf1	0.008246984	-0.007866887	Proteome
TRINITY_DN8738_c0_g1_i1_orf1	0.010044445	0.016039234	Proteome
TRINITY_DN100885_c0_g2_i1_orfp1	0.005337173	0.013329935	Proteome
TRINITY_DN13732_c0_g2_i3_orf1	-0.011782173	-0.004103804	Proteome
TRINITY_DN7313_c1_g1_i2_orf1	-0.010139849	0.017829649	Proteome
TRINITY_DN17864_c0_g1_i1_orf1	0.003110435	-0.014328982	Proteome
TRINITY_DN3934_c0_g1_i9_orf1	-0.042080585	-0.112381309	Proteome
TRINITY_DN29217_c0_g1_i3_orf1	-0.009851253	0.003497422	Proteome
TRINITY_DN10539_c0_g1_i1_orf1	-0.021159572	-0.02575322	Proteome
TRINITY_DN92232_c0_g1_i1_orf1	0.005986518	-0.012784876	Proteome
TRINITY_DN17505_c0_g1_i15_orf1	0.040654746	0.008762729	Proteome
TRINITY_DN37821_c0_g1_i6_orf1	0.015508754	-0.011341799	Proteome
TRINITY_DN2346_c0_g2_i1_orf1	0.010863197	-0.003403258	Proteome

TRINITY_DN2623_c1_g1_i3_orf1	-0.001903029	0.017423928	Proteome
TRINITY_DN33089_c0_g1_i1_orf1	0.011805989	0.020641925	Proteome
TRINITY_DN12317_c0_g1_i1_orf1	0.00706211	-0.001389368	Proteome
TRINITY_DN87170_c0_g1_i3_orf1	-0.006448607	0.009051608	Proteome
TRINITY_DN76307_c0_g1_i1_orf1	0.013500019	0.007929371	Proteome
TRINITY_DN81258_c0_g1_i2_orf1	0.009446108	0.016628459	Proteome
TRINITY_DN60358_c0_g1_i3_orf1	-0.014690314	-0.010253045	Proteome
TRINITY_DN7900_c0_g1_i4_orf1	-0.029472266	-0.027159715	Proteome
TRINITY_DN14269_c0_g1_i5_orf1	0.006928062	-0.012361545	Proteome
TRINITY_DN6392_c0_g1_i9_orf1	-0.009169238	0.022023906	Proteome
TRINITY_DN6711_c0_g1_i1_orf1	0.000303964	-0.007434482	Proteome
TRINITY_DN26186_c0_g1_i7_orf1	0.009094263	-0.003265079	Proteome
TRINITY_DN49956_c0_g1_i1_orf1	-0.007641579	-0.033257248	Proteome
TRINITY_DN13530_c0_g1_i1_orf1	0.007791029	-0.00713518	Proteome
TRINITY_DN30233_c0_g1_i2_orf1	0.010864534	0.001108505	Proteome
TRINITY_DN69691_c0_g1_i1_orf1	-0.019358173	-0.057970072	Proteome
TRINITY_DN11375_c0_g1_i6_orf1	0.006751863	0.021585932	Proteome
TRINITY_DN670_c0_g1_i3_orf1	-0.001876139	0.021232008	Proteome
TRINITY_DN13177_c0_g1_i9_orf1	-0.002761431	0.005975344	Proteome
TRINITY_DN46633_c0_g1_i4_orf1	-0.016586654	0.016855683	Proteome
TRINITY_DN12748_c2_g1_i1_orfp1	0.010353204	0.004030622	Proteome
TRINITY_DN880_c0_g1_i6_orf1	0.009947365	0.007981454	Proteome
TRINITY_DN5334_c0_g1_i6_orf1	0.009416805	0.010955305	Proteome
TRINITY_DN5531_c7_g1_i2_orf1	-0.015957602	-0.054990441	Proteome
TRINITY_DN10220_c1_g1_i7_orf1	0.018656538	-0.020266097	Proteome
TRINITY_DN11856_c0_g1_i4_orf1	-0.015934967	0.014628268	Proteome
TRINITY_DN69049_c0_g2_i1_orf1	-0.015273677	-0.001025393	Proteome
TRINITY_DN42275_c0_g1_i1_orfp1	-0.039194765	0.037251576	Proteome
TRINITY_DN12686_c0_g1_i4_orf1	-0.013905347	-0.011852008	Proteome
TRINITY_DN38498_c0_g3_i1_orf1	0.014694706	-0.002086463	Proteome
TRINITY_DN56270_c0_g1_i1_orf1	-0.015388914	0.009260371	Proteome
TRINITY_DN9790_c0_g1_i4_orf1	0.010976669	-0.00458776	Proteome
PC(18:3/0:0)	-0.016526179	0.000316034	Metabolome
PC(18:2/0:0)	-0.022184346	0.001107697	Metabolome
7,8-Dihydrovomifoliol 9-[rhamnosyl-(1	0.053909826	0.03940677	Metabolome
Didemethylcitalopram	0.037771899	0.023287818	Metabolome
LysoPE(18:3(9Z,12Z,15Z)/0:0)	-0.018979252	-0.001062406	Metabolome
Norvaline	0.013244529	-0.000818834	Metabolome
PE(18:3/0:0)	-0.022623172	-0.000663832	Metabolome
LysoPC(18:1(11Z)/0:0)	-0.023406795	0.002150093	Metabolome
P-Coumaraldehyde	0.017830618	0.000416839	Metabolome
PE(18:1(9Z)/0:0)	-0.008738499	-0.003248021	Metabolome
PC(18:1/0:0)	-0.022156724	0.005319125	Metabolome
Corchoroside A	0.004124116	0.002548194	Metabolome
1-O-Isopentyl-3-O-octadec-2-enoyl	0.012908873	0.00115393	Metabolome
Deoxycholic acid 3-glucuronide	0.018826688	0.002943574	Metabolome
Indoleacrylic acid	0.023399217	0.000197163	Metabolome
PE(18:2/0:0)	-0.023169996	0.000852098	Metabolome
Cyclohexane	0.009165883	-0.001928143	Metabolome
PE(18:1/0:0)	0.012256616	-0.003072225	Metabolome
LysoPC(18:1(9Z)/0:0)	-0.028331968	-0.002000854	Metabolome
LysoPC(20:4(8Z,11Z,14Z,17Z)/0:0)	-0.028895781	0.000714736	Metabolome
PC(16:0/18:3(6Z,9Z,12Z))	-0.025112185	-0.008540483	Metabolome
Gluconic Acid	-0.026406297	-0.002769113	Metabolome
LysoPC(18:3(6Z,9Z,12Z)/0:0)	-0.012036635	-0.00297157	Metabolome
N6-(1,2-Dicarboxyethyl)-AMP	0.041448011	-0.004197961	Metabolome
Adenylosuccinate	0.03901265	-0.002806328	Metabolome
Fenvalerate	0.019838623	-0.001067571	Metabolome

Cer(t18:0/LTE4)	-0.029291485	-0.006768854	Metabolome
(+/-)-(Z)-2-(5-Tetradecenyl)cyclobuta	0.014254592	-0.008402362	Metabolome
Methyltestosterone	9.08E-05	0.011435871	Metabolome
Succinyladenosine	0.0209924	-0.007225676	Metabolome
Malic acid	0.023866726	-0.001993815	Metabolome
Vindoline	-0.008683496	3.51E-05	Metabolome
Cyclopentanol	0.011485604	-0.006514966	Metabolome
(R)-1-Octen-3-ol	-0.005853807	-0.002465935	Metabolome
Adenosine 3'-monophosphate	0.032143551	0.000765711	Metabolome
Uridine	0.015872314	-0.006622969	Metabolome
Hydroxydeoxyguanosine	0.016676812	-0.002010455	Metabolome
Betaine	0.026135033	0.000224737	Metabolome
L-Glutamic acid	0.019809961	0.0009632	Metabolome
9H-Purine-9-ol	-0.006414532	-0.003170571	Metabolome
TG(15:0/22:6(4Z,7Z,10Z,13Z,16Z,19Z)/1	0.03202586	0.004521585	Metabolome
3-Methylcrotonylglycine	-0.002735003	0.005078101	Metabolome
LysoPE(18:2(9Z,12Z)/0:0)	-0.008402072	-0.003641036	Metabolome
PE(18:0/0:0)	-0.025315625	0.001024644	Metabolome
PE(16:0/0:0)	-0.02358033	-0.001836933	Metabolome
Pseudouridine 5'-phosphate	-0.021198725	-0.001519653	Metabolome
20alpha-Dihydroprogesterone	0.016802039	0.033896665	Metabolome
N-Docosahexaenoyl Proline	-0.019011943	-0.00963248	Metabolome
Azimsulfuron	-0.01697193	-0.0139864	Metabolome
Ceanothine B	0.003970841	0.002613222	Metabolome
16-Hydroxyhexadecanoic acid	0.039671031	-0.000225375	Metabolome
DG(20:4(5Z,8Z,11Z,14Z)-OH(18R)/0:0/2	0.025299906	-0.01002995	Metabolome
A-L-Fucopyranosyl-(1->2)-b-D-galac	0.019499607	-0.018129156	Metabolome
Feruloyltartaric acid	0.024801886	-0.031235281	Metabolome
3-Methyl-2-Oxovaleric Acid	0.009431238	-0.003884115	Metabolome
Edrophonium	0.114917619	-0.003471304	Metabolome
6-[3-(carboxymethyl)phenoxy]-3,4,5-t	0.01209686	-0.013710837	Metabolome
Pyroglutamic Acid	0.027748766	0.000476863	Metabolome
PC(18:0/0:0)	0.007727165	0.006894241	Metabolome
3'-Adenylic Acid	0.031171774	-0.001346104	Metabolome
9,10-Dihydroxystearic acid	0.006193194	-0.016155174	Metabolome
LysoPI(16:0/0:0)	0.010539373	0.017622582	Metabolome
SM(d18:1/14:0)	-0.016668559	0.004653376	Metabolome
PE(LTE4/20:1(11Z))	-0.029321085	-0.000493088	Metabolome
4-Oxo-L-proline	0.022686613	-0.001448587	Metabolome
Guanidylic acid (guanosine monophos	0.011709584	-2.29E-05	Metabolome
LysoPC(16:1(9Z)/0:0)	-0.03912905	-0.000842644	Metabolome
2,4,12-Octadecatrienoic acid piperidid	-0.016362372	-0.003979817	Metabolome
LysoPC(18:0/0:0)	-0.037480402	-0.003078838	Metabolome
PS(22:1(13Z)/18:1(11Z))	0.044331053	-0.073303962	Metabolome
(2R,3Z)-Phycocyanobilin	0.048293123	-0.068157485	Metabolome
Hypoxanthine	-0.012088998	-0.003772735	Metabolome
CMP-N-glycoloylneuraminate	0.003970841	0.002613222	Metabolome
LysoPA(18:1(9Z)/0:0)	0.002793658	-0.0055659	Metabolome
Isonicotinic acid	-0.013286756	0.002672192	Metabolome
5'-Guanylic Acid	0.010991444	-0.003163257	Metabolome
Prostaglandin D1	0.027668116	0.029929076	Metabolome
Xanthine	0.008312227	-0.004770511	Metabolome
Pheophorbide a	0.004392439	0.002741425	Metabolome
Buprenorphine	-0.030311036	0.006166481	Metabolome
Inosine	-0.011203939	-0.010092004	Metabolome
LysoPG(16:0/0:0)	0.009972919	0.017169541	Metabolome
Trigonelline	-0.020455342	0.003734056	Metabolome
N-Ethyl trans-2-cis-6-nonadienamide	0.031342272	0.012930762	Metabolome

Guanosine	0.015271862	-0.007212827	Metabolome
Riboflavin (Vitamin B2)	0.015556716	-0.026918696	Metabolome
Pyropheophorbide a	0.002511936	-0.012807738	Metabolome
LysoPE(P-18:0/0:0)	-0.029485614	0.001440943	Metabolome
Rocuronium	0.008308646	0.008216653	Metabolome
Dihydroferulic acid 4-O-glucuronide	-0.021339695	0.010185537	Metabolome
Chlorophyllide a	0.004124116	0.002548194	Metabolome
N-Acetyl-D-tryptophan	0.029891045	-0.002848602	Metabolome
Cilengitide	0.030879562	0.006397504	Metabolome
Indinavir	-0.014468957	-0.008838677	Metabolome
PI(20:5(5Z,8Z,11Z,14Z,17Z)/0:0)	0.012497577	-0.003403793	Metabolome
13(S)-Hydroperoxylinolenic acid	0.009856933	0.015402859	Metabolome
Farnesyl acetone	0.017907801	-0.008119302	Metabolome
6-Gingerol	0.032558154	0.029003744	Metabolome
Aminomalonic acid	0.015372961	-0.005787125	Metabolome
SM(d18:0/16:1(9Z))	-0.025657534	-0.004538628	Metabolome
3-[[(2S)-2,4-Dihydroxy-3,3-dimethylb	0.011201315	-0.005086039	Metabolome
Neoporrigenin B	-0.023139556	0.00258677	Metabolome
PC(18:1(6Z)/0:0)	-0.019686891	0.00224889	Metabolome
Uracil	0.007084012	-0.005567645	Metabolome
L-3-Cyanoalanine	0.007849833	-0.002663801	Metabolome
Ryanodine	0.004097317	0.002566255	Metabolome
Dihomo-gamma-linolenic acid	0.067313169	0.000625643	Metabolome
17-hydroxyprogesterone caproate	-0.021717528	-0.007007334	Metabolome
Succinic Acid	-0.017113289	0.006113481	Metabolome
Leukotriene B4 ethanolamide	-0.018910655	-0.002532052	Metabolome
PE(20:5(5Z,8Z,11Z,14Z,17Z)/18:0)	-0.02267541	0.005601352	Metabolome
N-Linoleoyl Threonine	-0.025618968	0.024338738	Metabolome
Ticlopidine	0.003970841	0.002613222	Metabolome
2-Hydroxycinnamic acid	0.023994042	-0.00223645	Metabolome
N-Palmitoyl Serine	0.018123513	0.001318456	Metabolome
SM(d16:1/16:0)	-0.019186182	0.000859045	Metabolome
2-Hydroxychlorpropamide	0.024463429	-0.032475619	Metabolome
Contignasterol	-0.034118643	0.018244843	Metabolome
PE(P-18:0/0:0)	-0.039572671	-0.00549429	Metabolome
Pregnenolone	0.022826895	0.028776104	Metabolome
N-Acetyl-D-phenylalanine	0.02400012	-0.004419799	Metabolome
Palmitoylcarnitine	-0.016240905	-0.002356579	Metabolome
1,3-Butadiene	0.014893382	0.000644586	Metabolome
Cholyltyrosine	-0.002055569	-0.00170558	Metabolome
Ganoderic acid F	0.04013701	-0.034046852	Metabolome
13(S)-HpODE	-0.000270146	-0.005316057	Metabolome
Val-Cit	-0.015617783	0.038257419	Metabolome
(S)-10,16-Dihydroxyhexadecanoic acic	0.045466814	-0.003541174	Metabolome
1-Hexadecanoyl-sn-Glycero-3-Phosp	-0.020510002	-0.001381316	Metabolome
RIOPROSTIL	-0.020467996	0.000411022	Metabolome
Ferulic Acid	-0.002091381	-0.005714621	Metabolome
Retaspimycin	-0.016526822	0.003029945	Metabolome
Sorbitan laurate	-0.023144127	0.002168345	Metabolome
Alpha-Linolenoyl Ethanolamide	0.014502929	-0.025037351	Metabolome
[(2R,3S,4R,5R)-5-(4-Amino-2-oxopyrir	0.001295543	-0.004439874	Metabolome
PI(20:1(11Z)/PGF1alpha)	-0.016439702	-0.001111557	Metabolome
O-Stearoylcarnitine	-0.015194187	-0.000503364	Metabolome
LysoPE(18:0/0:0)	-0.022464377	0.001983636	Metabolome
Valylglutamic acid	-0.019796033	-0.00012583	Metabolome
Vanilloylglycine	-0.016293784	-0.005628346	Metabolome
Beta-tocotrienol	0.029492271	0.009218885	Metabolome
Trihydroxystearic acid	0.025636694	-0.004070663	Metabolome

N-Linoleoyl Phenylalanine	-0.030278424	-0.014163455	Metabolome
Naphthionic acid	0.017917609	0.013465385	Metabolome
Gamma-Glu-Leu	-0.014003807	0.000150257	Metabolome
LysoPC(18:2(9Z,12Z)/0:0)	-0.023804116	0.009792854	Metabolome
LysoPA(22:4(7Z,10Z,13Z,16Z)/0:0)	-0.015104102	0.002935184	Metabolome
Adenosine 5'-Monophosphate	0.030280086	-0.001428187	Metabolome
Oxoglutaric acid	0.022794142	-0.001709081	Metabolome
Cytidine 5'-monophosphate-N-acetyl	0.003970841	0.002613222	Metabolome
UDP-N-acetyl-D-mannosamine	-0.022141038	-0.078833295	Metabolome
TG(18:3(6Z,9Z,12Z)/18:4(6Z,9Z,12Z,15Z	0.071296611	0.028476026	Metabolome
2-Amino-3-Methylsuccinic Acid	0.010513436	-0.003977187	Metabolome
N-Stearoyl Proline	-0.031689435	0.002050073	Metabolome
Vulgarin	0.004124116	0.002548194	Metabolome
Glycerol 3-Phosphate	0.02159323	-0.00298579	Metabolome
13-Dihydrodaunorubicin	0.004392439	0.002741425	Metabolome
Inecalcitol	0.036678398	-0.016689181	Metabolome
Tyrosylhydroxyproline	0.008443272	-0.003406289	Metabolome
2-Hydroxyadipic acid	-0.010978445	0.003209178	Metabolome
(R)-1-O-[b-D-Glucopyranosyl-(1->6)	0.00127257	-0.004911958	Metabolome
N-Palmitoyl Valine	-0.031877011	0.002227551	Metabolome
Quercetin 3-O-xylosyl-rutinoside	0.001296209	-0.004352213	Metabolome
Succinic anhydride	0.004899339	-0.012611559	Metabolome
CMP-3-deoxy-D-manno-octulosonat	0.004097317	0.002566255	Metabolome
L-alpha-Aminobutyric acid	-0.010671296	-0.002138199	Metabolome
D-Glycerate 3-phosphate	0.029221781	0.001155502	Metabolome
9(S)-HpOTrE	-0.010036932	-0.000186286	Metabolome
Bipindogulomethyloside	0.004097317	0.002566255	Metabolome
PC(16:0/18:3(9Z,12Z,15Z))	-0.027506866	-0.003472894	Metabolome
Butylisopropylamine	0.01085412	-0.003817431	Metabolome
Dihydro-5-pentyl-2(3H)-furanone	0.012887527	0.000900963	Metabolome
Phytosphingosine	-0.025343296	-0.001639812	Metabolome
MJDBISSN00000003	0.006106245	-0.006089133	Metabolome
Cytidylyl-(5'-3')-5'-inosinic acid homo	0.004228875	0.001833583	Metabolome
2-Aminomuconic acid	0.032046466	-0.008146858	Metabolome
SM(d18:2(4E,14Z)/18:1(12Z)-2OH(9,10	-0.031595832	0.005745806	Metabolome
Furaneol 4-(6-malonylglucoside)	-0.015151003	0.004215943	Metabolome
Linolenic Acid	0.008809611	-0.011908824	Metabolome
(R)-Pterosin B	-0.019259637	-0.000758902	Metabolome
Gamma-Glutamylvaline	-0.005709108	-0.011582839	Metabolome
Hexadecenal	0.016478062	-0.007464498	Metabolome
Batrachotoxinin A 20-alpha-benzoate	-0.009503943	0.001653291	Metabolome
LysoPS(18:0/0:0)	0.019676247	-0.003860453	Metabolome
Choline	0.012986814	0.007457015	Metabolome
(2E,6E)-2,6-bis(2-phenylhydrazono)cy	0.011683045	-0.009564258	Metabolome
Physagulin B	0.004228875	0.001833583	Metabolome
9,10-Epoxy-18-hydroxy-octadecanoic	0.000875102	0.018293601	Metabolome
Boc-val-pro-arg-mca	-0.013098338	-0.013774576	Metabolome
Cis-Hydroxy Perhexiline	-0.032266914	0.000569291	Metabolome
Canavalioside	0.001179529	-0.004363427	Metabolome
2-Hydroxy-2-ethylsuccinic acid	0.001774246	-0.013945551	Metabolome
PG(i-12:0/a-17:0)	0.015709995	-0.003443317	Metabolome
Subaphylline	0.004097317	0.002566255	Metabolome
N-Palmitoyl Glycine	-0.03039861	0.007862987	Metabolome
L-Glutamine	0.029665452	0.000108498	Metabolome
N,N-Dimethylsphingosine	0.016060466	-0.004210971	Metabolome
4-Hydroxy-3-methoxy-cinnamoylglyc	0.042892453	0.031356725	Metabolome
LysoPS(18:2(9Z,12Z)/0:0)	-0.012580673	0.005933202	Metabolome
15-Methylpalmitate	0.000293618	-0.008270361	Metabolome

Docosapentaenoic acid (22n-3)	0.060549066	0.02485867	Metabolome
Pantothenic Acid	0.010337126	-0.000232501	Metabolome
PE(O-18:0/0:0)	-0.017795551	-0.001557073	Metabolome
Palmitoleic acid	-0.006196715	0.003694871	Metabolome
(2S,4S)-Pinnatanine	0.004097317	0.002566255	Metabolome
Indole-3-Carboxaldehyde	0.023542473	-0.003215669	Metabolome
LysoPC(16:0/0:0)	-0.016823601	0.008049422	Metabolome
8-Oxohexadecanoic acid	0.031741335	0.009473448	Metabolome
Cucurbic acid	0.012959796	0.002565549	Metabolome
Diadenosine diphosphate	0.003970841	0.002613222	Metabolome
Ganoderic acid H	0.019599659	0.016056088	Metabolome
Indole	0.018694528	-0.003718297	Metabolome
13,14-Dihydro PGF-1a	0.025403509	-0.007667503	Metabolome
D-Digitoxose	-0.019430095	0.001937097	Metabolome
Gamma-Glutamylphenylalanine	0.00516698	-0.000568306	Metabolome
Beta-Aspartylhydroxamate	0.010538907	0.012632004	Metabolome
Fenoprofen	0.02415634	0.019948245	Metabolome
PI(18:1(9Z)/0:0)	0.027112083	0.017189408	Metabolome
PE(20:5/0:0)	-0.014913776	-0.003809176	Metabolome
Coproporphyrin III	0.004097317	0.002566255	Metabolome
2,4-Dihydroxybenzoic acid	0.024310202	-0.004862298	Metabolome
Pipecolic Acid	0.00543201	0.002191288	Metabolome
Uridine diphosphate-N-acetylglucosar	-0.00315232	0.016712404	Metabolome
Avocadyne 2-acetate	0.052601842	-0.004785486	Metabolome
Glycerophosphocholine	0.019254958	0.00218448	Metabolome
L-Carnitine	0.001168036	-0.002459045	Metabolome
Caffeic acid 4-O-glucuronide	0.001275852	-0.004231972	Metabolome
PC(18:3(9Z,12Z,15Z)/16:0)	-0.031072837	-0.004161256	Metabolome
Riboflavin	0.01914533	-0.020019144	Metabolome
D-Arabinono-1,4-lactone	-0.009590765	0.001094933	Metabolome
L-Threonine	0.013136364	-0.000896996	Metabolome
Isoschaftoside	0.014909693	-0.019283062	Metabolome
3-Ketosphingosine	-0.012900195	0.000686395	Metabolome
N-Palmitoyl Threonine	-0.03581534	0.004608388	Metabolome
9,10,13-Trihydroxystearic acid	-0.017746821	-0.004803927	Metabolome
MJDBISSN00000001	-0.000961402	-0.006968059	Metabolome
Oxalic Acid	0.034888744	0.021760862	Metabolome
S-Nitrosoglutathione	0.004249863	0.046622373	Metabolome
5,12-Naphthacenedione, 10-((4-aminc	-0.021882969	0.003132243	Metabolome
Kanokoside D	-0.010734626	0.072027477	Metabolome
(3b,20R,22R)-3,20,27-Trihydroxy-1-ox	0.051280678	0.007389318	Metabolome
B-D-Xylopyranosyl-(1->4)-a-L-rhamr	-0.039589556	0.005382893	Metabolome
16-Hydroxy-10-oxohexadecanoic acic	0.020474825	0.004414125	Metabolome
Astromicin	0.00646244	-0.014807099	Metabolome
Avocadyne 4-acetate	0.01703461	0.012416572	Metabolome
Fexofenadine	-0.042179996	0.040868355	Metabolome
LysoPS(16:0/0:0)	-0.009032391	0.004204866	Metabolome
2-Aminopurine	0.011903921	0.001515383	Metabolome
LysoPE(0:0/18:2(9Z,12Z))	-0.027255664	-0.002196822	Metabolome
Adenosine	0.03119214	0.002585106	Metabolome
Terbutaline	-0.017541961	0.001926532	Metabolome
PC(17:1/0:0)	-0.015965883	-0.007536611	Metabolome
O-Acetylcarnitine	0.013270195	-0.000586582	Metabolome
7-Aminocephalosporanic acid	0.004228875	0.001833583	Metabolome
2-Naphthylamine	0.023886924	-0.002229806	Metabolome
Glu-val-phe	-0.042643904	0.000122669	Metabolome
Deltaline	-0.000794588	-0.003053227	Metabolome
(4E,7E,10E,13E)-Hexadeca-4,7,10,13-te	-0.035477803	0.152977974	Metabolome

3-Methylbut-2-enoylcarnitine	-6.37E-07	0.013366172	Metabolome
Kojibiose	-0.01843655	0.00050868	Metabolome
Floionolic acid	0.011627879	0.001483446	Metabolome
PS(22:2(13Z,16Z)/15:0)	-0.018798812	-0.003703166	Metabolome
Hippuryl-L-lysine	-0.011707671	-0.00528563	Metabolome
12S-HHT	-0.011534767	0.024659771	Metabolome
L-Alanine	0.006304613	-0.003560659	Metabolome
Thymidine	0.01694796	-0.005475734	Metabolome
Ifosfamide	0.017926165	0.003085378	Metabolome
Cytidylyl-(3',5')-guanosine	-0.034182724	-0.103133991	Metabolome
LysoPC(14:0/0:0)	-0.019333993	0.00450505	Metabolome
(1R,6R)-6-hydroxy-2-succinylcyclohex	0.037985451	0.035118377	Metabolome
Concanamycin a	-0.026190643	0.019240373	Metabolome
5'-Thymidylic Acid	-0.03844383	-0.0013492	Metabolome
N(6)-L-Homocysteinyl-L-lysine	0.011771101	-0.000190412	Metabolome
PC(20:4(8Z,11Z,14Z,17Z)/20:0)	0.054122872	-0.014495868	Metabolome
9,10,13-TriHOME	-0.021881629	0.000870535	Metabolome
LysoPA(i-19:0/0:0)	0.00474955	-0.005108098	Metabolome
GPCho(18:1/16:0)	-0.031497808	8.42E-05	Metabolome
Trans-4-Carboxymethylenebut-2-en-4	0.007403829	-0.011823757	Metabolome
N-Palmitoyl Leucine	-0.030242219	2.21E-06	Metabolome
10'-Apo-beta-carotenal	0.001419228	-0.004401436	Metabolome
Tigemonam	0.021731519	0.054854923	Metabolome
1-Acetyl-3,27-dihydroxywitha-5,24-di	0.000297649	-0.000932781	Metabolome
Alcesefoliside	0.001419228	-0.004401436	Metabolome
Penitrem B	0.001419228	-0.004401436	Metabolome
7Z,10Z-Hexadecadienoic acid	-0.00211144	-0.021899846	Metabolome
Novobiocin	0.004097317	0.002566255	Metabolome
4-hydroxyheptanoyl-CoA	0.004392439	0.002741425	Metabolome
Octadecanamide	0.014927327	-0.007280216	Metabolome
Feruloyl Malate	-0.02669892	0.127028602	Metabolome
P-Hydroxyl-ethotoin	0.020006198	0.040812059	Metabolome
4-[10,15,20-Tris(4-sulfophenyl)-21,22,	0.026183866	0.018173302	Metabolome
Benzyl sulfate	0.00690553	-0.005042372	Metabolome
Nonadecanoic acid	0.002713958	-0.009399687	Metabolome
N-Oleoyl Alanine	-0.026308948	0.002328624	Metabolome
Glycylprolylhydroxyproline	0.004097317	0.002566255	Metabolome
Propionylcarnitine	0.007008354	0.002518404	Metabolome
SM(d16:1/14:0)	-0.008862401	0.024573631	Metabolome
L-Tryptophan	0.024567089	-0.002859075	Metabolome
5,6-epoxy,18R-HEPE	0.012912163	-0.00970477	Metabolome
PC(22:5/0:0)	0.047062757	-0.085618451	Metabolome
Dimethyl phthalate	0.038270358	0.020294512	Metabolome
N,N-Dimethylarginine	-0.008342407	0.006762841	Metabolome
Sebacic acid	0.012632067	0.000312295	Metabolome
Corchoionol C 9-glucoside	0.041406834	-0.002862538	Metabolome
Beta-D-Xylopyranosyl-(1->4)-alpha-L	-0.018924295	-0.002791709	Metabolome
Banoxantrone	0.024993352	0.001357447	Metabolome
Isoferulic acid	-0.023567327	-0.074087886	Metabolome
13,16,19-Docosatrienoic acid	0.001417844	-0.004874306	Metabolome
Vilaprisan	0.003970841	0.002613222	Metabolome
Oxypurinol	0.02266041	0.000414023	Metabolome
GPCho(16:0/20:3)	-0.025854649	0.021495235	Metabolome
15-Hydroxynorandrostene-3,17-dione	0.001295543	-0.004439874	Metabolome
2-Chloro-1-[(2S,3S,5S,10S,13S)-3-hyd	0.001179529	-0.004363427	Metabolome
6-Hydroxyhexanoic Acid	-0.006913753	-0.010836894	Metabolome
7-[(1R,2R,3R)-3-Hydroxy-2-[(1E,3S)-3	-0.02477413	0.007577776	Metabolome
N-Oleoyl Phenylalanine	-0.043372258	-0.017455467	Metabolome

3-Hydroxychlorpropamide	0.030874916	0.029769536	Metabolome
(16-Benzyl-5,12-dihydroxy-5,7,14-trin	0.004097317	0.002566255	Metabolome
1-Oleoylglycerophosphoserine	0.006983175	-0.003857913	Metabolome
DTDP-4-acetamido-4,6-dideoxy-D-g	-0.024035787	-0.028322362	Metabolome
(3S,3'S,5R,5'R,6R)-3,6-Epoxy-5,6-dihyc	0.016465098	0.003193104	Metabolome
Dhurrin 6'-glucoside	-0.012109349	0.001543884	Metabolome
Aspartylglycosamine	0.018113229	0.034051113	Metabolome
PC(24:1(15Z)/15:0)	0.050553406	0.002397596	Metabolome
Oxepin	0.017338287	-0.000609081	Metabolome
2-Isopropylmalic acid	0.012108683	-0.00690442	Metabolome
Tyr-Gly-Gly-Trp-Leu	0.010791197	0.015952309	Metabolome
Indolepyruvate	0.003970841	0.002613222	Metabolome
8(R)-Hydroperoxylinoleic acid	0.019063684	0.00159133	Metabolome
Quercetin	-0.045255722	0.017905941	Metabolome
Deoxyadenosine monophosphate	0.021468924	-0.011293202	Metabolome
FAD	-0.013136659	-0.00487189	Metabolome
Lefamulin	0.021026776	0.000482701	Metabolome
Xi-7-Hydroxyhexadecanedioic acid	-0.004803396	0.019716285	Metabolome
Aminodeoxykanamycin	0.004392439	0.002741425	Metabolome
Traumatic Acid	-0.014525427	-0.001346347	Metabolome
Allantoic Acid	-0.026897319	-0.006234278	Metabolome
Phaeophorbide b	0.00127257	-0.004911958	Metabolome
Chenodeoxycholylarginine	0.016348678	-0.002906115	Metabolome
Dynorphin A (6-8)	0.004228875	0.001833583	Metabolome
Paramethasone	0.004228875	0.001833583	Metabolome
L-Lactic acid	-0.014727688	-0.00310074	Metabolome
16-B1-phytoprostane	0.013750236	0.03369122	Metabolome
SM(d18:0/PGJ2)	-0.041715275	0.005647633	Metabolome
5-Hydroxyindoleacetic acid	0.016310674	-0.001735377	Metabolome
Tetraazacyclododecanetetraacetic acid	-0.018028961	-0.002066008	Metabolome
4,4'-Dioctyldiphenylamine	0.009427903	-0.002862025	Metabolome
PE(16:1/0:0)	-0.033729031	0.001376785	Metabolome
Thermophillin	-0.024521918	0.005146788	Metabolome
Sugeonol	-0.03428557	0.006299321	Metabolome
5-Hydroxy-L-tryptophan	0.032535087	0.037641406	Metabolome
Blumenol C O-[apiosyl-(1->6)-glucosi	0.034276752	0.07285688	Metabolome
LysoPE(14:0/0:0)	-0.057112716	-0.007822856	Metabolome
TG(16:1(9Z)/14:1(9Z)/20:2n6)	0.002787938	-0.013080795	Metabolome
Hexanal	0.007089279	-0.007826025	Metabolome
7b-Hydroxy-3-oxo-5b-cholanoic acid	0.023033431	-0.002377994	Metabolome
Cycloheximide	0.012201671	-0.002326763	Metabolome
Sphinganine	-0.028486368	-0.003112148	Metabolome
LysoPE(18:4(6Z,9Z,12Z,15Z)/0:0)	-0.02330531	-0.030733144	Metabolome
Ecgonine	-0.017359728	-0.000568878	Metabolome
N-Myristoyl Arginine	-0.007520292	-0.008218443	Metabolome
9-Octadecenamide	-0.021428823	0.003985036	Metabolome
3H-Pyrido(1,2-c)pyrimidin-3-one, 4-(2	0.01827872	-0.003410643	Metabolome
Oxaprozin glucuronide	0.01440924	0.006635341	Metabolome
PE(P-18:0/PGE1)	-0.019796084	-0.012591501	Metabolome
Clozapine	0.002311027	-0.023625864	Metabolome
Manoalide	-0.010309508	-0.008242222	Metabolome
Ganosporeric acid A	0.001296209	-0.004352213	Metabolome
15-Deacetylneosolaniol	-0.0104125	0.005717729	Metabolome
Adenylsuccinic acid	0.003970841	0.002613222	Metabolome
N-Stearoyl Glutamine	0.033780815	-5.09E-05	Metabolome
N(1)-Acetylsulfamethoxazole	0.001125047	-0.003684307	Metabolome
Ciprostene	0.024647973	0.014898036	Metabolome
PS(15:0/22:2(13Z,16Z))	-0.030288812	-0.004894318	Metabolome

25-Acetyl-6,7-didehydrofevicordin F 3	-0.01258714	0.095310156	Metabolome
1-(Hydroxymethyl)-5,5-dimethyl-2,4-i	0.012382635	-0.006210928	Metabolome
Asparaginylhydroxyproline	-0.012141409	0.055539325	Metabolome
Mepivacaine	0.003970841	0.002613222	Metabolome
2-Hydroxy-L-methionine	0.003530614	-0.010423983	Metabolome
PI(TXB2/20:4(8Z,11Z,14Z,17Z))	-0.020178553	-0.013239214	Metabolome
Grayanotoxin	0.012229986	-0.004228233	Metabolome
Asparaginyl-Phenylalanine	0.009679533	0.003195692	Metabolome
Epothilone D	-0.034271711	-0.001409026	Metabolome
(1,10,11,12,14,23-Hexahydroxy-6,10,19	-0.033425575	-0.001934801	Metabolome
Plumieride	-0.027771502	0.002713208	Metabolome
5alpha-Cholest-8-en-3beta-ol	0.008119615	-0.02239424	Metabolome
Salicylhydroxamic acid	-0.01768563	-0.014719101	Metabolome
4-Fluorobenzoic acid	0.020208416	0.002920479	Metabolome
Fleroxacin N-oxide	0.00127257	-0.004911958	Metabolome
(3beta,5beta,8beta,22E,24xi)-Ergosta-6	0.005758135	-0.009325648	Metabolome
Oligoadenylate	0.011300156	-0.062203207	Metabolome
(1R)-5-[2-[(1S,7As)-1-[(1S)-1-(3-hydr	0.028289454	-0.025727355	Metabolome
Trans-EKODE-(E)-Ib	-0.029717614	0.009950459	Metabolome
Phenylacetylglutamine	0.01091784	0.023666977	Metabolome
Azelaic Acid	0.010834105	-0.012811058	Metabolome
Corchorifatty acid D	-0.000876316	0.012649578	Metabolome
3-butenylglucosinolate	0.001326115	-0.004319813	Metabolome
TG(18:2(9Z,12Z)/18:2(9Z,12Z)/20:1(11Z	-0.028891135	0.001301855	Metabolome
C-2 Ceramide	-0.057209402	0.002084275	Metabolome
4-Trimethylammoniobutanoic acid	-0.023335265	-0.00073101	Metabolome
Solutol HS 15	-0.02774586	-0.00431064	Metabolome
Vicenin 2	0.062265966	0.017843359	Metabolome
9,12,13-TriHOME	0.004061435	-0.052211942	Metabolome
Prenyl glucoside	-0.00157502	-0.043193181	Metabolome
Starch, pregelatinized	0.004124116	0.002548194	Metabolome
Linoleoyl Ethanolamide	0.011741811	-0.010209628	Metabolome
Biochanin A 7-(6-malonylglucoside)	0.065592266	-0.166558148	Metabolome
7-Methylguanine	0.019872059	-0.001750788	Metabolome
LysoPC(20:5(5Z,8Z,11Z,14Z,17Z)/0:0)	-0.008525366	-0.00573278	Metabolome
Jervine	0.004097317	0.002566255	Metabolome
LysoPE(0:0/18:4(6Z,9Z,12Z,15Z))	-0.016291277	-0.010588914	Metabolome
Sinapoyl-(S)-malate	-0.025263267	0.048379815	Metabolome
DG(22:6(4Z,7Z,10Z,13Z,16Z,19Z)/22:5(4	-0.023640789	0.000637566	Metabolome
PS(14:0/14:1(9Z))	0.001296209	-0.004352213	Metabolome
Gamma-Linolenic Acid	-0.001401746	0.004660953	Metabolome
Phenylalanylphenylalanine	-0.007433746	0.093373488	Metabolome
Fe(II)-nicotianamine	0.00127257	-0.004911958	Metabolome
Coformycin	0.024267612	-0.006844466	Metabolome
Asn Ile Thr Glu	0.00127257	-0.004911958	Metabolome
3-(2-Furoyl)quinoline-2-carboxaldehy	0.006067642	-0.058891196	Metabolome
Carboprost methyl	0.00950056	0.000107103	Metabolome
Glyceric Acid	-0.027030668	-0.000868984	Metabolome
25-O-Desacetyl rifabutin	0.004097317	0.002566255	Metabolome
2-(3,4-Dihydroxybenzoyloxy)-4,6-dihy	0.013998579	-0.002142973	Metabolome
8-Hydroxyluteolin 8-sulfate	-0.008554649	-0.013575641	Metabolome
Homomethionine	0.060035009	-0.001272504	Metabolome
Cyclosquamosin A	-0.024065015	-0.003888773	Metabolome
LysoPI(0:0/18:0)	0.003285362	0.007196287	Metabolome
(Z)-7-[(1R,2R,3R,5S)-2-(1,2-Dihydroxy	0.013052024	0.003226882	Metabolome
3,4,5-trihydroxy-6-({7-oxo-7H-furo[3,	0.001419228	-0.004401436	Metabolome
3-Methyl-1-(2,4,6-trihydroxyphenyl)-1	0.018561	-0.017718782	Metabolome
Tetranorprostanedioic acid	-0.024586064	-0.004715437	Metabolome

Urobilinogen	0.007322925	-0.001248059	Metabolome
LysoPE(18:3(6Z,9Z,12Z)/0:0)	-0.026906046	-0.01513389	Metabolome
MG(22:6(4Z,7Z,10Z,13Z,16Z,19Z)/0:0/0	0.02031211	0.003967538	Metabolome
5-Acetylamino-6-formylamino-3-met	0.017784135	-0.002538402	Metabolome
Lithocholic acid	0.004097317	0.002566255	Metabolome
9-Tetradecenoic acid	-0.02048754	9.88E-05	Metabolome
Thiodiacetic acid	0.027481895	0.011712768	Metabolome
Thienamycin	-0.0055428	0.017055692	Metabolome
[(2R,5R)-5-(2-Amino-6-oxo-1H-purin	0.004097317	0.002566255	Metabolome
Tsangane L 3-glucoside	0.011404019	-0.000194298	Metabolome
2-Phenylacetamide	0.017115967	-0.005113424	Metabolome
Mepartricin	0.019828872	-0.015591342	Metabolome
Bis(carboxyethyl)carboxyfluorescein ac	0.001296209	-0.004352213	Metabolome
Setipiprant	-0.026016394	0.000446646	Metabolome
DG(8:0/12:0/0:0)	0.002036	0.021205373	Metabolome
D-Ornithine	0.015124719	-0.000417751	Metabolome
PC(14:0/0:0)	-0.008183321	-0.022245184	Metabolome
Ganglioside GD1b (d18:1/18:1(11Z))	0.025223398	0.01120833	Metabolome
UDP-D-Galactose	0.034155777	0.001943704	Metabolome
PS(20:0/20:0)	0.001419228	-0.004401436	Metabolome
Taxine B	0.00431055	0.00158541	Metabolome
(5E,7E)-Undeca-2,5,7-trienedioylcarnit	-0.010757941	-0.023276818	Metabolome
Xanthopterin	0.012824078	0.003453899	Metabolome
Gamma-Aminobutyric acid	-0.002417081	0.012808107	Metabolome
Vaccinoside	0.001179529	-0.004363427	Metabolome
Quercetin 7-glucuronide 3-rhamnosid	0.004124116	0.002548194	Metabolome
Enol-phenylpyruvate	-0.004794267	0.048046136	Metabolome
N-arachidonylethanolamine	0.012140003	-0.003761153	Metabolome
Adenosine 2'-phosphate	0.020814477	0.009597388	Metabolome
Glu-Gln	0.019181765	-0.00932233	Metabolome
Skimmin	-0.052762269	-0.024081807	Metabolome
(-)-Epigallocatechin 3'-glucuronide	-0.020114562	0.011138132	Metabolome
4-amino-4-deoxychorismate	-0.028167193	0.017891261	Metabolome
Gardenin B	-0.017223175	-0.000961229	Metabolome
Gymnodimine	-0.028339499	-0.001167479	Metabolome
N-Palmitoyl Histidine	-0.016556241	-0.000938949	Metabolome
Nona-2,6-dienedioyl-CoA	0.023596865	-0.00304493	Metabolome
Choline Phosphate	-0.011356067	0.001950597	Metabolome
N-Oleoyl Leucine	-0.029317721	0.002973351	Metabolome
Phytuberin	0.011620338	0.007829698	Metabolome
Aminocaproic acid	0.025873156	0.070330923	Metabolome
Deoxycholylarginine	-0.011988464	0.005828413	Metabolome
Daphnoretin	0.00127257	-0.004911958	Metabolome
LysoPE(20:2(11Z,14Z)/0:0)	0.009843587	-0.081863238	Metabolome
Hydroxytyrosyl acetate	-0.031738479	-0.000308106	Metabolome
Inosine 5'-Phosphate	-0.024135812	-0.001102144	Metabolome
Hexadecanedioic acid	0.030961175	-0.008687387	Metabolome
2'-Deoxyadenosine 5'-phosphate	0.009756378	-0.021547416	Metabolome
Polyoxyethylene 40 monostearate	-0.03028982	-0.006972966	Metabolome
Pangamic acid	0.034917967	-0.000631521	Metabolome
Isoxanthopterin	0.014586969	-0.007254699	Metabolome
Ethylenediamine-N,N'-diacetic acid	-0.018624908	0.014032679	Metabolome
5'-CMP	-0.032110961	0.016512888	Metabolome
6-Hydroxypentadecanedioic acid	0.011781659	-0.000323857	Metabolome
DG(22:6(4Z,7Z,11E,13Z,15E,19Z)-2OH(	-0.022692021	-0.025959488	Metabolome
4-(2-Nitroethyl)phenyl primeveroside	-0.021834416	0.002183843	Metabolome
(11R,16S)-misoprostol	0.006454018	0.007732386	Metabolome
4,5-Diacetyl-6,7-dichloro-3',6'-dihydr	0.072660062	-0.020225312	Metabolome

2-Hydroxymyristic Acid	0.041800003	0.002133631	Metabolome
Di-deacetyl adrogolide	-0.056585991	-0.000338122	Metabolome
Methidathion	-0.008035889	0.000572528	Metabolome
Palatinose	-0.017354956	-0.002526329	Metabolome
3-Hydroxyanthranilic acid	-0.010551743	-0.036760326	Metabolome
Aminohippuric acid	-0.014198779	-0.01822013	Metabolome
LysoPC(20:3(5Z,8Z,11Z)/0:0)	-0.034300665	-0.003384537	Metabolome
ADP	0.028428308	-0.004626213	Metabolome
2-[(2,6-Dioxocyclohexyl)methyl]cycloh	0.011298943	0.019988135	Metabolome
Muzanzagenin	-0.015571597	0.004257589	Metabolome
1-Heneicosanoyl-glycero-3-phospho:	-0.05655711	-0.001686452	Metabolome
(3alpha,5beta,7alpha)-23-Carboxy-7-l	-0.010469325	-0.021144358	Metabolome
Inosine 2'-phosphate	0.002958515	-0.074978044	Metabolome
CDP-DG(LTE4/i-21:0)	0.004124116	0.002548194	Metabolome
Bacoside A	0.004097317	0.002566255	Metabolome
N,N'-Dicyclohexylurea	0.007930578	0.001268089	Metabolome
Cytidine 3'-Phosphate	-0.023875133	-0.003273594	Metabolome
(+)-gamma-Hydroxy-L-homoarginine	0.001417844	-0.004874306	Metabolome
Pseudouridine	0.019488395	0.006674266	Metabolome
4,1-Benzoxazepine	-0.007776191	-0.005392338	Metabolome
Biliverdin	0.004124116	0.002548194	Metabolome
9,10-DHOME	0.038005912	0.028434114	Metabolome
Uridine Diphosphate Glucuronic Acid	-0.050371163	-0.030548533	Metabolome
PI(16:2(9Z,12Z)/6 keto-PGF1alpha)	-0.008416565	-0.012589588	Metabolome
Tazobactam	0.017297326	-0.009145184	Metabolome
Acetyl-DL-Leucine	0.015378757	-0.004961482	Metabolome
Taurochenodesoxycholic acid	-0.008675927	-0.007138387	Metabolome
LysoPC(22:5(7Z,10Z,13Z,16Z,19Z)/0:0)	0.004097317	0.002566255	Metabolome
7-[(1R,2R,5S)-5-Hydroxy-2-[(1E,3S)-3	-0.026879763	-0.005132549	Metabolome
Sclareol	-0.034197575	-0.03201361	Metabolome
2,3-Dihydroxypropanamide	0.01829436	0.000183889	Metabolome
2-[3-Methoxy-2-propoxy-5-[5-(3,4,5-	0.001417844	-0.004874306	Metabolome
(5E)-7-[3,5-Dihydroxy-2-(3-hydroxyo	0.000217654	-0.001754295	Metabolome
D-Ribulose 5-Phosphate	0.001157701	-0.026232223	Metabolome
6-[4-(carboxymethyl)phenoxy]-3,4,5-t	0.035968327	0.025783056	Metabolome
Kessyl glycol	-0.026316305	-0.034574872	Metabolome
Pentose	0.0064474	-0.003446612	Metabolome
1-(4-Aminophenyl)-3-acetyl-4-methy	0.011311719	-0.073383259	Metabolome
Dopaxanthin quinone	0.001419228	-0.004401436	Metabolome
Capecitabine	0.004228875	0.001833583	Metabolome
4-Benzofuranacetamide, N-methyl-N-	0.001962163	5.70E-05	Metabolome
Acetyl adenylate	0.027080205	0.003395708	Metabolome
Cystathionine	0.01726075	0.004289673	Metabolome
1-O-Hexadecyl-sn-glycero-3-phosph	-0.028950935	0.003705217	Metabolome
Polypodine B	0.030957504	0.016116006	Metabolome
Corchoionoside B	0.024650359	-0.006944774	Metabolome
Murnac-tripeptide	0.010768341	-0.004727561	Metabolome
10-Acetyl-3,7-dihydroxyphenoxazine	0.031904694	-0.047692891	Metabolome
Golotimod	0.025723034	-0.024207399	Metabolome
Glucosamine	0.01372993	-0.003027362	Metabolome
Leu-Arg-Asn-Arg	-0.024934273	-0.000314939	Metabolome
15-hydroxyicosanoic acid	-0.022374572	0.003133214	Metabolome
Adipic Acid	-0.018891151	-0.017353692	Metabolome
Glycerophosphoinositol	-0.025385905	0.00062374	Metabolome
Capsicoside A1	0.001419228	-0.004401436	Metabolome
Dihomolinoleic acid	-0.022229661	0.003492487	Metabolome
Laminine	-0.021872966	0.00555529	Metabolome
DG(20:5(7Z,9Z,11E,13E,17Z)-3OH(5,6,1	0.001419228	-0.004401436	Metabolome

Pretyrosine	0.027340872	-0.006331077	Metabolome
4-Methoxyestrone	0.004097317	0.002566255	Metabolome
SM(d18:1/16:0)	-0.026375904	0.001665542	Metabolome
3-(6-Aminopurin-9-yl)-5-(hydroxyme	0.009429337	-0.000442767	Metabolome
Sapienic acid	-0.007241252	0.003839477	Metabolome
Cropropamide	-0.024093823	0.003091812	Metabolome
N-Acetylmannosamine	-0.022044519	-0.004170481	Metabolome
2-Azaspiro[4.5]decan-3-one	0.006065779	-0.00568615	Metabolome
N-Arachidonoyl Lysine	-0.032058318	0.030583875	Metabolome
Tricholomic acid	-0.002089993	0.093890814	Metabolome
D-Tartaric Acid	0.011617182	-0.010265677	Metabolome
Persenone B	-0.043809916	-0.04911232	Metabolome
Acetoacetic acid	-0.020350771	-0.012376965	Metabolome
Ricinoleic acid	0.013909986	-0.006636514	Metabolome
PS(20:4(8Z,11Z,14Z,17Z)/20:3(8Z,11Z,1	0.038034854	-0.093101495	Metabolome
3,4-Dihydroxybenzaldehyde	-0.024456327	0.013040933	Metabolome
2-([1,1'-Biphenyl]-2-yl)acetic acid	0.01211544	-0.021980831	Metabolome
3'-Sialyllactose	0.031074556	-0.00012558	Metabolome
Quinol glucuronide	0.051764621	-0.07680582	Metabolome
MG(PGF1alpha/0:0/0:0)	0.008993849	0.004237075	Metabolome
Apigenin 7-glucuronosyl-glucoside	0.004228875	0.001833583	Metabolome
P-Anisaldehyde	0.006757353	0.000812386	Metabolome
Prostaglandin A1	0.011627856	0.009805103	Metabolome
Ruscogenin	-0.041714894	-0.000850481	Metabolome
Pectenotoxin 1	-0.014797163	-0.010865269	Metabolome
L-Asparagine	0.009841087	-0.005618658	Metabolome
7-Amino-4-methylcoumarin	0.027339689	-0.002466272	Metabolome
Beta-tyvelose	0.010927173	0.006672608	Metabolome
Rutin	0.036821507	-0.008026125	Metabolome
PE(18:3(9Z,12Z,15Z)/16:0)	-0.003534793	0.004749502	Metabolome
N-Stearoyl Threonine	0.001417844	-0.004874306	Metabolome
Dodecyl-beta-D-maltoside	0.039431477	-0.01168853	Metabolome
DG(8:0/a-13:0/0:0)	-0.033293764	-0.004271005	Metabolome
LysoPC(20:2(11Z,14Z)/0:0)	-0.041607128	-0.014739602	Metabolome
Secalonic acid A	0.004124116	0.002548194	Metabolome
12-Hydroxyjasmonic acid glucoside	-0.007916466	-0.02882143	Metabolome
5-Aminovaleric Acid	0.01399265	-0.002567881	Metabolome
Lamivudine sulfoxide	-0.008822339	-0.002643856	Metabolome
8-Hydroxyquinoline	-0.025692137	0.007031648	Metabolome
Tulipanin	-0.027994024	0.004386255	Metabolome
4-Hydroxystyrene	-0.009180648	-0.007749535	Metabolome
PIP(6 keto-PGF1alpha/20:1(11Z))	-0.030259383	-0.041526138	Metabolome
Cyanidin 3-triglucoside	-0.009096329	0.009960938	Metabolome
Vanillic acid 4-beta-D-glucoside	0.024305308	-0.004591645	Metabolome
Cmp-nana	0.004228875	0.001833583	Metabolome
Pantetheine 4'-phosphate	-0.034816069	-0.002654802	Metabolome
Efavirenz	0.001179529	-0.004363427	Metabolome
2-(1-Ethoxyethoxy)propanoic acid	0.018900026	0.009036204	Metabolome
Lubiprostone	0.020626432	0.006835424	Metabolome
N-Phenylacetylphenylalanine	0.004228875	0.001833583	Metabolome
Risbitin	0.00843787	-0.005973134	Metabolome
Curdione	0.004124116	0.002548194	Metabolome
3-Hydroxybutanoic Acid	-0.00115201	-0.012700803	Metabolome
Biflorin	0.00127257	-0.004911958	Metabolome
Melezitose	-0.005533244	0.009408618	Metabolome
2-Pyrocatechuic Acid	0.010144305	-0.00688779	Metabolome
Ganglioside GT3 (d18:1/22:1(13Z))	0.004097317	0.002566255	Metabolome
4-(2-Aminophenyl)-2,4-dioxobutanoi	0.02201944	-0.021569696	Metabolome

PGP(20:4(5Z,8Z,11Z,13E)-OH(15S)/i-2(	-0.015345127	-0.000691249	Metabolome
Eriojaposide A	0.001296209	-0.004352213	Metabolome
2-Amino-3-phosphonopropionic acid	-0.005817894	0.031746802	Metabolome
PS(18:1(9Z)/0:0)	-0.000286038	-0.015889345	Metabolome
P-Coumaric acid glucuronide	0.001295543	-0.004439874	Metabolome
5,9,11-trihydroxyprosta-6E,14Z-dien-:	-0.019597125	0.000881315	Metabolome
Heptylmalonic acid	-0.003678167	-0.077049183	Metabolome
GPCho(16:0/20:5)	-0.02705021	-0.015184877	Metabolome
Ruberythric acid	0.055124048	-0.020091471	Metabolome
Glutarate semialdehyde	-0.016405321	-0.000777617	Metabolome
Zidovudine	0.030078438	0.004403755	Metabolome
Shanzhiside	-0.059515017	0.122244959	Metabolome
Valtrate	0.034287892	0.04397019	Metabolome
Sterculic acid	0.004097317	0.002566255	Metabolome
Linatine	0.03990821	0.020571294	Metabolome
Pro Val	-0.014264474	-0.001820064	Metabolome
Dimethylethanolamine	-0.00887242	-0.000379011	Metabolome
2-Amino-4-oxo-6-(1',2'-dioxoprolyl)-	0.019600607	-0.031987827	Metabolome
UDP-D-apiose	0.000117496	0.049959975	Metabolome
PA(5-iso PGF2VI/18:1(11Z))	0.00771423	-0.003301541	Metabolome
PS(PGJ2/22:5(4Z,7Z,10Z,13Z,16Z))	0.004097317	0.002566255	Metabolome
Adenosine diphosphate ribose	0.005469271	-0.010020221	Metabolome
SM(d18:2(4E,14Z)/16:0)	-0.027615065	0.001253065	Metabolome
PE(17:0/0:0)	-0.026690648	-0.00021002	Metabolome
Vellein	-0.007759144	-0.018682994	Metabolome
DG(PGD2/2:0/0:0)	0.001419228	-0.004401436	Metabolome
Sarcosine	0.004311346	-0.000170899	Metabolome
N4-Acetylcytidine	0.004228875	0.001833583	Metabolome
4-Methyl-1H-pyrrole-3-carboxamide	-0.016746125	-0.008951858	Metabolome
Prostaglandin F1a	0.005354876	-0.020159867	Metabolome
Vanillic acid 4-O-sulfate	0.018229258	-0.003408843	Metabolome
Norclobazam	0.003970841	0.002613222	Metabolome
Sulfadimidine	0.009738217	-0.016023801	Metabolome
5'-Phosphoribosyl-N-formylglycinami	0.004228875	0.001833583	Metabolome
1-(9Z-Nonadecenoyl)-glycero-3-pho:	-0.019486522	-0.003286943	Metabolome
4-Propylphenol	0.021082714	-0.00197297	Metabolome
2-Hydroxyfelbamate	0.015537305	0.008121075	Metabolome
Vicenin	-0.029867748	-0.004698472	Metabolome
Trans-5,6-Dihydro-5,6-dihydroxy-7,1:	0.001148631	-0.003864389	Metabolome
PI(TXB2/18:0)	-0.041236529	-0.109755948	Metabolome
Oryzalide B	0.004097317	0.002566255	Metabolome
Sulbactam	0.003970841	0.002613222	Metabolome
Methyl acrylate	-0.02145919	0.002051607	Metabolome
Adenosine 3',5'-diphosphate	0.02723129	0.004746	Metabolome
(10E,12E,14E)-16-Hydroxy-9-oxoocta(	0.001419228	-0.004401436	Metabolome
Spongothymidine	-0.02410035	-0.030717699	Metabolome
Hyaluronan biosynthesis, precursor 1	-0.032690882	0.001458494	Metabolome
Calcitroic acid	0.020700657	-0.008853311	Metabolome
1,1,1-Trifluoro-2,4-pentanedione	-0.02930393	0.006393153	Metabolome
Glucocochlearin	-0.005291236	0.000761158	Metabolome
N,n-Dimethylguanosine	-0.01968205	-0.008257587	Metabolome
(+/-)-Ribaline	0.001692464	-0.010414389	Metabolome
2-O-(6-Phospho-alpha-mannosyl)-D	-0.02485382	-0.043444627	Metabolome
[(1S,2S)-2-[[2,2-Dimethylpropyl(nonyl)	0.004097317	0.002566255	Metabolome
Exherin	0.018206212	-0.008045238	Metabolome
DG(8:0/8:0/0:0)	0.004097317	0.002566255	Metabolome
Oxytetracycline	0.027810621	0.029184728	Metabolome
(+)-15,16-Dihydroxyoctadecanoic acic	0.003970841	0.002613222	Metabolome

Queuine	0.02449439	-0.001857362	Metabolome
Clothianidin	0.001179529	-0.004363427	Metabolome
7-Hexadecynoic acid	0.004392439	0.002741425	Metabolome
(Z)-Farnesol	0.001296209	-0.004352213	Metabolome
9-Oxo-nonanoic acid	0.030667824	0.098713233	Metabolome
Gamma-Glutamylthreonine	-0.025759663	0.065978734	Metabolome
1-(5Z,8Z,11Z,14Z-eicosatetraenoyl)-sn	-0.02615399	-0.011360519	Metabolome
Delta7-Dafachronic acid	-0.015833463	0.015263719	Metabolome
Isoleucyl-prolyl-arginine-4-nitroanilid	0.011789317	0.034459698	Metabolome
P-Menthane-3,8-diol	0.01224984	0.021199811	Metabolome
N,N,N',N',N'',N''-Hexakis(methoxymeth	0.004189872	0.006737628	Metabolome
4-Hydroxybenzaldehyde	0.023488486	0.000420342	Metabolome
Prenalterol	0.004151694	0.090735068	Metabolome
Amotosalen	0.004124116	0.002548194	Metabolome
PL	-0.056010255	-0.121406894	Metabolome
(4aS,5aS,12aS)-7-chloro-4-(dimethyla	0.022984126	-0.000815115	Metabolome
4-Allylpyrocatechol sulfate	0.007225615	0.013256128	Metabolome
Nintedanib	-0.010636022	-0.005698917	Metabolome
Resolvin E2	-0.025499328	0.079450061	Metabolome
Cyclic ADP-Ribose	0.014320294	-0.001498539	Metabolome
N-acetylaspartate	-0.041430608	-0.002315037	Metabolome
Bolton-Hunter reagent	-0.014033489	0.060395892	Metabolome
GPEtn(14:0/22:6)	-0.003362623	0.007346022	Metabolome
Ketamine	0.001419228	-0.004401436	Metabolome
PS(5-iso PGF2VI/14:0)	0.037572088	-0.010289088	Metabolome
10-hydroxy-(2E,8E)-decadien-4-ynoic	-0.003911518	-0.003080685	Metabolome
Gamma-Glutamylglutamine	-0.004462146	-0.012798353	Metabolome
3,4-Dihydroxymandelic Acid	-0.00115779	-0.006414213	Metabolome
Hydroxyethyl methacrylic acid	-0.001475039	0.003713674	Metabolome
Beta-nicotinate D-ribonucleotide	0.008132679	-0.017412902	Metabolome
2-Aminobenzoic Acid	-0.012069026	-0.0450126	Metabolome
Imidazoleacetic acid riboside	0.027144485	-0.008724751	Metabolome
2'-Deoxyuridine	-0.022416886	0.004522182	Metabolome
L-Furosine	0.004124116	0.002548194	Metabolome
Desmethyl Fluvoxamine	0.004097317	0.002566255	Metabolome
Violanthin	0.045826524	0.004169312	Metabolome
Imidazolone A	0.001419228	-0.004401436	Metabolome
Aspartic Acid	0.021469482	0.000112535	Metabolome
5,6-Dihydro-5-hydroxy-6-methyl-2H-	0.021719931	0.015708008	Metabolome
Serylcysteine	-0.019568852	-0.018521603	Metabolome
13E-Docosenamide	-0.007388578	-0.018970845	Metabolome
D-Biotin	-0.004815685	-0.022159852	Metabolome
3-Phenyl-2-thioxoimidazolidin-4-one	-0.024926729	-0.014971575	Metabolome
3-Keto-b-D-galactose	-0.025887284	-0.002501034	Metabolome
Gamma-Glutamylglutamic acid	0.007718386	0.005111033	Metabolome
Methionine sulfoxide	-0.024118426	-0.041952986	Metabolome
LysoPI(20:4(5Z,8Z,11Z,14Z)/0:0)	0.003970841	0.002613222	Metabolome
Palmitoyl Ethanolamide	-0.010174566	0.079222269	Metabolome
Cephalosporanic acid	-0.023138153	0.071522368	Metabolome
6-Thioinosine-5'-monophosphate	-0.013725463	0.000159044	Metabolome
Styrene	0.018914082	-0.007651921	Metabolome
Linustatin	-0.030774629	0.000235167	Metabolome
Ecdysone	-0.028686922	0.011915117	Metabolome
5-Pentyltetrahydro-2-oxo-3-furancarl	0.013653312	-0.014650389	Metabolome
Sorbinil	0.001419228	-0.004401436	Metabolome
Isoflupredone acetate	0.025620808	-0.021407748	Metabolome
Pelargonic acid	0.001296209	-0.004352213	Metabolome
Morin	0.004392439	0.002741425	Metabolome

Indoline	0.018494553	-0.001001384	Metabolome
1,2-Cyclohexanediol, 1-methyl-4-(1-r	-0.006107966	-0.003618103	Metabolome
3,7-Dimethylquercetin	0.005527857	-0.008691611	Metabolome
(+)-cis-abscisic aldehyde	0.003958764	-0.015156153	Metabolome
Embelin	-0.01442292	-0.003314582	Metabolome
D-Glucoside	-0.027265938	-0.063666258	Metabolome
Galactonolactone	-0.008234253	-0.005281681	Metabolome
6-Hydroxy flavin adenine dinucleotide	0.00127257	-0.004911958	Metabolome
Geneticin	0.004097317	0.002566255	Metabolome
5-Hydroxy-2-[2-methyl-3-(trifluorom	0.001296209	-0.004352213	Metabolome
4-Nitrophenyl beta-D-xyloside	-0.020830308	0.004152226	Metabolome
Isopropyl beta-D-glucoside	-0.012274489	0.014990406	Metabolome
Pyrroline hydroxycarboxylic acid	0.025034483	0.006559143	Metabolome
N-Isobutyloctadeca-trans-2-trans-4-(	0.005758165	-0.020469706	Metabolome
3-((((-Oxoheptyl)amino)acetyl)amino)r	0.001295543	-0.004439874	Metabolome
Acetyl-L-tyrosine	-0.029162421	-0.010300937	Metabolome
Epothilone C	0.046088167	0.0615779	Metabolome
Tuliposide A	-0.028765755	0.005836567	Metabolome
Jasmolone	0.008239272	0.027480055	Metabolome
MG(20:4(5Z,8Z,11Z,14Z)-OH(17)/0:0/0	0.004228875	0.001833583	Metabolome
Glycyl-Gamma-glutamate	0.019622738	0.000579829	Metabolome
Isoleucylhydroxyproline	-0.060488145	-0.116058441	Metabolome
(2R,3S)-2,3-dimethylmalate	-0.007542378	-0.016429809	Metabolome
CDP-DG(a-25:0/20:3(8Z,11Z,14Z)-2OH	0.025104906	-0.082400304	Metabolome
Coumarinic acid	0.030110157	-0.038310849	Metabolome
Stachyose	0.011386253	0.003219251	Metabolome
Lauroylcarnitine	0.017983999	-0.007063904	Metabolome
Prolylhydroxyproline	0.03102269	-0.003142603	Metabolome
(3R)-Sophorol	0.008312603	-0.00781426	Metabolome
Estriol	0.023235502	0.007529246	Metabolome
5-Hydroxytryptophol glucuronide	-0.006654512	0.006307094	Metabolome
FAPy-adenine	0.017015509	0.001108549	Metabolome
DG(18:3(9Z,12Z,15Z)/20:1(11Z)/0:0)	0.001295543	-0.004439874	Metabolome
Linoleic Acid	0.016614281	0.01259924	Metabolome
3',5'-Cyclic GMP	0.032237659	0.033469878	Metabolome
Dmg-mino	0.011482354	-0.012263044	Metabolome
Sesartemin	0.016564507	-0.023918404	Metabolome
Fasoracetam	0.021136252	-0.005105283	Metabolome
Uridine 2'-phosphate	-0.022344004	-0.008361725	Metabolome
Gamma-Glutaminyl-4-hydroxybenzen	0.005509311	0.022460133	Metabolome
N2-Succinyl-L-glutamic acid 5-semial	-0.000425017	-0.021549306	Metabolome
Danielone	-0.012985356	-0.024486691	Metabolome
Glutamylphenylalanine	0.025432498	-0.00480988	Metabolome
Dihydrozeatin	0.01137744	-0.008921625	Metabolome
Homocarnosine	0.001417844	-0.004874306	Metabolome
LysoPA(18:2(9Z,12Z)/0:0)	-0.007515305	0.000201241	Metabolome
Acotiamide	0.004097317	0.002566255	Metabolome
Barbituric acid	0.004097317	0.002566255	Metabolome
Fluvoxamino acid	-0.032125516	0.001407248	Metabolome
Methylisocitric acid	-0.018784063	0.023067772	Metabolome
Vinclozolin M2	0.031273079	0.005954246	Metabolome
Piperonyl Butoxide	0.012218321	0.01375214	Metabolome
2-Hydroxypropyl octanoate	0.008979844	0.007538929	Metabolome
Carteolol	0.004228875	0.001833583	Metabolome
6-Epi-7-isocucurbic acid glucoside	0.024649629	9.82E-05	Metabolome
N-Acetyl-DL-Valine	0.018179699	-0.001859571	Metabolome
N-[1'-(6-Cyano-1,2,3,4-tetrahydronap	-0.024842353	-0.003782616	Metabolome
SM(d18:2(4E,14Z)/14:0)	-0.02521351	-0.01760969	Metabolome

(2R)-2-[[(2S,3R)-3-Amino-2-hydroxy-	0.001419228	-0.004401436	Metabolome
Dihydroneopterin phosphate	0.004228875	0.001833583	Metabolome
Lichenin	0.012311997	-0.004949687	Metabolome
2-Methylbenzaldehyde	0.015344622	-0.000999573	Metabolome
Gabazine	0.022119922	-0.003963779	Metabolome
Guanosine diphosphate adenosine	0.001419228	-0.004401436	Metabolome
Taraxinic acid glucosyl ester	0.009966833	-0.008212214	Metabolome
(2s,3r,4s,5r)-3,4,5-Trihydroxy-6-oxopi	-0.029073257	-0.00350233	Metabolome
2-Phenylethanol glucuronide	-0.021865817	0.002292676	Metabolome
Esculetin	-0.014723541	0.002691211	Metabolome
7-[(7S)-7-Amino-5-azaspiro[2.4]hepta	0.020663178	-0.000393126	Metabolome
(Z)-6-Tetradecene-1,3-diyne-5,8-diol	-0.037050898	-0.005031346	Metabolome
Acarbose	0.004097317	0.002566255	Metabolome
Alloside B	0.004228875	0.001833583	Metabolome
G-Nitro-l-arginine methyl ester	0.003970841	0.002613222	Metabolome
N-Acetyl-L-phenylalanine	-0.018835919	-0.001536815	Metabolome
Quercetin 3-O-sophoroside	0.040788526	0.000232562	Metabolome
Finafloxacin	-0.014585911	0.006546562	Metabolome
Neopterin	-0.007780993	-0.014500635	Metabolome
Ginsenoside Rb1	0.004097317	0.002566255	Metabolome
2''-O-alpha-L-Rhamnosyl-6-C-quino	0.010795509	-0.01204017	Metabolome
Asiaticoside	0.014981956	0.013808501	Metabolome
Taraxacoside	0.001296209	-0.004352213	Metabolome
1-Kestose	0.003110975	0.004116999	Metabolome
(3Z)-phytochromobilin	0.004097317	0.002566255	Metabolome
N-Oxalylglycine	0.004097317	0.002566255	Metabolome
Diphenylacetic acid	0.020818367	-0.008756119	Metabolome
LysoPE(18:1(9Z)/0:0)	0.008731689	-0.022279931	Metabolome
S-Glutaryldihydrolipoamide	-0.00185594	-0.024150283	Metabolome
Physagulin A	0.004097317	0.002566255	Metabolome
D-Galactaric acid	-0.039182029	0.001705352	Metabolome
3-Methyloxindole	0.002901788	-0.005294603	Metabolome
(2S)-3-Hydroxy-1-[(2S)-pyrrolidine-2-	0.023404838	-0.003746985	Metabolome
Sumiki's acid	-0.026903952	0.017011272	Metabolome
3-O-Methyl-a-methyldopa	0.013586572	-0.014330282	Metabolome
Estrone glucuronide	0.004097317	0.002566255	Metabolome
PE-NMe(18:2(9Z,12Z)/20:3(5Z,8Z,11Z))	-0.028035476	-0.016150464	Metabolome
Asparaginyl-Proline	0.031214659	0.08765147	Metabolome
DL-Lanthionine	-0.033303063	-0.01001195	Metabolome
Osmundalactone	-0.00616252	-0.004363144	Metabolome
Kainic acid	-0.01010282	0.004587299	Metabolome
(11E)-Octadec-11-enedioylcarnitine	-0.01226339	0.00095552	Metabolome
CDP-DG(i-22:0/20:3(6,8,11)-OH(5))	0.004097317	0.002566255	Metabolome
TG(8:0/8:0/8:0)	0.01767318	0.006146456	Metabolome
Doramectin	0.004124116	0.002548194	Metabolome
2-(Difluoromethyl)arginine	0.024002993	0.000665885	Metabolome
2-Furamide	0.015675864	-0.004975097	Metabolome
Dopamine quinone	0.009915266	-0.020919826	Metabolome
Melatonin glucuronide	-0.023170022	-0.007779135	Metabolome
N-Acetyl-L-Tyrosine	0.016311705	-0.000487807	Metabolome
2-Hydroxy-3-(4-hydroxyphenyl)prope	-0.006861228	0.019434641	Metabolome
(1Ar,2Z,4E,14R,15aR)-8-chloro-9,11-d	-0.02872192	-0.00822528	Metabolome
LysoPA(0:0/16:0)	6.49E-05	0.013168261	Metabolome
Ancymidol	0.037440432	8.32E-05	Metabolome
PE(18:3(9Z,12Z,15Z)/18:2(9Z,12Z))	-0.007899945	-0.008126495	Metabolome
Pregnanediol 3-O-glucuronide	0.004124116	0.002548194	Metabolome
Galactosylhydroxylysine	-0.020575488	-0.006304435	Metabolome
7',8'-Dihydro-8'-hydroxyreticulataxant	0.012769944	0.007894004	Metabolome

6b-Angeloyl-3b,8b,9b-trihydroxy-7(1:	0.012667296	-0.002633175	Metabolome
Tetranor-12(R)-HETE	-0.024792914	0.066972428	Metabolome
Dibutyl Phthalate	-0.003580722	0.021135863	Metabolome
Cartap	0.040475702	0.009306216	Metabolome
Acacetin 7-[apiosyl(1->6)-glucoside]	0.023690158	0.000169476	Metabolome
P-Hydroxyfelbamate	-0.004523265	-0.139572604	Metabolome
Linoleic Acid ethyl ester	0.004228875	0.001833583	Metabolome
4-Hydroxybenzeneacetonitrile	0.017767542	0.002237591	Metabolome
1-Naphthylamine	0.026154665	-0.003177652	Metabolome
CDP-DG(PGE1/i-22:0)	0.027001759	0.019636959	Metabolome
Valinomycin	0.017432802	-0.069775816	Metabolome
Isorhamnetin 4'-O-glucuronide	0.001419228	-0.004401436	Metabolome
Alpha-Bixin	-0.001837203	0.00131887	Metabolome
Betanin	0.000529731	0.057627871	Metabolome
Fructose lactate	-0.006138441	-0.016142695	Metabolome
Propenoylcarnitine	0.024352809	-0.000978373	Metabolome
3-Methyl-2(3H)-benzothiazolone	-0.011979387	-0.020059728	Metabolome
Tyrosine-tyrosinate	-0.025495628	0.008356786	Metabolome
Temsirolimus	0.004124116	0.002548194	Metabolome
DTDP-D-galactose	0.014558859	0.008406205	Metabolome
Pyrodone	0.002757861	-0.00637718	Metabolome
PE(18:4/0:0)	-0.029445121	0.003197099	Metabolome
Prostaglandin J2	-0.003430484	-0.010581165	Metabolome
Dihomo-alpha-linolenic acid	-0.01208966	0.01329269	Metabolome
Amastatin	-0.041950345	0.009542746	Metabolome
3,4-Methylenesebacic acid	0.001623291	0.010413628	Metabolome
N-Docosahexaenoyl Valine	0.003970841	0.002613222	Metabolome
3,5-Dihydroxy-6,7-megastigmadien-9	0.042228402	-0.016793597	Metabolome
1-Arachidonoylglycerol	0.01780544	-0.015195678	Metabolome
Histidylvaline	0.038072411	-0.00034663	Metabolome
Dodecyl Hydrogen Sulfate	0.014186453	0.005277512	Metabolome
Imidazolelactic acid	0.001198608	-0.004359817	Metabolome
PE(18:1(9Z)/18:2(9Z,12Z))	-0.020401565	-0.007414581	Metabolome
19(S)-HETE	-0.059497459	0.017587511	Metabolome
8-oxo-dGDP	0.00127257	-0.004911958	Metabolome
PC(18:1(11Z)/18:3(9Z,12Z,15Z))	-0.027802013	-0.005396821	Metabolome
L-Proline	-0.022338251	-0.007920787	Metabolome
L-Serine	0.021532241	0.00045367	Metabolome
Glucotropaeolin	0.047294003	0.077957746	Metabolome
(2S)-3-(4H-Imidazol-4-yl)-2-[[(2S)-5-	-0.027244907	0.064494294	Metabolome
PA(PGF1alpha/2:0)	0.007268979	0.023703228	Metabolome
1-Methylguanosine	-0.005243977	0.000780379	Metabolome
Milbemycin alpha9	-0.008563292	0.041476888	Metabolome
Cis-Zeatin O-glucoside	-0.008390732	0.007235255	Metabolome
Dimethylglycine	0.006286642	-0.008161956	Metabolome
1-(2,3,8,8-Tetramethyl-1,2,3,4,5,6,7,8-(	0.004097317	0.002566255	Metabolome
Adenine	0.018561275	0.003157785	Metabolome
N-Gluconyl ethanolamine	0.02097085	0.002260253	Metabolome
PG(i-12:0/22:6(4Z,8Z,10Z,13Z,16Z,19Z)	0.003970841	0.002613222	Metabolome
D-Arabinose 5-phosphate	0.016671478	-0.012873746	Metabolome
MG(0:0/20:3(6,8,11)-OH(5)/0:0)	0.00127257	-0.004911958	Metabolome
4-Nitrophenol	0.00183562	0.000184268	Metabolome
Serylisoleucine	0.03608443	0.006407777	Metabolome
2,4-Dinitrophenylhydrazone	-0.025654782	-0.013332488	Metabolome
1-Nonanol	-0.035774572	0.001700226	Metabolome
22-Hydroxydocosanoic acid	0.001179529	-0.004363427	Metabolome
Stearaldehyde	-0.025028505	0.001890887	Metabolome
Ginkgoic acid	0.004097317	0.002566255	Metabolome

25-Ethoxy-24-methoxycylcoartanol	0.004124116	0.002548194	Metabolome
3-[(3-(2-Carboxyethyl)-4-methylpyrrc	0.014910978	-0.001607581	Metabolome
GSK264220A	-0.010407736	0.068583617	Metabolome
SM(d16:2(4E,8Z)/PGJ2)	0.004124116	0.002548194	Metabolome
Cytidine monophosphate	0.001419228	-0.004401436	Metabolome
Arg Leu	-0.012511767	0.015359363	Metabolome
Deoxyinosine	-0.026100696	-0.000438086	Metabolome
Benzoic acid, 2-(acetyloxy)-5-amino-	0.004097317	0.002566255	Metabolome
Sativic acid	0.00701815	-0.033755457	Metabolome
Isorheagenine	-0.008841217	0.001725811	Metabolome
3-Hexen-1-ol	0.061232287	0.028190736	Metabolome
Umbelliferone	0.001179529	-0.004363427	Metabolome
Nitrofurazone	-0.021262698	-0.004604833	Metabolome
Aconine	0.001295543	-0.004439874	Metabolome
Eicosapentaenoic Acid	-0.021281711	-0.007071511	Metabolome
SCHEMBL13960539	0.010307039	0.016132462	Metabolome
N-Epsilon-Acetyl-L-Lysine	-0.047453768	0.000422892	Metabolome
2-(Methylthio)benzothiazole	0.010290427	0.000975793	Metabolome
Visnagin	-0.023999157	-0.006750631	Metabolome
2,4,5,7alpha-Tetrahydro-1,4,4,7a-tetra	-0.046207762	-0.032915501	Metabolome
(+)-marmesin	-0.029762159	-0.043263732	Metabolome
Trioxsalen	0.001417844	-0.004874306	Metabolome
Benzoquinoneacetic acid	-0.008795333	0.003422181	Metabolome
(3S,5R,6R,7E)-3,5,6-Trihydroxy-7-meg	0.001296209	-0.004352213	Metabolome
Imperatorin	0.004097317	0.002566255	Metabolome
S-Adenosylmethionine	-0.00748668	0.102136179	Metabolome
Angelicin	0.027156745	0.002537123	Metabolome
5-Hydroxylysinonorleucine	0.000877563	-0.00270856	Metabolome
Mimosine	-0.034017892	0.003883024	Metabolome
Caffeic acid sulfate	-0.007781616	0.010232106	Metabolome
D-Sorbitol	0.022269762	0.002559554	Metabolome
(3R-(3alpha,4alpha(2R*,3R*),5beta,6be	0.001417844	-0.004874306	Metabolome
Ginsenoside Ra3	0.004228875	0.001833583	Metabolome
Tuberose lactone	-0.020385119	-0.001950067	Metabolome
Alpha-Eleostearic acid	0.004124116	0.002548194	Metabolome
Dehydrotremetone	-0.019948302	0.011160272	Metabolome
Lappaol F	0.039182188	-0.037614827	Metabolome
GDP-L-fucose	-0.046099149	-0.010303845	Metabolome
Acetylbalchanolide	-0.069815804	-0.005959571	Metabolome
Valyltyrosine	0.014611182	0.000258771	Metabolome
Sinapic acid 4-O-sulfate	0.03153377	-0.01158974	Metabolome
6-[(2R,4S,5R)-4-Hydroxy-5-(hydroxym	-0.003575278	0.008434358	Metabolome
Jubanine B	0.004097317	0.002566255	Metabolome
Sanfetrinem	-0.02190446	-0.003355218	Metabolome
Nalfurafine	0.001419228	-0.004401436	Metabolome
2,6-diamino-4-hydroxy-5-formamido	0.015384541	-0.006469162	Metabolome
5-Hydroxymethyl-2-furancarboxaldeh	-0.02231264	0.002489206	Metabolome
(R)-Methysticin	-0.002331312	-0.014309104	Metabolome
Spisulosine	-0.00526498	0.016898198	Metabolome
8-Amino-7-oxononanoic acid	0.02867728	-0.004443502	Metabolome
Virginiamycin m1	0.004392439	0.002741425	Metabolome
Nodularin-R	0.008527031	0.074894092	Metabolome
Monopropionylcadaverine	-0.002647967	0.011620415	Metabolome
Coniferin	0.003670161	-0.011875318	Metabolome
Arctiopicrin	0.00309808	0.00100478	Metabolome
4-Methoxyindoxyl sulfate	0.005063067	-0.021077292	Metabolome
2-Amino-6-methoxypurine	0.013429502	-0.006067281	Metabolome
L-arginino-succinate	0.013563335	-0.013838362	Metabolome

LysoPC(20:3(8Z,11Z,14Z)/0:0)	-0.043487341	-0.016421929	Metabolome
2-(Malonylamino)benzoic acid	0.029113284	0.005270049	Metabolome
Guanine	0.001419228	-0.004401436	Metabolome
(23R)-1alpha-Hydroxy-25,27-didehyd	0.00127257	-0.004911958	Metabolome
Lucidenic acid C	0.0054106	-0.000464942	Metabolome
N-Acetyl-D-mannosamine 6-phospha	0.021694805	-0.020146033	Metabolome
P-Coumaric Acid	-0.004935288	0.00363556	Metabolome
Nalpha-Acetyl-L-arginine	0.016996143	0.037967452	Metabolome
Alanylleucine	0.00482179	-0.008644594	Metabolome
4,5-Dihydro-1-(3-(trifluoromethyl)phe	0.014353544	0.008529737	Metabolome
Fusarin C	0.006832735	-0.005058618	Metabolome
N-(5-Amino-2-hydroxybenzoyl)glycin	0.008589871	-0.01582056	Metabolome
DL-2,5-Dihydrophenylalanine	0.01524102	0.008662717	Metabolome
Dihydroxylysinonorleucine	-0.00735766	-0.002645993	Metabolome
Homogentisic Acid	-0.01975409	-0.00016838	Metabolome
(+/-)-2-Butylthiazolidine	0.010692464	-0.007292272	Metabolome
P-Tolualdehyde	0.018118196	0.000802515	Metabolome
SA-beta-Gal	-0.006784405	0.002148398	Metabolome
Cotinine glucuronide	-0.005508643	-0.020921497	Metabolome
PI(22:2(13Z,16Z)/PGF2alpha)	0.015331315	0.002537687	Metabolome
D-Octopine	0.002563302	0.009692146	Metabolome
Pro Gln Ser	0.004228875	0.001833583	Metabolome
5-Amino-1-[(2R,3R,4S,5R)-3,4-dihydro	0.006712109	0.022552176	Metabolome
Bardoxolone methyl	-0.061631461	-0.015421362	Metabolome
Indole-3-acetamide	0.013218986	0.064305252	Metabolome
4-Aminophenylmannoside	0.004097317	0.002566255	Metabolome
Seselin	-0.057397966	-0.001113357	Metabolome
Threonic Acid	0.013170509	0.022194007	Metabolome
Zeatin	-0.0015261	-0.011294809	Metabolome
1-Nonadecanoyl-glycero-3-phosphoe	-0.034557554	-0.009667287	Metabolome
Serylasparagine	-0.013083527	0.056194971	Metabolome
9-Ethylguanine	0.005529269	0.030103315	Metabolome
3a,12b-Dihydroxy-5b-cholanoic acid	0.001296209	-0.004352213	Metabolome
2,5-diamino-6-(5-phospho-D-ribityla	0.017616412	0.002819384	Metabolome
Ethyl Myristate	-0.006816956	-0.005849942	Metabolome
Arctiol	0.020583412	-0.008606312	Metabolome
4-Hydroxy-6-methyl-3-(1H-pyrazol-3	-0.018106436	0.034771314	Metabolome
PE(20:3(8Z,11Z,14Z)/P-16:0)	0.001417844	-0.004874306	Metabolome
1-Methyl-2-undecylquinolin-4(1H)-or	0.008524008	-0.008772133	Metabolome
Alanylhydroxyproline	-0.026001168	0.004079797	Metabolome
N-Methyl-14-O-demethylepiporphyro	0.00861694	0.01122933	Metabolome
Diacetoxyscirpenol	0.001295543	-0.004439874	Metabolome
8,8'-Diferulic acid	0.001296209	-0.004352213	Metabolome
Isoquinoline	0.020295775	-0.010907728	Metabolome
2-Chloro-4-nitrophenyl a-D-maltotric	-0.023124265	0.001909411	Metabolome
5,7-Dimethoxyflavone	0.006830217	-0.019134107	Metabolome
Salicyluric Acid	-0.011928843	0.004403529	Metabolome
Fructosamine	-0.008638687	0.003614209	Metabolome
Cytosine	-0.011327926	0.027080921	Metabolome
Diosgenin	0.022182617	0.015118733	Metabolome
LysoPA(P-16:0/0:0)	-0.012202728	0.004102446	Metabolome
Gamma-Glutamyl-beta-cyanoalanine	-0.001758273	0.058400675	Metabolome
Glutamylhistidine	-0.019944098	-0.017013607	Metabolome
(2R,3R,4S,5R)-2-Amino-3,4,5,6-tetrahy	0.003149587	-0.00584988	Metabolome
Leonurine	0.037243955	0.013226753	Metabolome
2-Furoic Acid	0.022446661	-0.001079014	Metabolome
2,8-Quinolinediol	0.013563333	-0.008712993	Metabolome
DG(20:4(8Z,11Z,14Z,17Z)/24:1(15Z)/0:0	-0.027433406	0.007564938	Metabolome

2-Methyl-1,3-cyclohexadiene	0.010618052	-0.016742783	Metabolome
5-Fluorodeoxyuridine monophosphate	0.004155801	-0.009031585	Metabolome
2-Mercaptobenzothiazole	-0.001103081	-0.000215854	Metabolome
Cytidine	0.011313275	-0.000604393	Metabolome
D-Glucuronic Acid	-0.024898177	-0.015490003	Metabolome
Decanedioic acid	-0.010284705	-0.001432319	Metabolome
Gamma-Glutamylhistidine	-0.022236354	0.059722408	Metabolome
8-Hydroxyacyclovir	0.001295543	-0.004439874	Metabolome
Homoanserine	-0.006180541	0.064242081	Metabolome
Indole-3-carboxylic acid	0.006777438	-0.037850139	Metabolome
Iminoerythrose 4-phosphate	-0.009893993	-0.015637869	Metabolome
Budralazine	0.001417844	-0.004874306	Metabolome
PS(22:6(4Z,7Z,10Z,13Z,16Z,19Z)/19:0)	-0.036120591	-0.005603106	Metabolome
Protoapigenone	0.015760256	-0.00569233	Metabolome
3,4-Methylenepimelic acid	-0.01867518	-0.039632082	Metabolome
N-Acetyldopamine	-0.024465928	-0.004841735	Metabolome
HMDB0127639	0.000701879	0.019409624	Metabolome
4-Hydroxyquinoline	-0.001654159	0.01358273	Metabolome
Arbutin	-0.063025914	-0.013205728	Metabolome
2-Piperidinone	0.013070713	0.003062297	Metabolome
Candicine	0.001295543	-0.004439874	Metabolome
3,4-Dihydroxyphenylacetaldehyde	0.004062373	-0.00850944	Metabolome
12-Hydroxydodecanoic acid	0.003263778	0.035840621	Metabolome
4-Guanidinobutanoic Acid	-0.016271196	0.00245439	Metabolome
Glycyl-Phenylalanine	0.01551877	-0.016262108	Metabolome
2-Hydroxy-p-mentha-1,8-dien-6-one	0.011426401	-0.010733991	Metabolome
Glucose lactate acetate	-0.004624864	0.008664811	Metabolome
3-Gonal	0.020071025	0.022148935	Metabolome
Uric Acid	-0.019557176	-0.005982056	Metabolome
Phaseolic acid	0.017956731	0.045382458	Metabolome
Biopterin	-0.018656027	0.010315407	Metabolome
N-Acetyl-b-glucosaminylamine	0.002959688	0.014574834	Metabolome
Vanillylmandelic acid	-0.009535021	0.011150846	Metabolome
CPA(18:1(9Z)/0:0)	-0.03267391	0.035077132	Metabolome
Deoxyguanosine	-0.019416454	-0.069779247	Metabolome
7-Aminomethyl-7-carbaguanine	-0.01973188	0.010092838	Metabolome
6-Aminopurin-8-one	0.007080186	-0.00969876	Metabolome
Triethanolamine	0.012582329	-0.00721217	Metabolome
Homophytanic acid	0.00127257	-0.004911958	Metabolome
All-trans-13,14-dihydroretinol	-0.020747402	7.81E-05	Metabolome
Eugenol	0.013665025	0.009912783	Metabolome
Cinnavalininate	0.004124116	0.002548194	Metabolome
2-Methoxy-4-vinylphenol	0.00161279	0.105506788	Metabolome
1-Hydroxy-2,5-dioxopyrrolidine-3-sul	-0.014137367	-0.01634335	Metabolome
DG(PGD2/0:0/13:0)	0.004228875	0.001833583	Metabolome
Medphalan	-0.039243205	0.03129236	Metabolome
Ethynodiol Diacetate	-0.016698695	-0.014406363	Metabolome
N-(4-(2,5-Dihydro-5-oxo-3-furanyl)p	0.019278709	0.012630059	Metabolome
Actarit	0.010681	-0.012142807	Metabolome
PE(22:2(13Z,16Z)/18:2(9Z,11E)+=O(13)	0.001326115	-0.004319813	Metabolome
Curcumenol	0.004097317	0.002566255	Metabolome
3(2H)-Pyridazinone	0.02129455	0.04273146	Metabolome
Tetradecanedioic acid	-0.006875953	-0.017997416	Metabolome
PC(18:1(12Z)-O(9S,10R)/17:0)	0.001419228	-0.004401436	Metabolome
Alpha-Hydroxyisobutyric Acid	0.007383906	0.00167939	Metabolome
Sterigmatocystin	0.015788039	-0.025180976	Metabolome
Trehalose	-0.030384113	-0.000897876	Metabolome
Juglone	0.009216409	0.010622974	Metabolome

Ethyl 3-hydroxydodecanoate	-0.023415554	0.003003355	Metabolome
Glycine, N-[3-(4-hydroxy-3-methoxyp	0.001417844	-0.004874306	Metabolome
Delta-Tocotrienol	0.004097317	0.002566255	Metabolome
Citramalic Acid	0.000282137	0.01229099	Metabolome
1,6-anhydro-N-acetyl-beta-muramat	0.004097317	0.002566255	Metabolome
2-(3-Methylpiperidin-1-yl)[1,3]thiazol	0.010913222	0.00409445	Metabolome
Isoquercitrin	-0.032603504	0.00174478	Metabolome
(6R)-Folinic acid	0.013444593	-0.002206914	Metabolome
2-Cyclohexylidenecyclohexanone	0.004097317	0.002566255	Metabolome
Alpha-Methylene-Gamma-Butyrolact	-0.008731454	0.00860835	Metabolome
2-Ketobutyric acid	-0.040047368	0.00773219	Metabolome
2-[(2R)-2-Aminopropyl]-5-hydroxybe	-0.033481162	-0.001147686	Metabolome
3-Oxohexanoic acid	0.00884713	-0.001272604	Metabolome
3-[[5-Methyl-2-(1-methylethyl)cycloh	0.001417844	-0.004874306	Metabolome
Nalidixic Acid	0.01660756	-0.009706608	Metabolome
Laninamivir	-0.012125306	0.014191424	Metabolome
Piperacillin	0.03089928	0.000788432	Metabolome
L-Tyrosine	-0.002513493	0.006986433	Metabolome
Vanylglycol	0.007948358	0.020956668	Metabolome
Pentostatin	0.030035662	0.001617311	Metabolome
Nigakihemiacetal B	0.012850304	-0.016516183	Metabolome
3'-Methoxyfukiic acid	0.008977225	-0.029124699	Metabolome
Glutamyl pyruvate	0.001296209	-0.004352213	Metabolome
Cinnamaldehyde	0.008802902	0.005101047	Metabolome
Chavicol	0.020182247	0.004975403	Metabolome
Cidofovir	0.006475079	-0.016619163	Metabolome
1alpha,3beta,22R-Trihydroxyergosta-5	0.003970841	0.002613222	Metabolome
Oxazepam	-0.000368765	0.120864136	Metabolome
4-Hydroxyphenytoin glucuronide	0.01275575	-0.002058853	Metabolome
(S)-a-Amino-2,5-dihydro-5-oxo-4-is	0.003255548	-0.008552451	Metabolome
6-[3-(2-carboxyeth-1-en-1-yl)pheno	-0.032669057	0.016446776	Metabolome
1,3-Propanediamine	0.004124116	0.002548194	Metabolome
THIARABINE	0.003653663	-0.004797503	Metabolome
Adipate semialdehyde	0.020690331	0.004535521	Metabolome
Chondroitin sulfate E (GalNAc4,6diS-G	-0.002965185	-0.017879477	Metabolome
N-Oleoyl Asparagine	0.00127257	-0.004911958	Metabolome
P-cresol	0.018088442	0.000953265	Metabolome
16-Oxoestrone	0.015430612	-0.024761141	Metabolome
12-Hydroxydodecanoylcarnitine	-0.021005082	-0.007687987	Metabolome
Para-Phenylenediamine	0.013761696	-0.007981521	Metabolome
Elsamitrucin	0.011786757	0.016793522	Metabolome
Thr Leu	0.034508992	-0.05731867	Metabolome
Lauroyl diethanolamide	0.004348038	-0.011895346	Metabolome
Tricetin	0.013603852	-0.050938828	Metabolome
H-Tyr-gly-gly-OH	0.004004832	0.002848038	Metabolome
DG(22:0/20:5(5Z,8Z,11Z,14Z,17Z)/0:0)	-0.03146268	-0.003332027	Metabolome
Hispidin	0.001296209	-0.004352213	Metabolome
QUISQUALIC ACID	0.007038356	0.0344957	Metabolome
L-2,3-Dihydrodipicolinate	0.003970841	0.002613222	Metabolome
D-Fructose	-0.002219419	-0.024914131	Metabolome
Butyl 4-aminobenzoate	0.001179529	-0.004363427	Metabolome
11-(3,4-Dimethyl-5-pentylfuran-2-yl)	0.002995292	0.02678533	Metabolome
Nepsilon-Acetyl-L-lysine	-0.035164903	0.00352789	Metabolome
7-Epi-12-hydroxyjasmonic acid	-0.022739428	0.006730165	Metabolome
Gamma-Glutamylserine	0.01267331	0.03044819	Metabolome
5-(4-Carboxybutylperoxy)pentanoic a	0.005101494	0.001191176	Metabolome
4-Hydroxy-8-methoxy-2H-furo[2,3-h	0.007563189	-0.014576891	Metabolome
Ritipenem	0.014160644	-0.000856411	Metabolome

(R)-Menthone 8-thioacetate	0.001417844	-0.004874306	Metabolome
Nicotinic Acid Mononucleotide	0.003072328	0.042860649	Metabolome
Thiamphenicol glycinate	-0.008270775	0.000155879	Metabolome
Tobramycin	0.004392439	0.002741425	Metabolome
(Z)-2-decenal	0.001419228	-0.004401436	Metabolome
(1S,2S,3S,4R)-3-(1-Acetamido-2-ethyl	0.001417844	-0.004874306	Metabolome
TG(16:1(9Z)/16:1(9Z)/20:4(5Z,8Z,11Z,14	-0.005043246	-0.024341087	Metabolome
Allysine	0.011263437	0.021041682	Metabolome
3-Hydroxy-L-proline	-0.008231958	-0.007767567	Metabolome
4-Methylpyrimidine	0.004635634	0.003215392	Metabolome
Palmitic Acid ethyl ester	0.001027539	0.007870886	Metabolome
Miglitol	-0.000464363	0.055731878	Metabolome
Citrulline	0.001417844	-0.004874306	Metabolome
Tetrahydrodipicolinate	0.014033683	-0.005439536	Metabolome
Eriojaposide B	0.001419228	-0.004401436	Metabolome
Threonolactone	-0.010158031	-0.000374391	Metabolome
11-HydroxyNonadecanoylcarnitine	-0.022250014	0.00311156	Metabolome
Demethylated antipyrine	0.014512222	-0.011066564	Metabolome
9,10,16-trihydroxy palmitic acid	0.007918626	0.069507175	Metabolome
N-(L-Arginino)succinate	0.022636263	0.014926241	Metabolome
Gingerol	-0.038853398	0.048436662	Metabolome
L-Tyrosine Methyl Ester	-0.023422947	-0.006120833	Metabolome
1-(6-Hydroxy-2-azabicyclo[2.2.1]hept	0.003970841	0.002613222	Metabolome
Oleuropein	-0.015485219	-0.009050842	Metabolome
Deoxyguanylic Acid	0.032044895	0.013375407	Metabolome
Sakuranetin	-0.015660591	0.003733439	Metabolome
N-Stearoyl Isoleucine	0.00127257	-0.004911958	Metabolome
Cyclo(Ac-cys-asn-dmt-amf-gly-asp-c	0.007935513	0.00876918	Metabolome
3,5-dihydroxy-4-(sulfooxy)benzoic aci	0.016399757	-0.003204597	Metabolome
Melevodopa	0.023630534	-0.004818462	Metabolome
3-dehydroshikimate	-0.034166249	-0.002229746	Metabolome
Homocitric acid	0.011325074	-0.003814908	Metabolome
Aceglutamide	0.034800557	-0.004837188	Metabolome
4-Amino-1-[(2R,5R)-5-(hydroxymethy	-0.005785132	0.011134732	Metabolome
Carboxyifosfamide	0.00127257	-0.004911958	Metabolome
Capsaicin	0.004124116	0.002548194	Metabolome
Ozolinone	-0.023731917	-0.025717492	Metabolome
Acetophenone	0.00442394	-0.005360627	Metabolome
PE(20:0/0:0)	-0.019628702	0.005363159	Metabolome
Swertiamarin	-0.002014329	0.05337603	Metabolome
6-hydroxymethyl-7,8-dihydropterin	0.021005427	-0.006884891	Metabolome
(1r,3r)-1-Aminocyclopentane-1,3-dica	0.016242752	-0.001959408	Metabolome
Delpazolid	-0.068952358	0.002317156	Metabolome
N-acetylmuramyl-l-alanine	0.003970841	0.002613222	Metabolome
Carbamazepine-O-quinone	0.023692247	0.00167615	Metabolome
2-Keto-glutaramic acid	0.015109996	-0.002158149	Metabolome
2-Hydroxydecanedioic acid	0.028388826	-0.004733606	Metabolome
8-[(Aminomethyl)sulfanyl]-6-sulfanylo	0.001296209	-0.004352213	Metabolome
9-beta-d-Arabinofuranosylguanine	0.001179529	-0.004363427	Metabolome
(1R,4E,5'R,6R,6'R,7S,8S,10S,11S,12R,14S	0.004124116	0.002548194	Metabolome
1-(9Z,12Z,15Z-octadecatrienoyl)-glyce	-0.014943433	-0.006768022	Metabolome
4-(5H-Dibenzo[a,d]cyclohepten-5-ylic	-0.043221288	-0.004105379	Metabolome
6-Aminopenicillanic acid	-0.00610159	-0.025921045	Metabolome
Ala-Gly-OH	-0.016452125	-0.060016694	Metabolome
Quercetin 3-sulfate	0.001417844	-0.004874306	Metabolome
Arecaidine	0.00127257	-0.004911958	Metabolome
Capsidiol	0.00037245	-0.001148944	Metabolome
Ethenyl acetate	-0.012182881	0.00127077	Metabolome

N-Acetyllactosamine	-0.017445245	0.033315941	Metabolome
3-Hydroxybenzaldehyde	-0.018297596	0.000846447	Metabolome
Isokobusone	0.000318631	0.006306386	Metabolome
Edulitine	0.004413717	-0.048643169	Metabolome
Guanosine diphosphate	0.03159049	0.046219995	Metabolome
Daucic acid	0.00127257	-0.004911958	Metabolome
Delimotecan	0.00350776	0.012976162	Metabolome
N-Succinyl-L,L-2,6-diaminopimelate	0.000777299	-0.002480584	Metabolome
Tyrosylglycine	0.033186747	0.01721937	Metabolome
PC(20:5(5Z,8Z,11Z,14Z,16E)-OH(18)/2:(	-0.007210502	0.011194761	Metabolome
2-Hydroxy-3-Methylbutyric Acid	-0.004722759	-0.026587057	Metabolome
PI(22:2(13Z,16Z)/PGD2)	0.010295597	-0.008271621	Metabolome
Ile Phe	0.047490208	0.007438692	Metabolome
6-Hydroxy-R-acenocoumarol	0.02684415	-0.003232285	Metabolome
(3R)-3-Hydroxy-N-[(3S)-2-oxooxolan	0.044797011	-0.008643574	Metabolome
PG(16:0/0:0)[U]	0.015476943	0.0210123	Metabolome
Malonic Acid	-0.016864938	-0.037661676	Metabolome
Tranexamic Acid	0.021045304	-0.005460978	Metabolome
Dibenzo[a,c]cyclooctene, 5,6,7,8-tetrah	-0.005170964	-0.018702443	Metabolome
(1S,2R,4R)-p-Menth-8-ene-2,10-diol	-0.023225746	-0.002756287	Metabolome
Quinoline-4,8-diol	0.015764502	-0.001284569	Metabolome
Glutamyltyrosine	-0.010786592	-0.006630754	Metabolome
Propyl gallate	0.004124116	0.002548194	Metabolome
Sparstolonin B	0.031146282	-0.00540758	Metabolome
MJDBISSN00000009	-0.002393735	-0.018529377	Metabolome
Homovanillin	-0.034624556	-0.001651033	Metabolome
Zalcitabine	-0.002182781	0.087647553	Metabolome
Phosphate	0.006963131	0.003109724	Metabolome
Dihydrocitrinone	-0.030419395	-0.002288202	Metabolome
N-lactoyl-phenylalanine	0.014760423	-0.001567641	Metabolome
Hexahydropyrrolo[1,2-a]pyrazine-1,4-	0.001296209	-0.004352213	Metabolome
1-(2-Hydroxyethyl)-5-nitropyrrole-2-(	-0.008255873	-0.013541629	Metabolome
NeuNGc	-0.005735417	0.00917944	Metabolome
Nevirapine	0.021833251	-0.014346324	Metabolome
N-Myristoyl Serine	0.028299855	0.000393443	Metabolome
4-Decan-4-Ylbenzenesulfonic Acid	0.018103612	-0.004018276	Metabolome
Hydroxymethylcytosine	0.001417844	-0.004874306	Metabolome
Benzene	0.018185203	-0.010711133	Metabolome
N(2)-phenylacetyl-L-glutaminate	-0.020809837	0.000147825	Metabolome
3-(4-Hydroxyphenyl)lactate	0.00863577	-0.002207751	Metabolome
L-Aspartate-semialdehyde	0.016508467	-0.010683771	Metabolome
Bioepiderm	0.004097317	0.002566255	Metabolome
PI(6 keto-PGF1alpha/22:3(10Z,13Z,16Z	-0.019077333	-0.00801014	Metabolome
Methyl 2,6-dimethyl-5-nitro-4-[2-(trif	0.03008882	-0.024410148	Metabolome
Dehydroxyzyleuton	0.002100523	-0.002525477	Metabolome
Blepharin	-0.025586573	-0.004275572	Metabolome
Methylmalonic acid	-0.048000664	0.00328221	Metabolome
Pimelic acid	-0.003549656	-0.004627688	Metabolome
Finrozole	0.004097317	0.002566255	Metabolome
Menthyl pyrrolidone carboxylate	0.015239024	-0.013401913	Metabolome
Phosphoric acid	-0.000337779	-0.006508815	Metabolome
Isoleucyl-Glutamate	-0.005921832	-0.004084093	Metabolome
Caffeic acid	0.0189935	-0.018643749	Metabolome
Glu-Thr	0.029008661	-0.026057855	Metabolome
3-(6-((4-(Trifluoromethoxy)phenyl)am	0.007637304	-0.01281853	Metabolome
Fumigaclavine A	-0.014192881	0.06404707	Metabolome
(5R)-3-Ethylsulfanyl-6-(1-hydroxyethy	0.007375684	0.002389225	Metabolome
15-Demethyl plumieride	0.005504592	0.000392955	Metabolome

Olprinone	0.017865025	0.010903603	Metabolome
TIARAMIDE	0.005050103	0.008741618	Metabolome
5-(2'-Carboxyethyl)-4,6-Dihydroxypic	0.00127257	-0.004911958	Metabolome
Ophthalmic acid	-0.005074327	0.003625148	Metabolome
6,8-Dihydroxypurine	0.017601717	-0.002070166	Metabolome
6-Carboxy-5,6,7,8-tetrahydropterin	0.004097317	0.002566255	Metabolome
Neosaxitoxin	-0.06351223	0.005080315	Metabolome
Retinyl ester	-0.01217967	-0.009404501	Metabolome
N-Benzyl-9-(tetrahydro-2H-pyran-2-	0.031357446	-0.082908372	Metabolome
Leukotriene B4 dimethylamide	-0.012732624	-0.004166698	Metabolome
(+)-taxifolin	0.003970841	0.002613222	Metabolome
1-(4-Carboxyphenyl)-3,3-dimethyltria	-0.044757217	0.013173421	Metabolome
Casuarine 6-alpha-D-glucoside	-0.021867843	-0.001039913	Metabolome
Lysyl-Glycine	-0.021639016	0.000250196	Metabolome
Djenkolic acid	0.024893737	-0.02106098	Metabolome
Suberic Acid	0.002748685	-0.009564829	Metabolome
SM(d18:0/12:0)	-0.032103194	-0.025987359	Metabolome
Flunixin	-0.061439469	-0.003422081	Metabolome
3-Methoxytyramine	0.030350631	-0.000697064	Metabolome
Ajmalicine	0.00127257	-0.004911958	Metabolome
[(2S,3R,4R,5R)-5-(6-Aminopurin-9-yl)-	0.001295543	-0.004439874	Metabolome
Deoxyuridine	0.020192557	-0.002887891	Metabolome
1-Pyrroline-5-carboxylic acid	0.020580059	0.001454037	Metabolome
PC(17:2(9Z,12Z)/0:0)	-0.002530319	-0.003514201	Metabolome
3-(Trimethylsilyl)propionic Acid	0.027555235	0.004518517	Metabolome
Cis-4-Hydroxy-D-proline	-0.018902161	0.005412242	Metabolome
Artemidinol	0.002187294	0.007633905	Metabolome
SM(d16:1/18:1)	-0.040493529	-0.000344406	Metabolome
Alogliptin	0.038133972	-0.024299573	Metabolome
2,2-Difluoro-n-(2-hydroxyethyl)-3-(2-	-0.015555926	0.006796436	Metabolome
3-Indolebutyric acid	0.00431055	0.00158541	Metabolome
4-(2,6-Dioxo-1-propyl-3,7-dihydropu	-0.008068921	-0.023423083	Metabolome
Nicotinamide riboside	-0.011181822	-0.001125358	Metabolome
7-Methylinosine	0.019699877	-0.004657509	Metabolome
18-Nitrooctadeca-9,12-dienoic acid	0.001419228	-0.004401436	Metabolome
Aniline	0.015815234	-0.002984798	Metabolome
Molinate	-0.024871726	0.013758175	Metabolome
Lactucin	0.012178598	0.00427574	Metabolome
2-Aminopyridine	-0.011328217	-0.005155949	Metabolome
Glycyl-leucine	0.004339288	-0.010704418	Metabolome
(-)-3-(4-(2-(Phenoxazin-10-yl)ethoxy)	0.018961178	0.014172734	Metabolome
L-Phenylalanine	0.021335345	-0.006801667	Metabolome
Arkofix	0.001179529	-0.004363427	Metabolome
2-Methylglutaric Acid	-0.007325145	-0.00304464	Metabolome
L-3-Hydroxykynurenine	0.019617355	-0.026666982	Metabolome
3-(2-Hydroxyethyl)histidine	0.001419228	-0.004401436	Metabolome
(-)-Jasmonic acid	0.011425287	-0.000574938	Metabolome
Lipoamide	-0.01030921	-0.060633218	Metabolome
Kynurenic Acid	0.02664261	0.002063042	Metabolome
3-Hydroxyflavone	0.026418205	-0.002430028	Metabolome
Stachyoside A	-0.018091932	-0.010539154	Metabolome
Aminoadipic acid	0.023827137	0.01673472	Metabolome
L-Glutamic gamma-semialdehyde	-0.007712375	0.01430849	Metabolome
Osmundalin	-0.006531909	-0.01000907	Metabolome
CGMP	0.003970841	0.002613222	Metabolome
Niacinamide	-0.008814103	-0.006632409	Metabolome
Pirprofen	0.00127257	-0.004911958	Metabolome
Midodrine	0.005637272	-0.009030051	Metabolome

Daucol	0.005795133	-0.005421634	Metabolome
8-Hydroxyguanosine	-0.03104188	-0.068511705	Metabolome
Tanacetol B	-0.051578238	-0.003559434	Metabolome
Undecylenic acid	-0.023947716	-0.012011617	Metabolome
4-Hydroxymandelonitrile	-0.008278603	0.010015865	Metabolome
Alpha-Ketoglutarate cyanohydrin	-0.020016465	0.005938285	Metabolome
Ochratoxin B	0.015399902	-0.003487571	Metabolome
2-Cyclopentene-1,4-dione	-0.010288841	-0.002086888	Metabolome
Neryl formate	-0.016699939	-0.015045642	Metabolome
Lappaol B	-0.032591662	-0.004334959	Metabolome
Bindarit	0.00127257	-0.004911958	Metabolome
Threonylhistidine	0.001417844	-0.004874306	Metabolome
Gluconolactone	-0.008180039	-0.022402046	Metabolome
2'-Fluoro-2',3'-dideoxyinosine	0.010649241	0.021480371	Metabolome
Abscisic Acid	0.028278001	0.111840736	Metabolome
P-Benzosemiquinone	0.009698274	-0.012799701	Metabolome
2-Geranyl-2',3,4,4'-tetrahydroxydihydı	-0.039702654	-0.00803413	Metabolome
L-a-Lysophosphatidylserine	-0.019536098	0.007029571	Metabolome
N-Succinyl-2-amino-6-ketopimelate	0.004097317	0.002566255	Metabolome
Myristoylglycine	-0.007190747	-0.030339696	Metabolome
Mafenide	-0.01038721	-0.01637346	Metabolome
3-Methyldioxyindole	-0.007967539	-0.016207709	Metabolome
(2R,3R)-(-)-2,3-Butanediol	0.00856078	-0.014353207	Metabolome
Icariside B8	0.01533061	-0.002359694	Metabolome
Methyl n-acetylanthranilate	0.020245329	-0.002319425	Metabolome
Azimexon	-0.006888828	-0.00276184	Metabolome
Salsoline-1-carboxylate	0.020558139	-0.003870304	Metabolome
3,4,5-trihydroxy-6-{4-[(E)-2-(3-hydro:	-0.001131636	-0.009343937	Metabolome
Diaminopimelic acid	-0.014337259	0.011170062	Metabolome
2',2'-Difluorodeoxyuridine	0.004097317	0.002566255	Metabolome
O-Beta-D-Glucosylzeatin	-0.005904135	0.008678042	Metabolome
12-Hydroxynevirapine glucuronide	-0.022123015	-0.058222921	Metabolome
P-Aminophenazone	0.006690473	-0.008053872	Metabolome
(2R,3R)-3-[[(2S)-1-[4-(Diaminomethyl	0.001417844	-0.004874306	Metabolome
N-Formyl-L-aspartate	0.001419228	-0.004401436	Metabolome
MeOSuc-AAPA-CMK	-0.07640834	-0.001660196	Metabolome
Gentian Violet Cation	0.01405713	-1.35E-05	Metabolome
(2R)-2-Acetamido-6-hydroxy-2-(sulfa	-0.007103419	-0.002167482	Metabolome
Geniposide	0.001295543	-0.004439874	Metabolome
MG(10:0/0:0/0:0)	0.00036217	0.014064799	Metabolome
(2R,3S)-Piscidic acid	-0.020881506	0.009988374	Metabolome
Phlorisobutyrophenone	0.009792613	0.013749548	Metabolome
6-Hydroxy-1H-indole-3-acetamide	0.010976234	-0.002786914	Metabolome
Pinolenic Acid	-0.021269969	-0.034182607	Metabolome
Isocolumbin	0.004097317	0.002566255	Metabolome
Glucose-6-phosphate lactate	-0.022375102	-1.47E-05	Metabolome
O-Phospho-L-serine	0.001103713	-0.078281875	Metabolome
2,5-Dioxopyrrolidin-1-yl 2-(acetylthio)	0.003581947	0.03260792	Metabolome
Cyclocalopin D	0.02027947	-0.014014389	Metabolome
2-Imidazolidinone, 4-hydroxy-1-(5-ni	-0.020124882	0.004462848	Metabolome
Phaseic acid	0.001295543	-0.004439874	Metabolome
Vanillin Acetate	-0.006424592	-0.000312429	Metabolome
Neohesperidoside	0.028684429	0.003356983	Metabolome
Dopamine 3-O-sulfate	-0.019205825	-0.009242394	Metabolome
Thiamine	0.000567036	-2.22E-05	Metabolome
5'-(3',4'-Dihydroxyphenyl)-gamma-va	0.011037414	-0.00800565	Metabolome
4-Oxo-1,4-dihydroquinoline-3-carbo:	0.026159129	-0.000158634	Metabolome
Pyrocatechol	-0.022163084	-0.006777139	Metabolome

(2E)-4-Hydroxy-3-methylpent-2-enec	0.005671349	-0.014314612	Metabolome
2-(2-Hydroxyethoxy)-6-(2H-tetrazol-!	-0.008970063	-0.009121334	Metabolome
SM(d17:2(4E,8Z)/16:0)	-0.03079315	-0.01910118	Metabolome
L-Glutamic acid 5-phosphate	0.011589335	-0.010684989	Metabolome
Cafestol	-0.013626378	-0.0160667	Metabolome
DL-p-Chlorophenylalanine methyl este	0.006248438	0.026311344	Metabolome
N-Acetyl-L-Histidine	0.007825523	0.025228928	Metabolome
Salicylamide glucuronide	0.004097317	0.002566255	Metabolome
Phenylpropionylglycine	0.001296209	-0.004352213	Metabolome
Methylone	-0.023315253	0.01469972	Metabolome
Ibuprofen	0.0129429	0.009379411	Metabolome
Phenylalanylglutamic acid	0.008216556	0.05681863	Metabolome
Glaucarubol 15-O-beta-D-glucopyrar	0.004951783	0.004281734	Metabolome
Phosphoribosylformylglycinamidine	-0.01707502	-0.001734479	Metabolome
5-Acetyl-3,4-dihydro-2H-pyrrole	0.025054852	-0.005807764	Metabolome
His Leu	0.015410447	-0.000965677	Metabolome
Maleamic Acid	0.00127257	-0.004911958	Metabolome
O-Desmethylcarvedilol	0.00855537	8.60E-05	Metabolome
N-carboxymethyllysine	0.004392439	0.002741425	Metabolome
Arabinosylhypoxanthine	-0.0110342	-0.0082584	Metabolome
Taurine aspartate	0.003970841	0.002613222	Metabolome
Vidarabine	0.001419228	-0.004401436	Metabolome
N-Methyl-L-Threonine	0.022329218	-0.0012839	Metabolome
Deoxyribose	-0.01122931	-0.004035031	Metabolome
1-Carboxyethyltyrosine	0.02015112	0.005004752	Metabolome
1-Methoxy-4-(2-propenyl)benzene	0.005863059	-0.01205278	Metabolome
N-Acetylputrescine	-0.016969247	0.036724805	Metabolome
Imidazole-4-Acetic Acid	-0.00504039	-0.003012699	Metabolome
Peridinin	0.022405752	0.012704248	Metabolome
2,5-Dihydroxy-1H-pyrrole-3-sulfonic	0.001417844	-0.004874306	Metabolome
1-Ethylpiperidine	0.003970841	0.002613222	Metabolome
2,4-Diacetylphloroglucinol	0.001401008	-0.005417817	Metabolome
(E)-3-(2,3-Dihydroxyphenyl)-2-proper	-0.035910646	0.01618307	Metabolome
Kynurenine	0.017532132	-0.009001478	Metabolome
Quinic Acid	0.009172523	0.02327415	Metabolome
Alpha-D-GalpNAc-(1->3)-[alpha-L-F	-0.054201333	-0.004162963	Metabolome
(+/-)-Tryptophan	0.004124116	0.002548194	Metabolome
4-Nitrophenyl 4-guanidinobenzoate	0.021329125	0.012040912	Metabolome
Sabiporide	-0.064745738	-0.000725208	Metabolome
KAPA	0.028730556	0.002882096	Metabolome
Jasmonic acid	-0.038397273	-0.010123431	Metabolome
Xanthurenic Acid	0.037120392	0.00035674	Metabolome
Acipimox	0.001295543	-0.004439874	Metabolome
Biotin	0.001791229	-0.013023788	Metabolome
Tyrosyl-Isoleucine	0.003970841	0.002613222	Metabolome
Pro-Ile	-0.017121675	0.002025643	Metabolome
2-[(4-{2-[(4-Cyclohexylbutyl)(cyclohex	0.004124116	0.002548194	Metabolome
5-Hydroxy-N-formylkynurenine	-0.022617662	-0.006727551	Metabolome
LysoPE(0:0/16:1(9Z))	-0.030023154	-0.002172703	Metabolome
1,4-Benzothiazine-O-quinonimine	0.004392439	0.002741425	Metabolome
Porphobilinogen	0.01835778	-0.005604951	Metabolome
2-carboxy-L-xylonolactone	0.009877192	-0.003940527	Metabolome
LysoPC(18:4(6Z,9Z,12Z,15Z)/0:0)	-0.012035014	0.002126062	Metabolome
4-Hydroxyhippuric Acid	0.027279287	-0.002980128	Metabolome
2-Naphthol	-0.032320665	0.00025218	Metabolome
(S,S)-Nt-Histidinylalanine	0.015953595	0.068493463	Metabolome
L-Hypoglycin A	0.014006854	-0.003687078	Metabolome
Lucuminamide	-0.064541455	0.00157226	Metabolome

2,4-dimethylhexanedioylcarnitine	-0.037415998	-0.000868417	Metabolome
Vanillin	-0.01550845	-0.018944533	Metabolome
2-Oxoarginine	-0.011522517	-0.000575592	Metabolome
(2S)-1-[2-[[(2S)-Pyrrolidine-2-carbony	0.001179529	-0.004363427	Metabolome
4-Methyl-1H-Benzotriazole	0.002474724	0.017549821	Metabolome
2-Hydroxycarbamazepine	-0.016365392	0.004999723	Metabolome
Vomifoliol	0.003970841	0.002613222	Metabolome
Hexadecanedioic acid mono-L-carnitir	0.025636567	-0.00785165	Metabolome
Gln Val	0.033394166	-0.013876203	Metabolome
5-[(2R)-2-Aminopropyl]-2-hydroxybe	-0.030076073	0.003856958	Metabolome
Fluorescein 5-maleimide	0.00127257	-0.004911958	Metabolome
8-Epiloganic acid	0.025392853	-0.029946414	Metabolome
Glycyl-L-Tyrosine	-0.000205283	-0.024647183	Metabolome
N-alpha-Acetyl-L-lysine	-0.013539313	-0.039162049	Metabolome
Adrenochrome	-0.021326548	0.002253874	Metabolome
Val Ala Phe Asp	0.004228875	0.001833583	Metabolome
(S)-(-)-Perillyl alcohol	0.013107959	-0.079584178	Metabolome
1-(3-Methyl-2-butenoyl)-6-apiosylglu	-0.014002638	0.033131033	Metabolome
Dethiobiotin	0.017461351	-0.04175095	Metabolome
Alpelisib	0.004043287	-0.011936169	Metabolome
Trilostane	-0.009943115	0.003159792	Metabolome
Glucosyl (2E,6E,10x)-10,11-dihydroxy-	0.004124116	0.002548194	Metabolome
Glutaminylphenylalanine	0.032407609	0.008919332	Metabolome
3-Hydroxybenzyl Alcohol	0.024185695	-0.01309202	Metabolome
4-Hydroxynonenal	-0.027901121	-0.007052536	Metabolome
Gamma-Glutamylalanine	0.023432467	0.011141514	Metabolome
HBOA trihexose	0.017045008	-0.009794975	Metabolome
2-Amino-3-methylbenzoate	-0.0063774	-0.001229483	Metabolome
Halleridone	-0.020349335	0.024742165	Metabolome
5,6,8-Trihydroxy-2-methylbenzo[g]chi	-0.021164427	0.007203072	Metabolome
Beta-nicotinamide adenine dinucleotic	0.009183739	0.001535392	Metabolome
Proline betaine	0.017378494	-0.0012865	Metabolome
Hydroxyphenylacetylglycine	0.035501817	0.00442843	Metabolome
Aspartame	-0.029001987	-0.002401455	Metabolome
Simmondsin	-0.050423981	-0.00042445	Metabolome
Triton X-100	-0.001298457	0.007120316	Metabolome
Cyclohexyladenosine	0.018389102	0.002390656	Metabolome
11-Hydroxy-9-tridecenoic acid	0.004228875	0.001833583	Metabolome
Hydroxyprolyl-Lysine	0.042450015	0.014991209	Metabolome
1-(3-Carboxypropylcarbamoyl)-5-fluc	-0.007575699	0.046713563	Metabolome
5-Methoxytryptophan	0.016310731	-0.001716369	Metabolome
Histidylproline	0.001419228	-0.004401436	Metabolome
Mepenzolate	0.06712427	-0.001372312	Metabolome
Salicylic Acid	-0.017272883	-0.051522728	Metabolome
Benzo[d]isoxazol-3-ol	0.011571061	0.005295501	Metabolome
Glucosyl 6-hydroxy-2,6-dimethyl-2E,7	0.040898806	-0.00561035	Metabolome
Nitrosobenzene	-0.009487832	0.016822515	Metabolome
Methionylhydroxyproline	-0.003269567	-0.012907733	Metabolome
ADP-ribose 2'-phosphate	0.028836427	0.024712816	Metabolome
Bialaphos	0.002318311	0.010237685	Metabolome
Cerulenin	-0.012764777	0.002796276	Metabolome
Pro-Pro-Pro	-0.007724479	0.038121335	Metabolome
3-Amino-2,3-dihydrobenzoic acid	0.012406597	0.0017617	Metabolome
3-Dehydroquinic acid	-0.015687069	-0.008138363	Metabolome
5-hydroxyindole thiazolidine carboxyla	-0.012850422	-0.016512971	Metabolome
Pregnanetriol 3a-O-b-D-glucuronide	-0.022590266	-0.001888405	Metabolome
4-[2-(4-Isopropylbenzamido)ethoxy]b	0.038468907	-0.034567378	Metabolome
(-)-trans-Carveol	-0.02263837	-0.012631485	Metabolome

Raffinose	-0.006859698	0.011038018	Metabolome
Lawsone	-0.01235291	0.017552653	Metabolome
3-Chlorotyrosine	0.004097317	0.002566255	Metabolome
Maltotriose	0.009807051	0.002622458	Metabolome
Germacrone	0.001296209	-0.004352213	Metabolome
Polyethylene, oxidized	0.040628008	5.10E-05	Metabolome
Histamine	0.003561738	-0.021038501	Metabolome
Lucuminoside	0.039957338	0.000258615	Metabolome
Benzaldehyde	0.001993527	0.000761879	Metabolome
Nornicotine	0.023962623	0.001110244	Metabolome
SM(d18:0/16:1(9Z)(OH))	-0.03133782	-0.01686307	Metabolome
2,5-Diamino-4,5-diketopyrimidine	0.014504856	-0.009435008	Metabolome
2-(2,6-Dihydroxy-4-methoxycarbonyl	0.004097317	0.002566255	Metabolome
3,3-Bis(carboxymethyl)hexadecanedioi	0.014474556	-0.00462177	Metabolome
Uridine-5'-Monophosphate	-0.028235658	-0.018693493	Metabolome
Vasicinone	0.004228875	0.001833583	Metabolome
Beta-Thujaplicin	0.018066105	0.003701667	Metabolome
2',5'-Dideoxyadenosine	-0.028538128	-0.003259396	Metabolome
5-Hydroxyindoleacetate	0.018810026	-0.0070898	Metabolome
1,5-Naphthalene diisocyanate	-0.010148964	-0.014011443	Metabolome
Phosphoribulosylformimino-AICAR-P	-0.007955198	-0.015867336	Metabolome
Ceranapril	0.001419228	-0.004401436	Metabolome
Etiracetam	0.004326821	0.027344599	Metabolome
Trans-Cinnamic Acid	0.01055597	-0.023776012	Metabolome
1-Acetyl-2-phenylhydrazine	-0.017859244	0.022672656	Metabolome
Indolepropionylglycine	-0.002073558	0.0138777	Metabolome
Imazapyr	0.004228875	0.001833583	Metabolome
Trans-cinnamoyl-beta-D-glucoside	-0.025363945	0.004329664	Metabolome
1H-Imidazole-1-ethanol, 2-nitro-alph	-0.007305805	0.007333184	Metabolome
C.I. Solvent Red 80	-0.001818864	-0.037152374	Metabolome
Methylnoradrenaline	-0.019093588	-0.006225305	Metabolome
7-Methylxanthosine	-0.053930893	-0.01017896	Metabolome
Necatorine	0.02007117	0.014821451	Metabolome
2-[4-(sulfooxy)phenyl]acetic acid	0.053866293	-0.001702839	Metabolome
6-Hydroxymelatonin	-0.010440756	0.04001952	Metabolome
2,2':5',2''-Terthiophene	0.00127257	-0.004911958	Metabolome
9(S)-HpODE	0.004594147	0.043444028	Metabolome
Tert-Butyl 3-((3S,6S,12aS)-6-isobutyl-	0.004228875	0.001833583	Metabolome
Phenacemide	-0.006754117	0.018306386	Metabolome
P-CHLOROPHENYLALANINE	-0.015716208	0.007260542	Metabolome
Ginnalin B	0.029763287	-0.002162954	Metabolome
Phenacetyl-carnitine	-0.029358745	-0.024364215	Metabolome
N-Acetyl-L-Glutamic Acid	-0.033211999	0.000394733	Metabolome
Maltohexaose	-0.013274674	0.007913025	Metabolome
Hexafluoroacetylacetone	-0.023186488	0.00143303	Metabolome
3-Methylpyridazine	-0.012036098	0.011522464	Metabolome
Uridine, 2'-deoxy-2'-fluoro-2'-methyl	-0.03991581	-0.003632862	Metabolome
Penicillin X	-0.014862548	-0.028881549	Metabolome
N-Eicosapentaenoyl Glutamine	0.001417844	-0.004874306	Metabolome
Scytalone	0.004097317	0.002566255	Metabolome
PIP(PGF2alpha/18:1(15Z))	0.003970841	0.002613222	Metabolome
4-Hydroxychalcone	-0.026680087	0.000992465	Metabolome
Phenylacetohydroximoyl-glutathione	0.035375447	-0.096232138	Metabolome
NADP	0.029183282	0.000615216	Metabolome
Isopropyl 4-hydroxybenzoate	-0.010995462	-0.00085176	Metabolome
12-[Methyl-(4-nitro-2,1,3-benzoxadia	0.00827972	0.011656975	Metabolome
L-DOPA 3'-glucoside	-0.011270853	0.038043888	Metabolome
Maltotetraose	0.014743592	0.001805372	Metabolome

Pravastatin	0.003970841	0.002613222	Metabolome
Diethyl Phthalate	-0.00352874	0.015405106	Metabolome
3-Isopropyl-2-methoxy-5-methylpyra	0.010659443	-0.021247301	Metabolome
7-[2,5-Dihydroxy-2-[3-hydroxy-4-[3-	0.022684946	0.000707967	Metabolome
Difluoromethyl (2R)-2,5-diaminopenta	-0.019630978	1.33E-05	Metabolome
Methylthiouracil	0.003163848	-0.059986846	Metabolome
6-Hydroxynicotinic Acid	0.008573309	0.097016268	Metabolome
3-Hydroxykynurenic acid	0.037602052	-0.000861324	Metabolome
Metyrapone	0.004228875	0.001833583	Metabolome
1,5-Benzothiazepin-4(5H)-one, 2,3-di	-0.043960517	0.001721432	Metabolome
8-Methoxykynurenate	0.014284689	-0.039200354	Metabolome
4-methoxy-3-(sulfooxy)benzoic acid	0.014125571	0.015322429	Metabolome
Dihydromaleimide beta-D-glucoside	0.025186928	-0.004276404	Metabolome
DG(20:4(6E,8Z,11Z,14Z)-OH(5S)/0:0/2:	-0.018163114	-0.015868645	Metabolome
6-(4-carboxy-2,6-dihydroxyphenoxy)-	0.016519134	0.006180744	Metabolome
N-(2-benzoyl-4-chlorophenyl)-1-met	0.011867579	-0.011348674	Metabolome
Ethotoin	0.017276992	-0.0232418	Metabolome
1,3,5-Triazine-2,4(1H,3H)-dione	-0.026705605	0.05902533	Metabolome
3-Hydroxy-2-oxoindole	-0.011502101	-0.010953483	Metabolome
Amoxicillin	0.022581598	-0.011501343	Metabolome
Malvidin 3-glucoside	-0.01789609	-0.005623099	Metabolome
DIMBOA trihexose	0.008317465	-0.003441146	Metabolome
1-[(3R,4R,5R)-3-Fluoro-3,4-dihydroxy	0.032831326	-0.004123916	Metabolome
Indolelactic Acid	0.018903068	0.003016531	Metabolome
Trans-Dodec-2-enoic acid	-0.013678337	-0.008466751	Metabolome
LysoPA(16:0/0:0)	-0.019519595	-0.006681198	Metabolome
3-Methoxyphenol sulfate	0.02540165	-0.003679296	Metabolome
2-Stearoylglycerophosphoglycerol	0.009702935	-0.011506061	Metabolome
Genipic acid	0.012205375	-0.035847815	Metabolome
(2R,3S)-3-Hydroxy-1,1-dimethylpyrrol	0.012658695	-0.002583783	Metabolome
4-Amino-1-piperidinecarboxylic acid	0.005565394	0.034880144	Metabolome
Gamma-L-Glutamyl-L-methionine sul	0.035590608	-0.008140626	Metabolome
(2R,4Z)-4-Hepten-2-ol	0.00127257	-0.004911958	Metabolome
(R)-1-O-b-D-glucopyranosyl-1,3-octa	0.021279436	-0.006921243	Metabolome
Zierin	0.026964728	-0.008151676	Metabolome
Spermine	0.022351821	0.004079111	Metabolome
4-Methylumbelliferone	0.02199186	0.003882058	Metabolome
LysoPI(18:0/0:0)	0.016641427	0.048668311	Metabolome
Citrinin	0.017520192	-0.007053487	Metabolome
Orsellinic acid 2-O-beta-D-glucoside	0.023526746	-0.000960484	Metabolome
10-Deacetyltaxol	-0.011854718	-0.00695136	Metabolome
Urea glutamate	0.024072366	0.00122414	Metabolome
Lumichrome	-0.010899367	0.051110584	Metabolome
Coniferyl acetate	0.012129106	-0.007098831	Metabolome
3-Hydroxyphenylacetic Acid	0.015121326	-0.012240768	Metabolome
4,6-Dihydroxy-2-quinolinecarboxylic a	0.016767383	-0.006706735	Metabolome
6-Cyano-7-nitroquinoxaline-2,3-dion	0.001295543	-0.004439874	Metabolome
Urocanic Acid	0.010134639	0.001311643	Metabolome
Histidylhistidine	0.020376714	-0.002121006	Metabolome
(1E)-1-Phenyltriaz-1-ene	-0.012941884	0.055591169	Metabolome
(S,S)-Butane-2,3-diol	-0.000595908	-0.002025987	Metabolome
Cndac	-0.04977306	0.001032928	Metabolome
SM(d18:1/14:1(9Z))	-0.029315703	-0.020692642	Metabolome
Ricinine	0.01890722	-0.00322305	Metabolome
Beta-D-ribosylnicotinate	0.042829713	-0.004725526	Metabolome
PI(TXB2/20:1(11Z))	0.020355917	-0.022728106	Metabolome
Histidylglutamine	0.025708043	0.010887198	Metabolome
Gluten exorphin B4	0.024435052	-0.02935186	Metabolome

Quercetin 3,3'-diglucoside	-0.035183472	0.002214253	Metabolome
Aspirin	0.014582279	-0.03676429	Metabolome
3-methylthiopropyl-desulfoglucosinol	-0.003187028	0.002137954	Metabolome
Pteroside Z	-0.058409943	-0.000434571	Metabolome
N-Docosahexaenoyl Glutamine	-0.040748965	-0.001010527	Metabolome
Gentianine	-0.013113439	-0.000171626	Metabolome
N-Acetylmuramate	0.010965532	-0.015214405	Metabolome
Fluazifop	0.014531314	-0.059828694	Metabolome
Aciclovir	0.023742472	-0.006278996	Metabolome
Isoketocamphoric acid	-0.015740331	-0.005915182	Metabolome
8-Acetoxypinoresinol	-0.017749772	0.007166448	Metabolome
2,4-Quinolinediol	0.001955858	0.017781711	Metabolome
Palmitic acid	0.02020152	0.0543151	Metabolome
4-Imidazolone-5-propionic acid	-0.010078932	0.007203921	Metabolome
Sulfamethoxazole	0.006345095	0.027127323	Metabolome
Sufentanil	-0.006538005	-0.04452433	Metabolome
2-Methylbenzoic acid	-0.038337238	0.001011654	Metabolome
Fraxin	0.025884112	0.000265596	Metabolome
Tryptophol	0.001624989	0.005814096	Metabolome
Morph	-0.011091408	-0.003379699	Metabolome
Tridecanoic acid	-0.045313779	0.019247454	Metabolome
N-Acetyl-D-mannosamine	-0.041144782	-0.045237227	Metabolome
Caldine	0.036543693	-0.004718646	Metabolome
Hydantoin-5-propionic acid	0.004097317	0.002566255	Metabolome
Ethyl glucuronide	0.004761645	-0.008876613	Metabolome
Verbascose	0.010301812	-0.007120236	Metabolome
Epsilon-caprolactam	0.011106137	0.016917033	Metabolome
2-Hydroxyacetaminophen sulfate	0.03882892	-0.003206407	Metabolome
2-(2-Hydroxyethyl)phenyl hydrogen su	0.040326095	-0.017488084	Metabolome
3,5-Dihydroxyphenylglycine	0.023200664	0.002007555	Metabolome
3-Pyridinecarboxaldehyde	0.008427803	-0.003942196	Metabolome
Beta-D-3-Ribofuranosyluric acid	-0.020109187	-0.002265029	Metabolome
N-Acetyl-S-benzyl-L-cysteine	-0.016597923	0.013434208	Metabolome
Gallic acid	0.043180228	-0.004487286	Metabolome
N-Eicosapentaenoyl Tryptophan	-0.005295956	-0.007673151	Metabolome
Cinncassiol D2 glucoside	0.004228875	0.001833583	Metabolome
3'-Hydroxy-HT2 toxin	0.004228875	0.001833583	Metabolome
4-Acetamido-2-aminobutanoic acid	0.006297836	0.031202279	Metabolome
4,5-Dihydro-4-((1-piperazinyl)acetyl)-	-0.040663731	0.000835626	Metabolome
Gibberellin A44	-0.021082419	0.033232612	Metabolome
PG(5-iso PGF2VI/a-25:0)	0.006254939	0.052420475	Metabolome
Rafabegron	-0.006418277	-0.04336645	Metabolome
Penicillic Acid	-0.011648459	-0.008547675	Metabolome
(2Z)-2-(3-Phenylpropoxyimino)butanc	-0.016866334	-0.010201278	Metabolome
5-Methoxyindoleacetate	0.018110625	0.002822679	Metabolome
Dopamine	-0.040482285	-0.000582073	Metabolome
Petrosaspongiolide m	0.026195218	-0.004775005	Metabolome
4-(Glutamylamino) butanoate	-0.005104825	0.009926306	Metabolome
[D-Lys3]-GHRP-6	0.033129284	0.009407848	Metabolome
Secologanate	-0.007801951	0.043241287	Metabolome
Hepoxilin A3	-0.016522986	0.057023024	Metabolome
Pyrimidin-2-ol	0.033120163	-0.010903664	Metabolome
2,5-Furandicarboxaldehyde	0.005657748	-0.003011119	Metabolome
Benzo[b]thiophene-2-carboxylic acid	-0.010082989	0.002524243	Metabolome
Citrusin III	0.01668294	0.006716667	Metabolome
Tetranor 12-HETE	-0.042675168	0.010350323	Metabolome
Canavanine	-0.021172274	-0.038630789	Metabolome
Imidaprilat	0.037437275	-0.011576831	Metabolome

Ciadox	-0.026205464	-0.004837221	Metabolome
Mycorradicin	0.004124116	0.002548194	Metabolome
Herniarin	0.020294276	-0.012286383	Metabolome
O-Adipoylcarnitine	-0.036648132	0.001691245	Metabolome
5-hydroxy-2-oxo-4-ureido-2,5-dihyd	0.022294925	-0.020909315	Metabolome
Lusitanicoside	0.02710335	-0.002467097	Metabolome
Cyanidin 3-(6''-acetyl-galactoside)	0.034551257	-0.084817512	Metabolome
Eugenin	-0.015664778	0.022758637	Metabolome
Isoguvacine	-0.003598657	-0.043623281	Metabolome
Propafenone glucuronide	-0.03521017	0.007956599	Metabolome
O-Succinyl-L-homoserine	0.014741583	-0.009543863	Metabolome
4-Ethoxy-4-oxobutanoic acid	-0.014791591	0.00027521	Metabolome
2,6-Dimethoxy-1,4-benzoquinone	0.000836058	-0.002419247	Metabolome
1,3-Propanediol, 2,2-diethyl-, dicarba	0.00966374	0.004837556	Metabolome
24-Epibrassinolide	0.081267735	-0.002820308	Metabolome
Vanillic acid	0.019578109	-0.012694334	Metabolome
Hydromorphone-3-glucoside	0.013739801	-0.013541016	Metabolome
5'-Carboxy meloxicam	-0.018808377	-0.013885837	Metabolome
N-hydroxy-L-tryptophan	0.011541394	0.009897637	Metabolome
N-Lactoylvaline	0.015559835	-0.011099846	Metabolome
M-Xylene	0.008863162	0.038422726	Metabolome
(5S,6R)-Methyl 5,6,7-trihydroxyheptan	0.03353227	0.04423645	Metabolome
Carboxyethyllysine	0.005322737	0.014863602	Metabolome
N-Dodecylsarcosinate	-0.002864015	-0.012004571	Metabolome
3-Methoxyanthranilate	0.006530379	-0.004461578	Metabolome
Syrups, hydrolyzed starch, hydrogenat	-0.012041289	-0.004270613	Metabolome
Brisoral	-0.01685952	-0.000311315	Metabolome
5-Hydroxytryptophol sulfate	0.030476475	-0.004969054	Metabolome
3-(L-Menthoxy)-2-methylpropane-1,2	-0.020458302	0.004075014	Metabolome
5-Aminoisoquinolin-1(2H)-one	0.036327099	-0.006562055	Metabolome
4-Acetamido-2-amino-6-nitrotoluene	0.029069633	-0.007596041	Metabolome
Heptaethylene glycol monododecyl et	0.027853163	0.005637034	Metabolome
Formyl-5-hydroxykynurenamine	0.029921166	0.010919376	Metabolome
Policapram	-0.003994157	-0.026157718	Metabolome
CDP-DG(i-20:0/i-12:0)	0.031909225	0.009252278	Metabolome
Tasquinimod	-0.000335073	-0.005196002	Metabolome
4-Hydroxy-5-phenyltetrahydro-1,3-o	0.029756165	-0.017923011	Metabolome
Alpha-Hydroxyhippuric Acid	0.020600235	-0.005770913	Metabolome
Aminophenazone	-0.004700613	0.027578359	Metabolome
Cytosine deoxyribonucleoside	0.028819503	-0.037156447	Metabolome
(2S)-2-Amino-3-[(2S,3R)-2-amino-3-	-0.007166726	-0.008669262	Metabolome
Monotropein	-0.009535494	-0.007648666	Metabolome
4,5-seco-dopa	0.032046444	-0.007302383	Metabolome
Didanosine	-0.030811676	0.002786294	Metabolome
1-Deoxynojirimycin	0.010884871	-0.000650264	Metabolome
DTDP-4-dehydro-6-deoxy-beta-L-m	0.008059307	-0.001658307	Metabolome
Glycinamide, glycyl-L-prolyl-	0.031833492	-0.005168295	Metabolome
8-Hydroxy-3,4,5-trimethyl-6-oxo-4,6-	0.00070989	0.013548794	Metabolome
4-(2-Amino-3-hydroxyphenyl)-2,4-di	0.015973045	-0.009485446	Metabolome
N-(2,3-Dihydroxypropyl)valine	-0.012790011	0.005497307	Metabolome
1-Amino-2-methylanthraquinone	-0.030881497	-0.002031305	Metabolome
Lactulose	-0.022197971	0.015843597	Metabolome
10-EdAM	-0.001629955	-0.001324181	Metabolome
Isovaleric Acid	0.036798469	0.001066637	Metabolome
L-2-Aminoethyl seryl phosphate	0.004828611	0.006746737	Metabolome
Antramycin	-0.034170013	-0.083163634	Metabolome
(Z)-N-Coumaroyl-5-hydroxyanthranili	-0.011384165	0.013655012	Metabolome
PE(20:5(7Z,9Z,11E,13E,17Z)-3OH(5,6,1!	-0.043393257	-0.003219286	Metabolome

Histidinal	-0.004060851	-0.022476246	Metabolome
Phenyl-Alanine	0.018054603	-0.007985789	Metabolome
N6-[2-(4-Aminophenyl)ethyl]adenosir	-0.055182812	0.000283309	Metabolome
Trp Gly Asp	-0.005443249	0.050123089	Metabolome
5'-Deoxy-5-fluorouridine	0.01143081	0.009022397	Metabolome
P-Hydroxymandelic acid	-0.025248237	0.006765626	Metabolome
Isoformononetin	-0.021890177	-0.019122789	Metabolome
(4Z,10Z,12E)-3-Hydroxytetradeca-4,1(	0.021971884	-0.001481266	Metabolome
Leonuriside A	-0.015819824	-0.008754923	Metabolome
N-(2-Hydroxyethyl)nicotinamide	-0.038054481	0.017735029	Metabolome
Aminosalicylic Acid	-0.025901399	0.009051605	Metabolome
L-Pyridosine	0.009798506	0.043973767	Metabolome
Folinic acid	0.031669113	-0.008727083	Metabolome
Questiomycin A	-0.043024908	0.002093043	Metabolome
(-)-Aspidospermine	0.034175182	-0.012114298	Metabolome
S-(6-Purinyl)cysteine	-0.042824691	-0.10642999	Metabolome
Fosteabine	0.024051225	-0.025064215	Metabolome
Valaciclovir	-0.005200141	-0.005618471	Metabolome
1H-Imidazole-1-ethanol, alpha-(1-azi	0.016597835	0.02080895	Metabolome
Omega-Salicoyisalicin	0.016848832	0.03252399	Metabolome
Mesylate	-0.018983989	0.048022024	Metabolome
Gentisic acid	0.014876307	-0.011608343	Metabolome
Lysopc(18:3)	-0.011316303	0.004920556	Metabolome
1-(3-Fluoro-4-hydroxy-5-mercaptom	-0.027025156	0.003129529	Metabolome
Oxoadipic acid	-0.016721794	0.007096443	Metabolome
3-Hydroxyphloretin 2'-O-xylosyl-gluc	0.008935851	-0.005397784	Metabolome
Glucitol-lysine	-0.024520675	-0.006025638	Metabolome
Antiarrhythmic peptide	-0.016438619	-0.000451341	Metabolome
3-Feruloylquinic acid	-0.015235198	0.027602099	Metabolome
1,2-Dehydrosalsolinol	0.01570753	-0.02147197	Metabolome
SM(d15:1/18:1)	-0.041432122	-0.026236271	Metabolome
PS(22:6(4Z,7Z,10Z,13Z,16Z,19Z)/PGJ2)	0.003970841	0.002613222	Metabolome
Gamma-linolenyl carnitine	-0.016574697	-0.001733567	Metabolome
L-beta-aspartyl-L-leucine	-0.044761098	-0.012493505	Metabolome
2-(3-Mercaptopropyl)pentanedioic aci	-0.001623402	0.011519826	Metabolome
Pyridoxal	0.004336129	0.00750361	Metabolome
LysoPE(22:4(7Z,10Z,13Z,16Z)/0:0)	-0.000855437	0.053930905	Metabolome
Cordycepin	-0.016757211	0.003019551	Metabolome
Icofungipen	0.034725481	0.000519007	Metabolome
Neuraminic acid	0.034287809	-0.023583897	Metabolome
Endomorphin-2	-0.002216854	-0.034484645	Metabolome
5-(5-(2,6-Dichloro-4-(4,5-Dihydro-2-	0.013475464	-0.006124218	Metabolome
Tiglic acid	-0.004940013	-0.012746381	Metabolome
4-Hydroxymethylpyrazole	0.003228347	0.007754606	Metabolome
N2-Succinyl-L-ornithine	-0.016870116	-0.002866815	Metabolome
2-Amino-4-[carbamimidoyl(methyl)an	0.013425405	0.035663484	Metabolome
SM(d17:1/16:0)	-0.040503506	0.000271713	Metabolome
5-Hydroxyindoleacetylglycine	0.003297529	-0.026680743	Metabolome
Pyrazosulfuron-ethyl	-0.022265682	-0.017377499	Metabolome
4-Amino-3-hydroxybutyrate	-0.002851108	-0.007657989	Metabolome
Reproterol	0.057721909	0.025043625	Metabolome
2-(5'-methylthio)pentylmalate	0.003996607	0.001243762	Metabolome
Cuminaldehyde	0.023570269	0.00455019	Metabolome
1-(gamma-Glutamylamino)cyclopropa	0.011237041	0.017818661	Metabolome
Monoglutamyl folic acid	-0.039627316	-0.005691968	Metabolome
Alpha-Fluoro-beta-ureidopropionic a	-0.03639177	-0.024184136	Metabolome
Guanethidine	0.017069631	-0.02802054	Metabolome
2-Pyrrolidinone	-0.082728608	-0.005336543	Metabolome

Isonicophen	0.009754658	0.006299419	Metabolome
Hydroxyethylflurazepam	-0.021887975	-0.004317825	Metabolome
Pilaralisib	0.014052895	0.013400594	Metabolome
N-[(4-Amino-2-methylpyrimidin-5-yl	0.019402181	-0.003624549	Metabolome
3,5-Dichloro-2,6-dihydroxybenzoic ac	-0.019477728	-0.004520844	Metabolome
Luteolin 7-glucuronide	-0.023121437	0.020276606	Metabolome
Glucosamine, N-carbamoyl-(6CI)	0.007422212	-0.001125825	Metabolome
3-Methylpyrazole	-0.002672789	-0.023090635	Metabolome
Butyric Acid	0.02387555	-0.003039343	Metabolome
Xanthosine	0.010219053	-0.002130166	Metabolome
Physcion 8-gentiobioside	0.024685473	0.003764315	Metabolome
Uric acid lactate	0.018033934	-0.012181093	Metabolome
1-O-Sinapoyl-beta-D-glucose	0.029873948	-0.005970881	Metabolome
Narceine	-0.001450654	-0.025078798	Metabolome
Methyl 20-dihydroprednisolonate	-0.043514149	-0.020174863	Metabolome
N-methyl-L-glutamic Acid	-0.010470771	0.001876193	Metabolome
N-Oleoyl Glycine	-0.079519366	-0.000469476	Metabolome
GDP-Glucose	0.019082414	0.018116647	Metabolome
Cynaroside A	-0.000131274	-0.006193211	Metabolome
Chrysophanol	-0.060255564	0.001026746	Metabolome
Lysylglycine	-0.024208543	0.008920175	Metabolome
(4S,4As,5aR,12aS)-4,7-bis(dimethylami	0.004736877	-0.002768553	Metabolome
Dodecanoic acid	0.024907255	-0.003349673	Metabolome
N-Acetyl-L-methionine	0.01298832	-0.001526234	Metabolome
(S)-Isowillardiine	-0.025080217	0.006645648	Metabolome
Gamma-Glutamyl-2-aminobutyrate	0.020137674	-0.019257091	Metabolome
Ethyl 2-hydroxy-3-(3-indolyl)propano	-0.028291558	-0.002495202	Metabolome
Gastrodin	0.025068054	-0.020956309	Metabolome
CDP-DG(20:4(8Z,11Z,14Z,17Z)/20:1(11	0.003877468	0.002907899	Metabolome
N-Acetylserotonin	0.015725106	0.008677713	Metabolome
N-Lauroyl Proline	-0.049143291	0.005376054	Metabolome
Agmatine	-0.033929539	-0.007253414	Metabolome
Citalopram propionic acid	0.012311325	0.064364104	Metabolome
Amylopectin	0.003478405	0.005757215	Metabolome
2,3-Diphenylbenzofuran	0.014093466	0.005494771	Metabolome
Portulacaxanthin II	0.02316583	-0.032183739	Metabolome
Heliotridine	0.011194803	0.00173438	Metabolome
1-Carboxyethylisoleucine	0.020247217	0.006480718	Metabolome
3-Hydroxyindolin-2-one-sulfate	0.023504044	-0.00574327	Metabolome
Lavoltidine	0.022825623	-0.007774946	Metabolome
2,3-dinor-6-oxoprostaglandin F1alpha	-0.005929082	-0.000229001	Metabolome
Imidazole-4-acetaldehyde	0.024558921	-0.006518954	Metabolome
Columbianadin	0.026968967	0.007980445	Metabolome
4-hydroxy-3-nitrophenylacetate	0.019148869	-0.01015082	Metabolome
DG(2:0/0:0/20:5(7Z,9Z,11E,13E,17Z)-3C	-0.017465523	0.04222963	Metabolome
10-Formyldihydrofolate	-0.003662001	-0.013343062	Metabolome
Octadecenoylcarnitine	-0.019888476	-0.006761704	Metabolome
Gly Ala His	-0.032036865	0.008682672	Metabolome
5-Hydroxypentanoic acid	-0.012590295	0.005308445	Metabolome
(6Z,9Z)-Octadeca-6,9-dienoylcarnitine	-0.011131874	-0.011894485	Metabolome
Gamma-L-Glutamyl-S-(2-carboxy-1-|	0.014421779	-0.017065999	Metabolome
Beta-Hydroxy-gamma-trimethylaminc	0.004124116	0.002548194	Metabolome
2,3-dinor Fluprostenol	0.012435517	0.004657179	Metabolome
N-Arachidonoyl Glycine	-0.018072177	0.009581581	Metabolome
Aesculin	-0.037783091	0.002841277	Metabolome
Salidroside	0.015063943	0.010600158	Metabolome
Quercetin 3-galactoside	-0.019428963	-0.011477771	Metabolome
4-Methylumbelliferone glucuronide	0.019819654	-0.001555526	Metabolome

L-Agaridoxin	0.020383622	-0.005200559	Metabolome
Taurine	-0.003917147	-0.006864921	Metabolome
B-D-Glucuronopyranosyl-(1->3)-a-D	-0.021537387	-0.006005461	Metabolome
N-Eicosapentaenoyl Serine	-0.018256266	0.003696952	Metabolome
Lignan	0.018182557	-0.012025012	Metabolome
Noradrenochrome	0.011205767	-0.004055134	Metabolome
Glycerol glutamate	0.047609943	-0.021305936	Metabolome
Heliangin	0.022411568	0.002833763	Metabolome
Salsolinol	-0.040397595	-0.027504603	Metabolome
2'',6''-O-Diacetyloninin	-0.016663478	-0.009970952	Metabolome
Betalamic acid	0.001505671	-0.015860031	Metabolome
Salicylaldehyde	0.021413246	-0.000804468	Metabolome
Hydroxykynurenine	0.023236641	-0.021882507	Metabolome
1,3-Benzodioxole-5,6-diamine	-0.024981649	-0.008061427	Metabolome
5-trans Fluprostenol	0.02477282	0.005920207	Metabolome
Suspensolide F	-0.011740488	-0.026430921	Metabolome
Flindersine	-0.021885258	0.002959017	Metabolome
2-Hydroxynicotinic acid	0.031812379	-0.01021603	Metabolome
N-gamma-Glutamylcysteine	0.035590445	-0.005662402	Metabolome
Hibifolin	0.010002905	0.010076729	Metabolome
Isopropyl tartaric acid	-0.013923664	-0.008135792	Metabolome
Alpha-CYANO-3-HYDROXYCINNAMI(	0.017929596	0.008648642	Metabolome
Sweroside	-0.03397744	-0.00451582	Metabolome
Pantetheine	0.015251437	0.004791308	Metabolome
7-Methylxanthine	0.034400658	0.103427746	Metabolome
4-Hydroxy-3-methoxybenzenemethar	0.003567286	0.036146681	Metabolome
Fructosylvaline	0.04623044	0.023677569	Metabolome
Trehalose 6-Phosphate	0.012532782	-0.004080102	Metabolome
Gemcitabine	-0.017500625	0.002428458	Metabolome
4-Methylcatechol	-0.017393821	-0.003189174	Metabolome
(2S)-2-Amino-3-[4-[(2S)-2-amino-3-	-0.008100856	0.024322005	Metabolome
Dihydrolipoate	-0.024553013	-0.012522232	Metabolome
Dihydro-3-hydroxy-4,4-dimethyl-2(3I	0.021657342	0.005375649	Metabolome
(2,6-Dioxo-3H-purin-9-yl) pyridine-3	0.006704565	0.01759415	Metabolome
Asparaginyl-Gamma-glutamate	0.023645083	-0.002492984	Metabolome
Pentisomide	0.02165097	-0.006299206	Metabolome
Helenalin	-0.016705426	5.57E-05	Metabolome
HYPEROSIDE	-0.036528364	0.014677877	Metabolome
Trp Pro Glu	-0.028680948	-0.003688107	Metabolome
Decanoylcarnitine	0.022005656	-0.007579796	Metabolome
3,4-Methylenedioxybenzaldehyde	-0.006402006	-0.018975352	Metabolome
12-oxo-PDA	0.004392439	0.002741425	Metabolome
Floridin	0.001179529	-0.004363427	Metabolome
Gamma-Aminobutyric acid-betaxanth	0.037657298	-0.005079223	Metabolome
Tyramine glucuronide	0.014529907	-0.008614441	Metabolome
6-Methoxymellein	0.005502641	-0.011265081	Metabolome
7-Methoxycoumarin-3-carbonyl azide	-0.023952703	0.007039214	Metabolome
[8]-Shogaol	-0.033738121	-0.021740659	Metabolome
Isopropylmaleic acid	0.029173159	-0.004304415	Metabolome
Clofibryl glucuronide	0.029521676	-6.76E-05	Metabolome
Minocycline	0.02112907	0.0028097	Metabolome
Vanilloyl glucose	0.001796822	-0.003981025	Metabolome
Salannin	0.022878018	0.002909272	Metabolome
Histidylglycine	0.024075866	-0.018859868	Metabolome
Harmol glucuronide	0.008472417	-0.021459052	Metabolome
5-(3-Pyridyl)-2-hydroxytetrahydrofura	0.009082146	-0.007817778	Metabolome
Ala-Phe-Ala	0.030308522	-0.005054771	Metabolome
6-Hydroxyluteolin	0.011201613	0.040668193	Metabolome

(6-Aminoquinolin-2-yl) N-(2,5-dihydr	0.013883051	-0.01073006	Metabolome
3-Demethylubiquinone-9	0.009402349	0.063761285	Metabolome
Acetylcysteine	0.038373651	-0.000929144	Metabolome
Patulin	0.017410833	-0.00363158	Metabolome
Austinol	0.023286124	-0.000317734	Metabolome
Phenol	-0.016339998	-0.001216646	Metabolome
Threonylhydroxyproline	0.004291165	0.013640071	Metabolome
N-Acetyl-L-glutamate 5-semialdehyd	0.007328259	-0.004682113	Metabolome
Hypoglycin B	0.014904251	-0.006929502	Metabolome
Phenethylamine glucuronide	-0.031926262	0.023248473	Metabolome
(E)-indol-3-ylacetaldoxime	-0.009597703	0.003585557	Metabolome
2-Hydroxyethanesulfonate	0.019821458	-0.021492107	Metabolome
Liriodenine	0.039434645	-0.001442526	Metabolome
Serine-glyoxylate	-0.048892951	0.009898514	Metabolome
Natura	0.050458716	-0.114090065	Metabolome
5-Phenyl-1,3-oxazinane-2,4-dione	0.02322056	0.020942775	Metabolome
Kaempferol	-0.027715607	-0.003272994	Metabolome
Omadacycline	-0.022982559	0.010718994	Metabolome
(-)-cis-Rotenolone	-0.012014358	-0.023122162	Metabolome
Furfural	0.018881709	0.009207586	Metabolome
Xanthomicrol	-0.01610984	0.010891864	Metabolome
L-Histidine	-0.004595771	0.033807156	Metabolome
Polyribophosphate	0.003970841	0.002613222	Metabolome
Mivazerol	0.005634127	-0.019129089	Metabolome
Arginylaspartic acid	-0.017574219	-0.024033199	Metabolome
Sarmentosin	0.018071172	0.003950922	Metabolome
(+)-Neomenthol	0.006984975	-0.006156043	Metabolome
Zanamivir	0.020752834	-0.00853992	Metabolome
D-4'-Phosphopantothenate	0.013365375	-0.004504236	Metabolome
Valyllysine	-0.013949749	-0.011290974	Metabolome
VINBARBITAL	0.030591126	0.01748034	Metabolome
PI(22:3(10Z,13Z,16Z)/TXB2)	0.014630755	0.024793927	Metabolome
3-Hydroxymelatonin	0.015680789	-0.001939287	Metabolome
Crocin 5	-0.014973204	-0.015062339	Metabolome
D-Galactose	-0.018730263	0.005913033	Metabolome
Iodinated glycerol	0.012354003	-0.014962496	Metabolome
Imidazoleacetic acid ribotide	-0.032822266	0.005425015	Metabolome
Methyl (3x,10R)-dihydroxy-11-dodece	0.017977639	-0.008126825	Metabolome
Histidyltryptophan	-0.024242798	-0.002493766	Metabolome
(2R,3S)-2-methylisocitrate	-0.015588734	0.002591483	Metabolome
2-succinylbenzoate	-0.041414231	-0.001881535	Metabolome
Glutarimide	-0.005800716	-0.009513825	Metabolome
Lenticin	-0.025247871	0.002440366	Metabolome
Glutaminylhydroxyproline	-0.013849101	0.04364677	Metabolome
Ethyl 7-epi-12-hydroxyjasmonate gluc	0.000637302	0.009792801	Metabolome
Sec-o-Glucosylhamaudol	0.001419228	-0.004401436	Metabolome
2-Furanmethanol	-0.014210259	0.002625611	Metabolome
Quercetin 3-O-glucoside	0.01135914	-0.007409482	Metabolome
17-beta-Estradiol glucuronide	0.038704528	0.006470153	Metabolome
Cadaverine	-0.019413311	0.013695819	Metabolome
N-[1-(cyclobutanecarbonyl)piperidin-4	-0.057713089	-0.005897907	Metabolome
Integerrimine	0.023213257	-0.091662821	Metabolome
Glycyl-Histidine	0.011855013	-0.003524501	Metabolome
Trovafloxacin	0.01670502	-0.004067076	Metabolome
1-Methyl-3-(2-oxopropylidene)indol-	-0.018790875	-0.009494973	Metabolome
Xylobiose	-0.014686823	-0.00436225	Metabolome
1-Methyl-5-nitro-1H-imidazole-2-me	-0.017089174	0.020725812	Metabolome
3-Hydroxycinnamoylglycine sulfate	0.010493071	0.016143705	Metabolome

L-histidinol-phosphate	0.008032101	0.063118019	Metabolome
Fenoxaprop	0.003288382	0.005972172	Metabolome
4-Hydroxy-4-(methylnitrosoamino)-1-	2.50E-05	-0.002734653	Metabolome
Picraquassioside D	0.034446802	-0.003890897	Metabolome
Coniferaldehyde	-0.022513515	-0.001851633	Metabolome
Cyclo(D-trp-D-asp-pro-D-val-leu)	-0.025584964	-0.000518511	Metabolome
10-Acetoxytoxol	-0.022895045	-0.00648628	Metabolome
Houttuynin	0.033980288	0.000170895	Metabolome
Piridronic acid	0.060957632	0.027351414	Metabolome
Glycinexylidide	-0.056448559	-0.000964716	Metabolome
7-Hydroxyflumequine	-0.001566516	-0.005843711	Metabolome
Parvaquone	-0.007979527	0.025639208	Metabolome
Saligenin-Beta-D-Glucopyranoside	0.03627165	-0.000668377	Metabolome
1,7-Dimethylguanosine	0.0180492	-0.003924283	Metabolome
Benzoyl glucuronide (Benzoic acid)	-0.015076256	-0.020073311	Metabolome
L-beta-aspartyl-L-phenylalanine	0.011102582	-0.007565072	Metabolome
Senkyunolide	-0.033502162	-0.054288431	Metabolome
6-Aminoindazole	-0.006130009	-0.00449695	Metabolome
N-[[(2S,3S,4R,5R)-5-(6-Aminopurin-9-	0.003443189	-0.012149827	Metabolome
Methionyl-Aspartate	-0.041272993	-0.000181868	Metabolome
(1S,2R,4R,8S)-p-Menthane-2,8,9-triol	-0.059125466	-0.000449268	Metabolome
3,5-Dichloro-L-tyrosine	0.061543506	0.035034071	Metabolome
Lotaustralin	0.014188103	-0.006864111	Metabolome
Tafamidis	0.029195441	0.001154903	Metabolome
Menthyl ethylene glycol carbonate	-0.048032481	0.019656631	Metabolome
N-[(1-Chloro-4-hydroxyisoquinolin-3	-0.007693262	0.001181233	Metabolome
N6-Methyl-2'-deoxyadenosine	-0.013186526	-0.003776596	Metabolome
7,8-dihydromonapterin	0.051799646	-0.000661229	Metabolome
4-Oxo-1-(3-pyridyl)-1-butanone	0.014219204	0.006903805	Metabolome
Aminofructose 6-phosphate	0.02502977	-0.003967021	Metabolome
3-Chloro-L-Tyrosine	-0.015941916	0.070310493	Metabolome
1-(3-Ethyl-2,4-dihydroxy-6-methoxyp	-0.012753629	-0.004012076	Metabolome
4-Methylbenzoic Acid	-0.017436798	-0.07365577	Metabolome
Thiamylal	-0.015416831	0.023883094	Metabolome
N-lactoyl-Tryptophan	0.029576948	0.011368901	Metabolome
1-Piperideine-2-carboxylic acid	0.023664596	0.02291028	Metabolome
2beta,9xi-Dihydroxy-8-oxo-1(10),4,11	0.07061787	-0.002759433	Metabolome
Hept-4-enedioylcarnitine	-0.01615779	-0.013400101	Metabolome
2,3,4,5-Tetrahydro-2-pyridinecarboxyl	-0.005289223	0.041045312	Metabolome
1-O-Caffeoylglucose	-0.01955604	-0.004297223	Metabolome
6-Amino-2-methyl-2H-1,3-benzoxazi	0.026338942	0.045995989	Metabolome
Ellagic acid acetyl-arabinoside	-0.000364424	-0.002380202	Metabolome
Fluprostenol	0.026882221	-0.00706519	Metabolome
1-Naphthyl sulfate	0.032741469	-0.006617222	Metabolome
4-[(4-methylphenyl)methyl]-N-(2-phe	-0.025345054	-0.010126109	Metabolome
2,3,4,5-Tetrahydroxy-6-(1,2,3,4-tetrah	-0.008057501	-0.006010834	Metabolome
(1R)-Glutathionyl-(2R)-hydroxy-1,2-di	0.009182489	-0.004654231	Metabolome
Domoic acid	-0.009670797	-0.019972274	Metabolome
Cis-p-Coumaric acid sulfate	0.003970841	0.002613222	Metabolome
N-(2,6-Difluorophenyl)-2-oxoglycyl-N	0.007505407	-0.003490389	Metabolome
O-Ethyl-4-[(2',3',4'-tri-O-acetyl-alpha	0.02350776	-0.010019495	Metabolome
L-cis-Cyclo(aspartylphenylalanyl)	0.001419228	-0.004401436	Metabolome
5-Aminolevulinic acid	-0.007161389	-0.012096502	Metabolome
N-(2-Pyrimidinyl)formamide	0.020577315	-0.006819319	Metabolome
Gamma-L-Glutamyl-L-pipecolic acid	0.036383236	0.012431657	Metabolome
Epicylindrospermopsin	0.049780068	-0.019447871	Metabolome
Methionyl-Methionine	-0.023378739	-0.002534548	Metabolome
(2E,8E)-10-hydroxydeca-2,8-dien-4-y	0.001179529	-0.004363427	Metabolome

DIMBOA-Glc	0.025532434	-0.008681202	Metabolome
1-(beta-D-Ribofuranosyl)-1,4-dihydrc	0.04871723	-0.006468992	Metabolome
Quinoneimine	-0.065667395	-0.026134184	Metabolome
(6S)-Tetrahydro-L-biopterin	0.04894435	-0.008624182	Metabolome
1-Deoxy-1-morpholino-D-fructose	0.019405152	-0.006581229	Metabolome
Gallocatechin	0.012555893	-0.008462135	Metabolome
Linocinnamarin	-0.025522891	-0.01343671	Metabolome
Citalopram aldehyde	0.025053498	-0.019589018	Metabolome
Mergepta	0.093207279	-0.001047085	Metabolome
1-Methyluric acid	0.011379537	-0.016951543	Metabolome
N-Palmitoyl Cysteine	0.003877468	0.002907899	Metabolome
8-Hydroxy-7-methylguanine	0.009393083	-0.026335315	Metabolome
N-Acetylneuraminic acid	0.021832277	-0.003619959	Metabolome
Plumbagin	0.021265114	0.020515556	Metabolome
Neolinustatin	-0.035649087	-0.001984203	Metabolome
L-Dopa	0.017821338	-0.0408649	Metabolome
Libenzapril	-0.040749684	-0.001831154	Metabolome
Urolithin B 3-O-glucuronide	0.019373616	-0.003690126	Metabolome
(1R)-Hydroxy-(2R)-glutathionyl-1,2-d	-0.070922977	-0.000108575	Metabolome
Furylacryloylalanyllysine	-0.039522104	-0.00578037	Metabolome
Nicotinuric Acid	0.025372286	-0.004661244	Metabolome
Imipenem	-0.037777949	0.058402558	Metabolome
Acetamidopropanal	0.00095855	-0.00309189	Metabolome
Salicin	0.012258134	-0.009295597	Metabolome
Citreoviridin	0.028958537	-0.004744473	Metabolome
