# Supplementary material for: Comparative Proteomics and Metabonomics Analysis of Different Diapause Stages Revealed a New Regulation Mechanism of Diapause in Loxostege sticticalis (Lepidoptera: Pyralidae)
Source: Molecules. 2024 Jul 25;29(15):3472. doi: 10.3390/molecules29153472 (PMC11314584; doi:10.3390/molecules29153472)
Supplement: Supplementary file 1 [file molecules-29-03472-s001.zip › analysis process/Joint analysis of proteomics and metabolomics/CTvsD.paths.Venn.pdf]

Pathway_Id	proteome	metabolome
map00010	1	1
map00020	1	1
map00030	1	1
map00040	1	1
map00051	1	1
map00052	1	1
map00053	1	1
map00061	1	1
map00062	1	1
map00071	1	1
map00073	1	1
map00120	1	0
map00130	1	1
map00140	1	1
map00190	1	1
map00220	0	1
map00230	1	1
map00232	1	1
map00240	1	1
map00250	1	1
map00260	1	1
map00261	0	1
map00270	1	1
map00280	1	1
map00290	0	1
map00300	1	1
map00310	1	1
map00311	0	1
map00330	1	1
map00340	1	1
map00350	1	1
map00360	1	1
map00380	1	1
map00400	0	1
map00410	1	1
map00430	0	1
map00450	1	0
map00470	0	1
map00480	1	1
map00500	1	1
map00510	1	0
map00511	1	0
map00512	1	0
map00514	1	0
map00520	1	1
map00531	1	0
map00532	1	1
map00534	0	1
map00561	1	1
map00562	1	1
map00563	0	1
map00564	1	1
map00565	0	1
map00590	1	1
map00591	0	1
map00592	1	1
map00600	1	1

map00620	1	1
map00630	1	1
map00640	1	1
map00650	1	1
map00660	0	1
map00670	1	1
map00730	1	0
map00740	1	1
map00750	1	1
map00760	1	1
map00770	1	1
map00780	0	1
map00785	0	1
map00790	1	1
map00830	1	0
map00860	1	1
map00900	1	0
map00910	0	1
map00920	1	1
map00970	1	1
map00980	1	1
map00981	1	1
map00982	1	1
map00983	1	1
map01040	1	1
map01232	1	1
map01240	1	1
map01250	1	1
map01521	1	1
map01523	1	1
map01524	1	0
map02010	1	1
map03008	1	0
map03010	1	0
map03013	1	0
map03015	1	0
map03018	1	0
map03020	1	0
map03030	1	0
map03040	1	0
map03050	1	0
map03250	1	0
map03320	1	0
map03410	1	0
map03420	1	0
map03430	1	0
map03440	1	0
map03450	1	0
map04010	1	1
map04012	0	1
map04014	1	1
map04015	1	1
map04020	1	1
map04022	1	1
map04024	1	1
map04062	1	1
map04064	1	1
map04066	1	1

map04068	0	1
map04070	1	0
map04071	1	1
map04072	1	1
map04080	1	1
map04110	1	0
map04114	1	0
map04120	1	0
map04130	1	0
map04136	1	1
map04137	1	0
map04140	1	1
map04141	1	0
map04142	1	1
map04144	1	1
map04145	1	0
map04146	1	0
map04150	1	1
map04151	1	1
map04152	1	1
map04210	1	0
map04211	1	1
map04212	1	0
map04213	1	0
map04214	1	1
map04215	1	0
map04216	1	1
map04217	1	0
map04260	1	0
map04261	1	1
map04270	1	1
map04320	1	0
map04340	1	0
map04341	1	0
map04350	1	0
map04360	1	0
map04361	1	1
map04370	0	1
map04371	1	1
map04380	1	0
map04390	1	0
map04391	1	0
map04510	1	0
map04512	1	0
map04514	1	0
map04520	1	0
map04530	1	0
map04540	1	1
map04611	1	1
map04612	1	0
map04613	1	1
map04614	1	0
map04620	1	0
map04621	1	0
map04622	1	0
map04623	1	0
map04624	1	0
map04625	1	1

map04640	1	0
map04650	0	1
map04657	1	0
map04658	1	1
map04659	1	1
map04660	1	1
map04662	1	1
map04664	0	1
map04666	1	1
map04668	1	0
map04670	1	0
map04711	1	0
map04713	1	1
map04714	1	1
map04720	1	1
map04721	1	1
map04722	1	1
map04723	1	1
map04724	1	1
map04725	1	1
map04726	1	1
map04727	0	1
map04728	1	1
map04730	1	1
map04740	0	1
map04742	0	1
map04745	1	1
map04750	1	1
map04810	1	0
map04910	1	0
map04911	1	1
map04912	1	1
map04913	1	0
map04915	1	1
map04916	1	1
map04917	0	1
map04918	1	1
map04919	1	1
map04920	1	1
map04921	1	1
map04922	1	1
map04923	1	1
map04924	1	1
map04925	1	1
map04926	1	1
map04927	1	1
map04928	1	1
map04929	1	1
map04930	1	0
map04931	1	1
map04932	1	0
map04933	1	1
map04934	1	1
map04935	1	1
map04936	1	1
map04940	1	0
map04960	1	0
map04961	1	1

map04962	1	0
map04964	1	1
map04970	1	1
map04971	1	1
map04972	1	1
map04973	1	1
map04974	1	1
map04975	1	1
map04976	1	1
map04977	1	1
map04978	1	1
map04979	1	1
map05010	1	0
map05012	1	1
map05014	1	1
map05016	1	0
map05017	1	1
map05020	1	0
map05022	1	1
map05030	0	1
map05031	0	1
map05032	1	1
map05034	1	1
map05100	1	0
map05110	1	1
map05120	1	0
map05130	1	1
map05131	1	1
map05132	1	0
map05133	1	0
map05134	1	0
map05135	1	0
map05140	1	1
map05142	1	0
map05143	1	1
map05144	1	0
map05145	1	0
map05146	1	1
map05152	1	0
map05160	1	0
map05161	1	0
map05162	1	0
map05163	1	1
map05164	1	0
map05165	1	0
map05166	1	0
map05167	1	1
map05168	1	0
map05169	1	0
map05170	1	1
map05171	1	1
map05200	1	1
map05202	1	0
map05203	1	0
map05204	1	1
map05205	1	0
map05206	1	0
map05207	1	0

map05208	1	1
map05211	1	1
map05214	0	1
map05215	1	0
map05220	1	0
map05221	1	0
map05222	1	0
map05223	0	1
map05225	1	1
map05226	1	0
map05230	1	1
map05231	0	1
map05235	1	1
map05322	1	1
map05323	1	0
map05340	1	0
map05410	1	0
map05412	1	0
map05414	1	0
map05415	1	1
map05417	1	1
map05418	1	0
