# Supplementary material for: Comparative Proteomics and Metabonomics Analysis of Different Diapause Stages Revealed a New Regulation Mechanism of Diapause in Loxostege sticticalis (Lepidoptera: Pyralidae)
Source: Molecules. 2024 Jul 25;29(15):3472. doi: 10.3390/molecules29153472 (PMC11314584; doi:10.3390/molecules29153472)
Supplement: Supplementary file 1 [file molecules-29-03472-s001.zip › analysis process/Joint analysis of proteomics and metabolomics/CTvsD.pathway.detail.pdf]

Pathway_Id	Pathway_definition	PathwaytypeII	PathwaytypeI	number_of_pros	number_of_metas
map03010	Ribosome	Translation	Genetic Information Processing	23	0
map05171	Coronavirus disease - COVID-19	Infectious disease: viral	Human Diseases	21	1
map00480	Glutathione metabolism	Metabolism of other amino acids	Metabolism	19	6
map04142	Lysosome	Transport and catabolism	Cellular Processes	19	1
map00980	Metabolism of xenobiotics by cytochrome P450	Xenobiotics biodegradation and metabolism	Metabolism	18	4
map00983	Drug metabolism - other enzymes	Xenobiotics biodegradation and metabolism	Metabolism	17	2
map00982	Drug metabolism - cytochrome P450	Xenobiotics biodegradation and metabolism	Metabolism	17	5
map05204	Chemical carcinogenesis - DNA adducts	Cancer: overview	Human Diseases	16	1
map05200	Pathways in cancer	Cancer: overview	Human Diseases	16	3
map05014	Amyotrophic lateral sclerosis	Neurodegenerative disease	Human Diseases	16	1
map05022	Pathways of neurodegeneration - multiple diseases	Neurodegenerative disease	Human Diseases	15	3
map05208	Chemical carcinogenesis - reactive oxygen species	Cancer: overview	Human Diseases	14	1
map03320	PPAR signaling pathway	Endocrine system	Organismal Systems	13	0
map04212	Longevity regulating pathway - worm	Aging	Organismal Systems	13	0
map05207	Chemical carcinogenesis - receptor activation	Cancer: overview	Human Diseases	13	0
map00040	Pentose and glucuronate interconversions	Carbohydrate metabolism	Metabolism	13	5
map04141	Protein processing in endoplasmic reticulum	Folding, sorting and degradation	Genetic Information Processing	12	0
map05010	Alzheimer disease	Neurodegenerative disease	Human Diseases	12	0
map05016	Huntington disease	Neurodegenerative disease	Human Diseases	12	0
map04936	Alcoholic liver disease	Endocrine and metabolic disease	Human Diseases	11	1
map03040	Spliceosome	Transcription	Genetic Information Processing	11	0
map04976	Bile secretion	Digestive system	Organismal Systems	10	8
map05132	Salmonella infection	Infectious disease: bacterial	Human Diseases	10	0
map04146	Peroxisome	Transport and catabolism	Cellular Processes	10	0
map05012	Parkinson disease	Neurodegenerative disease	Human Diseases	10	6
map00590	Arachidonic acid metabolism	Lipid metabolism	Metabolism	10	3
map05131	Shigellosis	Infectious disease: bacterial	Human Diseases	9	1
map05225	Hepatocellular carcinoma	Cancer: specific types	Human Diseases	9	2
map01524	Platinum drug resistance	Drug resistance: antineoplastic	Human Diseases	9	0
map00860	Porphyrin metabolism	Metabolism of cofactors and vitamins	Metabolism	9	1
map05165	Human papillomavirus infection	Infectious disease: viral	Human Diseases	9	0
map04714	Thermogenesis	Environmental adaptation	Organismal Systems	9	3
map00053	Ascorbate and aldarate metabolism	Carbohydrate metabolism	Metabolism	9	6
map04974	Protein digestion and absorption	Digestive system	Organismal Systems	9	7
map05418	Fluid shear stress and atherosclerosis	Cardiovascular disease	Human Diseases	9	0
map04152	AMPK signaling pathway	Signal transduction	Environmental Information Proc	9	3
map00230	Purine metabolism	Nucleotide metabolism	Metabolism	8	16
map04213	Longevity regulating pathway - multiple species	Aging	Organismal Systems	8	0
map03013	Nucleocytoplasmic transport	Translation	Genetic Information Processing	8	0
map04144	Endocytosis	Transport and catabolism	Cellular Processes	8	1
map00350	Tyrosine metabolism	Amino acid metabolism	Metabolism	8	5
map04145	Phagosome	Transport and catabolism	Cellular Processes	7	0
map05164	Influenza A	Infectious disease: viral	Human Diseases	7	0
map04624	Toll and Imd signaling pathway	Immune system	Organismal Systems	7	0

map04910	Insulin signaling pathway	Endocrine system	Organismal Systems	7	0
map00561	Glycerolipid metabolism	Lipid metabolism	Metabolism	7	4
map00030	Pentose phosphate pathway	Carbohydrate metabolism	Metabolism	7	3
map05415	Diabetic cardiomyopathy	Cardiovascular disease	Human Diseases	7	3
map00790	Folate biosynthesis	Metabolism of cofactors and vitamins	Metabolism	7	2
map04614	Renin-angiotensin system	Endocrine system	Organismal Systems	7	0
map05203	Viral carcinogenesis	Cancer: overview	Human Diseases	6	0
map05202	Transcriptional misregulation in cancer	Cancer: overview	Human Diseases	6	0
map00071	Fatty acid degradation	Lipid metabolism	Metabolism	6	1
map05206	MicroRNAs in cancer	Cancer: overview	Human Diseases	6	0
map00310	Lysine degradation	Amino acid metabolism	Metabolism	6	6
map04151	PI3K-Akt signaling pathway	Signal transduction	Environmental Information Proc	6	1
map00630	Glyoxylate and dicarboxylate metabolism	Carbohydrate metabolism	Metabolism	6	6
map00051	Fructose and mannose metabolism	Carbohydrate metabolism	Metabolism	6	2
map03018	RNA degradation	Folding, sorting and degradation	Genetic Information Processing	6	0
map00830	Retinol metabolism	Metabolism of cofactors and vitamins	Metabolism	6	0
map05020	Prion disease	Neurodegenerative disease	Human Diseases	6	0
map00010	Glycolysis / Gluconeogenesis	Carbohydrate metabolism	Metabolism	6	2
map00981	Insect hormone biosynthesis	Metabolism of terpenoids and polyketides	Metabolism	6	1
map05130	Pathogenic Escherichia coli infection	Infectious disease: bacterial	Human Diseases	6	1
map00260	Glycine, serine and threonine metabolism	Amino acid metabolism	Metabolism	6	6
map05146	Amoebiasis	Infectious disease: parasitic	Human Diseases	6	2
map01040	Biosynthesis of unsaturated fatty acids	Lipid metabolism	Metabolism	6	2
map00531	Glycosaminoglycan degradation	Glycan biosynthesis and metabolism	Metabolism	6	0
map04640	Hematopoietic cell lineage	Immune system	Organismal Systems	6	0
map04510	Focal adhesion	Cellular community - eukaryotes	Cellular Processes	5	0
map05205	Proteoglycans in cancer	Cancer: overview	Human Diseases	5	0
map00190	Oxidative phosphorylation	Energy metabolism	Metabolism	5	1
map00620	Pyruvate metabolism	Carbohydrate metabolism	Metabolism	5	2
map00330	Arginine and proline metabolism	Amino acid metabolism	Metabolism	5	4
map04922	Glucagon signaling pathway	Endocrine system	Organismal Systems	5	3
map04217	Necroptosis	Cell growth and death	Cellular Processes	5	0
map05017	Spinocerebellar ataxia	Neurodegenerative disease	Human Diseases	5	1
map05168	Herpes simplex virus 1 infection	Infectious disease: viral	Human Diseases	5	0
map01232	Nucleotide metabolism	Global and overview maps	Metabolism	5	13
map05160	Hepatitis C	Infectious disease: viral	Human Diseases	5	0
map05166	Human T-cell leukemia virus 1 infection	Infectious disease: viral	Human Diseases	5	0
map04611	Platelet activation	Immune system	Organismal Systems	5	2
map04150	mTOR signaling pathway	Signal transduction	Environmental Information Proc	5	1
map04621	NOD-like receptor signaling pathway	Immune system	Organismal Systems	5	0
map00130	Ubiquinone and other terpenoid-quinone biosynthe	Metabolism of cofactors and vitamins	Metabolism	5	3
map00760	Nicotinate and nicotinamide metabolism	Metabolism of cofactors and vitamins	Metabolism	5	2
map00600	Sphingolipid metabolism	Lipid metabolism	Metabolism	5	3
map00140	Steroid hormone biosynthesis	Lipid metabolism	Metabolism	5	1
map04216	Ferroptosis	Cell growth and death	Cellular Processes	5	1

map05170	Human immunodeficiency virus 1 infection	Infectious disease: viral	Human Diseases	4	1
map05135	Yersinia infection	Infectious disease: bacterial	Human Diseases	4	0
map04066	HIF-1 signaling pathway	Signal transduction	Environmental Information Proc	4	2
map04925	Aldosterone synthesis and secretion	Endocrine system	Organismal Systems	4	3
map04530	Tight junction	Cellular community - eukaryotes	Cellular Processes	4	0
map03008	Ribosome biogenesis in eukaryotes	Translation	Genetic Information Processing	4	0
map00380	Tryptophan metabolism	Amino acid metabolism	Metabolism	4	10
map05134	Legionellosis	Infectious disease: bacterial	Human Diseases	4	0
map00410	beta-Alanine metabolism	Metabolism of other amino acids	Metabolism	4	1
map00052	Galactose metabolism	Carbohydrate metabolism	Metabolism	4	2
map04391	Hippo signaling pathway - fly	Signal transduction	Environmental Information Proc	4	0
map04972	Pancreatic secretion	Digestive system	Organismal Systems	4	1
map00500	Starch and sucrose metabolism	Carbohydrate metabolism	Metabolism	4	1
map05417	Lipid and atherosclerosis	Cardiovascular disease	Human Diseases	4	1
map05163	Human cytomegalovirus infection	Infectious disease: viral	Human Diseases	4	1
map00564	Glycerophospholipid metabolism	Lipid metabolism	Metabolism	4	12
map04210	Apoptosis	Cell growth and death	Cellular Processes	4	0
map04371	Apelin signaling pathway	Signal transduction	Environmental Information Proc	4	1
map04015	Rap1 signaling pathway	Signal transduction	Environmental Information Proc	4	2
map04979	Cholesterol metabolism	Digestive system	Organismal Systems	4	1
map05145	Toxoplasmosis	Infectious disease: parasitic	Human Diseases	4	0
map05162	Measles	Infectious disease: viral	Human Diseases	4	0
map05152	Tuberculosis	Infectious disease: bacterial	Human Diseases	4	0
map00970	Aminoacyl-tRNA biosynthesis	Translation	Genetic Information Processing	4	4
map04918	Thyroid hormone synthesis	Endocrine system	Organismal Systems	4	2
map04512	ECM-receptor interaction	Signaling molecules and interaction	Environmental Information Proc	4	0
map00340	Histidine metabolism	Amino acid metabolism	Metabolism	4	4
map04612	Antigen processing and presentation	Immune system	Organismal Systems	4	0
map00061	Fatty acid biosynthesis	Lipid metabolism	Metabolism	4	1
map05222	Small cell lung cancer	Cancer: specific types	Human Diseases	4	0
map04622	RIG-I-like receptor signaling pathway	Immune system	Organismal Systems	4	0
map05142	Chagas disease	Infectious disease: parasitic	Human Diseases	4	0
map04810	Regulation of actin cytoskeleton	Cell motility	Cellular Processes	3	0
map00270	Cysteine and methionine metabolism	Amino acid metabolism	Metabolism	3	4
map05161	Hepatitis B	Infectious disease: viral	Human Diseases	3	0
map05169	Epstein-Barr virus infection	Infectious disease: viral	Human Diseases	3	0
map05410	Hypertrophic cardiomyopathy	Cardiovascular disease	Human Diseases	3	0
map04723	Retrograde endocannabinoid signaling	Nervous system	Organismal Systems	3	4
map04062	Chemokine signaling pathway	Immune system	Organismal Systems	3	1
map04975	Fat digestion and absorption	Digestive system	Organismal Systems	3	2
map04970	Salivary secretion	Digestive system	Organismal Systems	3	1
map03250	Viral life cycle - HIV-1	Information processing in viruses	Genetic Information Processing	3	0
map04932	Non-alcoholic fatty liver disease	Endocrine and metabolic disease	Human Diseases	3	0
map04390	Hippo signaling pathway	Signal transduction	Environmental Information Proc	3	0
map00240	Pyrimidine metabolism	Nucleotide metabolism	Metabolism	3	6

map00640	Propanoate metabolism	Carbohydrate metabolism	Metabolism	3	3
map00280	Valine, leucine and isoleucine degradation	Amino acid metabolism	Metabolism	3	1
map04024	cAMP signaling pathway	Signal transduction	Environmental Information Proc	3	4
map05034	Alcoholism	Substance dependence	Human Diseases	3	3
map03015	mRNA surveillance pathway	Translation	Genetic Information Processing	3	0
map00250	Alanine, aspartate and glutamate metabolism	Amino acid metabolism	Metabolism	3	5
map04140	Autophagy - animal	Transport and catabolism	Cellular Processes	3	2
map04120	Ubiquitin mediated proteolysis	Folding, sorting and degradation	Genetic Information Processing	3	0
map04260	Cardiac muscle contraction	Circulatory system	Organismal Systems	3	0
map01250	Biosynthesis of nucleotide sugars	Global and overview maps	Metabolism	3	6
map05167	Kaposi sarcoma-associated herpesvirus infection	Infectious disease: viral	Human Diseases	3	2
map04926	Relaxin signaling pathway	Endocrine system	Organismal Systems	3	1
map04668	TNF signaling pathway	Signal transduction	Environmental Information Proc	3	0
map04934	Cushing syndrome	Endocrine and metabolic disease	Human Diseases	3	1
map04920	Adipocytokine signaling pathway	Endocrine system	Organismal Systems	3	1
map00360	Phenylalanine metabolism	Amino acid metabolism	Metabolism	3	3
map05140	Leishmaniasis	Infectious disease: parasitic	Human Diseases	3	1
map04613	Neutrophil extracellular trap formation	Immune system	Organismal Systems	3	1
map04927	Cortisol synthesis and secretion	Endocrine system	Organismal Systems	3	1
map00670	One carbon pool by folate	Metabolism of cofactors and vitamins	Metabolism	3	1
map04014	Ras signaling pathway	Signal transduction	Environmental Information Proc	2	2
map04722	Neurotrophin signaling pathway	Nervous system	Organismal Systems	2	1
map04670	Leukocyte transendothelial migration	Immune system	Organismal Systems	2	0
map04931	Insulin resistance	Endocrine and metabolic disease	Human Diseases	2	2
map04261	Adrenergic signaling in cardiomyocytes	Circulatory system	Organismal Systems	2	1
map05120	Epithelial cell signaling in Helicobacter pylori infecti	Infectious disease: bacterial	Human Diseases	2	0
map04921	Oxytocin signaling pathway	Endocrine system	Organismal Systems	2	1
map04915	Estrogen signaling pathway	Endocrine system	Organismal Systems	2	1
map00520	Amino sugar and nucleotide sugar metabolism	Carbohydrate metabolism	Metabolism	2	6
map04214	Apoptosis - fly	Cell growth and death	Cellular Processes	2	1
map04919	Thyroid hormone signaling pathway	Endocrine system	Organismal Systems	2	1
map04071	Sphingolipid signaling pathway	Signal transduction	Environmental Information Proc	2	4
map05100	Bacterial invasion of epithelial cells	Infectious disease: bacterial	Human Diseases	2	0
map04022	cGMP-PKG signaling pathway	Signal transduction	Environmental Information Proc	2	2
map05414	Dilated cardiomyopathy	Cardiovascular disease	Human Diseases	2	0
map00562	Inositol phosphate metabolism	Carbohydrate metabolism	Metabolism	2	1
map04270	Vascular smooth muscle contraction	Circulatory system	Organismal Systems	2	2
map04971	Gastric acid secretion	Digestive system	Organismal Systems	2	1
map04020	Calcium signaling pathway	Signal transduction	Environmental Information Proc	2	1
map04620	Toll-like receptor signaling pathway	Immune system	Organismal Systems	2	0
map04924	Renin secretion	Endocrine system	Organismal Systems	2	3
map02010	ABC transporters	Membrane transport	Environmental Information Proc	2	13
map05322	Systemic lupus erythematosus	Immune disease	Human Diseases	2	1
map05230	Central carbon metabolism in cancer	Cancer: overview	Human Diseases	2	7
map05323	Rheumatoid arthritis	Immune disease	Human Diseases	2	0

map04913	Ovarian steroidogenesis	Endocrine system	Organismal Systems	2	0
map04136	Autophagy - other	Transport and catabolism	Cellular Processes	2	1
map05226	Gastric cancer	Cancer: specific types	Human Diseases	2	0
map04341	Hedgehog signaling pathway - fly	Signal transduction	Environmental Information Proc	2	0
map04725	Cholinergic synapse	Nervous system	Organismal Systems	2	1
map00120	Primary bile acid biosynthesis	Lipid metabolism	Metabolism	2	0
map00514	Other types of O-glycan biosynthesis	Glycan biosynthesis and metabolism	Metabolism	2	0
map04977	Vitamin digestion and absorption	Digestive system	Organismal Systems	2	2
map05144	Malaria	Infectious disease: parasitic	Human Diseases	2	0
map00511	Other glycan degradation	Glycan biosynthesis and metabolism	Metabolism	2	0
map04750	Inflammatory mediator regulation of TRP channels	Sensory system	Organismal Systems	2	1
map04933	AGE-RAGE signaling pathway in diabetic complicati	Endocrine and metabolic disease	Human Diseases	2	1
map04514	Cell adhesion molecules	Signaling molecules and interaction	Environmental Information Proc	2	0
map00920	Sulfur metabolism	Energy metabolism	Metabolism	2	2
map00750	Vitamin B6 metabolism	Metabolism of cofactors and vitamins	Metabolism	2	1
map04961	Endocrine and other factor-regulated calcium reabs	Excretory system	Organismal Systems	2	1
map04962	Vasopressin-regulated water reabsorption	Excretory system	Organismal Systems	2	0
map04978	Mineral absorption	Digestive system	Organismal Systems	2	4
map04657	IL-17 signaling pathway	Immune system	Organismal Systems	2	0
map04911	Insulin secretion	Endocrine system	Organismal Systems	2	1
map03030	DNA replication	Replication and repair	Genetic Information Processing	2	0
map04010	MAPK signaling pathway	Signal transduction	Environmental Information Proc	1	1
map04728	Dopaminergic synapse	Nervous system	Organismal Systems	1	4
map05110	Vibrio cholerae infection	Infectious disease: bacterial	Human Diseases	1	1
map00770	Pantothenate and CoA biosynthesis	Metabolism of cofactors and vitamins	Metabolism	1	3
map00510	N-Glycan biosynthesis	Glycan biosynthesis and metabolism	Metabolism	1	0
map04114	Oocyte meiosis	Cell growth and death	Cellular Processes	1	0
map04520	Adherens junction	Cellular community - eukaryotes	Cellular Processes	1	0
map04721	Synaptic vesicle cycle	Nervous system	Organismal Systems	1	1
map00020	Citrate cycle (TCA cycle)	Carbohydrate metabolism	Metabolism	1	2
map04360	Axon guidance	Development and regeneration	Organismal Systems	1	0
map03050	Proteasome	Folding, sorting and degradation	Genetic Information Processing	1	0
map04666	Fc gamma R-mediated phagocytosis	Immune system	Organismal Systems	1	1
map04072	Phospholipase D signaling pathway	Signal transduction	Environmental Information Proc	1	1
map04916	Melanogenesis	Endocrine system	Organismal Systems	1	1
map03450	Non-homologous end-joining	Replication and repair	Genetic Information Processing	1	0
map05340	Primary immunodeficiency	Immune disease	Human Diseases	1	0
map05220	Chronic myeloid leukemia	Cancer: specific types	Human Diseases	1	0
map04711	Circadian rhythm - fly	Environmental adaptation	Organismal Systems	1	0
map04912	GnRH signaling pathway	Endocrine system	Organismal Systems	1	1
map01521	EGFR tyrosine kinase inhibitor resistance	Drug resistance: antineoplastic	Human Diseases	1	1
map03420	Nucleotide excision repair	Replication and repair	Genetic Information Processing	1	0
map05412	Arrhythmogenic right ventricular cardiomyopathy	Cardiovascular disease	Human Diseases	1	0
map00730	Thiamine metabolism	Metabolism of cofactors and vitamins	Metabolism	1	0
map00740	Riboflavin metabolism	Metabolism of cofactors and vitamins	Metabolism	1	1

map05143	African trypanosomiasis	Infectious disease: parasitic	Human Diseases	1	2
map04960	Aldosterone-regulated sodium reabsorption	Excretory system	Organismal Systems	1	0
map05215	Prostate cancer	Cancer: specific types	Human Diseases	1	0
map04940	Type I diabetes mellitus	Endocrine and metabolic disease	Human Diseases	1	0
map01523	Antifolate resistance	Drug resistance: antineoplastic	Human Diseases	1	3
map03410	Base excision repair	Replication and repair	Genetic Information Processing	1	0
map04930	Type II diabetes mellitus	Endocrine and metabolic disease	Human Diseases	1	0
map05221	Acute myeloid leukemia	Cancer: specific types	Human Diseases	1	0
map04923	Regulation of lipolysis in adipocytes	Endocrine system	Organismal Systems	1	4
map00512	Mucin type O-glycan biosynthesis	Glycan biosynthesis and metabolism	Metabolism	1	0
map04625	C-type lectin receptor signaling pathway	Immune system	Organismal Systems	1	1
map00450	Selenocompound metabolism	Metabolism of other amino acids	Metabolism	1	0
map00232	Caffeine metabolism	Biosynthesis of other secondary metabolites	Metabolism	1	1
map04080	Neuroactive ligand-receptor interaction	Signaling molecules and interaction	Environmental Information Proc	1	3
map05133	Pertussis	Infectious disease: bacterial	Human Diseases	1	0
map04350	TGF-beta signaling pathway	Signal transduction	Environmental Information Proc	1	0
map04935	Growth hormone synthesis, secretion and action	Endocrine system	Organismal Systems	1	1
map04973	Carbohydrate digestion and absorption	Digestive system	Organismal Systems	1	2
map04215	Apoptosis - multiple species	Cell growth and death	Cellular Processes	1	0
map00062	Fatty acid elongation	Lipid metabolism	Metabolism	1	1
map04720	Long-term potentiation	Nervous system	Organismal Systems	1	1
map04130	SNARE interactions in vesicular transport	Folding, sorting and degradation	Genetic Information Processing	1	0
map04713	Circadian entrainment	Environmental adaptation	Organismal Systems	1	1
map04137	Mitophagy - animal	Transport and catabolism	Cellular Processes	1	0
map04110	Cell cycle	Cell growth and death	Cellular Processes	1	0
map05235	PD-L1 expression and PD-1 checkpoint pathway in	Cancer: overview	Human Diseases	1	1
map04964	Proximal tubule bicarbonate reclamation	Excretory system	Organismal Systems	1	3
map04623	Cytosolic DNA-sensing pathway	Immune system	Organismal Systems	1	0
map04064	NF-kappa B signaling pathway	Signal transduction	Environmental Information Proc	1	1
map05032	Morphine addiction	Substance dependence	Human Diseases	1	3
map04660	T cell receptor signaling pathway	Immune system	Organismal Systems	1	1
map04662	B cell receptor signaling pathway	Immune system	Organismal Systems	1	1
map04724	Glutamatergic synapse	Nervous system	Organismal Systems	1	2
map00073	Cutin, suberine and wax biosynthesis	Lipid metabolism	Metabolism	1	3
map03430	Mismatch repair	Replication and repair	Genetic Information Processing	1	0
map04929	GnRH secretion	Endocrine system	Organismal Systems	1	1
map04070	Phosphatidylinositol signaling system	Signal transduction	Environmental Information Proc	1	0
map04730	Long-term depression	Nervous system	Organismal Systems	1	1
map04361	Axon regeneration	Development and regeneration	Organismal Systems	1	3
map04340	Hedgehog signaling pathway	Signal transduction	Environmental Information Proc	1	0
map04928	Parathyroid hormone synthesis, secretion and actior	Endocrine system	Organismal Systems	1	2
map00900	Terpenoid backbone biosynthesis	Metabolism of terpenoids and polyketides	Metabolism	1	0
map00300	Lysine biosynthesis	Amino acid metabolism	Metabolism	1	1
map04211	Longevity regulating pathway	Aging	Organismal Systems	1	1
map04659	Th17 cell differentiation	Immune system	Organismal Systems	1	1

map04726	Serotonergic synapse	Nervous system	Organismal Systems	1	3
map00532	Glycosaminoglycan biosynthesis - chondroitin sulfat	Glycan biosynthesis and metabolism	Metabolism	1	1
map04540	Gap junction	Cellular community - eukaryotes	Cellular Processes	1	2
map00650	Butanoate metabolism	Carbohydrate metabolism	Metabolism	1	2
map05211	Renal cell carcinoma	Cancer: specific types	Human Diseases	1	1
map04745	Phototransduction - fly	Sensory system	Organismal Systems	1	1
map04380	Osteoclast differentiation	Development and regeneration	Organismal Systems	1	0
map03020	RNA polymerase	Transcription	Genetic Information Processing	1	0
map04658	Th1 and Th2 cell differentiation	Immune system	Organismal Systems	1	1
map03440	Homologous recombination	Replication and repair	Genetic Information Processing	1	0
map00592	alpha-Linolenic acid metabolism	Lipid metabolism	Metabolism	1	4
map04320	Dorso-ventral axis formation	Development and regeneration	Organismal Systems	1	0
map05231	Choline metabolism in cancer	Cancer: overview	Human Diseases	0	10
map00470	D-Amino acid metabolism	Metabolism of other amino acids	Metabolism	0	6
map04742	Taste transduction	Sensory system	Organismal Systems	0	6
map00591	Linoleic acid metabolism	Lipid metabolism	Metabolism	0	4
map00400	Phenylalanine, tyrosine and tryptophan biosynthesis	Amino acid metabolism	Metabolism	0	3
map00220	Arginine biosynthesis	Amino acid metabolism	Metabolism	0	3
map00290	Valine, leucine and isoleucine biosynthesis	Amino acid metabolism	Metabolism	0	2
map04727	GABAergic synapse	Nervous system	Organismal Systems	0	2
map00780	Biotin metabolism	Metabolism of cofactors and vitamins	Metabolism	0	2
map05030	Cocaine addiction	Substance dependence	Human Diseases	0	2
map05031	Amphetamine addiction	Substance dependence	Human Diseases	0	2
map04068	FoxO signaling pathway	Signal transduction	Environmental Information Proc	0	2
map00660	C5-Branched dibasic acid metabolism	Carbohydrate metabolism	Metabolism	0	2
map04917	Prolactin signaling pathway	Endocrine system	Organismal Systems	0	2
map00261	Monobactam biosynthesis	Biosynthesis of other secondary metabolites	Metabolism	0	1
map00430	Taurine and hypotaurine metabolism	Metabolism of other amino acids	Metabolism	0	1
map00565	Ether lipid metabolism	Lipid metabolism	Metabolism	0	1
map00311	Penicillin and cephalosporin biosynthesis	Biosynthesis of other secondary metabolites	Metabolism	0	1
map04740	Olfactory transduction	Sensory system	Organismal Systems	0	1
map00563	Glycosylphosphatidylinositol (GPI)-anchor biosynthe	Glycan biosynthesis and metabolism	Metabolism	0	1
map04664	Fc epsilon RI signaling pathway	Immune system	Organismal Systems	0	1
map00910	Nitrogen metabolism	Energy metabolism	Metabolism	0	1
map05223	Non-small cell lung cancer	Cancer: specific types	Human Diseases	0	1
map04650	Natural killer cell mediated cytotoxicity	Immune system	Organismal Systems	0	1
map00534	Glycosaminoglycan biosynthesis - heparan sulfate /	Glycan biosynthesis and metabolism	Metabolism	0	1
map05214	Glioma	Cancer: specific types	Human Diseases	0	1
map04012	ErbB signaling pathway	Signal transduction	Environmental Information Proc	0	1
map00785	Lipoic acid metabolism	Metabolism of cofactors and vitamins	Metabolism	0	1
map04370	VEGF signaling pathway	Signal transduction	Environmental Information Proc	0	1
