# Supplementary material for: Comparative Proteomics and Metabonomics Analysis of Different Diapause Stages Revealed a New Regulation Mechanism of Diapause in Loxostege sticticalis (Lepidoptera: Pyralidae)
Source: Molecules. 2024 Jul 25;29(15):3472. doi: 10.3390/molecules29153472 (PMC11314584; doi:10.3390/molecules29153472)
Supplement: Supplementary file 1 [file molecules-29-03472-s001.zip › analysis process/metabolic/Data preprocessing/pos.pdf]

ID	Metabolite	Metab ID	Library ID	level	KEGG Compound ID
pos_1	3-[[(2S)-2,4-Dihydroxy-3,3-dimethylbutanoyl]amino]propanoic acid	metab_0	HMDB0250782	B(ii)	-
pos_1003	2-Hydroxy-2-ethylsuccinic acid	metab_6	HMDB0059758	B(ii)	C02488
pos_102	D-Ornithine	metab_24	HMDB0003374;PW_C001864	B(i)	C00515
pos_1035	N-alpha-Acetyl-L-lysine	metab_41	HMDB0000446	B(ii)	C12989
pos_1044	3-Pyridinecarboxaldehyde	metab_51	HMDB0245978	B(ii)	C07327
pos_1048	Policapram	metab_55	HMDB0256664	B(ii)	C19492
pos_105	Farnesyl acetone	metab_57	-	B(i)	-
pos_1057	Acetoacetic acid	metab_64	HMDB0000060;HMDB0304256;PW_C000042;LMFA01060003	B(ii)	C00164
pos_106	PC(18:2/0:0)	metab_67	LMGP01050034;LMGP01050035	B(i)	-
pos_1074	1,3-Propanediamine	metab_83	HMDB0000002	B(i)	C00986
pos_1078	Beta-nicotinate D-ribonucleotide	metab_87	HMDB0304543;HMDB0001132	B(ii)	C01185
pos_1084	(3R)-Sophorol	metab_94	HMDB0301810;LMPK12050461	B(ii)	C16228
pos_1086	Pipecolic Acid	metab_96	HMDB0000716;HMDB0000070;MJDBOTE0000664	B(i)	C00408
pos_1088	Glycyl-Gamma-glutamate	metab_98	HMDB0028855	B(ii)	-
pos_109	PC(16:0/18:3(6Z,9Z,12Z))	metab_100	PW_C004023;HMDB0007974;LMGP01010598	B(ii)	C00157
pos_1097	Penicillin X	metab_108	HMDB0256233	B(ii)	C17404
pos_110	PE(O-18:0/0:0)	metab_112	LMGP02060003	B(i)	-
pos_111	Sebacic acid	metab_121	HMDB0000792;LMFA01170006	B(i)	C08277
pos_1113	Agmatine	metab_124	HMDB0001432;HMDB0246298;MJDBOTE0000469	B(i)	C00179
pos_1115	Phenacetyl-carnitine	metab_126	HMDB0242104	B(ii)	-
pos_1137	Laminine	metab_149	-	B(i)	-
pos_1165	Cyclopentanol	metab_178	HMDB0302463	B(ii)	C02020
pos_1170	N-gamma-Glutamylcysteine	metab_184	HMDB0303807;HMDB0001049	B(ii)	C00669
pos_1176	Taurine aspartate	metab_190	HMDB0258737	B(ii)	-
pos_1177	15-Deacetylneosolaniol	metab_191	HMDB0036157	B(ii)	C02951
pos_1183	Choline Phosphate	metab_197	HMDB0001565	B(i)	C00588
pos_1184	Glycerophosphocholine	metab_198	HMDB0008720;HMDB0008620;HMDB0008587;HMDB0008818;HI	B(i)	C00670
pos_1185	1-Hydroxy-2,5-dioxopyrrolidine-3-sulfonic acid	metab_199	HMDB0243897	B(ii)	-
pos_1186	L-Threonine	metab_200	HMDB0000167;PW_C000109;MJDBOTE0000464	B(i)	C00188
pos_1187	L-Carnitine	metab_201	HMDB0000062;PW_C000044;MJDBOTE0000806	B(i)	C00318
pos_119	Dihydro-5-pentyl-2(3H)-furanone	metab_203	HMDB0031531	B(ii)	C08501
pos_1194	Imidazole-4-Acetic Acid	metab_208	HMDB0002024;MJDBOTE0000591	B(i)	C02835
pos_1197	Trigonelline	metab_210	HMDB0000875	B(i)	C01004
pos_1198	N-[1'-(6-Cyano-1,2,3,4-tetrahydronaphthalen-2-yl)-4-hydroxyspiro[3,4-dihydrochromene-2,4'-piperidine]-6-	metab_211	HMDB0250034	B(ii)	-
pos_1201	5'-Phosphoribosyl-N-formylglycinamidine	metab_216	HMDB0002305	B(ii)	C04640
pos_121	(+/-)-(Z)-2-(5-Tetradecenyl)cyclobutanone	metab_223	HMDB0037543	B(ii)	-
pos_1210	N-Acetyl-L-Histidine	metab_224	HMDB0032055	B(i)	C02997
pos_1211	1-Deoxynojirimycin	metab_225	HMDB0035359	B(ii)	C16843
pos_1212	Glucitol-lysine	metab_226	HMDB0252764	B(ii)	-
pos_1216	L-Glutamic gamma-semialdehyde	metab_229	PW_C001415;HMDB0002104	B(ii)	C01165
pos_1217	Tricholomic acid	metab_230	HMDB0030412	B(ii)	C08298
pos_1227	N-Acetylmannosamine	metab_237	PW_C000883;HMDB0001129	B(ii)	C00645
pos_123	PC(18:3/0:0)	metab_239	HMDB0010388;LMGP01050038	B(i)	-
pos_1234	2-Oxoarginine	metab_242	HMDB0004225;PW_C002094	B(ii)	C03771
pos_1236	Biopiderm	metab_244	HMDB0000030;HMDB0249220	B(ii)	C00120
pos_1239	Beta-D-Xylopyranosyl-(1->4)-alpha-L-rhamnopyranosyl-(1->2)-D-fucose	metab_246	HMDB0041225	B(ii)	-
pos_1246	N-Methyl-L-Threonine	metab_251	-	B(i)	-
pos_1247	Cerulenin	metab_252	HMDB0015168;LMFA08010013	B(ii)	C12058
pos_1248	1,3-Butadiene	metab_253	HMDB0041792	B(ii)	C16450
pos_1250	Lysylglycine	metab_256	HMDB0028951;HMDB0304784	B(ii)	-
pos_1270	Adenosine 3',5'-diphosphate	metab_274	HMDB0000061;PW_C000043	B(ii)	C00054
pos_1278	Beta-Aspartylhydroxamate	metab_282	HMDB0249105;HMDB0032332	B(ii)	C03124
pos_1280	Gamma-Glutamylglutamic acid	metab_284	HMDB0011737	B(ii)	C05282
pos_1291	2-Hydroxyadipic acid	metab_296	HMDB0000321;LMFA01170049	B(ii)	C02360
pos_1292	Betalamic acid	metab_297	HMDB0304273;HMDB0029842	B(ii)	C08538
pos_1293	Cyclic ADP-Ribose	metab_298	HMDB0249529	B(i)	C13050
pos_1295	1-(4-Carboxyphenyl)-3,3-dimethyltriazene	metab_300	HMDB0247318	B(ii)	-
pos_1304	Benzaldehyde	metab_311	HMDB0006115	B(ii)	C00261
pos_1322	Benzoic acid, 2-(acetyloxy)-5-amino-	metab_331	HMDB0247698	B(ii)	-
pos_1327	S-Adenosylmethionine	metab_336	HMDB0001185;PW_C000921	B(ii)	C00019
pos_1329	Paramethasone	metab_338	HMDB0015462	B(ii)	C07413
pos_1332	DL-2,5-Dihydrophenylalanine	metab_342	HMDB0251507	B(ii)	-
pos_1334	Deoxyuridine	metab_344	HMDB0000012;PW_C000007;HMDB0245528;MJDBOTE0000510	B(ii)	C00526
pos_1335	Gamma-L-Glutamyl-L-methionine sulfoxide	metab_345	HMDB0038613	B(ii)	-
pos_1348	2,3,4,5-Tetrahydro-2-pyridinecarboxylic acid	metab_359	HMDB0012130	B(ii)	C00450
pos_1353	Uracil	metab_364	PW_C000204;HMDB0000300;MJDBOTE0000838	B(i)	C00106
pos_1356	Gln Val	metab_367	-	B(i)	-
pos_136	Ricinoleic acid	metab_370	LMFA02000184;HMDB0034297	B(ii)	C08365
pos_1360	5-Hydroxy-L-tryptophan	metab_371	PW_C000358;HMDB0000472	B(ii)	C00643
pos_1362	3-Feruloylquinic acid	metab_373	HMDB0030669	B(ii)	C02572
pos_1363	7-Methylxanthosine	metab_374	HMDB0301918	B(ii)	C16352
pos_1364	Adipate semialdehyde	metab_375	HMDB0012882	B(ii)	C06102
pos_1368	L-3-Hydroxykynurenine	metab_378	HMDB0011631;PW_C007219	B(ii)	C03227
pos_1369	Nicotinuric Acid	metab_379	HMDB0003269	B(i)	C05380
pos_1370	Urea glutamate	metab_381	HMDB0259716	B(ii)	-
pos_1371	NeuNGc	metab_382	HMDB0062502	B(ii)	C03410
pos_1372	Cytidine 5'-monophosphate-N-acetylneuraminic acid	metab_383	HMDB0001176	B(ii)	C00128
pos_1378	Biopterin	metab_388	HMDB0000468;HMDB0000633;HMDB0000817;MJDBOTE000116(	B(i)	C06313
pos_1379	Arg Leu	metab_389	-	B(i)	-
pos_1381	Lucuminamide	metab_392	HMDB0031697	B(ii)	-
pos_1384	Phenethylamine glucuronide	metab_394	HMDB0010323	B(ii)	C03033
pos_1385	Cis-p-Coumaric acid sulfate	metab_395	HMDB0240710	B(ii)	-
pos_1386	Xanthopterin	metab_396	HMDB0259923	B(i)	-
pos_1387	Propionylcarnitine	metab_397	LMFA07070105;HMDB0000824;PW_C000658	B(i)	C03017
pos_1392	CGMP	metab_403	HMDB0001314	B(i)	C00942
pos_1398	5-Amino-1-[(2R,3R,4S,5R)-3,4-dihydroxy-5-(hydroxymethyl)oxolan-2-yl]oxyimidazole-4-carboxamide	metab_408	HMDB0257644	B(ii)	-
pos_1399	(S,S)-Butane-2,3-diol	metab_409	-	B(i)	C03046
pos_1403	Lysyl-Glycine	metab_415	HMDB0028951;HMDB0304784	B(ii)	-
pos_1405	2-Hydroxyfelbamate	metab_417	HMDB0060670;PW_C040524	B(ii)	C16582
pos_1406	A-L-Fucopyranosyl-(1->2)-b-D-galactopyranosyl-(1->2)-D-xylose	metab_418	HMDB0041222	B(ii)	-
pos_1415	Valyltyrosine	metab_427	HMDB0029139	B(ii)	-
pos_1416	5-(4-Carboxybutylperoxy)pentanoic acid	metab_428	HMDB0260291	B(ii)	-
pos_1420	Dodecyl-beta-D-maltoside	metab_433	HMDB0247203	B(ii)	-
pos_1421	Aminocaproic acid	metab_434	PW_C001268;HMDB0001901	B(ii)	C02378
pos_1427	2-Cyclopentene-1,4-dione	metab_439	-	B(i)	-
pos_1430	2-(1-Ethoxyethoxy)propanoic acid	metab_443	HMDB0059918	B(ii)	-
pos_1432	Glycyl-leucine	metab_444	HMDB0000759;MJDBOTE0000578	B(i)	C02155
pos_1434	Kainic acid	metab_446	HMDB0253757	B(ii)	C12819
pos_1435	N4-Acetylcytidine	metab_447	HMDB0005923	B(ii)	C22293
pos_1437	Ala-Phe-Ala	metab_449	HMDB0304795	B(ii)	-
pos_1445	Pro-Pro-Pro	metab_457	HMDB0256689	B(ii)	-
pos_1448	[(2R,5R)-5-(2-Amino-6-oxo-1H-purin-9-yl)-3,4-dihydroxyoxolan-2-yl]methyl [(2R,5R)-5-(4-amino-2-oxopyrimidin-1-yl)-3,4-dihydroxyoxolan-2-yl]methyl hydrogen phosphate	metab_460	HMDB0260224	B(ii)	-
pos_1451	Sulbactam	metab_464	HMDB0258566	B(ii)	C07770
pos_1459	2,5-Diamino-4,5-diketopyrimidine	metab_472	HMDB0251499	B(ii)	-
pos_1461	Brisoral	metab_475	HMDB0259678;HMDB0015281	B(ii)	C06888
pos_1466	Linustatin	metab_480	HMDB0303673	B(ii)	C08333
pos_1477	N-Acetyllactosamine	metab_491	HMDB0001542	B(ii)	C00611
pos_1481	Lenticin	metab_495	HMDB0061115	B(ii)	C09213
pos_1482	Glycylprolylhydroxyproline	metab_496	HMDB0002171	B(ii)	-
pos_1491	Linatine	metab_505	HMDB0038516	B(ii)	C05939
pos_1500	L-2,3-Dihydrodipicolinate	metab_515	HMDB0012247;HMDB0303995	B(ii)	C03340
pos_1504	Benzene	metab_519	HMDB0001505	B(ii)	C01407
pos_1505	Indoline	metab_520	HMDB0253472	B(ii)	-
pos_1509	6-Aminopenicillanic acid	metab_524	HMDB0060618	B(ii)	C02954
pos_1511	Methylmalonic acid	metab_527	HMDB0000202;LMFA01170118;PW_C000130	B(ii)	C02170
pos_1512	Citalopram propionic acid	metab_528	PW_C040620;HMDB0060463	B(ii)	C16610
pos_1516	P-Aminophenazone	metab_531	HMDB0256031	B(ii)	-
pos_1518	Mycorradicin	metab_532	HMDB0302033	B(ii)	C20426
pos_1521	Ticlopidine	metab_536	HMDB0014353;PW_C008752	B(ii)	C07140
pos_1527	CMP-3-deoxy-D-manno-octulosonate	metab_542	HMDB0304292	B(ii)	C04121
pos_1532	Prenalterol	metab_548	HMDB0243809;HMDB0256762	B(ii)	C07533
pos_1551	Arctiopicrin	metab_566	HMDB0301857	B(ii)	C09297
pos_1557	[(1S,2S)-2-[[2,2-Dimethylpropyl(nonyl)carbamoyl]amino]cyclohexyl] 3-[[(4R)-2,2,5,5-tetramethyl-1,3-dioxane-4-carbonyl]amino]propanoate	metab_572	HMDB0252152	B(ii)	-
pos_1558	2-Amino-3-phosphonopropionic acid	metab_573	HMDB0000370	B(ii)	C05672
pos_1560	Norclobazam	metab_576	HMDB0060970	B(ii)	-
pos_1565	6-[(2R,4S,5R)-4-Hydroxy-5-(hydroxymethyl)oxolan-2-yl]-7,8-dihydroimidazo[1,2-c]pyrimidin-5-one	metab_580	HMDB0260369	B(ii)	-
pos_1594	Uridine, 2'-deoxy-2'-fluoro-2'-methyl-, (2'R)-	metab_608	HMDB0247529	B(ii)	-
pos_1596	Fusarin C	metab_609	HMDB0033337	B(ii)	C19243
pos_1622	Hept-4-enedioylcarnitine	metab_636	HMDB0241688	B(ii)	-
pos_1633	Gamma-Glutamylhistidine	metab_648	HMDB0029151	B(ii)	-
pos_1635	Glutarate semialdehyde	metab_650	HMDB0012233	B(ii)	C03273
pos_1646	Tobramycin	metab_661	HMDB0014822;PW_C009161	B(ii)	C00397
pos_1652	Diphenylacetic acid	metab_667	HMDB0251455	B(ii)	-
pos_1658	7-Aminomethyl-7-carbaguanine	metab_669	HMDB0011690	B(ii)	C16675
pos_1660	Beta-Hydroxy-gamma-trimethylaminobutyric acid	metab_672	HMDB0244982	B(ii)	-
pos_1663	6-Carboxy-5,6,7,8-tetrahydropterin	metab_675	HMDB0060410	B(ii)	C20239
pos_1664	Chondroitin sulfate E (GalNAc4,6diS-GlcA), precursor 3	metab_676	HMDB0062463	B(ii)	-
pos_1668	Salicylhydroxamic acid	metab_680	HMDB0257460	B(ii)	C11343
pos_1670	Mepivacaine	metab_682	HMDB0015096;HMDB0253607;PW_C009402;MJDBOTE0001525	B(ii)	C07528
pos_1672	LysoPE(20:2(11Z,14Z)/0:0)	metab_684	HMDB0011513	B(ii)	-
pos_1675	Crocin 5	metab_687	HMDB0250541	B(ii)	-
pos_1678	Sanfetrinem	metab_690	HMDB0257478	B(ii)	-
pos_1692	2-succinylbenzoate	metab_706	HMDB0304087	B(ii)	C02730
pos_1694	Osmundalin	metab_708	HMDB0031304	B(ii)	-
pos_17	5-Hydroxyindoleacetic acid	metab_714	HMDB0000763;PW_C000609	B(i)	C05635
pos_1704	S-Glutaryldihydrolipoamide	metab_720	HMDB0006832	B(ii)	C06157
pos_1706	Cynaroside A	metab_722	HMDB0040532	B(ii)	-
pos_1709	Carteolol	metab_725	PW_C009020;HMDB0014662	B(ii)	C06874
pos_1715	PIP(6 keto-PGF1alpha/20:1(11Z))	metab_732	HMDB0279585	B(ii)	-
pos_1718	Plumbagin	metab_735	HMDB0256646;MJDBOTE0001003	B(ii)	C10387
pos_1721	Salicylaldehyde	metab_738	HMDB0034170	B(i)	C06202
pos_1722	Fraxin	metab_739	HMDB0252486	B(ii)	C09266
pos_173	Pro-Ile	metab_747	HMDB0304810	B(ii)	-
pos_1735	2-Methylbenzoic acid	metab_751	HMDB0002340	B(i)	C07215
pos_1738	Cyanidin 3-(6''-acetyl-galactoside)	metab_753	HMDB0029236	B(ii)	-
pos_1743	7-Methylxanthine	metab_757	PW_C001335;HMDB0001991	B(ii)	C16353
pos_1744	Leonurine	metab_758	HMDB0254014	B(ii)	C16985
pos_1749	Sterigmatocystin	metab_763	HMDB0030588;LMPK10000001	B(ii)	C00961
pos_1754	Piperacillin	metab_767	HMDB0014464	B(ii)	C14034
pos_1761	Acotiamide	metab_772	HMDB0247965	B(ii)	C14127
pos_1762	Desmethyl Fluvoxamine	metab_773	HMDB0251053	B(ii)	-
pos_1766	Plumieride	metab_776	HMDB0256647	B(ii)	C09797
pos_1774	5-Hydroxymethyl-2-furancarboxaldehyde	metab_782	HMDB0034355	B(ii)	C11101
pos_1775	Isoquinoline	metab_783	HMDB0034244	B(i)	C06323
pos_1777	Quinoneimine	metab_784	HMDB0257052	B(ii)	-
pos_178	L-Glutamic acid	metab_787	HMDB0003339;HMDB0060475;HMDB0000148;PW_C000095	B(i)	C00025
pos_1782	2-(5'-methylthio)pentylmalate	metab_789	HMDB0304052	B(ii)	C17222
pos_1783	N-Phenylacetylphenylalanine	metab_790	HMDB0002372	B(ii)	-
pos_1784	CDP-DG(LTE4/i-21:0)	metab_791	HMDB0294228	B(ii)	-
pos_1787	4-amino-4-deoxychorismate	metab_794	HMDB0304170	B(ii)	C11355
pos_1792	Thermophillin	metab_799	HMDB0034335	B(ii)	C01692
pos_1796	Methylthiouracil	metab_802	HMDB0254667	B(ii)	C19265
pos_1797	Cyclo(D-trp-D-asp-pro-D-val-leu)	metab_803	HMDB0249361	B(ii)	-
pos_1802	Propyl gallate	metab_810	HMDB0033835	B(ii)	C11155
pos_1805	Isoleucylhydroxyproline	metab_813	HMDB0028908	B(ii)	-
pos_1806	Gamma-Aminobutyric acid-betaxanthin	metab_814	HMDB0304751	B(ii)	-
pos_1813	PGP(20:4(5Z,8Z,11Z,13E)-OH(15S)/i-20:0)	metab_821	HMDB0275631	B(ii)	-
pos_1824	2',2'-Difluorodeoxyuridine	metab_832	HMDB0060727	B(ii)	-
pos_1825	[8]-Shogaol	metab_833	HMDB0031463	B(ii)	C10494
pos_1831	1-(3-Fluoro-4-hydroxy-5-mercaptomethyltetrahydrofuran-2-yl)-5-methylpyrimidine-2,4(1H,3H)-dione	metab_839	HMDB0243787	B(ii)	-
pos_184	Kojibiose	metab_848	HMDB0011742	B(ii)	C19632
pos_185	2-Piperidinone	metab_859	HMDB0011749	B(ii)	-
pos_187	1-Kestose	metab_877	HMDB0011729;MJDBOTE0001231	B(i)	C03661
pos_1874	4-Hydroxyquinoline	metab_882	HMDB0246466	B(i)	C06343

ID	M/Z	Retention time	Mode	Adducts	Formula	Fragmentation Score	Theoretical Fragmentation Score	Mass Error	CAS ID	RSD	ND1	ND2	ND3	ND4	ND5	ND6	PreD1	PreD2	PreD3	PreD4	PreD5	PreD6	D1	D2	D3	D4	D5	D6	CT1	CT2	CT3	CT4	CT5	CT6	RD1	RD2	RD3	RD4	RD5	RD6	OC01	OC02	OC03
pos_1	220.1178	2.7919	pos	M+H, M+Na, 2M+K, 2M+Na, M+H-H2O, M+H-2H2C	C9H17NO5	0	30.6	-0.892514355	-	0.00601851	7.07	7.079	7.062	7.053	7.075	7.052	7.601	7.601	7.593	7.599	7.602	7.603	7.645	7.638	7.631	7.643	7.646	7.629	7.616	7.603	7.589	7.601	7.595	7.605	7.775	7.758	7.761	7.773	7.757	7.761	7.554	7.549	7.551
pos_1003	145.0494	0.3855	pos	M+H-H2O, M+NH4	C6H10O5	0	51.8	-1.031497904	-	0.03260468	6.766	6.786	6.829	6.821	6.848	6.844	6.768	6.874	6.932	6.953	6.911	6.915	6.998	6.95	6.936	6.945	6.972	6.952	6.949	6.98	6.932	6.999	6.994	7.019	7.091	7.028	7.057	7.06	7.024	7.013	6.841	6.866	6.866
pos_102	133.0971	0.4864	pos	M+H-H2O, M+H, M+	C5H12N2O2	72.4	0	-0.732010516	348-66-3	0.00927529	6.102	6.113	6.107	6.094	6.1	6.085	5.915	5.898	5.899	5.88	5.895	5.913	5.63	5.639	5.615	5.625	5.622	5.583	5.587	5.559	5.556	5.575	5.548	5.58	5.571	5.582	5.596	5.604	5.628	5.57	5.923	5.916	5.917
pos_1035	153.1019	0.0323	pos	M+H-2H2O	C8H16N2O3	0	53.6	-1.592984613	1946-82-3	0.02015469	4.685	4.605	4.523	4.415	4.385	4.397	4.368	4.8	4.972	4.889	4.916	4.981	4.814	4.941	4.984	4.98	4.994	5.041	5.024	5.06	4.903	4.982	4.941	4.981	4.958	4.86	4.93	4.89	4.792	4.791	4.703	4.719	4.704
pos_1044	108.0446	0.2281	pos	M+H	C6H5NO	0	39.7	1.838433344	500-22-1	0.00514108	4.264	4.29	4.207	4.249	4.217	4.19	4.217	4.196	4.073	4.236	4.273	4.287	4.373	4.37	4.399	4.393	4.398	4.401	4.366	4.353	4.35	4.389	4.413	4.44	4.424	4.393	4.383	4.411	4.373	4.364	4.329	4.327	4.331
pos_1048	98.0967	0.3065	pos	M+H-H2O	C6H13NO	0	44.1	2.093035005	25038-54-4	0.0344897	4.06	4.038	4.038	4.086	4.096	4.103	4.147	4.196	4.156	4.184	4.132	4.123	4.196	4.255	4.265	4.223	4.318	4.163	4.192	4.323	4.241	4.248	4.267	4.289	4.357	4.281	4.207	4.288	4.258	4.216	4.12	4.146	4.147
pos_105	280.2629	6.7033	pos	M+H, M+NH4, M+H-H2O	C18H30O	85	0	-2.441197181	-	0.00192201	7.105	7.085	7.096	7.112	7.116	7.098	7.213	7.227	7.213	7.198	7.2	7.202	7.447	7.47	7.452	7.458	7.43	7.445	7.373	7.383	7.356	7.381	7.372	7.371	7.193	7.174	7.184	7.18	7.18	7.186	7.237	7.236	7.238
pos_1057	103.0392	0.4012	pos	M+H	C4H6O3	0	32.6	2.276863696	541-50-4	0.02674584	5.907	5.926	5.862	5.823	5.814	5.806	5.775	6.068	6.077	6.12	6.155	6.197	6.217	6.162	6.249	6.281	6.303	6.301	6.312	6.351	6.303	6.354	6.334	6.358	6.369	6.284	6.289	6.266	6.225	6.206	5.956	5.979	5.969
pos_106	520.3389	6.7577	pos	M+H-H2O, M+H, 2M+H	C26H50NO7P	85.2	0	-1.67951239	-	0.00294591	9.101	9.11	9.112	9.114	9.116	9.112	9.169	9.143	9.133	9.126	9.124	9.123	8.766	8.743	8.732	8.723	8.715	8.724	8.874	8.877	8.874	8.882	8.874	8.874	9.149	9.148	9.159	9.158	9.158	9.164	9.073	9.07	9.071
pos_1074	75.092	0.4712	pos	M+H	C3H10N2	95.6	0	4.026175195	109-76-2	0.02057658	5.206	5.205	5.204	5.197	5.199	5.192	2.147	2.204	2.154	2.145	2.905	2.153	2.231	2.237	2.238	2.234	2.24	2.244	2.229	2.228	2.232	2.237	2.231	2.241	2.148	3.037	3.166	2.526	3.129	2.158	4.811	4.794	4.808
pos_1078	397.0426	0.4788	pos	M+ACN+Na	C11H12NO9P-2	0	53	5.533604627	321-02-8	0.01140692	5.724	5.737	5.693	5.744	5.718	5.69	5.845	5.818	5.859	5.818	5.86	5.901	6.03	6.013	6.042	6.015	6.089	6.03	6.015	6.013	5.973	6.047	6.032	6.068	5.697	5.708	5.803	5.792	5.814	5.749	5.916	5.913	5.907
pos_1084	301.0726	0.4864	pos	M+H	C16H12O6	0	50	6.523861011	133067-72-8	0.02242501	5.657	5.678	5.683	5.68	5.676	5.66	5.73	5.752	5.751	5.725	5.788	5.783	5.935	5.935	5.967	5.941	5.971	5.899	5.917	5.927	5.916	5.909	5.931	5.907	5.71	5.719	5.732	5.749	5.734	5.706	5.864	5.847	5.848
pos_1086	130.0862	0.4941	pos	M+H	C6H11NO2	49.9	0	-0.409718672	3105-95-1:535-75-1	0.00535204	6.66	6.646	6.661	6.649	6.645	6.639	6.736	6.725	6.741	6.728	6.74	6.748	6.908	6.924	6.917	6.925	6.928	6.914	6.91	6.902	6.907	6.905	6.905	6.911	6.794	6.808	6.805	6.792	6.804	6.796	6.797	6.797	6.793

pos_1889	1-(4-Aminophenyl)-3-acetyl-4-methyl-3,4-dihydro-7,8-methylenedioxy-5H-2,3-benzodiazepine	metab_898	HMDB0252996	B(ii)	-	716.3224	3.3683	pos	[illegible]	[illegible]
pos_1904	Domoic acid	metab_913	HMDB0033939	B(ii)	C13732	375.1548	3.4404	pos	[illegible]	[illegible]
pos_1905	Physcion 8-gentiobioside	metab_914	HMDB0036009;LMPK13040012	B(ii)	C10382	591.1688	3.4404	pos	[illegible]	[illegible]
pos_1909	Glutaminylhydroxyproline	metab_918	HMDB0028798	B(ii)	-	259.1149	3.4561	pos	M+	[illegible]
pos_191	Ethylenediamine-N,N'-diacetic acid	metab_919	HMDB0246968	B(ii)	C21397	159.0761	0.6632	pos	M+H-H2O	[illegible]
pos_1911	Helenalin	metab_921	HMDB0253066	B(ii)	C09473	280.1542	3.464	pos	M+NH4	[illegible]
pos_1913	Alloside B	metab_923	HMDB0303716	B(ii)	-	391.2172	3.4797	pos	M+H+Na	[illegible]
pos_1925	H-Tyr-gly-gly-OH	metab_934	HMDB0247865	B(ii)	-	313.15	3.5121	pos	M+NH4	[illegible]
pos_1952	3-Hydroxyanthranilic acid	metab_958	PW_C001137:HMDB0001476	B(ii)	C00632	307.092	3.6385	pos	2M+H	[illegible]
pos_1955	(-)-cis-Rotenolone	metab_961	HMDB0034145	B(ii)	C10464	375.1186	3.6464	pos	M+H-2H2O	[illegible]
pos_1967	Amotosalen	metab_973	HMDB0248350	B(ii)	-	365.1453	3.7021	pos	M+ACN+H	[illegible]
pos_1999	Indole-3-carboxylic acid	metab_1003	HMDB0003320	B(ii)	C19837	323.1024	3.8206	pos	2M+H	[illegible]
pos_2	8-Oxohexadecanoic acid	metab_1004	HMDB0030972	B(ii)	-	293.2104	6.3755	pos	M+H-2H2O	[illegible]
pos_2002	PG(i-12:0/22:6(4Z,8Z,10Z,13Z,16Z,19Z)-OH(7))	metab_1009	HMDB0271127	B(ii)	-	389.216	3.8284	pos	M+H+Na	[illegible]
pos_2017	1,5-Benzothiazepin-4(5H)-one, 2,3-dihydro-3-hydroxy-2-(4-methoxyphenyl)-5-(2-(methylamino)ethyl)-, (2S,3S)-	metab_1021	HMDB0247530	B(ii)	-	381.1224	3.8761	pos	M+Na	[illegible]
pos_2025	Vomifoliol	metab_1029	HMDB0303570	B(ii)	C04166	189.1271	3.8996	pos	M+H-2H2O	[illegible]
pos_203	Dhurrin 6'-glucoside	metab_1034	HMDB0029776	B(ii)	-	506.1868	0.6787	pos	M+CH3OH+H	[illegible]
pos_2036	Vicenin 2	metab_1041	HMDB0030708;LMPK12110218	B(i)	C10195	595.1656	3.9467	pos	M+H	[illegible]
pos_204	1,4-Benzothiazine-O-quinonimine	metab_1045	HMDB0012491	B(ii)	-	329.0798	0.7172	pos	M+H-2H2O	[illegible]
pos_2043	N-Myristoyl Arginine	metab_1049	HMDB0242046	B(ii)	-	385.3171	3.9782	pos	M+H	[illegible]
pos_2049	Golotimod	metab_1055	HMDB0252908	B(ii)	-	334.1393	4.0016	pos	M+H	[illegible]
pos_2056	3,5-Dihydroxy-6,7-megastigmadien-9-one	metab_1063	HMDB0303443	B(ii)	-	207.1376	4.0257	pos	M+H-H2O	[illegible]
pos_2059	Genipic acid	metab_1066	HMDB0036072	B(ii)	-	149.0596	4.0413	pos	M+H-2H2O	[illegible]
pos_2061	2-Naphthol	metab_1069	HMDB0012322	B(i)	C11713	145.0646	4.0492	pos	M+H	[illegible]
pos_207	Coformycin	metab_1076	HMDB0250398	B(ii)	C01677	267.1083	0.6787	pos	M+H-H2O	[illegible]
pos_2072	PI(TXB2/18:0)	metab_1079	HMDB0276379	B(ii)	-	488.2786	4.0962	pos	M+H+Na	[illegible]
pos_2076	Isoferulic acid	metab_1083	HMDB0259693:HMDB0000955	B(ii)	C10470	177.0544	4.1119	pos	M+H-H2O	[illegible]
pos_2086	Dynorphin A (6-8)	metab_1094	HMDB0012932	B(ii)	-	443.2973	4.1822	pos	M+	[illegible]
pos_2094	3,4-Methylenesebacic acid	metab_1102	HMDB0059729	B(ii)	-	209.1169	4.2292	pos	M+H-H2O	[illegible]
pos_2101	2-Aminobenzoic Acid	metab_1110	PW_C000877:HMDB0001123	B(i)	C00108	120.0445	4.2655	pos	M+H-H2O	[illegible]
pos_2102	Biochanin A 7-(6-malonylglucoside)	metab_1111	HMDB0030640	B(ii)	C12625	565.1552	4.2655	pos	M+CH3OH+H	[illegible]
pos_2103	Gamma-Glutamylthreonine	metab_1112	HMDB0029159	B(i)	-	249.1093	4.2733	pos	M+H	[illegible]
pos_2114	Ile Phe	metab_1124	-	B(i)	-	279.1696	4.3189	pos	M+H	[illegible]
pos_2117	Epsilon-caprolactam	metab_1127	HMDB0062769	B(ii)	C06593	249.157	4.3389	pos	2M+Na	[illegible]
pos_2125	Icariside B8	metab_1135	HMDB0036846	B(ii)	-	411.1989	4.3682	pos	M+Na	[illegible]
pos_2154	Coniferaldehyde	metab_1162	HMDB0141782	B(ii)	C02666	161.0594	4.4622	pos	M+H-H2O	[illegible]
pos_2155	Isoquercitrin	metab_1163	LMPK12112086:HMDB0037362:MJDBOTE0000951	B(i)	C05623	465.1028	4.4622	pos	M+H	[illegible]
pos_216	10-Acetyl-3,7-dihydroxyphenoxazine	metab_1166	HMDB0244436	B(ii)	-	515.1439	0.7638	pos	[illegible]	[illegible]
pos_2160	N-Palmitoyl Cysteine	metab_1167	HMDB0241923	B(ii)	-	237.1481	4.47	pos	M+H-H2O	[illegible]
pos_2173	Valinomycin	metab_1179	HMDB0259750	B(ii)	C06684	556.3214	4.4856	pos	M+2H	[illegible]
pos_2174	Tulipanin	metab_1180	LMPK12010282	B(i)	C16315	611.1601	4.4856	pos	M+	[illegible]
pos_2180	Methylone	metab_1186	HMDB0254656	B(ii)	C20126	432.2127	4.4935	pos	2M+NH4	[illegible]
pos_2181	2,3-Diphenylbenzofuran	metab_1187	HMDB0245414	B(ii)	-	604.2262	4.4935	pos	2M+ACN+H	[illegible]
pos_2185	Quercetin	metab_1190	LMPK12110004:HMDB0005794:MJDBOTE0001650	B(i)	C00389	303.0494	4.5013	pos	M+H	[illegible]
pos_23	Linolenic Acid	metab_1281	HMDB0001388	B(i)	C06427	279.2316	0.3143	pos	M+H-H2O	[illegible]
pos_232	Pseudouridine	metab_1303	HMDB0000767	B(i)	C02067	245.0765	0.9983	pos	[illegible]	[illegible]
pos_2339	2-[(2R)-2-Aminopropyl]-5-hydroxybenzoic acid	metab_1324	HMDB0257865	B(ii)	-	432.2125	4.6895	pos	2M+ACN+H	[illegible]
pos_235	Guanidylic acid (guanosine monophosphate)	metab_1336	-	B(i)	-	364.0649	1.0374	pos	[illegible]	[illegible]
pos_238	9H-Purine-9-ol	metab_1368	HMDB0247704	B(ii)	-	137.0457	1.0921	pos	[illegible]	[illegible]
pos_2384	Vasicinone	metab_1373	HMDB0259770	B(i)	C10744	185.0706	4.7294	pos	M+H-H2O	[illegible]
pos_239	Beta-nicotinamide adenine dinucleotide	metab_1379	HMDB0000902:HMDB0249146	B(ii)	C00003	664.1164	1.0921	pos	[illegible]	[illegible]
pos_242	Di-deacetyl adrogolide	metab_1410	HMDB0247722	B(ii)	-	316.1388	1.1468	pos	[illegible]	[illegible]
pos_245	Propenoylcarnitine	metab_1437	HMDB0013124;LMFA07070104	B(ii)	-	248.1489	1.1938	pos	[illegible]	[illegible]
pos_2453	Temsirolimus	metab_1440	HMDB0015632:MJDBOTE0000851	B(ii)	C15182	515.81	4.7772	pos	M+2H	[illegible]
pos_250	2,8-Quinolinediol	metab_1490	HMDB0240311	B(ii)	C06342	162.0547	1.2642	pos	[illegible]	[illegible]
pos_2506	Indolepyruvate	metab_1497	HMDB0060484	B(ii)	C00331	245.0916	4.8167	pos	M+ACN+H	[illegible]
pos_2521	Morin	metab_1513	HMDB0030796;LMPK12112517:MJDBOTE0001649	B(ii)	C10105	303.0493	4.8247	pos	M+H	[illegible]
pos_2534	(+/-)-Tryptophan	metab_1525	HMDB0030396	B(ii)	C00806	227.0811	4.8328	pos	M+Na	[illegible]
pos_2546	5-[(2R)-2-Aminopropyl]-2-hydroxybenzoic acid	metab_1538	HMDB0257825	B(ii)	-	432.2127	4.8408	pos	2M+ACN+H	[illegible]
pos_255	4-Amino-1-[(2R,5R)-5-(hydroxymethyl)-4-sulfanyloxolan-2-	metab_1542	HMDB0257848	B(ii)	-	528.1687	1.389	pos	M+ACN+H	[illegible]
pos_2559	4-Methylumbelliferone	metab_1552	HMDB0059622;MJDBOTE0001043	B(i)	C03081	209.0805	4.8486	pos	M+CH3OH+H	[illegible]
pos_2570	Salidroside	metab_1565	HMDB0257463;MJDBOTE0000899	B(ii)	C06046	265.1065	4.8567	pos	M+H-2H2O	[illegible]
pos_2578	Coniferyl acetate	metab_1573	HMDB0304294	B(ii)	C20225	205.0856	4.8646	pos	M+H-H2O	[illegible]
pos_2588	Citrinin	metab_1584	HMDB0041857	B(ii)	C16765	233.0804	4.8726	pos	M+H-H2O	[illegible]
pos_262	P-cresol	metab_1616	HMDB0001858	B(i)	C01468	91.0545	1.3499	pos	M+H-H2O	[illegible]
pos_266	Linoleic Acid	metab_1654	HMDB0247706;PW_C000530:HMDB0006270:HMDB0000673:LMF	B(i)	C01595	263.2365	1.8158	pos	M+H-H2O	[illegible]
pos_2676	2-(2,6-Dihydroxy-4-methoxycarbonylbenzoyl)-3-Hydroxybenzoic Acid	metab_1669	-	B(i)	-	355.0392	4.9843	pos	M+Na	[illegible]
pos_2682	Deoxycholylarginine	metab_1676	HMDB0242418	B(ii)	-	566.4275	4.9922	pos	M+NH4	[illegible]
pos_2684	PS(5-iso PGE2VI/14:0)	metab_1678	HMDB0280844	B(ii)	-	760.4337	4.9922	pos	M+H-H2O	[illegible]
pos_2689	3-Hydroxyphloretin 2'-O-xylosyl-glucoside	metab_1683	HMDB0301671	B(ii)	-	602.2108	5.008	pos	M+NH4	[illegible]
pos_269	Acetophenone	metab_1684	HMDB0033910	B(i)	C07113	138.091	1.8551	pos	[illegible]	[illegible]
pos_271	12-Hydroxynevirapine glucuronide	metab_1705	HMDB0060705;PW_C040606	B(ii)	-	459.1545	1.8629	pos	[illegible]	[illegible]
pos_2726	5-Methoxyindoleacetate	metab_1721	PW_C002060:HMDB0004096	B(ii)	C05660	238.107	5.0792	pos	M+CH3OH+H	[illegible]
pos_273	P-Hydroxyl-ethotoin	metab_1725	HMDB0061174	B(ii)	-	221.0917	1.9255	pos	M+H-H2O	[illegible]
pos_2768	Citrusin III	metab_1756	HMDB0303682	B(ii)	-	710.3897	5.1897	pos	M+H-H2O	[illegible]
pos_2778	Lactucin	metab_1765	HMDB0035814	B(ii)	C09489	309.1328	5.2368	pos	M+CH3OH+H	[illegible]
pos_2787	11-Hydroxy-9-tridecenoic acid	metab_1774	LMFA01050437:HMDB0035881	B(ii)	-	211.1689	5.2762	pos	M+H-H2O	[illegible]
pos_279	Trp Pro Glu	metab_1777	-	B(i)	-	431.1925	1.9725	pos	[illegible]	[illegible]
pos_2805	HYPEROSIDE	metab_1793	HMDB0030775	B(i)	C10073	465.1026	5.3776	pos	M+H	[illegible]
pos_2816	Cordycepin	metab_1802	HMDB0250451:MJDBOTE0001321	B(ii)	C08431	293.1379	5.4319	pos	M+ACN+H	[illegible]
pos_2822	Eugenin	metab_1809	HMDB0036627	B(ii)	C20210	189.0544	5.463	pos	M+H-H2O	[illegible]
pos_2851	Beta-Thujaplicin	metab_1837	HMDB0035213	B(ii)	C09904	165.0909	5.6027	pos	M+H	[illegible]
pos_287	Melevodopa	metab_1856	HMDB0254424	B(ii)	-	194.0809	2.0429	pos	M+H-H2O	[illegible]
pos_2879	Isocolumbin	metab_1864	HMDB0036837	B(ii)	C17508	376.1728	5.7875	pos	M+NH4	[illegible]
pos_292	Didanosine	metab_1905	HMDB0015037;PW_C009350	B(ii)	C06953	236.0913	2.2387	pos	[illegible]	[illegible]
pos_2922	2-Azaspiro[4.5]decan-3-one	metab_1907	HMDB0247166	B(ii)	-	171.1489	5.8603	pos	M+NH4	[illegible]
pos_2943	(1,10,11,12,14,23-Hexahydroxy-6,10,19-trimethyl-24-oxa-4-azaheptacyclo[12.12.0.02,11.04,9.015,25.018,23.019,25]hexacosan-22-yl) 3,4-dimethoxybenzoate	metab_1929	HMDB0242290	B(ii)	-	718.323	5.8841	pos	M+2Na-H	[illegible]
pos_296	N6-(1,2-Dicarboxyethyl)-AMP	metab_1946	-	B(i)	C03794	464.0813	2.3713	pos	[illegible]	[illegible]
pos_2961	Laninamivir	metab_1948	HMDB0253972	B(ii)	C21557	379.1833	5.9154	pos	M+CH3OH+H	[illegible]
pos_2966	Finrozole	metab_1953	HMDB0252263	B(ii)	-	645.2547	5.9232	pos	2M+H	[illegible]
pos_298	Oxepin	metab_1967	HMDB0255979	B(ii)	-	95.0494	2.4791	pos	M+H-H2O	[illegible]
pos_2981	Juglone	metab_1969	HMDB0030773;MJDBOTE0001015	B(ii)	C03840	216.0652	5.9624	pos	M+ACN+H	[illegible]
pos_3	PE(18:1/0:0)	metab_1989	LMGP02050004	B(i)	-	480.3079	6.6877	pos	[illegible]	[illegible]
pos_3000	12-oxo-PDA	metab_1992	LMFA02010001:HMDB0301804:HMDB0302724	B(ii)	C01226	257.1895	6.0171	pos	M+H-2H2O	[illegible]
pos_3003	4-Methyl-1H-Benzotriazole	metab_1995	-	B(i)	-	134.0712	6.0249	pos	M+H	[illegible]
pos_3005	2-[(4-{2-[(4-Cyclohexylbutyl)(cyclohexylcarbamoyl)amino]ethyl}phenyl)sulfanyl]-2-	metab_1997	HMDB0252992	B(ii)	C15622	502.3276	6.0249	pos	M+	[illegible]
pos_3008	Tridecanoic acid	metab_1999	LMFA01010013:HMDB0000910	B(ii)	C17076	232.2265	6.0327	pos	M+NH4	[illegible]
pos_3009	Pregnanediol 3-O-glucuronide	metab_2000	HMDB0010318	B(ii)	C03033	529.3343	6.0327	pos	M+CH3OH+H	[illegible]
pos_3012	9,10-DHOME	metab_2004	HMDB0004704	B(ii)	C14828	337.2347	6.0405	pos	M+Na	[illegible]
pos_3020	9-Tetradecenoic acid	metab_2012	HMDB0258881;HMDB0002000	B(ii)	C08322	244.2267	6.0871	pos	M+NH4	[illegible]
pos_3021	Solutol HS 15	metab_2013	HMDB0258368	B(ii)	C11317	362.326	6.0871	pos	M+NH4	[illegible]
pos_3023	Geneticin	metab_2015	HMDB0242565	B(ii)	C17703	461.2618	6.0871	pos	M+H-2H2O	[illegible]
pos_3024	Taxine B	metab_2016	HMDB0258750	B(ii)	C19989	566.3084	6.0871	pos	M+H-H2O	[illegible]
pos_3025	Delta7-Dafachronic acid	metab_2017	-	B(i)	C21077	415.3202	6.0949	pos	M+H	[illegible]
pos_3027	Curcumenol	metab_2019	HMDB0033960;MJDBOTE0000956	B(i)	C16942	235.1687	6.1104	pos	M+H	[illegible]
pos_3028	Sapienic acid	metab_2020	HMDB0257483;LMFA01030267	B(ii)	C21942	272.2579	6.1104	pos	M+NH4	[illegible]
pos_3031	Hexadecanedioic acid mono-L-carnitine ester	metab_2024	LMFA07070007;HMDB0000712	B(ii)	-	394.2948	6.1182	pos	M+H-2H2O	[illegible]
pos_3040	(+)-cis-abscisic aldehyde	metab_2033	HMDB0303940	B(ii)	C13455	266.1745	6.1572	pos	M+NH4	[illegible]
pos_3041	Nonadecanoic acid	metab_2034	HMDB0000772;LMFA01010019	B(ii)	C16535	316.3205	6.1572	pos	M+NH4	[illegible]
pos_3046	Sulfadimidine	metab_2038	HMDB0015522	B(i)	C19530	279.0928	6.1648	pos	M+H	[illegible]
pos_3048	Nevirapine	metab_2040	HMDB0014383;PW_C008780	B(ii)	C07263	267.1222	6.1805	pos	M+H	[illegible]
pos_3049	Neryl formate	metab_2041	HMDB0035156	B(ii)	C12294	387.2495	6.1805	pos	2M+Na	[illegible]
pos_3056	Polypodine B	metab_2048	HMDB0302993;LMST01010198	B(ii)	C08834	535.2696	6.2118	pos	M+K	[illegible]
pos_3057	Spisulosine	metab_2049	HMDB0258433;LMSP01080032	B(ii)	-	327.3365	6.2196	pos	M+ACN+H	[illegible]
pos_3058	N-Isobutyloctadeca-trans-2-trans-4-dienamide	metab_2050	HMDB0302880	B(ii)	-	353.3521	6.2272	pos	M+NH4	[illegible]
pos_3060	Fexofenadine	metab_2053	PW_C002373:HMDB0005030	B(ii)	C06999	534.3186	6.2349	pos	M+CH3OH+H	[illegible]
pos_3064	4-Methoxyestrone	metab_2057	HMDB0060088	B(ii)	C03958	318.2056	6.2584	pos	M+NH4	[illegible]
pos_3067	Chavicol	metab_2060	HMDB0034107	B(i)	C16930	135.0803	6.2818	pos	M+H	[illegible]
pos_3072	4-Propylphenol	metab_2066	HMDB0032625	B(ii)	C14311	119.0856	6.2973	pos	M+H-H2O	[illegible]
pos_3073	5,6-epoxy-18R-HEPE	metab_2067	HMDB0062410	B(ii)	C18175	333.2029	6.2973	pos	M+H	[illegible]
pos_3079	3-(6-Aminopurin-9-yl)-5-(hydroxymethyl)cyclopentane-1,2-diol	metab_2073	HMDB0244841	B(ii)	-	283.1507	6.313	pos	M+NH4	[illegible]
pos_3087	Phytuberin	metab_2082	HMDB0035754	B(ii)	C09709	327.2139	6.3443	pos	M+CH3OH+H	[illegible]
pos_3092	Buprenorphine	metab_2087	HMDB0015057;PW_C009368	B(ii)	C08007	490.2925	6.36	pos	M+Na	[illegible]
pos_3101	2-Aminopurine	metab_2096	HMDB0245025	B(ii)	-	177.088	6.3833	pos	M+ACN+H	[illegible]
pos_3102	Dihydrozeatin	metab_2097	HMDB0012215;MJDBZSJ0000131	B(ii)	C02029	239.1609	6.3833	pos	M+NH4	[illegible]
pos_3113	(Z)-6-Tetradecene-1,3-diyne-5,8-diol	metab_2107	HMDB0038996	B(ii)	-	504.3081	6.4067	pos	2M+ACN+H	[illegible]
pos_3115	9,12,13-TriHOME	metab_2109	LMFA02000014;HMDB0004708	B(ii)	C14833	295.226	6.4224	pos	M+H-2H2O	[illegible]
pos_3116	Methyltestosterone	metab_2110	HMDB0259471;HMDB0015655	B(ii)	C07198	335.2575	6.4224	pos	M+CH3OH+H	[illegible]
pos_3118	Manoalide	metab_2112	LMPR0105020001:HMDB0254329	B(ii)	C17156	455.2166	6.4379	pos	M+K	[illegible]
pos_3122	(3Z)-phytochromobilin	metab_2117	HMDB0303975	B(ii)	C05913	565.243	6.4535	pos	M+H-H2O	[illegible]
pos_3123	19(S)-HETE	metab_2118	PW_C006775:HMDB0011136	B(ii)	C14749	353.2679	6.4613	pos	M+CH3OH+H	[illegible]
pos_3127	Piperonyl Butoxide	metab_2120	HMDB0256579	B(i)	C18880	339.2138	6.4925	pos	M+H	[illegible]
pos_3130	Cilengitide	metab_2124	HMDB0250242	B(ii)	-	611.2855	6.516	pos	M+Na	[illegible]
pos_3135	Alpha-Linolenoyl Ethanolamide	metab_2129	HMDB0013624	B(i)	C13828	322.2733	6.5549	pos	M+H	[illegible]
pos_3138	DG(8:0/8:0/0:0)	metab_2131	PW_C062137:HMDB0116368	B(ii)	C00165	309.2417	6.5628	pos	M+H-2H2O	[illegible]
pos_3139	LysoPE(18:2(9Z,12Z)/0:0)	metab_2132	HMDB0011507	B(ii)	-	500.2747	6.5628	pos	M+Na	[illegible]
pos_3141	SM(d18:2(4E,14Z)/14:0)	metab_2135	HMDB0240637	B(ii)	-	673.5271	6.5783	pos	M+H	[illegible]
pos_3145	3-Gonal	metab_2138	HMDB0245878	B(ii)	-	278.2473	6.5939	pos	M+NH4	[illegible]
pos_3150	Prostaglandin A1	metab_2144	HMDB0002656;LMFA03010005	B(ii)	C04685	369.2603	6.6096	pos	M+CH3OH+H	[illegible]
pos_3151	Ceanothine B	metab_2145	HMDB0029339	B(ii)	C10001	549.2486	6.6096	pos	M+2Na-H	[illegible]
pos_3153	7',8'-Dihydro-8'-hydroxyreticulataxanthin	metab_2147	HMDB0039090	B(ii)	-	473.3442	6.6252	pos	M+H-H2O	[illegible]
pos_3156	PI(20:1(11Z)/PGF1alpha)	metab_2150	HMDB0277163	B(ii)	-	997.6214	6.633	pos	M+CH3OH+H	[illegible]
pos_3157	PI(22:2(13Z,16Z)/PGD2)	metab_2151	HMDB0277717	B(ii)	-	1019.6034	6.633	pos	M+CH3OH+H	[illegible]
pos_3160	Linoleoyl Ethanolamide	metab_2155	HMDB0012252;HMDB0013624	B(i)	C13828	324.2889	6.6486	pos	M+H	[illegible]
pos_3163	Bipindogulomethyloside	metab_2158	HMDB0030623	B(ii)	-	591.2595	6.6564	pos	M+K	[illegible]
pos_3168	Subaphylline	metab_2163	HMDB0033463	B(ii)	C10497	567.2595	6.6877	pos	2M+K	[illegible]
pos_3172	Chlorophyllide a	metab_2168	HMDB0304288	B(ii)	C02139	623.2853	6.6955	pos	M+CH3OH+H	[illegible]
pos_3174	SM(d18:0/12:0)	metab_2169	LMSP03010079;HMDB0012084	B(ii)	C00550	695.5096	6.6955	pos	M+2Na-H	[illegible]
pos_3175	PC(16:0/18:3(9Z,12Z,15Z))	metab_2170	LMGP01010601;PW_C004024;HMDB0007975	B(ii)	C00157	778.5356	6.6955	pos	M+Na	[illegible]
pos_3177	Terbutaline	metab_2171	HMDB0015009	B(ii)	C07129	468.3078	6.7111	pos	2M+NH4	[illegible]
pos_3182	PC(18:0/0:0)	metab_2174	HMDB0010384;LMGP01050026	B(i)	C04230	546.3526	6.7268	pos	M+Na	[illegible]
pos_3184	LysoPE(22:4(7Z,10Z,13Z,16Z)/0:0)	metab_2175	HMDB0011523	B(ii)	-	512.3111	6.7423	pos	M+H-H2O	[illegible]
pos_3185	Palmitoyl Ethanolamide	metab_2176	HMDB0002100;LMFA08040013;LMFA08040057	B(i)	C16512	300.289	6.7501	pos	M+H	[illegible]
pos_3190	PI(22:2(13Z,16Z)/PGF2alpha)	metab_2180	HMDB0277721	B(ii)	-	1021.62	6.7656	pos	M+CH3OH+H	[illegible]
pos_3194	Linoleic Acid ethyl ester	metab_2183	-	B(i)	-	309.2779	6.789	pos	M+H	[illegible]
pos_3196	Guanethidine	metab_2185	HMDB0015301;MJDBOTE0001576	B(ii)	C07036	198.185	6.7965	pos	M+	[illegible]
pos_3197	MG(20:4(5Z,8Z,11Z,14Z)-OH(17)/0:0/0:0)	metab_2186	HMDB0260471	B(ii)	-	395.2758	6.7965	pos	M+H	[illegible]
pos_3199	Pheophorbide a	metab_2188	HMDB0256449	B(ii)	C18021	615.2567	6.7965	pos	M+Na	[illegible]
pos_3204	Novobiocin	metab_2195	HMDB0015185;MJDBOTE0001496	B(ii)	C05080	645.2671	6.8121	pos	M+CH3OH+H	[illegible]
pos_3212	7-Hexadecynoic acid	metab_2202	HMDB0247253;LMFA01030496	B(ii)	-	285.2417	6.851	pos	M+CH3OH+H	[illegible]
pos_3215	20alpha-Dihydroprogesterone	metab_2204	LMST02030169;HMDB0003069	B(ii)	C04042	349.273	6.8666	pos	M+CH3OH+H	[illegible]
pos_323	(2Z)-2-(3-Phenylpropoxyimino)butanoic acid	metab_2215	HMDB0249299	B(ii)	-	274.0819	2.682	pos	[illegible]	[illegible]
pos_3239	N,N-Dimethylsphingosine	metab_2221	LMSP01070001;HMDB0013645	B(ii)	C13914	310.3098	6.968	pos	M+H-H2O	[illegible]
pos_324	Gluten exorphin B4	metab_2222	HMDB0059794	B(ii)	-	530.1919	2.682	pos	[illegible]	[illegible]
pos_3240	Lubiprostone	metab_2223	HMDB0015180	B(ii)	C13707	413.2113	6.968	pos	M+Na	[illegible]
pos_3241	Cholyltyrosine	metab_2224	HMDB0242377	B(ii)	-	610.3088	6.968	pos	M+K	[illegible]
pos_3242	SM(d18:0/16:1(9Z))	metab_2225	HMDB0013464	B(ii)	C00550	725.5562	6.968	pos	M+Na	[illegible]
pos_3244	PC(20:4(8Z,11Z,14Z,17Z)/20:0)	metab_2226	LMGP01012213;PW_C004519;HMDB0008471	B(ii)	C00157	879.6515	7.007	pos	M+ACN+H	[illegible]
pos_3248	Docosapentaenoic acid (22n-3)	metab_2230	HMDB0006528;PW_C002795	B(ii)	C16513	363.2887	7.0461	pos	M+CH3OH+H	[illegible]
pos_3250	PG(i-12:0/a-17:0)	metab_2233	HMDB0116657;PW_C076275	B(ii)	C00344	663.4533	7.0617	pos	M+H-H2O	[illegible]
pos_3251	Midodrine	metab_2234	HMDB0014356	B(ii)	C07890	526.2883	7.0695	pos	2M+NH4	[illegible]
pos_3254	LysoPC(20:4(8Z,11Z,14Z,17Z)/0:0)	metab_2237	HMDB0010396	B(ii)	C04230	544.3367	7.1007	pos	M+H	[illegible]
pos_3257	TG(18:3(6Z,9Z,12Z)/18:4(6Z,9Z,12Z,15Z)/22:6(4Z,7Z,10Z,13Z,16Z,19Z))	metab_2239	HMDB0053193;PW_C033714	B(ii)	-	953.7251	7.1164	pos	M+CH3OH+H	[illegible]
pos_3260	13E-Docosenamide	metab_2243	-	B(i)	-	338.3412	7.1711	pos	M+H	[illegible]
pos_3264	25-Ethoxy-24-methoxycycloartanol	metab_2246	HMDB0302828	B(ii)	-	506.4563	7.1868	pos	M+NH4	[illegible]
pos_3266	Carboprost methyl	metab_2248	HMDB0249645	B(ii)	-	415.3022	7.2024	pos	M+CH3OH+H	[illegible]
pos_3272	Cyclohexane	metab_2255	HMDB0029597	B(ii)	C11249	102.128	7.2416	pos	M+NH4	[illegible]
pos_3276	Floionolic acid	metab_2259	HMDB0034295;LMFA02000147	B(ii)	C19621	315.2524	7.2963	pos	M+H-H2O	[illegible]
pos_3282	4,4'-Dioctyldiphenylamine	metab_2266	-	B(i)	-	394.3464	7.3276	pos	M+H	[illegible]
pos_3297	N-Arachidonoyl Lysine	metab_2279	HMDB0241999	B(ii)	-	865.6717	7.4371	pos	2M+H	[illegible]
pos_3298	Butylisopropylamine	metab_2280	-	B(i)	-	116.1435	7.445	pos	M+H	[illegible]
pos_330	Phenylalanylglutamic acid	metab_2283	HMDB0028994	B(ii)	-	317.113	2.6977	pos	[illegible]	[illegible]
pos_3304	Calcitroic acid	metab_2288	HMDB0006472	B(ii)	C18230	413.2114	7.4919	pos	M+K	[illegible]
pos_3321	PS(22:6(4Z,7Z,10Z,13Z,16Z,19Z)/19:0)	metab_2305	LMGP03010833;HMDB0256888	B(ii)	-	872.5378	7.5701	pos	M+Na	[illegible]
pos_3327	Neoporrigenin B	metab_2311	HMDB0032680	B(ii)	-	485.2633	7.6014	pos	M+K	[illegible]
pos_3328	LysoPC(18:1(11Z)/0:0)	metab_2312	HMDB0010385	B(ii)	C04230	544.3372	7.6014	pos	M+Na	[illegible]
pos_3333	SM(d17:1/16:0)	metab_2317	-	B(i)	-	711.5396	7.617	pos	M+Na	[illegible]
pos_3336	LysoPC(18:2(9Z,12Z)/0:0)	metab_2320	HMDB0010386	B(ii)	-	1039.6683	7.617	pos	2M+H	[illegible]

pos_3337	Muzanzagenin	metab_2321	HMDB0032601	B(ii)	-	481.2322	7.6326	pos	M+K	C27H38O5	0	45.3	-6.451893094	197080-19-6		0.00698905	6.02	5.986	5.954	6.006	5.99	[illegible]
pos_3338	LysoPI(18:0/0:0)	metab_2322	HMDB0240261	B(ii)	-	623.316	7.6326	pos	M+Na	C27H53O12P	0	72.1	-1.153980972	327620-46-2		0.13914443	4.392	5.3	5.026	4.894	4.831	[illegible]
pos_3341	PC(18:3(9Z,12Z,15Z)/16:0)	metab_2325	HMDB0008199;LMGP01011677;PW_C004247	B(ii)	C00157	756.5534	7.6481	pos	M+H	C42H78NO8P	0	74.5	-0.519468789	-		0.01423554	6.641	7.063	6.39	5.526	2.608	[illegible]
pos_3351	PC(20:5(5Z,8Z,11Z,14Z,16E)-OH(18)/2:0)	metab_2333	HMDB0288865	B(ii)	-	564.3133	7.6871	pos	M+H-2H2O	C30H50NO9P	0	62.6	8.139926497	-		0.03964696	5.06	5.191	5.09	5.028	4.953	[illegible]
pos_336	O-Ethyl-4-[(2',3',4'-tri-O-acetyl-alpha-L-	metab_2342	HMDB0031949	B(ii)	-	509.2134	2.7056	pos	M+NH4, M+ACN+H	C22H29NO10	0	37.6	0.839843887	159733-93-4		0.09379378	2.119	2.126	2.146	2.136	2.142	[illegible]
pos_338	Halleridone	metab_2363	HMDB0302803	B(ii)	-	137.0596	2.7134	pos	M+H-H2O, M+Na	C8H10O3	0	31.6	-0.787053454	-		0.02829213	4.659	4.611	4.634	4.596	4.581	[illegible]
pos_34	4-Oxo-L-proline	metab_2385	HMDB0304793;HMDB0246561	B(ii)	C01877	130.0499	1.1234	pos	M+H, 2M+H, M+Na	C5H7NO3	0	40.1	0.138132434	4347-18-6		0.00479293	7.277	7.261	7.26	7.253	7.258	[illegible]
pos_344	Serylisoleucine	metab_2429	HMDB0029042	B(ii)	-	260.16	2.7212	pos	M+H, M+ACN+H	C9H18N2O4	0	49.3	-2.299367049	91086-51-0		0.01413191	5.483	5.485	5.475	5.479	5.426	[illegible]
pos_3441	10-hydroxy-(2E,8E)-decadien-4-ynoic Acid	metab_2431	HMDB0062773	B(ii)	-	222.1122	7.9063	pos	M+ACN+H	C10H12O3	0	49.5	-1.469677238	-		0.01110078	5.727	5.704	5.712	5.698	5.667	[illegible]
pos_3443	Dimethylethanolamine	metab_2433	HMDB0032231	B(ii)	C04308	90.0916	7.9298	pos	M+H	C4H11NO	0	72.1	2.977187467	108-01-0		0.03226304	5.826	5.813	5.823	5.828	5.821	[illegible]
pos_3449	Dibutyl Phthalate	metab_2439	HMDB0033244;MJDBOTE0001756	B(i)	C14214	279.1588	8.0393	pos	M+H	C16H22O4	90	0	-0.936310593	84-74-2		0.01799792	5.549	5.554	5.555	5.54	5.575	[illegible]
pos_3458	4-Amino-1-piperidinecarboxylic acid	metab_2449	HMDB0060385	B(ii)	C16837	109.0762	8.1802	pos	M+H-2H2O	C6H12N2O2	0	60.9	1.563317647	959317-82-9		0.01806611	4.422	4.356	4.415	4.226	4.363	[illegible]
pos_3466	Etiracetam	metab_2458	HMDB0252105;HMDB0015333	B(ii)	C07841	153.102	8.1879	pos	M+H-H2O	C8H14N2O2	0	53.3	-1.344431032	102767-28-2		0.01453748	4.552	4.394	4.439	4.196	4.33	[illegible]
pos_348	N,N-Dimethylguanosine	metab_2473	-	B(i)	-	311.1233	2.7446	pos	M+, M+H	C12H17N5O5	39	0	1.096281571	-		0.00388392	5.764	5.816	5.783	5.783	5.792	[illegible]
pos_349	3-Methoxytyramine	metab_2484	HMDB0000022;PW_C000015;HMDB0259535	B(ii)	C05587	209.1282	2.7525	pos	M+ACN+H, 2M+ACN+Na	C9H13NO2	0	46.4	-1.677569318	554-52-9		0.00083421	4.975	4.925	4.942	4.92	4.928	[illegible]
pos_3494	Palmitic acid	metab_2489	HMDB0303203;HMDB0000220;PW_C000145;LMFA01010001	B(ii)	C00249	274.2739	7.961	pos	M+NH4	C16H32O2	0	66.2	-0.557758075		1957-10-3	0.16263778	4.321	3.833	3.735	4.275	3.164	[illegible]
pos_3499	2-Aminopyridine	metab_2494	HMDB0245026	B(i)	-	112.0871	7.9063	pos	M+NH4	C5H6N2	30.8	0	1.954055115	-		0.00343026	4.952	4.875	4.83	4.791	4.748	[illegible]
pos_35	4-(2-Aminophenyl)-2,4-dioxobutanoic acid	metab_2495	HMDB0000978;PW_C000778	B(ii)	C01252	225.0866	1.272	pos	M+H-H2O, M+NH4, M+H	C10H9NO4	0	43.5	-1.66255573	90924-76-8		0.00652484	5.851	5.846	5.848	5.842	5.827	[illegible]
pos_357	Isonicophen	metab_2569	HMDB0247959	B(ii)	-	299.091	2.7998	pos	M+H-2H2O, M+	C15H13N3O4	0	31.1	1.418464626	-		0.01394132	3.77	3.336	3.356	3.793	3.352	[illegible]
pos_358	(2S)-3-Hydroxy-1-[(2S)-pyrrolidine-2-carbonyl]pyrrolidine-2-carboxylic	metab_2579	HMDB0257791	B(ii)	-	229.118	2.7998	pos	M+H, M+Na	C10H16N2O4	0	44.4	-1.35799786	-		0.00411943	5.582	5.475	5.471	5.468	5.489	[illegible]
pos_3595	Cyclosquamosin A	metab_2594	HMDB0303367	B(ii)	-	606.3079	7.7027	pos	M+H-2H2O	C31H43N7O8	0	73.7	6.935241569	-		0.0108374	6.134	6.113	6.141	6.129	6.156	[illegible]
pos_3597	LysoPE(18:3(6Z,9Z,12Z)/0:0)	metab_2596	HMDB0011508	B(ii)	-	498.2587	7.7027	pos	M+Na	C23H42NO7P	0	39.1	-0.868661621	-		0.00846863	6.126	6.093	6.076	6.064	6.027	[illegible]
pos_3629	(3alpha,5beta,7alpha)-23-Carboxy-7-hydroxy-24-norcholan-3-yl-beta-D-Glucopyranosiduronic acid	metab_2629	LMST05010028;HMDB0243600	B(ii)	-	607.2916	7.6794	pos	M+K	C30H48O10	0	42.6	6.447347413	-		0.06050084	6.022	6.194	6.005	5.97	5.965	[illegible]
pos_3634	Sugeonol	metab_2635	HMDB0041036;LMPR0103450003	B(ii)	C17504	510.3554	7.6716	pos	2M+ACN+H	C15H22O2	0	42.8	-5.17807828	19419-58-0		0.01658634	6.31	6.299	6.232	6.295	6.235	[illegible]
pos_3641	1-O-Hexadecyl-sn-glycero-3-phosphocholine	metab_2643	HMDB0243890;LMGP01060010	B(ii)	C13903	504.3438	7.6638	pos	M+Na	C24H52NO6P	0	41.5	2.854098545	-		0.0252296	5.955	5.911	5.958	5.979	5.968	[illegible]
pos_3643	Contignasterol	metab_2645	HMDB0250439	B(ii)	C19902	1017.6866	7.6559	pos	2M+H	C29H48O7	0	36.8	-0.64173076	137571-30-3		0.06116192	7.014	6.928	6.96	6.954	6.951	[illegible]
pos_3645	SM(d17:2(4E,8Z)/16:0)	metab_2647	HMDB0240677	B(ii)	-	687.5434	7.6559	pos	M+H	C38H75N2O6P	0	30.6	-0.168831554	-		0.06862287	4.815	4.969	4.842	4.832	4.838	[illegible]
pos_3646	Ruscogenin	metab_2648	HMDB0257364;LMST01080038	B(ii)	C08909	494.324	7.6559	pos	M+ACN+Na	C27H42O4	0	57.7	-0.295883034	472-11-7		0.00854074	5.894	5.888	5.894	5.924	5.911	[illegible]
pos_3648	LysoPC(20:2(11Z,14Z)/0:0)	metab_2650	HMDB0010392	B(ii)	C04230	548.3703	7.6559	pos	M+H	C28H54NO7P	0	62.3	-1.40446381	-		0.04584218	5.874	5.836	5.916	5.865	5.861	[illegible]
pos_3650	4-Trimethylammoniobutanoic acid	metab_2653	PW_C000899;HMDB0001161	B(ii)	C01181	184.073	7.6559	pos	M+K	C7H15NO2	0	34	-2.805009462	407-64-7		0.01452812	6.195	6.151	6.151	6.176	6.169	[illegible]
pos_3651	GPCho(18:1/16:0)	metab_2654	HMDB0011239;HMDB0011305;HMDB0011272;HMDB0008067;HM	B(i)	C00157	782.5681	7.6481	pos	M+Na	C42H82NO8P	58.1	0	1.458402006	-		0.03827494	6.494	6.727	6.196	5.182	4.703	[illegible]
pos_3654	1-(5Z,8Z,11Z,14Z-eicosatetraenoyl)-sn-glycero-3-phosphate	metab_2657	HMDB0062312;LMGP10050013	B(ii)	-	500.2746	7.6403	pos	M+ACN+H	C23H39O7P	0	54.8	-5.627697121	-		0.01401765	5.751	5.803	5.801	5.776	5.757	[illegible]
pos_3657	1-Heneicosanoyl-glycero-3-phosphoserine	metab_2660	HMDB0243883;LMGP03050026	B(ii)	-	532.3364	7.6326	pos	M+H-2H2O	C27H54NO9P	0	62.8	-5.908575487	-		0.03580042	6.024	5.943	5.947	5.953	5.933	[illegible]
pos_3659	Tyr-Gly-Gly-Trp-Leu	metab_2662	HMDB0259342	B(ii)	-	617.2695	7.6248	pos	M+Na	C30H38N6O7	0	50	0.1937878	-		0.21182347	6.357	6.769	6.807	6.804	6.777	[illegible]
pos_366	Dibenzo[a,c]cyclooctene, 5,6,7,8-tetrahydro-	metab_2663	HMDB0244111	B(ii)	-	231.1125	2.8474	pos	M+Na, M+CH3OH+H	C16H16	0	34.8	-9.1629402	-		0.00182002	5.046	5.11	5.071	5.089	5.095	[illegible]
pos_3664	PC(18:1(6Z)/0:0)	metab_2667	LMGP01050029;HMDB0256159	B(ii)	-	560.3111	7.6092	pos	M+K	C26H52NO7P	0	89.8	-0.424236289	-		0.00800384	7.076	7.042	7.073	7.078	7.069	[illegible]
pos_3665	LysoPC(16:0/0:0)	metab_2668	HMDB0010382	B(ii)	C04230	534.2953	7.6092	pos	M+K	C24H50NO7P	0	75.9	-0.78678228	17364-16-8		0.01583018	6.734	6.699	6.732	6.729	6.718	[illegible]
pos_3666	Amastatin	metab_2669	HMDB0248277	B(ii)	C01552	516.3062	7.6092	pos	M+ACN+H	C21H38N4O8	0	69.9	7.126143362	67655-94-1:69400-55-1		0.03766705	5.51	5.491	5.542	5.546	5.493	[illegible]
pos_3669	SM(d15:1/18:1)	metab_2672	-	B(i)	-	709.5252	7.6014	pos	M+Na	C38H75N2O6P	91.4	0	-0.38034879	-		0.04818205	3.885	4.743	3.912	3.902	3.908	[illegible]
pos_3672	(R)-Pterosin B	metab_2676	HMDB0030759	B(ii)	-	459.2475	7.6014	pos	2M+Na	C14H18O2	0	30.4	-7.011305414	34175-96-7		0.00395801	6.803	6.768	6.772	6.793	6.784	[illegible]
pos_3678	SM(d18:2(4E,14Z)/16:0)	metab_2681	HMDB0240638	B(ii)	-	723.5405	7.5858	pos	M+Na	C39H77N2O6P	0	75.7	-0.902096587	2292199-28-9		0.09850595	5.819	5.166	5.481	5.177	5.361	[illegible]
pos_3680	CPA(18:1(9Z)/0:0)	metab_2684	HMDB0007006	B(ii)	-	463.2189	7.5779	pos	M+2Na-H	C21H39O6P	0	32.5	-1.606156615	-		0.02047409	5.304	5.145	5.299	5.189	5.326	[illegible]
pos_3681	Leukotriene B4 dimethylamide	metab_2685	HMDB0005085	B(ii)	-	765.5125	7.5701	pos	2M+K	C22H37NO3	0	39.7	-7.417467387	83024-92-4		0.16618704	4.991	5.662	5.206	5.271	5.104	[illegible]
pos_3685	Retaspimycin	metab_2689	HMDB0257169	B(ii)	-	610.3089	7.5623	pos	M+Na	C31H45N3O8	0	70.2	-1.599712858	-		0.00245464	6.969	6.93	6.965	6.973	6.989	[illegible]
pos_3712	Triethanolamine	metab_2716	HMDB0032538	B(i)	C06771	150.1122	7.4841	pos	M+H	C6H15NO3	83.3	0	-1.502314731	102-71-6		0.02199826	5.302	5.192	5.254	5.243	5.244	[illegible]
pos_3737	(3S,3'S,5R,5'R,6R)-3,6-Epoxy-5,6-dihydro-3',5,8'-trihydroxy-beta,kappa-caroten-6'-one	metab_2741	HMDB0037795	B(ii)	-	634.452	7.3745	pos	M+NH4	C40H56O5	0	37.2	8.700960781	256505-53-0		0.00736949	6.373	6.29	6.326	6.332	6.277	[illegible]
pos_3745	2-Hydroxy-p-mentha-1,8-dien-6-one	metab_2748	HMDB0037012	B(ii)	-	149.0959	7.3041	pos	M+H-H2O	C10H14O2	0	36.4	-1.313019441	51200-86-3		0.00952315	5.315	5.278	5.339	5.326	5.345	[illegible]
pos_3749	Cucurbic acid	metab_2752	HMDB0029388	B(ii)	C08482	245.1742	7.2963	pos	M+CH3OH+H	C12H20O3	0	73.1	-2.401062406	58240-50-9		0.0084209	6.732	6.716	6.732	6.738	6.736	[illegible]
pos_3750	2-Methyl-1,3-cyclohexadiene	metab_2754	HMDB0032395	B(ii)	-	95.0857	7.2885	pos	M+H	C7H10	0	41.7	2.102091382	1489-57-2		0.01130978	5.345	5.302	5.329	5.326	5.325	[illegible]
pos_3753	TG(16:1(9Z)/14:1(9Z)/20:2n6)	metab_2756	HMDB0048576	B(ii)	-	865.6723	7.2572	pos	M+K	C53H94O6	0	47.9	4.908459986	-		0.03851923	6.303	6.294	6.267	6.394	6.34	[illegible]
pos_3755	Chenodeoxycholylarginine	metab_2758	HMDB0242396	B(ii)	-	590.4263	7.2494	pos	M+ACN+H	C30H52N4O5	0	43.1	-2.440033461	-		0.00352566	6.345	6.288	6.331	6.342	6.325	[illegible]
pos_3757	Cycloheximide	metab_2759	HMDB0250657	B(ii)	C06685	281.1643	7.2416	pos	M+	C15H23NO4	0	49.7	5.75023499	66-81-9		0.00926179	6.295	6.259	6.283	6.273	6.29	[illegible]
pos_3763	Monopropionylcadaverine	metab_2764	HMDB0254871	B(ii)	-	355.2446	7.2181	pos	2M+K	C8H18N2O	0	32	-7.462378103	-		0.01106087	5.411	5.378	5.43	5.44	5.418	[illegible]
pos_3767	TG(8:0/8:0/8:0)	metab_2768	PW_C006819;HMDB0011187	B(ii)	C13044	435.3443	7.2024	pos	M+H-2H2O	C27H50O6	0	64.8	-5.468557207	538-23-8		0.03706383	5.569	5.594	5.576	5.574	5.599	[illegible]
pos_3781	Prostaglandin F1a	metab_2782	HMDB0002685	B(ii)	C06475	357.2604	7.1555	pos	M+H	C20H36O5	0	40	-8.8321982	745-62-0		0.01713532	5.79	5.754	5.768	5.769	5.748	[illegible]
pos_3790	LysoPC(18:1(9Z)/0:0)	metab_2789	HMDB0002815	B(ii)	-	522.3552	7.1086	pos	M+H	C26H52NO7P	0	82.2	-0.469017534	19420-56-5		0.01158809	8.136	8.107	8.083	8.104	8.096	[illegible]
pos_3794	PC(24:1(15Z)/15:0)	metab_2793	PW_C004838;HMDB0008790	B(ii)	C00157	893.6673	7.0773	pos	M+ACN+Na	C47H92NO8P	0	52.3	-5.450380124	-		0.01621701	6.364	6.332	6.387	6.424	6.432	[illegible]
pos_38	Histidylhistidine	metab_2799	HMDB0028887	B(ii)	-	257.1128	1.9725	pos	M+, 2M+Na, M+H-2H2O	C12H16N6O3	0	36.5	-5.939070106	306-14-9		0.00520492	4.371	4.472	4.488	4.423	4.577	[illegible]
pos_3801	Rishitin	metab_2802	HMDB0302980	B(ii)	C09715	445.3275	7.0382	pos	2M+H	C14H22O2	0	58.2	-8.330098729	-		0.1043544	5.858	5.979	5.987	5.968	5.992	[illegible]
pos_3805	Ganoderic acid H	metab_2803	HMDB0035987	B(ii)	-	617.2692	7.007	pos	M+2Na-H	C32H44O9	0	30.3	-0.824768944	98665-19-1		0.2194623	6.721	7.242	7.25	7.299	7.284	[illegible]
pos_381	(4Z,10Z,12E)-3-Hydroxytetradeca-4,10,12-trienoylcarnitine	metab_2807	HMDB0241414	B(ii)	-	420.2129	2.9341	pos	M+K, M+2Na-H	C21H35NO5	0	55.9	-4.675270266	-		0.00933904	3.979	3.542	4.151	4.112	4.028	[illegible]
pos_3812	LysoPC(22:5(7Z,10Z,13Z,16Z,19Z)/0:0)	metab_2810	HMDB0010403	B(ii)	C04230	592.3362	6.968	pos	M+Na	C30H52NO7P	0	60.6	-1.980322334	-		0.07743597	5.98	5.94	5.953	5.94	5.942	[illegible]
pos_3815	Octadecanamide	metab_2813	HMDB0034146;LMFA08010003	B(ii)	C13846	284.2941	6.968	pos	M+H	C18H37NO	0	32.4	-2.481196937	124-26-5		0.00210347	6.458	6.465	6.446	6.446	6.451	[illegible]
pos_3817	PE(20:5(5Z,8Z,11Z,14Z,17Z)/18:0)	metab_2815	PW_C005501;LMGP02010973;HMDB0009453	B(ii)	C00350	807.569	6.9601	pos	M+ACN+H	C43H76NO8P	0	84.3	5.615331155	-		0.25466983	7.04	7	6.766	6.495	6.069	[illegible]
pos_3818	Avocadyne 4-acetate	metab_2816	LMFA05000645;HMDB0031049	B(ii)	-	653.4957	6.9601	pos	2M+H	C19H34O4	0	63	-4.653562809	28884-46-0		0.0197241	6.581	6.565	6.575	6.66	6.63	[illegible]
pos_3820	7b-Hydroxy-3-oxo-5b-cholanoic acid	metab_2819	HMDB0000541	B(ii)	-	441.2427	6.9601	pos	M+K	C25H38O4	0	45	6.2933209	77060-26-5		0.00953542	6.29	6.333	6.375	6.381	6.401	[illegible]
pos_3829	Aminodeoxykanamycin	metab_2825	HMDB0015303;HMDB0242522	B(ii)	C01822	547.2688	6.9369	pos	M+ACN+Na	C18H37N5O10	0	49.2	-2.134663349		8063-7-8	0.01849048	6.348	6.34	6.387	6.396	6.381	[illegible]
pos_3836	LysoPE(18:0/0:0)	metab_2831	HMDB0011130	B(ii)	-	482.3238	6.8979	pos	M+H	C23H48NO7P	0	47.7	-0.609407465	69747-55-3		0.00313755	6.953	6.941	6.951	6.948	6.945	[illegible]
pos_3839	(+/-)-15,16-Dihydroxyoctadecanoic acid	metab_2834	HMDB0031008	B(ii)	-	299.2574	6.89	pos	M+H-H2O	C18H36O4	0	31.5	-2.269649765	-		0.02901127	5.757	5.783	5.767	5.752	5.773	[illegible]
pos_3840	1-(2,3,8,8-Tetramethyl-1,2,3,4,5,6,7,8-octahydronaphthalen-2-	metab_2836	-	B(i)	-	235.2051	6.89	pos	M+H	C16H26O	79.1	0	-2.308801549	-		0.08395355	5.488	5.464	5.427	5.463	5.471	[illegible]
pos_3847	DG(20:4(5Z,8Z,11Z,14Z)-OH(18R)/0:0/2:0)	metab_2842	HMDB0296878	B(ii)	-	437.2854	6.851	pos	M+H	C25H40O6	0	84.6	-9.947021627	-		0.00635038	7.422	7.418	7.427	7.44	7.444	[illegible]
pos_3849	Heptaethylene glycol monododecyl ether	metab_2844	HMDB0251574	B(ii)	-	539.349	6.8434	pos	M+2Na-H	C26H54O8	0	40.8	-8.089196868	-		0.026849	4.078	4.084	4.104	4.094	4.1	[illegible]
pos_3852	Ryanodine	metab_2847	HMDB0257371	B(ii)	C08705	557.2518	6.8355	pos	M+ACN+Na	C25H35NO9	0	60.1	9.834975543	15662-33-6		0.01814714	7.066	7.053	7.047	7.068	7.081	[illegible]
pos_3854	Avocadyne 2-acetate	metab_2849	HMDB0031047	B(ii)	-	653.4953	6.8277	pos	2M+H	C19H34O4	0	60.7	-5.148270083	37139-04-1		0.0094191	6.654	6.654	6.632	6.578	6.646	[illegible]
pos_3857	Dihomo-gamma-linolenic acid	metab_2852	HMDB0247333;HMDB0002925;LMFA01030158	B(ii)	C03242	651.4794	6.8121	pos	2M+K	C20H34O2	0	52.2	7.341257246	1783-84-2		0.01153991	7.065	7.051	7.058	7.081	7.083	[illegible]
pos_3859	Physagulin A	metab_2854	LMST01160042;LMST01160045;HMDB0041047	B(ii)	-	533.2545	6.8121	pos	M+Na	C30H38O7	0	84.8	6.857896635	146713-98-6		0.0530696	5.585	5.652	5.532	5.561	5.656	[illegible]
pos_3874	Sclareol	metab_2868	HMDB0036827;LMPR0104030010;MJDBOTE0001479	B(ii)	C09183	326.3046	6.7812	pos	M+NH4	C20H36O2	0	43.5	-2.542952729	515-03-7		0.00747813	5.981	5.997	5.92	6.008	5.974	[illegible]
pos_3875	Hexadecenal	metab_2869	HMDB0060482;HMDB0253123	B(ii)	C06123	256.2629	6.7812	pos	M+NH4	C16H30O	0	72.4	-2.600368739	-		0.01048494	6.796	6.793	6.793	6.815	6.811	[illegible]
pos_3879	Deoxycholic acid 3-glucuronide	metab_2871	HMDB0002596;LMST05010046	B(ii)	C03033	607.2908	6.7656	pos	M+K	C30H48O10	0	41.4	5.069897262	72504-58-6		0.0057908	8.332	8.402	8.414	8.422	8.411	[illegible]
pos_3886	SM(d16:1/18:1)	metab_2878	LMSP03010040	B(i)	-	723.5402	6.7268	pos	M+Na	C39H77N2O6P	93.5	0	-1.288648236	-		0.02667082	4.965	3.89	3.91	3.9	3.906	[illegible]
pos_3889	17-hydroxyprogesterone caproate	metab_2880	HMDB0244774	B(ii)	-	483.2475	6.7268	pos	M+K	C27H40O5	0	38.6	-7.229914316	-		0.01060716	7.065	7.036	7.015	7.04	7.055	[illegible]
pos_3890	GPCho(16:0/20:3)	metab_2882	HMDB0039527;HMDB0007980;HMDB0007981;HMDB0011219;HM	B(i)	C00157	806.5657	6.719	pos	M+Na	C44H82NO8P	86.5	0	-1.75544091	155575-01-2		0.11545433	6.395	5.502	4.906	4.98	4.902	[illegible]
pos_3894	PC(14:0/0:0)	metab_2886	LMGP01050012;HMDB0010379	B(i)	-	490.29	6.7111	pos	M+Na	C22H46NO7P	78.7	0	-0.841607973	20559-16-4		0.01938941	6.101	6.103	6.044	6.074	6.176	[illegible]
pos_3895	Vulgarin	metab_2887	HMDB0036130	B(ii)	C09600	551.2641	6.7033	pos	2M+Na	C15H20O4	0	32.8	4.859633645	3162-56-9		0.01175975	6.889	6.948	6.946	6.956	6.963	[illegible]
pos_3897	SM(d18:2(4E,14Z)/18:1(12Z)-2OH(9,10))	metab_2888	HMDB0290583	B(ii)	-	759.572	6.6955	pos	M+H	C41H79N2O8P	0	33.8	9.665447141	-		0.08847761	6.809	6.178	6.256	5.589	4.924	[illegible]
pos_3899	LysoPA(18:1(9Z)/0:0)	metab_2889	HMDB0007855	B(ii)	-	481.2319	6.6955	pos	M+2Na-H	C21H41O7P	0	41.5	4.008807862	-		0.01753165	7.247	7.235	7.237	7.255	7.263	[illegible]
pos_390	Orsellinic acid 2-O-beta-D-glucoside	metab_2891	HMDB0302998	B(ii)	-	348.1287	2.9341	pos	M+NH4, 2M+K	C14H18O9	0	42.1	-0.770549741	-		0.00541731	4.386	3.582	3.443	3.433	3.95	[illegible]
pos_3900	LysoPA(16:0/0:0)	metab_2892	HMDB0007853	B(ii)	C04036	433.2321	6.6955	pos	M+Na	C19H39O7P	0	89.8	-1.185246205	-		0.0531057	4.429	4.435	4.456	4.445	4.451	[illegible]
pos_3902	Didemethylcitalopram	metab_2894	PW_C040559;HMDB0060472	B(ii)	C16609	593.2751	6.6877	pos	2M+H	C18H17FN2O	0	31.2	4.818392104	62498-69-5		0.00169064	8.804	8.849	8.856	8.864	8.856	[illegible]
pos_3903	PC(22:5/0:0)	metab_2895	HMDB0010403	B(i)	C04230	570.354	6.6877	pos	M+H	C30H52NO7P	76	0	-2.482994249	-		0.01868575	6.424	6.414	6.381	6.422	6.415	[illegible]
pos_3904	LysoPC(16:1(9Z)/0:0)	metab_2896	HMDB0010383	B(ii)	C04230	516.3059	6.6877	pos	M+Na	C24H48NO7P	0	83.2	-0.238642886	76790-27-7		0.01480825	7.272	7.253	7.262	7.269	7.271	[illegible]
pos_3905	1-Hexadecanoyl-sn-Glycero-3-Phosphoethanolamine	metab_2897	LMGP02050002;HMDB0011503	B(i)	-	454.292	6.6877	pos	M+H	C21H44NO7P	81	0	-1.728707093	53862-35-4		0.0055629	6.97	6.954	6.955	6.951	6.964	[illegible]
pos_3906	Dihomolinoleic acid	metab_2898	LMFA01031043;HMDB0061864	B(ii)	C16525	313.273	6.6877	pos	M+CH3OH+H	C18H32O2	0	32.7	-2.740281177	7307-45-1		0.02382083	5.932	5.939	5.908	5.906	5.956	[illegible]
pos_3913	Arctiol	metab_2906	HMDB0301955	B(ii)	-	254.2472	6.6642	pos	M+NH4	C16H28O	0	59.2	-2.74689311	-		0.01511157	5.366	5.503	5.388	5.417	5.428	[illegible]
pos_392	5-trans Fluprostenol	metab_2913	-	B(i)	-	441.1865	2.9966	pos	M+H-H2O, M+Na	C23H29F3O6	31.3	0	-4.06269325	-		0.00540265	3.483	3.455	3.863	3.465	3.547	[illegible]
pos_3926	2-Hydroxypropyl octanoate	metab_2920	HMDB0245164	B(ii)	-	427.3023	6.6252	pos	2M+Na	C11H22O3	0	32.1	-1.768396117	-		0.03878043	5.632	5.512	5.538	5.708	5.536	[illegible]
pos_3929	(2R,3Z)-Phycocyanobilin	metab_2922	HMDB0242598	B(ii)	-	609.2698	6.6096	pos	M+Na	C33H38N4O6	0	46.9	2.411359532	-		0.00774674	7.257	7.287	7.283	7.255	7.27	[illegible]
pos_3935	Aminophenazone	metab_2928	HMDB0015493	B(ii)	C07539	526.2895	6.5939	pos	2M+ACN+Na	C13H17N3O	0	36.7	-1.326239337	58-15-1		0.14656996	4.051	4.058	4.078	4.068	4.074	[illegible]
pos_3938	Bardoxolone methyl	metab_2930	HMDB0248872	B(ii)	-	1011.639	6.5861	pos	2M+H	C32H43NO4	0	40.9	-6.579494781	-		0.26612739	5.377	5.355	5.201	4.255	4.261	[illegible]
pos_3940	Corchoroside A	metab_2933	HMDB0033846	B(ii)	-	579.2594	6.5783	pos	M+2Na-H	C29H42O9	0	82	9.942536418	508-76-9		0.00462274	8.364	8.382	8.38	8.383	8.385	[illegible]
pos_3941	LysoPA(18:2(9Z,12Z)/0:0)	metab_2934	HMDB0007856	B(ii)	-	457.2319	6.5783	pos	M+Na	C21H39O7P	0	82.6	-1.513776296	-		0.00404418	5.639	5.543	5.519	5.681	5.784	[illegible]
pos_3942	LysoPE(0:0/16:1(9Z))	metab_2935	HMDB0011474	B(ii)	-	474.2588	6.5628	pos	M+Na	C21H42NO7P	0	83.7	-0.72850072	-		0.14886703	4.73	4.736	4.757	4.746	4.752	[illegible]
pos_3943	All-trans-13,14-dihydroretinol	metab_2936	HMDB0011618;PW_C007207;LMPR01090047	B(ii)	C15492	306.2786	6.5628	pos	M+NH4	C20H32O	0	32.2	-2.038401166	115797-14-3		0.05059018	5.289	5.263	5.306	5.204	5.357	[illegible]
pos_3948	Lucidenic acid C	metab_2938	HMDB0037609	B(ii)	-	975.5428	6.5472	pos	2M+Na	C27H40O7	0	71.5	-1.253708942	95311-96-9		0.03218879	5.397	5.731	5.729	5.648	5.639	[illegible]
pos_3949	Pregnenolone	metab_2939	HMDB0244633;PW_C000173;LMST02030088;HMDB0000253;MJD	B(ii)	C01953	349.273	6.5472	pos	M+CH3OH+H	C21H32O2	0	52.7	-2.353343362	145-13-1		0.00622356	7.002	7.052	7.052	7.057	7.083	[illegible]
pos_3956	Zeatin	metab_2946	HMDB0012204;MJDBZSJ0000114	B(ii)	C00371	237.1452	6.516	pos	M+NH4	C10H13N5O	0	39.4	-2.890085398	1637-39-4		0.01702391	5.374	5.279	5.251	5.258	5.255	[illegible]
pos_3964	Kanokoside D	metab_2955	HMDB0036105	B(ii)	C17431	607.2544	6.4848	pos	M+H-H2O	C27H44O16	0	66	-8.395412335	64703-88-4		0.01027634	6.605	6.67	6.676	6.672	6.674	[illegible]
pos_3966	PA(5-iso PGF2VI/18:1(11Z))	metab_2956	HMDB0263726	B(ii)	-	727.4596	6.4613	pos	M+H-H2O	C39H69O11P	0	35.8	6.904672353	-		0.03198955	5.824	5.68	5.899	5.725	5.744	[illegible]
pos_3971	P-Menthane-3,8-diol	metab_2962	HMDB0036145;LMPR0102090063;HMDB0035595	B(ii)	C02904	367.2833	6.4613	pos	2M+Na	C10H20O2	0	62.4	4.083644271	565-50-4:91739-72-9:4		0.01355075	5.747	5.71	5.721	5.758	5.753	[illegible]
pos_3975	Methyl acrylate	metab_2966	HMDB0033977	B(ii)	C19443	124.9998	6.4535	pos	M+K	C4H6O2	0	43.9	-1.48918973	96-33-3		0.01595959	5.772	5.765	5.766	5.738	5.779	[illegible]
pos_3979	Vindoline	metab_2969	HMDB0259814;MJDBOTE0000982	B(ii)	C01626	498.2584	6.4301	pos	M+ACN+H	C25H32N2O6	0	71.5	-3.13521196	2182-14-1		0.00897116	7.717	7.7	7.704	7.704	7.699	[illegible]
pos_3981	9-Octadecenamide	metab_2971	HMDB0247607;HMDB0002117	B(ii)	C19670	304.2628	6.4301	pos	M+Na	C18H35NO	0	61.9	6.0879763	301-02-0		0.01295769	6.283	6.294	6.31	6.301	6.291	[illegible]
pos_3982	2-Cyclohexylidenecyclohexanone	metab_2972	HMDB0245090	B(ii)	-	179.1426	6.4301	pos	M+H	C12H18O	0	33.2	-2.500555099	-		0.09497714	5.237	5.218	5.21	5.159	5.229	[illegible]
pos_3983	DG(22:6(4Z,7Z,11E,13Z,15E,19Z)-2OH(10S,17)/0:0/2:0)	metab_2973	HMDB0297026	B(ii)	-	975.5437	6.4224	pos	2M+Na	C27H40O7	0	66.5	-0.384153217	-		0.04013198	6.046	6.117	6.009	5.971	6.076	[illegible]
pos_3999	SM(d18:1/14:0)	metab_2987	LMSP03010028;HMDB0012097	B(ii)	-	675.5427	6.3911	pos	M+H	C37H75N2O6P	0	55.2	-1.28511744	121999-55-1		0.0403002	7.295	5.008	7.245	6.596	6.522	[illegible]
pos_4	LysoPE(18:3(9Z,12Z,15Z)/0:0)	metab_2988	HMDB0011509	B(ii)	-	476.2767	6.4146	pos	M+2Na-H, 2M+H, 2M+Na, M+H, M+H-H2O	C23H42NO7P	0	55.3	-1.006922019	-		0.00369069	8.719	8.711	8.708	8.707	8.709	[illegible]
pos_4002	2-(Methylthio)benzothiazole	metab_2993	HMDB0303335	B(i)	C10910	182.0089	6.3911	pos	M+H	C8H7NS2	88.3	0	-2.286601664	-		0.00062936	5.453	5.459	5.444	5.469	5.436	[illegible]
pos_4008	Tsangane L 3-glucoside	metab_2999	HMDB0040824	B(ii)	-	357.2238	6.3833	pos	M+H-H2O	C19H34O7	0	57.6	-8.90027614	143775-68-2		0.00266822	6.117	6.059	6.107	6.08	6.096	[illegible]
pos_4009	6-Hydroxypentadecanedioic acid	metab_3000	HMDB0031885	B(ii)	-	311.182	6.3833	pos	M+Na	C15H28O5	0	43.9	-3.118688565	38076-52-7		0.0061958	6.047	5.989	6.041	6.042	6.039	[illegible]
pos_4010	N-Acetyl-b-glucosaminylamine	metab_3002	HMDB0001104	B(ii)	C01239	253.1402	6.3833	pos	M+CH3OH+H	C8H16N2O5	0	81.2	3.495632583	-		0.01430293	5.306	5.269	5.356	5.316	5.261	[illegible]
pos_4011	2-Amino-6-methoxypurine	metab_3003	HMDB0245002	B(ii)	-	207.0985	6.3833	pos	M+ACN+H	C6H7N5O	0	65.3	-2.301295024	-		0.00413562	5.403	5.359	5.456	5.417	5.359	[illegible]
pos_4015	SM(d16:1/14:0)	metab_3007	-	B(i)	-	669.4957	6.3755	pos	M+Na	C35H71N2O6P	48.2	0	2.266029799	-		0.27127668	6.431	6.009	5.997	5.933	5.934	[illegible]
pos_4016	(11R,16S)-misoprostol	metab_3008	HMDB0242321	B(ii)	-	415.3022	6.3755	pos	M+CH3OH+H	C22H38O5	0	50.7	-8.344653195	-		0.01515673	6.041	6.042	6.059	6.027	6.057	[illegible]
pos_402	Banoxantrone	metab_3012	HMDB0248859	B(ii)	-	409.1868	3.0825	pos	M+H-2H2O, M+H	C22H28N4O6	0	33.7	-0.523782492	-		0.00771597	6.41	6.402	6.406	6.403	6.41	[illegible]
pos_4021	Curdione	metab_3014	HMDB0034714	B(ii)	C17493	219.1738	6.3755	pos	M+H-H2O	C15H24O2	0	80.5	-2.172650699	13657-68-6		0.00768745	5.856	5.8	5.817	5.818	5.827	[illegible]
pos_4023	GPCho(16:0/20:5)	metab_3015	HMDB0039528;HMDB0007984	B(i)	C00157	802.5352	6.3676	pos	M+Na	C44H78NO8P	93.5	0	-0.670008555	132196-28-2		0.07155369	5.836	6.668	5.303	4.165	4.171	[illegible]
pos_4024	DG(22:6(4Z,7Z,10Z,13Z,16Z,19Z)/22:5(4Z,7Z,10Z,13Z,16Z)/0:0)	metab_3016	PW_C003837;HMDB0007786	B(ii)	C00165	697.5251	6.3676	pos	M+H-H2O	C47H70O5	0	63.1	8.52594768	-		0.05372668	6.169	4.392	6.052	4.442	4.408	[illegible]
pos_4027	Grayanotoxin	metab_3019	HMDB0246239	B(ii)	-	355.2446	6.3676	pos	M+H	C20H34O5	0	43.7	-9.193859949	-		0.00939443	6.238	6.206	6.218	6.212	6.219	[illegible]
pos_403	Ethotoin	metab_3022	HMDB0014892	B(ii)	C07839	249.0607	3.114	pos	M+K, M+2Na-H	C11H12N2O2	0	43.7	-1.673534869	86-35-1		0.10176422	4.441	4.694	4.405	4.382	4.525	[illegible]
pos_4047	(3beta,5beta,8beta,22E,24xi)-Ergosta-6,22-diene-3,5,8-triol	metab_3038	HMDB0038353	B(ii)	-	430.3461	6.3286	pos	M+	C28H46O3	0	55.7	3.164711512	288847-52-9		0.01310001	6.21	6.167	6.171	6.158	6.166	[illegible]
pos_4049	Physagulin B	metab_3040	HMDB0041048;LMST01160044	B(ii)	-	585.1978	6.3208	pos	M+K	C30H39ClO7	0	91.6	-6.869575566	146713-97-5		0.00905265	6.789	6.722	6.743	6.72	6.741	[illegible]
pos_4053	8(R)-Hydroperoxylinoleic acid	metab_3045	HMDB0004706	B(ii)	C14831	335.2191	6.313	pos	M+Na	C18H32O4	0	49.2	-0.616939576	143343-95-7		0.0064046	6.356	6.292	6.293	6.308	6.301	[illegible]
pos_4057	Prostaglandin D1	metab_3049	LMFA03010049;HMDB0005102	B(ii)	C06438	337.2363	6.3052	pos	M+H-H2O	C20H34O5	0	84.9	-2.984573456	17968-82-0		0.00526501	7.234	7.215	7.231	7.202	7.215	[illegible]
pos_4059	P-Anisaldehyde	metab_3051	HMDB0029686	B(i)	C10761	137.0595	6.3052	pos	M+H	C8H8O2	52.5	0	-1.698505318	123-11-5		0.01307289	5.89	5.867	5.903	5.865	5.881	[illegible]
pos_4069	Jasmolone	metab_3061	HMDB0030039	B(ii)	-	181.1219	6.2896	pos	M+H	C11H16O2	0	60.4	-2.27979523	54383-66-3		0.00566379	5.669	5.665	5.682	5.654	5.636	[illegible]
pos_4071	Astromicin	metab_3064	HMDB0248678	B(ii)	C17708	447.2924	6.2818	pos	M+ACN+H	C17H35N5O6	0	36.3	-0.507161728	55779-06-1		0.00552693	6.594	6.547	6.57	6.562	6.582	[illegible]
pos_4075	O-Stearoylcarnitine	metab_3068	-	B(i)	-	428.3734	6.274	pos	M+H	C25H49NO4	59.3	0	-0.032641684	-		0.00289954	6.955	6.97	6.993	6.978	6.987	[illegible]
pos_4078	N-Palmitoyl Histidine	metab_3071	HMDB0241926;LMFA08020132	B(ii)	-	394.3058	6.2662	pos	M+H	C22H39N3O3	0	71.9	-1.68528802	-		0.00499404	6.078	6.163	6.15	6.171	6.151	[illegible]
pos_4083	N,N'-Dicyclohexylurea	metab_3077	HMDB0244166	B(i)	-	225.1957	6.2584	pos	M+H	C13H24N2O	39.5	0	-2.004735885	-		0.00300688	6.011	5.963	5.994	5.966	5.976	[illegible]
pos_4088	Pyrodone	metab_3081	HMDB0256988	B(ii)	C18795	339.2057	6.2349	pos	M+ACN+Na	C17H25NO2	0	51.6	4.912801222	-		0.02580644	5.522	5.485	5.475	5.425	5.449	[illegible]
pos_4092	Palmitoylcarnitine	metab_3086	HMDB0000222;HMDB0240783;LMFA07070004	B(i)	C02990	400.3418	6.2272	pos	M+H	C23H45NO4	81.2	0	-0.953904033	2364-67-2		0.00928136	6.994	7.037	7.039	7.026	7.044	[illegible]
pos_4093	LysoPE(18:4(6Z,9Z,12Z,15Z)/0:0)	metab_3087	HMDB0011510	B(ii)	-	474.2612	6.2196	pos	M+H	C23H40NO7P	0	73	-0.595573639	-		0.0113876	6.289	6.159	6.16	6.132	6.146	[illegible]
pos_4102	1-Nonanol	metab_3097	LMFA05000092;HMDB0031265	B(ii)	C14696	330.3361	6.1961	pos	2M+ACN+H	C9H20O	0	31.4	-1.892284028	143-08-8		0.02129931	5.478	5.465	5.489	5.44	5.464	[illegible]
pos_4106	N-arachidonylethanolamine	metab_3100	HMDB0004080;HMDB0255084	B(ii)	C11695	351.3365	6.1883	pos	M+NH4	C22H39NO	0	42.3	-1.357848029	94421-68-8		0.00983336	6.087	6.07	6.064	6.061	6.05	[illegible]
pos_4108	16-B1-phytoprostane	metab_3102	HMDB0304634	B(ii)	-	291.1948	6.1883	pos	M+H-H2O	C18H28O4	0	63.9	-2.252942949	-		0.00277396	6.338	6.31	6.314	6.281	6.307	[illegible]
pos_4117	P-Tolualdehyde	metab_3112	HMDB0029638	B(i)	C06758	121.0649	6.1648	pos	M+H	C8H8O	88.6	0	0.751982058	104-87-0		0.00138083	5.381	5.367	5.382	5.339	5.373	[illegible]
pos_4120	Ethynodiol Diacetate	metab_3116	HMDB0014961;LMST02030124	B(ii)	C12724	385.2345	6.1572	pos	M+H	C24H32O4	0	36.9	-7.332900333	297-76-7		0.04607865	5.276	5.283	5.303	5.293	5.299	[illegible]
pos_4122	Polyoxyethylene 40 monostearate	metab_3118	HMDB0032477	B(ii)	-	346.3311	6.1572	pos	M+NH4	C20H40O3	0	75.1	-1.574200469	9004-99-3		0.00339091	6.052	6.028	6.042	6.04	6.051	[illegible]
pos_4124	Sphinganine	metab_3120	LMSP01020001;PW_C000184;HMDB0000269;HMDB0242634	B(i)	C00836	302.3048	6.1572	pos	M+H	C18H39NO2	79.3	0	-1.874047338	764-22-7		0.00889599	6.289	6.259	6.265	6.267	6.257	[illegible]
pos_4129	1-Arachidonoylglycerol	metab_3125	HMDB0011578;HMDB0243830;LMGL01010032	B(ii)	C13857	396.3106	6.1494	pos	M+NH4	C23H38O4	0	71.4	-0.667095099	124511-15-5		0.06652239	5.511	5.489	5.487	5.494	5.488	[illegible]
pos_4144	Lauroylcarnitine	metab_3141	LMFA07070090;HMDB0002250	B(i)	-	344.279	6.1104	pos	M+H	C19H37NO4	84.8	0	-1.492671129	25518-54-1		0.00612217	5.658	5.664	5.684	5.674	5.68	[illegible]
pos_4145	15-Methylpalmitate	metab_3142	HMDB0241917;HMDB0061709	B(ii)	-	288.2892	6.1104	pos	M+NH4	C17H34O2	0	62.2	-1.847288128	-		0.00639516	6.748	6.755	6.747	6.71	6.704	[illegible]
pos_4146	Lauroyl diethanolamide	metab_3143	HMDB0032358;LMFA08040058	B(ii)	-	252.2318	6.1104	pos	M+H-2H2O	C16H33NO3	0	47.4	-1.506186022	120-40-1		0.00202705	5.194	5.161	5.138	5.156	5.152	[illegible]
pos_415	Methyl 20-dihydroprednisolonate	metab_3147	HMDB0254566	B(ii)	-	435.1733	3.1452	pos	M+H, M+2Na-H	C22H30O6	0	56.5	-5.313716097	-		0.0994263	3.732	3.738	3.759	3.748	3.754	[illegible]
pos_4153	Delta-Tocotrienol	metab_3151	LMPR02020056;HMDB0030008	B(ii)	C14156	397.3095	6.0949	pos	M+H	C27H40O2	0	80.2	-1.504840514	25612-59-3		0.05715433	5.246	5.221	5.258	5.216	5.267	[illegible]
pos_4154	(R)-1-Octen-3-ol	metab_3152	HMDB0031299	B(ii)	C14272	274.2737	6.0949	pos	2M+NH4	C8H16O	0	64.1	-1.388023324	-		0.00753285	7.679	7.69	7.684	7.661	7.645	[illegible]
pos_4166	(S)-(-)-Perillyl alcohol	metab_3163	HMDB0003634	B(i)	C02452	135.1167	6.0715	pos	M+H-H2O	C10H16O	59.9	0	-1.136571528	536-59-4		0.01283362	4.679	4.686	4.732	4.725	4.673	[illegible]
pos_4167	Ancymidol	metab_3164	HMDB0248390	B(ii)	C18774	257.1279	6.0637	pos	M+H	C15H16N2O2	0	35.1	-2.148279476	12771-68-5		0.01953936	5.558	5.394	5.389	5.392	5.394	[illegible]
pos_4172	Starch, pregelatinized	metab_3169	HMDB0303308	B(ii)	-	675.2658	6.0405	pos	M+H-H2O	C27H48O20	0	86.4	-6.987852764	-		0.01933805	6.185	6.059	6.052	6.058	6.091	[illegible]
pos_4173	Decanoylcarnitine	metab_3170	LMFA07070059;HMDB0000651	B(i)	C03299	316.2479	6.0405	pos	M+H	C17H33NO4	89.2	0	-1.159572761	1492-27-9		0.01219742	3.345	3.351	3.371	3.361	3.367	[illegible]
pos_4176	Alpha-Bixin	metab_3173	HMDB0035317	B(ii)	C08582	359.2034	6.0327	pos	M+H-2H2O	C25H30O4	0	45.2	7.13548681	6983-79-5		0.01060784	5.531	5.543	5.513	5.481	5.469	[illegible]
pos_4183	(+/-)-2-Butylthiazolidine	metab_3181	HMDB0040116	B(ii)	-	187.126	6.0249	pos	M+ACN+H	C7H15NS	0	31.3	-2.062521741	-		0.01092049	5.383	5.403	5.417	5.38	5.431	[illegible]
pos_4188	Nornicotine	metab_3185	PW_C000880;HMDB0001126	B(ii)	C06524	314.2321	6.0171	pos	2M+NH4	C9H12N2	0	32.2	-6.014465141	494-97-3		0.00429289	4.573	4.579	4.599	4.589	4.595	[illegible]
pos_4189	MG(10:0/0:0/0:0)	metab_3186	HMDB0072866	B(ii)	-	211.1688	6.0171	pos	M+H-2H2O	C13H26O4	0	31.7	-1.853556156	-		0.04127032	4.845	4.83	4.767	4.831	4.857	[illegible]
pos_4193	Ginkgoic acid	metab_3190	HMDB0033897	B(ii)	C10794	364.2839	6.0015	pos	M+NH4	C22H34O3	0	61.6	-2.028125327	22910-60-7		0.0138872	5.469	5.43	5.36	5.385	5.423	[illegible]
pos_4197	Gentian Violet Cation	metab_3194	HMDB0014550	B(i)	-	372.2431	5.9936	pos	M+	C25H30N3+	90.7	0	-2.44643389	14426-25-6		0.02253539	4.848	4.955	4.844	5.03	4.938	[illegible]
pos_4202	Biliverdin	metab_3201	PW_C000797;HMDB0001008	B(ii)	C00500	615.2805	5.9781	pos	M+CH3OH+H	C33H34N4O6	0	74.5	-1.423656568	114-25-0		0.01295291	6.001	6.092	6.132	6.106	6.097	[illegible]
pos_4204	Mepenzolate	metab_3203	HMDB0015591	B(ii)	C07818	358.2273	5.9781	pos	M+NH4	C21H26NO3+	0	74.1	6.529812476	25990-43-6		0.01160705	4.6	4.616	4.729	4.803	4.669	[illegible]
pos_4206	Acarbose	metab_3205	HMDB0248185;HMDB0014429;MJDBOTE0001373	B(ii)	C06802	628.2408	5.9703	pos	M+H-H2O	C25H43NO18	0	91.4	-6.151264522	56180-94-0		0.11224767	5.606	5.514	5.423	5.399	5.463	[illegible]
pos_4210	DG(2:0/0:0/20:5(7Z,9Z,11E,13E,17Z)-3OH(5,6,15))	metab_3210	HMDB0296997	B(ii)	-	499.2875	5.9624	pos	M+CH3OH+H	C25H38O8	0	30.2	-5.60879389	-		0.01248528	3.607	3.403	3.424	3.413	3.419	[illegible]
pos_4211	Ecdysone	metab_3211	LMST01010210;HMDB0251684	B(ii)	C00477	429.2994	5.9624	pos	M+H-2H2O	C27H44O6	0	48.1	-1.240421272	3604-87-3		0.01311909	5.695	5.631	5.633	5.632	5.645	[illegible]
pos_4212	4-[2-(4-Isopropylbenzamido)ethoxy]benzoic acid	metab_3212	HMDB0255770	B(ii)	-	292.1361	5.9624	pos	M+H-2H2O	C19H21NO4	0	30.7	8.94384325	-		0.02458389	4.594	4.697	4.663	4.699	4.667	[illegible]
pos_4213	Artemidinol	metab_3213	HMDB0030647	B(ii)	-	280.0941	5.9624	pos	M+ACN+Na	C13H12O3	0	54.5	-1.426157743	62268-43-3		0.02005782	4.966	4.829	4.87	4.889	4.944	[illegible]
pos_4214	(-)-Jasmonic acid	metab_3214	HMDB0032797;LMFA02020001	B(i)	C08491	211.1326	5.9624	pos	M+H	C12H18O3	38.7	0	-1.212786854	6894-38-8:59366-47-1		0.0854095	4.936	4.953	4.887	4.854	4.854	[illegible]
pos_4215	(+)-Neomenthol	metab_3215	LMPR0102090003;HMDB0035763	B(ii)	C00553	198.185	5.9624	pos	M+ACN+H	C10H20O	0	34	-1.397586118	2216-52-6		0.02002172	3.074	3.08	3.1	3.09	3.096	[illegible]
pos_4217	(+/-)-Ribaline	metab_3217	HMDB0033345	B(ii)	-	258.112	5.9546	pos	M+H-H2O	C15H17NO4	0	64.3	-1.664717375	41234-32-6		0.00963827	5.762	5.718	5.701	5.693	5.729	[illegible]
pos_4218	N-Docosahexaenoyl Valine	metab_3218	HMDB0242025	B(ii)	-	450.2962	5.9467	pos	M+Na	C27H41NO3	0	84.5	-3.806215118	-		0.03179914	5.512	5.561	5.54	5.565	5.523	[illegible]
pos_4221	Cinncassiol D2 glucoside	metab_3222	HMDB0034679	B(ii)	-	513.2669	5.9389	pos	M+H-H2O	C26H42O11	0	35.5	-4.683937326	-		0.07211558	4.259	4.165	2.71	4.439	3.831	[illegible]
pos_4225	Heliangin	metab_3226	HMDB0036692	B(ii)	C09474	380.204	5.9311	pos	M+NH4	C20H26O6	0	54.2	-7.739889072	13323-48-3		0.03505318	3.506	1.364	1.383	3.483	1.379	[illegible]
pos_4242	PS(20:4(8Z,11Z,14Z,17Z)/20:3(8Z,11Z,14Z)-2OH(5,6))	metab_3245	HMDB0282711	B(ii)	-	898.5387	5.8998	pos	M+CH3OH+H	C46H76NO12P	0	53.4	-6.079817624	-		0.04167965	5.901	5.898	5.912	5.872	5.896	[illegible]
pos_4243	Jervine	metab_3246	HMDB0253731;MJDBOTE0000999	B(ii)	C10811	448.2801	5.8998	pos	M+Na	C27H39NO3	0	73.5	-4.893716649	469-59-0		0.06657324	6.176	6.196	6.199	6.179	6.203	[illegible]
pos_4274	TG(16:1(9Z)/16:1(9Z)/20:4(5Z,8Z,11Z,14Z))	metab_3279	HMDB0005436	B(ii)	C00422	868.7414	5.8603	pos	M+NH4	C55H94O6	0	33.1	3.024762356	-		0.07444343	5.165	5.204	5.304	5.359	5.467	[illegible]
pos_4283	Cyanidin 3-triglucoside	metab_3289	HMDB0302726	B(ii)	-	791.2534	5.8526	pos	M+NH4	C33H41O21+	0	51.7	7.104529369	-		0.03181112	5.865	5.878	5.81	5.802	5.76	[illegible]
pos_43	4-hydroxyheptanoyl-CoA	metab_3305	HMDB0301436	B(ii)	-	459.5999	2.5568	pos	M+H+Na, M+Na, M+K	C28H48N7O18P3	0	34.4	4.887475391	-		0.02324452	6.462	6.467	6.475	6.478	6.476	[illegible]
pos_4309	HMDB0127639	metab_3316	-	B(ii)	-	968.5056	5.8218	pos	M+K	C51H79NO14	0	54.8	-8.19451335	-		0.04963866	5.322	5.353	5.332	5.218	5.155	[illegible]
pos_4312	Capecitabine	metab_3320	PW_C009523;HMDB0015233	B(ii)	C12650	423.1621	5.8218	pos	M+ACN+Na	C15H22FN3O6	0	56.2	-8.182934553	154361-50-9		0.03911015	5.961	5.95	5.966	5.905	5.945	[illegible]
pos_4323	N-acetylmuramyl-l-alanine	metab_3332	HMDB0255071	B(ii)	-	397.1827	5.7875	pos	M+CH3OH+H	C14H24N2O9	0	42.8	2.846814254	-		0.08713242	5.103	5.072	5.054	5.027	5.073	[illegible]
pos_433	O-Beta-D-Glucosylzeatin	metab_3339	HMDB0302640	B(i)	C03423	404.1527	2.7842	pos	M+H, M+Na	C16H23N5O6	36.7	0	-3.561455554	-		0.00421541	4.863	4.913	4.6	4.811	4.866	[illegible]
pos_4347	PI(22:3(10Z,13Z,16Z)/TXB2)	metab_3353	HMDB0277833	B(ii)	-	1034.616	5.6364	pos	M+NH4	C52H89O17P	0	51	-1.567085309	-		0.10899409	3.046	3.006	3.026	3.678	3.022	[illegible]
pos_4355	Pyridoxal	metab_3361	HMDB0001545;PW_C001188	B(i)	C00250	150.0548	5.5949	pos	M+H-H2O	C8H9NO3	42.3	0	-0.65619533	66-72-8		0.0258595	3.868	3.799	3.884	3.923	3.864	[illegible]
pos_4357	N-(2-Hydroxyethyl)nicotinamide	metab_3363	HMDB0254988	B(ii)	-	396.165	5.5871	pos	2M+ACN+Na	C8H10N2O2	0	44.1	2.318748267	-		0.14058133	3.976	3.989	4.054	4.137	4.098	[illegible]

pos_4378	Ethyl 3-hydroxydodecanoate	metab_3379	HMDB0059850	B(ii)	-	262.2373	5.4855	pos	[illegible]
pos_4384	Suspensolide F	metab_3386	HMDB0031918	B(ii)	C17429	517.1659	5.4475	pos	[illegible]
pos_4385	2-Furanmethanol	metab_3387	HMDB0013742	B(ii)	C20441	197.0806	5.4475	pos	[illegible]
pos_439	3-Hydroxykynurenic acid	metab_3392	HMDB0246172	B(ii)	-	206.0446	3.4	pos	[illegible]
pos_4394	LysoPI(20:4(5Z,8Z,11Z,14Z)/0:0)	metab_3396	HMDB0061690	B(ii)	-	603.2983	5.4011	pos	[illegible]
pos_44	P-Coumaraldehyde	metab_3401	HMDB0040986	B(ii)	C05608	166.0861	2.4634	pos	[illegible]
pos_4408	Gamma-Glutamyl-beta-cyanoalanine	metab_3410	HMDB0060478	B(ii)	C05711	243.0873	5.3232	pos	[illegible]
pos_441	(2E)-4-Hydroxy-3-methylpent-2-enedioylcarnitine	metab_3412	HMDB0241661	B(ii)	-	326.1209	3.4158	pos	[illegible]
pos_4416	Indolepropionylglycine	metab_3419	HMDB0253470	B(ii)	-	247.1074	5.284	pos	[illegible]
pos_4428	Tryptophol	metab_3430	HMDB0003447;MJDBOTE0000453	B(i)	C00955	144.0806	5.2368	pos	[illegible]
pos_4475	Trp Gly Asp	metab_3474	-	B(i)	-	377.1453	5.1109	pos	[illegible]
pos_45	Valylglutamic acid	metab_3497	HMDB0029126	B(ii)	-	247.1285	2.4481	pos	[illegible]
pos_4503	(R)-1-O-b-D-glucopyranosyl-1,3-octanediol	metab_3501	HMDB0029362	B(ii)	-	639.3506	5.0555	pos	[illegible]
pos_4510	Eugenol	metab_3508	HMDB0005809;MJDBOTE0000375	B(ii)	C10453	197.1169	5.0475	pos	[illegible]
pos_452	N-(2-benzoyl-4-chlorophenyl)-1-methylpyrazole-3-carboxamide	metab_3517	-	B(i)	-	340.0814	3.5516	pos	[illegible]
pos_4528	Tasquinimod	metab_3525	HMDB0258735	B(ii)	-	407.1211	5.008	pos	[illegible]
pos_454	Tyrosylhydroxyproline	metab_3536	HMDB0029106	B(ii)	-	295.1286	3.5751	pos	[illegible]
pos_459	PS(22:6(4Z,7Z,10Z,13Z,16Z,19Z)/PGJ2)	metab_3585	HMDB0283532	B(ii)	-	443.7429	3.5356	pos	[illegible]
pos_4614	25-Acetyl-6,7-didehydrofevicordin F 3-[glucosyl-(1->6)-glucoside]	metab_3609	HMDB0036339	B(ii)	-	866.3921	4.8968	pos	[illegible]
pos_4648	Val Ala Phe Asp	metab_3644	-	B(i)	-	451.2186	4.8726	pos	[illegible]
pos_4685	Gastrodin	metab_3683	HMDB0252648;MJDBOTE0000895	B(ii)	C16964	251.0909	4.8486	pos	[illegible]
pos_470	Riboflavin (Vitamin B2)	metab_3699	-	B(i)	-	377.1455	3.7971	pos	[illegible]
pos_4714	Dihydro-3-hydroxy-4,4-dimethyl-2(3H)-Furanone	metab_3712	-	B(ii)	-	283.1171	4.8328	pos	[illegible]
pos_472	Polyribophosphate	metab_3718	HMDB0256690	B(ii)	-	327.0481	3.8284	pos	[illegible]
pos_4723	TIARAMIDE	metab_3721	HMDB0259071	B(ii)	-	711.1647	4.8247	pos	[illegible]
pos_473	Cuminaldehyde	metab_3726	HMDB0002214	B(i)	C06577	149.0959	3.8284	pos	[illegible]
pos_476	4-[(4-methylphenyl)methyl]-N-(2-phenylethyl)thieno[3,2-b]pyrrole-5-	metab_3755	-	B(i)	-	375.1547	3.8761	pos	[illegible]
pos_4761	Estrone glucuronide	metab_3757	PW_C002143;HMDB0004483	B(ii)	C11133	491.1632	4.8088	pos	[illegible]
pos_4774	PG(5-iso PGF2VI/a-25:0)	metab_3769	HMDB0270990	B(ii)	-	936.6463	4.7931	pos	[illegible]
pos_4822	CDP-DG(i-20:0/i-12:0)	metab_3820	HMDB0116301;PW_C076637	B(ii)	C00269	936.5028	4.761	pos	[illegible]
pos_483	Swertiamarin	metab_3828	HMDB0258644;MJDBOTE0000976	B(ii)	C09800	375.1298	3.9782	pos	[illegible]
pos_4836	3-Demethylubiquinone-9	metab_3835	LMPR02010036;HMDB0060370	B(ii)	C03226	819.5666	4.7532	pos	[illegible]
pos_4866	Integerrimine	metab_3865	HMDB0242680	B(ii)	C10328	712.385	4.7373	pos	[illegible]
pos_4879	Scytalone	metab_3878	-	B(i)	C00779	195.0649	4.7373	pos	[illegible]
pos_488	(3b,20R,22R)-3,20,27-Trihydroxy-1-oxowitha-5,24-dienolide 3-glucoside	metab_3879	HMDB0033573	B(ii)	-	635.34	4.0335	pos	[illegible]
pos_4932	5-Phenyl-1,3-oxazinane-2,4-dione	metab_3934	PW_C040533;HMDB0060400	B(ii)	C16596	209.0916	4.7057	pos	[illegible]
pos_4949	1-(3-Carboxypropylcarbamoyl)-5-fluorouracil	metab_3952	HMDB0243786	B(ii)	-	277.0966	4.6977	pos	[illegible]
pos_496	Isorheagenine	metab_3964	HMDB0029360	B(ii)	-	411.1527	4.151	pos	[illegible]
pos_4962	Ibuprofen	metab_3966	PW_C001284;HMDB0001925;MJDBOTE0001402	B(i)	C01588	207.1376	4.6895	pos	[illegible]
pos_50	2,3-dinor Fluprostenol	metab_4003	-	B(i)	-	413.1554	2.8474	pos	[illegible]
pos_5073	KAPA	metab_4075	HMDB0240687	B(i)	C01092	188.1278	4.6109	pos	[illegible]
pos_5077	Fosteabine	metab_4078	HMDB0252470	B(ii)	C21596	639.3496	4.6031	pos	[illegible]
pos_509	Quercetin 3,3'-diglucoside	metab_4087	HMDB0303591;LMPK12112103	B(ii)	-	649.1376	4.3453	pos	[illegible]
pos_5126	Enol-phenylpyruvate	metab_4116	HMDB0012225	B(ii)	C02763	206.0809	4.517	pos	[illegible]
pos_5135	Cropropamide	metab_4123	HMDB0250543	B(ii)	-	498.401	4.5013	pos	[illegible]
pos_5141	Sakuranetin	metab_4129	LMPK12140571;HMDB0030090	B(ii)	C09833	328.1173	4.4935	pos	[illegible]
pos_5150	L-Pyridosine	metab_4137	HMDB0029443	B(ii)	-	293.0914	4.4856	pos	[illegible]
pos_5172	Queuine	metab_4156	HMDB0001495	B(ii)	C01449	277.1179	4.4622	pos	[illegible]
pos_5185	3-Oxohexanoic acid	metab_4166	HMDB0010717;PW_C006725	B(ii)	C02122	172.0965	4.4231	pos	[illegible]
pos_5210	5-Hydroxyindoleacetylglycine	metab_4193	HMDB0004185	B(ii)	C05832	281.1129	4.3609	pos	[illegible]
pos_5216	Glucosyl (2E,6E,10x)-10,11-dihydroxy-2,6-farnesadienoate	metab_4199	HMDB0037823	B(ii)	-	433.2443	4.3453	pos	[illegible]
pos_5217	Virginiamycin m1	metab_4200	HMDB0259836	B(ii)	C11299	490.2295	4.3389	pos	[illegible]
pos_522	Ozolinone	metab_4203	HMDB0256028	B(ii)	-	298.1213	2.7134	pos	[illegible]
pos_5228	Polyethylene, oxidized	metab_4212	HMDB0032472	B(ii)	-	227.1274	4.289	pos	[illegible]
pos_5229	CDP-DG(a-25:0/20:3(8Z,11Z,14Z)-2OH(5,6))	metab_4213	HMDB0293197	B(ii)	-	603.816	4.2812	pos	[illegible]
pos_5237	6-Epi-7-isocucurbic acid glucoside	metab_4222	HMDB0029782	B(ii)	-	407.2261	4.2576	pos	[illegible]
pos_5239	Asiaticoside	metab_4224	HMDB0036656;MJDBOTE0000921	B(ii)	C15428	480.2688	4.2498	pos	[illegible]
pos_5259	Ganglioside GD1b (d18:1/18:1(11Z))	metab_4244	HMDB0011826	B(ii)	-	917.9923	4.1589	pos	[illegible]
pos_5261	2'',6''-O-Diacetylonitin	metab_4247	HMDB0302015	B(ii)	-	434.2284	4.1589	pos	[illegible]
pos_5263	CDP-DG(i-22:0/20:3(6,8,11)-OH(5))	metab_4249	HMDB0294395	B(ii)	-	563.811	4.151	pos	[illegible]
pos_5266	PE(20:5(7Z,9Z,11E,13E,17Z)-3OH(5,6,15)/22:2(13Z,16Z))	metab_4252	HMDB0283993	B(ii)	-	455.766	4.1432	pos	[illegible]
pos_5267	Fluazifop	metab_4253	HMDB0252322	B(i)	C18527	328.0813	4.1432	pos	[illegible]
pos_5271	Mafenide	metab_4258	HMDB0254296	B(ii)	C07106	395.0848	4.1041	pos	[illegible]
pos_5273	3,4-Dihydroxyphenylacetaldehyde	metab_4260	HMDB0003791;PW_C001969	B(ii)	C04043	194.0809	4.0962	pos	[illegible]
pos_5274	Monoglutamyl folic acid	metab_4261	HMDB0254856;HMDB0254857	B(ii)	-	571.1898	4.0884	pos	[illegible]
pos_5275	Flindersine	metab_4262	-	B(i)	C10679	245.1281	4.0884	pos	[illegible]
pos_5276	CDP-DG(PGE1/i-22:0)	metab_4263	HMDB0294390	B(ii)	-	579.8032	4.0805	pos	[illegible]
pos_5298	Citreoviridin	metab_4283	HMDB0250305	B(ii)	C16766	441.1636	4.0179	pos	[illegible]
pos_5306	Ginsenoside Rb1	metab_4293	LMPR0106080013;HMDB0035892	B(ii)	C20713	566.2979	3.9937	pos	[illegible]
pos_5326	Portulacaxanthin II	metab_4312	HMDB0012281	B(ii)	C08565	339.0972	3.9233	pos	[illegible]
pos_533	4-Hydroxy-3-methoxy-cinnamoylglycine	metab_4316	HMDB0059747	B(ii)	C02564	252.0862	4.517	pos	[illegible]
pos_5361	(E)-indol-3-ylacetaldoxime	metab_4348	HMDB0303989	B(ii)	C02937	175.0863	3.8284	pos	[illegible]
pos_5363	M-Xylene	metab_4350	HMDB0059810	B(i)	C07208	107.0857	3.8284	pos	[illegible]
pos_5369	Mepartricin	metab_4356	HMDB0253564;HMDB0254449;HMDB0254450	B(ii)	-	571.309	3.8127	pos	[illegible]
pos_5374	8-Methoxykynurenate	metab_4360	HMDB0060426	B(ii)	C05830	461.098	3.7971	pos	[illegible]
pos_5377	DL-p-Chlorophenylalanine methyl ester hydrochloride	metab_4363	-	B(i)	-	214.0626	3.7892	pos	[illegible]
pos_5387	2,4-Quinolinediol	metab_4373	HMDB0245468	B(ii)	C16716	203.0813	3.7571	pos	[illegible]
pos_5389	Lappaol B	metab_4375	HMDB0302315	B(ii)	C17685	573.2058	3.7492	pos	[illegible]
pos_5391	1H-Imidazole-1-ethanol, alpha-(1-aziridinylmethyl)-2-nitro-	metab_4378	HMDB0257342	B(ii)	-	212.0891	3.7492	pos	[illegible]
pos_5397	7-Amino-4-methylcoumarin	metab_4383	HMDB0247230	B(ii)	C01386	217.0968	3.7257	pos	[illegible]
pos_5404	Valtrate	metab_4391	HMDB0034493	B(ii)	C09801	177.0544	3.7021	pos	[illegible]
pos_5410	[D-Lys3]-GHRP-6	metab_4398	HMDB0243762	B(ii)	-	487.7435	3.6702	pos	[illegible]
pos_5413	PIP(PGF2alpha/18:1(15Z))	metab_4401	HMDB0278965	B(ii)	-	508.2643	3.6623	pos	[illegible]
pos_5423	Cephalosporanic acid	metab_4412	HMDB0249809	B(ii)	-	240.0321	3.623	pos	[illegible]
pos_5426	N-Succinyl-2-amino-6-ketopimelate	metab_4415	HMDB0012266	B(ii)	C04462	289.0815	3.6151	pos	[illegible]
pos_5431	1-Naphthylamine	metab_4421	HMDB0243964	B(i)	C14790	144.0806	3.5994	pos	[illegible]
pos_5453	Kynurenic Acid	metab_4443	PW_C000566;HMDB0000715;MJDBOTE0000367	B(i)	C01717	190.0496	3.5199	pos	[illegible]
pos_5456	Ganglioside GT3 (d18:1/22:1(13Z))	metab_4446	HMDB0012075	B(ii)	-	621.323	3.5037	pos	[illegible]
pos_5465	D-Glucoside	metab_4456	HMDB0250756	B(ii)	C01798	728.4297	3.488	pos	[illegible]
pos_5467	Ginsenoside Ra3	metab_4458	HMDB0039551	B(ii)	-	643.3058	3.488	pos	[illegible]
pos_5478	Milbemycin alpha9	metab_4469	HMDB0254730;HMDB0254721;HMDB0254726;HMDB0242623	B(ii)	-	637.3188	3.4482	pos	[illegible]
pos_548	Beta-D-3-Ribofuranosyluric acid	metab_4471	HMDB0029920	B(ii)	C05513	301.0778	2.6899	pos	[illegible]
pos_5480	Tyrosyl-Isoleucine	metab_4472	HMDB0029108	B(ii)	-	295.1646	3.4482	pos	[illegible]
pos_5482	Oryzalide B	metab_4474	HMDB0037592	B(ii)	-	384.2127	3.4404	pos	[illegible]
pos_5486	(5S,6R)-Methyl 5,6,7-trihydroxyheptanoate	metab_4478	HMDB0247393	B(ii)	-	234.1333	3.4326	pos	[illegible]
pos_5499	3,7-Dimethylquercetin	metab_4490	HMDB0029263	B(ii)	C01265	348.1075	3.4	pos	[illegible]
pos_5502	Harmol glucuronide	metab_4495	HMDB0253051	B(ii)	-	375.1186	3.3844	pos	[illegible]
pos_5503	2,4-dimethylhexanedioylcarnitine	metab_4496	HMDB0241047	B(ii)	-	318.1908	3.3844	pos	[illegible]
pos_5518	Jubanine B	metab_4510	HMDB0030206	B(ii)	-	694.3405	3.3446	pos	[illegible]
pos_5523	Pro Gln Ser	metab_4516	-	B(i)	-	331.1609	3.3359	pos	[illegible]
pos_5524	Neuraminic acid	metab_4517	HMDB0000830	B(ii)	C06469	267.0971	3.3359	pos	[illegible]
pos_5531	Doramectin	metab_4525	HMDB0242638	B(ii)	-	450.2633	3.3203	pos	[illegible]
pos_5535	Pravastatin	metab_4528	HMDB0256737;PW_C002369;LMFA05000695;HMDB0005022;MJI	B(ii)	C01844	425.2506	3.3119	pos	[illegible]
pos_5543	L-histidinol-phosphate	metab_4536	HMDB0304403	B(ii)	C01100	239.0924	3.2961	pos	[illegible]
pos_5548	Lucuminoside	metab_4541	HMDB0029900	B(ii)	C08335	427.1474	3.288	pos	[illegible]
pos_5562	(1R,4E,5'R,6R,6'R,7S,8S,10S,11S,12R,14S,15R,16S,18E,20E,22S,25R,27S,29S)-22-Ethyl-7,11,14,15-tetrahydroxy-6'-(2-hydroxypropyl)-5',6,8,10,12,14,16,29-octamethylspiro[2,26-dioxabicyclo[23.3.1]nonacosa-4,18,20-triene-27,2'-oxane]-3,9,13-trione	metab_4557	HMDB0242333	B(ii)	-	400.2554	3.2644	pos	[illegible]
pos_5569	1,6-anhydro-N-acetyl-beta-muramate	metab_4564	HMDB0304012	B(ii)	C19769	275.1022	3.2477	pos	[illegible]
pos_5572	Quinoline-4,8-diol	metab_4568	HMDB0060289	B(ii)	C05637	194.081	3.2399	pos	[illegible]
pos_558	Aniline	metab_4576	HMDB0003012	B(i)	C00292	94.0654	2.2231	pos	[illegible]
pos_5580	1alpha,3beta,22R-Trihydroxyergosta-5,24E-dien-26-oic acid 3-O-b-D-glucoside 26-O-[b-D-glucosyl-(1->2)-6-acetyl-b-D-glucosyl] ester	metab_4577	HMDB0040400	B(ii)	-	495.2482	3.2239	pos	[illegible]
pos_5587	Imipenem	metab_4583	HMDB0015536	B(ii)	C06665	322.0853	3.216	pos	[illegible]
pos_5588	Gemcitabine	metab_4584	PW_C008951;HMDB0014584	B(ii)	C07650	296.1059	3.216	pos	[illegible]
pos_5594	Serine-glyoxylate	metab_4590	HMDB0258250	B(ii)	-	340.0959	3.2081	pos	[illegible]
pos_5595	L-beta-aspartyl-L-leucine	metab_4591	HMDB0011166	B(ii)	-	211.1073	3.2081	pos	[illegible]
pos_5606	6-Hydroxyluteolin	metab_4604	HMDB0036632;LMPK12111229	B(ii)	C10072	344.0762	3.1922	pos	[illegible]
pos_5614	L-Tyrosine Methyl Ester	metab_4611	HMDB0029217	B(i)	C03404	196.0966	3.1767	pos	[illegible]
pos_5619	3-Methylcrotonylglycine	metab_4615	HMDB0000459	B(ii)	C20828	202.0439	3.161	pos	[illegible]
pos_5625	Bacoside A	metab_4622	HMDB0248842	B(ii)	-	813.4448	3.1452	pos	[illegible]
pos_5629	(3R)-3-Hydroxy-N-[(3S)-2-oxooxolan-3-yl]butanamide	metab_4625	HMDB0243607	B(ii)	-	229.1179	3.1374	pos	[illegible]
pos_5633	Dihydroneopterin phosphate	metab_4630	HMDB0006824	B(ii)	C05925	380.0365	3.1296	pos	[illegible]
pos_5642	2-Naphthylamine	metab_4640	HMDB0041802	B(i)	C02227	144.0806	3.0749	pos	[illegible]
pos_5645	2-(Difluoromethyl)arginine	metab_4643	HMDB0247212	B(ii)	-	207.1031	3.0671	pos	[illegible]
pos_5649	Tyrosine-tyrosinate	metab_4647	HMDB0259353	B(ii)	-	345.1441	3.0514	pos	[illegible]
pos_565	Hydroxydeoxyguanosine	metab_4648	HMDB0253263	B(ii)	-	284.0987	1.9646	pos	[illegible]
pos_5651	Chrysophanol	metab_4650	HMDB0030670;LMPK13040006	B(ii)	C10315	272.0912	3.0436	pos	[illegible]
pos_5653	Gibberellin A44	metab_4652	LMPR0104170030;HMDB0036901	B(ii)	C12308	391.1496	3.0357	pos	[illegible]
pos_5656	3-Indolebutyric acid	metab_4655	HMDB0002096	B(ii)	C11284	236.1275	3.0279	pos	[illegible]
pos_5661	Alpha-D-GalpNAc-(1->3)-[alpha-L-Fucp-(1->2)]-D-Galp	metab_4660	HMDB0247725	B(ii)	-	562.2394	3.0044	pos	[illegible]
pos_5662	Antramycin	metab_4661	HMDB0248464	B(ii)	-	280.111	3.0044	pos	[illegible]
pos_567	Indole-3-acetamide	metab_4667	HMDB0029739	B(i)	C02693	175.0863	1.9255	pos	[illegible]
pos_5671	SCHEMBL13960539	metab_4669	HMDB0257632	B(ii)	-	526.2394	2.9888	pos	[illegible]
pos_5674	Cotinine glucuronide	metab_4671	PW_C000801;HMDB0001013	B(ii)	C00190	375.1183	2.981	pos	[illegible]
pos_5695	Furfural	metab_4688	HMDB0032914	B(i)	C14279	97.0286	2.9419	pos	[illegible]
pos_5698	Ethyl 7-epi-12-hydroxyjasmonate glucoside	metab_4691	HMDB0036340	B(ii)	-	417.2128	2.9341	pos	[illegible]
pos_5699	5-Hydroxytryptophol glucuronide	metab_4692	HMDB0013200	B(ii)	-	403.1473	2.9341	pos	[illegible]
pos_5702	Amoxicillin	metab_4697	HMDB0015193;HMDB0243588;MJDBOTE0001515	B(ii)	C06827	205.5411	2.9341	pos	[illegible]
pos_5711	17-beta-Estradiol glucuronide	metab_4706	LMST05050028;HMDB0010317	B(ii)	C11237	449.2141	2.9178	pos	[illegible]
pos_5715	(+)-taxifolin	metab_4710	HMDB0303943	B(ii)	C01617	327.0497	2.91	pos	[illegible]
pos_5717	N-lactoyl-phenylalanine	metab_4712	HMDB0062175	B(i)	-	238.107	2.91	pos	[illegible]
pos_5738	Isoformononetin	metab_4730	HMDB0033994;LMPK12050039	B(ii)	C12125	286.107	2.8865	pos	[illegible]
pos_574	Diadenosine diphosphate	metab_4732	HMDB0001312	B(ii)	-	677.123	1.8707	pos	[illegible]
pos_5766	Alpha-Hydroxyhippuric Acid	metab_4759	HMDB0002404	B(i)	-	196.0603	2.8552	pos	[illegible]
pos_5768	Aspartylglycosamine	metab_4761	HMDB0000489	B(ii)	C04540	335.1346	2.8474	pos	[illegible]
pos_577	(1r,3r)-1-Aminocyclopentane-1,3-dicarboxylic acid	metab_4763	HMDB0242328	B(ii)	-	174.0758	1.1234	pos	[illegible]
pos_5772	2beta,9xi-Dihydroxy-8-oxo-1(10),4,11(13)-germacratrien-12,6alpha-	metab_4766	HMDB0036662	B(ii)	-	296.1489	2.8389	pos	[illegible]
pos_5775	Blumenol C O-[apiosyl-(1->6)-glucoside]	metab_4769	HMDB0031936	B(ii)	-	546.2884	2.8311	pos	[illegible]
pos_5777	12-Hydroxydodecanoic acid	metab_4771	HMDB0304547;HMDB0002059	B(ii)	C08317	261.1441	2.8311	pos	[illegible]
pos_5781	Patulin	metab_4775	HMDB0034299	B(ii)	C16748	155.0337	2.8233	pos	[illegible]
pos_5784	Fenvalerate	metab_4778	HMDB0031791	B(ii)	C10988	384.1148	2.8154	pos	[illegible]
pos_58	Isoleucyl-prolyl-arginine-4-nitroanilide	metab_4793	HMDB0250210	B(ii)	-	546.3193	6.3521	pos	[illegible]
pos_5801	6-hydroxymethyl-7,8-dihydropterin	metab_4796	HMDB0304227	B(ii)	C01300	196.0827	2.7919	pos	[illegible]
pos_5815	Thr Leu	metab_4809	-	B(i)	-	233.1493	2.7685	pos	[illegible]
pos_5820	PS(PGJ2/22:5(4Z,7Z,10Z,13Z,16Z))	metab_4814	HMDB0283325	B(ii)	-	466.7324	2.7607	pos	[illegible]
pos_5831	9-Ethylguanine	metab_4826	HMDB0247591	B(ii)	-	180.0874	2.7525	pos	[illegible]
pos_5832	Acacetin 7-[apiosyl(1->6)-glucoside]	metab_4827	HMDB0035023	B(ii)	C01470	620.1946	2.7446	pos	[illegible]
pos_5833	Tert-Butyl 3-((3S,6S,12aS)-6-isobutyl-9-methoxy-1,4-dioxo-1,2,3,4,6,7,12,12a-octahydropyrazino[1',2':1,6]pyrido[3,4-b]indol-3-	metab_4828	HMDB0253814	B(ii)	-	452.2505	2.7446	pos	[illegible]
pos_5837	Alanylleucine	metab_4832	HMDB0028691;HMDB0251509	B(ii)	-	203.1387	2.7446	pos	[illegible]
pos_5859	4-Ethoxy-4-oxobutanoic acid	metab_4850	HMDB0061930	B(ii)	-	188.0914	2.7212	pos	[illegible]
pos_5861	5-Hydroxy-N-formylkynurenine	metab_4853	HMDB0004086;PW_C002055	B(ii)	C05648	235.071	2.7212	pos	[illegible]
pos_587	Furaneol 4-(6-malonylglucoside)	metab_4862	HMDB0029778	B(ii)	-	409.1366	4.6977	pos	[illegible]
pos_5870	Folinic acid	metab_4863	HMDB0001562;HMDB0247218	B(ii)	C03479	496.1582	2.7134	pos	[illegible]
pos_5875	P-Hydroxymandelic acid	metab_4867	HMDB0000822	B(ii)	C11527	151.0387	2.7134	pos	[illegible]
pos_5886	MeOSuc-AAPA-CMK	metab_4877	HMDB0254447	B(ii)	-	516.2191	2.7056	pos	[illegible]
pos_5896	Vanilloyl glucose	metab_4887	HMDB0302470	B(ii)	C20470	331.102	2.7056	pos	[illegible]
pos_5898	Hypoglycin B	metab_4889	HMDB0029428	B(ii)	C08280	293.1128	2.7056	pos	[illegible]
pos_5899	Questiomycin A	metab_4890	HMDB0030483	B(ii)	C02161	230.0921	2.7056	pos	[illegible]
pos_5911	4-(Glutamylamino) butanoate	metab_4901	HMDB0012161	B(ii)	C15767	232.1078	2.6977	pos	[illegible]
pos_5921	Fumigaclavine A	metab_4911	HMDB0030202	B(ii)	C20436	321.1556	2.6899	pos	[illegible]
pos_5923	Dehydroxyzileuton	metab_4913	HMDB0013970	B(ii)	-	221.0738	2.6899	pos	[illegible]
pos_5926	Isoleucyl-Glutamate	metab_4915	HMDB0028906	B(ii)	-	243.1336	2.6899	pos	[illegible]
pos_5927	4-(2-Amino-3-hydroxyphenyl)-2,4-dioxobutanoic acid	metab_4916	PW_C002053;HMDB0004083	B(ii)	C05645	206.0445	2.6899	pos	[illegible]
pos_5935	DG(PGD2/0:0/13:0)	metab_4924	HMDB0294876	B(ii)	-	334.2101	2.682	pos	[illegible]
pos_5939	25-O-Desacetyl rifabutin	metab_4928	HMDB0060732	B(ii)	-	745.3732	2.6742	pos	[illegible]
pos_5941	4-Hydroxyphenytoin glucuronide	metab_4930	PW_C040701;HMDB0060769	B(ii)	-	486.1542	2.6742	pos	[illegible]
pos_5945	1-(6-Hydroxy-2-azabicyclo[2.2.1]heptane-3-carbonyl)pyrrolidine-2-	metab_4934	HMDB0260235	B(ii)	-	277.1656	2.6742	pos	[illegible]
pos_5950	CDP-DG(20:4(8Z,11Z,14Z,17Z)/20:1(11Z))	metab_4940	HMDB0116003;PW_C063146	B(ii)	C00269	550.7652	2.6664	pos	[illegible]
pos_5962	Nodularin-R	metab_4952	HMDB0255693	B(ii)	-	406.2238	2.6507	pos	[illegible]
pos_5967	Difluoromethyl (2R)-2,5-diaminopentanoate	metab_4956	HMDB0258025	B(ii)	-	428.1916	2.6429	pos	[illegible]
pos_5969	L-2-Aminoethyl seryl phosphate	metab_4958	HMDB0031950	B(ii)	C03872	261.0866	2.6429	pos	[illegible]
pos_5974	Pteroside Z	metab_4964	HMDB0032587	B(ii)	-	458.2134	2.6351	pos	[illegible]
pos_5975	VINBARBITAL	metab_4965	HMDB0259808	B(ii)	-	449.2396	2.6351	pos	[illegible]
pos_5977	SM(d16:2(4E,8Z)/PGJ2)	metab_4967	HMDB0290312	B(ii)	-	387.2474	2.6351	pos	[illegible]
pos_5996	5'-Deoxy-5-fluorouridine	metab_4981	PW_C040484;HMDB0060406	B(ii)	C12739	279.0976	2.6194	pos	[illegible]
pos_5997	Zidovudine	metab_4982	PW_C008998;HMDB0014638;HMDB0246088	B(ii)	C07210	250.0931	2.6194	pos	[illegible]
pos_6001	Glucosamine, N-carbamoyl-(6CI)	metab_4988	HMDB0248482	B(ii)	-	467.1631	2.6037	pos	[illegible]
pos_6006	1-Methylguanosine	metab_4993	HMDB0001563	B(i)	C04545	298.1142	2.5959	pos	[illegible]
pos_6011	O-Adipoylcarnitine	metab_4999	HMDB0061677;LMFA07070087	B(ii)	-	290.1595	2.5803	pos	[illegible]
pos_6014	2,5-diamino-6-(5-phospho-D-ribitylamino)pyrimidin-4(3H)-one	metab_5002	HMDB0304043	B(ii)	C18910	378.0807	2.5646	pos	[illegible]

pos_6016	Imidaprilat	metab 5004	HMDB0244459:HMDB0041908	B(ii)	C21519	[illegible]
pos_6018	3-Chlorotyrosine	metab 5006	HMDB0001885	B(i)	-	[illegible]
pos_6022	Salicylamide glucuronide	metab 5009	HMDB0257458	B(ii)	-	[illegible]
pos_6026	Cinnavalininate	metab 5013	PW C002050:HMDB0004078	B(ii)	C05640	[illegible]
pos_6027	Cytosine	metab 5014	HMDB0000630:MJDBOTE0000458	B(i)	C00380	[illegible]
pos_6032	5-(3-Pyridyl)-2-hydroxytetrahydrofuran	metab 5019	HMDB0062407	B(ii)	C19578	[illegible]
pos_6034	3-methylthiopropyl-desulfoglucosinolate	metab 5021	HMDB0304142	B(ii)	C17244	[illegible]
pos_6035	2-Phenylacetamide	metab 5022	HMDB0010715	B(i)	C02505	[illegible]
pos_6037	Secalonic acid A	metab_5024	HMDB0258200	B(ii)	-	[illegible]
pos_6039	2-Methylbenzaldehyde	metab 5026	HMDB0029636:HMDB0303310	B(ii)	C07214	[illegible]
pos_6044	Oxypurinol	metab 5032	HMDB0000786	B(i)	C07599	[illegible]
pos_6053	Adenylsuccinic acid	metab 5041	PW C000413:HMDB0013204:HMDB0000536	B(ii)	C03794	[illegible]
pos_6054	(2S,4S)-Pinnatanine	metab 5042	LMFA01050445:HMDB0029439	B(ii)	-	[illegible]
pos_6056	Capsaicin	metab 5044	LMFA08020085:HMDB0002227:MJDBOTE0001453	B(ii)	C06866	[illegible]
pos_6060	N-Oxalylglycine	metab 5049	HMDB0251540:HMDB0255221	B(ii)	-	[illegible]
pos_6062	P-Hydroxyfelbamate	metab 5050	PW C040523:HMDB0060669	B(ii)	C16584	[illegible]
pos_6076	3-Methoxyanthranilate	metab 5063	HMDB0060374	B(ii)	C05831	[illegible]
pos_6078	1-Piperideine-2-carboxylic acid	metab_5065	HMDB0001084	B(ii)	C04092	[illegible]
pos_6080	Fenoprofen	metab 5068	PW C009066:HMDB0014713	B(ii)	C06997	[illegible]
pos_6084	Gamma-Glutamylserine	metab 5072	HMDB0029158	B(ii)	-	[illegible]
pos_6087	Pyrimidin-2-ol	metab 5075	HMDB0256975	B(ii)	-	[illegible]
pos_6090	Phenacemide	metab 5079	HMDB0015253	B(ii)	C07428	[illegible]
pos_6091	3-Methyloxindole	metab 5080	HMDB0304943	B(i)	C02366	[illegible]
pos_6094	3'-Hydroxy-HT2 toxin	metab 5083	HMDB0036162	B(ii)	-	[illegible]
pos_6098	Cytidylyl-(5'-3')-5'-inosinic acid homopolymer	metab 5087	HMDB0256667	B(ii)	-	[illegible]
pos_61	PI(20:5(5Z,8Z,11Z,14Z,17Z)/0:0)	metab 5089	LMGP06050026:HMDB0256524	B(ii)	-	[illegible]
pos_6105	Thienamycin	metab_5096	HMDB0258982	B(ii)	C06664	[illegible]
pos_6106	Cinnamaldehyde	metab 5097	HMDB0003441:HMDB0245299:HMDB0258628:MJDBOTE000108	B(i)	C00903	[illegible]
pos_6107	2,5-Furandicarboxaldehyde	metab 5098	-	B(i)	C20899	[illegible]
pos_6111	2-Hydroxy-3-(4-hydroxyphenyl)propenoic acid	metab 5103	HMDB0006915:PW C003007	B(ii)	C05350	[illegible]
pos_6113	Para-Phenylenediamine	metab 5105	HMDB0256055	B(i)	C19499	[illegible]
pos_6115	Imperatorin	metab 5106	HMDB0034376:MJDBOTE0000939	B(ii)	C09269	[illegible]
pos_6119	Vanillin Acetate	metab 5110	HMDB0029663	B(i)	-	[illegible]
pos_6120	Vilaprisan	metab 5112	HMDB0259806	B(ii)	-	[illegible]
pos_6134	Phenylacetohydroximoyl-glutathione	metab 5127	HMDB0304460	B(ii)	-	[illegible]
pos_6136	Neolinustatin	metab_5129	HMDB0038482	B(ii)	C08336	[illegible]
pos_6146	Trilostane	metab 5140	HMDB0015240:MJDBOTE0000867	B(ii)	C12580	[illegible]
pos_6149	Hypoxanthine	metab 5143	HMDB0000157:PW C000102	B(i)	C00262	[illegible]
pos_6151	3-(2-Furoyl)quinoline-2-carboxaldehyde	metab 5146	HMDB0245772	B(ii)	-	[illegible]
pos_6152	D-4'-Phosphopantothenate	metab 5147	HMDB0001016:PW C000804	B(ii)	C03492	[illegible]
pos_6155	2-([1,1'-Biphenyl]-2-yl)acetic acid	metab 5150	HMDB0244909	B(ii)	-	[illegible]
pos_6157	3-Methyl-2(3H)-benzothiazolone	metab 5152	HMDB0245729	B(ii)	-	[illegible]
pos_6158	Pimelic acid	metab 5153	LMFA01170051:HMDB0000857	B(ii)	C02656	[illegible]
pos_6162	Apigenin 7-glucuronosyl-glucoside	metab 5158	HMDB0302596	B(ii)	-	[illegible]
pos_6168	Ophthalmic acid	metab_5163	HMDB0005765	B(ii)	-	[illegible]
pos_6169	Asparaginyl-Proline	metab 5164	HMDB0028739	B(ii)	-	[illegible]
pos_6171	Isopropyl beta-D-glucoside	metab 5167	HMDB0032705	B(ii)	-	[illegible]
pos_6178	Adenosine	metab 5174	PW C000034:HMDB0247583:HMDB0000050:MJDBOTE0000767	B(i)	C00212	[illegible]
pos_6184	Daucol	metab 5180	HMDB0035287	B(ii)	C09652	[illegible]
pos_6186	N-methyl-L-glutamic Acid	metab 5182	HMDB0062660	B(ii)	C01046	[illegible]
pos_6187	L-Tyrosine	metab 5183	HMDB0000158:PW C000103:MJDBOTE0000356	B(i)	C00082	[illegible]
pos_6189	Phlorisobutyrophenone	metab 5185	HMDB0304461	B(ii)	C07351	[illegible]
pos_6193	Neohesperidoside	metab 5190	HMDB0302540	B(ii)	C08244	[illegible]
pos_6213	Omega-Salicoylsalicin	metab_5211	HMDB0031423	B(ii)	-	[illegible]
pos_6214	L-alpha-Aminobutyric acid	metab 5212	HMDB0000452	B(ii)	C02356	[illegible]
pos_6218	Pro Val	metab 5216	-	B(i)	-	[illegible]
pos_6219	7-Aminocephalosporanic acid	metab 5217	HMDB0247232	B(ii)	C07756	[illegible]
pos_6222	Nigakihemiacetal B	metab 5221	HMDB0035922	B(ii)	C08771	[illegible]
pos_6225	Sulfamethoxazole	metab 5224	HMDB0015150:MJDBOTE0001709	B(ii)	C07315	[illegible]
pos_6230	QUISQUALIC ACID	metab 5229	HMDB0257060	B(ii)	C08296	[illegible]
pos_6232	2-Hydroxydecanedioic acid	metab 5231	HMDB0000424	B(ii)	-	[illegible]
pos_6236	CMP-N-glycoloylneuraminate	metab 5234	HMDB0012206	B(ii)	C03691	[illegible]
pos_6238	4-Hydroxybenzaldehyde	metab_5235	HMDB0011718;MJDBOTE0000554	B(i)	C00633	[illegible]
pos_6240	Morph	metab 5238	HMDB0014440:HMDB0254890	B(ii)	C01516	[illegible]
pos_6241	Demethylated antipyrine	metab 5239	HMDB0006240	B(ii)	C13008	[illegible]
pos_6248	2-(Malonylamino)benzoic acid	metab 5243	HMDB0039495	B(ii)	C03147	[illegible]
pos_6250	Adrenochrome	metab 5246	HMDB0012884	B(i)	-	[illegible]
pos_6260	3-dehydroshikimate	metab 5256	HMDB0304122	B(ii)	C02637	[illegible]
pos_6269	N2-Succinyl-L-ornithine	metab 5265	HMDB0001199	B(ii)	C03415	[illegible]
pos_6281	Acetyl-L-tyrosine	metab 5277	HMDB0000866:HMDB0244966	B(i)	C01657	[illegible]
pos_6283	His Leu	metab 5279	-	B(i)	-	[illegible]
pos_6287	Asparaginylhydroxyproline	metab_5282	HMDB0028732	B(ii)	-	[illegible]
pos_6288	G-Nitro-L-arginine methyl ester	metab 5283	HMDB0252563	B(ii)	-	[illegible]
pos_6290	Deoxyadenosine monophosphate	metab 5286	HMDB0000905:PW C000724	B(i)	C00360	[illegible]
pos_6293	1-(beta-D-Ribofuranosyl)-1,4-dihydronicotinamide	metab 5289	HMDB0011648:HMDB0011643	B(ii)	C15497	[illegible]
pos_6296	Glycinamide, glycyl-L-prolyl-	metab 5292	HMDB0253016	B(ii)	-	[illegible]
pos_6297	Glucose-6-phosphate lactate	metab 5293	HMDB0252789	B(ii)	-	[illegible]
pos_6300	Beta-tyvelose	metab 5298	HMDB0249152	B(ii)	-	[illegible]
pos_6303	Syrups, hydrolyzed starch, hydrogenated	metab 5301	HMDB0258656	B(ii)	-	[illegible]
pos_6308	Niacinamide	metab 5306	HMDB0001406:PW C001086	B(i)	C00153	[illegible]
pos_6315	Histidylglycine	metab_5312	HMDB0028885	B(ii)	-	[illegible]
pos_6317	(4S,4As,5aR,12aS)-4,7-bis(dimethylamino)-10,12a-dihydroxy-1,3,11,12-tetraoxo-4,4a,5,5a,6,11a-hexahydrotetracene-2-carboxamide	metab_5314	HMDB0248915	B(ii)	-	[illegible]
pos_6327	2-Chloro-4-nitrophenyl a-D-maltotrioside	metab 5325	HMDB0244430	B(ii)	-	[illegible]
pos_6328	Coniferin	metab 5326	HMDB0013682	B(ii)	C00761	[illegible]
pos_6333	4-Methyl-1H-pyrrole-3-carboxamide	metab 5331	HMDB0246501	B(ii)	-	[illegible]
pos_6334	3-Hydroxyphenylacetic Acid	metab 5332	HMDB0000440	B(i)	C05593	[illegible]
pos_6339	Dopamine quinone	metab 5336	HMDB0012219	B(ii)	C17755	[illegible]
pos_634	Hydroxyethyl methacrylic acid	metab 5337	HMDB0245159:HMDB0253265	B(ii)	C14530	[illegible]
pos_6343	Mimosine	metab_5341	HMDB0031398;HMDB0015188	B(ii)	C04771	[illegible]
pos_6344	3-(Trimethylsilyl)propionic Acid	metab 5342	-	B(i)	-	[illegible]
pos_6347	Pentostatin	metab 5345	HMDB0014692:HMDB0245535	B(ii)	C02267	[illegible]
pos_6348	3,4-Methylenepimelic acid	metab 5346	HMDB0059730	B(ii)	-	[illegible]
pos_6357	9,10-Epoxy-18-hydroxy-octadecanoic acid	metab 5355	HMDB0302107	B(ii)	C19620	[illegible]
pos_6362	O-Acetylcarnitine	metab 5361	HMDB0240773:HMDB0000201	B(i)	C02571	[illegible]
pos_6363	Ecgonine	metab 5362	HMDB0006548	B(i)	C10858	[illegible]
pos_6364	Uridine 2'-phosphate	metab 5363	HMDB0011641	B(ii)	C03031	[illegible]
pos_6365	Nalpha-Acetyl-L-arginine	metab 5364	-	B(i)	-	[illegible]
pos_6366	Aminomalonic acid	metab_5365	HMDB0001147	B(ii)	C00872	[illegible]
pos_6371	Cytidine	metab 5370	PW C000061:HMDB0000089:HMDB0248553:MJDBOTE0000789	B(i)	C00475	[illegible]
pos_6379	(6R)-Folinic acid	metab 5377	HMDB0247218	B(ii)	C03479	[illegible]
pos_6389	Raffinose	metab 5387	HMDB0003213:PW C001805:MJDBOTE0001734	B(i)	C00492	[illegible]
pos_6398	Melezitose	metab 5395	HMDB0011730:MJDBOTE0000489	B(i)	C08243	[illegible]
pos_6401	Thiodiacetic acid	metab 5400	HMDB0042032	B(ii)	C14872	[illegible]
pos_6402	Stachyose	metab 5401	HMDB0003553:PW C001923	B(i)	C01613	[illegible]
pos_6408	Asparaginyl-Phenylalanine	metab 5407	HMDB0028738	B(ii)	-	[illegible]
pos_6411	Norvaline	metab 5410	HMDB0013716:MJDBOTE0000679	B(i)	C01799	[illegible]
pos_6415	Homocitric acid	metab_5414	HMDB0003518	B(ii)	C01251	[illegible]
pos_6422	Pretyrosine	metab 5421	HMDB0304809	B(ii)	C00826	[illegible]
pos_6426	Quercetin 7-glucuronide 3-rhamnoside	metab 5424	HMDB0036264	B(ii)	-	[illegible]
pos_6428	Cmp-nana	metab 5426	HMDB0001176:HMDB0250377	B(ii)	C00128	[illegible]
pos_6429	Phosphoribulosylformimino-AICAR-P	metab 5427	HMDB0012278	B(ii)	C04916	[illegible]
pos_6433	Pantetheine 4'-phosphate	metab 5432	PW C001093:HMDB0001416	B(ii)	C01134	[illegible]
pos_6434	Neosaxitoxin	metab 5433	HMDB0029369	B(ii)	C17208	[illegible]
pos_6440	3-Hydroxy-L-proline	metab 5437	HMDB0002113	B(ii)	C04397	[illegible]
pos_6441	4-Guanidinobutanoic Acid	metab 5438	HMDB0031842:HMDB0003464:MJDBOTE0001114	B(i)	C01035	[illegible]
pos_6443	3-Hydroxybenzaldehyde	metab_5440	-	B(i)	C03067	[illegible]
pos_6445	N2-Succinyl-L-glutamic acid 5-semialdehyde	metab 5441	HMDB0001180	B(ii)	C05932	[illegible]
pos_6452	N-Methyl-14-O-demethylepiporphyroxine	metab 5446	HMDB0030171	B(ii)	-	[illegible]
pos_6456	N-Acetyl-D-mannosamine	metab 5450	HMDB0001129	B(i)	C00645	[illegible]
pos_6460	Vellein	metab 5455	HMDB0303360	B(ii)	-	[illegible]
pos_6465	D-Octopine	metab 5459	-	B(i)	C04137	[illegible]
pos_6475	Carboxyethyllysine	metab 5466	HMDB0249659	B(ii)	-	[illegible]
pos_6479	Nepsilon-Acetyl-L-lysine	metab 5469	HMDB0000206	B(i)	C02727	[illegible]
pos_6480	4-Imidazolone-5-propionic acid	metab 5471	HMDB0001014:PW C000802	B(ii)	C03680	[illegible]
pos_6481	Barbituric acid	metab_5472	HMDB0041833	B(ii)	C00813	[illegible]
pos_6482	3(2H)-Pyridazinone	metab 5473	HMDB0246116	B(ii)	-	[illegible]
pos_6484	5'-CMP	metab 5475	HMDB0000095	B(i)	C00055	[illegible]
pos_6487	N-Acetylputrescine	metab 5477	HMDB0002064	B(i)	C02714	[illegible]
pos_6489	Ochratoxin B	metab 5479	HMDB0029401	B(ii)	-	[illegible]
pos_649	Dimethyl phthalate	metab 5480	HMDB0251388	B(ii)	C11233	[illegible]
pos_6493	Oxoadipic acid	metab 5482	HMDB0000225:PW C000150:LMFA01170121	B(ii)	C00322	[illegible]
pos_6495	N-[1-(cyclobutanecarbonyl)piperidin-4-yl]-4-	metab_5484	-	B(i)	-	[illegible]
pos_6498	4-Amino-3-hydroxybutyrate	metab 5486	HMDB0061877	B(ii)	C03678	[illegible]
pos_6502	4-Aminophenylmannoside	metab_5491	HMDB0246178	B(ii)	-	[illegible]
pos_6503	Gamma-Glutamylglutamine	metab 5492	HMDB0011738:HMDB0029147	B(i)	C05283	[illegible]
pos_6504	Aminosalicylic Acid	metab 5493	HMDB0014378	B(ii)	C02518	[illegible]
pos_6507	L-Proline	metab 5496	HMDB0000162:PW C000106:HMDB0251528:MJDBOTE0000527	B(i)	C00148	[illegible]
pos_6511	L-Furosine	metab 5501	HMDB0029390	B(ii)	-	[illegible]
pos_6512	Diaminopimelic acid	metab 5502	HMDB0001370:HMDB0242532	B(i)	C00666	[illegible]
pos_6516	Gamma-Aminobutyric acid	metab 5505	HMDB0000112:PW C000071	B(ii)	C00334	[illegible]
pos_6517	4-Hydroxymethylpyrazole	metab 5506	HMDB0060768	B(ii)	-	[illegible]
pos_6519	Hydantoin-5-propionic acid	metab 5508	HMDB0001212	B(ii)	C05565	[illegible]
pos_6520	N,N-Dimethylarginine	metab_5510	HMDB0001539	B(i)	C03626	[illegible]
pos_6523	Betaine	metab 5513	PW C000030:HMDB0000043:MJDBTMA0000064	B(i)	C00719	[illegible]
pos_6526	L-3-Cyanoalanine	metab 5516	HMDB0060245	B(ii)	C02512	[illegible]
pos_6528	4-Methylpyrimidine	metab 5518	-	B(i)	-	[illegible]
pos_6531	Cis-4-Hydroxy-D-proline	metab 5521	HMDB0060460	B(i)	C03440	[illegible]
pos_6532	Pyroglutamic Acid	metab 5522	HMDB0000267:PW C000182:MJDBOTE0000549	B(i)	C01879	[illegible]
pos_6533	Ethenyl acetate	metab 5523	HMDB0031209	B(ii)	C19309	[illegible]
pos_6534	L-Alanine	metab 5524	PW C000105:HMDB0062251:HMDB0000161:MJDBAA00000185	B(i)	C00041	[illegible]
pos_6537	Alpha-Fluoro-beta-ureidopropionic acid	metab 5527	HMDB0060435	B(ii)	C16631	[illegible]
pos_6538	Val-Cit	metab_5528	HMDB0259738	B(ii)	-	[illegible]
pos_6546	3-Methylpyrazole	metab 5537	-	B(i)	-	[illegible]
pos_6551	Thiamine	metab 5543	HMDB0303167:HMDB0302835:HMDB0000235:PW C000158:MJD	B(i)	C00378	[illegible]
pos_6558	Histidinal	metab 5550	HMDB0012234	B(ii)	C01929	[illegible]
pos_6583	Valyllysine	metab 5578	HMDB0029132	B(ii)	-	[illegible]
pos_659	Vanylglycol	metab 5585	HMDB0001490:PW C001147	B(ii)	C05594	[illegible]
pos_6594	Gly Ala His	metab 5589	-	B(i)	-	[illegible]
pos_6618	Homomethionine	metab_5615	HMDB0030406	B(ii)	C17213	[illegible]
pos_664	Adenosine 3'-monophosphate	metab 5639	HMDB0003540	B(i)	C01367	[illegible]
pos_6658	Finafloxacin	metab_5658	HMDB0252260	B(ii)	-	[illegible]
pos_666	Alpha-Methylene-Gamma-Butyrolactone	metab 5660	HMDB0246945	B(i)	C20578	[illegible]
pos_6660	8-Hydroxyluteolin 8-sulfate	metab 5661	HMDB0037597	B(ii)	-	[illegible]
pos_6662	Monotropein	metab 5663	HMDB0035608:LMPR0102070012	B(ii)	C09788	[illegible]
pos_6681	Imazapyr	metab 5683	HMDB0253405	B(ii)	C18864	[illegible]
pos_6687	(2R,3R,4S,5R)-2-Amino-3,4,5,6-tetrahydroxyhexanal	metab 5689	HMDB0242589	B(ii)	-	[illegible]
pos_6689	1-Ethylpiperidine	metab 5691	HMDB0243877	B(ii)	-	[illegible]
pos_6690	3-Methylpyridazine	metab_5693	-	B(i)	-	[illegible]
pos_6692	3-(L-Menthoxy)-2-methylpropane-1,2-diol	metab 5695	HMDB0032417	B(ii)	-	[illegible]
pos_6695	Spermine	metab_5698	PW_C000970;HMDB0001256	B(i)	C00750	[illegible]
pos_6698	D-Arabinose 5-phosphate	metab 5701	HMDB0011734	B(ii)	C01112	[illegible]
pos_6700	Oxazepam	metab 5705	HMDB0014980	B(i)	C07359	[illegible]
pos_6702	Cadaverine	metab 5707	HMDB0002322:MJDBOTE0000688	B(i)	C01672	[illegible]
pos_6717	N-carboxymethyllysine	metab 5722	HMDB0255097	B(ii)	-	[illegible]
pos_6734	Caldine	metab 5741	HMDB0011634	B(i)	C03375	[illegible]
pos_6756	(2R,3R)-(-)-2,3-Butanediol	metab 5761	HMDB0033007	B(i)	C03044	[illegible]
pos_6761	5alpha-Cholest-8-en-3beta-ol	metab_5767	LMST01010096;HMDB0006841	B(ii)	C03845	[illegible]
pos_677	Feruloyl Malate	metab 5776	-	B(i)	-	[illegible]
pos_6776	Clozapine	metab_5783	HMDB0014507	B(ii)	C06924	[illegible]
pos_679	1-Carboxyethylisoleucine	metab 5798	HMDB0242158	B(ii)	-	[illegible]
pos_6828	P-CHLOROPHENYLALANINE	metab 5841	HMDB0033261	B(i)	-	[illegible]
pos_701	4-Acetamido-2-aminobutanoic acid	metab 5864	HMDB0031411	B(ii)	C06442	[illegible]
pos_702	Concanamycin a	metab 5865	HMDB0250430	B(ii)	C15761	[illegible]
pos_705	1-(Hydroxymethyl)-5,5-dimethyl-2,4-imidazolidinedione	metab 5868	HMDB0031670	B(ii)	-	[illegible]
pos_709	N-Acetyl-L-phenylalanine	metab 5872	HMDB0000512	B(ii)	C03519	[illegible]
pos_71	Indoleacrylic acid	metab_5873	HMDB0000734	B(ii)	-	[illegible]
pos_711	Cndac	metab 5875	HMDB0250381	B(ii)	-	[illegible]
pos_718	Methionine sulfoxide	metab_5882	HMDB0002005;PW_C001342	B(ii)	C02989	[illegible]
pos_73	Austinol	metab 5895	HMDB0034464	B(ii)	-	[illegible]
pos_76	N6-[2-(4-Aminophenyl)ethyl]adenosine	metab 5928	HMDB0248518	B(ii)	-	[illegible]
pos_767	Ifosfamide	metab 5936	HMDB0015312:PW C009594	B(ii)	C07047	[illegible]
pos_78	Formyl-5-hydroxykynurenamine	metab 5950	PW C008135:HMDB0012948	B(ii)	C05647	[illegible]

pos_783	N,N,N',N',N'',N''-Hexakis(methoxymethyl)-1,3,5-triazine-2,4,6-triamine	metab_5954	-	B(i)	-	413.2118	6.0171	pos	M+H, M+Na	C15H30N6O6	45.3	0	-0.321583676	-	0.00654817	5.746	5.662	5.685	5.666	5.674	5.648	5.784	5.853	5.826	5.838	5.849	5.833	5.959	5.983	5.972	5.997	5.95	5.956	5.98	5.945	5.961	5.972	5.963	5.964	5.868	5.862	5.847	5.844	5.832	5.881	5.919	5.914	5.914
pos_789	Phytosphingosine	metab_5960	LMSP01030001:HMDB0004610	B(ii)	C12144	318.2998	6.0949	pos	M+H, M+Na	C18H39NO3	0	36.3	-1.400333643	554-62-1	0.01118476	6.821	6.797	6.798	6.789	6.807	6.788	6.249	6.268	6.276	6.259	6.284	6.272	6.753	6.768	6.751	6.759	6.762	6.765	6.933	6.924	6.914	6.923	6.928	6.927	6.684	6.66	6.682	6.672	6.665	6.677	6.781	6.778	6.788
pos_79	D-Biotin	metab_5961	HMDB0000030	B(i)	C00120	245.0951	3.8604	pos	M+H, M+Na, M+H-H2O	C10H16N2O3S	63.7	0	-1.496854296	58-85-5	0.04760797	5.707	5.712	5.715	5.718	5.692	5.694	6.106	6.103	6.099	6.093	6.093	6.103	6.247	6.247	6.24	6.241	6.233	6.233	6.261	6.279	6.231	6.25	6.262	6.255	6.625	6.621	6.63	6.617	6.628	6.625	5.771	5.736	5.736
pos_790	11-(3,4-Dimethyl-5-pentylfuran-2-yl)undecanoylcarnitine	metab_5962	HMDB0241910	B(ii)	-	532.3372	6.1027	pos	M+H-2H2O, M+K	C29H51NO5	0	61.5	-5.500651659	-	0.01787132	5.183	5.541	5.69	5.699	5.649	5.66	6.073	6.076	6.047	6.038	6.043	6.039	5.496	5.087	4.845	4.639	4.889	5.048	5.16	5.064	5.142	5.135	5.114	5.261	4.638	5.051	4.648	4.617	5.076	5.084	5.228	5.214	5.214
pos_803	Batrachotoxinin A 20-alpha-benzoate	metab_5977	HMDB0248883	B(ii)	-	522.2828	6.274	pos	M+H, M+Na	C31H39NO6	0	34.2	-4.170337145	-	0.0752082	6.794	6.2	6.182	6.168	6.177	6.154	6.122	7.19	7.207	7.203	7.187	7.195	6.938	6.938	6.93	6.918	6.934	6.914	6.889	7.024	7.026	7.019	7.034	7.017	6.99	6.825	6.812	6.842	6.819	6.825	5.393	5.447	5.39
pos_806	6-Gingerol	metab_5980	HMDB0005783:HMDB0252720:MJDBOTE0000947	B(i)	C10462	277.1792	6.3052	pos	M+H-H2O, M+H	C17H26O4	41.6	0	-2.059125042	58253-27-3:23513-14-6	0.00400385	7.101	7.085	7.101	7.082	7.089	7.098	4.162	4.392	4.46	4.299	4.34	4.4	4.678	4.748	4.606	4.741	4.555	4.694	4.61	4.11	4.337	4.788	4.708	4.377	4.349	4.099	4.459	3.723	4.495	4.077	6.627	6.629	6.625
pos_809	16-Hydroxy-10-oxohexadecanoic acid	metab_5983	HMDB0041287	B(ii)	-	309.2048	6.3755	pos	M+H-H2O, M+Na	C16H30O4	0	82.2	4.177581068	53833-25-3	0.00359559	6.594	6.566	6.564	6.535	6.562	6.571	5.06	4.987	5.141	4.973	4.845	4.976	5.254	5.188	5.164	5.26	5.275	5.227	5.138	5.049	5.055	5.204	5.073	5.194	5.229	5.268	5.199	5.077	5.239	5.22	6.173	6.17	6.173
pos_813	Cer(t18:0/LTE4)	metab_5988	HMDB0290166	B(ii)	-	780.5527	6.3911	pos	M+H, M+NH4, M+ACN+H	C41H74N2O7S	0	37.7	-3.824095506	-	0.01912069	7.867	7.897	7.694	6.802	5.538	4.482	5.107	5.554	8.027	8.257	8.289	8.285	8.149	8.107	8.39	8.492	8.503	8.51	8.48	8.47	8.468	8.49	8.502	8.503	8.506	8.496	8.414	8.367	8.361	8.359	8.283	8.297	8.298
pos_816	Gamma-Linolenic Acid	metab_5991	LMFA01030141;PW_C001772;HMDB0003073;MJDBOTE0001062	B(i)	C06426	261.2207	6.4379	pos	M+H-H2O, M+NH4	C18H30O2	55.4	0	-2.175080666	506-26-3	0.01776203	6.164	6.198	6.198	6.236	6.21	6.229	5.883	5.771	5.745	5.751	5.709	5.745	5.846	5.862	5.79	5.759	5.749	5.767	5.819	5.847	5.848	5.859	5.834	5.878	5.93	5.958	5.918	5.943	5.95	5.962	6.057	6.07	6.07
pos_82	8-Epiloganic acid	metab_5995	HMDB0247433	B(ii)	-	415.1018	3.8049	pos	M+K, 2M+Na, M+2Na-H	C16H24O10	0	37.9	4.478136162	-	0.13552786	4.692	4.64	4.732	4.798	4.766	4.754	5.311	5.317	5.252	5.28	5.249	5.262	5.844	5.884	5.868	5.868	5.832	5.808	5.706	5.735	5.575	5.558	5.624	5.619	5.004	4.826	4.911	5.036	5.011	4.897	5.572	5.473	5.474
pos_823	RIOPROSTIL	metab_5999	HMDB0257238	B(ii)	-	337.273	6.5394	pos	M+H-H2O, M+	C21H38O4	0	73.3	-2.165820653	-	0.00404112	6.975	6.956	6.975	6.98	6.994	6.971	7.361	7.369	7.364	7.356	7.357	7.351	7.212	7.217	7.189	7.193	7.179	7.197	7.312	7.319	7.315	7.328	7.318	7.318	7.468	7.472	7.475	7.482	7.47	7.478	7.297	7.298	7.301
pos_827	MG(PGF1alpha/0:0/0:0)	metab_6003	HMDB0260511	B(ii)	-	413.2862	6.6096	pos	M+H-H2O, M+H	C23H42O7	0	41.3	-8.190330201	-	0.00374725	5.895	6.021	6.024	6.044	6.062	6.085	5.909	5.888	5.919	5.904	5.871	5.956	6.174	6.128	6.158	6.176	6.153	6.16	6.135	6.115	6.123	6.112	6.095	6.126	6.067	6.026	6.029	6.057	6.068	6.055	6.121	6.124	6.123
pos_837	Sterculic acid	metab_6012	HMDB0304490:LMFA01140018:HMDB0258495	B(ii)	C08366	295.2625	6.7423	pos	M+H, M+CH3OH+H	C19H34O2	0	37.2	-2.348376927	738-87-4	0.023413	5.827	5.871	5.803	5.867	5.819	5.894	5.384	5.389	5.391	5.382	5.383	5.39	5.469	5.474	5.476	5.472	5.478	5.482	5.466	5.466	5.47	5.475	5.469	5.478	5.385	5.399	5.402	5.393	5.399	5.396	5.389	5.407	5.406
pos_84	8-Hydroxyquinoline	metab_6015	-	B(i)	C19434	178.086	2.2231	pos	M+H, M+NH4, M+CH3OH+H	C9H7NO	66.1	0	-1.862240574	148-24-3	0.00146531	5.868	5.841	5.829	5.825	5.818	5.832	6.922	6.931	6.947	6.925	6.948	6.931	6.772	6.775	6.771	6.771	6.78	6.787	6.945	6.929	6.962	6.979	6.951	6.969	7.162	7.155	7.166	7.148	7.161	7.167	6.797	6.796	6.797
pos_840	Alpha-Eleostearic acid	metab_6016	HMDB0248208:LMFA01030147	B(ii)	C08315	311.2573	6.7812	pos	M+H-H2O, M+CH3OH+H	C18H30O2	0	34	-2.756868454	506-23-0	0.04810277	5.427	5.45	5.446	5.441	5.463	5.307	3.561	3.565	3.567	3.772	3.56	3.566	3.645	3.651	3.652	3.648	3.654	3.658	3.643	3.642	3.646	3.651	3.645	3.655	3.561	3.576	3.579	3.569	3.575	3.988	4.535	4.497	4.531
pos_846	7,8-Dihydrovomifoliol 9-[rhamnosyl-(1->6)-glucoside]	metab_6022	HMDB0029770	B(ii)	-	535.2701	6.8434	pos	M+, M+H	C25H42O12	0	72.6	-9.031400145	177261-69-7	0.00377961	8.938	8.967	8.966	8.967	8.969	8.958	6.195	4.753	0.6782	1.792	0.6721	1.192	5.684	0.7454	0.7466	0.7436	0.7485	0.7518	0.739	0.7386	0.7417	0.7458	0.7409	1.578	3.137	3.48	3.008	3.08	3.045	3.531	8.471	8.469	8.473
pos_851	3,3-Bis(carboxymethyl)hexadecanedioic acid	metab_6028	HMDB0257897	B(ii)	-	425.2137	6.4691	pos	M+H, M+Na	C20H34O8	0	31.2	-2.154087897	-	0.04439795	4.564	5.119	5.255	4.58	5.139	5.057	5.285	5.54	5.587	5.51	5.422	5.397	5.99	6.121	6.011	6.031	6.027	6	6.027	5.935	5.921	5.933	5.864	5.928	5.751	5.806	5.656	5.899	5.717	5.694	5.538	5.573	5.571
pos_853	Lefamulin	metab_6030	HMDB0254002	B(ii)	-	525.3362	6.9601	pos	M+H-H2O, M+NH4	C28H45NO5S	0	41.7	0.995441336	-	0.03462787	6.349	6.39	6.385	6.415	6.428	6.423	6.319	6.342	6.192	6.164	6.135	6.12	6.506	6.463	6.334	6.305	6.347	6.304	6.318	6.299	6.271	6.322	6.313	6.301	6.31	6.31	6.348	6.329	6.411	6.427	6.357	6.383	6.384
pos_856	PE(P-18:0/0:0)	metab_6033	HMDB0240598:LMGP02070002	B(i)	-	466.329	7.007	pos	M+H, M+Na	C23H48NO6P	73.9	0	-0.474263532	174062-73-8	0.00415675	7.007	7.001	7.015	7.029	7.029	7.025	6.19	6.146	6.119	6.125	6.074	6.076	5.483	5.222	5.374	5.441	5.344	5.409	5.796	5.807	5.739	5.759	5.845	5.754	5.484	5.384	5.463	5.42	5.45	5.351	6.655	6.656	6.658
pos_861	TG(15:0/22:6(4Z,7Z,10Z,13Z,16Z,19Z)/18:4(6Z,9Z,12Z,15Z))	metab_6039	HMDB0043795:PW_C024498	B(ii)	-	907.6837	7.1555	pos	M+H, M+Na	C58H92O6	0	30.8	5.771123689	-	0.00570713	7.489	7.483	7.509	7.556	7.534	7.542	4.275	4.294	4.281	4.273	4.273	4.28	6.688	6.648	6.555	6.542	6.503	6.456	6.31	6.372	6.378	6.266	6.302	6.316	4.275	4.29	4.293	4.283	4.289	4.286	6.986	6.983	6.988
pos_864	(Z)-7-[(1R,2R,3R,5S)-2-(1,2-Dihydroxy-3-oxooctyl)-3,5-dihydroxycyclopentyl]hept-5-enoic acid	metab_6042	HMDB0244520	B(ii)	-	419.2612	7.2259	pos	M+H, M+CH3OH+H	C20H34O7	0	35	-7.176397084	-	0.00585246	6.133	6.109	6.114	6.102	6.106	6.113	6.251	6.257	6.23	6.202	6.24	6.22	6.43	6.425	6.427	6.423	6.422	6.431	6.383	6.38	6.388	6.376	6.376	6.392	6.268	6.293	6.28	6.311	6.281	6.261	6.269	6.266	6.272
pos_865	1-O-Isopentyl-3-O-octadec-2-enoyl glycerol	metab_6043	HMDB0302897	B(ii)	-	427.3779	7.2963	pos	M+H, M+NH4	C26H50O4	0	33.7	-0.777624451	-	0.00358319	8.349	8.325	8.339	8.339	8.339	8.332	8.36	8.364	8.347	8.337	8.342	8.343	8.507	8.496	8.5	8.466	8.481	8.475	8.454	8.458	8.46	8.446	8.464	8.468	8.434	8.431	8.433	8.438	8.443	8.454	8.488	8.485	8.488
pos_868	GPEtn(14:0/22:6)	metab_6046	HMDB0008847	B(i)	C00350	736.4907	7.2572	pos	M+H, M+Na	C41H70NO8P	80.1	0	-0.698309254	-	0.05784852	5.728	5.735	5.755	5.745	5.751	5.74	6.089	6.079	6.062	6.007	6.051	5.962	6.494	6.498	6.513	6.439	6.437	6.442	6.466	6.524	6.546	6.47	6.502	6.503	6.22	6.164	6.141	6.231	6.173	6.235	5.928	5.883	5.926
pos_884	SM(d18:0/16:1(9Z)(OH))	metab_6064	HMDB0013463	B(ii)	C00550	739.5364	7.5936	pos	M+H-H2O, M+Na	C39H77N2O7P	0	76.2	0.431650706	-	0.07777965	4.567	6.076	5.448	5.336	5.419	5.414	5.249	5.512	6.312	6.32	6.323	6.383	5.43	6.239	6.497	6.548	6.545	6.541	6.497	6.483	6.5	6.486	6.511	6.514	6.48	6.457	6.063	6.123	6.063	6.051	6.334	6.32	6.268
pos_89	Aspirin	metab_6070	HMDB0001879:PW_C001251:MJDBOTE0001516	B(ii)	C01405	198.0759	1.0219	pos	M+H, M+NH4, M+H-H2O	C9H8O4	0	52.4	-1.239662255	50-78-2	0.00524889	4.34	4.476	4.475	4.538	4.315	4.018	6.906	6.895	6.869	6.906	6.862	6.894	6.733	6.748	6.764	6.75	6.746	6.72	6.693	6.72	6.57	6.591	6.65	6.628	7.485	7.485	7.482	7.49	7.496	7.483	6.817	6.813	6.814
pos_897	LysoPE(0:0/18:2(9Z,12Z))	metab_6078	HMDB0011477	B(ii)	-	478.2928	7.695	pos	M+, M+H	C23H44NO7P	0	82.4	-0.016867063	-	0.02256168	6.56	6.545	6.503	6.492	6.518	6.476	6.266	6.207	6.212	6.241	6.307	6.23	5.89	5.887	5.889	5.936	5.919	5.921	6.143	6.07	6.057	6.103	6.022	6.046	6.291	6.296	6.326	6.257	6.279	6.346	6.317	6.333	6.315
pos_93	(-)-Epigallocatechin 3'-glucuronide	metab_6115	HMDB0041638	B(ii)	-	521.0664	0.5097	pos	M+Na, M+K, M+H-H2O	C21H22O13	0	45.4	-5.816061694	-	0.02020435	6.083	6.018	5.996	6.004	6.034	6.011	5.675	5.686	5.55	5.711	5.606	5.731	5.801	5.791	5.79	5.833	5.83	5.789	5.928	5.875	5.918	5.919	5.922	5.895	5.426	5.562	5.523	5.516	5.501	5.507	5.947	5.96	5.944
pos_932	Ganoderic acid F	metab_6118	HMDB0035988	B(ii)	-	535.2703	7.6871	pos	M+H-2H2O, M+Na	C32H42O9	0	77.1	2.233388348	98665-15-7	0.07471287	6.989	7.052	6.866	6.785	6.883	6.799	5.614	5.083	4.105	3.879	4.538	3.817	4.139	4.258	3.859	3.75	3.517	3.576	3.614	3.756	3.396	3.762	3.808	3.663	4.4	4.201	4.236	4.22	4.472	4.466	6.239	6.29	6.231
pos_935	PC(18:1/0:0)	metab_6121	LMGP01050030:LMGP01050029:LMGP01050032	B(i)	-	522.3553	7.6638	pos	M+H, 2M+H	C26H52NO7P	87.9	0	-0.178865081	-	0.00072149	8.46	8.439	8.462	8.474	8.458	8.477	8.15	8.129	8.111	8.088	8.097	8.104	7.892	7.871	7.856	7.85	7.854	7.861	8.005	7.994	8.009	7.998	7.991	7.999	8.201	8.205	8.219	8.204	8.218	8.221	8.259	8.259	8.259
pos_94	L-Serine	metab_6126	PW_C000120:HMDB0000187:MJDBOTE0001776	B(i)	C00065	106.0501	0.5716	pos	M+ACN+Na, 2M+K, M+H	C3H7NO3	93.4	0	1.76557808	56-45-1	0.00840743	5.498	5.523	5.503	5.526	5.5	5.49	6.163	6.173	6.156	6.156	6.206	6.155	6.296	6.329	6.31	6.318	6.33	6.302	6.204	6.184	6.19	6.211	6.16	6.186	5.818	5.813	5.837	5.804	5.849	5.811	5.969	5.961	5.966
pos_944	MG(22:6(4Z,7Z,10Z,13Z,16Z,19Z)/0:0/0:0)	metab_6131	HMDB0011587	B(ii)	-	441.2426	7.4606	pos	M+H-H2O, M+K	C25H38O4	0	37	6.083860529	1044598-64-2	0.02621195	6.12	6.201	6.247	6.268	6.308	6.286	6.418	6.288	6.221	6.181	6.204	6.204	6.489	6.442	6.417	6.386	6.383	6.369	6.327	6.339	6.343	6.31	6.345	6.329	6.245	6.234	6.238	6.249	6.261	6.284	6.274	6.271	6.292
pos_948	SM(d16:1/16:0)	metab_6135	LMSP03010035:HMDB0240616	B(ii)	-	697.525	6.9601	pos	M+H, M+Na	C37H75N2O6P	0	90.2	-0.782023215	121999-53-9	0.06424624	7.027	7.827	7.366	7.266	7.235	7.235	7.159	7.168	8.096	8.154	8.134	8.135	7.535	7.866	7.961	7.941	7.935	7.937	7.937	7.929	8.012	8.029	8.028	8.023	8.1	8.095	8.144	8.179	8.18	8.182	8.053	8.102	8.056
pos_950	PC(17:1/0:0)	metab_6138	LMGP01050002	B(i)	-	508.3385	6.9525	pos	M+H, M+Na	C25H50NO7P	58.4	0	-2.591703478	-	0.06417368	6.559	6.585	6.588	6.564	6.608	6.545	6.573	6.554	6.515	6.509	6.49	6.474	6.396	6.358	6.314	6.254	6.279	6.293	6.446	6.452	6.408	6.403	6.431	6.406	6.562	6.555	6.571	6.588	6.621	6.596	6.519	6.516	6.565
pos_951	LysoPA(22:4(7Z,10Z,13Z,16Z)/0:0)	metab_6139	HMDB0114752	B(ii)	-	504.3057	6.9525	pos	M+H-H2O, M+NH4	C25H43O7P	0	72.4	-5.674438542	-	0.01321084	6.928	6.9	6.927	6.952	6.94	6.953	7.409	7.418	7.368	7.377	7.346	7.34	7.319	7.317	7.278	7.266	7.254	7.265	7.375	7.367	7.368	7.367	7.36	7.363	7.423	7.415	7.421	7.44	7.446	7.443	7.287	7.293	7.298
pos_954	Ciprostene	metab_6142	HMDB0250270	B(ii)	-	365.2678	6.4146	pos	M+H-H2O, M+H	C22H36O4	0	72.9	-2.243900483	-	0.01172585	6.253	6.211	6.238	6.191	6.241	6.241	4.037	4.041	4.043	4.035	4.035	4.042	4.785	4.327	4.128	4.373	4.314	4.61	4.205	4.118	4.122	4.127	4.121	4.131	4.037	4.052	4.055	4.045	4.051	4.048	5.838	5.847	5.847
pos_958	LysoPA(i-19:0/0:0)	metab_6146	HMDB0114766	B(ii)	-	516.3084	6.3911	pos	M+H-2H2O, M+ACN+Na	C22H45O7P	0	49.8	5.217763592	-	0.00753632	6.497	6.527	6.531	6.519	6.48	6.536	6.327	6.287	6.299	6.321	6.311	6.299	6.262	6.296	6.258	6.254	6.237	6.225	6.244	6.271	6.259	6.283	6.261	6.281	6.395	6.404	6.413	6.445	6.42	6.404	6.436	6.429	6.433
pos_962	N(6)-L-Homocysteinyl-L-lysine	metab_6151	HMDB0255539	B(ii)	-	281.1649	6.3755	pos	M+NH4, 2M+Na	C10H21N3O3S	0	38.3	2.615405293	-	0.00092323	6.505	6.543	6.554	6.542	6.555	6.536	6.232	6.232	6.255	6.274	6.263	6.276	6.465	6.475	6.465	6.459	6.466	6.473	6.436	6.431	6.431	6.445	6.44	6.445	6.391	6.371	6.38	6.4	6.376	6.395	6.357	6.356	6.357
pos_963	Palmitoleic acid	metab_6152	HMDB0003229:LMFA01030056:HMDB0012328	B(ii)	C08362	277.2156	6.3755	pos	M+H-H2O, M+Na	C16H30O2	0	68.9	7.119048997	10030-73-6:373-49-9	0.00300609	6.737	6.7	6.715	6.692	6.703	6.7	5.97	5.977	5.987	5.937	5.97	5.946	5.856	5.886	5.857	5.868	5.903	5.846	5.88	5.881	5.888	5.869	5.883	5.873	6.566	6.536	6.564	6.54	6.543	6.546	6.566	6.567	6.569
pos_964	7Z,10Z-Hexadecadienoic acid	metab_6153	HMDB0000477:LMFA01030807	B(ii)	-	275.1999	6.3755	pos	M+Na, M+H-2H2O	C16H28O2	0	50.9	6.997065359	28290-73-5	0.00489204	6.466	6.432	6.45	6.432	6.434	6.449	4.4	4.474	4.574	4.435	4.634	4.525	3.762	4.3	4.2	3.905	4.029	4.15	3.75	4.318	4.209	4.088	4.056	3.753	4.774	4.627	4.735	4.591	4.577	4.601	6.084	6.088	6.088
pos_966	(4E,7E,10E,13E)-Hexadeca-4,7,10,13-tetraenoic acid	metab_6155	HMDB0253125	B(ii)	-	249.1843	6.313	pos	M+H-H2O, M+H	C16H24O2	0	70.9	-2.380362644	-	0.01511816	6.541	6.524	6.541	6.515	6.533	6.547	3.553	1.087	1.089	1.081	3.898	1.088	1.161	1.166	1.167	3.613	1.169	1.173	1.159	1.158	3.594	1.166	1.161	1.17	4.346	1.097	3.952	3.621	1.096	3.643	6.062	6.074	6.074
pos_968	LysoPC(14:0/0:0)	metab_6157	PW_C006388:HMDB0010379	B(ii)	C04230	512.275	6.3521	pos	M+, M+2Na-H	C22H46NO7P	0	71.6	5.658503924	20559-16-4	0.0026277	6.517	6.39	6.419	6.383	6.414	6.411	5.781	5.912	5.915	5.823	5.77	5.741	5.555	5.674	5.612	5.541	5.503	5.462	5.781	5.7	5.731	5.706	5.779	5.721	5.72	5.598	5.621	5.664	5.717	5.704	6.319	6.317	6.316
pos_979	(16-Benzyl-5,12-dihydroxy-5,7,14-trimethyl-13-methylidene-6,18-dioxo-17-azatricyclo[9.7.0.01,15]octadeca-3,9-dien-2-yl) acetate	metab_6169	HMDB0242294	B(ii)	-	490.256	6.2272	pos	M+H-H2O, M+H	C30H37NO6	0	38.2	-5.498975033	-	0.01398699	6.378	6.246	6.221	6.255	6.263	6.281	5.185	4.106	4.108	4.099	4.1	4.107	4.186	4.191	4.193	4.189	4.195	4.199	4.183	4.183	4.187	4.192	4.186	4.195	4.102	4.117	4.119	4.11	4.116	4.113	5.878	5.866	5.875
pos_989	Trans-EKODE-(E)-Ib	metab_6180	-	B(i)	-	275.2	6.1182	pos	M+H-2H2O, M+H	C18H30O4	58.7	0	-1.815392491	-	0.00740796	6.211	6.17	6.185	6.167	6.171	6.198	5.344	5.457	5.434	5.461	5.442	5.439	4.695	4.433	4.752	4.799	4.788	4.524	5.064	4.815	4.901	4.91	5.087	4.982	6.44	6.424	6.432	6.437	6.444	6.425	6.12	6.119	6.114
pos_990	N-Dodecylsarcosinate	metab_6182	HMDB0255129	B(ii)	-	240.2318	6.1182	pos	M+H-H2O, M+H	C15H31NO2	0	60.3	-1.614480916	-	0.02009071	4.121	3.732	3.55	4.112	3.6	3.535	5.179	5.2	5.208	5.244	5.18	5.152	5.604	5.653	5.614	5.604	5.636	5.685	5.638	5.642	5.617	5.653	5.627	5.657	5.838	5.828	5.844	5.805	5.839	5.851	5.506	5.49	5.504
pos_5878	1-Pyrroline-5-carboxylic acid	metab_6191	HMDB0001301:PW_C001003	B(ii)	C03912	227.1023	2.7134	pos	2M+H	C5H7NO2	0	44	-1.436481617	2906-39-0:64199-88-8	0.01259093	4.968	5.003	4.946	4.937	4.961	4.963	4.768	4.744	4.756	4.724	4.762	4.715	5.386	5.411	5.413	5.415	5.367	5.376	5.293	5.288	5.3	5.288	5.293	5.31	5.767	5.761	5.761	5.79	5.756	5.746	5.378	5.367	5.375
pos_3869	13-Dihydrodaunorubicin	metab_6192	LMPK13050004:HMDB0244506	B(ii)	C12430:C12433	571.2301	6.7965	pos	M+ACN+H	C27H31NO10	0	79.8	2.879912773	28008-55-1	0.00950373	6.888	6.842	6.859	6.895	6.876	6.883	6.663	6.668	6.669	6.661	6.662	6.669	6.748	6.753	6.754	6.75	6.757	6.761	6.745	6.744	6.748	6.753	6.747	6.757	6.663	6.678	6.681	6.671	6.677	6.674	6.668	6.672	6.676
pos_36	2-Hydroxycinnamic acid	metab_6193	HMDB0041592:HMDB0002641	B(i)	C01772:C05838	182.081	1.3577	pos	M+H, M+NH4, M+H-H2O	C9H8O3	71.6	0	-1.102577553	495-79-4:614-60-8	0.00213563	7.029	7.016	7.021	7.015	7.01	7.012	7.45	7.447	7.451	7.449	7.454	7.456	7.416	7.424	7.42	7.419	7.421	7.422	7.271	7.276	7.268	7.273	7.273	7.275	8.338	8.335	8.34	8.332	8.336	8.332	7.813	7.812	7.812
pos_2179	4-Hydroxymandelonitrile	metab_6194	HMDB0060318	B(i)	C03742	150.0547	4.4935	pos	M+H	C8H7NO2	77.9	0	-1.83599501	71807-09-5	0.00749936	4.906	4.907	4.917	4.969	4.965	4.924	5.443	5.431	5.466	5.45	5.43	5.467	5.55	5.54	5.535	5.524	5.525	5.542	5.587	5.549	5.569	5.554	5.602	5.578	4.857	4.877	4.877	4.883	4.916	4.837	5.339	5.334	5.334
pos_6313	7-Methylguanine	metab_6195	HMDB0000897:HMDB0006040	B(i)	C02242	166.0721	1.0687	pos	M+H	C6H7N5O	82.7	0	-1.34590954	578-76-7:10030-78-1	0.01357363	6.177	6.17	6.164	6.161	6.156	6.152	6.584	6.58	6.587	6.572	6.585	6.577	6.781	6.783	6.784	6.779	6.782	6.784	6.682	6.683	6.678	6.689	6.682	6.68	6.512	6.505	6.525	6.506	6.511	6.512	6.627	6.616	6.617
pos_5736	Acetylcysteine	metab_6196	HMDB0000574:HMDB0001890:MJDBOTE0001037	B(ii)	C06809	368.0975	2.8865	pos	2M+ACN+H	C5H9NO3S	0	36.8	9.485491145	52-90-4:616-91-1	0.0086598	3.23	3.236	3.257	3.246	3.252	3.242	4.927	4.854	4.875	4.836	4.872	4.889	5.874	5.844	5.835	5.852	5.874	5.853	5.475	5.476	5.466	5.484	5.478	5.486	5.207	5.195	5.218	5.221	5.209	5.216	5.076	5.082	5.083
pos_6449	Adenosine 2'-phosphate	metab_6197	HMDB0003540:HMDB0000045:HMDB0011617	B(ii)	C00946	386.0257	0.7328	pos	M+K	C10H14N5O7P	0	37.6	-1.471164966	61-19-8:130-49-4:84-2	0.05112014	6.084	6.073	6.085	6.062	6.058	6.076	4.625	4.263	4.235	4.584	4.622	4.266	4.747	4.442	4.413	4.469	4.685	4.489	4.454	4.31	4.314	4.319	4.313	4.322	4.229	4.244	4.246	4.237	4.243	4.24	5.134	5.177	5.149
pos_6331	Allysine	metab_6198	HMDB0001263;HMDB0303320;PW_C000976	B(ii)	C04076	187.1074	1.0296	pos	M+ACN+H	C6H11NO3	0	50.3	-1.930497783	1962-83-0;6665-12-9	0.01262348	5.165	5.117	5.06	5.068	5.038	5.07	4.547	4.581	4.655	4.633	4.571	4.67	4.766	4.817	4.844	4.863	4.8	4.896	4.768	4.71	4.799	4.822	4.775	4.805	5.594	5.612	5.627	5.598	5.586	5.58	5.216	5.206	5.207
pos_6244	Caffeic acid	metab_6199	HMDB0001964:HMDB0003501	B(ii)	C01197	222.0757	1.3033	pos	M+ACN+H	C9H8O4	0	31	-1.905800458	4361-87-9:331-39-5:50	0.03148124	5.012	4.986	5.016	5.059	4.983	5.01	5.978	5.961	5.973	5.973	6.002	5.99	5.796	5.813	5.842	5.835	5.82	5.834	5.717	5.741	5.675	5.628	5.69	5.649	6.413	6.419	6.418	6.401	6.402	6.39	5.836	5.86	5.86
pos_3314	Choline	metab_6200	PW_C000065:HMDB0250194:HMDB0000097:MJDBOTE0000238	B(i)	C00114	104.1072	7.5389	pos	M+	C5H14NO+	90.8	0	-3.413321829	62-49-7	0.01429957	6.789	6.784	6.811	6.821	6.821	6.834	6.782	6.728	6.648	6.633	6.606	6.61	6.693	6.63	6.561	6.534	6.513	6.525	6.544	6.569	6.573	6.553	6.541	6.555	6.633	6.665	6.664	6.686	6.674	6.675	6.701	6.69	6.69
pos_3954	Coproporphyrin III	metab_6201	PW_C000445:HMDB0001261:HMDB0000570:HMDB0000916	B(ii)	C05770	637.2638	6.516	pos	M+H-H2O	C36H38N4O8	0	46.1	-2.9240199	2624-63-7:14643-66-4:	0.03246089	6.668	6.687	6.676	6.644	6.687	6.675	4.261	4.266	4.268	4.259	4.26	4.267	4.346	4.351	4.353	4.349	4.355	4.359	4.343	4.343	4.347	4.352	4.346	4.355	4.262	4.276	4.279	4.27	4.276	4.272	5.27	5.268	5.293
pos_1055	D-Fructose	metab_6202	HMDB0000660:PW_C000517:MJDBOTE0000506	B(i)	C02336:C10906:C0009	163.0598	0.3777	pos	M+H-H2O	C6H12O6	33.4	0	-1.75362736	53188-23-1;57-48-7	0.01864046	5.18	5.154	5.18	5.134	5.112	5.13	5.115	5.201	5.265	5.254	5.308	5.326	5.372	5.364	5.397	5.368	5.372	5.395	5.391	5.405	5.352	5.403	5.37	5.416	5.468	5.38	5.436	5.416	5.393	5.353	5.252	5.266	5.266
pos_2913	Dodecanoic acid	metab_6203	HMDB0002262:HMDB0000638:HMDB0303347:HMDB0303348:PW	B(ii)	C02679	242.2111	5.8526	pos	M+ACN+H	C12H24O2	0	38.8	-1.852459286	1908-11-8;143-07-7	0.03720565	3.701	3.707	3.728	3.718	3.723	3.713	4.08	4.072	4.161	3.852	3.942	3.895	4.639	4.71	4.654	4.706	4.631	4.622	4.509	4.508	4.496	4.544	4.388	4.418	4.751	4.858	4.808	4.838	4.838	4.799	4.315	4.287	4.287
pos_1928	Gallocatechin	metab_6204	HMDB0038361:HMDB0038365:MJDBOTE0000270	B(i)	C12136:C12127	306.0751	3.5199	pos	M+	C15H14O7	38	0	3.663391058	970-74-1:970-73-0	0.01723187	1.461	0.4557	2.071	1.077	0.8408	0.9959	3.122	0.5598	3.63	0.555	3.118	0.5606	5.266	5.273	5.316	5.354	5.308	5.278	5.253	5.232	5.217	5.154	5.28	5.262	0.5569	3.152	0.5696	3.221	0.5669	3.547	4.483	4.497	4.485
pos_6423	Glucosamine	metab_6205	HMDB0242589:HMDB0001514:HMDB0030091:HMDB0245095:PW	B(ii)	C00329	162.0758	0.756	pos	M+H-H2O	C6H13NO5	0	71.2	-1.581680897	14257-69-3;3416-24-8	0.00634285	5.944	5.919	5.915	5.911	5.922	5.917	5.808	5.825	5.832	5.829	5.831	5.842	5.883	5.863	5.872	5.857	5.881	5.872	5.815	5.829	5.819	5.813	5.816	5.832	5.699	5.727	5.752	5.746	5.699	5.708	5.887	5.891	5.892
pos_4903	Herniarin	metab_6206	HMDB0029758:HMDB0247248:MJDBOTE0001736	B(i)	C11052	209.0805	4.7214	pos	M+CH3OH+H	C10H8O3	78.7	0	-1.627545912	531-59-9	0.01349528	4.19	4.239	4.428	4.168	4.291	4.315	4.639	4.48	4.686	4.662	4.55	4.692	5.12	5.177	5.123	5.143	5.162	5.182	5.041	5.042	4.992	5.049	5.059	5.092	5.447	5.47	5.373	5.4	5.481	5.376	5.066	5.063	5.055
pos_3761	Hexanal	metab_6207	HMDB0005994;HMDB0031498;LMFA06000109	B(ii)	C02373	142.1224	7.2259	pos	M+ACN+H	C6H12O	0	30.6	-2.005722329	6789-80-6;66-25-1	0.00568391	6.299	6.291	6.31	6.299	6.298	6.298	6.348	6.351	6.353	6.346	6.351	6.353	6.488	6.506	6.496	6.493	6.495	6.487	6.486	6.483	6.471	6.476	6.486	6.479	6.43	6.43	6.428	6.423	6.432	6.435	6.412	6.416	6.417
pos_406	Indole	metab_6208	HMDB0000738:HMDB0035924	B(i)	C00463	118.0653	3.0749	pos	M+, M+H	C8H7N	75.8	0	1.420515516	120-72-9:109-97-7	0.00728058	6.715	6.694	6.698	6.696	6.707	6.682	6.671	6.667	6.672	6.669	6.681	6.688	6.831	6.839	6.832	6.824	6.837	6.827	6.757	6.743	6.731	6.75	6.743	6.746	6.75	6.736	6.754	6.739	6.741	6.739	6.752	6.746	6.747
pos_1776	Indole-3-Carboxaldehyde	metab_6209	HMDB0000197:HMDB0029737:HMDB0000195:MJDBSJD0000088	B(i)	C08493	146.0598	3.0749	pos	M+H	C9H7NO	98.6	0	-1.537343647	87-51-4:58-63-9:487-8	0.00579156	6.734	6.727	6.726	6.727	6.728	6.717	6.693	6.687	6.686	6.68	6.697	6.702	6.889	6.892	6.883	6.886	6.887	6.885	6.752	6.749	6.735	6.752	6.74	6.747	6.766	6.751	6.767	6.763	6.755	6.763	6.775	6.77	6.771
pos_1862	Isoguvacine	metab_6210	HMDB0030352:HMDB0253644	B(ii)	C10128	160.0966	3.304	pos	M+CH3OH+H	C6H9NO2	0	32.5	-1.904312889	499-04-7:64603-90-3	0.05047412	4.175	4.253	4.139	4.125	4.228	4.261	4.023	4.169	4.376	4.292	4.272	4.297	4.295	4.378	4.431	4.327	4.632	4.18	4.447	4.44	4.271	4.479	4.202	4.392	4.243	4.049	4.209	4.226	4.293	4.073	4.243	4.282	4.281
pos_6335	Isonicotinic acid	metab_6211	HMDB0060665:HMDB0041912	B(i)	C07446	124.0394	1.0296	pos	M+H	C6H5NO2	74.2	0	0.910597091	2015-20-5:55-22-1	0.00494829	7.246	7.23	7.234	7.219	7.217	7.221	7.386	7.388	7.387	7.38	7.383	7.386	7.346	7.346	7.338	7.341	7.336	7.341	7.393	7.386	7.391	7.392	7.39	7.392	7.534	7.536	7.537	7.526	7.531	7.528	7.404	7.4	7.401
pos_5142	Lawsone	metab_6212	HMDB0253997:HMDB0030773	B(ii)	C10368	192.0652	4.4935	pos	M+NH4	C10H6O3	0	60.2	-1.744431217	481-39-0:83-72-7	0.01452659	4.592	4.466	4.614	4.514	4.543	4.48	4.562	4.667	4.623	4.68	4.669	4.633	4.779	4.744	4.777	4.699	4.714	4.795	4.808	4.796	4.848	4.842	4.847	4.769	4.148	4.165	4.275	4.206	4.176	4.242	4.657	4.645	4.655
pos_4086	Lithocholic acid	metab_6213	PW_C000608:HMDB0000717:HMDB0000761:LMST04010003	B(ii)	C03990	421.269	6.2427	pos	M+2Na-H	C24H40O3	0	33.8	0.198708179	1534-35-6:434-13-9	0.02696499	6.122	6.096	6.093	6.118	6.088	6.077	3.876	3.881	3.883	3.874	3.875	3.882	3.961	3.966	3.968	3.964	3.97	3.974	3.958	3.958	3.962	3.967	3.961	3.97	3.877	3.891	3.894	3.885	3.966	3.887	5.516	5.514	5.535
pos_3822	LysoPC(18:0/0:0)	metab_6214	HMDB0011149:HMDB0010384	B(ii)	C04317:C04230	568.3366	6.9525	pos	M+2Na-H	C26H54NO7P	0	86	3.163011972	74430-89-0:19420-57-6	0.03168294	7.265	7.256	7.255	7.278	7.28	7.295	6.763	6.788	6.724	6.725	6.692	6.691	6.111	6.041	6.072	6.023	6	6.04	6.447	6.447	6.414	6.42	6.406	6.4	7.855	7.852	7.872	7.877	7.882	7.884	7.334	7.347	7.361
pos_5924	Medphalan	metab_6215	HMDB0015176:HMDB0244533	B(ii)	C07122	269.0587	2.6899	pos	M+H-2H2O	C13H18Cl2N2O2	0	32.8	-6.490181687	148-82-3:13045-94-8	0.00388314	5.278	5.286	5.255	5.263	5.244	5.279	3.218	3.357	3.224	3.275	4.013	3.224	3.794	4.191	3.915	4.205	3.439	4.139	4.435	4.314	4.612	4.431	4.542	4.519	5.698	5.672	5.712	5.687	5.681	5.688	5.652	5.653	5.649
pos_4500	Methylnoradrenaline	metab_6216	HMDB0002832;HMDB0015652	B(ii)	C17925;C11768	148.0754	5.0634	pos	M+H-2H2O	C9H13NO3	0	45.2	-1.33773329	6539-57-7;829-74-3	0.01815509	4.5	4.414	4.452	4.366	4.503	4.439	4.786	4.739	4.733	4.756	4.804	4.77	5.173	5.095	5.14	5.11	5.088	5.2	5.261	5.245	5.213	5.208	5.257	5.206	5.115	5.057	5.115	5.088	5.057	5.107	4.99	4.975	4.988
pos_4158	Metyrapone	metab_6217	HMDB0000692:HMDB0015146	B(ii)	C07205	470.259	6.0871	pos	2M+NH4	C14H14N2O	0	59.3	8.629758188	54-36-4:15438-31-0	0.14318212	4.459	4.71	4.565	4.59	4.769	4.377	4.046	3.805	3.576	3.567	3.635	3.575	3.654	3.659	3.66	3.657	3.663	3.667	3.651	3.651	3.654	3.659	3.653	3.663	5.768	5.755	5.711	5.697	5.764	5.759	4.792	4.678	4.79
pos_1243	N-Epsilon-Acetyl-L-Lysine	metab_6218	HMDB0000197:HMDB0000206:MJDBOTE0000583	B(i)	C02727	189.123	0.756	pos	M+H	C8H16N2O3	78.4	0	-1.736692282	87-51-4:692-04-6	0.01100684	5.45	5.443	5.468	5.425	5.415	5.454	5.413	5.442	5.468	5.492	5.39	5.491	5.434	5.46	5.457	5.425	5.502	5.492	6.033	6.026	6.028	6.027	6.024	6.03	5.399	5.436	5.369	5.376	5.404	5.38	5.846	5.836	5.843
pos_631	P-Coumaric Acid	metab_6219	HMDB0030677:HMDB0002035:HMDB0003654:MJDBOTE000071	B(i)	C00811	182.081	1.0374	pos	M+H-H2O, M+NH4	C9H8O3	57.7	0	-1.124685604	7400-08-0:501-98-4:50	0.00403034	5.397	5.297	5.329	5.296	5.314	5.285	6.981	6.981	6.996	6.976	6.99	6.995	6.846	6.854	6.857	6.857	6.862	6.854	6.866	6.856	6.863	6.868	6.868	6.865	7.087	7.086	7.111	7.086	7.097	7.086	6.818	6.815	6.815
pos_4390	Penicillic Acid	metab_6220	HMDB0256217	B(i)	C16804:C19495	153.0544	5.4319	pos	M+H-H2O	C8H10O4	59.3	0	-1.241097101	90-65-3:17397-87-4	0.01627711	4.239	4.245	4.265	4.255	4.261	4.251	4.457	4.473	4.398	4.439	4.493	4.506	4.787	4.793	4.732	4.836	4.805	4.827	4.825	4.834	4.797	4.828	4.831	4.84	5.058	5.063	5.106	5.063	5.045	5.037	4.71	4.697	4.698
pos_6444	Phenol	metab_6221	PW_C000153:HMDB0013674:HMDB0000228	B(i)	C00146	95.0494	0.7482	pos	M+H	C6H6O	71.8	0	2.44335135	108-95-2:87-66-1	0.02586254	3.231	3.238	3.258	3.248	3.254	3.243	4.929	4.939	4.955	4.944	4.924	4.937	4.68	4.769	4.751	4.721	4.723	4.686	4.792	4.825	4.832	4.783	4.804	4.843	5.159	5.127	5.115	5.129	5.114	5.121	4.762	4.76	4.78
pos_5496	Salicylic Acid	metab_6222	HMDB0001895:HMDB0302214:HMDB0000840:PW_C001263:MJD	B(i)	C00805	139.0388	3.408	pos	M+H	C7H6O3	74.3	0	-1.074917595	487-54-7;69-72-7	0.03237024	4.639	4.634	4.621	4.634	4.632	4.637	3.77	3.78	3.828	3.843	3.713	3.849	3.938	3.893	3.845	3.81	4.072	4.152	4.054	4.155	3.842	4.14	4.089	4.116	3.681	3.57	3.921	3.526	3.664	3.682	4.278	4.277	4.302
pos_3044	Styrene	metab_6223	HMDB0034240	B(i)	C07083	105.0701	6.1648	pos	M+H	C8H8	71.7	0	1.790397174	100-42-5	0.00506319	5.697	5.658	5.685	5.668	5.68	5.672	5.469	5.494	5.501	5.475	5.461	5.448	5.62	5.703	5.672	5.651	5.672	5.641	5.571	5.569	5.549	5.552	5.571	5.552	5.676	5.628	5.64	5.638	5.637	5.643	5.748	5.743	5.745
pos_446	Urobilinogen	metab_6224	HMDB0001898:HMDB0004158	B(ii)	C05791	591.3135	3.488	pos	M+H, M+Na	C33H42N4O6	0	39.9	-7.11160688	14684-37-8:17208-65-(	0.02944059	6.128	6.096	6.141	6.121	6.103	6.127	1.807	1.812	1.814	1.805	1.806	1.813	1.891	3.683	1.897	1.894	1.9	1.904	3.488	1.888	1.892	3.331	1.891	1.9	1.808	3.662	1.825	1.816	3.594	1.818	5.181	5.178	5.157
