# Supplementary material for: Comparative Proteomics and Metabonomics Analysis of Different Diapause Stages Revealed a New Regulation Mechanism of Diapause in Loxostege sticticalis (Lepidoptera: Pyralidae)
Source: Molecules. 2024 Jul 25;29(15):3472. doi: 10.3390/molecules29153472 (PMC11314584; doi:10.3390/molecules29153472)
Supplement: Supplementary file 1 [file molecules-29-03472-s001.zip › analysis process/metabolic/HMDB compound classification.pdf]

Superclass	Number
Lipids and lipid-like molecules	483
Not Available	60
Benzenoids	175
Organic 1,3-dipolar compounds	2
Organic acids and derivatives	412
Organic oxygen compounds	204
Phenylpropanoids and polyketides	132
Organoheterocyclic compounds	306
Hydrocarbon derivatives	1
Organic nitrogen compounds	31
Lignans, neolignans and related compounds	4
Homogeneous metal compounds	1
Hydrocarbons	3
Organosulfur compounds	2
Nucleosides, nucleotides, and analogues	95
Homogeneous non-metal compounds	2
Alkaloids and derivatives	17
