# Supplementary material for: Comparative Proteomics and Metabonomics Analysis of Different Diapause Stages Revealed a New Regulation Mechanism of Diapause in Loxostege sticticalis (Lepidoptera: Pyralidae)
Source: Molecules. 2024 Jul 25;29(15):3472. doi: 10.3390/molecules29153472 (PMC11314584; doi:10.3390/molecules29153472)
Supplement: Supplementary file 1 [file molecules-29-03472-s001.zip › analysis process/metabolic/KEGG compound classification.pdf]

First Category	Second Category	Number
Antibiotics	Polyketides and nonribosomal peptides	1
Antibiotics	beta-Lactams	2
Antibiotics	Quinolones	1
Hormones and transmitters	Peptide hormones	1
Hormones and transmitters	Neurotransmitters	7
Lipids	Fatty acids	10
Peptides	Amino acids	19
Steroids	27-Carbon atoms	2
Hormones and transmitters	Other hormones	2
Carbohydrates	Monosaccharides	13
Steroids	21-Carbon atoms	1
Antibiotics	Others	2
Lipids	Eicosanoids	4
Lipids	Phospholipids	37
Hormones and transmitters	Steroid hormones	3
Nucleic acids	Nucleotides	11
Vitamins and cofactors	Cofactors	7
Organic acids	Carboxylic acids	15
Nucleic acids	Bases	4
Nucleic acids	Cyclic nucleotides	2
Nucleic acids	Nucleosides	9
Peptides	Amines	3
Steroids	24-Carbon atoms	1
Lipids	Fats	2
Steroids	18-Carbon atoms	1
Vitamins and cofactors	Vitamins	5
Carbohydrates	Oligosaccharides	4
