# Supplementary material for: Comparative Proteomics and Metabonomics Analysis of Different Diapause Stages Revealed a New Regulation Mechanism of Diapause in Loxostege sticticalis (Lepidoptera: Pyralidae)
Source: Molecules. 2024 Jul 25;29(15):3472. doi: 10.3390/molecules29153472 (PMC11314584; doi:10.3390/molecules29153472)
Supplement: Supplementary file 1 [file molecules-29-03472-s001.zip › analysis process/metabolic/KEGG enrichment analysis.pdf]

| Num | First Category | Second Category | Pathway Desciption | 通路描述 | Pathway_ID | Database | Ratio_in_study | Ratio_in_pop | P_value | P_adjust |
|---|---|---|---|---|---|---|---|---|---|---|
| 1 | Cellular Processes | Cell growth and death | Apoptosis - fly | 凋亡-苍蝇 | map04214 | KEGG PATHWAY | 1/179 | 1/4493 | 0.03984 | 0.1779 |
| 1 | Human Diseases | Drug resistance: antineoplastic | EGFR tyrosine kinase inhibitor resistance | EGFR酪氨酸激酶抑制剂耐药性 | map01521 | KEGG PATHWAY | 1/179 | 2/4493 | 0.0781 | 0.2678 |
| 1 | Cellular Processes | Transport and catabolism | Autophagy - other | 自噬-其他 | map04136 | KEGG PATHWAY | 1/179 | 3/4493 | 0.1148 | 0.3341 |
| 1 | Organismal Systems | Immune system | Th1 and Th2 cell differentiation | Th1和Th2细胞分化 | map04658 | KEGG PATHWAY | 1/179 | 3/4493 | 0.1148 | 0.3341 |
| 1 | Human Diseases | Infectious disease: bacterial | Pathogenic Escherichia coli infection | 致病性大肠杆菌感染 | map05130 | KEGG PATHWAY | 1/179 | 3/4493 | 0.1148 | 0.3341 |
| 1 | Environmental Information Processing | Signal transduction | NF-kappa B signaling pathway | NF-κB信号通路 | map04064 | KEGG PATHWAY | 1/179 | 3/4493 | 0.1148 | 0.3341 |
| 1 | Human Diseases | Cancer: overview | PD-L1 expression and PD-1 checkpoint pathway in cancer | PD-L1在癌症中的表达和PD-1检查点通路 | map05235 | KEGG PATHWAY | 1/179 | 4/4493 | 0.1501 | 0.3793 |
| 1 | Organismal Systems | Immune system | Natural killer cell mediated cytotoxicity | 自然杀伤细胞介导的细胞毒性 | map04650 | KEGG PATHWAY | 1/179 | 4/4493 | 0.1501 | 0.3793 |
| 1 | Environmental Information Processing | Signal transduction | ErbB signaling pathway | ErbB信号通路 | map04012 | KEGG PATHWAY | 1/179 | 4/4493 | 0.1501 | 0.3793 |
| 1 | Organismal Systems | Immune system | B cell receptor signaling pathway | B细胞受体信号通路 | map04662 | KEGG PATHWAY | 1/179 | 4/4493 | 0.1501 | 0.3793 |
| 1 | Organismal Systems | Immune system | T cell receptor signaling pathway | T细胞受体信号通路 | map04660 | KEGG PATHWAY | 1/179 | 4/4493 | 0.1501 | 0.3793 |
| 1 | Organismal Systems | Immune system | Th17 cell differentiation | Th17细胞分化 | map04659 | KEGG PATHWAY | 1/179 | 4/4493 | 0.1501 | 0.3793 |
| 1 | Human Diseases | Cancer: specific types | Glioma | 神经胶质瘤 | map05214 | KEGG PATHWAY | 1/179 | 4/4493 | 0.1501 | 0.3793 |
| 1 | Organismal Systems | Endocrine system | Growth hormone synthesis, secretion and action | 生长激素的合成、分泌和作用 | map04935 | KEGG PATHWAY | 1/179 | 4/4493 | 0.1501 | 0.3793 |
| 1 | Organismal Systems | Nervous system | Neurotrophin signaling pathway | 神经营养素信号通路 | map04722 | KEGG PATHWAY | 1/179 | 5/4493 | 0.184 | 0.4108 |
| 1 | Environmental Information Processing | Signal transduction | Rap1 signaling pathway | Rap1信号通路 | map04015 | KEGG PATHWAY | 1/179 | 5/4493 | 0.184 | 0.4108 |
| 1 | Environmental Information Processing | Signal transduction | MAPK signaling pathway | MAPK信号通路 | map04010 | KEGG PATHWAY | 1/179 | 5/4493 | 0.184 | 0.4108 |
| 1 | Environmental Information Processing | Signal transduction | FoxO signaling pathway | FoxO信号通路 | map04068 | KEGG PATHWAY | 1/179 | 5/4493 | 0.184 | 0.4108 |
| 1 | Organismal Systems | Immune system | Chemokine signaling pathway | 趋化因子信号通路 | map04062 | KEGG PATHWAY | 1/179 | 5/4493 | 0.184 | 0.4108 |
| 1 | Metabolism | Glycan biosynthesis and metabolism | Glycosaminoglycan biosynthesis - heparan sulfate / hepari | 糖胺聚糖生物合成-硫酸乙酰肝素/肝素 | map00534 | KEGG PATHWAY | 1/179 | 5/4493 | 0.184 | 0.4108 |
| 1 | Human Diseases | Infectious disease: viral | Human immunodeficiency virus 1 infection | 人类免疫缺陷病毒1型感染 | map05170 | KEGG PATHWAY | 1/179 | 5/4493 | 0.184 | 0.4108 |
| 1 | Human Diseases | Cancer: specific types | Non-small cell lung cancer | 非小细胞肺癌 | map05223 | KEGG PATHWAY | 1/179 | 6/4493 | 0.2166 | 0.4331 |
| 1 | Human Diseases | Infectious disease: viral | Human cytomegalovirus infection | 人巨细胞病毒感染 | map05163 | KEGG PATHWAY | 1/179 | 6/4493 | 0.2166 | 0.4331 |
| 1 | Environmental Information Processing | Signal transduction | VEGF signaling pathway | VEGF信号通路 | map04370 | KEGG PATHWAY | 1/179 | 6/4493 | 0.2166 | 0.4331 |
| 1 | Human Diseases | Neurodegenerative disease | Huntington disease | 亨廷顿病 | map05016 | KEGG PATHWAY | 1/179 | 6/4493 | 0.2166 | 0.4331 |
| 1 | Cellular Processes | Transport and catabolism | Autophagy - animal | 自噬-动物 | map04140 | KEGG PATHWAY | 1/179 | 6/4493 | 0.2166 | 0.4331 |
| 1 | Organismal Systems | Endocrine system | Relaxin signaling pathway | 松弛素信号通路 | map04926 | KEGG PATHWAY | 1/179 | 6/4493 | 0.2166 | 0.4331 |
| 1 | Organismal Systems | Endocrine system | GnRH signaling pathway | GnRH信号通路 | map04912 | KEGG PATHWAY | 1/179 | 6/4493 | 0.2166 | 0.4331 |
| 1 | Organismal Systems | Endocrine system | Adipocytokine signaling pathway | 脂肪细胞因子信号通路 | map04920 | KEGG PATHWAY | 1/179 | 7/4493 | 0.2478 | 0.4489 |
| 1 | Metabolism | Glycan biosynthesis and metabolism | Glycosylphosphatidylinositol (GPI)-anchor biosynthesis | 糖基磷脂酰肌醇（GPI）-锚生物合成 | map00563 | KEGG PATHWAY | 1/179 | 7/4493 | 0.2478 | 0.4489 |
| 1 | Environmental Information Processing | Signal transduction | Ras signaling pathway | Ras信号通路 | map04014 | KEGG PATHWAY | 1/179 | 7/4493 | 0.2478 | 0.4489 |
| 1 | Organismal Systems | Immune system | Fc gamma R-mediated phagocytosis | FcγR介导的吞噬作用 | map04666 | KEGG PATHWAY | 1/179 | 8/4493 | 0.2778 | 0.4849 |
| 1 | Human Diseases | Infectious disease: parasitic | African trypanosomiasis | 非洲锥虫病 | map05143 | KEGG PATHWAY | 1/179 | 8/4493 | 0.2778 | 0.4849 |
| 1 | Organismal Systems | Excretory system | Endocrine and other factor-regulated calcium reabsorptior | 内分泌和其他因素调节钙的再吸收 | map04961 | KEGG PATHWAY | 1/179 | 8/4493 | 0.2778 | 0.4849 |
| 1 | Metabolism | Glycan biosynthesis and metabolism | Glycosaminoglycan biosynthesis - chondroitin sulfate / der | 糖胺聚糖生物合成-硫酸软骨素/硫酸皮肤 | map00532 | KEGG PATHWAY | 1/179 | 8/4493 | 0.2778 | 0.4849 |
| 1 | Organismal Systems | Aging | Longevity regulating pathway | 寿命调节途径 | map04211 | KEGG PATHWAY | 1/179 | 8/4493 | 0.2778 | 0.4849 |
| 1 | Human Diseases | Infectious disease: bacterial | Vibrio cholerae infection | 霍乱弧菌感染 | map05110 | KEGG PATHWAY | 1/179 | 9/4493 | 0.3067 | 0.5076 |
| 1 | Environmental Information Processing | Signal transduction | Apelin signaling pathway | Apelin信号通路 | map04371 | KEGG PATHWAY | 1/179 | 9/4493 | 0.3067 | 0.5076 |
| 1 | Human Diseases | Endocrine and metabolic disease | AGE-RAGE signaling pathway in diabetic complications | 糖尿病并发症中的AGE-RAGE信号通路 | map04933 | KEGG PATHWAY | 1/179 | 9/4493 | 0.3067 | 0.5076 |
| 1 | Organismal Systems | Endocrine system | Parathyroid hormone synthesis, secretion and action | 甲状旁腺激素的合成、分泌和作用 | map04928 | KEGG PATHWAY | 1/179 | 10/4493 | 0.3343 | 0.5394 |
| 1 | Organismal Systems | Circulatory system | Adrenergic signaling in cardiomyocytes | 心肌细胞中的肾上腺素能信号传导 | map04261 | KEGG PATHWAY | 1/179 | 10/4493 | 0.3343 | 0.5394 |
| 1 | Organismal Systems | Digestive system | Cholesterol metabolism | 胆固醇代谢 | map04979 | KEGG PATHWAY | 1/179 | 10/4493 | 0.3343 | 0.5394 |
| 1 | Human Diseases | Infectious disease: viral | Coronavirus disease - COVID-19 | | map05171 | KEGG PATHWAY | 1/179 | 10/4493 | 0.3343 | 0.5394 |
| 1 | Environmental Information Processing | Signal transduction | cGMP-PKG signaling pathway | cGMP-PKG信号通路 | map04022 | KEGG PATHWAY | 1/179 | 10/4493 | 0.3343 | 0.5394 |
| 1 | Organismal Systems | Endocrine system | Cortisol synthesis and secretion | 皮质醇的合成和分泌 | map04927 | KEGG PATHWAY | 1/179 | 12/4493 | 0.3864 | 0.5456 |
| 1 | Organismal Systems | Endocrine system | Insulin secretion | 胰岛素分泌 | map04911 | KEGG PATHWAY | 1/179 | 12/4493 | 0.3864 | 0.5456 |
| 1 | Organismal Systems | Immune system | C-type lectin receptor signaling pathway | C型凝集素受体信号通路 | map04625 | KEGG PATHWAY | 1/179 | 11/4493 | 0.3609 | 0.5456 |
| 1 | Organismal Systems | Sensory system | Phototransduction - fly | 光转导-苍蝇 | map04745 | KEGG PATHWAY | 1/179 | 11/4493 | 0.3609 | 0.5456 |
| 1 | Genetic Information Processing | Folding, sorting and degradation | Sulfur relay system | 硫磺继电器系统 | map04122 | KEGG PATHWAY | 1/179 | 11/4493 | 0.3609 | 0.5456 |
| 1 | Organismal Systems | Immune system | Fc epsilon RI signaling pathway | FcεRI信号通路 | map04664 | KEGG PATHWAY | 1/179 | 11/4493 | 0.3609 | 0.5456 |
| 1 | Organismal Systems | Endocrine system | Thyroid hormone signaling pathway | 甲状腺激素信号通路 | map04919 | KEGG PATHWAY | 1/179 | 11/4493 | 0.3609 | 0.5456 |
| 1 | Organismal Systems | Endocrine system | Prolactin signaling pathway | 催乳素信号通路 | map04917 | KEGG PATHWAY | 1/179 | 11/4493 | 0.3609 | 0.5456 |
| 1 | Human Diseases | Endocrine and metabolic disease | Alcoholic liver disease | | map04936 | KEGG PATHWAY | 1/179 | 11/4493 | 0.3609 | 0.5456 |
| 1 | Human Diseases | Infectious disease: parasitic | Amoebiasis | 阿米巴病 | map05146 | KEGG PATHWAY | 1/179 | 13/4493 | 0.4109 | 0.5636 |
| 1 | Human Diseases | Endocrine and metabolic disease | Cushing syndrome | 库欣综合征 | map04934 | KEGG PATHWAY | 1/179 | 13/4493 | 0.4109 | 0.5636 |
| 1 | Organismal Systems | Immune system | Neutrophil extracellular trap formation | | map04613 | KEGG PATHWAY | 1/179 | 13/4493 | 0.4109 | 0.5636 |
| 1 | Human Diseases | Infectious disease: bacterial | Shigellosis | 志贺氏菌病 | map05131 | KEGG PATHWAY | 1/179 | 14/4493 | 0.4345 | 0.5834 |
| 1 | Human Diseases | Cancer: specific types | Hepatocellular carcinoma | 肝细胞癌 | map05225 | KEGG PATHWAY | 1/179 | 14/4493 | 0.4345 | 0.5834 |
| 1 | Human Diseases | Cardiovascular disease | Lipid and atherosclerosis | | map05417 | KEGG PATHWAY | 1/179 | 14/4493 | 0.4345 | 0.5834 |
| 1 | Organismal Systems | Digestive system | Gastric acid secretion | 胃酸分泌 | map04971 | KEGG PATHWAY | 1/179 | 14/4493 | 0.4345 | 0.5834 |
| 1 | Human Diseases | Neurodegenerative disease | Amyotrophic lateral sclerosis | 肌萎缩侧索硬化症（ALS） | map05014 | KEGG PATHWAY | 1/179 | 14/4493 | 0.4345 | 0.5834 |
| 1 | Organismal Systems | Immune system | Platelet activation | 血小板活化 | map04611 | KEGG PATHWAY | 1/179 | 14/4493 | 0.4345 | 0.5834 |
| 1 | Environmental Information Processing | Signal transduction | HIF-1 signaling pathway | HIF-1信号通路 | map04066 | KEGG PATHWAY | 1/179 | 15/4493 | 0.4571 | 0.589 |
| 1 | Metabolism | Energy metabolism | Oxidative phosphorylation | 氧化磷酸化 | map00190 | KEGG PATHWAY | 1/179 | 16/4493 | 0.4788 | 0.6088 |
| 1 | Organismal Systems | Endocrine system | Renin secretion | 肾素分泌 | map04924 | KEGG PATHWAY | 1/179 | 17/4493 | 0.4996 | 0.627 |
| 1 | Organismal Systems | Excretory system | Proximal tubule bicarbonate reclamation | 近端小管碳酸氢盐回收 | map04964 | KEGG PATHWAY | 1/179 | 17/4493 | 0.4996 | 0.627 |
| 1 | Metabolism | Energy metabolism | Nitrogen metabolism | 氮代谢 | map00910 | KEGG PATHWAY | 1/179 | 19/4493 | 0.5388 | 0.659 |
| 1 | Organismal Systems | Endocrine system | Thyroid hormone synthesis | 甲状腺激素合成 | map04918 | KEGG PATHWAY | 1/179 | 21/4493 | 0.575 | 0.6988 |

| | | | | | | | | | | |
|---|---|---|---|---|---|---|---|---|---|---|
| 1 | Metabolism | Biosynthesis of other secondary meta | Caffeine metabolism | 咖啡因代谢 | map00232 | KEGG PATHWAY | 1/179 | 22/4493 | 0.592 | 0.7149 |
| 1 | Metabolism | Amino acid metabolism | Valine, leucine and isoleucine biosynthesis | 缬氨酸、亮氨酸和异亮氨酸生物合成 | map00290 | KEGG PATHWAY | 1/179 | 23/4493 | 0.6084 | 0.7255 |
| 1 | Metabolism | Amino acid metabolism | Arginine biosynthesis | 精氨酸生物合成 | map00220 | KEGG PATHWAY | 1/179 | 23/4493 | 0.6084 | 0.7255 |
| 1 | Organismal Systems | Endocrine system | Ovarian steroidogenesis | 卵巢类固醇生成 | map04913 | KEGG PATHWAY | 1/179 | 24/4493 | 0.624 | 0.7351 |
| 1 | Metabolism | Metabolism of terpenoids and polyke | Insect hormone biosynthesis | 昆虫激素生物合成 | map00981 | KEGG PATHWAY | 1/179 | 25/4493 | 0.6391 | 0.7482 |
| 1 | Metabolism | Metabolism of cofactors and vitamins | Retinol metabolism | 视黄醇代谢 | map00830 | KEGG PATHWAY | 1/179 | 25/4493 | 0.6391 | 0.7482 |
| 1 | Metabolism | Metabolism of other amino acids | Selenocompound metabolism | 硒化合物代谢 | map00450 | KEGG PATHWAY | 1/179 | 27/4493 | 0.6674 | 0.772 |
| 1 | Metabolism | Metabolism of cofactors and vitamins | Vitamin B6 metabolism | 维生素B6代谢 | map00750 | KEGG PATHWAY | 1/179 | 29/4493 | 0.6936 | 0.788 |
| 1 | Cellular Processes | Cell growth and death | Ferroptosis | 铁死亡 | map04216 | KEGG PATHWAY | 1/179 | 29/4493 | 0.6936 | 0.788 |
| 1 | Human Diseases | Cancer: overview | Pathways in cancer | 癌症的途径 | map05200 | KEGG PATHWAY | 1/179 | 31/4493 | 0.7177 | 0.8011 |
| 1 | Metabolism | Carbohydrate metabolism | Pyruvate metabolism | 丙酮酸代谢 | map00620 | KEGG PATHWAY | 1/179 | 32/4493 | 0.729 | 0.809 |
| 1 | Metabolism | Carbohydrate metabolism | C5-Branched dibasic acid metabolism | C5支化二元酸代谢 | map00660 | KEGG PATHWAY | 1/179 | 34/4493 | 0.7503 | 0.8279 |
| 1 | Metabolism | Carbohydrate metabolism | Starch and sucrose metabolism | 淀粉和蔗糖代谢 | map00500 | KEGG PATHWAY | 1/179 | 37/4493 | 0.7792 | 0.85 |
| 1 | Metabolism | Lipid metabolism | Glycerolipid metabolism | 甘油酯代谢 | map00561 | KEGG PATHWAY | 1/179 | 38/4493 | 0.7881 | 0.85 |
| 1 | Metabolism | Lipid metabolism | Fatty acid elongation | 脂肪酸伸长 | map00062 | KEGG PATHWAY | 1/179 | 40/4493 | 0.8047 | 0.8632 |
| 1 | Metabolism | Carbohydrate metabolism | Propanoate metabolism | 丙酸代谢 | map00640 | KEGG PATHWAY | 1/179 | 40/4493 | 0.8047 | 0.8632 |
| 1 | Metabolism | Carbohydrate metabolism | Galactose metabolism | 半乳糖代谢 | map00052 | KEGG PATHWAY | 1/179 | 46/4493 | 0.8474 | 0.8989 |
| 1 | Metabolism | Carbohydrate metabolism | Fructose and mannose metabolism | 果糖和甘露糖代谢 | map00051 | KEGG PATHWAY | 1/179 | 54/4493 | 0.8902 | 0.9339 |
| 1 | Metabolism | Carbohydrate metabolism | Pentose and glucuronate interconversions | 戊糖和葡萄糖醛酸的相互转化 | map00040 | KEGG PATHWAY | 1/179 | 58/4493 | 0.9068 | 0.9361 |
| 1 | Metabolism | Carbohydrate metabolism | Ascorbate and aldarate metabolism | 抗坏血酸和醛酸代谢 | map00053 | KEGG PATHWAY | 1/179 | 57/4493 | 0.9029 | 0.9422 |
| 1 | Metabolism | Lipid metabolism | Steroid biosynthesis | 类固醇生物合成 | map00100 | KEGG PATHWAY | 1/179 | 57/4493 | 0.9029 | 0.9422 |
| 1 | Metabolism | Biosynthesis of other secondary meta | Neomycin, kanamycin and gentamicin biosynthesis | 新霉素、卡那霉素和庆大霉素生物合成 | map00524 | KEGG PATHWAY | 1/179 | 81/4493 | 0.964 | 0.969 |
| 1 | Human Diseases | Cancer: overview | Chemical carcinogenesis - DNA adducts | 化学致癌 | map05204 | KEGG PATHWAY | 1/179 | 77/4493 | 0.9575 | 0.9779 |
| 2 | Human Diseases | Infectious disease: viral | Kaposi sarcoma-associated herpesvirus infection | 卡波西肉瘤相关疱疹病毒感染 | map05167 | KEGG PATHWAY | 2/179 | 5/4493 | 0.01458 | 0.1166 |
| 2 | Organismal Systems | Endocrine system | Melanogenesis | 黑素生成 | map04916 | KEGG PATHWAY | 2/179 | 6/4493 | 0.0213 | 0.1278 |
| 2 | Human Diseases | Substance dependence | Nicotine addiction | 尼古丁成瘾 | map05033 | KEGG PATHWAY | 2/179 | 7/4493 | 0.02905 | 0.164 |
| 2 | Human Diseases | Substance dependence | Cocaine addiction | 可卡因成瘾 | map05030 | KEGG PATHWAY | 2/179 | 7/4493 | 0.02905 | 0.164 |
| 2 | Organismal Systems | Nervous system | Long-term potentiation | 长时程增强 | map04720 | KEGG PATHWAY | 2/179 | 7/4493 | 0.02905 | 0.164 |
| 2 | Human Diseases | Neurodegenerative disease | Spinocerebellar ataxia | 脊髓小脑共济失调 | map05017 | KEGG PATHWAY | 2/179 | 7/4493 | 0.02905 | 0.164 |
| 2 | Organismal Systems | Nervous system | Glutamatergic synapse | 谷氨酸能突触 | map04724 | KEGG PATHWAY | 2/179 | 8/4493 | 0.03773 | 0.1811 |
| 2 | Human Diseases | Substance dependence | Morphine addiction | 吗啡成瘾 | map05032 | KEGG PATHWAY | 2/179 | 8/4493 | 0.03773 | 0.1811 |
| 2 | Organismal Systems | Endocrine system | Estrogen signaling pathway | 雌激素信号通路 | map04915 | KEGG PATHWAY | 2/179 | 8/4493 | 0.03773 | 0.1811 |
| 2 | Organismal Systems | Endocrine system | GnRH secretion | 促性腺激素释放激素分泌 | map04929 | KEGG PATHWAY | 2/179 | 9/4493 | 0.04726 | 0.2016 |
| 2 | Organismal Systems | Nervous system | GABAergic synapse | GABA能突触 | map04727 | KEGG PATHWAY | 2/179 | 9/4493 | 0.04726 | 0.2016 |
| 2 | Organismal Systems | Nervous system | Long-term depression | 长期抑郁症 | map04730 | KEGG PATHWAY | 2/179 | 9/4493 | 0.04726 | 0.2016 |
| 2 | Human Diseases | Substance dependence | Amphetamine addiction | 安非他明成瘾 | map05031 | KEGG PATHWAY | 2/179 | 9/4493 | 0.04726 | 0.2016 |
| 2 | Organismal Systems | Environmental adaptation | Circadian entrainment | 昼夜夹带 | map04713 | KEGG PATHWAY | 2/179 | 9/4493 | 0.04726 | 0.2016 |
| 2 | Environmental Information Processing | Signal transduction | Phospholipase D signaling pathway | 磷脂酶D信号通路 | map04072 | KEGG PATHWAY | 2/179 | 11/4493 | 0.06854 | 0.2531 |
| 2 | Cellular Processes | Cellular community - eukaryotes | Gap junction | 缝隙连接 | map04540 | KEGG PATHWAY | 2/179 | 11/4493 | 0.06854 | 0.2531 |
| 2 | Environmental Information Processing | Signal transduction | Calcium signaling pathway | 钙信号通路 | map04020 | KEGG PATHWAY | 2/179 | 11/4493 | 0.06854 | 0.2531 |
| 2 | Organismal Systems | Endocrine system | Oxytocin signaling pathway | 催产素信号通路 | map04921 | KEGG PATHWAY | 2/179 | 12/4493 | 0.08015 | 0.27 |
| 2 | Organismal Systems | Nervous system | Cholinergic synapse | 胆碱能突触 | map04725 | KEGG PATHWAY | 2/179 | 12/4493 | 0.08015 | 0.27 |
| 2 | Organismal Systems | Nervous system | Synaptic vesicle cycle | 突触囊泡周期 | map04721 | KEGG PATHWAY | 2/179 | 12/4493 | 0.08015 | 0.27 |
| 2 | Organismal Systems | Digestive system | Fat digestion and absorption | 脂肪的消化和吸收 | map04975 | KEGG PATHWAY | 2/179 | 13/4493 | 0.09232 | 0.2859 |
| 2 | Organismal Systems | Digestive system | Pancreatic secretion | 胰腺分泌 | map04972 | KEGG PATHWAY | 2/179 | 15/4493 | 0.1181 | 0.3193 |
| 2 | Organismal Systems | Circulatory system | Vascular smooth muscle contraction | 血管平滑肌收缩 | map04270 | KEGG PATHWAY | 2/179 | 16/4493 | 0.1316 | 0.346 |
| 2 | Organismal Systems | Digestive system | Salivary secretion | 唾液分泌 | map04970 | KEGG PATHWAY | 2/179 | 17/4493 | 0.1454 | 0.3721 |
| 2 | Environmental Information Processing | Signal transduction | AMPK signaling pathway | AMPK信号通路 | map04152 | KEGG PATHWAY | 2/179 | 22/4493 | 0.2178 | 0.406 |
| 2 | Metabolism | Metabolism of other amino acids | Taurine and hypotaurine metabolism | 牛磺酸和低牛磺酸代谢 | map00430 | KEGG PATHWAY | 2/179 | 24/4493 | 0.2476 | 0.4528 |
| 2 | Metabolism | Lipid metabolism | Ether lipid metabolism | 乙醚脂质代谢 | map00565 | KEGG PATHWAY | 2/179 | 25/4493 | 0.2626 | 0.4626 |
| 2 | Organismal Systems | Digestive system | Carbohydrate digestion and absorption | 碳水化合物的消化和吸收 | map04973 | KEGG PATHWAY | 2/179 | 27/4493 | 0.2926 | 0.4885 |
| 2 | Human Diseases | Neurodegenerative disease | Pathways of neurodegeneration - multiple diseases | | map05022 | KEGG PATHWAY | 2/179 | 32/4493 | 0.3665 | 0.5252 |
| 2 | Metabolism | Metabolism of cofactors and vitamins | Thiamine metabolism | 硫胺素代谢 | map00730 | KEGG PATHWAY | 2/179 | 31/4493 | 0.3519 | 0.5363 |
| 2 | Metabolism | Energy metabolism | Sulfur metabolism | 硫代谢 | map00920 | KEGG PATHWAY | 2/179 | 33/4493 | 0.381 | 0.5418 |
| 2 | Metabolism | Amino acid metabolism | Lysine biosynthesis | 赖氨酸生物合成 | map00300 | KEGG PATHWAY | 2/179 | 35/4493 | 0.4095 | 0.5656 |
| 2 | Metabolism | Amino acid metabolism | Valine, leucine and isoleucine degradation | 缬氨酸、亮氨酸和异亮氨酸降解 | map00280 | KEGG PATHWAY | 2/179 | 42/4493 | 0.5037 | 0.6239 |
| 2 | Metabolism | Lipid metabolism | Fatty acid degradation | 脂肪酸降解 | map00071 | KEGG PATHWAY | 2/179 | 50/4493 | 0.5988 | 0.7185 |
| 2 | Human Diseases | Cancer: overview | Chemical carcinogenesis - reactive oxygen species | | map05208 | KEGG PATHWAY | 2/179 | 57/4493 | 0.6703 | 0.7706 |
| 2 | Metabolism | Lipid metabolism | Fatty acid biosynthesis | 脂肪酸生物合成 | map00061 | KEGG PATHWAY | 2/179 | 58/4493 | 0.6796 | 0.7767 |
| 2 | Metabolism | Carbohydrate metabolism | Glyoxylate and dicarboxylate metabolism | 乙醛酸和二羧酸代谢 | map00630 | KEGG PATHWAY | 2/179 | 62/4493 | 0.7148 | 0.8026 |
| 2 | Metabolism | Metabolism of cofactors and vitamins | Ubiquinone and other terpenoid-quinone biosynthesis | 泛醌和其他萜类醌生物合成 | map00130 | KEGG PATHWAY | 2/179 | 71/4493 | 0.7823 | 0.8486 |
| 2 | Metabolism | Xenobiotics biodegradation and meta | Metabolism of xenobiotics by cytochrome P450 | 细胞色素P450对外源性物质的代谢 | map00980 | KEGG PATHWAY | 2/179 | 121/4493 | 0.9579 | 0.9732 |
| 2 | Metabolism | Metabolism of cofactors and vitamins | Porphyrin metabolism | 卟啉与叶绿素代谢 | map00860 | KEGG PATHWAY | 2/179 | 148/4493 | 0.9838 | 0.9838 |
| 3 | Organismal Systems | Development and regeneration | Axon regeneration | 轴突再生 | map04361 | KEGG PATHWAY | 3/179 | 7/4493 | 0.001933 | 0.03712 |
| 3 | Human Diseases | Substance dependence | Alcoholism | 酗酒 | map05034 | KEGG PATHWAY | 3/179 | 10/4493 | 0.006066 | 0.06851 |
| 3 | Organismal Systems | Aging | Longevity regulating pathway - worm | 长寿调节途径——蠕虫 | map04212 | KEGG PATHWAY | 3/179 | 10/4493 | 0.006066 | 0.06851 |
| 3 | Organismal Systems | Nervous system | Dopaminergic synapse | 多巴胺能突触 | map04728 | KEGG PATHWAY | 3/179 | 12/4493 | 0.01049 | 0.1007 |
| 3 | Organismal Systems | Endocrine system | Regulation of lipolysis in adipocytes | 脂肪细胞脂解的调节 | map04923 | KEGG PATHWAY | 3/179 | 14/4493 | 0.01636 | 0.1122 |
| 3 | Organismal Systems | Endocrine system | Aldosterone synthesis and secretion | 醛固酮的合成和分泌 | map04925 | KEGG PATHWAY | 3/179 | 22/4493 | 0.0549 | 0.2108 |

| | | | | | | | | | | |
|---|---|---|---|---|---|---|---|---|---|---|
| 3 | Environmental Information Processing | Signal transduction | cAMP signaling pathway | cAMP信号通路 | map04024 | KEGG PATHWAY | 3/179 | 25/4493 | 0.07524 | 0.2627 |
| 3 | Metabolism | Lipid metabolism | Cutin, suberine and wax biosynthesis | 角质、木栓碱和蜡的生物合成 | map00073 | KEGG PATHWAY | 3/179 | 27/4493 | 0.09039 | 0.2893 |
| 3 | Metabolism | Metabolism of cofactors and vitamins | Biotin metabolism | 生物素代谢 | map00780 | KEGG PATHWAY | 3/179 | 29/4493 | 0.1067 | 0.3152 |
| 3 | Organismal Systems | Sensory system | Taste transduction | 味觉传导 | map04742 | KEGG PATHWAY | 3/179 | 32/4493 | 0.1331 | 0.3453 |
| 3 | Organismal Systems | Sensory system | Inflammatory mediator regulation of TRP channels | TRP通道的炎症介质调节 | map04750 | KEGG PATHWAY | 3/179 | 35/4493 | 0.1615 | 0.3691 |
| 3 | Metabolism | Metabolism of other amino acids | Glutathione metabolism | 谷胱甘肽代谢 | map00480 | KEGG PATHWAY | 3/179 | 38/4493 | 0.1915 | 0.3954 |
| 3 | Metabolism | Biosynthesis of other secondary meta | Monobactam biosynthesis | 单巴坦生物合成 | map00261 | KEGG PATHWAY | 3/179 | 39/4493 | 0.2018 | 0.4122 |
| 3 | Human Diseases | Cardiovascular disease | Diabetic cardiomyopathy | | map05415 | KEGG PATHWAY | 3/179 | 39/4493 | 0.2018 | 0.4122 |
| 3 | Organismal Systems | Nervous system | Serotonergic synapse | 5-羟色胺能突触 | map04726 | KEGG PATHWAY | 3/179 | 42/4493 | 0.2335 | 0.431 |
| 3 | Metabolism | Xenobiotics biodegradation and meta | Drug metabolism - other enzymes | 药物代谢-其他酶 | map00983 | KEGG PATHWAY | 3/179 | 52/4493 | 0.3431 | 0.527 |
| 3 | Cellular Processes | Cell growth and death | Necroptosis | 坏死性下垂 | map04217 | KEGG PATHWAY | 1/179 | 10/4493 | 0.3343 | 0.5394 |
| 3 | Metabolism | Metabolism of cofactors and vitamins | Folate biosynthesis | 叶酸合成 | map00790 | KEGG PATHWAY | 3/179 | 58/4493 | 0.4089 | 0.5689 |
| 3 | Metabolism | Amino acid metabolism | Cysteine and methionine metabolism | 半胱氨酸和蛋氨酸代谢 | map00270 | KEGG PATHWAY | 3/179 | 66/4493 | 0.4932 | 0.623 |
| 3 | Metabolism | Lipid metabolism | Steroid hormone biosynthesis | 类固醇激素生物合成 | map00140 | KEGG PATHWAY | 3/179 | 99/4493 | 0.7628 | 0.8369 |
| 4 | Human Diseases | Endocrine and metabolic disease | Insulin resistance | 胰岛素抵抗 | map04931 | KEGG PATHWAY | 4/179 | 19/4493 | 0.005901 | 0.07554 |
| 4 | Organismal Systems | Environmental adaptation | Thermogenesis | 产热 | map04714 | KEGG PATHWAY | 4/179 | 23/4493 | 0.01191 | 0.1089 |
| 4 | Organismal Systems | Digestive system | Mineral absorption | 矿物吸收 | map04978 | KEGG PATHWAY | 4/179 | 29/4493 | 0.02655 | 0.1545 |
| 4 | Metabolism | Metabolism of other amino acids | beta-Alanine metabolism | β-丙氨酸代谢 | map00410 | KEGG PATHWAY | 4/179 | 32/4493 | 0.03667 | 0.1853 |
| 4 | Metabolism | Amino acid metabolism | Histidine metabolism | 组氨酸代谢 | map00340 | KEGG PATHWAY | 4/179 | 47/4493 | 0.1157 | 0.3173 |
| 4 | Metabolism | Amino acid metabolism | Glycine, serine and threonine metabolism | 甘氨酸、丝氨酸和苏氨酸代谢 | map00260 | KEGG PATHWAY | 4/179 | 48/4493 | 0.1225 | 0.3267 |
| 4 | Metabolism | Xenobiotics biodegradation and meta | Drug metabolism - cytochrome P450 | 药物代谢-细胞色素P450 | map00982 | KEGG PATHWAY | 4/179 | 87/4493 | 0.459 | 0.5875 |
| 4 | Metabolism | Carbohydrate metabolism | Amino sugar and nucleotide sugar metabolism | 氨基糖和核苷酸糖代谢 | map00520 | KEGG PATHWAY | 3/179 | 118/4493 | 0.8568 | 0.9039 |
| 5 | Human Diseases | Neurodegenerative disease | Parkinson disease | 帕金森病 | map05012 | KEGG PATHWAY | 5/179 | 26/4493 | 0.003156 | 0.05508 |
| 5 | Metabolism | Amino acid metabolism | Alanine, aspartate and glutamate metabolism | 丙氨酸、天冬氨酸和谷氨酸代谢 | map00250 | KEGG PATHWAY | 5/179 | 28/4493 | 0.004421 | 0.06529 |
| 5 | Metabolism | Metabolism of cofactors and vitamins | Pantothenate and CoA biosynthesis | 泛酸和辅酶A生物合成 | map00770 | KEGG PATHWAY | 5/179 | 30/4493 | 0.006011 | 0.07213 |
| 5 | Metabolism | Amino acid metabolism | Phenylalanine, tyrosine and tryptophan biosynthesis | 苯丙氨酸、酪氨酸和色氨酸生物合成 | map00400 | KEGG PATHWAY | 5/179 | 34/4493 | 0.01032 | 0.1043 |
| 5 | Human Diseases | Cancer: overview | Central carbon metabolism in cancer | 癌症的中枢碳代谢 | map05230 | KEGG PATHWAY | 5/179 | 37/4493 | 0.01469 | 0.1128 |
| 5 | Organismal Systems | Digestive system | Vitamin digestion and absorption | 维生素的消化和吸收 | map04977 | KEGG PATHWAY | 5/179 | 39/4493 | 0.01821 | 0.1205 |
| 5 | Metabolism | Carbohydrate metabolism | Butanoate metabolism | 丁酸代谢 | map00650 | KEGG PATHWAY | 5/179 | 47/4493 | 0.03764 | 0.1853 |
| 5 | Metabolism | Metabolism of cofactors and vitamins | Nicotinate and nicotinamide metabolism | 烟酸和烟酰胺代谢 | map00760 | KEGG PATHWAY | 5/179 | 54/4493 | 0.06228 | 0.2344 |
| 5 | Metabolism | Lipid metabolism | Biosynthesis of unsaturated fatty acids | 不饱和脂肪酸的生物合成 | map01040 | KEGG PATHWAY | 5/179 | 74/4493 | 0.171 | 0.3863 |
| 5 | Metabolism | Global and overview maps | Biosynthesis of nucleotide sugars | | map01250 | KEGG PATHWAY | 4/179 | 200/4493 | 0.9627 | 0.9728 |
| 5 | Metabolism | Lipid metabolism | Arachidonic acid metabolism | 花生四烯酸代谢 | map00590 | KEGG PATHWAY | 1/179 | 75/4493 | 0.9538 | 0.9793 |
| 6 | Metabolism | Amino acid metabolism | Phenylalanine metabolism | 苯丙氨酸代谢 | map00360 | KEGG PATHWAY | 6/179 | 49/4493 | 0.01238 | 0.108 |
| 6 | Genetic Information Processing | Translation | Aminoacyl-tRNA biosynthesis | 氨酰tRNA生物合成 | map00970 | KEGG PATHWAY | 6/179 | 52/4493 | 0.01634 | 0.1206 |
| 6 | Environmental Information Processing | Signaling molecules and interaction | Neuroactive ligand-receptor interaction | 神经活性配体受体相互作用 | map04080 | KEGG PATHWAY | 6/179 | 52/4493 | 0.01634 | 0.1206 |
| 6 | Metabolism | Lipid metabolism | Sphingolipid metabolism | 鞘脂代谢 | map00600 | KEGG PATHWAY | 4/179 | 27/4493 | 0.02086 | 0.1292 |
| 6 | Metabolism | Nucleotide metabolism | Pyrimidine metabolism | 嘧啶代谢 | map00240 | KEGG PATHWAY | 6/179 | 64/4493 | 0.04076 | 0.1779 |
| 6 | Metabolism | Lipid metabolism | alpha-Linolenic acid metabolism | α-亚麻酸代谢 | map00592 | KEGG PATHWAY | 2/179 | 44/4493 | 0.5288 | 0.6508 |
| 7 | Environmental Information Processing | Signal transduction | Sphingolipid signaling pathway | 鞘脂信号通路 | map04071 | KEGG PATHWAY | 5/179 | 15/4493 | 0.000206 | 0.006587 |
| 7 | Metabolism | Amino acid metabolism | Lysine degradation | 赖氨酸降解 | map00310 | KEGG PATHWAY | 6/179 | 50/4493 | 0.01361 | 0.1136 |
| 7 | Organismal Systems | Digestive system | Bile secretion | 胆汁分泌 | map04976 | KEGG PATHWAY | 7/179 | 97/4493 | 0.09053 | 0.285 |
| 7 | Metabolism | Nucleotide metabolism | Purine metabolism | 嘌呤代谢 | map00230 | KEGG PATHWAY | 7/179 | 101/4493 | 0.1065 | 0.3195 |
| 8 | Organismal Systems | Digestive system | Protein digestion and absorption | 蛋白质消化吸收 | map04974 | KEGG PATHWAY | 8/179 | 47/4493 | 0.00045 | 0.0108 |
| 8 | Metabolism | Global and overview maps | Nucleotide metabolism | | map01232 | KEGG PATHWAY | 8/179 | 58/4493 | 0.001886 | 0.04024 |
| 9 | Metabolism | Amino acid metabolism | Tyrosine metabolism | 酪氨酸代谢 | map00350 | KEGG PATHWAY | 9/179 | 78/4493 | 0.003513 | 0.05621 |
| 9 | Metabolism | Lipid metabolism | Linoleic acid metabolism | 亚油酸 | map00591 | KEGG PATHWAY | 5/179 | 28/4493 | 0.004421 | 0.06529 |
| 10 | Organismal Systems | Nervous system | Retrograde endocannabinoid signaling | 逆行内源性大麻素信号 | map04723 | KEGG PATHWAY | 6/179 | 19/4493 | 6.47E-05 | 0.002485 |
| 10 | Metabolism | Metabolism of other amino acids | D-Amino acid metabolism | | map00470 | KEGG PATHWAY | 10/179 | 66/4493 | 0.000237 | 0.006511 |
| 11 | Environmental Information Processing | Membrane transport | ABC transporters | ABC转运蛋白 | map02010 | KEGG PATHWAY | 11/179 | 138/4493 | 0.02081 | 0.1332 |
| 13 | Metabolism | Amino acid metabolism | Arginine and proline metabolism | 精氨酸和脯氨酸代谢 | map00330 | KEGG PATHWAY | 13/179 | 69/4493 | 2.26E-06 | 0.000144 |
| 17 | Metabolism | Amino acid metabolism | Tryptophan metabolism | 色氨酸代谢 | map00380 | KEGG PATHWAY | 17/179 | 83/4493 | 1.54E-08 | 2.95E-06 |
| 17 | Human Diseases | Cancer: overview | Choline metabolism in cancer | 癌症中的胆碱代谢 | map05231 | KEGG PATHWAY | 6/179 | 11/4493 | 1.44E-06 | 0.000138 |
| 18 | Metabolism | Global and overview maps | Biosynthesis of cofactors | | map01240 | KEGG PATHWAY | 18/179 | 328/4493 | 0.1004 | 0.3061 |
| 21 | Metabolism | Lipid metabolism | Glycerophospholipid metabolism | 甘油磷脂代谢 | map00564 | KEGG PATHWAY | 10/179 | 56/4493 | 5.66E-05 | 0.002719 |
