# Supplementary material for: Comparative Proteomics and Metabonomics Analysis of Different Diapause Stages Revealed a New Regulation Mechanism of Diapause in Loxostege sticticalis (Lepidoptera: Pyralidae)
Source: Molecules. 2024 Jul 25;29(15):3472. doi: 10.3390/molecules29153472 (PMC11314584; doi:10.3390/molecules29153472)
Supplement: Supplementary file 1 [file molecules-29-03472-s001.zip › analysis process/metabolic/sample information.pdf]

Sample Initial Name	Sample Analysis Name	Group Name	Sample Description
C_1_1	ND1	CK	ND
C_1_2	ND2	CK	ND
C_1_3	ND3	CK	ND
C_1_4	ND4	CK	ND
C_1_5	ND5	CK	ND
C_1_6	ND6	CK	ND
C_2_1	PreD1	ZYQ	PreD
C_2_2	PreD2	ZYQ	PreD
C_2_3	PreD3	ZYQ	PreD
C_2_4	PreD4	ZYQ	PreD
C_2_5	PreD5	ZYQ	PreD
C_2_6	PreD6	ZYQ	PreD
C_3_1	D1	ZY	D
C_3_2	D2	ZY	D
C_3_3	D3	ZY	D
C_3_4	D4	ZY	D
C_3_5	D5	ZY	D
C_3_6	D6	ZY	D
C_4_1	CT1	LCL	CT
C_4_2	CT2	LCL	CT
C_4_3	CT3	LCL	CT
C_4_4	CT4	LCL	CT
C_4_5	CT5	LCL	CT
C_4_6	CT6	LCL	CT
C_5_1	RD1	JCZY	RD
C_5_2	RD2	JCZY	RD
C_5_3	RD3	JCZY	RD
C_5_4	RD4	JCZY	RD
C_5_5	RD5	JCZY	RD
C_5_6	RD6	JCZY	RD
QC01	QC01	QC	
QC02	QC02	QC	
QC03	QC03	QC	
