# Supplementary material for: Comparative Proteomics and Metabonomics Analysis of Different Diapause Stages Revealed a New Regulation Mechanism of Diapause in Loxostege sticticalis (Lepidoptera: Pyralidae)
Source: Molecules. 2024 Jul 25;29(15):3472. doi: 10.3390/molecules29153472 (PMC11314584; doi:10.3390/molecules29153472)
Supplement: Supplementary file 1 [file molecules-29-03472-s001.zip › analysis process/metabolic/software information.pdf]

分析项	软件	软件版本	软件参数
表达量数据预处理	美吉自有软件	无	无
PCA分析	ropls(R packages)	Version1.6.2	默认参数
差异代谢物分析-多元统计	ropls(R packages)	Version1.6.2	默认参数
KEGG化合物分类	KEGG kegg_v20221012	Release 2017-05-01	无
KEGG功能通路	KEGG kegg_v20221012	Release 2017-05-01	无
Venn图	VennDiagram (R packages)	Version1.6.20	无
KEGG通路富集	scipy(Python)	Version1.0.0	pvalue < 0.05
Heatmap热图	scipy(Python)	Version1.0.0	无
聚类分析	scipy(Python)	Version1.0.0	无
相关性分析	scipy(Python)	Version1.0.0	无
Vip分析	ropls(R);scipy(Python)	Version1.6.2;Version1.0.0	无
iPath代谢通路分析	iPath3.0	Version3.0	无
时序表达趋势分析	STEM	Version1.3.11	无
时序差异分析	maSigPro(R packages)	Version1.56.0	无
WGCNA（加权基因共表达网络分析）	wgcna(R packages)	Version1.68	无
多因素相关性网络图	scipy(Python)	Version1.0.0	无
MIMOSA2	mimosa(R packages)	Version2.0.0	无
随机森林	sklearn(Python)	Version0.19.1	无
支持向量机	sklearn(Python)	Version0.19.1	无
LASSO	glmnet(R packages)	Version4.1.7	无
Logistic	stats(R packages)	Version4.1.2	无
