# Supplementary material for: Comparative Proteomics and Metabonomics Analysis of Different Diapause Stages Revealed a New Regulation Mechanism of Diapause in Loxostege sticticalis (Lepidoptera: Pyralidae)
Source: Molecules. 2024 Jul 25;29(15):3472. doi: 10.3390/molecules29153472 (PMC11314584; doi:10.3390/molecules29153472)
Supplement: Supplementary file 1 [file molecules-29-03472-s001.zip › analysis process/metabolic/Time series expression trend analysis.pdf]

Profile id	Member	Cluster id	P_value
0	6	-1	0.75
1	12	3	6.60E-04
2	3	-1	0.78
3	63	1	6.60E-22
6	1	-1	0.95
7	1	-1	0.99
8	1	-1	1
9	4	-1	1
10	8	4	0.05
12	1	-1	0.99
14	1	-1	1
16	21	-1	0.13
21	1	-1	1
24	3	-1	0.99
25	4	-1	1
26	1	-1	1
27	20	0	3.50E-05
28	60	0	1.70E-22
29	61	2	2.70E-36
