# Supplementary material for: Comparative Proteomics and Metabonomics Analysis of Different Diapause Stages Revealed a New Regulation Mechanism of Diapause in Loxostege sticticalis (Lepidoptera: Pyralidae)
Source: Molecules. 2024 Jul 25;29(15):3472. doi: 10.3390/molecules29153472 (PMC11314584; doi:10.3390/molecules29153472)
Supplement: Supplementary file 1 [file molecules-29-03472-s001.zip › analysis process/proteomic/analysis software.pdf]

软件	软件版本	分析项	信息来源
GO 数据库	Version 2022.0915	全蛋白功能注释	http://www.geneontology.org/
KEGG 数据库	Version 2022.10	全蛋白功能注释	http://www.genome.jp/kegg/
eggNOG 数据库	Version 2020.06	全蛋白功能注释	http://eggnogdb.embl.de/#/app/home
Pfam 数据库	Version 35.0	全蛋白功能注释	http://pfam.xfam.org/
Swiss-prot 数据库	Version 2022.10	全蛋白功能注释	ftp://ftp.uniprot.org/pub/databases/uniprot/current_release/knowledgebase/complete/uniprot_sprot.fasta.gz
NCBI 物种分类数据库	Version 2021.09	全蛋白功能注释	ftp://ftp.ncbi.nlm.nih.gov/pub/taxonomy/accession2taxid/nucl_gb.accession2taxid.gz
Uniprot 数据库	Version 2022.09	全蛋白功能注释	ftp://ftp.uniprot.org/pub/databases/uniprot/current_release
NR 数据库	Version 2022.10	全蛋白功能注释	https://www.ncbi.nlm.nih.gov/public/
PIR idmapping 数据库	Version 2022.10	全蛋白功能注释	ftp://ftp.pir.georgetown.edu/databases/idmapping/idmapping.tb.gz
美吉自编软件	无	数据质控	--
美吉自编软件	无	表达量分析	--
BLAST2GO	2.5.0	全蛋白功能注释_GO	https://www.blast2go.com/
eggNOG 数据库	Version 2020.06	全蛋白功能注释_EggNOG	http://eggnogdb.embl.de/#/app/home
HMMER	3.1b2	全蛋白功能注释_Pfam	https://www.ebi.ac.uk/Tools/hmmer
MultiLoc2	无	全蛋白功能注释_亚细胞定位（真核	https://pubmed.ncbi.nlm.nih.gov/19723330/
ngloc	无	全蛋白功能注释_亚细胞定位（原核	https://pubmed.ncbi.nlm.nih.gov/17472741/
DIAMOND	v0.8.37.99	全蛋白功能注释_NR_SwissProt	https://github.com/bbuchfink/diamond
Signalp	v6.0	全蛋白功能注释_信号肽注释	https://services.healthtech.dtu.dk/services/SignalP-6.0/
AnimalTFDB数据库	Version 4.0	全蛋白功能注释_转录因子	http://bioinfo.life.hust.edu.cn/AnimalTFDB4/#/
PlantTFDB数据库	Version 5.0	全蛋白功能注释_转录因子	http://planttfdb.gao-lab.org/
R	无	差异蛋白分析	https://www.r-project.org/
goatools	0.6.5	蛋白集分析（GO富集）	https://www.ncbi.nlm.nih.gov/pmc/articles/PMC6052049/
Python	无	蛋白集分析（KEGG富集）	https://www.python.org/
Ipath 数据库	Version 3	iPath代谢通路分析	http://pathways.embl.de
STRING 数据库	v12.0	蛋白互作网络分析	https://string-db.org/
WGCNA	1.68	WGCNA分析	https://cran.r-project.org/web/packages/WGCNA/index.html
GSEA	Version 4.3.2	GSEA分析	http://software.broadinstitute.org/gsea/index.jsp
Mfuzz	Version 2.6.0	Mfuzz时序分析	https://www.bioconductor.org/packages/release/bioc/html/Mfuzz.html
JASPAR数据库	Version 2024	全蛋白功能注释_转录因子	https://jaspar.elixir.no/
MSigDB 数据库	Version 2023.2	GSEA分析	https://docs.gsea-msigdb.org/#MSigDB/Release_Notes/MSigDB_Latest/
