# Supplementary material for: Comparative Proteomics and Metabonomics Analysis of Different Diapause Stages Revealed a New Regulation Mechanism of Diapause in Loxostege sticticalis (Lepidoptera: Pyralidae)
Source: Molecules. 2024 Jul 25;29(15):3472. doi: 10.3390/molecules29153472 (PMC11314584; doi:10.3390/molecules29153472)
Supplement: Supplementary file 1 [file molecules-29-03472-s001.zip › analysis process/proteomic/Cluster analysis of expression patterns/Down/CTvsD down.pdf]

Accession	Symbol	Protein Na	Entrez ID	Description	ND	RD	CT	D	PreD
TRINITY_DN971_c0_g1_i10_orfp1	-	-	-	TRINITY_DN971_c0_g1_i10_m.54268 TRINITY_DN971_c0_g1::TRINITY_DN971_c0_g1_i10::g.54268 ORF type:internal len:187 (+),score=141.68 TRINITY_DN971_c0_g1_i10:1-558(+)	-1.8853986	-0.108296909	0.425573874	0.676991073	0.891130596
TRINITY_DN3166_c1_g1_i6_orf1	-	-	-	hypothetical protein evm_013813 [Chilo suppressalis]	-1.7427023	-0.315989973	0.476800368	1.229471667	0.352420199
TRINITY_DN15202_c0_g1_i6_orf1	-	-	-	uncharacterized protein LOC114364499 isoform X2 [Ostrinia furnacalis]	-1.6714932	-0.535354716	0.764066655	1.105382122	0.337399175
TRINITY_DN36476_c1_g1_i1_orfp1	-	-	-	TRINITY_DN36476_c1_g1_i1_m.70910 TRINITY_DN36476_c1_g1::TRINITY_DN36476_c1_g1_i1::g.70910 ORF type:5prime_partial len:88 (-),score=0.50 TRINITY_DN36476_c1_g1_i1:49-	-1.6206469	-0.666693601	0.424577073	0.848273758	1.014489655
TRINITY_DN6330_c0_g1_i1_orfp1	-	-	-	TRINITY_DN6330_c0_g1_i1_m.42332 TRINITY_DN6330_c0_g1::TRINITY_DN6330_c0_g1_i1::g.42332 ORF type:3prime_partial len:51 (-),score=33.30 TRINITY_DN6330_c0_g1_i1:3-	-1.7299384	-0.475322263	0.409328755	0.874027884	0.921904039
TRINITY_DN5177_c0_g1_i2_orf1	-	-	-	hemolin-like isoform X1 [Ostrinia furnacalis]	-1.7365932	-0.025540713	0.770966852	1.165656579	-0.174489521
TRINITY_DN14904_c0_g1_i1_orf1	-	-	-	attacin [Ostrinia furnacalis]	-1.2886583	-0.270159115	-0.375625656	1.759131965	0.17531109
TRINITY_DN30510_c0_g1_i6_orf1	-	-	-	spodomicin-like [Ostrinia furnacalis]	-1.2995914	-1.059094767	0.37534477	0.789587698	1.193753729
TRINITY_DN86772_c0_g1_i3_orfp1	-	-	-	x-tox [Spodoptera exigua]	-1.786531	-0.305895145	0.296281792	0.814750672	0.981393686
TRINITY_DN9608_c0_g1_i3_orf1	-	-	-	cytochrome P450 monooxygenase CYP9G18 [Cnaphalocrocis medinalis]	-1.5218854	-0.53723669	0.578781935	1.434630186	0.045709926
TRINITY_DN20344_c0_g1_i5_orf1	-	-	-	uncharacterized protein LOC114351483 [Ostrinia furnacalis]	-1.1412645	-1.233631826	0.821767485	1.160140716	0.392988097
TRINITY_DN29026_c0_g1_i4_orf1	-	-	-	TIL [Ostrinia furnacalis]	-1.7124548	-0.211598993	0.418374517	1.350398089	0.155281172
TRINITY_DN3439_c0_g2_i2_orf1	-	-	-	histone H2A.Z-specific chaperone CHZ1-like [Ostrinia furnacalis]	-1.4622912	-0.868902624	0.350509736	1.095938207	0.884745833
TRINITY_DN8595_c0_g1_i3_orf1	-	-	-	aldose reductase-like isoform X4 [Trichoplusia ni]	-1.6374891	-0.654032915	0.632406597	1.06893236	0.590183026
TRINITY_DN14328_c0_g1_i12_orf1	-	-	-	larval cuticle protein LCP-30-like [Ostrinia furnacalis]	-1.2255064	-1.199733982	0.629382424	1.056196346	0.739661657
TRINITY_DN4802_c0_g1_i4_orf1	-	-	-	uncharacterized protein LOC114366345 isoform X2 [Ostrinia furnacalis]	-1.4897718	-0.507142917	0.016889199	1.520507892	0.459517652
TRINITY_DN5439_c0_g1_i2_orf1	-	-	-	uncharacterized protein LOC114353087 [Ostrinia furnacalis]	-1.8092522	-0.177048118	0.279551472	1.136760776	0.56998806
TRINITY_DN6415_c0_g2_i1_orf1	-	-	-	D-arabinitol dehydrogenase 1-like [Ostrinia furnacalis]	-1.3916503	-0.922919034	0.616745307	1.290416131	0.407407935
TRINITY_DN30177_c0_g2_i1_orf1	-	-	-	uncharacterized protein LOC114365032 [Ostrinia furnacalis]	-1.2123891	-0.092030003	-0.091058337	1.824608926	-0.429131511
TRINITY_DN47784_c0_g2_i1_orfp1	-	-	-	arylphorin subunit alpha-like [Ostrinia furnacalis]	-1.6683058	-0.181031074	-0.082005587	0.570565659	1.360776789
TRINITY_DN4068_c0_g2_i4_orf1	-	-	-	larval cuticle protein LCP-17-like precursor [Papilio polytes] >BAM18876.1 cuticular protein PpolCPR2 [Papilio polytes]	-0.8187749	-1.462106793	0.852375454	1.185948181	0.242558047
TRINITY_DN581_c3_g2_i1_orf1	-	-	-	uncharacterized protein LOC114364499 isoform X3 [Ostrinia furnacalis]	-1.5300708	-0.430618613	0.656118093	1.424298246	-0.119726935
TRINITY_DN394_c0_g1_i2_orf1	-	-	-	uncharacterized protein LOC114351483 [Ostrinia furnacalis]	-1.07239	-1.251812354	0.275719359	1.257461284	0.791021744
TRINITY_DN710_c0_g1_i11_orfp1	-	-	-	TRINITY_DN710_c0_g1_i11_m.67699 TRINITY_DN710_c0_g1::TRINITY_DN710_c0_g1_i11::g.67699 ORF type:complete len:194 (-),score=34.99,Collagen PF01391.19 0.00029 TRINITY_DN710_c0_g1_i11:1283-1864(-)	-1.3438399	-0.996754392	0.283021227	1.071626733	0.985946324
TRINITY_DN69_c0_g1_i1_orf1	-	-	-	glycerol-3-phosphate dehydrogenase [NAD(+)], cytoplasmic isoform X1 [Ostrinia furnacalis]	-1.8571074	-0.19637542	0.467698928	0.927968642	0.657815212
TRINITY_DN31286_c0_g1_i6_orfp1	-	-	-	TRINITY_DN31286_c0_g1_i6_m.28438 TRINITY_DN31286_c0_g1::TRINITY_DN31286_c0_g1_i6::g.28438 ORF type:internal len:92 (-),score=3.10,Perilipin PF03036.17 2e-05 TRINITY_DN31286_c0_g1_i6:1-273(-)	-1.8258532	-0.193184415	0.412497544	1.093356334	0.513183744
TRINITY_DN135780_c0_g1_i1_orf1	-	-	-	flotillin-1 isoform X1 [Pectinophora gossypiella]	-1.8716746	-0.079172943	0.266052873	0.858450056	0.826344575
TRINITY_DN17247_c0_g1_i14_orf1	-	-	-	uncharacterized protein LOC114356308 [Ostrinia furnacalis]	-1.8769059	0.1812934	-0.003014088	0.820316872	0.878309741
TRINITY_DN20658_c0_g2_i3_orf1	-	-	-	prostaglandin reductase 1-like [Ostrinia furnacalis]	-1.8077393	-0.190831025	0.305550739	1.137321839	0.555697734
TRINITY_DN335_c1_g1_i5_orf1	-	-	-	PREDICTED: perilipin-4 isoform X14 [Papilio polytes]	-1.3938798	-0.911760287	0.237514143	0.909952914	1.158173078
TRINITY_DN7740_c0_g1_i2_orf1	-	-	-	D-arabinitol dehydrogenase 1 [Eumeta japonica]	-1.5608573	-0.768009645	0.489839244	1.066026053	0.773001669
TRINITY_DN36434_c0_g2_i3_orf1	-	-	-	clotting factor B isoform X1 [Ostrinia furnacalis]	-1.5088135	-0.717469002	0.511598891	1.345663778	0.369019831
TRINITY_DN130575_c0_g1_i1_orfp1	-	-	-	TRINITY_DN130575_c0_g1_i1_m.77798 TRINITY_DN130575_c0_g1::TRINITY_DN130575_c0_g1_i1::g.77798 ORF type:internal len:70 (+),score=15.12 TRINITY_DN130575_c0_g1_i1:3-209(+)	-1.809118	-0.293946207	0.379524281	0.937115393	0.786424572
TRINITY_DN8853_c0_g1_i4_orf1	-	-	-	uncharacterized protein LOC114351488 isoform X1 [Ostrinia furnacalis]	-0.9976736	-1.125930021	0.171106228	1.609308143	0.343189278
TRINITY_DN1226_c0_g1_i11_orfp1	-	-	-	TRINITY_DN1226_c0_g1_i11_m.52385 TRINITY_DN1226_c0_g1::TRINITY_DN1226_c0_g1_i11::g.52385 ORF type:internal len:92 (-),score=5.77 TRINITY_DN1226_c0_g1_i11:2-274(-)	-1.193786	-1.119073386	0.26019784	1.298486087	0.754175482
TRINITY_DN24_c0_g1_i1_orf1	-	-	-	hypothetical protein evm_007803 [Chilo suppressalis]	-1.7481077	-0.039433056	-0.165275463	0.911204502	1.041611701
TRINITY_DN20558_c0_g1_i2_orf1	-	-	-	Transient receptor potential channel pyrexia [Operophtera brumata]	-1.5675928	-0.146913744	-0.218105553	1.516762471	0.415849583

TRINITY_DN4080_c0_g1_i8_orf1	-	-	-	AMP deaminase 2 isoform X3 [Ostrinia furnacalis] >XP_028163647.1 AMP deaminase 2 isoform X3 [Ostrinia furnacalis] >XP_028163648.1 AMP deaminase 2 isoform X3 [Ostrinia furnacalis]	-1.5053468	-0.262217134	0.585074689	1.492277442	-0.309788155
TRINITY_DN15755_c0_g1_i1_orf1	-	-	-	cytochrome P450 monooxygenase CYP6AB141 [Ostrinia furnacalis]	-1.0622211	-1.119640668	-0.078667797	1.298944554	0.961584961
TRINITY_DN448_c0_g1_i20_orf1	-	-	-	probable cytochrome P450 9f2 isoform X1 [Ostrinia furnacalis]	-1.5752093	-0.652139334	0.54576487	1.277660613	0.403923178
TRINITY_DN66453_c0_g1_i4_orfp1	-	-	-	TRINITY_DN66453_c0_g1_i4_m.7345 TRINITY_DN66453_c0_g1::TRINITY_DN66453_c0_g1_i4::g.7345 ORF type:internal len:93 (+),score=24.53 TRINITY_DN66453_c0_g1_i4:3-278(+)	-1.1438504	-1.112650816	0.077310677	1.280908922	0.898281606
TRINITY_DN4343_c0_g1_i2_orf1	-	-	-	uncharacterized protein LOC114365231 isoform X3 [Ostrinia furnacalis]	-1.8216652	-0.058886957	0.062088092	1.011231364	0.80723266
TRINITY_DN29555_c0_g1_i8_orf1	-	-	-	fasciclin-1 [Ostrinia furnacalis]	-1.9093913	0.271430835	0.061812518	0.656528703	0.919619224
TRINITY_DN1230_c1_g1_i5_orf1	-	-	-	uncharacterized protein LOC114353440 [Ostrinia furnacalis]	-1.3732674	-1.016045331	0.466477092	1.049563712	0.873271885
TRINITY_DN136031_c0_g1_i7_orf1	-	-	-	ferritin, lower subunit isoform X3 [Spodoptera litura]	-0.6313721	-0.809501066	-0.926427656	0.805660749	1.561640103
TRINITY_DN3616_c0_g2_i2_orf1	-	-	-	conotoxin ArMKLT2-032-like [Ostrinia furnacalis]	-1.1894978	-1.177774874	0.466078675	1.245081762	0.656112275
TRINITY_DN24121_c1_g1_i6_orf1	-	-	-	serine protease persephone-like [Ostrinia furnacalis]	-0.850006	-1.188332652	-0.311974943	1.120203086	1.230110539
TRINITY_DN3593_c0_g1_i3_orfp1	-	-	-	TRINITY_DN3593_c0_g1_i3_m.43968 TRINITY_DN3593_c0_g1::TRINITY_DN3593_c0_g1_i3::g.43968 ORF type:5prime_partial len:72 (-),score=1.41 TRINITY_DN3593_c0_g1_i3:138-	-0.4814543	-1.213645901	-0.40848576	1.72850112	0.375084845
TRINITY_DN2442_c0_g1_i6_orf1	-	-	-	cytochrome P450 6B5-like [Ostrinia furnacalis]	-1.8206828	0.029642801	0.044103991	1.153108524	0.593827471
TRINITY_DN701_c0_g1_i1_orf1	-	-	-	venom protease-like isoform X3 [Ostrinia furnacalis]	-1.3194333	-1.014066267	0.436974365	1.295692238	0.600833003
TRINITY_DN2392_c0_g2_i1_orf1	-	-	-	cytochrome P450 9e2-like [Ostrinia furnacalis] >QPF77612.1 cytochrome P450 monooxygenase CYP9A185 [Ostrinia furnacalis]	-1.4716697	-0.496794429	0.622468271	1.477381043	-0.131385173
TRINITY_DN3433_c0_g1_i15_orf1	-	-	-	cytosolic purine 5'-nucleotidase isoform X3 [Ostrinia furnacalis] >XP_028162965.1 cytosolic purine 5'-nucleotidase isoform X3 [Ostrinia furnacalis] >XP_028162966.1 cytosolic purine 5'-nucleotidase isoform X3 [Ostrinia furnacalis]	-1.6978764	-0.396817569	0.208001059	1.204767711	0.681925177
TRINITY_DN84938_c0_g1_i4_orf1	-	-	-	vinculin-like isoform X2 [Ostrinia furnacalis]	-1.1677761	-0.662531879	-0.500034338	0.824532977	1.505809294
TRINITY_DN4069_c0_g1_i5_orf1	-	-	-	putative sulfiredoxin [Ostrinia furnacalis]	-1.5380666	-0.605880157	0.015512676	1.095068771	1.033365359
TRINITY_DN1592_c0_g1_i1_orf1	-	-	-	serine protease 7-like isoform X2 [Ostrinia furnacalis]	-1.807214	-0.088907709	0.05712062	0.793310719	1.045690364
TRINITY_DN2207_c0_g1_i4_orf1	-	-	-	methionine-R-sulfoxide reductase B1 isoform X4 [Pectinophora gossypiella] >XP_049887601.1 methionine-R-sulfoxide reductase B1 isoform X4 [Pectinophora gossypiella]	-0.6902428	-1.452561161	0.310294038	1.48118961	0.351320283
TRINITY_DN3732_c1_g1_i5_orf1	-	-	-	cytochrome P450 6B2-like [Ostrinia furnacalis]	-1.1152026	-0.767649614	-0.501412404	1.383527351	1.000737226
TRINITY_DN140_c0_g1_i5_orf1	-	-	-	calcyphosin-like protein isoform X4 [Helicoverpa armigera]	-0.760485	-0.79938214	-0.883818824	1.133201557	1.310484382
TRINITY_DN57900_c0_g1_i2_orf1	-	-	-	hypothetical protein SFRURICE_000634 [Spodoptera frugiperda]	-0.7392841	-1.408036991	0.256963233	1.501117047	0.389240789
TRINITY_DN91946_c0_g1_i1_orf1	-	-	-	protein catecholamines up [Ostrinia furnacalis]	-1.4671856	-0.769680546	0.139533627	1.182992306	0.914340176
TRINITY_DN31225_c0_g1_i1_orf1	-	-	-	ribosome biogenesis protein BMS1 homolog [Ostrinia furnacalis]	-0.9014776	-1.33417466	0.043744483	1.056126212	1.135781613
TRINITY_DN14754_c0_g1_i6_orf1	-	-	-	cathepsin L [Papilio xuthus]	-0.8132115	-1.111718289	-0.229215319	0.472895672	1.681249408
TRINITY_DN12336_c0_g1_i1_orfp1	-	-	-	TRINITY_DN12336_c0_g1_i1_m.30792 TRINITY_DN12336_c0_g1::TRINITY_DN12336_c0_g1_i1::g.30792 ORF type:internal len:86 (-),score=13.56 TRINITY_DN12336_c0_g1_i1:3-257(-)	-0.8159666	-1.230938939	-0.285427096	1.260058605	1.07227401
TRINITY_DN5880_c0_g2_i2_orf1	-	-	-	macrophage mannose receptor 1 [Bombyx mori]	-1.1019015	-0.999737922	-0.082692269	1.535926247	0.648405474
TRINITY_DN11231_c1_g1_i1_orfp1	-	-	-	TRINITY_DN11231_c1_g1_i1_m.13377 TRINITY_DN11231_c1_g1::TRINITY_DN11231_c1_g1_i1::g.13377 ORF type:internal len:76 (-),score=1.43 TRINITY_DN11231_c1_g1_i1:1-225(-)	-1.4070671	-0.826943311	0.075256976	1.062714766	1.096038646
TRINITY_DN5696_c0_g1_i4_orf1	-	-	-	serine protease snake-like isoform X1 [Ostrinia furnacalis]	-1.2980992	-0.663316507	-0.356891811	1.332795381	0.985512185
TRINITY_DN2847_c0_g1_i20_orf1	-	-	-	uncharacterized protein LOC114352221 [Ostrinia furnacalis]	-1.4772397	-0.468712397	0.590425551	1.493466368	-0.137939808
TRINITY_DN34134_c0_g2_i1_orf1	-	-	-	THUMP domain-containing protein 1 homolog [Ostrinia furnacalis]	-1.5265722	-0.665181219	0.209159204	1.32146658	0.661127641
TRINITY_DN566_c0_g1_i13_orf1	-	-	-	uncharacterized protein LOC114355567 isoform X2 [Ostrinia furnacalis]	-1.3931261	-0.886107041	0.244626067	1.28596592	0.748641187
TRINITY_DN6698_c0_g2_i2_orf1	-	-	-	protein mesh isoform X1 [Ostrinia furnacalis]	-1.8620284	0.144211472	-0.019854419	0.837946438	0.899724899
TRINITY_DN6908_c0_g1_i3_orf1	-	-	-	serine--pyruvate aminotransferase, mitochondrial [Ostrinia furnacalis] >XP_028157324.1 serine--pyruvate aminotransferase, mitochondrial [Ostrinia furnacalis] >XP_028157325.1 serine--pyruvate aminotransferase, mitochondrial [Ostrinia furnacalis]	-1.6844664	0.384422499	-0.308329592	1.363781394	0.244592143
TRINITY_DN89483_c0_g1_i1_orf1	-	-	-	mitochondrial enolase superfamily member 1-like isoform X2 [Maniola	-1.1892011	-0.940048139	0.11360631	1.581317561	0.434325397
TRINITY_DN1008_c0_g1_i2_orf1	-	-	-	integrin beta-PS [Ostrinia furnacalis]	-1.5583158	-0.590843228	0.048474914	0.967367409	1.133316681
TRINITY_DN6415_c0_g1_i1_orf1	-	-	-	D-arabinitol dehydrogenase 1-like [Ostrinia furnacalis]	-0.4836986	-1.58940114	0.646028931	1.347657131	0.079413703

TRINITY_DN11108_c0_g1_i4_orf1	-	-	-	peroxisomal leader peptide-processing protease [Ostrinia furnacalis] >XP_028165527.1 peroxisomal leader peptide-processing protease [Ostrinia furnacalis]	-1.4749023	-0.595817041	-0.035685036	0.699703811	1.406700554
TRINITY_DN4123_c0_g1_i1_orf1	-	-	-	uncharacterized protein LOC114355030 [Ostrinia furnacalis]	-1.0625974	-1.145746614	-0.047635584	1.202690024	1.053289596
TRINITY_DN8651_c0_g1_i16_orf1	-	-	-	glutathione S-transferase theta 2 [Conogethes punctiferalis]	-1.3177462	-0.75746975	0.11151627	1.593932581	0.369767062
TRINITY_DN31609_c0_g1_i3_orf1	-	-	-	sorbitol dehydrogenase-like [Ostrinia furnacalis]	-1.5264377	-0.037782129	0.171738924	1.609904678	-0.217423762
TRINITY_DN3991_c0_g1_i6_orf1	-	-	-	acetyl-CoA carboxylase isoform X3 [Trichoplusia ni]	-1.2791789	-0.939514013	0.443587258	1.482844835	0.292260794
TRINITY_DN12969_c0_g1_i3_orf1	-	-	-	queuosine salvage protein [Ostrinia furnacalis] >XP_028167327.1 queuosine salvage protein [Ostrinia furnacalis]	-1.3324362	-0.115833661	-0.568655454	0.355073965	1.66185133
TRINITY_DN22242_c0_g1_i1_orf1	-	-	-	juvenile hormone epoxide hydrolase-like [Ostrinia furnacalis] >XP_028170526.1 juvenile hormone epoxide hydrolase-like [Ostrinia	-1.7236472	0.028195784	-0.161205324	1.30160511	0.55505163
TRINITY_DN14239_c0_g1_i5_orf1	-	-	-	uncharacterized protein LOC114352770 [Ostrinia furnacalis] >XP_028160292.1 uncharacterized protein LOC114352770 [Ostrinia	-1.3432395	-0.087552021	0.083152106	1.739901779	-0.392262336
TRINITY_DN15157_c0_g1_i1_orf1	-	-	-	UDP-glycosyltransferase UGT40AM2 [Ostrinia furnacalis]	-1.5472731	-0.397078987	0.025839209	1.510283043	0.408229861
TRINITY_DN230_c2_g1_i5_orf1	-	-	-	6-pyruvoyl tetrahydrobiopterin synthase [Ostrinia furnacalis]	-1.0854014	-0.843550612	-0.245047786	0.500851416	1.6731484
TRINITY_DN3486_c0_g1_i5_orf1	-	-	-	uncharacterized protein LOC114360519 [Ostrinia furnacalis]	-0.949615	-0.913560468	-0.561172858	1.141494042	1.282854258
TRINITY_DN452_c0_g1_i4_orf1	-	-	-	protein CREG1 [Ostrinia furnacalis] >XP_028170592.1 protein CREG1 [Ostrinia furnacalis]	-1.3344374	-0.394849844	-0.556032668	0.87540112	1.409918788
TRINITY_DN1543_c0_g2_i2_orf1	-	-	-	regulation of enolase protein 1-like isoform X3 [Ostrinia furnacalis] >XP_028161334.1 regulation of enolase protein 1-like isoform X3 [Ostrinia furnacalis]	-1.1261333	-0.250776795	-0.280878856	1.881488829	-0.223699874
TRINITY_DN23564_c0_g1_i7_orf1	-	-	-	cytochrome P450 6B6-like [Ostrinia furnacalis]	-1.6112168	-0.374171015	-0.124033726	1.163711553	0.945710018
TRINITY_DN829_c0_g1_i8_orf1	-	-	-	cytochrome P450 6B6-like [Ostrinia furnacalis]	-0.6868883	-1.469322479	0.330195287	1.457562935	0.368452553
TRINITY_DN19098_c0_g1_i4_orf1	-	-	-	L-xylulose reductase-like [Ostrinia furnacalis]	-1.171293	-0.79961838	-0.002723139	1.708294582	0.265339964
TRINITY_DN27833_c0_g2_i1_orf1	-	-	-	uncharacterized protein LOC114359161 [Ostrinia furnacalis]	-1.5502961	0.182442863	-0.148355028	1.592323461	-0.076115243
TRINITY_DN36632_c0_g1_i1_orf1	-	-	-	hypothetical protein evm_011159, partial [Chilo suppressalis]	-1.1940196	-0.638956286	-0.55039316	1.298678586	1.084690452
TRINITY_DN5174_c0_g3_i1_orf1	-	-	-	protein odr-4 homolog [Ostrinia furnacalis]	-1.1056712	-0.583153697	0.18361707	1.81817474	-0.312966873
TRINITY_DN22046_c1_g1_i5_orf1	-	-	-	uncharacterized protein LOC114351208 [Ostrinia furnacalis]	-1.251486	-0.661346993	0.020848725	1.722576366	0.169407868
TRINITY_DN5661_c0_g1_i5_orf1	-	-	-	cytochrome P450 6B7-like [Ostrinia furnacalis]	-0.7346773	-1.415117245	-0.04144769	0.929483439	1.26175875
TRINITY_DN8621_c0_g1_i4_orf1	-	-	-	aminopeptidase N-like isoform X2 [Ostrinia furnacalis]	-1.2502653	-0.647014094	-0.346063047	0.684545998	1.558796485
TRINITY_DN1622_c0_g1_i6_orf1	-	-	-	unnamed protein product [Parnassius apollo]	-1.1834198	-0.878188491	-0.279233484	1.243087696	1.097754117
TRINITY_DN47151_c0_g1_i1_orf1	-	-	-	unnamed protein product [Danaus chrysippus]	-0.5393994	-1.384844187	-0.369620313	1.255858186	1.038005728
TRINITY_DN4341_c0_g1_i4_orf1	-	-	-	uncharacterized protein LOC114354354 [Ostrinia furnacalis]	-1.0688814	-0.943193797	0.141359327	1.709343293	0.161372559
TRINITY_DN804_c0_g1_i7_orf1	-	-	-	hypothetical protein HF086_004695 [Spodoptera exigua] >CAH0695017.1 unnamed protein product [Spodoptera exigua]	-0.4700522	-1.421472	-0.310720859	0.756899416	1.445345663
TRINITY_DN3298_c0_g2_i4_orf1	-	-	-	macrophage mannose receptor 1-like [Ostrinia furnacalis]	-0.5133459	-1.386779387	-0.032431597	1.653620814	0.278936059
TRINITY_DN13563_c0_g1_i1_orf1	-	-	-	Golgi resident protein GCP60 isoform X1 [Ostrinia furnacalis]	-0.9277742	-0.842927982	-0.669573102	1.182008063	1.258267188
TRINITY_DN4064_c0_g2_i1_orf1	-	-	-	disintegrin and metalloproteinase domain-containing protein 12 isoform X1 [Ostrinia furnacalis] >XP_028158112.1 disintegrin and metalloproteinase domain-containing protein 12 isoform X2 [Ostrinia furnacalis]	-1.2858248	-0.084533788	-0.433431804	1.775054879	0.028735491
TRINITY_DN10430_c0_g1_i4_orf1	-	-	-	fatty acid synthase [Ostrinia furnacalis] >XP_028160534.1 fatty acid synthase [Ostrinia furnacalis] >XP_028160535.1 fatty acid synthase [Ostrinia furnacalis] >XP_028160536.1 fatty acid synthase [Ostrinia furnacalis]	-0.7410166	-1.323431795	-0.131998281	1.465517604	0.730929065
TRINITY_DN2844_c0_g1_i2_orf1	-	-	-	glutathione S-transferase 9 [Streltzoviella insularis]	-1.0559565	-1.074491181	-0.130984945	1.410205915	0.85122668
TRINITY_DN77318_c0_g2_i1_orf1	-	-	-	uncharacterized protein LOC114351191 [Ostrinia furnacalis]	-1.023297	-0.45071917	-0.89906333	0.935893338	1.437186204
TRINITY_DN4793_c0_g1_i7_orf1	-	-	-	probable hydroxyacid-oxoacid transhydrogenase, mitochondrial isoform X3 [Ostrinia furnacalis] >XP_028159821.1 probable hydroxyacid-oxoacid transhydrogenase, mitochondrial isoform X4 [Ostrinia furnacalis]	-0.5974611	-1.416308256	-0.234936843	1.288023514	0.960682699
TRINITY_DN2887_c0_g1_i1_orf1	-	-	-	F-box/LRR-repeat protein 4-like isoform X1 [Ostrinia furnacalis] >XP_028177606.1 F-box/LRR-repeat protein 4-like isoform X1 [Ostrinia furnacalis]	-0.7321557	-1.314614134	-0.261960944	1.18595136	1.122779398
TRINITY_DN8716_c0_g1_i3_orf1	-	-	-	aspartate--tRNA ligase, cytoplasmic isoform X1 [Ostrinia furnacalis] >XP_028161705.1 aspartate--tRNA ligase, cytoplasmic isoform X2 [Ostrinia furnacalis] >XP_028161706.1 aspartate--tRNA ligase, cytoplasmic isoform X1 [Ostrinia furnacalis] >XP_028161707.1 aspartate--tRNA ligase, cytoplasmic isoform X1 [Ostrinia furnacalis]	-0.9954607	-0.858331104	-0.518429203	1.494201855	0.878019167
TRINITY_DN54612_c0_g1_i3_orf1	-	-	-	hydroxyacid oxidase 1 isoform X1 [Ostrinia furnacalis]	-0.7347315	-1.002887244	-0.434965841	1.759021644	0.413562969

TRINITY_DN33995_c0_g1_i5_orf1	-	-	-	unnamed protein product [Spodoptera exigua]	-0.4275328	-1.06253344	-0.7481203	0.560606594	1.67757999
TRINITY_DN40434_c0_g1_i2_orf1	-	-	-	deoxyribodipyrimidine photo-lyase [Ostrinia furnacalis]	-0.6830869	-1.036024168	-0.534597686	0.563538428	1.690170375
TRINITY_DN85476_c0_g1_i1_orf1	-	-	-	iron-sulfur protein NUBPL-like [Ostrinia furnacalis]	-0.7796733	-1.127044349	-0.425964709	0.834881295	1.497801029
TRINITY_DN22824_c0_g1_i4_orf1	-	-	-	LIM domain and actin-binding protein 1 [Ostrinia furnacalis]	-0.6583197	-0.419625147	-1.111475945	0.478930656	1.710490148
TRINITY_DN131603_c0_g1_i4_orfp1	-	-	-	TRINITY_DN131603_c0_g1_i4_m.86149 TRINITY_DN131603_c0_g1::TRINITY_DN131603_c0_g1_i4::g.86149 ORF type:internal len:112 (-),score=8.40 TRINITY_DN131603_c0_g1_i4:2-334(-)	-0.3829089	-0.8336654	-0.664540983	1.927337937	-0.046222654
TRINITY_DN4142_c0_g1_i5_orf1	-	-	-	GDP-fucose protein O-fucosyltransferase 1 [Ostrinia furnacalis]	-0.3326936	-0.530891676	-1.405636867	1.303385248	0.965836888
TRINITY_DN2299_c0_g1_i3_orf1	-	-	-	DNA-directed RNA polymerase II subunit RPB1 [Ostrinia furnacalis]	0.00728084	-1.302634116	-0.828533923	1.486899166	0.636988036
TRINITY_DN17409_c0_g1_i5_orf1	-	-	-	integrin beta-6-like [Ostrinia furnacalis]	0.42408042	-0.85868187	-1.046260054	1.712975287	-0.232113782
TRINITY_DN20960_c0_g1_i1_orf1	-	-	-	aldo-keto reductase AKR2E4-like [Ostrinia furnacalis]	0.34999612	-1.229784053	-1.054813664	1.403855699	0.530745901
TRINITY_DN7291_c0_g1_i5_orf1	-	-	-	dynamin-1-like protein isoform X3 [Ostrinia furnacalis]	0.40714985	-0.759519986	-1.379159628	1.520050542	0.211479222
TRINITY_DN56998_c0_g1_i2_orf1	-	-	-	glycerate kinase [Ostrinia furnacalis]	-0.0698991	-0.902877717	-0.916804936	1.826305217	0.063276493
TRINITY_DN42185_c0_g1_i7_orf1	-	-	-	ADP-ribosylation factor-like protein 13B isoform X2 [Ostrinia furnacalis]	-0.1590272	-0.597828753	-1.405211878	0.690251273	1.471816531
TRINITY_DN41645_c0_g1_i1_orf1	-	-	-	60S acidic ribosomal protein P2 [Ostrinia furnacalis]	-0.3061409	-0.847585843	-1.135876942	1.516633112	0.7729706
TRINITY_DN57749_c0_g1_i4_orf1	-	-	-	LOW QUALITY PROTEIN: DENN domain-containing protein Crag [Ostrinia furnacalis]	-0.1709611	-0.438268729	-1.249982592	1.79213184	0.067080593
TRINITY_DN11172_c0_g1_i4_orf1	-	-	-	juvenile hormone epoxide hydrolase-like isoform X1 [Ostrinia furnacalis] >XP_028170522.1 juvenile hormone epoxide hydrolase-like isoform X2 [Ostrinia furnacalis]	0.17446862	-0.907368659	-1.212122265	1.599187524	0.345834781
TRINITY_DN2072_c0_g1_i1_orf1	-	-	-	dnaJ homolog subfamily C member 3 [Helicoverpa zea]	0.06884398	-0.913587146	-1.333548575	1.019182399	1.159109344
TRINITY_DN70236_c0_g1_i1_orf1	-	-	-	NAD-dependent protein deacetylase sirtuin-2-like [Ostrinia furnacalis]	0.51737009	-0.771907581	-1.569489506	0.842542907	0.981484089
TRINITY_DN6358_c0_g1_i5_orf1	-	-	-	histone H1B-like [Ostrinia furnacalis]	0.24240781	-0.581227046	-1.628965108	0.900830032	1.066954312
TRINITY_DN2770_c0_g2_i4_orf1	-	-	-	phosphatidylinositol 4-phosphate 3-kinase C2 domain-containing subunit alpha isoform X1 [Ostrinia furnacalis]	0.15459822	-0.40859934	-1.574013138	0.339198233	1.48881603
TRINITY_DN467_c3_g1_i5_orf1	-	-	-	lysozyme precursor [Loxostege sticticalis]	-1.9234217	0.735275062	0.37480948	0.786523082	0.026814037
TRINITY_DN12009_c0_g1_i1_orf1	-	-	-	uncharacterized protein LOC114365631 [Ostrinia furnacalis]	-1.8356627	0.992810085	0.017677902	0.802396022	0.022778646
TRINITY_DN75086_c0_g1_i5_orf1	-	-	-	lysosome membrane protein 2-like [Ostrinia furnacalis]	-1.9635779	0.751707553	0.169365065	0.581270292	0.461235029
TRINITY_DN2813_c0_g1_i7_orf1	-	-	-	arylphorin subunit alpha-like [Ostrinia furnacalis]	-1.8742067	0.645859638	-0.175482108	0.537518008	0.866311156
TRINITY_DN26209_c0_g1_i6_orf1	-	-	-	uncharacterized protein LOC114352357 [Ostrinia furnacalis]	-1.9449747	0.437305623	0.391514666	0.911385233	0.204769182
TRINITY_DN20560_c0_g1_i6_orf1	-	-	-	pupal cuticle protein C1B-like [Ostrinia furnacalis]	-1.8301935	1.162480051	-0.092483193	0.352446953	0.407749707
TRINITY_DN5310_c2_g1_i2_orf1	-	-	-	serine protease persephone-like [Ostrinia furnacalis]	-1.9061004	0.518664828	0.030334959	0.975187183	0.381913417
TRINITY_DN15247_c0_g1_i2_orf1	-	-	-	probable G-protein coupled receptor Mth-like 3 isoform X1 [Ostrinia	-1.868721	0.969644798	-0.134096062	0.605935249	0.427237024
TRINITY_DN45948_c1_g1_i1_orf1	-	-	-	unnamed protein product [Leptidea sinapis]	-1.7854652	1.185706483	-0.197165773	0.240672392	0.556252144
TRINITY_DN15400_c0_g1_i1_orf1	-	-	-	uncharacterized protein LOC114366781 [Ostrinia furnacalis]	-1.3647668	1.209989753	-0.997584051	0.491936221	0.660424921
TRINITY_DN5408_c0_g1_i5_orf1	-	-	-	uncharacterized protein LOC114359912 [Ostrinia furnacalis]	-1.4088941	1.520010402	-0.62288499	0.560553389	-0.048784689
TRINITY_DN3609_c0_g1_i6_orf1	-	-	-	leukocyte elastase inhibitor-like [Ostrinia furnacalis]	-1.8015062	1.054448722	-0.182253832	0.762722157	0.166589105
TRINITY_DN95558_c0_g3_i1_orf1	-	-	-	cytochrome P450 monooxygenase CYP9G19 [Cnaphalocrocis medinalis]	-1.9572982	0.432820535	0.152339592	0.778430943	0.59370711
TRINITY_DN18338_c0_g1_i6_orf1	-	-	-	aquaporin AQPAn.G isoform X1 [Ostrinia furnacalis]	-1.5681095	1.535257455	-0.338955398	0.184192785	0.187614698
TRINITY_DN2650_c0_g1_i1_orf1	-	-	-	hypothetical protein HW555_002849 [Spodoptera exigua] >CAH0691914.1 unnamed protein product [Spodoptera exigua]	-1.8289418	0.308889102	0.251461708	1.222370641	0.046220381
TRINITY_DN41_c0_g1_i3_orf1	-	-	-	uncharacterized protein LOC114359035 isoform X3 [Ostrinia furnacalis]	-1.82118	1.19311545	-0.080965065	0.385026574	0.324003077
TRINITY_DN955_c0_g1_i2_orf1	-	-	-	gloverin-like [Ostrinia furnacalis] >XP_028168251.1 gloverin-like [Ostrinia furnacalis] >AYM26645.1 gloverin [Ostrinia furnacalis]	-1.5242297	0.625898338	-0.838967961	1.058390976	0.678908314
TRINITY_DN34406_c0_g2_i9_orfp1	-	-	-	TRINITY_DN34406_c0_g2_i9_m.33755 TRINITY_DN34406_c0_g2::TRINITY_DN34406_c0_g2_i9::g.33755 ORF type:internal len:82 (-),score=12.88 TRINITY_DN34406_c0_g2_i9:3-245(-)	-1.3356961	0.758426048	-0.983494165	0.303175434	1.257588821
TRINITY_DN7534_c0_g1_i15_orf1	-	-	-	protein-glucosylgalactosylhydroxylysine glucosidase isoform X2 [Ostrinia furnacalis]	-1.8690888	0.684608642	-0.173575379	0.472228049	0.885827486
TRINITY_DN1672_c0_g1_i6_orf1	-	-	-	cystinosin homolog isoform X1 [Ostrinia furnacalis] >XP_028162341.1 cystinosin homolog isoform X1 [Ostrinia furnacalis] >XP_028162342.1 cystinosin homolog isoform X1 [Ostrinia furnacalis] >XP_028162343.1 cystinosin homolog isoform X1 [Ostrinia furnacalis] >XP_028162344.1 cystinosin homolog isoform X1 [Ostrinia furnacalis]	-1.8624064	0.607457928	-0.068356553	1.037282984	0.286022053
TRINITY_DN15597_c0_g1_i1_orf1	-	-	-	microsomal glutathione S-transferase 1-like [Ostrinia furnacalis]	-1.902097	0.76094586	0.051619515	0.866312372	0.223219221
TRINITY_DN16840_c1_g1_i1_orf1	-	-	-	attacin-like [Ostrinia furnacalis]	-1.5865353	0.494019131	-0.633895475	1.279606289	0.446805376
TRINITY_DN1012_c0_g1_i2_orf1	-	-	-	teneurin-a isoform X1 [Ostrinia furnacalis]	-1.479824	1.420801128	-0.678850421	0.539785455	0.198087856

TRINITY_DN892_c7_g1_i2_orf1	-	-	-	unnamed protein product [Diatraea saccharalis]	-1.8428629	0.458568829	-0.210792851	0.993739784	0.601347178
TRINITY_DN20680_c0_g1_i5_orf1	-	-	-	tsukushin isoform X2 [Ostrinia furnacalis]	-1.6882001	1.407010135	-0.180339763	0.355816029	0.105713709
TRINITY_DN52761_c0_g1_i2_orf1	-	-	-	atlastin isoform X4 [Ostrinia furnacalis]	-1.7121349	0.120247144	-0.365693345	1.025380278	0.932200823
TRINITY_DN125150_c0_g1_i1_orf1	-	-	-	aldehyde dehydrogenase, dimeric NADP-preferring isoform X5 [Ostrinia furnacalis]	-1.8976411	0.888083257	0.020203223	0.740082179	0.249272403
TRINITY_DN2311_c0_g3_i1_orf1	-	-	-	uncharacterized protein LOC114364231 isoform X1 [Ostrinia furnacalis] >XP_028176108.1 uncharacterized protein LOC114364231 isoform X2 [Ostrinia furnacalis]	-1.2124908	1.750833843	-0.362805718	0.310610667	-0.486147947
TRINITY_DN1759_c0_g1_i4_orf1	-	-	-	protein PFC0760c-like isoform X1 [Ostrinia furnacalis]	-1.6889955	0.433785026	-0.553012638	1.000200605	0.808022538
TRINITY_DN17505_c0_g1_i15_orf1	-	-	-	unnamed protein product [Chilo suppressalis]	-0.8674421	-0.219868978	-0.673628436	1.928079553	-0.167140033
TRINITY_DN2343_c1_g1_i2_orf1	-	-	-	receptor expression-enhancing protein 5-like isoform X1 [Ostrinia furnacalis] >XP_028170586.1 receptor expression-enhancing protein 5-like isoform X1 [Ostrinia furnacalis]	-1.0937706	0.225934082	-1.238578232	1.058306858	1.048107908
TRINITY_DN8766_c0_g1_i1_orf1	-	-	-	prolow-density lipoprotein receptor-related protein 1, partial [Ostrinia furnacalis]	-1.844384	0.90035527	-0.178863584	0.811618494	0.311273796
TRINITY_DN5170_c0_g1_i5_orf1	-	-	-	hemolymph lipopolysaccharide-binding protein-like isoform X2 [Leguminivora glycinivorella]	-1.1440056	0.606035874	-1.283990088	0.822740855	0.999218987
TRINITY_DN858_c0_g1_i3_orf1	-	-	-	uncharacterized protein LOC114351944 [Ostrinia furnacalis]	-1.2453405	1.23973485	-1.090513575	0.300848965	0.795270211
TRINITY_DN1465_c2_g1_i2_orf1	-	-	-	transcription initiation factor TFIID subunit 1-like [Ostrinia furnacalis]	-1.1895824	1.784503373	-0.454889394	0.233062283	-0.373093859
TRINITY_DN10231_c0_g2_i1_orf1	-	-	-	uncharacterized protein LOC114361472 [Ostrinia furnacalis]	-1.3533016	1.292377462	-0.8841286	0.840010757	0.105041982
TRINITY_DN52761_c0_g2_i1_orf1	-	-	-	atlastin-like isoform X4 [Ostrinia furnacalis]	-1.4085927	-0.20443252	-0.610007197	1.367473864	0.855558545
TRINITY_DN1116_c0_g1_i6_orf1	-	-	-	RNA exonuclease 4-like [Ostrinia furnacalis] >QEE79882.1 REX4 [Ostrinia furnacalis]	-0.8740486	-0.160933215	-1.003658432	1.769247512	0.269392776
TRINITY_DN96739_c0_g1_i1_orf1	-	-	-	annexin A6, isoform CRA_b [Homo sapiens]	-1.5769495	1.150346277	-0.724881501	0.6033272	0.548157559
TRINITY_DN28802_c0_g1_i1_orf1	-	-	-	apolipoprotein D-like [Ostrinia furnacalis]	-1.6778736	1.143035287	-0.317197787	0.881327474	-0.029291381
TRINITY_DN98016_c0_g1_i1_orf1	-	-	-	methanethiol oxidase [Ostrinia furnacalis]	-1.4113895	1.628487875	-0.444159964	0.37135863	-0.14429705
TRINITY_DN53281_c0_g1_i11_orf1	-	-	-	larval cuticle protein LCP-17-like [Ostrinia furnacalis]	-1.4750559	0.02060517	-0.651285514	1.3549856	0.750750688
TRINITY_DN9457_c0_g1_i9_orf1	-	-	-	plexin domain-containing protein 2 [Spodoptera litura]	-1.6381015	1.162409469	-0.606570992	0.618114104	0.464148934
TRINITY_DN10373_c0_g1_i1_orf1	-	-	-	homocysteine S-methyltransferase 1-like [Ostrinia furnacalis] >XP_028162778.1 homocysteine S-methyltransferase 1-like [Ostrinia	-1.5007194	0.816978409	-0.757668661	0.237230458	1.204179183
TRINITY_DN2416_c0_g1_i5_orf1	-	-	-	somatomedin-B and thrombospondin type-1 domain-containing protein [Ostrinia furnacalis] >XP_028177886.1 somatomedin-B and thrombospondin type-1 domain-containing protein [Ostrinia furnacalis]	-1.30679	1.61888216	-0.698264491	0.426947911	-0.040775588
TRINITY_DN8694_c1_g1_i4_orf1	-	-	-	sodium/potassium-transporting ATPase subunit beta-2-like isoform X2 [Ostrinia furnacalis]	-1.4520855	1.152192509	-0.764950616	0.986186502	0.078657078
TRINITY_DN483_c0_g1_i6_orf1	-	-	-	uncharacterized protein LOC114354803 isoform X1 [Ostrinia furnacalis] >XP_028163172.1 uncharacterized protein LOC114354803 isoform X2 [Ostrinia furnacalis]	-0.9261562	0.0033191	-0.897364781	1.826723201	-0.006521348
TRINITY_DN104_c0_g1_i4_orf1	-	-	-	PREDICTED: heparan-alpha-glucosaminide N-acetyltransferase [Amyelois transitella]	-1.2805165	1.670673928	-0.680473521	-0.033643588	0.323959725
TRINITY_DN9926_c1_g1_i1_orf1	-	-	-	rab GTPase-activating protein 1-like isoform X6 [Ostrinia furnacalis]	-1.098583	0.582061518	-1.295410543	1.16546782	0.646464233
TRINITY_DN8979_c0_g1_i5_orf1	-	-	-	ras-related protein Rab-5B [Vanessa cardui] >XP_046961939.1 ras-related protein Rab-5B [Vanessa cardui] >XP_046961940.1 ras-related protein Rab-5B [Vanessa cardui] >XP_046961941.1 ras-related protein Rab-5B [Vanessa cardui] >XP_047545265.1 ras-related protein Rab-5B [Vanessa atalanta] >XP_047545266.1 ras-related protein Rab-5B [Vanessa atalanta] >XP_047545267.1 ras-related protein Rab-5B [Vanessa atalanta]	-1.5281691	1.220488994	-0.768240385	0.593232234	0.48268828
TRINITY_DN51498_c0_g1_i1_orf1	-	-	-	delta-aminolevulinic acid dehydratase isoform X3 [Ostrinia furnacalis]	-0.8093473	0.100810228	-0.636382695	1.90237995	-0.557460219
TRINITY_DN40945_c0_g1_i1_orf1	-	-	-	RNA exonuclease 4-like [Ostrinia furnacalis] >QEE79882.1 REX4 [Ostrinia furnacalis]	-1.1918749	0.475706102	-1.13405012	1.346662039	0.503556901
TRINITY_DN37821_c0_g1_i6_orf1	-	-	-	uncharacterized protein LOC114350690 [Ostrinia furnacalis]	-1.7212586	0.758720941	-0.554688171	0.720237864	0.796987966
TRINITY_DN96801_c0_g1_i1_orf1	-	-	-	histone H4 isoform X2 [Gracilinanus agilis]	-0.9480191	-0.131153407	-1.152564052	1.480100901	0.75163561
TRINITY_DN61048_c0_g1_i2_orf1	-	-	-	PREDICTED: protein THEM6-like [Amyelois transitella]	-1.4994634	0.905563721	-0.85698295	0.456541805	0.994340821
TRINITY_DN701_c1_g1_i4_orf1	-	-	-	venom serine protease Bi-VSP-like [Ostrinia furnacalis]	-0.9559346	-0.106943868	-1.215059835	1.094922951	1.183015365
TRINITY_DN896_c0_g1_i2_orf1	-	-	-	uncharacterized protein LOC114356314 isoform X2 [Ostrinia furnacalis]	-1.1738324	1.214326903	-0.999048298	-0.108097549	1.066651343

TRINITY_DN4817_c0_g1_i4_orf1	-	-	-	palmitoyl-protein thioesterase 1 isoform X1 [Ostrinia furnacalis] >XP_028170290.1 palmitoyl-protein thioesterase 1 isoform X4 [Ostrinia furnacalis]	-1.2858213	1.012083412	-0.981553688	0.093312309	1.161979289
TRINITY_DN3276_c0_g1_i4_orf1	-	-	-	hypothetical protein B5X24_HaOG201803 [Helicoverpa armigera]	-1.3745575	0.929734683	-0.975217098	1.088754665	0.331285272
TRINITY_DN29144_c0_g3_i1_orf1	-	-	-	PREDICTED: ADP-ribosylation factor 6 [Papilio polytes] >XP_013133321.1 PREDICTED: ADP-ribosylation factor 6 [Papilio polytes] >XP_013177129.1 PREDICTED: ADP-ribosylation factor 6 [Papilio xuthus] >XP_013177130.1 PREDICTED: ADP-ribosylation factor 6 [Papilio xuthus] >XP_014356507.1 ADP-ribosylation factor 6 [Papilio machaon] >XP_021185579.1 ADP-ribosylation factor 6 [Helicoverpa armigera] >XP_021185581.1 ADP-ribosylation factor 6 [Helicoverpa armigera] >XP_022130228.1 ADP-ribosylation factor 6 [Pieris rapae] >XP_022822139.1 ADP-ribosylation factor 6 [Spodoptera litura] >XP_022822140.1 ADP-ribosylation factor 6 [Spodoptera litura] >XP_028159104.1 ADP-ribosylation factor 6 [Ostrinia furnacalis] >XP_028159105.1 ADP-ribosylation factor 6 [Ostrinia furnacalis] >XP_028159106.1 ADP-ribosylation factor 6 [Ostrinia furnacalis] >XP_028159107.1 ADP-ribosylation factor 6 [Ostrinia furnacalis] >XP_028163222.1 ADP-ribosylation factor 6 [Ostrinia furnacalis] >XP_030022165.1 ADP-ribosylation factor 6 [Manduca sexta] >XP_030022166.1 ADP-ribosylation factor 6 [Manduca sexta] >XP_030022167.1 ADP-ribosylation factor 6 [Manduca sexta] >XP_035444169.1 ADP-ribosylation factor 6 [Spodoptera frugiperda] >XP_035444175.1 ADP-ribosylation factor 6 [Spodoptera frugiperda] >XP_038207597.1 ADP-ribosylation factor 6 [Zerene cesonia] >XP_038207598.1 ADP-ribosylation factor 6 [Zerene cesonia] >XP_045510541.1 ADP-ribosylation factor 6 [Colias croceus] >XP_045510551.1 ADP-ribosylation factor 6 [Colias croceus] >XP_045527300.1 ADP-ribosylation factor 6 [Pieris brassicae] >XP_045527302.1 ADP-ribosylation factor 6 [Pieris brassicae] >XP_047029519.1 ADP-ribosylation factor 6 [Helicoverpa zea] >XP_047029551.1 ADP-ribosylation factor 6 [Helicoverpa zea] >XP_047504621.1 ADP-ribosylation factor 6 [Pieris napi] >XP_047504631.1 ADP-ribosylation factor 6 [Pieris napi] >XP_047504640.1 ADP-ribosylation factor 6 [Pieris napi] >XP_047504648.1 ADP-ribosylation factor 6 [Pieris	-0.316234	0.385356064	-1.630445477	1.441826915	0.119496547
TRINITY_DN4596_c0_g1_i14_orf1	-	-	-	aldehyde dehydrogenase, dimeric NADP-preferring isoform X7 [Ostrinia furnacalis]	-1.2031398	0.640223205	-1.139311023	1.295945183	0.406282413
TRINITY_DN4679_c0_g2_i13_orf1	-	-	-	GATOR complex protein MIOS [Ostrinia furnacalis]	-1.5349857	0.365140243	-0.779299262	0.932135584	1.017009103
TRINITY_DN4813_c0_g1_i5_orf1	-	-	-	piwi-like protein Siwi [Ostrinia furnacalis]	-0.9176005	0.795761105	-1.237485163	1.41093595	-0.051611352
TRINITY_DN1978_c0_g1_i4_orf1	-	-	-	RNA exonuclease 4-like [Ostrinia furnacalis]	-0.8072262	0.373096211	-1.431583021	1.390738943	0.474974052
TRINITY_DN22009_c0_g1_i1_orf1	-	-	-	armadillo repeat-containing protein 8-like [Ostrinia furnacalis]	-0.327426	0.325412156	-1.462271449	1.620004021	-0.155718721
TRINITY_DN5055_c0_g1_i12_orf1	-	-	-	probable peroxisomal acyl-coenzyme A oxidase 1 [Ostrinia furnacalis]	-1.0346134	0.233809148	-1.292376364	1.130163726	0.963016928
TRINITY_DN4628_c0_g1_i1_orf1	-	-	-	guanine nucleotide-binding protein G(q) subunit alpha isoform X1 [Ostrinia furnacalis] >XP_028160094.1 guanine nucleotide-binding protein G(q) subunit alpha isoform X1 [Ostrinia furnacalis]	-0.7706142	-0.068433954	-0.884038668	1.894846857	-0.171759985
TRINITY_DN3219_c0_g1_i6_orf1	-	-	-	non-specific lipid-transfer protein [Ostrinia furnacalis]	-0.7897206	0.161530849	-1.449529697	0.825958543	1.25176089
TRINITY_DN51197_c0_g1_i3_orf1	-	-	-	microtubule-actin cross-linking factor 1 isoform X15 [Ostrinia furnacalis]	-1.0146405	0.113864809	-1.222600738	1.384506276	0.738870199
TRINITY_DN39725_c0_g1_i4_orf1	-	-	-	lysosome membrane protein 2-like [Ostrinia furnacalis]	-0.4189053	0.77301957	-1.561003429	1.332120322	-0.125231177
TRINITY_DN2881_c0_g1_i7_orf1	-	-	-	5'-3' exoribonuclease 1 [Ostrinia furnacalis]	-0.2281071	0.485688521	-1.783534926	1.191234829	0.334718648
TRINITY_DN20442_c0_g2_i1_orf1	-	-	-	hypothetical protein evm_008218 [Chilo suppressalis]	-1.4546287	1.101528059	0.223047547	0.962936383	-0.832883269
TRINITY_DN12526_c0_g1_i5_orf1	-	-	-	uncharacterized protein LOC114359035 isoform X3 [Ostrinia furnacalis]	-1.6672271	1.175556879	0.20090453	0.760112407	-0.469346723
TRINITY_DN47842_c0_g1_i1_orf1	-	-	-	protein lethal(2)essential for life-like [Helicoverpa armigera] >PZC74790.1 hypothetical protein B5X24_HaOG207163 [Helicoverpa armigera]	-1.5629241	0.975453317	0.299622407	1.00257347	-0.714725109
TRINITY_DN307_c1_g1_i1_orf1	-	-	-	uncharacterized protein LOC114356704 [Ostrinia furnacalis]	-1.3735945	0.400108329	0.498515433	1.375869154	-0.900898465
TRINITY_DN1897_c0_g2_i4_orf1	-	-	-	phenoloxidase-activating factor 2-like [Hyposmocoma kahamanoa]	-1.3519957	0.875215393	-0.410437342	1.403835151	-0.51661747
TRINITY_DN29190_c0_g1_i4_orf1	-	-	-	gloverin-like [Ostrinia furnacalis]	-0.4414459	0.179820617	-0.698175704	1.862783676	-0.902982676
TRINITY_DN38392_c0_g1_i1_orf1	-	-	-	enoyl-CoA hydratase domain-containing protein 3 [Agrotis segetum]	-0.8409409	0.708859333	-0.172604637	1.515084389	-1.210398208
TRINITY_DN1493_c0_g1_i5_orf1	-	-	-	uncharacterized protein LOC114350869 [Ostrinia furnacalis]	-1.0667827	-0.063471822	0.387419633	1.681369772	-0.938534931
TRINITY_DN35147_c0_g1_i1_orf1	-	-	-	collagen alpha-2(IV) chain isoform X2 [Ostrinia furnacalis]	-1.3919369	0.538648076	-0.316894891	1.581907409	-0.411723644

TRINITY_DN810_c0_g1_i4_orf1	-	-	-	dicer 2 [Ostrinia nubilalis]	-1.5079698	0.735782328	-0.177139433	1.397339536	-0.448012645
TRINITY_DN225_c0_g1_i6_orf1	-	-	-	glutathione S-transferase delta 3 [Ostrinia furnacalis]	-1.2299734	0.577262842	-0.320054469	1.621803648	-0.64903864
TRINITY_DN962_c5_g1_i1_orf1	-	-	-	histone deacetylase 5 isoform X5 [Pectinophora gossypiella]	-0.937889	1.044619471	-0.676836632	1.382439985	-0.812333792
TRINITY_DN34465_c0_g1_i1_orf1	-	-	-	putative peptidyl-tRNA hydrolase PTRHD1 [Ostrinia furnacalis]	-0.2704361	0.169863427	-0.444140263	1.781063528	-1.236350635
TRINITY_DN26089_c0_g1_i1_orf1	-	-	-	putative neuropeptide precursor protein isoform X1 [Ostrinia furnacalis]	-0.8714793	1.044131754	-0.741952193	1.388680792	-0.819381017
TRINITY_DN48497_c0_g1_i1_orf1	-	-	-	unnamed protein product [Chrysodeixis includens]	-0.0703955	0.488829513	-0.789020609	1.610928146	-1.240341554
TRINITY_DN7388_c0_g1_i7_orf1	-	-	-	ras-related protein Rab-18A isoform X1 [Ostrinia furnacalis]	-1.42031	0.764082133	-0.569330004	1.426340672	-0.200782843
TRINITY_DN115210_c0_g4_i1_orf1	-	-	-	PREDICTED: CAD protein [Microplitis demolitor]	-0.5620425	0.984953616	-1.035683165	1.413993851	-0.801221795
TRINITY_DN2758_c0_g1_i7_orf1	-	-	-	hypothetical protein evm_012952 [Chilo suppressalis]	-1.4333478	0.751699276	-0.743262656	1.349965161	0.074946041
TRINITY_DN6595_c1_g1_i6_orf1	-	-	-	methenyltetrahydrofolate synthase domain-containing protein [Ostrinia furnacalis]	-1.3127353	0.434421561	-0.79827644	1.561234036	0.115356112
TRINITY_DN83150_c0_g1_i1_orf1	-	-	-	fructose-bisphosphate aldolase-like isoform X1 [Ostrinia furnacalis] >XP_028178678.1 fructose-bisphosphate aldolase-like isoform X1 [Ostrinia furnacalis]	-0.9551664	0.703200415	-0.364756105	1.58702172	-0.970299619
TRINITY_DN30037_c0_g1_i5_orf1	-	-	-	cytoglobin-1-like isoform X2 [Ostrinia furnacalis]	0.11870863	-0.330538636	0.301488761	1.501414081	-1.591072832
TRINITY_DN3209_c0_g2_i6_orf1	-	-	-	coatomer subunit beta, partial [Ostrinia furnacalis]	-0.6254986	0.615141189	-0.605983612	1.663382403	-1.047041392
TRINITY_DN51938_c0_g3_i1_orf1	-	-	-	unnamed protein product [Mus musculus]	0.27061953	0.070945394	-0.551912362	1.620924615	-1.410577173
TRINITY_DN27247_c0_g2_i1_orfp1	-	-	-	TRINITY_DN27247_c0_g2_i1_m.23157 TRINITY_DN27247_c0_g2::TRINITY_DN27247_c0_g2_i1::g.23157 ORF type:5prime_partial len:70 (+),score=25.00 TRINITY_DN27247_c0_g2_i1:2-211(+)	-0.8241252	-0.945188963	0.503376422	1.722176956	-0.456239258
TRINITY_DN9400_c0_g1_i1_orf1	-	-	-	lysophosphatidylserine lipase ABHD12 isoform X2 [Maniola hyperantus]	-0.352197	-0.77844223	0.574443564	1.653838137	-1.097642459
TRINITY_DN15000_c0_g1_i4_orf1	-	-	-	15-hydroxyprostaglandin dehydrogenase [NAD(+)]-like [Ostrinia furnacalis]	0.02635241	-1.260748691	-0.213246254	1.800043797	-0.352401266
TRINITY_DN11649_c0_g1_i4_orf1	-	-	-	ubiquitin carboxyl-terminal hydrolase 32-like, partial [Ostrinia furnacalis]	-0.4288392	-1.17132496	0.507920863	1.684017395	-0.591774124
TRINITY_DN41280_c0_g1_i2_orf1	-	-	-	unnamed protein product [Plutella xylostella]	0.17401271	-1.257953816	0.204904335	1.655851638	-0.776814868
TRINITY_DN5126_c0_g2_i1_orf1	-	-	-	cytochrome P450 monooxygenase CYP4L47 [Ostrinia furnacalis]	0.3498629	-0.947101529	-0.592613228	1.803547142	-0.61369529
TRINITY_DN920_c0_g1_i6_orf1	-	-	-	glutathione S-transferase omega 2 [Ostrinia furnacalis]	0.51925809	-1.136946624	0.044357805	1.565610581	-0.992279851
TRINITY_DN436_c0_g2_i5_orfp1	-	-	-	TRINITY_DN436_c0_g2_i5_m.4776 TRINITY_DN436_c0_g2::TRINITY_DN436_c0_g2_i5::g.4776 ORF type:complete len:158 (-),score=33.76 TRINITY_DN436_c0_g2_i5:320-772(-)	-0.0469832	-1.147290122	-0.248423428	1.856935626	-0.414238862
TRINITY_DN1292_c0_g1_i3_orf1	-	-	-	uncharacterized protein LOC114360660 [Ostrinia furnacalis]	0.18198764	-1.456444551	0.326820393	1.543825436	-0.596188917
TRINITY_DN7861_c0_g1_i5_orf1	-	-	-	cytochrome b5-related protein-like [Ostrinia furnacalis]	0.08506864	-1.400657492	0.487520658	1.52092096	-0.692852767
TRINITY_DN4321_c0_g1_i1_orf1	-	-	-	acyl-CoA Delta(11) desaturase isoform X1 [Ostrinia furnacalis]	0.21858881	-0.974301042	-0.499981705	1.845342569	-0.589648634
TRINITY_DN27903_c0_g1_i1_orf1	-	-	-	desaturase MPVE [Helicoverpa assulta]	0.44003092	-1.228819872	-0.079102171	1.640904518	-0.7730134
TRINITY_DN124711_c0_g1_i1_orf1	-	-	-	muskelin isoform X1 [Ostrinia furnacalis] >XP_028163274.1 muskelin isoform X2 [Ostrinia furnacalis]	0.59179694	-0.873017595	-0.737018267	1.697819482	-0.679580562
TRINITY_DN41708_c0_g1_i1_orf1	-	-	-	facilitated trehalose transporter Tret1-like [Ostrinia furnacalis]	0.51419653	-1.766587889	0.058180064	1.267297386	-0.073086091
TRINITY_DN1039_c0_g1_i5_orf1	-	-	-	WD repeat-containing protein 26 [Ostrinia furnacalis]	-0.0398961	-0.665351312	-0.721845629	1.941625824	-0.514532778
TRINITY_DN32_c0_g1_i4_orf1	-	-	-	epidermal growth factor receptor substrate 15 homolog [Ostrinia furnacalis]	0.53951956	-1.506101514	0.242552889	1.391489138	-0.667460074
TRINITY_DN1914_c0_g1_i4_orf1	-	-	-	loricrin-like [Ostrinia furnacalis]	0.8124631	-1.526211971	-0.144896588	1.329586497	-0.470941037
TRINITY_DN1329_c0_g1_i5_orf1	-	-	-	neurogenic locus notch homolog protein 3 [Ostrinia furnacalis] >XP_028157678.1 neurogenic locus notch homolog protein 3 [Ostrinia furnacalis]	-0.6347367	-1.632293499	0.407180216	1.066692271	0.793157702
TRINITY_DN9820_c0_g1_i1_orf1	-	-	-	endocuticle structural glycoprotein SgAbd-2-like [Ostrinia furnacalis]	-0.3193809	-1.803818464	0.409817487	0.791707737	0.921674163
TRINITY_DN120500_c0_g1_i1_orf1	-	-	-	cytochrome P450 6B5-like [Ostrinia furnacalis]	-0.1080675	-1.486156263	-0.145365576	1.658922123	0.080667256
TRINITY_DN13119_c0_g1_i4_orf1	-	-	-	endocuticle structural glycoprotein ABD-5-like [Bicyclus anynana]	0.04679148	-1.928098512	0.370866328	0.786758313	0.723682394
TRINITY_DN98995_c0_g1_i2_orf1	-	-	-	hypothetical protein HF086_008399, partial [Spodoptera exigua]	-0.2572105	-1.658832361	-0.15655531	1.106216242	0.966381923
TRINITY_DN2350_c0_g1_i6_orf1	-	-	-	protein yellow-like isoform X2 [Ostrinia furnacalis]	-0.1226912	-1.712595545	0.368883199	1.381553097	0.084850437
TRINITY_DN565_c0_g2_i1_orf1	-	-	-	uncharacterized protein LOC114362323 [Ostrinia furnacalis]	0.06477701	-1.602763964	0.073315728	1.553598519	-0.08892729
TRINITY_DN779_c0_g1_i12_orf1	-	-	-	unnamed protein product [Chilo suppressalis]	-0.0112062	-1.882277853	0.37718491	1.045425153	0.470873973

TRINITY_DN2193_c0_g1_i7_orf1	-	-	-	long-chain-fatty-acid--CoA ligase 5 isoform X1 [Ostrinia furnacalis] >XP_028176293.1 long-chain-fatty-acid--CoA ligase 5 isoform X1 [Ostrinia furnacalis] >XP_028176294.1 long-chain-fatty-acid--CoA ligase 5 isoform X1 [Ostrinia furnacalis] >XP_028176295.1 long-chain-fatty-acid--CoA ligase 5 isoform X1 [Ostrinia furnacalis] >XP_028176296.1 long-chain-fatty-acid--CoA ligase 5 isoform X1 [Ostrinia furnacalis] >XP_028176297.1 long-chain-fatty-acid--CoA ligase 5 isoform X1 [Ostrinia furnacalis] >XP_028176298.1 long-chain-fatty-acid--CoA ligase 5 isoform X2 [Ostrinia	-0.527675	-1.528960046	-0.118826205	1.046471421	1.12898987
TRINITY_DN57856_c0_g2_i1_orf1	-	-	-	cytochrome P450 6B2-like [Ostrinia furnacalis]	-0.1669238	-1.557679281	-0.432095621	1.207559517	0.949139139
TRINITY_DN8651_c0_g1_i18_orf1	-	-	-	glutathione S-transferase theta 2 [Conogethes punctiferalis]	-0.0162567	-1.727929151	-0.203128275	0.777800823	1.169513297
TRINITY_DN135077_c0_g1_i1_orf1	-	-	-	hypothetical protein KR038_001662 [Drosophila bunnanda]	0.22072821	-1.847943423	0.222012573	1.202499985	0.202702652
TRINITY_DN592_c0_g1_i6_orf1	-	-	-	PDZ and LIM domain protein Zasp isoform X4 [Pectinophora gossypiella]	0.15657155	-1.89198095	0.084054708	0.938243064	0.713111627
TRINITY_DN4053_c0_g1_i5_orf1	-	-	-	uncharacterized protein LOC114358355 [Ostrinia furnacalis]	-0.0309779	-1.767568698	0.078625167	1.301262453	0.418659022
TRINITY_DN101995_c0_g1_i1_orf1	-	-	-	microtubule-actin cross-linking factor 1 isoform X15 [Ostrinia furnacalis]	0.10654643	-1.843353597	-0.016012537	1.041107329	0.711712376
TRINITY_DN46216_c0_g3_i1_orf1	-	-	-	unnamed protein product, partial [Brenthis ino]	0.3519425	-1.877153748	-0.098342047	0.923124024	0.700429267
TRINITY_DN4744_c0_g1_i7_orf1	-	-	-	glutaryl-CoA dehydrogenase, mitochondrial [Ostrinia furnacalis]	0.03378886	-1.600809788	-0.512331881	1.116904425	0.962448381
TRINITY_DN12690_c0_g1_i1_orf1	-	-	-	ELAV-like protein 1 [Ostrinia furnacalis]	0.29718189	-1.825174083	-0.219858164	0.825995817	0.921854538
TRINITY_DN11448_c0_g1_i11_orf1	-	-	-	hypothetical protein B5X24_HaOG201808 [Helicoverpa armigera]	0.10217543	-1.501161946	-0.422074873	0.239787262	1.581274127
TRINITY_DN6612_c0_g1_i4_orf1	-	-	-	hypothetical protein O3G_MSEX008151 [Manduca sexta]	0.23766123	-1.750601941	-0.243857501	0.506731558	1.250066651
TRINITY_DN3010_c0_g1_i4_orf1	-	-	-	inositol oxygenase-like [Ostrinia furnacalis]	0.46717883	-1.700893401	-0.541603313	0.788930437	0.98638745
TRINITY_DN3332_c0_g1_i9_orf1	-	-	-	glutathione S-transferase sigma3 [Glyphodes pyloalis]	0.63613196	-1.771401773	-0.401158957	1.00929186	0.527136912
TRINITY_DN1104_c0_g1_i1_orfp1	-	-	-	TRINITY_DN1104_c0_g1_i1_m.5521 TRINITY_DN1104_c0_g1::TRINITY_DN1104_c0_g1_i1::g.5521 ORF type:5prime_partial len:204 (+),score=39.47 TRINITY_DN1104_c0_g1_i1:3-614(+)	-0.0267254	-1.522456616	-0.552456705	0.761198518	1.34044023
TRINITY_DN1814_c0_g1_i11_orf1	-	-	-	titin-like, partial [Ostrinia furnacalis]	0.77792588	-1.876008842	-0.188676921	0.566089345	0.720670534
TRINITY_DN27045_c0_g1_i1_orf1	-	-	-	cytochrome P450 6B5-like [Galleria mellonella]	0.92164184	-1.755952164	-0.312035461	0.968639616	0.177706168
TRINITY_DN76529_c0_g1_i1_orfp1	-	-	-	TRINITY_DN76529_c0_g1_i1_m.64079 TRINITY_DN76529_c0_g1::TRINITY_DN76529_c0_g1_i1::g.64079 ORF type:internal len:70 (+),score=14.68 TRINITY_DN76529_c0_g1_i1:3-209(+)	0.25284095	-1.565070788	-0.36920311	0.156353422	1.525079524
TRINITY_DN3906_c0_g1_i5_orf1	-	-	-	ejaculatory bulb-specific protein 3-like [Ostrinia furnacalis]	0.41694602	-1.676648219	-0.484651242	0.512499446	1.231853991
TRINITY_DN107962_c0_g1_i1_orf1	-	-	-	unnamed protein product [Euphydryas editha]	0.80478207	-1.762994567	-0.416906049	0.937449064	0.437669483
TRINITY_DN3529_c0_g1_i7_orf1	-	-	-	putative fatty acyl-CoA reductase CG5065 [Ostrinia furnacalis]	0.9779344	-1.408554436	-1.010447395	0.732304502	0.708762927
TRINITY_DN2254_c0_g1_i4_orf1	-	-	-	vigilin [Ostrinia furnacalis]	0.74369874	-1.477440868	-0.909346201	0.612617717	1.030470612
TRINITY_DN8964_c0_g1_i4_orf1	-	-	-	hypothetical protein evm_010115 [Chilo suppressalis]	0.86214965	-1.743076572	-0.495596489	0.575661045	0.800862367
TRINITY_DN920_c0_g1_i4_orf1	-	-	-	glutathione S-transferase omega 2 [Ostrinia furnacalis]	0.44001216	-1.165100476	-1.07519466	0.320323746	1.47995923
TRINITY_DN1125_c0_g1_i4_orf1	-	-	-	hypothetical protein evm_001907 [Chilo suppressalis] >CAH2985359.1 unnamed protein product [Chilo suppressalis]	1.10140849	-1.666650862	-0.56124814	0.735994099	0.390496408
TRINITY_DN15318_c0_g1_i1_orf1	-	-	-	hepatoma-derived growth factor-related protein 2-like [Ostrinia furnacalis]	0.9990972	-1.28317834	-1.092932813	0.362414748	1.014599208
TRINITY_DN111110_c0_g1_i1_orf1	-	-	-	NAD-dependent protein deacylase-like [Ostrinia furnacalis]	0.80908114	-1.690335477	-0.590032831	0.565831959	0.905455209
TRINITY_DN4408_c6_g1_i1_orf1	-	-	-	polyprotein, partial [Bemisia tabaci]	0.70323344	-1.605212257	-0.750086112	0.853123977	0.798940954
TRINITY_DN4133_c0_g1_i2_orfp2	-	-	-	unnamed protein product [Spodoptera exigua]	0.53025628	-1.342091152	-1.059632973	0.788082927	1.083384919
TRINITY_DN7580_c0_g1_i1_orf1	-	-	-	cytochrome P450 monooxygenase CYP6AB141 [Ostrinia furnacalis]	0.84873532	-1.668361248	-0.602329036	0.958405123	0.463549839
TRINITY_DN6365_c0_g1_i4_orf1	-	-	-	40S ribosomal protein S21 [Helicoverpa armigera] >XP_047038308.1 40S ribosomal protein S21 isoform X2 [Helicoverpa zea] >KAI5643652.1 ribosomal protein s21e domain-containing protein [Phthorimaea operculella] >PZC73652.1 hypothetical protein B5X24_HaOG209026	1.31418522	-1.635902944	-0.481590919	0.546837953	0.256470694
TRINITY_DN38366_c0_g1_i4_orfp1	-	-	-	TRINITY_DN38366_c0_g1_i4_m.10666 TRINITY_DN38366_c0_g1::TRINITY_DN38366_c0_g1_i4::g.10666 ORF type:internal len:143 (+),score=71.68 TRINITY_DN38366_c0_g1_i4:3-428(+)	0.82029016	-1.867467846	-0.213054458	0.640194856	0.620037284
TRINITY_DN27276_c0_g1_i5_orf1	-	-	-	probable small nuclear ribonucleoprotein Sm D1 [Ostrinia furnacalis] >CAG9751027.1 unnamed protein product [Diatraea saccharalis] >CAG9789712.1 unnamed protein product [Diatraea saccharalis]	1.03255596	-1.473788255	-0.851690701	0.963143031	0.32977997
TRINITY_DN27500_c0_g1_i4_orf1	-	-	-	hemicentin-1-like [Ostrinia furnacalis]	0.50167872	-1.73576758	-0.376086215	0.419304802	1.190870276
TRINITY_DN12508_c0_g1_i1_orf1	-	-	-	uncharacterized protein LOC114350091 [Ostrinia furnacalis]	1.19246205	-1.570173431	-0.71665983	0.538125666	0.556245542
TRINITY_DN42461_c0_g1_i4_orf1	-	-	-	obscurin [Ostrinia furnacalis]	0.77503743	-1.908693177	-0.030991546	0.386051891	0.778595397
TRINITY_DN50676_c0_g1_i1_orf1	-	-	-	uncharacterized protein LOC114360659 [Ostrinia furnacalis]	1.29888374	-1.555590842	-0.611267061	0.701102385	0.166871778

TRINITY_DN78492_c0_g1_i1_orf1	-	-	-	uncharacterized protein LOC114354775 [Ostrinia furnacalis]	1.41603566	-1.266117758	-0.990521869	0.589635586	0.250968377
TRINITY_DN11637_c0_g1_i1_orf1	-	-	-	ribosome-binding protein 1 isoform X4 [Colias croceus]	1.08046569	-0.635437526	-1.615643636	0.327103945	0.843511527
TRINITY_DN23474_c1_g1_i1_orf1	-	-	-	unnamed protein product [Chrysodeixis includens]	0.37894192	-1.312662922	-0.781014463	0.131703686	1.583031782
TRINITY_DN4822_c0_g1_i9_orf1	-	-	-	homogentisate 1,2-dioxygenase [Ostrinia furnacalis]	0.63413405	-0.750984917	-1.535591834	0.432159205	1.220283491
TRINITY_DN89613_c0_g1_i13_orf1	-	-	-	PREDICTED: uncharacterized protein LOC106137743 [Amyelois transitella]	1.31464752	-1.497519781	-0.760181878	0.414149543	0.528904594
TRINITY_DN21215_c0_g1_i7_orf1	-	-	-	phytanoyl-CoA dioxygenase, peroxisomal-like [Ostrinia furnacalis]	1.04467598	-0.648902514	-1.599953754	0.283734581	0.920445711
TRINITY_DN76815_c0_g1_i3_orf1	-	-	-	5-formyltetrahydrofolate cyclo-ligase [Ostrinia furnacalis]	0.7456015	-1.324074916	-1.098800966	1.034744026	0.642530357
TRINITY_DN7909_c0_g2_i1_orf1	-	-	-	aldehyde oxidase 3 [Ostrinia furnacalis]	1.24355954	-0.871349313	-1.321823844	-0.023293965	0.972907582
TRINITY_DN106534_c0_g1_i1_orf1	-	-	-	nucleolar complex protein 2 homolog [Ostrinia furnacalis]	1.42780183	-1.487097246	-0.667282723	0.220636353	0.50594179
TRINITY_DN21035_c0_g1_i14_orf1	-	-	-	mitochondrial amidoxime reducing component 2-like [Ostrinia furnacalis]	1.13267547	-1.166277163	-1.207762037	0.368033132	0.873330601
TRINITY_DN15903_c0_g1_i2_orf1	-	-	-	proton-coupled amino acid transporter-like protein pathetic isoform X1 [Ostrinia furnacalis]	1.19060787	-0.891582732	-0.991732248	-0.537976336	1.230683444
TRINITY_DN42964_c0_g1_i1_orf1	-	-	-	protein lethal(2)essential for life-like [Galleria mellonella]	-0.482095	1.46892825	-0.543639559	0.83103793	-1.274231604
TRINITY_DN95850_c0_g1_i1_orf1	-	-	-	alpha,alpha-trehalose-phosphate synthase [UDP-forming] isoform X2 [Chelonus insularis]	-1.0468411	1.088369009	-1.305971956	0.95979215	0.304651938
TRINITY_DN2002_c0_g1_i5_orfp1	-	-	-	TRINITY_DN2002_c0_g1_i5_m.4230 TRINITY_DN2002_c0_g1::TRINITY_DN2002_c0_g1_i5::g.4230 ORF type:3prime_partial len:259 (+),score=29.98,Peptidase_C39 PF03412.16 1.7,Peptidase_C39|PF03412.16|0.049 TRINITY_DN2002_c0_g1_i5:65-838(+)	-0.9271864	1.878110785	-0.770710255	-0.127473004	-0.052741143
TRINITY_DN98147_c0_g2_i1_orf1	-	-	-	hypothetical protein evm_002297 [Chilo suppressalis]	-0.488012	1.966085801	-0.779896275	-0.2001143	-0.498063214
TRINITY_DN8660_c0_g1_i1_orf1	-	-	-	chondroitin sulfate synthase 2 [Trichoplusia ni]	-0.8671331	1.325290258	-1.369590139	0.77238165	0.139051374
TRINITY_DN29879_c0_g1_i3_orf1	-	-	-	uncharacterized protein LOC114350556 isoform X1 [Ostrinia furnacalis] >XP_028157201.1 uncharacterized protein LOC114350556 isoform X2 [Ostrinia furnacalis] >XP_028157202.1 uncharacterized protein LOC114350556 isoform X3 [Ostrinia furnacalis]	-0.507021	1.695279265	-0.911358406	0.568673481	-0.845573304
TRINITY_DN661_c0_g1_i1_orf1	-	-	-	hypothetical protein evm_002822 [Chilo suppressalis]	-0.7302546	1.923667839	-0.782224471	-0.392432683	-0.018756126
TRINITY_DN49785_c1_g1_i3_orf1	-	-	-	uncharacterized protein LOC114365444, partial [Ostrinia furnacalis]	-0.651798	1.971880036	-0.705794902	-0.31086692	-0.303420245
TRINITY_DN15222_c0_g1_i4_orf1	-	-	-	lysosomal alpha-mannosidase-like [Ostrinia furnacalis]	-0.5228514	1.899751545	-0.863187977	0.089934133	-0.603646265
TRINITY_DN11666_c0_g1_i6_orf1	-	-	-	P protein-like [Ostrinia furnacalis] >XP_028167089.1 P protein-like [Ostrinia furnacalis]	-0.6415018	1.654109175	-1.28925023	0.41397201	-0.137329154
TRINITY_DN5422_c0_g1_i1_orf1	-	-	-	nitrilase and fragile histidine triad fusion protein NitFhit isoform X1 [Ostrinia furnacalis]	-0.1838559	1.06481431	-1.808729425	0.719312438	0.208458574
TRINITY_DN9109_c0_g1_i1_orf1	-	-	-	unnamed protein product [Chrysodeixis includens]	-0.6163857	1.60753098	-0.919294687	0.734862886	-0.806713478
TRINITY_DN36987_c0_g1_i1_orf1	-	-	-	basement membrane-specific heparan sulfate proteoglycan core protein isoform X13 [Ostrinia furnacalis]	0.14623327	1.318280183	-1.638738307	0.606781938	-0.432557085
TRINITY_DN36324_c0_g1_i12_orf1	-	-	-	motile sperm domain-containing protein 1-like [Ostrinia furnacalis]	-0.6248175	1.350369007	-1.559004848	0.481101516	0.352351848
TRINITY_DN23183_c1_g1_i2_orf1	-	-	-	myotubularin-related protein 9 [Ostrinia furnacalis]	-0.4286841	1.800424959	-1.224938052	-0.2515182	0.104715357
TRINITY_DN52553_c0_g2_i1_orf1	-	-	-	hemocyte protein-glutamine gamma-glutamyltransferase-like [Ostrinia furnacalis]	-0.4197176	1.992744112	-0.609772799	-0.389278839	-0.573974879
TRINITY_DN1260_c0_g2_i1_orf1	-	-	-	vegetative cell wall protein gp1 [Ostrinia furnacalis]	-0.1039819	1.091673859	-0.997667901	1.188635902	-1.178660007
TRINITY_DN71832_c0_g1_i1_orf1	-	-	-	basement membrane-specific heparan sulfate proteoglycan core protein isoform X13 [Ostrinia furnacalis]	-0.1808372	1.424186878	-1.649398599	0.4638271	-0.057778139
TRINITY_DN2904_c0_g1_i4_orf1	-	-	-	ATP-dependent DNA/RNA helicase DHX36 isoform X1 [Ostrinia furnacalis]	-0.7820812	1.522329463	-1.194198344	0.747415714	-0.293465643
TRINITY_DN13419_c0_g1_i5_orf1	-	-	-	atrial natriuretic peptide-converting enzyme-like [Ostrinia furnacalis]	-0.3270924	1.434197548	-1.537472139	0.650878061	-0.220511099
TRINITY_DN4456_c0_g1_i1_orf1	-	-	-	uncharacterized protein LOC114353382 isoform X1 [Ostrinia furnacalis]	-0.2123881	1.336538587	-1.42115723	0.891726593	-0.594719865
TRINITY_DN13167_c0_g1_i1_orf1	-	-	-	selenoprotein M-like [Ostrinia furnacalis]	-0.1674894	1.729205763	-1.06498762	0.353394052	-0.850122779
TRINITY_DN8306_c0_g1_i4_orf1	-	-	-	NAD(P) transhydrogenase, mitochondrial-like [Ostrinia furnacalis] >XP_028175067.1 NAD(P) transhydrogenase, mitochondrial-like [Ostrinia furnacalis] >XP_028175068.1 NAD(P) transhydrogenase, mitochondrial-like [Ostrinia furnacalis] >XP_028175069.1 NAD(P) transhydrogenase, mitochondrial-like [Ostrinia furnacalis]	-0.5342053	1.758237971	-1.272412729	0.063045151	-0.014665069
TRINITY_DN2438_c0_g1_i1_orf1	-	-	-	dystrophin, isoforms A/C/F/G/H-like [Ostrinia furnacalis]	-0.1579108	1.616337548	-1.017649583	0.563755582	-1.004532771
TRINITY_DN3177_c0_g1_i1_orf1	-	-	-	Pupal cuticle protein PCP52 [Papilio xuthus]	-0.511925	1.767914653	-1.122440388	0.347725337	-0.481274643
TRINITY_DN51829_c0_g1_i1_orf1	-	-	-	FAS-associated factor 2 [Ostrinia furnacalis]	0.18960889	1.238072743	-1.74858053	0.561823608	-0.240924708
TRINITY_DN8846_c0_g1_i1_orf1	-	-	-	PREDICTED: synapse-associated protein of 47 kDa-like isoform X2 [Papilio xuthus]	0.63415029	0.675323926	-1.395359086	1.089621929	-1.003737057
TRINITY_DN1749_c0_g2_i2_orf1	-	-	-	putative GPI-anchored protein pfl2 isoform X1 [Ostrinia furnacalis]	-0.3790747	1.978905312	-0.773826479	-0.401016518	-0.424987572

TRINITY_DN11566_c0_g1_i6_orf1	-	-	-	lens fiber major intrinsic protein-like isoform X1 [Ostrinia furnacalis]	0.32665766	1.233426598	-0.937722459	0.760957874	-1.383319669
TRINITY_DN62729_c0_g1_i13_orf1	-	-	-	G protein-coupled receptor kinase 2 isoform X3 [Manduca sexta]	-0.43621	1.343196306	-1.644381591	0.490512697	0.246882578
TRINITY_DN2201_c0_g1_i1_orf1	-	-	-	pleiotropic regulator 1 [Ostrinia furnacalis]	0.28405798	1.324472388	-0.769921737	0.634420567	-1.473029199
TRINITY_DN667_c0_g1_i5_orf1	-	-	-	unnamed protein product [Arctia plantaginis]	0.08972287	1.518923419	-1.622741453	0.167410134	-0.153314965
TRINITY_DN27456_c0_g2_i1_orf1	-	-	-	organic cation transporter-like protein [Ostrinia furnacalis]	-0.3188574	1.623813327	-1.481229991	0.2493538	-0.073079765
TRINITY_DN10630_c0_g1_i2_orf1	-	-	-	J domain-containing protein [Ostrinia furnacalis]	-0.1129353	1.553486135	-1.171408226	0.628812704	-0.897955347
TRINITY_DN37585_c0_g2_i1_orf1	-	-	-	cuticle protein 19.8-like [Ostrinia furnacalis]	-0.094652	1.925234834	-0.898522137	-0.319740871	-0.612319832
TRINITY_DN10415_c0_g1_i5_orf1	-	-	-	hypothetical protein evm_000184 [Chilo suppressalis]	0.36515397	1.098825784	-1.669359908	0.755143704	-0.549763548
TRINITY_DN4401_c0_g2_i1_orf1	-	-	-	hypothetical protein evm_003554 [Chilo suppressalis]	0.28225316	1.338090611	-0.859346213	0.644638314	-1.405635871
TRINITY_DN6362_c0_g1_i4_orf1	-	-	-	sodium/hydrogen exchanger 7 isoform X4 [Galleria mellonella]	0.31527451	1.49113776	-0.985828189	0.416924324	-1.237508406
TRINITY_DN4571_c0_g1_i4_orf1	-	-	-	PREDICTED: nuclear factor NF-kappa-B p105 subunit [Microplitis demolitor] >KAG6558391.1 viral ankyrin V1 [Microplitis demolitor]	0.3337348	1.598020761	-1.292299407	0.160095266	-0.799551423
TRINITY_DN13576_c0_g1_i1_orf1	-	-	-	uncharacterized protein LOC114350099 [Ostrinia furnacalis]	-0.388032	1.985197351	-0.728759256	-0.445779276	-0.422626817
TRINITY_DN206_c0_g1_i8_orf1	-	-	-	A-kinase anchor protein 200-like [Ostrinia furnacalis] >XP_028173114.1 A-kinase anchor protein 200-like [Ostrinia furnacalis] >XP_028173115.1 A-kinase anchor protein 200-like [Ostrinia furnacalis]	-0.1943836	1.942838439	-0.909922115	-0.355756536	-0.482776188
TRINITY_DN381_c0_g1_i1_orf1	-	-	-	cuticle protein 8-like [Ostrinia furnacalis]	-0.4126604	1.991366357	-0.675439948	-0.45357797	-0.449688006
TRINITY_DN21125_c0_g1_i1_orf1	-	-	-	protein angel homolog 1 isoform X3 [Ostrinia furnacalis]	0.56426887	1.140142555	-1.528842944	0.629468398	-0.805036883
TRINITY_DN7803_c0_g1_i2_orf1	-	-	-	membrane-associated protein Hem [Ostrinia furnacalis]	0.09822117	1.86127425	-0.901717694	-0.25187883	-0.805898891
TRINITY_DN12222_c0_g1_i1_orf1	-	-	-	unnamed protein product [Chilo suppressalis]	0.01492407	1.807032128	-1.241312247	-0.178558008	-0.40208594
TRINITY_DN5074_c0_g1_i7_orf1	-	-	-	zonadhesin-like [Ostrinia furnacalis]	-0.2287865	1.913831675	-1.029486268	-0.399138895	-0.256420053
TRINITY_DN33893_c0_g1_i1_orf1	-	-	-	high mobility group protein I-like [Ostrinia furnacalis]	0.22333636	1.634191288	-1.462856909	-0.372986833	-0.021683905
TRINITY_DN5829_c0_g2_i1_orf1	-	-	-	uncharacterized protein LOC114365758 isoform X2 [Ostrinia furnacalis]	0.40148008	1.600422635	-1.204936177	0.105506865	-0.902473404
TRINITY_DN27723_c0_g1_i1_orf1	-	-	-	putative uncharacterized protein DDB_G0282133 isoform X1 [Ostrinia	0.42956736	1.724556399	-1.076412942	-0.313043558	-0.76466726
TRINITY_DN1124_c0_g1_i7_orf1	-	-	-	PREDICTED: cuticle protein 18.6, isoform B [Amyelois transitella]	-0.2822424	1.964655506	-0.840022954	-0.424262169	-0.418128009
TRINITY_DN778_c0_g1_i1_orf1	-	-	-	uncharacterized protein LOC114363281 [Ostrinia furnacalis]	-0.0995364	1.938477789	-0.862066279	-0.410669986	-0.566205146
TRINITY_DN64769_c0_g1_i3_orf1	-	-	-	procollagen-lysine,2-oxoglutarate 5-dioxygenase isoform X2 [Ostrinia furnacalis]	0.78737056	1.526171171	-0.990326219	-0.349635417	-0.973580098
TRINITY_DN147458_c0_g1_i1_orf1	-	-	-	60S ribosomal protein L5, partial [Cotesia chilonis]	0.25867312	1.777832217	-1.164819412	-0.569406403	-0.302279524
TRINITY_DN644_c0_g1_i1_orf1	-	-	-	cuticle protein 19-like [Ostrinia furnacalis]	-0.08346	1.946384667	-0.787208145	-0.481548743	-0.59416773
TRINITY_DN9282_c0_g1_i2_orf1	-	-	-	uncharacterized protein LOC114363102 isoform X2 [Ostrinia furnacalis]	-0.0400434	1.932711101	-0.845284134	-0.52115567	-0.526227926
TRINITY_DN7247_c0_g1_i6_orf1	-	-	-	pyruvate kinase-like isoform X2 [Ostrinia furnacalis]	0.35025588	-0.011987778	-1.252687143	1.659264207	-0.744845169
TRINITY_DN22_c0_g1_i3_orf1	-	-	-	uncharacterized protein LOC114362831 [Ostrinia furnacalis]	0.59659526	-0.243547765	-1.014461114	1.622390446	-0.96097683
TRINITY_DN5962_c0_g1_i1_orf1	-	-	-	tRNA (cytosine(34)-C(5))-methyltransferase [Ostrinia furnacalis]	0.86401296	-1.115250354	-0.497370033	1.488331262	-0.739723837
TRINITY_DN5991_c0_g1_i6_orf1	-	-	-	uncharacterized protein LOC114357071 [Ostrinia furnacalis]	1.04545774	-0.851339524	-1.283770944	1.230574039	-0.140921316
TRINITY_DN1153_c1_g1_i1_orf1	-	-	-	gamma-butyrobetaine dioxygenase [Ostrinia furnacalis]	1.20122144	-0.660343764	-0.881042553	1.23940959	-0.899244714
TRINITY_DN20527_c0_g1_i1_orf1	-	-	-	dihydrofolate reductase [Ostrinia furnacalis]	1.13099665	-0.594394285	-0.340593049	1.173279844	-1.369289158
TRINITY_DN3732_c0_g1_i2_orf1	-	-	-	cytochrome P450 monooxygenase CYP6AB141 [Ostrinia furnacalis]	0.87240372	-0.260950025	-0.916977962	1.434030203	-1.128505932
TRINITY_DN1416_c0_g2_i1_orf1	-	-	-	uncharacterized protein LOC114352565 [Ostrinia furnacalis]	1.18041851	-0.904040395	-0.836252695	1.263114473	-0.703239898
TRINITY_DN12293_c0_g1_i1_orf1	-	-	-	hypothetical protein evm_011848 [Chilo suppressalis]	1.25536507	-1.202152996	-0.857412369	1.080466452	-0.276266157
TRINITY_DN1753_c1_g1_i8_orf1	-	-	-	serine/threonine-protein kinase WNK1-like isoform X15 [Ostrinia furnacalis]	1.28556176	-1.075700926	-0.717572372	1.133174501	-0.625462963
TRINITY_DN5210_c0_g1_i3_orf1	-	-	-	uncharacterized protein LOC114358376 isoform X2 [Ostrinia furnacalis] >XP_028168126.1 uncharacterized protein LOC114358376 isoform X2 [Ostrinia furnacalis] >XP_028168127.1 uncharacterized protein LOC114358376 isoform X2 [Ostrinia furnacalis] >XP_028168128.1 uncharacterized protein LOC114358376 isoform X2 [Ostrinia furnacalis]	1.38636685	-0.865019554	-1.129824256	0.963064392	-0.354587436
TRINITY_DN14935_c0_g1_i1_orf1	-	-	-	kynurenine/alpha-aminoadipate aminotransferase, mitochondrial [Ostrinia furnacalis]	1.21738682	-1.224801903	-0.207215096	1.095206403	-0.88057622
TRINITY_DN16354_c0_g1_i2_orf1	-	-	-	uncharacterized protein LOC114349750 isoform X1 [Ostrinia furnacalis]	1.54926905	-1.298844323	-0.268259109	0.657240736	-0.639406357
TRINITY_DN135679_c0_g1_i1_orfp1	-	-	-	TRINITY_DN135679_c0_g1_i1_m.85524 TRINITY_DN135679_c0_g1::TRINITY_DN135679_c0_g1_i1::g.85524 ORF type:5prime_partial len:55 (+),score=3.74,Toxin_2 PF00451.20 1.9e-06 TRINITY_DN135679_c0_g1_i1:3-167(+)	0.97951517	-0.628927537	-1.565596133	1.084885703	0.130122796
TRINITY_DN5161_c0_g1_i5_orf1	-	-	-	glyoxylate reductase/hydroxypyruvate reductase-like isoform X1 [Ostrinia furnacalis]	1.27964512	-1.272986898	-0.891032682	0.969925041	-0.085550581
TRINITY_DN36496_c0_g1_i1_orf1	-	-	-	unnamed protein product [Parnassius apollo]	1.1106168	-0.036896101	-1.127718992	1.143234109	-1.089235812
TRINITY_DN6994_c0_g1_i4_orf1	-	-	-	C-type mannose receptor 2-like isoform X1 [Ostrinia furnacalis]	1.60833861	-0.990038602	-0.637184524	0.72599235	-0.707107831

TRINITY_DN3964_c1_g1_i2_orf1	-	-	-	phosphoinositide 3-kinase regulatory subunit 4 isoform X1 [Ostrinia furnacalis] >XP_028172384.1 phosphoinositide 3-kinase regulatory subunit 4 isoform X5 [Ostrinia furnacalis]	1.23653952	-0.998154874	-1.15903609	1.055091501	-0.134440057
TRINITY_DN2749_c0_g2_i3_orf1	-	-	-	RNA exonuclease 4-like [Ostrinia furnacalis] >QEE79882.1 REX4 [Ostrinia furnacalis]	1.21071772	-0.694860372	-0.474876692	1.167992755	-1.208973413
TRINITY_DN25901_c0_g1_i2_orf1	-	-	-	short-chain specific acyl-CoA dehydrogenase, mitochondrial-like isoform X2 [Ostrinia furnacalis]	1.64097534	-1.165171012	-0.544085499	0.604761413	-0.536480241
TRINITY_DN1999_c0_g1_i9_orf1	-	-	-	acyl-CoA Delta(11) desaturase-like [Ostrinia furnacalis] >XP_028172986.1 acyl-CoA Delta(11) desaturase-like [Ostrinia furnacalis] >AAL27034.1 acyl-CoA delta-9 desaturase [Ostrinia furnacalis] >AAL29454.1 acyl-CoA delta-9 desaturase [Ostrinia nubilalis]	1.40037308	-0.323728874	-1.052253022	0.943477196	-0.967868382
TRINITY_DN14597_c0_g1_i5_orf1	-	-	-	UDP-glucuronosyltransferase 2B1-like isoform X3 [Ostrinia furnacalis]	1.57602216	-1.409323626	-0.454922892	0.519258533	-0.231034179
TRINITY_DN1656_c2_g1_i5_orf1	-	-	-	15-hydroxyprostaglandin dehydrogenase [NAD(+)]-like [Ostrinia furnacalis]	1.45586697	-1.328276825	-0.425607318	0.816310362	-0.518293193
TRINITY_DN15513_c0_g1_i6_orf1	-	-	-	uncharacterized protein LOC114350859 [Ostrinia furnacalis]	1.63254133	-1.335141852	-0.246829705	0.469690603	-0.52026038
TRINITY_DN2924_c0_g1_i2_orf1	-	-	-	cuticular protein RR-2 [Spodoptera litura]	1.36973869	-1.552351512	-0.420334897	0.72311726	-0.120169537
TRINITY_DN3949_c1_g1_i1_orf1	-	-	-	probable cytochrome P450 304a1 isoform X2 [Ostrinia furnacalis]	1.59506813	-0.959420309	-0.647300406	0.752888747	-0.741236162
TRINITY_DN2172_c0_g2_i5_orf1	-	-	-	4-hydroxyphenylpyruvate dioxygenase [Ostrinia furnacalis]	1.67312897	-1.383592187	-0.417266398	0.291947514	-0.164217898
TRINITY_DN51934_c0_g2_i1_orf1	-	-	-	SCAN domain-containing protein 3-like [Pieris napi] >XP_047520696.1 SCAN domain-containing protein 3-like [Pieris napi]	1.76621174	-1.190977915	-0.618024173	0.220390526	-0.17760018
TRINITY_DN3836_c0_g1_i4_orf1	-	-	-	2-oxoisovalerate dehydrogenase subunit alpha, mitochondrial [Ostrinia furnacalis]	1.77592757	-1.09053427	-0.738484726	0.261116852	-0.208025425
TRINITY_DN113626_c0_g1_i3_orfp1	-	-	-	TRINITY_DN113626_c0_g1_i3_m.80721 TRINITY_DN113626_c0_g1::TRINITY_DN113626_c0_g1_i3::g.80721 ORF type:internal len:118 (-),score=87.34 TRINITY_DN113626_c0_g1_i3:2-352(-)	1.53333417	-0.992625545	-0.972236965	0.774883017	-0.34335468
TRINITY_DN33452_c0_g1_i1_orf1	-	-	-	lethal(2) giant larvae protein isoform X8 [Ostrinia furnacalis]	1.40147841	-0.790385331	0.148319049	0.645183525	-1.404595658
TRINITY_DN32479_c0_g1_i8_orf1	-	-	-	hypothetical protein evm_009815 [Chilo suppressalis] >CAB3525305.1 unnamed protein product [Chilo suppressalis] >CAH0402632.1 unnamed protein product [Chilo suppressalis]	1.74660393	-1.284638764	-0.540039743	0.085873326	-0.007798745
TRINITY_DN3929_c0_g3_i3_orf1	-	-	-	Glutathione S-transferase 1, isoform D [Papilio machaon]	1.77880623	-1.051743205	-0.728480601	0.316144708	-0.314727132
TRINITY_DN2695_c0_g1_i14_orfp1	-	-	-	TRINITY_DN2695_c0_g1_i14_m.44485 TRINITY_DN2695_c0_g1::TRINITY_DN2695_c0_g1_i14::g.44485 ORF type:3prime_partial len:698 (+),score=187.51 TRINITY_DN2695_c0_g1_i14:101-2092(+)	1.68849855	-1.216419438	-0.51811986	0.470115621	-0.424074876
TRINITY_DN868_c0_g1_i4_orf1	-	-	-	uncharacterized protein LOC114359357 isoform X1 [Ostrinia furnacalis]	1.68483514	-0.878269755	-0.88888616	0.587267445	-0.504946669
TRINITY_DN26411_c0_g1_i2_orfp1	-	-	-	TRINITY_DN26411_c0_g1_i2_m.24123 TRINITY_DN26411_c0_g1::TRINITY_DN26411_c0_g1_i2::g.24123 ORF type:internal len:115 (-),score=72.83 TRINITY_DN26411_c0_g1_i2:3-344(-)	1.55132957	-0.715607498	-0.920538673	0.826721403	-0.741904805
TRINITY_DN578_c0_g1_i3_orf1	-	-	-	charged multivesicular body protein 7 [Ostrinia furnacalis]	-0.3277062	0.879289146	-1.814152019	0.506242019	0.756327101
TRINITY_DN11942_c0_g1_i1_orf1	-	-	-	hypothetical protein B5X24_HaOG213660 [Helicoverpa armigera]	-0.0779124	0.376723172	-1.84236602	0.406801324	1.136753906
TRINITY_DN1492_c0_g1_i4_orf1	-	-	-	sarcoplasmic calcium-binding protein isoform X2 [Ostrinia furnacalis]	-0.0262254	0.970402917	-1.888121537	0.318491912	0.625452132
TRINITY_DN4257_c0_g1_i2_orf1	-	-	-	dynactin subunit 1 [Ostrinia furnacalis]	0.52777206	0.507951229	-1.959839111	0.774690151	0.14942567
TRINITY_DN3209_c0_g1_i1_orf1	-	-	-	coatomer subunit beta [Helicoverpa armigera]	0.29805142	0.550675461	-1.933552843	0.16766533	0.917160635
TRINITY_DN2782_c0_g1_i7_orf1	-	-	-	CDK5 regulatory subunit-associated protein 3 [Ostrinia furnacalis]	0.17231417	0.880456313	-1.902166957	0.0959672	0.753429273
TRINITY_DN1520_c0_g1_i9_orf1	-	-	-	adipocyte plasma membrane-associated protein-like [Ostrinia furnacalis] >XP_028176496.1 adipocyte plasma membrane-associated protein-like [Ostrinia furnacalis]	0.44121024	0.730000058	-1.982555343	0.32577459	0.485570454
TRINITY_DN5475_c0_g1_i3_orf1	-	-	-	traB domain-containing protein-like isoform X1 [Ostrinia furnacalis] >XP_028169655.1 traB domain-containing protein-like isoform X1 [Ostrinia furnacalis]	0.33559961	1.185301204	-1.850515651	0.121779432	0.207835408
TRINITY_DN33024_c0_g1_i1_orf1	-	-	-	hypothetical protein evm_000959 [Chilo suppressalis]	0.70644477	-0.015010016	-1.798844721	1.124533213	-0.017123247
TRINITY_DN84669_c0_g1_i1_orf1	-	-	-	PREDICTED: microtubule-actin cross-linking factor 1, isoforms 1/2/3/5 [Amyelois transitella]	0.86185776	0.395610222	-1.932250685	0.601432249	0.073350451
TRINITY_DN10183_c0_g2_i3_orf1	-	-	-	uncharacterized protein LOC114360370 isoform X1 [Ostrinia furnacalis]	0.35447044	1.160304934	-1.845910999	-0.015857587	0.346993211
TRINITY_DN35865_c0_g1_i1_orf1	-	-	-	uncharacterized protein LOC114354496 isoform X1 [Ostrinia furnacalis] >XP_028162709.1 uncharacterized protein LOC114354496 isoform X1 [Ostrinia furnacalis]	0.58930763	0.759692717	-1.860305786	-0.236347915	0.747653359

TRINITY_DN19303_c0_g1_i5_orf1	-	-	-	lipopolysaccharide-induced tumor necrosis factor-alpha factor-like [Ostrinia furnacalis]	0.15728743	1.187038754	-1.589062392	-0.588663709	0.833399912
TRINITY_DN6588_c0_g1_i4_orf1	-	-	-	uncharacterized protein LOC114350845 [Ostrinia furnacalis]	1.67358396	0.105831392	-1.130668889	0.26684092	-0.915587378
TRINITY_DN5670_c0_g1_i2_orf1	-	-	-	DNA polymerase alpha subunit B [Ostrinia furnacalis]	1.10752473	-0.241433187	-1.800658685	0.601483817	0.33308332
TRINITY_DN1652_c0_g1_i12_orf1	-	-	-	synaptotagmin 1 isoform X1 [Ostrinia furnacalis] >XP_028161222.1 synaptotagmin 1 isoform X1 [Ostrinia furnacalis]	1.66766126	-0.410265645	-1.291566019	0.454041397	-0.419870994
TRINITY_DN2441_c0_g1_i1_orf1	-	-	-	protein RFT1 homolog [Ostrinia furnacalis]	1.71224715	-0.077598389	-1.352308119	0.169950897	-0.452291539
TRINITY_DN103118_c0_g1_i4_orf1	-	-	-	hypothetical protein evm_006930 [Chilo suppressalis]	0.90539904	0.92990873	-1.741851456	0.325500803	-0.418957117
TRINITY_DN8701_c0_g1_i3_orf1	-	-	-	putative uncharacterized protein DDB_G0282133 [Ostrinia furnacalis]	0.78370534	1.310814963	-1.555162354	-0.042097533	-0.497260417
TRINITY_DN143496_c0_g1_i1_orf1	-	-	-	cullin-3 [Diachasma alloeum]	1.20079189	-0.103945734	-1.793724568	0.13984086	0.557037555
TRINITY_DN3411_c0_g2_i1_orf1	-	-	-	putative U5 small nuclear ribonucleoprotein 200 kDa helicase, partial [Ostrinia furnacalis]	1.3568183	0.594727961	-1.553672639	0.196216562	-0.594090184
TRINITY_DN277_c1_g1_i1_orf1	-	-	-	uncharacterized protein LOC114363802 isoform X4 [Ostrinia furnacalis]	0.6983605	1.260862282	-1.695072383	-0.04733789	-0.216812506
TRINITY_DN28376_c0_g1_i15_orfp1	-	-	-	TRINITY_DN28376_c0_g1_i15_m.40022 TRINITY_DN28376_c0_g1::TRINITY_DN28376_c0_g1_i15::g.40022 ORF type:internal len:273 (+),score=43.58 TRINITY_DN28376_c0_g1_i15:3-818(+)	1.32346829	-0.434811274	-1.633761174	0.609833739	0.135270423
TRINITY_DN77572_c0_g1_i1_orf1	-	-	-	steroid receptor RNA activator 1 [Ostrinia furnacalis]	1.01773668	-0.144501391	-1.843059725	0.289742386	0.680082046
TRINITY_DN2224_c0_g2_i1_orf1	-	-	-	tumor susceptibility gene 101 protein [Ostrinia furnacalis]	1.33572084	0.430512438	-1.720605583	-0.208534562	0.162906869
TRINITY_DN11125_c0_g1_i1_orf1	-	-	-	LOW QUALITY PROTEIN: nuclear receptor-binding protein homolog [Ostrinia furnacalis]	1.12811983	0.487387163	-1.85929111	0.082391264	0.161392853
TRINITY_DN1351_c0_g1_i1_orf1	-	-	-	PREDICTED: flavin reductase (NADPH) [Microplitis demolitor] >XP_008553603.1 PREDICTED: flavin reductase (NADPH) [Microplitis	1.5471292	-0.745062143	-1.372195808	0.339050115	0.231078639
TRINITY_DN15339_c0_g1_i6_orf1	-	-	-	nuclear pore glycoprotein p62 isoform X3 [Ostrinia furnacalis]	1.62540925	-0.275951707	-1.285925044	0.527820206	-0.591352709
TRINITY_DN3029_c4_g1_i1_orf1	-	-	-	proliferation marker protein Ki-67-like, partial [Ostrinia furnacalis]	1.36742972	0.07963674	-1.736733174	0.325938891	-0.036272177
TRINITY_DN7329_c0_g1_i6_orf1	-	-	-	serine hydrolase-like protein 2 isoform X2 [Ostrinia furnacalis]	1.69781837	-0.623318311	-0.581716502	0.550186901	-1.042970454
TRINITY_DN6985_c0_g1_i5_orf1	-	-	-	LYR motif-containing protein 4B [Ostrinia furnacalis]	1.60403259	0.13255023	-1.33188694	0.323816338	-0.728512217
TRINITY_DN10672_c0_g1_i3_orf1	-	-	-	neurofilament heavy polypeptide-like isoform X10 [Ostrinia furnacalis]	1.57004662	0.06424399	-1.577524212	0.114151857	-0.17091825
TRINITY_DN1425_c0_g1_i4_orf1	-	-	-	fibulin-2-like [Ostrinia furnacalis]	1.1188549	1.09303771	-1.521013534	-0.32001171	-0.370867369
TRINITY_DN31119_c0_g1_i1_orf1	-	-	-	transforming acidic coiled-coil-containing protein 3-like [Ostrinia furnacalis] >XP_028170476.1 transforming acidic coiled-coil-containing protein 3-like [Ostrinia furnacalis] >XP_028170477.1 transforming acidic coiled-coil-containing protein 3-like [Ostrinia furnacalis] >XP_028170480.1 transforming acidic coiled-coil-containing protein 3-like [Ostrinia	1.54239489	0.190142787	-1.604703556	-0.035461589	-0.092372536
TRINITY_DN31431_c0_g1_i1_orf1	-	-	-	carnosine N-methyltransferase [Ostrinia furnacalis]	1.12375116	0.485848183	-1.808538207	-0.224980947	0.423919815
TRINITY_DN36006_c0_g1_i5_orf1	-	-	-	pro-resilin-like [Ostrinia furnacalis]	1.28383189	0.719377844	-1.606491014	0.097245895	-0.493964619
TRINITY_DN126648_c0_g1_i1_orf1	-	-	-	elongation factor 1 alpha, partial [Spodoptera exigua] >QYQ52647.1 elongation factor 1 alpha, partial [Spodoptera exigua]	1.48813676	-0.845028642	-1.349372673	0.32900603	0.377258523
TRINITY_DN9759_c0_g1_i1_orf1	-	-	-	iroquois-class homeodomain protein IRX-1-like isoform X1 [Ostrinia	1.58184952	-0.05249374	-1.557074151	-0.173400569	0.201118937
TRINITY_DN3450_c0_g1_i3_orf1	-	-	-	hypothetical protein evm_008214 [Chilo suppressalis]	1.55858352	0.453609239	-1.415586629	0.004352802	-0.600958928
TRINITY_DN23783_c0_g2_i1_orf1	-	-	-	cytochrome b5 [Ostrinia furnacalis]	1.60444352	0.422423535	-1.12169698	0.085675499	-0.990845573
TRINITY_DN49872_c0_g2_i1_orf1	-	-	-	NIF3-like protein 1 [Ostrinia furnacalis] >XP_028165862.1 NIF3-like protein 1 [Ostrinia furnacalis] >XP_028165864.1 NIF3-like protein 1 [Ostrinia	1.5054547	0.106556524	-1.561517552	0.350678675	-0.401172348
TRINITY_DN6602_c0_g1_i4_orf1	-	-	-	PREDICTED: E3 ubiquitin-protein ligase RNF181-like [Amyelois transitella]	0.99430192	0.799402759	-1.766807173	-0.367249259	0.340351752
TRINITY_DN73945_c0_g5_i3_orf1	-	-	-	cyclin-dependent kinase 12 isoform X1 [Diachasma alloeum] >XP_015114851.1 cyclin-dependent kinase 12 isoform X1 [Diachasma alloeum] >XP_015114852.1 cyclin-dependent kinase 12 isoform X1 [Diachasma alloeum] >XP_015114853.1 cyclin-dependent kinase 12 isoform X1 [Diachasma alloeum] >XP_015114854.1 cyclin-dependent kinase 12	1.6820554	-0.551636342	-1.27534741	0.41111876	-0.266190406
TRINITY_DN72816_c0_g1_i2_orf1	-	-	-	Golgi apparatus protein 1 [Ostrinia furnacalis]	0.56740432	1.582431051	-1.35651383	-0.494525476	-0.298796065
TRINITY_DN25997_c1_g2_i4_orf1	-	-	-	ribokinase-like [Ostrinia furnacalis]	1.41008355	0.3456329	-0.789457569	0.466115241	-1.432374119
TRINITY_DN140538_c0_g2_i1_orf1	-	-	-	peptidyl-prolyl cis-trans isomerase NIMA-interacting 1 [Urocitellus parryii]	1.52430514	-0.112248804	-1.548108834	-0.29113802	0.427190517
TRINITY_DN95414_c0_g1_i1_orf1	-	-	-	protein arginine N-methyltransferase 5 [Ostrinia furnacalis]	1.61276847	-1.041762137	-1.041762137	-0.00714597	0.477901777
TRINITY_DN1875_c0_g1_i1_orf1	-	-	-	uncharacterized protein LOC114366320 isoform X1 [Ostrinia furnacalis] >XP_028178963.1 uncharacterized protein LOC114366320 isoform X1 [Ostrinia furnacalis] >XP_028178964.1 uncharacterized protein LOC114366320 isoform X2 [Ostrinia furnacalis]	1.49010707	0.26170055	-1.57320216	0.242494454	-0.421099916
TRINITY_DN30097_c0_g1_i2_orf1	-	-	-	unnamed protein product [Chilo suppressalis]	1.77888348	0.22874907	-1.096439307	-0.167558673	-0.743634568

TRINITY_DN143_c0_g3_i1_orf1	-	-	-	Ubiquitin-60S ribosomal protein L40, partial [Cotesia chilonis] >UDP69015.1 egg surface protein ES-53, partial [Cotesia chilonis]	1.76155524	-0.572814733	-1.199091118	0.261039853	-0.250689244
TRINITY_DN1763_c0_g3_i2_orf1	-	-	-	heterogeneous nuclear ribonucleoprotein H-like isoform X2 [Ostrinia	1.22534616	0.840713976	-1.384648054	0.226001707	-0.907413792
TRINITY_DN4132_c0_g1_i14_orf1	-	-	-	thyroid receptor-interacting protein 11-like isoform X1 [Ostrinia furnacalis]	1.38801473	-0.008402425	-1.73238794	0.246925831	0.105849809
TRINITY_DN146718_c0_g1_i1_orf1	-	-	-	40S ribosomal protein S6 [Diachasma alloeum]	1.76300894	-0.534435993	-1.189805732	0.28866552	-0.327432732
TRINITY_DN81312_c0_g1_i1_orf1	-	-	-	atlastin isoform X4 [Ostrinia furnacalis]	1.61405083	-0.829514159	-1.224257738	-0.016001569	0.455722638
TRINITY_DN79803_c0_g1_i7_orf1	-	-	-	dnaJ homolog subfamily C member 22 [Ostrinia furnacalis]	0.87974641	1.291228567	-1.480157675	-0.091150866	-0.599666431
TRINITY_DN3909_c0_g2_i2_orf1	-	-	-	ribosomal protein L24 [Loxostege sticticalis]	1.70979426	-0.747206507	-1.199390402	-0.042395062	0.279197715
TRINITY_DN20984_c0_g1_i4_orf1	-	-	-	NADPH--cytochrome P450 reductase isoform X2 [Ostrinia furnacalis]	1.75683106	-0.66178909	-1.179948521	0.242073875	-0.15716732
TRINITY_DN9931_c0_g1_i1_orf1	-	-	-	syntaxin-18 [Ostrinia furnacalis]	1.82464714	-0.079073214	-1.220213385	-0.125772098	-0.399588442
TRINITY_DN51239_c0_g1_i5_orf1	-	-	-	regulatory-associated protein of mTOR [Ostrinia furnacalis]	1.7842711	-0.484856348	-1.06478027	0.340944291	-0.575578775
TRINITY_DN2577_c0_g1_i1_orf1	-	-	-	unnamed protein product [Diatraea saccharalis]	1.55824661	-0.76773169	-1.326148463	0.067399504	0.46823404
TRINITY_DN146119_c0_g1_i1_orf1	-	-	-	protein SEC13 homolog [Ostrinia furnacalis]	1.41703991	0.780663614	-1.361613733	-0.118839961	-0.717249831
TRINITY_DN1298_c0_g1_i3_orf1	-	-	-	ras GTPase-activating protein-binding protein 2-like, partial [Ostrinia	1.48261707	0.371720565	-1.549770329	-0.480537947	0.175970646
TRINITY_DN6556_c0_g1_i7_orf1	-	-	-	NFX1-type zinc finger-containing protein 1-like isoform X1 [Ostrinia furnacalis] >XP_028173496.1 NFX1-type zinc finger-containing protein 1-like isoform X1 [Ostrinia furnacalis] >XP_028173497.1 NFX1-type zinc finger-containing protein 1-like isoform X1 [Ostrinia furnacalis]	1.80196575	-0.347591096	-0.997883002	0.287572487	-0.74406414
TRINITY_DN78686_c0_g1_i1_orf1	-	-	-	myosin-2 essential light chain isoform X2 [Harpegnathos saltator] >XP_012170910.1 myosin-2 essential light chain isoform X2 [Bombus terrestris] >XP_033185931.1 myosin-2 essential light chain isoform X2 [Bombus vancouverensis nearcticus] >XP_033319091.1 myosin-2 essential light chain isoform X2 [Bombus bifarius] >XP_033349866.1 myosin-2 essential light chain isoform X2 [Bombus vosnesenskii] >XP_043597873.1 myosin-2 essential light chain isoform X2 [Bombus pyrosoma]	0.96003139	1.265543374	-1.477546686	-0.290880154	-0.457147924
TRINITY_DN57496_c0_g1_i1_orf1	-	-	-	NADPH:adrenodoxin oxidoreductase, mitochondrial [Ostrinia furnacalis]	1.57793953	0.302495588	-0.732944008	0.208248186	-1.355739295
TRINITY_DN5112_c0_g1_i1_orf1	-	-	-	unnamed protein product, partial [Iphiclides podalirius]	1.74195264	-0.127087094	-1.375322601	0.001156614	-0.24069956
TRINITY_DN1427_c0_g1_i7_orf1	-	-	-	SAFB-like transcription modulator isoform X1 [Ostrinia furnacalis] >XP_028158609.1 SAFB-like transcription modulator isoform X2 [Ostrinia furnacalis]	1.68154599	0.001107587	-1.451491169	-0.254967722	0.023805311
TRINITY_DN26824_c0_g1_i1_orf1	-	-	-	60S ribosomal protein L6 [Hyposmocoma kahamanoa]	1.48416371	-0.45044481	-1.543722094	0.456530795	0.053472404
TRINITY_DN29521_c0_g1_i1_orf1	-	-	-	density-regulated protein homolog [Ostrinia furnacalis]	1.52672976	-0.779248976	-1.28940092	-0.084338277	0.626258416
TRINITY_DN23004_c0_g1_i1_orf1	-	-	-	uncharacterized protein LOC114365313 [Ostrinia furnacalis]	1.77701206	-0.844535239	-1.053258416	-0.018161707	0.138943305
TRINITY_DN7868_c0_g1_i8_orf1	-	-	-	uncharacterized protein LOC114353432 isoform X4 [Ostrinia furnacalis]	1.69943323	-0.104363626	-1.444893244	-0.107041084	-0.043135276
TRINITY_DN3343_c0_g2_i1_orf1	-	-	-	AFG3-like protein 2 [Ostrinia furnacalis]	1.65844392	0.474427589	-1.268186925	-0.286835494	-0.577849086
TRINITY_DN8958_c0_g1_i1_orf1	-	-	-	nuclear cap-binding protein subunit 1 [Galleria mellonella]	1.38613726	0.660068474	-1.593934638	-0.230066303	-0.222204793
TRINITY_DN4309_c0_g1_i1_orf1	-	-	-	NEDD8-conjugating enzyme Ubc12, partial [Cotesia chilonis]	1.88897397	-0.218568156	-1.005267894	-0.05667267	-0.608465252
TRINITY_DN43293_c0_g1_i2_orf1	-	-	-	egl nine homolog 1 isoform X2 [Helicoverpa armigera]	1.3277747	0.481529432	-1.525954696	0.422445193	-0.705794634
TRINITY_DN22572_c0_g1_i1_orf1	-	-	-	eukaryotic translation elongation factor 1 epsilon-1 [Ostrinia furnacalis]	1.60220474	0.191303966	-1.525898995	-0.006983012	-0.260626694
TRINITY_DN9309_c0_g1_i5_orf1	-	-	-	uncharacterized protein LOC114361160 [Ostrinia furnacalis]	1.73158411	0.376451737	-1.109603204	-0.24402287	-0.754409778
TRINITY_DN18933_c0_g1_i3_orf1	-	-	-	PREDICTED: protein BUD31 homolog [Papilio xuthus] >XP_014361644.1 protein BUD31 homolog [Papilio machaon] >XP_026750578.1 protein BUD31 homolog [Galleria mellonella] >XP_047995610.1 protein BUD31 homolog [Leguminivora glycinivorella] >XP_049869593.1 protein BUD31 homolog [Pectinophora gossypiella] >KAI5652084.1 g10 protein domain-containing protein [Phthorimaea operculella] >CAB3251981.1 unnamed protein product [Arctia plantaginis] >CAB3520382.1 unnamed protein product [Chilo suppressalis] >CAG9747228.1 unnamed protein product [Diatraea saccharalis] >CAH2037008.1 unnamed protein product, partial	1.17208894	1.059560484	-1.47872928	-0.505793837	-0.247126305
TRINITY_DN3649_c0_g1_i6_orf1	-	-	-	unnamed protein product [Chilo suppressalis]	1.18087748	0.792963573	-1.663954214	0.127880375	-0.437767213
TRINITY_DN12134_c0_g1_i4_orf1	-	-	-	glutathione S-transferase 1-1 [Ostrinia furnacalis] >XP_028161942.1 glutathione S-transferase 1-1 [Ostrinia furnacalis] >XP_028161943.1 glutathione S-transferase 1-1 [Ostrinia furnacalis]	0.74393216	1.484997876	-1.364447398	-0.377631688	-0.486850954
TRINITY_DN3702_c0_g1_i1_orf1	-	-	-	U4/U6.U5 tri-snRNP-associated protein 2 [Ostrinia furnacalis]	1.22194432	0.360000578	-1.794667529	-0.152283875	0.36500651
TRINITY_DN3747_c1_g1_i3_orf1	-	-	-	unnamed protein product, partial [Iphiclides podalirius]	1.87287263	-0.481703495	-1.059606137	0.037415056	-0.368978054
TRINITY_DN8824_c0_g2_i1_orf1	-	-	-	60S ribosomal protein L34-like [Ostrinia furnacalis]	1.76330459	-0.700048475	-1.167318781	-0.075721166	0.179783833

TRINITY_DN97589_c0_g1_i3_orf1	-	-	-	ribosomal protein L37A [Bombyx mori] >XP_013189707.1 PREDICTED: 60S ribosomal protein L37a [Amyelois transitella] >XP_021198447.1 60S ribosomal protein L37a [Helicoverpa armigera] >XP_022122377.1 60S ribosomal protein L37a [Pieris rapae] >XP_022822835.1 60S ribosomal protein L37a [Spodoptera litura] >XP_023937141.1 60S ribosomal protein L37a [Bicyclus anynana] >XP_026321523.1 60S ribosomal protein L37a [Hyposmocoma kahamanoa] >XP_026495655.1 60S ribosomal protein L37a [Vanessa tameamea] >XP_026746489.1 60S ribosomal protein L37a [Trichoplusia ni] >XP_026756267.1 60S ribosomal protein L37a [Galleria mellonella] >XP_028041705.1 60S ribosomal protein L37a [Bombyx mandarina] >XP_028161757.1 60S ribosomal protein L37a [Ostrinia furnacalis] >XP_030020263.1 LOW QUALITY PROTEIN: 60S ribosomal protein L37a [Manduca sexta] >XP_032518929.1 60S ribosomal protein L37a [Danaus plexippus plexippus] >XP_034834514.1 60S ribosomal protein L37a [Maniola hyperantus] >XP_035444256.1 60S ribosomal protein L37a [Spodoptera frugiperda] >XP_038222439.1 60S ribosomal protein L37a [Zerene cesonia] >XP_039756348.1 60S ribosomal protein L37a [Pararge aegeria] >XP_041981914.1 60S ribosomal protein L37a [Aricia agestis] >XP_045451710.1 60S ribosomal protein L37a [Melitaea cinxia] >XP_045500579.1 60S ribosomal protein L37a [Colias croceus] >XP_045517305.1 60S ribosomal protein L37a [Pieris brassicae] >XP_045775103.1 60S ribosomal protein L37a [Maniola jurtina] >XP_046969745.1 60S ribosomal protein L37a [Vanessa cardui] >XP_047032252.1 60S ribosomal protein L37a [Helicoverpa zea] >XP_047525321.1 60S ribosomal protein L37a [Pieris napi] >XP_047535357.1 60S ribosomal protein L37a [Vanessa atalanta] >XP_049875744.1 60S ribosomal protein L37a [Pectinophora gossypiella] >XP_050348149.1 60S ribosomal protein L37a [Nymphalis io] >ADO95156.1 ribosomal protein L37A [Antheraea yamamai] >ADT80705.1 ribosomal protein L37A [Euphydryas aurinia] >AFL28885.1 ribosomal protein L37A	1.65906443	-1.175431526	-0.873510068	0.079085225	0.310791938
TRINITY_DN24318_c0_g1_i1_orf1	-	-	-	60S ribosomal protein L29 [Ostrinia furnacalis]	1.63854083	-0.784241314	-1.212931168	-0.107598063	0.466229713
TRINITY_DN2168_c0_g1_i2_orf1	-	-	-	protein arginine methyltransferase NDUFAF7 homolog, mitochondrial [Ostrinia furnacalis]	1.90435758	-0.656310406	-0.930495466	-0.043803033	-0.273748676
TRINITY_DN15737_c0_g1_i7_orf1	-	-	-	UPF0160 protein C27H6.8 [Ostrinia furnacalis]	1.65987323	-0.762827968	-1.222840979	-0.07637485	0.402170564
TRINITY_DN883_c0_g1_i8_orf1	-	-	-	diacylglycerol O-acyltransferase 1 isoform X1 [Ostrinia furnacalis]	1.7875657	-0.605172449	-1.091541045	0.302989066	-0.393841273
TRINITY_DN21619_c0_g1_i1_orf1	-	-	-	28S ribosomal protein S15, mitochondrial [Ostrinia furnacalis]	1.85975108	-0.421404662	-0.833143048	0.190592571	-0.795795939
TRINITY_DN147596_c0_g1_i1_orf1	-	-	-	activator of basal transcription 1 [Diachasma alloeum]	1.77480041	-0.862840145	-0.978278229	0.303679814	-0.237361855
TRINITY_DN43369_c0_g2_i1_orf1	-	-	-	cytochrome P450 monooxygenase 304 [Glyphodes pyloalis]	1.35977978	0.325363353	-0.760206458	0.547350919	-1.472287593
TRINITY_DN1882_c0_g1_i4_orf1	-	-	-	zinc transporter ZIP13 homolog [Ostrinia furnacalis]	1.26847451	-0.239019784	-1.706660573	0.028887114	0.64831873
TRINITY_DN131371_c0_g1_i1_orf1	-	-	-	golgin subfamily B member 1-like [Ostrinia furnacalis]	1.44940932	-0.152031766	-1.662772831	0.033485345	0.33190993
TRINITY_DN79734_c0_g2_i3_orf1	-	-	-	60S ribosomal protein L27a [Ostrinia furnacalis]	1.83283318	-0.70563191	-1.068067742	-0.041588675	-0.017544854
TRINITY_DN4497_c0_g1_i4_orf1	-	-	-	cytochrome P450 9e2-like [Ostrinia furnacalis] >QPF77612.1 cytochrome P450 monooxygenase CYP9A185 [Ostrinia furnacalis]	1.80482539	-0.558348841	-0.873042189	0.36051339	-0.733947753
TRINITY_DN19493_c0_g1_i5_orf1	-	-	-	zinc finger MYM-type protein 3 isoform X1 [Ostrinia furnacalis] >XP_028159738.1 zinc finger MYM-type protein 3 isoform X2 [Ostrinia furnacalis]	1.29325577	1.066145182	-1.255058862	-0.498016289	-0.606325805
TRINITY_DN1791_c0_g1_i3_orf1	-	-	-	succinate dehydrogenase assembly factor 2-B, mitochondrial-like [Ostrinia furnacalis]	1.70393072	-0.892522272	-1.086463545	-0.06470177	0.339756862
TRINITY_DN4156_c0_g1_i2_orf1	-	-	-	calcium channel flower [Ostrinia furnacalis]	0.86119411	1.399018047	-1.360853341	-0.599259238	-0.300099579
TRINITY_DN108354_c0_g1_i1_orf1	-	-	-	WD repeat-containing protein 61-like [Ostrinia furnacalis]	1.83227307	-0.0559155	-1.159722284	-0.07965466	-0.53698063
TRINITY_DN19187_c0_g1_i1_orf1	-	-	-	fumarylacetoacetase [Chelonus insularis]	1.80232842	-0.125554298	-1.287855996	-0.223152713	-0.165765416
TRINITY_DN37165_c0_g1_i4_orf1	-	-	-	pyridoxine-5'-phosphate oxidase-like [Ostrinia furnacalis]	1.88839597	-0.244618359	-1.016355104	-0.045089493	-0.582333018
TRINITY_DN11820_c0_g1_i1_orf1	-	-	-	hypothetical protein evm_000341 [Chilo suppressalis]	1.22439901	0.890003628	-1.592130097	-0.398026833	-0.124245711
TRINITY_DN81926_c0_g1_i1_orf1	-	-	-	membrane-bound alkaline phosphatase-like isoform X3 [Ostrinia furnacalis]	1.87205966	-0.82759592	-0.725611518	0.181991664	-0.500843883

TRINITY_DN42646_c0_g2_i1_orf1	-	-	-	40S ribosomal protein S3 [Helicoverpa armigera] >XP_026740562.1 40S ribosomal protein S3 [Trichoplusia ni] >XP_026751545.1 40S ribosomal protein S3 [Galleria mellonella] >XP_047027704.1 40S ribosomal protein S3 [Helicoverpa zea] >CAH0591481.1 unnamed protein product [Chrysodeixis includens] >AIR07416.1 ribosomal protein S3 [Helicoverpa armigera] >AND95944.1 ribosomal protein S3 [Helicoverpa armigera] >AXY94820.1 ribosomal ribosomal protein S3 [Galleria mellonella] >PZC80336.1 hypothetical protein B5X24_HaOG214853 [Helicoverpa armigera]	1.80977245	-0.922568419	-0.902056188	-0.165454688	0.180306841
TRINITY_DN1266_c2_g1_i1_orf1	-	-	-	serine/threonine-protein kinase RIO3 [Ostrinia furnacalis]	1.6267596	0.365607854	-0.947289923	0.100571328	-1.14564886
TRINITY_DN5841_c0_g1_i2_orf1	-	-	-	hypothetical protein evm_011295 [Chilo suppressalis]	1.46850523	0.749304574	-1.393504334	-0.397304082	-0.427001387
TRINITY_DN33_c0_g1_i1_orf1	-	-	-	uncharacterized protein CG45076-like isoform X2 [Ostrinia furnacalis]	1.80666851	-0.752479655	-1.074750684	-0.074639132	0.095200959
TRINITY_DN3534_c0_g1_i2_orf1	-	-	-	guanine nucleotide-binding protein subunit beta-like protein [Ostrinia furnacalis]	1.92426264	-0.633865075	-0.904415614	-0.154456966	-0.231524981
TRINITY_DN8949_c0_g1_i2_orf1	-	-	-	unnamed protein product [Arctia plantaginis]	1.81594153	-0.618419679	-1.118999512	-0.21908146	0.14055912
TRINITY_DN4262_c0_g1_i16_orf1	-	-	-	sperm-associated antigen 7 homolog [Ostrinia furnacalis]	1.39662458	0.789592459	-1.421095946	-0.621060742	-0.144060356
TRINITY_DN21357_c0_g1_i5_orf1	-	-	-	40S ribosomal protein S11 isoform X2 [Ostrinia furnacalis]	1.82676589	-0.785241636	-1.001322032	-0.166504722	0.1263025
TRINITY_DN31327_c0_g2_i1_orf1	-	-	-	multidrug resistance protein 1A isoform X1 [Ostrinia furnacalis]	1.90275069	-0.344589505	-1.06201018	-0.177899459	-0.318251546
TRINITY_DN5686_c0_g1_i4_orf1	-	-	-	FACT complex subunit spt16 isoform X2 [Ostrinia furnacalis]	1.89080423	-0.244113723	-1.05937003	-0.105870017	-0.481450458
TRINITY_DN4194_c0_g1_i1_orf1	-	-	-	hornerin-like [Ostrinia furnacalis]	1.06502523	1.06502523	-1.298137559	0.175753724	-1.007666624
TRINITY_DN4116_c0_g1_i3_orf1	-	-	-	transmembrane protein 131 homolog [Ostrinia furnacalis]	1.41140059	0.495054417	-1.489640526	-0.686410086	0.269595603
TRINITY_DN21609_c0_g2_i1_orf1	-	-	-	translation initiation factor eIF-2B subunit epsilon [Ostrinia furnacalis]	1.77610407	0.063881117	-1.269491989	-0.468320868	-0.102172331
TRINITY_DN12323_c0_g2_i2_orf1	-	-	-	polyribonucleotide nucleotidyltransferase 1, mitochondrial [Ostrinia	1.92852559	-0.487160195	-0.858761521	-0.03026649	-0.552337384
TRINITY_DN24142_c0_g1_i1_orf1	-	-	-	arylalkylamine N-acetyltransferase [Chilo suppressalis]	1.81164696	-0.8302248	-1.012519627	-0.023068345	0.054165814
TRINITY_DN38424_c0_g1_i1_orf1	-	-	-	glucose dehydrogenase [FAD, quinone]-like [Ostrinia furnacalis]	1.00216876	0.802291962	-1.705343644	-0.518000823	0.418883747
TRINITY_DN30131_c0_g1_i1_orf1	-	-	-	PREDICTED: 60S ribosomal protein L44 [Amyelois transitella] >XP_021198018.1 60S ribosomal protein L44 [Helicoverpa armigera] >XP_022814294.1 60S ribosomal protein L44 [Spodoptera litura] >XP_026732397.1 60S ribosomal protein L44 [Trichoplusia ni] >XP_026752106.1 60S ribosomal protein L44 [Galleria mellonella] >XP_028158932.1 60S ribosomal protein L44 [Ostrinia furnacalis] >XP_035434364.1 60S ribosomal protein L44 [Spodoptera frugiperda] >XP_035434370.1 60S ribosomal protein L44 [Spodoptera frugiperda] >XP_047019234.1 60S ribosomal protein L44 [Helicoverpa zea] >XP_049868501.1 60S ribosomal protein L44 [Pectinophora gossypiella] >AAM53948.1 ribosomal protein L44 [Choristoneura parallela] >KAF9418375.1 hypothetical protein HW555_004805 [Spodoptera exigua] >RVE50750.1 hypothetical protein evm_004660 [Chilo suppressalis] >CAB3235328.1 unnamed protein product [Arctia plantaginis] >CAB3516516.1 unnamed protein product [Spodoptera littoralis] >CAG9747186.1 unnamed protein product [Diatraea saccharalis]	1.82558752	-0.998221826	-0.813053707	-0.076526964	0.062214981
TRINITY_DN42082_c0_g2_i2_orfp1	-	-	-	TRINITY_DN42082_c0_g2_i2_m.7835 TRINITY_DN42082_c0_g2::TRINITY_DN42082_c0_g2_i2::g.7835 ORF type:internal len:133 (+),score=75.81 TRINITY_DN42082_c0_g2_i2:1-396(+)	1.80352344	-0.622427556	-1.101104021	-0.308564114	0.228572255
TRINITY_DN4025_c0_g1_i13_orf1	-	-	-	tetratricopeptide repeat protein 14 homolog isoform X2 [Ostrinia furnacalis]	1.6011542	0.094006689	-1.523857278	-0.29855379	0.127250181
TRINITY_DN16965_c0_g2_i1_orf1	-	-	-	hypothetical protein evm_007405 [Chilo suppressalis]	1.6203353	0.57413975	-1.270465695	-0.417860873	-0.506148479
TRINITY_DN2062_c0_g1_i11_orf1	-	-	-	uncharacterized protein LOC114350846 [Ostrinia furnacalis]	1.89974488	-0.200804415	-1.054955871	-0.198791228	-0.445193368
TRINITY_DN64472_c0_g2_i1_orf1	-	-	-	repressed by EFG1 protein 1-like isoform X3 [Ostrinia furnacalis]	1.81303756	-0.857257851	-0.938102595	0.212307415	-0.229984526
TRINITY_DN23926_c0_g1_i4_orf1	-	-	-	programmed cell death protein 10 [Ostrinia furnacalis]	1.46054402	0.602301811	-1.538273043	-0.253027959	-0.271544827
TRINITY_DN129226_c0_g1_i2_orf1	-	-	-	hypothetical protein evm_000268 [Chilo suppressalis]	1.36996672	0.909602379	-1.361723783	-0.358167897	-0.559677414
TRINITY_DN47389_c0_g1_i2_orf1	-	-	-	non-specific lipid-transfer protein-like [Ostrinia furnacalis]	1.89455276	-0.875497741	-0.56477211	0.106039836	-0.560322741
TRINITY_DN14937_c0_g1_i7_orf1	-	-	-	multidrug resistance protein homolog 49-like [Ostrinia furnacalis] >XP_028159925.1 multidrug resistance protein homolog 49-like [Ostrinia furnacalis]	1.89399277	-0.41107477	-0.676139752	0.076818321	-0.88359657
TRINITY_DN27087_c0_g1_i1_orf1	-	-	-	2',5'-phosphodiesterase 12 [Ostrinia furnacalis]	1.90818828	-0.66891628	-0.901852503	-0.025349025	-0.312070473

TRINITY_DN14063_c0_g1_i7_orf1	-	-	-	probable phosphorylase b kinase regulatory subunit beta isoform X1 [Ostrinia furnacalis] >XP_028175664.1 probable phosphorylase b kinase regulatory subunit beta isoform X2 [Ostrinia furnacalis] >XP_028175665.1 probable phosphorylase b kinase regulatory subunit beta isoform X3	1.93882808	-0.737772018	-0.680459298	-0.038822911	-0.481773854
TRINITY_DN10662_c0_g1_i4_orf1	-	-	-	HD domain-containing protein 2 [Ostrinia furnacalis]	1.77121226	0.329432522	-1.134654066	-0.471323341	-0.494667372
TRINITY_DN3647_c2_g1_i3_orf1	-	-	-	unnamed protein product, partial [Iphiclides podalirius]	1.54416515	0.761163198	-1.134165526	-0.406637354	-0.76452547
TRINITY_DN25783_c0_g1_i2_orf1	-	-	-	SET and MYND domain-containing protein 4 [Ostrinia furnacalis]	1.58294526	0.378170369	-1.470008081	-0.432308154	-0.058799396
TRINITY_DN620_c0_g1_i4_orf1	-	-	-	lysine--tRNA ligase isoform X1 [Ostrinia furnacalis]	1.92092469	-0.637540066	-0.894394988	-0.312799794	-0.076189843
TRINITY_DN69236_c0_g1_i1_orf1	-	-	-	peroxiredoxin [Ostrinia furnacalis]	1.46662288	0.754851319	-1.371639777	-0.28943318	-0.560401247
TRINITY_DN100821_c0_g1_i1_orf1	-	-	-	putative GMP synthase, partial [Operophtera brumata]	1.86537039	-0.45413107	-1.06273248	-0.423280982	0.074774143
TRINITY_DN4036_c0_g2_i1_orf1	-	-	-	microvitellogenin-like [Ostrinia furnacalis]	1.65041923	-0.357044665	-1.454402003	0.181472573	-0.020445133
TRINITY_DN8536_c0_g1_i2_orf1	-	-	-	PC4 and SFRS1-interacting protein isoform X4 [Galleria mellonella]	1.52606495	0.716479893	-1.294508176	-0.60177621	-0.346260458
TRINITY_DN1239_c0_g1_i3_orf1	-	-	-	uncharacterized protein LOC114355269 [Ostrinia furnacalis] >XP_028163822.1 uncharacterized protein LOC114355269 [Ostrinia	1.89950888	-0.653178145	-0.959791162	-0.104716104	-0.181823473
TRINITY_DN2062_c0_g1_i9_orf1	-	-	-	uncharacterized protein LOC114350846 [Ostrinia furnacalis]	1.79554165	0.17204021	-1.206868072	-0.36363734	-0.397076445
TRINITY_DN19814_c0_g1_i4_orf1	-	-	-	general odorant-binding protein 28a-like [Ostrinia furnacalis]	1.59116619	-1.30763613	-0.752359837	0.031520947	0.437308829
TRINITY_DN5578_c0_g1_i4_orf1	-	-	-	chromatin modification-related protein eaf-1-like [Ostrinia furnacalis]	1.74918675	0.318668898	-1.175594779	-0.617460549	-0.274800324
TRINITY_DN76377_c0_g1_i1_orf1	-	-	-	uncharacterized protein LOC111357764, partial [Spodoptera litura]	1.19673056	1.111839432	-1.313403272	-0.261529862	-0.733636854
TRINITY_DN6231_c0_g1_i6_orf1	-	-	-	ran-binding protein 3 isoform X1 [Ostrinia furnacalis] >XP_028166372.1 ran-binding protein 3 isoform X2 [Ostrinia furnacalis]	1.83660811	0.06153481	-1.133933358	-0.532556004	-0.231653556
TRINITY_DN1706_c0_g1_i7_orf1	-	-	-	LOW QUALITY PROTEIN: RNA polymerase-associated protein CTR9 homolog [Ostrinia furnacalis]	1.72481661	0.3981267	-1.175265792	-0.608387616	-0.339289898
TRINITY_DN39673_c0_g1_i1_orf1	-	-	-	uncharacterized protein LOC114359357 isoform X1 [Ostrinia furnacalis]	1.92667184	-0.641990288	-0.464727079	-0.007702342	-0.812252128
TRINITY_DN430_c0_g1_i5_orf1	-	-	-	hypothetical protein NE865_02252 [Phthorimaea operculella]	1.89012499	-0.545046073	-1.031177388	-0.251180297	-0.062721233
TRINITY_DN30233_c0_g1_i2_orf1	-	-	-	39S ribosomal protein L10, mitochondrial [Ostrinia furnacalis]	1.91141186	-0.207492114	-1.017547496	-0.215719692	-0.470652559
TRINITY_DN8833_c0_g1_i1_orf1	-	-	-	nucleolar protein 16 [Ostrinia furnacalis]	1.97215995	-0.467327013	-0.720256508	-0.211123847	-0.573452586
TRINITY_DN24873_c0_g1_i4_orf1	-	-	-	uncharacterized protein LOC114365742 [Ostrinia furnacalis]	1.94186612	-0.745739085	-0.760162704	-0.209339668	-0.226624668
TRINITY_DN753_c0_g1_i4_orf1	-	-	-	venom dipeptidyl peptidase 4-like isoform X2 [Ostrinia furnacalis]	1.93818965	-0.291497819	-0.953996134	-0.281433981	-0.411261714
TRINITY_DN42759_c0_g2_i1_orf1	-	-	-	fatty acid synthase-like [Ostrinia furnacalis]	1.93056039	-0.475317669	-0.534032187	-0.049810367	-0.871400166
TRINITY_DN279_c0_g1_i10_orf1	-	-	-	RE1-silencing transcription factor-like isoform X1 [Ostrinia furnacalis]	1.10248479	1.254131998	-1.247656986	-0.696142056	-0.41281775
TRINITY_DN3759_c0_g1_i1_orf1	-	-	-	uncharacterized protein LOC114350416 [Ostrinia furnacalis] >XP_028157016.1 uncharacterized protein LOC114350416 [Ostrinia furnacalis] >XP_028157017.1 uncharacterized protein LOC114350416 [Ostrinia furnacalis] >XP_028157018.1 uncharacterized protein LOC114350416 [Ostrinia furnacalis] >XP_028157019.1 uncharacterized	1.50071995	0.852299936	-1.122805999	-0.570284917	-0.659928972
TRINITY_DN2425_c0_g1_i1_orf1	-	-	-	thyroid receptor-interacting protein 11 [Ostrinia furnacalis]	1.69329722	-0.740732993	-1.241270001	0.115783375	0.1729224
TRINITY_DN747_c0_g1_i4_orf1	-	-	-	trypsin, alkaline C-like [Ostrinia furnacalis]	1.95185667	-0.698816527	-0.639784349	-0.077995369	-0.535260423
TRINITY_DN3733_c0_g1_i1_orf1	-	-	-	60S ribosomal protein L37, partial [Papilio machaon]	1.81846207	-0.206507947	-1.243173078	-0.048245131	-0.320535915
TRINITY_DN48020_c0_g1_i1_orf1	-	-	-	aminopeptidase N4 [Cnaphalocrocis medinalis]	1.94389021	-0.645252508	-0.520959297	-0.048876268	-0.728802141
TRINITY_DN9464_c0_g1_i1_orf1	-	-	-	angio-associated migratory cell protein [Ostrinia furnacalis] >XP_028162594.1 angio-associated migratory cell protein [Ostrinia	1.9653555	-0.632843178	-0.754617717	-0.304084859	-0.273809745
TRINITY_DN3062_c0_g1_i1_orf1	-	-	-	HEAT repeat-containing protein 1 [Ostrinia furnacalis]	1.91580079	-0.068795117	-0.956019658	-0.529376612	-0.361609407
TRINITY_DN50787_c0_g2_i2_orf1	-	-	-	40S ribosomal protein S29 [Hyposmocoma kahamanoa] >XP_028176503.1 40S ribosomal protein S29 [Ostrinia furnacalis] >XP_049877832.1 40S ribosomal protein S29 [Pectinophora gossypiella] >ADT80654.1 ribosomal protein S29 [Euphydryas aurinia] >CAB3523209.1 unnamed protein product [Chilo suppressalis] >CAH0400531.1 unnamed protein product [Chilo	1.80174242	-0.433102485	-1.226130662	-0.233420685	0.090911415
TRINITY_DN15900_c0_g1_i6_orf1	-	-	-	unnamed protein product [Diatraea saccharalis]	1.86898988	0.130274371	-0.983072526	-0.448300356	-0.567891371
TRINITY_DN2894_c0_g2_i3_orf1	-	-	-	myrosinase 1-like isoform X1 [Ostrinia furnacalis]	1.21583933	0.935226335	-1.388948804	-0.843387637	0.081270777
TRINITY_DN28592_c0_g1_i2_orf1	-	-	-	UDP-glucuronosyltransferase 2B14-like isoform X1 [Ostrinia furnacalis] >XP_028167291.1 UDP-glucuronosyltransferase 2B14-like isoform X2 [Ostrinia furnacalis]	1.94236487	-0.160998651	-0.901828123	-0.415166833	-0.464371267
TRINITY_DN13783_c0_g4_i2_orf1	-	-	-	hypothetical protein evm_010131 [Chilo suppressalis]	1.97724266	-0.502264088	-0.734376063	-0.259197704	-0.481404808
TRINITY_DN109733_c0_g1_i1_orf1	-	-	-	uncharacterized protein LOC112452128 [Temnothorax curvispinosus]	1.95164564	-0.442233553	-0.683320108	-0.106967226	-0.719124755
TRINITY_DN22664_c0_g1_i1_orf1	-	-	-	larval cuticle protein LCP-14-like [Ostrinia furnacalis]	1.6282264	-1.253496285	-0.75535729	-0.06913073	0.449757908
TRINITY_DN5238_c0_g1_i2_orf1	-	-	-	DNA-(apurinic or apyrimidinic site) lyase [Ostrinia furnacalis]	1.73569822	0.335259728	-1.173527464	-0.666769306	-0.230661181
TRINITY_DN657_c0_g1_i2_orf1	-	-	-	cytochrome c-type heme lyase [Ostrinia furnacalis]	1.95734106	-0.644005051	-0.729416306	-0.131422947	-0.452496759

TRINITY_DN11069_c0_g2_i1_orf1	-	-	-	fat storage-inducing transmembrane protein [Ostrinia furnacalis]	1.92311962	-0.244951404	-1.004243767	-0.391812622	-0.282111823
TRINITY_DN47114_c0_g1_i5_orf1	-	-	-	nucleolar protein dao-5 isoform X2 [Ostrinia furnacalis]	1.94890696	-0.465647616	-0.884250713	-0.408198769	-0.190809862
TRINITY_DN64403_c0_g2_i1_orf1	-	-	-	carboxylesterase [Ostrinia furnacalis]	1.90027123	-0.809457468	-0.562021718	0.108477224	-0.637269273
TRINITY_DN3235_c0_g1_i1_orf1	-	-	-	SPARC [Trichoplusia ni]	1.42772679	0.984989369	-0.976359642	-0.660933312	-0.775423209
TRINITY_DN16931_c0_g1_i1_orf1	-	-	-	pancreatic triacylglycerol lipase-like [Ostrinia furnacalis]	1.89796627	-0.685022484	-0.36042788	0.040201193	-0.892717103
TRINITY_DN13651_c0_g1_i2_orf1	-	-	-	40S ribosomal protein S12, mitochondrial [Ostrinia furnacalis]	1.92569532	-0.065558629	-0.906601797	-0.550450486	-0.403084409
TRINITY_DN19244_c0_g1_i7_orf1	-	-	-	uncharacterized protein LOC114350218 [Ostrinia furnacalis]	1.73855308	0.326024238	-1.18005187	-0.651339662	-0.233185787
TRINITY_DN2343_c1_g1_i8_orf1	-	-	-	receptor expression-enhancing protein 5-like isoform X3 [Ostrinia	1.96638018	-0.625185283	-0.46783749	-0.169991753	-0.703365652
TRINITY_DN2986_c1_g1_i1_orf1	-	-	-	Troponin C, isoform 1 [Papilio xuthus]	1.64357581	-1.248610172	-0.709993312	-0.147379149	0.46240682
TRINITY_DN3194_c0_g1_i6_orf1	-	-	-	uncharacterized protein LOC114361386 [Ostrinia furnacalis]	1.97415194	-0.305610964	-0.617604744	-0.330972702	-0.719963534
TRINITY_DN6140_c0_g3_i3_orf1	-	-	-	CD63 antigen-like [Ostrinia furnacalis]	1.84374897	-0.263378131	-0.571994753	0.08557145	-1.093947539
TRINITY_DN64446_c0_g1_i1_orf1	-	-	-	uncharacterized protein LOC114364307 [Ostrinia furnacalis]	1.94820437	-0.208273085	-0.868905584	-0.319792859	-0.551232847
TRINITY_DN75188_c0_g1_i1_orf1	-	-	-	fatty acid-binding protein 1-like [Ostrinia furnacalis]	1.94334287	-0.157195228	-0.886089496	-0.515225761	-0.384832386
TRINITY_DN35051_c0_g1_i1_orf1	-	-	-	uncharacterized protein LOC114364307 [Ostrinia furnacalis]	1.98253139	-0.495930595	-0.511627545	-0.279432583	-0.695540668
TRINITY_DN3862_c0_g1_i7_orf1	-	-	-	venom acid phosphatase Acph-1-like [Ostrinia furnacalis]	1.98688866	-0.452186259	-0.713992213	-0.385519987	-0.435190199
TRINITY_DN41086_c0_g1_i4_orf1	-	-	-	collagenase-like [Pectinophora gossypiella]	1.96440927	-0.522772657	-0.456906767	-0.197242274	-0.787487569
TRINITY_DN2490_c0_g2_i1_orfp1	-	-	-	TRINITY_DN2490_c0_g2_i1_m.56872 TRINITY_DN2490_c0_g2::TRINITY_DN2490_c0_g2_i1::g.56872 ORF type:internal len:359 (-),score=123.59 TRINITY_DN2490_c0_g2_i1:2-1075(-)	1.96023103	-0.224246135	-0.581869289	-0.350513465	-0.803602144
