# Supplementary material for: Comparative Proteomics and Metabonomics Analysis of Different Diapause Stages Revealed a New Regulation Mechanism of Diapause in Loxostege sticticalis (Lepidoptera: Pyralidae)
Source: Molecules. 2024 Jul 25;29(15):3472. doi: 10.3390/molecules29153472 (PMC11314584; doi:10.3390/molecules29153472)
Supplement: Supplementary file 1 [file molecules-29-03472-s001.zip › analysis process/proteomic/Cluster analysis of expression patterns/Down/CTvsND down.pdf]

| Accession | Symbol | Protein Na | Entrez ID | Description | ND | RD | CT | D | PreD |
|---|---|---|---|---|---|---|---|---|---|
| TRINITY_DN77642_c0_g1_i1_orf1 | - | - | - | peritrophic membrane chitin binding protein [Loxostege sticticalis] | 0.15185 | 1.49569 | -0.9903 | -1.207 | 0.54978 |
| TRINITY_DN12951_c1_g1_i5_orf1 | - | - | - | ADP-ribosylation factor-like protein 2 isoform X1 [Hyposmocoma kahamanoa] | 0.79668 | 1.10213 | -1.7679 | 0.02531 | -0.1562 |
| TRINITY_DN14843_c0_g1_i1_orf1 | - | - | - | myotubularin-related protein 13 [Ostrinia furnacalis] | 1.55298 | 0.30754 | -1.3177 | -0.8237 | 0.28088 |
| TRINITY_DN2874_c0_g1_i4_orf1 | - | - | - | ATP-binding cassette sub-family G member 1-like [Ostrinia furnacalis] | 1.45112 | 0.9309 | -0.8608 | -0.506 | -1.0152 |
| TRINITY_DN11159_c0_g2_i1_orf1 | - | - | - | sphingosine-1-phosphate lyase isoform X1 [Ostrinia furnacalis] | 1.96068 | -0.2186 | -0.4814 | -0.8322 | -0.4284 |
| TRINITY_DN12787_c1_g1_i1_orf1 | - | - | - | unnamed protein product [Parnassius apollo] | 1.91648 | -0.0272 | -0.7392 | -0.8186 | -0.3315 |
| TRINITY_DN6130_c0_g1_i6_orf1 | - | - | - | tryptophan--tRNA ligase, mitochondrial [Ostrinia furnacalis] | 0.98977 | 1.39087 | -1.1021 | -0.4752 | -0.8033 |
| TRINITY_DN12113_c0_g1_i1_orf1 | - | - | - | WD repeat-containing protein 5 [Helicoverpa armigera] >XP_022823790.1 WD repeat-containing protein 5 [Spodoptera litura] >XP_028176937.1 WD repeat-containing protein 5 [Ostrinia furnacalis] >XP_035429818.1 WD repeat-containing protein 5 [Spodoptera frugiperda] >XP_047030799.1 WD repeat-containing protein 5 [Helicoverpa zea] >CAB3513875.1 unnamed protein product [Spodoptera littoralis] >KAF9795358.1 hypothetical protein SFRURICE_004730 [Spodoptera frugiperda] >KAG8117399.1 hypothetical protein SFRUCORN_019548 [Spodoptera frugiperda] >PZC79830.1 hypothetical protein B5X24_HaOG215819 [Helicoverpa armigera] >CAH1643659.1 unnamed | 1.78547 | -0.7568 | 0.3877 | -0.5008 | -0.9156 |
| TRINITY_DN33485_c0_g1_i4_orf1 | - | - | - | luciferin 4-monooxygenase-like [Ostrinia furnacalis] | 1.28525 | 0.82107 | -0.5651 | -1.5345 | -0.0067 |
| TRINITY_DN747_c0_g1_i1_orf1 | - | - | - | trypsin, alkaline C-like [Ostrinia furnacalis] | 1.62949 | -0.8449 | 0.65242 | -1.0124 | -0.4246 |
| TRINITY_DN91198_c0_g2_i1_orf1 | - | - | - | unc-112-related protein-like [Pectinophora gossypiella] | 1.59061 | -0.1842 | -1.4473 | -0.3923 | 0.4331 |
| TRINITY_DN56121_c0_g1_i4_orf1 | - | - | - | uncharacterized protein LOC114361939 [Ostrinia furnacalis] | 1.88028 | -0.0744 | -0.1296 | -0.9748 | -0.7014 |
| TRINITY_DN11392_c0_g1_i4_orf1 | - | - | - | chondroitin sulfate synthase 1 isoform X1 [Ostrinia furnacalis] >XP_028178735.1 chondroitin sulfate synthase 1 isoform X2 [Ostrinia furnacalis] | 1.93474 | -0.0908 | -0.5861 | -0.3902 | -0.8676 |
| TRINITY_DN28938_c0_g1_i1_orf1 | - | - | - | uncharacterized protein LOC114354070 isoform X3 [Ostrinia furnacalis] | 1.48486 | 0.25385 | -1.5021 | -0.5908 | 0.35416 |
| TRINITY_DN12769_c0_g1_i5_orf1 | - | - | - | uncharacterized protein LOC114365633 [Ostrinia furnacalis] | 0.88953 | 1.39876 | -1.3353 | -0.3895 | -0.5635 |
| TRINITY_DN51441_c0_g1_i5_orf1 | - | - | - | uncharacterized protein LOC114353529 isoform X2 [Ostrinia furnacalis] | 1.23561 | 1.1438 | -1.2143 | -0.6591 | -0.506 |
| TRINITY_DN38720_c0_g1_i3_orf1 | - | - | - | transmembrane protein 214 [Ostrinia furnacalis] | 1.07989 | -0.2228 | -1.5183 | -0.4633 | 1.12447 |
| TRINITY_DN56379_c0_g1_i1_orf1 | - | - | - | alpha-tocopherol transfer protein-like [Ostrinia furnacalis] | 1.69748 | 0.12299 | -0.3107 | -0.0964 | -1.4134 |
| TRINITY_DN9555_c0_g1_i1_orf1 | - | - | - | pyridoxal kinase [Ostrinia furnacalis] | 1.83055 | -0.0063 | -0.2791 | -0.3382 | -1.207 |
| TRINITY_DN96884_c0_g1_i1_orf1 | - | - | - | hypothetical protein evm_003360 [Chilo suppressalis] | 1.96581 | -0.6671 | -0.72 | -0.2406 | -0.3382 |
| TRINITY_DN2441_c0_g1_i1_orf1 | - | - | - | protein RFT1 homolog [Ostrinia furnacalis] | 1.71225 | -0.0776 | -1.3523 | 0.16995 | -0.4523 |
| TRINITY_DN29707_c0_g1_i2_orf1 | - | - | - | 2-(3-amino-3-carboxypropyl)histidine synthase subunit 2 [Ostrinia furnacalis] | 1.93788 | -0.7059 | -0.7389 | -0.445 | -0.0481 |
| TRINITY_DN18035_c0_g1_i7_orf1 | - | - | - | mitochondrial ornithine transporter 1 [Ostrinia furnacalis] | 1.94937 | -0.8114 | -0.1988 | -0.6443 | -0.2949 |
| TRINITY_DN3971_c0_g1_i1_orf1 | - | - | - | L-asparaginase-like isoform X1 [Ostrinia furnacalis] | 1.9426 | -0.5835 | -0.1128 | -0.4035 | -0.8428 |
| TRINITY_DN4766_c0_g1_i4_orf1 | - | - | - | 26S proteasome non-ATPase regulatory subunit 12 [Ostrinia furnacalis] | 0.91402 | 0.96354 | -1.4936 | -0.8744 | 0.4905 |
| TRINITY_DN139438_c0_g1_i1_orf1 | - | - | - | T-complex protein 1 subunit delta [Ostrinia furnacalis] | 1.89516 | -0.175 | -0.9384 | -0.0814 | -0.7004 |
| TRINITY_DN294_c0_g1_i2_orf1 | - | - | - | chloride intracellular channel isoform 1 [Phthorimaea operculella] | 1.64711 | 0.07714 | -0.7527 | -1.274 | 0.30245 |
| TRINITY_DN107_c0_g1_i1_orf1 | - | - | - | dynein light chain 2, cytoplasmic isoform X1 [Pectinophora gossypiella] | 1.76695 | 0.40492 | -0.8896 | -0.8652 | -0.4171 |
| TRINITY_DN12823_c0_g1_i1_orf1 | - | - | - | malectin-A [Ostrinia furnacalis] | 1.37886 | 0.99931 | -0.9842 | -0.9798 | -0.4142 |
| TRINITY_DN20215_c0_g2_i1_orf1 | - | - | - | unnamed protein product [Spodoptera littoralis] >CAH1638553.1 unnamed protein product [Spodoptera littoralis] | 1.0028 | 1.36626 | -0.467 | -1.1754 | -0.7266 |
| TRINITY_DN57150_c0_g2_i1_orf1 | - | - | - | vacuolar protein-sorting-associated protein 25-like isoform X2 [Ostrinia | 1.72776 | -0.6168 | -1.2594 | -0.0623 | 0.21076 |
| TRINITY_DN13598_c1_g1_i1_orf1 | - | - | - | transport and Golgi organization protein 1-like [Ostrinia furnacalis] | 1.65337 | -0.9768 | -1.0191 | -0.1565 | 0.49905 |
| TRINITY_DN27723_c0_g1_i2_orf1 | - | - | - | putative uncharacterized protein DDB_G0282133 isoform X1 [Ostrinia furnacalis] | 1.92026 | -0.5852 | -0.3544 | -0.0639 | -0.9168 |
| TRINITY_DN11188_c0_g1_i2_orf1 | - | - | - | testin [Ostrinia furnacalis] >XP_028170617.1 testin [Ostrinia furnacalis] | 1.23278 | 0.94847 | -1.3885 | -0.8078 | 0.01505 |
| TRINITY_DN24024_c0_g1_i1_orf1 | - | - | - | dolichyl-diphosphooligosaccharide--protein glycosyltransferase subunit 1 [Ostrinia furnacalis] | 1.36536 | 0.61045 | -1.2298 | -1.0703 | 0.32431 |
| TRINITY_DN8640_c0_g1_i4_orf1 | - | - | - | glutathione S-transferase E14-like isoform X1 [Ostrinia furnacalis] | 1.96318 | -0.282 | -0.2738 | -0.6801 | -0.7272 |
| TRINITY_DN8553_c0_g1_i4_orf1 | - | - | - | coiled-coil-helix-coiled-coil-helix domain-containing protein 7 isoform X2 [Ostrinia furnacalis] | 1.35655 | 1.0587 | -1.0115 | -0.5783 | -0.8254 |

| | | | | | | | | | |
|---|---|---|---|---|---|---|---|---|---|
| TRINITY_DN56270_c0_g1_i1_orf1 | - | - | - | PREDICTED: putative elongator complex protein 1 [Microplitis demolitor] >XP_008554512.1 PREDICTED: putative elongator complex protein 1 [Microplitis demolitor] | 1.20812 | -1.2095 | 0.43416 | -1.1638 | 0.73109 |
| TRINITY_DN70409_c0_g1_i3_orf1 | - | - | - | tyrosine-protein phosphatase non-receptor type 9 isoform X3 [Ostrinia | 1.29361 | 0.53072 | -1.5626 | -0.6642 | 0.40246 |
| TRINITY_DN3664_c0_g1_i8_orf1 | - | - | - | hypothetical protein evm_011332 [Chilo suppressalis] >CAB3530638.1 unnamed protein product [Chilo suppressalis] >CAH0407229.1 unnamed protein product [Chilo suppressalis] | 1.76256 | 0.01865 | -1.0936 | -0.8237 | 0.13613 |
| TRINITY_DN471_c0_g1_i6_orf1 | - | - | - | ATP-dependent RNA helicase DBP2-A-like [Ostrinia furnacalis] | 1.08344 | 1.23758 | -0.4577 | -1.3494 | -0.5139 |
| TRINITY_DN19651_c0_g1_i1_orf1 | - | - | - | cytosolic non-specific dipeptidase [Ostrinia furnacalis] | 0.86787 | 1.37145 | -0.0968 | -1.2465 | -0.8959 |
| TRINITY_DN1273_c0_g1_i4_orf1 | - | - | - | hypothetical protein evm_006077 [Chilo suppressalis] >CAB3531025.1 unnamed protein product [Chilo suppressalis] >CAH0407617.1 unnamed protein product [Chilo suppressalis] | 1.72695 | 0.32766 | -1.159 | -0.7359 | -0.1597 |
| TRINITY_DN69697_c0_g1_i1_orf1 | - | - | - | PREDICTED: uncharacterized protein LOC103573287 [Microplitis demolitor] | 1.74578 | -1.0809 | 0.06254 | -0.8714 | 0.14394 |
| TRINITY_DN1652_c0_g1_i12_orf1 | - | - | - | synaptotagmin 1 isoform X1 [Ostrinia furnacalis] >XP_028161222.1 synaptotagmin 1 isoform X1 [Ostrinia furnacalis] | 1.66766 | -0.4103 | -1.2916 | 0.45404 | -0.4199 |
| TRINITY_DN8405_c0_g1_i4_orf1 | - | - | - | clathrin heavy chain isoform X1 [Ostrinia furnacalis] >XP_028169033.1 clathrin heavy chain isoform X2 [Ostrinia furnacalis] >XP_028169034.1 clathrin heavy chain isoform X3 [Ostrinia furnacalis] >XP_028169036.1 clathrin heavy chain isoform X5 [Ostrinia furnacalis] | 0.21104 | 1.09792 | -1.6654 | -0.4974 | 0.85385 |
| TRINITY_DN68725_c0_g1_i1_orf1 | - | - | - | hydroxymethylglutaryl-CoA synthase 1 [Ostrinia furnacalis] | 1.54231 | 0.80387 | -0.9627 | -0.9054 | -0.4781 |
| TRINITY_DN6503_c0_g1_i8_orf1 | - | - | - | uncharacterized protein LOC114354432 [Ostrinia furnacalis] | 1.80705 | -0.3407 | -0.6682 | -1.0523 | 0.25421 |
| TRINITY_DN629_c0_g1_i6_orf1 | - | - | - | annexin B9-like isoform X1 [Ostrinia furnacalis] | 0.65815 | 1.05934 | -0.8442 | -1.5207 | 0.64748 |
| TRINITY_DN6262_c0_g1_i2_orf1 | - | - | - | ADAMTS-like protein 4 isoform X2 [Ostrinia furnacalis] >XP_028169930.1 ADAMTS-like protein 4 isoform X2 [Ostrinia furnacalis] | 1.37985 | 0.42798 | -1.359 | -0.9191 | 0.47029 |
| TRINITY_DN2876_c0_g1_i5_orf1 | - | - | - | long-chain fatty acid transport protein 4-like isoform X1 [Ostrinia furnacalis] | 1.83157 | -0.667 | 0.1344 | -1.061 | -0.2379 |
| TRINITY_DN12576_c0_g1_i2_orf1 | - | - | - | eukaryotic translation initiation factor 4E transporter-like isoform X5 [Hyposmocoma kahamanoa] | 1.09421 | 1.32888 | -1.0258 | -0.6342 | -0.7631 |
| TRINITY_DN2795_c0_g1_i1_orf1 | - | - | - | hypothetical protein B5X24_HaOG209451 [Helicoverpa armigera] | 1.45232 | 0.59433 | -1.4652 | 0.04226 | -0.6237 |
| TRINITY_DN1601_c0_g1_i4_orf1 | - | - | - | cytoplasmic dynein 1 intermediate chain isoform X4 [Ostrinia furnacalis] | 0.95213 | 1.43349 | -0.568 | -0.7299 | -1.0878 |
| TRINITY_DN5834_c0_g1_i2_orf1 | - | - | - | CD2 antigen cytoplasmic tail-binding protein 2 homolog [Ostrinia furnacalis] | 1.49226 | 0.84169 | -0.7929 | -0.418 | -1.1231 |
| TRINITY_DN48536_c0_g1_i3_orf1 | - | - | - | unnamed protein product [Chilo suppressalis] | 1.90466 | -0.0084 | -0.3175 | -0.6771 | -0.9016 |
| TRINITY_DN11876_c0_g1_i2_orf1 | - | - | - | protein TAPT1 homolog [Ostrinia furnacalis] | 1.56643 | 0.80148 | -0.713 | -0.942 | -0.713 |
| TRINITY_DN5243_c1_g1_i1_orf1 | - | - | - | 14-3-3 protein epsilon [Maniola hyperantus] >XP_045770909.1 14-3-3 protein epsilon [Maniola jurtina] | 1.07721 | 0.46717 | -1.8871 | 0.19804 | 0.14471 |
| TRINITY_DN40211_c0_g1_i1_orf1 | - | - | - | rho GTPase-activating protein 44-like [Ostrinia furnacalis] | 1.63112 | 0.61594 | -0.6191 | -1.1687 | -0.4593 |
| TRINITY_DN892_c0_g1_i9_orf1 | - | - | - | calcyphosin-like protein isoform X2 [Ostrinia furnacalis] | 0.42712 | 1.216 | -1.7311 | -0.3674 | 0.45532 |
| TRINITY_DN1866_c0_g1_i4_orf1 | - | - | - | unnamed protein product [Chrysodeixis includens] | 1.81416 | -0.5419 | -0.0753 | -0.0098 | -1.1872 |
| TRINITY_DN40823_c0_g1_i1_orf1 | - | - | - | nucleolar protein 10 [Ostrinia furnacalis] | 1.83914 | 0.19955 | -0.9929 | -0.673 | -0.3728 |
| TRINITY_DN5008_c0_g1_i1_orf1 | - | - | - | LOW QUALITY PROTEIN: integrator complex subunit 14 [Ostrinia furnacalis] | 1.41345 | -0.4067 | -1.5048 | -0.2245 | 0.7225 |
| TRINITY_DN3562_c0_g1_i4_orf1 | - | - | - | peroxisomal membrane protein PEX14-like isoform X2 [Ostrinia furnacalis] | 1.82952 | -0.2603 | 0.17958 | -0.7656 | -0.9832 |
| TRINITY_DN2177_c0_g1_i1_orf1 | - | - | - | uncharacterized protein LOC114349824 isoform X1 [Ostrinia furnacalis] >XP_028156186.1 uncharacterized protein LOC114349824 isoform X1 [Ostrinia furnacalis] | 1.76414 | -1.053 | 0.3613 | -0.3446 | -0.7278 |
| TRINITY_DN1198_c0_g1_i1_orf1 | - | - | - | protein suppressor of sable isoform X1 [Ostrinia furnacalis] | 1.79112 | -0.6999 | -0.9871 | -0.4535 | 0.34937 |
| TRINITY_DN11464_c0_g1_i3_orf1 | - | - | - | unnamed protein product [Spodoptera littoralis] >CAH1635924.1 unnamed protein product [Spodoptera littoralis] | 1.13619 | 1.29919 | -0.9595 | -0.8136 | -0.6623 |
| TRINITY_DN6163_c0_g1_i4_orf1 | - | - | - | CTD nuclear envelope phosphatase 1 homolog [Ostrinia furnacalis] | 1.10781 | 1.18014 | -1.3741 | -0.2642 | -0.6497 |
| TRINITY_DN41108_c0_g1_i1_orf1 | - | - | - | coatomer subunit epsilon-like [Ostrinia furnacalis] | 1.21245 | 1.04343 | -1.0222 | -0.0532 | -1.1805 |

| | | | | | | | | | |
|---|---|---|---|---|---|---|---|---|---|
| TRINITY_DN18863_c0_g1_i3_orf1 | - | - | - | splicing factor 3A subunit 1 isoform X1 [Ostrinia furnacalis] >XP_028161155.1 splicing factor 3A subunit 1 isoform X2 [Ostrinia furnacalis] >XP_028161156.1 splicing factor 3A subunit 1 isoform X3 [Ostrinia furnacalis] >XP_028161157.1 splicing factor 3A subunit 1 isoform X4 [Ostrinia furnacalis] | 1.79571 | 0.13139 | -0.9893 | -0.881 | -0.0568 |
| TRINITY_DN1639_c0_g2_i2_orf1 | - | - | - | longitudinals lacking protein-like [Plutella xylostella] >XP_021192239.1 longitudinals lacking protein-like [Helicoverpa armigera] >XP_022818289.1 longitudinals lacking protein-like [Spodoptera litura] >XP_022818290.1 longitudinals lacking protein-like [Spodoptera litura] >XP_023945541.1 longitudinals lacking protein-like [Bicyclus anynana] >XP_026320461.1 longitudinals lacking protein-like [Hyposmocoma kahamanoa] >XP_026320471.1 longitudinals lacking protein-like [Hyposmocoma kahamanoa] >XP_026499391.1 longitudinals lacking protein-like [Vanessa tameamea] >XP_026499392.1 longitudinals lacking protein-like [Vanessa tameamea] >XP_026728238.1 longitudinals lacking protein-like isoform X2 [Trichoplusia ni] >XP_026750598.1 longitudinals lacking protein-like [Galleria mellonella] >XP_028032853.1 longitudinals lacking protein-like [Bombyx mandarina] >XP_028169299.1 longitudinals lacking protein-like [Ostrinia furnacalis] >XP_030036777.1 longitudinals lacking protein-like [Manduca sexta] >XP_034826408.1 longitudinals lacking protein-like [Maniola hyperantus] >XP_034826409.1 longitudinals lacking protein-like [Maniola hyperantus] >XP_035452035.1 longitudinals lacking protein-like [Spodoptera frugiperda] >XP_035452036.1 longitudinals lacking protein-like [Spodoptera frugiperda] >XP_035452270.1 longitudinals lacking protein-like [Spodoptera frugiperda] >XP_035452271.1 longitudinals lacking protein-like [Spodoptera frugiperda] >XP_037874172.1 longitudinals lacking protein-like [Bombyx mori] >XP_038218308.1 longitudinals lacking protein-like [Zerene cesonia] >XP_039758692.1 longitudinals lacking protein-like [Pararge aegeria] >XP_041981066.1 longitudinals lacking protein-like [Aricia agestis] >XP_041981074.1 longitudinals lacking protein-like [Aricia agestis] >XP_045455814.1 longitudinals lacking protein-like [Melitaea cinxia] >XP_045505524.1 longitudinals lacking protein-like [Colias croceus] >XP_045524236.1 longitudinals lacking protein-like [Pieris brassicae] >XP_045762005.1 longitudinals lacking protein-like [Maniola jurtina] >XP_045762006.1 longitudinals lacking protein-like [Maniola jurtina] | 1.73196 | 0.492 | -0.9203 | -0.4764 | -0.8273 |
| TRINITY_DN10785_c0_g1_i4_orf1 | - | - | - | alkylglycerol monooxygenase-like [Ostrinia furnacalis] >XP_028171363.1 alkylglycerol monooxygenase-like [Ostrinia furnacalis] | 1.76129 | -1.162 | -0.6231 | -0.2702 | 0.29397 |
| TRINITY_DN1816_c0_g1_i5_orf1 | - | - | - | nuclear pore complex protein Nup205 [Ostrinia furnacalis] | 1.89934 | -0.4302 | -0.8643 | -0.6748 | 0.07006 |
| TRINITY_DN43637_c0_g1_i1_orf1 | - | - | - | sarcolemmal membrane-associated protein [Ostrinia furnacalis] | 1.22684 | 1.01843 | -1.4596 | -0.2964 | -0.4893 |
| TRINITY_DN11973_c0_g1_i1_orf1 | - | - | - | nuclear pore complex protein Nup93-like [Ostrinia furnacalis] | 1.8962 | -0.3811 | -1.0267 | -0.4514 | -0.037 |
| TRINITY_DN2930_c0_g1_i8_orf1 | - | - | - | hypothetical protein evm_000171 [Chilo suppressalis] >CAB3523059.1 unnamed protein product [Chilo suppressalis] >CAH0400381.1 unnamed protein product [Chilo suppressalis] | 1.71533 | 0.54411 | -0.5556 | -0.8256 | -0.8782 |
| TRINITY_DN46_c0_g1_i2_orf1 | - | - | - | disks large 1 tumor suppressor protein isoform X12 [Ostrinia furnacalis] | 1.48818 | 0.82754 | -0.5314 | -1.2285 | -0.5558 |
| TRINITY_DN21559_c0_g1_i2_orf1 | - | - | - | protein bicaudal D [Ostrinia furnacalis] | 1.3571 | 0.89701 | -1.4131 | -0.4604 | -0.3806 |
| TRINITY_DN6169_c0_g1_i15_orf1 | - | - | - | cullin-2 isoform X1 [Ostrinia furnacalis] >XP_028165424.1 cullin-2 isoform X2 [Ostrinia furnacalis] | 1.48258 | 0.08065 | -1.455 | -0.634 | 0.52578 |
| TRINITY_DN33867_c0_g1_i9_orf1 | - | - | - | uncharacterized protein LOC114357513 [Ostrinia furnacalis] | 1.82335 | -0.9078 | 0.21427 | -0.854 | -0.2758 |
| TRINITY_DN38667_c0_g1_i9_orf1 | - | - | - | signal transducer and activator of transcription 5B-like [Melitaea cinxia] | 1.3957 | 0.8085 | -0.9025 | -1.2577 | -0.044 |
| TRINITY_DN1498_c0_g1_i2_orf1 | - | - | - | ran GTPase-activating protein 1 [Ostrinia furnacalis] | 1.82728 | 0.27922 | -0.5632 | -0.5779 | -0.9653 |
| TRINITY_DN60821_c0_g1_i1_orf1 | - | - | - | nucleolar GTP-binding protein 2 [Ostrinia furnacalis] | 1.36182 | 0.44893 | 0.2656 | -0.4391 | -1.6372 |
| TRINITY_DN54477_c0_g1_i1_orf1 | - | - | - | rho-associated protein kinase 2 isoform X11 [Ostrinia furnacalis] >XP_028155846.1 rho-associated protein kinase 2 isoform X12 [Ostrinia | 1.01972 | 0.01567 | -1.8092 | -0.0532 | 0.827 |

| | | | | | | | | |
|---|---|---|---|---|---|---|---|---|
| TRINITY_DN16385_c0_g1_i4_orf1 | - | - | - | cytosolic Fe-S cluster assembly factor NUBP1 homolog [Ostrinia furnacalis] | 1.62205 | -0.1579 | -1.5093 | 0.20301 | -0.1579 |
| TRINITY_DN41997_c0_g1_i2_orf1 | - | - | - | 39S ribosomal protein L23, mitochondrial [Ostrinia furnacalis] | 1.98878 | -0.3348 | -0.6565 | -0.5445 | -0.453 |
| TRINITY_DN57765_c0_g1_i1_orf1 | - | - | - | cytochrome P450 6B6-like [Ostrinia furnacalis] | 1.70765 | -0.5832 | 0.00472 | 0.17915 | -1.3083 |
| TRINITY_DN39490_c0_g1_i1_orf1 | - | - | - | unnamed protein product [Parnassius apollo] | 1.78242 | -0.5464 | -1.1136 | -0.4333 | 0.31085 |
| TRINITY_DN44407_c0_g4_i2_orf1 | - | - | - | eukaryotic translation initiation factor 5A [Antheraea pernyi] | 1.47109 | 0.53299 | -1.1934 | -1.0374 | 0.22673 |
| TRINITY_DN3244_c0_g1_i4_orf1 | - | - | - | protein ABHD16A isoform X1 [Ostrinia furnacalis] >XP_028156316.1 protein ABHD16A isoform X2 [Ostrinia furnacalis] | 1.60451 | 0.58429 | -0.4873 | -0.404 | -1.2975 |
| TRINITY_DN4159_c1_g1_i1_orf1 | - | - | - | F-actin-capping protein subunit beta [Ostrinia furnacalis] | 1.21434 | 1.02992 | -1.144 | -1.0748 | -0.0254 |
| TRINITY_DN4770_c0_g1_i4_orf1 | - | - | - | transportin-3 isoform X1 [Ostrinia furnacalis] | 1.64401 | 0.37129 | -1.3229 | -0.6374 | -0.055 |
| TRINITY_DN7836_c0_g1_i2_orf1 | - | - | - | uncharacterized protein LOC114353624 [Ostrinia furnacalis] | 1.92739 | -0.7067 | -0.6054 | 0.03142 | -0.6467 |
| TRINITY_DN121047_c0_g1_i3_orf1 | - | - | - | unnamed protein product [Parnassius apollo] | 1.67143 | 0.5392 | -0.336 | -1.0883 | -0.7864 |
| TRINITY_DN2542_c0_g2_i1_orf1 | - | - | - | peroxiredoxin-2-like [Ostrinia furnacalis] | 1.38369 | 0.61003 | -0.0555 | -0.3242 | -1.614 |
| TRINITY_DN6299_c0_g1_i1_orf1 | - | - | - | death-inducer obliterator 1 isoform X2 [Ostrinia furnacalis] | 1.78244 | -0.6637 | -0.7818 | 0.42921 | -0.7661 |
| TRINITY_DN1354_c0_g1_i6_orf1 | - | - | - | elongator complex protein 2 isoform X1 [Pectinophora gossypiella] | 1.90908 | -0.5886 | -0.5496 | 0.06719 | -0.8381 |
| TRINITY_DN4002_c0_g1_i1_orf1 | - | - | - | activating signal cointegrator 1 complex subunit 3 [Ostrinia furnacalis] | 1.68077 | -0.7777 | -0.0374 | 0.33986 | -1.2055 |
| TRINITY_DN145227_c0_g1_i1_orf1 | - | - | - | 26S protease regulatory subunit 7, partial [Cotesia chilonis] | 1.26518 | 0.58158 | -1.3474 | -0.9983 | 0.49895 |
| TRINITY_DN24266_c0_g2_i2_orf1 | - | - | - | chromobox-like protein 5 [Helicoverpa armigera] | 0.72452 | 1.21237 | -1.3264 | -1.0331 | 0.42261 |
| TRINITY_DN1921_c1_g1_i5_orf1 | - | - | - | hypothetical protein evm_002627 [Chilo suppressalis] >CAB3527269.1 unnamed protein product [Chilo suppressalis] | 1.22575 | 1.21824 | -0.7254 | -0.787 | -0.9316 |
| TRINITY_DN1552_c0_g1_i3_orf1 | - | - | - | casein kinase II subunit alpha isoform X3 [Galleria mellonella] | 1.19655 | 1.18128 | -0.9361 | -1.0795 | -0.3622 |
| TRINITY_DN32359_c0_g2_i1_orf1 | - | - | - | PREDICTED: 26S proteasome non-ATPase regulatory subunit 4 isoform X2 [Fopius arisanus] | 0.66803 | 1.13558 | -1.4814 | -0.8745 | 0.55225 |
| TRINITY_DN131662_c0_g1_i4_orf1 | - | - | - | splicing factor 3A subunit 1 isoform X1 [Ostrinia furnacalis] >XP_028161155.1 splicing factor 3A subunit 1 isoform X2 [Ostrinia furnacalis] >XP_028161156.1 splicing factor 3A subunit 1 isoform X3 [Ostrinia furnacalis] >XP_028161157.1 splicing factor 3A subunit 1 isoform X4 [Ostrinia furnacalis] | 1.82389 | 0.07239 | -0.9891 | -0.8267 | -0.0805 |
| TRINITY_DN5670_c0_g1_i2_orf1 | - | - | - | DNA polymerase alpha subunit B [Ostrinia furnacalis] | 1.10752 | -0.2414 | -1.8007 | 0.60148 | 0.33308 |
| TRINITY_DN24539_c0_g1_i4_orf1 | - | - | - | low molecular weight phosphotyrosine protein phosphatase 1-like isoform X2 [Ostrinia furnacalis] | 1.06588 | 1.25319 | -0.7428 | -1.2878 | -0.2885 |
| TRINITY_DN14183_c0_g1_i3_orf1 | - | - | - | multiple epidermal growth factor-like domains protein 6 [Ostrinia furnacalis] | 1.90114 | -0.1768 | -0.469 | -0.2114 | -1.0439 |
| TRINITY_DN972_c0_g1_i6_orf1 | - | - | - | DNA damage-binding protein 1 [Ostrinia furnacalis] | 1.51473 | 0.75729 | -1.1105 | -0.9157 | -0.2458 |
| TRINITY_DN119797_c0_g1_i1_orf1 | - | - | - | hypothetical protein evm_013979 [Chilo suppressalis] | 1.51008 | -0.6703 | -1.3072 | -0.2419 | 0.70933 |
| TRINITY_DN2999_c1_g2_i1_orf1 | - | - | - | uncharacterized protein C05D11.1-like [Ostrinia furnacalis] | 1.55302 | 0.09574 | -1.372 | -0.7121 | 0.43533 |
| TRINITY_DN2808_c0_g1_i8_orf1 | - | - | - | uncharacterized protein LOC114353011 isoform X2 [Ostrinia furnacalis] | 0.63079 | 1.39915 | -1.5968 | -0.2391 | -0.194 |
| TRINITY_DN2615_c0_g1_i1_orf1 | - | - | - | 14 kDa phosphohistidine phosphatase-like [Ostrinia furnacalis] | 1.44498 | 0.53247 | -1.5476 | -0.4806 | 0.05072 |
| TRINITY_DN3482_c0_g2_i1_orf1 | - | - | - | transcription elongation factor B polypeptide 3-like isoform X2 [Ostrinia | 0.77094 | 0.97949 | -1.6658 | -0.6201 | 0.53547 |
| TRINITY_DN8659_c0_g1_i1_orf1 | - | - | - | ubiquitin-like modifier-activating enzyme 1 [Manduca sexta] | 0.89739 | 1.47845 | -0.9652 | -0.9082 | -0.5024 |
| TRINITY_DN1641_c0_g1_i6_orf1 | - | - | - | rhodanese domain-containing protein CG4456-like [Ostrinia furnacalis] | 1.54613 | -0.2432 | -0.1086 | 0.35812 | -1.5525 |
| TRINITY_DN5562_c1_g2_i1_orf1 | - | - | - | cell division cycle and apoptosis regulator protein 1-like [Ostrinia furnacalis] | 1.9064 | 0.02615 | -0.3674 | -0.732 | -0.8332 |
| TRINITY_DN81084_c0_g3_i1_orf1 | - | - | - | unnamed protein product [Diatraea saccharalis] | 1.51509 | -0.2158 | -1.3381 | -0.6389 | 0.67767 |
| TRINITY_DN429_c0_g1_i12_orf1 | - | - | - | hypothetical protein SFRUCORN_009336 [Spodoptera frugiperda] | 1.53469 | 0.81796 | -0.8787 | -0.4948 | -0.9791 |
| TRINITY_DN30178_c0_g1_i3_orf1 | - | - | - | LOW QUALITY PROTEIN: fibrillin-2-like [Bicyclus anynana] | 0.60576 | 1.47091 | -1.5203 | -0.2376 | -0.3188 |
| TRINITY_DN20323_c0_g1_i1_orf1 | - | - | - | cuticle protein 7 [Ostrinia furnacalis] | 1.22029 | -0.027 | -1.7806 | 0.00469 | 0.58267 |
| TRINITY_DN13018_c0_g1_i1_orf1 | - | - | - | uncharacterized protein LOC114354768 isoform X2 [Ostrinia furnacalis] | 1.49702 | 0.58632 | -0.0841 | -0.547 | -1.4522 |
| TRINITY_DN25686_c0_g1_i4_orf1 | - | - | - | exocyst complex component 3 [Ostrinia furnacalis] | 1.1787 | 1.26353 | -0.8595 | -0.6843 | -0.8984 |
| TRINITY_DN84_c0_g1_i4_orf1 | - | - | - | aspartyl/asparaginyl beta-hydroxylase isoform X2 [Ostrinia furnacalis] | 1.88754 | -0.8975 | -0.7258 | 0.055 | -0.3193 |
| TRINITY_DN21961_c0_g2_i5_orf1 | - | - | - | hypothetical protein HW555_000844 [Spodoptera exigua] >KAH9635498.1 hypothetical protein HF086_014991 [Spodoptera exigua] >CAH0692121.1 unnamed protein product [Spodoptera exigua] | 0.98821 | -0.42 | -1.7111 | 0.93606 | 0.20689 |
| TRINITY_DN16539_c0_g1_i7_orf1 | - | - | - | dolichyl-diphosphooligosaccharide--protein glycosyltransferase 48 kDa subunit [Ostrinia furnacalis] | 1.55919 | -0.3222 | -1.5502 | 0.23703 | 0.07616 |

| | | | | | | | | | |
|---|---|---|---|---|---|---|---|---|---|
| TRINITY_DN8076_c0_g1_i6_orf1 | - | - | - | hypothetical protein evm_001812 [Chilo suppressalis] | 1.4213 | -0.8683 | -1.1771 | -0.2561 | 0.8802 |
| TRINITY_DN2160_c0_g1_i13_orf1 | - | - | - | unnamed protein product [Spodoptera exigua] | 1.32306 | 1.08311 | -0.9994 | -0.4935 | -0.9132 |
| TRINITY_DN14286_c0_g1_i5_orf1 | - | - | - | insulin-like growth factor 2 mRNA-binding protein 1 isoform X1 [Galleria mellonella] | 1.89124 | -0.224 | -0.7264 | -0.0217 | -0.9192 |
| TRINITY_DN766_c0_g1_i1_orf1 | - | - | - | nucleolar complex protein 3 homolog [Ostrinia furnacalis] | 1.76746 | -0.1383 | -0.9832 | -0.9067 | 0.26085 |
| TRINITY_DN19942_c0_g1_i2_orf1 | - | - | - | ribosomal protein l1p/L10e family domain-containing protein [Phthorimaea operculella] | 1.93017 | -0.8759 | -0.5024 | -0.5024 | -0.0495 |
| TRINITY_DN15904_c0_g1_i1_orf1 | - | - | - | papilin isoform X8 [Ostrinia furnacalis] | 1.42334 | 0.60088 | -1.5677 | -0.0682 | -0.3883 |
| TRINITY_DN41736_c0_g2_i1_orf1 | - | - | - | calcyclin-binding protein [Ostrinia furnacalis] | 1.39246 | 1.03607 | -0.7041 | -0.9123 | -0.8121 |
| TRINITY_DN19262_c0_g1_i1_orf1 | - | - | - | tRNA (guanine-N(7)-)-methyltransferase non-catalytic subunit wdr4 [Ostrinia furnacalis] | 1.86299 | -0.955 | -0.7087 | 0.11845 | -0.3177 |
| TRINITY_DN3260_c0_g1_i6_orf1 | - | - | - | vesicular integral-membrane protein VIP36 isoform X1 [Ostrinia furnacalis] >XP_028171839.1 vesicular integral-membrane protein VIP36 isoform X2 [Ostrinia furnacalis] | 1.36804 | -0.1403 | -1.4317 | -0.6185 | 0.82247 |
| TRINITY_DN42903_c0_g1_i4_orf1 | - | - | - | rab GTPase-binding effector protein 1 isoform X1 [Ostrinia furnacalis] >XP_028174977.1 rab GTPase-binding effector protein 1 isoform X2 [Ostrinia furnacalis] >XP_028174983.1 rab GTPase-binding effector protein 1 isoform X3 [Ostrinia furnacalis] >XP_028174990.1 rab GTPase-binding effector protein 1 isoform X4 [Ostrinia furnacalis] >XP_028174996.1 rab GTPase-binding effector protein 1 isoform X5 [Ostrinia furnacalis] | 1.71324 | 0.4611 | -1.123 | -0.6644 | -0.387 |
| TRINITY_DN23946_c0_g1_i1_orf1 | - | - | - | ubiquitin-conjugating enzyme E2L [Bombyx mori] >XP_013145013.1 PREDICTED: ubiquitin-conjugating enzyme E2 L3 [Papilio polytes] >XP_013145026.1 PREDICTED: ubiquitin-conjugating enzyme E2 L3 [Papilio polytes] >XP_013167448.1 PREDICTED: ubiquitin-conjugating enzyme E2 L3 [Papilio xuthus] >XP_013167449.1 PREDICTED: ubiquitin-conjugating enzyme E2 L3 [Papilio xuthus] >XP_014356324.1 ubiquitin-conjugating enzyme E2 L3 [Papilio machaon] >XP_021182538.1 ubiquitin-conjugating enzyme E2 L3 [Helicoverpa armigera] >XP_022831771.1 ubiquitin-conjugating enzyme E2 L3 [Spodoptera litura] >XP_023943862.1 ubiquitin-conjugating enzyme E2 L3 [Bicyclus anynana] >XP_026501320.1 ubiquitin-conjugating enzyme E2 L3 [Vanessa tameamea] >XP_026740672.1 ubiquitin-conjugating enzyme E2 L3 [Trichoplusia ni] >XP_030021931.1 ubiquitin-conjugating enzyme E2 L3 [Manduca sexta] >XP_035448309.1 ubiquitin-conjugating enzyme E2 L3 [Spodoptera frugiperda] >XP_038214502.1 ubiquitin-conjugating enzyme E2 L3 [Zerene cesonia] >XP_045453873.1 ubiquitin-conjugating enzyme E2 L3 [Melitaea cinxia] >XP_045503683.1 ubiquitin-conjugating enzyme E2 L3 [Colias croceus] >XP_045771134.1 ubiquitin-conjugating enzyme E2 L3 [Maniola jurtina] >XP_046963941.1 ubiquitin-conjugating enzyme E2 L3 [Vanessa cardui] >XP_047032812.1 ubiquitin-conjugating enzyme E2 L3 [Helicoverpa zea] >XP_047538650.1 ubiquitin-conjugating enzyme E2 L3 [Vanessa atalanta] >XP_050352992.1 ubiquitin-conjugating enzyme E2 L3 [Nymphalis io] >KAF9417333.1 hypothetical protein HW555_005549 [Spodoptera exigua] >CAB3511518.1 unnamed protein product [Spodoptera littoralis] >CAH0595358.1 unnamed protein product [Chrysodeixis includens] >ABB36655.1 ubiquitin-conjugating enzyme E2I [Bombyx mori] >ABF51360.1 ubiquitin-conjugating enzyme [Bombyx mori] | 1.25849 | 1.18842 | -0.7467 | -0.8286 | -0.8717 |
| TRINITY_DN17071_c0_g1_i6_orf1 | - | - | - | serine/arginine-rich splicing factor 7-like [Ostrinia furnacalis] | 1.33927 | 0.10698 | -1.7197 | -0.1795 | 0.45301 |
| TRINITY_DN2438_c0_g1_i4_orf1 | - | - | - | dystrophin, isoforms A/C/F/G/H isoform X2 [Manduca sexta] | 1.01924 | 0.95312 | -1.6465 | -0.5427 | 0.21692 |
| TRINITY_DN12320_c0_g1_i1_orf1 | - | - | - | brefeldin A-inhibited guanine nucleotide-exchange protein 1 [Ostrinia | 1.60058 | 0.66927 | -1.1505 | -0.7013 | -0.418 |
| TRINITY_DN14987_c0_g1_i3_orf1 | - | - | - | hypothetical protein evm_009121 [Chilo suppressalis] | 0.87372 | 1.31866 | -1.3684 | -0.79 | -0.034 |

| | | | | | | | | |
|---|---|---|---|---|---|---|---|---|
| TRINITY_DN3835_c0_g1_i4_orf1 | - | - | - | protein ERGIC-53 isoform X1 [Ostrinia furnacalis] >XP_028177940.1 protein ERGIC-53 isoform X2 [Ostrinia furnacalis] >XP_028177941.1 protein ERGIC-53 isoform X3 [Ostrinia furnacalis] | 1.522 | 0.05352 | -0.9587 | -1.1948 | 0.57796 |
| TRINITY_DN6247_c0_g1_i2_orf1 | - | - | - | innexin inx3 [Ostrinia furnacalis] | 1.87764 | -0.3047 | -0.7442 | 0.07775 | -0.9065 |
| TRINITY_DN4257_c0_g1_i2_orf1 | - | - | - | dynactin subunit 1 [Ostrinia furnacalis] | 0.52777 | 0.50795 | -1.9598 | 0.77469 | 0.14943 |
| TRINITY_DN6642_c0_g2_i1_orf1 | - | - | - | protein purity of essence [Ostrinia furnacalis] | 1.50429 | 0.66414 | -1.1156 | -1.025 | -0.0278 |
| TRINITY_DN11069_c0_g1_i6_orf1 | - | - | - | ganglioside-induced differentiation-associated protein 1 [Ostrinia furnacalis] | 1.83961 | -0.6809 | -0.3462 | 0.18632 | -0.9988 |
| TRINITY_DN13384_c0_g1_i1_orf1 | - | - | - | 26S proteasome regulatory subunit S3 [Aphelinus abdominalis] | 1.15716 | 0.5043 | -1.8105 | 0.31719 | -0.1682 |
| TRINITY_DN8729_c0_g1_i7_orf1 | - | - | - | myosin heavy chain 95F isoform X1 [Ostrinia furnacalis] >XP_028177153.1 myosin heavy chain 95F isoform X2 [Ostrinia furnacalis] >XP_028177154.1 myosin heavy chain 95F isoform X3 [Ostrinia furnacalis] >XP_028177155.1 myosin heavy chain 95F isoform X4 [Ostrinia furnacalis] >XP_028177156.1 myosin heavy chain 95F isoform X5 [Ostrinia furnacalis] >XP_028177158.1 myosin heavy chain 95F isoform X6 [Ostrinia furnacalis] | 1.86386 | -0.217 | 0.0205 | -0.6233 | -1.044 |
| TRINITY_DN20067_c0_g1_i6_orf1 | - | - | - | hypothetical protein evm_010712 [Chilo suppressalis] >CAB3527462.1 unnamed protein product [Chilo suppressalis] >CAH0401768.1 unnamed protein product [Chilo suppressalis] | 0.99633 | 1.40701 | -0.847 | -0.5555 | -1.0009 |
| TRINITY_DN9711_c0_g1_i10_orf1 | - | - | - | cAMP-specific 3',5'-cyclic phosphodiesterase isoform X2 [Ostrinia furnacalis] | 1.82156 | -0.8579 | -0.7757 | -0.4981 | 0.3101 |
| TRINITY_DN1266_c6_g1_i1_orf1 | - | - | - | transmembrane 9 superfamily member 2 [Ostrinia furnacalis] | 1.75146 | -0.953 | -0.9961 | 0.01956 | 0.17803 |
| TRINITY_DN2478_c0_g1_i1_orf1 | - | - | - | apoptosis inhibitor 5 [Ostrinia furnacalis] | 1.64167 | 0.6139 | -1.1223 | -0.4518 | -0.6814 |
| TRINITY_DN4538_c0_g1_i4_orf1 | - | - | - | 2-acylglycerol O-acyltransferase 1-like [Ostrinia furnacalis] | 1.92194 | -0.9191 | -0.371 | -0.0668 | -0.5651 |
| TRINITY_DN6545_c0_g1_i6_orf1 | - | - | - | organic cation transporter protein-like [Ostrinia furnacalis] | 1.68091 | 0.21481 | 0.04846 | -1.3157 | -0.6285 |
| TRINITY_DN13139_c0_g1_i1_orf1 | - | - | - | AP-1 complex subunit mu-1 [Ostrinia furnacalis] | 1.51992 | 0.84233 | -0.7906 | -0.5406 | -1.031 |
| TRINITY_DN13118_c0_g1_i6_orf1 | - | - | - | AP-1 complex subunit beta-1 [Helicoverpa armigera] >XP_021189434.2 AP-1 complex subunit beta-1 [Helicoverpa armigera] | 0.86374 | 1.23238 | -1.5178 | -0.6527 | 0.07436 |
| TRINITY_DN227_c0_g1_i1_orf1 | - | - | - | double-stranded ribonuclease 2 [Ostrinia nubilalis] | 1.65806 | 0.1485 | 0.23838 | -0.8211 | -1.2238 |
| TRINITY_DN3283_c0_g2_i1_orf1 | - | - | - | dipeptidyl peptidase 9 isoform X2 [Manduca sexta] | 1.43719 | -0.2637 | -1.4152 | -0.5244 | 0.76618 |
| TRINITY_DN19250_c0_g2_i2_orf1 | - | - | - | uncharacterized protein LOC114351683 isoform X8 [Ostrinia furnacalis] | 1.56326 | 0.05466 | -0.6869 | 0.44223 | -1.3733 |
| TRINITY_DN47914_c0_g2_i1_orf1 | - | - | - | UBX domain-containing protein 1-A-like [Ostrinia furnacalis] | 0.72875 | 1.54901 | -0.9218 | -1.0655 | -0.2904 |
| TRINITY_DN4301_c2_g2_i4_orf1 | - | - | - | stress-induced-phosphoprotein 1-like [Ostrinia furnacalis] | 1.71291 | 0.20419 | -1.1264 | 0.07522 | -0.8659 |
| TRINITY_DN12767_c0_g1_i1_orf1 | - | - | - | coatomer subunit alpha [Ostrinia furnacalis] | 1.77804 | -0.925 | -0.8792 | 0.33674 | -0.3105 |
| TRINITY_DN442_c0_g1_i10_orf1 | - | - | - | tuberin isoform X4 [Helicoverpa armigera] | 1.17697 | 0.69661 | -1.6894 | 0.26739 | -0.4516 |
| TRINITY_DN1334_c0_g1_i2_orf1 | - | - | - | phosphoenolpyruvate carboxykinase [GTP]-like [Ostrinia furnacalis] | 1.84559 | -0.1907 | -0.1787 | -1.205 | -0.2712 |
| TRINITY_DN6381_c0_g1_i2_orf1 | - | - | - | solute carrier family 12 member 8 [Ostrinia furnacalis] | 1.56448 | -0.1691 | -1.1847 | -0.8463 | 0.63561 |
| TRINITY_DN1771_c0_g2_i1_orf1 | - | - | - | eukaryotic peptide chain release factor subunit 1 isoform X1 [Danaus plexippus plexippus] >CAG9574852.1 unnamed protein product [Danaus chrysippus] | 1.64349 | 0.30808 | 0.08959 | -0.7831 | -1.2581 |
| TRINITY_DN147427_c0_g1_i1_orf1 | - | - | - | importin subunit alpha-4 [Ostrinia furnacalis] | 1.7712 | 0.43243 | -0.7229 | -0.5716 | -0.9091 |
| TRINITY_DN31399_c0_g1_i3_orf1 | - | - | - | ATP-dependent zinc metalloprotease YME1 homolog [Ostrinia furnacalis] | 1.91977 | -0.1636 | -0.9715 | -0.258 | -0.5266 |
| TRINITY_DN12608_c0_g1_i1_orf1 | - | - | - | centrosome-associated zinc finger protein CP190 [Ostrinia furnacalis] >XP_028173286.1 centrosome-associated zinc finger protein CP190 [Ostrinia furnacalis] | 1.70888 | -0.3261 | -1.3387 | -0.3222 | 0.27816 |
| TRINITY_DN7294_c0_g2_i4_orf1 | - | - | - | hypothetical protein evm_003455 [Chilo suppressalis] >CAB3524298.1 unnamed protein product [Chilo suppressalis] | 1.87368 | -0.9933 | -0.6915 | -0.1524 | -0.0365 |
| TRINITY_DN1673_c0_g1_i2_orf1 | - | - | - | hypothetical protein evm_006080 [Chilo suppressalis] | 1.80455 | 0.15825 | -1.0686 | -0.7444 | -0.1497 |
| TRINITY_DN4012_c0_g4_i2_orf1 | - | - | - | uncharacterized protein LOC114362418 [Ostrinia furnacalis] | 1.41159 | 0.84186 | -0.5953 | -0.2954 | -1.3627 |
| TRINITY_DN23444_c0_g1_i11_orf1 | - | - | - | serrate RNA effector molecule homolog isoform X3 [Ostrinia furnacalis] | 1.1842 | 0.8074 | -1.5952 | -0.5999 | 0.20343 |
| TRINITY_DN9146_c0_g1_i1_orf1 | - | - | - | drebrin-like protein [Ostrinia furnacalis] | 1.2249 | 1.21321 | -0.9674 | -0.8075 | -0.6632 |
| TRINITY_DN23432_c0_g1_i1_orf1 | - | - | - | 7-methylguanosine phosphate-specific 5'-nucleotidase-like isoform X2 [Ostrinia furnacalis] | 1.96532 | -0.6887 | -0.6957 | -0.3628 | -0.2181 |
| TRINITY_DN6262_c0_g2_i1_orf1 | - | - | - | thrombospondin type-1 domain-containing protein 4-like [Ostrinia furnacalis] | 1.3861 | -0.0914 | -1.5749 | -0.3846 | 0.66489 |
| TRINITY_DN45859_c0_g1_i1_orf1 | - | - | - | nuclear valosin-containing protein-like [Ostrinia furnacalis] | 1.55436 | 0.60693 | -0.3853 | -0.393 | -1.383 |

| | | | | | | | | | |
|---|---|---|---|---|---|---|---|---|---|
| TRINITY_DN2953_c1_g1_i11_orf1 | - | - | - | methionine--tRNA ligase, cytoplasmic isoform X2 [Ostrinia furnacalis] >XP_028156683.1 methionine--tRNA ligase, cytoplasmic isoform X4 [Ostrinia furnacalis] >XP_028156684.1 methionine--tRNA ligase, cytoplasmic isoform X5 [Ostrinia furnacalis] | 1.74772 | 0.43177 | -0.4155 | -1.0056 | -0.7584 |
| TRINITY_DN3111_c0_g1_i5_orf1 | - | - | - | CCAAT/enhancer-binding protein zeta-like [Ostrinia furnacalis] | 1.88096 | 0.10224 | -0.45 | -0.9585 | -0.5747 |
| TRINITY_DN9724_c0_g1_i4_orf1 | - | - | - | ras family domain-containing protein [Phthorimaea operculella] | 1.17337 | -0.5041 | -1.5002 | -0.2064 | 1.03729 |
| TRINITY_DN5562_c1_g1_i3_orf1 | - | - | - | cell division cycle and apoptosis regulator protein 1-like [Ostrinia furnacalis] | 1.96855 | -0.2521 | -0.6456 | -0.7239 | -0.3469 |
| TRINITY_DN21559_c0_g2_i1_orf1 | - | - | - | protein bicaudal D isoform X3 [Galleria mellonella] | 1.18586 | 1.24411 | -0.7024 | -0.6901 | -1.0375 |
| TRINITY_DN8133_c0_g1_i4_orf1 | - | - | - | protein sel-1 homolog 1 isoform X2 [Ostrinia furnacalis] | 0.95314 | 0.87433 | -1.8005 | -0.2195 | 0.19255 |
| TRINITY_DN535_c3_g2_i1_orf1 | - | - | - | PSME3-interacting protein isoform X2 [Ostrinia furnacalis] | 1.83107 | -0.1772 | -0.579 | -1.1303 | 0.05537 |
| TRINITY_DN16899_c0_g2_i1_orf1 | - | - | - | serine/threonine-protein kinase GA29083 [Ostrinia furnacalis] | 1.18215 | 0.02872 | -1.6406 | 0.85432 | -0.4246 |
| TRINITY_DN90321_c0_g2_i1_orf1 | - | - | - | pre-mRNA-processing-splicing factor 8 [Apis dorsata] >XP_016768675.1 pre-mRNA-processing-splicing factor 8 [Apis mellifera] >XP_016911911.1 pre-mRNA-processing-splicing factor 8 [Apis cerana] >XP_016911918.1 pre-mRNA-processing-splicing factor 8 [Apis cerana] >XP_031366255.1 pre-mRNA-processing-splicing factor 8 [Apis dorsata] >XP_043784871.1 pre-mRNA-processing-splicing factor 8 [Apis laboriosa] >XP_043784872.1 pre-mRNA-processing-splicing factor 8 [Apis laboriosa] >XP_624014.2 pre-mRNA-processing-splicing factor 8 [Apis mellifera] >KAG6797633.1 pre-mRNA-processing-splicing factor 8 [Apis mellifera caucasica] >PBC26212.1 Pre-mRNA-processing-splicing factor [Apis cerana cerana] | 1.50612 | 0.66449 | -1.3279 | -0.1284 | -0.7143 |
| TRINITY_DN6312_c0_g1_i1_orf1 | - | - | - | cytochrome c oxidase assembly protein COX19 [Ostrinia furnacalis] | 0.75724 | 1.49211 | -1.2078 | -0.2053 | -0.8362 |
| TRINITY_DN11552_c0_g1_i4_orf1 | - | - | - | glutathione hydrolase 1 proenzyme-like isoform X3 [Ostrinia furnacalis] | 1.95937 | -0.741 | -0.6062 | -0.1373 | -0.4749 |
| TRINITY_DN20321_c0_g1_i5_orf1 | - | - | - | uncharacterized protein LOC114350467 isoform X3 [Ostrinia furnacalis] | 1.74211 | -0.8112 | 0.13548 | 0.06683 | -1.1333 |
| TRINITY_DN5234_c0_g1_i2_orf1 | - | - | - | proline dehydrogenase 1, mitochondrial isoform X2 [Ostrinia furnacalis] | 1.83815 | -0.817 | 0.28934 | -0.7304 | -0.5801 |
| TRINITY_DN48638_c0_g1_i5_orf1 | - | - | - | NADH dehydrogenase [ubiquinone] flavoprotein 2, mitochondrial [Ostrinia furnacalis] >ALD03682.1 mitochondrial complex I NDUFV2 subunit [Ostrinia nubilalis] | 1.73386 | -0.9518 | -0.2189 | -0.9453 | 0.38225 |
| TRINITY_DN3411_c0_g1_i2_orf1 | - | - | - | unnamed protein product [Chilo suppressalis] | 1.88515 | 0.12552 | -0.8639 | -0.6887 | -0.458 |
| TRINITY_DN41664_c0_g1_i4_orf1 | - | - | - | uncharacterized protein LOC114356631 [Ostrinia furnacalis] | 1.08079 | 0.40242 | -1.409 | -0.9541 | 0.87992 |
| TRINITY_DN13114_c0_g1_i1_orf1 | - | - | - | nicalin-1 [Helicoverpa zea] | 1.91032 | -0.0014 | -0.8416 | -0.7245 | -0.3428 |
| TRINITY_DN35099_c0_g1_i1_orf1 | - | - | - | transmembrane emp24 domain-containing protein 2 [Ostrinia furnacalis] | 0.50837 | 1.11302 | -1.7002 | -0.5125 | 0.59125 |
| TRINITY_DN14477_c0_g1_i12_orf1 | - | - | - | PREDICTED: phosphoribosyl pyrophosphate synthase-associated protein 2 isoform X2 [Amyelois transitella] | 1.16886 | 1.22245 | -0.5907 | -0.6094 | -1.1913 |
| TRINITY_DN5525_c0_g1_i4_orf1 | - | - | - | probable glucosamine 6-phosphate N-acetyltransferase [Ostrinia furnacalis] | 1.63912 | 0.62837 | -0.415 | -1.0493 | -0.8032 |
| TRINITY_DN2907_c0_g2_i4_orf1 | - | - | - | nucleoporin NUP188 homolog isoform X1 [Ostrinia furnacalis] | 1.4395 | -0.2808 | -1.6515 | 0.25275 | 0.24003 |
| TRINITY_DN89829_c0_g1_i1_orf1 | - | - | - | PREDICTED: ubiquitin-conjugating enzyme E2 T [Microplitis demolitor] | 1.33535 | 1.0219 | -1.1785 | -0.8002 | -0.3785 |
| TRINITY_DN12767_c0_g1_i2_orf1 | - | - | - | coatomer subunit alpha [Ostrinia furnacalis] | 1.37848 | 0.84604 | -1.4124 | -0.2338 | -0.5783 |
| TRINITY_DN67716_c0_g1_i1_orf1 | - | - | - | unnamed protein product [Spodoptera exigua] | 1.9718 | -0.7227 | -0.2081 | -0.5681 | -0.4729 |
| TRINITY_DN3638_c0_g1_i1_orf1 | - | - | - | DNA replication licensing factor Mcm3 [Ostrinia furnacalis] | 1.94714 | -0.1712 | -0.3843 | -0.5178 | -0.8738 |
| TRINITY_DN35377_c0_g1_i3_orf1 | - | - | - | unnamed protein product [Chilo suppressalis] | 1.41846 | 0.73945 | -0.1385 | -0.5722 | -1.4473 |
| TRINITY_DN38211_c0_g1_i1_orf1 | - | - | - | Golgi reassembly-stacking protein 2 [Ostrinia furnacalis] | 0.96706 | 1.36317 | -1.1028 | -0.2694 | -0.9581 |
| TRINITY_DN22871_c0_g2_i1_orf1 | - | - | - | mitochondrial import inner membrane translocase subunit Tim17-B [Ostrinia furnacalis] | 1.79149 | -0.4655 | 0.23795 | -0.3984 | -1.1656 |
| TRINITY_DN31815_c0_g1_i4_orf1 | - | - | - | E3 ubiquitin-protein ligase listerin-like [Ostrinia furnacalis] | 1.94618 | -0.6141 | -0.8118 | -0.403 | -0.1172 |
| TRINITY_DN30097_c0_g1_i2_orf1 | - | - | - | unnamed protein product [Chilo suppressalis] | 1.77888 | 0.22875 | -1.0964 | -0.1676 | -0.7436 |
| TRINITY_DN7674_c0_g1_i2_orf1 | - | - | - | prefoldin subunit 2 [Ostrinia furnacalis] | 1.37756 | -0.5039 | -1.1578 | -0.7145 | 0.99867 |
| TRINITY_DN11322_c0_g1_i2_orf1 | - | - | - | CRAL-TRIO domain-containing protein C3H8.02 [Ostrinia furnacalis] | 1.86519 | -0.1005 | -0.0468 | -0.7301 | -0.9878 |
| TRINITY_DN15458_c0_g1_i3_orf1 | - | - | - | integrin alpha-PS1 isoform X1 [Ostrinia furnacalis] | 1.42029 | 0.59268 | -1.5856 | -0.3279 | -0.0995 |
| TRINITY_DN181_c0_g1_i3_orf1 | - | - | - | hypothetical protein evm_003589 [Chilo suppressalis] | 1.49287 | 0.4884 | -1.4399 | -0.6663 | 0.12489 |
| TRINITY_DN38650_c0_g1_i2_orf1 | - | - | - | elongator complex protein 1 [Ostrinia furnacalis] | 1.92405 | -0.0821 | -0.6047 | -0.3356 | -0.9017 |

| | | | | | | | | | |
|---|---|---|---|---|---|---|---|---|---|
| TRINITY_DN72859_c0_g1_i1_orf1 | - | - | - | hypothetical protein evm_010574 [Chilo suppressalis] | 1.47401 | 0.78382 | -1.3361 | -0.4224 | -0.4993 |
| TRINITY_DN1045_c0_g1_i6_orf1 | - | - | - | ornithine decarboxylase 1-like isoform X1 [Ostrinia furnacalis] | 1.40277 | 0.95949 | -1.0703 | -0.3896 | -0.9024 |
| TRINITY_DN1888_c0_g2_i1_orf1 | - | - | - | peptidyl-prolyl cis-trans isomerase FKBP8 [Ostrinia furnacalis] | 1.76913 | 0.27945 | -1.1744 | -0.5612 | -0.313 |
| TRINITY_DN41546_c0_g1_i15_orf1 | - | - | - | monocarboxylate transporter 12 isoform X8 [Ostrinia furnacalis] | 1.95834 | -0.4664 | -0.6419 | -0.1301 | -0.7201 |
| TRINITY_DN500_c0_g1_i1_orf1 | - | - | - | splicing factor U2AF 50 kDa subunit isoform X2 [Manduca sexta] | 1.75346 | -0.604 | 0.2836 | -1.1926 | -0.2405 |
| TRINITY_DN21126_c0_g1_i1_orf1 | - | - | - | serine/threonine-protein kinase unc-51 isoform X5 [Ostrinia furnacalis] | 1.69278 | 0.54159 | -1.0876 | -0.5541 | -0.5926 |
| TRINITY_DN3801_c0_g1_i9_orf1 | - | - | - | claspin-like isoform X2 [Ostrinia furnacalis] | 1.71972 | 0.50071 | -0.9955 | -0.7716 | -0.4534 |
| TRINITY_DN1856_c0_g1_i3_orf1 | - | - | - | hypothetical protein evm_006253 [Chilo suppressalis] | 1.57101 | 0.72649 | -1.1695 | -0.5541 | -0.5739 |
| TRINITY_DN1405_c0_g1_i1_orf1 | - | - | - | cyclin-dependent kinase 10 isoform X1 [Ostrinia furnacalis] >XP_028178194.1 cyclin-dependent kinase 10 isoform X2 [Ostrinia furnacalis] | 0.83562 | 1.46784 | -1.1636 | -0.8378 | -0.302 |
| TRINITY_DN25681_c0_g1_i5_orf1 | - | - | - | hypothetical protein evm_005766 [Chilo suppressalis] >CAB3520395.1 unnamed protein product [Chilo suppressalis] >CAH0397716.1 unnamed protein product [Chilo suppressalis] | 1.60363 | -1.29 | 0.5836 | -0.3456 | -0.5517 |
| TRINITY_DN6317_c1_g2_i3_orf1 | - | - | - | nucleosome assembly protein 1-like 1 isoform X2 [Ostrinia furnacalis] | 1.67762 | 0.26932 | -1.3263 | -0.0263 | -0.5943 |
| TRINITY_DN8406_c0_g1_i3_orfp1 | - | - | - | TRINITY_DN8406_c0_g1_i3_m.76210 TRINITY_DN8406_c0_g1::TRINITY_DN8406_c0_g1_i3::g.76210 ORF type:internal len:154 (+),score=51.92 TRINITY_DN8406_c0_g1_i3:2-460(+) | 1.16416 | -0.5752 | -1.6564 | 0.5147 | 0.55269 |
| TRINITY_DN86580_c0_g1_i1_orf1 | - | - | - | microtubule-actin cross-linking factor 1 isoform X15 [Ostrinia furnacalis] | 1.45266 | 0.50898 | -1.5164 | 0.1181 | -0.5634 |
| TRINITY_DN57_c0_g2_i3_orf1 | - | - | - | transcriptional repressor CTCF-like [Ostrinia furnacalis] >XP_028163401.1 transcriptional repressor CTCF-like [Ostrinia furnacalis] | 1.07848 | 0.33862 | -1.7372 | -0.4115 | 0.73154 |
| TRINITY_DN50225_c0_g1_i1_orf1 | - | - | - | SRSF protein kinase 3 [Galleria mellonella] | 1.26827 | 1.14749 | -0.812 | -1.0562 | -0.5475 |
| TRINITY_DN15916_c0_g1_i1_orf1 | - | - | - | balbiani ring protein 3-like [Bicyclus anynana] >XP_023946842.1 balbiani ring protein 3-like [Bicyclus anynana] | 1.66258 | -0.5876 | -0.1879 | 0.41136 | -1.2985 |
| TRINITY_DN3132_c0_g1_i10_orf1 | - | - | - | oxysterol-binding protein 1-like [Ostrinia furnacalis] | 1.36831 | -0.2594 | -1.6453 | -0.0555 | 0.59188 |
| TRINITY_DN280_c0_g1_i12_orf1 | - | - | - | tubulin beta-1 chain-like [Leguminivora glycinivorella] | 1.8287 | 0.07998 | -0.9858 | -0.8163 | -0.1066 |
| TRINITY_DN351_c14_g1_i2_orf1 | - | - | - | calnexin [Ostrinia furnacalis] >XP_028173720.1 calnexin [Ostrinia furnacalis] | 1.13928 | 0.09245 | -1.7458 | -0.2498 | 0.76379 |
| TRINITY_DN18558_c0_g1_i7_orf1 | - | - | - | cytosolic Fe-S cluster assembly factor NUBP2 homolog [Ostrinia furnacalis] | 1.78688 | 0.4018 | -0.8277 | -0.5494 | -0.8116 |
| TRINITY_DN5908_c0_g1_i2_orf1 | - | - | - | ATP-binding cassette sub-family B member 10, mitochondrial-like [Ostrinia furnacalis] | 1.86838 | -0.3658 | 0.14333 | -0.8113 | -0.8346 |
| TRINITY_DN130778_c0_g1_i1_orf1 | - | - | - | 26S proteasome non-ATPase regulatory subunit 8 [Ostrinia furnacalis] | 0.91288 | 0.71462 | -1.7061 | -0.5699 | 0.64846 |
| TRINITY_DN20426_c0_g2_i1_orf1 | - | - | - | prolyl 3-hydroxylase sudestada1 [Ostrinia furnacalis] | 0.84937 | 0.15897 | -1.8732 | 0.00204 | 0.86281 |
| TRINITY_DN19260_c0_g1_i5_orf1 | - | - | - | probable 26S proteasome non-ATPase regulatory subunit 3 [Ostrinia furnacalis] | 0.80779 | 0.84136 | -1.4761 | -0.9367 | 0.76368 |
| TRINITY_DN14572_c0_g1_i1_orf1 | - | - | - | ras-related protein Rab-11A [Ostrinia furnacalis] | 1.33541 | 0.91308 | -1.4293 | -0.4573 | -0.3619 |
| TRINITY_DN1554_c0_g1_i9_orf1 | - | - | - | LOW QUALITY PROTEIN: puff-specific protein Bx42 [Ostrinia furnacalis] | 1.18985 | 1.22435 | -0.512 | -0.8679 | -1.0344 |
| TRINITY_DN18300_c0_g1_i17_orf1 | - | - | - | RNA-binding protein lark isoform X1 [Helicoverpa armigera] >XP_047032035.1 RNA-binding protein lark isoform X1 [Helicoverpa zea] >PZC74210.1 hypothetical protein B5X24_HaOG208200 [Helicoverpa armigera] | 1.91004 | -0.3191 | -0.7888 | -0.7922 | -0.0099 |
| TRINITY_DN10871_c0_g1_i3_orf1 | - | - | - | hypothetical protein HF086_013701 [Spodoptera exigua] | 1.37258 | 0.62705 | -1.2126 | -1.0801 | 0.29305 |
| TRINITY_DN15762_c0_g1_i2_orf1 | - | - | - | YTH domain-containing family protein 3 isoform X3 [Maniola hyperantus] | 1.45166 | 0.49457 | -1.2076 | -1.0464 | 0.30781 |
| TRINITY_DN65604_c0_g1_i2_orf1 | - | - | - | LOW QUALITY PROTEIN: ankyrin repeat domain-containing protein 17 [Ostrinia furnacalis] | 1.94207 | -0.2546 | -0.2823 | -0.4828 | -0.9224 |
| TRINITY_DN3753_c0_g1_i7_orf1 | - | - | - | very-long-chain 3-oxoacyl-CoA reductase isoform X2 [Ostrinia furnacalis] | 1.87177 | 0.12321 | -0.4226 | -0.9686 | -0.6038 |
| TRINITY_DN4403_c0_g1_i3_orf1 | - | - | - | AP-1 complex subunit gamma-1 [Ostrinia furnacalis] | 1.43708 | 0.98402 | -0.7216 | -0.8786 | -0.8208 |
| TRINITY_DN14220_c0_g1_i1_orf1 | - | - | - | U3 small nucleolar ribonucleoprotein protein IMP4 [Ostrinia furnacalis] | 1.90784 | -0.6631 | -0.0211 | -0.3191 | -0.9045 |
| TRINITY_DN10716_c1_g1_i1_orf1 | - | - | - | apoptosis-inducing factor 3-like [Ostrinia furnacalis] | 1.96116 | -0.8155 | -0.5545 | -0.3529 | -0.2382 |
| TRINITY_DN47605_c0_g2_i1_orf1 | - | - | - | hypothetical protein evm_002030 [Chilo suppressalis] | 1.9376 | -0.8775 | -0.6029 | -0.1665 | -0.2907 |
| TRINITY_DN7064_c0_g1_i6_orf1 | - | - | - | unnamed protein product [Chilo suppressalis] | 1.86021 | -0.0514 | -1.0487 | -0.1078 | -0.6524 |
| TRINITY_DN45000_c0_g1_i5_orf1 | - | - | - | PREDICTED: ATP synthase subunit beta, mitochondrial, partial [Papilio polytes] | 1.96465 | -0.4043 | -0.282 | -0.8392 | -0.4391 |
| TRINITY_DN6308_c0_g1_i3_orf1 | - | - | - | myc box-dependent-interacting protein 1 isoform X4 [Pectinophora | 1.8201 | -1.1729 | -0.5541 | -0.0411 | -0.0519 |
| TRINITY_DN10257_c0_g1_i2_orf1 | - | - | - | prefoldin subunit domain-containing protein [Phthorimaea operculella] | 1.86415 | 0.1645 | -0.4237 | -0.6792 | -0.9258 |
| TRINITY_DN14298_c0_g1_i1_orf1 | - | - | - | kinesin heavy chain [Ostrinia furnacalis] | 0.99878 | 1.2973 | -1.3206 | -0.711 | -0.2645 |

| | | | | | | | | | |
|---|---|---|---|---|---|---|---|---|---|
| TRINITY_DN33967_c0_g1_i1_orf1 | - | - | - | PREDICTED: elongation factor 1-alpha [Microplitis demolitor] >XP_008547401.1 PREDICTED: elongation factor 1-alpha [Microplitis demolitor] | 1.69897 | -0.7767 | -1.057 | -0.3708 | 0.50552 |
| TRINITY_DN71494_c0_g1_i2_orf1 | - | - | - | hypothetical protein O3G_MSEX005876 [Manduca sexta] | 1.79285 | 0.08033 | -0.8851 | 0.00977 | -0.9979 |
| TRINITY_DN129259_c0_g2_i1_orf1 | - | - | - | spectrin alpha chain-like isoform X3 [Helicoverpa armigera] | 1.50753 | -0.884 | -1.2769 | 0.10084 | 0.55252 |
| TRINITY_DN802_c0_g1_i2_orf1 | - | - | - | active breakpoint cluster region-related protein [Ostrinia furnacalis] | 1.27153 | 1.07602 | -0.9137 | -1.1427 | -0.2911 |
| TRINITY_DN53400_c0_g1_i1_orf1 | - | - | - | hypothetical protein evm_004547 [Chilo suppressalis] | 1.91295 | -0.2528 | -0.3656 | -0.2568 | -1.0379 |
| TRINITY_DN44335_c0_g1_i7_orf1 | - | - | - | hypothetical protein SFRURICE_000584 [Spodoptera frugiperda] | 1.54291 | 0.79142 | -1.1208 | -0.6229 | -0.5906 |
| TRINITY_DN49527_c0_g1_i1_orf1 | - | - | - | Protein lin-7 homolog B [Eumeta japonica] | 1.99038 | -0.4399 | -0.6423 | -0.5539 | -0.3543 |
| TRINITY_DN2611_c0_g1_i10_orf1 | - | - | - | ancient ubiquitous protein 1-like [Hyposmocoma kahamanoa] | 1.53376 | 0.17281 | -1.5152 | -0.4852 | 0.29388 |
| TRINITY_DN42205_c0_g1_i4_orf1 | - | - | - | eukaryotic translation initiation factor 4H [Ostrinia furnacalis] | 1.51424 | 0.86669 | -0.897 | -0.5834 | -0.9005 |
| TRINITY_DN3245_c2_g1_i4_orf1 | - | - | - | membrane-associated progesterone receptor component 1-like [Ostrinia | 1.85485 | -1.0832 | -0.5749 | -0.233 | 0.03632 |
| TRINITY_DN51737_c0_g1_i3_orf1 | - | - | - | N-alpha-acetyltransferase 40 [Ostrinia furnacalis] | 0.97785 | 1.4392 | -0.8915 | -0.6789 | -0.8467 |
| TRINITY_DN1781_c0_g1_i8_orf1 | - | - | - | transportin-1 [Pectinophora gossypiella] | 0.60887 | 1.2295 | -1.002 | -1.3573 | 0.52092 |
| TRINITY_DN934_c2_g1_i7_orf1 | - | - | - | ubiquitin-40S ribosomal protein S27a [Ostrinia furnacalis] | 1.21232 | 1.16847 | -1.1889 | -0.7396 | -0.4524 |
| TRINITY_DN101358_c0_g2_i1_orf1 | - | - | - | glycylpeptide N-tetradecanoyltransferase 2 [Ostrinia furnacalis] | 1.5537 | 0.54797 | -0.8921 | -1.2206 | 0.01098 |
| TRINITY_DN1383_c0_g1_i2_orf1 | - | - | - | uncharacterized protein LOC114353133 isoform X1 [Ostrinia furnacalis] >XP_028160773.1 uncharacterized protein LOC114353133 isoform X2 [Ostrinia furnacalis] | 1.64306 | -1.107 | 0.41655 | -0.0032 | -0.9495 |
| TRINITY_DN4546_c0_g1_i3_orf1 | - | - | - | telomerase Cajal body protein 1 homolog [Ostrinia furnacalis] | 1.52045 | 0.53321 | -0.5589 | -1.4454 | -0.0494 |
| TRINITY_DN6313_c0_g1_i4_orf1 | - | - | - | pyruvate dehydrogenase E1 component subunit beta, mitochondrial isoform X1 [Ostrinia furnacalis] >XP_028161232.1 pyruvate dehydrogenase E1 component subunit beta, mitochondrial isoform X2 [Ostrinia furnacalis] >XP_028161233.1 pyruvate dehydrogenase E1 component subunit beta, mitochondrial isoform X3 [Ostrinia furnacalis] >XP_028161234.1 pyruvate dehydrogenase E1 component subunit beta, mitochondrial isoform X4 [Ostrinia furnacalis] | 1.9123 | -0.9929 | -0.0839 | -0.4421 | -0.3934 |
| TRINITY_DN18329_c0_g1_i2_orf1 | - | - | - | bromodomain-containing protein 3 [Ostrinia furnacalis] >XP_028169629.1 bromodomain-containing protein 3 [Ostrinia furnacalis] >XP_028169637.1 bromodomain-containing protein 3 [Ostrinia furnacalis] >XP_028169644.1 bromodomain-containing protein 3 [Ostrinia furnacalis] | 1.42205 | 0.62222 | -1.5734 | -0.1905 | -0.2803 |
| TRINITY_DN2649_c0_g1_i3_orf1 | - | - | - | GPI inositol-deacylase isoform X1 [Ostrinia furnacalis] | 1.29359 | 0.55941 | -0.8628 | 0.44803 | -1.4383 |
| TRINITY_DN20966_c0_g1_i6_orf1 | - | - | - | clavesin-1-like [Ostrinia furnacalis] | 1.90096 | -0.8987 | -0.0544 | -0.7255 | -0.2224 |
| TRINITY_DN2812_c0_g1_i5_orf1 | - | - | - | myotubularin-related protein 2 [Ostrinia furnacalis] >XP_028170267.1 myotubularin-related protein 2 [Ostrinia furnacalis] | 1.39192 | 0.99009 | -0.6807 | -1.1436 | -0.5577 |
| TRINITY_DN1494_c0_g1_i3_orf1 | - | - | - | dihydropyrimidine dehydrogenase [NADP(+)] [Ostrinia furnacalis] | 1.96587 | -0.2995 | -0.6969 | -0.6969 | -0.2726 |
| TRINITY_DN164_c0_g1_i11_orf1 | - | - | - | hypothetical protein evm_000323 [Chilo suppressalis] >CAB3530114.1 unnamed protein product [Chilo suppressalis] >CAH0406706.1 unnamed protein product [Chilo suppressalis] | 1.89919 | 0.08119 | -0.551 | -0.8898 | -0.5397 |
| TRINITY_DN2019_c0_g1_i4_orf1 | - | - | - | sushi, von Willebrand factor type A, EGF and pentraxin domain-containing protein 1 [Ostrinia furnacalis] >XP_028159318.1 sushi, von Willebrand factor type A, EGF and pentraxin domain-containing protein 1 [Ostrinia furnacalis] | 1.61289 | 0.72238 | -0.9413 | -0.5993 | -0.7946 |
| TRINITY_DN441_c0_g2_i1_orf1 | - | - | - | guanine nucleotide-binding protein subunit beta-like protein [Diachasma | 1.65796 | -0.5174 | -1.3246 | -0.2336 | 0.41762 |
| TRINITY_DN27398_c0_g1_i3_orf1 | - | - | - | lissencephaly-1 homolog [Helicoverpa armigera] >XP_021185428.1 lissencephaly-1 homolog [Helicoverpa armigera] >XP_047030140.1 lissencephaly-1 homolog [Helicoverpa zea] >XP_047030141.1 lissencephaly-1 | 0.97683 | 0.79864 | -1.1197 | -1.3126 | 0.65681 |
| TRINITY_DN129207_c0_g1_i1_orf1 | - | - | - | U3 small nucleolar RNA-interacting protein 2 [Ostrinia furnacalis] | 1.92081 | -0.0561 | -0.4039 | -0.9269 | -0.534 |
| TRINITY_DN3471_c0_g1_i1_orf1 | - | - | - | EH domain-containing protein 3 [Ostrinia furnacalis] | 1.85683 | -0.3136 | -0.6913 | -0.9796 | 0.1277 |

| | | | | | | | | | |
|---|---|---|---|---|---|---|---|---|---|
| TRINITY_DN47666_c0_g1_i4_orf1 | - | - | - | PREDICTED: probable splicing factor 3B subunit 5 [Amyelois transitella] >XP_021194781.1 splicing factor 3B subunit 5 [Helicoverpa armigera] >XP_022127816.1 splicing factor 3B subunit 5 [Pieris rapae] >XP_022818375.1 probable splicing factor 3B subunit 5 [Spodoptera litura] >XP_026319816.1 splicing factor 3B subunit 5 [Hyposmocoma kahamanoa] >XP_026728249.1 splicing factor 3B subunit 5 [Trichoplusia ni] >XP_026750486.1 splicing factor 3B subunit 5 [Galleria mellonella] >XP_028161523.1 splicing factor 3B subunit 5 [Ostrinia furnacalis] >XP_030037274.1 splicing factor 3B subunit 5 [Manduca sexta] >XP_035451936.1 splicing factor 3B subunit 5 [Spodoptera frugiperda] >XP_047035127.1 splicing factor 3B subunit 5 [Helicoverpa zea] >XP_047519960.1 splicing factor 3B subunit 5 [Pieris napi] >XP_049880275.1 splicing factor 3B subunit 5 [Pectinophora gossypiella] >KAF9410749.1 hypothetical protein HW555_010266 [Spodoptera exigua] >CAB3229682.1 unnamed protein product [Arctia plantaginis] >CAF4874265.1 unnamed protein product [Pieris macdunnoughi] >CAG9753950.1 unnamed protein product [Diatraea saccharalis] >CAH0596996.1 unnamed protein product [Chrysodeixis includens] >CAH0717822.1 unnamed protein product, partial [Brenthis ino] >CAH2007681.1 unnamed protein product [Euphydryas editha] | 1.56195 | 0.71027 | -1.2059 | -0.662 | -0.4044 |
| TRINITY_DN6239_c0_g1_i1_orf1 | - | - | - | eukaryotic translation initiation factor 6 [Ostrinia furnacalis] | 1.14951 | 1.24456 | -0.4174 | -1.0188 | -0.9579 |
| TRINITY_DN48460_c0_g1_i1_orf1 | - | - | - | PREDICTED: signal recognition particle 54 kDa protein [Fopius arisanus] | 1.39219 | 0.7369 | -1.5141 | -0.444 | -0.1711 |
| TRINITY_DN1469_c0_g1_i1_orf1 | - | - | - | kynurenine--oxoglutarate transaminase 3 isoform X4 [Orussus abietinus] >XP_012270451.1 kynurenine--oxoglutarate transaminase 3 isoform X4 [Orussus abietinus] >XP_012270459.1 kynurenine--oxoglutarate transaminase 3 isoform X4 [Orussus abietinus] >XP_012270468.1 kynurenine--oxoglutarate transaminase 3 isoform X4 [Orussus abietinus] | 1.45913 | 0.13149 | -1.4697 | -0.6463 | 0.52534 |
| TRINITY_DN3179_c0_g1_i1_orf1 | - | - | - | diamine acetyltransferase 2-like [Ostrinia furnacalis] | 1.56226 | 0.71553 | -1.139 | -0.7949 | -0.344 |
| TRINITY_DN6876_c0_g2_i1_orf1 | - | - | - | serine/threonine-protein phosphatase 5 [Spodoptera litura] >CAB3508320.1 unnamed protein product [Spodoptera littoralis] >CAH1637875.1 unnamed protein product [Spodoptera littoralis] | 1.91561 | -0.9339 | -0.2352 | -0.6226 | -0.124 |
| TRINITY_DN18912_c1_g1_i1_orf1 | - | - | - | engulfment and cell motility protein 1 [Ostrinia furnacalis] | 1.57404 | 0.75227 | -1.0502 | -0.4978 | -0.7783 |
| TRINITY_DN8980_c0_g1_i2_orf1 | - | - | - | putative ATP-dependent RNA helicase me31b [Ostrinia furnacalis] >XP_028162602.1 putative ATP-dependent RNA helicase me31b [Ostrinia furnacalis] >XP_028162603.1 putative ATP-dependent RNA helicase me31b [Ostrinia furnacalis] | 1.7828 | 0.39757 | -0.5686 | -0.9497 | -0.662 |
| TRINITY_DN3597_c0_g1_i10_orf1 | - | - | - | unnamed protein product [Diatraea saccharalis] | 1.23923 | 0.60941 | -1.6576 | -0.5 | 0.30892 |
| TRINITY_DN101325_c0_g1_i4_orf1 | - | - | - | endonuclease G, mitochondrial [Ostrinia furnacalis] | 1.88813 | -0.62 | -0.0997 | -1.0073 | -0.1612 |
| TRINITY_DN3805_c0_g1_i2_orf1 | - | - | - | heat shock protein 89 [Glyphodes pyloalis] | 1.17752 | -0.3906 | -1.6208 | -0.0761 | 0.90998 |
| TRINITY_DN5982_c0_g1_i3_orf1 | - | - | - | coatomer subunit gamma [Ostrinia furnacalis] | 1.69769 | 0.5167 | -1.1179 | -0.5449 | -0.5516 |
| TRINITY_DN13259_c0_g1_i2_orf1 | - | - | - | mitotic spindle assembly checkpoint protein MAD1 [Pectinophora gossypiella] | 1.84129 | -0.6047 | -1.1079 | -0.1288 | 9.93E-05 |
| TRINITY_DN5092_c0_g1_i2_orf1 | - | - | - | peroxisomal acyl-coenzyme A oxidase 3 isoform X3 [Ostrinia furnacalis] | 1.9413 | -0.5441 | -0.8968 | -0.1957 | -0.3047 |
| TRINITY_DN61711_c0_g1_i1_orf1 | - | - | - | mitochondrial carnitine/acylcarnitine translocase [Loxostege sticticalis] | 1.67717 | -0.6181 | -1.0936 | -0.5343 | 0.56884 |
| TRINITY_DN17861_c0_g1_i5_orf1 | - | - | - | acyl-CoA-binding protein homolog isoform X1 [Ostrinia furnacalis] | 1.62408 | 0.70078 | -0.9892 | -0.6483 | -0.6873 |
| TRINITY_DN4859_c0_g1_i5_orf1 | - | - | - | importin-11, partial [Ostrinia furnacalis] | 1.42603 | 0.65088 | -1.5497 | -0.2975 | -0.2297 |
| TRINITY_DN7560_c0_g1_i4_orf1 | - | - | - | unnamed protein product [Chilo suppressalis] | 1.36737 | 0.84005 | -1.1166 | -1.0852 | -0.0056 |
| TRINITY_DN13093_c0_g1_i2_orf1 | - | - | - | enhancer of mRNA-decapping protein 4 [Ostrinia furnacalis] | 1.43816 | 0.43281 | -1.6046 | 0.12587 | -0.3923 |
| TRINITY_DN5133_c0_g1_i7_orf1 | - | - | - | epimerase family protein SDR39U1 [Ostrinia furnacalis] | 1.85668 | -0.2387 | -0.6732 | 0.07363 | -1.0184 |
| TRINITY_DN4026_c0_g1_i4_orf1 | - | - | - | serine protease [Ostrinia furnacalis] | 1.78896 | 0.40209 | -0.7954 | -0.8231 | -0.5725 |
| TRINITY_DN4207_c0_g1_i1_orf1 | - | - | - | mitochondrial import inner membrane translocase subunit Tim21 [Ostrinia furnacalis] | 1.45218 | 0.00876 | -0.2606 | 0.42519 | -1.6256 |
| TRINITY_DN1947_c0_g1_i6_orf1 | - | - | - | insulin-degrading enzyme [Ostrinia furnacalis] >XP_028163443.1 insulin-degrading enzyme [Ostrinia furnacalis] | 1.87786 | 0.028 | -1.0297 | -0.3182 | -0.558 |
| TRINITY_DN24325_c0_g1_i12_orf1 | - | - | - | MICOS complex subunit MIC10-like [Ostrinia furnacalis] | 1.9268 | -0.9611 | -0.1501 | -0.3422 | -0.4734 |

| | | | | | | | | | |
|---|---|---|---|---|---|---|---|---|---|
| TRINITY_DN4300_c0_g1_i5_orf1 | - | - | - | replication factor C subunit 3 [Ostrinia furnacalis] | 1.9804 | -0.2977 | -0.7308 | -0.5094 | -0.4426 |
| TRINITY_DN518_c0_g1_i1_orf1 | - | - | - | unnamed protein product [Arctia plantaginis] | 1.25132 | 1.1675 | -1.0733 | -0.7563 | -0.5892 |
| TRINITY_DN13944_c0_g1_i1_orf1 | - | - | - | vacuolar protein sorting-associated protein 52 homolog [Ostrinia furnacalis] | 1.96507 | -0.4955 | -0.3608 | -0.2823 | -0.8265 |
| TRINITY_DN4814_c0_g1_i6_orf1 | - | - | - | vesicle transport protein GOT1B [Pectinophora gossypiella] | 1.65615 | 0.10567 | -0.3878 | 0.07183 | -1.4458 |
| TRINITY_DN1391_c1_g2_i4_orf1 | - | - | - | hypothetical protein SFRURICE_002236 [Spodoptera frugiperda] | 1.74661 | -0.8493 | -1.0978 | 0.06261 | 0.13786 |
| TRINITY_DN4439_c0_g1_i2_orf1 | - | - | - | cytoplasmic FMR1-interacting protein isoform X1 [Ostrinia furnacalis] >XP_028169436.1 cytoplasmic FMR1-interacting protein isoform X2 [Ostrinia furnacalis] | 1.23427 | 1.11537 | -0.9859 | -1.0884 | -0.2753 |
| TRINITY_DN28428_c0_g1_i2_orf1 | - | - | - | unnamed protein product [Chrysodeixis includens] | 0.93955 | 1.2707 | -1.4865 | -0.4863 | -0.2375 |
| TRINITY_DN9437_c0_g1_i1_orf1 | - | - | - | glyoxylate reductase/hydroxypyruvate reductase-like [Ostrinia furnacalis] | 1.90286 | -0.0109 | -0.6185 | -0.3321 | -0.9414 |
| TRINITY_DN8076_c0_g1_i5_orf1 | - | - | - | hypothetical protein evm_001812 [Chilo suppressalis] | 1.8774 | -0.024 | -0.4913 | -0.29 | -1.0721 |
| TRINITY_DN3584_c0_g1_i3_orf1 | - | - | - | paired amphipathic helix protein Sin3b [Ostrinia furnacalis] | 1.5281 | 0.29383 | -1.5809 | -0.279 | 0.03798 |
| TRINITY_DN2984_c0_g1_i3_orf1 | - | - | - | connectin-like [Ostrinia furnacalis] | 1.18247 | 1.22168 | -1.1152 | -0.7765 | -0.5124 |
| TRINITY_DN655_c0_g1_i3_orf1 | - | - | - | moesin/ezrin/radixin homolog 1 isoform X2 [Bombyx mori] >XP_028038189.1 moesin/ezrin/radixin homolog 1 isoform X2 [Bombyx mandarina] | 1.76794 | 0.08956 | -1.1416 | -0.7496 | 0.03375 |
| TRINITY_DN30027_c0_g1_i1_orf1 | - | - | - | 40S ribosomal protein S14 [Plutella xylostella] >NP_[illegible] 40S ribosomal protein S14 [Papilio polytes] >NP_001299342.1 40S ribosomal protein S14 [Papilio xuthus] >XP_013200267.1 PREDICTED: 40S ribosomal protein S14 [Amyelois transitella] >XP_013200268.1 PREDICTED: 40S ribosomal protein S14 [Amyelois transitella] >XP_014369569.1 40S ribosomal protein S14 [Papilio machaon] >XP_021200686.1 40S ribosomal protein S14 [Helicoverpa armigera] >XP_026737481.1 40S ribosomal protein S14 [Trichoplusia ni] >XP_028029011.1 40S ribosomal protein S14 [Bombyx mandarina] >XP_028179467.1 40S ribosomal protein S14 [Ostrinia furnacalis] >XP_028179468.1 40S ribosomal protein S14 [Ostrinia furnacalis] >XP_030030611.1 40S ribosomal protein S14 [Manduca sexta] >XP_034829960.1 40S ribosomal protein S14 [Maniola hyperantus] >XP_034829961.1 40S ribosomal protein S14 [Maniola hyperantus] >XP_047022662.1 40S ribosomal protein S14 [Helicoverpa zea] >XP_047984027.1 40S ribosomal protein S14 [Leguminivora glycinivorella] >XP_049869822.1 40S ribosomal protein S14 [Pectinophora gossypiella] >Q5UAM9.1 RecName: Full=40S ribosomal protein S14 [Bombyx mori] >CAH0605581.1 unnamed protein product [Chrysodeixis includens] >AAV34871.1 ribosomal protein S14 [Bombyx mori] >ACY95302.1 ribosomal protein S14 [Manduca sexta] >KAG6456546.1 hypothetical protein O3G_MSEX009812 [Manduca sexta] >KAG6456547.1 hypothetical protein O3G_MSEX009812 [Manduca sexta] | 1.68007 | -0.1568 | -1.4351 | -0.2556 | 0.16736 |
| TRINITY_DN3791_c0_g1_i2_orf1 | - | - | - | transmembrane protein 19 [Ostrinia furnacalis] | 1.92982 | -0.0258 | -0.8295 | -0.4672 | -0.6073 |
| TRINITY_DN54586_c1_g1_i1_orf1 | - | - | - | protein YIPF5 [Ostrinia furnacalis] | 0.60293 | 1.213 | -1.2963 | -1.0813 | 0.56167 |
| TRINITY_DN5442_c0_g1_i4_orf1 | - | - | - | hypothetical protein evm_004688 [Chilo suppressalis] | 1.47048 | 0.92953 | -0.7777 | -0.9738 | -0.6486 |
| TRINITY_DN19920_c1_g1_i2_orf1 | - | - | - | probable ATP-dependent RNA helicase DDX10 [Ostrinia furnacalis] | 1.94347 | -0.8113 | -0.1232 | -0.6468 | -0.3621 |
| TRINITY_DN4808_c0_g1_i3_orf1 | - | - | - | kinesin light chain [Ostrinia furnacalis] | 1.0505 | 1.28242 | -1.1353 | -0.9494 | -0.2482 |
| TRINITY_DN3454_c0_g1_i1_orf1 | - | - | - | MICOS complex subunit MIC13 homolog QIL1 [Ostrinia furnacalis] | 1.88442 | -0.9138 | 0.11648 | -0.4748 | -0.6123 |
| TRINITY_DN8701_c0_g1_i3_orf1 | - | - | - | putative uncharacterized protein DDB_G0282133 [Ostrinia furnacalis] | 0.78371 | 1.31081 | -1.5552 | -0.0421 | -0.4973 |
| TRINITY_DN6063_c1_g2_i1_orf1 | - | - | - | short-chain specific acyl-CoA dehydrogenase, mitochondrial [Ostrinia | 1.83703 | -0.2581 | -0.8042 | -0.9404 | 0.16578 |
| TRINITY_DN42705_c0_g1_i3_orf1 | - | - | - | multiple inositol polyphosphate phosphatase 1 isoform X1 [Ostrinia furnacalis] | 1.50866 | -0.3383 | -1.0165 | -0.9614 | 0.80749 |
| TRINITY_DN32509_c0_g1_i3_orf1 | - | - | - | heat shock 70 kDa protein 4 isoform X1 [Ostrinia furnacalis] | 1.93903 | -0.2455 | -0.9097 | -0.2417 | -0.5422 |
| TRINITY_DN143496_c0_g1_i1_orf1 | - | - | - | cullin-3 [Diachasma alloeum] | 1.20079 | -0.1039 | -1.7937 | 0.13984 | 0.55704 |
| TRINITY_DN4929_c0_g1_i1_orf1 | - | - | - | unnamed protein product [Danaus chrysippus] | 1.26549 | 0.35409 | -0.2719 | 0.39646 | -1.7441 |
| TRINITY_DN31058_c0_g1_i6_orf1 | - | - | - | HIV Tat-specific factor 1 homolog [Ostrinia furnacalis] | 1.65485 | 0.3267 | -0.997 | -1.0737 | 0.08912 |
| TRINITY_DN7942_c0_g1_i1_orf1 | - | - | - | hypothetical protein evm_012160 [Chilo suppressalis] >CAB3521803.1 unnamed protein product [Chilo suppressalis] >CAH0399125.1 unnamed protein product [Chilo suppressalis] | 1.85122 | 0.25917 | -0.6339 | -0.6548 | -0.8218 |

| | | | | | | | | | |
|---|---|---|---|---|---|---|---|---|---|
| TRINITY_DN12820_c0_g1_i1_orf1 | - | - | - | chromodomain-helicase-DNA-binding protein 7 [Ostrinia furnacalis] >XP_028176739.1 chromodomain-helicase-DNA-binding protein 7 [Ostrinia furnacalis] | 1.45459 | 0.84067 | -1.2244 | -0.3064 | -0.7645 |
| TRINITY_DN14301_c0_g1_i1_orf1 | - | - | - | apoptosis-inducing factor 1, mitochondrial-like [Ostrinia furnacalis] | 1.82684 | -1.0086 | -0.2498 | 0.17481 | -0.7432 |
| TRINITY_DN37141_c0_g1_i2_orf1 | - | - | - | dnaJ homolog shv [Ostrinia furnacalis] | 1.64942 | 0.61666 | -1.1046 | -0.5336 | -0.6278 |
| TRINITY_DN1459_c1_g1_i1_orf1 | - | - | - | reticulon-1 isoform X2 [Ostrinia furnacalis] | 1.65387 | 0.55093 | -0.6651 | -1.1782 | -0.3615 |
| TRINITY_DN1567_c0_g1_i15_orf1 | - | - | - | probable dual specificity protein kinase madd-3 isoform X1 [Ostrinia furnacalis] | 1.95837 | -0.3732 | -0.5219 | -0.8381 | -0.2251 |
| TRINITY_DN81719_c0_g1_i1_orf1 | - | - | - | tyrosine 3-monooxygenase isoform X1 [Ostrinia furnacalis] >ARE68330.1 tyrosin hydroxylase [Ostrinia furnacalis] | 1.92948 | -0.1356 | -0.303 | -0.5788 | -0.9121 |
| TRINITY_DN2859_c0_g1_i7_orf1 | - | - | - | cleavage and polyadenylation specificity factor subunit 5 [Ostrinia furnacalis] | 1.99559 | -0.4052 | -0.568 | -0.5751 | -0.4473 |
| TRINITY_DN848_c0_g1_i1_orf1 | - | - | - | actin-interacting protein 1 isoform X2 [Ostrinia furnacalis] | 1.75452 | -0.8786 | -0.9998 | -0.2048 | 0.32875 |
| TRINITY_DN2172_c0_g2_i8_orf1 | - | - | - | hypothetical protein evm_003685 [Chilo suppressalis] | 0.86022 | 1.2087 | -1.5516 | -0.6176 | 0.10032 |
| TRINITY_DN3434_c0_g1_i1_orf1 | - | - | - | coiled-coil domain-containing protein 47 [Ostrinia furnacalis] >XP_028161458.1 coiled-coil domain-containing protein 47 [Ostrinia furnacalis] | 1.87746 | -0.9405 | -0.7547 | -0.1387 | -0.0437 |
| TRINITY_DN2973_c1_g1_i9_orf1 | - | - | - | unconventional myosin ID [Ostrinia furnacalis] | 1.98159 | -0.7413 | -0.3333 | -0.48 | -0.427 |
| TRINITY_DN15753_c0_g1_i1_orf1 | - | - | - | uncharacterized protein LOC114366450 [Ostrinia furnacalis] | 1.28076 | 1.04971 | -0.8339 | -1.2186 | -0.2779 |
| TRINITY_DN133760_c0_g1_i1_orf1 | - | - | - | THO complex subunit 7 homolog [Ostrinia furnacalis] | 1.78809 | 0.39056 | -0.5299 | -0.753 | -0.8958 |
| TRINITY_DN26503_c0_g1_i1_orf1 | - | - | - | ruvB-like 2 isoform X1 [Ostrinia furnacalis] >XP_028160979.1 ruvB-like 2 isoform X2 [Ostrinia furnacalis] | 1.83136 | 0.06752 | -1.1426 | -0.5364 | -0.2199 |
| TRINITY_DN38693_c0_g1_i4_orf1 | - | - | - | protein RER1 [Ostrinia furnacalis] >XP_028157304.1 protein RER1 [Ostrinia furnacalis] >XP_028157310.1 protein RER1 [Ostrinia furnacalis] | 1.90092 | 0.00412 | -0.9598 | -0.3765 | -0.5687 |
| TRINITY_DN40197_c0_g1_i1_orf1 | - | - | - | UDP-N-acetylglucosamine--dolichyl-phosphate N-acetylglucosaminephosphotransferase-like [Ostrinia furnacalis] | 1.7187 | 0.48878 | -1.0668 | -0.6666 | -0.4741 |
| TRINITY_DN7603_c0_g1_i5_orf1 | - | - | - | tetratricopeptide repeat protein 1-like [Ostrinia furnacalis] | 0.87622 | 1.46348 | -1.107 | -0.8457 | -0.387 |
| TRINITY_DN10287_c0_g1_i1_orf1 | - | - | - | nibrin [Ostrinia furnacalis] | 1.93448 | -0.8598 | -0.5806 | -0.4194 | -0.0747 |
| TRINITY_DN4752_c0_g1_i3_orf1 | - | - | - | thioredoxin domain-containing protein 9 [Ostrinia furnacalis] >XP_028172592.1 thioredoxin domain-containing protein 9 [Ostrinia furnacalis] | 1.93867 | -0.2401 | -0.6542 | -0.1977 | -0.8467 |
| TRINITY_DN18909_c0_g1_i6_orf1 | - | - | - | unnamed protein product [Euphydryas editha] | 1.57483 | -0.9876 | -0.0949 | 0.59478 | -1.0871 |
| TRINITY_DN28759_c0_g1_i1_orf1 | - | - | - | innexin inx2 [Ostrinia furnacalis] | 0.98902 | 1.3934 | -1.0363 | -0.4491 | -0.8971 |
| TRINITY_DN146758_c0_g1_i1_orf1 | - | - | - | PREDICTED: mitochondrial import inner membrane translocase subunit Tim16-like [Fopius arisanus] | 1.88956 | -0.8735 | -0.0101 | -0.2197 | -0.7862 |
| TRINITY_DN36592_c0_g1_i1_orf1 | - | - | - | uncharacterized protein LOC114359903 [Ostrinia furnacalis] | 1.88825 | -0.9864 | -0.4706 | -0.4868 | 0.05552 |
| TRINITY_DN2778_c0_g1_i5_orf1 | - | - | - | hypothetical protein evm_001346 [Chilo suppressalis] | 1.90349 | -0.088 | -0.92 | -0.6944 | -0.2012 |
| TRINITY_DN28622_c0_g1_i1_orf1 | - | - | - | actin-related protein 3 [Ostrinia furnacalis] | 1.06613 | 1.31412 | -1.1903 | -0.6715 | -0.5185 |
| TRINITY_DN26168_c0_g1_i1_orf1 | - | - | - | ATP-dependent RNA helicase Ddx1-like [Ostrinia furnacalis] | 1.5892 | 0.66818 | -1.2008 | -0.6462 | -0.4104 |
| TRINITY_DN14677_c0_g2_i3_orf1 | - | - | - | AP-3 complex subunit beta-2 [Ostrinia furnacalis] | 0.51627 | 1.15998 | -1.5438 | -0.7718 | 0.6394 |
| TRINITY_DN9085_c0_g1_i1_orf1 | - | - | - | golgin subfamily A member 2-like [Ostrinia furnacalis] | 1.89096 | -0.3343 | -1.0898 | -0.3226 | -0.1443 |
| TRINITY_DN84669_c0_g1_i1_orf1 | - | - | - | PREDICTED: microtubule-actin cross-linking factor 1, isoforms 1/2/3/5 [Amyelois transitella] | 0.86186 | 0.39561 | -1.9323 | 0.60143 | 0.07335 |
| TRINITY_DN17935_c0_g1_i1_orf1 | - | - | - | NEDD8-conjugating enzyme Ubc12 [Ostrinia furnacalis] | 1.46637 | 0.90037 | -0.9648 | -0.4504 | -0.9515 |
| TRINITY_DN6084_c0_g1_i4_orf1 | - | - | - | unconventional myosin-Va isoform X1 [Manduca sexta] | 1.6882 | 0.27426 | -0.6853 | -0.0102 | -1.2669 |
| TRINITY_DN35245_c0_g1_i1_orf1 | - | - | - | ras GTPase-activating protein-binding protein 2 isoform X1 [Nymphalis io] >XP_050349014.1 ras GTPase-activating protein-binding protein 2 isoform X1 [Nymphalis io] >XP_050349015.1 ras GTPase-activating protein-binding protein 2 isoform X2 [Nymphalis io] | 1.67913 | 0.28353 | -0.7821 | -1.2194 | 0.03885 |
| TRINITY_DN21218_c0_g1_i4_orf1 | - | - | - | leukotriene A-4 hydrolase isoform X2 [Ostrinia furnacalis] | 1.5094 | 0.83107 | -0.4105 | -0.9735 | -0.9565 |
| TRINITY_DN19687_c0_g1_i1_orf1 | - | - | - | probable ribosome production factor 1 [Ostrinia furnacalis] | 1.65503 | -0.1577 | 0.21897 | -0.2602 | -1.4562 |
| TRINITY_DN10110_c1_g2_i1_orf1 | - | - | - | venom allergen 3-like [Ostrinia furnacalis] | 0.51602 | 0.94586 | -1.644 | -0.6571 | 0.83929 |
| TRINITY_DN1790_c1_g1_i3_orf1 | - | - | - | unnamed protein product [Diatraea saccharalis] | 1.83992 | -0.5712 | -0.6734 | -0.8711 | 0.27582 |
| TRINITY_DN2265_c0_g1_i5_orf1 | - | - | - | elongation factor G, mitochondrial [Ostrinia furnacalis] | 1.67708 | -0.8562 | 0.21469 | 0.14253 | -1.1781 |
| TRINITY_DN12858_c0_g1_i5_orf1 | - | - | - | unnamed protein product, partial [Iphiclides podalirius] | 0.99682 | 1.3553 | -1.1376 | -0.8698 | -0.3448 |

| | | | | | | | | | |
|---|---|---|---|---|---|---|---|---|---|
| TRINITY_DN23790_c0_g1_i1_orf1 | - | - | - | wiskott-Aldrich syndrome protein family member 2 [Ostrinia furnacalis] | 1.37348 | 0.89355 | -1.3879 | -0.4741 | -0.405 |
| TRINITY_DN19155_c0_g1_i1_orf1 | - | - | - | cleavage and polyadenylation specificity factor 73 [Ostrinia furnacalis] | 1.70178 | 0.51656 | -0.7088 | -1.0673 | -0.4423 |
| TRINITY_DN2919_c0_g1_i5_orf1 | - | - | - | nidogen-1 [Ostrinia furnacalis] | 1.18571 | 0.80799 | -1.3334 | -1.0177 | 0.35735 |
| TRINITY_DN34479_c0_g1_i2_orf1 | - | - | - | PREDICTED: 26S protease regulatory subunit 4 [Amyelois transitella] >XP_021186380.1 26S proteasome regulatory subunit 4 [Helicoverpa armigera] >XP_022116536.1 26S proteasome regulatory subunit 4 [Pieris rapae] >XP_022817854.1 26S proteasome regulatory subunit 4 [Spodoptera litura] >XP_026745369.1 26S proteasome regulatory subunit 4 [Trichoplusia ni] >XP_026760570.1 26S proteasome regulatory subunit 4 [Galleria mellonella] >XP_028176505.1 26S proteasome regulatory subunit 4 [Ostrinia furnacalis] >XP_030038234.1 26S proteasome regulatory subunit 4 [Manduca sexta] >XP_035449919.1 26S proteasome regulatory subunit 4 [Spodoptera frugiperda] >XP_038206559.1 26S proteasome regulatory subunit 4 [Zerene cesonia] >XP_045502541.1 26S proteasome regulatory subunit 4 [Colias croceus] >XP_045532999.1 26S proteasome regulatory subunit 4 [Pieris brassicae] >XP_047033702.1 26S proteasome regulatory subunit 4 [Helicoverpa zea] >XP_047994509.1 26S proteasome regulatory subunit 4 [Leguminivora glycinivorella] >XP_049877826.1 26S proteasome regulatory subunit 4 [Pectinophora gossypiella] >KAH9639287.1 hypothetical protein HF086_014151 [Spodoptera exigua] >KAI5631153.1 ATPase family associated with various cellular activities (AAA) domain-containing protein [Phthorimaea operculella] >RVE50066.1 hypothetical protein evm_005272 [Chilo suppressalis] >CAB3245712.1 unnamed protein product [Arctia plantaginis] >KAF9801312.1 hypothetical protein SFRURICE_000406 [Spodoptera frugiperda] | 1.09943 | 0.52597 | -1.4898 | -0.8697 | 0.73405 |
| TRINITY_DN6696_c0_g1_i4_orf1 | - | - | - | mitochondrial 2-oxodicarboxylate carrier [Ostrinia furnacalis] | 1.81398 | 0.00062 | -0.7082 | -1.099 | -0.0074 |
| TRINITY_DN24164_c0_g1_i1_orf1 | - | - | - | PREDICTED: ras-related protein Rab6 [Microplitis demolitor] >XP_034947251.1 ras-related protein Rab6 isoform X2 [Chelonus insularis] >XP_044581106.1 ras-related protein Rab6 isoform X2 [Cotesia glomerata] >KAH0553942.1 Ras-protein Rab6 [Cotesia glomerata] >CAG5097549.1 Similar to Rab6: Ras-related protein Rab6 (Drosophila melanogaster) [Cotesia congregata] | 1.87496 | -0.759 | -0.8068 | 0.16923 | -0.4783 |
| TRINITY_DN93566_c0_g2_i1_orf1 | - | - | - | 60S acidic ribosomal protein P1 [Manduca sexta] >ACY95374.1 ribosomal protein P1 [Manduca sexta] >KAG6447985.1 hypothetical protein O3G_MSEX005254 [Manduca sexta] >KAG6447986.1 hypothetical protein O3G_MSEX005254 [Manduca sexta] | 1.82484 | -0.9643 | -0.7596 | -0.3316 | 0.23062 |
| TRINITY_DN21492_c0_g1_i1_orf1 | - | - | - | isocitrate dehydrogenase [NAD] subunit gamma, mitochondrial [Chelonus | 1.93121 | -0.4555 | -0.2855 | -0.2256 | -0.9647 |
| TRINITY_DN1227_c0_g1_i1_orf1 | - | - | - | uncharacterized protein LOC114356076 [Ostrinia furnacalis] | 1.87613 | 0.13814 | -0.4025 | -0.7979 | -0.8139 |
| TRINITY_DN146493_c0_g1_i1_orf1 | - | - | - | anaphase-promoting complex subunit 1 [Chelonus insularis] | 1.13254 | 0.9 | -1.5013 | -0.7717 | 0.24049 |
| TRINITY_DN1268_c0_g1_i1_orf1 | - | - | - | nuclear pore complex protein Nup154 [Ostrinia furnacalis] | 1.86347 | -1.0407 | -0.6562 | -0.0817 | -0.0849 |
| TRINITY_DN6901_c0_g1_i4_orf1 | - | - | - | (E3-independent) E2 ubiquitin-conjugating enzyme isoform X1 [Ostrinia | 1.45687 | 0.07566 | -1.3988 | 0.60625 | -0.7399 |
| TRINITY_DN7573_c0_g2_i1_orf1 | - | - | - | nucleolar protein 56 [Ostrinia furnacalis] | 1.94497 | -0.6233 | -0.5829 | -0.6979 | -0.0408 |
| TRINITY_DN3619_c0_g2_i1_orf1 | - | - | - | transport and Golgi organization protein 11 [Ostrinia furnacalis] | 1.80987 | 0.32895 | -0.8742 | -0.794 | -0.4706 |
| TRINITY_DN7839_c0_g1_i4_orf1 | - | - | - | elongin-C [Ostrinia furnacalis] >XP_028163922.1 elongin-C [Ostrinia furnacalis] | 1.80881 | -0.0973 | -1.234 | -0.4411 | -0.0364 |
| TRINITY_DN11639_c0_g1_i1_orf1 | - | - | - | cysteine--tRNA ligase, cytoplasmic isoform X1 [Ostrinia furnacalis] >XP_028156309.1 cysteine--tRNA ligase, cytoplasmic isoform X2 [Ostrinia furnacalis] >XP_028156310.1 cysteine--tRNA ligase, cytoplasmic isoform X3 [Ostrinia furnacalis] >XP_028156311.1 cysteine--tRNA ligase, cytoplasmic isoform X4 [Ostrinia furnacalis] | 1.97714 | -0.2156 | -0.509 | -0.6475 | -0.605 |
| TRINITY_DN37830_c0_g1_i1_orf1 | - | - | - | 60S ribosomal protein L18a [Galleria mellonella] >AXY94862.1 ribosomal protein L18A [Galleria mellonella] | 1.87016 | -0.7184 | -0.8647 | -0.4578 | 0.17073 |
| TRINITY_DN53115_c0_g1_i1_orf1 | - | - | - | small glutamine-rich tetratricopeptide repeat-containing protein beta-like [Ostrinia furnacalis] | 1.69032 | 0.582 | -0.8699 | -0.8801 | -0.5224 |

| | | | | | | | | | |
|---|---|---|---|---|---|---|---|---|---|
| TRINITY_DN6307_c0_g1_i5_orf1 | - | - | - | putative helicase mov-10-B.1 [Ostrinia furnacalis] | 1.9495 | -0.6574 | -0.2487 | -0.8055 | -0.2379 |
| TRINITY_DN59291_c0_g1_i1_orf1 | - | - | - | ATP-dependent RNA helicase vasa [Ostrinia furnacalis] | 1.17592 | 1.01904 | -1.5092 | -0.5246 | -0.1612 |
| TRINITY_DN17651_c0_g1_i2_orf1 | - | - | - | transmembrane protein 70 homolog, mitochondrial [Ostrinia furnacalis] | 1.9117 | -0.4126 | -0.1618 | -0.3117 | -1.0256 |
| TRINITY_DN11178_c0_g1_i1_orf1 | - | - | - | hypoxia up-regulated protein 1 [Ostrinia furnacalis] | 1.40009 | -0.3065 | -1.4987 | -0.3532 | 0.75832 |
| TRINITY_DN18593_c0_g1_i1_orf1 | - | - | - | 60S ribosomal protein L22-like [Ostrinia furnacalis] | 1.60541 | -0.863 | -0.9293 | -0.5377 | 0.72465 |
| TRINITY_DN383_c0_g1_i1_orf1 | - | - | - | probable Golgi SNAP receptor complex member 2 [Ostrinia furnacalis] | 1.33763 | 1.0129 | -1.2124 | -0.3858 | -0.7523 |
| TRINITY_DN3953_c0_g1_i2_orf1 | - | - | - | TP53-binding protein 1-like [Ostrinia furnacalis] | 0.98281 | 1.40753 | -0.4837 | -0.9792 | -0.9274 |
| TRINITY_DN3355_c0_g1_i1_orf1 | - | - | - | UDP-glucuronosyltransferase 2B2-like [Ostrinia furnacalis] | 1.77458 | -0.4064 | 0.24889 | -0.4109 | -1.2062 |
| TRINITY_DN19303_c0_g1_i5_orf1 | - | - | - | lipopolysaccharide-induced tumor necrosis factor-alpha factor-like [Ostrinia furnacalis] | 0.15729 | 1.18704 | -1.5891 | -0.5887 | 0.8334 |
| TRINITY_DN13186_c0_g1_i1_orf1 | - | - | - | NADH dehydrogenase [ubiquinone] iron-sulfur protein 5-like [Bicyclus | 1.83473 | -0.7678 | 0.27476 | -0.8561 | -0.4855 |
| TRINITY_DN40028_c0_g1_i1_orf1 | - | - | - | signal recognition particle receptor subunit alpha homolog [Ostrinia furnacalis] | 1.69412 | -0.3317 | -1.3849 | -0.2143 | 0.23677 |
| TRINITY_DN6567_c0_g1_i5_orf1 | - | - | - | dymeclin isoform X1 [Ostrinia furnacalis] >XP_028159584.1 dymeclin isoform X2 [Ostrinia furnacalis] | 1.30383 | 0.4583 | -1.737 | -0.2031 | 0.17794 |
| TRINITY_DN8908_c0_g1_i1_orf1 | - | - | - | unnamed protein product [Spodoptera littoralis] >CAH1641822.1 unnamed protein product [Spodoptera littoralis] | 1.75468 | -1.0164 | -0.5738 | -0.6043 | 0.4399 |
| TRINITY_DN35865_c0_g1_i1_orf1 | - | - | - | uncharacterized protein LOC114354496 isoform X1 [Ostrinia furnacalis] >XP_028162709.1 uncharacterized protein LOC114354496 isoform X1 [Ostrinia furnacalis] | 0.58931 | 0.75969 | -1.8603 | -0.2363 | 0.74765 |
| TRINITY_DN42269_c2_g1_i1_orf1 | - | - | - | probable enoyl-CoA hydratase, mitochondrial [Ostrinia furnacalis] | 1.8586 | -0.7145 | 0.01612 | -0.1548 | -1.0054 |
| TRINITY_DN5593_c0_g1_i1_orf1 | - | - | - | PREDICTED: leucine-rich repeat-containing protein 47-like [Fopius arisanus] | 1.68177 | -0.2982 | -0.3213 | -1.3723 | 0.31009 |
| TRINITY_DN6103_c0_g1_i6_orf1 | - | - | - | sedoheptulokinase-like [Ostrinia furnacalis] | 1.66278 | 0.56554 | -1.1528 | -0.4743 | -0.6012 |
| TRINITY_DN15811_c0_g1_i7_orf1 | - | - | - | mitochondrial import inner membrane translocase subunit Tim10-like [Ostrinia furnacalis] >XP_028174557.1 mitochondrial import inner membrane translocase subunit Tim10 [Ostrinia furnacalis] >XP_028174558.1 mitochondrial import inner membrane translocase subunit Tim10 [Ostrinia furnacalis] >XP_028174559.1 mitochondrial import inner membrane translocase subunit Tim10 [Ostrinia furnacalis] | 1.64591 | -1.1447 | -0.3005 | -0.76 | 0.55926 |
| TRINITY_DN54134_c0_g1_i1_orf1 | - | - | - | NFU1 iron-sulfur cluster scaffold homolog, mitochondrial-like [Ostrinia | 1.73374 | 0.37978 | -0.5724 | -1.1797 | -0.3614 |
| TRINITY_DN4429_c0_g1_i5_orf1 | - | - | - | FACT complex subunit Ssrp1 isoform X1 [Ostrinia furnacalis] >XP_028173375.1 FACT complex subunit Ssrp1 isoform X2 [Ostrinia furnacalis] >XP_028173376.1 FACT complex subunit Ssrp1 isoform X3 [Ostrinia furnacalis] | 1.78272 | 0.32037 | -0.3508 | -0.7017 | -1.0506 |
| TRINITY_DN43076_c0_g1_i6_orf1 | - | - | - | protein argonaute-2 isoform X2 [Pectinophora gossypiella] | 1.30249 | 0.99997 | -1.2656 | -0.805 | -0.2318 |
| TRINITY_DN64810_c0_g1_i1_orf1 | - | - | - | arginine--tRNA ligase, cytoplasmic [Ostrinia furnacalis] | 1.95154 | -0.7937 | -0.4745 | -0.5684 | -0.1148 |
| TRINITY_DN122867_c1_g1_i1_orf1 | - | - | - | nuclear migration protein nudC [Ostrinia furnacalis] | 1.42466 | 0.91471 | -0.9272 | -0.334 | -1.0781 |
| TRINITY_DN13347_c0_g1_i1_orf1 | - | - | - | endothelial differentiation-related factor 1 homolog [Ostrinia furnacalis] | 1.96592 | -0.7289 | -0.6612 | -0.3094 | -0.2665 |
| TRINITY_DN547_c0_g1_i1_orf1 | - | - | - | WD repeat-containing protein 43 [Ostrinia furnacalis] | 1.85489 | -0.572 | -0.1712 | -0.0151 | -1.0967 |
| TRINITY_DN121_c0_g1_i9_orf1 | - | - | - | lethal(2) giant larvae protein homolog 1 isoform X1 [Ostrinia furnacalis] | 1.99465 | -0.4104 | -0.532 | -0.6165 | -0.4358 |
| TRINITY_DN7451_c0_g1_i10_orf1 | - | - | - | huntingtin-interacting protein 1 isoform X4 [Pectinophora gossypiella] | 1.36859 | 0.72144 | -1.4419 | -0.7224 | 0.07426 |
| TRINITY_DN46022_c0_g1_i1_orf1 | - | - | - | mRNA-decapping enzyme 1A [Ostrinia furnacalis] | 1.70451 | 0.54563 | -0.9918 | -0.5253 | -0.7331 |
| TRINITY_DN6380_c0_g1_i1_orf1 | - | - | - | THAP domain-containing protein 1-like isoform X1 [Ostrinia furnacalis] | 1.55023 | 0.76833 | -1.0632 | -0.8376 | -0.4178 |
| TRINITY_DN7247_c0_g1_i7_orf1 | - | - | - | pyruvate kinase-like isoform X3 [Ostrinia furnacalis] | 1.44685 | 0.80446 | -1.3649 | -0.4001 | -0.4864 |
| TRINITY_DN37856_c0_g1_i5_orf1 | - | - | - | spermine synthase [Ostrinia furnacalis] | 1.71114 | -0.6958 | -1.0427 | -0.4864 | 0.51381 |

| | | | | | | | | | |
|---|---|---|---|---|---|---|---|---|---|
| TRINITY_DN2571_c0_g2_i1_orf1 | - | - | - | PREDICTED: huntingtin-interacting protein K isoform X1 [Amyelois transitella] >XP_013190319.1 PREDICTED: huntingtin-interacting protein K isoform X2 [Amyelois transitella] >XP_021200735.1 huntingtin-interacting protein K [Helicoverpa armigera] >XP_026737126.1 huntingtin-interacting protein K [Trichoplusia ni] >XP_041970494.1 huntingtin-interacting protein K [Aricia agestis] >XP_047022511.1 huntingtin-interacting protein K [Helicoverpa zea] >XP_047984206.1 huntingtin-interacting protein K [Leguminivora glycinivorella] >RVE51917.1 hypothetical protein evm_003383 [Chilo suppressalis] >PZC85551.1 hypothetical protein B5X24_HaOG216659 [Helicoverpa armigera] >CAB3530729.1 unnamed protein product [Chilo suppressalis] >CAH0407320.1 unnamed protein product [Chilo suppressalis] | 1.95339 | -0.7105 | -0.3291 | -0.7341 | -0.1798 |
| TRINITY_DN21218_c0_g2_i3_orf1 | - | - | - | leukotriene A-4 hydrolase isoform X2 [Ostrinia furnacalis] | 1.78678 | 0.39419 | -0.7154 | -0.9149 | -0.5507 |
| TRINITY_DN23089_c0_g1_i1_orf1 | - | - | - | integrator complex subunit 3 homolog [Ostrinia furnacalis] | 1.63143 | 0.01287 | -1.1357 | -0.9322 | 0.4236 |
| TRINITY_DN2642_c0_g1_i5_orf1 | - | - | - | protein LSM12 homolog [Ostrinia furnacalis] | 1.79136 | -1.0478 | -0.7933 | -0.152 | 0.20174 |
| TRINITY_DN7647_c0_g1_i4_orf1 | - | - | - | E3 ubiquitin-protein ligase Bre1 isoform X6 [Ostrinia furnacalis] | 1.74752 | 0.01027 | -1.0585 | -0.8886 | 0.18939 |
| TRINITY_DN3588_c0_g1_i1_orf1 | - | - | - | probable peroxisomal acyl-coenzyme A oxidase 1 [Ostrinia furnacalis] | 1.84004 | -1.0336 | -0.6112 | -0.3745 | 0.17927 |
| TRINITY_DN493_c0_g1_i4_orf1 | - | - | - | ADP-ribosylation factor GTPase-activating protein 3 [Ostrinia furnacalis] | 1.637 | 0.43613 | -1.2187 | -0.8012 | -0.0532 |
| TRINITY_DN7770_c0_g1_i4_orf1 | - | - | - | presequence protease, mitochondrial [Ostrinia furnacalis] | 1.92349 | -0.208 | -0.3926 | -0.324 | -0.9989 |
| TRINITY_DN605_c0_g1_i4_orf1 | - | - | - | dnaJ homolog subfamily C member 7 [Ostrinia furnacalis] | 1.96236 | -0.1367 | -0.5395 | -0.7215 | -0.5647 |
| TRINITY_DN33178_c0_g1_i1_orf1 | - | - | - | synaptojanin-1 [Ostrinia furnacalis] | 1.84277 | 0.12121 | -1.0747 | -0.5845 | -0.3048 |
| TRINITY_DN54711_c0_g1_i1_orf1 | - | - | - | 39S ribosomal protein L50, mitochondrial [Ostrinia furnacalis] | 1.92362 | -0.7354 | -0.834 | -0.1598 | -0.1945 |
| TRINITY_DN9062_c0_g2_i3_orf1 | - | - | - | ubiquitin conjugation factor E4 B isoform X2 [Ostrinia furnacalis] | 1.23562 | 1.205 | -0.9678 | -0.7375 | -0.7353 |
| TRINITY_DN10399_c0_g1_i2_orf1 | - | - | - | unnamed protein product [Chilo suppressalis] | 1.93833 | -0.2938 | -0.936 | -0.4757 | -0.2329 |
| TRINITY_DN63719_c0_g1_i5_orf1 | - | - | - | eukaryotic peptide chain release factor GTP-binding subunit ERF3A isoform X2 [Manduca sexta] >KAG6462744.1 hypothetical protein O3G_MSEX013441 [Manduca sexta] | 1.88792 | -0.3454 | -1.0797 | -0.0837 | -0.3791 |
| TRINITY_DN3924_c0_g1_i5_orf1 | - | - | - | SH3 domain-containing kinase-binding protein 1-like isoform X1 [Ostrinia furnacalis] | 1.75811 | 0.06345 | -1.2953 | -0.4738 | -0.0525 |
| TRINITY_DN3113_c1_g2_i1_orf1 | - | - | - | short-chain dehydrogenase/reductase family 16C member 6-like [Ostrinia furnacalis] >XP_028174076.1 short-chain dehydrogenase/reductase family 16C member 6-like [Ostrinia furnacalis] | 1.68137 | 0.49419 | -1.1145 | -0.7793 | -0.2818 |
| TRINITY_DN4810_c0_g1_i3_orf1 | - | - | - | clathrin interactor 1 isoform X2 [Maniola jurtina] | 1.92375 | -0.1265 | -0.955 | -0.5121 | -0.3302 |
| TRINITY_DN3411_c0_g2_i1_orf1 | - | - | - | putative U5 small nuclear ribonucleoprotein 200 kDa helicase, partial [Ostrinia furnacalis] | 1.35682 | 0.59473 | -1.5537 | 0.19622 | -0.5941 |
| TRINITY_DN34689_c0_g1_i4_orf1 | - | - | - | [illegible] >XP_049707197.1 uncharacterized protein DDB_G0283357 isoform X14 [Helicoverpa armigera] >XP_049707198.1 uncharacterized protein DDB_G0283357 isoform X15 [Helicoverpa armigera] >XP_049707199.1 uncharacterized protein DDB_G0283357 isoform X16 [Helicoverpa armigera] >XP_049707200.1 uncharacterized protein DDB_G0283357 isoform X17 [Helicoverpa armigera] >XP_049707201.1 uncharacterized protein DDB_G0283357 isoform X18 [Helicoverpa armigera] >XP_049707202.1 uncharacterized protein DDB_G0283357 isoform X19 [Helicoverpa armigera] >XP_049707203.1 uncharacterized protein DDB_G0283357 isoform X20 [Helicoverpa armigera] >XP_049707204.1 uncharacterized protein DDB_G0283357 isoform X21 [Helicoverpa armigera] >XP_049707205.1 uncharacterized protein DDB_G0283357 isoform X22 [Helicoverpa armigera] >XP_049707206.1 uncharacterized protein DDB_G0283357 isoform X23 [Helicoverpa armigera] >XP_049707207.1 uncharacterized protein [illegible] | 1.6624 | 0.64305 | -0.9152 | -0.7924 | -0.5979 |
| TRINITY_DN17726_c0_g1_i1_orf1 | - | - | - | ubiquitin conjugation factor E4 A isoform X1 [Ostrinia furnacalis] >XP_028165923.1 ubiquitin conjugation factor E4 A isoform X2 [Ostrinia | 1.71835 | 0.40813 | -1.0476 | -0.8566 | -0.2223 |

| | | | | | | | | | |
|---|---|---|---|---|---|---|---|---|---|
| TRINITY_DN9765_c0_g1_i6_orf1 | - | - | - | hypothetical protein evm_005049 [Chilo suppressalis] >CAB3525510.1 unnamed protein product [Chilo suppressalis] >CAH0402837.1 unnamed protein product [Chilo suppressalis] | 1.0182 | 1.28814 | -1.2969 | -0.742 | -0.2675 |
| TRINITY_DN65988_c0_g1_i4_orf1 | - | - | - | uncharacterized protein LOC114354070 isoform X3 [Ostrinia furnacalis] | 1.83783 | -0.3495 | -1.117 | -0.4887 | 0.11735 |
| TRINITY_DN1826_c0_g2_i4_orf1 | - | - | - | glutaminyl-peptide cyclotransferase-like [Ostrinia furnacalis] | 1.95417 | -0.1198 | -0.5029 | -0.7831 | -0.5483 |
| TRINITY_DN12777_c0_g1_i5_orf1 | - | - | - | clathrin light chain isoform X2 [Ostrinia furnacalis] | 0.33592 | 1.25539 | -1.5815 | -0.6412 | 0.63144 |
| TRINITY_DN10266_c0_g1_i5_orf1 | - | - | - | armadillo repeat-containing protein 6 homolog [Ostrinia furnacalis] | 1.6233 | -0.0482 | -1.4722 | -0.3595 | 0.25663 |
| TRINITY_DN5346_c0_g1_i5_orf1 | - | - | - | syntaxin-1A isoform X2 [Pectinophora gossypiella] | 1.04862 | 0.87033 | -1.209 | -1.2003 | 0.49038 |
| TRINITY_DN47723_c0_g1_i1_orf1 | - | - | - | dnaJ homolog subfamily C member 21 [Ostrinia furnacalis] | 1.49127 | 0.90825 | -0.6962 | -0.9407 | -0.7626 |
| TRINITY_DN6572_c0_g1_i2_orf1 | - | - | - | zinc finger protein 330 homolog [Ostrinia furnacalis] | 1.65946 | 0.46676 | -1.1728 | -0.7916 | -0.1618 |
| TRINITY_DN100885_c0_g2_i1_orfp1 | - | - | - | CCHC-type zinc finger, partial [Cricetulus griseus] | 1.95976 | -0.4507 | -0.7994 | -0.1741 | -0.5356 |
| TRINITY_DN25997_c1_g1_i1_orf1 | - | - | - | uncharacterized protein LOC114366342 [Ostrinia furnacalis] | 1.81469 | 0.1646 | -0.5594 | -1.1335 | -0.2864 |
| TRINITY_DN11124_c0_g1_i4_orf1 | - | - | - | hypothetical protein O3G_MSEX014253 [Manduca sexta] | 1.97311 | -0.2283 | -0.5342 | -0.7405 | -0.47 |
| TRINITY_DN821_c0_g1_i8_orf1 | - | - | - | nuclear pore complex protein Nup153 isoform X2 [Ostrinia furnacalis] | 1.9718 | -0.2767 | -0.4771 | -0.7908 | -0.4273 |
| TRINITY_DN2826_c0_g1_i7_orf1 | - | - | - | ATP-binding cassette subfamily D member 1 [Chilo suppressalis] >CAB3531327.1 unnamed protein product [Chilo suppressalis] >CAH0407919.1 unnamed protein product [Chilo suppressalis] | 1.63828 | 0.22299 | -0.6346 | 0.13208 | -1.3587 |
| TRINITY_DN7670_c0_g1_i1_orf1 | - | - | - | striatin-interacting protein 1 [Ostrinia furnacalis] | 1.29118 | 1.14576 | -0.8642 | -0.6513 | -0.9214 |
| TRINITY_DN43355_c0_g1_i1_orf1 | - | - | - | uncharacterized protein CG16817-like [Ostrinia furnacalis] | 1.76682 | 0.40952 | -1.0203 | -0.6047 | -0.5513 |
| TRINITY_DN16933_c0_g1_i10_orf1 | - | - | - | uridine phosphorylase 1-like [Ostrinia furnacalis] >XP_028164122.1 uridine phosphorylase 1-like [Ostrinia furnacalis] | 1.87393 | -0.3258 | -1.0564 | 0.02373 | -0.5155 |
| TRINITY_DN437_c0_g1_i1_orf1 | - | - | - | LOW QUALITY PROTEIN: fibronectin type-III domain-containing protein 3A-like [Ostrinia furnacalis] | 1.75865 | -0.1531 | -0.8239 | -1.0614 | 0.27975 |
| TRINITY_DN26429_c0_g1_i4_orf1 | - | - | - | zinc transporter 9 [Ostrinia furnacalis] | 1.78489 | 0.37631 | -0.9608 | -0.7206 | -0.4799 |
| TRINITY_DN33024_c0_g1_i1_orf1 | - | - | - | hypothetical protein evm_000959 [Chilo suppressalis] | 0.70644 | -0.015 | -1.7988 | 1.12453 | -0.0171 |
| TRINITY_DN19079_c0_g1_i5_orf1 | - | - | - | unnamed protein product [Euphydryas editha] | 1.63999 | 0.54989 | -1.0459 | -0.9324 | -0.2116 |
| TRINITY_DN48096_c0_g2_i2_orf1 | - | - | - | eukaryotic translation initiation factor 4E-like [Ostrinia furnacalis] | 1.54646 | -0.2639 | -1.5035 | -0.2459 | 0.46683 |
| TRINITY_DN2047_c0_g1_i1_orf1 | - | - | - | carboxylesterase CXE17 [Ostrinia furnacalis] | 1.98788 | -0.4797 | -0.4537 | -0.6952 | -0.3593 |
| TRINITY_DN397_c0_g1_i1_orf1 | - | - | - | striatin-3 isoform X1 [Ostrinia furnacalis] | 1.16791 | 1.22499 | -1.1843 | -0.639 | -0.5696 |
| TRINITY_DN11616_c0_g1_i3_orf1 | - | - | - | coiled-coil domain-containing protein 6-like [Ostrinia furnacalis] | 1.6739 | 0.56926 | -1.0088 | -0.3998 | -0.8345 |
| TRINITY_DN3299_c0_g1_i2_orf1 | - | - | - | metaxin-1 isoform X3 [Ostrinia furnacalis] >XP_028170907.1 metaxin-1 isoform X4 [Ostrinia furnacalis] | 1.87783 | -0.5157 | -0.4914 | -0.9773 | 0.10648 |
| TRINITY_DN4861_c0_g1_i7_orf1 | - | - | - | 2-hydroxyacyl-CoA lyase 1 isoform X1 [Ostrinia furnacalis] | 1.96999 | -0.8021 | -0.297 | -0.3709 | -0.5 |
| TRINITY_DN115_c0_g1_i6_orf1 | - | - | - | basigin [Ostrinia furnacalis] | 1.85199 | 0.22669 | -0.9168 | -0.5405 | -0.6214 |
| TRINITY_DN88640_c0_g1_i1_orf1 | - | - | - | tetratricopeptide repeat protein 27-like [Ostrinia furnacalis] | 1.88364 | -0.4904 | -0.6242 | 0.12837 | -0.8973 |
| TRINITY_DN1170_c0_g1_i8_orf1 | - | - | - | titin homolog [Trichoplusia ni] | 1.64949 | 0.53376 | -1.037 | -0.9351 | -0.2112 |
| TRINITY_DN40281_c0_g2_i1_orf1 | - | - | - | glyoxylate reductase/hydroxypyruvate reductase [Ostrinia furnacalis] | 1.84791 | -0.7791 | -0.6354 | 0.27349 | -0.7068 |
| TRINITY_DN1066_c0_g1_i8_orf1 | - | - | - | hypothetical protein evm_012420 [Chilo suppressalis] | 0.13392 | 1.40167 | -1.2174 | -1.0207 | 0.70251 |
| TRINITY_DN20776_c0_g1_i3_orf1 | - | - | - | hypothetical protein evm_003273 [Chilo suppressalis] >CAH2981954.1 unnamed protein product [Chilo suppressalis] | 1.97598 | -0.3411 | -0.7735 | -0.3605 | -0.5009 |
| TRINITY_DN37366_c0_g1_i7_orf1 | - | - | - | juvenile hormone epoxide hydrolase-like [Ostrinia furnacalis] | 1.81773 | -0.098 | -0.9459 | 0.1091 | -0.8829 |
| TRINITY_DN2783_c1_g1_i2_orf1 | - | - | - | proline-rich extensin-like protein EPR1 isoform X1 [Ostrinia furnacalis] >XP_028168549.1 proline-rich extensin-like protein EPR1 isoform X2 [Ostrinia furnacalis] >XP_028168550.1 proline-rich extensin-like protein EPR1 isoform X2 [Ostrinia furnacalis] | 1.79178 | 0.40543 | -0.6445 | -0.7299 | -0.8229 |
| TRINITY_DN9558_c0_g1_i2_orf1 | - | - | - | NADH dehydrogenase [ubiquinone] iron-sulfur protein 4, mitochondrial-like [Ostrinia furnacalis] | 1.90297 | -0.6197 | 0.11139 | -0.6276 | -0.767 |
| TRINITY_DN31751_c0_g1_i5_orf1 | - | - | - | THO complex subunit 3 [Ostrinia furnacalis] | 1.91037 | 0.06919 | -0.6182 | -0.8165 | -0.5449 |
| TRINITY_DN4152_c0_g1_i1_orf1 | - | - | - | importin subunit beta-1 isoform X2 [Ostrinia furnacalis] | 1.49974 | 0.87763 | -0.8199 | -0.9972 | -0.5603 |

| | | | | | | | | | |
|---|---|---|---|---|---|---|---|---|---|
| TRINITY_DN1661_c0_g1_i1_orf1 | - | - | - | NAD(P) transhydrogenase, mitochondrial-like [Ostrinia furnacalis] >XP_028175067.1 NAD(P) transhydrogenase, mitochondrial-like [Ostrinia furnacalis] >XP_028175068.1 NAD(P) transhydrogenase, mitochondrial-like [Ostrinia furnacalis] >XP_028175069.1 NAD(P) transhydrogenase, | 1.18743 | 1.17694 | -1.2652 | -0.5545 | -0.5447 |
| TRINITY_DN11612_c0_g3_i1_orf1 | - | - | - | hypothetical protein O3G_MSEX005294 [Manduca sexta] | 1.80689 | 0.11843 | -1.1977 | -0.2591 | -0.4686 |
| TRINITY_DN27771_c0_g1_i1_orf1 | - | - | - | glycine--tRNA ligase [Ostrinia furnacalis] | 1.36704 | 0.41561 | -1.4953 | -0.7274 | 0.43999 |
| TRINITY_DN3472_c1_g1_i4_orf1 | - | - | - | Krueppel homolog 2-like [Ostrinia furnacalis] | 1.18275 | 1.06893 | -1.4623 | -0.3288 | -0.4606 |
| TRINITY_DN10007_c0_g1_i1_orf1 | - | - | - | 28S ribosomal protein S31, mitochondrial [Ostrinia furnacalis] | 1.82583 | -0.9314 | 0.18159 | -0.2323 | -0.8438 |
| TRINITY_DN639_c0_g1_i10_orf1 | - | - | - | unnamed protein product, partial [Iphiclides podalirius] | 1.69596 | -0.8821 | -0.9395 | -0.4143 | 0.53985 |
| TRINITY_DN1494_c0_g2_i1_orf1 | - | - | - | dihydropyrimidine dehydrogenase [NADP(+)] [Ostrinia furnacalis] | 1.97751 | -0.2573 | -0.5044 | -0.7298 | -0.486 |
| TRINITY_DN2876_c0_g1_i3_orf1 | - | - | - | long-chain fatty acid transport protein 4-like isoform X1 [Ostrinia furnacalis] | 1.78276 | 0.31322 | -0.2852 | -0.9435 | -0.8673 |
| TRINITY_DN9156_c0_g1_i1_orf1 | - | - | - | GTP:AMP phosphotransferase AK3, mitochondrial [Ostrinia furnacalis] | 1.89235 | -0.3352 | -0.2977 | -0.1688 | -1.0907 |
| TRINITY_DN16187_c0_g1_i1_orf1 | - | - | - | eIF-2-alpha kinase activator GCN1 [Colias croceus] | 1.61431 | -0.6773 | -1.3086 | -0.0916 | 0.46317 |
| TRINITY_DN10385_c0_g1_i5_orf1 | - | - | - | unnamed protein product [Arctia plantaginis] >CAB3259747.1 unnamed protein product [Arctia plantaginis] | 1.10536 | 1.19196 | -1.3957 | -0.4891 | -0.4125 |
| TRINITY_DN57462_c0_g1_i1_orf1 | - | - | - | glutathione S transferase-S5 [Glyphodes pyloalis] | 1.67025 | 0.25295 | -0.2913 | -0.2119 | -1.4201 |
| TRINITY_DN22941_c0_g1_i1_orf1 | - | - | - | protein CWC15 homolog [Ostrinia furnacalis] | 1.91417 | -0.9673 | -0.51 | -0.3682 | -0.0687 |
| TRINITY_DN48838_c0_g1_i6_orf1 | - | - | - | merlin-like [Ostrinia furnacalis] | 1.76654 | 0.37802 | -1.0638 | -0.6423 | -0.4385 |
| TRINITY_DN332_c0_g1_i6_orf1 | - | - | - | dolichyl-diphosphooligosaccharide--protein glycosyltransferase subunit STT3B isoform X2 [Ostrinia furnacalis] | 1.78072 | -0.6519 | -1.09 | 0.30859 | -0.3474 |
| TRINITY_DN36682_c0_g1_i1_orf1 | - | - | - | uncharacterized protein DDB_G0287625-like [Ostrinia furnacalis] | 1.91691 | -0.7495 | -0.8222 | -0.054 | -0.2912 |
| TRINITY_DN607_c0_g1_i16_orf1 | - | - | - | protein muscleblind isoform X1 [Ostrinia furnacalis] >XP_028162801.1 protein muscleblind isoform X1 [Ostrinia furnacalis] >XP_028162802.1 protein muscleblind isoform X1 [Ostrinia furnacalis] >XP_028162803.1 protein muscleblind isoform X1 [Ostrinia furnacalis] >XP_028162804.1 protein muscleblind isoform X1 [Ostrinia furnacalis] >XP_028162806.1 protein | 1.70602 | 0.48259 | -1.1412 | -0.5773 | -0.4701 |
| TRINITY_DN30224_c0_g1_i1_orf1 | - | - | - | PREDICTED: serine--tRNA ligase, mitochondrial [Amyelois transitella] | 1.83442 | -0.8185 | -0.9086 | 0.20486 | -0.3122 |
| TRINITY_DN11409_c0_g1_i4_orf1 | - | - | - | nodal modulator 1 [Ostrinia furnacalis] | 1.87914 | -0.1258 | -1.0037 | -0.6618 | -0.0879 |
| TRINITY_DN24323_c0_g1_i3_orf1 | - | - | - | ubiquitin-protein ligase E3A [Ostrinia furnacalis] | 1.13278 | 0.70077 | -1.7512 | -0.3201 | 0.23779 |
| TRINITY_DN51050_c0_g1_i3_orf1 | - | - | - | GRB10-interacting GYF protein 2 isoform X1 [Ostrinia furnacalis] | 1.58586 | 0.55788 | -1.3687 | -0.3926 | -0.3824 |
| TRINITY_DN14705_c0_g2_i1_orf1 | - | - | - | coiled-coil-helix-coiled-coil-helix domain-containing protein 10, mitochondrial [Ostrinia furnacalis] | 1.68348 | -0.0345 | 0.36458 | -1.0545 | -0.9591 |
| TRINITY_DN5559_c0_g1_i1_orf1 | - | - | - | ferrochelatase, mitochondrial isoform X2 [Ostrinia furnacalis] | 1.95003 | -0.6331 | -0.2314 | -0.2655 | -0.8201 |
| TRINITY_DN6668_c0_g1_i4_orf1 | - | - | - | UBX domain-containing protein 4 isoform X1 [Ostrinia furnacalis] >XP_028156702.1 UBX domain-containing protein 4 isoform X2 [Ostrinia furnacalis] | 1.78787 | -1.0165 | 0.23601 | -0.8255 | -0.1819 |
| TRINITY_DN28509_c0_g1_i1_orf1 | - | - | - | 39S ribosomal protein L43, mitochondrial [Ostrinia furnacalis] | 1.82792 | -0.0755 | -0.0412 | -0.5496 | -1.1616 |
| TRINITY_DN2258_c0_g2_i1_orf1 | - | - | - | 60S ribosomal protein L14 [Ostrinia furnacalis] | 1.91725 | -0.3188 | -0.6574 | -0.8874 | -0.0536 |
| TRINITY_DN2497_c0_g1_i1_orf1 | - | - | - | protein stunted-like isoform X2 [Vanessa tameamea] >XP_046960183.1 protein stunted-like isoform X2 [Vanessa cardui] >XP_047527093.1 protein stunted-like isoform X2 [Vanessa atalanta] | 1.24143 | 0.58633 | -0.2191 | 0.13726 | -1.7459 |
| TRINITY_DN5512_c0_g1_i8_orf1 | - | - | - | peroxisomal multifunctional enzyme type 2-like isoform X1 [Ostrinia furnacalis] | 1.86661 | -0.0781 | -1.1344 | -0.3936 | -0.2605 |
| TRINITY_DN2802_c1_g1_i1_orf1 | - | - | - | psi [Ostrinia furnacalis] | 1.25928 | 0.99697 | -0.9897 | -1.1984 | -0.0682 |
| TRINITY_DN69049_c0_g2_i1_orf1 | - | - | - | membrane alanyl aminopeptidase-like [Ostrinia furnacalis] | 1.63304 | 0.17825 | 0.02351 | -1.4731 | -0.3617 |
| TRINITY_DN3488_c0_g1_i2_orf1 | - | - | - | hsp70-binding protein 1 isoform X1 [Ostrinia furnacalis] >XP_028170128.1 hsp70-binding protein 1 isoform X2 [Ostrinia furnacalis] | 1.82638 | 0.13335 | -0.5823 | -1.1123 | -0.2651 |
| TRINITY_DN1368_c0_g1_i6_orf1 | - | - | - | hypothetical protein SFRUCORN_009408 [Spodoptera frugiperda] | 1.80408 | 0.11754 | -1.0951 | -0.1045 | -0.722 |
| TRINITY_DN2967_c0_g1_i4_orf1 | - | - | - | UDP-glycosyltransferase UGT41G1 [Ostrinia furnacalis] | 1.87142 | -0.7238 | -0.1241 | -0.0455 | -0.978 |
| TRINITY_DN10058_c0_g1_i1_orf1 | - | - | - | dolichyl-diphosphooligosaccharide--protein glycosyltransferase subunit DAD1 [Ostrinia furnacalis] | 1.94212 | -0.7221 | -0.7509 | -0.1063 | -0.3629 |

| | | | | | | | | | |
|---|---|---|---|---|---|---|---|---|---|
| TRINITY_DN18696_c0_g1_i1_orf1 | - | - | - | rap guanine nucleotide exchange factor 2 [Ostrinia furnacalis] >XP_028159576.1 rap guanine nucleotide exchange factor 2 [Ostrinia furnacalis] | 1.9374 | -0.7403 | -0.4904 | -0.676 | -0.0307 |
| TRINITY_DN30950_c0_g1_i13_orf1 | - | - | - | unnamed protein product [Chilo suppressalis] | 1.64044 | 0.37533 | -1.1569 | -0.9095 | 0.05061 |
| TRINITY_DN11121_c0_g1_i5_orf1 | - | - | - | unnamed protein product [Chilo suppressalis] | 1.88124 | -0.8591 | -0.7818 | -0.3237 | 0.0833 |
| TRINITY_DN2356_c2_g1_i6_orf1 | - | - | - | ER membrane protein complex subunit 3 [Ostrinia furnacalis] | 1.59098 | -0.1359 | -1.07 | -0.9772 | 0.59208 |
| TRINITY_DN14475_c0_g1_i1_orf1 | - | - | - | NPC intracellular cholesterol transporter 1 homolog 1b-like [Ostrinia furnacalis] | 1.83988 | -0.7807 | -0.2725 | 0.16416 | -0.9509 |
| TRINITY_DN5976_c0_g1_i1_orf1 | - | - | - | ribosomal protein L32 [Bombyx mori] >XP_028034407.1 60S ribosomal protein L32 [Bombyx mandarina] >AAV34844.1 ribosomal protein L32 [Bombyx mori] | 1.9104 | -0.812 | -0.6804 | -0.4743 | 0.05624 |
| TRINITY_DN19413_c0_g1_i2_orf1 | - | - | - | U4/U6 small nuclear ribonucleoprotein Prp4 [Papilio xuthus] | 1.94203 | -0.692 | -0.713 | -0.0482 | -0.4889 |
| TRINITY_DN31303_c0_g1_i4_orf1 | - | - | - | tRNA pseudouridine synthase A isoform X1 [Ostrinia furnacalis] | 1.95065 | -0.1958 | -0.6146 | -0.8239 | -0.3164 |
| TRINITY_DN38341_c0_g2_i2_orf1 | - | - | - | delta(24)-sterol reductase-like [Ostrinia furnacalis] | 1.97622 | -0.7087 | -0.2878 | -0.6114 | -0.3683 |
| TRINITY_DN17212_c0_g1_i6_orf1 | - | - | - | putative deoxyribonuclease TATDN1 [Ostrinia furnacalis] | 1.82688 | 0.31153 | -0.7116 | -0.5699 | -0.8569 |
| TRINITY_DN1772_c1_g3_i1_orf1 | - | - | - | protein Red isoform X1 [Ostrinia furnacalis] >XP_028157673.1 protein Red isoform X2 [Ostrinia furnacalis] >XP_028157674.1 protein Red isoform X3 [Ostrinia furnacalis] >XP_028157675.1 protein Red isoform X4 [Ostrinia | 1.7625 | -0.2952 | -1.1243 | -0.6632 | 0.32019 |
| TRINITY_DN2630_c0_g3_i3_orf1 | - | - | - | eukaryotic translation initiation factor 4E-binding protein 2 [Ostrinia furnacalis] | 1.81823 | -0.3579 | 0.18898 | -1.1163 | -0.533 |
| TRINITY_DN32822_c0_g1_i1_orf1 | - | - | - | eukaryotic translation initiation factor 2 subunit 1 [Ostrinia furnacalis] | 1.96211 | -0.5648 | -0.8109 | -0.2647 | -0.3218 |
| TRINITY_DN31232_c1_g1_i9_orf1 | - | - | - | PREDICTED: elongation factor 1-alpha 1, partial [Haliaeetus albicilla] | 1.93574 | -0.7574 | -0.0493 | -0.705 | -0.424 |
| TRINITY_DN749_c0_g1_i1_orf1 | - | - | - | serine/threonine-protein phosphatase 4 regulatory subunit 3 isoform X3 [Ostrinia furnacalis] | 1.2095 | 1.2125 | -1.0408 | -0.5684 | -0.8128 |
| TRINITY_DN277_c1_g1_i1_orf1 | - | - | - | uncharacterized protein LOC114363802 isoform X4 [Ostrinia furnacalis] | 0.69836 | 1.26086 | -1.6951 | -0.0473 | -0.2168 |
| TRINITY_DN17446_c0_g1_i1_orf1 | - | - | - | eukaryotic translation initiation factor 3 subunit E [Ostrinia furnacalis] | 1.93567 | -0.7033 | -0.6366 | -0.5944 | -0.0014 |
| TRINITY_DN4272_c0_g1_i1_orf1 | - | - | - | MICOS complex subunit MIC19-like [Ostrinia furnacalis] | 1.83045 | -0.6584 | -1.091 | 0.06524 | -0.1463 |
| TRINITY_DN817_c0_g1_i3_orf1 | - | - | - | phenylalanine--tRNA ligase beta subunit [Ostrinia furnacalis] | 1.58954 | -0.8479 | -1.1131 | -0.2867 | 0.65817 |

| | | | | | | | | | |
|---|---|---|---|---|---|---|---|---|---|
| TRINITY_DN50724_c0_g2_i1_orf1 | - | - | - | 40S ribosomal protein S13 [Papilio polytes] >NP_001299165.1 40S ribosomal protein S13 [Papilio xuthus] >XP_013193651.1 PREDICTED: 40S ribosomal protein S13 [Amyelois transitella] >XP_014356884.1 40S ribosomal protein S13 [Papilio machaon] >XP_021184589.1 40S ribosomal protein S13 [Helicoverpa armigera] >XP_022827875.1 40S ribosomal protein S13 [Spodoptera litura] >XP_023936121.1 40S ribosomal protein S13 [Bicyclus anynana] >XP_026318936.1 40S ribosomal protein S13 [Hyposmocoma kahamanoa] >XP_026488656.1 40S ribosomal protein S13 [Vanessa tameamea] >XP_026736523.1 40S ribosomal protein S13 [Trichoplusia ni] >XP_028172792.1 40S ribosomal protein S13 [Ostrinia furnacalis] >XP_032516773.1 40S ribosomal protein S13 [Danaus plexippus plexippus] >XP_034829282.1 40S ribosomal protein S13 [Maniola hyperantus] >XP_035450454.1 40S ribosomal protein S13 [Spodoptera frugiperda] >XP_039754671.1 40S ribosomal protein S13 [Pararge aegeria] >XP_045449727.1 40S ribosomal protein S13 [Melitaea cinxia] >XP_046977814.1 40S ribosomal protein S13 [Vanessa cardui] >XP_047024943.1 40S ribosomal protein S13 [Helicoverpa zea] >XP_047531020.1 40S ribosomal protein S13 [Vanessa atalanta] >XP_047990290.1 40S ribosomal protein S13 [Leguminivora glycinivorella] >XP_050348531.1 40S ribosomal protein S13 [Nymphalis io] >Q962R6.3 RecName: Full=40S ribosomal protein S13 [Spodoptera frugiperda] >ADT80641.1 ribosomal protein S13 [Euphydryas aurinia] >ATG34155.1 ribosomal protein S13 [Epirrita autumnata] >KAF9422710.1 hypothetical protein HW555_001704 [Spodoptera exigua] >KAG7298871.1 ribosomal 40S subunit protein S13 [Plutella xylostella] >KAI5637622.1 ribosomal protein s15 domain-containing protein [Phthorimaea operculella] >RVE47566.1 hypothetical protein evm_007764 [Chilo suppressalis] >UNW37540.1 ribosomal protein S13 [Sesamia inferens] >CAB3507114.1 unnamed protein product [Spodoptera littoralis] >CAD0201945.1 unnamed protein product [Chrysodeixis includens] >CAG4974609.1 unnamed protein product [Parnassius apollo] >CAG9562339.1 unnamed protein product [Danaus chrysippus] >CAG9749269.1 unnamed | 1.93018 | -0.6421 | -0.7882 | -0.4906 | -0.0092 |
| TRINITY_DN13233_c0_g1_i3_orf1 | - | - | - | 60S ribosomal protein L31 [Galleria mellonella] >XP_028158009.1 60S ribosomal protein L31 [Ostrinia furnacalis] >XP_030037192.1 60S ribosomal protein L31 [Manduca sexta] >XP_046978528.1 60S ribosomal protein L31 [Vanessa cardui] >XP_047545474.1 60S ribosomal protein L31 [Vanessa atalanta] >XP_050342244.1 60S ribosomal protein L31 [Nymphalis io] >GBP35474.1 60S ribosomal protein L31 [Eumeta japonica] >ACY95330.1 ribosomal protein L31 [Manduca sexta] >KAG6463984.1 hypothetical protein O3G_MSEX014198 [Manduca sexta] >KAG6463985.1 hypothetical protein O3G_MSEX014198 | 1.73542 | -1.0013 | -0.7569 | -0.4428 | 0.46558 |
| TRINITY_DN647_c4_g1_i1_orf1 | - | - | - | coiled-coil domain-containing protein 51-like isoform X2 [Ostrinia furnacalis] | 1.84751 | -0.0865 | -1.0851 | -0.6324 | -0.0436 |
| TRINITY_DN20279_c0_g1_i1_orf1 | - | - | - | NADH dehydrogenase [ubiquinone] iron-sulfur protein 3, mitochondrial [Ostrinia furnacalis] | 1.97801 | -0.7421 | -0.2827 | -0.5124 | -0.4408 |
| TRINITY_DN8012_c0_g1_i3_orf1 | - | - | - | uncharacterized protein LOC114354053 [Ostrinia furnacalis] | 1.89163 | 0.06858 | -0.6358 | -0.4016 | -0.9227 |
| TRINITY_DN7228_c0_g1_i6_orf1 | - | - | - | neutral alpha-glucosidase AB [Ostrinia furnacalis] | 1.74987 | 0.4062 | -0.7908 | -0.3543 | -1.011 |
| TRINITY_DN101922_c0_g1_i1_orf1 | - | - | - | PREDICTED: uncharacterized protein LOC103569676 [Microplitis demolitor] | 1.75806 | -0.3851 | 0.2 | -0.2946 | -1.2783 |
| TRINITY_DN942_c0_g1_i1_orf1 | - | - | - | probable nuclear transport factor 2 isoform X1 [Ostrinia furnacalis] | 1.13645 | 1.27419 | -1.1122 | -0.5995 | -0.6989 |
| TRINITY_DN5756_c0_g1_i4_orf1 | - | - | - | leucine--tRNA ligase, cytoplasmic [Ostrinia furnacalis] | 1.8363 | -0.7905 | -0.8929 | -0.388 | 0.2351 |
| TRINITY_DN16886_c0_g1_i4_orf1 | - | - | - | ER membrane protein complex subunit 1 [Ostrinia furnacalis] | 1.97012 | -0.7396 | -0.5539 | -0.4724 | -0.2042 |
| TRINITY_DN1824_c0_g2_i2_orf1 | - | - | - | branched-chain-amino-acid aminotransferase, cytosolic [Manduca sexta] >KAG6453781.1 hypothetical protein O3G_MSEX008327 [Manduca sexta] | 1.84705 | -0.3769 | -0.448 | 0.0903 | -1.1124 |
| TRINITY_DN63561_c1_g1_i2_orf1 | - | - | - | unnamed protein product [Diatraea saccharalis] | 1.69554 | 0.50402 | -1.1236 | -0.66 | -0.416 |
| TRINITY_DN20499_c0_g1_i1_orf1 | - | - | - | exosome RNA helicase MTR4 isoform X2 [Ostrinia furnacalis] | 1.98885 | -0.3007 | -0.6122 | -0.5527 | -0.5232 |

| | | | | | | | | | |
|---|---|---|---|---|---|---|---|---|---|
| TRINITY_DN659_c0_g1_i3_orf1 | - | - | - | probable malonyl-CoA-acyl carrier protein transacylase, mitochondrial [Ostrinia furnacalis] | 1.94117 | -0.2748 | -0.635 | -0.1831 | -0.8483 |
| TRINITY_DN40508_c0_g1_i1_orf1 | - | - | - | mRNA turnover protein 4 homolog [Ostrinia furnacalis] | 1.87457 | -0.3468 | -0.1155 | -0.2848 | -1.1275 |
| TRINITY_DN5952_c0_g1_i6_orf1 | - | - | - | LOW QUALITY PROTEIN: phosphoacetylglucosamine mutase [Ostrinia | 1.41135 | 0.98347 | -1.0805 | -0.7265 | -0.5878 |
| TRINITY_DN1443_c0_g1_i4_orf1 | - | - | - | ATP-dependent RNA helicase DDX3X isoform X1 [Ostrinia furnacalis] | 1.86376 | -0.1906 | -1.1559 | -0.158 | -0.3593 |
| TRINITY_DN2738_c1_g1_i3_orf1 | - | - | - | uridine-cytidine kinase isoform X1 [Helicoverpa zea] >XP_049697747.1 uridine-cytidine kinase-like isoform X1 [Helicoverpa armigera] >XP_049698409.1 uridine-cytidine kinase isoform X1 [Helicoverpa armigera] | 1.80845 | 0.35777 | -0.8628 | -0.7135 | -0.5899 |
| TRINITY_DN7161_c0_g1_i7_orf1 | - | - | - | ATP-binding cassette sub-family E member 1 [Ostrinia furnacalis] | 1.8556 | -0.2056 | -1.0816 | -0.5868 | 0.01835 |
| TRINITY_DN10502_c0_g1_i4_orf1 | - | - | - | interleukin enhancer-binding factor 2 homolog [Ostrinia furnacalis] | 1.61346 | 0.70871 | -1.0157 | -0.7219 | -0.5847 |
| TRINITY_DN6071_c0_g1_i1_orf1 | - | - | - | transcription initiation factor IIB isoform X1 [Manduca sexta] >XP_038208211.1 transcription initiation factor IIB isoform X1 [Zerene cesonia] >XP_045510964.1 transcription initiation factor IIB isoform X1 [Colias croceus] >XP_049872283.1 transcription initiation factor IIB [Pectinophora gossypiella] | 1.09227 | 0.7522 | -0.9879 | -1.4025 | 0.54597 |
| TRINITY_DN26650_c0_g1_i1_orfp1 | - | - | - | TRINITY_DN26650_c0_g1_i1_m.72504 TRINITY_DN26650_c0_g1::TRINITY_DN26650_c0_g1_i1::g.72504 ORF type:5prime_partial len:72 (-),score=2.71 TRINITY_DN26650_c0_g1_i1:317-532(- | 1.93774 | -0.6583 | -0.6152 | -0.6583 | -0.006 |
| TRINITY_DN1316_c0_g1_i1_orf1 | - | - | - | mitochondrial import receptor subunit TOM70 [Ostrinia furnacalis] | 1.95829 | -0.2687 | -0.2585 | -0.6834 | -0.7477 |
| TRINITY_DN8290_c0_g1_i3_orf1 | - | - | - | zinc finger CCHC domain-containing protein 8 homolog [Ostrinia furnacalis] | 1.59511 | 0.49477 | -1.3079 | -0.7028 | -0.0792 |
| TRINITY_DN7828_c0_g1_i2_orf1 | - | - | - | alpha-N-acetylgalactosaminidase-like isoform X1 [Ostrinia furnacalis] >XP_028171449.1 alpha-N-acetylgalactosaminidase-like isoform X2 [Ostrinia furnacalis] | 1.28233 | 1.15496 | -0.9675 | -0.6823 | -0.7875 |
| TRINITY_DN20339_c0_g1_i3_orf1 | - | - | - | ecto-NOX disulfide-thiol exchanger 2-like [Ostrinia furnacalis] | 1.35072 | 0.80429 | -1.2661 | -0.9595 | 0.07061 |
| TRINITY_DN98723_c1_g1_i1_orf1 | - | - | - | uncharacterized protein LOC114362777 [Ostrinia furnacalis] | 1.77842 | -1.0013 | -0.8817 | -0.1084 | 0.21299 |
| TRINITY_DN1884_c0_g2_i2_orf1 | - | - | - | phosphotriesterase-related protein [Ostrinia furnacalis] | 1.75551 | -0.8105 | 0.05385 | 0.11683 | -1.1157 |
| TRINITY_DN115498_c0_g1_i1_orf1 | - | - | - | fatty acid synthase-like [Ostrinia furnacalis] | 1.93161 | -0.89 | -0.448 | -0.0733 | -0.5202 |
| TRINITY_DN6535_c0_g1_i3_orf1 | - | - | - | mRNA export factor [Ostrinia furnacalis] | 1.85815 | -0.4875 | -0.9364 | -0.6285 | 0.19425 |
| TRINITY_DN2224_c0_g2_i1_orf1 | - | - | - | tumor susceptibility gene 101 protein [Ostrinia furnacalis] | 1.33572 | 0.43051 | -1.7206 | -0.2085 | 0.16291 |
| TRINITY_DN5169_c0_g1_i5_orf1 | - | - | - | ero1-like protein isoform X1 [Ostrinia furnacalis] | 1.99294 | -0.5872 | -0.5872 | -0.4516 | -0.3669 |
| TRINITY_DN23801_c0_g1_i2_orf1 | - | - | - | signal recognition particle subunit SRP72 [Pectinophora gossypiella] | 1.68095 | -0.385 | -1.3697 | -0.2345 | 0.3083 |
| TRINITY_DN344_c1_g1_i1_orf1 | - | - | - | chymotrypsin-like serine protease 16 [Ostrinia nubilalis] | 1.52301 | -1.1001 | 0.82822 | -0.5969 | -0.6543 |
| TRINITY_DN3335_c0_g1_i1_orf1 | - | - | - | unnamed protein product [Pieris macdunnoughi] | 0.50244 | 0.83144 | -1.5196 | -0.8372 | 1.02287 |
| TRINITY_DN14498_c0_g1_i1_orf1 | - | - | - | eukaryotic translation initiation factor 2 subunit 2 [Ostrinia furnacalis] | 1.75972 | -1.0276 | -0.7603 | -0.3525 | 0.38076 |
| TRINITY_DN140538_c0_g2_i1_orf1 | - | - | - | peptidyl-prolyl cis-trans isomerase NIMA-interacting 1 [Urocitellus parryii] | 1.52431 | -0.1122 | -1.5481 | -0.2911 | 0.42719 |
| TRINITY_DN4451_c0_g1_i1_orf1 | - | - | - | uncharacterized protein LOC114361986 isoform X1 [Ostrinia furnacalis] >XP_028173022.1 uncharacterized protein LOC114361986 isoform X2 [Ostrinia furnacalis] | 1.86483 | -0.557 | 0.21827 | -0.7596 | -0.7666 |
| TRINITY_DN31433_c0_g1_i1_orf1 | - | - | - | notchless protein homolog 1 [Ostrinia furnacalis] | 1.92904 | -0.3749 | -0.6135 | -0.0706 | -0.87 |
| TRINITY_DN3176_c0_g1_i2_orf1 | - | - | - | dnaJ homolog subfamily B member 6 isoform X2 [Ostrinia furnacalis] | 1.61141 | 0.74072 | -0.8475 | -0.7995 | -0.7051 |
| TRINITY_DN2365_c0_g1_i6_orf1 | - | - | - | carnitine O-acetyltransferase isoform X2 [Ostrinia furnacalis] | 1.81968 | 0.31489 | -0.5987 | -0.6071 | -0.9288 |
| TRINITY_DN25285_c0_g1_i1_orf1 | - | - | - | pancreatic triacylglycerol lipase-like [Ostrinia furnacalis] | 1.46218 | 0.66654 | -0.8266 | 0.0148 | -1.3169 |
| TRINITY_DN371_c0_g1_i6_orf1 | - | - | - | trypsin beta-like [Ostrinia furnacalis] | 1.94819 | -0.8199 | -0.1179 | -0.442 | -0.5685 |
| TRINITY_DN132043_c0_g1_i1_orf1 | - | - | - | ankyrin repeat and MYND domain-containing protein 2 [Ostrinia furnacalis] | 1.67492 | 0.57654 | -1.0042 | -0.8186 | -0.4287 |
| TRINITY_DN5562_c0_g1_i3_orf1 | - | - | - | cell division cycle and apoptosis regulator protein 1-like [Ostrinia furnacalis] | 1.67789 | 0.63079 | -0.7882 | -0.6909 | -0.8295 |
| TRINITY_DN24469_c0_g2_i2_orf1 | - | - | - | unnamed protein product, partial [Brenthis ino] | 0.89057 | 1.4866 | -0.9513 | -0.5188 | -0.9071 |
| TRINITY_DN19727_c0_g1_i7_orf1 | - | - | - | dihydrolipoyllysine-residue succinyltransferase component of 2-oxoglutarate dehydrogenase complex, mitochondrial-like [Ostrinia furnacalis] >XP_028160614.1 dihydrolipoyllysine-residue succinyltransferase component of 2-oxoglutarate dehydrogenase complex, mitochondrial-like [Ostrinia furnacalis] >XP_028160615.1 dihydrolipoyllysine-residue succinyltransferase component of 2-oxoglutarate dehydrogenase complex, mitochondrial-like [Ostrinia furnacalis] | 1.9144 | -0.8166 | -0.1307 | -0.1807 | -0.7864 |

| | | | | | | | | | |
|---|---|---|---|---|---|---|---|---|---|
| TRINITY_DN649_c1_g1_i13_orf1 | - | - | - | U1 small nuclear ribonucleoprotein 70 kDa isoform X2 [Ostrinia furnacalis] | 1.80467 | -0.0101 | -1.0607 | -0.7844 | 0.05064 |
| TRINITY_DN32161_c0_g1_i1_orf1 | - | - | - | uncharacterized protein LOC114352518 [Ostrinia furnacalis] | 1.53445 | 0.30418 | 0.02329 | -0.291 | -1.5709 |
| TRINITY_DN95665_c0_g1_i1_orf1 | - | - | - | hypothetical protein HW555_003264 [Spodoptera exigua] >KAH9639693.1 hypothetical protein HF086_017083 [Spodoptera exigua] >CAH0696396.1 unnamed protein product [Spodoptera exigua] | 1.95485 | -0.3512 | -0.2832 | -0.8874 | -0.433 |
| TRINITY_DN44119_c0_g1_i1_orf1 | - | - | - | PREDICTED: GTP-binding protein 128up [Fopius arisanus] | 1.96049 | -0.8418 | -0.242 | -0.3882 | -0.4886 |
| TRINITY_DN22156_c0_g1_i1_orf1 | - | - | - | trafficking protein particle complex subunit 8 [Ostrinia furnacalis] | 1.4157 | 0.79849 | -1.1371 | -0.046 | -1.0311 |
| TRINITY_DN23824_c0_g1_i1_orf1 | - | - | - | 28S ribosomal protein S30, mitochondrial [Ostrinia furnacalis] | 1.95569 | -0.5466 | -0.1827 | -0.3998 | -0.8266 |
| TRINITY_DN87170_c0_g1_i3_orf1 | - | - | - | uncharacterized protein LOC114360175 [Ostrinia furnacalis] | 1.10539 | 1.14557 | -0.9188 | -1.272 | -0.0602 |
| TRINITY_DN7655_c0_g1_i3_orf1 | - | - | - | hypothetical protein evm_001118 [Chilo suppressalis] >CAB3522191.1 unnamed protein product [Chilo suppressalis] | 1.87657 | 0.14751 | -0.918 | -0.5188 | -0.5873 |
| TRINITY_DN4944_c0_g1_i5_orf1 | - | - | - | bifunctional glutamate/proline--tRNA ligase [Ostrinia furnacalis] | 1.89392 | -0.2179 | -0.5601 | -1.0212 | -0.0947 |
| TRINITY_DN14073_c0_g1_i1_orf1 | - | - | - | cytochrome c oxidase subunit 4 isoform 1, mitochondrial-like [Ostrinia furnacalis] >XP_028164918.1 cytochrome c oxidase subunit 4 isoform 1, mitochondrial-like [Ostrinia furnacalis] | 1.88216 | -0.5038 | 0.13622 | -0.6193 | -0.8953 |
| TRINITY_DN12584_c0_g1_i1_orf1 | - | - | - | carnitine O-palmitoyltransferase 1, liver isoform [Ostrinia furnacalis] | 1.88712 | -0.9968 | -0.5144 | 0.04646 | -0.4223 |
| TRINITY_DN4439_c0_g2_i1_orf1 | - | - | - | unnamed protein product, partial [Brenthis ino] | 1.02798 | 1.35833 | -1.1544 | -0.6742 | -0.5577 |
| TRINITY_DN4183_c0_g1_i8_orf1 | - | - | - | histidine--tRNA ligase, cytoplasmic isoform X3 [Ostrinia furnacalis] | 1.95333 | -0.8438 | -0.5367 | -0.3875 | -0.1854 |
| TRINITY_DN1820_c0_g1_i6_orf1 | - | - | - | 3-hydroxyisobutyryl-CoA hydrolase, mitochondrial [Ostrinia furnacalis] | 1.97937 | -0.3932 | -0.6042 | -0.2906 | -0.6914 |
| TRINITY_DN12476_c0_g1_i4_orf1 | - | - | - | guanine nucleotide-binding protein-like 3 homolog [Ostrinia furnacalis] | 1.80719 | -0.9881 | 0.15807 | -0.1313 | -0.8459 |
| TRINITY_DN452_c1_g1_i3_orf1 | - | - | - | ruvB-like helicase 1 [Colias croceus] | 1.84154 | 0.14708 | -1.076 | -0.3762 | -0.5363 |
| TRINITY_DN157_c0_g1_i4_orf1 | - | - | - | ATP-binding cassette sub-family A member 1-like [Ostrinia furnacalis] | 1.94634 | -0.7506 | -0.5924 | -0.0614 | -0.5419 |
| TRINITY_DN11886_c0_g1_i1_orf1 | - | - | - | glycerophosphodiester phosphodiesterase GDPD6-like [Ostrinia furnacalis] >XP_028159459.1 glycerophosphodiester phosphodiesterase GDPD6-like [Ostrinia furnacalis] | 1.80878 | -0.664 | -0.7968 | -0.718 | 0.37005 |
| TRINITY_DN6785_c0_g1_i1_orf1 | - | - | - | Similar to CG4038: Probable H/ACA ribonucleoprotein complex subunit 1 (Drosophila melanogaster) [Cotesia congregata] | 1.83302 | -0.6153 | -0.9027 | 0.288 | -0.603 |
| TRINITY_DN141_c0_g1_i1_orf1 | - | - | - | hypothetical protein evm_010402 [Chilo suppressalis] | 1.72258 | 0.36495 | -0.9562 | -0.9811 | -0.1502 |
| TRINITY_DN4463_c0_g1_i2_orf1 | - | - | - | uncharacterized protein LOC113521486 isoform X3 [Galleria mellonella] | 1.45421 | 0.04818 | -1.4106 | 0.6208 | -0.7126 |
| TRINITY_DN124300_c0_g1_i2_orf1 | - | - | - | protein transport protein Sec23A isoform X1 [Venturia canescens] | 1.94385 | -0.3476 | -0.881 | -0.5429 | -0.1723 |
| TRINITY_DN46140_c0_g1_i1_orf1 | - | - | - | protein PRRC2A-like isoform X4 [Ostrinia furnacalis] | 1.55354 | 0.79543 | -0.5072 | -0.9366 | -0.9052 |
| TRINITY_DN152_c0_g1_i4_orf1 | - | - | - | LOW QUALITY PROTEIN: protein tyrosine phosphatase type IVA 3 [Ostrinia furnacalis] | 1.22746 | 0.26945 | -1.6272 | -0.5532 | 0.68343 |
| TRINITY_DN20185_c0_g1_i6_orf1 | - | - | - | zinc finger protein on ecdysone puffs [Ostrinia furnacalis] | 1.39723 | 0.94178 | -1.0227 | -1.0106 | -0.3056 |
| TRINITY_DN15244_c0_g1_i5_orf1 | - | - | - | titin homolog [Ostrinia furnacalis] | 1.84716 | -0.0298 | -0.963 | -0.8111 | -0.0432 |
| TRINITY_DN1772_c7_g1_i7_orf1 | - | - | - | sulfotransferase family cytosolic 1B member 1-like [Ostrinia furnacalis] | 1.90117 | -0.2332 | -0.3978 | -0.2071 | -1.063 |
| TRINITY_DN4381_c0_g2_i1_orf1 | - | - | - | eukaryotic initiation factor 4A [Glyphodes caesalis] | 1.77432 | -0.2648 | -1.0622 | -0.7497 | 0.30234 |
| TRINITY_DN21123_c0_g1_i1_orf1 | - | - | - | maternal protein exuperantia [Ostrinia furnacalis] | 1.86553 | -0.4142 | -1.1008 | -0.369 | 0.01845 |
| TRINITY_DN2954_c0_g1_i1_orf1 | - | - | - | unnamed protein product [Diatraea saccharalis] | 1.79788 | -0.6812 | -1.0728 | -0.2972 | 0.25342 |
| TRINITY_DN3321_c0_g1_i3_orf1 | - | - | - | peroxidasin [Ostrinia furnacalis] | 1.67555 | 0.24956 | -1.304 | -0.6548 | 0.03366 |
| TRINITY_DN113778_c0_g2_i1_orf1 | - | - | - | metastasis-associated protein MTA3 [Galleria mellonella] | 0.81493 | 1.26352 | -1.5219 | -0.6445 | 0.08799 |
| TRINITY_DN10297_c0_g1_i1_orf1 | - | - | - | polyglutamine-binding protein 1 [Ostrinia furnacalis] | 1.27596 | 1.08533 | -0.334 | -1.1309 | -0.8964 |
| TRINITY_DN29034_c0_g1_i1_orf1 | - | - | - | trypsin-like serine protease [Ostrinia nubilalis] | 1.58967 | 0.70294 | -0.5563 | -0.5836 | -1.1527 |
| TRINITY_DN57454_c0_g1_i4_orf1 | - | - | - | translation machinery-associated protein 7 homolog [Zerene cesonia] | 1.8469 | 0.16092 | -0.3394 | -0.6667 | -1.0017 |
| TRINITY_DN31967_c0_g1_i5_orf1 | - | - | - | N-acetylgalactosamine kinase [Ostrinia furnacalis] | 1.35619 | 1.07664 | -0.7734 | -0.7143 | -0.9451 |
| TRINITY_DN48970_c0_g1_i1_orf1 | - | - | - | uncharacterized protein LOC114356431 isoform X2 [Ostrinia furnacalis] | 1.29554 | 1.03333 | -1.2897 | -0.6785 | -0.3606 |
| TRINITY_DN211_c1_g1_i10_orf1 | - | - | - | protein hu-li tai shao isoform X5 [Galleria mellonella] | 1.77798 | -1.0343 | -0.8218 | -0.1739 | 0.252 |
| TRINITY_DN7464_c1_g1_i1_orf1 | - | - | - | T-complex protein 1 subunit theta [Ostrinia furnacalis] | 1.84894 | 0.25609 | -0.8504 | -0.5744 | -0.6802 |
| TRINITY_DN5873_c0_g4_i1_orf1 | - | - | - | hypothetical protein evm_003048 [Chilo suppressalis] | 1.69254 | -1.0346 | -0.9507 | -0.0968 | 0.38956 |
| TRINITY_DN3520_c0_g1_i4_orf1 | - | - | - | protein KRTCAP2 homolog [Ostrinia furnacalis] | 1.25987 | 0.47413 | -1.7699 | -0.1473 | 0.1832 |

| | | | | | | | | | |
|---|---|---|---|---|---|---|---|---|---|
| TRINITY_DN3985_c0_g2_i1_orf1 | - | - | - | hypothetical protein evm_012077 [Chilo suppressalis] >CAB3529218.1 unnamed protein product [Chilo suppressalis] >CAH0405810.1 unnamed protein product [Chilo suppressalis] | 1.82501 | -0.9492 | -0.7141 | -0.4311 | 0.26937 |
| TRINITY_DN1447_c0_g1_i5_orf1 | - | - | - | PREDICTED: coatomer subunit beta' [Amyelois transitella] | 1.66532 | 0.36019 | -1.3631 | -0.4299 | -0.2325 |
| TRINITY_DN30070_c0_g1_i6_orf1 | - | - | - | uncharacterized protein LOC114361440 isoform X1 [Ostrinia furnacalis] >XP_028172261.1 uncharacterized protein LOC114361440 isoform X2 [Ostrinia furnacalis] | 1.89111 | -0.3331 | -0.1778 | -0.2847 | -1.0955 |
| TRINITY_DN3838_c0_g1_i8_orf1 | - | - | - | ER membrane protein complex subunit 2-like isoform X1 [Ostrinia furnacalis] >XP_028161204.1 ER membrane protein complex subunit 2-like isoform X2 [Ostrinia furnacalis] >XP_028161205.1 ER membrane protein complex subunit 2-like isoform X3 [Ostrinia furnacalis] | 1.29027 | 0.8283 | -1.3904 | -0.8388 | 0.11066 |
| TRINITY_DN108818_c0_g1_i5_orf1 | - | - | - | hypothetical protein O3G_MSEX012842 [Manduca sexta] | 1.72959 | 0.09409 | -1.1255 | -0.8437 | 0.14548 |
| TRINITY_DN11215_c0_g1_i1_orf1 | - | - | - | dnaJ homolog subfamily C member 2 [Ostrinia furnacalis] | 1.89292 | 0.13827 | -0.5555 | -0.7508 | -0.7249 |
| TRINITY_DN3618_c0_g1_i4_orf1 | - | - | - | WD repeat-containing protein 74-like isoform X1 [Ostrinia furnacalis] >XP_028161051.1 WD repeat-containing protein 74-like isoform X2 [Ostrinia furnacalis] | 1.27091 | 0.35691 | -0.3899 | 0.46253 | -1.7004 |
| TRINITY_DN4533_c0_g1_i1_orf1 | - | - | - | neurofilament heavy polypeptide-like isoform X2 [Ostrinia furnacalis] | 1.20226 | 1.0719 | -1.416 | -0.5561 | -0.3021 |
| TRINITY_DN2593_c0_g3_i1_orf1 | - | - | - | midgut carboxypeptidase [Loxostege sticticalis] | 1.9491 | -0.8482 | -0.3312 | -0.1904 | -0.5793 |
| TRINITY_DN48983_c0_g1_i2_orf1 | - | - | - | E3 ubiquitin-protein ligase Su(dx) [Ostrinia furnacalis] >XP_028176753.1 E3 ubiquitin-protein ligase Su(dx) [Ostrinia furnacalis] >XP_028176754.1 E3 ubiquitin-protein ligase Su(dx) [Ostrinia furnacalis] | 1.36155 | 0.44716 | -1.6296 | 0.28094 | -0.46 |
| TRINITY_DN8598_c0_g1_i2_orf1 | - | - | - | tyrosine--tRNA ligase, mitochondrial [Ostrinia furnacalis] | 1.99097 | -0.3186 | -0.5382 | -0.596 | -0.5382 |
| TRINITY_DN33038_c0_g1_i1_orf1 | - | - | - | 39S ribosomal protein L46, mitochondrial [Ostrinia furnacalis] | 1.87296 | -0.6383 | 0.06287 | -0.3032 | -0.9944 |
| TRINITY_DN18681_c0_g1_i7_orf1 | - | - | - | fragile X mental retardation syndrome-related protein 1 isoform X3 [Ostrinia furnacalis] | 1.48159 | 0.76072 | -1.1499 | -0.9382 | -0.1542 |
| TRINITY_DN937_c0_g1_i2_orf1 | - | - | - | protein brunelleschi [Ostrinia furnacalis] | 1.67961 | -0.7751 | -0.9419 | -0.5688 | 0.60619 |
| TRINITY_DN4686_c0_g2_i1_orf1 | - | - | - | lysophospholipase-like protein 1 [Ostrinia furnacalis] | 1.24847 | 1.16903 | -1.0964 | -0.6618 | -0.6593 |
| TRINITY_DN4151_c1_g1_i4_orf1 | - | - | - | 5-methylcytosine rRNA methyltransferase NSUN4 isoform X1 [Ostrinia furnacalis] >XP_028161245.1 5-methylcytosine rRNA methyltransferase NSUN4 isoform X2 [Ostrinia furnacalis] | 1.75582 | 0.32684 | -1.075 | -0.2327 | -0.775 |
| TRINITY_DN31503_c0_g1_i4_orf1 | - | - | - | hypothetical protein evm_001345 [Chilo suppressalis] >CAB3523265.1 unnamed protein product [Chilo suppressalis] >CAH0400587.1 unnamed protein product [Chilo suppressalis] | 1.68292 | 0.61791 | -0.6522 | -0.8527 | -0.7959 |
| TRINITY_DN90289_c0_g1_i5_orf1 | - | - | - | 40S ribosomal protein S25 [Eumeta japonica] | 1.94241 | -0.1763 | -0.4289 | -0.9114 | -0.4258 |
| TRINITY_DN72934_c0_g1_i1_orf1 | - | - | - | carboxylesterase 8 [Streltzoviella insularis] | 1.98675 | -0.3085 | -0.4482 | -0.6262 | -0.6038 |
| TRINITY_DN3878_c0_g1_i4_orf1 | - | - | - | eukaryotic translation initiation factor 3 subunit J [Ostrinia furnacalis] | 1.96699 | -0.4465 | -0.6069 | -0.7269 | -0.1867 |
| TRINITY_DN5472_c0_g1_i1_orf1 | - | - | - | serine/threonine-protein kinase 26 isoform X3 [Ostrinia furnacalis] | 1.49695 | 0.75298 | -1.2067 | -0.2119 | -0.8313 |
| TRINITY_DN2300_c0_g1_i1_orf1 | - | - | - | ATP synthase subunit beta, mitochondrial isoform X4 [Ostrinia furnacalis] | 1.93186 | -0.9574 | -0.3412 | -0.4476 | -0.1856 |

| | | | | | | | | | |
|---|---|---|---|---|---|---|---|---|---|
| TRINITY_DN7613_c1_g2_i1_orf1 | - | - | - | 60S ribosomal protein L19 [Helicoverpa armigera] >XP_[illegible] 60S ribosomal protein L19 [Pieris rapae] >XP_022821503.1 60S ribosomal protein L19 [Spodoptera litura] >XP_026731717.1 60S ribosomal protein L19 [Trichoplusia ni] >XP_035451321.1 60S ribosomal protein L19-like [Spodoptera frugiperda] >XP_035452592.1 60S ribosomal protein L19-like [Spodoptera frugiperda] >XP_041975187.1 60S ribosomal protein L19 [Aricia agestis] >XP_045524933.1 60S ribosomal protein L19 [Pieris brassicae] >XP_047023231.1 60S ribosomal protein L19 [Helicoverpa zea] >XP_047984679.1 60S ribosomal protein L19 [Leguminivora glycinivorella] >XP_049874324.1 60S ribosomal protein L19 [Pectinophora gossypiella] >ACY95336.1 ribosomal protein L19 [Manduca sexta] >KAF9423217.1 hypothetical protein HW555_001286 [Spodoptera exigua] >KAI5632448.1 ribosomal protein l19e domain-containing protein [Phthorimaea operculella] >RVE50663.1 hypothetical protein evm_004695 [Chilo suppressalis] >CAB3239671.1 unnamed protein product [Arctia plantaginis] >CAB3509883.1 unnamed protein product [Spodoptera littoralis] >CAG4986349.1 unnamed protein product [Parnassius apollo] >CAG9758258.1 unnamed protein product [Diatraea saccharalis] >CAH2049991.1 unnamed protein product, partial [Iphiclides podalirius] | 1.77643 | -0.868 | -0.9808 | -0.2151 | 0.28748 |
| TRINITY_DN2173_c0_g1_i1_orf1 | - | - | - | heat shock 70 kDa protein cognate 4 [Cephus cinctus] | 1.87908 | 0.02221 | -0.6296 | -0.9989 | -0.2728 |
| TRINITY_DN9637_c0_g1_i14_orf1 | - | - | - | zinc finger protein swm isoform X3 [Ostrinia furnacalis] | 1.43399 | 0.98239 | -0.8727 | -0.8848 | -0.6589 |
| TRINITY_DN14298_c0_g3_i1_orf1 | - | - | - | kinesin heavy chain [Ostrinia furnacalis] | 0.91447 | 1.25916 | -1.4649 | -0.6553 | -0.0534 |
| TRINITY_DN15845_c0_g1_i1_orf1 | - | - | - | probable ATP-dependent RNA helicase DDX28 isoform X1 [Ostrinia furnacalis] >XP_028162024.1 probable ATP-dependent RNA helicase DDX28 isoform X2 [Ostrinia furnacalis] >XP_028162026.1 probable ATP-dependent RNA helicase DDX28 isoform X3 [Ostrinia furnacalis] >XP_028162027.1 probable ATP-dependent RNA helicase DDX28 isoform X3 [Ostrinia furnacalis] | 1.79207 | 0.23552 | -1.1314 | -0.6083 | -0.2879 |
| TRINITY_DN3343_c0_g1_i4_orf1 | - | - | - | AFG3-like protein 2 [Ostrinia furnacalis] | 1.99748 | -0.506 | -0.5811 | -0.4867 | -0.4238 |
| TRINITY_DN3856_c0_g1_i7_orf1 | - | - | - | uncharacterized protein LOC114355702 [Ostrinia furnacalis] | 1.67893 | 0.52315 | -1.1724 | -0.4774 | -0.5522 |
| TRINITY_DN23444_c0_g1_i10_orf1 | - | - | - | serrate RNA effector molecule homolog isoform X2 [Ostrinia furnacalis] | 1.82534 | 0.10153 | -1.0477 | -0.7342 | -0.1449 |
| TRINITY_DN4494_c0_g1_i1_orf1 | - | - | - | venom serine carboxypeptidase [Ostrinia furnacalis] | 0.84329 | 1.34211 | -1.0325 | -1.1916 | 0.03876 |
| TRINITY_DN2403_c0_g1_i3_orf1 | - | - | - | FAD-dependent oxidoreductase domain-containing protein 1 [Ostrinia | 1.29116 | 1.06312 | -1.1286 | -0.911 | -0.3147 |
| TRINITY_DN10106_c0_g2_i1_orf1 | - | - | - | arrestin domain-containing protein 2-like isoform X3 [Ostrinia furnacalis] | 1.94849 | -0.5938 | -0.3771 | -0.8285 | -0.1491 |
| TRINITY_DN31520_c1_g1_i1_orf1 | - | - | - | probable DNA-directed RNA polymerase III subunit RPC6 [Ostrinia furnacalis] | 1.67694 | 0.46317 | -1.2148 | -0.2762 | -0.6492 |
| TRINITY_DN4233_c0_g2_i2_orf1 | - | - | - | actin-related protein 2/3 complex subunit 5-B [Ostrinia furnacalis] | 1.46555 | 0.87448 | -1.1923 | -0.634 | -0.5137 |
| TRINITY_DN22951_c0_g1_i1_orf1 | - | - | - | TAR DNA-binding protein 43-like [Ostrinia furnacalis] | 1.46304 | 0.12128 | -1.5446 | 0.45882 | -0.4985 |
| TRINITY_DN49742_c0_g1_i4_orf1 | - | - | - | neuroglobin-like [Ostrinia furnacalis] | 1.95808 | -0.4232 | -0.836 | -0.4972 | -0.2016 |
| TRINITY_DN42171_c0_g1_i1_orf1 | - | - | - | amyloid-beta-like protein isoform X1 [Manduca sexta] >AAY25024.3 beta amyloid protein precursor-like protein [Manduca sexta] | 1.01318 | 1.39305 | -1.0483 | -0.7564 | -0.6016 |
| TRINITY_DN1337_c0_g2_i1_orf1 | - | - | - | minor histocompatibility antigen H13 [Ostrinia furnacalis] | 1.75279 | -0.1434 | -1.3688 | -0.1683 | -0.0723 |
| TRINITY_DN7037_c0_g1_i4_orf1 | - | - | - | unnamed protein product [Chilo suppressalis] | 1.58868 | 0.67107 | -0.5006 | -0.5421 | -1.2171 |
| TRINITY_DN2783_c0_g1_i22_orf1 | - | - | - | methionine aminopeptidase 1-like [Pectinophora gossypiella] >XP_049887084.1 methionine aminopeptidase 1-like [Pectinophora gossypiella] | 1.73659 | -0.8483 | -1.1129 | 0.13489 | 0.08965 |
| TRINITY_DN1425_c0_g1_i4_orf1 | - | - | - | fibulin-2-like [Ostrinia furnacalis] | 1.11885 | 1.09304 | -1.521 | -0.32 | -0.3709 |
| TRINITY_DN7991_c0_g1_i9_orf1 | - | - | - | hypothetical protein evm_006720 [Chilo suppressalis] >CAB3528247.1 unnamed protein product [Chilo suppressalis] >CAH0404834.1 unnamed protein product [Chilo suppressalis] | 1.8517 | -0.8561 | -0.8533 | -0.2949 | 0.15259 |
| TRINITY_DN32487_c0_g1_i1_orf1 | - | - | - | heat shock protein 75 kDa, mitochondrial [Ostrinia furnacalis] | 1.99965 | -0.5201 | -0.51 | -0.4647 | -0.5049 |
| TRINITY_DN16128_c0_g1_i5_orf1 | - | - | - | probable prefoldin subunit 4 [Ostrinia furnacalis] | 1.69768 | -0.1679 | -1.3429 | -0.4602 | 0.27326 |
| TRINITY_DN19810_c1_g1_i7_orf1 | - | - | - | RNA-binding protein spenito [Ostrinia furnacalis] >XP_028167555.1 RNA-binding protein spenito [Ostrinia furnacalis] | 1.0377 | 1.18446 | -1.4816 | -0.5298 | -0.2108 |
| TRINITY_DN2914_c0_g1_i1_orf1 | - | - | - | U1 small nuclear ribonucleoprotein A [Ostrinia furnacalis] | 1.6458 | 0.52472 | -1.1983 | -0.7179 | -0.2543 |

| | | | | | | | | | |
|---|---|---|---|---|---|---|---|---|---|
| TRINITY_DN1313_c0_g1_i2_orf1 | - | - | - | 39S ribosomal protein L40, mitochondrial [Ostrinia furnacalis] | 0.82255 | 1.51007 | -0.396 | -1.0424 | -0.8942 |
| TRINITY_DN1572_c0_g1_i6_orf1 | - | - | - | eukaryotic translation initiation factor 3 subunit D [Ostrinia furnacalis] | 1.96765 | -0.4139 | -0.7393 | -0.6057 | -0.2087 |
| TRINITY_DN3551_c0_g1_i4_orf1 | - | - | - | carnitine O-palmitoyltransferase 2, mitochondrial [Ostrinia furnacalis] | 1.98232 | -0.7225 | -0.3563 | -0.5333 | -0.3702 |
| TRINITY_DN18794_c0_g1_i5_orf1 | - | - | - | hypothetical protein evm_012380 [Chilo suppressalis] >CAB3520845.1 unnamed protein product [Chilo suppressalis] >CAH0398166.1 unnamed protein product [Chilo suppressalis] | 1.74616 | 0.32501 | -0.77 | -1.1011 | -0.2001 |
| TRINITY_DN14035_c0_g1_i1_orf1 | - | - | - | protein takeout-like [Ostrinia furnacalis] | 1.9664 | -0.7956 | -0.5364 | -0.2421 | -0.3923 |
| TRINITY_DN7405_c0_g1_i3_orf1 | - | - | - | hexokinase-2-like [Ostrinia furnacalis] >XP_028178415.1 hexokinase-2-like [Ostrinia furnacalis] | 1.92113 | -0.7869 | -0.1566 | -0.1826 | -0.7951 |
| TRINITY_DN43656_c0_g1_i1_orf1 | - | - | - | GPI ethanolamine phosphate transferase 3 isoform X2 [Ostrinia furnacalis] | 1.95163 | -0.7371 | -0.503 | -0.6219 | -0.0896 |
| TRINITY_DN43792_c0_g1_i1_orf1 | - | - | - | 40S ribosomal protein S3a [Spodoptera litura] >XP_022824163.1 40S ribosomal protein S3a [Spodoptera litura] >XP_026734591.1 40S ribosomal protein S3a [Trichoplusia ni] >XP_035456172.1 40S ribosomal protein S3a [Spodoptera frugiperda] >Q95V35.1 RecName: Full=40S ribosomal protein S3a [Spodoptera frugiperda] >CAB3514148.1 unnamed protein product [Spodoptera littoralis] >CAH0602005.1 unnamed protein product [Chrysodeixis includens] >AAL26579.1 ribosomal protein S3A [Spodoptera frugiperda] >CAH1642305.1 unnamed protein product [Spodoptera littoralis] | 1.83643 | -0.4416 | -1.1879 | -0.1062 | -0.1007 |
| TRINITY_DN207_c0_g2_i3_orf1 | - | - | - | JNK-interacting protein 3 isoform X2 [Ostrinia furnacalis] | 1.98884 | -0.2932 | -0.5568 | -0.5752 | -0.5637 |
| TRINITY_DN4757_c0_g1_i3_orf1 | - | - | - | melanotransferrin isoform X1 [Ostrinia furnacalis] >XP_028175370.1 melanotransferrin isoform X2 [Ostrinia furnacalis] >XP_028175371.1 melanotransferrin isoform X3 [Ostrinia furnacalis] | 1.1888 | 1.25286 | -0.8711 | -0.6736 | -0.897 |
| TRINITY_DN142485_c0_g1_i1_orf1 | - | - | - | uncharacterized protein CG16817-like [Ostrinia furnacalis] | 1.73629 | -0.0543 | -1.0292 | -0.9223 | 0.26944 |
| TRINITY_DN29743_c0_g1_i9_orf1 | - | - | - | polyadenylate-binding protein 2 isoform X1 [Ostrinia furnacalis] >XP_028168980.1 polyadenylate-binding protein 2 isoform X2 [Ostrinia | 1.31884 | 0.956 | -1.2389 | -0.8889 | -0.147 |
| TRINITY_DN3628_c0_g1_i5_orf1 | - | - | - | palmitoyltransferase Hip14 isoform X2 [Ostrinia furnacalis] | 1.92769 | 0.0156 | -0.7075 | -0.717 | -0.5188 |
| TRINITY_DN10521_c0_g1_i7_orf1 | - | - | - | tubulin beta chain-like [Ostrinia furnacalis] | 1.95466 | -0.8942 | -0.3922 | -0.3703 | -0.2979 |
| TRINITY_DN3976_c0_g1_i6_orf1 | - | - | - | grpE protein homolog, mitochondrial [Ostrinia furnacalis] | 1.93445 | -0.9413 | -0.1752 | -0.3515 | -0.4665 |
| TRINITY_DN19628_c1_g1_i1_orf1 | - | - | - | transcription factor BTF3 homolog 4-like [Ostrinia furnacalis] | 1.9541 | -0.1761 | -0.727 | -0.7122 | -0.3389 |
| TRINITY_DN14934_c0_g1_i17_orf1 | - | - | - | putative tricarboxylate transport protein, mitochondrial isoform X1 [Ostrinia furnacalis] >XP_028177526.1 putative tricarboxylate transport protein, mitochondrial isoform X2 [Ostrinia furnacalis] | 1.85251 | -1.1405 | -0.3679 | 0.01887 | -0.363 |
| TRINITY_DN49047_c0_g1_i2_orf1 | - | - | - | unnamed protein product [Parnassius apollo] | 1.00968 | 0.67305 | -1.6794 | -0.5963 | 0.59295 |
| TRINITY_DN33183_c0_g1_i4_orf1 | - | - | - | glutamate--cysteine ligase regulatory subunit [Ostrinia furnacalis] | 1.89956 | 0.09378 | -0.6808 | -0.486 | -0.8266 |
| TRINITY_DN1528_c0_g1_i4_orf1 | - | - | - | uncharacterized protein LOC114353202 [Ostrinia furnacalis] | 0.73503 | 1.47924 | -1.3418 | -0.2234 | -0.6491 |
| TRINITY_DN8343_c0_g1_i2_orf1 | - | - | - | multidrug resistance-associated protein 1 isoform X4 [Ostrinia furnacalis] | 1.88418 | -0.5465 | -0.7298 | -0.7696 | 0.1618 |
| TRINITY_DN31637_c0_g1_i3_orf1 | - | - | - | protein CDV3 homolog isoform X1 [Ostrinia furnacalis] >XP_028156793.1 protein CDV3 homolog isoform X2 [Ostrinia furnacalis] >XP_028156794.1 protein CDV3 homolog isoform X3 [Ostrinia furnacalis] >XP_028156795.1 protein CDV3 homolog isoform X1 [Ostrinia furnacalis] | 1.8268 | 0.07321 | -1.0829 | -0.6833 | -0.1338 |
| TRINITY_DN5233_c0_g1_i1_orf1 | - | - | - | pre-mRNA-splicing factor 38-like [Ostrinia furnacalis] | 1.81581 | 0.03343 | -1.0722 | -0.0348 | -0.7422 |
| TRINITY_DN4944_c1_g1_i4_orf1 | - | - | - | bifunctional glutamate/proline--tRNA ligase [Ostrinia furnacalis] | 1.9249 | -0.8318 | -0.7049 | -0.318 | -0.0702 |
| TRINITY_DN40015_c0_g1_i2_orf1 | - | - | - | PREDICTED: 60S ribosomal protein L18 [Amyelois transitella] | 1.86058 | -0.7817 | -0.8952 | -0.3252 | 0.14153 |
| TRINITY_DN9874_c0_g1_i7_orf1 | - | - | - | hypothetical protein evm_013697 [Chilo suppressalis] | 1.90448 | -0.8569 | -0.7479 | -0.2812 | -0.0185 |
| TRINITY_DN3450_c0_g1_i3_orf1 | - | - | - | hypothetical protein evm_008214 [Chilo suppressalis] | 1.55858 | 0.45361 | -1.4156 | 0.00435 | -0.601 |
| TRINITY_DN2802_c0_g1_i1_orf1 | - | - | - | far upstream element-binding protein 1 isoform X3 [Ostrinia furnacalis] | 1.52149 | 0.78872 | -1.137 | -0.7892 | -0.3841 |
| TRINITY_DN31585_c0_g1_i1_orf1 | - | - | - | transcription elongation factor SPT5 [Ostrinia furnacalis] | 1.74102 | 0.49754 | -0.9288 | -0.6766 | -0.6332 |
| TRINITY_DN135679_c0_g1_i1_orfp1 | - | - | - | TRINITY_DN135679_c0_g1_i1_m.85524 TRINITY_DN135679_c0_g1::TRINITY_DN135679_c0_g1_i1::g.85524 ORF type:5prime_partial len:55 (+),score=3.74,Toxin_2 PF00451.20 1.9e-06 TRINITY_DN135679_c0_g1_i1:3-167(+) | 0.97952 | -0.6289 | -1.5656 | 1.08489 | 0.13012 |

| | | | | | | | | |
|---|---|---|---|---|---|---|---|---|
| TRINITY_DN170_c1_g1_i5_orf1 | - | - | - | regulator of chromosome condensation isoform X2 [Helicoverpa zea] | 1.69088 | 0.29151 | -1.1308 | -0.8812 | 0.02956 |
| TRINITY_DN147517_c0_g1_i1_orf1 | - | - | - | eukaryotic translation initiation factor 4 gamma 3-like isoform X5 [Ostrinia furnacalis] | 1.89939 | -0.5211 | -1.0157 | -0.2897 | -0.0729 |
| TRINITY_DN57798_c0_g1_i1_orf1 | - | - | - | ubiquitin carboxyl-terminal hydrolase 36 [Ostrinia furnacalis] | 1.78163 | 0.40968 | -0.7708 | -0.5446 | -0.876 |
| TRINITY_DN15376_c0_g1_i1_orf1 | - | - | - | peptidyl-prolyl cis-trans isomerase isoform X1 [Ostrinia furnacalis] | 1.98501 | -0.4488 | -0.5586 | -0.6738 | -0.3039 |
| TRINITY_DN16077_c0_g1_i13_orf1 | - | - | - | dynamin-like 120 kDa protein, mitochondrial [Ostrinia furnacalis] | 1.98864 | -0.4586 | -0.3372 | -0.6667 | -0.5261 |
| TRINITY_DN91_c0_g1_i9_orf1 | - | - | - | ribosome-binding protein 1 isoform X8 [Helicoverpa armigera] | 1.68505 | 0.29122 | -1.3648 | -0.4203 | -0.1912 |
| TRINITY_DN15339_c0_g1_i6_orf1 | - | - | - | nuclear pore glycoprotein p62 isoform X3 [Ostrinia furnacalis] | 1.62541 | -0.276 | -1.2859 | 0.52782 | -0.5914 |
| TRINITY_DN69170_c0_g2_i1_orf1 | - | - | - | stromal membrane-associated protein 1-like [Pectinophora gossypiella] | 1.71305 | 0.48607 | -1.0853 | -0.6814 | -0.4324 |
| TRINITY_DN4380_c0_g1_i9_orf1 | - | - | - | hypothetical protein evm_012370 [Chilo suppressalis] | 1.75183 | 0.24933 | -1.0778 | -0.8365 | -0.0869 |
| TRINITY_DN288_c0_g1_i9_orf1 | - | - | - | unnamed protein product [Chilo suppressalis] | 1.41743 | 0.96763 | -1.0943 | -0.5357 | -0.7551 |
| TRINITY_DN16816_c0_g1_i1_orf1 | - | - | - | ATP-binding cassette sub-family F member 1 [Ostrinia furnacalis] >XP_028179049.1 ATP-binding cassette sub-family F member 1 [Ostrinia | 1.87396 | -0.6863 | -0.9783 | -0.2431 | 0.0337 |
| TRINITY_DN14501_c0_g1_i1_orf1 | - | - | - | 28S ribosomal protein S28, mitochondrial [Ostrinia furnacalis] | 1.73508 | 0.48086 | -0.4558 | -0.8526 | -0.9075 |
| TRINITY_DN53311_c0_g2_i1_orf1 | - | - | - | transcription elongation factor S-II [Chelonus insularis] | 1.10479 | 1.27903 | -1.0853 | -0.4017 | -0.8968 |
| TRINITY_DN12826_c0_g1_i1_orf1 | - | - | - | uncharacterized protein LOC114363296 [Ostrinia furnacalis] | 1.83205 | -0.2077 | -1.1316 | 0.06866 | -0.5614 |
| TRINITY_DN2977_c0_g1_i3_orf1 | - | - | - | transmembrane 9 superfamily member 3 [Ostrinia furnacalis] | 1.56014 | 0.80761 | -0.9532 | -0.7554 | -0.6591 |
| TRINITY_DN95414_c0_g1_i1_orf1 | - | - | - | protein arginine N-methyltransferase 5 [Ostrinia furnacalis] | 1.61277 | -1.0418 | -1.0418 | -0.0071 | 0.4779 |
| TRINITY_DN642_c0_g1_i6_orf1 | - | - | - | reticulon-3-B isoform X5 [Ostrinia furnacalis] | 1.82887 | -0.8109 | -0.9365 | -0.283 | 0.2015 |
| TRINITY_DN972_c0_g2_i1_orf1 | - | - | - | DNA damage-binding protein 1 [Ostrinia furnacalis] | 1.63958 | 0.56121 | -1.2306 | -0.4084 | -0.5618 |
| TRINITY_DN2497_c0_g1_i2_orf1 | - | - | - | protein stunted-like isoform X1 [Colias croceus] | 1.74511 | -1.0496 | -0.206 | -0.8325 | 0.34292 |
| TRINITY_DN11584_c0_g1_i2_orf1 | - | - | - | L-threonine 3-dehydrogenase, mitochondrial [Ostrinia furnacalis] | 1.95391 | -0.4644 | -0.1077 | -0.6799 | -0.7019 |
| TRINITY_DN2668_c0_g1_i6_orf1 | - | - | - | pancreatic triacylglycerol lipase-like [Spodoptera litura] | 1.94052 | -0.1271 | -0.3791 | -0.5657 | -0.8686 |
| TRINITY_DN10672_c0_g1_i3_orf1 | - | - | - | neurofilament heavy polypeptide-like isoform X10 [Ostrinia furnacalis] | 1.57005 | 0.06424 | -1.5775 | 0.11415 | -0.1709 |
| TRINITY_DN62_c0_g1_i18_orf1 | - | - | - | hypothetical protein evm_002481 [Chilo suppressalis] >CAB3531063.1 unnamed protein product [Chilo suppressalis] >CAH0407655.1 unnamed protein product [Chilo suppressalis] | 1.66094 | 0.58861 | -0.9643 | -0.9063 | -0.379 |
| TRINITY_DN10548_c0_g2_i1_orf1 | - | - | - | uridine 5'-monophosphate synthase-like [Ostrinia furnacalis] | 1.54083 | 0.35536 | -1.4345 | -0.6403 | 0.17858 |
| TRINITY_DN18728_c0_g1_i2_orf1 | - | - | - | H/ACA ribonucleoprotein complex subunit 3 [Galleria mellonella] | 1.97301 | -0.5995 | -0.7284 | -0.3903 | -0.2548 |
| TRINITY_DN4156_c0_g1_i2_orf1 | - | - | - | calcium channel flower [Ostrinia furnacalis] | 0.86119 | 1.39902 | -1.3609 | -0.5993 | -0.3001 |
| TRINITY_DN2927_c0_g1_i2_orf1 | - | - | - | unnamed protein product [Spodoptera littoralis] >CAH1641960.1 unnamed protein product [Spodoptera littoralis] | 1.87981 | 0.12669 | -0.9375 | -0.536 | -0.533 |
| TRINITY_DN15448_c0_g1_i1_orf1 | - | - | - | ragulator complex protein LAMTOR1-like [Ostrinia furnacalis] | 1.97384 | -0.2992 | -0.3356 | -0.7209 | -0.6181 |
| TRINITY_DN3063_c0_g1_i5_orf1 | - | - | - | mini-chromosome maintenance complex-binding protein [Ostrinia furnacalis] | 1.82422 | -0.3091 | -1.1065 | -0.571 | 0.16233 |
| TRINITY_DN41842_c0_g1_i2_orf1 | - | - | - | importin-5 [Ostrinia furnacalis] | 1.98934 | -0.4514 | -0.5473 | -0.6511 | -0.3395 |
| TRINITY_DN70382_c0_g1_i10_orf1 | - | - | - | TGF-beta receptor type-1 isoform X4 [Ostrinia furnacalis] | 1.58601 | -0.7308 | -1.2939 | -0.0807 | 0.5194 |
| TRINITY_DN66822_c0_g1_i1_orf1 | - | - | - | heterogeneous nuclear ribonucleoprotein 27C isoform X6 [Pieris rapae] | 1.9427 | -0.2182 | -0.9242 | -0.4443 | -0.356 |
| TRINITY_DN14464_c0_g1_i1_orf1 | - | - | - | GMP synthase [glutamine-hydrolyzing] [Chelonus insularis] | 1.89024 | -0.6865 | -0.8613 | -0.4497 | 0.10736 |
| TRINITY_DN33883_c0_g1_i1_orf1 | - | - | - | probable 28S ribosomal protein S6, mitochondrial [Ostrinia furnacalis] | 1.92992 | -0.0707 | -0.4005 | -0.5777 | -0.8811 |
| TRINITY_DN28376_c0_g1_i15_orfp1 | - | - | - | TRINITY_DN28376_c0_g1_i15_m.40022 TRINITY_DN28376_c0_g1::TRINITY_DN28376_c0_g1_i15::g.40022 ORF type:internal len:273 (+),score=43.58 TRINITY_DN28376_c0_g1_i15:3-818(+) | 1.32347 | -0.4348 | -1.6338 | 0.60983 | 0.13527 |
| TRINITY_DN3312_c0_g1_i10_orf1 | - | - | - | glycerol-3-phosphate dehydrogenase, mitochondrial-like isoform X3 [Ostrinia furnacalis] | 1.94945 | -0.4206 | -0.2422 | -0.9044 | -0.3823 |
| TRINITY_DN92153_c0_g2_i2_orf1 | - | - | - | methylenetetrahydrofolate reductase [Ostrinia furnacalis] | 1.20021 | 0.24122 | -1.3785 | -0.9256 | 0.86269 |
| TRINITY_DN27852_c0_g1_i1_orf1 | - | - | - | baculoviral IAP repeat-containing protein 6-like [Ostrinia furnacalis] | 1.76179 | 0.30854 | -0.3628 | -1.1789 | -0.5287 |
| TRINITY_DN31119_c0_g1_i1_orf1 | - | - | - | transforming acidic coiled-coil-containing protein 3-like [Ostrinia furnacalis] >XP_028170476.1 transforming acidic coiled-coil-containing protein 3-like [Ostrinia furnacalis] >XP_028170477.1 transforming acidic coiled-coil-containing protein 3-like [Ostrinia furnacalis] >XP_028170480.1 transforming acidic coiled-coil-containing protein 3-like [Ostrinia furnacalis] | 1.54239 | 0.19014 | -1.6047 | -0.0355 | -0.0924 |

| | | | | | | | | |
|---|---|---|---|---|---|---|---|---|
| TRINITY_DN11457_c0_g1_i5_orf1 | - | - | - | uncharacterized protein LOC114352268 [Ostrinia furnacalis] | 1.92678 | -0.0722 | -0.8417 | -0.6809 | -0.332 |
| TRINITY_DN3889_c0_g1_i7_orfp1 | - | - | - | TRINITY_DN3889_c0_g1_i7_m.1657 TRINITY_DN3889_c0_g1::TRINITY_DN3889_c0_g1_i7::g.1657 ORF type:5prime_partial len:235 (+),score=70.90 TRINITY_DN3889_c0_g1_i7:1-705(+) | 1.93936 | -0.0897 | -0.5153 | -0.4735 | -0.8609 |
| TRINITY_DN23783_c0_g2_i1_orf1 | - | - | - | cytochrome b5 [Ostrinia furnacalis] | 1.60444 | 0.42242 | -1.1217 | 0.08568 | -0.9908 |
| TRINITY_DN57074_c0_g2_i1_orf1 | - | - | - | ribosomal protein l36e domain-containing protein [Phthorimaea operculella] | 1.91454 | -0.7722 | -0.7007 | -0.4944 | 0.05281 |
| TRINITY_DN11396_c0_g1_i1_orf1 | - | - | - | uncharacterized protein LOC114352414 isoform X1 [Ostrinia furnacalis] | 1.87321 | 0.17091 | -0.5529 | -0.5968 | -0.8945 |
| TRINITY_DN18249_c0_g1_i1_orf1 | - | - | - | 60S ribosomal protein L13 [Pectinophora gossypiella] | 1.52785 | -0.7061 | -1.2573 | -0.278 | 0.71351 |
| TRINITY_DN756_c0_g1_i11_orf1 | - | - | - | calcium-binding protein E63-1 isoform X1 [Ostrinia furnacalis] | 1.81429 | -0.8618 | -0.9457 | -0.1922 | 0.18537 |
| TRINITY_DN4950_c0_g1_i2_orf1 | - | - | - | unnamed protein product [Diatraea saccharalis] | 1.5643 | 0.73399 | -1.1756 | -0.5925 | -0.5301 |
| TRINITY_DN73945_c0_g5_i3_orf1 | - | - | - | cyclin-dependent kinase 12 isoform X1 [Diachasma alloeum] >XP_015114851.1 cyclin-dependent kinase 12 isoform X1 [Diachasma alloeum] >XP_015114852.1 cyclin-dependent kinase 12 isoform X1 [Diachasma alloeum] >XP_015114853.1 cyclin-dependent kinase 12 isoform X1 [Diachasma alloeum] >XP_015114854.1 cyclin-dependent kinase 12 isoform X1 [Diachasma alloeum] | 1.68206 | -0.5516 | -1.2753 | 0.41112 | -0.2662 |
| TRINITY_DN3832_c0_g1_i1_orf1 | - | - | - | serine-threonine kinase receptor-associated protein [Galleria mellonella] | 1.74565 | 0.48879 | -0.6151 | -0.9199 | -0.6994 |
| TRINITY_DN14487_c0_g1_i4_orf1 | - | - | - | hypothetical protein HW555_009956 [Spodoptera exigua] >KAH9643419.1 hypothetical protein HF086_016708 [Spodoptera exigua] >CAH0702087.1 unnamed protein product [Spodoptera exigua] | 1.76746 | 0.4153 | -0.4858 | -0.9663 | -0.7307 |
| TRINITY_DN17215_c0_g1_i4_orf1 | - | - | - | 28S ribosomal protein S29, mitochondrial [Ostrinia furnacalis] | 1.97604 | -0.7394 | -0.2868 | -0.5622 | -0.3877 |
| TRINITY_DN33249_c0_g1_i1_orf1 | - | - | - | eukaryotic translation initiation factor 2 subunit 3-like isoform X2 [Spodoptera frugiperda] >CAH0683085.1 unnamed protein product [Spodoptera exigua] | 1.91348 | 0.04603 | -0.8277 | -0.6397 | -0.4921 |
| TRINITY_DN7407_c0_g1_i9_orf1 | - | - | - | sec1 family domain-containing protein 2-like [Ostrinia furnacalis] | 1.95807 | -0.801 | -0.5143 | -0.4846 | -0.1582 |
| TRINITY_DN401_c0_g1_i15_orf1 | - | - | - | cholinephosphotransferase 1 isoform X2 [Ostrinia furnacalis] | 1.99516 | -0.4886 | -0.6179 | -0.4886 | -0.4001 |
| TRINITY_DN517_c0_g1_i5_orf1 | - | - | - | putative pre-mRNA-splicing factor ATP-dependent RNA helicase PRP1 [Helicoverpa zea] >XP_049708007.1 putative pre-mRNA-splicing factor ATP-dependent RNA helicase PRP1 isoform X1 [Helicoverpa armigera] | 1.97186 | -0.2664 | -0.785 | -0.4825 | -0.438 |
| TRINITY_DN6185_c0_g1_i12_orf1 | - | - | - | mitogen-activated protein kinase 1 [Ostrinia furnacalis] >AXF67444.1 mitogen-activated protein kinase 1 [Ostrinia furnacalis] | 1.51045 | 0.83695 | -0.4732 | -0.7995 | -1.0747 |
| TRINITY_DN17417_c0_g1_i11_orf1 | - | - | - | sodium/hydrogen exchanger 9B2-like isoform X4 [Ostrinia furnacalis] | 1.87281 | -1.032 | -0.2453 | 0.01055 | -0.6061 |
| TRINITY_DN23004_c0_g1_i1_orf1 | - | - | - | uncharacterized protein LOC114365313 [Ostrinia furnacalis] | 1.77701 | -0.8445 | -1.0533 | -0.0182 | 0.13894 |
| TRINITY_DN4731_c0_g2_i1_orf1 | - | - | - | gelsolin-like [Ostrinia furnacalis] | 1.75947 | -0.6596 | 0.04859 | 0.06113 | -1.2096 |
| TRINITY_DN14730_c0_g1_i7_orf1 | - | - | - | titin homolog [Ostrinia furnacalis] | 1.98257 | -0.54 | -0.6727 | -0.5052 | -0.2646 |
| TRINITY_DN6406_c0_g1_i1_orf1 | - | - | - | protein FAM98A-like [Ostrinia furnacalis] | 1.8752 | -0.8014 | -0.3984 | 0.13906 | -0.8145 |
| TRINITY_DN145666_c0_g1_i1_orf1 | - | - | - | PREDICTED: 40S ribosomal protein S29 [Microplitis demolitor] >XP_044581406.1 40S ribosomal protein S29 [Cotesia glomerata] | 1.89912 | -0.1692 | -0.9544 | -0.6644 | -0.111 |
| TRINITY_DN7241_c0_g2_i2_orf1 | - | - | - | 40S ribosomal protein S10 [Zerene cesonia] >XP_045492164.1 40S ribosomal protein S10 [Colias croceus] | 1.77353 | -0.7413 | -0.9072 | -0.5494 | 0.42443 |
| TRINITY_DN43576_c0_g1_i3_orf1 | - | - | - | regulator of microtubule dynamics protein 1-like [Ostrinia furnacalis] | 1.87449 | -0.6388 | -1.0231 | -0.1731 | -0.0395 |
| TRINITY_DN53810_c0_g1_i1_orf1 | - | - | - | 39S ribosomal protein L53, mitochondrial [Pectinophora gossypiella] | 1.87508 | 0.16597 | -0.5052 | -0.6634 | -0.8725 |
| TRINITY_DN25210_c0_g1_i1_orf1 | - | - | - | mitochondrial import receptor subunit TOM22 homolog [Ostrinia furnacalis] | 1.88304 | -0.0079 | -1.0217 | -0.5785 | -0.275 |
| TRINITY_DN21984_c0_g1_i6_orf1 | - | - | - | venom serine protease 34-like [Ostrinia furnacalis] | 1.88274 | -0.522 | -0.0938 | -0.2025 | -1.0644 |
| TRINITY_DN19286_c0_g1_i1_orf1 | - | - | - | signal recognition particle 9 kDa protein [Ostrinia furnacalis] | 1.96119 | -0.3972 | -0.3444 | -0.8683 | -0.3513 |
| TRINITY_DN10455_c0_g1_i2_orf1 | - | - | - | actin-related protein 2/3 complex subunit 4 [Plutella xylostella] >XP_013184242.1 PREDICTED: actin-related protein 2/3 complex subunit 4 [Amyelois transitella] >XP_026754865.1 actin-related protein 2/3 complex subunit 4 [Galleria mellonella] >XP_028168998.1 actin-related protein 2/3 complex subunit 4 [Ostrinia furnacalis] >KAI5632346.1 ARP2/3 complex 20 kDa subunit (ARPC4) domain-containing protein [Phthorimaea operculella] >KAG7303373.1 Actin- protein 2/3 complex subunit 4 [Plutella xylostella] >CAG9104981.1 unnamed protein product [Plutella xylostella] | 1.71043 | 0.21104 | -1.0612 | -0.9467 | 0.08644 |

| | | | | | | | | | |
|---|---|---|---|---|---|---|---|---|---|
| TRINITY_DN42824_c0_g1_i5_orf1 | - | - | - | prefoldin subunit 3 [Ostrinia furnacalis] | 1.75123 | 0.43445 | -0.8139 | -0.9515 | -0.4202 |
| TRINITY_DN3027_c0_g1_i4_orf1 | - | - | - | ribosome biogenesis protein BOP1 homolog [Ostrinia furnacalis] | 1.98703 | -0.6953 | -0.4765 | -0.4743 | -0.3409 |
| TRINITY_DN12964_c0_g1_i1_orf1 | - | - | - | dnaJ homolog subfamily A member 1 [Ostrinia furnacalis] | 1.85985 | 0.13648 | -0.9582 | -0.3383 | -0.6999 |
| TRINITY_DN36045_c0_g1_i2_orf1 | - | - | - | hypothetical protein evm_012355 [Chilo suppressalis] >CAB3522006.1 unnamed protein product [Chilo suppressalis] >CAH0399328.1 unnamed protein product [Chilo suppressalis] | 1.91109 | -0.2291 | -0.242 | -0.4038 | -1.0362 |
| TRINITY_DN50820_c0_g1_i2_orf1 | - | - | - | transmembrane protein 135-like isoform X2 [Ostrinia furnacalis] | 1.65968 | 0.65101 | -0.6485 | -0.9302 | -0.7319 |
| TRINITY_DN9309_c0_g1_i5_orf1 | - | - | - | uncharacterized protein LOC114361160 [Ostrinia furnacalis] | 1.73158 | 0.37645 | -1.1096 | -0.244 | -0.7544 |
| TRINITY_DN19122_c0_g1_i7_orf1 | - | - | - | phosphatidylserine decarboxylase proenzyme, mitochondrial [Ostrinia | 1.95483 | -0.6229 | -0.3037 | -0.2228 | -0.8055 |
| TRINITY_DN41179_c0_g1_i1_orf1 | - | - | - | RNA-binding protein NOB1 [Ostrinia furnacalis] | 1.88441 | -0.1281 | -0.44 | -0.2265 | -1.0898 |
| TRINITY_DN1437_c0_g1_i6_orf1 | - | - | - | nucleoprotein TPR isoform X1 [Ostrinia furnacalis] | 1.58465 | 0.68596 | -1.1463 | -0.7524 | -0.372 |
| TRINITY_DN2627_c0_g1_i2_orf1 | - | - | - | probable cytosolic oligopeptidase A [Ostrinia furnacalis] | 1.96244 | -0.1991 | -0.433 | -0.5295 | -0.8009 |
| TRINITY_DN4025_c0_g1_i1_orf1 | - | - | - | unnamed protein product [Chilo suppressalis] | 1.8797 | -0.1619 | -1.1197 | -0.3622 | -0.2359 |
| TRINITY_DN298_c0_g1_i4_orf1 | - | - | - | luc7-like protein 3 isoform X1 [Ostrinia furnacalis] >XP_028160033.1 luc7-like protein 3 isoform X1 [Ostrinia furnacalis] | 1.58972 | 0.68426 | -0.9157 | -1.0282 | -0.3301 |
| TRINITY_DN9938_c0_g2_i1_orf1 | - | - | - | hypothetical protein E2986_04423 [Frieseomelitta varia] | 1.57887 | 0.60334 | -1.3201 | -0.5514 | -0.3107 |
| TRINITY_DN2168_c0_g1_i2_orf1 | - | - | - | protein arginine methyltransferase NDUFAF7 homolog, mitochondrial [Ostrinia furnacalis] | 1.90436 | -0.6563 | -0.9305 | -0.0438 | -0.2737 |
| TRINITY_DN21181_c0_g1_i6_orf1 | - | - | - | unnamed protein product, partial [Brenthis ino] | 1.9961 | -0.5824 | -0.5264 | -0.3906 | -0.4967 |
| TRINITY_DN2748_c0_g1_i6_orf1 | - | - | - | uncharacterized protein LOC114352811 [Ostrinia furnacalis] | 1.85398 | -0.9908 | -0.1216 | -0.7525 | 0.01085 |
| TRINITY_DN82810_c0_g1_i1_orf1 | - | - | - | putative carbonic anhydrase 3 [Ostrinia furnacalis] | 1.75805 | 0.24728 | -1.0819 | -0.1073 | -0.8161 |
| TRINITY_DN31216_c0_g1_i2_orf1 | - | - | - | uncharacterized protein LOC114361092 [Ostrinia furnacalis] | 1.70196 | -0.0426 | -1.4309 | -0.2326 | 0.00412 |
| TRINITY_DN13174_c0_g1_i4_orf1 | - | - | - | N-alpha-acetyltransferase 35, NatC auxiliary subunit [Ostrinia furnacalis] | 1.9634 | -0.7353 | -0.284 | -0.6696 | -0.2745 |
| TRINITY_DN2430_c0_g1_i1_orf1 | - | - | - | glutathione S-transferase omega 1 [Ostrinia furnacalis] | 1.7935 | 0.12416 | -1.1798 | -0.141 | -0.5969 |
| TRINITY_DN3057_c0_g2_i1_orf1 | - | - | - | chromodomain-helicase-DNA-binding protein Mi-2 homolog isoform X3 [Chelonus insularis] | 1.72374 | 0.52805 | -0.8677 | -0.5522 | -0.8319 |
| TRINITY_DN41311_c0_g2_i3_orf1 | - | - | - | ras-related protein Rab-8A isoform X2 [Ostrinia furnacalis] | 1.68424 | 0.59886 | -0.9724 | -0.6464 | -0.6643 |
| TRINITY_DN1665_c1_g1_i2_orf1 | - | - | - | translation elongation factor 2 [Melitaea cinxia] | 1.64329 | -0.8635 | -1.0745 | -0.2745 | 0.56923 |
| TRINITY_DN9759_c0_g1_i1_orf1 | - | - | - | iroquois-class homeodomain protein IRX-1-like isoform X1 [Ostrinia furnacalis] | 1.58185 | -0.0525 | -1.5571 | -0.1734 | 0.20112 |
| TRINITY_DN5129_c0_g3_i3_orf1 | - | - | - | probable citrate synthase 2, mitochondrial [Ostrinia furnacalis] | 1.98822 | -0.6519 | -0.3214 | -0.4646 | -0.5504 |
| TRINITY_DN143_c0_g3_i1_orf1 | - | - | - | Ubiquitin-60S ribosomal protein L40, partial [Cotesia chilonis] >UDP69015.1 egg surface protein ES-53, partial [Cotesia chilonis] | 1.76156 | -0.5728 | -1.1991 | 0.26104 | -0.2507 |
| TRINITY_DN36006_c0_g1_i5_orf1 | - | - | - | pro-resilin-like [Ostrinia furnacalis] | 1.28383 | 0.71938 | -1.6065 | 0.09725 | -0.494 |
| TRINITY_DN71840_c0_g1_i1_orf1 | - | - | - | 60S ribosomal protein L7 [Ostrinia furnacalis] >XP_028162266.1 60S ribosomal protein L7 [Ostrinia furnacalis] | 1.89498 | -0.7207 | -0.9093 | -0.2503 | -0.0147 |
| TRINITY_DN21782_c0_g1_i8_orf1 | - | - | - | atypical kinase COQ8B, mitochondrial [Ostrinia furnacalis] | 1.93291 | 0.00735 | -0.7215 | -0.6258 | -0.593 |
| TRINITY_DN72056_c0_g1_i1_orf1 | - | - | - | protein PBDC1 [Ostrinia furnacalis] | 1.93096 | -0.1518 | -0.9008 | -0.5992 | -0.2792 |
| TRINITY_DN81312_c0_g1_i1_orf1 | - | - | - | atlastin isoform X4 [Ostrinia furnacalis] | 1.61405 | -0.8295 | -1.2243 | -0.016 | 0.45572 |
| TRINITY_DN27960_c0_g1_i1_orf1 | - | - | - | ATP synthase mitochondrial F1 complex assembly factor 1 [Ostrinia furnacalis] | 1.87978 | 0.18868 | -0.6536 | -0.6706 | -0.7443 |
| TRINITY_DN79000_c1_g1_i1_orf1 | - | - | - | AT15141p, partial [Drosophila melanogaster] | 1.85593 | 0.19842 | -0.9003 | -0.7179 | -0.4361 |
| TRINITY_DN2997_c0_g1_i6_orf1 | - | - | - | titin-like [Ostrinia furnacalis] | 1.72922 | 0.06768 | -0.9238 | -1.0573 | 0.18419 |
| TRINITY_DN36701_c0_g1_i4_orf1 | - | - | - | hypothetical protein SFRURICE_004895 [Spodoptera frugiperda] >KAG8116760.1 hypothetical protein SFRUCORN_001970 [Spodoptera | 1.93264 | -0.6664 | -0.7812 | -0.4579 | -0.0271 |
| TRINITY_DN17208_c0_g1_i2_orf1 | - | - | - | integrator complex subunit 11 [Ostrinia furnacalis] | 1.88827 | -0.3226 | -1.1068 | -0.2365 | -0.2223 |
| TRINITY_DN198_c0_g1_i2_orf1 | - | - | - | retinol dehydrogenase 13-like [Ostrinia furnacalis] | 0.59399 | 0.79039 | -1.2198 | -1.205 | 1.04043 |
| TRINITY_DN44877_c0_g1_i2_orf1 | - | - | - | U6 snRNA-associated Sm-like protein LSm7 [Diachasma alloeum] | 1.46174 | 0.81448 | -1.2132 | -0.817 | -0.246 |
| TRINITY_DN4469_c0_g1_i2_orf1 | - | - | - | metal transporter CNNM4-like [Ostrinia furnacalis] | 1.77798 | -0.8411 | -1.0037 | -0.2132 | 0.28006 |
| TRINITY_DN120979_c0_g1_i1_orf1 | - | - | - | la-related protein 1-like isoform X2 [Ostrinia furnacalis] | 1.86695 | 0.06198 | -0.8143 | -0.8938 | -0.2209 |
| TRINITY_DN60680_c0_g1_i2_orf1 | - | - | - | unnamed protein product [Euphydryas editha] | 1.92244 | -0.4736 | -0.8788 | -0.5544 | -0.0156 |
| TRINITY_DN18569_c0_g2_i1_orf1 | - | - | - | stomatin-like protein 2, mitochondrial [Ostrinia furnacalis] | 1.92474 | -0.1249 | -0.9602 | -0.4715 | -0.3682 |
| TRINITY_DN77572_c0_g1_i1_orf1 | - | - | - | steroid receptor RNA activator 1 [Ostrinia furnacalis] | 1.01774 | -0.1445 | -1.8431 | 0.28974 | 0.68008 |

| | | | | | | | | |
|---|---|---|---|---|---|---|---|---|
| TRINITY_DN31253_c0_g1_i2_orf1 | - | - | - | hypothetical protein evm_009655 [Chilo suppressalis] | 1.83411 | -1.1099 | -0.5887 | -0.2235 | 0.08793 |
| TRINITY_DN94337_c0_g1_i1_orf1 | - | - | - | hypothetical protein evm_006136 [Chilo suppressalis] | 1.89166 | -0.9838 | -0.2039 | -0.0652 | -0.6386 |
| TRINITY_DN5630_c4_g1_i2_orf1 | - | - | - | PREDICTED: stress-associated endoplasmic reticulum protein 2 [Amyelois transitella] >XP_014371593.1 stress-associated endoplasmic reticulum protein 2 [Papilio machaon] >XP_022818474.1 stress-associated endoplasmic reticulum protein 2 [Spodoptera litura] >XP_028162992.1 stress-associated endoplasmic reticulum protein 2 [Ostrinia furnacalis] >XP_028162993.1 stress-associated endoplasmic reticulum protein 2 [Ostrinia furnacalis] >XP_031767943.1 stress-associated endoplasmic reticulum protein 2 [Galleria mellonella] >XP_035452408.1 stress-associated endoplasmic reticulum protein 2-like [Spodoptera frugiperda] >XP_035452409.1 stress-associated endoplasmic reticulum protein 2-like [Spodoptera frugiperda] >XP_035452411.1 stress-associated endoplasmic reticulum protein 2-like [Spodoptera frugiperda] >XP_045455924.1 stress-associated endoplasmic reticulum protein 2 [Melitaea cinxia] >KPJ00707.1 Stress-associated endoplasmic reticulum protein 2 [Papilio xuthus] >CAB3510969.1 unnamed protein product [Spodoptera littoralis] >AXY94738.1 stress-associated endoplasmic reticulum protein 2 [Galleria mellonella] >KAF9797689.1 hypothetical protein SFRURICE_017884 [Spodoptera frugiperda] >KAG8114722.1 hypothetical protein SFRUCORN_004134 [Spodoptera frugiperda] | 1.78955 | -0.1782 | -0.7456 | -1.079 | 0.21329 |
| TRINITY_DN10030_c0_g1_i2_orf1 | - | - | - | uncharacterized protein LOC114360702 [Ostrinia furnacalis] >XP_028171286.1 uncharacterized protein LOC114360702 [Ostrinia furnacalis] >XP_028171287.1 uncharacterized protein LOC114360702 [Ostrinia furnacalis] | 1.9829 | -0.2854 | -0.6145 | -0.4352 | -0.6478 |
| TRINITY_DN10831_c1_g1_i1_orf1 | - | - | - | 40S ribosomal protein S16 [Ostrinia furnacalis] | 1.71519 | -1.0443 | -0.8619 | -0.2259 | 0.41686 |
| TRINITY_DN107288_c0_g1_i2_orf1 | - | - | - | methionine-tRNA synthetase, partial [Papilio xuthus] | 1.68864 | -1.0387 | 0.39716 | -0.95 | -0.0971 |
| TRINITY_DN59042_c1_g1_i1_orf1 | - | - | - | nuclear pore complex protein Nup50 [Ostrinia furnacalis] | 1.83065 | 0.16184 | -1.0105 | -0.2468 | -0.7352 |
| TRINITY_DN20130_c0_g1_i1_orf1 | - | - | - | uncharacterized protein LOC114354518 isoform X1 [Ostrinia furnacalis] | 1.85346 | -0.5199 | -1.1053 | -0.2667 | 0.03847 |
| TRINITY_DN122423_c0_g5_i1_orf1 | - | - | - | PREDICTED: dynein heavy chain, cytoplasmic isoform X3 [Fopius arisanus] | 1.61973 | 0.20951 | -1.4656 | 0.0616 | -0.4252 |
| TRINITY_DN20_c0_g1_i11_orf1 | - | - | - | plasma membrane calcium-transporting ATPase 2 [Ostrinia furnacalis] | 1.92258 | -0.9055 | -0.5378 | -0.4393 | -0.04 |
| TRINITY_DN11065_c0_g2_i1_orf1 | - | - | - | ribosomal protein s6e domain-containing protein [Phthorimaea operculella] | 1.66419 | -0.9793 | -1.0154 | -0.1395 | 0.47003 |
| TRINITY_DN14996_c0_g1_i2_orf1 | - | - | - | 40S ribosomal protein S17 [Ostrinia furnacalis] | 1.82364 | -1.0961 | -0.6849 | -0.0592 | 0.01655 |
| TRINITY_DN740_c0_g1_i1_orf1 | - | - | - | GTP-binding nuclear protein Ran [Pieris rapae] >XP_028162165.1 GTP-binding nuclear protein Ran [Ostrinia furnacalis] >XP_028162166.1 GTP-binding nuclear protein Ran [Ostrinia furnacalis] >XP_028162167.1 GTP-binding nuclear protein Ran [Ostrinia furnacalis] >XP_045532338.1 GTP-binding nuclear protein Ran [Pieris brassicae] >XP_045532339.1 GTP-binding nuclear protein Ran [Pieris brassicae] >CAG9745207.1 unnamed protein product [Diatraea saccharalis] >CAG9783892.1 unnamed protein product [Diatraea saccharalis] | 1.94779 | -0.0864 | -0.7912 | -0.5351 | -0.5351 |
| TRINITY_DN13160_c0_g1_i1_orf1 | - | - | - | serine/threonine-protein kinase 10-like, partial [Ostrinia furnacalis] | 1.92124 | -0.6284 | -0.6349 | -0.7126 | 0.05469 |
| TRINITY_DN37923_c0_g1_i1_orf1 | - | - | - | hypothetical protein NE865_05974 [Phthorimaea operculella] | 1.32131 | 1.01026 | -1.1651 | -0.8961 | -0.2704 |
| TRINITY_DN21981_c0_g1_i8_orf1 | - | - | - | dihydroorotate dehydrogenase (quinone), mitochondrial [Ostrinia furnacalis] | 1.93679 | -0.0856 | -0.8728 | -0.5127 | -0.4657 |
| TRINITY_DN2593_c0_g1_i1_orf1 | - | - | - | midgut carboxypeptidase [Loxostege sticticalis] | 1.90855 | -0.5508 | -0.2116 | -0.1533 | -0.9929 |

| | | | | | | | | | |
|---|---|---|---|---|---|---|---|---|---|
| TRINITY_DN4707_c0_g1_i1_orf1 | - | - | - | PREDICTED: DNA-directed RNA polymerases I, II, and III subunit RPABC1 [Papilio xuthus] >XP_013187738.1 PREDICTED: DNA-directed RNA polymerases I, II, and III subunit RPABC1 [Amyelois transitella] >XP_028158146.1 DNA-directed RNA polymerases I, II, and III subunit RPABC1 [Ostrinia furnacalis] >XP_045537534.1 DNA-directed RNA polymerases I, II, and III subunit RPABC1 [Papilio machaon] >XP_049876738.1 DNA-directed RNA polymerases I, II, and III subunit RPABC1 [Pectinophora gossypiella] >KAG6452000.1 hypothetical protein O3G_MSEX007416 [Manduca sexta] >RVE48301.1 hypothetical protein evm_007052 [Chilo suppressalis] >CAG5049330.1 unnamed protein product [Parnassius apollo] >CAG9757053.1 unnamed protein product [Diatraea saccharalis] >CAH2042370.1 unnamed protein product, partial [Iphiclides | 1.82999 | 0.2733 | -0.9588 | -0.6053 | -0.5392 |
| TRINITY_DN29156_c0_g1_i1_orf1 | - | - | - | protein FAM136A [Ostrinia furnacalis] | 1.9195 | -0.8426 | -0.6366 | -0.4475 | 0.00718 |
| TRINITY_DN391_c1_g2_i1_orf1 | - | - | - | NADH dehydrogenase [ubiquinone] 1 alpha subcomplex subunit 13 [Ostrinia furnacalis] | 1.98187 | -0.5771 | -0.465 | -0.6716 | -0.2682 |
| TRINITY_DN33619_c0_g1_i1_orf1 | - | - | - | eukaryotic translation initiation factor 3 subunit C [Ostrinia furnacalis] >XP_028176017.1 eukaryotic translation initiation factor 3 subunit C [Ostrinia furnacalis] | 1.9076 | -0.3819 | -0.8555 | -0.6947 | 0.02459 |
| TRINITY_DN3029_c4_g1_i1_orf1 | - | - | - | proliferation marker protein Ki-67-like, partial [Ostrinia furnacalis] | 1.36743 | 0.07964 | -1.7367 | 0.32594 | -0.0363 |
| TRINITY_DN37599_c0_g1_i1_orf1 | - | - | - | bmp-2 protein isoform X3 [Bombyx mori] >XP_028041166.1 RNA-binding protein 4.1-like isoform X2 [Bombyx mandarina] | 1.65039 | 0.5519 | -1.08 | -0.8573 | -0.265 |
| TRINITY_DN46367_c0_g1_i2_orf1 | - | - | - | T-complex protein 1 subunit zeta [Ostrinia furnacalis] | 1.92953 | 0.02282 | -0.5888 | -0.6871 | -0.6764 |
| TRINITY_DN13368_c0_g1_i1_orf1 | - | - | - | isoleucine--tRNA ligase, cytoplasmic [Ostrinia furnacalis] | 1.94859 | -0.6359 | -0.6527 | -0.6082 | -0.0518 |
| TRINITY_DN172_c1_g1_i3_orf1 | - | - | - | galactose oxidase, central domain-containing protein [Phthorimaea operculella] | 1.54055 | 0.75578 | -0.7567 | -0.384 | -1.1556 |
| TRINITY_DN1875_c0_g1_i1_orf1 | - | - | - | uncharacterized protein LOC114366320 isoform X1 [Ostrinia furnacalis] >XP_028178963.1 uncharacterized protein LOC114366320 isoform X1 [Ostrinia furnacalis] >XP_028178964.1 uncharacterized protein LOC114366320 isoform X2 [Ostrinia furnacalis] | 1.49011 | 0.2617 | -1.5732 | 0.24249 | -0.4211 |
| TRINITY_DN9931_c0_g1_i1_orf1 | - | - | - | syntaxin-18 [Ostrinia furnacalis] | 1.82465 | -0.0791 | -1.2202 | -0.1258 | -0.3996 |
| TRINITY_DN2456_c0_g1_i2_orf1 | - | - | - | glycerol-3-phosphate phosphatase isoform X1 [Ostrinia furnacalis] | 1.89915 | 0.13164 | -0.6566 | -0.6704 | -0.7038 |
| TRINITY_DN82008_c0_g1_i1_orf1 | - | - | - | GMP reductase 1-like [Ostrinia furnacalis] | 1.94219 | -0.677 | -0.8133 | -0.1711 | -0.2808 |
| TRINITY_DN271_c0_g2_i6_orf1 | - | - | - | hypothetical protein NE865_03378 [Phthorimaea operculella] | 1.85446 | -0.7174 | -0.7199 | -0.6781 | 0.26101 |
| TRINITY_DN257_c0_g1_i7_orf1 | - | - | - | zinc finger RNA-binding protein 2 [Ostrinia furnacalis] | 1.49379 | 0.70089 | -1.3049 | -0.7438 | -0.1459 |
| TRINITY_DN2266_c0_g1_i6_orf1 | - | - | - | bilin-binding protein-like [Ostrinia furnacalis] | 1.73985 | -0.6203 | 0.5073 | -0.8746 | -0.7522 |
| TRINITY_DN344_c0_g1_i1_orf1 | - | - | - | chymotrypsin-like serine protease 16 [Ostrinia nubilalis] | 1.99405 | -0.4777 | -0.6376 | -0.4011 | -0.4777 |
| TRINITY_DN4836_c0_g1_i4_orf1 | - | - | - | hypothetical protein O3G_MSEX014157 [Manduca sexta] >KAG6463927.1 hypothetical protein O3G_MSEX014157 [Manduca sexta] | 1.62699 | 0.13708 | -1.1375 | -0.9629 | 0.33629 |
| TRINITY_DN12806_c0_g2_i1_orf1 | - | - | - | inactive pancreatic lipase-related protein 1-like isoform X2 [Ostrinia furnacalis] | 1.59852 | -0.9115 | -1.0677 | -0.2576 | 0.63834 |
| TRINITY_DN127151_c0_g1_i1_orf1 | - | - | - | 3-oxoacyl-[acyl-carrier-protein] synthase, mitochondrial [Ostrinia furnacalis] | 1.97184 | -0.7355 | -0.2108 | -0.4963 | -0.5293 |
| TRINITY_DN3008_c0_g1_i12_orf1 | - | - | - | reticulon-4-interacting protein 1 homolog, mitochondrial [Ostrinia furnacalis] | 1.92333 | -0.9319 | -0.0722 | -0.5078 | -0.4114 |
| TRINITY_DN37729_c0_g1_i8_orf1 | - | - | - | adenylyltransferase and sulfurtransferase MOCS3 isoform X1 [Ostrinia furnacalis] | 1.87635 | -0.0578 | -1.0949 | -0.4492 | -0.2744 |
| TRINITY_DN2623_c0_g1_i3_orf1 | - | - | - | unnamed protein product [Chilo suppressalis] | 1.641 | 0.59352 | -0.9625 | -0.9673 | -0.3048 |
| TRINITY_DN40345_c0_g1_i6_orf1 | - | - | - | 60S ribosomal protein L28 [Ostrinia furnacalis] | 1.86517 | -0.645 | -0.8851 | -0.5318 | 0.19684 |
| TRINITY_DN4497_c0_g1_i4_orf1 | - | - | - | cytochrome P450 9e2-like [Ostrinia furnacalis] >QPF77612.1 cytochrome P450 monooxygenase CYP9A185 [Ostrinia furnacalis] | 1.80483 | -0.5583 | -0.873 | 0.36051 | -0.7339 |
| TRINITY_DN2064_c1_g1_i1_orf1 | - | - | - | hypothetical protein evm_007509 [Chilo suppressalis] >CAB3521498.1 unnamed protein product [Chilo suppressalis] | 1.94339 | -0.9425 | -0.3208 | -0.3593 | -0.3208 |
| TRINITY_DN6396_c0_g1_i1_orf1 | - | - | - | PR domain zinc finger protein 10-like [Ostrinia furnacalis] | 1.70947 | -0.2483 | -1.2653 | -0.5429 | 0.34701 |
| TRINITY_DN3343_c0_g2_i1_orf1 | - | - | - | AFG3-like protein 2 [Ostrinia furnacalis] | 1.65844 | 0.47443 | -1.2682 | -0.2868 | -0.5778 |
| TRINITY_DN31431_c0_g1_i1_orf1 | - | - | - | carnosine N-methyltransferase [Ostrinia furnacalis] | 1.12375 | 0.48585 | -1.8085 | -0.225 | 0.42392 |
| TRINITY_DN2374_c0_g1_i1_orf1 | - | - | - | uncharacterized protein LOC114357127 [Ostrinia furnacalis] | 1.58972 | 0.3792 | -1.1666 | -0.9696 | 0.16727 |
| TRINITY_DN11375_c0_g1_i6_orf1 | - | - | - | uncharacterized protein LOC114363514 isoform X2 [Ostrinia furnacalis] | 1.18958 | 1.19351 | -0.9108 | -0.3847 | -1.0876 |

| | | | | | | | | | |
|---|---|---|---|---|---|---|---|---|---|
| TRINITY_DN53684_c0_g1_i1_orf1 | - | - | - | eukaryotic translation initiation factor 3 subunit M-like [Ostrinia furnacalis] | 1.93099 | -0.0314 | -0.786 | -0.684 | -0.4296 |
| TRINITY_DN5818_c1_g1_i2_orf1 | - | - | - | unnamed protein product [Chrysodeixis includens] | 1.79349 | 0.18925 | -0.8883 | -0.9712 | -0.1232 |
| TRINITY_DN13972_c0_g1_i5_orf1 | - | - | - | myelin expression factor 2-like [Ostrinia furnacalis] >XP_028173185.1 myelin expression factor 2-like [Ostrinia furnacalis] | 1.67138 | 0.50184 | -1.0354 | -0.9119 | -0.2259 |
| TRINITY_DN12101_c0_g1_i2_orf1 | - | - | - | UPF0545 protein C22orf39 homolog [Ostrinia furnacalis] | 1.76554 | 0.28157 | -0.166 | -0.8826 | -0.9986 |
| TRINITY_DN20007_c0_g1_i1_orf1 | - | - | - | hypothetical protein evm_011958 [Chilo suppressalis] >CAB3521085.1 unnamed protein product [Chilo suppressalis] | 1.77689 | 0.43211 | -0.8691 | -0.7085 | -0.6314 |
| TRINITY_DN24490_c0_g1_i6_orf1 | - | - | - | E3 ubiquitin-protein ligase Hakai [Ostrinia furnacalis] | 1.31083 | 1.07522 | -1.1028 | -0.4342 | -0.849 |
| TRINITY_DN5648_c0_g1_i5_orf1 | - | - | - | protein tumorous imaginal discs, mitochondrial-like isoform X2 [Ostrinia | 1.89242 | 0.14415 | -0.7652 | -0.5897 | -0.6817 |
| TRINITY_DN3909_c0_g2_i2_orf1 | - | - | - | ribosomal protein L24 [Loxostege sticticalis] | 1.70979 | -0.7472 | -1.1994 | -0.0424 | 0.2792 |
| TRINITY_DN26243_c0_g1_i2_orf1 | - | - | - | dynein heavy chain 6, axonemal [Ostrinia furnacalis] | 1.80807 | -0.0614 | -1.0582 | 0.08605 | -0.7746 |
| TRINITY_DN21251_c1_g1_i1_orf1 | - | - | - | 60S ribosomal protein L4 [Ostrinia furnacalis] | 1.79477 | -0.7547 | -1.0759 | -0.1416 | 0.17752 |
| TRINITY_DN11402_c0_g1_i1_orf1 | - | - | - | constitutive coactivator of PPAR-gamma-like protein 1 isoform X1 [Ostrinia furnacalis] >XP_028158538.1 constitutive coactivator of PPAR-gamma-like protein 1 isoform X2 [Ostrinia furnacalis] | 1.51015 | 0.88295 | -0.8613 | -0.8773 | -0.6545 |
| TRINITY_DN932_c0_g1_i4_orf1 | - | - | - | SXSS-APN2 [Ostrinia furnacalis] | 1.97865 | -0.6369 | -0.3886 | -0.2842 | -0.669 |
| TRINITY_DN1298_c0_g1_i3_orf1 | - | - | - | ras GTPase-activating protein-binding protein 2-like, partial [Ostrinia furnacalis] | 1.48262 | 0.37172 | -1.5498 | -0.4805 | 0.17597 |
| TRINITY_DN26355_c0_g1_i4_orf1 | - | - | - | small integral membrane protein 12 [Ostrinia furnacalis] | 1.7215 | 0.54774 | -0.846 | -0.7744 | -0.6488 |
| TRINITY_DN3835_c0_g1_i3_orf1 | - | - | - | protein ERGIC-53 isoform X1 [Ostrinia furnacalis] >XP_028177940.1 protein ERGIC-53 isoform X2 [Ostrinia furnacalis] >XP_028177941.1 protein ERGIC-53 isoform X3 [Ostrinia furnacalis] | 1.75628 | 0.39057 | -1.1016 | -0.5623 | -0.483 |
| TRINITY_DN32769_c1_g1_i5_orf1 | - | - | - | large subunit GTPase 1 homolog [Ostrinia furnacalis] | 1.89065 | -1.0284 | -0.5577 | -0.0803 | -0.2242 |
| TRINITY_DN5086_c0_g1_i1_orf1 | - | - | - | unnamed protein product [Diatraea saccharalis] | 1.72463 | 0.12954 | -1.0907 | -0.8954 | 0.13197 |
| TRINITY_DN42373_c0_g4_i1_orf1 | - | - | - | unnamed protein product [Spodoptera exigua] | 1.5308 | -0.9564 | -1.0869 | -0.2071 | 0.71957 |
| TRINITY_DN1666_c0_g1_i2_orf1 | - | - | - | putative defense protein Hdd11 [Ostrinia furnacalis] >XP_028179344.1 putative defense protein Hdd11 [Ostrinia furnacalis] >AGV28583.1 immune-induced protein [Ostrinia furnacalis] | 1.65913 | -0.9765 | -0.0628 | 0.43094 | -1.0508 |
| TRINITY_DN22842_c0_g1_i4_orf1 | - | - | - | MICOS complex subunit MIC27-like [Ostrinia furnacalis] >XP_028158921.1 MICOS complex subunit MIC27-like [Ostrinia furnacalis] | 1.9877 | -0.367 | -0.5591 | -0.6691 | -0.3925 |
| TRINITY_DN16408_c0_g1_i1_orf1 | - | - | - | ABC transporter G family member 20 isoform X1 [Ostrinia furnacalis] >XP_028158027.1 ABC transporter G family member 20 isoform X1 [Ostrinia furnacalis] >XP_028158037.1 ABC transporter G family member 20 isoform X2 [Ostrinia furnacalis] >XP_028158043.1 ABC transporter G family member 20 isoform X3 [Ostrinia furnacalis] >XP_028158060.1 ABC transporter G family member 20 isoform X5 [Ostrinia furnacalis] >XP_028158070.1 ABC transporter G family member 20 isoform X6 [Ostrinia furnacalis] | 1.78832 | -0.5458 | -1.1605 | -0.3184 | 0.23641 |
| TRINITY_DN2913_c0_g1_i5_orf1 | - | - | - | aquaporin-11 isoform X1 [Spodoptera litura] | 1.85269 | -0.8865 | -0.8431 | 0.11627 | -0.2393 |
| TRINITY_DN25373_c0_g1_i1_orf1 | - | - | - | epidermal growth factor receptor substrate 15-like 1 [Ostrinia furnacalis] | 1.85898 | 0.22905 | -0.7589 | -0.5371 | -0.792 |
| TRINITY_DN45446_c0_g1_i2_orf1 | - | - | - | peptide transporter family 1-like isoform X1 [Ostrinia furnacalis] | 1.92942 | -0.91 | -0.0845 | -0.4174 | -0.5176 |
| TRINITY_DN18869_c0_g1_i1_orf1 | - | - | - | unnamed protein product [Parnassius apollo] | 1.84753 | -1.1036 | -0.5495 | -0.2518 | 0.05739 |
| TRINITY_DN25916_c0_g1_i1_orf1 | - | - | - | uncharacterized protein LOC125063950 [Vanessa atalanta] | 1.23467 | 0.39166 | -1.4978 | -0.7958 | 0.66733 |
| TRINITY_DN36928_c0_g1_i2_orf1 | - | - | - | actin-interacting protein 1 isoform X2 [Ostrinia furnacalis] | 1.53363 | -0.5385 | -1.29 | -0.4227 | 0.71768 |
| TRINITY_DN34726_c0_g2_i1_orf1 | - | - | - | heat shock factor-binding protein 1 [Ostrinia furnacalis] | 1.74004 | 0.4341 | -0.9706 | -0.3594 | -0.8442 |
| TRINITY_DN14301_c0_g2_i1_orf1 | - | - | - | unnamed protein product [Chrysodeixis includens] | 1.93678 | -0.3985 | -0.4165 | -0.1818 | -0.94 |
| TRINITY_DN8958_c0_g1_i1_orf1 | - | - | - | nuclear cap-binding protein subunit 1 [Galleria mellonella] | 1.38614 | 0.66007 | -1.5939 | -0.2301 | -0.2222 |
| TRINITY_DN5262_c0_g1_i7_orf1 | - | - | - | T-complex protein 1 subunit beta [Ostrinia furnacalis] | 1.88441 | 0.04275 | -0.8931 | -0.7567 | -0.2774 |

| | | | | | | | | |
|---|---|---|---|---|---|---|---|---|
| TRINITY_DN1509_c0_g1_i1_orf1 | - | - | - | ribosomal protein S15A [Bombyx mori] >XP_011566807.1 40S ribosomal protein S15Aa [Plutella xylostella] >XP_013186470.1 PREDICTED: 40S ribosomal protein S15Aa [Amyelois transitella] >XP_021181353.1 40S ribosomal protein S15Aa [Helicoverpa armigera] >XP_022114309.1 40S ribosomal protein S15Aa [Pieris rapae] >XP_022820057.1 40S ribosomal protein S15Aa [Spodoptera litura] >XP_023947206.1 40S ribosomal protein S15Aa [Bicyclus anynana] >XP_026329660.1 40S ribosomal protein S15Aa [Hyposmocoma kahamanoa] >XP_026495126.1 40S ribosomal protein S15Aa [Vanessa tameamea] >XP_026745810.1 40S ribosomal protein S15Aa [Trichoplusia ni] >XP_026757934.1 40S ribosomal protein S15Aa [Galleria mellonella] >XP_028041686.1 40S ribosomal protein S15Aa [Bombyx mandarina] >XP_028161999.1 40S ribosomal protein S15Aa [Ostrinia furnacalis] >XP_028162000.1 40S ribosomal protein S15Aa [Ostrinia furnacalis] >XP_030024299.1 40S ribosomal protein S15Aa [Manduca sexta] >XP_032514052.1 40S ribosomal protein S15Aa [Danaus plexippus plexippus] >XP_034834075.1 40S ribosomal protein S15Aa [Maniola hyperantus] >XP_034840269.1 40S ribosomal protein S15Aa [Maniola hyperantus] >XP_035436795.1 40S ribosomal protein S15Aa [Spodoptera frugiperda] >XP_037869057.1 ribosomal protein S15A isoform X1 [Bombyx mori] >XP_039747368.1 40S ribosomal protein S15Aa [Pararge aegeria] >XP_045445511.1 40S ribosomal protein S15Aa [Melitaea cinxia] >XP_045491780.1 40S ribosomal protein S15Aa [Colias croceus] >XP_045519936.1 40S ribosomal protein S15Aa [Pieris brassicae] >XP_045785214.1 40S ribosomal protein S15Aa [Maniola jurtina] >XP_046965230.1 40S ribosomal protein S15Aa [Vanessa cardui] >XP_046965231.1 40S ribosomal protein S15Aa [Vanessa cardui] >XP_047021729.1 40S ribosomal protein S15Aa [Helicoverpa zea] >XP_047509814.1 40S ribosomal protein S15Aa [Pieris napi] >XP_047527633.1 40S ribosomal protein S15Aa [Vanessa atalanta] >XP_047527634.1 40S ribosomal protein S15Aa [Vanessa atalanta] >XP_047988443.1 40S ribosomal | 1.86766 | -0.9696 | -0.7423 | -0.1435 | -0.0123 |
| TRINITY_DN14336_c0_g3_i2_orf1 | - | - | - | calbindin-32 isoform X1 [Ostrinia furnacalis] | 1.97368 | -0.6947 | -0.2567 | -0.6406 | -0.3817 |
| TRINITY_DN12579_c0_g1_i1_orf1 | - | - | - | uncharacterized protein LOC114354070 isoform X3 [Ostrinia furnacalis] | 1.90869 | -0.6675 | -0.9277 | -0.1851 | -0.1284 |
| TRINITY_DN9316_c1_g1_i1_orf1 | - | - | - | peptidyl-tRNA hydrolase 2, mitochondrial-like [Ostrinia furnacalis] | 1.97911 | -0.7414 | -0.4577 | -0.2906 | -0.4893 |
| TRINITY_DN41922_c0_g3_i1_orf1 | - | - | - | influenza virus NS1A-binding protein-like [Ostrinia furnacalis] | 1.9412 | -0.7965 | -0.5873 | -0.4989 | -0.0585 |
| TRINITY_DN11825_c0_g1_i4_orf1 | - | - | - | 39S ribosomal protein L22, mitochondrial [Ostrinia furnacalis] | 1.97699 | -0.5933 | -0.7209 | -0.3231 | -0.3397 |
| TRINITY_DN27556_c0_g1_i1_orf1 | - | - | - | bystin [Ostrinia furnacalis] | 1.83926 | 0.28267 | -0.5723 | -0.8418 | -0.7078 |
| TRINITY_DN825_c2_g1_i5_orf1 | - | - | - | hypothetical protein SFRURICE_018365 [Spodoptera frugiperda] >KAG8107343.1 hypothetical protein SFRUCORN_012069 [Spodoptera frugiperda] >CAB3509414.1 unnamed protein product [Spodoptera littoralis] >CAH1638995.1 unnamed protein product [Spodoptera littoralis] | 1.95156 | -0.2719 | -0.2297 | -0.8071 | -0.6429 |
| TRINITY_DN36648_c0_g1_i1_orf1 | - | - | - | UMP-CMP kinase [Ostrinia furnacalis] | 1.98716 | -0.4385 | -0.7138 | -0.4385 | -0.3963 |
| TRINITY_DN4835_c0_g1_i2_orf1 | - | - | - | ribonucleoside-diphosphate reductase large subunit [Ostrinia furnacalis] | 1.12323 | 1.14202 | -1.2435 | -0.9386 | -0.0832 |
| TRINITY_DN146718_c0_g1_i1_orf1 | - | - | - | 40S ribosomal protein S6 [Diachasma alloeum] | 1.76301 | -0.5344 | -1.1898 | 0.28867 | -0.3274 |
| TRINITY_DN3582_c0_g1_i2_orf1 | - | - | - | uncharacterized protein LOC114357129 [Ostrinia furnacalis] | 1.66722 | 0.62536 | -0.9794 | -0.7194 | -0.5938 |
| TRINITY_DN91989_c0_g1_i1_orf1 | - | - | - | protein l(2)37Cc [Pectinophora gossypiella] | 1.96927 | -0.2125 | -0.4366 | -0.7461 | -0.5741 |
| TRINITY_DN11928_c0_g1_i3_orf1 | - | - | - | leucyl-cystinyl aminopeptidase-like isoform X4 [Ostrinia furnacalis] | 1.91148 | 0.06676 | -0.5389 | -0.6314 | -0.8078 |
| TRINITY_DN57496_c0_g1_i1_orf1 | - | - | - | NADPH:adrenodoxin oxidoreductase, mitochondrial [Ostrinia furnacalis] | 1.57794 | 0.3025 | -0.7329 | 0.20825 | -1.3557 |
| TRINITY_DN7289_c0_g1_i1_orf1 | - | - | - | nuclear cap-binding protein subunit 2 [Ostrinia furnacalis] | 1.77377 | -0.4491 | -0.8562 | -0.8704 | 0.40185 |

| | | | | | | | | | |
|---|---|---|---|---|---|---|---|---|---|
| TRINITY_DN4141_c0_g1_i9_orf1 | - | - | - | la-related protein 1 isoform X2 [Ostrinia furnacalis] >XP_028160900.1 la-related protein 1 isoform X3 [Ostrinia furnacalis] >XP_028160902.1 la-related protein 1 isoform X2 [Ostrinia furnacalis] >XP_028160903.1 la-related protein 1 isoform X2 [Ostrinia furnacalis] >XP_028160904.1 la-related protein 1 isoform X2 [Ostrinia furnacalis] >XP_028160905.1 la-related protein 1 isoform X4 [Ostrinia furnacalis] >XP_028160906.1 la-related protein 1 isoform X2 [Ostrinia furnacalis] >XP_028160908.1 la-related protein 1 isoform X2 [Ostrinia furnacalis] >XP_028160909.1 la-related protein 1 isoform X2 [Ostrinia furnacalis] >XP_028160910.1 la-related protein 1 isoform X2 [Ostrinia furnacalis] | 1.96866 | -0.6419 | -0.7298 | -0.2704 | -0.3265 |
| TRINITY_DN58261_c0_g1_i1_orf1 | - | - | - | 15-hydroxyprostaglandin dehydrogenase [NAD(+)]-like [Ostrinia furnacalis] | 1.57736 | -0.398 | -1.3547 | -0.4137 | 0.5891 |
| TRINITY_DN88539_c0_g2_i1_orf1 | - | - | - | uncharacterized protein LOC114352312 isoform X1 [Ostrinia furnacalis] >XP_028159669.1 uncharacterized protein LOC114352312 isoform X1 [Ostrinia furnacalis] | 1.76558 | -0.147 | -0.9179 | -0.972 | 0.27142 |
| TRINITY_DN71465_c0_g1_i1_orf1 | - | - | - | adenylate kinase isoenzyme 6 [Ostrinia furnacalis] | 1.98406 | -0.4628 | -0.6343 | -0.2797 | -0.6073 |
| TRINITY_DN9094_c0_g1_i1_orf1 | - | - | - | uncharacterized protein LOC114356316 [Ostrinia furnacalis] | 1.95968 | -0.7469 | -0.3142 | -0.6721 | -0.2265 |
| TRINITY_DN60048_c0_g2_i1_orf1 | - | - | - | facilitated trehalose transporter Tret1-like [Venturia canescens] >XP_043281789.1 facilitated trehalose transporter Tret1-like [Venturia canescens] >XP_043282611.1 facilitated trehalose transporter Tret1-like [Venturia canescens] >XP_043283442.1 facilitated trehalose transporter Tret1- | 1.92602 | -0.2986 | -0.9803 | -0.4477 | -0.1994 |
| TRINITY_DN2026_c0_g1_i4_orf1 | - | - | - | 60S ribosomal protein L35a [Ostrinia furnacalis] >XP_028167523.1 60S ribosomal protein L35a [Ostrinia furnacalis] | 1.89889 | -0.9822 | -0.4251 | -0.4987 | 0.00716 |
| TRINITY_DN10429_c0_g1_i2_orf1 | - | - | - | lon protease homolog, mitochondrial isoform X1 [Ostrinia furnacalis] >XP_028176557.1 lon protease homolog, mitochondrial isoform X2 [Ostrinia furnacalis] | 1.97655 | -0.7079 | -0.567 | -0.4615 | -0.2402 |
| TRINITY_DN2682_c0_g1_i4_orf1 | - | - | - | 40S ribosomal protein S5 [Manduca sexta] >ACY95347.1 ribosomal protein S5 [Manduca sexta] >KAG6447616.1 hypothetical protein O3G_MSEX005033 [Manduca sexta] >KAG6447617.1 hypothetical protein O3G_MSEX005033 [Manduca sexta] | 1.68329 | -0.7908 | -1.0676 | -0.3519 | 0.52697 |
| TRINITY_DN27_c0_g1_i1_orf1 | - | - | - | THO complex subunit 4-A [Ostrinia furnacalis] | 1.74515 | 0.34532 | -1.1063 | -0.744 | -0.2401 |
| TRINITY_DN3366_c0_g1_i6_orf1 | - | - | - | eukaryotic translation initiation factor 3 subunit K [Helicoverpa zea] | 1.90351 | -0.0997 | -0.9596 | -0.6337 | -0.2105 |
| TRINITY_DN82324_c0_g1_i4_orf1 | - | - | - | hypothetical protein evm_001824 [Chilo suppressalis] >CAG9754426.1 unnamed protein product [Diatraea saccharalis] >CAG9793111.1 unnamed protein product [Diatraea saccharalis] | 1.81148 | -0.6998 | -0.9511 | -0.475 | 0.3143 |
| TRINITY_DN21214_c0_g2_i1_orf1 | - | - | - | heat shock protein family A (Hsp70) member 1A [Homo sapiens] >KAI4017664.1 heat shock protein family A (Hsp70) member 1A [Homo sapiens] >PNI76655.1 HSPA1A isoform 2 [Pan troglodytes] | 1.95414 | -0.1978 | -0.5773 | -0.8282 | -0.3509 |
| TRINITY_DN54150_c0_g1_i1_orf1 | - | - | - | uncharacterized protein LOC114351648 [Ostrinia furnacalis] | 1.95554 | -0.3589 | -0.2553 | -0.4678 | -0.8735 |
| TRINITY_DN10458_c0_g1_i1_orf1 | - | - | - | V-type proton ATPase 21 kDa proteolipid subunit [Ostrinia furnacalis] | 1.89603 | -0.897 | -0.2813 | 0.00428 | -0.722 |
| TRINITY_DN37393_c0_g1_i1_orf1 | - | - | - | protein melted [Pectinophora gossypiella] | 1.93614 | -0.2943 | -0.9348 | -0.4966 | -0.2104 |
| TRINITY_DN1081_c0_g1_i7_orf1 | - | - | - | 3-ketoacyl-CoA thiolase, mitochondrial-like [Ostrinia furnacalis] | 1.91851 | -0.7658 | -0.4562 | 0.02735 | -0.7239 |
| TRINITY_DN15706_c0_g2_i5_orf1 | - | - | - | cdc42 homolog [Galleria mellonella] >XP_028178764.1 cdc42 homolog [Ostrinia furnacalis] >XP_028178765.1 cdc42 homolog [Ostrinia furnacalis] | 1.78845 | 0.37911 | -0.8739 | -0.4776 | -0.8161 |
| TRINITY_DN48641_c0_g1_i4_orf1 | - | - | - | RNA-binding protein 45-like [Galleria mellonella] | 0.85224 | 1.45476 | -0.9484 | -1.0886 | -0.27 |
| TRINITY_DN20984_c0_g1_i4_orf1 | - | - | - | NADPH--cytochrome P450 reductase isoform X2 [Ostrinia furnacalis] | 1.75683 | -0.6618 | -1.1799 | 0.24207 | -0.1572 |
| TRINITY_DN25896_c0_g1_i6_orf1 | - | - | - | 60S ribosomal export protein NMD3 [Ostrinia furnacalis] | 1.97272 | -0.4878 | -0.2357 | -0.7561 | -0.493 |
| TRINITY_DN4514_c0_g1_i1_orf1 | - | - | - | enoyl-CoA delta isomerase 1, mitochondrial-like isoform X1 [Ostrinia furnacalis] >XP_028158560.1 enoyl-CoA delta isomerase 1, mitochondrial-like isoform X2 [Ostrinia furnacalis] | 1.55263 | -1.2947 | 0.00521 | -0.7943 | 0.53118 |

| | | | | | | | | |
|---|---|---|---|---|---|---|---|---|
| TRINITY_DN6711_c0_g1_i1_orf1 | - | - | - | mitochondrial uncoupling protein 4 [Ostrinia furnacalis] >XP_028158360.1 mitochondrial uncoupling protein 4 [Ostrinia furnacalis] >XP_028158361.1 mitochondrial uncoupling protein 4 [Ostrinia furnacalis] >XP_028158362.1 mitochondrial uncoupling protein 4 [Ostrinia furnacalis] | 1.99855 | -0.4501 | -0.5468 | -0.5365 | -0.4652 |
| TRINITY_DN1444_c1_g1_i5_orf1 | - | - | - | spondin-1 isoform X1 [Ostrinia furnacalis] >XP_028167312.1 spondin-1 isoform X1 [Ostrinia furnacalis] >XP_028167313.1 spondin-1 isoform X1 [Ostrinia furnacalis] >XP_028167314.1 spondin-1 isoform X2 [Ostrinia furnacalis] | 1.23855 | 1.15787 | -1.1687 | -0.6678 | -0.5599 |
| TRINITY_DN35277_c0_g1_i1_orf1 | - | - | - | luciferin 4-monooxygenase-like, partial [Ostrinia furnacalis] | 1.91541 | -0.7257 | -0.3485 | -0.0149 | -0.8264 |
| TRINITY_DN2196_c0_g1_i2_orf1 | - | - | - | HIRA-interacting protein 3-like [Ostrinia furnacalis] | 1.63265 | 0.67497 | -0.5159 | -0.9575 | -0.8342 |
| TRINITY_DN42310_c0_g1_i1_orf1 | - | - | - | uncharacterized protein LOC114349955 [Ostrinia furnacalis] | 1.91249 | -0.8517 | -0.7245 | -0.3016 | -0.0347 |
| TRINITY_DN19135_c0_g1_i1_orf1 | - | - | - | ER membrane protein complex subunit 10 [Ostrinia furnacalis] | 1.79137 | 0.13694 | -1.2382 | -0.3668 | -0.3233 |
| TRINITY_DN141738_c0_g1_i1_orf1 | - | - | - | PREDICTED: fibroblast growth factor 1 [Microplitis demolitor] | 1.98928 | -0.621 | -0.6022 | -0.3605 | -0.4056 |
| TRINITY_DN23264_c0_g1_i1_orf1 | - | - | - | U5 small nuclear ribonucleoprotein 40 kDa protein [Ostrinia furnacalis] | 1.63633 | 0.03669 | -1.2538 | -0.7846 | 0.36537 |
| TRINITY_DN24281_c0_g1_i1_orf1 | - | - | - | elongin-B isoform X1 [Maniola jurtina] | 1.91925 | -0.7239 | -0.5438 | -0.7029 | 0.05137 |
| TRINITY_DN48554_c0_g1_i1_orf1 | - | - | - | 39S ribosomal protein L39, mitochondrial [Ostrinia furnacalis] | 1.98343 | -0.5442 | -0.4158 | -0.7031 | -0.3203 |
| TRINITY_DN235_c0_g3_i1_orf1 | - | - | - | actin, muscle-type A2 [Bombyx mori] >XP_013199497.1 PREDICTED: actin, muscle-type A2 [Amyelois transitella] >XP_021196684.1 actin, muscle-type A2 [Helicoverpa armigera] >XP_022837900.1 actin, muscle-type A2 [Spodoptera litura] >XP_026314060.1 actin, muscle-type A2 [Hyposmocoma kahamanoa] >XP_026738711.1 actin, muscle-type A2 [Trichoplusia ni] >XP_028179440.1 actin, muscle-type A2 [Ostrinia furnacalis] >XP_030030527.1 actin, muscle-type A2 [Manduca sexta] >XP_035439272.1 actin, muscle-type A2 [Spodoptera frugiperda] >XP_047029939.1 actin, muscle-type A2 [Helicoverpa zea] >XP_049873365.1 actin, muscle-type A2 [Pectinophora gossypiella] >P07837.1 RecName: Full=Actin, muscle-type A2; Flags: Precursor [Bombyx mori] >KAF9423784.1 hypothetical protein HW555_000842 [Spodoptera exigua] >QLI62214.1 actin [Streltzoviella insularis] >CAB3227390.1 unnamed protein product [Arctia plantaginis] >CAB3508892.1 unnamed protein product [Spodoptera littoralis] >CAB3520808.1 unnamed protein product [Chilo suppressalis] >CAG9748331.1 unnamed protein product [Diatraea saccharalis] >CAH0585396.1 unnamed protein product [Chrysodeixis includens] >CBB21118.1 Actin, muscle-type A2 [Eumeta japonica] | 1.45453 | 0.8164 | -1.2914 | -0.6774 | -0.3021 |
| TRINITY_DN64769_c0_g1_i3_orf1 | - | - | - | procollagen-lysine,2-oxoglutarate 5-dioxygenase isoform X2 [Ostrinia | 0.78737 | 1.52617 | -0.9903 | -0.3496 | -0.9736 |
| TRINITY_DN40562_c0_g2_i1_orf1 | - | - | - | dual specificity protein phosphatase 23-like isoform X2 [Ostrinia furnacalis] | 1.69783 | 0.53325 | -0.7823 | -0.4315 | -1.0173 |
| TRINITY_DN16487_c0_g1_i1_orf1 | - | - | - | p21-activated protein kinase-interacting protein 1-like [Ostrinia furnacalis] | 1.75995 | -0.6728 | 0.18336 | -0.0833 | -1.1871 |
| TRINITY_DN24693_c1_g1_i1_orf1 | - | - | - | ubiquitin-conjugating enzyme E2 G2 isoform X2 [Ostrinia furnacalis] | 1.98006 | -0.5155 | -0.4822 | -0.2692 | -0.7132 |
| TRINITY_DN413_c0_g1_i11_orf1 | - | - | - | regulator of nonsense transcripts 1 [Helicoverpa armigera] >XP_047028926.1 regulator of nonsense transcripts 1 [Helicoverpa zea] | 1.94498 | -0.0607 | -0.4975 | -0.7395 | -0.6473 |
| TRINITY_DN146119_c0_g1_i1_orf1 | - | - | - | protein SEC13 homolog [Ostrinia furnacalis] | 1.41704 | 0.78066 | -1.3616 | -0.1188 | -0.7172 |
| TRINITY_DN3814_c1_g1_i1_orf1 | - | - | - | 39S ribosomal protein L11, mitochondrial [Ostrinia furnacalis] | 1.9726 | -0.491 | -0.7934 | -0.317 | -0.3712 |
| TRINITY_DN84357_c0_g1_i1_orf1 | - | - | - | 4-coumarate--CoA ligase 1-like [Ostrinia furnacalis] | 1.39544 | -1.3008 | 0.08216 | -0.9064 | 0.72962 |
| TRINITY_DN6602_c0_g1_i4_orf1 | - | - | - | PREDICTED: E3 ubiquitin-protein ligase RNF181-like [Amyelois transitella] | 0.9943 | 0.7994 | -1.7668 | -0.3672 | 0.34035 |
| TRINITY_DN4631_c0_g1_i7_orf1 | - | - | - | uncharacterized protein LOC114366284 [Ostrinia furnacalis] | 1.96727 | -0.3787 | -0.3405 | -0.4099 | -0.8381 |
| TRINITY_DN8971_c1_g1_i4_orf1 | - | - | - | synaptosomal-associated protein 25 isoform X1 [Bombyx mori] | 1.64573 | 0.33507 | -1.4185 | -0.2135 | -0.3489 |
| TRINITY_DN49409_c0_g1_i2_orf1 | - | - | - | proliferation-associated protein 2G4 [Ostrinia furnacalis] | 1.95241 | -0.3756 | -0.9011 | -0.2797 | -0.396 |
| TRINITY_DN1132_c0_g1_i5_orf1 | - | - | - | unnamed protein product [Spodoptera exigua] | 1.908 | -0.2604 | -0.7675 | -0.8371 | -0.0429 |
| TRINITY_DN2167_c0_g1_i6_orf1 | - | - | - | retinol dehydrogenase 13-like [Ostrinia furnacalis] | 1.98084 | -0.7388 | -0.3708 | -0.3537 | -0.5176 |
| TRINITY_DN5857_c0_g1_i13_orf1 | - | - | - | uncharacterized protein LOC114353981 isoform X1 [Ostrinia furnacalis] | 1.86415 | -0.881 | -0.8349 | -0.2168 | 0.06857 |
| TRINITY_DN45633_c0_g1_i1_orf1 | - | - | - | ubiquitin thioesterase otubain-like [Ostrinia furnacalis] | 1.51959 | 0.83544 | -1.0515 | -0.5145 | -0.789 |
| TRINITY_DN5457_c0_g1_i4_orf1 | - | - | - | unnamed protein product [Chrysodeixis includens] | 1.52048 | 0.73871 | -1.1474 | -0.8784 | -0.2335 |
| TRINITY_DN110888_c0_g1_i2_orf1 | - | - | - | uncharacterized protein LOC114364502 [Ostrinia furnacalis] | 1.77636 | -0.8885 | 0.41555 | -0.5233 | -0.7801 |
| TRINITY_DN3045_c0_g1_i7_orf1 | - | - | - | hypothetical protein evm_007836 [Chilo suppressalis] | 1.40999 | 0.14412 | -1.2686 | -0.9616 | 0.67609 |

| | | | | | | | | | |
|---|---|---|---|---|---|---|---|---|---|
| TRINITY_DN141352_c0_g1_i1_orf1 | - | - | - | carboxy-terminal domain RNA polymerase II polypeptide A small phosphatase 1 isoform X1 [Ostrinia furnacalis] >XP_028156862.1 carboxy-terminal domain RNA polymerase II polypeptide A small phosphatase 1 isoform X2 [Ostrinia furnacalis] >XP_028156863.1 carboxy-terminal domain RNA polymerase II polypeptide A small phosphatase 1 isoform X3 [Ostrinia furnacalis] | 1.96058 | -0.3693 | -0.6268 | -0.1978 | -0.7666 |
| TRINITY_DN28806_c0_g1_i1_orf1 | - | - | - | ATP-dependent RNA helicase WM6 [Ostrinia furnacalis] | 1.89686 | -0.0325 | -0.9947 | -0.3059 | -0.5637 |
| TRINITY_DN21506_c0_g1_i4_orf1 | - | - | - | glutamate dehydrogenase, mitochondrial isoform X2 [Ostrinia furnacalis] | 1.95455 | -0.8987 | -0.3543 | -0.366 | -0.3356 |
| TRINITY_DN2252_c0_g1_i4_orfp1 | - | - | - | TRINITY_DN2252_c0_g1_i4_m.69997 TRINITY_DN2252_c0_g1::TRINITY_DN2252_c0_g1_i4::g.69997 ORF type:5prime_partial len:168 (-),score=65.67 TRINITY_DN2252_c0_g1_i4:244- | 1.50545 | 0.80946 | -1.2085 | -0.6076 | -0.4988 |
| TRINITY_DN2577_c0_g1_i1_orf1 | - | - | - | unnamed protein product [Diatraea saccharalis] | 1.55825 | -0.7677 | -1.3261 | 0.0674 | 0.46823 |
| TRINITY_DN43942_c0_g1_i1_orf1 | - | - | - | LOW QUALITY PROTEIN: caprin homolog [Ostrinia furnacalis] | 1.89025 | 0.03485 | -0.425 | -0.995 | -0.5051 |
| TRINITY_DN4905_c0_g1_i6_orf1 | - | - | - | uncharacterized protein LOC114351759 [Ostrinia furnacalis] | 1.94623 | -0.7309 | -0.1087 | -0.3892 | -0.7174 |
| TRINITY_DN130_c0_g1_i7_orf1 | - | - | - | RNA-binding protein fusilli isoform X1 [Bombyx mori] | 1.71697 | 0.09105 | -1.3651 | -0.4243 | -0.0187 |
| TRINITY_DN49872_c0_g2_i1_orf1 | - | - | - | NIF3-like protein 1 [Ostrinia furnacalis] >XP_028165862.1 NIF3-like protein 1 [Ostrinia furnacalis] >XP_028165864.1 NIF3-like protein 1 [Ostrinia furnacalis] | 1.50545 | 0.10656 | -1.5615 | 0.35068 | -0.4012 |
| TRINITY_DN4008_c0_g1_i7_orf1 | - | - | - | nuclear export mediator factor NEMF homolog isoform X1 [Ostrinia furnacalis] | 1.78132 | -0.7353 | -0.9524 | -0.4797 | 0.38607 |
| TRINITY_DN1965_c0_g1_i7_orf1 | - | - | - | CTP synthase isoform X1 [Ostrinia furnacalis] | 1.95972 | -0.2821 | -0.4213 | -0.8661 | -0.3902 |
| TRINITY_DN1578_c0_g3_i1_orf1 | - | - | - | S-adenosylmethionine synthase isoform X1 [Ostrinia furnacalis] | 1.95945 | -0.7377 | -0.1306 | -0.5013 | -0.5899 |
| TRINITY_DN112120_c0_g1_i1_orf1 | - | - | - | juvenile hormone esterase-like [Ostrinia furnacalis] | 1.96363 | -0.6102 | -0.1342 | -0.537 | -0.6822 |
| TRINITY_DN6985_c0_g1_i5_orf1 | - | - | - | LYR motif-containing protein 4B [Ostrinia furnacalis] | 1.60403 | 0.13255 | -1.3319 | 0.32382 | -0.7285 |
| TRINITY_DN4309_c0_g1_i1_orf1 | - | - | - | NEDD8-conjugating enzyme Ubc12, partial [Cotesia chilonis] | 1.88897 | -0.2186 | -1.0053 | -0.0567 | -0.6085 |
| TRINITY_DN880_c0_g1_i6_orf1 | - | - | - | cuticle protein 19-like [Ostrinia furnacalis] | 0.75837 | 1.52714 | -1.1631 | -0.3266 | -0.7958 |
| TRINITY_DN59335_c0_g1_i2_orf1 | - | - | - | peroxisomal acyl-coenzyme A oxidase 3 [Ostrinia furnacalis] | 1.9413 | -0.9336 | -0.309 | -0.249 | -0.4498 |
| TRINITY_DN27751_c0_g2_i1_orf1 | - | - | - | eukaryotic translation initiation factor 3 subunit I [Ostrinia furnacalis] | 1.89431 | -0.2529 | -1.0086 | -0.5714 | -0.0614 |
| TRINITY_DN11297_c0_g1_i1_orf1 | - | - | - | ribosomal protein L13 [Conogethes punctiferalis] >QEE82690.1 ribosomal protein L13 [Conogethes pinicolalis] | 1.834 | -0.9776 | -0.8196 | -0.0832 | 0.04634 |
| TRINITY_DN1427_c0_g1_i7_orf1 | - | - | - | SAFB-like transcription modulator isoform X1 [Ostrinia furnacalis] >XP_028158609.1 SAFB-like transcription modulator isoform X2 [Ostrinia | 1.68155 | 0.00111 | -1.4515 | -0.255 | 0.02381 |
| TRINITY_DN1791_c0_g1_i3_orf1 | - | - | - | succinate dehydrogenase assembly factor 2-B, mitochondrial-like [Ostrinia furnacalis] | 1.70393 | -0.8925 | -1.0865 | -0.0647 | 0.33976 |
| TRINITY_DN5919_c0_g1_i4_orf1 | - | - | - | esterase FE4-like [Ostrinia furnacalis] | 1.96011 | -0.5321 | -0.7798 | -0.1558 | -0.4925 |
| TRINITY_DN5349_c0_g1_i1_orf1 | - | - | - | glutamate-rich WD repeat-containing protein 1 [Galleria mellonella] | 1.97438 | -0.6583 | -0.6139 | -0.5011 | -0.201 |
| TRINITY_DN2103_c0_g1_i1_orf1 | - | - | - | probable aconitate hydratase, mitochondrial isoform X1 [Ostrinia furnacalis] >XP_028166656.1 probable aconitate hydratase, mitochondrial isoform X2 [Ostrinia furnacalis] | 1.99886 | -0.5162 | -0.4403 | -0.5435 | -0.4989 |
| TRINITY_DN2505_c0_g1_i1_orf1 | - | - | - | uncharacterized protein LOC114349853 [Ostrinia furnacalis] | 1.95631 | -0.8406 | -0.296 | -0.556 | -0.2638 |
| TRINITY_DN5775_c0_g1_i1_orf1 | - | - | - | proteasome assembly chaperone 2 [Ostrinia furnacalis] | 1.87118 | -0.1077 | -1.131 | -0.2533 | -0.3792 |
| TRINITY_DN4725_c0_g1_i4_orf1 | - | - | - | uncharacterized protein LOC114354375 [Ostrinia furnacalis] | 1.95127 | -0.2892 | -0.345 | -0.411 | -0.9061 |
| TRINITY_DN52893_c0_g1_i1_orf1 | - | - | - | growth arrest and DNA damage-inducible proteins-interacting protein 1 [Galleria mellonella] | 1.93128 | -0.1328 | -0.8961 | -0.5968 | -0.3056 |
| TRINITY_DN35669_c0_g1_i1_orf1 | - | - | - | unnamed protein product [Diatraea saccharalis] | 1.6974 | 0.55456 | -0.9165 | -0.8673 | -0.4682 |
| TRINITY_DN15370_c0_g1_i4_orf1 | - | - | - | DNA replication licensing factor Mcm5 [Spodoptera litura] | 1.93702 | -0.1288 | -0.4781 | -0.4219 | -0.9081 |
| TRINITY_DN6189_c0_g1_i1_orf1 | - | - | - | optic atrophy 3 protein homolog isoform X2 [Ostrinia furnacalis] | 1.50916 | 0.84481 | -0.6297 | -0.6115 | -1.1128 |
| TRINITY_DN24318_c0_g1_i1_orf1 | - | - | - | 60S ribosomal protein L29 [Ostrinia furnacalis] | 1.63854 | -0.7842 | -1.2129 | -0.1076 | 0.46623 |
| TRINITY_DN7464_c0_g1_i14_orf1 | - | - | - | 60S ribosomal protein L9 [Nymphalis io] | 1.76688 | -1.0556 | -0.7998 | -0.201 | 0.2895 |
| TRINITY_DN15896_c0_g1_i4_orf1 | - | - | - | phosphatidylinositol transfer protein alpha isoform [Ostrinia furnacalis] | 1.96084 | -0.7216 | -0.638 | -0.4533 | -0.148 |

| | | | | | | | | | |
|---|---|---|---|---|---|---|---|---|---|
| TRINITY_DN338_c2_g1_i2_orf1 | - | - | - | alpha-tocopherol transfer protein-like isoform X1 [Ostrinia furnacalis] >XP_028160444.1 alpha-tocopherol transfer protein-like isoform X1 [Ostrinia furnacalis] >XP_028160446.1 alpha-tocopherol transfer protein-like isoform X1 [Ostrinia furnacalis] >XP_028160447.1 alpha-tocopherol transfer protein-like isoform X1 [Ostrinia furnacalis] >XP_028160448.1 alpha-tocopherol transfer protein-like isoform X1 [Ostrinia furnacalis] >XP_028160449.1 alpha-tocopherol transfer protein-like isoform X1 [Ostrinia furnacalis] | 1.93925 | -0.0695 | -0.5003 | -0.8372 | -0.5322 |
| TRINITY_DN5704_c0_g1_i6_orf1 | - | - | - | 2-oxoglutarate dehydrogenase, mitochondrial isoform X2 [Ostrinia furnacalis] | 1.97594 | -0.7764 | -0.3286 | -0.4883 | -0.3826 |
| TRINITY_DN19493_c0_g1_i5_orf1 | - | - | - | zinc finger MYM-type protein 3 isoform X1 [Ostrinia furnacalis] >XP_028159738.1 zinc finger MYM-type protein 3 isoform X2 [Ostrinia | 1.29326 | 1.06615 | -1.2551 | -0.498 | -0.6063 |
| TRINITY_DN3673_c0_g1_i10_orf1 | - | - | - | hypothetical protein evm_008955 [Chilo suppressalis] >CAB3526829.1 unnamed protein product [Chilo suppressalis] >CAH0404157.1 unnamed protein product [Chilo suppressalis] | 1.75951 | 0.36921 | -0.3564 | -1.0736 | -0.6987 |
| TRINITY_DN1725_c0_g1_i7_orf1 | - | - | - | T-complex protein 1 subunit gamma isoform X1 [Ostrinia furnacalis] >XP_028159782.1 T-complex protein 1 subunit gamma isoform X2 [Ostrinia furnacalis] | 1.81854 | 0.18875 | -1.1256 | -0.4126 | -0.469 |
| TRINITY_DN83327_c0_g1_i1_orf1 | - | - | - | trypsin-like serine proteinase T22 [Ostrinia nubilalis] | 1.95835 | -0.7131 | -0.1475 | -0.6756 | -0.4222 |
| TRINITY_DN136028_c0_g2_i1_orf1 | - | - | - | cytochrome c oxidase subunit 5A, mitochondrial [Ostrinia furnacalis] | 1.85666 | 0.17946 | -0.7358 | -0.3915 | -0.9088 |
| TRINITY_DN3393_c0_g2_i1_orf1 | - | - | - | 40S ribosomal protein S8 [Ostrinia furnacalis] | 1.8958 | -0.6068 | -0.8967 | -0.4762 | 0.0838 |
| TRINITY_DN4779_c0_g1_i5_orf1 | - | - | - | T-complex protein 1 subunit epsilon isoform X1 [Ostrinia furnacalis] >XP_028156782.1 T-complex protein 1 subunit epsilon isoform X2 [Ostrinia furnacalis] | 1.89353 | 0.01937 | -0.9634 | -0.6076 | -0.342 |
| TRINITY_DN17299_c0_g1_i4_orf1 | - | - | - | RNA cytidine acetyltransferase isoform X1 [Ostrinia furnacalis] >XP_028171321.1 RNA cytidine acetyltransferase isoform X2 [Ostrinia furnacalis] >XP_028171329.1 RNA cytidine acetyltransferase isoform X3 [Ostrinia furnacalis] | 1.96382 | -0.813 | -0.4997 | -0.2235 | -0.4276 |
| TRINITY_DN33926_c0_g1_i1_orf1 | - | - | - | ribosomal protein S9 [Ailuropoda melanoleuca] >AEA39538.1 ribosomal protein S9 [Ailuropoda melanoleuca] | 1.8659 | -1.1349 | -0.3907 | -0.27 | -0.0703 |
| TRINITY_DN37218_c0_g1_i12_orf1 | - | - | - | protein white [Ostrinia furnacalis] | 1.93389 | -0.0798 | -0.8131 | -0.3612 | -0.6797 |
| TRINITY_DN9410_c0_g1_i4_orf1 | - | - | - | protein RRP5 homolog [Ostrinia furnacalis] | 1.8755 | -1.0968 | -0.2863 | -0.4417 | -0.0508 |
| TRINITY_DN8367_c0_g1_i1_orf1 | - | - | - | uncharacterized protein LOC114357075 [Ostrinia furnacalis] | 1.91652 | 0.0011 | -0.5488 | -0.4735 | -0.8953 |
| TRINITY_DN98538_c0_g1_i1_orf1 | - | - | - | ATP synthase subunit d, mitochondrial [Ostrinia furnacalis] | 1.98411 | -0.7079 | -0.4182 | -0.527 | -0.331 |
| TRINITY_DN13496_c0_g1_i7_orf1 | - | - | - | nucleolar protein 58 [Ostrinia furnacalis] | 1.89473 | -0.7971 | -0.7076 | -0.5096 | 0.11954 |
| TRINITY_DN36893_c0_g1_i1_orf1 | - | - | - | 40S ribosomal protein S15 [Bicyclus anynana] >XP_026325996.1 40S ribosomal protein S15 [Hyposmocoma kahamanoa] >XP_028175376.1 40S ribosomal protein S15 [Ostrinia furnacalis] >XP_030034906.1 40S ribosomal protein S15 [Manduca sexta] >XP_039758445.1 40S ribosomal protein S15 [Pararge aegeria] >XP_045775675.1 40S ribosomal protein S15 [Maniola jurtina] >CAH2267288.1 jg14755 [Pararge aegeria aegeria] >ACY95351.1 ribosomal protein S15 [Manduca sexta] >KAG6461386.1 hypothetical protein O3G_MSEX012590 [Manduca sexta] >KAG6461387.1 hypothetical protein O3G_MSEX012590 | 1.59632 | -0.7359 | -0.9892 | -0.6151 | 0.74388 |
| TRINITY_DN2691_c0_g1_i1_orf1 | - | - | - | WD repeat-containing protein 18 [Ostrinia furnacalis] | 1.98204 | -0.7441 | -0.4318 | -0.3462 | -0.4599 |
| TRINITY_DN8511_c0_g1_i1_orf1 | - | - | - | NADH dehydrogenase [ubiquinone] 1 beta subcomplex subunit 10 [Ostrinia furnacalis] | 1.97186 | -0.7044 | -0.5392 | -0.5367 | -0.1915 |
| TRINITY_DN31327_c0_g2_i1_orf1 | - | - | - | multidrug resistance protein 1A isoform X1 [Ostrinia furnacalis] | 1.90275 | -0.3446 | -1.062 | -0.1779 | -0.3183 |
| TRINITY_DN5112_c0_g1_i1_orf1 | - | - | - | unnamed protein product, partial [Iphiclides podalirius] | 1.74195 | -0.1271 | -1.3753 | 0.00116 | -0.2407 |
| TRINITY_DN42364_c0_g1_i4_orf1 | - | - | - | brain tumor protein [Ostrinia furnacalis] >XP_028157996.1 brain tumor protein [Ostrinia furnacalis] | 1.97966 | -0.357 | -0.7542 | -0.3728 | -0.4956 |
| TRINITY_DN10379_c0_g1_i3_orf1 | - | - | - | succinate dehydrogenase [ubiquinone] cytochrome b small subunit, mitochondrial [Ostrinia furnacalis] | 1.98882 | -0.5017 | -0.3063 | -0.5597 | -0.6212 |
| TRINITY_DN9770_c0_g1_i1_orf1 | - | - | - | flavin reductase (NADPH) [Ostrinia furnacalis] >XP_028160803.1 flavin reductase (NADPH) [Ostrinia furnacalis] | 1.91211 | -0.5429 | -0.9162 | 0.00486 | -0.4579 |

| | | | | | | | | |
|---|---|---|---|---|---|---|---|---|
| TRINITY_DN9101_c0_g2_i1_orf1 | - | - | - | 60S ribosomal protein L7a [Ostrinia furnacalis] | 1.88382 | -0.6962 | -0.8048 | -0.5418 | 0.15898 |
| TRINITY_DN81926_c0_g1_i1_orf1 | - | - | - | membrane-bound alkaline phosphatase-like isoform X3 [Ostrinia furnacalis] | 1.87206 | -0.8276 | -0.7256 | 0.18199 | -0.5008 |
| TRINITY_DN9741_c0_g1_i3_orf1 | - | - | - | metaxin-2 isoform X4 [Manduca sexta] >KAG6447312.1 hypothetical protein O3G_MSEX004872 [Manduca sexta] | 1.83667 | 0.17975 | -0.9992 | -0.3104 | -0.7069 |
| TRINITY_DN5653_c0_g1_i4_orf1 | - | - | - | hrp65 protein-like [Ostrinia furnacalis] | 1.59492 | 0.69613 | -0.8924 | -1.0133 | -0.3853 |
| TRINITY_DN17376_c0_g1_i2_orf1 | - | - | - | E3 UFM1-protein ligase 1 homolog [Ostrinia furnacalis] | 1.8361 | -0.4278 | -1.1799 | -0.2312 | 0.0029 |
| TRINITY_DN9498_c0_g1_i3_orf1 | - | - | - | eukaryotic translation initiation factor 4 gamma 3-like isoform X2 [Ostrinia furnacalis] | 1.91796 | -0.0403 | -0.9345 | -0.5011 | -0.442 |
| TRINITY_DN4532_c0_g1_i1_orf1 | - | - | - | 3-hydroxyacyl-CoA dehydrogenase type-2 [Ostrinia furnacalis] | 1.95004 | -0.8469 | -0.5406 | -0.1595 | -0.403 |
| TRINITY_DN5031_c0_g1_i1_orf1 | - | - | - | PREDICTED: 40S ribosomal protein S12 [Trachymyrmex septentrionalis] | 1.77055 | -0.8976 | -0.9577 | -0.2209 | 0.30567 |
| TRINITY_DN124950_c0_g2_i1_orf1 | - | - | - | TATA box-binding protein-like protein 1 [Ostrinia furnacalis] >XP_028155830.1 TATA box-binding protein-like protein 1 [Ostrinia furnacalis] | 1.55777 | 0.18518 | -0.9368 | -1.2206 | 0.4144 |
| TRINITY_DN9575_c0_g1_i1_orf1 | - | - | - | uncharacterized protein LOC114351119 [Ostrinia furnacalis] | 1.40814 | 0.67233 | -1.5512 | -0.3619 | -0.1674 |
| TRINITY_DN2232_c1_g1_i3_orf1 | - | - | - | protein FAM98A-like [Ostrinia furnacalis] | 1.26225 | 0.80632 | -1.2437 | -1.0719 | 0.24696 |
| TRINITY_DN103457_c0_g1_i1_orf1 | - | - | - | 28S ribosomal protein S22, mitochondrial [Ostrinia furnacalis] | 1.94612 | -0.3134 | -0.2664 | -0.4486 | -0.9177 |
| TRINITY_DN1266_c2_g1_i1_orf1 | - | - | - | serine/threonine-protein kinase RIO3 [Ostrinia furnacalis] | 1.62676 | 0.36561 | -0.9473 | 0.10057 | -1.1456 |
| TRINITY_DN107840_c1_g1_i1_orf1 | - | - | - | HEAT repeat-containing protein 3 [Ostrinia furnacalis] | 1.95603 | -0.0965 | -0.641 | -0.534 | -0.6845 |
| TRINITY_DN34509_c0_g1_i1_orf1 | - | - | - | transcription initiation factor IIA subunit 2 [Aphidius gifuensis] >KAF7997556.1 hypothetical protein HCN44_006127 [Aphidius gifuensis] | 1.77518 | 0.42004 | -0.7273 | -0.9155 | -0.5524 |
| TRINITY_DN1375_c0_g1_i5_orf1 | - | - | - | glutaminase [Chilo suppressalis] >CAB3528726.1 unnamed protein product [Chilo suppressalis] >CAH0405319.1 unnamed protein product [Chilo | 1.96849 | -0.654 | -0.3821 | -0.2247 | -0.7077 |
| TRINITY_DN106038_c0_g1_i1_orf1 | - | - | - | ankyrin-3-like isoform X1 [Galleria mellonella] | 1.79543 | 0.39636 | -0.8376 | -0.6979 | -0.6563 |
| TRINITY_DN32681_c0_g1_i3_orf1 | - | - | - | long-chain-fatty-acid--CoA ligase ACSBG2 isoform X2 [Ostrinia furnacalis] | 1.87188 | -1.0552 | -0.4254 | 0.05443 | -0.4457 |
| TRINITY_DN2803_c2_g1_i8_orf1 | - | - | - | trans-1,2-dihydrobenzene-1,2-diol dehydrogenase-like isoform X1 [Ostrinia furnacalis] | 1.9322 | -0.7454 | -0.6666 | -0.0039 | -0.5162 |
| TRINITY_DN7574_c0_g1_i10_orf1 | - | - | - | proteasome activator complex subunit 3 isoform X2 [Ostrinia furnacalis] | 1.74949 | 0.0671 | -1.1891 | -0.7162 | 0.08871 |
| TRINITY_DN29291_c0_g1_i1_orf1 | - | - | - | carboxylesterase [Ostrinia furnacalis] | 1.93913 | -0.3531 | -0.104 | -0.6859 | -0.7961 |
| TRINITY_DN1191_c0_g1_i4_orf1 | - | - | - | interferon-inducible double-stranded RNA-dependent protein kinase activator A homolog isoform X4 [Helicoverpa zea] >XP_047029704.1 interferon-inducible double-stranded RNA-dependent protein kinase activator A homolog isoform X4 [Helicoverpa zea] | 1.98947 | -0.4298 | -0.6443 | -0.3499 | -0.5655 |
| TRINITY_DN15388_c0_g1_i5_orf1 | - | - | - | RNA-binding protein 28-like isoform X1 [Ostrinia furnacalis] | 1.96473 | -0.5248 | -0.7367 | -0.1587 | -0.5445 |
| TRINITY_DN15667_c0_g1_i2_orf1 | - | - | - | coiled-coil domain-containing protein 25 [Ostrinia furnacalis] | 1.94392 | -0.0672 | -0.5469 | -0.7941 | -0.5357 |
| TRINITY_DN3539_c0_g1_i7_orf1 | - | - | - | transcription elongation regulator 1-like [Ostrinia furnacalis] | 1.70579 | 0.41319 | -1.0791 | -0.8473 | -0.1925 |
| TRINITY_DN2181_c1_g1_i8_orf1 | - | - | - | vacuolar protein sorting-associated protein 37B [Ostrinia furnacalis] | 1.46044 | 0.85635 | -1.1179 | -0.3119 | -0.887 |
| TRINITY_DN755_c0_g1_i3_orf1 | - | - | - | uncharacterized protein LOC114358844 [Ostrinia furnacalis] | 1.97946 | -0.2699 | -0.5274 | -0.4647 | -0.7175 |
| TRINITY_DN79868_c0_g1_i1_orf1 | - | - | - | lethal(2)neighbour of Tid protein [Ostrinia furnacalis] | 1.44478 | 0.56433 | -1.4631 | -0.6632 | 0.11714 |
| TRINITY_DN142588_c0_g1_i1_orf1 | - | - | - | peptidyl-prolyl cis-trans isomerase [Cotesia flavipes] | 1.38026 | 1.0107 | -1.0337 | -0.8832 | -0.474 |
| TRINITY_DN8944_c0_g1_i1_orf1 | - | - | - | actin, clone 403 [Trichonephila clavata] | 1.57473 | 0.75806 | -0.9999 | -0.8359 | -0.4969 |
| TRINITY_DN1956_c1_g1_i5_orf1 | - | - | - | uncharacterized protein LOC114356377 [Ostrinia furnacalis] | 1.94262 | -0.4482 | -0.9071 | -0.4163 | -0.171 |
| TRINITY_DN122786_c0_g2_i1_orf1 | - | - | - | glucose dehydrogenase [FAD, quinone]-like [Ostrinia furnacalis] | 1.96194 | -0.4846 | -0.6015 | -0.73 | -0.1459 |
| TRINITY_DN7251_c0_g1_i3_orf1 | - | - | - | hypothetical protein evm_008498 [Chilo suppressalis] >CAB3527693.1 unnamed protein product [Chilo suppressalis] >CAH0401999.1 unnamed protein product [Chilo suppressalis] | 1.5615 | 0.78477 | -0.9628 | -0.8677 | -0.5158 |
| TRINITY_DN37532_c0_g1_i1_orf1 | - | - | - | transcription elongation factor S-II [Ostrinia furnacalis] | 1.3182 | 0.9768 | -1.3474 | -0.6216 | -0.326 |
| TRINITY_DN45598_c0_g1_i2_orf1 | - | - | - | heat shock protein 60A-like [Ostrinia furnacalis] | 1.97478 | -0.723 | -0.518 | -0.2255 | -0.5083 |
| TRINITY_DN14372_c0_g2_i1_orf1 | - | - | - | 12 kDa FK506-binding protein-like [Ostrinia furnacalis] | 1.89341 | -0.0101 | -0.9084 | -0.7268 | -0.2482 |
| TRINITY_DN1348_c0_g1_i1_orf1 | - | - | - | hypothetical protein evm_002665 [Chilo suppressalis] | 1.98979 | -0.4271 | -0.676 | -0.5075 | -0.3792 |
| TRINITY_DN8824_c0_g2_i1_orf1 | - | - | - | 60S ribosomal protein L34-like [Ostrinia furnacalis] | 1.7633 | -0.7 | -1.1673 | -0.0757 | 0.17978 |

| | | | | | | | | | |
|---|---|---|---|---|---|---|---|---|---|
| TRINITY_DN6556_c0_g1_i7_orf1 | - | - | - | NFX1-type zinc finger-containing protein 1-like isoform X1 [Ostrinia furnacalis] >XP_028173496.1 NFX1-type zinc finger-containing protein 1-like isoform X1 [Ostrinia furnacalis] >XP_028173497.1 NFX1-type zinc finger-containing protein 1-like isoform X1 [Ostrinia furnacalis] | 1.80197 | -0.3476 | -0.9979 | 0.28757 | -0.7441 |
| TRINITY_DN4908_c1_g1_i5_orf1 | - | - | - | DNA topoisomerase 2 isoform X1 [Ostrinia furnacalis] | 1.15561 | 1.28543 | -0.9453 | -0.7522 | -0.7435 |
| TRINITY_DN8561_c0_g4_i1_orf1 | - | - | - | dynactin subunit 4 [Ostrinia furnacalis] | 1.69124 | 0.37702 | -1.1998 | -0.1335 | -0.735 |
| TRINITY_DN4820_c0_g1_i1_orf1 | - | - | - | tudor and KH domain-containing protein homolog [Galleria mellonella] | 1.9005 | -0.6142 | -0.9174 | -0.4095 | 0.04054 |
| TRINITY_DN4589_c0_g1_i1_orf1 | - | - | - | protein penguin [Ostrinia furnacalis] | 1.97446 | -0.5993 | -0.7276 | -0.3646 | -0.2831 |
| TRINITY_DN14953_c0_g1_i5_orf1 | - | - | - | EEF1A lysine methyltransferase 2 [Ostrinia furnacalis] | 1.96737 | -0.6613 | -0.4466 | -0.6781 | -0.1814 |
| TRINITY_DN21619_c0_g1_i1_orf1 | - | - | - | 28S ribosomal protein S15, mitochondrial [Ostrinia furnacalis] | 1.85975 | -0.4214 | -0.8331 | 0.19059 | -0.7958 |
| TRINITY_DN3370_c0_g1_i5_orf1 | - | - | - | unnamed protein product, partial [Brenthis ino] | 1.5837 | -0.35 | -0.9943 | -0.9419 | 0.70253 |
| TRINITY_DN21792_c0_g1_i1_orf1 | - | - | - | probable 28S ribosomal protein S25, mitochondrial [Ostrinia furnacalis] | 1.96375 | -0.282 | -0.3443 | -0.5086 | -0.8288 |
| TRINITY_DN108819_c0_g1_i1_orf1 | - | - | - | NADH dehydrogenase [ubiquinone] 1 beta subcomplex subunit 8, mitochondrial [Ostrinia furnacalis] | 1.94125 | -0.1902 | -0.9099 | -0.5033 | -0.3378 |
| TRINITY_DN14168_c0_g1_i1_orf1 | - | - | - | transmembrane 7 superfamily member 3-like [Ostrinia furnacalis] | 1.9769 | -0.5543 | -0.7259 | -0.435 | -0.2617 |
| TRINITY_DN143852_c0_g1_i1_orf1 | - | - | - | 60S ribosomal protein L10 [Cotesia glomerata] | 1.49273 | -1.0131 | -1.0655 | -0.1754 | 0.76123 |
| TRINITY_DN2084_c0_g1_i1_orf1 | - | - | - | 40S ribosomal protein S24 [Helicoverpa armigera] >XP_022830907.1 40S ribosomal protein S24 [Spodoptera litura] >XP_026729116.1 40S ribosomal protein S24 [Trichoplusia ni] >XP_035432908.1 40S ribosomal protein S24 [Spodoptera frugiperda] >XP_047024473.1 40S ribosomal protein S24 [Helicoverpa zea] >Q962Q6.1 RecName: Full=40S ribosomal protein S24 [Spodoptera frugiperda] >KAF9418537.1 hypothetical protein HW555_004686 [Spodoptera exigua] >CAB3515448.1 unnamed protein product [Spodoptera littoralis] >CAH0579501.1 unnamed protein product [Chrysodeixis includens] >AAK92192.1 ribosomal protein S24 [Spodoptera frugiperda] >KAF9808794.1 hypothetical protein SFRURICE_013056 [Spodoptera frugiperda] | 1.95615 | -0.8369 | -0.4692 | -0.1808 | -0.4692 |
| TRINITY_DN14507_c0_g1_i5_orf1 | - | - | - | PTB domain-containing adapter protein ced-6 [Ostrinia furnacalis] | 0.92095 | 1.24389 | -0.995 | -1.267 | 0.09709 |
| TRINITY_DN18159_c0_g1_i6_orf1 | - | - | - | zinc carboxypeptidase-like [Ostrinia furnacalis] | 1.5613 | -0.8859 | -1.1841 | -0.0963 | 0.60505 |
| TRINITY_DN2283_c0_g2_i1_orf1 | - | - | - | H/ACA ribonucleoprotein complex subunit 4 [Ostrinia furnacalis] | 1.91626 | -0.9468 | -0.5631 | -0.0771 | -0.3292 |
| TRINITY_DN2975_c0_g1_i4_orf1 | - | - | - | ubiquitin-like protein 4A [Ostrinia furnacalis] | 1.89304 | -0.0803 | -1.0466 | -0.2805 | -0.4856 |
| TRINITY_DN86090_c0_g1_i1_orf1 | - | - | - | ATP synthase subunit b, mitochondrial [Ostrinia furnacalis] | 1.9634 | -0.8246 | -0.4622 | -0.4444 | -0.2322 |
| TRINITY_DN18933_c0_g1_i3_orf1 | - | - | - | PREDICTED: protein BUD31 homolog [Papilio xuthus] >XP_014361644.1 protein BUD31 homolog [Papilio machaon] >XP_026750578.1 protein BUD31 homolog [Galleria mellonella] >XP_047995610.1 protein BUD31 homolog [Leguminivora glycinivorella] >XP_049869593.1 protein BUD31 homolog [Pectinophora gossypiella] >KAI5652084.1 g10 protein domain-containing protein [Phthorimaea operculella] >CAB3251981.1 unnamed protein product [Arctia plantaginis] >CAB3520382.1 unnamed protein product [Chilo suppressalis] >CAG9747228.1 unnamed protein product [Diatraea saccharalis] >CAH2037008.1 unnamed protein product, partial [Iphiclides podalirius] | 1.17209 | 1.05956 | -1.4787 | -0.5058 | -0.2471 |
| TRINITY_DN26251_c0_g1_i1_orf1 | - | - | - | serine/arginine-rich splicing factor 1A [Neodiprion lecontei] >XP_046417766.1 serine/arginine-rich splicing factor 1A [Neodiprion fabricii] >XP_046473571.1 serine/arginine-rich splicing factor 1A [Neodiprion pinetum] >XP_046610590.1 serine/arginine-rich splicing factor 1A [Neodiprion virginianus] >XP_046738887.1 serine/arginine-rich splicing factor 1A [Diprion similis] | 1.58375 | 0.4142 | -1.3438 | -0.7146 | 0.06047 |
| TRINITY_DN154_c0_g1_i4_orf1 | - | - | - | ER membrane protein complex subunit 4 [Ostrinia furnacalis] | 1.77325 | 0.37014 | -1.018 | -0.7192 | -0.4062 |
| TRINITY_DN12527_c0_g1_i4_orf1 | - | - | - | DNA-directed RNA polymerase III subunit RPC4 isoform X1 [Ostrinia furnacalis] | 1.69871 | 0.45903 | -1.1214 | -0.2845 | -0.7518 |
| TRINITY_DN9059_c0_g1_i1_orf1 | - | - | - | sulfotransferase family cytosolic 1B member 1-like [Ostrinia furnacalis] | 1.93917 | -0.0302 | -0.5122 | -0.7199 | -0.6769 |
| TRINITY_DN25997_c1_g2_i4_orf1 | - | - | - | ribokinase-like [Ostrinia furnacalis] | 1.41008 | 0.34563 | -0.7895 | 0.46612 | -1.4324 |
| TRINITY_DN6235_c0_g1_i5_orf1 | - | - | - | rRNA 2'-O-methyltransferase fibrillarin [Vanessa cardui] | 1.92191 | -0.5642 | -0.7628 | -0.6359 | 0.04101 |
| TRINITY_DN13530_c0_g1_i1_orf1 | - | - | - | unnamed protein product [Euphydryas editha] | 1.96611 | -0.4887 | -0.8272 | -0.3251 | -0.3251 |

| | | | | | | | | | |
|---|---|---|---|---|---|---|---|---|---|
| TRINITY_DN11799_c0_g1_i4_orf1 | - | - | - | V-type proton ATPase 116 kDa subunit a1 isoform X1 [Manduca sexta] | 1.93679 | -0.0639 | -0.4002 | -0.7437 | -0.7291 |
| TRINITY_DN29521_c0_g1_i1_orf1 | - | - | - | density-regulated protein homolog [Ostrinia furnacalis] | 1.52673 | -0.7792 | -1.2894 | -0.0843 | 0.62626 |
| TRINITY_DN50151_c0_g1_i1_orf1 | - | - | - | heat shock 70 kDa protein 14 [Ostrinia furnacalis] | 1.95408 | -0.2179 | -0.8621 | -0.5038 | -0.3703 |
| TRINITY_DN646_c0_g1_i5_orf1 | - | - | - | unnamed protein product [Diatraea saccharalis] | 1.89821 | 0.10418 | -0.8359 | -0.5247 | -0.6418 |
| TRINITY_DN25542_c0_g1_i1_orf1 | - | - | - | tRNA-dihydrouridine(47) synthase [NAD(P)(+)]-like [Ostrinia furnacalis] | 1.94109 | -0.4539 | -0.9341 | -0.2943 | -0.2588 |
| TRINITY_DN7512_c0_g1_i1_orf1 | - | - | - | hypothetical protein evm_010529 [Chilo suppressalis] >CAB3530682.1 unnamed protein product [Chilo suppressalis] >CAH0407273.1 unnamed protein product [Chilo suppressalis] | 1.92632 | -0.8885 | -0.2243 | -0.1632 | -0.6503 |
| TRINITY_DN2769_c0_g1_i1_orf1 | - | - | - | pseudouridylate synthase 7 homolog [Ostrinia furnacalis] | 1.80583 | 0.22357 | -1.0366 | -0.743 | -0.2498 |
| TRINITY_DN34432_c0_g1_i1_orf1 | - | - | - | 39S ribosomal protein L44, mitochondrial [Ostrinia furnacalis] | 1.78389 | 0.42504 | -0.7643 | -0.6536 | -0.7911 |
| TRINITY_DN3860_c0_g1_i5_orf1 | - | - | - | nucleoplasmin-like protein isoform X1 [Hyposmocoma kahamanoa] | 1.4081 | 0.41215 | -1.1636 | -1.1277 | 0.47099 |
| TRINITY_DN9164_c0_g1_i3_orf1 | - | - | - | unnamed protein product [Parnassius apollo] | 1.78025 | 0.18306 | -1.133 | -0.7057 | -0.1246 |
| TRINITY_DN5767_c0_g1_i4_orf1 | - | - | - | cell division cycle 5-like protein [Helicoverpa armigera] | 1.94936 | -0.0557 | -0.6591 | -0.6025 | -0.6321 |
| TRINITY_DN492_c0_g1_i4_orf1 | - | - | - | hypothetical protein evm_000589 [Chilo suppressalis] >CAB3530014.1 unnamed protein product [Chilo suppressalis] >CAH0406606.1 unnamed protein product [Chilo suppressalis] | 1.97483 | -0.5173 | -0.5936 | -0.6632 | -0.2008 |
| TRINITY_DN18242_c0_g1_i3_orf1 | - | - | - | CCHC-type zinc finger protein CG3800 [Papilio xuthus] | 1.70898 | 0.17094 | -1.0886 | -0.921 | 0.1297 |
| TRINITY_DN14107_c0_g1_i4_orf1 | - | - | - | bifunctional methylenetetrahydrofolate dehydrogenase/cyclohydrolase, mitochondrial isoform X1 [Ostrinia furnacalis] | 1.93663 | -0.1423 | -0.3158 | -0.6043 | -0.8742 |
| TRINITY_DN3474_c1_g2_i7_orf1 | - | - | - | LOW QUALITY PROTEIN: endoplasmic reticulum metallopeptidase 1-like [Ostrinia furnacalis] | 1.85238 | 0.06412 | -0.9929 | -0.186 | -0.7376 |
| TRINITY_DN30638_c0_g1_i1_orf1 | - | - | - | alanine--tRNA ligase, cytoplasmic [Ostrinia furnacalis] | 1.93608 | -0.3634 | -0.9131 | -0.5144 | -0.1451 |
| TRINITY_DN42646_c0_g2_i1_orf1 | - | - | - | 40S ribosomal protein S3 [Helicoverpa armigera] >XP_026740562.1 40S ribosomal protein S3 [Trichoplusia ni] >XP_026751545.1 40S ribosomal protein S3 [Galleria mellonella] >XP_047027704.1 40S ribosomal protein S3 [Helicoverpa zea] >CAH0591481.1 unnamed protein product [Chrysodeixis includens] >AIR07416.1 ribosomal protein S3 [Helicoverpa armigera] >AND95944.1 ribosomal protein S3 [Helicoverpa armigera] >AXY94820.1 ribosomal ribosomal protein S3 [Galleria mellonella] >PZC80336.1 hypothetical protein B5X24_HaOG214853 [Helicoverpa armigera] | 1.80977 | -0.9226 | -0.9021 | -0.1655 | 0.18031 |
| TRINITY_DN66596_c0_g1_i1_orf1 | - | - | - | CCR4-NOT transcription complex subunit 10 [Ostrinia furnacalis] | 1.94141 | -0.3853 | -0.9397 | -0.2396 | -0.3768 |
| TRINITY_DN4747_c0_g1_i4_orf1 | - | - | - | transcription factor A, mitochondrial [Ostrinia furnacalis] | 1.87947 | 0.07444 | -1.0029 | -0.4306 | -0.5204 |
| TRINITY_DN15737_c0_g1_i7_orf1 | - | - | - | UPF0160 protein C27H6.8 [Ostrinia furnacalis] | 1.65987 | -0.7628 | -1.2228 | -0.0764 | 0.40217 |
| TRINITY_DN15882_c0_g1_i1_orf1 | - | - | - | succinate--CoA ligase [ADP-forming] subunit beta, mitochondrial [Ostrinia furnacalis] | 1.98583 | -0.6955 | -0.5388 | -0.3769 | -0.3746 |
| TRINITY_DN24043_c0_g1_i1_orf1 | - | - | - | uncharacterized protein LOC110377964 [Helicoverpa armigera] >XP_047026962.1 cytochrome c1-2, heme protein, mitochondrial [Helicoverpa zea] >PZC76159.1 hypothetical protein B5X24_HaOG204935 [Helicoverpa | 1.97196 | -0.6504 | -0.3722 | -0.6974 | -0.252 |
| TRINITY_DN10520_c0_g1_i2_orf1 | - | - | - | probable 39S ribosomal protein L45, mitochondrial [Ostrinia furnacalis] | 1.9782 | -0.2349 | -0.5704 | -0.6823 | -0.4906 |
| TRINITY_DN144956_c0_g1_i1_orf1 | - | - | - | 40S ribosomal protein S18 [Cotesia glomerata] >CAD6216330.1 GSCOCG00004483001-RA-CDS [Cotesia congregata] >CAG5095266.1 Similar to RpS18: 40S ribosomal protein S18 (Spodoptera frugiperda) [Cotesia | 1.85941 | -0.7509 | -0.9701 | -0.1875 | 0.04915 |
| TRINITY_DN10644_c0_g1_i2_orf1 | - | - | - | carboxylesterase [Cnaphalocrocis medinalis] | 1.81656 | 0.32963 | -0.5631 | -0.6883 | -0.8947 |
| TRINITY_DN2070_c1_g1_i1_orf1 | - | - | - | mitochondrial carrier homolog 2-like [Helicoverpa zea] >PZC82360.1 hypothetical protein B5X24_HaOG210663 [Helicoverpa armigera] | 1.98856 | -0.6063 | -0.5893 | -0.4869 | -0.306 |
| TRINITY_DN7868_c0_g1_i8_orf1 | - | - | - | uncharacterized protein LOC114353432 isoform X4 [Ostrinia furnacalis] | 1.69943 | -0.1044 | -1.4449 | -0.107 | -0.0431 |
| TRINITY_DN4237_c1_g1_i5_orf1 | - | - | - | eukaryotic translation initiation factor 3 subunit A-like isoform X1 [Ostrinia furnacalis] >XP_028173593.1 eukaryotic translation initiation factor 3 subunit A-like isoform X2 [Ostrinia furnacalis] >XP_028173594.1 eukaryotic translation initiation factor 3 subunit A-like isoform X3 [Ostrinia furnacalis] >XP_028173595.1 eukaryotic translation initiation factor 3 subunit A-like | 1.88345 | -0.2463 | -1.0306 | -0.5733 | -0.0332 |

| | | | | | | | | | |
|---|---|---|---|---|---|---|---|---|---|
| TRINITY_DN33801_c0_g1_i1_orf1 | - | - | - | unnamed protein product [Diatraea saccharalis] | 1.79402 | 0.24141 | -1.1408 | -0.5513 | -0.3434 |
| TRINITY_DN35301_c0_g1_i3_orf1 | - | - | - | ATP synthase subunit gamma, mitochondrial-like [Ostrinia furnacalis] >XP_028164649.1 ATP synthase subunit gamma, mitochondrial-like [Ostrinia furnacalis] | 1.94941 | -0.9079 | -0.4401 | -0.3224 | -0.279 |
| TRINITY_DN24751_c0_g1_i1_orf1 | - | - | - | NADH dehydrogenase [ubiquinone] 1 beta subcomplex subunit 7-like [Ostrinia furnacalis] | 1.96916 | -0.4489 | -0.8151 | -0.2899 | -0.4152 |
| TRINITY_DN29038_c0_g2_i1_orf1 | - | - | - | PREDICTED: ATP synthase lipid-binding protein, mitochondrial [Fopius arisanus] >XP_011314178.1 PREDICTED: ATP synthase lipid-binding protein, mitochondrial [Fopius arisanus] >XP_011314186.1 PREDICTED: ATP synthase lipid-binding protein, mitochondrial [Fopius arisanus] | 1.95804 | -0.452 | -0.8704 | -0.3508 | -0.2848 |
| TRINITY_DN23586_c0_g1_i3_orf1 | - | - | - | myrosinase 1-like isoform X1 [Ostrinia furnacalis] | 1.97613 | -0.7219 | -0.3098 | -0.346 | -0.5984 |
| TRINITY_DN2718_c0_g1_i6_orf1 | - | - | - | cleavage stimulation factor subunit 2 isoform X1 [Ostrinia furnacalis] | 1.44914 | 0.89166 | -1.1171 | -0.8445 | -0.3792 |
| TRINITY_DN4132_c0_g1_i14_orf1 | - | - | - | thyroid receptor-interacting protein 11-like isoform X1 [Ostrinia furnacalis] | 1.38801 | -0.0084 | -1.7324 | 0.24693 | 0.10585 |
| TRINITY_DN1422_c0_g1_i4_orf1 | - | - | - | unnamed protein product [Chilo suppressalis] | 1.98452 | -0.6473 | -0.5031 | -0.5614 | -0.2727 |
| TRINITY_DN108354_c0_g1_i1_orf1 | - | - | - | WD repeat-containing protein 61-like [Ostrinia furnacalis] | 1.83227 | -0.0559 | -1.1597 | -0.0797 | -0.537 |
| TRINITY_DN825_c8_g1_i5_orf1 | - | - | - | ATP-binding cassette sub-family F member 2 [Ostrinia furnacalis] >XP_028169527.1 ATP-binding cassette sub-family F member 2 [Ostrinia | 1.96884 | -0.5884 | -0.7092 | -0.4924 | -0.1788 |
| TRINITY_DN959_c0_g1_i7_orf1 | - | - | - | Golgi to ER traffic protein 4 homolog [Ostrinia furnacalis] | 1.84772 | 0.18443 | -0.3671 | -0.7081 | -0.9569 |
| TRINITY_DN147475_c0_g1_i1_orf1 | - | - | - | casein kinase II subunit beta, partial [Rhincodon typus] | 1.19206 | 1.15026 | -1.205 | -0.849 | -0.2883 |
| TRINITY_DN1477_c0_g1_i5_orf1 | - | - | - | mitochondrial import inner membrane translocase subunit Tim9 [Ostrinia | 1.97814 | -0.4179 | -0.6813 | -0.6154 | -0.2635 |
| TRINITY_DN2593_c0_g2_i1_orf1 | - | - | - | midgut carboxypeptidase [Loxostege sticticalis] | 1.9548 | -0.751 | -0.2129 | -0.2974 | -0.6935 |
| TRINITY_DN38075_c0_g1_i1_orf1 | - | - | - | 60S ribosomal protein L26 [Ostrinia furnacalis] | 1.91826 | -0.4781 | -0.9167 | -0.5009 | -0.0226 |
| TRINITY_DN11655_c0_g1_i1_orf1 | - | - | - | ribosome biogenesis protein BRX1 homolog [Ostrinia furnacalis] | 1.9701 | -0.8022 | -0.2798 | -0.4085 | -0.4795 |
| TRINITY_DN22678_c0_g1_i4_orf1 | - | - | - | NADH-cytochrome b5 reductase 2 isoform X2 [Ostrinia furnacalis] >XP_028163866.1 NADH-cytochrome b5 reductase 2 isoform X2 [Ostrinia furnacalis] | 1.91384 | -0.7524 | -0.6674 | 0.07206 | -0.5661 |
| TRINITY_DN24163_c0_g1_i1_orf1 | - | - | - | luciferin 4-monooxygenase-like [Ostrinia furnacalis] | 1.83207 | 0.24932 | -0.9155 | -0.4047 | -0.7613 |
| TRINITY_DN101682_c0_g1_i1_orf1 | - | - | - | cysteine-rich with EGF-like domain protein 2 [Ostrinia furnacalis] | 1.65369 | -0.4215 | -1.4325 | 0.01162 | 0.18865 |
| TRINITY_DN37165_c0_g1_i4_orf1 | - | - | - | pyridoxine-5'-phosphate oxidase-like [Ostrinia furnacalis] | 1.8884 | -0.2446 | -1.0164 | -0.0451 | -0.5823 |
| TRINITY_DN16122_c0_g1_i4_orf1 | - | - | - | cytochrome P450 6k1-like [Ostrinia furnacalis] | 1.91122 | -0.6776 | -0.2186 | -0.1043 | -0.9108 |
| TRINITY_DN17045_c0_g2_i3_orf1 | - | - | - | unnamed protein product [Diatraea saccharalis] | 1.84678 | -0.2725 | -1.0239 | -0.6723 | 0.12187 |
| TRINITY_DN14429_c0_g1_i2_orf1 | - | - | - | NADH dehydrogenase [ubiquinone] 1 beta subcomplex subunit 11, mitochondrial [Ostrinia furnacalis] | 1.661 | 0.5428 | -0.4871 | -0.5151 | -1.2016 |
| TRINITY_DN17049_c0_g1_i6_orf1 | - | - | - | unnamed protein product [Arctia plantaginis] >CAB3248215.1 unnamed protein product [Arctia plantaginis] | 1.96642 | -0.391 | -0.7809 | -0.563 | -0.2315 |
| TRINITY_DN10131_c0_g1_i7_orf1 | - | - | - | aldo-keto reductase AKR2E4-like [Ostrinia furnacalis] | 1.04157 | 1.37075 | -1.0576 | -0.6712 | -0.6836 |
| TRINITY_DN50875_c0_g1_i3_orf1 | - | - | - | conserved oligomeric Golgi complex subunit 8 [Ostrinia furnacalis] | 1.15983 | 1.19502 | -1.2863 | -0.5096 | -0.5589 |
| TRINITY_DN3229_c0_g1_i1_orf1 | - | - | - | uncharacterized protein LOC114358442 isoform X1 [Ostrinia furnacalis] | 1.86854 | -1.1452 | -0.359 | -0.152 | -0.2124 |
| TRINITY_DN10476_c0_g1_i1_orf1 | - | - | - | prohibitin-2 [Ostrinia furnacalis] | 1.9722 | -0.5746 | -0.7335 | -0.4362 | -0.2279 |
| TRINITY_DN16258_c0_g1_i2_orf1 | - | - | - | uncharacterized protein LOC114359911 [Ostrinia furnacalis] | 1.76277 | 0.37106 | -0.9015 | -0.9186 | -0.3137 |
| TRINITY_DN142652_c0_g1_i1_orf1 | - | - | - | pre-mRNA-splicing factor RBM22 [Chelonus insularis] | 1.70224 | 0.12835 | -1.3849 | -0.4079 | -0.0377 |
| TRINITY_DN2535_c0_g1_i4_orf1 | - | - | - | ATP-dependent RNA helicase DDX24 [Ostrinia furnacalis] | 1.93773 | -0.7809 | -0.031 | -0.5812 | -0.5447 |
| TRINITY_DN143603_c0_g1_i1_orf1 | - | - | - | hypothetical protein KR044_005587 [Drosophila immigrans] | 1.96445 | -0.7315 | -0.514 | -0.1555 | -0.5634 |
| TRINITY_DN20499_c0_g3_i1_orf1 | - | - | - | exosome RNA helicase MTR4 isoform X2 [Ostrinia furnacalis] | 1.85433 | -0.0045 | -1.0146 | -0.7203 | -0.115 |
| TRINITY_DN51239_c0_g1_i5_orf1 | - | - | - | regulatory-associated protein of mTOR [Ostrinia furnacalis] | 1.78427 | -0.4849 | -1.0648 | 0.34094 | -0.5756 |
| TRINITY_DN40650_c0_g1_i1_orf1 | - | - | - | 60S ribosomal protein L12 [Zerene cesonia] | 1.7939 | -0.8969 | -0.9298 | -0.2207 | 0.2535 |
| TRINITY_DN61222_c0_g1_i1_orf1 | - | - | - | 60S ribosomal protein L38 [Bicyclus anynana] | 1.88546 | -0.4892 | -0.9651 | -0.5167 | 0.08556 |
| TRINITY_DN12134_c0_g1_i4_orf1 | - | - | - | glutathione S-transferase 1-1 [Ostrinia furnacalis] >XP_028161942.1 glutathione S-transferase 1-1 [Ostrinia furnacalis] >XP_028161943.1 glutathione S-transferase 1-1 [Ostrinia furnacalis] | 0.74393 | 1.485 | -1.3644 | -0.3776 | -0.4869 |
| TRINITY_DN3929_c0_g1_i1_orf1 | - | - | - | glutathione S-transferase 1-1-like [Ostrinia furnacalis] | 1.9385 | -0.8355 | -0.2679 | -0.1689 | -0.6662 |

| | | | | | | | | | |
|---|---|---|---|---|---|---|---|---|---|
| TRINITY_DN51568_c0_g1_i1_orf1 | - | - | - | splicing factor 3A subunit 2 [Ostrinia furnacalis] | 1.87945 | -0.1527 | -1.0528 | -0.5702 | -0.1037 |
| TRINITY_DN94625_c0_g1_i1_orf1 | - | - | - | uncharacterized protein LOC114354112 [Ostrinia furnacalis] | 1.99139 | -0.6638 | -0.5065 | -0.4161 | -0.405 |
| TRINITY_DN886_c0_g1_i1_orf1 | - | - | - | collagenase-like isoform X1 [Ostrinia furnacalis] | 1.92497 | -0.2934 | -0.2381 | -0.396 | -0.9974 |
| TRINITY_DN79734_c0_g2_i3_orf1 | - | - | - | 60S ribosomal protein L27a [Ostrinia furnacalis] | 1.83283 | -0.7056 | -1.0681 | -0.0416 | -0.0175 |
| TRINITY_DN17271_c0_g1_i1_orf1 | - | - | - | uncharacterized protein LOC114350693 [Ostrinia furnacalis] | 1.23102 | 1.14092 | -1.1671 | -0.8203 | -0.3845 |
| TRINITY_DN4956_c0_g1_i6_orf1 | - | - | - | nucleolar GTP-binding protein 1 [Ostrinia furnacalis] | 1.99475 | -0.5067 | -0.3644 | -0.5711 | -0.5525 |
| TRINITY_DN7787_c0_g1_i1_orf1 | - | - | - | trimeric intracellular cation channel type 1B.1 [Manduca sexta] >KAG6456518.1 hypothetical protein O3G_MSEX009773 [Manduca sexta] | 1.86323 | 0.0625 | -0.9339 | -0.2127 | -0.7791 |
| TRINITY_DN2879_c0_g1_i4_orf1 | - | - | - | nucleoporin Nup35 [Ostrinia furnacalis] | 1.06596 | 1.10363 | -1.4411 | -0.7538 | 0.02536 |
| TRINITY_DN78686_c0_g1_i1_orf1 | - | - | - | myosin-2 essential light chain isoform X2 [Harpegnathos saltator] >XP_012170910.1 myosin-2 essential light chain isoform X2 [Bombus terrestris] >XP_033185931.1 myosin-2 essential light chain isoform X2 [Bombus vancouverensis nearcticus] >XP_033319091.1 myosin-2 essential light chain isoform X2 [Bombus bifarius] >XP_033349866.1 myosin-2 essential light chain isoform X2 [Bombus vosnesenskii] >XP_043597873.1 myosin-2 essential light chain isoform X2 [Bombus pyrosoma] | 0.96003 | 1.26554 | -1.4775 | -0.2909 | -0.4571 |
| TRINITY_DN104507_c0_g1_i2_orf1 | - | - | - | replication protein A 32 kDa subunit [Ostrinia furnacalis] | 1.40976 | 0.80831 | -1.3638 | -0.6863 | -0.168 |
| TRINITY_DN799_c0_g1_i7_orf1 | - | - | - | hypothetical protein evm_002571 [Chilo suppressalis] >CAB3529880.1 unnamed protein product [Chilo suppressalis] >CAH0406472.1 unnamed protein product [Chilo suppressalis] | 1.75797 | -0.4851 | -1.1948 | -0.3881 | 0.30999 |
| TRINITY_DN13375_c0_g1_i6_orf1 | - | - | - | thioredoxin, mitochondrial isoform X2 [Ostrinia furnacalis] | 1.08572 | 1.18545 | -1.1735 | -0.0817 | -1.0159 |
| TRINITY_DN16343_c0_g1_i6_orf1 | - | - | - | aminopeptidase N6 [Ostrinia nubilalis] | 1.9887 | -0.6912 | -0.3873 | -0.4159 | -0.4944 |
| TRINITY_DN46778_c0_g1_i2_orf1 | - | - | - | Deoxycytidylate deaminase [Papilio xuthus] | 1.79746 | 0.33154 | -0.4688 | -0.6551 | -1.0052 |

| | | | | | | | | | |
|---|---|---|---|---|---|---|---|---|---|
| TRINITY_DN31663_c0_g1_i2_orf1 | - | - | - | PHD finger-like domain-containing protein 5A [Nasonia vitripennis] >XP_002427197.1 conserved hypothetical protein [Pediculus humanus corporis] >XP_003484388.1 PHD finger-like domain-containing protein 5A [Bombus impatiens] >XP_003701008.1 PREDICTED: PHD finger-like domain-containing protein 5A [Megachile rotundata] >XP_006623871.1 PHD finger-like domain-containing protein 5A [Apis dorsata] >XP_011068502.1 PREDICTED: PHD finger-like domain-containing protein 5A [Acromyrmex echinatior] >XP_011154391.1 PHD finger-like domain-containing protein 5A [Harpegnathos saltator] >XP_011164776.1 PHD finger-like domain-containing protein 5A [Solenopsis invicta] >XP_011262550.1 PHD finger-like domain-containing protein 5A [Camponotus floridanus] >XP_011297178.1 PREDICTED: PHD finger-like domain-containing protein 5A [Fopius arisanus] >XP_011334720.1 PHD finger-like domain-containing protein 5A [Ooceraea biroi] >XP_011506347.1 PREDICTED: PHD finger-like domain-containing protein 5A [Ceratosolen solmsi marchali] >XP_011506348.1 PREDICTED: PHD finger-like domain-containing protein 5A [Ceratosolen solmsi marchali] >XP_011638597.1 PHD finger-like domain-containing protein 5A isoform X2 [Pogonomyrmex barbatus] >XP_011686073.1 PREDICTED: PHD finger-like domain-containing protein 5A [Wasmannia auropunctata] >XP_011858255.1 PREDICTED: PHD finger-like domain-containing protein 5A [Vollenhovia emeryi] >XP_012058015.1 PREDICTED: PHD finger-like domain-containing protein 5A [Atta cephalotes] >XP_012135327.1 PREDICTED: PHD finger-like domain-containing protein 5A [Megachile rotundata] >XP_012135328.1 PREDICTED: PHD finger-like domain-containing protein 5A [Megachile rotundata] >XP_012222185.1 PREDICTED: PHD finger-like domain-containing protein 5A [Linepithema humile] >XP_012261946.1 PHD finger-like domain-containing protein 5A [Athalia rosae] >XP_012273120.1 PHD finger-like domain-containing protein 5A [Orussus abietinus] >XP_012526512.1 PHD finger-like domain-containing protein 5A [Monomorium pharaonis] >XP_014217558.1 PHD finger-like domain-containing protein 5A [Copidosoma floridanum] >XP_014484566.1 | 1.82142 | 0.2424 | -1.0421 | -0.5994 | -0.4223 |
| TRINITY_DN6147_c0_g1_i2_orf1 | - | - | - | uncharacterized protein LOC114352519 [Ostrinia furnacalis] | 1.95109 | -0.4867 | -0.1221 | -0.5286 | -0.8136 |
| TRINITY_DN26824_c0_g1_i1_orf1 | - | - | - | 60S ribosomal protein L6 [Hyposmocoma kahamanoa] | 1.48416 | -0.4504 | -1.5437 | 0.45653 | 0.05347 |
| TRINITY_DN86149_c0_g1_i1_orf1 | - | - | - | NADH dehydrogenase [ubiquinone] 1 alpha subcomplex subunit 8 [Galleria mellonella] | 1.94831 | -0.3272 | -0.2826 | -0.9156 | -0.4229 |
| TRINITY_DN28981_c0_g1_i1_orf1 | - | - | - | uncharacterized protein C6orf203 homolog [Ostrinia furnacalis] | 1.94767 | -0.1035 | -0.5835 | -0.4561 | -0.8046 |
| TRINITY_DN23360_c0_g1_i3_orf1 | - | - | - | protein PTCD3 homolog, mitochondrial [Ostrinia furnacalis] | 1.82748 | 0.32329 | -0.813 | -0.6701 | -0.6677 |
| TRINITY_DN2304_c0_g1_i4_orf1 | - | - | - | clustered mitochondria protein homolog isoform X2 [Ostrinia furnacalis] | 1.8968 | -0.7732 | -0.0174 | -0.2431 | -0.863 |
| TRINITY_DN10658_c0_g1_i1_orf1 | - | - | - | DNA-directed RNA polymerase II subunit RPB9 [Ostrinia furnacalis] >XP_030035935.1 DNA-directed RNA polymerase II subunit RPB9 [Manduca sexta] >XP_037301147.1 DNA-directed RNA polymerase II subunit RPB9-like [Manduca sexta] >KAG6462484.1 hypothetical protein O3G_MSEX013297 | 1.6503 | 0.35078 | -0.6318 | -0.0456 | -1.3237 |
| TRINITY_DN23502_c0_g1_i1_orf1 | - | - | - | small nuclear ribonucleoprotein F [Ostrinia furnacalis] | 1.39261 | 0.56962 | -1.4141 | -0.8154 | 0.26733 |
| TRINITY_DN76036_c0_g1_i1_orf1 | - | - | - | cytochrome c oxidase subunit 6A1, mitochondrial-like [Ostrinia furnacalis] | 1.93779 | -0.2306 | -0.3765 | -0.9523 | -0.3785 |
| TRINITY_DN25341_c0_g1_i1_orf1 | - | - | - | heat shock protein 90 [Loxostege sticticalis] | 1.79834 | 0.36705 | -0.9155 | -0.7023 | -0.5475 |
| TRINITY_DN22654_c0_g2_i4_orf1 | - | - | - | protein EFR3 homolog cmp44E isoform X1 [Ostrinia furnacalis] >XP_028166854.1 protein EFR3 homolog cmp44E isoform X2 [Ostrinia | 1.71172 | 0.54173 | -0.9652 | -0.7312 | -0.5571 |
| TRINITY_DN11820_c0_g1_i1_orf1 | - | - | - | hypothetical protein evm_000341 [Chilo suppressalis] | 1.2244 | 0.89 | -1.5921 | -0.398 | -0.1242 |
| TRINITY_DN12323_c0_g2_i2_orf1 | - | - | - | polyribonucleotide nucleotidyltransferase 1, mitochondrial [Ostrinia furnacalis] | 1.92853 | -0.4872 | -0.8588 | -0.0303 | -0.5523 |
| TRINITY_DN79803_c0_g1_i7_orf1 | - | - | - | dnaJ homolog subfamily C member 22 [Ostrinia furnacalis] | 0.87975 | 1.29123 | -1.4802 | -0.0912 | -0.5997 |
| TRINITY_DN2927_c0_g1_i6_orf1 | - | - | - | T-complex protein 1 subunit eta [Ostrinia furnacalis] | 1.95195 | -0.0844 | -0.6956 | -0.6641 | -0.5079 |
| TRINITY_DN47591_c1_g1_i1_orf1 | - | - | - | uncharacterized protein LOC114364828 [Ostrinia furnacalis] | 1.95535 | -0.1239 | -0.7561 | -0.6129 | -0.4624 |
| TRINITY_DN108200_c0_g1_i1_orf1 | - | - | - | uncharacterized protein LOC114350842 [Ostrinia furnacalis] | 1.9512 | -0.871 | -0.4492 | -0.1864 | -0.4446 |

| | | | | | | | | | |
|---|---|---|---|---|---|---|---|---|---|
| TRINITY_DN14565_c0_g1_i11_orf1 | - | - | - | 4-aminobutyrate aminotransferase, mitochondrial [Galleria mellonella] | 1.87449 | 0.02897 | -0.6757 | -0.2429 | -0.9848 |
| TRINITY_DN2265_c0_g2_i1_orf1 | - | - | - | LOW QUALITY PROTEIN: elongation factor G, mitochondrial-like [Leguminivora glycinivorella] | 1.90534 | 0.07001 | -0.6132 | -0.5064 | -0.8558 |
| TRINITY_DN11117_c0_g1_i1_orf1 | - | - | - | venom carboxylesterase-6-like [Ostrinia furnacalis] | 1.95808 | -0.7702 | -0.3421 | -0.2015 | -0.6443 |
| TRINITY_DN5046_c0_g3_i1_orf1 | - | - | - | uncharacterized protein LOC114358520 [Ostrinia furnacalis] | 1.46956 | 0.90814 | -1.0212 | -0.5159 | -0.8406 |
| TRINITY_DN8676_c0_g1_i1_orf1 | - | - | - | probable rRNA-processing protein EBP2 homolog [Ostrinia furnacalis] | 1.99292 | -0.3785 | -0.5163 | -0.461 | -0.6371 |
| TRINITY_DN631_c0_g1_i6_orf1 | - | - | - | cytosolic 10-formyltetrahydrofolate dehydrogenase isoform X1 [Ostrinia furnacalis] >XP_028172896.1 cytosolic 10-formyltetrahydrofolate dehydrogenase isoform X2 [Ostrinia furnacalis] | 1.91403 | -0.6506 | -0.9192 | -0.2395 | -0.1048 |
| TRINITY_DN33248_c0_g1_i1_orf1 | - | - | - | elongation factor Ts, mitochondrial isoform X3 [Ostrinia furnacalis] >XP_028155866.1 elongation factor Ts, mitochondrial isoform X3 [Ostrinia furnacalis] | 1.91583 | -0.6428 | -0.8946 | -0.3383 | -0.0401 |
| TRINITY_DN51968_c0_g1_i1_orf1 | - | - | - | splicing factor U2af 38 kDa subunit [Aphidius gifuensis] >KAF7990547.1 hypothetical protein HCN44_000352 [Aphidius gifuensis] | 1.49887 | 0.53877 | -1.2234 | -0.9705 | 0.1563 |
| TRINITY_DN10796_c0_g2_i1_orf1 | - | - | - | F-BAR domain only protein 2 [Ostrinia furnacalis] | 1.98113 | -0.2947 | -0.5959 | -0.4065 | -0.6841 |
| TRINITY_DN14920_c0_g1_i1_orf1 | - | - | - | anamorsin homolog [Ostrinia furnacalis] | 1.75126 | 0.44196 | -0.797 | -0.9509 | -0.4453 |
| TRINITY_DN58636_c0_g1_i1_orf1 | - | - | - | uncharacterized protein LOC114363665 [Ostrinia furnacalis] | 1.88658 | 0.03452 | -0.9128 | -0.7257 | -0.2827 |
| TRINITY_DN58413_c0_g1_i4_orf1 | - | - | - | cysteine and histidine-rich protein 1 isoform X1 [Ostrinia furnacalis] | 1.9781 | -0.77 | -0.4589 | -0.3361 | -0.4132 |
| TRINITY_DN146264_c0_g1_i1_orf1 | - | - | - | PREDICTED: protein preli-like [Fopius arisanus] | 1.81755 | -0.3614 | -1.1636 | -0.4371 | 0.14453 |
| TRINITY_DN5009_c0_g1_i2_orf1 | - | - | - | GSCOCG00009487001-RA-CDS [Cotesia congregata] >CAG5088842.1 Similar to RpL18: 60S ribosomal protein L18 (Timarcha balearica) [Cotesia congregata] | 1.70898 | -0.6292 | -1.1831 | -0.3214 | 0.42474 |
| TRINITY_DN2184_c0_g1_i4_orf1 | - | - | - | uncharacterized protein LOC114359356 [Ostrinia furnacalis] | 1.82781 | -0.7069 | -0.193 | 0.12413 | -1.052 |
| TRINITY_DN4213_c0_g1_i4_orf1 | - | - | - | nardilysin-like isoform X1 [Ostrinia furnacalis] >XP_028157649.1 nardilysin-like isoform X2 [Ostrinia furnacalis] >XP_028157650.1 nardilysin-like isoform X3 [Ostrinia furnacalis] >XP_028157651.1 nardilysin-like isoform X4 [Ostrinia | 1.70573 | 0.58386 | -0.7277 | -0.793 | -0.7689 |
| TRINITY_DN2885_c1_g1_i2_orf1 | - | - | - | ubiquitin-like-specific protease ESD4 [Ostrinia furnacalis] | 1.37053 | 0.8457 | -1.2616 | -0.901 | -0.0536 |
| TRINITY_DN5678_c0_g2_i3_orf1 | - | - | - | coiled-coil domain-containing protein 115 [Ostrinia furnacalis] | 1.96454 | -0.4117 | -0.5625 | -0.2086 | -0.7818 |
| TRINITY_DN53136_c0_g1_i1_orf1 | - | - | - | glutathione S transferase-E4 [Glyphodes pyloalis] | 1.96634 | -0.6691 | -0.3848 | -0.7027 | -0.2098 |
| TRINITY_DN36788_c0_g1_i2_orf1 | - | - | - | isocitrate dehydrogenase [NADP] cytoplasmic-like [Bicyclus anynana] | 1.72924 | 0.3014 | -1.1898 | -0.6941 | -0.1468 |
| TRINITY_DN9862_c0_g2_i1_orf1 | - | - | - | 40S ribosomal protein S4 [Manduca sexta] >ACY95325.1 ribosomal protein S4 [Manduca sexta] >KAG6465430.1 hypothetical protein O3G_MSEX015149 [Manduca sexta] | 1.80675 | -0.8348 | -0.8736 | -0.4172 | 0.3188 |
| TRINITY_DN19187_c0_g1_i1_orf1 | - | - | - | fumarylacetoacetase [Chelonus insularis] | 1.80233 | -0.1256 | -1.2879 | -0.2232 | -0.1658 |
| TRINITY_DN7122_c0_g1_i1_orf1 | - | - | - | hypothetical protein evm_003965 [Chilo suppressalis] | 1.39704 | 0.56424 | -1.1876 | 0.32739 | -1.1011 |
| TRINITY_DN38301_c0_g1_i2_orf1 | - | - | - | gamma-taxilin [Ostrinia furnacalis] | 1.92305 | 0.04108 | -0.6225 | -0.5912 | -0.7504 |
| TRINITY_DN26649_c0_g1_i2_orf1 | - | - | - | ATP synthase-coupling factor 6, mitochondrial [Ostrinia furnacalis] | 1.98906 | -0.6461 | -0.401 | -0.5752 | -0.3667 |
| TRINITY_DN5697_c0_g1_i1_orf1 | - | - | - | GPI ethanolamine phosphate transferase 2-like [Ostrinia furnacalis] | 1.63305 | 0.54176 | -0.9966 | -1.0088 | -0.1694 |
| TRINITY_DN754_c1_g1_i8_orf1 | - | - | - | lysophospholipid acyltransferase 5 [Ostrinia furnacalis] >XP_028169982.1 lysophospholipid acyltransferase 5 [Ostrinia furnacalis] | 1.84154 | 0.26538 | -0.7589 | -0.51 | -0.838 |
| TRINITY_DN4121_c0_g1_i1_orf1 | - | - | - | uncharacterized protein LOC114358001, partial [Ostrinia furnacalis] | 1.90952 | -0.8746 | -0.6282 | -0.4393 | 0.03268 |
| TRINITY_DN107617_c3_g1_i1_orf1 | - | - | - | NADH dehydrogenase [ubiquinone] 1 alpha subcomplex subunit 9, mitochondrial [Ostrinia furnacalis] | 1.91995 | -0.9883 | -0.464 | -0.3126 | -0.1551 |
| TRINITY_DN108051_c0_g1_i2_orf1 | - | - | - | uncharacterized protein LOC114351921 [Ostrinia furnacalis] | 1.99497 | -0.3836 | -0.5423 | -0.5953 | -0.4737 |
| TRINITY_DN4262_c0_g1_i16_orf1 | - | - | - | sperm-associated antigen 7 homolog [Ostrinia furnacalis] | 1.39662 | 0.78959 | -1.4211 | -0.6211 | -0.1441 |
| TRINITY_DN48413_c1_g1_i2_orf1 | - | - | - | probable protein phosphatase 2C 11 isoform X1 [Manduca sexta] >KAG6442694.1 hypothetical protein O3G_MSEX002471 [Manduca sexta] | 1.49262 | 0.88952 | -0.8872 | -0.9427 | -0.5522 |
| TRINITY_DN49936_c0_g2_i1_orf1 | - | - | - | 39S ribosomal protein L20, mitochondrial [Ostrinia furnacalis] | 1.87814 | -0.1224 | -0.3281 | -0.307 | -1.1206 |
| TRINITY_DN237_c1_g1_i1_orf1 | - | - | - | PREDICTED: cytoplasmic protein NCK1 isoform X1 [Microplitis demolitor] | 1.64735 | 0.6462 | -0.5816 | -1.0363 | -0.6757 |
| TRINITY_DN3534_c0_g1_i2_orf1 | - | - | - | guanine nucleotide-binding protein subunit beta-like protein [Ostrinia | 1.92426 | -0.6339 | -0.9044 | -0.1545 | -0.2315 |
| TRINITY_DN36718_c0_g1_i1_orf1 | - | - | - | unnamed protein product [Chilo suppressalis] | 1.99819 | -0.4441 | -0.4934 | -0.5752 | -0.4856 |
| TRINITY_DN14018_c0_g1_i4_orf1 | - | - | - | chitobiosyldiphosphodolichol beta-mannosyltransferase [Ostrinia furnacalis] | 1.84839 | 0.24599 | -0.6667 | -0.8861 | -0.5416 |

| | | | | | | | | | |
|---|---|---|---|---|---|---|---|---|---|
| TRINITY_DN4025_c0_g1_i13_orf1 | - | - | - | tetratricopeptide repeat protein 14 homolog isoform X2 [Ostrinia furnacalis] | 1.60115 | 0.09401 | -1.5239 | -0.2986 | 0.12725 |
| TRINITY_DN783_c0_g1_i7_orf1 | - | - | - | microtubule-associated protein Jupiter isoform X4 [Helicoverpa armigera] | 1.14845 | 1.28544 | -1.0004 | -0.7325 | -0.701 |
| TRINITY_DN4659_c0_g1_i2_orf1 | - | - | - | uncharacterized protein LOC114351134 [Ostrinia furnacalis] | 1.17886 | 1.26323 | -0.7607 | -0.735 | -0.9464 |
| TRINITY_DN4135_c0_g1_i5_orf1 | - | - | - | probable small nuclear ribonucleoprotein Sm D2 [Manduca sexta] >KAG6451233.1 hypothetical protein O3G_MSEX007016 [Manduca sexta] | 1.86562 | -0.0594 | -1.1106 | -0.4903 | -0.2053 |
| TRINITY_DN38540_c0_g1_i1_orf1 | - | - | - | GSCOCG00000129001-RA-CDS [Cotesia congregata] >CAG5101050.1 Similar to LUC7L2: Putative RNA-binding protein Luc7-like 2 (Homo sapiens) [Cotesia congregata] | 1.74162 | 0.43194 | -1.0944 | -0.55 | -0.5292 |
| TRINITY_DN1109_c0_g1_i6_orf1 | - | - | - | 1-phosphatidylinositol phosphodiesterase-like [Cotesia glomerata] | 1.7203 | 0.37742 | -0.2687 | -0.6376 | -1.1914 |
| TRINITY_DN17394_c0_g1_i1_orf1 | - | - | - | monocarboxylate transporter 14-like [Ostrinia furnacalis] | 1.70966 | -0.2468 | -1.0632 | -0.8347 | 0.43498 |
| TRINITY_DN57105_c0_g1_i2_orf1 | - | - | - | transmembrane protein 161B isoform X1 [Galleria mellonella] | 1.47487 | 0.88298 | -0.8369 | -1.0671 | -0.4539 |
| TRINITY_DN8754_c0_g1_i2_orf1 | - | - | - | dnaJ homolog subfamily C member 11 [Ostrinia furnacalis] | 1.99755 | -0.5257 | -0.4789 | -0.4219 | -0.5711 |
| TRINITY_DN3893_c0_g2_i3_orf1 | - | - | - | cleavage and polyadenylation specificity factor subunit CG7185 isoform X2 [Ostrinia furnacalis] | 1.44358 | 0.79885 | -1.3053 | -0.7284 | -0.2088 |
| TRINITY_DN16174_c0_g1_i2_orf1 | - | - | - | LOW QUALITY PROTEIN: ATP-dependent RNA helicase SUV3 homolog, mitochondrial [Ostrinia furnacalis] | 1.88592 | -0.2105 | -1.0941 | -0.4189 | -0.1624 |
| TRINITY_DN105359_c0_g2_i5_orf1 | - | - | - | uncharacterized protein LOC114359499 [Ostrinia furnacalis] | 0.79801 | 1.02222 | -1.6942 | -0.532 | 0.40598 |
| TRINITY_DN8949_c0_g1_i2_orf1 | - | - | - | unnamed protein product [Arctia plantaginis] | 1.81594 | -0.6184 | -1.119 | -0.2191 | 0.14056 |
| TRINITY_DN391_c5_g1_i1_orf1 | - | - | - | hypothetical protein B5X24_HaOG210395 [Helicoverpa armigera] | 1.9946 | -0.5332 | -0.5733 | -0.5285 | -0.3597 |
| TRINITY_DN2062_c0_g1_i11_orf1 | - | - | - | uncharacterized protein LOC114350846 [Ostrinia furnacalis] | 1.89974 | -0.2008 | -1.055 | -0.1988 | -0.4452 |
| TRINITY_DN27994_c0_g1_i1_orf1 | - | - | - | uncharacterized protein LOC114364076 [Ostrinia furnacalis] | 1.84165 | 0.25415 | -0.5797 | -0.9289 | -0.5873 |
| TRINITY_DN816_c0_g1_i3_orf1 | - | - | - | calcium-binding mitochondrial carrier protein SCaMC-2 isoform X1 [Ostrinia furnacalis] | 1.882 | 0.04566 | -1.0128 | -0.5339 | -0.3809 |
| TRINITY_DN147596_c0_g1_i1_orf1 | - | - | - | activator of basal transcription 1 [Diachasma alloeum] | 1.7748 | -0.8628 | -0.9783 | 0.30368 | -0.2374 |
| TRINITY_DN3836_c0_g1_i4_orf1 | - | - | - | 2-oxoisovalerate dehydrogenase subunit alpha, mitochondrial [Ostrinia | 1.77593 | -1.0905 | -0.7385 | 0.26112 | -0.208 |
| TRINITY_DN3773_c0_g1_i4_orf1 | - | - | - | peptidyl-prolyl cis-trans isomerase G isoform X2 [Ostrinia furnacalis] | 1.82875 | 0.02927 | -1.1799 | -0.467 | -0.2111 |
| TRINITY_DN27641_c0_g1_i1_orf1 | - | - | - | succinate dehydrogenase [ubiquinone] iron-sulfur subunit, mitochondrial [Ostrinia furnacalis] | 1.99562 | -0.5549 | -0.4512 | -0.5855 | -0.4041 |
| TRINITY_DN3702_c0_g1_i1_orf1 | - | - | - | U4/U6.U5 tri-snRNP-associated protein 2 [Ostrinia furnacalis] | 1.22194 | 0.36 | -1.7947 | -0.1523 | 0.36501 |
| TRINITY_DN20582_c0_g1_i1_orf1 | - | - | - | 2-oxoglutarate dehydrogenase, mitochondrial isoform X3 [Ostrinia furnacalis] >XP_028167081.1 2-oxoglutarate dehydrogenase, mitochondrial isoform X3 [Ostrinia furnacalis] | 1.98158 | -0.3999 | -0.5792 | -0.6955 | -0.307 |
| TRINITY_DN123139_c0_g1_i1_orfp1 | - | - | - | TRINITY_DN123139_c0_g1_i1_m.79879 TRINITY_DN123139_c0_g1::TRINITY_DN123139_c0_g1_i1::g.79879 ORF type:3prime_partial len:76 (+),score=3.83 TRINITY_DN123139_c0_g1_i1:25- | 1.86415 | -0.3568 | -1.0589 | -0.5207 | 0.07227 |
| TRINITY_DN22572_c0_g1_i1_orf1 | - | - | - | eukaryotic translation elongation factor 1 epsilon-1 [Ostrinia furnacalis] | 1.6022 | 0.1913 | -1.5259 | -0.007 | -0.2606 |
| TRINITY_DN1633_c0_g1_i1_orf1 | - | - | - | collagen alpha-2(I) chain-like isoform X1 [Ostrinia furnacalis] | 1.98487 | -0.4028 | -0.4752 | -0.3808 | -0.7261 |
| TRINITY_DN20369_c0_g1_i2_orf1 | - | - | - | uncharacterized protein LOC114366225 [Ostrinia furnacalis] | 1.91805 | 0.02527 | -0.8305 | -0.6323 | -0.4805 |
| TRINITY_DN45227_c0_g1_i3_orf1 | - | - | - | uncharacterized protein LOC114359191 [Ostrinia furnacalis] | 1.95953 | -0.2765 | -0.7783 | -0.6378 | -0.2669 |
| TRINITY_DN21341_c0_g1_i4_orf1 | - | - | - | FAST kinase domain-containing protein 4 isoform X6 [Ostrinia furnacalis] >XP_028160336.1 FAST kinase domain-containing protein 4 isoform X7 [Ostrinia furnacalis] >XP_028160337.1 FAST kinase domain-containing protein 4 isoform X8 [Ostrinia furnacalis] | 1.96619 | -0.1652 | -0.6657 | -0.4695 | -0.6657 |
| TRINITY_DN19115_c0_g1_i1_orf1 | - | - | - | putative ATP synthase subunit f, mitochondrial [Ostrinia furnacalis] | 1.88046 | -0.0443 | -0.3469 | -1.0857 | -0.4035 |
| TRINITY_DN6671_c0_g1_i6_orf1 | - | - | - | hypothetical protein evm_013656 [Chilo suppressalis] >CAB3521812.1 unnamed protein product [Chilo suppressalis] >CAH0399134.1 unnamed protein product [Chilo suppressalis] | 1.81837 | 0.17026 | -1.1425 | -0.4484 | -0.3977 |
| TRINITY_DN5686_c0_g1_i4_orf1 | - | - | - | FACT complex subunit spt16 isoform X2 [Ostrinia furnacalis] | 1.8908 | -0.2441 | -1.0594 | -0.1059 | -0.4815 |
| TRINITY_DN2780_c0_g1_i5_orf1 | - | - | - | probable ATP-dependent RNA helicase DDX27 [Ostrinia furnacalis] | 1.98621 | -0.3726 | -0.3619 | -0.5965 | -0.6553 |
| TRINITY_DN16749_c0_g1_i1_orf1 | - | - | - | uncharacterized protein LOC114353228 [Ostrinia furnacalis] >XP_028176007.1 uncharacterized protein LOC114364183 [Ostrinia furnacalis] | 1.96422 | -0.6089 | -0.601 | -0.6275 | -0.1268 |

| | | | | | | | | | |
|---|---|---|---|---|---|---|---|---|---|
| TRINITY_DN19829_c0_g2_i1_orf1 | - | - | - | 28S ribosomal protein S35, mitochondrial [Ostrinia furnacalis] | 1.9623 | -0.3786 | -0.4087 | -0.8602 | -0.3149 |
| TRINITY_DN4040_c0_g1_i10_orf1 | - | - | - | hypothetical protein evm_007488 [Chilo suppressalis] | 1.97503 | -0.3972 | -0.3494 | -0.4341 | -0.7943 |
| TRINITY_DN32997_c0_g1_i8_orf1 | - | - | - | RNA-binding protein squid isoform X1 [Ostrinia furnacalis] | 1.88669 | 0.09246 | -0.8212 | -0.7876 | -0.3703 |
| TRINITY_DN3127_c0_g1_i9_orf1 | - | - | - | RNA-binding protein 1 isoform X1 [Galleria mellonella] | 1.84986 | 0.09022 | -0.6598 | -1.037 | -0.2433 |
| TRINITY_DN44557_c0_g1_i4_orf1 | - | - | - | serine hydrolase-like protein [Ostrinia furnacalis] | 1.80962 | -0.9153 | -0.8806 | -0.2436 | 0.2298 |
| TRINITY_DN2089_c0_g1_i5_orf1 | - | - | - | eukaryotic translation initiation factor 4B [Ostrinia furnacalis] | 1.70868 | 0.24467 | -1.165 | -0.814 | 0.02568 |
| TRINITY_DN2274_c0_g1_i6_orf1 | - | - | - | membrane alanyl aminopeptidase-like [Ostrinia furnacalis] | 1.9819 | -0.5588 | -0.3245 | -0.3895 | -0.7091 |
| TRINITY_DN96080_c0_g2_i1_orf1 | - | - | - | ATP synthase subunit delta, mitochondrial [Ostrinia furnacalis] | 1.94281 | -0.8875 | -0.3387 | -0.5405 | -0.1761 |
| TRINITY_DN48619_c0_g1_i1_orf1 | - | - | - | PREDICTED: lysine--tRNA ligase isoform X2 [Fopius arisanus] | 1.73373 | -1.0391 | -0.8975 | -0.1088 | 0.31167 |
| TRINITY_DN26963_c0_g1_i1_orf1 | - | - | - | aminoacyl tRNA synthase complex-interacting multifunctional protein 1 isoform X2 [Ostrinia furnacalis] | 1.92648 | -0.1089 | -0.9424 | -0.3838 | -0.4913 |
| TRINITY_DN43293_c0_g1_i2_orf1 | - | - | - | egl nine homolog 1 isoform X2 [Helicoverpa armigera] | 1.32777 | 0.48153 | -1.526 | 0.42245 | -0.7058 |
| TRINITY_DN51252_c0_g2_i1_orf1 | - | - | - | peroxidase-like [Ostrinia furnacalis] | 1.75855 | 0.36234 | -0.6792 | -0.3496 | -1.0921 |
| TRINITY_DN42738_c0_g1_i1_orf1 | - | - | - | arf-GAP domain and FG repeat-containing protein 1 [Ostrinia furnacalis] | 1.90403 | -0.5772 | -0.9805 | -0.0726 | -0.2737 |
| TRINITY_DN19829_c0_g1_i1_orf1 | - | - | - | 28S ribosomal protein S35, mitochondrial [Ostrinia furnacalis] | 1.90567 | -0.8512 | -0.7735 | -0.0857 | -0.1952 |
| TRINITY_DN1386_c0_g1_i6_orf1 | - | - | - | ras-related protein Rab-36 [Ostrinia furnacalis] | 1.83623 | 0.20399 | -0.8193 | -0.3189 | -0.9021 |
| TRINITY_DN30_c0_g1_i6_orf1 | - | - | - | casein kinase I-like isoform X1 [Hyposmocoma kahamanoa] | 1.7836 | 0.35447 | -0.9968 | -0.4153 | -0.7259 |
| TRINITY_DN83005_c0_g1_i1_orf1 | - | - | - | ATP synthase subunit O, mitochondrial [Danaus plexippus plexippus] >OWR53927.1 H+ transporting ATP synthase O subunit [Danaus plexippus plexippus] | 1.9867 | -0.7183 | -0.4423 | -0.4019 | -0.4243 |
| TRINITY_DN16349_c0_g1_i10_orf1 | - | - | - | protein lingerer-like isoform X1 [Nymphalis io] >XP_050356663.1 protein lingerer-like isoform X1 [Nymphalis io] >XP_050356664.1 protein lingerer-like isoform X1 [Nymphalis io] | 1.55748 | 0.74044 | -1.1667 | -0.6773 | -0.4539 |
| TRINITY_DN80560_c0_g1_i1_orf1 | - | - | - | ATP synthase subunit alpha, mitochondrial [Ostrinia furnacalis] | 1.97507 | -0.7978 | -0.372 | -0.4049 | -0.4005 |
| TRINITY_DN905_c0_g1_i4_orf1 | - | - | - | (11Z)-hexadec-11-enoyl-CoA conjugase-like [Ostrinia furnacalis] >XP_028172978.1 (11Z)-hexadec-11-enoyl-CoA conjugase-like [Ostrinia furnacalis] | 1.96637 | -0.2085 | -0.585 | -0.7595 | -0.4134 |
| TRINITY_DN50085_c0_g1_i1_orf1 | - | - | - | hypothetical protein evm_013997 [Chilo suppressalis] | 1.86999 | 0.07178 | -0.9466 | -0.7286 | -0.2666 |
| TRINITY_DN4938_c0_g1_i13_orf1 | - | - | - | peroxisomal biogenesis factor 19 [Ostrinia furnacalis] | 1.82356 | -0.9713 | -0.5204 | -0.6161 | 0.28426 |
| TRINITY_DN2238_c0_g2_i1_orf1 | - | - | - | mitochondrial import inner membrane translocase subunit Tim8 [Ostrinia | 1.94674 | -0.352 | -0.1657 | -0.5771 | -0.8519 |
| TRINITY_DN1504_c0_g1_i1_orf1 | - | - | - | uncharacterized protein LOC114352862 [Ostrinia furnacalis] >XP_028160407.1 uncharacterized protein LOC114352862 [Ostrinia furnacalis] | 0.9835 | 1.27102 | -1.3897 | -0.6692 | -0.1957 |
| TRINITY_DN2065_c1_g2_i1_orf1 | - | - | - | 2-amino-3-ketobutyrate coenzyme A ligase, mitochondrial [Ostrinia furnacalis] | 1.66607 | 0.65242 | -0.7458 | -0.733 | -0.8397 |
| TRINITY_DN21609_c0_g2_i1_orf1 | - | - | - | translation initiation factor eIF-2B subunit epsilon [Ostrinia furnacalis] | 1.7761 | 0.06388 | -1.2695 | -0.4683 | -0.1022 |
| TRINITY_DN8173_c0_g1_i3_orf1 | - | - | - | dihydroceramide fatty acyl 2-hydroxylase FAH1 [Ostrinia furnacalis] | 1.92512 | -0.597 | -0.5006 | 0.0013 | -0.8288 |
| TRINITY_DN106476_c0_g1_i3_orf1 | - | - | - | mitochondrial import inner membrane translocase subunit TIM44 [Ostrinia furnacalis] | 1.93872 | -0.6308 | -0.8547 | -0.2982 | -0.155 |
| TRINITY_DN12683_c0_g1_i3_orf1 | - | - | - | sulfated surface glycoprotein 185-like [Ostrinia furnacalis] | 1.93505 | -0.9573 | -0.4401 | -0.2896 | -0.248 |
| TRINITY_DN78873_c0_g1_i4_orf1 | - | - | - | hypothetical protein evm_008224 [Chilo suppressalis] | 1.74306 | -0.2606 | -1.2866 | -0.4291 | 0.23325 |
| TRINITY_DN14235_c0_g1_i1_orf1 | - | - | - | maltase A1 [Helicoverpa armigera] | 1.91931 | -0.6428 | -0.7358 | -0.5986 | 0.05788 |
| TRINITY_DN1465_c0_g2_i1_orf1 | - | - | - | unnamed protein product, partial [Iphiclides podalirius] | 1.97016 | -0.6998 | -0.2309 | -0.387 | -0.6524 |
| TRINITY_DN536_c0_g1_i7_orf1 | - | - | - | polymerase delta-interacting protein 2 isoform X3 [Ostrinia furnacalis] | 1.96825 | -0.7752 | -0.4085 | -0.551 | -0.2335 |
| TRINITY_DN4143_c0_g1_i1_orf1 | - | - | - | zinc finger protein 530-like isoform X8 [Ostrinia furnacalis] | 1.82158 | 0.21863 | -0.2764 | -0.9146 | -0.8492 |
| TRINITY_DN15959_c0_g1_i1_orf1 | - | - | - | dnaJ homolog subfamily A member 2-like [Ostrinia furnacalis] | 1.94385 | -0.0729 | -0.7575 | -0.6627 | -0.4507 |
| TRINITY_DN7626_c0_g1_i1_orf1 | - | - | - | NADH dehydrogenase [ubiquinone] flavoprotein 1, mitochondrial isoform X1 [Ostrinia furnacalis] | 1.9898 | -0.5175 | -0.5739 | -0.5906 | -0.3078 |
| TRINITY_DN45477_c0_g1_i1_orf1 | - | - | - | putative E3 ubiquitin-protein ligase UBR7 [Ostrinia furnacalis] | 1.82918 | 0.21356 | -0.7566 | -0.3198 | -0.9663 |
| TRINITY_DN24142_c0_g1_i1_orf1 | - | - | - | arylalkylamine N-acetyltransferase [Chilo suppressalis] | 1.81165 | -0.8302 | -1.0125 | -0.0231 | 0.05417 |
| TRINITY_DN33_c0_g1_i1_orf1 | - | - | - | uncharacterized protein CG45076-like isoform X2 [Ostrinia furnacalis] | 1.80667 | -0.7525 | -1.0748 | -0.0746 | 0.0952 |
| TRINITY_DN478_c0_g1_i16_orf1 | - | - | - | lipid storage droplets surface-binding protein 2 isoform X1 [Ostrinia furnacalis] | 1.75638 | -0.0341 | -0.6241 | -1.2279 | 0.12968 |
| TRINITY_DN6563_c0_g1_i1_orf1 | - | - | - | cytochrome c oxidase assembly protein COX15 homolog [Ostrinia furnacalis] | 1.80849 | 0.36817 | -0.81 | -0.6396 | -0.7271 |

| | | | | | | | | | |
|---|---|---|---|---|---|---|---|---|---|
| TRINITY_DN18860_c0_g1_i1_orf1 | - | - | - | DDB1- and CUL4-associated factor 13 [Ostrinia furnacalis] | 1.97374 | -0.2054 | -0.4777 | -0.6221 | -0.6685 |
| TRINITY_DN19186_c0_g1_i1_orf1 | - | - | - | 39S ribosomal protein L9, mitochondrial [Ostrinia furnacalis] | 1.93985 | -0.0983 | -0.7125 | -0.3617 | -0.7673 |
| TRINITY_DN1066_c0_g1_i4_orf1 | - | - | - | ribosome biogenesis protein WDR12 homolog [Ostrinia furnacalis] | 1.97275 | -0.3317 | -0.7508 | -0.5834 | -0.3068 |
| TRINITY_DN3649_c0_g1_i6_orf1 | - | - | - | unnamed protein product [Chilo suppressalis] | 1.18088 | 0.79296 | -1.664 | 0.12788 | -0.4378 |
| TRINITY_DN754_c1_g1_i6_orf1 | - | - | - | 28S ribosomal protein S2, mitochondrial [Ostrinia furnacalis] | 1.95042 | -0.3723 | -0.8604 | -0.1865 | -0.5313 |
| TRINITY_DN4056_c0_g1_i8_orf1 | - | - | - | uncharacterized protein LOC114349672 [Ostrinia furnacalis] >XP_028155936.1 uncharacterized protein LOC114349672 [Ostrinia furnacalis] >XP_028155937.1 uncharacterized protein LOC114349672 [Ostrinia furnacalis] >XP_028155939.1 uncharacterized protein LOC114349672 [Ostrinia furnacalis] | 1.55546 | 0.07599 | -1.4031 | -0.6528 | 0.42436 |
| TRINITY_DN43328_c0_g1_i1_orf1 | - | - | - | tubulin--tyrosine ligase-like protein 12 [Ostrinia furnacalis] | 1.14772 | 1.27984 | -1.0402 | -0.6811 | -0.7062 |
| TRINITY_DN11894_c1_g1_i5_orf1 | - | - | - | 39S ribosomal protein L33, mitochondrial [Ostrinia furnacalis] | 1.92046 | -0.4946 | -0.9723 | -0.1295 | -0.324 |
| TRINITY_DN131371_c0_g1_i1_orf1 | - | - | - | golgin subfamily B member 1-like [Ostrinia furnacalis] | 1.44941 | -0.152 | -1.6628 | 0.03349 | 0.33191 |
| TRINITY_DN27979_c0_g1_i2_orf1 | - | - | - | zinc finger CCCH domain-containing protein 15 homolog [Ostrinia furnacalis] | 1.97679 | -0.2092 | -0.6577 | -0.5538 | -0.5561 |
| TRINITY_DN81715_c0_g1_i1_orf1 | - | - | - | gamma-interferon-inducible lysosomal thiol reductase-like [Ostrinia furnacalis] | 1.4531 | 0.93771 | -1.0528 | -0.7222 | -0.6158 |
| TRINITY_DN5275_c0_g1_i1_orf1 | - | - | - | paraplegin [Ostrinia furnacalis] | 1.65307 | 0.49639 | -1.1985 | -0.7327 | -0.2182 |
| TRINITY_DN5417_c0_g1_i1_orf1 | - | - | - | NADH dehydrogenase [ubiquinone] 1 alpha subcomplex subunit 10, mitochondrial isoform X1 [Ostrinia furnacalis] >XP_028175885.1 NADH dehydrogenase [ubiquinone] 1 alpha subcomplex subunit 10, mitochondrial isoform X2 [Ostrinia furnacalis] | 1.99214 | -0.4691 | -0.5945 | -0.58 | -0.3485 |
| TRINITY_DN109144_c0_g1_i5_orf1 | - | - | - | uncharacterized protein LOC126369165 [Pectinophora gossypiella] | 1.92086 | -0.5106 | -0.4332 | -0.05 | -0.9271 |
| TRINITY_DN45924_c0_g1_i14_orf1 | - | - | - | adenylosuccinate synthetase isoform X1 [Ostrinia furnacalis] >XP_028166048.1 adenylosuccinate synthetase isoform X2 [Ostrinia furnacalis] | 1.92542 | -0.3549 | -0.9059 | -0.5826 | -0.082 |
| TRINITY_DN17559_c0_g1_i4_orf1 | - | - | - | GDP-mannose 4,6 dehydratase isoform X2 [Ostrinia furnacalis] >XP_028166204.1 GDP-mannose 4,6 dehydratase isoform X2 [Ostrinia | 1.99223 | -0.4159 | -0.4007 | -0.6445 | -0.5312 |
| TRINITY_DN4434_c0_g1_i7_orf1 | - | - | - | V-type proton ATPase catalytic subunit A [Ostrinia furnacalis] >XP_028155921.1 V-type proton ATPase catalytic subunit A [Ostrinia furnacalis] >XP_028155922.1 V-type proton ATPase catalytic subunit A [Ostrinia furnacalis] >ADP23923.1 V-ATPase subunit A [Ostrinia furnacalis] >ADT80587.1 V-type proton ATPase catalytic subunit A [Ostrinia furnacalis] >CBY05457.1 V-type proton ATPase catalytic subunit A [Ostrinia furnacalis] | 1.99235 | -0.5678 | -0.3791 | -0.4279 | -0.6175 |
| TRINITY_DN11013_c0_g1_i3_orf1 | - | - | - | glutamine:fructose-6-phosphate aminotransferase 1 [Heortia vitessoides] | 1.88702 | 0.11683 | -0.9033 | -0.5946 | -0.506 |
| TRINITY_DN21357_c0_g1_i5_orf1 | - | - | - | 40S ribosomal protein S11 isoform X2 [Ostrinia furnacalis] | 1.82677 | -0.7852 | -1.0013 | -0.1665 | 0.1263 |
| TRINITY_DN9647_c0_g1_i1_orf1 | - | - | - | cytochrome P450 6B2-like [Ostrinia furnacalis] | 1.91207 | -1.0207 | -0.4458 | -0.177 | -0.2686 |
| TRINITY_DN107261_c0_g1_i1_orf1 | - | - | - | ATP synthase subunit g, mitochondrial [Ostrinia furnacalis] | 1.93474 | -0.8575 | -0.1025 | -0.6214 | -0.3534 |
| TRINITY_DN9002_c0_g1_i1_orf1 | - | - | - | membrane magnesium transporter 1 [Ostrinia furnacalis] | 1.89276 | -0.5302 | -0.9348 | -0.5063 | 0.07853 |
| TRINITY_DN3292_c2_g1_i4_orf1 | - | - | - | ribosome biogenesis regulatory protein homolog [Ostrinia furnacalis] | 1.95364 | -0.5583 | -0.3168 | -0.2314 | -0.8472 |
| TRINITY_DN42506_c0_g1_i1_orf1 | - | - | - | 28S ribosomal protein S7, mitochondrial [Ostrinia furnacalis] | 1.88675 | 0.10564 | -0.8039 | -0.3986 | -0.7899 |
| TRINITY_DN886_c0_g2_i4_orf1 | - | - | - | collagenase-like [Ostrinia furnacalis] | 1.85128 | 0.24103 | -0.8835 | -0.5625 | -0.6464 |
| TRINITY_DN63914_c0_g1_i1_orf1 | - | - | - | myophilin-like [Ostrinia furnacalis] | 1.98468 | -0.5822 | -0.3623 | -0.3623 | -0.6779 |
| TRINITY_DN2807_c0_g1_i4_orf1 | - | - | - | FK506-binding protein 59 isoform X1 [Ostrinia furnacalis] | 1.86907 | 0.04397 | -0.9422 | -0.7552 | -0.2156 |
| TRINITY_DN53167_c0_g1_i2_orf1 | - | - | - | uncharacterized protein LOC114359219 [Ostrinia furnacalis] | 1.97755 | -0.3207 | -0.3448 | -0.5973 | -0.7146 |

| | | | | | | | | | |
|---|---|---|---|---|---|---|---|---|---|
| TRINITY_DN30131_c0_g1_i1_orf1 | - | - | - | PREDICTED: 60S ribosomal protein L44 [Amyelois transitella] >XP_021198018.1 60S ribosomal protein L44 [Helicoverpa armigera] >XP_022814294.1 60S ribosomal protein L44 [Spodoptera litura] >XP_026732397.1 60S ribosomal protein L44 [Trichoplusia ni] >XP_026752106.1 60S ribosomal protein L44 [Galleria mellonella] >XP_028158932.1 60S ribosomal protein L44 [Ostrinia furnacalis] >XP_035434364.1 60S ribosomal protein L44 [Spodoptera frugiperda] >XP_035434370.1 60S ribosomal protein L44 [Spodoptera frugiperda] >XP_047019234.1 60S ribosomal protein L44 [Helicoverpa zea] >XP_049868501.1 60S ribosomal protein L44 [Pectinophora gossypiella] >AAM53948.1 ribosomal protein L44 [Choristoneura parallela] >KAF9418375.1 hypothetical protein HW555_004805 [Spodoptera exigua] >RVE50750.1 hypothetical protein evm_004660 [Chilo suppressalis] >CAB3235328.1 unnamed protein product [Arctia plantaginis] >CAB3516516.1 unnamed protein product [Spodoptera littoralis] >CAG9747186.1 unnamed protein product [Diatraea saccharalis] >CAH0581656.1 unnamed protein product [Chrysodeixis includens] | 1.82559 | -0.9982 | -0.8131 | -0.0765 | 0.06221 |
| TRINITY_DN55160_c0_g2_i1_orf1 | - | - | - | esterase FE4-like isoform X2 [Ostrinia furnacalis] | 1.86703 | -0.3335 | 0.05259 | -0.5263 | -1.0598 |
| TRINITY_DN4085_c0_g1_i1_orf1 | - | - | - | Protein TSSC1 [Papilio machaon] | 1.04631 | 1.26488 | -1.1393 | -0.9864 | -0.1856 |
| TRINITY_DN16900_c0_g2_i1_orf1 | - | - | - | uncharacterized oxidoreductase dhs-27-like [Ostrinia furnacalis] | 1.93946 | -0.414 | -0.3416 | -0.2386 | -0.9452 |
| TRINITY_DN19092_c0_g1_i2_orf1 | - | - | - | eukaryotic translation initiation factor 3 subunit L [Ostrinia furnacalis] | 1.94717 | -0.3675 | -0.912 | -0.4309 | -0.2368 |
| TRINITY_DN1803_c0_g1_i3_orf1 | - | - | - | translocator protein-like isoform X1 [Ostrinia furnacalis] >XP_028178947.1 translocator protein-like isoform X1 [Ostrinia furnacalis] | 1.85109 | 0.26992 | -0.695 | -0.6924 | -0.7336 |
| TRINITY_DN1330_c0_g1_i1_orf1 | - | - | - | pancreatic triacylglycerol lipase-like [Ostrinia furnacalis] | 1.93792 | -0.2109 | -0.266 | -0.5546 | -0.9065 |
| TRINITY_DN38424_c0_g1_i1_orf1 | - | - | - | glucose dehydrogenase [FAD, quinone]-like [Ostrinia furnacalis] | 1.00217 | 0.80229 | -1.7053 | -0.518 | 0.41888 |
| TRINITY_DN137_c0_g1_i1_orf1 | - | - | - | 60S ribosomal protein L8 [Cotesia glomerata] >XP_044597650.1 60S ribosomal protein L8 [Cotesia glomerata] >KAG8034499.1 hypothetical protein G9C98_007575 [Cotesia typhae] >CAD6216378.1 GSCOCG00004534001-RA-CDS [Cotesia congregata] >KAH0544237.1 60S ribosomal protein L8 [Cotesia glomerata] >KAH0564528.1 60S ribosomal protein L8 [Cotesia glomerata] >CAG5095185.1 Similar to RpL8: 60S ribosomal protein L8 (Spodoptera frugiperda) [Cotesia congregata] | 1.79823 | -0.589 | -0.9565 | -0.6125 | 0.35974 |
| TRINITY_DN27704_c0_g1_i1_orf1 | - | - | - | PREDICTED: tRNA (guanine-N(7)-)-methyltransferase [Amyelois transitella] | 1.95523 | -0.1843 | -0.8247 | -0.3857 | -0.5605 |
| TRINITY_DN2716_c0_g2_i1_orf1 | - | - | - | eukaryotic translation initiation factor 1A, X-chromosomal [Ostrinia furnacalis] >XP_045445466.1 eukaryotic translation initiation factor 1A, X-chromosomal [Melitaea cinxia] >XP_049867692.1 eukaryotic translation initiation factor 1A, X-chromosomal [Pectinophora gossypiella] >KOB79530.1 Eukaryotic translation initiation factor 1A [Operophtera brumata] >CAH2086435.1 unnamed protein product [Euphydryas editha] >KOB79531.1 Eukaryotic translation initiation factor 1A [Operophtera brumata] | 1.77574 | 0.03682 | -1.2209 | -0.5956 | 0.00396 |
| TRINITY_DN3598_c0_g1_i1_orf1 | - | - | - | esterase FE4-like [Ostrinia furnacalis] | 1.98066 | -0.6502 | -0.3475 | -0.3327 | -0.6502 |
| TRINITY_DN29448_c0_g1_i1_orf1 | - | - | - | 28S ribosomal protein S9, mitochondrial [Ostrinia furnacalis] | 1.8526 | 0.25036 | -0.8406 | -0.6612 | -0.6011 |
| TRINITY_DN76283_c0_g2_i1_orf1 | - | - | - | fatty acid synthase-like [Ostrinia furnacalis] | 1.90023 | -0.9308 | -0.3473 | 0.0119 | -0.634 |
| TRINITY_DN3747_c1_g1_i3_orf1 | - | - | - | unnamed protein product, partial [Iphiclides podalirius] | 1.87287 | -0.4817 | -1.0596 | 0.03742 | -0.369 |
| TRINITY_DN10701_c0_g2_i2_orf1 | - | - | - | synaptosomal-associated protein 29 [Ostrinia furnacalis] | 1.73784 | 0.34735 | -1.0122 | -0.8964 | -0.1766 |
| TRINITY_DN3665_c0_g1_i2_orf1 | - | - | - | regucalcin-like isoform X2 [Ostrinia furnacalis] >XP_028175354.1 regucalcin-like isoform X2 [Ostrinia furnacalis] | 1.98763 | -0.4438 | -0.6631 | -0.5518 | -0.329 |
| TRINITY_DN44219_c0_g1_i1_orf1 | - | - | - | mitochondrial import inner membrane translocase subunit TIM50-C-like [Ostrinia furnacalis] | 1.87094 | 0.11557 | -0.8574 | -0.803 | -0.3262 |
| TRINITY_DN5554_c0_g1_i2_orf1 | - | - | - | double-stranded RNA-binding protein Staufen homolog 2 isoform X5 [Pectinophora gossypiella] | 1.91455 | 0.0332 | -0.5573 | -0.5277 | -0.8627 |
| TRINITY_DN10637_c0_g1_i4_orf1 | - | - | - | V-type proton ATPase subunit d [Bombyx mandarina] | 1.99853 | -0.5455 | -0.4432 | -0.4732 | -0.5366 |
| TRINITY_DN34821_c0_g1_i4_orf1 | - | - | - | acetylcholine receptor subunit alpha-L1-like [Ostrinia furnacalis] | 1.19076 | 1.17849 | -0.5268 | -0.5907 | -1.2517 |

| | | | | | | | | | |
|---|---|---|---|---|---|---|---|---|---|
| TRINITY_DN41697_c0_g1_i1_orf1 | - | - | - | 5-formyltetrahydrofolate cyclo-ligase [Ostrinia furnacalis] | 1.36771 | 0.49628 | 0.36306 | -1.4819 | -0.7452 |
| TRINITY_DN15965_c0_g1_i1_orf1 | - | - | - | U3 small nucleolar RNA-associated protein 15 homolog [Ostrinia furnacalis] | 1.9835 | -0.6068 | -0.5403 | -0.2512 | -0.5853 |
| TRINITY_DN883_c0_g1_i8_orf1 | - | - | - | diacylglycerol O-acyltransferase 1 isoform X1 [Ostrinia furnacalis] | 1.78757 | -0.6052 | -1.0915 | 0.30299 | -0.3938 |
| TRINITY_DN12227_c0_g2_i3_orf1 | - | - | - | exonuclease 3'-5' domain-containing protein 2 [Ostrinia furnacalis] | 1.09503 | 0.60142 | -1.6725 | -0.5784 | 0.55443 |
| TRINITY_DN5087_c0_g1_i6_orf1 | - | - | - | nascent polypeptide-associated complex subunit alpha [Ostrinia furnacalis] >XP_028156807.1 nascent polypeptide-associated complex subunit alpha [Ostrinia furnacalis] >XP_028156808.1 nascent polypeptide-associated complex subunit alpha [Ostrinia furnacalis] | 1.93149 | -0.0137 | -0.6973 | -0.7483 | -0.4722 |
| TRINITY_DN58207_c0_g1_i1_orf1 | - | - | - | 60S ribosomal protein L6 [Ostrinia furnacalis] >XP_028170357.1 60S ribosomal protein L6 [Ostrinia furnacalis] | 1.82444 | -0.9521 | -0.8162 | -0.2486 | 0.19242 |
| TRINITY_DN86956_c0_g5_i1_orf1 | - | - | - | PREDICTED: protein sly1 homolog [Microplitis demolitor] | 1.92897 | 0.00041 | -0.7559 | -0.6845 | -0.4889 |
| TRINITY_DN1617_c0_g1_i5_orf1 | - | - | - | hypothetical protein evm_009822 [Chilo suppressalis] >CAB3525311.1 unnamed protein product [Chilo suppressalis] >CAH0402638.1 unnamed protein product [Chilo suppressalis] | 1.94395 | -0.5586 | -0.4014 | -0.1288 | -0.8552 |
| TRINITY_DN2993_c0_g1_i4_orf1 | - | - | - | heat shock 70 kDa protein cognate 5 [Ostrinia furnacalis] | 1.98517 | -0.29 | -0.6254 | -0.6112 | -0.4586 |
| TRINITY_DN6933_c1_g1_i1_orf1 | - | - | - | Chlorophyll a-b binding protein 40, chloroplastic [Trichinella nelsoni] >KRY99282.1 Chlorophyll a-b binding protein 40, chloroplastic [Trichinella | 1.96756 | -0.1755 | -0.5392 | -0.7321 | -0.5208 |
| TRINITY_DN47389_c0_g1_i2_orf1 | - | - | - | non-specific lipid-transfer protein-like [Ostrinia furnacalis] | 1.89455 | -0.8755 | -0.5648 | 0.10604 | -0.5603 |
| TRINITY_DN28638_c0_g1_i1_orf1 | - | - | - | uncharacterized protein LOC114364075 [Ostrinia furnacalis] | 1.89526 | -1.0128 | -0.4171 | -0.0091 | -0.4562 |
| TRINITY_DN75746_c0_g1_i1_orfp1 | - | - | - | TRINITY_DN75746_c0_g1_i1_m.54871 TRINITY_DN75746_c0_g1::TRINITY_DN75746_c0_g1_i1::g.54871 ORF type:internal len:83 (+),score=-1.09,Polyhedrin PF00738.19 1.7e-25 TRINITY_DN75746_c0_g1_i1:2-247(+) | 1.99033 | -0.4083 | -0.5058 | -0.4023 | -0.674 |
| TRINITY_DN45449_c0_g1_i1_orf1 | - | - | - | ATP-dependent helicase brm [Ostrinia furnacalis] | 1.5129 | 0.42963 | -1.2694 | 0.25022 | -0.9234 |
| TRINITY_DN4977_c0_g1_i2_orf1 | - | - | - | manganese-transporting ATPase 13A1 [Ostrinia furnacalis] | 1.94224 | -0.0579 | -0.7282 | -0.6916 | -0.4645 |
| TRINITY_DN698_c0_g1_i5_orf1 | - | - | - | PREDICTED: small nuclear ribonucleoprotein Sm D3 [Amyelois transitella] | 1.90997 | 0.02404 | -0.849 | -0.6874 | -0.3976 |
| TRINITY_DN10662_c0_g1_i4_orf1 | - | - | - | HD domain-containing protein 2 [Ostrinia furnacalis] | 1.77121 | 0.32943 | -1.1347 | -0.4713 | -0.4947 |
| TRINITY_DN106730_c0_g1_i1_orf1 | - | - | - | Photosystem I reaction center subunit II, chloroplastic, partial [Trichinella zimbabwensis] | 1.93581 | -0.6696 | -0.4554 | -0.0392 | -0.7716 |
| TRINITY_DN9853_c0_g3_i1_orf1 | - | - | - | importin-7 isoform X1 [Ostrinia furnacalis] | 1.93457 | -0.3662 | -0.8898 | -0.5644 | -0.1141 |
| TRINITY_DN15624_c0_g1_i1_orf1 | - | - | - | LOW QUALITY PROTEIN: V-type proton ATPase subunit S1-like [Ostrinia | 1.84277 | 0.20432 | -0.3677 | -0.9322 | -0.7471 |
| TRINITY_DN10722_c0_g3_i1_orf1 | - | - | - | inositol-3-phosphate synthase [Ostrinia furnacalis] | 1.49096 | 0.67682 | -1.3351 | -0.7244 | -0.1083 |
| TRINITY_DN16965_c0_g2_i1_orf1 | - | - | - | hypothetical protein evm_007405 [Chilo suppressalis] | 1.62034 | 0.57414 | -1.2705 | -0.4179 | -0.5061 |
| TRINITY_DN12133_c0_g2_i1_orf1 | - | - | - | O-acyltransferase like protein-like [Ostrinia furnacalis] | 1.97868 | -0.3517 | -0.3188 | -0.61 | -0.6982 |
| TRINITY_DN4116_c0_g1_i3_orf1 | - | - | - | transmembrane protein 131 homolog [Ostrinia furnacalis] | 1.4114 | 0.49505 | -1.4896 | -0.6864 | 0.2696 |
| TRINITY_DN14601_c0_g1_i2_orf1 | - | - | - | unnamed protein product [Chilo suppressalis] | 1.9006 | 0.01421 | -0.9293 | -0.3543 | -0.6313 |
| TRINITY_DN4125_c1_g1_i5_orf1 | - | - | - | angiotensin-converting enzyme-like isoform X2 [Ostrinia furnacalis] | 1.89647 | 0.13945 | -0.692 | -0.7039 | -0.6401 |
| TRINITY_DN7964_c0_g1_i1_orfp1 | - | - | - | TRINITY_DN7964_c0_g1_i1_m.23483 TRINITY_DN7964_c0_g1::TRINITY_DN7964_c0_g1_i1::g.23483 ORF type:internal len:79 (+),score=44.25 TRINITY_DN7964_c0_g1_i1:3-236(+) | 1.96546 | -0.5155 | -0.4195 | -0.2286 | -0.8019 |
| TRINITY_DN14063_c0_g1_i7_orf1 | - | - | - | probable phosphorylase b kinase regulatory subunit beta isoform X1 [Ostrinia furnacalis] >XP_028175664.1 probable phosphorylase b kinase regulatory subunit beta isoform X2 [Ostrinia furnacalis] >XP_028175665.1 probable phosphorylase b kinase regulatory subunit beta isoform X3 [Ostrinia furnacalis] | 1.93883 | -0.7378 | -0.6805 | -0.0388 | -0.4818 |
| TRINITY_DN6535_c0_g2_i1_orf1 | - | - | - | NADH dehydrogenase [ubiquinone] 1 beta subcomplex subunit 3 [Ostrinia furnacalis] >XP_028166399.1 NADH dehydrogenase [ubiquinone] 1 beta subcomplex subunit 3 [Ostrinia furnacalis] | 1.96739 | -0.1887 | -0.5916 | -0.7335 | -0.4536 |
| TRINITY_DN4710_c0_g1_i1_orf1 | - | - | - | hypothetical protein evm_000671 [Chilo suppressalis] | 1.97817 | -0.2757 | -0.4956 | -0.4675 | -0.7393 |
| TRINITY_DN19000_c0_g1_i4_orf1 | - | - | - | NADH dehydrogenase [ubiquinone] 1 beta subcomplex subunit 9 [Ostrinia furnacalis] | 1.98541 | -0.5053 | -0.6986 | -0.4592 | -0.3223 |
| TRINITY_DN130075_c1_g2_i1_orf1 | - | - | - | 60S ribosomal protein L23 [Microtus ochrogaster] | 1.91553 | -0.7584 | 0.06256 | -0.6726 | -0.5472 |

| | | | | | | | | | |
|---|---|---|---|---|---|---|---|---|---|
| TRINITY_DN19361_c0_g1_i7_orf1 | - | - | - | hydroxylysine kinase [Ostrinia furnacalis] >XP_028168144.1 hydroxylysine kinase [Ostrinia furnacalis] | 1.91686 | 0.05746 | -0.659 | -0.765 | -0.5503 |
| TRINITY_DN334_c0_g1_i3_orf1 | - | - | - | chymotrypsin-like serine protease [Ostrinia nubilalis] >AAX62030.1 chymotrypsin-like serine protease [Ostrinia nubilalis] | 1.76288 | 0.47051 | -0.7195 | -0.8172 | -0.6967 |
| TRINITY_DN2082_c0_g1_i2_orf1 | - | - | - | choline-phosphate cytidylyltransferase B-like isoform X1 [Ostrinia furnacalis] | 1.92534 | -0.0191 | -0.8241 | -0.4227 | -0.6595 |
| TRINITY_DN14826_c0_g1_i1_orf1 | - | - | - | uncharacterized protein LOC114350939 [Ostrinia furnacalis] | 1.96595 | -0.505 | -0.7597 | -0.5195 | -0.1818 |
| TRINITY_DN12973_c0_g1_i1_orf1 | - | - | - | mitochondrial-processing peptidase subunit alpha [Ostrinia furnacalis] | 1.99173 | -0.3441 | -0.5218 | -0.6283 | -0.4976 |
| TRINITY_DN3749_c0_g1_i1_orf1 | - | - | - | cytochrome c oxidase subunit 6B1 [Ostrinia furnacalis] | 1.98605 | -0.4396 | -0.4723 | -0.7137 | -0.3605 |
| TRINITY_DN84322_c0_g2_i1_orf1 | - | - | - | alanyl-tRNA synthetase 1 [Homo sapiens] >KAI4055846.1 alanyl-tRNA synthetase 1 [Homo sapiens] | 1.82043 | -0.2294 | -1.1563 | -0.535 | 0.10028 |
| TRINITY_DN7583_c0_g1_i1_orf1 | - | - | - | 39S ribosomal protein L21, mitochondrial [Ostrinia furnacalis] | 1.80569 | 0.34421 | -0.5934 | -0.9478 | -0.6087 |
| TRINITY_DN105901_c0_g1_i2_orfp1 | - | - | - | contactin-like [Pectinophora gossypiella] | 1.90937 | -0.8373 | 0.06594 | -0.5431 | -0.5949 |
| TRINITY_DN4030_c0_g2_i1_orf1 | - | - | - | putative trypsin 6 [Ostrinia nubilalis] | 1.92009 | -0.4356 | -0.0728 | -0.4591 | -0.9526 |
| TRINITY_DN10558_c0_g1_i4_orf1 | - | - | - | unnamed protein product [Chrysodeixis includens] | 0.93463 | 1.04237 | -1.25 | -1.1423 | 0.41533 |
| TRINITY_DN21531_c0_g1_i1_orf1 | - | - | - | viral IAP-associated factor homolog [Ostrinia furnacalis] | 1.7378 | 0.48422 | -0.5588 | -0.99 | -0.6733 |
| TRINITY_DN32022_c0_g1_i1_orf1 | - | - | - | striatin isoform X1 [Diachasma alloeum] | 1.73489 | 0.14115 | -1.22 | -0.6932 | 0.03713 |
| TRINITY_DN42082_c0_g2_i2_orfp1 | - | - | - | TRINITY_DN42082_c0_g2_i2_m.7835 TRINITY_DN42082_c0_g2::TRINITY_DN42082_c0_g2_i2::g.7835 ORF type:internal len:133 (+),score=75.81 TRINITY_DN42082_c0_g2_i2:1-396(+) | 1.80352 | -0.6224 | -1.1011 | -0.3086 | 0.22857 |
| TRINITY_DN46173_c0_g3_i2_orf1 | - | - | - | tropomyosin-1, isoforms 9A/A/B isoform X33 [Aedes aegypti] >EAT46020.1 AAEL002761-PB [Aedes aegypti] | 1.91296 | 0.01061 | -0.8766 | -0.633 | -0.414 |
| TRINITY_DN5019_c0_g1_i2_orf1 | - | - | - | RRP12-like protein isoform X4 [Ostrinia furnacalis] >XP_028175539.1 RRP12-like protein isoform X5 [Ostrinia furnacalis] | 1.98634 | -0.3859 | -0.7192 | -0.4407 | -0.4407 |
| TRINITY_DN58531_c0_g1_i1_orf1 | - | - | - | uncharacterized protein LOC114357371 [Ostrinia furnacalis] >XP_028166768.1 uncharacterized protein LOC114357371 [Ostrinia furnacalis] | 1.52328 | 0.83072 | -1.0856 | -0.6913 | -0.577 |
| TRINITY_DN3028_c0_g1_i1_orf1 | - | - | - | pre-rRNA processing protein FTSJ3 [Ostrinia furnacalis] | 1.95518 | -0.688 | -0.6602 | -0.0988 | -0.5082 |
| TRINITY_DN3457_c0_g1_i4_orf1 | - | - | - | aryl hydrocarbon receptor nuclear translocator homolog [Ostrinia furnacalis] | 1.53826 | 0.3421 | -1.4541 | -0.6077 | 0.18147 |
| TRINITY_DN29934_c0_g1_i6_orf1 | - | - | - | sodium/potassium-transporting ATPase subunit beta-2-like [Ostrinia furnacalis] >XP_028176258.1 sodium/potassium-transporting ATPase subunit beta-2-like [Ostrinia furnacalis] | 1.5612 | 0.29549 | -1.1313 | -1.0455 | 0.32009 |
| TRINITY_DN969_c0_g1_i3_orf1 | - | - | - | protein UBASH3A homolog isoform X3 [Ostrinia furnacalis] | 1.64475 | 0.6359 | -1.0576 | -0.7211 | -0.5019 |
| TRINITY_DN47575_c0_g1_i1_orf1 | - | - | - | PREDICTED: splicing factor 1-like [Fopius arisanus] | 1.70474 | -0.7155 | -1.0387 | -0.476 | 0.52559 |
| TRINITY_DN5841_c0_g1_i2_orf1 | - | - | - | hypothetical protein evm_011295 [Chilo suppressalis] | 1.46851 | 0.7493 | -1.3935 | -0.3973 | -0.427 |
| TRINITY_DN21539_c0_g1_i1_orf1 | - | - | - | probable phenylalanine--tRNA ligase, mitochondrial [Ostrinia furnacalis] | 1.72611 | 0.50185 | -1.0135 | -0.562 | -0.6525 |
| TRINITY_DN21150_c0_g1_i4_orf1 | - | - | - | RNA-binding protein cabeza-like isoform X2 [Bicyclus anynana] | 1.7751 | 0.25961 | -1.1296 | -0.6717 | -0.2334 |
| TRINITY_DN5867_c0_g1_i1_orf1 | - | - | - | NADH dehydrogenase [ubiquinone] 1 alpha subcomplex subunit 7-like [Ostrinia furnacalis] | 1.9465 | -0.0465 | -0.6912 | -0.6229 | -0.5859 |
| TRINITY_DN50571_c1_g1_i1_orf1 | - | - | - | WD repeat-containing protein 46 [Ostrinia furnacalis] | 1.66286 | 0.58588 | -0.6175 | -0.5158 | -1.1155 |
| TRINITY_DN26130_c0_g1_i1_orf1 | - | - | - | membrane alanyl aminopeptidase-like [Ostrinia furnacalis] | 1.87724 | 0.07622 | -0.3258 | -0.6736 | -0.9541 |
| TRINITY_DN4070_c0_g1_i4_orf1 | - | - | - | alpha-N-acetylgalactosaminidase isoform X3 [Ostrinia furnacalis] | 0.96014 | 1.41896 | -1.0681 | -0.835 | -0.4759 |
| TRINITY_DN11657_c0_g1_i2_orf1 | - | - | - | trehalase-1 [Omphisa fuscidentalis] | 1.48657 | 0.89195 | -0.6532 | -1.0621 | -0.6632 |
| TRINITY_DN20118_c0_g1_i4_orfp1 | - | - | - | hypothetical protein HF086_007571 [Spodoptera exigua] | 1.38904 | 0.80564 | -1.4682 | -0.3956 | -0.3309 |
| TRINITY_DN47123_c0_g1_i1_orf1 | - | - | - | WD40 repeat-containing protein SMU1 [Ostrinia furnacalis] | 1.87622 | 0.02523 | -0.9275 | -0.7559 | -0.2181 |
| TRINITY_DN2668_c0_g1_i7_orf1 | - | - | - | unnamed protein product [Chrysodeixis includens] | 1.86427 | -1.159 | -0.3092 | -0.138 | -0.2581 |
| TRINITY_DN109540_c0_g1_i3_orf1 | - | - | - | 4-coumarate--CoA ligase 1-like isoform X4 [Ostrinia furnacalis] | 1.98969 | -0.6604 | -0.398 | -0.3851 | -0.5462 |
| TRINITY_DN18164_c0_g1_i7_orf1 | - | - | - | uncharacterized protein LOC114366518 isoform X5 [Ostrinia furnacalis] | 1.09892 | 1.23678 | -0.9433 | -1.1454 | -0.247 |
| TRINITY_DN37699_c0_g1_i3_orfp1 | - | - | - | TRINITY_DN37699_c0_g1_i3_m.58788 TRINITY_DN37699_c0_g1::TRINITY_DN37699_c0_g1_i3::g.58788 ORF type:internal len:122 (+),score=39.86 TRINITY_DN37699_c0_g1_i3:1-363(+) | 1.76243 | -1.0826 | -0.5884 | -0.4766 | 0.38519 |
| TRINITY_DN21971_c0_g1_i4_orf1 | - | - | - | 40S ribosomal protein S26 [Nymphalis io] | 1.81736 | -0.1169 | -1.195 | -0.5055 | 3.58E-06 |
| TRINITY_DN8019_c0_g1_i4_orf1 | - | - | - | deoxyhypusine hydroxylase [Ostrinia furnacalis] | 1.96715 | -0.2013 | -0.7356 | -0.61 | -0.4203 |

| | | | | | | | | | |
|---|---|---|---|---|---|---|---|---|---|
| TRINITY_DN5925_c0_g1_i5_orf1 | - | - | - | isocitrate dehydrogenase [NAD] subunit gamma, mitochondrial-like isoform X1 [Ostrinia furnacalis] | 1.95934 | -0.5797 | -0.6142 | -0.6608 | -0.1045 |
| TRINITY_DN36538_c0_g1_i2_orf1 | - | - | - | xaa-Pro dipeptidase isoform X1 [Ostrinia furnacalis] >XP_028156507.1 xaa-Pro dipeptidase isoform X2 [Ostrinia furnacalis] | 1.93845 | -0.0965 | -0.7421 | -0.7469 | -0.353 |
| TRINITY_DN17312_c0_g1_i1_orf1 | - | - | - | mRNA cap guanine-N7 methyltransferase [Ostrinia furnacalis] | 1.72448 | 0.40395 | -0.9457 | -0.9583 | -0.2245 |
| TRINITY_DN8261_c0_g1_i1_orf1 | - | - | - | UDP-N-acetylhexosamine pyrophosphorylase-like protein 1 [Ostrinia furnacalis] | 1.90062 | 0.12686 | -0.703 | -0.6733 | -0.6512 |
| TRINITY_DN27848_c0_g1_i2_orf1 | - | - | - | cystathionine beta-synthase-like [Ostrinia furnacalis] >XP_028159011.1 cystathionine beta-synthase-like [Ostrinia furnacalis] | 1.98574 | -0.6619 | -0.3687 | -0.3622 | -0.593 |
| TRINITY_DN389_c0_g1_i2_orf1 | - | - | - | uncharacterized protein LOC118068293 isoform X2 [Chelonus insularis] | 1.41807 | 1.01043 | -0.7755 | -0.8424 | -0.8106 |
| TRINITY_DN1445_c0_g1_i1_orf1 | - | - | - | leucine-rich PPR motif-containing protein, mitochondrial [Ostrinia furnacalis] | 1.95138 | -0.0672 | -0.6487 | -0.6604 | -0.5751 |
| TRINITY_DN12442_c0_g1_i4_orf1 | - | - | - | midasin-like [Ostrinia furnacalis] | 1.77141 | -0.4939 | -0.9434 | 0.41275 | -0.7469 |
| TRINITY_DN1294_c0_g1_i3_orf1 | - | - | - | 46 kDa FK506-binding nuclear protein-like isoform X1 [Ostrinia furnacalis] >XP_028157904.1 46 kDa FK506-binding nuclear protein-like isoform X2 [Ostrinia furnacalis] | 1.97293 | -0.213 | -0.7178 | -0.5655 | -0.4766 |
| TRINITY_DN16482_c0_g1_i6_orf1 | - | - | - | transmembrane protein 120 homolog isoform X2 [Ostrinia furnacalis] | 1.93117 | -0.2009 | -0.9105 | -0.5904 | -0.2293 |
| TRINITY_DN35635_c0_g1_i1_orf1 | - | - | - | probable NADH dehydrogenase [ubiquinone] 1 alpha subcomplex subunit 12 [Ostrinia furnacalis] | 1.88856 | -0.0226 | -0.5404 | -1.0249 | -0.3006 |
| TRINITY_DN3053_c0_g1_i2_orf1 | - | - | - | prostaglandin reductase 1-like [Ostrinia furnacalis] | 1.9485 | -0.7315 | -0.7192 | -0.3653 | -0.1325 |
| TRINITY_DN13216_c0_g1_i5_orf1 | - | - | - | uncharacterized protein LOC114358344 isoform X1 [Ostrinia furnacalis] | 1.3129 | 1.13177 | -0.7701 | -0.827 | -0.8476 |
| TRINITY_DN4944_c0_g1_i2_orf1 | - | - | - | bifunctional glutamate/proline--tRNA ligase [Ostrinia furnacalis] | 1.95492 | -0.2292 | -0.7613 | -0.6837 | -0.2808 |
| TRINITY_DN129226_c0_g1_i2_orf1 | - | - | - | hypothetical protein evm_000268 [Chilo suppressalis] | 1.36997 | 0.9096 | -1.3617 | -0.3582 | -0.5597 |
| TRINITY_DN21909_c0_g1_i1_orf1 | - | - | - | complement component 1 Q subcomponent-binding protein, mitochondrial [Ostrinia furnacalis] | 1.99365 | -0.4053 | -0.6127 | -0.5575 | -0.4181 |
| TRINITY_DN2704_c0_g1_i5_orf1 | - | - | - | hypothetical protein evm_009002 [Chilo suppressalis] | 1.94253 | -0.1027 | -0.447 | -0.5444 | -0.8484 |
| TRINITY_DN43369_c0_g2_i1_orf1 | - | - | - | cytochrome P450 monooxygenase 304 [Glyphodes pyloalis] | 1.35978 | 0.32536 | -0.7602 | 0.54735 | -1.4723 |
| TRINITY_DN44557_c0_g2_i1_orf1 | - | - | - | serine hydrolase-like protein [Ostrinia furnacalis] | 1.85566 | 0.03643 | -1.1188 | -0.4337 | -0.3395 |
| TRINITY_DN6248_c0_g1_i1_orf1 | - | - | - | DNA topoisomerase I, mitochondrial [Ostrinia furnacalis] | 1.77612 | 0.44497 | -0.7712 | -0.753 | -0.6968 |
| TRINITY_DN22815_c0_g1_i2_orf1 | - | - | - | acyl carrier protein, mitochondrial isoform X1 [Ostrinia furnacalis] | 1.98357 | -0.6872 | -0.5099 | -0.5026 | -0.2838 |
| TRINITY_DN620_c0_g1_i4_orf1 | - | - | - | lysine--tRNA ligase isoform X1 [Ostrinia furnacalis] | 1.92092 | -0.6375 | -0.8944 | -0.3128 | -0.0762 |
| TRINITY_DN20749_c0_g1_i3_orf1 | - | - | - | protein arginine N-methyltransferase 1-like [Ostrinia furnacalis] | 1.94267 | -0.5412 | -0.872 | -0.3925 | -0.1371 |
| TRINITY_DN47_c0_g1_i2_orf1 | - | - | - | uncharacterized protein LOC114356437 isoform X1 [Ostrinia furnacalis] | 1.87847 | -0.8521 | -0.0785 | -0.0932 | -0.8547 |
| TRINITY_DN7112_c0_g1_i1_orf1 | - | - | - | heterogeneous nuclear ribonucleoprotein K isoform X2 [Ostrinia furnacalis] | 1.44205 | 0.65261 | -1.286 | -0.9112 | 0.10254 |
| TRINITY_DN24399_c0_g1_i1_orf1 | - | - | - | retinol-binding protein pinta-like [Ostrinia furnacalis] | 1.87922 | -0.7622 | -0.4453 | -0.8174 | 0.14563 |
| TRINITY_DN1079_c0_g1_i4_orf1 | - | - | - | CD109 antigen-like [Ostrinia furnacalis] | 1.98283 | -0.3983 | -0.5975 | -0.3124 | -0.6746 |
| TRINITY_DN51045_c0_g1_i1_orf1 | - | - | - | cell growth-regulating nucleolar protein [Ostrinia furnacalis] | 1.95359 | -0.0762 | -0.6324 | -0.5849 | -0.66 |
| TRINITY_DN100821_c0_g1_i1_orf1 | - | - | - | putative GMP synthase, partial [Operophtera brumata] | 1.86537 | -0.4541 | -1.0627 | -0.4233 | 0.07477 |
| TRINITY_DN1239_c0_g1_i3_orf1 | - | - | - | uncharacterized protein LOC114355269 [Ostrinia furnacalis] >XP_028163822.1 uncharacterized protein LOC114355269 [Ostrinia furnacalis] | 1.89951 | -0.6532 | -0.9598 | -0.1047 | -0.1818 |
| TRINITY_DN3401_c0_g1_i1_orf1 | - | - | - | 28S ribosomal protein S5, mitochondrial [Ostrinia furnacalis] | 1.98161 | -0.5513 | -0.5319 | -0.2458 | -0.6527 |
| TRINITY_DN11153_c0_g1_i1_orf1 | - | - | - | unnamed protein product [Chilo suppressalis] | 1.96363 | -0.4768 | -0.8392 | -0.2864 | -0.3612 |
| TRINITY_DN14967_c0_g2_i1_orf1 | - | - | - | glyceraldehyde-3-phosphate dehydrogenase 2 [Holotrichia oblita] | 1.98902 | -0.5264 | -0.3562 | -0.4354 | -0.671 |
| TRINITY_DN86844_c0_g2_i1_orf1 | - | - | - | spermine oxidase-like isoform X1 [Ostrinia furnacalis] | 1.93378 | -0.8474 | -0.4475 | -0.5821 | -0.0567 |
| TRINITY_DN3503_c0_g1_i1_orfp1 | - | - | - | uncharacterized protein LOC114356429 [Ostrinia furnacalis] | 1.96203 | -0.2999 | -0.6276 | -0.2608 | -0.7737 |
| TRINITY_DN39532_c0_g1_i1_orf1 | - | - | - | hypothetical protein evm_009649 [Chilo suppressalis] | 1.73233 | 0.36931 | -1.1996 | -0.3595 | -0.5425 |
| TRINITY_DN760_c1_g2_i6_orf1 | - | - | - | ADP,ATP carrier protein [Pieris napi] | 1.97327 | -0.6802 | -0.2227 | -0.6378 | -0.4326 |
| TRINITY_DN92232_c0_g1_i1_orf1 | - | - | - | protein SDA1 homolog [Ostrinia furnacalis] | 1.94163 | -0.4361 | -0.5285 | -0.1121 | -0.8648 |
| TRINITY_DN1557_c0_g1_i9_orf1 | - | - | - | carboxylesterase CXE18 [Ostrinia furnacalis] | 1.91218 | -0.8637 | -0.6434 | -0.428 | 0.02286 |
| TRINITY_DN4842_c0_g1_i5_orf1 | - | - | - | cytochrome c oxidase assembly factor 4 homolog, mitochondrial isoform X1 [Ostrinia furnacalis] >XP_028162331.1 cytochrome c oxidase assembly factor 4 homolog, mitochondrial isoform X2 [Ostrinia furnacalis] | 1.92861 | -0.0615 | -0.6086 | -0.8684 | -0.3902 |
| TRINITY_DN1882_c0_g1_i4_orf1 | - | - | - | zinc transporter ZIP13 homolog [Ostrinia furnacalis] | 1.26847 | -0.239 | -1.7067 | 0.02889 | 0.64832 |

| | | | | | | | | |
|---|---|---|---|---|---|---|---|---|
| TRINITY_DN2117_c0_g1_i1_orf1 | - | - | - | BUB3-interacting and GLEBS motif-containing protein ZNF207 [Chelonus | 1.75429 | 0.30715 | -1.0418 | -0.8438 | -0.1759 |
| TRINITY_DN20294_c0_g2_i1_orf1 | - | - | - | cytochrome b-c1 complex subunit 2, mitochondrial isoform X1 [Ostrinia furnacalis] >XP_028170208.1 cytochrome b-c1 complex subunit 2, mitochondrial isoform X2 [Ostrinia furnacalis] | 1.98754 | -0.39 | -0.5705 | -0.6631 | -0.364 |
| TRINITY_DN20346_c0_g1_i1_orf1 | - | - | - | NADH dehydrogenase [ubiquinone] 1 alpha subcomplex subunit 6 [Ostrinia furnacalis] | 1.97405 | -0.2693 | -0.5776 | -0.3879 | -0.7393 |
| TRINITY_DN14937_c0_g1_i7_orf1 | - | - | - | multidrug resistance protein homolog 49-like [Ostrinia furnacalis] >XP_028159925.1 multidrug resistance protein homolog 49-like [Ostrinia furnacalis] | 1.89399 | -0.4111 | -0.6761 | 0.07682 | -0.8836 |
| TRINITY_DN52861_c0_g1_i1_orf1 | - | - | - | protein MAK16 homolog A [Ostrinia furnacalis] | 1.97575 | -0.3298 | -0.7439 | -0.568 | -0.334 |
| TRINITY_DN96170_c0_g1_i1_orf1 | - | - | - | uncharacterized protein LOC114355569 [Ostrinia furnacalis] | 1.98188 | -0.6467 | -0.5858 | -0.2523 | -0.4971 |
| TRINITY_DN3647_c2_g1_i3_orf1 | - | - | - | unnamed protein product, partial [Iphiclides podalirius] | 1.54417 | 0.76116 | -1.1342 | -0.4066 | -0.7645 |
| TRINITY_DN787_c0_g1_i7_orf1 | - | - | - | YLP motif-containing protein 1-like isoform X1 [Ostrinia furnacalis] | 1.65675 | 0.61119 | -1.0159 | -0.4447 | -0.8073 |
| TRINITY_DN2673_c0_g3_i1_orf1 | - | - | - | uncharacterized protein LOC114361372 [Ostrinia furnacalis] | 1.78896 | 0.021 | -1.2088 | -0.0201 | -0.5811 |
| TRINITY_DN6439_c0_g1_i1_orf1 | - | - | - | GPI mannosyltransferase 3 isoform X4 [Ostrinia furnacalis] >XP_028164836.1 GPI mannosyltransferase 3 isoform X5 [Ostrinia furnacalis] | 1.63769 | 0.56421 | -1.208 | -0.6494 | -0.3445 |
| TRINITY_DN7920_c0_g1_i2_orf1 | - | - | - | uncharacterized protein LOC114357268 [Ostrinia furnacalis] >XP_028166599.1 uncharacterized protein LOC114357268 [Ostrinia furnacalis] | 1.88788 | -0.344 | -0.2908 | -0.1532 | -1.0998 |
| TRINITY_DN3428_c0_g1_i1_orf1 | - | - | - | 10 kDa heat shock protein, mitochondrial [Ostrinia furnacalis] | 1.92255 | -0.12 | -0.8791 | -0.6742 | -0.2493 |
| TRINITY_DN8366_c0_g1_i4_orf1 | - | - | - | luciferin 4-monooxygenase-like [Ostrinia furnacalis] | 1.93493 | -0.6615 | -0.3388 | -0.102 | -0.8326 |
| TRINITY_DN25856_c0_g1_i1_orf1 | - | - | - | myrosinase 1-like [Ostrinia furnacalis] | 1.97517 | -0.7425 | -0.5373 | -0.4398 | -0.2556 |
| TRINITY_DN37538_c0_g4_i1_orf1 | - | - | - | esterase FE4-like [Ostrinia furnacalis] | 1.89464 | 0.13013 | -0.7164 | -0.544 | -0.7643 |
| TRINITY_DN3476_c0_g1_i5_orf1 | - | - | - | maltase A1-like [Ostrinia furnacalis] | 1.96415 | -0.8013 | -0.2413 | -0.3682 | -0.5534 |
| TRINITY_DN37538_c0_g2_i1_orf1 | - | - | - | esterase FE4-like [Ostrinia furnacalis] | 1.97287 | -0.2306 | -0.4557 | -0.545 | -0.7415 |
| TRINITY_DN4895_c0_g1_i2_orf1 | - | - | - | coiled-coil domain-containing protein 86 [Ostrinia furnacalis] | 1.96505 | -0.8374 | -0.4703 | -0.3359 | -0.3214 |
| TRINITY_DN145647_c0_g1_i1_orf1 | - | - | - | PREDICTED: U6 snRNA-associated Sm-like protein LSm5 isoform X1 [Fopius arisanus] | 1.97577 | -0.2413 | -0.6288 | -0.4274 | -0.6783 |
| TRINITY_DN3847_c1_g1_i1_orf1 | - | - | - | ribosome production factor 2 homolog [Ostrinia furnacalis] | 1.97149 | -0.3562 | -0.3434 | -0.4633 | -0.8085 |
| TRINITY_DN12242_c0_g1_i5_orf1 | - | - | - | heterogeneous nuclear ribonucleoprotein 87F-like isoform X1 [Vanessa tameamea] >XP_046967652.1 heterogeneous nuclear ribonucleoprotein 87F-like isoform X1 [Vanessa cardui] >XP_047532045.1 heterogeneous nuclear ribonucleoprotein 87F-like [Vanessa atalanta] | 1.60535 | 0.71703 | -0.888 | -0.931 | -0.5033 |
| TRINITY_DN2600_c0_g1_i7_orf1 | - | - | - | mucin-5AC isoform X2 [Ostrinia furnacalis] | 1.12898 | 1.29529 | -1.0483 | -0.6562 | -0.7197 |
| TRINITY_DN10636_c0_g1_i1_orf1 | - | - | - | unnamed protein product [Arctia plantaginis] >CAB3253774.1 unnamed protein product [Arctia plantaginis] | 1.94263 | -0.724 | -0.6281 | -0.5532 | -0.0373 |
| TRINITY_DN27087_c0_g1_i1_orf1 | - | - | - | 2',5'-phosphodiesterase 12 [Ostrinia furnacalis] | 1.90819 | -0.6689 | -0.9019 | -0.0253 | -0.3121 |
| TRINITY_DN15607_c0_g1_i6_orf1 | - | - | - | protein artichoke-like [Ostrinia furnacalis] | 1.92895 | -0.489 | -0.803 | -0.6286 | -0.0083 |
| TRINITY_DN12495_c0_g1_i2_orf1 | - | - | - | probable ATP-dependent RNA helicase pitchoune [Manduca sexta] >KAG6441249.1 hypothetical protein O3G_MSEX001749 [Manduca sexta] | 1.98431 | -0.607 | -0.619 | -0.4873 | -0.271 |
| TRINITY_DN23926_c0_g1_i4_orf1 | - | - | - | programmed cell death protein 10 [Ostrinia furnacalis] | 1.46054 | 0.6023 | -1.5383 | -0.253 | -0.2715 |
| TRINITY_DN279_c0_g1_i10_orf1 | - | - | - | RE1-silencing transcription factor-like isoform X1 [Ostrinia furnacalis] | 1.10248 | 1.25413 | -1.2477 | -0.6961 | -0.4128 |
| TRINITY_DN10530_c0_g1_i1_orf1 | - | - | - | cytochrome c oxidase subunit NDUFA4 [Ostrinia furnacalis] | 1.99387 | -0.4433 | -0.5228 | -0.6289 | -0.3989 |
| TRINITY_DN1173_c1_g1_i9_orf1 | - | - | - | hypothetical protein evm_001011 [Chilo suppressalis] | 1.9302 | -0.8788 | -0.6485 | -0.1908 | -0.212 |
| TRINITY_DN3014_c0_g1_i4_orf1 | - | - | - | putative inorganic phosphate cotransporter isoform X1 [Ostrinia furnacalis] | 1.82151 | -1.2258 | -0.3304 | 4.67E-05 | -0.2654 |
| TRINITY_DN327_c1_g1_i4_orf1 | - | - | - | mitochondrial import receptor subunit TOM40 homolog 1-like [Ostrinia | 1.98302 | -0.3525 | -0.7373 | -0.4666 | -0.4266 |
| TRINITY_DN7336_c0_g1_i13_orf1 | - | - | - | PREDICTED: calcium-transporting ATPase sarcoplasmic/endoplasmic reticulum type isoform X2 [Amyelois transitella] | 1.87001 | -1.0784 | -0.5051 | -0.2914 | 0.0049 |
| TRINITY_DN2579_c0_g1_i7_orf1 | - | - | - | aminopeptidase N5 [Ostrinia nubilalis] | 1.99861 | -0.4514 | -0.5528 | -0.5271 | -0.4673 |
| TRINITY_DN53167_c0_g1_i3_orf1 | - | - | - | uncharacterized protein LOC114359219 [Ostrinia furnacalis] | 1.97968 | -0.7265 | -0.5184 | -0.4522 | -0.2826 |
| TRINITY_DN8536_c0_g1_i2_orf1 | - | - | - | PC4 and SFRS1-interacting protein isoform X4 [Galleria mellonella] | 1.52606 | 0.71648 | -1.2945 | -0.6018 | -0.3463 |
| TRINITY_DN24317_c0_g1_i7_orf1 | - | - | - | peptidyl-tRNA hydrolase ICT1, mitochondrial [Ostrinia furnacalis] | 1.94474 | -0.1791 | -0.862 | -0.3203 | -0.5833 |

| | | | | | | | | | |
|---|---|---|---|---|---|---|---|---|---|
| TRINITY_DN28152_c0_g1_i1_orf1 | - | - | - | mitochondrial import inner membrane translocase subunit Tim29 [Ostrinia furnacalis] | 1.99052 | -0.3924 | -0.5645 | -0.3924 | -0.6413 |
| TRINITY_DN948_c0_g1_i1_orf1 | - | - | - | mitochondrial-processing peptidase subunit beta [Ostrinia furnacalis] | 1.98539 | -0.3907 | -0.6831 | -0.5641 | -0.3475 |
| TRINITY_DN9119_c0_g1_i3_orf1 | - | - | - | actin-binding Rho-activating protein [Helicoverpa armigera] >XP_047029227.1 actin-binding Rho-activating protein-like [Helicoverpa zea] >PZC85229.1 hypothetical protein B5X24_HaOG202414 [Helicoverpa armigera] | 1.97468 | -0.3148 | -0.3193 | -0.6932 | -0.6474 |
| TRINITY_DN63662_c0_g4_i1_orf1 | - | - | - | polyadenylate-binding protein 1 [Ostrinia furnacalis] | 1.91891 | 0.00959 | -0.8612 | -0.5906 | -0.4768 |
| TRINITY_DN6710_c0_g1_i6_orf1 | - | - | - | multiple C2 and transmembrane domain-containing protein-like [Ostrinia furnacalis] | 1.40229 | 0.8962 | -0.9766 | -1.1098 | -0.2121 |
| TRINITY_DN2062_c0_g1_i9_orf1 | - | - | - | uncharacterized protein LOC114350846 [Ostrinia furnacalis] | 1.79554 | 0.17204 | -1.2069 | -0.3636 | -0.3971 |
| TRINITY_DN46409_c0_g1_i1_orf1 | - | - | - | unnamed protein product [Heterotrigona itama] | 1.901 | -0.0372 | -0.2325 | -0.8067 | -0.8247 |
| TRINITY_DN32601_c0_g1_i2_orf1 | - | - | - | uncharacterized protein LOC114363197 [Ostrinia furnacalis] | 1.08832 | 1.33724 | -1.0091 | -0.6537 | -0.7628 |
| TRINITY_DN8833_c0_g1_i1_orf1 | - | - | - | nucleolar protein 16 [Ostrinia furnacalis] | 1.97216 | -0.4673 | -0.7203 | -0.2111 | -0.5735 |
| TRINITY_DN220_c0_g1_i3_orf1 | - | - | - | serine-arginine protein 55 isoform X6 [Pieris brassicae] | 1.52846 | 0.67164 | -1.1942 | -0.8775 | -0.1284 |
| TRINITY_DN54543_c0_g5_i2_orf1 | - | - | - | reactive oxygen species modulator 1 [Papilio machaon] >XP_022123076.1 reactive oxygen species modulator 1 [Pieris rapae] >XP_022820703.1 reactive oxygen species modulator 1 [Spodoptera litura] >XP_028176790.1 reactive oxygen species modulator 1 [Ostrinia furnacalis] >XP_030021418.1 reactive oxygen species modulator 1 [Manduca sexta] >XP_034836896.1 reactive oxygen species modulator 1 [Maniola hyperantus] >XP_035446679.1 reactive oxygen species modulator 1-like [Spodoptera frugiperda] >XP_045458971.1 reactive oxygen species modulator 1 [Melitaea cinxia] >XP_045521377.1 reactive oxygen species modulator 1 [Pieris brassicae] >XP_045765649.1 reactive oxygen species modulator 1 [Maniola jurtina] >XP_047042173.1 reactive oxygen species modulator 1 [Helicoverpa zea] >XP_049691668.1 reactive oxygen species modulator 1 [Helicoverpa armigera] >KAF9414699.1 hypothetical protein HW555_007477 [Spodoptera exigua] >KPI93862.1 Reactive oxygen species modulator 1 [Papilio xuthus] >CAB3515149.1 unnamed protein product [Spodoptera littoralis] >CAG5021695.1 unnamed protein product [Parnassius apollo] >KAF9823017.1 hypothetical protein SFRURICE_018191 [Spodoptera frugiperda] | 1.98059 | -0.2781 | -0.5416 | -0.4526 | -0.7084 |
| TRINITY_DN17738_c0_g1_i2_orf1 | - | - | - | unnamed protein product [Diatraea saccharalis] | 1.97855 | -0.4225 | -0.7416 | -0.5194 | -0.295 |
| TRINITY_DN18036_c0_g1_i7_orf1 | - | - | - | pentatricopeptide repeat-containing protein 2, mitochondrial-like [Ostrinia furnacalis] | 1.96512 | -0.1594 | -0.7207 | -0.5917 | -0.4932 |
| TRINITY_DN17905_c0_g3_i1_orf1 | - | - | - | zinc finger protein 706-like [Ostrinia furnacalis] >XP_028176219.1 zinc finger protein 706-like [Ostrinia furnacalis] >XP_028176220.1 zinc finger protein 706-like [Ostrinia furnacalis] >XP_028176221.1 zinc finger protein 706-like [Ostrinia furnacalis] | 1.87486 | 0.20371 | -0.65 | -0.7297 | -0.6989 |
| TRINITY_DN24873_c0_g1_i4_orf1 | - | - | - | uncharacterized protein LOC114365742 [Ostrinia furnacalis] | 1.94187 | -0.7457 | -0.7602 | -0.2093 | -0.2266 |
| TRINITY_DN121893_c0_g1_i1_orf1 | - | - | - | hypothetical protein, partial [Ostrinia furnacalis] | 1.60456 | -0.6275 | -1.3873 | 0.09828 | 0.31197 |
| TRINITY_DN1707_c0_g1_i1_orf1 | - | - | - | inositol oxygenase-like [Ostrinia furnacalis] | 1.91496 | -0.5994 | -0.064 | -0.3205 | -0.9311 |
| TRINITY_DN69236_c0_g1_i1_orf1 | - | - | - | peroxiredoxin [Ostrinia furnacalis] | 1.46662 | 0.75485 | -1.3716 | -0.2894 | -0.5604 |
| TRINITY_DN49265_c0_g3_i2_orf1 | - | - | - | cytochrome c [Ostrinia furnacalis] >XP_028160278.1 cytochrome c [Ostrinia furnacalis] | 1.96888 | -0.5521 | -0.3943 | -0.7763 | -0.2462 |
| TRINITY_DN21494_c0_g1_i2_orf1 | - | - | - | pancreatic triacylglycerol lipase-like [Ostrinia furnacalis] | 1.972 | -0.2397 | -0.7201 | -0.6131 | -0.3991 |
| TRINITY_DN6308_c0_g1_i6_orf1 | - | - | - | myc box-dependent-interacting protein 1 isoform X2 [Ostrinia furnacalis] | 1.92338 | -0.0288 | -0.8524 | -0.6437 | -0.3984 |
| TRINITY_DN5956_c1_g1_i5_orf1 | - | - | - | uncharacterized protein DDB_G0286299-like [Ostrinia furnacalis] >XP_028170990.1 uncharacterized protein DDB_G0286299-like [Ostrinia furnacalis] >XP_028170991.1 uncharacterized protein DDB_G0286299-like | 1.97889 | -0.6636 | -0.5616 | -0.2282 | -0.5256 |
| TRINITY_DN259_c0_g1_i8_orf1 | - | - | - | hypothetical protein evm_000095 [Chilo suppressalis] | 1.86926 | 0.07717 | -1.044 | -0.4886 | -0.4138 |
| TRINITY_DN2594_c0_g2_i4_orf1 | - | - | - | isocitrate dehydrogenase [NAD] subunit beta, mitochondrial isoform X2 [Ostrinia furnacalis] | 1.95928 | -0.1497 | -0.5339 | -0.7794 | -0.4963 |

| | | | | | | | | | |
|---|---|---|---|---|---|---|---|---|---|
| TRINITY_DN7073_c0_g1_i1_orf1 | - | - | - | unnamed protein product, partial [Brenthis ino] | 1.95935 | -0.6757 | -0.74 | -0.3391 | -0.2046 |
| TRINITY_DN3082_c1_g1_i7_orf1 | - | - | - | ribosomal RNA processing protein 1 homolog [Ostrinia furnacalis] | 1.96907 | -0.2038 | -0.5882 | -0.4428 | -0.7343 |
| TRINITY_DN501_c1_g1_i1_orf1 | - | - | - | sodium- and chloride-dependent GABA transporter ine isoform X1 [Ostrinia furnacalis] | 1.96809 | -0.4233 | -0.8267 | -0.4146 | -0.3035 |
| TRINITY_DN79804_c0_g1_i1_orf1 | - | - | - | zinc finger protein on ecdysone puffs-like [Ostrinia furnacalis] | 1.88466 | 0.08592 | -0.9569 | -0.5818 | -0.4319 |
| TRINITY_DN64510_c0_g1_i1_orf1 | - | - | - | 39S ribosomal protein L15, mitochondrial [Ostrinia furnacalis] | 1.98979 | -0.4094 | -0.6869 | -0.4139 | -0.4796 |
| TRINITY_DN15046_c0_g1_i8_orf1 | - | - | - | epidermal retinol dehydrogenase 2-like isoform X1 [Ostrinia furnacalis] >XP_028169999.1 epidermal retinol dehydrogenase 2-like isoform X2 [Ostrinia furnacalis] | 1.9074 | -0.7147 | -0.0023 | -0.3283 | -0.8621 |
| TRINITY_DN14313_c0_g1_i1_orf1 | - | - | - | 25S rRNA (cytosine-C(5))-methyltransferase nop2 [Ostrinia furnacalis] | 1.93983 | -0.0294 | -0.7472 | -0.6076 | -0.5556 |
| TRINITY_DN7964_c0_g1_i6_orfp1 | - | - | - | TRINITY_DN7964_c0_g1_i6_m.23478 TRINITY_DN7964_c0_g1::TRINITY_DN7964_c0_g1_i6::g.23478 ORF type:internal len:87 (+),score=67.94 TRINITY_DN7964_c0_g1_i6:2-259(+) | 1.93157 | -0.9043 | -0.0885 | -0.5066 | -0.4321 |
| TRINITY_DN25783_c0_g1_i2_orf1 | - | - | - | SET and MYND domain-containing protein 4 [Ostrinia furnacalis] | 1.58295 | 0.37817 | -1.47 | -0.4323 | -0.0588 |
| TRINITY_DN29440_c1_g1_i4_orf1 | - | - | - | neutral lipase [Helicoverpa armigera] | 1.89651 | 0.04622 | -0.339 | -0.7916 | -0.8122 |
| TRINITY_DN11347_c0_g1_i1_orf1 | - | - | - | N(4)-(Beta-N-acetylglucosaminyl)-L-asparaginase-like [Ostrinia furnacalis] | 1.95032 | -0.2416 | -0.3113 | -0.519 | -0.8784 |
| TRINITY_DN14436_c0_g1_i7_orf1 | - | - | - | V-type proton ATPase subunit C [Vanessa cardui] | 1.95242 | -0.0831 | -0.7087 | -0.5285 | -0.6322 |
| TRINITY_DN1775_c0_g1_i3_orf1 | - | - | - | ATP-dependent RNA helicase dbp2-like isoform X1 [Ostrinia furnacalis] | 1.74393 | 0.50522 | -0.6691 | -0.7289 | -0.8511 |
| TRINITY_DN56110_c0_g1_i1_orf1 | - | - | - | pescadillo homolog [Ostrinia furnacalis] | 1.98418 | -0.5831 | -0.6297 | -0.2653 | -0.5061 |
| TRINITY_DN3134_c0_g1_i1_orf1 | - | - | - | cytochrome c oxidase subunit 6C-1 isoform X1 [Hyposmocoma kahamanoa] | 1.99001 | -0.4663 | -0.5063 | -0.6645 | -0.3529 |
| TRINITY_DN1505_c0_g1_i1_orf1 | - | - | - | uncharacterized protein LOC114362816 isoform X1 [Ostrinia furnacalis] >XP_028174154.1 uncharacterized protein LOC114362816 isoform X2 [Ostrinia furnacalis] | 1.98192 | -0.2387 | -0.617 | -0.5701 | -0.5562 |
| TRINITY_DN2574_c0_g1_i5_orf1 | - | - | - | prion-like-(Q/N-rich) domain-bearing protein 25 [Ostrinia furnacalis] >XP_028158239.1 prion-like-(Q/N-rich) domain-bearing protein 25 [Ostrinia furnacalis] >XP_028158240.1 prion-like-(Q/N-rich) domain-bearing protein 25 [Ostrinia furnacalis] >XP_028158241.1 prion-like-(Q/N-rich) domain-bearing protein 25 [Ostrinia furnacalis] | 1.9608 | -0.776 | -0.4478 | -0.1673 | -0.5697 |
| TRINITY_DN3929_c0_g3_i3_orf1 | - | - | - | Glutathione S-transferase 1, isoform D [Papilio machaon] | 1.77881 | -1.0517 | -0.7285 | 0.31614 | -0.3147 |
| TRINITY_DN6685_c0_g1_i8_orf1 | - | - | - | cleft lip and palate transmembrane protein 1 homolog [Ostrinia furnacalis] | 1.85157 | 0.08722 | -0.9576 | -0.7784 | -0.2027 |
| TRINITY_DN56910_c0_g2_i1_orf1 | - | - | - | mitochondrial ribonuclease P protein 1 homolog [Ostrinia furnacalis] | 1.9406 | -0.0833 | -0.7537 | -0.7101 | -0.3935 |
| TRINITY_DN4923_c0_g1_i4_orf1 | - | - | - | O-acyltransferase like protein-like [Ostrinia furnacalis] | 1.96916 | -0.6126 | -0.5331 | -0.1628 | -0.6606 |
| TRINITY_DN17351_c0_g1_i3_orf1 | - | - | - | V-type proton ATPase subunit F [Ostrinia furnacalis] | 1.9829 | -0.7036 | -0.4691 | -0.2938 | -0.5163 |
| TRINITY_DN34536_c0_g1_i6_orf1 | - | - | - | clustered mitochondria protein homolog isoform X2 [Ostrinia furnacalis] | 1.98501 | -0.3087 | -0.6907 | -0.4642 | -0.5215 |
| TRINITY_DN747_c0_g1_i4_orf1 | - | - | - | trypsin, alkaline C-like [Ostrinia furnacalis] | 1.95186 | -0.6988 | -0.6398 | -0.078 | -0.5353 |
| TRINITY_DN108122_c0_g1_i9_orf1 | - | - | - | hypothetical protein SFRUCORN_003152 [Spodoptera frugiperda] | 1.93048 | -0.585 | -0.9152 | -0.1944 | -0.2359 |
| TRINITY_DN1154_c0_g1_i1_orf1 | - | - | - | calexcitin-1-like [Ostrinia furnacalis] >ADK94879.2 juvenile hormone diol kinase [Ostrinia furnacalis] | 1.88964 | -0.9055 | -0.0092 | -0.2286 | -0.7463 |
| TRINITY_DN430_c0_g1_i5_orf1 | - | - | - | hypothetical protein NE865_02252 [Phthorimaea operculella] | 1.89012 | -0.545 | -1.0312 | -0.2512 | -0.0627 |
| TRINITY_DN6231_c0_g1_i6_orf1 | - | - | - | ran-binding protein 3 isoform X1 [Ostrinia furnacalis] >XP_028166372.1 ran-binding protein 3 isoform X2 [Ostrinia furnacalis] | 1.83661 | 0.06153 | -1.1339 | -0.5326 | -0.2317 |
| TRINITY_DN753_c0_g1_i4_orf1 | - | - | - | venom dipeptidyl peptidase 4-like isoform X2 [Ostrinia furnacalis] | 1.93819 | -0.2915 | -0.954 | -0.2814 | -0.4113 |
| TRINITY_DN9790_c0_g1_i4_orf1 | - | - | - | protein IWS1 homolog [Ostrinia furnacalis] | 1.65464 | 0.17202 | -1.46 | -0.3135 | -0.0532 |
| TRINITY_DN3959_c1_g2_i1_orf1 | - | - | - | PREDICTED: probable isocitrate dehydrogenase [NAD] subunit alpha, mitochondrial isoform X3 [Papilio xuthus] >XP_014363280.1 probable isocitrate dehydrogenase [NAD] subunit alpha, mitochondrial isoform X3 [Papilio | 1.99848 | -0.472 | -0.4541 | -0.5037 | -0.5686 |
| TRINITY_DN23266_c0_g2_i1_orf1 | - | - | - | medium-chain acyl-CoA ligase ACSF2, mitochondrial [Chelonus insularis] | 1.98917 | -0.4197 | -0.621 | -0.6 | -0.3485 |
| TRINITY_DN85319_c0_g1_i1_orf1 | - | - | - | cholinesterase 2-like [Ostrinia furnacalis] | 1.97336 | -0.3763 | -0.808 | -0.3891 | -0.3999 |
| TRINITY_DN3332_c0_g1_i2_orf1 | - | - | - | glutathione S-transferase sigma3 [Glyphodes pyloalis] | 1.98589 | -0.3396 | -0.4822 | -0.4578 | -0.7063 |
| TRINITY_DN1277_c4_g1_i5_orf1 | - | - | - | GTP cyclohydrolase 1 isoform X1 [Ostrinia furnacalis] >XP_028166842.1 GTP cyclohydrolase 1 isoform X1 [Ostrinia furnacalis] | 1.98061 | -0.451 | -0.6117 | -0.261 | -0.6568 |

| | | | | | | | | | |
|---|---|---|---|---|---|---|---|---|---|
| TRINITY_DN5578_c0_g1_i4_orf1 | - | - | - | chromatin modification-related protein eaf-1-like [Ostrinia furnacalis] | 1.74919 | 0.31867 | -1.1756 | -0.6175 | -0.2748 |
| TRINITY_DN2270_c0_g2_i1_orf1 | - | - | - | integrin beta-nu [Ostrinia furnacalis] | 1.98803 | -0.4623 | -0.5719 | -0.6383 | -0.3156 |
| TRINITY_DN18404_c0_g1_i5_orf1 | - | - | - | periodic tryptophan protein 1 homolog isoform X1 [Ostrinia furnacalis] >XP_028157695.1 periodic tryptophan protein 1 homolog isoform X2 [Ostrinia furnacalis] | 1.96772 | -0.2086 | -0.4482 | -0.5451 | -0.7659 |
| TRINITY_DN42759_c0_g3_i1_orf1 | - | - | - | fatty acid synthase-like [Ostrinia furnacalis] | 1.97291 | -0.7901 | -0.288 | -0.4407 | -0.4541 |
| TRINITY_DN37538_c0_g3_i1_orf1 | - | - | - | esterase FE4-like [Ostrinia furnacalis] | 1.95277 | -0.8333 | -0.1673 | -0.4021 | -0.5501 |
| TRINITY_DN1901_c0_g1_i6_orf1 | - | - | - | mitochondrial intermembrane space import and assembly protein 40 [Ostrinia furnacalis] >XP_028171079.1 mitochondrial intermembrane space import and assembly protein 40 [Ostrinia furnacalis] >XP_028171080.1 mitochondrial intermembrane space import and assembly protein 40 [Ostrinia furnacalis] | 1.98519 | -0.2654 | -0.5645 | -0.6136 | -0.5416 |
| TRINITY_DN43391_c0_g1_i5_orf1 | - | - | - | myrosinase 1-like isoform X2 [Ostrinia furnacalis] | 1.98323 | -0.5429 | -0.3334 | -0.398 | -0.7088 |
| TRINITY_DN628_c0_g1_i7_orf1 | - | - | - | prostamide/prostaglandin F synthase-like [Ostrinia furnacalis] | 1.86953 | 0.15754 | -0.461 | -0.9266 | -0.6395 |
| TRINITY_DN791_c0_g1_i2_orf1 | - | - | - | peroxiredoxin 1 isoform X1 [Maniola jurtina] | 1.94682 | -0.1408 | -0.839 | -0.58 | -0.387 |
| TRINITY_DN16830_c0_g1_i5_orf1 | - | - | - | adrenodoxin [Ostrinia furnacalis] | 1.97053 | -0.2871 | -0.7983 | -0.3899 | -0.4953 |
| TRINITY_DN2852_c0_g1_i9_orf1 | - | - | - | golgin subfamily A member 4-like [Ostrinia furnacalis] | 1.93839 | -0.1861 | -0.8696 | -0.6178 | -0.2649 |
| TRINITY_DN25975_c0_g3_i2_orf1 | - | - | - | V-type proton ATPase subunit D isoform X2 [Ostrinia furnacalis] | 1.94714 | -0.0935 | -0.6084 | -0.4576 | -0.7877 |
| TRINITY_DN40_c0_g2_i1_orf1 | - | - | - | trypsin CFT-1-like [Ostrinia furnacalis] | 1.96834 | -0.8182 | -0.4619 | -0.2891 | -0.3991 |
| TRINITY_DN64472_c0_g2_i1_orf1 | - | - | - | repressed by EFG1 protein 1-like isoform X3 [Ostrinia furnacalis] | 1.81304 | -0.8573 | -0.9381 | 0.21231 | -0.23 |
| TRINITY_DN94248_c0_g2_i3_orf1 | - | - | - | uncharacterized protein LOC114357292 isoform X4 [Ostrinia furnacalis] | 1.9633 | -0.7177 | -0.2255 | -0.3381 | -0.682 |
| TRINITY_DN3332_c0_g1_i11_orf1 | - | - | - | glutathione S-transferase sigma3 [Glyphodes pyloalis] | 1.95148 | -0.2303 | -0.2788 | -0.6193 | -0.8231 |
| TRINITY_DN2083_c0_g1_i4_orf1 | - | - | - | uncharacterized protein LOC114359113 [Ostrinia furnacalis] | 1.92437 | -0.237 | -0.1691 | -0.5867 | -0.9316 |
| TRINITY_DN32306_c0_g1_i3_orf1 | - | - | - | acetyl-coenzyme A transporter 1 [Ostrinia furnacalis] | 1.70732 | -0.057 | -1.3903 | -0.3698 | 0.10981 |
| TRINITY_DN13055_c0_g1_i5_orf1 | - | - | - | 116 kDa U5 small nuclear ribonucleoprotein component isoform X1 [Ostrinia furnacalis] >XP_028159219.1 116 kDa U5 small nuclear ribonucleoprotein component isoform X2 [Ostrinia furnacalis] | 1.70039 | 0.33338 | -1.143 | -0.8289 | -0.0618 |
| TRINITY_DN146544_c0_g1_i1_orf1 | - | - | - | UPF0047 protein YjbQ [Aphidius gifuensis] >KAF7996225.1 hypothetical protein HCN44_001857 [Aphidius gifuensis] | 1.99669 | -0.432 | -0.5326 | -0.588 | -0.4441 |
| TRINITY_DN679_c0_g1_i2_orf1 | - | - | - | cytochrome b-c1 complex subunit 7-like [Ostrinia furnacalis] | 1.98776 | -0.4777 | -0.5699 | -0.6342 | -0.306 |
| TRINITY_DN12497_c0_g1_i1_orf1 | - | - | - | probable N-acetyltransferase san [Ostrinia furnacalis] | 1.96488 | -0.1774 | -0.7645 | -0.5093 | -0.5137 |
| TRINITY_DN6418_c0_g1_i28_orf1 | - | - | - | peritrophic membrane chitin binding protein [Loxostege sticticalis] | 1.96479 | -0.8459 | -0.3072 | -0.4179 | -0.3939 |
| TRINITY_DN1427_c0_g1_i9_orf1 | - | - | - | SAFB-like transcription modulator isoform X3 [Ostrinia furnacalis] | 1.59647 | 0.3578 | -1.2408 | -0.8711 | 0.15764 |
| TRINITY_DN12474_c0_g1_i6_orf1 | - | - | - | aromatic-L-amino-acid decarboxylase [Ostrinia furnacalis] | 1.99967 | -0.5256 | -0.5095 | -0.4936 | -0.471 |
| TRINITY_DN1285_c0_g1_i6_orf1 | - | - | - | bifunctional 3'-phosphoadenosine 5'-phosphosulfate synthase isoform X3 [Ostrinia furnacalis] | 1.9501 | -0.3272 | -0.8959 | -0.4712 | -0.2558 |
| TRINITY_DN16894_c0_g1_i5_orf1 | - | - | - | dnaJ homolog subfamily C member 5 isoform X1 [Colias croceus] | 1.75811 | 0.30809 | -1.1741 | -0.3088 | -0.5833 |
| TRINITY_DN40_c0_g1_i3_orf1 | - | - | - | trypsin CFT-1-like [Ostrinia furnacalis] | 1.98792 | -0.3453 | -0.4388 | -0.525 | -0.6788 |
| TRINITY_DN40704_c0_g1_i2_orf1 | - | - | - | COX assembly mitochondrial protein homolog [Ostrinia furnacalis] | 1.83491 | 0.25315 | -0.9737 | -0.5628 | -0.5516 |
| TRINITY_DN1366_c0_g1_i5_orf1 | - | - | - | unnamed protein product, partial [Iphiclides podalirius] | 1.99748 | -0.5784 | -0.4441 | -0.4486 | -0.5265 |
| TRINITY_DN23343_c0_g1_i9_orf1 | - | - | - | pre-mRNA-processing factor 6 isoform X1 [Ostrinia furnacalis] >XP_028175021.1 pre-mRNA-processing factor 6 isoform X2 [Ostrinia | 1.88981 | -0.1081 | -1.0805 | -0.3928 | -0.3084 |
| TRINITY_DN43412_c0_g1_i2_orf1 | - | - | - | U1 small nuclear ribonucleoprotein C [Ostrinia furnacalis] | 1.54844 | 0.54035 | -1.2843 | -0.813 | 0.00847 |
| TRINITY_DN4345_c0_g1_i9_orf1 | - | - | - | uncharacterized protein LOC114357127 [Ostrinia furnacalis] | 1.49897 | 0.20499 | -1.4264 | -0.7035 | 0.42598 |
| TRINITY_DN96566_c0_g1_i1_orf1 | - | - | - | NADH-ubiquinone oxidoreductase subunit 8-like [Ostrinia furnacalis] | 1.9381 | -0.7314 | -0.1421 | -0.2854 | -0.7792 |
| TRINITY_DN26186_c0_g1_i7_orf1 | - | - | - | sodium- and chloride-dependent glycine transporter 1-like [Ostrinia furnacalis] | 1.81414 | 0.08256 | -0.779 | -0.0738 | -1.0439 |
| TRINITY_DN42759_c0_g2_i1_orf1 | - | - | - | fatty acid synthase-like [Ostrinia furnacalis] | 1.93056 | -0.4753 | -0.534 | -0.0498 | -0.8714 |
| TRINITY_DN73923_c0_g1_i1_orf1 | - | - | - | protein obstructor-E-like [Ostrinia furnacalis] | 0.81498 | 1.56249 | -0.7678 | -0.7363 | -0.8734 |
| TRINITY_DN66302_c0_g1_i1_orf1 | - | - | - | carboxypeptidase B-like [Ostrinia furnacalis] | 1.95823 | -0.5012 | -0.4899 | -0.1623 | -0.8049 |
| TRINITY_DN96170_c0_g2_i1_orf1 | - | - | - | uncharacterized protein LOC114355569 [Ostrinia furnacalis] | 1.9696 | -0.3318 | -0.6054 | -0.2805 | -0.7519 |
| TRINITY_DN1285_c0_g2_i1_orf1 | - | - | - | bifunctional 3'-phosphoadenosine 5'-phosphosulfate synthase isoform X3 [Ostrinia furnacalis] | 1.99544 | -0.5243 | -0.6112 | -0.4288 | -0.4311 |

| | | | | | | | | | |
|---|---|---|---|---|---|---|---|---|---|
| TRINITY_DN2243_c0_g1_i4_orf1 | - | - | - | WD repeat-containing protein 75 [Ostrinia furnacalis] | 1.97576 | -0.6942 | -0.6383 | -0.3288 | -0.3145 |
| TRINITY_DN4550_c1_g1_i5_orfp2 | - | - | - | TRINITY_DN4550_c1_g1_i5_m.14710 TRINITY_DN4550_c1_g1::TRINITY_DN4550_c1_g1_i5::g.14710 ORF type:5prime_partial len:168 (+),score=78.12 TRINITY_DN4550_c1_g1_i5:3-506(+) | 1.93484 | -0.7498 | -0.7347 | -0.3877 | -0.0625 |
| TRINITY_DN76377_c0_g1_i1_orf1 | - | - | - | uncharacterized protein LOC111357764, partial [Spodoptera litura] | 1.19673 | 1.11184 | -1.3134 | -0.2615 | -0.7336 |
| TRINITY_DN35725_c0_g1_i1_orf1 | - | - | - | mitochondrial import inner membrane translocase subunit Tim13-like [Bicyclus anynana] >CAG9745432.1 unnamed protein product [Diatraea saccharalis] >CAG9784117.1 unnamed protein product [Diatraea saccharalis] | 1.86993 | 0.21306 | -0.6715 | -0.632 | -0.7795 |
| TRINITY_DN107035_c0_g1_i1_orf1 | - | - | - | splicing factor 3A subunit 3 [Ostrinia furnacalis] | 1.94443 | -0.0774 | -0.8058 | -0.5102 | -0.5509 |
| TRINITY_DN3759_c0_g1_i1_orf1 | - | - | - | uncharacterized protein LOC114350416 [Ostrinia furnacalis] >XP_028157016.1 uncharacterized protein LOC114350416 [Ostrinia furnacalis] >XP_028157017.1 uncharacterized protein LOC114350416 [Ostrinia furnacalis] >XP_028157018.1 uncharacterized protein LOC114350416 [Ostrinia furnacalis] >XP_028157019.1 uncharacterized protein LOC114350416 [Ostrinia furnacalis] | 1.50072 | 0.8523 | -1.1228 | -0.5703 | -0.6599 |
| TRINITY_DN8369_c0_g1_i1_orf1 | - | - | - | 39S ribosomal protein L37, mitochondrial [Ostrinia furnacalis] | 1.91852 | 0.01225 | -0.5307 | -0.5307 | -0.8694 |
| TRINITY_DN2894_c0_g3_i1_orf1 | - | - | - | lactase-phlorizin hydrolase-like [Ostrinia furnacalis] | 1.99781 | -0.5302 | -0.4724 | -0.5656 | -0.4296 |
| TRINITY_DN39673_c0_g1_i1_orf1 | - | - | - | uncharacterized protein LOC114359357 isoform X1 [Ostrinia furnacalis] | 1.92667 | -0.642 | -0.4647 | -0.0077 | -0.8123 |
| TRINITY_DN6087_c0_g1_i7_orf1 | - | - | - | uncharacterized protein LOC114355564 [Ostrinia furnacalis] | 1.9731 | -0.2381 | -0.4877 | -0.4924 | -0.7549 |
| TRINITY_DN4036_c0_g2_i1_orf1 | - | - | - | microvitellogenin-like [Ostrinia furnacalis] | 1.65042 | -0.357 | -1.4544 | 0.18147 | -0.0204 |
| TRINITY_DN72707_c0_g1_i1_orf1 | - | - | - | uncharacterized protein LOC114357549 [Ostrinia furnacalis] | 1.98506 | -0.629 | -0.2678 | -0.5477 | -0.5405 |
| TRINITY_DN52768_c0_g1_i1_orf1 | - | - | - | carboxypeptidase Q-like isoform X2 [Ostrinia furnacalis] | 1.99152 | -0.5129 | -0.4991 | -0.3449 | -0.6346 |
| TRINITY_DN9464_c0_g1_i1_orf1 | - | - | - | angio-associated migratory cell protein [Ostrinia furnacalis] >XP_028162594.1 angio-associated migratory cell protein [Ostrinia furnacalis] | 1.96536 | -0.6328 | -0.7546 | -0.3041 | -0.2738 |
| TRINITY_DN542_c0_g1_i4_orf1 | - | - | - | uncharacterized protein LOC114364889 [Ostrinia furnacalis] | 1.96751 | -0.8383 | -0.3609 | -0.4029 | -0.3653 |
| TRINITY_DN7556_c0_g1_i1_orf1 | - | - | - | venom carboxylesterase-6-like [Ostrinia furnacalis] | 1.97098 | -0.1742 | -0.5667 | -0.555 | -0.675 |
| TRINITY_DN43420_c0_g2_i1_orf1 | - | - | - | collagenase-like [Ostrinia furnacalis] | 1.9946 | -0.4186 | -0.6099 | -0.5452 | -0.4209 |
| TRINITY_DN31047_c0_g1_i4_orf1 | - | - | - | 4-coumarate--CoA ligase 1-like isoform X1 [Ostrinia furnacalis] >XP_028160248.1 4-coumarate--CoA ligase 1-like isoform X1 [Ostrinia furnacalis] >XP_028160249.1 4-coumarate--CoA ligase 1-like isoform X1 [Ostrinia furnacalis] >XP_028160250.1 4-coumarate--CoA ligase 1-like isoform X1 [Ostrinia furnacalis] >XP_028160251.1 4-coumarate--CoA ligase 1-like isoform X1 [Ostrinia furnacalis] >XP_028160253.1 4-coumarate--CoA ligase 1- | 1.93642 | -0.7307 | -0.6957 | -0.4812 | -0.0289 |
| TRINITY_DN18909_c0_g1_i8_orf1 | - | - | - | unnamed protein product [Euphydryas editha] | 1.92933 | -0.6272 | -0.4712 | -0.0173 | -0.8136 |
| TRINITY_DN79319_c0_g1_i8_orfp1 | - | - | - | TRINITY_DN79319_c0_g1_i8_m.49956 TRINITY_DN79319_c0_g1::TRINITY_DN79319_c0_g1_i8::g.49956 ORF type:5prime_partial len:84 (+),score=1.39 TRINITY_DN79319_c0_g1_i8:1-252(+) | 1.95994 | -0.6544 | -0.2656 | -0.2759 | -0.7641 |
| TRINITY_DN2825_c0_g1_i3_orf1 | - | - | - | carbonic anhydrase 2-like [Ostrinia furnacalis] | 1.96978 | -0.8123 | -0.3121 | -0.4749 | -0.3705 |
| TRINITY_DN37699_c0_g1_i4_orfp1 | - | - | - | TRINITY_DN37699_c0_g1_i4_m.58777 TRINITY_DN37699_c0_g1::TRINITY_DN37699_c0_g1_i4::g.58777 ORF type:internal len:122 (+),score=34.90 TRINITY_DN37699_c0_g1_i4:1-363(+) | 1.72563 | -0.8212 | -1.1057 | -0.1285 | 0.32975 |
| TRINITY_DN6221_c0_g1_i5_orf1 | - | - | - | unnamed protein product [Diatraea saccharalis] | 1.98169 | -0.7138 | -0.3527 | -0.5582 | -0.357 |
| TRINITY_DN25779_c0_g1_i6_orf1 | - | - | - | aldo-keto reductase AKR2E4-like [Ostrinia furnacalis] | 1.5711 | 0.78433 | -0.794 | -0.9637 | -0.5977 |
| TRINITY_DN2425_c0_g1_i1_orf1 | - | - | - | thyroid receptor-interacting protein 11 [Ostrinia furnacalis] | 1.6933 | -0.7407 | -1.2413 | 0.11578 | 0.17292 |
| TRINITY_DN1706_c0_g1_i7_orf1 | - | - | - | LOW QUALITY PROTEIN: RNA polymerase-associated protein CTR9 homolog [Ostrinia furnacalis] | 1.72482 | 0.39813 | -1.1753 | -0.6084 | -0.3393 |
| TRINITY_DN36262_c0_g1_i1_orf1 | - | - | - | trypsin, alkaline C-like [Maniola jurtina] | 1.9644 | -0.1722 | -0.7199 | -0.4405 | -0.6317 |
| TRINITY_DN1318_c0_g1_i5_orf1 | - | - | - | uncharacterized protein LOC114360956 [Ostrinia furnacalis] | 1.97485 | -0.7734 | -0.4412 | -0.4753 | -0.2849 |

| | | | | | | | | | |
|---|---|---|---|---|---|---|---|---|---|
| TRINITY_DN144258_c0_g1_i1_orf1 | - | - | - | PREDICTED: enhancer of rudimentary homolog [Microplitis demolitor] >XP_044577051.1 enhancer of rudimentary homolog [Cotesia glomerata] >KAG8041963.1 hypothetical protein G9C98_007267 [Cotesia typhae] >KAH0539785.1 hypothetical protein KQX54_008036 [Cotesia glomerata] >CAD6227368.1 GSCOCG00006137001-RA-CDS [Cotesia congregata] | 1.12581 | 1.04677 | -1.3406 | -0.9128 | 0.08076 |
| TRINITY_DN3062_c0_g1_i1_orf1 | - | - | - | HEAT repeat-containing protein 1 [Ostrinia furnacalis] | 1.9158 | -0.0688 | -0.956 | -0.5294 | -0.3616 |
| TRINITY_DN2918_c0_g1_i1_orf1 | - | - | - | 28S ribosomal protein S10, mitochondrial [Ostrinia furnacalis] >XP_028175147.1 28S ribosomal protein S10, mitochondrial [Ostrinia furnacalis] | 1.85829 | 0.24932 | -0.655 | -0.7422 | -0.7104 |
| TRINITY_DN1044_c0_g1_i2_orf1 | - | - | - | V-type proton ATPase subunit H isoform X3 [Ostrinia furnacalis] >QRR19186.1 V-type proton ATPase subunit H [Ostrinia nubilalis] | 1.99773 | -0.477 | -0.5075 | -0.4335 | -0.5797 |
| TRINITY_DN28577_c0_g1_i6_orf1 | - | - | - | delta-1-pyrroline-5-carboxylate dehydrogenase, mitochondrial [Nymphalis io] | 1.99081 | -0.3495 | -0.6189 | -0.4447 | -0.5777 |
| TRINITY_DN2267_c0_g1_i1_orf1 | - | - | - | hypothetical protein evm_006312 [Chilo suppressalis] | 1.99321 | -0.564 | -0.458 | -0.6031 | -0.3681 |
| TRINITY_DN76283_c0_g6_i1_orf1 | - | - | - | fatty acid synthase-like [Ostrinia furnacalis] | 1.99531 | -0.5552 | -0.5124 | -0.37 | -0.5576 |
| TRINITY_DN140669_c0_g1_i1_orf1 | - | - | - | S-methyl-5'-thioadenosine phosphorylase-like isoform X1 [Hyposmocoma kahamanoa] | 1.93978 | -0.1711 | -0.4874 | -0.3671 | -0.9141 |
| TRINITY_DN7213_c0_g1_i2_orf1 | - | - | - | probable ATP-dependent RNA helicase CG8611 [Ostrinia furnacalis] | 1.96319 | -0.3013 | -0.761 | -0.6377 | -0.2631 |
| TRINITY_DN7966_c0_g1_i4_orf1 | - | - | - | leucine-rich repeat neuronal protein 1-like [Ostrinia furnacalis] | 1.98771 | -0.3036 | -0.6486 | -0.5212 | -0.5142 |
| TRINITY_DN1073_c0_g1_i4_orf1 | - | - | - | carboxylesterase [Loxostege sticticalis] | 1.98645 | -0.5306 | -0.5473 | -0.2808 | -0.6278 |
| TRINITY_DN35662_c0_g1_i5_orf1 | - | - | - | hypothetical protein evm_006436 [Chilo suppressalis] >CAB3522373.1 unnamed protein product [Chilo suppressalis] >CAH0399695.1 unnamed protein product [Chilo suppressalis] | 1.97199 | -0.1731 | -0.5584 | -0.6165 | -0.6239 |
| TRINITY_DN2953_c1_g1_i2_orf1 | - | - | - | methionine--tRNA ligase, cytoplasmic isoform X6 [Ostrinia furnacalis] | 1.78577 | 0.38621 | -0.634 | -0.5725 | -0.9655 |
| TRINITY_DN4476_c0_g1_i5_orf1 | - | - | - | trypsin, alkaline C-like isoform X1 [Ostrinia furnacalis] | 1.95827 | -0.2223 | -0.8342 | -0.3694 | -0.5323 |
| TRINITY_DN1249_c0_g1_i10_orf1 | - | - | - | venom carboxylesterase-6-like [Ostrinia furnacalis] | 1.89215 | -0.7956 | -0.084 | -0.1408 | -0.8717 |
| TRINITY_DN30233_c0_g1_i2_orf1 | - | - | - | 39S ribosomal protein L10, mitochondrial [Ostrinia furnacalis] | 1.91141 | -0.2075 | -1.0175 | -0.2157 | -0.4707 |
| TRINITY_DN29229_c0_g1_i5_orfp1 | - | - | - | TRINITY_DN29229_c0_g1_i5_m.11187 TRINITY_DN29229_c0_g1::TRINITY_DN29229_c0_g1_i5::g.11187 ORF type:internal len:133 (+),score=48.64 TRINITY_DN29229_c0_g1_i5:3-398(+) | 1.84242 | -1.0188 | -0.7236 | 0.08953 | -0.1896 |
| TRINITY_DN5740_c0_g1_i4_orf1 | - | - | - | unconventional myosin IC isoform X1 [Ostrinia furnacalis] | 1.9892 | -0.4343 | -0.4001 | -0.4598 | -0.6949 |
| TRINITY_DN15900_c0_g1_i6_orf1 | - | - | - | unnamed protein product [Diatraea saccharalis] | 1.86899 | 0.13027 | -0.9831 | -0.4483 | -0.5679 |
| TRINITY_DN7024_c0_g1_i1_orf1 | - | - | - | uncharacterized protein LOC114363116 [Ostrinia furnacalis] | 1.98129 | -0.3777 | -0.4252 | -0.4208 | -0.7576 |
| TRINITY_DN8543_c0_g1_i1_orf1 | - | - | - | 39S ribosomal protein L38, mitochondrial [Ostrinia furnacalis] | 1.87421 | -0.4232 | -0.9174 | -0.6694 | 0.13584 |
| TRINITY_DN122170_c0_g1_i2_orfp1 | - | - | - | TRINITY_DN122170_c0_g1_i2_m.81408 TRINITY_DN122170_c0_g1::TRINITY_DN122170_c0_g1_i2::g.81408 ORF type:internal len:87 (+),score=9.58,Baculo_E25 PF05274.12 1.7e-07 TRINITY_DN122170_c0_g1_i2:2-259(+) | 1.99404 | -0.5629 | -0.6008 | -0.4404 | -0.39 |
| TRINITY_DN79210_c0_g1_i1_orf1 | - | - | - | V-type proton ATPase 16 kDa proteolipid subunit [Frieseomelitta varia] | 1.99567 | -0.4546 | -0.4038 | -0.5477 | -0.5896 |
| TRINITY_DN30663_c0_g1_i1_orf1 | - | - | - | surfeit locus protein 6 homolog [Ostrinia furnacalis] | 1.95041 | -0.7084 | -0.2058 | -0.7578 | -0.2784 |
| TRINITY_DN18172_c0_g1_i6_orf1 | - | - | - | digestive cysteine proteinase 2-like [Ostrinia furnacalis] | 1.9582 | -0.1669 | -0.5283 | -0.457 | -0.806 |
| TRINITY_DN9715_c0_g1_i1_orf1 | - | - | - | V-type proton ATPase subunit E [Manduca sexta] >P31402.1 RecName: Full=V-type proton ATPase subunit E; Short=V-ATPase subunit E; AltName: Full=V-ATPase 26 kDa subunit; AltName: Full=Vacuolar proton pump subunit E [Manduca sexta] >KAG6457535.1 hypothetical protein O3G_MSEX010354 [Manduca sexta] >CAA47610.1 H(+)-transporting ATPase [Manduca sexta] | 1.99798 | -0.4842 | -0.4912 | -0.4424 | -0.5802 |
| TRINITY_DN3733_c0_g1_i1_orf1 | - | - | - | 60S ribosomal protein L37, partial [Papilio machaon] | 1.81846 | -0.2065 | -1.2432 | -0.0482 | -0.3205 |
| TRINITY_DN117_c0_g1_i5_orf1 | - | - | - | lipase member I-like [Ostrinia furnacalis] | 1.97747 | -0.7112 | -0.4321 | -0.2603 | -0.5739 |
| TRINITY_DN3614_c0_g2_i1_orf1 | - | - | - | PC4 and SFRS1-interacting protein isoform X4 [Galleria mellonella] | 1.17881 | 0.83727 | -1.3439 | -0.9985 | 0.32627 |
| TRINITY_DN700_c0_g1_i3_orf1 | - | - | - | V-type proton ATPase subunit H isoform X1 [Chelonus insularis] | 1.9787 | -0.3131 | -0.3924 | -0.5311 | -0.7421 |
| TRINITY_DN109733_c0_g1_i1_orf1 | - | - | - | uncharacterized protein LOC112452128 [Temnothorax curvispinosus] | 1.95165 | -0.4422 | -0.6833 | -0.107 | -0.7191 |
| TRINITY_DN108573_c0_g1_i1_orf1 | - | - | - | uncharacterized protein LOC114366171 [Ostrinia furnacalis] | 1.97779 | -0.3539 | -0.3188 | -0.5824 | -0.7227 |
| TRINITY_DN97680_c0_g1_i1_orf1 | - | - | - | 39S ribosomal protein L52, mitochondrial [Ostrinia furnacalis] | 1.89732 | -0.1391 | -0.4759 | -1.0487 | -0.2335 |

| | | | | | | | | | |
|---|---|---|---|---|---|---|---|---|---|
| TRINITY_DN49508_c0_g2_i8_orf1 | - | - | - | putative fatty acyl-CoA reductase CG5065 [Ostrinia furnacalis] | 1.93716 | -0.8654 | -0.3673 | -0.1119 | -0.5925 |
| TRINITY_DN44288_c0_g1_i2_orf1 | - | - | - | ATP-dependent RNA helicase p62 [Ostrinia furnacalis] | 1.92291 | -0.0317 | -0.5001 | -0.4909 | -0.9002 |
| TRINITY_DN6059_c0_g1_i1_orf1 | - | - | - | brachyurin-like [Ostrinia furnacalis] | 1.97075 | -0.8181 | -0.3936 | -0.3356 | -0.4235 |
| TRINITY_DN468_c0_g1_i3_orf1 | - | - | - | transmembrane protein 41 homolog isoform X2 [Ostrinia furnacalis] | 1.94776 | -0.4302 | -0.4032 | -0.2124 | -0.9019 |
| TRINITY_DN24476_c0_g1_i1_orf1 | - | - | - | ensconsin-like isoform X1 [Ostrinia furnacalis] | 1.88645 | 0.06018 | -0.7041 | -0.9128 | -0.3297 |
| TRINITY_DN26375_c0_g1_i1_orf1 | - | - | - | hypothetical protein O3G_MSEX007366 [Manduca sexta] | 1.9375 | -0.094 | -0.6371 | -0.3756 | -0.8309 |
| TRINITY_DN5107_c0_g1_i4_orf1 | - | - | - | peptide methionine sulfoxide reductase [Ostrinia furnacalis] | 1.99571 | -0.594 | -0.498 | -0.5149 | -0.3888 |
| TRINITY_DN82320_c0_g1_i2_orf1 | - | - | - | glutathione S-transferase sigma3 [Glyphodes pyloalis] | 1.87062 | -0.4251 | 0.02908 | -0.3967 | -1.0779 |
| TRINITY_DN19160_c0_g1_i1_orf1 | - | - | - | alkyldihydroxyacetonephosphate synthase [Ostrinia furnacalis] | 1.36627 | 1.0667 | -0.7921 | -0.9248 | -0.716 |
| TRINITY_DN48237_c0_g1_i5_orf1 | - | - | - | myogenesis-regulating glycosidase-like [Ostrinia furnacalis] | 1.96421 | -0.2462 | -0.7039 | -0.3179 | -0.6962 |
| TRINITY_DN98091_c0_g1_i3_orf1 | - | - | - | UDP-glycosyltransferase UGT40AP2, partial [Ostrinia furnacalis] | 1.96671 | -0.3577 | -0.2586 | -0.5624 | -0.788 |
| TRINITY_DN52296_c0_g1_i6_orf1 | - | - | - | protein takeout-like [Ostrinia furnacalis] | 1.83707 | -0.3426 | -1.0236 | -0.6532 | 0.18238 |
| TRINITY_DN139212_c0_g1_i4_orf1 | - | - | - | uncharacterized protein LOC114350112 [Ostrinia furnacalis] | 1.52551 | 0.63873 | -1.1009 | -1.0254 | -0.0379 |
| TRINITY_DN334_c0_g1_i2_orf1 | - | - | - | chymotrypsin-like serine protease, partial [Ostrinia nubilalis] | 1.98973 | -0.6138 | -0.4348 | -0.3435 | -0.5976 |
| TRINITY_DN1353_c0_g1_i1_orf1 | - | - | - | UDP-glucose 4-epimerase-like [Ostrinia furnacalis] | 1.82726 | 0.3063 | -0.6118 | -0.6283 | -0.8934 |
| TRINITY_DN15870_c0_g1_i3_orf1 | - | - | - | PREDICTED: mitochondrial import inner membrane translocase subunit Tim23 isoform X1 [Fopius arisanus] | 1.98405 | -0.2553 | -0.6107 | -0.5736 | -0.5444 |
| TRINITY_DN18773_c0_g1_i3_orf1 | - | - | - | keratin, type II cytoskeletal 68 kDa, component IB-like [Ostrinia furnacalis] | 1.87494 | -1.0847 | -0.5175 | -0.1755 | -0.0973 |
| TRINITY_DN6747_c0_g1_i7_orf1 | - | - | - | retinol dehydrogenase 12-like [Ostrinia furnacalis] | 1.92317 | -0.3023 | -0.8981 | -0.6281 | -0.0947 |
| TRINITY_DN11069_c0_g2_i1_orf1 | - | - | - | fat storage-inducing transmembrane protein [Ostrinia furnacalis] | 1.92312 | -0.245 | -1.0042 | -0.3918 | -0.2821 |
| TRINITY_DN5238_c0_g1_i2_orf1 | - | - | - | DNA-(apurinic or apyrimidinic site) lyase [Ostrinia furnacalis] | 1.7357 | 0.33526 | -1.1735 | -0.6668 | -0.2307 |
| TRINITY_DN36061_c0_g4_i2_orf1 | - | - | - | putative GPI-anchored protein pfl2 [Ostrinia furnacalis] >XP_028163002.1 putative GPI-anchored protein pfl2 [Ostrinia furnacalis] | 0.95821 | 1.45831 | -0.7428 | -0.7998 | -0.8739 |
| TRINITY_DN141462_c0_g1_i1_orf1 | - | - | - | mitochondrial-processing peptidase subunit beta [Diachasma alloeum] >THK33262.1 core protein 1, ubiquinol-cytochrome c reductase [Diachasma alloeum] | 1.98764 | -0.5948 | -0.5998 | -0.4997 | -0.2934 |
| TRINITY_DN8838_c0_g1_i1_orf1 | - | - | - | mannose-P-dolichol utilization defect 1 protein homolog [Ostrinia furnacalis] | 1.84227 | -0.1007 | -1.0501 | 0.0107 | -0.7022 |
| TRINITY_DN13783_c0_g4_i2_orf1 | - | - | - | hypothetical protein evm_010131 [Chilo suppressalis] | 1.97724 | -0.5023 | -0.7344 | -0.2592 | -0.4814 |
| TRINITY_DN17825_c1_g1_i1_orf1 | - | - | - | 39S ribosomal protein L1, mitochondrial [Ostrinia furnacalis] | 1.98 | -0.4479 | -0.7622 | -0.4138 | -0.3561 |
| TRINITY_DN5459_c0_g1_i1_orf1 | - | - | - | protein takeout-like isoform X2 [Ostrinia furnacalis] | 1.46689 | 0.94817 | -0.7533 | -0.8536 | -0.8082 |
| TRINITY_DN2394_c0_g1_i4_orf1 | - | - | - | uncharacterized protein LOC114363116 [Ostrinia furnacalis] | 1.98741 | -0.4512 | -0.448 | -0.38 | -0.7081 |
| TRINITY_DN29034_c0_g1_i2_orf1 | - | - | - | trypsin-like serine protease [Ostrinia nubilalis] | 1.95549 | -0.8612 | -0.5006 | -0.3584 | -0.2353 |
| TRINITY_DN9003_c0_g1_i20_orf1 | - | - | - | RNA-binding protein Nova-2 isoform X4 [Ostrinia furnacalis] | 1.90354 | -0.4719 | -0.8985 | -0.5863 | 0.05316 |
| TRINITY_DN8087_c0_g1_i9_orf1 | - | - | - | cysteine-rich with EGF-like domain protein 2 isoform X1 [Ostrinia furnacalis] | 1.84593 | 0.17227 | -0.3031 | -0.8687 | -0.8464 |
| TRINITY_DN3159_c0_g1_i4_orf1 | - | - | - | uncharacterized protein LOC114362782 [Ostrinia furnacalis] | 1.97857 | -0.2439 | -0.4919 | -0.5454 | -0.6974 |
| TRINITY_DN4731_c0_g1_i1_orf1 | - | - | - | gelsolin-like [Ostrinia furnacalis] | 1.98032 | -0.5952 | -0.4391 | -0.2684 | -0.6777 |
| TRINITY_DN5891_c0_g2_i4_orf1 | - | - | - | amino acid transporter AVT1A-like [Ostrinia furnacalis] >XP_028156666.1 amino acid transporter AVT1A-like [Ostrinia furnacalis] | 1.98661 | -0.5411 | -0.3928 | -0.3648 | -0.6879 |
| TRINITY_DN657_c0_g1_i2_orf1 | - | - | - | cytochrome c-type heme lyase [Ostrinia furnacalis] | 1.95734 | -0.644 | -0.7294 | -0.1314 | -0.4525 |
| TRINITY_DN15380_c0_g1_i1_orf1 | - | - | - | 39S ribosomal protein L32, mitochondrial [Ostrinia furnacalis] | 1.98899 | -0.3213 | -0.5945 | -0.6121 | -0.4611 |
| TRINITY_DN27725_c0_g1_i2_orf1 | - | - | - | BRISC complex subunit FAM175B-like [Ostrinia furnacalis] | 1.12913 | 1.10729 | -0.9973 | -1.2264 | -0.0127 |
| TRINITY_DN17864_c0_g1_i1_orf1 | - | - | - | PREDICTED: erlin-2-B [Microplitis demolitor] | 1.9959 | -0.5149 | -0.6072 | -0.4168 | -0.457 |
| TRINITY_DN21722_c0_g1_i3_orf1 | - | - | - | V-type proton ATPase 116 kDa subunit a isoform X1 [Ostrinia furnacalis] >XP_028177509.1 V-type proton ATPase 116 kDa subunit a isoform X1 [Ostrinia furnacalis] | 1.99575 | -0.5696 | -0.478 | -0.3887 | -0.5595 |
| TRINITY_DN47114_c0_g1_i5_orf1 | - | - | - | nucleolar protein dao-5 isoform X2 [Ostrinia furnacalis] | 1.94891 | -0.4656 | -0.8843 | -0.4082 | -0.1908 |
| TRINITY_DN8691_c0_g1_i3_orf1 | - | - | - | nucleolin-like [Melitaea cinxia] | 1.70085 | 0.51566 | -0.8562 | -0.9826 | -0.3777 |
| TRINITY_DN4762_c0_g1_i2_orf1 | - | - | - | ATPase family AAA domain-containing protein 1 isoform X2 [Ostrinia furnacalis] | 1.72993 | 0.49044 | -1.0169 | -0.5365 | -0.667 |
| TRINITY_DN11259_c0_g1_i1_orf1 | - | - | - | uncharacterized protein LOC114357075 [Ostrinia furnacalis] | 1.96195 | -0.6976 | -0.3495 | -0.209 | -0.7059 |

| | | | | | | | | | |
|---|---|---|---|---|---|---|---|---|---|
| TRINITY_DN10332_c0_g1_i2_orfp1 | - | - | - | TRINITY_DN10332_c0_g1_i2_m.42894 TRINITY_DN10332_c0_g1::TRINITY_DN10332_c0_g1_i2::g.42894 ORF type:3prime_partial len:77 (+),score=1.70 TRINITY_DN10332_c0_g1_i2:1005-1232(+) | 1.80891 | -0.012 | -1.1539 | -0.6293 | -0.0137 |
| TRINITY_DN28592_c0_g1_i2_orf1 | - | - | - | UDP-glucuronosyltransferase 2B14-like isoform X1 [Ostrinia furnacalis] >XP_028167291.1 UDP-glucuronosyltransferase 2B14-like isoform X2 [Ostrinia furnacalis] | 1.94236 | -0.161 | -0.9018 | -0.4152 | -0.4644 |
| TRINITY_DN48020_c0_g1_i1_orf1 | - | - | - | aminopeptidase N4 [Cnaphalocrocis medinalis] | 1.94389 | -0.6453 | -0.521 | -0.0489 | -0.7288 |
| TRINITY_DN2178_c0_g1_i1_orf1 | - | - | - | carboxypeptidase B-like [Ostrinia furnacalis] | 1.95396 | -0.4934 | -0.1997 | -0.4026 | -0.8583 |
| TRINITY_DN26688_c0_g1_i2_orf1 | - | - | - | myogenesis-regulating glycosidase-like [Ostrinia furnacalis] | 1.96274 | -0.1346 | -0.6997 | -0.6046 | -0.5238 |
| TRINITY_DN48410_c0_g2_i1_orf1 | - | - | - | alpha-amylase 2-like isoform X3 [Ostrinia furnacalis] | 1.98929 | -0.5705 | -0.3715 | -0.3995 | -0.6478 |
| TRINITY_DN43611_c0_g1_i1_orf1 | - | - | - | 39S ribosomal protein L41, mitochondrial [Ostrinia furnacalis] | 1.9732 | -0.2946 | -0.7533 | -0.5724 | -0.3529 |
| TRINITY_DN69713_c0_g1_i1_orf1 | - | - | - | membrane-bound alkaline phosphatase-like [Ostrinia furnacalis] | 1.92969 | -0.925 | -0.291 | -0.1552 | -0.5585 |
| TRINITY_DN123396_c0_g1_i1_orf1 | - | - | - | PREDICTED: delta-1-pyrroline-5-carboxylate dehydrogenase, mitochondrial isoform X1 [Megachile rotundata] | 1.98339 | -0.3864 | -0.5551 | -0.7025 | -0.3393 |
| TRINITY_DN36494_c0_g1_i1_orf1 | - | - | - | MKI67 FHA domain-interacting nucleolar phosphoprotein-like [Ostrinia | 1.93828 | -0.8079 | -0.6539 | -0.3953 | -0.0812 |
| TRINITY_DN82801_c0_g1_i1_orf1 | - | - | - | uncharacterized protein LOC114364712 [Ostrinia furnacalis] | 1.46569 | 0.93012 | -0.688 | -0.688 | -1.0197 |
| TRINITY_DN23175_c0_g1_i6_orf1 | - | - | - | myb-binding protein 1A-like protein [Ostrinia furnacalis] | 1.97042 | -0.1627 | -0.6049 | -0.5723 | -0.6304 |
| TRINITY_DN311_c0_g1_i4_orfp1 | - | - | - | TRINITY_DN311_c0_g1_i4_m.65135 TRINITY_DN311_c0_g1::TRINITY_DN311_c0_g1_i4::g.65135 ORF type:5prime_partial len:126 (+),score=71.21 TRINITY_DN311_c0_g1_i4:1-378(+) | 1.85821 | -0.4413 | -0.095 | -0.1764 | -1.1455 |
| TRINITY_DN2343_c1_g1_i8_orf1 | - | - | - | receptor expression-enhancing protein 5-like isoform X3 [Ostrinia furnacalis] | 1.96638 | -0.6252 | -0.4678 | -0.17 | -0.7034 |
| TRINITY_DN3235_c0_g1_i1_orf1 | - | - | - | SPARC [Trichoplusia ni] | 1.42773 | 0.98499 | -0.9764 | -0.6609 | -0.7754 |
| TRINITY_DN1310_c0_g1_i4_orf1 | - | - | - | trypsin-like isoform X1 [Ostrinia furnacalis] >XP_028159118.1 trypsin-like isoform X2 [Ostrinia furnacalis] | 1.9794 | -0.4018 | -0.3563 | -0.4561 | -0.7652 |
| TRINITY_DN29018_c0_g1_i4_orf1 | - | - | - | prostaglandin reductase 1-like isoform X1 [Ostrinia furnacalis] >XP_028178925.1 prostaglandin reductase 1-like isoform X2 [Ostrinia | 1.95403 | -0.7823 | -0.1212 | -0.566 | -0.4845 |
| TRINITY_DN96_c0_g1_i1_orf1 | - | - | - | collagenase-like [Ostrinia furnacalis] | 1.97496 | -0.6858 | -0.3028 | -0.3333 | -0.6529 |
| TRINITY_DN16939_c0_g1_i4_orf1 | - | - | - | 39S ribosomal protein L17, mitochondrial [Ostrinia furnacalis] | 1.98296 | -0.5243 | -0.6968 | -0.4738 | -0.2881 |
| TRINITY_DN11376_c0_g2_i1_orf1 | - | - | - | cathepsin K-like [Ostrinia furnacalis] | 1.98721 | -0.459 | -0.3753 | -0.4444 | -0.7085 |
| TRINITY_DN334_c0_g1_i1_orf1 | - | - | - | putative chymotrypsin 12 [Ostrinia nubilalis] | 1.99049 | -0.5561 | -0.4034 | -0.3842 | -0.6469 |
| TRINITY_DN6074_c0_g1_i1_orf1 | - | - | - | uncharacterized protein C1683.06c-like isoform X1 [Ostrinia furnacalis] | 1.9738 | -0.2446 | -0.3986 | -0.6723 | -0.6583 |
| TRINITY_DN2894_c0_g2_i3_orf1 | - | - | - | myrosinase 1-like isoform X1 [Ostrinia furnacalis] | 1.21584 | 0.93523 | -1.3889 | -0.8434 | 0.08127 |
| TRINITY_DN47731_c0_g1_i2_orf1 | - | - | - | nucleolar GTP-binding protein 2 [Ostrinia furnacalis] | 1.73001 | 0.5288 | -0.6405 | -0.7468 | -0.8715 |
| TRINITY_DN5578_c0_g1_i10_orf1 | - | - | - | unnamed protein product [Chilo suppressalis] | 1.83907 | 0.17567 | -0.331 | -0.6687 | -1.015 |
| TRINITY_DN16931_c0_g1_i1_orf1 | - | - | - | pancreatic triacylglycerol lipase-like [Ostrinia furnacalis] | 1.89797 | -0.685 | -0.3604 | 0.0402 | -0.8927 |
| TRINITY_DN57202_c0_g1_i1_orf1 | - | - | - | PREDICTED: U4/U6 small nuclear ribonucleoprotein Prp31 [Amyelois transitella] | 1.99171 | -0.5351 | -0.5427 | -0.3254 | -0.5885 |
| TRINITY_DN1199_c0_g1_i1_orf1 | - | - | - | pupal cuticle protein 36a-like [Ostrinia furnacalis] | 1.96703 | -0.676 | -0.6841 | -0.4073 | -0.1995 |
| TRINITY_DN2894_c0_g1_i2_orf1 | - | - | - | myrosinase 1-like isoform X1 [Ostrinia furnacalis] | 1.99831 | -0.458 | -0.5554 | -0.5344 | -0.4506 |
| TRINITY_DN67623_c0_g1_i1_orf1 | - | - | - | maltase A1-like [Ostrinia furnacalis] | 1.99635 | -0.5232 | -0.5205 | -0.3865 | -0.5662 |
| TRINITY_DN6693_c0_g1_i1_orf1 | - | - | - | uncharacterized protein LOC114356358 [Ostrinia furnacalis] | 1.98013 | -0.2338 | -0.522 | -0.5768 | -0.6474 |
| TRINITY_DN69049_c0_g1_i2_orf1 | - | - | - | membrane alanyl aminopeptidase-like [Ostrinia furnacalis] | 1.98407 | -0.5803 | -0.6763 | -0.4127 | -0.3148 |
| TRINITY_DN23570_c0_g1_i2_orf1 | - | - | - | putative trypsin 6 [Ostrinia nubilalis] | 1.98817 | -0.3368 | -0.6133 | -0.616 | -0.4221 |
| TRINITY_DN10792_c0_g2_i5_orf1 | - | - | - | uncharacterized protein LOC114366171 [Ostrinia furnacalis] | 1.97999 | -0.4242 | -0.3773 | -0.4118 | -0.7666 |
| TRINITY_DN713_c0_g1_i4_orf1 | - | - | - | periodic tryptophan protein 2 homolog isoform X1 [Ostrinia furnacalis] >XP_028176443.1 periodic tryptophan protein 2 homolog isoform X2 [Ostrinia furnacalis] >XP_028176445.1 periodic tryptophan protein 2 homolog isoform X3 [Ostrinia furnacalis] | 1.97719 | -0.3122 | -0.7697 | -0.4429 | -0.4524 |
| TRINITY_DN8116_c0_g1_i1_orf1 | - | - | - | uncharacterized protein LOC114350845 [Ostrinia furnacalis] | 1.97465 | -0.1921 | -0.6138 | -0.5392 | -0.6296 |
| TRINITY_DN43942_c0_g2_i1_orf1 | - | - | - | LOW QUALITY PROTEIN: caprin homolog [Ostrinia furnacalis] | 1.79832 | 0.32132 | -1.0377 | -0.5226 | -0.5593 |

| | | | | | | | | | |
|---|---|---|---|---|---|---|---|---|---|
| TRINITY_DN50787_c0_g2_i2_orf1 | - | - | - | 40S ribosomal protein S29 [Hyposmocoma kahamanoa] >XP_028176503.1 40S ribosomal protein S29 [Ostrinia furnacalis] >XP_049877832.1 40S ribosomal protein S29 [Pectinophora gossypiella] >ADT80654.1 ribosomal protein S29 [Euphydryas aurinia] >CAB3523209.1 unnamed protein product [Chilo suppressalis] >CAH0400531.1 unnamed protein product [Chilo suppressalis] | 1.80174 | -0.4331 | -1.2261 | -0.2334 | 0.09091 |
| TRINITY_DN8116_c0_g1_i2_orf1 | - | - | - | uncharacterized protein LOC114350845 [Ostrinia furnacalis] | 1.98321 | -0.4507 | -0.5494 | -0.2932 | -0.6898 |
| TRINITY_DN717_c0_g1_i2_orfp1 | - | - | - | TRINITY_DN717_c0_g1_i2_m.67915 TRINITY_DN717_c0_g1::TRINITY_DN717_c0_g1_i2::g.67915 ORF type:internal len:868 (+),score=265.71,Collagen PF01391.19 0.11,Collagen\|PF01391.19\|0.039,Collagen\|PF01391.19\|0.00054,Collagen\|PF01391.19\|0.0019,Collagen\|PF01391.19\|0.0005,Collagen\|PF01391.19\|9.9e-05,Collagen\|PF01391.19\|1.7e-07 TRINITY_DN717_c0_g1_i2:3-2603(+) | 1.94223 | -0.0993 | -0.4451 | -0.5527 | -0.8451 |
| TRINITY_DN3194_c0_g1_i6_orf1 | - | - | - | uncharacterized protein LOC114361386 [Ostrinia furnacalis] | 1.97415 | -0.3056 | -0.6176 | -0.331 | -0.72 |
| TRINITY_DN7047_c0_g1_i1_orf1 | - | - | - | hypothetical protein G9C98_004728 [Cotesia typhae] | 1.95743 | -0.1383 | -0.7154 | -0.4326 | -0.6711 |
| TRINITY_DN51766_c0_g1_i2_orf1 | - | - | - | facilitated trehalose transporter Tret1-like [Ostrinia furnacalis] | 1.89175 | 0.14687 | -0.7774 | -0.6265 | -0.6347 |
| TRINITY_DN27721_c1_g1_i2_orf1 | - | - | - | mitochondrial import receptor subunit TOM20 homolog [Ostrinia furnacalis] | 1.95503 | -0.1406 | -0.806 | -0.5029 | -0.5055 |
| TRINITY_DN542_c0_g2_i1_orf1 | - | - | - | uncharacterized protein LOC114364889 [Ostrinia furnacalis] | 1.98647 | -0.3804 | -0.4783 | -0.4148 | -0.7131 |
| TRINITY_DN26010_c0_g1_i2_orf1 | - | - | - | cytochrome b-c1 complex subunit 10-like [Ostrinia furnacalis] | 1.99481 | -0.5674 | -0.4327 | -0.5891 | -0.4056 |
| TRINITY_DN13651_c0_g1_i2_orf1 | - | - | - | 40S ribosomal protein S12, mitochondrial [Ostrinia furnacalis] | 1.9257 | -0.0656 | -0.9066 | -0.5505 | -0.4031 |
| TRINITY_DN144807_c0_g1_i1_orf1 | - | - | - | hypothetical protein G9C98_004245 [Cotesia typhae] | 1.98871 | -0.6784 | -0.5117 | -0.4447 | -0.354 |
| TRINITY_DN64403_c0_g2_i1_orf1 | - | - | - | carboxylesterase [Ostrinia furnacalis] | 1.90027 | -0.8095 | -0.562 | 0.10848 | -0.6373 |
| TRINITY_DN20682_c0_g2_i1_orf1 | - | - | - | glutathione S-transferase delta3 [Glyphodes pyloalis] | 1.95126 | -0.134 | -0.6929 | -0.3854 | -0.7389 |
| TRINITY_DN2114_c0_g1_i5_orf1 | - | - | - | vegetative cell wall protein gp1-like isoform X1 [Ostrinia furnacalis] | 1.96068 | -0.8281 | -0.3548 | -0.2446 | -0.5332 |
| TRINITY_DN61112_c0_g1_i4_orfp1 | - | - | - | TRINITY_DN61112_c0_g1_i4_m.53012 TRINITY_DN61112_c0_g1::TRINITY_DN61112_c0_g1_i4::g.53012 ORF type:3prime_partial len:87 (-),score=12.29,HMMR_N PF15905.6 0.0011 TRINITY_DN61112_c0_g1_i4:2-232(-) | 1.99368 | -0.4419 | -0.4189 | -0.4865 | -0.6464 |
| TRINITY_DN30932_c0_g1_i2_orf1 | - | - | - | delta(24)-sterol reductase-like isoform X2 [Ostrinia furnacalis] | 1.85642 | 0.17885 | -0.5036 | -0.5477 | -0.9839 |
| TRINITY_DN104586_c0_g1_i1_orf1 | - | - | - | Chlorophyll a-b binding protein 37, chloroplastic, partial [Trichinella | 1.98704 | -0.5776 | -0.3819 | -0.365 | -0.6625 |
| TRINITY_DN7688_c0_g1_i10_orf1 | - | - | - | uncharacterized protein LOC114352518 [Ostrinia furnacalis] | 1.97232 | -0.5614 | -0.4262 | -0.2391 | -0.7456 |
| TRINITY_DN4612_c0_g1_i1_orf1 | - | - | - | uncharacterized protein LOC114362092 [Ostrinia furnacalis] | 1.92561 | -0.7785 | -0.1382 | -0.2234 | -0.7855 |
| TRINITY_DN57904_c0_g2_i1_orf1 | - | - | - | cuticle protein 19 [Plutella xylostella] >CAG9138481.1 unnamed protein product [Plutella xylostella] | 0.95237 | 1.4628 | -0.882 | -0.7657 | -0.7675 |
| TRINITY_DN23734_c0_g1_i1_orf1 | - | - | - | histone-lysine N-methyltransferase SMYD3 [Ostrinia furnacalis] | 1.78797 | -0.0405 | -1.3045 | -0.1929 | -0.25 |
| TRINITY_DN48410_c0_g1_i1_orf1 | - | - | - | alpha-amylase 1-like [Ostrinia furnacalis] | 1.98615 | -0.4851 | -0.3894 | -0.3971 | -0.7145 |
| TRINITY_DN61674_c0_g1_i2_orf1 | - | - | - | fatty acid-binding protein 1-like [Ostrinia furnacalis] | 1.98771 | -0.3915 | -0.4369 | -0.4515 | -0.7079 |
| TRINITY_DN29120_c0_g1_i6_orf1 | - | - | - | putative inorganic phosphate cotransporter [Ostrinia furnacalis] | 1.85722 | 0.22275 | -0.7199 | -0.5098 | -0.8503 |
| TRINITY_DN22983_c0_g1_i2_orfp1 | - | - | - | TRINITY_DN22983_c0_g1_i2_m.10495 TRINITY_DN22983_c0_g1::TRINITY_DN22983_c0_g1_i2::g.10495 ORF type:internal len:79 (-),score=15.95,Polyhedrin PF00738.19 7.6e-40 TRINITY_DN22983_c0_g1_i2:2-235(-) | 1.9854 | -0.3579 | -0.6968 | -0.5409 | -0.3898 |
| TRINITY_DN117_c0_g1_i6_orf1 | - | - | - | lipase member I-like [Ostrinia furnacalis] | 1.89133 | -0.0346 | -0.3199 | -0.5054 | -1.0315 |
| TRINITY_DN3784_c0_g1_i1_orf1 | - | - | - | pancreatic triacylglycerol lipase-like [Ostrinia furnacalis] | 1.96291 | -0.2267 | -0.4207 | -0.4944 | -0.8211 |
| TRINITY_DN4869_c0_g1_i10_orf1 | - | - | - | estrogen sulfotransferase-like isoform X1 [Ostrinia furnacalis] | 1.99157 | -0.455 | -0.4579 | -0.4067 | -0.672 |
| TRINITY_DN26408_c0_g1_i7_orf1 | - | - | - | venom carboxylesterase-6-like [Ostrinia furnacalis] | 1.98747 | -0.5164 | -0.6625 | -0.4978 | -0.3108 |
| TRINITY_DN19244_c0_g1_i7_orf1 | - | - | - | uncharacterized protein LOC114350218 [Ostrinia furnacalis] | 1.73855 | 0.32602 | -1.1801 | -0.6513 | -0.2332 |
| TRINITY_DN1914_c0_g1_i6_orf1 | - | - | - | loricrin-like [Ostrinia furnacalis] | 1.91171 | 0.02288 | -0.6115 | -0.4429 | -0.8802 |

| | | | | | | | | | |
|---|---|---|---|---|---|---|---|---|---|
| TRINITY_DN11587_c0_g1_i7_orf1 | - | - | - | elongation of very long chain fatty acids protein AAEL008004-like isoform X1 [Danaus plexippus plexippus] >XP_032511006.1 elongation of very long chain fatty acids protein AAEL008004-like isoform X1 [Danaus plexippus plexippus] >XP_032511007.1 elongation of very long chain fatty acids protein AAEL008004-like isoform X1 [Danaus plexippus plexippus] >XP_032511008.1 elongation of very long chain fatty acids protein AAEL008004-like isoform X1 [Danaus plexippus plexippus] >XP_032511009.1 elongation of very long chain fatty acids protein AAEL008004-like isoform X1 [Danaus plexippus plexippus] | 1.97115 | -0.1799 | -0.6858 | -0.5321 | -0.5733 |
| TRINITY_DN41259_c0_g1_i6_orf1 | - | - | - | endocuticle structural glycoprotein SgAbd-8 [Ostrinia furnacalis] | 0.99293 | 1.43186 | -0.855 | -0.7657 | -0.8041 |
| TRINITY_DN6044_c0_g1_i4_orf1 | - | - | - | acyl-CoA-binding protein-like [Ostrinia furnacalis] | 1.99594 | -0.4325 | -0.4235 | -0.5632 | -0.5767 |
| TRINITY_DN7688_c0_g1_i2_orf1 | - | - | - | uncharacterized protein LOC114352518 [Ostrinia furnacalis] | 1.96166 | -0.607 | -0.2993 | -0.2659 | -0.7894 |
| TRINITY_DN334_c0_g1_i4_orf1 | - | - | - | collagenase-like [Ostrinia furnacalis] | 1.89551 | -1.0204 | -0.4359 | -0.0204 | -0.4188 |
| TRINITY_DN75188_c0_g1_i1_orf1 | - | - | - | fatty acid-binding protein 1-like [Ostrinia furnacalis] | 1.94334 | -0.1572 | -0.8861 | -0.5152 | -0.3848 |
| TRINITY_DN3862_c0_g1_i7_orf1 | - | - | - | venom acid phosphatase Acph-1-like [Ostrinia furnacalis] | 1.98689 | -0.4522 | -0.714 | -0.3855 | -0.4352 |
| TRINITY_DN35051_c0_g1_i1_orf1 | - | - | - | uncharacterized protein LOC114364307 [Ostrinia furnacalis] | 1.98253 | -0.4959 | -0.5116 | -0.2794 | -0.6955 |
| TRINITY_DN14679_c0_g1_i1_orf1 | - | - | - | hypothetical protein evm_003043 [Chilo suppressalis] | 1.95596 | -0.5376 | -0.2789 | -0.2885 | -0.851 |
| TRINITY_DN78546_c0_g5_i1_orf1 | - | - | - | kinesin-like protein KIF13A isoform X9 [Cephus cinctus] | 1.69459 | -0.1153 | -1.4027 | -0.3453 | 0.16859 |
| TRINITY_DN2815_c0_g1_i3_orf1 | - | - | - | uncharacterized protein LOC114364075 [Ostrinia furnacalis] | 1.98924 | -0.4125 | -0.6185 | -0.3544 | -0.6039 |
| TRINITY_DN64446_c0_g1_i1_orf1 | - | - | - | uncharacterized protein LOC114364307 [Ostrinia furnacalis] | 1.9482 | -0.2083 | -0.8689 | -0.3198 | -0.5512 |
| TRINITY_DN81803_c0_g2_i1_orf1 | - | - | - | cathepsin K-like [Ostrinia furnacalis] | 1.93601 | -0.1101 | -0.4883 | -0.4378 | -0.8998 |
| TRINITY_DN22430_c0_g3_i1_orf1 | - | - | - | V-type proton ATPase 16 kDa proteolipid subunit c [Homo sapiens] >NP_001685.1 V-type proton ATPase 16 kDa proteolipid subunit c [Homo sapiens] >P27449.1 RecName: Full=V-type proton ATPase 16 kDa proteolipid subunit c; Short=V-ATPase 16 kDa proteolipid subunit c; AltName: Full=Vacuolar proton pump 16 kDa proteolipid subunit c [Homo sapiens] >6WLW_1 The Vo region of human V-ATPase in state 1 (focused refinement) [Homo sapiens] >6WLW_2 The Vo region of human V-ATPase in state 1 (focused refinement) [Homo sapiens] >6WLW_3 The Vo region of human V-ATPase in state 1 (focused refinement) [Homo sapiens] >6WLW_4 The Vo region of human V-ATPase in state 1 (focused refinement) [Homo sapiens] >6WLW_5 The Vo region of human V-ATPase in state 1 (focused refinement) [Homo sapiens] >6WLW_6 The Vo region of human V-ATPase in state 1 (focused refinement) [Homo sapiens] >6WLW_7 The Vo region of human V-ATPase in state 1 (focused refinement) [Homo sapiens] >6WLW_8 The Vo region of human V-ATPase in state 1 (focused refinement) [Homo sapiens] >6WLW_9 The Vo region of human V-ATPase in state 1 (focused refinement) [Homo sapiens] >6WM2_1 Human V-ATPase in state 1 with SidK and ADP [Homo sapiens] >6WM2_2 Human V-ATPase in state 1 with SidK and ADP [Homo sapiens] >6WM2_3 Human V-ATPase in state 1 with SidK and ADP [Homo sapiens] >6WM2_4 Human V-ATPase in state 1 with SidK and ADP [Homo sapiens] >6WM2_5 Human V-ATPase in state 1 with SidK and ADP [Homo sapiens] >6WM2_6 Human V-ATPase in state 1 with SidK and ADP [Homo sapiens] >6WM2_7 Human V-ATPase in state 1 with SidK and ADP [Homo sapiens] >6WM2_8 Human V-ATPase in state 1 with SidK and ADP [Homo sapiens] >6WM2_9 Human V-ATPase in state 1 with SidK and ADP [Homo sapiens] >6WM3_1 Human V-ATPase in state 2 with SidK and ADP [Homo sapiens] >6WM3_2 Human V-ATPase in state 2 with SidK and ADP [Homo sapiens] >6WM3_3 Human V-ATPase in state 2 with SidK and ADP [Homo sapiens] >6WM3_4 Human V-ATPase in state 2 with SidK and ADP [Homo sapiens] >6WM3_5 Human V-ATPase in state 2 with SidK and ADP | 1.99836 | -0.44 | -0.5588 | -0.476 | -0.5236 |
| TRINITY_DN37538_c0_g1_i1_orf1 | - | - | - | esterase FE4-like [Ostrinia furnacalis] | 1.98456 | -0.494 | -0.348 | -0.4235 | -0.719 |
| TRINITY_DN41086_c0_g1_i4_orf1 | - | - | - | collagenase-like [Pectinophora gossypiella] | 1.96441 | -0.5228 | -0.4569 | -0.1972 | -0.7875 |

| | | | | | | | | | |
|---|---|---|---|---|---|---|---|---|---|
| TRINITY_DN348_c0_g2_i3_orf1 | - | - | - | pancreatic triacylglycerol lipase-like [Ostrinia furnacalis] | 1.97794 | -0.3502 | -0.4295 | -0.422 | -0.7762 |
| TRINITY_DN6933_c0_g1_i2_orf1 | - | - | - | Chlorophyll a-b binding protein 40, chloroplastic [Trichinella nelsoni] >KRY99282.1 Chlorophyll a-b binding protein 40, chloroplastic [Trichinella | 1.99176 | -0.4515 | -0.3871 | -0.4919 | -0.6612 |
| TRINITY_DN39200_c0_g1_i5_orf1 | - | - | - | juvenile hormone epoxide hydrolase-like [Ostrinia furnacalis] | 1.99574 | -0.3947 | -0.4806 | -0.596 | -0.5244 |
| TRINITY_DN6140_c0_g3_i3_orf1 | - | - | - | CD63 antigen-like [Ostrinia furnacalis] | 1.84375 | -0.2634 | -0.572 | 0.08557 | -1.0939 |
| TRINITY_DN36199_c0_g1_i1_orf1 | - | - | - | fatty acid-binding protein 1-like [Ostrinia furnacalis] | 1.99314 | -0.4516 | -0.3693 | -0.5861 | -0.5861 |
| TRINITY_DN94755_c0_g1_i5_orfp1 | - | - | - | TRINITY_DN94755_c0_g1_i5_m.62794 TRINITY_DN94755_c0_g1::TRINITY_DN94755_c0_g1_i5::g.62794 ORF type:internal len:70 (+),score=5.17 TRINITY_DN94755_c0_g1_i5:2-208(+) | 1.999 | -0.5278 | -0.5399 | -0.4543 | -0.477 |
| TRINITY_DN94355_c0_g1_i2_orf1 | - | - | - | uncharacterized protein LOC126369488 [Pectinophora gossypiella] | 1.88694 | -0.5148 | -0.7059 | -0.8097 | 0.14341 |
| TRINITY_DN2490_c0_g2_i1_orfp1 | - | - | - | TRINITY_DN2490_c0_g2_i1_m.56872 TRINITY_DN2490_c0_g2::TRINITY_DN2490_c0_g2_i1::g.56872 ORF type:internal len:359 (-),score=123.59 TRINITY_DN2490_c0_g2_i1:2-1075(-) | 1.96023 | -0.2242 | -0.5819 | -0.3505 | -0.8036 |
| TRINITY_DN4959_c0_g1_i1_orf1 | - | - | - | pancreatic triacylglycerol lipase-like [Ostrinia furnacalis] | 1.80133 | -0.0878 | -0.253 | -0.1748 | -1.2857 |
| TRINITY_DN117_c0_g1_i4_orf1 | - | - | - | lipase member I-like [Ostrinia furnacalis] | 1.85689 | 0.1922 | -0.579 | -0.5123 | -0.9577 |
| TRINITY_DN1249_c0_g1_i6_orf1 | - | - | - | venom carboxylesterase-6-like [Ostrinia furnacalis] | 1.52609 | 0.81961 | -1.0348 | -0.8416 | -0.4693 |
| TRINITY_DN144190_c0_g1_i1_orf1 | - | - | - | PREDICTED: uncharacterized protein LOC103572804 isoform X2 [Microplitis demolitor] | 1.92351 | -0.4019 | -0.2012 | -0.3237 | -0.9966 |
| TRINITY_DN19917_c0_g1_i1_orf1 | - | - | - | synaptic vesicle glycoprotein 2B-like isoform X4 [Ostrinia furnacalis] | 1.99479 | -0.3814 | -0.5808 | -0.4668 | -0.5658 |
| TRINITY_DN14134_c0_g2_i3_orf1 | - | - | - | anoctamin-8-like isoform X2 [Helicoverpa zea] | 1.67408 | -1.3328 | -0.5709 | 0.30022 | -0.0706 |
| TRINITY_DN46372_c0_g2_i1_orf1 | - | - | - | basic salivary proline-rich protein 1 isoform X2 [Ostrinia furnacalis] | 1.03398 | -1.651 | -0.4068 | 0.00416 | 1.01964 |
| TRINITY_DN17189_c0_g1_i2_orf1 | - | - | - | fibroin heavy chain [Haritalodes derogata] | 1.56661 | -1.0493 | -0.637 | -0.6584 | 0.77809 |
| TRINITY_DN18009_c0_g1_i1_orf1 | - | - | - | pre-mRNA-splicing factor ISY1 homolog [Ostrinia furnacalis] | 0.68825 | -1.8369 | -0.2051 | 0.36577 | 0.98802 |
| TRINITY_DN640_c0_g1_i2_orf1 | - | - | - | pancreatic triacylglycerol lipase-like [Ostrinia furnacalis] | 1.7156 | -1.3375 | -0.249 | 0.24969 | -0.3788 |
| TRINITY_DN4323_c0_g1_i1_orf1 | - | - | - | uncharacterized protein LOC114364842 [Ostrinia furnacalis] | 1.63057 | -1.4809 | -0.356 | 0.10919 | 0.09714 |
| TRINITY_DN19080_c0_g1_i4_orf1 | - | - | - | synaptic vesicle 2-related protein-like isoform X1 [Ostrinia furnacalis] >XP_028161172.1 synaptic vesicle 2-related protein-like isoform X1 [Ostrinia furnacalis] | 1.31761 | -1.7808 | 0.12577 | 0.26885 | 0.06853 |
| TRINITY_DN1814_c0_g1_i11_orf1 | - | - | - | titin-like, partial [Ostrinia furnacalis] | 0.77793 | -1.876 | -0.1887 | 0.56609 | 0.72067 |
| TRINITY_DN47257_c0_g1_i4_orf1 | - | - | - | PREDICTED: microtubule-actin cross-linking factor 1-like, partial [Amyelois transitella] | 1.24326 | -1.7005 | -0.4193 | 0.40184 | 0.47467 |
| TRINITY_DN72541_c0_g1_i2_orf1 | - | - | - | xaa-Pro aminopeptidase ApepP-like isoform X2 [Ostrinia furnacalis] | 1.47835 | -1.5439 | -0.147 | -0.3335 | 0.546 |
| TRINITY_DN12250_c0_g1_i4_orf1 | - | - | - | ubiquinone biosynthesis monooxygenase COQ6, mitochondrial isoform X1 [Ostrinia furnacalis] >XP_028160041.1 ubiquinone biosynthesis monooxygenase COQ6, mitochondrial isoform X2 [Ostrinia furnacalis] | 1.76456 | -1.0738 | -0.0168 | 0.16598 | -0.84 |
| TRINITY_DN110132_c0_g1_i1_orf1 | - | - | - | uncharacterized protein LOC114361588 isoform X14 [Ostrinia furnacalis] | 1.18274 | -1.4883 | -0.8282 | 0.73623 | 0.39756 |
| TRINITY_DN15988_c0_g1_i1_orf1 | - | - | - | uncharacterized protein LOC114351163 [Ostrinia furnacalis] | 1.35583 | -0.8415 | -1.4209 | 0.56245 | 0.34409 |
| TRINITY_DN76529_c0_g1_i1_orfp1 | - | - | - | TRINITY_DN76529_c0_g1_i1_m.64079 TRINITY_DN76529_c0_g1::TRINITY_DN76529_c0_g1_i1::g.64079 ORF type:internal len:70 (+),score=14.68 TRINITY_DN76529_c0_g1_i1:3-209(+) | 0.25284 | -1.5651 | -0.3692 | 0.15635 | 1.52508 |
| TRINITY_DN18338_c0_g1_i7_orf1 | - | - | - | aquaporin AQPAn.G isoform X2 [Ostrinia furnacalis] | 1.62115 | -1.4382 | -0.5013 | 0.13131 | 0.18703 |
| TRINITY_DN348_c0_g2_i1_orf1 | - | - | - | pancreatic triacylglycerol lipase-like [Ostrinia furnacalis] | 1.61119 | -1.5326 | -0.0881 | 0.15891 | -0.1495 |
| TRINITY_DN640_c0_g1_i5_orf1 | - | - | - | pancreatic triacylglycerol lipase-like [Ostrinia furnacalis] | 1.6233 | -1.4113 | -0.002 | 0.31397 | -0.524 |
| TRINITY_DN21596_c0_g1_i1_orf1 | - | - | - | peptidyl-prolyl cis-trans isomerase NIMA-interacting 4 [Zerene cesonia] >XP_038208701.1 peptidyl-prolyl cis-trans isomerase NIMA-interacting 4 [Zerene cesonia] | 1.2445 | -1.454 | -0.8528 | 0.38006 | 0.68224 |
| TRINITY_DN2146_c0_g1_i1_orf1 | - | - | - | heat shock protein 68-like [Ostrinia furnacalis] | 1.45083 | -1.6406 | -0.1221 | 0.4206 | -0.1088 |
| TRINITY_DN7686_c0_g1_i4_orf1 | - | - | - | eIF-2-alpha kinase activator GCN1 [Ostrinia furnacalis] | 1.4808 | -1.4846 | -0.3313 | -0.3001 | 0.63517 |
| TRINITY_DN8940_c0_g1_i4_orf1 | - | - | - | probable ATP-dependent RNA helicase DDX23 [Ostrinia furnacalis] | 1.81428 | -1.1285 | -0.4313 | 0.20174 | -0.4562 |

| | | | | | | | | | |
|---|---|---|---|---|---|---|---|---|---|
| TRINITY_DN117707_c0_g1_i3_orf1 | - | - | - | acyl-CoA synthetase family member 2, mitochondrial isoform X1 [Ostrinia furnacalis] >XP_028172249.1 acyl-CoA synthetase family member 2, mitochondrial isoform X2 [Ostrinia furnacalis] >XP_028172258.1 acyl-CoA synthetase family member 2, mitochondrial isoform X3 [Ostrinia furnacalis] | 1.84559 | -1.1897 | -0.1688 | -0.1185 | -0.3685 |
| TRINITY_DN13718_c0_g1_i7_orf1 | - | - | - | immulectin-4 [Ostrinia furnacalis] | 1.4608 | -1.6406 | 0.35763 | 0.03503 | -0.2128 |
| TRINITY_DN8406_c0_g1_i4_orf1 | - | - | - | titin [Ostrinia furnacalis] | 1.05529 | -1.6064 | -0.3165 | -0.2101 | 1.07771 |
| TRINITY_DN1515_c0_g1_i2_orf1 | - | - | - | unnamed protein product [Diatraea saccharalis] | 1.57177 | -0.9144 | -1.1835 | 0.54088 | -0.0148 |
| TRINITY_DN2318_c1_g1_i1_orf1 | - | - | - | transcription factor SPT20 homolog [Ostrinia furnacalis] | 1.2347 | -1.6751 | -0.1939 | -0.1469 | 0.78124 |
| TRINITY_DN121156_c0_g2_i1_orf1 | - | - | - | terminal nucleotidyltransferase 5C isoform X1 [Ostrinia furnacalis] >XP_028163344.1 terminal nucleotidyltransferase 5C isoform X1 [Ostrinia furnacalis] >XP_028163346.1 terminal nucleotidyltransferase 5C isoform X1 | 1.38243 | -1.3005 | -0.9385 | 0.70216 | 0.1544 |
| TRINITY_DN659_c0_g2_i1_orf1 | - | - | - | unnamed protein product [Diatraea saccharalis] | 1.26783 | -1.347 | -0.7639 | -0.1438 | 0.98688 |
| TRINITY_DN23204_c0_g1_i1_orf1 | - | - | - | LOW QUALITY PROTEIN: uncharacterized protein LOC114350452 [Ostrinia furnacalis] | 1.50835 | -1.5018 | 0.26138 | 0.29342 | -0.5614 |
| TRINITY_DN3760_c0_g1_i1_orf1 | - | - | - | something about silencing protein 10 [Ostrinia furnacalis] | 1.77445 | -1.2395 | 0.19763 | -0.4277 | -0.3049 |
| TRINITY_DN3529_c0_g1_i7_orf1 | - | - | - | putative fatty acyl-CoA reductase CG5065 [Ostrinia furnacalis] | 0.97793 | -1.4086 | -1.0104 | 0.7323 | 0.70876 |
| TRINITY_DN8454_c0_g1_i4_orf1 | - | - | - | translocon-associated protein subunit alpha [Ostrinia furnacalis] | 0.85199 | -1.6767 | -0.5213 | 0.29554 | 1.0505 |
| TRINITY_DN38562_c0_g1_i3_orf1 | - | - | - | persulfide dioxygenase ETHE1, mitochondrial isoform X1 [Ostrinia furnacalis] | 1.44782 | -1.5107 | 0.45489 | 0.21504 | -0.6071 |
| TRINITY_DN27045_c0_g1_i1_orf1 | - | - | - | cytochrome P450 6B5-like [Galleria mellonella] | 0.92164 | -1.756 | -0.312 | 0.96864 | 0.17771 |
| TRINITY_DN1982_c0_g1_i17_orf1 | - | - | - | unnamed protein product, partial [Iphiclides podalirius] | 0.73087 | -1.9434 | 0.04109 | 0.56205 | 0.6094 |
| TRINITY_DN5721_c0_g1_i5_orf1 | - | - | - | fumarate hydratase, mitochondrial-like isoform X2 [Ostrinia furnacalis] | 1.80896 | -1.1376 | 0.20812 | -0.3981 | -0.4814 |
| TRINITY_DN12_c0_g1_i5_orf1 | - | - | - | cAMP-dependent protein kinase type II regulatory subunit isoform X1 [Ostrinia furnacalis] >XP_028175270.1 cAMP-dependent protein kinase type II regulatory subunit isoform X1 [Ostrinia furnacalis] | 1.06841 | -1.4124 | -0.9771 | 0.52659 | 0.79459 |
| TRINITY_DN7335_c0_g1_i1_orf1 | - | - | - | probable methylmalonate-semialdehyde dehydrogenase [acylating], mitochondrial [Bicyclus anynana] | 1.66909 | -1.421 | 0.13425 | -0.4189 | 0.03657 |
| TRINITY_DN8724_c0_g1_i5_orf1 | - | - | - | vesicle-associated membrane protein/synaptobrevin-binding protein isoform X1 [Pectinophora gossypiella] | 1.24579 | -0.8307 | -1.4801 | 0.53642 | 0.52863 |
| TRINITY_DN19830_c0_g1_i1_orf1 | - | - | - | macrophage migration inhibitory factor-like [Ostrinia furnacalis] | 1.48518 | -1.6401 | 0.30154 | -0.037 | -0.1096 |
| TRINITY_DN61_c0_g2_i3_orf1 | - | - | - | mitochondrial dicarboxylate carrier [Ostrinia furnacalis] >XP_028161565.1 mitochondrial dicarboxylate carrier [Ostrinia furnacalis] >XP_028161566.1 mitochondrial dicarboxylate carrier [Ostrinia furnacalis] | 1.77967 | -1.2337 | -0.5093 | 0.14098 | -0.1776 |
| TRINITY_DN25492_c0_g1_i1_orf1 | - | - | - | PREDICTED: myrosinase 1-like [Amyelois transitella] | 1.20647 | -1.8119 | 0.11147 | -0.0051 | 0.49903 |
| TRINITY_DN8964_c0_g1_i4_orf1 | - | - | - | hypothetical protein evm_010115 [Chilo suppressalis] | 0.86215 | -1.7431 | -0.4956 | 0.57566 | 0.80086 |
| TRINITY_DN5218_c0_g1_i4_orf1 | - | - | - | threonine--tRNA ligase, cytoplasmic isoform X1 [Trichoplusia ni] | 1.6852 | -1.4428 | -0.2163 | -0.1379 | 0.11185 |
| TRINITY_DN146126_c0_g1_i1_orf1 | - | - | - | malate dehydrogenase, mitochondrial [Chelonus insularis] | 1.43713 | -1.694 | 0.04612 | 0.24783 | -0.037 |
| TRINITY_DN1697_c0_g1_i1_orf1 | - | - | - | mitogen-activated protein kinase-binding protein 1 [Ostrinia furnacalis] | 1.57083 | -1.411 | -0.6485 | 0.21603 | 0.27266 |
| TRINITY_DN48851_c0_g1_i2_orf1 | - | - | - | translationally-controlled tumor protein homolog [Ostrinia furnacalis] | 1.30789 | -1.3943 | -0.6501 | -0.2024 | 0.93896 |
| TRINITY_DN136906_c0_g1_i1_orf1 | - | - | - | translational elongation factor-1alpha, partial [Ethmia eupostica] | 1.49127 | -1.4208 | -0.4704 | -0.2775 | 0.67752 |
| TRINITY_DN21872_c0_g1_i2_orf1 | - | - | - | facilitated trehalose transporter Tret1-2 homolog [Ostrinia furnacalis] >XP_028178438.1 facilitated trehalose transporter Tret1-2 homolog [Ostrinia furnacalis] >XP_028178439.1 facilitated trehalose transporter Tret1-2 homolog [Ostrinia furnacalis] | 1.49891 | -1.5298 | -0.0152 | -0.4306 | 0.47672 |
| TRINITY_DN16673_c0_g1_i1_orf1 | - | - | - | myosin heavy chain, partial [Drosophila virilis] | 1.40382 | -1.6773 | -0.1907 | 0.0425 | 0.4217 |
| TRINITY_DN6365_c0_g1_i4_orf1 | - | - | - | 40S ribosomal protein S21 [Helicoverpa armigera] >XP_047038308.1 40S ribosomal protein S21 isoform X2 [Helicoverpa zea] >KAI5643652.1 ribosomal protein s21e domain-containing protein [Phthorimaea operculella] >PZC73652.1 hypothetical protein B5X24_HaOG209026 [Helicoverpa armigera] | 1.31419 | -1.6359 | -0.4816 | 0.54684 | 0.25647 |
| TRINITY_DN3906_c0_g1_i5_orf1 | - | - | - | ejaculatory bulb-specific protein 3-like [Ostrinia furnacalis] | 0.41695 | -1.6766 | -0.4847 | 0.5125 | 1.23185 |
| TRINITY_DN3970_c0_g1_i1_orf1 | - | - | - | hypothetical protein evm_002369 [Chilo suppressalis] | 1.48325 | -1.3716 | -0.7168 | -0.0305 | 0.6356 |
| TRINITY_DN235_c0_g1_i2_orf1 | - | - | - | unnamed protein product [Parnassius apollo] | 0.88794 | -1.9049 | -0.0548 | 0.48361 | 0.58814 |

| | | | | | | | | |
|---|---|---|---|---|---|---|---|---|
| TRINITY_DN42461_c0_g1_i4_orf1 | - | - | - | obscurin [Ostrinia furnacalis] | 0.77504 | -1.9087 | -0.031 | 0.38605 | 0.7786 |
| TRINITY_DN1369_c0_g2_i3_orf1 | - | - | - | ATP-dependent Clp protease ATP-binding subunit clpX-like, mitochondrial isoform X2 [Helicoverpa zea] | 1.36079 | -1.1275 | -0.9287 | -0.2739 | 0.96929 |
| TRINITY_DN11050_c0_g1_i8_orf1 | - | - | - | uncharacterized protein LOC114360965, partial [Ostrinia furnacalis] | 1.51599 | -1.5122 | -0.3813 | 0.50378 | -0.1263 |
| TRINITY_DN38435_c0_g1_i1_orf1 | - | - | - | UDP-glucuronosyltransferase 2B20-like [Ostrinia furnacalis] | 1.26559 | -1.7565 | 0.05592 | -0.1106 | 0.54559 |
| TRINITY_DN107962_c0_g1_i1_orf1 | - | - | - | unnamed protein product [Euphydryas editha] | 0.80478 | -1.763 | -0.4169 | 0.93745 | 0.43767 |
| TRINITY_DN22747_c0_g1_i5_orf1 | - | - | - | hypothetical protein HF086_001789 [Spodoptera exigua] | 1.52591 | -1.1303 | -1.0777 | 0.33931 | 0.34276 |
| TRINITY_DN2254_c0_g1_i4_orf1 | - | - | - | vigilin [Ostrinia furnacalis] | 0.7437 | -1.4774 | -0.9093 | 0.61262 | 1.03047 |
| TRINITY_DN496_c0_g1_i7_orf1 | - | - | - | unnamed protein product [Diatraea saccharalis] | 1.27702 | -1.4026 | -0.7733 | 0.00223 | 0.89663 |
| TRINITY_DN41166_c0_g1_i1_orf1 | - | - | - | arginine kinase isoform X1 [Ostrinia furnacalis] | 1.33646 | -1.4921 | -0.5359 | -0.1344 | 0.82594 |
| TRINITY_DN37986_c0_g1_i2_orf1 | - | - | - | unnamed protein product [Diatraea saccharalis] | 1.54504 | -1.1332 | -0.9567 | -0.0915 | 0.63637 |
| TRINITY_DN6916_c0_g1_i4_orf1 | - | - | - | isovaleryl-CoA dehydrogenase, mitochondrial [Ostrinia furnacalis] | 1.54485 | -1.5622 | -0.1359 | -0.1905 | 0.34377 |
| TRINITY_DN5111_c0_g1_i2_orf1 | - | - | - | uncharacterized protein LOC126368598 [Pectinophora gossypiella] | 1.77498 | -1.2237 | 0.22496 | -0.3821 | -0.3941 |
| TRINITY_DN139326_c0_g1_i1_orf1 | - | - | - | 40S ribosomal protein S13 [Gallus gallus] >NP_001008.1 40S ribosomal protein S13 [Homo sapiens] >NP_001020513.1 40S ribosomal protein S13 [Bos taurus] >NP_001231758.1 40S ribosomal protein S13 [Sus scrofa] >NP_001232227.1 40S ribosomal protein S13 [Taeniopygia guttata] >NP_001252846.1 40S ribosomal protein S13 [Macaca mulatta] >NP_080809.1 40S ribosomal protein S13 [Mus musculus] >NP_569116.1 40S ribosomal protein S13 [Rattus norvegicus] >XP_001504999.3 40S ribosomal protein S13 [Equus caballus] >XP_002721403.1 PREDICTED: 40S ribosomal protein S13 [Oryctolagus cuniculus] >XP_002925190.2 40S ribosomal protein S13 [Ailuropoda melanoleuca] >XP_003254322.1 40S ribosomal protein S13 [Nomascus leucogenys] >XP_003312983.1 40S ribosomal protein S13 [Pan troglodytes] >XP_003412037.1 40S ribosomal protein S13 [Loxodonta africana] >XP_003777851.1 40S ribosomal protein S13 [Pongo abelii] >XP_003781188.1 40S ribosomal protein S13 [Otolemur garnettii] >XP_003818255.1 40S ribosomal protein S13 [Pan paniscus] >XP_003910218.1 40S ribosomal protein S13 [Papio anubis] >XP_003993072.1 40S ribosomal protein S13 [Felis catus] >XP_004050806.1 40S ribosomal protein S13 [Gorilla gorilla gorilla] >XP_004285569.1 40S ribosomal protein S13 [Orcinus orca] >XP_004312593.1 40S ribosomal protein S13 [Tursiops truncatus] >XP_004369736.1 40S ribosomal protein S13 [Trichechus manatus latirostris] >XP_004415573.1 PREDICTED: 40S ribosomal protein S13 [Odobenus rosmarus divergens] >XP_004418532.1 PREDICTED: 40S ribosomal protein S13 [Ceratotherium simum simum] >XP_004472558.1 40S ribosomal protein S13 [Dasypus novemcinctus] >XP_004593806.1 40S ribosomal protein S13 [Ochotona princeps] >XP_004632754.1 40S ribosomal protein S13 [Octodon degus] >XP_004634969.1 40S ribosomal protein S13 isoform X1 [Octodon degus] >XP_004650891.1 40S ribosomal protein S13 [Jaculus jaculus] >XP_004683069.1 PREDICTED: 40S ribosomal protein S13 [Condylura cristata] >XP_004711628.1 40S ribosomal protein S13 [Echinops telfairi] >XP_004752124.1 40S ribosomal protein S13 [Mustela putorius furo] >XP_004851550.1 40S ribosomal protein | 1.50962 | -1.2109 | -0.9164 | -0.0261 | 0.64371 |
| TRINITY_DN3511_c0_g2_i1_orf1 | - | - | - | eukaryotic translation initiation factor 2A [Ostrinia furnacalis] | 1.47409 | -1.1207 | -1.0037 | -0.0946 | 0.74484 |
| TRINITY_DN110376_c0_g1_i1_orf1 | - | - | - | nucleolysin TIAR [Osmia bicornis bicornis] >XP_034192702.1 nucleolysin TIAR [Osmia lignaria] | 1.44204 | -1.3238 | -0.9129 | 0.49453 | 0.30015 |

| | | | | | | | | | |
|---|---|---|---|---|---|---|---|---|---|
| TRINITY_DN15234_c0_g1_i3_orf1 | - | - | - | 60S ribosomal protein L30 [Papilio polytes] >XP_[illegible].1 60S ribosomal protein L30 [Papilio machaon] >XP_026485186.1 60S ribosomal protein L30 isoform X1 [Vanessa tameamea] >XP_028160279.1 60S ribosomal protein L30 [Ostrinia furnacalis] >XP_030027999.1 60S ribosomal protein L30 [Manduca sexta] >XP_032515151.1 60S ribosomal protein L30 [Danaus plexippus plexippus] >XP_034840952.1 60S ribosomal protein L30 [Maniola hyperantus] >XP_037301873.1 60S ribosomal protein L30 [Manduca sexta] >XP_039745408.1 60S ribosomal protein L30 [Pararge aegeria] >XP_041974708.1 60S ribosomal protein L30 [Aricia agestis] >XP_045455248.1 60S ribosomal protein L30 [Melitaea cinxia] >XP_045457914.1 60S ribosomal protein L30 [Melitaea cinxia] >XP_046969892.1 60S ribosomal protein L30 [Vanessa cardui] >XP_047539529.1 60S ribosomal protein L30 [Vanessa atalanta] >XP_049887645.1 60S ribosomal protein L30 [Pectinophora gossypiella] >XP_050360253.1 60S ribosomal protein L30 [Nymphalis io] >ADT80684.1 ribosomal protein L30 [Euphydryas aurinia] >CAG9575798.1 unnamed protein product [Danaus chrysippus] >CAH0722581.1 unnamed protein product, partial [Brenthis ino] >CAH2099946.1 unnamed protein product [Euphydryas editha] >CAH2267204.1 jg2932 [Pararge aegeria aegeria] >[illegible] 60S ribosomal protein L30 [Eumeta japonica] | 1.70537 | -1.3776 | -0.414 | -0.0537 | 0.13996 |
| TRINITY_DN79673_c0_g1_i1_orf1 | - | - | - | thioredoxin, mitochondrial-like [Ostrinia furnacalis] | 1.3876 | -1.6977 | 0.01057 | 0.42126 | -0.1217 |
| TRINITY_DN2224_c0_g1_i1_orf1 | - | - | - | serine--tRNA ligase, cytoplasmic [Ostrinia furnacalis] | 1.4605 | -1.5702 | -0.3722 | -0.0301 | 0.51197 |
| TRINITY_DN5753_c0_g1_i10_orf1 | - | - | - | ryanodine receptor [Ostrinia furnacalis] | 1.46929 | -1.569 | -0.4124 | 0.45372 | 0.05845 |
| TRINITY_DN1125_c0_g1_i4_orf1 | - | - | - | hypothetical protein evm_001907 [Chilo suppressalis] >CAH2985359.1 unnamed protein product [Chilo suppressalis] | 1.10141 | -1.6667 | -0.5612 | 0.73599 | 0.3905 |
| TRINITY_DN8584_c0_g1_i6_orf1 | - | - | - | uncharacterized protein LOC114354070 isoform X3 [Ostrinia furnacalis] | 1.69836 | -1.4119 | -0.265 | 0.14985 | -0.1713 |
| TRINITY_DN8352_c0_g1_i3_orf1 | - | - | - | TRPL translocation defect protein 14 isoform X1 [Ostrinia furnacalis] | 1.77547 | -1.1973 | 0.18885 | -0.1781 | -0.589 |
| TRINITY_DN1752_c0_g1_i18_orf1 | - | - | - | titin isoform X1 [Ostrinia furnacalis] | 1.55201 | -1.5723 | -0.2745 | 0.16025 | 0.13451 |
| TRINITY_DN9248_c0_g1_i10_orf1 | - | - | - | unnamed protein product [Arctia plantaginis] | 1.65725 | -1.4378 | -0.3354 | -0.125 | 0.24101 |
| TRINITY_DN32896_c0_g3_i1_orf1 | - | - | - | PREDICTED: calcium-binding mitochondrial carrier protein Aralar1 isoform X1 [Microplitis demolitor] | 1.53048 | -1.4846 | -0.3637 | 0.52686 | -0.209 |
| TRINITY_DN5765_c0_g2_i3_orf1 | - | - | - | unnamed protein product [Diatraea saccharalis] | 1.68375 | -1.3547 | -0.4696 | -0.1525 | 0.29307 |
| TRINITY_DN14701_c0_g1_i2_orf1 | - | - | - | staphylococcal nuclease domain-containing protein 1 [Ostrinia furnacalis] | 1.57356 | -1.2722 | -0.8345 | 0.08367 | 0.44949 |
| TRINITY_DN130159_c0_g2_i1_orf1 | - | - | - | lachesin-like [Chelonus insularis] >XP_034946935.1 lachesin-like [Chelonus | 1.54254 | -1.4711 | -0.5094 | -0.0059 | 0.44383 |
| TRINITY_DN13371_c0_g1_i4_orf1 | - | - | - | ATP synthase mitochondrial F1 complex assembly factor 2 [Ostrinia furnacalis] | 1.78988 | -1.2425 | -0.3549 | -0.3285 | 0.13611 |
| TRINITY_DN83542_c0_g1_i1_orf1 | - | - | - | PREDICTED: WASH complex subunit strumpellin [Microplitis demolitor] | 1.7063 | -1.3855 | 0.18853 | -0.2116 | -0.2977 |
| TRINITY_DN129808_c0_g1_i1_orf1 | - | - | - | uncharacterized protein LOC114354070 isoform X3 [Ostrinia furnacalis] | 1.47857 | -1.1164 | -1.0455 | -0.0053 | 0.68871 |
| TRINITY_DN111110_c0_g1_i1_orf1 | - | - | - | NAD-dependent protein deacylase-like [Ostrinia furnacalis] | 0.80908 | -1.6903 | -0.59 | 0.56583 | 0.90546 |
| TRINITY_DN12508_c0_g1_i1_orf1 | - | - | - | uncharacterized protein LOC114350091 [Ostrinia furnacalis] | 1.19246 | -1.5702 | -0.7167 | 0.53813 | 0.55625 |
| TRINITY_DN38366_c0_g1_i4_orfp1 | - | - | - | TRINITY_DN38366_c0_g1_i4_m.10666 TRINITY_DN38366_c0_g1::TRINITY_DN38366_c0_g1_i4::g.10666 ORF type:internal len:143 (+),score=71.68 TRINITY_DN38366_c0_g1_i4:3-428(+) | 0.82029 | -1.8675 | -0.2131 | 0.64019 | 0.62004 |
| TRINITY_DN83948_c0_g1_i3_orf1 | - | - | - | carbonyl reductase [NADPH] 1-like [Ostrinia furnacalis] | 1.87114 | -1.0408 | 0.07439 | -0.4335 | -0.4712 |
| TRINITY_DN11448_c0_g1_i4_orf1 | - | - | - | uncharacterized protein LOC114364760 isoform X5 [Ostrinia furnacalis] | 1.35317 | -1.7358 | -0.1178 | 0.15818 | 0.34225 |
| TRINITY_DN133228_c0_g1_i3_orf1 | - | - | - | microtubule-actin cross-linking factor 1 isoform X15 [Ostrinia furnacalis] | 1.54778 | -1.5603 | -0.1274 | 0.3382 | -0.1983 |
| TRINITY_DN110460_c0_g2_i1_orf1 | - | - | - | Similar to chaf1a-b: Chromatin assembly factor 1 subunit A-B (Xenopus laevis) [Cotesia congregata] | 1.34595 | -1.7647 | 0.22627 | 0.1429 | 0.04963 |
| TRINITY_DN22956_c0_g1_i1_orf1 | - | - | - | lipoamide acyltransferase component of branched-chain alpha-keto acid dehydrogenase complex, mitochondrial [Ostrinia furnacalis] | 1.51832 | -1.5424 | -0.0857 | 0.44362 | -0.3338 |
| TRINITY_DN20763_c0_g1_i2_orf1 | - | - | - | uncharacterized protein LOC114355848 [Ostrinia furnacalis] | 1.39256 | -1.52 | -0.4692 | -0.1215 | 0.71807 |
| TRINITY_DN4451_c0_g2_i4_orf1 | - | - | - | uncharacterized protein LOC114361986 isoform X1 [Ostrinia furnacalis] >XP_028173022.1 uncharacterized protein LOC114361986 isoform X2 [Ostrinia furnacalis] | 1.63047 | -1.4371 | 0.14913 | -0.4839 | 0.14136 |

| | | | | | | | | | |
|---|---|---|---|---|---|---|---|---|---|
| TRINITY_DN38835_c0_g3_i1_orf1 | - | - | - | protein transport protein Sec61 subunit alpha [Spodoptera litura] >XP_035429226.1 protein transport protein Sec61 subunit alpha [Spodoptera frugiperda] >XP_047985890.1 protein transport protein Sec61 subunit alpha [Leguminivora glycinivorella] >KAF9413961.1 hypothetical protein HW555_007991 [Spodoptera exigua] >CAB3514725.1 unnamed protein product [Spodoptera littoralis] >KAF9810869.1 hypothetical protein SFRURICE_005295 [Spodoptera frugiperda] >KAG8115796.1 hypothetical protein SFRUCORN_012373 [Spodoptera frugiperda] >CAH0700181.1 unnamed protein | 1.41453 | -1.6051 | -0.4544 | 0.38651 | 0.2585 |
| TRINITY_DN1445_c0_g2_i4_orf1 | - | - | - | leucine-rich PPR motif-containing protein, mitochondrial [Ostrinia furnacalis] | 1.66909 | -1.4737 | -0.0087 | -0.2045 | 0.01793 |
| TRINITY_DN15362_c0_g1_i1_orf1 | - | - | - | probable elongation factor 1-delta isoform X1 [Ostrinia furnacalis] | 1.47499 | -1.1195 | -0.8951 | -0.2889 | 0.82852 |
| TRINITY_DN25960_c0_g1_i1_orf1 | - | - | - | protein mini spindles [Ostrinia furnacalis] | 1.5777 | -1.0917 | -1.0705 | 0.26002 | 0.32455 |
| TRINITY_DN9965_c0_g1_i1_orf1 | - | - | - | dihydrolipoyl dehydrogenase [Ostrinia furnacalis] | 1.80322 | -1.2659 | -0.3207 | -0.2076 | -0.0091 |
| TRINITY_DN7808_c0_g1_i1_orf1 | - | - | - | probable pyruvate dehydrogenase E1 component subunit alpha, mitochondrial isoform X1 [Ostrinia furnacalis] >XP_028158738.1 probable pyruvate dehydrogenase E1 component subunit alpha, mitochondrial isoform X2 [Ostrinia furnacalis] >XP_028158739.1 probable pyruvate dehydrogenase E1 component subunit alpha, mitochondrial isoform X3 [Ostrinia furnacalis] >XP_028158740.1 probable pyruvate dehydrogenase E1 component subunit alpha, mitochondrial isoform X4 [Ostrinia furnacalis] | 1.66559 | -1.476 | -0.0627 | 0.06916 | -0.196 |
| TRINITY_DN1757_c0_g1_i4_orf1 | - | - | - | F-box/LRR-repeat protein 2 isoform X1 [Ostrinia furnacalis] | 1.75521 | -1.3265 | -0.215 | 0.10592 | -0.3196 |
| TRINITY_DN50676_c0_g1_i1_orf1 | - | - | - | uncharacterized protein LOC114360659 [Ostrinia furnacalis] | 1.29888 | -1.5556 | -0.6113 | 0.7011 | 0.16687 |
| TRINITY_DN105055_c0_g1_i1_orfp1 | - | - | - | unnamed protein product [Euphydryas editha] | 1.87362 | -1.0753 | -0.1253 | -0.123 | -0.55 |
| TRINITY_DN120439_c1_g1_i1_orf1 | - | - | - | myosin heavy chain variant, partial [Bombyx mori] | 1.15961 | -1.8054 | -0.1495 | 0.22845 | 0.56686 |
| TRINITY_DN1173_c1_g1_i10_orf1 | - | - | - | hypothetical protein evm_001011 [Chilo suppressalis] | 0.96313 | -1.818 | -0.0739 | 0.05792 | 0.87085 |
| TRINITY_DN36817_c0_g1_i1_orf1 | - | - | - | uncharacterized protein LOC114357350 [Ostrinia furnacalis] | 1.19413 | -1.8316 | 0.0963 | 0.09229 | 0.44889 |
| TRINITY_DN7134_c0_g1_i1_orf1 | - | - | - | phosphatidylglycerophosphatase and protein-tyrosine phosphatase 1 [Ostrinia furnacalis] | 1.84737 | -1.1144 | 0.01316 | -0.1903 | -0.5558 |
| TRINITY_DN63533_c0_g1_i2_orf1 | - | - | - | glutathione S-transferase sigma3 [Glyphodes pyloalis] | 0.94663 | -1.8136 | -0.3032 | 0.72337 | 0.44681 |
| TRINITY_DN78492_c0_g1_i1_orf1 | - | - | - | uncharacterized protein LOC114354775 [Ostrinia furnacalis] | 1.41604 | -1.2661 | -0.9905 | 0.58964 | 0.25097 |
| TRINITY_DN57918_c0_g1_i1_orf1 | - | - | - | PREDICTED: serine--tRNA ligase, cytoplasmic [Fopius arisanus] | 1.41489 | -1.6412 | -0.2486 | -0.0176 | 0.49249 |
| TRINITY_DN4385_c0_g2_i1_orf1 | - | - | - | LOW QUALITY PROTEIN: carbonic anhydrase 1-like [Ostrinia furnacalis] | 1.50947 | -1.3891 | -0.6849 | -0.0037 | 0.56818 |
| TRINITY_DN1351_c0_g1_i1_orf1 | - | - | - | PREDICTED: flavin reductase (NADPH) [Microplitis demolitor] >XP_008553603.1 PREDICTED: flavin reductase (NADPH) [Microplitis demolitor] | 1.54713 | -0.7451 | -1.3722 | 0.33905 | 0.23108 |
| TRINITY_DN7267_c1_g1_i4_orf1 | - | - | - | probable pseudouridine-5'-phosphatase [Ostrinia furnacalis] | 1.72758 | -1.3158 | -0.516 | 0.13094 | -0.0267 |
| TRINITY_DN21451_c0_g1_i3_orf1 | - | - | - | gelsolin-like [Ostrinia furnacalis] | 1.61632 | -1.4443 | -0.0201 | 0.30454 | -0.4565 |
| TRINITY_DN2186_c0_g1_i17_orf1 | - | - | - | paxillin isoform X6 [Leguminivora glycinivorella] | 0.84617 | -1.9173 | 0.02583 | 0.34836 | 0.69698 |
| TRINITY_DN9117_c0_g1_i1_orf1 | - | - | - | spherulin-2A-like [Ostrinia furnacalis] | 1.78555 | -1.3126 | -0.1246 | -0.2538 | -0.0945 |
| TRINITY_DN27500_c0_g1_i4_orf1 | - | - | - | hemicentin-1-like [Ostrinia furnacalis] | 0.50168 | -1.7358 | -0.3761 | 0.4193 | 1.19087 |
| TRINITY_DN29229_c0_g1_i4_orf1 | - | - | - | uncharacterized protein LOC114351433 isoform X1 [Ostrinia furnacalis] | 1.55509 | -1.1708 | -0.6953 | -0.4272 | 0.73821 |
| TRINITY_DN34399_c0_g1_i1_orf1 | - | - | - | cysteine synthase-like [Ostrinia furnacalis] | 1.48356 | -1.3839 | -0.7308 | 0.04109 | 0.59004 |
| TRINITY_DN5597_c0_g1_i2_orf1 | - | - | - | monocarboxylate transporter 9-like [Ostrinia furnacalis] >XP_028156211.1 monocarboxylate transporter 9-like [Ostrinia furnacalis] | 1.84346 | -1.0666 | -0.0255 | -0.0748 | -0.6766 |
| TRINITY_DN34347_c0_g1_i1_orf1 | - | - | - | nesprin-1-like isoform X8 [Bombyx mandarina] | 1.36056 | -1.2947 | -0.7858 | -0.1858 | 0.90579 |
| TRINITY_DN4920_c0_g1_i5_orf1 | - | - | - | titin homolog [Ostrinia furnacalis] | 1.08524 | -1.8415 | -0.0659 | 0.20018 | 0.62195 |
| TRINITY_DN116951_c0_g3_i2_orf1 | - | - | - | spermine oxidase-like isoform X2 [Ostrinia furnacalis] | 1.01154 | -1.902 | 0.09775 | 0.52927 | 0.26347 |
| TRINITY_DN135_c0_g1_i1_orf1 | - | - | - | 60S ribosomal protein L11 [Nymphalis io] | 1.69197 | -1.454 | -0.133 | -0.0512 | -0.0537 |
| TRINITY_DN1232_c0_g1_i1_orf1 | - | - | - | acanthoscurrin-2-like isoform X1 [Ostrinia furnacalis] | 1.01414 | -1.8039 | -0.2617 | 0.30617 | 0.74523 |
| TRINITY_DN129869_c0_g4_i1_orf1 | - | - | - | putative myosin heavy chain, muscle, partial [Cotesia chilonis] | 1.27825 | -1.779 | -0.0875 | 0.19268 | 0.39557 |
| TRINITY_DN46216_c0_g3_i1_orf1 | - | - | - | unnamed protein product, partial [Brenthis ino] | 0.35194 | -1.8772 | -0.0983 | 0.92312 | 0.70043 |
| TRINITY_DN20957_c0_g1_i1_orf1 | - | - | - | adenylate kinase isoenzyme 1 isoform X2 [Ostrinia furnacalis] | 1.26355 | -1.7615 | 0.00595 | -0.0537 | 0.54568 |

| | | | | | | | | | |
|---|---|---|---|---|---|---|---|---|---|
| TRINITY_DN27276_c0_g1_i5_orf1 | - | - | - | probable small nuclear ribonucleoprotein Sm D1 [Ostrinia furnacalis] >CAG9751027.1 unnamed protein product [Diatraea saccharalis] >CAG9789712.1 unnamed protein product [Diatraea saccharalis] | 1.03256 | -1.4738 | -0.8517 | 0.96314 | 0.32978 |
| TRINITY_DN7414_c0_g1_i1_orf1 | - | - | - | uncharacterized protein LOC114357447 [Ostrinia furnacalis] | 1.65158 | -1.2852 | -0.7366 | 0.11572 | 0.25443 |
| TRINITY_DN1952_c0_g1_i2_orf1 | - | - | - | uncharacterized protein LOC114354403 [Ostrinia furnacalis] >AYE20402.1 RNAi efficiency-related nuclease REase [Ostrinia furnacalis] | 1.49918 | -1.632 | -0.0535 | 0.27855 | -0.0922 |
| TRINITY_DN147676_c0_g1_i1_orf1 | - | - | - | PREDICTED: 60S ribosomal protein L23 [Microplitis demolitor] >XP_044591174.1 60S ribosomal protein L23 [Cotesia glomerata] >KAG8035666.1 hypothetical protein G9C98_001094 [Cotesia typhae] >KAH0547433.1 60S ribosomal protein L23A [Cotesia glomerata] | 1.5449 | -1.2928 | -0.8229 | 0.05967 | 0.51112 |
| TRINITY_DN4622_c0_g1_i1_orf1 | - | - | - | keratin-associated protein 19-2-like [Ostrinia furnacalis] | 1.66637 | -1.4417 | -0.1569 | -0.2764 | 0.20873 |
| TRINITY_DN74889_c0_g1_i1_orf1 | - | - | - | probable 28S ribosomal protein S23, mitochondrial [Ostrinia furnacalis] | 1.80761 | -1.1594 | -0.1582 | 0.10376 | -0.5937 |
| TRINITY_DN434_c0_g1_i4_orf1 | - | - | - | uncharacterized protein LOC126367148 [Pectinophora gossypiella] | 1.44835 | -1.6193 | -0.2067 | 0.47703 | -0.0994 |
| TRINITY_DN80245_c0_g1_i1_orf1 | - | - | - | peroxisomal membrane protein 2 [Ostrinia furnacalis] | 1.58988 | -1.5158 | -0.3146 | 0.27309 | -0.0325 |
| TRINITY_DN11194_c0_g1_i4_orf1 | - | - | - | ATPase family AAA domain-containing protein 3A homolog [Ostrinia furnacalis] | 1.70742 | -1.3619 | -0.4496 | 0.15816 | -0.0541 |
| TRINITY_DN12372_c0_g1_i4_orf1 | - | - | - | WD repeat-containing protein 44 isoform X4 [Ostrinia furnacalis] | 1.50603 | -1.1483 | -1.0637 | 0.22526 | 0.48071 |
| TRINITY_DN4016_c0_g1_i1_orf1 | - | - | - | 60S acidic ribosomal protein P0 [Homo sapiens] >NP_444505.1 60S acidic ribosomal protein P0 [Homo sapiens] >XP_002823894.1 60S acidic ribosomal protein P0 [Pongo abelii] >XP_003280010.1 60S acidic ribosomal protein P0 [Nomascus leucogenys] >XP_004054038.1 60S acidic ribosomal protein P0 [Gorilla gorilla gorilla] >XP_004054039.1 60S acidic ribosomal protein P0 [Gorilla gorilla gorilla] >XP_008956032.1 60S acidic ribosomal protein P0 [Pan paniscus] >XP_008956033.1 60S acidic ribosomal protein P0 [Pan paniscus] >XP_012611945.1 60S acidic ribosomal protein P0 [Microcebus murinus] >XP_016802006.1 60S acidic ribosomal protein P0 [Pan troglodytes] >XP_016802007.1 60S acidic ribosomal protein P0 [Pan troglodytes] >XP_025256707.1 60S acidic ribosomal protein P0 isoform X1 [Theropithecus gelada] >XP_025256708.1 60S acidic ribosomal protein P0 isoform X1 [Theropithecus gelada] >XP_032024425.1 60S acidic ribosomal protein P0 [Hylobates moloch] >XP_032657670.1 60S acidic ribosomal protein P0 [Chelonoidis abingdonii] >XP_045390642.1 60S acidic ribosomal protein P0 [Lemur catta] >P05388.1 RecName: Full=60S acidic ribosomal protein P0; AltName: Full=60S ribosomal protein L10E; AltName: Full=Large ribosomal subunit protein uL10 [Homo sapiens] >3J92_s Structure and assembly pathway of the ribosome quality control complex [Oryctolagus cuniculus] >4V5Z_Bg Chain Bg, 60S acidic ribosomal protein P0 [Canis lupus familiaris] >4V6X_Cq Chain Cq, 60S acidic ribosomal protein P0 [Homo sapiens] >5AJ0_AK Chain AK, 60S acidic ribosomal protein P0 [Homo sapiens] >6ZM7_Ls Chain Ls, 60S acidic ribosomal protein P0 [Homo sapiens] >6ZME_Ls Chain Ls, 60S acidic ribosomal protein P0 [Homo sapiens] >6ZMI_Ls Chain Ls, 60S acidic ribosomal protein P0 [Homo sapiens] >6ZMO_Ls Chain Ls, 60S acidic ribosomal protein P0 [Homo sapiens] >ABM82739.1 ribosomal protein, large, P0 [synthetic construct] >SJX33952.1 unnamed protein product, partial [Human ORFeome Gateway entry vector] >AAA36470.1 acidic ribosomal phosphoprotein (P0) [Homo sapiens] >AAC05176.1 60S ACIDIC RIBOSOMAL PROTEIN; match to P05388 (PID:g133041) [Homo sapiens] >AAH00087.1 Ribosomal protein, large, P0 | 1.26996 | -1.7953 | -0.0438 | 0.27196 | 0.29713 |
| TRINITY_DN7580_c0_g1_i1_orf1 | - | - | - | cytochrome P450 monooxygenase CYP6AB141 [Ostrinia furnacalis] | 0.84874 | -1.6684 | -0.6023 | 0.95841 | 0.46355 |
| TRINITY_DN3461_c0_g1_i1_orf1 | - | - | - | protein SCO1 homolog, mitochondrial [Ostrinia furnacalis] | 1.71859 | -1.4052 | -0.2311 | 0.04586 | -0.1282 |
| TRINITY_DN14597_c0_g1_i5_orf1 | - | - | - | UDP-glucuronosyltransferase 2B1-like isoform X3 [Ostrinia furnacalis] | 1.57602 | -1.4093 | -0.4549 | 0.51926 | -0.231 |
| TRINITY_DN4408_c6_g1_i1_orf1 | - | - | - | polyprotein, partial [Bemisia tabaci] | 0.70323 | -1.6052 | -0.7501 | 0.85312 | 0.79894 |
| TRINITY_DN89613_c0_g1_i13_orf1 | - | - | - | PREDICTED: uncharacterized protein LOC106137743 [Amyelois transitella] | 1.31465 | -1.4975 | -0.7602 | 0.41415 | 0.5289 |
| TRINITY_DN96557_c0_g1_i1_orf1 | - | - | - | charged multivesicular body protein 4B [Phyllostomus discolor] | 1.21615 | -1.8149 | -0.0245 | 0.18456 | 0.43867 |

| | | | | | | | | | |
|---|---|---|---|---|---|---|---|---|---|
| TRINITY_DN126648_c0_g1_i1_orf1 | - | - | - | elongation factor 1 alpha, partial [Spodoptera exigua] >QYQ52647.1 elongation factor 1 alpha, partial [Spodoptera exigua] | 1.48814 | -0.845 | -1.3494 | 0.32901 | 0.37726 |
| TRINITY_DN1173_c0_g1_i12_orf1 | - | - | - | obscurin [Ostrinia furnacalis] | 1.63042 | -1.4058 | -0.5469 | 0.24624 | 0.07601 |
| TRINITY_DN10629_c0_g1_i1_orf1 | - | - | - | caspase-1-like [Ostrinia furnacalis] | 1.30104 | -1.7101 | -0.2387 | 0.5646 | 0.08321 |
| TRINITY_DN1123_c2_g1_i5_orf1 | - | - | - | troponin I isoform X4 [Leguminivora glycinivorella] | 1.10226 | -1.7624 | -0.098 | -0.0581 | 0.81617 |
| TRINITY_DN4133_c0_g1_i2_orfp2 | - | - | - | unnamed protein product [Spodoptera exigua] | 0.53026 | -1.3421 | -1.0596 | 0.78808 | 1.08338 |
| TRINITY_DN3355_c0_g2_i4_orf1 | - | - | - | UDP-glycosyltransferase UGT33AL1 [Ostrinia furnacalis] | 1.83471 | -1.0723 | -0.01 | -0.0593 | -0.6932 |
| TRINITY_DN115082_c0_g1_i5_orf1 | - | - | - | protein dj-1beta-like isoform X2 [Ostrinia furnacalis] | 1.01295 | -1.4333 | -0.2235 | -0.5897 | 1.2336 |
| TRINITY_DN4501_c0_g1_i3_orf1 | - | - | - | methylcrotonoyl-CoA carboxylase subunit alpha, mitochondrial [Ostrinia | 1.35524 | -1.6488 | -0.3995 | 0.19675 | 0.49637 |
| TRINITY_DN55160_c0_g1_i1_orf1 | - | - | - | esterase FE4-like isoform X2 [Ostrinia furnacalis] | 1.84094 | -1.155 | -0.1618 | -0.0241 | -0.5001 |
| TRINITY_DN4145_c0_g1_i1_orf1 | - | - | - | uncharacterized protein LOC114353175 isoform X1 [Ostrinia furnacalis] | 1.43662 | -1.6926 | -0.0692 | 0.08066 | 0.24454 |
| TRINITY_DN106534_c0_g1_i1_orf1 | - | - | - | nucleolar complex protein 2 homolog [Ostrinia furnacalis] | 1.4278 | -1.4871 | -0.6673 | 0.22064 | 0.50594 |
| TRINITY_DN97589_c0_g1_i3_orf1 | - | - | - | ribosomal protein L37A [Bombyx mori] >XP_013189707.1 PREDICTED: 60S ribosomal protein L37a [Amyelois transitella] >XP_021198447.1 60S ribosomal protein L37a [Helicoverpa armigera] >XP_022122377.1 60S ribosomal protein L37a [Pieris rapae] >XP_022822835.1 60S ribosomal protein L37a [Spodoptera litura] >XP_023937141.1 60S ribosomal protein L37a [Bicyclus anynana] >XP_026321523.1 60S ribosomal protein L37a [Hyposmocoma kahamanoa] >XP_026495655.1 60S ribosomal protein L37a [Vanessa tameamea] >XP_026746489.1 60S ribosomal protein L37a [Trichoplusia ni] >XP_026756267.1 60S ribosomal protein L37a [Galleria mellonella] >XP_028041705.1 60S ribosomal protein L37a [Bombyx mandarina] >XP_028161757.1 60S ribosomal protein L37a [Ostrinia furnacalis] >XP_030020263.1 LOW QUALITY PROTEIN: 60S ribosomal protein L37a [Manduca sexta] >XP_032518929.1 60S ribosomal protein L37a [Danaus plexippus plexippus] >XP_034834514.1 60S ribosomal protein L37a [Maniola hyperantus] >XP_035444256.1 60S ribosomal protein L37a [Spodoptera frugiperda] >XP_038222439.1 60S ribosomal protein L37a [Zerene cesonia] >XP_039756348.1 60S ribosomal protein L37a [Pararge aegeria] >XP_041981914.1 60S ribosomal protein L37a [Aricia agestis] >XP_045451710.1 60S ribosomal protein L37a [Melitaea cinxia] >XP_045500579.1 60S ribosomal protein L37a [Colias croceus] >XP_045517305.1 60S ribosomal protein L37a [Pieris brassicae] >XP_045775103.1 60S ribosomal protein L37a [Maniola jurtina] >XP_046969745.1 60S ribosomal protein L37a [Vanessa cardui] >XP_047032252.1 60S ribosomal protein L37a [Helicoverpa zea] >XP_047525321.1 60S ribosomal protein L37a [Pieris napi] >XP_047535357.1 60S ribosomal protein L37a [Vanessa atalanta] >XP_049875744.1 60S ribosomal protein L37a [Pectinophora gossypiella] >XP_050348149.1 60S ribosomal protein L37a [Nymphalis io] >ADO95156.1 ribosomal protein L37A [Antheraea yamamai] >ADT80705.1 ribosomal protein L37A [Euphydryas aurinia] >AEL28885.1 ribosomal protein L37A [Heliconius melpomene cythera] >KAF9418899.1 hypothetical protein HW555_004419 [Spodoptera exigua] | 1.65906 | -1.1754 | -0.8735 | 0.07909 | 0.31079 |
| TRINITY_DN2172_c0_g2_i5_orf1 | - | - | - | 4-hydroxyphenylpyruvate dioxygenase [Ostrinia furnacalis] | 1.67313 | -1.3836 | -0.4173 | 0.29195 | -0.1642 |
| TRINITY_DN51934_c0_g2_i1_orf1 | - | - | - | SCAN domain-containing protein 3-like [Pieris napi] >XP_047520696.1 SCAN domain-containing protein 3-like [Pieris napi] | 1.76621 | -1.191 | -0.618 | 0.22039 | -0.1776 |
| TRINITY_DN6351_c0_g1_i4_orf1 | - | - | - | cytochrome P450 CYP12A2-like [Ostrinia furnacalis] | 1.88994 | -1.1052 | -0.2373 | -0.2894 | -0.258 |
| TRINITY_DN3504_c0_g1_i3_orfp2 | - | - | - | TRINITY_DN3504_c0_g1_i3_m.43947 TRINITY_DN3504_c0_g1::TRINITY_DN3504_c0_g1_i3::g.43947 ORF type:5prime_partial len:208 (-),score=77.75 TRINITY_DN3504_c0_g1_i3:185- | 1.45307 | -1.605 | -0.3986 | 0.24413 | 0.30648 |
| TRINITY_DN20682_c0_g1_i2_orf1 | - | - | - | hypothetical protein B5X24_HaOG200252 [Helicoverpa armigera] | 1.77466 | -1.282 | -0.4386 | -0.108 | 0.05401 |
| TRINITY_DN2924_c0_g1_i2_orf1 | - | - | - | cuticular protein RR-2 [Spodoptera litura] | 1.36974 | -1.5524 | -0.4203 | 0.72312 | -0.1202 |
| TRINITY_DN18396_c0_g1_i1_orf1 | - | - | - | uncharacterized protein LOC114359424 [Ostrinia furnacalis] | 1.44257 | -1.5131 | -0.4827 | -0.0722 | 0.62547 |

| | | | | | | | | | |
|---|---|---|---|---|---|---|---|---|---|
| TRINITY_DN2709_c0_g1_i4_orf1 | - | - | - | ATP-dependent RNA helicase dbp2-like [Ostrinia furnacalis] | 1.74613 | -1.3414 | 0.11917 | -0.2541 | -0.2697 |
| TRINITY_DN34040_c0_g2_i1_orf1 | - | - | - | uncharacterized protein LOC114352849 [Ostrinia furnacalis] | 1.76021 | -1.2874 | -0.077 | 0.08499 | -0.4809 |
| TRINITY_DN6143_c0_g2_i1_orf1 | - | - | - | uncharacterized protein LOC114365036 [Ostrinia furnacalis] | 1.72925 | -1.3804 | -0.2763 | -0.1488 | 0.0762 |
| TRINITY_DN83295_c0_g1_i3_orf1 | - | - | - | SSSX-APN4 [Ostrinia furnacalis] | 1.86128 | -1.162 | -0.1389 | -0.3477 | -0.2126 |
| TRINITY_DN23167_c0_g1_i4_orf1 | - | - | - | uncharacterized protein LOC114363065 [Ostrinia furnacalis] | 1.54021 | -1.3277 | -0.6191 | -0.2434 | 0.64999 |
| TRINITY_DN8621_c0_g1_i5_orf1 | - | - | - | aminopeptidase N-like isoform X2 [Ostrinia furnacalis] | 1.85298 | -1.1793 | -0.1061 | -0.3246 | -0.243 |
| TRINITY_DN5012_c0_g1_i6_orf1 | - | - | - | putative serine protease K12H4.7 [Ostrinia furnacalis] | 1.78253 | -1.2725 | -0.0734 | 0.00821 | -0.4448 |
| TRINITY_DN3135_c0_g1_i6_orf1 | - | - | - | acanthoscurrin-1-like [Ostrinia furnacalis] | 1.47513 | -1.6658 | -0.043 | 0.21712 | 0.01649 |
| TRINITY_DN4929_c1_g2_i5_orf1 | - | - | - | guanylate kinase isoform X2 [Ostrinia furnacalis] | 1.65908 | -1.2004 | -0.6886 | -0.2762 | 0.50607 |
| TRINITY_DN32479_c0_g1_i8_orf1 | - | - | - | hypothetical protein evm_009815 [Chilo suppressalis] >CAB3525305.1 unnamed protein product [Chilo suppressalis] >CAH0402632.1 unnamed protein product [Chilo suppressalis] | 1.7466 | -1.2846 | -0.54 | 0.08587 | -0.0078 |
| TRINITY_DN53462_c0_g1_i1_orf1 | - | - | - | uncharacterized protein LOC118072968 isoform X1 [Chelonus insularis] >XP_034949073.1 uncharacterized protein LOC118072968 isoform X1 [Chelonus insularis] | 1.4066 | -1.2337 | -0.8025 | -0.2583 | 0.88802 |
| TRINITY_DN313_c0_g1_i5_orf1 | - | - | - | collagen alpha-1(X) chain-like [Ostrinia furnacalis] | 1.71349 | -1.3114 | -0.5737 | 0.09654 | 0.07511 |
| TRINITY_DN19814_c0_g1_i4_orf1 | - | - | - | general odorant-binding protein 28a-like [Ostrinia furnacalis] | 1.59117 | -1.3076 | -0.7524 | 0.03152 | 0.43731 |
| TRINITY_DN5244_c0_g1_i1_orf1 | - | - | - | eukaryotic peptide chain release factor GTP-binding subunit-like [Ostrinia | 1.74602 | -1.3691 | -0.2541 | -0.0126 | -0.1102 |
| TRINITY_DN130051_c0_g1_i1_orf1 | - | - | - | 5-methyltetrahydropteroyltriglutamate--homocysteine S-methyltransferase-like protein [Leptotrombidium deliense] | 1.74656 | -1.2497 | -0.3954 | 0.28569 | -0.3872 |
| TRINITY_DN10940_c0_g1_i10_orfp1 | - | - | - | TRINITY_DN10940_c0_g1_i10_m.52163 TRINITY_DN10940_c0_g1::TRINITY_DN10940_c0_g1_i10::g.52163 ORF type:5prime_partial len:248 (-),score=128.24 TRINITY_DN10940_c0_g1_i10:121-864(-) | 1.80827 | -1.279 | -0.1611 | -0.167 | -0.2012 |
| TRINITY_DN22664_c0_g1_i1_orf1 | - | - | - | larval cuticle protein LCP-14-like [Ostrinia furnacalis] | 1.62823 | -1.2535 | -0.7554 | -0.0691 | 0.44976 |
| TRINITY_DN30704_c0_g1_i1_orf1 | - | - | - | cytochrome P450 monooxygenase CYP6AE134v2 [Ostrinia furnacalis] | 1.79163 | -1.2332 | -0.2129 | 0.11383 | -0.4594 |
| TRINITY_DN3504_c0_g1_i4_orfp1 | - | - | - | TRINITY_DN3504_c0_g1_i4_m.43930 TRINITY_DN3504_c0_g1::TRINITY_DN3504_c0_g1_i4::g.43930 ORF type:internal len:196 (-),score=84.82 TRINITY_DN3504_c0_g1_i4:3-587(-) | 1.59652 | -1.5395 | -0.2232 | 0.17611 | -0.0099 |
| TRINITY_DN336_c0_g1_i6_orfp1 | - | - | - | TRINITY_DN336_c0_g1_i6_m.64791 TRINITY_DN336_c0_g1::TRINITY_DN336_c0_g1_i6::g.64791 ORF type:complete len:61 (-),score=19.53 TRINITY_DN336_c0_g1_i6:236-418(-) | 1.84736 | -1.0979 | 0.10981 | -0.4487 | -0.4106 |
| TRINITY_DN38498_c0_g3_i1_orf1 | - | - | - | unnamed protein product [Parnassius apollo] | 1.49356 | -1.4275 | -0.715 | 0.39392 | 0.25504 |
| TRINITY_DN2986_c1_g1_i1_orf1 | - | - | - | Troponin C, isoform 1 [Papilio xuthus] | 1.64358 | -1.2486 | -0.71 | -0.1474 | 0.46241 |
| TRINITY_DN76815_c0_g1_i3_orf1 | - | - | - | 5-formyltetrahydrofolate cyclo-ligase [Ostrinia furnacalis] | 0.7456 | -1.3241 | -1.0988 | 1.03474 | 0.64253 |
| TRINITY_DN36883_c0_g1_i1_orf1 | - | - | - | PREDICTED: importin subunit alpha-4 [Microplitis demolitor] | 0.50366 | 1.51373 | -1.5113 | -0.3993 | -0.1068 |
| TRINITY_DN30273_c1_g1_i1_orf1 | - | - | - | uncharacterized protein LOC114358591 isoform X2 [Ostrinia furnacalis] | 0.20152 | 1.72656 | -1.2877 | -0.5602 | -0.0802 |
| TRINITY_DN2061_c0_g1_i3_orf1 | - | - | - | uncharacterized protein LOC114357318 isoform X1 [Ostrinia furnacalis] >XP_028166680.1 uncharacterized protein LOC114357318 isoform X2 [Ostrinia furnacalis] | 0.33181 | 1.74218 | -0.9652 | -0.1617 | -0.9471 |
| TRINITY_DN1749_c0_g2_i2_orf1 | - | - | - | putative GPI-anchored protein pfl2 isoform X1 [Ostrinia furnacalis] | -0.3791 | 1.97891 | -0.7738 | -0.401 | -0.425 |
| TRINITY_DN10745_c0_g1_i14_orf1 | - | - | - | septin-1 [Ostrinia furnacalis] | 0.63822 | 1.66325 | -0.799 | -0.5793 | -0.9232 |
| TRINITY_DN1233_c0_g2_i1_orf1 | - | - | - | unnamed protein product [Spodoptera exigua] | 0.27197 | 1.81938 | -0.9077 | -0.8056 | -0.378 |
| TRINITY_DN32956_c0_g1_i4_orf1 | - | - | - | inositol-trisphosphate 3-kinase A isoform X1 [Vanessa tameamea] >XP_047534115.1 inositol-trisphosphate 3-kinase A isoform X1 [Vanessa atalanta] >XP_047534116.1 inositol-trisphosphate 3-kinase A isoform X1 [Vanessa atalanta] >XP_047534117.1 inositol-trisphosphate 3-kinase A isoform | 0.35912 | 1.75816 | -1.0643 | -0.7418 | -0.3111 |
| TRINITY_DN37585_c0_g2_i1_orf1 | - | - | - | cuticle protein 19.8-like [Ostrinia furnacalis] | -0.0947 | 1.92523 | -0.8985 | -0.3197 | -0.6123 |
| TRINITY_DN2638_c0_g1_i7_orf1 | - | - | - | structural maintenance of chromosomes protein 1A [Trichoplusia ni] | 0.51516 | 1.65422 | -1.0334 | -0.9455 | -0.1905 |
| TRINITY_DN14009_c0_g1_i1_orf1 | - | - | - | proline-rich extensin-like protein EPR1 [Manduca sexta] | -0.1996 | 1.9602 | -0.815 | -0.5297 | -0.4159 |
| TRINITY_DN54524_c0_g1_i6_orf1 | - | - | - | serine protease inhibitor dipetalogastin [Ostrinia furnacalis] | -0.3108 | 1.98843 | -0.6461 | -0.5101 | -0.5214 |

| | | | | | | | | | |
|---|---|---|---|---|---|---|---|---|---|
| TRINITY_DN19923_c0_g1_i1_orf1 | - | - | - | uncharacterized protein LOC114350958 [Ostrinia furnacalis] | -0.0245 | 1.94062 | -0.5786 | -0.6231 | -0.7144 |
| TRINITY_DN9100_c0_g1_i5_orf1 | - | - | - | microtubule-associated protein futsch-like isoform X6 [Ostrinia furnacalis] | 0.26785 | 1.84266 | -0.7338 | -0.5355 | -0.8412 |
| TRINITY_DN6656_c0_g1_i1_orf1 | - | - | - | sorting and assembly machinery component 50 homolog isoform X9 [Ostrinia furnacalis] >XP_028169233.1 sorting and assembly machinery component 50 homolog isoform X10 [Ostrinia furnacalis] >XP_028169234.1 sorting and assembly machinery component 50 homolog isoform X11 [Ostrinia furnacalis] | 0.09235 | 1.87477 | -0.9822 | -0.3764 | -0.6086 |
| TRINITY_DN40669_c0_g2_i1_orf1 | - | - | - | uncharacterized protein LOC114355531 [Ostrinia furnacalis] | -0.4265 | 1.99572 | -0.6175 | -0.4538 | -0.4979 |
| TRINITY_DN46090_c0_g2_i1_orf1 | - | - | - | inactive tyrosine-protein kinase 7-like, partial [Ostrinia furnacalis] | -0.0198 | 1.90951 | -0.9165 | -0.6278 | -0.3454 |
| TRINITY_DN5840_c0_g1_i6_orf1 | - | - | - | catenin alpha isoform X2 [Ostrinia furnacalis] | 0.45809 | 1.75117 | -0.7722 | -0.9362 | -0.5009 |
| TRINITY_DN206_c0_g1_i8_orf1 | - | - | - | A-kinase anchor protein 200-like [Ostrinia furnacalis] >XP_028173114.1 A-kinase anchor protein 200-like [Ostrinia furnacalis] >XP_028173115.1 A-kinase anchor protein 200-like [Ostrinia furnacalis] | -0.1944 | 1.94284 | -0.9099 | -0.3558 | -0.4828 |
| TRINITY_DN120593_c0_g1_i1_orf1 | - | - | - | SUMO-activating enzyme subunit 1 [Ostrinia furnacalis] | 0.5269 | 1.69562 | -1.1043 | -0.5945 | -0.5237 |
| TRINITY_DN110534_c0_g1_i3_orf1 | - | - | - | unnamed protein product [Euphydryas editha] | 0.5383 | 1.67097 | -0.6304 | -1.1597 | -0.4191 |
| TRINITY_DN25870_c0_g2_i6_orf1 | - | - | - | homeobox protein extradenticle isoform X3 [Ostrinia furnacalis] | 0.40838 | 1.58092 | -1.3043 | -0.7887 | 0.10365 |
| TRINITY_DN668_c0_g1_i4_orf1 | - | - | - | fatty acid synthase-like isoform X1 [Ostrinia furnacalis] | -0.1118 | 1.95841 | -0.685 | -0.6451 | -0.5165 |
| TRINITY_DN9000_c0_g2_i1_orf1 | - | - | - | uncharacterized protein LOC114356585 [Ostrinia furnacalis] | 0.32542 | 1.82252 | -0.7712 | -0.5657 | -0.811 |
| TRINITY_DN2946_c0_g1_i1_orf1 | - | - | - | histidine-rich glycoprotein [Ostrinia furnacalis] | -0.3101 | 1.98559 | -0.6359 | -0.429 | -0.6107 |
| TRINITY_DN7735_c1_g1_i1_orf1 | - | - | - | cuticular protein CPH [Spodoptera litura] | -0.2785 | 1.95789 | -0.8515 | -0.3107 | -0.5172 |
| TRINITY_DN1293_c0_g1_i4_orf1 | - | - | - | putative fatty acyl-CoA reductase CG5065 [Ostrinia furnacalis] | -0.0377 | 1.9038 | -0.8903 | -0.7175 | -0.2584 |
| TRINITY_DN13576_c0_g1_i1_orf1 | - | - | - | uncharacterized protein LOC114350099 [Ostrinia furnacalis] | -0.388 | 1.9852 | -0.7288 | -0.4458 | -0.4226 |
| TRINITY_DN35633_c0_g2_i1_orf1 | - | - | - | uncharacterized protein LOC114353024 [Ostrinia furnacalis] | 0.62328 | 1.679 | -0.6615 | -0.7539 | -0.8869 |
| TRINITY_DN20793_c0_g2_i1_orf1 | - | - | - | mucin-2-like [Ostrinia furnacalis] | -0.0551 | 1.94798 | -0.6181 | -0.6996 | -0.5752 |
| TRINITY_DN7803_c0_g1_i2_orf1 | - | - | - | membrane-associated protein Hem [Ostrinia furnacalis] | 0.09822 | 1.86127 | -0.9017 | -0.2519 | -0.8059 |
| TRINITY_DN1133_c0_g1_i6_orf1 | - | - | - | zinc finger protein 391-like [Ostrinia furnacalis] >XP_028169193.1 zinc finger protein 391-like [Ostrinia furnacalis] | 0.34322 | 1.78491 | -1.0256 | -0.4159 | -0.6866 |
| TRINITY_DN20244_c0_g1_i1_orfp1 | - | - | - | uncharacterized protein LOC125235519 [Leguminivora glycinivorella] | 0.39736 | 1.77601 | -0.4517 | -0.8312 | -0.8905 |
| TRINITY_DN1280_c0_g1_i1_orf1 | - | - | - | coronin-7 isoform X1 [Ostrinia furnacalis] >XP_028164815.1 coronin-7 isoform X2 [Ostrinia furnacalis] >XP_028164817.1 coronin-7 isoform X3 [Ostrinia furnacalis] >XP_028164818.1 coronin-7 isoform X4 [Ostrinia furnacalis] >XP_028164820.1 coronin-7 isoform X6 [Ostrinia furnacalis] >XP_028164821.1 coronin-7 isoform X7 [Ostrinia furnacalis] >XP_028164822.1 coronin-7 isoform X1 [Ostrinia furnacalis] >XP_028164823.1 coronin-7 isoform X8 [Ostrinia furnacalis] >XP_028164824.1 coronin-7 isoform X9 [Ostrinia furnacalis] >XP_028164825.1 coronin-7 isoform X10 [Ostrinia furnacalis] | 0.47808 | 1.75435 | -0.8383 | -0.7949 | -0.5992 |
| TRINITY_DN4021_c0_g1_i1_orf1 | - | - | - | leech-derived tryptase inhibitor C-like [Ostrinia furnacalis] | -0.4082 | 1.9664 | -0.8414 | -0.3884 | -0.3284 |
| TRINITY_DN5595_c0_g1_i1_orf1 | - | - | - | keratin, type I cytoskeletal 10-like [Ostrinia furnacalis] | -0.4201 | 1.98264 | -0.7499 | -0.4149 | -0.3978 |
| TRINITY_DN5200_c0_g1_i2_orf1 | - | - | - | uncharacterized protein LOC114351644 [Ostrinia furnacalis] | 0.35279 | 1.59155 | -1.4325 | -0.5383 | 0.02647 |
| TRINITY_DN4898_c0_g1_i7_orf1 | - | - | - | annulin-like isoform X3 [Ostrinia furnacalis] | 0.24566 | 1.85648 | -0.7939 | -0.7138 | -0.5944 |
| TRINITY_DN18922_c0_g1_i1_orf1 | - | - | - | LOW QUALITY PROTEIN: CCR4-NOT transcription complex subunit 6 [Ostrinia furnacalis] | 0.17705 | 1.4704 | -1.391 | -0.7759 | 0.51941 |
| TRINITY_DN2054_c0_g1_i1_orf1 | - | - | - | macrophage mannose receptor 1-like [Ostrinia furnacalis] | 0.49434 | 1.59174 | -0.9341 | -1.1616 | 0.00965 |
| TRINITY_DN1935_c0_g1_i1_orf1 | - | - | - | adult-specific cuticular protein ACP-22-like [Ostrinia furnacalis] | -0.2259 | 1.97884 | -0.6113 | -0.6333 | -0.5083 |
| TRINITY_DN806_c0_g2_i1_orf1 | - | - | - | uncharacterized protein LOC114355167 [Ostrinia furnacalis] | -0.1207 | 1.93624 | -0.8625 | -0.615 | -0.3381 |
| TRINITY_DN9028_c0_g1_i5_orf1 | - | - | - | decaprenyl-diphosphate synthase subunit 2-like [Ostrinia furnacalis] | -0.137 | 1.91759 | -0.9615 | -0.2611 | -0.5581 |
| TRINITY_DN2450_c0_g1_i6_orf1 | - | - | - | oxysterol-binding protein-related protein 9 [Manduca sexta] | 0.74341 | 1.6106 | -0.8465 | -0.7685 | -0.739 |
| TRINITY_DN486_c0_g1_i5_orf1 | - | - | - | adaptor complexes medium subunit family domain-containing protein [Phthorimaea operculella] | 0.48981 | 1.39775 | -1.5421 | -0.6021 | 0.25662 |
| TRINITY_DN2936_c0_g1_i1_orf1 | - | - | - | myosin heavy chain, non-muscle isoform X1 [Hyposmocoma kahamanoa] | 0.68481 | 1.6374 | -0.9602 | -0.6667 | -0.6953 |
| TRINITY_DN13395_c0_g1_i1_orf1 | - | - | - | cytoplasmic dynein 1 light intermediate chain 2 [Galleria mellonella] | 0.40297 | 1.78199 | -0.7786 | -0.8862 | -0.5202 |

| | | | | | | | | |
|---|---|---|---|---|---|---|---|---|
| TRINITY_DN2345_c0_g1_i4_orf1 | - | - | - | chromobox protein homolog 3-like [Ostrinia furnacalis] >XP_028157236.1 chromobox protein homolog 3-like [Ostrinia furnacalis] | 0.29718 | 1.71118 | -1.1252 | -0.8462 | -0.0369 |
| TRINITY_DN14443_c0_g1_i1_orf1 | - | - | - | SUMO-activating enzyme subunit 2 [Ostrinia furnacalis] | 0.66177 | 1.60715 | -1.1279 | -0.7377 | -0.4032 |
| TRINITY_DN56993_c0_g1_i4_orf1 | - | - | - | polypyrimidine tract-binding protein 1 isoform X11 [Helicoverpa zea] | 0.47794 | 1.67294 | -0.9313 | -0.1845 | -1.0351 |
| TRINITY_DN4144_c0_g1_i7_orf1 | - | - | - | uncharacterized protein LOC114350172 [Ostrinia furnacalis] | 0.16822 | 1.87969 | -0.5632 | -0.84 | -0.6447 |
| TRINITY_DN381_c0_g1_i1_orf1 | - | - | - | cuticle protein 8-like [Ostrinia furnacalis] | -0.4127 | 1.99137 | -0.6754 | -0.4536 | -0.4497 |
| TRINITY_DN9354_c0_g1_i7_orf1 | - | - | - | hypothetical protein evm_012205 [Chilo suppressalis] >CAB3527181.1 unnamed protein product [Chilo suppressalis] >CAH0404510.1 unnamed protein product [Chilo suppressalis] | 0.54897 | 1.60529 | -0.4274 | -1.3367 | -0.3902 |
| TRINITY_DN5954_c0_g1_i2_orf1 | - | - | - | myosin-VIIa [Ostrinia furnacalis] >XP_028155907.1 myosin-VIIa [Ostrinia | 0.10325 | 1.90547 | -0.6794 | -0.7465 | -0.5828 |
| TRINITY_DN4782_c0_g1_i1_orf1 | - | - | - | patched domain-containing protein 3-like [Ostrinia furnacalis] | 0.25743 | 1.47416 | -1.4602 | -0.6795 | 0.40815 |
| TRINITY_DN143497_c0_g1_i1_orf1 | - | - | - | fibroin heavy chain-like [Ostrinia furnacalis] | -0.1805 | 1.97035 | -0.6986 | -0.5169 | -0.5744 |
| TRINITY_DN14721_c0_g1_i2_orf1 | - | - | - | protein masquerade-like isoform X2 [Ostrinia furnacalis] | 0.31441 | 1.8303 | -0.6418 | -0.8218 | -0.681 |
| TRINITY_DN123184_c0_g1_i1_orf1 | - | - | - | double-strand break repair protein MRE11 [Ostrinia furnacalis] | 0.36647 | 1.61415 | -1.2518 | -0.8268 | 0.09798 |
| TRINITY_DN12222_c0_g1_i1_orf1 | - | - | - | unnamed protein product [Chilo suppressalis] | 0.01492 | 1.80703 | -1.2413 | -0.1786 | -0.4021 |
| TRINITY_DN5074_c0_g1_i7_orf1 | - | - | - | zonadhesin-like [Ostrinia furnacalis] | -0.2288 | 1.91383 | -1.0295 | -0.3991 | -0.2564 |
| TRINITY_DN8738_c0_g1_i1_orf1 | - | - | - | unnamed protein product [Plutella xylostella] | 0.60316 | 1.4732 | -1.4798 | -0.5197 | -0.0769 |
| TRINITY_DN17655_c0_g1_i1_orf1 | - | - | - | BRISC and BRCA1-A complex member 1-like [Ostrinia furnacalis] | 0.3707 | 1.69511 | -0.6585 | -1.2353 | -0.172 |
| TRINITY_DN15865_c0_g1_i1_orf1 | - | - | - | carboxylesterase, partial [Ostrinia furnacalis] | 0.14796 | 1.86531 | -0.8385 | -0.3576 | -0.8171 |
| TRINITY_DN286_c0_g1_i2_orf1 | - | - | - | uncharacterized protein LOC114361329 [Ostrinia furnacalis] | 0.55637 | 1.69285 | -0.661 | -0.5426 | -1.0457 |
| TRINITY_DN5568_c0_g2_i2_orf1 | - | - | - | carboxypeptidase D isoform X5 [Ostrinia furnacalis] | 0.23837 | 1.83727 | -0.936 | -0.4247 | -0.7149 |
| TRINITY_DN33893_c0_g1_i1_orf1 | - | - | - | high mobility group protein I-like [Ostrinia furnacalis] | 0.22334 | 1.63419 | -1.4629 | -0.373 | -0.0217 |
| TRINITY_DN5235_c0_g1_i7_orf1 | - | - | - | peptidoglycan-recognition protein SA-like [Ostrinia furnacalis] | 0.46261 | 1.73819 | -0.7522 | -0.4507 | -0.9978 |
| TRINITY_DN67649_c0_g1_i1_orf1 | - | - | - | proliferating cell nuclear antigen [Ostrinia furnacalis] >XP_028174842.1 proliferating cell nuclear antigen [Ostrinia furnacalis] | 0.84402 | 1.52844 | -0.8546 | -0.9449 | -0.5729 |
| TRINITY_DN535_c1_g1_i2_orf1 | - | - | - | protein tramtrack, beta isoform isoform X24 [Bicyclus anynana] | 0.39845 | 1.71138 | -0.9725 | -0.9686 | -0.1687 |
| TRINITY_DN33008_c0_g1_i1_orf1 | - | - | - | double-stranded RNA-binding protein Staufen homolog 2 isoform X3 [Helicoverpa armigera] | 0.64299 | 1.6487 | -0.7939 | -0.5077 | -0.9901 |
| TRINITY_DN661_c1_g2_i1_orf1 | - | - | - | larval/pupal cuticle protein H1C-like [Ostrinia furnacalis] | 0.02299 | 1.91021 | -0.9071 | -0.5386 | -0.4875 |
| TRINITY_DN4937_c0_g1_i2_orf1 | - | - | - | zinc finger protein 778-like [Ostrinia furnacalis] | 0.49078 | 1.36061 | -1.5931 | -0.5393 | 0.28103 |
| TRINITY_DN978_c9_g2_i1_orf1 | - | - | - | hypothetical protein evm_000959 [Chilo suppressalis] | 0.26639 | 1.84075 | -0.8889 | -0.6744 | -0.5439 |
| TRINITY_DN54366_c0_g1_i1_orf1 | - | - | - | protein obstructor-E-like [Ostrinia furnacalis] | 0.09528 | 1.90817 | -0.735 | -0.5838 | -0.6847 |
| TRINITY_DN1853_c0_g1_i3_orf1 | - | - | - | trans-Golgi network integral membrane protein TGN38-like isoform X1 [Ostrinia furnacalis] | 0.40468 | 1.67924 | -1.3018 | -0.4803 | -0.3019 |
| TRINITY_DN17003_c0_g1_i1_orf1 | - | - | - | mucin-5AC [Ostrinia furnacalis] | 0.62395 | 1.65808 | -0.9257 | -0.884 | -0.4724 |
| TRINITY_DN5211_c0_g1_i1_orf1 | - | - | - | elongation of very long chain fatty acids protein AAEL008004-like [Ostrinia furnacalis] | 0.67653 | 1.64229 | -0.9413 | -0.7607 | -0.6168 |
| TRINITY_DN1326_c0_g1_i1_orf1 | - | - | - | cuticle protein 7-like [Ostrinia furnacalis] | -0.0884 | 1.94666 | -0.4804 | -0.7953 | -0.5825 |
| TRINITY_DN3310_c0_g1_i1_orf1 | - | - | - | hypothetical protein evm_010516 [Chilo suppressalis] | -0.1063 | 1.96004 | -0.6037 | -0.6012 | -0.6489 |
| TRINITY_DN27321_c0_g1_i1_orf1 | - | - | - | ras-related protein Rap-2c [Bicyclus anynana] >XP_026492616.1 ras-related protein Rap-2c [Vanessa tameamea] >XP_034838061.1 ras-related protein Rap-2c [Maniola hyperantus] >XP_039759141.1 ras-related protein Rap-2c [Pararge aegeria] >XP_045498804.1 ras-related protein Rap-2c [Colias croceus] >XP_046959644.1 ras-related protein Rap-2c [Vanessa cardui] >XP_047530248.1 ras-related protein Rap-2c [Vanessa atalanta] >CAH2268047.1 jg10357 [Pararge aegeria aegeria] | 0.36854 | 1.74117 | -1.1835 | -0.4248 | -0.5015 |
| TRINITY_DN467_c0_g3_i1_orf1 | - | - | - | histone-lysine N-methyltransferase 2B-like, partial [Ostrinia furnacalis] | 0.0258 | 1.92398 | -0.7922 | -0.5836 | -0.574 |
| TRINITY_DN650_c0_g1_i3_orf1 | - | - | - | chitinase 7 [Glyphodes pyloalis] | 0.28102 | 1.80654 | -0.7603 | -0.3485 | -0.9787 |
| TRINITY_DN73224_c0_g4_i2_orf1 | - | - | - | PREDICTED: poly(rC)-binding protein 3 isoform X2 [Vollenhovia emeryi] | 0.62198 | 1.67414 | -0.8232 | -0.5816 | -0.8913 |
| TRINITY_DN34745_c0_g2_i1_orf1 | - | - | - | GSK3-beta interaction protein-like [Galleria mellonella] | 0.77465 | 1.57009 | -1.0273 | -0.5827 | -0.7347 |
| TRINITY_DN9311_c0_g1_i1_orf1 | - | - | - | cuticle protein 8-like [Ostrinia furnacalis] | -0.1998 | 1.96093 | -0.7592 | -0.6361 | -0.3658 |

| | | | | | | | | |
|---|---|---|---|---|---|---|---|---|
| TRINITY_DN2004_c0_g1_i20_orf1 | - | - | - | hypothetical protein evm_006436 [Chilo suppressalis] >CAB3522373.1 unnamed protein product [Chilo suppressalis] >CAH0399695.1 unnamed protein product [Chilo suppressalis] | 0.29388 | 1.56354 | -1.3209 | -0.8069 | 0.27041 |
| TRINITY_DN24218_c0_g1_i1_orf1 | - | - | - | uncharacterized protein LOC114362624 [Ostrinia furnacalis] | 0.25686 | 1.48548 | -1.4585 | -0.6711 | 0.3872 |
| TRINITY_DN44256_c0_g1_i1_orf1 | - | - | - | essential MCU regulator, mitochondrial [Cotesia glomerata] | 0.6127 | 1.63641 | -1.0918 | -0.3724 | -0.7849 |
| TRINITY_DN27723_c0_g1_i1_orf1 | - | - | - | putative uncharacterized protein DDB_G0282133 isoform X1 [Ostrinia furnacalis] | 0.42957 | 1.72456 | -1.0764 | -0.313 | -0.7647 |
| TRINITY_DN267_c0_g1_i1_orf1 | - | - | - | keratin, type I cytoskeletal 9-like [Ostrinia furnacalis] | -0.271 | 1.95928 | -0.817 | -0.5783 | -0.293 |
| TRINITY_DN44658_c0_g1_i2_orf1 | - | - | - | lipase 3-like [Ostrinia furnacalis] | 0.44793 | 1.55827 | -1.4609 | -0.4828 | -0.0625 |
| TRINITY_DN14532_c0_g1_i1_orf1 | - | - | - | pupal cuticle protein-like [Trichoplusia ni] | 0.09595 | 1.89689 | -0.7332 | -0.4542 | -0.8054 |
| TRINITY_DN34703_c0_g1_i4_orf1 | - | - | - | gamma-tubulin complex component 3 homolog [Ostrinia furnacalis] | 0.26529 | 1.63962 | -1.4628 | -0.1778 | -0.2643 |
| TRINITY_DN7549_c0_g1_i1_orf1 | - | - | - | uncharacterized protein LOC114355006 [Ostrinia furnacalis] | -0.2098 | 1.96663 | -0.7847 | -0.4801 | -0.4921 |
| TRINITY_DN1231_c0_g1_i4_orf1 | - | - | - | AN1-type zinc finger protein 6 isoform X1 [Galleria mellonella] | 0.54684 | 1.66429 | -1.0861 | -0.3189 | -0.8061 |
| TRINITY_DN6436_c0_g1_i1_orf1 | - | - | - | serine/threonine-protein kinase PAK 3 isoform X1 [Ostrinia furnacalis] >XP_028164178.1 serine/threonine-protein kinase PAK 3 isoform X2 [Ostrinia furnacalis] >XP_028164179.1 serine/threonine-protein kinase PAK 3 isoform X3 [Ostrinia furnacalis] | 0.64813 | 1.64997 | -0.7112 | -1.0086 | -0.5783 |
| TRINITY_DN7785_c0_g1_i1_orf1 | - | - | - | uncharacterized protein LOC114364098 [Ostrinia furnacalis] | -0.0375 | 1.93856 | -0.7398 | -0.6782 | -0.4829 |
| TRINITY_DN98242_c0_g1_i1_orf1 | - | - | - | adenosine deaminase 2-A-like [Galleria mellonella] | 0.21135 | 1.82519 | -0.9611 | -0.7848 | -0.2907 |
| TRINITY_DN31619_c0_g1_i2_orf1 | - | - | - | endocuticle structural glycoprotein ABD-4-like [Ostrinia furnacalis] | 0.11217 | 1.89043 | -0.8432 | -0.7035 | -0.456 |
| TRINITY_DN6586_c0_g1_i1_orf1 | - | - | - | fatty acyl-CoA reductase wat-like isoform X1 [Ostrinia furnacalis] | 0.49127 | 1.75321 | -0.8028 | -0.7452 | -0.6965 |
| TRINITY_DN1868_c0_g1_i1_orf1 | - | - | - | protein obstructor-E isoform X1 [Ostrinia furnacalis] | 0.47972 | 1.73191 | -0.4808 | -0.7217 | -1.0091 |
| TRINITY_DN147458_c0_g1_i1_orf1 | - | - | - | 60S ribosomal protein L5, partial [Cotesia chilonis] | 0.25867 | 1.77783 | -1.1648 | -0.5694 | -0.3023 |
| TRINITY_DN2101_c0_g1_i6_orf1 | - | - | - | protein obstructor-E-like [Ostrinia furnacalis] | -0.1741 | 1.96441 | -0.7602 | -0.5494 | -0.4807 |
| TRINITY_DN1124_c0_g1_i7_orf1 | - | - | - | PREDICTED: cuticle protein 18.6, isoform B [Amyelois transitella] | -0.2822 | 1.96466 | -0.84 | -0.4243 | -0.4181 |
| TRINITY_DN778_c0_g1_i1_orf1 | - | - | - | uncharacterized protein LOC114363281 [Ostrinia furnacalis] | -0.0995 | 1.93848 | -0.8621 | -0.4107 | -0.5662 |
| TRINITY_DN72816_c0_g1_i2_orf1 | - | - | - | Golgi apparatus protein 1 [Ostrinia furnacalis] | 0.5674 | 1.58243 | -1.3565 | -0.4945 | -0.2988 |
| TRINITY_DN9916_c0_g1_i1_orf1 | - | - | - | PREDICTED: dynein light chain Tctex-type [Amyelois transitella] >XP_021195381.1 dynein light chain Tctex-type [Helicoverpa armigera] >XP_022815696.1 dynein light chain Tctex-type [Spodoptera litura] >XP_028156399.1 dynein light chain Tctex-type [Ostrinia furnacalis] >XP_035458261.1 dynein light chain Tctex-type-like [Spodoptera frugiperda] >XP_047034788.1 dynein light chain Tctex-type [Helicoverpa zea] >CAB3233358.1 unnamed protein product [Arctia plantaginis] >CAB3506583.1 unnamed protein product [Spodoptera littoralis] >CAG9754627.1 unnamed protein product [Diatraea saccharalis] >CAH0596395.1 unnamed protein product [Chrysodeixis includens] >KAF9808454.1 hypothetical protein | 0.54546 | 1.68231 | -1.1065 | -0.4623 | -0.659 |
| TRINITY_DN1074_c0_g1_i7_orf1 | - | - | - | eukaryotic translation initiation factor 4E type 2 [Ostrinia furnacalis] | 0.74375 | 1.58005 | -1.0795 | -0.5486 | -0.6957 |
| TRINITY_DN3255_c0_g1_i1_orf1 | - | - | - | uncharacterized protein LOC114351042 [Ostrinia furnacalis] | 0.03529 | 1.91909 | -0.82 | -0.5715 | -0.5628 |
| TRINITY_DN10057_c0_g2_i1_orf1 | - | - | - | cell wall protein DAN4 [Ostrinia furnacalis] | 0.50736 | 1.74491 | -0.809 | -0.6911 | -0.7522 |
| TRINITY_DN4125_c0_g1_i14_orf1 | - | - | - | angiotensin-converting enzyme-like isoform X1 [Ostrinia furnacalis] | 0.12597 | 1.88712 | -0.8866 | -0.5291 | -0.5973 |
| TRINITY_DN113272_c0_g1_i1_orf1 | - | - | - | altered inheritance of mitochondria protein 3-like [Ostrinia furnacalis] | 0.12477 | 1.89062 | -0.857 | -0.5313 | -0.6271 |
| TRINITY_DN72999_c0_g1_i1_orf1 | - | - | - | protein obstructor-E-like isoform X1 [Ostrinia furnacalis] >XP_028169319.1 protein obstructor-E-like isoform X2 [Ostrinia furnacalis] | 0.33894 | 1.81549 | -0.875 | -0.6896 | -0.5898 |
| TRINITY_DN21124_c0_g1_i4_orf1 | - | - | - | calsyntenin-1 [Ostrinia furnacalis] | 0.69195 | 1.64206 | -0.7804 | -0.7098 | -0.8438 |
| TRINITY_DN59829_c0_g1_i1_orf1 | - | - | - | putative mediator of RNA polymerase II transcription subunit 12 [Ostrinia | 0.78609 | 1.56881 | -0.9035 | -0.8859 | -0.5655 |
| TRINITY_DN619_c0_g1_i1_orf1 | - | - | - | putative uncharacterized protein DDB_G0271606 [Ostrinia furnacalis] | -0.0382 | 1.93351 | -0.8003 | -0.6478 | -0.4472 |
| TRINITY_DN20767_c0_g2_i1_orf1 | - | - | - | glycosylated lysosomal membrane protein B-like [Vanessa atalanta] | 0.80863 | 1.53935 | -1.0848 | -0.6623 | -0.6009 |
| TRINITY_DN48590_c0_g1_i1_orf1 | - | - | - | acyl-CoA Delta(11) desaturase isoform X1 [Ostrinia furnacalis] >XP_028172999.1 acyl-CoA Delta(11) desaturase isoform X2 [Ostrinia furnacalis] >XP_028173000.1 acyl-CoA Delta(11) desaturase isoform X1 [Ostrinia furnacalis] | 0.24769 | 1.85729 | -0.7892 | -0.6396 | -0.6761 |
| TRINITY_DN2840_c0_g1_i5_orf1 | - | - | - | hypothetical protein evm_002181 [Chilo suppressalis] | 0.66718 | 1.65421 | -0.8062 | -0.656 | -0.8592 |

| | | | | | | | | | |
|---|---|---|---|---|---|---|---|---|---|
| TRINITY_DN18502_c0_g1_i1_orf1 | - | - | - | uncharacterized protein LOC114359515 [Ostrinia furnacalis] | 0.22858 | 1.86481 | -0.7772 | -0.6549 | -0.6613 |
| TRINITY_DN644_c0_g1_i1_orf1 | - | - | - | cuticle protein 19-like [Ostrinia furnacalis] | -0.0835 | 1.94638 | -0.7872 | -0.4815 | -0.5942 |
| TRINITY_DN10479_c0_g1_i6_orf1 | - | - | - | unnamed protein product [Chrysodeixis includens] | 0.52515 | 1.70627 | -1.0434 | -0.4979 | -0.6901 |
| TRINITY_DN9282_c0_g1_i2_orf1 | - | - | - | uncharacterized protein LOC114363102 isoform X2 [Ostrinia furnacalis] | -0.04 | 1.93271 | -0.8453 | -0.5212 | -0.5262 |
| TRINITY_DN42337_c0_g1_i6_orf1 | - | - | - | cuticle protein 8-like [Leguminivora glycinivorella] | 0.4195 | 1.75362 | -1.0084 | -0.4185 | -0.7463 |
| TRINITY_DN7291_c0_g1_i5_orf1 | - | - | - | dynamin-1-like protein isoform X3 [Ostrinia furnacalis] | 0.40715 | -0.7595 | -1.3792 | 1.52005 | 0.21148 |
| TRINITY_DN2299_c0_g1_i3_orf1 | - | - | - | DNA-directed RNA polymerase II subunit RPB1 [Ostrinia furnacalis] | 0.00728 | -1.3026 | -0.8285 | 1.4869 | 0.63699 |
| TRINITY_DN11172_c0_g1_i4_orf1 | - | - | - | juvenile hormone epoxide hydrolase-like isoform X1 [Ostrinia furnacalis] >XP_028170522.1 juvenile hormone epoxide hydrolase-like isoform X2 [Ostrinia furnacalis] | 0.17447 | -0.9074 | -1.2121 | 1.59919 | 0.34583 |
| TRINITY_DN131603_c0_g1_i4_orfp1 | - | - | - | TRINITY_DN131603_c0_g1_i4_m.86149 TRINITY_DN131603_c0_g1::TRINITY_DN131603_c0_g1_i4::g.86149 ORF type:internal len:112 (-),score=8.40 TRINITY_DN131603_c0_g1_i4:2-334(-) | -0.3829 | -0.8337 | -0.6645 | 1.92734 | -0.0462 |
| TRINITY_DN19584_c0_g1_i2_orf1 | - | - | - | protein NDUFAF4 homolog [Ostrinia furnacalis] | 1.16079 | 0.86017 | -1.103 | 0.33938 | -1.2574 |
| TRINITY_DN9661_c0_g1_i1_orf1 | - | - | - | tetratricopeptide repeat protein 37 [Ostrinia furnacalis] | 0.43134 | 0.63665 | -1.3473 | 1.26979 | -0.9905 |
| TRINITY_DN8846_c0_g1_i1_orf1 | - | - | - | PREDICTED: synapse-associated protein of 47 kDa-like isoform X2 [Papilio | 0.63415 | 0.67532 | -1.3954 | 1.08962 | -1.0037 |
| TRINITY_DN13287_c0_g1_i5_orf1 | - | - | - | dystrophin-like, partial [Ostrinia furnacalis] | 1.22639 | 0.76592 | -1.2892 | 0.35559 | -1.0587 |
| TRINITY_DN11566_c0_g1_i6_orf1 | - | - | - | lens fiber major intrinsic protein-like isoform X1 [Ostrinia furnacalis] | 0.32666 | 1.23343 | -0.9377 | 0.76096 | -1.3833 |
| TRINITY_DN4571_c0_g1_i4_orf1 | - | - | - | PREDICTED: nuclear factor NF-kappa-B p105 subunit [Microplitis demolitor] >KAG6558391.1 viral ankyrin V1 [Microplitis demolitor] | 0.33373 | 1.59802 | -1.2923 | 0.1601 | -0.7996 |
| TRINITY_DN4732_c0_g1_i2_orf1 | - | - | - | reversion-inducing cysteine-rich protein with Kazal motifs [Ostrinia furnacalis] | 0.67687 | 0.93126 | -0.6773 | 0.71414 | -1.6449 |
| TRINITY_DN2201_c0_g1_i1_orf1 | - | - | - | pleiotropic regulator 1 [Ostrinia furnacalis] | 0.28406 | 1.32447 | -0.7699 | 0.63442 | -1.473 |
| TRINITY_DN6362_c0_g1_i4_orf1 | - | - | - | sodium/hydrogen exchanger 7 isoform X4 [Galleria mellonella] | 0.31527 | 1.49114 | -0.9858 | 0.41692 | -1.2375 |
| TRINITY_DN7391_c0_g1_i2_orf1 | - | - | - | hypothetical protein evm_000945 [Chilo suppressalis] >CAB3528924.1 unnamed protein product [Chilo suppressalis] >CAH0405517.1 unnamed protein product [Chilo suppressalis] | 1.10889 | 1.10367 | -0.6213 | -0.1248 | -1.4665 |
| TRINITY_DN10415_c0_g1_i5_orf1 | - | - | - | hypothetical protein evm_000184 [Chilo suppressalis] | 0.36515 | 1.09883 | -1.6694 | 0.75514 | -0.5498 |
| TRINITY_DN111_c0_g2_i2_orf1 | - | - | - | hypothetical protein O3G_MSEX007696 [Manduca sexta] >KAG6452639.1 hypothetical protein O3G_MSEX007696 [Manduca sexta] | 0.69763 | 0.44591 | -1.6492 | 1.10919 | -0.6035 |
| TRINITY_DN87603_c0_g2_i1_orf1 | - | - | - | 40S ribosomal protein S3-3, partial [Trichinella patagoniensis] | 1.15059 | 0.78355 | -0.8812 | 0.40394 | -1.4569 |
| TRINITY_DN103118_c0_g1_i4_orf1 | - | - | - | hypothetical protein evm_006930 [Chilo suppressalis] | 0.9054 | 0.92991 | -1.7419 | 0.3255 | -0.419 |
| TRINITY_DN21125_c0_g1_i1_orf1 | - | - | - | protein angel homolog 1 isoform X3 [Ostrinia furnacalis] | 0.56427 | 1.14014 | -1.5288 | 0.62947 | -0.805 |
| TRINITY_DN5829_c0_g2_i1_orf1 | - | - | - | uncharacterized protein LOC114365758 isoform X2 [Ostrinia furnacalis] | 0.40148 | 1.60042 | -1.2049 | 0.10551 | -0.9025 |
| TRINITY_DN8596_c0_g1_i2_orf1 | - | - | - | SWI/SNF-related matrix-associated actin-dependent regulator of chromatin subfamily E member 1-like isoform X2 [Ostrinia furnacalis] | 1.05962 | 1.24594 | -0.5865 | -0.3579 | -1.3612 |
| TRINITY_DN10630_c0_g1_i2_orf1 | - | - | - | J domain-containing protein [Ostrinia furnacalis] | -0.1129 | 1.55349 | -1.1714 | 0.62881 | -0.898 |
| TRINITY_DN467_c4_g1_i2_orf1 | - | - | - | GRIP and coiled-coil domain-containing protein 1 [Ostrinia furnacalis] | 0.66761 | 1.50173 | -0.872 | -0.0583 | -1.2391 |
| TRINITY_DN14019_c0_g1_i5_orf1 | - | - | - | hypothetical protein evm_009768 [Chilo suppressalis] | 0.73439 | 1.31237 | -0.7174 | 0.15388 | -1.4833 |
| TRINITY_DN1628_c0_g1_i1_orf1 | - | - | - | uncharacterized protein LOC114363979 [Ostrinia furnacalis] | 0.75716 | 1.38104 | -1.215 | 0.09384 | -1.017 |
| TRINITY_DN1763_c0_g3_i2_orf1 | - | - | - | heterogeneous nuclear ribonucleoprotein H-like isoform X2 [Ostrinia furnacalis] | 1.22535 | 0.84071 | -1.3846 | 0.226 | -0.9074 |
| TRINITY_DN4194_c0_g1_i1_orf1 | - | - | - | hornerin-like [Ostrinia furnacalis] | 1.06503 | 1.06503 | -1.2981 | 0.17575 | -1.0077 |
| TRINITY_DN5422_c0_g1_i1_orf1 | - | - | - | nitrilase and fragile histidine triad fusion protein NitFhit isoform X1 [Ostrinia furnacalis] | -0.1839 | 1.06481 | -1.8087 | 0.71931 | 0.20846 |
| TRINITY_DN11942_c0_g1_i1_orf1 | - | - | - | hypothetical protein B5X24_HaOG213660 [Helicoverpa armigera] | -0.0779 | 0.37672 | -1.8424 | 0.4068 | 1.13675 |
| TRINITY_DN578_c0_g1_i3_orf1 | - | - | - | charged multivesicular body protein 7 [Ostrinia furnacalis] | -0.3277 | 0.87929 | -1.8142 | 0.50624 | 0.75633 |
| TRINITY_DN667_c0_g1_i5_orf1 | - | - | - | unnamed protein product [Arctia plantaginis] | 0.08972 | 1.51892 | -1.6227 | 0.16741 | -0.1533 |
| TRINITY_DN13419_c0_g1_i5_orf1 | - | - | - | atrial natriuretic peptide-converting enzyme-like [Ostrinia furnacalis] | -0.3271 | 1.4342 | -1.5375 | 0.65088 | -0.2205 |
| TRINITY_DN1492_c0_g1_i4_orf1 | - | - | - | sarcoplasmic calcium-binding protein isoform X2 [Ostrinia furnacalis] | -0.0262 | 0.9704 | -1.8881 | 0.31849 | 0.62545 |
| TRINITY_DN3209_c0_g1_i1_orf1 | - | - | - | coatomer subunit beta [Helicoverpa armigera] | 0.29805 | 0.55068 | -1.9336 | 0.16767 | 0.91716 |
| TRINITY_DN2782_c0_g1_i7_orf1 | - | - | - | CDK5 regulatory subunit-associated protein 3 [Ostrinia furnacalis] | 0.17231 | 0.88046 | -1.9022 | 0.09597 | 0.75343 |
| TRINITY_DN53760_c0_g1_i1_orf1 | - | - | - | unnamed protein product [Parnassius apollo] | 0.49156 | 0.41814 | -1.8693 | -0.081 | 1.04056 |

| | | | | | | | | |
|---|---|---|---|---|---|---|---|---|
| TRINITY_DN5475_c0_g1_i3_orf1 | - | - | - | traB domain-containing protein-like isoform X1 [Ostrinia furnacalis] >XP_028169655.1 traB domain-containing protein-like isoform X1 [Ostrinia furnacalis] | 0.3356 | 1.1853 | -1.8505 | 0.12178 | 0.20784 |
| TRINITY_DN1520_c0_g1_i9_orf1 | - | - | - | adipocyte plasma membrane-associated protein-like [Ostrinia furnacalis] >XP_028176496.1 adipocyte plasma membrane-associated protein-like [Ostrinia furnacalis] | 0.44121 | 0.73 | -1.9826 | 0.32577 | 0.48557 |
| TRINITY_DN95056_c0_g2_i2_orf1 | - | - | - | 40S ribosomal protein S18 [Halotydeus destructor] | 0.60105 | 0.39375 | -1.9836 | 0.32086 | 0.66793 |
| TRINITY_DN10183_c0_g2_i3_orf1 | - | - | - | uncharacterized protein LOC114360370 isoform X1 [Ostrinia furnacalis] | 0.35447 | 1.1603 | -1.8459 | -0.0159 | 0.34699 |
| TRINITY_DN27456_c0_g2_i1_orf1 | - | - | - | organic cation transporter-like protein [Ostrinia furnacalis] | -0.3189 | 1.62381 | -1.4812 | 0.24935 | -0.0731 |
| TRINITY_DN71832_c0_g1_i1_orf1 | - | - | - | basement membrane-specific heparan sulfate proteoglycan core protein isoform X13 [Ostrinia furnacalis] | -0.1808 | 1.42419 | -1.6494 | 0.46383 | -0.0578 |
| TRINITY_DN14347_c0_g1_i1_orf1 | - | - | - | putative nuclease HARBI1 [Ostrinia furnacalis] | 0.54349 | 0.57108 | -1.9671 | 0.15653 | 0.69603 |
| TRINITY_DN29144_c0_g3_i1_orf1 | - | - | - | PREDICTED: ADP-ribosylation factor 6 [Papilio polytes] >XP_013133321.1 PREDICTED: ADP-ribosylation factor 6 [Papilio polytes] >XP_013177129.1 PREDICTED: ADP-ribosylation factor 6 [Papilio xuthus] >XP_013177130.1 PREDICTED: ADP-ribosylation factor 6 [Papilio xuthus] >XP_014356507.1 ADP-ribosylation factor 6 [Papilio machaon] >XP_021185579.1 ADP-ribosylation factor 6 [Helicoverpa armigera] >XP_021185581.1 ADP-ribosylation factor 6 [Helicoverpa armigera] >XP_022130228.1 ADP-ribosylation factor 6 [Pieris rapae] >XP_022822139.1 ADP-ribosylation factor 6 [Spodoptera litura] >XP_022822140.1 ADP-ribosylation factor 6 [Spodoptera litura] >XP_028159104.1 ADP-ribosylation factor 6 [Ostrinia furnacalis] >XP_028159105.1 ADP-ribosylation factor 6 [Ostrinia furnacalis] >XP_028159106.1 ADP-ribosylation factor 6 [Ostrinia furnacalis] >XP_028159107.1 ADP-ribosylation factor 6 [Ostrinia furnacalis] >XP_028163222.1 ADP-ribosylation factor 6 [Ostrinia furnacalis] >XP_030022165.1 ADP-ribosylation factor 6 [Manduca sexta] >XP_030022166.1 ADP-ribosylation factor 6 [Manduca sexta] >XP_030022167.1 ADP-ribosylation factor 6 [Manduca sexta] >XP_035444169.1 ADP-ribosylation factor 6 [Spodoptera frugiperda] >XP_035444175.1 ADP-ribosylation factor 6 [Spodoptera frugiperda] >XP_038207597.1 ADP-ribosylation factor 6 [Zerene cesonia] >XP_038207598.1 ADP-ribosylation factor 6 [Zerene cesonia] >XP_045510541.1 ADP-ribosylation factor 6 [Colias croceus] >XP_045510551.1 ADP-ribosylation factor 6 [Colias croceus] >XP_045527300.1 ADP-ribosylation factor 6 [Pieris brassicae] >XP_045527302.1 ADP-ribosylation factor 6 [Pieris brassicae] >XP_047029519.1 ADP-ribosylation factor 6 [Helicoverpa zea] >XP_047029551.1 ADP-ribosylation factor 6 [Helicoverpa zea] >XP_047504621.1 ADP-ribosylation factor 6 [Pieris napi] >XP_047504631.1 ADP-ribosylation factor 6 [Pieris napi] >XP_047504640.1 ADP-ribosylation factor 6 [Pieris napi] >XP_047504648.1 ADP-ribosylation factor 6 [Pieris napi] >XP_047504657.1 ADP-ribosylation factor 6 [Pieris napi] >XP_048489067.1 ADP-ribosylation factor 6 [Plutella xylostella] >XP_048489068.1 ADP- | -0.3162 | 0.38536 | -1.6304 | 1.44183 | 0.1195 |
| TRINITY_DN23962_c0_g1_i3_orf1 | - | - | - | uncharacterized protein LOC124629606 [Helicoverpa zea] | 1.60846 | -0.7917 | -0.9891 | 0.71582 | -0.5435 |
| TRINITY_DN49204_c0_g1_i1_orf1 | - | - | - | uncharacterized protein C05D11.1-like [Chelonus insularis] | 1.2065 | -1.2498 | -0.15 | 1.08182 | -0.8885 |
| TRINITY_DN9198_c0_g1_i4_orf1 | - | - | - | 4-coumarate--CoA ligase 1-like isoform X1 [Ostrinia furnacalis] | 0.97603 | -1.5703 | -0.1389 | 1.17075 | -0.4375 |
| TRINITY_DN72285_c1_g1_i1_orf1 | - | - | - | hypothetical protein HF086_001747 [Spodoptera exigua] | 1.34585 | -0.8105 | -1.2555 | 0.95006 | -0.2299 |
| TRINITY_DN22836_c0_g1_i5_orf1 | - | - | - | AP-3 complex subunit beta-2 [Ostrinia furnacalis] | 1.72058 | -0.8773 | -0.9096 | 0.50242 | -0.4361 |
| TRINITY_DN16354_c0_g1_i2_orf1 | - | - | - | uncharacterized protein LOC114349750 isoform X1 [Ostrinia furnacalis] | 1.54927 | -1.2988 | -0.2683 | 0.65724 | -0.6394 |
| TRINITY_DN1153_c1_g1_i1_orf1 | - | - | - | gamma-butyrobetaine dioxygenase [Ostrinia furnacalis] | 1.20122 | -0.6603 | -0.881 | 1.23941 | -0.8992 |
| TRINITY_DN667_c0_g1_i13_orf1 | - | - | - | enoyl-CoA hydratase domain-containing protein 2, mitochondrial [Ostrinia furnacalis] | 1.78304 | -0.9898 | -0.4798 | 0.37326 | -0.6867 |
| TRINITY_DN22_c0_g1_i3_orf1 | - | - | - | uncharacterized protein LOC114362831 [Ostrinia furnacalis] | 0.5966 | -0.2435 | -1.0145 | 1.62239 | -0.961 |

| | | | | | | | | | |
|---|---|---|---|---|---|---|---|---|---|
| TRINITY_DN20527_c0_g1_i1_orf1 | - | - | - | dihydrofolate reductase [Ostrinia furnacalis] | 1.131 | -0.5944 | -0.3406 | 1.17328 | -1.3693 |
| TRINITY_DN12293_c0_g1_i1_orf1 | - | - | - | hypothetical protein evm_011848 [Chilo suppressalis] | 1.25537 | -1.2022 | -0.8574 | 1.08047 | -0.2763 |
| TRINITY_DN7247_c0_g1_i6_orf1 | - | - | - | pyruvate kinase-like isoform X2 [Ostrinia furnacalis] | 0.35026 | -0.012 | -1.2527 | 1.65926 | -0.7448 |
| TRINITY_DN5991_c0_g1_i6_orf1 | - | - | - | uncharacterized protein LOC114357071 [Ostrinia furnacalis] | 1.04546 | -0.8513 | -1.2838 | 1.23057 | -0.1409 |
| TRINITY_DN11746_c0_g2_i1_orf1 | - | - | - | splicing factor 3B subunit 1 isoform X1 [Diprion similis] | 1.63677 | -0.9197 | -0.9466 | 0.6405 | -0.411 |
| TRINITY_DN141396_c0_g1_i1_orf1 | - | - | - | pre-rRNA 2'-O-ribose RNA methyltransferase FTSJ3 [Diachasma alloeum] | 1.45945 | -0.6821 | -1.2013 | 0.87218 | -0.4482 |
| TRINITY_DN5210_c0_g1_i3_orf1 | - | - | - | uncharacterized protein LOC114358376 isoform X2 [Ostrinia furnacalis] >XP_028168126.1 uncharacterized protein LOC114358376 isoform X2 [Ostrinia furnacalis] >XP_028168127.1 uncharacterized protein LOC114358376 isoform X2 [Ostrinia furnacalis] >XP_028168128.1 uncharacterized protein LOC114358376 isoform X2 [Ostrinia furnacalis] | 1.38637 | -0.865 | -1.1298 | 0.96306 | -0.3546 |
| TRINITY_DN2473_c0_g1_i2_orf1 | - | - | - | translation initiation factor eIF-2B subunit delta [Ostrinia furnacalis] | 1.17463 | -1.0895 | -1.222 | 0.95167 | 0.18519 |
| TRINITY_DN32420_c0_g1_i2_orf1 | - | - | - | PREDICTED: plectin-like, partial [Papilio polytes] | 1.35326 | -1.398 | -0.1274 | 0.85525 | -0.6831 |
| TRINITY_DN863_c0_g1_i6_orf1 | - | - | - | protein henna [Galleria mellonella] | 1.48487 | -1.3493 | -0.0211 | 0.63836 | -0.7528 |
| TRINITY_DN1753_c1_g1_i8_orf1 | - | - | - | serine/threonine-protein kinase WNK1-like isoform X15 [Ostrinia furnacalis] | 1.28556 | -1.0757 | -0.7176 | 1.13317 | -0.6255 |
| TRINITY_DN1416_c0_g2_i1_orf1 | - | - | - | uncharacterized protein LOC114352565 [Ostrinia furnacalis] | 1.18042 | -0.904 | -0.8363 | 1.26311 | -0.7032 |
| TRINITY_DN2120_c0_g1_i2_orf1 | - | - | - | cullin-4A [Ostrinia furnacalis] | 1.65547 | -1.1259 | -0.5409 | 0.59693 | -0.5856 |
| TRINITY_DN124711_c0_g1_i1_orf1 | - | - | - | muskelin isoform X1 [Ostrinia furnacalis] >XP_028163274.1 muskelin isoform X2 [Ostrinia furnacalis] | 0.5918 | -0.873 | -0.737 | 1.69782 | -0.6796 |
| TRINITY_DN14935_c0_g1_i1_orf1 | - | - | - | kynurenine/alpha-aminoadipate aminotransferase, mitochondrial [Ostrinia furnacalis] | 1.21739 | -1.2248 | -0.2072 | 1.09521 | -0.8806 |
| TRINITY_DN8717_c0_g1_i5_orf1 | - | - | - | hypothetical protein evm_006607 [Chilo suppressalis] >CAG9745590.1 unnamed protein product [Diatraea saccharalis] >CAG9784275.1 unnamed protein product [Diatraea saccharalis] | 1.75836 | -0.9412 | -0.5416 | 0.45001 | -0.7256 |
| TRINITY_DN3732_c0_g1_i2_orf1 | - | - | - | cytochrome P450 monooxygenase CYP6AB141 [Ostrinia furnacalis] | 0.8724 | -0.261 | -0.917 | 1.43403 | -1.1285 |
| TRINITY_DN5161_c0_g1_i5_orf1 | - | - | - | glyoxylate reductase/hydroxypyruvate reductase-like isoform X1 [Ostrinia | 1.27965 | -1.273 | -0.891 | 0.96993 | -0.0856 |
| TRINITY_DN6994_c0_g1_i4_orf1 | - | - | - | C-type mannose receptor 2-like isoform X1 [Ostrinia furnacalis] | 1.60834 | -0.99 | -0.6372 | 0.72599 | -0.7071 |
| TRINITY_DN25901_c0_g1_i2_orf1 | - | - | - | short-chain specific acyl-CoA dehydrogenase, mitochondrial-like isoform X2 [Ostrinia furnacalis] | 1.64098 | -1.1652 | -0.5441 | 0.60476 | -0.5365 |
| TRINITY_DN7329_c0_g1_i6_orf1 | - | - | - | serine hydrolase-like protein 2 isoform X2 [Ostrinia furnacalis] | 1.69782 | -0.6233 | -0.5817 | 0.55019 | -1.043 |
| TRINITY_DN1914_c0_g1_i4_orf1 | - | - | - | loricrin-like [Ostrinia furnacalis] | 0.81246 | -1.5262 | -0.1449 | 1.32959 | -0.4709 |
| TRINITY_DN15513_c0_g1_i6_orf1 | - | - | - | uncharacterized protein LOC114350859 [Ostrinia furnacalis] | 1.63254 | -1.3351 | -0.2468 | 0.46969 | -0.5203 |
| TRINITY_DN5962_c0_g1_i1_orf1 | - | - | - | tRNA (cytosine(34)-C(5))-methyltransferase [Ostrinia furnacalis] | 0.86401 | -1.1153 | -0.4974 | 1.48833 | -0.7397 |
| TRINITY_DN5126_c0_g2_i1_orf1 | - | - | - | cytochrome P450 monooxygenase CYP4L47 [Ostrinia furnacalis] | 0.34986 | -0.9471 | -0.5926 | 1.80355 | -0.6137 |
| TRINITY_DN36496_c0_g1_i1_orf1 | - | - | - | unnamed protein product [Parnassius apollo] | 1.11062 | -0.0369 | -1.1277 | 1.14323 | -1.0892 |
| TRINITY_DN1999_c0_g1_i9_orf1 | - | - | - | acyl-CoA Delta(11) desaturase-like [Ostrinia furnacalis] >XP_028172986.1 acyl-CoA Delta(11) desaturase-like [Ostrinia furnacalis] >AAL27034.1 acyl-CoA delta-9 desaturase [Ostrinia furnacalis] >AAL29454.1 acyl-CoA delta-9 desaturase [Ostrinia nubilalis] | 1.40037 | -0.3237 | -1.0523 | 0.94348 | -0.9679 |
| TRINITY_DN3964_c1_g1_i2_orf1 | - | - | - | phosphoinositide 3-kinase regulatory subunit 4 isoform X1 [Ostrinia furnacalis] >XP_028172384.1 phosphoinositide 3-kinase regulatory subunit 4 isoform X5 [Ostrinia furnacalis] | 1.23654 | -0.9982 | -1.159 | 1.05509 | -0.1344 |
| TRINITY_DN3949_c1_g1_i1_orf1 | - | - | - | probable cytochrome P450 304a1 isoform X2 [Ostrinia furnacalis] | 1.59507 | -0.9594 | -0.6473 | 0.75289 | -0.7412 |
| TRINITY_DN2749_c0_g2_i3_orf1 | - | - | - | RNA exonuclease 4-like [Ostrinia furnacalis] >QEE79882.1 REX4 [Ostrinia | 1.21072 | -0.6949 | -0.4749 | 1.16799 | -1.209 |
| TRINITY_DN1656_c2_g1_i5_orf1 | - | - | - | 15-hydroxyprostaglandin dehydrogenase [NAD(+)]-like [Ostrinia furnacalis] | 1.45587 | -1.3283 | -0.4256 | 0.81631 | -0.5183 |
| TRINITY_DN4795_c0_g1_i2_orf1 | - | - | - | D-amino-acid oxidase isoform X1 [Ostrinia furnacalis] | 1.69032 | -0.7555 | -1.0052 | 0.56396 | -0.4937 |
| TRINITY_DN114198_c0_g1_i1_orf1 | - | - | - | catalase isozyme 1, partial [Sturnira hondurensis] | 1.63158 | -0.6661 | -0.5478 | 0.6592 | -1.0769 |
| TRINITY_DN113626_c0_g1_i3_orfp1 | - | - | - | TRINITY_DN113626_c0_g1_i3_m.80721 TRINITY_DN113626_c0_g1::TRINITY_DN113626_c0_g1_i3::g.80721 ORF type:internal len:118 (-),score=87.34 TRINITY_DN113626_c0_g1_i3:2-352(-) | 1.53333 | -0.9926 | -0.9722 | 0.77488 | -0.3434 |

| | | | | | | | | | |
|---|---|---|---|---|---|---|---|---|---|
| TRINITY_DN2695_c0_g1_i14_orfp1 | - | - | - | TRINITY_DN2695_c0_g1_i14_m.44485 TRINITY_DN2695_c0_g1::TRINITY_DN2695_c0_g1_i14::g.44485 ORF type:3prime_partial len:698 (+),score=187.51 TRINITY_DN2695_c0_g1_i14:101-2092(+) | 1.6885 | -1.2164 | -0.5181 | 0.47012 | -0.4241 |
| TRINITY_DN868_c0_g1_i4_orf1 | - | - | - | uncharacterized protein LOC114359357 isoform X1 [Ostrinia furnacalis] | 1.68484 | -0.8783 | -0.8889 | 0.58727 | -0.5049 |
| TRINITY_DN26411_c0_g1_i2_orfp1 | - | - | - | TRINITY_DN26411_c0_g1_i2_m.24123 TRINITY_DN26411_c0_g1::TRINITY_DN26411_c0_g1_i2::g.24123 ORF type:internal len:115 (-),score=72.83 TRINITY_DN26411_c0_g1_i2:3-344(-) | 1.55133 | -0.7156 | -0.9205 | 0.82672 | -0.7419 |
| TRINITY_DN27300_c0_g1_i6_orfp1 | - | - | - | TRINITY_DN27300_c0_g1_i6_m.71140 TRINITY_DN27300_c0_g1::TRINITY_DN27300_c0_g1_i6::g.71140 ORF type:internal len:129 (-),score=20.75 TRINITY_DN27300_c0_g1_i6:1-384(-) | 0.03732 | -0.9817 | -0.6308 | -0.307 | 1.8822 |
| TRINITY_DN146236_c0_g1_i1_orf1 | - | - | - | vesicle-fusing ATPase 1-like [Chelonus insularis] | 0.80665 | -1.505 | -0.6279 | 0.02648 | 1.29977 |
| TRINITY_DN8046_c0_g1_i5_orf1 | - | - | - | tumor protein D53 homolog isoform X2 [Ostrinia furnacalis] | 0.9673 | -0.0018 | -1.4685 | -0.6922 | 1.19525 |
| TRINITY_DN14209_c0_g1_i1_orf1 | - | - | - | unnamed protein product [Diatraea saccharalis] | 1.07168 | -1.0766 | -1.2566 | 0.23227 | 1.02927 |
| TRINITY_DN240_c0_g1_i4_orf1 | - | - | - | unnamed protein product [Chilo suppressalis] | 1.42432 | -0.5717 | -1.4909 | -0.011 | 0.64931 |
| TRINITY_DN40440_c0_g1_i1_orf1 | - | - | - | Na(+)/H(+) exchange regulatory cofactor NHE-RF1 [Helicoverpa zea] >XP_049705606.1 Na(+)/H(+) exchange regulatory cofactor NHE-RF1 [Helicoverpa armigera] | 0.56712 | 0.32759 | -1.4984 | -0.7335 | 1.33714 |
| TRINITY_DN70236_c0_g1_i1_orf1 | - | - | - | NAD-dependent protein deacetylase sirtuin-2-like [Ostrinia furnacalis] | 0.51737 | -0.7719 | -1.5695 | 0.84254 | 0.98148 |
| TRINITY_DN43881_c0_g1_i2_orf1 | - | - | - | estradiol 17-beta-dehydrogenase 8-like [Ostrinia furnacalis] | 0.54187 | -1.2754 | -0.8676 | 0.07773 | 1.52344 |
| TRINITY_DN36612_c0_g1_i1_orf1 | - | - | - | lamin-C isoform X1 [Ostrinia furnacalis] >XP_028158861.1 lamin-C isoform X2 [Ostrinia furnacalis] >XP_028158863.1 lamin-C isoform X3 [Ostrinia furnacalis] >XP_028158864.1 lamin-C isoform X4 [Ostrinia furnacalis] | 1.08175 | -0.8918 | -1.4306 | 0.29014 | 0.95057 |
| TRINITY_DN119893_c0_g2_i3_orf1 | - | - | - | ATP-binding cassette sub-family F member 3 isoform X1 [Ostrinia furnacalis] >XP_028168051.1 ATP-binding cassette sub-family F member 3 isoform X2 [Ostrinia furnacalis] | 0.84223 | -1.3736 | -0.8365 | 0.06416 | 1.30379 |
| TRINITY_DN19939_c0_g1_i4_orf1 | - | - | - | unnamed protein product [Chilo suppressalis] | 1.13816 | -0.7946 | -0.6183 | -1.0144 | 1.28914 |
| TRINITY_DN15265_c0_g1_i1_orf1 | - | - | - | cullin-5 [Ostrinia furnacalis] | 0.98562 | 0.19845 | -1.4083 | -0.883 | 1.10728 |
| TRINITY_DN102260_c0_g1_i1_orf1 | - | - | - | unnamed protein product [Diatraea saccharalis] | 1.22515 | -0.9468 | -0.8109 | -0.6833 | 1.21581 |
| TRINITY_DN1272_c1_g1_i4_orf1 | - | - | - | E3 ubiquitin-protein transferase MAEA [Ostrinia furnacalis] >XP_028157102.1 E3 ubiquitin-protein transferase MAEA [Ostrinia furnacalis] | 0.03772 | -0.1668 | -1.3477 | -0.2774 | 1.75426 |
| TRINITY_DN9979_c0_g1_i1_orf1 | - | - | - | ADP-dependent glucokinase [Ostrinia furnacalis] | 0.14275 | -0.5179 | -1.3003 | -0.0614 | 1.73688 |
| TRINITY_DN9510_c0_g2_i1_orf1 | - | - | - | RNA polymerase II transcriptional coactivator [Ostrinia furnacalis] | 0.9068 | -0.4656 | -1.0841 | -0.8142 | 1.45701 |
| TRINITY_DN112706_c0_g1_i2_orf1 | - | - | - | alaserpin-like isoform X4 [Ostrinia furnacalis] | 0.9862 | -0.9542 | -1.1848 | -0.1478 | 1.30053 |
| TRINITY_DN28039_c0_g1_i1_orf1 | - | - | - | translation elongation factor 2 [Athalia rosae] | 0.84919 | -0.8229 | -0.9669 | -0.5843 | 1.52491 |
| TRINITY_DN1710_c0_g2_i2_orf1 | - | - | - | relish [Ostrinia furnacalis] | 0.35518 | 0.14414 | -1.4595 | -0.5834 | 1.54357 |
| TRINITY_DN42177_c0_g1_i4_orf1 | - | - | - | androgen-dependent TFPI-regulating protein-like [Ostrinia furnacalis] | 0.9853 | -1.3439 | -0.8187 | -0.0669 | 1.2443 |
| TRINITY_DN2873_c0_g1_i7_orf1 | - | - | - | transitional endoplasmic reticulum ATPase TER94 [Galleria mellonella] | 0.67532 | -0.4737 | -1.4806 | -0.1697 | 1.44866 |
| TRINITY_DN115658_c0_g1_i1_orf1 | - | - | - | hypothetical protein B5X24_HaOG203018 [Helicoverpa armigera] | 1.13535 | -1.1679 | -0.9209 | -0.2458 | 1.19932 |

| | | | | | | | | | |
|---|---|---|---|---|---|---|---|---|---|
| TRINITY_DN66442_c0_g2_i3_orf1 | - | - | - | ubiquitin-conjugating enzyme E2 G1 [Papilio polytes] >XP_013167686.1 PREDICTED: ubiquitin-conjugating enzyme E2 G1 isoform X2 [Papilio xuthus] >XP_014357225.1 ubiquitin-conjugating enzyme E2 G1 isoform X2 [Papilio machaon] >XP_021182486.1 ubiquitin-conjugating enzyme E2 G1 isoform X2 [Helicoverpa armigera] >XP_022831375.1 ubiquitin-conjugating enzyme E2 G1 isoform X2 [Spodoptera litura] >XP_023943756.1 ubiquitin-conjugating enzyme E2 G1 isoform X2 [Bicyclus anynana] >XP_026488925.1 ubiquitin-conjugating enzyme E2 G1 isoform X2 [Vanessa tameamea] >XP_026740300.1 ubiquitin-conjugating enzyme E2 G1-like isoform X2 [Trichoplusia ni] >XP_026754924.1 ubiquitin-conjugating enzyme E2 G1 isoform X2 [Galleria mellonella] >XP_028172940.1 ubiquitin-conjugating enzyme E2 G1 isoform X2 [Ostrinia furnacalis] >XP_030034967.1 ubiquitin-conjugating enzyme E2 G1 isoform X2 [Manduca sexta] >XP_032526880.1 ubiquitin-conjugating enzyme E2 G1 isoform X2 [Danaus plexippus plexippus] >XP_034828280.1 ubiquitin-conjugating enzyme E2 G1 isoform X1 [Maniola hyperantus] >XP_035448963.1 ubiquitin-conjugating enzyme E2 G1-like isoform X2 [Spodoptera frugiperda] >XP_039752058.1 ubiquitin-conjugating enzyme E2 G1 isoform X2 [Pararge aegeria] >XP_041971665.1 ubiquitin-conjugating enzyme E2 G1 isoform X2 [Aricia agestis] >XP_045454385.1 ubiquitin-conjugating enzyme E2 G1 isoform X1 [Melitaea cinxia] >XP_045771256.1 ubiquitin-conjugating enzyme E2 G1 isoform X1 [Maniola jurtina] >XP_046964023.1 ubiquitin-conjugating enzyme E2 G1 isoform X2 [Vanessa cardui] >XP_047032842.1 ubiquitin-conjugating enzyme E2 G1 isoform X2 [Helicoverpa zea] >XP_047537963.1 ubiquitin-conjugating enzyme E2 G1 isoform X2 [Vanessa atalanta] >XP_047999421.1 ubiquitin-conjugating enzyme E2 G1 isoform X2 [Leguminivora glycinivorella] >XP_048482262.1 ubiquitin-conjugating enzyme E2 G1 isoform X2 [Plutella xylostella] >XP_049879261.1 ubiquitin-conjugating enzyme E2 G1 isoform X2 [Pectinophora gossypiella] >XP_050353097.1 ubiquitin-conjugating enzyme E2 G1 isoform X2 [Nymphalis io] >RVE50249.1 hypothetical protein evm_005084 [Chilo suppressalis] >CAB3220594.1 unnamed protein product [Arctia | 0.64571 | 0.51778 | -1.6304 | -0.6462 | 1.11315 |
| TRINITY_DN21000_c0_g1_i1_orf1 | - | - | - | elongation factor-1 alpha, partial [Loxostege sticticalis] >QCO92153.1 elongation factor-1 alpha, partial [Sitochroa umbrosalis] | 1.32035 | -0.9949 | -0.7644 | -0.6711 | 1.11004 |
| TRINITY_DN15318_c0_g1_i1_orf1 | - | - | - | hepatoma-derived growth factor-related protein 2-like [Ostrinia furnacalis] | 0.9991 | -1.2832 | -1.0929 | 0.36241 | 1.0146 |
| TRINITY_DN7570_c0_g1_i18_orf1 | - | - | - | sodium/potassium-transporting ATPase subunit alpha isoform X4 [Trichoplusia ni] >XP_026734855.1 sodium/potassium-transporting ATPase subunit alpha isoform X4 [Trichoplusia ni] | 0.30603 | -0.2893 | -1.2491 | -0.5026 | 1.7349 |
| TRINITY_DN4742_c0_g1_i1_orf1 | - | - | - | ribosomal protein S6 kinase beta-1 [Ostrinia furnacalis] | 0.39927 | 0.04659 | -1.7528 | -0.0218 | 1.32877 |
| TRINITY_DN920_c0_g1_i4_orf1 | - | - | - | glutathione S-transferase omega 2 [Ostrinia furnacalis] | 0.44001 | -1.1651 | -1.0752 | 0.32032 | 1.47996 |
| TRINITY_DN103107_c0_g1_i2_orf1 | - | - | - | superoxide dismutase [Cu-Zn] [Ostrinia furnacalis] >XP_028177872.1 superoxide dismutase [Cu-Zn] [Ostrinia furnacalis] | -0.2 | -0.3184 | -0.7482 | -0.689 | 1.95555 |
| TRINITY_DN40911_c0_g1_i1_orf1 | - | - | - | peroxisomal membrane protein PEX16 [Ostrinia furnacalis] | 0.37412 | -0.3876 | -0.6856 | -1.0638 | 1.76295 |
| TRINITY_DN19659_c1_g1_i1_orf1 | - | - | - | elongation factor 1-gamma [Ostrinia furnacalis] | 1.10681 | -1.3118 | -0.7359 | -0.2611 | 1.2019 |
| TRINITY_DN21170_c0_g1_i5_orf1 | - | - | - | twitchin-like [Ostrinia furnacalis] | 0.10922 | -1.2331 | -0.478 | -0.1881 | 1.78992 |
| TRINITY_DN14855_c0_g1_i1_orf1 | - | - | - | neurochondrin homolog [Ostrinia furnacalis] | 1.32496 | -0.8721 | -1.3531 | 0.098 | 0.8022 |
| TRINITY_DN8241_c0_g1_i3_orf1 | - | - | - | transforming growth factor beta-1-induced transcript 1 protein [Ostrinia | 0.97058 | -1.1496 | -1.0004 | -0.1315 | 1.31088 |
| TRINITY_DN32687_c0_g1_i2_orf1 | - | - | - | protein D2-like isoform X2 [Ostrinia furnacalis] >XP_028164613.1 protein D2-like isoform X2 [Ostrinia furnacalis] | 0.92504 | -0.9597 | -0.6265 | -0.8122 | 1.47343 |
| TRINITY_DN8024_c0_g1_i6_orf1 | - | - | - | zinc finger CCHC domain-containing protein 24-like isoform X2 [Pararge | 1.14609 | -1.1087 | -1.1274 | 0.00077 | 1.08919 |
| TRINITY_DN6358_c0_g1_i5_orf1 | - | - | - | histone H1B-like [Ostrinia furnacalis] | 0.24241 | -0.5812 | -1.629 | 0.90083 | 1.06695 |
| TRINITY_DN136031_c0_g1_i7_orf1 | - | - | - | ferritin, lower subunit isoform X3 [Spodoptera litura] | -0.6314 | -0.8095 | -0.9264 | 0.80566 | 1.56164 |
| TRINITY_DN4741_c0_g1_i4_orf1 | - | - | - | PREDICTED: 23 kDa integral membrane protein-like [Papilio xuthus] | 0.84247 | 0.14075 | -1.6083 | -0.5502 | 1.17525 |

| | | | | | | | | | |
|---|---|---|---|---|---|---|---|---|---|
| TRINITY_DN2031_c11_g1_i2_orfp1 | - | - | - | TRINITY_DN2031_c11_g1_i2_m.4044 TRINITY_DN2031_c11_g1::TRINITY_DN2031_c11_g1_i2::g.4044 ORF type:3prime_partial len:149 (-),score=79.84 TRINITY_DN2031_c11_g1_i2:2-445(- | 0.88276 | -1.1954 | -1.2328 | 0.57568 | 0.96978 |
| TRINITY_DN2770_c0_g2_i4_orf1 | - | - | - | phosphatidylinositol 4-phosphate 3-kinase C2 domain-containing subunit alpha isoform X1 [Ostrinia furnacalis] | 0.1546 | -0.4086 | -1.574 | 0.3392 | 1.48882 |
| TRINITY_DN34413_c0_g1_i1_orf1 | - | - | - | LOW QUALITY PROTEIN: probable RNA methyltransferase bin3 [Ostrinia | 1.25314 | -0.7186 | -1.4982 | 0.16228 | 0.80141 |
| TRINITY_DN11637_c0_g1_i1_orf1 | - | - | - | ribosome-binding protein 1 isoform X4 [Colias croceus] | 1.08047 | -0.6354 | -1.6156 | 0.3271 | 0.84351 |
| TRINITY_DN11612_c0_g2_i1_orf1 | - | - | - | eukaryotic translation initiation factor 5B [Manduca sexta] | 0.23217 | -0.0508 | -1.2131 | -0.6964 | 1.72823 |
| TRINITY_DN23474_c1_g1_i1_orf1 | - | - | - | unnamed protein product [Chrysodeixis includens] | 0.37894 | -1.3127 | -0.781 | 0.1317 | 1.58303 |
| TRINITY_DN7909_c0_g2_i1_orf1 | - | - | - | aldehyde oxidase 3 [Ostrinia furnacalis] | 1.24356 | -0.8713 | -1.3218 | -0.0233 | 0.97291 |
| TRINITY_DN21215_c0_g1_i7_orf1 | - | - | - | phytanoyl-CoA dioxygenase, peroxisomal-like [Ostrinia furnacalis] | 1.04468 | -0.6489 | -1.6 | 0.28373 | 0.92045 |
| TRINITY_DN4822_c0_g1_i9_orf1 | - | - | - | homogentisate 1,2-dioxygenase [Ostrinia furnacalis] | 0.63413 | -0.751 | -1.5356 | 0.43216 | 1.22028 |
| TRINITY_DN69334_c0_g1_i1_orf1 | - | - | - | PREDICTED: 15-hydroxyprostaglandin dehydrogenase [NAD(+)]-like [Papilio | 0.93755 | -0.6506 | -0.9199 | -0.8348 | 1.46781 |
| TRINITY_DN195_c8_g1_i1_orf1 | - | - | - | hypothetical protein evm_009768 [Chilo suppressalis] | 0.53551 | -0.1835 | -1.0497 | -0.9423 | 1.64002 |
| TRINITY_DN195_c4_g1_i1_orf1 | - | - | - | beta-1,3-glucan-binding protein 1 [Ostrinia furnacalis] | 1.10521 | -0.8555 | -0.8124 | -0.7742 | 1.33689 |
| TRINITY_DN21035_c0_g1_i14_orf1 | - | - | - | mitochondrial amidoxime reducing component 2-like [Ostrinia furnacalis] | 1.13268 | -1.1663 | -1.2078 | 0.36803 | 0.87333 |
| TRINITY_DN27592_c0_g1_i1_orf1 | - | - | - | D-arabinitol dehydrogenase 1-like [Ostrinia furnacalis] | 1.36271 | -0.8912 | -0.5719 | -0.954 | 1.05436 |
| TRINITY_DN467_c9_g1_i2_orf1 | - | - | - | band 4.1-like protein 5 [Ostrinia furnacalis] | 1.34127 | -0.1828 | -0.7248 | -1.3456 | 0.91194 |
| TRINITY_DN3307_c1_g1_i2_orf1 | - | - | - | BTB/POZ domain-containing protein 2-like [Ostrinia furnacalis] | 0.71451 | -0.1915 | -0.6489 | -1.3555 | 1.48134 |
| TRINITY_DN6016_c0_g1_i8_orf1 | - | - | - | hypothetical protein evm_010883 [Chilo suppressalis] | 1.08612 | 0.45713 | -0.5758 | -1.6694 | 0.70201 |
| TRINITY_DN556_c0_g2_i1_orf1 | - | - | - | serine protease inhibitor dipetalogastin-like [Ostrinia furnacalis] | 0.97254 | 0.54584 | -0.4111 | -1.7731 | 0.66585 |
| TRINITY_DN33452_c0_g1_i3_orf1 | - | - | - | lethal(2) giant larvae protein isoform X8 [Ostrinia furnacalis] | 1.00146 | -0.173 | -0.5771 | -1.4678 | 1.21644 |
| TRINITY_DN1013_c0_g1_i3_orf1 | - | - | - | TELO2-interacting protein 1 homolog isoform X2 [Ostrinia furnacalis] | 1.01458 | 0.55579 | -0.8272 | -1.5337 | 0.7906 |
| TRINITY_DN2749_c0_g1_i4_orf1 | - | - | - | RNA exonuclease 4-like [Ostrinia furnacalis] >QEE79882.1 REX4 [Ostrinia | 1.36027 | -0.9203 | 0.62305 | 0.28993 | -1.3529 |
| TRINITY_DN4356_c0_g1_i6_orf1 | - | - | - | mulatexin-like [Ostrinia furnacalis] | 1.2879 | -0.3049 | 0.57028 | 0.14983 | -1.7031 |
| TRINITY_DN33452_c0_g1_i1_orf1 | - | - | - | lethal(2) giant larvae protein isoform X8 [Ostrinia furnacalis] | 1.40148 | -0.7904 | 0.14832 | 0.64518 | -1.4046 |
