# Supplementary material for: Comparative Proteomics and Metabonomics Analysis of Different Diapause Stages Revealed a New Regulation Mechanism of Diapause in Loxostege sticticalis (Lepidoptera: Pyralidae)
Source: Molecules. 2024 Jul 25;29(15):3472. doi: 10.3390/molecules29153472 (PMC11314584; doi:10.3390/molecules29153472)
Supplement: Supplementary file 1 [file molecules-29-03472-s001.zip › analysis process/proteomic/Cluster analysis of expression patterns/Down/CTvsPreD down.pdf]

Accession	Symbol	Protein Na	Entrez ID	Description	ND	PreD	RD	CT	D
TRINITY_DN971_c0_g1_i10_orfp1	-	-	-	TRINITY_DN971_c0_g1_i10_m.54268 TRINITY_DN971_c0_g1::TRINITY_DN971_c0_g1_i10::g.54268 ORF type:internal len:187	-1.8854	0.89113	-0.1083	0.42557	0.67699
TRINITY_DN10877_c0_g1_i1_orf1	-	-	-	spodomicin-like [Ostrinia furnacalis]	-1.5409	1.42832	-0.39422	0.64179	-0.13499
TRINITY_DN27300_c0_g1_i1_orfp1	-	-	-	TRINITY_DN27300_c0_g1_i1_m.71142 TRINITY_DN27300_c0_g1::TRINITY_DN27300_c0_g1_i1::g.71142 ORF type:internal len:82 (-),score=9.46 TRINITY_DN27300_c0_g1_i1:3-245(-)	-1.16404	1.67074	-0.88268	0.23183	0.14416
TRINITY_DN36476_c1_g1_i1_orfp1	-	-	-	TRINITY_DN36476_c1_g1_i1_m.70910 TRINITY_DN36476_c1_g1::TRINITY_DN36476_c1_g1_i1::g.70910 ORF type:5prime_partial len:88 (-),score=0.50 TRINITY_DN36476_c1_g1_i1:49-312(-)	-1.62065	1.01449	-0.66669	0.42458	0.84827
TRINITY_DN30510_c0_g1_i6_orf1	-	-	-	spodomicin-like [Ostrinia furnacalis]	-1.29959	1.19375	-1.05909	0.37534	0.78959
TRINITY_DN6330_c0_g1_i1_orfp1	-	-	-	TRINITY_DN6330_c0_g1_i1_m.42332 TRINITY_DN6330_c0_g1::TRINITY_DN6330_c0_g1_i1::g.42332 ORF type:3prime_partial len:51 (-	-1.72994	0.9219	-0.47532	0.40933	0.87403
TRINITY_DN28501_c0_g1_i2_orfp1	-	-	-	TRINITY_DN28501_c0_g1_i2_m.58934 TRINITY_DN28501_c0_g1::TRINITY_DN28501_c0_g1_i2::g.58934 ORF type:internal len:98 (+),score=13.70 TRINITY_DN28501_c0_g1_i2:3-293(+)	-0.9138	1.52418	-1.27054	0.26055	0.39961
TRINITY_DN86772_c0_g1_i3_orfp1	-	-	-	x-tox [Spodoptera exigua]	-1.78653	0.98139	-0.3059	0.29628	0.81475
TRINITY_DN47784_c0_g2_i1_orfp1	-	-	-	arylphorin subunit alpha-like [Ostrinia furnacalis]	-1.66831	1.36078	-0.18103	-0.08201	0.57057
TRINITY_DN2457_c0_g1_i8_orf1	-	-	-	uncharacterized protein LOC114355596 [Ostrinia furnacalis]	-1.35161	1.4347	-0.88645	0.34564	0.45773
TRINITY_DN609_c0_g1_i1_orf1	-	-	-	zonadhesin-like isoform X1 [Ostrinia furnacalis]	-1.14528	1.40622	-1.1348	0.58059	0.29327
TRINITY_DN30306_c0_g2_i1_orf1	-	-	-	perilipin-4-like isoform X3 [Ostrinia furnacalis]	-1.64237	1.14023	-0.58492	0.35707	0.72998
TRINITY_DN3275_c0_g2_i3_orf1	-	-	-	hypothetical protein B5X24_HaOG216046 [Helicoverpa armigera]	-1.74395	1.18595	-0.23787	0.09888	0.69698
TRINITY_DN1149_c0_g1_i4_orf1	-	-	-	circadian clock-controlled protein-like [Ostrinia furnacalis]	-1.45568	1.26362	-0.82548	0.71512	0.30242
TRINITY_DN24789_c0_g1_i9_orfp1	-	-	-	TRINITY_DN24789_c0_g1_i9_m.25888 TRINITY_DN24789_c0_g1::TRINITY_DN24789_c0_g1_i9::g.25888 ORF type:internal len:114 (-),score=12.65 TRINITY_DN24789_c0_g1_i9:2-340(-)	-1.01474	1.69065	-1.02403	0.21118	0.13694
TRINITY_DN3439_c0_g2_i2_orf1	-	-	-	histone H2A.Z-specific chaperone CHZ1-like [Ostrinia furnacalis]	-1.46229	0.88475	-0.8689	0.35051	1.09594
TRINITY_DN136031_c0_g1_i7_orf1	-	-	-	ferritin, lower subunit isoform X3 [Spodoptera litura]	-0.63137	1.56164	-0.8095	-0.92643	0.80566
TRINITY_DN14754_c0_g1_i6_orf1	-	-	-	cathepsin L [Papilio xuthus]	-0.81321	1.68125	-1.11172	-0.22922	0.4729
TRINITY_DN11981_c0_g1_i7_orf1	-	-	-	luciferin 4-monooxygenase-like isoform X2 [Ostrinia furnacalis]	-1.64747	1.1955	-0.55027	0.66168	0.34057
TRINITY_DN5198_c0_g1_i5_orfp1	-	-	-	TRINITY_DN5198_c0_g1_i5_m.8637 TRINITY_DN5198_c0_g1::TRINITY_DN5198_c0_g1_i5::g.8637 ORF type:complete len:223 (-),score=54.99 TRINITY_DN5198_c0_g1_i5:319-987(-)	-1.36167	1.31173	-0.9491	0.38687	0.61217
TRINITY_DN710_c0_g1_i11_orfp1	-	-	-	TRINITY_DN710_c0_g1_i11_m.67699 TRINITY_DN710_c0_g1::TRINITY_DN710_c0_g1_i11::g.67699 ORF type:complete len:194 (-),score=34.99,Collagen PF01391.19 0.00029 TRINITY_DN710_c0_g1_i11:1283-1864(-)	-1.34384	0.98595	-0.99675	0.28302	1.07163
TRINITY_DN5153_c1_g1_i1_orf1	-	-	-	nose resistant to fluoxetine protein 6-like isoform X1 [Ostrinia furnacalis]	-1.64455	1.19455	-0.57412	0.42701	0.59712
TRINITY_DN1363_c0_g1_i11_orf1	-	-	-	cytochrome P450 CYP12A2-like isoform X1 [Ostrinia furnacalis] >QPF77619.1 cytochrome P450 monooxygenase CYP333A20 [Ostrinia furnacalis]	-1.81711	0.96686	-0.29938	0.49475	0.65489
TRINITY_DN15545_c0_g1_i1_orf1	-	-	-	larval cuticle protein LCP-14-like [Ostrinia furnacalis]	-1.0717	0.95578	-1.35118	0.59929	0.86782
TRINITY_DN27300_c0_g1_i7_orfp1	-	-	-	TRINITY_DN27300_c0_g1_i7_m.71141 TRINITY_DN27300_c0_g1::TRINITY_DN27300_c0_g1_i7::g.71141 ORF type:internal len:82 (-),score=6.59 TRINITY_DN27300_c0_g1_i7:3-245(-)	-1.49298	1.51949	-0.49544	0.46551	0.00342
TRINITY_DN135780_c0_g1_i1_orf1	-	-	-	flotillin-1 isoform X1 [Pectinophora gossypiella]	-1.87167	0.82634	-0.07917	0.26605	0.85845
TRINITY_DN17247_c0_g1_i14_orf1	-	-	-	uncharacterized protein LOC114356308 [Ostrinia furnacalis]	-1.87691	0.87831	0.18129	-0.00301	0.82032
TRINITY_DN15685_c0_g1_i5_orf1	-	-	-	uncharacterized protein LOC114352354 [Ostrinia furnacalis]	-1.12233	1.09574	-1.2913	0.61491	0.70297
TRINITY_DN57111_c0_g1_i1_orf1	-	-	-	trypsin-like serine proteinase T26 protein, partial [Chilo infuscatellus]	-0.54962	1.38376	-1.58185	0.39994	0.34777
TRINITY_DN64719_c0_g1_i2_orfp1	-	-	-	TRINITY_DN64719_c0_g1_i2_m.37745 TRINITY_DN64719_c0_g1::TRINITY_DN64719_c0_g1_i2::g.37745 ORF type:internal len:91 (+),score=41.89 TRINITY_DN64719_c0_g1_i2:1-270(+)	-0.81997	1.57752	-1.29225	0.15284	0.38186
TRINITY_DN1593_c0_g1_i1_orf1	-	-	-	chemosensory protein csp11 [Helopeltis theivora]	-1.25187	1.31471	-1.08597	0.53995	0.48319
TRINITY_DN8685_c0_g1_i5_orf1	-	-	-	macrophage mannose receptor 1-like [Zerene cesonia]	-1.43299	1.3785	-0.78348	0.62066	0.21731
TRINITY_DN394_c0_g1_i2_orf1	-	-	-	uncharacterized protein LOC114351483 [Ostrinia furnacalis]	-1.07239	0.79102	-1.25181	0.27572	1.25746
TRINITY_DN214_c0_g1_i4_orf1	-	-	-	uncharacterized protein LOC114352813 [Ostrinia furnacalis]	-1.00733	1.4236	-1.24034	0.61285	0.21121
TRINITY_DN335_c1_g1_i5_orf1	-	-	-	PREDICTED: perilipin-4 isoform X14 [Papilio polytes]	-1.39388	1.15817	-0.91176	0.23751	0.90995

TRINITY_DN5748_c0_g1_i6_orf1	-	-	-	glycine N-methyltransferase isoform X1 [Ostrinia furnacalis] >XP_028165118.1 glycine N-methyltransferase isoform X2 [Ostrinia furnacalis] >XP_028165119.1 glycine N-methyltransferase isoform X1 [Ostrinia furnacalis] >XP_028165120.1 glycine N-methyltransferase isoform X2 [Ostrinia furnacalis]	-1.42537	1.38264	-0.82201	0.37303	0.4917
TRINITY_DN24_c0_g1_i1_orf1	-	-	-	hypothetical protein evm_007803 [Chilo suppressalis]	-1.74811	1.04161	-0.03943	-0.16528	0.9112
TRINITY_DN13236_c0_g1_i4_orf1	-	-	-	zonadhesin-like isoform X4 [Ostrinia furnacalis]	-0.9254	1.4502	-1.31082	0.31118	0.47484
TRINITY_DN14670_c0_g1_i1_orf1	-	-	-	heat shock protein beta-1 isoform X1 [Helicoverpa armigera] >XP_022829066.1 heat shock protein beta-1 isoform X1 [Spodoptera litura] >XP_026747148.1 heat shock protein beta-1 isoform X3 [Trichoplusia ni] >XP_026748187.1 heat shock protein beta-1 isoform X2 [Galleria mellonella] >XP_028167756.1 heat shock protein beta-1 isoform X2 [Ostrinia furnacalis] >XP_035431734.1 heat shock protein beta-1-like isoform X3 [Spodoptera frugiperda] >XP_047023072.1 heat shock protein beta-1 isoform X1 [Helicoverpa zea] >XP_049865086.1 heat shock protein beta-1 [Pectinophora gossypiella] >KAH9640995.1 hypothetical protein HF086_015091 [Spodoptera exigua] >QGZ00460.1 heat shock protein 21.4 [Glyphodes pyloalis] >QKR72095.1 heat-shock protein 21.4 [Mythimna separata] >CAB3228281.1 unnamed protein product [Arctia plantaginis] >CAH0628881.1 unnamed protein product [Chrysodeixis includens]	-1.47876	1.2992	-0.80182	0.46987	0.51152
TRINITY_DN38230_c0_g1_i4_orf1	-	-	-	hypothetical protein evm_007803 [Chilo suppressalis]	-0.78664	1.40858	-1.40544	0.15204	0.63146
TRINITY_DN214_c0_g1_i3_orf1	-	-	-	uncharacterized protein LOC114352813 [Ostrinia furnacalis]	-1.15132	1.36991	-1.14001	0.65265	0.26877
TRINITY_DN4068_c1_g2_i1_orf1	-	-	-	larval cuticle protein LCP-17 [Helicoverpa armigera] >PZC82071.1 hypothetical protein B5X24_HaOG211161 [Helicoverpa armigera] >PZC87412.1 hypothetical protein B5X24_HaOG216859 [Helicoverpa armigera]	-0.96395	1.34151	-1.34265	0.51963	0.44546
TRINITY_DN37307_c0_g1_i4_orf1	-	-	-	superoxide dismutase [Mn], mitochondrial [Ostrinia furnacalis]	-1.70829	1.38045	-0.18805	0.19596	0.31994
TRINITY_DN1108_c1_g2_i1_orfp1	-	-	-	TRINITY_DN1108_c1_g2_i1_m.5565 TRINITY_DN1108_c1_g2::TRINITY_DN1108_c1_g2_i1::g.5565 ORF type:internal len:205 (-),score=147.90 TRINITY_DN1108_c1_g2_i1:2-613(-)	-1.54217	1.50535	-0.47359	0.23202	0.27838
TRINITY_DN84938_c0_g1_i4_orf1	-	-	-	vinculin-like isoform X2 [Ostrinia furnacalis]	-1.16778	1.50581	-0.66253	-0.50003	0.82453
TRINITY_DN1226_c0_g1_i11_orfp1	-	-	-	TRINITY_DN1226_c0_g1_i11_m.52385 TRINITY_DN1226_c0_g1::TRINITY_DN1226_c0_g1_i11::g.52385 ORF type:internal len:92 (-),score=5.77 TRINITY_DN1226_c0_g1_i11:2-274(-)	-1.19379	0.75418	-1.11907	0.2602	1.29849
TRINITY_DN4343_c0_g1_i2_orf1	-	-	-	uncharacterized protein LOC114365231 isoform X3 [Ostrinia furnacalis]	-1.82167	0.80723	-0.05889	0.06209	1.01123
TRINITY_DN24121_c1_g1_i6_orf1	-	-	-	serine protease persephone-like [Ostrinia furnacalis]	-0.85001	1.23011	-1.18833	-0.31197	1.1202
TRINITY_DN15755_c0_g1_i1_orf1	-	-	-	cytochrome P450 monooxygenase CYP6AB141 [Ostrinia furnacalis]	-1.06222	0.96158	-1.11964	-0.07867	1.29894
TRINITY_DN28503_c0_g1_i6_orf1	-	-	-	uncharacterized protein LOC114363584 [Ostrinia furnacalis] >AXY94663.1 seroin transcript 3 [Ostrinia nubilalis]	-0.46922	1.62986	-1.436	0.02959	0.24578
TRINITY_DN4062_c0_g2_i1_orf1	-	-	-	venom peptide BmKAPI-like isoform X2 [Ostrinia furnacalis]	-1.78841	1.21185	-0.15121	0.21333	0.51443
TRINITY_DN66453_c0_g1_i4_orfp1	-	-	-	TRINITY_DN66453_c0_g1_i4_m.7345 TRINITY_DN66453_c0_g1::TRINITY_DN66453_c0_g1_i4::g.7345 ORF type:internal len:93	-1.14385	0.89828	-1.11265	0.07731	1.28091
TRINITY_DN140_c0_g1_i5_orf1	-	-	-	calcyphosin-like protein isoform X4 [Helicoverpa armigera]	-0.76048	1.31048	-0.79938	-0.88382	1.1332
TRINITY_DN12013_c0_g1_i6_orf1	-	-	-	tudor domain-containing protein 7 isoform X3 [Ostrinia furnacalis]	-1.00998	1.79872	-0.85777	0.09119	-0.02217
TRINITY_DN22875_c0_g1_i6_orf1	-	-	-	microtubule-actin cross-linking factor 1 isoform X15 [Ostrinia furnacalis]	-1.63237	1.06815	-0.58475	0.2643	0.88467
TRINITY_DN1592_c0_g1_i1_orf1	-	-	-	serine protease 7-like isoform X2 [Ostrinia furnacalis]	-1.80721	1.04569	-0.08891	0.05712	0.79331
TRINITY_DN14611_c0_g1_i5_orf1	-	-	-	hsc70-interacting protein-like [Galleria mellonella]	-1.72201	1.23601	-0.35729	0.31274	0.53055
TRINITY_DN230_c2_g1_i5_orf1	-	-	-	6-pyruvoyl tetrahydrobiopterin synthase [Ostrinia furnacalis]	-1.0854	1.67315	-0.84355	-0.24505	0.50085
TRINITY_DN3029_c1_g2_i1_orf1	-	-	-	unnamed protein product [Plutella xylostella]	-1.56698	1.49097	-0.39192	0.40499	0.06294
TRINITY_DN38225_c0_g2_i1_orf1	-	-	-	uncharacterized protein LOC114354273 [Ostrinia furnacalis]	-1.35795	1.38972	-0.90341	0.31622	0.55542
TRINITY_DN4069_c0_g1_i5_orf1	-	-	-	putative sulfiredoxin [Ostrinia furnacalis]	-1.53807	1.03337	-0.60588	0.01551	1.09507
TRINITY_DN31225_c0_g1_i1_orf1	-	-	-	ribosome biogenesis protein BMS1 homolog [Ostrinia furnacalis]	-0.90148	1.13578	-1.33417	0.04374	1.05613
TRINITY_DN11108_c0_g1_i4_orf1	-	-	-	peroxisomal leader peptide-processing protease [Ostrinia furnacalis] >XP_028165527.1 peroxisomal leader peptide-processing protease [Ostrinia furnacalis]	-1.4749	1.4067	-0.59582	-0.03569	0.6997
TRINITY_DN58751_c0_g1_i2_orf1	-	-	-	FK506-binding protein 2 isoform X1 [Vanessa tameamea] >XP_046977568.1 FK506-binding protein 2 isoform X1 [Vanessa cardui]	-1.81915	1.02808	-0.06837	0.06933	0.79011
TRINITY_DN11231_c1_g1_i1_orfp1	-	-	-	TRINITY_DN11231_c1_g1_i1_m.13377 TRINITY_DN11231_c1_g1::TRINITY_DN11231_c1_g1_i1::g.13377 ORF type:internal len:76 (-),score=1.43 TRINITY_DN11231_c1_g1_i1:1-225(-)	-1.40707	1.09604	-0.82694	0.07526	1.06271
TRINITY_DN91946_c0_g1_i1_orf1	-	-	-	protein catecholamines up [Ostrinia furnacalis]	-1.46719	0.91434	-0.76968	0.13953	1.18299

TRINITY_DN12545_c0_g1_i7_orf1	-	-	-	hypothetical protein evm_003491 [Chilo suppressalis]	-1.1449	1.2189	-1.18171	0.24279	0.86493
TRINITY_DN6698_c0_g2_i2_orf1	-	-	-	protein mesh isoform X1 [Ostrinia furnacalis]	-1.86203	0.89972	0.14421	-0.01985	0.83795
TRINITY_DN452_c9_g1_i1_orf1	-	-	-	epidermal retinol dehydrogenase 2-like [Ostrinia furnacalis]	-1.53889	1.35867	-0.58882	0.11673	0.6523
TRINITY_DN33995_c0_g1_i5_orf1	-	-	-	unnamed protein product [Spodoptera exigua]	-0.42753	1.67758	-1.06253	-0.74812	0.56061
TRINITY_DN12336_c0_g1_i1_orfp1	-	-	-	TRINITY_DN12336_c0_g1_i1_m.30792 TRINITY_DN12336_c0_g1::TRINITY_DN12336_c0_g1_i1::g.30792 ORF type:internal len:86 (-),score=13.56 TRINITY_DN12336_c0_g1_i1:3-257(-)	-0.81597	1.07227	-1.23094	-0.28543	1.26006
TRINITY_DN56708_c0_g3_i1_orfp1	-	-	-	TRINITY_DN56708_c0_g3_i1_m.56611 TRINITY_DN56708_c0_g3::TRINITY_DN56708_c0_g3_i1::g.56611 ORF type:internal len:69 (-),score=2.50 TRINITY_DN56708_c0_g3_i1:1-204(-)	-0.85903	1.45736	-1.35329	0.27311	0.48185
TRINITY_DN1008_c0_g1_i2_orf1	-	-	-	integrin beta-PS [Ostrinia furnacalis]	-1.55832	1.13332	-0.59084	0.04847	0.96737
TRINITY_DN3732_c1_g1_i5_orf1	-	-	-	cytochrome P450 6B2-like [Ostrinia furnacalis]	-1.1152	1.00074	-0.76765	-0.50141	1.38353
TRINITY_DN33_c0_g1_i14_orf1	-	-	-	uncharacterized protein CG45076-like isoform X1 [Ostrinia furnacalis]	-1.44015	1.08428	-0.88362	0.30246	0.93703
TRINITY_DN62707_c0_g1_i1_orf1	-	-	-	uncharacterized protein LOC114362831 [Ostrinia furnacalis]	-1.15593	1.49791	-1.03975	0.56749	0.13028
TRINITY_DN10441_c0_g1_i3_orf1	-	-	-	zonadhesin-like [Ostrinia furnacalis]	-0.78268	1.38412	-1.45224	0.40694	0.44386
TRINITY_DN452_c0_g1_i4_orf1	-	-	-	protein CREG1 [Ostrinia furnacalis] >XP_028170592.1 protein CREG1 [Ostrinia furnacalis]	-1.33444	1.40992	-0.39485	-0.55603	0.8754
TRINITY_DN8621_c0_g1_i4_orf1	-	-	-	aminopeptidase N-like isoform X2 [Ostrinia furnacalis]	-1.25027	1.5588	-0.64701	-0.34606	0.68455
TRINITY_DN5696_c0_g1_i4_orf1	-	-	-	serine protease snake-like isoform X1 [Ostrinia furnacalis]	-1.2981	0.98551	-0.66332	-0.35689	1.3328
TRINITY_DN16400_c0_g2_i1_orf1	-	-	-	superoxide dismutase [Cu-Zn]-like isoform X1 [Ostrinia furnacalis]	-0.6059	1.48134	-1.49795	0.29009	0.33243
TRINITY_DN1267_c0_g2_i10_orf1	-	-	-	secretory phospholipase A2 receptor-like [Ostrinia furnacalis]	-0.53484	1.44259	-1.52985	0.53347	0.08863
TRINITY_DN3766_c0_g1_i10_orf1	-	-	-	circadian clock-controlled protein-like [Ostrinia furnacalis]	-1.80718	1.14064	-0.0108	0.01967	0.65766
TRINITY_DN44110_c0_g1_i4_orf1	-	-	-	putative phosphatidate phosphatase [Ostrinia furnacalis]	-1.30686	1.63223	-0.40229	-0.44276	0.51967
TRINITY_DN804_c0_g1_i7_orf1	-	-	-	hypothetical protein HF086_004695 [Spodoptera exigua] >CAH0695017.1 unnamed protein product [Spodoptera exigua]	-0.47005	1.44535	-1.42147	-0.31072	0.7569
TRINITY_DN4123_c0_g1_i1_orf1	-	-	-	uncharacterized protein LOC114355030 [Ostrinia furnacalis]	-1.0626	1.05329	-1.14575	-0.04764	1.20269
TRINITY_DN3486_c0_g1_i5_orf1	-	-	-	uncharacterized protein LOC114360519 [Ostrinia furnacalis]	-0.94961	1.28285	-0.91356	-0.56117	1.14149
TRINITY_DN5880_c0_g2_i2_orf1	-	-	-	macrophage mannose receptor 1 [Bombyx mori]	-1.1019	0.64841	-0.99974	-0.08269	1.53593
TRINITY_DN6994_c0_g1_i3_orf1	-	-	-	C-type mannose receptor 2-like isoform X1 [Ostrinia furnacalis]	-1.04889	1.73871	-0.90284	0.24601	-0.03299
TRINITY_DN19116_c0_g1_i3_orf1	-	-	-	UDP-glucose 4-epimerase isoform X1 [Ostrinia furnacalis]	-1.56725	1.16601	-0.53648	-0.00898	0.94671
TRINITY_DN5661_c0_g1_i5_orf1	-	-	-	cytochrome P450 6B7-like [Ostrinia furnacalis]	-0.73468	1.26176	-1.41512	-0.04145	0.92948
TRINITY_DN46372_c0_g1_i1_orf1	-	-	-	unnamed protein product [Chilo suppressalis]	-0.26928	1.37572	-1.69094	0.24345	0.34105
TRINITY_DN60792_c0_g1_i2_orf1	-	-	-	ATP-binding cassette sub-family D member 2 [Ostrinia furnacalis] >XP_028165108.1 ATP-binding cassette sub-family D member 2 [Ostrinia furnacalis]	-0.71242	1.70042	-1.21134	-0.12151	0.34485
TRINITY_DN19521_c0_g1_i1_orf1	-	-	-	sodium/potassium-transporting ATPase subunit beta-1-like isoform X1 [Ostrinia furnacalis]	-1.7714	1.26149	-0.01482	0.00458	0.52014
TRINITY_DN2559_c0_g1_i4_orf1	-	-	-	uricase [Ostrinia furnacalis]	-0.56656	1.38525	-1.56077	0.2149	0.52718
TRINITY_DN23564_c0_g1_i7_orf1	-	-	-	cytochrome P450 6B6-like [Ostrinia furnacalis]	-1.61122	0.94571	-0.37417	-0.12403	1.16371
TRINITY_DN2302_c0_g1_i1_orf1	-	-	-	enoyl-CoA hydratase domain-containing protein 3, mitochondrial [Ostrinia furnacalis]	-1.2374	1.56134	-0.86378	0.00609	0.53375
TRINITY_DN40434_c0_g1_i2_orf1	-	-	-	deoxyribodipyrimidine photo-lyase [Ostrinia furnacalis]	-0.68309	1.69017	-1.03602	-0.5346	0.56354
TRINITY_DN3374_c0_g1_i7_orf1	-	-	-	TPPP family protein CG45057 [Ostrinia furnacalis] >XP_028172578.1 TPPP family protein CG45057 [Ostrinia furnacalis]	-1.13836	1.29926	-1.11887	0.08827	0.86971
TRINITY_DN36632_c0_g1_i1_orf1	-	-	-	hypothetical protein evm_011159, partial [Chilo suppressalis]	-1.19402	1.08469	-0.63896	-0.55039	1.29868
TRINITY_DN1622_c0_g1_i6_orf1	-	-	-	unnamed protein product [Parnassius apollo]	-1.18342	1.09775	-0.87819	-0.27923	1.24309
TRINITY_DN10652_c0_g1_i4_orf1	-	-	-	uncharacterized protein LOC114364166 [Ostrinia furnacalis]	-0.95303	1.81415	-0.88484	0.10499	-0.08127
TRINITY_DN52761_c0_g2_i1_orf1	-	-	-	atlastin-like isoform X4 [Ostrinia furnacalis]	-1.40859	0.85556	-0.20443	-0.61001	1.36747
TRINITY_DN47151_c0_g1_i1_orf1	-	-	-	unnamed protein product [Danaus chrysippus]	-0.5394	1.03801	-1.38484	-0.36962	1.25586
TRINITY_DN5558_c0_g1_i4_orf1	-	-	-	unnamed protein product [Parnassius apollo]	-1.45795	1.46717	-0.34131	-0.35843	0.69053
TRINITY_DN3433_c0_g1_i6_orf1	-	-	-	cytosolic purine 5'-nucleotidase isoform X2 [Ostrinia furnacalis] >XP_028162963.1 cytosolic purine 5'-nucleotidase isoform X2 [Ostrinia furnacalis]	-1.23385	1.65249	-0.807	0.09365	0.29471
TRINITY_DN26993_c1_g1_i8_orf1	-	-	-	endocuticle structural glycoprotein ABD-4-like [Ostrinia furnacalis]	-0.89398	1.48352	-1.25735	0.02083	0.64699
TRINITY_DN77318_c0_g2_i1_orf1	-	-	-	uncharacterized protein LOC114351191 [Ostrinia furnacalis]	-1.0233	1.43719	-0.45072	-0.89906	0.93589
TRINITY_DN11263_c0_g1_i5_orf1	-	-	-	SET domain-containing protein SmydA-8 [Ostrinia furnacalis]	-1.54261	1.59509	-0.24114	0.10122	0.08745
TRINITY_DN98995_c0_g1_i2_orf1	-	-	-	hypothetical protein HF086_008399, partial [Spodoptera exigua]	-0.25721	0.96638	-1.65883	-0.15656	1.10622
TRINITY_DN1575_c0_g1_i7_orf1	-	-	-	adenine phosphoribosyltransferase [Ostrinia furnacalis]	-1.41719	1.37685	-0.58185	-0.21983	0.84201
TRINITY_DN13563_c0_g1_i1_orf1	-	-	-	Golgi resident protein GCP60 isoform X1 [Ostrinia furnacalis]	-0.92777	1.25827	-0.84293	-0.66957	1.18201

TRINITY_DN3055_c0_g1_i9_orf1	-	-	-	WAP four-disulfide core domain protein 2-like [Ostrinia furnacalis]	-0.79956	1.75267	-0.83316	-0.60196	0.48201
TRINITY_DN842_c0_g1_i9_orf1	-	-	-	hypothetical protein evm_011651 [Chilo suppressalis]	-0.69645	1.878	-0.81157	-0.54624	0.17626
TRINITY_DN1993_c0_g1_i1_orf1	-	-	-	6-phosphofructo-2-kinase/fructose-2,6-bisphosphatase isoform X1 [Ostrinia furnacalis]	-0.38654	1.65878	-1.43298	-0.04749	0.20822
TRINITY_DN2193_c0_g1_i7_orf1	-	-	-	long-chain-fatty-acid--CoA ligase 5 isoform X1 [Ostrinia furnacalis] >XP_028176293.1 long-chain-fatty-acid--CoA ligase 5 isoform X1 [Ostrinia furnacalis] >XP_028176294.1 long-chain-fatty-acid--CoA ligase 5 isoform X1 [Ostrinia furnacalis] >XP_028176295.1 long-chain-fatty-acid--CoA ligase 5 isoform X1 [Ostrinia furnacalis] >XP_028176296.1 long-chain-fatty-acid--CoA ligase 5 isoform X1 [Ostrinia furnacalis] >XP_028176297.1 long-chain-fatty-acid--CoA ligase 5 isoform X1 [Ostrinia furnacalis] >XP_028176298.1 long-chain-fatty-acid--CoA ligase 5 isoform X2 [Ostrinia furnacalis]	-0.52768	1.12899	-1.52896	-0.11883	1.04647
TRINITY_DN85476_c0_g1_i1_orf1	-	-	-	iron-sulfur protein NUBPL-like [Ostrinia furnacalis]	-0.77967	1.4978	-1.12704	-0.42596	0.83488
TRINITY_DN19251_c0_g1_i8_orf1	-	-	-	succinate--CoA ligase [GDP-forming] subunit beta, mitochondrial [Ostrinia furnacalis]	-0.40389	1.55326	-1.53168	0.14039	0.24192
TRINITY_DN4793_c0_g1_i7_orf1	-	-	-	probable hydroxyacid-oxoacid transhydrogenase, mitochondrial isoform X3 [Ostrinia furnacalis] >XP_028159821.1 probable hydroxyacid-oxoacid transhydrogenase, mitochondrial isoform X4 [Ostrinia furnacalis]	-0.59746	0.96068	-1.41631	-0.23494	1.28802
TRINITY_DN25423_c0_g1_i1_orf1	-	-	-	uncharacterized protein LOC114353174 [Ostrinia furnacalis]	-1.23763	1.62219	-0.37545	-0.59438	0.58527
TRINITY_DN7477_c0_g1_i1_orf1	-	-	-	ethanolamine kinase [Ostrinia furnacalis]	-1.02364	1.46845	-0.60865	-0.75831	0.92214
TRINITY_DN4424_c0_g1_i1_orf1	-	-	-	calreticulin [Ostrinia furnacalis]	-1.00529	1.63555	-0.73784	-0.56434	0.67192
TRINITY_DN57856_c0_g2_i1_orf1	-	-	-	cytochrome P450 6B2-like [Ostrinia furnacalis]	-0.16692	0.94914	-1.55768	-0.4321	1.20756
TRINITY_DN77480_c0_g1_i2_orf1	-	-	-	hypothetical protein evm_008422 [Chilo suppressalis]	0.10078	1.31077	-1.56245	-0.56451	0.71541
TRINITY_DN27491_c0_g1_i1_orf1	-	-	-	prolactin regulatory element-binding protein [Galleria mellonella]	-0.31218	1.45882	-1.5147	-0.27003	0.63808
TRINITY_DN1104_c0_g1_i1_orfp1	-	-	-	TRINITY_DN1104_c0_g1_i1_m.5521 TRINITY_DN1104_c0_g1::TRINITY_DN1104_c0_g1_i1::g.5521 ORF type:5prime_partial len:204 (+),score=39.47 TRINITY_DN1104_c0_g1_i1:3-614(+)	-0.02673	1.34044	-1.52246	-0.55246	0.7612
TRINITY_DN20356_c0_g1_i5_orf1	-	-	-	uncharacterized protein LOC114362428 [Ostrinia furnacalis]	-0.50714	1.92876	-0.1106	-0.38067	-0.93034
TRINITY_DN2407_c0_g1_i2_orf1	-	-	-	uncharacterized protein LOC114366345 isoform X2 [Ostrinia furnacalis]	-1.02281	1.70113	-0.7624	0.52949	-0.44541
TRINITY_DN103107_c0_g1_i2_orf1	-	-	-	superoxide dismutase [Cu-Zn] [Ostrinia furnacalis] >XP_028177872.1 superoxide dismutase [Cu-Zn] [Ostrinia furnacalis]	-0.20002	1.95555	-0.31837	-0.74816	-0.689
TRINITY_DN184_c0_g1_i1_orf1	-	-	-	macrophage mannose receptor 1-like isoform X1 [Maniola jurtina]	-0.96923	1.73147	-0.04771	0.27718	-0.99172
TRINITY_DN66287_c0_g1_i1_orfp1	-	-	-	tenascin-like isoform X4 [Trichoplusia ni]	-0.24384	1.98087	-0.64446	-0.49866	-0.5939
TRINITY_DN12920_c0_g3_i1_orf1	-	-	-	zonadhesin-like isoform X4 [Ostrinia furnacalis]	-0.86972	1.93764	-0.25238	-0.6233	-0.19224
TRINITY_DN14874_c0_g1_i6_orf1	-	-	-	uncharacterized protein LOC114358148 [Ostrinia furnacalis]	-1.20115	1.69405	-0.25373	0.42561	-0.66478
TRINITY_DN50725_c0_g1_i6_orf1	-	-	-	BTB/POZ domain-containing protein 2-like [Ostrinia furnacalis]	-0.21857	1.96068	-0.53514	-0.38764	-0.81933
TRINITY_DN27300_c0_g1_i6_orfp1	-	-	-	TRINITY_DN27300_c0_g1_i6_m.71140 TRINITY_DN27300_c0_g1::TRINITY_DN27300_c0_g1_i6::g.71140 ORF type:internal len:129 (-),score=20.75 TRINITY_DN27300_c0_g1_i6:1-384(-)	0.03732	1.8822	-0.98174	-0.63077	-0.30702
TRINITY_DN2049_c1_g1_i2_orf1	-	-	-	luciferin 4-monooxygenase-like [Ostrinia furnacalis]	-1.20165	1.79769	0.01861	-0.0474	-0.56725
TRINITY_DN6325_c0_g1_i8_orf1	-	-	-	unnamed protein product [Pieris macdunnoughi]	-0.09141	1.73952	-1.38833	-0.08246	-0.17733
TRINITY_DN21170_c0_g1_i5_orf1	-	-	-	twitchin-like [Ostrinia furnacalis]	0.10922	1.78992	-1.23306	-0.47797	-0.1881
TRINITY_DN1272_c1_g1_i4_orf1	-	-	-	E3 ubiquitin-protein transferase MAEA [Ostrinia furnacalis] >XP_028157102.1 E3 ubiquitin-protein transferase MAEA [Ostrinia furnacalis]	0.03772	1.75426	-0.16684	-1.34772	-0.27742
TRINITY_DN3627_c0_g1_i7_orf1	-	-	-	spermine oxidase-like isoform X2 [Ostrinia furnacalis]	-1.00952	1.84876	0.14822	-0.65719	-0.33028
TRINITY_DN29969_c0_g1_i5_orf1	-	-	-	twitchin-like [Ostrinia furnacalis]	-0.18881	1.72402	-1.39292	0.07332	-0.21561
TRINITY_DN30509_c0_g1_i9_orf1	-	-	-	prostatic acid phosphatase [Ostrinia furnacalis]	-0.29101	1.98093	-0.72261	-0.50839	-0.45893
TRINITY_DN81181_c0_g1_i6_orfp1	-	-	-	C-type lectin domain family 4 member E [Pieris rapae]	-0.13551	1.76548	-0.87189	0.26017	-1.01825
TRINITY_DN2343_c1_g1_i12_orf1	-	-	-	receptor expression-enhancing protein 5-like isoform X3 [Ostrinia furnacalis]	-0.68684	1.89017	-0.46255	-0.85371	0.11293
TRINITY_DN36581_c0_g1_i5_orf1	-	-	-	enoyl-CoA delta isomerase 1, mitochondrial-like isoform X1 [Ostrinia furnacalis] >XP_028158560.1 enoyl-CoA delta isomerase 1, mitochondrial-like isoform X2 [Ostrinia	-0.47281	1.78089	-0.9674	0.38238	-0.72306
TRINITY_DN22824_c0_g1_i4_orf1	-	-	-	LIM domain and actin-binding protein 1 [Ostrinia furnacalis]	-0.65832	1.71049	-0.41963	-1.11148	0.47893
TRINITY_DN3687_c0_g1_i1_orf1	-	-	-	acyl-CoA-binding domain-containing protein 5 isoform X2 [Manduca sexta]	-0.83242	1.65227	0.01078	-1.20018	0.36954
TRINITY_DN11962_c0_g1_i2_orf1	-	-	-	BTB/POZ domain-containing protein 2-like [Ostrinia furnacalis]	-0.05575	1.93919	-0.70795	-0.43885	-0.73664
TRINITY_DN36230_c0_g1_i1_orf1	-	-	-	sorting nexin-2 [Ostrinia furnacalis]	-0.995	1.5471	0.19682	-1.17984	0.43093
TRINITY_DN18216_c0_g1_i4_orf1	-	-	-	golgin subfamily A member 7 [Ostrinia furnacalis]	-0.64346	1.74534	-0.29992	-1.15206	0.35009
TRINITY_DN42185_c0_g1_i7_orf1	-	-	-	ADP-ribosylation factor-like protein 13B isoform X2 [Ostrinia furnacalis]	-0.15903	1.47182	-0.59783	-1.40521	0.69025

TRINITY_DN14409_c0_g1_i1_orf1	-	-	-	endoplasmic reticulum mannosyl-oligosaccharide 1,2-alpha-mannosidase [Ostrinia furnacalis] >XP_028171972.1 endoplasmic reticulum mannosyl-oligosaccharide 1,2-alpha-mannosidase [Ostrinia furnacalis]	-0.29897	1.69452	-0.70073	-1.15575	0.46093
TRINITY_DN2694_c0_g1_i3_orf1	-	-	-	cubilin homolog [Ostrinia furnacalis]	0.21487	1.86278	-0.81317	-0.52454	-0.73993
TRINITY_DN2719_c1_g1_i6_orf1	-	-	-	unnamed protein product [Chrysodeixis includens]	-0.00589	1.62811	-1.28481	-0.73508	0.39767
TRINITY_DN3588_c0_g1_i4_orf1	-	-	-	probable peroxisomal acyl-coenzyme A oxidase 1 isoform X1 [Ostrinia furnacalis] >XP_028165840.1 probable peroxisomal acyl-coenzyme A oxidase 1 isoform X2 [Ostrinia furnacalis]	-0.52656	1.74072	0.25195	-1.25956	-0.20656
TRINITY_DN2346_c0_g2_i1_orf1	-	-	-	uncharacterized protein LOC113514389 isoform X1 [Galleria mellonella]	-0.8757	1.69141	-0.18634	-1.06951	0.44013
TRINITY_DN825_c23_g1_i5_orf1	-	-	-	methionine-tRNA synthetase, partial [Papilio xuthus]	-0.17469	1.8174	-0.47551	-1.19974	0.03255
TRINITY_DN13385_c0_g1_i4_orf1	-	-	-	putative aminopeptidase W07G4.4 [Ostrinia furnacalis]	-0.14971	1.5366	-1.07983	-0.99117	0.68412
TRINITY_DN11942_c0_g1_i1_orf1	-	-	-	hypothetical protein B5X24_HaOG213660 [Helicoverpa armigera]	-0.07791	1.13675	0.37672	-1.84237	0.4068
TRINITY_DN17896_c0_g1_i1_orf1	-	-	-	probable aminopeptidase NPEPL1 isoform X1 [Ostrinia furnacalis] >XP_028174563.1 probable aminopeptidase NPEPL1 isoform X2 [Ostrinia furnacalis] >XP_028174564.1 probable aminopeptidase NPEPL1 isoform X3 [Ostrinia furnacalis]	-0.05332	1.56142	-1.18253	-0.90701	0.58144
TRINITY_DN82_c0_g1_i1_orf1	-	-	-	vacuolar protein sorting-associated protein 26B-like [Ostrinia furnacalis]	-0.46424	1.60921	0.51226	-1.35745	-0.29978
TRINITY_DN5432_c1_g1_i3_orf1	-	-	-	electron transfer flavoprotein-ubiquinone oxidoreductase, mitochondrial [Ostrinia furnacalis]	0.44171	1.74159	-1.07625	-0.57191	-0.53514
TRINITY_DN7570_c0_g1_i18_orf1	-	-	-	sodium/potassium-transporting ATPase subunit alpha isoform X4 [Trichoplusia ni] >XP_026734855.1 sodium/potassium-transporting ATPase subunit alpha isoform X4 [Trichoplusia ni]	0.30603	1.7349	-0.28929	-1.24909	-0.50256
TRINITY_DN6684_c0_g1_i4_orf1	-	-	-	26S proteasome non-ATPase regulatory subunit 6 isoform X1 [Ostrinia furnacalis]	-0.02764	1.59817	0.21863	-1.52575	-0.26341
TRINITY_DN359_c0_g1_i5_orf1	-	-	-	eukaryotic translation initiation factor 5 isoform X1 [Ostrinia furnacalis] >XP_028162054.1 eukaryotic translation initiation factor 5 isoform X1 [Ostrinia furnacalis] >XP_028162062.1 eukaryotic translation initiation factor 5 isoform X1 [Ostrinia furnacalis] >XP_028162073.1 eukaryotic translation initiation factor 5 isoform X1 [Ostrinia furnacalis] >XP_028162080.1 eukaryotic translation initiation factor 5 isoform X1 [Ostrinia furnacalis] >XP_028162091.1 eukaryotic translation initiation factor 5 isoform X1 [Ostrinia furnacalis] >XP_028162099.1 eukaryotic translation initiation factor 5 isoform X1 [Ostrinia furnacalis] >XP_028162107.1 eukaryotic translation initiation factor 5 isoform X1 [Ostrinia furnacalis]	-0.47545	1.26184	0.65405	-1.64664	0.20619
TRINITY_DN4636_c0_g3_i1_orf1	-	-	-	microtubule-associated serine/threonine-protein kinase 4 [Ostrinia furnacalis]	-0.06881	1.79815	-0.67237	-1.14136	0.08438
TRINITY_DN9979_c0_g1_i1_orf1	-	-	-	ADP-dependent glucokinase [Ostrinia furnacalis]	0.14275	1.73688	-0.51791	-1.30034	-0.06138
TRINITY_DN11612_c0_g2_i1_orf1	-	-	-	eukaryotic translation initiation factor 5B [Manduca sexta]	0.23217	1.72823	-0.05084	-1.21314	-0.69643
TRINITY_DN40176_c0_g1_i1_orf1	-	-	-	transcription elongation factor 1 homolog [Plutella xylostella] >XP_014365734.1 transcription elongation factor 1 homolog [Papilio machaon] >XP_021188366.1 transcription elongation factor 1 homolog [Helicoverpa armigera] >XP_022815988.1 transcription elongation factor 1 homolog [Spodoptera litura] >XP_022815989.1 transcription elongation factor 1 homolog [Spodoptera litura] >XP_026739943.1 transcription elongation factor 1 homolog [Trichoplusia ni] >XP_026739944.1 transcription elongation factor 1 homolog [Trichoplusia ni] >XP_028167273.1 transcription elongation factor 1 homolog [Ostrinia furnacalis] >XP_035429172.1 transcription elongation factor 1 homolog [Spodoptera frugiperda] >XP_038210306.1 transcription elongation factor 1 homolog [Zerene cesonia] >XP_045492874.1 transcription elongation factor 1 homolog [Colias croceus] >XP_045492875.1 transcription elongation factor 1 homolog [Colias croceus] >XP_047019949.1 transcription elongation factor 1 homolog [Helicoverpa zea] >KAF9414193.1 hypothetical protein HW555_007806 [Spodoptera exigua] >KPJ01541.1 Transcription elongation factor 1-like [Papilio xuthus] >CAB3243126.1 unnamed protein product [Arctia plantaginis] >CAB3514670.1 unnamed protein product [Spodoptera littoralis] >CAH0626924.1 unnamed protein product [Chrysodeixis includens] >CAH2075456.1 unnamed protein product, partial [Iphiclides	0.01773	1.28593	0.69774	-1.65508	-0.34632
TRINITY_DN140613_c0_g1_i1_orf1	-	-	-	hypothetical protein M0804_013066 [Polistes exclamans]	-0.06232	1.49046	-0.8668	-1.24652	0.68519
TRINITY_DN61536_c0_g3_i1_orf1	-	-	-	cubilin homolog [Ostrinia furnacalis]	-0.12303	1.9076	-0.4805	-0.28819	-1.01588
TRINITY_DN63492_c0_g1_i1_orf1	-	-	-	unnamed protein product [Chilo suppressalis]	0.46718	1.28769	-0.39129	-1.69194	0.32836
TRINITY_DN1954_c0_g1_i4_orf1	-	-	-	unnamed protein product [Diatraea saccharalis]	0.2687	1.56666	-0.48557	-1.48933	0.13955
TRINITY_DN2873_c0_g1_i7_orf1	-	-	-	transitional endoplasmic reticulum ATPase TER94 [Galleria mellonella]	0.67532	1.44866	-0.47368	-1.4806	-0.1697
TRINITY_DN4742_c0_g1_i1_orf1	-	-	-	ribosomal protein S6 kinase beta-1 [Ostrinia furnacalis]	0.39927	1.32877	0.04659	-1.7528	-0.02184

TRINITY_DN2770_c0_g2_i4_orf1	-	-	-	phosphatidylinositol 4-phosphate 3-kinase C2 domain-containing subunit alpha isoform X1 [Ostrinia furnacalis]	0.1546	1.48882	-0.4086	-1.57401	0.3392
TRINITY_DN2813_c0_g1_i10_orf1	-	-	-	arylphorin subunit alpha-like [Ostrinia furnacalis]	-1.90318	1.03526	0.46591	0.1367	0.26532
TRINITY_DN34406_c0_g2_i9_orfp1	-	-	-	TRINITY_DN34406_c0_g2_i9_m.33755 TRINITY_DN34406_c0_g2::TRINITY_DN34406_c0_g2_i9::g.33755 ORF type:internal len:82 (-),score=12.88 TRINITY_DN34406_c0_g2_i9:3-245(-)	-1.3357	1.25759	0.75843	-0.98349	0.30318
TRINITY_DN5406_c0_g2_i1_orf1	-	-	-	uncharacterized protein LOC114350326 [Ostrinia furnacalis]	-1.87232	0.7566	0.94781	0.153	0.01491
TRINITY_DN2813_c0_g1_i7_orf1	-	-	-	arylphorin subunit alpha-like [Ostrinia furnacalis]	-1.87421	0.86631	0.64586	-0.17548	0.53752
TRINITY_DN9044_c0_g1_i2_orf1	-	-	-	unnamed protein product [Euphydryas editha]	-1.6119	1.42082	0.34158	0.28184	-0.43234
TRINITY_DN15578_c0_g2_i1_orfp1	-	-	-	uncharacterized protein LOC125235519 [Leguminivora glycinivorella]	-1.88831	0.8435	0.71887	0.43946	-0.11351
TRINITY_DN25234_c0_g1_i1_orf1	-	-	-	uncharacterized protein LOC114353853 [Ostrinia furnacalis]	-1.75627	1.28922	0.3191	0.33917	-0.19121
TRINITY_DN98692_c0_g3_i1_orf1	-	-	-	fatty acyl-CoA hydrolase precursor, medium chain [Ostrinia furnacalis]	-1.93298	0.67659	0.85037	0.21462	0.19139
TRINITY_DN20560_c0_g1_i6_orf1	-	-	-	pupal cuticle protein C1B-like [Ostrinia furnacalis]	-1.83019	0.40775	1.16248	-0.09248	0.35245
TRINITY_DN45948_c1_g1_i1_orf1	-	-	-	unnamed protein product [Leptidea sinapis]	-1.78547	0.55625	1.18571	-0.19717	0.24067
TRINITY_DN15400_c0_g1_i1_orf1	-	-	-	uncharacterized protein LOC114366781 [Ostrinia furnacalis]	-1.36477	0.66042	1.20999	-0.99758	0.49194
TRINITY_DN2406_c0_g1_i6_orf1	-	-	-	uncharacterized protein LOC114361672 [Ostrinia furnacalis]	-1.53018	1.51432	0.43542	-0.00028	-0.41928
TRINITY_DN15247_c0_g1_i2_orf1	-	-	-	probable G-protein coupled receptor Mth-like 3 isoform X1 [Ostrinia furnacalis]	-1.86872	0.42724	0.96964	-0.1341	0.60594
TRINITY_DN801_c0_g1_i2_orf1	-	-	-	cathepsin L [Ostrinia furnacalis] >XP_028165920.1 cathepsin L [Ostrinia furnacalis] >UKI61015.1 cathepsin L [Ostrinia furnacalis]	-1.65486	0.53302	1.39293	-0.14842	-0.12267
TRINITY_DN4802_c0_g1_i4_orf1	-	-	-	uncharacterized protein LOC114366345 isoform X2 [Ostrinia furnacalis]	-1.48977	0.45952	-0.50714	0.01689	1.52051
TRINITY_DN14904_c0_g1_i1_orf1	-	-	-	attacin [Ostrinia furnacalis]	-1.28866	0.17531	-0.27016	-0.37563	1.75913
TRINITY_DN960_c1_g1_i6_orf1	-	-	-	hypothetical protein evm_007130 [Chilo suppressalis]	-1.95407	0.85884	0.51431	0.21719	0.36374
TRINITY_DN364_c1_g1_i2_orf1	-	-	-	talin-2-like, partial [Ostrinia furnacalis]	-1.83063	0.99666	0.79969	0.10478	-0.07049
TRINITY_DN7534_c0_g1_i15_orf1	-	-	-	protein-glucosylgalactosylhydroxylysine glucosidase isoform X2 [Ostrinia furnacalis]	-1.86909	0.88583	0.68461	-0.17358	0.47223
TRINITY_DN4443_c0_g1_i4_orf1	-	-	-	lysosome-associated membrane glycoprotein 1-like isoform X4 [Ostrinia furnacalis]	-1.90562	0.9771	0.56689	0.29729	0.06433
TRINITY_DN29555_c0_g1_i8_orf1	-	-	-	fasciclin-1 [Ostrinia furnacalis]	-1.90939	0.91962	0.27143	0.06181	0.65653
TRINITY_DN20717_c0_g1_i1_orf1	-	-	-	putative uncharacterized protein DDB_G0282133 isoform X1 [Ostrinia furnacalis]	-1.40657	0.49019	1.5924	-0.43032	-0.24569
TRINITY_DN11060_c0_g1_i6_orf1	-	-	-	extracellular matrix protein A-like isoform X3 [Ostrinia furnacalis]	-1.79233	0.95414	0.93331	-0.0756	-0.01952
TRINITY_DN59388_c0_g1_i1_orf1	-	-	-	uncharacterized protein LOC114353759 [Ostrinia furnacalis]	-1.44043	0.48286	1.5435	-0.03031	-0.55562
TRINITY_DN22962_c0_g1_i1_orf1	-	-	-	lysosomal acid glucosylceramidase-like isoform X2 [Ostrinia furnacalis]	-1.51127	1.62574	-0.01972	-0.23198	0.13723
TRINITY_DN97883_c0_g1_i2_orf1	-	-	-	talin-2-like, partial [Ostrinia furnacalis]	-1.56012	1.25497	0.69155	0.27487	-0.66127
TRINITY_DN12969_c0_g1_i3_orf1	-	-	-	queuosine salvage protein [Ostrinia furnacalis] >XP_028167327.1 queuosine salvage protein [Ostrinia furnacalis]	-1.33244	1.66185	-0.11583	-0.56866	0.35507
TRINITY_DN5408_c0_g1_i5_orf1	-	-	-	uncharacterized protein LOC114359912 [Ostrinia furnacalis]	-1.40889	-0.04878	1.52001	-0.62288	0.56055
TRINITY_DN4565_c0_g2_i1_orf1	-	-	-	acid phosphatase type 7 isoform X2 [Ostrinia furnacalis]	-1.90357	1.03612	0.43712	0.31314	0.1172
TRINITY_DN2880_c0_g1_i2_orf1	-	-	-	sialomucin core protein 24 [Pectinophora gossypiella]	-1.86566	0.84819	0.87065	-0.0512	0.19803
TRINITY_DN184_c0_g1_i10_orf1	-	-	-	C-type mannose receptor 2-like isoform X1 [Leguminivora glycinivorella]	-1.69794	1.27613	0.66493	-0.02989	-0.21323
TRINITY_DN955_c0_g1_i2_orf1	-	-	-	gloverin-like [Ostrinia furnacalis] >XP_028168251.1 gloverin-like [Ostrinia furnacalis] >AYM26645.1 gloverin [Ostrinia furnacalis]	-1.52423	0.67891	0.6259	-0.83897	1.05839
TRINITY_DN52761_c0_g1_i2_orf1	-	-	-	atlastin isoform X4 [Ostrinia furnacalis]	-1.71213	0.9322	0.12025	-0.36569	1.02538
TRINITY_DN12014_c0_g1_i2_orf1	-	-	-	unnamed protein product [Chilo suppressalis]	-1.50932	1.33928	0.83407	-0.40995	-0.25408
TRINITY_DN20558_c0_g1_i2_orf1	-	-	-	Transient receptor potential channel pyrexia [Operophtera brumata]	-1.56759	0.41585	-0.14691	-0.21811	1.51676
TRINITY_DN1491_c0_g1_i4_orf1	-	-	-	GILT-like protein 2 isoform X1 [Ostrinia furnacalis] >XP_028156245.1 GILT-like protein 2 isoform X2 [Ostrinia furnacalis] >XP_028156247.1 GILT-like protein 2 isoform X3 [Ostrinia	-1.04608	0.74159	1.43535	0.00733	-1.13819
TRINITY_DN892_c7_g1_i2_orf1	-	-	-	unnamed protein product [Diatraea saccharalis]	-1.84286	0.60135	0.45857	-0.21079	0.99374
TRINITY_DN42856_c0_g1_i1_orf1	-	-	-	GSCOCG00007769001-RA-CDS [Cotesia congregata] >CAG5103393.1 Similar to GLDC: Glycine dehydrogenase (decarboxylating) [Cotesia congregata]	-1.9232	0.90353	0.44652	0.04032	0.53283
TRINITY_DN1012_c0_g1_i2_orf1	-	-	-	teneurin-a isoform X1 [Ostrinia furnacalis]	-1.47982	0.19809	1.4208	-0.67885	0.53979
TRINITY_DN858_c0_g1_i3_orf1	-	-	-	uncharacterized protein LOC114351944 [Ostrinia furnacalis]	-1.24534	0.79527	1.23973	-1.09051	0.30085
TRINITY_DN2141_c0_g1_i1_orf1	-	-	-	low density lipoprotein receptor adapter protein 1-like [Ostrinia furnacalis]	-1.56092	1.2249	0.88735	-0.02687	-0.52446
TRINITY_DN6822_c0_g2_i4_orf1	-	-	-	gelsolin-like [Bicyclus anynana]	-1.82891	1.15038	0.57086	0.05857	0.04909
TRINITY_DN3647_c1_g1_i5_orf1	-	-	-	unnamed protein product, partial [Iphiclides podalirius]	-1.62344	0.94416	1.1311	-0.43985	-0.01197
TRINITY_DN16840_c1_g1_i1_orf1	-	-	-	attacin-like [Ostrinia furnacalis]	-1.58654	0.44681	0.49402	-0.6339	1.27961
TRINITY_DN1759_c0_g1_i4_orf1	-	-	-	protein PFC0760c-like isoform X1 [Ostrinia furnacalis]	-1.689	0.80802	0.43379	-0.55301	1.0002

TRINITY_DN3712_c0_g1_i1_orf1	-	-	-	ribonuclease Oy [Ostrinia furnacalis]	-1.74258	1.32457	0.2393	-0.17111	0.34981
TRINITY_DN28741_c0_g1_i3_orf1	-	-	-	uncharacterized protein LOC114351652 [Ostrinia furnacalis]	-1.1431	1.72204	0.43471	-0.39464	-0.61901
TRINITY_DN2343_c1_g1_i2_orf1	-	-	-	receptor expression-enhancing protein 5-like isoform X1 [Ostrinia furnacalis] >XP_028170586.1 receptor expression-enhancing protein 5-like isoform X1 [Ostrinia furnacalis]	-1.09377	1.04811	0.22593	-1.23858	1.05831
TRINITY_DN9420_c0_g1_i2_orf1	-	-	-	protein PFC0760c-like isoform X2 [Ostrinia furnacalis]	-1.70113	1.34286	0.45636	0.16283	-0.26093
TRINITY_DN9383_c0_g1_i3_orf1	-	-	-	uncharacterized protein LOC114361502 [Ostrinia furnacalis]	-1.38247	0.83188	1.4029	-0.2453	-0.607
TRINITY_DN3109_c0_g1_i5_orf1	-	-	-	protein takeout isoform X2 [Ostrinia furnacalis]	-1.80446	0.7659	1.07515	-0.03722	0.00062
TRINITY_DN2227_c0_g1_i5_orf1	-	-	-	protein 60A [Ostrinia furnacalis]	-1.65243	0.33267	1.37725	-0.38952	0.33204
TRINITY_DN12661_c0_g1_i3_orf1	-	-	-	T-complex protein 11-like protein 1 [Ostrinia furnacalis]	-1.63723	1.24283	0.81103	-0.33136	-0.08528
TRINITY_DN5170_c0_g1_i5_orf1	-	-	-	hemolymph lipopolysaccharide-binding protein-like isoform X2 [Leguminivora glycinivorella]	-1.14401	0.99922	0.60604	-1.28399	0.82274
TRINITY_DN8083_c0_g1_i1_orf1	-	-	-	solute carrier family 35 member F6 [Ostrinia furnacalis]	-1.53889	0.75293	1.36715	-0.40663	-0.17457
TRINITY_DN10373_c0_g1_i1_orf1	-	-	-	homocysteine S-methyltransferase 1-like [Ostrinia furnacalis] >XP_028162778.1 homocysteine S-methyltransferase 1-like [Ostrinia furnacalis]	-1.50072	1.20418	0.81698	-0.75767	0.23723
TRINITY_DN364_c0_g1_i2_orf1	-	-	-	uncharacterized protein LOC114366657, partial [Ostrinia furnacalis]	-1.77715	1.14092	0.7245	-0.11902	0.03075
TRINITY_DN827_c1_g1_i1_orf1	-	-	-	peptidoglycan recognition protein 4-like isoform X1 [Ostrinia furnacalis]	-1.66902	1.42598	0.22671	-0.24621	0.26254
TRINITY_DN8783_c0_g1_i5_orf1	-	-	-	luciferin 4-monooxygenase-like [Ostrinia furnacalis] >XP_028165580.1 luciferin 4-monooxygenase-like [Ostrinia furnacalis]	-1.1785	1.77891	-0.24787	-0.57817	0.22563
TRINITY_DN21533_c0_g1_i7_orf1	-	-	-	annexin B9 isoform X1 [Ostrinia furnacalis]	-1.46475	1.31632	0.90783	-0.44791	-0.31149
TRINITY_DN11868_c0_g1_i2_orf1	-	-	-	uncharacterized protein LOC114361308 [Ostrinia furnacalis]	-1.55467	0.98066	1.07345	-0.66462	0.16518
TRINITY_DN65299_c0_g4_i1_orf1	-	-	-	LOW QUALITY PROTEIN: signal transducing adapter molecule 2 [Ostrinia furnacalis]	-1.7275	1.02117	0.95975	-0.22598	-0.02744
TRINITY_DN11370_c0_g1_i6_orf1	-	-	-	protein MEMO1 [Hyposmocoma kahamanoa]	-1.76716	1.23361	0.54338	-0.17819	0.16836
TRINITY_DN230_c1_g1_i3_orf1	-	-	-	protein artichoke-like [Ostrinia furnacalis]	-1.62147	1.53238	0.1444	-0.03927	-0.01605
TRINITY_DN24668_c0_g1_i8_orf1	-	-	-	uncharacterized protein LOC114364067 isoform X3 [Ostrinia furnacalis]	-1.31482	1.25614	1.05858	-0.30909	-0.69081
TRINITY_DN1054_c0_g1_i8_orf1	-	-	-	tubulin-specific chaperone D [Ostrinia furnacalis]	-1.83962	1.01264	0.36117	-0.18626	0.65208
TRINITY_DN1091_c0_g1_i1_orf1	-	-	-	macrophage mannose receptor 1-like [Pararge aegeria]	-1.3514	0.73937	1.49356	-0.50319	-0.37834
TRINITY_DN8644_c0_g1_i3_orf1	-	-	-	SEC14-like protein 2 [Ostrinia furnacalis]	-1.31186	1.65491	0.40408	-0.59484	-0.1523
TRINITY_DN5064_c0_g1_i4_orf1	-	-	-	sortilin-related receptor-like [Ostrinia furnacalis]	-1.64119	0.42395	1.38948	-0.38718	0.21493
TRINITY_DN2743_c0_g1_i5_orf1	-	-	-	regucalcin-like [Ostrinia furnacalis]	-1.46811	0.15847	0.11422	-0.42503	1.62045
TRINITY_DN896_c0_g1_i2_orf1	-	-	-	uncharacterized protein LOC114356314 isoform X2 [Ostrinia furnacalis]	-1.17383	1.06665	1.21433	-0.99905	-0.1081
TRINITY_DN1194_c0_g1_i5_orf1	-	-	-	sequestosome-1-like isoform X4 [Ostrinia furnacalis]	-1.22234	0.83189	1.48736	-0.54737	-0.54955
TRINITY_DN10900_c0_g1_i7_orf1	-	-	-	fatty acid synthase-like [Ostrinia furnacalis]	-1.4691	1.53432	0.54662	-0.33445	-0.27739
TRINITY_DN96739_c0_g1_i1_orf1	-	-	-	annexin A6, isoform CRA_b [Homo sapiens]	-1.57695	0.54816	1.15035	-0.72488	0.60333
TRINITY_DN3300_c0_g2_i1_orf1	-	-	-	annexin B10 isoform X9 [Ostrinia furnacalis] >XP_028177766.1 annexin B10 isoform X10 [Ostrinia furnacalis]	-1.69953	1.34882	0.46646	-0.24225	0.12651
TRINITY_DN9_c0_g1_i7_orf1	-	-	-	heterogeneous nuclear ribonucleoprotein R isoform X1 [Ostrinia furnacalis]	-1.17972	1.74219	0.01346	-0.73909	0.16315
TRINITY_DN140_c1_g1_i2_orf1	-	-	-	modular serine protease-like isoform X1 [Ostrinia furnacalis]	-1.36051	0.57878	1.57972	-0.39091	-0.40708
TRINITY_DN9457_c0_g1_i9_orf1	-	-	-	plexin domain-containing protein 2 [Spodoptera litura]	-1.6381	0.46415	1.16241	-0.60657	0.61811
TRINITY_DN53427_c0_g1_i2_orf1	-	-	-	heparanase-like [Ostrinia furnacalis]	-1.10802	0.50923	1.70369	-0.55385	-0.55106
TRINITY_DN4817_c0_g1_i4_orf1	-	-	-	palmitoyl-protein thioesterase 1 isoform X1 [Ostrinia furnacalis] >XP_028170290.1 palmitoyl-protein thioesterase 1 isoform X4 [Ostrinia furnacalis]	-1.28582	1.16198	1.01208	-0.98155	0.09331
TRINITY_DN9316_c0_g3_i1_orf1	-	-	-	cytochrome P450 6k1-like isoform X1 [Ostrinia furnacalis] >XP_028171821.1 cytochrome P450 6k1-like isoform X1 [Ostrinia furnacalis] >QPF77617.1 cytochrome P450 monooxygenase CYP324A34 [Ostrinia furnacalis]	-1.56479	1.45986	0.31574	-0.49162	0.2808
TRINITY_DN10231_c0_g2_i1_orf1	-	-	-	uncharacterized protein LOC114361472 [Ostrinia furnacalis]	-1.3533	0.10504	1.29238	-0.88413	0.84001
TRINITY_DN61048_c0_g1_i2_orf1	-	-	-	PREDICTED: protein THEM6-like [Amyelois transitella]	-1.49946	0.99434	0.90556	-0.85698	0.45654
TRINITY_DN37821_c0_g1_i6_orf1	-	-	-	uncharacterized protein LOC114350690 [Ostrinia furnacalis]	-1.72126	0.79699	0.75872	-0.55469	0.72024
TRINITY_DN119265_c0_g2_i1_orf1	-	-	-	PREDICTED: sorting nexin-12 [Fopius arisanus]	-1.45276	0.9467	1.2959	-0.36351	-0.42633
TRINITY_DN4108_c0_g1_i6_orf1	-	-	-	chromobox protein homolog 1-like [Ostrinia furnacalis]	-1.43671	0.77701	1.19366	-0.88534	0.35138
TRINITY_DN9926_c1_g1_i1_orf1	-	-	-	rab GTPase-activating protein 1-like isoform X6 [Ostrinia furnacalis]	-1.09858	0.64646	0.58206	-1.29541	1.16547
TRINITY_DN10650_c0_g1_i1_orf1	-	-	-	protein Skeletor, isoforms B/C-like [Ostrinia furnacalis]	-1.4576	1.46271	0.25467	-0.69437	0.4346
TRINITY_DN3158_c0_g1_i5_orf1	-	-	-	neuroglian [Ostrinia furnacalis]	-1.50228	1.22329	0.87885	-0.68361	0.08374
TRINITY_DN22928_c0_g1_i6_orf1	-	-	-	hypothetical protein B5X24_HaOG209714 [Helicoverpa armigera]	-1.51523	0.6141	1.2076	-0.79422	0.48775
TRINITY_DN95850_c0_g1_i1_orf1	-	-	-	alpha,alpha-trehalose-phosphate synthase [UDP-forming] isoform X2 [Chelonus insularis]	-1.04684	0.30465	1.08837	-1.30597	0.95979
TRINITY_DN2432_c0_g1_i1_orf1	-	-	-	unconventional myosin-XV-like [Ostrinia furnacalis]	-1.05809	0.92967	1.44025	-0.85765	-0.45418

TRINITY_DN96801_c0_g1_i1_orf1	-	-	-	histone H4 isoform X2 [Gracilinanus agilis]	-0.94802	0.75164	-0.13115	-1.15256	1.4801
TRINITY_DN11693_c0_g1_i6_orf1	-	-	-	sorting nexin-32 isoform X1 [Ostrinia furnacalis] >XP_028166096.1 sorting nexin-32 isoform X2 [Ostrinia furnacalis]	-1.08412	0.91288	1.30829	-1.13122	-0.00583
TRINITY_DN855_c0_g1_i5_orf1	-	-	-	hypothetical protein HF086_009970 [Spodoptera exigua] >CAH0702798.1 unnamed protein product [Spodoptera exigua]	-1.09784	1.26591	0.91383	-1.16219	0.08029
TRINITY_DN11133_c0_g1_i5_orf1	-	-	-	UHRF1-binding protein 1-like isoform X6 [Helicoverpa zea]	-1.35711	1.5232	0.15249	-0.77739	0.45881
TRINITY_DN40945_c0_g1_i1_orf1	-	-	-	RNA exonuclease 4-like [Ostrinia furnacalis] >QEE79882.1 REX4 [Ostrinia furnacalis]	-1.19187	0.50356	0.47571	-1.13405	1.34666
TRINITY_DN15114_c0_g2_i1_orf1	-	-	-	uncharacterized protein LOC114359552 [Ostrinia furnacalis]	-1.09499	0.61875	1.63855	-0.75118	-0.41113
TRINITY_DN2326_c0_g1_i1_orf1	-	-	-	transmembrane protein 256 homolog isoform X1 [Ostrinia furnacalis]	-1.13511	0.91188	1.25263	-1.13964	0.11024
TRINITY_DN4679_c0_g2_i13_orf1	-	-	-	GATOR complex protein MIOS [Ostrinia furnacalis]	-1.53499	1.01701	0.36514	-0.7793	0.93214
TRINITY_DN3219_c0_g1_i6_orf1	-	-	-	non-specific lipid-transfer protein [Ostrinia furnacalis]	-0.78972	1.25176	0.16153	-1.44953	0.82596
TRINITY_DN7170_c0_g1_i11_orf1	-	-	-	sulfite oxidase isoform X1 [Ostrinia furnacalis]	-1.19255	1.34099	0.62931	-1.12409	0.34635
TRINITY_DN2941_c0_g1_i1_orf1	-	-	-	FAS-associated factor 1 [Ostrinia furnacalis]	-1.40075	1.54356	0.14162	-0.68758	0.40315
TRINITY_DN2855_c0_g1_i6_orf1	-	-	-	uncharacterized protein LOC114359301 [Ostrinia furnacalis]	-1.07502	0.99007	0.98432	-1.31305	0.41367
TRINITY_DN5055_c0_g1_i12_orf1	-	-	-	probable peroxisomal acyl-coenzyme A oxidase 1 [Ostrinia furnacalis]	-1.03461	0.96302	0.23381	-1.29238	1.13016
TRINITY_DN51197_c0_g1_i3_orf1	-	-	-	microtubule-actin cross-linking factor 1 isoform X15 [Ostrinia furnacalis]	-1.01464	0.73887	0.11386	-1.2226	1.38451
TRINITY_DN1978_c0_g1_i4_orf1	-	-	-	RNA exonuclease 4-like [Ostrinia furnacalis]	-0.80723	0.47497	0.3731	-1.43158	1.39074
TRINITY_DN48694_c0_g1_i1_orfp1	-	-	-	TRINITY_DN48694_c0_g1_i1_m.75338 TRINITY_DN48694_c0_g1::TRINITY_DN48694_c0_g1_i1::g.75338 ORF type:internal len:84 (+),score=16.02 TRINITY_DN48694_c0_g1_i1:2-250(+)	-0.53088	1.04725	0.87221	0.27953	-1.6681
TRINITY_DN325_c0_g1_i15_orf1	-	-	-	protein draper-like [Ostrinia furnacalis]	-0.95404	1.6192	0.67937	-0.89874	-0.44579
TRINITY_DN26985_c0_g1_i5_orf1	-	-	-	secretory phospholipase A2 receptor-like [Helicoverpa zea]	-0.57368	1.03384	1.36674	-0.73275	-1.09415
TRINITY_DN38106_c0_g1_i6_orf1	-	-	-	unnamed protein product [Pieris macdunnoughi]	-0.82534	1.44438	0.90834	-1.11086	-0.41652
TRINITY_DN1293_c1_g1_i4_orf1	-	-	-	putative fatty acyl-CoA reductase CG5065 [Ostrinia furnacalis]	-0.61234	1.62698	0.69473	-0.7236	-0.98576
TRINITY_DN80424_c0_g1_i1_orf1	-	-	-	PREDICTED: cytoplasmic FMR1-interacting protein [Dufourea novaeangliae] >KZC10094.1 Cytoplasmic FMR1-interacting protein [Dufourea novaeangliae]	-0.55388	1.70473	0.54726	-1.00044	-0.69767
TRINITY_DN919_c0_g1_i7_orf1	-	-	-	facilitated trehalose transporter Tret1-like [Ostrinia furnacalis] >XP_028161733.1 facilitated trehalose transporter Tret1-like [Ostrinia furnacalis]	-0.70451	1.79098	0.41212	-0.79017	-0.70842
TRINITY_DN132_c0_g2_i2_orf1	-	-	-	alpha-tocopherol transfer protein-like isoform X1 [Ostrinia furnacalis]	-1.19574	1.40088	0.95664	-0.67501	-0.48678
TRINITY_DN12432_c0_g1_i2_orf1	-	-	-	sorting nexin-8 isoform X3 [Ostrinia furnacalis]	-0.70298	1.50509	0.65342	-0.11366	-1.34187
TRINITY_DN52244_c1_g1_i1_orf1	-	-	-	triokinase/FMN cyclase-like isoform X2 [Ostrinia furnacalis]	0.03859	1.39091	0.39129	-0.11883	-1.70195
TRINITY_DN6205_c0_g1_i4_orfp1	-	-	-	TRINITY_DN6205_c0_g1_i4_m.72677 TRINITY_DN6205_c0_g1::TRINITY_DN6205_c0_g1_i4::g.72677 ORF type:internal len:68 (-	-0.61883	1.04104	1.37868	-0.97437	-0.82651
TRINITY_DN7316_c0_g2_i1_orf1	-	-	-	ubiquitin-protein ligase E3C [Ostrinia furnacalis]	-0.67841	1.15401	1.26443	-1.08841	-0.65163
TRINITY_DN526_c0_g1_i1_orf1	-	-	-	secretory phospholipase A2 receptor-like [Ostrinia furnacalis]	-0.24343	1.41687	0.47858	-0.0076	-1.64442
TRINITY_DN60949_c0_g1_i4_orf1	-	-	-	aldo-keto reductase AKR2E4-like [Galleria mellonella]	-0.70658	1.29785	1.09138	-1.16503	-0.51762
TRINITY_DN18136_c0_g1_i1_orf1	-	-	-	proteoglycan 4-like [Ostrinia furnacalis]	-0.82774	1.13269	1.3127	-0.79659	-0.82105
TRINITY_DN6205_c0_g1_i8_orf1	-	-	-	phenoloxidase-activating factor 2-like [Ostrinia furnacalis]	-0.38258	1.49705	0.83882	-0.95243	-1.00087
TRINITY_DN3324_c0_g1_i3_orf1	-	-	-	uncharacterized protein LOC114363957 isoform X2 [Ostrinia furnacalis]	-0.56868	1.87863	0.09918	-0.97352	-0.43562
TRINITY_DN51658_c0_g1_i1_orf1	-	-	-	E3 ubiquitin-protein ligase synoviolin B [Ostrinia furnacalis]	-0.11768	1.68187	0.42032	-1.06867	-0.91584
TRINITY_DN11204_c0_g1_i3_orf1	-	-	-	spermosin-like [Ostrinia furnacalis]	-0.6099	1.00776	1.37642	-0.61893	-1.15534
TRINITY_DN26488_c0_g1_i6_orf1	-	-	-	phosphatidate phosphatase LPIN2 isoform X1 [Ostrinia furnacalis] >XP_028176373.1 phosphatidate phosphatase LPIN2 isoform X1 [Ostrinia furnacalis] >XP_028176374.1 phosphatidate phosphatase LPIN2 isoform X2 [Ostrinia furnacalis] >XP_028176375.1 phosphatidate phosphatase LPIN2 isoform X3 [Ostrinia furnacalis] >XP_028176376.1 phosphatidate phosphatase LPIN2 isoform X4 [Ostrinia furnacalis]	-0.99485	1.51799	0.81292	-0.94425	-0.39181
TRINITY_DN31342_c2_g2_i1_orf1	-	-	-	26S proteasome non-ATPase regulatory subunit 2 isoform X2 [Ostrinia furnacalis]	-0.66864	1.34896	1.08326	-0.93001	-0.83357
TRINITY_DN14952_c0_g3_i1_orf1	-	-	-	protein DEK isoform X3 [Ostrinia furnacalis] >XP_028162408.1 protein DEK isoform X4 [Ostrinia furnacalis]	-0.44858	1.34707	0.92699	-1.39163	-0.43385
TRINITY_DN46202_c0_g1_i1_orf1	-	-	-	alpha-tubulin N-acetyltransferase 1-like isoform X2 [Ostrinia furnacalis]	-0.59262	1.1076	1.28871	-1.16127	-0.64243
TRINITY_DN9560_c0_g1_i5_orf1	-	-	-	uncharacterized protein LOC114357350 [Ostrinia furnacalis]	-0.38926	1.10851	-1.75571	0.51552	0.52095
TRINITY_DN9820_c0_g1_i1_orf1	-	-	-	endocuticle structural glycoprotein SgAbd-2-like [Ostrinia furnacalis]	-0.31938	0.92167	-1.80382	0.40982	0.79171
TRINITY_DN109931_c0_g1_i1_orf1	-	-	-	hydroxymethylglutaryl-CoA lyase, mitochondrial isoform X1 [Ostrinia furnacalis]	-0.17976	0.93436	-1.85689	0.41132	0.69097
TRINITY_DN52395_c0_g2_i2_orf1	-	-	-	twitchin isoform X20 [Zerene cesonia]	0.0613	1.25425	-1.816	0.2495	0.25095

TRINITY_DN13119_c0_g1_i4_orf1	-	-	-	endocuticle structural glycoprotein ABD-5-like [Bicyclus anynana]	0.04679	0.72368	-1.9281	0.37087	0.78676
TRINITY_DN76529_c0_g1_i1_orfp1	-	-	-	TRINITY_DN76529_c0_g1_i1_m.64079 TRINITY_DN76529_c0_g1::TRINITY_DN76529_c0_g1_i1::g.64079 ORF type:internal len:70 (+),score=14.68 TRINITY_DN76529_c0_g1_i1:3-209(+)	0.25284	1.52508	-1.56507	-0.3692	0.15635
TRINITY_DN31645_c0_g1_i3_orf1	-	-	-	dystonin isoform X43 [Helicoverpa armigera]	-0.09128	1.01227	-1.85056	0.22986	0.69971
TRINITY_DN11448_c0_g1_i11_orf1	-	-	-	hypothetical protein B5X24_HaOG201808 [Helicoverpa armigera]	0.10218	1.58127	-1.50116	-0.42207	0.23979
TRINITY_DN8651_c0_g1_i18_orf1	-	-	-	glutathione S-transferase theta 2 [Conogethes punctiferalis]	-0.01626	1.16951	-1.72793	-0.20313	0.7778
TRINITY_DN30208_c0_g1_i3_orf1	-	-	-	unnamed protein product [Timema cristinae]	0.07254	0.95265	-1.904	0.24468	0.63413
TRINITY_DN9871_c0_g1_i11_orf1	-	-	-	PEST proteolytic signal-containing nuclear protein-like [Ostrinia furnacalis]	0.29687	1.03905	-1.90861	0.17347	0.39922
TRINITY_DN122393_c0_g1_i1_orf1	-	-	-	microtubule-associated protein futsch isoform X4 [Ostrinia furnacalis] >XP_028162562.1 microtubule-associated protein futsch isoform X4 [Ostrinia furnacalis]	0.15849	1.25611	-1.7969	-0.02698	0.40928
TRINITY_DN4010_c0_g2_i1_orf1	-	-	-	myophilin [Ostrinia furnacalis]	0.29356	1.09712	-1.87995	0.07692	0.41235
TRINITY_DN77830_c0_g2_i2_orf1	-	-	-	prostaglandin reductase 1-like [Leguminivora glycinivorella] >XP_047994907.1 prostaglandin reductase 1-like [Leguminivora glycinivorella]	0.43876	1.58216	-1.41907	-0.06705	-0.53479
TRINITY_DN1455_c0_g1_i8_orf1	-	-	-	troponin T, skeletal muscle isoform X1 [Galleria mellonella]	0.32021	0.86653	-1.95623	0.27585	0.49364
TRINITY_DN48610_c0_g1_i2_orf1	-	-	-	hypothetical protein evm_002298 [Chilo suppressalis] >CAH0682062.1 unnamed protein product [Chilo suppressalis]	0.3218	0.99689	-1.91586	0.13453	0.46265
TRINITY_DN920_c0_g1_i4_orf1	-	-	-	glutathione S-transferase omega 2 [Ostrinia furnacalis]	0.44001	1.47996	-1.1651	-1.07519	0.32032
TRINITY_DN3105_c0_g1_i4_orf1	-	-	-	nose resistant to fluoxetine protein 6-like [Ostrinia furnacalis]	0.6636	1.37471	-1.60174	-0.28511	-0.15147
TRINITY_DN6612_c0_g1_i4_orf1	-	-	-	hypothetical protein O3G_MSEX008151 [Manduca sexta]	0.23766	1.25007	-1.7506	-0.24386	0.50673
TRINITY_DN27500_c0_g1_i4_orf1	-	-	-	hemicentin-1-like [Ostrinia furnacalis]	0.50168	1.19087	-1.73577	-0.37609	0.4193
TRINITY_DN2958_c0_g1_i2_orf1	-	-	-	uncharacterized protein LOC114356495 [Ostrinia furnacalis]	0.50797	1.39986	-1.63754	-0.31445	0.04416
TRINITY_DN1895_c0_g1_i2_orf1	-	-	-	unnamed protein product [Chrysodeixis includens]	0.17634	0.91711	-1.86979	-0.01825	0.79459
TRINITY_DN3732_c0_g1_i6_orf1	-	-	-	cytochrome P450 monooxygenase CYP6AB141 [Ostrinia furnacalis]	0.22348	1.69073	-1.14727	-0.87397	0.10704
TRINITY_DN11448_c0_g1_i15_orf1	-	-	-	unnamed protein product [Chilo suppressalis]	0.46384	0.96015	-1.92068	0.08926	0.40743
TRINITY_DN12690_c0_g1_i1_orf1	-	-	-	ELAV-like protein 1 [Ostrinia furnacalis]	0.29718	0.92185	-1.82517	-0.21986	0.826
TRINITY_DN1123_c2_g1_i3_orf1	-	-	-	troponin I isoform X8 [Ostrinia furnacalis]	0.47159	0.82936	-1.95538	0.16581	0.48863
TRINITY_DN23474_c1_g1_i1_orf1	-	-	-	unnamed protein product [Chrysodeixis includens]	0.37894	1.58303	-1.31266	-0.78101	0.1317
TRINITY_DN68401_c1_g1_i1_orf1	-	-	-	endoplasmic reticulum chaperone BiP isoform X1 [Ostrinia furnacalis]	0.26043	1.70844	-1.17528	-0.79506	0.00147
TRINITY_DN1982_c0_g1_i24_orf1	-	-	-	uncharacterized protein LOC114361215 isoform X5 [Ostrinia furnacalis]	0.43783	1.09342	-1.88007	0.08157	0.26725
TRINITY_DN43881_c0_g1_i2_orf1	-	-	-	estradiol 17-beta-dehydrogenase 8-like [Ostrinia furnacalis]	0.54187	1.52344	-1.27544	-0.86759	0.07773
TRINITY_DN114344_c0_g1_i4_orf1	-	-	-	microtubule-actin cross-linking factor 1 isoform X15 [Ostrinia furnacalis]	0.46892	1.13914	-1.81698	-0.17771	0.38664
TRINITY_DN18009_c0_g1_i1_orf1	-	-	-	pre-mRNA-splicing factor ISY1 homolog [Ostrinia furnacalis]	0.68825	0.98802	-1.83692	-0.20511	0.36577
TRINITY_DN3906_c0_g1_i5_orf1	-	-	-	ejaculatory bulb-specific protein 3-like [Ostrinia furnacalis]	0.41695	1.23185	-1.67665	-0.48465	0.5125
TRINITY_DN2621_c0_g1_i1_orf1	-	-	-	GPN-loop GTPase 1 [Ostrinia furnacalis]	0.65138	1.53456	-1.34119	-0.603	-0.24174
TRINITY_DN8406_c0_g1_i2_orf1	-	-	-	titin [Ostrinia furnacalis]	0.55246	1.41155	-1.56365	-0.49783	0.09747
TRINITY_DN4501_c0_g2_i1_orf1	-	-	-	methylcrotonoyl-CoA carboxylase subunit alpha, mitochondrial [Ostrinia furnacalis]	0.83942	1.43812	-1.32312	-0.57987	-0.37456
TRINITY_DN248_c0_g1_i12_orf1	-	-	-	twitchin-like [Ostrinia furnacalis]	0.66343	1.19718	-1.74055	0.15197	-0.27205
TRINITY_DN416_c0_g1_i1_orf1	-	-	-	unnamed protein product [Diatraea saccharalis]	0.55715	0.87594	-1.92964	0.05396	0.44259
TRINITY_DN3010_c0_g1_i4_orf1	-	-	-	inositol oxygenase-like [Ostrinia furnacalis]	0.46718	0.98639	-1.70089	-0.5416	0.78893
TRINITY_DN46372_c0_g2_i1_orf1	-	-	-	basic salivary proline-rich protein 1 isoform X2 [Ostrinia furnacalis]	1.03398	1.01964	-1.65097	-0.40681	0.00416
TRINITY_DN146236_c0_g1_i1_orf1	-	-	-	vesicle-fusing ATPase 1-like [Chelonus insularis]	0.80665	1.29977	-1.50496	-0.62794	0.02648
TRINITY_DN119893_c0_g2_i3_orf1	-	-	-	ATP-binding cassette sub-family F member 3 isoform X1 [Ostrinia furnacalis] >XP_028168051.1 ATP-binding cassette sub-family F member 3 isoform X2 [Ostrinia furnacalis]	0.84223	1.30379	-1.37363	-0.83654	0.06416
TRINITY_DN46216_c0_g3_i1_orf1	-	-	-	unnamed protein product, partial [Brenthis ino]	0.35194	0.70043	-1.87715	-0.09834	0.92312
TRINITY_DN140423_c0_g1_i2_orf1	-	-	-	obscurin [Ostrinia furnacalis]	0.46313	1.37623	-0.97774	-1.31521	0.45359
TRINITY_DN1814_c0_g1_i11_orf1	-	-	-	titin-like, partial [Ostrinia furnacalis]	0.77793	0.72067	-1.87601	-0.18868	0.56609
TRINITY_DN1197_c0_g1_i6_orf1	-	-	-	tensin-2-like isoform X6 [Ostrinia furnacalis] >XP_028159238.1 tensin-2-like isoform X6 [Ostrinia furnacalis] >XP_028159239.1 tensin-2-like isoform X6 [Ostrinia furnacalis] >XP_028159240.1 tensin-2-like isoform X6 [Ostrinia furnacalis]	1.01303	1.21212	-1.41881	-0.69164	-0.11471
TRINITY_DN8454_c0_g1_i4_orf1	-	-	-	translocon-associated protein subunit alpha [Ostrinia furnacalis]	0.85199	1.0505	-1.67674	-0.52129	0.29554
TRINITY_DN42177_c0_g1_i4_orf1	-	-	-	androgen-dependent TFPI-regulating protein-like [Ostrinia furnacalis]	0.9853	1.2443	-1.34392	-0.81873	-0.06695
TRINITY_DN7778_c0_g1_i1_orf1	-	-	-	peroxiredoxin-2 [Cotesia glomerata] >KAH0561449.1 Peroxiredoxin-4 [Cotesia glomerata]	0.66419	1.03895	-1.83438	-0.16445	0.2957
TRINITY_DN2254_c0_g1_i4_orf1	-	-	-	vigilin [Ostrinia furnacalis]	0.7437	1.03047	-1.47744	-0.90935	0.61262

TRINITY_DN115658_c0_g1_i1_orf1	-	-	-	hypothetical protein B5X24_HaOG203018 [Helicoverpa armigera]	1.13535	1.19932	-1.16794	-0.92094	-0.24578
TRINITY_DN8964_c0_g1_i4_orf1	-	-	-	hypothetical protein evm_010115 [Chilo suppressalis]	0.86215	0.80086	-1.74308	-0.4956	0.57566
TRINITY_DN42461_c0_g1_i4_orf1	-	-	-	obscurin [Ostrinia furnacalis]	0.77504	0.7786	-1.90869	-0.03099	0.38605
TRINITY_DN3529_c0_g1_i7_orf1	-	-	-	putative fatty acyl-CoA reductase CG5065 [Ostrinia furnacalis]	0.97793	0.70876	-1.40855	-1.01045	0.7323
TRINITY_DN8241_c0_g1_i3_orf1	-	-	-	transforming growth factor beta-1-induced transcript 1 protein [Ostrinia furnacalis]	0.97058	1.31088	-1.14956	-1.00039	-0.1315
TRINITY_DN4133_c0_g1_i2_orfp2	-	-	-	unnamed protein product [Spodoptera exigua]	0.53026	1.08338	-1.34209	-1.05963	0.78808
TRINITY_DN111110_c0_g1_i1_orf1	-	-	-	NAD-dependent protein deacylase-like [Ostrinia furnacalis]	0.80908	0.90546	-1.69034	-0.59003	0.56583
TRINITY_DN19659_c1_g1_i1_orf1	-	-	-	elongation factor 1-gamma [Ostrinia furnacalis]	1.10681	1.2019	-1.31178	-0.73585	-0.26108
TRINITY_DN115082_c0_g1_i5_orf1	-	-	-	protein dj-1beta-like isoform X2 [Ostrinia furnacalis]	1.01295	1.2336	-1.43334	-0.2235	-0.58971
TRINITY_DN4408_c6_g1_i1_orf1	-	-	-	polyprotein, partial [Bemisia tabaci]	0.70323	0.79894	-1.60521	-0.75009	0.85312
TRINITY_DN38366_c0_g1_i4_orfp1	-	-	-	TRINITY_DN38366_c0_g1_i4_m.10666 TRINITY_DN38366_c0_g1::TRINITY_DN38366_c0_g1_i4::g.10666 ORF type:internal len:143 (+),score=71.68 TRINITY_DN38366_c0_g1_i4:3-428(+)	0.82029	0.62004	-1.86747	-0.21305	0.64019
TRINITY_DN2186_c0_g1_i17_orf1	-	-	-	paxillin isoform X6 [Leguminivora glycinivorella]	0.84617	0.69698	-1.91734	0.02583	0.34836
TRINITY_DN1232_c0_g1_i1_orf1	-	-	-	acanthoscurrin-2-like isoform X1 [Ostrinia furnacalis]	1.01414	0.74523	-1.80387	-0.26167	0.30617
TRINITY_DN4920_c0_g1_i5_orf1	-	-	-	titin homolog [Ostrinia furnacalis]	1.08524	0.62195	-1.84147	-0.0659	0.20018
TRINITY_DN120439_c1_g1_i1_orf1	-	-	-	myosin heavy chain variant, partial [Bombyx mori]	1.15961	0.56686	-1.80539	-0.14953	0.22845
TRINITY_DN1123_c2_g1_i5_orf1	-	-	-	troponin I isoform X4 [Leguminivora glycinivorella]	1.10226	0.81617	-1.76236	-0.09796	-0.05811
TRINITY_DN7580_c0_g1_i1_orf1	-	-	-	cytochrome P450 monooxygenase CYP6AB141 [Ostrinia furnacalis]	0.84874	0.46355	-1.66836	-0.60233	0.95841
TRINITY_DN27276_c0_g1_i5_orf1	-	-	-	probable small nuclear ribonucleoprotein Sm D1 [Ostrinia furnacalis] >CAG9751027.1 unnamed protein product [Diatraea saccharalis] >CAG9789712.1 unnamed protein product [Diatraea saccharalis]	1.03256	0.32978	-1.47379	-0.85169	0.96314
TRINITY_DN20957_c0_g1_i1_orf1	-	-	-	adenylate kinase isoenzyme 1 isoform X2 [Ostrinia furnacalis]	1.26355	0.54568	-1.76146	0.00595	-0.05373
TRINITY_DN96557_c0_g1_i1_orf1	-	-	-	charged multivesicular body protein 4B [Phyllostomus discolor]	1.21615	0.43867	-1.81491	-0.02448	0.18456
TRINITY_DN76815_c0_g1_i3_orf1	-	-	-	5-formyltetrahydrofolate cyclo-ligase [Ostrinia furnacalis]	0.7456	0.64253	-1.32407	-1.0988	1.03474
TRINITY_DN2442_c0_g1_i2_orf1	-	-	-	digestive cysteine proteinase 2 [Ostrinia furnacalis]	-1.25582	0.1596	1.77788	-0.38702	-0.29464
TRINITY_DN110231_c0_g1_i1_orf1	-	-	-	protein singed [Ostrinia furnacalis] >XP_028161434.1 protein singed [Ostrinia furnacalis]	-1.09849	0.27123	1.80145	-0.48644	-0.48775
TRINITY_DN843_c0_g1_i2_orf1	-	-	-	unnamed protein product [Diatraea saccharalis]	-0.72084	0.60207	1.68204	-0.59892	-0.96434
TRINITY_DN4676_c0_g1_i16_orf1	-	-	-	meiosis-specific nuclear structural protein 1-like isoform X2 [Ostrinia furnacalis]	-0.83685	0.10404	1.89343	-0.45735	-0.70327
TRINITY_DN4189_c0_g2_i1_orf1	-	-	-	unnamed protein product [Chilo suppressalis]	-1.08714	0.21088	1.81843	-0.5786	-0.36358
TRINITY_DN104_c0_g1_i4_orf1	-	-	-	PREDICTED: heparan-alpha-glucosaminide N-acetyltransferase [Amyelois transitella]	-1.28052	0.32396	1.67067	-0.68047	-0.03364
TRINITY_DN661_c0_g1_i1_orf1	-	-	-	hypothetical protein evm_002822 [Chilo suppressalis]	-0.73025	-0.01876	1.92367	-0.78222	-0.39243
TRINITY_DN2367_c1_g1_i20_orf1	-	-	-	RNA exonuclease 4-like [Ostrinia furnacalis]	-0.42037	1.08679	1.02945	-1.60438	-0.09149
TRINITY_DN10824_c0_g1_i3_orf1	-	-	-	endochitinase isoform X2 [Ostrinia furnacalis]	-0.69765	-0.1412	1.96431	-0.58999	-0.53547
TRINITY_DN1196_c0_g1_i4_orf1	-	-	-	glucosamine-6-phosphate isomerase isoform X2 [Ostrinia furnacalis]	-0.65979	0.42066	1.7745	-0.94114	-0.59424
TRINITY_DN2002_c0_g1_i5_orfp1	-	-	-	TRINITY_DN2002_c0_g1_i5_m.4230 TRINITY_DN2002_c0_g1::TRINITY_DN2002_c0_g1_i5::g.4230 ORF type:3prime_partial len:259 (+),score=29.98,Peptidase_C39 PF03412.16 1.7,Peptidase_C39|PF03412.16|0.049 TRINITY_DN2002_c0_g1_i5:65-838(+)	-0.92719	-0.05274	1.87811	-0.77071	-0.12747
TRINITY_DN11670_c0_g1_i1_orf1	-	-	-	teneurin-m isoform X1 [Ostrinia furnacalis]	-0.93952	-0.01638	1.89127	-0.69428	-0.24108
TRINITY_DN14856_c0_g1_i1_orf1	-	-	-	upstream activation factor subunit spp27 [Ostrinia furnacalis]	-1.03317	0.04922	1.84638	-0.70427	-0.15816
TRINITY_DN10766_c0_g1_i1_orf1	-	-	-	hypothetical protein evm_008559 [Chilo suppressalis]	-0.89548	-0.1069	1.89928	-0.74611	-0.15079
TRINITY_DN2290_c0_g1_i2_orfp1	-	-	-	TRINITY_DN2290_c0_g1_i2_m.69732 TRINITY_DN2290_c0_g1::TRINITY_DN2290_c0_g1_i2::g.69732 ORF type:complete len:234	-0.57686	0.35091	1.81174	-0.74624	-0.83954
TRINITY_DN4013_c0_g1_i4_orf1	-	-	-	uncharacterized protein LOC114353190 isoform X1 [Ostrinia furnacalis] >XP_028160984.1 uncharacterized protein LOC114353190 isoform X2 [Ostrinia furnacalis] >XP_028161062.1 uncharacterized protein LOC114353190 isoform X1 [Ostrinia furnacalis] >XP_028161142.1 uncharacterized protein LOC114353190 isoform X1 [Ostrinia furnacalis]	-0.82999	0.25227	1.85295	-0.61603	-0.6592
TRINITY_DN14774_c0_g1_i4_orf1	-	-	-	aminopeptidase N-like [Ostrinia furnacalis]	-0.57826	0.51519	1.73297	-0.87197	-0.79793
TRINITY_DN7711_c1_g1_i3_orf1	-	-	-	long-chain fatty acid transport protein 1-like [Ostrinia furnacalis]	-0.86408	0.42684	1.76844	-0.8355	-0.4957
TRINITY_DN49785_c1_g1_i3_orf1	-	-	-	uncharacterized protein LOC114365444, partial [Ostrinia furnacalis]	-0.6518	-0.30342	1.97188	-0.70579	-0.31087
TRINITY_DN110523_c0_g2_i1_orf1	-	-	-	uncharacterized protein LOC107036393 [Diachasma alloeum]	-0.35668	0.3944	1.72681	-0.58347	-1.18106
TRINITY_DN23183_c1_g1_i2_orf1	-	-	-	myotubularin-related protein 9 [Ostrinia furnacalis]	-0.42868	0.10472	1.80042	-1.22494	-0.25152
TRINITY_DN77642_c0_g1_i1_orf1	-	-	-	peritrophic membrane chitin binding protein [Loxostege sticticalis]	0.15185	0.54978	1.49569	-0.99029	-1.20704
TRINITY_DN36324_c0_g1_i12_orf1	-	-	-	motile sperm domain-containing protein 1-like [Ostrinia furnacalis]	-0.62482	0.35235	1.35037	-1.559	0.4811

TRINITY_DN1099_c1_g1_i2_orf1	-	-	-	dehydrogenase/reductase SDR family protein 7-like [Ostrinia furnacalis]	0.06806	0.61882	1.20324	-1.77522	-0.11491
TRINITY_DN8569_c1_g2_i7_orf1	-	-	-	furin-like protease 1, partial [Ostrinia furnacalis]	-0.40637	-0.05131	1.8892	-1.06112	-0.3704
TRINITY_DN210_c0_g1_i9_orf1	-	-	-	glycine-rich cell wall structural protein-like [Ostrinia furnacalis]	-0.11427	0.81109	1.2355	-1.65015	-0.28216
TRINITY_DN25251_c0_g2_i1_orf1	-	-	-	dolichyl-phosphate beta-glucosyltransferase [Ostrinia furnacalis]	0.55127	1.2307	0.58199	-1.33793	-1.02602
TRINITY_DN1196_c0_g1_i5_orf1	-	-	-	glucosamine-6-phosphate isomerase isoform X1 [Ostrinia furnacalis]	-0.49814	-0.11311	1.93157	-0.92138	-0.39893
TRINITY_DN5422_c0_g1_i1_orf1	-	-	-	nitrilase and fragile histidine triad fusion protein NitFhit isoform X1 [Ostrinia furnacalis]	-0.18386	0.20846	1.06481	-1.80873	0.71931
TRINITY_DN578_c0_g1_i3_orf1	-	-	-	charged multivesicular body protein 7 [Ostrinia furnacalis]	-0.32771	0.75633	0.87929	-1.81415	0.50624
TRINITY_DN34830_c0_g1_i1_orf1	-	-	-	ubiquitin-like domain-containing CTD phosphatase 1 [Ostrinia furnacalis]	-0.48672	0.23935	1.82679	-1.03608	-0.54333
TRINITY_DN552_c0_g1_i3_orf1	-	-	-	patronin isoform X9 [Ostrinia furnacalis]	0.72143	-0.03165	1.45941	-1.21447	-0.93472
TRINITY_DN4798_c0_g1_i3_orf1	-	-	-	unnamed protein product [Spodoptera exigua]	0.03117	0.86152	1.2871	-0.75487	-1.42491
TRINITY_DN4711_c0_g1_i2_orf1	-	-	-	xanthine dehydrogenase-like isoform X1 [Ostrinia furnacalis] >XP_028179066.1 xanthine dehydrogenase-like isoform X1 [Ostrinia furnacalis] >XP_028179067.1 xanthine dehydrogenase-like isoform X1 [Ostrinia furnacalis] >XP_028179068.1 xanthine dehydrogenase-like isoform X1 [Ostrinia furnacalis] >XP_028179069.1 xanthine	-0.47421	0.45437	1.62517	-1.36789	-0.23743
TRINITY_DN23020_c0_g1_i1_orf1	-	-	-	actin-related protein 2/3 complex subunit 1A-A [Ostrinia furnacalis] >XP_028170151.1 actin-related protein 2/3 complex subunit 1A-A [Ostrinia furnacalis]	-0.46889	0.74652	1.33731	-1.55928	-0.05565
TRINITY_DN81791_c0_g2_i2_orf1	-	-	-	UDP-N-acetylglucosamine--peptide N-acetylglucosaminyltransferase 110 kDa subunit isoform X2 [Diachasma alloeum]	-0.01426	1.03764	1.20168	-1.1549	-1.07017
TRINITY_DN5666_c0_g1_i2_orf1	-	-	-	protein ROP isoform X2 [Ostrinia furnacalis]	0.01256	0.72774	1.31166	-1.59366	-0.45831
TRINITY_DN3667_c0_g1_i4_orf1	-	-	-	coronin-2B-like isoform X2 [Ostrinia furnacalis]	0.31086	1.2289	0.7544	-0.83683	-1.45732
TRINITY_DN4540_c0_g1_i9_orf1	-	-	-	dihydropyrimidinase isoform X2 [Manduca sexta]	-0.06717	0.69687	1.40787	-1.49414	-0.54344
TRINITY_DN8306_c0_g1_i4_orf1	-	-	-	NAD(P) transhydrogenase, mitochondrial-like [Ostrinia furnacalis] >XP_028175067.1 NAD(P) transhydrogenase, mitochondrial-like [Ostrinia furnacalis] >XP_028175068.1 NAD(P) transhydrogenase, mitochondrial-like [Ostrinia furnacalis] >XP_028175069.1 NAD(P) transhydrogenase, mitochondrial-like [Ostrinia furnacalis]	-0.53421	-0.01467	1.75824	-1.27241	0.06305
TRINITY_DN14865_c0_g1_i2_orf1	-	-	-	ubiquilin-1 [Ostrinia furnacalis]	-0.01562	0.42	1.59965	-1.36007	-0.64396
TRINITY_DN1492_c0_g1_i4_orf1	-	-	-	sarcoplasmic calcium-binding protein isoform X2 [Ostrinia furnacalis]	-0.02623	0.62545	0.9704	-1.88812	0.31849
TRINITY_DN8405_c0_g1_i4_orf1	-	-	-	clathrin heavy chain isoform X1 [Ostrinia furnacalis] >XP_028169033.1 clathrin heavy chain isoform X2 [Ostrinia furnacalis] >XP_028169034.1 clathrin heavy chain isoform X3 [Ostrinia furnacalis] >XP_028169036.1 clathrin heavy chain isoform X5 [Ostrinia furnacalis]	0.21104	0.85385	1.09792	-1.6654	-0.49741
TRINITY_DN30273_c1_g1_i1_orf1	-	-	-	uncharacterized protein LOC114358591 isoform X2 [Ostrinia furnacalis]	0.20152	-0.08021	1.72656	-1.28769	-0.56017
TRINITY_DN1749_c0_g2_i2_orf1	-	-	-	putative GPI-anchored protein pfl2 isoform X1 [Ostrinia furnacalis]	-0.37907	-0.42499	1.97891	-0.77383	-0.40102
TRINITY_DN3209_c0_g1_i1_orf1	-	-	-	coatomer subunit beta [Helicoverpa armigera]	0.29805	0.91716	0.55068	-1.93355	0.16767
TRINITY_DN2782_c0_g1_i7_orf1	-	-	-	CDK5 regulatory subunit-associated protein 3 [Ostrinia furnacalis]	0.17231	0.75343	0.88046	-1.90217	0.09597
TRINITY_DN8044_c0_g1_i2_orf1	-	-	-	protein FRG1 homolog [Pectinophora gossypiella]	0.49112	0.89987	0.81061	-1.75975	-0.44184
TRINITY_DN5162_c0_g1_i3_orf1	-	-	-	steroidogenic acute regulatory protein-like [Ostrinia furnacalis]	0.57223	0.70765	1.12297	-1.31071	-1.09214
TRINITY_DN53760_c0_g1_i1_orf1	-	-	-	unnamed protein product [Parnassius apollo]	0.49156	1.04056	0.41814	-1.86928	-0.08098
TRINITY_DN1066_c0_g1_i8_orf1	-	-	-	hypothetical protein evm_012420 [Chilo suppressalis]	0.13392	0.70251	1.40167	-1.21736	-1.02073
TRINITY_DN19303_c0_g1_i5_orf1	-	-	-	lipopolysaccharide-induced tumor necrosis factor-alpha factor-like [Ostrinia furnacalis]	0.15729	0.8334	1.18704	-1.58906	-0.58866
TRINITY_DN629_c0_g1_i6_orf1	-	-	-	annexin B9-like isoform X1 [Ostrinia furnacalis]	0.65815	0.64748	1.05934	-0.84425	-1.52073
TRINITY_DN14944_c0_g1_i7_orf1	-	-	-	alpha-tocopherol transfer protein-like isoform X1 [Ostrinia furnacalis] >XP_028158173.1 alpha-tocopherol transfer protein-like isoform X1 [Ostrinia furnacalis] >XP_028158174.1 alpha-tocopherol transfer protein-like isoform X1 [Ostrinia furnacalis]	0.57795	0.66834	1.14713	-1.05677	-1.33664
TRINITY_DN892_c0_g1_i9_orf1	-	-	-	calcyphosin-like protein isoform X2 [Ostrinia furnacalis]	0.42712	0.45532	1.216	-1.73108	-0.36737
TRINITY_DN4021_c0_g1_i1_orf1	-	-	-	leech-derived tryptase inhibitor C-like [Ostrinia furnacalis]	-0.40822	-0.32842	1.9664	-0.84138	-0.38839
TRINITY_DN13419_c0_g1_i5_orf1	-	-	-	atrial natriuretic peptide-converting enzyme-like [Ostrinia furnacalis]	-0.32709	-0.22051	1.4342	-1.53747	0.65088
TRINITY_DN2342_c0_g1_i1_orf1	-	-	-	BRCA1-associated protein [Ostrinia furnacalis]	0.27006	1.03409	1.0306	-1.35453	-0.98023
TRINITY_DN667_c0_g1_i5_orf1	-	-	-	unnamed protein product [Arctia plantaginis]	0.08972	-0.15331	1.51892	-1.62274	0.16741
TRINITY_DN18922_c0_g1_i1_orf1	-	-	-	LOW QUALITY PROTEIN: CCR4-NOT transcription complex subunit 6 [Ostrinia furnacalis]	0.17705	0.51941	1.4704	-1.39097	-0.77588
TRINITY_DN32359_c0_g2_i1_orf1	-	-	-	PREDICTED: 26S proteasome non-ATPase regulatory subunit 4 isoform X2 [Fopius arisanus]	0.66803	0.55225	1.13558	-1.4814	-0.87446
TRINITY_DN198_c0_g1_i2_orf1	-	-	-	retinol dehydrogenase 13-like [Ostrinia furnacalis]	0.59399	1.04043	0.79039	-1.21981	-1.20501
TRINITY_DN1520_c0_g1_i9_orf1	-	-	-	adipocyte plasma membrane-associated protein-like [Ostrinia furnacalis] >XP_028176496.1 adipocyte plasma membrane-associated protein-like [Ostrinia furnacalis]	0.44121	0.48557	0.73	-1.98256	0.32577
TRINITY_DN35099_c0_g1_i1_orf1	-	-	-	transmembrane emp24 domain-containing protein 2 [Ostrinia furnacalis]	0.50837	0.59125	1.11302	-1.70016	-0.51248

TRINITY_DN5475_c0_g1_i3_orf1	-	-	-	traB domain-containing protein-like isoform X1 [Ostrinia furnacalis] >XP_028169655.1 traB domain-containing protein-like isoform X1 [Ostrinia furnacalis]	0.3356	0.20784	1.1853	-1.85052	0.12178
TRINITY_DN3335_c0_g1_i1_orf1	-	-	-	unnamed protein product [Pieris macdunnoughi]	0.50244	1.02287	0.83144	-1.5196	-0.83715
TRINITY_DN27456_c0_g2_i1_orf1	-	-	-	organic cation transporter-like protein [Ostrinia furnacalis]	-0.31886	-0.07308	1.62381	-1.48123	0.24935
TRINITY_DN71832_c0_g1_i1_orf1	-	-	-	basement membrane-specific heparan sulfate proteoglycan core protein isoform X13 [Ostrinia furnacalis]	-0.18084	-0.05778	1.42419	-1.6494	0.46383
TRINITY_DN13576_c0_g1_i1_orf1	-	-	-	uncharacterized protein LOC114350099 [Ostrinia furnacalis]	-0.38803	-0.42263	1.9852	-0.72876	-0.44578
TRINITY_DN10110_c1_g2_i1_orf1	-	-	-	venom allergen 3-like [Ostrinia furnacalis]	0.51602	0.83929	0.94586	-1.64402	-0.65715
TRINITY_DN95056_c0_g2_i2_orf1	-	-	-	40S ribosomal protein S18 [Halotydeus destructor]	0.60105	0.66793	0.39375	-1.98359	0.32086
TRINITY_DN25870_c0_g2_i6_orf1	-	-	-	homeobox protein extradenticle isoform X3 [Ostrinia furnacalis]	0.40838	0.10365	1.58092	-1.30428	-0.78868
TRINITY_DN19260_c0_g1_i5_orf1	-	-	-	probable 26S proteasome non-ATPase regulatory subunit 3 [Ostrinia furnacalis]	0.80779	0.76368	0.84136	-1.47615	-0.93668
TRINITY_DN4782_c0_g1_i1_orf1	-	-	-	patched domain-containing protein 3-like [Ostrinia furnacalis]	0.25743	0.40815	1.47416	-1.46022	-0.67952
TRINITY_DN12777_c0_g1_i5_orf1	-	-	-	clathrin light chain isoform X2 [Ostrinia furnacalis]	0.33592	0.63144	1.25539	-1.58154	-0.64121
TRINITY_DN10183_c0_g2_i3_orf1	-	-	-	uncharacterized protein LOC114360370 isoform X1 [Ostrinia furnacalis]	0.35447	0.34699	1.1603	-1.84591	-0.01586
TRINITY_DN1781_c0_g1_i8_orf1	-	-	-	transportin-1 [Pectinophora gossypiella]	0.60887	0.52092	1.2295	-1.00197	-1.35732
TRINITY_DN14677_c0_g2_i3_orf1	-	-	-	AP-3 complex subunit beta-2 [Ostrinia furnacalis]	0.51627	0.6394	1.15998	-1.5438	-0.77185
TRINITY_DN1293_c0_g1_i4_orf1	-	-	-	putative fatty acyl-CoA reductase CG5065 [Ostrinia furnacalis]	-0.03767	-0.25839	1.9038	-0.89027	-0.71748
TRINITY_DN24266_c0_g2_i2_orf1	-	-	-	chromobox-like protein 5 [Helicoverpa armigera]	0.72452	0.42261	1.21237	-1.32638	-1.03312
TRINITY_DN35865_c0_g1_i1_orf1	-	-	-	uncharacterized protein LOC114354496 isoform X1 [Ostrinia furnacalis] >XP_028162709.1 uncharacterized protein LOC114354496 isoform X1 [Ostrinia furnacalis]	0.58931	0.74765	0.75969	-1.86031	-0.23635
TRINITY_DN54586_c1_g1_i1_orf1	-	-	-	protein YIPF5 [Ostrinia furnacalis]	0.60293	0.56167	1.213	-1.29629	-1.08132
TRINITY_DN486_c0_g1_i5_orf1	-	-	-	adaptor complexes medium subunit family domain-containing protein [Phthorimaea	0.48981	0.25662	1.39775	-1.54206	-0.60214
TRINITY_DN9243_c0_g1_i4_orf1	-	-	-	copper-transporting ATPase 1 [Ostrinia furnacalis]	0.02802	0.42462	1.48827	-1.57063	-0.37027
TRINITY_DN5074_c0_g1_i7_orf1	-	-	-	zonadhesin-like [Ostrinia furnacalis]	-0.22879	-0.25642	1.91383	-1.02949	-0.39914
TRINITY_DN206_c0_g1_i8_orf1	-	-	-	A-kinase anchor protein 200-like [Ostrinia furnacalis] >XP_028173114.1 A-kinase anchor protein 200-like [Ostrinia furnacalis] >XP_028173115.1 A-kinase anchor protein 200-like [Ostrinia furnacalis]	-0.19438	-0.48278	1.94284	-0.90992	-0.35576
TRINITY_DN123184_c0_g1_i1_orf1	-	-	-	double-strand break repair protein MRE11 [Ostrinia furnacalis]	0.36647	0.09798	1.61415	-1.25182	-0.82679
TRINITY_DN24218_c0_g1_i1_orf1	-	-	-	uncharacterized protein LOC114362624 [Ostrinia furnacalis]	0.25686	0.3872	1.48548	-1.45845	-0.67109
TRINITY_DN2054_c0_g1_i1_orf1	-	-	-	macrophage mannose receptor 1-like [Ostrinia furnacalis]	0.49434	0.00965	1.59174	-0.93413	-1.1616
TRINITY_DN33893_c0_g1_i1_orf1	-	-	-	high mobility group protein I-like [Ostrinia furnacalis]	0.22334	-0.02168	1.63419	-1.46286	-0.37299
TRINITY_DN4937_c0_g1_i2_orf1	-	-	-	zinc finger protein 778-like [Ostrinia furnacalis]	0.49078	0.28103	1.36061	-1.59312	-0.5393
TRINITY_DN267_c0_g1_i1_orf1	-	-	-	keratin, type I cytoskeletal 9-like [Ostrinia furnacalis]	-0.27097	-0.293	1.95928	-0.81698	-0.57835
TRINITY_DN14347_c0_g1_i1_orf1	-	-	-	putative nuclease HARBI1 [Ostrinia furnacalis]	0.54349	0.69603	0.57108	-1.96711	0.15653
TRINITY_DN44658_c0_g1_i2_orf1	-	-	-	lipase 3-like [Ostrinia furnacalis]	0.44793	-0.06249	1.55827	-1.46087	-0.48284
TRINITY_DN9311_c0_g1_i1_orf1	-	-	-	cuticle protein 8-like [Ostrinia furnacalis]	-0.19981	-0.3658	1.96093	-0.75924	-0.63607
TRINITY_DN4494_c0_g1_i1_orf1	-	-	-	venom serine carboxypeptidase [Ostrinia furnacalis]	0.84329	0.03876	1.34211	-1.03254	-1.19162
TRINITY_DN129835_c0_g1_i2_orf1	-	-	-	alpha-tocopherol transfer protein-like [Chelonus insularis]	-0.22438	-0.16582	1.93008	-0.69468	-0.8452
TRINITY_DN661_c1_g2_i1_orf1	-	-	-	larval/pupal cuticle protein H1C-like [Ostrinia furnacalis]	0.02299	-0.48752	1.91021	-0.90706	-0.53862
TRINITY_DN7549_c0_g1_i1_orf1	-	-	-	uncharacterized protein LOC114355006 [Ostrinia furnacalis]	-0.20979	-0.49208	1.96663	-0.78469	-0.48008
TRINITY_DN1124_c0_g1_i7_orf1	-	-	-	PREDICTED: cuticle protein 18.6, isoform B [Amyelois transitella]	-0.28224	-0.41813	1.96466	-0.84002	-0.42426
TRINITY_DN72816_c0_g1_i2_orf1	-	-	-	Golgi apparatus protein 1 [Ostrinia furnacalis]	0.5674	-0.2988	1.58243	-1.35651	-0.49453
TRINITY_DN98242_c0_g1_i1_orf1	-	-	-	adenosine deaminase 2-A-like [Galleria mellonella]	0.21135	-0.29072	1.82519	-0.96106	-0.78476
TRINITY_DN2101_c0_g1_i6_orf1	-	-	-	protein obstructor-E-like [Ostrinia furnacalis]	-0.17414	-0.48073	1.96441	-0.76018	-0.54936
TRINITY_DN147458_c0_g1_i1_orf1	-	-	-	60S ribosomal protein L5, partial [Cotesia chilonis]	0.25867	-0.30228	1.77783	-1.16482	-0.56941
TRINITY_DN14507_c0_g1_i5_orf1	-	-	-	PTB domain-containing adapter protein ced-6 [Ostrinia furnacalis]	0.92095	0.09709	1.24389	-0.99497	-1.26696
TRINITY_DN619_c0_g1_i1_orf1	-	-	-	putative uncharacterized protein DDB_G0271606 [Ostrinia furnacalis]	-0.03825	-0.44717	1.93351	-0.80031	-0.64778
TRINITY_DN12134_c0_g1_i4_orf1	-	-	-	glutathione S-transferase 1-1 [Ostrinia furnacalis] >XP_028161942.1 glutathione S-transferase 1-1 [Ostrinia furnacalis] >XP_028161943.1 glutathione S-transferase 1-1 [Ostrinia furnacalis]	0.74393	-0.48685	1.485	-1.36445	-0.37763
TRINITY_DN48641_c0_g1_i4_orf1	-	-	-	RNA-binding protein 45-like [Galleria mellonella]	0.85224	-0.27004	1.45476	-0.94836	-1.0886
TRINITY_DN9282_c0_g1_i2_orf1	-	-	-	uncharacterized protein LOC114363102 isoform X2 [Ostrinia furnacalis]	-0.04004	-0.52623	1.93271	-0.84528	-0.52116
TRINITY_DN4070_c0_g1_i4_orf1	-	-	-	alpha-N-acetylgalactosaminidase isoform X3 [Ostrinia furnacalis]	0.96014	-0.47594	1.41896	-1.06811	-0.83504
TRINITY_DN20238_c0_g1_i7_orf1	-	-	-	zinc finger FYVE domain-containing protein 26 [Ostrinia furnacalis]	1.17226	1.05159	0.01401	-1.03018	-1.20769
TRINITY_DN40911_c0_g1_i1_orf1	-	-	-	peroxisomal membrane protein PEX16 [Ostrinia furnacalis]	0.37412	1.76295	-0.38765	-0.68562	-1.06381

TRINITY_DN1860_c0_g1_i1_orf1	-	-	-	26S proteasome non-ATPase regulatory subunit 5 [Ostrinia furnacalis] >XP_028175444.1 26S proteasome non-ATPase regulatory subunit 5 [Ostrinia furnacalis]	0.18737	1.67179	0.15319	-0.75942	-1.25293
TRINITY_DN195_c8_g1_i1_orf1	-	-	-	hypothetical protein evm_009768 [Chilo suppressalis]	0.53551	1.64002	-0.18349	-1.04975	-0.94229
TRINITY_DN35002_c0_g2_i2_orf1	-	-	-	sulfite oxidase, mitochondrial isoform X3 [Leguminivora glycinivorella]	0.18581	1.83944	-0.2974	-0.8449	-0.88295
TRINITY_DN3307_c1_g1_i2_orf1	-	-	-	BTB/POZ domain-containing protein 2-like [Ostrinia furnacalis]	0.71451	1.48134	-0.19145	-0.6489	-1.3555
TRINITY_DN8136_c0_g1_i1_orf1	-	-	-	HIG1 domain family member 2A, mitochondrial [Ostrinia furnacalis]	0.40848	1.48866	0.10459	-0.45194	-1.54979
TRINITY_DN3797_c0_g2_i3_orf1	-	-	-	3'(2'),5'-bisphosphate nucleotidase 1 isoform X2 [Ostrinia furnacalis]	0.76343	1.57037	-0.59902	-1.0766	-0.65818
TRINITY_DN49221_c0_g1_i1_orf1	-	-	-	Similar to ND-23: NADH dehydrogenase (ubiquinone) 23 kDa subunit (Drosophila melanogaster) [Cotesia congregata]	0.69168	1.32379	0.19768	-0.73171	-1.48145
TRINITY_DN1710_c0_g2_i2_orf1	-	-	-	relish [Ostrinia furnacalis]	0.35518	1.54357	0.14414	-1.4595	-0.58338
TRINITY_DN2813_c0_g1_i3_orf1	-	-	-	arylphorin subunit alpha-like [Ostrinia furnacalis]	0.69086	1.59195	-0.90312	-0.3574	-1.02229
TRINITY_DN40440_c0_g1_i1_orf1	-	-	-	Na(+)/H(+) exchange regulatory cofactor NHE-RF1 [Helicoverpa zea] >XP_049705606.1 Na(+)/H(+) exchange regulatory cofactor NHE-RF1 [Helicoverpa armigera]	0.56712	1.33714	0.32759	-1.49837	-0.73348
TRINITY_DN19995_c0_g1_i2_orf1	-	-	-	E3 ubiquitin-protein ligase ZNF598 [Ostrinia furnacalis]	0.95055	1.42586	-0.67594	-0.56678	-1.13369
TRINITY_DN32687_c0_g1_i2_orf1	-	-	-	protein D2-like isoform X2 [Ostrinia furnacalis] >XP_028164613.1 protein D2-like isoform X2 [Ostrinia furnacalis]	0.92504	1.47343	-0.95974	-0.62653	-0.81219
TRINITY_DN60903_c0_g1_i1_orf1	-	-	-	26S proteasome non-ATPase regulatory subunit 11 [Ostrinia furnacalis]	0.57454	1.24876	0.47767	-1.49333	-0.80764
TRINITY_DN82017_c0_g1_i5_orf1	-	-	-	carboxylesterase CXE17 [Ostrinia furnacalis]	0.89939	1.44366	-0.70859	-0.45042	-1.18404
TRINITY_DN28039_c0_g1_i1_orf1	-	-	-	translation elongation factor 2 [Athalia rosae]	0.84919	1.52491	-0.82294	-0.96691	-0.58426
TRINITY_DN9510_c0_g2_i1_orf1	-	-	-	RNA polymerase II transcriptional coactivator [Ostrinia furnacalis]	0.9068	1.45701	-0.46556	-1.08405	-0.8142
TRINITY_DN19939_c0_g1_i4_orf1	-	-	-	unnamed protein product [Chilo suppressalis]	1.13816	1.28914	-0.79461	-0.61828	-1.0144
TRINITY_DN15265_c0_g1_i1_orf1	-	-	-	cullin-5 [Ostrinia furnacalis]	0.98562	1.10728	0.19845	-1.40832	-0.88303
TRINITY_DN102260_c0_g1_i1_orf1	-	-	-	unnamed protein product [Diatraea saccharalis]	1.22515	1.21581	-0.94678	-0.81092	-0.68326
TRINITY_DN27958_c0_g1_i1_orf1	-	-	-	UV excision repair protein RAD23 homolog A [Ostrinia furnacalis]	1.11624	1.19539	-0.17121	-1.1204	-1.02002
TRINITY_DN4741_c0_g1_i4_orf1	-	-	-	PREDICTED: 23 kDa integral membrane protein-like [Papilio xuthus]	0.84247	1.17525	0.14075	-1.60825	-0.55022
TRINITY_DN41664_c0_g1_i4_orf1	-	-	-	uncharacterized protein LOC114356631 [Ostrinia furnacalis]	1.08079	0.87992	0.40242	-1.40902	-0.95412
TRINITY_DN69334_c0_g1_i1_orf1	-	-	-	PREDICTED: 15-hydroxyprostaglandin dehydrogenase [NAD(+)]-like [Papilio xuthus]	0.93755	1.46781	-0.65057	-0.91995	-0.83485
TRINITY_DN7674_c0_g1_i2_orf1	-	-	-	prefoldin subunit 2 [Ostrinia furnacalis]	1.37756	0.99867	-0.5039	-1.1578	-0.71454
TRINITY_DN21000_c0_g1_i1_orf1	-	-	-	elongation factor-1 alpha, partial [Loxostege sticticalis] >QCO92153.1 elongation factor-1 alpha, partial [Sitochroa umbrosalis]	1.32035	1.11004	-0.99489	-0.76444	-0.67105
TRINITY_DN6016_c0_g1_i8_orf1	-	-	-	hypothetical protein evm_010883 [Chilo suppressalis]	1.08612	0.70201	0.45713	-0.57584	-1.66942
TRINITY_DN34479_c0_g1_i2_orf1	-	-	-	PREDICTED: 26S protease regulatory subunit 4 [Amyelois transitella] >XP_021186380.1 26S proteasome regulatory subunit 4 [Helicoverpa armigera] >XP_022116536.1 26S proteasome regulatory subunit 4 [Pieris rapae] >XP_022817854.1 26S proteasome regulatory subunit 4 [Spodoptera litura] >XP_026745369.1 26S proteasome regulatory subunit 4 [Trichoplusia ni] >XP_026760570.1 26S proteasome regulatory subunit 4 [Galleria mellonella] >XP_028176505.1 26S proteasome regulatory subunit 4 [Ostrinia furnacalis] >XP_030038234.1 26S proteasome regulatory subunit 4 [Manduca sexta] >XP_035449919.1 26S proteasome regulatory subunit 4 [Spodoptera frugiperda] >XP_038206559.1 26S proteasome regulatory subunit 4 [Zerene cesonia] >XP_045502541.1 26S proteasome regulatory subunit 4 [Colias croceus] >XP_045532999.1 26S proteasome regulatory subunit 4 [Pieris brassicae] >XP_047033702.1 26S proteasome regulatory subunit 4 [Helicoverpa zea] >XP_047994509.1 26S proteasome regulatory subunit 4 [Leguminivora glycinivorella] >XP_049877826.1 26S proteasome regulatory subunit 4 [Pectinophora gossypiella] >KAH9639287.1 hypothetical protein HF086_014151 [Spodoptera exigua] >KAI5631153.1 ATPase family associated with various cellular activities (AAA) domain-containing protein [Phthorimaea operculella] >RVE50066.1 hypothetical protein evm_005272 [Chilo suppressalis] >CAB3245712.1 unnamed protein product [Arctia plantaginis] >KAF9801312.1 hypothetical protein SFRURICE_000406	1.09943	0.73405	0.52597	-1.48979	-0.86966
TRINITY_DN6381_c0_g1_i2_orf1	-	-	-	solute carrier family 12 member 8 [Ostrinia furnacalis]	1.56448	0.63561	-0.16907	-1.18475	-0.84628
TRINITY_DN33452_c0_g1_i3_orf1	-	-	-	lethal(2) giant larvae protein isoform X8 [Ostrinia furnacalis]	1.00146	1.21644	-0.17303	-0.57709	-1.46778
TRINITY_DN42705_c0_g1_i3_orf1	-	-	-	multiple inositol polyphosphate phosphatase 1 isoform X1 [Ostrinia furnacalis]	1.50866	0.80749	-0.33829	-1.01649	-0.96137
TRINITY_DN556_c0_g2_i1_orf1	-	-	-	serine protease inhibitor dipetalogastin-like [Ostrinia furnacalis]	0.97254	0.66585	0.54584	-0.41115	-1.77308
TRINITY_DN92153_c0_g2_i2_orf1	-	-	-	methylenetetrahydrofolate reductase [Ostrinia furnacalis]	1.20021	0.86269	0.24122	-1.37854	-0.92558
TRINITY_DN25916_c0_g1_i1_orf1	-	-	-	uncharacterized protein LOC125063950 [Vanessa atalanta]	1.23467	0.66733	0.39166	-1.49784	-0.79582

TRINITY_DN2356_c2_g1_i6_orf1	-	-	-	ER membrane protein complex subunit 3 [Ostrinia furnacalis]	1.59098	0.59208	-0.13588	-1.06998	-0.97719
TRINITY_DN3045_c0_g1_i7_orf1	-	-	-	hypothetical protein evm_007836 [Chilo suppressalis]	1.40999	0.67609	0.14412	-1.26861	-0.9616
TRINITY_DN3370_c0_g1_i5_orf1	-	-	-	unnamed protein product, partial [Brenthis ino]	1.5837	0.70253	-0.35004	-0.99434	-0.94186
TRINITY_DN195_c4_g1_i1_orf1	-	-	-	beta-1,3-glucan-binding protein 1 [Ostrinia furnacalis]	1.10521	1.33689	-0.85546	-0.81243	-0.7742
TRINITY_DN3860_c0_g1_i5_orf1	-	-	-	nucleoplasmin-like protein isoform X1 [Hyposmocoma kahamanoa]	1.4081	0.47099	0.41215	-1.16359	-1.12766
TRINITY_DN1013_c0_g1_i3_orf1	-	-	-	TELO2-interacting protein 1 homolog isoform X2 [Ostrinia furnacalis]	1.01458	0.7906	0.55579	-0.82725	-1.53373
TRINITY_DN27592_c0_g1_i1_orf1	-	-	-	D-arabinitol dehydrogenase 1-like [Ostrinia furnacalis]	1.36271	1.05436	-0.89118	-0.57188	-0.95399
TRINITY_DN1116_c0_g1_i6_orf1	-	-	-	RNA exonuclease 4-like [Ostrinia furnacalis] >QEE79882.1 REX4 [Ostrinia furnacalis]	-0.87405	0.26939	-0.16093	-1.00366	1.76925
TRINITY_DN131603_c0_g1_i4_orfp1	-	-	-	TRINITY_DN131603_c0_g1_i4_m.86149 TRINITY_DN131603_c0_g1::TRINITY_DN131603_c0_g1_i4::g.86149 ORF type:internal len:112 (-),score=8.40 TRINITY_DN131603_c0_g1_i4:2-334(-)	-0.38291	-0.04622	-0.83367	-0.66454	1.92734
TRINITY_DN2299_c0_g1_i3_orf1	-	-	-	DNA-directed RNA polymerase II subunit RPB1 [Ostrinia furnacalis]	0.00728	0.63699	-1.30263	-0.82853	1.4869
TRINITY_DN41645_c0_g1_i1_orf1	-	-	-	60S acidic ribosomal protein P2 [Ostrinia furnacalis]	-0.30614	0.77297	-0.84759	-1.13588	1.51663
TRINITY_DN11172_c0_g1_i4_orf1	-	-	-	juvenile hormone epoxide hydrolase-like isoform X1 [Ostrinia furnacalis] >XP_028170522.1 juvenile hormone epoxide hydrolase-like isoform X2 [Ostrinia furnacalis]	0.17447	0.34583	-0.90737	-1.21212	1.59919
TRINITY_DN29144_c0_g3_i1_orf1	-	-	-	PREDICTED: ADP-ribosylation factor 6 [Papilio polytes] >XP_013133321.1 PREDICTED: ADP-ribosylation factor 6 [Papilio polytes] >XP_013177129.1 PREDICTED: ADP-ribosylation factor 6 [Papilio xuthus] >XP_013177130.1 PREDICTED: ADP-ribosylation factor 6 [Papilio xuthus] >XP_014356507.1 ADP-ribosylation factor 6 [Papilio machaon] >XP_021185579.1 ADP-ribosylation factor 6 [Helicoverpa armigera] >XP_021185581.1 ADP-ribosylation factor 6 [Helicoverpa armigera] >XP_022130228.1 ADP-ribosylation factor 6 [Pieris rapae] >XP_022822139.1 ADP-ribosylation factor 6 [Spodoptera litura] >XP_022822140.1 ADP-ribosylation factor 6 [Spodoptera litura] >XP_028159104.1 ADP-ribosylation factor 6 [Ostrinia furnacalis] >XP_028159105.1 ADP-ribosylation factor 6 [Ostrinia furnacalis] >XP_028159106.1 ADP-ribosylation factor 6 [Ostrinia furnacalis] >XP_028159107.1 ADP-ribosylation factor 6 [Ostrinia furnacalis] >XP_028163222.1 ADP-ribosylation factor 6 [Ostrinia furnacalis] >XP_030022165.1 ADP-ribosylation factor 6 [Manduca sexta] >XP_030022166.1 ADP-ribosylation factor 6 [Manduca sexta] >XP_030022167.1 ADP-ribosylation factor 6 [Manduca sexta] >XP_035444169.1 ADP-ribosylation factor 6 [Spodoptera frugiperda] >XP_035444175.1 ADP-ribosylation factor 6 [Spodoptera frugiperda] >XP_038207597.1 ADP-ribosylation factor 6 [Zerene cesonia] >XP_038207598.1 ADP-ribosylation factor 6 [Zerene cesonia] >XP_045510541.1 ADP-ribosylation factor 6 [Colias croceus] >XP_045510551.1 ADP-ribosylation factor 6 [Colias croceus] >XP_045527300.1 ADP-ribosylation factor 6 [Pieris brassicae] >XP_045527302.1 ADP-ribosylation factor 6 [Pieris brassicae] >XP_047029519.1 ADP-ribosylation factor 6 [Helicoverpa zea] >XP_047029551.1 ADP-ribosylation factor 6 [Helicoverpa zea] >XP_047504621.1 ADP-ribosylation factor 6 [Pieris napi] >XP_047504631.1 ADP-ribosylation factor 6 [Pieris napi] >XP_047504640.1 ADP-ribosylation factor 6 [Pieris napi] >XP_047504648.1 ADP-ribosylation factor 6 [Pieris napi] >XP_047504657.1 ADP-ribosylation factor 6 [Pieris napi] >XP_048489067.1 ADP-ribosylation factor 6 [Plutella xylostella] >XP_048489068.1 ADP-ribosylation factor 6 [Plutella xylostella] >XP_048489069.1 ADP-ribosylation factor 6 [Plutella xylostella] >XP_049883531.1 ADP-ribosylation factor 6 [Pectinophora gossypiella] >XP_049883539.1 ADP-ribosylation factor 6 [Pectinophora gossypiella] >KAG5678369.1 hypothetical protein PVAND_008051 [Polypedilum vanderplanki] >RVE51130.1 hypothetical protein evm_004273 [Chilo suppressalis] >CAB3510283.1 unnamed protein product [Spodoptera littoralis] >CAF4796780.1 unnamed protein product [Pieris	-0.31623	0.1195	0.38536	-1.63045	1.44183
TRINITY_DN33024_c0_g1_i1_orf1	-	-	-	hypothetical protein evm_000959 [Chilo suppressalis]	0.70644	-0.01712	-0.01501	-1.79884	1.12453
TRINITY_DN111_c0_g2_i2_orf1	-	-	-	hypothetical protein O3G_MSEX007696 [Manduca sexta] >KAG6452639.1 hypothetical protein O3G_MSEX007696 [Manduca sexta]	0.69763	-0.60353	0.44591	-1.64922	1.10919
TRINITY_DN5991_c0_g1_i6_orf1	-	-	-	uncharacterized protein LOC114357071 [Ostrinia furnacalis]	1.04546	-0.14092	-0.85134	-1.28377	1.23057
TRINITY_DN135679_c0_g1_i1_orfp1	-	-	-	TRINITY_DN135679_c0_g1_i1_m.85524 TRINITY_DN135679_c0_g1::TRINITY_DN135679_c0_g1_i1::g.85524 ORF type:5prime_partial len:55 (+),score=3.74,Toxin_2 PF00451.20 1.9e-06 TRINITY_DN135679_c0_g1_i1:3-167(+)	0.97952	0.13012	-0.62893	-1.5656	1.08489
TRINITY_DN3698_c0_g1_i4_orf1	-	-	-	neuroblastoma-amplified sequence-like [Ostrinia furnacalis]	0.89753	1.25084	-1.09457	-1.18891	0.13511
TRINITY_DN2072_c0_g1_i1_orf1	-	-	-	dnaJ homolog subfamily C member 3 [Helicoverpa zea]	0.06884	1.15911	-0.91359	-1.33355	1.01918

TRINITY_DN670_c0_g1_i15_orf1	-	-	-	hypothetical protein evm_002669 [Chilo suppressalis]	1.08123	0.59046	0.15157	-1.85957	0.03631
TRINITY_DN38720_c0_g1_i3_orf1	-	-	-	transmembrane protein 214 [Ostrinia furnacalis]	1.07989	1.12447	-0.22278	-1.51826	-0.46333
TRINITY_DN70236_c0_g1_i1_orf1	-	-	-	NAD-dependent protein deacetylase sirtuin-2-like [Ostrinia furnacalis]	0.51737	0.98148	-0.77191	-1.56949	0.84254
TRINITY_DN6358_c0_g1_i5_orf1	-	-	-	histone H1B-like [Ostrinia furnacalis]	0.24241	1.06695	-0.58123	-1.62897	0.90083
TRINITY_DN8107_c0_g1_i1_orf1	-	-	-	protein pelota [Ostrinia furnacalis]	0.71056	0.93641	-0.89039	-1.50507	0.74849
TRINITY_DN36612_c0_g1_i1_orf1	-	-	-	lamin-C isoform X1 [Ostrinia furnacalis] >XP_028158861.1 lamin-C isoform X2 [Ostrinia furnacalis] >XP_028158863.1 lamin-C isoform X3 [Ostrinia furnacalis] >XP_028158864.1 lamin-C isoform X4 [Ostrinia furnacalis]	1.08175	0.95057	-0.89184	-1.43062	0.29014
TRINITY_DN54477_c0_g1_i1_orf1	-	-	-	rho-associated protein kinase 2 isoform X11 [Ostrinia furnacalis] >XP_028155846.1 rho-associated protein kinase 2 isoform X12 [Ostrinia furnacalis]	1.01972	0.827	0.01567	-1.80919	-0.0532
TRINITY_DN17189_c0_g1_i2_orf1	-	-	-	fibroin heavy chain [Haritalodes derogata]	1.56661	0.77809	-1.04932	-0.63696	-0.65842
TRINITY_DN28938_c0_g1_i1_orf1	-	-	-	uncharacterized protein LOC114354070 isoform X3 [Ostrinia furnacalis]	1.48486	0.35416	0.25385	-1.5021	-0.59078
TRINITY_DN659_c0_g2_i1_orf1	-	-	-	unnamed protein product [Diatraea saccharalis]	1.26783	0.98688	-1.34699	-0.76394	-0.14377
TRINITY_DN9724_c0_g1_i4_orf1	-	-	-	ras family domain-containing protein [Phthorimaea operculella]	1.17337	1.03729	-0.50413	-1.50016	-0.20637
TRINITY_DN15318_c0_g1_i1_orf1	-	-	-	hepatoma-derived growth factor-related protein 2-like [Ostrinia furnacalis]	0.9991	1.0146	-1.28318	-1.09293	0.36241
TRINITY_DN20426_c0_g2_i1_orf1	-	-	-	prolyl 3-hydroxylase sudestada1 [Ostrinia furnacalis]	0.84937	0.86281	0.15897	-1.87319	0.00204
TRINITY_DN20323_c0_g1_i1_orf1	-	-	-	cuticle protein 7 [Ostrinia furnacalis]	1.22029	0.58267	-0.02702	-1.78063	0.00469
TRINITY_DN5008_c0_g1_i1_orf1	-	-	-	LOW QUALITY PROTEIN: integrator complex subunit 14 [Ostrinia furnacalis]	1.41345	0.7225	-0.40672	-1.50477	-0.22447
TRINITY_DN3260_c0_g1_i6_orf1	-	-	-	vesicular integral-membrane protein VIP36 isoform X1 [Ostrinia furnacalis] >XP_028171839.1 vesicular integral-membrane protein VIP36 isoform X2 [Ostrinia furnacalis]	1.36804	0.82247	-0.14033	-1.43172	-0.61846
TRINITY_DN130778_c0_g1_i1_orf1	-	-	-	26S proteasome non-ATPase regulatory subunit 8 [Ostrinia furnacalis]	0.91288	0.64846	0.71462	-1.70608	-0.56989
TRINITY_DN351_c14_g1_i2_orf1	-	-	-	calnexin [Ostrinia furnacalis] >XP_028173720.1 calnexin [Ostrinia furnacalis]	1.13928	0.76379	0.09245	-1.74577	-0.24975
TRINITY_DN57_c0_g2_i3_orf1	-	-	-	transcriptional repressor CTCF-like [Ostrinia furnacalis] >XP_028163401.1 transcriptional repressor CTCF-like [Ostrinia furnacalis]	1.07848	0.73154	0.33862	-1.73717	-0.41148
TRINITY_DN48851_c0_g1_i2_orf1	-	-	-	translationally-controlled tumor protein homolog [Ostrinia furnacalis]	1.30789	0.93896	-1.39434	-0.65008	-0.20244
TRINITY_DN1369_c0_g2_i3_orf1	-	-	-	ATP-dependent Clp protease ATP-binding subunit clpX-like, mitochondrial isoform X2 [Helicoverpa zea]	1.36079	0.96929	-1.1275	-0.92869	-0.2739
TRINITY_DN3805_c0_g1_i2_orf1	-	-	-	heat shock protein 89 [Glyphodes pyloalis]	1.17752	0.90998	-0.39056	-1.62082	-0.07611
TRINITY_DN3283_c0_g2_i1_orf1	-	-	-	dipeptidyl peptidase 9 isoform X2 [Manduca sexta]	1.43719	0.76618	-0.26374	-1.41523	-0.52441
TRINITY_DN6262_c0_g2_i1_orf1	-	-	-	thrombospondin type-1 domain-containing protein 4-like [Ostrinia furnacalis]	1.3861	0.66489	-0.09143	-1.57491	-0.38465
TRINITY_DN2438_c0_g1_i4_orf1	-	-	-	dystrophin, isoforms A/C/F/G/H isoform X2 [Manduca sexta]	1.01924	0.21692	0.95312	-1.64654	-0.54273
TRINITY_DN13118_c0_g1_i6_orf1	-	-	-	AP-1 complex subunit beta-1 [Helicoverpa armigera] >XP_021189434.2 AP-1 complex subunit beta-1 [Helicoverpa armigera]	0.86374	0.07436	1.23238	-1.51778	-0.65269
TRINITY_DN2031_c11_g1_i2_orfp1	-	-	-	TRINITY_DN2031_c11_g1_i2_m.4044 TRINITY_DN2031_c11_g1::TRINITY_DN2031_c11_g1_i2::g.4044 ORF type:3prime_partial len:149	0.88276	0.96978	-1.1954	-1.23283	0.57568
TRINITY_DN3132_c0_g1_i10_orf1	-	-	-	oxysterol-binding protein 1-like [Ostrinia furnacalis]	1.36831	0.59188	-0.25937	-1.64532	-0.0555
TRINITY_DN496_c0_g1_i7_orf1	-	-	-	unnamed protein product [Diatraea saccharalis]	1.27702	0.89663	-1.40262	-0.77326	0.00223
TRINITY_DN8024_c0_g1_i6_orf1	-	-	-	zinc finger CCHC domain-containing protein 24-like isoform X2 [Pararge aegeria]	1.14609	1.08919	-1.10866	-1.12739	0.00077
TRINITY_DN41166_c0_g1_i1_orf1	-	-	-	arginine kinase isoform X1 [Ostrinia furnacalis]	1.33646	0.82594	-1.49214	-0.53588	-0.13439
TRINITY_DN5670_c0_g1_i2_orf1	-	-	-	DNA polymerase alpha subunit B [Ostrinia furnacalis]	1.10752	0.33308	-0.24143	-1.80066	0.60148
TRINITY_DN14855_c0_g1_i1_orf1	-	-	-	neurochondrin homolog [Ostrinia furnacalis]	1.32496	0.8022	-0.87211	-1.35306	0.098
TRINITY_DN4822_c0_g1_i9_orf1	-	-	-	homogentisate 1,2-dioxygenase [Ostrinia furnacalis]	0.63413	1.22028	-0.75098	-1.53559	0.43216
TRINITY_DN3970_c0_g1_i1_orf1	-	-	-	hypothetical protein evm_002369 [Chilo suppressalis]	1.48325	0.6356	-1.3716	-0.71678	-0.03047
TRINITY_DN136906_c0_g1_i1_orf1	-	-	-	translational elongation factor-1alpha, partial [Ethmia eupostica]	1.49127	0.67752	-1.42084	-0.4704	-0.27754
TRINITY_DN44407_c0_g4_i2_orf1	-	-	-	eukaryotic translation initiation factor 5A [Antheraea pernyi]	1.47109	0.22673	0.53299	-1.19336	-1.03745
TRINITY_DN129808_c0_g1_i1_orf1	-	-	-	uncharacterized protein LOC114354070 isoform X3 [Ostrinia furnacalis]	1.47857	0.68871	-1.1164	-1.04554	-0.00535
TRINITY_DN37986_c0_g1_i2_orf1	-	-	-	unnamed protein product [Diatraea saccharalis]	1.54504	0.63637	-1.13321	-0.95675	-0.09146
TRINITY_DN11178_c0_g1_i1_orf1	-	-	-	hypoxia up-regulated protein 1 [Ostrinia furnacalis]	1.40009	0.75832	-0.30651	-1.49866	-0.35324
TRINITY_DN2172_c0_g2_i8_orf1	-	-	-	hypothetical protein evm_003685 [Chilo suppressalis]	0.86022	0.10032	1.2087	-1.55163	-0.6176
TRINITY_DN143496_c0_g1_i1_orf1	-	-	-	cullin-3 [Diachasma alloeum]	1.20079	0.55704	-0.10395	-1.79372	0.13984
TRINITY_DN11637_c0_g1_i1_orf1	-	-	-	ribosome-binding protein 1 isoform X4 [Colias croceus]	1.08047	0.84351	-0.63544	-1.61564	0.3271
TRINITY_DN61711_c0_g1_i1_orf1	-	-	-	mitochondrial carnitine/acylcarnitine translocase [Loxostege sticticalis]	1.67717	0.56884	-0.61807	-1.09364	-0.53429
TRINITY_DN3597_c0_g1_i10_orf1	-	-	-	unnamed protein product [Diatraea saccharalis]	1.23923	0.30892	0.60941	-1.65755	-0.50001
TRINITY_DN12508_c0_g1_i1_orf1	-	-	-	uncharacterized protein LOC114350091 [Ostrinia furnacalis]	1.19246	0.55625	-1.57017	-0.71666	0.53813

TRINITY_DN5346_c0_g1_i5_orf1	-	-	-	syntaxin-1A isoform X2 [Pectinophora gossypiella]	1.04862	0.49038	0.87033	-1.20903	-1.2003
TRINITY_DN33967_c0_g1_i1_orf1	-	-	-	PREDICTED: elongation factor 1-alpha [Microplitis demolitor] >XP_008547401.1 PREDICTED: elongation factor 1-alpha [Microplitis demolitor]	1.69897	0.50552	-0.77673	-1.05695	-0.37081
TRINITY_DN24323_c0_g1_i3_orf1	-	-	-	ubiquitin-protein ligase E3A [Ostrinia furnacalis]	1.13278	0.23779	0.70077	-1.75121	-0.32013
TRINITY_DN2919_c0_g1_i5_orf1	-	-	-	nidogen-1 [Ostrinia furnacalis]	1.18571	0.35735	0.80799	-1.33338	-1.01767
TRINITY_DN15362_c0_g1_i1_orf1	-	-	-	probable elongation factor 1-delta isoform X1 [Ostrinia furnacalis]	1.47499	0.82852	-1.11953	-0.89505	-0.28893
TRINITY_DN6567_c0_g1_i5_orf1	-	-	-	dymeclin isoform X1 [Ostrinia furnacalis] >XP_028159584.1 dymeclin isoform X2 [Ostrinia furnacalis]	1.30383	0.17794	0.4583	-1.73697	-0.20311
TRINITY_DN18593_c0_g1_i1_orf1	-	-	-	60S ribosomal protein L22-like [Ostrinia furnacalis]	1.60541	0.72465	-0.86303	-0.9293	-0.53772
TRINITY_DN27771_c0_g1_i1_orf1	-	-	-	glycine--tRNA ligase [Ostrinia furnacalis]	1.36704	0.43999	0.41561	-1.49526	-0.72738
TRINITY_DN48096_c0_g2_i2_orf1	-	-	-	eukaryotic translation initiation factor 4E-like [Ostrinia furnacalis]	1.54646	0.46683	-0.26389	-1.50347	-0.24593
TRINITY_DN152_c0_g1_i4_orf1	-	-	-	LOW QUALITY PROTEIN: protein tyrosine phosphatase type IVA 3 [Ostrinia furnacalis]	1.22746	0.68343	0.26945	-1.62717	-0.55316
TRINITY_DN77572_c0_g1_i1_orf1	-	-	-	steroid receptor RNA activator 1 [Ostrinia furnacalis]	1.01774	0.68008	-0.1445	-1.84306	0.28974
TRINITY_DN49047_c0_g1_i2_orf1	-	-	-	unnamed protein product [Parnassius apollo]	1.00968	0.59295	0.67305	-1.67941	-0.59627
TRINITY_DN34399_c0_g1_i1_orf1	-	-	-	cysteine synthase-like [Ostrinia furnacalis]	1.48356	0.59004	-1.38389	-0.7308	0.04109
TRINITY_DN817_c0_g1_i3_orf1	-	-	-	phenylalanine--tRNA ligase beta subunit [Ostrinia furnacalis]	1.58954	0.65817	-0.84789	-1.11314	-0.28667
TRINITY_DN30027_c0_g1_i1_orf1	-	-	-	40S ribosomal protein S14 [Plutella xylostella] >NP_001298660.1 40S ribosomal protein S14 [Papilio polytes] >NP_001299342.1 40S ribosomal protein S14 [Papilio xuthus] >XP_013200267.1 PREDICTED: 40S ribosomal protein S14 [Amyelois transitella] >XP_013200268.1 PREDICTED: 40S ribosomal protein S14 [Amyelois transitella] >XP_014369569.1 40S ribosomal protein S14 [Papilio machaon] >XP_021200686.1 40S ribosomal protein S14 [Helicoverpa armigera] >XP_026737481.1 40S ribosomal protein S14 [Trichoplusia ni] >XP_028029011.1 40S ribosomal protein S14 [Bombyx mandarina] >XP_028179467.1 40S ribosomal protein S14 [Ostrinia furnacalis] >XP_028179468.1 40S ribosomal protein S14 [Ostrinia furnacalis] >XP_030030611.1 40S ribosomal protein S14 [Manduca sexta] >XP_034829960.1 40S ribosomal protein S14 [Maniola hyperantus] >XP_034829961.1 40S ribosomal protein S14 [Maniola hyperantus] >XP_047022662.1 40S ribosomal protein S14 [Helicoverpa zea] >XP_047984027.1 40S ribosomal protein S14 [Leguminivora glycinivorella] >XP_049869822.1 40S ribosomal protein S14 [Pectinophora gossypiella] >Q5UAM9.1 RecName: Full=40S ribosomal protein S14 [Bombyx mori] >CAH0605581.1 unnamed protein product [Chrysodeixis includens] >AAV34871.1 ribosomal protein S14 [Bombyx mori] >ACY95302.1 ribosomal protein S14 [Manduca sexta] >KAG6456546.1 hypothetical protein O3G_MSEX009812 [Manduca sexta] >KAG6456547.1	1.68007	0.16736	-0.15677	-1.43508	-0.25558
TRINITY_DN10785_c0_g1_i4_orf1	-	-	-	alkylglycerol monooxygenase-like [Ostrinia furnacalis] >XP_028171363.1 alkylglycerol monooxygenase-like [Ostrinia furnacalis]	1.76129	0.29397	-1.16197	-0.62308	-0.27022
TRINITY_DN25960_c0_g1_i1_orf1	-	-	-	protein mini spindles [Ostrinia furnacalis]	1.5777	0.32455	-1.09175	-1.07052	0.26002
TRINITY_DN841_c0_g1_i8_orf1	-	-	-	uncharacterized protein LOC114354070 isoform X3 [Ostrinia furnacalis]	1.09875	0.97596	-0.87676	-1.42177	0.22382
TRINITY_DN16187_c0_g1_i1_orf1	-	-	-	eIF-2-alpha kinase activator GCN1 [Colias croceus]	1.61431	0.46317	-0.67728	-1.30857	-0.09163
TRINITY_DN37856_c0_g1_i5_orf1	-	-	-	spermine synthase [Ostrinia furnacalis]	1.71114	0.51381	-0.69585	-1.0427	-0.4864
TRINITY_DN103118_c0_g1_i4_orf1	-	-	-	hypothetical protein evm_006930 [Chilo suppressalis]	0.9054	-0.41896	0.92991	-1.74185	0.3255
TRINITY_DN21215_c0_g1_i7_orf1	-	-	-	phytanoyl-CoA dioxygenase, peroxisomal-like [Ostrinia furnacalis]	1.04468	0.92045	-0.6489	-1.59995	0.28373
TRINITY_DN2224_c0_g2_i1_orf1	-	-	-	tumor susceptibility gene 101 protein [Ostrinia furnacalis]	1.33572	0.16291	0.43051	-1.72061	-0.20853
TRINITY_DN59291_c0_g1_i1_orf1	-	-	-	ATP-dependent RNA helicase vasa [Ostrinia furnacalis]	1.17592	-0.16122	1.01904	-1.50916	-0.52459
TRINITY_DN7909_c0_g2_i1_orf1	-	-	-	aldehyde oxidase 3 [Ostrinia furnacalis]	1.24356	0.97291	-0.87135	-1.32182	-0.02329
TRINITY_DN277_c1_g1_i1_orf1	-	-	-	uncharacterized protein LOC114363802 isoform X4 [Ostrinia furnacalis]	0.69836	-0.21681	1.26086	-1.69507	-0.04734
TRINITY_DN113778_c0_g2_i1_orf1	-	-	-	metastasis-associated protein MTA3 [Galleria mellonella]	0.81493	0.08799	1.26352	-1.52193	-0.64452
TRINITY_DN78492_c0_g1_i1_orf1	-	-	-	uncharacterized protein LOC114354775 [Ostrinia furnacalis]	1.41604	0.25097	-1.26612	-0.99052	0.58964
TRINITY_DN23801_c0_g1_i2_orf1	-	-	-	signal recognition particle subunit SRP72 [Pectinophora gossypiella]	1.68095	0.3083	-0.38499	-1.36972	-0.23454
TRINITY_DN937_c0_g1_i2_orf1	-	-	-	protein brunelleschi [Ostrinia furnacalis]	1.67961	0.60619	-0.77512	-0.94185	-0.56882
TRINITY_DN18249_c0_g1_i1_orf1	-	-	-	60S ribosomal protein L13 [Pectinophora gossypiella]	1.52785	0.71351	-0.70612	-1.25726	-0.27797
TRINITY_DN4385_c0_g2_i1_orf1	-	-	-	LOW QUALITY PROTEIN: carbonic anhydrase 1-like [Ostrinia furnacalis]	1.50947	0.56818	-1.3891	-0.68487	-0.00369
TRINITY_DN31431_c0_g1_i1_orf1	-	-	-	carnosine N-methyltransferase [Ostrinia furnacalis]	1.12375	0.42392	0.48585	-1.80854	-0.22498
TRINITY_DN20339_c0_g1_i3_orf1	-	-	-	ecto-NOX disulfide-thiol exchanger 2-like [Ostrinia furnacalis]	1.35072	0.07061	0.80429	-1.26609	-0.95953

TRINITY_DN147676_c0_g1_i1_orf1	-	-	-	PREDICTED: 60S ribosomal protein L23 [Microplitis demolitor] >XP_044591174.1 60S ribosomal protein L23 [Cotesia glomerata] >KAG8035666.1 hypothetical protein G9C98_001094 [Cotesia typhae] >KAH0547433.1 60S ribosomal protein L23A [Cotesia glomerata]	1.5449	0.51112	-1.29281	-0.82288	0.05967
TRINITY_DN6602_c0_g1_i4_orf1	-	-	-	PREDICTED: E3 ubiquitin-protein ligase RNF181-like [Amyelois transitella]	0.9943	0.34035	0.7994	-1.76681	-0.36725
TRINITY_DN140538_c0_g2_i1_orf1	-	-	-	peptidyl-prolyl cis-trans isomerase NIMA-interacting 1 [Urocitellus parryii]	1.52431	0.42719	-0.11225	-1.54811	-0.29114
TRINITY_DN1351_c0_g1_i1_orf1	-	-	-	PREDICTED: flavin reductase (NADPH) [Microplitis demolitor] >XP_008553603.1 PREDICTED: flavin reductase (NADPH) [Microplitis demolitor]	1.54713	0.23108	-0.74506	-1.3722	0.33905
TRINITY_DN1772_c1_g3_i1_orf1	-	-	-	protein Red isoform X1 [Ostrinia furnacalis] >XP_028157673.1 protein Red isoform X2 [Ostrinia furnacalis] >XP_028157674.1 protein Red isoform X3 [Ostrinia furnacalis] >XP_028157675.1 protein Red isoform X4 [Ostrinia furnacalis]	1.7625	0.32019	-0.29517	-1.12431	-0.66321
TRINITY_DN3838_c0_g1_i8_orf1	-	-	-	ER membrane protein complex subunit 2-like isoform X1 [Ostrinia furnacalis] >XP_028161204.1 ER membrane protein complex subunit 2-like isoform X2 [Ostrinia furnacalis] >XP_028161205.1 ER membrane protein complex subunit 2-like isoform X3 [Ostrinia furnacalis]	1.29027	0.11066	0.8283	-1.39042	-0.83881
TRINITY_DN65988_c0_g1_i4_orf1	-	-	-	uncharacterized protein LOC114354070 isoform X3 [Ostrinia furnacalis]	1.83783	0.11735	-0.34951	-1.11699	-0.48867
TRINITY_DN13233_c0_g1_i3_orf1	-	-	-	60S ribosomal protein L31 [Galleria mellonella] >XP_028158009.1 60S ribosomal protein L31 [Ostrinia furnacalis] >XP_030037192.1 60S ribosomal protein L31 [Manduca sexta] >XP_046978528.1 60S ribosomal protein L31 [Vanessa cardui] >XP_047545474.1 60S ribosomal protein L31 [Vanessa atalanta] >XP_050342244.1 60S ribosomal protein L31 [Nymphalis io] >GBP35474.1 60S ribosomal protein L31 [Eumeta japonica] >ACY95330.1 ribosomal protein L31 [Manduca sexta] >KAG6463984.1 hypothetical protein O3G_MSEX014198 [Manduca sexta] >KAG6463985.1 hypothetical protein O3G_MSEX014198 [Manduca sexta]	1.73542	0.46558	-1.00127	-0.75693	-0.4428
TRINITY_DN14298_c0_g3_i1_orf1	-	-	-	kinesin heavy chain [Ostrinia furnacalis]	0.91447	-0.05341	1.25916	-1.46492	-0.6553
TRINITY_DN89613_c0_g1_i13_orf1	-	-	-	PREDICTED: uncharacterized protein LOC106137743 [Amyelois transitella]	1.31465	0.5289	-1.49752	-0.76018	0.41415
TRINITY_DN29229_c0_g1_i4_orf1	-	-	-	uncharacterized protein LOC114351433 isoform X1 [Ostrinia furnacalis]	1.55509	0.73821	-1.17083	-0.69528	-0.42719
TRINITY_DN36928_c0_g1_i2_orf1	-	-	-	actin-interacting protein 1 isoform X2 [Ostrinia furnacalis]	1.53363	0.71768	-0.53853	-1.29004	-0.42274
TRINITY_DN441_c0_g2_i1_orf1	-	-	-	guanine nucleotide-binding protein subunit beta-like protein [Diachasma alloeum]	1.65796	0.41762	-0.5174	-1.32457	-0.2336
TRINITY_DN30950_c0_g1_i13_orf1	-	-	-	unnamed protein product [Chilo suppressalis]	1.64044	0.05061	0.37533	-1.15685	-0.90952
TRINITY_DN11886_c0_g1_i1_orf1	-	-	-	glycerophosphodiester phosphodiesterase GDPD6-like [Ostrinia furnacalis] >XP_028159459.1 glycerophosphodiester phosphodiesterase GDPD6-like [Ostrinia furnacalis]	1.80878	0.37005	-0.66403	-0.79682	-0.71799
TRINITY_DN7613_c1_g2_i1_orf1	-	-	-	60S ribosomal protein L19 [Helicoverpa armigera] >XP_022126104.1 60S ribosomal protein L19 [Pieris rapae] >XP_022821503.1 60S ribosomal protein L19 [Spodoptera litura] >XP_026731717.1 60S ribosomal protein L19 [Trichoplusia ni] >XP_035451321.1 60S ribosomal protein L19-like [Spodoptera frugiperda] >XP_035452592.1 60S ribosomal protein L19-like [Spodoptera frugiperda] >XP_041975187.1 60S ribosomal protein L19 [Aricia agestis] >XP_045524933.1 60S ribosomal protein L19 [Pieris brassicae] >XP_047023231.1 60S ribosomal protein L19 [Helicoverpa zea] >XP_047984679.1 60S ribosomal protein L19 [Leguminivora glycinivorella] >XP_049874324.1 60S ribosomal protein L19 [Pectinophora gossypiella] >ACY95336.1 ribosomal protein L19 [Manduca sexta] >KAF9423217.1 hypothetical protein HW555_001286 [Spodoptera exigua] >KAI5632448.1 ribosomal protein l19e domain-containing protein [Phthorimaea operculella] >RVE50663.1 hypothetical protein evm_004695 [Chilo suppressalis] >CAB3239671.1 unnamed protein product [Arctia plantaginis] >CAB3509883.1 unnamed protein product [Spodoptera littoralis] >CAG4986349.1 unnamed protein product [Parnassius apollo] >CAG9758258.1 unnamed protein product [Diatraea saccharalis] >CAH2049991.1 unnamed protein product, partial [Iphiclides podalirius]	1.77643	0.28748	-0.868	-0.98078	-0.21513
TRINITY_DN5873_c0_g4_i1_orf1	-	-	-	hypothetical protein evm_003048 [Chilo suppressalis]	1.69254	0.38956	-1.03461	-0.95065	-0.09684
TRINITY_DN4381_c0_g2_i1_orf1	-	-	-	eukaryotic initiation factor 4A [Glyphodes caesalis]	1.77432	0.30234	-0.26477	-1.06218	-0.74971
TRINITY_DN9759_c0_g1_i1_orf1	-	-	-	iroquois-class homeodomain protein IRX-1-like isoform X1 [Ostrinia furnacalis]	1.58185	0.20112	-0.05249	-1.55707	-0.1734
TRINITY_DN19810_c1_g1_i7_orf1	-	-	-	RNA-binding protein spenito [Ostrinia furnacalis] >XP_028167555.1 RNA-binding protein spenito [Ostrinia furnacalis]	1.0377	-0.21075	1.18446	-1.48159	-0.52981
TRINITY_DN14498_c0_g1_i1_orf1	-	-	-	eukaryotic translation initiation factor 2 subunit 2 [Ostrinia furnacalis]	1.75972	0.38076	-1.02763	-0.76033	-0.35252
TRINITY_DN16128_c0_g1_i5_orf1	-	-	-	probable prefoldin subunit 4 [Ostrinia furnacalis]	1.69768	0.27326	-0.16789	-1.34286	-0.46018
TRINITY_DN143852_c0_g1_i1_orf1	-	-	-	60S ribosomal protein L10 [Cotesia glomerata]	1.49273	0.76123	-1.01306	-1.06548	-0.17542
TRINITY_DN95414_c0_g1_i1_orf1	-	-	-	protein arginine N-methyltransferase 5 [Ostrinia furnacalis]	1.61277	0.4779	-1.04176	-1.04176	-0.00715

TRINITY_DN4836_c0_g1_i4_orf1	-	-	-	hypothetical protein O3G_MSEX014157 [Manduca sexta] >KAG6463927.1 hypothetical protein O3G_MSEX014157 [Manduca sexta]	1.62699	0.33629	0.13708	-1.13748	-0.96289
TRINITY_DN10548_c0_g2_i1_orf1	-	-	-	uridine 5'-monophosphate synthase-like [Ostrinia furnacalis]	1.54083	0.17858	0.35536	-1.43447	-0.6403
TRINITY_DN1425_c0_g1_i4_orf1	-	-	-	fibulin-2-like [Ostrinia furnacalis]	1.11885	-0.37087	1.09304	-1.52101	-0.32001
TRINITY_DN3321_c0_g1_i3_orf1	-	-	-	peroxidasin [Ostrinia furnacalis]	1.67555	0.03366	0.24956	-1.30396	-0.65481
TRINITY_DN126648_c0_g1_i1_orf1	-	-	-	elongation factor 1 alpha, partial [Spodoptera exigua] >QYQ52647.1 elongation factor 1 alpha, partial [Spodoptera exigua]	1.48814	0.37726	-0.84503	-1.34937	0.32901
TRINITY_DN1665_c1_g1_i2_orf1	-	-	-	translation elongation factor 2 [Melitaea cinxia]	1.64329	0.56923	-0.86346	-1.07454	-0.27452
TRINITY_DN36893_c0_g1_i1_orf1	-	-	-	40S ribosomal protein S15 [Bicyclus anynana] >XP_026325996.1 40S ribosomal protein S15 [Hyposmocoma kahamanoa] >XP_028175376.1 40S ribosomal protein S15 [Ostrinia furnacalis] >XP_030034906.1 40S ribosomal protein S15 [Manduca sexta] >XP_039758445.1 40S ribosomal protein S15 [Pararge aegeria] >XP_045775675.1 40S ribosomal protein S15 [Maniola jurtina] >CAH2267288.1 jg14755 [Pararge aegeria aegeria] >ACY95351.1 ribosomal protein S15 [Manduca sexta] >KAG6461386.1 hypothetical protein O3G_MSEX012590 [Manduca sexta] >KAG6461387.1 hypothetical protein O3G_MSEX012590 [Manduca sexta]	1.59632	0.74388	-0.73593	-0.98916	-0.61511
TRINITY_DN58261_c0_g1_i1_orf1	-	-	-	15-hydroxyprostaglandin dehydrogenase [NAD(+)]-like [Ostrinia furnacalis]	1.57736	0.5891	-0.39798	-1.35474	-0.41375
TRINITY_DN42373_c0_g4_i1_orf1	-	-	-	unnamed protein product [Spodoptera exigua]	1.5308	0.71957	-0.95641	-1.08685	-0.20711
TRINITY_DN12806_c0_g2_i1_orf1	-	-	-	inactive pancreatic lipase-related protein 1-like isoform X2 [Ostrinia furnacalis]	1.59852	0.63834	-0.91151	-1.06772	-0.25763
TRINITY_DN3029_c4_g1_i1_orf1	-	-	-	proliferation marker protein Ki-67-like, partial [Ostrinia furnacalis]	1.36743	-0.03627	0.07964	-1.73673	0.32594
TRINITY_DN31119_c0_g1_i1_orf1	-	-	-	transforming acidic coiled-coil-containing protein 3-like [Ostrinia furnacalis] >XP_028170476.1 transforming acidic coiled-coil-containing protein 3-like [Ostrinia furnacalis] >XP_028170477.1 transforming acidic coiled-coil-containing protein 3-like [Ostrinia furnacalis] >XP_028170480.1 transforming acidic coiled-coil-containing protein 3-like [Ostrinia furnacalis]	1.54239	-0.09237	0.19014	-1.6047	-0.03546
TRINITY_DN4533_c0_g1_i1_orf1	-	-	-	neurofilament heavy polypeptide-like isoform X2 [Ostrinia furnacalis]	1.20226	-0.30206	1.0719	-1.41604	-0.55606
TRINITY_DN6535_c0_g1_i3_orf1	-	-	-	mRNA export factor [Ostrinia furnacalis]	1.85815	0.19425	-0.4875	-0.93645	-0.62845
TRINITY_DN81312_c0_g1_i1_orf1	-	-	-	atlastin isoform X4 [Ostrinia furnacalis]	1.61405	0.45572	-0.82951	-1.22426	-0.016
TRINITY_DN142485_c0_g1_i1_orf1	-	-	-	uncharacterized protein CG16817-like [Ostrinia furnacalis]	1.73629	0.26944	-0.05431	-1.02915	-0.92228
TRINITY_DN10672_c0_g1_i3_orf1	-	-	-	neurofilament heavy polypeptide-like isoform X10 [Ostrinia furnacalis]	1.57005	-0.17092	0.06424	-1.57752	0.11415
TRINITY_DN18159_c0_g1_i6_orf1	-	-	-	zinc carboxypeptidase-like [Ostrinia furnacalis]	1.5613	0.60505	-0.88592	-1.18411	-0.09632
TRINITY_DN16408_c0_g1_i1_orf1	-	-	-	ABC transporter G family member 20 isoform X1 [Ostrinia furnacalis] >XP_028158027.1 ABC transporter G family member 20 isoform X1 [Ostrinia furnacalis] >XP_028158037.1 ABC transporter G family member 20 isoform X2 [Ostrinia furnacalis] >XP_028158043.1 ABC transporter G family member 20 isoform X3 [Ostrinia furnacalis] >XP_028158060.1 ABC transporter G family member 20 isoform X5 [Ostrinia furnacalis] >XP_028158070.1 ABC transporter G family member 20 isoform X6 [Ostrinia furnacalis]	1.78832	0.23641	-0.54582	-1.16047	-0.31844
TRINITY_DN11065_c0_g2_i1_orf1	-	-	-	ribosomal protein s6e domain-containing protein [Phthorimaea operculella]	1.66419	0.47003	-0.97932	-1.01539	-0.1395
TRINITY_DN1298_c0_g1_i3_orf1	-	-	-	ras GTPase-activating protein-binding protein 2-like, partial [Ostrinia furnacalis]	1.48262	0.17597	0.37172	-1.54977	-0.48054
TRINITY_DN1447_c0_g1_i5_orf1	-	-	-	PREDICTED: coatomer subunit beta' [Amyelois transitella]	1.66532	-0.23249	0.36019	-1.36313	-0.4299
TRINITY_DN29521_c0_g1_i1_orf1	-	-	-	density-regulated protein homolog [Ostrinia furnacalis]	1.52673	0.62626	-0.77925	-1.2894	-0.08434
TRINITY_DN2577_c0_g1_i1_orf1	-	-	-	unnamed protein product [Diatraea saccharalis]	1.55825	0.46823	-0.76773	-1.32615	0.0674
TRINITY_DN170_c1_g1_i5_orf1	-	-	-	regulator of chromosome condensation isoform X2 [Helicoverpa zea]	1.69088	0.02956	0.29151	-1.13075	-0.88118
TRINITY_DN3909_c0_g2_i2_orf1	-	-	-	ribosomal protein L24 [Loxostege sticticalis]	1.70979	0.2792	-0.74721	-1.19939	-0.0424
TRINITY_DN2682_c0_g1_i4_orf1	-	-	-	40S ribosomal protein S5 [Manduca sexta] >ACY95347.1 ribosomal protein S5 [Manduca sexta] >KAG6447616.1 hypothetical protein O3G_MSEX005033 [Manduca sexta] >KAG6447617.1 hypothetical protein O3G_MSEX005033 [Manduca sexta]	1.68329	0.52697	-0.79078	-1.06755	-0.35193
TRINITY_DN40015_c0_g1_i2_orf1	-	-	-	PREDICTED: 60S ribosomal protein L18 [Amyelois transitella]	1.86058	0.14153	-0.7817	-0.8952	-0.3252
TRINITY_DN10455_c0_g1_i2_orf1	-	-	-	actin-related protein 2/3 complex subunit 4 [Plutella xylostella] >XP_013184242.1 PREDICTED: actin-related protein 2/3 complex subunit 4 [Amyelois transitella] >XP_026754865.1 actin-related protein 2/3 complex subunit 4 [Galleria mellonella] >XP_028168998.1 actin-related protein 2/3 complex subunit 4 [Ostrinia furnacalis] >KAI5632346.1 ARP2/3 complex 20 kDa subunit (ARPC4) domain-containing protein [Phthorimaea operculella] >KAG7303373.1 Actin-protein 2/3 complex subunit 4 [Plutella xylostella] >CAG9104981.1 unnamed protein product	1.71043	0.08644	0.21104	-1.06125	-0.94666
TRINITY_DN105359_c0_g2_i5_orf1	-	-	-	uncharacterized protein LOC114359499 [Ostrinia furnacalis]	0.79801	0.40598	1.02222	-1.69423	-0.53198
TRINITY_DN2232_c1_g1_i3_orf1	-	-	-	protein FAM98A-like [Ostrinia furnacalis]	1.26225	0.24696	0.80632	-1.24367	-1.07186

TRINITY_DN3063_c0_g1_i5_orf1	-	-	-	mini-chromosome maintenance complex-binding protein [Ostrinia furnacalis]	1.82422	0.16233	-0.30907	-1.1065	-0.57097
TRINITY_DN4501_c0_g1_i3_orf1	-	-	-	methylcrotonoyl-CoA carboxylase subunit alpha, mitochondrial [Ostrinia furnacalis]	1.35524	0.49637	-1.64882	-0.39954	0.19675
TRINITY_DN7241_c0_g2_i2_orf1	-	-	-	40S ribosomal protein S10 [Zerene cesonia] >XP_045492164.1 40S ribosomal protein S10 [Colias croceus]	1.77353	0.42443	-0.74128	-0.90723	-0.54944
TRINITY_DN5630_c4_g1_i2_orf1	-	-	-	PREDICTED: stress-associated endoplasmic reticulum protein 2 [Amyelois transitella] >XP_014371593.1 stress-associated endoplasmic reticulum protein 2 [Papilio machaon] >XP_022818474.1 stress-associated endoplasmic reticulum protein 2 [Spodoptera litura] >XP_028162992.1 stress-associated endoplasmic reticulum protein 2 [Ostrinia furnacalis] >XP_028162993.1 stress-associated endoplasmic reticulum protein 2 [Ostrinia furnacalis] >XP_031767943.1 stress-associated endoplasmic reticulum protein 2 [Galleria mellonella] >XP_035452408.1 stress-associated endoplasmic reticulum protein 2-like [Spodoptera frugiperda] >XP_035452409.1 stress-associated endoplasmic reticulum protein 2-like [Spodoptera frugiperda] >XP_035452411.1 stress-associated endoplasmic reticulum protein 2-like [Spodoptera frugiperda] >XP_045455924.1 stress-associated endoplasmic reticulum protein 2 [Melitaea cinxia] >KPJ00707.1 Stress-associated endoplasmic reticulum protein 2 [Papilio xuthus] >CAB3510969.1 unnamed protein product [Spodoptera littoralis] >AXY94738.1 stress-associated endoplasmic reticulum protein 2 [Galleria mellonella] >KAF9797689.1 hypothetical protein SFRURICE_017884 [Spodoptera frugiperda] >KAG8114722.1 hypothetical	1.78955	0.21329	-0.1782	-0.74563	-1.07902
TRINITY_DN7991_c0_g1_i9_orf1	-	-	-	hypothetical protein evm_006720 [Chilo suppressalis] >CAB3528247.1 unnamed protein product [Chilo suppressalis] >CAH0404834.1 unnamed protein product [Chilo suppressalis]	1.8517	0.15259	-0.85606	-0.85329	-0.29494
TRINITY_DN4469_c0_g1_i2_orf1	-	-	-	metal transporter CNNM4-like [Ostrinia furnacalis]	1.77798	0.28006	-0.84112	-1.00368	-0.21324
TRINITY_DN12227_c0_g2_i3_orf1	-	-	-	exonuclease 3'-5' domain-containing protein 2 [Ostrinia furnacalis]	1.09503	0.55443	0.60142	-1.67252	-0.57836
TRINITY_DN2374_c0_g1_i1_orf1	-	-	-	uncharacterized protein LOC114357127 [Ostrinia furnacalis]	1.58972	0.16727	0.3792	-1.16655	-0.96963
TRINITY_DN7647_c0_g1_i4_orf1	-	-	-	E3 ubiquitin-protein ligase Bre1 isoform X6 [Ostrinia furnacalis]	1.74752	0.18939	0.01027	-1.05855	-0.88864
TRINITY_DN4380_c0_g1_i9_orf1	-	-	-	hypothetical protein evm_012370 [Chilo suppressalis]	1.75183	-0.08687	0.24933	-1.07783	-0.83646
TRINITY_DN4835_c0_g1_i2_orf1	-	-	-	ribonucleoside-diphosphate reductase large subunit [Ostrinia furnacalis]	1.12323	-0.08316	1.14202	-1.24349	-0.9386
TRINITY_DN21251_c1_g1_i1_orf1	-	-	-	60S ribosomal protein L4 [Ostrinia furnacalis]	1.79477	0.17752	-0.75475	-1.07591	-0.14164
TRINITY_DN79868_c0_g1_i1_orf1	-	-	-	lethal(2)neighbour of Tid protein [Ostrinia furnacalis]	1.44478	0.11714	0.56433	-1.4631	-0.66315
TRINITY_DN20130_c0_g1_i1_orf1	-	-	-	uncharacterized protein LOC114354518 isoform X1 [Ostrinia furnacalis]	1.85346	0.03847	-0.51992	-1.10534	-0.26666
TRINITY_DN40345_c0_g1_i6_orf1	-	-	-	60S ribosomal protein L28 [Ostrinia furnacalis]	1.86517	0.19684	-0.64504	-0.88512	-0.53185
TRINITY_DN14464_c0_g1_i1_orf1	-	-	-	GMP synthase [glutamine-hydrolyzing] [Chelonus insularis]	1.89024	0.10736	-0.68655	-0.8613	-0.44975
TRINITY_DN130_c0_g1_i7_orf1	-	-	-	RNA-binding protein fusilli isoform X1 [Bombyx mori]	1.71697	-0.0187	0.09105	-1.36505	-0.42426
TRINITY_DN23264_c0_g1_i1_orf1	-	-	-	U5 small nuclear ribonucleoprotein 40 kDa protein [Ostrinia furnacalis]	1.63633	0.36537	0.03669	-1.25376	-0.78463
TRINITY_DN4132_c0_g1_i14_orf1	-	-	-	thyroid receptor-interacting protein 11-like isoform X1 [Ostrinia furnacalis]	1.38801	0.10585	-0.0084	-1.73239	0.24693
TRINITY_DN642_c0_g1_i6_orf1	-	-	-	reticulon-3-B isoform X5 [Ostrinia furnacalis]	1.82887	0.2015	-0.81085	-0.93652	-0.283
TRINITY_DN10831_c1_g1_i1_orf1	-	-	-	40S ribosomal protein S16 [Ostrinia furnacalis]	1.71519	0.41686	-1.04425	-0.86188	-0.22593
TRINITY_DN2954_c0_g1_i1_orf1	-	-	-	unnamed protein product [Diatraea saccharalis]	1.79788	0.25342	-0.68125	-1.07282	-0.29724
TRINITY_DN1427_c0_g1_i7_orf1	-	-	-	SAFB-like transcription modulator isoform X1 [Ostrinia furnacalis] >XP_028158609.1 SAFB-like transcription modulator isoform X2 [Ostrinia furnacalis]	1.68155	0.02381	0.00111	-1.45149	-0.25497
TRINITY_DN257_c0_g1_i7_orf1	-	-	-	zinc finger RNA-binding protein 2 [Ostrinia furnacalis]	1.49379	-0.14595	0.70089	-1.30489	-0.74385
TRINITY_DN2879_c0_g1_i4_orf1	-	-	-	nucleoporin Nup35 [Ostrinia furnacalis]	1.06596	0.02536	1.10363	-1.44112	-0.75383
TRINITY_DN6396_c0_g1_i1_orf1	-	-	-	PR domain zinc finger protein 10-like [Ostrinia furnacalis]	1.70947	0.34701	-0.24835	-1.26526	-0.54288
TRINITY_DN5031_c0_g1_i1_orf1	-	-	-	PREDICTED: 40S ribosomal protein S12 [Trachymyrmex septentrionalis]	1.77055	0.30567	-0.89764	-0.95768	-0.22089
TRINITY_DN26251_c0_g1_i1_orf1	-	-	-	serine/arginine-rich splicing factor 1A [Neodiprion lecontei] >XP_046417766.1 serine/arginine-rich splicing factor 1A [Neodiprion fabricii] >XP_046473571.1 serine/arginine-rich splicing factor 1A [Neodiprion pinetum] >XP_046610590.1 serine/arginine-rich splicing factor 1A [Neodiprion virginianus] >XP_046738887.1 serine/arginine-rich splicing factor 1A [Diprion	1.58375	0.06047	0.4142	-1.34382	-0.71459
TRINITY_DN3702_c0_g1_i1_orf1	-	-	-	U4/U6.U5 tri-snRNP-associated protein 2 [Ostrinia furnacalis]	1.22194	0.36501	0.36	-1.79467	-0.15228
TRINITY_DN23004_c0_g1_i1_orf1	-	-	-	uncharacterized protein LOC114365313 [Ostrinia furnacalis]	1.77701	0.13894	-0.84454	-1.05326	-0.01816
TRINITY_DN24318_c0_g1_i1_orf1	-	-	-	60S ribosomal protein L29 [Ostrinia furnacalis]	1.63854	0.46623	-0.78424	-1.21293	-0.1076

TRINITY_DN235_c0_g3_i1_orf1	-	-	-	actin, muscle-type A2 [Bombyx mori] >XP_013199497.1 PREDICTED: actin, muscle-type A2 [Amyelois transitella] >XP_021196684.1 actin, muscle-type A2 [Helicoverpa armigera] >XP_022837900.1 actin, muscle-type A2 [Spodoptera litura] >XP_026314060.1 actin, muscle-type A2 [Hyposmocoma kahamanoa] >XP_026738711.1 actin, muscle-type A2 [Trichoplusia ni] >XP_028179440.1 actin, muscle-type A2 [Ostrinia furnacalis] >XP_030030527.1 actin, muscle-type A2 [Manduca sexta] >XP_035439272.1 actin, muscle-type A2 [Spodoptera frugiperda] >XP_047029939.1 actin, muscle-type A2 [Helicoverpa zea] >XP_049873365.1 actin, muscle-type A2 [Pectinophora gossypiella] >P07837.1 RecName: Full=Actin, muscle-type A2; Flags: Precursor [Bombyx mori] >KAF9423784.1 hypothetical protein HW555_000842 [Spodoptera exigua] >QLI62214.1 actin [Streltzoviella insularis] >CAB3227390.1 unnamed protein product [Arctia plantaginis] >CAB3508892.1 unnamed protein product [Spodoptera littoralis] >CAB3520808.1 unnamed protein product [Chilo suppressalis] >CAG9748331.1 unnamed protein product [Diatraea saccharalis] >CAH0585396.1 unnamed protein product [Chrysodeixis includens] >GBP21118.1 Actin, muscle-type A2 [Eumeta japonica]	1.45453	-0.30213	0.8164	-1.29136	-0.67745
TRINITY_DN37532_c0_g1_i1_orf1	-	-	-	transcription elongation factor S-II [Ostrinia furnacalis]	1.3182	-0.32598	0.9768	-1.34743	-0.62159
TRINITY_DN49872_c0_g2_i1_orf1	-	-	-	NIF3-like protein 1 [Ostrinia furnacalis] >XP_028165862.1 NIF3-like protein 1 [Ostrinia furnacalis] >XP_028165864.1 NIF3-like protein 1 [Ostrinia furnacalis]	1.50545	-0.40117	0.10656	-1.56152	0.35068
TRINITY_DN10558_c0_g1_i4_orf1	-	-	-	unnamed protein product [Chrysodeixis includens]	0.93463	0.41533	1.04237	-1.25005	-1.14229
TRINITY_DN4008_c0_g1_i7_orf1	-	-	-	nuclear export mediator factor NEMF homolog isoform X1 [Ostrinia furnacalis]	1.78132	0.38607	-0.73531	-0.95239	-0.47969
TRINITY_DN17376_c0_g1_i2_orf1	-	-	-	E3 UFM1-protein ligase 1 homolog [Ostrinia furnacalis]	1.8361	0.0029	-0.42784	-1.17991	-0.23125
TRINITY_DN20984_c0_g1_i4_orf1	-	-	-	NADPH--cytochrome P450 reductase isoform X2 [Ostrinia furnacalis]	1.75683	-0.15717	-0.66179	-1.17995	0.24207
TRINITY_DN1882_c0_g1_i4_orf1	-	-	-	zinc transporter ZIP13 homolog [Ostrinia furnacalis]	1.26847	0.64832	-0.23902	-1.70666	0.02889
TRINITY_DN143_c0_g3_i1_orf1	-	-	-	Ubiquitin-60S ribosomal protein L40, partial [Cotesia chilonis] >UDP69015.1 egg surface protein ES-53, partial [Cotesia chilonis]	1.76156	-0.25069	-0.57281	-1.19909	0.26104
TRINITY_DN3393_c0_g2_i1_orf1	-	-	-	40S ribosomal protein S8 [Ostrinia furnacalis]	1.8958	0.0838	-0.60679	-0.89665	-0.47616
TRINITY_DN7868_c0_g1_i8_orf1	-	-	-	uncharacterized protein LOC114353432 isoform X4 [Ostrinia furnacalis]	1.69943	-0.04314	-0.10436	-1.44489	-0.10704
TRINITY_DN7464_c0_g1_i14_orf1	-	-	-	60S ribosomal protein L9 [Nymphalis io]	1.76688	0.2895	-1.05558	-0.79977	-0.20103
TRINITY_DN38424_c0_g1_i1_orf1	-	-	-	glucose dehydrogenase [FAD, quinone]-like [Ostrinia furnacalis]	1.00217	0.41888	0.80229	-1.70534	-0.518
TRINITY_DN271_c0_g2_i6_orf1	-	-	-	hypothetical protein NE865_03378 [Phthorimaea operculella]	1.85446	0.26101	-0.71745	-0.71992	-0.6781
TRINITY_DN106534_c0_g1_i1_orf1	-	-	-	nucleolar complex protein 2 homolog [Ostrinia furnacalis]	1.4278	0.50594	-1.4871	-0.66728	0.22064
TRINITY_DN15737_c0_g1_i7_orf1	-	-	-	UPF0160 protein C27H6.8 [Ostrinia furnacalis]	1.65987	0.40217	-0.76283	-1.22284	-0.07637
TRINITY_DN18933_c0_g1_i3_orf1	-	-	-	PREDICTED: protein BUD31 homolog [Papilio xuthus] >XP_014361644.1 protein BUD31 homolog [Papilio machaon] >XP_026750578.1 protein BUD31 homolog [Galleria mellonella] >XP_047995610.1 protein BUD31 homolog [Leguminivora glycinivorella] >XP_049869593.1 protein BUD31 homolog [Pectinophora gossypiella] >KAI5652084.1 g10 protein domain-containing protein [Phthorimaea operculella] >CAB3251981.1 unnamed protein product [Arctia plantaginis] >CAB3520382.1 unnamed protein product [Chilo suppressalis] >CAG9747228.1 unnamed protein product [Diatraea saccharalis] >CAH2037008.1 unnamed protein product, partial [Iphiclides podalirius]	1.17209	-0.24713	1.05956	-1.47873	-0.50579
TRINITY_DN8958_c0_g1_i1_orf1	-	-	-	nuclear cap-binding protein subunit 1 [Galleria mellonella]	1.38614	-0.2222	0.66007	-1.59393	-0.23007
TRINITY_DN79803_c0_g1_i7_orf1	-	-	-	dnaJ homolog subfamily C member 22 [Ostrinia furnacalis]	0.87975	-0.59967	1.29123	-1.48016	-0.09115
TRINITY_DN9101_c0_g2_i1_orf1	-	-	-	60S ribosomal protein L7a [Ostrinia furnacalis]	1.88382	0.15898	-0.69617	-0.80482	-0.54181
TRINITY_DN23502_c0_g1_i1_orf1	-	-	-	small nuclear ribonucleoprotein F [Ostrinia furnacalis]	1.39261	0.26733	0.56962	-1.41415	-0.81541
TRINITY_DN5457_c0_g1_i4_orf1	-	-	-	unnamed protein product [Chrysodeixis includens]	1.52048	-0.23348	0.73871	-1.14736	-0.87835
TRINITY_DN1875_c0_g1_i1_orf1	-	-	-	uncharacterized protein LOC114366320 isoform X1 [Ostrinia furnacalis] >XP_028178963.1 uncharacterized protein LOC114366320 isoform X1 [Ostrinia furnacalis] >XP_028178964.1 uncharacterized protein LOC114366320 isoform X2 [Ostrinia furnacalis]	1.49011	-0.4211	0.2617	-1.5732	0.24249
TRINITY_DN82324_c0_g1_i4_orf1	-	-	-	hypothetical protein evm_001824 [Chilo suppressalis] >CAG9754426.1 unnamed protein product [Diatraea saccharalis] >CAG9793111.1 unnamed protein product [Diatraea saccharalis]	1.81148	0.3143	-0.69976	-0.95105	-0.47496
TRINITY_DN799_c0_g1_i7_orf1	-	-	-	hypothetical protein evm_002571 [Chilo suppressalis] >CAB3529880.1 unnamed protein product [Chilo suppressalis] >CAH0406472.1 unnamed protein product [Chilo suppressalis]	1.75797	0.30999	-0.48509	-1.19479	-0.38808
TRINITY_DN5112_c0_g1_i1_orf1	-	-	-	unnamed protein product, partial [Iphiclides podalirius]	1.74195	-0.2407	-0.12709	-1.37532	0.00116
TRINITY_DN146264_c0_g1_i1_orf1	-	-	-	PREDICTED: protein preli-like [Fopius arisanus]	1.81755	0.14453	-0.36139	-1.16361	-0.43708
TRINITY_DN8824_c0_g2_i1_orf1	-	-	-	60S ribosomal protein L34-like [Ostrinia furnacalis]	1.7633	0.17978	-0.70005	-1.16732	-0.07572

TRINITY_DN27751_c0_g2_i1_orf1	-	-	-	eukaryotic translation initiation factor 3 subunit I [Ostrinia furnacalis]	1.89431	-0.06137	-0.25289	-1.00862	-0.57144
TRINITY_DN4056_c0_g1_i8_orf1	-	-	-	uncharacterized protein LOC114349672 [Ostrinia furnacalis] >XP_028155936.1 uncharacterized protein LOC114349672 [Ostrinia furnacalis] >XP_028155937.1 uncharacterized protein LOC114349672 [Ostrinia furnacalis] >XP_028155939.1 uncharacterized protein LOC114349672 [Ostrinia furnacalis]	1.55546	0.42436	0.07599	-1.40306	-0.65275
TRINITY_DN97589_c0_g1_i3_orf1	-	-	-	ribosomal protein L37A [Bombyx mori] >XP_013189707.1 PREDICTED: 60S ribosomal protein L37a [Amyelois transitella] >XP_021198447.1 60S ribosomal protein L37a [Helicoverpa armigera] >XP_022122377.1 60S ribosomal protein L37a [Pieris rapae] >XP_022822835.1 60S ribosomal protein L37a [Spodoptera litura] >XP_023937141.1 60S ribosomal protein L37a [Bicyclus anynana] >XP_026321523.1 60S ribosomal protein L37a [Hyposmocoma kahamanoa] >XP_026495655.1 60S ribosomal protein L37a [Vanessa tameamea] >XP_026746489.1 60S ribosomal protein L37a [Trichoplusia ni] >XP_026756267.1 60S ribosomal protein L37a [Galleria mellonella] >XP_028041705.1 60S ribosomal protein L37a [Bombyx mandarina] >XP_028161757.1 60S ribosomal protein L37a [Ostrinia furnacalis] >XP_030020263.1 LOW QUALITY PROTEIN: 60S ribosomal protein L37a [Manduca sexta] >XP_032518929.1 60S ribosomal protein L37a [Danaus plexippus plexippus] >XP_034834514.1 60S ribosomal protein L37a [Maniola hyperantus] >XP_035444256.1 60S ribosomal protein L37a [Spodoptera frugiperda] >XP_038222439.1 60S ribosomal protein L37a [Zerene cesonia] >XP_039756348.1 60S ribosomal protein L37a [Pararge aegeria] >XP_041981914.1 60S ribosomal protein L37a [Aricia agestis] >XP_045451710.1 60S ribosomal protein L37a [Melitaea cinxia] >XP_045500579.1 60S ribosomal protein L37a [Colias croceus] >XP_045517305.1 60S ribosomal protein L37a [Pieris brassicae] >XP_045775103.1 60S ribosomal protein L37a [Maniola jurtina] >XP_046969745.1 60S ribosomal protein L37a [Vanessa cardui] >XP_047032252.1 60S ribosomal protein L37a [Helicoverpa zea] >XP_047525321.1 60S ribosomal protein L37a [Pieris napi] >XP_047535357.1 60S ribosomal protein L37a [Vanessa atalanta] >XP_049875744.1 60S ribosomal protein L37a [Pectinophora gossypiella] >XP_050348149.1 60S ribosomal protein L37a [Nymphalis io] >ADO95156.1 ribosomal protein L37A [Antheraea yamamai] >ADT80705.1 ribosomal protein L37A [Euphydryas aurinia] >AEL28885.1 ribosomal protein L37A [Heliconius melpomene cythera] >KAF9418899.1 hypothetical protein HW555_004419 [Spodoptera exigua] >KOB75009.1 Ribosomal protein L37A [Operophtera brumata] >RVE49828.1 hypothetical protein evm_005558 [Chilo suppressalis] >CAB3234150.1 unnamed protein product [Arctia plantaginis] >CAB3509616.1 unnamed protein product [Spodoptera littoralis] >CAF4811073.1 unnamed protein product [Pieris macdunnoughi] >CAG4956733.1 unnamed protein product [Parnassius apollo] >CAG9564640.1 unnamed protein product [Danaus chrysippus] >CAG9750098.1 unnamed	1.65906	0.31079	-1.17543	-0.87351	0.07909
TRINITY_DN4156_c0_g1_i2_orf1	-	-	-	calcium channel flower [Ostrinia furnacalis]	0.86119	-0.3001	1.39902	-1.36085	-0.59926
TRINITY_DN1791_c0_g1_i3_orf1	-	-	-	succinate dehydrogenase assembly factor 2-B, mitochondrial-like [Ostrinia furnacalis]	1.70393	0.33976	-0.89252	-1.08646	-0.0647
TRINITY_DN40650_c0_g1_i1_orf1	-	-	-	60S ribosomal protein L12 [Zerene cesonia]	1.7939	0.2535	-0.89694	-0.92978	-0.22068
TRINITY_DN9862_c0_g2_i1_orf1	-	-	-	40S ribosomal protein S4 [Manduca sexta] >ACY95325.1 ribosomal protein S4 [Manduca sexta] >KAG6465430.1 hypothetical protein O3G_MSEX015149 [Manduca sexta]	1.80675	0.3188	-0.83477	-0.87357	-0.4172
TRINITY_DN131371_c0_g1_i1_orf1	-	-	-	golgin subfamily B member 1-like [Ostrinia furnacalis]	1.44941	0.33191	-0.15203	-1.66277	0.03349
TRINITY_DN17394_c0_g1_i1_orf1	-	-	-	monocarboxylate transporter 14-like [Ostrinia furnacalis]	1.70966	0.43498	-0.24684	-1.06315	-0.83465
TRINITY_DN9164_c0_g1_i3_orf1	-	-	-	unnamed protein product [Parnassius apollo]	1.78025	-0.12464	0.18306	-1.13299	-0.70569
TRINITY_DN27_c0_g1_i1_orf1	-	-	-	THO complex subunit 4-A [Ostrinia furnacalis]	1.74515	-0.24011	0.34532	-1.10631	-0.74405
TRINITY_DN4116_c0_g1_i3_orf1	-	-	-	transmembrane protein 131 homolog [Ostrinia furnacalis]	1.4114	0.2696	0.49505	-1.48964	-0.68641
TRINITY_DN144956_c0_g1_i1_orf1	-	-	-	40S ribosomal protein S18 [Cotesia glomerata] >CAD6216330.1 GSCOCG00004483001-RA-CDS [Cotesia congregata] >CAG5095266.1 Similar to RpS18: 40S ribosomal protein S18 (Spodoptera frugiperda) [Cotesia congregata]	1.85941	0.04915	-0.75095	-0.97011	-0.18749
TRINITY_DN5857_c0_g1_i13_orf1	-	-	-	uncharacterized protein LOC114353981 isoform X1 [Ostrinia furnacalis]	1.86415	0.06857	-0.88097	-0.83492	-0.21683
TRINITY_DN47575_c0_g1_i1_orf1	-	-	-	PREDICTED: splicing factor 1-like [Fopius arisanus]	1.70474	0.52559	-0.71555	-1.03875	-0.47604
TRINITY_DN26824_c0_g1_i1_orf1	-	-	-	60S ribosomal protein L6 [Hyposmocoma kahamanoa]	1.48416	0.05347	-0.45044	-1.54372	0.45653
TRINITY_DN17045_c0_g2_i3_orf1	-	-	-	unnamed protein product [Diatraea saccharalis]	1.84678	0.12187	-0.27247	-1.0239	-0.67227
TRINITY_DN5009_c0_g1_i2_orf1	-	-	-	GSCOCG00009487001-RA-CDS [Cotesia congregata] >CAG5088842.1 Similar to RpL18: 60S ribosomal protein L18 (Timarcha balearica) [Cotesia congregata]	1.70898	0.42474	-0.62917	-1.18314	-0.32142

TRINITY_DN2885_c1_g1_i2_orf1	-	-	-	ubiquitin-like-specific protease ESD4 [Ostrinia furnacalis]	1.37053	-0.05359	0.8457	-1.26163	-0.90102
TRINITY_DN18396_c0_g1_i1_orf1	-	-	-	uncharacterized protein LOC114359424 [Ostrinia furnacalis]	1.44257	0.62547	-1.51312	-0.48273	-0.07218
TRINITY_DN53462_c0_g1_i1_orf1	-	-	-	uncharacterized protein LOC118072968 isoform X1 [Chelonus insularis] >XP_034949073.1 uncharacterized protein LOC118072968 isoform X1 [Chelonus insularis]	1.4066	0.88802	-1.23373	-0.80254	-0.25835
TRINITY_DN18242_c0_g1_i3_orf1	-	-	-	CCHC-type zinc finger protein CG3800 [Papilio xuthus]	1.70898	0.1297	0.17094	-1.08865	-0.92097
TRINITY_DN11820_c0_g1_i1_orf1	-	-	-	hypothetical protein evm_000341 [Chilo suppressalis]	1.2244	-0.12425	0.89	-1.59213	-0.39803
TRINITY_DN22572_c0_g1_i1_orf1	-	-	-	eukaryotic translation elongation factor 1 epsilon-1 [Ostrinia furnacalis]	1.6022	-0.26063	0.1913	-1.5259	-0.00698
TRINITY_DN42646_c0_g2_i1_orf1	-	-	-	40S ribosomal protein S3 [Helicoverpa armigera] >XP_026740562.1 40S ribosomal protein S3 [Trichoplusia ni] >XP_026751545.1 40S ribosomal protein S3 [Galleria mellonella] >XP_047027704.1 40S ribosomal protein S3 [Helicoverpa zea] >CAH0591481.1 unnamed protein product [Chrysodeixis includens] >AIR07416.1 ribosomal protein S3 [Helicoverpa armigera] >AND95944.1 ribosomal protein S3 [Helicoverpa armigera] >AXY94820.1 ribosomal protein S3 [Galleria mellonella] >PZC80336.1 hypothetical protein B5X24_HaOG214853 [Helicoverpa armigera]	1.80977	0.18031	-0.92257	-0.90206	-0.16545
TRINITY_DN51568_c0_g1_i1_orf1	-	-	-	splicing factor 3A subunit 2 [Ostrinia furnacalis]	1.87945	-0.1037	-0.15273	-1.05284	-0.57018
TRINITY_DN61222_c0_g1_i1_orf1	-	-	-	60S ribosomal protein L38 [Bicyclus anynana]	1.88546	0.08556	-0.48925	-0.9651	-0.51667
TRINITY_DN4237_c1_g1_i5_orf1	-	-	-	eukaryotic translation initiation factor 3 subunit A-like isoform X1 [Ostrinia furnacalis] >XP_028173593.1 eukaryotic translation initiation factor 3 subunit A-like isoform X2 [Ostrinia furnacalis] >XP_028173594.1 eukaryotic translation initiation factor 3 subunit A-like isoform X3 [Ostrinia furnacalis] >XP_028173595.1 eukaryotic translation initiation factor 3 subunit A-like isoform X4 [Ostrinia furnacalis]	1.88345	-0.03325	-0.24629	-1.03064	-0.57328
TRINITY_DN23167_c0_g1_i4_orf1	-	-	-	uncharacterized protein LOC114363065 [Ostrinia furnacalis]	1.54021	0.64999	-1.32769	-0.61907	-0.24345
TRINITY_DN79734_c0_g2_i3_orf1	-	-	-	60S ribosomal protein L27a [Ostrinia furnacalis]	1.83283	-0.01754	-0.70563	-1.06807	-0.04159
TRINITY_DN6235_c0_g1_i5_orf1	-	-	-	rRNA 2'-O-methyltransferase fibrillarin [Vanessa cardui]	1.92191	0.04101	-0.56421	-0.76278	-0.63594
TRINITY_DN44557_c0_g1_i4_orf1	-	-	-	serine hydrolase-like protein [Ostrinia furnacalis]	1.80962	0.2298	-0.91526	-0.88059	-0.24356
TRINITY_DN20499_c0_g3_i1_orf1	-	-	-	exosome RNA helicase MTR4 isoform X2 [Ostrinia furnacalis]	1.85433	-0.11497	-0.00448	-1.01461	-0.72027
TRINITY_DN19187_c0_g1_i1_orf1	-	-	-	fumarylacetoacetase [Chelonus insularis]	1.80233	-0.16577	-0.12555	-1.28786	-0.22315
TRINITY_DN4262_c0_g1_i16_orf1	-	-	-	sperm-associated antigen 7 homolog [Ostrinia furnacalis]	1.39662	-0.14406	0.78959	-1.4211	-0.62106
TRINITY_DN4820_c0_g1_i1_orf1	-	-	-	tudor and KH domain-containing protein homolog [Galleria mellonella]	1.9005	0.04054	-0.61415	-0.91737	-0.40951
TRINITY_DN21971_c0_g1_i4_orf1	-	-	-	40S ribosomal protein S26 [Nymphalis io]	1.81736	3.58E-06	-0.11689	-1.195	-0.50548
TRINITY_DN36788_c0_g1_i2_orf1	-	-	-	isocitrate dehydrogenase [NADP] cytoplasmic-like [Bicyclus anynana]	1.72924	-0.14679	0.3014	-1.18979	-0.69406
TRINITY_DN2769_c0_g1_i1_orf1	-	-	-	pseudouridylate synthase 7 homolog [Ostrinia furnacalis]	1.80583	-0.2498	0.22357	-1.03662	-0.74298
TRINITY_DN51968_c0_g1_i1_orf1	-	-	-	splicing factor U2af 38 kDa subunit [Aphidius gifuensis] >KAF7990547.1 hypothetical protein HCN44_000352 [Aphidius gifuensis]	1.49887	0.1563	0.53877	-1.22345	-0.97049
TRINITY_DN8949_c0_g1_i2_orf1	-	-	-	unnamed protein product [Arctia plantaginis]	1.81594	0.14056	-0.61842	-1.119	-0.21908
TRINITY_DN104507_c0_g1_i2_orf1	-	-	-	replication protein A 32 kDa subunit [Ostrinia furnacalis]	1.40976	-0.16798	0.80831	-1.36383	-0.68626
TRINITY_DN101682_c0_g1_i1_orf1	-	-	-	cysteine-rich with EGF-like domain protein 2 [Ostrinia furnacalis]	1.65369	0.18865	-0.4215	-1.43246	0.01162
TRINITY_DN3893_c0_g2_i3_orf1	-	-	-	cleavage and polyadenylation specificity factor subunit CG7185 isoform X2 [Ostrinia furnacalis]	1.44358	-0.20876	0.79885	-1.30531	-0.72835
TRINITY_DN4938_c0_g1_i13_orf1	-	-	-	peroxisomal biogenesis factor 19 [Ostrinia furnacalis]	1.82356	0.28426	-0.97127	-0.5204	-0.61615
TRINITY_DN48619_c0_g1_i1_orf1	-	-	-	PREDICTED: lysine--tRNA ligase isoform X2 [Fopius arisanus]	1.73373	0.31167	-1.03907	-0.89752	-0.10882
TRINITY_DN121893_c0_g1_i1_orf1	-	-	-	hypothetical protein, partial [Ostrinia furnacalis]	1.60456	0.31197	-0.62747	-1.38734	0.09828
TRINITY_DN137_c0_g1_i1_orf1	-	-	-	60S ribosomal protein L8 [Cotesia glomerata] >XP_044597650.1 60S ribosomal protein L8 [Cotesia glomerata] >KAG8034499.1 hypothetical protein G9C98_007575 [Cotesia typhae] >CAD6216378.1 GSCOCG00004534001-RA-CDS [Cotesia congregata] >KAH0544237.1 60S ribosomal protein L8 [Cotesia glomerata] >KAH0564528.1 60S ribosomal protein L8 [Cotesia glomerata] >CAG5095185.1 Similar to RpL8: 60S ribosomal protein L8 (Spodoptera frugiperda) [Cotesia congregata]	1.79823	0.35974	-0.58898	-0.95652	-0.61247
TRINITY_DN33_c0_g1_i1_orf1	-	-	-	uncharacterized protein CG45076-like isoform X2 [Ostrinia furnacalis]	1.80667	0.0952	-0.75248	-1.07475	-0.07464
TRINITY_DN33801_c0_g1_i1_orf1	-	-	-	unnamed protein product [Diatraea saccharalis]	1.79402	-0.34335	0.24141	-1.14078	-0.5513
TRINITY_DN7574_c0_g1_i10_orf1	-	-	-	proteasome activator complex subunit 3 isoform X2 [Ostrinia furnacalis]	1.74949	0.08871	0.0671	-1.18911	-0.71619
TRINITY_DN1504_c0_g1_i1_orf1	-	-	-	uncharacterized protein LOC114352862 [Ostrinia furnacalis] >XP_028160407.1 uncharacterized protein LOC114352862 [Ostrinia furnacalis]	0.9835	-0.19569	1.27102	-1.38966	-0.66917
TRINITY_DN2089_c0_g1_i5_orf1	-	-	-	eukaryotic translation initiation factor 4B [Ostrinia furnacalis]	1.70868	0.02568	0.24467	-1.16501	-0.81403
TRINITY_DN21357_c0_g1_i5_orf1	-	-	-	40S ribosomal protein S11 isoform X2 [Ostrinia furnacalis]	1.82677	0.1263	-0.78524	-1.00132	-0.1665

TRINITY_DN4135_c0_g1_i5_orf1	-	-	-	probable small nuclear ribonucleoprotein Sm D2 [Manduca sexta] >KAG6451233.1 hypothetical protein O3G_MSEX007016 [Manduca sexta]	1.86562	-0.20531	-0.05941	-1.11056	-0.49034
TRINITY_DN5841_c0_g1_i2_orf1	-	-	-	hypothetical protein evm_011295 [Chilo suppressalis]	1.46851	-0.427	0.7493	-1.3935	-0.3973
TRINITY_DN42082_c0_g2_i2_orfp1	-	-	-	TRINITY_DN42082_c0_g2_i2_m.7835 TRINITY_DN42082_c0_g2::TRINITY_DN42082_c0_g2_i2::g.7835 ORF type:internal len:133	1.80352	0.22857	-0.62243	-1.1011	-0.30856
TRINITY_DN21609_c0_g2_i1_orf1	-	-	-	translation initiation factor eIF-2B subunit epsilon [Ostrinia furnacalis]	1.7761	-0.10217	0.06388	-1.26949	-0.46832
TRINITY_DN3649_c0_g1_i6_orf1	-	-	-	unnamed protein product [Chilo suppressalis]	1.18088	-0.43777	0.79296	-1.66395	0.12788
TRINITY_DN21035_c0_g1_i14_orf1	-	-	-	mitochondrial amidoxime reducing component 2-like [Ostrinia furnacalis]	1.13268	0.87333	-1.16628	-1.20776	0.36803
TRINITY_DN17271_c0_g1_i1_orf1	-	-	-	uncharacterized protein LOC114350693 [Ostrinia furnacalis]	1.23102	-0.38453	1.14092	-1.1671	-0.8203
TRINITY_DN32022_c0_g1_i1_orf1	-	-	-	striatin isoform X1 [Diachasma alloeum]	1.73489	0.03713	0.14115	-1.21998	-0.69319
TRINITY_DN4025_c0_g1_i13_orf1	-	-	-	tetratricopeptide repeat protein 14 homolog isoform X2 [Ostrinia furnacalis]	1.60115	0.12725	0.09401	-1.52386	-0.29855
TRINITY_DN5275_c0_g1_i1_orf1	-	-	-	paraplegin [Ostrinia furnacalis]	1.65307	-0.21822	0.49639	-1.19855	-0.73269
TRINITY_DN2716_c0_g2_i1_orf1	-	-	-	eukaryotic translation initiation factor 1A, X-chromosomal [Ostrinia furnacalis] >XP_045445466.1 eukaryotic translation initiation factor 1A, X-chromosomal [Melitaea cinxia] >XP_049867692.1 eukaryotic translation initiation factor 1A, X-chromosomal [Pectinophora gossypiella] >KOB79530.1 Eukaryotic translation initiation factor 1A [Operophtera brumata] >CAH2086435.1 unnamed protein product [Euphydryas editha] >KOB79531.1 Eukaryotic translation initiation factor 1A [Operophtera brumata]	1.77574	0.00396	0.03682	-1.22091	-0.59561
TRINITY_DN33248_c0_g1_i1_orf1	-	-	-	elongation factor Ts, mitochondrial isoform X3 [Ostrinia furnacalis] >XP_028155866.1 elongation factor Ts, mitochondrial isoform X3 [Ostrinia furnacalis]	1.91583	-0.04009	-0.6428	-0.89463	-0.33831
TRINITY_DN4929_c1_g2_i5_orf1	-	-	-	guanylate kinase isoform X2 [Ostrinia furnacalis]	1.65908	0.50607	-1.20038	-0.68858	-0.2762
TRINITY_DN2718_c0_g1_i6_orf1	-	-	-	cleavage stimulation factor subunit 2 isoform X1 [Ostrinia furnacalis]	1.44914	-0.3792	0.89166	-1.11714	-0.84447
TRINITY_DN631_c0_g1_i6_orf1	-	-	-	cytosolic 10-formyltetrahydrofolate dehydrogenase isoform X1 [Ostrinia furnacalis] >XP_028172896.1 cytosolic 10-formyltetrahydrofolate dehydrogenase isoform X2 [Ostrinia furnacalis]	1.91403	-0.10477	-0.65059	-0.91919	-0.23949
TRINITY_DN4121_c0_g1_i1_orf1	-	-	-	uncharacterized protein LOC114358001, partial [Ostrinia furnacalis]	1.90952	0.03268	-0.87462	-0.62825	-0.43934
TRINITY_DN5697_c0_g1_i1_orf1	-	-	-	GPI ethanolamine phosphate transferase 2-like [Ostrinia furnacalis]	1.63305	-0.16938	0.54176	-0.9966	-1.00883
TRINITY_DN123139_c0_g1_i1_orfp1	-	-	-	TRINITY_DN123139_c0_g1_i1_m.79879 TRINITY_DN123139_c0_g1::TRINITY_DN123139_c0_g1_i1::g.79879 ORF type:3prime_partial len:76 (+),score=3.83 TRINITY_DN123139_c0_g1_i1:25-225(+)	1.86415	0.07227	-0.35681	-1.0589	-0.5207
TRINITY_DN478_c0_g1_i16_orf1	-	-	-	lipid storage droplets surface-binding protein 2 isoform X1 [Ostrinia furnacalis]	1.75638	0.12968	-0.03412	-0.62406	-1.22788
TRINITY_DN58207_c0_g1_i1_orf1	-	-	-	60S ribosomal protein L6 [Ostrinia furnacalis] >XP_028170357.1 60S ribosomal protein L6 [Ostrinia furnacalis]	1.82444	0.19242	-0.95206	-0.81619	-0.24861
TRINITY_DN7112_c0_g1_i1_orf1	-	-	-	heterogeneous nuclear ribonucleoprotein K isoform X2 [Ostrinia furnacalis]	1.44205	0.10254	0.65261	-1.28599	-0.91121
TRINITY_DN84322_c0_g2_i1_orf1	-	-	-	alanyl-tRNA synthetase 1 [Homo sapiens] >KAI4055846.1 alanyl-tRNA synthetase 1 [Homo sapiens]	1.82043	0.10028	-0.22937	-1.15634	-0.535
TRINITY_DN24142_c0_g1_i1_orf1	-	-	-	arylalkylamine N-acetyltransferase [Chilo suppressalis]	1.81165	0.05417	-0.83022	-1.01252	-0.02307
TRINITY_DN30131_c0_g1_i1_orf1	-	-	-	PREDICTED: 60S ribosomal protein L44 [Amyelois transitella] >XP_021198018.1 60S ribosomal protein L44 [Helicoverpa armigera] >XP_022814294.1 60S ribosomal protein L44 [Spodoptera litura] >XP_026732397.1 60S ribosomal protein L44 [Trichoplusia ni] >XP_026752106.1 60S ribosomal protein L44 [Galleria mellonella] >XP_028158932.1 60S ribosomal protein L44 [Ostrinia furnacalis] >XP_035434364.1 60S ribosomal protein L44 [Spodoptera frugiperda] >XP_035434370.1 60S ribosomal protein L44 [Spodoptera frugiperda] >XP_047019234.1 60S ribosomal protein L44 [Helicoverpa zea] >XP_049868501.1 60S ribosomal protein L44 [Pectinophora gossypiella] >AAM53948.1 ribosomal protein L44 [Choristoneura parallela] >KAF9418375.1 hypothetical protein HW555_004805 [Spodoptera exigua] >RVE50750.1 hypothetical protein evm_004660 [Chilo suppressalis] >CAB3235328.1 unnamed protein product [Arctia plantaginis] >CAB3516516.1 unnamed protein product [Spodoptera littoralis] >CAG9747186.1 unnamed protein product [Diatraea saccharalis] >CAH0581656.1 unnamed protein product [Chrysodeixis includens]	1.82559	0.06221	-0.99822	-0.81305	-0.07653
TRINITY_DN31327_c0_g2_i1_orf1	-	-	-	multidrug resistance protein 1A isoform X1 [Ostrinia furnacalis]	1.90275	-0.31825	-0.34459	-1.06201	-0.1779

TRINITY_DN144258_c0_g1_i1_orf1	-	-	-	PREDICTED: enhancer of rudimentary homolog [Microplitis demolitor] >XP_044577051.1 enhancer of rudimentary homolog [Cotesia glomerata] >KAG8041963.1 hypothetical protein G9C98_007267 [Cotesia typhae] >KAH0539785.1 hypothetical protein KQX54_008036 [Cotesia glomerata] >CAD6227368.1 GSCOCG00006137001-RA-CDS [Cotesia congregata]	1.12581	0.08076	1.04677	-1.34058	-0.91277
TRINITY_DN50085_c0_g1_i1_orf1	-	-	-	hypothetical protein evm_013997 [Chilo suppressalis]	1.86999	-0.26656	0.07178	-0.94665	-0.72856
TRINITY_DN4345_c0_g1_i9_orf1	-	-	-	uncharacterized protein LOC114357127 [Ostrinia furnacalis]	1.49897	0.42598	0.20499	-1.42642	-0.70351
TRINITY_DN9853_c0_g3_i1_orf1	-	-	-	importin-7 isoform X1 [Ostrinia furnacalis]	1.93457	-0.11414	-0.36623	-0.88981	-0.56439
TRINITY_DN16349_c0_g1_i10_orf1	-	-	-	protein lingerer-like isoform X1 [Nymphalis io] >XP_050356663.1 protein lingerer-like isoform X1 [Nymphalis io] >XP_050356664.1 protein lingerer-like isoform X1 [Nymphalis io]	1.55748	-0.45391	0.74044	-1.16672	-0.67729
TRINITY_DN106476_c0_g1_i3_orf1	-	-	-	mitochondrial import inner membrane translocase subunit TIM44 [Ostrinia furnacalis]	1.93872	-0.155	-0.63078	-0.85471	-0.29823
TRINITY_DN16965_c0_g2_i1_orf1	-	-	-	hypothetical protein evm_007405 [Chilo suppressalis]	1.62034	-0.50615	0.57414	-1.27047	-0.41786
TRINITY_DN45924_c0_g1_i14_orf1	-	-	-	adenylosuccinate synthetase isoform X1 [Ostrinia furnacalis] >XP_028166048.1 adenylosuccinate synthetase isoform X2 [Ostrinia furnacalis]	1.92542	-0.08204	-0.35488	-0.90587	-0.58264
TRINITY_DN9002_c0_g1_i1_orf1	-	-	-	membrane magnesium transporter 1 [Ostrinia furnacalis]	1.89276	0.07853	-0.53017	-0.93479	-0.50633
TRINITY_DN220_c0_g1_i3_orf1	-	-	-	serine-arginine protein 55 isoform X6 [Pieris brassicae]	1.52846	-0.12835	0.67164	-1.19421	-0.87755
TRINITY_DN32306_c0_g1_i3_orf1	-	-	-	acetyl-coenzyme A transporter 1 [Ostrinia furnacalis]	1.70732	0.10981	-0.05704	-1.39033	-0.36975
TRINITY_DN100821_c0_g1_i1_orf1	-	-	-	putative GMP synthase, partial [Operophtera brumata]	1.86537	0.07477	-0.45413	-1.06273	-0.42328
TRINITY_DN23926_c0_g1_i4_orf1	-	-	-	programmed cell death protein 10 [Ostrinia furnacalis]	1.46054	-0.27154	0.6023	-1.53827	-0.25303
TRINITY_DN19814_c0_g1_i4_orf1	-	-	-	general odorant-binding protein 28a-like [Ostrinia furnacalis]	1.59117	0.43731	-1.30764	-0.75236	0.03152
TRINITY_DN2807_c0_g1_i4_orf1	-	-	-	FK506-binding protein 59 isoform X1 [Ostrinia furnacalis]	1.86907	-0.21559	0.04397	-0.94224	-0.75521
TRINITY_DN2117_c0_g1_i1_orf1	-	-	-	BUB3-interacting and GLEBS motif-containing protein ZNF207 [Chelonus insularis]	1.75429	-0.17592	0.30715	-1.04175	-0.84376
TRINITY_DN21150_c0_g1_i4_orf1	-	-	-	RNA-binding protein cabeza-like isoform X2 [Bicyclus anynana]	1.7751	-0.23341	0.25961	-1.12957	-0.67173
TRINITY_DN3614_c0_g2_i1_orf1	-	-	-	PC4 and SFRS1-interacting protein isoform X4 [Galleria mellonella]	1.17881	0.32627	0.83727	-1.34388	-0.99847
TRINITY_DN14235_c0_g1_i1_orf1	-	-	-	maltase A1 [Helicoverpa armigera]	1.91931	0.05788	-0.64282	-0.73578	-0.59859
TRINITY_DN620_c0_g1_i4_orf1	-	-	-	lysine--tRNA ligase isoform X1 [Ostrinia furnacalis]	1.92092	-0.07619	-0.63754	-0.89439	-0.3128
TRINITY_DN43412_c0_g1_i2_orf1	-	-	-	U1 small nuclear ribonucleoprotein C [Ostrinia furnacalis]	1.54844	0.00847	0.54035	-1.28428	-0.81297
TRINITY_DN13055_c0_g1_i5_orf1	-	-	-	116 kDa U5 small nuclear ribonucleoprotein component isoform X1 [Ostrinia furnacalis] >XP_028159219.1 116 kDa U5 small nuclear ribonucleoprotein component isoform X2 [Ostrinia furnacalis]	1.70039	-0.06178	0.33338	-1.14305	-0.82895
TRINITY_DN313_c0_g1_i5_orf1	-	-	-	collagen alpha-1(X) chain-like [Ostrinia furnacalis]	1.71349	0.07511	-1.31143	-0.57371	0.09654
TRINITY_DN8536_c0_g1_i2_orf1	-	-	-	PC4 and SFRS1-interacting protein isoform X4 [Galleria mellonella]	1.52606	-0.34626	0.71648	-1.29451	-0.60178
TRINITY_DN8971_c1_g1_i4_orf1	-	-	-	synaptosomal-associated protein 25 isoform X1 [Bombyx mori]	1.64573	-0.34888	0.33507	-1.41846	-0.21346
TRINITY_DN142652_c0_g1_i1_orf1	-	-	-	pre-mRNA-splicing factor RBM22 [Chelonus insularis]	1.70224	-0.03772	0.12835	-1.38494	-0.40794
TRINITY_DN10662_c0_g1_i4_orf1	-	-	-	HD domain-containing protein 2 [Ostrinia furnacalis]	1.77121	-0.49467	0.32943	-1.13465	-0.47132
TRINITY_DN29934_c0_g1_i6_orf1	-	-	-	sodium/potassium-transporting ATPase subunit beta-2-like [Ostrinia furnacalis] >XP_028176258.1 sodium/potassium-transporting ATPase subunit beta-2-like [Ostrinia	1.5612	0.32009	0.29549	-1.13126	-1.04552
TRINITY_DN47123_c0_g1_i1_orf1	-	-	-	WD40 repeat-containing protein SMU1 [Ostrinia furnacalis]	1.87622	-0.21806	0.02523	-0.92746	-0.75593
TRINITY_DN3053_c0_g1_i2_orf1	-	-	-	prostaglandin reductase 1-like [Ostrinia furnacalis]	1.9485	-0.1325	-0.73149	-0.71924	-0.36526
TRINITY_DN1427_c0_g1_i9_orf1	-	-	-	SAFB-like transcription modulator isoform X3 [Ostrinia furnacalis]	1.59647	0.15764	0.3578	-1.24079	-0.87112
TRINITY_DN15607_c0_g1_i6_orf1	-	-	-	protein artichoke-like [Ostrinia furnacalis]	1.92895	-0.0083	-0.48904	-0.80296	-0.62865
TRINITY_DN3428_c0_g1_i1_orf1	-	-	-	10 kDa heat shock protein, mitochondrial [Ostrinia furnacalis]	1.92255	-0.24928	-0.12003	-0.87906	-0.67419
TRINITY_DN1557_c0_g1_i9_orf1	-	-	-	carboxylesterase CXE18 [Ostrinia furnacalis]	1.91218	0.02286	-0.86369	-0.64338	-0.42796
TRINITY_DN2062_c0_g1_i9_orf1	-	-	-	uncharacterized protein LOC114350846 [Ostrinia furnacalis]	1.79554	-0.39708	0.17204	-1.20687	-0.36364
TRINITY_DN6231_c0_g1_i6_orf1	-	-	-	ran-binding protein 3 isoform X1 [Ostrinia furnacalis] >XP_028166372.1 ran-binding protein 3 isoform X2 [Ostrinia furnacalis]	1.83661	-0.23165	0.06153	-1.13393	-0.53256
TRINITY_DN1239_c0_g1_i3_orf1	-	-	-	uncharacterized protein LOC114355269 [Ostrinia furnacalis] >XP_028163822.1 uncharacterized protein LOC114355269 [Ostrinia furnacalis]	1.89951	-0.18182	-0.65318	-0.95979	-0.10472
TRINITY_DN69236_c0_g1_i1_orf1	-	-	-	peroxiredoxin [Ostrinia furnacalis]	1.46662	-0.5604	0.75485	-1.37164	-0.28943
TRINITY_DN4036_c0_g2_i1_orf1	-	-	-	microvitellogenin-like [Ostrinia furnacalis]	1.65042	-0.02045	-0.35704	-1.4544	0.18147
TRINITY_DN32479_c0_g1_i8_orf1	-	-	-	hypothetical protein evm_009815 [Chilo suppressalis] >CAB3525305.1 unnamed protein product [Chilo suppressalis] >CAH0402632.1 unnamed protein product [Chilo suppressalis]	1.7466	-0.0078	-1.28464	-0.54004	0.08587
TRINITY_DN1706_c0_g1_i7_orf1	-	-	-	LOW QUALITY PROTEIN: RNA polymerase-associated protein CTR9 homolog [Ostrinia	1.72482	-0.33929	0.39813	-1.17527	-0.60839
TRINITY_DN24399_c0_g1_i1_orf1	-	-	-	retinol-binding protein pinta-like [Ostrinia furnacalis]	1.87922	0.14563	-0.76217	-0.4453	-0.81738
TRINITY_DN2852_c0_g1_i9_orf1	-	-	-	golgin subfamily A member 4-like [Ostrinia furnacalis]	1.93839	-0.26489	-0.18613	-0.86959	-0.61778

TRINITY_DN7336_c0_g1_i13_orf1	-	-	-	PREDICTED: calcium-transporting ATPase sarcoplasmic/endoplasmic reticulum type isoform X2 [Amyelois transitella]	1.87001	0.0049	-1.07845	-0.50508	-0.29138
TRINITY_DN52296_c0_g1_i6_orf1	-	-	-	protein takeout-like [Ostrinia furnacalis]	1.83707	0.18238	-0.34257	-1.02364	-0.65324
TRINITY_DN1285_c0_g1_i6_orf1	-	-	-	bifunctional 3'-phosphoadenosine 5'-phosphosulfate synthase isoform X3 [Ostrinia furnacalis]	1.9501	-0.2558	-0.32725	-0.89588	-0.47116
TRINITY_DN430_c0_g1_i5_orf1	-	-	-	hypothetical protein NE865_02252 [Phthorimaea operculella]	1.89012	-0.06272	-0.54505	-1.03118	-0.25118
TRINITY_DN78546_c0_g5_i1_orf1	-	-	-	kinesin-like protein KIF13A isoform X9 [Cephus cinctus]	1.69459	0.16859	-0.11528	-1.40265	-0.34526
TRINITY_DN37699_c0_g1_i4_orfp1	-	-	-	TRINITY_DN37699_c0_g1_i4_m.58777 TRINITY_DN37699_c0_g1::TRINITY_DN37699_c0_g1_i4::g.58777 ORF type:internal len:122 (+),score=34.90 TRINITY_DN37699_c0_g1_i4:1-363(+)	1.72563	0.32975	-0.82117	-1.10573	-0.12849
TRINITY_DN4550_c1_g1_i5_orfp2	-	-	-	TRINITY_DN4550_c1_g1_i5_m.14710 TRINITY_DN4550_c1_g1::TRINITY_DN4550_c1_g1_i5::g.14710 ORF type:5prime_partial len:168	1.93484	-0.06251	-0.74985	-0.73475	-0.38774
TRINITY_DN2894_c0_g2_i3_orf1	-	-	-	myrosinase 1-like isoform X1 [Ostrinia furnacalis]	1.21584	0.08127	0.93523	-1.38895	-0.84339
TRINITY_DN279_c0_g1_i10_orf1	-	-	-	RE1-silencing transcription factor-like isoform X1 [Ostrinia furnacalis]	1.10248	-0.41282	1.25413	-1.24766	-0.69614
TRINITY_DN6685_c0_g1_i8_orf1	-	-	-	cleft lip and palate transmembrane protein 1 homolog [Ostrinia furnacalis]	1.85157	-0.20273	0.08722	-0.95762	-0.77844
TRINITY_DN31047_c0_g1_i4_orf1	-	-	-	4-coumarate--CoA ligase 1-like isoform X1 [Ostrinia furnacalis] >XP_028160248.1 4-coumarate--CoA ligase 1-like isoform X1 [Ostrinia furnacalis] >XP_028160249.1 4-coumarate--CoA ligase 1-like isoform X1 [Ostrinia furnacalis] >XP_028160250.1 4-coumarate--CoA ligase 1-like isoform X1 [Ostrinia furnacalis] >XP_028160251.1 4-coumarate--CoA ligase 1-like isoform X1 [Ostrinia furnacalis] >XP_028160253.1 4-coumarate--CoA ligase 1-like isoform X2	1.93642	-0.02886	-0.73072	-0.69565	-0.48119
TRINITY_DN24873_c0_g1_i4_orf1	-	-	-	uncharacterized protein LOC114365742 [Ostrinia furnacalis]	1.94187	-0.22662	-0.74574	-0.76016	-0.20934
TRINITY_DN8543_c0_g1_i1_orf1	-	-	-	39S ribosomal protein L38, mitochondrial [Ostrinia furnacalis]	1.87421	0.13584	-0.42322	-0.91741	-0.66942
TRINITY_DN7073_c0_g1_i1_orf1	-	-	-	unnamed protein product, partial [Brenthis ino]	1.95935	-0.20459	-0.67566	-0.73997	-0.33913
TRINITY_DN10332_c0_g1_i2_orfp1	-	-	-	TRINITY_DN10332_c0_g1_i2_m.42894 TRINITY_DN10332_c0_g1::TRINITY_DN10332_c0_g1_i2::g.42894 ORF type:3prime_partial len:77 (+),score=1.70 TRINITY_DN10332_c0_g1_i2:1005-1232(+)	1.80891	-0.01375	-0.01195	-1.15388	-0.62934
TRINITY_DN753_c0_g1_i4_orf1	-	-	-	venom dipeptidyl peptidase 4-like isoform X2 [Ostrinia furnacalis]	1.93819	-0.41126	-0.2915	-0.954	-0.28143
TRINITY_DN2425_c0_g1_i1_orf1	-	-	-	thyroid receptor-interacting protein 11 [Ostrinia furnacalis]	1.6933	0.17292	-0.74073	-1.24127	0.11578
TRINITY_DN30233_c0_g1_i2_orf1	-	-	-	39S ribosomal protein L10, mitochondrial [Ostrinia furnacalis]	1.91141	-0.47065	-0.20749	-1.01755	-0.21572
TRINITY_DN3929_c0_g3_i3_orf1	-	-	-	Glutathione S-transferase 1, isoform D [Papilio machaon]	1.77881	-0.31473	-1.05174	-0.72848	0.31614
TRINITY_DN7213_c0_g1_i2_orf1	-	-	-	probable ATP-dependent RNA helicase CG8611 [Ostrinia furnacalis]	1.96319	-0.26313	-0.30132	-0.76099	-0.63775
TRINITY_DN3759_c0_g1_i1_orf1	-	-	-	uncharacterized protein LOC114350416 [Ostrinia furnacalis] >XP_028157016.1 uncharacterized protein LOC114350416 [Ostrinia furnacalis] >XP_028157017.1 uncharacterized protein LOC114350416 [Ostrinia furnacalis] >XP_028157018.1 uncharacterized protein LOC114350416 [Ostrinia furnacalis] >XP_028157019.1 uncharacterized protein LOC114350416 [Ostrinia	1.50072	-0.65993	0.8523	-1.12281	-0.57028
TRINITY_DN9003_c0_g1_i20_orf1	-	-	-	RNA-binding protein Nova-2 isoform X4 [Ostrinia furnacalis]	1.90354	0.05316	-0.47193	-0.89848	-0.58629
TRINITY_DN76377_c0_g1_i1_orf1	-	-	-	uncharacterized protein LOC111357764, partial [Spodoptera litura]	1.19673	-0.73364	1.11184	-1.3134	-0.26153
TRINITY_DN22664_c0_g1_i1_orf1	-	-	-	larval cuticle protein LCP-14-like [Ostrinia furnacalis]	1.62823	0.44976	-1.2535	-0.75536	-0.06913
TRINITY_DN50787_c0_g2_i2_orf1	-	-	-	40S ribosomal protein S29 [Hyposmocoma kahamanoa] >XP_028176503.1 40S ribosomal protein S29 [Ostrinia furnacalis] >XP_049877832.1 40S ribosomal protein S29 [Pectinophora gossypiella] >ADT80654.1 ribosomal protein S29 [Euphydryas aurinia] >CAB3523209.1 unnamed protein product [Chilo suppressalis] >CAH0400531.1 unnamed protein product [Chilo	1.80174	0.09091	-0.4331	-1.22613	-0.23342
TRINITY_DN6747_c0_g1_i7_orf1	-	-	-	retinol dehydrogenase 12-like [Ostrinia furnacalis]	1.92317	-0.09468	-0.30232	-0.89806	-0.62812
TRINITY_DN9464_c0_g1_i1_orf1	-	-	-	angio-associated migratory cell protein [Ostrinia furnacalis] >XP_028162594.1 angio-associated migratory cell protein [Ostrinia furnacalis]	1.96536	-0.27381	-0.63284	-0.75462	-0.30408
TRINITY_DN3733_c0_g1_i1_orf1	-	-	-	60S ribosomal protein L37, partial [Papilio machaon]	1.81846	-0.32054	-0.20651	-1.24317	-0.04825
TRINITY_DN37699_c0_g1_i3_orfp1	-	-	-	TRINITY_DN37699_c0_g1_i3_m.58788 TRINITY_DN37699_c0_g1::TRINITY_DN37699_c0_g1_i3::g.58788 ORF type:internal len:122 (+),score=39.86 TRINITY_DN37699_c0_g1_i3:1-363(+)	1.76243	0.38519	-1.08264	-0.58841	-0.47658
TRINITY_DN3062_c0_g1_i1_orf1	-	-	-	HEAT repeat-containing protein 1 [Ostrinia furnacalis]	1.9158	-0.36161	-0.0688	-0.95602	-0.52938
TRINITY_DN139212_c0_g1_i4_orf1	-	-	-	uncharacterized protein LOC114350112 [Ostrinia furnacalis]	1.52551	-0.03795	0.63873	-1.10087	-1.02542
TRINITY_DN5238_c0_g1_i2_orf1	-	-	-	DNA-(apurinic or apyrimidinic site) lyase [Ostrinia furnacalis]	1.7357	-0.23066	0.33526	-1.17353	-0.66677
TRINITY_DN28592_c0_g1_i2_orf1	-	-	-	UDP-glucuronosyltransferase 2B14-like isoform X1 [Ostrinia furnacalis] >XP_028167291.1 UDP-glucuronosyltransferase 2B14-like isoform X2 [Ostrinia furnacalis]	1.94236	-0.46437	-0.161	-0.90183	-0.41517
TRINITY_DN16939_c0_g1_i4_orf1	-	-	-	39S ribosomal protein L17, mitochondrial [Ostrinia furnacalis]	1.98296	-0.28812	-0.52425	-0.69677	-0.47382

TRINITY_DN11069_c0_g2_i1_orf1	-	-	-	fat storage-inducing transmembrane protein [Ostrinia furnacalis]	1.92312	-0.28211	-0.24495	-1.00424	-0.39181
TRINITY_DN2986_c1_g1_i1_orf1	-	-	-	Troponin C, isoform 1 [Papilio xuthus]	1.64358	0.46241	-1.24861	-0.70999	-0.14738
TRINITY_DN47114_c0_g1_i5_orf1	-	-	-	nucleolar protein dao-5 isoform X2 [Ostrinia furnacalis]	1.94891	-0.19081	-0.46565	-0.88425	-0.4082
TRINITY_DN36494_c0_g1_i1_orf1	-	-	-	MKI67 FHA domain-interacting nucleolar phosphoprotein-like [Ostrinia furnacalis]	1.93828	-0.08124	-0.80786	-0.65389	-0.39529
TRINITY_DN8691_c0_g1_i3_orf1	-	-	-	nucleolin-like [Melitaea cinxia]	1.70085	-0.37775	0.51566	-0.85615	-0.98261
TRINITY_DN19244_c0_g1_i7_orf1	-	-	-	uncharacterized protein LOC114350218 [Ostrinia furnacalis]	1.73855	-0.23319	0.32602	-1.18005	-0.65134
TRINITY_DN1199_c0_g1_i1_orf1	-	-	-	pupal cuticle protein 36a-like [Ostrinia furnacalis]	1.96703	-0.19955	-0.67603	-0.68413	-0.40732
TRINITY_DN13651_c0_g1_i2_orf1	-	-	-	40S ribosomal protein S12, mitochondrial [Ostrinia furnacalis]	1.9257	-0.40308	-0.06556	-0.9066	-0.55045
TRINITY_DN75188_c0_g1_i1_orf1	-	-	-	fatty acid-binding protein 1-like [Ostrinia furnacalis]	1.94334	-0.38483	-0.1572	-0.88609	-0.51523
TRINITY_DN94355_c0_g1_i2_orf1	-	-	-	uncharacterized protein LOC126369488 [Pectinophora gossypiella]	1.88694	0.14341	-0.51477	-0.70593	-0.80966
TRINITY_DN84357_c0_g1_i1_orf1	-	-	-	4-coumarate--CoA ligase 1-like [Ostrinia furnacalis]	1.39544	0.72962	-1.30077	0.08216	-0.90645
