# Supplementary material for: Comparative Proteomics and Metabonomics Analysis of Different Diapause Stages Revealed a New Regulation Mechanism of Diapause in Loxostege sticticalis (Lepidoptera: Pyralidae)
Source: Molecules. 2024 Jul 25;29(15):3472. doi: 10.3390/molecules29153472 (PMC11314584; doi:10.3390/molecules29153472)
Supplement: Supplementary file 1 [file molecules-29-03472-s001.zip › analysis process/proteomic/Cluster analysis of expression patterns/Down/DvsND down.pdf]

Accession	Symbol	Protein Na	Entrez ID	Description	ND	RD	PreD	CT	D
TRINITY_DN2441_c0_g1_i1_orf1	-	-	-	protein RFT1 homolog [Ostrinia furnacalis]	1.712247	-0.0776	-0.45229	-1.35231	0.169951
TRINITY_DN91198_c0_g2_i1_orf1	-	-	-	unc-112-related protein-like [Pectinophora gossypiella]	1.590613	-0.18418	0.433104	-1.44726	-0.39227
TRINITY_DN139438_c0_g1_i1_orf1	-	-	-	T-complex protein 1 subunit delta [Ostrinia furnacalis]	1.895163	-0.17502	-0.70035	-0.93841	-0.08139
TRINITY_DN12787_c1_g1_i1_orf1	-	-	-	unnamed protein product [Parnassius apollo]	1.916476	-0.02722	-0.33146	-0.73919	-0.8186
TRINITY_DN6299_c0_g1_i1_orf1	-	-	-	death-inducer obliterator 1 isoform X2 [Ostrinia furnacalis]	1.782439	-0.66371	-0.76614	-0.7818	0.429208
TRINITY_DN11392_c0_g1_i4_orf1	-	-	-	chondroitin sulfate synthase 1 isoform X1 [Ostrinia furnacalis] >XP_028178735.1 chondroitin sulfate synthase 1 isoform X2 [Ostrinia furnacalis]	1.934738	-0.09079	-0.86761	-0.58611	-0.39023
TRINITY_DN11159_c0_g2_i1_orf1	-	-	-	sphingosine-1-phosphate lyase isoform X1 [Ostrinia furnacalis]	1.960684	-0.2186	-0.4284	-0.48145	-0.83223
TRINITY_DN29707_c0_g1_i2_orf1	-	-	-	2-(3-amino-3-carboxypropyl)histidine synthase subunit 2 [Ostrinia furnacalis]	1.937884	-0.70593	-0.0481	-0.73889	-0.44497
TRINITY_DN56379_c0_g1_i1_orf1	-	-	-	alpha-tocopherol transfer protein-like [Ostrinia furnacalis]	1.697481	0.12299	-1.41337	-0.31067	-0.09643
TRINITY_DN30097_c0_g1_i2_orf1	-	-	-	unnamed protein product [Chilo suppressalis]	1.778883	0.228749	-0.74363	-1.09644	-0.16756
TRINITY_DN84_c0_g1_i4_orf1	-	-	-	aspartyl/asparaginyl beta-hydroxylase isoform X2 [Ostrinia furnacalis]	1.88754	-0.89746	-0.31927	-0.72581	0.055003
TRINITY_DN441_c0_g2_i1_orf1	-	-	-	guanine nucleotide-binding protein subunit beta-like protein [Diachasma alloeum]	1.657957	-0.5174	0.417622	-1.32457	-0.2336
TRINITY_DN9555_c0_g1_i1_orf1	-	-	-	pyridoxal kinase [Ostrinia furnacalis]	1.830554	-0.00634	-1.20698	-0.27907	-0.33816
TRINITY_DN1198_c0_g1_i1_orf1	-	-	-	protein suppressor of sable isoform X1 [Ostrinia furnacalis]	1.791116	-0.69988	0.349366	-0.98709	-0.45352
TRINITY_DN1639_c0_g2_i2_orf1	-	-	-	longitudinals lacking protein-like [Plutella xylostella] >XP_021192239.1 longitudinals lacking protein-like [Helicoverpa armigera] >XP_022818289.1 longitudinals lacking protein-like [Spodoptera litura] >XP_022818290.1 longitudinals lacking protein-like [Spodoptera litura] >XP_023945541.1 longitudinals lacking protein-like [Bicyclus anynana] >XP_026320461.1 longitudinals lacking protein-like [Hyposmocoma kahamanoa] >XP_026320471.1 longitudinals lacking protein-like [Hyposmocoma kahamanoa] >XP_026499391.1 longitudinals lacking protein-like [Vanessa tameamea] >XP_026499392.1 longitudinals lacking protein-like [Vanessa tameamea] >XP_026728238.1 longitudinals lacking protein-like isoform X2 [Trichoplusia ni] >XP_026750598.1 longitudinals lacking protein-like [Galleria mellonella] >XP_028032853.1 longitudinals lacking protein-like [Bombyx mandarina] >XP_028169299.1 longitudinals lacking protein-like [Ostrinia furnacalis] >XP_030036777.1 longitudinals lacking protein-like [Manduca sexta] >XP_034826408.1 longitudinals lacking protein-like [Maniola hyperantus] >XP_034826409.1 longitudinals lacking protein-like [Maniola hyperantus] >XP_035452035.1 longitudinals lacking protein-like [Spodoptera frugiperda] >XP_035452036.1 longitudinals lacking protein-like [Spodoptera frugiperda] >XP_035452270.1 longitudinals lacking protein-like [Spodoptera frugiperda] >XP_035452271.1 longitudinals lacking protein-like [Spodoptera frugiperda] >XP_037874172.1 longitudinals lacking protein-like [Bombyx mori] >XP_038218308.1 longitudinals lacking protein-like [Zerene cesonia] >XP_039758692.1 longitudinals lacking protein-like [Pararge aegeria] >XP_041981066.1 longitudinals lacking protein-like [Aricia agestis] >XP_041981074.1 longitudinals lacking protein-like [Aricia agestis] >XP_045455814.1 longitudinals lacking protein-like [Melitaea cinxia] >XP_045505524.1 longitudinals lacking protein-like [Colias croceus] >XP_045524236.1 longitudinals lacking protein-like [Pieris brassicae] >XP_045762005.1 longitudinals lacking protein-like [Maniola jurtina] >XP_045762006.1 longitudinals lacking protein-like [Maniola jurtina] >XP_046973407.1 longitudinals lacking protein-like [Vanessa cardui] >XP_046973408.1 longitudinals lacking protein-like [Vanessa cardui] >XP_047035325.1 longitudinals lacking protein-like [Helicoverpa zea] >XP_047035326.1 longitudinals lacking protein-like [Helicoverpa zea] >XP_047520236.1 longitudinals lacking protein-like [Pieris napi] >XP_047540188.1 longitudinals lacking protein-like [Vanessa atalanta] >XP_048001034.1 longitudinals lacking protein-like [Leguminivora	1.73196	0.492	-0.8273	-0.92025	-0.47641
TRINITY_DN10785_c0_g1_i4_orf1	-	-	-	alkylglycerol monooxygenase-like [Ostrinia furnacalis] >XP_028171363.1 alkylglycerol monooxygenase-like [Ostrinia furnacalis]	1.761294	-1.16197	0.293974	-0.62308	-0.27022
TRINITY_DN4770_c0_g1_i4_orf1	-	-	-	transportin-3 isoform X1 [Ostrinia furnacalis]	1.644013	0.371294	-0.05501	-1.3229	-0.6374
TRINITY_DN107_c0_g1_i1_orf1	-	-	-	dynein light chain 2, cytoplasmic isoform X1 [Pectinophora gossypiella]	1.766955	0.404917	-0.41706	-0.88959	-0.86522
TRINITY_DN19262_c0_g1_i1_orf1	-	-	-	tRNA (guanine-N(7)-)-methyltransferase non-catalytic subunit wdr4 [Ostrinia furnacalis]	1.862989	-0.95501	-0.31774	-0.70868	0.118449
TRINITY_DN1351_c0_g1_i1_orf1	-	-	-	PREDICTED: flavin reductase (NADPH) [Microplitis demolitor] >XP_008553603.1 PREDICTED: flavin reductase (NADPH) [Microplitis demolitor]	1.547129	-0.74506	0.231079	-1.3722	0.33905
TRINITY_DN3664_c0_g1_i8_orf1	-	-	-	hypothetical protein evm_011332 [Chilo suppressalis] >CAB3530638.1 unnamed protein product [Chilo suppressalis] >CAH0407229.1 unnamed protein product [Chilo suppressalis]	1.762558	0.018646	0.136132	-1.09362	-0.82372

TRINITY_DN16084_c0_g1_i4_orf1	-	-	-	synaptobrevin homolog YKT6 [Ostrinia furnacalis]	1.323376	-0.12166	-0.62141	0.867288	-1.4476
TRINITY_DN11973_c0_g1_i1_orf1	-	-	-	nuclear pore complex protein Nup93-like [Ostrinia furnacalis]	1.896204	-0.38115	-0.03703	-1.02666	-0.45137
TRINITY_DN40823_c0_g1_i1_orf1	-	-	-	nucleolar protein 10 [Ostrinia furnacalis]	1.839139	0.19955	-0.37281	-0.99291	-0.67297
TRINITY_DN39490_c0_g1_i1_orf1	-	-	-	unnamed protein product [Parnassius apollo]	1.782417	-0.54643	0.31085	-1.11358	-0.43326
TRINITY_DN3283_c0_g2_i1_orf1	-	-	-	dipeptidyl peptidase 9 isoform X2 [Manduca sexta]	1.437194	-0.26374	0.766181	-1.41523	-0.52441
TRINITY_DN11069_c0_g1_i6_orf1	-	-	-	ganglioside-induced differentiation-associated protein 1 [Ostrinia furnacalis]	1.839612	-0.68093	-0.99879	-0.34621	0.186322
TRINITY_DN1354_c0_g1_i6_orf1	-	-	-	elongator complex protein 2 isoform X1 [Pectinophora gossypiella]	1.909082	-0.58858	-0.8381	-0.54959	0.067192
TRINITY_DN3260_c0_g1_i6_orf1	-	-	-	vesicular integral-membrane protein VIP36 isoform X1 [Ostrinia furnacalis] >XP_028171839.1 vesicular integral-membrane protein VIP36 isoform X2 [Ostrinia furnacalis]	1.368039	-0.14033	0.822471	-1.43172	-0.61846
TRINITY_DN12608_c0_g1_i1_orf1	-	-	-	centrosome-associated zinc finger protein CP190 [Ostrinia furnacalis] >XP_028173286.1 centrosome-associated zinc finger protein CP190 [Ostrinia furnacalis]	1.708882	-0.32613	0.278163	-1.33872	-0.32219
TRINITY_DN6247_c0_g1_i2_orf1	-	-	-	innexin inx3 [Ostrinia furnacalis]	1.877637	-0.30467	-0.90651	-0.74421	0.077754
TRINITY_DN15154_c0_g1_i5_orf1	-	-	-	motile sperm domain-containing protein 2-like [Ostrinia furnacalis]	1.644828	0.314456	0.124102	-1.11244	-0.97095
TRINITY_DN15916_c0_g1_i1_orf1	-	-	-	balbiani ring protein 3-like [Bicyclus anynana] >XP_023946842.1 balbiani ring protein 3-like [Bicyclus anynana]	1.662578	-0.5876	-1.29848	-0.18786	0.411364
TRINITY_DN68725_c0_g1_i1_orf1	-	-	-	hydroxymethylglutaryl-CoA synthase 1 [Ostrinia furnacalis]	1.542309	0.803874	-0.47812	-0.96267	-0.90539
TRINITY_DN3971_c0_g1_i1_orf1	-	-	-	L-asparaginase-like isoform X1 [Ostrinia furnacalis]	1.942605	-0.58352	-0.84277	-0.11284	-0.40347
TRINITY_DN57765_c0_g1_i1_orf1	-	-	-	cytochrome P450 6B6-like [Ostrinia furnacalis]	1.707646	-0.58318	-1.30833	0.004719	0.179146
TRINITY_DN1866_c0_g1_i4_orf1	-	-	-	unnamed protein product [Chrysodeixis includens]	1.814164	-0.54191	-1.18717	-0.07525	-0.00983
TRINITY_DN18035_c0_g1_i7_orf1	-	-	-	mitochondrial ornithine transporter 1 [Ostrinia furnacalis]	1.949369	-0.81136	-0.29489	-0.19877	-0.64435
TRINITY_DN18863_c0_g1_i3_orf1	-	-	-	splicing factor 3A subunit 1 isoform X1 [Ostrinia furnacalis] >XP_028161155.1 splicing factor 3A subunit 1 isoform X2 [Ostrinia furnacalis] >XP_028161156.1 splicing factor 3A subunit 1 isoform X3 [Ostrinia furnacalis] >XP_028161157.1 splicing factor 3A subunit 1 isoform X4 [Ostrinia furnacalis]	1.795708	0.131388	-0.05676	-0.98931	-0.88103
TRINITY_DN73945_c0_g5_i3_orf1	-	-	-	cyclin-dependent kinase 12 isoform X1 [Diachasma alloeum] >XP_015114851.1 cyclin-dependent kinase 12 isoform X1 [Diachasma alloeum] >XP_015114852.1 cyclin-dependent kinase 12 isoform X1 [Diachasma alloeum] >XP_015114853.1 cyclin-dependent kinase 12 isoform X1 [Diachasma alloeum] >XP_015114854.1 cyclin-dependent kinase 12 isoform X1	1.682055	-0.55164	-0.26619	-1.27535	0.411119
TRINITY_DN30027_c0_g1_i1_orf1	-	-	-	40S ribosomal protein S14 [Plutella xylostella] >NP_001298660.1 40S ribosomal protein S14 [Papilio polytes] >NP_001299342.1 40S ribosomal protein S14 [Papilio xuthus] >XP_013200267.1 PREDICTED: 40S ribosomal protein S14 [Amyelois transitella] >XP_013200268.1 PREDICTED: 40S ribosomal protein S14 [Amyelois transitella] >XP_014369569.1 40S ribosomal protein S14 [Papilio machaon] >XP_021200686.1 40S ribosomal protein S14 [Helicoverpa armigera] >XP_026737481.1 40S ribosomal protein S14 [Trichoplusia ni] >XP_028029011.1 40S ribosomal protein S14 [Bombyx mandarina] >XP_028179467.1 40S ribosomal protein S14 [Ostrinia furnacalis] >XP_028179468.1 40S ribosomal protein S14 [Ostrinia furnacalis] >XP_030030611.1 40S ribosomal protein S14 [Manduca sexta] >XP_034829960.1 40S ribosomal protein S14 [Maniola hyperantus] >XP_034829961.1 40S ribosomal protein S14 [Maniola hyperantus] >XP_047022662.1 40S ribosomal protein S14 [Helicoverpa zea] >XP_047984027.1 40S ribosomal protein S14 [Leguminivora glycinivorella] >XP_049869822.1 40S ribosomal protein S14 [Pectinophora gossypiella] >Q5UAM9.1 RecName: Full=40S ribosomal protein S14 [Bombyx mori] >CAH0605581.1 unnamed protein product [Chrysodeixis includens] >AAV34871.1 ribosomal protein S14 [Bombyx mori] >ACY95302.1 ribosomal protein S14 [Manduca sexta] >KAG6456546.1 hypothetical protein O3G_MSEX009812 [Manduca sexta] >KAG6456547.1	1.680073	-0.15677	0.167356	-1.43508	-0.25558
TRINITY_DN1391_c1_g2_i4_orf1	-	-	-	hypothetical protein SFRURICE_002236 [Spodoptera frugiperda]	1.746607	-0.84927	0.137864	-1.09781	0.062606
TRINITY_DN1875_c0_g1_i1_orf1	-	-	-	uncharacterized protein LOC114366320 isoform X1 [Ostrinia furnacalis] >XP_028178963.1 uncharacterized protein LOC114366320 isoform X1 [Ostrinia furnacalis] >XP_028178964.1 uncharacterized protein LOC114366320 isoform X2 [Ostrinia furnacalis]	1.490107	0.261701	-0.4211	-1.5732	0.242494
TRINITY_DN31399_c0_g1_i3_orf1	-	-	-	ATP-dependent zinc metalloprotease YME1 homolog [Ostrinia furnacalis]	1.919768	-0.16364	-0.52657	-0.97152	-0.25804
TRINITY_DN11552_c0_g1_i4_orf1	-	-	-	glutathione hydrolase 1 proenzyme-like isoform X3 [Ostrinia furnacalis]	1.959371	-0.74095	-0.47485	-0.60622	-0.13734
TRINITY_DN2478_c0_g1_i1_orf1	-	-	-	apoptosis inhibitor 5 [Ostrinia furnacalis]	1.641666	0.613896	-0.68145	-1.1223	-0.45181
TRINITY_DN41546_c0_g1_i15_orf1	-	-	-	monocarboxylate transporter 12 isoform X8 [Ostrinia furnacalis]	1.958344	-0.46637	-0.72007	-0.64186	-0.13005
TRINITY_DN14183_c0_g1_i3_orf1	-	-	-	multiple epidermal growth factor-like domains protein 6 [Ostrinia furnacalis]	1.901138	-0.17676	-1.0439	-0.46903	-0.21144

TRINITY_DN56121_c0_g1_i4_orf1	-	-	-	uncharacterized protein LOC114361939 [Ostrinia furnacalis]	1.880277	-0.07442	-0.70142	-0.12964	-0.9748
TRINITY_DN41997_c0_g1_i2_orf1	-	-	-	39S ribosomal protein L23, mitochondrial [Ostrinia furnacalis]	1.988776	-0.33477	-0.45297	-0.65653	-0.5445
TRINITY_DN8640_c0_g1_i4_orf1	-	-	-	glutathione S-transferase E14-like isoform X1 [Ostrinia furnacalis]	1.963179	-0.28203	-0.72723	-0.27381	-0.68012
TRINITY_DN3254_c0_g1_i1_orf1	-	-	-	ATP-dependent (S)-NAD(P)H-hydrate dehydratase [Ostrinia furnacalis]	1.693689	-0.01193	-1.37698	0.154815	-0.45959
TRINITY_DN12320_c0_g1_i1_orf1	-	-	-	brefeldin A-inhibited guanine nucleotide-exchange protein 1 [Ostrinia furnacalis]	1.600575	0.669267	-0.41804	-1.15051	-0.7013
TRINITY_DN131662_c0_g1_i4_orf1	-	-	-	splicing factor 3A subunit 1 isoform X1 [Ostrinia furnacalis] >XP_028161155.1 splicing factor 3A subunit 1 isoform X2 [Ostrinia furnacalis] >XP_028161156.1 splicing factor 3A subunit 1 isoform X3 [Ostrinia furnacalis] >XP_028161157.1 splicing factor 3A subunit 1 isoform X4 [Ostrinia furnacalis]	1.823886	0.072386	-0.08049	-0.98912	-0.82666
TRINITY_DN42903_c0_g1_i4_orf1	-	-	-	rab GTPase-binding effector protein 1 isoform X1 [Ostrinia furnacalis] >XP_028174977.1 rab GTPase-binding effector protein 1 isoform X2 [Ostrinia furnacalis] >XP_028174983.1 rab GTPase-binding effector protein 1 isoform X3 [Ostrinia furnacalis] >XP_028174990.1 rab GTPase-binding effector protein 1 isoform X4 [Ostrinia furnacalis] >XP_028174996.1 rab GTPase-binding effector protein 1 isoform X5 [Ostrinia furnacalis]	1.713243	0.461095	-0.38703	-1.12296	-0.66435
TRINITY_DN13259_c0_g1_i2_orf1	-	-	-	mitotic spindle assembly checkpoint protein MAD1 [Pectinophora gossypiella]	1.841293	-0.60467	9.93E-05	-1.10789	-0.12884
TRINITY_DN1498_c0_g1_i2_orf1	-	-	-	ran GTPase-activating protein 1 [Ostrinia furnacalis]	1.827279	0.279216	-0.96532	-0.56324	-0.57794
TRINITY_DN1856_c0_g1_i3_orf1	-	-	-	hypothetical protein evm_006253 [Chilo suppressalis]	1.571012	0.726494	-0.57393	-1.1695	-0.55407
TRINITY_DN3949_c1_g1_i1_orf1	-	-	-	probable cytochrome P450 304a1 isoform X2 [Ostrinia furnacalis]	1.595068	-0.95942	-0.74124	-0.6473	0.752889
TRINITY_DN5133_c0_g1_i7_orf1	-	-	-	epimerase family protein SDR39U1 [Ostrinia furnacalis]	1.856676	-0.23872	-1.01843	-0.67316	0.073634
TRINITY_DN33967_c0_g1_i1_orf1	-	-	-	PREDICTED: elongation factor 1-alpha [Microplitis demolitor] >XP_008547401.1 PREDICTED: elongation factor 1-alpha [Microplitis demolitor]	1.698966	-0.77673	0.505525	-1.05695	-0.37081
TRINITY_DN11876_c0_g1_i2_orf1	-	-	-	protein TAPT1 homolog [Ostrinia furnacalis]	1.566425	0.801478	-0.71297	-0.71297	-0.94196
TRINITY_DN12113_c0_g1_i1_orf1	-	-	-	WD repeat-containing protein 5 [Helicoverpa armigera] >XP_022823790.1 WD repeat-containing protein 5 [Spodoptera litura] >XP_028176937.1 WD repeat-containing protein 5 [Ostrinia furnacalis] >XP_035429818.1 WD repeat-containing protein 5 [Spodoptera frugiperda] >XP_047030799.1 WD repeat-containing protein 5 [Helicoverpa zea] >CAB3513875.1 unnamed protein product [Spodoptera littoralis] >KAF9795358.1 hypothetical protein SFRURICE_004730 [Spodoptera frugiperda] >KAG8117399.1 hypothetical protein SFRUCORN_019548 [Spodoptera frugiperda] >PZC79830.1 hypothetical protein B5X24_HaOG215819 [Helicoverpa armigera] >CAH1643659.1 unnamed protein product	1.785465	-0.75677	-0.91558	0.3877	-0.50082
TRINITY_DN30224_c0_g1_i1_orf1	-	-	-	PREDICTED: serine--tRNA ligase, mitochondrial [Amyelois transitella]	1.834421	-0.81853	-0.31219	-0.90856	0.204856
TRINITY_DN9711_c0_g1_i10_orf1	-	-	-	cAMP-specific 3',5'-cyclic phosphodiesterase isoform X2 [Ostrinia furnacalis]	1.821561	-0.85786	0.310099	-0.77574	-0.49807
TRINITY_DN4497_c0_g1_i4_orf1	-	-	-	cytochrome P450 9e2-like [Ostrinia furnacalis] >QPF77612.1 cytochrome P450 monooxygenase CYP9A185 [Ostrinia furnacalis]	1.804825	-0.55835	-0.73395	-0.87304	0.360513
TRINITY_DN21126_c0_g1_i1_orf1	-	-	-	serine/threonine-protein kinase unc-51 isoform X5 [Ostrinia furnacalis]	1.692778	0.541595	-0.59261	-1.08762	-0.55414
TRINITY_DN23432_c0_g1_i1_orf1	-	-	-	7-methylguanosine phosphate-specific 5'-nucleotidase-like isoform X2 [Ostrinia furnacalis]	1.965324	-0.68866	-0.21813	-0.69574	-0.3628
TRINITY_DN140538_c0_g2_i1_orf1	-	-	-	peptidyl-prolyl cis-trans isomerase NIMA-interacting 1 [Urocitellus parryii]	1.524305	-0.11225	0.427191	-1.54811	-0.29114
TRINITY_DN1888_c0_g2_i1_orf1	-	-	-	peptidyl-prolyl cis-trans isomerase FKBP8 [Ostrinia furnacalis]	1.769134	0.279446	-0.31302	-1.17438	-0.56118
TRINITY_DN6503_c0_g1_i8_orf1	-	-	-	uncharacterized protein LOC114354432 [Ostrinia furnacalis]	1.807048	-0.34072	0.254206	-0.66824	-1.0523
TRINITY_DN332_c0_g1_i6_orf1	-	-	-	dolichyl-diphosphooligosaccharide--protein glycosyltransferase subunit STT3B isoform X2 [Ostrinia furnacalis]	1.780724	-0.65192	-0.34738	-1.09001	0.308589
TRINITY_DN11178_c0_g1_i1_orf1	-	-	-	hypoxia up-regulated protein 1 [Ostrinia furnacalis]	1.400087	-0.30651	0.758319	-1.49866	-0.35324
TRINITY_DN2930_c0_g1_i8_orf1	-	-	-	hypothetical protein evm_000171 [Chilo suppressalis] >CAB3523059.1 unnamed protein product [Chilo suppressalis] >CAH0400381.1 unnamed protein product [Chilo suppressalis]	1.715332	0.54411	-0.87815	-0.55565	-0.82564
TRINITY_DN1673_c0_g1_i2_orf1	-	-	-	hypothetical protein evm_006080 [Chilo suppressalis]	1.804549	0.158247	-0.14975	-1.06864	-0.7444
TRINITY_DN126648_c0_g1_i1_orf1	-	-	-	elongation factor 1 alpha, partial [Spodoptera exigua] >QYQ52647.1 elongation factor 1 alpha, partial [Spodoptera exigua]	1.488137	-0.84503	0.377259	-1.34937	0.329006
TRINITY_DN6855_c1_g1_i3_orf1	-	-	-	developmentally-regulated GTP-binding protein 2 [Ostrinia furnacalis] >XP_028162614.1 developmentally-regulated GTP-binding protein 2 [Ostrinia furnacalis]	1.893341	-0.66126	-0.78697	0.137731	-0.58284
TRINITY_DN5312_c4_g1_i2_orf1	-	-	-	unnamed protein product [Chilo suppressalis]	1.958418	-0.56161	-0.82235	-0.22423	-0.35024
TRINITY_DN766_c0_g1_i1_orf1	-	-	-	nucleolar complex protein 3 homolog [Ostrinia furnacalis]	1.76746	-0.13835	0.260851	-0.98323	-0.90673
TRINITY_DN5092_c0_g1_i2_orf1	-	-	-	peroxisomal acyl-coenzyme A oxidase 3 isoform X3 [Ostrinia furnacalis]	1.941296	-0.54408	-0.30472	-0.89676	-0.19573
TRINITY_DN8717_c0_g1_i5_orf1	-	-	-	hypothetical protein evm_006607 [Chilo suppressalis] >CAG9745590.1 unnamed protein product [Diatraea saccharalis] >CAG9784275.1 unnamed protein product [Diatraea	1.758362	-0.94121	-0.72559	-0.54158	0.450008

TRINITY_DN4199_c0_g1_i1_orf1	-	-	-	plasminogen activator inhibitor 1 RNA-binding protein-like [Ostrinia furnacalis]	1.966136	-0.34232	-0.77878	-0.25883	-0.58621
TRINITY_DN26824_c0_g1_i1_orf1	-	-	-	60S ribosomal protein L6 [Hyposmocoma kahamanoa]	1.484164	-0.45044	0.053472	-1.54372	0.456531
TRINITY_DN16187_c0_g1_i1_orf1	-	-	-	eIF-2-alpha kinase activator GCN1 [Colias croceus]	1.614313	-0.67728	0.463171	-1.30857	-0.09163
TRINITY_DN48536_c0_g1_i3_orf1	-	-	-	unnamed protein product [Chilo suppressalis]	1.904656	-0.0084	-0.9016	-0.31753	-0.67713
TRINITY_DN7674_c0_g1_i2_orf1	-	-	-	prefoldin subunit 2 [Ostrinia furnacalis]	1.377564	-0.5039	0.998671	-1.1578	-0.71454
TRINITY_DN31815_c0_g1_i4_orf1	-	-	-	E3 ubiquitin-protein ligase listerin-like [Ostrinia furnacalis]	1.946184	-0.61408	-0.11722	-0.81184	-0.40303
TRINITY_DN7329_c0_g1_i6_orf1	-	-	-	serine hydrolase-like protein 2 isoform X2 [Ostrinia furnacalis]	1.697818	-0.62332	-1.04297	-0.58172	0.550187
TRINITY_DN19942_c0_g1_i2_orf1	-	-	-	ribosomal protein l1p/L10e family domain-containing protein [Phthorimaea operculella]	1.930171	-0.87586	-0.04946	-0.50243	-0.50243
TRINITY_DN24164_c0_g1_i1_orf1	-	-	-	PREDICTED: ras-related protein Rab6 [Microplitis demolitor] >XP_034947251.1 ras-related protein Rab6 isoform X2 [Chelonus insularis] >XP_044581106.1 ras-related protein Rab6 isoform X2 [Cotesia glomerata] >KAH0553942.1 Ras- protein Rab6 [Cotesia glomerata] >CAG5097549.1 Similar to Rab6: Ras-related protein Rab6 (Drosophila melanogaster) [Cotesia	1.874956	-0.75904	-0.47832	-0.80682	0.169231
TRINITY_DN6308_c0_g1_i3_orf1	-	-	-	myc box-dependent-interacting protein 1 isoform X4 [Pectinophora gossypiella]	1.820098	-1.17294	-0.05189	-0.55414	-0.04113
TRINITY_DN40028_c0_g1_i1_orf1	-	-	-	signal recognition particle receptor subunit alpha homolog [Ostrinia furnacalis]	1.694116	-0.33165	0.236772	-1.38491	-0.21432
TRINITY_DN40281_c0_g2_i1_orf1	-	-	-	glyoxylate reductase/hydroxypyruvate reductase [Ostrinia furnacalis]	1.84791	-0.77913	-0.70682	-0.63544	0.273488
TRINITY_DN40211_c0_g1_i1_orf1	-	-	-	rho GTPase-activating protein 44-like [Ostrinia furnacalis]	1.631125	0.615937	-0.45925	-0.61912	-1.16869
TRINITY_DN44335_c0_g1_i7_orf1	-	-	-	hypothetical protein SFRURICE_000584 [Spodoptera frugiperda]	1.542913	0.791422	-0.59063	-1.12082	-0.62288
TRINITY_DN10266_c0_g1_i5_orf1	-	-	-	armadillo repeat-containing protein 6 homolog [Ostrinia furnacalis]	1.623303	-0.04819	0.256629	-1.47223	-0.35952
TRINITY_DN147427_c0_g1_i1_orf1	-	-	-	importin subunit alpha-4 [Ostrinia furnacalis]	1.771204	0.432435	-0.90913	-0.7229	-0.57161
TRINITY_DN848_c0_g1_i1_orf1	-	-	-	actin-interacting protein 1 isoform X2 [Ostrinia furnacalis]	1.754516	-0.87862	0.328749	-0.99984	-0.2048
TRINITY_DN38650_c0_g1_i2_orf1	-	-	-	elongator complex protein 1 [Ostrinia furnacalis]	1.924048	-0.08207	-0.90165	-0.60473	-0.3356
TRINITY_DN9234_c0_g1_i5_orf1	-	-	-	glutathione S-transferase 1-like [Ostrinia furnacalis] >QIC35738.1 glutathione S-transferase delta 2 [Ostrinia furnacalis]	1.690026	-0.32051	-0.2703	0.276192	-1.3754
TRINITY_DN48096_c0_g2_i2_orf1	-	-	-	eukaryotic translation initiation factor 4E-like [Ostrinia furnacalis]	1.546457	-0.26389	0.466831	-1.50347	-0.24593
TRINITY_DN69535_c0_g1_i2_orf1	-	-	-	juvenile hormone esterase-like [Ostrinia furnacalis]	1.795422	-0.6386	-1.10912	0.238225	-0.28593
TRINITY_DN47666_c0_g1_i4_orf1	-	-	-	PREDICTED: probable splicing factor 3B subunit 5 [Amyelois transitella] >XP_021194781.1 splicing factor 3B subunit 5 [Helicoverpa armigera] >XP_022127816.1 splicing factor 3B subunit 5 [Pieris rapae] >XP_022818375.1 probable splicing factor 3B subunit 5 [Spodoptera litura] >XP_026319816.1 splicing factor 3B subunit 5 [Hyposmocoma kahamanoa] >XP_026728249.1 splicing factor 3B subunit 5 [Trichoplusia ni] >XP_026750486.1 splicing factor 3B subunit 5 [Galleria mellonella] >XP_028161523.1 splicing factor 3B subunit 5 [Ostrinia furnacalis] >XP_030037274.1 splicing factor 3B subunit 5 [Manduca sexta] >XP_035451936.1 splicing factor 3B subunit 5 [Spodoptera frugiperda] >XP_047035127.1 splicing factor 3B subunit 5 [Helicoverpa zea] >XP_047519960.1 splicing factor 3B subunit 5 [Pieris napi] >XP_049880275.1 splicing factor 3B subunit 5 [Pectinophora gossypiella] >KAF9410749.1 hypothetical protein HW555_010266 [Spodoptera exigua] >CAB3229682.1 unnamed protein product [Arctia plantaginis] >CAF4874265.1 unnamed protein product [Pieris macdunnoughi] >CAG9753950.1 unnamed protein product [Diatraea saccharalis] >CAH0596996.1 unnamed protein product [Chrysodeixis includens] >CAH0717822.1 unnamed protein product, partial [Brenthis ino] >CAH2097681.1 unnamed protein product [Euphydryas	1.561947	0.710265	-0.40437	-1.20587	-0.66198
TRINITY_DN1947_c0_g1_i6_orf1	-	-	-	insulin-degrading enzyme [Ostrinia furnacalis] >XP_028163443.1 insulin-degrading enzyme [Ostrinia furnacalis]	1.87786	0.027998	-0.55801	-1.02969	-0.31816
TRINITY_DN4814_c0_g1_i6_orf1	-	-	-	vesicle transport protein GOT1B [Pectinophora gossypiella]	1.656153	0.105667	-1.44583	-0.38782	0.071832
TRINITY_DN10672_c0_g1_i3_orf1	-	-	-	neurofilament heavy polypeptide-like isoform X10 [Ostrinia furnacalis]	1.570047	0.064244	-0.17092	-1.57752	0.114152
TRINITY_DN3801_c0_g1_i9_orf1	-	-	-	claspin-like isoform X2 [Ostrinia furnacalis]	1.719722	0.500706	-0.45338	-0.99548	-0.77156
TRINITY_DN5982_c0_g1_i3_orf1	-	-	-	coatomer subunit gamma [Ostrinia furnacalis]	1.697687	0.516702	-0.55161	-1.11791	-0.54487
TRINITY_DN143_c0_g3_i1_orf1	-	-	-	Ubiquitin-60S ribosomal protein L40, partial [Cotesia chilonis] >UDP69015.1 egg surface protein ES-53, partial [Cotesia chilonis]	1.761555	-0.57281	-0.25069	-1.19909	0.26104
TRINITY_DN9356_c0_g1_i1_orf1	-	-	-	vacuolar protein sorting-associated protein 33B [Ostrinia furnacalis]	1.825466	0.328764	-0.79949	-0.64036	-0.71438
TRINITY_DN16933_c0_g1_i10_orf1	-	-	-	uridine phosphorylase 1-like [Ostrinia furnacalis] >XP_028164122.1 uridine phosphorylase 1-like [Ostrinia furnacalis]	1.873925	-0.32578	-0.51546	-1.05641	0.023731
TRINITY_DN3411_c0_g1_i2_orf1	-	-	-	unnamed protein product [Chilo suppressalis]	1.885145	0.125523	-0.45804	-0.8639	-0.68873
TRINITY_DN3434_c0_g1_i1_orf1	-	-	-	coiled-coil domain-containing protein 47 [Ostrinia furnacalis] >XP_028161458.1 coiled-coil domain-containing protein 47 [Ostrinia furnacalis]	1.877464	-0.94046	-0.04367	-0.75467	-0.13866

TRINITY_DN13114_c0_g1_i1_orf1	-	-	-	nicalin-1 [Helicoverpa zea]	1.910319	-0.00141	-0.34284	-0.8416	-0.72446
TRINITY_DN18558_c0_g1_i7_orf1	-	-	-	cytosolic Fe-S cluster assembly factor NUBP2 homolog [Ostrinia furnacalis]	1.786884	0.401796	-0.81159	-0.82772	-0.54937
TRINITY_DN37366_c0_g1_i7_orf1	-	-	-	juvenile hormone epoxide hydrolase-like [Ostrinia furnacalis]	1.81773	-0.09797	-0.88294	-0.94592	0.109101
TRINITY_DN49872_c0_g2_i1_orf1	-	-	-	NIF3-like protein 1 [Ostrinia furnacalis] >XP_028165862.1 NIF3-like protein 1 [Ostrinia furnacalis] >XP_028165864.1 NIF3-like protein 1 [Ostrinia furnacalis]	1.505455	0.106557	-0.40117	-1.56152	0.350679
TRINITY_DN38693_c0_g1_i4_orf1	-	-	-	protein RER1 [Ostrinia furnacalis] >XP_028157304.1 protein RER1 [Ostrinia furnacalis] >XP_028157310.1 protein RER1 [Ostrinia furnacalis]	1.900925	0.004123	-0.56873	-0.95983	-0.37649
TRINITY_DN61711_c0_g1_i1_orf1	-	-	-	mitochondrial carnitine/acylcarnitine translocase [Loxostege sticticalis]	1.677167	-0.61807	0.568839	-1.09364	-0.53429
TRINITY_DN32509_c0_g1_i3_orf1	-	-	-	heat shock 70 kDa protein 4 isoform X1 [Ostrinia furnacalis]	1.939032	-0.24546	-0.54217	-0.90969	-0.24172
TRINITY_DN63719_c0_g1_i5_orf1	-	-	-	eukaryotic peptide chain release factor GTP-binding subunit ERF3A isoform X2 [Manduca sexta] >KAG6462744.1 hypothetical protein O3G_MSEX013441 [Manduca sexta]	1.887921	-0.34539	-0.37914	-1.07968	-0.08371
TRINITY_DN41736_c0_g2_i1_orf1	-	-	-	calcyclin-binding protein [Ostrinia furnacalis]	1.392462	1.036071	-0.8121	-0.70411	-0.91232
TRINITY_DN13941_c0_g1_i6_orf1	-	-	-	tryptophan 2,3-dioxygenase [Ostrinia furnacalis]	1.73665	-0.17229	-0.37194	0.147118	-1.33955
TRINITY_DN4272_c0_g1_i1_orf1	-	-	-	MICOS complex subunit MIC19-like [Ostrinia furnacalis]	1.830453	-0.65839	-0.14629	-1.09101	0.065238
TRINITY_DN3791_c0_g1_i2_orf1	-	-	-	transmembrane protein 19 [Ostrinia furnacalis]	1.929824	-0.02585	-0.60729	-0.82946	-0.46722
TRINITY_DN7839_c0_g1_i4_orf1	-	-	-	elongin-C [Ostrinia furnacalis] >XP_028163922.1 elongin-C [Ostrinia furnacalis]	1.808813	-0.09733	-0.03637	-1.23404	-0.44108
TRINITY_DN5562_c1_g2_i1_orf1	-	-	-	cell division cycle and apoptosis regulator protein 1-like [Ostrinia furnacalis]	1.906405	0.026152	-0.83317	-0.36743	-0.73195
TRINITY_DN9085_c0_g1_i1_orf1	-	-	-	golgin subfamily A member 2-like [Ostrinia furnacalis]	1.890964	-0.33433	-0.14429	-1.08977	-0.32258
TRINITY_DN3179_c0_g1_i1_orf1	-	-	-	diamine acetyltransferase 2-like [Ostrinia furnacalis]	1.562259	0.71553	-0.34396	-1.13897	-0.79485
TRINITY_DN280_c0_g1_i12_orf1	-	-	-	tubulin beta-1 chain-like [Leguminivora glycinivorella]	1.828697	0.079976	-0.10659	-0.98578	-0.8163
TRINITY_DN11612_c0_g3_i1_orf1	-	-	-	hypothetical protein O3G_MSEX005294 [Manduca sexta]	1.806894	0.118425	-0.46859	-1.19767	-0.25905
TRINITY_DN5562_c1_g1_i3_orf1	-	-	-	cell division cycle and apoptosis regulator protein 1-like [Ostrinia furnacalis]	1.96855	-0.25206	-0.34692	-0.64564	-0.72392
TRINITY_DN2019_c0_g1_i4_orf1	-	-	-	sushi, von Willebrand factor type A, EGF and pentraxin domain-containing protein 1 [Ostrinia furnacalis] >XP_028159318.1 sushi, von Willebrand factor type A, EGF and pentraxin domain-containing protein 1 [Ostrinia furnacalis]	1.612895	0.722384	-0.79464	-0.94134	-0.5993
TRINITY_DN3245_c2_g1_i4_orf1	-	-	-	membrane-associated progesterone receptor component 1-like [Ostrinia furnacalis]	1.854847	-1.08324	0.036324	-0.57489	-0.23304
TRINITY_DN31119_c0_g1_i1_orf1	-	-	-	transforming acidic coiled-coil-containing protein 3-like [Ostrinia furnacalis] >XP_028170476.1 transforming acidic coiled-coil-containing protein 3-like [Ostrinia furnacalis] >XP_028170477.1 transforming acidic coiled-coil-containing protein 3-like [Ostrinia furnacalis] >XP_028170480.1 transforming acidic coiled-coil-containing protein 3-	1.542395	0.190143	-0.09237	-1.6047	-0.03546
TRINITY_DN10716_c1_g1_i1_orf1	-	-	-	apoptosis-inducing factor 3-like [Ostrinia furnacalis]	1.961162	-0.81552	-0.23822	-0.55449	-0.35293
TRINITY_DN26168_c0_g1_i1_orf1	-	-	-	ATP-dependent RNA helicase Ddx1-like [Ostrinia furnacalis]	1.589203	0.668179	-0.41035	-1.20084	-0.64619
TRINITY_DN88640_c0_g1_i1_orf1	-	-	-	tetratricopeptide repeat protein 27-like [Ostrinia furnacalis]	1.883642	-0.49042	-0.89734	-0.62425	0.128367
TRINITY_DN1368_c0_g1_i6_orf1	-	-	-	hypothetical protein SFRUCORN_009408 [Spodoptera frugiperda]	1.804076	0.117536	-0.72197	-1.09514	-0.1045
TRINITY_DN54711_c0_g1_i1_orf1	-	-	-	39S ribosomal protein L50, mitochondrial [Ostrinia furnacalis]	1.923623	-0.73542	-0.19446	-0.83395	-0.15979
TRINITY_DN26503_c0_g1_i1_orf1	-	-	-	ruvB-like 2 isoform X1 [Ostrinia furnacalis] >XP_028160979.1 ruvB-like 2 isoform X2 [Ostrinia furnacalis]	1.831357	0.06752	-0.21992	-1.1426	-0.53635
TRINITY_DN3924_c0_g1_i5_orf1	-	-	-	SH3 domain-containing kinase-binding protein 1-like isoform X1 [Ostrinia furnacalis]	1.75811	0.063448	-0.05248	-1.2953	-0.47377
TRINITY_DN37141_c0_g1_i2_orf1	-	-	-	dnaJ homolog shv [Ostrinia furnacalis]	1.64942	0.616664	-0.62782	-1.10464	-0.53362
TRINITY_DN20321_c0_g1_i5_orf1	-	-	-	uncharacterized protein LOC114350467 isoform X3 [Ostrinia furnacalis]	1.742105	-0.81116	-1.13325	0.135484	0.066826
TRINITY_DN51050_c0_g1_i3_orf1	-	-	-	GRB10-interacting GYF protein 2 isoform X1 [Ostrinia furnacalis]	1.585863	0.557879	-0.38239	-1.36874	-0.39262
TRINITY_DN1443_c0_g1_i4_orf1	-	-	-	ATP-dependent RNA helicase DDX3X isoform X1 [Ostrinia furnacalis]	1.863759	-0.19062	-0.35929	-1.15586	-0.15798
TRINITY_DN1268_c0_g1_i1_orf1	-	-	-	nuclear pore complex protein Nup154 [Ostrinia furnacalis]	1.863474	-1.04068	-0.0849	-0.65616	-0.08172
TRINITY_DN6103_c0_g1_i6_orf1	-	-	-	sedoheptulokinase-like [Ostrinia furnacalis]	1.66278	0.565535	-0.60123	-1.15277	-0.47432
TRINITY_DN655_c0_g1_i3_orf1	-	-	-	moesin/ezrin/radixin homolog 1 isoform X2 [Bombyx mori] >XP_028038189.1 moesin/ezrin/radixin homolog 1 isoform X2 [Bombyx mandarina]	1.767936	0.089558	0.033753	-1.14163	-0.74962
TRINITY_DN2826_c0_g1_i7_orf1	-	-	-	ATP-binding cassette subfamily D member 1 [Chilo suppressalis] >CAB3531327.1 unnamed protein product [Chilo suppressalis] >CAH0407919.1 unnamed protein product [Chilo suppressalis]	1.638279	0.22299	-1.35872	-0.63462	0.132077
TRINITY_DN65988_c0_g1_i4_orf1	-	-	-	uncharacterized protein LOC114354070 isoform X3 [Ostrinia furnacalis]	1.837825	-0.34951	0.117347	-1.11699	-0.48867
TRINITY_DN23801_c0_g1_i2_orf1	-	-	-	signal recognition particle subunit SRP72 [Pectinophora gossypiella]	1.680948	-0.38499	0.308303	-1.36972	-0.23454
TRINITY_DN9437_c0_g1_i1_orf1	-	-	-	glyoxylate reductase/hydroxypyruvate reductase-like [Ostrinia furnacalis]	1.902863	-0.01091	-0.94136	-0.61852	-0.33207
TRINITY_DN2642_c0_g1_i5_orf1	-	-	-	protein LSM12 homolog [Ostrinia furnacalis]	1.791355	-1.04784	0.201739	-0.79327	-0.15198

TRINITY_DN10058_c0_g1_i1_orf1	-	-	-	dolichyl-diphosphooligosaccharide--protein glycosyltransferase subunit DAD1 [Ostrinia furnacalis]	1.942116	-0.72208	-0.36289	-0.75087	-0.10628
TRINITY_DN4280_c0_g1_i8_orf1	-	-	-	uncharacterized protein LOC114354853 [Ostrinia furnacalis]	1.828458	-0.59509	-0.01463	-0.08035	-1.13839
TRINITY_DN4752_c0_g1_i3_orf1	-	-	-	thioredoxin domain-containing protein 9 [Ostrinia furnacalis] >XP_028172592.1 thioredoxin domain-containing protein 9 [Ostrinia furnacalis]	1.93867	-0.24008	-0.84665	-0.65422	-0.19771
TRINITY_DN53400_c0_g1_i1_orf1	-	-	-	hypothetical protein evm_004547 [Chilo suppressalis]	1.912954	-0.25276	-1.03786	-0.36557	-0.25677
TRINITY_DN18300_c0_g1_i17_orf1	-	-	-	RNA-binding protein lark isoform X1 [Helicoverpa armigera] >XP_047032035.1 RNA-binding protein lark isoform X1 [Helicoverpa zea] >PZC74210.1 hypothetical protein B5X24_HaOG208200 [Helicoverpa armigera]	1.910037	-0.31905	-0.00993	-0.78884	-0.79221
TRINITY_DN49527_c0_g1_i1_orf1	-	-	-	Protein lin-7 homolog B [Eumeta japonica]	1.990384	-0.43992	-0.35429	-0.64228	-0.5539
TRINITY_DN17861_c0_g1_i5_orf1	-	-	-	acyl-CoA-binding protein homolog isoform X1 [Ostrinia furnacalis]	1.624079	0.700777	-0.68733	-0.98922	-0.64831
TRINITY_DN23783_c0_g2_i1_orf1	-	-	-	cytochrome b5 [Ostrinia furnacalis]	1.604444	0.422424	-0.99085	-1.1217	0.085675
TRINITY_DN11616_c0_g1_i3_orf1	-	-	-	coiled-coil domain-containing protein 6-like [Ostrinia furnacalis]	1.673902	0.569258	-0.8345	-1.00883	-0.39983
TRINITY_DN37856_c0_g1_i5_orf1	-	-	-	spermine synthase [Ostrinia furnacalis]	1.711136	-0.69585	0.513811	-1.0427	-0.4864
TRINITY_DN40197_c0_g1_i1_orf1	-	-	-	UDP-N-acetylglucosamine--dolichyl-phosphate N-acetylglucosaminephosphotransferase-like [Ostrinia furnacalis]	1.718697	0.488776	-0.47409	-1.06683	-0.66655
TRINITY_DN5828_c0_g1_i5_orf1	-	-	-	unnamed protein product [Diatraea saccharalis]	1.51826	-0.30757	-0.12099	0.453129	-1.54283
TRINITY_DN4300_c0_g1_i5_orf1	-	-	-	replication factor C subunit 3 [Ostrinia furnacalis]	1.980404	-0.29768	-0.44255	-0.73081	-0.50936
TRINITY_DN2954_c0_g1_i1_orf1	-	-	-	unnamed protein product [Diatraea saccharalis]	1.797884	-0.68125	0.253421	-1.07282	-0.29724
TRINITY_DN146718_c0_g1_i1_orf1	-	-	-	40S ribosomal protein S6 [Diachasma alloeum]	1.763009	-0.53444	-0.32743	-1.18981	0.288666
TRINITY_DN817_c0_g1_i3_orf1	-	-	-	phenylalanine--tRNA ligase beta subunit [Ostrinia furnacalis]	1.589537	-0.84789	0.658171	-1.11314	-0.28667
TRINITY_DN2783_c0_g1_i22_orf1	-	-	-	methionine aminopeptidase 1-like [Pectinophora gossypiella] >XP_049887084.1 methionine aminopeptidase 1-like [Pectinophora gossypiella]	1.73659	-0.84826	0.089649	-1.11287	0.13489
TRINITY_DN23004_c0_g1_i1_orf1	-	-	-	uncharacterized protein LOC114365313 [Ostrinia furnacalis]	1.777012	-0.84454	0.138943	-1.05326	-0.01816
TRINITY_DN10399_c0_g1_i2_orf1	-	-	-	unnamed protein product [Chilo suppressalis]	1.938328	-0.29382	-0.23286	-0.93599	-0.47565
TRINITY_DN4132_c0_g1_i14_orf1	-	-	-	thyroid receptor-interacting protein 11-like isoform X1 [Ostrinia furnacalis]	1.388015	-0.0084	0.10585	-1.73239	0.246926
TRINITY_DN81312_c0_g1_i1_orf1	-	-	-	atlastin isoform X4 [Ostrinia furnacalis]	1.614051	-0.82951	0.455723	-1.22426	-0.016
TRINITY_DN3638_c0_g1_i1_orf1	-	-	-	DNA replication licensing factor Mcm3 [Ostrinia furnacalis]	1.947138	-0.17122	-0.87378	-0.38431	-0.51783
TRINITY_DN3562_c0_g1_i4_orf1	-	-	-	peroxisomal membrane protein PEX14-like isoform X2 [Ostrinia furnacalis]	1.829516	-0.26026	-0.9832	0.179583	-0.76563
TRINITY_DN93566_c0_g2_i1_orf1	-	-	-	60S acidic ribosomal protein P1 [Manduca sexta] >ACY95374.1 ribosomal protein P1 [Manduca sexta] >KAG6447985.1 hypothetical protein O3G_MSEX005254 [Manduca sexta] >KAG6447986.1 hypothetical protein O3G_MSEX005254 [Manduca sexta]	1.824844	-0.96429	0.230623	-0.75956	-0.33162
TRINITY_DN36682_c0_g1_i1_orf1	-	-	-	uncharacterized protein DDB_G0287625-like [Ostrinia furnacalis]	1.916907	-0.74954	-0.29117	-0.82216	-0.05403
TRINITY_DN46022_c0_g1_i1_orf1	-	-	-	mRNA-decapping enzyme 1A [Ostrinia furnacalis]	1.704514	0.545626	-0.73306	-0.99176	-0.52532
TRINITY_DN6125_c0_g1_i2_orf1	-	-	-	uncharacterized protein LOC114350989 [Ostrinia furnacalis] >XP_028157805.1 uncharacterized protein LOC114350989 [Ostrinia furnacalis]	1.756517	-0.03383	0.04904	-0.47259	-1.29914
TRINITY_DN8076_c0_g1_i5_orf1	-	-	-	hypothetical protein evm_001812 [Chilo suppressalis]	1.877403	-0.02404	-1.07205	-0.49129	-0.29003
TRINITY_DN6572_c0_g1_i2_orf1	-	-	-	zinc finger protein 330 homolog [Ostrinia furnacalis]	1.659456	0.466764	-0.16177	-1.1728	-0.79165
TRINITY_DN1337_c0_g2_i1_orf1	-	-	-	minor histocompatibility antigen H13 [Ostrinia furnacalis]	1.752791	-0.14339	-0.07232	-1.3688	-0.16828
TRINITY_DN37830_c0_g1_i1_orf1	-	-	-	60S ribosomal protein L18a [Galleria mellonella] >AXY94862.1 ribosomal protein L18A [Galleria mellonella]	1.870161	-0.71836	0.170729	-0.86469	-0.45784
TRINITY_DN18593_c0_g1_i1_orf1	-	-	-	60S ribosomal protein L22-like [Ostrinia furnacalis]	1.605405	-0.86303	0.724645	-0.9293	-0.53772
TRINITY_DN5512_c0_g1_i8_orf1	-	-	-	peroxisomal multifunctional enzyme type 2-like isoform X1 [Ostrinia furnacalis]	1.866611	-0.0781	-0.26051	-1.13441	-0.3936
TRINITY_DN4810_c0_g1_i3_orf1	-	-	-	clathrin interactor 1 isoform X2 [Maniola jurtina]	1.923747	-0.12654	-0.33018	-0.95497	-0.51205
TRINITY_DN33178_c0_g1_i1_orf1	-	-	-	synaptojanin-1 [Ostrinia furnacalis]	1.842773	0.12121	-0.30481	-1.0747	-0.58448
TRINITY_DN43792_c0_g1_i1_orf1	-	-	-	40S ribosomal protein S3a [Spodoptera litura] >XP_022824163.1 40S ribosomal protein S3a [Spodoptera litura] >XP_026734591.1 40S ribosomal protein S3a [Trichoplusia ni] >XP_035456172.1 40S ribosomal protein S3a [Spodoptera frugiperda] >Q95V35.1 RecName: Full=40S ribosomal protein S3a [Spodoptera frugiperda] >CAB3514148.1 unnamed protein product [Spodoptera littoralis] >CAH0602005.1 unnamed protein product [Chrysodeixis includens] >AAL26579.1 ribosomal protein S3A [Spodoptera frugiperda] >CAH1642305.1 unnamed protein product [Spodoptera littoralis]	1.836429	-0.44164	-0.10071	-1.18788	-0.10619
TRINITY_DN13944_c0_g1_i1_orf1	-	-	-	vacuolar protein sorting-associated protein 52 homolog [Ostrinia furnacalis]	1.965072	-0.4955	-0.82652	-0.36077	-0.28229
TRINITY_DN1494_c0_g1_i3_orf1	-	-	-	dihydropyrimidine dehydrogenase [NADP(+)] [Ostrinia furnacalis]	1.965871	-0.29947	-0.27258	-0.69691	-0.69691

TRINITY_DN67716_c0_g1_i1_orf1	-	-	-	unnamed protein product [Spodoptera exigua]	1.9718	-0.72274	-0.47287	-0.20809	-0.5681
TRINITY_DN1447_c0_g1_i5_orf1	-	-	-	PREDICTED: coatomer subunit beta' [Amyelois transitella]	1.665317	0.360194	-0.23249	-1.36313	-0.4299
TRINITY_DN2778_c0_g1_i5_orf1	-	-	-	hypothetical protein evm_001346 [Chilo suppressalis]	1.903494	-0.08796	-0.2012	-0.91998	-0.69435
TRINITY_DN493_c0_g1_i4_orf1	-	-	-	ADP-ribosylation factor GTPase-activating protein 3 [Ostrinia furnacalis]	1.637001	0.436127	-0.05322	-1.21874	-0.80117
TRINITY_DN4403_c0_g1_i3_orf1	-	-	-	AP-1 complex subunit gamma-1 [Ostrinia furnacalis]	1.437076	0.984021	-0.82083	-0.72162	-0.87864
TRINITY_DN50225_c0_g1_i1_orf1	-	-	-	SRSF protein kinase 3 [Galleria mellonella]	1.268268	1.147487	-0.54749	-0.81204	-1.05623
TRINITY_DN65604_c0_g1_i2_orf1	-	-	-	LOW QUALITY PROTEIN: ankyrin repeat domain-containing protein 17 [Ostrinia furnacalis]	1.942069	-0.2546	-0.92237	-0.28232	-0.48278
TRINITY_DN98723_c1_g1_i1_orf1	-	-	-	uncharacterized protein LOC114362777 [Ostrinia furnacalis]	1.778422	-1.00132	0.212992	-0.88175	-0.10835
TRINITY_DN82810_c0_g1_i1_orf1	-	-	-	putative carbonic anhydrase 3 [Ostrinia furnacalis]	1.758048	0.24728	-0.81609	-1.08195	-0.10729
TRINITY_DN100885_c0_g2_i1_orfp1	-	-	-	CCHC-type zinc finger, partial [Cricetulus griseus]	1.959757	-0.45067	-0.5356	-0.79943	-0.17406
TRINITY_DN607_c0_g1_i16_orf1	-	-	-	protein muscleblind isoform X1 [Ostrinia furnacalis] >XP_028162801.1 protein muscleblind isoform X1 [Ostrinia furnacalis] >XP_028162802.1 protein muscleblind isoform X1 [Ostrinia furnacalis] >XP_028162803.1 protein muscleblind isoform X1 [Ostrinia furnacalis] >XP_028162804.1 protein muscleblind isoform X1 [Ostrinia furnacalis] >XP_028162806.1 protein muscleblind isoform X1 [Ostrinia furnacalis]	1.706023	0.482594	-0.4701	-1.14117	-0.57735
TRINITY_DN101682_c0_g1_i1_orf1	-	-	-	cysteine-rich with EGF-like domain protein 2 [Ostrinia furnacalis]	1.653687	-0.4215	0.188652	-1.43246	0.011618
TRINITY_DN19413_c0_g1_i2_orf1	-	-	-	U4/U6 small nuclear ribonucleoprotein Prp4 [Papilio xuthus]	1.942031	-0.69198	-0.48892	-0.71296	-0.04817
TRINITY_DN8290_c0_g1_i3_orf1	-	-	-	zinc finger CCHC domain-containing protein 8 homolog [Ostrinia furnacalis]	1.595111	0.494775	-0.07916	-1.30788	-0.70285
TRINITY_DN20984_c0_g1_i4_orf1	-	-	-	NADPH--cytochrome P450 reductase isoform X2 [Ostrinia furnacalis]	1.756831	-0.66179	-0.15717	-1.17995	0.242074
TRINITY_DN43355_c0_g1_i1_orf1	-	-	-	uncharacterized protein CG16817-like [Ostrinia furnacalis]	1.766819	0.409517	-0.5513	-1.0203	-0.60474
TRINITY_DN1824_c0_g2_i2_orf1	-	-	-	branched-chain-amino-acid aminotransferase, cytosolic [Manduca sexta] >KAG6453781.1 hypothetical protein O3G_MSEX008327 [Manduca sexta]	1.847051	-0.37691	-1.11241	-0.44802	0.090298
TRINITY_DN2168_c0_g1_i2_orf1	-	-	-	protein arginine methyltransferase NDUFAF7 homolog, mitochondrial [Ostrinia furnacalis]	1.904358	-0.65631	-0.27375	-0.9305	-0.0438
TRINITY_DN659_c0_g1_i3_orf1	-	-	-	probable malonyl-CoA-acyl carrier protein transacylase, mitochondrial [Ostrinia furnacalis]	1.941172	-0.27477	-0.84827	-0.63503	-0.1831
TRINITY_DN10257_c0_g1_i2_orf1	-	-	-	prefoldin subunit domain-containing protein [Phthorimaea operculella]	1.864154	0.164498	-0.92579	-0.4237	-0.67916
TRINITY_DN10287_c0_g1_i1_orf1	-	-	-	nibrin [Ostrinia furnacalis]	1.934484	-0.85976	-0.07471	-0.58061	-0.4194
TRINITY_DN8729_c0_g1_i7_orf1	-	-	-	myosin heavy chain 95F isoform X1 [Ostrinia furnacalis] >XP_028177153.1 myosin heavy chain 95F isoform X2 [Ostrinia furnacalis] >XP_028177154.1 myosin heavy chain 95F isoform X3 [Ostrinia furnacalis] >XP_028177155.1 myosin heavy chain 95F isoform X4 [Ostrinia furnacalis] >XP_028177156.1 myosin heavy chain 95F isoform X5 [Ostrinia furnacalis] >XP_028177158.1 myosin heavy chain 95F isoform X6 [Ostrinia furnacalis]	1.863856	-0.217	-1.04402	0.020502	-0.62334
TRINITY_DN639_c0_g1_i10_orf1	-	-	-	unnamed protein product, partial [Iphiclides podalirius]	1.695958	-0.88206	0.539852	-0.93949	-0.41426
TRINITY_DN2876_c0_g1_i5_orf1	-	-	-	long-chain fatty acid transport protein 4-like isoform X1 [Ostrinia furnacalis]	1.831567	-0.66701	-0.23793	0.134402	-1.06103
TRINITY_DN9759_c0_g1_i1_orf1	-	-	-	iroquois-class homeodomain protein IRX-1-like isoform X1 [Ostrinia furnacalis]	1.58185	-0.05249	0.201119	-1.55707	-0.1734
TRINITY_DN48838_c0_g1_i6_orf1	-	-	-	merlin-like [Ostrinia furnacalis]	1.766538	0.378023	-0.43849	-1.06378	-0.64229
TRINITY_DN3113_c1_g2_i1_orf1	-	-	-	short-chain dehydrogenase/reductase family 16C member 6-like [Ostrinia furnacalis] >XP_028174076.1 short-chain dehydrogenase/reductase family 16C member 6-like [Ostrinia furnacalis]	1.681374	0.494188	-0.28179	-1.11447	-0.7793
TRINITY_DN5105_c0_g1_i10_orf1	-	-	-	poly(U)-binding-splicing factor half pint [Ostrinia furnacalis]	1.990324	-0.38887	-0.53007	-0.66314	-0.40824
TRINITY_DN4026_c0_g1_i4_orf1	-	-	-	serine protease [Ostrinia furnacalis]	1.788958	0.402085	-0.57254	-0.79543	-0.82308
TRINITY_DN13347_c0_g1_i1_orf1	-	-	-	endothelial differentiation-related factor 1 homolog [Ostrinia furnacalis]	1.965917	-0.72891	-0.26647	-0.66117	-0.30937
TRINITY_DN2577_c0_g1_i1_orf1	-	-	-	unnamed protein product [Diatraea saccharalis]	1.558247	-0.76773	0.468234	-1.32615	0.0674
TRINITY_DN14220_c0_g1_i1_orf1	-	-	-	U3 small nucleolar ribonucleoprotein protein IMP4 [Ostrinia furnacalis]	1.907839	-0.6631	-0.90454	-0.02111	-0.3191
TRINITY_DN157_c0_g1_i4_orf1	-	-	-	ATP-binding cassette sub-family A member 1-like [Ostrinia furnacalis]	1.946337	-0.75057	-0.5419	-0.59245	-0.06143
TRINITY_DN1266_c2_g1_i1_orf1	-	-	-	serine/threonine-protein kinase RIO3 [Ostrinia furnacalis]	1.62676	0.365608	-1.14565	-0.94729	0.100571
TRINITY_DN535_c3_g2_i1_orf1	-	-	-	PSME3-interacting protein isoform X2 [Ostrinia furnacalis]	1.83107	-0.1772	0.055375	-0.57899	-1.13026
TRINITY_DN31433_c0_g1_i1_orf1	-	-	-	notchless protein homolog 1 [Ostrinia furnacalis]	1.929043	-0.37491	-0.86996	-0.61353	-0.07065
TRINITY_DN20776_c0_g1_i3_orf1	-	-	-	hypothetical protein evm_003273 [Chilo suppressalis] >CAH2981954.1 unnamed protein product [Chilo suppressalis]	1.975979	-0.34109	-0.50093	-0.7735	-0.36046
TRINITY_DN32822_c0_g1_i1_orf1	-	-	-	eukaryotic translation initiation factor 2 subunit 1 [Ostrinia furnacalis]	1.962114	-0.56478	-0.32177	-0.81089	-0.26468
TRINITY_DN2953_c1_g1_i11_orf1	-	-	-	methionine--tRNA ligase, cytoplasmic isoform X2 [Ostrinia furnacalis] >XP_028156683.1 methionine--tRNA ligase, cytoplasmic isoform X4 [Ostrinia furnacalis] >XP_028156684.1 methionine--tRNA ligase, cytoplasmic isoform X5 [Ostrinia furnacalis]	1.747723	0.431774	-0.75844	-0.41548	-1.00557
TRINITY_DN3111_c0_g1_i5_orf1	-	-	-	CCAAT/enhancer-binding protein zeta-like [Ostrinia furnacalis]	1.880963	0.102239	-0.57473	-0.44996	-0.95851

TRINITY_DN9309_c0_g1_i5_orf1	-	-	-	uncharacterized protein LOC114361160 [Ostrinia furnacalis]	1.731584	0.376452	-0.75441	-1.1096	-0.24402
TRINITY_DN452_c1_g1_i3_orf1	-	-	-	ruvB-like helicase 1 [Colias croceus]	1.841542	0.147084	-0.53634	-1.07605	-0.37624
TRINITY_DN2497_c0_g1_i1_orf1	-	-	-	protein stunted-like isoform X2 [Vanessa tameamea] >XP_046960183.1 protein stunted-like isoform X2 [Vanessa cardui] >XP_047527093.1 protein stunted-like isoform X2 [Vanessa	1.241433	0.586328	-1.74591	-0.21911	0.137265
TRINITY_DN31520_c1_g1_i1_orf1	-	-	-	probable DNA-directed RNA polymerase III subunit RPC6 [Ostrinia furnacalis]	1.676941	0.463168	-0.64916	-1.21477	-0.27618
TRINITY_DN11121_c0_g1_i5_orf1	-	-	-	unnamed protein product [Chilo suppressalis]	1.88124	-0.85909	0.083302	-0.7818	-0.32365
TRINITY_DN6380_c0_g1_i1_orf1	-	-	-	THAP domain-containing protein 1-like isoform X1 [Ostrinia furnacalis]	1.550227	0.768331	-0.41777	-1.06316	-0.83763
TRINITY_DN12826_c0_g1_i1_orf1	-	-	-	uncharacterized protein LOC114363296 [Ostrinia furnacalis]	1.832051	-0.20765	-0.56145	-1.13161	0.068656
TRINITY_DN2859_c0_g1_i7_orf1	-	-	-	cleavage and polyadenylation specificity factor subunit 5 [Ostrinia furnacalis]	1.99559	-0.40517	-0.44731	-0.56797	-0.57513
TRINITY_DN7647_c0_g1_i4_orf1	-	-	-	E3 ubiquitin-protein ligase Bre1 isoform X6 [Ostrinia furnacalis]	1.747521	0.010267	0.189394	-1.05855	-0.88864
TRINITY_DN3588_c0_g1_i1_orf1	-	-	-	probable peroxisomal acyl-coenzyme A oxidase 1 [Ostrinia furnacalis]	1.840039	-1.03358	0.179274	-0.61119	-0.37455
TRINITY_DN7942_c0_g1_i1_orf1	-	-	-	hypothetical protein evm_012160 [Chilo suppressalis] >CAB3521803.1 unnamed protein product [Chilo suppressalis] >CAH0399125.1 unnamed protein product [Chilo suppressalis]	1.851225	0.259169	-0.82176	-0.63385	-0.65478
TRINITY_DN9207_c0_g1_i1_orf1	-	-	-	RNA polymerases N / 8 kDa subunit domain-containing protein [Phthorimaea operculella]	1.790198	-0.8447	-1.03573	0.094451	-0.00422
TRINITY_DN115_c0_g1_i6_orf1	-	-	-	basigin [Ostrinia furnacalis]	1.85199	0.226686	-0.62137	-0.91677	-0.54054
TRINITY_DN16128_c0_g1_i5_orf1	-	-	-	probable prefoldin subunit 4 [Ostrinia furnacalis]	1.697677	-0.16789	0.273256	-1.34286	-0.46018
TRINITY_DN57496_c0_g1_i1_orf1	-	-	-	NADPH:adrenodoxin oxidoreductase, mitochondrial [Ostrinia furnacalis]	1.57794	0.302496	-1.35574	-0.73294	0.208248
TRINITY_DN547_c0_g1_i1_orf1	-	-	-	WD repeat-containing protein 43 [Ostrinia furnacalis]	1.854891	-0.57197	-1.09668	-0.17116	-0.01508
TRINITY_DN5756_c0_g1_i4_orf1	-	-	-	leucine--tRNA ligase, cytoplasmic [Ostrinia furnacalis]	1.8363	-0.79053	0.235103	-0.8929	-0.38797
TRINITY_DN3471_c0_g1_i1_orf1	-	-	-	EH domain-containing protein 3 [Ostrinia furnacalis]	1.856833	-0.31363	0.127702	-0.69132	-0.97958
TRINITY_DN31058_c0_g1_i6_orf1	-	-	-	HIV Tat-specific factor 1 homolog [Ostrinia furnacalis]	1.654854	0.326699	0.089118	-0.99697	-1.0737
TRINITY_DN91_c0_g1_i9_orf1	-	-	-	ribosome-binding protein 1 isoform X8 [Helicoverpa armigera]	1.685049	0.291223	-0.19119	-1.36476	-0.42032
TRINITY_DN647_c4_g1_i1_orf1	-	-	-	coiled-coil domain-containing protein 51-like isoform X2 [Ostrinia furnacalis]	1.847512	-0.08646	-0.04357	-1.08506	-0.63243
TRINITY_DN21492_c0_g1_i1_orf1	-	-	-	isocitrate dehydrogenase [NAD] subunit gamma, mitochondrial [Chelonus insularis]	1.931208	-0.45549	-0.96467	-0.28548	-0.22556
TRINITY_DN4151_c1_g1_i4_orf1	-	-	-	5-methylcytosine rRNA methyltransferase NSUN4 isoform X1 [Ostrinia furnacalis] >XP_028161245.1 5-methylcytosine rRNA methyltransferase NSUN4 isoform X2 [Ostrinia furnacalis]	1.755816	0.326838	-0.77497	-1.07497	-0.23272
TRINITY_DN14475_c0_g1_i1_orf1	-	-	-	NPC intracellular cholesterol transporter 1 homolog 1b-like [Ostrinia furnacalis]	1.839881	-0.78072	-0.95086	-0.27246	0.164159
TRINITY_DN164_c0_g1_i11_orf1	-	-	-	hypothetical protein evm_000323 [Chilo suppressalis] >CAB3530114.1 unnamed protein product [Chilo suppressalis] >CAH0406706.1 unnamed protein product [Chilo suppressalis]	1.899194	0.08119	-0.53965	-0.55097	-0.88976
TRINITY_DN3619_c0_g2_i1_orf1	-	-	-	transport and Golgi organization protein 11 [Ostrinia furnacalis]	1.809869	0.328946	-0.47058	-0.87425	-0.79399
TRINITY_DN36592_c0_g1_i1_orf1	-	-	-	uncharacterized protein LOC114359903 [Ostrinia furnacalis]	1.888251	-0.98643	0.055522	-0.47058	-0.48677
TRINITY_DN21123_c0_g1_i1_orf1	-	-	-	maternal protein exuperantia [Ostrinia furnacalis]	1.865534	-0.41418	0.018452	-1.10078	-0.36903
TRINITY_DN7613_c1_g2_i1_orf1	-	-	-	60S ribosomal protein L19 [Helicoverpa armigera] >XP_022126104.1 60S ribosomal protein L19 [Pieris rapae] >XP_022821503.1 60S ribosomal protein L19 [Spodoptera litura] >XP_026731717.1 60S ribosomal protein L19 [Trichoplusia ni] >XP_035451321.1 60S ribosomal protein L19-like [Spodoptera frugiperda] >XP_035452592.1 60S ribosomal protein L19-like [Spodoptera frugiperda] >XP_041975187.1 60S ribosomal protein L19 [Aricia agestis] >XP_045524933.1 60S ribosomal protein L19 [Pieris brassicae] >XP_047023231.1 60S ribosomal protein L19 [Helicoverpa zea] >XP_047984679.1 60S ribosomal protein L19 [Leguminivora glycinivorella] >XP_049874324.1 60S ribosomal protein L19 [Pectinophora gossypiella] >ACY95336.1 ribosomal protein L19 [Manduca sexta] >KAF9423217.1 hypothetical protein HW555_001286 [Spodoptera exigua] >KAI5632448.1 ribosomal protein l19e domain-containing protein [Phthorimaea operculella] >RVE50663.1 hypothetical protein evm_004695 [Chilo suppressalis] >CAB3239671.1 unnamed protein product [Arctia plantaginis] >CAB3509883.1 unnamed protein product [Spodoptera littoralis] >CAG4986349.1 unnamed protein product [Parnassius apollo] >CAG9758258.1 unnamed protein product [Diatraea saccharalis] >CAH2049991.1 unnamed protein product, partial [Iphiclides podalirius]	1.776434	-0.868	0.287477	-0.98078	-0.21513
TRINITY_DN26429_c0_g1_i4_orf1	-	-	-	zinc transporter 9 [Ostrinia furnacalis]	1.78489	0.376306	-0.47985	-0.96076	-0.72058
TRINITY_DN11409_c0_g1_i4_orf1	-	-	-	nodal modulator 1 [Ostrinia furnacalis]	1.879138	-0.12581	-0.0879	-1.00368	-0.66175
TRINITY_DN7228_c0_g1_i6_orf1	-	-	-	neutral alpha-glucosidase AB [Ostrinia furnacalis]	1.749874	0.406197	-1.01096	-0.7908	-0.35431
TRINITY_DN3856_c0_g1_i7_orf1	-	-	-	uncharacterized protein LOC114355702 [Ostrinia furnacalis]	1.67893	0.523153	-0.55221	-1.17245	-0.47742
TRINITY_DN6063_c1_g2_i1_orf1	-	-	-	short-chain specific acyl-CoA dehydrogenase, mitochondrial [Ostrinia furnacalis]	1.837026	-0.25813	0.165776	-0.80424	-0.94044
TRINITY_DN3909_c0_g2_i2_orf1	-	-	-	ribosomal protein L24 [Loxostege sticticalis]	1.709794	-0.74721	0.279198	-1.19939	-0.0424

TRINITY_DN2430_c0_g1_i1_orf1	-	-	-	glutathione S-transferase omega 1 [Ostrinia furnacalis]	1.793497	0.124162	-0.59686	-1.17975	-0.14104
TRINITY_DN211_c1_g1_i10_orf1	-	-	-	protein hu-li tai shao isoform X5 [Galleria mellonella]	1.777981	-1.03426	0.251996	-0.82179	-0.17393
TRINITY_DN6313_c0_g1_i4_orf1	-	-	-	pyruvate dehydrogenase E1 component subunit beta, mitochondrial isoform X1 [Ostrinia furnacalis] >XP_028161232.1 pyruvate dehydrogenase E1 component subunit beta, mitochondrial isoform X2 [Ostrinia furnacalis] >XP_028161233.1 pyruvate dehydrogenase E1 component subunit beta, mitochondrial isoform X3 [Ostrinia furnacalis] >XP_028161234.1 pyruvate dehydrogenase E1 component subunit beta, mitochondrial isoform X4 [Ostrinia	1.912305	-0.99289	-0.39338	-0.08391	-0.44214
TRINITY_DN3321_c0_g1_i3_orf1	-	-	-	peroxidasin [Ostrinia furnacalis]	1.675555	0.249561	0.033661	-1.30396	-0.65481
TRINITY_DN24318_c0_g1_i1_orf1	-	-	-	60S ribosomal protein L29 [Ostrinia furnacalis]	1.638541	-0.78424	0.46623	-1.21293	-0.1076
TRINITY_DN7161_c0_g1_i7_orf1	-	-	-	ATP-binding cassette sub-family E member 1 [Ostrinia furnacalis]	1.855598	-0.20556	0.018346	-1.08159	-0.5868
TRINITY_DN18249_c0_g1_i1_orf1	-	-	-	60S ribosomal protein L13 [Pectinophora gossypiella]	1.527846	-0.70612	0.713513	-1.25726	-0.27797
TRINITY_DN34689_c0_g1_i4_orf1	-	-	-	uncharacterized protein DDB_G0283357 isoform X13 [Helicoverpa armigera] >XP_049707197.1 uncharacterized protein DDB_G0283357 isoform X14 [Helicoverpa armigera] >XP_049707198.1 uncharacterized protein DDB_G0283357 isoform X15 [Helicoverpa armigera] >XP_049707199.1 uncharacterized protein DDB_G0283357 isoform X16 [Helicoverpa armigera] >XP_049707200.1 uncharacterized protein DDB_G0283357 isoform X17 [Helicoverpa armigera] >XP_049707201.1 uncharacterized protein DDB_G0283357 isoform X18 [Helicoverpa armigera] >XP_049707202.1 uncharacterized protein DDB_G0283357 isoform X19 [Helicoverpa armigera] >XP_049707203.1 uncharacterized protein DDB_G0283357 isoform X20 [Helicoverpa armigera] >XP_049707204.1 uncharacterized protein DDB_G0283357 isoform X21 [Helicoverpa armigera] >XP_049707205.1 uncharacterized protein DDB_G0283357 isoform X22 [Helicoverpa armigera] >XP_049707206.1 uncharacterized protein DDB_G0283357 isoform	1.662405	0.64305	-0.5979	-0.9152	-0.79235
TRINITY_DN24325_c0_g1_i12_orf1	-	-	-	MICOS complex subunit MIC10-like [Ostrinia furnacalis]	1.926804	-0.96108	-0.47338	-0.15011	-0.34224
TRINITY_DN17726_c0_g1_i1_orf1	-	-	-	ubiquitin conjugation factor E4 A isoform X1 [Ostrinia furnacalis] >XP_028165923.1 ubiquitin conjugation factor E4 A isoform X2 [Ostrinia furnacalis]	1.718353	0.408133	-0.22225	-1.04763	-0.85661
TRINITY_DN3343_c0_g2_i1_orf1	-	-	-	AFG3-like protein 2 [Ostrinia furnacalis]	1.658444	0.474428	-0.57785	-1.26819	-0.28684
TRINITY_DN31216_c0_g1_i2_orf1	-	-	-	uncharacterized protein LOC114361092 [Ostrinia furnacalis]	1.701957	-0.04263	0.004118	-1.43088	-0.23256
TRINITY_DN1334_c0_g1_i2_orf1	-	-	-	phosphoenolpyruvate carboxykinase [GTP]-like [Ostrinia furnacalis]	1.845592	-0.19071	-0.27124	-0.17869	-1.20496
TRINITY_DN1791_c0_g1_i3_orf1	-	-	-	succinate dehydrogenase assembly factor 2-B, mitochondrial-like [Ostrinia furnacalis]	1.703931	-0.89252	0.339757	-1.08646	-0.0647
TRINITY_DN14498_c0_g1_i1_orf1	-	-	-	eukaryotic translation initiation factor 2 subunit 2 [Ostrinia furnacalis]	1.759721	-1.02763	0.380763	-0.76033	-0.35252
TRINITY_DN26243_c0_g1_i2_orf1	-	-	-	dynein heavy chain 6, axonemal [Ostrinia furnacalis]	1.80807	-0.06137	-0.77457	-1.05819	0.086052
TRINITY_DN13233_c0_g1_i3_orf1	-	-	-	60S ribosomal protein L31 [Galleria mellonella] >XP_028158009.1 60S ribosomal protein L31 [Ostrinia furnacalis] >XP_030037192.1 60S ribosomal protein L31 [Manduca sexta] >XP_046978528.1 60S ribosomal protein L31 [Vanessa cardui] >XP_047545474.1 60S ribosomal protein L31 [Vanessa atalanta] >XP_050342244.1 60S ribosomal protein L31 [Nymphalis io] >GBP35474.1 60S ribosomal protein L31 [Eumeta japonica] >ACY95330.1 ribosomal protein L31 [Manduca sexta] >KAG6463984.1 hypothetical protein O3G_MSEX014198 [Manduca sexta] >KAG6463985.1 hypothetical protein O3G_MSEX014198	1.73542	-1.00127	0.465581	-0.75693	-0.4428
TRINITY_DN63561_c1_g1_i2_orf1	-	-	-	unnamed protein product [Diatraea saccharalis]	1.695542	0.504024	-0.41599	-1.1236	-0.65998
TRINITY_DN9931_c0_g1_i1_orf1	-	-	-	syntaxin-18 [Ostrinia furnacalis]	1.824647	-0.07907	-0.39959	-1.22021	-0.12577
TRINITY_DN53115_c0_g1_i1_orf1	-	-	-	small glutamine-rich tetratricopeptide repeat-containing protein beta-like [Ostrinia furnacalis]	1.690325	0.581998	-0.52238	-0.86988	-0.88006
TRINITY_DN24917_c0_g2_i1_orf1	-	-	-	hypothetical protein L3Q82_022586 [Scortum barcoo]	1.731256	-0.18108	-1.19856	0.309783	-0.6614
TRINITY_DN2812_c0_g1_i5_orf1	-	-	-	myotubularin-related protein 2 [Ostrinia furnacalis] >XP_028170267.1 myotubularin-related protein 2 [Ostrinia furnacalis]	1.391922	0.990095	-0.55767	-0.68072	-1.14363
TRINITY_DN5112_c0_g1_i1_orf1	-	-	-	unnamed protein product, partial [Iphiclides podalirius]	1.741953	-0.12709	-0.2407	-1.37532	0.001157
TRINITY_DN7770_c0_g1_i4_orf1	-	-	-	presequence protease, mitochondrial [Ostrinia furnacalis]	1.923489	-0.20796	-0.99891	-0.39261	-0.32401
TRINITY_DN147517_c0_g1_i1_orf1	-	-	-	eukaryotic translation initiation factor 4 gamma 3-like isoform X5 [Ostrinia furnacalis]	1.899386	-0.52107	-0.07294	-1.01566	-0.28972
TRINITY_DN5442_c0_g1_i4_orf1	-	-	-	hypothetical protein evm_004688 [Chilo suppressalis]	1.47048	0.929532	-0.64856	-0.77766	-0.97379
TRINITY_DN2973_c1_g1_i9_orf1	-	-	-	unconventional myosin ID [Ostrinia furnacalis]	1.981586	-0.74128	-0.42698	-0.33331	-0.48002
TRINITY_DN972_c0_g2_i1_orf1	-	-	-	DNA damage-binding protein 1 [Ostrinia furnacalis]	1.639579	0.561205	-0.56177	-1.23064	-0.40837
TRINITY_DN6876_c0_g2_i1_orf1	-	-	-	serine/threonine-protein phosphatase 5 [Spodoptera litura] >CAB3508320.1 unnamed protein product [Spodoptera littoralis] >CAH1637875.1 unnamed protein product [Spodoptera	1.91561	-0.93389	-0.12396	-0.23517	-0.62259

TRINITY_DN16816_c0_g1_i1_orf1	-	-	-	ATP-binding cassette sub-family F member 1 [Ostrinia furnacalis] >XP_028179049.1 ATP-binding cassette sub-family F member 1 [Ostrinia furnacalis]	1.873963	-0.68626	0.033697	-0.9783	-0.2431
TRINITY_DN642_c0_g1_i6_orf1	-	-	-	reticulon-3-B isoform X5 [Ostrinia furnacalis]	1.828869	-0.81085	0.201501	-0.93652	-0.283
TRINITY_DN8908_c0_g1_i1_orf1	-	-	-	unnamed protein product [Spodoptera littoralis] >CAH1641822.1 unnamed protein product [Spodoptera littoralis]	1.754678	-1.01641	0.439899	-0.57384	-0.60433
TRINITY_DN8980_c0_g1_i2_orf1	-	-	-	putative ATP-dependent RNA helicase me31b [Ostrinia furnacalis] >XP_028162602.1 putative ATP-dependent RNA helicase me31b [Ostrinia furnacalis] >XP_028162603.1 putative ATP-dependent RNA helicase me31b [Ostrinia furnacalis]	1.782798	0.397572	-0.66203	-0.56863	-0.94971
TRINITY_DN9156_c0_g1_i1_orf1	-	-	-	GTP:AMP phosphotransferase AK3, mitochondrial [Ostrinia furnacalis]	1.892352	-0.33521	-1.09066	-0.29769	-0.1688
TRINITY_DN1665_c1_g1_i2_orf1	-	-	-	translation elongation factor 2 [Melitaea cinxia]	1.643293	-0.86346	0.56923	-1.07454	-0.27452
TRINITY_DN5952_c0_g1_i6_orf1	-	-	-	LOW QUALITY PROTEIN: phosphoacetylglucosamine mutase [Ostrinia furnacalis]	1.411353	0.983471	-0.58776	-1.08055	-0.72651
TRINITY_DN11322_c0_g1_i2_orf1	-	-	-	CRAL-TRIO domain-containing protein C3H8.02 [Ostrinia furnacalis]	1.865186	-0.10045	-0.9878	-0.04683	-0.73011
TRINITY_DN50724_c0_g2_i1_orf1	-	-	-	40S ribosomal protein S13 [Papilio polytes] >NP_001299165.1 40S ribosomal protein S13 [Papilio xuthus] >XP_013193651.1 PREDICTED: 40S ribosomal protein S13 [Amyelois transitella] >XP_014356884.1 40S ribosomal protein S13 [Papilio machaon] >XP_021184589.1 40S ribosomal protein S13 [Helicoverpa armigera] >XP_022827875.1 40S ribosomal protein S13 [Spodoptera litura] >XP_023936121.1 40S ribosomal protein S13 [Bicyclus anynana] >XP_026318936.1 40S ribosomal protein S13 [Hyposmocoma kahamanoa] >XP_026488656.1 40S ribosomal protein S13 [Vanessa tameamea] >XP_026736523.1 40S ribosomal protein S13 [Trichoplusia ni] >XP_028172792.1 40S ribosomal protein S13 [Ostrinia furnacalis] >XP_032516773.1 40S ribosomal protein S13 [Danaus plexippus plexippus] >XP_034829282.1 40S ribosomal protein S13 [Maniola hyperantus] >XP_035450454.1 40S ribosomal protein S13 [Spodoptera frugiperda] >XP_039754671.1 40S ribosomal protein S13 [Pararge aegeria] >XP_045449727.1 40S ribosomal protein S13 [Melitaea cinxia] >XP_046977814.1 40S ribosomal protein S13 [Vanessa cardui] >XP_047024943.1 40S ribosomal protein S13 [Helicoverpa zea] >XP_047531020.1 40S ribosomal protein S13 [Vanessa atalanta] >XP_047990290.1 40S ribosomal protein S13 [Leguminivora glycinivorella] >XP_050348531.1 40S ribosomal protein S13 [Nymphalis io] >Q962R6.3 RecName: Full=40S ribosomal protein S13 [Spodoptera frugiperda] >ADT80641.1 ribosomal protein S13 [Euphydryas aurinia] >ATG34155.1 ribosomal protein S13 [Epirrita autumnata] >KAF9422710.1 hypothetical protein HW555_001704 [Spodoptera exigua] >KAG7298871.1 ribosomal 40S subunit protein S13 [Plutella xylostella] >KAI5637622.1 ribosomal protein s15 domain-containing protein [Phthorimaea operculella] >RVE47566.1 hypothetical protein evm_007764 [Chilo suppressalis] >UNW37540.1 ribosomal protein S13 [Sesamia inferens] >CAB3507114.1 unnamed protein product [Spodoptera littoralis] >CAD0201945.1 unnamed protein product [Chrysodeixis includens] >CAG4974609.1 unnamed protein product [Parnassius apollo] >CAG9562339.1 unnamed protein product [Danaus chrysippus] >CAG9749269.1 unnamed protein product [Diatraea saccharalis] >CAH0720232.1 unnamed protein product, partial [Brenthis ino] >CAH2046814.1 unnamed protein product, partial [Iphiclides podalirius] >CAH2085029.1	1.930181	-0.64208	-0.00923	-0.78825	-0.49062
TRINITY_DN30950_c0_g1_i13_orf1	-	-	-	unnamed protein product [Chilo suppressalis]	1.640437	0.37533	0.050605	-1.15685	-0.90952
TRINITY_DN2265_c0_g1_i5_orf1	-	-	-	elongation factor G, mitochondrial [Ostrinia furnacalis]	1.677075	-0.85617	-1.17813	0.214692	0.14253
TRINITY_DN19079_c0_g1_i5_orf1	-	-	-	unnamed protein product [Euphydryas editha]	1.639991	0.549892	-0.21158	-1.04589	-0.93242
TRINITY_DN7991_c0_g1_i9_orf1	-	-	-	hypothetical protein evm_006720 [Chilo suppressalis] >CAB3528247.1 unnamed protein product [Chilo suppressalis] >CAH0404834.1 unnamed protein product [Chilo suppressalis]	1.851699	-0.85606	0.152587	-0.85329	-0.29494
TRINITY_DN42373_c0_g4_i1_orf1	-	-	-	unnamed protein product [Spodoptera exigua]	1.530804	-0.95641	0.719567	-1.08685	-0.20711
TRINITY_DN8012_c0_g1_i3_orf1	-	-	-	uncharacterized protein LOC114354053 [Ostrinia furnacalis]	1.891628	0.068575	-0.92275	-0.63581	-0.40164
TRINITY_DN133760_c0_g1_i1_orf1	-	-	-	THO complex subunit 7 homolog [Ostrinia furnacalis]	1.788091	0.390564	-0.89579	-0.5299	-0.75297
TRINITY_DN43576_c0_g1_i3_orf1	-	-	-	regulator of microtubule dynamics protein 1-like [Ostrinia furnacalis]	1.874487	-0.63878	-0.03952	-1.0231	-0.17308
TRINITY_DN5976_c0_g1_i1_orf1	-	-	-	ribosomal protein L32 [Bombyx mori] >XP_028034407.1 60S ribosomal protein L32 [Bombyx mandarina] >AAV34844.1 ribosomal protein L32 [Bombyx mori]	1.910404	-0.81203	0.056236	-0.68036	-0.47425
TRINITY_DN7573_c0_g2_i1_orf1	-	-	-	nucleolar protein 56 [Ostrinia furnacalis]	1.944971	-0.6233	-0.04081	-0.58293	-0.69793
TRINITY_DN3753_c0_g1_i7_orf1	-	-	-	very-long-chain 3-oxoacyl-CoA reductase isoform X2 [Ostrinia furnacalis]	1.871766	0.123207	-0.60382	-0.42257	-0.96858
TRINITY_DN57462_c0_g1_i1_orf1	-	-	-	glutathione S transferase-S5 [Glyphodes pyloalis]	1.670249	0.252951	-1.42006	-0.29127	-0.21187
TRINITY_DN10502_c0_g1_i4_orf1	-	-	-	interleukin enhancer-binding factor 2 homolog [Ostrinia furnacalis]	1.613465	0.708709	-0.58465	-1.01565	-0.72187

TRINITY_DN6556_c0_g1_i7_orf1	-	-	-	NFX1-type zinc finger-containing protein 1-like isoform X1 [Ostrinia furnacalis] >XP_028173496.1 NFX1-type zinc finger-containing protein 1-like isoform X1 [Ostrinia furnacalis] >XP_028173497.1 NFX1-type zinc finger-containing protein 1-like isoform X1	1.801966	-0.34759	-0.74406	-0.99788	0.287572
TRINITY_DN1170_c0_g1_i8_orf1	-	-	-	titin homolog [Trichoplusia ni]	1.649494	0.533755	-0.21121	-1.03699	-0.93505
TRINITY_DN22941_c0_g1_i1_orf1	-	-	-	protein CWC15 homolog [Ostrinia furnacalis]	1.914166	-0.96726	-0.06872	-0.50999	-0.3682
TRINITY_DN131371_c0_g1_i1_orf1	-	-	-	golgin subfamily B member 1-like [Ostrinia furnacalis]	1.449409	-0.15203	0.33191	-1.66277	0.033485
TRINITY_DN1790_c1_g1_i3_orf1	-	-	-	unnamed protein product [Diatraea saccharalis]	1.839921	-0.5712	0.275817	-0.67341	-0.87113
TRINITY_DN4469_c0_g1_i2_orf1	-	-	-	metal transporter CNNM4-like [Ostrinia furnacalis]	1.777983	-0.84112	0.280063	-1.00368	-0.21324
TRINITY_DN129207_c0_g1_i1_orf1	-	-	-	U3 small nucleolar RNA-interacting protein 2 [Ostrinia furnacalis]	1.920809	-0.05609	-0.53398	-0.40385	-0.92688
TRINITY_DN756_c0_g1_i11_orf1	-	-	-	calcium-binding protein E63-1 isoform X1 [Ostrinia furnacalis]	1.814291	-0.86176	0.185368	-0.94574	-0.19215
TRINITY_DN22871_c0_g2_i1_orf1	-	-	-	mitochondrial import inner membrane translocase subunit Tim17-B [Ostrinia furnacalis]	1.791495	-0.46552	-1.16556	0.237947	-0.39836
TRINITY_DN6535_c0_g1_i3_orf1	-	-	-	mRNA export factor [Ostrinia furnacalis]	1.858149	-0.4875	0.194254	-0.93645	-0.62845
TRINITY_DN7655_c0_g1_i3_orf1	-	-	-	hypothetical protein evm_001118 [Chilo suppressalis] >CAB3522191.1 unnamed protein product [Chilo suppressalis]	1.876567	0.147511	-0.58729	-0.91795	-0.51884
TRINITY_DN11639_c0_g1_i1_orf1	-	-	-	cysteine--tRNA ligase, cytoplasmic isoform X1 [Ostrinia furnacalis] >XP_028156309.1 cysteine--tRNA ligase, cytoplasmic isoform X2 [Ostrinia furnacalis] >XP_028156310.1 cysteine--tRNA ligase, cytoplasmic isoform X3 [Ostrinia furnacalis] >XP_028156311.1 cysteine--tRNA ligase, cytoplasmic isoform X4 [Ostrinia furnacalis]	1.977138	-0.21556	-0.60504	-0.50901	-0.64753
TRINITY_DN15845_c0_g1_i1_orf1	-	-	-	probable ATP-dependent RNA helicase DDX28 isoform X1 [Ostrinia furnacalis] >XP_028162024.1 probable ATP-dependent RNA helicase DDX28 isoform X2 [Ostrinia furnacalis] >XP_028162026.1 probable ATP-dependent RNA helicase DDX28 isoform X3 [Ostrinia furnacalis] >XP_028162027.1 probable ATP-dependent RNA helicase DDX28 isoform	1.792072	0.23552	-0.2879	-1.13143	-0.60827
TRINITY_DN81926_c0_g1_i1_orf1	-	-	-	membrane-bound alkaline phosphatase-like isoform X3 [Ostrinia furnacalis]	1.87206	-0.8276	-0.50084	-0.72561	0.181992
TRINITY_DN12806_c0_g2_i1_orf1	-	-	-	inactive pancreatic lipase-related protein 1-like isoform X2 [Ostrinia furnacalis]	1.598521	-0.91151	0.638342	-1.06772	-0.25763
TRINITY_DN64810_c0_g1_i1_orf1	-	-	-	arginine--tRNA ligase, cytoplasmic [Ostrinia furnacalis]	1.951539	-0.79375	-0.11485	-0.47455	-0.56839
TRINITY_DN15836_c0_g1_i1_orf1	-	-	-	DNA-directed RNA polymerase I subunit RPA49-like [Ostrinia furnacalis]	1.766173	0.409689	-0.7165	-0.47031	-0.98905
TRINITY_DN9938_c0_g2_i1_orf1	-	-	-	hypothetical protein E2986_04423 [Frieseomelitta varia]	1.578868	0.603338	-0.31074	-1.32006	-0.55141
TRINITY_DN121_c0_g1_i9_orf1	-	-	-	lethal(2) giant larvae protein homolog 1 isoform X1 [Ostrinia furnacalis]	1.994647	-0.4104	-0.43581	-0.53198	-0.61647
TRINITY_DN21251_c1_g1_i1_orf1	-	-	-	60S ribosomal protein L4 [Ostrinia furnacalis]	1.794772	-0.75475	0.177525	-1.07591	-0.14164
TRINITY_DN20130_c0_g1_i1_orf1	-	-	-	uncharacterized protein LOC114354518 isoform X1 [Ostrinia furnacalis]	1.853459	-0.51992	0.038468	-1.10534	-0.26666
TRINITY_DN17208_c0_g1_i2_orf1	-	-	-	integrator complex subunit 11 [Ostrinia furnacalis]	1.888269	-0.32263	-0.22231	-1.10679	-0.23654
TRINITY_DN4025_c0_g1_i1_orf1	-	-	-	unnamed protein product [Chilo suppressalis]	1.879703	-0.16194	-0.23586	-1.11966	-0.36224
TRINITY_DN146758_c0_g1_i1_orf1	-	-	-	PREDICTED: mitochondrial import inner membrane translocase subunit Tim16-like [Fopius arisanus]	1.889561	-0.87352	-0.78622	-0.0101	-0.21972
TRINITY_DN4233_c0_g2_i2_orf1	-	-	-	actin-related protein 2/3 complex subunit 5-B [Ostrinia furnacalis]	1.46555	0.874477	-0.51369	-1.19229	-0.63405
TRINITY_DN40015_c0_g1_i2_orf1	-	-	-	PREDICTED: 60S ribosomal protein L18 [Amyelois transitella]	1.860582	-0.7817	0.141528	-0.8952	-0.3252
TRINITY_DN4950_c0_g1_i2_orf1	-	-	-	unnamed protein product [Diatraea saccharalis]	1.564297	0.733985	-0.53014	-1.17564	-0.59251
TRINITY_DN17651_c0_g1_i2_orf1	-	-	-	transmembrane protein 70 homolog, mitochondrial [Ostrinia furnacalis]	1.911701	-0.41261	-1.02557	-0.16179	-0.31173
TRINITY_DN17212_c0_g1_i6_orf1	-	-	-	putative deoxyribonuclease TATDN1 [Ostrinia furnacalis]	1.826881	0.311528	-0.8569	-0.71163	-0.56988
TRINITY_DN2967_c0_g1_i4_orf1	-	-	-	UDP-glycosyltransferase UGT41G1 [Ostrinia furnacalis]	1.871421	-0.72378	-0.97798	-0.12413	-0.04553
TRINITY_DN6406_c0_g1_i1_orf1	-	-	-	protein FAM98A-like [Ostrinia furnacalis]	1.875198	-0.80138	-0.81446	-0.39842	0.139061
TRINITY_DN2913_c0_g1_i5_orf1	-	-	-	aquaporin-11 isoform X1 [Spodoptera litura]	1.852687	-0.88649	-0.23933	-0.84315	0.116272
TRINITY_DN1820_c0_g1_i6_orf1	-	-	-	3-hydroxyisobutyryl-CoA hydrolase, mitochondrial [Ostrinia furnacalis]	1.979369	-0.39322	-0.69139	-0.60417	-0.2906
TRINITY_DN124300_c0_g1_i2_orf1	-	-	-	protein transport protein Sec23A isoform X1 [Venturia canescens]	1.943854	-0.34763	-0.17234	-0.88099	-0.54289
TRINITY_DN45000_c0_g1_i5_orf1	-	-	-	PREDICTED: ATP synthase subunit beta, mitochondrial, partial [Papilio polytes]	1.964654	-0.40431	-0.43912	-0.28198	-0.83924
TRINITY_DN21619_c0_g1_i1_orf1	-	-	-	28S ribosomal protein S15, mitochondrial [Ostrinia furnacalis]	1.859751	-0.4214	-0.7958	-0.83314	0.190593
TRINITY_DN81719_c0_g1_i1_orf1	-	-	-	tyrosine 3-monooxygenase isoform X1 [Ostrinia furnacalis] >ARE68330.1 tyrosin hydroxylase [Ostrinia furnacalis]	1.92948	-0.13563	-0.9121	-0.30297	-0.57878
TRINITY_DN1427_c0_g1_i7_orf1	-	-	-	SAFB-like transcription modulator isoform X1 [Ostrinia furnacalis] >XP_028158609.1 SAFB-like transcription modulator isoform X2 [Ostrinia furnacalis]	1.681546	0.001108	0.023805	-1.45149	-0.25497
TRINITY_DN9874_c0_g1_i7_orf1	-	-	-	hypothetical protein evm_013697 [Chilo suppressalis]	1.904476	-0.8569	-0.01853	-0.74785	-0.28119
TRINITY_DN2914_c0_g1_i1_orf1	-	-	-	U1 small nuclear ribonucleoprotein A [Ostrinia furnacalis]	1.645804	0.524719	-0.2543	-1.1983	-0.71792
TRINITY_DN49742_c0_g1_i4_orf1	-	-	-	neuroglobin-like [Ostrinia furnacalis]	1.958077	-0.42322	-0.2016	-0.83603	-0.49723
TRINITY_DN42269_c2_g1_i1_orf1	-	-	-	probable enoyl-CoA hydratase, mitochondrial [Ostrinia furnacalis]	1.858598	-0.71448	-1.00544	0.016125	-0.1548

TRINITY_DN6696_c0_g1_i4_orf1	-	-	-	mitochondrial 2-oxodicarboxylate carrier [Ostrinia furnacalis]	1.813981	0.000618	-0.00737	-0.70818	-1.09905
TRINITY_DN6396_c0_g1_i1_orf1	-	-	-	PR domain zinc finger protein 10-like [Ostrinia furnacalis]	1.709474	-0.24835	0.347015	-1.26526	-0.54288
TRINITY_DN20966_c0_g1_i6_orf1	-	-	-	clavesin-1-like [Ostrinia furnacalis]	1.900961	-0.89868	-0.22237	-0.05444	-0.72547
TRINITY_DN1567_c0_g1_i15_orf1	-	-	-	probable dual specificity protein kinase madd-3 isoform X1 [Ostrinia furnacalis]	1.958366	-0.37321	-0.22514	-0.5219	-0.83813
TRINITY_DN2738_c1_g1_i3_orf1	-	-	-	uridine-cytidine kinase isoform X1 [Helicoverpa zea] >XP_049697747.1 uridine-cytidine kinase-like isoform X1 [Helicoverpa armigera] >XP_049698409.1 uridine-cytidine kinase isoform X1 [Helicoverpa armigera]	1.80845	0.357772	-0.58994	-0.86278	-0.7135
TRINITY_DN59042_c1_g1_i1_orf1	-	-	-	nuclear pore complex protein Nup50 [Ostrinia furnacalis]	1.830648	0.161836	-0.7352	-1.0105	-0.24678
TRINITY_DN4861_c0_g1_i7_orf1	-	-	-	2-hydroxyacyl-CoA lyase 1 isoform X1 [Ostrinia furnacalis]	1.969991	-0.80209	-0.50004	-0.297	-0.37085
TRINITY_DN21218_c0_g2_i3_orf1	-	-	-	leukotriene A-4 hydrolase isoform X2 [Ostrinia furnacalis]	1.786775	0.394187	-0.5507	-0.71538	-0.91488
TRINITY_DN8824_c0_g2_i1_orf1	-	-	-	60S ribosomal protein L34-like [Ostrinia furnacalis]	1.763305	-0.70005	0.179784	-1.16732	-0.07572
TRINITY_DN1666_c0_g1_i2_orf1	-	-	-	putative defense protein Hdd11 [Ostrinia furnacalis] >XP_028179344.1 putative defense protein Hdd11 [Ostrinia furnacalis] >AGV28583.1 immune-induced protein [Ostrinia	1.659134	-0.97649	-1.05076	-0.06283	0.430937
TRINITY_DN605_c0_g1_i4_orf1	-	-	-	dnaJ homolog subfamily C member 7 [Ostrinia furnacalis]	1.96236	-0.1367	-0.5647	-0.53947	-0.72149
TRINITY_DN2783_c1_g1_i2_orf1	-	-	-	proline-rich extensin-like protein EPR1 isoform X1 [Ostrinia furnacalis] >XP_028168549.1 proline-rich extensin-like protein EPR1 isoform X2 [Ostrinia furnacalis] >XP_028168550.1 proline-rich extensin-like protein EPR1 isoform X2 [Ostrinia furnacalis]	1.791775	0.405432	-0.82288	-0.64446	-0.72987
TRINITY_DN5908_c0_g1_i2_orf1	-	-	-	ATP-binding cassette sub-family B member 10, mitochondrial-like [Ostrinia furnacalis]	1.868378	-0.36576	-0.83463	0.143329	-0.81131
TRINITY_DN6871_c0_g1_i3_orf1	-	-	-	1-acyl-sn-glycerol-3-phosphate acyltransferase gamma-like [Ostrinia furnacalis] >XP_028169689.1 1-acyl-sn-glycerol-3-phosphate acyltransferase gamma-like [Ostrinia furnacalis]	1.612139	0.430511	-0.73711	-0.01241	-1.29313
TRINITY_DN71840_c0_g1_i1_orf1	-	-	-	60S ribosomal protein L7 [Ostrinia furnacalis] >XP_028162266.1 60S ribosomal protein L7 [Ostrinia furnacalis]	1.894975	-0.72073	-0.01468	-0.90926	-0.25031
TRINITY_DN649_c1_g1_i13_orf1	-	-	-	U1 small nuclear ribonucleoprotein 70 kDa isoform X2 [Ostrinia furnacalis]	1.80467	-0.01015	0.050645	-1.06075	-0.78442
TRINITY_DN58261_c0_g1_i1_orf1	-	-	-	15-hydroxyprostaglandin dehydrogenase [NAD(+)]-like [Ostrinia furnacalis]	1.57736	-0.39798	0.589104	-1.35474	-0.41375
TRINITY_DN437_c0_g1_i1_orf1	-	-	-	LOW QUALITY PROTEIN: fibronectin type-III domain-containing protein 3A-like [Ostrinia furnacalis]	1.758647	-0.15305	0.27975	-0.8239	-1.06144
TRINITY_DN5559_c0_g1_i1_orf1	-	-	-	ferrochelatase, mitochondrial isoform X2 [Ostrinia furnacalis]	1.950031	-0.63313	-0.82008	-0.23136	-0.26546
TRINITY_DN3063_c0_g1_i5_orf1	-	-	-	mini-chromosome maintenance complex-binding protein [Ostrinia furnacalis]	1.824217	-0.30907	0.16233	-1.1065	-0.57097
TRINITY_DN12964_c0_g1_i1_orf1	-	-	-	dnaJ homolog subfamily A member 1 [Ostrinia furnacalis]	1.859848	0.136479	-0.69989	-0.95815	-0.33829
TRINITY_DN15737_c0_g1_i7_orf1	-	-	-	UPF0160 protein C27H6.8 [Ostrinia furnacalis]	1.659873	-0.76283	0.402171	-1.22284	-0.07637
TRINITY_DN19155_c0_g1_i1_orf1	-	-	-	cleavage and polyadenylation specificity factor 73 [Ostrinia furnacalis]	1.701781	0.516555	-0.44225	-0.70875	-1.06733
TRINITY_DN31327_c0_g2_i1_orf1	-	-	-	multidrug resistance protein 1A isoform X1 [Ostrinia furnacalis]	1.902751	-0.34459	-0.31825	-1.06201	-0.1779
TRINITY_DN16886_c0_g1_i4_orf1	-	-	-	ER membrane protein complex subunit 1 [Ostrinia furnacalis]	1.970117	-0.73956	-0.20424	-0.5539	-0.47242
TRINITY_DN17446_c0_g1_i1_orf1	-	-	-	eukaryotic translation initiation factor 3 subunit E [Ostrinia furnacalis]	1.935666	-0.7033	-0.00137	-0.63661	-0.5944
TRINITY_DN19135_c0_g1_i1_orf1	-	-	-	ER membrane protein complex subunit 10 [Ostrinia furnacalis]	1.791371	0.136935	-0.32328	-1.23822	-0.36681
TRINITY_DN132043_c0_g1_i1_orf1	-	-	-	ankyrin repeat and MYND domain-containing protein 2 [Ostrinia furnacalis]	1.674916	0.576544	-0.42867	-1.00416	-0.81863
TRINITY_DN14996_c0_g1_i2_orf1	-	-	-	40S ribosomal protein S17 [Ostrinia furnacalis]	1.823644	-1.09609	0.016547	-0.68494	-0.05916
TRINITY_DN41602_c0_g3_i1_orf1	-	-	-	CCR4-NOT transcription complex subunit 3 [Cotesia glomerata] >KAH0561609.1 CCR4-NOT transcription complex, subunit 3 [Cotesia glomerata]	1.823045	-0.0233	-0.54593	-0.08289	-1.17092
TRINITY_DN937_c0_g1_i2_orf1	-	-	-	protein brunelleschi [Ostrinia furnacalis]	1.679607	-0.77512	0.606188	-0.94185	-0.56882
TRINITY_DN5169_c0_g1_i5_orf1	-	-	-	ero1-like protein isoform X1 [Ostrinia furnacalis]	1.992938	-0.58721	-0.36695	-0.58721	-0.45156
TRINITY_DN7464_c1_g1_i1_orf1	-	-	-	T-complex protein 1 subunit theta [Ostrinia furnacalis]	1.848939	0.256088	-0.68025	-0.85043	-0.57435
TRINITY_DN36928_c0_g1_i2_orf1	-	-	-	actin-interacting protein 1 isoform X2 [Ostrinia furnacalis]	1.533631	-0.53853	0.717675	-1.29004	-0.42274
TRINITY_DN29521_c0_g1_i1_orf1	-	-	-	density-regulated protein homolog [Ostrinia furnacalis]	1.52673	-0.77925	0.626258	-1.2894	-0.08434
TRINITY_DN20499_c0_g1_i1_orf1	-	-	-	exosome RNA helicase MTR4 isoform X2 [Ostrinia furnacalis]	1.988847	-0.30069	-0.52319	-0.61225	-0.55272
TRINITY_DN35245_c0_g1_i1_orf1	-	-	-	ras GTPase-activating protein-binding protein 2 isoform X1 [Nymphalis io] >XP_050349014.1 ras GTPase-activating protein-binding protein 2 isoform X1 [Nymphalis io] >XP_050349015.1 ras GTPase-activating protein-binding protein 2 isoform X2 [Nymphalis io]	1.679129	0.283532	0.038853	-0.78213	-1.21938
TRINITY_DN31751_c0_g1_i5_orf1	-	-	-	THO complex subunit 3 [Ostrinia furnacalis]	1.910372	0.069188	-0.54494	-0.61816	-0.81646
TRINITY_DN1826_c0_g2_i4_orf1	-	-	-	glutaminyl-peptide cyclotransferase-like [Ostrinia furnacalis]	1.95417	-0.1198	-0.54833	-0.50293	-0.78312
TRINITY_DN1772_c7_g1_i7_orf1	-	-	-	sulfotransferase family cytosolic 1B member 1-like [Ostrinia furnacalis]	1.901168	-0.2332	-1.06302	-0.39782	-0.20712
TRINITY_DN12579_c0_g1_i1_orf1	-	-	-	uncharacterized protein LOC114354070 isoform X3 [Ostrinia furnacalis]	1.908687	-0.66748	-0.1284	-0.9277	-0.1851
TRINITY_DN1884_c0_g2_i2_orf1	-	-	-	phosphotriesterase-related protein [Ostrinia furnacalis]	1.755506	-0.81053	-1.11566	0.053848	0.116834

TRINITY_DN11124_c0_g1_i4_orf1	-	-	-	hypothetical protein O3G_MSEX014253 [Manduca sexta]	1.973113	-0.22835	-0.47002	-0.53424	-0.74051
TRINITY_DN14035_c0_g1_i1_orf1	-	-	-	protein takeout-like [Ostrinia furnacalis]	1.966403	-0.79564	-0.39225	-0.53643	-0.24208
TRINITY_DN108818_c0_g1_i5_orf1	-	-	-	hypothetical protein O3G_MSEX012842 [Manduca sexta]	1.72959	0.094086	0.14548	-1.12549	-0.84366
TRINITY_DN3755_c0_g1_i3_orf1	-	-	-	caspase-1-like isoform X2 [Ostrinia furnacalis]	1.685284	-0.26713	-1.33162	0.351387	-0.43793
TRINITY_DN4944_c1_g1_i4_orf1	-	-	-	bifunctional glutamate/proline--tRNA ligase [Ostrinia furnacalis]	1.924901	-0.83177	-0.07025	-0.70488	-0.31801
TRINITY_DN4152_c0_g1_i1_orf1	-	-	-	importin subunit beta-1 isoform X2 [Ostrinia furnacalis]	1.49974	0.877631	-0.56026	-0.81993	-0.99718
TRINITY_DN82008_c0_g1_i1_orf1	-	-	-	GMP reductase 1-like [Ostrinia furnacalis]	1.942186	-0.67703	-0.28078	-0.81329	-0.17109
TRINITY_DN3985_c0_g2_i1_orf1	-	-	-	hypothetical protein evm_012077 [Chilo suppressalis] >CAB3529218.1 unnamed protein product [Chilo suppressalis] >CAH0405810.1 unnamed protein product [Chilo suppressalis]	1.825013	-0.94923	0.269368	-0.71409	-0.43107
TRINITY_DN4183_c0_g1_i8_orf1	-	-	-	histidine--tRNA ligase, cytoplasmic isoform X3 [Ostrinia furnacalis]	1.953333	-0.84377	-0.18541	-0.53668	-0.38747
TRINITY_DN4309_c0_g1_i1_orf1	-	-	-	NEDD8-conjugating enzyme Ubc12, partial [Cotesia chilonis]	1.888974	-0.21857	-0.60847	-1.00527	-0.05667
TRINITY_DN1227_c0_g1_i1_orf1	-	-	-	uncharacterized protein LOC114356076 [Ostrinia furnacalis]	1.876128	0.138135	-0.81395	-0.40245	-0.79786
TRINITY_DN66822_c0_g1_i1_orf1	-	-	-	heterogeneous nuclear ribonucleoprotein 27C isoform X6 [Pieris rapae]	1.942705	-0.21816	-0.35602	-0.92418	-0.44435
TRINITY_DN43369_c0_g2_i1_orf1	-	-	-	cytochrome P450 monooxygenase 304 [Glyphodes pyloalis]	1.35978	0.325363	-1.47229	-0.76021	0.547351
TRINITY_DN31637_c0_g1_i3_orf1	-	-	-	protein CDV3 homolog isoform X1 [Ostrinia furnacalis] >XP_028156793.1 protein CDV3 homolog isoform X2 [Ostrinia furnacalis] >XP_028156794.1 protein CDV3 homolog isoform X3 [Ostrinia furnacalis] >XP_028156795.1 protein CDV3 homolog isoform X1 [Ostrinia furnacalis]	1.826799	0.073214	-0.13384	-1.08289	-0.68329
TRINITY_DN5562_c0_g1_i3_orf1	-	-	-	cell division cycle and apoptosis regulator protein 1-like [Ostrinia furnacalis]	1.677889	0.630788	-0.82952	-0.78825	-0.69091
TRINITY_DN19920_c1_g1_i2_orf1	-	-	-	probable ATP-dependent RNA helicase DDX10 [Ostrinia furnacalis]	1.943472	-0.81133	-0.36213	-0.12322	-0.64679
TRINITY_DN23444_c0_g1_i10_orf1	-	-	-	serrate RNA effector molecule homolog isoform X2 [Ostrinia furnacalis]	1.825345	0.101532	-0.14493	-1.04772	-0.73422
TRINITY_DN15244_c0_g1_i5_orf1	-	-	-	titin homolog [Ostrinia furnacalis]	1.847155	-0.02985	-0.0432	-0.96299	-0.81111
TRINITY_DN69170_c0_g2_i1_orf1	-	-	-	stromal membrane-associated protein 1-like [Pectinophora gossypiella]	1.713054	0.486071	-0.43239	-1.08529	-0.68144
TRINITY_DN4429_c0_g1_i5_orf1	-	-	-	FACT complex subunit Ssrp1 isoform X1 [Ostrinia furnacalis] >XP_028173375.1 FACT complex subunit Ssrp1 isoform X2 [Ostrinia furnacalis] >XP_028173376.1 FACT complex subunit Ssrp1 isoform X3 [Ostrinia furnacalis]	1.782725	0.320369	-1.05064	-0.35075	-0.7017
TRINITY_DN1459_c1_g1_i1_orf1	-	-	-	reticulon-1 isoform X2 [Ostrinia furnacalis]	1.653873	0.550931	-0.36152	-0.6651	-1.17819
TRINITY_DN3176_c0_g1_i2_orf1	-	-	-	dnaJ homolog subfamily B member 6 isoform X2 [Ostrinia furnacalis]	1.611414	0.740718	-0.70506	-0.84752	-0.79955
TRINITY_DN4025_c0_g1_i13_orf1	-	-	-	tetratricopeptide repeat protein 14 homolog isoform X2 [Ostrinia furnacalis]	1.601154	0.094007	0.12725	-1.52386	-0.29855
TRINITY_DN14464_c0_g1_i1_orf1	-	-	-	GMP synthase [glutamine-hydrolyzing] [Chelonus insularis]	1.890239	-0.68655	0.107364	-0.8613	-0.44975
TRINITY_DN142652_c0_g1_i1_orf1	-	-	-	pre-mRNA-splicing factor RBM22 [Chelonus insularis]	1.702242	0.128354	-0.03772	-1.38494	-0.40794
TRINITY_DN57798_c0_g1_i1_orf1	-	-	-	ubiquitin carboxyl-terminal hydrolase 36 [Ostrinia furnacalis]	1.781632	0.409678	-0.87596	-0.77076	-0.54459
TRINITY_DN2802_c0_g1_i1_orf1	-	-	-	far upstream element-binding protein 1 isoform X3 [Ostrinia furnacalis]	1.52149	0.788719	-0.38408	-1.13697	-0.78916
TRINITY_DN18918_c0_g1_i3_orf1	-	-	-	myrosinase 1-like isoform X2 [Ostrinia furnacalis]	1.597342	-0.69075	-0.64719	0.741752	-1.00116
TRINITY_DN2047_c0_g1_i1_orf1	-	-	-	carboxylesterase CXE17 [Ostrinia furnacalis]	1.987875	-0.4797	-0.35927	-0.45368	-0.69523
TRINITY_DN821_c0_g1_i8_orf1	-	-	-	nuclear pore complex protein Nup153 isoform X2 [Ostrinia furnacalis]	1.971796	-0.27668	-0.42727	-0.47709	-0.79075
TRINITY_DN147596_c0_g1_i1_orf1	-	-	-	activator of basal transcription 1 [Diachasma alloeum]	1.7748	-0.86284	-0.23736	-0.97828	0.30368
TRINITY_DN17828_c0_g1_i1_orf1	-	-	-	ER membrane protein complex subunit 8/9 homolog [Ostrinia furnacalis]	1.478173	0.134457	-0.42164	0.381726	-1.57271
TRINITY_DN7868_c0_g1_i8_orf1	-	-	-	uncharacterized protein LOC114353432 isoform X4 [Ostrinia furnacalis]	1.699433	-0.10436	-0.04314	-1.44489	-0.10704
TRINITY_DN1494_c0_g2_i1_orf1	-	-	-	dihydropyrimidine dehydrogenase [NADP(+)] [Ostrinia furnacalis]	1.977508	-0.25728	-0.48598	-0.50441	-0.72984
TRINITY_DN5121_c0_g1_i1_orf1	-	-	-	unnamed protein product [Parnassius apollo]	1.744685	0.250006	-1.25951	-0.23165	-0.50353
TRINITY_DN11886_c0_g1_i1_orf1	-	-	-	glycerophosphodiester phosphodiesterase GDPD6-like [Ostrinia furnacalis] >XP_028159459.1 glycerophosphodiester phosphodiesterase GDPD6-like [Ostrinia furnacalis]	1.808784	-0.66403	0.370051	-0.79682	-0.71799
TRINITY_DN26650_c0_g1_i1_orfp1	-	-	-	TRINITY_DN26650_c0_g1_i1_m.72504 TRINITY_DN26650_c0_g1::TRINITY_DN26650_c0_g1_i1::g.72504 ORF type:5prime_partial len:72 (-),score=2.71 TRINITY_DN26650_c0_g1_i1:317-532(-)	1.937741	-0.65829	-0.00599	-0.61516	-0.65829
TRINITY_DN16408_c0_g1_i1_orf1	-	-	-	ABC transporter G family member 20 isoform X1 [Ostrinia furnacalis] >XP_028158027.1 ABC transporter G family member 20 isoform X1 [Ostrinia furnacalis] >XP_028158037.1 ABC transporter G family member 20 isoform X2 [Ostrinia furnacalis] >XP_028158043.1 ABC transporter G family member 20 isoform X3 [Ostrinia furnacalis] >XP_028158060.1 ABC transporter G family member 20 isoform X5 [Ostrinia furnacalis] >XP_028158070.1 ABC transporter G family member 20 isoform X6 [Ostrinia furnacalis]	1.788323	-0.54582	0.236409	-1.16047	-0.31844
TRINITY_DN2927_c0_g1_i2_orf1	-	-	-	unnamed protein product [Spodoptera littoralis] >CAH1641960.1 unnamed protein product [Spodoptera littoralis]	1.879808	0.12669	-0.53302	-0.93748	-0.536
TRINITY_DN2258_c0_g2_i1_orf1	-	-	-	60S ribosomal protein L14 [Ostrinia furnacalis]	1.917248	-0.31883	-0.05362	-0.65744	-0.88736

TRINITY_DN18728_c0_g1_i2_orf1	-	-	-	H/ACA ribonucleoprotein complex subunit 3 [Galleria mellonella]	1.973011	-0.59952	-0.25481	-0.72839	-0.3903
TRINITY_DN2365_c0_g1_i6_orf1	-	-	-	carnitine O-acetyltransferase isoform X2 [Ostrinia furnacalis]	1.819678	0.314885	-0.92877	-0.59872	-0.60707
TRINITY_DN2682_c0_g1_i4_orf1	-	-	-	40S ribosomal protein S5 [Manduca sexta] >ACY95347.1 ribosomal protein S5 [Manduca sexta] >KAG6447616.1 hypothetical protein O3G_MSEX005033 [Manduca sexta] >KAG6447617.1 hypothetical protein O3G_MSEX005033 [Manduca sexta]	1.683287	-0.79078	0.526969	-1.06755	-0.35193
TRINITY_DN25210_c0_g1_i1_orf1	-	-	-	mitochondrial import receptor subunit TOM22 homolog [Ostrinia furnacalis]	1.883044	-0.00791	-0.27497	-1.02168	-0.57848
TRINITY_DN108354_c0_g1_i1_orf1	-	-	-	WD repeat-containing protein 61-like [Ostrinia furnacalis]	1.832273	-0.05592	-0.53698	-1.15972	-0.07965
TRINITY_DN22572_c0_g1_i1_orf1	-	-	-	eukaryotic translation elongation factor 1 epsilon-1 [Ostrinia furnacalis]	1.602205	0.191304	-0.26063	-1.5259	-0.00698
TRINITY_DN130_c0_g1_i7_orf1	-	-	-	RNA-binding protein fusilli isoform X1 [Bombyx mori]	1.716971	0.091048	-0.0187	-1.36505	-0.42426
TRINITY_DN47723_c0_g1_i1_orf1	-	-	-	dnaJ homolog subfamily C member 21 [Ostrinia furnacalis]	1.49127	0.90825	-0.76258	-0.69624	-0.9407
TRINITY_DN7241_c0_g2_i2_orf1	-	-	-	40S ribosomal protein S10 [Zerene cesonia] >XP_045492164.1 40S ribosomal protein S10 [Colias croceus]	1.77353	-0.74128	0.424425	-0.90723	-0.54944
TRINITY_DN6545_c0_g1_i6_orf1	-	-	-	organic cation transporter protein-like [Ostrinia furnacalis]	1.68091	0.21481	-0.62848	0.048457	-1.3157
TRINITY_DN2571_c0_g2_i1_orf1	-	-	-	PREDICTED: huntingtin-interacting protein K isoform X1 [Amyelois transitella] >XP_013190319.1 PREDICTED: huntingtin-interacting protein K isoform X2 [Amyelois transitella] >XP_021200735.1 huntingtin-interacting protein K [Helicoverpa armigera] >XP_026737126.1 huntingtin-interacting protein K [Trichoplusia ni] >XP_041970494.1 huntingtin-interacting protein K [Aricia agestis] >XP_047022511.1 huntingtin-interacting protein K [Helicoverpa zea] >XP_047984206.1 huntingtin-interacting protein K [Leguminivora glycinivorella] >RVE51917.1 hypothetical protein evm_003383 [Chilo suppressalis] >PZC85551.1 hypothetical protein B5X24_HaOG216659 [Helicoverpa armigera] >CAB3530729.1 unnamed protein product [Chilo suppressalis] >CAH0407320.1 unnamed	1.953393	-0.71047	-0.17978	-0.32905	-0.73409
TRINITY_DN5775_c0_g1_i1_orf1	-	-	-	proteasome assembly chaperone 2 [Ostrinia furnacalis]	1.871178	-0.10771	-0.3792	-1.131	-0.25328
TRINITY_DN18569_c0_g2_i1_orf1	-	-	-	stomatin-like protein 2, mitochondrial [Ostrinia furnacalis]	1.92474	-0.12492	-0.36815	-0.96017	-0.4715
TRINITY_DN34726_c0_g2_i1_orf1	-	-	-	heat shock factor-binding protein 1 [Ostrinia furnacalis]	1.740038	0.434096	-0.8442	-0.97057	-0.35936
TRINITY_DN18159_c0_g1_i6_orf1	-	-	-	zinc carboxypeptidase-like [Ostrinia furnacalis]	1.561303	-0.88592	0.60505	-1.18411	-0.09632

TRINITY_DN1509_c0_g1_i1_orf1	-	-	-	ribosomal protein S15A [Bombyx mori] >XP_011566807.1 40S ribosomal protein S15Aa [Plutella xylostella] >XP_013186470.1 PREDICTED: 40S ribosomal protein S15Aa [Amyelois transitella] >XP_021181353.1 40S ribosomal protein S15Aa [Helicoverpa armigera] >XP_022114309.1 40S ribosomal protein S15Aa [Pieris rapae] >XP_022820057.1 40S ribosomal protein S15Aa [Spodoptera litura] >XP_023947206.1 40S ribosomal protein S15Aa [Bicyclus anynana] >XP_026329660.1 40S ribosomal protein S15Aa [Hyposmocoma kahamanoa] >XP_026495126.1 40S ribosomal protein S15Aa [Vanessa tameamea] >XP_026745810.1 40S ribosomal protein S15Aa [Trichoplusia ni] >XP_026757934.1 40S ribosomal protein S15Aa [Galleria mellonella] >XP_028041686.1 40S ribosomal protein S15Aa [Bombyx mandarina] >XP_028161999.1 40S ribosomal protein S15Aa [Ostrinia furnacalis] >XP_028162000.1 40S ribosomal protein S15Aa [Ostrinia furnacalis] >XP_030024299.1 40S ribosomal protein S15Aa [Manduca sexta] >XP_032514052.1 40S ribosomal protein S15Aa [Danaus plexippus plexippus] >XP_034834075.1 40S ribosomal protein S15Aa [Maniola hyperantus] >XP_034840269.1 40S ribosomal protein S15Aa [Maniola hyperantus] >XP_035436795.1 40S ribosomal protein S15Aa [Spodoptera frugiperda] >XP_037869057.1 ribosomal protein S15A isoform X1 [Bombyx mori] >XP_039747368.1 40S ribosomal protein S15Aa [Pararge aegeria] >XP_045445511.1 40S ribosomal protein S15Aa [Melitaea cinxia] >XP_045491780.1 40S ribosomal protein S15Aa [Colias croceus] >XP_045519936.1 40S ribosomal protein S15Aa [Pieris brassicae] >XP_045785214.1 40S ribosomal protein S15Aa [Maniola jurtina] >XP_046965230.1 40S ribosomal protein S15Aa [Vanessa cardui] >XP_046965231.1 40S ribosomal protein S15Aa [Vanessa cardui] >XP_047021729.1 40S ribosomal protein S15Aa [Helicoverpa zea] >XP_047509814.1 40S ribosomal protein S15Aa [Pieris napi] >XP_047527633.1 40S ribosomal protein S15Aa [Vanessa atalanta] >XP_047527634.1 40S ribosomal protein S15Aa [Vanessa atalanta] >XP_047988443.1 40S ribosomal protein S15Aa [Leguminivora glycinivorella] >XP_050344874.1 40S ribosomal protein S15Aa [Nymphalis io] >XP_050344875.1 40S ribosomal protein S15Aa [Nymphalis io] >ADP21467.1 ribosomal protein S15A [Antheraea yamamai] >ADT80666.1 ribosomal protein S15A [Euphydryas aurinia] >AEL28847.1 ribosomal protein S15A [Heliconius melpomene cythera] >KAF9418418.1 hypothetical protein HW555_004706 [Spodoptera exigua] >KAI5633077.1 ribosomal protein s8 domain-containing protein [Phthorimaea operculella] >KOB75105.1 Ribosomal protein S15A, partial [Operophtera	1.867661	-0.96957	-0.01229	-0.74232	-0.14348
TRINITY_DN31585_c0_g1_i1_orf1	-	-	-	transcription elongation factor SPT5 [Ostrinia furnacalis]	1.741018	0.497535	-0.63322	-0.92877	-0.67657
TRINITY_DN1437_c0_g1_i6_orf1	-	-	-	nucleoprotein TPR isoform X1 [Ostrinia furnacalis]	1.584652	0.685959	-0.37198	-1.14626	-0.75237
TRINITY_DN51239_c0_g1_i5_orf1	-	-	-	regulatory-associated protein of mTOR [Ostrinia furnacalis]	1.784271	-0.48486	-0.57558	-1.06478	0.340944
TRINITY_DN31303_c0_g1_i4_orf1	-	-	-	tRNA pseudouridine synthase A isoform X1 [Ostrinia furnacalis]	1.950654	-0.19578	-0.31639	-0.61461	-0.82388
TRINITY_DN19687_c0_g1_i1_orf1	-	-	-	probable ribosome production factor 1 [Ostrinia furnacalis]	1.655027	-0.15769	-1.45616	0.21897	-0.26015
TRINITY_DN170_c1_g1_i5_orf1	-	-	-	regulator of chromosome condensation isoform X2 [Helicoverpa zea]	1.690876	0.291506	0.029556	-1.13075	-0.88118
TRINITY_DN19727_c0_g1_i7_orf1	-	-	-	dihydrolipoyllysine-residue succinyltransferase component of 2-oxoglutarate dehydrogenase complex, mitochondrial-like [Ostrinia furnacalis] >XP_028160614.1 dihydrolipoyllysine-residue succinyltransferase component of 2-oxoglutarate dehydrogenase complex, mitochondrial-like [Ostrinia furnacalis] >XP_028160615.1 dihydrolipoyllysine-residue succinyltransferase component of 2-oxoglutarate dehydrogenase complex, mitochondrial-like [Ostrinia	1.9144	-0.81662	-0.78644	-0.13068	-0.18066
TRINITY_DN3454_c0_g1_i1_orf1	-	-	-	MICOS complex subunit MIC13 homolog QIL1 [Ostrinia furnacalis]	1.884419	-0.91382	-0.61231	0.11648	-0.47477
TRINITY_DN41311_c0_g2_i3_orf1	-	-	-	ras-related protein Rab-8A isoform X2 [Ostrinia furnacalis]	1.684236	0.598862	-0.66429	-0.97244	-0.64637
TRINITY_DN4380_c0_g1_i9_orf1	-	-	-	hypothetical protein evm_012370 [Chilo suppressalis]	1.751827	0.249333	-0.08687	-1.07783	-0.83646
TRINITY_DN33183_c0_g1_i4_orf1	-	-	-	glutamate--cysteine ligase regulatory subunit [Ostrinia furnacalis]	1.899559	0.093782	-0.82659	-0.68079	-0.48596
TRINITY_DN141_c0_g1_i1_orf1	-	-	-	hypothetical protein evm_010402 [Chilo suppressalis]	1.722576	0.364945	-0.15016	-0.95624	-0.98113
TRINITY_DN9770_c0_g1_i1_orf1	-	-	-	flavin reductase (NADPH) [Ostrinia furnacalis] >XP_028160803.1 flavin reductase (NADPH) [Ostrinia furnacalis]	1.912113	-0.54292	-0.45786	-0.91619	0.004859
TRINITY_DN17376_c0_g1_i2_orf1	-	-	-	E3 UFM1-protein ligase 1 homolog [Ostrinia furnacalis]	1.836104	-0.42784	0.002896	-1.17991	-0.23125
TRINITY_DN2977_c0_g1_i3_orf1	-	-	-	transmembrane 9 superfamily member 3 [Ostrinia furnacalis]	1.560138	0.807614	-0.6591	-0.95325	-0.75541
TRINITY_DN18696_c0_g1_i1_orf1	-	-	-	rap guanine nucleotide exchange factor 2 [Ostrinia furnacalis] >XP_028159576.1 rap guanine nucleotide exchange factor 2 [Ostrinia furnacalis]	1.937403	-0.74032	-0.03069	-0.49042	-0.67598
TRINITY_DN5234_c0_g1_i2_orf1	-	-	-	proline dehydrogenase 1, mitochondrial isoform X2 [Ostrinia furnacalis]	1.838152	-0.81702	-0.58012	0.289341	-0.73036
TRINITY_DN3343_c0_g1_i4_orf1	-	-	-	AFG3-like protein 2 [Ostrinia furnacalis]	1.99748	-0.50596	-0.42378	-0.58107	-0.48667
TRINITY_DN5857_c0_g1_i13_orf1	-	-	-	uncharacterized protein LOC114353981 isoform X1 [Ostrinia furnacalis]	1.864154	-0.88097	0.068571	-0.83492	-0.21683

TRINITY_DN37729_c0_g1_i8_orf1	-	-	-	adenylyltransferase and sulfurtransferase MOCS3 isoform X1 [Ostrinia furnacalis]	1.876348	-0.0578	-0.27444	-1.09495	-0.44916
TRINITY_DN6307_c0_g1_i5_orf1	-	-	-	putative helicase mov-10-B.1 [Ostrinia furnacalis]	1.949503	-0.65736	-0.23789	-0.24874	-0.80551
TRINITY_DN2252_c0_g1_i4_orfp1	-	-	-	TRINITY_DN2252_c0_g1_i4_m.69997 TRINITY_DN2252_c0_g1::TRINITY_DN2252_c0_g1_i4::g.69997 ORF type:5prime_partial len:168	1.505447	0.809463	-0.49883	-1.20845	-0.60763
TRINITY_DN1572_c0_g1_i6_orf1	-	-	-	eukaryotic translation initiation factor 3 subunit D [Ostrinia furnacalis]	1.967647	-0.41391	-0.2087	-0.73931	-0.60572
TRINITY_DN11297_c0_g1_i1_orf1	-	-	-	ribosomal protein L13 [Conogethes punctiferalis] >QEE82690.1 ribosomal protein L13 [Conogethes pinicolalis]	1.834	-0.97759	0.046344	-0.81956	-0.08319
TRINITY_DN40508_c0_g1_i1_orf1	-	-	-	mRNA turnover protein 4 homolog [Ostrinia furnacalis]	1.874571	-0.34675	-1.12752	-0.11552	-0.28478
TRINITY_DN54134_c0_g1_i1_orf1	-	-	-	NFU1 iron-sulfur cluster scaffold homolog, mitochondrial-like [Ostrinia furnacalis]	1.733738	0.37978	-0.36141	-0.57244	-1.17967
TRINITY_DN517_c0_g1_i5_orf1	-	-	-	putative pre-mRNA-splicing factor ATP-dependent RNA helicase PRP1 [Helicoverpa zea] >XP_049708007.1 putative pre-mRNA-splicing factor ATP-dependent RNA helicase PRP1 isoform X1 [Helicoverpa armigera]	1.971857	-0.26638	-0.43803	-0.78495	-0.48249
TRINITY_DN3057_c0_g2_i1_orf1	-	-	-	chromodomain-helicase-DNA-binding protein Mi-2 homolog isoform X3 [Chelonus insularis]	1.72374	0.528052	-0.83192	-0.86769	-0.55218
TRINITY_DN37165_c0_g1_i4_orf1	-	-	-	pyridoxine-5'-phosphate oxidase-like [Ostrinia furnacalis]	1.888396	-0.24462	-0.58233	-1.01636	-0.04509
TRINITY_DN31253_c0_g1_i2_orf1	-	-	-	hypothetical protein evm_009655 [Chilo suppressalis]	1.834114	-1.10986	0.087934	-0.58868	-0.2235
TRINITY_DN145666_c0_g1_i1_orf1	-	-	-	PREDICTED: 40S ribosomal protein S29 [Microplitis demolitor] >XP_044581406.1 40S ribosomal protein S29 [Cotesia glomerata]	1.899117	-0.16924	-0.11103	-0.95442	-0.66443
TRINITY_DN20279_c0_g1_i1_orf1	-	-	-	NADH dehydrogenase [ubiquinone] iron-sulfur protein 3, mitochondrial [Ostrinia furnacalis]	1.978009	-0.7421	-0.44078	-0.28275	-0.51238
TRINITY_DN79734_c0_g2_i3_orf1	-	-	-	60S ribosomal protein L27a [Ostrinia furnacalis]	1.832833	-0.70563	-0.01754	-1.06807	-0.04159
TRINITY_DN142485_c0_g1_i1_orf1	-	-	-	uncharacterized protein CG16817-like [Ostrinia furnacalis]	1.73629	-0.05431	0.269443	-1.02915	-0.92228
TRINITY_DN3835_c0_g1_i3_orf1	-	-	-	protein ERGIC-53 isoform X1 [Ostrinia furnacalis] >XP_028177940.1 protein ERGIC-53 isoform X2 [Ostrinia furnacalis] >XP_028177941.1 protein ERGIC-53 isoform X3 [Ostrinia furnacalis]	1.75628	0.390565	-0.48296	-1.1016	-0.56228
TRINITY_DN3814_c1_g1_i1_orf1	-	-	-	39S ribosomal protein L11, mitochondrial [Ostrinia furnacalis]	1.972599	-0.49096	-0.37123	-0.79343	-0.31699
TRINITY_DN8561_c0_g4_i1_orf1	-	-	-	dynactin subunit 4 [Ostrinia furnacalis]	1.691238	0.377016	-0.73498	-1.19982	-0.13346
TRINITY_DN2803_c2_g1_i8_orf1	-	-	-	trans-1,2-dihydrobenzene-1,2-diol dehydrogenase-like isoform X1 [Ostrinia furnacalis]	1.932203	-0.74543	-0.51625	-0.66663	-0.00389
TRINITY_DN28806_c0_g1_i1_orf1	-	-	-	ATP-dependent RNA helicase WM6 [Ostrinia furnacalis]	1.896856	-0.03246	-0.56373	-0.99474	-0.30593
TRINITY_DN11457_c0_g1_i5_orf1	-	-	-	uncharacterized protein LOC114352268 [Ostrinia furnacalis]	1.926776	-0.07218	-0.33197	-0.84171	-0.68092
TRINITY_DN3836_c0_g1_i4_orf1	-	-	-	2-oxoisovalerate dehydrogenase subunit alpha, mitochondrial [Ostrinia furnacalis]	1.775928	-1.09053	-0.20803	-0.73848	0.261117
TRINITY_DN49409_c0_g1_i2_orf1	-	-	-	proliferation-associated protein 2G4 [Ostrinia furnacalis]	1.952408	-0.37557	-0.396	-0.9011	-0.27974
TRINITY_DN4707_c0_g1_i1_orf1	-	-	-	PREDICTED: DNA-directed RNA polymerases I, II, and III subunit RPABC1 [Papilio xuthus] >XP_013187738.1 PREDICTED: DNA-directed RNA polymerases I, II, and III subunit RPABC1 [Amyelois transitella] >XP_028158146.1 DNA-directed RNA polymerases I, II, and III subunit RPABC1 [Ostrinia furnacalis] >XP_045537534.1 DNA-directed RNA polymerases I, II, and III subunit RPABC1 [Papilio machaon] >XP_049876738.1 DNA-directed RNA polymerases I, II, and III subunit RPABC1 [Pectinophora gossypiella] >KAG6452000.1 hypothetical protein O3G_MSEX007416 [Manduca sexta] >RVE48301.1 hypothetical protein evm_007052 [Chilo suppressalis] >CAG5049330.1 unnamed protein product [Parnassius apollo] >CAG9757053.1 unnamed protein product [Diatraea saccharalis] >CAH2042370.1 unnamed protein product, partial [Iphiclides podalirius]	1.829994	0.273297	-0.5392	-0.95882	-0.60527
TRINITY_DN21981_c0_g1_i8_orf1	-	-	-	dihydroorotate dehydrogenase (quinone), mitochondrial [Ostrinia furnacalis]	1.936786	-0.08557	-0.46567	-0.8728	-0.51274
TRINITY_DN32487_c0_g1_i1_orf1	-	-	-	heat shock protein 75 kDa, mitochondrial [Ostrinia furnacalis]	1.999646	-0.52007	-0.50491	-0.50996	-0.46471
TRINITY_DN57074_c0_g2_i1_orf1	-	-	-	ribosomal protein l36e domain-containing protein [Phthorimaea operculella]	1.914537	-0.77222	0.052807	-0.70069	-0.49443
TRINITY_DN2975_c0_g1_i4_orf1	-	-	-	ubiquitin-like protein 4A [Ostrinia furnacalis]	1.893042	-0.08028	-0.48559	-1.04664	-0.28054
TRINITY_DN36701_c0_g1_i4_orf1	-	-	-	hypothetical protein SFRURICE_004895 [Spodoptera frugiperda] >KAG8116760.1 hypothetical protein SFRUCORN_001970 [Spodoptera frugiperda]	1.932645	-0.66644	-0.02706	-0.78124	-0.4579
TRINITY_DN1081_c0_g1_i7_orf1	-	-	-	3-ketoacyl-CoA thiolase, mitochondrial-like [Ostrinia furnacalis]	1.918511	-0.76584	-0.72386	-0.45616	0.027349
TRINITY_DN38341_c0_g2_i2_orf1	-	-	-	delta(24)-sterol reductase-like [Ostrinia furnacalis]	1.976217	-0.70874	-0.36831	-0.2878	-0.61138
TRINITY_DN14730_c0_g1_i7_orf1	-	-	-	titin homolog [Ostrinia furnacalis]	1.98257	-0.54004	-0.26463	-0.67267	-0.50523
TRINITY_DN60048_c0_g2_i1_orf1	-	-	-	facilitated trehalose transporter Tret1-like [Venturia canescens] >XP_043281789.1 facilitated trehalose transporter Tret1-like [Venturia canescens] >XP_043282611.1 facilitated trehalose transporter Tret1-like [Venturia canescens] >XP_043283442.1 facilitated trehalose transporter Tret1-like [Venturia canescens]	1.926023	-0.29858	-0.19936	-0.98034	-0.44775
TRINITY_DN82324_c0_g1_i4_orf1	-	-	-	hypothetical protein evm_001824 [Chilo suppressalis] >CAG9754426.1 unnamed protein product [Diatraea saccharalis] >CAG9793111.1 unnamed protein product [Diatraea	1.811481	-0.69976	0.314297	-0.95105	-0.47496

TRINITY_DN41179_c0_g1_i1_orf1	-	-	-	RNA-binding protein NOB1 [Ostrinia furnacalis]	1.884408	-0.1281	-1.08981	-0.44002	-0.22648
TRINITY_DN44119_c0_g1_i1_orf1	-	-	-	PREDICTED: GTP-binding protein 128up [Fopius arisanus]	1.960487	-0.84176	-0.48855	-0.242	-0.38817
TRINITY_DN8598_c0_g1_i2_orf1	-	-	-	tyrosine--tRNA ligase, mitochondrial [Ostrinia furnacalis]	1.990974	-0.31857	-0.53821	-0.53821	-0.59598
TRINITY_DN9637_c0_g1_i14_orf1	-	-	-	zinc finger protein swm isoform X3 [Ostrinia furnacalis]	1.433987	0.982389	-0.65888	-0.87272	-0.88478
TRINITY_DN60680_c0_g1_i2_orf1	-	-	-	unnamed protein product [Euphydryas editha]	1.922441	-0.47357	-0.01564	-0.87883	-0.5544
TRINITY_DN12442_c0_g1_i4_orf1	-	-	-	midasin-like [Ostrinia furnacalis]	1.771413	-0.49391	-0.74687	-0.94338	0.412751
TRINITY_DN5031_c0_g1_i1_orf1	-	-	-	PREDICTED: 40S ribosomal protein S12 [Trachymyrmex septentrionalis]	1.770552	-0.89764	0.305666	-0.95768	-0.22089
TRINITY_DN72056_c0_g1_i1_orf1	-	-	-	protein PBDC1 [Ostrinia furnacalis]	1.93096	-0.15181	-0.27922	-0.90077	-0.59916
TRINITY_DN33249_c0_g1_i1_orf1	-	-	-	eukaryotic translation initiation factor 2 subunit 3-like isoform X2 [Spodoptera frugiperda] >CAH0683085.1 unnamed protein product [Spodoptera exigua]	1.913482	0.046031	-0.49212	-0.82768	-0.63971
TRINITY_DN9741_c0_g1_i3_orf1	-	-	-	metaxin-2 isoform X4 [Manduca sexta] >KAG6447312.1 hypothetical protein O3G_MSEX004872 [Manduca sexta]	1.83667	0.179752	-0.70686	-0.99917	-0.3104
TRINITY_DN5919_c0_g1_i4_orf1	-	-	-	esterase FE4-like [Ostrinia furnacalis]	1.960108	-0.53206	-0.49248	-0.7798	-0.15576
TRINITY_DN7464_c0_g1_i14_orf1	-	-	-	60S ribosomal protein L9 [Nymphalis io]	1.766885	-1.05558	0.289503	-0.79977	-0.20103
TRINITY_DN4141_c0_g1_i9_orf1	-	-	-	la-related protein 1 isoform X2 [Ostrinia furnacalis] >XP_028160900.1 la-related protein 1 isoform X3 [Ostrinia furnacalis] >XP_028160902.1 la-related protein 1 isoform X2 [Ostrinia furnacalis] >XP_028160903.1 la-related protein 1 isoform X2 [Ostrinia furnacalis] >XP_028160904.1 la-related protein 1 isoform X2 [Ostrinia furnacalis] >XP_028160905.1 la-related protein 1 isoform X4 [Ostrinia furnacalis] >XP_028160906.1 la-related protein 1 isoform X2 [Ostrinia furnacalis] >XP_028160908.1 la-related protein 1 isoform X2 [Ostrinia furnacalis] >XP_028160909.1 la-related protein 1 isoform X2 [Ostrinia furnacalis] >XP_028160910.1 la-related protein 1 isoform X2 [Ostrinia furnacalis]	1.968661	-0.64191	-0.32654	-0.72977	-0.27044
TRINITY_DN10521_c0_g1_i7_orf1	-	-	-	tubulin beta chain-like [Ostrinia furnacalis]	1.954661	-0.89418	-0.29795	-0.39224	-0.3703
TRINITY_DN371_c0_g1_i6_orf1	-	-	-	trypsin beta-like [Ostrinia furnacalis]	1.948187	-0.81986	-0.56849	-0.11788	-0.44196
TRINITY_DN1882_c0_g1_i4_orf1	-	-	-	zinc transporter ZIP13 homolog [Ostrinia furnacalis]	1.268475	-0.23902	0.648319	-1.70666	0.028887
TRINITY_DN1725_c0_g1_i7_orf1	-	-	-	T-complex protein 1 subunit gamma isoform X1 [Ostrinia furnacalis] >XP_028159782.1 T-complex protein 1 subunit gamma isoform X2 [Ostrinia furnacalis]	1.818537	0.188751	-0.46903	-1.12563	-0.41262
TRINITY_DN19628_c1_g1_i1_orf1	-	-	-	transcription factor BTF3 homolog 4-like [Ostrinia furnacalis]	1.954101	-0.17612	-0.33885	-0.72695	-0.71217
TRINITY_DN40345_c0_g1_i6_orf1	-	-	-	60S ribosomal protein L28 [Ostrinia furnacalis]	1.865173	-0.64504	0.196838	-0.88512	-0.53185
TRINITY_DN25997_c1_g1_i1_orf1	-	-	-	uncharacterized protein LOC114366342 [Ostrinia furnacalis]	1.814692	0.164597	-0.28639	-0.55938	-1.13353
TRINITY_DN21181_c0_g1_i6_orf1	-	-	-	unnamed protein product, partial [Brenthis ino]	1.996104	-0.5824	-0.49667	-0.52641	-0.39062
TRINITY_DN3628_c0_g1_i5_orf1	-	-	-	palmitoyltransferase Hip14 isoform X2 [Ostrinia furnacalis]	1.927688	0.015603	-0.51883	-0.70745	-0.71701
TRINITY_DN7405_c0_g1_i3_orf1	-	-	-	hexokinase-2-like [Ostrinia furnacalis] >XP_028178415.1 hexokinase-2-like [Ostrinia	1.921134	-0.78691	-0.79509	-0.15656	-0.18257
TRINITY_DN143852_c0_g1_i1_orf1	-	-	-	60S ribosomal protein L10 [Cotesia glomerata]	1.492727	-1.01306	0.761228	-1.06548	-0.17542
TRINITY_DN11825_c0_g1_i4_orf1	-	-	-	39S ribosomal protein L22, mitochondrial [Ostrinia furnacalis]	1.976995	-0.59327	-0.33966	-0.72092	-0.32314
TRINITY_DN207_c0_g2_i3_orf1	-	-	-	JNK-interacting protein 3 isoform X2 [Ostrinia furnacalis]	1.988836	-0.2932	-0.56367	-0.55678	-0.57519
TRINITY_DN71465_c0_g1_i1_orf1	-	-	-	adenylate kinase isoenzyme 6 [Ostrinia furnacalis]	1.984059	-0.46283	-0.60728	-0.63425	-0.27969
TRINITY_DN30070_c0_g1_i6_orf1	-	-	-	uncharacterized protein LOC114361440 isoform X1 [Ostrinia furnacalis] >XP_028172261.1 uncharacterized protein LOC114361440 isoform X2 [Ostrinia furnacalis]	1.891106	-0.33311	-1.09548	-0.17777	-0.28475
TRINITY_DN401_c0_g1_i15_orf1	-	-	-	cholinephosphotransferase 1 isoform X2 [Ostrinia furnacalis]	1.995164	-0.48858	-0.40006	-0.61794	-0.48858
TRINITY_DN883_c0_g1_i8_orf1	-	-	-	diacylglycerol O-acyltransferase 1 isoform X1 [Ostrinia furnacalis]	1.787566	-0.60517	-0.39384	-1.09154	0.302989
TRINITY_DN2300_c0_g1_i1_orf1	-	-	-	ATP synthase subunit beta, mitochondrial isoform X4 [Ostrinia furnacalis]	1.931858	-0.95745	-0.18558	-0.34124	-0.44759
TRINITY_DN740_c0_g1_i1_orf1	-	-	-	GTP-binding nuclear protein Ran [Pieris rapae] >XP_028162165.1 GTP-binding nuclear protein Ran [Ostrinia furnacalis] >XP_028162166.1 GTP-binding nuclear protein Ran [Ostrinia furnacalis] >XP_028162167.1 GTP-binding nuclear protein Ran [Ostrinia furnacalis] >XP_045532338.1 GTP-binding nuclear protein Ran [Pieris brassicae] >XP_045532339.1 GTP-binding nuclear protein Ran [Pieris brassicae] >CAG9745207.1 unnamed protein product [Diatraea saccharalis] >CAG9783892.1 unnamed protein product [Diatraea saccharalis]	1.947789	-0.08641	-0.53507	-0.79123	-0.53507
TRINITY_DN62_c0_g1_i18_orf1	-	-	-	hypothetical protein evm_002481 [Chilo suppressalis] >CAB3531063.1 unnamed protein product [Chilo suppressalis] >CAH0407655.1 unnamed protein product [Chilo suppressalis]	1.660939	0.588609	-0.37898	-0.96427	-0.9063

TRINITY_DN42646_c0_g2_i1_orf1	-	-	-	40S ribosomal protein S3 [Helicoverpa armigera] >XP_026740562.1 40S ribosomal protein S3 [Trichoplusia ni] >XP_026751545.1 40S ribosomal protein S3 [Galleria mellonella] >XP_047027704.1 40S ribosomal protein S3 [Helicoverpa zea] >CAH0591481.1 unnamed protein product [Chrysodeixis includens] >AIR07416.1 ribosomal protein S3 [Helicoverpa armigera] >AND95944.1 ribosomal protein S3 [Helicoverpa armigera] >AXY94820.1 ribosomal ribosomal protein S3 [Galleria mellonella] >PZC80336.1 hypothetical protein B5X24_HaOG214853 [Helicoverpa armigera]	1.809772	-0.92257	0.180307	-0.90206	-0.16545
TRINITY_DN3878_c0_g1_i4_orf1	-	-	-	eukaryotic translation initiation factor 3 subunit J [Ostrinia furnacalis]	1.966987	-0.44648	-0.18666	-0.60693	-0.72692
TRINITY_DN37393_c0_g1_i1_orf1	-	-	-	protein melted [Pectinophora gossypiella]	1.936135	-0.2943	-0.21042	-0.93481	-0.49661
TRINITY_DN8343_c0_g1_i2_orf1	-	-	-	multidrug resistance-associated protein 1 isoform X4 [Ostrinia furnacalis]	1.884184	-0.54654	0.161803	-0.72981	-0.76964
TRINITY_DN23824_c0_g1_i1_orf1	-	-	-	28S ribosomal protein S30, mitochondrial [Ostrinia furnacalis]	1.955695	-0.54664	-0.82657	-0.18267	-0.39982
TRINITY_DN3889_c0_g1_i7_orfp1	-	-	-	TRINITY_DN3889_c0_g1_i7_m.1657 TRINITY_DN3889_c0_g1::TRINITY_DN3889_c0_g1_i7::g.1657 ORF type:5prime_partial len:235 (+),score=70.90 TRINITY_DN3889_c0_g1_i7:1-705(+)	1.93936	-0.08969	-0.86089	-0.51526	-0.47352
TRINITY_DN79000_c1_g1_i1_orf1	-	-	-	AT15141p, partial [Drosophila melanogaster]	1.855926	0.198417	-0.4361	-0.90032	-0.71792
TRINITY_DN37599_c0_g1_i1_orf1	-	-	-	bmp-2 protein isoform X3 [Bombyx mori] >XP_028041166.1 RNA-binding protein 4.1-like isoform X2 [Bombyx mandarina]	1.650395	0.551904	-0.265	-1.08003	-0.85726
TRINITY_DN10455_c0_g1_i2_orf1	-	-	-	actin-related protein 2/3 complex subunit 4 [Plutella xylostella] >XP_013184242.1 PREDICTED: actin-related protein 2/3 complex subunit 4 [Amyelois transitella] >XP_026754865.1 actin-related protein 2/3 complex subunit 4 [Galleria mellonella] >XP_028168998.1 actin-related protein 2/3 complex subunit 4 [Ostrinia furnacalis] >KAI5632346.1 ARP2/3 complex 20 kDa subunit (ARPC4) domain-containing protein [Phthorimaea operculella] >KAG7303373.1 Actin-protein 2/3 complex subunit 4 [Plutella xylostella] >CAG9104981.1 unnamed protein product [Plutella xylostella]	1.71043	0.21104	0.08644	-1.06125	-0.94666
TRINITY_DN4008_c0_g1_i7_orf1	-	-	-	nuclear export mediator factor NEMF homolog isoform X1 [Ostrinia furnacalis]	1.781324	-0.73531	0.38607	-0.95239	-0.47969
TRINITY_DN11215_c0_g1_i1_orf1	-	-	-	dnaJ homolog subfamily C member 2 [Ostrinia furnacalis]	1.892916	0.138271	-0.72491	-0.55547	-0.75081
TRINITY_DN15706_c0_g2_i5_orf1	-	-	-	cdc42 homolog [Galleria mellonella] >XP_028178764.1 cdc42 homolog [Ostrinia furnacalis] >XP_028178765.1 cdc42 homolog [Ostrinia furnacalis]	1.788446	0.379109	-0.81609	-0.87386	-0.47761
TRINITY_DN101922_c0_g1_i1_orf1	-	-	-	PREDICTED: uncharacterized protein LOC103569676 [Microplitis demolitor]	1.758062	-0.38515	-1.27831	0.200004	-0.29461
TRINITY_DN3488_c0_g1_i2_orf1	-	-	-	hsp70-binding protein 1 isoform X1 [Ostrinia furnacalis] >XP_028170128.1 hsp70-binding protein 1 isoform X2 [Ostrinia furnacalis]	1.826376	0.133345	-0.26515	-0.58226	-1.11231
TRINITY_DN29156_c0_g1_i1_orf1	-	-	-	protein FAM136A [Ostrinia furnacalis]	1.919504	-0.84257	0.007175	-0.63661	-0.4475
TRINITY_DN10030_c0_g1_i2_orf1	-	-	-	uncharacterized protein LOC114360702 [Ostrinia furnacalis] >XP_028171286.1 uncharacterized protein LOC114360702 [Ostrinia furnacalis] >XP_028171287.1 uncharacterized protein LOC114360702 [Ostrinia furnacalis]	1.982902	-0.28544	-0.64782	-0.61449	-0.43516
TRINITY_DN12323_c0_g2_i2_orf1	-	-	-	polyribonucleotide nucleotidyltransferase 1, mitochondrial [Ostrinia furnacalis]	1.928526	-0.48716	-0.55234	-0.85876	-0.03027
TRINITY_DN15388_c0_g1_i5_orf1	-	-	-	RNA-binding protein 28-like isoform X1 [Ostrinia furnacalis]	1.96473	-0.52478	-0.54449	-0.73673	-0.15872
TRINITY_DN344_c0_g1_i1_orf1	-	-	-	chymotrypsin-like serine protease 16 [Ostrinia nubilalis]	1.994051	-0.47771	-0.47771	-0.63758	-0.40106
TRINITY_DN19122_c0_g1_i7_orf1	-	-	-	phosphatidylserine decarboxylase proenzyme, mitochondrial [Ostrinia furnacalis]	1.954826	-0.6229	-0.80549	-0.30368	-0.22277
TRINITY_DN101325_c0_g1_i4_orf1	-	-	-	endonuclease G, mitochondrial [Ostrinia furnacalis]	1.88813	-0.62002	-0.16116	-0.09966	-1.00729
TRINITY_DN35277_c0_g1_i1_orf1	-	-	-	luciferin 4-monooxygenase-like, partial [Ostrinia furnacalis]	1.915413	-0.72567	-0.82641	-0.34847	-0.01487
TRINITY_DN12527_c0_g1_i4_orf1	-	-	-	DNA-directed RNA polymerase III subunit RPC4 isoform X1 [Ostrinia furnacalis]	1.698712	0.459027	-0.75183	-1.12136	-0.28454
TRINITY_DN3474_c1_g2_i7_orf1	-	-	-	LOW QUALITY PROTEIN: endoplasmic reticulum metallopeptidase 1-like [Ostrinia furnacalis]	1.852382	0.06412	-0.73762	-0.99293	-0.18595
TRINITY_DN144956_c0_g1_i1_orf1	-	-	-	40S ribosomal protein S18 [Cotesia glomerata] >CAD6216330.1 GSCOCG00004483001-RA-CDS [Cotesia congregata] >CAG5095266.1 Similar to RpS18: 40S ribosomal protein S18 (Spodoptera frugiperda) [Cotesia congregata]	1.859405	-0.75095	0.049149	-0.97011	-0.18749
TRINITY_DN43656_c0_g1_i1_orf1	-	-	-	GPI ethanolamine phosphate transferase 3 isoform X2 [Ostrinia furnacalis]	1.951627	-0.73714	-0.0896	-0.50303	-0.62186
TRINITY_DN19187_c0_g1_i1_orf1	-	-	-	fumarylacetoacetase [Chelonus insularis]	1.802328	-0.12555	-0.16577	-1.28786	-0.22315
TRINITY_DN27_c0_g1_i1_orf1	-	-	-	THO complex subunit 4-A [Ostrinia furnacalis]	1.745154	0.345321	-0.24011	-1.10631	-0.74405
TRINITY_DN28509_c0_g1_i1_orf1	-	-	-	39S ribosomal protein L43, mitochondrial [Ostrinia furnacalis]	1.827922	-0.07553	-1.16158	-0.04124	-0.54957
TRINITY_DN7407_c0_g1_i9_orf1	-	-	-	sec1 family domain-containing protein 2-like [Ostrinia furnacalis]	1.958069	-0.80101	-0.15817	-0.51426	-0.48463
TRINITY_DN42310_c0_g1_i1_orf1	-	-	-	uncharacterized protein LOC114349955 [Ostrinia furnacalis]	1.912488	-0.85167	-0.03473	-0.72448	-0.3016
TRINITY_DN747_c0_g1_i1_orf1	-	-	-	trypsin, alkaline C-like [Ostrinia furnacalis]	1.629487	-0.84487	-0.4246	0.65242	-1.01244
TRINITY_DN31503_c0_g1_i4_orf1	-	-	-	hypothetical protein evm_001345 [Chilo suppressalis] >CAB3523265.1 unnamed protein product [Chilo suppressalis] >CAH0400587.1 unnamed protein product [Chilo suppressalis]	1.682923	0.617911	-0.79594	-0.65224	-0.85266

TRINITY_DN15376_c0_g1_i1_orf1	-	-	-	peptidyl-prolyl cis-trans isomerase isoform X1 [Ostrinia furnacalis]	1.985012	-0.44876	-0.30388	-0.5586	-0.67377
TRINITY_DN22678_c0_g1_i4_orf1	-	-	-	NADH-cytochrome b5 reductase 2 isoform X2 [Ostrinia furnacalis] >XP_028163866.1 NADH-cytochrome b5 reductase 2 isoform X2 [Ostrinia furnacalis]	1.913842	-0.75245	-0.56606	-0.66739	0.072058
TRINITY_DN1316_c0_g1_i1_orf1	-	-	-	mitochondrial import receptor subunit TOM70 [Ostrinia furnacalis]	1.958294	-0.2687	-0.74765	-0.25852	-0.68342
TRINITY_DN4731_c0_g2_i1_orf1	-	-	-	gelsolin-like [Ostrinia furnacalis]	1.759468	-0.65959	-1.2096	0.04859	0.061125
TRINITY_DN18869_c0_g1_i1_orf1	-	-	-	unnamed protein product [Parnassius apollo]	1.847531	-1.10361	0.057395	-0.54953	-0.25179
TRINITY_DN3299_c0_g1_i2_orf1	-	-	-	metaxin-1 isoform X3 [Ostrinia furnacalis] >XP_028170907.1 metaxin-1 isoform X4 [Ostrinia furnacalis]	1.877829	-0.51566	0.106484	-0.49138	-0.97727
TRINITY_DN14301_c0_g2_i1_orf1	-	-	-	unnamed protein product [Chrysodeixis includens]	1.936775	-0.39853	-0.94	-0.41645	-0.18179
TRINITY_DN33619_c0_g1_i1_orf1	-	-	-	eukaryotic translation initiation factor 3 subunit C [Ostrinia furnacalis] >XP_028176017.1 eukaryotic translation initiation factor 3 subunit C [Ostrinia furnacalis]	1.907605	-0.38195	0.02459	-0.85551	-0.69474
TRINITY_DN40562_c0_g2_i1_orf1	-	-	-	dual specificity protein phosphatase 23-like isoform X2 [Ostrinia furnacalis]	1.697829	0.53325	-1.01729	-0.78226	-0.43153
TRINITY_DN20_c0_g1_i11_orf1	-	-	-	plasma membrane calcium-transporting ATPase 2 [Ostrinia furnacalis]	1.922583	-0.90548	-0.04005	-0.5378	-0.43925
TRINITY_DN141352_c0_g1_i1_orf1	-	-	-	carboxy-terminal domain RNA polymerase II polypeptide A small phosphatase 1 isoform X1 [Ostrinia furnacalis] >XP_028156862.1 carboxy-terminal domain RNA polymerase II polypeptide A small phosphatase 1 isoform X2 [Ostrinia furnacalis] >XP_028156863.1 carboxy-terminal domain RNA polymerase II polypeptide A small phosphatase 1 isoform X3 [Ostrinia	1.960581	-0.36934	-0.76662	-0.62679	-0.19783
TRINITY_DN21782_c0_g1_i8_orf1	-	-	-	atypical kinase COQ8B, mitochondrial [Ostrinia furnacalis]	1.932912	0.007354	-0.59297	-0.72149	-0.6258
TRINITY_DN2374_c0_g1_i1_orf1	-	-	-	uncharacterized protein LOC114357127 [Ostrinia furnacalis]	1.589716	0.379198	0.167272	-1.16655	-0.96963
TRINITY_DN500_c0_g1_i1_orf1	-	-	-	splicing factor U2AF 50 kDa subunit isoform X2 [Manduca sexta]	1.753458	-0.60395	-0.24047	0.283598	-1.19263
TRINITY_DN3551_c0_g1_i4_orf1	-	-	-	carnitine O-palmitoyltransferase 2, mitochondrial [Ostrinia furnacalis]	1.982319	-0.72246	-0.37017	-0.35634	-0.53334
TRINITY_DN5009_c0_g1_i2_orf1	-	-	-	GSCOCG00009487001-RA-CDS [Cotesia congregata] >CAG5088842.1 Similar to RpL18: 60S ribosomal protein L18 (Timarcha balearica) [Cotesia congregata]	1.708984	-0.62917	0.424737	-1.18314	-0.32142
TRINITY_DN3366_c0_g1_i6_orf1	-	-	-	eukaryotic translation initiation factor 3 subunit K [Helicoverpa zea]	1.903506	-0.09968	-0.21051	-0.95958	-0.63373
TRINITY_DN7574_c0_g1_i10_orf1	-	-	-	proteasome activator complex subunit 3 isoform X2 [Ostrinia furnacalis]	1.749492	0.067096	0.08871	-1.18911	-0.71619
TRINITY_DN72934_c0_g1_i1_orf1	-	-	-	carboxylesterase 8 [Streltzoviella insularis]	1.986748	-0.30845	-0.60383	-0.44824	-0.62623
TRINITY_DN11396_c0_g1_i1_orf1	-	-	-	uncharacterized protein LOC114352414 isoform X1 [Ostrinia furnacalis]	1.873208	0.170909	-0.89446	-0.55287	-0.59679
TRINITY_DN3027_c0_g1_i4_orf1	-	-	-	ribosome biogenesis protein BOP1 homolog [Ostrinia furnacalis]	1.987032	-0.69533	-0.34092	-0.47648	-0.47431
TRINITY_DN25373_c0_g1_i1_orf1	-	-	-	epidermal growth factor receptor substrate 15-like 1 [Ostrinia furnacalis]	1.858981	0.229049	-0.79201	-0.75894	-0.53708
TRINITY_DN36648_c0_g1_i1_orf1	-	-	-	UMP-CMP kinase [Ostrinia furnacalis]	1.987162	-0.43851	-0.39633	-0.71381	-0.43851
TRINITY_DN25542_c0_g1_i1_orf1	-	-	-	tRNA-dihydrouridine(47) synthase [NAD(P)(+)]-like [Ostrinia furnacalis]	1.941088	-0.45392	-0.2588	-0.93412	-0.29425
TRINITY_DN27751_c0_g2_i1_orf1	-	-	-	eukaryotic translation initiation factor 3 subunit I [Ostrinia furnacalis]	1.894315	-0.25289	-0.06137	-1.00862	-0.57144
TRINITY_DN5648_c0_g1_i5_orf1	-	-	-	protein tumorous imaginal discs, mitochondrial-like isoform X2 [Ostrinia furnacalis]	1.892423	0.144154	-0.68171	-0.76516	-0.5897
TRINITY_DN3582_c0_g1_i2_orf1	-	-	-	uncharacterized protein LOC114357129 [Ostrinia furnacalis]	1.667219	0.625361	-0.59381	-0.97935	-0.71942
TRINITY_DN41842_c0_g1_i2_orf1	-	-	-	importin-5 [Ostrinia furnacalis]	1.989339	-0.45139	-0.33952	-0.54734	-0.6511
TRINITY_DN24142_c0_g1_i1_orf1	-	-	-	arylalkylamine N-acetyltransferase [Chilo suppressalis]	1.811647	-0.83022	0.054166	-1.01252	-0.02307
TRINITY_DN5686_c0_g1_i4_orf1	-	-	-	FACT complex subunit spt16 isoform X2 [Ostrinia furnacalis]	1.890804	-0.24411	-0.48145	-1.05937	-0.10587
TRINITY_DN4820_c0_g1_i1_orf1	-	-	-	tudor and KH domain-containing protein homolog [Galleria mellonella]	1.900501	-0.61415	0.040537	-0.91737	-0.40951
TRINITY_DN57454_c0_g1_i4_orf1	-	-	-	translation machinery-associated protein 7 homolog [Zerene cesonia]	1.846898	0.160924	-1.00168	-0.33944	-0.66671
TRINITY_DN2563_c0_g1_i4_orf1	-	-	-	trypsin, alkaline B-like [Ostrinia furnacalis]	1.851038	-0.36331	-1.07938	0.106656	-0.51501
TRINITY_DN3976_c0_g1_i6_orf1	-	-	-	grpE protein homolog, mitochondrial [Ostrinia furnacalis]	1.934449	-0.94132	-0.46646	-0.17516	-0.35151
TRINITY_DN20007_c0_g1_i1_orf1	-	-	-	hypothetical protein evm_011958 [Chilo suppressalis] >CAB3521085.1 unnamed protein product [Chilo suppressalis]	1.776887	0.432111	-0.63143	-0.86906	-0.7085
TRINITY_DN2593_c0_g1_i1_orf1	-	-	-	midgut carboxypeptidase [Loxostege sticticalis]	1.90855	-0.55079	-0.99288	-0.21157	-0.15332
TRINITY_DN3355_c0_g1_i1_orf1	-	-	-	UDP-glucuronosyltransferase 2B2-like [Ostrinia furnacalis]	1.77458	-0.40641	-1.20621	0.248894	-0.41085
TRINITY_DN42738_c0_g1_i1_orf1	-	-	-	arf-GAP domain and FG repeat-containing protein 1 [Ostrinia furnacalis]	1.904032	-0.57721	-0.27374	-0.98045	-0.07264
TRINITY_DN13530_c0_g1_i1_orf1	-	-	-	unnamed protein product [Euphydryas editha]	1.966109	-0.48867	-0.32514	-0.82715	-0.32514
TRINITY_DN799_c0_g1_i7_orf1	-	-	-	hypothetical protein evm_002571 [Chilo suppressalis] >CAB3529880.1 unnamed protein product [Chilo suppressalis] >CAH0406472.1 unnamed protein product [Chilo suppressalis]	1.757972	-0.48509	0.309985	-1.19479	-0.38808
TRINITY_DN2062_c0_g1_i11_orf1	-	-	-	uncharacterized protein LOC114350846 [Ostrinia furnacalis]	1.899745	-0.2008	-0.44519	-1.05496	-0.19879
TRINITY_DN5086_c0_g1_i1_orf1	-	-	-	unnamed protein product [Diatraea saccharalis]	1.724629	0.129539	0.131973	-1.09075	-0.89539
TRINITY_DN13972_c0_g1_i5_orf1	-	-	-	myelin expression factor 2-like [Ostrinia furnacalis] >XP_028173185.1 myelin expression factor 2-like [Ostrinia furnacalis]	1.671382	0.501842	-0.22595	-1.03543	-0.91185
TRINITY_DN2173_c0_g1_i1_orf1	-	-	-	heat shock 70 kDa protein cognate 4 [Cephus cinctus]	1.879078	0.022212	-0.27282	-0.62959	-0.99888

TRINITY_DN26251_c0_g1_i1_orf1	-	-	-	serine/arginine-rich splicing factor 1A [Neodiprion lecontei] >XP_046417766.1 serine/arginine-rich splicing factor 1A [Neodiprion fabricii] >XP_046473571.1 serine/arginine-rich splicing factor 1A [Neodiprion pinetum] >XP_046610590.1 serine/arginine-rich splicing factor 1A [Neodiprion virginianus] >XP_046738887.1 serine/arginine-rich splicing factor 1A	1.583753	0.414197	0.060469	-1.34382	-0.71459
TRINITY_DN27960_c0_g1_i1_orf1	-	-	-	ATP synthase mitochondrial F1 complex assembly factor 1 [Ostrinia furnacalis]	1.87978	0.188684	-0.74427	-0.6536	-0.6706
TRINITY_DN9498_c0_g1_i3_orf1	-	-	-	eukaryotic translation initiation factor 4 gamma 3-like isoform X2 [Ostrinia furnacalis]	1.917958	-0.04033	-0.44196	-0.93453	-0.50113
TRINITY_DN4944_c0_g1_i5_orf1	-	-	-	bifunctional glutamate/proline--tRNA ligase [Ostrinia furnacalis]	1.893919	-0.2179	-0.09469	-0.5601	-1.02123
TRINITY_DN2876_c0_g1_i3_orf1	-	-	-	long-chain fatty acid transport protein 4-like isoform X1 [Ostrinia furnacalis]	1.78276	0.313224	-0.86729	-0.28524	-0.94345
TRINITY_DN40650_c0_g1_i1_orf1	-	-	-	60S ribosomal protein L12 [Zerene cesonia]	1.793895	-0.89694	0.253497	-0.92978	-0.22068
TRINITY_DN13368_c0_g1_i1_orf1	-	-	-	isoleucine--tRNA ligase, cytoplasmic [Ostrinia furnacalis]	1.948594	-0.63594	-0.05179	-0.65267	-0.6082
TRINITY_DN8949_c0_g1_i2_orf1	-	-	-	unnamed protein product [Arctia plantaginis]	1.815942	-0.61842	0.140559	-1.119	-0.21908
TRINITY_DN7787_c0_g1_i1_orf1	-	-	-	trimeric intracellular cation channel type 1B.1 [Manduca sexta] >KAG6456518.1 hypothetical protein O3G_MSEX009773 [Manduca sexta]	1.863234	0.062504	-0.77907	-0.93394	-0.21272
TRINITY_DN18794_c0_g1_i5_orf1	-	-	-	hypothetical protein evm_012380 [Chilo suppressalis] >CAB3520845.1 unnamed protein product [Chilo suppressalis] >CAH0398166.1 unnamed protein product [Chilo suppressalis]	1.746159	0.325007	-0.20009	-0.77	-1.10108
TRINITY_DN53684_c0_g1_i1_orf1	-	-	-	eukaryotic translation initiation factor 3 subunit M-like [Ostrinia furnacalis]	1.930993	-0.03136	-0.42956	-0.78604	-0.68404
TRINITY_DN66596_c0_g1_i1_orf1	-	-	-	CCR4-NOT transcription complex subunit 10 [Ostrinia furnacalis]	1.941411	-0.38532	-0.3768	-0.93972	-0.23956
TRINITY_DN4532_c0_g1_i1_orf1	-	-	-	3-hydroxyacyl-CoA dehydrogenase type-2 [Ostrinia furnacalis]	1.950041	-0.84688	-0.40305	-0.54059	-0.15953
TRINITY_DN9316_c1_g1_i1_orf1	-	-	-	peptidyl-tRNA hydrolase 2, mitochondrial-like [Ostrinia furnacalis]	1.979107	-0.74145	-0.48927	-0.45774	-0.29065
TRINITY_DN3393_c0_g2_i1_orf1	-	-	-	40S ribosomal protein S8 [Ostrinia furnacalis]	1.895803	-0.60679	0.083801	-0.89665	-0.47616
TRINITY_DN2668_c0_g1_i6_orf1	-	-	-	pancreatic triacylglycerol lipase-like [Spodoptera litura]	1.940517	-0.12706	-0.86861	-0.37913	-0.56571
TRINITY_DN271_c0_g2_i6_orf1	-	-	-	hypothetical protein NE865_03378 [Phthorimaea operculella]	1.854464	-0.71745	0.261006	-0.71992	-0.6781
TRINITY_DN932_c0_g1_i4_orf1	-	-	-	SXSS-APN2 [Ostrinia furnacalis]	1.978655	-0.6369	-0.66897	-0.38856	-0.28423
TRINITY_DN1956_c1_g1_i5_orf1	-	-	-	uncharacterized protein LOC114356377 [Ostrinia furnacalis]	1.942617	-0.44819	-0.17104	-0.90709	-0.41629
TRINITY_DN2627_c0_g1_i2_orf1	-	-	-	probable cytosolic oligopeptidase A [Ostrinia furnacalis]	1.962435	-0.1991	-0.80086	-0.433	-0.52948
TRINITY_DN52893_c0_g1_i1_orf1	-	-	-	growth arrest and DNA damage-inducible proteins-interacting protein 1 [Galleria mellonella]	1.931284	-0.13284	-0.30556	-0.89607	-0.59681
TRINITY_DN33_c0_g1_i1_orf1	-	-	-	uncharacterized protein CG45076-like isoform X2 [Ostrinia furnacalis]	1.806669	-0.75248	0.095201	-1.07475	-0.07464
TRINITY_DN5262_c0_g1_i7_orf1	-	-	-	T-complex protein 1 subunit beta [Ostrinia furnacalis]	1.884414	0.042753	-0.27737	-0.89311	-0.75669
TRINITY_DN298_c0_g1_i4_orf1	-	-	-	luc7-like protein 3 isoform X1 [Ostrinia furnacalis] >XP_028160033.1 luc7-like protein 3 isoform X1 [Ostrinia furnacalis]	1.589716	0.684256	-0.33011	-0.91571	-1.02816
TRINITY_DN21984_c0_g1_i6_orf1	-	-	-	venom serine protease 34-like [Ostrinia furnacalis]	1.882736	-0.52199	-1.06444	-0.09381	-0.20251
TRINITY_DN42824_c0_g1_i5_orf1	-	-	-	prefoldin subunit 3 [Ostrinia furnacalis]	1.751226	0.434451	-0.42023	-0.81393	-0.95152
TRINITY_DN37218_c0_g1_i12_orf1	-	-	-	protein white [Ostrinia furnacalis]	1.933888	-0.0798	-0.67975	-0.81313	-0.36121
TRINITY_DN42364_c0_g1_i4_orf1	-	-	-	brain tumor protein [Ostrinia furnacalis] >XP_028157996.1 brain tumor protein [Ostrinia	1.979664	-0.35702	-0.49563	-0.75421	-0.3728
TRINITY_DN33801_c0_g1_i1_orf1	-	-	-	unnamed protein product [Diatraea saccharalis]	1.794021	0.241412	-0.34335	-1.14078	-0.5513
TRINITY_DN141738_c0_g1_i1_orf1	-	-	-	PREDICTED: fibroblast growth factor 1 [Microplitis demolitor]	1.989282	-0.62101	-0.40558	-0.60222	-0.36047
TRINITY_DN4779_c0_g1_i5_orf1	-	-	-	T-complex protein 1 subunit epsilon isoform X1 [Ostrinia furnacalis] >XP_028156782.1 T-complex protein 1 subunit epsilon isoform X2 [Ostrinia furnacalis]	1.893535	0.019366	-0.34197	-0.96336	-0.60757
TRINITY_DN136028_c0_g2_i1_orf1	-	-	-	cytochrome c oxidase subunit 5A, mitochondrial [Ostrinia furnacalis]	1.856659	0.179459	-0.90882	-0.73578	-0.39152
TRINITY_DN33883_c0_g1_i1_orf1	-	-	-	probable 28S ribosomal protein S6, mitochondrial [Ostrinia furnacalis]	1.92992	-0.07066	-0.88107	-0.4005	-0.5777
TRINITY_DN9558_c0_g1_i2_orf1	-	-	-	NADH dehydrogenase [ubiquinone] iron-sulfur protein 4, mitochondrial-like [Ostrinia	1.902972	-0.61971	-0.76702	0.111394	-0.62763
TRINITY_DN10106_c0_g2_i1_orf1	-	-	-	arrestin domain-containing protein 2-like isoform X3 [Ostrinia furnacalis]	1.948489	-0.59381	-0.14913	-0.37709	-0.82846
TRINITY_DN24751_c0_g1_i1_orf1	-	-	-	NADH dehydrogenase [ubiquinone] 1 beta subcomplex subunit 7-like [Ostrinia furnacalis]	1.96916	-0.44889	-0.41518	-0.81515	-0.28994
TRINITY_DN2456_c0_g1_i2_orf1	-	-	-	glycerol-3-phosphate phosphatase isoform X1 [Ostrinia furnacalis]	1.899155	0.131638	-0.70377	-0.6566	-0.67042
TRINITY_DN26355_c0_g1_i4_orf1	-	-	-	small integral membrane protein 12 [Ostrinia furnacalis]	1.721501	0.54774	-0.64881	-0.84601	-0.77441
TRINITY_DN90289_c0_g1_i5_orf1	-	-	-	40S ribosomal protein S25 [Eumeta japonica]	1.942412	-0.17628	-0.42582	-0.42889	-0.91142
TRINITY_DN120979_c0_g1_i1_orf1	-	-	-	la-related protein 1-like isoform X2 [Ostrinia furnacalis]	1.866949	0.061979	-0.22088	-0.81429	-0.89376
TRINITY_DN36893_c0_g1_i1_orf1	-	-	-	40S ribosomal protein S15 [Bicyclus anynana] >XP_026325996.1 40S ribosomal protein S15 [Hyposmocoma kahamanoa] >XP_028175376.1 40S ribosomal protein S15 [Ostrinia furnacalis] >XP_030034906.1 40S ribosomal protein S15 [Manduca sexta] >XP_039758445.1 40S ribosomal protein S15 [Pararge aegeria] >XP_045775675.1 40S ribosomal protein S15 [Maniola jurtina] >CAH2267288.1 jg14755 [Pararge aegeria aegeria] >ACY95351.1 ribosomal protein S15 [Manduca sexta] >KAG6461386.1 hypothetical protein O3G_MSEX012590 [Manduca sexta] >KAG6461387.1 hypothetical protein O3G_MSEX012590 [Manduca sexta]	1.596322	-0.73593	0.743881	-0.98916	-0.61511

TRINITY_DN53810_c0_g1_i1_orf1	-	-	-	39S ribosomal protein L53, mitochondrial [Pectinophora gossypiella]	1.87508	0.165972	-0.87251	-0.50518	-0.66337
TRINITY_DN2623_c0_g1_i3_orf1	-	-	-	unnamed protein product [Chilo suppressalis]	1.640999	0.593519	-0.30477	-0.96248	-0.96727
TRINITY_DN13160_c0_g1_i1_orf1	-	-	-	serine/threonine-protein kinase 10-like, partial [Ostrinia furnacalis]	1.921244	-0.62845	0.054692	-0.63488	-0.71261
TRINITY_DN4747_c0_g1_i4_orf1	-	-	-	transcription factor A, mitochondrial [Ostrinia furnacalis]	1.879471	0.074436	-0.52038	-1.00291	-0.43062
TRINITY_DN36045_c0_g1_i2_orf1	-	-	-	hypothetical protein evm_012355 [Chilo suppressalis] >CAB3522006.1 unnamed protein product [Chilo suppressalis] >CAH0399328.1 unnamed protein product [Chilo suppressalis]	1.911086	-0.22914	-1.03616	-0.24196	-0.40383
TRINITY_DN24693_c1_g1_i1_orf1	-	-	-	ubiquitin-conjugating enzyme E2 G2 isoform X2 [Ostrinia furnacalis]	1.98006	-0.51545	-0.71325	-0.48217	-0.26919
TRINITY_DN17299_c0_g1_i4_orf1	-	-	-	RNA cytidine acetyltransferase isoform X1 [Ostrinia furnacalis] >XP_028171321.1 RNA cytidine acetyltransferase isoform X2 [Ostrinia furnacalis] >XP_028171329.1 RNA cytidine acetyltransferase isoform X3 [Ostrinia furnacalis]	1.963815	-0.81296	-0.4276	-0.49971	-0.22355
TRINITY_DN5129_c0_g3_i3_orf1	-	-	-	probable citrate synthase 2, mitochondrial [Ostrinia furnacalis]	1.988221	-0.65188	-0.55035	-0.32139	-0.4646
TRINITY_DN16077_c0_g1_i13_orf1	-	-	-	dynamin-like 120 kDa protein, mitochondrial [Ostrinia furnacalis]	1.988637	-0.45863	-0.52611	-0.3372	-0.6667
TRINITY_DN4589_c0_g1_i1_orf1	-	-	-	protein penguin [Ostrinia furnacalis]	1.974462	-0.59927	-0.28307	-0.72755	-0.36456
TRINITY_DN41922_c0_g3_i1_orf1	-	-	-	influenza virus NS1A-binding protein-like [Ostrinia furnacalis]	1.941204	-0.79653	-0.05846	-0.58734	-0.49888
TRINITY_DN2997_c0_g1_i6_orf1	-	-	-	titin-like [Ostrinia furnacalis]	1.729222	0.067679	0.184195	-0.92378	-1.05731
TRINITY_DN10429_c0_g1_i2_orf1	-	-	-	lon protease homolog, mitochondrial isoform X1 [Ostrinia furnacalis] >XP_028176557.1 lon protease homolog, mitochondrial isoform X2 [Ostrinia furnacalis]	1.976551	-0.70791	-0.24019	-0.56697	-0.46148
TRINITY_DN631_c0_g1_i6_orf1	-	-	-	cytosolic 10-formyltetrahydrofolate dehydrogenase isoform X1 [Ostrinia furnacalis] >XP_028172896.1 cytosolic 10-formyltetrahydrofolate dehydrogenase isoform X2 [Ostrinia furnacalis]	1.914028	-0.65059	-0.10477	-0.91919	-0.23949
TRINITY_DN85490_c0_g2_i1_orf1	-	-	-	venom carboxylesterase-6-like [Ostrinia furnacalis]	1.839056	-0.60611	-1.10676	0.030803	-0.15699
TRINITY_DN108819_c0_g1_i1_orf1	-	-	-	NADH dehydrogenase [ubiquinone] 1 beta subcomplex subunit 8, mitochondrial [Ostrinia furnacalis]	1.941255	-0.19022	-0.33784	-0.90988	-0.50332
TRINITY_DN3534_c0_g1_i2_orf1	-	-	-	guanine nucleotide-binding protein subunit beta-like protein [Ostrinia furnacalis]	1.924263	-0.63387	-0.23152	-0.90442	-0.15446
TRINITY_DN29038_c0_g2_i1_orf1	-	-	-	PREDICTED: ATP synthase lipid-binding protein, mitochondrial [Fopius arisanus] >XP_011314178.1 PREDICTED: ATP synthase lipid-binding protein, mitochondrial [Fopius arisanus] >XP_011314186.1 PREDICTED: ATP synthase lipid-binding protein, mitochondrial [Fopius arisanus]	1.958036	-0.45197	-0.28481	-0.8704	-0.35085
TRINITY_DN95665_c0_g1_i1_orf1	-	-	-	hypothetical protein HW555_003264 [Spodoptera exigua] >KAH9639693.1 hypothetical protein HF086_017083 [Spodoptera exigua] >CAH0696396.1 unnamed protein product [Spodoptera exigua]	1.954849	-0.35119	-0.433	-0.28324	-0.88742
TRINITY_DN2064_c1_g1_i1_orf1	-	-	-	hypothetical protein evm_007509 [Chilo suppressalis] >CAB3521498.1 unnamed protein product [Chilo suppressalis]	1.94339	-0.94251	-0.32077	-0.32077	-0.35933
TRINITY_DN17215_c0_g1_i4_orf1	-	-	-	28S ribosomal protein S29, mitochondrial [Ostrinia furnacalis]	1.976038	-0.73936	-0.38771	-0.28678	-0.5622
TRINITY_DN24163_c0_g1_i1_orf1	-	-	-	luciferin 4-monooxygenase-like [Ostrinia furnacalis]	1.832071	0.24932	-0.76126	-0.91547	-0.40466
TRINITY_DN46367_c0_g1_i2_orf1	-	-	-	T-complex protein 1 subunit zeta [Ostrinia furnacalis]	1.929532	0.022825	-0.67641	-0.5888	-0.68715
TRINITY_DN4036_c0_g2_i1_orf1	-	-	-	microvitellogenin-like [Ostrinia furnacalis]	1.650419	-0.35704	-0.02045	-1.4544	0.181473
TRINITY_DN11402_c0_g1_i1_orf1	-	-	-	constitutive coactivator of PPAR-gamma-like protein 1 isoform X1 [Ostrinia furnacalis] >XP_028158538.1 constitutive coactivator of PPAR-gamma-like protein 1 isoform X2 [Ostrinia furnacalis]	1.510149	0.882947	-0.65451	-0.86126	-0.87733
TRINITY_DN33038_c0_g1_i1_orf1	-	-	-	39S ribosomal protein L46, mitochondrial [Ostrinia furnacalis]	1.872964	-0.63826	-0.99435	0.062866	-0.30322
TRINITY_DN1191_c0_g1_i4_orf1	-	-	-	interferon-inducible double-stranded RNA-dependent protein kinase activator A homolog isoform X4 [Helicoverpa zea] >XP_047029704.1 interferon-inducible double-stranded RNA-dependent protein kinase activator A homolog isoform X4 [Helicoverpa zea]	1.989465	-0.42979	-0.56549	-0.64428	-0.34991
TRINITY_DN45598_c0_g1_i2_orf1	-	-	-	heat shock protein 60A-like [Ostrinia furnacalis]	1.974776	-0.72299	-0.5083	-0.51802	-0.22547
TRINITY_DN78873_c0_g1_i4_orf1	-	-	-	hypothetical protein evm_008224 [Chilo suppressalis]	1.743064	-0.26063	0.233254	-1.28656	-0.42913
TRINITY_DN5818_c1_g1_i2_orf1	-	-	-	unnamed protein product [Chrysodeixis includens]	1.793493	0.189252	-0.1232	-0.8883	-0.97124
TRINITY_DN11894_c1_g1_i5_orf1	-	-	-	39S ribosomal protein L33, mitochondrial [Ostrinia furnacalis]	1.920465	-0.49461	-0.324	-0.97233	-0.12953
TRINITY_DN9101_c0_g2_i1_orf1	-	-	-	60S ribosomal protein L7a [Ostrinia furnacalis]	1.88382	-0.69617	0.158981	-0.80482	-0.54181
TRINITY_DN3832_c0_g1_i1_orf1	-	-	-	serine-threonine kinase receptor-associated protein [Galleria mellonella]	1.745655	0.488789	-0.69945	-0.61512	-0.91988
TRINITY_DN14168_c0_g1_i1_orf1	-	-	-	transmembrane 7 superfamily member 3-like [Ostrinia furnacalis]	1.976899	-0.5543	-0.26174	-0.72588	-0.43498
TRINITY_DN15896_c0_g1_i4_orf1	-	-	-	phosphatidylinositol transfer protein alpha isoform [Ostrinia furnacalis]	1.960839	-0.72163	-0.14797	-0.63799	-0.45326

TRINITY_DN4237_c1_g1_i5_orf1	-	-	-	eukaryotic translation initiation factor 3 subunit A-like isoform X1 [Ostrinia furnacalis] >XP_028173593.1 eukaryotic translation initiation factor 3 subunit A-like isoform X2 [Ostrinia furnacalis] >XP_028173594.1 eukaryotic translation initiation factor 3 subunit A-like isoform X3 [Ostrinia furnacalis] >XP_028173595.1 eukaryotic translation initiation factor 3 subunit A-like isoform X4 [Ostrinia furnacalis]	1.883448	-0.24629	-0.03325	-1.03064	-0.57328
TRINITY_DN2084_c0_g1_i1_orf1	-	-	-	40S ribosomal protein S24 [Helicoverpa armigera] >XP_022830907.1 40S ribosomal protein S24 [Spodoptera litura] >XP_026729116.1 40S ribosomal protein S24 [Trichoplusia ni] >XP_035432908.1 40S ribosomal protein S24 [Spodoptera frugiperda] >XP_047024473.1 40S ribosomal protein S24 [Helicoverpa zea] >Q962Q6.1 RecName: Full=40S ribosomal protein S24 [Spodoptera frugiperda] >KAF9418537.1 hypothetical protein HW555_004686 [Spodoptera exigua] >CAB3515448.1 unnamed protein product [Spodoptera littoralis] >CAH0579501.1 unnamed protein product [Chrysodeixis includens] >AAK92192.1 ribosomal protein S24 [Spodoptera frugiperda] >KAF9808794.1 hypothetical protein SFRURICE_013056 [Spodoptera frugiperda]	1.956147	-0.83695	-0.46922	-0.46922	-0.18077
TRINITY_DN3773_c0_g1_i4_orf1	-	-	-	peptidyl-prolyl cis-trans isomerase G isoform X2 [Ostrinia furnacalis]	1.828751	0.029271	-0.21111	-1.17989	-0.46702
TRINITY_DN9164_c0_g1_i3_orf1	-	-	-	unnamed protein product [Parnassius apollo]	1.780254	0.183059	-0.12464	-1.13299	-0.70569
TRINITY_DN50151_c0_g1_i1_orf1	-	-	-	heat shock 70 kDa protein 14 [Ostrinia furnacalis]	1.954082	-0.21795	-0.37029	-0.86206	-0.50379
TRINITY_DN14501_c0_g1_i1_orf1	-	-	-	28S ribosomal protein S28, mitochondrial [Ostrinia furnacalis]	1.735082	0.480857	-0.90747	-0.45584	-0.85263
TRINITY_DN10658_c0_g1_i1_orf1	-	-	-	DNA-directed RNA polymerase II subunit RPB9 [Ostrinia furnacalis] >XP_030035935.1 DNA-directed RNA polymerase II subunit RPB9 [Manduca sexta] >XP_037301147.1 DNA-directed RNA polymerase II subunit RPB9-like [Manduca sexta] >KAG6462484.1 hypothetical protein O3G_MSEX013297 [Manduca sexta]	1.650303	0.350781	-1.32371	-0.63179	-0.04558
TRINITY_DN13496_c0_g1_i7_orf1	-	-	-	nucleolar protein 58 [Ostrinia furnacalis]	1.894731	-0.79708	0.119545	-0.70756	-0.50964
TRINITY_DN7289_c0_g1_i1_orf1	-	-	-	nuclear cap-binding protein subunit 2 [Ostrinia furnacalis]	1.773768	-0.44906	0.401853	-0.85616	-0.8704
TRINITY_DN143603_c0_g1_i1_orf1	-	-	-	hypothetical protein KR044_005587 [Drosophila immigrans]	1.964445	-0.73152	-0.56343	-0.51402	-0.15548
TRINITY_DN146264_c0_g1_i1_orf1	-	-	-	PREDICTED: protein preli-like [Fopius arisanus]	1.817549	-0.36139	0.144533	-1.16361	-0.43708
TRINITY_DN5349_c0_g1_i1_orf1	-	-	-	glutamate-rich WD repeat-containing protein 1 [Galleria mellonella]	1.974385	-0.65831	-0.20103	-0.61391	-0.50113
TRINITY_DN6711_c0_g1_i1_orf1	-	-	-	mitochondrial uncoupling protein 4 [Ostrinia furnacalis] >XP_028158360.1 mitochondrial uncoupling protein 4 [Ostrinia furnacalis] >XP_028158361.1 mitochondrial uncoupling protein 4 [Ostrinia furnacalis] >XP_028158362.1 mitochondrial uncoupling protein 4 [Ostrinia	1.998554	-0.45007	-0.46519	-0.54679	-0.5365
TRINITY_DN15448_c0_g1_i1_orf1	-	-	-	ragulator complex protein LAMTOR1-like [Ostrinia furnacalis]	1.973837	-0.29915	-0.61811	-0.33563	-0.72095
TRINITY_DN31232_c1_g1_i9_orf1	-	-	-	PREDICTED: elongation factor 1-alpha 1, partial [Haliaeetus albicilla]	1.935737	-0.75741	-0.42398	-0.04932	-0.70502
TRINITY_DN391_c1_g2_i1_orf1	-	-	-	NADH dehydrogenase [ubiquinone] 1 alpha subcomplex subunit 13 [Ostrinia furnacalis]	1.981868	-0.57709	-0.26821	-0.465	-0.67157
TRINITY_DN646_c0_g1_i5_orf1	-	-	-	unnamed protein product [Diatraea saccharalis]	1.89821	0.104177	-0.64178	-0.83593	-0.52468
TRINITY_DN33248_c0_g1_i1_orf1	-	-	-	elongation factor Ts, mitochondrial isoform X3 [Ostrinia furnacalis] >XP_028155866.1 elongation factor Ts, mitochondrial isoform X3 [Ostrinia furnacalis]	1.915826	-0.6428	-0.04009	-0.89463	-0.33831
TRINITY_DN754_c1_g1_i6_orf1	-	-	-	28S ribosomal protein S2, mitochondrial [Ostrinia furnacalis]	1.950419	-0.37231	-0.53129	-0.86036	-0.18646
TRINITY_DN121893_c0_g1_i1_orf1	-	-	-	hypothetical protein, partial [Ostrinia furnacalis]	1.604555	-0.62747	0.311971	-1.38734	0.098279
TRINITY_DN88539_c0_g2_i1_orf1	-	-	-	uncharacterized protein LOC114352312 isoform X1 [Ostrinia furnacalis] >XP_028159669.1 uncharacterized protein LOC114352312 isoform X1 [Ostrinia furnacalis]	1.76558	-0.14704	0.271422	-0.91795	-0.97201
TRINITY_DN154_c0_g1_i4_orf1	-	-	-	ER membrane protein complex subunit 4 [Ostrinia furnacalis]	1.773248	0.370145	-0.40617	-1.018	-0.71923
TRINITY_DN22842_c0_g1_i4_orf1	-	-	-	MICOS complex subunit MIC27-like [Ostrinia furnacalis] >XP_028158921.1 MICOS complex subunit MIC27-like [Ostrinia furnacalis]	1.9877	-0.367	-0.39248	-0.55908	-0.66914
TRINITY_DN3539_c0_g1_i7_orf1	-	-	-	transcription elongation regulator 1-like [Ostrinia furnacalis]	1.705789	0.413185	-0.19251	-1.07914	-0.84732
TRINITY_DN21357_c0_g1_i5_orf1	-	-	-	40S ribosomal protein S11 isoform X2 [Ostrinia furnacalis]	1.826766	-0.78524	0.126302	-1.00132	-0.1665
TRINITY_DN33926_c0_g1_i1_orf1	-	-	-	ribosomal protein S9 [Ailuropoda melanoleuca] >AEA39538.1 ribosomal protein S9 [Ailuropoda melanoleuca]	1.865904	-1.13486	-0.07034	-0.3907	-0.27001
TRINITY_DN19829_c0_g1_i1_orf1	-	-	-	28S ribosomal protein S35, mitochondrial [Ostrinia furnacalis]	1.905666	-0.85125	-0.19518	-0.77355	-0.08569
TRINITY_DN30638_c0_g1_i1_orf1	-	-	-	alanine--tRNA ligase, cytoplasmic [Ostrinia furnacalis]	1.936079	-0.36341	-0.14509	-0.91315	-0.51443
TRINITY_DN1375_c0_g1_i5_orf1	-	-	-	glutaminase [Chilo suppressalis] >CAB3528726.1 unnamed protein product [Chilo suppressalis] >CAH0405319.1 unnamed protein product [Chilo suppressalis]	1.968485	-0.65401	-0.70773	-0.38208	-0.22467
TRINITY_DN8944_c0_g1_i1_orf1	-	-	-	actin, clone 403 [Trichonephila clavata]	1.574732	0.758057	-0.49693	-0.99991	-0.83594
TRINITY_DN2167_c0_g1_i6_orf1	-	-	-	retinol dehydrogenase 13-like [Ostrinia furnacalis]	1.980836	-0.73882	-0.51757	-0.37076	-0.35369
TRINITY_DN13186_c0_g1_i1_orf1	-	-	-	NADH dehydrogenase [ubiquinone] iron-sulfur protein 5-like [Bicyclus anynana]	1.834734	-0.76784	-0.48555	0.274762	-0.85611

TRINITY_DN2026_c0_g1_i4_orf1	-	-	-	60S ribosomal protein L35a [Ostrinia furnacalis] >XP_028167523.1 60S ribosomal protein L35a [Ostrinia furnacalis]	1.89889	-0.98219	0.007157	-0.42513	-0.49872
TRINITY_DN3747_c1_g1_i3_orf1	-	-	-	unnamed protein product, partial [Iphiclides podalirius]	1.872873	-0.4817	-0.36898	-1.05961	0.037415
TRINITY_DN61222_c0_g1_i1_orf1	-	-	-	60S ribosomal protein L38 [Bicyclus anynana]	1.885457	-0.48925	0.085563	-0.9651	-0.51667
TRINITY_DN31663_c0_g1_i2_orf1	-	-	-	[illegible] conserved hypothetical protein [Pediculus humanus corporis] >XP_003484388.1 PHD finger-like domain-containing protein 5A [Bombus impatiens] >XP_003701008.1 PREDICTED: PHD finger-like domain-containing protein 5A [Megachile rotundata] >XP_006623871.1 PHD finger-like domain-containing protein 5A [Apis dorsata] >XP_011068502.1 PREDICTED: PHD finger-like domain-containing protein 5A [Acromyrmex echinatior] >XP_011154391.1 PHD finger-like domain-containing protein 5A [Harpegnathos saltator] >XP_011164776.1 PHD finger-like domain-containing protein 5A [Solenopsis invicta] >XP_011262550.1 PHD finger-like domain-containing protein 5A [Camponotus floridanus] >XP_011297178.1 PREDICTED: PHD finger-like domain-containing protein 5A [Fopius arisanus] >XP_011334720.1 PHD finger-like domain-containing protein 5A [Ooceraea biroi] >XP_011506347.1 PREDICTED: PHD finger-like domain-containing protein 5A [Ceratosolen solmsi marchali] >XP_011506348.1 PREDICTED: PHD finger-like domain-containing protein 5A [Ceratosolen solmsi marchali] >XP_011638597.1 PHD finger-like domain-containing protein 5A isoform X2 [Pogonomyrmex barbatus] >XP_011686073.1 PREDICTED: PHD finger-like domain-containing protein 5A [Wasmannia auropunctata] >XP_011858255.1 PREDICTED: PHD finger-like domain-containing protein 5A [Vollenhovia emeryi] >XP_012058015.1 PREDICTED: PHD finger-like domain-containing protein 5A [Atta cephalotes] >XP_012135327.1 PREDICTED: PHD finger-like domain-containing protein 5A [Megachile rotundata] >XP_012135328.1 PREDICTED: PHD finger-like domain-containing protein 5A [Megachile rotundata] >XP_012222185.1 PREDICTED: PHD finger-like domain-containing protein 5A [Linepithema humile] >XP_012261946.1 PHD finger-like domain-containing protein 5A [Athalia rosae] >XP_012273120.1 PHD finger-like domain-containing protein 5A [Orussus abietinus] >XP_012526512.1 PHD finger-like domain-containing protein 5A [Monomorium pharaonis] >XP_014217558.1 PHD finger-like domain-containing protein 5A [Copidosoma floridanum] >XP_014484566.1 PREDICTED: PHD finger-like domain-containing protein 5A [Dinoponera quadriceps] >XP_014611099.1 PREDICTED: PHD finger-like domain-containing protein 5A [Polistes canadensis] >XP_015122018.1 PHD finger-like domain-containing protein 5A [Diachasma alloeum] >XP_015174163.1 PREDICTED: PHD finger-like domain-containing [illegible]	1.821423	0.242398	-0.42225	-1.04213	-0.59944
TRINITY_DN11928_c0_g1_i3_orf1	-	-	-	leucyl-cystinyl aminopeptidase-like isoform X4 [Ostrinia furnacalis]	1.911476	0.066759	-0.80785	-0.53894	-0.63145
TRINITY_DN106038_c0_g1_i1_orf1	-	-	-	ankyrin-3-like isoform X1 [Galleria mellonella]	1.795425	0.396362	-0.65632	-0.83759	-0.69788
TRINITY_DN51568_c0_g1_i1_orf1	-	-	-	splicing factor 3A subunit 2 [Ostrinia furnacalis]	1.879452	-0.15273	-0.1037	-1.05284	-0.57018
TRINITY_DN81248_c0_g1_i1_orf1	-	-	-	39S ribosomal protein L12, mitochondrial [Ostrinia furnacalis]	1.89412	-0.4697	-0.88588	0.092596	-0.63113
TRINITY_DN2769_c0_g1_i1_orf1	-	-	-	pseudouridylate synthase 7 homolog [Ostrinia furnacalis]	1.805829	0.223569	-0.2498	-1.03662	-0.74298
TRINITY_DN5630_c4_g1_i2_orf1	-	-	-	PREDICTED: stress-associated endoplasmic reticulum protein 2 [Amyelois transitella] >XP_014371593.1 stress-associated endoplasmic reticulum protein 2 [Papilio machaon] >XP_022818474.1 stress-associated endoplasmic reticulum protein 2 [Spodoptera litura] >XP_028162992.1 stress-associated endoplasmic reticulum protein 2 [Ostrinia furnacalis] >XP_028162993.1 stress-associated endoplasmic reticulum protein 2 [Ostrinia furnacalis] >XP_031767943.1 stress-associated endoplasmic reticulum protein 2 [Galleria mellonella] >XP_035452408.1 stress-associated endoplasmic reticulum protein 2-like [Spodoptera frugiperda] >XP_035452409.1 stress-associated endoplasmic reticulum protein 2-like [Spodoptera frugiperda] >XP_035452411.1 stress-associated endoplasmic reticulum protein 2-like [Spodoptera frugiperda] >XP_045455924.1 stress-associated endoplasmic reticulum protein 2 [Melitaea cinxia] >KPJ00707.1 Stress-associated endoplasmic reticulum protein 2 [Papilio xuthus] >CAB3510969.1 unnamed protein product [Spodoptera littoralis] >AXY94738.1 stress-associated endoplasmic reticulum protein 2 [Galleria mellonella] >KAF9797689.1 hypothetical protein SFRURICE_017884 [Spodoptera frugiperda] >KAG8114722.1 hypothetical protein SFRUCORN_004134 [Spodoptera frugiperda]	1.789554	-0.1782	0.213293	-0.74563	-1.07902
TRINITY_DN21609_c0_g2_i1_orf1	-	-	-	translation initiation factor eIF-2B subunit epsilon [Ostrinia furnacalis]	1.776104	0.063881	-0.10217	-1.26949	-0.46832

TRINITY_DN14372_c0_g2_i1_orf1	-	-	-	12 kDa FK506-binding protein-like [Ostrinia furnacalis]	1.893413	-0.01014	-0.24815	-0.90836	-0.72677
TRINITY_DN15370_c0_g1_i4_orf1	-	-	-	DNA replication licensing factor Mcm5 [Spodoptera litura]	1.937023	-0.12885	-0.90815	-0.47814	-0.42188
TRINITY_DN30131_c0_g1_i1_orf1	-	-	-	PREDICTED: 60S ribosomal protein L44 [Amyelois transitella] >XP_021198018.1 60S ribosomal protein L44 [Helicoverpa armigera] >XP_022814294.1 60S ribosomal protein L44 [Spodoptera litura] >XP_026732397.1 60S ribosomal protein L44 [Trichoplusia ni] >XP_026752106.1 60S ribosomal protein L44 [Galleria mellonella] >XP_028158932.1 60S ribosomal protein L44 [Ostrinia furnacalis] >XP_035434364.1 60S ribosomal protein L44 [Spodoptera frugiperda] >XP_035434370.1 60S ribosomal protein L44 [Spodoptera frugiperda] >XP_047019234.1 60S ribosomal protein L44 [Helicoverpa zea] >XP_049868501.1 60S ribosomal protein L44 [Pectinophora gossypiella] >AAM53948.1 ribosomal protein L44 [Choristoneura parallela] >KAF9418375.1 hypothetical protein HW555_004805 [Spodoptera exigua] >RVE50750.1 hypothetical protein evm_004660 [Chilo suppressalis] >CAB3235328.1 unnamed protein product [Arctia plantaginis] >CAB3516516.1 unnamed protein product [Spodoptera littoralis] >CAG9747186.1 unnamed protein product [Diatraea saccharalis] >CAH0581656.1 unnamed protein product [Chrysodeixis includens]	1.825588	-0.99822	0.062215	-0.81305	-0.07653
TRINITY_DN38540_c0_g1_i1_orf1	-	-	-	GSCOCG00000129001-RA-CDS [Cotesia congregata] >CAG5101050.1 Similar to LUC7L2: Putative RNA-binding protein Luc7-like 2 (Homo sapiens) [Cotesia congregata]	1.741623	0.43194	-0.52923	-1.09436	-0.54998
TRINITY_DN2691_c0_g1_i1_orf1	-	-	-	WD repeat-containing protein 18 [Ostrinia furnacalis]	1.982035	-0.74411	-0.45994	-0.43176	-0.34622
TRINITY_DN1348_c0_g1_i1_orf1	-	-	-	hypothetical protein evm_002665 [Chilo suppressalis]	1.989789	-0.42711	-0.37915	-0.676	-0.50752
TRINITY_DN6671_c0_g1_i6_orf1	-	-	-	hypothetical protein evm_013656 [Chilo suppressalis] >CAB3521812.1 unnamed protein product [Chilo suppressalis] >CAH0399134.1 unnamed protein product [Chilo suppressalis]	1.818374	0.170261	-0.39774	-1.14248	-0.44842
TRINITY_DN35669_c0_g1_i1_orf1	-	-	-	unnamed protein product [Diatraea saccharalis]	1.697402	0.554556	-0.46823	-0.91647	-0.86726
TRINITY_DN38075_c0_g1_i1_orf1	-	-	-	60S ribosomal protein L26 [Ostrinia furnacalis]	1.918263	-0.47813	-0.02259	-0.91665	-0.50089
TRINITY_DN8367_c0_g1_i1_orf1	-	-	-	uncharacterized protein LOC114357075 [Ostrinia furnacalis]	1.916516	0.001096	-0.89531	-0.54882	-0.47349
TRINITY_DN21506_c0_g1_i4_orf1	-	-	-	glutamate dehydrogenase, mitochondrial isoform X2 [Ostrinia furnacalis]	1.954555	-0.89868	-0.33561	-0.35428	-0.36599
TRINITY_DN16174_c0_g1_i2_orf1	-	-	-	LOW QUALITY PROTEIN: ATP-dependent RNA helicase SUV3 homolog, mitochondrial [Ostrinia furnacalis]	1.885922	-0.21052	-0.16237	-1.09414	-0.4189
TRINITY_DN44557_c0_g1_i4_orf1	-	-	-	serine hydrolase-like protein [Ostrinia furnacalis]	1.809617	-0.91526	0.229803	-0.88059	-0.24356
TRINITY_DN107840_c1_g1_i1_orf1	-	-	-	HEAT repeat-containing protein 3 [Ostrinia furnacalis]	1.95603	-0.09648	-0.68453	-0.641	-0.53401
TRINITY_DN14565_c0_g1_i11_orf1	-	-	-	4-aminobutyrate aminotransferase, mitochondrial [Galleria mellonella]	1.874486	0.028975	-0.98482	-0.6757	-0.24294
TRINITY_DN4135_c0_g1_i5_orf1	-	-	-	probable small nuclear ribonucleoprotein Sm D2 [Manduca sexta] >KAG6451233.1 hypothetical protein O3G_MSEX007016 [Manduca sexta]	1.865623	-0.05941	-0.20531	-1.11056	-0.49034
TRINITY_DN13174_c0_g1_i4_orf1	-	-	-	N-alpha-acetyltransferase 35, NatC auxiliary subunit [Ostrinia furnacalis]	1.963398	-0.73531	-0.27448	-0.28398	-0.66963
TRINITY_DN30_c0_g1_i6_orf1	-	-	-	casein kinase I-like isoform X1 [Hyposmocoma kahamanoa]	1.7836	0.354468	-0.72594	-0.99682	-0.41531
TRINITY_DN1132_c0_g1_i5_orf1	-	-	-	unnamed protein product [Spodoptera exigua]	1.908005	-0.26043	-0.04294	-0.76751	-0.83713
TRINITY_DN4631_c0_g1_i7_orf1	-	-	-	uncharacterized protein LOC114366284 [Ostrinia furnacalis]	1.967271	-0.37868	-0.83811	-0.34053	-0.40995
TRINITY_DN17045_c0_g2_i3_orf1	-	-	-	unnamed protein product [Diatraea saccharalis]	1.846776	-0.27247	0.121871	-1.0239	-0.67227
TRINITY_DN3929_c0_g3_i3_orf1	-	-	-	Glutathione S-transferase 1, isoform D [Papilio machaon]	1.778806	-1.05174	-0.31473	-0.72848	0.316145
TRINITY_DN16965_c0_g2_i1_orf1	-	-	-	hypothetical protein evm_007405 [Chilo suppressalis]	1.620335	0.57414	-0.50615	-1.27047	-0.41786
TRINITY_DN47389_c0_g1_i2_orf1	-	-	-	non-specific lipid-transfer protein-like [Ostrinia furnacalis]	1.894553	-0.8755	-0.56032	-0.56477	0.10604
TRINITY_DN14487_c0_g1_i4_orf1	-	-	-	hypothetical protein HW555_009956 [Spodoptera exigua] >KAH9643419.1 hypothetical protein HF086_016708 [Spodoptera exigua] >CAH0702087.1 unnamed protein product [Spodoptera exigua]	1.767459	0.4153	-0.73072	-0.48575	-0.96628
TRINITY_DN127151_c0_g1_i1_orf1	-	-	-	3-oxoacyl-[acyl-carrier-protein] synthase, mitochondrial [Ostrinia furnacalis]	1.97184	-0.73554	-0.52926	-0.21076	-0.49628
TRINITY_DN8173_c0_g1_i3_orf1	-	-	-	dihydroceramide fatty acyl 2-hydroxylase FAH1 [Ostrinia furnacalis]	1.925117	-0.59698	-0.82879	-0.50065	0.001299
TRINITY_DN7251_c0_g1_i3_orf1	-	-	-	hypothetical protein evm_008498 [Chilo suppressalis] >CAB3527693.1 unnamed protein product [Chilo suppressalis] >CAH0401999.1 unnamed protein product [Chilo suppressalis]	1.561498	0.784767	-0.51581	-0.96277	-0.86768
TRINITY_DN10476_c0_g1_i1_orf1	-	-	-	prohibitin-2 [Ostrinia furnacalis]	1.972205	-0.5746	-0.22788	-0.7335	-0.43623
TRINITY_DN36788_c0_g1_i2_orf1	-	-	-	isocitrate dehydrogenase [NADP] cytoplasmic-like [Bicyclus anynana]	1.729245	0.301398	-0.14679	-1.18979	-0.69406
TRINITY_DN755_c0_g1_i3_orf1	-	-	-	uncharacterized protein LOC114358844 [Ostrinia furnacalis]	1.979461	-0.26988	-0.71753	-0.52738	-0.46467
TRINITY_DN123139_c0_g1_i1_orfp1	-	-	-	TRINITY_DN123139_c0_g1_i1_m.79879 TRINITY_DN123139_c0_g1::TRINITY_DN123139_c0_g1_i1::g.79879 ORF type:3prime_partial len:76 (+),score=3.83 TRINITY_DN123139_c0_g1_i1:25-225(+)	1.86415	-0.35681	0.07227	-1.0589	-0.5207
TRINITY_DN8511_c0_g1_i1_orf1	-	-	-	NADH dehydrogenase [ubiquinone] 1 beta subcomplex subunit 10 [Ostrinia furnacalis]	1.971864	-0.70445	-0.19146	-0.53922	-0.53674

TRINITY_DN2673_c0_g3_i1_orf1	-	-	-	uncharacterized protein LOC114361372 [Ostrinia furnacalis]	1.788959	0.021003	-0.58111	-1.20875	-0.02009
TRINITY_DN2184_c0_g1_i4_orf1	-	-	-	uncharacterized protein LOC114359356 [Ostrinia furnacalis]	1.82781	-0.70694	-1.05199	-0.19301	0.124134
TRINITY_DN4356_c0_g1_i6_orf1	-	-	-	mulatexin-like [Ostrinia furnacalis]	1.287896	-0.30486	-1.70315	0.570281	0.149829
TRINITY_DN6235_c0_g1_i5_orf1	-	-	-	rRNA 2'-O-methyltransferase fibrillarin [Vanessa cardui]	1.92191	-0.56421	0.041015	-0.76278	-0.63594
TRINITY_DN4725_c0_g1_i4_orf1	-	-	-	uncharacterized protein LOC114354375 [Ostrinia furnacalis]	1.95127	-0.2892	-0.90606	-0.34496	-0.41105
TRINITY_DN24281_c0_g1_i1_orf1	-	-	-	elongin-B isoform X1 [Maniola jurtina]	1.919247	-0.72391	0.051372	-0.54378	-0.70293
TRINITY_DN9862_c0_g2_i1_orf1	-	-	-	40S ribosomal protein S4 [Manduca sexta] >ACY95325.1 ribosomal protein S4 [Manduca sexta] >KAG6465430.1 hypothetical protein O3G_MSEX015149 [Manduca sexta]	1.806747	-0.83477	0.318798	-0.87357	-0.4172
TRINITY_DN825_c8_g1_i5_orf1	-	-	-	ATP-binding cassette sub-family F member 2 [Ostrinia furnacalis] >XP_028169527.1 ATP-binding cassette sub-family F member 2 [Ostrinia furnacalis]	1.968844	-0.58843	-0.1788	-0.7092	-0.49241
TRINITY_DN18242_c0_g1_i3_orf1	-	-	-	CCHC-type zinc finger protein CG3800 [Papilio xuthus]	1.708982	0.170938	0.129698	-1.08865	-0.92097
TRINITY_DN2425_c0_g1_i1_orf1	-	-	-	thyroid receptor-interacting protein 11 [Ostrinia furnacalis]	1.693297	-0.74073	0.172922	-1.24127	0.115783
TRINITY_DN5678_c0_g2_i3_orf1	-	-	-	coiled-coil domain-containing protein 115 [Ostrinia furnacalis]	1.964543	-0.41166	-0.78176	-0.56254	-0.20858
TRINITY_DN26963_c0_g1_i1_orf1	-	-	-	aminoacyl tRNA synthase complex-interacting multifunctional protein 1 isoform X2 [Ostrinia furnacalis]	1.926479	-0.10895	-0.4913	-0.94239	-0.38384
TRINITY_DN20499_c0_g3_i1_orf1	-	-	-	exosome RNA helicase MTR4 isoform X2 [Ostrinia furnacalis]	1.854332	-0.00448	-0.11497	-1.01461	-0.72027
TRINITY_DN27556_c0_g1_i1_orf1	-	-	-	bystin [Ostrinia furnacalis]	1.839264	0.282672	-0.70785	-0.57226	-0.84183
TRINITY_DN15882_c0_g1_i1_orf1	-	-	-	succinate--CoA ligase [ADP-forming] subunit beta, mitochondrial [Ostrinia furnacalis]	1.985831	-0.69555	-0.37455	-0.53884	-0.37688
TRINITY_DN14073_c0_g1_i1_orf1	-	-	-	cytochrome c oxidase subunit 4 isoform 1, mitochondrial-like [Ostrinia furnacalis] >XP_028164918.1 cytochrome c oxidase subunit 4 isoform 1, mitochondrial-like [Ostrinia furnacalis]	1.882162	-0.50375	-0.89532	0.136223	-0.61932
TRINITY_DN54150_c0_g1_i1_orf1	-	-	-	uncharacterized protein LOC114351648 [Ostrinia furnacalis]	1.955541	-0.35894	-0.8735	-0.25529	-0.46781
TRINITY_DN2103_c0_g1_i1_orf1	-	-	-	probable aconitate hydratase, mitochondrial isoform X1 [Ostrinia furnacalis] >XP_028166656.1 probable aconitate hydratase, mitochondrial isoform X2 [Ostrinia furnacalis]	1.998857	-0.51618	-0.49892	-0.4403	-0.54346
TRINITY_DN17049_c0_g1_i6_orf1	-	-	-	unnamed protein product [Arctia plantaginis] >CAB3248215.1 unnamed protein product [Arctia plantaginis]	1.966418	-0.39105	-0.23155	-0.78086	-0.56297
TRINITY_DN34432_c0_g1_i1_orf1	-	-	-	39S ribosomal protein L44, mitochondrial [Ostrinia furnacalis]	1.78389	0.425035	-0.79108	-0.76429	-0.65356
TRINITY_DN106476_c0_g1_i3_orf1	-	-	-	mitochondrial import inner membrane translocase subunit TIM44 [Ostrinia furnacalis]	1.938717	-0.63078	-0.155	-0.85471	-0.29823
TRINITY_DN19286_c0_g1_i1_orf1	-	-	-	signal recognition particle 9 kDa protein [Ostrinia furnacalis]	1.961191	-0.3972	-0.35127	-0.34443	-0.8683
TRINITY_DN21214_c0_g2_i1_orf1	-	-	-	heat shock protein family A (Hsp70) member 1A [Homo sapiens] >KAI4017664.1 heat shock protein family A (Hsp70) member 1A [Homo sapiens] >PNI76655.1 HSPA1A isoform 2 [Pan troglodytes]	1.954138	-0.19778	-0.35094	-0.57726	-0.82815
TRINITY_DN16349_c0_g1_i10_orf1	-	-	-	protein lingerer-like isoform X1 [Nymphalis io] >XP_050356663.1 protein lingerer-like isoform X1 [Nymphalis io] >XP_050356664.1 protein lingerer-like isoform X1 [Nymphalis io]	1.557485	0.740437	-0.45391	-1.16672	-0.67729
TRINITY_DN1386_c0_g1_i6_orf1	-	-	-	ras-related protein Rab-36 [Ostrinia furnacalis]	1.836235	0.203993	-0.90205	-0.81929	-0.31888
TRINITY_DN11584_c0_g1_i2_orf1	-	-	-	L-threonine 3-dehydrogenase, mitochondrial [Ostrinia furnacalis]	1.953905	-0.46437	-0.70195	-0.10767	-0.67992
TRINITY_DN91989_c0_g1_i1_orf1	-	-	-	protein l(2)37Cc [Pectinophora gossypiella]	1.969269	-0.21254	-0.57406	-0.43655	-0.74611
TRINITY_DN413_c0_g1_i11_orf1	-	-	-	regulator of nonsense transcripts 1 [Helicoverpa armigera] >XP_047028926.1 regulator of nonsense transcripts 1 [Helicoverpa zea]	1.944977	-0.06067	-0.64728	-0.4975	-0.73954
TRINITY_DN3312_c0_g1_i10_orf1	-	-	-	glycerol-3-phosphate dehydrogenase, mitochondrial-like isoform X3 [Ostrinia furnacalis]	1.949453	-0.42057	-0.38229	-0.24218	-0.90441
TRINITY_DN35301_c0_g1_i3_orf1	-	-	-	ATP synthase subunit gamma, mitochondrial-like [Ostrinia furnacalis] >XP_028164649.1 ATP synthase subunit gamma, mitochondrial-like [Ostrinia furnacalis]	1.949408	-0.90794	-0.27897	-0.44014	-0.32236
TRINITY_DN86090_c0_g1_i1_orf1	-	-	-	ATP synthase subunit b, mitochondrial [Ostrinia furnacalis]	1.963402	-0.82465	-0.23216	-0.46218	-0.44441
TRINITY_DN2070_c1_g1_i1_orf1	-	-	-	mitochondrial carrier homolog 2-like [Helicoverpa zea] >PZC82360.1 hypothetical protein B5X24_HaOG210663 [Helicoverpa armigera]	1.98856	-0.60629	-0.30604	-0.58933	-0.48689
TRINITY_DN98538_c0_g1_i1_orf1	-	-	-	ATP synthase subunit d, mitochondrial [Ostrinia furnacalis]	1.984115	-0.70786	-0.33103	-0.41821	-0.52702
TRINITY_DN108200_c0_g1_i1_orf1	-	-	-	uncharacterized protein LOC114350842 [Ostrinia furnacalis]	1.951196	-0.871	-0.44457	-0.44924	-0.18639
TRINITY_DN14063_c0_g1_i7_orf1	-	-	-	probable phosphorylase b kinase regulatory subunit beta isoform X1 [Ostrinia furnacalis] >XP_028175664.1 probable phosphorylase b kinase regulatory subunit beta isoform X2 [Ostrinia furnacalis] >XP_028175665.1 probable phosphorylase b kinase regulatory subunit beta isoform X3 [Ostrinia furnacalis]	1.938828	-0.73777	-0.48177	-0.68046	-0.03882
TRINITY_DN1239_c0_g1_i3_orf1	-	-	-	uncharacterized protein LOC114355269 [Ostrinia furnacalis] >XP_028163822.1 uncharacterized protein LOC114355269 [Ostrinia furnacalis]	1.899509	-0.65318	-0.18182	-0.95979	-0.10472
TRINITY_DN70_c2_g1_i1_orf1	-	-	-	inositol-tetrakisphosphate 1-kinase-like [Ostrinia furnacalis]	1.659968	-0.40418	-0.96356	0.598869	-0.89109

TRINITY_DN816_c0_g1_i3_orf1	-	-	-	calcium-binding mitochondrial carrier protein SCaMC-2 isoform X1 [Ostrinia furnacalis]	1.881999	0.045659	-0.3809	-1.01284	-0.53393
TRINITY_DN9094_c0_g1_i1_orf1	-	-	-	uncharacterized protein LOC114356316 [Ostrinia furnacalis]	1.959684	-0.74691	-0.22648	-0.31415	-0.67214
TRINITY_DN45477_c0_g1_i1_orf1	-	-	-	putative E3 ubiquitin-protein ligase UBR7 [Ostrinia furnacalis]	1.829175	0.213561	-0.9663	-0.75664	-0.3198
TRINITY_DN5767_c0_g1_i4_orf1	-	-	-	cell division cycle 5-like protein [Helicoverpa armigera]	1.949358	-0.05571	-0.63205	-0.65905	-0.60254
TRINITY_DN9410_c0_g1_i4_orf1	-	-	-	protein RRP5 homolog [Ostrinia furnacalis]	1.875498	-1.09676	-0.05075	-0.2863	-0.44169
TRINITY_DN5653_c0_g1_i4_orf1	-	-	-	hrp65 protein-like [Ostrinia furnacalis]	1.594916	0.696132	-0.38534	-0.89244	-1.01326
TRINITY_DN45446_c0_g1_i2_orf1	-	-	-	peptide transporter family 1-like isoform X1 [Ostrinia furnacalis]	1.929422	-0.91002	-0.51756	-0.08448	-0.41737
TRINITY_DN5704_c0_g1_i6_orf1	-	-	-	2-oxoglutarate dehydrogenase, mitochondrial isoform X2 [Ostrinia furnacalis]	1.975941	-0.77645	-0.38265	-0.32859	-0.48826
TRINITY_DN10662_c0_g1_i4_orf1	-	-	-	HD domain-containing protein 2 [Ostrinia furnacalis]	1.771212	0.329433	-0.49467	-1.13465	-0.47132
TRINITY_DN16122_c0_g1_i4_orf1	-	-	-	cytochrome P450 6k1-like [Ostrinia furnacalis]	1.911221	-0.67755	-0.91077	-0.21858	-0.10432
TRINITY_DN48554_c0_g1_i1_orf1	-	-	-	39S ribosomal protein L39, mitochondrial [Ostrinia furnacalis]	1.983428	-0.54418	-0.3203	-0.41583	-0.70312
TRINITY_DN26411_c0_g1_i2_orfp1	-	-	-	TRINITY_DN26411_c0_g1_i2_m.24123 TRINITY_DN26411_c0_g1::TRINITY_DN26411_c0_g1_i2::g.24123 ORF type:internal len:115 (-),score=72.83 TRINITY_DN26411_c0_g1_i2:3-344(-)	1.55133	-0.71561	-0.7419	-0.92054	0.826721
TRINITY_DN33452_c0_g1_i1_orf1	-	-	-	lethal(2) giant larvae protein isoform X8 [Ostrinia furnacalis]	1.401478	-0.79039	-1.4046	0.148319	0.645184
TRINITY_DN2716_c0_g2_i1_orf1	-	-	-	eukaryotic translation initiation factor 1A, X-chromosomal [Ostrinia furnacalis] >XP_045445466.1 eukaryotic translation initiation factor 1A, X-chromosomal [Melitaea cinxia] >XP_049867692.1 eukaryotic translation initiation factor 1A, X-chromosomal [Pectinophora gossypiella] >KOB79530.1 Eukaryotic translation initiation factor 1A [Operophtera brumata] >CAH2086435.1 unnamed protein product [Euphydryas editha] >KOB79531.1 Eukaryotic translation initiation factor 1A [Operophtera brumata]	1.775743	0.036816	0.003955	-1.22091	-0.59561
TRINITY_DN3008_c0_g1_i12_orf1	-	-	-	reticulon-4-interacting protein 1 homolog, mitochondrial [Ostrinia furnacalis]	1.92333	-0.93192	-0.41136	-0.07222	-0.50783
TRINITY_DN25341_c0_g1_i1_orf1	-	-	-	heat shock protein 90 [Loxostege sticticalis]	1.798339	0.367052	-0.54753	-0.91552	-0.70233
TRINITY_DN42082_c0_g2_i2_orfp1	-	-	-	TRINITY_DN42082_c0_g2_i2_m.7835 TRINITY_DN42082_c0_g2::TRINITY_DN42082_c0_g2_i2::g.7835 ORF type:internal len:133 (+),score=75.81 TRINITY_DN42082_c0_g2_i2:1-396(+)	1.803523	-0.62243	0.228572	-1.1011	-0.30856
TRINITY_DN58207_c0_g1_i1_orf1	-	-	-	60S ribosomal protein L6 [Ostrinia furnacalis] >XP_028170357.1 60S ribosomal protein L6 [Ostrinia furnacalis]	1.824443	-0.95206	0.192417	-0.81619	-0.24861
TRINITY_DN1477_c0_g1_i5_orf1	-	-	-	mitochondrial import inner membrane translocase subunit Tim9 [Ostrinia furnacalis]	1.978137	-0.41792	-0.26353	-0.68129	-0.6154
TRINITY_DN868_c0_g1_i4_orf1	-	-	-	uncharacterized protein LOC114359357 isoform X1 [Ostrinia furnacalis]	1.684835	-0.87827	-0.50495	-0.88889	0.587267
TRINITY_DN122786_c0_g2_i1_orf1	-	-	-	glucose dehydrogenase [FAD, quinone]-like [Ostrinia furnacalis]	1.961944	-0.4846	-0.14587	-0.6015	-0.72997
TRINITY_DN14336_c0_g3_i2_orf1	-	-	-	calbindin-32 isoform X1 [Ostrinia furnacalis]	1.973678	-0.69474	-0.38165	-0.25666	-0.64062
TRINITY_DN22654_c0_g2_i4_orf1	-	-	-	protein EFR3 homolog cmp44E isoform X1 [Ostrinia furnacalis] >XP_028166854.1 protein EFR3 homolog cmp44E isoform X2 [Ostrinia furnacalis]	1.711721	0.541725	-0.55707	-0.96517	-0.73121
TRINITY_DN16487_c0_g1_i1_orf1	-	-	-	p21-activated protein kinase-interacting protein 1-like [Ostrinia furnacalis]	1.759949	-0.67282	-1.18715	0.183362	-0.08334
TRINITY_DN51252_c0_g2_i1_orf1	-	-	-	peroxidase-like [Ostrinia furnacalis]	1.758549	0.362343	-1.09211	-0.67917	-0.34961
TRINITY_DN2630_c0_g3_i3_orf1	-	-	-	eukaryotic translation initiation factor 4E-binding protein 2 [Ostrinia furnacalis]	1.81823	-0.35787	-0.53304	0.188978	-1.1163
TRINITY_DN754_c1_g1_i8_orf1	-	-	-	lysophospholipid acyltransferase 5 [Ostrinia furnacalis] >XP_028169982.1 lysophospholipid acyltransferase 5 [Ostrinia furnacalis]	1.841542	0.265378	-0.83801	-0.7589	-0.51002
TRINITY_DN34509_c0_g1_i1_orf1	-	-	-	transcription initiation factor IIA subunit 2 [Aphidius gifuensis] >KAF7997556.1 hypothetical protein HCN44_006127 [Aphidius gifuensis]	1.775184	0.420035	-0.55238	-0.72734	-0.91551
TRINITY_DN94625_c0_g1_i1_orf1	-	-	-	uncharacterized protein LOC114354112 [Ostrinia furnacalis]	1.991394	-0.66383	-0.40497	-0.5065	-0.41609
TRINITY_DN28981_c0_g1_i1_orf1	-	-	-	uncharacterized protein C6orf203 homolog [Ostrinia furnacalis]	1.947674	-0.10348	-0.80458	-0.5835	-0.45611
TRINITY_DN2505_c0_g1_i1_orf1	-	-	-	uncharacterized protein LOC114349853 [Ostrinia furnacalis]	1.956312	-0.84056	-0.26382	-0.29595	-0.55598
TRINITY_DN58636_c0_g1_i1_orf1	-	-	-	uncharacterized protein LOC114363665 [Ostrinia furnacalis]	1.88658	0.034516	-0.28267	-0.91276	-0.72567
TRINITY_DN23360_c0_g1_i3_orf1	-	-	-	protein PTCD3 homolog, mitochondrial [Ostrinia furnacalis]	1.82748	0.323292	-0.66768	-0.81303	-0.67006
TRINITY_DN103457_c0_g1_i1_orf1	-	-	-	28S ribosomal protein S22, mitochondrial [Ostrinia furnacalis]	1.946125	-0.31337	-0.91768	-0.26644	-0.44865
TRINITY_DN10796_c0_g2_i1_orf1	-	-	-	F-BAR domain only protein 2 [Ostrinia furnacalis]	1.981127	-0.29466	-0.68415	-0.59585	-0.40647
TRINITY_DN31851_c0_g1_i2_orf1	-	-	-	CDGSH iron-sulfur domain-containing protein 2 homolog [Helicoverpa armigera] >PZC85510.1 hypothetical protein B5X24_HaOG216618 [Helicoverpa armigera]	1.76477	-0.04025	-0.33768	-0.0577	-1.32914
TRINITY_DN2089_c0_g1_i5_orf1	-	-	-	eukaryotic translation initiation factor 4B [Ostrinia furnacalis]	1.708682	0.24467	0.025683	-1.16501	-0.81403
TRINITY_DN492_c0_g1_i4_orf1	-	-	-	hypothetical protein evm_000589 [Chilo suppressalis] >CAB3530014.1 unnamed protein product [Chilo suppressalis] >CAH0406606.1 unnamed protein product [Chilo suppressalis]	1.974825	-0.51727	-0.20076	-0.59359	-0.66321
TRINITY_DN14601_c0_g1_i2_orf1	-	-	-	unnamed protein product [Chilo suppressalis]	1.900598	0.014205	-0.63126	-0.92926	-0.35429

TRINITY_DN5275_c0_g1_i1_orf1	-	-	-	paraplegin [Ostrinia furnacalis]	1.653065	0.496392	-0.21822	-1.19855	-0.73269
TRINITY_DN27852_c0_g1_i1_orf1	-	-	-	baculoviral IAP repeat-containing protein 6-like [Ostrinia furnacalis]	1.761786	0.308545	-0.52867	-0.36275	-1.17891
TRINITY_DN39532_c0_g1_i1_orf1	-	-	-	hypothetical protein evm_009649 [Chilo suppressalis]	1.732328	0.369309	-0.54249	-1.19963	-0.35952
TRINITY_DN9002_c0_g1_i1_orf1	-	-	-	membrane magnesium transporter 1 [Ostrinia furnacalis]	1.892763	-0.53017	0.078525	-0.93479	-0.50633
TRINITY_DN42506_c0_g1_i1_orf1	-	-	-	28S ribosomal protein S7, mitochondrial [Ostrinia furnacalis]	1.886747	0.105638	-0.78988	-0.80386	-0.39864
TRINITY_DN10379_c0_g1_i3_orf1	-	-	-	succinate dehydrogenase [ubiquinone] cytochrome b small subunit, mitochondrial [Ostrinia furnacalis]	1.988825	-0.50166	-0.62115	-0.30633	-0.55968
TRINITY_DN14937_c0_g1_i7_orf1	-	-	-	multidrug resistance protein homolog 49-like [Ostrinia furnacalis] >XP_028159925.1 multidrug resistance protein homolog 49-like [Ostrinia furnacalis]	1.893993	-0.41107	-0.8836	-0.67614	0.076818
TRINITY_DN4121_c0_g1_i1_orf1	-	-	-	uncharacterized protein LOC114358001, partial [Ostrinia furnacalis]	1.909519	-0.87462	0.032684	-0.62825	-0.43934
TRINITY_DN21792_c0_g1_i1_orf1	-	-	-	probable 28S ribosomal protein S25, mitochondrial [Ostrinia furnacalis]	1.963749	-0.282	-0.82881	-0.34434	-0.5086
TRINITY_DN58413_c0_g1_i4_orf1	-	-	-	cysteine and histidine-rich protein 1 isoform X1 [Ostrinia furnacalis]	1.978101	-0.77001	-0.41317	-0.45887	-0.33605
TRINITY_DN47591_c1_g1_i1_orf1	-	-	-	uncharacterized protein LOC114364828 [Ostrinia furnacalis]	1.955352	-0.12385	-0.46245	-0.75611	-0.61295
TRINITY_DN81715_c0_g1_i1_orf1	-	-	-	gamma-interferon-inducible lysosomal thiol reductase-like [Ostrinia furnacalis]	1.453103	0.937706	-0.61581	-1.05283	-0.72217
TRINITY_DN2265_c0_g2_i1_orf1	-	-	-	LOW QUALITY PROTEIN: elongation factor G, mitochondrial-like [Leguminivora glycinivorella]	1.905337	0.070007	-0.85581	-0.61318	-0.50636
TRINITY_DN23586_c0_g1_i3_orf1	-	-	-	myrosinase 1-like isoform X1 [Ostrinia furnacalis]	1.976127	-0.7219	-0.59839	-0.30981	-0.34603
TRINITY_DN2748_c0_g1_i6_orf1	-	-	-	uncharacterized protein LOC114352811 [Ostrinia furnacalis]	1.853981	-0.99077	0.010848	-0.12157	-0.75249
TRINITY_DN84322_c0_g2_i1_orf1	-	-	-	alanyl-tRNA synthetase 1 [Homo sapiens] >KAI4055846.1 alanyl-tRNA synthetase 1 [Homo sapiens]	1.820435	-0.22937	0.100281	-1.15634	-0.535
TRINITY_DN19092_c0_g1_i2_orf1	-	-	-	eukaryotic translation initiation factor 3 subunit L [Ostrinia furnacalis]	1.947172	-0.36751	-0.23677	-0.91199	-0.4309
TRINITY_DN27087_c0_g1_i1_orf1	-	-	-	2',5'-phosphodiesterase 12 [Ostrinia furnacalis]	1.908188	-0.66892	-0.31207	-0.90185	-0.02535
TRINITY_DN4905_c0_g1_i6_orf1	-	-	-	uncharacterized protein LOC114351759 [Ostrinia furnacalis]	1.946229	-0.73087	-0.71744	-0.10871	-0.38921
TRINITY_DN338_c2_g1_i2_orf1	-	-	-	alpha-tocopherol transfer protein-like isoform X1 [Ostrinia furnacalis] >XP_028160444.1 alpha-tocopherol transfer protein-like isoform X1 [Ostrinia furnacalis] >XP_028160446.1 alpha-tocopherol transfer protein-like isoform X1 [Ostrinia furnacalis] >XP_028160447.1 alpha-tocopherol transfer protein-like isoform X1 [Ostrinia furnacalis] >XP_028160448.1 alpha-tocopherol transfer protein-like isoform X1 [Ostrinia furnacalis] >XP_028160449.1 alpha-tocopherol transfer protein-like isoform X1 [Ostrinia furnacalis]	1.939251	-0.06955	-0.5322	-0.50026	-0.83724
TRINITY_DN64472_c0_g2_i1_orf1	-	-	-	repressed by EFG1 protein 1-like isoform X3 [Ostrinia furnacalis]	1.813038	-0.85726	-0.22998	-0.9381	0.212307
TRINITY_DN14953_c0_g1_i5_orf1	-	-	-	EEF1A lysine methyltransferase 2 [Ostrinia furnacalis]	1.967369	-0.66132	-0.18139	-0.4466	-0.67806
TRINITY_DN1422_c0_g1_i4_orf1	-	-	-	unnamed protein product [Chilo suppressalis]	1.984518	-0.64734	-0.27268	-0.50306	-0.56144
TRINITY_DN107617_c3_g1_i1_orf1	-	-	-	NADH dehydrogenase [ubiquinone] 1 alpha subcomplex subunit 9, mitochondrial [Ostrinia furnacalis]	1.919952	-0.98831	-0.15509	-0.46396	-0.31259
TRINITY_DN27704_c0_g1_i1_orf1	-	-	-	PREDICTED: tRNA (guanine-N(7)-)-methyltransferase [Amyelois transitella]	1.955232	-0.18429	-0.56052	-0.82471	-0.3857
TRINITY_DN16258_c0_g1_i2_orf1	-	-	-	uncharacterized protein LOC114359911 [Ostrinia furnacalis]	1.762766	0.371058	-0.31371	-0.90148	-0.91864
TRINITY_DN15667_c0_g1_i2_orf1	-	-	-	coiled-coil domain-containing protein 25 [Ostrinia furnacalis]	1.943916	-0.06723	-0.53568	-0.54689	-0.79413
TRINITY_DN106730_c0_g1_i1_orf1	-	-	-	Photosystem I reaction center subunit II, chloroplastic, partial [Trichinella zimbabwensis]	1.935809	-0.66958	-0.77158	-0.45545	-0.0392
TRINITY_DN1073_c0_g1_i1_orf1	-	-	-	carboxylesterase [Loxostege sticticalis]	1.872227	-0.85019	-0.65247	0.188002	-0.55757
TRINITY_DN19186_c0_g1_i1_orf1	-	-	-	39S ribosomal protein L9, mitochondrial [Ostrinia furnacalis]	1.939855	-0.09829	-0.76734	-0.71249	-0.36174
TRINITY_DN10520_c0_g1_i2_orf1	-	-	-	probable 39S ribosomal protein L45, mitochondrial [Ostrinia furnacalis]	1.978199	-0.23492	-0.49064	-0.57035	-0.68229
TRINITY_DN20369_c0_g1_i2_orf1	-	-	-	uncharacterized protein LOC114366225 [Ostrinia furnacalis]	1.918047	0.025273	-0.4805	-0.83054	-0.63227
TRINITY_DN9059_c0_g1_i1_orf1	-	-	-	sulfotransferase family cytosolic 1B member 1-like [Ostrinia furnacalis]	1.939166	-0.03022	-0.67689	-0.51219	-0.71986
TRINITY_DN112120_c0_g1_i1_orf1	-	-	-	juvenile hormone esterase-like [Ostrinia furnacalis]	1.96363	-0.61019	-0.68223	-0.1342	-0.537
TRINITY_DN25896_c0_g1_i6_orf1	-	-	-	60S ribosomal export protein NMD3 [Ostrinia furnacalis]	1.972722	-0.48785	-0.493	-0.23573	-0.75615
TRINITY_DN8676_c0_g1_i1_orf1	-	-	-	probable rRNA-processing protein EBP2 homolog [Ostrinia furnacalis]	1.992923	-0.37851	-0.63707	-0.51631	-0.46102
TRINITY_DN2593_c0_g2_i1_orf1	-	-	-	midgut carboxypeptidase [Loxostege sticticalis]	1.954801	-0.75102	-0.69352	-0.21289	-0.29737
TRINITY_DN10644_c0_g1_i2_orf1	-	-	-	carboxylesterase [Cnaphalocrocis medinalis]	1.816556	0.329632	-0.89475	-0.56311	-0.68833
TRINITY_DN1965_c0_g1_i7_orf1	-	-	-	CTP synthase isoform X1 [Ostrinia furnacalis]	1.959717	-0.28206	-0.39021	-0.42132	-0.86613
TRINITY_DN9790_c0_g1_i4_orf1	-	-	-	protein IWS1 homolog [Ostrinia furnacalis]	1.654635	0.172016	-0.0532	-1.45996	-0.31349
TRINITY_DN3028_c0_g1_i1_orf1	-	-	-	pre-rRNA processing protein FTSJ3 [Ostrinia furnacalis]	1.955182	-0.68798	-0.5082	-0.66025	-0.09876
TRINITY_DN25783_c0_g1_i2_orf1	-	-	-	SET and MYND domain-containing protein 4 [Ostrinia furnacalis]	1.582945	0.37817	-0.0588	-1.47001	-0.43231
TRINITY_DN4451_c0_g1_i1_orf1	-	-	-	uncharacterized protein LOC114361986 isoform X1 [Ostrinia furnacalis] >XP_028173022.1 uncharacterized protein LOC114361986 isoform X2 [Ostrinia furnacalis]	1.864831	-0.55698	-0.76655	0.218266	-0.75957
TRINITY_DN109144_c0_g1_i5_orf1	-	-	-	uncharacterized protein LOC126369165 [Pectinophora gossypiella]	1.920858	-0.51058	-0.92707	-0.43318	-0.05002

TRINITY_DN2927_c0_g1_i6_orf1	-	-	-	T-complex protein 1 subunit eta [Ostrinia furnacalis]	1.951947	-0.08441	-0.50787	-0.69557	-0.6641
TRINITY_DN8079_c0_g1_i2_orf1	-	-	-	hypothetical protein evm_000040 [Chilo suppressalis]	1.730102	-0.8194	-0.32699	0.435484	-1.0192
TRINITY_DN21341_c0_g1_i4_orf1	-	-	-	FAST kinase domain-containing protein 4 isoform X6 [Ostrinia furnacalis] >XP_028160336.1 FAST kinase domain-containing protein 4 isoform X7 [Ostrinia furnacalis] >XP_028160337.1 FAST kinase domain-containing protein 4 isoform X8 [Ostrinia furnacalis]	1.966188	-0.16525	-0.66572	-0.66572	-0.4695
TRINITY_DN1578_c0_g3_i1_orf1	-	-	-	S-adenosylmethionine synthase isoform X1 [Ostrinia furnacalis]	1.959454	-0.7377	-0.58994	-0.13055	-0.50126
TRINITY_DN45924_c0_g1_i14_orf1	-	-	-	adenylosuccinate synthetase isoform X1 [Ostrinia furnacalis] >XP_028166048.1 adenylosuccinate synthetase isoform X2 [Ostrinia furnacalis]	1.925425	-0.35488	-0.08204	-0.90587	-0.58264
TRINITY_DN16343_c0_g1_i6_orf1	-	-	-	aminopeptidase N6 [Ostrinia nubilalis]	1.988704	-0.69116	-0.49436	-0.38727	-0.41591
TRINITY_DN11655_c0_g1_i1_orf1	-	-	-	ribosome biogenesis protein BRX1 homolog [Ostrinia furnacalis]	1.970096	-0.80225	-0.47954	-0.27982	-0.40848
TRINITY_DN45227_c0_g1_i3_orf1	-	-	-	uncharacterized protein LOC114359191 [Ostrinia furnacalis]	1.959526	-0.27652	-0.26687	-0.77829	-0.63784
TRINITY_DN886_c0_g2_i4_orf1	-	-	-	collagenase-like [Ostrinia furnacalis]	1.851275	0.241031	-0.64635	-0.88348	-0.56248
TRINITY_DN825_c2_g1_i5_orf1	-	-	-	hypothetical protein SFRURICE_018365 [Spodoptera frugiperda] >KAG8107343.1 hypothetical protein SFRUCORN_012069 [Spodoptera frugiperda] >CAB3509414.1 unnamed protein product [Spodoptera littoralis] >CAH1638995.1 unnamed protein product [Spodoptera	1.951555	-0.27186	-0.6429	-0.22967	-0.80713
TRINITY_DN11013_c0_g1_i3_orf1	-	-	-	glutamine:fructose-6-phosphate aminotransferase 1 [Heortia vitessoides]	1.887023	0.116833	-0.50599	-0.90331	-0.59456
TRINITY_DN12101_c0_g1_i2_orf1	-	-	-	UPF0545 protein C22orf39 homolog [Ostrinia furnacalis]	1.76554	0.28157	-0.99855	-0.16599	-0.88257
TRINITY_DN137_c0_g1_i1_orf1	-	-	-	60S ribosomal protein L8 [Cotesia glomerata] >XP_044597650.1 60S ribosomal protein L8 [Cotesia glomerata] >KAG8034499.1 hypothetical protein G9C98_007575 [Cotesia typhae] >CAD6216378.1 GSCOCG00004534001-RA-CDS [Cotesia congregata] >KAH0544237.1 60S ribosomal protein L8 [Cotesia glomerata] >KAH0564528.1 60S ribosomal protein L8 [Cotesia glomerata] >CAG5095185.1 Similar to RpL8: 60S ribosomal protein L8 (Spodoptera frugiperda) [Cotesia congregata]	1.798233	-0.58898	0.35974	-0.95652	-0.61247
TRINITY_DN2062_c0_g1_i9_orf1	-	-	-	uncharacterized protein LOC114350846 [Ostrinia furnacalis]	1.795542	0.17204	-0.39708	-1.20687	-0.36364
TRINITY_DN50085_c0_g1_i1_orf1	-	-	-	hypothetical protein evm_013997 [Chilo suppressalis]	1.869987	0.071783	-0.26656	-0.94665	-0.72856
TRINITY_DN38301_c0_g1_i2_orf1	-	-	-	gamma-taxilin [Ostrinia furnacalis]	1.923053	0.041084	-0.75044	-0.62248	-0.59122
TRINITY_DN100821_c0_g1_i1_orf1	-	-	-	putative GMP synthase, partial [Operophtera brumata]	1.86537	-0.45413	0.074774	-1.06273	-0.42328
TRINITY_DN8754_c0_g1_i2_orf1	-	-	-	dnaJ homolog subfamily C member 11 [Ostrinia furnacalis]	1.997547	-0.52573	-0.57107	-0.47887	-0.42189
TRINITY_DN44557_c0_g2_i1_orf1	-	-	-	serine hydrolase-like protein [Ostrinia furnacalis]	1.85566	0.036426	-0.33954	-1.11885	-0.4337
TRINITY_DN47575_c0_g1_i1_orf1	-	-	-	PREDICTED: splicing factor 1-like [Fopius arisanus]	1.704738	-0.71555	0.525594	-1.03875	-0.47604
TRINITY_DN49936_c0_g2_i1_orf1	-	-	-	39S ribosomal protein L20, mitochondrial [Ostrinia furnacalis]	1.878141	-0.12241	-1.12056	-0.32813	-0.30703
TRINITY_DN1633_c0_g1_i1_orf1	-	-	-	collagen alpha-2(I) chain-like isoform X1 [Ostrinia furnacalis]	1.984872	-0.40281	-0.72613	-0.47517	-0.38077
TRINITY_DN32022_c0_g1_i1_orf1	-	-	-	striatin isoform X1 [Diachasma alloeum]	1.734888	0.141154	0.037127	-1.21998	-0.69319
TRINITY_DN15965_c0_g1_i1_orf1	-	-	-	U3 small nucleolar RNA-associated protein 15 homolog [Ostrinia furnacalis]	1.983504	-0.6068	-0.58526	-0.54028	-0.25118
TRINITY_DN2082_c0_g1_i2_orf1	-	-	-	choline-phosphate cytidylyltransferase B-like isoform X1 [Ostrinia furnacalis]	1.925345	-0.01909	-0.65949	-0.82407	-0.42269
TRINITY_DN620_c0_g1_i4_orf1	-	-	-	lysine--tRNA ligase isoform X1 [Ostrinia furnacalis]	1.920925	-0.63754	-0.07619	-0.89439	-0.3128
TRINITY_DN5697_c0_g1_i1_orf1	-	-	-	GPI ethanolamine phosphate transferase 2-like [Ostrinia furnacalis]	1.633051	0.541762	-0.16938	-0.9966	-1.00883
TRINITY_DN58531_c0_g1_i1_orf1	-	-	-	uncharacterized protein LOC114357371 [Ostrinia furnacalis] >XP_028166768.1 uncharacterized protein LOC114357371 [Ostrinia furnacalis]	1.52328	0.830722	-0.57703	-1.08564	-0.69133
TRINITY_DN14107_c0_g1_i4_orf1	-	-	-	bifunctional methylenetetrahydrofolate dehydrogenase/cyclohydrolase, mitochondrial isoform X1 [Ostrinia furnacalis]	1.936631	-0.1423	-0.87425	-0.31577	-0.60431
TRINITY_DN391_c5_g1_i1_orf1	-	-	-	hypothetical protein B5X24_HaOG210395 [Helicoverpa armigera]	1.994602	-0.53316	-0.3597	-0.57327	-0.52848
TRINITY_DN1617_c0_g1_i5_orf1	-	-	-	hypothetical protein evm_009822 [Chilo suppressalis] >CAB3525311.1 unnamed protein product [Chilo suppressalis] >CAH0402638.1 unnamed protein product [Chilo suppressalis]	1.943946	-0.55855	-0.85522	-0.40138	-0.12879
TRINITY_DN12683_c0_g1_i3_orf1	-	-	-	sulfated surface glycoprotein 185-like [Ostrinia furnacalis]	1.935049	-0.95734	-0.24797	-0.44013	-0.2896
TRINITY_DN21971_c0_g1_i4_orf1	-	-	-	40S ribosomal protein S26 [Nymphalis io]	1.817363	-0.11689	3.58E-06	-1.195	-0.50548
TRINITY_DN6563_c0_g1_i1_orf1	-	-	-	cytochrome c oxidase assembly protein COX15 homolog [Ostrinia furnacalis]	1.808492	0.36817	-0.72712	-0.80998	-0.63957
TRINITY_DN886_c0_g1_i1_orf1	-	-	-	collagenase-like isoform X1 [Ostrinia furnacalis]	1.924972	-0.29344	-0.99744	-0.23813	-0.39596
TRINITY_DN21539_c0_g1_i1_orf1	-	-	-	probable phenylalanine--tRNA ligase, mitochondrial [Ostrinia furnacalis]	1.726106	0.501852	-0.65251	-1.01346	-0.56198
TRINITY_DN43942_c0_g1_i1_orf1	-	-	-	LOW QUALITY PROTEIN: caprin homolog [Ostrinia furnacalis]	1.890249	0.034846	-0.50507	-0.42505	-0.99498
TRINITY_DN1066_c0_g1_i4_orf1	-	-	-	ribosome biogenesis protein WDR12 homolog [Ostrinia furnacalis]	1.972753	-0.33171	-0.30685	-0.75082	-0.58337
TRINITY_DN14235_c0_g1_i1_orf1	-	-	-	maltase A1 [Helicoverpa armigera]	1.91931	-0.64282	0.057878	-0.73578	-0.59859
TRINITY_DN11799_c0_g1_i4_orf1	-	-	-	V-type proton ATPase 116 kDa subunit a1 isoform X1 [Manduca sexta]	1.936794	-0.06385	-0.7291	-0.40019	-0.74365

TRINITY_DN4213_c0_g1_i4_orf1	-	-	-	nardilysin-like isoform X1 [Ostrinia furnacalis] >XP_028157649.1 nardilysin-like isoform X2 [Ostrinia furnacalis] >XP_028157650.1 nardilysin-like isoform X3 [Ostrinia furnacalis] >XP_028157651.1 nardilysin-like isoform X4 [Ostrinia furnacalis]	1.705731	0.583862	-0.76888	-0.72775	-0.79297
TRINITY_DN21150_c0_g1_i4_orf1	-	-	-	RNA-binding protein cabeza-like isoform X2 [Bicyclus anynana]	1.775099	0.259613	-0.23341	-1.12957	-0.67173
TRINITY_DN9853_c0_g3_i1_orf1	-	-	-	importin-7 isoform X1 [Ostrinia furnacalis]	1.934569	-0.36623	-0.11414	-0.88981	-0.56439
TRINITY_DN108051_c0_g1_i2_orf1	-	-	-	uncharacterized protein LOC114351921 [Ostrinia furnacalis]	1.994972	-0.38361	-0.47372	-0.54232	-0.59532
TRINITY_DN11153_c0_g1_i1_orf1	-	-	-	unnamed protein product [Chilo suppressalis]	1.963628	-0.47685	-0.36116	-0.83925	-0.28637
TRINITY_DN32997_c0_g1_i8_orf1	-	-	-	RNA-binding protein squid isoform X1 [Ostrinia furnacalis]	1.886686	0.092463	-0.37032	-0.82122	-0.78761
TRINITY_DN2807_c0_g1_i4_orf1	-	-	-	FK506-binding protein 59 isoform X1 [Ostrinia furnacalis]	1.869072	0.04397	-0.21559	-0.94224	-0.75521
TRINITY_DN48413_c1_g1_i2_orf1	-	-	-	probable protein phosphatase 2C 11 isoform X1 [Manduca sexta] >KAG6442694.1 hypothetical protein O3G_MSEX002471 [Manduca sexta]	1.492624	0.889518	-0.55217	-0.88723	-0.94275
TRINITY_DN8536_c0_g1_i2_orf1	-	-	-	PC4 and SFRS1-interacting protein isoform X4 [Galleria mellonella]	1.526065	0.71648	-0.34626	-1.29451	-0.60178
TRINITY_DN24043_c0_g1_i1_orf1	-	-	-	uncharacterized protein LOC110377964 [Helicoverpa armigera] >XP_047026962.1 cytochrome c1-2, heme protein, mitochondrial [Helicoverpa zea] >PZC76159.1 hypothetical protein B5X24_HaOG204935 [Helicoverpa armigera]	1.97196	-0.65039	-0.25202	-0.37217	-0.69738
TRINITY_DN4956_c0_g1_i6_orf1	-	-	-	nucleolar GTP-binding protein 1 [Ostrinia furnacalis]	1.994747	-0.5067	-0.55254	-0.36443	-0.57107
TRINITY_DN69049_c0_g2_i1_orf1	-	-	-	membrane alanyl aminopeptidase-like [Ostrinia furnacalis]	1.63304	0.178253	-0.3617	0.023508	-1.4731
TRINITY_DN36718_c0_g1_i1_orf1	-	-	-	unnamed protein product [Chilo suppressalis]	1.998193	-0.44407	-0.48556	-0.49339	-0.57518
TRINITY_DN83005_c0_g1_i1_orf1	-	-	-	ATP synthase subunit O, mitochondrial [Danaus plexippus plexippus] >OWR53927.1 H+ transporting ATP synthase O subunit [Danaus plexippus plexippus]	1.9867	-0.71825	-0.42427	-0.44225	-0.40193
TRINITY_DN46778_c0_g1_i2_orf1	-	-	-	Deoxycytidylate deaminase [Papilio xuthus]	1.797458	0.331542	-1.00518	-0.46876	-0.65506
TRINITY_DN32306_c0_g1_i3_orf1	-	-	-	acetyl-coenzyme A transporter 1 [Ostrinia furnacalis]	1.707321	-0.05704	0.109807	-1.39033	-0.36975
TRINITY_DN959_c0_g1_i7_orf1	-	-	-	Golgi to ER traffic protein 4 homolog [Ostrinia furnacalis]	1.847719	0.184427	-0.95693	-0.36708	-0.70813
TRINITY_DN15959_c0_g1_i1_orf1	-	-	-	dnaJ homolog subfamily A member 2-like [Ostrinia furnacalis]	1.943852	-0.07293	-0.45071	-0.75746	-0.66274
TRINITY_DN29448_c0_g1_i1_orf1	-	-	-	28S ribosomal protein S9, mitochondrial [Ostrinia furnacalis]	1.852599	0.250356	-0.60108	-0.84063	-0.66124
TRINITY_DN27979_c0_g1_i2_orf1	-	-	-	zinc finger CCCH domain-containing protein 15 homolog [Ostrinia furnacalis]	1.976791	-0.20915	-0.55613	-0.6577	-0.55381
TRINITY_DN14920_c0_g1_i1_orf1	-	-	-	anamorsin homolog [Ostrinia furnacalis]	1.751258	0.441963	-0.44529	-0.79699	-0.95094
TRINITY_DN787_c0_g1_i7_orf1	-	-	-	YLP motif-containing protein 1-like isoform X1 [Ostrinia furnacalis]	1.656752	0.611187	-0.80732	-1.01592	-0.4447
TRINITY_DN16749_c0_g1_i1_orf1	-	-	-	uncharacterized protein LOC114353228 [Ostrinia furnacalis] >XP_028176007.1 uncharacterized protein LOC114364183 [Ostrinia furnacalis]	1.964223	-0.60892	-0.1268	-0.60096	-0.62755
TRINITY_DN37699_c0_g1_i4_orfp1	-	-	-	TRINITY_DN37699_c0_g1_i4_m.58777 TRINITY_DN37699_c0_g1::TRINITY_DN37699_c0_g1_i4::g.58777 ORF type:internal len:122 (+),score=34.90 TRINITY_DN37699_c0_g1_i4:1-363(+)	1.725632	-0.82117	0.329749	-1.10573	-0.12849
TRINITY_DN3673_c0_g1_i10_orf1	-	-	-	hypothetical protein evm_008955 [Chilo suppressalis] >CAB3526829.1 unnamed protein product [Chilo suppressalis] >CAH0404157.1 unnamed protein product [Chilo suppressalis]	1.759512	0.369211	-0.69875	-0.35643	-1.07355
TRINITY_DN16900_c0_g2_i1_orf1	-	-	-	uncharacterized oxidoreductase dhs-27-like [Ostrinia furnacalis]	1.93946	-0.41404	-0.94522	-0.34161	-0.23859
TRINITY_DN3733_c0_g1_i1_orf1	-	-	-	60S ribosomal protein L37, partial [Papilio machaon]	1.818462	-0.20651	-0.32054	-1.24317	-0.04825
TRINITY_DN92232_c0_g1_i1_orf1	-	-	-	protein SDA1 homolog [Ostrinia furnacalis]	1.94163	-0.43614	-0.86485	-0.5285	-0.11213
TRINITY_DN969_c0_g1_i3_orf1	-	-	-	protein UBASH3A homolog isoform X3 [Ostrinia furnacalis]	1.644747	0.635897	-0.50194	-1.05758	-0.72112
TRINITY_DN6439_c0_g1_i1_orf1	-	-	-	GPI mannosyltransferase 3 isoform X4 [Ostrinia furnacalis] >XP_028164836.1 GPI mannosyltransferase 3 isoform X5 [Ostrinia furnacalis]	1.637695	0.564214	-0.34452	-1.20796	-0.64942
TRINITY_DN3292_c2_g1_i4_orf1	-	-	-	ribosome biogenesis regulatory protein homolog [Ostrinia furnacalis]	1.953638	-0.55831	-0.84717	-0.3168	-0.23136
TRINITY_DN20749_c0_g1_i3_orf1	-	-	-	protein arginine N-methyltransferase 1-like [Ostrinia furnacalis]	1.942673	-0.54116	-0.13706	-0.87197	-0.39248
TRINITY_DN430_c0_g1_i5_orf1	-	-	-	hypothetical protein NE865_02252 [Phthorimaea operculella]	1.890125	-0.54505	-0.06272	-1.03118	-0.25118
TRINITY_DN7964_c0_g1_i1_orfp1	-	-	-	TRINITY_DN7964_c0_g1_i1_m.23483 TRINITY_DN7964_c0_g1::TRINITY_DN7964_c0_g1_i1::g.23483 ORF type:internal len:79	1.965463	-0.51547	-0.80188	-0.41947	-0.22864
TRINITY_DN2065_c1_g2_i1_orf1	-	-	-	2-amino-3-ketobutyrate coenzyme A ligase, mitochondrial [Ostrinia furnacalis]	1.666066	0.65242	-0.83966	-0.74583	-0.733
TRINITY_DN19000_c0_g1_i4_orf1	-	-	-	NADH dehydrogenase [ubiquinone] 1 beta subcomplex subunit 9 [Ostrinia furnacalis]	1.985415	-0.5053	-0.32229	-0.6986	-0.45923
TRINITY_DN26649_c0_g1_i2_orf1	-	-	-	ATP synthase-coupling factor 6, mitochondrial [Ostrinia furnacalis]	1.989059	-0.64613	-0.36673	-0.40096	-0.57524
TRINITY_DN3665_c0_g1_i2_orf1	-	-	-	regucalcin-like isoform X2 [Ostrinia furnacalis] >XP_028175354.1 regucalcin-like isoform X2 [Ostrinia furnacalis]	1.987629	-0.44375	-0.32896	-0.66308	-0.55184
TRINITY_DN14826_c0_g1_i1_orf1	-	-	-	uncharacterized protein LOC114350939 [Ostrinia furnacalis]	1.965949	-0.50503	-0.18177	-0.75965	-0.51949
TRINITY_DN80560_c0_g1_i1_orf1	-	-	-	ATP synthase subunit alpha, mitochondrial [Ostrinia furnacalis]	1.975075	-0.79776	-0.40045	-0.37201	-0.40485

TRINITY_DN5019_c0_g1_i2_orf1	-	-	-	RRP12-like protein isoform X4 [Ostrinia furnacalis] >XP_028175539.1 RRP12-like protein isoform X5 [Ostrinia furnacalis]	1.986341	-0.38586	-0.44065	-0.71917	-0.44065
TRINITY_DN2304_c0_g1_i4_orf1	-	-	-	clustered mitochondria protein homolog isoform X2 [Ostrinia furnacalis]	1.896802	-0.77324	-0.86303	-0.01739	-0.24314
TRINITY_DN2274_c0_g1_i6_orf1	-	-	-	membrane alanyl aminopeptidase-like [Ostrinia furnacalis]	1.9819	-0.55883	-0.70905	-0.32448	-0.38954
TRINITY_DN4040_c0_g1_i10_orf1	-	-	-	hypothetical protein evm_007488 [Chilo suppressalis]	1.975029	-0.39719	-0.79434	-0.34941	-0.43408
TRINITY_DN83327_c0_g1_i1_orf1	-	-	-	trypsin-like serine proteinase T22 [Ostrinia nubilalis]	1.958351	-0.71308	-0.42218	-0.14753	-0.67556
TRINITY_DN20582_c0_g1_i1_orf1	-	-	-	2-oxoglutarate dehydrogenase, mitochondrial isoform X3 [Ostrinia furnacalis] >XP_028167081.1 2-oxoglutarate dehydrogenase, mitochondrial isoform X3 [Ostrinia	1.98158	-0.39994	-0.30697	-0.57917	-0.6955
TRINITY_DN10701_c0_g2_i2_orf1	-	-	-	synaptosomal-associated protein 29 [Ostrinia furnacalis]	1.737842	0.34735	-0.1766	-1.01218	-0.89641
TRINITY_DN108122_c0_g1_i9_orf1	-	-	-	hypothetical protein SFRUCORN_003152 [Spodoptera frugiperda]	1.930481	-0.58502	-0.23588	-0.91519	-0.19439
TRINITY_DN14018_c0_g1_i4_orf1	-	-	-	chitobiosyldiphosphodolichol beta-mannosyltransferase [Ostrinia furnacalis]	1.848388	0.245993	-0.54162	-0.66668	-0.88608
TRINITY_DN698_c0_g1_i5_orf1	-	-	-	PREDICTED: small nuclear ribonucleoprotein Sm D3 [Amyelois transitella]	1.909975	0.024035	-0.3976	-0.849	-0.68741
TRINITY_DN24873_c0_g1_i4_orf1	-	-	-	uncharacterized protein LOC114365742 [Ostrinia furnacalis]	1.941866	-0.74574	-0.22662	-0.76016	-0.20934
TRINITY_DN3053_c0_g1_i2_orf1	-	-	-	prostaglandin reductase 1-like [Ostrinia furnacalis]	1.948496	-0.73149	-0.1325	-0.71924	-0.36526
TRINITY_DN5417_c0_g1_i1_orf1	-	-	-	NADH dehydrogenase [ubiquinone] 1 alpha subcomplex subunit 10, mitochondrial isoform X1 [Ostrinia furnacalis] >XP_028175885.1 NADH dehydrogenase [ubiquinone] 1 alpha subcomplex subunit 10, mitochondrial isoform X2 [Ostrinia furnacalis]	1.992139	-0.46913	-0.34848	-0.59448	-0.58004
TRINITY_DN27641_c0_g1_i1_orf1	-	-	-	succinate dehydrogenase [ubiquinone] iron-sulfur subunit, mitochondrial [Ostrinia furnacalis]	1.995617	-0.55486	-0.40405	-0.45124	-0.58546
TRINITY_DN7626_c0_g1_i1_orf1	-	-	-	NADH dehydrogenase [ubiquinone] flavoprotein 1, mitochondrial isoform X1 [Ostrinia	1.989798	-0.51747	-0.30782	-0.57392	-0.59058
TRINITY_DN46173_c0_g3_i2_orf1	-	-	-	tropomyosin-1, isoforms 9A/A/B isoform X33 [Aedes aegypti] >EAT46020.1 AAEL002761-PB [Aedes aegypti]	1.912958	0.010613	-0.41397	-0.87658	-0.63302
TRINITY_DN75746_c0_g1_i1_orfp1	-	-	-	TRINITY_DN75746_c0_g1_i1_m.54871 TRINITY_DN75746_c0_g1::TRINITY_DN75746_c0_g1_i1::g.54871 ORF type:internal len:83 (+),score=-1.09,Polyhedrin PF00738.19 1.7e-25 TRINITY_DN75746_c0_g1_i1:2-247(+)	1.99033	-0.40831	-0.67397	-0.50577	-0.40228
TRINITY_DN57105_c0_g1_i2_orf1	-	-	-	transmembrane protein 161B isoform X1 [Galleria mellonella]	1.474873	0.882984	-0.45388	-0.83685	-1.06713
TRINITY_DN44219_c0_g1_i1_orf1	-	-	-	mitochondrial import inner membrane translocase subunit TIM50-C-like [Ostrinia furnacalis]	1.870945	0.115566	-0.32619	-0.85736	-0.80296
TRINITY_DN8833_c0_g1_i1_orf1	-	-	-	nucleolar protein 16 [Ostrinia furnacalis]	1.97216	-0.46733	-0.57345	-0.72026	-0.21112
TRINITY_DN63914_c0_g1_i1_orf1	-	-	-	myophilin-like [Ostrinia furnacalis]	1.984683	-0.5822	-0.67793	-0.36228	-0.36228
TRINITY_DN29291_c0_g1_i1_orf1	-	-	-	carboxylesterase [Ostrinia furnacalis]	1.939127	-0.35313	-0.79609	-0.10396	-0.68595
TRINITY_DN86956_c0_g5_i1_orf1	-	-	-	PREDICTED: protein sly1 homolog [Microplitis demolitor]	1.928974	0.000406	-0.48891	-0.75594	-0.68453
TRINITY_DN2535_c0_g1_i4_orf1	-	-	-	ATP-dependent RNA helicase DDX24 [Ostrinia furnacalis]	1.937733	-0.78085	-0.54473	-0.03097	-0.58118
TRINITY_DN4938_c0_g1_i13_orf1	-	-	-	peroxisomal biogenesis factor 19 [Ostrinia furnacalis]	1.82356	-0.97127	0.284259	-0.5204	-0.61615
TRINITY_DN24317_c0_g1_i7_orf1	-	-	-	peptidyl-tRNA hydrolase ICT1, mitochondrial [Ostrinia furnacalis]	1.944742	-0.17914	-0.58334	-0.86199	-0.32027
TRINITY_DN3598_c0_g1_i1_orf1	-	-	-	esterase FE4-like [Ostrinia furnacalis]	1.980658	-0.65019	-0.65019	-0.34754	-0.33273
TRINITY_DN3503_c0_g1_i1_orfp1	-	-	-	uncharacterized protein LOC114356429 [Ostrinia furnacalis]	1.96203	-0.29991	-0.77366	-0.62762	-0.26084
TRINITY_DN5554_c0_g1_i2_orf1	-	-	-	double-stranded RNA-binding protein Staufen homolog 2 isoform X5 [Pectinophora	1.914552	0.033204	-0.86275	-0.55728	-0.52773
TRINITY_DN1803_c0_g1_i3_orf1	-	-	-	translocator protein-like isoform X1 [Ostrinia furnacalis] >XP_028178947.1 translocator protein-like isoform X1 [Ostrinia furnacalis]	1.851088	0.269919	-0.73356	-0.695	-0.69244
TRINITY_DN16482_c0_g1_i6_orf1	-	-	-	transmembrane protein 120 homolog isoform X2 [Ostrinia furnacalis]	1.931168	-0.20092	-0.22933	-0.91054	-0.59037
TRINITY_DN753_c0_g1_i4_orf1	-	-	-	venom dipeptidyl peptidase 4-like isoform X2 [Ostrinia furnacalis]	1.93819	-0.2915	-0.41126	-0.954	-0.28143
TRINITY_DN26186_c0_g1_i7_orf1	-	-	-	sodium- and chloride-dependent glycine transporter 1-like [Ostrinia furnacalis]	1.814136	0.082557	-1.04395	-0.77899	-0.07375
TRINITY_DN4434_c0_g1_i7_orf1	-	-	-	V-type proton ATPase catalytic subunit A [Ostrinia furnacalis] >XP_028155921.1 V-type proton ATPase catalytic subunit A [Ostrinia furnacalis] >XP_028155922.1 V-type proton ATPase catalytic subunit A [Ostrinia furnacalis] >ADP23923.1 V-ATPase subunit A [Ostrinia furnacalis] >ADT80587.1 V-type proton ATPase catalytic subunit A [Ostrinia furnacalis] >CBY05457.1 V-type proton ATPase catalytic subunit A [Ostrinia furnacalis]	1.992345	-0.56778	-0.61753	-0.3791	-0.42793
TRINITY_DN16894_c0_g1_i5_orf1	-	-	-	dnaJ homolog subfamily C member 5 isoform X1 [Colias croceus]	1.758111	0.308087	-0.5833	-1.17411	-0.30879
TRINITY_DN1079_c0_g1_i4_orf1	-	-	-	CD109 antigen-like [Ostrinia furnacalis]	1.982832	-0.39826	-0.67465	-0.5975	-0.31242
TRINITY_DN96170_c0_g1_i1_orf1	-	-	-	uncharacterized protein LOC114355569 [Ostrinia furnacalis]	1.981885	-0.64667	-0.49714	-0.5858	-0.25227
TRINITY_DN747_c0_g1_i4_orf1	-	-	-	trypsin, alkaline C-like [Ostrinia furnacalis]	1.951857	-0.69882	-0.53526	-0.63978	-0.078
TRINITY_DN53136_c0_g1_i1_orf1	-	-	-	glutathione S transferase-E4 [Glyphodes pyloalis]	1.966339	-0.6691	-0.2098	-0.38476	-0.70268
TRINITY_DN2993_c0_g1_i4_orf1	-	-	-	heat shock 70 kDa protein cognate 5 [Ostrinia furnacalis]	1.985174	-0.28997	-0.45863	-0.62542	-0.61116
TRINITY_DN3401_c0_g1_i1_orf1	-	-	-	28S ribosomal protein S5, mitochondrial [Ostrinia furnacalis]	1.981614	-0.55127	-0.65271	-0.53186	-0.24577
TRINITY_DN4977_c0_g1_i2_orf1	-	-	-	manganese-transporting ATPase 13A1 [Ostrinia furnacalis]	1.94224	-0.05792	-0.46454	-0.7282	-0.69157

TRINITY_DN6147_c0_g1_i2_orf1	-	-	-	uncharacterized protein LOC114352519 [Ostrinia furnacalis]	1.95109	-0.48668	-0.81364	-0.12212	-0.52865
TRINITY_DN10637_c0_g1_i4_orf1	-	-	-	V-type proton ATPase subunit d [Bombyx mandarina]	1.998527	-0.54552	-0.53664	-0.44316	-0.47321
TRINITY_DN536_c0_g1_i7_orf1	-	-	-	polymerase delta-interacting protein 2 isoform X3 [Ostrinia furnacalis]	1.968246	-0.77524	-0.23346	-0.40855	-0.55099
TRINITY_DN96080_c0_g2_i1_orf1	-	-	-	ATP synthase subunit delta, mitochondrial [Ostrinia furnacalis]	1.942806	-0.88746	-0.17608	-0.33873	-0.54053
TRINITY_DN905_c0_g1_i4_orf1	-	-	-	(11Z)-hexadec-11-enoyl-CoA conjugase-like [Ostrinia furnacalis] >XP_028172978.1 (11Z)-hexadec-11-enoyl-CoA conjugase-like [Ostrinia furnacalis]	1.966371	-0.20854	-0.41336	-0.58499	-0.75949
TRINITY_DN2780_c0_g1_i5_orf1	-	-	-	probable ATP-dependent RNA helicase DDX27 [Ostrinia furnacalis]	1.986214	-0.3726	-0.65529	-0.36185	-0.59647
TRINITY_DN18860_c0_g1_i1_orf1	-	-	-	DDB1- and CUL4-associated factor 13 [Ostrinia furnacalis]	1.973744	-0.20539	-0.66848	-0.47775	-0.62212
TRINITY_DN47123_c0_g1_i1_orf1	-	-	-	WD40 repeat-containing protein SMU1 [Ostrinia furnacalis]	1.876221	0.02523	-0.21806	-0.92746	-0.75593
TRINITY_DN5087_c0_g1_i6_orf1	-	-	-	nascent polypeptide-associated complex subunit alpha [Ostrinia furnacalis] >XP_028156807.1 nascent polypeptide-associated complex subunit alpha [Ostrinia furnacalis] >XP_028156808.1 nascent polypeptide-associated complex subunit alpha [Ostrinia furnacalis]	1.931491	-0.01371	-0.47215	-0.69731	-0.74832
TRINITY_DN27994_c0_g1_i1_orf1	-	-	-	uncharacterized protein LOC114364076 [Ostrinia furnacalis]	1.841654	0.254154	-0.58725	-0.57966	-0.9289
TRINITY_DN1465_c0_g2_i1_orf1	-	-	-	unnamed protein product, partial [Iphiclides podalirius]	1.970156	-0.69982	-0.6524	-0.2309	-0.38705
TRINITY_DN4710_c0_g1_i1_orf1	-	-	-	hypothetical protein evm_000671 [Chilo suppressalis]	1.978169	-0.27568	-0.73935	-0.49563	-0.46751
TRINITY_DN4125_c1_g1_i5_orf1	-	-	-	angiotensin-converting enzyme-like isoform X2 [Ostrinia furnacalis]	1.89647	0.139449	-0.64008	-0.69196	-0.70388
TRINITY_DN6231_c0_g1_i6_orf1	-	-	-	ran-binding protein 3 isoform X1 [Ostrinia furnacalis] >XP_028166372.1 ran-binding protein 3 isoform X2 [Ostrinia furnacalis]	1.836608	0.061535	-0.23165	-1.13393	-0.53256
TRINITY_DN1557_c0_g1_i9_orf1	-	-	-	carboxylesterase CXE18 [Ostrinia furnacalis]	1.912177	-0.86369	0.022858	-0.64338	-0.42796
TRINITY_DN37699_c0_g1_i3_orfp1	-	-	-	TRINITY_DN37699_c0_g1_i3_m.58788 TRINITY_DN37699_c0_g1::TRINITY_DN37699_c0_g1_i3::g.58788 ORF type:internal len:122 (+),score=39.86 TRINITY_DN37699_c0_g1_i3:1-363(+)	1.762435	-1.08264	0.385189	-0.58841	-0.47658
TRINITY_DN1294_c0_g1_i3_orf1	-	-	-	46 kDa FK506-binding nuclear protein-like isoform X1 [Ostrinia furnacalis] >XP_028157904.1 46 kDa FK506-binding nuclear protein-like isoform X2 [Ostrinia furnacalis]	1.97293	-0.21303	-0.4766	-0.71777	-0.56553
TRINITY_DN8019_c0_g1_i4_orf1	-	-	-	deoxyhypusine hydroxylase [Ostrinia furnacalis]	1.967146	-0.20125	-0.42035	-0.73559	-0.60995
TRINITY_DN23343_c0_g1_i9_orf1	-	-	-	pre-mRNA-processing factor 6 isoform X1 [Ostrinia furnacalis] >XP_028175021.1 pre-mRNA-processing factor 6 isoform X2 [Ostrinia furnacalis]	1.889805	-0.10811	-0.30838	-1.08055	-0.39277
TRINITY_DN7073_c0_g1_i1_orf1	-	-	-	unnamed protein product, partial [Brenthis ino]	1.959352	-0.67566	-0.20459	-0.73997	-0.33913
TRINITY_DN5867_c0_g1_i1_orf1	-	-	-	NADH dehydrogenase [ubiquinone] 1 alpha subcomplex subunit 7-like [Ostrinia furnacalis]	1.946498	-0.04654	-0.58591	-0.69116	-0.62288
TRINITY_DN259_c0_g1_i8_orf1	-	-	-	hypothetical protein evm_000095 [Chilo suppressalis]	1.869259	0.077168	-0.41381	-1.044	-0.48862
TRINITY_DN3127_c0_g1_i9_orf1	-	-	-	RNA-binding protein 1 isoform X1 [Galleria mellonella]	1.84986	0.090224	-0.2433	-0.6598	-1.03699
TRINITY_DN8366_c0_g1_i4_orf1	-	-	-	luciferin 4-monooxygenase-like [Ostrinia furnacalis]	1.934927	-0.66152	-0.83262	-0.33882	-0.10197
TRINITY_DN20346_c0_g1_i1_orf1	-	-	-	NADH dehydrogenase [ubiquinone] 1 alpha subcomplex subunit 6 [Ostrinia furnacalis]	1.974049	-0.26927	-0.73931	-0.57755	-0.38791
TRINITY_DN2117_c0_g1_i1_orf1	-	-	-	BUB3-interacting and GLEBS motif-containing protein ZNF207 [Chelonus insularis]	1.754288	0.307149	-0.17592	-1.04175	-0.84376
TRINITY_DN501_c1_g1_i1_orf1	-	-	-	sodium- and chloride-dependent GABA transporter ine isoform X1 [Ostrinia furnacalis]	1.968086	-0.42332	-0.30346	-0.82672	-0.41458
TRINITY_DN5956_c1_g1_i5_orf1	-	-	-	uncharacterized protein DDB_G0286299-like [Ostrinia furnacalis] >XP_028170990.1 uncharacterized protein DDB_G0286299-like [Ostrinia furnacalis] >XP_028170991.1 uncharacterized protein DDB_G0286299-like [Ostrinia furnacalis]	1.978892	-0.66357	-0.52561	-0.56155	-0.22816
TRINITY_DN1109_c0_g1_i6_orf1	-	-	-	1-phosphatidylinositol phosphodiesterase-like [Cotesia glomerata]	1.720299	0.377422	-1.19136	-0.26874	-0.63761
TRINITY_DN29229_c0_g1_i5_orfp1	-	-	-	TRINITY_DN29229_c0_g1_i5_m.11187 TRINITY_DN29229_c0_g1::TRINITY_DN29229_c0_g1_i5::g.11187 ORF type:internal len:133 (+),score=48.64 TRINITY_DN29229_c0_g1_i5:3-398(+)	1.842417	-1.01883	-0.18956	-0.72356	0.089533
TRINITY_DN5578_c0_g1_i4_orf1	-	-	-	chromatin modification-related protein eaf-1-like [Ostrinia furnacalis]	1.749187	0.318669	-0.2748	-1.17559	-0.61746
TRINITY_DN17559_c0_g1_i4_orf1	-	-	-	GDP-mannose 4,6 dehydratase isoform X2 [Ostrinia furnacalis] >XP_028166204.1 GDP-mannose 4,6 dehydratase isoform X2 [Ostrinia furnacalis]	1.99223	-0.41586	-0.53119	-0.4007	-0.64449
TRINITY_DN747_c0_g2_i1_orf1	-	-	-	trypsin, alkaline C-like [Ostrinia furnacalis]	1.722146	-0.50607	-1.21349	0.389572	-0.39216
TRINITY_DN52861_c0_g1_i1_orf1	-	-	-	protein MAK16 homolog A [Ostrinia furnacalis]	1.975754	-0.32983	-0.33397	-0.74391	-0.56804
TRINITY_DN3428_c0_g1_i1_orf1	-	-	-	10 kDa heat shock protein, mitochondrial [Ostrinia furnacalis]	1.922547	-0.12003	-0.24928	-0.87906	-0.67419
TRINITY_DN334_c0_g1_i3_orf1	-	-	-	chymotrypsin-like serine protease [Ostrinia nubilalis] >AAX62030.1 chymotrypsin-like serine protease [Ostrinia nubilalis]	1.762877	0.470514	-0.69668	-0.71948	-0.81723
TRINITY_DN327_c1_g1_i4_orf1	-	-	-	mitochondrial import receptor subunit TOM40 homolog 1-like [Ostrinia furnacalis]	1.983024	-0.35253	-0.42656	-0.73734	-0.46659
TRINITY_DN4944_c0_g1_i2_orf1	-	-	-	bifunctional glutamate/proline--tRNA ligase [Ostrinia furnacalis]	1.954918	-0.22916	-0.28083	-0.76126	-0.68366
TRINITY_DN109540_c0_g1_i3_orf1	-	-	-	4-coumarate--CoA ligase 1-like isoform X4 [Ostrinia furnacalis]	1.989685	-0.66041	-0.54617	-0.39798	-0.38513
TRINITY_DN12973_c0_g1_i1_orf1	-	-	-	mitochondrial-processing peptidase subunit alpha [Ostrinia furnacalis]	1.991734	-0.34405	-0.49756	-0.52183	-0.62829

TRINITY_DN19361_c0_g1_i7_orf1	-	-	-	hydroxylysine kinase [Ostrinia furnacalis] >XP_028168144.1 hydroxylysine kinase [Ostrinia furnacalis]	1.916856	0.057463	-0.5503	-0.65903	-0.76499
TRINITY_DN79804_c0_g1_i1_orf1	-	-	-	zinc finger protein on ecdysone puffs-like [Ostrinia furnacalis]	1.884662	0.085917	-0.43186	-0.95689	-0.58184
TRINITY_DN145647_c0_g1_i1_orf1	-	-	-	PREDICTED: U6 snRNA-associated Sm-like protein LSm5 isoform X1 [Fopius arisanus]	1.975765	-0.24125	-0.67827	-0.62881	-0.42743
TRINITY_DN76036_c0_g1_i1_orf1	-	-	-	cytochrome c oxidase subunit 6A1, mitochondrial-like [Ostrinia furnacalis]	1.937791	-0.23055	-0.37851	-0.37646	-0.95227
TRINITY_DN53167_c0_g1_i2_orf1	-	-	-	uncharacterized protein LOC114359219 [Ostrinia furnacalis]	1.977546	-0.32072	-0.71464	-0.34483	-0.59734
TRINITY_DN7920_c0_g1_i2_orf1	-	-	-	uncharacterized protein LOC114357268 [Ostrinia furnacalis] >XP_028166599.1 uncharacterized protein LOC114357268 [Ostrinia furnacalis]	1.887882	-0.34404	-1.09976	-0.29084	-0.15324
TRINITY_DN21909_c0_g1_i1_orf1	-	-	-	complement component 1 Q subcomponent-binding protein, mitochondrial [Ostrinia	1.993654	-0.40526	-0.41815	-0.6127	-0.55755
TRINITY_DN63662_c0_g4_i1_orf1	-	-	-	polyadenylate-binding protein 1 [Ostrinia furnacalis]	1.918914	0.009585	-0.47678	-0.86117	-0.59055
TRINITY_DN6933_c1_g1_i1_orf1	-	-	-	Chlorophyll a-b binding protein 40, chloroplastic [Trichinella nelsoni] >KRY99282.1 Chlorophyll a-b binding protein 40, chloroplastic [Trichinella zimbabwensis]	1.967555	-0.17545	-0.52081	-0.53919	-0.7321
TRINITY_DN56110_c0_g1_i1_orf1	-	-	-	pescadillo homolog [Ostrinia furnacalis]	1.984184	-0.58311	-0.50606	-0.62968	-0.26533
TRINITY_DN4923_c0_g1_i4_orf1	-	-	-	O-acyltransferase like protein-like [Ostrinia furnacalis]	1.969158	-0.6126	-0.66062	-0.53311	-0.16283
TRINITY_DN7336_c0_g1_i13_orf1	-	-	-	PREDICTED: calcium-transporting ATPase sarcoplasmic/endoplasmic reticulum type isoform X2 [Amyelois transitella]	1.870006	-1.07845	0.004897	-0.50508	-0.29138
TRINITY_DN8261_c0_g1_i1_orf1	-	-	-	UDP-N-acetylhexosamine pyrophosphorylase-like protein 1 [Ostrinia furnacalis]	1.900616	0.126856	-0.65117	-0.70302	-0.67329
TRINITY_DN19829_c0_g2_i1_orf1	-	-	-	28S ribosomal protein S35, mitochondrial [Ostrinia furnacalis]	1.962302	-0.37863	-0.31486	-0.40865	-0.86016
TRINITY_DN6535_c0_g2_i1_orf1	-	-	-	NADH dehydrogenase [ubiquinone] 1 beta subcomplex subunit 3 [Ostrinia furnacalis] >XP_028166399.1 NADH dehydrogenase [ubiquinone] 1 beta subcomplex subunit 3 [Ostrinia furnacalis]	1.967386	-0.18867	-0.45361	-0.59158	-0.73353
TRINITY_DN37538_c0_g4_i1_orf1	-	-	-	esterase FE4-like [Ostrinia furnacalis]	1.894639	0.130132	-0.76433	-0.71641	-0.54404
TRINITY_DN86149_c0_g1_i1_orf1	-	-	-	NADH dehydrogenase [ubiquinone] 1 alpha subcomplex subunit 8 [Galleria mellonella]	1.948313	-0.32723	-0.42288	-0.28262	-0.91558
TRINITY_DN27848_c0_g1_i2_orf1	-	-	-	cystathionine beta-synthase-like [Ostrinia furnacalis] >XP_028159011.1 cystathionine beta-synthase-like [Ostrinia furnacalis]	1.985742	-0.6619	-0.59296	-0.36866	-0.36223
TRINITY_DN51045_c0_g1_i1_orf1	-	-	-	cell growth-regulating nucleolar protein [Ostrinia furnacalis]	1.953586	-0.07625	-0.66003	-0.63243	-0.58487
TRINITY_DN36538_c0_g1_i2_orf1	-	-	-	xaa-Pro dipeptidase isoform X1 [Ostrinia furnacalis] >XP_028156507.1 xaa-Pro dipeptidase isoform X2 [Ostrinia furnacalis]	1.938455	-0.09652	-0.353	-0.74206	-0.74687
TRINITY_DN17312_c0_g1_i1_orf1	-	-	-	mRNA cap guanine-N7 methyltransferase [Ostrinia furnacalis]	1.724481	0.403954	-0.22448	-0.94569	-0.95827
TRINITY_DN85319_c0_g1_i1_orf1	-	-	-	cholinesterase 2-like [Ostrinia furnacalis]	1.973361	-0.37632	-0.39988	-0.80802	-0.38914
TRINITY_DN3759_c0_g1_i1_orf1	-	-	-	uncharacterized protein LOC114350416 [Ostrinia furnacalis] >XP_028157016.1 uncharacterized protein LOC114350416 [Ostrinia furnacalis] >XP_028157017.1 uncharacterized protein LOC114350416 [Ostrinia furnacalis] >XP_028157018.1 uncharacterized protein LOC114350416 [Ostrinia furnacalis] >XP_028157019.1	1.50072	0.8523	-0.65993	-1.12281	-0.57028
TRINITY_DN6248_c0_g1_i1_orf1	-	-	-	DNA topoisomerase I, mitochondrial [Ostrinia furnacalis]	1.776118	0.444965	-0.69683	-0.77124	-0.75301
TRINITY_DN42759_c0_g2_i1_orf1	-	-	-	fatty acid synthase-like [Ostrinia furnacalis]	1.93056	-0.47532	-0.8714	-0.53403	-0.04981
TRINITY_DN4895_c0_g1_i2_orf1	-	-	-	coiled-coil domain-containing protein 86 [Ostrinia furnacalis]	1.965048	-0.83741	-0.32143	-0.4703	-0.33591
TRINITY_DN8838_c0_g1_i1_orf1	-	-	-	mannose-P-dolichol utilization defect 1 protein homolog [Ostrinia furnacalis]	1.842272	-0.10067	-0.70222	-1.05008	0.010701
TRINITY_DN15607_c0_g1_i6_orf1	-	-	-	protein artichoke-like [Ostrinia furnacalis]	1.928946	-0.48904	-0.0083	-0.80296	-0.62865
TRINITY_DN30233_c0_g1_i2_orf1	-	-	-	39S ribosomal protein L10, mitochondrial [Ostrinia furnacalis]	1.911412	-0.20749	-0.47065	-1.01755	-0.21572
TRINITY_DN1445_c0_g1_i1_orf1	-	-	-	leucine-rich PPR motif-containing protein, mitochondrial [Ostrinia furnacalis]	1.95138	-0.06724	-0.57511	-0.64867	-0.66035
TRINITY_DN22815_c0_g1_i2_orf1	-	-	-	acyl carrier protein, mitochondrial isoform X1 [Ostrinia furnacalis]	1.983569	-0.68723	-0.28384	-0.50994	-0.50255
TRINITY_DN5925_c0_g1_i5_orf1	-	-	-	isocitrate dehydrogenase [NAD] subunit gamma, mitochondrial-like isoform X1 [Ostrinia furnacalis]	1.959337	-0.57975	-0.10454	-0.6142	-0.66085
TRINITY_DN17738_c0_g1_i2_orf1	-	-	-	unnamed protein product [Diatraea saccharalis]	1.978552	-0.42254	-0.29498	-0.7416	-0.51943
TRINITY_DN28152_c0_g1_i1_orf1	-	-	-	mitochondrial import inner membrane translocase subunit Tim29 [Ostrinia furnacalis]	1.990523	-0.39237	-0.64129	-0.56451	-0.39237
TRINITY_DN1330_c0_g1_i1_orf1	-	-	-	pancreatic triacylglycerol lipase-like [Ostrinia furnacalis]	1.937923	-0.2109	-0.90647	-0.26599	-0.55456
TRINITY_DN25856_c0_g1_i1_orf1	-	-	-	myrosinase 1-like [Ostrinia furnacalis]	1.975167	-0.74245	-0.25558	-0.53735	-0.43979
TRINITY_DN2574_c0_g1_i5_orf1	-	-	-	prion-like-(Q/N-rich) domain-bearing protein 25 [Ostrinia furnacalis] >XP_028158239.1 prion-like-(Q/N-rich) domain-bearing protein 25 [Ostrinia furnacalis] >XP_028158240.1 prion-like-(Q/N-rich) domain-bearing protein 25 [Ostrinia furnacalis] >XP_028158241.1 prion-like-(Q/N-rich) domain-bearing protein 25 [Ostrinia furnacalis]	1.960801	-0.776	-0.5697	-0.44782	-0.16728
TRINITY_DN2238_c0_g2_i1_orf1	-	-	-	mitochondrial import inner membrane translocase subunit Tim8 [Ostrinia furnacalis]	1.946737	-0.35204	-0.85195	-0.16567	-0.57708
TRINITY_DN16830_c0_g1_i5_orf1	-	-	-	adrenodoxin [Ostrinia furnacalis]	1.970528	-0.28706	-0.49528	-0.79832	-0.38987

TRINITY_DN47_c0_g1_i2_orf1	-	-	-	uncharacterized protein LOC114356437 isoform X1 [Ostrinia furnacalis]	1.878469	-0.85208	-0.85467	-0.07854	-0.09318
TRINITY_DN64510_c0_g1_i1_orf1	-	-	-	39S ribosomal protein L15, mitochondrial [Ostrinia furnacalis]	1.989789	-0.40943	-0.4796	-0.68686	-0.4139
TRINITY_DN1277_c4_g1_i5_orf1	-	-	-	GTP cyclohydrolase 1 isoform X1 [Ostrinia furnacalis] >XP_028166842.1 GTP cyclohydrolase 1 isoform X1 [Ostrinia furnacalis]	1.980605	-0.45105	-0.65682	-0.61174	-0.261
TRINITY_DN12495_c0_g1_i2_orf1	-	-	-	probable ATP-dependent RNA helicase pitchoune [Manduca sexta] >KAG6441249.1 hypothetical protein O3G_MSEX001749 [Manduca sexta]	1.984311	-0.60698	-0.27102	-0.619	-0.48731
TRINITY_DN6308_c0_g1_i6_orf1	-	-	-	myc box-dependent-interacting protein 1 isoform X2 [Ostrinia furnacalis]	1.923382	-0.02879	-0.39844	-0.85244	-0.64371
TRINITY_DN389_c0_g1_i2_orf1	-	-	-	uncharacterized protein LOC118068293 isoform X2 [Chelonus insularis]	1.418066	1.010433	-0.81064	-0.77545	-0.84241
TRINITY_DN43412_c0_g1_i2_orf1	-	-	-	U1 small nuclear ribonucleoprotein C [Ostrinia furnacalis]	1.548438	0.540345	0.008466	-1.28428	-0.81297
TRINITY_DN3749_c0_g1_i1_orf1	-	-	-	cytochrome c oxidase subunit 6B1 [Ostrinia furnacalis]	1.986054	-0.43955	-0.36054	-0.47228	-0.71368
TRINITY_DN39673_c0_g1_i1_orf1	-	-	-	uncharacterized protein LOC114359357 isoform X1 [Ostrinia furnacalis]	1.926672	-0.64199	-0.81225	-0.46473	-0.0077
TRINITY_DN948_c0_g1_i1_orf1	-	-	-	mitochondrial-processing peptidase subunit beta [Ostrinia furnacalis]	1.985395	-0.39074	-0.3475	-0.68305	-0.5641
TRINITY_DN10636_c0_g1_i1_orf1	-	-	-	unnamed protein product [Arctia plantaginis] >CAB3253774.1 unnamed protein product [Arctia plantaginis]	1.942632	-0.72404	-0.03728	-0.62814	-0.55318
TRINITY_DN12133_c0_g2_i1_orf1	-	-	-	O-acyltransferase like protein-like [Ostrinia furnacalis]	1.978677	-0.35173	-0.69815	-0.31884	-0.60996
TRINITY_DN1285_c0_g1_i6_orf1	-	-	-	bifunctional 3'-phosphoadenosine 5'-phosphosulfate synthase isoform X3 [Ostrinia furnacalis]	1.950096	-0.32725	-0.2558	-0.89588	-0.47116
TRINITY_DN18036_c0_g1_i7_orf1	-	-	-	pentatricopeptide repeat-containing protein 2, mitochondrial-like [Ostrinia furnacalis]	1.965117	-0.15939	-0.49324	-0.72074	-0.59175
TRINITY_DN2704_c0_g1_i5_orf1	-	-	-	hypothetical protein evm_009002 [Chilo suppressalis]	1.942531	-0.10269	-0.84845	-0.44702	-0.54437
TRINITY_DN19115_c0_g1_i1_orf1	-	-	-	putative ATP synthase subunit f, mitochondrial [Ostrinia furnacalis]	1.880457	-0.04432	-0.40352	-0.34693	-1.08569
TRINITY_DN40704_c0_g1_i2_orf1	-	-	-	COX assembly mitochondrial protein homolog [Ostrinia furnacalis]	1.834907	0.25315	-0.55156	-0.97368	-0.56282
TRINITY_DN18909_c0_g1_i8_orf1	-	-	-	unnamed protein product [Euphydryas editha]	1.929332	-0.62718	-0.81361	-0.47124	-0.01731
TRINITY_DN53167_c0_g1_i3_orf1	-	-	-	uncharacterized protein LOC114359219 [Ostrinia furnacalis]	1.979679	-0.72651	-0.28259	-0.51836	-0.45222
TRINITY_DN14967_c0_g2_i1_orf1	-	-	-	glyceraldehyde-3-phosphate dehydrogenase 2 [Holotrichia oblita]	1.98902	-0.52637	-0.67101	-0.35624	-0.4354
TRINITY_DN3082_c1_g1_i7_orf1	-	-	-	ribosomal RNA processing protein 1 homolog [Ostrinia furnacalis]	1.969069	-0.20377	-0.73427	-0.58824	-0.44278
TRINITY_DN54543_c0_g5_i2_orf1	-	-	-	reactive oxygen species modulator 1 [Papilio machaon] >XP_022123076.1 reactive oxygen species modulator 1 [Pieris rapae] >XP_022820703.1 reactive oxygen species modulator 1 [Spodoptera litura] >XP_028176790.1 reactive oxygen species modulator 1 [Ostrinia furnacalis] >XP_030021418.1 reactive oxygen species modulator 1 [Manduca sexta] >XP_034836896.1 reactive oxygen species modulator 1 [Maniola hyperantus] >XP_035446679.1 reactive oxygen species modulator 1-like [Spodoptera frugiperda] >XP_045458971.1 reactive oxygen species modulator 1 [Melitaea cinxia] >XP_045521377.1 reactive oxygen species modulator 1 [Pieris brassicae] >XP_045765649.1 reactive oxygen species modulator 1 [Maniola jurtina] >XP_047042173.1 reactive oxygen species modulator 1 [Helicoverpa zea] >XP_049691668.1 reactive oxygen species modulator 1 [Helicoverpa armigera] >KAF9414699.1 hypothetical protein HW555_007477 [Spodoptera exigua] >KPI93862.1 Reactive oxygen species modulator 1 [Papilio xuthus] >CAB3515149.1 unnamed protein product [Spodoptera littoralis] >CAG5021695.1 unnamed protein product [Parnassius apollo] >KAF9823017.1 hypothetical protein SFRURICE_018191 [Spodoptera frugiperda]	1.980595	-0.27807	-0.70838	-0.54157	-0.45258
TRINITY_DN1427_c0_g1_i9_orf1	-	-	-	SAFB-like transcription modulator isoform X3 [Ostrinia furnacalis]	1.596468	0.357804	0.15764	-1.24079	-0.87112
TRINITY_DN34536_c0_g1_i6_orf1	-	-	-	clustered mitochondria protein homolog isoform X2 [Ostrinia furnacalis]	1.985015	-0.30869	-0.52148	-0.69067	-0.46418
TRINITY_DN21494_c0_g1_i2_orf1	-	-	-	pancreatic triacylglycerol lipase-like [Ostrinia furnacalis]	1.971999	-0.23966	-0.39908	-0.72013	-0.61314
TRINITY_DN9464_c0_g1_i1_orf1	-	-	-	angio-associated migratory cell protein [Ostrinia furnacalis] >XP_028162594.1 angio-associated migratory cell protein [Ostrinia furnacalis]	1.965355	-0.63284	-0.27381	-0.75462	-0.30408
TRINITY_DN1706_c0_g1_i7_orf1	-	-	-	LOW QUALITY PROTEIN: RNA polymerase-associated protein CTR9 homolog [Ostrinia	1.724817	0.398127	-0.33929	-1.17527	-0.60839
TRINITY_DN14436_c0_g1_i7_orf1	-	-	-	V-type proton ATPase subunit C [Vanessa cardui]	1.952418	-0.08308	-0.63219	-0.70866	-0.52848
TRINITY_DN20294_c0_g2_i1_orf1	-	-	-	cytochrome b-c1 complex subunit 2, mitochondrial isoform X1 [Ostrinia furnacalis] >XP_028170208.1 cytochrome b-c1 complex subunit 2, mitochondrial isoform X2 [Ostrinia furnacalis]	1.98754	-0.39003	-0.36398	-0.57047	-0.66305
TRINITY_DN110888_c0_g1_i2_orf1	-	-	-	uncharacterized protein LOC114364502 [Ostrinia furnacalis]	1.77636	-0.88854	-0.78009	0.415551	-0.52329
TRINITY_DN2579_c0_g1_i7_orf1	-	-	-	aminopeptidase N5 [Ostrinia nubilalis]	1.998607	-0.45139	-0.46727	-0.55284	-0.52711
TRINITY_DN66302_c0_g1_i1_orf1	-	-	-	carboxypeptidase B-like [Ostrinia furnacalis]	1.95823	-0.50118	-0.80488	-0.48989	-0.16229
TRINITY_DN17351_c0_g1_i3_orf1	-	-	-	V-type proton ATPase subunit F [Ostrinia furnacalis]	1.982905	-0.70363	-0.51632	-0.4691	-0.29385
TRINITY_DN6685_c0_g1_i8_orf1	-	-	-	cleft lip and palate transmembrane protein 1 homolog [Ostrinia furnacalis]	1.851567	0.087216	-0.20273	-0.95762	-0.77844
TRINITY_DN86844_c0_g2_i1_orf1	-	-	-	spermine oxidase-like isoform X1 [Ostrinia furnacalis]	1.933779	-0.84742	-0.05669	-0.44753	-0.58214

TRINITY_DN14313_c0_g1_i1_orf1	-	-	-	25S rRNA (cytosine-C(5))-methyltransferase nop2 [Ostrinia furnacalis]	1.939825	-0.0294	-0.55558	-0.7472	-0.60764
TRINITY_DN12242_c0_g1_i5_orf1	-	-	-	heterogeneous nuclear ribonucleoprotein 87F-like isoform X1 [Vanessa tameamea] >XP_046967652.1 heterogeneous nuclear ribonucleoprotein 87F-like isoform X1 [Vanessa cardui] >XP_047532045.1 heterogeneous nuclear ribonucleoprotein 87F-like [Vanessa	1.605351	0.717025	-0.50334	-0.88803	-0.93101
TRINITY_DN13055_c0_g1_i5_orf1	-	-	-	116 kDa U5 small nuclear ribonucleoprotein component isoform X1 [Ostrinia furnacalis] >XP_028159219.1 116 kDa U5 small nuclear ribonucleoprotein component isoform X2 [Ostrinia furnacalis]	1.70039	0.333385	-0.06178	-1.14305	-0.82895
TRINITY_DN107261_c0_g1_i1_orf1	-	-	-	ATP synthase subunit g, mitochondrial [Ostrinia furnacalis]	1.934745	-0.85746	-0.35342	-0.10248	-0.62138
TRINITY_DN7583_c0_g1_i1_orf1	-	-	-	39S ribosomal protein L21, mitochondrial [Ostrinia furnacalis]	1.805694	0.344213	-0.60871	-0.59338	-0.94782
TRINITY_DN478_c0_g1_i16_orf1	-	-	-	lipid storage droplets surface-binding protein 2 isoform X1 [Ostrinia furnacalis]	1.756385	-0.03412	0.129676	-0.62406	-1.22788
TRINITY_DN791_c0_g1_i2_orf1	-	-	-	peroxiredoxin 1 isoform X1 [Maniola jurtina]	1.946816	-0.14082	-0.38703	-0.839	-0.57997
TRINITY_DN96170_c0_g2_i1_orf1	-	-	-	uncharacterized protein LOC114355569 [Ostrinia furnacalis]	1.969602	-0.33176	-0.75195	-0.60538	-0.28051
TRINITY_DN4550_c1_g1_i5_orfp2	-	-	-	TRINITY_DN4550_c1_g1_i5_m.14710 TRINITY_DN4550_c1_g1::TRINITY_DN4550_c1_g1_i5::g.14710 ORF type:5prime_partial len:168	1.934844	-0.74985	-0.06251	-0.73475	-0.38774
TRINITY_DN2852_c0_g1_i9_orf1	-	-	-	golgin subfamily A member 4-like [Ostrinia furnacalis]	1.938389	-0.18613	-0.26489	-0.86959	-0.61778
TRINITY_DN109733_c0_g1_i1_orf1	-	-	-	uncharacterized protein LOC112452128 [Temnothorax curvispinosus]	1.951646	-0.44223	-0.71912	-0.68332	-0.10697
TRINITY_DN2243_c0_g1_i4_orf1	-	-	-	WD repeat-containing protein 75 [Ostrinia furnacalis]	1.975764	-0.6942	-0.31448	-0.63833	-0.32875
TRINITY_DN12497_c0_g1_i1_orf1	-	-	-	probable N-acetyltransferase san [Ostrinia furnacalis]	1.964883	-0.17735	-0.51374	-0.76453	-0.50926
TRINITY_DN37538_c0_g2_i1_orf1	-	-	-	esterase FE4-like [Ostrinia furnacalis]	1.972874	-0.23062	-0.74155	-0.45572	-0.54499
TRINITY_DN15900_c0_g1_i6_orf1	-	-	-	unnamed protein product [Diatraea saccharalis]	1.86899	0.130274	-0.56789	-0.98307	-0.4483
TRINITY_DN40_c0_g2_i1_orf1	-	-	-	trypsin CFT-1-like [Ostrinia furnacalis]	1.968337	-0.81816	-0.39909	-0.46194	-0.28915
TRINITY_DN3847_c1_g1_i1_orf1	-	-	-	ribosome production factor 2 homolog [Ostrinia furnacalis]	1.971494	-0.35623	-0.80853	-0.34344	-0.4633
TRINITY_DN3476_c0_g1_i5_orf1	-	-	-	maltase A1-like [Ostrinia furnacalis]	1.964154	-0.80132	-0.55336	-0.24131	-0.36816
TRINITY_DN10530_c0_g1_i1_orf1	-	-	-	cytochrome c oxidase subunit NDUFA4 [Ostrinia furnacalis]	1.993874	-0.44326	-0.39886	-0.52284	-0.62891
TRINITY_DN3062_c0_g1_i1_orf1	-	-	-	HEAT repeat-containing protein 1 [Ostrinia furnacalis]	1.915801	-0.0688	-0.36161	-0.95602	-0.52938
TRINITY_DN25975_c0_g3_i2_orf1	-	-	-	V-type proton ATPase subunit D isoform X2 [Ostrinia furnacalis]	1.947135	-0.09349	-0.78765	-0.60835	-0.45764
TRINITY_DN107035_c0_g1_i1_orf1	-	-	-	splicing factor 3A subunit 3 [Ostrinia furnacalis]	1.944426	-0.07745	-0.55091	-0.80583	-0.51023
TRINITY_DN1505_c0_g1_i1_orf1	-	-	-	uncharacterized protein LOC114362816 isoform X1 [Ostrinia furnacalis] >XP_028174154.1 uncharacterized protein LOC114362816 isoform X2 [Ostrinia furnacalis]	1.981924	-0.23866	-0.5562	-0.61698	-0.57009
TRINITY_DN26130_c0_g1_i1_orf1	-	-	-	membrane alanyl aminopeptidase-like [Ostrinia furnacalis]	1.877242	0.076223	-0.9541	-0.32579	-0.67358
TRINITY_DN4842_c0_g1_i5_orf1	-	-	-	cytochrome c oxidase assembly factor 4 homolog, mitochondrial isoform X1 [Ostrinia furnacalis] >XP_028162331.1 cytochrome c oxidase assembly factor 4 homolog, mitochondrial isoform X2 [Ostrinia furnacalis]	1.928611	-0.06148	-0.39018	-0.60858	-0.86837
TRINITY_DN17905_c0_g3_i1_orf1	-	-	-	zinc finger protein 706-like [Ostrinia furnacalis] >XP_028176219.1 zinc finger protein 706-like [Ostrinia furnacalis] >XP_028176220.1 zinc finger protein 706-like [Ostrinia furnacalis] >XP_028176221.1 zinc finger protein 706-like [Ostrinia furnacalis]	1.874863	0.203713	-0.69891	-0.64999	-0.72968
TRINITY_DN21531_c0_g1_i1_orf1	-	-	-	viral IAP-associated factor homolog [Ostrinia furnacalis]	1.737804	0.484217	-0.6733	-0.55876	-0.98996
TRINITY_DN56910_c0_g2_i1_orf1	-	-	-	mitochondrial ribonuclease P protein 1 homolog [Ostrinia furnacalis]	1.940598	-0.08329	-0.39352	-0.7537	-0.71008
TRINITY_DN4143_c0_g1_i1_orf1	-	-	-	zinc finger protein 530-like isoform X8 [Ostrinia furnacalis]	1.821578	0.218632	-0.84925	-0.27639	-0.91457
TRINITY_DN4030_c0_g2_i1_orf1	-	-	-	putative trypsin 6 [Ostrinia nubilalis]	1.920093	-0.43558	-0.9526	-0.07282	-0.45909
TRINITY_DN4476_c0_g1_i5_orf1	-	-	-	trypsin, alkaline C-like isoform X1 [Ostrinia furnacalis]	1.958268	-0.22228	-0.53233	-0.83421	-0.36944
TRINITY_DN1285_c0_g2_i1_orf1	-	-	-	bifunctional 3'-phosphoadenosine 5'-phosphosulfate synthase isoform X3 [Ostrinia furnacalis]	1.995445	-0.5243	-0.43113	-0.61117	-0.42884
TRINITY_DN11657_c0_g1_i2_orf1	-	-	-	trehalase-1 [Omphisa fuscidentalis]	1.486567	0.89195	-0.66323	-0.65324	-1.06205
TRINITY_DN23266_c0_g2_i1_orf1	-	-	-	medium-chain acyl-CoA ligase ACSF2, mitochondrial [Chelonus insularis]	1.989169	-0.4197	-0.34849	-0.62101	-0.59997
TRINITY_DN3332_c0_g1_i2_orf1	-	-	-	glutathione S-transferase sigma3 [Glyphodes pyloalis]	1.985887	-0.33964	-0.70628	-0.48221	-0.45776
TRINITY_DN15624_c0_g1_i1_orf1	-	-	-	LOW QUALITY PROTEIN: V-type proton ATPase subunit S1-like [Ostrinia furnacalis]	1.84277	0.204317	-0.74714	-0.36775	-0.9322
TRINITY_DN55160_c0_g2_i1_orf1	-	-	-	esterase FE4-like isoform X2 [Ostrinia furnacalis]	1.86703	-0.33349	-1.05982	0.052587	-0.52631
TRINITY_DN1775_c0_g1_i3_orf1	-	-	-	ATP-dependent RNA helicase dbp2-like isoform X1 [Ostrinia furnacalis]	1.743933	0.505222	-0.85115	-0.66914	-0.72887
TRINITY_DN36262_c0_g1_i1_orf1	-	-	-	trypsin, alkaline C-like [Maniola jurtina]	1.964403	-0.1722	-0.63174	-0.71994	-0.44052
TRINITY_DN24399_c0_g1_i1_orf1	-	-	-	retinol-binding protein pinta-like [Ostrinia furnacalis]	1.879224	-0.76217	0.145627	-0.4453	-0.81738
TRINITY_DN11069_c0_g2_i1_orf1	-	-	-	fat storage-inducing transmembrane protein [Ostrinia furnacalis]	1.92312	-0.24495	-0.28211	-1.00424	-0.39181

TRINITY_DN50787_c0_g2_i2_orf1	-	-	-	40S ribosomal protein S29 [Hyposmocoma kahamanoa] >XP_028176503.1 40S ribosomal protein S29 [Ostrinia furnacalis] >XP_049877832.1 40S ribosomal protein S29 [Pectinophora gossypiella] >ADT80654.1 ribosomal protein S29 [Euphydryas aurinia] >CAB3523209.1 unnamed protein product [Chilo suppressalis] >CAH0400531.1 unnamed protein product	1.801742	-0.4331	0.090911	-1.22613	-0.23342
TRINITY_DN31047_c0_g1_i4_orf1	-	-	-	4-coumarate--CoA ligase 1-like isoform X1 [Ostrinia furnacalis] >XP_028160248.1 4-coumarate--CoA ligase 1-like isoform X1 [Ostrinia furnacalis] >XP_028160249.1 4-coumarate--CoA ligase 1-like isoform X1 [Ostrinia furnacalis] >XP_028160250.1 4-coumarate--CoA ligase 1-like isoform X1 [Ostrinia furnacalis] >XP_028160251.1 4-coumarate--CoA ligase 1-like isoform X1 [Ostrinia furnacalis] >XP_028160253.1 4-	1.936423	-0.73072	-0.02886	-0.69565	-0.48119
TRINITY_DN657_c0_g1_i2_orf1	-	-	-	cytochrome c-type heme lyase [Ostrinia furnacalis]	1.957341	-0.64401	-0.4525	-0.72942	-0.13142
TRINITY_DN52768_c0_g1_i1_orf1	-	-	-	carboxypeptidase Q-like isoform X2 [Ostrinia furnacalis]	1.991522	-0.51291	-0.63458	-0.49909	-0.34495
TRINITY_DN3959_c1_g2_i1_orf1	-	-	-	PREDICTED: probable isocitrate dehydrogenase [NAD] subunit alpha, mitochondrial isoform X3 [Papilio xuthus] >XP_014363280.1 probable isocitrate dehydrogenase [NAD] subunit alpha, mitochondrial isoform X3 [Papilio machaon]	1.998479	-0.47204	-0.56857	-0.45413	-0.50374
TRINITY_DN35635_c0_g1_i1_orf1	-	-	-	probable NADH dehydrogenase [ubiquinone] 1 alpha subcomplex subunit 12 [Ostrinia	1.888557	-0.02263	-0.30062	-0.54039	-1.02491
TRINITY_DN760_c1_g2_i6_orf1	-	-	-	ADP,ATP carrier protein [Pieris napi]	1.973275	-0.68023	-0.43259	-0.22268	-0.63777
TRINITY_DN12474_c0_g1_i6_orf1	-	-	-	aromatic-L-amino-acid decarboxylase [Ostrinia furnacalis]	1.999675	-0.52558	-0.47101	-0.50952	-0.49357
TRINITY_DN2266_c0_g1_i6_orf1	-	-	-	bilin-binding protein-like [Ostrinia furnacalis]	1.739847	-0.62034	-0.75217	0.507304	-0.87464
TRINITY_DN1901_c0_g1_i6_orf1	-	-	-	mitochondrial intermembrane space import and assembly protein 40 [Ostrinia furnacalis] >XP_028171079.1 mitochondrial intermembrane space import and assembly protein 40 [Ostrinia furnacalis] >XP_028171080.1 mitochondrial intermembrane space import and assembly protein 40 [Ostrinia furnacalis]	1.98519	-0.26544	-0.54156	-0.56455	-0.61365
TRINITY_DN1073_c0_g1_i4_orf1	-	-	-	carboxylesterase [Loxostege sticticalis]	1.986449	-0.53055	-0.62784	-0.54728	-0.28078
TRINITY_DN2270_c0_g2_i1_orf1	-	-	-	integrin beta-nu [Ostrinia furnacalis]	1.988025	-0.46231	-0.31555	-0.57187	-0.63829
TRINITY_DN3134_c0_g1_i1_orf1	-	-	-	cytochrome c oxidase subunit 6C-1 isoform X1 [Hyposmocoma kahamanoa]	1.990007	-0.46632	-0.35291	-0.50628	-0.66449
TRINITY_DN1366_c0_g1_i5_orf1	-	-	-	unnamed protein product, partial [Iphiclides podalirius]	1.997476	-0.57838	-0.52645	-0.44408	-0.44857
TRINITY_DN2594_c0_g2_i4_orf1	-	-	-	isocitrate dehydrogenase [NAD] subunit beta, mitochondrial isoform X2 [Ostrinia furnacalis]	1.959284	-0.14971	-0.49627	-0.53391	-0.77939
TRINITY_DN43391_c0_g1_i5_orf1	-	-	-	myrosinase 1-like isoform X2 [Ostrinia furnacalis]	1.983228	-0.54293	-0.70881	-0.33345	-0.39804
TRINITY_DN23734_c0_g1_i1_orf1	-	-	-	histone-lysine N-methyltransferase SMYD3 [Ostrinia furnacalis]	1.787969	-0.04048	-0.25005	-1.30453	-0.19292
TRINITY_DN5238_c0_g1_i2_orf1	-	-	-	DNA-(apurinic or apyrimidinic site) lyase [Ostrinia furnacalis]	1.735698	0.33526	-0.23066	-1.17353	-0.66677
TRINITY_DN35725_c0_g1_i1_orf1	-	-	-	mitochondrial import inner membrane translocase subunit Tim13-like [Bicyclus anynana] >CAG9745432.1 unnamed protein product [Diatraea saccharalis] >CAG9784117.1 unnamed protein product [Diatraea saccharalis]	1.869933	0.213061	-0.77952	-0.67147	-0.632
TRINITY_DN13783_c0_g4_i2_orf1	-	-	-	hypothetical protein evm_010131 [Chilo suppressalis]	1.977243	-0.50226	-0.4814	-0.73438	-0.2592
TRINITY_DN146544_c0_g1_i1_orf1	-	-	-	UPF0047 protein YjbQ [Aphidius gifuensis] >KAF7996225.1 hypothetical protein HCN44_001857 [Aphidius gifuensis]	1.996688	-0.43202	-0.4441	-0.53258	-0.58799
TRINITY_DN18404_c0_g1_i5_orf1	-	-	-	periodic tryptophan protein 1 homolog isoform X1 [Ostrinia furnacalis] >XP_028157695.1 periodic tryptophan protein 1 homolog isoform X2 [Ostrinia furnacalis]	1.967725	-0.20859	-0.76588	-0.44817	-0.54509
TRINITY_DN679_c0_g1_i2_orf1	-	-	-	cytochrome b-c1 complex subunit 7-like [Ostrinia furnacalis]	1.987762	-0.47773	-0.30596	-0.56986	-0.63421
TRINITY_DN105901_c0_g1_i2_orfp1	-	-	-	contactin-like [Pectinophora gossypiella]	1.909371	-0.83731	-0.59492	0.065941	-0.54308
TRINITY_DN1707_c0_g1_i1_orf1	-	-	-	inositol oxygenase-like [Ostrinia furnacalis]	1.914957	-0.59936	-0.93106	-0.06401	-0.32052
TRINITY_DN11347_c0_g1_i1_orf1	-	-	-	N(4)-(Beta-N-acetylglucosaminyl)-L-asparaginase-like [Ostrinia furnacalis]	1.950322	-0.2416	-0.87841	-0.31133	-0.51897
TRINITY_DN48237_c0_g1_i5_orf1	-	-	-	myogenesis-regulating glycosidase-like [Ostrinia furnacalis]	1.964214	-0.24621	-0.69617	-0.70395	-0.31789
TRINITY_DN8543_c0_g1_i1_orf1	-	-	-	39S ribosomal protein L38, mitochondrial [Ostrinia furnacalis]	1.874212	-0.42322	0.135835	-0.91741	-0.66942
TRINITY_DN28577_c0_g1_i6_orf1	-	-	-	delta-1-pyrroline-5-carboxylate dehydrogenase, mitochondrial [Nymphalis io]	1.990806	-0.34947	-0.57768	-0.61892	-0.44474
TRINITY_DN43420_c0_g2_i1_orf1	-	-	-	collagenase-like [Ostrinia furnacalis]	1.994595	-0.41864	-0.42088	-0.60989	-0.54517
TRINITY_DN9119_c0_g1_i3_orf1	-	-	-	actin-binding Rho-activating protein [Helicoverpa armigera] >XP_047029227.1 actin-binding Rho-activating protein-like [Helicoverpa zea] >PZC85229.1 hypothetical protein B5X24_HaOG202414 [Helicoverpa armigera]	1.97468	-0.31484	-0.64737	-0.31932	-0.69316
TRINITY_DN130075_c1_g2_i1_orf1	-	-	-	60S ribosomal protein L23 [Microtus ochrogaster]	1.915527	-0.75837	-0.54715	0.06256	-0.67256
TRINITY_DN94248_c0_g2_i3_orf1	-	-	-	uncharacterized protein LOC114357292 isoform X4 [Ostrinia furnacalis]	1.963303	-0.71772	-0.68205	-0.22545	-0.33808
TRINITY_DN8369_c0_g1_i1_orf1	-	-	-	39S ribosomal protein L37, mitochondrial [Ostrinia furnacalis]	1.91852	0.012247	-0.86943	-0.53067	-0.53067
TRINITY_DN40_c0_g1_i3_orf1	-	-	-	trypsin CFT-1-like [Ostrinia furnacalis]	1.987921	-0.34534	-0.67877	-0.4388	-0.52501
TRINITY_DN48020_c0_g1_i1_orf1	-	-	-	aminopeptidase N4 [Cnaphalocrocis medinalis]	1.94389	-0.64525	-0.7288	-0.52096	-0.04888

TRINITY_DN42759_c0_g3_i1_orf1	-	-	-	fatty acid synthase-like [Ostrinia furnacalis]	1.972906	-0.79011	-0.45411	-0.288	-0.44068
TRINITY_DN76283_c0_g6_i1_orf1	-	-	-	fatty acid synthase-like [Ostrinia furnacalis]	1.995309	-0.55524	-0.55764	-0.51245	-0.36998
TRINITY_DN6087_c0_g1_i7_orf1	-	-	-	uncharacterized protein LOC114355564 [Ostrinia furnacalis]	1.973096	-0.2381	-0.75494	-0.48768	-0.49237
TRINITY_DN7556_c0_g1_i1_orf1	-	-	-	venom carboxylesterase-6-like [Ostrinia furnacalis]	1.97098	-0.1742	-0.67503	-0.56671	-0.55504
TRINITY_DN6418_c0_g1_i28_orf1	-	-	-	peritrophic membrane chitin binding protein [Loxostege sticticalis]	1.964791	-0.84586	-0.39388	-0.30718	-0.41786
TRINITY_DN49265_c0_g3_i2_orf1	-	-	-	cytochrome c [Ostrinia furnacalis] >XP_028160278.1 cytochrome c [Ostrinia furnacalis]	1.968878	-0.55206	-0.2462	-0.39431	-0.77631
TRINITY_DN52296_c0_g1_i6_orf1	-	-	-	protein takeout-like [Ostrinia furnacalis]	1.83707	-0.34257	0.182378	-1.02364	-0.65324
TRINITY_DN4762_c0_g1_i2_orf1	-	-	-	ATPase family AAA domain-containing protein 1 isoform X2 [Ostrinia furnacalis]	1.729932	0.490438	-0.66695	-1.01694	-0.53647
TRINITY_DN10332_c0_g1_i2_orfp1	-	-	-	TRINITY_DN10332_c0_g1_i2_m.42894 TRINITY_DN10332_c0_g1::TRINITY_DN10332_c0_g1_i2::g.42894 ORF type:3prime_partial len:77 (+),score=1.70 TRINITY_DN10332_c0_g1_i2:1005-1232(+)	1.808914	-0.01195	-0.01375	-1.15388	-0.62934
TRINITY_DN18773_c0_g1_i3_orf1	-	-	-	keratin, type II cytoskeletal 68 kDa, component IB-like [Ostrinia furnacalis]	1.874936	-1.08471	-0.0973	-0.51747	-0.17546
TRINITY_DN140669_c0_g1_i1_orf1	-	-	-	S-methyl-5'-thioadenosine phosphorylase-like isoform X1 [Hyposmocoma kahamanoa]	1.939776	-0.17115	-0.91415	-0.48742	-0.36706
TRINITY_DN47114_c0_g1_i5_orf1	-	-	-	nucleolar protein dao-5 isoform X2 [Ostrinia furnacalis]	1.948907	-0.46565	-0.19081	-0.88425	-0.4082
TRINITY_DN7966_c0_g1_i4_orf1	-	-	-	leucine-rich repeat neuronal protein 1-like [Ostrinia furnacalis]	1.98771	-0.30363	-0.51425	-0.6486	-0.52124
TRINITY_DN7213_c0_g1_i2_orf1	-	-	-	probable ATP-dependent RNA helicase CG8611 [Ostrinia furnacalis]	1.963195	-0.30132	-0.26313	-0.76099	-0.63775
TRINITY_DN28592_c0_g1_i2_orf1	-	-	-	UDP-glucuronosyltransferase 2B14-like isoform X1 [Ostrinia furnacalis] >XP_028167291.1 UDP-glucuronosyltransferase 2B14-like isoform X2 [Ostrinia furnacalis]	1.942365	-0.161	-0.46437	-0.90183	-0.41517
TRINITY_DN122170_c0_g1_i2_orfp1	-	-	-	TRINITY_DN122170_c0_g1_i2_m.81408 TRINITY_DN122170_c0_g1::TRINITY_DN122170_c0_g1_i2::g.81408 ORF type:internal len:87 (+),score=9.58,Baculo_E25 PF05274.12 1.7e-07 TRINITY_DN122170_c0_g1_i2:2-259(+)	1.994041	-0.56287	-0.39003	-0.60078	-0.44036
TRINITY_DN1154_c0_g1_i1_orf1	-	-	-	calexcitin-1-like [Ostrinia furnacalis] >ADK94879.2 juvenile hormone diol kinase [Ostrinia furnacalis]	1.889639	-0.90553	-0.74629	-0.00924	-0.22857
TRINITY_DN117_c0_g1_i5_orf1	-	-	-	lipase member I-like [Ostrinia furnacalis]	1.977467	-0.71122	-0.57388	-0.4321	-0.26026
TRINITY_DN542_c0_g1_i4_orf1	-	-	-	uncharacterized protein LOC114364889 [Ostrinia furnacalis]	1.967512	-0.83835	-0.36532	-0.36094	-0.40291
TRINITY_DN79319_c0_g1_i8_orfp1	-	-	-	TRINITY_DN79319_c0_g1_i8_m.49956 TRINITY_DN79319_c0_g1::TRINITY_DN79319_c0_g1_i8::g.49956 ORF type:5prime_partial len:84 (+),score=1.39 TRINITY_DN79319_c0_g1_i8:1-252(+)	1.959937	-0.65436	-0.76406	-0.26557	-0.27594
TRINITY_DN1044_c0_g1_i2_orf1	-	-	-	V-type proton ATPase subunit H isoform X3 [Ostrinia furnacalis] >QRR19186.1 V-type proton ATPase subunit H [Ostrinia nubilalis]	1.997728	-0.47704	-0.57972	-0.50745	-0.43352
TRINITY_DN26375_c0_g1_i1_orf1	-	-	-	hypothetical protein O3G_MSEX007366 [Manduca sexta]	1.9375	-0.09399	-0.83085	-0.6371	-0.37556
TRINITY_DN468_c0_g1_i3_orf1	-	-	-	transmembrane protein 41 homolog isoform X2 [Ostrinia furnacalis]	1.94776	-0.4302	-0.90192	-0.40322	-0.21243
TRINITY_DN17825_c1_g1_i1_orf1	-	-	-	39S ribosomal protein L1, mitochondrial [Ostrinia furnacalis]	1.980003	-0.44787	-0.3561	-0.76218	-0.41385
TRINITY_DN2894_c0_g3_i1_orf1	-	-	-	lactase-phlorizin hydrolase-like [Ostrinia furnacalis]	1.997814	-0.53017	-0.42961	-0.47244	-0.56559
TRINITY_DN37538_c0_g3_i1_orf1	-	-	-	esterase FE4-like [Ostrinia furnacalis]	1.952768	-0.83332	-0.55014	-0.16725	-0.40206
TRINITY_DN96566_c0_g1_i1_orf1	-	-	-	NADH-ubiquinone oxidoreductase subunit 8-like [Ostrinia furnacalis]	1.938102	-0.73142	-0.7792	-0.14212	-0.28536
TRINITY_DN64403_c0_g2_i1_orf1	-	-	-	carboxylesterase [Ostrinia furnacalis]	1.900271	-0.80946	-0.63727	-0.56202	0.108477
TRINITY_DN15046_c0_g1_i8_orf1	-	-	-	epidermal retinol dehydrogenase 2-like isoform X1 [Ostrinia furnacalis] >XP_028169999.1 epidermal retinol dehydrogenase 2-like isoform X2 [Ostrinia furnacalis]	1.907397	-0.71469	-0.86215	-0.0023	-0.32826
TRINITY_DN2953_c1_g1_i2_orf1	-	-	-	methionine--tRNA ligase, cytoplasmic isoform X6 [Ostrinia furnacalis]	1.785769	0.386209	-0.96549	-0.63403	-0.57246
TRINITY_DN1318_c0_g1_i5_orf1	-	-	-	uncharacterized protein LOC114360956 [Ostrinia furnacalis]	1.974851	-0.77344	-0.28486	-0.44121	-0.47534
TRINITY_DN9003_c0_g1_i20_orf1	-	-	-	RNA-binding protein Nova-2 isoform X4 [Ostrinia furnacalis]	1.903539	-0.47193	0.053158	-0.89848	-0.58629
TRINITY_DN18172_c0_g1_i6_orf1	-	-	-	digestive cysteine proteinase 2-like [Ostrinia furnacalis]	1.958197	-0.16688	-0.80601	-0.52832	-0.45698
TRINITY_DN6747_c0_g1_i7_orf1	-	-	-	retinol dehydrogenase 12-like [Ostrinia furnacalis]	1.923173	-0.30232	-0.09468	-0.89806	-0.62812
TRINITY_DN29440_c1_g1_i4_orf1	-	-	-	neutral lipase [Helicoverpa armigera]	1.896505	0.04622	-0.8122	-0.33897	-0.79156
TRINITY_DN9715_c0_g1_i1_orf1	-	-	-	V-type proton ATPase subunit E [Manduca sexta] >P31402.1 RecName: Full=V-type proton ATPase subunit E; Short=V-ATPase subunit E; AltName: Full=V-ATPase 26 kDa subunit; AltName: Full=Vacuolar proton pump subunit E [Manduca sexta] >KAG6457535.1 hypothetical protein O3G_MSEX010354 [Manduca sexta] >CAA47610.1 H(+)-transporting ATPase	1.997984	-0.48417	-0.5802	-0.49122	-0.44239
TRINITY_DN7024_c0_g1_i1_orf1	-	-	-	uncharacterized protein LOC114363116 [Ostrinia furnacalis]	1.981293	-0.37766	-0.7576	-0.42524	-0.4208
TRINITY_DN2918_c0_g1_i1_orf1	-	-	-	28S ribosomal protein S10, mitochondrial [Ostrinia furnacalis] >XP_028175147.1 28S ribosomal protein S10, mitochondrial [Ostrinia furnacalis]	1.858295	0.249322	-0.71036	-0.65501	-0.74224
TRINITY_DN7964_c0_g1_i6_orfp1	-	-	-	TRINITY_DN7964_c0_g1_i6_m.23478 TRINITY_DN7964_c0_g1::TRINITY_DN7964_c0_g1_i6::g.23478 ORF type:internal len:87	1.931574	-0.90434	-0.4321	-0.08853	-0.5066

TRINITY_DN16931_c0_g1_i1_orf1	-	-	-	pancreatic triacylglycerol lipase-like [Ostrinia furnacalis]	1.897966	-0.68502	-0.89272	-0.36043	0.040201
TRINITY_DN6059_c0_g1_i1_orf1	-	-	-	brachyurin-like [Ostrinia furnacalis]	1.970749	-0.8181	-0.42347	-0.39363	-0.33555
TRINITY_DN3332_c0_g1_i11_orf1	-	-	-	glutathione S-transferase sigma3 [Glyphodes pyloalis]	1.951476	-0.23028	-0.82308	-0.27884	-0.61928
TRINITY_DN2825_c0_g1_i3_orf1	-	-	-	carbonic anhydrase 2-like [Ostrinia furnacalis]	1.969776	-0.81226	-0.37047	-0.31213	-0.47492
TRINITY_DN46409_c0_g1_i1_orf1	-	-	-	unnamed protein product [Heterotrigona itama]	1.900997	-0.03715	-0.82466	-0.23253	-0.80666
TRINITY_DN35662_c0_g1_i5_orf1	-	-	-	hypothetical protein evm_006436 [Chilo suppressalis] >CAB3522373.1 unnamed protein product [Chilo suppressalis] >CAH0399695.1 unnamed protein product [Chilo suppressalis]	1.971994	-0.17306	-0.62394	-0.55845	-0.61654
TRINITY_DN25779_c0_g1_i6_orf1	-	-	-	aldo-keto reductase AKR2E4-like [Ostrinia furnacalis]	1.571105	0.78433	-0.5977	-0.79402	-0.96371
TRINITY_DN5740_c0_g1_i4_orf1	-	-	-	unconventional myosin IC isoform X1 [Ostrinia furnacalis]	1.9892	-0.43432	-0.6949	-0.40014	-0.45983
TRINITY_DN1249_c0_g1_i10_orf1	-	-	-	venom carboxylesterase-6-like [Ostrinia furnacalis]	1.892155	-0.79563	-0.87169	-0.08405	-0.14079
TRINITY_DN2343_c1_g1_i8_orf1	-	-	-	receptor expression-enhancing protein 5-like isoform X3 [Ostrinia furnacalis]	1.96638	-0.62519	-0.70337	-0.46784	-0.16999
TRINITY_DN4731_c0_g1_i1_orf1	-	-	-	gelsolin-like [Ostrinia furnacalis]	1.980319	-0.59518	-0.67773	-0.43906	-0.26836
TRINITY_DN44288_c0_g1_i2_orf1	-	-	-	ATP-dependent RNA helicase p62 [Ostrinia furnacalis]	1.922906	-0.0317	-0.90017	-0.5001	-0.49093
TRINITY_DN6221_c0_g1_i5_orf1	-	-	-	unnamed protein product [Diatraea saccharalis]	1.981687	-0.71379	-0.35703	-0.35269	-0.55818
TRINITY_DN334_c0_g1_i2_orf1	-	-	-	chymotrypsin-like serine protease, partial [Ostrinia nubilalis]	1.989731	-0.61384	-0.59763	-0.43476	-0.3435
TRINITY_DN29034_c0_g1_i2_orf1	-	-	-	trypsin-like serine protease [Ostrinia nubilalis]	1.955487	-0.86121	-0.23532	-0.50056	-0.3584
TRINITY_DN2267_c0_g1_i1_orf1	-	-	-	hypothetical protein evm_006312 [Chilo suppressalis]	1.99321	-0.56404	-0.36809	-0.45796	-0.60312
TRINITY_DN36494_c0_g1_i1_orf1	-	-	-	MKI67 FHA domain-interacting nucleolar phosphoprotein-like [Ostrinia furnacalis]	1.938281	-0.80786	-0.08124	-0.65389	-0.39529
TRINITY_DN17864_c0_g1_i1_orf1	-	-	-	PREDICTED: erlin-2-B [Microplitis demolitor]	1.9959	-0.51491	-0.45698	-0.60719	-0.41682
TRINITY_DN628_c0_g1_i7_orf1	-	-	-	prostamide/prostaglandin F synthase-like [Ostrinia furnacalis]	1.869528	0.157538	-0.63948	-0.46096	-0.92662
TRINITY_DN72707_c0_g1_i1_orf1	-	-	-	uncharacterized protein LOC114357549 [Ostrinia furnacalis]	1.985059	-0.62902	-0.54051	-0.2678	-0.54773
TRINITY_DN141462_c0_g1_i1_orf1	-	-	-	mitochondrial-processing peptidase subunit beta [Diachasma alloeum] >THK33262.1 core protein 1, ubiquinol-cytochrome c reductase [Diachasma alloeum]	1.987642	-0.59479	-0.29338	-0.5998	-0.49967
TRINITY_DN43942_c0_g2_i1_orf1	-	-	-	LOW QUALITY PROTEIN: caprin homolog [Ostrinia furnacalis]	1.798322	0.321319	-0.55929	-1.0377	-0.52265
TRINITY_DN2083_c0_g1_i4_orf1	-	-	-	uncharacterized protein LOC114359113 [Ostrinia furnacalis]	1.924366	-0.23699	-0.93159	-0.1691	-0.58669
TRINITY_DN79210_c0_g1_i1_orf1	-	-	-	V-type proton ATPase 16 kDa proteolipid subunit [Frieseomelitta varia]	1.995672	-0.45459	-0.58959	-0.40378	-0.54771
TRINITY_DN11259_c0_g1_i1_orf1	-	-	-	uncharacterized protein LOC114357075 [Ostrinia furnacalis]	1.961951	-0.69761	-0.70589	-0.34948	-0.20897
TRINITY_DN5107_c0_g1_i4_orf1	-	-	-	peptide methionine sulfoxide reductase [Ostrinia furnacalis]	1.995711	-0.59403	-0.38881	-0.49801	-0.51486
TRINITY_DN15870_c0_g1_i3_orf1	-	-	-	PREDICTED: mitochondrial import inner membrane translocase subunit Tim23 isoform X1 [Fopius arisanus]	1.984049	-0.25534	-0.54444	-0.61067	-0.5736
TRINITY_DN43611_c0_g1_i1_orf1	-	-	-	39S ribosomal protein L41, mitochondrial [Ostrinia furnacalis]	1.973204	-0.29458	-0.35289	-0.75334	-0.57239
TRINITY_DN2394_c0_g1_i4_orf1	-	-	-	uncharacterized protein LOC114363116 [Ostrinia furnacalis]	1.987408	-0.45122	-0.70814	-0.44802	-0.38002
TRINITY_DN700_c0_g1_i3_orf1	-	-	-	V-type proton ATPase subunit H isoform X1 [Chelonus insularis]	1.978701	-0.31314	-0.74205	-0.39245	-0.53106
TRINITY_DN21722_c0_g1_i3_orf1	-	-	-	V-type proton ATPase 116 kDa subunit a isoform X1 [Ostrinia furnacalis] >XP_028177509.1 V-type proton ATPase 116 kDa subunit a isoform X1 [Ostrinia furnacalis]	1.995745	-0.56958	-0.55946	-0.47805	-0.38866
TRINITY_DN1353_c0_g1_i1_orf1	-	-	-	UDP-glucose 4-epimerase-like [Ostrinia furnacalis]	1.827257	0.306303	-0.89342	-0.61181	-0.62833
TRINITY_DN5891_c0_g2_i4_orf1	-	-	-	amino acid transporter AVT1A-like [Ostrinia furnacalis] >XP_028156666.1 amino acid transporter AVT1A-like [Ostrinia furnacalis]	1.986613	-0.54108	-0.6879	-0.39282	-0.36481
TRINITY_DN16939_c0_g1_i4_orf1	-	-	-	39S ribosomal protein L17, mitochondrial [Ostrinia furnacalis]	1.982964	-0.52425	-0.28812	-0.69677	-0.47382
TRINITY_DN1199_c0_g1_i1_orf1	-	-	-	pupal cuticle protein 36a-like [Ostrinia furnacalis]	1.967032	-0.67603	-0.19955	-0.68413	-0.40732
TRINITY_DN26688_c0_g1_i2_orf1	-	-	-	myogenesis-regulating glycosidase-like [Ostrinia furnacalis]	1.962743	-0.13462	-0.52382	-0.69973	-0.60457
TRINITY_DN78546_c0_g5_i1_orf1	-	-	-	kinesin-like protein KIF13A isoform X9 [Cephus cinctus]	1.694595	-0.11528	0.168593	-1.40265	-0.34526
TRINITY_DN69049_c0_g1_i2_orf1	-	-	-	membrane alanyl aminopeptidase-like [Ostrinia furnacalis]	1.984066	-0.58026	-0.31483	-0.67626	-0.41272
TRINITY_DN57202_c0_g1_i1_orf1	-	-	-	PREDICTED: U4/U6 small nuclear ribonucleoprotein Prp31 [Amyelois transitella]	1.991713	-0.53511	-0.58853	-0.54265	-0.32542
TRINITY_DN713_c0_g1_i4_orf1	-	-	-	periodic tryptophan protein 2 homolog isoform X1 [Ostrinia furnacalis] >XP_028176443.1 periodic tryptophan protein 2 homolog isoform X2 [Ostrinia furnacalis] >XP_028176445.1 periodic tryptophan protein 2 homolog isoform X3 [Ostrinia furnacalis]	1.977189	-0.3122	-0.45243	-0.7697	-0.44286
TRINITY_DN19160_c0_g1_i1_orf1	-	-	-	alkyldihydroxyacetonephosphate synthase [Ostrinia furnacalis]	1.366273	1.066699	-0.71602	-0.79212	-0.92483
TRINITY_DN15380_c0_g1_i1_orf1	-	-	-	39S ribosomal protein L32, mitochondrial [Ostrinia furnacalis]	1.988989	-0.32134	-0.46106	-0.59445	-0.61214
TRINITY_DN3159_c0_g1_i4_orf1	-	-	-	uncharacterized protein LOC114362782 [Ostrinia furnacalis]	1.978573	-0.2439	-0.69738	-0.49189	-0.54541
TRINITY_DN24476_c0_g1_i1_orf1	-	-	-	ensconsin-like isoform X1 [Ostrinia furnacalis]	1.886446	0.060184	-0.32972	-0.70407	-0.91283
TRINITY_DN5459_c0_g1_i1_orf1	-	-	-	protein takeout-like isoform X2 [Ostrinia furnacalis]	1.466891	0.948167	-0.8082	-0.7533	-0.85356
TRINITY_DN108573_c0_g1_i1_orf1	-	-	-	uncharacterized protein LOC114366171 [Ostrinia furnacalis]	1.977792	-0.35394	-0.72267	-0.31878	-0.5824
TRINITY_DN23175_c0_g1_i6_orf1	-	-	-	myb-binding protein 1A-like protein [Ostrinia furnacalis]	1.970421	-0.16271	-0.63043	-0.60495	-0.57232

TRINITY_DN13651_c0_g1_i2_orf1	-	-	-	40S ribosomal protein S12, mitochondrial [Ostrinia furnacalis]	1.925695	-0.06556	-0.40308	-0.9066	-0.55045
TRINITY_DN48410_c0_g2_i1_orf1	-	-	-	alpha-amylase 2-like isoform X3 [Ostrinia furnacalis]	1.989293	-0.57046	-0.64781	-0.37149	-0.39953
TRINITY_DN8116_c0_g1_i2_orf1	-	-	-	uncharacterized protein LOC114350845 [Ostrinia furnacalis]	1.983209	-0.45074	-0.68984	-0.54943	-0.2932
TRINITY_DN3194_c0_g1_i6_orf1	-	-	-	uncharacterized protein LOC114361386 [Ostrinia furnacalis]	1.974152	-0.30561	-0.71996	-0.6176	-0.33097
TRINITY_DN7047_c0_g1_i1_orf1	-	-	-	hypothetical protein G9C98_004728 [Cotesia typhae]	1.957433	-0.13834	-0.67115	-0.71537	-0.43257
TRINITY_DN20682_c0_g2_i1_orf1	-	-	-	glutathione S-transferase delta3 [Glyphodes pyloalis]	1.951257	-0.13403	-0.73886	-0.69294	-0.38543
TRINITY_DN67623_c0_g1_i1_orf1	-	-	-	maltase A1-like [Ostrinia furnacalis]	1.996351	-0.52317	-0.56619	-0.52052	-0.38647
TRINITY_DN27721_c1_g1_i2_orf1	-	-	-	mitochondrial import receptor subunit TOM20 homolog [Ostrinia furnacalis]	1.955031	-0.1406	-0.50552	-0.80597	-0.50294
TRINITY_DN8691_c0_g1_i3_orf1	-	-	-	nucleolin-like [Melitaea cinxia]	1.700847	0.515663	-0.37775	-0.85615	-0.98261
TRINITY_DN334_c0_g1_i1_orf1	-	-	-	putative chymotrypsin 12 [Ostrinia nubilalis]	1.99049	-0.55605	-0.64687	-0.40342	-0.38415
TRINITY_DN19244_c0_g1_i7_orf1	-	-	-	uncharacterized protein LOC114350218 [Ostrinia furnacalis]	1.738553	0.326024	-0.23319	-1.18005	-0.65134
TRINITY_DN96_c0_g1_i1_orf1	-	-	-	collagenase-like [Ostrinia furnacalis]	1.974959	-0.68584	-0.65295	-0.30285	-0.33333
TRINITY_DN311_c0_g1_i4_orfp1	-	-	-	TRINITY_DN311_c0_g1_i4_m.65135 TRINITY_DN311_c0_g1::TRINITY_DN311_c0_g1_i4::g.65135 ORF type:5prime_partial len:126 (+),score=71.21 TRINITY_DN311_c0_g1_i4:1-378(+)	1.858215	-0.44135	-1.14547	-0.09502	-0.17637
TRINITY_DN98091_c0_g1_i3_orf1	-	-	-	UDP-glycosyltransferase UGT40AP2, partial [Ostrinia furnacalis]	1.966706	-0.35773	-0.78802	-0.25859	-0.56237
TRINITY_DN123396_c0_g1_i1_orf1	-	-	-	PREDICTED: delta-1-pyrroline-5-carboxylate dehydrogenase, mitochondrial isoform X1 [Megachile rotundata]	1.983393	-0.38643	-0.33934	-0.55509	-0.70253
TRINITY_DN1310_c0_g1_i4_orf1	-	-	-	trypsin-like isoform X1 [Ostrinia furnacalis] >XP_028159118.1 trypsin-like isoform X2 [Ostrinia furnacalis]	1.979404	-0.40175	-0.76521	-0.35633	-0.45612
TRINITY_DN2894_c0_g1_i2_orf1	-	-	-	myrosinase 1-like isoform X1 [Ostrinia furnacalis]	1.998308	-0.458	-0.45056	-0.55538	-0.53437
TRINITY_DN11376_c0_g2_i1_orf1	-	-	-	cathepsin K-like [Ostrinia furnacalis]	1.98721	-0.45903	-0.70848	-0.37531	-0.44439
TRINITY_DN8116_c0_g1_i1_orf1	-	-	-	uncharacterized protein LOC114350845 [Ostrinia furnacalis]	1.974649	-0.19205	-0.62961	-0.61379	-0.5392
TRINITY_DN51766_c0_g1_i2_orf1	-	-	-	facilitated trehalose transporter Tret1-like [Ostrinia furnacalis]	1.891749	0.146866	-0.63467	-0.77741	-0.62654
TRINITY_DN2178_c0_g1_i1_orf1	-	-	-	carboxypeptidase B-like [Ostrinia furnacalis]	1.953964	-0.49342	-0.85825	-0.1997	-0.40259
TRINITY_DN23570_c0_g1_i2_orf1	-	-	-	putative trypsin 6 [Ostrinia nubilalis]	1.988174	-0.33683	-0.42206	-0.61333	-0.61595
TRINITY_DN7688_c0_g1_i10_orf1	-	-	-	uncharacterized protein LOC114352518 [Ostrinia furnacalis]	1.972317	-0.56143	-0.74564	-0.42618	-0.23907
TRINITY_DN47731_c0_g1_i2_orf1	-	-	-	nucleolar GTP-binding protein 2 [Ostrinia furnacalis]	1.730005	0.5288	-0.87146	-0.64053	-0.74682
TRINITY_DN30663_c0_g1_i1_orf1	-	-	-	surfeit locus protein 6 homolog [Ostrinia furnacalis]	1.950409	-0.70836	-0.27843	-0.20584	-0.75779
TRINITY_DN10792_c0_g2_i5_orf1	-	-	-	uncharacterized protein LOC114366171 [Ostrinia furnacalis]	1.979987	-0.42425	-0.76664	-0.37734	-0.41176
TRINITY_DN6693_c0_g1_i1_orf1	-	-	-	uncharacterized protein LOC114356358 [Ostrinia furnacalis]	1.980128	-0.23384	-0.64743	-0.52201	-0.57684
TRINITY_DN97680_c0_g1_i1_orf1	-	-	-	39S ribosomal protein L52, mitochondrial [Ostrinia furnacalis]	1.897316	-0.13914	-0.23354	-0.47594	-1.0487
TRINITY_DN542_c0_g2_i1_orf1	-	-	-	uncharacterized protein LOC114364889 [Ostrinia furnacalis]	1.986473	-0.38035	-0.71308	-0.47826	-0.41478
TRINITY_DN144807_c0_g1_i1_orf1	-	-	-	hypothetical protein G9C98_004245 [Cotesia typhae]	1.98871	-0.67835	-0.354	-0.51169	-0.44467
TRINITY_DN82320_c0_g1_i2_orf1	-	-	-	glutathione S-transferase sigma3 [Glyphodes pyloalis]	1.870615	-0.42513	-1.07791	0.029075	-0.39665
TRINITY_DN104586_c0_g1_i1_orf1	-	-	-	Chlorophyll a-b binding protein 37, chloroplastic, partial [Trichinella patagoniensis]	1.987045	-0.57764	-0.66251	-0.38187	-0.36503
TRINITY_DN64446_c0_g1_i1_orf1	-	-	-	uncharacterized protein LOC114364307 [Ostrinia furnacalis]	1.948204	-0.20827	-0.55123	-0.86891	-0.31979
TRINITY_DN6074_c0_g1_i1_orf1	-	-	-	uncharacterized protein C1683.06c-like isoform X1 [Ostrinia furnacalis]	1.973805	-0.24458	-0.65832	-0.39857	-0.67233
TRINITY_DN29120_c0_g1_i6_orf1	-	-	-	putative inorganic phosphate cotransporter [Ostrinia furnacalis]	1.85722	0.222754	-0.85027	-0.7199	-0.50981
TRINITY_DN75188_c0_g1_i1_orf1	-	-	-	fatty acid-binding protein 1-like [Ostrinia furnacalis]	1.943343	-0.1572	-0.38483	-0.88609	-0.51523
TRINITY_DN717_c0_g1_i2_orfp1	-	-	-	TRINITY_DN717_c0_g1_i2_m.67915 TRINITY_DN717_c0_g1::TRINITY_DN717_c0_g1_i2::g.67915 ORF type:internal len:868 (+),score=265.71,Collagen PF01391.19 0.11,Collagen|PF01391.19|0.039,Collagen|PF01391.19|0.00054,Collagen|PF01391.19|0.0019,Collagen|PF01391.19|0.0005,Collagen|PF01391.19|9.9e-05,Collagen|PF01391.19|1.7e-07 TRINITY_DN717_c0_g1_i2:3-2603(+)	1.942234	-0.09928	-0.84514	-0.44507	-0.55274
TRINITY_DN30932_c0_g1_i2_orf1	-	-	-	delta(24)-sterol reductase-like isoform X2 [Ostrinia furnacalis]	1.856418	0.178851	-0.98391	-0.50365	-0.54771
TRINITY_DN61112_c0_g1_i4_orfp1	-	-	-	TRINITY_DN61112_c0_g1_i4_m.53012 TRINITY_DN61112_c0_g1::TRINITY_DN61112_c0_g1_i4::g.53012 ORF type:3prime_partial len:87 (-),score=12.29,HMMR_N PF15905.6 0.0011 TRINITY_DN61112_c0_g1_i4:2-232(-)	1.993679	-0.44193	-0.64638	-0.41886	-0.48651
TRINITY_DN3862_c0_g1_i7_orf1	-	-	-	venom acid phosphatase Acph-1-like [Ostrinia furnacalis]	1.986889	-0.45219	-0.43519	-0.71399	-0.38552
TRINITY_DN22983_c0_g1_i2_orfp1	-	-	-	TRINITY_DN22983_c0_g1_i2_m.10495 TRINITY_DN22983_c0_g1::TRINITY_DN22983_c0_g1_i2::g.10495 ORF type:internal len:79 (-),score=15.95,Polyhedrin PF00738.19 7.6e-40 TRINITY_DN22983_c0_g1_i2:2-235(-)	1.985405	-0.35789	-0.38977	-0.69683	-0.54092
TRINITY_DN26408_c0_g1_i7_orf1	-	-	-	venom carboxylesterase-6-like [Ostrinia furnacalis]	1.987474	-0.51641	-0.31081	-0.6625	-0.49775
TRINITY_DN48410_c0_g1_i1_orf1	-	-	-	alpha-amylase 1-like [Ostrinia furnacalis]	1.986149	-0.48513	-0.71452	-0.38938	-0.39711

TRINITY_DN8087_c0_g1_i9_orf1	-	-	-	cysteine-rich with EGF-like domain protein 2 isoform X1 [Ostrinia furnacalis]	1.845929	0.172266	-0.8464	-0.30312	-0.86868
TRINITY_DN1914_c0_g1_i6_orf1	-	-	-	loricrin-like [Ostrinia furnacalis]	1.911713	0.022879	-0.88019	-0.61151	-0.4429
TRINITY_DN61674_c0_g1_i2_orf1	-	-	-	fatty acid-binding protein 1-like [Ostrinia furnacalis]	1.987707	-0.39147	-0.70785	-0.43691	-0.45147
TRINITY_DN5578_c0_g1_i10_orf1	-	-	-	unnamed protein product [Chilo suppressalis]	1.839069	0.175669	-1.01499	-0.33103	-0.66871
TRINITY_DN26010_c0_g1_i2_orf1	-	-	-	cytochrome b-c1 complex subunit 10-like [Ostrinia furnacalis]	1.994807	-0.56741	-0.40555	-0.43271	-0.58913
TRINITY_DN11587_c0_g1_i7_orf1	-	-	-	elongation of very long chain fatty acids protein AAEL008004-like isoform X1 [Danaus plexippus plexippus] >XP_032511006.1 elongation of very long chain fatty acids protein AAEL008004-like isoform X1 [Danaus plexippus plexippus] >XP_032511007.1 elongation of very long chain fatty acids protein AAEL008004-like isoform X1 [Danaus plexippus plexippus] >XP_032511008.1 elongation of very long chain fatty acids protein AAEL008004-like isoform X1 [Danaus plexippus plexippus] >XP_032511009.1 elongation of very long chain fatty acids protein AAEL008004-like isoform X1 [Danaus plexippus plexippus]	1.971153	-0.17991	-0.57335	-0.68583	-0.53207
TRINITY_DN35051_c0_g1_i1_orf1	-	-	-	uncharacterized protein LOC114364307 [Ostrinia furnacalis]	1.982531	-0.49593	-0.69554	-0.51163	-0.27943
TRINITY_DN6140_c0_g3_i3_orf1	-	-	-	CD63 antigen-like [Ostrinia furnacalis]	1.843749	-0.26338	-1.09395	-0.57199	0.085571
TRINITY_DN4612_c0_g1_i1_orf1	-	-	-	uncharacterized protein LOC114362092 [Ostrinia furnacalis]	1.925608	-0.77846	-0.78551	-0.13825	-0.2234
TRINITY_DN4869_c0_g1_i10_orf1	-	-	-	estrogen sulfotransferase-like isoform X1 [Ostrinia furnacalis]	1.991572	-0.45499	-0.67196	-0.45788	-0.40674
TRINITY_DN29018_c0_g1_i4_orf1	-	-	-	prostaglandin reductase 1-like isoform X1 [Ostrinia furnacalis] >XP_028178925.1 prostaglandin reductase 1-like isoform X2 [Ostrinia furnacalis]	1.954029	-0.78229	-0.48451	-0.12124	-0.566
TRINITY_DN2815_c0_g1_i3_orf1	-	-	-	uncharacterized protein LOC114364075 [Ostrinia furnacalis]	1.989243	-0.41253	-0.60386	-0.61845	-0.35439
TRINITY_DN7688_c0_g1_i2_orf1	-	-	-	uncharacterized protein LOC114352518 [Ostrinia furnacalis]	1.96166	-0.607	-0.78937	-0.29935	-0.26594
TRINITY_DN3784_c0_g1_i1_orf1	-	-	-	pancreatic triacylglycerol lipase-like [Ostrinia furnacalis]	1.96291	-0.22673	-0.82106	-0.42073	-0.4944
TRINITY_DN6044_c0_g1_i4_orf1	-	-	-	acyl-CoA-binding protein-like [Ostrinia furnacalis]	1.995938	-0.43251	-0.57675	-0.42349	-0.5632
TRINITY_DN117_c0_g1_i6_orf1	-	-	-	lipase member I-like [Ostrinia furnacalis]	1.891329	-0.03461	-1.03148	-0.31985	-0.50539
TRINITY_DN14679_c0_g1_i1_orf1	-	-	-	hypothetical protein evm_003043 [Chilo suppressalis]	1.955962	-0.53757	-0.85102	-0.27887	-0.2885
TRINITY_DN81803_c0_g2_i1_orf1	-	-	-	cathepsin K-like [Ostrinia furnacalis]	1.936008	-0.11015	-0.89984	-0.48826	-0.43776
TRINITY_DN41086_c0_g1_i4_orf1	-	-	-	collagenase-like [Pectinophora gossypiella]	1.964409	-0.52277	-0.78749	-0.45691	-0.19724

TRINITY_DN22430_c0_g3_i1_orf1	-	-	-	V-type proton ATPase 16 kDa proteolipid subunit c [Homo sapiens] >NP_001685.1 V-type proton ATPase 16 kDa proteolipid subunit c [Homo sapiens] >P27449.1 RecName: Full=V-type proton ATPase 16 kDa proteolipid subunit c; Short=V-ATPase 16 kDa proteolipid subunit c; AltName: Full=Vacuolar proton pump 16 kDa proteolipid subunit c [Homo sapiens] >6WLW_1 The Vo region of human V-ATPase in state 1 (focused refinement) [Homo sapiens] >6WLW_2 The Vo region of human V-ATPase in state 1 (focused refinement) [Homo sapiens] >6WLW_3 The Vo region of human V-ATPase in state 1 (focused refinement) [Homo sapiens] >6WLW_4 The Vo region of human V-ATPase in state 1 (focused refinement) [Homo sapiens] >6WLW_5 The Vo region of human V-ATPase in state 1 (focused refinement) [Homo sapiens] >6WLW_6 The Vo region of human V-ATPase in state 1 (focused refinement) [Homo sapiens] >6WLW_7 The Vo region of human V-ATPase in state 1 (focused refinement) [Homo sapiens] >6WLW_8 The Vo region of human V-ATPase in state 1 (focused refinement) [Homo sapiens] >6WLW_9 The Vo region of human V-ATPase in state 1 (focused refinement) [Homo sapiens] >6WM2_1 Human V-ATPase in state 1 with SidK and ADP [Homo sapiens] >6WM2_2 Human V-ATPase in state 1 with SidK and ADP [Homo sapiens] >6WM2_3 Human V-ATPase in state 1 with SidK and ADP [Homo sapiens] >6WM2_4 Human V-ATPase in state 1 with SidK and ADP [Homo sapiens] >6WM2_5 Human V-ATPase in state 1 with SidK and ADP [Homo sapiens] >6WM2_6 Human V-ATPase in state 1 with SidK and ADP [Homo sapiens] >6WM2_7 Human V-ATPase in state 1 with SidK and ADP [Homo sapiens] >6WM2_8 Human V-ATPase in state 1 with SidK and ADP [Homo sapiens] >6WM2_9 Human V-ATPase in state 1 with SidK and ADP [Homo sapiens] >6WM3_1 Human V-ATPase in state 2 with SidK and ADP [Homo sapiens] >6WM3_2 Human V-ATPase in state 2 with SidK and ADP [Homo sapiens] >6WM3_3 Human V-ATPase in state 2 with SidK and ADP [Homo sapiens] >6WM3_4 Human V-ATPase in state 2 with SidK and ADP [Homo sapiens] >6WM3_5 Human V-ATPase in state 2 with SidK and ADP [Homo sapiens] >6WM3_6 Human V-ATPase in state 2 with SidK and ADP [Homo sapiens] >6WM3_7 Human V-ATPase in state 2 with SidK and ADP [Homo sapiens] >6WM3_8 Human V-ATPase in state 2 with SidK and ADP [Homo sapiens] >6WM3_9 Human V-ATPase in state 2 with SidK and ADP [Homo sapiens] >6WM4_1 Human V-ATPase in state 3 with SidK and ADP [Homo sapiens] >6WM4_2 Human V-ATPase in state 3 with SidK and ADP [Homo sapiens] >6WM4_3 Human V-ATPase in state 3 with SidK and ADP [Homo sapiens] >6WM4_4 Human	1.998363	-0.44	-0.52357	-0.55875	-0.47603
TRINITY_DN348_c0_g2_i3_orf1	-	-	-	pancreatic triacylglycerol lipase-like [Ostrinia furnacalis]	1.97794	-0.35016	-0.77625	-0.42951	-0.42203
TRINITY_DN37538_c0_g1_i1_orf1	-	-	-	esterase FE4-like [Ostrinia furnacalis]	1.984561	-0.49402	-0.71902	-0.34798	-0.42354
TRINITY_DN2490_c0_g2_i1_orfp1	-	-	-	TRINITY_DN2490_c0_g2_i1_m.56872 TRINITY_DN2490_c0_g2::TRINITY_DN2490_c0_g2_i1::g.56872 ORF type:internal len:359 (-	1.960231	-0.22425	-0.8036	-0.58187	-0.35051
TRINITY_DN94755_c0_g1_i5_orfp1	-	-	-	TRINITY_DN94755_c0_g1_i5_m.62794 TRINITY_DN94755_c0_g1::TRINITY_DN94755_c0_g1_i5::g.62794 ORF type:internal len:70 (+),score=5.17 TRINITY_DN94755_c0_g1_i5:2-208(+)	1.999002	-0.52782	-0.47696	-0.53992	-0.4543
TRINITY_DN1249_c0_g1_i6_orf1	-	-	-	venom carboxylesterase-6-like [Ostrinia furnacalis]	1.526086	0.819609	-0.46934	-1.0348	-0.84156
TRINITY_DN6933_c0_g1_i2_orf1	-	-	-	Chlorophyll a-b binding protein 40, chloroplastic [Trichinella nelsoni] >KRY99282.1 Chlorophyll a-b binding protein 40, chloroplastic [Trichinella zimbabwensis]	1.991756	-0.4515	-0.66123	-0.38712	-0.49191
TRINITY_DN4959_c0_g1_i1_orf1	-	-	-	pancreatic triacylglycerol lipase-like [Ostrinia furnacalis]	1.801331	-0.08783	-1.28566	-0.25301	-0.17483
TRINITY_DN39200_c0_g1_i5_orf1	-	-	-	juvenile hormone epoxide hydrolase-like [Ostrinia furnacalis]	1.995743	-0.39473	-0.5244	-0.48062	-0.59599
TRINITY_DN117_c0_g1_i4_orf1	-	-	-	lipase member I-like [Ostrinia furnacalis]	1.856894	0.192203	-0.95772	-0.57903	-0.51235
TRINITY_DN94355_c0_g1_i2_orf1	-	-	-	uncharacterized protein LOC126369488 [Pectinophora gossypiella]	1.886944	-0.51477	0.143415	-0.70593	-0.80966
TRINITY_DN36199_c0_g1_i1_orf1	-	-	-	fatty acid-binding protein 1-like [Ostrinia furnacalis]	1.993144	-0.45163	-0.58609	-0.36935	-0.58609
TRINITY_DN19917_c0_g1_i1_orf1	-	-	-	synaptic vesicle glycoprotein 2B-like isoform X4 [Ostrinia furnacalis]	1.994789	-0.38139	-0.56578	-0.58081	-0.46681
TRINITY_DN144190_c0_g1_i1_orf1	-	-	-	PREDICTED: uncharacterized protein LOC103572804 isoform X2 [Microplitis demolitor]	1.923509	-0.40193	-0.99663	-0.20121	-0.32374
TRINITY_DN15904_c0_g1_i1_orf1	-	-	-	papilin isoform X8 [Ostrinia furnacalis]	1.423344	0.600879	-0.38834	-1.56767	-0.06822
TRINITY_DN32956_c0_g1_i4_orf1	-	-	-	inositol-trisphosphate 3-kinase A isoform X1 [Vanessa tameamea] >XP_047534115.1 inositol-trisphosphate 3-kinase A isoform X1 [Vanessa atalanta] >XP_047534116.1 inositol-trisphosphate 3-kinase A isoform X1 [Vanessa atalanta] >XP_047534117.1 inositol-trisphosphate 3-kinase A isoform X1 [Vanessa atalanta]	0.359123	1.758158	-0.31111	-1.06433	-0.74183
TRINITY_DN21559_c0_g1_i2_orf1	-	-	-	protein bicaudal D [Ostrinia furnacalis]	1.357103	0.897013	-0.38062	-1.41307	-0.46042
TRINITY_DN10745_c0_g1_i14_orf1	-	-	-	septin-1 [Ostrinia furnacalis]	0.638224	1.663254	-0.92319	-0.79901	-0.57928
TRINITY_DN11188_c0_g1_i2_orf1	-	-	-	testin [Ostrinia furnacalis] >XP_028170617.1 testin [Ostrinia furnacalis]	1.232781	0.948468	0.015045	-1.38847	-0.80782

TRINITY_DN670_c0_g1_i3_orf1	-	-	-	myrosinase 1-like [Ostrinia furnacalis]	1.489065	0.865849	-1.11543	-0.51815	-0.72133
TRINITY_DN8553_c0_g1_i4_orf1	-	-	-	coiled-coil-helix-coiled-coil-helix domain-containing protein 7 isoform X2 [Ostrinia	1.35655	1.0587	-0.82544	-1.0115	-0.57831
TRINITY_DN23714_c0_g1_i4_orf1	-	-	-	DNA-directed RNA polymerases I, II, and III subunit RPABC4 [Ostrinia furnacalis]	1.656148	0.408787	-1.02766	-0.02063	-1.01664
TRINITY_DN2638_c0_g1_i7_orf1	-	-	-	structural maintenance of chromosomes protein 1A [Trichoplusia ni]	0.515157	1.654223	-0.19049	-1.03341	-0.94549
TRINITY_DN9455_c0_g1_i6_orf1	-	-	-	uncharacterized protein LOC114360866 isoform X4 [Ostrinia furnacalis]	0.376636	1.785979	-0.99009	-0.60553	-0.56699
TRINITY_DN25534_c0_g1_i1_orf1	-	-	-	venom serine carboxypeptidase-like [Ostrinia furnacalis]	-0.2934	1.975751	-0.71297	-0.35791	-0.61148
TRINITY_DN12576_c0_g1_i2_orf1	-	-	-	eukaryotic translation initiation factor 4E transporter-like isoform X5 [Hyposmocoma	1.094206	1.328885	-0.76307	-1.0258	-0.63422
TRINITY_DN5834_c0_g1_i2_orf1	-	-	-	CD2 antigen cytoplasmic tail-binding protein 2 homolog [Ostrinia furnacalis]	1.492259	0.841694	-1.12309	-0.7929	-0.41796
TRINITY_DN3499_c0_g1_i8_orf1	-	-	-	modular serine protease-like [Ostrinia furnacalis]	0.521181	1.597964	-0.31791	-0.42543	-1.3758
TRINITY_DN6914_c0_g1_i2_orf1	-	-	-	atypical protein kinase C isoform X2 [Spodoptera litura] >XP_035444071.1 LOW QUALITY PROTEIN: atypical protein kinase C-like [Spodoptera frugiperda]	1.34177	1.064055	-1.0519	-0.52798	-0.82595
TRINITY_DN12823_c0_g1_i1_orf1	-	-	-	malectin-A [Ostrinia furnacalis]	1.378858	0.999306	-0.41421	-0.98416	-0.9798
TRINITY_DN25870_c0_g2_i6_orf1	-	-	-	homeobox protein extradenticle isoform X3 [Ostrinia furnacalis]	0.408383	1.580925	0.103649	-1.30428	-0.78868
TRINITY_DN486_c0_g1_i5_orf1	-	-	-	adaptor complexes medium subunit family domain-containing protein [Phthorimaea	0.489813	1.397755	0.256625	-1.54206	-0.60214
TRINITY_DN1641_c0_g1_i8_orf1	-	-	-	rhodanese domain-containing protein CG4456-like isoform X1 [Ostrinia furnacalis]	1.577058	0.675668	-0.82381	-0.29208	-1.13683
TRINITY_DN72859_c0_g1_i1_orf1	-	-	-	hypothetical protein evm_010574 [Chilo suppressalis]	1.474011	0.783815	-0.4993	-1.33611	-0.42241
TRINITY_DN1045_c0_g1_i6_orf1	-	-	-	ornithine decarboxylase 1-like isoform X1 [Ostrinia furnacalis]	1.402767	0.959487	-0.90237	-1.07033	-0.38955
TRINITY_DN2160_c0_g1_i13_orf1	-	-	-	unnamed protein product [Spodoptera exigua]	1.323064	1.083107	-0.91318	-0.99945	-0.49354
TRINITY_DN879_c0_g1_i2_orf1	-	-	-	DNA-directed RNA polymerase I subunit RPA12 [Ostrinia furnacalis]	1.427637	0.832391	-1.25275	-0.19356	-0.81372
TRINITY_DN14987_c0_g1_i3_orf1	-	-	-	hypothetical protein evm_009121 [Chilo suppressalis]	0.87372	1.318664	-0.03401	-1.3684	-0.78997
TRINITY_DN11464_c0_g1_i3_orf1	-	-	-	unnamed protein product [Spodoptera littoralis] >CAH1635924.1 unnamed protein product [Spodoptera littoralis]	1.136185	1.299189	-0.6623	-0.95952	-0.81355
TRINITY_DN2623_c1_g1_i3_orf1	-	-	-	COPII coat assembly protein sec16-like [Ostrinia furnacalis]	0.160976	1.883056	-0.55389	-0.67048	-0.81967
TRINITY_DN4859_c0_g1_i5_orf1	-	-	-	importin-11, partial [Ostrinia furnacalis]	1.426032	0.650881	-0.22973	-1.54968	-0.29751
TRINITY_DN806_c0_g2_i1_orf1	-	-	-	uncharacterized protein LOC114355167 [Ostrinia furnacalis]	-0.12068	1.936238	-0.33807	-0.86252	-0.61497
TRINITY_DN1293_c0_g1_i4_orf1	-	-	-	putative fatty acyl-CoA reductase CG5065 [Ostrinia furnacalis]	-0.03767	1.903802	-0.25839	-0.89027	-0.71748
TRINITY_DN9000_c0_g2_i1_orf1	-	-	-	uncharacterized protein LOC114356585 [Ostrinia furnacalis]	0.325424	1.822517	-0.81099	-0.77121	-0.56574
TRINITY_DN1716_c0_g1_i14_orf1	-	-	-	putative gamma-glutamylcyclotransferase CG2811 isoform X3 [Ostrinia furnacalis]	0.31781	1.767948	-1.05244	-0.25989	-0.77343
TRINITY_DN28428_c0_g1_i2_orf1	-	-	-	unnamed protein product [Chrysodeixis includens]	0.939555	1.270701	-0.23749	-1.48651	-0.48625
TRINITY_DN19923_c0_g1_i1_orf1	-	-	-	uncharacterized protein LOC114350958 [Ostrinia furnacalis]	-0.02453	1.940621	-0.71441	-0.57856	-0.62312
TRINITY_DN1601_c0_g1_i4_orf1	-	-	-	cytoplasmic dynein 1 intermediate chain isoform X4 [Ostrinia furnacalis]	0.952128	1.433488	-1.08778	-0.56795	-0.72988
TRINITY_DN48460_c0_g1_i1_orf1	-	-	-	PREDICTED: signal recognition particle 54 kDa protein [Fopius arisanus]	1.392189	0.736899	-0.17105	-1.51408	-0.44395
TRINITY_DN172_c8_g2_i1_orf1	-	-	-	sialin [Ostrinia furnacalis]	0.197874	1.558338	0.415998	-1.07822	-1.09399
TRINITY_DN3244_c0_g1_i4_orf1	-	-	-	protein ABHD16A isoform X1 [Ostrinia furnacalis] >XP_028156316.1 protein ABHD16A isoform X2 [Ostrinia furnacalis]	1.604509	0.584291	-1.29748	-0.48728	-0.40403
TRINITY_DN1831_c0_g1_i3_orf1	-	-	-	hypothetical protein O3G_MSEX014886 [Manduca sexta]	1.141982	1.241586	-0.74571	-0.46048	-1.17738
TRINITY_DN5182_c0_g1_i5_orf1	-	-	-	rab proteins geranylgeranyltransferase component A 1 isoform X1 [Ostrinia furnacalis]	0.251994	1.844142	-0.91482	-0.56843	-0.61289
TRINITY_DN20067_c0_g1_i6_orf1	-	-	-	hypothetical protein evm_010712 [Chilo suppressalis] >CAB3527462.1 unnamed protein product [Chilo suppressalis] >CAH0401768.1 unnamed protein product [Chilo suppressalis]	0.996325	1.407006	-1.00087	-0.84697	-0.55549
TRINITY_DN4159_c1_g1_i1_orf1	-	-	-	F-actin-capping protein subunit beta [Ostrinia furnacalis]	1.214342	1.029923	-0.02544	-1.14401	-1.07482
TRINITY_DN1181_c0_g1_i1_orf1	-	-	-	cytochrome c oxidase assembly factor 5 [Ostrinia furnacalis]	1.418688	0.517596	-1.46781	0.242852	-0.71133
TRINITY_DN972_c0_g1_i6_orf1	-	-	-	DNA damage-binding protein 1 [Ostrinia furnacalis]	1.51473	0.757286	-0.24583	-1.11048	-0.91571
TRINITY_DN12820_c0_g1_i1_orf1	-	-	-	chromodomain-helicase-DNA-binding protein 7 [Ostrinia furnacalis] >XP_028176739.1 chromodomain-helicase-DNA-binding protein 7 [Ostrinia furnacalis]	1.454588	0.840667	-0.76449	-1.2244	-0.30637
TRINITY_DN668_c0_g1_i4_orf1	-	-	-	fatty acid synthase-like isoform X1 [Ostrinia furnacalis]	-0.11183	1.958409	-0.5165	-0.68499	-0.64509
TRINITY_DN778_c0_g1_i1_orf1	-	-	-	uncharacterized protein LOC114363281 [Ostrinia furnacalis]	-0.09954	1.938478	-0.56621	-0.86207	-0.41067
TRINITY_DN5568_c0_g2_i2_orf1	-	-	-	carboxypeptidase D isoform X5 [Ostrinia furnacalis]	0.238366	1.837271	-0.71493	-0.93602	-0.42469
TRINITY_DN2172_c0_g2_i8_orf1	-	-	-	hypothetical protein evm_003685 [Chilo suppressalis]	0.860217	1.208703	0.100315	-1.55163	-0.6176
TRINITY_DN7247_c0_g1_i7_orf1	-	-	-	pyruvate kinase-like isoform X3 [Ostrinia furnacalis]	1.446846	0.804457	-0.48635	-1.36488	-0.40007
TRINITY_DN8659_c0_g1_i1_orf1	-	-	-	ubiquitin-like modifier-activating enzyme 1 [Manduca sexta]	0.897387	1.478451	-0.50245	-0.96522	-0.90817
TRINITY_DN13139_c0_g1_i1_orf1	-	-	-	AP-1 complex subunit mu-1 [Ostrinia furnacalis]	1.519917	0.842332	-1.03103	-0.79058	-0.54064
TRINITY_DN4012_c0_g4_i2_orf1	-	-	-	uncharacterized protein LOC114362418 [Ostrinia furnacalis]	1.411589	0.841858	-1.36274	-0.59526	-0.29545
TRINITY_DN14298_c0_g1_i1_orf1	-	-	-	kinesin heavy chain [Ostrinia furnacalis]	0.998781	1.297301	-0.26447	-1.32058	-0.71104
TRINITY_DN36006_c0_g1_i5_orf1	-	-	-	pro-resilin-like [Ostrinia furnacalis]	1.283832	0.719378	-0.49396	-1.60649	0.097246

TRINITY_DN25136_c0_g1_i1_orf1	-	-	-	jg9843 [Pararge aegeria aegeria]	1.683156	0.213407	-1.15285	0.135994	-0.8797
TRINITY_DN59291_c0_g1_i1_orf1	-	-	-	ATP-dependent RNA helicase vasa [Ostrinia furnacalis]	1.175922	1.019044	-0.16122	-1.50916	-0.52459
TRINITY_DN23790_c0_g1_i1_orf1	-	-	-	wiskott-Aldrich syndrome protein family member 2 [Ostrinia furnacalis]	1.373482	0.893548	-0.40505	-1.38792	-0.47406
TRINITY_DN89829_c0_g1_i1_orf1	-	-	-	PREDICTED: ubiquitin-conjugating enzyme E2 T [Microplitis demolitor]	1.335352	1.021903	-0.37852	-1.17854	-0.80019
TRINITY_DN1921_c1_g1_i5_orf1	-	-	-	hypothetical protein evm_002627 [Chilo suppressalis] >CAB3527269.1 unnamed protein product [Chilo suppressalis]	1.225748	1.218235	-0.93164	-0.72536	-0.78699
TRINITY_DN143497_c0_g1_i1_orf1	-	-	-	fibroin heavy chain-like [Ostrinia furnacalis]	-0.1805	1.970354	-0.57436	-0.69863	-0.51687
TRINITY_DN661_c1_g2_i1_orf1	-	-	-	larval/pupal cuticle protein H1C-like [Ostrinia furnacalis]	0.022991	1.910214	-0.48752	-0.90706	-0.53862
TRINITY_DN9536_c0_g1_i4_orf1	-	-	-	adrenodoxin-like protein, mitochondrial isoform X1 [Ostrinia furnacalis]	0.056315	1.901412	-0.58745	-0.46431	-0.90597
TRINITY_DN5840_c0_g1_i6_orf1	-	-	-	catenin alpha isoform X2 [Ostrinia furnacalis]	0.458091	1.751166	-0.50092	-0.77218	-0.93616
TRINITY_DN446_c0_g1_i20_orf1	-	-	-	uncharacterized protein LOC114356043 [Ostrinia furnacalis]	1.718844	-0.04438	-0.96193	0.301172	-1.0137
TRINITY_DN64627_c0_g1_i1_orf1	-	-	-	probable 3-hydroxyacyl-CoA dehydrogenase B0272.3 [Ostrinia furnacalis]	1.505212	0.745113	-0.64561	-0.31513	-1.28958
TRINITY_DN1552_c0_g1_i3_orf1	-	-	-	casein kinase II subunit alpha isoform X3 [Galleria mellonella]	1.196545	1.181277	-0.36218	-0.93613	-1.07952
TRINITY_DN6642_c0_g2_i1_orf1	-	-	-	protein purity of essence [Ostrinia furnacalis]	1.504287	0.664143	-0.0278	-1.11562	-1.02502
TRINITY_DN9146_c0_g1_i1_orf1	-	-	-	drebrin-like protein [Ostrinia furnacalis]	1.224895	1.213213	-0.66325	-0.96737	-0.80749
TRINITY_DN6202_c0_g1_i2_orf1	-	-	-	PREDICTED: serine/threonine-protein phosphatase PP1-beta catalytic subunit [Papilio polytes] >XP_013173027.1 PREDICTED: serine/threonine-protein phosphatase PP1-beta catalytic subunit [Papilio xuthus] >XP_013196427.1 PREDICTED: serine/threonine-protein phosphatase PP1-beta catalytic subunit isoform X1 [Amyelois transitella] >XP_014358529.1 serine/threonine-protein phosphatase PP1-beta catalytic subunit isoform X2 [Papilio machaon] >XP_021196714.1 serine/threonine-protein phosphatase PP1-beta catalytic subunit isoform X3 [Helicoverpa armigera] >XP_022826582.1 serine/threonine-protein phosphatase PP1-beta catalytic subunit [Spodoptera litura] >XP_023952136.1 serine/threonine-protein phosphatase PP1-beta catalytic subunit isoform X2 [Bicyclus anynana] >XP_026730418.1 serine/threonine-protein phosphatase PP1-beta catalytic subunit [Trichoplusia ni] >XP_028156492.1 serine/threonine-protein phosphatase PP1-beta catalytic subunit [Ostrinia furnacalis] >XP_030022932.1 serine/threonine-protein phosphatase PP1-beta catalytic subunit [Manduca sexta] >XP_034832911.1 serine/threonine-protein phosphatase PP1-beta catalytic subunit isoform X2 [Maniola hyperantus] >XP_039756948.1 serine/threonine-protein phosphatase PP1-beta catalytic subunit [Pararge aegeria] >XP_045452222.1 serine/threonine-protein phosphatase PP1-beta catalytic subunit [Melitaea cinxia] >XP_045773496.1 serine/threonine-protein phosphatase PP1-beta catalytic subunit isoform X2 [Maniola jurtina] >XP_047028552.1 serine/threonine-protein phosphatase PP1-beta catalytic subunit [Helicoverpa zea] >XP_047997696.1 serine/threonine-protein phosphatase PP1-beta catalytic subunit isoform X1 [Leguminivora glycinivorella] >CAB3247342.1 unnamed protein product [Arctia plantaginis] >CAB3522774.1 unnamed protein product [Chilo suppressalis] >CAD0197791.1 unnamed protein product [Chrysodeixis includens] >CAG4945530.1 unnamed protein product [Parnassius apollo] >CAG9749509.1 unnamed protein product [Diatraea saccharalis] >CAH0686192.1 unnamed protein product [Spodoptera exigua] >CAH0727047.1 unnamed protein product, partial [Brenthis ino] >CAH2090384.1 unnamed protein product	1.051464	1.332734	-0.90241	-0.41992	-1.06187
TRINITY_DN18912_c1_g1_i1_orf1	-	-	-	engulfment and cell motility protein 1 [Ostrinia furnacalis]	1.574041	0.75227	-0.77835	-1.05017	-0.49779
TRINITY_DN3472_c1_g1_i4_orf1	-	-	-	Krueppel homolog 2-like [Ostrinia furnacalis]	1.182747	1.068926	-0.46063	-1.46227	-0.32878
TRINITY_DN662_c0_g1_i1_orf1	-	-	-	PREDICTED: actin-related protein 2 [Amyelois transitella]	1.417551	0.874479	-0.63103	-0.35712	-1.30388
TRINITY_DN383_c0_g1_i1_orf1	-	-	-	probable Golgi SNAP receptor complex member 2 [Ostrinia furnacalis]	1.337634	1.012901	-0.75235	-1.2124	-0.38579
TRINITY_DN43293_c0_g1_i2_orf1	-	-	-	egl nine homolog 1 isoform X2 [Helicoverpa armigera]	1.327775	0.481529	-0.70579	-1.52595	0.422445
TRINITY_DN14443_c0_g1_i1_orf1	-	-	-	SUMO-activating enzyme subunit 2 [Ostrinia furnacalis]	0.66177	1.607149	-0.40322	-1.12795	-0.73775
TRINITY_DN1405_c0_g1_i1_orf1	-	-	-	cyclin-dependent kinase 10 isoform X1 [Ostrinia furnacalis] >XP_028178194.1 cyclin-dependent kinase 10 isoform X2 [Ostrinia furnacalis]	0.835618	1.46784	-0.302	-1.16362	-0.83784
TRINITY_DN20215_c0_g2_i1_orf1	-	-	-	unnamed protein product [Spodoptera littoralis] >CAH1638553.1 unnamed protein product [Spodoptera littoralis]	1.002796	1.36626	-0.72662	-0.46698	-1.17545
TRINITY_DN3450_c0_g1_i3_orf1	-	-	-	hypothetical protein evm_008214 [Chilo suppressalis]	1.558584	0.453609	-0.60096	-1.41559	0.004353
TRINITY_DN2936_c0_g1_i1_orf1	-	-	-	myosin heavy chain, non-muscle isoform X1 [Hyposmocoma kahamanoa]	0.684813	1.6374	-0.69533	-0.96023	-0.66665

TRINITY_DN23946_c0_g1_i1_orf1	-	-	-	ubiquitin-conjugating enzyme E2L [Bombyx mori] >XP_013145013.1 PREDICTED: ubiquitin-conjugating enzyme E2 L3 [Papilio polytes] >XP_013145026.1 PREDICTED: ubiquitin-conjugating enzyme E2 L3 [Papilio polytes] >XP_013167448.1 PREDICTED: ubiquitin-conjugating enzyme E2 L3 [Papilio xuthus] >XP_013167449.1 PREDICTED: ubiquitin-conjugating enzyme E2 L3 [Papilio xuthus] >XP_014356324.1 ubiquitin-conjugating enzyme E2 L3 [Papilio machaon] >XP_021182538.1 ubiquitin-conjugating enzyme E2 L3 [Helicoverpa armigera] >XP_022831771.1 ubiquitin-conjugating enzyme E2 L3 [Spodoptera litura] >XP_023943862.1 ubiquitin-conjugating enzyme E2 L3 [Bicyclus anynana] >XP_026501320.1 ubiquitin-conjugating enzyme E2 L3 [Vanessa tameamea] >XP_026740672.1 ubiquitin-conjugating enzyme E2 L3 [Trichoplusia ni] >XP_030021931.1 ubiquitin-conjugating enzyme E2 L3 [Manduca sexta] >XP_035448309.1 ubiquitin-conjugating enzyme E2 L3 [Spodoptera frugiperda] >XP_038214502.1 ubiquitin-conjugating enzyme E2 L3 [Zerene cesonia] >XP_045453873.1 ubiquitin-conjugating enzyme E2 L3 [Melitaea cinxia] >XP_045503683.1 ubiquitin-conjugating enzyme E2 L3 [Colias croceus] >XP_045771134.1 ubiquitin-conjugating enzyme E2 L3 [Maniola jurtina] >XP_046963941.1 ubiquitin-conjugating enzyme E2 L3 [Vanessa cardui] >XP_047032812.1 ubiquitin-conjugating enzyme E2 L3 [Helicoverpa zea] >XP_047538650.1 ubiquitin-conjugating enzyme E2 L3 [Vanessa atalanta] >XP_050352992.1 ubiquitin-conjugating enzyme E2 L3 [Nymphalis io] >KAF9417333.1 hypothetical protein HW555_005549 [Spodoptera exigua] >CAB3511518.1 unnamed protein product [Spodoptera littoralis] >CAH0595358.1 unnamed protein product [Chrysodeixis includens] >ABB36655.1 ubiquitin-conjugating enzyme E2I [Bombyx mori] >ABF51360.1 ubiquitin-conjugating enzyme	1.258494	1.188419	-0.87165	-0.74667	-0.82859
TRINITY_DN4898_c0_g1_i7_orf1	-	-	-	annulin-like isoform X3 [Ostrinia furnacalis]	0.245659	1.856475	-0.59439	-0.7939	-0.71384
TRINITY_DN28759_c0_g1_i1_orf1	-	-	-	innexin inx2 [Ostrinia furnacalis]	0.989019	1.393401	-0.89707	-1.03628	-0.44907
TRINITY_DN26293_c0_g1_i4_orf1	-	-	-	UDP-glucose 6-dehydrogenase [Ostrinia furnacalis]	0.434403	1.724338	-1.07776	-0.32587	-0.75512
TRINITY_DN2542_c0_g2_i1_orf1	-	-	-	peroxiredoxin-2-like [Ostrinia furnacalis]	1.383687	0.61003	-1.61404	-0.05552	-0.32416
TRINITY_DN48713_c0_g1_i1_orf1	-	-	-	sphingomyelin phosphodiesterase 4 [Ostrinia furnacalis]	1.478706	0.293763	-1.49037	0.341726	-0.62383
TRINITY_DN2591_c0_g1_i4_orf1	-	-	-	26S proteasome non-ATPase regulatory subunit 13 isoform X1 [Ostrinia furnacalis]	0.385749	1.699011	-0.19297	-0.68333	-1.20846
TRINITY_DN1763_c0_g3_i2_orf1	-	-	-	heterogeneous nuclear ribonucleoprotein H-like isoform X2 [Ostrinia furnacalis]	1.225346	0.840714	-0.90741	-1.38465	0.226002
TRINITY_DN146493_c0_g1_i1_orf1	-	-	-	anaphase-promoting complex subunit 1 [Chelonus insularis]	1.132542	0.899999	0.240489	-1.50133	-0.7717
TRINITY_DN45859_c0_g1_i1_orf1	-	-	-	nuclear valosin-containing protein-like [Ostrinia furnacalis]	1.554364	0.606929	-1.383	-0.38532	-0.39297
TRINITY_DN21559_c0_g2_i1_orf1	-	-	-	protein bicaudal D isoform X3 [Galleria mellonella]	1.185859	1.244107	-1.03746	-0.70237	-0.69014
TRINITY_DN1691_c0_g1_i3_orf1	-	-	-	uncharacterized protein LOC114354192 isoform X2 [Ostrinia furnacalis]	1.29639	1.146355	-0.85313	-0.87839	-0.71122
TRINITY_DN147458_c0_g1_i1_orf1	-	-	-	60S ribosomal protein L5, partial [Cotesia chilonis]	0.258673	1.777832	-0.30228	-1.16482	-0.56941
TRINITY_DN934_c2_g1_i7_orf1	-	-	-	ubiquitin-40S ribosomal protein S27a [Ostrinia furnacalis]	1.212319	1.168466	-0.45237	-1.18886	-0.73956
TRINITY_DN22156_c0_g1_i1_orf1	-	-	-	trafficking protein particle complex subunit 8 [Ostrinia furnacalis]	1.415701	0.798494	-1.03108	-1.13708	-0.04604
TRINITY_DN1280_c0_g1_i1_orf1	-	-	-	coronin-7 isoform X1 [Ostrinia furnacalis] >XP_028164815.1 coronin-7 isoform X2 [Ostrinia furnacalis] >XP_028164817.1 coronin-7 isoform X3 [Ostrinia furnacalis] >XP_028164818.1 coronin-7 isoform X4 [Ostrinia furnacalis] >XP_028164820.1 coronin-7 isoform X6 [Ostrinia furnacalis] >XP_028164821.1 coronin-7 isoform X7 [Ostrinia furnacalis] >XP_028164822.1 coronin-7 isoform X1 [Ostrinia furnacalis] >XP_028164823.1 coronin-7 isoform X8 [Ostrinia furnacalis] >XP_028164824.1 coronin-7 isoform X9 [Ostrinia furnacalis] >XP_028164825.1 coronin-7 isoform X10 [Ostrinia furnacalis]	0.478075	1.754352	-0.59921	-0.83833	-0.79488
TRINITY_DN123184_c0_g1_i1_orf1	-	-	-	double-strand break repair protein MRE11 [Ostrinia furnacalis]	0.366467	1.614151	0.097984	-1.25182	-0.82679
TRINITY_DN42205_c0_g1_i4_orf1	-	-	-	eukaryotic translation initiation factor 4H [Ostrinia furnacalis]	1.514237	0.86669	-0.90054	-0.89702	-0.58337
TRINITY_DN46_c0_g1_i2_orf1	-	-	-	disks large 1 tumor suppressor protein isoform X12 [Ostrinia furnacalis]	1.488182	0.827542	-0.55581	-0.53145	-1.22846
TRINITY_DN10385_c0_g1_i5_orf1	-	-	-	unnamed protein product [Arctia plantaginis] >CAB3259747.1 unnamed protein product [Arctia plantaginis]	1.105357	1.191962	-0.41251	-1.39572	-0.48909
TRINITY_DN4156_c0_g1_i2_orf1	-	-	-	calcium channel flower [Ostrinia furnacalis]	0.861194	1.399018	-0.3001	-1.36085	-0.59926
TRINITY_DN471_c0_g1_i6_orf1	-	-	-	ATP-dependent RNA helicase DBP2-A-like [Ostrinia furnacalis]	1.083444	1.237576	-0.51387	-0.4577	-1.34944
TRINITY_DN47914_c0_g2_i1_orf1	-	-	-	UBX domain-containing protein 1-A-like [Ostrinia furnacalis]	0.728745	1.549012	-0.29037	-0.92184	-1.06555
TRINITY_DN14477_c0_g1_i12_orf1	-	-	-	PREDICTED: phosphoribosyl pyrophosphate synthase-associated protein 2 isoform X2 [Amyelois transitella]	1.168859	1.222454	-1.19129	-0.59065	-0.60937
TRINITY_DN28622_c0_g1_i1_orf1	-	-	-	actin-related protein 3 [Ostrinia furnacalis]	1.066131	1.314117	-0.5185	-1.19026	-0.67149
TRINITY_DN51737_c0_g1_i3_orf1	-	-	-	N-alpha-acetyltransferase 40 [Ostrinia furnacalis]	0.97785	1.439202	-0.84666	-0.89151	-0.67888

TRINITY_DN376_c0_g1_i1_orf1	-	-	-	thioredoxin reductase 1, mitochondrial isoform X4 [Helicoverpa zea]	1.542361	0.587772	-1.28594	-0.05763	-0.78656
TRINITY_DN122867_c1_g1_i1_orf1	-	-	-	nuclear migration protein nudC [Ostrinia furnacalis]	1.424663	0.91471	-1.07809	-0.92724	-0.33404
TRINITY_DN1425_c0_g1_i4_orf1	-	-	-	fibulin-2-like [Ostrinia furnacalis]	1.118855	1.093038	-0.37087	-1.52101	-0.32001
TRINITY_DN2450_c0_g1_i6_orf1	-	-	-	oxysterol-binding protein-related protein 9 [Manduca sexta]	0.743414	1.6106	-0.73898	-0.84653	-0.7685
TRINITY_DN650_c0_g1_i3_orf1	-	-	-	chitinase 7 [Glyphodes pyloalis]	0.281018	1.806545	-0.9787	-0.76033	-0.34854
TRINITY_DN17935_c0_g1_i1_orf1	-	-	-	NEDD8-conjugating enzyme Ubc12 [Ostrinia furnacalis]	1.466368	0.900375	-0.95149	-0.96481	-0.45045
TRINITY_DN8958_c0_g1_i1_orf1	-	-	-	nuclear cap-binding protein subunit 1 [Galleria mellonella]	1.386137	0.660068	-0.2222	-1.59393	-0.23007
TRINITY_DN35633_c0_g2_i1_orf1	-	-	-	uncharacterized protein LOC114353024 [Ostrinia furnacalis]	0.623276	1.678998	-0.88685	-0.66148	-0.75394
TRINITY_DN121047_c0_g1_i3_orf1	-	-	-	unnamed protein product [Parnassius apollo]	1.671434	0.539199	-0.78636	-0.33601	-1.08826
TRINITY_DN24218_c0_g1_i1_orf1	-	-	-	uncharacterized protein LOC114362624 [Ostrinia furnacalis]	0.256864	1.48548	0.3872	-1.45845	-0.67109
TRINITY_DN113778_c0_g2_i1_orf1	-	-	-	metastasis-associated protein MTA3 [Galleria mellonella]	0.814929	1.263522	0.087992	-1.52193	-0.64452
TRINITY_DN5235_c0_g1_i7_orf1	-	-	-	peptidoglycan-recognition protein SA-like [Ostrinia furnacalis]	0.462614	1.73819	-0.99782	-0.75224	-0.45074
TRINITY_DN518_c0_g1_i1_orf1	-	-	-	unnamed protein product [Arctia plantaginis]	1.251318	1.167504	-0.5892	-1.07328	-0.75634
TRINITY_DN24539_c0_g1_i4_orf1	-	-	-	low molecular weight phosphotyrosine protein phosphatase 1-like isoform X2 [Ostrinia	1.065882	1.253191	-0.28849	-0.74276	-1.28782
TRINITY_DN2984_c0_g1_i3_orf1	-	-	-	connectin-like [Ostrinia furnacalis]	1.182469	1.221685	-0.51243	-1.11521	-0.77651
TRINITY_DN13018_c0_g1_i1_orf1	-	-	-	uncharacterized protein LOC114354768 isoform X2 [Ostrinia furnacalis]	1.497021	0.586323	-1.45218	-0.08412	-0.54705
TRINITY_DN10174_c0_g1_i4_orf1	-	-	-	protein D2-like isoform X2 [Aricia agestis] >XP_041972210.1 protein D2-like isoform X2 [Aricia agestis]	0.917832	1.343357	-1.3301	-0.19137	-0.73971
TRINITY_DN1935_c0_g1_i1_orf1	-	-	-	adult-specific cuticular protein ACP-22-like [Ostrinia furnacalis]	-0.22591	1.978837	-0.50828	-0.61132	-0.63334
TRINITY_DN23616_c0_g1_i4_orf1	-	-	-	ribosome biogenesis protein NSA2 homolog [Ostrinia furnacalis] >CAG9749295.1 unnamed protein product [Diatraea saccharalis] >CAG9787980.1 unnamed protein product [Diatraea saccharalis]	1.622234	0.352933	-1.08997	0.133584	-1.01878
TRINITY_DN1661_c0_g1_i1_orf1	-	-	-	NAD(P) transhydrogenase, mitochondrial-like [Ostrinia furnacalis] >XP_028175067.1 NAD(P) transhydrogenase, mitochondrial-like [Ostrinia furnacalis] >XP_028175068.1 NAD(P) transhydrogenase, mitochondrial-like [Ostrinia furnacalis] >XP_028175069.1 NAD(P) transhydrogenase, mitochondrial-like [Ostrinia furnacalis]	1.187432	1.176937	-0.54466	-1.26517	-0.55454
TRINITY_DN1231_c0_g1_i4_orf1	-	-	-	AN1-type zinc finger protein 6 isoform X1 [Galleria mellonella]	0.546836	1.664292	-0.80614	-1.08606	-0.31893
TRINITY_DN7603_c0_g1_i5_orf1	-	-	-	tetratricopeptide repeat protein 1-like [Ostrinia furnacalis]	0.87622	1.463483	-0.387	-1.10704	-0.84567
TRINITY_DN12064_c0_g2_i1_orf1	-	-	-	ras-related protein Rab-35 [Ostrinia furnacalis]	1.074992	0.920412	-0.10127	-0.1747	-1.71944
TRINITY_DN397_c0_g1_i1_orf1	-	-	-	striatin-3 isoform X1 [Ostrinia furnacalis]	1.167914	1.224992	-0.56959	-1.18433	-0.63898
TRINITY_DN43076_c0_g1_i6_orf1	-	-	-	protein argonaute-2 isoform X2 [Pectinophora gossypiella]	1.302492	0.999972	-0.23183	-1.2656	-0.80504
TRINITY_DN978_c9_g2_i1_orf1	-	-	-	hypothetical protein evm_000959 [Chilo suppressalis]	0.266385	1.840747	-0.54388	-0.88887	-0.67438
TRINITY_DN12858_c0_g1_i5_orf1	-	-	-	unnamed protein product, partial [Iphiclides podalirius]	0.996824	1.355303	-0.34475	-1.13759	-0.86978
TRINITY_DN4808_c0_g1_i3_orf1	-	-	-	kinesin light chain [Ostrinia furnacalis]	1.050497	1.282416	-0.24822	-1.1353	-0.94939
TRINITY_DN14298_c0_g3_i1_orf1	-	-	-	kinesin heavy chain [Ostrinia furnacalis]	0.914473	1.25916	-0.05341	-1.46492	-0.6553
TRINITY_DN54366_c0_g1_i1_orf1	-	-	-	protein obstructor-E-like [Ostrinia furnacalis]	0.095282	1.908173	-0.68469	-0.73496	-0.5838
TRINITY_DN53311_c0_g2_i1_orf1	-	-	-	transcription elongation factor S-II [Chelonus insularis]	1.104787	1.279033	-0.89678	-1.08533	-0.40171
TRINITY_DN20793_c0_g2_i1_orf1	-	-	-	mucin-2-like [Ostrinia furnacalis]	-0.05511	1.947984	-0.57519	-0.61807	-0.69962
TRINITY_DN12771_c0_g1_i1_orf1	-	-	-	histone acetyltransferase type B catalytic subunit [Ostrinia furnacalis]	0.732459	1.436408	-0.82605	-0.03254	-1.31027
TRINITY_DN19810_c1_g1_i7_orf1	-	-	-	RNA-binding protein spenito [Ostrinia furnacalis] >XP_028167555.1 RNA-binding protein spenito [Ostrinia furnacalis]	1.037698	1.184461	-0.21075	-1.48159	-0.52981
TRINITY_DN4533_c0_g1_i1_orf1	-	-	-	neurofilament heavy polypeptide-like isoform X2 [Ostrinia furnacalis]	1.202263	1.071895	-0.30206	-1.41604	-0.55606
TRINITY_DN3649_c0_g1_i6_orf1	-	-	-	unnamed protein product [Chilo suppressalis]	1.180877	0.792964	-0.43777	-1.66395	0.12788
TRINITY_DN44256_c0_g1_i1_orf1	-	-	-	essential MCU regulator, mitochondrial [Cotesia glomerata]	0.612703	1.636405	-0.78491	-1.09179	-0.37241
TRINITY_DN7560_c0_g1_i4_orf1	-	-	-	unnamed protein product [Chilo suppressalis]	1.367366	0.840052	-0.00556	-1.11664	-1.08522
TRINITY_DN2475_c0_g2_i1_orf1	-	-	-	uncharacterized protein LOC114363767 [Ostrinia furnacalis]	1.264508	1.1455	-1.0831	-0.53041	-0.79649
TRINITY_DN2054_c0_g1_i1_orf1	-	-	-	macrophage mannose receptor 1-like [Ostrinia furnacalis]	0.494336	1.591739	0.009652	-0.93413	-1.1616
TRINITY_DN13395_c0_g1_i1_orf1	-	-	-	cytoplasmic dynein 1 light intermediate chain 2 [Galleria mellonella]	0.402973	1.781989	-0.52018	-0.7786	-0.88618
TRINITY_DN286_c0_g1_i2_orf1	-	-	-	uncharacterized protein LOC114361329 [Ostrinia furnacalis]	0.556365	1.692853	-1.04568	-0.66096	-0.54258
TRINITY_DN44792_c0_g1_i1_orf1	-	-	-	parafibromin [Ostrinia furnacalis]	0.50034	1.557891	-1.18979	0.080532	-0.94898
TRINITY_DN9062_c0_g2_i3_orf1	-	-	-	ubiquitin conjugation factor E4 B isoform X2 [Ostrinia furnacalis]	1.235621	1.204999	-0.73534	-0.96776	-0.73752
TRINITY_DN942_c0_g1_i1_orf1	-	-	-	probable nuclear transport factor 2 isoform X1 [Ostrinia furnacalis]	1.136454	1.274186	-0.69892	-1.11222	-0.59949
TRINITY_DN467_c0_g3_i1_orf1	-	-	-	histone-lysine N-methyltransferase 2B-like, partial [Ostrinia furnacalis]	0.025796	1.923982	-0.57397	-0.79222	-0.58359
TRINITY_DN2101_c0_g1_i6_orf1	-	-	-	protein obstructor-E-like [Ostrinia furnacalis]	-0.17414	1.964409	-0.48073	-0.76018	-0.54936

TRINITY_DN72816_c0_g1_i2_orf1	-	-	-	Golgi apparatus protein 1 [Ostrinia furnacalis]	0.567404	1.582431	-0.2988	-1.35651	-0.49453
TRINITY_DN867_c0_g1_i1_orf1	-	-	-	hemicentin-2-like isoform X1 [Ostrinia furnacalis]	-0.07052	1.897185	-0.46884	-0.32251	-1.03533
TRINITY_DN5525_c0_g1_i4_orf1	-	-	-	probable glucosamine 6-phosphate N-acetyltransferase [Ostrinia furnacalis]	1.639123	0.628374	-0.80321	-0.41497	-1.04933
TRINITY_DN24469_c0_g2_i2_orf1	-	-	-	unnamed protein product, partial [Brenthis ino]	0.890571	1.486603	-0.90711	-0.95125	-0.51881
TRINITY_DN2004_c0_g1_i20_orf1	-	-	-	hypothetical protein evm_006436 [Chilo suppressalis] >CAB3522373.1 unnamed protein product [Chilo suppressalis] >CAH0399695.1 unnamed protein product [Chilo suppressalis]	0.293876	1.563536	0.270409	-1.32087	-0.80695
TRINITY_DN146119_c0_g1_i1_orf1	-	-	-	protein SEC13 homolog [Ostrinia furnacalis]	1.41704	0.780664	-0.71725	-1.36161	-0.11884
TRINITY_DN9311_c0_g1_i1_orf1	-	-	-	cuticle protein 8-like [Ostrinia furnacalis]	-0.19981	1.960926	-0.3658	-0.75924	-0.63607
TRINITY_DN32700_c0_g1_i2_orf1	-	-	-	ribosomal protein S6 kinase 2 beta [Ostrinia furnacalis]	0.934923	1.460782	-0.9475	-0.57306	-0.87515
TRINITY_DN14532_c0_g1_i1_orf1	-	-	-	pupal cuticle protein-like [Trichoplusia ni]	0.095949	1.89689	-0.80543	-0.73319	-0.45422
TRINITY_DN4439_c0_g1_i2_orf1	-	-	-	cytoplasmic FMR1-interacting protein isoform X1 [Ostrinia furnacalis] >XP_028169436.1 cytoplasmic FMR1-interacting protein isoform X2 [Ostrinia furnacalis]	1.234268	1.115373	-0.27527	-0.98593	-1.08844
TRINITY_DN802_c0_g1_i2_orf1	-	-	-	active breakpoint cluster region-related protein [Ostrinia furnacalis]	1.271528	1.076018	-0.29113	-0.9137	-1.14272
TRINITY_DN7670_c0_g1_i1_orf1	-	-	-	striatin-interacting protein 1 [Ostrinia furnacalis]	1.291184	1.145759	-0.92143	-0.8642	-0.65131
TRINITY_DN110534_c0_g1_i3_orf1	-	-	-	unnamed protein product [Euphydryas editha]	0.538298	1.670972	-0.4191	-0.63043	-1.15975
TRINITY_DN35377_c0_g1_i3_orf1	-	-	-	unnamed protein product [Chilo suppressalis]	1.418465	0.739448	-1.44728	-0.13845	-0.57218
TRINITY_DN5954_c0_g1_i2_orf1	-	-	-	myosin-VIIa [Ostrinia furnacalis] >XP_028155907.1 myosin-VIIa [Ostrinia furnacalis]	0.103249	1.905471	-0.58283	-0.67944	-0.74645
TRINITY_DN34745_c0_g2_i1_orf1	-	-	-	GSK3-beta interaction protein-like [Galleria mellonella]	0.774648	1.570089	-0.73473	-1.02732	-0.58269
TRINITY_DN1554_c0_g1_i9_orf1	-	-	-	LOW QUALITY PROTEIN: puff-specific protein Bx42 [Ostrinia furnacalis]	1.189854	1.22435	-1.03435	-0.51197	-0.86788
TRINITY_DN48970_c0_g1_i1_orf1	-	-	-	uncharacterized protein LOC114356431 isoform X2 [Ostrinia furnacalis]	1.295537	1.033329	-0.36064	-1.28973	-0.6785
TRINITY_DN4439_c0_g2_i1_orf1	-	-	-	unnamed protein product, partial [Brenthis ino]	1.027983	1.358331	-0.55773	-1.15436	-0.67422
TRINITY_DN48973_c0_g1_i5_orf1	-	-	-	uncharacterized protein LOC114351683 isoform X8 [Ostrinia furnacalis]	1.420333	0.549576	-0.54014	0.111779	-1.54155
TRINITY_DN749_c0_g1_i1_orf1	-	-	-	serine/threonine-protein phosphatase 4 regulatory subunit 3 isoform X3 [Ostrinia furnacalis]	1.209496	1.212498	-0.81276	-1.04081	-0.56843
TRINITY_DN5211_c0_g1_i1_orf1	-	-	-	elongation of very long chain fatty acids protein AAEL008004-like [Ostrinia furnacalis]	0.676528	1.642294	-0.61681	-0.94129	-0.76072
TRINITY_DN1616_c0_g1_i3_orf1	-	-	-	U4/U6 small nuclear ribonucleoprotein Prp3 isoform X1 [Ostrinia furnacalis] >XP_028161035.1 U4/U6 small nuclear ribonucleoprotein Prp3 isoform X2 [Ostrinia furnacalis] >XP_028161037.1 U4/U6 small nuclear ribonucleoprotein Prp3 isoform X3 [Ostrinia furnacalis]	0.606702	1.649413	-0.52134	-0.60775	-1.12703
TRINITY_DN1771_c0_g2_i1_orf1	-	-	-	eukaryotic peptide chain release factor subunit 1 isoform X1 [Danaus plexippus plexippus] >CAG9574852.1 unnamed protein product [Danaus chrysippus]	1.64349	0.308079	-1.2581	0.089593	-0.78307
TRINITY_DN73224_c0_g4_i2_orf1	-	-	-	PREDICTED: poly(rC)-binding protein 3 isoform X2 [Vollenhovia emeryi]	0.621982	1.674145	-0.89131	-0.82322	-0.5816
TRINITY_DN3838_c0_g1_i8_orf1	-	-	-	ER membrane protein complex subunit 2-like isoform X1 [Ostrinia furnacalis] >XP_028161204.1 ER membrane protein complex subunit 2-like isoform X2 [Ostrinia furnacalis] >XP_028161205.1 ER membrane protein complex subunit 2-like isoform X3	1.290271	0.828303	0.110656	-1.39042	-0.83881
TRINITY_DN39170_c0_g1_i4_orf1	-	-	-	unnamed protein product [Arctia plantaginis]	1.376845	0.629009	-1.12199	0.285797	-1.16966
TRINITY_DN9575_c0_g1_i1_orf1	-	-	-	uncharacterized protein LOC114351119 [Ostrinia furnacalis]	1.408141	0.672328	-0.16738	-1.55116	-0.36193
TRINITY_DN60821_c0_g1_i1_orf1	-	-	-	nucleolar GTP-binding protein 2 [Ostrinia furnacalis]	1.36182	0.448929	-1.63724	0.265595	-0.4391
TRINITY_DN987_c0_g1_i11_orf1	-	-	-	macrophage mannose receptor 1-like [Pieris napi]	1.073137	0.412207	-0.22341	0.559054	-1.82099
TRINITY_DN7828_c0_g1_i2_orf1	-	-	-	alpha-N-acetylgalactosaminidase-like isoform X1 [Ostrinia furnacalis] >XP_028171449.1 alpha-N-acetylgalactosaminidase-like isoform X2 [Ostrinia furnacalis]	1.282328	1.154955	-0.78753	-0.96747	-0.68229
TRINITY_DN8596_c0_g1_i2_orf1	-	-	-	SWI/SNF-related matrix-associated actin-dependent regulator of chromatin subfamily E member 1-like isoform X2 [Ostrinia furnacalis]	1.05962	1.245943	-1.36116	-0.58651	-0.35789
TRINITY_DN2474_c0_g1_i5_orf1	-	-	-	glucose-6-phosphate 1-epimerase [Galleria mellonella]	0.47666	1.646827	-0.6091	-0.2361	-1.27828
TRINITY_DN535_c1_g1_i2_orf1	-	-	-	protein tramtrack, beta isoform isoform X24 [Bicyclus anynana]	0.398448	1.711375	-0.16871	-0.97251	-0.9686
TRINITY_DN834_c0_g1_i1_orf1	-	-	-	tissue inhibitor of metalloproteinase [Ostrinia furnacalis] >XP_028174266.1 tissue inhibitor of metalloproteinase [Ostrinia furnacalis]	1.525252	-0.01035	-1.17276	0.616201	-0.95835
TRINITY_DN20339_c0_g1_i3_orf1	-	-	-	ecto-NOX disulfide-thiol exchanger 2-like [Ostrinia furnacalis]	1.350721	0.804291	0.070611	-1.26609	-0.95953
TRINITY_DN4686_c0_g2_i1_orf1	-	-	-	lysophospholipase-like protein 1 [Ostrinia furnacalis]	1.248467	1.169026	-0.6593	-1.09636	-0.66183
TRINITY_DN12134_c0_g1_i4_orf1	-	-	-	glutathione S-transferase 1-1 [Ostrinia furnacalis] >XP_028161942.1 glutathione S-transferase 1-1 [Ostrinia furnacalis] >XP_028161943.1 glutathione S-transferase 1-1 [Ostrinia furnacalis]	0.743932	1.484998	-0.48685	-1.36445	-0.37763
TRINITY_DN19651_c0_g1_i1_orf1	-	-	-	cytosolic non-specific dipeptidase [Ostrinia furnacalis]	0.86787	1.371447	-0.89593	-0.09684	-1.24654
TRINITY_DN17003_c0_g1_i1_orf1	-	-	-	mucin-5AC [Ostrinia furnacalis]	0.623947	1.658083	-0.47236	-0.92571	-0.88396
TRINITY_DN15753_c0_g1_i1_orf1	-	-	-	uncharacterized protein LOC114366450 [Ostrinia furnacalis]	1.280757	1.049711	-0.27789	-0.83393	-1.21865

TRINITY_DN9916_c0_g1_i1_orf1	-	-	-	PREDICTED: dynein light chain Tctex-type [Amyelois transitella] >XP_021195381.1 dynein light chain Tctex-type [Helicoverpa armigera] >XP_022815696.1 dynein light chain Tctex-type [Spodoptera litura] >XP_028156399.1 dynein light chain Tctex-type [Ostrinia furnacalis] >XP_035458261.1 dynein light chain Tctex-type-like [Spodoptera frugiperda] >XP_047034788.1 dynein light chain Tctex-type [Helicoverpa zea] >CAB3233358.1 unnamed protein product [Arctia plantaginis] >CAB3506583.1 unnamed protein product [Spodoptera littoralis] >CAG9754627.1 unnamed protein product [Diatraea saccharalis] >CAH0596395.1 unnamed protein product [Chrysodeixis includens] >KAF9808454.1 hypothetical protein SFRURICE_008507 [Spodoptera frugiperda]	0.545463	1.682307	-0.65896	-1.10651	-0.4623
TRINITY_DN64769_c0_g1_i3_orf1	-	-	-	procollagen-lysine,2-oxoglutarate 5-dioxygenase isoform X2 [Ostrinia furnacalis]	0.787371	1.526171	-0.97358	-0.99033	-0.34964
TRINITY_DN67649_c0_g1_i1_orf1	-	-	-	proliferating cell nuclear antigen [Ostrinia furnacalis] >XP_028174842.1 proliferating cell nuclear antigen [Ostrinia furnacalis]	0.844019	1.528445	-0.57293	-0.85459	-0.94494
TRINITY_DN288_c0_g1_i9_orf1	-	-	-	unnamed protein product [Chilo suppressalis]	1.417426	0.967632	-0.75511	-1.09429	-0.53566
TRINITY_DN4194_c0_g1_i1_orf1	-	-	-	hornerin-like [Ostrinia furnacalis]	1.065025	1.065025	-1.00767	-1.29814	0.175754
TRINITY_DN4125_c0_g1_i14_orf1	-	-	-	angiotensin-converting enzyme-like isoform X1 [Ostrinia furnacalis]	0.125972	1.887121	-0.59731	-0.88665	-0.52913
TRINITY_DN4144_c0_g1_i7_orf1	-	-	-	uncharacterized protein LOC114350172 [Ostrinia furnacalis]	0.168221	1.87969	-0.64469	-0.56318	-0.84004
TRINITY_DN11820_c0_g1_i1_orf1	-	-	-	hypothetical protein evm_000341 [Chilo suppressalis]	1.224399	0.890004	-0.12425	-1.59213	-0.39803
TRINITY_DN6239_c0_g1_i1_orf1	-	-	-	eukaryotic translation initiation factor 6 [Ostrinia furnacalis]	1.149511	1.244556	-0.95786	-0.41739	-1.01882
TRINITY_DN14721_c0_g1_i2_orf1	-	-	-	protein masquerade-like isoform X2 [Ostrinia furnacalis]	0.314407	1.830295	-0.68105	-0.64183	-0.82183
TRINITY_DN42171_c0_g1_i1_orf1	-	-	-	amyloid-beta-like protein isoform X1 [Manduca sexta] >AAY25024.3 beta amyloid protein precursor-like protein [Manduca sexta]	1.013178	1.393052	-0.60158	-1.04827	-0.75638
TRINITY_DN24490_c0_g1_i6_orf1	-	-	-	E3 ubiquitin-protein ligase Hakai [Ostrinia furnacalis]	1.310834	1.075223	-0.84903	-1.10283	-0.4342
TRINITY_DN3310_c0_g1_i1_orf1	-	-	-	hypothetical protein evm_010516 [Chilo suppressalis]	-0.10627	1.960045	-0.64886	-0.60367	-0.60124
TRINITY_DN113272_c0_g1_i1_orf1	-	-	-	altered inheritance of mitochondria protein 3-like [Ostrinia furnacalis]	0.124765	1.890625	-0.62715	-0.85699	-0.53125
TRINITY_DN13375_c0_g1_i6_orf1	-	-	-	thioredoxin, mitochondrial isoform X2 [Ostrinia furnacalis]	1.085717	1.18545	-1.01591	-1.17353	-0.08173
TRINITY_DN11375_c0_g1_i6_orf1	-	-	-	uncharacterized protein LOC114363514 isoform X2 [Ostrinia furnacalis]	1.189579	1.193506	-1.0876	-0.91083	-0.38465
TRINITY_DN19493_c0_g1_i5_orf1	-	-	-	zinc finger MYM-type protein 3 isoform X1 [Ostrinia furnacalis] >XP_028159738.1 zinc finger MYM-type protein 3 isoform X2 [Ostrinia furnacalis]	1.293256	1.066145	-0.60633	-1.25506	-0.49802
TRINITY_DN78686_c0_g1_i1_orf1	-	-	-	myosin-2 essential light chain isoform X2 [Harpegnathos saltator] >XP_012170910.1 myosin-2 essential light chain isoform X2 [Bombus terrestris] >XP_033185931.1 myosin-2 essential light chain isoform X2 [Bombus vancouverensis nearcticus] >XP_033319091.1 myosin-2 essential light chain isoform X2 [Bombus bifarius] >XP_033349866.1 myosin-2 essential light chain isoform X2 [Bombus vosnesenskii] >XP_043597873.1 myosin-2 essential light chain isoform X2 [Bombus pyrosoma]	0.960031	1.265543	-0.45715	-1.47755	-0.29088
TRINITY_DN18933_c0_g1_i3_orf1	-	-	-	PREDICTED: protein BUD31 homolog [Papilio xuthus] >XP_014361644.1 protein BUD31 homolog [Papilio machaon] >XP_026750578.1 protein BUD31 homolog [Galleria mellonella] >XP_047995610.1 protein BUD31 homolog [Leguminivora glycinivorella] >XP_049869593.1 protein BUD31 homolog [Pectinophora gossypiella] >KAI5652084.1 g10 protein domain-containing protein [Phthorimaea operculella] >CAB3251981.1 unnamed protein product [Arctia plantaginis] >CAB3520382.1 unnamed protein product [Chilo suppressalis] >CAG9747228.1 unnamed protein product [Diatraea saccharalis] >CAH2037008.1 unnamed protein product, partial [Iphiclides podalirius]	1.172089	1.05956	-0.24713	-1.47873	-0.50579
TRINITY_DN18681_c0_g1_i7_orf1	-	-	-	fragile X mental retardation syndrome-related protein 1 isoform X3 [Ostrinia furnacalis]	1.481591	0.760715	-0.15425	-1.14989	-0.93817
TRINITY_DN98242_c0_g1_i1_orf1	-	-	-	adenosine deaminase 2-A-like [Galleria mellonella]	0.21135	1.825191	-0.29072	-0.96106	-0.78476
TRINITY_DN29743_c0_g1_i9_orf1	-	-	-	polyadenylate-binding protein 2 isoform X1 [Ostrinia furnacalis] >XP_028168980.1 polyadenylate-binding protein 2 isoform X2 [Ostrinia furnacalis]	1.31884	0.956003	-0.147	-1.23893	-0.88891
TRINITY_DN257_c0_g1_i7_orf1	-	-	-	zinc finger RNA-binding protein 2 [Ostrinia furnacalis]	1.493791	0.700889	-0.14595	-1.30489	-0.74385
TRINITY_DN1074_c0_g1_i7_orf1	-	-	-	eukaryotic translation initiation factor 4E type 2 [Ostrinia furnacalis]	0.743748	1.580049	-0.69569	-1.07951	-0.5486
TRINITY_DN4757_c0_g1_i3_orf1	-	-	-	melanotransferrin isoform X1 [Ostrinia furnacalis] >XP_028175370.1 melanotransferrin isoform X2 [Ostrinia furnacalis] >XP_028175371.1 melanotransferrin isoform X3 [Ostrinia furnacalis]	1.188798	1.252861	-0.897	-0.87108	-0.67358
TRINITY_DN14734_c0_g1_i2_orf1	-	-	-	SET and MYND domain-containing protein 5 isoform X1 [Ostrinia furnacalis] >XP_028173610.1 SET and MYND domain-containing protein 5 isoform X2 [Ostrinia	1.242099	0.890874	-0.7624	0.070706	-1.44128
TRINITY_DN20244_c0_g1_i1_orfp1	-	-	-	uncharacterized protein LOC125235519 [Leguminivora glycinivorella]	0.397365	1.77601	-0.8905	-0.45168	-0.83119
TRINITY_DN2403_c0_g1_i3_orf1	-	-	-	FAD-dependent oxidoreductase domain-containing protein 1 [Ostrinia furnacalis]	1.291161	1.063119	-0.31468	-1.1286	-0.91099

TRINITY_DN98814_c0_g1_i2_orf1	-	-	-	PREDICTED: chaoptin [Amyelois transitella]	0.51035	1.698063	-0.75089	-0.38957	-1.06795
TRINITY_DN2802_c1_g1_i1_orf1	-	-	-	psi [Ostrinia furnacalis]	1.259282	0.996972	-0.06818	-0.98971	-1.19837
TRINITY_DN31967_c0_g1_i5_orf1	-	-	-	N-acetylgalactosamine kinase [Ostrinia furnacalis]	1.356187	1.076638	-0.94506	-0.77344	-0.71433
TRINITY_DN31619_c0_g1_i2_orf1	-	-	-	endocuticle structural glycoprotein ABD-4-like [Ostrinia furnacalis]	0.112171	1.890427	-0.45596	-0.84318	-0.70345
TRINITY_DN20185_c0_g1_i6_orf1	-	-	-	zinc finger protein on ecdysone puffs [Ostrinia furnacalis]	1.397228	0.94178	-0.30562	-1.02274	-1.01065
TRINITY_DN3255_c0_g1_i1_orf1	-	-	-	uncharacterized protein LOC114351042 [Ostrinia furnacalis]	0.035289	1.919087	-0.56282	-0.82003	-0.57152
TRINITY_DN21218_c0_g1_i4_orf1	-	-	-	leukotriene A-4 hydrolase isoform X2 [Ostrinia furnacalis]	1.509399	0.831068	-0.95648	-0.4105	-0.97349
TRINITY_DN30150_c0_g1_i7_orf1	-	-	-	unnamed protein product [Chrysodeixis includens]	1.382257	0.898133	-0.97915	-0.16206	-1.13918
TRINITY_DN1444_c1_g1_i5_orf1	-	-	-	spondin-1 isoform X1 [Ostrinia furnacalis] >XP_028167312.1 spondin-1 isoform X1 [Ostrinia furnacalis] >XP_028167313.1 spondin-1 isoform X1 [Ostrinia furnacalis] >XP_028167314.1 spondin-1 isoform X2 [Ostrinia furnacalis]	1.238547	1.157865	-0.55992	-1.16873	-0.66776
TRINITY_DN3953_c0_g1_i2_orf1	-	-	-	TP53-binding protein 1-like [Ostrinia furnacalis]	0.982807	1.407531	-0.92745	-0.48369	-0.9792
TRINITY_DN129226_c0_g4_i1_orf1	-	-	-	hypothetical protein evm_000268 [Chilo suppressalis]	1.414494	0.485277	-1.60752	0.115499	-0.40775
TRINITY_DN29034_c0_g1_i1_orf1	-	-	-	trypsin-like serine protease [Ostrinia nubilalis]	1.589669	0.702937	-1.15274	-0.55628	-0.58359
TRINITY_DN45633_c0_g1_i1_orf1	-	-	-	ubiquitin thioesterase otubain-like [Ostrinia furnacalis]	1.519589	0.835439	-0.78901	-1.05148	-0.51454
TRINITY_DN17655_c0_g1_i1_orf1	-	-	-	BRISC and BRCA1-A complex member 1-like [Ostrinia furnacalis]	0.370696	1.695115	-0.17201	-0.6585	-1.2353
TRINITY_DN44877_c0_g1_i2_orf1	-	-	-	U6 snRNA-associated Sm-like protein LSm7 [Diachasma alloeum]	1.461735	0.814479	-0.24596	-1.21324	-0.81701
TRINITY_DN644_c0_g1_i1_orf1	-	-	-	cuticle protein 19-like [Ostrinia furnacalis]	-0.08346	1.946385	-0.59417	-0.78721	-0.48155
TRINITY_DN235_c0_g3_i1_orf1	-	-	-	actin, muscle-type A2 [Bombyx mori] >XP_013199497.1 PREDICTED: actin, muscle-type A2 [Amyelois transitella] >XP_021196684.1 actin, muscle-type A2 [Helicoverpa armigera] >XP_022837900.1 actin, muscle-type A2 [Spodoptera litura] >XP_026314060.1 actin, muscle-type A2 [Hyposmocoma kahamanoa] >XP_026738711.1 actin, muscle-type A2 [Trichoplusia ni] >XP_028179440.1 actin, muscle-type A2 [Ostrinia furnacalis] >XP_030030527.1 actin, muscle-type A2 [Manduca sexta] >XP_035439272.1 actin, muscle-type A2 [Spodoptera frugiperda] >XP_047029939.1 actin, muscle-type A2 [Helicoverpa zea] >XP_049873365.1 actin, muscle-type A2 [Pectinophora gossypiella] >P07837.1 RecName: Full=Actin, muscle-type A2; Flags: Precursor [Bombyx mori] >KAF9423784.1 hypothetical protein HW555_000842 [Spodoptera exigua] >QLI62214.1 actin [Streltzoviella insularis] >CAB3227390.1 unnamed protein product [Arctia plantaginis] >CAB3508892.1 unnamed protein product [Spodoptera littoralis] >CAB3520808.1 unnamed protein product [Chilo suppressalis] >CAG9748331.1 unnamed protein product [Diatraea saccharalis] >CAH0585396.1 unnamed protein product [Chrysodeixis includens] >GBP21118.1 Actin, muscle-type A2 [Eumeta japonica]	1.454532	0.816403	-0.30213	-1.29136	-0.67745
TRINITY_DN7785_c0_g1_i1_orf1	-	-	-	uncharacterized protein LOC114364098 [Ostrinia furnacalis]	-0.03754	1.938556	-0.48295	-0.73982	-0.67824
TRINITY_DN2181_c1_g1_i8_orf1	-	-	-	vacuolar protein sorting-associated protein 37B [Ostrinia furnacalis]	1.460441	0.856346	-0.88699	-1.11793	-0.31186
TRINITY_DN7037_c0_g1_i4_orf1	-	-	-	unnamed protein product [Chilo suppressalis]	1.58868	0.671074	-1.2171	-0.50056	-0.5421
TRINITY_DN37923_c0_g1_i1_orf1	-	-	-	hypothetical protein NE865_05974 [Phthorimaea operculella]	1.321309	1.010261	-0.27039	-1.16509	-0.89609
TRINITY_DN227_c0_g1_i1_orf1	-	-	-	double-stranded ribonuclease 2 [Ostrinia nubilalis]	1.658062	0.148497	-1.22381	0.238376	-0.82113
TRINITY_DN172_c1_g1_i3_orf1	-	-	-	galactose oxidase, central domain-containing protein [Phthorimaea operculella]	1.540553	0.755782	-1.1556	-0.7567	-0.38404
TRINITY_DN37532_c0_g1_i1_orf1	-	-	-	transcription elongation factor S-II [Ostrinia furnacalis]	1.318202	0.976798	-0.32598	-1.34743	-0.62159
TRINITY_DN6586_c0_g1_i1_orf1	-	-	-	fatty acyl-CoA reductase wat-like isoform X1 [Ostrinia furnacalis]	0.491275	1.753212	-0.69654	-0.80278	-0.74516
TRINITY_DN4835_c0_g1_i2_orf1	-	-	-	ribonucleoside-diphosphate reductase large subunit [Ostrinia furnacalis]	1.123226	1.142023	-0.08316	-1.24349	-0.9386
TRINITY_DN4494_c0_g1_i1_orf1	-	-	-	venom serine carboxypeptidase [Ostrinia furnacalis]	0.843291	1.342112	0.038756	-1.03254	-1.19162
TRINITY_DN87170_c0_g1_i3_orf1	-	-	-	uncharacterized protein LOC114360175 [Ostrinia furnacalis]	1.105386	1.145573	-0.06018	-0.91875	-1.27203
TRINITY_DN32161_c0_g1_i1_orf1	-	-	-	uncharacterized protein LOC114352518 [Ostrinia furnacalis]	1.534448	0.304177	-1.5709	0.023288	-0.29102
TRINITY_DN9354_c0_g1_i7_orf1	-	-	-	hypothetical protein evm_012205 [Chilo suppressalis] >CAB3527181.1 unnamed protein product [Chilo suppressalis] >CAH0404510.1 unnamed protein product [Chilo suppressalis]	0.548972	1.605294	-0.39016	-0.42741	-1.3367
TRINITY_DN18937_c0_g1_i1_orf1	-	-	-	uncharacterized protein LOC114351683 isoform X7 [Ostrinia furnacalis]	1.219321	0.484588	-0.02988	0.131585	-1.80561
TRINITY_DN104507_c0_g1_i2_orf1	-	-	-	replication protein A 32 kDa subunit [Ostrinia furnacalis]	1.409763	0.808306	-0.16798	-1.36383	-0.68626
TRINITY_DN72999_c0_g1_i1_orf1	-	-	-	protein obstructor-E-like isoform X1 [Ostrinia furnacalis] >XP_028169319.1 protein obstructor-E-like isoform X2 [Ostrinia furnacalis]	0.338945	1.815492	-0.58976	-0.87502	-0.68965
TRINITY_DN2879_c0_g1_i4_orf1	-	-	-	nucleoporin Nup35 [Ostrinia furnacalis]	1.065957	1.103632	0.025362	-1.44112	-0.75383
TRINITY_DN4262_c0_g1_i16_orf1	-	-	-	sperm-associated antigen 7 homolog [Ostrinia furnacalis]	1.396625	0.789592	-0.14406	-1.4211	-0.62106
TRINITY_DN5457_c0_g1_i4_orf1	-	-	-	unnamed protein product [Chrysodeixis includens]	1.520483	0.738709	-0.23348	-1.14736	-0.87835

TRINITY_DN1504_c0_g1_i1_orf1	-	-	-	uncharacterized protein LOC114352862 [Ostrinia furnacalis] >XP_028160407.1 uncharacterized protein LOC114352862 [Ostrinia furnacalis]	0.983497	1.271024	-0.19569	-1.38966	-0.66917
TRINITY_DN50875_c0_g1_i3_orf1	-	-	-	conserved oligomeric Golgi complex subunit 8 [Ostrinia furnacalis]	1.159827	1.195023	-0.55892	-1.28632	-0.50961
TRINITY_DN10057_c0_g2_i1_orf1	-	-	-	cell wall protein DAN4 [Ostrinia furnacalis]	0.507359	1.744915	-0.75219	-0.80897	-0.69112
TRINITY_DN46140_c0_g1_i1_orf1	-	-	-	protein PRRC2A-like isoform X4 [Ostrinia furnacalis]	1.553541	0.795426	-0.9052	-0.50719	-0.93658
TRINITY_DN14046_c0_g1_i1_orf1	-	-	-	retinol dehydrogenase 14 [Ostrinia furnacalis] >XP_028165567.1 retinol dehydrogenase 14 [Ostrinia furnacalis] >XP_028165568.1 retinol dehydrogenase 14 [Ostrinia furnacalis]	0.546856	1.722036	-0.73361	-0.67066	-0.86463
TRINITY_DN619_c0_g1_i1_orf1	-	-	-	putative uncharacterized protein DDB_G0271606 [Ostrinia furnacalis]	-0.03825	1.93351	-0.44717	-0.80031	-0.64778
TRINITY_DN6185_c0_g1_i12_orf1	-	-	-	mitogen-activated protein kinase 1 [Ostrinia furnacalis] >AXF67444.1 mitogen-activated protein kinase 1 [Ostrinia furnacalis]	1.510447	0.836945	-1.0747	-0.47322	-0.79947
TRINITY_DN20767_c0_g2_i1_orf1	-	-	-	glycosylated lysosomal membrane protein B-like [Vanessa atalanta]	0.808634	1.539353	-0.6009	-1.08481	-0.66228
TRINITY_DN50820_c0_g1_i2_orf1	-	-	-	transmembrane protein 135-like isoform X2 [Ostrinia furnacalis]	1.659683	0.651005	-0.73194	-0.6485	-0.93024
TRINITY_DN23926_c0_g1_i4_orf1	-	-	-	programmed cell death protein 10 [Ostrinia furnacalis]	1.460544	0.602302	-0.27154	-1.53827	-0.25303
TRINITY_DN10131_c0_g1_i7_orf1	-	-	-	aldo-keto reductase AKR2E4-like [Ostrinia furnacalis]	1.041565	1.37075	-0.68358	-1.05755	-0.67119
TRINITY_DN4908_c1_g1_i5_orf1	-	-	-	DNA topoisomerase 2 isoform X1 [Ostrinia furnacalis]	1.155609	1.285429	-0.7435	-0.94529	-0.75225
TRINITY_DN147475_c0_g1_i1_orf1	-	-	-	casein kinase II subunit beta, partial [Rhincodon typus]	1.192061	1.150255	-0.28833	-1.20496	-0.84903
TRINITY_DN129226_c0_g1_i2_orf1	-	-	-	hypothetical protein evm_000268 [Chilo suppressalis]	1.369967	0.909602	-0.55968	-1.36172	-0.35817
TRINITY_DN5046_c0_g3_i1_orf1	-	-	-	uncharacterized protein LOC114358520 [Ostrinia furnacalis]	1.469563	0.908142	-0.84059	-1.02125	-0.51586
TRINITY_DN6436_c0_g1_i1_orf1	-	-	-	serine/threonine-protein kinase PAK 3 isoform X1 [Ostrinia furnacalis] >XP_028164178.1 serine/threonine-protein kinase PAK 3 isoform X2 [Ostrinia furnacalis] >XP_028164179.1 serine/threonine-protein kinase PAK 3 isoform X3 [Ostrinia furnacalis]	0.648131	1.649968	-0.5783	-0.71116	-1.00864
TRINITY_DN10297_c0_g1_i1_orf1	-	-	-	polyglutamine-binding protein 1 [Ostrinia furnacalis]	1.275961	1.08533	-0.89644	-0.334	-1.13085
TRINITY_DN142588_c0_g1_i1_orf1	-	-	-	peptidyl-prolyl cis-trans isomerase [Cotesia flavipes]	1.380264	1.010703	-0.47403	-1.03373	-0.8832
TRINITY_DN17271_c0_g1_i1_orf1	-	-	-	uncharacterized protein LOC114350693 [Ostrinia furnacalis]	1.231016	1.140924	-0.38453	-1.1671	-0.8203
TRINITY_DN3893_c0_g2_i3_orf1	-	-	-	cleavage and polyadenylation specificity factor subunit CG7185 isoform X2 [Ostrinia furnacalis]	1.443581	0.798846	-0.20876	-1.30531	-0.72835
TRINITY_DN2718_c0_g1_i6_orf1	-	-	-	cleavage stimulation factor subunit 2 isoform X1 [Ostrinia furnacalis]	1.449145	0.891663	-0.3792	-1.11714	-0.84447
TRINITY_DN6189_c0_g1_i1_orf1	-	-	-	optic atrophy 3 protein homolog isoform X2 [Ostrinia furnacalis]	1.509155	0.844808	-1.11282	-0.62967	-0.61147
TRINITY_DN21124_c0_g1_i4_orf1	-	-	-	calsyntenin-1 [Ostrinia furnacalis]	0.691948	1.642058	-0.84379	-0.78044	-0.70977
TRINITY_DN48590_c0_g1_i1_orf1	-	-	-	acyl-CoA Delta(11) desaturase isoform X1 [Ostrinia furnacalis] >XP_028172999.1 acyl-CoA Delta(11) desaturase isoform X2 [Ostrinia furnacalis] >XP_028173000.1 acyl-CoA Delta(11) desaturase isoform X1 [Ostrinia furnacalis]	0.247686	1.857285	-0.67612	-0.78924	-0.6396
TRINITY_DN69236_c0_g1_i1_orf1	-	-	-	peroxiredoxin [Ostrinia furnacalis]	1.466623	0.754851	-0.5604	-1.37164	-0.28943
TRINITY_DN2257_c0_g1_i4_orf1	-	-	-	protein phosphatase 1 catalytic subunit [Bombyx mori] >NP_001296033.1 serine/threonine-protein phosphatase alpha-2 isoform [Plutella xylostella] >XP_013188351.1 PREDICTED: serine/threonine-protein phosphatase alpha-2 isoform [Amyelois transitella] >XP_021183772.1 serine/threonine-protein phosphatase alpha-2 isoform isoform X2 [Helicoverpa armigera] >XP_022831789.1 serine/threonine-protein phosphatase alpha-2 isoform [Spodoptera litura] >XP_026314622.1 serine/threonine-protein phosphatase alpha-2 isoform isoform X2 [Hyposmocoma kahamanoa] >XP_026755085.1 serine/threonine-protein phosphatase alpha-2 isoform [Galleria mellonella] >XP_028026894.1 serine/threonine-protein phosphatase alpha-2 isoform isoform X2 [Bombyx mandarina] >XP_028168459.1 serine/threonine-protein phosphatase alpha-2 isoform [Ostrinia furnacalis] >XP_035448597.1 serine/threonine-protein phosphatase alpha-2 isoform isoform X2 [Spodoptera frugiperda] >XP_047033174.1 serine/threonine-protein phosphatase alpha-2 isoform-like [Helicoverpa zea] >XP_049878952.1 serine/threonine-protein phosphatase alpha-2 isoform isoform X2 [Pectinophora gossypiella] >RVE45538.1 hypothetical protein evm_009803 [Chilo suppressalis] >CAH0595519.1 unnamed protein product [Chrysodeixis includens] >CAH0714008.1 unnamed protein product, partial [Brenthis ino] >ABF51476.1 protein phosphatase 1 catalytic subunit [Bombyx mori] >AHF45925.1 protein phosphatase 1 [Plutella xylostella]	1.62133	-0.02368	-1.1109	0.470125	-0.95687
TRINITY_DN48641_c0_g1_i4_orf1	-	-	-	RNA-binding protein 45-like [Galleria mellonella]	0.85224	1.45476	-0.27004	-0.94836	-1.0886
TRINITY_DN2885_c1_g1_i2_orf1	-	-	-	ubiquitin-like-specific protease ESD4 [Ostrinia furnacalis]	1.370534	0.845705	-0.05359	-1.26163	-0.90102
TRINITY_DN5841_c0_g1_i2_orf1	-	-	-	hypothetical protein evm_011295 [Chilo suppressalis]	1.468505	0.749305	-0.427	-1.3935	-0.3973
TRINITY_DN9282_c0_g1_i2_orf1	-	-	-	uncharacterized protein LOC114363102 isoform X2 [Ostrinia furnacalis]	-0.04004	1.932711	-0.52623	-0.84528	-0.52116
TRINITY_DN2840_c0_g1_i5_orf1	-	-	-	hypothetical protein evm_002181 [Chilo suppressalis]	0.667178	1.654206	-0.85915	-0.8062	-0.65603

TRINITY_DN783_c0_g1_i7_orf1	-	-	-	microtubule-associated protein Jupiter isoform X4 [Helicoverpa armigera]	1.148446	1.285436	-0.70103	-1.0004	-0.73245
TRINITY_DN1868_c0_g1_i1_orf1	-	-	-	protein obstructor-E isoform X1 [Ostrinia furnacalis]	0.47972	1.731908	-1.00914	-0.48081	-0.72168
TRINITY_DN10479_c0_g1_i6_orf1	-	-	-	unnamed protein product [Chrysodeixis includens]	0.525153	1.706272	-0.69007	-1.04341	-0.49794
TRINITY_DN76377_c0_g1_i1_orf1	-	-	-	uncharacterized protein LOC111357764, partial [Spodoptera litura]	1.196731	1.111839	-0.73364	-1.3134	-0.26153
TRINITY_DN43328_c0_g1_i1_orf1	-	-	-	tubulin--tyrosine ligase-like protein 12 [Ostrinia furnacalis]	1.147724	1.279837	-0.70623	-1.04024	-0.68109
TRINITY_DN18502_c0_g1_i1_orf1	-	-	-	uncharacterized protein LOC114359515 [Ostrinia furnacalis]	0.228585	1.864808	-0.66128	-0.7772	-0.65491
TRINITY_DN59829_c0_g1_i1_orf1	-	-	-	putative mediator of RNA polymerase II transcription subunit 12 [Ostrinia furnacalis]	0.786086	1.568805	-0.56548	-0.90348	-0.88593
TRINITY_DN34159_c0_g2_i1_orf1	-	-	-	guanine nucleotide exchange factor subunit Rich isoform X1 [Ostrinia furnacalis]	1.670394	0.20861	-0.89932	0.172578	-1.15226
TRINITY_DN10722_c0_g3_i1_orf1	-	-	-	inositol-3-phosphate synthase [Ostrinia furnacalis]	1.490964	0.676822	-0.1083	-1.33509	-0.7244
TRINITY_DN2196_c0_g1_i2_orf1	-	-	-	HIRA-interacting protein 3-like [Ostrinia furnacalis]	1.632649	0.674972	-0.83422	-0.51592	-0.95749
TRINITY_DN14429_c0_g1_i2_orf1	-	-	-	NADH dehydrogenase [ubiquinone] 1 beta subcomplex subunit 11, mitochondrial [Ostrinia furnacalis]	1.660996	0.542802	-1.20162	-0.48705	-0.51513
TRINITY_DN3647_c2_g1_i3_orf1	-	-	-	unnamed protein product, partial [Iphiclides podalirius]	1.544165	0.761163	-0.76453	-1.13417	-0.40664
TRINITY_DN1326_c0_g1_i1_orf1	-	-	-	cuticle protein 7-like [Ostrinia furnacalis]	-0.08838	1.946663	-0.58254	-0.48042	-0.79532
TRINITY_DN4659_c0_g1_i2_orf1	-	-	-	uncharacterized protein LOC114351134 [Ostrinia furnacalis]	1.178855	1.263232	-0.94641	-0.7607	-0.73498
TRINITY_DN279_c0_g1_i10_orf1	-	-	-	RE1-silencing transcription factor-like isoform X1 [Ostrinia furnacalis]	1.102485	1.254132	-0.41282	-1.24766	-0.69614
TRINITY_DN14507_c0_g1_i5_orf1	-	-	-	PTB domain-containing adapter protein ced-6 [Ostrinia furnacalis]	0.920947	1.243894	0.097093	-0.99497	-1.26696
TRINITY_DN1313_c0_g1_i2_orf1	-	-	-	39S ribosomal protein L40, mitochondrial [Ostrinia furnacalis]	0.822551	1.510073	-0.89421	-0.39597	-1.04244
TRINITY_DN4085_c0_g1_i1_orf1	-	-	-	Protein TSSC1 [Papilio machaon]	1.046314	1.264885	-0.18556	-1.13927	-0.98637
TRINITY_DN14705_c0_g2_i1_orf1	-	-	-	coiled-coil-helix-coiled-coil-helix domain-containing protein 10, mitochondrial [Ostrinia furnacalis]	1.683475	-0.03447	-0.9591	0.364576	-1.05448
TRINITY_DN33_c0_g1_i8_orf1	-	-	-	uncharacterized protein CG45076-like isoform X2 [Ostrinia furnacalis]	1.648517	0.552996	-0.73985	-0.30599	-1.15567
TRINITY_DN4070_c0_g1_i4_orf1	-	-	-	alpha-N-acetylgalactosaminidase isoform X3 [Ostrinia furnacalis]	0.960137	1.418962	-0.47594	-1.06811	-0.83504
TRINITY_DN2600_c0_g1_i7_orf1	-	-	-	mucin-5AC isoform X2 [Ostrinia furnacalis]	1.128976	1.295294	-0.71968	-1.04835	-0.65625
TRINITY_DN7112_c0_g1_i1_orf1	-	-	-	heterogeneous nuclear ribonucleoprotein K isoform X2 [Ostrinia furnacalis]	1.442054	0.652608	0.102538	-1.28599	-0.91121
TRINITY_DN8432_c0_g2_i1_orf1	-	-	-	unnamed protein product [Chrysodeixis includens]	1.370521	0.975471	-0.6849	-0.42965	-1.23144
TRINITY_DN32601_c0_g1_i2_orf1	-	-	-	uncharacterized protein LOC114363197 [Ostrinia furnacalis]	1.088315	1.337237	-0.76277	-1.00905	-0.65372
TRINITY_DN50571_c1_g1_i1_orf1	-	-	-	WD repeat-containing protein 46 [Ostrinia furnacalis]	1.662861	0.585882	-1.1155	-0.61749	-0.51575
TRINITY_DN34821_c0_g1_i4_orf1	-	-	-	acetylcholine receptor subunit alpha-L1-like [Ostrinia furnacalis]	1.190755	1.178485	-1.25173	-0.52684	-0.59068
TRINITY_DN220_c0_g1_i3_orf1	-	-	-	serine-arginine protein 55 isoform X6 [Pieris brassicae]	1.528463	0.671645	-0.12835	-1.19421	-0.87755
TRINITY_DN237_c1_g1_i1_orf1	-	-	-	PREDICTED: cytoplasmic protein NCK1 isoform X1 [Microplitis demolitor]	1.647346	0.646202	-0.6757	-0.58156	-1.03628
TRINITY_DN13216_c0_g1_i5_orf1	-	-	-	uncharacterized protein LOC114358344 isoform X1 [Ostrinia furnacalis]	1.312902	1.131775	-0.84762	-0.77007	-0.82698
TRINITY_DN18164_c0_g1_i7_orf1	-	-	-	uncharacterized protein LOC114366518 isoform X5 [Ostrinia furnacalis]	1.098915	1.236784	-0.24701	-0.9433	-1.14539
TRINITY_DN144258_c0_g1_i1_orf1	-	-	-	PREDICTED: enhancer of rudimentary homolog [Microplitis demolitor] >XP_044577051.1 enhancer of rudimentary homolog [Cotesia glomerata] >KAG8041963.1 hypothetical protein G9C98_007267 [Cotesia typhae] >KAH0539785.1 hypothetical protein KQX54_008036 [Cotesia glomerata] >CAD6227368.1 GSCOCG00006137001-RA-CDS [Cotesia congregata]	1.125813	1.04677	0.080763	-1.34058	-0.91277
TRINITY_DN129835_c0_g1_i2_orf1	-	-	-	alpha-tocopherol transfer protein-like [Chelonus insularis]	-0.22438	1.930081	-0.16582	-0.69468	-0.8452
TRINITY_DN6710_c0_g1_i6_orf1	-	-	-	multiple C2 and transmembrane domain-containing protein-like [Ostrinia furnacalis]	1.402293	0.896198	-0.21208	-0.97663	-1.10979
TRINITY_DN73923_c0_g1_i1_orf1	-	-	-	protein obstructor-E-like [Ostrinia furnacalis]	0.81498	1.562491	-0.87337	-0.76778	-0.73633
TRINITY_DN2894_c0_g2_i3_orf1	-	-	-	myrosinase 1-like isoform X1 [Ostrinia furnacalis]	1.215839	0.935226	0.081271	-1.38895	-0.84339
TRINITY_DN3235_c0_g1_i1_orf1	-	-	-	SPARC [Trichoplusia ni]	1.427727	0.984989	-0.77542	-0.97636	-0.66093
TRINITY_DN139212_c0_g1_i4_orf1	-	-	-	uncharacterized protein LOC114350112 [Ostrinia furnacalis]	1.525507	0.638732	-0.03795	-1.10087	-1.02542
TRINITY_DN36061_c0_g4_i2_orf1	-	-	-	putative GPI-anchored protein pfl2 [Ostrinia furnacalis] >XP_028163002.1 putative GPI-anchored protein pfl2 [Ostrinia furnacalis]	0.958213	1.458308	-0.87392	-0.74281	-0.79979
TRINITY_DN82801_c0_g1_i1_orf1	-	-	-	uncharacterized protein LOC114364712 [Ostrinia furnacalis]	1.465685	0.930118	-1.01974	-0.68803	-0.68803
TRINITY_DN27725_c0_g1_i2_orf1	-	-	-	BRISC complex subunit FAM175B-like [Ostrinia furnacalis]	1.129126	1.107295	-0.01265	-0.99734	-1.22642
TRINITY_DN57904_c0_g2_i1_orf1	-	-	-	cuticle protein 19 [Plutella xylostella] >CAG9138481.1 unnamed protein product [Plutella xylostella]	0.952365	1.462801	-0.76747	-0.88196	-0.76574
TRINITY_DN41259_c0_g1_i6_orf1	-	-	-	endocuticle structural glycoprotein SgAbd-8 [Ostrinia furnacalis]	0.992926	1.431861	-0.80414	-0.85496	-0.76569
TRINITY_DN41697_c0_g1_i1_orf1	-	-	-	5-formyltetrahydrofolate cyclo-ligase [Ostrinia furnacalis]	1.367713	0.496278	-0.74517	0.363061	-1.48188
TRINITY_DN8430_c0_g1_i1_orf1	-	-	-	transducin beta-like protein 3 [Ostrinia furnacalis]	1.582865	-1.08886	-0.46504	0.724518	-0.75348
TRINITY_DN5298_c0_g1_i3_orf1	-	-	-	uncharacterized protein LOC114352519 [Ostrinia furnacalis]	1.40394	-0.18573	-1.41407	0.796382	-0.60052

TRINITY_DN46633_c0_g1_i4_orf1	-	-	-	uncharacterized protein LOC114365425 [Ostrinia furnacalis] >QKV49448.1 fas-associated death domain protein [Ostrinia furnacalis]	0.884343	-1.72154	0.250766	1.007152	-0.42072
TRINITY_DN2338_c0_g2_i2_orf1	-	-	-	prophenoloxidase PPO3 [Ostrinia furnacalis]	1.430749	-1.29773	-0.78431	0.795556	-0.14427
TRINITY_DN34056_c0_g1_i4_orf1	-	-	-	hypothetical protein evm_001944 [Chilo suppressalis]	0.951198	-1.19725	-0.58397	1.409074	-0.57906
TRINITY_DN39813_c0_g1_i1_orf1	-	-	-	nucleoside diphosphate kinase [Ostrinia furnacalis]	1.157801	-1.49223	-0.01909	1.004371	-0.65085
TRINITY_DN29_c0_g1_i4_orf1	-	-	-	sodium-dependent nutrient amino acid transporter 1-like [Ostrinia furnacalis] >XP_028167707.1 sodium-dependent nutrient amino acid transporter 1-like [Ostrinia	1.002285	-1.61535	-0.42557	1.096194	-0.05756
TRINITY_DN67495_c0_g1_i1_orf1	-	-	-	hypothetical protein KGM_200102A, partial [Danaus plexippus plexippus]	1.333821	-1.36859	-0.67462	0.920891	-0.2115
TRINITY_DN3918_c0_g1_i1_orf1	-	-	-	odorant binding protein 3 [Ostrinia furnacalis]	0.775753	-1.89299	0.446486	0.777249	-0.1065
TRINITY_DN9506_c0_g1_i2_orf1	-	-	-	glutathione S-transferase sigma 4 [Conogethes punctiferalis]	1.221177	-1.6444	0.284889	0.666637	-0.5283
TRINITY_DN6660_c0_g1_i5_orf1	-	-	-	pre-mRNA-splicing factor SYF1 [Ostrinia furnacalis]	1.530858	-0.96926	-1.01186	0.769086	-0.31882
TRINITY_DN3456_c0_g2_i1_orf1	-	-	-	protein purity of essence [Ostrinia furnacalis]	1.147264	-0.25462	-0.90564	1.189436	-1.17644
TRINITY_DN840_c5_g1_i11_orf1	-	-	-	unnamed protein product [Diatraea saccharalis]	0.964074	-0.66542	-1.11354	1.421293	-0.6064
TRINITY_DN14094_c0_g1_i1_orfp1	-	-	-	TRINITY_DN14094_c0_g1_i1_m.76391 TRINITY_DN14094_c0_g1::TRINITY_DN14094_c0_g1_i1::g.76391 ORF type:5prime_partial len:120 (+),score=12.94 TRINITY_DN14094_c0_g1_i1:3-362(+)	0.899952	-1.78252	-0.18959	0.984416	0.087736
TRINITY_DN13094_c0_g1_i1_orf1	-	-	-	probable ATP-dependent RNA helicase DDX56 [Ostrinia furnacalis]	1.515926	-1.06868	-1.07966	0.627851	0.004565
TRINITY_DN13923_c0_g2_i1_orf1	-	-	-	sideroflexin-2 [Zerene cesonia]	1.361032	-0.17831	-1.06046	0.934798	-1.05706
TRINITY_DN5354_c0_g1_i4_orf1	-	-	-	NADP-dependent malic enzyme-like [Ostrinia furnacalis]	1.531794	-1.21312	-0.78709	0.709991	-0.24158
TRINITY_DN940_c0_g1_i4_orf1	-	-	-	uncharacterized protein LOC114357075 [Ostrinia furnacalis]	1.452121	-0.93731	-1.16093	0.800712	-0.15459
TRINITY_DN3092_c0_g1_i2_orf1	-	-	-	replication factor C subunit 1 isoform X1 [Ostrinia furnacalis] >XP_028157702.1 replication factor C subunit 1 isoform X2 [Ostrinia furnacalis]	0.480896	-0.7081	-0.63257	1.752306	-0.89253
TRINITY_DN2749_c0_g1_i4_orf1	-	-	-	RNA exonuclease 4-like [Ostrinia furnacalis] >QEE79882.1 REX4 [Ostrinia furnacalis]	1.360273	-0.92033	-1.35292	0.623052	0.289927
TRINITY_DN33009_c0_g1_i2_orfp1	-	-	-	TRINITY_DN33009_c0_g1_i2_m.55538 TRINITY_DN33009_c0_g1::TRINITY_DN33009_c0_g1_i2::g.55538 ORF type:complete len:172 (+),score=11.82,fn3 PF00041.22 1.4e-05 TRINITY_DN33009_c0_g1_i2:54-518(+)	1.093802	0.057728	-1.24424	1.106504	-1.01379
TRINITY_DN18918_c0_g1_i2_orf1	-	-	-	myrosinase 1-like isoform X2 [Ostrinia furnacalis]	1.497528	-1.17635	-0.64731	0.834414	-0.50828
TRINITY_DN8771_c0_g2_i1_orf1	-	-	-	regucalcin-like [Ostrinia furnacalis]	0.560955	-0.40398	-0.60057	1.647116	-1.20352
TRINITY_DN99_c0_g1_i3_orf1	-	-	-	uncharacterized protein LOC126375979 [Pectinophora gossypiella] >XP_049879066.1 uncharacterized protein LOC126375979 [Pectinophora gossypiella]	1.53317	-1.0207	-0.84205	0.814482	-0.4849
TRINITY_DN344_c1_g1_i1_orf1	-	-	-	chymotrypsin-like serine protease 16 [Ostrinia nubilalis]	1.523007	-1.10006	-0.65427	0.828224	-0.5969
TRINITY_DN2695_c0_g1_i8_orfp1	-	-	-	TRINITY_DN2695_c0_g1_i8_m.44478 TRINITY_DN2695_c0_g1::TRINITY_DN2695_c0_g1_i8::g.44478 ORF type:3prime_partial len:532	1.227826	-1.16317	-0.85962	1.133873	-0.33891
TRINITY_DN15338_c0_g1_i7_orf1	-	-	-	methyltransferase-like protein 17, mitochondrial [Ostrinia furnacalis]	1.367183	-0.39892	-1.10519	1.001431	-0.8645
TRINITY_DN17189_c0_g1_i2_orf1	-	-	-	fibroin heavy chain [Haritalodes derogata]	1.566614	-1.04932	0.778086	-0.63696	-0.65842
TRINITY_DN640_c0_g1_i5_orf1	-	-	-	pancreatic triacylglycerol lipase-like [Ostrinia furnacalis]	1.623298	-1.4113	-0.52399	-0.00197	0.313965
TRINITY_DN348_c0_g2_i1_orf1	-	-	-	pancreatic triacylglycerol lipase-like [Ostrinia furnacalis]	1.611186	-1.53256	-0.14948	-0.08806	0.158909
TRINITY_DN12250_c0_g1_i4_orf1	-	-	-	ubiquinone biosynthesis monooxygenase COQ6, mitochondrial isoform X1 [Ostrinia furnacalis] >XP_028160041.1 ubiquinone biosynthesis monooxygenase COQ6, mitochondrial isoform X2 [Ostrinia furnacalis]	1.764563	-1.07375	-0.83997	-0.01682	0.165977
TRINITY_DN115658_c0_g1_i1_orf1	-	-	-	hypothetical protein B5X24_HaOG203018 [Helicoverpa armigera]	1.135347	-1.16794	1.199317	-0.92094	-0.24578
TRINITY_DN1697_c0_g1_i1_orf1	-	-	-	mitogen-activated protein kinase-binding protein 1 [Ostrinia furnacalis]	1.57083	-1.41098	0.272664	-0.64854	0.216029
TRINITY_DN61_c0_g2_i3_orf1	-	-	-	mitochondrial dicarboxylate carrier [Ostrinia furnacalis] >XP_028161565.1 mitochondrial dicarboxylate carrier [Ostrinia furnacalis] >XP_028161566.1 mitochondrial dicarboxylate carrier [Ostrinia furnacalis]	1.779669	-1.23366	-0.17765	-0.50935	0.140982
TRINITY_DN27885_c0_g1_i3_orf1	-	-	-	ubiquinone biosynthesis protein COQ9-B, mitochondrial-like isoform X2 [Ostrinia furnacalis]	1.683347	-1.25911	-0.67686	0.33955	-0.08693
TRINITY_DN64759_c0_g1_i1_orf1	-	-	-	mitochondrial inner membrane protein OXA1L-like [Ostrinia furnacalis]	1.460837	-1.67257	-0.00879	-0.0382	0.258723
TRINITY_DN496_c0_g1_i7_orf1	-	-	-	unnamed protein product [Diatraea saccharalis]	1.27702	-1.40262	0.896631	-0.77326	0.002226
TRINITY_DN36699_c0_g1_i3_orf1	-	-	-	pyridoxal phosphate homeostasis protein [Ostrinia furnacalis]	1.561879	-1.18296	0.703311	-0.34002	-0.74221

TRINITY_DN38835_c0_g3_i1_orf1	-	-	-	protein transport protein Sec61 subunit alpha [Spodoptera litura] >XP_035429226.1 protein transport protein Sec61 subunit alpha [Spodoptera frugiperda] >XP_047985890.1 protein transport protein Sec61 subunit alpha [Leguminivora glycinivorella] >KAF9413961.1 hypothetical protein HW555_007991 [Spodoptera exigua] >CAB3514725.1 unnamed protein product [Spodoptera littoralis] >KAF9810869.1 hypothetical protein SFRURICE_005295 [Spodoptera frugiperda] >KAG8115796.1 hypothetical protein SFRUCORN_012373 [Spodoptera frugiperda] >CAH0700181.1 unnamed protein product [Spodoptera exigua]	1.414528	-1.60512	0.258504	-0.45442	0.386507
TRINITY_DN3970_c0_g1_i1_orf1	-	-	-	hypothetical protein evm_002369 [Chilo suppressalis]	1.483247	-1.3716	0.635601	-0.71678	-0.03047
TRINITY_DN14306_c0_g1_i1_orf1	-	-	-	prostaglandin E synthase 2 [Galleria mellonella]	1.523452	-1.34979	0.670941	-0.26368	-0.58093
TRINITY_DN37986_c0_g1_i2_orf1	-	-	-	unnamed protein product [Diatraea saccharalis]	1.545042	-1.13321	0.636367	-0.95675	-0.09146
TRINITY_DN7686_c0_g1_i4_orf1	-	-	-	eIF-2-alpha kinase activator GCN1 [Ostrinia furnacalis]	1.480797	-1.48459	0.635167	-0.33127	-0.30011
TRINITY_DN8406_c0_g1_i4_orf1	-	-	-	titin [Ostrinia furnacalis]	1.055293	-1.60643	1.077707	-0.3165	-0.21008
TRINITY_DN117707_c0_g1_i3_orf1	-	-	-	acyl-CoA synthetase family member 2, mitochondrial isoform X1 [Ostrinia furnacalis] >XP_028172249.1 acyl-CoA synthetase family member 2, mitochondrial isoform X2 [Ostrinia furnacalis] >XP_028172258.1 acyl-CoA synthetase family member 2, mitochondrial isoform X3 [Ostrinia furnacalis]	1.845591	-1.18972	-0.36853	-0.16879	-0.11855
TRINITY_DN12401_c0_g2_i4_orf1	-	-	-	hydroxysteroid dehydrogenase-like protein 2 [Ostrinia furnacalis]	1.528208	-1.42634	0.287306	0.291138	-0.68031
TRINITY_DN72541_c0_g1_i2_orf1	-	-	-	xaa-Pro aminopeptidase ApepP-like isoform X2 [Ostrinia furnacalis]	1.478351	-1.54388	0.545997	-0.147	-0.33348
TRINITY_DN4014_c0_g1_i1_orf1	-	-	-	cyclin-Q [Ostrinia furnacalis]	1.135314	-0.98774	1.045823	0.085059	-1.27845
TRINITY_DN48851_c0_g1_i2_orf1	-	-	-	translationally-controlled tumor protein homolog [Ostrinia furnacalis]	1.307892	-1.39434	0.938963	-0.65008	-0.20244
TRINITY_DN2318_c1_g1_i1_orf1	-	-	-	transcription factor SPT20 homolog [Ostrinia furnacalis]	1.234698	-1.67511	0.781238	-0.19392	-0.1469
TRINITY_DN25901_c0_g1_i2_orf1	-	-	-	short-chain specific acyl-CoA dehydrogenase, mitochondrial-like isoform X2 [Ostrinia	1.640975	-1.16517	-0.53648	-0.54409	0.604761
TRINITY_DN1369_c0_g2_i3_orf1	-	-	-	ATP-dependent Clp protease ATP-binding subunit clpX-like, mitochondrial isoform X2 [Helicoverpa zea]	1.360786	-1.1275	0.969294	-0.92869	-0.2739
TRINITY_DN4538_c0_g1_i4_orf1	-	-	-	2-acylglycerol O-acyltransferase 1-like [Ostrinia furnacalis]	1.921938	-0.91909	-0.56509	-0.371	-0.06675
TRINITY_DN14701_c0_g1_i2_orf1	-	-	-	staphylococcal nuclease domain-containing protein 1 [Ostrinia furnacalis]	1.573558	-1.27217	0.449487	-0.83454	0.083667
TRINITY_DN357_c0_g1_i8_orf1	-	-	-	trifunctional enzyme subunit alpha, mitochondrial [Ostrinia furnacalis]	1.59976	-1.1827	0.643915	-0.35086	-0.71012
TRINITY_DN16673_c0_g1_i1_orf1	-	-	-	myosin heavy chain, partial [Drosophila virilis]	1.403822	-1.67728	0.421695	-0.19074	0.042503
TRINITY_DN146126_c0_g1_i1_orf1	-	-	-	malate dehydrogenase, mitochondrial [Chelonus insularis]	1.437125	-1.69403	-0.03705	0.046121	0.247834
TRINITY_DN79673_c0_g1_i1_orf1	-	-	-	thioredoxin, mitochondrial-like [Ostrinia furnacalis]	1.387605	-1.69769	-0.12175	0.010573	0.421261
TRINITY_DN41166_c0_g1_i1_orf1	-	-	-	arginine kinase isoform X1 [Ostrinia furnacalis]	1.336461	-1.49214	0.82594	-0.53588	-0.13439
TRINITY_DN136906_c0_g1_i1_orf1	-	-	-	translational elongation factor-1alpha, partial [Ethmia eupostica]	1.491267	-1.42084	0.677518	-0.4704	-0.27754
TRINITY_DN277_c0_g1_i5_orf1	-	-	-	uncharacterized protein LOC114363802 isoform X2 [Ostrinia furnacalis]	1.768941	-0.91934	0.286286	-0.18156	-0.95433
TRINITY_DN5218_c0_g1_i4_orf1	-	-	-	threonine--tRNA ligase, cytoplasmic isoform X1 [Trichoplusia ni]	1.685201	-1.44284	0.111845	-0.21635	-0.13786
TRINITY_DN10071_c0_g1_i2_orf1	-	-	-	trypsin, alkaline C-like [Ostrinia furnacalis]	1.590843	-1.22749	-0.08496	0.537749	-0.81614
TRINITY_DN1752_c0_g1_i18_orf1	-	-	-	titin isoform X1 [Ostrinia furnacalis]	1.552012	-1.57231	0.13451	-0.27446	0.16025
TRINITY_DN130159_c0_g2_i1_orf1	-	-	-	lachesin-like [Chelonus insularis] >XP_034946935.1 lachesin-like [Chelonus insularis]	1.542543	-1.47105	0.443833	-0.50945	-0.00587
TRINITY_DN22956_c0_g1_i1_orf1	-	-	-	lipoamide acyltransferase component of branched-chain alpha-keto acid dehydrogenase complex, mitochondrial [Ostrinia furnacalis]	1.518321	-1.54244	-0.33384	-0.08567	0.443625
TRINITY_DN2186_c0_g1_i17_orf1	-	-	-	paxillin isoform X6 [Leguminivora glycinivorella]	0.846168	-1.91734	0.69698	0.025832	0.348361
TRINITY_DN19659_c1_g1_i1_orf1	-	-	-	elongation factor 1-gamma [Ostrinia furnacalis]	1.106814	-1.31178	1.201896	-0.73585	-0.26108
TRINITY_DN10066_c0_g2_i2_orf1	-	-	-	inositol polyphosphate 5-phosphatase K isoform X2 [Manduca sexta]	1.718081	-1.18569	-0.0451	0.267033	-0.75432
TRINITY_DN47605_c0_g2_i1_orf1	-	-	-	hypothetical protein evm_002030 [Chilo suppressalis]	1.937603	-0.87746	-0.29067	-0.60295	-0.16653
TRINITY_DN2224_c0_g1_i1_orf1	-	-	-	serine--tRNA ligase, cytoplasmic [Ostrinia furnacalis]	1.4605	-1.57016	0.511975	-0.37218	-0.03014

TRINITY_DN15234_c0_g1_i3_orf1	-	-	-	60S ribosomal protein L30 [Papilio polytes] >XP_014360326.1 60S ribosomal protein L30 [Papilio machaon] >XP_026485186.1 60S ribosomal protein L30 isoform X1 [Vanessa tameamea] >XP_028160279.1 60S ribosomal protein L30 [Ostrinia furnacalis] >XP_030027999.1 60S ribosomal protein L30 [Manduca sexta] >XP_032515151.1 60S ribosomal protein L30 [Danaus plexippus plexippus] >XP_034840952.1 60S ribosomal protein L30 [Maniola hyperantus] >XP_037301873.1 60S ribosomal protein L30 [Manduca sexta] >XP_039745408.1 60S ribosomal protein L30 [Pararge aegeria] >XP_041974708.1 60S ribosomal protein L30 [Aricia agestis] >XP_045455248.1 60S ribosomal protein L30 [Melitaea cinxia] >XP_045457914.1 60S ribosomal protein L30 [Melitaea cinxia] >XP_046969892.1 60S ribosomal protein L30 [Vanessa cardui] >XP_047539529.1 60S ribosomal protein L30 [Vanessa atalanta] >XP_049887645.1 60S ribosomal protein L30 [Pectinophora gossypiella] >XP_050360253.1 60S ribosomal protein L30 [Nymphalis io] >ADT80684.1 ribosomal protein L30 [Euphydryas aurinia] >CAG9575798.1 unnamed protein product [Danaus chrysippus] >CAH0722581.1 unnamed protein product, partial [Brenthis ino] >CAH2099946.1 unnamed protein product [Euphydryas editha] >CAH2267204.1 jg2932 [Pararge aegeria aegeria]	1.705374	-1.37761	0.139958	-0.41402	-0.0537
TRINITY_DN13718_c0_g1_i7_orf1	-	-	-	immulectin-4 [Ostrinia furnacalis]	1.4608	-1.64062	-0.21285	0.357633	0.035033
TRINITY_DN8584_c0_g1_i6_orf1	-	-	-	uncharacterized protein LOC114354070 isoform X3 [Ostrinia furnacalis]	1.698355	-1.41194	-0.17129	-0.26498	0.149852
TRINITY_DN116951_c0_g3_i2_orf1	-	-	-	spermine oxidase-like isoform X2 [Ostrinia furnacalis]	1.011536	-1.90202	0.263472	0.097748	0.529268
TRINITY_DN89613_c0_g1_i13_orf1	-	-	-	PREDICTED: uncharacterized protein LOC106137743 [Amyelois transitella]	1.314648	-1.49752	0.528905	-0.76018	0.41415
TRINITY_DN25492_c0_g1_i1_orf1	-	-	-	PREDICTED: myrosinase 1-like [Amyelois transitella]	1.206473	-1.81189	0.499033	0.111468	-0.00509
TRINITY_DN1656_c2_g1_i5_orf1	-	-	-	15-hydroxyprostaglandin dehydrogenase [NAD(+)]-like [Ostrinia furnacalis]	1.455867	-1.32828	-0.51829	-0.42561	0.81631
TRINITY_DN7908_c0_g1_i5_orf1	-	-	-	protein transport protein Sec24A [Helicoverpa zea]	1.645282	-1.23319	-0.52987	0.551126	-0.43335
TRINITY_DN14301_c0_g1_i1_orf1	-	-	-	apoptosis-inducing factor 1, mitochondrial-like [Ostrinia furnacalis]	1.826843	-1.00864	-0.74317	-0.24984	0.174813
TRINITY_DN38562_c0_g1_i3_orf1	-	-	-	persulfide dioxygenase ETHE1, mitochondrial isoform X1 [Ostrinia furnacalis]	1.447818	-1.51066	-0.6071	0.454895	0.21504
TRINITY_DN120439_c1_g1_i1_orf1	-	-	-	myosin heavy chain variant, partial [Bombyx mori]	1.159612	-1.80539	0.566858	-0.14953	0.22845
TRINITY_DN15362_c0_g1_i1_orf1	-	-	-	probable elongation factor 1-delta isoform X1 [Ostrinia furnacalis]	1.474994	-1.11953	0.828518	-0.89505	-0.28893
TRINITY_DN1232_c0_g1_i1_orf1	-	-	-	acanthoscurrin-2-like isoform X1 [Ostrinia furnacalis]	1.014137	-1.80387	0.745229	-0.26167	0.30617
TRINITY_DN5765_c0_g2_i3_orf1	-	-	-	unnamed protein product [Diatraea saccharalis]	1.683753	-1.35475	0.293066	-0.46956	-0.15251
TRINITY_DN2924_c0_g1_i2_orf1	-	-	-	cuticular protein RR-2 [Spodoptera litura]	1.369739	-1.55235	-0.12017	-0.42033	0.723117
TRINITY_DN2177_c0_g1_i1_orf1	-	-	-	uncharacterized protein LOC114349824 isoform X1 [Ostrinia furnacalis] >XP_028156186.1 uncharacterized protein LOC114349824 isoform X1 [Ostrinia furnacalis]	1.764145	-1.05296	-0.72783	0.3613	-0.34465
TRINITY_DN9248_c0_g1_i10_orf1	-	-	-	unnamed protein product [Arctia plantaginis]	1.657246	-1.43781	0.241015	-0.33542	-0.12502
TRINITY_DN4385_c0_g2_i1_orf1	-	-	-	LOW QUALITY PROTEIN: carbonic anhydrase 1-like [Ostrinia furnacalis]	1.509474	-1.3891	0.568184	-0.68487	-0.00369
TRINITY_DN14597_c0_g1_i5_orf1	-	-	-	UDP-glucuronosyltransferase 2B1-like isoform X3 [Ostrinia furnacalis]	1.576022	-1.40932	-0.23103	-0.45492	0.519259
TRINITY_DN7267_c1_g1_i4_orf1	-	-	-	probable pseudouridine-5'-phosphatase [Ostrinia furnacalis]	1.727576	-1.31582	-0.02671	-0.51599	0.130941
TRINITY_DN1757_c0_g1_i4_orf1	-	-	-	F-box/LRR-repeat protein 2 isoform X1 [Ostrinia furnacalis]	1.755205	-1.32652	-0.31964	-0.21497	0.105924
TRINITY_DN11448_c0_g1_i4_orf1	-	-	-	uncharacterized protein LOC114364760 isoform X5 [Ostrinia furnacalis]	1.353168	-1.73577	0.342247	-0.11782	0.158179
TRINITY_DN6916_c0_g1_i4_orf1	-	-	-	isovaleryl-CoA dehydrogenase, mitochondrial [Ostrinia furnacalis]	1.544847	-1.56221	0.343772	-0.13592	-0.19049
TRINITY_DN7335_c0_g1_i1_orf1	-	-	-	probable methylmalonate-semialdehyde dehydrogenase [acylating], mitochondrial [Bicyclus anynana]	1.669091	-1.42102	0.036568	0.134249	-0.41888
TRINITY_DN15513_c0_g1_i6_orf1	-	-	-	uncharacterized protein LOC114350859 [Ostrinia furnacalis]	1.632541	-1.33514	-0.52026	-0.24683	0.469691
TRINITY_DN19830_c0_g1_i1_orf1	-	-	-	macrophage migration inhibitory factor-like [Ostrinia furnacalis]	1.485178	-1.64011	-0.10957	0.301538	-0.03705
TRINITY_DN95414_c0_g1_i1_orf1	-	-	-	protein arginine N-methyltransferase 5 [Ostrinia furnacalis]	1.612768	-1.04176	0.477902	-1.04176	-0.00715
TRINITY_DN5873_c0_g4_i1_orf1	-	-	-	hypothetical protein evm_003048 [Chilo suppressalis]	1.692544	-1.03461	0.38956	-0.95065	-0.09684
TRINITY_DN5721_c0_g1_i5_orf1	-	-	-	fumarate hydratase, mitochondrial-like isoform X2 [Ostrinia furnacalis]	1.80896	-1.1376	-0.48143	0.208116	-0.39805
TRINITY_DN34399_c0_g1_i1_orf1	-	-	-	cysteine synthase-like [Ostrinia furnacalis]	1.483563	-1.38389	0.590036	-0.7308	0.041089
TRINITY_DN54336_c0_g1_i1_orf1	-	-	-	basement membrane-specific heparan sulfate proteoglycan core protein isoform X13 [Ostrinia furnacalis]	1.780225	-1.12383	-0.24884	0.256025	-0.66358
TRINITY_DN38435_c0_g1_i1_orf1	-	-	-	UDP-glucuronosyltransferase 2B20-like [Ostrinia furnacalis]	1.265592	-1.75649	0.545589	0.055915	-0.11061
TRINITY_DN147676_c0_g1_i1_orf1	-	-	-	PREDICTED: 60S ribosomal protein L23 [Microplitis demolitor] >XP_044591174.1 60S ribosomal protein L23 [Cotesia glomerata] >KAG8035666.1 hypothetical protein G9C98_001094 [Cotesia typhae] >KAH0547433.1 60S ribosomal protein L23A [Cotesia	1.544897	-1.29281	0.511122	-0.82288	0.059672
TRINITY_DN7414_c0_g1_i1_orf1	-	-	-	uncharacterized protein LOC114357447 [Ostrinia furnacalis]	1.651577	-1.28517	0.254429	-0.73656	0.115717
TRINITY_DN13371_c0_g1_i4_orf1	-	-	-	ATP synthase mitochondrial F1 complex assembly factor 2 [Ostrinia furnacalis]	1.789882	-1.24253	0.136114	-0.35491	-0.32855

TRINITY_DN21872_c0_g1_i2_orf1	-	-	-	facilitated trehalose transporter Tret1-2 homolog [Ostrinia furnacalis] >XP_028178438.1 facilitated trehalose transporter Tret1-2 homolog [Ostrinia furnacalis] >XP_028178439.1 facilitated trehalose transporter Tret1-2 homolog [Ostrinia furnacalis]	1.498906	-1.52984	0.476721	-0.01523	-0.43056
TRINITY_DN3760_c0_g1_i1_orf1	-	-	-	something about silencing protein 10 [Ostrinia furnacalis]	1.774454	-1.2395	-0.30486	0.197633	-0.42772
TRINITY_DN34347_c0_g1_i1_orf1	-	-	-	nesprin-1-like isoform X8 [Bombyx mandarina]	1.360564	-1.29474	0.905789	-0.78585	-0.18577
TRINITY_DN12584_c0_g1_i1_orf1	-	-	-	carnitine O-palmitoyltransferase 1, liver isoform [Ostrinia furnacalis]	1.887118	-0.9968	-0.42235	-0.51443	0.046459
TRINITY_DN1173_c1_g1_i10_orf1	-	-	-	hypothetical protein evm_001011 [Chilo suppressalis]	0.963132	-1.81802	0.870849	-0.07389	0.057925
TRINITY_DN69697_c0_g1_i1_orf1	-	-	-	PREDICTED: uncharacterized protein LOC103573287 [Microplitis demolitor]	1.745783	-1.08088	0.143943	0.062537	-0.87138
TRINITY_DN7909_c0_g2_i1_orf1	-	-	-	aldehyde oxidase 3 [Ostrinia furnacalis]	1.24356	-0.87135	0.972908	-1.32182	-0.02329
TRINITY_DN104139_c0_g1_i1_orf1	-	-	-	cytochrome c oxidase assembly factor 7 homolog isoform X2 [Ostrinia furnacalis]	1.677975	-1.30622	-0.56325	0.362771	-0.17128
TRINITY_DN115498_c0_g1_i1_orf1	-	-	-	fatty acid synthase-like [Ostrinia furnacalis]	1.931605	-0.89002	-0.52024	-0.44803	-0.07331
TRINITY_DN4920_c0_g1_i5_orf1	-	-	-	titin homolog [Ostrinia furnacalis]	1.085239	-1.84147	0.621955	-0.0659	0.200176
TRINITY_DN129869_c0_g4_i1_orf1	-	-	-	putative myosin heavy chain, muscle, partial [Cotesia chilonis]	1.278248	-1.77899	0.39557	-0.08751	0.192682
TRINITY_DN2200_c0_g1_i4_orf1	-	-	-	uncharacterized protein LOC114363443 [Ostrinia furnacalis]	0.946269	-1.85039	0.624643	0.494275	-0.21479
TRINITY_DN21451_c0_g1_i3_orf1	-	-	-	gelsolin-like [Ostrinia furnacalis]	1.616321	-1.44428	-0.45651	-0.02007	0.304541
TRINITY_DN7808_c0_g1_i1_orf1	-	-	-	probable pyruvate dehydrogenase E1 component subunit alpha, mitochondrial isoform X1 [Ostrinia furnacalis] >XP_028158738.1 probable pyruvate dehydrogenase E1 component subunit alpha, mitochondrial isoform X2 [Ostrinia furnacalis] >XP_028158739.1 probable pyruvate dehydrogenase E1 component subunit alpha, mitochondrial isoform X3 [Ostrinia furnacalis] >XP_028158740.1 probable pyruvate dehydrogenase E1 component subunit alpha, mitochondrial isoform X4 [Ostrinia furnacalis]	1.665586	-1.47604	-0.19602	-0.06268	0.069155
TRINITY_DN928_c0_g1_i3_orf1	-	-	-	fasciclin-2-like [Ostrinia furnacalis]	0.802601	-1.89232	0.81685	0.324184	-0.05132
TRINITY_DN33867_c0_g1_i9_orf1	-	-	-	uncharacterized protein LOC114357513 [Ostrinia furnacalis]	1.823352	-0.9078	-0.27584	0.21427	-0.85398
TRINITY_DN57918_c0_g1_i1_orf1	-	-	-	PREDICTED: serine--tRNA ligase, cytoplasmic [Fopius arisanus]	1.414885	-1.64118	0.492491	-0.24857	-0.01763
TRINITY_DN11194_c0_g1_i4_orf1	-	-	-	ATPase family AAA domain-containing protein 3A homolog [Ostrinia furnacalis]	1.707417	-1.36186	-0.05414	-0.44957	0.158157
TRINITY_DN9965_c0_g1_i1_orf1	-	-	-	dihydrolipoyl dehydrogenase [Ostrinia furnacalis]	1.80322	-1.26585	-0.00908	-0.32066	-0.20763
TRINITY_DN29229_c0_g1_i4_orf1	-	-	-	uncharacterized protein LOC114351433 isoform X1 [Ostrinia furnacalis]	1.55509	-1.17083	0.738208	-0.69528	-0.42719
TRINITY_DN5281_c0_g2_i3_orf1	-	-	-	serine/threonine-protein kinase RIO2 isoform X2 [Ostrinia furnacalis]	1.284925	-1.7295	0.581219	-0.14128	0.00464
TRINITY_DN11065_c0_g2_i1_orf1	-	-	-	ribosomal protein s6e domain-containing protein [Phthorimaea operculella]	1.664185	-0.97932	0.470034	-1.01539	-0.1395
TRINITY_DN106534_c0_g1_i1_orf1	-	-	-	nucleolar complex protein 2 homolog [Ostrinia furnacalis]	1.427802	-1.4871	0.505942	-0.66728	0.220636
TRINITY_DN8352_c0_g1_i3_orf1	-	-	-	TRPL translocation defect protein 14 isoform X1 [Ostrinia furnacalis]	1.775472	-1.19725	-0.58899	0.188854	-0.17809
TRINITY_DN48638_c0_g1_i5_orf1	-	-	-	NADH dehydrogenase [ubiquinone] flavoprotein 2, mitochondrial [Ostrinia furnacalis] >ALD03682.1 mitochondrial complex I NDUFV2 subunit [Ostrinia nubilalis]	1.733864	-0.95183	0.38225	-0.21893	-0.94535
TRINITY_DN80245_c0_g1_i1_orf1	-	-	-	peroxisomal membrane protein 2 [Ostrinia furnacalis]	1.589881	-1.5158	-0.03254	-0.31463	0.273091
TRINITY_DN1952_c0_g1_i2_orf1	-	-	-	uncharacterized protein LOC114354403 [Ostrinia furnacalis] >AYE20402.1 RNAi efficiency-related nuclease REase [Ostrinia furnacalis]	1.499181	-1.63202	-0.0922	-0.05351	0.278549
TRINITY_DN5382_c0_g2_i1_orf1	-	-	-	protein seele [Ostrinia furnacalis]	1.512193	-1.21673	0.282399	0.413255	-0.99112
TRINITY_DN1173_c0_g1_i12_orf1	-	-	-	obscurin [Ostrinia furnacalis]	1.630416	-1.40579	0.076015	-0.54689	0.246245
TRINITY_DN110460_c0_g2_i1_orf1	-	-	-	Similar to chaf1a-b: Chromatin assembly factor 1 subunit A-B (Xenopus laevis) [Cotesia congregata]	1.345948	-1.76475	0.04963	0.226271	0.1429
TRINITY_DN10831_c1_g1_i1_orf1	-	-	-	40S ribosomal protein S16 [Ostrinia furnacalis]	1.715194	-1.04425	0.416861	-0.86188	-0.22593
TRINITY_DN105055_c0_g1_i1_orfp1	-	-	-	unnamed protein product [Euphydryas editha]	1.873616	-1.07529	-0.54998	-0.12531	-0.12304
TRINITY_DN2038_c0_g1_i2_orf1	-	-	-	tryptophan--tRNA ligase, cytoplasmic-like [Ostrinia furnacalis]	1.713792	-0.93445	-0.01093	0.284511	-1.05292
TRINITY_DN9280_c0_g1_i1_orf1	-	-	-	stromal cell-derived factor 2 [Ostrinia furnacalis]	1.281661	-0.96913	0.90652	0.043662	-1.26271
TRINITY_DN5111_c0_g1_i2_orf1	-	-	-	uncharacterized protein LOC126368598 [Pectinophora gossypiella]	1.774979	-1.22374	-0.39409	0.224957	-0.38211
TRINITY_DN248_c0_g1_i12_orf1	-	-	-	twitchin-like [Ostrinia furnacalis]	0.663435	-1.74055	1.197185	0.151972	-0.27205
TRINITY_DN36817_c0_g1_i1_orf1	-	-	-	uncharacterized protein LOC114357350 [Ostrinia furnacalis]	1.194126	-1.8316	0.448893	0.096298	0.092285
TRINITY_DN1383_c0_g1_i2_orf1	-	-	-	uncharacterized protein LOC114353133 isoform X1 [Ostrinia furnacalis] >XP_028160773.1 uncharacterized protein LOC114353133 isoform X2 [Ostrinia furnacalis]	1.64306	-1.10696	-0.94945	0.416553	-0.0032
TRINITY_DN14934_c0_g1_i17_orf1	-	-	-	putative tricarboxylate transport protein, mitochondrial isoform X1 [Ostrinia furnacalis] >XP_028177526.1 putative tricarboxylate transport protein, mitochondrial isoform X2 [Ostrinia furnacalis]	1.852509	-1.14051	-0.36296	-0.36791	0.018869
TRINITY_DN4501_c0_g1_i3_orf1	-	-	-	methylcrotonoyl-CoA carboxylase subunit alpha, mitochondrial [Ostrinia furnacalis]	1.355238	-1.64882	0.496372	-0.39954	0.196746

TRINITY_DN4016_c0_g1_i1_orf1	-	-	-	60S acidic ribosomal protein P0 [Homo sapiens] >NP_444505.1 60S acidic ribosomal protein P0 [Homo sapiens] >XP_002823894.1 60S acidic ribosomal protein P0 [Pongo abelii] >XP_003280010.1 60S acidic ribosomal protein P0 [Nomascus leucogenys] >XP_004054038.1 60S acidic ribosomal protein P0 [Gorilla gorilla gorilla] >XP_004054039.1 60S acidic ribosomal protein P0 [Gorilla gorilla gorilla] >XP_008956032.1 60S acidic ribosomal protein P0 [Pan paniscus] >XP_008956033.1 60S acidic ribosomal protein P0 [Pan paniscus] >XP_012611945.1 60S acidic ribosomal protein P0 [Microcebus murinus] >XP_016802006.1 60S acidic ribosomal protein P0 [Pan troglodytes] >XP_016802007.1 60S acidic ribosomal protein P0 [Pan troglodytes] >XP_025256707.1 60S acidic ribosomal protein P0 isoform X1 [Theropithecus gelada] >XP_025256708.1 60S acidic ribosomal protein P0 isoform X1 [Theropithecus gelada] >XP_032024425.1 60S acidic ribosomal protein P0 [Hylobates moloch] >XP_032657670.1 60S acidic ribosomal protein P0 [Chelonoidis abingdonii] >XP_045390642.1 60S acidic ribosomal protein P0 [Lemur catta] >P05388.1 RecName: Full=60S acidic ribosomal protein P0; AltName: Full=60S ribosomal protein L10E; AltName: Full=Large ribosomal subunit protein uL10 [Homo sapiens] >3J92_s Structure and assembly pathway of the ribosome quality control complex [Oryctolagus cuniculus] >4V5Z_Bg Chain Bg, 60S acidic ribosomal protein P0 [Canis lupus familiaris] >4V6X_Cq Chain Cq, 60S acidic ribosomal protein P0 [Homo sapiens] >5AJ0_AK Chain AK, 60S acidic ribosomal protein P0 [Homo sapiens] >6ZM7_Ls Chain Ls, 60S acidic ribosomal protein P0 [Homo sapiens] >6ZME_Ls Chain Ls, 60S acidic ribosomal protein P0 [Homo sapiens] >6ZMI_Ls Chain Ls, 60S acidic ribosomal protein P0 [Homo sapiens] >6ZMO_Ls Chain Ls, 60S acidic ribosomal protein P0 [Homo sapiens] >ABM82739.1 ribosomal protein, large, P0 [synthetic construct] >SJX33952.1 unnamed protein product, partial [Human ORFeome Gateway entry vector] >AAA36470.1 acidic ribosomal phosphoprotein (P0) [Homo sapiens] >AAC05176.1 60S ACIDIC RIBOSOMAL PROTEIN; match to P05388 (PID:g133041) [Homo sapiens] >AAH00087.1 Ribosomal protein, large, P0 [Homo sapiens]	1.269962	-1.79528	0.297134	-0.04378	0.271962
TRINITY_DN1445_c0_g2_i4_orf1	-	-	-	leucine-rich PPR motif-containing protein, mitochondrial [Ostrinia furnacalis]	1.669088	-1.47374	0.01793	-0.00873	-0.20455
TRINITY_DN83542_c0_g1_i1_orf1	-	-	-	PREDICTED: WASH complex subunit strumpellin [Microplitis demolitor]	1.706301	-1.38549	-0.29772	0.18853	-0.21162

TRINITY_DN97589_c0_g1_i3_orf1	-	-	-	ribosomal protein L37A [Bombyx mori] >XP_013189707.1 PREDICTED: 60S ribosomal protein L37a [Amyelois transitella] >XP_021198447.1 60S ribosomal protein L37a [Helicoverpa armigera] >XP_022122377.1 60S ribosomal protein L37a [Pieris rapae] >XP_022822835.1 60S ribosomal protein L37a [Spodoptera litura] >XP_023937141.1 60S ribosomal protein L37a [Bicyclus anynana] >XP_026321523.1 60S ribosomal protein L37a [Hyposmocoma kahamanoa] >XP_026495655.1 60S ribosomal protein L37a [Vanessa tameamea] >XP_026746489.1 60S ribosomal protein L37a [Trichoplusia ni] >XP_026756267.1 60S ribosomal protein L37a [Galleria mellonella] >XP_028041705.1 60S ribosomal protein L37a [Bombyx mandarina] >XP_028161757.1 60S ribosomal protein L37a [Ostrinia furnacalis] >XP_030020263.1 LOW QUALITY PROTEIN: 60S ribosomal protein L37a [Manduca sexta] >XP_032518929.1 60S ribosomal protein L37a [Danaus plexippus plexippus] >XP_034834514.1 60S ribosomal protein L37a [Maniola hyperantus] >XP_035444256.1 60S ribosomal protein L37a [Spodoptera frugiperda] >XP_038222439.1 60S ribosomal protein L37a [Zerene cesonia] >XP_039756348.1 60S ribosomal protein L37a [Pararge aegeria] >XP_041981914.1 60S ribosomal protein L37a [Aricia agestis] >XP_045451710.1 60S ribosomal protein L37a [Melitaea cinxia] >XP_045500579.1 60S ribosomal protein L37a [Colias croceus] >XP_045517305.1 60S ribosomal protein L37a [Pieris brassicae] >XP_045775103.1 60S ribosomal protein L37a [Maniola jurtina] >XP_046969745.1 60S ribosomal protein L37a [Vanessa cardui] >XP_047032252.1 60S ribosomal protein L37a [Helicoverpa zea] >XP_047525321.1 60S ribosomal protein L37a [Pieris napi] >XP_047535357.1 60S ribosomal protein L37a [Vanessa atalanta] >XP_049875744.1 60S ribosomal protein L37a [Pectinophora gossypiella] >XP_050348149.1 60S ribosomal protein L37a [Nymphalis io] >ADO95156.1 ribosomal protein L37A [Antheraea yamamai] >ADT80705.1 ribosomal protein L37A [Euphydryas aurinia] >AEL28885.1 ribosomal protein L37A [Heliconius melpomene cythera] >KAF9418899.1 hypothetical protein HW555_004419 [Spodoptera exigua] >KOB75009.1 Ribosomal protein L37A [Operophtera brumata] >RVE49828.1 hypothetical protein evm_005558 [Chilo suppressalis] >CAB3234150.1 unnamed protein product [Arctia plantaginis] >CAB3509616.1 unnamed protein product [Spodoptera littoralis] >CAF4811073.1 unnamed protein product [Pieris macdunnoughi] >CAG4956733.1 unnamed protein product [Parnassius apollo] >CAG9564640.1 unnamed protein product [Danaus chrysippus] >CAG9750098.1 unnamed	1.659064	-1.17543	0.310792	-0.87351	0.079085
TRINITY_DN74889_c0_g1_i1_orf1	-	-	-	probable 28S ribosomal protein S23, mitochondrial [Ostrinia furnacalis]	1.807606	-1.15941	-0.59373	-0.15822	0.103759
TRINITY_DN135_c0_g1_i1_orf1	-	-	-	60S ribosomal protein L11 [Nymphalis io]	1.691973	-1.45396	-0.05375	-0.13305	-0.05121
TRINITY_DN2593_c0_g3_i1_orf1	-	-	-	midgut carboxypeptidase [Loxostege sticticalis]	1.949101	-0.84824	-0.57925	-0.33124	-0.19036
TRINITY_DN96557_c0_g1_i1_orf1	-	-	-	charged multivesicular body protein 4B [Phyllostomus discolor]	1.216149	-1.81491	0.438673	-0.02448	0.184563
TRINITY_DN3504_c0_g1_i3_orfp2	-	-	-	TRINITY_DN3504_c0_g1_i3_m.43947 TRINITY_DN3504_c0_g1::TRINITY_DN3504_c0_g1_i3::g.43947 ORF type:5prime_partial len:208	1.453067	-1.60504	0.306482	-0.39864	0.244129
TRINITY_DN7325_c0_g1_i1_orf1	-	-	-	collagenase-like [Ostrinia furnacalis]	1.642265	-1.19013	-0.88231	0.303306	0.126867
TRINITY_DN51934_c0_g2_i1_orf1	-	-	-	SCAN domain-containing protein 3-like [Pieris napi] >XP_047520696.1 SCAN domain-containing protein 3-like [Pieris napi]	1.766212	-1.19098	-0.1776	-0.61802	0.220391
TRINITY_DN7134_c0_g1_i1_orf1	-	-	-	phosphatidylglycerophosphatase and protein-tyrosine phosphatase 1 [Ostrinia furnacalis]	1.847371	-1.1144	-0.55583	0.013162	-0.1903
TRINITY_DN17417_c0_g1_i11_orf1	-	-	-	sodium/hydrogen exchanger 9B2-like isoform X4 [Ostrinia furnacalis]	1.872808	-1.03196	-0.60611	-0.24529	0.010553
TRINITY_DN3461_c0_g1_i1_orf1	-	-	-	protein SCO1 homolog, mitochondrial [Ostrinia furnacalis]	1.718594	-1.40517	-0.12822	-0.23107	0.045863
TRINITY_DN15811_c0_g1_i7_orf1	-	-	-	mitochondrial import inner membrane translocase subunit Tim10-like [Ostrinia furnacalis] >XP_028174557.1 mitochondrial import inner membrane translocase subunit Tim10 [Ostrinia furnacalis] >XP_028174558.1 mitochondrial import inner membrane translocase subunit Tim10 [Ostrinia furnacalis] >XP_028174559.1 mitochondrial import inner membrane translocase subunit Tim10 [Ostrinia furnacalis]	1.645911	-1.14466	0.559265	-0.30047	-0.76004
TRINITY_DN5597_c0_g1_i2_orf1	-	-	-	monocarboxylate transporter 9-like [Ostrinia furnacalis] >XP_028156211.1 monocarboxylate transporter 9-like [Ostrinia furnacalis]	1.843463	-1.06658	-0.67662	-0.02548	-0.07479
TRINITY_DN2172_c0_g2_i5_orf1	-	-	-	4-hydroxyphenylpyruvate dioxygenase [Ostrinia furnacalis]	1.673129	-1.38359	-0.16422	-0.41727	0.291948
TRINITY_DN9117_c0_g1_i1_orf1	-	-	-	spherulin-2A-like [Ostrinia furnacalis]	1.785551	-1.3126	-0.09454	-0.12461	-0.2538
TRINITY_DN23740_c1_g1_i1_orf1	-	-	-	NADH dehydrogenase [ubiquinone] iron-sulfur protein 6, mitochondrial isoform X1 [Ostrinia furnacalis]	1.445005	-1.4821	0.129257	0.543148	-0.63531
TRINITY_DN32769_c1_g1_i5_orf1	-	-	-	large subunit GTPase 1 homolog [Ostrinia furnacalis]	1.890646	-1.02845	-0.22421	-0.55771	-0.08029
TRINITY_DN83948_c0_g1_i3_orf1	-	-	-	carbonyl reductase [NADPH] 1-like [Ostrinia furnacalis]	1.871143	-1.04084	-0.47122	0.07439	-0.43348

TRINITY_DN25681_c0_g1_i5_orf1	-	-	-	hypothetical protein evm_005766 [Chilo suppressalis] >CAB3520395.1 unnamed protein product [Chilo suppressalis] >CAH0397716.1 unnamed protein product [Chilo suppressalis]	1.603631	-1.28996	-0.5517	0.583597	-0.34557
TRINITY_DN6027_c0_g1_i13_orf1	-	-	-	5-demethoxyubiquinone hydroxylase, mitochondrial [Ostrinia furnacalis] >XP_028160430.1 5-demethoxyubiquinone hydroxylase, mitochondrial [Ostrinia furnacalis]	1.438938	-1.65023	0.073257	0.378294	-0.24026
TRINITY_DN20957_c0_g1_i1_orf1	-	-	-	adenylate kinase isoenzyme 1 isoform X2 [Ostrinia furnacalis]	1.263552	-1.76146	0.545682	0.005954	-0.05373
TRINITY_DN10007_c0_g1_i1_orf1	-	-	-	28S ribosomal protein S31, mitochondrial [Ostrinia furnacalis]	1.825834	-0.93138	-0.84376	0.181595	-0.23229
TRINITY_DN94337_c0_g1_i1_orf1	-	-	-	hypothetical protein evm_006136 [Chilo suppressalis]	1.891657	-0.98384	-0.63862	-0.20394	-0.06525
TRINITY_DN10458_c0_g1_i1_orf1	-	-	-	V-type proton ATPase 21 kDa proteolipid subunit [Ostrinia furnacalis]	1.896026	-0.89704	-0.72199	-0.28127	0.004275
TRINITY_DN32681_c0_g1_i3_orf1	-	-	-	long-chain-fatty-acid--CoA ligase ACSBG2 isoform X2 [Ostrinia furnacalis]	1.871884	-1.05521	-0.44572	-0.42538	0.054431
TRINITY_DN4622_c0_g1_i1_orf1	-	-	-	keratin-associated protein 19-2-like [Ostrinia furnacalis]	1.666368	-1.44174	0.20873	-0.15691	-0.27645
TRINITY_DN4451_c0_g2_i4_orf1	-	-	-	uncharacterized protein LOC114361986 isoform X1 [Ostrinia furnacalis] >XP_028173022.1 uncharacterized protein LOC114361986 isoform X2 [Ostrinia furnacalis]	1.630471	-1.43708	0.14136	0.149126	-0.48388
TRINITY_DN12476_c0_g1_i4_orf1	-	-	-	guanine nucleotide-binding protein-like 3 homolog [Ostrinia furnacalis]	1.807188	-0.9881	-0.84588	0.158065	-0.13127
TRINITY_DN2283_c0_g2_i1_orf1	-	-	-	H/ACA ribonucleoprotein complex subunit 4 [Ostrinia furnacalis]	1.916264	-0.94685	-0.32917	-0.56313	-0.07712
TRINITY_DN1123_c2_g1_i5_orf1	-	-	-	troponin I isoform X4 [Leguminivora glycinivorella]	1.102257	-1.76236	0.816173	-0.09796	-0.05811
TRINITY_DN15160_c0_g1_i1_orf1	-	-	-	tyrosine--tRNA ligase, cytoplasmic [Ostrinia furnacalis]	1.442352	-1.20706	-0.00538	0.73244	-0.96235
TRINITY_DN48619_c0_g1_i1_orf1	-	-	-	PREDICTED: lysine--tRNA ligase isoform X2 [Fopius arisanus]	1.73373	-1.03907	0.311674	-0.89752	-0.10882
TRINITY_DN21035_c0_g1_i14_orf1	-	-	-	mitochondrial amidoxime reducing component 2-like [Ostrinia furnacalis]	1.132675	-1.16628	0.873331	-1.20776	0.368033
TRINITY_DN55160_c0_g1_i1_orf1	-	-	-	esterase FE4-like isoform X2 [Ostrinia furnacalis]	1.840943	-1.15504	-0.50006	-0.16178	-0.02406
TRINITY_DN56270_c0_g1_i1_orf1	-	-	-	PREDICTED: putative elongator complex protein 1 [Microplitis demolitor] >XP_008554512.1 PREDICTED: putative elongator complex protein 1 [Microplitis demolitor]	1.208119	-1.20952	0.731087	0.434155	-1.16384
TRINITY_DN4145_c0_g1_i1_orf1	-	-	-	uncharacterized protein LOC114353175 isoform X1 [Ostrinia furnacalis]	1.436618	-1.69264	0.244541	-0.06918	0.08066
TRINITY_DN3355_c0_g2_i4_orf1	-	-	-	UDP-glycosyltransferase UGT33AL1 [Ostrinia furnacalis]	1.834711	-1.07225	-0.69318	-0.01	-0.05928
TRINITY_DN6668_c0_g1_i4_orf1	-	-	-	UBX domain-containing protein 4 isoform X1 [Ostrinia furnacalis] >XP_028156702.1 UBX domain-containing protein 4 isoform X2 [Ostrinia furnacalis]	1.787874	-1.01648	-0.18187	0.236006	-0.82553
TRINITY_DN18396_c0_g1_i1_orf1	-	-	-	uncharacterized protein LOC114359424 [Ostrinia furnacalis]	1.44257	-1.51312	0.625465	-0.48273	-0.07218
TRINITY_DN59335_c0_g1_i2_orf1	-	-	-	peroxisomal acyl-coenzyme A oxidase 3 [Ostrinia furnacalis]	1.941302	-0.9336	-0.44975	-0.30898	-0.24896
TRINITY_DN20682_c0_g1_i2_orf1	-	-	-	hypothetical protein B5X24_HaOG200252 [Helicoverpa armigera]	1.774663	-1.28203	0.054011	-0.4386	-0.10804
TRINITY_DN2695_c0_g1_i14_orfp1	-	-	-	TRINITY_DN2695_c0_g1_i14_m.44485 TRINITY_DN2695_c0_g1::TRINITY_DN2695_c0_g1_i14::g.44485 ORF type:3prime_partial len:698 (+),score=187.51 TRINITY_DN2695_c0_g1_i14:101-2092(+)	1.688499	-1.21642	-0.42407	-0.51812	0.470116
TRINITY_DN3229_c0_g1_i1_orf1	-	-	-	uncharacterized protein LOC114358442 isoform X1 [Ostrinia furnacalis]	1.868537	-1.14521	-0.21238	-0.35898	-0.15197
TRINITY_DN32479_c0_g1_i8_orf1	-	-	-	hypothetical protein evm_009815 [Chilo suppressalis] >CAB3525305.1 unnamed protein product [Chilo suppressalis] >CAH0402632.1 unnamed protein product [Chilo suppressalis]	1.746604	-1.28464	-0.0078	-0.54004	0.085873
TRINITY_DN7512_c0_g1_i1_orf1	-	-	-	hypothetical protein evm_010529 [Chilo suppressalis] >CAB3530682.1 unnamed protein product [Chilo suppressalis] >CAH0407273.1 unnamed protein product [Chilo suppressalis]	1.926319	-0.88853	-0.65029	-0.22429	-0.16322
TRINITY_DN117362_c0_g1_i5_orf1	-	-	-	uncharacterized protein LOC114354191 [Ostrinia furnacalis]	1.793134	-1.05744	-0.81178	0.086063	-0.00998
TRINITY_DN19814_c0_g1_i4_orf1	-	-	-	general odorant-binding protein 28a-like [Ostrinia furnacalis]	1.591166	-1.30764	0.437309	-0.75236	0.031521
TRINITY_DN3929_c0_g1_i1_orf1	-	-	-	glutathione S-transferase 1-1-like [Ostrinia furnacalis]	1.938498	-0.83552	-0.66623	-0.26788	-0.16887
TRINITY_DN2497_c0_g1_i2_orf1	-	-	-	protein stunted-like isoform X1 [Colias croceus]	1.745109	-1.04956	0.342922	-0.20602	-0.83246
TRINITY_DN23167_c0_g1_i4_orf1	-	-	-	uncharacterized protein LOC114363065 [Ostrinia furnacalis]	1.540209	-1.32769	0.649994	-0.61907	-0.24345
TRINITY_DN11117_c0_g1_i1_orf1	-	-	-	venom carboxylesterase-6-like [Ostrinia furnacalis]	1.958077	-0.7702	-0.64429	-0.34206	-0.20153
TRINITY_DN313_c0_g1_i5_orf1	-	-	-	collagen alpha-1(X) chain-like [Ostrinia furnacalis]	1.71349	-1.31143	0.075109	-0.57371	0.096542
TRINITY_DN76283_c0_g2_i1_orf1	-	-	-	fatty acid synthase-like [Ostrinia furnacalis]	1.900228	-0.93082	-0.63397	-0.34734	0.0119
TRINITY_DN28638_c0_g1_i1_orf1	-	-	-	uncharacterized protein LOC114364075 [Ostrinia furnacalis]	1.895258	-1.01283	-0.45617	-0.41713	-0.00913
TRINITY_DN4929_c1_g2_i5_orf1	-	-	-	guanylate kinase isoform X2 [Ostrinia furnacalis]	1.659081	-1.20038	0.506075	-0.68858	-0.2762
TRINITY_DN3135_c0_g1_i6_orf1	-	-	-	acanthoscurrin-1-like [Ostrinia furnacalis]	1.475125	-1.66576	0.016489	-0.04298	0.21712
TRINITY_DN6351_c0_g1_i4_orf1	-	-	-	cytochrome P450 CYP12A2-like [Ostrinia furnacalis]	1.889945	-1.10519	-0.25804	-0.23728	-0.28944
TRINITY_DN53462_c0_g1_i1_orf1	-	-	-	uncharacterized protein LOC118072968 isoform X1 [Chelonus insularis] >XP_034949073.1 uncharacterized protein LOC118072968 isoform X1 [Chelonus insularis]	1.406599	-1.23373	0.888019	-0.80254	-0.25835
TRINITY_DN9647_c0_g1_i1_orf1	-	-	-	cytochrome P450 6B2-like [Ostrinia furnacalis]	1.912071	-1.02066	-0.2686	-0.44585	-0.17696
TRINITY_DN34040_c0_g2_i1_orf1	-	-	-	uncharacterized protein LOC114352849 [Ostrinia furnacalis]	1.760214	-1.28735	-0.48085	-0.07701	0.084995
TRINITY_DN6143_c0_g2_i1_orf1	-	-	-	uncharacterized protein LOC114365036 [Ostrinia furnacalis]	1.729254	-1.38036	0.076204	-0.27626	-0.14884
TRINITY_DN5012_c0_g1_i6_orf1	-	-	-	putative serine protease K12H4.7 [Ostrinia furnacalis]	1.782533	-1.2725	-0.44481	-0.07343	0.008208

TRINITY_DN1173_c1_g1_i9_orf1	-	-	-	hypothetical protein evm_001011 [Chilo suppressalis]	1.930201	-0.87884	-0.212	-0.64855	-0.19082
TRINITY_DN2668_c0_g1_i7_orf1	-	-	-	unnamed protein product [Chrysodeixis includens]	1.864269	-1.15898	-0.25808	-0.30924	-0.13797
TRINITY_DN3014_c0_g1_i4_orf1	-	-	-	putative inorganic phosphate cotransporter isoform X1 [Ostrinia furnacalis]	1.82151	-1.22577	-0.26541	-0.33038	4.67E-05
TRINITY_DN130051_c0_g1_i1_orf1	-	-	-	5-methyltetrahydropteroyltriglutamate--homocysteine S-methyltransferase-like protein [Leptotrombidium deliense]	1.746562	-1.24966	-0.38724	-0.39535	0.285687
TRINITY_DN4514_c0_g1_i1_orf1	-	-	-	enoyl-CoA delta isomerase 1, mitochondrial-like isoform X1 [Ostrinia furnacalis] >XP_028158560.1 enoyl-CoA delta isomerase 1, mitochondrial-like isoform X2 [Ostrinia furnacalis]	1.552625	-1.29469	0.531185	0.005206	-0.79433
TRINITY_DN83295_c0_g1_i3_orf1	-	-	-	SSSX-APN4 [Ostrinia furnacalis]	1.861278	-1.16198	-0.21264	-0.13891	-0.34775
TRINITY_DN38498_c0_g3_i1_orf1	-	-	-	unnamed protein product [Parnassius apollo]	1.493562	-1.42753	0.255041	-0.71499	0.393916
TRINITY_DN8621_c0_g1_i5_orf1	-	-	-	aminopeptidase N-like isoform X2 [Ostrinia furnacalis]	1.852984	-1.17932	-0.24301	-0.10607	-0.32459
TRINITY_DN2709_c0_g1_i4_orf1	-	-	-	ATP-dependent RNA helicase dbp2-like [Ostrinia furnacalis]	1.746134	-1.34145	-0.26973	0.119173	-0.25413
TRINITY_DN107288_c0_g1_i2_orf1	-	-	-	methionine-tRNA synthetase, partial [Papilio xuthus]	1.68864	-1.0387	-0.09713	0.397156	-0.94996
TRINITY_DN84357_c0_g1_i1_orf1	-	-	-	4-coumarate--CoA ligase 1-like [Ostrinia furnacalis]	1.395442	-1.30077	0.729618	0.082157	-0.90645
TRINITY_DN22664_c0_g1_i1_orf1	-	-	-	larval cuticle protein LCP-14-like [Ostrinia furnacalis]	1.628226	-1.2535	0.449758	-0.75536	-0.06913
TRINITY_DN5244_c0_g1_i1_orf1	-	-	-	eukaryotic peptide chain release factor GTP-binding subunit-like [Ostrinia furnacalis]	1.746016	-1.36914	-0.11017	-0.25412	-0.01258
TRINITY_DN49508_c0_g2_i8_orf1	-	-	-	putative fatty acyl-CoA reductase CG5065 [Ostrinia furnacalis]	1.93716	-0.86536	-0.59254	-0.36734	-0.11192
TRINITY_DN3504_c0_g1_i4_orfp1	-	-	-	TRINITY_DN3504_c0_g1_i4_m.43930 TRINITY_DN3504_c0_g1::TRINITY_DN3504_c0_g1_i4::g.43930 ORF type:internal len:196 (-	1.59652	-1.53955	-0.00991	-0.22318	0.176111
TRINITY_DN30704_c0_g1_i1_orf1	-	-	-	cytochrome P450 monooxygenase CYP6AE134v2 [Ostrinia furnacalis]	1.791626	-1.23319	-0.4594	-0.21286	0.11383
TRINITY_DN10940_c0_g1_i10_orfp1	-	-	-	TRINITY_DN10940_c0_g1_i10_m.52163 TRINITY_DN10940_c0_g1::TRINITY_DN10940_c0_g1_i10::g.52163 ORF type:5prime_partial len:248 (-),score=128.24 TRINITY_DN10940_c0_g1_i10:121-864(-)	1.808268	-1.279	-0.20121	-0.16106	-0.16699
TRINITY_DN69713_c0_g1_i1_orf1	-	-	-	membrane-bound alkaline phosphatase-like [Ostrinia furnacalis]	1.929689	-0.925	-0.55851	-0.29096	-0.15523
TRINITY_DN2986_c1_g1_i1_orf1	-	-	-	Troponin C, isoform 1 [Papilio xuthus]	1.643576	-1.24861	0.462407	-0.70999	-0.14738
TRINITY_DN2114_c0_g1_i5_orf1	-	-	-	vegetative cell wall protein gp1-like isoform X1 [Ostrinia furnacalis]	1.960682	-0.82807	-0.53323	-0.35476	-0.24462
TRINITY_DN334_c0_g1_i4_orf1	-	-	-	collagenase-like [Ostrinia furnacalis]	1.895514	-1.0204	-0.41884	-0.43585	-0.02042
TRINITY_DN336_c0_g1_i6_orfp1	-	-	-	TRINITY_DN336_c0_g1_i6_m.64791 TRINITY_DN336_c0_g1::TRINITY_DN336_c0_g1_i6::g.64791 ORF type:complete len:61 (-),score=19.53 TRINITY_DN336_c0_g1_i6:236-418(-)	1.847356	-1.09785	-0.41063	0.109813	-0.44869
TRINITY_DN70409_c0_g1_i3_orf1	-	-	-	tyrosine-protein phosphatase non-receptor type 9 isoform X3 [Ostrinia furnacalis]	1.29361	0.530722	0.402462	-1.56263	-0.66416
TRINITY_DN6262_c0_g1_i2_orf1	-	-	-	ADAMTS-like protein 4 isoform X2 [Ostrinia furnacalis] >XP_028169930.1 ADAMTS-like protein 4 isoform X2 [Ostrinia furnacalis]	1.379845	0.427984	0.470294	-1.35901	-0.91911
TRINITY_DN24024_c0_g1_i1_orf1	-	-	-	dolichyl-diphosphooligosaccharide--protein glycosyltransferase subunit 1 [Ostrinia furnacalis]	1.365355	0.610455	0.324306	-1.22977	-1.07035
TRINITY_DN32359_c0_g2_i1_orf1	-	-	-	PREDICTED: 26S proteasome non-ATPase regulatory subunit 4 isoform X2 [Fopius arisanus]	0.668028	1.135578	0.552251	-1.4814	-0.87446
TRINITY_DN24266_c0_g2_i2_orf1	-	-	-	chromobox-like protein 5 [Helicoverpa armigera]	0.724521	1.212373	0.422606	-1.32638	-1.03312
TRINITY_DN2999_c1_g2_i1_orf1	-	-	-	uncharacterized protein C05D11.1-like [Ostrinia furnacalis]	1.553024	0.095739	0.435334	-1.37198	-0.71212
TRINITY_DN3597_c0_g1_i10_orf1	-	-	-	unnamed protein product [Diatraea saccharalis]	1.23923	0.609412	0.308921	-1.65755	-0.50001
TRINITY_DN294_c0_g1_i2_orf1	-	-	-	chloride intracellular channel isoform 1 [Phthorimaea operculella]	1.647109	0.077141	0.302451	-0.75273	-1.27397
TRINITY_DN44407_c0_g4_i2_orf1	-	-	-	eukaryotic translation initiation factor 5A [Antheraea pernyi]	1.471091	0.532986	0.226732	-1.19336	-1.03745
TRINITY_DN145227_c0_g1_i1_orf1	-	-	-	26S protease regulatory subunit 7, partial [Cotesia chilonis]	1.265182	0.581579	0.498954	-1.34741	-0.99831
TRINITY_DN10110_c1_g2_i1_orf1	-	-	-	venom allergen 3-like [Ostrinia furnacalis]	0.516019	0.945859	0.839295	-1.64402	-0.65715
TRINITY_DN181_c0_g1_i3_orf1	-	-	-	hypothetical protein evm_003589 [Chilo suppressalis]	1.492871	0.4884	0.124892	-1.43989	-0.66627
TRINITY_DN41664_c0_g1_i4_orf1	-	-	-	uncharacterized protein LOC114356631 [Ostrinia furnacalis]	1.080793	0.402424	0.879922	-1.40902	-0.95412
TRINITY_DN6381_c0_g1_i2_orf1	-	-	-	solute carrier family 12 member 8 [Ostrinia furnacalis]	1.564479	-0.16907	0.635615	-1.18475	-0.84628
TRINITY_DN33485_c0_g1_i4_orf1	-	-	-	luciferin 4-monooxygenase-like [Ostrinia furnacalis]	1.285251	0.82107	-0.00673	-0.56514	-1.53445
TRINITY_DN146364_c0_g1_i1_orf1	-	-	-	pupal cuticle protein 20-like [Ostrinia furnacalis]	1.282343	0.028405	0.635685	-0.24644	-1.69999
TRINITY_DN461_c0_g1_i5_orf1	-	-	-	cullin-1 isoform X1 [Ostrinia furnacalis] >XP_028173060.1 cullin-1 isoform X2 [Ostrinia furnacalis] >XP_028173061.1 cullin-1 isoform X1 [Ostrinia furnacalis] >XP_028173062.1 cullin-1 isoform X3 [Ostrinia furnacalis] >XP_028173063.1 cullin-1 isoform X4 [Ostrinia furnacalis]	1.426288	0.658802	0.123383	-0.90703	-1.30144
TRINITY_DN14944_c0_g1_i7_orf1	-	-	-	alpha-tocopherol transfer protein-like isoform X1 [Ostrinia furnacalis] >XP_028158173.1 alpha-tocopherol transfer protein-like isoform X1 [Ostrinia furnacalis] >XP_028158174.1 alpha-tocopherol transfer protein-like isoform X1 [Ostrinia furnacalis]	0.577949	1.147132	0.668337	-1.05677	-1.33664
TRINITY_DN19260_c0_g1_i5_orf1	-	-	-	probable 26S proteasome non-ATPase regulatory subunit 3 [Ostrinia furnacalis]	0.807792	0.841358	0.763679	-1.47615	-0.93668
TRINITY_DN14677_c0_g2_i3_orf1	-	-	-	AP-3 complex subunit beta-2 [Ostrinia furnacalis]	0.516267	1.159983	0.6394	-1.5438	-0.77185

TRINITY_DN38667_c0_g1_i9_orf1	-	-	-	signal transducer and activator of transcription 5B-like [Melitaea cinxia]	1.395701	0.808502	-0.04396	-0.90253	-1.25771
TRINITY_DN2224_c0_g2_i1_orf1	-	-	-	tumor susceptibility gene 101 protein [Ostrinia furnacalis]	1.335721	0.430512	0.162907	-1.72061	-0.20853
TRINITY_DN3835_c0_g1_i4_orf1	-	-	-	protein ERGIC-53 isoform X1 [Ostrinia furnacalis] >XP_028177940.1 protein ERGIC-53 isoform X2 [Ostrinia furnacalis] >XP_028177941.1 protein ERGIC-53 isoform X3 [Ostrinia furnacalis]	1.522003	0.053517	0.577964	-0.95873	-1.19475
TRINITY_DN629_c0_g1_i6_orf1	-	-	-	annexin B9-like isoform X1 [Ostrinia furnacalis]	0.658154	1.059344	0.647484	-0.84425	-1.52073
TRINITY_DN61744_c0_g1_i1_orf1	-	-	-	diphosphoinositol polyphosphate phosphohydrolase 2 [Spodoptera litura] >XP_026733652.1 diphosphoinositol polyphosphate phosphohydrolase 2 [Trichoplusia ni] >XP_035444979.1 diphosphoinositol polyphosphate phosphohydrolase 2-like [Spodoptera frugiperda] >KAF9413641.1 hypothetical protein HW555_008219 [Spodoptera exigua] >CAD0201055.1 unnamed protein product [Chrysodeixis includens] >CAH1638489.1 unnamed protein product [Spodoptera littoralis] >KAF9791543.1 hypothetical protein SFRURICE_003239 [Spodoptera frugiperda] >KAG8120910.1 hypothetical protein SFRUCORN_006455 [Spodoptera frugiperda]	1.463708	0.241908	0.276668	-0.37561	-1.60668
TRINITY_DN15762_c0_g1_i2_orf1	-	-	-	YTH domain-containing family protein 3 isoform X3 [Maniola hyperantus]	1.451657	0.494573	0.307812	-1.20761	-1.04643
TRINITY_DN34479_c0_g1_i2_orf1	-	-	-	PREDICTED: 26S protease regulatory subunit 4 [Amyelois transitella] >XP_021186380.1 26S proteasome regulatory subunit 4 [Helicoverpa armigera] >XP_022116536.1 26S proteasome regulatory subunit 4 [Pieris rapae] >XP_022817854.1 26S proteasome regulatory subunit 4 [Spodoptera litura] >XP_026745369.1 26S proteasome regulatory subunit 4 [Trichoplusia ni] >XP_026760570.1 26S proteasome regulatory subunit 4 [Galleria mellonella] >XP_028176505.1 26S proteasome regulatory subunit 4 [Ostrinia furnacalis] >XP_030038234.1 26S proteasome regulatory subunit 4 [Manduca sexta] >XP_035449919.1 26S proteasome regulatory subunit 4 [Spodoptera frugiperda] >XP_038206559.1 26S proteasome regulatory subunit 4 [Zerene cesonia] >XP_045502541.1 26S proteasome regulatory subunit 4 [Colias croceus] >XP_045532999.1 26S proteasome regulatory subunit 4 [Pieris brassicae] >XP_047033702.1 26S proteasome regulatory subunit 4 [Helicoverpa zea] >XP_047994509.1 26S proteasome regulatory subunit 4 [Leguminivora glycinivorella] >XP_049877826.1 26S proteasome regulatory subunit 4 [Pectinophora gossypiella] >KAH9639287.1 hypothetical protein HF086_014151 [Spodoptera exigua] >KAI5631153.1 ATPase family associated with various cellular activities (AAA) domain-containing protein [Phthorimaea operculella] >RVE50066.1 hypothetical protein evm_005272 [Chilo suppressalis] >CAB3245712.1 unnamed protein product [Arctia plantaginis] >KAF9801312.1 hypothetical protein SFRURICE_000406	1.099429	0.525973	0.734049	-1.48979	-0.86966
TRINITY_DN10871_c0_g1_i3_orf1	-	-	-	hypothetical protein HF086_013701 [Spodoptera exigua]	1.37258	0.627047	0.293046	-1.21261	-1.08006
TRINITY_DN3335_c0_g1_i1_orf1	-	-	-	unnamed protein product [Pieris macdunnoughi]	0.502443	0.831441	1.022867	-1.5196	-0.83715
TRINITY_DN7451_c0_g1_i10_orf1	-	-	-	huntingtin-interacting protein 1 isoform X4 [Pectinophora gossypiella]	1.368591	0.721441	0.074261	-1.44193	-0.72237
TRINITY_DN152_c0_g1_i4_orf1	-	-	-	LOW QUALITY PROTEIN: protein tyrosine phosphatase type IVA 3 [Ostrinia furnacalis]	1.227456	0.269454	0.683425	-1.62717	-0.55316
TRINITY_DN54586_c1_g1_i1_orf1	-	-	-	protein YIPF5 [Ostrinia furnacalis]	0.602935	1.213003	0.561672	-1.29629	-1.08132
TRINITY_DN27771_c0_g1_i1_orf1	-	-	-	glycine--tRNA ligase [Ostrinia furnacalis]	1.367041	0.415609	0.439989	-1.49526	-0.72738
TRINITY_DN31431_c0_g1_i1_orf1	-	-	-	carnosine N-methyltransferase [Ostrinia furnacalis]	1.123751	0.485848	0.42392	-1.80854	-0.22498
TRINITY_DN49047_c0_g1_i2_orf1	-	-	-	unnamed protein product [Parnassius apollo]	1.009677	0.673053	0.59295	-1.67941	-0.59627
TRINITY_DN2919_c0_g1_i5_orf1	-	-	-	nidogen-1 [Ostrinia furnacalis]	1.185707	0.807995	0.357345	-1.33338	-1.01767
TRINITY_DN42705_c0_g1_i3_orf1	-	-	-	multiple inositol polyphosphate phosphatase 1 isoform X1 [Ostrinia furnacalis]	1.508657	-0.33829	0.80749	-1.01649	-0.96137
TRINITY_DN101358_c0_g2_i1_orf1	-	-	-	glycylpeptide N-tetradecanoyltransferase 2 [Ostrinia furnacalis]	1.553702	0.547972	0.010975	-0.89205	-1.2206
TRINITY_DN1772_c1_g3_i1_orf1	-	-	-	protein Red isoform X1 [Ostrinia furnacalis] >XP_028157673.1 protein Red isoform X2 [Ostrinia furnacalis] >XP_028157674.1 protein Red isoform X3 [Ostrinia furnacalis] >XP_028157675.1 protein Red isoform X4 [Ostrinia furnacalis]	1.762504	-0.29517	0.320192	-1.12431	-0.66321
TRINITY_DN27398_c0_g1_i3_orf1	-	-	-	lissencephaly-1 homolog [Helicoverpa armigera] >XP_021185428.1 lissencephaly-1 homolog [Helicoverpa armigera] >XP_047030140.1 lissencephaly-1 homolog [Helicoverpa zea] >XP_047030141.1 lissencephaly-1 homolog [Helicoverpa zea]	0.97683	0.798635	0.656806	-1.11965	-1.31262
TRINITY_DN23089_c0_g1_i1_orf1	-	-	-	integrator complex subunit 3 homolog [Ostrinia furnacalis]	1.631434	0.012869	0.423601	-1.1357	-0.9322
TRINITY_DN6602_c0_g1_i4_orf1	-	-	-	PREDICTED: E3 ubiquitin-protein ligase RNF181-like [Amyelois transitella]	0.994302	0.799403	0.340352	-1.76681	-0.36725
TRINITY_DN1781_c0_g1_i8_orf1	-	-	-	transportin-1 [Pectinophora gossypiella]	0.608869	1.229497	0.52092	-1.00197	-1.35732
TRINITY_DN3702_c0_g1_i1_orf1	-	-	-	U4/U6.U5 tri-snRNP-associated protein 2 [Ostrinia furnacalis]	1.221944	0.360001	0.365007	-1.79467	-0.15228
TRINITY_DN10548_c0_g2_i1_orf1	-	-	-	uridine 5'-monophosphate synthase-like [Ostrinia furnacalis]	1.540828	0.355358	0.178578	-1.43447	-0.6403
TRINITY_DN1298_c0_g1_i3_orf1	-	-	-	ras GTPase-activating protein-binding protein 2-like, partial [Ostrinia furnacalis]	1.482617	0.371721	0.175971	-1.54977	-0.48054
TRINITY_DN5346_c0_g1_i5_orf1	-	-	-	syntaxin-1A isoform X2 [Pectinophora gossypiella]	1.048616	0.870325	0.490384	-1.20903	-1.2003

TRINITY_DN2356_c2_g1_i6_orf1	-	-	-	ER membrane protein complex subunit 3 [Ostrinia furnacalis]	1.590977	-0.13588	0.59208	-1.06998	-0.97719
TRINITY_DN105359_c0_g2_i5_orf1	-	-	-	uncharacterized protein LOC114359499 [Ostrinia furnacalis]	0.798008	1.022221	0.405982	-1.69423	-0.53198
TRINITY_DN4381_c0_g2_i1_orf1	-	-	-	eukaryotic initiation factor 4A [Glyphodes caesalis]	1.774319	-0.26477	0.302337	-1.06218	-0.74971
TRINITY_DN92153_c0_g2_i2_orf1	-	-	-	methylenetetrahydrofolate reductase [Ostrinia furnacalis]	1.200213	0.241218	0.862689	-1.37854	-0.92558
TRINITY_DN25916_c0_g1_i1_orf1	-	-	-	uncharacterized protein LOC125063950 [Vanessa atalanta]	1.23467	0.391661	0.66733	-1.49784	-0.79582
TRINITY_DN4546_c0_g1_i3_orf1	-	-	-	telomerase Cajal body protein 1 homolog [Ostrinia furnacalis]	1.52045	0.533207	-0.04937	-0.55892	-1.44537
TRINITY_DN38424_c0_g1_i1_orf1	-	-	-	glucose dehydrogenase [FAD, quinone]-like [Ostrinia furnacalis]	1.002169	0.802292	0.418884	-1.70534	-0.518
TRINITY_DN6071_c0_g1_i1_orf1	-	-	-	transcription initiation factor IIB isoform X1 [Manduca sexta] >XP_038208211.1 transcription initiation factor IIB isoform X1 [Zerene cesonia] >XP_045510964.1 transcription initiation factor IIB isoform X1 [Colias croceus] >XP_049872283.1 transcription initiation factor IIB [Pectinophora gossypiella]	1.092273	0.752198	0.545971	-0.98792	-1.40252
TRINITY_DN79868_c0_g1_i1_orf1	-	-	-	lethal(2)neighbour of Tid protein [Ostrinia furnacalis]	1.444778	0.564331	0.117142	-1.4631	-0.66315
TRINITY_DN23264_c0_g1_i1_orf1	-	-	-	U5 small nuclear ribonucleoprotein 40 kDa protein [Ostrinia furnacalis]	1.636333	0.036692	0.365371	-1.25376	-0.78463
TRINITY_DN6016_c0_g1_i8_orf1	-	-	-	hypothetical protein evm_010883 [Chilo suppressalis]	1.086116	0.457133	0.702013	-0.57584	-1.66942
TRINITY_DN4836_c0_g1_i4_orf1	-	-	-	hypothetical protein O3G_MSEX014157 [Manduca sexta] >KAG6463927.1 hypothetical protein O3G_MSEX014157 [Manduca sexta]	1.626992	0.137084	0.336293	-1.13748	-0.96289
TRINITY_DN198_c0_g1_i2_orf1	-	-	-	retinol dehydrogenase 13-like [Ostrinia furnacalis]	0.593993	0.790391	1.040427	-1.21981	-1.20501
TRINITY_DN3045_c0_g1_i7_orf1	-	-	-	hypothetical protein evm_007836 [Chilo suppressalis]	1.409993	0.144123	0.676093	-1.26861	-0.9616
TRINITY_DN12227_c0_g2_i3_orf1	-	-	-	exonuclease 3'-5' domain-containing protein 2 [Ostrinia furnacalis]	1.095027	0.601419	0.554435	-1.67252	-0.57836
TRINITY_DN23502_c0_g1_i1_orf1	-	-	-	small nuclear ribonucleoprotein F [Ostrinia furnacalis]	1.392613	0.569616	0.267326	-1.41415	-0.81541
TRINITY_DN2232_c1_g1_i3_orf1	-	-	-	protein FAM98A-like [Ostrinia furnacalis]	1.262248	0.80632	0.246958	-1.24367	-1.07186
TRINITY_DN4056_c0_g1_i8_orf1	-	-	-	uncharacterized protein LOC114349672 [Ostrinia furnacalis] >XP_028155936.1 uncharacterized protein LOC114349672 [Ostrinia furnacalis] >XP_028155937.1 uncharacterized protein LOC114349672 [Ostrinia furnacalis] >XP_028155939.1 uncharacterized protein LOC114349672 [Ostrinia furnacalis]	1.555463	0.075992	0.424362	-1.40306	-0.65275
TRINITY_DN3457_c0_g1_i4_orf1	-	-	-	aryl hydrocarbon receptor nuclear translocator homolog [Ostrinia furnacalis]	1.538264	0.342103	0.181474	-1.45411	-0.60773
TRINITY_DN3370_c0_g1_i5_orf1	-	-	-	unnamed protein product, partial [Brenthis ino]	1.583704	-0.35004	0.702533	-0.99434	-0.94186
TRINITY_DN4116_c0_g1_i3_orf1	-	-	-	transmembrane protein 131 homolog [Ostrinia furnacalis]	1.411401	0.495054	0.269596	-1.48964	-0.68641
TRINITY_DN3860_c0_g1_i5_orf1	-	-	-	nucleoplasmin-like protein isoform X1 [Hyposmocoma kahamanoa]	1.408103	0.412146	0.470995	-1.16359	-1.12766
TRINITY_DN51968_c0_g1_i1_orf1	-	-	-	splicing factor U2af 38 kDa subunit [Aphidius gifuensis] >KAF7990547.1 hypothetical protein HCN44_000352 [Aphidius gifuensis]	1.498871	0.538772	0.156296	-1.22345	-0.97049
TRINITY_DN17394_c0_g1_i1_orf1	-	-	-	monocarboxylate transporter 14-like [Ostrinia furnacalis]	1.709657	-0.24684	0.434983	-1.06315	-0.83465
TRINITY_DN124950_c0_g2_i1_orf1	-	-	-	TATA box-binding protein-like protein 1 [Ostrinia furnacalis] >XP_028155830.1 TATA box-binding protein-like protein 1 [Ostrinia furnacalis]	1.557767	0.185178	0.4144	-0.93676	-1.22058
TRINITY_DN10558_c0_g1_i4_orf1	-	-	-	unnamed protein product [Chrysodeixis includens]	0.934631	1.042369	0.415332	-1.25005	-1.14229
TRINITY_DN556_c0_g2_i1_orf1	-	-	-	serine protease inhibitor dipetalogastin-like [Ostrinia furnacalis]	0.972543	0.54584	0.665846	-0.41115	-1.77308
TRINITY_DN4345_c0_g1_i9_orf1	-	-	-	uncharacterized protein LOC114357127 [Ostrinia furnacalis]	1.498971	0.204987	0.425977	-1.42642	-0.70351
TRINITY_DN29934_c0_g1_i6_orf1	-	-	-	sodium/potassium-transporting ATPase subunit beta-2-like [Ostrinia furnacalis] >XP_028176258.1 sodium/potassium-transporting ATPase subunit beta-2-like [Ostrinia furnacalis]	1.561204	0.295491	0.320088	-1.13126	-1.04552
TRINITY_DN3614_c0_g2_i1_orf1	-	-	-	PC4 and SFRS1-interacting protein isoform X4 [Galleria mellonella]	1.178807	0.837267	0.326272	-1.34388	-0.99847
TRINITY_DN1013_c0_g1_i3_orf1	-	-	-	TELO2-interacting protein 1 homolog isoform X2 [Ostrinia furnacalis]	1.014583	0.555792	0.790603	-0.82725	-1.53373
TRINITY_DN37336_c1_g1_i1_orf1	-	-	-	actin-related protein 2/3 complex subunit 2 [Ostrinia furnacalis]	1.738191	-0.32607	0.26692	-0.39707	-1.28198
TRINITY_DN20878_c0_g4_i2_orf1	-	-	-	GTP 3',8-cyclase, mitochondrial isoform X1 [Ostrinia furnacalis]	1.477726	-0.43862	0.668137	-0.25354	-1.4537
TRINITY_DN49221_c0_g1_i1_orf1	-	-	-	Similar to ND-23: NADH dehydrogenase (ubiquinone) 23 kDa subunit (Drosophila melanogaster) [Cotesia congregata]	0.69168	0.197683	1.323791	-0.73171	-1.48145
TRINITY_DN102260_c0_g1_i1_orf1	-	-	-	unnamed protein product [Diatraea saccharalis]	1.225147	-0.94678	1.21581	-0.81092	-0.68326
TRINITY_DN129_c0_g1_i6_orf1	-	-	-	xanthine dehydrogenase [Ostrinia furnacalis]	0.970941	-0.19543	0.944596	0.047534	-1.76764
TRINITY_DN9510_c0_g2_i1_orf1	-	-	-	RNA polymerase II transcriptional coactivator [Ostrinia furnacalis]	0.906802	-0.46556	1.457009	-1.08405	-0.8142
TRINITY_DN19995_c0_g1_i2_orf1	-	-	-	E3 ubiquitin-protein ligase ZNF598 [Ostrinia furnacalis]	0.950549	-0.67594	1.425858	-0.56678	-1.13369
TRINITY_DN21000_c0_g1_i1_orf1	-	-	-	elongation factor-1 alpha, partial [Loxostege sticticalis] >QCO92153.1 elongation factor-1 alpha, partial [Sitochroa umbrosalis]	1.32035	-0.99489	1.110038	-0.76444	-0.67105
TRINITY_DN2813_c0_g1_i3_orf1	-	-	-	arylphorin subunit alpha-like [Ostrinia furnacalis]	0.690856	-0.90312	1.591946	-0.3574	-1.02229
TRINITY_DN95713_c0_g1_i1_orf1	-	-	-	SH3 domain-binding glutamic acid-rich protein homolog [Zerene cesonia]	1.680386	-0.47865	0.404702	-0.30668	-1.29975

TRINITY_DN19939_c0_g1_i4_orf1	-	-	-	unnamed protein product [Chilo suppressalis]	1.13816	-0.79461	1.289143	-0.61828	-1.0144
TRINITY_DN8136_c0_g1_i1_orf1	-	-	-	HIG1 domain family member 2A, mitochondrial [Ostrinia furnacalis]	0.408475	0.104594	1.488659	-0.45194	-1.54979
TRINITY_DN467_c9_g1_i2_orf1	-	-	-	band 4.1-like protein 5 [Ostrinia furnacalis]	1.341269	-0.18281	0.911939	-0.72481	-1.34558
TRINITY_DN6638_c0_g1_i1_orf1	-	-	-	ubiquinone biosynthesis protein COQ4 homolog, mitochondrial [Ostrinia furnacalis]	1.291865	-0.58307	0.851645	-0.05623	-1.50421
TRINITY_DN1760_c0_g1_i4_orf1	-	-	-	uncharacterized protein LOC114357676 [Ostrinia furnacalis]	1.041268	-0.09613	0.466117	0.454857	-1.86611
TRINITY_DN2532_c0_g3_i1_orf1	-	-	-	hypothetical protein O3G_MSEX013489 [Manduca sexta]	1.221202	-0.05216	0.272243	0.373244	-1.81453
TRINITY_DN3307_c1_g1_i2_orf1	-	-	-	BTB/POZ domain-containing protein 2-like [Ostrinia furnacalis]	0.714513	-0.19145	1.48134	-0.6489	-1.3555
TRINITY_DN32687_c0_g1_i2_orf1	-	-	-	protein D2-like isoform X2 [Ostrinia furnacalis] >XP_028164613.1 protein D2-like isoform X2 [Ostrinia furnacalis]	0.925043	-0.95974	1.473426	-0.62653	-0.81219
TRINITY_DN40911_c0_g1_i1_orf1	-	-	-	peroxisomal membrane protein PEX16 [Ostrinia furnacalis]	0.374119	-0.38765	1.762954	-0.68562	-1.06381
TRINITY_DN5593_c0_g1_i1_orf1	-	-	-	PREDICTED: leucine-rich repeat-containing protein 47-like [Fopius arisanus]	1.681768	-0.29818	0.310089	-0.32133	-1.37235
TRINITY_DN69334_c0_g1_i1_orf1	-	-	-	PREDICTED: 15-hydroxyprostaglandin dehydrogenase [NAD(+)]-like [Papilio xuthus]	0.937551	-0.65057	1.467815	-0.91995	-0.83485
TRINITY_DN91877_c0_g1_i1_orf1	-	-	-	NADH dehydrogenase [ubiquinone] 1 alpha subcomplex assembly factor 2 [Ostrinia furnacalis]	0.975401	-0.44823	0.905131	0.284517	-1.71682
TRINITY_DN115082_c0_g1_i5_orf1	-	-	-	protein dj-1beta-like isoform X2 [Ostrinia furnacalis]	1.012946	-1.43334	1.233602	-0.2235	-0.58971
TRINITY_DN195_c8_g1_i1_orf1	-	-	-	hypothetical protein evm_009768 [Chilo suppressalis]	0.535507	-0.18349	1.640023	-1.04975	-0.94229
TRINITY_DN33452_c0_g1_i3_orf1	-	-	-	lethal(2) giant larvae protein isoform X8 [Ostrinia furnacalis]	1.001461	-0.17303	1.216438	-0.57709	-1.46778
TRINITY_DN195_c4_g1_i1_orf1	-	-	-	beta-1,3-glucan-binding protein 1 [Ostrinia furnacalis]	1.105209	-0.85546	1.336887	-0.81243	-0.7742
TRINITY_DN27592_c0_g1_i1_orf1	-	-	-	D-arabinitol dehydrogenase 1-like [Ostrinia furnacalis]	1.362706	-0.89118	1.054356	-0.57188	-0.95399
TRINITY_DN33408_c0_g1_i1_orf1	-	-	-	hypothetical protein HF086_017664 [Spodoptera exigua]	-0.00132	0.345798	-0.28069	1.517235	-1.58102
TRINITY_DN6203_c0_g1_i1_orfp1	-	-	-	TRINITY_DN6203_c0_g1_i1_m.72736 TRINITY_DN6203_c0_g1::TRINITY_DN6203_c0_g1_i1::g.72736 ORF type:internal len:93	-0.07726	0.41175	-1.01625	1.674922	-0.99316
TRINITY_DN128231_c0_g1_i5_orf1	-	-	-	glutathione S-transferase sigma3 [Glyphodes pyloalis]	-0.42858	-0.19122	-0.39614	1.940932	-0.92499
TRINITY_DN52887_c0_g1_i1_orf1	-	-	-	cytochrome P450 6B5-like [Ostrinia furnacalis]	0.242648	-0.58719	0.236942	1.559521	-1.45193
TRINITY_DN102712_c0_g1_i1_orf1	-	-	-	transmembrane protein 177 [Ostrinia furnacalis]	0.533195	-0.25587	-0.05061	1.406834	-1.63355
TRINITY_DN3322_c0_g1_i2_orf1	-	-	-	myrosinase 1-like isoform X2 [Ostrinia furnacalis]	0.988429	0.715541	-1.57527	0.650231	-0.77893
TRINITY_DN11856_c0_g1_i4_orf1	-	-	-	cartilage-associated protein-like [Ostrinia furnacalis]	0.578674	0.984745	-1.47725	0.82566	-0.91183
TRINITY_DN32780_c0_g1_i2_orf1	-	-	-	renin receptor [Ostrinia furnacalis]	0.435011	1.130015	-0.77514	0.749836	-1.53972
TRINITY_DN3478_c0_g1_i10_orf1	-	-	-	acyl-CoA dehydrogenase family member 9, mitochondrial [Ostrinia furnacalis]	0.982924	0.46842	-1.74662	0.747704	-0.45242
TRINITY_DN2109_c0_g1_i4_orf1	-	-	-	mucin-2-like isoform X2 [Ostrinia furnacalis]	-0.30812	1.709622	-1.02869	0.467261	-0.84007
TRINITY_DN13221_c0_g1_i3_orf1	-	-	-	fasciclin-3-like [Ostrinia furnacalis]	-0.01932	1.490172	-1.55856	0.459815	-0.3721
TRINITY_DN1322_c0_g1_i4_orf1	-	-	-	putative histone-binding protein Caf1 [Papilio machaon]	0.631976	1.032552	-0.75556	0.674778	-1.58374
TRINITY_DN25345_c0_g1_i1_orf1	-	-	-	chromodomain-helicase-DNA-binding protein 1 isoform X3 [Ostrinia furnacalis]	-0.25722	1.835843	-0.96462	0.164445	-0.77845
TRINITY_DN23582_c0_g1_i1_orf1	-	-	-	unnamed protein product [Diatraea saccharalis]	-0.16339	1.744339	-1.05162	0.320395	-0.84972
TRINITY_DN21380_c0_g1_i1_orf1	-	-	-	ankyrin repeat domain-containing protein 13C [Ostrinia furnacalis]	0.034251	1.450412	-0.6795	0.624371	-1.42954
TRINITY_DN23838_c0_g1_i4_orf1	-	-	-	ubiquitin domain-containing protein 2 isoform X1 [Ostrinia furnacalis] >XP_028177862.1 ubiquitin domain-containing protein 2 isoform X2 [Ostrinia furnacalis] >XP_028177863.1 ubiquitin domain-containing protein 2 isoform X1 [Ostrinia furnacalis]	0.804977	0.770324	-1.13792	0.86945	-1.30683
TRINITY_DN13999_c0_g1_i4_orf1	-	-	-	LOW QUALITY PROTEIN: succinate--hydroxymethylglutarate CoA-transferase-like [Ostrinia furnacalis]	1.034411	0.580163	-1.33029	0.802137	-1.08642
TRINITY_DN14217_c0_g1_i1_orf1	-	-	-	putative serine protease K12H4.7 [Ostrinia furnacalis] >XP_028166339.1 putative serine protease K12H4.7 [Ostrinia furnacalis]	0.826851	1.032655	-1.3884	0.542688	-1.01379
TRINITY_DN526_c0_g1_i1_orf1	-	-	-	secretory phospholipase A2 receptor-like [Ostrinia furnacalis]	-0.24343	0.478584	1.41687	-0.0076	-1.64442
TRINITY_DN110523_c0_g2_i1_orf1	-	-	-	uncharacterized protein LOC107036393 [Diachasma alloeum]	-0.35668	1.726811	0.394405	-0.58347	-1.18106
TRINITY_DN4798_c0_g1_i3_orf1	-	-	-	unnamed protein product [Spodoptera exigua]	0.031166	1.287096	0.861517	-0.75487	-1.42491
TRINITY_DN2745_c0_g1_i2_orf1	-	-	-	PREDICTED: tubulin alpha-1A chain-like [Papilio polytes] >XP_013164648.1 PREDICTED: tubulin alpha-1A chain-like [Papilio xuthus]	0.074167	1.608109	0.291133	-0.5528	-1.42061
TRINITY_DN2499_c0_g1_i4_orf1	-	-	-	WD repeat-containing protein 92 isoform X1 [Ostrinia furnacalis]	-0.21588	0.3365	1.500007	-0.01127	-1.60935
TRINITY_DN26985_c0_g1_i5_orf1	-	-	-	secretory phospholipase A2 receptor-like [Helicoverpa zea]	-0.57368	1.366742	1.033837	-0.73275	-1.09415
TRINITY_DN1436_c0_g1_i5_orf1	-	-	-	vacuolar protein sorting-associated protein 27-like [Trichoplusia ni]	0.129728	1.496428	0.262195	-0.27627	-1.61208
TRINITY_DN100208_c0_g1_i1_orf1	-	-	-	neurofilament heavy polypeptide-like isoform X2 [Ostrinia furnacalis]	-0.1176	1.130128	0.674427	0.113393	-1.80035
TRINITY_DN52244_c1_g1_i1_orf1	-	-	-	triokinase/FMN cyclase-like isoform X2 [Ostrinia furnacalis]	0.038587	0.391289	1.390911	-0.11883	-1.70195
TRINITY_DN48694_c0_g1_i1_orfp1	-	-	-	TRINITY_DN48694_c0_g1_i1_m.75338 TRINITY_DN48694_c0_g1::TRINITY_DN48694_c0_g1_i1::g.75338 ORF type:internal len:84 (+),score=16.02 TRINITY_DN48694_c0_g1_i1:2-250(+)	-0.53088	0.872206	1.047248	0.279529	-1.6681

TRINITY_DN5770_c0_g1_i4_orf1	-	-	-	rotatin-like [Ostrinia furnacalis]	-0.40245	-0.56363	1.550677	0.693824	-1.27842
TRINITY_DN50725_c0_g1_i6_orf1	-	-	-	BTB/POZ domain-containing protein 2-like [Ostrinia furnacalis]	-0.21857	-0.53514	1.960683	-0.38764	-0.81933
TRINITY_DN1380_c0_g1_i5_orf1	-	-	-	ubiquitin-fold modifier-conjugating enzyme 1 [Ostrinia furnacalis]	-0.25459	-0.59099	1.499664	0.703252	-1.35734
