# Supplementary material for: Comparative Proteomics and Metabonomics Analysis of Different Diapause Stages Revealed a New Regulation Mechanism of Diapause in Loxostege sticticalis (Lepidoptera: Pyralidae)
Source: Molecules. 2024 Jul 25;29(15):3472. doi: 10.3390/molecules29153472 (PMC11314584; doi:10.3390/molecules29153472)
Supplement: Supplementary file 1 [file molecules-29-03472-s001.zip › analysis process/proteomic/Cluster analysis of expression patterns/Down/DvsPreD down.pdf]

Accession	Symbol	Protein Na	Entrez ID	Description	ND	PreD	RD	CT	D
TRINITY_DN2396_c0_g1_i9_orfp1	-	-	-	TRINITY_DN2396_c0_g1_i9_m.39038 TRINITY_DN2396_c0_g1::TRINITY_DN2396_c0_g1_i9::g.39038 ORF type:5prime_partial len:181 (+),score=90.33 TRINITY_DN2396_c0_g1_i9:3-545(+)	-1.92051	0.75417	-0.04221	0.67782	0.53072
TRINITY_DN971_c0_g1_i10_orfp1	-	-	-	TRINITY_DN971_c0_g1_i10_m.54268 TRINITY_DN971_c0_g1::TRINITY_DN971_c0_g1_i10::g.54268 ORF type:internal len:187 (+),score=141.68 TRINITY_DN971_c0_g1_i10:1-558(+)	-1.8854	0.89113	-0.1083	0.42557	0.67699
TRINITY_DN14242_c0_g1_i2_orfp1	-	-	-	TRINITY_DN14242_c0_g1_i2_m.18449 TRINITY_DN14242_c0_g1::TRINITY_DN14242_c0_g1_i2::g.18449 ORF type:internal len:148 (-),score=102.30 TRINITY_DN14242_c0_g1_i2:2-442(-)	-1.88284	0.95197	0.08701	0.72609	0.11777
TRINITY_DN10877_c0_g1_i1_orf1	-	-	-	spodomicin-like [Ostrinia furnacalis]	-1.5409	1.42832	-0.39422	0.64179	-0.13499
TRINITY_DN1108_c3_g1_i1_orfp1	-	-	-	TRINITY_DN1108_c3_g1_i1_m.5561 TRINITY_DN1108_c3_g1::TRINITY_DN1108_c3_g1_i1::g.5561 ORF type:internal len:113 (+),score=89.07 TRINITY_DN1108_c3_g1_i1:1-336(+)	-1.90698	0.7938	0.17271	0.83166	0.10881
TRINITY_DN30510_c0_g1_i6_orf1	-	-	-	spodomicin-like [Ostrinia furnacalis]	-1.29959	1.19375	-1.05909	0.37534	0.78959
TRINITY_DN27300_c0_g1_i1_orfp1	-	-	-	TRINITY_DN27300_c0_g1_i1_m.71142 TRINITY_DN27300_c0_g1::TRINITY_DN27300_c0_g1_i1::g.71142 ORF type:internal len:82 (-),score=9.46 TRINITY_DN27300_c0_g1_i1:3-245(-)	-1.16404	1.67074	-0.88268	0.23183	0.14416
TRINITY_DN35809_c0_g1_i1_orf1	-	-	-	spodomicin-like [Ostrinia furnacalis] >QKV49445.1 diapausin [Ostrinia furnacalis]	-1.6706	0.99078	-0.59427	0.81406	0.46002
TRINITY_DN28501_c0_g1_i2_orfp1	-	-	-	TRINITY_DN28501_c0_g1_i2_m.58934 TRINITY_DN28501_c0_g1::TRINITY_DN28501_c0_g1_i2::g.58934 ORF type:internal len:98 (+),score=13.70 TRINITY_DN28501_c0_g1_i2:3-293(+)	-0.9138	1.52418	-1.27054	0.26055	0.39961
TRINITY_DN47784_c0_g2_i1_orfp1	-	-	-	arylphorin subunit alpha-like [Ostrinia furnacalis]	-1.66831	1.36078	-0.18103	-0.08201	0.57057
TRINITY_DN2457_c0_g1_i8_orf1	-	-	-	uncharacterized protein LOC114355596 [Ostrinia furnacalis]	-1.35161	1.4347	-0.88645	0.34564	0.45773
TRINITY_DN3275_c0_g2_i3_orf1	-	-	-	hypothetical protein B5X24_HaOG216046 [Helicoverpa armigera]	-1.74395	1.18595	-0.23787	0.09888	0.69698
TRINITY_DN136031_c0_g1_i7_orf1	-	-	-	ferritin, lower subunit isoform X3 [Spodoptera litura]	-0.63137	1.56164	-0.8095	-0.92643	0.80566
TRINITY_DN30306_c0_g2_i1_orf1	-	-	-	perilipin-4-like isoform X3 [Ostrinia furnacalis]	-1.64237	1.14023	-0.58492	0.35707	0.72998
TRINITY_DN59429_c0_g1_i6_orf1	-	-	-	uncharacterized protein LOC114366345 isoform X2 [Ostrinia furnacalis]	-1.52804	1.06094	-0.79362	0.8701	0.39062
TRINITY_DN2407_c0_g1_i2_orf1	-	-	-	uncharacterized protein LOC114366345 isoform X2 [Ostrinia furnacalis]	-1.02281	1.70113	-0.7624	0.52949	-0.44541
TRINITY_DN609_c0_g1_i1_orf1	-	-	-	zonadhesin-like isoform X1 [Ostrinia furnacalis]	-1.14528	1.40622	-1.1348	0.58059	0.29327
TRINITY_DN24789_c0_g1_i9_orfp1	-	-	-	TRINITY_DN24789_c0_g1_i9_m.25888 TRINITY_DN24789_c0_g1::TRINITY_DN24789_c0_g1_i9::g.25888 ORF type:internal len:114 (-),score=12.65 TRINITY_DN24789_c0_g1_i9:2-340(-)	-1.01474	1.69065	-1.02403	0.21118	0.13694
TRINITY_DN14754_c0_g1_i6_orf1	-	-	-	cathepsin L [Papilio xuthus]	-0.81321	1.68125	-1.11172	-0.22922	0.4729
TRINITY_DN1149_c0_g1_i4_orf1	-	-	-	circadian clock-controlled protein-like [Ostrinia furnacalis]	-1.45568	1.26362	-0.82548	0.71512	0.30242
TRINITY_DN7226_c0_g1_i2_orf1	-	-	-	chemosensory protein [Conogethes punctiferalis]	-1.7465	0.82251	-0.44449	0.94813	0.42034
TRINITY_DN7226_c0_g1_i5_orf1	-	-	-	chemosensory protein [Dioryctria abietella]	-1.79643	0.94469	-0.34679	0.74398	0.45455
TRINITY_DN5198_c0_g1_i5_orfp1	-	-	-	TRINITY_DN5198_c0_g1_i5_m.8637 TRINITY_DN5198_c0_g1::TRINITY_DN5198_c0_g1_i5::g.8637 ORF type:complete len:223 (-),score=54.99 TRINITY_DN5198_c0_g1_i5:319-987(-)	-1.36167	1.31173	-0.9491	0.38687	0.61217
TRINITY_DN71698_c0_g1_i1_orfp1	-	-	-	TRINITY_DN71698_c0_g1_i1_m.1194 TRINITY_DN71698_c0_g1::TRINITY_DN71698_c0_g1_i1::g.1194 ORF type:internal len:134 (+),score=19.66,Toxin_2 PF00451.20 4.3e-05,Toxin_2|PF00451.20|0.037,Toxin_2|PF00451.20|7.5e-05,Gamma-thionin|PF00304.21|0.017,Gamma-thionin|PF00304.21|0.05,Gamma-thionin|PF00304.21|0.021,Toxin_38|PF14866.7|0.13,Toxin_38|PF14866.7|0.15,Toxin_38|PF14866.7|0.15,Defensin_2|PF01097.19|0.053,Defensin_2|PF01097.19|0.34,Defensin_2|PF01097.19|0.092 TRINITY_DN71698_c0_g1_i1:3-401(+)	-1.67369	0.86061	-0.57881	0.9738	0.41809
TRINITY_DN5153_c1_g1_i1_orf1	-	-	-	nose resistant to fluoxetine protein 6-like isoform X1 [Ostrinia furnacalis]	-1.64455	1.19455	-0.57412	0.42701	0.59712
TRINITY_DN12586_c0_g1_i4_orf1	-	-	-	zonadhesin-like isoform X4 [Ostrinia furnacalis]	-1.11446	1.07618	-1.25954	0.9415	0.35631
TRINITY_DN11981_c0_g1_i7_orf1	-	-	-	luciferin 4-monooxygenase-like isoform X2 [Ostrinia furnacalis]	-1.64747	1.1955	-0.55027	0.66168	0.34057

TRINITY_DN64719_c0_g1_i2_orfp1	-	-	-	TRINITY_DN64719_c0_g1_i2_m.37745 TRINITY_DN64719_c0_g1::TRINITY_DN64719_c0_g1_i2::g.37745 ORF type:internal len:91 (+),score=41.89 TRINITY_DN64719_c0_g1_i2:1-270(+)	-0.81997	1.57752	-1.29225	0.15284	0.38186
TRINITY_DN15685_c0_g1_i5_orf1	-	-	-	uncharacterized protein LOC114352354 [Ostrinia furnacalis]	-1.12233	1.09574	-1.2913	0.61491	0.70297
TRINITY_DN27300_c0_g1_i7_orfp1	-	-	-	TRINITY_DN27300_c0_g1_i7_m.71141 TRINITY_DN27300_c0_g1::TRINITY_DN27300_c0_g1_i7::g.71141 ORF type:internal len:82 (-),score=6.59 TRINITY_DN27300_c0_g1_i7:3-245(-)	-1.49298	1.51949	-0.49544	0.46551	0.00342
TRINITY_DN57111_c0_g1_i1_orf1	-	-	-	trypsin-like serine proteinase T26 protein, partial [Chilo infuscatellus]	-0.54962	1.38376	-1.58185	0.39994	0.34777
TRINITY_DN1593_c0_g1_i1_orf1	-	-	-	chemosensory protein csp11 [Helopeltis theivora]	-1.25187	1.31471	-1.08597	0.53995	0.48319
TRINITY_DN5748_c0_g1_i6_orf1	-	-	-	glycine N-methyltransferase isoform X1 [Ostrinia furnacalis] >XP_028165118.1 glycine N-methyltransferase isoform X2 [Ostrinia furnacalis] >XP_028165119.1 glycine N-methyltransferase isoform X1 [Ostrinia furnacalis] >XP_028165120.1 glycine N-methyltransferase isoform X2 [Ostrinia furnacalis]	-1.42537	1.38264	-0.82201	0.37303	0.4917
TRINITY_DN8685_c0_g1_i5_orf1	-	-	-	macrophage mannose receptor 1-like [Zerene cesonia]	-1.43299	1.3785	-0.78348	0.62066	0.21731
TRINITY_DN84938_c0_g1_i4_orf1	-	-	-	vinculin-like isoform X2 [Ostrinia furnacalis]	-1.16778	1.50581	-0.66253	-0.50003	0.82453
TRINITY_DN85290_c0_g2_i1_orf1	-	-	-	unnamed protein product, partial [Brenthis ino]	-1.31486	1.02085	-0.21394	1.26744	-0.75949
TRINITY_DN214_c0_g1_i4_orf1	-	-	-	uncharacterized protein LOC114352813 [Ostrinia furnacalis]	-1.00733	1.4236	-1.24034	0.61285	0.21121
TRINITY_DN13236_c0_g1_i4_orf1	-	-	-	zonadhesin-like isoform X4 [Ostrinia furnacalis]	-0.9254	1.4502	-1.31082	0.31118	0.47484
TRINITY_DN61042_c0_g2_i2_orfp1	-	-	-	TRINITY_DN61042_c0_g2_i2_m.5292 TRINITY_DN61042_c0_g2::TRINITY_DN61042_c0_g2_i2::g.5292 ORF type:5prime_partial len:63 (-),score=21.86 TRINITY_DN61042_c0_g2_i2:77-265(-)	-1.38151	1.35313	-0.57132	0.91395	-0.31425
TRINITY_DN350_c0_g1_i5_orf1	-	-	-	tau-like protein isoform X6 [Bombyx mori]	-1.62296	1.16233	-0.63224	0.64153	0.45133
TRINITY_DN14670_c0_g1_i1_orf1	-	-	-	heat shock protein beta-1 isoform X1 [Helicoverpa armigera] >XP_022829066.1 heat shock protein beta-1 isoform X1 [Spodoptera litura] >XP_026747148.1 heat shock protein beta-1 isoform X3 [Trichoplusia ni] >XP_026748187.1 heat shock protein beta-1 isoform X2 [Galleria mellonella] >XP_028167756.1 heat shock protein beta-1 isoform X2 [Ostrinia furnacalis] >XP_035431734.1 heat shock protein beta-1-like isoform X3 [Spodoptera frugiperda] >XP_047023072.1 heat shock protein beta-1 isoform X1 [Helicoverpa zea] >XP_049865086.1 heat shock protein beta-1 [Pectinophora gossypiella] >KAH9640995.1 hypothetical protein HF086_015091 [Spodoptera exigua] >QGZ00460.1 heat shock protein 21.4 [Glyphodes pyloalis] >QKR72095.1 heat-shock protein 21.4 [Mythimna separata] >CAB3228281.1 unnamed protein product [Arctia plantaginis] >CAH0628881.1 unnamed protein product [Chrysodeixis includens]	-1.47876	1.2992	-0.80182	0.46987	0.51152
TRINITY_DN14185_c0_g1_i1_orf1	-	-	-	uncharacterized protein LOC114358675 [Ostrinia furnacalis] >XP_028168498.1 uncharacterized protein LOC114358675 [Ostrinia furnacalis]	-1.4804	1.02381	-0.83945	0.97643	0.31961
TRINITY_DN12969_c0_g1_i3_orf1	-	-	-	queuosine salvage protein [Ostrinia furnacalis] >XP_028167327.1 queuosine salvage protein [Ostrinia furnacalis]	-1.33244	1.66185	-0.11583	-0.56866	0.35507
TRINITY_DN37307_c0_g1_i4_orf1	-	-	-	superoxide dismutase [Mn], mitochondrial [Ostrinia furnacalis]	-1.70829	1.38045	-0.18805	0.19596	0.31994
TRINITY_DN4068_c1_g2_i1_orf1	-	-	-	larval cuticle protein LCP-17 [Helicoverpa armigera] >PZC82071.1 hypothetical protein B5X24_HaOG211161 [Helicoverpa armigera] >PZC87412.1 hypothetical protein B5X24_HaOG216859 [Helicoverpa armigera]	-0.96395	1.34151	-1.34265	0.51963	0.44546
TRINITY_DN1503_c0_g1_i6_orf1	-	-	-	ecdysteroid-regulated 16 kDa protein [Ostrinia furnacalis]	-1.83672	1.08672	-0.11707	0.60066	0.26641
TRINITY_DN1108_c1_g2_i1_orfp1	-	-	-	TRINITY_DN1108_c1_g2_i1_m.5565 TRINITY_DN1108_c1_g2::TRINITY_DN1108_c1_g2_i1::g.5565 ORF type:internal len:205 (-),score=147.90 TRINITY_DN1108_c1_g2_i1:2-613(-)	-1.54217	1.50535	-0.47359	0.23202	0.27838
TRINITY_DN121802_c0_g1_i6_orfp1	-	-	-	TRINITY_DN121802_c0_g1_i6_m.78506 TRINITY_DN121802_c0_g1::TRINITY_DN121802_c0_g1_i6::g.78506 ORF type:3prime_partial len:123 (+),score=6.04 TRINITY_DN121802_c0_g1_i6:35-367(+)	-1.11741	1.3535	-0.94365	0.97773	-0.27017
TRINITY_DN22962_c0_g1_i1_orf1	-	-	-	lysosomal acid glucosylceramidase-like isoform X2 [Ostrinia furnacalis]	-1.51127	1.62574	-0.01972	-0.23198	0.13723
TRINITY_DN214_c0_g1_i3_orf1	-	-	-	uncharacterized protein LOC114352813 [Ostrinia furnacalis]	-1.15132	1.36991	-1.14001	0.65265	0.26877
TRINITY_DN5682_c0_g1_i6_orf1	-	-	-	spodomicin-like [Ostrinia furnacalis] >QKV49445.1 diapausin [Ostrinia furnacalis]	-1.8746	1.11851	0.0889	0.28709	0.3801
TRINITY_DN14262_c0_g1_i5_orf1	-	-	-	cytochrome P450 monooxygenase CYP9G18 [Cnaphalocrocis medinalis]	-1.78657	0.95254	-0.18652	0.92577	0.09478

TRINITY_DN15858_c0_g1_i2_orf1	-	-	-	15-hydroxyprostaglandin dehydrogenase [NAD(+)]-like [Ostrinia furnacalis]	-1.55418	1.12761	-0.74927	0.7616	0.41423
TRINITY_DN28503_c0_g1_i6_orf1	-	-	-	uncharacterized protein LOC114363584 [Ostrinia furnacalis] >AXY94663.1 seroin transcript 3 [Ostrinia nubilalis]	-0.46922	1.62986	-1.436	0.02959	0.24578
TRINITY_DN4062_c0_g2_i1_orf1	-	-	-	venom peptide BmKAPI-like isoform X2 [Ostrinia furnacalis]	-1.78841	1.21185	-0.15121	0.21333	0.51443
TRINITY_DN230_c2_g1_i5_orf1	-	-	-	6-pyruvoyl tetrahydrobiopterin synthase [Ostrinia furnacalis]	-1.0854	1.67315	-0.84355	-0.24505	0.50085
TRINITY_DN33995_c0_g1_i5_orf1	-	-	-	unnamed protein product [Spodoptera exigua]	-0.42753	1.67758	-1.06253	-0.74812	0.56061
TRINITY_DN27021_c0_g1_i1_orf1	-	-	-	rab3 GTPase-activating protein catalytic subunit isoform X1 [Ostrinia furnacalis]	-1.15998	1.52958	-0.66673	0.79128	-0.49414
TRINITY_DN184_c0_g1_i1_orf1	-	-	-	macrophage mannose receptor 1-like isoform X1 [Maniola jurtina]	-0.96923	1.73147	-0.04771	0.27718	-0.99172
TRINITY_DN11108_c0_g1_i4_orf1	-	-	-	peroxisomal leader peptide-processing protease [Ostrinia furnacalis] >XP_028165527.1 peroxisomal leader peptide-processing protease [Ostrinia furnacalis]	-1.4749	1.4067	-0.59582	-0.03569	0.6997
TRINITY_DN38225_c0_g2_i1_orf1	-	-	-	uncharacterized protein LOC114354273 [Ostrinia furnacalis]	-1.35795	1.38972	-0.90341	0.31622	0.55542
TRINITY_DN12013_c0_g1_i6_orf1	-	-	-	tudor domain-containing protein 7 isoform X3 [Ostrinia furnacalis]	-1.00998	1.79872	-0.85777	0.09119	-0.02217
TRINITY_DN14611_c0_g1_i5_orf1	-	-	-	hsc70-interacting protein-like [Galleria mellonella]	-1.72201	1.23601	-0.35729	0.31274	0.53055
TRINITY_DN9560_c0_g1_i5_orf1	-	-	-	uncharacterized protein LOC114357350 [Ostrinia furnacalis]	-0.38926	1.10851	-1.75571	0.51552	0.52095
TRINITY_DN3712_c0_g1_i1_orf1	-	-	-	ribonuclease Oy [Ostrinia furnacalis]	-1.74258	1.32457	0.2393	-0.17111	0.34981
TRINITY_DN9340_c0_g1_i4_orf1	-	-	-	sarcosine dehydrogenase, mitochondrial [Ostrinia furnacalis]	-1.695	1.1941	-0.45698	0.60837	0.3495
TRINITY_DN3029_c1_g2_i1_orf1	-	-	-	unnamed protein product [Plutella xylostella]	-1.56698	1.49097	-0.39192	0.40499	0.06294
TRINITY_DN804_c0_g1_i7_orf1	-	-	-	hypothetical protein HF086_004695 [Spodoptera exigua] >CAH0695017.1 unnamed protein product [Spodoptera exigua]	-0.47005	1.44535	-1.42147	-0.31072	0.7569
TRINITY_DN15682_c0_g1_i4_orf1	-	-	-	seroin transcript 1B [Ostrinia nubilalis]	-1.12092	0.68044	-0.56477	1.61085	-0.60559
TRINITY_DN56708_c0_g3_i1_orfp1	-	-	-	TRINITY_DN56708_c0_g3_i1_m.56611 TRINITY_DN56708_c0_g3::TRINITY_DN56708_c0_g3_i1::g.56611 ORF type:internal len:69 (-),score=2.50 TRINITY_DN56708_c0_g3_i1:1-204(-)	-0.85903	1.45736	-1.35329	0.27311	0.48185
TRINITY_DN76633_c0_g1_i1_orfp1	-	-	-	TRINITY_DN76633_c0_g1_i1_m.53394 TRINITY_DN76633_c0_g1::TRINITY_DN76633_c0_g1_i1::g.53394 ORF type:internal len:164 (-),score=26.46,Toxin_2 PF00451.20 0.00022,Toxin_2|PF00451.20|0.00056,Toxin_2|PF00451.20|0.0002,Toxin_2|PF00451.20|5.9e-06,Gamma-thionin|PF00304.21|2,Gamma-thionin|PF00304.21|0.024,Gamma-thionin|PF00304.21|0.027,Gamma-thionin|PF00304.21|0.066,Toxin_38|PF14866.7|0.27,Toxin_38|PF14866.7|0.054,Toxin_38|PF14866.7|0.14,Defensin_2|PF01097.19|1.7,Defensin_2|PF01097.19|0.055,Defensin_2|PF01097.19|1.2,Defensin_2|PF01097.19|0.15 TRINITY_DN76633_c0_g1_i1:1-489(-)	-0.89206	0.97157	-1.31831	1.23382	0.00498
TRINITY_DN8783_c0_g1_i5_orf1	-	-	-	luciferin 4-monooxygenase-like [Ostrinia furnacalis] >XP_028165580.1 luciferin 4-monooxygenase-like [Ostrinia furnacalis]	-1.1785	1.77891	-0.24787	-0.57817	0.22563
TRINITY_DN44110_c0_g1_i4_orf1	-	-	-	putative phosphatidate phosphatase [Ostrinia furnacalis]	-1.30686	1.63223	-0.40229	-0.44276	0.51967
TRINITY_DN10441_c0_g1_i3_orf1	-	-	-	zonadhesin-like [Ostrinia furnacalis]	-0.78268	1.38412	-1.45224	0.40694	0.44386
TRINITY_DN827_c1_g1_i1_orf1	-	-	-	peptidoglycan recognition protein 4-like isoform X1 [Ostrinia furnacalis]	-1.66902	1.42598	0.22671	-0.24621	0.26254
TRINITY_DN16400_c0_g2_i1_orf1	-	-	-	superoxide dismutase [Cu-Zn]-like isoform X1 [Ostrinia furnacalis]	-0.6059	1.48134	-1.49795	0.29009	0.33243
TRINITY_DN40434_c0_g1_i2_orf1	-	-	-	deoxyribodipyrimidine photo-lyase [Ostrinia furnacalis]	-0.68309	1.69017	-1.03602	-0.5346	0.56354
TRINITY_DN62707_c0_g1_i1_orf1	-	-	-	uncharacterized protein LOC114362831 [Ostrinia furnacalis]	-1.15593	1.49791	-1.03975	0.56749	0.13028
TRINITY_DN14874_c0_g1_i6_orf1	-	-	-	uncharacterized protein LOC114358148 [Ostrinia furnacalis]	-1.20115	1.69405	-0.25373	0.42561	-0.66478
TRINITY_DN39933_c0_g1_i2_orf1	-	-	-	thioredoxin domain-containing protein 11 isoform X4 [Ostrinia furnacalis]	-1.02996	1.34664	-1.26484	0.66955	0.27861
TRINITY_DN230_c1_g1_i3_orf1	-	-	-	protein artichoke-like [Ostrinia furnacalis]	-1.62147	1.53238	0.1444	-0.03927	-0.01605
TRINITY_DN16147_c0_g1_i4_orf1	-	-	-	aldo-keto reductase AKR2E4-like isoform X1 [Ostrinia furnacalis]	-1.75971	1.05503	-0.0005	0.87301	-0.16784
TRINITY_DN46132_c0_g2_i2_orf1	-	-	-	hypothetical protein evm_003834 [Chilo suppressalis]	-0.87124	1.52698	-1.29297	0.18736	0.44987
TRINITY_DN46372_c0_g1_i1_orf1	-	-	-	unnamed protein product [Chilo suppressalis]	-0.26928	1.37572	-1.69094	0.24345	0.34105
TRINITY_DN11798_c0_g2_i1_orf1	-	-	-	N-acetylglucosamine-6-sulfatase-like isoform X2 [Ostrinia furnacalis]	-1.60112	1.08465	-0.44496	1.02835	-0.06692
TRINITY_DN2559_c0_g1_i4_orf1	-	-	-	uricase [Ostrinia furnacalis]	-0.56656	1.38525	-1.56077	0.2149	0.52718
TRINITY_DN2175_c0_g1_i4_orf1	-	-	-	uncharacterized protein LOC114353827 [Ostrinia furnacalis]	-0.898	1.33908	-1.36488	0.69734	0.22646
TRINITY_DN1267_c0_g2_i10_orf1	-	-	-	secretory phospholipase A2 receptor-like [Ostrinia furnacalis]	-0.53484	1.44259	-1.52985	0.53347	0.08863
TRINITY_DN9_c0_g1_i7_orf1	-	-	-	heterogeneous nuclear ribonucleoprotein R isoform X1 [Ostrinia furnacalis]	-1.17972	1.74219	0.01346	-0.73909	0.16315
TRINITY_DN6994_c0_g1_i3_orf1	-	-	-	C-type mannose receptor 2-like isoform X1 [Ostrinia furnacalis]	-1.04889	1.73871	-0.90284	0.24601	-0.03299

TRINITY_DN3975_c0_g1_i7_orf1	-	-	-	low-density lipoprotein receptor domain class A domain-containing protein [Phthorimaea operculella]	-1.35064	1.73351	-0.06657	0.08293	-0.39922
TRINITY_DN2688_c0_g1_i3_orf1	-	-	-	mitochondrial amidoxime reducing component 2-like [Ostrinia furnacalis]	-1.19192	1.49357	-0.94582	0.67316	-0.029
TRINITY_DN699_c0_g1_i5_orf1	-	-	-	TPA_exp: putative parasitoid killing factor [Trichoplusia ni]	-1.42816	0.9652	-0.6251	1.26723	-0.17917
TRINITY_DN2049_c1_g1_i2_orf1	-	-	-	luciferin 4-monooxygenase-like [Ostrinia furnacalis]	-1.20165	1.79769	0.01861	-0.0474	-0.56725
TRINITY_DN45949_c0_g1_i1_orf1	-	-	-	uncharacterized protein LOC114355167 [Ostrinia furnacalis]	-0.11719	0.86444	-1.86967	0.79938	0.32304
TRINITY_DN22824_c0_g1_i4_orf1	-	-	-	LIM domain and actin-binding protein 1 [Ostrinia furnacalis]	-0.65832	1.71049	-0.41963	-1.11148	0.47893
TRINITY_DN3433_c0_g1_i6_orf1	-	-	-	cytosolic purine 5'-nucleotidase isoform X2 [Ostrinia furnacalis] >XP_028162963.1 cytosolic purine 5'-nucleotidase isoform X2 [Ostrinia furnacalis]	-1.23385	1.65249	-0.807	0.09365	0.29471
TRINITY_DN22797_c0_g1_i5_orf1	-	-	-	phenoloxidase-activating factor 2-like isoform X1 [Ostrinia furnacalis]	-0.42154	1.3784	-1.63398	0.44627	0.23085
TRINITY_DN3377_c0_g1_i1_orf1	-	-	-	calcyphosin-like protein isoform X3 [Helicoverpa armigera] >XP_047020698.1 calcyphosin-like protein isoform X2 [Helicoverpa zea]	-1.26918	1.30544	-0.81388	0.98891	-0.21129
TRINITY_DN1789_c0_g1_i5_orf1	-	-	-	uncharacterized protein LOC114365543 [Ostrinia furnacalis]	-1.19746	1.60329	-0.54979	0.65633	-0.51237
TRINITY_DN10652_c0_g1_i4_orf1	-	-	-	uncharacterized protein LOC114364166 [Ostrinia furnacalis]	-0.95303	1.81415	-0.88484	0.10499	-0.08127
TRINITY_DN2343_c1_g1_i12_orf1	-	-	-	receptor expression-enhancing protein 5-like isoform X3 [Ostrinia furnacalis]	-0.68684	1.89017	-0.46255	-0.85371	0.11293
TRINITY_DN11263_c0_g1_i5_orf1	-	-	-	SET domain-containing protein SmydA-8 [Ostrinia furnacalis]	-1.54261	1.59509	-0.24114	0.10122	0.08745
TRINITY_DN842_c0_g1_i9_orf1	-	-	-	hypothetical protein evm_011651 [Chilo suppressalis]	-0.69645	1.878	-0.81157	-0.54624	0.17626
TRINITY_DN17657_c0_g1_i1_orf1	-	-	-	alcohol dehydrogenase 18, partial [Helicoverpa assulta]	-0.25167	1.05774	-1.69284	0.97204	-0.08527
TRINITY_DN7022_c0_g1_i7_orf1	-	-	-	fatty-acid amide hydrolase 2-A-like [Ostrinia furnacalis]	-0.83906	1.55714	-1.09664	0.73563	-0.35707
TRINITY_DN1993_c0_g1_i1_orf1	-	-	-	6-phosphofructo-2-kinase/fructose-2,6-bisphosphatase isoform X1 [Ostrinia	-0.38654	1.65878	-1.43298	-0.04749	0.20822
TRINITY_DN12003_c0_g1_i1_orf1	-	-	-	hypothetical protein, partial [Ostrinia furnacalis]	-1.43533	1.62026	0.11923	0.20438	-0.50854
TRINITY_DN31390_c0_g1_i2_orf1	-	-	-	UDP-glucuronosyltransferase 2B20-like [Ostrinia furnacalis]	-0.35698	1.09817	-1.59727	1.03994	-0.18387
TRINITY_DN37418_c0_g1_i4_orf1	-	-	-	NADH dehydrogenase [ubiquinone] 1 alpha subcomplex assembly factor 3 isoform X1 [Ostrinia furnacalis] >XP_028169243.1 NADH dehydrogenase [ubiquinone] 1 alpha subcomplex assembly factor 3 isoform X1 [Ostrinia furnacalis] >XP_028169244.1 NADH dehydrogenase [ubiquinone] 1 alpha subcomplex assembly factor 3 isoform X2 [Ostrinia furnacalis] >XP_028169245.1 NADH dehydrogenase [ubiquinone] 1 alpha subcomplex assembly factor 3 isoform X2 [Ostrinia furnacalis]	-0.90133	1.66999	-0.65466	0.63713	-0.75114
TRINITY_DN84478_c0_g1_i8_orf1	-	-	-	uncharacterized protein LOC114359035 isoform X1 [Ostrinia furnacalis]	-1.96218	0.50348	0.47386	0.79809	0.18675
TRINITY_DN2813_c0_g1_i10_orf1	-	-	-	arylphorin subunit alpha-like [Ostrinia furnacalis]	-1.90318	1.03526	0.46591	0.1367	0.26532
TRINITY_DN34406_c0_g2_i9_orfp1	-	-	-	TRINITY_DN34406_c0_g2_i9_m.33755 TRINITY_DN34406_c0_g2::TRINITY_DN34406_c0_g2_i9::g.33755 ORF type:internal len:82 (-),score=12.88 TRINITY_DN34406_c0_g2_i9:3-245(-)	-1.3357	1.25759	0.75843	-0.98349	0.30318
TRINITY_DN2813_c0_g1_i7_orf1	-	-	-	arylphorin subunit alpha-like [Ostrinia furnacalis]	-1.87421	0.86631	0.64586	-0.17548	0.53752
TRINITY_DN64297_c0_g1_i1_orf1	-	-	-	vanin-like protein 2 isoform X2 [Ostrinia furnacalis]	-1.56091	0.25072	1.33079	0.59363	-0.61424
TRINITY_DN5406_c0_g2_i1_orf1	-	-	-	uncharacterized protein LOC114350326 [Ostrinia furnacalis]	-1.87232	0.7566	0.94781	0.153	0.01491
TRINITY_DN64181_c0_g1_i1_orf1	-	-	-	uncharacterized protein LOC114356431 isoform X2 [Ostrinia furnacalis]	-1.55545	0.54647	1.46639	-0.11249	-0.34493
TRINITY_DN9044_c0_g1_i2_orf1	-	-	-	unnamed protein product [Euphydryas editha]	-1.6119	1.42082	0.34158	0.28184	-0.43234
TRINITY_DN129863_c0_g1_i1_orf1	-	-	-	protein PFC0760c-like isoform X2 [Ostrinia furnacalis]	-1.88125	1.09705	0.35248	0.3572	0.07453
TRINITY_DN15578_c0_g2_i1_orfp1	-	-	-	uncharacterized protein LOC125235519 [Leguminivora glycinivorella]	-1.88831	0.8435	0.71887	0.43946	-0.11351
TRINITY_DN98692_c0_g3_i1_orf1	-	-	-	fatty acyl-CoA hydrolase precursor, medium chain [Ostrinia furnacalis]	-1.93298	0.67659	0.85037	0.21462	0.19139
TRINITY_DN636_c1_g1_i9_orf1	-	-	-	secretory phospholipase A2 receptor-like [Ostrinia furnacalis]	-1.83278	0.26549	0.77582	0.96816	-0.17669
TRINITY_DN25234_c0_g1_i1_orf1	-	-	-	uncharacterized protein LOC114353853 [Ostrinia furnacalis]	-1.75627	1.28922	0.3191	0.33917	-0.19121
TRINITY_DN42719_c0_g2_i1_orf1	-	-	-	inter-alpha-trypsin inhibitor heavy chain H4-like isoform X11 [Ostrinia furnacalis]	-1.87198	0.36021	0.91789	0.71352	-0.11964
TRINITY_DN2406_c0_g1_i6_orf1	-	-	-	uncharacterized protein LOC114361672 [Ostrinia furnacalis]	-1.53018	1.51432	0.43542	-0.00028	-0.41928
TRINITY_DN801_c0_g1_i2_orf1	-	-	-	cathepsin L [Ostrinia furnacalis] >XP_028165920.1 cathepsin L [Ostrinia furnacalis] >UKI61015.1 cathepsin L [Ostrinia furnacalis]	-1.65486	0.53302	1.39293	-0.14842	-0.12267
TRINITY_DN1575_c0_g1_i10_orf1	-	-	-	uncharacterized protein LOC114359245 [Ostrinia furnacalis]	-1.37917	1.18157	0.42138	0.7539	-0.97768
TRINITY_DN418_c1_g1_i3_orf1	-	-	-	hypothetical protein evm_003996 [Chilo suppressalis]	-1.71255	0.27099	0.69259	1.15985	-0.41088
TRINITY_DN364_c1_g1_i2_orf1	-	-	-	talin-2-like, partial [Ostrinia furnacalis]	-1.83063	0.99666	0.79969	0.10478	-0.07049

TRINITY_DN13760_c1_g1_i1_orf1	-	-	-	pre-mRNA-processing factor 40 homolog A isoform X1 [Ostrinia furnacalis] >XP_028162665.1 pre-mRNA-processing factor 40 homolog A isoform X2 [Ostrinia furnacalis] >XP_028162667.1 pre-mRNA-processing factor 40 homolog A isoform X3 [Ostrinia furnacalis]	-1.68891	0.25016	1.32162	0.46566	-0.34853
TRINITY_DN20717_c0_g1_i1_orf1	-	-	-	putative uncharacterized protein DDB_G0282133 isoform X1 [Ostrinia furnacalis]	-1.40657	0.49019	1.5924	-0.43032	-0.24569
TRINITY_DN858_c0_g1_i3_orf1	-	-	-	uncharacterized protein LOC114351944 [Ostrinia furnacalis]	-1.24534	0.79527	1.23973	-1.09051	0.30085
TRINITY_DN4443_c0_g1_i4_orf1	-	-	-	lysosome-associated membrane glycoprotein 1-like isoform X4 [Ostrinia furnacalis]	-1.90562	0.9771	0.56689	0.29729	0.06433
TRINITY_DN11060_c0_g1_i6_orf1	-	-	-	extracellular matrix protein A-like isoform X3 [Ostrinia furnacalis]	-1.79233	0.95414	0.93331	-0.0756	-0.01952
TRINITY_DN1048_c0_g1_i6_orf1	-	-	-	uncharacterized protein LOC114360661 [Ostrinia furnacalis]	-1.83455	0.37292	0.41615	1.1455	-0.10002
TRINITY_DN2880_c0_g1_i2_orf1	-	-	-	sialomucin core protein 24 [Pectinophora gossypiella]	-1.86566	0.84819	0.87065	-0.0512	0.19803
TRINITY_DN2024_c0_g1_i12_orfp1	-	-	-	unnamed protein product, partial [Brenthis ino]	-1.70772	0.40147	1.32399	0.28217	-0.2999
TRINITY_DN4565_c0_g2_i1_orf1	-	-	-	acid phosphatase type 7 isoform X2 [Ostrinia furnacalis]	-1.90357	1.03612	0.43712	0.31314	0.1172
TRINITY_DN184_c0_g1_i10_orf1	-	-	-	C-type mannose receptor 2-like isoform X1 [Leguminivora glycinivorella]	-1.69794	1.27613	0.66493	-0.02989	-0.21323
TRINITY_DN8037_c0_g2_i1_orf1	-	-	-	2-oxo-4-hydroxy-4-carboxy-5-ureidoimidazoline decarboxylase-like [Ostrinia	-1.90407	0.95886	0.4802	0.47375	-0.00874
TRINITY_DN12014_c0_g1_i2_orf1	-	-	-	unnamed protein product [Chilo suppressalis]	-1.50932	1.33928	0.83407	-0.40995	-0.25408
TRINITY_DN59388_c0_g1_i1_orf1	-	-	-	uncharacterized protein LOC114353759 [Ostrinia furnacalis]	-1.44043	0.48286	1.5435	-0.03031	-0.55562
TRINITY_DN3616_c0_g1_i4_orf1	-	-	-	conotoxin ArMKLT2-032-like [Ostrinia furnacalis]	-1.42733	-0.04762	1.43024	0.69833	-0.65362
TRINITY_DN97883_c0_g1_i2_orf1	-	-	-	talin-2-like, partial [Ostrinia furnacalis]	-1.56012	1.25497	0.69155	0.27487	-0.66127
TRINITY_DN3647_c1_g1_i5_orf1	-	-	-	unnamed protein product, partial [Iphiclides podalirius]	-1.62344	0.94416	1.1311	-0.43985	-0.01197
TRINITY_DN811_c0_g1_i15_orf1	-	-	-	uncharacterized protein LOC114364160 [Ostrinia furnacalis]	-1.84474	1.10487	0.3387	0.50145	-0.10028
TRINITY_DN44073_c0_g1_i3_orf1	-	-	-	inter-alpha-trypsin inhibitor heavy chain H4-like isoform X11 [Ostrinia furnacalis]	-1.65582	0.32426	1.3692	0.3538	-0.39144
TRINITY_DN10373_c0_g1_i1_orf1	-	-	-	homocysteine S-methyltransferase 1-like [Ostrinia furnacalis] >XP_028162778.1 homocysteine S-methyltransferase 1-like [Ostrinia furnacalis]	-1.50072	1.20418	0.81698	-0.75767	0.23723
TRINITY_DN6822_c0_g2_i4_orf1	-	-	-	gelsolin-like [Bicyclus anynana]	-1.82891	1.15038	0.57086	0.05857	0.04909
TRINITY_DN1466_c0_g1_i4_orf1	-	-	-	insecticyanin-A-like [Ostrinia furnacalis]	-1.65595	0.84162	0.46605	0.97152	-0.62325
TRINITY_DN2141_c0_g1_i1_orf1	-	-	-	low density lipoprotein receptor adapter protein 1-like [Ostrinia furnacalis]	-1.56092	1.2249	0.88735	-0.02687	-0.52446
TRINITY_DN11868_c0_g1_i2_orf1	-	-	-	uncharacterized protein LOC114361308 [Ostrinia furnacalis]	-1.55467	0.98066	1.07345	-0.66462	0.16518
TRINITY_DN28741_c0_g1_i3_orf1	-	-	-	uncharacterized protein LOC114351652 [Ostrinia furnacalis]	-1.1431	1.72204	0.43471	-0.39464	-0.61901
TRINITY_DN3109_c0_g1_i5_orf1	-	-	-	protein takeout isoform X2 [Ostrinia furnacalis]	-1.80446	0.7659	1.07515	-0.03722	0.00062
TRINITY_DN6205_c0_g1_i1_orf1	-	-	-	phenoloxidase-activating factor 2-like [Ostrinia furnacalis]	-1.67876	0.29831	0.96468	0.94128	-0.5255
TRINITY_DN8083_c0_g1_i1_orf1	-	-	-	solute carrier family 35 member F6 [Ostrinia furnacalis]	-1.53889	0.75293	1.36715	-0.40663	-0.17457
TRINITY_DN106479_c1_g1_i1_orf1	-	-	-	secretory phospholipase A2 receptor-like [Ostrinia furnacalis]	-1.9021	0.8137	0.52408	0.66033	-0.096
TRINITY_DN12661_c0_g1_i3_orf1	-	-	-	T-complex protein 11-like protein 1 [Ostrinia furnacalis]	-1.63723	1.24283	0.81103	-0.33136	-0.08528
TRINITY_DN9420_c0_g1_i2_orf1	-	-	-	protein PFC0760c-like isoform X2 [Ostrinia furnacalis]	-1.70113	1.34286	0.45636	0.16283	-0.26093
TRINITY_DN364_c0_g1_i2_orf1	-	-	-	uncharacterized protein LOC114366657, partial [Ostrinia furnacalis]	-1.77715	1.14092	0.7245	-0.11902	0.03075
TRINITY_DN896_c0_g1_i2_orf1	-	-	-	uncharacterized protein LOC114356314 isoform X2 [Ostrinia furnacalis]	-1.17383	1.06665	1.21433	-0.99905	-0.1081
TRINITY_DN9383_c0_g1_i3_orf1	-	-	-	uncharacterized protein LOC114361502 [Ostrinia furnacalis]	-1.38247	0.83188	1.4029	-0.2453	-0.607
TRINITY_DN130439_c0_g1_i1_orf1	-	-	-	DDRGK domain-containing protein 1-like [Ostrinia furnacalis]	-1.43774	1.14758	1.09269	-0.62482	-0.17771
TRINITY_DN21533_c0_g1_i7_orf1	-	-	-	annexin B9 isoform X1 [Ostrinia furnacalis]	-1.46475	1.31632	0.90783	-0.44791	-0.31149
TRINITY_DN65299_c0_g4_i1_orf1	-	-	-	LOW QUALITY PROTEIN: signal transducing adapter molecule 2 [Ostrinia furnacalis]	-1.7275	1.02117	0.95975	-0.22598	-0.02744
TRINITY_DN8644_c0_g1_i3_orf1	-	-	-	SEC14-like protein 2 [Ostrinia furnacalis]	-1.31186	1.65491	0.40408	-0.59484	-0.1523
TRINITY_DN1091_c0_g1_i1_orf1	-	-	-	macrophage mannose receptor 1-like [Pararge aegeria]	-1.3514	0.73937	1.49356	-0.50319	-0.37834
TRINITY_DN1491_c0_g1_i4_orf1	-	-	-	GILT-like protein 2 isoform X1 [Ostrinia furnacalis] >XP_028156245.1 GILT-like protein 2 isoform X2 [Ostrinia furnacalis] >XP_028156247.1 GILT-like protein 2 isoform X3 [Ostrinia furnacalis]	-1.04608	0.74159	1.43535	0.00733	-1.13819
TRINITY_DN110231_c0_g1_i1_orf1	-	-	-	protein singed [Ostrinia furnacalis] >XP_028161434.1 protein singed [Ostrinia	-1.09849	0.27123	1.80145	-0.48644	-0.48775
TRINITY_DN661_c0_g1_i1_orf1	-	-	-	hypothetical protein evm_002822 [Chilo suppressalis]	-0.73025	-0.01876	1.92367	-0.78222	-0.39243
TRINITY_DN1497_c0_g2_i6_orf1	-	-	-	unnamed protein product [Chilo suppressalis]	-1.78816	1.22265	0.45609	0.27106	-0.16164
TRINITY_DN24668_c0_g1_i8_orf1	-	-	-	uncharacterized protein LOC114364067 isoform X3 [Ostrinia furnacalis]	-1.31482	1.25614	1.05858	-0.30909	-0.69081
TRINITY_DN48602_c0_g1_i6_orf1	-	-	-	amidophosphoribosyltransferase-like isoform X1 [Ostrinia furnacalis] >XP_028157758.1 amidophosphoribosyltransferase-like isoform X1 [Ostrinia furnacalis] >XP_028157759.1 amidophosphoribosyltransferase-like isoform X1 [Ostrinia furnacalis]	-1.65412	1.07896	0.35507	0.79904	-0.57895

TRINITY_DN1194_c0_g1_i5_orf1	-	-	-	sequestosome-1-like isoform X4 [Ostrinia furnacalis]	-1.22234	0.83189	1.48736	-0.54737	-0.54955
TRINITY_DN48765_c0_g1_i7_orf1	-	-	-	uncharacterized protein LOC114352307 [Ostrinia furnacalis]	-1.58697	0.74182	1.16355	0.35398	-0.67238
TRINITY_DN4817_c0_g1_i4_orf1	-	-	-	palmitoyl-protein thioesterase 1 isoform X1 [Ostrinia furnacalis] >XP_028170290.1 palmitoyl-protein thioesterase 1 isoform X4 [Ostrinia furnacalis]	-1.28582	1.16198	1.01208	-0.98155	0.09331
TRINITY_DN10900_c0_g1_i7_orf1	-	-	-	fatty acid synthase-like [Ostrinia furnacalis]	-1.4691	1.53432	0.54662	-0.33445	-0.27739
TRINITY_DN5433_c0_g1_i5_orf1	-	-	-	uncharacterized protein LOC114351067 [Ostrinia furnacalis]	-1.75433	0.88859	0.39484	0.89346	-0.42256
TRINITY_DN16316_c0_g1_i7_orf1	-	-	-	exocyst complex component 6 isoform X1 [Ostrinia furnacalis] >XP_028166671.1 exocyst complex component 6 isoform X2 [Ostrinia furnacalis]	-1.51888	0.81025	0.83452	0.75319	-0.87908
TRINITY_DN843_c0_g1_i2_orf1	-	-	-	unnamed protein product [Diatraea saccharalis]	-0.72084	0.60207	1.68204	-0.59892	-0.96434
TRINITY_DN21533_c0_g1_i6_orf1	-	-	-	annexin B9 isoform X2 [Ostrinia furnacalis]	-1.84372	0.87886	0.82198	0.33833	-0.19545
TRINITY_DN10824_c0_g1_i3_orf1	-	-	-	endochitinase isoform X2 [Ostrinia furnacalis]	-0.69765	-0.1412	1.96431	-0.58999	-0.53547
TRINITY_DN140_c1_g1_i2_orf1	-	-	-	modular serine protease-like isoform X1 [Ostrinia furnacalis]	-1.36051	0.57878	1.57972	-0.39091	-0.40708
TRINITY_DN53427_c0_g1_i2_orf1	-	-	-	heparanase-like [Ostrinia furnacalis]	-1.10802	0.50923	1.70369	-0.55385	-0.55106
TRINITY_DN4676_c0_g1_i16_orf1	-	-	-	meiosis-specific nuclear structural protein 1-like isoform X2 [Ostrinia furnacalis]	-0.83685	0.10404	1.89343	-0.45735	-0.70327
TRINITY_DN1196_c0_g1_i4_orf1	-	-	-	glucosamine-6-phosphate isomerase isoform X2 [Ostrinia furnacalis]	-0.65979	0.42066	1.7745	-0.94114	-0.59424
TRINITY_DN4276_c0_g1_i6_orf1	-	-	-	arylsulfatase B [Ostrinia furnacalis]	-1.42492	0.38713	1.53834	0.15467	-0.65522
TRINITY_DN261_c0_g1_i5_orfp1	-	-	-	TRINITY_DN261_c0_g1_i5_m.18559 TRINITY_DN261_c0_g1::TRINITY_DN261_c0_g1_i5::g.18559 ORF type:internal len:190 (+),score=69.81 TRINITY_DN261_c0_g1_i5:3-569(+)	-0.90157	0.07345	1.81538	-0.04707	-0.94019
TRINITY_DN119265_c0_g2_i1_orf1	-	-	-	PREDICTED: sorting nexin-12 [Fopius arisanus]	-1.45276	0.9467	1.2959	-0.36351	-0.42633
TRINITY_DN5462_c0_g2_i1_orf1	-	-	-	acyl-protein thioesterase 1 [Ostrinia furnacalis] >XP_028165602.1 acyl-protein thioesterase 1 [Ostrinia furnacalis] >XP_028165603.1 acyl-protein thioesterase 1 [Ostrinia furnacalis] >XP_028165604.1 acyl-protein thioesterase 1 [Ostrinia furnacalis] >XP_028165606.1 acyl-protein thioesterase 1 [Ostrinia furnacalis] >XP_028165607.1 acyl-protein thioesterase 1 [Ostrinia furnacalis] >XP_028165608.1 acyl-protein thioesterase 1 [Ostrinia furnacalis] >XP_028165609.1 acyl-protein thioesterase 1 [Ostrinia furnacalis] >XP_028165610.1 acyl-protein thioesterase 1 [Ostrinia furnacalis]	-1.05784	0.91049	1.01257	0.47634	-1.34156
TRINITY_DN7711_c1_g1_i3_orf1	-	-	-	long-chain fatty acid transport protein 1-like [Ostrinia furnacalis]	-0.86408	0.42684	1.76844	-0.8355	-0.4957
TRINITY_DN14774_c0_g1_i4_orf1	-	-	-	aminopeptidase N-like [Ostrinia furnacalis]	-0.57826	0.51519	1.73297	-0.87197	-0.79793
TRINITY_DN12673_c3_g1_i2_orf1	-	-	-	unnamed protein product [Chilo suppressalis]	-0.917	0.19573	1.85975	-0.48536	-0.65312
TRINITY_DN2290_c0_g1_i2_orfp1	-	-	-	TRINITY_DN2290_c0_g1_i2_m.69732 TRINITY_DN2290_c0_g1::TRINITY_DN2290_c0_g1_i2::g.69732 ORF type:complete len:234 (+),score=14.43 TRINITY_DN2290_c0_g1_i2:60-761(+)	-0.57686	0.35091	1.81174	-0.74624	-0.83954
TRINITY_DN1091_c0_g2_i10_orf1	-	-	-	macrophage mannose receptor 1-like isoform X2 [Maniola hyperantus]	-0.95099	0.48995	1.44873	0.30271	-1.2904
TRINITY_DN56690_c0_g1_i4_orf1	-	-	-	hypothetical protein evm_002209, partial [Chilo suppressalis]	-1.20149	0.22576	1.28358	0.79752	-1.10537
TRINITY_DN1362_c0_g1_i4_orf1	-	-	-	heparan-alpha-glucosaminide N-acetyltransferase [Helicoverpa armigera]	-0.7674	0.33687	1.79745	-0.426	-0.94092
TRINITY_DN2058_c0_g1_i2_orf1	-	-	-	proteasomal ubiquitin receptor ADRM1 [Ostrinia furnacalis]	-0.85566	-0.09902	1.81574	0.11281	-0.97387
TRINITY_DN585_c0_g1_i5_orf1	-	-	-	very low-density lipoprotein receptor isoform X2 [Galleria mellonella]	-0.76484	-0.10784	1.93387	-0.30645	-0.75474
TRINITY_DN23978_c0_g1_i2_orf1	-	-	-	insulin-like growth factor-binding protein complex acid labile subunit [Ostrinia	-0.89383	-0.09579	1.08126	1.18271	-1.27435
TRINITY_DN110523_c0_g2_i1_orf1	-	-	-	uncharacterized protein LOC107036393 [Diachasma alloeum]	-0.35668	0.3944	1.72681	-0.58347	-1.18106
TRINITY_DN20356_c0_g1_i5_orf1	-	-	-	uncharacterized protein LOC114362428 [Ostrinia furnacalis]	-0.50714	1.92876	-0.1106	-0.38067	-0.93034
TRINITY_DN103107_c0_g1_i2_orf1	-	-	-	superoxide dismutase [Cu-Zn] [Ostrinia furnacalis] >XP_028177872.1 superoxide dismutase [Cu-Zn] [Ostrinia furnacalis]	-0.20002	1.95555	-0.31837	-0.74816	-0.689
TRINITY_DN66287_c0_g1_i1_orfp1	-	-	-	tenascin-like isoform X4 [Trichoplusia ni]	-0.24384	1.98087	-0.64446	-0.49866	-0.5939
TRINITY_DN12920_c0_g3_i1_orf1	-	-	-	zonadhesin-like isoform X4 [Ostrinia furnacalis]	-0.86972	1.93764	-0.25238	-0.6233	-0.19224
TRINITY_DN48694_c0_g1_i1_orfp1	-	-	-	TRINITY_DN48694_c0_g1_i1_m.75338 TRINITY_DN48694_c0_g1::TRINITY_DN48694_c0_g1_i1::g.75338 ORF type:internal len:84 (+),score=16.02 TRINITY_DN48694_c0_g1_i1:2-250(+)	-0.53088	1.04725	0.87221	0.27953	-1.6681
TRINITY_DN325_c0_g1_i15_orf1	-	-	-	protein draper-like [Ostrinia furnacalis]	-0.95404	1.6192	0.67937	-0.89874	-0.44579
TRINITY_DN50725_c0_g1_i6_orf1	-	-	-	BTB/POZ domain-containing protein 2-like [Ostrinia furnacalis]	-0.21857	1.96068	-0.53514	-0.38764	-0.81933
TRINITY_DN38106_c0_g1_i6_orf1	-	-	-	unnamed protein product [Pieris macdunnoughi]	-0.82534	1.44438	0.90834	-1.11086	-0.41652

TRINITY_DN80424_c0_g1_i1_orf1	-	-	-	PREDICTED: cytoplasmic FMR1-interacting protein [Dufourea novaeangliae] >KZC10094.1 Cytoplasmic FMR1-interacting protein [Dufourea novaeangliae]	-0.55388	1.70473	0.54726	-1.00044	-0.69767
TRINITY_DN26985_c0_g1_i5_orf1	-	-	-	secretory phospholipase A2 receptor-like [Helicoverpa zea]	-0.57368	1.03384	1.36674	-0.73275	-1.09415
TRINITY_DN1293_c1_g1_i4_orf1	-	-	-	putative fatty acyl-CoA reductase CG5065 [Ostrinia furnacalis]	-0.61234	1.62698	0.69473	-0.7236	-0.98576
TRINITY_DN919_c0_g1_i7_orf1	-	-	-	facilitated trehalose transporter Tret1-like [Ostrinia furnacalis] >XP_028161733.1 facilitated trehalose transporter Tret1-like [Ostrinia furnacalis]	-0.70451	1.79098	0.41212	-0.79017	-0.70842
TRINITY_DN132_c0_g2_i2_orf1	-	-	-	alpha-tocopherol transfer protein-like isoform X1 [Ostrinia furnacalis]	-1.19574	1.40088	0.95664	-0.67501	-0.48678
TRINITY_DN3627_c0_g1_i7_orf1	-	-	-	spermine oxidase-like isoform X2 [Ostrinia furnacalis]	-1.00952	1.84876	0.14822	-0.65719	-0.33028
TRINITY_DN7316_c0_g2_i1_orf1	-	-	-	ubiquitin-protein ligase E3C [Ostrinia furnacalis]	-0.67841	1.15401	1.26443	-1.08841	-0.65163
TRINITY_DN6205_c0_g1_i4_orfp1	-	-	-	TRINITY_DN6205_c0_g1_i4_m.72677 TRINITY_DN6205_c0_g1::TRINITY_DN6205_c0_g1_i4::g.72677 ORF type:internal len:68 (-),score=1.69 TRINITY_DN6205_c0_g1_i4:2-202(-)	-0.61883	1.04104	1.37868	-0.97437	-0.82651
TRINITY_DN30509_c0_g1_i9_orf1	-	-	-	prostatic acid phosphatase [Ostrinia furnacalis]	-0.29101	1.98093	-0.72261	-0.50839	-0.45893
TRINITY_DN60949_c0_g1_i4_orf1	-	-	-	aldo-keto reductase AKR2E4-like [Galleria mellonella]	-0.70658	1.29785	1.09138	-1.16503	-0.51762
TRINITY_DN12432_c0_g1_i2_orf1	-	-	-	sorting nexin-8 isoform X3 [Ostrinia furnacalis]	-0.70298	1.50509	0.65342	-0.11366	-1.34187
TRINITY_DN825_c23_g1_i5_orf1	-	-	-	methionine-tRNA synthetase, partial [Papilio xuthus]	-0.17469	1.8174	-0.47551	-1.19974	0.03255
TRINITY_DN18136_c0_g1_i1_orf1	-	-	-	proteoglycan 4-like [Ostrinia furnacalis]	-0.82774	1.13269	1.3127	-0.79659	-0.82105
TRINITY_DN3324_c0_g1_i3_orf1	-	-	-	uncharacterized protein LOC114363957 isoform X2 [Ostrinia furnacalis]	-0.56868	1.87863	0.09918	-0.97352	-0.43562
TRINITY_DN11962_c0_g1_i2_orf1	-	-	-	BTB/POZ domain-containing protein 2-like [Ostrinia furnacalis]	-0.05575	1.93919	-0.70795	-0.43885	-0.73664
TRINITY_DN3588_c0_g1_i4_orf1	-	-	-	probable peroxisomal acyl-coenzyme A oxidase 1 isoform X1 [Ostrinia furnacalis] >XP_028165840.1 probable peroxisomal acyl-coenzyme A oxidase 1 isoform X2 [Ostrinia furnacalis]	-0.52656	1.74072	0.25195	-1.25956	-0.20656
TRINITY_DN51658_c0_g1_i1_orf1	-	-	-	E3 ubiquitin-protein ligase synoviolin B [Ostrinia furnacalis]	-0.11768	1.68187	0.42032	-1.06867	-0.91584
TRINITY_DN6205_c0_g1_i8_orf1	-	-	-	phenoloxidase-activating factor 2-like [Ostrinia furnacalis]	-0.38258	1.49705	0.83882	-0.95243	-1.00087
TRINITY_DN26488_c0_g1_i6_orf1	-	-	-	phosphatidate phosphatase LPIN2 isoform X1 [Ostrinia furnacalis] >XP_028176373.1 phosphatidate phosphatase LPIN2 isoform X1 [Ostrinia furnacalis] >XP_028176374.1 phosphatidate phosphatase LPIN2 isoform X2 [Ostrinia furnacalis] >XP_028176375.1 phosphatidate phosphatase LPIN2 isoform X3 [Ostrinia furnacalis] >XP_028176376.1 phosphatidate phosphatase LPIN2 isoform X4 [Ostrinia furnacalis]	-0.99485	1.51799	0.81292	-0.94425	-0.39181
TRINITY_DN195_c8_g1_i1_orf1	-	-	-	hypothetical protein evm_009768 [Chilo suppressalis]	0.53551	1.64002	-0.18349	-1.04975	-0.94229
TRINITY_DN40911_c0_g1_i1_orf1	-	-	-	peroxisomal membrane protein PEX16 [Ostrinia furnacalis]	0.37412	1.76295	-0.38765	-0.68562	-1.06381
TRINITY_DN2694_c0_g1_i3_orf1	-	-	-	cubilin homolog [Ostrinia furnacalis]	0.21487	1.86278	-0.81317	-0.52454	-0.73993
TRINITY_DN6684_c0_g1_i4_orf1	-	-	-	26S proteasome non-ATPase regulatory subunit 6 isoform X1 [Ostrinia furnacalis]	-0.02764	1.59817	0.21863	-1.52575	-0.26341
TRINITY_DN7570_c0_g1_i18_orf1	-	-	-	sodium/potassium-transporting ATPase subunit alpha isoform X4 [Trichoplusia ni] >XP_026734855.1 sodium/potassium-transporting ATPase subunit alpha isoform X4 [Trichoplusia ni]	0.30603	1.7349	-0.28929	-1.24909	-0.50256
TRINITY_DN31342_c2_g2_i1_orf1	-	-	-	26S proteasome non-ATPase regulatory subunit 2 isoform X2 [Ostrinia furnacalis]	-0.66864	1.34896	1.08326	-0.93001	-0.83357
TRINITY_DN9979_c0_g1_i1_orf1	-	-	-	ADP-dependent glucokinase [Ostrinia furnacalis]	0.14275	1.73688	-0.51791	-1.30034	-0.06138
TRINITY_DN526_c0_g1_i1_orf1	-	-	-	secretory phospholipase A2 receptor-like [Ostrinia furnacalis]	-0.24343	1.41687	0.47858	-0.0076	-1.64442
TRINITY_DN52244_c1_g1_i1_orf1	-	-	-	triokinase/FMN cyclase-like isoform X2 [Ostrinia furnacalis]	0.03859	1.39091	0.39129	-0.11883	-1.70195
TRINITY_DN35002_c0_g2_i2_orf1	-	-	-	sulfite oxidase, mitochondrial isoform X3 [Leguminivora glycinivorella]	0.18581	1.83944	-0.2974	-0.8449	-0.88295
TRINITY_DN11204_c0_g1_i3_orf1	-	-	-	spermosin-like [Ostrinia furnacalis]	-0.6099	1.00776	1.37642	-0.61893	-1.15534

TRINITY_DN40176_c0_g1_i1_orf1	-	-	-	transcription elongation factor 1 homolog [Plutella xylostella] >XP_014365734.1 transcription elongation factor 1 homolog [Papilio machaon] >XP_021188366.1 transcription elongation factor 1 homolog [Helicoverpa armigera] >XP_022815988.1 transcription elongation factor 1 homolog [Spodoptera litura] >XP_022815989.1 transcription elongation factor 1 homolog [Spodoptera litura] >XP_026739943.1 transcription elongation factor 1 homolog [Trichoplusia ni] >XP_026739944.1 transcription elongation factor 1 homolog [Trichoplusia ni] >XP_028167273.1 transcription elongation factor 1 homolog [Ostrinia furnacalis] >XP_035429172.1 transcription elongation factor 1 homolog [Spodoptera frugiperda] >XP_038210306.1 transcription elongation factor 1 homolog [Zerene cesonia] >XP_045492874.1 transcription elongation factor 1 homolog [Colias croceus] >XP_045492875.1 transcription elongation factor 1 homolog [Colias croceus] >XP_047019949.1 transcription elongation factor 1 homolog [Helicoverpa zea] >KAF9414193.1 hypothetical protein HW555_007806 [Spodoptera exigua] >KPJ01541.1 Transcription elongation factor 1-like [Papilio xuthus] >CAB3243126.1 unnamed protein product [Arctia plantaginis] >CAB3514670.1 unnamed protein product [Spodoptera littoralis] >CAH0626924.1 unnamed protein product [Chrysodeixis includens] >CAH2075456.1	0.01773	1.28593	0.69774	-1.65508	-0.34632
TRINITY_DN11612_c0_g2_i1_orf1	-	-	-	eukaryotic translation initiation factor 5B [Manduca sexta]	0.23217	1.72823	-0.05084	-1.21314	-0.69643
TRINITY_DN10871_c0_g2_i1_orf1	-	-	-	eukaryotic translation initiation factor 4 gamma 2 [Ostrinia furnacalis] >XP_028157481.1 eukaryotic translation initiation factor 4 gamma 2 [Ostrinia furnacalis]	0.0842	1.86011	-0.38681	-0.48687	-1.07063
TRINITY_DN1710_c0_g2_i2_orf1	-	-	-	relish [Ostrinia furnacalis]	0.35518	1.54357	0.14414	-1.4595	-0.58338
TRINITY_DN40440_c0_g1_i1_orf1	-	-	-	Na(+)/H(+) exchange regulatory cofactor NHE-RF1 [Helicoverpa zea] >XP_049705606.1 Na(+)/H(+) exchange regulatory cofactor NHE-RF1 [Helicoverpa armigera]	0.56712	1.33714	0.32759	-1.49837	-0.73348
TRINITY_DN2499_c0_g1_i4_orf1	-	-	-	WD repeat-containing protein 92 isoform X1 [Ostrinia furnacalis]	-0.21588	1.50001	0.3365	-0.01127	-1.60935
TRINITY_DN100208_c0_g1_i1_orf1	-	-	-	neurofilament heavy polypeptide-like isoform X2 [Ostrinia furnacalis]	-0.1176	0.67443	1.13013	0.11339	-1.80035
TRINITY_DN61536_c0_g3_i1_orf1	-	-	-	cubilin homolog [Ostrinia furnacalis]	-0.12303	1.9076	-0.4805	-0.28819	-1.01588
TRINITY_DN4360_c0_g1_i4_orf1	-	-	-	glucose-6-phosphate isomerase-like, partial [Bicyclus anynana]	-0.96938	1.3734	-0.40961	1.00436	-0.99878
TRINITY_DN5770_c0_g1_i4_orf1	-	-	-	rotatin-like [Ostrinia furnacalis]	-0.40245	1.55068	-0.56363	0.69382	-1.27842
TRINITY_DN311_c0_g1_i8_orfp1	-	-	-	TRINITY_DN311_c0_g1_i8_m.65152 TRINITY_DN311_c0_g1::TRINITY_DN311_c0_g1_i8::g.65152 ORF type:5prime_partial len:138 (+),score=74.30 TRINITY_DN311_c0_g1_i8:2-415(+)	-0.60118	1.0148	-0.76826	1.39298	-1.03834
TRINITY_DN5852_c0_g1_i6_orf1	-	-	-	probable maltase isoform X6 [Ostrinia furnacalis]	-0.7384	1.40678	-0.5155	0.97196	-1.12485
TRINITY_DN36581_c0_g1_i5_orf1	-	-	-	enoyl-CoA delta isomerase 1, mitochondrial-like isoform X1 [Ostrinia furnacalis] >XP_028158560.1 enoyl-CoA delta isomerase 1, mitochondrial-like isoform X2 [Ostrinia furnacalis]	-0.47281	1.78089	-0.9674	0.38238	-0.72306
TRINITY_DN81181_c0_g1_i6_orfp1	-	-	-	C-type lectin domain family 4 member E [Pieris rapae]	-0.13551	1.76548	-0.87189	0.26017	-1.01825
TRINITY_DN1380_c0_g1_i5_orf1	-	-	-	ubiquitin-fold modifier-conjugating enzyme 1 [Ostrinia furnacalis]	-0.25459	1.49966	-0.59099	0.70325	-1.35734
TRINITY_DN3058_c0_g1_i1_orf1	-	-	-	mesencephalic astrocyte-derived neurotrophic factor homolog [Ostrinia furnacalis]	0.09992	1.42458	-1.12276	0.69894	-1.10068
TRINITY_DN3126_c0_g1_i4_orf1	-	-	-	unnamed protein product, partial [Iphiclides podalirius]	-0.00418	0.8056	-1.05098	1.39271	-1.14315
TRINITY_DN1968_c0_g1_i3_orf1	-	-	-	protein ABHD4 isoform X3 [Ostrinia furnacalis]	0.25084	1.16861	-1.27194	0.91182	-1.05933
TRINITY_DN8046_c0_g1_i4_orf1	-	-	-	tumor protein D54 isoform X3 [Hyposmocoma kahamanoa]	-0.0898	1.32677	-0.59619	0.83522	-1.476
TRINITY_DN27300_c0_g1_i6_orfp1	-	-	-	TRINITY_DN27300_c0_g1_i6_m.71140 TRINITY_DN27300_c0_g1::TRINITY_DN27300_c0_g1_i6::g.71140 ORF type:internal len:129 (-),score=20.75 TRINITY_DN27300_c0_g1_i6:1-384(-)	0.03732	1.8822	-0.98174	-0.63077	-0.30702
TRINITY_DN52395_c0_g2_i2_orf1	-	-	-	twitchin isoform X20 [Zerene cesonia]	0.0613	1.25425	-1.816	0.2495	0.25095
TRINITY_DN76529_c0_g1_i1_orfp1	-	-	-	TRINITY_DN76529_c0_g1_i1_m.64079 TRINITY_DN76529_c0_g1::TRINITY_DN76529_c0_g1_i1::g.64079 ORF type:internal len:70 (+),score=14.68 TRINITY_DN76529_c0_g1_i1:3-209(+)	0.25284	1.52508	-1.56507	-0.3692	0.15635
TRINITY_DN21170_c0_g1_i5_orf1	-	-	-	twitchin-like [Ostrinia furnacalis]	0.10922	1.78992	-1.23306	-0.47797	-0.1881
TRINITY_DN6325_c0_g1_i8_orf1	-	-	-	unnamed protein product [Pieris macdunnoughi]	-0.09141	1.73952	-1.38833	-0.08246	-0.17733
TRINITY_DN11448_c0_g1_i11_orf1	-	-	-	hypothetical protein B5X24_HaOG201808 [Helicoverpa armigera]	0.10218	1.58127	-1.50116	-0.42207	0.23979
TRINITY_DN920_c0_g1_i4_orf1	-	-	-	glutathione S-transferase omega 2 [Ostrinia furnacalis]	0.44001	1.47996	-1.1651	-1.07519	0.32032

TRINITY_DN27500_c0_g1_i4_orf1	-	-	-	hemicentin-1-like [Ostrinia furnacalis]	0.50168	1.19087	-1.73577	-0.37609	0.4193
TRINITY_DN9871_c0_g1_i11_orf1	-	-	-	PEST proteolytic signal-containing nuclear protein-like [Ostrinia furnacalis]	0.29687	1.03905	-1.90861	0.17347	0.39922
TRINITY_DN29969_c0_g1_i5_orf1	-	-	-	twitchin-like [Ostrinia furnacalis]	-0.18881	1.72402	-1.39292	0.07332	-0.21561
TRINITY_DN4010_c0_g2_i1_orf1	-	-	-	myophilin [Ostrinia furnacalis]	0.29356	1.09712	-1.87995	0.07692	0.41235
TRINITY_DN2719_c1_g1_i6_orf1	-	-	-	unnamed protein product [Chrysodeixis includens]	-0.00589	1.62811	-1.28481	-0.73508	0.39767
TRINITY_DN48610_c0_g1_i2_orf1	-	-	-	hypothetical protein evm_002298 [Chilo suppressalis] >CAH0682062.1 unnamed protein product [Chilo suppressalis]	0.3218	0.99689	-1.91586	0.13453	0.46265
TRINITY_DN6612_c0_g1_i4_orf1	-	-	-	hypothetical protein O3G_MSEX008151 [Manduca sexta]	0.23766	1.25007	-1.7506	-0.24386	0.50673
TRINITY_DN24322_c0_g1_i4_orf1	-	-	-	unnamed protein product, partial [Brenthis ino]	0.16453	1.0696	-1.89292	0.44924	0.20956
TRINITY_DN23474_c1_g1_i1_orf1	-	-	-	unnamed protein product [Chrysodeixis includens]	0.37894	1.58303	-1.31266	-0.78101	0.1317
TRINITY_DN5149_c0_g1_i12_orfp1	-	-	-	TRINITY_DN5149_c0_g1_i12_m.8808 TRINITY_DN5149_c0_g1::TRINITY_DN5149_c0_g1_i12::g.8808 ORF type:internal len:254 (+),score=89.66 TRINITY_DN5149_c0_g1_i12:1-759(+)	0.30581	1.47212	-1.6471	-0.15985	0.02901
TRINITY_DN1459_c0_g1_i2_orf1	-	-	-	reticulon-1 isoform X1 [Ostrinia furnacalis]	-0.056	1.65667	-1.45875	0.16806	-0.30998
TRINITY_DN77830_c0_g2_i2_orf1	-	-	-	prostaglandin reductase 1-like [Leguminivora glycinivorella] >XP_047994907.1 prostaglandin reductase 1-like [Leguminivora glycinivorella]	0.43876	1.58216	-1.41907	-0.06705	-0.53479
TRINITY_DN3105_c0_g1_i4_orf1	-	-	-	nose resistant to fluoxetine protein 6-like [Ostrinia furnacalis]	0.6636	1.37471	-1.60174	-0.28511	-0.15147
TRINITY_DN2958_c0_g1_i2_orf1	-	-	-	uncharacterized protein LOC114356495 [Ostrinia furnacalis]	0.50797	1.39986	-1.63754	-0.31445	0.04416
TRINITY_DN43881_c0_g1_i2_orf1	-	-	-	estradiol 17-beta-dehydrogenase 8-like [Ostrinia furnacalis]	0.54187	1.52344	-1.27544	-0.86759	0.07773
TRINITY_DN5432_c1_g1_i3_orf1	-	-	-	electron transfer flavoprotein-ubiquinone oxidoreductase, mitochondrial [Ostrinia furnacalis]	0.44171	1.74159	-1.07625	-0.57191	-0.53514
TRINITY_DN119893_c0_g2_i3_orf1	-	-	-	ATP-binding cassette sub-family F member 3 isoform X1 [Ostrinia furnacalis] >XP_028168051.1 ATP-binding cassette sub-family F member 3 isoform X2 [Ostrinia furnacalis]	0.84223	1.30379	-1.37363	-0.83654	0.06416
TRINITY_DN3797_c0_g2_i3_orf1	-	-	-	3'(2'),5'-bisphosphate nucleotidase 1 isoform X2 [Ostrinia furnacalis]	0.76343	1.57037	-0.59902	-1.0766	-0.65818
TRINITY_DN146236_c0_g1_i1_orf1	-	-	-	vesicle-fusing ATPase 1-like [Chelonus insularis]	0.80665	1.29977	-1.50496	-0.62794	0.02648
TRINITY_DN4822_c0_g1_i9_orf1	-	-	-	homogentisate 1,2-dioxygenase [Ostrinia furnacalis]	0.63413	1.22028	-0.75098	-1.53559	0.43216
TRINITY_DN4501_c0_g2_i1_orf1	-	-	-	methylcrotonoyl-CoA carboxylase subunit alpha, mitochondrial [Ostrinia furnacalis]	0.83942	1.43812	-1.32312	-0.57987	-0.37456
TRINITY_DN28039_c0_g1_i1_orf1	-	-	-	translation elongation factor 2 [Athalia rosae]	0.84919	1.52491	-0.82294	-0.96691	-0.58426
TRINITY_DN115658_c0_g1_i1_orf1	-	-	-	hypothetical protein B5X24_HaOG203018 [Helicoverpa armigera]	1.13535	1.19932	-1.16794	-0.92094	-0.24578
TRINITY_DN9510_c0_g2_i1_orf1	-	-	-	RNA polymerase II transcriptional coactivator [Ostrinia furnacalis]	0.9068	1.45701	-0.46556	-1.08405	-0.8142
TRINITY_DN2813_c0_g1_i3_orf1	-	-	-	arylphorin subunit alpha-like [Ostrinia furnacalis]	0.69086	1.59195	-0.90312	-0.3574	-1.02229
TRINITY_DN32687_c0_g1_i2_orf1	-	-	-	protein D2-like isoform X2 [Ostrinia furnacalis] >XP_028164613.1 protein D2-like isoform X2 [Ostrinia furnacalis]	0.92504	1.47343	-0.95974	-0.62653	-0.81219
TRINITY_DN248_c0_g1_i12_orf1	-	-	-	twitchin-like [Ostrinia furnacalis]	0.66343	1.19718	-1.74055	0.15197	-0.27205
TRINITY_DN19995_c0_g1_i2_orf1	-	-	-	E3 ubiquitin-protein ligase ZNF598 [Ostrinia furnacalis]	0.95055	1.42586	-0.67594	-0.56678	-1.13369
TRINITY_DN8241_c0_g1_i3_orf1	-	-	-	transforming growth factor beta-1-induced transcript 1 protein [Ostrinia furnacalis]	0.97058	1.31088	-1.14956	-1.00039	-0.1315
TRINITY_DN102260_c0_g1_i1_orf1	-	-	-	unnamed protein product [Diatraea saccharalis]	1.22515	1.21581	-0.94678	-0.81092	-0.68326
TRINITY_DN19659_c1_g1_i1_orf1	-	-	-	elongation factor 1-gamma [Ostrinia furnacalis]	1.10681	1.2019	-1.31178	-0.73585	-0.26108
TRINITY_DN19939_c0_g1_i4_orf1	-	-	-	unnamed protein product [Chilo suppressalis]	1.13816	1.28914	-0.79461	-0.61828	-1.0144
TRINITY_DN82017_c0_g1_i5_orf1	-	-	-	carboxylesterase CXE17 [Ostrinia furnacalis]	0.89939	1.44366	-0.70859	-0.45042	-1.18404
TRINITY_DN17189_c0_g1_i2_orf1	-	-	-	fibroin heavy chain [Haritalodes derogata]	1.56661	0.77809	-1.04932	-0.63696	-0.65842
TRINITY_DN69334_c0_g1_i1_orf1	-	-	-	PREDICTED: 15-hydroxyprostaglandin dehydrogenase [NAD(+)]-like [Papilio xuthus]	0.93755	1.46781	-0.65057	-0.91995	-0.83485
TRINITY_DN21000_c0_g1_i1_orf1	-	-	-	elongation factor-1 alpha, partial [Loxostege sticticalis] >QCO92153.1 elongation factor-1 alpha, partial [Sitochroa umbrosalis]	1.32035	1.11004	-0.99489	-0.76444	-0.67105
TRINITY_DN928_c0_g1_i3_orf1	-	-	-	fasciclin-2-like [Ostrinia furnacalis]	0.8026	0.81685	-1.89232	0.32418	-0.05132
TRINITY_DN2186_c0_g1_i17_orf1	-	-	-	paxillin isoform X6 [Leguminivora glycinivorella]	0.84617	0.69698	-1.91734	0.02583	0.34836
TRINITY_DN1173_c1_g1_i10_orf1	-	-	-	hypothetical protein evm_001011 [Chilo suppressalis]	0.96313	0.87085	-1.81802	-0.07389	0.05792
TRINITY_DN2200_c0_g1_i4_orf1	-	-	-	uncharacterized protein LOC114363443 [Ostrinia furnacalis]	0.94627	0.62464	-1.85039	0.49428	-0.21479
TRINITY_DN115082_c0_g1_i5_orf1	-	-	-	protein dj-1beta-like isoform X2 [Ostrinia furnacalis]	1.01295	1.2336	-1.43334	-0.2235	-0.58971
TRINITY_DN42373_c0_g4_i1_orf1	-	-	-	unnamed protein product [Spodoptera exigua]	1.5308	0.71957	-0.95641	-1.08685	-0.20711
TRINITY_DN1123_c2_g1_i5_orf1	-	-	-	troponin I isoform X4 [Leguminivora glycinivorella]	1.10226	0.81617	-1.76236	-0.09796	-0.05811

TRINITY_DN48638_c0_g1_i5_orf1	-	-	-	NADH dehydrogenase [ubiquinone] flavoprotein 2, mitochondrial [Ostrinia furnacalis] >ALD03682.1 mitochondrial complex I NDUFV2 subunit [Ostrinia nubilalis]	1.73386	0.38225	-0.95183	-0.21893	-0.94535
TRINITY_DN29229_c0_g1_i4_orf1	-	-	-	uncharacterized protein LOC114351433 isoform X1 [Ostrinia furnacalis]	1.55509	0.73821	-1.17083	-0.69528	-0.42719
TRINITY_DN21035_c0_g1_i14_orf1	-	-	-	mitochondrial amidoxime reducing component 2-like [Ostrinia furnacalis]	1.13268	0.87333	-1.16628	-1.20776	0.36803
TRINITY_DN20957_c0_g1_i1_orf1	-	-	-	adenylate kinase isoenzyme 1 isoform X2 [Ostrinia furnacalis]	1.26355	0.54568	-1.76146	0.00595	-0.05373
TRINITY_DN195_c4_g1_i1_orf1	-	-	-	beta-1,3-glucan-binding protein 1 [Ostrinia furnacalis]	1.10521	1.33689	-0.85546	-0.81243	-0.7742
TRINITY_DN53462_c0_g1_i1_orf1	-	-	-	uncharacterized protein LOC118072968 isoform X1 [Chelonus insularis] >XP_034949073.1 uncharacterized protein LOC118072968 isoform X1 [Chelonus	1.4066	0.88802	-1.23373	-0.80254	-0.25835
TRINITY_DN18396_c0_g1_i1_orf1	-	-	-	uncharacterized protein LOC114359424 [Ostrinia furnacalis]	1.44257	0.62547	-1.51312	-0.48273	-0.07218
TRINITY_DN23167_c0_g1_i4_orf1	-	-	-	uncharacterized protein LOC114363065 [Ostrinia furnacalis]	1.54021	0.64999	-1.32769	-0.61907	-0.24345
TRINITY_DN4929_c1_g2_i5_orf1	-	-	-	guanylate kinase isoform X2 [Ostrinia furnacalis]	1.65908	0.50607	-1.20038	-0.68858	-0.2762
TRINITY_DN2497_c0_g1_i2_orf1	-	-	-	protein stunted-like isoform X1 [Colias croceus]	1.74511	0.34292	-1.04956	-0.20602	-0.83246
TRINITY_DN84357_c0_g1_i1_orf1	-	-	-	4-coumarate--CoA ligase 1-like [Ostrinia furnacalis]	1.39544	0.72962	-1.30077	0.08216	-0.90645
TRINITY_DN4514_c0_g1_i1_orf1	-	-	-	enoyl-CoA delta isomerase 1, mitochondrial-like isoform X1 [Ostrinia furnacalis] >XP_028158560.1 enoyl-CoA delta isomerase 1, mitochondrial-like isoform X2 [Ostrinia furnacalis]	1.55263	0.53118	-1.29469	0.00521	-0.79433
TRINITY_DN4938_c0_g1_i13_orf1	-	-	-	peroxisomal biogenesis factor 19 [Ostrinia furnacalis]	1.82356	0.28426	-0.97127	-0.5204	-0.61615
TRINITY_DN27592_c0_g1_i1_orf1	-	-	-	D-arabinitol dehydrogenase 1-like [Ostrinia furnacalis]	1.36271	1.05436	-0.89118	-0.57188	-0.95399
TRINITY_DN22664_c0_g1_i1_orf1	-	-	-	larval cuticle protein LCP-14-like [Ostrinia furnacalis]	1.62823	0.44976	-1.2535	-0.75536	-0.06913
TRINITY_DN37699_c0_g1_i3_orfp1	-	-	-	TRINITY_DN37699_c0_g1_i3_m.58788 TRINITY_DN37699_c0_g1::TRINITY_DN37699_c0_g1_i3::g.58788 ORF type:internal len:122 (+),score=39.86 TRINITY_DN37699_c0_g1_i3:1-363(+)	1.76243	0.38519	-1.08264	-0.58841	-0.47658
TRINITY_DN2986_c1_g1_i1_orf1	-	-	-	Troponin C, isoform 1 [Papilio xuthus]	1.64358	0.46241	-1.24861	-0.70999	-0.14738
TRINITY_DN20238_c0_g1_i7_orf1	-	-	-	zinc finger FYVE domain-containing protein 26 [Ostrinia furnacalis]	1.17226	1.05159	0.01401	-1.03018	-1.20769
TRINITY_DN3307_c1_g1_i2_orf1	-	-	-	BTB/POZ domain-containing protein 2-like [Ostrinia furnacalis]	0.71451	1.48134	-0.19145	-0.6489	-1.3555
TRINITY_DN49221_c0_g1_i1_orf1	-	-	-	Similar to ND-23: NADH dehydrogenase (ubiquinone) 23 kDa subunit (Drosophila melanogaster) [Cotesia congregata]	0.69168	1.32379	0.19768	-0.73171	-1.48145
TRINITY_DN8136_c0_g1_i1_orf1	-	-	-	HIG1 domain family member 2A, mitochondrial [Ostrinia furnacalis]	0.40848	1.48866	0.10459	-0.45194	-1.54979
TRINITY_DN4014_c0_g1_i1_orf1	-	-	-	cyclin-Q [Ostrinia furnacalis]	1.13531	1.04582	-0.98774	0.08506	-1.27845
TRINITY_DN129_c0_g1_i6_orf1	-	-	-	xanthine dehydrogenase [Ostrinia furnacalis]	0.97094	0.9446	-0.19543	0.04753	-1.76764
TRINITY_DN3469_c0_g1_i4_orf1	-	-	-	LOW QUALITY PROTEIN: cadherin-87A-like [Ostrinia furnacalis]	0.27493	1.41615	0.34348	-0.41261	-1.62195
TRINITY_DN146364_c0_g1_i1_orf1	-	-	-	pupal cuticle protein 20-like [Ostrinia furnacalis]	1.28234	0.63568	0.0284	-0.24644	-1.69999
TRINITY_DN6381_c0_g1_i2_orf1	-	-	-	solute carrier family 12 member 8 [Ostrinia furnacalis]	1.56448	0.63561	-0.16907	-1.18475	-0.84628
TRINITY_DN3835_c0_g1_i4_orf1	-	-	-	protein ERGIC-53 isoform X1 [Ostrinia furnacalis] >XP_028177940.1 protein ERGIC-53 isoform X2 [Ostrinia furnacalis] >XP_028177941.1 protein ERGIC-53 isoform X3 [Ostrinia furnacalis]	1.522	0.57796	0.05352	-0.95873	-1.19475
TRINITY_DN1760_c0_g1_i4_orf1	-	-	-	uncharacterized protein LOC114357676 [Ostrinia furnacalis]	1.04127	0.46612	-0.09613	0.45486	-1.86611
TRINITY_DN6638_c0_g1_i1_orf1	-	-	-	ubiquinone biosynthesis protein COQ4 homolog, mitochondrial [Ostrinia furnacalis]	1.29186	0.85165	-0.58307	-0.05623	-1.50421
TRINITY_DN42705_c0_g1_i3_orf1	-	-	-	multiple inositol polyphosphate phosphatase 1 isoform X1 [Ostrinia furnacalis]	1.50866	0.80749	-0.33829	-1.01649	-0.96137
TRINITY_DN6071_c0_g1_i1_orf1	-	-	-	transcription initiation factor IIB isoform X1 [Manduca sexta] >XP_038208211.1 transcription initiation factor IIB isoform X1 [Zerene cesonia] >XP_045510964.1 transcription initiation factor IIB isoform X1 [Colias croceus] >XP_049872283.1 transcription initiation factor IIB [Pectinophora gossypiella]	1.09227	0.54597	0.7522	-0.98792	-1.40252
TRINITY_DN6503_c0_g1_i8_orf1	-	-	-	uncharacterized protein LOC114354432 [Ostrinia furnacalis]	1.80705	0.25421	-0.34072	-0.66824	-1.0523
TRINITY_DN6016_c0_g1_i8_orf1	-	-	-	hypothetical protein evm_010883 [Chilo suppressalis]	1.08612	0.70201	0.45713	-0.57584	-1.66942
TRINITY_DN36928_c0_g1_i2_orf1	-	-	-	actin-interacting protein 1 isoform X2 [Ostrinia furnacalis]	1.53363	0.71768	-0.53853	-1.29004	-0.42274
TRINITY_DN58261_c0_g1_i1_orf1	-	-	-	15-hydroxyprostaglandin dehydrogenase [NAD(+)]-like [Ostrinia furnacalis]	1.57736	0.5891	-0.39798	-1.35474	-0.41375
TRINITY_DN33452_c0_g1_i3_orf1	-	-	-	lethal(2) giant larvae protein isoform X8 [Ostrinia furnacalis]	1.00146	1.21644	-0.17303	-0.57709	-1.46778
TRINITY_DN2356_c2_g1_i6_orf1	-	-	-	ER membrane protein complex subunit 3 [Ostrinia furnacalis]	1.59098	0.59208	-0.13588	-1.06998	-0.97719
TRINITY_DN91877_c0_g1_i1_orf1	-	-	-	NADH dehydrogenase [ubiquinone] 1 alpha subcomplex assembly factor 2 [Ostrinia furnacalis]	0.9754	0.90513	-0.44823	0.28452	-1.71682

TRINITY_DN36893_c0_g1_i1_orf1	-	-	-	40S ribosomal protein S15 [Bicyclus anynana] >XP_026325996.1 40S ribosomal protein S15 [Hyposmocoma kahamanoa] >XP_028175376.1 40S ribosomal protein S15 [Ostrinia furnacalis] >XP_030034906.1 40S ribosomal protein S15 [Manduca sexta] >XP_039758445.1 40S ribosomal protein S15 [Pararge aegeria] >XP_045775675.1 40S ribosomal protein S15 [Maniola jurtina] >CAH2267288.1 jg14755 [Pararge aegeria aegeria] >ACY95351.1 ribosomal protein S15 [Manduca sexta] >KAG6461386.1 hypothetical protein O3G_MSEX012590 [Manduca sexta] >KAG6461387.1 hypothetical protein O3G_MSEX012590 [Manduca sexta]	1.59632	0.74388	-0.73593	-0.98916	-0.61511
TRINITY_DN4836_c0_g1_i4_orf1	-	-	-	hypothetical protein O3G_MSEX014157 [Manduca sexta] >KAG6463927.1 hypothetical protein O3G_MSEX014157 [Manduca sexta]	1.62699	0.33629	0.13708	-1.13748	-0.96289
TRINITY_DN142485_c0_g1_i1_orf1	-	-	-	uncharacterized protein CG16817-like [Ostrinia furnacalis]	1.73629	0.26944	-0.05431	-1.02915	-0.92228
TRINITY_DN7241_c0_g2_i2_orf1	-	-	-	40S ribosomal protein S10 [Zerene cesonia] >XP_045492164.1 40S ribosomal protein S10 [Colias croceus]	1.77353	0.42443	-0.74128	-0.90723	-0.54944
TRINITY_DN6396_c0_g1_i1_orf1	-	-	-	PR domain zinc finger protein 10-like [Ostrinia furnacalis]	1.70947	0.34701	-0.24835	-1.26526	-0.54288
TRINITY_DN3370_c0_g1_i5_orf1	-	-	-	unnamed protein product, partial [Brenthis ino]	1.5837	0.70253	-0.35004	-0.99434	-0.94186
TRINITY_DN10455_c0_g1_i2_orf1	-	-	-	actin-related protein 2/3 complex subunit 4 [Plutella xylostella] >XP_013184242.1 PREDICTED: actin-related protein 2/3 complex subunit 4 [Amyelois transitella] >XP_026754865.1 actin-related protein 2/3 complex subunit 4 [Galleria mellonella] >XP_028168998.1 actin-related protein 2/3 complex subunit 4 [Ostrinia furnacalis] >KAI5632346.1 ARP2/3 complex 20 kDa subunit (ARPC4) domain-containing protein [Phthorimaea operculella] >KAG7303373.1 Actin- protein 2/3 complex subunit 4 [Plutella xylostella] >CAG9104981.1 unnamed protein product [Plutella xylostella]	1.71043	0.08644	0.21104	-1.06125	-0.94666
TRINITY_DN23264_c0_g1_i1_orf1	-	-	-	U5 small nuclear ribonucleoprotein 40 kDa protein [Ostrinia furnacalis]	1.63633	0.36537	0.03669	-1.25376	-0.78463
TRINITY_DN5593_c0_g1_i1_orf1	-	-	-	PREDICTED: leucine-rich repeat-containing protein 47-like [Fopius arisanus]	1.68177	0.31009	-0.29818	-0.32133	-1.37235
TRINITY_DN2374_c0_g1_i1_orf1	-	-	-	uncharacterized protein LOC114357127 [Ostrinia furnacalis]	1.58972	0.16727	0.3792	-1.16655	-0.96963
TRINITY_DN5086_c0_g1_i1_orf1	-	-	-	unnamed protein product [Diatraea saccharalis]	1.72463	0.13197	0.12954	-1.09075	-0.89539
TRINITY_DN7647_c0_g1_i4_orf1	-	-	-	E3 ubiquitin-protein ligase Bre1 isoform X6 [Ostrinia furnacalis]	1.74752	0.18939	0.01027	-1.05855	-0.88864
TRINITY_DN556_c0_g2_i1_orf1	-	-	-	serine protease inhibitor dipetalogastin-like [Ostrinia furnacalis]	0.97254	0.66585	0.54584	-0.41115	-1.77308
TRINITY_DN4008_c0_g1_i7_orf1	-	-	-	nuclear export mediator factor NEMF homolog isoform X1 [Ostrinia furnacalis]	1.78132	0.38607	-0.73531	-0.95239	-0.47969
TRINITY_DN88539_c0_g2_i1_orf1	-	-	-	uncharacterized protein LOC114352312 isoform X1 [Ostrinia furnacalis] >XP_028159669.1 uncharacterized protein LOC114352312 isoform X1 [Ostrinia	1.76558	0.27142	-0.14704	-0.91795	-0.97201
TRINITY_DN3860_c0_g1_i5_orf1	-	-	-	nucleoplasmin-like protein isoform X1 [Hyposmocoma kahamanoa]	1.4081	0.47099	0.41215	-1.16359	-1.12766
TRINITY_DN23502_c0_g1_i1_orf1	-	-	-	small nuclear ribonucleoprotein F [Ostrinia furnacalis]	1.39261	0.26733	0.56962	-1.41415	-0.81541
TRINITY_DN4056_c0_g1_i8_orf1	-	-	-	uncharacterized protein LOC114349672 [Ostrinia furnacalis] >XP_028155936.1 uncharacterized protein LOC114349672 [Ostrinia furnacalis] >XP_028155937.1 uncharacterized protein LOC114349672 [Ostrinia furnacalis] >XP_028155939.1 uncharacterized protein LOC114349672 [Ostrinia furnacalis]	1.55546	0.42436	0.07599	-1.40306	-0.65275
TRINITY_DN82324_c0_g1_i4_orf1	-	-	-	hypothetical protein evm_001824 [Chilo suppressalis] >CAG9754426.1 unnamed protein product [Diatraea saccharalis] >CAG9793111.1 unnamed protein product [Diatraea saccharalis]	1.81148	0.3143	-0.69976	-0.95105	-0.47496

TRINITY_DN5630_c4_g1_i2_orf1	-	-	-	PREDICTED: stress-associated endoplasmic reticulum protein 2 [Amyelois transitella] >XP_014371593.1 stress-associated endoplasmic reticulum protein 2 [Papilio machaon] >XP_022818474.1 stress-associated endoplasmic reticulum protein 2 [Spodoptera litura] >XP_028162992.1 stress-associated endoplasmic reticulum protein 2 [Ostrinia furnacalis] >XP_028162993.1 stress-associated endoplasmic reticulum protein 2 [Ostrinia furnacalis] >XP_031767943.1 stress-associated endoplasmic reticulum protein 2 [Galleria mellonella] >XP_035452408.1 stress-associated endoplasmic reticulum protein 2-like [Spodoptera frugiperda] >XP_035452409.1 stress-associated endoplasmic reticulum protein 2-like [Spodoptera frugiperda] >XP_035452411.1 stress-associated endoplasmic reticulum protein 2-like [Spodoptera frugiperda] >XP_045455924.1 stress-associated endoplasmic reticulum protein 2 [Melitaea cinxia] >KPJ00707.1 Stress-associated endoplasmic reticulum protein 2 [Papilio xuthus] >CAB3510969.1 unnamed protein product [Spodoptera littoralis] >AXY94738.1 stress-associated endoplasmic reticulum protein 2 [Galleria mellonella] >KAF9797689.1 hypothetical protein SFRURICE_017884 [Spodoptera frugiperda] >KAG8114722.1	1.78955	0.21329	-0.1782	-0.74563	-1.07902
TRINITY_DN271_c0_g2_i6_orf1	-	-	-	hypothetical protein NE865_03378 [Phthorimaea operculella]	1.85446	0.26101	-0.71745	-0.71992	-0.6781
TRINITY_DN4944_c0_g1_i5_orf1	-	-	-	bifunctional glutamate/proline--tRNA ligase [Ostrinia furnacalis]	1.89392	-0.09469	-0.2179	-0.5601	-1.02123
TRINITY_DN17394_c0_g1_i1_orf1	-	-	-	monocarboxylate transporter 14-like [Ostrinia furnacalis]	1.70966	0.43498	-0.24684	-1.06315	-0.83465
TRINITY_DN2885_c1_g1_i2_orf1	-	-	-	ubiquitin-like-specific protease ESD4 [Ostrinia furnacalis]	1.37053	-0.05359	0.8457	-1.26163	-0.90102
TRINITY_DN18242_c0_g1_i3_orf1	-	-	-	CCHC-type zinc finger protein CG3800 [Papilio xuthus]	1.70898	0.1297	0.17094	-1.08865	-0.92097
TRINITY_DN1013_c0_g1_i3_orf1	-	-	-	TELO2-interacting protein 1 homolog isoform X2 [Ostrinia furnacalis]	1.01458	0.7906	0.55579	-0.82725	-1.53373
TRINITY_DN7574_c0_g1_i10_orf1	-	-	-	proteasome activator complex subunit 3 isoform X2 [Ostrinia furnacalis]	1.74949	0.08871	0.0671	-1.18911	-0.71619
TRINITY_DN51968_c0_g1_i1_orf1	-	-	-	splicing factor U2af 38 kDa subunit [Aphidius gifuensis] >KAF7990547.1 hypothetical protein HCN44_000352 [Aphidius gifuensis]	1.49887	0.1563	0.53877	-1.22345	-0.97049
TRINITY_DN137_c0_g1_i1_orf1	-	-	-	60S ribosomal protein L8 [Cotesia glomerata] >XP_044597650.1 60S ribosomal protein L8 [Cotesia glomerata] >KAG8034499.1 hypothetical protein G9C98_007575 [Cotesia typhae] >CAD6216378.1 GSCOCG00004534001-RA-CDS [Cotesia congregata] >KAH0544237.1 60S ribosomal protein L8 [Cotesia glomerata] >KAH0564528.1 60S ribosomal protein L8 [Cotesia glomerata] >CAG5095185.1 Similar to RpL8: 60S ribosomal protein L8 (Spodoptera frugiperda) [Cotesia congregata]	1.79823	0.35974	-0.58898	-0.95652	-0.61247
TRINITY_DN2089_c0_g1_i5_orf1	-	-	-	eukaryotic translation initiation factor 4B [Ostrinia furnacalis]	1.70868	0.02568	0.24467	-1.16501	-0.81403
TRINITY_DN31851_c0_g1_i2_orf1	-	-	-	CDGSH iron-sulfur domain-containing protein 2 homolog [Helicoverpa armigera] >PZC85510.1 hypothetical protein B5X24_HaOG216618 [Helicoverpa armigera]	1.76477	-0.33768	-0.04025	-0.0577	-1.32914
TRINITY_DN124950_c0_g2_i1_orf1	-	-	-	TATA box-binding protein-like protein 1 [Ostrinia furnacalis] >XP_028155830.1 TATA box-binding protein-like protein 1 [Ostrinia furnacalis]	1.55777	0.4144	0.18518	-0.93676	-1.22058
TRINITY_DN4345_c0_g1_i9_orf1	-	-	-	uncharacterized protein LOC114357127 [Ostrinia furnacalis]	1.49897	0.42598	0.20499	-1.42642	-0.70351
TRINITY_DN7112_c0_g1_i1_orf1	-	-	-	heterogeneous nuclear ribonucleoprotein K isoform X2 [Ostrinia furnacalis]	1.44205	0.10254	0.65261	-1.28599	-0.91121
TRINITY_DN69049_c0_g2_i1_orf1	-	-	-	membrane alanyl aminopeptidase-like [Ostrinia furnacalis]	1.63304	-0.3617	0.17825	0.02351	-1.4731
TRINITY_DN5697_c0_g1_i1_orf1	-	-	-	GPI ethanolamine phosphate transferase 2-like [Ostrinia furnacalis]	1.63305	-0.16938	0.54176	-0.9966	-1.00883
TRINITY_DN3299_c0_g1_i2_orf1	-	-	-	metaxin-1 isoform X3 [Ostrinia furnacalis] >XP_028170907.1 metaxin-1 isoform X4 [Ostrinia furnacalis]	1.87783	0.10648	-0.51566	-0.49138	-0.97727
TRINITY_DN220_c0_g1_i3_orf1	-	-	-	serine-arginine protein 55 isoform X6 [Pieris brassicae]	1.52846	-0.12835	0.67164	-1.19421	-0.87755
TRINITY_DN3127_c0_g1_i9_orf1	-	-	-	RNA-binding protein 1 isoform X1 [Galleria mellonella]	1.84986	-0.2433	0.09022	-0.6598	-1.03699
TRINITY_DN43412_c0_g1_i2_orf1	-	-	-	U1 small nuclear ribonucleoprotein C [Ostrinia furnacalis]	1.54844	0.00847	0.54035	-1.28428	-0.81297
TRINITY_DN2117_c0_g1_i1_orf1	-	-	-	BUB3-interacting and GLEBS motif-containing protein ZNF207 [Chelonus insularis]	1.75429	-0.17592	0.30715	-1.04175	-0.84376
TRINITY_DN478_c0_g1_i16_orf1	-	-	-	lipid storage droplets surface-binding protein 2 isoform X1 [Ostrinia furnacalis]	1.75638	0.12968	-0.03412	-0.62406	-1.22788
TRINITY_DN14235_c0_g1_i1_orf1	-	-	-	maltase A1 [Helicoverpa armigera]	1.91931	0.05788	-0.64282	-0.73578	-0.59859
TRINITY_DN17312_c0_g1_i1_orf1	-	-	-	mRNA cap guanine-N7 methyltransferase [Ostrinia furnacalis]	1.72448	-0.22448	0.40395	-0.94569	-0.95827
TRINITY_DN1427_c0_g1_i9_orf1	-	-	-	SAFB-like transcription modulator isoform X3 [Ostrinia furnacalis]	1.59647	0.15764	0.3578	-1.24079	-0.87112
TRINITY_DN29934_c0_g1_i6_orf1	-	-	-	sodium/potassium-transporting ATPase subunit beta-2-like [Ostrinia furnacalis] >XP_028176258.1 sodium/potassium-transporting ATPase subunit beta-2-like [Ostrinia furnacalis]	1.5612	0.32009	0.29549	-1.13126	-1.04552

TRINITY_DN15607_c0_g1_i6_orf1	-	-	-	protein artichoke-like [Ostrinia furnacalis]	1.92895	-0.0083	-0.48904	-0.80296	-0.62865
TRINITY_DN52296_c0_g1_i6_orf1	-	-	-	protein takeout-like [Ostrinia furnacalis]	1.83707	0.18238	-0.34257	-1.02364	-0.65324
TRINITY_DN5925_c0_g1_i5_orf1	-	-	-	isocitrate dehydrogenase [NAD] subunit gamma, mitochondrial-like isoform X1 [Ostrinia furnacalis]	1.95934	-0.10454	-0.57975	-0.6142	-0.66085
TRINITY_DN76036_c0_g1_i1_orf1	-	-	-	cytochrome c oxidase subunit 6A1, mitochondrial-like [Ostrinia furnacalis]	1.93779	-0.37851	-0.23055	-0.37646	-0.95227
TRINITY_DN10332_c0_g1_i2_orfp1	-	-	-	TRINITY_DN10332_c0_g1_i2_m.42894 TRINITY_DN10332_c0_g1::TRINITY_DN10332_c0_g1_i2::g.42894 ORF type:3prime_partial len:77 (+),score=1.70 TRINITY_DN10332_c0_g1_i2:1005-1232(+)	1.80891	-0.01375	-0.01195	-1.15388	-0.62934
TRINITY_DN35635_c0_g1_i1_orf1	-	-	-	probable NADH dehydrogenase [ubiquinone] 1 alpha subcomplex subunit 12 [Ostrinia furnacalis]	1.88856	-0.30062	-0.02263	-0.54039	-1.02491
TRINITY_DN8543_c0_g1_i1_orf1	-	-	-	39S ribosomal protein L38, mitochondrial [Ostrinia furnacalis]	1.87421	0.13584	-0.42322	-0.91741	-0.66942
TRINITY_DN24399_c0_g1_i1_orf1	-	-	-	retinol-binding protein pinta-like [Ostrinia furnacalis]	1.87922	0.14563	-0.76217	-0.4453	-0.81738
TRINITY_DN9003_c0_g1_i20_orf1	-	-	-	RNA-binding protein Nova-2 isoform X4 [Ostrinia furnacalis]	1.90354	0.05316	-0.47193	-0.89848	-0.58629
TRINITY_DN49265_c0_g3_i2_orf1	-	-	-	cytochrome c [Ostrinia furnacalis] >XP_028160278.1 cytochrome c [Ostrinia furnacalis]	1.96888	-0.2462	-0.55206	-0.39431	-0.77631
TRINITY_DN5238_c0_g1_i2_orf1	-	-	-	DNA-(apurinic or apyrimidinic site) lyase [Ostrinia furnacalis]	1.7357	-0.23066	0.33526	-1.17353	-0.66677
TRINITY_DN6747_c0_g1_i7_orf1	-	-	-	retinol dehydrogenase 12-like [Ostrinia furnacalis]	1.92317	-0.09468	-0.30232	-0.89806	-0.62812
TRINITY_DN139212_c0_g1_i4_orf1	-	-	-	uncharacterized protein LOC114350112 [Ostrinia furnacalis]	1.52551	-0.03795	0.63873	-1.10087	-1.02542
TRINITY_DN25779_c0_g1_i6_orf1	-	-	-	aldo-keto reductase AKR2E4-like [Ostrinia furnacalis]	1.5711	-0.5977	0.78433	-0.79402	-0.96371
TRINITY_DN24476_c0_g1_i1_orf1	-	-	-	ensconsin-like isoform X1 [Ostrinia furnacalis]	1.88645	-0.32972	0.06018	-0.70407	-0.91283
TRINITY_DN8691_c0_g1_i3_orf1	-	-	-	nucleolin-like [Melitaea cinxia]	1.70085	-0.37775	0.51566	-0.85615	-0.98261
TRINITY_DN94355_c0_g1_i2_orf1	-	-	-	uncharacterized protein LOC126369488 [Pectinophora gossypiella]	1.88694	0.14341	-0.51477	-0.70593	-0.80966
TRINITY_DN77642_c0_g1_i1_orf1	-	-	-	peritrophic membrane chitin binding protein [Loxostege sticticalis]	0.15185	0.54978	1.49569	-0.99029	-1.20704
TRINITY_DN25251_c0_g2_i1_orf1	-	-	-	dolichyl-phosphate beta-glucosyltransferase [Ostrinia furnacalis]	0.55127	1.2307	0.58199	-1.33793	-1.02602
TRINITY_DN19303_c0_g1_i5_orf1	-	-	-	lipopolysaccharide-induced tumor necrosis factor-alpha factor-like [Ostrinia furnacalis]	0.15729	0.8334	1.18704	-1.58906	-0.58866
TRINITY_DN29633_c0_g1_i8_orf1	-	-	-	transmembrane protein 87A isoform X1 [Ostrinia furnacalis] >XP_028156931.1 transmembrane protein 87A isoform X2 [Ostrinia furnacalis] >XP_028156932.1 transmembrane protein 87A isoform X3 [Ostrinia furnacalis] >XP_028156933.1 transmembrane protein 87A isoform X4 [Ostrinia furnacalis] >XP_028156934.1 transmembrane protein 87A isoform X5 [Ostrinia furnacalis] >XP_028156935.1 transmembrane protein 87A isoform X6 [Ostrinia furnacalis]	0.12748	0.36902	1.61094	-1.17983	-0.9276
TRINITY_DN4798_c0_g1_i3_orf1	-	-	-	unnamed protein product [Spodoptera exigua]	0.03117	0.86152	1.2871	-0.75487	-1.42491
TRINITY_DN3667_c0_g1_i4_orf1	-	-	-	coronin-2B-like isoform X2 [Ostrinia furnacalis]	0.31086	1.2289	0.7544	-0.83683	-1.45732
TRINITY_DN1066_c0_g1_i8_orf1	-	-	-	hypothetical protein evm_012420 [Chilo suppressalis]	0.13392	0.70251	1.40167	-1.21736	-1.02073
TRINITY_DN10110_c1_g2_i1_orf1	-	-	-	venom allergen 3-like [Ostrinia furnacalis]	0.51602	0.83929	0.94586	-1.64402	-0.65715
TRINITY_DN12777_c0_g1_i5_orf1	-	-	-	clathrin light chain isoform X2 [Ostrinia furnacalis]	0.33592	0.63144	1.25539	-1.58154	-0.64121
TRINITY_DN3335_c0_g1_i1_orf1	-	-	-	unnamed protein product [Pieris macdunnoughi]	0.50244	1.02287	0.83144	-1.5196	-0.83715
TRINITY_DN8659_c0_g2_i1_orf1	-	-	-	ubiquitin-like modifier-activating enzyme 1 [Ostrinia furnacalis]	-0.3919	0.02678	1.79408	-0.16402	-1.26494
TRINITY_DN172_c8_g2_i1_orf1	-	-	-	sialin [Ostrinia furnacalis]	0.19787	0.416	1.55834	-1.07822	-1.09399
TRINITY_DN32359_c0_g2_i1_orf1	-	-	-	PREDICTED: 26S proteasome non-ATPase regulatory subunit 4 isoform X2 [Fopius	0.66803	0.55225	1.13558	-1.4814	-0.87446
TRINITY_DN2342_c0_g1_i1_orf1	-	-	-	BRCA1-associated protein [Ostrinia furnacalis]	0.27006	1.03409	1.0306	-1.35453	-0.98023
TRINITY_DN30673_c0_g1_i5_orf1	-	-	-	protein phosphatase 1 regulatory subunit 21 [Ostrinia furnacalis]	-0.06012	0.76862	1.39574	-0.70312	-1.40112
TRINITY_DN14677_c0_g2_i3_orf1	-	-	-	AP-3 complex subunit beta-2 [Ostrinia furnacalis]	0.51627	0.6394	1.15998	-1.5438	-0.77185
TRINITY_DN14944_c0_g1_i7_orf1	-	-	-	alpha-tocopherol transfer protein-like isoform X1 [Ostrinia furnacalis] >XP_028158173.1 alpha-tocopherol transfer protein-like isoform X1 [Ostrinia furnacalis] >XP_028158174.1 alpha-tocopherol transfer protein-like isoform X1	0.57795	0.66834	1.14713	-1.05677	-1.33664
TRINITY_DN2745_c0_g1_i2_orf1	-	-	-	PREDICTED: tubulin alpha-1A chain-like [Papilio polytes] >XP_013164648.1 PREDICTED: tubulin alpha-1A chain-like [Papilio xuthus]	0.07417	0.29113	1.60811	-0.5528	-1.42061
TRINITY_DN5460_c0_g1_i5_orf1	-	-	-	unnamed protein product [Parnassius apollo]	0.49346	1.1667	0.70581	-0.96136	-1.40461
TRINITY_DN19260_c0_g1_i5_orf1	-	-	-	probable 26S proteasome non-ATPase regulatory subunit 3 [Ostrinia furnacalis]	0.80779	0.76368	0.84136	-1.47615	-0.93668
TRINITY_DN41664_c0_g1_i4_orf1	-	-	-	uncharacterized protein LOC114356631 [Ostrinia furnacalis]	1.08079	0.87992	0.40242	-1.40902	-0.95412
TRINITY_DN198_c0_g1_i2_orf1	-	-	-	retinol dehydrogenase 13-like [Ostrinia furnacalis]	0.59399	1.04043	0.79039	-1.21981	-1.20501
TRINITY_DN24218_c0_g1_i1_orf1	-	-	-	uncharacterized protein LOC114362624 [Ostrinia furnacalis]	0.25686	0.3872	1.48548	-1.45845	-0.67109

TRINITY_DN24266_c0_g2_i2_orf1	-	-	-	chromobox-like protein 5 [Helicoverpa armigera]	0.72452	0.42261	1.21237	-1.32638	-1.03312
TRINITY_DN629_c0_g1_i6_orf1	-	-	-	annexin B9-like isoform X1 [Ostrinia furnacalis]	0.65815	0.64748	1.05934	-0.84425	-1.52073
TRINITY_DN54586_c1_g1_i1_orf1	-	-	-	protein YIPF5 [Ostrinia furnacalis]	0.60293	0.56167	1.213	-1.29629	-1.08132
TRINITY_DN34479_c0_g1_i2_orf1	-	-	-	PREDICTED: 26S protease regulatory subunit 4 [Amyelois transitella] >XP_021186380.1 26S proteasome regulatory subunit 4 [Helicoverpa armigera] >XP_022116536.1 26S proteasome regulatory subunit 4 [Pieris rapae] >XP_022817854.1 26S proteasome regulatory subunit 4 [Spodoptera litura] >XP_026745369.1 26S proteasome regulatory subunit 4 [Trichoplusia ni] >XP_026760570.1 26S proteasome regulatory subunit 4 [Galleria mellonella] >XP_028176505.1 26S proteasome regulatory subunit 4 [Ostrinia furnacalis] >XP_030038234.1 26S proteasome regulatory subunit 4 [Manduca sexta] >XP_035449919.1 26S proteasome regulatory subunit 4 [Spodoptera frugiperda] >XP_038206559.1 26S proteasome regulatory subunit 4 [Zerene cesonia] >XP_045502541.1 26S proteasome regulatory subunit 4 [Colias croceus] >XP_045532999.1 26S proteasome regulatory subunit 4 [Pieris brassicae] >XP_047033702.1 26S proteasome regulatory subunit 4 [Helicoverpa zea] >XP_047994509.1 26S proteasome regulatory subunit 4 [Leguminivora glycinivorella] >XP_049877826.1 26S proteasome regulatory subunit 4 [Pectinophora gossypiella] >KAH9639287.1 hypothetical protein HF086_014151 [Spodoptera exigua] >KAI5631153.1 ATPase family associated with various cellular activities (AAA) domain-containing protein [Phthorimaea operculella] >RVE50066.1 hypothetical protein evm_005272 [Chilo suppressalis] >CAB3245712.1 unnamed protein product [Arctia plantaginis] >KAF9801312.1 hypothetical protein SFRURICE_000406 [Spodoptera	1.09943	0.73405	0.52597	-1.48979	-0.86966
TRINITY_DN1781_c0_g1_i8_orf1	-	-	-	transportin-1 [Pectinophora gossypiella]	0.60887	0.52092	1.2295	-1.00197	-1.35732
TRINITY_DN49047_c0_g1_i2_orf1	-	-	-	unnamed protein product [Parnassius apollo]	1.00968	0.59295	0.67305	-1.67941	-0.59627
TRINITY_DN131264_c0_g1_i2_orf1	-	-	-	unnamed protein product [Parnassius apollo]	-0.00635	0.09463	1.73387	-0.50825	-1.31391
TRINITY_DN2054_c0_g1_i1_orf1	-	-	-	macrophage mannose receptor 1-like [Ostrinia furnacalis]	0.49434	0.00965	1.59174	-0.93413	-1.1616
TRINITY_DN2919_c0_g1_i5_orf1	-	-	-	nidogen-1 [Ostrinia furnacalis]	1.18571	0.35735	0.80799	-1.33338	-1.01767
TRINITY_DN5346_c0_g1_i5_orf1	-	-	-	syntaxin-1A isoform X2 [Pectinophora gossypiella]	1.04862	0.49038	0.87033	-1.20903	-1.2003
TRINITY_DN92153_c0_g2_i2_orf1	-	-	-	methylenetetrahydrofolate reductase [Ostrinia furnacalis]	1.20021	0.86269	0.24122	-1.37854	-0.92558
TRINITY_DN9311_c0_g1_i1_orf1	-	-	-	cuticle protein 8-like [Ostrinia furnacalis]	-0.19981	-0.3658	1.96093	-0.75924	-0.63607
TRINITY_DN535_c1_g1_i2_orf1	-	-	-	protein tramtrack, beta isoform isoform X24 [Bicyclus anynana]	0.39845	-0.16871	1.71138	-0.97251	-0.9686
TRINITY_DN25916_c0_g1_i1_orf1	-	-	-	uncharacterized protein LOC125063950 [Vanessa atalanta]	1.23467	0.66733	0.39166	-1.49784	-0.79582
TRINITY_DN17655_c0_g1_i1_orf1	-	-	-	BRISC and BRCA1-A complex member 1-like [Ostrinia furnacalis]	0.3707	-0.17201	1.69511	-0.6585	-1.2353
TRINITY_DN110534_c0_g1_i3_orf1	-	-	-	unnamed protein product [Euphydryas editha]	0.5383	-0.4191	1.67097	-0.63043	-1.15975
TRINITY_DN12227_c0_g2_i3_orf1	-	-	-	exonuclease 3'-5' domain-containing protein 2 [Ostrinia furnacalis]	1.09503	0.55443	0.60142	-1.67252	-0.57836
TRINITY_DN4494_c0_g1_i1_orf1	-	-	-	venom serine carboxypeptidase [Ostrinia furnacalis]	0.84329	0.03876	1.34211	-1.03254	-1.19162
TRINITY_DN38424_c0_g1_i1_orf1	-	-	-	glucose dehydrogenase [FAD, quinone]-like [Ostrinia furnacalis]	1.00217	0.41888	0.80229	-1.70534	-0.518
TRINITY_DN2802_c1_g1_i1_orf1	-	-	-	psi [Ostrinia furnacalis]	1.25928	-0.06818	0.99697	-0.98971	-1.19837
TRINITY_DN98242_c0_g1_i1_orf1	-	-	-	adenosine deaminase 2-A-like [Galleria mellonella]	0.21135	-0.29072	1.82519	-0.96106	-0.78476
TRINITY_DN129835_c0_g1_i2_orf1	-	-	-	alpha-tocopherol transfer protein-like [Chelonus insularis]	-0.22438	-0.16582	1.93008	-0.69468	-0.8452
TRINITY_DN87170_c0_g1_i3_orf1	-	-	-	uncharacterized protein LOC114360175 [Ostrinia furnacalis]	1.10539	-0.06018	1.14557	-0.91875	-1.27203
TRINITY_DN2232_c1_g1_i3_orf1	-	-	-	protein FAM98A-like [Ostrinia furnacalis]	1.26225	0.24696	0.80632	-1.24367	-1.07186
TRINITY_DN4835_c0_g1_i2_orf1	-	-	-	ribonucleoside-diphosphate reductase large subunit [Ostrinia furnacalis]	1.12323	-0.08316	1.14202	-1.24349	-0.9386
TRINITY_DN10558_c0_g1_i4_orf1	-	-	-	unnamed protein product [Chrysodeixis includens]	0.93463	0.41533	1.04237	-1.25005	-1.14229
TRINITY_DN14507_c0_g1_i5_orf1	-	-	-	PTB domain-containing adapter protein ced-6 [Ostrinia furnacalis]	0.92095	0.09709	1.24389	-0.99497	-1.26696
TRINITY_DN144258_c0_g1_i1_orf1	-	-	-	PREDICTED: enhancer of rudimentary homolog [Microplitis demolitor] >XP_044577051.1 enhancer of rudimentary homolog [Cotesia glomerata] >KAG8041963.1 hypothetical protein G9C98_007267 [Cotesia typhae] >KAH0539785.1 hypothetical protein KQX54_008036 [Cotesia glomerata] >CAD6227368.1 GSCOCG00006137001-RA-CDS [Cotesia congregata]	1.12581	0.08076	1.04677	-1.34058	-0.91277
TRINITY_DN48641_c0_g1_i4_orf1	-	-	-	RNA-binding protein 45-like [Galleria mellonella]	0.85224	-0.27004	1.45476	-0.94836	-1.0886
TRINITY_DN3614_c0_g2_i1_orf1	-	-	-	PC4 and SFRS1-interacting protein isoform X4 [Galleria mellonella]	1.17881	0.32627	0.83727	-1.34388	-0.99847

TRINITY_DN2894_c0_g2_i3_orf1	-	-	-	myrosinase 1-like isoform X1 [Ostrinia furnacalis]	1.21584	0.08127	0.93523	-1.38895	-0.84339
TRINITY_DN2338_c0_g1_i3_orf1	-	-	-	phenoloxidase subunit 1-like [Ostrinia furnacalis]	-0.07179	-0.02241	-0.61963	1.83265	-1.11883
TRINITY_DN128231_c0_g1_i5_orf1	-	-	-	glutathione S-transferase sigma3 [Glyphodes pyloalis]	-0.42858	-0.39614	-0.19122	1.94093	-0.92499
TRINITY_DN52887_c0_g1_i1_orf1	-	-	-	cytochrome P450 6B5-like [Ostrinia furnacalis]	0.24265	0.23694	-0.58719	1.55952	-1.45193
TRINITY_DN102712_c0_g1_i1_orf1	-	-	-	transmembrane protein 177 [Ostrinia furnacalis]	0.53319	-0.05061	-0.25587	1.40683	-1.63355
TRINITY_DN987_c0_g1_i11_orf1	-	-	-	macrophage mannose receptor 1-like [Pieris napi]	1.07314	-0.22341	0.41221	0.55905	-1.82099
TRINITY_DN18937_c0_g1_i1_orf1	-	-	-	uncharacterized protein LOC114351683 isoform X7 [Ostrinia furnacalis]	1.21932	-0.02988	0.48459	0.13158	-1.80561
TRINITY_DN41697_c0_g1_i1_orf1	-	-	-	5-formyltetrahydrofolate cyclo-ligase [Ostrinia furnacalis]	1.36771	-0.74517	0.49628	0.36306	-1.48188
TRINITY_DN15160_c0_g1_i1_orf1	-	-	-	tyrosine--tRNA ligase, cytoplasmic [Ostrinia furnacalis]	1.44235	-0.00538	-1.20706	0.73244	-0.96235
TRINITY_DN107288_c0_g1_i2_orf1	-	-	-	methionine-tRNA synthetase, partial [Papilio xuthus]	1.68864	-0.09713	-1.0387	0.39716	-0.94996
