# Supplementary material for: Comparative Proteomics and Metabonomics Analysis of Different Diapause Stages Revealed a New Regulation Mechanism of Diapause in Loxostege sticticalis (Lepidoptera: Pyralidae)
Source: Molecules. 2024 Jul 25;29(15):3472. doi: 10.3390/molecules29153472 (PMC11314584; doi:10.3390/molecules29153472)
Supplement: Supplementary file 1 [file molecules-29-03472-s001.zip › analysis process/proteomic/Cluster analysis of expression patterns/Down/PreDvsND down.pdf]

Accession	Symbol	Protein Na	Entrez ID	Description	ND	RD	CT	D	PreD
TRINITY_DN77642_c0_g1_i1_orf1	-	-	-	peritrophic membrane chitin binding protein [Loxostege sticticalis]	0.15185	1.49569	-0.99029	-1.20704	0.54978
TRINITY_DN12951_c1_g1_i5_orf1	-	-	-	ADP-ribosylation factor-like protein 2 isoform X1 [Hyposmocoma kahamanoa]	0.79668	1.10213	-1.76792	0.02531	-0.15621
TRINITY_DN14843_c0_g1_i1_orf1	-	-	-	myotubularin-related protein 13 [Ostrinia furnacalis]	1.55298	0.30754	-1.31769	-0.8237	0.28088
TRINITY_DN2874_c0_g1_i4_orf1	-	-	-	ATP-binding cassette sub-family G member 1-like [Ostrinia furnacalis]	1.45112	0.9309	-0.86079	-0.50603	-1.01521
TRINITY_DN11159_c0_g2_i1_orf1	-	-	-	sphingosine-1-phosphate lyase isoform X1 [Ostrinia furnacalis]	1.96068	-0.2186	-0.48145	-0.83223	-0.4284
TRINITY_DN12787_c1_g1_i1_orf1	-	-	-	unnamed protein product [Parnassius apollo]	1.91648	-0.02722	-0.73919	-0.8186	-0.33146
TRINITY_DN6130_c0_g1_i6_orf1	-	-	-	tryptophan--tRNA ligase, mitochondrial [Ostrinia furnacalis]	0.98977	1.39087	-1.10213	-0.47519	-0.80332
TRINITY_DN12113_c0_g1_i1_orf1	-	-	-	WD repeat-containing protein 5 [Helicoverpa armigera] >XP_022823790.1 WD repeat-containing protein 5 [Spodoptera litura] >XP_028176937.1 WD repeat-containing protein 5 [Ostrinia furnacalis] >XP_035429818.1 WD repeat-containing protein 5 [Spodoptera frugiperda] >XP_047030799.1 WD repeat-containing protein 5 [Helicoverpa zea] >CAB3513875.1 unnamed protein product [Spodoptera littoralis] >KAF9795358.1 hypothetical protein SFRURICE_004730 [Spodoptera frugiperda] >KAG8117399.1 hypothetical protein SFRUCORN_019548 [Spodoptera frugiperda] >PZC79830.1 hypothetical protein B5X24_HaOG215819 [Helicoverpa armigera] >CAH1643659.1 unnamed protein product	1.78547	-0.75677	0.3877	-0.50082	-0.91558
TRINITY_DN33485_c0_g1_i4_orf1	-	-	-	luciferin 4-monooxygenase-like [Ostrinia furnacalis]	1.28525	0.82107	-0.56514	-1.53445	-0.00673
TRINITY_DN747_c0_g1_i1_orf1	-	-	-	trypsin, alkaline C-like [Ostrinia furnacalis]	1.62949	-0.84487	0.65242	-1.01244	-0.4246
TRINITY_DN91198_c0_g2_i1_orf1	-	-	-	unc-112-related protein-like [Pectinophora gossypiella]	1.59061	-0.18418	-1.44726	-0.39227	0.4331
TRINITY_DN56121_c0_g1_i4_orf1	-	-	-	uncharacterized protein LOC114361939 [Ostrinia furnacalis]	1.88028	-0.07442	-0.12964	-0.9748	-0.70142
TRINITY_DN11392_c0_g1_i4_orf1	-	-	-	chondroitin sulfate synthase 1 isoform X1 [Ostrinia furnacalis] >XP_028178735.1 chondroitin sulfate synthase 1 isoform X2 [Ostrinia furnacalis]	1.93474	-0.09079	-0.58611	-0.39023	-0.86761
TRINITY_DN28938_c0_g1_i1_orf1	-	-	-	uncharacterized protein LOC114354070 isoform X3 [Ostrinia furnacalis]	1.48486	0.25385	-1.5021	-0.59078	0.35416
TRINITY_DN12769_c0_g1_i5_orf1	-	-	-	uncharacterized protein LOC114365633 [Ostrinia furnacalis]	0.88953	1.39876	-1.33528	-0.38953	-0.56349
TRINITY_DN51441_c0_g1_i5_orf1	-	-	-	uncharacterized protein LOC114353529 isoform X2 [Ostrinia furnacalis]	1.23561	1.1438	-1.21433	-0.65906	-0.50602
TRINITY_DN38720_c0_g1_i3_orf1	-	-	-	transmembrane protein 214 [Ostrinia furnacalis]	1.07989	-0.22278	-1.51826	-0.46333	1.12447
TRINITY_DN56379_c0_g1_i1_orf1	-	-	-	alpha-tocopherol transfer protein-like [Ostrinia furnacalis]	1.69748	0.12299	-0.31067	-0.09643	-1.41337
TRINITY_DN9555_c0_g1_i1_orf1	-	-	-	pyridoxal kinase [Ostrinia furnacalis]	1.83055	-0.00634	-0.27907	-0.33816	-1.20698
TRINITY_DN96884_c0_g1_i1_orf1	-	-	-	hypothetical protein evm_003360 [Chilo suppressalis]	1.96581	-0.66705	-0.71999	-0.24057	-0.3382
TRINITY_DN2441_c0_g1_i1_orf1	-	-	-	protein RFT1 homolog [Ostrinia furnacalis]	1.71225	-0.0776	-1.35231	0.16995	-0.45229
TRINITY_DN29707_c0_g1_i2_orf1	-	-	-	2-(3-amino-3-carboxypropyl)histidine synthase subunit 2 [Ostrinia furnacalis]	1.93788	-0.70593	-0.73889	-0.44497	-0.0481
TRINITY_DN18035_c0_g1_i7_orf1	-	-	-	mitochondrial ornithine transporter 1 [Ostrinia furnacalis]	1.94937	-0.81136	-0.19877	-0.64435	-0.29489
TRINITY_DN3971_c0_g1_i1_orf1	-	-	-	L-asparaginase-like isoform X1 [Ostrinia furnacalis]	1.9426	-0.58352	-0.11284	-0.40347	-0.84277
TRINITY_DN4766_c0_g1_i4_orf1	-	-	-	26S proteasome non-ATPase regulatory subunit 12 [Ostrinia furnacalis]	0.91402	0.96354	-1.49364	-0.87442	0.4905
TRINITY_DN139438_c0_g1_i1_orf1	-	-	-	T-complex protein 1 subunit delta [Ostrinia furnacalis]	1.89516	-0.17502	-0.93841	-0.08139	-0.70035
TRINITY_DN294_c0_g1_i2_orf1	-	-	-	chloride intracellular channel isoform 1 [Phthorimaea operculella]	1.64711	0.07714	-0.75273	-1.27397	0.30245
TRINITY_DN107_c0_g1_i1_orf1	-	-	-	dynein light chain 2, cytoplasmic isoform X1 [Pectinophora gossypiella]	1.76695	0.40492	-0.88959	-0.86522	-0.41706
TRINITY_DN12823_c0_g1_i1_orf1	-	-	-	malectin-A [Ostrinia furnacalis]	1.37886	0.99931	-0.98416	-0.9798	-0.41421
TRINITY_DN20215_c0_g2_i1_orf1	-	-	-	unnamed protein product [Spodoptera littoralis] >CAH1638553.1 unnamed protein product [Spodoptera littoralis]	1.0028	1.36626	-0.46698	-1.17545	-0.72662
TRINITY_DN57150_c0_g2_i1_orf1	-	-	-	vacuolar protein-sorting-associated protein 25-like isoform X2 [Ostrinia furnacalis]	1.72776	-0.6168	-1.25941	-0.06231	0.21076
TRINITY_DN13598_c1_g1_i1_orf1	-	-	-	transport and Golgi organization protein 1-like [Ostrinia furnacalis]	1.65337	-0.97682	-1.01915	-0.15645	0.49905
TRINITY_DN27723_c0_g1_i2_orf1	-	-	-	putative uncharacterized protein DDB_G0282133 isoform X1 [Ostrinia furnacalis]	1.92026	-0.58521	-0.35435	-0.06393	-0.91677
TRINITY_DN11188_c0_g1_i2_orf1	-	-	-	testin [Ostrinia furnacalis] >XP_028170617.1 testin [Ostrinia furnacalis]	1.23278	0.94847	-1.38847	-0.80782	0.01505
TRINITY_DN24024_c0_g1_i1_orf1	-	-	-	dolichyl-diphosphooligosaccharide--protein glycosyltransferase subunit 1 [Ostrinia furnacalis]	1.36536	0.61045	-1.22977	-1.07035	0.32431
TRINITY_DN8640_c0_g1_i4_orf1	-	-	-	glutathione S-transferase E14-like isoform X1 [Ostrinia furnacalis]	1.96318	-0.28203	-0.27381	-0.68012	-0.72723
TRINITY_DN8553_c0_g1_i4_orf1	-	-	-	coiled-coil-helix-coiled-coil-helix domain-containing protein 7 isoform X2 [Ostrinia furnacalis]	1.35655	1.0587	-1.0115	-0.57831	-0.82544
TRINITY_DN56270_c0_g1_i1_orf1	-	-	-	PREDICTED: putative elongator complex protein 1 [Microplitis demolitor] >XP_008554512.1 PREDICTED: putative elongator complex protein 1 [Microplitis demolitor]	1.20812	-1.20952	0.43416	-1.16384	0.73109
TRINITY_DN70409_c0_g1_i3_orf1	-	-	-	tyrosine-protein phosphatase non-receptor type 9 isoform X3 [Ostrinia furnacalis]	1.29361	0.53072	-1.56263	-0.66416	0.40246
TRINITY_DN3664_c0_g1_i8_orf1	-	-	-	hypothetical protein evm_011332 [Chilo suppressalis] >CAB3530638.1 unnamed protein product [Chilo suppressalis] >CAH0407229.1 unnamed protein product [Chilo suppressalis]	1.76256	0.01865	-1.09362	-0.82372	0.13613
TRINITY_DN471_c0_g1_i6_orf1	-	-	-	ATP-dependent RNA helicase DBP2-A-like [Ostrinia furnacalis]	1.08344	1.23758	-0.4577	-1.34944	-0.51387
TRINITY_DN19651_c0_g1_i1_orf1	-	-	-	cytosolic non-specific dipeptidase [Ostrinia furnacalis]	0.86787	1.37145	-0.09684	-1.24654	-0.89593

TRINITY_DN1273_c0_g1_i4_orf1	-	-	-	hypothetical protein evm_006077 [Chilo suppressalis] >CAB3531025.1 unnamed protein product [Chilo suppressalis] >CAH0407617.1 unnamed protein product [Chilo suppressalis]	1.72695	0.32766	-1.159	-0.73587	-0.15973
TRINITY_DN69697_c0_g1_i1_orf1	-	-	-	PREDICTED: uncharacterized protein LOC103573287 [Microplitis demolitor]	1.74578	-1.08088	0.06254	-0.87138	0.14394
TRINITY_DN1652_c0_g1_i12_orf1	-	-	-	synaptotagmin 1 isoform X1 [Ostrinia furnacalis] >XP_028161222.1 synaptotagmin 1 isoform X1 [Ostrinia furnacalis]	1.66766	-0.41027	-1.29157	0.45404	-0.41987
TRINITY_DN8405_c0_g1_i4_orf1	-	-	-	clathrin heavy chain isoform X1 [Ostrinia furnacalis] >XP_028169033.1 clathrin heavy chain isoform X2 [Ostrinia furnacalis] >XP_028169034.1 clathrin heavy chain isoform X3 [Ostrinia furnacalis] >XP_028169036.1 clathrin heavy chain isoform X5 [Ostrinia furnacalis]	0.21104	1.09792	-1.6654	-0.49741	0.85385
TRINITY_DN68725_c0_g1_i1_orf1	-	-	-	hydroxymethylglutaryl-CoA synthase 1 [Ostrinia furnacalis]	1.54231	0.80387	-0.96267	-0.90539	-0.47812
TRINITY_DN6503_c0_g1_i8_orf1	-	-	-	uncharacterized protein LOC114354432 [Ostrinia furnacalis]	1.80705	-0.34072	-0.66824	-1.0523	0.25421
TRINITY_DN629_c0_g1_i6_orf1	-	-	-	annexin B9-like isoform X1 [Ostrinia furnacalis]	0.65815	1.05934	-0.84425	-1.52073	0.64748
TRINITY_DN6262_c0_g1_i2_orf1	-	-	-	ADAMTS-like protein 4 isoform X2 [Ostrinia furnacalis] >XP_028169930.1 ADAMTS-like protein 4 isoform X2 [Ostrinia furnacalis]	1.37985	0.42798	-1.35901	-0.91911	0.47029
TRINITY_DN2876_c0_g1_i5_orf1	-	-	-	long-chain fatty acid transport protein 4-like isoform X1 [Ostrinia furnacalis]	1.83157	-0.66701	0.1344	-1.06103	-0.23793
TRINITY_DN12576_c0_g1_i2_orf1	-	-	-	eukaryotic translation initiation factor 4E transporter-like isoform X5 [Hyposmocoma	1.09421	1.32888	-1.0258	-0.63422	-0.76307
TRINITY_DN2795_c0_g1_i1_orf1	-	-	-	hypothetical protein B5X24_HaOG209451 [Helicoverpa armigera]	1.45232	0.59433	-1.46516	0.04226	-0.62374
TRINITY_DN1601_c0_g1_i4_orf1	-	-	-	cytoplasmic dynein 1 intermediate chain isoform X4 [Ostrinia furnacalis]	0.95213	1.43349	-0.56795	-0.72988	-1.08778
TRINITY_DN5834_c0_g1_i2_orf1	-	-	-	CD2 antigen cytoplasmic tail-binding protein 2 homolog [Ostrinia furnacalis]	1.49226	0.84169	-0.7929	-0.41796	-1.12309
TRINITY_DN48536_c0_g1_i3_orf1	-	-	-	unnamed protein product [Chilo suppressalis]	1.90466	-0.0084	-0.31753	-0.67713	-0.9016
TRINITY_DN11876_c0_g1_i2_orf1	-	-	-	protein TAPT1 homolog [Ostrinia furnacalis]	1.56643	0.80148	-0.71297	-0.94196	-0.71297
TRINITY_DN5243_c1_g1_i1_orf1	-	-	-	14-3-3 protein epsilon [Maniola hyperantus] >XP_045770909.1 14-3-3 protein epsilon [Maniola jurtina]	1.07721	0.46717	-1.88712	0.19804	0.14471
TRINITY_DN40211_c0_g1_i1_orf1	-	-	-	rho GTPase-activating protein 44-like [Ostrinia furnacalis]	1.63112	0.61594	-0.61912	-1.16869	-0.45925
TRINITY_DN892_c0_g1_i9_orf1	-	-	-	calcyphosin-like protein isoform X2 [Ostrinia furnacalis]	0.42712	1.216	-1.73108	-0.36737	0.45532
TRINITY_DN1866_c0_g1_i4_orf1	-	-	-	unnamed protein product [Chrysodeixis includens]	1.81416	-0.54191	-0.07525	-0.00983	-1.18717
TRINITY_DN40823_c0_g1_i1_orf1	-	-	-	nucleolar protein 10 [Ostrinia furnacalis]	1.83914	0.19955	-0.99291	-0.67297	-0.37281
TRINITY_DN5008_c0_g1_i1_orf1	-	-	-	LOW QUALITY PROTEIN: integrator complex subunit 14 [Ostrinia furnacalis]	1.41345	-0.40672	-1.50477	-0.22447	0.7225
TRINITY_DN3562_c0_g1_i4_orf1	-	-	-	peroxisomal membrane protein PEX14-like isoform X2 [Ostrinia furnacalis]	1.82952	-0.26026	0.17958	-0.76563	-0.9832
TRINITY_DN2177_c0_g1_i1_orf1	-	-	-	uncharacterized protein LOC114349824 isoform X1 [Ostrinia furnacalis] >XP_028156186.1 uncharacterized protein LOC114349824 isoform X1 [Ostrinia furnacalis]	1.76414	-1.05296	0.3613	-0.34465	-0.72783
TRINITY_DN1198_c0_g1_i1_orf1	-	-	-	protein suppressor of sable isoform X1 [Ostrinia furnacalis]	1.79112	-0.69988	-0.98709	-0.45352	0.34937
TRINITY_DN11464_c0_g1_i3_orf1	-	-	-	unnamed protein product [Spodoptera littoralis] >CAH1635924.1 unnamed protein product [Spodoptera littoralis]	1.13619	1.29919	-0.95952	-0.81355	-0.6623
TRINITY_DN6163_c0_g1_i4_orf1	-	-	-	CTD nuclear envelope phosphatase 1 homolog [Ostrinia furnacalis]	1.10781	1.18014	-1.3741	-0.26418	-0.64967
TRINITY_DN41108_c0_g1_i1_orf1	-	-	-	coatomer subunit epsilon-like [Ostrinia furnacalis]	1.21245	1.04343	-1.02222	-0.0532	-1.18046
TRINITY_DN18863_c0_g1_i3_orf1	-	-	-	splicing factor 3A subunit 1 isoform X1 [Ostrinia furnacalis] >XP_028161155.1 splicing factor 3A subunit 1 isoform X2 [Ostrinia furnacalis] >XP_028161156.1 splicing factor 3A subunit 1 isoform X3 [Ostrinia furnacalis] >XP_028161157.1 splicing factor 3A subunit 1 isoform X4 [Ostrinia furnacalis]	1.79571	0.13139	-0.98931	-0.88103	-0.05676

TRINITY_DN1639_c0_g2_i2_orf1	-	-	-	longitudinals lacking protein-like [Plutella xylostella] >XP_021192239.1 longitudinals lacking protein-like [Helicoverpa armigera] >XP_022818289.1 longitudinals lacking protein-like [Spodoptera litura] >XP_022818290.1 longitudinals lacking protein-like [Spodoptera litura] >XP_023945541.1 longitudinals lacking protein-like [Bicyclus anynana] >XP_026320461.1 longitudinals lacking protein-like [Hyposmocoma kahamanoa] >XP_026320471.1 longitudinals lacking protein-like [Hyposmocoma kahamanoa] >XP_026499391.1 longitudinals lacking protein-like [Vanessa tameamea] >XP_026499392.1 longitudinals lacking protein-like [Vanessa tameamea] >XP_026728238.1 longitudinals lacking protein-like isoform X2 [Trichoplusia ni] >XP_026750598.1 longitudinals lacking protein-like [Galleria mellonella] >XP_028032853.1 longitudinals lacking protein-like [Bombyx mandarina] >XP_028169299.1 longitudinals lacking protein-like [Ostrinia furnacalis] >XP_030036777.1 longitudinals lacking protein-like [Manduca sexta] >XP_034826408.1 longitudinals lacking protein-like [Maniola hyperantus] >XP_034826409.1 longitudinals lacking protein-like [Maniola hyperantus] >XP_035452035.1 longitudinals lacking protein-like [Spodoptera frugiperda] >XP_035452036.1 longitudinals lacking protein-like [Spodoptera frugiperda] >XP_035452270.1 longitudinals lacking protein-like [Spodoptera frugiperda] >XP_035452271.1 longitudinals lacking protein-like [Spodoptera frugiperda] >XP_037874172.1 longitudinals lacking protein-like [Bombyx mori] >XP_038218308.1 longitudinals lacking protein-like [Zerene cesonia] >XP_039758692.1 longitudinals lacking protein-like [Pararge aegeria] >XP_041981066.1 longitudinals lacking protein-like [Aricia agestis] >XP_041981074.1 longitudinals lacking protein-like [Aricia agestis] >XP_045455814.1 longitudinals lacking protein-like [Melitaea cinxia] >XP_045505524.1 longitudinals lacking protein-like [Colias croceus] >XP_045524236.1 longitudinals lacking protein-like [Pieris brassicae] >XP_045762005.1 longitudinals lacking protein-like [Maniola jurtina] >XP_045762006.1 longitudinals lacking protein-like [Maniola jurtina] >XP_046973407.1 longitudinals lacking protein-like [Vanessa cardui] >XP_046973408.1 longitudinals lacking protein-like [Vanessa cardui] >XP_047035325.1 longitudinals lacking protein-like [Helicoverpa zea] >XP_047035326.1 longitudinals lacking protein-like [Helicoverpa zea] >XP_047520236.1 longitudinals lacking protein-like [Pieris napi] >XP_047540188.1 longitudinals lacking protein-like [Vanessa atalanta] >XP_048001034.1 longitudinals lacking protein-like [Leguminivora glycinivorella] >XP_049880222.1 longitudinals lacking protein-like isoform X2 [Pectinophora	1.73196	0.492	-0.92025	-0.47641	-0.8273
TRINITY_DN10785_c0_g1_i4_orf1	-	-	-	alkylglycerol monooxygenase-like [Ostrinia furnacalis] >XP_028171363.1 alkylglycerol monooxygenase-like [Ostrinia furnacalis]	1.76129	-1.16197	-0.62308	-0.27022	0.29397
TRINITY_DN1816_c0_g1_i5_orf1	-	-	-	nuclear pore complex protein Nup205 [Ostrinia furnacalis]	1.89934	-0.43024	-0.86433	-0.67484	0.07006
TRINITY_DN43637_c0_g1_i1_orf1	-	-	-	sarcolemmal membrane-associated protein [Ostrinia furnacalis]	1.22684	1.01843	-1.45959	-0.29642	-0.48926
TRINITY_DN11973_c0_g1_i1_orf1	-	-	-	nuclear pore complex protein Nup93-like [Ostrinia furnacalis]	1.8962	-0.38115	-1.02666	-0.45137	-0.03703
TRINITY_DN2930_c0_g1_i8_orf1	-	-	-	hypothetical protein evm_000171 [Chilo suppressalis] >CAB3523059.1 unnamed protein product [Chilo suppressalis] >CAH0400381.1 unnamed protein product [Chilo suppressalis]	1.71533	0.54411	-0.55565	-0.82564	-0.87815
TRINITY_DN46_c0_g1_i2_orf1	-	-	-	disks large 1 tumor suppressor protein isoform X12 [Ostrinia furnacalis]	1.48818	0.82754	-0.53145	-1.22846	-0.55581
TRINITY_DN21559_c0_g1_i2_orf1	-	-	-	protein bicaudal D [Ostrinia furnacalis]	1.3571	0.89701	-1.41307	-0.46042	-0.38062
TRINITY_DN6169_c0_g1_i15_orf1	-	-	-	cullin-2 isoform X1 [Ostrinia furnacalis] >XP_028165424.1 cullin-2 isoform X2 [Ostrinia	1.48258	0.08065	-1.45501	-0.634	0.52578
TRINITY_DN33867_c0_g1_i9_orf1	-	-	-	uncharacterized protein LOC114357513 [Ostrinia furnacalis]	1.82335	-0.9078	0.21427	-0.85398	-0.27584
TRINITY_DN38667_c0_g1_i9_orf1	-	-	-	signal transducer and activator of transcription 5B-like [Melitaea cinxia]	1.3957	0.8085	-0.90253	-1.25771	-0.04396
TRINITY_DN1498_c0_g1_i2_orf1	-	-	-	ran GTPase-activating protein 1 [Ostrinia furnacalis]	1.82728	0.27922	-0.56324	-0.57794	-0.96532
TRINITY_DN60821_c0_g1_i1_orf1	-	-	-	nucleolar GTP-binding protein 2 [Ostrinia furnacalis]	1.36182	0.44893	0.2656	-0.4391	-1.63724
TRINITY_DN54477_c0_g1_i1_orf1	-	-	-	rho-associated protein kinase 2 isoform X11 [Ostrinia furnacalis] >XP_028155846.1 rho-associated protein kinase 2 isoform X12 [Ostrinia furnacalis]	1.01972	0.01567	-1.80919	-0.0532	0.827
TRINITY_DN16385_c0_g1_i4_orf1	-	-	-	cytosolic Fe-S cluster assembly factor NUBP1 homolog [Ostrinia furnacalis]	1.62205	-0.15789	-1.50927	0.20301	-0.15789
TRINITY_DN41997_c0_g1_i2_orf1	-	-	-	39S ribosomal protein L23, mitochondrial [Ostrinia furnacalis]	1.98878	-0.33477	-0.65653	-0.5445	-0.45297
TRINITY_DN57765_c0_g1_i1_orf1	-	-	-	cytochrome P450 6B6-like [Ostrinia furnacalis]	1.70765	-0.58318	0.00472	0.17915	-1.30833
TRINITY_DN39490_c0_g1_i1_orf1	-	-	-	unnamed protein product [Parnassius apollo]	1.78242	-0.54643	-1.11358	-0.43326	0.31085
TRINITY_DN44407_c0_g4_i2_orf1	-	-	-	eukaryotic translation initiation factor 5A [Antheraea pernyi]	1.47109	0.53299	-1.19336	-1.03745	0.22673
TRINITY_DN3244_c0_g1_i4_orf1	-	-	-	protein ABHD16A isoform X1 [Ostrinia furnacalis] >XP_028156316.1 protein ABHD16A isoform X2 [Ostrinia furnacalis]	1.60451	0.58429	-0.48728	-0.40403	-1.29748
TRINITY_DN4159_c1_g1_i1_orf1	-	-	-	F-actin-capping protein subunit beta [Ostrinia furnacalis]	1.21434	1.02992	-1.14401	-1.07482	-0.02544

TRINITY_DN4770_c0_g1_i4_orf1	-	-	-	transportin-3 isoform X1 [Ostrinia furnacalis]	1.64401	0.37129	-1.3229	-0.6374	-0.05501
TRINITY_DN7836_c0_g1_i2_orf1	-	-	-	uncharacterized protein LOC114353624 [Ostrinia furnacalis]	1.92739	-0.7067	-0.60539	0.03142	-0.64672
TRINITY_DN121047_c0_g1_i3_orf1	-	-	-	unnamed protein product [Parnassius apollo]	1.67143	0.5392	-0.33601	-1.08826	-0.78636
TRINITY_DN2542_c0_g2_i1_orf1	-	-	-	peroxiredoxin-2-like [Ostrinia furnacalis]	1.38369	0.61003	-0.05552	-0.32416	-1.61404
TRINITY_DN6299_c0_g1_i1_orf1	-	-	-	death-inducer obliterator 1 isoform X2 [Ostrinia furnacalis]	1.78244	-0.66371	-0.7818	0.42921	-0.76614
TRINITY_DN1354_c0_g1_i6_orf1	-	-	-	elongator complex protein 2 isoform X1 [Pectinophora gossypiella]	1.90908	-0.58858	-0.54959	0.06719	-0.8381
TRINITY_DN4002_c0_g1_i1_orf1	-	-	-	activating signal cointegrator 1 complex subunit 3 [Ostrinia furnacalis]	1.68077	-0.77775	-0.03739	0.33986	-1.2055
TRINITY_DN145227_c0_g1_i1_orf1	-	-	-	26S protease regulatory subunit 7, partial [Cotesia chilonis]	1.26518	0.58158	-1.34741	-0.99831	0.49895
TRINITY_DN24266_c0_g2_i2_orf1	-	-	-	chromobox-like protein 5 [Helicoverpa armigera]	0.72452	1.21237	-1.32638	-1.03312	0.42261
TRINITY_DN1921_c1_g1_i5_orf1	-	-	-	hypothetical protein evm_002627 [Chilo suppressalis] >CAB3527269.1 unnamed protein product [Chilo suppressalis]	1.22575	1.21824	-0.72536	-0.78699	-0.93164
TRINITY_DN1552_c0_g1_i3_orf1	-	-	-	casein kinase II subunit alpha isoform X3 [Galleria mellonella]	1.19655	1.18128	-0.93613	-1.07952	-0.36218
TRINITY_DN32359_c0_g2_i1_orf1	-	-	-	PREDICTED: 26S proteasome non-ATPase regulatory subunit 4 isoform X2 [Fopius arisanus]	0.66803	1.13558	-1.4814	-0.87446	0.55225
TRINITY_DN131662_c0_g1_i4_orf1	-	-	-	splicing factor 3A subunit 1 isoform X1 [Ostrinia furnacalis] >XP_028161155.1 splicing factor 3A subunit 1 isoform X2 [Ostrinia furnacalis] >XP_028161156.1 splicing factor 3A subunit 1 isoform X3 [Ostrinia furnacalis] >XP_028161157.1 splicing factor 3A subunit 1 isoform X4 [Ostrinia furnacalis]	1.82389	0.07239	-0.98912	-0.82666	-0.08049
TRINITY_DN5670_c0_g1_i2_orf1	-	-	-	DNA polymerase alpha subunit B [Ostrinia furnacalis]	1.10752	-0.24143	-1.80066	0.60148	0.33308
TRINITY_DN24539_c0_g1_i4_orf1	-	-	-	low molecular weight phosphotyrosine protein phosphatase 1-like isoform X2 [Ostrinia	1.06588	1.25319	-0.74276	-1.28782	-0.28849
TRINITY_DN14183_c0_g1_i3_orf1	-	-	-	multiple epidermal growth factor-like domains protein 6 [Ostrinia furnacalis]	1.90114	-0.17676	-0.46903	-0.21144	-1.0439
TRINITY_DN972_c0_g1_i6_orf1	-	-	-	DNA damage-binding protein 1 [Ostrinia furnacalis]	1.51473	0.75729	-1.11048	-0.91571	-0.24583
TRINITY_DN119797_c0_g1_i1_orf1	-	-	-	hypothetical protein evm_013979 [Chilo suppressalis]	1.51008	-0.67034	-1.30715	-0.24191	0.70933
TRINITY_DN2999_c1_g2_i1_orf1	-	-	-	uncharacterized protein C05D11.1-like [Ostrinia furnacalis]	1.55302	0.09574	-1.37198	-0.71212	0.43533
TRINITY_DN2808_c0_g1_i8_orf1	-	-	-	uncharacterized protein LOC114353011 isoform X2 [Ostrinia furnacalis]	0.63079	1.39915	-1.59676	-0.23913	-0.19404
TRINITY_DN2615_c0_g1_i1_orf1	-	-	-	14 kDa phosphohistidine phosphatase-like [Ostrinia furnacalis]	1.44498	0.53247	-1.54756	-0.48062	0.05072
TRINITY_DN3482_c0_g2_i1_orf1	-	-	-	transcription elongation factor B polypeptide 3-like isoform X2 [Ostrinia furnacalis]	0.77094	0.97949	-1.66584	-0.62007	0.53547
TRINITY_DN8659_c0_g1_i1_orf1	-	-	-	ubiquitin-like modifier-activating enzyme 1 [Manduca sexta]	0.89739	1.47845	-0.96522	-0.90817	-0.50245
TRINITY_DN1641_c0_g1_i6_orf1	-	-	-	rhodanese domain-containing protein CG4456-like [Ostrinia furnacalis]	1.54613	-0.24318	-0.10856	0.35812	-1.55252
TRINITY_DN5562_c1_g2_i1_orf1	-	-	-	cell division cycle and apoptosis regulator protein 1-like [Ostrinia furnacalis]	1.9064	0.02615	-0.36743	-0.73195	-0.83317
TRINITY_DN81084_c0_g3_i1_orf1	-	-	-	unnamed protein product [Diatraea saccharalis]	1.51509	-0.21577	-1.33811	-0.63888	0.67767
TRINITY_DN429_c0_g1_i12_orf1	-	-	-	hypothetical protein SFRUCORN_009336 [Spodoptera frugiperda]	1.53469	0.81796	-0.87871	-0.49482	-0.97912
TRINITY_DN30178_c0_g1_i3_orf1	-	-	-	LOW QUALITY PROTEIN: fibrillin-2-like [Bicyclus anynana]	0.60576	1.47091	-1.52034	-0.23756	-0.31877
TRINITY_DN20323_c0_g1_i1_orf1	-	-	-	cuticle protein 7 [Ostrinia furnacalis]	1.22029	-0.02702	-1.78063	0.00469	0.58267
TRINITY_DN13018_c0_g1_i1_orf1	-	-	-	uncharacterized protein LOC114354768 isoform X2 [Ostrinia furnacalis]	1.49702	0.58632	-0.08412	-0.54705	-1.45218
TRINITY_DN25686_c0_g1_i4_orf1	-	-	-	exocyst complex component 3 [Ostrinia furnacalis]	1.1787	1.26353	-0.85953	-0.68431	-0.89839
TRINITY_DN84_c0_g1_i4_orf1	-	-	-	aspartyl/asparaginyl beta-hydroxylase isoform X2 [Ostrinia furnacalis]	1.88754	-0.89746	-0.72581	0.055	-0.31927
TRINITY_DN21961_c0_g2_i5_orf1	-	-	-	hypothetical protein HW555_000844 [Spodoptera exigua] >KAH9635498.1 hypothetical protein HF086_014991 [Spodoptera exigua] >CAH0692121.1 unnamed protein product [Spodoptera exigua]	0.98821	-0.42001	-1.71115	0.93606	0.20689
TRINITY_DN16539_c0_g1_i7_orf1	-	-	-	dolichyl-diphosphooligosaccharide--protein glycosyltransferase 48 kDa subunit [Ostrinia furnacalis]	1.55919	-0.32216	-1.55022	0.23703	0.07616
TRINITY_DN8076_c0_g1_i6_orf1	-	-	-	hypothetical protein evm_001812 [Chilo suppressalis]	1.4213	-0.86833	-1.17711	-0.25605	0.8802
TRINITY_DN2160_c0_g1_i13_orf1	-	-	-	unnamed protein product [Spodoptera exigua]	1.32306	1.08311	-0.99945	-0.49354	-0.91318
TRINITY_DN14286_c0_g1_i5_orf1	-	-	-	insulin-like growth factor 2 mRNA-binding protein 1 isoform X1 [Galleria mellonella]	1.89124	-0.22395	-0.72636	-0.02169	-0.91924
TRINITY_DN766_c0_g1_i1_orf1	-	-	-	nucleolar complex protein 3 homolog [Ostrinia furnacalis]	1.76746	-0.13835	-0.98323	-0.90673	0.26085
TRINITY_DN19942_c0_g1_i2_orf1	-	-	-	ribosomal protein l1p/L10e family domain-containing protein [Phthorimaea operculella]	1.93017	-0.87586	-0.50243	-0.50243	-0.04946
TRINITY_DN15904_c0_g1_i1_orf1	-	-	-	papilin isoform X8 [Ostrinia furnacalis]	1.42334	0.60088	-1.56767	-0.06822	-0.38834
TRINITY_DN41736_c0_g2_i1_orf1	-	-	-	calcyclin-binding protein [Ostrinia furnacalis]	1.39246	1.03607	-0.70411	-0.91232	-0.8121
TRINITY_DN19262_c0_g1_i1_orf1	-	-	-	tRNA (guanine-N(7)-)-methyltransferase non-catalytic subunit wdr4 [Ostrinia furnacalis]	1.86299	-0.95501	-0.70868	0.11845	-0.31774
TRINITY_DN3260_c0_g1_i6_orf1	-	-	-	vesicular integral-membrane protein VIP36 isoform X1 [Ostrinia furnacalis] >XP_028171839.1 vesicular integral-membrane protein VIP36 isoform X2 [Ostrinia furnacalis]	1.36804	-0.14033	-1.43172	-0.61846	0.82247

TRINITY_DN42903_c0_g1_i4_orf1	-	-	-	rab GTPase-binding effector protein 1 isoform X1 [Ostrinia furnacalis] >XP_028174977.1 rab GTPase-binding effector protein 1 isoform X2 [Ostrinia furnacalis] >XP_028174983.1 rab GTPase-binding effector protein 1 isoform X3 [Ostrinia furnacalis] >XP_028174990.1 rab GTPase-binding effector protein 1 isoform X4 [Ostrinia furnacalis] >XP_028174996.1 rab GTPase-binding effector protein 1 isoform X5 [Ostrinia furnacalis]	1.71324	0.4611	-1.12296	-0.66435	-0.38703
TRINITY_DN23946_c0_g1_i1_orf1	-	-	-	ubiquitin-conjugating enzyme E2L [Bombyx mori] >XP_013145013.1 PREDICTED: ubiquitin-conjugating enzyme E2 L3 [Papilio polytes] >XP_013145026.1 PREDICTED: ubiquitin-conjugating enzyme E2 L3 [Papilio polytes] >XP_013167448.1 PREDICTED: ubiquitin-conjugating enzyme E2 L3 [Papilio xuthus] >XP_013167449.1 PREDICTED: ubiquitin-conjugating enzyme E2 L3 [Papilio xuthus] >XP_014356324.1 ubiquitin-conjugating enzyme E2 L3 [Papilio machaon] >XP_021182538.1 ubiquitin-conjugating enzyme E2 L3 [Helicoverpa armigera] >XP_022831771.1 ubiquitin-conjugating enzyme E2 L3 [Spodoptera litura] >XP_023943862.1 ubiquitin-conjugating enzyme E2 L3 [Bicyclus anynana] >XP_026501320.1 ubiquitin-conjugating enzyme E2 L3 [Vanessa tameamea] >XP_026740672.1 ubiquitin-conjugating enzyme E2 L3 [Trichoplusia ni] >XP_030021931.1 ubiquitin-conjugating enzyme E2 L3 [Manduca sexta] >XP_035448309.1 ubiquitin-conjugating enzyme E2 L3 [Spodoptera frugiperda] >XP_038214502.1 ubiquitin-conjugating enzyme E2 L3 [Zerene cesonia] >XP_045453873.1 ubiquitin-conjugating enzyme E2 L3 [Melitaea cinxia] >XP_045503683.1 ubiquitin-conjugating enzyme E2 L3 [Colias croceus] >XP_045771134.1 ubiquitin-conjugating enzyme E2 L3 [Maniola jurtina] >XP_046963941.1 ubiquitin-conjugating enzyme E2 L3 [Vanessa cardui] >XP_047032812.1 ubiquitin-conjugating enzyme E2 L3 [Helicoverpa zea] >XP_047538650.1 ubiquitin-conjugating enzyme E2 L3 [Vanessa atalanta] >XP_050352992.1 ubiquitin-conjugating enzyme E2 L3 [Nymphalis io] >KAF9417333.1 hypothetical protein HW555_005549 [Spodoptera exigua] >CAB3511518.1 unnamed protein product [Spodoptera littoralis] >CAH0595358.1 unnamed protein product [Chrysodeixis includens] >ABB36655.1 ubiquitin-conjugating enzyme E2I [Bombyx mori] >ABF51360.1 ubiquitin-conjugating enzyme	1.25849	1.18842	-0.74667	-0.82859	-0.87165
TRINITY_DN17071_c0_g1_i6_orf1	-	-	-	serine/arginine-rich splicing factor 7-like [Ostrinia furnacalis]	1.33927	0.10698	-1.71973	-0.17952	0.45301
TRINITY_DN2438_c0_g1_i4_orf1	-	-	-	dystrophin, isoforms A/C/F/G/H isoform X2 [Manduca sexta]	1.01924	0.95312	-1.64654	-0.54273	0.21692
TRINITY_DN12320_c0_g1_i1_orf1	-	-	-	brefeldin A-inhibited guanine nucleotide-exchange protein 1 [Ostrinia furnacalis]	1.60058	0.66927	-1.15051	-0.7013	-0.41804
TRINITY_DN14987_c0_g1_i3_orf1	-	-	-	hypothetical protein evm_009121 [Chilo suppressalis]	0.87372	1.31866	-1.3684	-0.78997	-0.03401
TRINITY_DN3835_c0_g1_i4_orf1	-	-	-	protein ERGIC-53 isoform X1 [Ostrinia furnacalis] >XP_028177940.1 protein ERGIC-53 isoform X2 [Ostrinia furnacalis] >XP_028177941.1 protein ERGIC-53 isoform X3 [Ostrinia furnacalis]	1.522	0.05352	-0.95873	-1.19475	0.57796
TRINITY_DN6247_c0_g1_i2_orf1	-	-	-	innexin inx3 [Ostrinia furnacalis]	1.87764	-0.30467	-0.74421	0.07775	-0.90651
TRINITY_DN4257_c0_g1_i2_orf1	-	-	-	dynactin subunit 1 [Ostrinia furnacalis]	0.52777	0.50795	-1.95984	0.77469	0.14943
TRINITY_DN6642_c0_g2_i1_orf1	-	-	-	protein purity of essence [Ostrinia furnacalis]	1.50429	0.66414	-1.11562	-1.02502	-0.0278
TRINITY_DN11069_c0_g1_i6_orf1	-	-	-	ganglioside-induced differentiation-associated protein 1 [Ostrinia furnacalis]	1.83961	-0.68093	-0.34621	0.18632	-0.99879
TRINITY_DN13384_c0_g1_i1_orf1	-	-	-	26S proteasome regulatory subunit S3 [Aphelinus abdominalis]	1.15716	0.5043	-1.81046	0.31719	-0.1682
TRINITY_DN8729_c0_g1_i7_orf1	-	-	-	myosin heavy chain 95F isoform X1 [Ostrinia furnacalis] >XP_028177153.1 myosin heavy chain 95F isoform X2 [Ostrinia furnacalis] >XP_028177154.1 myosin heavy chain 95F isoform X3 [Ostrinia furnacalis] >XP_028177155.1 myosin heavy chain 95F isoform X4 [Ostrinia furnacalis] >XP_028177156.1 myosin heavy chain 95F isoform X5 [Ostrinia furnacalis] >XP_028177158.1 myosin heavy chain 95F isoform X6 [Ostrinia furnacalis]	1.86386	-0.217	0.0205	-0.62334	-1.04402
TRINITY_DN20067_c0_g1_i6_orf1	-	-	-	hypothetical protein evm_010712 [Chilo suppressalis] >CAB3527462.1 unnamed protein product [Chilo suppressalis] >CAH0401768.1 unnamed protein product [Chilo suppressalis]	0.99633	1.40701	-0.84697	-0.55549	-1.00087
TRINITY_DN9711_c0_g1_i10_orf1	-	-	-	cAMP-specific 3',5'-cyclic phosphodiesterase isoform X2 [Ostrinia furnacalis]	1.82156	-0.85786	-0.77574	-0.49807	0.3101
TRINITY_DN1266_c6_g1_i1_orf1	-	-	-	transmembrane 9 superfamily member 2 [Ostrinia furnacalis]	1.75146	-0.95299	-0.99605	0.01956	0.17803
TRINITY_DN2478_c0_g1_i1_orf1	-	-	-	apoptosis inhibitor 5 [Ostrinia furnacalis]	1.64167	0.6139	-1.1223	-0.45181	-0.68145
TRINITY_DN4538_c0_g1_i4_orf1	-	-	-	2-acylglycerol O-acyltransferase 1-like [Ostrinia furnacalis]	1.92194	-0.91909	-0.371	-0.06675	-0.56509
TRINITY_DN6545_c0_g1_i6_orf1	-	-	-	organic cation transporter protein-like [Ostrinia furnacalis]	1.68091	0.21481	0.04846	-1.3157	-0.62848
TRINITY_DN13139_c0_g1_i1_orf1	-	-	-	AP-1 complex subunit mu-1 [Ostrinia furnacalis]	1.51992	0.84233	-0.79058	-0.54064	-1.03103
TRINITY_DN13118_c0_g1_i6_orf1	-	-	-	AP-1 complex subunit beta-1 [Helicoverpa armigera] >XP_021189434.2 AP-1 complex subunit beta-1 [Helicoverpa armigera]	0.86374	1.23238	-1.51778	-0.65269	0.07436
TRINITY_DN227_c0_g1_i1_orf1	-	-	-	double-stranded ribonuclease 2 [Ostrinia nubilalis]	1.65806	0.1485	0.23838	-0.82113	-1.22381
TRINITY_DN3283_c0_g2_i1_orf1	-	-	-	dipeptidyl peptidase 9 isoform X2 [Manduca sexta]	1.43719	-0.26374	-1.41523	-0.52441	0.76618

TRINITY_DN19250_c0_g2_i2_orf1	-	-	-	uncharacterized protein LOC114351683 isoform X8 [Ostrinia furnacalis]	1.56326	0.05466	-0.6869	0.44223	-1.37325
TRINITY_DN47914_c0_g2_i1_orf1	-	-	-	UBX domain-containing protein 1-A-like [Ostrinia furnacalis]	0.72875	1.54901	-0.92184	-1.06555	-0.29037
TRINITY_DN4301_c2_g2_i4_orf1	-	-	-	stress-induced-phosphoprotein 1-like [Ostrinia furnacalis]	1.71291	0.20419	-1.12638	0.07522	-0.86595
TRINITY_DN12767_c0_g1_i1_orf1	-	-	-	coatomer subunit alpha [Ostrinia furnacalis]	1.77804	-0.92505	-0.87923	0.33674	-0.31051
TRINITY_DN442_c0_g1_i10_orf1	-	-	-	tuberin isoform X4 [Helicoverpa armigera]	1.17697	0.69661	-1.6894	0.26739	-0.45157
TRINITY_DN1334_c0_g1_i2_orf1	-	-	-	phosphoenolpyruvate carboxykinase [GTP]-like [Ostrinia furnacalis]	1.84559	-0.19071	-0.17869	-1.20496	-0.27124
TRINITY_DN6381_c0_g1_i2_orf1	-	-	-	solute carrier family 12 member 8 [Ostrinia furnacalis]	1.56448	-0.16907	-1.18475	-0.84628	0.63561
TRINITY_DN1771_c0_g2_i1_orf1	-	-	-	eukaryotic peptide chain release factor subunit 1 isoform X1 [Danaus plexippus plexippus] >CAG9574852.1 unnamed protein product [Danaus chrysippus]	1.64349	0.30808	0.08959	-0.78307	-1.2581
TRINITY_DN147427_c0_g1_i1_orf1	-	-	-	importin subunit alpha-4 [Ostrinia furnacalis]	1.7712	0.43243	-0.7229	-0.57161	-0.90913
TRINITY_DN31399_c0_g1_i3_orf1	-	-	-	ATP-dependent zinc metalloprotease YME1 homolog [Ostrinia furnacalis]	1.91977	-0.16364	-0.97152	-0.25804	-0.52657
TRINITY_DN12608_c0_g1_i1_orf1	-	-	-	centrosome-associated zinc finger protein CP190 [Ostrinia furnacalis] >XP_028173286.1 centrosome-associated zinc finger protein CP190 [Ostrinia furnacalis]	1.70888	-0.32613	-1.33872	-0.32219	0.27816
TRINITY_DN7294_c0_g2_i4_orf1	-	-	-	hypothetical protein evm_003455 [Chilo suppressalis] >CAB3524298.1 unnamed protein product [Chilo suppressalis]	1.87368	-0.99327	-0.6915	-0.15243	-0.03648
TRINITY_DN1673_c0_g1_i2_orf1	-	-	-	hypothetical protein evm_006080 [Chilo suppressalis]	1.80455	0.15825	-1.06864	-0.7444	-0.14975
TRINITY_DN4012_c0_g4_i2_orf1	-	-	-	uncharacterized protein LOC114362418 [Ostrinia furnacalis]	1.41159	0.84186	-0.59526	-0.29545	-1.36274
TRINITY_DN23444_c0_g1_i11_orf1	-	-	-	serrate RNA effector molecule homolog isoform X3 [Ostrinia furnacalis]	1.1842	0.8074	-1.59517	-0.59986	0.20343
TRINITY_DN9146_c0_g1_i1_orf1	-	-	-	drebrin-like protein [Ostrinia furnacalis]	1.2249	1.21321	-0.96737	-0.80749	-0.66325
TRINITY_DN23432_c0_g1_i1_orf1	-	-	-	7-methylguanosine phosphate-specific 5'-nucleotidase-like isoform X2 [Ostrinia furnacalis]	1.96532	-0.68866	-0.69574	-0.3628	-0.21813
TRINITY_DN6262_c0_g2_i1_orf1	-	-	-	thrombospondin type-1 domain-containing protein 4-like [Ostrinia furnacalis]	1.3861	-0.09143	-1.57491	-0.38465	0.66489
TRINITY_DN45859_c0_g1_i1_orf1	-	-	-	nuclear valosin-containing protein-like [Ostrinia furnacalis]	1.55436	0.60693	-0.38532	-0.39297	-1.383
TRINITY_DN2953_c1_g1_i11_orf1	-	-	-	methionine--tRNA ligase, cytoplasmic isoform X2 [Ostrinia furnacalis] >XP_028156683.1 methionine--tRNA ligase, cytoplasmic isoform X4 [Ostrinia furnacalis] >XP_028156684.1 methionine--tRNA ligase, cytoplasmic isoform X5 [Ostrinia furnacalis]	1.74772	0.43177	-0.41548	-1.00557	-0.75844
TRINITY_DN3111_c0_g1_i5_orf1	-	-	-	CCAAT/enhancer-binding protein zeta-like [Ostrinia furnacalis]	1.88096	0.10224	-0.44996	-0.95851	-0.57473
TRINITY_DN9724_c0_g1_i4_orf1	-	-	-	ras family domain-containing protein [Phthorimaea operculella]	1.17337	-0.50413	-1.50016	-0.20637	1.03729
TRINITY_DN5562_c1_g1_i3_orf1	-	-	-	cell division cycle and apoptosis regulator protein 1-like [Ostrinia furnacalis]	1.96855	-0.25206	-0.64564	-0.72392	-0.34692
TRINITY_DN21559_c0_g2_i1_orf1	-	-	-	protein bicaudal D isoform X3 [Galleria mellonella]	1.18586	1.24411	-0.70237	-0.69014	-1.03746
TRINITY_DN8133_c0_g1_i4_orf1	-	-	-	protein sel-1 homolog 1 isoform X2 [Ostrinia furnacalis]	0.95314	0.87433	-1.80051	-0.21951	0.19255
TRINITY_DN535_c3_g2_i1_orf1	-	-	-	PSME3-interacting protein isoform X2 [Ostrinia furnacalis]	1.83107	-0.1772	-0.57899	-1.13026	0.05537
TRINITY_DN16899_c0_g2_i1_orf1	-	-	-	serine/threonine-protein kinase GA29083 [Ostrinia furnacalis]	1.18215	0.02872	-1.64061	0.85432	-0.42457
TRINITY_DN90321_c0_g2_i1_orf1	-	-	-	pre-mRNA-processing-splicing factor 8 [Apis dorsata] >XP_016768675.1 pre-mRNA-processing-splicing factor 8 [Apis mellifera] >XP_016911911.1 pre-mRNA-processing-splicing factor 8 [Apis cerana] >XP_016911918.1 pre-mRNA-processing-splicing factor 8 [Apis cerana] >XP_031366255.1 pre-mRNA-processing-splicing factor 8 [Apis dorsata] >XP_043784871.1 pre-mRNA-processing-splicing factor 8 [Apis laboriosa] >XP_043784872.1 pre-mRNA-processing-splicing factor 8 [Apis laboriosa] >XP_624014.2 pre-mRNA-processing-splicing factor 8 [Apis mellifera] >KAG6797633.1 pre-mRNA-processing-splicing factor 8 [Apis mellifera caucasica] >PBC26212.1 Pre-mRNA-processing-splicing factor [Apis cerana cerana]	1.50612	0.66449	-1.3279	-0.12838	-0.71433
TRINITY_DN6312_c0_g1_i1_orf1	-	-	-	cytochrome c oxidase assembly protein COX19 [Ostrinia furnacalis]	0.75724	1.49211	-1.20781	-0.20534	-0.8362
TRINITY_DN11552_c0_g1_i4_orf1	-	-	-	glutathione hydrolase 1 proenzyme-like isoform X3 [Ostrinia furnacalis]	1.95937	-0.74095	-0.60622	-0.13734	-0.47485
TRINITY_DN20321_c0_g1_i5_orf1	-	-	-	uncharacterized protein LOC114350467 isoform X3 [Ostrinia furnacalis]	1.74211	-0.81116	0.13548	0.06683	-1.13325
TRINITY_DN5234_c0_g1_i2_orf1	-	-	-	proline dehydrogenase 1, mitochondrial isoform X2 [Ostrinia furnacalis]	1.83815	-0.81702	0.28934	-0.73036	-0.58012
TRINITY_DN48638_c0_g1_i5_orf1	-	-	-	NADH dehydrogenase [ubiquinone] flavoprotein 2, mitochondrial [Ostrinia furnacalis] >ALD03682.1 mitochondrial complex I NDUFV2 subunit [Ostrinia nubilalis]	1.73386	-0.95183	-0.21893	-0.94535	0.38225
TRINITY_DN3411_c0_g1_i2_orf1	-	-	-	unnamed protein product [Chilo suppressalis]	1.88515	0.12552	-0.8639	-0.68873	-0.45804
TRINITY_DN41664_c0_g1_i4_orf1	-	-	-	uncharacterized protein LOC114356631 [Ostrinia furnacalis]	1.08079	0.40242	-1.40902	-0.95412	0.87992
TRINITY_DN13114_c0_g1_i1_orf1	-	-	-	nicalin-1 [Helicoverpa zea]	1.91032	-0.00141	-0.8416	-0.72446	-0.34284
TRINITY_DN35099_c0_g1_i1_orf1	-	-	-	transmembrane emp24 domain-containing protein 2 [Ostrinia furnacalis]	0.50837	1.11302	-1.70016	-0.51248	0.59125
TRINITY_DN14477_c0_g1_i12_orf1	-	-	-	PREDICTED: phosphoribosyl pyrophosphate synthase-associated protein 2 isoform X2 [Amyelois transitella]	1.16886	1.22245	-0.59065	-0.60937	-1.19129
TRINITY_DN5525_c0_g1_i4_orf1	-	-	-	probable glucosamine 6-phosphate N-acetyltransferase [Ostrinia furnacalis]	1.63912	0.62837	-0.41497	-1.04933	-0.80321
TRINITY_DN2907_c0_g2_i4_orf1	-	-	-	nucleoporin NUP188 homolog isoform X1 [Ostrinia furnacalis]	1.4395	-0.28077	-1.65152	0.25275	0.24003

TRINITY_DN89829_c0_g1_i1_orf1	-	-	-	PREDICTED: ubiquitin-conjugating enzyme E2 T [Microplitis demolitor]	1.33535	1.0219	-1.17854	-0.80019	-0.37852
TRINITY_DN12767_c0_g1_i2_orf1	-	-	-	coatomer subunit alpha [Ostrinia furnacalis]	1.37848	0.84604	-1.4124	-0.23378	-0.57834
TRINITY_DN67716_c0_g1_i1_orf1	-	-	-	unnamed protein product [Spodoptera exigua]	1.9718	-0.72274	-0.20809	-0.5681	-0.47287
TRINITY_DN3638_c0_g1_i1_orf1	-	-	-	DNA replication licensing factor Mcm3 [Ostrinia furnacalis]	1.94714	-0.17122	-0.38431	-0.51783	-0.87378
TRINITY_DN35377_c0_g1_i3_orf1	-	-	-	unnamed protein product [Chilo suppressalis]	1.41846	0.73945	-0.13845	-0.57218	-1.44728
TRINITY_DN38211_c0_g1_i1_orf1	-	-	-	Golgi reassembly-stacking protein 2 [Ostrinia furnacalis]	0.96706	1.36317	-1.10276	-0.26938	-0.95809
TRINITY_DN22871_c0_g2_i1_orf1	-	-	-	mitochondrial import inner membrane translocase subunit Tim17-B [Ostrinia furnacalis]	1.79149	-0.46552	0.23795	-0.39836	-1.16556
TRINITY_DN31815_c0_g1_i4_orf1	-	-	-	E3 ubiquitin-protein ligase listerin-like [Ostrinia furnacalis]	1.94618	-0.61408	-0.81184	-0.40303	-0.11722
TRINITY_DN30097_c0_g1_i2_orf1	-	-	-	unnamed protein product [Chilo suppressalis]	1.77888	0.22875	-1.09644	-0.16756	-0.74363
TRINITY_DN7674_c0_g1_i2_orf1	-	-	-	prefoldin subunit 2 [Ostrinia furnacalis]	1.37756	-0.5039	-1.1578	-0.71454	0.99867
TRINITY_DN11322_c0_g1_i2_orf1	-	-	-	CRAL-TRIO domain-containing protein C3H8.02 [Ostrinia furnacalis]	1.86519	-0.10045	-0.04683	-0.73011	-0.9878
TRINITY_DN15458_c0_g1_i3_orf1	-	-	-	integrin alpha-PS1 isoform X1 [Ostrinia furnacalis]	1.42029	0.59268	-1.58559	-0.32789	-0.09949
TRINITY_DN181_c0_g1_i3_orf1	-	-	-	hypothetical protein evm_003589 [Chilo suppressalis]	1.49287	0.4884	-1.43989	-0.66627	0.12489
TRINITY_DN38650_c0_g1_i2_orf1	-	-	-	elongator complex protein 1 [Ostrinia furnacalis]	1.92405	-0.08207	-0.60473	-0.3356	-0.90165
TRINITY_DN72859_c0_g1_i1_orf1	-	-	-	hypothetical protein evm_010574 [Chilo suppressalis]	1.47401	0.78382	-1.33611	-0.42241	-0.4993
TRINITY_DN1045_c0_g1_i6_orf1	-	-	-	ornithine decarboxylase 1-like isoform X1 [Ostrinia furnacalis]	1.40277	0.95949	-1.07033	-0.38955	-0.90237
TRINITY_DN1888_c0_g2_i1_orf1	-	-	-	peptidyl-prolyl cis-trans isomerase FKBP8 [Ostrinia furnacalis]	1.76913	0.27945	-1.17438	-0.56118	-0.31302
TRINITY_DN41546_c0_g1_i15_orf1	-	-	-	monocarboxylate transporter 12 isoform X8 [Ostrinia furnacalis]	1.95834	-0.46637	-0.64186	-0.13005	-0.72007
TRINITY_DN500_c0_g1_i1_orf1	-	-	-	splicing factor U2AF 50 kDa subunit isoform X2 [Manduca sexta]	1.75346	-0.60395	0.2836	-1.19263	-0.24047
TRINITY_DN21126_c0_g1_i1_orf1	-	-	-	serine/threonine-protein kinase unc-51 isoform X5 [Ostrinia furnacalis]	1.69278	0.54159	-1.08762	-0.55414	-0.59261
TRINITY_DN3801_c0_g1_i9_orf1	-	-	-	claspin-like isoform X2 [Ostrinia furnacalis]	1.71972	0.50071	-0.99548	-0.77156	-0.45338
TRINITY_DN1856_c0_g1_i3_orf1	-	-	-	hypothetical protein evm_006253 [Chilo suppressalis]	1.57101	0.72649	-1.1695	-0.55407	-0.57393
TRINITY_DN1405_c0_g1_i1_orf1	-	-	-	cyclin-dependent kinase 10 isoform X1 [Ostrinia furnacalis] >XP_028178194.1 cyclin-dependent kinase 10 isoform X2 [Ostrinia furnacalis]	0.83562	1.46784	-1.16362	-0.83784	-0.302
TRINITY_DN25681_c0_g1_i5_orf1	-	-	-	hypothetical protein evm_005766 [Chilo suppressalis] >CAB3520395.1 unnamed protein product [Chilo suppressalis] >CAH0397716.1 unnamed protein product [Chilo suppressalis]	1.60363	-1.28996	0.5836	-0.34557	-0.5517
TRINITY_DN6317_c1_g2_i3_orf1	-	-	-	nucleosome assembly protein 1-like 1 isoform X2 [Ostrinia furnacalis]	1.67762	0.26932	-1.32632	-0.02629	-0.59433
TRINITY_DN8406_c0_g1_i3_orfp1	-	-	-	TRINITY_DN8406_c0_g1_i3_m.76210 TRINITY_DN8406_c0_g1::TRINITY_DN8406_c0_g1_i3::g.76210 ORF type:internal len:154	1.16416	-0.5752	-1.65635	0.5147	0.55269
TRINITY_DN86580_c0_g1_i1_orf1	-	-	-	microtubule-actin cross-linking factor 1 isoform X15 [Ostrinia furnacalis]	1.45266	0.50898	-1.51638	0.1181	-0.56336
TRINITY_DN57_c0_g2_i3_orf1	-	-	-	transcriptional repressor CTCF-like [Ostrinia furnacalis] >XP_028163401.1 transcriptional repressor CTCF-like [Ostrinia furnacalis]	1.07848	0.33862	-1.73717	-0.41148	0.73154
TRINITY_DN50225_c0_g1_i1_orf1	-	-	-	SRSF protein kinase 3 [Galleria mellonella]	1.26827	1.14749	-0.81204	-1.05623	-0.54749
TRINITY_DN15916_c0_g1_i1_orf1	-	-	-	balbiani ring protein 3-like [Bicyclus anynana] >XP_023946842.1 balbiani ring protein 3-like [Bicyclus anynana]	1.66258	-0.5876	-0.18786	0.41136	-1.29848
TRINITY_DN3132_c0_g1_i10_orf1	-	-	-	oxysterol-binding protein 1-like [Ostrinia furnacalis]	1.36831	-0.25937	-1.64532	-0.0555	0.59188
TRINITY_DN280_c0_g1_i12_orf1	-	-	-	tubulin beta-1 chain-like [Leguminivora glycinivorella]	1.8287	0.07998	-0.98578	-0.8163	-0.10659
TRINITY_DN351_c14_g1_i2_orf1	-	-	-	calnexin [Ostrinia furnacalis] >XP_028173720.1 calnexin [Ostrinia furnacalis]	1.13928	0.09245	-1.74577	-0.24975	0.76379
TRINITY_DN18558_c0_g1_i7_orf1	-	-	-	cytosolic Fe-S cluster assembly factor NUBP2 homolog [Ostrinia furnacalis]	1.78688	0.4018	-0.82772	-0.54937	-0.81159
TRINITY_DN5908_c0_g1_i2_orf1	-	-	-	ATP-binding cassette sub-family B member 10, mitochondrial-like [Ostrinia furnacalis]	1.86838	-0.36576	0.14333	-0.81131	-0.83463
TRINITY_DN130778_c0_g1_i1_orf1	-	-	-	26S proteasome non-ATPase regulatory subunit 8 [Ostrinia furnacalis]	0.91288	0.71462	-1.70608	-0.56989	0.64846
TRINITY_DN20426_c0_g2_i1_orf1	-	-	-	prolyl 3-hydroxylase sudestada1 [Ostrinia furnacalis]	0.84937	0.15897	-1.87319	0.00204	0.86281
TRINITY_DN19260_c0_g1_i5_orf1	-	-	-	probable 26S proteasome non-ATPase regulatory subunit 3 [Ostrinia furnacalis]	0.80779	0.84136	-1.47615	-0.93668	0.76368
TRINITY_DN14572_c0_g1_i1_orf1	-	-	-	ras-related protein Rab-11A [Ostrinia furnacalis]	1.33541	0.91308	-1.4293	-0.45727	-0.36192
TRINITY_DN1554_c0_g1_i9_orf1	-	-	-	LOW QUALITY PROTEIN: puff-specific protein Bx42 [Ostrinia furnacalis]	1.18985	1.22435	-0.51197	-0.86788	-1.03435
TRINITY_DN18300_c0_g1_i17_orf1	-	-	-	RNA-binding protein lark isoform X1 [Helicoverpa armigera] >XP_047032035.1 RNA-binding protein lark isoform X1 [Helicoverpa zea] >PZC74210.1 hypothetical protein B5X24_HaOG208200 [Helicoverpa armigera]	1.91004	-0.31905	-0.78884	-0.79221	-0.00993
TRINITY_DN10871_c0_g1_i3_orf1	-	-	-	hypothetical protein HF086_013701 [Spodoptera exigua]	1.37258	0.62705	-1.21261	-1.08006	0.29305
TRINITY_DN15762_c0_g1_i2_orf1	-	-	-	YTH domain-containing family protein 3 isoform X3 [Maniola hyperantus]	1.45166	0.49457	-1.20761	-1.04643	0.30781
TRINITY_DN65604_c0_g1_i2_orf1	-	-	-	LOW QUALITY PROTEIN: ankyrin repeat domain-containing protein 17 [Ostrinia furnacalis]	1.94207	-0.2546	-0.28232	-0.48278	-0.92237
TRINITY_DN3753_c0_g1_i7_orf1	-	-	-	very-long-chain 3-oxoacyl-CoA reductase isoform X2 [Ostrinia furnacalis]	1.87177	0.12321	-0.42257	-0.96858	-0.60382
TRINITY_DN4403_c0_g1_i3_orf1	-	-	-	AP-1 complex subunit gamma-1 [Ostrinia furnacalis]	1.43708	0.98402	-0.72162	-0.87864	-0.82083
TRINITY_DN14220_c0_g1_i1_orf1	-	-	-	U3 small nucleolar ribonucleoprotein protein IMP4 [Ostrinia furnacalis]	1.90784	-0.6631	-0.02111	-0.3191	-0.90454

TRINITY_DN10716_c1_g1_i1_orf1	-	-	-	apoptosis-inducing factor 3-like [Ostrinia furnacalis]	1.96116	-0.81552	-0.55449	-0.35293	-0.23822
TRINITY_DN47605_c0_g2_i1_orf1	-	-	-	hypothetical protein evm_002030 [Chilo suppressalis]	1.9376	-0.87746	-0.60295	-0.16653	-0.29067
TRINITY_DN7064_c0_g1_i6_orf1	-	-	-	unnamed protein product [Chilo suppressalis]	1.86021	-0.05138	-1.0487	-0.10776	-0.65236
TRINITY_DN45000_c0_g1_i5_orf1	-	-	-	PREDICTED: ATP synthase subunit beta, mitochondrial, partial [Papilio polytes]	1.96465	-0.40431	-0.28198	-0.83924	-0.43912
TRINITY_DN6308_c0_g1_i3_orf1	-	-	-	myc box-dependent-interacting protein 1 isoform X4 [Pectinophora gossypiella]	1.8201	-1.17294	-0.55414	-0.04113	-0.05189
TRINITY_DN10257_c0_g1_i2_orf1	-	-	-	prefoldin subunit domain-containing protein [Phthorimaea operculella]	1.86415	0.1645	-0.4237	-0.67916	-0.92579
TRINITY_DN14298_c0_g1_i1_orf1	-	-	-	kinesin heavy chain [Ostrinia furnacalis]	0.99878	1.2973	-1.32058	-0.71104	-0.26447
TRINITY_DN33967_c0_g1_i1_orf1	-	-	-	PREDICTED: elongation factor 1-alpha [Microplitis demolitor] >XP_008547401.1 PREDICTED: elongation factor 1-alpha [Microplitis demolitor]	1.69897	-0.77673	-1.05695	-0.37081	0.50552
TRINITY_DN71494_c0_g1_i2_orf1	-	-	-	hypothetical protein O3G_MSEX005876 [Manduca sexta]	1.79285	0.08033	-0.88506	0.00977	-0.9979
TRINITY_DN129259_c0_g2_i1_orf1	-	-	-	spectrin alpha chain-like isoform X3 [Helicoverpa armigera]	1.50753	-0.88399	-1.2769	0.10084	0.55252
TRINITY_DN802_c0_g1_i2_orf1	-	-	-	active breakpoint cluster region-related protein [Ostrinia furnacalis]	1.27153	1.07602	-0.9137	-1.14272	-0.29113
TRINITY_DN53400_c0_g1_i1_orf1	-	-	-	hypothetical protein evm_004547 [Chilo suppressalis]	1.91295	-0.25276	-0.36557	-0.25677	-1.03786
TRINITY_DN44335_c0_g1_i7_orf1	-	-	-	hypothetical protein SFRURICE_000584 [Spodoptera frugiperda]	1.54291	0.79142	-1.12082	-0.62288	-0.59063
TRINITY_DN49527_c0_g1_i1_orf1	-	-	-	Protein lin-7 homolog B [Eumeta japonica]	1.99038	-0.43992	-0.64228	-0.5539	-0.35429
TRINITY_DN2611_c0_g1_i10_orf1	-	-	-	ancient ubiquitous protein 1-like [Hyposmocoma kahamanoa]	1.53376	0.17281	-1.51523	-0.48522	0.29388
TRINITY_DN42205_c0_g1_i4_orf1	-	-	-	eukaryotic translation initiation factor 4H [Ostrinia furnacalis]	1.51424	0.86669	-0.89702	-0.58337	-0.90054
TRINITY_DN3245_c2_g1_i4_orf1	-	-	-	membrane-associated progesterone receptor component 1-like [Ostrinia furnacalis]	1.85485	-1.08324	-0.57489	-0.23304	0.03632
TRINITY_DN51737_c0_g1_i3_orf1	-	-	-	N-alpha-acetyltransferase 40 [Ostrinia furnacalis]	0.97785	1.4392	-0.89151	-0.67888	-0.84666
TRINITY_DN1781_c0_g1_i8_orf1	-	-	-	transportin-1 [Pectinophora gossypiella]	0.60887	1.2295	-1.00197	-1.35732	0.52092
TRINITY_DN934_c2_g1_i7_orf1	-	-	-	ubiquitin-40S ribosomal protein S27a [Ostrinia furnacalis]	1.21232	1.16847	-1.18886	-0.73956	-0.45237
TRINITY_DN101358_c0_g2_i1_orf1	-	-	-	glycylpeptide N-tetradecanoyltransferase 2 [Ostrinia furnacalis]	1.5537	0.54797	-0.89205	-1.2206	0.01098
TRINITY_DN1383_c0_g1_i2_orf1	-	-	-	uncharacterized protein LOC114353133 isoform X1 [Ostrinia furnacalis] >XP_028160773.1 uncharacterized protein LOC114353133 isoform X2 [Ostrinia furnacalis]	1.64306	-1.10696	0.41655	-0.0032	-0.94945
TRINITY_DN4546_c0_g1_i3_orf1	-	-	-	telomerase Cajal body protein 1 homolog [Ostrinia furnacalis]	1.52045	0.53321	-0.55892	-1.44537	-0.04937
TRINITY_DN6313_c0_g1_i4_orf1	-	-	-	pyruvate dehydrogenase E1 component subunit beta, mitochondrial isoform X1 [Ostrinia furnacalis] >XP_028161232.1 pyruvate dehydrogenase E1 component subunit beta, mitochondrial isoform X2 [Ostrinia furnacalis] >XP_028161233.1 pyruvate dehydrogenase E1 component subunit beta, mitochondrial isoform X3 [Ostrinia furnacalis] >XP_028161234.1 pyruvate dehydrogenase E1 component subunit beta, mitochondrial isoform X4 [Ostrinia	1.9123	-0.99289	-0.08391	-0.44214	-0.39338
TRINITY_DN18329_c0_g1_i2_orf1	-	-	-	bromodomain-containing protein 3 [Ostrinia furnacalis] >XP_028169629.1 bromodomain-containing protein 3 [Ostrinia furnacalis] >XP_028169637.1 bromodomain-containing protein 3 [Ostrinia furnacalis] >XP_028169644.1 bromodomain-containing protein 3 [Ostrinia	1.42205	0.62222	-1.57345	-0.19053	-0.28029
TRINITY_DN2649_c0_g1_i3_orf1	-	-	-	GPI inositol-deacylase isoform X1 [Ostrinia furnacalis]	1.29359	0.55941	-0.86278	0.44803	-1.43825
TRINITY_DN20966_c0_g1_i6_orf1	-	-	-	clavesin-1-like [Ostrinia furnacalis]	1.90096	-0.89868	-0.05444	-0.72547	-0.22237
TRINITY_DN2812_c0_g1_i5_orf1	-	-	-	myotubularin-related protein 2 [Ostrinia furnacalis] >XP_028170267.1 myotubularin-related protein 2 [Ostrinia furnacalis]	1.39192	0.99009	-0.68072	-1.14363	-0.55767
TRINITY_DN1494_c0_g1_i3_orf1	-	-	-	dihydropyrimidine dehydrogenase [NADP(+)] [Ostrinia furnacalis]	1.96587	-0.29947	-0.69691	-0.69691	-0.27258
TRINITY_DN164_c0_g1_i11_orf1	-	-	-	hypothetical protein evm_000323 [Chilo suppressalis] >CAB3530114.1 unnamed protein product [Chilo suppressalis] >CAH0406706.1 unnamed protein product [Chilo suppressalis]	1.89919	0.08119	-0.55097	-0.88976	-0.53965
TRINITY_DN2019_c0_g1_i4_orf1	-	-	-	sushi, von Willebrand factor type A, EGF and pentraxin domain-containing protein 1 [Ostrinia furnacalis] >XP_028159318.1 sushi, von Willebrand factor type A, EGF and pentraxin domain-containing protein 1 [Ostrinia furnacalis]	1.61289	0.72238	-0.94134	-0.5993	-0.79464
TRINITY_DN441_c0_g2_i1_orf1	-	-	-	guanine nucleotide-binding protein subunit beta-like protein [Diachasma alloeum]	1.65796	-0.5174	-1.32457	-0.2336	0.41762
TRINITY_DN27398_c0_g1_i3_orf1	-	-	-	lissencephaly-1 homolog [Helicoverpa armigera] >XP_021185428.1 lissencephaly-1 homolog [Helicoverpa armigera] >XP_047030140.1 lissencephaly-1 homolog [Helicoverpa zea] >XP_047030141.1 lissencephaly-1 homolog [Helicoverpa zea]	0.97683	0.79864	-1.11965	-1.31262	0.65681
TRINITY_DN129207_c0_g1_i1_orf1	-	-	-	U3 small nucleolar RNA-interacting protein 2 [Ostrinia furnacalis]	1.92081	-0.05609	-0.40385	-0.92688	-0.53398
TRINITY_DN3471_c0_g1_i1_orf1	-	-	-	EH domain-containing protein 3 [Ostrinia furnacalis]	1.85683	-0.31363	-0.69132	-0.97958	0.1277

TRINITY_DN47666_c0_g1_i4_orf1	-	-	-	PREDICTED: probable splicing factor 3B subunit 5 [Amyelois transitella] >XP_021194781.1 splicing factor 3B subunit 5 [Helicoverpa armigera] >XP_022127816.1 splicing factor 3B subunit 5 [Pieris rapae] >XP_022818375.1 probable splicing factor 3B subunit 5 [Spodoptera litura] >XP_026319816.1 splicing factor 3B subunit 5 [Hyposmocoma kahamanoa] >XP_026728249.1 splicing factor 3B subunit 5 [Trichoplusia ni] >XP_026750486.1 splicing factor 3B subunit 5 [Galleria mellonella] >XP_028161523.1 splicing factor 3B subunit 5 [Ostrinia furnacalis] >XP_030037274.1 splicing factor 3B subunit 5 [Manduca sexta] >XP_035451936.1 splicing factor 3B subunit 5 [Spodoptera frugiperda] >XP_047035127.1 splicing factor 3B subunit 5 [Helicoverpa zea] >XP_047519960.1 splicing factor 3B subunit 5 [Pieris napi] >XP_049880275.1 splicing factor 3B subunit 5 [Pectinophora gossypiella] >KAF9410749.1 hypothetical protein HW555_010266 [Spodoptera exigua] >CAB3229682.1 unnamed protein product [Arctia plantaginis] >CAF4874265.1 unnamed protein product [Pieris macdunnoughi] >CAG9753950.1 unnamed protein product [Diatraea saccharalis] >CAH0596996.1 unnamed protein product [Chrysodeixis includens] >CAH0717822.1 unnamed protein product, partial [Brenthis ino] >CAH2097681.1 unnamed protein product [Euphydryas editha]	1.56195	0.71027	-1.20587	-0.66198	-0.40437
TRINITY_DN6239_c0_g1_i1_orf1	-	-	-	eukaryotic translation initiation factor 6 [Ostrinia furnacalis]	1.14951	1.24456	-0.41739	-1.01882	-0.95786
TRINITY_DN48460_c0_g1_i1_orf1	-	-	-	PREDICTED: signal recognition particle 54 kDa protein [Fopius arisanus]	1.39219	0.7369	-1.51408	-0.44395	-0.17105
TRINITY_DN1469_c0_g1_i1_orf1	-	-	-	kynurenine--oxoglutarate transaminase 3 isoform X4 [Orussus abietinus] >XP_012270451.1 kynurenine--oxoglutarate transaminase 3 isoform X4 [Orussus abietinus] >XP_012270459.1 kynurenine--oxoglutarate transaminase 3 isoform X4 [Orussus abietinus] >XP_012270468.1 kynurenine--oxoglutarate transaminase 3 isoform X4 [Orussus abietinus]	1.45913	0.13149	-1.4697	-0.64626	0.52534
TRINITY_DN3179_c0_g1_i1_orf1	-	-	-	diamine acetyltransferase 2-like [Ostrinia furnacalis]	1.56226	0.71553	-1.13897	-0.79485	-0.34396
TRINITY_DN6876_c0_g2_i1_orf1	-	-	-	serine/threonine-protein phosphatase 5 [Spodoptera litura] >CAB3508320.1 unnamed protein product [Spodoptera littoralis] >CAH1637875.1 unnamed protein product [Spodoptera	1.91561	-0.93389	-0.23517	-0.62259	-0.12396
TRINITY_DN18912_c1_g1_i1_orf1	-	-	-	engulfment and cell motility protein 1 [Ostrinia furnacalis]	1.57404	0.75227	-1.05017	-0.49779	-0.77835
TRINITY_DN8980_c0_g1_i2_orf1	-	-	-	putative ATP-dependent RNA helicase me31b [Ostrinia furnacalis] >XP_028162602.1 putative ATP-dependent RNA helicase me31b [Ostrinia furnacalis] >XP_028162603.1 putative ATP-dependent RNA helicase me31b [Ostrinia furnacalis]	1.7828	0.39757	-0.56863	-0.94971	-0.66203
TRINITY_DN3597_c0_g1_i10_orf1	-	-	-	unnamed protein product [Diatraea saccharalis]	1.23923	0.60941	-1.65755	-0.50001	0.30892
TRINITY_DN101325_c0_g1_i4_orf1	-	-	-	endonuclease G, mitochondrial [Ostrinia furnacalis]	1.88813	-0.62002	-0.09966	-1.00729	-0.16116
TRINITY_DN3805_c0_g1_i2_orf1	-	-	-	heat shock protein 89 [Glyphodes pyloalis]	1.17752	-0.39056	-1.62082	-0.07611	0.90998
TRINITY_DN5982_c0_g1_i3_orf1	-	-	-	coatomer subunit gamma [Ostrinia furnacalis]	1.69769	0.5167	-1.11791	-0.54487	-0.55161
TRINITY_DN13259_c0_g1_i2_orf1	-	-	-	mitotic spindle assembly checkpoint protein MAD1 [Pectinophora gossypiella]	1.84129	-0.60467	-1.10789	-0.12884	9.93E-05
TRINITY_DN5092_c0_g1_i2_orf1	-	-	-	peroxisomal acyl-coenzyme A oxidase 3 isoform X3 [Ostrinia furnacalis]	1.9413	-0.54408	-0.89676	-0.19573	-0.30472
TRINITY_DN61711_c0_g1_i1_orf1	-	-	-	mitochondrial carnitine/acylcarnitine translocase [Loxostege sticticalis]	1.67717	-0.61807	-1.09364	-0.53429	0.56884
TRINITY_DN17861_c0_g1_i5_orf1	-	-	-	acyl-CoA-binding protein homolog isoform X1 [Ostrinia furnacalis]	1.62408	0.70078	-0.98922	-0.64831	-0.68733
TRINITY_DN4859_c0_g1_i5_orf1	-	-	-	importin-11, partial [Ostrinia furnacalis]	1.42603	0.65088	-1.54968	-0.29751	-0.22973
TRINITY_DN7560_c0_g1_i4_orf1	-	-	-	unnamed protein product [Chilo suppressalis]	1.36737	0.84005	-1.11664	-1.08522	-0.00556
TRINITY_DN13093_c0_g1_i2_orf1	-	-	-	enhancer of mRNA-decapping protein 4 [Ostrinia furnacalis]	1.43816	0.43281	-1.60456	0.12587	-0.39228
TRINITY_DN5133_c0_g1_i7_orf1	-	-	-	epimerase family protein SDR39U1 [Ostrinia furnacalis]	1.85668	-0.23872	-0.67316	0.07363	-1.01843
TRINITY_DN4026_c0_g1_i4_orf1	-	-	-	serine protease [Ostrinia furnacalis]	1.78896	0.40209	-0.79543	-0.82308	-0.57254
TRINITY_DN4207_c0_g1_i1_orf1	-	-	-	mitochondrial import inner membrane translocase subunit Tim21 [Ostrinia furnacalis]	1.45218	0.00876	-0.26058	0.42519	-1.62555
TRINITY_DN1947_c0_g1_i6_orf1	-	-	-	insulin-degrading enzyme [Ostrinia furnacalis] >XP_028163443.1 insulin-degrading enzyme [Ostrinia furnacalis]	1.87786	0.028	-1.02969	-0.31816	-0.55801
TRINITY_DN24325_c0_g1_i12_orf1	-	-	-	MICOS complex subunit MIC10-like [Ostrinia furnacalis]	1.9268	-0.96108	-0.15011	-0.34224	-0.47338
TRINITY_DN4300_c0_g1_i5_orf1	-	-	-	replication factor C subunit 3 [Ostrinia furnacalis]	1.9804	-0.29768	-0.73081	-0.50936	-0.44255
TRINITY_DN518_c0_g1_i1_orf1	-	-	-	unnamed protein product [Arctia plantaginis]	1.25132	1.1675	-1.07328	-0.75634	-0.5892
TRINITY_DN13944_c0_g1_i1_orf1	-	-	-	vacuolar protein sorting-associated protein 52 homolog [Ostrinia furnacalis]	1.96507	-0.4955	-0.36077	-0.28229	-0.82652
TRINITY_DN4814_c0_g1_i6_orf1	-	-	-	vesicle transport protein GOT1B [Pectinophora gossypiella]	1.65615	0.10567	-0.38782	0.07183	-1.44583
TRINITY_DN1391_c1_g2_i4_orf1	-	-	-	hypothetical protein SFRURICE_002236 [Spodoptera frugiperda]	1.74661	-0.84927	-1.09781	0.06261	0.13786
TRINITY_DN4439_c0_g1_i2_orf1	-	-	-	cytoplasmic FMR1-interacting protein isoform X1 [Ostrinia furnacalis] >XP_028169436.1 cytoplasmic FMR1-interacting protein isoform X2 [Ostrinia furnacalis]	1.23427	1.11537	-0.98593	-1.08844	-0.27527
TRINITY_DN28428_c0_g1_i2_orf1	-	-	-	unnamed protein product [Chrysodeixis includens]	0.93955	1.2707	-1.48651	-0.48625	-0.23749
TRINITY_DN9437_c0_g1_i1_orf1	-	-	-	glyoxylate reductase/hydroxypyruvate reductase-like [Ostrinia furnacalis]	1.90286	-0.01091	-0.61852	-0.33207	-0.94136

TRINITY_DN8076_c0_g1_i5_orf1	-	-	-	hypothetical protein evm_001812 [Chilo suppressalis]	1.8774	-0.02404	-0.49129	-0.29003	-1.07205
TRINITY_DN3584_c0_g1_i3_orf1	-	-	-	paired amphipathic helix protein Sin3b [Ostrinia furnacalis]	1.5281	0.29383	-1.58092	-0.27899	0.03798
TRINITY_DN2984_c0_g1_i3_orf1	-	-	-	connectin-like [Ostrinia furnacalis]	1.18247	1.22168	-1.11521	-0.77651	-0.51243
TRINITY_DN655_c0_g1_i3_orf1	-	-	-	moesin/ezrin/radixin homolog 1 isoform X2 [Bombyx mori] >XP_028038189.1 moesin/ezrin/radixin homolog 1 isoform X2 [Bombyx mandarina]	1.76794	0.08956	-1.14163	-0.74962	0.03375
TRINITY_DN30027_c0_g1_i1_orf1	-	-	-	40S ribosomal protein S14 [Plutella xylostella] >NP_001298660.1 40S ribosomal protein S14 [Papilio polytes] >NP_001299342.1 40S ribosomal protein S14 [Papilio xuthus] >XP_013200267.1 PREDICTED: 40S ribosomal protein S14 [Amyelois transitella] >XP_013200268.1 PREDICTED: 40S ribosomal protein S14 [Amyelois transitella] >XP_014369569.1 40S ribosomal protein S14 [Papilio machaon] >XP_021200686.1 40S ribosomal protein S14 [Helicoverpa armigera] >XP_026737481.1 40S ribosomal protein S14 [Trichoplusia ni] >XP_028029011.1 40S ribosomal protein S14 [Bombyx mandarina] >XP_028179467.1 40S ribosomal protein S14 [Ostrinia furnacalis] >XP_028179468.1 40S ribosomal protein S14 [Ostrinia furnacalis] >XP_030030611.1 40S ribosomal protein S14 [Manduca sexta] >XP_034829960.1 40S ribosomal protein S14 [Maniola hyperantus] >XP_034829961.1 40S ribosomal protein S14 [Maniola hyperantus] >XP_047022662.1 40S ribosomal protein S14 [Helicoverpa zea] >XP_047984027.1 40S ribosomal protein S14 [Leguminivora glycinivorella] >XP_049869822.1 40S ribosomal protein S14 [Pectinophora gossypiella] >Q5UAM9.1 RecName: Full=40S ribosomal protein S14 [Bombyx mori] >CAH0605581.1 unnamed protein product [Chrysodeixis includens] >AAV34871.1 ribosomal protein S14 [Bombyx mori] >ACY95302.1 ribosomal protein S14 [Manduca sexta] >KAG6456546.1 hypothetical protein O3G_MSEX009812 [Manduca sexta] >KAG6456547.1	1.68007	-0.15677	-1.43508	-0.25558	0.16736
TRINITY_DN3791_c0_g1_i2_orf1	-	-	-	transmembrane protein 19 [Ostrinia furnacalis]	1.92982	-0.02585	-0.82946	-0.46722	-0.60729
TRINITY_DN54586_c1_g1_i1_orf1	-	-	-	protein YIPF5 [Ostrinia furnacalis]	0.60293	1.213	-1.29629	-1.08132	0.56167
TRINITY_DN5442_c0_g1_i4_orf1	-	-	-	hypothetical protein evm_004688 [Chilo suppressalis]	1.47048	0.92953	-0.77766	-0.97379	-0.64856
TRINITY_DN19920_c1_g1_i2_orf1	-	-	-	probable ATP-dependent RNA helicase DDX10 [Ostrinia furnacalis]	1.94347	-0.81133	-0.12322	-0.64679	-0.36213
TRINITY_DN4808_c0_g1_i3_orf1	-	-	-	kinesin light chain [Ostrinia furnacalis]	1.0505	1.28242	-1.1353	-0.94939	-0.24822
TRINITY_DN3454_c0_g1_i1_orf1	-	-	-	MICOS complex subunit MIC13 homolog QIL1 [Ostrinia furnacalis]	1.88442	-0.91382	0.11648	-0.47477	-0.61231
TRINITY_DN8701_c0_g1_i3_orf1	-	-	-	putative uncharacterized protein DDB_G0282133 [Ostrinia furnacalis]	0.78371	1.31081	-1.55516	-0.0421	-0.49726
TRINITY_DN6063_c1_g2_i1_orf1	-	-	-	short-chain specific acyl-CoA dehydrogenase, mitochondrial [Ostrinia furnacalis]	1.83703	-0.25813	-0.80424	-0.94044	0.16578
TRINITY_DN42705_c0_g1_i3_orf1	-	-	-	multiple inositol polyphosphate phosphatase 1 isoform X1 [Ostrinia furnacalis]	1.50866	-0.33829	-1.01649	-0.96137	0.80749
TRINITY_DN32509_c0_g1_i3_orf1	-	-	-	heat shock 70 kDa protein 4 isoform X1 [Ostrinia furnacalis]	1.93903	-0.24546	-0.90969	-0.24172	-0.54217
TRINITY_DN143496_c0_g1_i1_orf1	-	-	-	cullin-3 [Diachasma alloeum]	1.20079	-0.10395	-1.79372	0.13984	0.55704
TRINITY_DN4929_c0_g1_i1_orf1	-	-	-	unnamed protein product [Danaus chrysippus]	1.26549	0.35409	-0.27189	0.39646	-1.74415
TRINITY_DN31058_c0_g1_i6_orf1	-	-	-	HIV Tat-specific factor 1 homolog [Ostrinia furnacalis]	1.65485	0.3267	-0.99697	-1.0737	0.08912
TRINITY_DN7942_c0_g1_i1_orf1	-	-	-	hypothetical protein evm_012160 [Chilo suppressalis] >CAB3521803.1 unnamed protein product [Chilo suppressalis] >CAH0399125.1 unnamed protein product [Chilo suppressalis]	1.85122	0.25917	-0.63385	-0.65478	-0.82176
TRINITY_DN12820_c0_g1_i1_orf1	-	-	-	chromodomain-helicase-DNA-binding protein 7 [Ostrinia furnacalis] >XP_028176739.1 chromodomain-helicase-DNA-binding protein 7 [Ostrinia furnacalis]	1.45459	0.84067	-1.2244	-0.30637	-0.76449
TRINITY_DN14301_c0_g1_i1_orf1	-	-	-	apoptosis-inducing factor 1, mitochondrial-like [Ostrinia furnacalis]	1.82684	-1.00864	-0.24984	0.17481	-0.74317
TRINITY_DN37141_c0_g1_i2_orf1	-	-	-	dnaJ homolog shv [Ostrinia furnacalis]	1.64942	0.61666	-1.10464	-0.53362	-0.62782
TRINITY_DN1459_c1_g1_i1_orf1	-	-	-	reticulon-1 isoform X2 [Ostrinia furnacalis]	1.65387	0.55093	-0.6651	-1.17819	-0.36152
TRINITY_DN1567_c0_g1_i15_orf1	-	-	-	probable dual specificity protein kinase madd-3 isoform X1 [Ostrinia furnacalis]	1.95837	-0.37321	-0.5219	-0.83813	-0.22514
TRINITY_DN81719_c0_g1_i1_orf1	-	-	-	tyrosine 3-monooxygenase isoform X1 [Ostrinia furnacalis] >ARE68330.1 tyrosin hydroxylase [Ostrinia furnacalis]	1.92948	-0.13563	-0.30297	-0.57878	-0.9121
TRINITY_DN2859_c0_g1_i7_orf1	-	-	-	cleavage and polyadenylation specificity factor subunit 5 [Ostrinia furnacalis]	1.99559	-0.40517	-0.56797	-0.57513	-0.44731
TRINITY_DN848_c0_g1_i1_orf1	-	-	-	actin-interacting protein 1 isoform X2 [Ostrinia furnacalis]	1.75452	-0.87862	-0.99984	-0.2048	0.32875
TRINITY_DN2172_c0_g2_i8_orf1	-	-	-	hypothetical protein evm_003685 [Chilo suppressalis]	0.86022	1.2087	-1.55163	-0.6176	0.10032
TRINITY_DN3434_c0_g1_i1_orf1	-	-	-	coiled-coil domain-containing protein 47 [Ostrinia furnacalis] >XP_028161458.1 coiled-coil domain-containing protein 47 [Ostrinia furnacalis]	1.87746	-0.94046	-0.75467	-0.13866	-0.04367
TRINITY_DN2973_c1_g1_i9_orf1	-	-	-	unconventional myosin ID [Ostrinia furnacalis]	1.98159	-0.74128	-0.33331	-0.48002	-0.42698
TRINITY_DN15753_c0_g1_i1_orf1	-	-	-	uncharacterized protein LOC114366450 [Ostrinia furnacalis]	1.28076	1.04971	-0.83393	-1.21865	-0.27789
TRINITY_DN133760_c0_g1_i1_orf1	-	-	-	THO complex subunit 7 homolog [Ostrinia furnacalis]	1.78809	0.39056	-0.5299	-0.75297	-0.89579

TRINITY_DN26503_c0_g1_i1_orf1	-	-	-	ruvB-like 2 isoform X1 [Ostrinia furnacalis] >XP_028160979.1 ruvB-like 2 isoform X2 [Ostrinia furnacalis]	1.83136	0.06752	-1.1426	-0.53635	-0.21992
TRINITY_DN38693_c0_g1_i4_orf1	-	-	-	protein RER1 [Ostrinia furnacalis] >XP_028157304.1 protein RER1 [Ostrinia furnacalis] >XP_028157310.1 protein RER1 [Ostrinia furnacalis]	1.90092	0.00412	-0.95983	-0.37649	-0.56873
TRINITY_DN40197_c0_g1_i1_orf1	-	-	-	UDP-N-acetylglucosamine--dolichyl-phosphate N-acetylglucosaminephosphotransferase-like [Ostrinia furnacalis]	1.7187	0.48878	-1.06683	-0.66655	-0.47409
TRINITY_DN7603_c0_g1_i5_orf1	-	-	-	tetratricopeptide repeat protein 1-like [Ostrinia furnacalis]	0.87622	1.46348	-1.10704	-0.84567	-0.387
TRINITY_DN10287_c0_g1_i1_orf1	-	-	-	nibrin [Ostrinia furnacalis]	1.93448	-0.85976	-0.58061	-0.4194	-0.07471
TRINITY_DN4752_c0_g1_i3_orf1	-	-	-	thioredoxin domain-containing protein 9 [Ostrinia furnacalis] >XP_028172592.1 thioredoxin domain-containing protein 9 [Ostrinia furnacalis]	1.93867	-0.24008	-0.65422	-0.19771	-0.84665
TRINITY_DN18909_c0_g1_i6_orf1	-	-	-	unnamed protein product [Euphydryas editha]	1.57483	-0.98757	-0.0949	0.59478	-1.08713
TRINITY_DN28759_c0_g1_i1_orf1	-	-	-	innexin inx2 [Ostrinia furnacalis]	0.98902	1.3934	-1.03628	-0.44907	-0.89707
TRINITY_DN146758_c0_g1_i1_orf1	-	-	-	PREDICTED: mitochondrial import inner membrane translocase subunit Tim16-like [Fopius arisanus]	1.88956	-0.87352	-0.0101	-0.21972	-0.78622
TRINITY_DN36592_c0_g1_i1_orf1	-	-	-	uncharacterized protein LOC114359903 [Ostrinia furnacalis]	1.88825	-0.98643	-0.47058	-0.48677	0.05552
TRINITY_DN2778_c0_g1_i5_orf1	-	-	-	hypothetical protein evm_001346 [Chilo suppressalis]	1.90349	-0.08796	-0.91998	-0.69435	-0.2012
TRINITY_DN28622_c0_g1_i1_orf1	-	-	-	actin-related protein 3 [Ostrinia furnacalis]	1.06613	1.31412	-1.19026	-0.67149	-0.5185
TRINITY_DN26168_c0_g1_i1_orf1	-	-	-	ATP-dependent RNA helicase Ddx1-like [Ostrinia furnacalis]	1.5892	0.66818	-1.20084	-0.64619	-0.41035
TRINITY_DN14677_c0_g2_i3_orf1	-	-	-	AP-3 complex subunit beta-2 [Ostrinia furnacalis]	0.51627	1.15998	-1.5438	-0.77185	0.6394
TRINITY_DN9085_c0_g1_i1_orf1	-	-	-	golgin subfamily A member 2-like [Ostrinia furnacalis]	1.89096	-0.33433	-1.08977	-0.32258	-0.14429
TRINITY_DN84669_c0_g1_i1_orf1	-	-	-	PREDICTED: microtubule-actin cross-linking factor 1, isoforms 1/2/3/5 [Amyelois transitella]	0.86186	0.39561	-1.93225	0.60143	0.07335
TRINITY_DN17935_c0_g1_i1_orf1	-	-	-	NEDD8-conjugating enzyme Ubc12 [Ostrinia furnacalis]	1.46637	0.90037	-0.96481	-0.45045	-0.95149
TRINITY_DN6084_c0_g1_i4_orf1	-	-	-	unconventional myosin-Va isoform X1 [Manduca sexta]	1.6882	0.27426	-0.68534	-0.01024	-1.26687
TRINITY_DN35245_c0_g1_i1_orf1	-	-	-	ras GTPase-activating protein-binding protein 2 isoform X1 [Nymphalis io] >XP_050349014.1 ras GTPase-activating protein-binding protein 2 isoform X1 [Nymphalis io] >XP_050349015.1 ras GTPase-activating protein-binding protein 2 isoform X2 [Nymphalis io]	1.67913	0.28353	-0.78213	-1.21938	0.03885
TRINITY_DN21218_c0_g1_i4_orf1	-	-	-	leukotriene A-4 hydrolase isoform X2 [Ostrinia furnacalis]	1.5094	0.83107	-0.4105	-0.97349	-0.95648
TRINITY_DN19687_c0_g1_i1_orf1	-	-	-	probable ribosome production factor 1 [Ostrinia furnacalis]	1.65503	-0.15769	0.21897	-0.26015	-1.45616
TRINITY_DN10110_c1_g2_i1_orf1	-	-	-	venom allergen 3-like [Ostrinia furnacalis]	0.51602	0.94586	-1.64402	-0.65715	0.83929
TRINITY_DN1790_c1_g1_i3_orf1	-	-	-	unnamed protein product [Diatraea saccharalis]	1.83992	-0.5712	-0.67341	-0.87113	0.27582
TRINITY_DN2265_c0_g1_i5_orf1	-	-	-	elongation factor G, mitochondrial [Ostrinia furnacalis]	1.67708	-0.85617	0.21469	0.14253	-1.17813
TRINITY_DN12858_c0_g1_i5_orf1	-	-	-	unnamed protein product, partial [Iphiclides podalirius]	0.99682	1.3553	-1.13759	-0.86978	-0.34475
TRINITY_DN23790_c0_g1_i1_orf1	-	-	-	wiskott-Aldrich syndrome protein family member 2 [Ostrinia furnacalis]	1.37348	0.89355	-1.38792	-0.47406	-0.40505
TRINITY_DN19155_c0_g1_i1_orf1	-	-	-	cleavage and polyadenylation specificity factor 73 [Ostrinia furnacalis]	1.70178	0.51656	-0.70875	-1.06733	-0.44225
TRINITY_DN2919_c0_g1_i5_orf1	-	-	-	nidogen-1 [Ostrinia furnacalis]	1.18571	0.80799	-1.33338	-1.01767	0.35735
TRINITY_DN34479_c0_g1_i2_orf1	-	-	-	PREDICTED: 26S protease regulatory subunit 4 [Amyelois transitella] >XP_021186380.1 26S proteasome regulatory subunit 4 [Helicoverpa armigera] >XP_022116536.1 26S proteasome regulatory subunit 4 [Pieris rapae] >XP_022817854.1 26S proteasome regulatory subunit 4 [Spodoptera litura] >XP_026745369.1 26S proteasome regulatory subunit 4 [Trichoplusia ni] >XP_026760570.1 26S proteasome regulatory subunit 4 [Galleria mellonella] >XP_028176505.1 26S proteasome regulatory subunit 4 [Ostrinia furnacalis] >XP_030038234.1 26S proteasome regulatory subunit 4 [Manduca sexta] >XP_035449919.1 26S proteasome regulatory subunit 4 [Spodoptera frugiperda] >XP_038206559.1 26S proteasome regulatory subunit 4 [Zerene cesonia] >XP_045502541.1 26S proteasome regulatory subunit 4 [Colias croceus] >XP_045532999.1 26S proteasome regulatory subunit 4 [Pieris brassicae] >XP_047033702.1 26S proteasome regulatory subunit 4 [Helicoverpa zea] >XP_047994509.1 26S proteasome regulatory subunit 4 [Leguminivora glycinivorella] >XP_049877826.1 26S proteasome regulatory subunit 4 [Pectinophora gossypiella] >KAH9639287.1 hypothetical protein HF086_014151 [Spodoptera exigua] >KAI5631153.1 ATPase family associated with various cellular activities (AAA) domain-containing protein [Phthorimaea operculella] >RVE50066.1 hypothetical protein evm_005272 [Chilo suppressalis] >CAB3245712.1 unnamed protein product [Arctia plantaginis] >KAF9801312.1 hypothetical protein SFRURICE_000406	1.09943	0.52597	-1.48979	-0.86966	0.73405
TRINITY_DN6696_c0_g1_i4_orf1	-	-	-	mitochondrial 2-oxodicarboxylate carrier [Ostrinia furnacalis]	1.81398	0.00062	-0.70818	-1.09905	-0.00737

TRINITY_DN24164_c0_g1_i1_orf1	-	-	-	PREDICTED: ras-related protein Rab6 [Microplitis demolitor] >XP_034947251.1 ras-related protein Rab6 isoform X2 [Chelonus insularis] >XP_044581106.1 ras-related protein Rab6 isoform X2 [Cotesia glomerata] >KAH0553942.1 Ras- protein Rab6 [Cotesia glomerata] >CAG5097549.1 Similar to Rab6: Ras-related protein Rab6 (Drosophila melanogaster) [Cotesia	1.87496	-0.75904	-0.80682	0.16923	-0.47832
TRINITY_DN93566_c0_g2_i1_orf1	-	-	-	60S acidic ribosomal protein P1 [Manduca sexta] >ACY95374.1 ribosomal protein P1 [Manduca sexta] >KAG6447985.1 hypothetical protein O3G_MSEX005254 [Manduca sexta] >KAG6447986.1 hypothetical protein O3G_MSEX005254 [Manduca sexta]	1.82484	-0.96429	-0.75956	-0.33162	0.23062
TRINITY_DN21492_c0_g1_i1_orf1	-	-	-	isocitrate dehydrogenase [NAD] subunit gamma, mitochondrial [Chelonus insularis]	1.93121	-0.45549	-0.28548	-0.22556	-0.96467
TRINITY_DN1227_c0_g1_i1_orf1	-	-	-	uncharacterized protein LOC114356076 [Ostrinia furnacalis]	1.87613	0.13814	-0.40245	-0.79786	-0.81395
TRINITY_DN146493_c0_g1_i1_orf1	-	-	-	anaphase-promoting complex subunit 1 [Chelonus insularis]	1.13254	0.9	-1.50133	-0.7717	0.24049
TRINITY_DN1268_c0_g1_i1_orf1	-	-	-	nuclear pore complex protein Nup154 [Ostrinia furnacalis]	1.86347	-1.04068	-0.65616	-0.08172	-0.0849
TRINITY_DN6901_c0_g1_i4_orf1	-	-	-	(E3-independent) E2 ubiquitin-conjugating enzyme isoform X1 [Ostrinia furnacalis]	1.45687	0.07566	-1.39884	0.60625	-0.73994
TRINITY_DN7573_c0_g2_i1_orf1	-	-	-	nucleolar protein 56 [Ostrinia furnacalis]	1.94497	-0.6233	-0.58293	-0.69793	-0.04081
TRINITY_DN3619_c0_g2_i1_orf1	-	-	-	transport and Golgi organization protein 11 [Ostrinia furnacalis]	1.80987	0.32895	-0.87425	-0.79399	-0.47058
TRINITY_DN7839_c0_g1_i4_orf1	-	-	-	elongin-C [Ostrinia furnacalis] >XP_028163922.1 elongin-C [Ostrinia furnacalis]	1.80881	-0.09733	-1.23404	-0.44108	-0.03637
TRINITY_DN11639_c0_g1_i1_orf1	-	-	-	cysteine--tRNA ligase, cytoplasmic isoform X1 [Ostrinia furnacalis] >XP_028156309.1 cysteine--tRNA ligase, cytoplasmic isoform X2 [Ostrinia furnacalis] >XP_028156310.1 cysteine--tRNA ligase, cytoplasmic isoform X3 [Ostrinia furnacalis] >XP_028156311.1 cysteine--tRNA ligase, cytoplasmic isoform X4 [Ostrinia furnacalis]	1.97714	-0.21556	-0.50901	-0.64753	-0.60504
TRINITY_DN37830_c0_g1_i1_orf1	-	-	-	60S ribosomal protein L18a [Galleria mellonella] >AXY94862.1 ribosomal protein L18A [Galleria mellonella]	1.87016	-0.71836	-0.86469	-0.45784	0.17073
TRINITY_DN53115_c0_g1_i1_orf1	-	-	-	small glutamine-rich tetratricopeptide repeat-containing protein beta-like [Ostrinia furnacalis]	1.69032	0.582	-0.86988	-0.88006	-0.52238
TRINITY_DN6307_c0_g1_i5_orf1	-	-	-	putative helicase mov-10-B.1 [Ostrinia furnacalis]	1.9495	-0.65736	-0.24874	-0.80551	-0.23789
TRINITY_DN59291_c0_g1_i1_orf1	-	-	-	ATP-dependent RNA helicase vasa [Ostrinia furnacalis]	1.17592	1.01904	-1.50916	-0.52459	-0.16122
TRINITY_DN17651_c0_g1_i2_orf1	-	-	-	transmembrane protein 70 homolog, mitochondrial [Ostrinia furnacalis]	1.9117	-0.41261	-0.16179	-0.31173	-1.02557
TRINITY_DN11178_c0_g1_i1_orf1	-	-	-	hypoxia up-regulated protein 1 [Ostrinia furnacalis]	1.40009	-0.30651	-1.49866	-0.35324	0.75832
TRINITY_DN18593_c0_g1_i1_orf1	-	-	-	60S ribosomal protein L22-like [Ostrinia furnacalis]	1.60541	-0.86303	-0.9293	-0.53772	0.72465
TRINITY_DN383_c0_g1_i1_orf1	-	-	-	probable Golgi SNAP receptor complex member 2 [Ostrinia furnacalis]	1.33763	1.0129	-1.2124	-0.38579	-0.75235
TRINITY_DN3953_c0_g1_i2_orf1	-	-	-	TP53-binding protein 1-like [Ostrinia furnacalis]	0.98281	1.40753	-0.48369	-0.9792	-0.92745
TRINITY_DN3355_c0_g1_i1_orf1	-	-	-	UDP-glucuronosyltransferase 2B2-like [Ostrinia furnacalis]	1.77458	-0.40641	0.24889	-0.41085	-1.20621
TRINITY_DN19303_c0_g1_i5_orf1	-	-	-	lipopolysaccharide-induced tumor necrosis factor-alpha factor-like [Ostrinia furnacalis]	0.15729	1.18704	-1.58906	-0.58866	0.8334
TRINITY_DN13186_c0_g1_i1_orf1	-	-	-	NADH dehydrogenase [ubiquinone] iron-sulfur protein 5-like [Bicyclus anynana]	1.83473	-0.76784	0.27476	-0.85611	-0.48555
TRINITY_DN40028_c0_g1_i1_orf1	-	-	-	signal recognition particle receptor subunit alpha homolog [Ostrinia furnacalis]	1.69412	-0.33165	-1.38491	-0.21432	0.23677
TRINITY_DN6567_c0_g1_i5_orf1	-	-	-	dymeclin isoform X1 [Ostrinia furnacalis] >XP_028159584.1 dymeclin isoform X2 [Ostrinia furnacalis]	1.30383	0.4583	-1.73697	-0.20311	0.17794
TRINITY_DN8908_c0_g1_i1_orf1	-	-	-	unnamed protein product [Spodoptera littoralis] >CAH1641822.1 unnamed protein product [Spodoptera littoralis]	1.75468	-1.01641	-0.57384	-0.60433	0.4399
TRINITY_DN35865_c0_g1_i1_orf1	-	-	-	uncharacterized protein LOC114354496 isoform X1 [Ostrinia furnacalis] >XP_028162709.1 uncharacterized protein LOC114354496 isoform X1 [Ostrinia furnacalis]	0.58931	0.75969	-1.86031	-0.23635	0.74765
TRINITY_DN42269_c2_g1_i1_orf1	-	-	-	probable enoyl-CoA hydratase, mitochondrial [Ostrinia furnacalis]	1.8586	-0.71448	0.01612	-0.1548	-1.00544
TRINITY_DN5593_c0_g1_i1_orf1	-	-	-	PREDICTED: leucine-rich repeat-containing protein 47-like [Fopius arisanus]	1.68177	-0.29818	-0.32133	-1.37235	0.31009
TRINITY_DN6103_c0_g1_i6_orf1	-	-	-	sedoheptulokinase-like [Ostrinia furnacalis]	1.66278	0.56554	-1.15277	-0.47432	-0.60123
TRINITY_DN15811_c0_g1_i7_orf1	-	-	-	mitochondrial import inner membrane translocase subunit Tim10-like [Ostrinia furnacalis] >XP_028174557.1 mitochondrial import inner membrane translocase subunit Tim10 [Ostrinia furnacalis] >XP_028174558.1 mitochondrial import inner membrane translocase subunit Tim10 [Ostrinia furnacalis] >XP_028174559.1 mitochondrial import inner membrane translocase subunit Tim10 [Ostrinia furnacalis]	1.64591	-1.14466	-0.30047	-0.76004	0.55926
TRINITY_DN54134_c0_g1_i1_orf1	-	-	-	NFU1 iron-sulfur cluster scaffold homolog, mitochondrial-like [Ostrinia furnacalis]	1.73374	0.37978	-0.57244	-1.17967	-0.36141
TRINITY_DN4429_c0_g1_i5_orf1	-	-	-	FACT complex subunit Ssrp1 isoform X1 [Ostrinia furnacalis] >XP_028173375.1 FACT complex subunit Ssrp1 isoform X2 [Ostrinia furnacalis] >XP_028173376.1 FACT complex subunit Ssrp1 isoform X3 [Ostrinia furnacalis]	1.78272	0.32037	-0.35075	-0.7017	-1.05064
TRINITY_DN43076_c0_g1_i6_orf1	-	-	-	protein argonaute-2 isoform X2 [Pectinophora gossypiella]	1.30249	0.99997	-1.2656	-0.80504	-0.23183
TRINITY_DN64810_c0_g1_i1_orf1	-	-	-	arginine--tRNA ligase, cytoplasmic [Ostrinia furnacalis]	1.95154	-0.79375	-0.47455	-0.56839	-0.11485
TRINITY_DN122867_c1_g1_i1_orf1	-	-	-	nuclear migration protein nudC [Ostrinia furnacalis]	1.42466	0.91471	-0.92724	-0.33404	-1.07809

TRINITY_DN13347_c0_g1_i1_orf1	-	-	-	endothelial differentiation-related factor 1 homolog [Ostrinia furnacalis]	1.96592	-0.72891	-0.66117	-0.30937	-0.26647
TRINITY_DN547_c0_g1_i1_orf1	-	-	-	WD repeat-containing protein 43 [Ostrinia furnacalis]	1.85489	-0.57197	-0.17116	-0.01508	-1.09668
TRINITY_DN121_c0_g1_i9_orf1	-	-	-	lethal(2) giant larvae protein homolog 1 isoform X1 [Ostrinia furnacalis]	1.99465	-0.4104	-0.53198	-0.61647	-0.43581
TRINITY_DN7451_c0_g1_i10_orf1	-	-	-	huntingtin-interacting protein 1 isoform X4 [Pectinophora gossypiella]	1.36859	0.72144	-1.44193	-0.72237	0.07426
TRINITY_DN46022_c0_g1_i1_orf1	-	-	-	mRNA-decapping enzyme 1A [Ostrinia furnacalis]	1.70451	0.54563	-0.99176	-0.52532	-0.73306
TRINITY_DN6380_c0_g1_i1_orf1	-	-	-	THAP domain-containing protein 1-like isoform X1 [Ostrinia furnacalis]	1.55023	0.76833	-1.06316	-0.83763	-0.41777
TRINITY_DN7247_c0_g1_i7_orf1	-	-	-	pyruvate kinase-like isoform X3 [Ostrinia furnacalis]	1.44685	0.80446	-1.36488	-0.40007	-0.48635
TRINITY_DN37856_c0_g1_i5_orf1	-	-	-	spermine synthase [Ostrinia furnacalis]	1.71114	-0.69585	-1.0427	-0.4864	0.51381
TRINITY_DN2571_c0_g2_i1_orf1	-	-	-	PREDICTED: huntingtin-interacting protein K isoform X1 [Amyelois transitella] >XP_013190319.1 PREDICTED: huntingtin-interacting protein K isoform X2 [Amyelois transitella] >XP_021200735.1 huntingtin-interacting protein K [Helicoverpa armigera] >XP_026737126.1 huntingtin-interacting protein K [Trichoplusia ni] >XP_041970494.1 huntingtin-interacting protein K [Aricia agestis] >XP_047022511.1 huntingtin-interacting protein K [Helicoverpa zea] >XP_047984206.1 huntingtin-interacting protein K [Leguminivora glycinivorella] >RVE51917.1 hypothetical protein evm_003383 [Chilo suppressalis] >PZC85551.1 hypothetical protein B5X24_HaOG216659 [Helicoverpa armigera] >CAB3530729.1 unnamed protein product [Chilo suppressalis] >CAH0407320.1 unnamed	1.95339	-0.71047	-0.32905	-0.73409	-0.17978
TRINITY_DN21218_c0_g2_i3_orf1	-	-	-	leukotriene A-4 hydrolase isoform X2 [Ostrinia furnacalis]	1.78678	0.39419	-0.71538	-0.91488	-0.5507
TRINITY_DN23089_c0_g1_i1_orf1	-	-	-	integrator complex subunit 3 homolog [Ostrinia furnacalis]	1.63143	0.01287	-1.1357	-0.9322	0.4236
TRINITY_DN2642_c0_g1_i5_orf1	-	-	-	protein LSM12 homolog [Ostrinia furnacalis]	1.79136	-1.04784	-0.79327	-0.15198	0.20174
TRINITY_DN7647_c0_g1_i4_orf1	-	-	-	E3 ubiquitin-protein ligase Bre1 isoform X6 [Ostrinia furnacalis]	1.74752	0.01027	-1.05855	-0.88864	0.18939
TRINITY_DN3588_c0_g1_i1_orf1	-	-	-	probable peroxisomal acyl-coenzyme A oxidase 1 [Ostrinia furnacalis]	1.84004	-1.03358	-0.61119	-0.37455	0.17927
TRINITY_DN493_c0_g1_i4_orf1	-	-	-	ADP-ribosylation factor GTPase-activating protein 3 [Ostrinia furnacalis]	1.637	0.43613	-1.21874	-0.80117	-0.05322
TRINITY_DN7770_c0_g1_i4_orf1	-	-	-	presequence protease, mitochondrial [Ostrinia furnacalis]	1.92349	-0.20796	-0.39261	-0.32401	-0.99891
TRINITY_DN605_c0_g1_i4_orf1	-	-	-	dnaJ homolog subfamily C member 7 [Ostrinia furnacalis]	1.96236	-0.1367	-0.53947	-0.72149	-0.5647
TRINITY_DN33178_c0_g1_i1_orf1	-	-	-	synaptojanin-1 [Ostrinia furnacalis]	1.84277	0.12121	-1.0747	-0.58448	-0.30481
TRINITY_DN54711_c0_g1_i1_orf1	-	-	-	39S ribosomal protein L50, mitochondrial [Ostrinia furnacalis]	1.92362	-0.73542	-0.83395	-0.15979	-0.19446
TRINITY_DN9062_c0_g2_i3_orf1	-	-	-	ubiquitin conjugation factor E4 B isoform X2 [Ostrinia furnacalis]	1.23562	1.205	-0.96776	-0.73752	-0.73534
TRINITY_DN10399_c0_g1_i2_orf1	-	-	-	unnamed protein product [Chilo suppressalis]	1.93833	-0.29382	-0.93599	-0.47565	-0.23286
TRINITY_DN63719_c0_g1_i5_orf1	-	-	-	eukaryotic peptide chain release factor GTP-binding subunit ERF3A isoform X2 [Manduca sexta] >KAG6462744.1 hypothetical protein O3G_MSEX013441 [Manduca sexta]	1.88792	-0.34539	-1.07968	-0.08371	-0.37914
TRINITY_DN3924_c0_g1_i5_orf1	-	-	-	SH3 domain-containing kinase-binding protein 1-like isoform X1 [Ostrinia furnacalis]	1.75811	0.06345	-1.2953	-0.47377	-0.05248
TRINITY_DN3113_c1_g2_i1_orf1	-	-	-	short-chain dehydrogenase/reductase family 16C member 6-like [Ostrinia furnacalis] >XP_028174076.1 short-chain dehydrogenase/reductase family 16C member 6-like [Ostrinia furnacalis]	1.68137	0.49419	-1.11447	-0.7793	-0.28179
TRINITY_DN4810_c0_g1_i3_orf1	-	-	-	clathrin interactor 1 isoform X2 [Maniola jurtina]	1.92375	-0.12654	-0.95497	-0.51205	-0.33018
TRINITY_DN3411_c0_g2_i1_orf1	-	-	-	putative U5 small nuclear ribonucleoprotein 200 kDa helicase, partial [Ostrinia furnacalis]	1.35682	0.59473	-1.55367	0.19622	-0.59409
TRINITY_DN34689_c0_g1_i4_orf1	-	-	-	uncharacterized protein DDB_G0283357 isoform X13 [Helicoverpa armigera] >XP_049707197.1 uncharacterized protein DDB_G0283357 isoform X14 [Helicoverpa armigera] >XP_049707198.1 uncharacterized protein DDB_G0283357 isoform X15 [Helicoverpa armigera] >XP_049707199.1 uncharacterized protein DDB_G0283357 isoform X16 [Helicoverpa armigera] >XP_049707200.1 uncharacterized protein DDB_G0283357 isoform X17 [Helicoverpa armigera] >XP_049707201.1 uncharacterized protein DDB_G0283357 isoform X18 [Helicoverpa armigera] >XP_049707202.1 uncharacterized protein DDB_G0283357 isoform X19 [Helicoverpa armigera] >XP_049707203.1 uncharacterized protein DDB_G0283357 isoform X20 [Helicoverpa armigera] >XP_049707204.1 uncharacterized protein DDB_G0283357 isoform X21 [Helicoverpa armigera] >XP_049707205.1 uncharacterized protein DDB_G0283357 isoform X22 [Helicoverpa armigera] >XP_049707206.1 uncharacterized protein DDB_G0283357 isoform X23 [Helicoverpa armigera] >XP_049707207.1 uncharacterized protein DDB_G0283357 isoform X24 [Helicoverpa armigera]	1.6624	0.64305	-0.9152	-0.79235	-0.5979
TRINITY_DN17726_c0_g1_i1_orf1	-	-	-	ubiquitin conjugation factor E4 A isoform X1 [Ostrinia furnacalis] >XP_028165923.1 ubiquitin conjugation factor E4 A isoform X2 [Ostrinia furnacalis]	1.71835	0.40813	-1.04763	-0.85661	-0.22225
TRINITY_DN9765_c0_g1_i6_orf1	-	-	-	hypothetical protein evm_005049 [Chilo suppressalis] >CAB3525510.1 unnamed protein product [Chilo suppressalis] >CAH0402837.1 unnamed protein product [Chilo suppressalis]	1.0182	1.28814	-1.29686	-0.74201	-0.26747
TRINITY_DN65988_c0_g1_i4_orf1	-	-	-	uncharacterized protein LOC114354070 isoform X3 [Ostrinia furnacalis]	1.83783	-0.34951	-1.11699	-0.48867	0.11735

TRINITY_DN1826_c0_g2_i4_orf1	-	-	-	glutaminyl-peptide cyclotransferase-like [Ostrinia furnacalis]	1.95417	-0.1198	-0.50293	-0.78312	-0.54833
TRINITY_DN12777_c0_g1_i5_orf1	-	-	-	clathrin light chain isoform X2 [Ostrinia furnacalis]	0.33592	1.25539	-1.58154	-0.64121	0.63144
TRINITY_DN10266_c0_g1_i5_orf1	-	-	-	armadillo repeat-containing protein 6 homolog [Ostrinia furnacalis]	1.6233	-0.04819	-1.47223	-0.35952	0.25663
TRINITY_DN5346_c0_g1_i5_orf1	-	-	-	syntaxin-1A isoform X2 [Pectinophora gossypiella]	1.04862	0.87033	-1.20903	-1.2003	0.49038
TRINITY_DN47723_c0_g1_i1_orf1	-	-	-	dnaJ homolog subfamily C member 21 [Ostrinia furnacalis]	1.49127	0.90825	-0.69624	-0.9407	-0.76258
TRINITY_DN6572_c0_g1_i2_orf1	-	-	-	zinc finger protein 330 homolog [Ostrinia furnacalis]	1.65946	0.46676	-1.1728	-0.79165	-0.16177
TRINITY_DN100885_c0_g2_i1_orfp1	-	-	-	CCHC-type zinc finger, partial [Cricetulus griseus]	1.95976	-0.45067	-0.79943	-0.17406	-0.5356
TRINITY_DN25997_c1_g1_i1_orf1	-	-	-	uncharacterized protein LOC114366342 [Ostrinia furnacalis]	1.81469	0.1646	-0.55938	-1.13353	-0.28639
TRINITY_DN11124_c0_g1_i4_orf1	-	-	-	hypothetical protein O3G_MSEX014253 [Manduca sexta]	1.97311	-0.22835	-0.53424	-0.74051	-0.47002
TRINITY_DN821_c0_g1_i8_orf1	-	-	-	nuclear pore complex protein Nup153 isoform X2 [Ostrinia furnacalis]	1.9718	-0.27668	-0.47709	-0.79075	-0.42727
TRINITY_DN2826_c0_g1_i7_orf1	-	-	-	ATP-binding cassette subfamily D member 1 [Chilo suppressalis] >CAB3531327.1 unnamed protein product [Chilo suppressalis] >CAH0407919.1 unnamed protein product [Chilo suppressalis]	1.63828	0.22299	-0.63462	0.13208	-1.35872
TRINITY_DN7670_c0_g1_i1_orf1	-	-	-	striatin-interacting protein 1 [Ostrinia furnacalis]	1.29118	1.14576	-0.8642	-0.65131	-0.92143
TRINITY_DN43355_c0_g1_i1_orf1	-	-	-	uncharacterized protein CG16817-like [Ostrinia furnacalis]	1.76682	0.40952	-1.0203	-0.60474	-0.5513
TRINITY_DN16933_c0_g1_i10_orf1	-	-	-	uridine phosphorylase 1-like [Ostrinia furnacalis] >XP_028164122.1 uridine phosphorylase 1-like [Ostrinia furnacalis]	1.87393	-0.32578	-1.05641	0.02373	-0.51546
TRINITY_DN437_c0_g1_i1_orf1	-	-	-	LOW QUALITY PROTEIN: fibronectin type-III domain-containing protein 3A-like [Ostrinia furnacalis]	1.75865	-0.15305	-0.8239	-1.06144	0.27975
TRINITY_DN26429_c0_g1_i4_orf1	-	-	-	zinc transporter 9 [Ostrinia furnacalis]	1.78489	0.37631	-0.96076	-0.72058	-0.47985
TRINITY_DN33024_c0_g1_i1_orf1	-	-	-	hypothetical protein evm_000959 [Chilo suppressalis]	0.70644	-0.01501	-1.79884	1.12453	-0.01712
TRINITY_DN19079_c0_g1_i5_orf1	-	-	-	unnamed protein product [Euphydryas editha]	1.63999	0.54989	-1.04589	-0.93242	-0.21158
TRINITY_DN48096_c0_g2_i2_orf1	-	-	-	eukaryotic translation initiation factor 4E-like [Ostrinia furnacalis]	1.54646	-0.26389	-1.50347	-0.24593	0.46683
TRINITY_DN2047_c0_g1_i1_orf1	-	-	-	carboxylesterase CXE17 [Ostrinia furnacalis]	1.98788	-0.4797	-0.45368	-0.69523	-0.35927
TRINITY_DN397_c0_g1_i1_orf1	-	-	-	striatin-3 isoform X1 [Ostrinia furnacalis]	1.16791	1.22499	-1.18433	-0.63898	-0.56959
TRINITY_DN11616_c0_g1_i3_orf1	-	-	-	coiled-coil domain-containing protein 6-like [Ostrinia furnacalis]	1.6739	0.56926	-1.00883	-0.39983	-0.8345
TRINITY_DN3299_c0_g1_i2_orf1	-	-	-	metaxin-1 isoform X3 [Ostrinia furnacalis] >XP_028170907.1 metaxin-1 isoform X4 [Ostrinia furnacalis]	1.87783	-0.51566	-0.49138	-0.97727	0.10648
TRINITY_DN4861_c0_g1_i7_orf1	-	-	-	2-hydroxyacyl-CoA lyase 1 isoform X1 [Ostrinia furnacalis]	1.96999	-0.80209	-0.297	-0.37085	-0.50004
TRINITY_DN115_c0_g1_i6_orf1	-	-	-	basigin [Ostrinia furnacalis]	1.85199	0.22669	-0.91677	-0.54054	-0.62137
TRINITY_DN88640_c0_g1_i1_orf1	-	-	-	tetratricopeptide repeat protein 27-like [Ostrinia furnacalis]	1.88364	-0.49042	-0.62425	0.12837	-0.89734
TRINITY_DN1170_c0_g1_i8_orf1	-	-	-	titin homolog [Trichoplusia ni]	1.64949	0.53376	-1.03699	-0.93505	-0.21121
TRINITY_DN40281_c0_g2_i1_orf1	-	-	-	glyoxylate reductase/hydroxypyruvate reductase [Ostrinia furnacalis]	1.84791	-0.77913	-0.63544	0.27349	-0.70682
TRINITY_DN1066_c0_g1_i8_orf1	-	-	-	hypothetical protein evm_012420 [Chilo suppressalis]	0.13392	1.40167	-1.21736	-1.02073	0.70251
TRINITY_DN20776_c0_g1_i3_orf1	-	-	-	hypothetical protein evm_003273 [Chilo suppressalis] >CAH2981954.1 unnamed protein product [Chilo suppressalis]	1.97598	-0.34109	-0.7735	-0.36046	-0.50093
TRINITY_DN37366_c0_g1_i7_orf1	-	-	-	juvenile hormone epoxide hydrolase-like [Ostrinia furnacalis]	1.81773	-0.09797	-0.94592	0.1091	-0.88294
TRINITY_DN2783_c1_g1_i2_orf1	-	-	-	proline-rich extensin-like protein EPR1 isoform X1 [Ostrinia furnacalis] >XP_028168549.1 proline-rich extensin-like protein EPR1 isoform X2 [Ostrinia furnacalis] >XP_028168550.1 proline-rich extensin-like protein EPR1 isoform X2 [Ostrinia furnacalis]	1.79178	0.40543	-0.64446	-0.72987	-0.82288
TRINITY_DN9558_c0_g1_i2_orf1	-	-	-	NADH dehydrogenase [ubiquinone] iron-sulfur protein 4, mitochondrial-like [Ostrinia	1.90297	-0.61971	0.11139	-0.62763	-0.76702
TRINITY_DN31751_c0_g1_i5_orf1	-	-	-	THO complex subunit 3 [Ostrinia furnacalis]	1.91037	0.06919	-0.61816	-0.81646	-0.54494
TRINITY_DN4152_c0_g1_i1_orf1	-	-	-	importin subunit beta-1 isoform X2 [Ostrinia furnacalis]	1.49974	0.87763	-0.81993	-0.99718	-0.56026
TRINITY_DN1661_c0_g1_i1_orf1	-	-	-	NAD(P) transhydrogenase, mitochondrial-like [Ostrinia furnacalis] >XP_028175067.1 NAD(P) transhydrogenase, mitochondrial-like [Ostrinia furnacalis] >XP_028175068.1 NAD(P) transhydrogenase, mitochondrial-like [Ostrinia furnacalis] >XP_028175069.1 NAD(P) transhydrogenase, mitochondrial-like [Ostrinia furnacalis]	1.18743	1.17694	-1.26517	-0.55454	-0.54466
TRINITY_DN11612_c0_g3_i1_orf1	-	-	-	hypothetical protein O3G_MSEX005294 [Manduca sexta]	1.80689	0.11843	-1.19767	-0.25905	-0.46859
TRINITY_DN27771_c0_g1_i1_orf1	-	-	-	glycine--tRNA ligase [Ostrinia furnacalis]	1.36704	0.41561	-1.49526	-0.72738	0.43999
TRINITY_DN3472_c1_g1_i4_orf1	-	-	-	Krueppel homolog 2-like [Ostrinia furnacalis]	1.18275	1.06893	-1.46227	-0.32878	-0.46063
TRINITY_DN10007_c0_g1_i1_orf1	-	-	-	28S ribosomal protein S31, mitochondrial [Ostrinia furnacalis]	1.82583	-0.93138	0.18159	-0.23229	-0.84376
TRINITY_DN639_c0_g1_i10_orf1	-	-	-	unnamed protein product, partial [Iphiclides podalirius]	1.69596	-0.88206	-0.93949	-0.41426	0.53985
TRINITY_DN1494_c0_g2_i1_orf1	-	-	-	dihydropyrimidine dehydrogenase [NADP(+)] [Ostrinia furnacalis]	1.97751	-0.25728	-0.50441	-0.72984	-0.48598
TRINITY_DN2876_c0_g1_i3_orf1	-	-	-	long-chain fatty acid transport protein 4-like isoform X1 [Ostrinia furnacalis]	1.78276	0.31322	-0.28524	-0.94345	-0.86729

TRINITY_DN9156_c0_g1_i1_orf1	-	-	-	GTP:AMP phosphotransferase AK3, mitochondrial [Ostrinia furnacalis]	1.89235	-0.33521	-0.29769	-0.1688	-1.09066
TRINITY_DN16187_c0_g1_i1_orf1	-	-	-	eIF-2-alpha kinase activator GCN1 [Colias croceus]	1.61431	-0.67728	-1.30857	-0.09163	0.46317
TRINITY_DN10385_c0_g1_i5_orf1	-	-	-	unnamed protein product [Arctia plantaginis] >CAB3259747.1 unnamed protein product [Arctia plantaginis]	1.10536	1.19196	-1.39572	-0.48909	-0.41251
TRINITY_DN57462_c0_g1_i1_orf1	-	-	-	glutathione S transferase-S5 [Glyphodes pyloalis]	1.67025	0.25295	-0.29127	-0.21187	-1.42006
TRINITY_DN22941_c0_g1_i1_orf1	-	-	-	protein CWC15 homolog [Ostrinia furnacalis]	1.91417	-0.96726	-0.50999	-0.3682	-0.06872
TRINITY_DN48838_c0_g1_i6_orf1	-	-	-	merlin-like [Ostrinia furnacalis]	1.76654	0.37802	-1.06378	-0.64229	-0.43849
TRINITY_DN332_c0_g1_i6_orf1	-	-	-	dolichyl-diphosphooligosaccharide--protein glycosyltransferase subunit STT3B isoform X2 [Ostrinia furnacalis]	1.78072	-0.65192	-1.09001	0.30859	-0.34738
TRINITY_DN36682_c0_g1_i1_orf1	-	-	-	uncharacterized protein DDB_G0287625-like [Ostrinia furnacalis]	1.91691	-0.74954	-0.82216	-0.05403	-0.29117
TRINITY_DN607_c0_g1_i16_orf1	-	-	-	protein muscleblind isoform X1 [Ostrinia furnacalis] >XP_028162801.1 protein muscleblind isoform X1 [Ostrinia furnacalis] >XP_028162802.1 protein muscleblind isoform X1 [Ostrinia furnacalis] >XP_028162803.1 protein muscleblind isoform X1 [Ostrinia furnacalis] >XP_028162804.1 protein muscleblind isoform X1 [Ostrinia furnacalis] >XP_028162806.1 protein muscleblind isoform X1 [Ostrinia furnacalis]	1.70602	0.48259	-1.14117	-0.57735	-0.4701
TRINITY_DN30224_c0_g1_i1_orf1	-	-	-	PREDICTED: serine--tRNA ligase, mitochondrial [Amyelois transitella]	1.83442	-0.81853	-0.90856	0.20486	-0.31219
TRINITY_DN11409_c0_g1_i4_orf1	-	-	-	nodal modulator 1 [Ostrinia furnacalis]	1.87914	-0.12581	-1.00368	-0.66175	-0.0879
TRINITY_DN24323_c0_g1_i3_orf1	-	-	-	ubiquitin-protein ligase E3A [Ostrinia furnacalis]	1.13278	0.70077	-1.75121	-0.32013	0.23779
TRINITY_DN51050_c0_g1_i3_orf1	-	-	-	GRB10-interacting GYF protein 2 isoform X1 [Ostrinia furnacalis]	1.58586	0.55788	-1.36874	-0.39262	-0.38239
TRINITY_DN14705_c0_g2_i1_orf1	-	-	-	coiled-coil-helix-coiled-coil-helix domain-containing protein 10, mitochondrial [Ostrinia furnacalis]	1.68348	-0.03447	0.36458	-1.05448	-0.9591
TRINITY_DN5559_c0_g1_i1_orf1	-	-	-	ferrochelatase, mitochondrial isoform X2 [Ostrinia furnacalis]	1.95003	-0.63313	-0.23136	-0.26546	-0.82008
TRINITY_DN6668_c0_g1_i4_orf1	-	-	-	UBX domain-containing protein 4 isoform X1 [Ostrinia furnacalis] >XP_028156702.1 UBX domain-containing protein 4 isoform X2 [Ostrinia furnacalis]	1.78787	-1.01648	0.23601	-0.82553	-0.18187
TRINITY_DN28509_c0_g1_i1_orf1	-	-	-	39S ribosomal protein L43, mitochondrial [Ostrinia furnacalis]	1.82792	-0.07553	-0.04124	-0.54957	-1.16158
TRINITY_DN2258_c0_g2_i1_orf1	-	-	-	60S ribosomal protein L14 [Ostrinia furnacalis]	1.91725	-0.31883	-0.65744	-0.88736	-0.05362
TRINITY_DN2497_c0_g1_i1_orf1	-	-	-	protein stunted-like isoform X2 [Vanessa tameamea] >XP_046960183.1 protein stunted-like isoform X2 [Vanessa cardui] >XP_047527093.1 protein stunted-like isoform X2 [Vanessa	1.24143	0.58633	-0.21911	0.13726	-1.74591
TRINITY_DN5512_c0_g1_i8_orf1	-	-	-	peroxisomal multifunctional enzyme type 2-like isoform X1 [Ostrinia furnacalis]	1.86661	-0.0781	-1.13441	-0.3936	-0.26051
TRINITY_DN2802_c1_g1_i1_orf1	-	-	-	psi [Ostrinia furnacalis]	1.25928	0.99697	-0.98971	-1.19837	-0.06818
TRINITY_DN69049_c0_g2_i1_orf1	-	-	-	membrane alanyl aminopeptidase-like [Ostrinia furnacalis]	1.63304	0.17825	0.02351	-1.4731	-0.3617
TRINITY_DN3488_c0_g1_i2_orf1	-	-	-	hsp70-binding protein 1 isoform X1 [Ostrinia furnacalis] >XP_028170128.1 hsp70-binding protein 1 isoform X2 [Ostrinia furnacalis]	1.82638	0.13335	-0.58226	-1.11231	-0.26515
TRINITY_DN1368_c0_g1_i6_orf1	-	-	-	hypothetical protein SFRUCORN_009408 [Spodoptera frugiperda]	1.80408	0.11754	-1.09514	-0.1045	-0.72197
TRINITY_DN2967_c0_g1_i4_orf1	-	-	-	UDP-glycosyltransferase UGT41G1 [Ostrinia furnacalis]	1.87142	-0.72378	-0.12413	-0.04553	-0.97798
TRINITY_DN10058_c0_g1_i1_orf1	-	-	-	dolichyl-diphosphooligosaccharide--protein glycosyltransferase subunit DAD1 [Ostrinia	1.94212	-0.72208	-0.75087	-0.10628	-0.36289
TRINITY_DN18696_c0_g1_i1_orf1	-	-	-	rap guanine nucleotide exchange factor 2 [Ostrinia furnacalis] >XP_028159576.1 rap guanine nucleotide exchange factor 2 [Ostrinia furnacalis]	1.9374	-0.74032	-0.49042	-0.67598	-0.03069
TRINITY_DN30950_c0_g1_i13_orf1	-	-	-	unnamed protein product [Chilo suppressalis]	1.64044	0.37533	-1.15685	-0.90952	0.05061
TRINITY_DN11121_c0_g1_i5_orf1	-	-	-	unnamed protein product [Chilo suppressalis]	1.88124	-0.85909	-0.7818	-0.32365	0.0833
TRINITY_DN2356_c2_g1_i6_orf1	-	-	-	ER membrane protein complex subunit 3 [Ostrinia furnacalis]	1.59098	-0.13588	-1.06998	-0.97719	0.59208
TRINITY_DN14475_c0_g1_i1_orf1	-	-	-	NPC intracellular cholesterol transporter 1 homolog 1b-like [Ostrinia furnacalis]	1.83988	-0.78072	-0.27246	0.16416	-0.95086
TRINITY_DN5976_c0_g1_i1_orf1	-	-	-	ribosomal protein L32 [Bombyx mori] >XP_028034407.1 60S ribosomal protein L32 [Bombyx mandarina] >AAV34844.1 ribosomal protein L32 [Bombyx mori]	1.9104	-0.81203	-0.68036	-0.47425	0.05624
TRINITY_DN19413_c0_g1_i2_orf1	-	-	-	U4/U6 small nuclear ribonucleoprotein Prp4 [Papilio xuthus]	1.94203	-0.69198	-0.71296	-0.04817	-0.48892
TRINITY_DN31303_c0_g1_i4_orf1	-	-	-	tRNA pseudouridine synthase A isoform X1 [Ostrinia furnacalis]	1.95065	-0.19578	-0.61461	-0.82388	-0.31639
TRINITY_DN38341_c0_g2_i2_orf1	-	-	-	delta(24)-sterol reductase-like [Ostrinia furnacalis]	1.97622	-0.70874	-0.2878	-0.61138	-0.36831
TRINITY_DN17212_c0_g1_i6_orf1	-	-	-	putative deoxyribonuclease TATDN1 [Ostrinia furnacalis]	1.82688	0.31153	-0.71163	-0.56988	-0.8569
TRINITY_DN1772_c1_g3_i1_orf1	-	-	-	protein Red isoform X1 [Ostrinia furnacalis] >XP_028157673.1 protein Red isoform X2 [Ostrinia furnacalis] >XP_028157674.1 protein Red isoform X3 [Ostrinia furnacalis] >XP_028157675.1 protein Red isoform X4 [Ostrinia furnacalis]	1.7625	-0.29517	-1.12431	-0.66321	0.32019
TRINITY_DN2630_c0_g3_i3_orf1	-	-	-	eukaryotic translation initiation factor 4E-binding protein 2 [Ostrinia furnacalis]	1.81823	-0.35787	0.18898	-1.1163	-0.53304
TRINITY_DN32822_c0_g1_i1_orf1	-	-	-	eukaryotic translation initiation factor 2 subunit 1 [Ostrinia furnacalis]	1.96211	-0.56478	-0.81089	-0.26468	-0.32177
TRINITY_DN31232_c1_g1_i9_orf1	-	-	-	PREDICTED: elongation factor 1-alpha 1, partial [Haliaeetus albicilla]	1.93574	-0.75741	-0.04932	-0.70502	-0.42398

TRINITY_DN749_c0_g1_i1_orf1	-	-	-	serine/threonine-protein phosphatase 4 regulatory subunit 3 isoform X3 [Ostrinia furnacalis]	1.2095	1.2125	-1.04081	-0.56843	-0.81276
TRINITY_DN277_c1_g1_i1_orf1	-	-	-	uncharacterized protein LOC114363802 isoform X4 [Ostrinia furnacalis]	0.69836	1.26086	-1.69507	-0.04734	-0.21681
TRINITY_DN17446_c0_g1_i1_orf1	-	-	-	eukaryotic translation initiation factor 3 subunit E [Ostrinia furnacalis]	1.93567	-0.7033	-0.63661	-0.5944	-0.00137
TRINITY_DN4272_c0_g1_i1_orf1	-	-	-	MICOS complex subunit MIC19-like [Ostrinia furnacalis]	1.83045	-0.65839	-1.09101	0.06524	-0.14629
TRINITY_DN817_c0_g1_i3_orf1	-	-	-	phenylalanine--tRNA ligase beta subunit [Ostrinia furnacalis]	1.58954	-0.84789	-1.11314	-0.28667	0.65817
TRINITY_DN50724_c0_g2_i1_orf1	-	-	-	40S ribosomal protein S13 [Papilio polytes] >NP_001299165.1 40S ribosomal protein S13 [Papilio xuthus] >XP_013193651.1 PREDICTED: 40S ribosomal protein S13 [Amyelois transitella] >XP_014356884.1 40S ribosomal protein S13 [Papilio machaon] >XP_021184589.1 40S ribosomal protein S13 [Helicoverpa armigera] >XP_022827875.1 40S ribosomal protein S13 [Spodoptera litura] >XP_023936121.1 40S ribosomal protein S13 [Bicyclus anynana] >XP_026318936.1 40S ribosomal protein S13 [Hyposmocoma kahamanoa] >XP_026488656.1 40S ribosomal protein S13 [Vanessa tameamea] >XP_026736523.1 40S ribosomal protein S13 [Trichoplusia ni] >XP_028172792.1 40S ribosomal protein S13 [Ostrinia furnacalis] >XP_032516773.1 40S ribosomal protein S13 [Danaus plexippus plexippus] >XP_034829282.1 40S ribosomal protein S13 [Maniola hyperantus] >XP_035450454.1 40S ribosomal protein S13 [Spodoptera frugiperda] >XP_039754671.1 40S ribosomal protein S13 [Pararge aegeria] >XP_045449727.1 40S ribosomal protein S13 [Melitaea cinxia] >XP_046977814.1 40S ribosomal protein S13 [Vanessa cardui] >XP_047024943.1 40S ribosomal protein S13 [Helicoverpa zea] >XP_047531020.1 40S ribosomal protein S13 [Vanessa atalanta] >XP_047990290.1 40S ribosomal protein S13 [Leguminivora glycinivorella] >XP_050348531.1 40S ribosomal protein S13 [Nymphalis io] >Q962R6.3 RecName: Full=40S ribosomal protein S13 [Spodoptera frugiperda] >ADT80641.1 ribosomal protein S13 [Euphydryas aurinia] >ATG34155.1 ribosomal protein S13 [Epirrita autumnata] >KAF9422710.1 hypothetical protein HW555_001704 [Spodoptera exigua] >KAG7298871.1 ribosomal 40S subunit protein S13 [Plutella xylostella] >KAI5637622.1 ribosomal protein s15 domain-containing protein [Phthorimaea operculella] >RVE47566.1 hypothetical protein evm_007764 [Chilo suppressalis] >UNW37540.1 ribosomal protein S13 [Sesamia inferens] >CAB3507114.1 unnamed protein product [Spodoptera littoralis] >CAD0201945.1 unnamed protein product [Chrysodeixis includens] >CAG4974609.1 unnamed protein product [Parnassius apollo] >CAG9562339.1 unnamed protein product [Danaus chrysippus] >CAG9749269.1 unnamed protein product [Diatraea saccharalis] >CAH0720232.1 unnamed protein product, partial [Brenthis ino] >CAH2046814.1 unnamed protein product, partial [Iphiclides podalirius] >CAH2085029.1	1.93018	-0.64208	-0.78825	-0.49062	-0.00923
TRINITY_DN13233_c0_g1_i3_orf1	-	-	-	60S ribosomal protein L31 [Galleria mellonella] >XP_028158009.1 60S ribosomal protein L31 [Ostrinia furnacalis] >XP_030037192.1 60S ribosomal protein L31 [Manduca sexta] >XP_046978528.1 60S ribosomal protein L31 [Vanessa cardui] >XP_047545474.1 60S ribosomal protein L31 [Vanessa atalanta] >XP_050342244.1 60S ribosomal protein L31 [Nymphalis io] >GBP35474.1 60S ribosomal protein L31 [Eumeta japonica] >ACY95330.1 ribosomal protein L31 [Manduca sexta] >KAG6463984.1 hypothetical protein O3G_MSEX014198 [Manduca sexta] >KAG6463985.1 hypothetical protein O3G_MSEX014198	1.73542	-1.00127	-0.75693	-0.4428	0.46558
TRINITY_DN647_c4_g1_i1_orf1	-	-	-	coiled-coil domain-containing protein 51-like isoform X2 [Ostrinia furnacalis]	1.84751	-0.08646	-1.08506	-0.63243	-0.04357
TRINITY_DN20279_c0_g1_i1_orf1	-	-	-	NADH dehydrogenase [ubiquinone] iron-sulfur protein 3, mitochondrial [Ostrinia furnacalis]	1.97801	-0.7421	-0.28275	-0.51238	-0.44078
TRINITY_DN8012_c0_g1_i3_orf1	-	-	-	uncharacterized protein LOC114354053 [Ostrinia furnacalis]	1.89163	0.06858	-0.63581	-0.40164	-0.92275
TRINITY_DN7228_c0_g1_i6_orf1	-	-	-	neutral alpha-glucosidase AB [Ostrinia furnacalis]	1.74987	0.4062	-0.7908	-0.35431	-1.01096
TRINITY_DN101922_c0_g1_i1_orf1	-	-	-	PREDICTED: uncharacterized protein LOC103569676 [Microplitis demolitor]	1.75806	-0.38515	0.2	-0.29461	-1.27831
TRINITY_DN942_c0_g1_i1_orf1	-	-	-	probable nuclear transport factor 2 isoform X1 [Ostrinia furnacalis]	1.13645	1.27419	-1.11222	-0.59949	-0.69892
TRINITY_DN5756_c0_g1_i4_orf1	-	-	-	leucine--tRNA ligase, cytoplasmic [Ostrinia furnacalis]	1.8363	-0.79053	-0.8929	-0.38797	0.2351
TRINITY_DN16886_c0_g1_i4_orf1	-	-	-	ER membrane protein complex subunit 1 [Ostrinia furnacalis]	1.97012	-0.73956	-0.5539	-0.47242	-0.20424
TRINITY_DN1824_c0_g2_i2_orf1	-	-	-	branched-chain-amino-acid aminotransferase, cytosolic [Manduca sexta] >KAG6453781.1 hypothetical protein O3G_MSEX008327 [Manduca sexta]	1.84705	-0.37691	-0.44802	0.0903	-1.11241
TRINITY_DN63561_c1_g1_i2_orf1	-	-	-	unnamed protein product [Diatraea saccharalis]	1.69554	0.50402	-1.1236	-0.65998	-0.41599
TRINITY_DN20499_c0_g1_i1_orf1	-	-	-	exosome RNA helicase MTR4 isoform X2 [Ostrinia furnacalis]	1.98885	-0.30069	-0.61225	-0.55272	-0.52319
TRINITY_DN659_c0_g1_i3_orf1	-	-	-	probable malonyl-CoA-acyl carrier protein transacylase, mitochondrial [Ostrinia furnacalis]	1.94117	-0.27477	-0.63503	-0.1831	-0.84827
TRINITY_DN40508_c0_g1_i1_orf1	-	-	-	mRNA turnover protein 4 homolog [Ostrinia furnacalis]	1.87457	-0.34675	-0.11552	-0.28478	-1.12752
TRINITY_DN5952_c0_g1_i6_orf1	-	-	-	LOW QUALITY PROTEIN: phosphoacetylglucosamine mutase [Ostrinia furnacalis]	1.41135	0.98347	-1.08055	-0.72651	-0.58776

TRINITY_DN1443_c0_g1_i4_orf1	-	-	-	ATP-dependent RNA helicase DDX3X isoform X1 [Ostrinia furnacalis]	1.86376	-0.19062	-1.15586	-0.15798	-0.35929
TRINITY_DN2738_c1_g1_i3_orf1	-	-	-	uridine-cytidine kinase isoform X1 [Helicoverpa zea] >XP_049697747.1 uridine-cytidine kinase-like isoform X1 [Helicoverpa armigera] >XP_049698409.1 uridine-cytidine kinase isoform X1 [Helicoverpa armigera]	1.80845	0.35777	-0.86278	-0.7135	-0.58994
TRINITY_DN7161_c0_g1_i7_orf1	-	-	-	ATP-binding cassette sub-family E member 1 [Ostrinia furnacalis]	1.8556	-0.20556	-1.08159	-0.5868	0.01835
TRINITY_DN10502_c0_g1_i4_orf1	-	-	-	interleukin enhancer-binding factor 2 homolog [Ostrinia furnacalis]	1.61346	0.70871	-1.01565	-0.72187	-0.58465
TRINITY_DN6071_c0_g1_i1_orf1	-	-	-	transcription initiation factor IIB isoform X1 [Manduca sexta] >XP_038208211.1 transcription initiation factor IIB isoform X1 [Zerene cesonia] >XP_045510964.1 transcription initiation factor IIB isoform X1 [Colias croceus] >XP_049872283.1 transcription initiation factor IIB [Pectinophora gossypiella]	1.09227	0.7522	-0.98792	-1.40252	0.54597
TRINITY_DN26650_c0_g1_i1_orfp1	-	-	-	TRINITY_DN26650_c0_g1_i1_m.72504 TRINITY_DN26650_c0_g1::TRINITY_DN26650_c0_g1_i1::g.72504 ORF type:5prime_partial len:72 (-),score=2.71 TRINITY_DN26650_c0_g1_i1:317-532(-)	1.93774	-0.65829	-0.61516	-0.65829	-0.00599
TRINITY_DN1316_c0_g1_i1_orf1	-	-	-	mitochondrial import receptor subunit TOM70 [Ostrinia furnacalis]	1.95829	-0.2687	-0.25852	-0.68342	-0.74765
TRINITY_DN8290_c0_g1_i3_orf1	-	-	-	zinc finger CCHC domain-containing protein 8 homolog [Ostrinia furnacalis]	1.59511	0.49477	-1.30788	-0.70285	-0.07916
TRINITY_DN7828_c0_g1_i2_orf1	-	-	-	alpha-N-acetylgalactosaminidase-like isoform X1 [Ostrinia furnacalis] >XP_028171449.1 alpha-N-acetylgalactosaminidase-like isoform X2 [Ostrinia furnacalis]	1.28233	1.15496	-0.96747	-0.68229	-0.78753
TRINITY_DN20339_c0_g1_i3_orf1	-	-	-	ecto-NOX disulfide-thiol exchanger 2-like [Ostrinia furnacalis]	1.35072	0.80429	-1.26609	-0.95953	0.07061
TRINITY_DN98723_c1_g1_i1_orf1	-	-	-	uncharacterized protein LOC114362777 [Ostrinia furnacalis]	1.77842	-1.00132	-0.88175	-0.10835	0.21299
TRINITY_DN1884_c0_g2_i2_orf1	-	-	-	phosphotriesterase-related protein [Ostrinia furnacalis]	1.75551	-0.81053	0.05385	0.11683	-1.11566
TRINITY_DN115498_c0_g1_i1_orf1	-	-	-	fatty acid synthase-like [Ostrinia furnacalis]	1.93161	-0.89002	-0.44803	-0.07331	-0.52024
TRINITY_DN6535_c0_g1_i3_orf1	-	-	-	mRNA export factor [Ostrinia furnacalis]	1.85815	-0.4875	-0.93645	-0.62845	0.19425
TRINITY_DN2224_c0_g2_i1_orf1	-	-	-	tumor susceptibility gene 101 protein [Ostrinia furnacalis]	1.33572	0.43051	-1.72061	-0.20853	0.16291
TRINITY_DN5169_c0_g1_i5_orf1	-	-	-	ero1-like protein isoform X1 [Ostrinia furnacalis]	1.99294	-0.58721	-0.58721	-0.45156	-0.36695
TRINITY_DN23801_c0_g1_i2_orf1	-	-	-	signal recognition particle subunit SRP72 [Pectinophora gossypiella]	1.68095	-0.38499	-1.36972	-0.23454	0.3083
TRINITY_DN344_c1_g1_i1_orf1	-	-	-	chymotrypsin-like serine protease 16 [Ostrinia nubilalis]	1.52301	-1.10006	0.82822	-0.5969	-0.65427
TRINITY_DN3335_c0_g1_i1_orf1	-	-	-	unnamed protein product [Pieris macdunnoughi]	0.50244	0.83144	-1.5196	-0.83715	1.02287
TRINITY_DN14498_c0_g1_i1_orf1	-	-	-	eukaryotic translation initiation factor 2 subunit 2 [Ostrinia furnacalis]	1.75972	-1.02763	-0.76033	-0.35252	0.38076
TRINITY_DN140538_c0_g2_i1_orf1	-	-	-	peptidyl-prolyl cis-trans isomerase NIMA-interacting 1 [Urocitellus parryii]	1.52431	-0.11225	-1.54811	-0.29114	0.42719
TRINITY_DN4451_c0_g1_i1_orf1	-	-	-	uncharacterized protein LOC114361986 isoform X1 [Ostrinia furnacalis] >XP_028173022.1 uncharacterized protein LOC114361986 isoform X2 [Ostrinia furnacalis]	1.86483	-0.55698	0.21827	-0.75957	-0.76655
TRINITY_DN31433_c0_g1_i1_orf1	-	-	-	notchless protein homolog 1 [Ostrinia furnacalis]	1.92904	-0.37491	-0.61353	-0.07065	-0.86996
TRINITY_DN3176_c0_g1_i2_orf1	-	-	-	dnaJ homolog subfamily B member 6 isoform X2 [Ostrinia furnacalis]	1.61141	0.74072	-0.84752	-0.79955	-0.70506
TRINITY_DN2365_c0_g1_i6_orf1	-	-	-	carnitine O-acetyltransferase isoform X2 [Ostrinia furnacalis]	1.81968	0.31489	-0.59872	-0.60707	-0.92877
TRINITY_DN25285_c0_g1_i1_orf1	-	-	-	pancreatic triacylglycerol lipase-like [Ostrinia furnacalis]	1.46218	0.66654	-0.82661	0.0148	-1.31691
TRINITY_DN371_c0_g1_i6_orf1	-	-	-	trypsin beta-like [Ostrinia furnacalis]	1.94819	-0.81986	-0.11788	-0.44196	-0.56849
TRINITY_DN132043_c0_g1_i1_orf1	-	-	-	ankyrin repeat and MYND domain-containing protein 2 [Ostrinia furnacalis]	1.67492	0.57654	-1.00416	-0.81863	-0.42867
TRINITY_DN5562_c0_g1_i3_orf1	-	-	-	cell division cycle and apoptosis regulator protein 1-like [Ostrinia furnacalis]	1.67789	0.63079	-0.78825	-0.69091	-0.82952
TRINITY_DN24469_c0_g2_i2_orf1	-	-	-	unnamed protein product, partial [Brenthis ino]	0.89057	1.4866	-0.95125	-0.51881	-0.90711
TRINITY_DN19727_c0_g1_i7_orf1	-	-	-	dihydrolipoyllysine-residue succinyltransferase component of 2-oxoglutarate dehydrogenase complex, mitochondrial-like [Ostrinia furnacalis] >XP_028160614.1 dihydrolipoyllysine-residue succinyltransferase component of 2-oxoglutarate dehydrogenase complex, mitochondrial-like [Ostrinia furnacalis] >XP_028160615.1 dihydrolipoyllysine-residue succinyltransferase component of 2-oxoglutarate dehydrogenase complex, mitochondrial-like [Ostrinia furnacalis]	1.9144	-0.81662	-0.13068	-0.18066	-0.78644
TRINITY_DN649_c1_g1_i13_orf1	-	-	-	U1 small nuclear ribonucleoprotein 70 kDa isoform X2 [Ostrinia furnacalis]	1.80467	-0.01015	-1.06075	-0.78442	0.05064
TRINITY_DN32161_c0_g1_i1_orf1	-	-	-	uncharacterized protein LOC114352518 [Ostrinia furnacalis]	1.53445	0.30418	0.02329	-0.29102	-1.5709
TRINITY_DN95665_c0_g1_i1_orf1	-	-	-	hypothetical protein HW555_003264 [Spodoptera exigua] >KAH9639693.1 hypothetical protein HF086_017083 [Spodoptera exigua] >CAH0696396.1 unnamed protein product [Spodoptera exigua]	1.95485	-0.35119	-0.28324	-0.88742	-0.433
TRINITY_DN44119_c0_g1_i1_orf1	-	-	-	PREDICTED: GTP-binding protein 128up [Fopius arisanus]	1.96049	-0.84176	-0.242	-0.38817	-0.48855
TRINITY_DN22156_c0_g1_i1_orf1	-	-	-	trafficking protein particle complex subunit 8 [Ostrinia furnacalis]	1.4157	0.79849	-1.13708	-0.04604	-1.03108
TRINITY_DN23824_c0_g1_i1_orf1	-	-	-	28S ribosomal protein S30, mitochondrial [Ostrinia furnacalis]	1.95569	-0.54664	-0.18267	-0.39982	-0.82657
TRINITY_DN87170_c0_g1_i3_orf1	-	-	-	uncharacterized protein LOC114360175 [Ostrinia furnacalis]	1.10539	1.14557	-0.91875	-1.27203	-0.06018

TRINITY_DN7655_c0_g1_i3_orf1	-	-	-	hypothetical protein evm_001118 [Chilo suppressalis] >CAB3522191.1 unnamed protein product [Chilo suppressalis]	1.87657	0.14751	-0.91795	-0.51884	-0.58729
TRINITY_DN4944_c0_g1_i5_orf1	-	-	-	bifunctional glutamate/proline--tRNA ligase [Ostrinia furnacalis]	1.89392	-0.2179	-0.5601	-1.02123	-0.09469
TRINITY_DN14073_c0_g1_i1_orf1	-	-	-	cytochrome c oxidase subunit 4 isoform 1, mitochondrial-like [Ostrinia furnacalis] >XP_028164918.1 cytochrome c oxidase subunit 4 isoform 1, mitochondrial-like [Ostrinia furnacalis]	1.88216	-0.50375	0.13622	-0.61932	-0.89532
TRINITY_DN12584_c0_g1_i1_orf1	-	-	-	carnitine O-palmitoyltransferase 1, liver isoform [Ostrinia furnacalis]	1.88712	-0.9968	-0.51443	0.04646	-0.42235
TRINITY_DN4439_c0_g2_i1_orf1	-	-	-	unnamed protein product, partial [Brenthis ino]	1.02798	1.35833	-1.15436	-0.67422	-0.55773
TRINITY_DN4183_c0_g1_i8_orf1	-	-	-	histidine--tRNA ligase, cytoplasmic isoform X3 [Ostrinia furnacalis]	1.95333	-0.84377	-0.53668	-0.38747	-0.18541
TRINITY_DN1820_c0_g1_i6_orf1	-	-	-	3-hydroxyisobutyryl-CoA hydrolase, mitochondrial [Ostrinia furnacalis]	1.97937	-0.39322	-0.60417	-0.2906	-0.69139
TRINITY_DN12476_c0_g1_i4_orf1	-	-	-	guanine nucleotide-binding protein-like 3 homolog [Ostrinia furnacalis]	1.80719	-0.9881	0.15807	-0.13127	-0.84588
TRINITY_DN452_c1_g1_i3_orf1	-	-	-	ruvB-like helicase 1 [Colias croceus]	1.84154	0.14708	-1.07605	-0.37624	-0.53634
TRINITY_DN157_c0_g1_i4_orf1	-	-	-	ATP-binding cassette sub-family A member 1-like [Ostrinia furnacalis]	1.94634	-0.75057	-0.59245	-0.06143	-0.5419
TRINITY_DN11886_c0_g1_i1_orf1	-	-	-	glycerophosphodiester phosphodiesterase GDPD6-like [Ostrinia furnacalis] >XP_028159459.1 glycerophosphodiester phosphodiesterase GDPD6-like [Ostrinia furnacalis]	1.80878	-0.66403	-0.79682	-0.71799	0.37005
TRINITY_DN6785_c0_g1_i1_orf1	-	-	-	Similar to CG4038: Probable H/ACA ribonucleoprotein complex subunit 1 (Drosophila melanogaster) [Cotesia congregata]	1.83302	-0.61532	-0.90271	0.288	-0.60299
TRINITY_DN141_c0_g1_i1_orf1	-	-	-	hypothetical protein evm_010402 [Chilo suppressalis]	1.72258	0.36495	-0.95624	-0.98113	-0.15016
TRINITY_DN4463_c0_g1_i2_orf1	-	-	-	uncharacterized protein LOC113521486 isoform X3 [Galleria mellonella]	1.45421	0.04818	-1.41058	0.6208	-0.71261
TRINITY_DN124300_c0_g1_i2_orf1	-	-	-	protein transport protein Sec23A isoform X1 [Venturia canescens]	1.94385	-0.34763	-0.88099	-0.54289	-0.17234
TRINITY_DN46140_c0_g1_i1_orf1	-	-	-	protein PRRC2A-like isoform X4 [Ostrinia furnacalis]	1.55354	0.79543	-0.50719	-0.93658	-0.9052
TRINITY_DN152_c0_g1_i4_orf1	-	-	-	LOW QUALITY PROTEIN: protein tyrosine phosphatase type IVA 3 [Ostrinia furnacalis]	1.22746	0.26945	-1.62717	-0.55316	0.68343
TRINITY_DN20185_c0_g1_i6_orf1	-	-	-	zinc finger protein on ecdysone puffs [Ostrinia furnacalis]	1.39723	0.94178	-1.02274	-1.01065	-0.30562
TRINITY_DN15244_c0_g1_i5_orf1	-	-	-	titin homolog [Ostrinia furnacalis]	1.84716	-0.02985	-0.96299	-0.81111	-0.0432
TRINITY_DN1772_c7_g1_i7_orf1	-	-	-	sulfotransferase family cytosolic 1B member 1-like [Ostrinia furnacalis]	1.90117	-0.2332	-0.39782	-0.20712	-1.06302
TRINITY_DN4381_c0_g2_i1_orf1	-	-	-	eukaryotic initiation factor 4A [Glyphodes caesalis]	1.77432	-0.26477	-1.06218	-0.74971	0.30234
TRINITY_DN21123_c0_g1_i1_orf1	-	-	-	maternal protein exuperantia [Ostrinia furnacalis]	1.86553	-0.41418	-1.10078	-0.36903	0.01845
TRINITY_DN2954_c0_g1_i1_orf1	-	-	-	unnamed protein product [Diatraea saccharalis]	1.79788	-0.68125	-1.07282	-0.29724	0.25342
TRINITY_DN3321_c0_g1_i3_orf1	-	-	-	peroxidasin [Ostrinia furnacalis]	1.67555	0.24956	-1.30396	-0.65481	0.03366
TRINITY_DN113778_c0_g2_i1_orf1	-	-	-	metastasis-associated protein MTA3 [Galleria mellonella]	0.81493	1.26352	-1.52193	-0.64452	0.08799
TRINITY_DN10297_c0_g1_i1_orf1	-	-	-	polyglutamine-binding protein 1 [Ostrinia furnacalis]	1.27596	1.08533	-0.334	-1.13085	-0.89644
TRINITY_DN29034_c0_g1_i1_orf1	-	-	-	trypsin-like serine protease [Ostrinia nubilalis]	1.58967	0.70294	-0.55628	-0.58359	-1.15274
TRINITY_DN57454_c0_g1_i4_orf1	-	-	-	translation machinery-associated protein 7 homolog [Zerene cesonia]	1.8469	0.16092	-0.33944	-0.66671	-1.00168
TRINITY_DN31967_c0_g1_i5_orf1	-	-	-	N-acetylgalactosamine kinase [Ostrinia furnacalis]	1.35619	1.07664	-0.77344	-0.71433	-0.94506
TRINITY_DN48970_c0_g1_i1_orf1	-	-	-	uncharacterized protein LOC114356431 isoform X2 [Ostrinia furnacalis]	1.29554	1.03333	-1.28973	-0.6785	-0.36064
TRINITY_DN211_c1_g1_i10_orf1	-	-	-	protein hu-li tai shao isoform X5 [Galleria mellonella]	1.77798	-1.03426	-0.82179	-0.17393	0.252
TRINITY_DN7464_c1_g1_i1_orf1	-	-	-	T-complex protein 1 subunit theta [Ostrinia furnacalis]	1.84894	0.25609	-0.85043	-0.57435	-0.68025
TRINITY_DN5873_c0_g4_i1_orf1	-	-	-	hypothetical protein evm_003048 [Chilo suppressalis]	1.69254	-1.03461	-0.95065	-0.09684	0.38956
TRINITY_DN3520_c0_g1_i4_orf1	-	-	-	protein KRTCAP2 homolog [Ostrinia furnacalis]	1.25987	0.47413	-1.76994	-0.14726	0.1832
TRINITY_DN3985_c0_g2_i1_orf1	-	-	-	hypothetical protein evm_012077 [Chilo suppressalis] >CAB3529218.1 unnamed protein product [Chilo suppressalis] >CAH0405810.1 unnamed protein product [Chilo suppressalis]	1.82501	-0.94923	-0.71409	-0.43107	0.26937
TRINITY_DN1447_c0_g1_i5_orf1	-	-	-	PREDICTED: coatomer subunit beta' [Amyelois transitella]	1.66532	0.36019	-1.36313	-0.4299	-0.23249
TRINITY_DN30070_c0_g1_i6_orf1	-	-	-	uncharacterized protein LOC114361440 isoform X1 [Ostrinia furnacalis] >XP_028172261.1 uncharacterized protein LOC114361440 isoform X2 [Ostrinia furnacalis]	1.89111	-0.33311	-0.17777	-0.28475	-1.09548
TRINITY_DN3838_c0_g1_i8_orf1	-	-	-	ER membrane protein complex subunit 2-like isoform X1 [Ostrinia furnacalis] >XP_028161204.1 ER membrane protein complex subunit 2-like isoform X2 [Ostrinia furnacalis] >XP_028161205.1 ER membrane protein complex subunit 2-like isoform X3	1.29027	0.8283	-1.39042	-0.83881	0.11066
TRINITY_DN108818_c0_g1_i5_orf1	-	-	-	hypothetical protein O3G_MSEX012842 [Manduca sexta]	1.72959	0.09409	-1.12549	-0.84366	0.14548
TRINITY_DN11215_c0_g1_i1_orf1	-	-	-	dnaJ homolog subfamily C member 2 [Ostrinia furnacalis]	1.89292	0.13827	-0.55547	-0.75081	-0.72491
TRINITY_DN3618_c0_g1_i4_orf1	-	-	-	WD repeat-containing protein 74-like isoform X1 [Ostrinia furnacalis] >XP_028161051.1 WD repeat-containing protein 74-like isoform X2 [Ostrinia furnacalis]	1.27091	0.35691	-0.38992	0.46253	-1.70043
TRINITY_DN4533_c0_g1_i1_orf1	-	-	-	neurofilament heavy polypeptide-like isoform X2 [Ostrinia furnacalis]	1.20226	1.0719	-1.41604	-0.55606	-0.30206
TRINITY_DN2593_c0_g3_i1_orf1	-	-	-	midgut carboxypeptidase [Loxostege sticticalis]	1.9491	-0.84824	-0.33124	-0.19036	-0.57925

TRINITY_DN48983_c0_g1_i2_orf1	-	-	-	E3 ubiquitin-protein ligase Su(dx) [Ostrinia furnacalis] >XP_028176753.1 E3 ubiquitin-protein ligase Su(dx) [Ostrinia furnacalis] >XP_028176754.1 E3 ubiquitin-protein ligase Su(dx) [Ostrinia furnacalis]	1.36155	0.44716	-1.62961	0.28094	-0.46005
TRINITY_DN8598_c0_g1_i2_orf1	-	-	-	tyrosine--tRNA ligase, mitochondrial [Ostrinia furnacalis]	1.99097	-0.31857	-0.53821	-0.59598	-0.53821
TRINITY_DN33038_c0_g1_i1_orf1	-	-	-	39S ribosomal protein L46, mitochondrial [Ostrinia furnacalis]	1.87296	-0.63826	0.06287	-0.30322	-0.99435
TRINITY_DN18681_c0_g1_i7_orf1	-	-	-	fragile X mental retardation syndrome-related protein 1 isoform X3 [Ostrinia furnacalis]	1.48159	0.76072	-1.14989	-0.93817	-0.15425
TRINITY_DN937_c0_g1_i2_orf1	-	-	-	protein brunelleschi [Ostrinia furnacalis]	1.67961	-0.77512	-0.94185	-0.56882	0.60619
TRINITY_DN4686_c0_g2_i1_orf1	-	-	-	lysophospholipase-like protein 1 [Ostrinia furnacalis]	1.24847	1.16903	-1.09636	-0.66183	-0.6593
TRINITY_DN4151_c1_g1_i4_orf1	-	-	-	5-methylcytosine rRNA methyltransferase NSUN4 isoform X1 [Ostrinia furnacalis] >XP_028161245.1 5-methylcytosine rRNA methyltransferase NSUN4 isoform X2 [Ostrinia furnacalis]	1.75582	0.32684	-1.07497	-0.23272	-0.77497
TRINITY_DN31503_c0_g1_i4_orf1	-	-	-	hypothetical protein evm_001345 [Chilo suppressalis] >CAB3523265.1 unnamed protein product [Chilo suppressalis] >CAH0400587.1 unnamed protein product [Chilo suppressalis]	1.68292	0.61791	-0.65224	-0.85266	-0.79594
TRINITY_DN90289_c0_g1_i5_orf1	-	-	-	40S ribosomal protein S25 [Eumeta japonica]	1.94241	-0.17628	-0.42889	-0.91142	-0.42582
TRINITY_DN72934_c0_g1_i1_orf1	-	-	-	carboxylesterase 8 [Streltzoviella insularis]	1.98675	-0.30845	-0.44824	-0.62623	-0.60383
TRINITY_DN3878_c0_g1_i4_orf1	-	-	-	eukaryotic translation initiation factor 3 subunit J [Ostrinia furnacalis]	1.96699	-0.44648	-0.60693	-0.72692	-0.18666
TRINITY_DN5472_c0_g1_i1_orf1	-	-	-	serine/threonine-protein kinase 26 isoform X3 [Ostrinia furnacalis]	1.49695	0.75298	-1.20674	-0.21189	-0.83129
TRINITY_DN2300_c0_g1_i1_orf1	-	-	-	ATP synthase subunit beta, mitochondrial isoform X4 [Ostrinia furnacalis]	1.93186	-0.95745	-0.34124	-0.44759	-0.18558
TRINITY_DN7613_c1_g2_i1_orf1	-	-	-	60S ribosomal protein L19 [Helicoverpa armigera] >XP_022126104.1 60S ribosomal protein L19 [Pieris rapae] >XP_022821503.1 60S ribosomal protein L19 [Spodoptera litura] >XP_026731717.1 60S ribosomal protein L19 [Trichoplusia ni] >XP_035451321.1 60S ribosomal protein L19-like [Spodoptera frugiperda] >XP_035452592.1 60S ribosomal protein L19-like [Spodoptera frugiperda] >XP_041975187.1 60S ribosomal protein L19 [Aricia agestis] >XP_045524933.1 60S ribosomal protein L19 [Pieris brassicae] >XP_047023231.1 60S ribosomal protein L19 [Helicoverpa zea] >XP_047984679.1 60S ribosomal protein L19 [Leguminivora glycinivorella] >XP_049874324.1 60S ribosomal protein L19 [Pectinophora gossypiella] >ACY95336.1 ribosomal protein L19 [Manduca sexta] >KAF9423217.1 hypothetical protein HW555_001286 [Spodoptera exigua] >KAI5632448.1 ribosomal protein l19e domain-containing protein [Phthorimaea operculella] >RVE50663.1 hypothetical protein evm_004695 [Chilo suppressalis] >CAB3239671.1 unnamed protein product [Arctia plantaginis] >CAB3509883.1 unnamed protein product [Spodoptera littoralis] >CAG4986349.1 unnamed protein product [Parnassius apollo] >CAG9758258.1 unnamed protein product [Diatraea saccharalis] >CAH2049991.1 unnamed protein product, partial [Iphiclides podalirius]	1.77643	-0.868	-0.98078	-0.21513	0.28748
TRINITY_DN2173_c0_g1_i1_orf1	-	-	-	heat shock 70 kDa protein cognate 4 [Cephus cinctus]	1.87908	0.02221	-0.62959	-0.99888	-0.27282
TRINITY_DN9637_c0_g1_i14_orf1	-	-	-	zinc finger protein swm isoform X3 [Ostrinia furnacalis]	1.43399	0.98239	-0.87272	-0.88478	-0.65888
TRINITY_DN14298_c0_g3_i1_orf1	-	-	-	kinesin heavy chain [Ostrinia furnacalis]	0.91447	1.25916	-1.46492	-0.6553	-0.05341
TRINITY_DN15845_c0_g1_i1_orf1	-	-	-	probable ATP-dependent RNA helicase DDX28 isoform X1 [Ostrinia furnacalis] >XP_028162024.1 probable ATP-dependent RNA helicase DDX28 isoform X2 [Ostrinia furnacalis] >XP_028162026.1 probable ATP-dependent RNA helicase DDX28 isoform X3 [Ostrinia furnacalis] >XP_028162027.1 probable ATP-dependent RNA helicase DDX28 isoform	1.79207	0.23552	-1.13143	-0.60827	-0.2879
TRINITY_DN3343_c0_g1_i4_orf1	-	-	-	AFG3-like protein 2 [Ostrinia furnacalis]	1.99748	-0.50596	-0.58107	-0.48667	-0.42378
TRINITY_DN3856_c0_g1_i7_orf1	-	-	-	uncharacterized protein LOC114355702 [Ostrinia furnacalis]	1.67893	0.52315	-1.17245	-0.47742	-0.55221
TRINITY_DN23444_c0_g1_i10_orf1	-	-	-	serrate RNA effector molecule homolog isoform X2 [Ostrinia furnacalis]	1.82534	0.10153	-1.04772	-0.73422	-0.14493
TRINITY_DN4494_c0_g1_i1_orf1	-	-	-	venom serine carboxypeptidase [Ostrinia furnacalis]	0.84329	1.34211	-1.03254	-1.19162	0.03876
TRINITY_DN2403_c0_g1_i3_orf1	-	-	-	FAD-dependent oxidoreductase domain-containing protein 1 [Ostrinia furnacalis]	1.29116	1.06312	-1.1286	-0.91099	-0.31468
TRINITY_DN10106_c0_g2_i1_orf1	-	-	-	arrestin domain-containing protein 2-like isoform X3 [Ostrinia furnacalis]	1.94849	-0.59381	-0.37709	-0.82846	-0.14913
TRINITY_DN31520_c1_g1_i1_orf1	-	-	-	probable DNA-directed RNA polymerase III subunit RPC6 [Ostrinia furnacalis]	1.67694	0.46317	-1.21477	-0.27618	-0.64916
TRINITY_DN4233_c0_g2_i2_orf1	-	-	-	actin-related protein 2/3 complex subunit 5-B [Ostrinia furnacalis]	1.46555	0.87448	-1.19229	-0.63405	-0.51369
TRINITY_DN22951_c0_g1_i1_orf1	-	-	-	TAR DNA-binding protein 43-like [Ostrinia furnacalis]	1.46304	0.12128	-1.54458	0.45882	-0.49855
TRINITY_DN49742_c0_g1_i4_orf1	-	-	-	neuroglobin-like [Ostrinia furnacalis]	1.95808	-0.42322	-0.83603	-0.49723	-0.2016
TRINITY_DN42171_c0_g1_i1_orf1	-	-	-	amyloid-beta-like protein isoform X1 [Manduca sexta] >AAY25024.3 beta amyloid protein precursor-like protein [Manduca sexta]	1.01318	1.39305	-1.04827	-0.75638	-0.60158
TRINITY_DN1337_c0_g2_i1_orf1	-	-	-	minor histocompatibility antigen H13 [Ostrinia furnacalis]	1.75279	-0.14339	-1.3688	-0.16828	-0.07232
TRINITY_DN7037_c0_g1_i4_orf1	-	-	-	unnamed protein product [Chilo suppressalis]	1.58868	0.67107	-0.50056	-0.5421	-1.2171

TRINITY_DN2783_c0_g1_i22_orf1	-	-	-	methionine aminopeptidase 1-like [Pectinophora gossypiella] >XP_049887084.1 methionine aminopeptidase 1-like [Pectinophora gossypiella]	1.73659	-0.84826	-1.11287	0.13489	0.08965
TRINITY_DN1425_c0_g1_i4_orf1	-	-	-	fibulin-2-like [Ostrinia furnacalis]	1.11885	1.09304	-1.52101	-0.32001	-0.37087
TRINITY_DN7991_c0_g1_i9_orf1	-	-	-	hypothetical protein evm_006720 [Chilo suppressalis] >CAB3528247.1 unnamed protein product [Chilo suppressalis] >CAH0404834.1 unnamed protein product [Chilo suppressalis]	1.8517	-0.85606	-0.85329	-0.29494	0.15259
TRINITY_DN32487_c0_g1_i1_orf1	-	-	-	heat shock protein 75 kDa, mitochondrial [Ostrinia furnacalis]	1.99965	-0.52007	-0.50996	-0.46471	-0.50491
TRINITY_DN16128_c0_g1_i5_orf1	-	-	-	probable prefoldin subunit 4 [Ostrinia furnacalis]	1.69768	-0.16789	-1.34286	-0.46018	0.27326
TRINITY_DN19810_c1_g1_i7_orf1	-	-	-	RNA-binding protein spenito [Ostrinia furnacalis] >XP_028167555.1 RNA-binding protein spenito [Ostrinia furnacalis]	1.0377	1.18446	-1.48159	-0.52981	-0.21075
TRINITY_DN2914_c0_g1_i1_orf1	-	-	-	U1 small nuclear ribonucleoprotein A [Ostrinia furnacalis]	1.6458	0.52472	-1.1983	-0.71792	-0.2543
TRINITY_DN1313_c0_g1_i2_orf1	-	-	-	39S ribosomal protein L40, mitochondrial [Ostrinia furnacalis]	0.82255	1.51007	-0.39597	-1.04244	-0.89421
TRINITY_DN1572_c0_g1_i6_orf1	-	-	-	eukaryotic translation initiation factor 3 subunit D [Ostrinia furnacalis]	1.96765	-0.41391	-0.73931	-0.60572	-0.2087
TRINITY_DN3551_c0_g1_i4_orf1	-	-	-	carnitine O-palmitoyltransferase 2, mitochondrial [Ostrinia furnacalis]	1.98232	-0.72246	-0.35634	-0.53334	-0.37017
TRINITY_DN18794_c0_g1_i5_orf1	-	-	-	hypothetical protein evm_012380 [Chilo suppressalis] >CAB3520845.1 unnamed protein product [Chilo suppressalis] >CAH0398166.1 unnamed protein product [Chilo suppressalis]	1.74616	0.32501	-0.77	-1.10108	-0.20009
TRINITY_DN14035_c0_g1_i1_orf1	-	-	-	protein takeout-like [Ostrinia furnacalis]	1.9664	-0.79564	-0.53643	-0.24208	-0.39225
TRINITY_DN7405_c0_g1_i3_orf1	-	-	-	hexokinase-2-like [Ostrinia furnacalis] >XP_028178415.1 hexokinase-2-like [Ostrinia furnacalis]	1.92113	-0.78691	-0.15656	-0.18257	-0.79509
TRINITY_DN43656_c0_g1_i1_orf1	-	-	-	GPI ethanolamine phosphate transferase 3 isoform X2 [Ostrinia furnacalis]	1.95163	-0.73714	-0.50303	-0.62186	-0.0896
TRINITY_DN43792_c0_g1_i1_orf1	-	-	-	40S ribosomal protein S3a [Spodoptera litura] >XP_022824163.1 40S ribosomal protein S3a [Spodoptera litura] >XP_026734591.1 40S ribosomal protein S3a [Trichoplusia ni] >XP_035456172.1 40S ribosomal protein S3a [Spodoptera frugiperda] >Q95V35.1 RecName: Full=40S ribosomal protein S3a [Spodoptera frugiperda] >CAB3514148.1 unnamed protein product [Spodoptera littoralis] >CAH0602005.1 unnamed protein product [Chrysodeixis includens] >AAL26579.1 ribosomal protein S3A [Spodoptera frugiperda] >CAH1642305.1 unnamed protein product [Spodoptera littoralis]	1.83643	-0.44164	-1.18788	-0.10619	-0.10071
TRINITY_DN207_c0_g2_i3_orf1	-	-	-	JNK-interacting protein 3 isoform X2 [Ostrinia furnacalis]	1.98884	-0.2932	-0.55678	-0.57519	-0.56367
TRINITY_DN4757_c0_g1_i3_orf1	-	-	-	melanotransferrin isoform X1 [Ostrinia furnacalis] >XP_028175370.1 melanotransferrin isoform X2 [Ostrinia furnacalis] >XP_028175371.1 melanotransferrin isoform X3 [Ostrinia furnacalis]	1.1888	1.25286	-0.87108	-0.67358	-0.897
TRINITY_DN142485_c0_g1_i1_orf1	-	-	-	uncharacterized protein CG16817-like [Ostrinia furnacalis]	1.73629	-0.05431	-1.02915	-0.92228	0.26944
TRINITY_DN29743_c0_g1_i9_orf1	-	-	-	polyadenylate-binding protein 2 isoform X1 [Ostrinia furnacalis] >XP_028168980.1 polyadenylate-binding protein 2 isoform X2 [Ostrinia furnacalis]	1.31884	0.956	-1.23893	-0.88891	-0.147
TRINITY_DN3628_c0_g1_i5_orf1	-	-	-	palmitoyltransferase Hip14 isoform X2 [Ostrinia furnacalis]	1.92769	0.0156	-0.70745	-0.71701	-0.51883
TRINITY_DN10521_c0_g1_i7_orf1	-	-	-	tubulin beta chain-like [Ostrinia furnacalis]	1.95466	-0.89418	-0.39224	-0.3703	-0.29795
TRINITY_DN3976_c0_g1_i6_orf1	-	-	-	grpE protein homolog, mitochondrial [Ostrinia furnacalis]	1.93445	-0.94132	-0.17516	-0.35151	-0.46646
TRINITY_DN19628_c1_g1_i1_orf1	-	-	-	transcription factor BTF3 homolog 4-like [Ostrinia furnacalis]	1.9541	-0.17612	-0.72695	-0.71217	-0.33885
TRINITY_DN14934_c0_g1_i17_orf1	-	-	-	putative tricarboxylate transport protein, mitochondrial isoform X1 [Ostrinia furnacalis] >XP_028177526.1 putative tricarboxylate transport protein, mitochondrial isoform X2 [Ostrinia furnacalis]	1.85251	-1.14051	-0.36791	0.01887	-0.36296
TRINITY_DN49047_c0_g1_i2_orf1	-	-	-	unnamed protein product [Parnassius apollo]	1.00968	0.67305	-1.67941	-0.59627	0.59295
TRINITY_DN33183_c0_g1_i4_orf1	-	-	-	glutamate--cysteine ligase regulatory subunit [Ostrinia furnacalis]	1.89956	0.09378	-0.68079	-0.48596	-0.82659
TRINITY_DN1528_c0_g1_i4_orf1	-	-	-	uncharacterized protein LOC114353202 [Ostrinia furnacalis]	0.73503	1.47924	-1.34178	-0.22342	-0.64907
TRINITY_DN8343_c0_g1_i2_orf1	-	-	-	multidrug resistance-associated protein 1 isoform X4 [Ostrinia furnacalis]	1.88418	-0.54654	-0.72981	-0.76964	0.1618
TRINITY_DN31637_c0_g1_i3_orf1	-	-	-	protein CDV3 homolog isoform X1 [Ostrinia furnacalis] >XP_028156793.1 protein CDV3 homolog isoform X2 [Ostrinia furnacalis] >XP_028156794.1 protein CDV3 homolog isoform X3 [Ostrinia furnacalis] >XP_028156795.1 protein CDV3 homolog isoform X1 [Ostrinia furnacalis]	1.8268	0.07321	-1.08289	-0.68329	-0.13384
TRINITY_DN5233_c0_g1_i1_orf1	-	-	-	pre-mRNA-splicing factor 38-like [Ostrinia furnacalis]	1.81581	0.03343	-1.0722	-0.0348	-0.74224
TRINITY_DN4944_c1_g1_i4_orf1	-	-	-	bifunctional glutamate/proline--tRNA ligase [Ostrinia furnacalis]	1.9249	-0.83177	-0.70488	-0.31801	-0.07025
TRINITY_DN40015_c0_g1_i2_orf1	-	-	-	PREDICTED: 60S ribosomal protein L18 [Amyelois transitella]	1.86058	-0.7817	-0.8952	-0.3252	0.14153
TRINITY_DN9874_c0_g1_i7_orf1	-	-	-	hypothetical protein evm_013697 [Chilo suppressalis]	1.90448	-0.8569	-0.74785	-0.28119	-0.01853
TRINITY_DN3450_c0_g1_i3_orf1	-	-	-	hypothetical protein evm_008214 [Chilo suppressalis]	1.55858	0.45361	-1.41559	0.00435	-0.60096
TRINITY_DN2802_c0_g1_i1_orf1	-	-	-	far upstream element-binding protein 1 isoform X3 [Ostrinia furnacalis]	1.52149	0.78872	-1.13697	-0.78916	-0.38408
TRINITY_DN31585_c0_g1_i1_orf1	-	-	-	transcription elongation factor SPT5 [Ostrinia furnacalis]	1.74102	0.49754	-0.92877	-0.67657	-0.63322

TRINITY_DN135679_c0_g1_i1_orfp1	-	-	-	TRINITY_DN135679_c0_g1_i1_m.85524 TRINITY_DN135679_c0_g1::TRINITY_DN135679_c0_g1_i1::g.85524 ORF type:5prime_partial len:55 (+),score=3.74,Toxin_2 PF00451.20 1.9e-06 TRINITY_DN135679_c0_g1_i1:3-167(+)	0.97952	-0.62893	-1.5656	1.08489	0.13012
TRINITY_DN170_c1_g1_i5_orf1	-	-	-	regulator of chromosome condensation isoform X2 [Helicoverpa zea]	1.69088	0.29151	-1.13075	-0.88118	0.02956
TRINITY_DN147517_c0_g1_i1_orf1	-	-	-	eukaryotic translation initiation factor 4 gamma 3-like isoform X5 [Ostrinia furnacalis]	1.89939	-0.52107	-1.01566	-0.28972	-0.07294
TRINITY_DN57798_c0_g1_i1_orf1	-	-	-	ubiquitin carboxyl-terminal hydrolase 36 [Ostrinia furnacalis]	1.78163	0.40968	-0.77076	-0.54459	-0.87596
TRINITY_DN15376_c0_g1_i1_orf1	-	-	-	peptidyl-prolyl cis-trans isomerase isoform X1 [Ostrinia furnacalis]	1.98501	-0.44876	-0.5586	-0.67377	-0.30388
TRINITY_DN16077_c0_g1_i13_orf1	-	-	-	dynamin-like 120 kDa protein, mitochondrial [Ostrinia furnacalis]	1.98864	-0.45863	-0.3372	-0.6667	-0.52611
TRINITY_DN91_c0_g1_i9_orf1	-	-	-	ribosome-binding protein 1 isoform X8 [Helicoverpa armigera]	1.68505	0.29122	-1.36476	-0.42032	-0.19119
TRINITY_DN15339_c0_g1_i6_orf1	-	-	-	nuclear pore glycoprotein p62 isoform X3 [Ostrinia furnacalis]	1.62541	-0.27595	-1.28593	0.52782	-0.59135
TRINITY_DN69170_c0_g2_i1_orf1	-	-	-	stromal membrane-associated protein 1-like [Pectinophora gossypiella]	1.71305	0.48607	-1.08529	-0.68144	-0.43239
TRINITY_DN4380_c0_g1_i9_orf1	-	-	-	hypothetical protein evm_012370 [Chilo suppressalis]	1.75183	0.24933	-1.07783	-0.83646	-0.08687
TRINITY_DN288_c0_g1_i9_orf1	-	-	-	unnamed protein product [Chilo suppressalis]	1.41743	0.96763	-1.09429	-0.53566	-0.75511
TRINITY_DN16816_c0_g1_i1_orf1	-	-	-	ATP-binding cassette sub-family F member 1 [Ostrinia furnacalis] >XP_028179049.1 ATP-binding cassette sub-family F member 1 [Ostrinia furnacalis]	1.87396	-0.68626	-0.9783	-0.2431	0.0337
TRINITY_DN14501_c0_g1_i1_orf1	-	-	-	28S ribosomal protein S28, mitochondrial [Ostrinia furnacalis]	1.73508	0.48086	-0.45584	-0.85263	-0.90747
TRINITY_DN53311_c0_g2_i1_orf1	-	-	-	transcription elongation factor S-II [Chelonus insularis]	1.10479	1.27903	-1.08533	-0.40171	-0.89678
TRINITY_DN12826_c0_g1_i1_orf1	-	-	-	uncharacterized protein LOC114363296 [Ostrinia furnacalis]	1.83205	-0.20765	-1.13161	0.06866	-0.56145
TRINITY_DN2977_c0_g1_i3_orf1	-	-	-	transmembrane 9 superfamily member 3 [Ostrinia furnacalis]	1.56014	0.80761	-0.95325	-0.75541	-0.6591
TRINITY_DN95414_c0_g1_i1_orf1	-	-	-	protein arginine N-methyltransferase 5 [Ostrinia furnacalis]	1.61277	-1.04176	-1.04176	-0.00715	0.4779
TRINITY_DN642_c0_g1_i6_orf1	-	-	-	reticulon-3-B isoform X5 [Ostrinia furnacalis]	1.82887	-0.81085	-0.93652	-0.283	0.2015
TRINITY_DN972_c0_g2_i1_orf1	-	-	-	DNA damage-binding protein 1 [Ostrinia furnacalis]	1.63958	0.56121	-1.23064	-0.40837	-0.56177
TRINITY_DN2497_c0_g1_i2_orf1	-	-	-	protein stunted-like isoform X1 [Colias croceus]	1.74511	-1.04956	-0.20602	-0.83246	0.34292
TRINITY_DN11584_c0_g1_i2_orf1	-	-	-	L-threonine 3-dehydrogenase, mitochondrial [Ostrinia furnacalis]	1.95391	-0.46437	-0.10767	-0.67992	-0.70195
TRINITY_DN2668_c0_g1_i6_orf1	-	-	-	pancreatic triacylglycerol lipase-like [Spodoptera litura]	1.94052	-0.12706	-0.37913	-0.56571	-0.86861
TRINITY_DN10672_c0_g1_i3_orf1	-	-	-	neurofilament heavy polypeptide-like isoform X10 [Ostrinia furnacalis]	1.57005	0.06424	-1.57752	0.11415	-0.17092
TRINITY_DN62_c0_g1_i18_orf1	-	-	-	hypothetical protein evm_002481 [Chilo suppressalis] >CAB3531063.1 unnamed protein product [Chilo suppressalis] >CAH0407655.1 unnamed protein product [Chilo suppressalis]	1.66094	0.58861	-0.96427	-0.9063	-0.37898
TRINITY_DN10548_c0_g2_i1_orf1	-	-	-	uridine 5'-monophosphate synthase-like [Ostrinia furnacalis]	1.54083	0.35536	-1.43447	-0.6403	0.17858
TRINITY_DN18728_c0_g1_i2_orf1	-	-	-	H/ACA ribonucleoprotein complex subunit 3 [Galleria mellonella]	1.97301	-0.59952	-0.72839	-0.3903	-0.25481
TRINITY_DN4156_c0_g1_i2_orf1	-	-	-	calcium channel flower [Ostrinia furnacalis]	0.86119	1.39902	-1.36085	-0.59926	-0.3001
TRINITY_DN2927_c0_g1_i2_orf1	-	-	-	unnamed protein product [Spodoptera littoralis] >CAH1641960.1 unnamed protein product [Spodoptera littoralis]	1.87981	0.12669	-0.93748	-0.536	-0.53302
TRINITY_DN15448_c0_g1_i1_orf1	-	-	-	ragulator complex protein LAMTOR1-like [Ostrinia furnacalis]	1.97384	-0.29915	-0.33563	-0.72095	-0.61811
TRINITY_DN3063_c0_g1_i5_orf1	-	-	-	mini-chromosome maintenance complex-binding protein [Ostrinia furnacalis]	1.82422	-0.30907	-1.1065	-0.57097	0.16233
TRINITY_DN41842_c0_g1_i2_orf1	-	-	-	importin-5 [Ostrinia furnacalis]	1.98934	-0.45139	-0.54734	-0.6511	-0.33952
TRINITY_DN70382_c0_g1_i10_orf1	-	-	-	TGF-beta receptor type-1 isoform X4 [Ostrinia furnacalis]	1.58601	-0.73084	-1.29389	-0.08069	0.5194
TRINITY_DN66822_c0_g1_i1_orf1	-	-	-	heterogeneous nuclear ribonucleoprotein 27C isoform X6 [Pieris rapae]	1.9427	-0.21816	-0.92418	-0.44435	-0.35602
TRINITY_DN14464_c0_g1_i1_orf1	-	-	-	GMP synthase [glutamine-hydrolyzing] [Chelonus insularis]	1.89024	-0.68655	-0.8613	-0.44975	0.10736
TRINITY_DN33883_c0_g1_i1_orf1	-	-	-	probable 28S ribosomal protein S6, mitochondrial [Ostrinia furnacalis]	1.92992	-0.07066	-0.4005	-0.5777	-0.88107
TRINITY_DN28376_c0_g1_i15_orfp1	-	-	-	TRINITY_DN28376_c0_g1_i15_m.40022 TRINITY_DN28376_c0_g1::TRINITY_DN28376_c0_g1_i15::g.40022 ORF type:internal len:273 (+),score=43.58 TRINITY_DN28376_c0_g1_i15:3-818(+)	1.32347	-0.43481	-1.63376	0.60983	0.13527
TRINITY_DN3312_c0_g1_i10_orf1	-	-	-	glycerol-3-phosphate dehydrogenase, mitochondrial-like isoform X3 [Ostrinia furnacalis]	1.94945	-0.42057	-0.24218	-0.90441	-0.38229
TRINITY_DN92153_c0_g2_i2_orf1	-	-	-	methylenetetrahydrofolate reductase [Ostrinia furnacalis]	1.20021	0.24122	-1.37854	-0.92558	0.86269
TRINITY_DN27852_c0_g1_i1_orf1	-	-	-	baculoviral IAP repeat-containing protein 6-like [Ostrinia furnacalis]	1.76179	0.30854	-0.36275	-1.17891	-0.52867
TRINITY_DN31119_c0_g1_i1_orf1	-	-	-	transforming acidic coiled-coil-containing protein 3-like [Ostrinia furnacalis] >XP_028170476.1 transforming acidic coiled-coil-containing protein 3-like [Ostrinia furnacalis] >XP_028170477.1 transforming acidic coiled-coil-containing protein 3-like [Ostrinia furnacalis] >XP_028170480.1 transforming acidic coiled-coil-containing protein 3-like [Ostrinia furnacalis]	1.54239	0.19014	-1.6047	-0.03546	-0.09237
TRINITY_DN11457_c0_g1_i5_orf1	-	-	-	uncharacterized protein LOC114352268 [Ostrinia furnacalis]	1.92678	-0.07218	-0.84171	-0.68092	-0.33197
TRINITY_DN3889_c0_g1_i7_orfp1	-	-	-	TRINITY_DN3889_c0_g1_i7_m.1657 TRINITY_DN3889_c0_g1::TRINITY_DN3889_c0_g1_i7::g.1657 ORF type:5prime_partial len:235 (+),score=70.90 TRINITY_DN3889_c0_g1_i7:1-705(+)	1.93936	-0.08969	-0.51526	-0.47352	-0.86089
TRINITY_DN23783_c0_g2_i1_orf1	-	-	-	cytochrome b5 [Ostrinia furnacalis]	1.60444	0.42242	-1.1217	0.08568	-0.99085

TRINITY_DN57074_c0_g2_i1_orf1	-	-	-	ribosomal protein l36e domain-containing protein [Phthorimaea operculella]	1.91454	-0.77222	-0.70069	-0.49443	0.05281
TRINITY_DN11396_c0_g1_i1_orf1	-	-	-	uncharacterized protein LOC114352414 isoform X1 [Ostrinia furnacalis]	1.87321	0.17091	-0.55287	-0.59679	-0.89446
TRINITY_DN18249_c0_g1_i1_orf1	-	-	-	60S ribosomal protein L13 [Pectinophora gossypiella]	1.52785	-0.70612	-1.25726	-0.27797	0.71351
TRINITY_DN756_c0_g1_i11_orf1	-	-	-	calcium-binding protein E63-1 isoform X1 [Ostrinia furnacalis]	1.81429	-0.86176	-0.94574	-0.19215	0.18537
TRINITY_DN4950_c0_g1_i2_orf1	-	-	-	unnamed protein product [Diatraea saccharalis]	1.5643	0.73399	-1.17564	-0.59251	-0.53014
TRINITY_DN73945_c0_g5_i3_orf1	-	-	-	cyclin-dependent kinase 12 isoform X1 [Diachasma alloeum] >XP_015114851.1 cyclin-dependent kinase 12 isoform X1 [Diachasma alloeum] >XP_015114852.1 cyclin-dependent kinase 12 isoform X1 [Diachasma alloeum] >XP_015114853.1 cyclin-dependent kinase 12 isoform X1 [Diachasma alloeum] >XP_015114854.1 cyclin-dependent kinase 12 isoform X1	1.68206	-0.55164	-1.27535	0.41112	-0.26619
TRINITY_DN3832_c0_g1_i1_orf1	-	-	-	serine-threonine kinase receptor-associated protein [Galleria mellonella]	1.74565	0.48879	-0.61512	-0.91988	-0.69945
TRINITY_DN14487_c0_g1_i4_orf1	-	-	-	hypothetical protein HW555_009956 [Spodoptera exigua] >KAH9643419.1 hypothetical protein HF086_016708 [Spodoptera exigua] >CAH0702087.1 unnamed protein product [Spodoptera exigua]	1.76746	0.4153	-0.48575	-0.96628	-0.73072
TRINITY_DN17215_c0_g1_i4_orf1	-	-	-	28S ribosomal protein S29, mitochondrial [Ostrinia furnacalis]	1.97604	-0.73936	-0.28678	-0.5622	-0.38771
TRINITY_DN33249_c0_g1_i1_orf1	-	-	-	eukaryotic translation initiation factor 2 subunit 3-like isoform X2 [Spodoptera frugiperda] >CAH0683085.1 unnamed protein product [Spodoptera exigua]	1.91348	0.04603	-0.82768	-0.63971	-0.49212
TRINITY_DN7407_c0_g1_i9_orf1	-	-	-	sec1 family domain-containing protein 2-like [Ostrinia furnacalis]	1.95807	-0.80101	-0.51426	-0.48463	-0.15817
TRINITY_DN401_c0_g1_i15_orf1	-	-	-	cholinephosphotransferase 1 isoform X2 [Ostrinia furnacalis]	1.99516	-0.48858	-0.61794	-0.48858	-0.40006
TRINITY_DN517_c0_g1_i5_orf1	-	-	-	putative pre-mRNA-splicing factor ATP-dependent RNA helicase PRP1 [Helicoverpa zea] >XP_049708007.1 putative pre-mRNA-splicing factor ATP-dependent RNA helicase PRP1 isoform X1 [Helicoverpa armigera]	1.97186	-0.26638	-0.78495	-0.48249	-0.43803
TRINITY_DN6185_c0_g1_i12_orf1	-	-	-	mitogen-activated protein kinase 1 [Ostrinia furnacalis] >AXF67444.1 mitogen-activated protein kinase 1 [Ostrinia furnacalis]	1.51045	0.83695	-0.47322	-0.79947	-1.0747
TRINITY_DN17417_c0_g1_i11_orf1	-	-	-	sodium/hydrogen exchanger 9B2-like isoform X4 [Ostrinia furnacalis]	1.87281	-1.03196	-0.24529	0.01055	-0.60611
TRINITY_DN23004_c0_g1_i1_orf1	-	-	-	uncharacterized protein LOC114365313 [Ostrinia furnacalis]	1.77701	-0.84454	-1.05326	-0.01816	0.13894
TRINITY_DN4731_c0_g2_i1_orf1	-	-	-	gelsolin-like [Ostrinia furnacalis]	1.75947	-0.65959	0.04859	0.06113	-1.2096
TRINITY_DN14730_c0_g1_i7_orf1	-	-	-	titin homolog [Ostrinia furnacalis]	1.98257	-0.54004	-0.67267	-0.50523	-0.26463
TRINITY_DN6406_c0_g1_i1_orf1	-	-	-	protein FAM98A-like [Ostrinia furnacalis]	1.8752	-0.80138	-0.39842	0.13906	-0.81446
TRINITY_DN145666_c0_g1_i1_orf1	-	-	-	PREDICTED: 40S ribosomal protein S29 [Microplitis demolitor] >XP_044581406.1 40S ribosomal protein S29 [Cotesia glomerata]	1.89912	-0.16924	-0.95442	-0.66443	-0.11103
TRINITY_DN7241_c0_g2_i2_orf1	-	-	-	40S ribosomal protein S10 [Zerene cesonia] >XP_045492164.1 40S ribosomal protein S10 [Colias croceus]	1.77353	-0.74128	-0.90723	-0.54944	0.42443
TRINITY_DN43576_c0_g1_i3_orf1	-	-	-	regulator of microtubule dynamics protein 1-like [Ostrinia furnacalis]	1.87449	-0.63878	-1.0231	-0.17308	-0.03952
TRINITY_DN53810_c0_g1_i1_orf1	-	-	-	39S ribosomal protein L53, mitochondrial [Pectinophora gossypiella]	1.87508	0.16597	-0.50518	-0.66337	-0.87251
TRINITY_DN25210_c0_g1_i1_orf1	-	-	-	mitochondrial import receptor subunit TOM22 homolog [Ostrinia furnacalis]	1.88304	-0.00791	-1.02168	-0.57848	-0.27497
TRINITY_DN21984_c0_g1_i6_orf1	-	-	-	venom serine protease 34-like [Ostrinia furnacalis]	1.88274	-0.52199	-0.09381	-0.20251	-1.06444
TRINITY_DN19286_c0_g1_i1_orf1	-	-	-	signal recognition particle 9 kDa protein [Ostrinia furnacalis]	1.96119	-0.3972	-0.34443	-0.8683	-0.35127
TRINITY_DN10455_c0_g1_i2_orf1	-	-	-	actin-related protein 2/3 complex subunit 4 [Plutella xylostella] >XP_013184242.1 PREDICTED: actin-related protein 2/3 complex subunit 4 [Amyelois transitella] >XP_026754865.1 actin-related protein 2/3 complex subunit 4 [Galleria mellonella] >XP_028168998.1 actin-related protein 2/3 complex subunit 4 [Ostrinia furnacalis] >KAI5632346.1 ARP2/3 complex 20 kDa subunit (ARPC4) domain-containing protein [Phthorimaea operculella] >KAG7303373.1 Actin-protein 2/3 complex subunit 4 [Plutella xylostella] >CAG9104981.1 unnamed protein product	1.71043	0.21104	-1.06125	-0.94666	0.08644
TRINITY_DN42824_c0_g1_i5_orf1	-	-	-	prefoldin subunit 3 [Ostrinia furnacalis]	1.75123	0.43445	-0.81393	-0.95152	-0.42023
TRINITY_DN3027_c0_g1_i4_orf1	-	-	-	ribosome biogenesis protein BOP1 homolog [Ostrinia furnacalis]	1.98703	-0.69533	-0.47648	-0.47431	-0.34092
TRINITY_DN12964_c0_g1_i1_orf1	-	-	-	dnaJ homolog subfamily A member 1 [Ostrinia furnacalis]	1.85985	0.13648	-0.95815	-0.33829	-0.69989
TRINITY_DN36045_c0_g1_i2_orf1	-	-	-	hypothetical protein evm_012355 [Chilo suppressalis] >CAB3522006.1 unnamed protein product [Chilo suppressalis] >CAH0399328.1 unnamed protein product [Chilo suppressalis]	1.91109	-0.22914	-0.24196	-0.40383	-1.03616
TRINITY_DN50820_c0_g1_i2_orf1	-	-	-	transmembrane protein 135-like isoform X2 [Ostrinia furnacalis]	1.65968	0.65101	-0.6485	-0.93024	-0.73194
TRINITY_DN9309_c0_g1_i5_orf1	-	-	-	uncharacterized protein LOC114361160 [Ostrinia furnacalis]	1.73158	0.37645	-1.1096	-0.24402	-0.75441
TRINITY_DN19122_c0_g1_i7_orf1	-	-	-	phosphatidylserine decarboxylase proenzyme, mitochondrial [Ostrinia furnacalis]	1.95483	-0.6229	-0.30368	-0.22277	-0.80549
TRINITY_DN41179_c0_g1_i1_orf1	-	-	-	RNA-binding protein NOB1 [Ostrinia furnacalis]	1.88441	-0.1281	-0.44002	-0.22648	-1.08981
TRINITY_DN1437_c0_g1_i6_orf1	-	-	-	nucleoprotein TPR isoform X1 [Ostrinia furnacalis]	1.58465	0.68596	-1.14626	-0.75237	-0.37198
TRINITY_DN2627_c0_g1_i2_orf1	-	-	-	probable cytosolic oligopeptidase A [Ostrinia furnacalis]	1.96244	-0.1991	-0.433	-0.52948	-0.80086

TRINITY_DN4025_c0_g1_i1_orf1	-	-	-	unnamed protein product [Chilo suppressalis]	1.8797	-0.16194	-1.11966	-0.36224	-0.23586
TRINITY_DN298_c0_g1_i4_orf1	-	-	-	luc7-like protein 3 isoform X1 [Ostrinia furnacalis] >XP_028160033.1 luc7-like protein 3 isoform X1 [Ostrinia furnacalis]	1.58972	0.68426	-0.91571	-1.02816	-0.33011
TRINITY_DN9938_c0_g2_i1_orf1	-	-	-	hypothetical protein E2986_04423 [Frieseomelitta varia]	1.57887	0.60334	-1.32006	-0.55141	-0.31074
TRINITY_DN2168_c0_g1_i2_orf1	-	-	-	protein arginine methyltransferase NDUFAF7 homolog, mitochondrial [Ostrinia furnacalis]	1.90436	-0.65631	-0.9305	-0.0438	-0.27375
TRINITY_DN21181_c0_g1_i6_orf1	-	-	-	unnamed protein product, partial [Brenthis ino]	1.9961	-0.5824	-0.52641	-0.39062	-0.49667
TRINITY_DN2748_c0_g1_i6_orf1	-	-	-	uncharacterized protein LOC114352811 [Ostrinia furnacalis]	1.85398	-0.99077	-0.12157	-0.75249	0.01085
TRINITY_DN82810_c0_g1_i1_orf1	-	-	-	putative carbonic anhydrase 3 [Ostrinia furnacalis]	1.75805	0.24728	-1.08195	-0.10729	-0.81609
TRINITY_DN31216_c0_g1_i2_orf1	-	-	-	uncharacterized protein LOC114361092 [Ostrinia furnacalis]	1.70196	-0.04263	-1.43088	-0.23256	0.00412
TRINITY_DN13174_c0_g1_i4_orf1	-	-	-	N-alpha-acetyltransferase 35, NatC auxiliary subunit [Ostrinia furnacalis]	1.9634	-0.73531	-0.28398	-0.66963	-0.27448
TRINITY_DN2430_c0_g1_i1_orf1	-	-	-	glutathione S-transferase omega 1 [Ostrinia furnacalis]	1.7935	0.12416	-1.17975	-0.14104	-0.59686
TRINITY_DN3057_c0_g2_i1_orf1	-	-	-	chromodomain-helicase-DNA-binding protein Mi-2 homolog isoform X3 [Chelonus insularis]	1.72374	0.52805	-0.86769	-0.55218	-0.83192
TRINITY_DN41311_c0_g2_i3_orf1	-	-	-	ras-related protein Rab-8A isoform X2 [Ostrinia furnacalis]	1.68424	0.59886	-0.97244	-0.64637	-0.66429
TRINITY_DN1665_c1_g1_i2_orf1	-	-	-	translation elongation factor 2 [Melitaea cinxia]	1.64329	-0.86346	-1.07454	-0.27452	0.56923
TRINITY_DN9759_c0_g1_i1_orf1	-	-	-	iroquois-class homeodomain protein IRX-1-like isoform X1 [Ostrinia furnacalis]	1.58185	-0.05249	-1.55707	-0.1734	0.20112
TRINITY_DN5129_c0_g3_i3_orf1	-	-	-	probable citrate synthase 2, mitochondrial [Ostrinia furnacalis]	1.98822	-0.65188	-0.32139	-0.4646	-0.55035
TRINITY_DN143_c0_g3_i1_orf1	-	-	-	Ubiquitin-60S ribosomal protein L40, partial [Cotesia chilonis] >UDP69015.1 egg surface protein ES-53, partial [Cotesia chilonis]	1.76156	-0.57281	-1.19909	0.26104	-0.25069
TRINITY_DN36006_c0_g1_i5_orf1	-	-	-	pro-resilin-like [Ostrinia furnacalis]	1.28383	0.71938	-1.60649	0.09725	-0.49396
TRINITY_DN71840_c0_g1_i1_orf1	-	-	-	60S ribosomal protein L7 [Ostrinia furnacalis] >XP_028162266.1 60S ribosomal protein L7 [Ostrinia furnacalis]	1.89498	-0.72073	-0.90926	-0.25031	-0.01468
TRINITY_DN21782_c0_g1_i8_orf1	-	-	-	atypical kinase COQ8B, mitochondrial [Ostrinia furnacalis]	1.93291	0.00735	-0.72149	-0.6258	-0.59297
TRINITY_DN72056_c0_g1_i1_orf1	-	-	-	protein PBDC1 [Ostrinia furnacalis]	1.93096	-0.15181	-0.90077	-0.59916	-0.27922
TRINITY_DN81312_c0_g1_i1_orf1	-	-	-	atlastin isoform X4 [Ostrinia furnacalis]	1.61405	-0.82951	-1.22426	-0.016	0.45572
TRINITY_DN27960_c0_g1_i1_orf1	-	-	-	ATP synthase mitochondrial F1 complex assembly factor 1 [Ostrinia furnacalis]	1.87978	0.18868	-0.6536	-0.6706	-0.74427
TRINITY_DN79000_c1_g1_i1_orf1	-	-	-	AT15141p, partial [Drosophila melanogaster]	1.85593	0.19842	-0.90032	-0.71792	-0.4361
TRINITY_DN2997_c0_g1_i6_orf1	-	-	-	titin-like [Ostrinia furnacalis]	1.72922	0.06768	-0.92378	-1.05731	0.18419
TRINITY_DN36701_c0_g1_i4_orf1	-	-	-	hypothetical protein SFRURICE_004895 [Spodoptera frugiperda] >KAG8116760.1 hypothetical protein SFRUCORN_001970 [Spodoptera frugiperda]	1.93264	-0.66644	-0.78124	-0.4579	-0.02706
TRINITY_DN17208_c0_g1_i2_orf1	-	-	-	integrator complex subunit 11 [Ostrinia furnacalis]	1.88827	-0.32263	-1.10679	-0.23654	-0.22231
TRINITY_DN198_c0_g1_i2_orf1	-	-	-	retinol dehydrogenase 13-like [Ostrinia furnacalis]	0.59399	0.79039	-1.21981	-1.20501	1.04043
TRINITY_DN44877_c0_g1_i2_orf1	-	-	-	U6 snRNA-associated Sm-like protein LSm7 [Diachasma alloeum]	1.46174	0.81448	-1.21324	-0.81701	-0.24596
TRINITY_DN4469_c0_g1_i2_orf1	-	-	-	metal transporter CNNM4-like [Ostrinia furnacalis]	1.77798	-0.84112	-1.00368	-0.21324	0.28006
TRINITY_DN120979_c0_g1_i1_orf1	-	-	-	la-related protein 1-like isoform X2 [Ostrinia furnacalis]	1.86695	0.06198	-0.81429	-0.89376	-0.22088
TRINITY_DN60680_c0_g1_i2_orf1	-	-	-	unnamed protein product [Euphydryas editha]	1.92244	-0.47357	-0.87883	-0.5544	-0.01564
TRINITY_DN18569_c0_g2_i1_orf1	-	-	-	stomatin-like protein 2, mitochondrial [Ostrinia furnacalis]	1.92474	-0.12492	-0.96017	-0.4715	-0.36815
TRINITY_DN77572_c0_g1_i1_orf1	-	-	-	steroid receptor RNA activator 1 [Ostrinia furnacalis]	1.01774	-0.1445	-1.84306	0.28974	0.68008
TRINITY_DN31253_c0_g1_i2_orf1	-	-	-	hypothetical protein evm_009655 [Chilo suppressalis]	1.83411	-1.10986	-0.58868	-0.2235	0.08793
TRINITY_DN94337_c0_g1_i1_orf1	-	-	-	hypothetical protein evm_006136 [Chilo suppressalis]	1.89166	-0.98384	-0.20394	-0.06525	-0.63862
TRINITY_DN5630_c4_g1_i2_orf1	-	-	-	PREDICTED: stress-associated endoplasmic reticulum protein 2 [Amyelois transitella] >XP_014371593.1 stress-associated endoplasmic reticulum protein 2 [Papilio machaon] >XP_022818474.1 stress-associated endoplasmic reticulum protein 2 [Spodoptera litura] >XP_028162992.1 stress-associated endoplasmic reticulum protein 2 [Ostrinia furnacalis] >XP_028162993.1 stress-associated endoplasmic reticulum protein 2 [Ostrinia furnacalis] >XP_031767943.1 stress-associated endoplasmic reticulum protein 2 [Galleria mellonella] >XP_035452408.1 stress-associated endoplasmic reticulum protein 2-like [Spodoptera frugiperda] >XP_035452409.1 stress-associated endoplasmic reticulum protein 2-like [Spodoptera frugiperda] >XP_035452411.1 stress-associated endoplasmic reticulum protein 2-like [Spodoptera frugiperda] >XP_045455924.1 stress-associated endoplasmic reticulum protein 2 [Melitaea cinxia] >KPJ00707.1 Stress-associated endoplasmic reticulum protein 2 [Papilio xuthus] >CAB3510969.1 unnamed protein product [Spodoptera littoralis] >AXY94738.1 stress-associated endoplasmic reticulum protein 2 [Galleria mellonella] >KAF9797689.1 hypothetical protein SFRURICE_017884 [Spodoptera frugiperda] >KAG8114722.1 hypothetical protein SFRUCORN_004134 [Spodoptera frugiperda]	1.78955	-0.1782	-0.74563	-1.07902	0.21329

TRINITY_DN10030_c0_g1_i2_orf1	-	-	-	uncharacterized protein LOC114360702 [Ostrinia furnacalis] >XP_028171286.1 uncharacterized protein LOC114360702 [Ostrinia furnacalis] >XP_028171287.1 uncharacterized protein LOC114360702 [Ostrinia furnacalis]	1.9829	-0.28544	-0.61449	-0.43516	-0.64782
TRINITY_DN10831_c1_g1_i1_orf1	-	-	-	40S ribosomal protein S16 [Ostrinia furnacalis]	1.71519	-1.04425	-0.86188	-0.22593	0.41686
TRINITY_DN107288_c0_g1_i2_orf1	-	-	-	methionine-tRNA synthetase, partial [Papilio xuthus]	1.68864	-1.0387	0.39716	-0.94996	-0.09713
TRINITY_DN59042_c1_g1_i1_orf1	-	-	-	nuclear pore complex protein Nup50 [Ostrinia furnacalis]	1.83065	0.16184	-1.0105	-0.24678	-0.7352
TRINITY_DN20130_c0_g1_i1_orf1	-	-	-	uncharacterized protein LOC114354518 isoform X1 [Ostrinia furnacalis]	1.85346	-0.51992	-1.10534	-0.26666	0.03847
TRINITY_DN122423_c0_g5_i1_orf1	-	-	-	PREDICTED: dynein heavy chain, cytoplasmic isoform X3 [Fopius arisanus]	1.61973	0.20951	-1.46559	0.0616	-0.42524
TRINITY_DN20_c0_g1_i11_orf1	-	-	-	plasma membrane calcium-transporting ATPase 2 [Ostrinia furnacalis]	1.92258	-0.90548	-0.5378	-0.43925	-0.04005
TRINITY_DN11065_c0_g2_i1_orf1	-	-	-	ribosomal protein s6e domain-containing protein [Phthorimaea operculella]	1.66419	-0.97932	-1.01539	-0.1395	0.47003
TRINITY_DN14996_c0_g1_i2_orf1	-	-	-	40S ribosomal protein S17 [Ostrinia furnacalis]	1.82364	-1.09609	-0.68494	-0.05916	0.01655
TRINITY_DN740_c0_g1_i1_orf1	-	-	-	GTP-binding nuclear protein Ran [Pieris rapae] >XP_028162165.1 GTP-binding nuclear protein Ran [Ostrinia furnacalis] >XP_028162166.1 GTP-binding nuclear protein Ran [Ostrinia furnacalis] >XP_028162167.1 GTP-binding nuclear protein Ran [Ostrinia furnacalis] >XP_045532338.1 GTP-binding nuclear protein Ran [Pieris brassicae] >XP_045532339.1 GTP-binding nuclear protein Ran [Pieris brassicae] >CAG9745207.1 unnamed protein product [Diatraea saccharalis] >CAG9783892.1 unnamed protein product [Diatraea saccharalis]	1.94779	-0.08641	-0.79123	-0.53507	-0.53507
TRINITY_DN13160_c0_g1_i1_orf1	-	-	-	serine/threonine-protein kinase 10-like, partial [Ostrinia furnacalis]	1.92124	-0.62845	-0.63488	-0.71261	0.05469
TRINITY_DN37923_c0_g1_i1_orf1	-	-	-	hypothetical protein NE865_05974 [Phthorimaea operculella]	1.32131	1.01026	-1.16509	-0.89609	-0.27039
TRINITY_DN21981_c0_g1_i8_orf1	-	-	-	dihydroorotate dehydrogenase (quinone), mitochondrial [Ostrinia furnacalis]	1.93679	-0.08557	-0.8728	-0.51274	-0.46567
TRINITY_DN2593_c0_g1_i1_orf1	-	-	-	midgut carboxypeptidase [Loxostege sticticalis]	1.90855	-0.55079	-0.21157	-0.15332	-0.99288
TRINITY_DN4707_c0_g1_i1_orf1	-	-	-	PREDICTED: DNA-directed RNA polymerases I, II, and III subunit RPABC1 [Papilio xuthus] >XP_013187738.1 PREDICTED: DNA-directed RNA polymerases I, II, and III subunit RPABC1 [Amyelois transitella] >XP_028158146.1 DNA-directed RNA polymerases I, II, and III subunit RPABC1 [Ostrinia furnacalis] >XP_045537534.1 DNA-directed RNA polymerases I, II, and III subunit RPABC1 [Papilio machaon] >XP_049876738.1 DNA-directed RNA polymerases I, II, and III subunit RPABC1 [Pectinophora gossypiella] >KAG6452000.1 hypothetical protein O3G_MSEX007416 [Manduca sexta] >RVE48301.1 hypothetical protein evm_007052 [Chilo suppressalis] >CAG5049330.1 unnamed protein product [Parnassius apollo] >CAG9757053.1 unnamed protein product [Diatraea saccharalis] >CAH2042370.1 unnamed protein product, partial [Iphiclides podalirius]	1.82999	0.2733	-0.95882	-0.60527	-0.5392
TRINITY_DN29156_c0_g1_i1_orf1	-	-	-	protein FAM136A [Ostrinia furnacalis]	1.9195	-0.84257	-0.63661	-0.4475	0.00718
TRINITY_DN391_c1_g2_i1_orf1	-	-	-	NADH dehydrogenase [ubiquinone] 1 alpha subcomplex subunit 13 [Ostrinia furnacalis]	1.98187	-0.57709	-0.465	-0.67157	-0.26821
TRINITY_DN33619_c0_g1_i1_orf1	-	-	-	eukaryotic translation initiation factor 3 subunit C [Ostrinia furnacalis] >XP_028176017.1 eukaryotic translation initiation factor 3 subunit C [Ostrinia furnacalis]	1.9076	-0.38195	-0.85551	-0.69474	0.02459
TRINITY_DN3029_c4_g1_i1_orf1	-	-	-	proliferation marker protein Ki-67-like, partial [Ostrinia furnacalis]	1.36743	0.07964	-1.73673	0.32594	-0.03627
TRINITY_DN37599_c0_g1_i1_orf1	-	-	-	bmp-2 protein isoform X3 [Bombyx mori] >XP_028041166.1 RNA-binding protein 4.1-like isoform X2 [Bombyx mandarina]	1.65039	0.5519	-1.08003	-0.85726	-0.265
TRINITY_DN46367_c0_g1_i2_orf1	-	-	-	T-complex protein 1 subunit zeta [Ostrinia furnacalis]	1.92953	0.02282	-0.5888	-0.68715	-0.67641
TRINITY_DN13368_c0_g1_i1_orf1	-	-	-	isoleucine--tRNA ligase, cytoplasmic [Ostrinia furnacalis]	1.94859	-0.63594	-0.65267	-0.6082	-0.05179
TRINITY_DN172_c1_g1_i3_orf1	-	-	-	galactose oxidase, central domain-containing protein [Phthorimaea operculella]	1.54055	0.75578	-0.7567	-0.38404	-1.1556
TRINITY_DN1875_c0_g1_i1_orf1	-	-	-	uncharacterized protein LOC114366320 isoform X1 [Ostrinia furnacalis] >XP_028178963.1 uncharacterized protein LOC114366320 isoform X1 [Ostrinia furnacalis] >XP_028178964.1 uncharacterized protein LOC114366320 isoform X2 [Ostrinia furnacalis]	1.49011	0.2617	-1.5732	0.24249	-0.4211
TRINITY_DN9931_c0_g1_i1_orf1	-	-	-	syntaxin-18 [Ostrinia furnacalis]	1.82465	-0.07907	-1.22021	-0.12577	-0.39959
TRINITY_DN2456_c0_g1_i2_orf1	-	-	-	glycerol-3-phosphate phosphatase isoform X1 [Ostrinia furnacalis]	1.89915	0.13164	-0.6566	-0.67042	-0.70377
TRINITY_DN82008_c0_g1_i1_orf1	-	-	-	GMP reductase 1-like [Ostrinia furnacalis]	1.94219	-0.67703	-0.81329	-0.17109	-0.28078
TRINITY_DN271_c0_g2_i6_orf1	-	-	-	hypothetical protein NE865_03378 [Phthorimaea operculella]	1.85446	-0.71745	-0.71992	-0.6781	0.26101
TRINITY_DN257_c0_g1_i7_orf1	-	-	-	zinc finger RNA-binding protein 2 [Ostrinia furnacalis]	1.49379	0.70089	-1.30489	-0.74385	-0.14595
TRINITY_DN2266_c0_g1_i6_orf1	-	-	-	bilin-binding protein-like [Ostrinia furnacalis]	1.73985	-0.62034	0.5073	-0.87464	-0.75217
TRINITY_DN344_c0_g1_i1_orf1	-	-	-	chymotrypsin-like serine protease 16 [Ostrinia nubilalis]	1.99405	-0.47771	-0.63758	-0.40106	-0.47771
TRINITY_DN4836_c0_g1_i4_orf1	-	-	-	hypothetical protein O3G_MSEX014157 [Manduca sexta] >KAG6463927.1 hypothetical protein O3G_MSEX014157 [Manduca sexta]	1.62699	0.13708	-1.13748	-0.96289	0.33629
TRINITY_DN12806_c0_g2_i1_orf1	-	-	-	inactive pancreatic lipase-related protein 1-like isoform X2 [Ostrinia furnacalis]	1.59852	-0.91151	-1.06772	-0.25763	0.63834

TRINITY_DN127151_c0_g1_i1_orf1	-	-	-	3-oxoacyl-[acyl-carrier-protein] synthase, mitochondrial [Ostrinia furnacalis]	1.97184	-0.73554	-0.21076	-0.49628	-0.52926
TRINITY_DN3008_c0_g1_i12_orf1	-	-	-	reticulon-4-interacting protein 1 homolog, mitochondrial [Ostrinia furnacalis]	1.92333	-0.93192	-0.07222	-0.50783	-0.41136
TRINITY_DN37729_c0_g1_i8_orf1	-	-	-	adenylyltransferase and sulfurtransferase MOCS3 isoform X1 [Ostrinia furnacalis]	1.87635	-0.0578	-1.09495	-0.44916	-0.27444
TRINITY_DN2623_c0_g1_i3_orf1	-	-	-	unnamed protein product [Chilo suppressalis]	1.641	0.59352	-0.96248	-0.96727	-0.30477
TRINITY_DN40345_c0_g1_i6_orf1	-	-	-	60S ribosomal protein L28 [Ostrinia furnacalis]	1.86517	-0.64504	-0.88512	-0.53185	0.19684
TRINITY_DN4497_c0_g1_i4_orf1	-	-	-	cytochrome P450 9e2-like [Ostrinia furnacalis] >QPF77612.1 cytochrome P450 monooxygenase CYP9A185 [Ostrinia furnacalis]	1.80483	-0.55835	-0.87304	0.36051	-0.73395
TRINITY_DN2064_c1_g1_i1_orf1	-	-	-	hypothetical protein evm_007509 [Chilo suppressalis] >CAB3521498.1 unnamed protein product [Chilo suppressalis]	1.94339	-0.94251	-0.32077	-0.35933	-0.32077
TRINITY_DN6396_c0_g1_i1_orf1	-	-	-	PR domain zinc finger protein 10-like [Ostrinia furnacalis]	1.70947	-0.24835	-1.26526	-0.54288	0.34701
TRINITY_DN3343_c0_g2_i1_orf1	-	-	-	AFG3-like protein 2 [Ostrinia furnacalis]	1.65844	0.47443	-1.26819	-0.28684	-0.57785
TRINITY_DN31431_c0_g1_i1_orf1	-	-	-	carnosine N-methyltransferase [Ostrinia furnacalis]	1.12375	0.48585	-1.80854	-0.22498	0.42392
TRINITY_DN2374_c0_g1_i1_orf1	-	-	-	uncharacterized protein LOC114357127 [Ostrinia furnacalis]	1.58972	0.3792	-1.16655	-0.96963	0.16727
TRINITY_DN11375_c0_g1_i6_orf1	-	-	-	uncharacterized protein LOC114363514 isoform X2 [Ostrinia furnacalis]	1.18958	1.19351	-0.91083	-0.38465	-1.0876
TRINITY_DN53684_c0_g1_i1_orf1	-	-	-	eukaryotic translation initiation factor 3 subunit M-like [Ostrinia furnacalis]	1.93099	-0.03136	-0.78604	-0.68404	-0.42956
TRINITY_DN5818_c1_g1_i2_orf1	-	-	-	unnamed protein product [Chrysodeixis includens]	1.79349	0.18925	-0.8883	-0.97124	-0.1232
TRINITY_DN13972_c0_g1_i5_orf1	-	-	-	myelin expression factor 2-like [Ostrinia furnacalis] >XP_028173185.1 myelin expression factor 2-like [Ostrinia furnacalis]	1.67138	0.50184	-1.03543	-0.91185	-0.22595
TRINITY_DN12101_c0_g1_i2_orf1	-	-	-	UPF0545 protein C22orf39 homolog [Ostrinia furnacalis]	1.76554	0.28157	-0.16599	-0.88257	-0.99855
TRINITY_DN20007_c0_g1_i1_orf1	-	-	-	hypothetical protein evm_011958 [Chilo suppressalis] >CAB3521085.1 unnamed protein product [Chilo suppressalis]	1.77689	0.43211	-0.86906	-0.7085	-0.63143
TRINITY_DN24490_c0_g1_i6_orf1	-	-	-	E3 ubiquitin-protein ligase Hakai [Ostrinia furnacalis]	1.31083	1.07522	-1.10283	-0.4342	-0.84903
TRINITY_DN5648_c0_g1_i5_orf1	-	-	-	protein tumorous imaginal discs, mitochondrial-like isoform X2 [Ostrinia furnacalis]	1.89242	0.14415	-0.76516	-0.5897	-0.68171
TRINITY_DN3909_c0_g2_i2_orf1	-	-	-	ribosomal protein L24 [Loxostege sticticalis]	1.70979	-0.74721	-1.19939	-0.0424	0.2792
TRINITY_DN26243_c0_g1_i2_orf1	-	-	-	dynein heavy chain 6, axonemal [Ostrinia furnacalis]	1.80807	-0.06137	-1.05819	0.08605	-0.77457
TRINITY_DN21251_c1_g1_i1_orf1	-	-	-	60S ribosomal protein L4 [Ostrinia furnacalis]	1.79477	-0.75475	-1.07591	-0.14164	0.17752
TRINITY_DN11402_c0_g1_i1_orf1	-	-	-	constitutive coactivator of PPAR-gamma-like protein 1 isoform X1 [Ostrinia furnacalis] >XP_028158538.1 constitutive coactivator of PPAR-gamma-like protein 1 isoform X2 [Ostrinia furnacalis]	1.51015	0.88295	-0.86126	-0.87733	-0.65451
TRINITY_DN932_c0_g1_i4_orf1	-	-	-	SXSS-APN2 [Ostrinia furnacalis]	1.97865	-0.6369	-0.38856	-0.28423	-0.66897
TRINITY_DN1298_c0_g1_i3_orf1	-	-	-	ras GTPase-activating protein-binding protein 2-like, partial [Ostrinia furnacalis]	1.48262	0.37172	-1.54977	-0.48054	0.17597
TRINITY_DN26355_c0_g1_i4_orf1	-	-	-	small integral membrane protein 12 [Ostrinia furnacalis]	1.7215	0.54774	-0.84601	-0.77441	-0.64881
TRINITY_DN3835_c0_g1_i3_orf1	-	-	-	protein ERGIC-53 isoform X1 [Ostrinia furnacalis] >XP_028177940.1 protein ERGIC-53 isoform X2 [Ostrinia furnacalis] >XP_028177941.1 protein ERGIC-53 isoform X3 [Ostrinia furnacalis]	1.75628	0.39057	-1.1016	-0.56228	-0.48296
TRINITY_DN32769_c1_g1_i5_orf1	-	-	-	large subunit GTPase 1 homolog [Ostrinia furnacalis]	1.89065	-1.02845	-0.55771	-0.08029	-0.22421
TRINITY_DN5086_c0_g1_i1_orf1	-	-	-	unnamed protein product [Diatraea saccharalis]	1.72463	0.12954	-1.09075	-0.89539	0.13197
TRINITY_DN42373_c0_g4_i1_orf1	-	-	-	unnamed protein product [Spodoptera exigua]	1.5308	-0.95641	-1.08685	-0.20711	0.71957
TRINITY_DN1666_c0_g1_i2_orf1	-	-	-	putative defense protein Hdd11 [Ostrinia furnacalis] >XP_028179344.1 putative defense protein Hdd11 [Ostrinia furnacalis] >AGV28583.1 immune-induced protein [Ostrinia furnacalis]	1.65913	-0.97649	-0.06283	0.43094	-1.05076
TRINITY_DN22842_c0_g1_i4_orf1	-	-	-	MICOS complex subunit MIC27-like [Ostrinia furnacalis] >XP_028158921.1 MICOS complex subunit MIC27-like [Ostrinia furnacalis]	1.9877	-0.367	-0.55908	-0.66914	-0.39248
TRINITY_DN16408_c0_g1_i1_orf1	-	-	-	ABC transporter G family member 20 isoform X1 [Ostrinia furnacalis] >XP_028158027.1 ABC transporter G family member 20 isoform X1 [Ostrinia furnacalis] >XP_028158037.1 ABC transporter G family member 20 isoform X2 [Ostrinia furnacalis] >XP_028158043.1 ABC transporter G family member 20 isoform X3 [Ostrinia furnacalis] >XP_028158060.1 ABC transporter G family member 20 isoform X5 [Ostrinia furnacalis] >XP_028158070.1 ABC transporter G family member 20 isoform X6 [Ostrinia furnacalis]	1.78832	-0.54582	-1.16047	-0.31844	0.23641
TRINITY_DN2913_c0_g1_i5_orf1	-	-	-	aquaporin-11 isoform X1 [Spodoptera litura]	1.85269	-0.88649	-0.84315	0.11627	-0.23933
TRINITY_DN25373_c0_g1_i1_orf1	-	-	-	epidermal growth factor receptor substrate 15-like 1 [Ostrinia furnacalis]	1.85898	0.22905	-0.75894	-0.53708	-0.79201
TRINITY_DN45446_c0_g1_i2_orf1	-	-	-	peptide transporter family 1-like isoform X1 [Ostrinia furnacalis]	1.92942	-0.91002	-0.08448	-0.41737	-0.51756
TRINITY_DN18869_c0_g1_i1_orf1	-	-	-	unnamed protein product [Parnassius apollo]	1.84753	-1.10361	-0.54953	-0.25179	0.05739
TRINITY_DN25916_c0_g1_i1_orf1	-	-	-	uncharacterized protein LOC125063950 [Vanessa atalanta]	1.23467	0.39166	-1.49784	-0.79582	0.66733
TRINITY_DN36928_c0_g1_i2_orf1	-	-	-	actin-interacting protein 1 isoform X2 [Ostrinia furnacalis]	1.53363	-0.53853	-1.29004	-0.42274	0.71768
TRINITY_DN34726_c0_g2_i1_orf1	-	-	-	heat shock factor-binding protein 1 [Ostrinia furnacalis]	1.74004	0.4341	-0.97057	-0.35936	-0.8442

TRINITY_DN14301_c0_g2_i1_orf1	-	-	-	unnamed protein product [Chrysodeixis includens]	1.93678	-0.39853	-0.41645	-0.18179	-0.94
TRINITY_DN8958_c0_g1_i1_orf1	-	-	-	nuclear cap-binding protein subunit 1 [Galleria mellonella]	1.38614	0.66007	-1.59393	-0.23007	-0.2222
TRINITY_DN5262_c0_g1_i7_orf1	-	-	-	T-complex protein 1 subunit beta [Ostrinia furnacalis]	1.88441	0.04275	-0.89311	-0.75669	-0.27737
TRINITY_DN1509_c0_g1_i1_orf1	-	-	-	ribosomal protein S15A [Bombyx mori] >XP_011566807.1 40S ribosomal protein S15Aa [Plutella xylostella] >XP_013186470.1 PREDICTED: 40S ribosomal protein S15Aa [Amyelois transitella] >XP_021181353.1 40S ribosomal protein S15Aa [Helicoverpa armigera] >XP_022114309.1 40S ribosomal protein S15Aa [Pieris rapae] >XP_022820057.1 40S ribosomal protein S15Aa [Spodoptera litura] >XP_023947206.1 40S ribosomal protein S15Aa [Bicyclus anynana] >XP_026329660.1 40S ribosomal protein S15Aa [Hyposmocoma kahamanoa] >XP_026495126.1 40S ribosomal protein S15Aa [Vanessa tameamea] >XP_026745810.1 40S ribosomal protein S15Aa [Trichoplusia ni] >XP_026757934.1 40S ribosomal protein S15Aa [Galleria mellonella] >XP_028041686.1 40S ribosomal protein S15Aa [Bombyx mandarina] >XP_028161999.1 40S ribosomal protein S15Aa [Ostrinia furnacalis] >XP_028162000.1 40S ribosomal protein S15Aa [Ostrinia furnacalis] >XP_030024299.1 40S ribosomal protein S15Aa [Manduca sexta] >XP_032514052.1 40S ribosomal protein S15Aa [Danaus plexippus plexippus] >XP_034834075.1 40S ribosomal protein S15Aa [Maniola hyperantus] >XP_034840269.1 40S ribosomal protein S15Aa [Maniola hyperantus] >XP_035436795.1 40S ribosomal protein S15Aa [Spodoptera frugiperda] >XP_037869057.1 ribosomal protein S15A isoform X1 [Bombyx mori] >XP_039747368.1 40S ribosomal protein S15Aa [Pararge aegeria] >XP_045445511.1 40S ribosomal protein S15Aa [Melitaea cinxia] >XP_045491780.1 40S ribosomal protein S15Aa [Colias croceus] >XP_045519936.1 40S ribosomal protein S15Aa [Pieris brassicae] >XP_045785214.1 40S ribosomal protein S15Aa [Maniola jurtina] >XP_046965230.1 40S ribosomal protein S15Aa [Vanessa cardui] >XP_046965231.1 40S ribosomal protein S15Aa [Vanessa cardui] >XP_047021729.1 40S ribosomal protein S15Aa [Helicoverpa zea] >XP_047509814.1 40S ribosomal protein S15Aa [Pieris napi] >XP_047527633.1 40S ribosomal protein S15Aa [Vanessa atalanta] >XP_047527634.1 40S ribosomal protein S15Aa [Vanessa atalanta] >XP_047988443.1 40S ribosomal protein S15Aa [Leguminivora glycinivorella] >XP_050344874.1 40S ribosomal protein S15Aa [Nymphalis io] >XP_050344875.1 40S ribosomal protein S15Aa [Nymphalis io] >ADP21467.1 ribosomal protein S15A [Antheraea yamamai] >ADT80666.1 ribosomal protein S15A [Euphydryas aurinia] >AEL28847.1 ribosomal protein S15A [Heliconius melpomene cythera] >KAF9418418.1 hypothetical protein HW555_004706 [Spodoptera exigua] >KAI5633077.1 ribosomal protein s8 domain-containing protein [Phthorimaea operculella] >KOB75105.1 Ribosomal protein S15A, partial [Operophtera	1.86766	-0.96957	-0.74232	-0.14348	-0.01229
TRINITY_DN14336_c0_g3_i2_orf1	-	-	-	calbindin-32 isoform X1 [Ostrinia furnacalis]	1.97368	-0.69474	-0.25666	-0.64062	-0.38165
TRINITY_DN12579_c0_g1_i1_orf1	-	-	-	uncharacterized protein LOC114354070 isoform X3 [Ostrinia furnacalis]	1.90869	-0.66748	-0.9277	-0.1851	-0.1284
TRINITY_DN9316_c1_g1_i1_orf1	-	-	-	peptidyl-tRNA hydrolase 2, mitochondrial-like [Ostrinia furnacalis]	1.97911	-0.74145	-0.45774	-0.29065	-0.48927
TRINITY_DN41922_c0_g3_i1_orf1	-	-	-	influenza virus NS1A-binding protein-like [Ostrinia furnacalis]	1.9412	-0.79653	-0.58734	-0.49888	-0.05846
TRINITY_DN11825_c0_g1_i4_orf1	-	-	-	39S ribosomal protein L22, mitochondrial [Ostrinia furnacalis]	1.97699	-0.59327	-0.72092	-0.32314	-0.33966
TRINITY_DN27556_c0_g1_i1_orf1	-	-	-	bystin [Ostrinia furnacalis]	1.83926	0.28267	-0.57226	-0.84183	-0.70785
TRINITY_DN825_c2_g1_i5_orf1	-	-	-	hypothetical protein SFRURICE_018365 [Spodoptera frugiperda] >KAG8107343.1 hypothetical protein SFRUCORN_012069 [Spodoptera frugiperda] >CAB3509414.1 unnamed protein product [Spodoptera littoralis] >CAH1638995.1 unnamed protein product [Spodoptera	1.95156	-0.27186	-0.22967	-0.80713	-0.6429
TRINITY_DN36648_c0_g1_i1_orf1	-	-	-	UMP-CMP kinase [Ostrinia furnacalis]	1.98716	-0.43851	-0.71381	-0.43851	-0.39633
TRINITY_DN4835_c0_g1_i2_orf1	-	-	-	ribonucleoside-diphosphate reductase large subunit [Ostrinia furnacalis]	1.12323	1.14202	-1.24349	-0.9386	-0.08316
TRINITY_DN146718_c0_g1_i1_orf1	-	-	-	40S ribosomal protein S6 [Diachasma alloeum]	1.76301	-0.53444	-1.18981	0.28867	-0.32743
TRINITY_DN3582_c0_g1_i2_orf1	-	-	-	uncharacterized protein LOC114357129 [Ostrinia furnacalis]	1.66722	0.62536	-0.97935	-0.71942	-0.59381
TRINITY_DN91989_c0_g1_i1_orf1	-	-	-	protein l(2)37Cc [Pectinophora gossypiella]	1.96927	-0.21254	-0.43655	-0.74611	-0.57406
TRINITY_DN11928_c0_g1_i3_orf1	-	-	-	leucyl-cystinyl aminopeptidase-like isoform X4 [Ostrinia furnacalis]	1.91148	0.06676	-0.53894	-0.63145	-0.80785
TRINITY_DN57496_c0_g1_i1_orf1	-	-	-	NADPH:adrenodoxin oxidoreductase, mitochondrial [Ostrinia furnacalis]	1.57794	0.3025	-0.73294	0.20825	-1.35574
TRINITY_DN7289_c0_g1_i1_orf1	-	-	-	nuclear cap-binding protein subunit 2 [Ostrinia furnacalis]	1.77377	-0.44906	-0.85616	-0.8704	0.40185

TRINITY_DN4141_c0_g1_i9_orf1	-	-	-	la-related protein 1 isoform X2 [Ostrinia furnacalis] >XP_028160900.1 la-related protein 1 isoform X3 [Ostrinia furnacalis] >XP_028160902.1 la-related protein 1 isoform X2 [Ostrinia furnacalis] >XP_028160903.1 la-related protein 1 isoform X2 [Ostrinia furnacalis] >XP_028160904.1 la-related protein 1 isoform X2 [Ostrinia furnacalis] >XP_028160905.1 la-related protein 1 isoform X4 [Ostrinia furnacalis] >XP_028160906.1 la-related protein 1 isoform X2 [Ostrinia furnacalis] >XP_028160908.1 la-related protein 1 isoform X2 [Ostrinia furnacalis] >XP_028160909.1 la-related protein 1 isoform X2 [Ostrinia furnacalis] >XP_028160910.1 la-related protein 1 isoform X2 [Ostrinia furnacalis]	1.96866	-0.64191	-0.72977	-0.27044	-0.32654
TRINITY_DN58261_c0_g1_i1_orf1	-	-	-	15-hydroxyprostaglandin dehydrogenase [NAD(+)]-like [Ostrinia furnacalis]	1.57736	-0.39798	-1.35474	-0.41375	0.5891
TRINITY_DN88539_c0_g2_i1_orf1	-	-	-	uncharacterized protein LOC114352312 isoform X1 [Ostrinia furnacalis] >XP_028159669.1 uncharacterized protein LOC114352312 isoform X1 [Ostrinia furnacalis]	1.76558	-0.14704	-0.91795	-0.97201	0.27142
TRINITY_DN71465_c0_g1_i1_orf1	-	-	-	adenylate kinase isoenzyme 6 [Ostrinia furnacalis]	1.98406	-0.46283	-0.63425	-0.27969	-0.60728
TRINITY_DN9094_c0_g1_i1_orf1	-	-	-	uncharacterized protein LOC114356316 [Ostrinia furnacalis]	1.95968	-0.74691	-0.31415	-0.67214	-0.22648
TRINITY_DN60048_c0_g2_i1_orf1	-	-	-	facilitated trehalose transporter Tret1-like [Venturia canescens] >XP_043281789.1 facilitated trehalose transporter Tret1-like [Venturia canescens] >XP_043282611.1 facilitated trehalose transporter Tret1-like [Venturia canescens] >XP_043283442.1 facilitated trehalose transporter Tret1-like [Venturia canescens]	1.92602	-0.29858	-0.98034	-0.44775	-0.19936
TRINITY_DN2026_c0_g1_i4_orf1	-	-	-	60S ribosomal protein L35a [Ostrinia furnacalis] >XP_028167523.1 60S ribosomal protein L35a [Ostrinia furnacalis]	1.89889	-0.98219	-0.42513	-0.49872	0.00716
TRINITY_DN10429_c0_g1_i2_orf1	-	-	-	lon protease homolog, mitochondrial isoform X1 [Ostrinia furnacalis] >XP_028176557.1 lon protease homolog, mitochondrial isoform X2 [Ostrinia furnacalis]	1.97655	-0.70791	-0.56697	-0.46148	-0.24019
TRINITY_DN2682_c0_g1_i4_orf1	-	-	-	40S ribosomal protein S5 [Manduca sexta] >ACY95347.1 ribosomal protein S5 [Manduca sexta] >KAG6447616.1 hypothetical protein O3G_MSEX005033 [Manduca sexta] >KAG6447617.1 hypothetical protein O3G_MSEX005033 [Manduca sexta]	1.68329	-0.79078	-1.06755	-0.35193	0.52697
TRINITY_DN27_c0_g1_i1_orf1	-	-	-	THO complex subunit 4-A [Ostrinia furnacalis]	1.74515	0.34532	-1.10631	-0.74405	-0.24011
TRINITY_DN3366_c0_g1_i6_orf1	-	-	-	eukaryotic translation initiation factor 3 subunit K [Helicoverpa zea]	1.90351	-0.09968	-0.95958	-0.63373	-0.21051
TRINITY_DN82324_c0_g1_i4_orf1	-	-	-	hypothetical protein evm_001824 [Chilo suppressalis] >CAG9754426.1 unnamed protein product [Diatraea saccharalis] >CAG9793111.1 unnamed protein product [Diatraea saccharalis]	1.81148	-0.69976	-0.95105	-0.47496	0.3143
TRINITY_DN21214_c0_g2_i1_orf1	-	-	-	heat shock protein family A (Hsp70) member 1A [Homo sapiens] >KAI4017664.1 heat shock protein family A (Hsp70) member 1A [Homo sapiens] >PNI76655.1 HSPA1A isoform 2 [Pan troglodytes]	1.95414	-0.19778	-0.57726	-0.82815	-0.35094
TRINITY_DN54150_c0_g1_i1_orf1	-	-	-	uncharacterized protein LOC114351648 [Ostrinia furnacalis]	1.95554	-0.35894	-0.25529	-0.46781	-0.8735
TRINITY_DN10458_c0_g1_i1_orf1	-	-	-	V-type proton ATPase 21 kDa proteolipid subunit [Ostrinia furnacalis]	1.89603	-0.89704	-0.28127	0.00428	-0.72199
TRINITY_DN37393_c0_g1_i1_orf1	-	-	-	protein melted [Pectinophora gossypiella]	1.93614	-0.2943	-0.93481	-0.49661	-0.21042
TRINITY_DN1081_c0_g1_i7_orf1	-	-	-	3-ketoacyl-CoA thiolase, mitochondrial-like [Ostrinia furnacalis]	1.91851	-0.76584	-0.45616	0.02735	-0.72386
TRINITY_DN15706_c0_g2_i5_orf1	-	-	-	cdc42 homolog [Galleria mellonella] >XP_028178764.1 cdc42 homolog [Ostrinia furnacalis] >XP_028178765.1 cdc42 homolog [Ostrinia furnacalis]	1.78845	0.37911	-0.87386	-0.47761	-0.81609
TRINITY_DN48641_c0_g1_i4_orf1	-	-	-	RNA-binding protein 45-like [Galleria mellonella]	0.85224	1.45476	-0.94836	-1.0886	-0.27004
TRINITY_DN20984_c0_g1_i4_orf1	-	-	-	NADPH--cytochrome P450 reductase isoform X2 [Ostrinia furnacalis]	1.75683	-0.66179	-1.17995	0.24207	-0.15717
TRINITY_DN25896_c0_g1_i6_orf1	-	-	-	60S ribosomal export protein NMD3 [Ostrinia furnacalis]	1.97272	-0.48785	-0.23573	-0.75615	-0.493
TRINITY_DN4514_c0_g1_i1_orf1	-	-	-	enoyl-CoA delta isomerase 1, mitochondrial-like isoform X1 [Ostrinia furnacalis] >XP_028158560.1 enoyl-CoA delta isomerase 1, mitochondrial-like isoform X2 [Ostrinia furnacalis]	1.55263	-1.29469	0.00521	-0.79433	0.53118
TRINITY_DN6711_c0_g1_i1_orf1	-	-	-	mitochondrial uncoupling protein 4 [Ostrinia furnacalis] >XP_028158360.1 mitochondrial uncoupling protein 4 [Ostrinia furnacalis] >XP_028158361.1 mitochondrial uncoupling protein 4 [Ostrinia furnacalis] >XP_028158362.1 mitochondrial uncoupling protein 4 [Ostrinia	1.99855	-0.45007	-0.54679	-0.5365	-0.46519
TRINITY_DN1444_c1_g1_i5_orf1	-	-	-	spondin-1 isoform X1 [Ostrinia furnacalis] >XP_028167312.1 spondin-1 isoform X1 [Ostrinia furnacalis] >XP_028167313.1 spondin-1 isoform X1 [Ostrinia furnacalis] >XP_028167314.1 spondin-1 isoform X2 [Ostrinia furnacalis]	1.23855	1.15787	-1.16873	-0.66776	-0.55992
TRINITY_DN35277_c0_g1_i1_orf1	-	-	-	luciferin 4-monooxygenase-like, partial [Ostrinia furnacalis]	1.91541	-0.72567	-0.34847	-0.01487	-0.82641
TRINITY_DN2196_c0_g1_i2_orf1	-	-	-	HIRA-interacting protein 3-like [Ostrinia furnacalis]	1.63265	0.67497	-0.51592	-0.95749	-0.83422
TRINITY_DN42310_c0_g1_i1_orf1	-	-	-	uncharacterized protein LOC114349955 [Ostrinia furnacalis]	1.91249	-0.85167	-0.72448	-0.3016	-0.03473
TRINITY_DN19135_c0_g1_i1_orf1	-	-	-	ER membrane protein complex subunit 10 [Ostrinia furnacalis]	1.79137	0.13694	-1.23822	-0.36681	-0.32328
TRINITY_DN141738_c0_g1_i1_orf1	-	-	-	PREDICTED: fibroblast growth factor 1 [Microplitis demolitor]	1.98928	-0.62101	-0.60222	-0.36047	-0.40558

TRINITY_DN23264_c0_g1_i1_orf1	-	-	-	U5 small nuclear ribonucleoprotein 40 kDa protein [Ostrinia furnacalis]	1.63633	0.03669	-1.25376	-0.78463	0.36537
TRINITY_DN24281_c0_g1_i1_orf1	-	-	-	elongin-B isoform X1 [Maniola jurtina]	1.91925	-0.72391	-0.54378	-0.70293	0.05137
TRINITY_DN48554_c0_g1_i1_orf1	-	-	-	39S ribosomal protein L39, mitochondrial [Ostrinia furnacalis]	1.98343	-0.54418	-0.41583	-0.70312	-0.3203
TRINITY_DN235_c0_g3_i1_orf1	-	-	-	actin, muscle-type A2 [Bombyx mori] >XP_013199497.1 PREDICTED: actin, muscle-type A2 [Amyelois transitella] >XP_021196684.1 actin, muscle-type A2 [Helicoverpa armigera] >XP_022837900.1 actin, muscle-type A2 [Spodoptera litura] >XP_026314060.1 actin, muscle-type A2 [Hyposmocoma kahamanoa] >XP_026738711.1 actin, muscle-type A2 [Trichoplusia ni] >XP_028179440.1 actin, muscle-type A2 [Ostrinia furnacalis] >XP_030030527.1 actin, muscle-type A2 [Manduca sexta] >XP_035439272.1 actin, muscle-type A2 [Spodoptera frugiperda] >XP_047029939.1 actin, muscle-type A2 [Helicoverpa zea] >XP_049873365.1 actin, muscle-type A2 [Pectinophora gossypiella] >P07837.1 RecName: Full=Actin, muscle-type A2; Flags: Precursor [Bombyx mori] >KAF9423784.1 hypothetical protein HW555_000842 [Spodoptera exigua] >QLI62214.1 actin [Streltzoviella insularis] >CAB3227390.1 unnamed protein product [Arctia plantaginis] >CAB3508892.1 unnamed protein product [Spodoptera littoralis] >CAB3520808.1 unnamed protein product [Chilo suppressalis] >CAG9748331.1 unnamed protein product [Diatraea saccharalis] >CAH0585396.1 unnamed protein product [Chrysodeixis includens] >GBP21118.1 Actin, muscle-type A2 [Eumeta japonica]	1.45453	0.8164	-1.29136	-0.67745	-0.30213
TRINITY_DN64769_c0_g1_i3_orf1	-	-	-	procollagen-lysine,2-oxoglutarate 5-dioxygenase isoform X2 [Ostrinia furnacalis]	0.78737	1.52617	-0.99033	-0.34964	-0.97358
TRINITY_DN40562_c0_g2_i1_orf1	-	-	-	dual specificity protein phosphatase 23-like isoform X2 [Ostrinia furnacalis]	1.69783	0.53325	-0.78226	-0.43153	-1.01729
TRINITY_DN16487_c0_g1_i1_orf1	-	-	-	p21-activated protein kinase-interacting protein 1-like [Ostrinia furnacalis]	1.75995	-0.67282	0.18336	-0.08334	-1.18715
TRINITY_DN24693_c1_g1_i1_orf1	-	-	-	ubiquitin-conjugating enzyme E2 G2 isoform X2 [Ostrinia furnacalis]	1.98006	-0.51545	-0.48217	-0.26919	-0.71325
TRINITY_DN413_c0_g1_i11_orf1	-	-	-	regulator of nonsense transcripts 1 [Helicoverpa armigera] >XP_047028926.1 regulator of nonsense transcripts 1 [Helicoverpa zea]	1.94498	-0.06067	-0.4975	-0.73954	-0.64728
TRINITY_DN146119_c0_g1_i1_orf1	-	-	-	protein SEC13 homolog [Ostrinia furnacalis]	1.41704	0.78066	-1.36161	-0.11884	-0.71725
TRINITY_DN3814_c1_g1_i1_orf1	-	-	-	39S ribosomal protein L11, mitochondrial [Ostrinia furnacalis]	1.9726	-0.49096	-0.79343	-0.31699	-0.37123
TRINITY_DN84357_c0_g1_i1_orf1	-	-	-	4-coumarate--CoA ligase 1-like [Ostrinia furnacalis]	1.39544	-1.30077	0.08216	-0.90645	0.72962
TRINITY_DN6602_c0_g1_i4_orf1	-	-	-	PREDICTED: E3 ubiquitin-protein ligase RNF181-like [Amyelois transitella]	0.9943	0.7994	-1.76681	-0.36725	0.34035
TRINITY_DN4631_c0_g1_i7_orf1	-	-	-	uncharacterized protein LOC114366284 [Ostrinia furnacalis]	1.96727	-0.37868	-0.34053	-0.40995	-0.83811
TRINITY_DN8971_c1_g1_i4_orf1	-	-	-	synaptosomal-associated protein 25 isoform X1 [Bombyx mori]	1.64573	0.33507	-1.41846	-0.21346	-0.34888
TRINITY_DN49409_c0_g1_i2_orf1	-	-	-	proliferation-associated protein 2G4 [Ostrinia furnacalis]	1.95241	-0.37557	-0.9011	-0.27974	-0.396
TRINITY_DN1132_c0_g1_i5_orf1	-	-	-	unnamed protein product [Spodoptera exigua]	1.908	-0.26043	-0.76751	-0.83713	-0.04294
TRINITY_DN2167_c0_g1_i6_orf1	-	-	-	retinol dehydrogenase 13-like [Ostrinia furnacalis]	1.98084	-0.73882	-0.37076	-0.35369	-0.51757
TRINITY_DN5857_c0_g1_i13_orf1	-	-	-	uncharacterized protein LOC114353981 isoform X1 [Ostrinia furnacalis]	1.86415	-0.88097	-0.83492	-0.21683	0.06857
TRINITY_DN45633_c0_g1_i1_orf1	-	-	-	ubiquitin thioesterase otubain-like [Ostrinia furnacalis]	1.51959	0.83544	-1.05148	-0.51454	-0.78901
TRINITY_DN5457_c0_g1_i4_orf1	-	-	-	unnamed protein product [Chrysodeixis includens]	1.52048	0.73871	-1.14736	-0.87835	-0.23348
TRINITY_DN110888_c0_g1_i2_orf1	-	-	-	uncharacterized protein LOC114364502 [Ostrinia furnacalis]	1.77636	-0.88854	0.41555	-0.52329	-0.78009
TRINITY_DN3045_c0_g1_i7_orf1	-	-	-	hypothetical protein evm_007836 [Chilo suppressalis]	1.40999	0.14412	-1.26861	-0.9616	0.67609
TRINITY_DN141352_c0_g1_i1_orf1	-	-	-	carboxy-terminal domain RNA polymerase II polypeptide A small phosphatase 1 isoform X1 [Ostrinia furnacalis] >XP_028156862.1 carboxy-terminal domain RNA polymerase II polypeptide A small phosphatase 1 isoform X2 [Ostrinia furnacalis] >XP_028156863.1 carboxy-terminal domain RNA polymerase II polypeptide A small phosphatase 1 isoform X3 [Ostrinia	1.96058	-0.36934	-0.62679	-0.19783	-0.76662
TRINITY_DN28806_c0_g1_i1_orf1	-	-	-	ATP-dependent RNA helicase WM6 [Ostrinia furnacalis]	1.89686	-0.03246	-0.99474	-0.30593	-0.56373
TRINITY_DN21506_c0_g1_i4_orf1	-	-	-	glutamate dehydrogenase, mitochondrial isoform X2 [Ostrinia furnacalis]	1.95455	-0.89868	-0.35428	-0.36599	-0.33561
TRINITY_DN2252_c0_g1_i4_orfp1	-	-	-	TRINITY_DN2252_c0_g1_i4_m.69997 TRINITY_DN2252_c0_g1::TRINITY_DN2252_c0_g1_i4::g.69997 ORF type:5prime_partial len:168	1.50545	0.80946	-1.20845	-0.60763	-0.49883
TRINITY_DN2577_c0_g1_i1_orf1	-	-	-	unnamed protein product [Diatraea saccharalis]	1.55825	-0.76773	-1.32615	0.0674	0.46823
TRINITY_DN43942_c0_g1_i1_orf1	-	-	-	LOW QUALITY PROTEIN: caprin homolog [Ostrinia furnacalis]	1.89025	0.03485	-0.42505	-0.99498	-0.50507
TRINITY_DN4905_c0_g1_i6_orf1	-	-	-	uncharacterized protein LOC114351759 [Ostrinia furnacalis]	1.94623	-0.73087	-0.10871	-0.38921	-0.71744
TRINITY_DN130_c0_g1_i7_orf1	-	-	-	RNA-binding protein fusilli isoform X1 [Bombyx mori]	1.71697	0.09105	-1.36505	-0.42426	-0.0187
TRINITY_DN49872_c0_g2_i1_orf1	-	-	-	NIF3-like protein 1 [Ostrinia furnacalis] >XP_028165862.1 NIF3-like protein 1 [Ostrinia furnacalis] >XP_028165864.1 NIF3-like protein 1 [Ostrinia furnacalis]	1.50545	0.10656	-1.56152	0.35068	-0.40117
TRINITY_DN4008_c0_g1_i7_orf1	-	-	-	nuclear export mediator factor NEMF homolog isoform X1 [Ostrinia furnacalis]	1.78132	-0.73531	-0.95239	-0.47969	0.38607
TRINITY_DN1965_c0_g1_i7_orf1	-	-	-	CTP synthase isoform X1 [Ostrinia furnacalis]	1.95972	-0.28206	-0.42132	-0.86613	-0.39021
TRINITY_DN1578_c0_g3_i1_orf1	-	-	-	S-adenosylmethionine synthase isoform X1 [Ostrinia furnacalis]	1.95945	-0.7377	-0.13055	-0.50126	-0.58994

TRINITY_DN112120_c0_g1_i1_orf1	-	-	-	juvenile hormone esterase-like [Ostrinia furnacalis]	1.96363	-0.61019	-0.1342	-0.537	-0.68223
TRINITY_DN6985_c0_g1_i5_orf1	-	-	-	LYR motif-containing protein 4B [Ostrinia furnacalis]	1.60403	0.13255	-1.33189	0.32382	-0.72851
TRINITY_DN4309_c0_g1_i1_orf1	-	-	-	NEDD8-conjugating enzyme Ubc12, partial [Cotesia chilonis]	1.88897	-0.21857	-1.00527	-0.05667	-0.60847
TRINITY_DN880_c0_g1_i6_orf1	-	-	-	cuticle protein 19-like [Ostrinia furnacalis]	0.75837	1.52714	-1.16305	-0.32664	-0.79582
TRINITY_DN59335_c0_g1_i2_orf1	-	-	-	peroxisomal acyl-coenzyme A oxidase 3 [Ostrinia furnacalis]	1.9413	-0.9336	-0.30898	-0.24896	-0.44975
TRINITY_DN27751_c0_g2_i1_orf1	-	-	-	eukaryotic translation initiation factor 3 subunit I [Ostrinia furnacalis]	1.89431	-0.25289	-1.00862	-0.57144	-0.06137
TRINITY_DN11297_c0_g1_i1_orf1	-	-	-	ribosomal protein L13 [Conogethes punctiferalis] >QEE82690.1 ribosomal protein L13 [Conogethes pinicolalis]	1.834	-0.97759	-0.81956	-0.08319	0.04634
TRINITY_DN1427_c0_g1_i7_orf1	-	-	-	SAFB-like transcription modulator isoform X1 [Ostrinia furnacalis] >XP_028158609.1 SAFB-like transcription modulator isoform X2 [Ostrinia furnacalis]	1.68155	0.00111	-1.45149	-0.25497	0.02381
TRINITY_DN1791_c0_g1_i3_orf1	-	-	-	succinate dehydrogenase assembly factor 2-B, mitochondrial-like [Ostrinia furnacalis]	1.70393	-0.89252	-1.08646	-0.0647	0.33976
TRINITY_DN5919_c0_g1_i4_orf1	-	-	-	esterase FE4-like [Ostrinia furnacalis]	1.96011	-0.53206	-0.7798	-0.15576	-0.49248
TRINITY_DN5349_c0_g1_i1_orf1	-	-	-	glutamate-rich WD repeat-containing protein 1 [Galleria mellonella]	1.97438	-0.65831	-0.61391	-0.50113	-0.20103
TRINITY_DN2103_c0_g1_i1_orf1	-	-	-	probable aconitate hydratase, mitochondrial isoform X1 [Ostrinia furnacalis] >XP_028166656.1 probable aconitate hydratase, mitochondrial isoform X2 [Ostrinia furnacalis]	1.99886	-0.51618	-0.4403	-0.54346	-0.49892
TRINITY_DN2505_c0_g1_i1_orf1	-	-	-	uncharacterized protein LOC114349853 [Ostrinia furnacalis]	1.95631	-0.84056	-0.29595	-0.55598	-0.26382
TRINITY_DN5775_c0_g1_i1_orf1	-	-	-	proteasome assembly chaperone 2 [Ostrinia furnacalis]	1.87118	-0.10771	-1.131	-0.25328	-0.3792
TRINITY_DN4725_c0_g1_i4_orf1	-	-	-	uncharacterized protein LOC114354375 [Ostrinia furnacalis]	1.95127	-0.2892	-0.34496	-0.41105	-0.90606
TRINITY_DN52893_c0_g1_i1_orf1	-	-	-	growth arrest and DNA damage-inducible proteins-interacting protein 1 [Galleria mellonella]	1.93128	-0.13284	-0.89607	-0.59681	-0.30556
TRINITY_DN35669_c0_g1_i1_orf1	-	-	-	unnamed protein product [Diatraea saccharalis]	1.6974	0.55456	-0.91647	-0.86726	-0.46823
TRINITY_DN15370_c0_g1_i4_orf1	-	-	-	DNA replication licensing factor Mcm5 [Spodoptera litura]	1.93702	-0.12885	-0.47814	-0.42188	-0.90815
TRINITY_DN6189_c0_g1_i1_orf1	-	-	-	optic atrophy 3 protein homolog isoform X2 [Ostrinia furnacalis]	1.50916	0.84481	-0.62967	-0.61147	-1.11282
TRINITY_DN24318_c0_g1_i1_orf1	-	-	-	60S ribosomal protein L29 [Ostrinia furnacalis]	1.63854	-0.78424	-1.21293	-0.1076	0.46623
TRINITY_DN7464_c0_g1_i14_orf1	-	-	-	60S ribosomal protein L9 [Nymphalis io]	1.76688	-1.05558	-0.79977	-0.20103	0.2895
TRINITY_DN15896_c0_g1_i4_orf1	-	-	-	phosphatidylinositol transfer protein alpha isoform [Ostrinia furnacalis]	1.96084	-0.72163	-0.63799	-0.45326	-0.14797
TRINITY_DN338_c2_g1_i2_orf1	-	-	-	alpha-tocopherol transfer protein-like isoform X1 [Ostrinia furnacalis] >XP_028160444.1 alpha-tocopherol transfer protein-like isoform X1 [Ostrinia furnacalis] >XP_028160446.1 alpha-tocopherol transfer protein-like isoform X1 [Ostrinia furnacalis] >XP_028160447.1 alpha-tocopherol transfer protein-like isoform X1 [Ostrinia furnacalis] >XP_028160448.1 alpha-tocopherol transfer protein-like isoform X1 [Ostrinia furnacalis] >XP_028160449.1 alpha-tocopherol transfer protein-like isoform X1 [Ostrinia furnacalis]	1.93925	-0.06955	-0.50026	-0.83724	-0.5322
TRINITY_DN5704_c0_g1_i6_orf1	-	-	-	2-oxoglutarate dehydrogenase, mitochondrial isoform X2 [Ostrinia furnacalis]	1.97594	-0.77645	-0.32859	-0.48826	-0.38265
TRINITY_DN19493_c0_g1_i5_orf1	-	-	-	zinc finger MYM-type protein 3 isoform X1 [Ostrinia furnacalis] >XP_028159738.1 zinc finger MYM-type protein 3 isoform X2 [Ostrinia furnacalis]	1.29326	1.06615	-1.25506	-0.49802	-0.60633
TRINITY_DN3673_c0_g1_i10_orf1	-	-	-	hypothetical protein evm_008955 [Chilo suppressalis] >CAB3526829.1 unnamed protein product [Chilo suppressalis] >CAH0404157.1 unnamed protein product [Chilo suppressalis]	1.75951	0.36921	-0.35643	-1.07355	-0.69875
TRINITY_DN1725_c0_g1_i7_orf1	-	-	-	T-complex protein 1 subunit gamma isoform X1 [Ostrinia furnacalis] >XP_028159782.1 T-complex protein 1 subunit gamma isoform X2 [Ostrinia furnacalis]	1.81854	0.18875	-1.12563	-0.41262	-0.46903
TRINITY_DN83327_c0_g1_i1_orf1	-	-	-	trypsin-like serine proteinase T22 [Ostrinia nubilalis]	1.95835	-0.71308	-0.14753	-0.67556	-0.42218
TRINITY_DN136028_c0_g2_i1_orf1	-	-	-	cytochrome c oxidase subunit 5A, mitochondrial [Ostrinia furnacalis]	1.85666	0.17946	-0.73578	-0.39152	-0.90882
TRINITY_DN3393_c0_g2_i1_orf1	-	-	-	40S ribosomal protein S8 [Ostrinia furnacalis]	1.8958	-0.60679	-0.89665	-0.47616	0.0838
TRINITY_DN4779_c0_g1_i5_orf1	-	-	-	T-complex protein 1 subunit epsilon isoform X1 [Ostrinia furnacalis] >XP_028156782.1 T-complex protein 1 subunit epsilon isoform X2 [Ostrinia furnacalis]	1.89353	0.01937	-0.96336	-0.60757	-0.34197
TRINITY_DN17299_c0_g1_i4_orf1	-	-	-	RNA cytidine acetyltransferase isoform X1 [Ostrinia furnacalis] >XP_028171321.1 RNA cytidine acetyltransferase isoform X2 [Ostrinia furnacalis] >XP_028171329.1 RNA cytidine acetyltransferase isoform X3 [Ostrinia furnacalis]	1.96382	-0.81296	-0.49971	-0.22355	-0.4276
TRINITY_DN33926_c0_g1_i1_orf1	-	-	-	ribosomal protein S9 [Ailuropoda melanoleuca] >AEA39538.1 ribosomal protein S9 [Ailuropoda melanoleuca]	1.8659	-1.13486	-0.3907	-0.27001	-0.07034
TRINITY_DN37218_c0_g1_i12_orf1	-	-	-	protein white [Ostrinia furnacalis]	1.93389	-0.0798	-0.81313	-0.36121	-0.67975
TRINITY_DN9410_c0_g1_i4_orf1	-	-	-	protein RRP5 homolog [Ostrinia furnacalis]	1.8755	-1.09676	-0.2863	-0.44169	-0.05075
TRINITY_DN8367_c0_g1_i1_orf1	-	-	-	uncharacterized protein LOC114357075 [Ostrinia furnacalis]	1.91652	0.0011	-0.54882	-0.47349	-0.89531
TRINITY_DN98538_c0_g1_i1_orf1	-	-	-	ATP synthase subunit d, mitochondrial [Ostrinia furnacalis]	1.98411	-0.70786	-0.41821	-0.52702	-0.33103
TRINITY_DN13496_c0_g1_i7_orf1	-	-	-	nucleolar protein 58 [Ostrinia furnacalis]	1.89473	-0.79708	-0.70756	-0.50964	0.11954

TRINITY_DN36893_c0_g1_i1_orf1	-	-	-	40S ribosomal protein S15 [Bicyclus anynana] >XP_026325996.1 40S ribosomal protein S15 [Hyposmocoma kahamanoa] >XP_028175376.1 40S ribosomal protein S15 [Ostrinia furnacalis] >XP_030034906.1 40S ribosomal protein S15 [Manduca sexta] >XP_039758445.1 40S ribosomal protein S15 [Pararge aegeria] >XP_045775675.1 40S ribosomal protein S15 [Maniola jurtina] >CAH2267288.1 jg14755 [Pararge aegeria aegeria] >ACY95351.1 ribosomal protein S15 [Manduca sexta] >KAG6461386.1 hypothetical protein O3G_MSEX012590 [Manduca sexta] >KAG6461387.1 hypothetical protein O3G_MSEX012590 [Manduca sexta]	1.59632	-0.73593	-0.98916	-0.61511	0.74388
TRINITY_DN2691_c0_g1_i1_orf1	-	-	-	WD repeat-containing protein 18 [Ostrinia furnacalis]	1.98204	-0.74411	-0.43176	-0.34622	-0.45994
TRINITY_DN8511_c0_g1_i1_orf1	-	-	-	NADH dehydrogenase [ubiquinone] 1 beta subcomplex subunit 10 [Ostrinia furnacalis]	1.97186	-0.70445	-0.53922	-0.53674	-0.19146
TRINITY_DN31327_c0_g2_i1_orf1	-	-	-	multidrug resistance protein 1A isoform X1 [Ostrinia furnacalis]	1.90275	-0.34459	-1.06201	-0.1779	-0.31825
TRINITY_DN5112_c0_g1_i1_orf1	-	-	-	unnamed protein product, partial [Iphiclides podalirius]	1.74195	-0.12709	-1.37532	0.00116	-0.2407
TRINITY_DN42364_c0_g1_i4_orf1	-	-	-	brain tumor protein [Ostrinia furnacalis] >XP_028157996.1 brain tumor protein [Ostrinia	1.97966	-0.35702	-0.75421	-0.3728	-0.49563
TRINITY_DN10379_c0_g1_i3_orf1	-	-	-	succinate dehydrogenase [ubiquinone] cytochrome b small subunit, mitochondrial [Ostrinia furnacalis]	1.98882	-0.50166	-0.30633	-0.55968	-0.62115
TRINITY_DN9770_c0_g1_i1_orf1	-	-	-	flavin reductase (NADPH) [Ostrinia furnacalis] >XP_028160803.1 flavin reductase (NADPH) [Ostrinia furnacalis]	1.91211	-0.54292	-0.91619	0.00486	-0.45786
TRINITY_DN9101_c0_g2_i1_orf1	-	-	-	60S ribosomal protein L7a [Ostrinia furnacalis]	1.88382	-0.69617	-0.80482	-0.54181	0.15898
TRINITY_DN81926_c0_g1_i1_orf1	-	-	-	membrane-bound alkaline phosphatase-like isoform X3 [Ostrinia furnacalis]	1.87206	-0.8276	-0.72561	0.18199	-0.50084
TRINITY_DN9741_c0_g1_i3_orf1	-	-	-	metaxin-2 isoform X4 [Manduca sexta] >KAG6447312.1 hypothetical protein O3G_MSEX004872 [Manduca sexta]	1.83667	0.17975	-0.99917	-0.3104	-0.70686
TRINITY_DN5653_c0_g1_i4_orf1	-	-	-	hrp65 protein-like [Ostrinia furnacalis]	1.59492	0.69613	-0.89244	-1.01326	-0.38534
TRINITY_DN17376_c0_g1_i2_orf1	-	-	-	E3 UFM1-protein ligase 1 homolog [Ostrinia furnacalis]	1.8361	-0.42784	-1.17991	-0.23125	0.0029
TRINITY_DN9498_c0_g1_i3_orf1	-	-	-	eukaryotic translation initiation factor 4 gamma 3-like isoform X2 [Ostrinia furnacalis]	1.91796	-0.04033	-0.93453	-0.50113	-0.44196
TRINITY_DN4532_c0_g1_i1_orf1	-	-	-	3-hydroxyacyl-CoA dehydrogenase type-2 [Ostrinia furnacalis]	1.95004	-0.84688	-0.54059	-0.15953	-0.40305
TRINITY_DN5031_c0_g1_i1_orf1	-	-	-	PREDICTED: 40S ribosomal protein S12 [Trachymyrmex septentrionalis]	1.77055	-0.89764	-0.95768	-0.22089	0.30567
TRINITY_DN124950_c0_g2_i1_orf1	-	-	-	TATA box-binding protein-like protein 1 [Ostrinia furnacalis] >XP_028155830.1 TATA box-binding protein-like protein 1 [Ostrinia furnacalis]	1.55777	0.18518	-0.93676	-1.22058	0.4144
TRINITY_DN9575_c0_g1_i1_orf1	-	-	-	uncharacterized protein LOC114351119 [Ostrinia furnacalis]	1.40814	0.67233	-1.55116	-0.36193	-0.16738
TRINITY_DN2232_c1_g1_i3_orf1	-	-	-	protein FAM98A-like [Ostrinia furnacalis]	1.26225	0.80632	-1.24367	-1.07186	0.24696
TRINITY_DN103457_c0_g1_i1_orf1	-	-	-	28S ribosomal protein S22, mitochondrial [Ostrinia furnacalis]	1.94612	-0.31337	-0.26644	-0.44865	-0.91768
TRINITY_DN1266_c2_g1_i1_orf1	-	-	-	serine/threonine-protein kinase RIO3 [Ostrinia furnacalis]	1.62676	0.36561	-0.94729	0.10057	-1.14565
TRINITY_DN107840_c1_g1_i1_orf1	-	-	-	HEAT repeat-containing protein 3 [Ostrinia furnacalis]	1.95603	-0.09648	-0.641	-0.53401	-0.68453
TRINITY_DN34509_c0_g1_i1_orf1	-	-	-	transcription initiation factor IIA subunit 2 [Aphidius gifuensis] >KAF7997556.1 hypothetical protein HCN44_006127 [Aphidius gifuensis]	1.77518	0.42004	-0.72734	-0.91551	-0.55238
TRINITY_DN1375_c0_g1_i5_orf1	-	-	-	glutaminase [Chilo suppressalis] >CAB3528726.1 unnamed protein product [Chilo suppressalis] >CAH0405319.1 unnamed protein product [Chilo suppressalis]	1.96849	-0.65401	-0.38208	-0.22467	-0.70773
TRINITY_DN106038_c0_g1_i1_orf1	-	-	-	ankyrin-3-like isoform X1 [Galleria mellonella]	1.79543	0.39636	-0.83759	-0.69788	-0.65632
TRINITY_DN32681_c0_g1_i3_orf1	-	-	-	long-chain-fatty-acid--CoA ligase ACSBG2 isoform X2 [Ostrinia furnacalis]	1.87188	-1.05521	-0.42538	0.05443	-0.44572
TRINITY_DN2803_c2_g1_i8_orf1	-	-	-	trans-1,2-dihydrobenzene-1,2-diol dehydrogenase-like isoform X1 [Ostrinia furnacalis]	1.9322	-0.74543	-0.66663	-0.00389	-0.51625
TRINITY_DN7574_c0_g1_i10_orf1	-	-	-	proteasome activator complex subunit 3 isoform X2 [Ostrinia furnacalis]	1.74949	0.0671	-1.18911	-0.71619	0.08871
TRINITY_DN29291_c0_g1_i1_orf1	-	-	-	carboxylesterase [Ostrinia furnacalis]	1.93913	-0.35313	-0.10396	-0.68595	-0.79609
TRINITY_DN1191_c0_g1_i4_orf1	-	-	-	interferon-inducible double-stranded RNA-dependent protein kinase activator A homolog isoform X4 [Helicoverpa zea] >XP_047029704.1 interferon-inducible double-stranded RNA-dependent protein kinase activator A homolog isoform X4 [Helicoverpa zea]	1.98947	-0.42979	-0.64428	-0.34991	-0.56549
TRINITY_DN15388_c0_g1_i5_orf1	-	-	-	RNA-binding protein 28-like isoform X1 [Ostrinia furnacalis]	1.96473	-0.52478	-0.73673	-0.15872	-0.54449
TRINITY_DN15667_c0_g1_i2_orf1	-	-	-	coiled-coil domain-containing protein 25 [Ostrinia furnacalis]	1.94392	-0.06723	-0.54689	-0.79413	-0.53568
TRINITY_DN3539_c0_g1_i7_orf1	-	-	-	transcription elongation regulator 1-like [Ostrinia furnacalis]	1.70579	0.41319	-1.07914	-0.84732	-0.19251
TRINITY_DN2181_c1_g1_i8_orf1	-	-	-	vacuolar protein sorting-associated protein 37B [Ostrinia furnacalis]	1.46044	0.85635	-1.11793	-0.31186	-0.88699
TRINITY_DN755_c0_g1_i3_orf1	-	-	-	uncharacterized protein LOC114358844 [Ostrinia furnacalis]	1.97946	-0.26988	-0.52738	-0.46467	-0.71753
TRINITY_DN79868_c0_g1_i1_orf1	-	-	-	lethal(2)neighbour of Tid protein [Ostrinia furnacalis]	1.44478	0.56433	-1.4631	-0.66315	0.11714
TRINITY_DN142588_c0_g1_i1_orf1	-	-	-	peptidyl-prolyl cis-trans isomerase [Cotesia flavipes]	1.38026	1.0107	-1.03373	-0.8832	-0.47403
TRINITY_DN8944_c0_g1_i1_orf1	-	-	-	actin, clone 403 [Trichonephila clavata]	1.57473	0.75806	-0.99991	-0.83594	-0.49693
TRINITY_DN1956_c1_g1_i5_orf1	-	-	-	uncharacterized protein LOC114356377 [Ostrinia furnacalis]	1.94262	-0.44819	-0.90709	-0.41629	-0.17104
TRINITY_DN122786_c0_g2_i1_orf1	-	-	-	glucose dehydrogenase [FAD, quinone]-like [Ostrinia furnacalis]	1.96194	-0.4846	-0.6015	-0.72997	-0.14587

TRINITY_DN7251_c0_g1_i3_orf1	-	-	-	hypothetical protein evm_008498 [Chilo suppressalis] >CAB3527693.1 unnamed protein product [Chilo suppressalis] >CAH0401999.1 unnamed protein product [Chilo suppressalis]	1.5615	0.78477	-0.96277	-0.86768	-0.51581
TRINITY_DN37532_c0_g1_i1_orf1	-	-	-	transcription elongation factor S-II [Ostrinia furnacalis]	1.3182	0.9768	-1.34743	-0.62159	-0.32598
TRINITY_DN45598_c0_g1_i2_orf1	-	-	-	heat shock protein 60A-like [Ostrinia furnacalis]	1.97478	-0.72299	-0.51802	-0.22547	-0.5083
TRINITY_DN14372_c0_g2_i1_orf1	-	-	-	12 kDa FK506-binding protein-like [Ostrinia furnacalis]	1.89341	-0.01014	-0.90836	-0.72677	-0.24815
TRINITY_DN1348_c0_g1_i1_orf1	-	-	-	hypothetical protein evm_002665 [Chilo suppressalis]	1.98979	-0.42711	-0.676	-0.50752	-0.37915
TRINITY_DN8824_c0_g2_i1_orf1	-	-	-	60S ribosomal protein L34-like [Ostrinia furnacalis]	1.7633	-0.70005	-1.16732	-0.07572	0.17978
TRINITY_DN6556_c0_g1_i7_orf1	-	-	-	NFX1-type zinc finger-containing protein 1-like isoform X1 [Ostrinia furnacalis] >XP_028173496.1 NFX1-type zinc finger-containing protein 1-like isoform X1 [Ostrinia furnacalis] >XP_028173497.1 NFX1-type zinc finger-containing protein 1-like isoform X1	1.80197	-0.34759	-0.99788	0.28757	-0.74406
TRINITY_DN4908_c1_g1_i5_orf1	-	-	-	DNA topoisomerase 2 isoform X1 [Ostrinia furnacalis]	1.15561	1.28543	-0.94529	-0.75225	-0.7435
TRINITY_DN8561_c0_g4_i1_orf1	-	-	-	dynactin subunit 4 [Ostrinia furnacalis]	1.69124	0.37702	-1.19982	-0.13346	-0.73498
TRINITY_DN4820_c0_g1_i1_orf1	-	-	-	tudor and KH domain-containing protein homolog [Galleria mellonella]	1.9005	-0.61415	-0.91737	-0.40951	0.04054
TRINITY_DN4589_c0_g1_i1_orf1	-	-	-	protein penguin [Ostrinia furnacalis]	1.97446	-0.59927	-0.72755	-0.36456	-0.28307
TRINITY_DN14953_c0_g1_i5_orf1	-	-	-	EEF1A lysine methyltransferase 2 [Ostrinia furnacalis]	1.96737	-0.66132	-0.4466	-0.67806	-0.18139
TRINITY_DN21619_c0_g1_i1_orf1	-	-	-	28S ribosomal protein S15, mitochondrial [Ostrinia furnacalis]	1.85975	-0.4214	-0.83314	0.19059	-0.7958
TRINITY_DN3370_c0_g1_i5_orf1	-	-	-	unnamed protein product, partial [Brenthis ino]	1.5837	-0.35004	-0.99434	-0.94186	0.70253
TRINITY_DN21792_c0_g1_i1_orf1	-	-	-	probable 28S ribosomal protein S25, mitochondrial [Ostrinia furnacalis]	1.96375	-0.282	-0.34434	-0.5086	-0.82881
TRINITY_DN108819_c0_g1_i1_orf1	-	-	-	NADH dehydrogenase [ubiquinone] 1 beta subcomplex subunit 8, mitochondrial [Ostrinia furnacalis]	1.94125	-0.19022	-0.90988	-0.50332	-0.33784
TRINITY_DN14168_c0_g1_i1_orf1	-	-	-	transmembrane 7 superfamily member 3-like [Ostrinia furnacalis]	1.9769	-0.5543	-0.72588	-0.43498	-0.26174
TRINITY_DN143852_c0_g1_i1_orf1	-	-	-	60S ribosomal protein L10 [Cotesia glomerata]	1.49273	-1.01306	-1.06548	-0.17542	0.76123
TRINITY_DN2084_c0_g1_i1_orf1	-	-	-	40S ribosomal protein S24 [Helicoverpa armigera] >XP_022830907.1 40S ribosomal protein S24 [Spodoptera litura] >XP_026729116.1 40S ribosomal protein S24 [Trichoplusia ni] >XP_035432908.1 40S ribosomal protein S24 [Spodoptera frugiperda] >XP_047024473.1 40S ribosomal protein S24 [Helicoverpa zea] >Q962Q6.1 RecName: Full=40S ribosomal protein S24 [Spodoptera frugiperda] >KAF9418537.1 hypothetical protein HW555_004686 [Spodoptera exigua] >CAB3515448.1 unnamed protein product [Spodoptera littoralis] >CAH0579501.1 unnamed protein product [Chrysodeixis includens] >AAK92192.1 ribosomal protein S24 [Spodoptera frugiperda] >KAF9808794.1 hypothetical protein SFRURICE_013056 [Spodoptera frugiperda]	1.95615	-0.83695	-0.46922	-0.18077	-0.46922
TRINITY_DN14507_c0_g1_i5_orf1	-	-	-	PTB domain-containing adapter protein ced-6 [Ostrinia furnacalis]	0.92095	1.24389	-0.99497	-1.26696	0.09709
TRINITY_DN18159_c0_g1_i6_orf1	-	-	-	zinc carboxypeptidase-like [Ostrinia furnacalis]	1.5613	-0.88592	-1.18411	-0.09632	0.60505
TRINITY_DN2283_c0_g2_i1_orf1	-	-	-	H/ACA ribonucleoprotein complex subunit 4 [Ostrinia furnacalis]	1.91626	-0.94685	-0.56313	-0.07712	-0.32917
TRINITY_DN2975_c0_g1_i4_orf1	-	-	-	ubiquitin-like protein 4A [Ostrinia furnacalis]	1.89304	-0.08028	-1.04664	-0.28054	-0.48559
TRINITY_DN86090_c0_g1_i1_orf1	-	-	-	ATP synthase subunit b, mitochondrial [Ostrinia furnacalis]	1.9634	-0.82465	-0.46218	-0.44441	-0.23216
TRINITY_DN18933_c0_g1_i3_orf1	-	-	-	PREDICTED: protein BUD31 homolog [Papilio xuthus] >XP_014361644.1 protein BUD31 homolog [Papilio machaon] >XP_026750578.1 protein BUD31 homolog [Galleria mellonella] >XP_047995610.1 protein BUD31 homolog [Leguminivora glycinivorella] >XP_049869593.1 protein BUD31 homolog [Pectinophora gossypiella] >KAI5652084.1 g10 protein domain-containing protein [Phthorimaea operculella] >CAB3251981.1 unnamed protein product [Arctia plantaginis] >CAB3520382.1 unnamed protein product [Chilo suppressalis] >CAG9747228.1 unnamed protein product [Diatraea saccharalis] >CAH2037008.1 unnamed protein product, partial [Iphiclides podalirius]	1.17209	1.05956	-1.47873	-0.50579	-0.24713
TRINITY_DN26251_c0_g1_i1_orf1	-	-	-	serine/arginine-rich splicing factor 1A [Neodiprion lecontei] >XP_046417766.1 serine/arginine-rich splicing factor 1A [Neodiprion fabricii] >XP_046473571.1 serine/arginine-rich splicing factor 1A [Neodiprion pinetum] >XP_046610590.1 serine/arginine-rich splicing factor 1A [Neodiprion virginianus] >XP_046738887.1 serine/arginine-rich splicing factor 1A [Diprion	1.58375	0.4142	-1.34382	-0.71459	0.06047
TRINITY_DN154_c0_g1_i4_orf1	-	-	-	ER membrane protein complex subunit 4 [Ostrinia furnacalis]	1.77325	0.37014	-1.018	-0.71923	-0.40617
TRINITY_DN12527_c0_g1_i4_orf1	-	-	-	DNA-directed RNA polymerase III subunit RPC4 isoform X1 [Ostrinia furnacalis]	1.69871	0.45903	-1.12136	-0.28454	-0.75183
TRINITY_DN9059_c0_g1_i1_orf1	-	-	-	sulfotransferase family cytosolic 1B member 1-like [Ostrinia furnacalis]	1.93917	-0.03022	-0.51219	-0.71986	-0.67689
TRINITY_DN25997_c1_g2_i4_orf1	-	-	-	ribokinase-like [Ostrinia furnacalis]	1.41008	0.34563	-0.78946	0.46612	-1.43237
TRINITY_DN6235_c0_g1_i5_orf1	-	-	-	rRNA 2'-O-methyltransferase fibrillarin [Vanessa cardui]	1.92191	-0.56421	-0.76278	-0.63594	0.04101
TRINITY_DN13530_c0_g1_i1_orf1	-	-	-	unnamed protein product [Euphydryas editha]	1.96611	-0.48867	-0.82715	-0.32514	-0.32514

TRINITY_DN11799_c0_g1_i4_orf1	-	-	-	V-type proton ATPase 116 kDa subunit a1 isoform X1 [Manduca sexta]	1.93679	-0.06385	-0.40019	-0.74365	-0.7291
TRINITY_DN29521_c0_g1_i1_orf1	-	-	-	density-regulated protein homolog [Ostrinia furnacalis]	1.52673	-0.77925	-1.2894	-0.08434	0.62626
TRINITY_DN50151_c0_g1_i1_orf1	-	-	-	heat shock 70 kDa protein 14 [Ostrinia furnacalis]	1.95408	-0.21795	-0.86206	-0.50379	-0.37029
TRINITY_DN646_c0_g1_i5_orf1	-	-	-	unnamed protein product [Diatraea saccharalis]	1.89821	0.10418	-0.83593	-0.52468	-0.64178
TRINITY_DN25542_c0_g1_i1_orf1	-	-	-	tRNA-dihydrouridine(47) synthase [NAD(P)(+)]-like [Ostrinia furnacalis]	1.94109	-0.45392	-0.93412	-0.29425	-0.2588
TRINITY_DN7512_c0_g1_i1_orf1	-	-	-	hypothetical protein evm_010529 [Chilo suppressalis] >CAB3530682.1 unnamed protein product [Chilo suppressalis] >CAH0407273.1 unnamed protein product [Chilo suppressalis]	1.92632	-0.88853	-0.22429	-0.16322	-0.65029
TRINITY_DN2769_c0_g1_i1_orf1	-	-	-	pseudouridylate synthase 7 homolog [Ostrinia furnacalis]	1.80583	0.22357	-1.03662	-0.74298	-0.2498
TRINITY_DN34432_c0_g1_i1_orf1	-	-	-	39S ribosomal protein L44, mitochondrial [Ostrinia furnacalis]	1.78389	0.42504	-0.76429	-0.65356	-0.79108
TRINITY_DN3860_c0_g1_i5_orf1	-	-	-	nucleoplasmin-like protein isoform X1 [Hyposmocoma kahamanoa]	1.4081	0.41215	-1.16359	-1.12766	0.47099
TRINITY_DN9164_c0_g1_i3_orf1	-	-	-	unnamed protein product [Parnassius apollo]	1.78025	0.18306	-1.13299	-0.70569	-0.12464
TRINITY_DN5767_c0_g1_i4_orf1	-	-	-	cell division cycle 5-like protein [Helicoverpa armigera]	1.94936	-0.05571	-0.65905	-0.60254	-0.63205
TRINITY_DN492_c0_g1_i4_orf1	-	-	-	hypothetical protein evm_000589 [Chilo suppressalis] >CAB3530014.1 unnamed protein product [Chilo suppressalis] >CAH0406606.1 unnamed protein product [Chilo suppressalis]	1.97483	-0.51727	-0.59359	-0.66321	-0.20076
TRINITY_DN18242_c0_g1_i3_orf1	-	-	-	CCHC-type zinc finger protein CG3800 [Papilio xuthus]	1.70898	0.17094	-1.08865	-0.92097	0.1297
TRINITY_DN14107_c0_g1_i4_orf1	-	-	-	bifunctional methylenetetrahydrofolate dehydrogenase/cyclohydrolase, mitochondrial isoform X1 [Ostrinia furnacalis]	1.93663	-0.1423	-0.31577	-0.60431	-0.87425
TRINITY_DN3474_c1_g2_i7_orf1	-	-	-	LOW QUALITY PROTEIN: endoplasmic reticulum metallopeptidase 1-like [Ostrinia furnacalis]	1.85238	0.06412	-0.99293	-0.18595	-0.73762
TRINITY_DN30638_c0_g1_i1_orf1	-	-	-	alanine--tRNA ligase, cytoplasmic [Ostrinia furnacalis]	1.93608	-0.36341	-0.91315	-0.51443	-0.14509
TRINITY_DN42646_c0_g2_i1_orf1	-	-	-	40S ribosomal protein S3 [Helicoverpa armigera] >XP_026740562.1 40S ribosomal protein S3 [Trichoplusia ni] >XP_026751545.1 40S ribosomal protein S3 [Galleria mellonella] >XP_047027704.1 40S ribosomal protein S3 [Helicoverpa zea] >CAH0591481.1 unnamed protein product [Chrysodeixis includens] >AIR07416.1 ribosomal protein S3 [Helicoverpa armigera] >AND95944.1 ribosomal protein S3 [Helicoverpa armigera] >AXY94820.1 ribosomal ribosomal protein S3 [Galleria mellonella] >PZC80336.1 hypothetical protein B5X24_HaOG214853 [Helicoverpa armigera]	1.80977	-0.92257	-0.90206	-0.16545	0.18031
TRINITY_DN66596_c0_g1_i1_orf1	-	-	-	CCR4-NOT transcription complex subunit 10 [Ostrinia furnacalis]	1.94141	-0.38532	-0.93972	-0.23956	-0.3768
TRINITY_DN4747_c0_g1_i4_orf1	-	-	-	transcription factor A, mitochondrial [Ostrinia furnacalis]	1.87947	0.07444	-1.00291	-0.43062	-0.52038
TRINITY_DN15737_c0_g1_i7_orf1	-	-	-	UPF0160 protein C27H6.8 [Ostrinia furnacalis]	1.65987	-0.76283	-1.22284	-0.07637	0.40217
TRINITY_DN15882_c0_g1_i1_orf1	-	-	-	succinate--CoA ligase [ADP-forming] subunit beta, mitochondrial [Ostrinia furnacalis]	1.98583	-0.69555	-0.53884	-0.37688	-0.37455
TRINITY_DN24043_c0_g1_i1_orf1	-	-	-	uncharacterized protein LOC110377964 [Helicoverpa armigera] >XP_047026962.1 cytochrome c1-2, heme protein, mitochondrial [Helicoverpa zea] >PZC76159.1 hypothetical protein B5X24_HaOG204935 [Helicoverpa armigera]	1.97196	-0.65039	-0.37217	-0.69738	-0.25202
TRINITY_DN10520_c0_g1_i2_orf1	-	-	-	probable 39S ribosomal protein L45, mitochondrial [Ostrinia furnacalis]	1.9782	-0.23492	-0.57035	-0.68229	-0.49064
TRINITY_DN144956_c0_g1_i1_orf1	-	-	-	40S ribosomal protein S18 [Cotesia glomerata] >CAD6216330.1 GSCOCG00004483001-RA-CDS [Cotesia congregata] >CAG5095266.1 Similar to RpS18: 40S ribosomal protein S18 (Spodoptera frugiperda) [Cotesia congregata]	1.85941	-0.75095	-0.97011	-0.18749	0.04915
TRINITY_DN10644_c0_g1_i2_orf1	-	-	-	carboxylesterase [Cnaphalocrocis medinalis]	1.81656	0.32963	-0.56311	-0.68833	-0.89475
TRINITY_DN2070_c1_g1_i1_orf1	-	-	-	mitochondrial carrier homolog 2-like [Helicoverpa zea] >PZC82360.1 hypothetical protein B5X24_HaOG210663 [Helicoverpa armigera]	1.98856	-0.60629	-0.58933	-0.48689	-0.30604
TRINITY_DN7868_c0_g1_i8_orf1	-	-	-	uncharacterized protein LOC114353432 isoform X4 [Ostrinia furnacalis]	1.69943	-0.10436	-1.44489	-0.10704	-0.04314
TRINITY_DN4237_c1_g1_i5_orf1	-	-	-	eukaryotic translation initiation factor 3 subunit A-like isoform X1 [Ostrinia furnacalis] >XP_028173593.1 eukaryotic translation initiation factor 3 subunit A-like isoform X2 [Ostrinia furnacalis] >XP_028173594.1 eukaryotic translation initiation factor 3 subunit A-like isoform X3 [Ostrinia furnacalis] >XP_028173595.1 eukaryotic translation initiation factor 3 subunit A-like isoform X4 [Ostrinia furnacalis]	1.88345	-0.24629	-1.03064	-0.57328	-0.03325
TRINITY_DN33801_c0_g1_i1_orf1	-	-	-	unnamed protein product [Diatraea saccharalis]	1.79402	0.24141	-1.14078	-0.5513	-0.34335
TRINITY_DN35301_c0_g1_i3_orf1	-	-	-	ATP synthase subunit gamma, mitochondrial-like [Ostrinia furnacalis] >XP_028164649.1 ATP synthase subunit gamma, mitochondrial-like [Ostrinia furnacalis]	1.94941	-0.90794	-0.44014	-0.32236	-0.27897
TRINITY_DN24751_c0_g1_i1_orf1	-	-	-	NADH dehydrogenase [ubiquinone] 1 beta subcomplex subunit 7-like [Ostrinia furnacalis]	1.96916	-0.44889	-0.81515	-0.28994	-0.41518
TRINITY_DN29038_c0_g2_i1_orf1	-	-	-	PREDICTED: ATP synthase lipid-binding protein, mitochondrial [Fopius arisanus] >XP_011314178.1 PREDICTED: ATP synthase lipid-binding protein, mitochondrial [Fopius arisanus] >XP_011314186.1 PREDICTED: ATP synthase lipid-binding protein, mitochondrial	1.95804	-0.45197	-0.8704	-0.35085	-0.28481
TRINITY_DN23586_c0_g1_i3_orf1	-	-	-	myrosinase 1-like isoform X1 [Ostrinia furnacalis]	1.97613	-0.7219	-0.30981	-0.34603	-0.59839

TRINITY_DN2718_c0_g1_i6_orf1	-	-	-	cleavage stimulation factor subunit 2 isoform X1 [Ostrinia furnacalis]	1.44914	0.89166	-1.11714	-0.84447	-0.3792
TRINITY_DN4132_c0_g1_i14_orf1	-	-	-	thyroid receptor-interacting protein 11-like isoform X1 [Ostrinia furnacalis]	1.38801	-0.0084	-1.73239	0.24693	0.10585
TRINITY_DN1422_c0_g1_i4_orf1	-	-	-	unnamed protein product [Chilo suppressalis]	1.98452	-0.64734	-0.50306	-0.56144	-0.27268
TRINITY_DN108354_c0_g1_i1_orf1	-	-	-	WD repeat-containing protein 61-like [Ostrinia furnacalis]	1.83227	-0.05592	-1.15972	-0.07965	-0.53698
TRINITY_DN825_c8_g1_i5_orf1	-	-	-	ATP-binding cassette sub-family F member 2 [Ostrinia furnacalis] >XP_028169527.1 ATP-binding cassette sub-family F member 2 [Ostrinia furnacalis]	1.96884	-0.58843	-0.7092	-0.49241	-0.1788
TRINITY_DN959_c0_g1_i7_orf1	-	-	-	Golgi to ER traffic protein 4 homolog [Ostrinia furnacalis]	1.84772	0.18443	-0.36708	-0.70813	-0.95693
TRINITY_DN147475_c0_g1_i1_orf1	-	-	-	casein kinase II subunit beta, partial [Rhincodon typus]	1.19206	1.15026	-1.20496	-0.84903	-0.28833
TRINITY_DN1477_c0_g1_i5_orf1	-	-	-	mitochondrial import inner membrane translocase subunit Tim9 [Ostrinia furnacalis]	1.97814	-0.41792	-0.68129	-0.6154	-0.26353
TRINITY_DN2593_c0_g2_i1_orf1	-	-	-	midgut carboxypeptidase [Loxostege sticticalis]	1.9548	-0.75102	-0.21289	-0.29737	-0.69352
TRINITY_DN38075_c0_g1_i1_orf1	-	-	-	60S ribosomal protein L26 [Ostrinia furnacalis]	1.91826	-0.47813	-0.91665	-0.50089	-0.02259
TRINITY_DN11655_c0_g1_i1_orf1	-	-	-	ribosome biogenesis protein BRX1 homolog [Ostrinia furnacalis]	1.9701	-0.80225	-0.27982	-0.40848	-0.47954
TRINITY_DN22678_c0_g1_i4_orf1	-	-	-	NADH-cytochrome b5 reductase 2 isoform X2 [Ostrinia furnacalis] >XP_028163866.1 NADH-cytochrome b5 reductase 2 isoform X2 [Ostrinia furnacalis]	1.91384	-0.75245	-0.66739	0.07206	-0.56606
TRINITY_DN24163_c0_g1_i1_orf1	-	-	-	luciferin 4-monooxygenase-like [Ostrinia furnacalis]	1.83207	0.24932	-0.91547	-0.40466	-0.76126
TRINITY_DN101682_c0_g1_i1_orf1	-	-	-	cysteine-rich with EGF-like domain protein 2 [Ostrinia furnacalis]	1.65369	-0.4215	-1.43246	0.01162	0.18865
TRINITY_DN37165_c0_g1_i4_orf1	-	-	-	pyridoxine-5'-phosphate oxidase-like [Ostrinia furnacalis]	1.8884	-0.24462	-1.01636	-0.04509	-0.58233
TRINITY_DN16122_c0_g1_i4_orf1	-	-	-	cytochrome P450 6k1-like [Ostrinia furnacalis]	1.91122	-0.67755	-0.21858	-0.10432	-0.91077
TRINITY_DN17045_c0_g2_i3_orf1	-	-	-	unnamed protein product [Diatraea saccharalis]	1.84678	-0.27247	-1.0239	-0.67227	0.12187
TRINITY_DN14429_c0_g1_i2_orf1	-	-	-	NADH dehydrogenase [ubiquinone] 1 beta subcomplex subunit 11, mitochondrial [Ostrinia furnacalis]	1.661	0.5428	-0.48705	-0.51513	-1.20162
TRINITY_DN17049_c0_g1_i6_orf1	-	-	-	unnamed protein product [Arctia plantaginis] >CAB3248215.1 unnamed protein product [Arctia plantaginis]	1.96642	-0.39105	-0.78086	-0.56297	-0.23155
TRINITY_DN10131_c0_g1_i7_orf1	-	-	-	aldo-keto reductase AKR2E4-like [Ostrinia furnacalis]	1.04157	1.37075	-1.05755	-0.67119	-0.68358
TRINITY_DN50875_c0_g1_i3_orf1	-	-	-	conserved oligomeric Golgi complex subunit 8 [Ostrinia furnacalis]	1.15983	1.19502	-1.28632	-0.50961	-0.55892
TRINITY_DN3229_c0_g1_i1_orf1	-	-	-	uncharacterized protein LOC114358442 isoform X1 [Ostrinia furnacalis]	1.86854	-1.14521	-0.35898	-0.15197	-0.21238
TRINITY_DN10476_c0_g1_i1_orf1	-	-	-	prohibitin-2 [Ostrinia furnacalis]	1.9722	-0.5746	-0.7335	-0.43623	-0.22788
TRINITY_DN16258_c0_g1_i2_orf1	-	-	-	uncharacterized protein LOC114359911 [Ostrinia furnacalis]	1.76277	0.37106	-0.90148	-0.91864	-0.31371
TRINITY_DN142652_c0_g1_i1_orf1	-	-	-	pre-mRNA-splicing factor RBM22 [Chelonus insularis]	1.70224	0.12835	-1.38494	-0.40794	-0.03772
TRINITY_DN2535_c0_g1_i4_orf1	-	-	-	ATP-dependent RNA helicase DDX24 [Ostrinia furnacalis]	1.93773	-0.78085	-0.03097	-0.58118	-0.54473
TRINITY_DN143603_c0_g1_i1_orf1	-	-	-	hypothetical protein KR044_005587 [Drosophila immigrans]	1.96445	-0.73152	-0.51402	-0.15548	-0.56343
TRINITY_DN20499_c0_g3_i1_orf1	-	-	-	exosome RNA helicase MTR4 isoform X2 [Ostrinia furnacalis]	1.85433	-0.00448	-1.01461	-0.72027	-0.11497
TRINITY_DN51239_c0_g1_i5_orf1	-	-	-	regulatory-associated protein of mTOR [Ostrinia furnacalis]	1.78427	-0.48486	-1.06478	0.34094	-0.57558
TRINITY_DN40650_c0_g1_i1_orf1	-	-	-	60S ribosomal protein L12 [Zerene cesonia]	1.7939	-0.89694	-0.92978	-0.22068	0.2535
TRINITY_DN61222_c0_g1_i1_orf1	-	-	-	60S ribosomal protein L38 [Bicyclus anynana]	1.88546	-0.48925	-0.9651	-0.51667	0.08556
TRINITY_DN12134_c0_g1_i4_orf1	-	-	-	glutathione S-transferase 1-1 [Ostrinia furnacalis] >XP_028161942.1 glutathione S-transferase 1-1 [Ostrinia furnacalis] >XP_028161943.1 glutathione S-transferase 1-1 [Ostrinia furnacalis]	0.74393	1.485	-1.36445	-0.37763	-0.48685
TRINITY_DN3929_c0_g1_i1_orf1	-	-	-	glutathione S-transferase 1-1-like [Ostrinia furnacalis]	1.9385	-0.83552	-0.26788	-0.16887	-0.66623
TRINITY_DN51568_c0_g1_i1_orf1	-	-	-	splicing factor 3A subunit 2 [Ostrinia furnacalis]	1.87945	-0.15273	-1.05284	-0.57018	-0.1037
TRINITY_DN94625_c0_g1_i1_orf1	-	-	-	uncharacterized protein LOC114354112 [Ostrinia furnacalis]	1.99139	-0.66383	-0.5065	-0.41609	-0.40497
TRINITY_DN886_c0_g1_i1_orf1	-	-	-	collagenase-like isoform X1 [Ostrinia furnacalis]	1.92497	-0.29344	-0.23813	-0.39596	-0.99744
TRINITY_DN79734_c0_g2_i3_orf1	-	-	-	60S ribosomal protein L27a [Ostrinia furnacalis]	1.83283	-0.70563	-1.06807	-0.04159	-0.01754
TRINITY_DN17271_c0_g1_i1_orf1	-	-	-	uncharacterized protein LOC114350693 [Ostrinia furnacalis]	1.23102	1.14092	-1.1671	-0.8203	-0.38453
TRINITY_DN4956_c0_g1_i6_orf1	-	-	-	nucleolar GTP-binding protein 1 [Ostrinia furnacalis]	1.99475	-0.5067	-0.36443	-0.57107	-0.55254
TRINITY_DN7787_c0_g1_i1_orf1	-	-	-	trimeric intracellular cation channel type 1B.1 [Manduca sexta] >KAG6456518.1 hypothetical protein O3G_MSEX009773 [Manduca sexta]	1.86323	0.0625	-0.93394	-0.21272	-0.77907
TRINITY_DN2879_c0_g1_i4_orf1	-	-	-	nucleoporin Nup35 [Ostrinia furnacalis]	1.06596	1.10363	-1.44112	-0.75383	0.02536
TRINITY_DN78686_c0_g1_i1_orf1	-	-	-	myosin-2 essential light chain isoform X2 [Harpegnathos saltator] >XP_012170910.1 myosin-2 essential light chain isoform X2 [Bombus terrestris] >XP_033185931.1 myosin-2 essential light chain isoform X2 [Bombus vancouverensis nearcticus] >XP_033319091.1 myosin-2 essential light chain isoform X2 [Bombus bifarius] >XP_033349866.1 myosin-2 essential light chain isoform X2 [Bombus vosnesenskii] >XP_043597873.1 myosin-2 essential light chain isoform X2 [Bombus pyrosoma]	0.96003	1.26554	-1.47755	-0.29088	-0.45715
TRINITY_DN104507_c0_g1_i2_orf1	-	-	-	replication protein A 32 kDa subunit [Ostrinia furnacalis]	1.40976	0.80831	-1.36383	-0.68626	-0.16798

TRINITY_DN799_c0_g1_i7_orf1	-	-	-	hypothetical protein evm_002571 [Chilo suppressalis] >CAB3529880.1 unnamed protein product [Chilo suppressalis] >CAH0406472.1 unnamed protein product [Chilo suppressalis]	1.75797	-0.48509	-1.19479	-0.38808	0.30999
TRINITY_DN13375_c0_g1_i6_orf1	-	-	-	thioredoxin, mitochondrial isoform X2 [Ostrinia furnacalis]	1.08572	1.18545	-1.17353	-0.08173	-1.01591
TRINITY_DN16343_c0_g1_i6_orf1	-	-	-	aminopeptidase N6 [Ostrinia nubilalis]	1.9887	-0.69116	-0.38727	-0.41591	-0.49436
TRINITY_DN46778_c0_g1_i2_orf1	-	-	-	Deoxycytidylate deaminase [Papilio xuthus]	1.79746	0.33154	-0.46876	-0.65506	-1.00518
TRINITY_DN31663_c0_g1_i2_orf1	-	-	-	PHD finger-like domain-containing protein 5A [Nasonia vitripennis] >XP_002427197.1 conserved hypothetical protein [Pediculus humanus corporis] >XP_003484388.1 PHD finger-like domain-containing protein 5A [Bombus impatiens] >XP_003701008.1 PREDICTED: PHD finger-like domain-containing protein 5A [Megachile rotundata] >XP_006623871.1 PHD finger-like domain-containing protein 5A [Apis dorsata] >XP_011068502.1 PREDICTED: PHD finger-like domain-containing protein 5A [Acromyrmex echinatior] >XP_011154391.1 PHD finger-like domain-containing protein 5A [Harpegnathos saltator] >XP_011164776.1 PHD finger-like domain-containing protein 5A [Solenopsis invicta] >XP_011262550.1 PHD finger-like domain-containing protein 5A [Camponotus floridanus] >XP_011297178.1 PREDICTED: PHD finger-like domain-containing protein 5A [Fopius arisanus] >XP_011334720.1 PHD finger-like domain-containing protein 5A [Ooceraea biroi] >XP_011506347.1 PREDICTED: PHD finger-like domain-containing protein 5A [Ceratosolen solmsi marchali] >XP_011506348.1 PREDICTED: PHD finger-like domain-containing protein 5A [Ceratosolen solmsi marchali] >XP_011638597.1 PHD finger-like domain-containing protein 5A isoform X2 [Pogonomyrmex barbatus] >XP_011686073.1 PREDICTED: PHD finger-like domain-containing protein 5A [Wasmannia auropunctata] >XP_011858255.1 PREDICTED: PHD finger-like domain-containing protein 5A [Vollenhovia emeryi] >XP_012058015.1 PREDICTED: PHD finger-like domain-containing protein 5A [Atta cephalotes] >XP_012135327.1 PREDICTED: PHD finger-like domain-containing protein 5A [Megachile rotundata] >XP_012135328.1 PREDICTED: PHD finger-like domain-containing protein 5A [Megachile rotundata] >XP_012222185.1 PREDICTED: PHD finger-like domain-containing protein 5A [Linepithema humile] >XP_012261946.1 PHD finger-like domain-containing protein 5A [Athalia rosae] >XP_012273120.1 PHD finger-like domain-containing protein 5A [Orussus abietinus] >XP_012526512.1 PHD finger-like domain-containing protein 5A [Monomorium pharaonis] >XP_014217558.1 PHD finger-like domain-containing protein 5A [Copidosoma floridanum] >XP_014484566.1 PREDICTED: PHD finger-like domain-containing protein 5A [Dinoponera quadriceps] >XP_014611099.1 PREDICTED: PHD finger-like domain-containing protein 5A [Polistes canadensis] >XP_015122018.1 PHD finger-like domain-containing protein 5A [Diachasma alloeum] >XP_015174163.1 PREDICTED: PHD finger-like domain-containing protein 5A [Polistes dominula] >XP_015433827.1 PREDICTED: PHD finger-like domain	1.82142	0.2424	-1.04213	-0.59944	-0.42225
TRINITY_DN6147_c0_g1_i2_orf1	-	-	-	uncharacterized protein LOC114352519 [Ostrinia furnacalis]	1.95109	-0.48668	-0.12212	-0.52865	-0.81364
TRINITY_DN26824_c0_g1_i1_orf1	-	-	-	60S ribosomal protein L6 [Hyposmocoma kahamanoa]	1.48416	-0.45044	-1.54372	0.45653	0.05347
TRINITY_DN86149_c0_g1_i1_orf1	-	-	-	NADH dehydrogenase [ubiquinone] 1 alpha subcomplex subunit 8 [Galleria mellonella]	1.94831	-0.32723	-0.28262	-0.91558	-0.42288
TRINITY_DN28981_c0_g1_i1_orf1	-	-	-	uncharacterized protein C6orf203 homolog [Ostrinia furnacalis]	1.94767	-0.10348	-0.5835	-0.45611	-0.80458
TRINITY_DN23360_c0_g1_i3_orf1	-	-	-	protein PTCD3 homolog, mitochondrial [Ostrinia furnacalis]	1.82748	0.32329	-0.81303	-0.67006	-0.66768
TRINITY_DN2304_c0_g1_i4_orf1	-	-	-	clustered mitochondria protein homolog isoform X2 [Ostrinia furnacalis]	1.8968	-0.77324	-0.01739	-0.24314	-0.86303
TRINITY_DN10658_c0_g1_i1_orf1	-	-	-	DNA-directed RNA polymerase II subunit RPB9 [Ostrinia furnacalis] >XP_030035935.1 DNA-directed RNA polymerase II subunit RPB9 [Manduca sexta] >XP_037301147.1 DNA-directed RNA polymerase II subunit RPB9-like [Manduca sexta] >KAG6462484.1 hypothetical protein O3G_MSEX013297 [Manduca sexta]	1.6503	0.35078	-0.63179	-0.04558	-1.32371
TRINITY_DN23502_c0_g1_i1_orf1	-	-	-	small nuclear ribonucleoprotein F [Ostrinia furnacalis]	1.39261	0.56962	-1.41415	-0.81541	0.26733
TRINITY_DN76036_c0_g1_i1_orf1	-	-	-	cytochrome c oxidase subunit 6A1, mitochondrial-like [Ostrinia furnacalis]	1.93779	-0.23055	-0.37646	-0.95227	-0.37851
TRINITY_DN25341_c0_g1_i1_orf1	-	-	-	heat shock protein 90 [Loxostege sticticalis]	1.79834	0.36705	-0.91552	-0.70233	-0.54753
TRINITY_DN22654_c0_g2_i4_orf1	-	-	-	protein EFR3 homolog cmp44E isoform X1 [Ostrinia furnacalis] >XP_028166854.1 protein EFR3 homolog cmp44E isoform X2 [Ostrinia furnacalis]	1.71172	0.54173	-0.96517	-0.73121	-0.55707
TRINITY_DN11820_c0_g1_i1_orf1	-	-	-	hypothetical protein evm_000341 [Chilo suppressalis]	1.2244	0.89	-1.59213	-0.39803	-0.12425
TRINITY_DN12323_c0_g2_i2_orf1	-	-	-	polyribonucleotide nucleotidyltransferase 1, mitochondrial [Ostrinia furnacalis]	1.92853	-0.48716	-0.85876	-0.03027	-0.55234
TRINITY_DN79803_c0_g1_i7_orf1	-	-	-	dnaJ homolog subfamily C member 22 [Ostrinia furnacalis]	0.87975	1.29123	-1.48016	-0.09115	-0.59967
TRINITY_DN2927_c0_g1_i6_orf1	-	-	-	T-complex protein 1 subunit eta [Ostrinia furnacalis]	1.95195	-0.08441	-0.69557	-0.6641	-0.50787

TRINITY_DN47591_c1_g1_i1_orf1	-	-	-	uncharacterized protein LOC114364828 [Ostrinia furnacalis]	1.95535	-0.12385	-0.75611	-0.61295	-0.46245
TRINITY_DN108200_c0_g1_i1_orf1	-	-	-	uncharacterized protein LOC114350842 [Ostrinia furnacalis]	1.9512	-0.871	-0.44924	-0.18639	-0.44457
TRINITY_DN14565_c0_g1_i11_orf1	-	-	-	4-aminobutyrate aminotransferase, mitochondrial [Galleria mellonella]	1.87449	0.02897	-0.6757	-0.24294	-0.98482
TRINITY_DN2265_c0_g2_i1_orf1	-	-	-	LOW QUALITY PROTEIN: elongation factor G, mitochondrial-like [Leguminivora glycinivorella]	1.90534	0.07001	-0.61318	-0.50636	-0.85581
TRINITY_DN11117_c0_g1_i1_orf1	-	-	-	venom carboxylesterase-6-like [Ostrinia furnacalis]	1.95808	-0.7702	-0.34206	-0.20153	-0.64429
TRINITY_DN5046_c0_g3_i1_orf1	-	-	-	uncharacterized protein LOC114358520 [Ostrinia furnacalis]	1.46956	0.90814	-1.02125	-0.51586	-0.84059
TRINITY_DN8676_c0_g1_i1_orf1	-	-	-	probable rRNA-processing protein EBP2 homolog [Ostrinia furnacalis]	1.99292	-0.37851	-0.51631	-0.46102	-0.63707
TRINITY_DN631_c0_g1_i6_orf1	-	-	-	cytosolic 10-formyltetrahydrofolate dehydrogenase isoform X1 [Ostrinia furnacalis] >XP_028172896.1 cytosolic 10-formyltetrahydrofolate dehydrogenase isoform X2 [Ostrinia furnacalis]	1.91403	-0.65059	-0.91919	-0.23949	-0.10477
TRINITY_DN33248_c0_g1_i1_orf1	-	-	-	elongation factor Ts, mitochondrial isoform X3 [Ostrinia furnacalis] >XP_028155866.1 elongation factor Ts, mitochondrial isoform X3 [Ostrinia furnacalis]	1.91583	-0.6428	-0.89463	-0.33831	-0.04009
TRINITY_DN51968_c0_g1_i1_orf1	-	-	-	splicing factor U2af 38 kDa subunit [Aphidius gifuensis] >KAF7990547.1 hypothetical protein HCN44_000352 [Aphidius gifuensis]	1.49887	0.53877	-1.22345	-0.97049	0.1563
TRINITY_DN10796_c0_g2_i1_orf1	-	-	-	F-BAR domain only protein 2 [Ostrinia furnacalis]	1.98113	-0.29466	-0.59585	-0.40647	-0.68415
TRINITY_DN14920_c0_g1_i1_orf1	-	-	-	anamorsin homolog [Ostrinia furnacalis]	1.75126	0.44196	-0.79699	-0.95094	-0.44529
TRINITY_DN58636_c0_g1_i1_orf1	-	-	-	uncharacterized protein LOC114363665 [Ostrinia furnacalis]	1.88658	0.03452	-0.91276	-0.72567	-0.28267
TRINITY_DN58413_c0_g1_i4_orf1	-	-	-	cysteine and histidine-rich protein 1 isoform X1 [Ostrinia furnacalis]	1.9781	-0.77001	-0.45887	-0.33605	-0.41317
TRINITY_DN146264_c0_g1_i1_orf1	-	-	-	PREDICTED: protein preli-like [Fopius arisanus]	1.81755	-0.36139	-1.16361	-0.43708	0.14453
TRINITY_DN5009_c0_g1_i2_orf1	-	-	-	GSCOCG00009487001-RA-CDS [Cotesia congregata] >CAG5088842.1 Similar to RpL18: 60S ribosomal protein L18 (Timarcha balearica) [Cotesia congregata]	1.70898	-0.62917	-1.18314	-0.32142	0.42474
TRINITY_DN2184_c0_g1_i4_orf1	-	-	-	uncharacterized protein LOC114359356 [Ostrinia furnacalis]	1.82781	-0.70694	-0.19301	0.12413	-1.05199
TRINITY_DN4213_c0_g1_i4_orf1	-	-	-	nardilysin-like isoform X1 [Ostrinia furnacalis] >XP_028157649.1 nardilysin-like isoform X2 [Ostrinia furnacalis] >XP_028157650.1 nardilysin-like isoform X3 [Ostrinia furnacalis] >XP_028157651.1 nardilysin-like isoform X4 [Ostrinia furnacalis]	1.70573	0.58386	-0.72775	-0.79297	-0.76888
TRINITY_DN2885_c1_g1_i2_orf1	-	-	-	ubiquitin-like-specific protease ESD4 [Ostrinia furnacalis]	1.37053	0.8457	-1.26163	-0.90102	-0.05359
TRINITY_DN5678_c0_g2_i3_orf1	-	-	-	coiled-coil domain-containing protein 115 [Ostrinia furnacalis]	1.96454	-0.41166	-0.56254	-0.20858	-0.78176
TRINITY_DN53136_c0_g1_i1_orf1	-	-	-	glutathione S transferase-E4 [Glyphodes pyloalis]	1.96634	-0.6691	-0.38476	-0.70268	-0.2098
TRINITY_DN36788_c0_g1_i2_orf1	-	-	-	isocitrate dehydrogenase [NADP] cytoplasmic-like [Bicyclus anynana]	1.72924	0.3014	-1.18979	-0.69406	-0.14679
TRINITY_DN9862_c0_g2_i1_orf1	-	-	-	40S ribosomal protein S4 [Manduca sexta] >ACY95325.1 ribosomal protein S4 [Manduca sexta] >KAG6465430.1 hypothetical protein O3G_MSEX015149 [Manduca sexta]	1.80675	-0.83477	-0.87357	-0.4172	0.3188
TRINITY_DN19187_c0_g1_i1_orf1	-	-	-	fumarylacetoacetase [Chelonus insularis]	1.80233	-0.12555	-1.28786	-0.22315	-0.16577
TRINITY_DN7122_c0_g1_i1_orf1	-	-	-	hypothetical protein evm_003965 [Chilo suppressalis]	1.39704	0.56424	-1.18757	0.32739	-1.1011
TRINITY_DN38301_c0_g1_i2_orf1	-	-	-	gamma-taxilin [Ostrinia furnacalis]	1.92305	0.04108	-0.62248	-0.59122	-0.75044
TRINITY_DN26649_c0_g1_i2_orf1	-	-	-	ATP synthase-coupling factor 6, mitochondrial [Ostrinia furnacalis]	1.98906	-0.64613	-0.40096	-0.57524	-0.36673
TRINITY_DN5697_c0_g1_i1_orf1	-	-	-	GPI ethanolamine phosphate transferase 2-like [Ostrinia furnacalis]	1.63305	0.54176	-0.9966	-1.00883	-0.16938
TRINITY_DN754_c1_g1_i8_orf1	-	-	-	lysophospholipid acyltransferase 5 [Ostrinia furnacalis] >XP_028169982.1 lysophospholipid acyltransferase 5 [Ostrinia furnacalis]	1.84154	0.26538	-0.7589	-0.51002	-0.83801
TRINITY_DN4121_c0_g1_i1_orf1	-	-	-	uncharacterized protein LOC114358001, partial [Ostrinia furnacalis]	1.90952	-0.87462	-0.62825	-0.43934	0.03268
TRINITY_DN107617_c3_g1_i1_orf1	-	-	-	NADH dehydrogenase [ubiquinone] 1 alpha subcomplex subunit 9, mitochondrial [Ostrinia furnacalis]	1.91995	-0.98831	-0.46396	-0.31259	-0.15509
TRINITY_DN108051_c0_g1_i2_orf1	-	-	-	uncharacterized protein LOC114351921 [Ostrinia furnacalis]	1.99497	-0.38361	-0.54232	-0.59532	-0.47372
TRINITY_DN4262_c0_g1_i16_orf1	-	-	-	sperm-associated antigen 7 homolog [Ostrinia furnacalis]	1.39662	0.78959	-1.4211	-0.62106	-0.14406
TRINITY_DN48413_c1_g1_i2_orf1	-	-	-	probable protein phosphatase 2C 11 isoform X1 [Manduca sexta] >KAG6442694.1 hypothetical protein O3G_MSEX002471 [Manduca sexta]	1.49262	0.88952	-0.88723	-0.94275	-0.55217
TRINITY_DN49936_c0_g2_i1_orf1	-	-	-	39S ribosomal protein L20, mitochondrial [Ostrinia furnacalis]	1.87814	-0.12241	-0.32813	-0.30703	-1.12056
TRINITY_DN237_c1_g1_i1_orf1	-	-	-	PREDICTED: cytoplasmic protein NCK1 isoform X1 [Microplitis demolitor]	1.64735	0.6462	-0.58156	-1.03628	-0.6757
TRINITY_DN3534_c0_g1_i2_orf1	-	-	-	guanine nucleotide-binding protein subunit beta-like protein [Ostrinia furnacalis]	1.92426	-0.63387	-0.90442	-0.15446	-0.23152
TRINITY_DN36718_c0_g1_i1_orf1	-	-	-	unnamed protein product [Chilo suppressalis]	1.99819	-0.44407	-0.49339	-0.57518	-0.48556
TRINITY_DN14018_c0_g1_i4_orf1	-	-	-	chitobiosyldiphosphodolichol beta-mannosyltransferase [Ostrinia furnacalis]	1.84839	0.24599	-0.66668	-0.88608	-0.54162
TRINITY_DN4025_c0_g1_i13_orf1	-	-	-	tetratricopeptide repeat protein 14 homolog isoform X2 [Ostrinia furnacalis]	1.60115	0.09401	-1.52386	-0.29855	0.12725
TRINITY_DN783_c0_g1_i7_orf1	-	-	-	microtubule-associated protein Jupiter isoform X4 [Helicoverpa armigera]	1.14845	1.28544	-1.0004	-0.73245	-0.70103
TRINITY_DN4659_c0_g1_i2_orf1	-	-	-	uncharacterized protein LOC114351134 [Ostrinia furnacalis]	1.17886	1.26323	-0.7607	-0.73498	-0.94641

TRINITY_DN4135_c0_g1_i5_orf1	-	-	-	probable small nuclear ribonucleoprotein Sm D2 [Manduca sexta] >KAG6451233.1 hypothetical protein O3G_MSEX007016 [Manduca sexta]	1.86562	-0.05941	-1.11056	-0.49034	-0.20531
TRINITY_DN38540_c0_g1_i1_orf1	-	-	-	GSCOCG00000129001-RA-CDS [Cotesia congregata] >CAG5101050.1 Similar to LUC7L2: Putative RNA-binding protein Luc7-like 2 (Homo sapiens) [Cotesia congregata]	1.74162	0.43194	-1.09436	-0.54998	-0.52923
TRINITY_DN1109_c0_g1_i6_orf1	-	-	-	1-phosphatidylinositol phosphodiesterase-like [Cotesia glomerata]	1.7203	0.37742	-0.26874	-0.63761	-1.19136
TRINITY_DN17394_c0_g1_i1_orf1	-	-	-	monocarboxylate transporter 14-like [Ostrinia furnacalis]	1.70966	-0.24684	-1.06315	-0.83465	0.43498
TRINITY_DN57105_c0_g1_i2_orf1	-	-	-	transmembrane protein 161B isoform X1 [Galleria mellonella]	1.47487	0.88298	-0.83685	-1.06713	-0.45388
TRINITY_DN8754_c0_g1_i2_orf1	-	-	-	dnaJ homolog subfamily C member 11 [Ostrinia furnacalis]	1.99755	-0.52573	-0.47887	-0.42189	-0.57107
TRINITY_DN3893_c0_g2_i3_orf1	-	-	-	cleavage and polyadenylation specificity factor subunit CG7185 isoform X2 [Ostrinia furnacalis]	1.44358	0.79885	-1.30531	-0.72835	-0.20876
TRINITY_DN16174_c0_g1_i2_orf1	-	-	-	LOW QUALITY PROTEIN: ATP-dependent RNA helicase SUV3 homolog, mitochondrial [Ostrinia furnacalis]	1.88592	-0.21052	-1.09414	-0.4189	-0.16237
TRINITY_DN105359_c0_g2_i5_orf1	-	-	-	uncharacterized protein LOC114359499 [Ostrinia furnacalis]	0.79801	1.02222	-1.69423	-0.53198	0.40598
TRINITY_DN8949_c0_g1_i2_orf1	-	-	-	unnamed protein product [Arctia plantaginis]	1.81594	-0.61842	-1.119	-0.21908	0.14056
TRINITY_DN391_c5_g1_i1_orf1	-	-	-	hypothetical protein B5X24_HaOG210395 [Helicoverpa armigera]	1.9946	-0.53316	-0.57327	-0.52848	-0.3597
TRINITY_DN2062_c0_g1_i11_orf1	-	-	-	uncharacterized protein LOC114350846 [Ostrinia furnacalis]	1.89974	-0.2008	-1.05496	-0.19879	-0.44519
TRINITY_DN27994_c0_g1_i1_orf1	-	-	-	uncharacterized protein LOC114364076 [Ostrinia furnacalis]	1.84165	0.25415	-0.57966	-0.9289	-0.58725
TRINITY_DN816_c0_g1_i3_orf1	-	-	-	calcium-binding mitochondrial carrier protein SCaMC-2 isoform X1 [Ostrinia furnacalis]	1.882	0.04566	-1.01284	-0.53393	-0.3809
TRINITY_DN147596_c0_g1_i1_orf1	-	-	-	activator of basal transcription 1 [Diachasma alloeum]	1.7748	-0.86284	-0.97828	0.30368	-0.23736
TRINITY_DN3836_c0_g1_i4_orf1	-	-	-	2-oxoisovalerate dehydrogenase subunit alpha, mitochondrial [Ostrinia furnacalis]	1.77593	-1.09053	-0.73848	0.26112	-0.20803
TRINITY_DN3773_c0_g1_i4_orf1	-	-	-	peptidyl-prolyl cis-trans isomerase G isoform X2 [Ostrinia furnacalis]	1.82875	0.02927	-1.17989	-0.46702	-0.21111
TRINITY_DN27641_c0_g1_i1_orf1	-	-	-	succinate dehydrogenase [ubiquinone] iron-sulfur subunit, mitochondrial [Ostrinia furnacalis]	1.99562	-0.55486	-0.45124	-0.58546	-0.40405
TRINITY_DN3702_c0_g1_i1_orf1	-	-	-	U4/U6.U5 tri-snRNP-associated protein 2 [Ostrinia furnacalis]	1.22194	0.36	-1.79467	-0.15228	0.36501
TRINITY_DN20582_c0_g1_i1_orf1	-	-	-	2-oxoglutarate dehydrogenase, mitochondrial isoform X3 [Ostrinia furnacalis] >XP_028167081.1 2-oxoglutarate dehydrogenase, mitochondrial isoform X3 [Ostrinia	1.98158	-0.39994	-0.57917	-0.6955	-0.30697
TRINITY_DN123139_c0_g1_i1_orfp1	-	-	-	TRINITY_DN123139_c0_g1_i1_m.79879 TRINITY_DN123139_c0_g1::TRINITY_DN123139_c0_g1_i1::g.79879 ORF type:3prime_partial len:76 (+),score=3.83 TRINITY_DN123139_c0_g1_i1:25-225(+)	1.86415	-0.35681	-1.0589	-0.5207	0.07227
TRINITY_DN22572_c0_g1_i1_orf1	-	-	-	eukaryotic translation elongation factor 1 epsilon-1 [Ostrinia furnacalis]	1.6022	0.1913	-1.5259	-0.00698	-0.26063
TRINITY_DN1633_c0_g1_i1_orf1	-	-	-	collagen alpha-2(I) chain-like isoform X1 [Ostrinia furnacalis]	1.98487	-0.40281	-0.47517	-0.38077	-0.72613
TRINITY_DN20369_c0_g1_i2_orf1	-	-	-	uncharacterized protein LOC114366225 [Ostrinia furnacalis]	1.91805	0.02527	-0.83054	-0.63227	-0.4805
TRINITY_DN45227_c0_g1_i3_orf1	-	-	-	uncharacterized protein LOC114359191 [Ostrinia furnacalis]	1.95953	-0.27652	-0.77829	-0.63784	-0.26687
TRINITY_DN21341_c0_g1_i4_orf1	-	-	-	FAST kinase domain-containing protein 4 isoform X6 [Ostrinia furnacalis] >XP_028160336.1 FAST kinase domain-containing protein 4 isoform X7 [Ostrinia furnacalis] >XP_028160337.1 FAST kinase domain-containing protein 4 isoform X8 [Ostrinia furnacalis]	1.96619	-0.16525	-0.66572	-0.4695	-0.66572
TRINITY_DN19115_c0_g1_i1_orf1	-	-	-	putative ATP synthase subunit f, mitochondrial [Ostrinia furnacalis]	1.88046	-0.04432	-0.34693	-1.08569	-0.40352
TRINITY_DN6671_c0_g1_i6_orf1	-	-	-	hypothetical protein evm_013656 [Chilo suppressalis] >CAB3521812.1 unnamed protein product [Chilo suppressalis] >CAH0399134.1 unnamed protein product [Chilo suppressalis]	1.81837	0.17026	-1.14248	-0.44842	-0.39774
TRINITY_DN5686_c0_g1_i4_orf1	-	-	-	FACT complex subunit spt16 isoform X2 [Ostrinia furnacalis]	1.8908	-0.24411	-1.05937	-0.10587	-0.48145
TRINITY_DN2780_c0_g1_i5_orf1	-	-	-	probable ATP-dependent RNA helicase DDX27 [Ostrinia furnacalis]	1.98621	-0.3726	-0.36185	-0.59647	-0.65529
TRINITY_DN16749_c0_g1_i1_orf1	-	-	-	uncharacterized protein LOC114353228 [Ostrinia furnacalis] >XP_028176007.1 uncharacterized protein LOC114364183 [Ostrinia furnacalis]	1.96422	-0.60892	-0.60096	-0.62755	-0.1268
TRINITY_DN19829_c0_g2_i1_orf1	-	-	-	28S ribosomal protein S35, mitochondrial [Ostrinia furnacalis]	1.9623	-0.37863	-0.40865	-0.86016	-0.31486
TRINITY_DN4040_c0_g1_i10_orf1	-	-	-	hypothetical protein evm_007488 [Chilo suppressalis]	1.97503	-0.39719	-0.34941	-0.43408	-0.79434
TRINITY_DN32997_c0_g1_i8_orf1	-	-	-	RNA-binding protein squid isoform X1 [Ostrinia furnacalis]	1.88669	0.09246	-0.82122	-0.78761	-0.37032
TRINITY_DN3127_c0_g1_i9_orf1	-	-	-	RNA-binding protein 1 isoform X1 [Galleria mellonella]	1.84986	0.09022	-0.6598	-1.03699	-0.2433
TRINITY_DN44557_c0_g1_i4_orf1	-	-	-	serine hydrolase-like protein [Ostrinia furnacalis]	1.80962	-0.91526	-0.88059	-0.24356	0.2298
TRINITY_DN2089_c0_g1_i5_orf1	-	-	-	eukaryotic translation initiation factor 4B [Ostrinia furnacalis]	1.70868	0.24467	-1.16501	-0.81403	0.02568
TRINITY_DN2274_c0_g1_i6_orf1	-	-	-	membrane alanyl aminopeptidase-like [Ostrinia furnacalis]	1.9819	-0.55883	-0.32448	-0.38954	-0.70905
TRINITY_DN96080_c0_g2_i1_orf1	-	-	-	ATP synthase subunit delta, mitochondrial [Ostrinia furnacalis]	1.94281	-0.88746	-0.33873	-0.54053	-0.17608
TRINITY_DN48619_c0_g1_i1_orf1	-	-	-	PREDICTED: lysine--tRNA ligase isoform X2 [Fopius arisanus]	1.73373	-1.03907	-0.89752	-0.10882	0.31167
TRINITY_DN26963_c0_g1_i1_orf1	-	-	-	aminoacyl tRNA synthase complex-interacting multifunctional protein 1 isoform X2 [Ostrinia furnacalis]	1.92648	-0.10895	-0.94239	-0.38384	-0.4913
TRINITY_DN43293_c0_g1_i2_orf1	-	-	-	egl nine homolog 1 isoform X2 [Helicoverpa armigera]	1.32777	0.48153	-1.52595	0.42245	-0.70579
TRINITY_DN51252_c0_g2_i1_orf1	-	-	-	peroxidase-like [Ostrinia furnacalis]	1.75855	0.36234	-0.67917	-0.34961	-1.09211

TRINITY_DN42738_c0_g1_i1_orf1	-	-	-	arf-GAP domain and FG repeat-containing protein 1 [Ostrinia furnacalis]	1.90403	-0.57721	-0.98045	-0.07264	-0.27374
TRINITY_DN19829_c0_g1_i1_orf1	-	-	-	28S ribosomal protein S35, mitochondrial [Ostrinia furnacalis]	1.90567	-0.85125	-0.77355	-0.08569	-0.19518
TRINITY_DN1386_c0_g1_i6_orf1	-	-	-	ras-related protein Rab-36 [Ostrinia furnacalis]	1.83623	0.20399	-0.81929	-0.31888	-0.90205
TRINITY_DN30_c0_g1_i6_orf1	-	-	-	casein kinase I-like isoform X1 [Hyposmocoma kahamanoa]	1.7836	0.35447	-0.99682	-0.41531	-0.72594
TRINITY_DN83005_c0_g1_i1_orf1	-	-	-	ATP synthase subunit O, mitochondrial [Danaus plexippus plexippus] >OWR53927.1 H+ transporting ATP synthase O subunit [Danaus plexippus plexippus]	1.9867	-0.71825	-0.44225	-0.40193	-0.42427
TRINITY_DN16349_c0_g1_i10_orf1	-	-	-	protein lingerer-like isoform X1 [Nymphalis io] >XP_050356663.1 protein lingerer-like isoform X1 [Nymphalis io] >XP_050356664.1 protein lingerer-like isoform X1 [Nymphalis io]	1.55748	0.74044	-1.16672	-0.67729	-0.45391
TRINITY_DN80560_c0_g1_i1_orf1	-	-	-	ATP synthase subunit alpha, mitochondrial [Ostrinia furnacalis]	1.97507	-0.79776	-0.37201	-0.40485	-0.40045
TRINITY_DN905_c0_g1_i4_orf1	-	-	-	(11Z)-hexadec-11-enoyl-CoA conjugase-like [Ostrinia furnacalis] >XP_028172978.1 (11Z)-hexadec-11-enoyl-CoA conjugase-like [Ostrinia furnacalis]	1.96637	-0.20854	-0.58499	-0.75949	-0.41336
TRINITY_DN50085_c0_g1_i1_orf1	-	-	-	hypothetical protein evm_013997 [Chilo suppressalis]	1.86999	0.07178	-0.94665	-0.72856	-0.26656
TRINITY_DN4938_c0_g1_i13_orf1	-	-	-	peroxisomal biogenesis factor 19 [Ostrinia furnacalis]	1.82356	-0.97127	-0.5204	-0.61615	0.28426
TRINITY_DN2238_c0_g2_i1_orf1	-	-	-	mitochondrial import inner membrane translocase subunit Tim8 [Ostrinia furnacalis]	1.94674	-0.35204	-0.16567	-0.57708	-0.85195
TRINITY_DN1504_c0_g1_i1_orf1	-	-	-	uncharacterized protein LOC114352862 [Ostrinia furnacalis] >XP_028160407.1 uncharacterized protein LOC114352862 [Ostrinia furnacalis]	0.9835	1.27102	-1.38966	-0.66917	-0.19569
TRINITY_DN2065_c1_g2_i1_orf1	-	-	-	2-amino-3-ketobutyrate coenzyme A ligase, mitochondrial [Ostrinia furnacalis]	1.66607	0.65242	-0.74583	-0.733	-0.83966
TRINITY_DN21609_c0_g2_i1_orf1	-	-	-	translation initiation factor eIF-2B subunit epsilon [Ostrinia furnacalis]	1.7761	0.06388	-1.26949	-0.46832	-0.10217
TRINITY_DN8173_c0_g1_i3_orf1	-	-	-	dihydroceramide fatty acyl 2-hydroxylase FAH1 [Ostrinia furnacalis]	1.92512	-0.59698	-0.50065	0.0013	-0.82879
TRINITY_DN106476_c0_g1_i3_orf1	-	-	-	mitochondrial import inner membrane translocase subunit TIM44 [Ostrinia furnacalis]	1.93872	-0.63078	-0.85471	-0.29823	-0.155
TRINITY_DN12683_c0_g1_i3_orf1	-	-	-	sulfated surface glycoprotein 185-like [Ostrinia furnacalis]	1.93505	-0.95734	-0.44013	-0.2896	-0.24797
TRINITY_DN78873_c0_g1_i4_orf1	-	-	-	hypothetical protein evm_008224 [Chilo suppressalis]	1.74306	-0.26063	-1.28656	-0.42913	0.23325
TRINITY_DN14235_c0_g1_i1_orf1	-	-	-	maltase A1 [Helicoverpa armigera]	1.91931	-0.64282	-0.73578	-0.59859	0.05788
TRINITY_DN1465_c0_g2_i1_orf1	-	-	-	unnamed protein product, partial [Iphiclides podalirius]	1.97016	-0.69982	-0.2309	-0.38705	-0.6524
TRINITY_DN536_c0_g1_i7_orf1	-	-	-	polymerase delta-interacting protein 2 isoform X3 [Ostrinia furnacalis]	1.96825	-0.77524	-0.40855	-0.55099	-0.23346
TRINITY_DN4143_c0_g1_i1_orf1	-	-	-	zinc finger protein 530-like isoform X8 [Ostrinia furnacalis]	1.82158	0.21863	-0.27639	-0.91457	-0.84925
TRINITY_DN15959_c0_g1_i1_orf1	-	-	-	dnaJ homolog subfamily A member 2-like [Ostrinia furnacalis]	1.94385	-0.07293	-0.75746	-0.66274	-0.45071
TRINITY_DN7626_c0_g1_i1_orf1	-	-	-	NADH dehydrogenase [ubiquinone] flavoprotein 1, mitochondrial isoform X1 [Ostrinia	1.9898	-0.51747	-0.57392	-0.59058	-0.30782
TRINITY_DN45477_c0_g1_i1_orf1	-	-	-	putative E3 ubiquitin-protein ligase UBR7 [Ostrinia furnacalis]	1.82918	0.21356	-0.75664	-0.3198	-0.9663
TRINITY_DN24142_c0_g1_i1_orf1	-	-	-	arylalkylamine N-acetyltransferase [Chilo suppressalis]	1.81165	-0.83022	-1.01252	-0.02307	0.05417
TRINITY_DN33_c0_g1_i1_orf1	-	-	-	uncharacterized protein CG45076-like isoform X2 [Ostrinia furnacalis]	1.80667	-0.75248	-1.07475	-0.07464	0.0952
TRINITY_DN478_c0_g1_i16_orf1	-	-	-	lipid storage droplets surface-binding protein 2 isoform X1 [Ostrinia furnacalis]	1.75638	-0.03412	-0.62406	-1.22788	0.12968
TRINITY_DN6563_c0_g1_i1_orf1	-	-	-	cytochrome c oxidase assembly protein COX15 homolog [Ostrinia furnacalis]	1.80849	0.36817	-0.80998	-0.63957	-0.72712
TRINITY_DN18860_c0_g1_i1_orf1	-	-	-	DDB1- and CUL4-associated factor 13 [Ostrinia furnacalis]	1.97374	-0.20539	-0.47775	-0.62212	-0.66848
TRINITY_DN19186_c0_g1_i1_orf1	-	-	-	39S ribosomal protein L9, mitochondrial [Ostrinia furnacalis]	1.93985	-0.09829	-0.71249	-0.36174	-0.76734
TRINITY_DN1066_c0_g1_i4_orf1	-	-	-	ribosome biogenesis protein WDR12 homolog [Ostrinia furnacalis]	1.97275	-0.33171	-0.75082	-0.58337	-0.30685
TRINITY_DN3649_c0_g1_i6_orf1	-	-	-	unnamed protein product [Chilo suppressalis]	1.18088	0.79296	-1.66395	0.12788	-0.43777
TRINITY_DN754_c1_g1_i6_orf1	-	-	-	28S ribosomal protein S2, mitochondrial [Ostrinia furnacalis]	1.95042	-0.37231	-0.86036	-0.18646	-0.53129
TRINITY_DN4056_c0_g1_i8_orf1	-	-	-	uncharacterized protein LOC114349672 [Ostrinia furnacalis] >XP_028155936.1 uncharacterized protein LOC114349672 [Ostrinia furnacalis] >XP_028155937.1 uncharacterized protein LOC114349672 [Ostrinia furnacalis] >XP_028155939.1 uncharacterized protein LOC114349672 [Ostrinia furnacalis]	1.55546	0.07599	-1.40306	-0.65275	0.42436
TRINITY_DN43328_c0_g1_i1_orf1	-	-	-	tubulin--tyrosine ligase-like protein 12 [Ostrinia furnacalis]	1.14772	1.27984	-1.04024	-0.68109	-0.70623
TRINITY_DN11894_c1_g1_i5_orf1	-	-	-	39S ribosomal protein L33, mitochondrial [Ostrinia furnacalis]	1.92046	-0.49461	-0.97233	-0.12953	-0.324
TRINITY_DN131371_c0_g1_i1_orf1	-	-	-	golgin subfamily B member 1-like [Ostrinia furnacalis]	1.44941	-0.15203	-1.66277	0.03349	0.33191
TRINITY_DN27979_c0_g1_i2_orf1	-	-	-	zinc finger CCCH domain-containing protein 15 homolog [Ostrinia furnacalis]	1.97679	-0.20915	-0.6577	-0.55381	-0.55613
TRINITY_DN81715_c0_g1_i1_orf1	-	-	-	gamma-interferon-inducible lysosomal thiol reductase-like [Ostrinia furnacalis]	1.4531	0.93771	-1.05283	-0.72217	-0.61581
TRINITY_DN5275_c0_g1_i1_orf1	-	-	-	paraplegin [Ostrinia furnacalis]	1.65307	0.49639	-1.19855	-0.73269	-0.21822
TRINITY_DN5417_c0_g1_i1_orf1	-	-	-	NADH dehydrogenase [ubiquinone] 1 alpha subcomplex subunit 10, mitochondrial isoform X1 [Ostrinia furnacalis] >XP_028175885.1 NADH dehydrogenase [ubiquinone] 1 alpha subcomplex subunit 10, mitochondrial isoform X2 [Ostrinia furnacalis]	1.99214	-0.46913	-0.59448	-0.58004	-0.34848
TRINITY_DN109144_c0_g1_i5_orf1	-	-	-	uncharacterized protein LOC126369165 [Pectinophora gossypiella]	1.92086	-0.51058	-0.43318	-0.05002	-0.92707
TRINITY_DN45924_c0_g1_i14_orf1	-	-	-	adenylosuccinate synthetase isoform X1 [Ostrinia furnacalis] >XP_028166048.1 adenylosuccinate synthetase isoform X2 [Ostrinia furnacalis]	1.92542	-0.35488	-0.90587	-0.58264	-0.08204

TRINITY_DN17559_c0_g1_i4_orf1	-	-	-	GDP-mannose 4,6 dehydratase isoform X2 [Ostrinia furnacalis] >XP_028166204.1 GDP-mannose 4,6 dehydratase isoform X2 [Ostrinia furnacalis]	1.99223	-0.41586	-0.4007	-0.64449	-0.53119
TRINITY_DN4434_c0_g1_i7_orf1	-	-	-	V-type proton ATPase catalytic subunit A [Ostrinia furnacalis] >XP_028155921.1 V-type proton ATPase catalytic subunit A [Ostrinia furnacalis] >XP_028155922.1 V-type proton ATPase catalytic subunit A [Ostrinia furnacalis] >ADP23923.1 V-ATPase subunit A [Ostrinia furnacalis] >ADT80587.1 V-type proton ATPase catalytic subunit A [Ostrinia furnacalis] >CBY05457.1 V-type proton ATPase catalytic subunit A [Ostrinia furnacalis]	1.99235	-0.56778	-0.3791	-0.42793	-0.61753
TRINITY_DN11013_c0_g1_i3_orf1	-	-	-	glutamine:fructose-6-phosphate aminotransferase 1 [Heortia vitessoides]	1.88702	0.11683	-0.90331	-0.59456	-0.50599
TRINITY_DN21357_c0_g1_i5_orf1	-	-	-	40S ribosomal protein S11 isoform X2 [Ostrinia furnacalis]	1.82677	-0.78524	-1.00132	-0.1665	0.1263
TRINITY_DN9647_c0_g1_i1_orf1	-	-	-	cytochrome P450 6B2-like [Ostrinia furnacalis]	1.91207	-1.02066	-0.44585	-0.17696	-0.2686
TRINITY_DN107261_c0_g1_i1_orf1	-	-	-	ATP synthase subunit g, mitochondrial [Ostrinia furnacalis]	1.93474	-0.85746	-0.10248	-0.62138	-0.35342
TRINITY_DN9002_c0_g1_i1_orf1	-	-	-	membrane magnesium transporter 1 [Ostrinia furnacalis]	1.89276	-0.53017	-0.93479	-0.50633	0.07853
TRINITY_DN3292_c2_g1_i4_orf1	-	-	-	ribosome biogenesis regulatory protein homolog [Ostrinia furnacalis]	1.95364	-0.55831	-0.3168	-0.23136	-0.84717
TRINITY_DN42506_c0_g1_i1_orf1	-	-	-	28S ribosomal protein S7, mitochondrial [Ostrinia furnacalis]	1.88675	0.10564	-0.80386	-0.39864	-0.78988
TRINITY_DN886_c0_g2_i4_orf1	-	-	-	collagenase-like [Ostrinia furnacalis]	1.85128	0.24103	-0.88348	-0.56248	-0.64635
TRINITY_DN63914_c0_g1_i1_orf1	-	-	-	myophilin-like [Ostrinia furnacalis]	1.98468	-0.5822	-0.36228	-0.36228	-0.67793
TRINITY_DN2807_c0_g1_i4_orf1	-	-	-	FK506-binding protein 59 isoform X1 [Ostrinia furnacalis]	1.86907	0.04397	-0.94224	-0.75521	-0.21559
TRINITY_DN53167_c0_g1_i2_orf1	-	-	-	uncharacterized protein LOC114359219 [Ostrinia furnacalis]	1.97755	-0.32072	-0.34483	-0.59734	-0.71464
TRINITY_DN30131_c0_g1_i1_orf1	-	-	-	PREDICTED: 60S ribosomal protein L44 [Amyelois transitella] >XP_021198018.1 60S ribosomal protein L44 [Helicoverpa armigera] >XP_022814294.1 60S ribosomal protein L44 [Spodoptera litura] >XP_026732397.1 60S ribosomal protein L44 [Trichoplusia ni] >XP_026752106.1 60S ribosomal protein L44 [Galleria mellonella] >XP_028158932.1 60S ribosomal protein L44 [Ostrinia furnacalis] >XP_035434364.1 60S ribosomal protein L44 [Spodoptera frugiperda] >XP_035434370.1 60S ribosomal protein L44 [Spodoptera frugiperda] >XP_047019234.1 60S ribosomal protein L44 [Helicoverpa zea] >XP_049868501.1 60S ribosomal protein L44 [Pectinophora gossypiella] >AAM53948.1 ribosomal protein L44 [Choristoneura parallela] >KAF9418375.1 hypothetical protein HW555_004805 [Spodoptera exigua] >RVE50750.1 hypothetical protein evm_004660 [Chilo suppressalis] >CAB3235328.1 unnamed protein product [Arctia plantaginis] >CAB3516516.1 unnamed protein product [Spodoptera littoralis] >CAG9747186.1 unnamed protein product [Diatraea saccharalis] >CAH0581656.1 unnamed protein product [Chrysodeixis includens]	1.82559	-0.99822	-0.81305	-0.07653	0.06221
TRINITY_DN55160_c0_g2_i1_orf1	-	-	-	esterase FE4-like isoform X2 [Ostrinia furnacalis]	1.86703	-0.33349	0.05259	-0.52631	-1.05982
TRINITY_DN4085_c0_g1_i1_orf1	-	-	-	Protein TSSC1 [Papilio machaon]	1.04631	1.26488	-1.13927	-0.98637	-0.18556
TRINITY_DN16900_c0_g2_i1_orf1	-	-	-	uncharacterized oxidoreductase dhs-27-like [Ostrinia furnacalis]	1.93946	-0.41404	-0.34161	-0.23859	-0.94522
TRINITY_DN19092_c0_g1_i2_orf1	-	-	-	eukaryotic translation initiation factor 3 subunit L [Ostrinia furnacalis]	1.94717	-0.36751	-0.91199	-0.4309	-0.23677
TRINITY_DN1803_c0_g1_i3_orf1	-	-	-	translocator protein-like isoform X1 [Ostrinia furnacalis] >XP_028178947.1 translocator protein-like isoform X1 [Ostrinia furnacalis]	1.85109	0.26992	-0.695	-0.69244	-0.73356
TRINITY_DN1330_c0_g1_i1_orf1	-	-	-	pancreatic triacylglycerol lipase-like [Ostrinia furnacalis]	1.93792	-0.2109	-0.26599	-0.55456	-0.90647
TRINITY_DN38424_c0_g1_i1_orf1	-	-	-	glucose dehydrogenase [FAD, quinone]-like [Ostrinia furnacalis]	1.00217	0.80229	-1.70534	-0.518	0.41888
TRINITY_DN137_c0_g1_i1_orf1	-	-	-	60S ribosomal protein L8 [Cotesia glomerata] >XP_044597650.1 60S ribosomal protein L8 [Cotesia glomerata] >KAG8034499.1 hypothetical protein G9C98_007575 [Cotesia typhae] >CAD6216378.1 GSCOCG00004534001-RA-CDS [Cotesia congregata] >KAH0544237.1 60S ribosomal protein L8 [Cotesia glomerata] >KAH0564528.1 60S ribosomal protein L8 [Cotesia glomerata] >CAG5095185.1 Similar to RpL8: 60S ribosomal protein L8 (Spodoptera frugiperda) [Cotesia congregata]	1.79823	-0.58898	-0.95652	-0.61247	0.35974
TRINITY_DN27704_c0_g1_i1_orf1	-	-	-	PREDICTED: tRNA (guanine-N(7)-)-methyltransferase [Amyelois transitella]	1.95523	-0.18429	-0.82471	-0.3857	-0.56052
TRINITY_DN2716_c0_g2_i1_orf1	-	-	-	eukaryotic translation initiation factor 1A, X-chromosomal [Ostrinia furnacalis] >XP_045445466.1 eukaryotic translation initiation factor 1A, X-chromosomal [Melitaea cinxia] >XP_049867692.1 eukaryotic translation initiation factor 1A, X-chromosomal [Pectinophora gossypiella] >KOB79530.1 Eukaryotic translation initiation factor 1A [Operophtera brumata] >CAH2086435.1 unnamed protein product [Euphydryas editha] >KOB79531.1 Eukaryotic translation initiation factor 1A [Operophtera brumata]	1.77574	0.03682	-1.22091	-0.59561	0.00396
TRINITY_DN3598_c0_g1_i1_orf1	-	-	-	esterase FE4-like [Ostrinia furnacalis]	1.98066	-0.65019	-0.34754	-0.33273	-0.65019
TRINITY_DN29448_c0_g1_i1_orf1	-	-	-	28S ribosomal protein S9, mitochondrial [Ostrinia furnacalis]	1.8526	0.25036	-0.84063	-0.66124	-0.60108

TRINITY_DN76283_c0_g2_i1_orf1	-	-	-	fatty acid synthase-like [Ostrinia furnacalis]	1.90023	-0.93082	-0.34734	0.0119	-0.63397
TRINITY_DN3747_c1_g1_i3_orf1	-	-	-	unnamed protein product, partial [Iphiclides podalirius]	1.87287	-0.4817	-1.05961	0.03742	-0.36898
TRINITY_DN10701_c0_g2_i2_orf1	-	-	-	synaptosomal-associated protein 29 [Ostrinia furnacalis]	1.73784	0.34735	-1.01218	-0.89641	-0.1766
TRINITY_DN3665_c0_g1_i2_orf1	-	-	-	regucalcin-like isoform X2 [Ostrinia furnacalis] >XP_028175354.1 regucalcin-like isoform X2 [Ostrinia furnacalis]	1.98763	-0.44375	-0.66308	-0.55184	-0.32896
TRINITY_DN44219_c0_g1_i1_orf1	-	-	-	mitochondrial import inner membrane translocase subunit TIM50-C-like [Ostrinia furnacalis]	1.87094	0.11557	-0.85736	-0.80296	-0.32619
TRINITY_DN5554_c0_g1_i2_orf1	-	-	-	double-stranded RNA-binding protein Staufen homolog 2 isoform X5 [Pectinophora	1.91455	0.0332	-0.55728	-0.52773	-0.86275
TRINITY_DN10637_c0_g1_i4_orf1	-	-	-	V-type proton ATPase subunit d [Bombyx mandarina]	1.99853	-0.54552	-0.44316	-0.47321	-0.53664
TRINITY_DN34821_c0_g1_i4_orf1	-	-	-	acetylcholine receptor subunit alpha-L1-like [Ostrinia furnacalis]	1.19076	1.17849	-0.52684	-0.59068	-1.25173
TRINITY_DN41697_c0_g1_i1_orf1	-	-	-	5-formyltetrahydrofolate cyclo-ligase [Ostrinia furnacalis]	1.36771	0.49628	0.36306	-1.48188	-0.74517
TRINITY_DN15965_c0_g1_i1_orf1	-	-	-	U3 small nucleolar RNA-associated protein 15 homolog [Ostrinia furnacalis]	1.9835	-0.6068	-0.54028	-0.25118	-0.58526
TRINITY_DN883_c0_g1_i8_orf1	-	-	-	diacylglycerol O-acyltransferase 1 isoform X1 [Ostrinia furnacalis]	1.78757	-0.60517	-1.09154	0.30299	-0.39384
TRINITY_DN12227_c0_g2_i3_orf1	-	-	-	exonuclease 3'-5' domain-containing protein 2 [Ostrinia furnacalis]	1.09503	0.60142	-1.67252	-0.57836	0.55443
TRINITY_DN5087_c0_g1_i6_orf1	-	-	-	nascent polypeptide-associated complex subunit alpha [Ostrinia furnacalis] >XP_028156807.1 nascent polypeptide-associated complex subunit alpha [Ostrinia furnacalis] >XP_028156808.1 nascent polypeptide-associated complex subunit alpha [Ostrinia furnacalis]	1.93149	-0.01371	-0.69731	-0.74832	-0.47215
TRINITY_DN58207_c0_g1_i1_orf1	-	-	-	60S ribosomal protein L6 [Ostrinia furnacalis] >XP_028170357.1 60S ribosomal protein L6 [Ostrinia furnacalis]	1.82444	-0.95206	-0.81619	-0.24861	0.19242
TRINITY_DN86956_c0_g5_i1_orf1	-	-	-	PREDICTED: protein sly1 homolog [Microplitis demolitor]	1.92897	0.00041	-0.75594	-0.68453	-0.48891
TRINITY_DN1617_c0_g1_i5_orf1	-	-	-	hypothetical protein evm_009822 [Chilo suppressalis] >CAB3525311.1 unnamed protein product [Chilo suppressalis] >CAH0402638.1 unnamed protein product [Chilo suppressalis]	1.94395	-0.55855	-0.40138	-0.12879	-0.85522
TRINITY_DN2993_c0_g1_i4_orf1	-	-	-	heat shock 70 kDa protein cognate 5 [Ostrinia furnacalis]	1.98517	-0.28997	-0.62542	-0.61116	-0.45863
TRINITY_DN6933_c1_g1_i1_orf1	-	-	-	Chlorophyll a-b binding protein 40, chloroplastic [Trichinella nelsoni] >KRY99282.1 Chlorophyll a-b binding protein 40, chloroplastic [Trichinella zimbabwensis]	1.96756	-0.17545	-0.53919	-0.7321	-0.52081
TRINITY_DN47389_c0_g1_i2_orf1	-	-	-	non-specific lipid-transfer protein-like [Ostrinia furnacalis]	1.89455	-0.8755	-0.56477	0.10604	-0.56032
TRINITY_DN28638_c0_g1_i1_orf1	-	-	-	uncharacterized protein LOC114364075 [Ostrinia furnacalis]	1.89526	-1.01283	-0.41713	-0.00913	-0.45617
TRINITY_DN75746_c0_g1_i1_orfp1	-	-	-	TRINITY_DN75746_c0_g1_i1_m.54871 TRINITY_DN75746_c0_g1::TRINITY_DN75746_c0_g1_i1::g.54871 ORF type:internal len:83 (+),score=-1.09,Polyhedrin PF00738.19 1.7e-25 TRINITY_DN75746_c0_g1_i1:2-247(+)	1.99033	-0.40831	-0.50577	-0.40228	-0.67397
TRINITY_DN45449_c0_g1_i1_orf1	-	-	-	ATP-dependent helicase brm [Ostrinia furnacalis]	1.5129	0.42963	-1.26939	0.25022	-0.92336
TRINITY_DN4977_c0_g1_i2_orf1	-	-	-	manganese-transporting ATPase 13A1 [Ostrinia furnacalis]	1.94224	-0.05792	-0.7282	-0.69157	-0.46454
TRINITY_DN698_c0_g1_i5_orf1	-	-	-	PREDICTED: small nuclear ribonucleoprotein Sm D3 [Amyelois transitella]	1.90997	0.02404	-0.849	-0.68741	-0.3976
TRINITY_DN10662_c0_g1_i4_orf1	-	-	-	HD domain-containing protein 2 [Ostrinia furnacalis]	1.77121	0.32943	-1.13465	-0.47132	-0.49467
TRINITY_DN106730_c0_g1_i1_orf1	-	-	-	Photosystem I reaction center subunit II, chloroplastic, partial [Trichinella zimbabwensis]	1.93581	-0.66958	-0.45545	-0.0392	-0.77158
TRINITY_DN9853_c0_g3_i1_orf1	-	-	-	importin-7 isoform X1 [Ostrinia furnacalis]	1.93457	-0.36623	-0.88981	-0.56439	-0.11414
TRINITY_DN15624_c0_g1_i1_orf1	-	-	-	LOW QUALITY PROTEIN: V-type proton ATPase subunit S1-like [Ostrinia furnacalis]	1.84277	0.20432	-0.36775	-0.9322	-0.74714
TRINITY_DN10722_c0_g3_i1_orf1	-	-	-	inositol-3-phosphate synthase [Ostrinia furnacalis]	1.49096	0.67682	-1.33509	-0.7244	-0.1083
TRINITY_DN16965_c0_g2_i1_orf1	-	-	-	hypothetical protein evm_007405 [Chilo suppressalis]	1.62034	0.57414	-1.27047	-0.41786	-0.50615
TRINITY_DN12133_c0_g2_i1_orf1	-	-	-	O-acyltransferase like protein-like [Ostrinia furnacalis]	1.97868	-0.35173	-0.31884	-0.60996	-0.69815
TRINITY_DN4116_c0_g1_i3_orf1	-	-	-	transmembrane protein 131 homolog [Ostrinia furnacalis]	1.4114	0.49505	-1.48964	-0.68641	0.2696
TRINITY_DN14601_c0_g1_i2_orf1	-	-	-	unnamed protein product [Chilo suppressalis]	1.9006	0.01421	-0.92926	-0.35429	-0.63126
TRINITY_DN4125_c1_g1_i5_orf1	-	-	-	angiotensin-converting enzyme-like isoform X2 [Ostrinia furnacalis]	1.89647	0.13945	-0.69196	-0.70388	-0.64008
TRINITY_DN7964_c0_g1_i1_orfp1	-	-	-	TRINITY_DN7964_c0_g1_i1_m.23483 TRINITY_DN7964_c0_g1::TRINITY_DN7964_c0_g1_i1::g.23483 ORF type:internal len:79	1.96546	-0.51547	-0.41947	-0.22864	-0.80188
TRINITY_DN14063_c0_g1_i7_orf1	-	-	-	probable phosphorylase b kinase regulatory subunit beta isoform X1 [Ostrinia furnacalis] >XP_028175664.1 probable phosphorylase b kinase regulatory subunit beta isoform X2 [Ostrinia furnacalis] >XP_028175665.1 probable phosphorylase b kinase regulatory subunit beta isoform X3 [Ostrinia furnacalis]	1.93883	-0.73777	-0.68046	-0.03882	-0.48177
TRINITY_DN6535_c0_g2_i1_orf1	-	-	-	NADH dehydrogenase [ubiquinone] 1 beta subcomplex subunit 3 [Ostrinia furnacalis] >XP_028166399.1 NADH dehydrogenase [ubiquinone] 1 beta subcomplex subunit 3 [Ostrinia furnacalis]	1.96739	-0.18867	-0.59158	-0.73353	-0.45361
TRINITY_DN4710_c0_g1_i1_orf1	-	-	-	hypothetical protein evm_000671 [Chilo suppressalis]	1.97817	-0.27568	-0.49563	-0.46751	-0.73935
TRINITY_DN19000_c0_g1_i4_orf1	-	-	-	NADH dehydrogenase [ubiquinone] 1 beta subcomplex subunit 9 [Ostrinia furnacalis]	1.98541	-0.5053	-0.6986	-0.45923	-0.32229
TRINITY_DN130075_c1_g2_i1_orf1	-	-	-	60S ribosomal protein L23 [Microtus ochrogaster]	1.91553	-0.75837	0.06256	-0.67256	-0.54715

TRINITY_DN19361_c0_g1_i7_orf1	-	-	-	hydroxylysine kinase [Ostrinia furnacalis] >XP_028168144.1 hydroxylysine kinase [Ostrinia furnacalis]	1.91686	0.05746	-0.65903	-0.76499	-0.5503
TRINITY_DN334_c0_g1_i3_orf1	-	-	-	chymotrypsin-like serine protease [Ostrinia nubilalis] >AAX62030.1 chymotrypsin-like serine protease [Ostrinia nubilalis]	1.76288	0.47051	-0.71948	-0.81723	-0.69668
TRINITY_DN2082_c0_g1_i2_orf1	-	-	-	choline-phosphate cytidylyltransferase B-like isoform X1 [Ostrinia furnacalis]	1.92534	-0.01909	-0.82407	-0.42269	-0.65949
TRINITY_DN14826_c0_g1_i1_orf1	-	-	-	uncharacterized protein LOC114350939 [Ostrinia furnacalis]	1.96595	-0.50503	-0.75965	-0.51949	-0.18177
TRINITY_DN12973_c0_g1_i1_orf1	-	-	-	mitochondrial-processing peptidase subunit alpha [Ostrinia furnacalis]	1.99173	-0.34405	-0.52183	-0.62829	-0.49756
TRINITY_DN3749_c0_g1_i1_orf1	-	-	-	cytochrome c oxidase subunit 6B1 [Ostrinia furnacalis]	1.98605	-0.43955	-0.47228	-0.71368	-0.36054
TRINITY_DN84322_c0_g2_i1_orf1	-	-	-	alanyl-tRNA synthetase 1 [Homo sapiens] >KAI4055846.1 alanyl-tRNA synthetase 1 [Homo sapiens]	1.82043	-0.22937	-1.15634	-0.535	0.10028
TRINITY_DN7583_c0_g1_i1_orf1	-	-	-	39S ribosomal protein L21, mitochondrial [Ostrinia furnacalis]	1.80569	0.34421	-0.59338	-0.94782	-0.60871
TRINITY_DN105901_c0_g1_i2_orfp1	-	-	-	contactin-like [Pectinophora gossypiella]	1.90937	-0.83731	0.06594	-0.54308	-0.59492
TRINITY_DN4030_c0_g2_i1_orf1	-	-	-	putative trypsin 6 [Ostrinia nubilalis]	1.92009	-0.43558	-0.07282	-0.45909	-0.9526
TRINITY_DN10558_c0_g1_i4_orf1	-	-	-	unnamed protein product [Chrysodeixis includens]	0.93463	1.04237	-1.25005	-1.14229	0.41533
TRINITY_DN21531_c0_g1_i1_orf1	-	-	-	viral IAP-associated factor homolog [Ostrinia furnacalis]	1.7378	0.48422	-0.55876	-0.98996	-0.6733
TRINITY_DN32022_c0_g1_i1_orf1	-	-	-	striatin isoform X1 [Diachasma alloeum]	1.73489	0.14115	-1.21998	-0.69319	0.03713
TRINITY_DN42082_c0_g2_i2_orfp1	-	-	-	TRINITY_DN42082_c0_g2_i2_m.7835 TRINITY_DN42082_c0_g2::TRINITY_DN42082_c0_g2_i2::g.7835 ORF type:internal len:133 (+),score=75.81 TRINITY_DN42082_c0_g2_i2:1-396(+)	1.80352	-0.62243	-1.1011	-0.30856	0.22857
TRINITY_DN46173_c0_g3_i2_orf1	-	-	-	tropomyosin-1, isoforms 9A/A/B isoform X33 [Aedes aegypti] >EAT46020.1 AAEL002761-PB [Aedes aegypti]	1.91296	0.01061	-0.87658	-0.63302	-0.41397
TRINITY_DN5019_c0_g1_i2_orf1	-	-	-	RRP12-like protein isoform X4 [Ostrinia furnacalis] >XP_028175539.1 RRP12-like protein isoform X5 [Ostrinia furnacalis]	1.98634	-0.38586	-0.71917	-0.44065	-0.44065
TRINITY_DN58531_c0_g1_i1_orf1	-	-	-	uncharacterized protein LOC114357371 [Ostrinia furnacalis] >XP_028166768.1 uncharacterized protein LOC114357371 [Ostrinia furnacalis]	1.52328	0.83072	-1.08564	-0.69133	-0.57703
TRINITY_DN3028_c0_g1_i1_orf1	-	-	-	pre-rRNA processing protein FTSJ3 [Ostrinia furnacalis]	1.95518	-0.68798	-0.66025	-0.09876	-0.5082
TRINITY_DN3457_c0_g1_i4_orf1	-	-	-	aryl hydrocarbon receptor nuclear translocator homolog [Ostrinia furnacalis]	1.53826	0.3421	-1.45411	-0.60773	0.18147
TRINITY_DN29934_c0_g1_i6_orf1	-	-	-	sodium/potassium-transporting ATPase subunit beta-2-like [Ostrinia furnacalis] >XP_028176258.1 sodium/potassium-transporting ATPase subunit beta-2-like [Ostrinia	1.5612	0.29549	-1.13126	-1.04552	0.32009
TRINITY_DN969_c0_g1_i3_orf1	-	-	-	protein UBASH3A homolog isoform X3 [Ostrinia furnacalis]	1.64475	0.6359	-1.05758	-0.72112	-0.50194
TRINITY_DN47575_c0_g1_i1_orf1	-	-	-	PREDICTED: splicing factor 1-like [Fopius arisanus]	1.70474	-0.71555	-1.03875	-0.47604	0.52559
TRINITY_DN5841_c0_g1_i2_orf1	-	-	-	hypothetical protein evm_011295 [Chilo suppressalis]	1.46851	0.7493	-1.3935	-0.3973	-0.427
TRINITY_DN21539_c0_g1_i1_orf1	-	-	-	probable phenylalanine--tRNA ligase, mitochondrial [Ostrinia furnacalis]	1.72611	0.50185	-1.01346	-0.56198	-0.65251
TRINITY_DN21150_c0_g1_i4_orf1	-	-	-	RNA-binding protein cabeza-like isoform X2 [Bicyclus anynana]	1.7751	0.25961	-1.12957	-0.67173	-0.23341
TRINITY_DN5867_c0_g1_i1_orf1	-	-	-	NADH dehydrogenase [ubiquinone] 1 alpha subcomplex subunit 7-like [Ostrinia furnacalis]	1.9465	-0.04654	-0.69116	-0.62288	-0.58591
TRINITY_DN50571_c1_g1_i1_orf1	-	-	-	WD repeat-containing protein 46 [Ostrinia furnacalis]	1.66286	0.58588	-0.61749	-0.51575	-1.1155
TRINITY_DN26130_c0_g1_i1_orf1	-	-	-	membrane alanyl aminopeptidase-like [Ostrinia furnacalis]	1.87724	0.07622	-0.32579	-0.67358	-0.9541
TRINITY_DN4070_c0_g1_i4_orf1	-	-	-	alpha-N-acetylgalactosaminidase isoform X3 [Ostrinia furnacalis]	0.96014	1.41896	-1.06811	-0.83504	-0.47594
TRINITY_DN11657_c0_g1_i2_orf1	-	-	-	trehalase-1 [Omphisa fuscidentalis]	1.48657	0.89195	-0.65324	-1.06205	-0.66323
TRINITY_DN20118_c0_g1_i4_orfp1	-	-	-	hypothetical protein HF086_007571 [Spodoptera exigua]	1.38904	0.80564	-1.46817	-0.39561	-0.3309
TRINITY_DN47123_c0_g1_i1_orf1	-	-	-	WD40 repeat-containing protein SMU1 [Ostrinia furnacalis]	1.87622	0.02523	-0.92746	-0.75593	-0.21806
TRINITY_DN2668_c0_g1_i7_orf1	-	-	-	unnamed protein product [Chrysodeixis includens]	1.86427	-1.15898	-0.30924	-0.13797	-0.25808
TRINITY_DN109540_c0_g1_i3_orf1	-	-	-	4-coumarate--CoA ligase 1-like isoform X4 [Ostrinia furnacalis]	1.98969	-0.66041	-0.39798	-0.38513	-0.54617
TRINITY_DN18164_c0_g1_i7_orf1	-	-	-	uncharacterized protein LOC114366518 isoform X5 [Ostrinia furnacalis]	1.09892	1.23678	-0.9433	-1.14539	-0.24701
TRINITY_DN37699_c0_g1_i3_orfp1	-	-	-	TRINITY_DN37699_c0_g1_i3_m.58788 TRINITY_DN37699_c0_g1::TRINITY_DN37699_c0_g1_i3::g.58788 ORF type:internal len:122 (+),score=39.86 TRINITY_DN37699_c0_g1_i3:1-363(+)	1.76243	-1.08264	-0.58841	-0.47658	0.38519
TRINITY_DN21971_c0_g1_i4_orf1	-	-	-	40S ribosomal protein S26 [Nymphalis io]	1.81736	-0.11689	-1.195	-0.50548	3.58E-06
TRINITY_DN8019_c0_g1_i4_orf1	-	-	-	deoxyhypusine hydroxylase [Ostrinia furnacalis]	1.96715	-0.20125	-0.73559	-0.60995	-0.42035
TRINITY_DN5925_c0_g1_i5_orf1	-	-	-	isocitrate dehydrogenase [NAD] subunit gamma, mitochondrial-like isoform X1 [Ostrinia furnacalis]	1.95934	-0.57975	-0.6142	-0.66085	-0.10454
TRINITY_DN36538_c0_g1_i2_orf1	-	-	-	xaa-Pro dipeptidase isoform X1 [Ostrinia furnacalis] >XP_028156507.1 xaa-Pro dipeptidase isoform X2 [Ostrinia furnacalis]	1.93845	-0.09652	-0.74206	-0.74687	-0.353
TRINITY_DN17312_c0_g1_i1_orf1	-	-	-	mRNA cap guanine-N7 methyltransferase [Ostrinia furnacalis]	1.72448	0.40395	-0.94569	-0.95827	-0.22448

TRINITY_DN8261_c0_g1_i1_orf1	-	-	-	UDP-N-acetylhexosamine pyrophosphorylase-like protein 1 [Ostrinia furnacalis]	1.90062	0.12686	-0.70302	-0.67329	-0.65117
TRINITY_DN27848_c0_g1_i2_orf1	-	-	-	cystathionine beta-synthase-like [Ostrinia furnacalis] >XP_028159011.1 cystathionine beta-synthase-like [Ostrinia furnacalis]	1.98574	-0.6619	-0.36866	-0.36223	-0.59296
TRINITY_DN389_c0_g1_i2_orf1	-	-	-	uncharacterized protein LOC118068293 isoform X2 [Chelonus insularis]	1.41807	1.01043	-0.77545	-0.84241	-0.81064
TRINITY_DN1445_c0_g1_i1_orf1	-	-	-	leucine-rich PPR motif-containing protein, mitochondrial [Ostrinia furnacalis]	1.95138	-0.06724	-0.64867	-0.66035	-0.57511
TRINITY_DN12442_c0_g1_i4_orf1	-	-	-	midasin-like [Ostrinia furnacalis]	1.77141	-0.49391	-0.94338	0.41275	-0.74687
TRINITY_DN1294_c0_g1_i3_orf1	-	-	-	46 kDa FK506-binding nuclear protein-like isoform X1 [Ostrinia furnacalis] >XP_028157904.1 46 kDa FK506-binding nuclear protein-like isoform X2 [Ostrinia furnacalis]	1.97293	-0.21303	-0.71777	-0.56553	-0.4766
TRINITY_DN16482_c0_g1_i6_orf1	-	-	-	transmembrane protein 120 homolog isoform X2 [Ostrinia furnacalis]	1.93117	-0.20092	-0.91054	-0.59037	-0.22933
TRINITY_DN35635_c0_g1_i1_orf1	-	-	-	probable NADH dehydrogenase [ubiquinone] 1 alpha subcomplex subunit 12 [Ostrinia	1.88856	-0.02263	-0.54039	-1.02491	-0.30062
TRINITY_DN3053_c0_g1_i2_orf1	-	-	-	prostaglandin reductase 1-like [Ostrinia furnacalis]	1.9485	-0.73149	-0.71924	-0.36526	-0.1325
TRINITY_DN13216_c0_g1_i5_orf1	-	-	-	uncharacterized protein LOC114358344 isoform X1 [Ostrinia furnacalis]	1.3129	1.13177	-0.77007	-0.82698	-0.84762
TRINITY_DN4944_c0_g1_i2_orf1	-	-	-	bifunctional glutamate/proline--tRNA ligase [Ostrinia furnacalis]	1.95492	-0.22916	-0.76126	-0.68366	-0.28083
TRINITY_DN129226_c0_g1_i2_orf1	-	-	-	hypothetical protein evm_000268 [Chilo suppressalis]	1.36997	0.9096	-1.36172	-0.35817	-0.55968
TRINITY_DN21909_c0_g1_i1_orf1	-	-	-	complement component 1 Q subcomponent-binding protein, mitochondrial [Ostrinia	1.99365	-0.40526	-0.6127	-0.55755	-0.41815
TRINITY_DN2704_c0_g1_i5_orf1	-	-	-	hypothetical protein evm_009002 [Chilo suppressalis]	1.94253	-0.10269	-0.44702	-0.54437	-0.84845
TRINITY_DN43369_c0_g2_i1_orf1	-	-	-	cytochrome P450 monooxygenase 304 [Glyphodes pyloalis]	1.35978	0.32536	-0.76021	0.54735	-1.47229
TRINITY_DN44557_c0_g2_i1_orf1	-	-	-	serine hydrolase-like protein [Ostrinia furnacalis]	1.85566	0.03643	-1.11885	-0.4337	-0.33954
TRINITY_DN6248_c0_g1_i1_orf1	-	-	-	DNA topoisomerase I, mitochondrial [Ostrinia furnacalis]	1.77612	0.44497	-0.77124	-0.75301	-0.69683
TRINITY_DN22815_c0_g1_i2_orf1	-	-	-	acyl carrier protein, mitochondrial isoform X1 [Ostrinia furnacalis]	1.98357	-0.68723	-0.50994	-0.50255	-0.28384
TRINITY_DN620_c0_g1_i4_orf1	-	-	-	lysine--tRNA ligase isoform X1 [Ostrinia furnacalis]	1.92092	-0.63754	-0.89439	-0.3128	-0.07619
TRINITY_DN20749_c0_g1_i3_orf1	-	-	-	protein arginine N-methyltransferase 1-like [Ostrinia furnacalis]	1.94267	-0.54116	-0.87197	-0.39248	-0.13706
TRINITY_DN47_c0_g1_i2_orf1	-	-	-	uncharacterized protein LOC114356437 isoform X1 [Ostrinia furnacalis]	1.87847	-0.85208	-0.07854	-0.09318	-0.85467
TRINITY_DN7112_c0_g1_i1_orf1	-	-	-	heterogeneous nuclear ribonucleoprotein K isoform X2 [Ostrinia furnacalis]	1.44205	0.65261	-1.28599	-0.91121	0.10254
TRINITY_DN24399_c0_g1_i1_orf1	-	-	-	retinol-binding protein pinta-like [Ostrinia furnacalis]	1.87922	-0.76217	-0.4453	-0.81738	0.14563
TRINITY_DN1079_c0_g1_i4_orf1	-	-	-	CD109 antigen-like [Ostrinia furnacalis]	1.98283	-0.39826	-0.5975	-0.31242	-0.67465
TRINITY_DN51045_c0_g1_i1_orf1	-	-	-	cell growth-regulating nucleolar protein [Ostrinia furnacalis]	1.95359	-0.07625	-0.63243	-0.58487	-0.66003
TRINITY_DN100821_c0_g1_i1_orf1	-	-	-	putative GMP synthase, partial [Operophtera brumata]	1.86537	-0.45413	-1.06273	-0.42328	0.07477
TRINITY_DN1239_c0_g1_i3_orf1	-	-	-	uncharacterized protein LOC114355269 [Ostrinia furnacalis] >XP_028163822.1 uncharacterized protein LOC114355269 [Ostrinia furnacalis]	1.89951	-0.65318	-0.95979	-0.10472	-0.18182
TRINITY_DN3401_c0_g1_i1_orf1	-	-	-	28S ribosomal protein S5, mitochondrial [Ostrinia furnacalis]	1.98161	-0.55127	-0.53186	-0.24577	-0.65271
TRINITY_DN11153_c0_g1_i1_orf1	-	-	-	unnamed protein product [Chilo suppressalis]	1.96363	-0.47685	-0.83925	-0.28637	-0.36116
TRINITY_DN14967_c0_g2_i1_orf1	-	-	-	glyceraldehyde-3-phosphate dehydrogenase 2 [Holotrichia oblita]	1.98902	-0.52637	-0.35624	-0.4354	-0.67101
TRINITY_DN86844_c0_g2_i1_orf1	-	-	-	spermine oxidase-like isoform X1 [Ostrinia furnacalis]	1.93378	-0.84742	-0.44753	-0.58214	-0.05669
TRINITY_DN3503_c0_g1_i1_orfp1	-	-	-	uncharacterized protein LOC114356429 [Ostrinia furnacalis]	1.96203	-0.29991	-0.62762	-0.26084	-0.77366
TRINITY_DN39532_c0_g1_i1_orf1	-	-	-	hypothetical protein evm_009649 [Chilo suppressalis]	1.73233	0.36931	-1.19963	-0.35952	-0.54249
TRINITY_DN760_c1_g2_i6_orf1	-	-	-	ADP,ATP carrier protein [Pieris napi]	1.97327	-0.68023	-0.22268	-0.63777	-0.43259
TRINITY_DN92232_c0_g1_i1_orf1	-	-	-	protein SDA1 homolog [Ostrinia furnacalis]	1.94163	-0.43614	-0.5285	-0.11213	-0.86485
TRINITY_DN1557_c0_g1_i9_orf1	-	-	-	carboxylesterase CXE18 [Ostrinia furnacalis]	1.91218	-0.86369	-0.64338	-0.42796	0.02286
TRINITY_DN4842_c0_g1_i5_orf1	-	-	-	cytochrome c oxidase assembly factor 4 homolog, mitochondrial isoform X1 [Ostrinia furnacalis] >XP_028162331.1 cytochrome c oxidase assembly factor 4 homolog, mitochondrial isoform X2 [Ostrinia furnacalis]	1.92861	-0.06148	-0.60858	-0.86837	-0.39018
TRINITY_DN1882_c0_g1_i4_orf1	-	-	-	zinc transporter ZIP13 homolog [Ostrinia furnacalis]	1.26847	-0.23902	-1.70666	0.02889	0.64832
TRINITY_DN2117_c0_g1_i1_orf1	-	-	-	BUB3-interacting and GLEBS motif-containing protein ZNF207 [Chelonus insularis]	1.75429	0.30715	-1.04175	-0.84376	-0.17592
TRINITY_DN20294_c0_g2_i1_orf1	-	-	-	cytochrome b-c1 complex subunit 2, mitochondrial isoform X1 [Ostrinia furnacalis] >XP_028170208.1 cytochrome b-c1 complex subunit 2, mitochondrial isoform X2 [Ostrinia furnacalis]	1.98754	-0.39003	-0.57047	-0.66305	-0.36398
TRINITY_DN20346_c0_g1_i1_orf1	-	-	-	NADH dehydrogenase [ubiquinone] 1 alpha subcomplex subunit 6 [Ostrinia furnacalis]	1.97405	-0.26927	-0.57755	-0.38791	-0.73931
TRINITY_DN14937_c0_g1_i7_orf1	-	-	-	multidrug resistance protein homolog 49-like [Ostrinia furnacalis] >XP_028159925.1 multidrug resistance protein homolog 49-like [Ostrinia furnacalis]	1.89399	-0.41107	-0.67614	0.07682	-0.8836
TRINITY_DN52861_c0_g1_i1_orf1	-	-	-	protein MAK16 homolog A [Ostrinia furnacalis]	1.97575	-0.32983	-0.74391	-0.56804	-0.33397
TRINITY_DN96170_c0_g1_i1_orf1	-	-	-	uncharacterized protein LOC114355569 [Ostrinia furnacalis]	1.98188	-0.64667	-0.5858	-0.25227	-0.49714
TRINITY_DN3647_c2_g1_i3_orf1	-	-	-	unnamed protein product, partial [Iphiclides podalirius]	1.54417	0.76116	-1.13417	-0.40664	-0.76453
TRINITY_DN787_c0_g1_i7_orf1	-	-	-	YLP motif-containing protein 1-like isoform X1 [Ostrinia furnacalis]	1.65675	0.61119	-1.01592	-0.4447	-0.80732

TRINITY_DN2673_c0_g3_i1_orf1	-	-	-	uncharacterized protein LOC114361372 [Ostrinia furnacalis]	1.78896	0.021	-1.20875	-0.02009	-0.58111
TRINITY_DN6439_c0_g1_i1_orf1	-	-	-	GPI mannosyltransferase 3 isoform X4 [Ostrinia furnacalis] >XP_028164836.1 GPI mannosyltransferase 3 isoform X5 [Ostrinia furnacalis]	1.63769	0.56421	-1.20796	-0.64942	-0.34452
TRINITY_DN7920_c0_g1_i2_orf1	-	-	-	uncharacterized protein LOC114357268 [Ostrinia furnacalis] >XP_028166599.1 uncharacterized protein LOC114357268 [Ostrinia furnacalis]	1.88788	-0.34404	-0.29084	-0.15324	-1.09976
TRINITY_DN3428_c0_g1_i1_orf1	-	-	-	10 kDa heat shock protein, mitochondrial [Ostrinia furnacalis]	1.92255	-0.12003	-0.87906	-0.67419	-0.24928
TRINITY_DN8366_c0_g1_i4_orf1	-	-	-	luciferin 4-monooxygenase-like [Ostrinia furnacalis]	1.93493	-0.66152	-0.33882	-0.10197	-0.83262
TRINITY_DN25856_c0_g1_i1_orf1	-	-	-	myrosinase 1-like [Ostrinia furnacalis]	1.97517	-0.74245	-0.53735	-0.43979	-0.25558
TRINITY_DN37538_c0_g4_i1_orf1	-	-	-	esterase FE4-like [Ostrinia furnacalis]	1.89464	0.13013	-0.71641	-0.54404	-0.76433
TRINITY_DN3476_c0_g1_i5_orf1	-	-	-	maltase A1-like [Ostrinia furnacalis]	1.96415	-0.80132	-0.24131	-0.36816	-0.55336
TRINITY_DN37538_c0_g2_i1_orf1	-	-	-	esterase FE4-like [Ostrinia furnacalis]	1.97287	-0.23062	-0.45572	-0.54499	-0.74155
TRINITY_DN4895_c0_g1_i2_orf1	-	-	-	coiled-coil domain-containing protein 86 [Ostrinia furnacalis]	1.96505	-0.83741	-0.4703	-0.33591	-0.32143
TRINITY_DN145647_c0_g1_i1_orf1	-	-	-	PREDICTED: U6 snRNA-associated Sm-like protein LSm5 isoform X1 [Fopius arisanus]	1.97577	-0.24125	-0.62881	-0.42743	-0.67827
TRINITY_DN3847_c1_g1_i1_orf1	-	-	-	ribosome production factor 2 homolog [Ostrinia furnacalis]	1.97149	-0.35623	-0.34344	-0.4633	-0.80853
TRINITY_DN12242_c0_g1_i5_orf1	-	-	-	heterogeneous nuclear ribonucleoprotein 87F-like isoform X1 [Vanessa tameamea] >XP_046967652.1 heterogeneous nuclear ribonucleoprotein 87F-like isoform X1 [Vanessa cardui] >XP_047532045.1 heterogeneous nuclear ribonucleoprotein 87F-like [Vanessa	1.60535	0.71703	-0.88803	-0.93101	-0.50334
TRINITY_DN2600_c0_g1_i7_orf1	-	-	-	mucin-5AC isoform X2 [Ostrinia furnacalis]	1.12898	1.29529	-1.04835	-0.65625	-0.71968
TRINITY_DN10636_c0_g1_i1_orf1	-	-	-	unnamed protein product [Arctia plantaginis] >CAB3253774.1 unnamed protein product [Arctia plantaginis]	1.94263	-0.72404	-0.62814	-0.55318	-0.03728
TRINITY_DN27087_c0_g1_i1_orf1	-	-	-	2',5'-phosphodiesterase 12 [Ostrinia furnacalis]	1.90819	-0.66892	-0.90185	-0.02535	-0.31207
TRINITY_DN15607_c0_g1_i6_orf1	-	-	-	protein artichoke-like [Ostrinia furnacalis]	1.92895	-0.48904	-0.80296	-0.62865	-0.0083
TRINITY_DN12495_c0_g1_i2_orf1	-	-	-	probable ATP-dependent RNA helicase pitchoune [Manduca sexta] >KAG6441249.1 hypothetical protein O3G_MSEX001749 [Manduca sexta]	1.98431	-0.60698	-0.619	-0.48731	-0.27102
TRINITY_DN23926_c0_g1_i4_orf1	-	-	-	programmed cell death protein 10 [Ostrinia furnacalis]	1.46054	0.6023	-1.53827	-0.25303	-0.27154
TRINITY_DN279_c0_g1_i10_orf1	-	-	-	RE1-silencing transcription factor-like isoform X1 [Ostrinia furnacalis]	1.10248	1.25413	-1.24766	-0.69614	-0.41282
TRINITY_DN10530_c0_g1_i1_orf1	-	-	-	cytochrome c oxidase subunit NDUFA4 [Ostrinia furnacalis]	1.99387	-0.44326	-0.52284	-0.62891	-0.39886
TRINITY_DN1173_c1_g1_i9_orf1	-	-	-	hypothetical protein evm_001011 [Chilo suppressalis]	1.9302	-0.87884	-0.64855	-0.19082	-0.212
TRINITY_DN3014_c0_g1_i4_orf1	-	-	-	putative inorganic phosphate cotransporter isoform X1 [Ostrinia furnacalis]	1.82151	-1.22577	-0.33038	4.67E-05	-0.26541
TRINITY_DN327_c1_g1_i4_orf1	-	-	-	mitochondrial import receptor subunit TOM40 homolog 1-like [Ostrinia furnacalis]	1.98302	-0.35253	-0.73734	-0.46659	-0.42656
TRINITY_DN7336_c0_g1_i13_orf1	-	-	-	PREDICTED: calcium-transporting ATPase sarcoplasmic/endoplasmic reticulum type isoform X2 [Amyelois transitella]	1.87001	-1.07845	-0.50508	-0.29138	0.0049
TRINITY_DN2579_c0_g1_i7_orf1	-	-	-	aminopeptidase N5 [Ostrinia nubilalis]	1.99861	-0.45139	-0.55284	-0.52711	-0.46727
TRINITY_DN53167_c0_g1_i3_orf1	-	-	-	uncharacterized protein LOC114359219 [Ostrinia furnacalis]	1.97968	-0.72651	-0.51836	-0.45222	-0.28259
TRINITY_DN8536_c0_g1_i2_orf1	-	-	-	PC4 and SFRS1-interacting protein isoform X4 [Galleria mellonella]	1.52606	0.71648	-1.29451	-0.60178	-0.34626
TRINITY_DN24317_c0_g1_i7_orf1	-	-	-	peptidyl-tRNA hydrolase ICT1, mitochondrial [Ostrinia furnacalis]	1.94474	-0.17914	-0.86199	-0.32027	-0.58334
TRINITY_DN28152_c0_g1_i1_orf1	-	-	-	mitochondrial import inner membrane translocase subunit Tim29 [Ostrinia furnacalis]	1.99052	-0.39237	-0.56451	-0.39237	-0.64129
TRINITY_DN948_c0_g1_i1_orf1	-	-	-	mitochondrial-processing peptidase subunit beta [Ostrinia furnacalis]	1.98539	-0.39074	-0.68305	-0.5641	-0.3475
TRINITY_DN9119_c0_g1_i3_orf1	-	-	-	actin-binding Rho-activating protein [Helicoverpa armigera] >XP_047029227.1 actin-binding Rho-activating protein-like [Helicoverpa zea] >PZC85229.1 hypothetical protein B5X24_HaOG202414 [Helicoverpa armigera]	1.97468	-0.31484	-0.31932	-0.69316	-0.64737
TRINITY_DN63662_c0_g4_i1_orf1	-	-	-	polyadenylate-binding protein 1 [Ostrinia furnacalis]	1.91891	0.00959	-0.86117	-0.59055	-0.47678
TRINITY_DN6710_c0_g1_i6_orf1	-	-	-	multiple C2 and transmembrane domain-containing protein-like [Ostrinia furnacalis]	1.40229	0.8962	-0.97663	-1.10979	-0.21208
TRINITY_DN2062_c0_g1_i9_orf1	-	-	-	uncharacterized protein LOC114350846 [Ostrinia furnacalis]	1.79554	0.17204	-1.20687	-0.36364	-0.39708
TRINITY_DN46409_c0_g1_i1_orf1	-	-	-	unnamed protein product [Heterotrigona itama]	1.901	-0.03715	-0.23253	-0.80666	-0.82466
TRINITY_DN32601_c0_g1_i2_orf1	-	-	-	uncharacterized protein LOC114363197 [Ostrinia furnacalis]	1.08832	1.33724	-1.00905	-0.65372	-0.76277
TRINITY_DN8833_c0_g1_i1_orf1	-	-	-	nucleolar protein 16 [Ostrinia furnacalis]	1.97216	-0.46733	-0.72026	-0.21112	-0.57345
TRINITY_DN220_c0_g1_i3_orf1	-	-	-	serine-arginine protein 55 isoform X6 [Pieris brassicae]	1.52846	0.67164	-1.19421	-0.87755	-0.12835

TRINITY_DN54543_c0_g5_i2_orf1	-	-	-	reactive oxygen species modulator 1 [Papilio machaon] >XP_022123076.1 reactive oxygen species modulator 1 [Pieris rapae] >XP_022820703.1 reactive oxygen species modulator 1 [Spodoptera litura] >XP_028176790.1 reactive oxygen species modulator 1 [Ostrinia furnacalis] >XP_030021418.1 reactive oxygen species modulator 1 [Manduca sexta] >XP_034836896.1 reactive oxygen species modulator 1 [Maniola hyperantus] >XP_035446679.1 reactive oxygen species modulator 1-like [Spodoptera frugiperda] >XP_045458971.1 reactive oxygen species modulator 1 [Melitaea cinxia] >XP_045521377.1 reactive oxygen species modulator 1 [Pieris brassicae] >XP_045765649.1 reactive oxygen species modulator 1 [Maniola jurtina] >XP_047042173.1 reactive oxygen species modulator 1 [Helicoverpa zea] >XP_049691668.1 reactive oxygen species modulator 1 [Helicoverpa armigera] >KAF9414699.1 hypothetical protein HW555_007477 [Spodoptera exigua] >KPI93862.1 Reactive oxygen species modulator 1 [Papilio xuthus] >CAB3515149.1 unnamed protein product [Spodoptera littoralis] >CAG5021695.1 unnamed protein product [Parnassius apollo] >KAF9823017.1 hypothetical protein SFRURICE_018191 [Spodoptera frugiperda]	1.98059	-0.27807	-0.54157	-0.45258	-0.70838
TRINITY_DN17738_c0_g1_i2_orf1	-	-	-	unnamed protein product [Diatraea saccharalis]	1.97855	-0.42254	-0.7416	-0.51943	-0.29498
TRINITY_DN18036_c0_g1_i7_orf1	-	-	-	pentatricopeptide repeat-containing protein 2, mitochondrial-like [Ostrinia furnacalis]	1.96512	-0.15939	-0.72074	-0.59175	-0.49324
TRINITY_DN17905_c0_g3_i1_orf1	-	-	-	zinc finger protein 706-like [Ostrinia furnacalis] >XP_028176219.1 zinc finger protein 706-like [Ostrinia furnacalis] >XP_028176220.1 zinc finger protein 706-like [Ostrinia furnacalis] >XP_028176221.1 zinc finger protein 706-like [Ostrinia furnacalis]	1.87486	0.20371	-0.64999	-0.72968	-0.69891
TRINITY_DN24873_c0_g1_i4_orf1	-	-	-	uncharacterized protein LOC114365742 [Ostrinia furnacalis]	1.94187	-0.74574	-0.76016	-0.20934	-0.22662
TRINITY_DN121893_c0_g1_i1_orf1	-	-	-	hypothetical protein, partial [Ostrinia furnacalis]	1.60456	-0.62747	-1.38734	0.09828	0.31197
TRINITY_DN1707_c0_g1_i1_orf1	-	-	-	inositol oxygenase-like [Ostrinia furnacalis]	1.91496	-0.59936	-0.06401	-0.32052	-0.93106
TRINITY_DN69236_c0_g1_i1_orf1	-	-	-	peroxiredoxin [Ostrinia furnacalis]	1.46662	0.75485	-1.37164	-0.28943	-0.5604
TRINITY_DN49265_c0_g3_i2_orf1	-	-	-	cytochrome c [Ostrinia furnacalis] >XP_028160278.1 cytochrome c [Ostrinia furnacalis]	1.96888	-0.55206	-0.39431	-0.77631	-0.2462
TRINITY_DN21494_c0_g1_i2_orf1	-	-	-	pancreatic triacylglycerol lipase-like [Ostrinia furnacalis]	1.972	-0.23966	-0.72013	-0.61314	-0.39908
TRINITY_DN6308_c0_g1_i6_orf1	-	-	-	myc box-dependent-interacting protein 1 isoform X2 [Ostrinia furnacalis]	1.92338	-0.02879	-0.85244	-0.64371	-0.39844
TRINITY_DN5956_c1_g1_i5_orf1	-	-	-	uncharacterized protein DDB_G0286299-like [Ostrinia furnacalis] >XP_028170990.1 uncharacterized protein DDB_G0286299-like [Ostrinia furnacalis] >XP_028170991.1 uncharacterized protein DDB_G0286299-like [Ostrinia furnacalis]	1.97889	-0.66357	-0.56155	-0.22816	-0.52561
TRINITY_DN259_c0_g1_i8_orf1	-	-	-	hypothetical protein evm_000095 [Chilo suppressalis]	1.86926	0.07717	-1.044	-0.48862	-0.41381
TRINITY_DN2594_c0_g2_i4_orf1	-	-	-	isocitrate dehydrogenase [NAD] subunit beta, mitochondrial isoform X2 [Ostrinia furnacalis]	1.95928	-0.14971	-0.53391	-0.77939	-0.49627
TRINITY_DN7073_c0_g1_i1_orf1	-	-	-	unnamed protein product, partial [Brenthis ino]	1.95935	-0.67566	-0.73997	-0.33913	-0.20459
TRINITY_DN3082_c1_g1_i7_orf1	-	-	-	ribosomal RNA processing protein 1 homolog [Ostrinia furnacalis]	1.96907	-0.20377	-0.58824	-0.44278	-0.73427
TRINITY_DN501_c1_g1_i1_orf1	-	-	-	sodium- and chloride-dependent GABA transporter ine isoform X1 [Ostrinia furnacalis]	1.96809	-0.42332	-0.82672	-0.41458	-0.30346
TRINITY_DN79804_c0_g1_i1_orf1	-	-	-	zinc finger protein on ecdysone puffs-like [Ostrinia furnacalis]	1.88466	0.08592	-0.95689	-0.58184	-0.43186
TRINITY_DN64510_c0_g1_i1_orf1	-	-	-	39S ribosomal protein L15, mitochondrial [Ostrinia furnacalis]	1.98979	-0.40943	-0.68686	-0.4139	-0.4796
TRINITY_DN15046_c0_g1_i8_orf1	-	-	-	epidermal retinol dehydrogenase 2-like isoform X1 [Ostrinia furnacalis] >XP_028169999.1 epidermal retinol dehydrogenase 2-like isoform X2 [Ostrinia furnacalis]	1.9074	-0.71469	-0.0023	-0.32826	-0.86215
TRINITY_DN14313_c0_g1_i1_orf1	-	-	-	25S rRNA (cytosine-C(5))-methyltransferase nop2 [Ostrinia furnacalis]	1.93983	-0.0294	-0.7472	-0.60764	-0.55558
TRINITY_DN7964_c0_g1_i6_orfp1	-	-	-	TRINITY_DN7964_c0_g1_i6_m.23478 TRINITY_DN7964_c0_g1::TRINITY_DN7964_c0_g1_i6::g.23478 ORF type:internal len:87	1.93157	-0.90434	-0.08853	-0.5066	-0.4321
TRINITY_DN25783_c0_g1_i2_orf1	-	-	-	SET and MYND domain-containing protein 4 [Ostrinia furnacalis]	1.58295	0.37817	-1.47001	-0.43231	-0.0588
TRINITY_DN29440_c1_g1_i4_orf1	-	-	-	neutral lipase [Helicoverpa armigera]	1.89651	0.04622	-0.33897	-0.79156	-0.8122
TRINITY_DN11347_c0_g1_i1_orf1	-	-	-	N(4)-(Beta-N-acetylglucosaminyl)-L-asparaginase-like [Ostrinia furnacalis]	1.95032	-0.2416	-0.31133	-0.51897	-0.87841
TRINITY_DN14436_c0_g1_i7_orf1	-	-	-	V-type proton ATPase subunit C [Vanessa cardui]	1.95242	-0.08308	-0.70866	-0.52848	-0.63219
TRINITY_DN1775_c0_g1_i3_orf1	-	-	-	ATP-dependent RNA helicase dbp2-like isoform X1 [Ostrinia furnacalis]	1.74393	0.50522	-0.66914	-0.72887	-0.85115
TRINITY_DN56110_c0_g1_i1_orf1	-	-	-	pescadillo homolog [Ostrinia furnacalis]	1.98418	-0.58311	-0.62968	-0.26533	-0.50606
TRINITY_DN3134_c0_g1_i1_orf1	-	-	-	cytochrome c oxidase subunit 6C-1 isoform X1 [Hyposmocoma kahamanoa]	1.99001	-0.46632	-0.50628	-0.66449	-0.35291
TRINITY_DN1505_c0_g1_i1_orf1	-	-	-	uncharacterized protein LOC114362816 isoform X1 [Ostrinia furnacalis] >XP_028174154.1 uncharacterized protein LOC114362816 isoform X2 [Ostrinia furnacalis]	1.98192	-0.23866	-0.61698	-0.57009	-0.5562
TRINITY_DN2574_c0_g1_i5_orf1	-	-	-	prion-like-(Q/N-rich) domain-bearing protein 25 [Ostrinia furnacalis] >XP_028158239.1 prion-like-(Q/N-rich) domain-bearing protein 25 [Ostrinia furnacalis] >XP_028158240.1 prion-like-(Q/N-rich) domain-bearing protein 25 [Ostrinia furnacalis] >XP_028158241.1 prion-like-(Q/N-rich) domain-bearing protein 25 [Ostrinia furnacalis]	1.9608	-0.776	-0.44782	-0.16728	-0.5697

TRINITY_DN3929_c0_g3_i3_orf1	-	-	-	Glutathione S-transferase 1, isoform D [Papilio machaon]	1.77881	-1.05174	-0.72848	0.31614	-0.31473
TRINITY_DN6685_c0_g1_i8_orf1	-	-	-	cleft lip and palate transmembrane protein 1 homolog [Ostrinia furnacalis]	1.85157	0.08722	-0.95762	-0.77844	-0.20273
TRINITY_DN56910_c0_g2_i1_orf1	-	-	-	mitochondrial ribonuclease P protein 1 homolog [Ostrinia furnacalis]	1.9406	-0.08329	-0.7537	-0.71008	-0.39352
TRINITY_DN4923_c0_g1_i4_orf1	-	-	-	O-acyltransferase like protein-like [Ostrinia furnacalis]	1.96916	-0.6126	-0.53311	-0.16283	-0.66062
TRINITY_DN17351_c0_g1_i3_orf1	-	-	-	V-type proton ATPase subunit F [Ostrinia furnacalis]	1.9829	-0.70363	-0.4691	-0.29385	-0.51632
TRINITY_DN34536_c0_g1_i6_orf1	-	-	-	clustered mitochondria protein homolog isoform X2 [Ostrinia furnacalis]	1.98501	-0.30869	-0.69067	-0.46418	-0.52148
TRINITY_DN747_c0_g1_i4_orf1	-	-	-	trypsin, alkaline C-like [Ostrinia furnacalis]	1.95186	-0.69882	-0.63978	-0.078	-0.53526
TRINITY_DN108122_c0_g1_i9_orf1	-	-	-	hypothetical protein SFRUCORN_003152 [Spodoptera frugiperda]	1.93048	-0.58502	-0.91519	-0.19439	-0.23588
TRINITY_DN1154_c0_g1_i1_orf1	-	-	-	calexcitin-1-like [Ostrinia furnacalis] >ADK94879.2 juvenile hormone diol kinase [Ostrinia furnacalis]	1.88964	-0.90553	-0.00924	-0.22857	-0.74629
TRINITY_DN430_c0_g1_i5_orf1	-	-	-	hypothetical protein NE865_02252 [Phthorimaea operculella]	1.89012	-0.54505	-1.03118	-0.25118	-0.06272
TRINITY_DN6231_c0_g1_i6_orf1	-	-	-	ran-binding protein 3 isoform X1 [Ostrinia furnacalis] >XP_028166372.1 ran-binding protein 3 isoform X2 [Ostrinia furnacalis]	1.83661	0.06153	-1.13393	-0.53256	-0.23165
TRINITY_DN753_c0_g1_i4_orf1	-	-	-	venom dipeptidyl peptidase 4-like isoform X2 [Ostrinia furnacalis]	1.93819	-0.2915	-0.954	-0.28143	-0.41126
TRINITY_DN9790_c0_g1_i4_orf1	-	-	-	protein IWS1 homolog [Ostrinia furnacalis]	1.65464	0.17202	-1.45996	-0.31349	-0.0532
TRINITY_DN3959_c1_g2_i1_orf1	-	-	-	PREDICTED: probable isocitrate dehydrogenase [NAD] subunit alpha, mitochondrial isoform X3 [Papilio xuthus] >XP_014363280.1 probable isocitrate dehydrogenase [NAD] subunit alpha, mitochondrial isoform X3 [Papilio machaon]	1.99848	-0.47204	-0.45413	-0.50374	-0.56857
TRINITY_DN23266_c0_g2_i1_orf1	-	-	-	medium-chain acyl-CoA ligase ACSF2, mitochondrial [Chelonus insularis]	1.98917	-0.4197	-0.62101	-0.59997	-0.34849
TRINITY_DN85319_c0_g1_i1_orf1	-	-	-	cholinesterase 2-like [Ostrinia furnacalis]	1.97336	-0.37632	-0.80802	-0.38914	-0.39988
TRINITY_DN3332_c0_g1_i2_orf1	-	-	-	glutathione S-transferase sigma3 [Glyphodes pyloalis]	1.98589	-0.33964	-0.48221	-0.45776	-0.70628
TRINITY_DN1277_c4_g1_i5_orf1	-	-	-	GTP cyclohydrolase 1 isoform X1 [Ostrinia furnacalis] >XP_028166842.1 GTP cyclohydrolase 1 isoform X1 [Ostrinia furnacalis]	1.98061	-0.45105	-0.61174	-0.261	-0.65682
TRINITY_DN5578_c0_g1_i4_orf1	-	-	-	chromatin modification-related protein eaf-1-like [Ostrinia furnacalis]	1.74919	0.31867	-1.17559	-0.61746	-0.2748
TRINITY_DN2270_c0_g2_i1_orf1	-	-	-	integrin beta-nu [Ostrinia furnacalis]	1.98803	-0.46231	-0.57187	-0.63829	-0.31555
TRINITY_DN18404_c0_g1_i5_orf1	-	-	-	periodic tryptophan protein 1 homolog isoform X1 [Ostrinia furnacalis] >XP_028157695.1 periodic tryptophan protein 1 homolog isoform X2 [Ostrinia furnacalis]	1.96772	-0.20859	-0.44817	-0.54509	-0.76588
TRINITY_DN42759_c0_g3_i1_orf1	-	-	-	fatty acid synthase-like [Ostrinia furnacalis]	1.97291	-0.79011	-0.288	-0.44068	-0.45411
TRINITY_DN37538_c0_g3_i1_orf1	-	-	-	esterase FE4-like [Ostrinia furnacalis]	1.95277	-0.83332	-0.16725	-0.40206	-0.55014
TRINITY_DN1901_c0_g1_i6_orf1	-	-	-	mitochondrial intermembrane space import and assembly protein 40 [Ostrinia furnacalis] >XP_028171079.1 mitochondrial intermembrane space import and assembly protein 40 [Ostrinia furnacalis] >XP_028171080.1 mitochondrial intermembrane space import and assembly protein 40 [Ostrinia furnacalis]	1.98519	-0.26544	-0.56455	-0.61365	-0.54156
TRINITY_DN43391_c0_g1_i5_orf1	-	-	-	myrosinase 1-like isoform X2 [Ostrinia furnacalis]	1.98323	-0.54293	-0.33345	-0.39804	-0.70881
TRINITY_DN628_c0_g1_i7_orf1	-	-	-	prostamide/prostaglandin F synthase-like [Ostrinia furnacalis]	1.86953	0.15754	-0.46096	-0.92662	-0.63948
TRINITY_DN791_c0_g1_i2_orf1	-	-	-	peroxiredoxin 1 isoform X1 [Maniola jurtina]	1.94682	-0.14082	-0.839	-0.57997	-0.38703
TRINITY_DN16830_c0_g1_i5_orf1	-	-	-	adrenodoxin [Ostrinia furnacalis]	1.97053	-0.28706	-0.79832	-0.38987	-0.49528
TRINITY_DN2852_c0_g1_i9_orf1	-	-	-	golgin subfamily A member 4-like [Ostrinia furnacalis]	1.93839	-0.18613	-0.86959	-0.61778	-0.26489
TRINITY_DN25975_c0_g3_i2_orf1	-	-	-	V-type proton ATPase subunit D isoform X2 [Ostrinia furnacalis]	1.94714	-0.09349	-0.60835	-0.45764	-0.78765
TRINITY_DN40_c0_g2_i1_orf1	-	-	-	trypsin CFT-1-like [Ostrinia furnacalis]	1.96834	-0.81816	-0.46194	-0.28915	-0.39909
TRINITY_DN64472_c0_g2_i1_orf1	-	-	-	repressed by EFG1 protein 1-like isoform X3 [Ostrinia furnacalis]	1.81304	-0.85726	-0.9381	0.21231	-0.22998
TRINITY_DN94248_c0_g2_i3_orf1	-	-	-	uncharacterized protein LOC114357292 isoform X4 [Ostrinia furnacalis]	1.9633	-0.71772	-0.22545	-0.33808	-0.68205
TRINITY_DN3332_c0_g1_i11_orf1	-	-	-	glutathione S-transferase sigma3 [Glyphodes pyloalis]	1.95148	-0.23028	-0.27884	-0.61928	-0.82308
TRINITY_DN2083_c0_g1_i4_orf1	-	-	-	uncharacterized protein LOC114359113 [Ostrinia furnacalis]	1.92437	-0.23699	-0.1691	-0.58669	-0.93159
TRINITY_DN32306_c0_g1_i3_orf1	-	-	-	acetyl-coenzyme A transporter 1 [Ostrinia furnacalis]	1.70732	-0.05704	-1.39033	-0.36975	0.10981
TRINITY_DN13055_c0_g1_i5_orf1	-	-	-	116 kDa U5 small nuclear ribonucleoprotein component isoform X1 [Ostrinia furnacalis] >XP_028159219.1 116 kDa U5 small nuclear ribonucleoprotein component isoform X2 [Ostrinia furnacalis]	1.70039	0.33338	-1.14305	-0.82895	-0.06178
TRINITY_DN146544_c0_g1_i1_orf1	-	-	-	UPF0047 protein YjbQ [Aphidius gifuensis] >KAF7996225.1 hypothetical protein HCN44_001857 [Aphidius gifuensis]	1.99669	-0.43202	-0.53258	-0.58799	-0.4441
TRINITY_DN679_c0_g1_i2_orf1	-	-	-	cytochrome b-c1 complex subunit 7-like [Ostrinia furnacalis]	1.98776	-0.47773	-0.56986	-0.63421	-0.30596
TRINITY_DN12497_c0_g1_i1_orf1	-	-	-	probable N-acetyltransferase san [Ostrinia furnacalis]	1.96488	-0.17735	-0.76453	-0.50926	-0.51374
TRINITY_DN6418_c0_g1_i28_orf1	-	-	-	peritrophic membrane chitin binding protein [Loxostege sticticalis]	1.96479	-0.84586	-0.30718	-0.41786	-0.39388
TRINITY_DN1427_c0_g1_i9_orf1	-	-	-	SAFB-like transcription modulator isoform X3 [Ostrinia furnacalis]	1.59647	0.3578	-1.24079	-0.87112	0.15764

TRINITY_DN12474_c0_g1_i6_orf1	-	-	-	aromatic-L-amino-acid decarboxylase [Ostrinia furnacalis]	1.99967	-0.52558	-0.50952	-0.49357	-0.47101
TRINITY_DN1285_c0_g1_i6_orf1	-	-	-	bifunctional 3'-phosphoadenosine 5'-phosphosulfate synthase isoform X3 [Ostrinia furnacalis]	1.9501	-0.32725	-0.89588	-0.47116	-0.2558
TRINITY_DN16894_c0_g1_i5_orf1	-	-	-	dnaJ homolog subfamily C member 5 isoform X1 [Colias croceus]	1.75811	0.30809	-1.17411	-0.30879	-0.5833
TRINITY_DN40_c0_g1_i3_orf1	-	-	-	trypsin CFT-1-like [Ostrinia furnacalis]	1.98792	-0.34534	-0.4388	-0.52501	-0.67877
TRINITY_DN40704_c0_g1_i2_orf1	-	-	-	COX assembly mitochondrial protein homolog [Ostrinia furnacalis]	1.83491	0.25315	-0.97368	-0.56282	-0.55156
TRINITY_DN1366_c0_g1_i5_orf1	-	-	-	unnamed protein product, partial [Iphiclides podalirius]	1.99748	-0.57838	-0.44408	-0.44857	-0.52645
TRINITY_DN23343_c0_g1_i9_orf1	-	-	-	pre-mRNA-processing factor 6 isoform X1 [Ostrinia furnacalis] >XP_028175021.1 pre-mRNA-processing factor 6 isoform X2 [Ostrinia furnacalis]	1.88981	-0.10811	-1.08055	-0.39277	-0.30838
TRINITY_DN43412_c0_g1_i2_orf1	-	-	-	U1 small nuclear ribonucleoprotein C [Ostrinia furnacalis]	1.54844	0.54035	-1.28428	-0.81297	0.00847
TRINITY_DN4345_c0_g1_i9_orf1	-	-	-	uncharacterized protein LOC114357127 [Ostrinia furnacalis]	1.49897	0.20499	-1.42642	-0.70351	0.42598
TRINITY_DN96566_c0_g1_i1_orf1	-	-	-	NADH-ubiquinone oxidoreductase subunit 8-like [Ostrinia furnacalis]	1.9381	-0.73142	-0.14212	-0.28536	-0.7792
TRINITY_DN26186_c0_g1_i7_orf1	-	-	-	sodium- and chloride-dependent glycine transporter 1-like [Ostrinia furnacalis]	1.81414	0.08256	-0.77899	-0.07375	-1.04395
TRINITY_DN42759_c0_g2_i1_orf1	-	-	-	fatty acid synthase-like [Ostrinia furnacalis]	1.93056	-0.47532	-0.53403	-0.04981	-0.8714
TRINITY_DN73923_c0_g1_i1_orf1	-	-	-	protein obstructor-E-like [Ostrinia furnacalis]	0.81498	1.56249	-0.76778	-0.73633	-0.87337
TRINITY_DN66302_c0_g1_i1_orf1	-	-	-	carboxypeptidase B-like [Ostrinia furnacalis]	1.95823	-0.50118	-0.48989	-0.16229	-0.80488
TRINITY_DN96170_c0_g2_i1_orf1	-	-	-	uncharacterized protein LOC114355569 [Ostrinia furnacalis]	1.9696	-0.33176	-0.60538	-0.28051	-0.75195
TRINITY_DN1285_c0_g2_i1_orf1	-	-	-	bifunctional 3'-phosphoadenosine 5'-phosphosulfate synthase isoform X3 [Ostrinia furnacalis]	1.99544	-0.5243	-0.61117	-0.42884	-0.43113
TRINITY_DN2243_c0_g1_i4_orf1	-	-	-	WD repeat-containing protein 75 [Ostrinia furnacalis]	1.97576	-0.6942	-0.63833	-0.32875	-0.31448
TRINITY_DN4550_c1_g1_i5_orfp2	-	-	-	TRINITY_DN4550_c1_g1_i5_m.14710 TRINITY_DN4550_c1_g1::TRINITY_DN4550_c1_g1_i5::g.14710 ORF type:5prime_partial len:168	1.93484	-0.74985	-0.73475	-0.38774	-0.06251
TRINITY_DN76377_c0_g1_i1_orf1	-	-	-	uncharacterized protein LOC111357764, partial [Spodoptera litura]	1.19673	1.11184	-1.3134	-0.26153	-0.73364
TRINITY_DN35725_c0_g1_i1_orf1	-	-	-	mitochondrial import inner membrane translocase subunit Tim13-like [Bicyclus anynana] >CAG9745432.1 unnamed protein product [Diatraea saccharalis] >CAG9784117.1 unnamed protein product [Diatraea saccharalis]	1.86993	0.21306	-0.67147	-0.632	-0.77952
TRINITY_DN107035_c0_g1_i1_orf1	-	-	-	splicing factor 3A subunit 3 [Ostrinia furnacalis]	1.94443	-0.07745	-0.80583	-0.51023	-0.55091
TRINITY_DN3759_c0_g1_i1_orf1	-	-	-	uncharacterized protein LOC114350416 [Ostrinia furnacalis] >XP_028157016.1 uncharacterized protein LOC114350416 [Ostrinia furnacalis] >XP_028157017.1 uncharacterized protein LOC114350416 [Ostrinia furnacalis] >XP_028157018.1 uncharacterized protein LOC114350416 [Ostrinia furnacalis] >XP_028157019.1 uncharacterized protein LOC114350416 [Ostrinia	1.50072	0.8523	-1.12281	-0.57028	-0.65993
TRINITY_DN8369_c0_g1_i1_orf1	-	-	-	39S ribosomal protein L37, mitochondrial [Ostrinia furnacalis]	1.91852	0.01225	-0.53067	-0.53067	-0.86943
TRINITY_DN2894_c0_g3_i1_orf1	-	-	-	lactase-phlorizin hydrolase-like [Ostrinia furnacalis]	1.99781	-0.53017	-0.47244	-0.56559	-0.42961
TRINITY_DN39673_c0_g1_i1_orf1	-	-	-	uncharacterized protein LOC114359357 isoform X1 [Ostrinia furnacalis]	1.92667	-0.64199	-0.46473	-0.0077	-0.81225
TRINITY_DN6087_c0_g1_i7_orf1	-	-	-	uncharacterized protein LOC114355564 [Ostrinia furnacalis]	1.9731	-0.2381	-0.48768	-0.49237	-0.75494
TRINITY_DN4036_c0_g2_i1_orf1	-	-	-	microvitellogenin-like [Ostrinia furnacalis]	1.65042	-0.35704	-1.4544	0.18147	-0.02045
TRINITY_DN72707_c0_g1_i1_orf1	-	-	-	uncharacterized protein LOC114357549 [Ostrinia furnacalis]	1.98506	-0.62902	-0.2678	-0.54773	-0.54051
TRINITY_DN52768_c0_g1_i1_orf1	-	-	-	carboxypeptidase Q-like isoform X2 [Ostrinia furnacalis]	1.99152	-0.51291	-0.49909	-0.34495	-0.63458
TRINITY_DN9464_c0_g1_i1_orf1	-	-	-	angio-associated migratory cell protein [Ostrinia furnacalis] >XP_028162594.1 angio-associated migratory cell protein [Ostrinia furnacalis]	1.96536	-0.63284	-0.75462	-0.30408	-0.27381
TRINITY_DN542_c0_g1_i4_orf1	-	-	-	uncharacterized protein LOC114364889 [Ostrinia furnacalis]	1.96751	-0.83835	-0.36094	-0.40291	-0.36532
TRINITY_DN7556_c0_g1_i1_orf1	-	-	-	venom carboxylesterase-6-like [Ostrinia furnacalis]	1.97098	-0.1742	-0.56671	-0.55504	-0.67503
TRINITY_DN43420_c0_g2_i1_orf1	-	-	-	collagenase-like [Ostrinia furnacalis]	1.9946	-0.41864	-0.60989	-0.54517	-0.42088
TRINITY_DN31047_c0_g1_i4_orf1	-	-	-	4-coumarate--CoA ligase 1-like isoform X1 [Ostrinia furnacalis] >XP_028160248.1 4-coumarate--CoA ligase 1-like isoform X1 [Ostrinia furnacalis] >XP_028160249.1 4-coumarate--CoA ligase 1-like isoform X1 [Ostrinia furnacalis] >XP_028160250.1 4-coumarate--CoA ligase 1-like isoform X1 [Ostrinia furnacalis] >XP_028160251.1 4-coumarate--CoA ligase 1-like isoform X1 [Ostrinia furnacalis] >XP_028160253.1 4-coumarate--CoA ligase 1-like isoform X2	1.93642	-0.73072	-0.69565	-0.48119	-0.02886
TRINITY_DN18909_c0_g1_i8_orf1	-	-	-	unnamed protein product [Euphydryas editha]	1.92933	-0.62718	-0.47124	-0.01731	-0.81361
TRINITY_DN79319_c0_g1_i8_orfp1	-	-	-	TRINITY_DN79319_c0_g1_i8_m.49956 TRINITY_DN79319_c0_g1::TRINITY_DN79319_c0_g1_i8::g.49956 ORF type:5prime_partial len:84 (+),score=1.39 TRINITY_DN79319_c0_g1_i8:1-252(+)	1.95994	-0.65436	-0.26557	-0.27594	-0.76406
TRINITY_DN2825_c0_g1_i3_orf1	-	-	-	carbonic anhydrase 2-like [Ostrinia furnacalis]	1.96978	-0.81226	-0.31213	-0.47492	-0.37047
TRINITY_DN37699_c0_g1_i4_orfp1	-	-	-	TRINITY_DN37699_c0_g1_i4_m.58777 TRINITY_DN37699_c0_g1::TRINITY_DN37699_c0_g1_i4::g.58777 ORF type:internal len:122 (+),score=34.90 TRINITY_DN37699_c0_g1_i4:1-363(+)	1.72563	-0.82117	-1.10573	-0.12849	0.32975

TRINITY_DN6221_c0_g1_i5_orf1	-	-	-	unnamed protein product [Diatraea saccharalis]	1.98169	-0.71379	-0.35269	-0.55818	-0.35703
TRINITY_DN25779_c0_g1_i6_orf1	-	-	-	aldo-keto reductase AKR2E4-like [Ostrinia furnacalis]	1.5711	0.78433	-0.79402	-0.96371	-0.5977
TRINITY_DN2425_c0_g1_i1_orf1	-	-	-	thyroid receptor-interacting protein 11 [Ostrinia furnacalis]	1.6933	-0.74073	-1.24127	0.11578	0.17292
TRINITY_DN1706_c0_g1_i7_orf1	-	-	-	LOW QUALITY PROTEIN: RNA polymerase-associated protein CTR9 homolog [Ostrinia	1.72482	0.39813	-1.17527	-0.60839	-0.33929
TRINITY_DN36262_c0_g1_i1_orf1	-	-	-	trypsin, alkaline C-like [Maniola jurtina]	1.9644	-0.1722	-0.71994	-0.44052	-0.63174
TRINITY_DN1318_c0_g1_i5_orf1	-	-	-	uncharacterized protein LOC114360956 [Ostrinia furnacalis]	1.97485	-0.77344	-0.44121	-0.47534	-0.28486
TRINITY_DN144258_c0_g1_i1_orf1	-	-	-	PREDICTED: enhancer of rudimentary homolog [Microplitis demolitor] >XP_044577051.1 enhancer of rudimentary homolog [Cotesia glomerata] >KAG8041963.1 hypothetical protein G9C98_007267 [Cotesia typhae] >KAH0539785.1 hypothetical protein KQX54_008036 [Cotesia glomerata] >CAD6227368.1 GSCOCG00006137001-RA-CDS [Cotesia congregata]	1.12581	1.04677	-1.34058	-0.91277	0.08076
TRINITY_DN3062_c0_g1_i1_orf1	-	-	-	HEAT repeat-containing protein 1 [Ostrinia furnacalis]	1.9158	-0.0688	-0.95602	-0.52938	-0.36161
TRINITY_DN2918_c0_g1_i1_orf1	-	-	-	28S ribosomal protein S10, mitochondrial [Ostrinia furnacalis] >XP_028175147.1 28S ribosomal protein S10, mitochondrial [Ostrinia furnacalis]	1.85829	0.24932	-0.65501	-0.74224	-0.71036
TRINITY_DN1044_c0_g1_i2_orf1	-	-	-	V-type proton ATPase subunit H isoform X3 [Ostrinia furnacalis] >QRR19186.1 V-type proton ATPase subunit H [Ostrinia nubilalis]	1.99773	-0.47704	-0.50745	-0.43352	-0.57972
TRINITY_DN28577_c0_g1_i6_orf1	-	-	-	delta-1-pyrroline-5-carboxylate dehydrogenase, mitochondrial [Nymphalis io]	1.99081	-0.34947	-0.61892	-0.44474	-0.57768
TRINITY_DN2267_c0_g1_i1_orf1	-	-	-	hypothetical protein evm_006312 [Chilo suppressalis]	1.99321	-0.56404	-0.45796	-0.60312	-0.36809
TRINITY_DN76283_c0_g6_i1_orf1	-	-	-	fatty acid synthase-like [Ostrinia furnacalis]	1.99531	-0.55524	-0.51245	-0.36998	-0.55764
TRINITY_DN140669_c0_g1_i1_orf1	-	-	-	S-methyl-5'-thioadenosine phosphorylase-like isoform X1 [Hyposmocoma kahamanoa]	1.93978	-0.17115	-0.48742	-0.36706	-0.91415
TRINITY_DN7213_c0_g1_i2_orf1	-	-	-	probable ATP-dependent RNA helicase CG8611 [Ostrinia furnacalis]	1.96319	-0.30132	-0.76099	-0.63775	-0.26313
TRINITY_DN7966_c0_g1_i4_orf1	-	-	-	leucine-rich repeat neuronal protein 1-like [Ostrinia furnacalis]	1.98771	-0.30363	-0.6486	-0.52124	-0.51425
TRINITY_DN1073_c0_g1_i4_orf1	-	-	-	carboxylesterase [Loxostege sticticalis]	1.98645	-0.53055	-0.54728	-0.28078	-0.62784
TRINITY_DN35662_c0_g1_i5_orf1	-	-	-	hypothetical protein evm_006436 [Chilo suppressalis] >CAB3522373.1 unnamed protein product [Chilo suppressalis] >CAH0399695.1 unnamed protein product [Chilo suppressalis]	1.97199	-0.17306	-0.55845	-0.61654	-0.62394
TRINITY_DN2953_c1_g1_i2_orf1	-	-	-	methionine--tRNA ligase, cytoplasmic isoform X6 [Ostrinia furnacalis]	1.78577	0.38621	-0.63403	-0.57246	-0.96549
TRINITY_DN4476_c0_g1_i5_orf1	-	-	-	trypsin, alkaline C-like isoform X1 [Ostrinia furnacalis]	1.95827	-0.22228	-0.83421	-0.36944	-0.53233
TRINITY_DN1249_c0_g1_i10_orf1	-	-	-	venom carboxylesterase-6-like [Ostrinia furnacalis]	1.89215	-0.79563	-0.08405	-0.14079	-0.87169
TRINITY_DN30233_c0_g1_i2_orf1	-	-	-	39S ribosomal protein L10, mitochondrial [Ostrinia furnacalis]	1.91141	-0.20749	-1.01755	-0.21572	-0.47065
TRINITY_DN29229_c0_g1_i5_orfp1	-	-	-	TRINITY_DN29229_c0_g1_i5_m.11187 TRINITY_DN29229_c0_g1::TRINITY_DN29229_c0_g1_i5::g.11187 ORF type:internal len:133 (+),score=48.64 TRINITY_DN29229_c0_g1_i5:3-398(+)	1.84242	-1.01883	-0.72356	0.08953	-0.18956
TRINITY_DN5740_c0_g1_i4_orf1	-	-	-	unconventional myosin IC isoform X1 [Ostrinia furnacalis]	1.9892	-0.43432	-0.40014	-0.45983	-0.6949
TRINITY_DN15900_c0_g1_i6_orf1	-	-	-	unnamed protein product [Diatraea saccharalis]	1.86899	0.13027	-0.98307	-0.4483	-0.56789
TRINITY_DN7024_c0_g1_i1_orf1	-	-	-	uncharacterized protein LOC114363116 [Ostrinia furnacalis]	1.98129	-0.37766	-0.42524	-0.4208	-0.7576
TRINITY_DN8543_c0_g1_i1_orf1	-	-	-	39S ribosomal protein L38, mitochondrial [Ostrinia furnacalis]	1.87421	-0.42322	-0.91741	-0.66942	0.13584
TRINITY_DN122170_c0_g1_i2_orfp1	-	-	-	TRINITY_DN122170_c0_g1_i2_m.81408 TRINITY_DN122170_c0_g1::TRINITY_DN122170_c0_g1_i2::g.81408 ORF type:internal len:87 (+),score=9.58,Baculo_E25 PF05274.12 1.7e-07 TRINITY_DN122170_c0_g1_i2:2-259(+)	1.99404	-0.56287	-0.60078	-0.44036	-0.39003
TRINITY_DN79210_c0_g1_i1_orf1	-	-	-	V-type proton ATPase 16 kDa proteolipid subunit [Frieseomelitta varia]	1.99567	-0.45459	-0.40378	-0.54771	-0.58959
TRINITY_DN30663_c0_g1_i1_orf1	-	-	-	surfeit locus protein 6 homolog [Ostrinia furnacalis]	1.95041	-0.70836	-0.20584	-0.75779	-0.27843
TRINITY_DN18172_c0_g1_i6_orf1	-	-	-	digestive cysteine proteinase 2-like [Ostrinia furnacalis]	1.9582	-0.16688	-0.52832	-0.45698	-0.80601
TRINITY_DN9715_c0_g1_i1_orf1	-	-	-	V-type proton ATPase subunit E [Manduca sexta] >P31402.1 RecName: Full=V-type proton ATPase subunit E; Short=V-ATPase subunit E; AltName: Full=V-ATPase 26 kDa subunit; AltName: Full=Vacuolar proton pump subunit E [Manduca sexta] >KAG6457535.1 hypothetical protein O3G_MSEX010354 [Manduca sexta] >CAA47610.1 H(+)-transporting ATPase	1.99798	-0.48417	-0.49122	-0.44239	-0.5802
TRINITY_DN3733_c0_g1_i1_orf1	-	-	-	60S ribosomal protein L37, partial [Papilio machaon]	1.81846	-0.20651	-1.24317	-0.04825	-0.32054
TRINITY_DN117_c0_g1_i5_orf1	-	-	-	lipase member I-like [Ostrinia furnacalis]	1.97747	-0.71122	-0.4321	-0.26026	-0.57388
TRINITY_DN3614_c0_g2_i1_orf1	-	-	-	PC4 and SFRS1-interacting protein isoform X4 [Galleria mellonella]	1.17881	0.83727	-1.34388	-0.99847	0.32627
TRINITY_DN700_c0_g1_i3_orf1	-	-	-	V-type proton ATPase subunit H isoform X1 [Chelonus insularis]	1.9787	-0.31314	-0.39245	-0.53106	-0.74205
TRINITY_DN109733_c0_g1_i1_orf1	-	-	-	uncharacterized protein LOC112452128 [Temnothorax curvispinosus]	1.95165	-0.44223	-0.68332	-0.10697	-0.71912
TRINITY_DN108573_c0_g1_i1_orf1	-	-	-	uncharacterized protein LOC114366171 [Ostrinia furnacalis]	1.97779	-0.35394	-0.31878	-0.5824	-0.72267
TRINITY_DN97680_c0_g1_i1_orf1	-	-	-	39S ribosomal protein L52, mitochondrial [Ostrinia furnacalis]	1.89732	-0.13914	-0.47594	-1.0487	-0.23354
TRINITY_DN49508_c0_g2_i8_orf1	-	-	-	putative fatty acyl-CoA reductase CG5065 [Ostrinia furnacalis]	1.93716	-0.86536	-0.36734	-0.11192	-0.59254
TRINITY_DN44288_c0_g1_i2_orf1	-	-	-	ATP-dependent RNA helicase p62 [Ostrinia furnacalis]	1.92291	-0.0317	-0.5001	-0.49093	-0.90017

TRINITY_DN6059_c0_g1_i1_orf1	-	-	-	brachyurin-like [Ostrinia furnacalis]	1.97075	-0.8181	-0.39363	-0.33555	-0.42347
TRINITY_DN468_c0_g1_i3_orf1	-	-	-	transmembrane protein 41 homolog isoform X2 [Ostrinia furnacalis]	1.94776	-0.4302	-0.40322	-0.21243	-0.90192
TRINITY_DN24476_c0_g1_i1_orf1	-	-	-	ensconsin-like isoform X1 [Ostrinia furnacalis]	1.88645	0.06018	-0.70407	-0.91283	-0.32972
TRINITY_DN26375_c0_g1_i1_orf1	-	-	-	hypothetical protein O3G_MSEX007366 [Manduca sexta]	1.9375	-0.09399	-0.6371	-0.37556	-0.83085
TRINITY_DN5107_c0_g1_i4_orf1	-	-	-	peptide methionine sulfoxide reductase [Ostrinia furnacalis]	1.99571	-0.59403	-0.49801	-0.51486	-0.38881
TRINITY_DN82320_c0_g1_i2_orf1	-	-	-	glutathione S-transferase sigma3 [Glyphodes pyloalis]	1.87062	-0.42513	0.02908	-0.39665	-1.07791
TRINITY_DN19160_c0_g1_i1_orf1	-	-	-	alkyldihydroxyacetonephosphate synthase [Ostrinia furnacalis]	1.36627	1.0667	-0.79212	-0.92483	-0.71602
TRINITY_DN48237_c0_g1_i5_orf1	-	-	-	myogenesis-regulating glycosidase-like [Ostrinia furnacalis]	1.96421	-0.24621	-0.70395	-0.31789	-0.69617
TRINITY_DN98091_c0_g1_i3_orf1	-	-	-	UDP-glycosyltransferase UGT40AP2, partial [Ostrinia furnacalis]	1.96671	-0.35773	-0.25859	-0.56237	-0.78802
TRINITY_DN52296_c0_g1_i6_orf1	-	-	-	protein takeout-like [Ostrinia furnacalis]	1.83707	-0.34257	-1.02364	-0.65324	0.18238
TRINITY_DN139212_c0_g1_i4_orf1	-	-	-	uncharacterized protein LOC114350112 [Ostrinia furnacalis]	1.52551	0.63873	-1.10087	-1.02542	-0.03795
TRINITY_DN334_c0_g1_i2_orf1	-	-	-	chymotrypsin-like serine protease, partial [Ostrinia nubilalis]	1.98973	-0.61384	-0.43476	-0.3435	-0.59763
TRINITY_DN1353_c0_g1_i1_orf1	-	-	-	UDP-glucose 4-epimerase-like [Ostrinia furnacalis]	1.82726	0.3063	-0.61181	-0.62833	-0.89342
TRINITY_DN15870_c0_g1_i3_orf1	-	-	-	PREDICTED: mitochondrial import inner membrane translocase subunit Tim23 isoform X1 [Fopius arisanus]	1.98405	-0.25534	-0.61067	-0.5736	-0.54444
TRINITY_DN18773_c0_g1_i3_orf1	-	-	-	keratin, type II cytoskeletal 68 kDa, component IB-like [Ostrinia furnacalis]	1.87494	-1.08471	-0.51747	-0.17546	-0.0973
TRINITY_DN6747_c0_g1_i7_orf1	-	-	-	retinol dehydrogenase 12-like [Ostrinia furnacalis]	1.92317	-0.30232	-0.89806	-0.62812	-0.09468
TRINITY_DN11069_c0_g2_i1_orf1	-	-	-	fat storage-inducing transmembrane protein [Ostrinia furnacalis]	1.92312	-0.24495	-1.00424	-0.39181	-0.28211
TRINITY_DN5238_c0_g1_i2_orf1	-	-	-	DNA-(apurinic or apyrimidinic site) lyase [Ostrinia furnacalis]	1.7357	0.33526	-1.17353	-0.66677	-0.23066
TRINITY_DN36061_c0_g4_i2_orf1	-	-	-	putative GPI-anchored protein pfl2 [Ostrinia furnacalis] >XP_028163002.1 putative GPI-anchored protein pfl2 [Ostrinia furnacalis]	0.95821	1.45831	-0.74281	-0.79979	-0.87392
TRINITY_DN141462_c0_g1_i1_orf1	-	-	-	mitochondrial-processing peptidase subunit beta [Diachasma alloeum] >THK33262.1 core protein 1, ubiquinol-cytochrome c reductase [Diachasma alloeum]	1.98764	-0.59479	-0.5998	-0.49967	-0.29338
TRINITY_DN8838_c0_g1_i1_orf1	-	-	-	mannose-P-dolichol utilization defect 1 protein homolog [Ostrinia furnacalis]	1.84227	-0.10067	-1.05008	0.0107	-0.70222
TRINITY_DN13783_c0_g4_i2_orf1	-	-	-	hypothetical protein evm_010131 [Chilo suppressalis]	1.97724	-0.50226	-0.73438	-0.2592	-0.4814
TRINITY_DN17825_c1_g1_i1_orf1	-	-	-	39S ribosomal protein L1, mitochondrial [Ostrinia furnacalis]	1.98	-0.44787	-0.76218	-0.41385	-0.3561
TRINITY_DN5459_c0_g1_i1_orf1	-	-	-	protein takeout-like isoform X2 [Ostrinia furnacalis]	1.46689	0.94817	-0.7533	-0.85356	-0.8082
TRINITY_DN2394_c0_g1_i4_orf1	-	-	-	uncharacterized protein LOC114363116 [Ostrinia furnacalis]	1.98741	-0.45122	-0.44802	-0.38002	-0.70814
TRINITY_DN29034_c0_g1_i2_orf1	-	-	-	trypsin-like serine protease [Ostrinia nubilalis]	1.95549	-0.86121	-0.50056	-0.3584	-0.23532
TRINITY_DN9003_c0_g1_i20_orf1	-	-	-	RNA-binding protein Nova-2 isoform X4 [Ostrinia furnacalis]	1.90354	-0.47193	-0.89848	-0.58629	0.05316
TRINITY_DN8087_c0_g1_i9_orf1	-	-	-	cysteine-rich with EGF-like domain protein 2 isoform X1 [Ostrinia furnacalis]	1.84593	0.17227	-0.30312	-0.86868	-0.8464
TRINITY_DN3159_c0_g1_i4_orf1	-	-	-	uncharacterized protein LOC114362782 [Ostrinia furnacalis]	1.97857	-0.2439	-0.49189	-0.54541	-0.69738
TRINITY_DN4731_c0_g1_i1_orf1	-	-	-	gelsolin-like [Ostrinia furnacalis]	1.98032	-0.59518	-0.43906	-0.26836	-0.67773
TRINITY_DN5891_c0_g2_i4_orf1	-	-	-	amino acid transporter AVT1A-like [Ostrinia furnacalis] >XP_028156666.1 amino acid transporter AVT1A-like [Ostrinia furnacalis]	1.98661	-0.54108	-0.39282	-0.36481	-0.6879
TRINITY_DN657_c0_g1_i2_orf1	-	-	-	cytochrome c-type heme lyase [Ostrinia furnacalis]	1.95734	-0.64401	-0.72942	-0.13142	-0.4525
TRINITY_DN15380_c0_g1_i1_orf1	-	-	-	39S ribosomal protein L32, mitochondrial [Ostrinia furnacalis]	1.98899	-0.32134	-0.59445	-0.61214	-0.46106
TRINITY_DN27725_c0_g1_i2_orf1	-	-	-	BRISC complex subunit FAM175B-like [Ostrinia furnacalis]	1.12913	1.10729	-0.99734	-1.22642	-0.01265
TRINITY_DN17864_c0_g1_i1_orf1	-	-	-	PREDICTED: erlin-2-B [Microplitis demolitor]	1.9959	-0.51491	-0.60719	-0.41682	-0.45698
TRINITY_DN21722_c0_g1_i3_orf1	-	-	-	V-type proton ATPase 116 kDa subunit a isoform X1 [Ostrinia furnacalis] >XP_028177509.1 V-type proton ATPase 116 kDa subunit a isoform X1 [Ostrinia furnacalis]	1.99575	-0.56958	-0.47805	-0.38866	-0.55946
TRINITY_DN47114_c0_g1_i5_orf1	-	-	-	nucleolar protein dao-5 isoform X2 [Ostrinia furnacalis]	1.94891	-0.46565	-0.88425	-0.4082	-0.19081
TRINITY_DN8691_c0_g1_i3_orf1	-	-	-	nucleolin-like [Melitaea cinxia]	1.70085	0.51566	-0.85615	-0.98261	-0.37775
TRINITY_DN4762_c0_g1_i2_orf1	-	-	-	ATPase family AAA domain-containing protein 1 isoform X2 [Ostrinia furnacalis]	1.72993	0.49044	-1.01694	-0.53647	-0.66695
TRINITY_DN11259_c0_g1_i1_orf1	-	-	-	uncharacterized protein LOC114357075 [Ostrinia furnacalis]	1.96195	-0.69761	-0.34948	-0.20897	-0.70589
TRINITY_DN10332_c0_g1_i2_orfp1	-	-	-	TRINITY_DN10332_c0_g1_i2_m.42894 TRINITY_DN10332_c0_g1::TRINITY_DN10332_c0_g1_i2::g.42894 ORF type:3prime_partial len:77 (+),score=1.70 TRINITY_DN10332_c0_g1_i2:1005-1232(+)	1.80891	-0.01195	-1.15388	-0.62934	-0.01375
TRINITY_DN28592_c0_g1_i2_orf1	-	-	-	UDP-glucuronosyltransferase 2B14-like isoform X1 [Ostrinia furnacalis] >XP_028167291.1 UDP-glucuronosyltransferase 2B14-like isoform X2 [Ostrinia furnacalis]	1.94236	-0.161	-0.90183	-0.41517	-0.46437
TRINITY_DN48020_c0_g1_i1_orf1	-	-	-	aminopeptidase N4 [Cnaphalocrocis medinalis]	1.94389	-0.64525	-0.52096	-0.04888	-0.7288
TRINITY_DN2178_c0_g1_i1_orf1	-	-	-	carboxypeptidase B-like [Ostrinia furnacalis]	1.95396	-0.49342	-0.1997	-0.40259	-0.85825
TRINITY_DN26688_c0_g1_i2_orf1	-	-	-	myogenesis-regulating glycosidase-like [Ostrinia furnacalis]	1.96274	-0.13462	-0.69973	-0.60457	-0.52382
TRINITY_DN48410_c0_g2_i1_orf1	-	-	-	alpha-amylase 2-like isoform X3 [Ostrinia furnacalis]	1.98929	-0.57046	-0.37149	-0.39953	-0.64781

TRINITY_DN43611_c0_g1_i1_orf1	-	-	-	39S ribosomal protein L41, mitochondrial [Ostrinia furnacalis]	1.9732	-0.29458	-0.75334	-0.57239	-0.35289
TRINITY_DN69713_c0_g1_i1_orf1	-	-	-	membrane-bound alkaline phosphatase-like [Ostrinia furnacalis]	1.92969	-0.925	-0.29096	-0.15523	-0.55851
TRINITY_DN123396_c0_g1_i1_orf1	-	-	-	PREDICTED: delta-1-pyrroline-5-carboxylate dehydrogenase, mitochondrial isoform X1 [Megachile rotundata]	1.98339	-0.38643	-0.55509	-0.70253	-0.33934
TRINITY_DN36494_c0_g1_i1_orf1	-	-	-	MKI67 FHA domain-interacting nucleolar phosphoprotein-like [Ostrinia furnacalis]	1.93828	-0.80786	-0.65389	-0.39529	-0.08124
TRINITY_DN82801_c0_g1_i1_orf1	-	-	-	uncharacterized protein LOC114364712 [Ostrinia furnacalis]	1.46569	0.93012	-0.68803	-0.68803	-1.01974
TRINITY_DN23175_c0_g1_i6_orf1	-	-	-	myb-binding protein 1A-like protein [Ostrinia furnacalis]	1.97042	-0.16271	-0.60495	-0.57232	-0.63043
TRINITY_DN311_c0_g1_i4_orfp1	-	-	-	TRINITY_DN311_c0_g1_i4_m.65135 TRINITY_DN311_c0_g1::TRINITY_DN311_c0_g1_i4::g.65135 ORF type:5prime_partial len:126 (+),score=71.21 TRINITY_DN311_c0_g1_i4:1-378(+)	1.85821	-0.44135	-0.09502	-0.17637	-1.14547
TRINITY_DN2343_c1_g1_i8_orf1	-	-	-	receptor expression-enhancing protein 5-like isoform X3 [Ostrinia furnacalis]	1.96638	-0.62519	-0.46784	-0.16999	-0.70337
TRINITY_DN3235_c0_g1_i1_orf1	-	-	-	SPARC [Trichoplusia ni]	1.42773	0.98499	-0.97636	-0.66093	-0.77542
TRINITY_DN1310_c0_g1_i4_orf1	-	-	-	trypsin-like isoform X1 [Ostrinia furnacalis] >XP_028159118.1 trypsin-like isoform X2 [Ostrinia furnacalis]	1.9794	-0.40175	-0.35633	-0.45612	-0.76521
TRINITY_DN29018_c0_g1_i4_orf1	-	-	-	prostaglandin reductase 1-like isoform X1 [Ostrinia furnacalis] >XP_028178925.1 prostaglandin reductase 1-like isoform X2 [Ostrinia furnacalis]	1.95403	-0.78229	-0.12124	-0.566	-0.48451
TRINITY_DN96_c0_g1_i1_orf1	-	-	-	collagenase-like [Ostrinia furnacalis]	1.97496	-0.68584	-0.30285	-0.33333	-0.65295
TRINITY_DN16939_c0_g1_i4_orf1	-	-	-	39S ribosomal protein L17, mitochondrial [Ostrinia furnacalis]	1.98296	-0.52425	-0.69677	-0.47382	-0.28812
TRINITY_DN11376_c0_g2_i1_orf1	-	-	-	cathepsin K-like [Ostrinia furnacalis]	1.98721	-0.45903	-0.37531	-0.44439	-0.70848
TRINITY_DN334_c0_g1_i1_orf1	-	-	-	putative chymotrypsin 12 [Ostrinia nubilalis]	1.99049	-0.55605	-0.40342	-0.38415	-0.64687
TRINITY_DN6074_c0_g1_i1_orf1	-	-	-	uncharacterized protein C1683.06c-like isoform X1 [Ostrinia furnacalis]	1.9738	-0.24458	-0.39857	-0.67233	-0.65832
TRINITY_DN2894_c0_g2_i3_orf1	-	-	-	myrosinase 1-like isoform X1 [Ostrinia furnacalis]	1.21584	0.93523	-1.38895	-0.84339	0.08127
TRINITY_DN47731_c0_g1_i2_orf1	-	-	-	nucleolar GTP-binding protein 2 [Ostrinia furnacalis]	1.73001	0.5288	-0.64053	-0.74682	-0.87146
TRINITY_DN5578_c0_g1_i10_orf1	-	-	-	unnamed protein product [Chilo suppressalis]	1.83907	0.17567	-0.33103	-0.66871	-1.01499
TRINITY_DN16931_c0_g1_i1_orf1	-	-	-	pancreatic triacylglycerol lipase-like [Ostrinia furnacalis]	1.89797	-0.68502	-0.36043	0.0402	-0.89272
TRINITY_DN57202_c0_g1_i1_orf1	-	-	-	PREDICTED: U4/U6 small nuclear ribonucleoprotein Prp31 [Amyelois transitella]	1.99171	-0.53511	-0.54265	-0.32542	-0.58853
TRINITY_DN1199_c0_g1_i1_orf1	-	-	-	pupal cuticle protein 36a-like [Ostrinia furnacalis]	1.96703	-0.67603	-0.68413	-0.40732	-0.19955
TRINITY_DN2894_c0_g1_i2_orf1	-	-	-	myrosinase 1-like isoform X1 [Ostrinia furnacalis]	1.99831	-0.458	-0.55538	-0.53437	-0.45056
TRINITY_DN67623_c0_g1_i1_orf1	-	-	-	maltase A1-like [Ostrinia furnacalis]	1.99635	-0.52317	-0.52052	-0.38647	-0.56619
TRINITY_DN6693_c0_g1_i1_orf1	-	-	-	uncharacterized protein LOC114356358 [Ostrinia furnacalis]	1.98013	-0.23384	-0.52201	-0.57684	-0.64743
TRINITY_DN69049_c0_g1_i2_orf1	-	-	-	membrane alanyl aminopeptidase-like [Ostrinia furnacalis]	1.98407	-0.58026	-0.67626	-0.41272	-0.31483
TRINITY_DN23570_c0_g1_i2_orf1	-	-	-	putative trypsin 6 [Ostrinia nubilalis]	1.98817	-0.33683	-0.61333	-0.61595	-0.42206
TRINITY_DN10792_c0_g2_i5_orf1	-	-	-	uncharacterized protein LOC114366171 [Ostrinia furnacalis]	1.97999	-0.42425	-0.37734	-0.41176	-0.76664
TRINITY_DN713_c0_g1_i4_orf1	-	-	-	periodic tryptophan protein 2 homolog isoform X1 [Ostrinia furnacalis] >XP_028176443.1 periodic tryptophan protein 2 homolog isoform X2 [Ostrinia furnacalis] >XP_028176445.1 periodic tryptophan protein 2 homolog isoform X3 [Ostrinia furnacalis]	1.97719	-0.3122	-0.7697	-0.44286	-0.45243
TRINITY_DN8116_c0_g1_i1_orf1	-	-	-	uncharacterized protein LOC114350845 [Ostrinia furnacalis]	1.97465	-0.19205	-0.61379	-0.5392	-0.62961
TRINITY_DN43942_c0_g2_i1_orf1	-	-	-	LOW QUALITY PROTEIN: caprin homolog [Ostrinia furnacalis]	1.79832	0.32132	-1.0377	-0.52265	-0.55929
TRINITY_DN50787_c0_g2_i2_orf1	-	-	-	40S ribosomal protein S29 [Hyposmocoma kahamanoa] >XP_028176503.1 40S ribosomal protein S29 [Ostrinia furnacalis] >XP_049877832.1 40S ribosomal protein S29 [Pectinophora gossypiella] >ADT80654.1 ribosomal protein S29 [Euphydryas aurinia] >CAB3523209.1 unnamed protein product [Chilo suppressalis] >CAH0400531.1 unnamed protein product	1.80174	-0.4331	-1.22613	-0.23342	0.09091
TRINITY_DN8116_c0_g1_i2_orf1	-	-	-	uncharacterized protein LOC114350845 [Ostrinia furnacalis]	1.98321	-0.45074	-0.54943	-0.2932	-0.68984
TRINITY_DN717_c0_g1_i2_orfp1	-	-	-	TRINITY_DN717_c0_g1_i2_m.67915 TRINITY_DN717_c0_g1::TRINITY_DN717_c0_g1_i2::g.67915 ORF type:internal len:868 (+),score=265.71,Collagen PF01391.19 0.11,Collagen|PF01391.19|0.039,Collagen|PF01391.19|0.00054,Collagen|PF01391.19|0.0019,Collagen|PF01391.19|0.0005,Collagen|PF01391.19|9.9e-05,Collagen|PF01391.19|1.7e-07 TRINITY_DN717_c0_g1_i2:3-2603(+)	1.94223	-0.09928	-0.44507	-0.55274	-0.84514
TRINITY_DN3194_c0_g1_i6_orf1	-	-	-	uncharacterized protein LOC114361386 [Ostrinia furnacalis]	1.97415	-0.30561	-0.6176	-0.33097	-0.71996
TRINITY_DN7047_c0_g1_i1_orf1	-	-	-	hypothetical protein G9C98_004728 [Cotesia typhae]	1.95743	-0.13834	-0.71537	-0.43257	-0.67115
TRINITY_DN51766_c0_g1_i2_orf1	-	-	-	facilitated trehalose transporter Tret1-like [Ostrinia furnacalis]	1.89175	0.14687	-0.77741	-0.62654	-0.63467
TRINITY_DN27721_c1_g1_i2_orf1	-	-	-	mitochondrial import receptor subunit TOM20 homolog [Ostrinia furnacalis]	1.95503	-0.1406	-0.80597	-0.50294	-0.50552
TRINITY_DN542_c0_g2_i1_orf1	-	-	-	uncharacterized protein LOC114364889 [Ostrinia furnacalis]	1.98647	-0.38035	-0.47826	-0.41478	-0.71308
TRINITY_DN26010_c0_g1_i2_orf1	-	-	-	cytochrome b-c1 complex subunit 10-like [Ostrinia furnacalis]	1.99481	-0.56741	-0.43271	-0.58913	-0.40555
TRINITY_DN13651_c0_g1_i2_orf1	-	-	-	40S ribosomal protein S12, mitochondrial [Ostrinia furnacalis]	1.9257	-0.06556	-0.9066	-0.55045	-0.40308

TRINITY_DN144807_c0_g1_i1_orf1	-	-	-	hypothetical protein G9C98_004245 [Cotesia typhae]	1.98871	-0.67835	-0.51169	-0.44467	-0.354
TRINITY_DN64403_c0_g2_i1_orf1	-	-	-	carboxylesterase [Ostrinia furnacalis]	1.90027	-0.80946	-0.56202	0.10848	-0.63727
TRINITY_DN20682_c0_g2_i1_orf1	-	-	-	glutathione S-transferase delta3 [Glyphodes pyloalis]	1.95126	-0.13403	-0.69294	-0.38543	-0.73886
TRINITY_DN2114_c0_g1_i5_orf1	-	-	-	vegetative cell wall protein gp1-like isoform X1 [Ostrinia furnacalis]	1.96068	-0.82807	-0.35476	-0.24462	-0.53323
TRINITY_DN61112_c0_g1_i4_orfp1	-	-	-	TRINITY_DN61112_c0_g1_i4_m.53012 TRINITY_DN61112_c0_g1::TRINITY_DN61112_c0_g1_i4::g.53012 ORF type:3prime_partial len:87 (-),score=12.29,HMMR_N PF15905.6 0.0011 TRINITY_DN61112_c0_g1_i4:2-232(-)	1.99368	-0.44193	-0.41886	-0.48651	-0.64638
TRINITY_DN30932_c0_g1_i2_orf1	-	-	-	delta(24)-sterol reductase-like isoform X2 [Ostrinia furnacalis]	1.85642	0.17885	-0.50365	-0.54771	-0.98391
TRINITY_DN104586_c0_g1_i1_orf1	-	-	-	Chlorophyll a-b binding protein 37, chloroplastic, partial [Trichinella patagoniensis]	1.98704	-0.57764	-0.38187	-0.36503	-0.66251
TRINITY_DN7688_c0_g1_i10_orf1	-	-	-	uncharacterized protein LOC114352518 [Ostrinia furnacalis]	1.97232	-0.56143	-0.42618	-0.23907	-0.74564
TRINITY_DN4612_c0_g1_i1_orf1	-	-	-	uncharacterized protein LOC114362092 [Ostrinia furnacalis]	1.92561	-0.77846	-0.13825	-0.2234	-0.78551
TRINITY_DN57904_c0_g2_i1_orf1	-	-	-	cuticle protein 19 [Plutella xylostella] >CAG9138481.1 unnamed protein product [Plutella xylostella]	0.95237	1.4628	-0.88196	-0.76574	-0.76747
TRINITY_DN23734_c0_g1_i1_orf1	-	-	-	histone-lysine N-methyltransferase SMYD3 [Ostrinia furnacalis]	1.78797	-0.04048	-1.30453	-0.19292	-0.25005
TRINITY_DN48410_c0_g1_i1_orf1	-	-	-	alpha-amylase 1-like [Ostrinia furnacalis]	1.98615	-0.48513	-0.38938	-0.39711	-0.71452
TRINITY_DN61674_c0_g1_i2_orf1	-	-	-	fatty acid-binding protein 1-like [Ostrinia furnacalis]	1.98771	-0.39147	-0.43691	-0.45147	-0.70785
TRINITY_DN29120_c0_g1_i6_orf1	-	-	-	putative inorganic phosphate cotransporter [Ostrinia furnacalis]	1.85722	0.22275	-0.7199	-0.50981	-0.85027
TRINITY_DN22983_c0_g1_i2_orfp1	-	-	-	TRINITY_DN22983_c0_g1_i2_m.10495 TRINITY_DN22983_c0_g1::TRINITY_DN22983_c0_g1_i2::g.10495 ORF type:internal len:79 (-),score=15.95,Polyhedrin PF00738.19 7.6e-40 TRINITY_DN22983_c0_g1_i2:2-235(-)	1.9854	-0.35789	-0.69683	-0.54092	-0.38977
TRINITY_DN117_c0_g1_i6_orf1	-	-	-	lipase member I-like [Ostrinia furnacalis]	1.89133	-0.03461	-0.31985	-0.50539	-1.03148
TRINITY_DN3784_c0_g1_i1_orf1	-	-	-	pancreatic triacylglycerol lipase-like [Ostrinia furnacalis]	1.96291	-0.22673	-0.42073	-0.4944	-0.82106
TRINITY_DN4869_c0_g1_i10_orf1	-	-	-	estrogen sulfotransferase-like isoform X1 [Ostrinia furnacalis]	1.99157	-0.45499	-0.45788	-0.40674	-0.67196
TRINITY_DN26408_c0_g1_i7_orf1	-	-	-	venom carboxylesterase-6-like [Ostrinia furnacalis]	1.98747	-0.51641	-0.6625	-0.49775	-0.31081
TRINITY_DN19244_c0_g1_i7_orf1	-	-	-	uncharacterized protein LOC114350218 [Ostrinia furnacalis]	1.73855	0.32602	-1.18005	-0.65134	-0.23319
TRINITY_DN1914_c0_g1_i6_orf1	-	-	-	loricrin-like [Ostrinia furnacalis]	1.91171	0.02288	-0.61151	-0.4429	-0.88019
TRINITY_DN11587_c0_g1_i7_orf1	-	-	-	elongation of very long chain fatty acids protein AAEL008004-like isoform X1 [Danaus plexippus plexippus] >XP_032511006.1 elongation of very long chain fatty acids protein AAEL008004-like isoform X1 [Danaus plexippus plexippus] >XP_032511007.1 elongation of very long chain fatty acids protein AAEL008004-like isoform X1 [Danaus plexippus plexippus] >XP_032511008.1 elongation of very long chain fatty acids protein AAEL008004-like isoform X1 [Danaus plexippus plexippus] >XP_032511009.1 elongation of very long chain fatty acids protein AAEL008004-like isoform X1 [Danaus plexippus plexippus]	1.97115	-0.17991	-0.68583	-0.53207	-0.57335
TRINITY_DN41259_c0_g1_i6_orf1	-	-	-	endocuticle structural glycoprotein SgAbd-8 [Ostrinia furnacalis]	0.99293	1.43186	-0.85496	-0.76569	-0.80414
TRINITY_DN6044_c0_g1_i4_orf1	-	-	-	acyl-CoA-binding protein-like [Ostrinia furnacalis]	1.99594	-0.43251	-0.42349	-0.5632	-0.57675
TRINITY_DN7688_c0_g1_i2_orf1	-	-	-	uncharacterized protein LOC114352518 [Ostrinia furnacalis]	1.96166	-0.607	-0.29935	-0.26594	-0.78937
TRINITY_DN334_c0_g1_i4_orf1	-	-	-	collagenase-like [Ostrinia furnacalis]	1.89551	-1.0204	-0.43585	-0.02042	-0.41884
TRINITY_DN75188_c0_g1_i1_orf1	-	-	-	fatty acid-binding protein 1-like [Ostrinia furnacalis]	1.94334	-0.1572	-0.88609	-0.51523	-0.38483
TRINITY_DN3862_c0_g1_i7_orf1	-	-	-	venom acid phosphatase Acph-1-like [Ostrinia furnacalis]	1.98689	-0.45219	-0.71399	-0.38552	-0.43519
TRINITY_DN35051_c0_g1_i1_orf1	-	-	-	uncharacterized protein LOC114364307 [Ostrinia furnacalis]	1.98253	-0.49593	-0.51163	-0.27943	-0.69554
TRINITY_DN14679_c0_g1_i1_orf1	-	-	-	hypothetical protein evm_003043 [Chilo suppressalis]	1.95596	-0.53757	-0.27887	-0.2885	-0.85102
TRINITY_DN78546_c0_g5_i1_orf1	-	-	-	kinesin-like protein KIF13A isoform X9 [Cephus cinctus]	1.69459	-0.11528	-1.40265	-0.34526	0.16859
TRINITY_DN2815_c0_g1_i3_orf1	-	-	-	uncharacterized protein LOC114364075 [Ostrinia furnacalis]	1.98924	-0.41253	-0.61845	-0.35439	-0.60386
TRINITY_DN64446_c0_g1_i1_orf1	-	-	-	uncharacterized protein LOC114364307 [Ostrinia furnacalis]	1.9482	-0.20827	-0.86891	-0.31979	-0.55123
TRINITY_DN81803_c0_g2_i1_orf1	-	-	-	cathepsin K-like [Ostrinia furnacalis]	1.93601	-0.11015	-0.48826	-0.43776	-0.89984

TRINITY_DN22430_c0_g3_i1_orf1	-	-	-	V-type proton ATPase 16 kDa proteolipid subunit c [Homo sapiens] >NP_001685.1 V-type proton ATPase 16 kDa proteolipid subunit c [Homo sapiens] >P27449.1 RecName: Full=V-type proton ATPase 16 kDa proteolipid subunit c; Short=V-ATPase 16 kDa proteolipid subunit c; AltName: Full=Vacuolar proton pump 16 kDa proteolipid subunit c [Homo sapiens] >6WLW_1 The Vo region of human V-ATPase in state 1 (focused refinement) [Homo sapiens] >6WLW_2 The Vo region of human V-ATPase in state 1 (focused refinement) [Homo sapiens] >6WLW_3 The Vo region of human V-ATPase in state 1 (focused refinement) [Homo sapiens] >6WLW_4 The Vo region of human V-ATPase in state 1 (focused refinement) [Homo sapiens] >6WLW_5 The Vo region of human V-ATPase in state 1 (focused refinement) [Homo sapiens] >6WLW_6 The Vo region of human V-ATPase in state 1 (focused refinement) [Homo sapiens] >6WLW_7 The Vo region of human V-ATPase in state 1 (focused refinement) [Homo sapiens] >6WLW_8 The Vo region of human V-ATPase in state 1 (focused refinement) [Homo sapiens] >6WLW_9 The Vo region of human V-ATPase in state 1 (focused refinement) [Homo sapiens] >6WM2_1 Human V-ATPase in state 1 with SidK and ADP [Homo sapiens] >6WM2_2 Human V-ATPase in state 1 with SidK and ADP [Homo sapiens] >6WM2_3 Human V-ATPase in state 1 with SidK and ADP [Homo sapiens] >6WM2_4 Human V-ATPase in state 1 with SidK and ADP [Homo sapiens] >6WM2_5 Human V-ATPase in state 1 with SidK and ADP [Homo sapiens] >6WM2_6 Human V-ATPase in state 1 with SidK and ADP [Homo sapiens] >6WM2_7 Human V-ATPase in state 1 with SidK and ADP [Homo sapiens] >6WM2_8 Human V-ATPase in state 1 with SidK and ADP [Homo sapiens] >6WM2_9 Human V-ATPase in state 1 with SidK and ADP [Homo sapiens] >6WM3_1 Human V-ATPase in state 2 with SidK and ADP [Homo sapiens] >6WM3_2 Human V-ATPase in state 2 with SidK and ADP [Homo sapiens] >6WM3_3 Human V-ATPase in state 2 with SidK and ADP [Homo sapiens] >6WM3_4 Human V-ATPase in state 2 with SidK and ADP [Homo sapiens] >6WM3_5 Human V-ATPase in state 2 with SidK and ADP [Homo sapiens] >6WM3_6 Human V-ATPase in state 2 with SidK and ADP [Homo sapiens] >6WM3_7 Human V-ATPase in state 2 with SidK and ADP [Homo sapiens] >6WM3_8 Human V-ATPase in state 2 with SidK and ADP [Homo sapiens] >6WM3_9 Human V-ATPase in state 2 with SidK and ADP [Homo sapiens] >6WM4_1 Human V-ATPase in state 3 with SidK and ADP [Homo sapiens] >6WM4_2 Human V-ATPase in state 3 with SidK and ADP [Homo sapiens] >6WM4_3 Human V-ATPase in state 3 with SidK and ADP [Homo sapiens] >6WM4_4 Human V-ATPase	1.99836	-0.44	-0.55875	-0.47603	-0.52357
TRINITY_DN37538_c0_g1_i1_orf1	-	-	-	esterase FE4-like [Ostrinia furnacalis]	1.98456	-0.49402	-0.34798	-0.42354	-0.71902
TRINITY_DN41086_c0_g1_i4_orf1	-	-	-	collagenase-like [Pectinophora gossypiella]	1.96441	-0.52277	-0.45691	-0.19724	-0.78749
TRINITY_DN348_c0_g2_i3_orf1	-	-	-	pancreatic triacylglycerol lipase-like [Ostrinia furnacalis]	1.97794	-0.35016	-0.42951	-0.42203	-0.77625
TRINITY_DN6933_c0_g1_i2_orf1	-	-	-	Chlorophyll a-b binding protein 40, chloroplastic [Trichinella nelsoni] >KRY99282.1 Chlorophyll a-b binding protein 40, chloroplastic [Trichinella zimbabwensis]	1.99176	-0.4515	-0.38712	-0.49191	-0.66123
TRINITY_DN39200_c0_g1_i5_orf1	-	-	-	juvenile hormone epoxide hydrolase-like [Ostrinia furnacalis]	1.99574	-0.39473	-0.48062	-0.59599	-0.5244
TRINITY_DN6140_c0_g3_i3_orf1	-	-	-	CD63 antigen-like [Ostrinia furnacalis]	1.84375	-0.26338	-0.57199	0.08557	-1.09395
TRINITY_DN36199_c0_g1_i1_orf1	-	-	-	fatty acid-binding protein 1-like [Ostrinia furnacalis]	1.99314	-0.45163	-0.36935	-0.58609	-0.58609
TRINITY_DN94755_c0_g1_i5_orfp1	-	-	-	TRINITY_DN94755_c0_g1_i5_m.62794 TRINITY_DN94755_c0_g1::TRINITY_DN94755_c0_g1_i5::g.62794 ORF type:internal len:70 (+),score=5.17 TRINITY_DN94755_c0_g1_i5:2-208(+)	1.999	-0.52782	-0.53992	-0.4543	-0.47696
TRINITY_DN94355_c0_g1_i2_orf1	-	-	-	uncharacterized protein LOC126369488 [Pectinophora gossypiella]	1.88694	-0.51477	-0.70593	-0.80966	0.14341
TRINITY_DN2490_c0_g2_i1_orfp1	-	-	-	TRINITY_DN2490_c0_g2_i1_m.56872 TRINITY_DN2490_c0_g2::TRINITY_DN2490_c0_g2_i1::g.56872 ORF type:internal len:359 (-	1.96023	-0.22425	-0.58187	-0.35051	-0.8036
TRINITY_DN4959_c0_g1_i1_orf1	-	-	-	pancreatic triacylglycerol lipase-like [Ostrinia furnacalis]	1.80133	-0.08783	-0.25301	-0.17483	-1.28566
TRINITY_DN117_c0_g1_i4_orf1	-	-	-	lipase member I-like [Ostrinia furnacalis]	1.85689	0.1922	-0.57903	-0.51235	-0.95772
TRINITY_DN1249_c0_g1_i6_orf1	-	-	-	venom carboxylesterase-6-like [Ostrinia furnacalis]	1.52609	0.81961	-1.0348	-0.84156	-0.46934
TRINITY_DN144190_c0_g1_i1_orf1	-	-	-	PREDICTED: uncharacterized protein LOC103572804 isoform X2 [Microplitis demolitor]	1.92351	-0.40193	-0.20121	-0.32374	-0.99663
TRINITY_DN19917_c0_g1_i1_orf1	-	-	-	synaptic vesicle glycoprotein 2B-like isoform X4 [Ostrinia furnacalis]	1.99479	-0.38139	-0.58081	-0.46681	-0.56578
TRINITY_DN14134_c0_g2_i3_orf1	-	-	-	anoctamin-8-like isoform X2 [Helicoverpa zea]	1.67408	-1.33281	-0.57092	0.30022	-0.07057
TRINITY_DN46372_c0_g2_i1_orf1	-	-	-	basic salivary proline-rich protein 1 isoform X2 [Ostrinia furnacalis]	1.03398	-1.65097	-0.40681	0.00416	1.01964
TRINITY_DN17189_c0_g1_i2_orf1	-	-	-	fibroin heavy chain [Haritalodes derogata]	1.56661	-1.04932	-0.63696	-0.65842	0.77809
TRINITY_DN18009_c0_g1_i1_orf1	-	-	-	pre-mRNA-splicing factor ISY1 homolog [Ostrinia furnacalis]	0.68825	-1.83692	-0.20511	0.36577	0.98802
TRINITY_DN640_c0_g1_i2_orf1	-	-	-	pancreatic triacylglycerol lipase-like [Ostrinia furnacalis]	1.7156	-1.3375	-0.24897	0.24969	-0.37881

TRINITY_DN4323_c0_g1_i1_orf1	-	-	-	uncharacterized protein LOC114364842 [Ostrinia furnacalis]	1.63057	-1.48093	-0.35597	0.10919	0.09714
TRINITY_DN19080_c0_g1_i4_orf1	-	-	-	synaptic vesicle 2-related protein-like isoform X1 [Ostrinia furnacalis] >XP_028161172.1 synaptic vesicle 2-related protein-like isoform X1 [Ostrinia furnacalis]	1.31761	-1.78076	0.12577	0.26885	0.06853
TRINITY_DN1814_c0_g1_i11_orf1	-	-	-	titin-like, partial [Ostrinia furnacalis]	0.77793	-1.87601	-0.18868	0.56609	0.72067
TRINITY_DN47257_c0_g1_i4_orf1	-	-	-	PREDICTED: microtubule-actin cross-linking factor 1-like, partial [Amyelois transitella]	1.24326	-1.70051	-0.41926	0.40184	0.47467
TRINITY_DN72541_c0_g1_i2_orf1	-	-	-	xaa-Pro aminopeptidase ApepP-like isoform X2 [Ostrinia furnacalis]	1.47835	-1.54388	-0.147	-0.33348	0.546
TRINITY_DN12250_c0_g1_i4_orf1	-	-	-	ubiquinone biosynthesis monooxygenase COQ6, mitochondrial isoform X1 [Ostrinia furnacalis] >XP_028160041.1 ubiquinone biosynthesis monooxygenase COQ6, mitochondrial isoform X2 [Ostrinia furnacalis]	1.76456	-1.07375	-0.01682	0.16598	-0.83997
TRINITY_DN110132_c0_g1_i1_orf1	-	-	-	uncharacterized protein LOC114361588 isoform X14 [Ostrinia furnacalis]	1.18274	-1.4883	-0.82824	0.73623	0.39756
TRINITY_DN15988_c0_g1_i1_orf1	-	-	-	uncharacterized protein LOC114351163 [Ostrinia furnacalis]	1.35583	-0.84147	-1.42089	0.56245	0.34409
TRINITY_DN76529_c0_g1_i1_orfp1	-	-	-	TRINITY_DN76529_c0_g1_i1_m.64079 TRINITY_DN76529_c0_g1::TRINITY_DN76529_c0_g1_i1::g.64079 ORF type:internal len:70 (+),score=14.68 TRINITY_DN76529_c0_g1_i1:3-209(+)	0.25284	-1.56507	-0.3692	0.15635	1.52508
TRINITY_DN18338_c0_g1_i7_orf1	-	-	-	aquaporin AQPAn.G isoform X2 [Ostrinia furnacalis]	1.62115	-1.43817	-0.50132	0.13131	0.18703
TRINITY_DN348_c0_g2_i1_orf1	-	-	-	pancreatic triacylglycerol lipase-like [Ostrinia furnacalis]	1.61119	-1.53256	-0.08806	0.15891	-0.14948
TRINITY_DN640_c0_g1_i5_orf1	-	-	-	pancreatic triacylglycerol lipase-like [Ostrinia furnacalis]	1.6233	-1.4113	-0.00197	0.31397	-0.52399
TRINITY_DN21596_c0_g1_i1_orf1	-	-	-	peptidyl-prolyl cis-trans isomerase NIMA-interacting 4 [Zerene cesonia] >XP_038208701.1 peptidyl-prolyl cis-trans isomerase NIMA-interacting 4 [Zerene cesonia]	1.2445	-1.45396	-0.85284	0.38006	0.68224
TRINITY_DN2146_c0_g1_i1_orf1	-	-	-	heat shock protein 68-like [Ostrinia furnacalis]	1.45083	-1.64056	-0.12207	0.4206	-0.10881
TRINITY_DN7686_c0_g1_i4_orf1	-	-	-	eIF-2-alpha kinase activator GCN1 [Ostrinia furnacalis]	1.4808	-1.48459	-0.33127	-0.30011	0.63517
TRINITY_DN8940_c0_g1_i4_orf1	-	-	-	probable ATP-dependent RNA helicase DDX23 [Ostrinia furnacalis]	1.81428	-1.12851	-0.43126	0.20174	-0.45625
TRINITY_DN117707_c0_g1_i3_orf1	-	-	-	acyl-CoA synthetase family member 2, mitochondrial isoform X1 [Ostrinia furnacalis] >XP_028172249.1 acyl-CoA synthetase family member 2, mitochondrial isoform X2 [Ostrinia furnacalis] >XP_028172258.1 acyl-CoA synthetase family member 2, mitochondrial isoform X3 [Ostrinia furnacalis]	1.84559	-1.18972	-0.16879	-0.11855	-0.36853
TRINITY_DN13718_c0_g1_i7_orf1	-	-	-	immulectin-4 [Ostrinia furnacalis]	1.4608	-1.64062	0.35763	0.03503	-0.21285
TRINITY_DN8406_c0_g1_i4_orf1	-	-	-	titin [Ostrinia furnacalis]	1.05529	-1.60643	-0.3165	-0.21008	1.07771
TRINITY_DN1515_c0_g1_i2_orf1	-	-	-	unnamed protein product [Diatraea saccharalis]	1.57177	-0.91437	-1.18351	0.54088	-0.01477
TRINITY_DN2318_c1_g1_i1_orf1	-	-	-	transcription factor SPT20 homolog [Ostrinia furnacalis]	1.2347	-1.67511	-0.19392	-0.1469	0.78124
TRINITY_DN121156_c0_g2_i1_orf1	-	-	-	terminal nucleotidyltransferase 5C isoform X1 [Ostrinia furnacalis] >XP_028163344.1 terminal nucleotidyltransferase 5C isoform X1 [Ostrinia furnacalis] >XP_028163346.1 terminal nucleotidyltransferase 5C isoform X1 [Ostrinia furnacalis]	1.38243	-1.30046	-0.93853	0.70216	0.1544
TRINITY_DN659_c0_g2_i1_orf1	-	-	-	unnamed protein product [Diatraea saccharalis]	1.26783	-1.34699	-0.76394	-0.14377	0.98688
TRINITY_DN23204_c0_g1_i1_orf1	-	-	-	LOW QUALITY PROTEIN: uncharacterized protein LOC114350452 [Ostrinia furnacalis]	1.50835	-1.50178	0.26138	0.29342	-0.56136
TRINITY_DN3760_c0_g1_i1_orf1	-	-	-	something about silencing protein 10 [Ostrinia furnacalis]	1.77445	-1.2395	0.19763	-0.42772	-0.30486
TRINITY_DN3529_c0_g1_i7_orf1	-	-	-	putative fatty acyl-CoA reductase CG5065 [Ostrinia furnacalis]	0.97793	-1.40855	-1.01045	0.7323	0.70876
TRINITY_DN8454_c0_g1_i4_orf1	-	-	-	translocon-associated protein subunit alpha [Ostrinia furnacalis]	0.85199	-1.67674	-0.52129	0.29554	1.0505
TRINITY_DN38562_c0_g1_i3_orf1	-	-	-	persulfide dioxygenase ETHE1, mitochondrial isoform X1 [Ostrinia furnacalis]	1.44782	-1.51066	0.45489	0.21504	-0.6071
TRINITY_DN27045_c0_g1_i1_orf1	-	-	-	cytochrome P450 6B5-like [Galleria mellonella]	0.92164	-1.75595	-0.31204	0.96864	0.17771
TRINITY_DN1982_c0_g1_i17_orf1	-	-	-	unnamed protein product, partial [Iphiclides podalirius]	0.73087	-1.94342	0.04109	0.56205	0.6094
TRINITY_DN5721_c0_g1_i5_orf1	-	-	-	fumarate hydratase, mitochondrial-like isoform X2 [Ostrinia furnacalis]	1.80896	-1.1376	0.20812	-0.39805	-0.48143
TRINITY_DN12_c0_g1_i5_orf1	-	-	-	cAMP-dependent protein kinase type II regulatory subunit isoform X1 [Ostrinia furnacalis] >XP_028175270.1 cAMP-dependent protein kinase type II regulatory subunit isoform X1 [Ostrinia furnacalis]	1.06841	-1.41245	-0.97714	0.52659	0.79459
TRINITY_DN7335_c0_g1_i1_orf1	-	-	-	probable methylmalonate-semialdehyde dehydrogenase [acylating], mitochondrial [Bicyclus anynana]	1.66909	-1.42102	0.13425	-0.41888	0.03657
TRINITY_DN8724_c0_g1_i5_orf1	-	-	-	vesicle-associated membrane protein/synaptobrevin-binding protein isoform X1 [Pectinophora gossypiella]	1.24579	-0.83075	-1.48009	0.53642	0.52863
TRINITY_DN19830_c0_g1_i1_orf1	-	-	-	macrophage migration inhibitory factor-like [Ostrinia furnacalis]	1.48518	-1.64011	0.30154	-0.03705	-0.10957
TRINITY_DN61_c0_g2_i3_orf1	-	-	-	mitochondrial dicarboxylate carrier [Ostrinia furnacalis] >XP_028161565.1 mitochondrial dicarboxylate carrier [Ostrinia furnacalis] >XP_028161566.1 mitochondrial dicarboxylate carrier [Ostrinia furnacalis]	1.77967	-1.23366	-0.50935	0.14098	-0.17765
TRINITY_DN25492_c0_g1_i1_orf1	-	-	-	PREDICTED: myrosinase 1-like [Amyelois transitella]	1.20647	-1.81189	0.11147	-0.00509	0.49903

TRINITY_DN8964_c0_g1_i4_orf1	-	-	-	hypothetical protein evm_010115 [Chilo suppressalis]	0.86215	-1.74308	-0.4956	0.57566	0.80086
TRINITY_DN5218_c0_g1_i4_orf1	-	-	-	threonine--tRNA ligase, cytoplasmic isoform X1 [Trichoplusia ni]	1.6852	-1.44284	-0.21635	-0.13786	0.11185
TRINITY_DN146126_c0_g1_i1_orf1	-	-	-	malate dehydrogenase, mitochondrial [Chelonus insularis]	1.43713	-1.69403	0.04612	0.24783	-0.03705
TRINITY_DN1697_c0_g1_i1_orf1	-	-	-	mitogen-activated protein kinase-binding protein 1 [Ostrinia furnacalis]	1.57083	-1.41098	-0.64854	0.21603	0.27266
TRINITY_DN48851_c0_g1_i2_orf1	-	-	-	translationally-controlled tumor protein homolog [Ostrinia furnacalis]	1.30789	-1.39434	-0.65008	-0.20244	0.93896
TRINITY_DN136906_c0_g1_i1_orf1	-	-	-	translational elongation factor-1alpha, partial [Ethmia eupostica]	1.49127	-1.42084	-0.4704	-0.27754	0.67752
TRINITY_DN21872_c0_g1_i2_orf1	-	-	-	facilitated trehalose transporter Tret1-2 homolog [Ostrinia furnacalis] >XP_028178438.1 facilitated trehalose transporter Tret1-2 homolog [Ostrinia furnacalis] >XP_028178439.1 facilitated trehalose transporter Tret1-2 homolog [Ostrinia furnacalis]	1.49891	-1.52984	-0.01523	-0.43056	0.47672
TRINITY_DN16673_c0_g1_i1_orf1	-	-	-	myosin heavy chain, partial [Drosophila virilis]	1.40382	-1.67728	-0.19074	0.0425	0.4217
TRINITY_DN6365_c0_g1_i4_orf1	-	-	-	40S ribosomal protein S21 [Helicoverpa armigera] >XP_047038308.1 40S ribosomal protein S21 isoform X2 [Helicoverpa zea] >KAI5643652.1 ribosomal protein s21e domain-containing protein [Phthorimaea operculella] >PZC73652.1 hypothetical protein B5X24_HaOG209026 [Helicoverpa armigera]	1.31419	-1.6359	-0.48159	0.54684	0.25647
TRINITY_DN3906_c0_g1_i5_orf1	-	-	-	ejaculatory bulb-specific protein 3-like [Ostrinia furnacalis]	0.41695	-1.67665	-0.48465	0.5125	1.23185
TRINITY_DN3970_c0_g1_i1_orf1	-	-	-	hypothetical protein evm_002369 [Chilo suppressalis]	1.48325	-1.3716	-0.71678	-0.03047	0.6356
TRINITY_DN235_c0_g1_i2_orf1	-	-	-	unnamed protein product [Parnassius apollo]	0.88794	-1.90493	-0.05477	0.48361	0.58814
TRINITY_DN42461_c0_g1_i4_orf1	-	-	-	obscurin [Ostrinia furnacalis]	0.77504	-1.90869	-0.03099	0.38605	0.7786
TRINITY_DN1369_c0_g2_i3_orf1	-	-	-	ATP-dependent Clp protease ATP-binding subunit clpX-like, mitochondrial isoform X2 [Helicoverpa zea]	1.36079	-1.1275	-0.92869	-0.2739	0.96929
TRINITY_DN11050_c0_g1_i8_orf1	-	-	-	uncharacterized protein LOC114360965, partial [Ostrinia furnacalis]	1.51599	-1.51215	-0.38133	0.50378	-0.12629
TRINITY_DN38435_c0_g1_i1_orf1	-	-	-	UDP-glucuronosyltransferase 2B20-like [Ostrinia furnacalis]	1.26559	-1.75649	0.05592	-0.11061	0.54559
TRINITY_DN107962_c0_g1_i1_orf1	-	-	-	unnamed protein product [Euphydryas editha]	0.80478	-1.76299	-0.41691	0.93745	0.43767
TRINITY_DN22747_c0_g1_i5_orf1	-	-	-	hypothetical protein HF086_001789 [Spodoptera exigua]	1.52591	-1.13029	-1.07769	0.33931	0.34276
TRINITY_DN2254_c0_g1_i4_orf1	-	-	-	vigilin [Ostrinia furnacalis]	0.7437	-1.47744	-0.90935	0.61262	1.03047
TRINITY_DN496_c0_g1_i7_orf1	-	-	-	unnamed protein product [Diatraea saccharalis]	1.27702	-1.40262	-0.77326	0.00223	0.89663
TRINITY_DN41166_c0_g1_i1_orf1	-	-	-	arginine kinase isoform X1 [Ostrinia furnacalis]	1.33646	-1.49214	-0.53588	-0.13439	0.82594
TRINITY_DN37986_c0_g1_i2_orf1	-	-	-	unnamed protein product [Diatraea saccharalis]	1.54504	-1.13321	-0.95675	-0.09146	0.63637
TRINITY_DN6916_c0_g1_i4_orf1	-	-	-	isovaleryl-CoA dehydrogenase, mitochondrial [Ostrinia furnacalis]	1.54485	-1.56221	-0.13592	-0.19049	0.34377
TRINITY_DN5111_c0_g1_i2_orf1	-	-	-	uncharacterized protein LOC126368598 [Pectinophora gossypiella]	1.77498	-1.22374	0.22496	-0.38211	-0.39409

TRINITY_DN139326_c0_g1_i1_orf1	-	-	-	40S ribosomal protein S13 [Gallus gallus] >NP_001008.1 40S ribosomal protein S13 [Homo sapiens] >NP_001020513.1 40S ribosomal protein S13 [Bos taurus] >NP_001231758.1 40S ribosomal protein S13 [Sus scrofa] >NP_001232227.1 40S ribosomal protein S13 [Taeniopygia guttata] >NP_001252846.1 40S ribosomal protein S13 [Macaca mulatta] >NP_080809.1 40S ribosomal protein S13 [Mus musculus] >NP_569116.1 40S ribosomal protein S13 [Rattus norvegicus] >XP_001504999.3 40S ribosomal protein S13 [Equus caballus] >XP_002721403.1 PREDICTED: 40S ribosomal protein S13 [Oryctolagus cuniculus] >XP_002925190.2 40S ribosomal protein S13 [Ailuropoda melanoleuca] >XP_003254322.1 40S ribosomal protein S13 [Nomascus leucogenys] >XP_003312983.1 40S ribosomal protein S13 [Pan troglodytes] >XP_003412037.1 40S ribosomal protein S13 [Loxodonta africana] >XP_003777851.1 40S ribosomal protein S13 [Pongo abelii] >XP_003781188.1 40S ribosomal protein S13 [Otolemur garnettii] >XP_003818255.1 40S ribosomal protein S13 [Pan paniscus] >XP_003910218.1 40S ribosomal protein S13 [Papio anubis] >XP_003993072.1 40S ribosomal protein S13 [Felis catus] >XP_004050806.1 40S ribosomal protein S13 [Gorilla gorilla gorilla] >XP_004285569.1 40S ribosomal protein S13 [Orcinus orca] >XP_004312593.1 40S ribosomal protein S13 [Tursiops truncatus] >XP_004369736.1 40S ribosomal protein S13 [Trichechus manatus latirostris] >XP_004415573.1 PREDICTED: 40S ribosomal protein S13 [Odobenus rosmarus divergens] >XP_004418532.1 PREDICTED: 40S ribosomal protein S13 [Ceratotherium simum simum] >XP_004472558.1 40S ribosomal protein S13 [Dasypus novemcinctus] >XP_004593806.1 40S ribosomal protein S13 [Ochotona princeps] >XP_004632754.1 40S ribosomal protein S13 [Octodon degus] >XP_004634969.1 40S ribosomal protein S13 isoform X1 [Octodon degus] >XP_004650891.1 40S ribosomal protein S13 [Jaculus jaculus] >XP_004683069.1 PREDICTED: 40S ribosomal protein S13 [Condylura cristata] >XP_004711628.1 40S ribosomal protein S13 [Echinops telfairi] >XP_004752124.1 40S ribosomal protein S13 [Mustela putorius furo] >XP_004851550.1 40S ribosomal protein S13 [Heterocephalus glaber] >XP_005075688.1 40S ribosomal protein S13 [Mesocricetus auratus] >XP_005153329.1 40S ribosomal protein S13 [Melopsittacus undulatus] >XP_005308755.1 40S ribosomal protein S13 [Chrysemys picta bellii] >XP_005326819.1 40S ribosomal protein S13 [Ictidomys tridecemlineatus] >XP_005351096.1 40S ribosomal protein S13 [Microtus ochrogaster] >XP_005380432.1 PREDICTED: 40S ribosomal protein S13 [Chinchilla lanigera] >XP_005486670.1 40S ribosomal protein S13	1.50962	-1.21087	-0.9164	-0.02606	0.64371
TRINITY_DN3511_c0_g2_i1_orf1	-	-	-	eukaryotic translation initiation factor 2A [Ostrinia furnacalis]	1.47409	-1.12065	-1.00373	-0.09455	0.74484
TRINITY_DN110376_c0_g1_i1_orf1	-	-	-	nucleolysin TIAR [Osmia bicornis bicornis] >XP_034192702.1 nucleolysin TIAR [Osmia lignaria]	1.44204	-1.32382	-0.9129	0.49453	0.30015
TRINITY_DN15234_c0_g1_i3_orf1	-	-	-	60S ribosomal protein L30 [Papilio polytes] >XP_014360326.1 60S ribosomal protein L30 [Papilio machaon] >XP_026485186.1 60S ribosomal protein L30 isoform X1 [Vanessa tameamea] >XP_028160279.1 60S ribosomal protein L30 [Ostrinia furnacalis] >XP_030027999.1 60S ribosomal protein L30 [Manduca sexta] >XP_032515151.1 60S ribosomal protein L30 [Danaus plexippus plexippus] >XP_034840952.1 60S ribosomal protein L30 [Maniola hyperantus] >XP_037301873.1 60S ribosomal protein L30 [Manduca sexta] >XP_039745408.1 60S ribosomal protein L30 [Pararge aegeria] >XP_041974708.1 60S ribosomal protein L30 [Aricia agestis] >XP_045455248.1 60S ribosomal protein L30 [Melitaea cinxia] >XP_045457914.1 60S ribosomal protein L30 [Melitaea cinxia] >XP_046969892.1 60S ribosomal protein L30 [Vanessa cardui] >XP_047539529.1 60S ribosomal protein L30 [Vanessa atalanta] >XP_049887645.1 60S ribosomal protein L30 [Pectinophora gossypiella] >XP_050360253.1 60S ribosomal protein L30 [Nymphalis io] >ADT80684.1 ribosomal protein L30 [Euphydryas aurinia] >CAG9575798.1 unnamed protein product [Danaus chrysippus] >CAH0722581.1 unnamed protein product, partial [Brenthis ino] >CAH2099946.1 unnamed protein product [Euphydryas editha] >CAH2267204.1 jg2932 [Pararge aegeria aegeria]	1.70537	-1.37761	-0.41402	-0.0537	0.13996
TRINITY_DN79673_c0_g1_i1_orf1	-	-	-	thioredoxin, mitochondrial-like [Ostrinia furnacalis]	1.3876	-1.69769	0.01057	0.42126	-0.12175
TRINITY_DN2224_c0_g1_i1_orf1	-	-	-	serine--tRNA ligase, cytoplasmic [Ostrinia furnacalis]	1.4605	-1.57016	-0.37218	-0.03014	0.51197
TRINITY_DN5753_c0_g1_i10_orf1	-	-	-	ryanodine receptor [Ostrinia furnacalis]	1.46929	-1.56902	-0.41244	0.45372	0.05845
TRINITY_DN1125_c0_g1_i4_orf1	-	-	-	hypothetical protein evm_001907 [Chilo suppressalis] >CAH2985359.1 unnamed protein product [Chilo suppressalis]	1.10141	-1.66665	-0.56125	0.73599	0.3905
TRINITY_DN8584_c0_g1_i6_orf1	-	-	-	uncharacterized protein LOC114354070 isoform X3 [Ostrinia furnacalis]	1.69836	-1.41194	-0.26498	0.14985	-0.17129
TRINITY_DN8352_c0_g1_i3_orf1	-	-	-	TRPL translocation defect protein 14 isoform X1 [Ostrinia furnacalis]	1.77547	-1.19725	0.18885	-0.17809	-0.58899

TRINITY_DN1752_c0_g1_i18_orf1	-	-	-	titin isoform X1 [Ostrinia furnacalis]	1.55201	-1.57231	-0.27446	0.16025	0.13451
TRINITY_DN9248_c0_g1_i10_orf1	-	-	-	unnamed protein product [Arctia plantaginis]	1.65725	-1.43781	-0.33542	-0.12502	0.24101
TRINITY_DN32896_c0_g3_i1_orf1	-	-	-	PREDICTED: calcium-binding mitochondrial carrier protein Aralar1 isoform X1 [Microplitis demolitor]	1.53048	-1.48461	-0.36375	0.52686	-0.20898
TRINITY_DN5765_c0_g2_i3_orf1	-	-	-	unnamed protein product [Diatraea saccharalis]	1.68375	-1.35475	-0.46956	-0.15251	0.29307
TRINITY_DN14701_c0_g1_i2_orf1	-	-	-	staphylococcal nuclease domain-containing protein 1 [Ostrinia furnacalis]	1.57356	-1.27217	-0.83454	0.08367	0.44949
TRINITY_DN130159_c0_g2_i1_orf1	-	-	-	lachesin-like [Chelonus insularis] >XP_034946935.1 lachesin-like [Chelonus insularis]	1.54254	-1.47105	-0.50945	-0.00587	0.44383
TRINITY_DN13371_c0_g1_i4_orf1	-	-	-	ATP synthase mitochondrial F1 complex assembly factor 2 [Ostrinia furnacalis]	1.78988	-1.24253	-0.35491	-0.32855	0.13611
TRINITY_DN83542_c0_g1_i1_orf1	-	-	-	PREDICTED: WASH complex subunit strumpellin [Microplitis demolitor]	1.7063	-1.38549	0.18853	-0.21162	-0.29772
TRINITY_DN129808_c0_g1_i1_orf1	-	-	-	uncharacterized protein LOC114354070 isoform X3 [Ostrinia furnacalis]	1.47857	-1.1164	-1.04554	-0.00535	0.68871
TRINITY_DN111110_c0_g1_i1_orf1	-	-	-	NAD-dependent protein deacylase-like [Ostrinia furnacalis]	0.80908	-1.69034	-0.59003	0.56583	0.90546
TRINITY_DN12508_c0_g1_i1_orf1	-	-	-	uncharacterized protein LOC114350091 [Ostrinia furnacalis]	1.19246	-1.57017	-0.71666	0.53813	0.55625
TRINITY_DN38366_c0_g1_i4_orfp1	-	-	-	TRINITY_DN38366_c0_g1_i4_m.10666 TRINITY_DN38366_c0_g1::TRINITY_DN38366_c0_g1_i4::g.10666 ORF type:internal len:143 (+),score=71.68 TRINITY_DN38366_c0_g1_i4:3-428(+)	0.82029	-1.86747	-0.21305	0.64019	0.62004
TRINITY_DN83948_c0_g1_i3_orf1	-	-	-	carbonyl reductase [NADPH] 1-like [Ostrinia furnacalis]	1.87114	-1.04084	0.07439	-0.43348	-0.47122
TRINITY_DN11448_c0_g1_i4_orf1	-	-	-	uncharacterized protein LOC114364760 isoform X5 [Ostrinia furnacalis]	1.35317	-1.73577	-0.11782	0.15818	0.34225
TRINITY_DN133228_c0_g1_i3_orf1	-	-	-	microtubule-actin cross-linking factor 1 isoform X15 [Ostrinia furnacalis]	1.54778	-1.56027	-0.12744	0.3382	-0.19828
TRINITY_DN110460_c0_g2_i1_orf1	-	-	-	Similar to chaf1a-b: Chromatin assembly factor 1 subunit A-B (Xenopus laevis) [Cotesia congregata]	1.34595	-1.76475	0.22627	0.1429	0.04963
TRINITY_DN22956_c0_g1_i1_orf1	-	-	-	lipoamide acyltransferase component of branched-chain alpha-keto acid dehydrogenase complex, mitochondrial [Ostrinia furnacalis]	1.51832	-1.54244	-0.08567	0.44362	-0.33384
TRINITY_DN20763_c0_g1_i2_orf1	-	-	-	uncharacterized protein LOC114355848 [Ostrinia furnacalis]	1.39256	-1.51997	-0.46916	-0.12149	0.71807
TRINITY_DN4451_c0_g2_i4_orf1	-	-	-	uncharacterized protein LOC114361986 isoform X1 [Ostrinia furnacalis] >XP_028173022.1 uncharacterized protein LOC114361986 isoform X2 [Ostrinia furnacalis]	1.63047	-1.43708	0.14913	-0.48388	0.14136
TRINITY_DN38835_c0_g3_i1_orf1	-	-	-	protein transport protein Sec61 subunit alpha [Spodoptera litura] >XP_035429226.1 protein transport protein Sec61 subunit alpha [Spodoptera frugiperda] >XP_047985890.1 protein transport protein Sec61 subunit alpha [Leguminivora glycinivorella] >KAF9413961.1 hypothetical protein HW555_007991 [Spodoptera exigua] >CAB3514725.1 unnamed protein product [Spodoptera littoralis] >KAF9810869.1 hypothetical protein SFRURICE_005295 [Spodoptera frugiperda] >KAG8115796.1 hypothetical protein SFRUCORN_012373 [Spodoptera frugiperda] >CAH0700181.1 unnamed protein product [Spodoptera exigua]	1.41453	-1.60512	-0.45442	0.38651	0.2585
TRINITY_DN1445_c0_g2_i4_orf1	-	-	-	leucine-rich PPR motif-containing protein, mitochondrial [Ostrinia furnacalis]	1.66909	-1.47374	-0.00873	-0.20455	0.01793
TRINITY_DN15362_c0_g1_i1_orf1	-	-	-	probable elongation factor 1-delta isoform X1 [Ostrinia furnacalis]	1.47499	-1.11953	-0.89505	-0.28893	0.82852
TRINITY_DN25960_c0_g1_i1_orf1	-	-	-	protein mini spindles [Ostrinia furnacalis]	1.5777	-1.09175	-1.07052	0.26002	0.32455
TRINITY_DN9965_c0_g1_i1_orf1	-	-	-	dihydrolipoyl dehydrogenase [Ostrinia furnacalis]	1.80322	-1.26585	-0.32066	-0.20763	-0.00908
TRINITY_DN7808_c0_g1_i1_orf1	-	-	-	probable pyruvate dehydrogenase E1 component subunit alpha, mitochondrial isoform X1 [Ostrinia furnacalis] >XP_028158738.1 probable pyruvate dehydrogenase E1 component subunit alpha, mitochondrial isoform X2 [Ostrinia furnacalis] >XP_028158739.1 probable pyruvate dehydrogenase E1 component subunit alpha, mitochondrial isoform X3 [Ostrinia furnacalis] >XP_028158740.1 probable pyruvate dehydrogenase E1 component subunit alpha, mitochondrial isoform X4 [Ostrinia furnacalis]	1.66559	-1.47604	-0.06268	0.06916	-0.19602
TRINITY_DN1757_c0_g1_i4_orf1	-	-	-	F-box/LRR-repeat protein 2 isoform X1 [Ostrinia furnacalis]	1.75521	-1.32652	-0.21497	0.10592	-0.31964
TRINITY_DN50676_c0_g1_i1_orf1	-	-	-	uncharacterized protein LOC114360659 [Ostrinia furnacalis]	1.29888	-1.55559	-0.61127	0.7011	0.16687
TRINITY_DN105055_c0_g1_i1_orfp1	-	-	-	unnamed protein product [Euphydryas editha]	1.87362	-1.07529	-0.12531	-0.12304	-0.54998
TRINITY_DN120439_c1_g1_i1_orf1	-	-	-	myosin heavy chain variant, partial [Bombyx mori]	1.15961	-1.80539	-0.14953	0.22845	0.56686
TRINITY_DN1173_c1_g1_i10_orf1	-	-	-	hypothetical protein evm_001011 [Chilo suppressalis]	0.96313	-1.81802	-0.07389	0.05792	0.87085
TRINITY_DN36817_c0_g1_i1_orf1	-	-	-	uncharacterized protein LOC114357350 [Ostrinia furnacalis]	1.19413	-1.8316	0.0963	0.09229	0.44889
TRINITY_DN7134_c0_g1_i1_orf1	-	-	-	phosphatidylglycerophosphatase and protein-tyrosine phosphatase 1 [Ostrinia furnacalis]	1.84737	-1.1144	0.01316	-0.1903	-0.55583
TRINITY_DN63533_c0_g1_i2_orf1	-	-	-	glutathione S-transferase sigma3 [Glyphodes pyloalis]	0.94663	-1.81357	-0.30325	0.72337	0.44681
TRINITY_DN78492_c0_g1_i1_orf1	-	-	-	uncharacterized protein LOC114354775 [Ostrinia furnacalis]	1.41604	-1.26612	-0.99052	0.58964	0.25097
TRINITY_DN57918_c0_g1_i1_orf1	-	-	-	PREDICTED: serine--tRNA ligase, cytoplasmic [Fopius arisanus]	1.41489	-1.64118	-0.24857	-0.01763	0.49249
TRINITY_DN4385_c0_g2_i1_orf1	-	-	-	LOW QUALITY PROTEIN: carbonic anhydrase 1-like [Ostrinia furnacalis]	1.50947	-1.3891	-0.68487	-0.00369	0.56818

TRINITY_DN1351_c0_g1_i1_orf1	-	-	-	PREDICTED: flavin reductase (NADPH) [Microplitis demolitor] >XP_008553603.1 PREDICTED: flavin reductase (NADPH) [Microplitis demolitor]	1.54713	-0.74506	-1.3722	0.33905	0.23108
TRINITY_DN7267_c1_g1_i4_orf1	-	-	-	probable pseudouridine-5'-phosphatase [Ostrinia furnacalis]	1.72758	-1.31582	-0.51599	0.13094	-0.02671
TRINITY_DN21451_c0_g1_i3_orf1	-	-	-	gelsolin-like [Ostrinia furnacalis]	1.61632	-1.44428	-0.02007	0.30454	-0.45651
TRINITY_DN2186_c0_g1_i17_orf1	-	-	-	paxillin isoform X6 [Leguminivora glycinivorella]	0.84617	-1.91734	0.02583	0.34836	0.69698
TRINITY_DN9117_c0_g1_i1_orf1	-	-	-	spherulin-2A-like [Ostrinia furnacalis]	1.78555	-1.3126	-0.12461	-0.2538	-0.09454
TRINITY_DN27500_c0_g1_i4_orf1	-	-	-	hemicentin-1-like [Ostrinia furnacalis]	0.50168	-1.73577	-0.37609	0.4193	1.19087
TRINITY_DN29229_c0_g1_i4_orf1	-	-	-	uncharacterized protein LOC114351433 isoform X1 [Ostrinia furnacalis]	1.55509	-1.17083	-0.69528	-0.42719	0.73821
TRINITY_DN34399_c0_g1_i1_orf1	-	-	-	cysteine synthase-like [Ostrinia furnacalis]	1.48356	-1.38389	-0.7308	0.04109	0.59004
TRINITY_DN5597_c0_g1_i2_orf1	-	-	-	monocarboxylate transporter 9-like [Ostrinia furnacalis] >XP_028156211.1 monocarboxylate transporter 9-like [Ostrinia furnacalis]	1.84346	-1.06658	-0.02548	-0.07479	-0.67662
TRINITY_DN34347_c0_g1_i1_orf1	-	-	-	nesprin-1-like isoform X8 [Bombyx mandarina]	1.36056	-1.29474	-0.78585	-0.18577	0.90579
TRINITY_DN4920_c0_g1_i5_orf1	-	-	-	titin homolog [Ostrinia furnacalis]	1.08524	-1.84147	-0.0659	0.20018	0.62195
TRINITY_DN116951_c0_g3_i2_orf1	-	-	-	spermine oxidase-like isoform X2 [Ostrinia furnacalis]	1.01154	-1.90202	0.09775	0.52927	0.26347
TRINITY_DN135_c0_g1_i1_orf1	-	-	-	60S ribosomal protein L11 [Nymphalis io]	1.69197	-1.45396	-0.13305	-0.05121	-0.05375
TRINITY_DN1232_c0_g1_i1_orf1	-	-	-	acanthoscurrin-2-like isoform X1 [Ostrinia furnacalis]	1.01414	-1.80387	-0.26167	0.30617	0.74523
TRINITY_DN129869_c0_g4_i1_orf1	-	-	-	putative myosin heavy chain, muscle, partial [Cotesia chilonis]	1.27825	-1.77899	-0.08751	0.19268	0.39557
TRINITY_DN46216_c0_g3_i1_orf1	-	-	-	unnamed protein product, partial [Brenthis ino]	0.35194	-1.87715	-0.09834	0.92312	0.70043
TRINITY_DN20957_c0_g1_i1_orf1	-	-	-	adenylate kinase isoenzyme 1 isoform X2 [Ostrinia furnacalis]	1.26355	-1.76146	0.00595	-0.05373	0.54568
TRINITY_DN27276_c0_g1_i5_orf1	-	-	-	probable small nuclear ribonucleoprotein Sm D1 [Ostrinia furnacalis] >CAG9751027.1 unnamed protein product [Diatraea saccharalis] >CAG9789712.1 unnamed protein product [Diatraea saccharalis]	1.03256	-1.47379	-0.85169	0.96314	0.32978
TRINITY_DN7414_c0_g1_i1_orf1	-	-	-	uncharacterized protein LOC114357447 [Ostrinia furnacalis]	1.65158	-1.28517	-0.73656	0.11572	0.25443
TRINITY_DN1952_c0_g1_i2_orf1	-	-	-	uncharacterized protein LOC114354403 [Ostrinia furnacalis] >AYE20402.1 RNAi efficiency-related nuclease REase [Ostrinia furnacalis]	1.49918	-1.63202	-0.05351	0.27855	-0.0922
TRINITY_DN147676_c0_g1_i1_orf1	-	-	-	PREDICTED: 60S ribosomal protein L23 [Microplitis demolitor] >XP_044591174.1 60S ribosomal protein L23 [Cotesia glomerata] >KAG8035666.1 hypothetical protein G9C98_001094 [Cotesia typhae] >KAH0547433.1 60S ribosomal protein L23A [Cotesia	1.5449	-1.29281	-0.82288	0.05967	0.51112
TRINITY_DN4622_c0_g1_i1_orf1	-	-	-	keratin-associated protein 19-2-like [Ostrinia furnacalis]	1.66637	-1.44174	-0.15691	-0.27645	0.20873
TRINITY_DN74889_c0_g1_i1_orf1	-	-	-	probable 28S ribosomal protein S23, mitochondrial [Ostrinia furnacalis]	1.80761	-1.15941	-0.15822	0.10376	-0.59373
TRINITY_DN434_c0_g1_i4_orf1	-	-	-	uncharacterized protein LOC126367148 [Pectinophora gossypiella]	1.44835	-1.6193	-0.20668	0.47703	-0.0994
TRINITY_DN80245_c0_g1_i1_orf1	-	-	-	peroxisomal membrane protein 2 [Ostrinia furnacalis]	1.58988	-1.5158	-0.31463	0.27309	-0.03254
TRINITY_DN11194_c0_g1_i4_orf1	-	-	-	ATPase family AAA domain-containing protein 3A homolog [Ostrinia furnacalis]	1.70742	-1.36186	-0.44957	0.15816	-0.05414
TRINITY_DN12372_c0_g1_i4_orf1	-	-	-	WD repeat-containing protein 44 isoform X4 [Ostrinia furnacalis]	1.50603	-1.14828	-1.06372	0.22526	0.48071

TRINITY_DN4016_c0_g1_i1_orf1	-	-	-	60S acidic ribosomal protein P0 [Homo sapiens] >NP_444505.1 60S acidic ribosomal protein P0 [Homo sapiens] >XP_002823894.1 60S acidic ribosomal protein P0 [Pongo abelii] >XP_003280010.1 60S acidic ribosomal protein P0 [Nomascus leucogenys] >XP_004054038.1 60S acidic ribosomal protein P0 [Gorilla gorilla gorilla] >XP_004054039.1 60S acidic ribosomal protein P0 [Gorilla gorilla gorilla] >XP_008956032.1 60S acidic ribosomal protein P0 [Pan paniscus] >XP_008956033.1 60S acidic ribosomal protein P0 [Pan paniscus] >XP_012611945.1 60S acidic ribosomal protein P0 [Microcebus murinus] >XP_016802006.1 60S acidic ribosomal protein P0 [Pan troglodytes] >XP_016802007.1 60S acidic ribosomal protein P0 [Pan troglodytes] >XP_025256707.1 60S acidic ribosomal protein P0 isoform X1 [Theropithecus gelada] >XP_025256708.1 60S acidic ribosomal protein P0 isoform X1 [Theropithecus gelada] >XP_032024425.1 60S acidic ribosomal protein P0 [Hylobates moloch] >XP_032657670.1 60S acidic ribosomal protein P0 [Chelonoidis abingdonii] >XP_045390642.1 60S acidic ribosomal protein P0 [Lemur catta] >P05388.1 RecName: Full=60S acidic ribosomal protein P0; AltName: Full=60S ribosomal protein L10E; AltName: Full=Large ribosomal subunit protein uL10 [Homo sapiens] >3J92_s Structure and assembly pathway of the ribosome quality control complex [Oryctolagus cuniculus] >4V5Z_Bg Chain Bg, 60S acidic ribosomal protein P0 [Canis lupus familiaris] >4V6X_Cq Chain Cq, 60S acidic ribosomal protein P0 [Homo sapiens] >5AJ0_AK Chain AK, 60S acidic ribosomal protein P0 [Homo sapiens] >6ZM7_Ls Chain Ls, 60S acidic ribosomal protein P0 [Homo sapiens] >6ZME_Ls Chain Ls, 60S acidic ribosomal protein P0 [Homo sapiens] >6ZMI_Ls Chain Ls, 60S acidic ribosomal protein P0 [Homo sapiens] >6ZMO_Ls Chain Ls, 60S acidic ribosomal protein P0 [Homo sapiens] >ABM82739.1 ribosomal protein, large, P0 [synthetic construct] >SJX33952.1 unnamed protein product, partial [Human ORFeome Gateway entry vector] >AAA36470.1 acidic ribosomal phosphoprotein (P0) [Homo sapiens] >AAC05176.1 60S ACIDIC RIBOSOMAL PROTEIN; match to P05388 (PID:g133041) [Homo sapiens] >AAH00087.1 Ribosomal protein, large, P0 [Homo sapiens]	1.26996	-1.79528	-0.04378	0.27196	0.29713
TRINITY_DN7580_c0_g1_i1_orf1	-	-	-	cytochrome P450 monooxygenase CYP6AB141 [Ostrinia furnacalis]	0.84874	-1.66836	-0.60233	0.95841	0.46355
TRINITY_DN3461_c0_g1_i1_orf1	-	-	-	protein SCO1 homolog, mitochondrial [Ostrinia furnacalis]	1.71859	-1.40517	-0.23107	0.04586	-0.12822
TRINITY_DN14597_c0_g1_i5_orf1	-	-	-	UDP-glucuronosyltransferase 2B1-like isoform X3 [Ostrinia furnacalis]	1.57602	-1.40932	-0.45492	0.51926	-0.23103
TRINITY_DN4408_c6_g1_i1_orf1	-	-	-	polyprotein, partial [Bemisia tabaci]	0.70323	-1.60521	-0.75009	0.85312	0.79894
TRINITY_DN89613_c0_g1_i13_orf1	-	-	-	PREDICTED: uncharacterized protein LOC106137743 [Amyelois transitella]	1.31465	-1.49752	-0.76018	0.41415	0.5289
TRINITY_DN96557_c0_g1_i1_orf1	-	-	-	charged multivesicular body protein 4B [Phyllostomus discolor]	1.21615	-1.81491	-0.02448	0.18456	0.43867
TRINITY_DN126648_c0_g1_i1_orf1	-	-	-	elongation factor 1 alpha, partial [Spodoptera exigua] >QYQ52647.1 elongation factor 1 alpha, partial [Spodoptera exigua]	1.48814	-0.84503	-1.34937	0.32901	0.37726
TRINITY_DN1173_c0_g1_i12_orf1	-	-	-	obscurin [Ostrinia furnacalis]	1.63042	-1.40579	-0.54689	0.24624	0.07601
TRINITY_DN10629_c0_g1_i1_orf1	-	-	-	caspase-1-like [Ostrinia furnacalis]	1.30104	-1.71015	-0.23871	0.5646	0.08321
TRINITY_DN1123_c2_g1_i5_orf1	-	-	-	troponin I isoform X4 [Leguminivora glycinivorella]	1.10226	-1.76236	-0.09796	-0.05811	0.81617
TRINITY_DN4133_c0_g1_i2_orfp2	-	-	-	unnamed protein product [Spodoptera exigua]	0.53026	-1.34209	-1.05963	0.78808	1.08338
TRINITY_DN3355_c0_g2_i4_orf1	-	-	-	UDP-glycosyltransferase UGT33AL1 [Ostrinia furnacalis]	1.83471	-1.07225	-0.01	-0.05928	-0.69318
TRINITY_DN115082_c0_g1_i5_orf1	-	-	-	protein dj-1beta-like isoform X2 [Ostrinia furnacalis]	1.01295	-1.43334	-0.2235	-0.58971	1.2336
TRINITY_DN4501_c0_g1_i3_orf1	-	-	-	methylcrotonoyl-CoA carboxylase subunit alpha, mitochondrial [Ostrinia furnacalis]	1.35524	-1.64882	-0.39954	0.19675	0.49637
TRINITY_DN55160_c0_g1_i1_orf1	-	-	-	esterase FE4-like isoform X2 [Ostrinia furnacalis]	1.84094	-1.15504	-0.16178	-0.02406	-0.50006
TRINITY_DN4145_c0_g1_i1_orf1	-	-	-	uncharacterized protein LOC114353175 isoform X1 [Ostrinia furnacalis]	1.43662	-1.69264	-0.06918	0.08066	0.24454
TRINITY_DN106534_c0_g1_i1_orf1	-	-	-	nucleolar complex protein 2 homolog [Ostrinia furnacalis]	1.4278	-1.4871	-0.66728	0.22064	0.50594

TRINITY_DN97589_c0_g1_i3_orf1	-	-	-	ribosomal protein L37A [Bombyx mori] >XP_013189707.1 PREDICTED: 60S ribosomal protein L37a [Amyelois transitella] >XP_021198447.1 60S ribosomal protein L37a [Helicoverpa armigera] >XP_022122377.1 60S ribosomal protein L37a [Pieris rapae] >XP_022822835.1 60S ribosomal protein L37a [Spodoptera litura] >XP_023937141.1 60S ribosomal protein L37a [Bicyclus anynana] >XP_026321523.1 60S ribosomal protein L37a [Hyposmocoma kahamanoa] >XP_026495655.1 60S ribosomal protein L37a [Vanessa tameamea] >XP_026746489.1 60S ribosomal protein L37a [Trichoplusia ni] >XP_026756267.1 60S ribosomal protein L37a [Galleria mellonella] >XP_028041705.1 60S ribosomal protein L37a [Bombyx mandarina] >XP_028161757.1 60S ribosomal protein L37a [Ostrinia furnacalis] >XP_030020263.1 LOW QUALITY PROTEIN: 60S ribosomal protein L37a [Manduca sexta] >XP_032518929.1 60S ribosomal protein L37a [Danaus plexippus plexippus] >XP_034834514.1 60S ribosomal protein L37a [Maniola hyperantus] >XP_035444256.1 60S ribosomal protein L37a [Spodoptera frugiperda] >XP_038222439.1 60S ribosomal protein L37a [Zerene cesonia] >XP_039756348.1 60S ribosomal protein L37a [Pararge aegeria] >XP_041981914.1 60S ribosomal protein L37a [Aricia agestis] >XP_045451710.1 60S ribosomal protein L37a [Melitaea cinxia] >XP_045500579.1 60S ribosomal protein L37a [Colias croceus] >XP_045517305.1 60S ribosomal protein L37a [Pieris brassicae] >XP_045775103.1 60S ribosomal protein L37a [Maniola jurtina] >XP_046969745.1 60S ribosomal protein L37a [Vanessa cardui] >XP_047032252.1 60S ribosomal protein L37a [Helicoverpa zea] >XP_047525321.1 60S ribosomal protein L37a [Pieris napi] >XP_047535357.1 60S ribosomal protein L37a [Vanessa atalanta] >XP_049875744.1 60S ribosomal protein L37a [Pectinophora gossypiella] >XP_050348149.1 60S ribosomal protein L37a [Nymphalis io] >ADO95156.1 ribosomal protein L37A [Antheraea yamamai] >ADT80705.1 ribosomal protein L37A [Euphydryas aurinia] >AEL28885.1 ribosomal protein L37A [Heliconius melpomene cythera] >KAF9418899.1 hypothetical protein HW555_004419 [Spodoptera exigua] >KOB75009.1 Ribosomal protein L37A [Operophtera brumata] >RVE49828.1 hypothetical protein evm_005558 [Chilo suppressalis] >CAB3234150.1 unnamed protein product [Arctia plantaginis] >CAB3509616.1 unnamed protein product [Spodoptera littoralis] >CAF4811073.1 unnamed protein product [Pieris macdunnoughi] >CAG4956733.1 unnamed protein product [Parnassius apollo] >CAG9564640.1 unnamed protein product [Danaus chrysippus] >CAG9750098.1 unnamed	1.65906	-1.17543	-0.87351	0.07909	0.31079
TRINITY_DN2172_c0_g2_i5_orf1	-	-	-	4-hydroxyphenylpyruvate dioxygenase [Ostrinia furnacalis]	1.67313	-1.38359	-0.41727	0.29195	-0.16422
TRINITY_DN51934_c0_g2_i1_orf1	-	-	-	SCAN domain-containing protein 3-like [Pieris napi] >XP_047520696.1 SCAN domain-containing protein 3-like [Pieris napi]	1.76621	-1.19098	-0.61802	0.22039	-0.1776
TRINITY_DN6351_c0_g1_i4_orf1	-	-	-	cytochrome P450 CYP12A2-like [Ostrinia furnacalis]	1.88994	-1.10519	-0.23728	-0.28944	-0.25804
TRINITY_DN3504_c0_g1_i3_orfp2	-	-	-	TRINITY_DN3504_c0_g1_i3_m.43947 TRINITY_DN3504_c0_g1::TRINITY_DN3504_c0_g1_i3::g.43947 ORF type:5prime_partial len:208	1.45307	-1.60504	-0.39864	0.24413	0.30648
TRINITY_DN20682_c0_g1_i2_orf1	-	-	-	hypothetical protein B5X24_HaOG200252 [Helicoverpa armigera]	1.77466	-1.28203	-0.4386	-0.10804	0.05401
TRINITY_DN2924_c0_g1_i2_orf1	-	-	-	cuticular protein RR-2 [Spodoptera litura]	1.36974	-1.55235	-0.42033	0.72312	-0.12017
TRINITY_DN18396_c0_g1_i1_orf1	-	-	-	uncharacterized protein LOC114359424 [Ostrinia furnacalis]	1.44257	-1.51312	-0.48273	-0.07218	0.62547
TRINITY_DN2709_c0_g1_i4_orf1	-	-	-	ATP-dependent RNA helicase dbp2-like [Ostrinia furnacalis]	1.74613	-1.34145	0.11917	-0.25413	-0.26973
TRINITY_DN34040_c0_g2_i1_orf1	-	-	-	uncharacterized protein LOC114352849 [Ostrinia furnacalis]	1.76021	-1.28735	-0.07701	0.08499	-0.48085
TRINITY_DN6143_c0_g2_i1_orf1	-	-	-	uncharacterized protein LOC114365036 [Ostrinia furnacalis]	1.72925	-1.38036	-0.27626	-0.14884	0.0762
TRINITY_DN83295_c0_g1_i3_orf1	-	-	-	SSSX-APN4 [Ostrinia furnacalis]	1.86128	-1.16198	-0.13891	-0.34775	-0.21264
TRINITY_DN23167_c0_g1_i4_orf1	-	-	-	uncharacterized protein LOC114363065 [Ostrinia furnacalis]	1.54021	-1.32769	-0.61907	-0.24345	0.64999
TRINITY_DN8621_c0_g1_i5_orf1	-	-	-	aminopeptidase N-like isoform X2 [Ostrinia furnacalis]	1.85298	-1.17932	-0.10607	-0.32459	-0.24301
TRINITY_DN5012_c0_g1_i6_orf1	-	-	-	putative serine protease K12H4.7 [Ostrinia furnacalis]	1.78253	-1.2725	-0.07343	0.00821	-0.44481
TRINITY_DN3135_c0_g1_i6_orf1	-	-	-	acanthoscurrin-1-like [Ostrinia furnacalis]	1.47513	-1.66576	-0.04298	0.21712	0.01649
TRINITY_DN4929_c1_g2_i5_orf1	-	-	-	guanylate kinase isoform X2 [Ostrinia furnacalis]	1.65908	-1.20038	-0.68858	-0.2762	0.50607
TRINITY_DN32479_c0_g1_i8_orf1	-	-	-	hypothetical protein evm_009815 [Chilo suppressalis] >CAB3525305.1 unnamed protein product [Chilo suppressalis] >CAH0402632.1 unnamed protein product [Chilo suppressalis]	1.7466	-1.28464	-0.54004	0.08587	-0.0078
TRINITY_DN53462_c0_g1_i1_orf1	-	-	-	uncharacterized protein LOC118072968 isoform X1 [Chelonus insularis] >XP_034949073.1 uncharacterized protein LOC118072968 isoform X1 [Chelonus insularis]	1.4066	-1.23373	-0.80254	-0.25835	0.88802
TRINITY_DN313_c0_g1_i5_orf1	-	-	-	collagen alpha-1(X) chain-like [Ostrinia furnacalis]	1.71349	-1.31143	-0.57371	0.09654	0.07511
TRINITY_DN19814_c0_g1_i4_orf1	-	-	-	general odorant-binding protein 28a-like [Ostrinia furnacalis]	1.59117	-1.30764	-0.75236	0.03152	0.43731

TRINITY_DN5244_c0_g1_i1_orf1	-	-	-	eukaryotic peptide chain release factor GTP-binding subunit-like [Ostrinia furnacalis]	1.74602	-1.36914	-0.25412	-0.01258	-0.11017
TRINITY_DN130051_c0_g1_i1_orf1	-	-	-	5-methyltetrahydropteroyltriglutamate--homocysteine S-methyltransferase-like protein [Leptotrombidium deliense]	1.74656	-1.24966	-0.39535	0.28569	-0.38724
TRINITY_DN10940_c0_g1_i10_orfp1	-	-	-	TRINITY_DN10940_c0_g1_i10_m.52163 TRINITY_DN10940_c0_g1::TRINITY_DN10940_c0_g1_i10::g.52163 ORF type:5prime_partial len:248 (-),score=128.24 TRINITY_DN10940_c0_g1_i10:121-864(-)	1.80827	-1.279	-0.16106	-0.16699	-0.20121
TRINITY_DN22664_c0_g1_i1_orf1	-	-	-	larval cuticle protein LCP-14-like [Ostrinia furnacalis]	1.62823	-1.2535	-0.75536	-0.06913	0.44976
TRINITY_DN30704_c0_g1_i1_orf1	-	-	-	cytochrome P450 monooxygenase CYP6AE134v2 [Ostrinia furnacalis]	1.79163	-1.23319	-0.21286	0.11383	-0.4594
TRINITY_DN3504_c0_g1_i4_orfp1	-	-	-	TRINITY_DN3504_c0_g1_i4_m.43930 TRINITY_DN3504_c0_g1::TRINITY_DN3504_c0_g1_i4::g.43930 ORF type:internal len:196 (-	1.59652	-1.53955	-0.22318	0.17611	-0.00991
TRINITY_DN336_c0_g1_i6_orfp1	-	-	-	TRINITY_DN336_c0_g1_i6_m.64791 TRINITY_DN336_c0_g1::TRINITY_DN336_c0_g1_i6::g.64791 ORF type:complete len:61 (-),score=19.53 TRINITY_DN336_c0_g1_i6:236-418(-)	1.84736	-1.09785	0.10981	-0.44869	-0.41063
TRINITY_DN38498_c0_g3_i1_orf1	-	-	-	unnamed protein product [Parnassius apollo]	1.49356	-1.42753	-0.71499	0.39392	0.25504
TRINITY_DN2986_c1_g1_i1_orf1	-	-	-	Troponin C, isoform 1 [Papilio xuthus]	1.64358	-1.24861	-0.70999	-0.14738	0.46241
TRINITY_DN76815_c0_g1_i3_orf1	-	-	-	5-formyltetrahydrofolate cyclo-ligase [Ostrinia furnacalis]	0.7456	-1.32407	-1.0988	1.03474	0.64253
TRINITY_DN36883_c0_g1_i1_orf1	-	-	-	PREDICTED: importin subunit alpha-4 [Microplitis demolitor]	0.50366	1.51373	-1.51133	-0.39926	-0.1068
TRINITY_DN30273_c1_g1_i1_orf1	-	-	-	uncharacterized protein LOC114358591 isoform X2 [Ostrinia furnacalis]	0.20152	1.72656	-1.28769	-0.56017	-0.08021
TRINITY_DN2061_c0_g1_i3_orf1	-	-	-	uncharacterized protein LOC114357318 isoform X1 [Ostrinia furnacalis] >XP_028166680.1 uncharacterized protein LOC114357318 isoform X2 [Ostrinia furnacalis]	0.33181	1.74218	-0.96522	-0.1617	-0.94706
TRINITY_DN1749_c0_g2_i2_orf1	-	-	-	putative GPI-anchored protein pfl2 isoform X1 [Ostrinia furnacalis]	-0.37907	1.97891	-0.77383	-0.40102	-0.42499
TRINITY_DN10745_c0_g1_i14_orf1	-	-	-	septin-1 [Ostrinia furnacalis]	0.63822	1.66325	-0.79901	-0.57928	-0.92319
TRINITY_DN1233_c0_g2_i1_orf1	-	-	-	unnamed protein product [Spodoptera exigua]	0.27197	1.81938	-0.90774	-0.80561	-0.378
TRINITY_DN32956_c0_g1_i4_orf1	-	-	-	inositol-trisphosphate 3-kinase A isoform X1 [Vanessa tameamea] >XP_047534115.1 inositol-trisphosphate 3-kinase A isoform X1 [Vanessa atalanta] >XP_047534116.1 inositol-trisphosphate 3-kinase A isoform X1 [Vanessa atalanta] >XP_047534117.1 inositol-trisphosphate 3-kinase A isoform X1 [Vanessa atalanta]	0.35912	1.75816	-1.06433	-0.74183	-0.31111
TRINITY_DN37585_c0_g2_i1_orf1	-	-	-	cuticle protein 19.8-like [Ostrinia furnacalis]	-0.09465	1.92523	-0.89852	-0.31974	-0.61232
TRINITY_DN2638_c0_g1_i7_orf1	-	-	-	structural maintenance of chromosomes protein 1A [Trichoplusia ni]	0.51516	1.65422	-1.03341	-0.94549	-0.19049
TRINITY_DN14009_c0_g1_i1_orf1	-	-	-	proline-rich extensin-like protein EPR1 [Manduca sexta]	-0.1996	1.9602	-0.81502	-0.52969	-0.41589
TRINITY_DN54524_c0_g1_i6_orf1	-	-	-	serine protease inhibitor dipetalogastin [Ostrinia furnacalis]	-0.3108	1.98843	-0.64609	-0.51015	-0.52139
TRINITY_DN19923_c0_g1_i1_orf1	-	-	-	uncharacterized protein LOC114350958 [Ostrinia furnacalis]	-0.02453	1.94062	-0.57856	-0.62312	-0.71441
TRINITY_DN9100_c0_g1_i5_orf1	-	-	-	microtubule-associated protein futsch-like isoform X6 [Ostrinia furnacalis]	0.26785	1.84266	-0.73382	-0.53547	-0.84122
TRINITY_DN6656_c0_g1_i1_orf1	-	-	-	sorting and assembly machinery component 50 homolog isoform X9 [Ostrinia furnacalis] >XP_028169233.1 sorting and assembly machinery component 50 homolog isoform X10 [Ostrinia furnacalis] >XP_028169234.1 sorting and assembly machinery component 50 homolog isoform X11 [Ostrinia furnacalis]	0.09235	1.87477	-0.98218	-0.37637	-0.60857
TRINITY_DN40669_c0_g2_i1_orf1	-	-	-	uncharacterized protein LOC114355531 [Ostrinia furnacalis]	-0.42646	1.99572	-0.61754	-0.45384	-0.49788
TRINITY_DN46090_c0_g2_i1_orf1	-	-	-	inactive tyrosine-protein kinase 7-like, partial [Ostrinia furnacalis]	-0.01977	1.90951	-0.91646	-0.62782	-0.34545
TRINITY_DN5840_c0_g1_i6_orf1	-	-	-	catenin alpha isoform X2 [Ostrinia furnacalis]	0.45809	1.75117	-0.77218	-0.93616	-0.50092
TRINITY_DN206_c0_g1_i8_orf1	-	-	-	A-kinase anchor protein 200-like [Ostrinia furnacalis] >XP_028173114.1 A-kinase anchor protein 200-like [Ostrinia furnacalis] >XP_028173115.1 A-kinase anchor protein 200-like [Ostrinia furnacalis]	-0.19438	1.94284	-0.90992	-0.35576	-0.48278
TRINITY_DN120593_c0_g1_i1_orf1	-	-	-	SUMO-activating enzyme subunit 1 [Ostrinia furnacalis]	0.5269	1.69562	-1.10433	-0.59452	-0.52368
TRINITY_DN110534_c0_g1_i3_orf1	-	-	-	unnamed protein product [Euphydryas editha]	0.5383	1.67097	-0.63043	-1.15975	-0.4191
TRINITY_DN25870_c0_g2_i6_orf1	-	-	-	homeobox protein extradenticle isoform X3 [Ostrinia furnacalis]	0.40838	1.58092	-1.30428	-0.78868	0.10365
TRINITY_DN668_c0_g1_i4_orf1	-	-	-	fatty acid synthase-like isoform X1 [Ostrinia furnacalis]	-0.11183	1.95841	-0.68499	-0.64509	-0.5165
TRINITY_DN9000_c0_g2_i1_orf1	-	-	-	uncharacterized protein LOC114356585 [Ostrinia furnacalis]	0.32542	1.82252	-0.77121	-0.56574	-0.81099
TRINITY_DN2946_c0_g1_i1_orf1	-	-	-	histidine-rich glycoprotein [Ostrinia furnacalis]	-0.31007	1.98559	-0.63591	-0.42896	-0.61066
TRINITY_DN7735_c1_g1_i1_orf1	-	-	-	cuticular protein CPH [Spodoptera litura]	-0.27846	1.95789	-0.8515	-0.3107	-0.51723
TRINITY_DN1293_c0_g1_i4_orf1	-	-	-	putative fatty acyl-CoA reductase CG5065 [Ostrinia furnacalis]	-0.03767	1.9038	-0.89027	-0.71748	-0.25839
TRINITY_DN13576_c0_g1_i1_orf1	-	-	-	uncharacterized protein LOC114350099 [Ostrinia furnacalis]	-0.38803	1.9852	-0.72876	-0.44578	-0.42263
TRINITY_DN35633_c0_g2_i1_orf1	-	-	-	uncharacterized protein LOC114353024 [Ostrinia furnacalis]	0.62328	1.679	-0.66148	-0.75394	-0.88685
TRINITY_DN20793_c0_g2_i1_orf1	-	-	-	mucin-2-like [Ostrinia furnacalis]	-0.05511	1.94798	-0.61807	-0.69962	-0.57519
TRINITY_DN7803_c0_g1_i2_orf1	-	-	-	membrane-associated protein Hem [Ostrinia furnacalis]	0.09822	1.86127	-0.90172	-0.25188	-0.8059

TRINITY_DN1133_c0_g1_i6_orf1	-	-	-	zinc finger protein 391-like [Ostrinia furnacalis] >XP_028169193.1 zinc finger protein 391-like [Ostrinia furnacalis]	0.34322	1.78491	-1.02561	-0.41591	-0.68661
TRINITY_DN20244_c0_g1_i1_orfp1	-	-	-	uncharacterized protein LOC125235519 [Leguminivora glycinivorella]	0.39736	1.77601	-0.45168	-0.83119	-0.8905
TRINITY_DN1280_c0_g1_i1_orf1	-	-	-	coronin-7 isoform X1 [Ostrinia furnacalis] >XP_028164815.1 coronin-7 isoform X2 [Ostrinia furnacalis] >XP_028164817.1 coronin-7 isoform X3 [Ostrinia furnacalis] >XP_028164818.1 coronin-7 isoform X4 [Ostrinia furnacalis] >XP_028164820.1 coronin-7 isoform X6 [Ostrinia furnacalis] >XP_028164821.1 coronin-7 isoform X7 [Ostrinia furnacalis] >XP_028164822.1 coronin-7 isoform X1 [Ostrinia furnacalis] >XP_028164823.1 coronin-7 isoform X8 [Ostrinia furnacalis] >XP_028164824.1 coronin-7 isoform X9 [Ostrinia furnacalis] >XP_028164825.1 coronin-7 isoform X10 [Ostrinia furnacalis]	0.47808	1.75435	-0.83833	-0.79488	-0.59921
TRINITY_DN4021_c0_g1_i1_orf1	-	-	-	leech-derived tryptase inhibitor C-like [Ostrinia furnacalis]	-0.40822	1.9664	-0.84138	-0.38839	-0.32842
TRINITY_DN5595_c0_g1_i1_orf1	-	-	-	keratin, type I cytoskeletal 10-like [Ostrinia furnacalis]	-0.42006	1.98264	-0.74987	-0.4149	-0.39781
TRINITY_DN5200_c0_g1_i2_orf1	-	-	-	uncharacterized protein LOC114351644 [Ostrinia furnacalis]	0.35279	1.59155	-1.4325	-0.53831	0.02647
TRINITY_DN4898_c0_g1_i7_orf1	-	-	-	annulin-like isoform X3 [Ostrinia furnacalis]	0.24566	1.85648	-0.7939	-0.71384	-0.59439
TRINITY_DN18922_c0_g1_i1_orf1	-	-	-	LOW QUALITY PROTEIN: CCR4-NOT transcription complex subunit 6 [Ostrinia furnacalis]	0.17705	1.4704	-1.39097	-0.77588	0.51941
TRINITY_DN2054_c0_g1_i1_orf1	-	-	-	macrophage mannose receptor 1-like [Ostrinia furnacalis]	0.49434	1.59174	-0.93413	-1.1616	0.00965
TRINITY_DN1935_c0_g1_i1_orf1	-	-	-	adult-specific cuticular protein ACP-22-like [Ostrinia furnacalis]	-0.22591	1.97884	-0.61132	-0.63334	-0.50828
TRINITY_DN806_c0_g2_i1_orf1	-	-	-	uncharacterized protein LOC114355167 [Ostrinia furnacalis]	-0.12068	1.93624	-0.86252	-0.61497	-0.33807
TRINITY_DN9028_c0_g1_i5_orf1	-	-	-	decaprenyl-diphosphate synthase subunit 2-like [Ostrinia furnacalis]	-0.13696	1.91759	-0.96151	-0.26105	-0.55807
TRINITY_DN2450_c0_g1_i6_orf1	-	-	-	oxysterol-binding protein-related protein 9 [Manduca sexta]	0.74341	1.6106	-0.84653	-0.7685	-0.73898
TRINITY_DN486_c0_g1_i5_orf1	-	-	-	adaptor complexes medium subunit family domain-containing protein [Phthorimaea	0.48981	1.39775	-1.54206	-0.60214	0.25662
TRINITY_DN2936_c0_g1_i1_orf1	-	-	-	myosin heavy chain, non-muscle isoform X1 [Hyposmocoma kahamanoa]	0.68481	1.6374	-0.96023	-0.66665	-0.69533
TRINITY_DN13395_c0_g1_i1_orf1	-	-	-	cytoplasmic dynein 1 light intermediate chain 2 [Galleria mellonella]	0.40297	1.78199	-0.7786	-0.88618	-0.52018
TRINITY_DN2345_c0_g1_i4_orf1	-	-	-	chromobox protein homolog 3-like [Ostrinia furnacalis] >XP_028157236.1 chromobox protein homolog 3-like [Ostrinia furnacalis]	0.29718	1.71118	-1.12523	-0.8462	-0.03694
TRINITY_DN14443_c0_g1_i1_orf1	-	-	-	SUMO-activating enzyme subunit 2 [Ostrinia furnacalis]	0.66177	1.60715	-1.12795	-0.73775	-0.40322
TRINITY_DN56993_c0_g1_i4_orf1	-	-	-	polypyrimidine tract-binding protein 1 isoform X11 [Helicoverpa zea]	0.47794	1.67294	-0.93131	-0.18445	-1.03512
TRINITY_DN4144_c0_g1_i7_orf1	-	-	-	uncharacterized protein LOC114350172 [Ostrinia furnacalis]	0.16822	1.87969	-0.56318	-0.84004	-0.64469
TRINITY_DN381_c0_g1_i1_orf1	-	-	-	cuticle protein 8-like [Ostrinia furnacalis]	-0.41266	1.99137	-0.67544	-0.45358	-0.44969
TRINITY_DN9354_c0_g1_i7_orf1	-	-	-	hypothetical protein evm_012205 [Chilo suppressalis] >CAB3527181.1 unnamed protein product [Chilo suppressalis] >CAH0404510.1 unnamed protein product [Chilo suppressalis]	0.54897	1.60529	-0.42741	-1.3367	-0.39016
TRINITY_DN5954_c0_g1_i2_orf1	-	-	-	myosin-VIIa [Ostrinia furnacalis] >XP_028155907.1 myosin-VIIa [Ostrinia furnacalis]	0.10325	1.90547	-0.67944	-0.74645	-0.58283
TRINITY_DN4782_c0_g1_i1_orf1	-	-	-	patched domain-containing protein 3-like [Ostrinia furnacalis]	0.25743	1.47416	-1.46022	-0.67952	0.40815
TRINITY_DN143497_c0_g1_i1_orf1	-	-	-	fibroin heavy chain-like [Ostrinia furnacalis]	-0.1805	1.97035	-0.69863	-0.51687	-0.57436
TRINITY_DN14721_c0_g1_i2_orf1	-	-	-	protein masquerade-like isoform X2 [Ostrinia furnacalis]	0.31441	1.8303	-0.64183	-0.82183	-0.68105
TRINITY_DN123184_c0_g1_i1_orf1	-	-	-	double-strand break repair protein MRE11 [Ostrinia furnacalis]	0.36647	1.61415	-1.25182	-0.82679	0.09798
TRINITY_DN12222_c0_g1_i1_orf1	-	-	-	unnamed protein product [Chilo suppressalis]	0.01492	1.80703	-1.24131	-0.17856	-0.40209
TRINITY_DN5074_c0_g1_i7_orf1	-	-	-	zonadhesin-like [Ostrinia furnacalis]	-0.22879	1.91383	-1.02949	-0.39914	-0.25642
TRINITY_DN8738_c0_g1_i1_orf1	-	-	-	unnamed protein product [Plutella xylostella]	0.60316	1.4732	-1.47983	-0.51968	-0.07685
TRINITY_DN17655_c0_g1_i1_orf1	-	-	-	BRISC and BRCA1-A complex member 1-like [Ostrinia furnacalis]	0.3707	1.69511	-0.6585	-1.2353	-0.17201
TRINITY_DN15865_c0_g1_i1_orf1	-	-	-	carboxylesterase, partial [Ostrinia furnacalis]	0.14796	1.86531	-0.83855	-0.35761	-0.81711
TRINITY_DN286_c0_g1_i2_orf1	-	-	-	uncharacterized protein LOC114361329 [Ostrinia furnacalis]	0.55637	1.69285	-0.66096	-0.54258	-1.04568
TRINITY_DN5568_c0_g2_i2_orf1	-	-	-	carboxypeptidase D isoform X5 [Ostrinia furnacalis]	0.23837	1.83727	-0.93602	-0.42469	-0.71493
TRINITY_DN33893_c0_g1_i1_orf1	-	-	-	high mobility group protein I-like [Ostrinia furnacalis]	0.22334	1.63419	-1.46286	-0.37299	-0.02168
TRINITY_DN5235_c0_g1_i7_orf1	-	-	-	peptidoglycan-recognition protein SA-like [Ostrinia furnacalis]	0.46261	1.73819	-0.75224	-0.45074	-0.99782
TRINITY_DN67649_c0_g1_i1_orf1	-	-	-	proliferating cell nuclear antigen [Ostrinia furnacalis] >XP_028174842.1 proliferating cell nuclear antigen [Ostrinia furnacalis]	0.84402	1.52844	-0.85459	-0.94494	-0.57293
TRINITY_DN535_c1_g1_i2_orf1	-	-	-	protein tramtrack, beta isoform isoform X24 [Bicyclus anynana]	0.39845	1.71138	-0.97251	-0.9686	-0.16871
TRINITY_DN33008_c0_g1_i1_orf1	-	-	-	double-stranded RNA-binding protein Staufen homolog 2 isoform X3 [Helicoverpa armigera]	0.64299	1.6487	-0.79389	-0.50768	-0.99012
TRINITY_DN661_c1_g2_i1_orf1	-	-	-	larval/pupal cuticle protein H1C-like [Ostrinia furnacalis]	0.02299	1.91021	-0.90706	-0.53862	-0.48752
TRINITY_DN4937_c0_g1_i2_orf1	-	-	-	zinc finger protein 778-like [Ostrinia furnacalis]	0.49078	1.36061	-1.59312	-0.5393	0.28103
TRINITY_DN978_c9_g2_i1_orf1	-	-	-	hypothetical protein evm_000959 [Chilo suppressalis]	0.26639	1.84075	-0.88887	-0.67438	-0.54388
TRINITY_DN54366_c0_g1_i1_orf1	-	-	-	protein obstructor-E-like [Ostrinia furnacalis]	0.09528	1.90817	-0.73496	-0.5838	-0.68469
TRINITY_DN1853_c0_g1_i3_orf1	-	-	-	trans-Golgi network integral membrane protein TGN38-like isoform X1 [Ostrinia furnacalis]	0.40468	1.67924	-1.30177	-0.48029	-0.30185

TRINITY_DN17003_c0_g1_i1_orf1	-	-	-	mucin-5AC [Ostrinia furnacalis]	0.62395	1.65808	-0.92571	-0.88396	-0.47236
TRINITY_DN5211_c0_g1_i1_orf1	-	-	-	elongation of very long chain fatty acids protein AAEL008004-like [Ostrinia furnacalis]	0.67653	1.64229	-0.94129	-0.76072	-0.61681
TRINITY_DN1326_c0_g1_i1_orf1	-	-	-	cuticle protein 7-like [Ostrinia furnacalis]	-0.08838	1.94666	-0.48042	-0.79532	-0.58254
TRINITY_DN3310_c0_g1_i1_orf1	-	-	-	hypothetical protein evm_010516 [Chilo suppressalis]	-0.10627	1.96004	-0.60367	-0.60124	-0.64886
TRINITY_DN27321_c0_g1_i1_orf1	-	-	-	ras-related protein Rap-2c [Bicyclus anynana] >XP_026492616.1 ras-related protein Rap-2c [Vanessa tameamea] >XP_034838061.1 ras-related protein Rap-2c [Maniola hyperantus] >XP_039759141.1 ras-related protein Rap-2c [Pararge aegeria] >XP_045498804.1 ras-related protein Rap-2c [Colias croceus] >XP_046959644.1 ras-related protein Rap-2c [Vanessa cardui] >XP_047530248.1 ras-related protein Rap-2c [Vanessa atalanta] >CAH2268047.1 jg10357 [Pararge aegeria aegeria]	0.36854	1.74117	-1.18346	-0.42477	-0.50147
TRINITY_DN467_c0_g3_i1_orf1	-	-	-	histone-lysine N-methyltransferase 2B-like, partial [Ostrinia furnacalis]	0.0258	1.92398	-0.79222	-0.58359	-0.57397
TRINITY_DN650_c0_g1_i3_orf1	-	-	-	chitinase 7 [Glyphodes pyloalis]	0.28102	1.80654	-0.76033	-0.34854	-0.9787
TRINITY_DN73224_c0_g4_i2_orf1	-	-	-	PREDICTED: poly(rC)-binding protein 3 isoform X2 [Vollenhovia emeryi]	0.62198	1.67414	-0.82322	-0.5816	-0.89131
TRINITY_DN34745_c0_g2_i1_orf1	-	-	-	GSK3-beta interaction protein-like [Galleria mellonella]	0.77465	1.57009	-1.02732	-0.58269	-0.73473
TRINITY_DN9311_c0_g1_i1_orf1	-	-	-	cuticle protein 8-like [Ostrinia furnacalis]	-0.19981	1.96093	-0.75924	-0.63607	-0.3658
TRINITY_DN2004_c0_g1_i20_orf1	-	-	-	hypothetical protein evm_006436 [Chilo suppressalis] >CAB3522373.1 unnamed protein product [Chilo suppressalis] >CAH0399695.1 unnamed protein product [Chilo suppressalis]	0.29388	1.56354	-1.32087	-0.80695	0.27041
TRINITY_DN24218_c0_g1_i1_orf1	-	-	-	uncharacterized protein LOC114362624 [Ostrinia furnacalis]	0.25686	1.48548	-1.45845	-0.67109	0.3872
TRINITY_DN44256_c0_g1_i1_orf1	-	-	-	essential MCU regulator, mitochondrial [Cotesia glomerata]	0.6127	1.63641	-1.09179	-0.37241	-0.78491
TRINITY_DN27723_c0_g1_i1_orf1	-	-	-	putative uncharacterized protein DDB_G0282133 isoform X1 [Ostrinia furnacalis]	0.42957	1.72456	-1.07641	-0.31304	-0.76467
TRINITY_DN267_c0_g1_i1_orf1	-	-	-	keratin, type I cytoskeletal 9-like [Ostrinia furnacalis]	-0.27097	1.95928	-0.81698	-0.57835	-0.293
TRINITY_DN44658_c0_g1_i2_orf1	-	-	-	lipase 3-like [Ostrinia furnacalis]	0.44793	1.55827	-1.46087	-0.48284	-0.06249
TRINITY_DN14532_c0_g1_i1_orf1	-	-	-	pupal cuticle protein-like [Trichoplusia ni]	0.09595	1.89689	-0.73319	-0.45422	-0.80543
TRINITY_DN34703_c0_g1_i4_orf1	-	-	-	gamma-tubulin complex component 3 homolog [Ostrinia furnacalis]	0.26529	1.63962	-1.46281	-0.1778	-0.26429
TRINITY_DN7549_c0_g1_i1_orf1	-	-	-	uncharacterized protein LOC114355006 [Ostrinia furnacalis]	-0.20979	1.96663	-0.78469	-0.48008	-0.49208
TRINITY_DN1231_c0_g1_i4_orf1	-	-	-	AN1-type zinc finger protein 6 isoform X1 [Galleria mellonella]	0.54684	1.66429	-1.08606	-0.31893	-0.80614
TRINITY_DN6436_c0_g1_i1_orf1	-	-	-	serine/threonine-protein kinase PAK 3 isoform X1 [Ostrinia furnacalis] >XP_028164178.1 serine/threonine-protein kinase PAK 3 isoform X2 [Ostrinia furnacalis] >XP_028164179.1 serine/threonine-protein kinase PAK 3 isoform X3 [Ostrinia furnacalis]	0.64813	1.64997	-0.71116	-1.00864	-0.5783
TRINITY_DN7785_c0_g1_i1_orf1	-	-	-	uncharacterized protein LOC114364098 [Ostrinia furnacalis]	-0.03754	1.93856	-0.73982	-0.67824	-0.48295
TRINITY_DN98242_c0_g1_i1_orf1	-	-	-	adenosine deaminase 2-A-like [Galleria mellonella]	0.21135	1.82519	-0.96106	-0.78476	-0.29072
TRINITY_DN31619_c0_g1_i2_orf1	-	-	-	endocuticle structural glycoprotein ABD-4-like [Ostrinia furnacalis]	0.11217	1.89043	-0.84318	-0.70345	-0.45596
TRINITY_DN6586_c0_g1_i1_orf1	-	-	-	fatty acyl-CoA reductase wat-like isoform X1 [Ostrinia furnacalis]	0.49127	1.75321	-0.80278	-0.74516	-0.69654
TRINITY_DN1868_c0_g1_i1_orf1	-	-	-	protein obstructor-E isoform X1 [Ostrinia furnacalis]	0.47972	1.73191	-0.48081	-0.72168	-1.00914
TRINITY_DN147458_c0_g1_i1_orf1	-	-	-	60S ribosomal protein L5, partial [Cotesia chilonis]	0.25867	1.77783	-1.16482	-0.56941	-0.30228
TRINITY_DN2101_c0_g1_i6_orf1	-	-	-	protein obstructor-E-like [Ostrinia furnacalis]	-0.17414	1.96441	-0.76018	-0.54936	-0.48073
TRINITY_DN1124_c0_g1_i7_orf1	-	-	-	PREDICTED: cuticle protein 18.6, isoform B [Amyelois transitella]	-0.28224	1.96466	-0.84002	-0.42426	-0.41813
TRINITY_DN778_c0_g1_i1_orf1	-	-	-	uncharacterized protein LOC114363281 [Ostrinia furnacalis]	-0.09954	1.93848	-0.86207	-0.41067	-0.56621
TRINITY_DN72816_c0_g1_i2_orf1	-	-	-	Golgi apparatus protein 1 [Ostrinia furnacalis]	0.5674	1.58243	-1.35651	-0.49453	-0.2988
TRINITY_DN9916_c0_g1_i1_orf1	-	-	-	PREDICTED: dynein light chain Tctex-type [Amyelois transitella] >XP_021195381.1 dynein light chain Tctex-type [Helicoverpa armigera] >XP_022815696.1 dynein light chain Tctex-type [Spodoptera litura] >XP_028156399.1 dynein light chain Tctex-type [Ostrinia furnacalis] >XP_035458261.1 dynein light chain Tctex-type-like [Spodoptera frugiperda] >XP_047034788.1 dynein light chain Tctex-type [Helicoverpa zea] >CAB3233358.1 unnamed protein product [Arctia plantaginis] >CAB3506583.1 unnamed protein product [Spodoptera littoralis] >CAG9754627.1 unnamed protein product [Diatraea saccharalis] >CAH0596395.1 unnamed protein product [Chrysodeixis includens] >KAF9808454.1 hypothetical protein SFRURICE_008507 [Spodoptera frugiperda]	0.54546	1.68231	-1.10651	-0.4623	-0.65896
TRINITY_DN1074_c0_g1_i7_orf1	-	-	-	eukaryotic translation initiation factor 4E type 2 [Ostrinia furnacalis]	0.74375	1.58005	-1.07951	-0.5486	-0.69569
TRINITY_DN3255_c0_g1_i1_orf1	-	-	-	uncharacterized protein LOC114351042 [Ostrinia furnacalis]	0.03529	1.91909	-0.82003	-0.57152	-0.56282
TRINITY_DN10057_c0_g2_i1_orf1	-	-	-	cell wall protein DAN4 [Ostrinia furnacalis]	0.50736	1.74491	-0.80897	-0.69112	-0.75219
TRINITY_DN4125_c0_g1_i14_orf1	-	-	-	angiotensin-converting enzyme-like isoform X1 [Ostrinia furnacalis]	0.12597	1.88712	-0.88665	-0.52913	-0.59731
TRINITY_DN113272_c0_g1_i1_orf1	-	-	-	altered inheritance of mitochondria protein 3-like [Ostrinia furnacalis]	0.12477	1.89062	-0.85699	-0.53125	-0.62715

TRINITY_DN72999_c0_g1_i1_orf1	-	-	-	protein obstructor-E-like isoform X1 [Ostrinia furnacalis] >XP_028169319.1 protein obstructor-E-like isoform X2 [Ostrinia furnacalis]	0.33894	1.81549	-0.87502	-0.68965	-0.58976
TRINITY_DN21124_c0_g1_i4_orf1	-	-	-	calsyntenin-1 [Ostrinia furnacalis]	0.69195	1.64206	-0.78044	-0.70977	-0.84379
TRINITY_DN59829_c0_g1_i1_orf1	-	-	-	putative mediator of RNA polymerase II transcription subunit 12 [Ostrinia furnacalis]	0.78609	1.56881	-0.90348	-0.88593	-0.56548
TRINITY_DN619_c0_g1_i1_orf1	-	-	-	putative uncharacterized protein DDB_G0271606 [Ostrinia furnacalis]	-0.03825	1.93351	-0.80031	-0.64778	-0.44717
TRINITY_DN20767_c0_g2_i1_orf1	-	-	-	glycosylated lysosomal membrane protein B-like [Vanessa atalanta]	0.80863	1.53935	-1.08481	-0.66228	-0.6009
TRINITY_DN48590_c0_g1_i1_orf1	-	-	-	acyl-CoA Delta(11) desaturase isoform X1 [Ostrinia furnacalis] >XP_028172999.1 acyl-CoA Delta(11) desaturase isoform X2 [Ostrinia furnacalis] >XP_028173000.1 acyl-CoA Delta(11) desaturase isoform X1 [Ostrinia furnacalis]	0.24769	1.85729	-0.78924	-0.6396	-0.67612
TRINITY_DN2840_c0_g1_i5_orf1	-	-	-	hypothetical protein evm_002181 [Chilo suppressalis]	0.66718	1.65421	-0.8062	-0.65603	-0.85915
TRINITY_DN18502_c0_g1_i1_orf1	-	-	-	uncharacterized protein LOC114359515 [Ostrinia furnacalis]	0.22858	1.86481	-0.7772	-0.65491	-0.66128
TRINITY_DN644_c0_g1_i1_orf1	-	-	-	cuticle protein 19-like [Ostrinia furnacalis]	-0.08346	1.94638	-0.78721	-0.48155	-0.59417
TRINITY_DN10479_c0_g1_i6_orf1	-	-	-	unnamed protein product [Chrysodeixis includens]	0.52515	1.70627	-1.04341	-0.49794	-0.69007
TRINITY_DN9282_c0_g1_i2_orf1	-	-	-	uncharacterized protein LOC114363102 isoform X2 [Ostrinia furnacalis]	-0.04004	1.93271	-0.84528	-0.52116	-0.52623
TRINITY_DN42337_c0_g1_i6_orf1	-	-	-	cuticle protein 8-like [Leguminivora glycinivorella]	0.4195	1.75362	-1.00835	-0.41849	-0.74628
TRINITY_DN7291_c0_g1_i5_orf1	-	-	-	dynamin-1-like protein isoform X3 [Ostrinia furnacalis]	0.40715	-0.75952	-1.37916	1.52005	0.21148
TRINITY_DN2299_c0_g1_i3_orf1	-	-	-	DNA-directed RNA polymerase II subunit RPB1 [Ostrinia furnacalis]	0.00728	-1.30263	-0.82853	1.4869	0.63699
TRINITY_DN11172_c0_g1_i4_orf1	-	-	-	juvenile hormone epoxide hydrolase-like isoform X1 [Ostrinia furnacalis] >XP_028170522.1 juvenile hormone epoxide hydrolase-like isoform X2 [Ostrinia furnacalis]	0.17447	-0.90737	-1.21212	1.59919	0.34583
TRINITY_DN131603_c0_g1_i4_orfp1	-	-	-	TRINITY_DN131603_c0_g1_i4_m.86149 TRINITY_DN131603_c0_g1::TRINITY_DN131603_c0_g1_i4::g.86149 ORF type:internal len:112 (-),score=8.40 TRINITY_DN131603_c0_g1_i4:2-334(-)	-0.38291	-0.83367	-0.66454	1.92734	-0.04622
TRINITY_DN19584_c0_g1_i2_orf1	-	-	-	protein NDUFAF4 homolog [Ostrinia furnacalis]	1.16079	0.86017	-1.10297	0.33938	-1.25736
TRINITY_DN9661_c0_g1_i1_orf1	-	-	-	tetratricopeptide repeat protein 37 [Ostrinia furnacalis]	0.43134	0.63665	-1.34729	1.26979	-0.99049
TRINITY_DN8846_c0_g1_i1_orf1	-	-	-	PREDICTED: synapse-associated protein of 47 kDa-like isoform X2 [Papilio xuthus]	0.63415	0.67532	-1.39536	1.08962	-1.00374
TRINITY_DN13287_c0_g1_i5_orf1	-	-	-	dystrophin-like, partial [Ostrinia furnacalis]	1.22639	0.76592	-1.28919	0.35559	-1.05871
TRINITY_DN11566_c0_g1_i6_orf1	-	-	-	lens fiber major intrinsic protein-like isoform X1 [Ostrinia furnacalis]	0.32666	1.23343	-0.93772	0.76096	-1.38332
TRINITY_DN4571_c0_g1_i4_orf1	-	-	-	PREDICTED: nuclear factor NF-kappa-B p105 subunit [Microplitis demolitor] >KAG6558391.1 viral ankyrin V1 [Microplitis demolitor]	0.33373	1.59802	-1.2923	0.1601	-0.79955
TRINITY_DN4732_c0_g1_i2_orf1	-	-	-	reversion-inducing cysteine-rich protein with Kazal motifs [Ostrinia furnacalis]	0.67687	0.93126	-0.67733	0.71414	-1.64494
TRINITY_DN2201_c0_g1_i1_orf1	-	-	-	pleiotropic regulator 1 [Ostrinia furnacalis]	0.28406	1.32447	-0.76992	0.63442	-1.47303
TRINITY_DN6362_c0_g1_i4_orf1	-	-	-	sodium/hydrogen exchanger 7 isoform X4 [Galleria mellonella]	0.31527	1.49114	-0.98583	0.41692	-1.23751
TRINITY_DN7391_c0_g1_i2_orf1	-	-	-	hypothetical protein evm_000945 [Chilo suppressalis] >CAB3528924.1 unnamed protein product [Chilo suppressalis] >CAH0405517.1 unnamed protein product [Chilo suppressalis]	1.10889	1.10367	-0.62127	-0.12475	-1.46654
TRINITY_DN10415_c0_g1_i5_orf1	-	-	-	hypothetical protein evm_000184 [Chilo suppressalis]	0.36515	1.09883	-1.66936	0.75514	-0.54976
TRINITY_DN111_c0_g2_i2_orf1	-	-	-	hypothetical protein O3G_MSEX007696 [Manduca sexta] >KAG6452639.1 hypothetical protein O3G_MSEX007696 [Manduca sexta]	0.69763	0.44591	-1.64922	1.10919	-0.60353
TRINITY_DN87603_c0_g2_i1_orf1	-	-	-	40S ribosomal protein S3-3, partial [Trichinella patagoniensis]	1.15059	0.78355	-0.88121	0.40394	-1.45687
TRINITY_DN103118_c0_g1_i4_orf1	-	-	-	hypothetical protein evm_006930 [Chilo suppressalis]	0.9054	0.92991	-1.74185	0.3255	-0.41896
TRINITY_DN21125_c0_g1_i1_orf1	-	-	-	protein angel homolog 1 isoform X3 [Ostrinia furnacalis]	0.56427	1.14014	-1.52884	0.62947	-0.80504
TRINITY_DN5829_c0_g2_i1_orf1	-	-	-	uncharacterized protein LOC114365758 isoform X2 [Ostrinia furnacalis]	0.40148	1.60042	-1.20494	0.10551	-0.90247
TRINITY_DN8596_c0_g1_i2_orf1	-	-	-	SWI/SNF-related matrix-associated actin-dependent regulator of chromatin subfamily E member 1-like isoform X2 [Ostrinia furnacalis]	1.05962	1.24594	-0.58651	-0.35789	-1.36116
TRINITY_DN10630_c0_g1_i2_orf1	-	-	-	J domain-containing protein [Ostrinia furnacalis]	-0.11294	1.55349	-1.17141	0.62881	-0.89796
TRINITY_DN467_c4_g1_i2_orf1	-	-	-	GRIP and coiled-coil domain-containing protein 1 [Ostrinia furnacalis]	0.66761	1.50173	-0.87197	-0.05826	-1.23911
TRINITY_DN14019_c0_g1_i5_orf1	-	-	-	hypothetical protein evm_009768 [Chilo suppressalis]	0.73439	1.31237	-0.71739	0.15388	-1.48325
TRINITY_DN1628_c0_g1_i1_orf1	-	-	-	uncharacterized protein LOC114363979 [Ostrinia furnacalis]	0.75716	1.38104	-1.21504	0.09384	-1.017
TRINITY_DN1763_c0_g3_i2_orf1	-	-	-	heterogeneous nuclear ribonucleoprotein H-like isoform X2 [Ostrinia furnacalis]	1.22535	0.84071	-1.38465	0.226	-0.90741
TRINITY_DN4194_c0_g1_i1_orf1	-	-	-	hornerin-like [Ostrinia furnacalis]	1.06503	1.06503	-1.29814	0.17575	-1.00767
TRINITY_DN5422_c0_g1_i1_orf1	-	-	-	nitrilase and fragile histidine triad fusion protein NitFhit isoform X1 [Ostrinia furnacalis]	-0.18386	1.06481	-1.80873	0.71931	0.20846
TRINITY_DN11942_c0_g1_i1_orf1	-	-	-	hypothetical protein B5X24_HaOG213660 [Helicoverpa armigera]	-0.07791	0.37672	-1.84237	0.4068	1.13675
TRINITY_DN578_c0_g1_i3_orf1	-	-	-	charged multivesicular body protein 7 [Ostrinia furnacalis]	-0.32771	0.87929	-1.81415	0.50624	0.75633
TRINITY_DN667_c0_g1_i5_orf1	-	-	-	unnamed protein product [Arctia plantaginis]	0.08972	1.51892	-1.62274	0.16741	-0.15331
TRINITY_DN13419_c0_g1_i5_orf1	-	-	-	atrial natriuretic peptide-converting enzyme-like [Ostrinia furnacalis]	-0.32709	1.4342	-1.53747	0.65088	-0.22051

TRINITY_DN1492_c0_g1_i4_orf1	-	-	-	sarcoplasmic calcium-binding protein isoform X2 [Ostrinia furnacalis]	-0.02623	0.9704	-1.88812	0.31849	0.62545
TRINITY_DN3209_c0_g1_i1_orf1	-	-	-	coatomer subunit beta [Helicoverpa armigera]	0.29805	0.55068	-1.93355	0.16767	0.91716
TRINITY_DN2782_c0_g1_i7_orf1	-	-	-	CDK5 regulatory subunit-associated protein 3 [Ostrinia furnacalis]	0.17231	0.88046	-1.90217	0.09597	0.75343
TRINITY_DN53760_c0_g1_i1_orf1	-	-	-	unnamed protein product [Parnassius apollo]	0.49156	0.41814	-1.86928	-0.08098	1.04056
TRINITY_DN5475_c0_g1_i3_orf1	-	-	-	traB domain-containing protein-like isoform X1 [Ostrinia furnacalis] >XP_028169655.1 traB domain-containing protein-like isoform X1 [Ostrinia furnacalis]	0.3356	1.1853	-1.85052	0.12178	0.20784
TRINITY_DN1520_c0_g1_i9_orf1	-	-	-	adipocyte plasma membrane-associated protein-like [Ostrinia furnacalis] >XP_028176496.1 adipocyte plasma membrane-associated protein-like [Ostrinia furnacalis]	0.44121	0.73	-1.98256	0.32577	0.48557
TRINITY_DN95056_c0_g2_i2_orf1	-	-	-	40S ribosomal protein S18 [Halotydeus destructor]	0.60105	0.39375	-1.98359	0.32086	0.66793
TRINITY_DN10183_c0_g2_i3_orf1	-	-	-	uncharacterized protein LOC114360370 isoform X1 [Ostrinia furnacalis]	0.35447	1.1603	-1.84591	-0.01586	0.34699
TRINITY_DN27456_c0_g2_i1_orf1	-	-	-	organic cation transporter-like protein [Ostrinia furnacalis]	-0.31886	1.62381	-1.48123	0.24935	-0.07308
TRINITY_DN71832_c0_g1_i1_orf1	-	-	-	basement membrane-specific heparan sulfate proteoglycan core protein isoform X13 [Ostrinia furnacalis]	-0.18084	1.42419	-1.6494	0.46383	-0.05778
TRINITY_DN14347_c0_g1_i1_orf1	-	-	-	putative nuclease HARBI1 [Ostrinia furnacalis]	0.54349	0.57108	-1.96711	0.15653	0.69603
TRINITY_DN29144_c0_g3_i1_orf1	-	-	-	PREDICTED: ADP-ribosylation factor 6 [Papilio polytes] >XP_013133321.1 PREDICTED: ADP-ribosylation factor 6 [Papilio polytes] >XP_013177129.1 PREDICTED: ADP-ribosylation factor 6 [Papilio xuthus] >XP_013177130.1 PREDICTED: ADP-ribosylation factor 6 [Papilio xuthus] >XP_014356507.1 ADP-ribosylation factor 6 [Papilio machaon] >XP_021185579.1 ADP-ribosylation factor 6 [Helicoverpa armigera] >XP_021185581.1 ADP-ribosylation factor 6 [Helicoverpa armigera] >XP_022130228.1 ADP-ribosylation factor 6 [Pieris rapae] >XP_022822139.1 ADP-ribosylation factor 6 [Spodoptera litura] >XP_022822140.1 ADP-ribosylation factor 6 [Spodoptera litura] >XP_028159104.1 ADP-ribosylation factor 6 [Ostrinia furnacalis] >XP_028159105.1 ADP-ribosylation factor 6 [Ostrinia furnacalis] >XP_028159106.1 ADP-ribosylation factor 6 [Ostrinia furnacalis] >XP_028159107.1 ADP-ribosylation factor 6 [Ostrinia furnacalis] >XP_028163222.1 ADP-ribosylation factor 6 [Ostrinia furnacalis] >XP_030022165.1 ADP-ribosylation factor 6 [Manduca sexta] >XP_030022166.1 ADP-ribosylation factor 6 [Manduca sexta] >XP_030022167.1 ADP-ribosylation factor 6 [Manduca sexta] >XP_035444169.1 ADP-ribosylation factor 6 [Spodoptera frugiperda] >XP_035444175.1 ADP-ribosylation factor 6 [Spodoptera frugiperda] >XP_038207597.1 ADP-ribosylation factor 6 [Zerene cesonia] >XP_038207598.1 ADP-ribosylation factor 6 [Zerene cesonia] >XP_045510541.1 ADP-ribosylation factor 6 [Colias croceus] >XP_045510551.1 ADP-ribosylation factor 6 [Colias croceus] >XP_045527300.1 ADP-ribosylation factor 6 [Pieris brassicae] >XP_045527302.1 ADP-ribosylation factor 6 [Pieris brassicae] >XP_047029519.1 ADP-ribosylation factor 6 [Helicoverpa zea] >XP_047029551.1 ADP-ribosylation factor 6 [Helicoverpa zea] >XP_047504621.1 ADP-ribosylation factor 6 [Pieris napi] >XP_047504631.1 ADP-ribosylation factor 6 [Pieris napi] >XP_047504640.1 ADP-ribosylation factor 6 [Pieris napi] >XP_047504648.1 ADP-ribosylation factor 6 [Pieris napi] >XP_047504657.1 ADP-ribosylation factor 6 [Pieris napi] >XP_048489067.1 ADP-ribosylation factor 6 [Plutella xylostella] >XP_048489068.1 ADP-ribosylation factor 6 [Plutella xylostella] >XP_048489069.1 ADP-ribosylation factor 6 [Plutella xylostella] >XP_049883531.1 ADP-ribosylation factor 6 [Pectinophora gossypiella] >XP_049883539.1 ADP-ribosylation factor 6 [Pectinophora gossypiella] >KAG5678369.1 hypothetical protein PVAND_008051 [Polypedilum vanderplanki] >RVE51130.1 hypothetical protein evm_004273 [Chilo suppressalis] >CAB3510283.1 unnamed protein product [Spodoptera littoralis] >CAF4796780.1 unnamed protein product [Pieris	-0.31623	0.38536	-1.63045	1.44183	0.1195
TRINITY_DN23962_c0_g1_i3_orf1	-	-	-	uncharacterized protein LOC124629606 [Helicoverpa zea]	1.60846	-0.79166	-0.9891	0.71582	-0.54353
TRINITY_DN49204_c0_g1_i1_orf1	-	-	-	uncharacterized protein C05D11.1-like [Chelonus insularis]	1.2065	-1.24982	-0.14998	1.08182	-0.88852
TRINITY_DN9198_c0_g1_i4_orf1	-	-	-	4-coumarate--CoA ligase 1-like isoform X1 [Ostrinia furnacalis]	0.97603	-1.57035	-0.13893	1.17075	-0.4375
TRINITY_DN72285_c1_g1_i1_orf1	-	-	-	hypothetical protein HF086_001747 [Spodoptera exigua]	1.34585	-0.81048	-1.25552	0.95006	-0.22991
TRINITY_DN22836_c0_g1_i5_orf1	-	-	-	AP-3 complex subunit beta-2 [Ostrinia furnacalis]	1.72058	-0.8773	-0.90956	0.50242	-0.43614
TRINITY_DN16354_c0_g1_i2_orf1	-	-	-	uncharacterized protein LOC114349750 isoform X1 [Ostrinia furnacalis]	1.54927	-1.29884	-0.26826	0.65724	-0.63941
TRINITY_DN1153_c1_g1_i1_orf1	-	-	-	gamma-butyrobetaine dioxygenase [Ostrinia furnacalis]	1.20122	-0.66034	-0.88104	1.23941	-0.89924
TRINITY_DN667_c0_g1_i13_orf1	-	-	-	enoyl-CoA hydratase domain-containing protein 2, mitochondrial [Ostrinia furnacalis]	1.78304	-0.98981	-0.4798	0.37326	-0.68669
TRINITY_DN22_c0_g1_i3_orf1	-	-	-	uncharacterized protein LOC114362831 [Ostrinia furnacalis]	0.5966	-0.24355	-1.01446	1.62239	-0.96098
TRINITY_DN20527_c0_g1_i1_orf1	-	-	-	dihydrofolate reductase [Ostrinia furnacalis]	1.131	-0.59439	-0.34059	1.17328	-1.36929

TRINITY_DN12293_c0_g1_i1_orf1	-	-	-	hypothetical protein evm_011848 [Chilo suppressalis]	1.25537	-1.20215	-0.85741	1.08047	-0.27627
TRINITY_DN7247_c0_g1_i6_orf1	-	-	-	pyruvate kinase-like isoform X2 [Ostrinia furnacalis]	0.35026	-0.01199	-1.25269	1.65926	-0.74485
TRINITY_DN5991_c0_g1_i6_orf1	-	-	-	uncharacterized protein LOC114357071 [Ostrinia furnacalis]	1.04546	-0.85134	-1.28377	1.23057	-0.14092
TRINITY_DN11746_c0_g2_i1_orf1	-	-	-	splicing factor 3B subunit 1 isoform X1 [Diprion similis]	1.63677	-0.91969	-0.94658	0.6405	-0.41099
TRINITY_DN141396_c0_g1_i1_orf1	-	-	-	pre-rRNA 2'-O-ribose RNA methyltransferase FTSJ3 [Diachasma alloeum]	1.45945	-0.68207	-1.20132	0.87218	-0.44824
TRINITY_DN5210_c0_g1_i3_orf1	-	-	-	uncharacterized protein LOC114358376 isoform X2 [Ostrinia furnacalis] >XP_028168126.1 uncharacterized protein LOC114358376 isoform X2 [Ostrinia furnacalis] >XP_028168127.1 uncharacterized protein LOC114358376 isoform X2 [Ostrinia furnacalis] >XP_028168128.1 uncharacterized protein LOC114358376 isoform X2 [Ostrinia furnacalis]	1.38637	-0.86502	-1.12982	0.96306	-0.35459
TRINITY_DN2473_c0_g1_i2_orf1	-	-	-	translation initiation factor eIF-2B subunit delta [Ostrinia furnacalis]	1.17463	-1.08948	-1.22201	0.95167	0.18519
TRINITY_DN32420_c0_g1_i2_orf1	-	-	-	PREDICTED: plectin-like, partial [Papilio polytes]	1.35326	-1.39797	-0.1274	0.85525	-0.68314
TRINITY_DN863_c0_g1_i6_orf1	-	-	-	protein henna [Galleria mellonella]	1.48487	-1.34925	-0.02115	0.63836	-0.75282
TRINITY_DN1753_c1_g1_i8_orf1	-	-	-	serine/threonine-protein kinase WNK1-like isoform X15 [Ostrinia furnacalis]	1.28556	-1.0757	-0.71757	1.13317	-0.62546
TRINITY_DN1416_c0_g2_i1_orf1	-	-	-	uncharacterized protein LOC114352565 [Ostrinia furnacalis]	1.18042	-0.90404	-0.83625	1.26311	-0.70324
TRINITY_DN2120_c0_g1_i2_orf1	-	-	-	cullin-4A [Ostrinia furnacalis]	1.65547	-1.12585	-0.54091	0.59693	-0.58563
TRINITY_DN124711_c0_g1_i1_orf1	-	-	-	muskelin isoform X1 [Ostrinia furnacalis] >XP_028163274.1 muskelin isoform X2 [Ostrinia furnacalis]	0.5918	-0.87302	-0.73702	1.69782	-0.67958
TRINITY_DN14935_c0_g1_i1_orf1	-	-	-	kynurenine/alpha-aminoadipate aminotransferase, mitochondrial [Ostrinia furnacalis]	1.21739	-1.2248	-0.20722	1.09521	-0.88058
TRINITY_DN8717_c0_g1_i5_orf1	-	-	-	hypothetical protein evm_006607 [Chilo suppressalis] >CAG9745590.1 unnamed protein product [Diatraea saccharalis] >CAG9784275.1 unnamed protein product [Diatraea saccharalis]	1.75836	-0.94121	-0.54158	0.45001	-0.72559
TRINITY_DN3732_c0_g1_i2_orf1	-	-	-	cytochrome P450 monooxygenase CYP6AB141 [Ostrinia furnacalis]	0.8724	-0.26095	-0.91698	1.43403	-1.12851
TRINITY_DN5161_c0_g1_i5_orf1	-	-	-	glyoxylate reductase/hydroxypyruvate reductase-like isoform X1 [Ostrinia furnacalis]	1.27965	-1.27299	-0.89103	0.96993	-0.08555
TRINITY_DN6994_c0_g1_i4_orf1	-	-	-	C-type mannose receptor 2-like isoform X1 [Ostrinia furnacalis]	1.60834	-0.99004	-0.63718	0.72599	-0.70711
TRINITY_DN25901_c0_g1_i2_orf1	-	-	-	short-chain specific acyl-CoA dehydrogenase, mitochondrial-like isoform X2 [Ostrinia	1.64098	-1.16517	-0.54409	0.60476	-0.53648
TRINITY_DN7329_c0_g1_i6_orf1	-	-	-	serine hydrolase-like protein 2 isoform X2 [Ostrinia furnacalis]	1.69782	-0.62332	-0.58172	0.55019	-1.04297
TRINITY_DN1914_c0_g1_i4_orf1	-	-	-	loricrin-like [Ostrinia furnacalis]	0.81246	-1.52621	-0.1449	1.32959	-0.47094
TRINITY_DN15513_c0_g1_i6_orf1	-	-	-	uncharacterized protein LOC114350859 [Ostrinia furnacalis]	1.63254	-1.33514	-0.24683	0.46969	-0.52026
TRINITY_DN5962_c0_g1_i1_orf1	-	-	-	tRNA (cytosine(34)-C(5))-methyltransferase [Ostrinia furnacalis]	0.86401	-1.11525	-0.49737	1.48833	-0.73972
TRINITY_DN5126_c0_g2_i1_orf1	-	-	-	cytochrome P450 monooxygenase CYP4L47 [Ostrinia furnacalis]	0.34986	-0.9471	-0.59261	1.80355	-0.6137
TRINITY_DN36496_c0_g1_i1_orf1	-	-	-	unnamed protein product [Parnassius apollo]	1.11062	-0.0369	-1.12772	1.14323	-1.08924
TRINITY_DN1999_c0_g1_i9_orf1	-	-	-	acyl-CoA Delta(11) desaturase-like [Ostrinia furnacalis] >XP_028172986.1 acyl-CoA Delta(11) desaturase-like [Ostrinia furnacalis] >AAL27034.1 acyl-CoA delta-9 desaturase [Ostrinia furnacalis] >AAL29454.1 acyl-CoA delta-9 desaturase [Ostrinia nubilalis]	1.40037	-0.32373	-1.05225	0.94348	-0.96787
TRINITY_DN3964_c1_g1_i2_orf1	-	-	-	phosphoinositide 3-kinase regulatory subunit 4 isoform X1 [Ostrinia furnacalis] >XP_028172384.1 phosphoinositide 3-kinase regulatory subunit 4 isoform X5 [Ostrinia	1.23654	-0.99815	-1.15904	1.05509	-0.13444
TRINITY_DN3949_c1_g1_i1_orf1	-	-	-	probable cytochrome P450 304a1 isoform X2 [Ostrinia furnacalis]	1.59507	-0.95942	-0.6473	0.75289	-0.74124
TRINITY_DN2749_c0_g2_i3_orf1	-	-	-	RNA exonuclease 4-like [Ostrinia furnacalis] >QEE79882.1 REX4 [Ostrinia furnacalis]	1.21072	-0.69486	-0.47488	1.16799	-1.20897
TRINITY_DN1656_c2_g1_i5_orf1	-	-	-	15-hydroxyprostaglandin dehydrogenase [NAD(+)]-like [Ostrinia furnacalis]	1.45587	-1.32828	-0.42561	0.81631	-0.51829
TRINITY_DN4795_c0_g1_i2_orf1	-	-	-	D-amino-acid oxidase isoform X1 [Ostrinia furnacalis]	1.69032	-0.75546	-1.00517	0.56396	-0.49365
TRINITY_DN114198_c0_g1_i1_orf1	-	-	-	catalase isozyme 1, partial [Sturnira hondurensis]	1.63158	-0.66612	-0.54779	0.6592	-1.07686
TRINITY_DN113626_c0_g1_i3_orfp1	-	-	-	TRINITY_DN113626_c0_g1_i3_m.80721 TRINITY_DN113626_c0_g1::TRINITY_DN113626_c0_g1_i3::g.80721 ORF type:internal len:118 (-),score=87.34 TRINITY_DN113626_c0_g1_i3:2-352(-)	1.53333	-0.99263	-0.97224	0.77488	-0.34335
TRINITY_DN2695_c0_g1_i14_orfp1	-	-	-	TRINITY_DN2695_c0_g1_i14_m.44485 TRINITY_DN2695_c0_g1::TRINITY_DN2695_c0_g1_i14::g.44485 ORF type:3prime_partial len:698 (+),score=187.51 TRINITY_DN2695_c0_g1_i14:101-2092(+)	1.6885	-1.21642	-0.51812	0.47012	-0.42407
TRINITY_DN868_c0_g1_i4_orf1	-	-	-	uncharacterized protein LOC114359357 isoform X1 [Ostrinia furnacalis]	1.68484	-0.87827	-0.88889	0.58727	-0.50495
TRINITY_DN26411_c0_g1_i2_orfp1	-	-	-	TRINITY_DN26411_c0_g1_i2_m.24123 TRINITY_DN26411_c0_g1::TRINITY_DN26411_c0_g1_i2::g.24123 ORF type:internal len:115 (-),score=72.83 TRINITY_DN26411_c0_g1_i2:3-344(-)	1.55133	-0.71561	-0.92054	0.82672	-0.7419
TRINITY_DN27300_c0_g1_i6_orfp1	-	-	-	TRINITY_DN27300_c0_g1_i6_m.71140 TRINITY_DN27300_c0_g1::TRINITY_DN27300_c0_g1_i6::g.71140 ORF type:internal len:129 (-),score=20.75 TRINITY_DN27300_c0_g1_i6:1-384(-)	0.03732	-0.98174	-0.63077	-0.30702	1.8822
TRINITY_DN146236_c0_g1_i1_orf1	-	-	-	vesicle-fusing ATPase 1-like [Chelonus insularis]	0.80665	-1.50496	-0.62794	0.02648	1.29977

TRINITY_DN8046_c0_g1_i5_orf1	-	-	-	tumor protein D53 homolog isoform X2 [Ostrinia furnacalis]	0.9673	-0.00184	-1.46855	-0.69216	1.19525
TRINITY_DN14209_c0_g1_i1_orf1	-	-	-	unnamed protein product [Diatraea saccharalis]	1.07168	-1.07661	-1.25661	0.23227	1.02927
TRINITY_DN240_c0_g1_i4_orf1	-	-	-	unnamed protein product [Chilo suppressalis]	1.42432	-0.5717	-1.49089	-0.01104	0.64931
TRINITY_DN40440_c0_g1_i1_orf1	-	-	-	Na(+)/H(+) exchange regulatory cofactor NHE-RF1 [Helicoverpa zea] >XP_049705606.1 Na(+)/H(+) exchange regulatory cofactor NHE-RF1 [Helicoverpa armigera]	0.56712	0.32759	-1.49837	-0.73348	1.33714
TRINITY_DN70236_c0_g1_i1_orf1	-	-	-	NAD-dependent protein deacetylase sirtuin-2-like [Ostrinia furnacalis]	0.51737	-0.77191	-1.56949	0.84254	0.98148
TRINITY_DN43881_c0_g1_i2_orf1	-	-	-	estradiol 17-beta-dehydrogenase 8-like [Ostrinia furnacalis]	0.54187	-1.27544	-0.86759	0.07773	1.52344
TRINITY_DN36612_c0_g1_i1_orf1	-	-	-	lamin-C isoform X1 [Ostrinia furnacalis] >XP_028158861.1 lamin-C isoform X2 [Ostrinia furnacalis] >XP_028158863.1 lamin-C isoform X3 [Ostrinia furnacalis] >XP_028158864.1 lamin-C isoform X4 [Ostrinia furnacalis]	1.08175	-0.89184	-1.43062	0.29014	0.95057
TRINITY_DN119893_c0_g2_i3_orf1	-	-	-	ATP-binding cassette sub-family F member 3 isoform X1 [Ostrinia furnacalis] >XP_028168051.1 ATP-binding cassette sub-family F member 3 isoform X2 [Ostrinia	0.84223	-1.37363	-0.83654	0.06416	1.30379
TRINITY_DN19939_c0_g1_i4_orf1	-	-	-	unnamed protein product [Chilo suppressalis]	1.13816	-0.79461	-0.61828	-1.0144	1.28914
TRINITY_DN15265_c0_g1_i1_orf1	-	-	-	cullin-5 [Ostrinia furnacalis]	0.98562	0.19845	-1.40832	-0.88303	1.10728
TRINITY_DN102260_c0_g1_i1_orf1	-	-	-	unnamed protein product [Diatraea saccharalis]	1.22515	-0.94678	-0.81092	-0.68326	1.21581
TRINITY_DN1272_c1_g1_i4_orf1	-	-	-	E3 ubiquitin-protein transferase MAEA [Ostrinia furnacalis] >XP_028157102.1 E3 ubiquitin-protein transferase MAEA [Ostrinia furnacalis]	0.03772	-0.16684	-1.34772	-0.27742	1.75426
TRINITY_DN9979_c0_g1_i1_orf1	-	-	-	ADP-dependent glucokinase [Ostrinia furnacalis]	0.14275	-0.51791	-1.30034	-0.06138	1.73688
TRINITY_DN9510_c0_g2_i1_orf1	-	-	-	RNA polymerase II transcriptional coactivator [Ostrinia furnacalis]	0.9068	-0.46556	-1.08405	-0.8142	1.45701
TRINITY_DN112706_c0_g1_i2_orf1	-	-	-	alaserpin-like isoform X4 [Ostrinia furnacalis]	0.9862	-0.95416	-1.1848	-0.14777	1.30053
TRINITY_DN28039_c0_g1_i1_orf1	-	-	-	translation elongation factor 2 [Athalia rosae]	0.84919	-0.82294	-0.96691	-0.58426	1.52491
TRINITY_DN1710_c0_g2_i2_orf1	-	-	-	relish [Ostrinia furnacalis]	0.35518	0.14414	-1.4595	-0.58338	1.54357
TRINITY_DN42177_c0_g1_i4_orf1	-	-	-	androgen-dependent TFPI-regulating protein-like [Ostrinia furnacalis]	0.9853	-1.34392	-0.81873	-0.06695	1.2443
TRINITY_DN2873_c0_g1_i7_orf1	-	-	-	transitional endoplasmic reticulum ATPase TER94 [Galleria mellonella]	0.67532	-0.47368	-1.4806	-0.1697	1.44866
TRINITY_DN115658_c0_g1_i1_orf1	-	-	-	hypothetical protein B5X24_HaOG203018 [Helicoverpa armigera]	1.13535	-1.16794	-0.92094	-0.24578	1.19932
TRINITY_DN66442_c0_g2_i3_orf1	-	-	-	ubiquitin-conjugating enzyme E2 G1 [Papilio polytes] >XP_013167686.1 PREDICTED: ubiquitin-conjugating enzyme E2 G1 isoform X2 [Papilio xuthus] >XP_014357225.1 ubiquitin-conjugating enzyme E2 G1 isoform X2 [Papilio machaon] >XP_021182486.1 ubiquitin-conjugating enzyme E2 G1 isoform X2 [Helicoverpa armigera] >XP_022831375.1 ubiquitin-conjugating enzyme E2 G1 isoform X2 [Spodoptera litura] >XP_023943756.1 ubiquitin-conjugating enzyme E2 G1 isoform X2 [Bicyclus anynana] >XP_026488925.1 ubiquitin-conjugating enzyme E2 G1 isoform X2 [Vanessa tameamea] >XP_026740300.1 ubiquitin-conjugating enzyme E2 G1-like isoform X2 [Trichoplusia ni] >XP_026754924.1 ubiquitin-conjugating enzyme E2 G1 isoform X2 [Galleria mellonella] >XP_028172940.1 ubiquitin-conjugating enzyme E2 G1 isoform X2 [Ostrinia furnacalis] >XP_030034967.1 ubiquitin-conjugating enzyme E2 G1 isoform X2 [Manduca sexta] >XP_032526880.1 ubiquitin-conjugating enzyme E2 G1 isoform X2 [Danaus plexippus plexippus] >XP_034828280.1 ubiquitin-conjugating enzyme E2 G1 isoform X1 [Maniola hyperantus] >XP_035448963.1 ubiquitin-conjugating enzyme E2 G1-like isoform X2 [Spodoptera frugiperda] >XP_039752058.1 ubiquitin-conjugating enzyme E2 G1 isoform X2 [Pararge aegeria] >XP_041971665.1 ubiquitin-conjugating enzyme E2 G1 isoform X2 [Aricia agestis] >XP_045454385.1 ubiquitin-conjugating enzyme E2 G1 isoform X1 [Melitaea cinxia] >XP_045771256.1 ubiquitin-conjugating enzyme E2 G1 isoform X1 [Maniola jurtina] >XP_046964023.1 ubiquitin-conjugating enzyme E2 G1 isoform X2 [Vanessa cardui] >XP_047032842.1 ubiquitin-conjugating enzyme E2 G1 isoform X2 [Helicoverpa zea] >XP_047537963.1 ubiquitin-conjugating enzyme E2 G1 isoform X2 [Vanessa atalanta] >XP_047999421.1 ubiquitin-conjugating enzyme E2 G1 isoform X2 [Leguminivora glycinivorella] >XP_048482262.1 ubiquitin-conjugating enzyme E2 G1 isoform X2 [Plutella xylostella] >XP_049879261.1 ubiquitin-conjugating enzyme E2 G1 isoform X2 [Pectinophora gossypiella] >XP_050353097.1 ubiquitin-conjugating enzyme E2 G1 isoform X2 [Nymphalis io] >RVE50249.1 hypothetical protein evm_005084 [Chilo suppressalis] >CAB3220594.1 unnamed protein product [Arctia plantaginis] >CAH0595405.1 unnamed protein product [Chrysodeixis includens] >CAH0713838.1 unnamed protein product, partial [Brenthis ino] >KAG6461453.1	0.64571	0.51778	-1.63042	-0.64621	1.11315

TRINITY_DN21000_c0_g1_i1_orf1	-	-	-	elongation factor-1 alpha, partial [Loxostege sticticalis] >QCO92153.1 elongation factor-1 alpha, partial [Sitochroa umbrosalis]	1.32035	-0.99489	-0.76444	-0.67105	1.11004
TRINITY_DN15318_c0_g1_i1_orf1	-	-	-	hepatoma-derived growth factor-related protein 2-like [Ostrinia furnacalis]	0.9991	-1.28318	-1.09293	0.36241	1.0146
TRINITY_DN7570_c0_g1_i18_orf1	-	-	-	sodium/potassium-transporting ATPase subunit alpha isoform X4 [Trichoplusia ni] >XP_026734855.1 sodium/potassium-transporting ATPase subunit alpha isoform X4 [Trichoplusia ni]	0.30603	-0.28929	-1.24909	-0.50256	1.7349
TRINITY_DN4742_c0_g1_i1_orf1	-	-	-	ribosomal protein S6 kinase beta-1 [Ostrinia furnacalis]	0.39927	0.04659	-1.7528	-0.02184	1.32877
TRINITY_DN920_c0_g1_i4_orf1	-	-	-	glutathione S-transferase omega 2 [Ostrinia furnacalis]	0.44001	-1.1651	-1.07519	0.32032	1.47996
TRINITY_DN103107_c0_g1_i2_orf1	-	-	-	superoxide dismutase [Cu-Zn] [Ostrinia furnacalis] >XP_028177872.1 superoxide dismutase [Cu-Zn] [Ostrinia furnacalis]	-0.20002	-0.31837	-0.74816	-0.689	1.95555
TRINITY_DN40911_c0_g1_i1_orf1	-	-	-	peroxisomal membrane protein PEX16 [Ostrinia furnacalis]	0.37412	-0.38765	-0.68562	-1.06381	1.76295
TRINITY_DN19659_c1_g1_i1_orf1	-	-	-	elongation factor 1-gamma [Ostrinia furnacalis]	1.10681	-1.31178	-0.73585	-0.26108	1.2019
TRINITY_DN21170_c0_g1_i5_orf1	-	-	-	twitchin-like [Ostrinia furnacalis]	0.10922	-1.23306	-0.47797	-0.1881	1.78992
TRINITY_DN14855_c0_g1_i1_orf1	-	-	-	neurochondrin homolog [Ostrinia furnacalis]	1.32496	-0.87211	-1.35306	0.098	0.8022
TRINITY_DN8241_c0_g1_i3_orf1	-	-	-	transforming growth factor beta-1-induced transcript 1 protein [Ostrinia furnacalis]	0.97058	-1.14956	-1.00039	-0.1315	1.31088
TRINITY_DN32687_c0_g1_i2_orf1	-	-	-	protein D2-like isoform X2 [Ostrinia furnacalis] >XP_028164613.1 protein D2-like isoform X2 [Ostrinia furnacalis]	0.92504	-0.95974	-0.62653	-0.81219	1.47343
TRINITY_DN8024_c0_g1_i6_orf1	-	-	-	zinc finger CCHC domain-containing protein 24-like isoform X2 [Pararge aegeria]	1.14609	-1.10866	-1.12739	0.00077	1.08919
TRINITY_DN6358_c0_g1_i5_orf1	-	-	-	histone H1B-like [Ostrinia furnacalis]	0.24241	-0.58123	-1.62897	0.90083	1.06695
TRINITY_DN136031_c0_g1_i7_orf1	-	-	-	ferritin, lower subunit isoform X3 [Spodoptera litura]	-0.63137	-0.8095	-0.92643	0.80566	1.56164
TRINITY_DN4741_c0_g1_i4_orf1	-	-	-	PREDICTED: 23 kDa integral membrane protein-like [Papilio xuthus]	0.84247	0.14075	-1.60825	-0.55022	1.17525
TRINITY_DN2031_c11_g1_i2_orfp1	-	-	-	TRINITY_DN2031_c11_g1_i2_m.4044 TRINITY_DN2031_c11_g1::TRINITY_DN2031_c11_g1_i2::g.4044 ORF type:3prime_partial len:149 (-),score=79.84 TRINITY_DN2031_c11_g1_i2:2-445(-)	0.88276	-1.1954	-1.23283	0.57568	0.96978
TRINITY_DN2770_c0_g2_i4_orf1	-	-	-	phosphatidylinositol 4-phosphate 3-kinase C2 domain-containing subunit alpha isoform X1 [Ostrinia furnacalis]	0.1546	-0.4086	-1.57401	0.3392	1.48882
TRINITY_DN34413_c0_g1_i1_orf1	-	-	-	LOW QUALITY PROTEIN: probable RNA methyltransferase bin3 [Ostrinia furnacalis]	1.25314	-0.71863	-1.4982	0.16228	0.80141
TRINITY_DN11637_c0_g1_i1_orf1	-	-	-	ribosome-binding protein 1 isoform X4 [Colias croceus]	1.08047	-0.63544	-1.61564	0.3271	0.84351
TRINITY_DN11612_c0_g2_i1_orf1	-	-	-	eukaryotic translation initiation factor 5B [Manduca sexta]	0.23217	-0.05084	-1.21314	-0.69643	1.72823
TRINITY_DN23474_c1_g1_i1_orf1	-	-	-	unnamed protein product [Chrysodeixis includens]	0.37894	-1.31266	-0.78101	0.1317	1.58303
TRINITY_DN7909_c0_g2_i1_orf1	-	-	-	aldehyde oxidase 3 [Ostrinia furnacalis]	1.24356	-0.87135	-1.32182	-0.02329	0.97291
TRINITY_DN21215_c0_g1_i7_orf1	-	-	-	phytanoyl-CoA dioxygenase, peroxisomal-like [Ostrinia furnacalis]	1.04468	-0.6489	-1.59995	0.28373	0.92045
TRINITY_DN4822_c0_g1_i9_orf1	-	-	-	homogentisate 1,2-dioxygenase [Ostrinia furnacalis]	0.63413	-0.75098	-1.53559	0.43216	1.22028
TRINITY_DN69334_c0_g1_i1_orf1	-	-	-	PREDICTED: 15-hydroxyprostaglandin dehydrogenase [NAD(+)]-like [Papilio xuthus]	0.93755	-0.65057	-0.91995	-0.83485	1.46781
TRINITY_DN195_c8_g1_i1_orf1	-	-	-	hypothetical protein evm_009768 [Chilo suppressalis]	0.53551	-0.18349	-1.04975	-0.94229	1.64002
TRINITY_DN195_c4_g1_i1_orf1	-	-	-	beta-1,3-glucan-binding protein 1 [Ostrinia furnacalis]	1.10521	-0.85546	-0.81243	-0.7742	1.33689
TRINITY_DN21035_c0_g1_i14_orf1	-	-	-	mitochondrial amidoxime reducing component 2-like [Ostrinia furnacalis]	1.13268	-1.16628	-1.20776	0.36803	0.87333
TRINITY_DN27592_c0_g1_i1_orf1	-	-	-	D-arabinitol dehydrogenase 1-like [Ostrinia furnacalis]	1.36271	-0.89118	-0.57188	-0.95399	1.05436
TRINITY_DN467_c9_g1_i2_orf1	-	-	-	band 4.1-like protein 5 [Ostrinia furnacalis]	1.34127	-0.18281	-0.72481	-1.34558	0.91194
TRINITY_DN3307_c1_g1_i2_orf1	-	-	-	BTB/POZ domain-containing protein 2-like [Ostrinia furnacalis]	0.71451	-0.19145	-0.6489	-1.3555	1.48134
TRINITY_DN6016_c0_g1_i8_orf1	-	-	-	hypothetical protein evm_010883 [Chilo suppressalis]	1.08612	0.45713	-0.57584	-1.66942	0.70201
TRINITY_DN556_c0_g2_i1_orf1	-	-	-	serine protease inhibitor dipetalogastin-like [Ostrinia furnacalis]	0.97254	0.54584	-0.41115	-1.77308	0.66585
TRINITY_DN33452_c0_g1_i3_orf1	-	-	-	lethal(2) giant larvae protein isoform X8 [Ostrinia furnacalis]	1.00146	-0.17303	-0.57709	-1.46778	1.21644
TRINITY_DN1013_c0_g1_i3_orf1	-	-	-	TELO2-interacting protein 1 homolog isoform X2 [Ostrinia furnacalis]	1.01458	0.55579	-0.82725	-1.53373	0.7906
TRINITY_DN2749_c0_g1_i4_orf1	-	-	-	RNA exonuclease 4-like [Ostrinia furnacalis] >QEE79882.1 REX4 [Ostrinia furnacalis]	1.36027	-0.92033	0.62305	0.28993	-1.35292
TRINITY_DN4356_c0_g1_i6_orf1	-	-	-	mulatexin-like [Ostrinia furnacalis]	1.2879	-0.30486	0.57028	0.14983	-1.70315
TRINITY_DN33452_c0_g1_i1_orf1	-	-	-	lethal(2) giant larvae protein isoform X8 [Ostrinia furnacalis]	1.40148	-0.79039	0.14832	0.64518	-1.4046
