# Supplementary material for: Comparative Proteomics and Metabonomics Analysis of Different Diapause Stages Revealed a New Regulation Mechanism of Diapause in Loxostege sticticalis (Lepidoptera: Pyralidae)
Source: Molecules. 2024 Jul 25;29(15):3472. doi: 10.3390/molecules29153472 (PMC11314584; doi:10.3390/molecules29153472)
Supplement: Supplementary file 1 [file molecules-29-03472-s001.zip › analysis process/proteomic/Cluster analysis of expression patterns/Down/RDvsCT down.pdf]

Accession	Symbol	Protein Na	Entrez ID	Description	RD	ND	PreD	CT	D
TRINITY_DN2396_c0_g1_i9_orfp1	-	-	-	TRINITY_DN2396_c0_g1_i9_m.39038 TRINITY_DN2396_c0_g1::TRINITY_DN2396_c0_g1_i9::g.39038 ORF type:5prime_partial len:181 (+),score=90.33 TRINITY_DN2396_c0_g1_i9:3-545(+)	-0.0422	-1.9205	0.75417	0.67782	0.53072
TRINITY_DN14242_c0_g1_i2_orfp1	-	-	-	TRINITY_DN14242_c0_g1_i2_m.18449 TRINITY_DN14242_c0_g1::TRINITY_DN14242_c0_g1_i2::g.18449 ORF type:internal len:148 (-),score=102.30 TRINITY_DN14242_c0_g1_i2:2-442(-)	0.08701	-1.8828	0.95197	0.72609	0.11777
TRINITY_DN971_c0_g1_i10_orfp1	-	-	-	TRINITY_DN971_c0_g1_i10_m.54268 TRINITY_DN971_c0_g1::TRINITY_DN971_c0_g1_i10::g.54268 ORF type:internal len:187 (+),score=141.68 TRINITY_DN971_c0_g1_i10:1-558(+)	-0.1083	-1.8854	0.89113	0.42557	0.67699
TRINITY_DN971_c0_g1_i5_orfp1	-	-	-	TRINITY_DN971_c0_g1_i5_m.54249 TRINITY_DN971_c0_g1::TRINITY_DN971_c0_g1_i5::g.54249 ORF type:internal len:108 (+),score=66.98 TRINITY_DN971_c0_g1_i5:1-321(+)	-0.3999	-1.7632	0.48896	1.04991	0.62427
TRINITY_DN1108_c3_g1_i1_orfp1	-	-	-	TRINITY_DN1108_c3_g1_i1_m.5561 TRINITY_DN1108_c3_g1::TRINITY_DN1108_c3_g1_i1::g.5561 ORF type:internal len:113 (+),score=89.07 TRINITY_DN1108_c3_g1_i1:1-336(+)	0.17271	-1.907	0.7938	0.83166	0.10881
TRINITY_DN295_c3_g1_i1_orfp1	-	-	-	TRINITY_DN295_c3_g1_i1_m.18839 TRINITY_DN295_c3_g1::TRINITY_DN295_c3_g1_i1::g.18839 ORF type:5prime_partial len:118 (+),score=46.25 TRINITY_DN295_c3_g1_i1:1-354(+)	-0.1243	-1.8977	0.58171	0.78172	0.65859
TRINITY_DN1506_c0_g1_i6_orfp1	-	-	-	TRINITY_DN1506_c0_g1_i6_m.57691 TRINITY_DN1506_c0_g1::TRINITY_DN1506_c0_g1_i6::g.57691 ORF type:5prime_partial len:173 (+),score=30.49 TRINITY_DN1506_c0_g1_i6:1-519(+)	-1.3243	-1.1155	0.70693	0.84806	0.88481
TRINITY_DN2044_c0_g1_i5_orfp1	-	-	-	TRINITY_DN2044_c0_g1_i5_m.4210 TRINITY_DN2044_c0_g1::TRINITY_DN2044_c0_g1_i5::g.4210 ORF type:complete len:151 (-),score=85.31 TRINITY_DN2044_c0_g1_i5:857-1309(-)	-0.4013	-1.7613	0.44879	1.03947	0.67434
TRINITY_DN34423_c0_g1_i3_orf1	-	-	-	THAP domain-containing protein 4-like [Ostrinia furnacalis]	0.07258	-1.9506	0.60276	0.71887	0.55645
TRINITY_DN57137_c0_g1_i1_orfp1	-	-	-	TRINITY_DN57137_c0_g1_i1_m.46420 TRINITY_DN57137_c0_g1::TRINITY_DN57137_c0_g1_i1::g.46420 ORF type:5prime_partial len:56 (-),score=4.65 TRINITY_DN57137_c0_g1_i1:82-249(-)	-0.2302	-1.8476	0.79953	0.44279	0.83552
TRINITY_DN35809_c0_g1_i1_orf1	-	-	-	spodomicin-like [Ostrinia furnacalis] >QKV49445.1 diapausin [Ostrinia furnacalis]	-0.5943	-1.6706	0.99078	0.81406	0.46002
TRINITY_DN56459_c0_g1_i2_orf1	-	-	-	aldo-keto reductase AKR2E4-like [Ostrinia furnacalis]	-0.6049	-1.693	0.70412	0.82862	0.76515
TRINITY_DN7064_c0_g1_i19_orf1	-	-	-	unnamed protein product [Chilo suppressalis]	-1.1627	-1.2318	0.44399	0.84973	1.10076
TRINITY_DN17615_c0_g1_i3_orf1	-	-	-	hypothetical protein SFRUCORN_008858 [Spodoptera frugiperda]	-0.0689	-1.9064	0.52512	0.59568	0.85456
TRINITY_DN3166_c1_g1_i6_orf1	-	-	-	hypothetical protein evm_013813 [Chilo suppressalis]	-0.316	-1.7427	0.35242	0.4768	1.22947
TRINITY_DN12534_c0_g1_i4_orf1	-	-	-	antibacterial protein [Heliothis virescens]	-0.5078	-1.7409	0.64548	0.87018	0.73311
TRINITY_DN15202_c0_g1_i6_orf1	-	-	-	uncharacterized protein LOC114364499 isoform X2 [Ostrinia furnacalis]	-0.5354	-1.6715	0.3374	0.76407	1.10538
TRINITY_DN380_c0_g2_i2_orf1	-	-	-	chemosensory protein 10 [Ostrinia furnacalis]	-0.349	-1.7971	0.4764	0.95239	0.71729
TRINITY_DN6330_c0_g1_i1_orfp1	-	-	-	TRINITY_DN6330_c0_g1_i1_m.42332 TRINITY_DN6330_c0_g1::TRINITY_DN6330_c0_g1_i1::g.42332 ORF type:3prime_partial len:51 (-),score=33.30 TRINITY_DN6330_c0_g1_i1:3-152(-)	-0.4753	-1.7299	0.9219	0.40933	0.87403
TRINITY_DN1597_c0_g1_i5_orfp1	-	-	-	TRINITY_DN1597_c0_g1_i5_m.57494 TRINITY_DN1597_c0_g1::TRINITY_DN1597_c0_g1_i5::g.57494 ORF type:complete len:86 (+),score=7.19 TRINITY_DN1597_c0_g1_i5:134-391(+)	0.1037	-1.8514	-0.004	0.75829	0.99332
TRINITY_DN36476_c1_g1_i1_orfp1	-	-	-	TRINITY_DN36476_c1_g1_i1_m.70910 TRINITY_DN36476_c1_g1::TRINITY_DN36476_c1_g1_i1::g.70910 ORF type:5prime_partial len:88 (-),score=0.50 TRINITY_DN36476_c1_g1_i1:49-312(-)	-0.6667	-1.6206	1.01449	0.42458	0.84827
TRINITY_DN114890_c0_g1_i4_orf1	-	-	-	chemosensory protein 10 [Ostrinia furnacalis]	-0.7429	-1.475	0.56866	1.36742	0.28177
TRINITY_DN31348_c0_g1_i1_orf1	-	-	-	protein lethal(2)essential for life [Bombyx mori]	-0.5548	-1.6407	0.45428	1.24357	0.4976
TRINITY_DN59429_c0_g1_i6_orf1	-	-	-	uncharacterized protein LOC114366345 isoform X2 [Ostrinia furnacalis]	-0.7936	-1.528	1.06094	0.8701	0.39062
TRINITY_DN86772_c0_g1_i3_orfp1	-	-	-	x-tox [Spodoptera exigua]	-0.3059	-1.7865	0.98139	0.29628	0.81475
TRINITY_DN24723_c2_g1_i1_orf1	-	-	-	hypothetical protein evm_001103 [Chilo suppressalis]	-0.4458	-1.7666	0.77289	0.87289	0.56665
TRINITY_DN8008_c0_g1_i6_orf1	-	-	-	uncharacterized protein LOC114357965 isoform X1 [Ostrinia furnacalis] >XP_028167599.1 uncharacterized protein LOC114357965 isoform X1 [Ostrinia furnacalis] >XP_028167600.1 uncharacterized protein LOC114357965 isoform X2 [Ostrinia furnacalis] >XP_028167601.1 uncharacterized protein LOC114357965 isoform X3 [Ostrinia furnacalis]	-0.8444	-1.4788	0.40514	1.17864	0.73942
TRINITY_DN1880_c0_g1_i4_orf1	-	-	-	serine protease inhibitor dipetalogastin-like [Helicoverpa zea]	-0.7326	-1.6005	0.69273	1.01569	0.62467
TRINITY_DN38506_c0_g1_i4_orf1	-	-	-	C-1-tetrahydrofolate synthase, cytoplasmic isoform X1 [Ostrinia furnacalis] >XP_028166137.1 C-1-tetrahydrofolate synthase, cytoplasmic isoform X2 [Ostrinia furnacalis] >XP_028166140.1 C-1-tetrahydrofolate synthase, cytoplasmic isoform X4 [Ostrinia furnacalis]	-0.918	-1.4898	0.82381	0.84048	0.74348
TRINITY_DN295_c5_g1_i2_orf1	-	-	-	unnamed protein product [Chilo suppressalis]	-0.3868	-1.729	0.54383	1.19189	0.3801
TRINITY_DN4816_c0_g2_i3_orf1	-	-	-	15-hydroxyprostaglandin dehydrogenase [NAD(+)]-like [Ostrinia furnacalis]	-0.1432	-1.8939	0.69379	0.73797	0.6054
TRINITY_DN9079_c0_g1_i5_orf1	-	-	-	UDP-glucuronosyltransferase-like [Ostrinia furnacalis]	-0.1702	-1.885	0.64225	0.76676	0.64616
TRINITY_DN71698_c0_g1_i1_orfp1	-	-	-	TRINITY_DN71698_c0_g1_i1_m.1194 TRINITY_DN71698_c0_g1::TRINITY_DN71698_c0_g1_i1::g.1194 ORF type:internal len:134 (+),score=19.66,Toxin_2 PF00451.20 4.3e-05,Toxin_2|PF00451.20|0.037,Toxin_2|PF00451.20|7.5e-05,Gamma-thionin|PF00304.21|0.017,Gamma-thionin|PF00304.21|0.05,Gamma-thionin|PF00304.21|0.021,Toxin_38|PF14866.7|0.13,Toxin_38|PF14866.7|0.15,Toxin_38|PF14866.7|0.15,Defensin_2|PF01097.19|0.053,Defensin_2|PF01097.19|0.34,Defensin_2|PF01097.19|0.092 TRINITY_DN71698_c0_g1_i1:3-401(+)	-0.5788	-1.6737	0.86061	0.9738	0.41809
TRINITY_DN19731_c0_g1_i1_orf1	-	-	-	allergen Tha p 1-like [Ostrinia furnacalis] >XP_028174916.1 allergen Tha p 1-like [Ostrinia furnacalis] >BAV56808.1 chemosensory protein 4 [Ostrinia furnacalis]	-0.1912	-1.8323	0.27496	0.90423	0.84429
TRINITY_DN29026_c0_g1_i4_orf1	-	-	-	TIL [Ostrinia furnacalis]	-0.2116	-1.7125	0.15528	0.41837	1.3504
TRINITY_DN628_c0_g1_i1_orf1	-	-	-	prostamide/prostaglandin F synthase-like [Ostrinia furnacalis]	0.10288	-1.9579	0.58343	0.69631	0.57525
TRINITY_DN30510_c0_g1_i6_orf1	-	-	-	spodomicin-like [Ostrinia furnacalis]	-1.0591	-1.2996	1.19375	0.37534	0.78959
TRINITY_DN7226_c0_g1_i5_orf1	-	-	-	chemosensory protein [Dioryctria abietella]	-0.3468	-1.7964	0.94469	0.74398	0.45455
TRINITY_DN5337_c0_g1_i6_orf1	-	-	-	epoxide hydrolase 1-like [Ostrinia furnacalis]	0.15109	-1.912	0.16032	0.71476	0.88586
TRINITY_DN5439_c0_g1_i2_orf1	-	-	-	uncharacterized protein LOC114353087 [Ostrinia furnacalis]	-0.177	-1.8093	0.56999	0.27955	1.13676
TRINITY_DN1772_c1_g2_i1_orf1	-	-	-	aldose reductase-like isoform X2 [Ostrinia furnacalis]	-0.8172	-1.483	0.2967	0.86385	1.13962
TRINITY_DN21420_c0_g1_i2_orf1	-	-	-	glutathione peroxidase, partial [Ostrinia furnacalis]	-0.0941	-1.84	0.44521	1.13098	0.35796
TRINITY_DN8595_c0_g1_i3_orf1	-	-	-	aldose reductase-like isoform X4 [Trichoplusia ni]	-0.654	-1.6375	0.59018	0.63241	1.06893
TRINITY_DN30306_c0_g2_i1_orf1	-	-	-	perilipin-4-like isoform X3 [Ostrinia furnacalis]	-0.5849	-1.6424	1.14023	0.35707	0.72998

TRINITY_DN13660_c0_g1_i1_orf1	-	-	-	Aliphatic nitrilase [Operophtera brumata]	-0.2362	-1.8389	0.5464	0.98319	0.54548
TRINITY_DN33346_c0_g1_i1_orf1	-	-	-	PREDICTED: U6 snRNA-associated Sm-like protein LSm3 [Papilio xuthus] >XP_028165558.1 U6 snRNA-associated Sm-like protein LSm3 [Ostrinia furnacalis] >KOB73597.1 LSM Sm-like protein family member [Operophtera brumata] >RVE45517.1 hypothetical protein evm_009856 [Chilo suppressalis] >CAB3523639.1 unnamed protein product [Chilo suppressalis] >CAH0400961.1 unnamed protein product [Chilo suppressalis]	0.02651	-1.9243	0.40817	0.64415	0.84548
TRINITY_DN3821_c1_g1_i7_orf1	-	-	-	mitochondrial carrier protein Rim2 isoform X1 [Ostrinia furnacalis]	-0.3225	-1.7959	0.37776	0.95202	0.78856
TRINITY_DN1209_c0_g1_i9_orf1	-	-	-	NADP-dependent malic enzyme-like isoform X1 [Ostrinia furnacalis] >XP_028161889.1 NADP-dependent malic enzyme-like isoform X1 [Ostrinia furnacalis] >XP_028161891.1 NADP-dependent malic enzyme-like isoform X3 [Ostrinia furnacalis]	-0.5154	-1.5853	0.27823	1.40264	0.41986
TRINITY_DN1149_c0_g1_i4_orf1	-	-	-	circadian clock-controlled protein-like [Ostrinia furnacalis]	-0.8255	-1.4557	1.26362	0.71512	0.30242
TRINITY_DN9608_c0_g1_i3_orf1	-	-	-	cytochrome P450 monooxygenase CYP9G18 [Cnaphalocrocis medinalis]	-0.5372	-1.5219	0.04571	0.57878	1.43463
TRINITY_DN69_c0_g1_i1_orf1	-	-	-	glycerol-3-phosphate dehydrogenase [NAD(+)], cytoplasmic isoform X1 [Ostrinia furnacalis]	-0.1964	-1.8571	0.65782	0.4677	0.92797
TRINITY_DN140_c0_g1_i1_orf1	-	-	-	calcyphosin-like protein [Ostrinia furnacalis]	-0.1325	-1.8274	0.24237	1.09271	0.62481
TRINITY_DN21367_c0_g1_i1_orf1	-	-	-	40S ribosomal protein S15 isoform 2 [Homo sapiens] >NP_001125055.1 40S ribosomal protein S15 [Pongo abelii] >NP_001252851.1 40S ribosomal protein S15 [Macaca mulatta] >NP_001268540.1 40S ribosomal protein S15 [Mesocricetus auratus] >NP_001291817.1 40S ribosomal protein S15 [Ailuropoda melanoleuca] >NP_033117.1 40S ribosomal protein S15 isoform 1 [Mus musculus] >NP_058847.1 40S ribosomal protein S15 [Rattus norvegicus] >NP_990793.1 40S ribosomal protein S15 [Gallus gallus] >NP_999499.1 40S ribosomal protein S15 [Sus scrofa] >XP_001513459.4 40S ribosomal protein S15 [Ornithorhynchus anatinus] >XP_003277039.1 40S ribosomal protein S15 isoform X2 [Nomascus leucogenys] >XP_003461016.1 40S ribosomal protein S15 [Cavia porcellus] >XP_004378568.1 40S ribosomal protein S15 [Trichechus manatus latirostris] >XP_004395466.1 PREDICTED: 40S ribosomal protein S15 [Odobenus rosmarus divergens] >XP_004441415.1 PREDICTED: 40S ribosomal protein S15 [Ceratotherium simum simum] >XP_004462792.1 40S ribosomal protein S15 [Dasypus novemcinctus] >XP_004632738.1 40S ribosomal protein S15 [Octodon degus] >XP_004866007.1 40S ribosomal protein S15 [Heterocephalus glaber] >XP_005359120.1 40S ribosomal protein S15 isoform X2 [Microtus ochrogaster] >XP_005406026.1 PREDICTED: 40S ribosomal protein S15 [Chinchilla lanigera] >XP_005531236.1 PREDICTED: 40S ribosomal protein S15 [Pseudopodoces humilis] >XP_005587458.1 40S ribosomal protein S15 isoform X4 [Macaca fascicularis] >XP_005866755.1 PREDICTED: 40S ribosomal protein S15 [Myotis brandtii] >XP_006206493.1 40S ribosomal protein S15 [Vicugna pacos] >XP_006764872.1 PREDICTED: 40S ribosomal protein S15 [Myotis davidii] >XP_006897998.1 PREDICTED: 40S ribosomal protein S15 [Elephantulus edwardii] >XP_006904116.1 40S ribosomal protein S15 [Pteropus alecto] >XP_006928238.1 40S ribosomal protein S15 [Felis catus] >XP_006978252.1 40S ribosomal protein S15 [Peromyscus maniculatus bairdii] >XP_007992808.1 40S ribosomal protein S15 isoform X2 [Chlorocebus sabaeus] >XP_008591572.1 PREDICTED: 40S ribosomal protein S15 [Galeopterus variegatus] >XP_008835382.1 40S ribosomal protein S15 isoform X3 [Nannospalax galili] >XP_009095157.1 40S ribosomal protein S15 [Serinus canaria] >XP_010373202.1 40S ribosomal protein S15 isoform X2 [Rhinopithecus roxellana] >XP_010561484.1 PREDICTED: 40S ribosomal protein S15 [Haliaeetus leucocephalus] >XP_010633228.1 40S ribosomal protein S15 [Fukomys damarensis] >XP_010723314.1 40S ribosomal protein S15 [Meleagris gallopavo] >XP_010964943.1 40S ribosomal protein S15 [Camelus bactrianus] >XP_010996087.1 40S ribosomal protein S15 [Camelus dromedarius] >XP_011747134.1 40S ribosomal protein S15 isoform X4 [Macaca nemestrina] >XP_011819405.1 PREDICTED: 40S ribosomal protein S15 isoform X2 [Colobus angolensis palliatus] >XP_011852804.1 PREDICTED: 40S ribosomal protein S15 isoform X2 [Mandrillus leucophaeus] >XP_011928279.1 PREDICTED: 40S ribosomal protein S15 isoform X4 [Cercocebus atys] >XP_012291424.2 40S ribosomal protein S15 isoform X1 [Aotus nancymaae] >XP_012394559.1 40S ribosomal protein S15 isoform X2 [Orcinus orca] >XP_012500924.1 PREDICTED: 40S ribosomal protein S15 [Propithecus coquereli] >XP_012601179.1 40S ribosomal protein S15 [Microcebus murinus] >XP_012659873.2 40S ribosomal protein S15 [Otolemur garnettii] >XP_012878256.1 PREDICTED: 40S ribosomal protein S15 [Dipodomys ordii] >XP_013800709.1 PREDICTED: 40S ribosomal protein S15 [Apteryx mantelli mantelli] >XP_014129960.1 40S ribosomal protein S15 [Zonotrichia albicollis] >XP_014701153.1 40S ribosomal protein S15 [Equus asinus] >XP_014738550.1 PREDICTED: 40S ribosomal protein S15 [Sturnus	-0.612	-1.3002	0.08012	1.70668	0.12542
TRINITY_DN9132_c0_g1_i5_orf1	-	-	-	ubiquitin-like-conjugating enzyme ATG3 [Spodoptera frugiperda]	-0.1254	-1.8371	0.23396	1.03876	0.68973
TRINITY_DN27300_c0_g1_i1_orfp1	-	-	-	TRINITY_DN27300_c0_g1_i1_m.71142 TRINITY_DN27300_c0_g1::TRINITY_DN27300_c0_g1_i1::g.71142 ORF type:internal len:82 (-),score=9.46 TRINITY_DN27300_c0_g1_i1:3-245(-)	-0.8827	-1.164	1.67074	0.23183	0.14416
TRINITY_DN1363_c0_g1_i11_orf1	-	-	-	cytochrome P450 CYP12A2-like isoform X1 [Ostrinia furnacalis] >QPF77619.1 cytochrome P450 monooxygenase CYP333A20 [Ostrinia furnacalis]	-0.2994	-1.8171	0.96686	0.49475	0.65489
TRINITY_DN581_c3_g2_i1_orf1	-	-	-	uncharacterized protein LOC114364499 isoform X3 [Ostrinia furnacalis]	-0.4306	-1.5301	-0.1197	0.65612	1.4243
TRINITY_DN4998_c0_g1_i21_orf1	-	-	-	phenoloxidase subunit 2-like [Ostrinia furnacalis]	0.01451	-1.9295	0.74996	0.71659	0.44839
TRINITY_DN20344_c0_g1_i5_orf1	-	-	-	uncharacterized protein LOC114351483 [Ostrinia furnacalis]	-1.2336	-1.1413	0.39299	0.82177	1.16014
TRINITY_DN6098_c1_g1_i5_orf1	-	-	-	unnamed protein product, partial [Iphiclides podalirius]	-0.5411	-1.7269	0.67888	0.82118	0.76796
TRINITY_DN40126_c0_g1_i1_orf1	-	-	-	aldehyde dehydrogenase X, mitochondrial-like [Ostrinia furnacalis]	0.0301	-1.94	0.57633	0.58228	0.75126
TRINITY_DN43350_c0_g3_i1_orf1	-	-	-	uncharacterized protein LOC114355190 [Ostrinia furnacalis]	-1.0414	-1.3572	0.50193	0.84143	1.05524
TRINITY_DN31286_c0_g1_i6_orfp1	-	-	-	TRINITY_DN31286_c0_g1_i6_m.28438 TRINITY_DN31286_c0_g1::TRINITY_DN31286_c0_g1_i6::g.28438 ORF type:internal len:92 (-),score=3.10,Perilipin PF03036.17 2e-05 TRINITY_DN31286_c0_g1_i6:1-273(-)	-0.1932	-1.8259	0.51318	0.4125	1.09336
TRINITY_DN2040_c0_g1_i6_orf1	-	-	-	trypsin-like serine proteinase T26 protein, partial [Chilo infuscatellus]	-0.6819	-1.6353	0.5554	0.86859	0.89324
TRINITY_DN1161_c0_g1_i2_orf1	-	-	-	fructose-1,6-bisphosphatase 1 [Ostrinia furnacalis]	0.0716	-1.9365	0.61007	0.83427	0.42051
TRINITY_DN2457_c0_g1_i8_orf1	-	-	-	uncharacterized protein LOC114355596 [Ostrinia furnacalis]	-0.8865	-1.3516	1.4347	0.34564	0.45773
TRINITY_DN3439_c0_g2_i2_orf1	-	-	-	histone H2A.Z-specific chaperone CHZ1-like [Ostrinia furnacalis]	-0.8689	-1.4623	0.88475	0.35051	1.09594
TRINITY_DN11981_c0_g1_i7_orf1	-	-	-	luciferin 4-monooxygenase-like isoform X2 [Ostrinia furnacalis]	-0.5503	-1.6475	1.1955	0.66168	0.34057
TRINITY_DN14328_c0_g1_i12_orf1	-	-	-	larval cuticle protein LCP-30-like [Ostrinia furnacalis]	-1.1997	-1.2255	0.73966	0.62938	1.0562
TRINITY_DN6415_c0_g2_i1_orf1	-	-	-	D-arabinitol dehydrogenase 1-like [Ostrinia furnacalis]	-0.9229	-1.3917	0.40741	0.61675	1.29042
TRINITY_DN609_c0_g1_i1_orf1	-	-	-	zonadhesin-like isoform X1 [Ostrinia furnacalis]	-1.1348	-1.1453	1.40622	0.58059	0.29327
TRINITY_DN10222_c0_g1_i2_orf1	-	-	-	glutathione S-transferase sigma 3 [Ostrinia furnacalis]	-0.9739	-1.3953	0.45478	1.13803	0.77637
TRINITY_DN4245_c0_g2_i1_orf1	-	-	-	long-chain fatty acid transport protein 4-like [Ostrinia furnacalis]	-0.1507	-1.8804	0.70272	0.48335	0.84497

TRINITY_DN1750_c1_g1_i5_orf1	-	-	-	lipid droplet localized protein-like [Ostrinia furnacalis] >XP_028161280.1 lipid droplet localized protein-like [Ostrinia furnacalis]	-0.2498	-1.8472	0.713	0.86401	0.52001
TRINITY_DN12586_c0_g1_i4_orf1	-	-	-	zonadhesin-like isoform X4 [Ostrinia furnacalis]	-1.2595	-1.1145	1.07618	0.9415	0.35631
TRINITY_DN2187_c0_g1_i1_orf1	-	-	-	flotillin-1 [Chelonus insularis] >XP_034947202.1 flotillin-1 [Chelonus insularis]	-0.1939	-1.8582	0.81471	0.41887	0.8186
TRINITY_DN7960_c0_g1_i2_orf1	-	-	-	uncharacterized protein LOC114364878 [Ostrinia furnacalis]	-0.7169	-1.5541	0.3175	1.15288	0.80058
TRINITY_DN11817_c0_g1_i4_orf1	-	-	-	glycogen phosphorylase [Heortia vitessoides]	-0.0665	-1.8942	0.74305	0.84752	0.37012
TRINITY_DN5153_c1_g1_i1_orf1	-	-	-	nose resistant to fluoxetine protein 6-like isoform X1 [Ostrinia furnacalis]	-0.5741	-1.6446	1.19455	0.42701	0.59712
TRINITY_DN1450_c0_g2_i1_orf1	-	-	-	death-associated protein 1 [Ostrinia furnacalis]	-0.6984	-1.618	0.99672	0.53741	0.7823
TRINITY_DN1352_c0_g1_i5_orf1	-	-	-	uncharacterized protein LOC113491815 [Trichoplusia ni]	-0.2263	-1.8482	0.52277	0.94302	0.60867
TRINITY_DN12775_c0_g1_i10_orfp1	-	-	-	TRINITY_DN12775_c0_g1_i10_m.21238 TRINITY_DN12775_c0_g1::TRINITY_DN12775_c0_g1_i10::g.21238 ORF type:5prime_partial len:67 (-),score=0.74 TRINITY_DN12775_c0_g1_i10:275-475(-)	-0.3412	-1.7247	0.1233	0.91368	1.02899
TRINITY_DN14262_c0_g1_i5_orf1	-	-	-	cytochrome P450 monooxygenase CYP9G18 [Cnaphalocrocis medinalis]	-0.1865	-1.7866	0.95254	0.92577	0.09478
TRINITY_DN73900_c0_g1_i1_orf1	-	-	-	carbonic anhydrase 7 [Ostrinia furnacalis]	-0.9749	-1.419	0.77584	1.05902	0.55906
TRINITY_DN122321_c0_g1_i1_orf1	-	-	-	uncharacterized protein LOC114356271 [Ostrinia furnacalis]	-0.3295	-1.7814	0.3795	0.67218	1.05927
TRINITY_DN5099_c0_g1_i3_orf1	-	-	-	trans-1,2-dihydrobenzene-1,2-diol dehydrogenase-like [Ostrinia furnacalis]	-0.168	-1.8503	0.44519	0.54908	1.02401
TRINITY_DN14185_c0_g1_i1_orf1	-	-	-	uncharacterized protein LOC114358675 [Ostrinia furnacalis] >XP_028168498.1 uncharacterized protein LOC114358675 [Ostrinia	-0.8394	-1.4804	1.02381	0.97643	0.31961
TRINITY_DN5274_c0_g2_i2_orf1	-	-	-	lopap-like [Ostrinia furnacalis]	-0.3785	-1.7814	0.93704	0.78099	0.44193
TRINITY_DN2464_c0_g1_i12_orf1	-	-	-	uncharacterized protein LOC114362996 isoform X1 [Ostrinia furnacalis]	-1.2101	-1.2387	0.81621	0.78066	0.8519
TRINITY_DN20658_c0_g1_i1_orf1	-	-	-	prostaglandin reductase 1-like [Ostrinia furnacalis]	-0.2979	-1.8214	0.83431	0.45259	0.83237
TRINITY_DN34423_c0_g1_i2_orf1	-	-	-	THAP domain-containing protein 4-like [Ostrinia furnacalis]	-0.0895	-1.8771	0.58271	0.98476	0.39909
TRINITY_DN4802_c0_g1_i4_orf1	-	-	-	uncharacterized protein LOC114366345 isoform X2 [Ostrinia furnacalis]	-0.5071	-1.4898	0.45952	0.01689	1.52051
TRINITY_DN130575_c0_g1_i1_orfp1	-	-	-	TRINITY_DN130575_c0_g1_i1_m.77798 TRINITY_DN130575_c0_g1::TRINITY_DN130575_c0_g1_i1::g.77798 ORF type:internal len:70 (+),score=15.12 TRINITY_DN130575_c0_g1_i1:3-209(+)	-0.2939	-1.8091	0.78642	0.37952	0.93712
TRINITY_DN51480_c0_g1_i1_orf1	-	-	-	uncharacterized protein LOC114361588 isoform X14 [Ostrinia furnacalis]	-0.3234	-1.7425	0.15079	0.92144	0.99368
TRINITY_DN1503_c0_g1_i6_orf1	-	-	-	ecdysteroid-regulated 16 kDa protein [Ostrinia furnacalis]	-0.1171	-1.8367	1.08672	0.60066	0.26641
TRINITY_DN49530_c0_g1_i1_orf1	-	-	-	ommochrome-binding protein-like [Ostrinia furnacalis]	-0.3339	-1.7712	0.27703	0.95688	0.87122
TRINITY_DN4068_c0_g2_i4_orf1	-	-	-	larval cuticle protein LCP-17-like precursor [Papilio polytes] >BAM18876.1 cuticular protein PpolCPR2 [Papilio polytes]	-1.4621	-0.8188	0.24256	0.85238	1.18595
TRINITY_DN1206_c0_g1_i6_orf1	-	-	-	sorbitol dehydrogenase-like [Spodoptera frugiperda] >KAG8104768.1 hypothetical protein SFRUCORN_013827 [Spodoptera	-0.6284	-1.6765	0.72804	0.89144	0.68542
TRINITY_DN40126_c0_g2_i1_orf1	-	-	-	aldehyde dehydrogenase X, mitochondrial-like [Ostrinia furnacalis]	-0.6328	-1.6368	1.09532	0.46233	0.71196
TRINITY_DN1960_c5_g1_i3_orf1	-	-	-	cytochrome P450 monooxygenase CYP9G18 [Cnaphalocrocis medinalis]	-0.5487	-1.6543	0.2563	1.00303	0.94367
TRINITY_DN350_c0_g1_i5_orf1	-	-	-	tau-like protein isoform X6 [Bombyx mori]	-0.6322	-1.623	1.16233	0.64153	0.45133
TRINITY_DN8685_c0_g1_i5_orf1	-	-	-	macrophage mannose receptor 1-like [Zerene cesonia]	-0.7835	-1.433	1.3785	0.62066	0.21731
TRINITY_DN18482_c0_g1_i3_orf1	-	-	-	calcyphosin-like protein isoform X3 [Helicoverpa armigera] >XP_047020698.1 calcyphosin-like protein isoform X2 [Helicoverpa zea]	-1.0723	-1.3109	0.42551	1.11023	0.84742
TRINITY_DN285_c0_g1_i4_orf1	-	-	-	catalase-like [Ostrinia furnacalis]	-0.997	-1.393	0.50637	0.80903	1.07456
TRINITY_DN11948_c0_g1_i8_orf1	-	-	-	cystathionine gamma-lyase [Ostrinia furnacalis]	-0.7467	-1.5905	0.84555	0.95209	0.53962
TRINITY_DN4497_c2_g1_i3_orf1	-	-	-	uncharacterized protein LOC114353086 [Ostrinia furnacalis]	-0.4487	-1.7108	0.30063	0.76547	1.09341
TRINITY_DN618_c0_g1_i3_orf1	-	-	-	triokinase/FMN cyclase-like isoform X1 [Ostrinia furnacalis]	-0.2351	-1.8535	0.67167	0.54344	0.87346
TRINITY_DN710_c0_g1_i11_orfp1	-	-	-	TRINITY_DN710_c0_g1_i11_m.67699 TRINITY_DN710_c0_g1::TRINITY_DN710_c0_g1_i11::g.67699 ORF type:complete len:194 (-),score=34.99,Collagen PF01391.19 0.00029 TRINITY_DN710_c0_g1_i11:1283-1864(-)	-0.9968	-1.3438	0.98595	0.28302	1.07163
TRINITY_DN9435_c0_g1_i7_orf1	-	-	-	uncharacterized protein LOC114350197 [Ostrinia furnacalis]	-0.7163	-1.4207	0.24495	1.50223	0.38978
TRINITY_DN812_c2_g1_i1_orf1	-	-	-	1,4-alpha-glucan-branching enzyme [Ostrinia furnacalis]	-0.5764	-1.7055	0.86719	0.76284	0.6519
TRINITY_DN10994_c0_g1_i4_orf1	-	-	-	trypsin inhibitor-like [Ostrinia furnacalis]	-0.7438	-1.5925	0.54119	0.95513	0.84
TRINITY_DN125521_c0_g2_i1_orf1	-	-	-	seroin transcript 1A2 [Ostrinia nubilalis]	-1.3903	-1.0033	0.50122	0.84879	1.04353
TRINITY_DN3175_c0_g1_i7_orf1	-	-	-	unnamed protein product, partial [Brenthis ino]	-0.2328	-1.86	0.60996	0.65608	0.82678
TRINITY_DN7740_c0_g1_i2_orf1	-	-	-	D-arabinitol dehydrogenase 1 [Eumeta japonica]	-0.768	-1.5609	0.773	0.48984	1.06603
TRINITY_DN394_c0_g1_i4_orf1	-	-	-	uncharacterized protein LOC114351483 [Ostrinia furnacalis]	-1.1552	-1.1528	0.15944	1.03687	1.11173
TRINITY_DN970_c0_g1_i4_orf1	-	-	-	spermine oxidase-like isoform X2 [Ostrinia furnacalis]	-0.4063	-1.7478	0.3543	0.76778	1.03205
TRINITY_DN1024_c0_g4_i1_orf1	-	-	-	superoxide dismutase [Cu-Zn]-like [Ostrinia furnacalis]	-0.7855	-1.5727	0.97735	0.67041	0.71049
TRINITY_DN82944_c0_g1_i4_orf1	-	-	-	senecionine N-oxygenase isoform X2 [Ostrinia furnacalis]	-0.3343	-1.8006	0.42139	0.84468	0.86884
TRINITY_DN15545_c0_g1_i1_orf1	-	-	-	larval cuticle protein LCP-14-like [Ostrinia furnacalis]	-1.3512	-1.0717	0.95578	0.59929	0.86782
TRINITY_DN54205_c0_g1_i1_orf1	-	-	-	aldo-keto reductase AKR2E4-like [Ostrinia furnacalis]	-0.3585	-1.7663	0.3096	0.84315	0.97204
TRINITY_DN28501_c0_g1_i2_orfp1	-	-	-	TRINITY_DN28501_c0_g1_i2_m.58934 TRINITY_DN28501_c0_g1::TRINITY_DN28501_c0_g1_i2::g.58934 ORF type:internal len:98 (+),score=13.70 TRINITY_DN28501_c0_g1_i2:3-293(+)	-1.2705	-0.9138	1.52418	0.26055	0.39961
TRINITY_DN8473_c0_g1_i6_orf1	-	-	-	serine/threonine-protein phosphatase 6 regulatory subunit 1 [Ostrinia furnacalis]	-0.8279	-1.403	0.06895	1.127	1.03499
TRINITY_DN1540_c0_g1_i14_orf1	-	-	-	alaserpin-like isoform X1 [Ostrinia furnacalis]	-0.5189	-1.5962	0.06926	0.81676	1.22916
TRINITY_DN4080_c0_g1_i8_orf1	-	-	-	AMP deaminase 2 isoform X3 [Ostrinia furnacalis] >XP_028163647.1 AMP deaminase 2 isoform X3 [Ostrinia furnacalis] >XP_028163648.1 AMP deaminase 2 isoform X3 [Ostrinia furnacalis]	-0.2622	-1.5053	-0.3098	0.58507	1.49228
TRINITY_DN15858_c0_g1_i2_orf1	-	-	-	15-hydroxyprostaglandin dehydrogenase [NAD(+)]-like [Ostrinia furnacalis]	-0.7493	-1.5542	1.12761	0.7616	0.41423
TRINITY_DN15327_c2_g1_i2_orf1	-	-	-	protein lethal(2)essential for life-like [Ostrinia furnacalis]	-1.4861	-0.907	0.64615	0.98654	0.76039
TRINITY_DN5748_c0_g1_i6_orf1	-	-	-	glycine N-methyltransferase isoform X1 [Ostrinia furnacalis] >XP_028165118.1 glycine N-methyltransferase isoform X2 [Ostrinia furnacalis] >XP_028165119.1 glycine N-methyltransferase isoform X1 [Ostrinia furnacalis] >XP_028165120.1 glycine N-methyltransferase isoform X2 [Ostrinia furnacalis]	-0.822	-1.4254	1.38264	0.37303	0.4917
TRINITY_DN55147_c0_g1_i1_orfp1	-	-	-	TRINITY_DN55147_c0_g1_i1_m.59251 TRINITY_DN55147_c0_g1::TRINITY_DN55147_c0_g1_i1::g.59251 ORF type:5prime_partial len:331 (-),score=113.97,Cuticle_3 PF11018.9 0.29,Cuticle_3|PF11018.9|2.9e-05,Cuticle_3|PF11018.9|0.00037 TRINITY_DN55147_c0_g1_i1:21-[illegible]	-1.3966	-1.0286	0.95132	0.72154	0.75231
TRINITY_DN335_c1_g1_i5_orf1	-	-	-	PREDICTED: perilipin-4 isoform X14 [Papilio polytes]	-0.9118	-1.3939	1.15817	0.23751	0.90995
TRINITY_DN1593_c0_g1_i1_orf1	-	-	-	chemosensory protein csp11 [Helopeltis theivora]	-1.086	-1.2519	1.31471	0.53995	0.48319

TRINITY_DN36434_c0_g2_i3_orf1	-	-	-	clotting factor B isoform X1 [Ostrinia furnacalis]	-0.7175	-1.5088	0.36902	0.5116	1.34566
TRINITY_DN33488_c0_g1_i2_orf1	-	-	-	semaphorin-1A isoform X3 [Trichoplusia ni]	-0.4462	-1.7632	0.93194	0.7001	0.57734
TRINITY_DN13322_c0_g1_i6_orf1	-	-	-	macrophage mannose receptor 1-like [Ostrinia furnacalis]	-0.465	-1.7368	0.41688	0.8753	0.90962
TRINITY_DN512_c0_g1_i10_orf1	-	-	-	uncharacterized protein LOC114366781 [Ostrinia furnacalis]	-0.1818	-1.7801	0.11295	0.72914	1.11975
TRINITY_DN5198_c0_g1_i5_orfp1	-	-	-	TRINITY_DN5198_c0_g1_i5_m.8637 TRINITY_DN5198_c0_g1::TRINITY_DN5198_c0_g1_i5::g.8637 ORF type:complete len:223 (-),score=54.99 TRINITY_DN5198_c0_g1_i5:319-987(-)	-0.9491	-1.3617	1.31173	0.38687	0.61217
TRINITY_DN9492_c1_g1_i1_orf1	-	-	-	aldo-keto reductase AKR2E4-like [Galleria mellonella]	-0.301	-1.7529	0.1917	0.74891	1.11323
TRINITY_DN2338_c0_g1_i5_orf1	-	-	-	prophenoloxidase PPO1b [Ostrinia furnacalis]	-0.0953	-1.8583	0.572	1.04866	0.33295
TRINITY_DN14670_c0_g1_i1_orf1	-	-	-	heat shock protein beta-1 isoform X1 [Helicoverpa armigera] >XP_022829066.1 heat shock protein beta-1 isoform X1 [Spodoptera litura] >XP_026747148.1 heat shock protein beta-1 isoform X3 [Trichoplusia ni] >XP_026748187.1 heat shock protein beta-1 isoform X2 [Galleria mellonella] >XP_028167756.1 heat shock protein beta-1 isoform X2 [Ostrinia furnacalis] >XP_035431734.1 heat shock protein beta-1-like isoform X3 [Spodoptera frugiperda] >XP_047023072.1 heat shock protein beta-1 isoform X1 [Helicoverpa zea] >XP_049865086.1 heat shock protein beta-1 [Pectinophora gossypiella] >KAH9640995.1 hypothetical protein HF086_015091 [Spodoptera exigua] >QGZ00460.1 heat shock protein 21.4 [Glyphodes pyloalis] >QKR72095.1 heat-shock protein 21.4 [Mythimna separata] >CAB3228281.1 unnamed protein product [Arctia plantaginis] >CAH0628881.1 unnamed protein product [Chrysodeixis includens]	-0.8018	-1.4788	1.2992	0.46987	0.51152
TRINITY_DN448_c0_g1_i20_orf1	-	-	-	probable cytochrome P450 9f2 isoform X1 [Ostrinia furnacalis]	-0.6521	-1.5752	0.40392	0.54576	1.27766
TRINITY_DN2255_c0_g1_i1_orf1	-	-	-	glutathione S-transferase sigma 3 [Ostrinia furnacalis]	-0.1037	-1.8726	0.32769	0.73486	0.91381
TRINITY_DN15685_c0_g1_i5_orf1	-	-	-	uncharacterized protein LOC114352354 [Ostrinia furnacalis]	-1.2913	-1.1223	1.09574	0.61491	0.70297
TRINITY_DN17326_c0_g1_i8_orf1	-	-	-	aminoacylase-1-like [Ostrinia furnacalis]	-0.4692	-1.7512	0.63097	0.63366	0.95575
TRINITY_DN4621_c0_g1_i4_orf1	-	-	-	uncharacterized protein LOC114358242 isoform X3 [Ostrinia furnacalis]	-0.6413	-1.6641	0.59062	0.86621	0.84863
TRINITY_DN1252_c0_g1_i1_orf1	-	-	-	WD repeat-containing protein 47 isoform X1 [Ostrinia furnacalis]	-1.4565	-0.92	1.04897	0.82292	0.50461
TRINITY_DN1175_c1_g1_i2_orf1	-	-	-	methanethiol oxidase [Ostrinia furnacalis]	-1.0471	-1.3329	0.93906	1.04224	0.39867
TRINITY_DN24789_c0_g1_i9_orfp1	-	-	-	TRINITY_DN24789_c0_g1_i9_m.25888 TRINITY_DN24789_c0_g1::TRINITY_DN24789_c0_g1_i9::g.25888 ORF type:internal len:114 (-),score=12.65 TRINITY_DN24789_c0_g1_i9:2-340(-)	-1.024	-1.0147	1.69065	0.21118	0.13694
TRINITY_DN1216_c0_g1_i4_orf1	-	-	-	bifunctional purine biosynthesis protein PURH isoform X1 [Ostrinia furnacalis] >XP_028176123.1 bifunctional purine biosynthesis protein PURH isoform X2 [Ostrinia furnacalis] >XP_028176129.1 bifunctional purine biosynthesis protein PURH isoform X3 [Ostrinia furnacalis]	-0.1232	-1.8287	0.20127	0.69093	1.05966
TRINITY_DN80660_c0_g1_i1_orf1	-	-	-	probable phospholipid hydroperoxide glutathione peroxidase isoform X1 [Pieris rapae]	-0.3136	-1.5991	0.56574	1.42089	-0.0739
TRINITY_DN2461_c0_g1_i5_orf1	-	-	-	secretory phospholipase A2 receptor [Vanessa cardui]	-0.9807	-1.4282	0.67942	0.72084	1.00862
TRINITY_DN15858_c0_g1_i1_orf1	-	-	-	15-hydroxyprostaglandin dehydrogenase [NAD(+)]-like [Ostrinia furnacalis]	-0.9075	-1.4782	0.5607	0.98784	0.83724
TRINITY_DN11383_c0_g2_i4_orf1	-	-	-	aminoacylase-1A-like [Ostrinia furnacalis]	-0.5964	-1.6337	0.29201	0.89144	1.04664
TRINITY_DN28299_c0_g1_i1_orf1	-	-	-	adenylosuccinate lyase isoform X1 [Ostrinia furnacalis]	-0.6274	-1.5833	0.20884	1.1626	0.83925
TRINITY_DN394_c0_g1_i2_orf1	-	-	-	uncharacterized protein LOC114351483 [Ostrinia furnacalis]	-1.2518	-1.0724	0.79102	0.27572	1.25746
TRINITY_DN8095_c0_g1_i3_orf1	-	-	-	circadian clock-controlled protein-like [Ostrinia furnacalis]	-1.36	-1.0745	0.89069	0.84661	0.69711
TRINITY_DN2618_c0_g1_i3_orf1	-	-	-	CDP-diacylglycerol--inositol 3-phosphatidyltransferase [Ostrinia furnacalis]	-0.262	-1.7362	0.07561	0.78345	1.13909
TRINITY_DN5914_c1_g1_i9_orf1	-	-	-	unnamed protein product [Chilo suppressalis]	-0.3929	-1.756	0.74252	0.37012	1.03624
TRINITY_DN9340_c0_g1_i4_orf1	-	-	-	sarcosine dehydrogenase, mitochondrial [Ostrinia furnacalis]	-0.457	-1.695	1.1941	0.60837	0.3495
TRINITY_DN22875_c0_g1_i6_orf1	-	-	-	microtubule-actin cross-linking factor 1 isoform X15 [Ostrinia furnacalis]	-0.5848	-1.6324	1.06815	0.2643	0.88467
TRINITY_DN4502_c0_g1_i3_orf1	-	-	-	kynurenine formamidase isoform X1 [Ostrinia furnacalis]	-0.5348	-1.7103	0.92838	0.48493	0.8318
TRINITY_DN26439_c0_g1_i2_orf1	-	-	-	uncharacterized protein LOC114351853 [Ostrinia furnacalis]	-0.9057	-1.4429	1.16983	0.45826	0.72055
TRINITY_DN3826_c0_g1_i1_orf1	-	-	-	39S ribosomal protein L18, mitochondrial [Ostrinia furnacalis]	-1.0952	-1.1241	0.47951	1.49986	0.23988
TRINITY_DN214_c0_g1_i4_orf1	-	-	-	uncharacterized protein LOC114352813 [Ostrinia furnacalis]	-1.2403	-1.0073	1.4236	0.61285	0.21121
TRINITY_DN2392_c0_g2_i1_orf1	-	-	-	cytochrome P450 9e2-like [Ostrinia furnacalis] >QPF77612.1 cytochrome P450 monooxygenase CYP9A185 [Ostrinia furnacalis]	-0.4968	-1.4717	-0.1314	0.62247	1.47738
TRINITY_DN11698_c0_g1_i1_orf1	-	-	-	hypothetical protein evm_015129 [Chilo suppressalis]	-0.8045	-1.5159	0.41208	0.77516	1.13319
TRINITY_DN3196_c0_g1_i1_orf1	-	-	-	organic cation transporter-like protein [Ostrinia furnacalis]	-0.4167	-1.6991	0.28531	0.61271	1.21769
TRINITY_DN5266_c0_g1_i1_orf1	-	-	-	malate dehydrogenase, cytoplasmic isoform X2 [Ostrinia furnacalis]	-0.6811	-1.6325	0.59319	0.71996	1.00045
TRINITY_DN6813_c1_g1_i1_orf1	-	-	-	pantothenate kinase 3 isoform X2 [Ostrinia furnacalis] >XP_028173241.1 pantothenate kinase 3 isoform X2 [Ostrinia furnacalis]	-0.8523	-1.4126	0.17222	1.21786	0.87482
TRINITY_DN214_c0_g1_i3_orf1	-	-	-	uncharacterized protein LOC114352813 [Ostrinia furnacalis]	-1.14	-1.1513	1.36991	0.65265	0.26877
TRINITY_DN138086_c0_g1_i1_orf1	-	-	-	hypothetical protein evm_000614 [Chilo suppressalis]	-0.9705	-1.4059	1.12567	0.49825	0.75248
TRINITY_DN12873_c0_g1_i3_orf1	-	-	-	proteoglycan 4-like [Ostrinia furnacalis]	-0.8395	-1.4375	0.22303	1.21369	0.84027
TRINITY_DN1226_c0_g1_i11_orfp1	-	-	-	TRINITY_DN1226_c0_g1_i11_m.52385 TRINITY_DN1226_c0_g1::TRINITY_DN1226_c0_g1_i11::g.52385 ORF type:internal len:92 (-),score=5.77 TRINITY_DN1226_c0_g1_i11:2-274(-)	-1.1191	-1.1938	0.75418	0.2602	1.29849
TRINITY_DN1230_c1_g1_i5_orf1	-	-	-	uncharacterized protein LOC114353440 [Ostrinia furnacalis]	-1.016	-1.3733	0.87327	0.46648	1.04956
TRINITY_DN6483_c0_g1_i6_orf1	-	-	-	transketolase-like protein 2 isoform X1 [Ostrinia furnacalis] >XP_028164795.1 transketolase-like protein 2 isoform X2 [Ostrinia	-0.4891	-1.7343	0.75333	0.50624	0.96388
TRINITY_DN103475_c0_g1_i4_orf1	-	-	-	lipid storage droplets surface-binding protein 1 isoform X3 [Ostrinia furnacalis]	-0.8857	-1.4875	1.03294	0.5326	0.80764
TRINITY_DN3758_c0_g1_i2_orf1	-	-	-	S-formylglutathione hydrolase isoform X1 [Spodoptera litura]	-0.1422	-1.8479	0.51393	1.06309	0.41314
TRINITY_DN67243_c0_g1_i1_orf1	-	-	-	39S ribosomal protein L3, mitochondrial [Ostrinia furnacalis]	-0.4647	-1.7652	0.6624	0.76647	0.8011
TRINITY_DN117844_c0_g1_i1_orf1	-	-	-	ATP-citrate synthase [Cotesia glomerata] >XP_044590631.1 ATP-citrate synthase [Cotesia glomerata] >KAH0546822.1 hypothetical protein KQX54_015428 [Cotesia glomerata]	-1.075	-1.3484	0.59378	0.91078	0.9188
TRINITY_DN135188_c0_g1_i2_orf1	-	-	-	proteasome inhibitor PI31 subunit [Ostrinia furnacalis]	-0.3741	-1.8071	0.65161	0.76706	0.76253
TRINITY_DN4314_c0_g1_i9_orf1	-	-	-	serine proteinase inhibitor 2 [Ostrinia furnacalis]	-1.1943	-1.1955	0.44553	1.13917	0.80511
TRINITY_DN29698_c0_g1_i3_orf1	-	-	-	15-hydroxyprostaglandin dehydrogenase [NAD(+)]-like [Ostrinia furnacalis]	-1.0878	-1.3142	1.13357	0.63215	0.63632
TRINITY_DN384_c0_g1_i8_orf1	-	-	-	unnamed protein product [Chilo suppressalis]	-0.8339	-1.5327	0.57016	0.80611	0.99028
TRINITY_DN29873_c0_g1_i1_orf1	-	-	-	glyceraldehyde-3-phosphate dehydrogenase 2 [Pectinophora gossypiella]	-0.4248	-1.7002	0.55244	1.22621	0.34641
TRINITY_DN7075_c0_g2_i1_orf1	-	-	-	retinal dehydrogenase 1-like [Ostrinia furnacalis]	-0.4074	-1.6216	-0.0528	0.90916	1.17263
TRINITY_DN24970_c0_g1_i4_orf1	-	-	-	pyrroline-5-carboxylate reductase-like isoform X1 [Ostrinia furnacalis]	-0.8727	-1.5205	0.85304	0.69033	0.84983

TRINITY_DN245_c0_g1_i4_orf1	-	-	-	ER lumen protein-retaining receptor [Ostrinia furnacalis]	-1.4415	-0.9378	0.47339	0.98805	0.91781
TRINITY_DN2182_c0_g1_i4_orf1	-	-	-	growth-blocking peptide, long form-like isoform X1 [Ostrinia furnacalis] >XP_028159332.1 growth-blocking peptide, long form-like isoform X1 [Ostrinia furnacalis] >QWX20072.1 growth-blocking peptide [Ostrinia furnacalis]	-0.4384	-1.7109	1.02537	0.87627	0.24765
TRINITY_DN625_c9_g1_i7_orf1	-	-	-	ecdysone 20-monooxygenase [Ostrinia furnacalis]	-0.711	-1.5979	0.49407	0.74877	1.06604
TRINITY_DN135679_c0_g1_i2_orfp1	-	-	-	TRINITY_DN135679_c0_g1_i2_m.85525 TRINITY_DN135679_c0_g1::TRINITY_DN135679_c0_g1_i2::g.85525 ORF type:5prime_partial len:55 (+),score=5.08,Toxin_2 PF00451.20 1.9e-06 TRINITY_DN135679_c0_g1_i2:3-167(+)	-1.3145	-1.042	0.61395	1.25296	0.48952
TRINITY_DN5001_c0_g1_i4_orf1	-	-	-	uncharacterized protein LOC114356665 [Ostrinia furnacalis]	-1.2161	-1.0354	0.05198	0.98282	1.21671
TRINITY_DN2772_c0_g1_i3_orf1	-	-	-	uncharacterized protein LOC114353284 isoform X4 [Ostrinia furnacalis] >XP_028161011.1 uncharacterized protein LOC114353284 isoform X4 [Ostrinia furnacalis] >XP_028161012.1 uncharacterized protein LOC114353284 isoform X4 [Ostrinia furnacalis]	-1.0565	-1.2004	0.21757	1.41714	0.62222
TRINITY_DN12666_c0_g1_i2_orf1	-	-	-	sodium- and chloride-dependent GABA transporter 1 [Ostrinia furnacalis]	-0.9833	-1.3174	0.77094	1.2812	0.24855
TRINITY_DN2514_c1_g1_i13_orf1	-	-	-	seroin transcript 1A2 [Ostrinia nubilalis]	-1.2286	-1.2016	0.76788	0.63721	1.02515
TRINITY_DN1034_c0_g2_i1_orf1	-	-	-	glycerol kinase isoform X7 [Ostrinia furnacalis] >XP_028168096.1 glycerol kinase isoform X8 [Ostrinia furnacalis]	-0.2765	-1.8283	0.466	0.9216	0.71719
TRINITY_DN1047_c0_g1_i6_orf1	-	-	-	mitochondrial genome maintenance exonuclease 1-like [Ostrinia furnacalis]	-0.5376	-1.7214	0.83014	0.57626	0.85252
TRINITY_DN8625_c0_g1_i1_orf1	-	-	-	GDP-L-fucose synthase [Ostrinia furnacalis]	-0.9143	-1.186	0.27083	1.62561	0.20384
TRINITY_DN1827_c0_g1_i4_orf1	-	-	-	phosphoglycerate mutase 1 [Ostrinia furnacalis]	-0.5543	-1.6558	0.54932	1.19794	0.46286
TRINITY_DN1084_c0_g1_i2_orf1	-	-	-	ATP-citrate synthase [Ostrinia furnacalis]	-0.6055	-1.5429	0.10453	1.29863	0.74526
TRINITY_DN4245_c0_g1_i5_orf1	-	-	-	long-chain fatty acid transport protein 4-like [Ostrinia furnacalis]	-0.8368	-1.5312	1.01665	0.61023	0.7411
TRINITY_DN7630_c0_g2_i1_orf1	-	-	-	flotillin-2 isoform X1 [Ostrinia furnacalis] >XP_028172931.1 flotillin-2 isoform X2 [Ostrinia furnacalis]	-0.524	-1.7047	0.84554	0.41968	0.96349
TRINITY_DN2847_c0_g1_i20_orf1	-	-	-	uncharacterized protein LOC114352221 [Ostrinia furnacalis]	-0.4687	-1.4772	-0.1379	0.59043	1.49347
TRINITY_DN701_c0_g1_i1_orf1	-	-	-	venom protease-like isoform X3 [Ostrinia furnacalis]	-1.0141	-1.3194	0.60083	0.43697	1.29569
TRINITY_DN2378_c0_g1_i5_orf1	-	-	-	integrin alpha-8-like isoform X1 [Ostrinia furnacalis]	-0.6393	-1.6147	0.68149	0.40985	1.16262
TRINITY_DN5081_c0_g1_i5_orf1	-	-	-	ester hydrolase C11orf54 homolog isoform X1 [Ostrinia furnacalis]	-0.4544	-1.6673	0.1437	0.85329	1.12471
TRINITY_DN8030_c0_g1_i2_orf1	-	-	-	chaoptin isoform X1 [Ostrinia furnacalis] >XP_028159953.1 chaoptin isoform X2 [Ostrinia furnacalis] >XP_028159954.1 chaoptin isoform X3 [Ostrinia furnacalis] >XP_028159955.1 chaoptin isoform X4 [Ostrinia furnacalis] >XP_028159956.1 chaoptin isoform X5 [Ostrinia furnacalis]	-0.9623	-1.3678	0.27146	0.9568	1.10181
TRINITY_DN3616_c0_g2_i2_orf1	-	-	-	conotoxin ArMKLT2-032-like [Ostrinia furnacalis]	-1.1778	-1.1895	0.65611	0.46608	1.24508
TRINITY_DN4068_c1_g2_i1_orf1	-	-	-	larval cuticle protein LCP-17 [Helicoverpa armigera] >PZC82071.1 hypothetical protein B5X24_HaOG211161 [Helicoverpa armigera] >PZC87412.1 hypothetical protein B5X24_HaOG216859 [Helicoverpa armigera]	-1.3426	-0.964	1.34151	0.51963	0.44546
TRINITY_DN34134_c0_g2_i1_orf1	-	-	-	THUMP domain-containing protein 1 homolog [Ostrinia furnacalis]	-0.6652	-1.5266	0.66113	0.20916	1.32147
TRINITY_DN91946_c0_g1_i1_orf1	-	-	-	protein catecholamines up [Ostrinia furnacalis]	-0.7697	-1.4672	0.91434	0.13953	1.18299
TRINITY_DN65518_c0_g1_i1_orf1	-	-	-	unc-112-related protein-like, partial [Ostrinia furnacalis]	-0.9369	-1.4615	0.59447	0.95666	0.84722
TRINITY_DN22513_c0_g1_i4_orf1	-	-	-	DNA-directed RNA polymerase II subunit RPB1 [Ostrinia furnacalis] >XP_028179194.1 DNA-directed RNA polymerase II subunit RPB1 [Ostrinia furnacalis] >XP_028179195.1 DNA-directed RNA polymerase II subunit RPB1 [Ostrinia furnacalis]	-1.4546	-0.9573	0.86804	0.66633	0.87756
TRINITY_DN8527_c0_g2_i1_orfp1	-	-	-	TRINITY_DN8527_c0_g2_i1_m.16937 TRINITY_DN8527_c0_g2::TRINITY_DN8527_c0_g2_i1::g.16937 ORF type:5prime_partial len:54 (-),score=0.36 TRINITY_DN8527_c0_g2_i1:195-356(-)	-1.3598	-1.047	0.52817	0.86394	1.01464
TRINITY_DN10403_c0_g1_i1_orf1	-	-	-	hypothetical protein evm_000264 [Chilo suppressalis] >CAH2987898.1 unnamed protein product [Chilo suppressalis]	-0.8494	-1.403	0.30404	0.5742	1.37408
TRINITY_DN6587_c0_g1_i3_orf1	-	-	-	phosphoribosylformylglycinamidine synthase isoform X2 [Ostrinia furnacalis]	-0.474	-1.6381	0.34297	1.33687	0.43235
TRINITY_DN8702_c0_g1_i1_orf1	-	-	-	programmed cell death protein 4 isoform X1 [Ostrinia furnacalis] >XP_028157160.1 programmed cell death protein 4 isoform X2 [Ostrinia furnacalis]	-0.7039	-1.603	0.79603	0.4728	1.0381
TRINITY_DN66453_c0_g1_i4_orfp1	-	-	-	TRINITY_DN66453_c0_g1_i4_m.7345 TRINITY_DN66453_c0_g1::TRINITY_DN66453_c0_g1_i4::g.7345 ORF type:internal len:93 (+),score=24.53 TRINITY_DN66453_c0_g1_i4:3-278(+)	-1.1127	-1.1439	0.89828	0.07731	1.28091
TRINITY_DN11231_c1_g1_i1_orfp1	-	-	-	TRINITY_DN11231_c1_g1_i1_m.13377 TRINITY_DN11231_c1_g1::TRINITY_DN11231_c1_g1_i1::g.13377 ORF type:internal len:76 (-),score=1.43 TRINITY_DN11231_c1_g1_i1:1-225(-)	-0.8269	-1.4071	1.09604	0.07526	1.06271
TRINITY_DN38225_c0_g2_i1_orf1	-	-	-	uncharacterized protein LOC114354273 [Ostrinia furnacalis]	-0.9034	-1.358	1.38972	0.31622	0.55542
TRINITY_DN511_c0_g2_i1_orf1	-	-	-	pyruvate carboxylase, mitochondrial isoform X1 [Manduca sexta] >XP_037293486.1 pyruvate carboxylase, mitochondrial isoform X1 [Manduca sexta]	-0.8281	-1.5454	0.96842	0.67785	0.72719

TRINITY_DN10070_c0_g1_i1_orf1	-	-	-	40S ribosomal protein SA isoform 1 [Homo sapiens] >XP_002813955.1 40S ribosomal protein SA [Pongo abelii] >XP_004033937.1 40S ribosomal protein SA [Gorilla gorilla gorilla] >XP_008949773.1 40S ribosomal protein SA [Pan paniscus] >XP_009237465.1 40S ribosomal protein SA [Pongo abelii] >XP_024211184.1 40S ribosomal protein SA [Pan troglodytes] >XP_032017897.1 40S ribosomal protein SA [Hylobates moloch] >XP_032017898.1 40S ribosomal protein SA [Hylobates moloch] >XP_032615302.1 40S ribosomal protein SA [Hylobates moloch] >XP_034820112.1 40S ribosomal protein SA [Pan paniscus] >P08865.4 RecName: Full=40S ribosomal protein SA; AltName: Full=37 kDa laminin receptor precursor; Short=37LRP; AltName: Full=37/67 kDa laminin receptor; Short=LRP/LR; AltName: Full=67 kDa laminin receptor; Short=67LR; AltName: Full=Colon carcinoma laminin-binding protein; AltName: Full=Laminin receptor 1; Short=LamR; AltName: Full=Laminin-binding protein precursor p40; Short=LBP/p40; AltName: Full=Multidrug resistance-associated protein MGr1-Ag; AltName: Full=NEM/1CHD4; AltName: Full=Small ribosomal subunit protein uS2 [Homo sapiens] >4D5L_A Cryo-EM structures of ribosomal 80S complexes with termination factors and cricket paralysis virus IRES reveal the IRES in the translocated state [Oryctolagus cuniculus] >4D61_A Cryo-EM structures of ribosomal 80S complexes with termination factors and cricket paralysis virus IRES reveal the IRES in the translocated state [Oryctolagus cuniculus] >4UG0_SA Chain SA, 40S RIBOSOMAL PROTEIN SA [Homo sapiens] >4UJD_CA Chain CA, 40S RIBOSOMAL PROTEIN US2 [Oryctolagus cuniculus] >4UJE_BA Chain BA, 40S RIBOSOMAL PROTEIN SA [Oryctolagus cuniculus] >4V6X_AA Chain AA, 40S ribosomal protein SA [Homo sapiens] >5A2Q_A Structure of the HCV IRES bound to the human ribosome [Homo sapiens] >5AJ0_BA Chain BA, 40S ribosomal protein SA [Homo sapiens] >5FLX_A Mammalian 40S HCV-IRES complex [Oryctolagus cuniculus] >5LKS_SA Chain SA, 40S ribosomal protein SA [Homo sapiens] >5OA3_A Human 40S-eIF2D-re-initiation complex [Homo sapiens] >5T2C_Ao Chain Ao, 40S ribosomal protein SA [Homo sapiens] >5VYC_A1 Chain A1, 40S ribosomal protein SA [Homo sapiens] >5VYC_A2 Chain A2, 40S ribosomal protein SA [Homo sapiens] >5VYC_A3 Chain A3, 40S ribosomal protein SA [Homo sapiens] >5VYC_A4 Chain A4, 40S ribosomal protein SA [Homo sapiens] >5VYC_A5 Chain A5, 40S ribosomal protein SA [Homo sapiens] >5VYC_A6 Chain A6, 40S ribosomal protein SA [Homo sapiens] >6EK0_SA Chain SA, 40S ribosomal protein SA [Homo sapiens] >6G18_A Cryo-EM structure of a late human pre-40S ribosomal subunit - State C [Homo sapiens] >6G4S_A Cryo-EM structure of a late human pre-40S ribosomal subunit - State B [Homo sapiens] >6G51_A Cryo-EM structure of a late human pre-40S ribosomal subunit - State D [Homo sapiens] >6G53_A Cryo-EM structure of a late human pre-40S ribosomal subunit - State E [Homo sapiens] >6G5H_A Cryo-EM structure of a late human pre-40S ribosomal subunit - Mature [Homo sapiens] >6G5I_A Cryo-EM structure of a late human pre-40S ribosomal subunit - State R [Homo sapiens] >6IP5_2n Chain 2n, 40S ribosomal protein SA [Homo sapiens] >6IP6_2n Chain 2n, 40S ribosomal protein SA [Homo sapiens] >6IP8_2n Chain 2n, 40S ribosomal protein SA [Homo sapiens] >6Y0G_SA Chain SA, 40S ribosomal protein SA [Homo sapiens] >6Y2L_SA Chain SA, 40S ribosomal protein SA [Homo sapiens] >6Y57_SA Chain SA, 40S ribosomal protein SA [Homo sapiens] >6YBD_N Structure of a human 48S translational initiation complex - eIF3 [Homo sapiens] >6YBW_N Structure of a human 48S translational initiation complex - 40S body [Homo sapiens] >6Z6L_SA Chain SA, 40S ribosomal protein SA [Homo sapiens] >6Z6M_SA Chain SA, 40S ribosomal	-1.374	-0.9218	0.29484	1.295	0.70598
TRINITY_DN15755_c0_g1_i1_orf1	-	-	-	cytochrome P450 monooxygenase CYP6AB141 [Ostrinia furnacalis]	-1.1196	-1.0622	0.96158	-0.0787	1.29894
TRINITY_DN9694_c0_g1_i1_orf1	-	-	-	larval cuticle protein LCP-17 [Helicoverpa armigera] >PZC82071.1 hypothetical protein B5X24_HaOG211161 [Helicoverpa armigera] >PZC87412.1 hypothetical protein B5X24_HaOG216859 [Helicoverpa armigera]	-1.5181	-0.8696	0.73023	0.70097	0.95642
TRINITY_DN1328_c0_g1_i6_orf1	-	-	-	fungal protease inhibitor-1-like [Ostrinia furnacalis]	-1.112	-1.2203	0.31708	1.27504	0.74022
TRINITY_DN44083_c0_g1_i2_orf1	-	-	-	putative alpha-ketoglutarate-dependent hypophosphite dioxygenase [Operophtera brumata]	-0.9269	-1.4462	0.91872	0.45545	0.99893
TRINITY_DN49038_c0_g4_i1_orf1	-	-	-	6-phosphogluconate dehydrogenase, decarboxylating [Ostrinia furnacalis]	-0.5405	-1.727	0.83303	0.75285	0.68164
TRINITY_DN3859_c0_g1_i5_orf1	-	-	-	D-aspartate oxidase [Ostrinia furnacalis] >XP_028166452.1 D-aspartate oxidase [Ostrinia furnacalis] >XP_028166454.1 D-aspartate oxidase [Ostrinia furnacalis]	-1.2222	-1.1492	0.96059	1.07118	0.33965
TRINITY_DN31163_c1_g1_i4_orf1	-	-	-	phenoloxidase subunit 2-like [Ostrinia furnacalis]	-0.8351	-1.2361	0.2996	1.6327	0.13885
TRINITY_DN12013_c0_g1_i6_orf1	-	-	-	tudor domain-containing protein 7 isoform X3 [Ostrinia furnacalis]	-0.8578	-1.01	1.79872	0.09119	-0.0222
TRINITY_DN350_c0_g1_i4_orf1	-	-	-	microtubule-associated protein tau-like isoform X6 [Ostrinia furnacalis]	-0.9066	-1.4769	0.92384	0.53198	0.92763
TRINITY_DN610_c0_g1_i1_orf1	-	-	-	CAP-Gly domain-containing linker protein 1 isoform X10 [Ostrinia furnacalis]	-1.1576	-1.232	0.96644	1.02417	0.399
TRINITY_DN10360_c0_g1_i16_orf1	-	-	-	H(+)/Cl(-) exchange transporter 3 isoform X1 [Ostrinia furnacalis]	-1.2384	-1.1457	0.62895	0.54719	1.20787
TRINITY_DN77559_c0_g1_i1_orf1	-	-	-	uncharacterized protein LOC114359392 isoform X2 [Ostrinia furnacalis]	-0.9177	-1.3714	0.2321	0.79649	1.26049
TRINITY_DN1038_c1_g1_i3_orf1	-	-	-	uncharacterized protein LOC114363583 [Ostrinia furnacalis]	-1.3507	-1.0305	1.10594	0.44096	0.83432
TRINITY_DN33_c0_g1_i14_orf1	-	-	-	uncharacterized protein CG45076-like isoform X1 [Ostrinia furnacalis]	-0.8836	-1.4402	1.08428	0.30246	0.93703
TRINITY_DN64719_c0_g1_i2_orfp1	-	-	-	TRINITY_DN64719_c0_g1_i2_m.37745 TRINITY_DN64719_c0_g1::TRINITY_DN64719_c0_g1_i2::g.37745 ORF type:internal len:91 (+),score=41.89 TRINITY_DN64719_c0_g1_i2:1-270(+)	-1.2922	-0.82	1.57752	0.15284	0.38186
TRINITY_DN566_c0_g1_i13_orf1	-	-	-	uncharacterized protein LOC114355567 isoform X2 [Ostrinia furnacalis]	-0.8861	-1.3931	0.74864	0.24463	1.28597
TRINITY_DN13236_c0_g1_i4_orf1	-	-	-	zonadhesin-like isoform X4 [Ostrinia furnacalis]	-1.3108	-0.9254	1.4502	0.31118	0.47484
TRINITY_DN39837_c0_g1_i1_orf1	-	-	-	unnamed protein product [Plutella xylostella]	-1.2523	-1.1769	0.90928	0.93988	0.58006
TRINITY_DN2596_c0_g1_i6_orf1	-	-	-	unnamed protein product [Arctia plantaginis]	-1.1681	-1.1127	0.11708	0.93568	1.22808
TRINITY_DN93764_c0_g1_i1_orf1	-	-	-	microtubule-associated protein futsch isoform X4 [Ostrinia furnacalis] >XP_028162562.1 microtubule-associated protein futsch isoform X4 [Ostrinia furnacalis]	-0.9674	-1.2589	1.36422	0.78227	0.07976
TRINITY_DN8853_c0_g1_i4_orf1	-	-	-	uncharacterized protein LOC114351488 isoform X1 [Ostrinia furnacalis]	-1.1259	-0.9977	0.34319	0.17111	1.60931
TRINITY_DN17759_c0_g1_i5_orf1	-	-	-	hypothetical protein evm_013530 [Chilo suppressalis]	-1.0755	-1.2977	1.22451	0.5659	0.58279
TRINITY_DN8854_c0_g1_i2_orf1	-	-	-	glutathione S-transferase epsilon 2 [Ostrinia furnacalis]	-0.6079	-1.5223	0.13375	1.38693	0.60955
TRINITY_DN12545_c0_g1_i7_orf1	-	-	-	hypothetical protein evm_003491 [Chilo suppressalis]	-1.1817	-1.1449	1.2189	0.24279	0.86493
TRINITY_DN51776_c0_g1_i1_orf1	-	-	-	unnamed protein product, partial [Iphiclides podalirius]	-0.9375	-1.3319	0.20557	0.73587	1.32793
TRINITY_DN1455_c0_g1_i4_orf1	-	-	-	troponin domain-containing protein [Phthorimaea operculella]	-1.212	-1.2165	1.02591	0.61468	0.78792
TRINITY_DN15040_c0_g4_i1_orf1	-	-	-	hypothetical protein O3G_MSEX013320 [Manduca sexta]	-1.1747	-1.0231	0.13527	0.56866	1.49388
TRINITY_DN64126_c0_g1_i1_orf1	-	-	-	senecionine N-oxygenase isoform X2 [Galleria mellonella]	-0.9441	-1.4023	0.40815	0.74859	1.18964
TRINITY_DN44491_c0_g1_i12_orf1	-	-	-	unnamed protein product [Chrysodeixis includens]	-1.067	-1.2839	1.04087	0.28643	1.02358
TRINITY_DN2688_c0_g1_i3_orf1	-	-	-	mitochondrial amidoxime reducing component 2-like [Ostrinia furnacalis]	-0.9458	-1.1919	1.49357	0.67316	-0.029
TRINITY_DN757_c3_g1_i2_orf1	-	-	-	PREDICTED: galectin-4-like [Amyelois transitella]	-0.7952	-1.5514	0.85701	0.50289	0.98666

TRINITY_DN32586_c0_g2_i1_orf1	-	-	-	unnamed protein product [Euphydryas editha]	-1.6179	-0.7164	0.67344	0.6958	0.96511
TRINITY_DN3991_c0_g1_i6_orf1	-	-	-	acetyl-CoA carboxylase isoform X3 [Trichoplusia ni]	-0.9395	-1.2792	0.29226	0.44359	1.48284
TRINITY_DN13711_c0_g1_i1_orf1	-	-	-	putative nuclease HARBI1 [Myzus persicae]	-1.1895	-1.1202	0.27853	1.32389	0.70721
TRINITY_DN1470_c0_g1_i8_orf1	-	-	-	oxidation resistance protein 1 isoform X5 [Ostrinia furnacalis]	-1.1742	-1.2008	1.23451	0.52121	0.61932
TRINITY_DN1034_c0_g1_i4_orf1	-	-	-	glycerol kinase isoform X4 [Ostrinia furnacalis]	-1.1034	-1.2657	0.88194	1.12886	0.3583
TRINITY_DN695_c0_g1_i5_orf1	-	-	-	uncharacterized protein LOC114363574 isoform X1 [Ostrinia furnacalis]	-1.2491	-1.1838	0.95322	0.87237	0.60728
TRINITY_DN5748_c0_g1_i5_orf1	-	-	-	glycine N-methyltransferase isoform X1 [Ostrinia furnacalis] >XP_028165118.1 glycine N-methyltransferase isoform X2 [Ostrinia furnacalis] >XP_028165119.1 glycine N-methyltransferase isoform X1 [Ostrinia furnacalis] >XP_028165120.1 glycine N-methyltransferase isoform X2 [Ostrinia furnacalis]	-0.9231	-1.4259	0.60185	0.53725	1.2099
TRINITY_DN14112_c0_g1_i3_orf1	-	-	-	uncharacterized protein LOC114350956 [Ostrinia furnacalis]	-0.968	-1.3602	0.44261	0.59709	1.28854
TRINITY_DN89483_c0_g1_i1_orf1	-	-	-	mitochondrial enolase superfamily member 1-like isoform X2 [Maniola jurtina]	-0.94	-1.1892	0.43433	0.11361	1.58132
TRINITY_DN23069_c0_g2_i3_orf1	-	-	-	uncharacterized protein LOC114364799 [Ostrinia furnacalis]	-1.3311	-1.0622	0.49467	0.78673	1.11189
TRINITY_DN6642_c0_g1_i2_orf1	-	-	-	protein purity of essence [Ostrinia furnacalis]	-0.9854	-1.1384	0.12537	0.39902	1.5994
TRINITY_DN38230_c0_g1_i4_orf1	-	-	-	hypothetical protein evm_007803 [Chilo suppressalis]	-1.4054	-0.7866	1.40858	0.15204	0.63146
TRINITY_DN62707_c0_g1_i1_orf1	-	-	-	uncharacterized protein LOC114362831 [Ostrinia furnacalis]	-1.0397	-1.1559	1.49791	0.56749	0.13028
TRINITY_DN50593_c0_g1_i1_orf1	-	-	-	uncharacterized protein LOC114361588 isoform X14 [Ostrinia furnacalis]	-1.5215	-0.8002	0.39622	0.83218	1.09327
TRINITY_DN8651_c0_g1_i16_orf1	-	-	-	glutathione S-transferase theta 2 [Conogethes punctiferalis]	-0.7575	-1.3177	0.36977	0.11152	1.59393
TRINITY_DN1172_c0_g1_i1_orf1	-	-	-	hypothetical protein O3G_MSEX009550 [Manduca sexta]	-1.075	-1.3136	0.50697	0.72707	1.1546
TRINITY_DN1607_c0_g1_i16_orf1	-	-	-	LOW QUALITY PROTEIN: asparagine--tRNA ligase, cytoplasmic [Ostrinia furnacalis]	-1.5134	-0.8426	1.08801	0.71103	0.55699
TRINITY_DN9569_c1_g1_i7_orf1	-	-	-	V-set and immunoglobulin domain-containing protein 1-like isoform X1 [Ostrinia furnacalis] >XP_028156015.1 V-set and immunoglobulin domain-containing protein 1-like isoform X2 [Ostrinia furnacalis]	-1.3108	-1.1286	0.8528	0.89809	0.68856
TRINITY_DN57111_c0_g1_i1_orf1	-	-	-	trypsin-like serine proteinase T26 protein, partial [Chilo infuscatellus]	-1.5818	-0.5496	1.38376	0.39994	0.34777
TRINITY_DN60787_c0_g1_i5_orf1	-	-	-	probable transaldolase [Ostrinia furnacalis]	-1.0854	-1.3473	0.71858	0.96002	0.7541
TRINITY_DN5880_c0_g2_i2_orf1	-	-	-	macrophage mannose receptor 1 [Bombyx mori]	-0.9997	-1.1019	0.64841	-0.0827	1.53593
TRINITY_DN1245_c0_g1_i4_orf1	-	-	-	nuclear RNA export factor 1 [Ostrinia furnacalis]	-1.1863	-0.9791	0.15762	0.45905	1.54872
TRINITY_DN928_c0_g2_i1_orf1	-	-	-	fasciclin-2-like [Ostrinia furnacalis]	-1.4422	-0.9507	0.70404	0.62693	1.0619
TRINITY_DN61335_c0_g2_i1_orf1	-	-	-	uncharacterized protein LOC114352149 [Ostrinia furnacalis]	-1.2101	-1.1272	1.32051	0.46154	0.55525
TRINITY_DN10889_c0_g1_i8_orf1	-	-	-	four and a half LIM domains protein 2 isoform X7 [Pectinophora gossypiella]	-1.6738	-0.6175	0.75432	0.58581	0.95123
TRINITY_DN5768_c0_g1_i2_orf1	-	-	-	adenosylhomocysteinase [Ostrinia furnacalis]	-1.4462	-0.9186	1.08293	0.82833	0.45358
TRINITY_DN2175_c0_g1_i4_orf1	-	-	-	uncharacterized protein LOC114353827 [Ostrinia furnacalis]	-1.3649	-0.898	1.33908	0.69734	0.22646
TRINITY_DN22597_c0_g1_i4_orf1	-	-	-	uncharacterized protein LOC114361588 isoform X16 [Ostrinia furnacalis]	-1.4774	-0.8672	1.11528	0.43337	0.79598
TRINITY_DN2207_c0_g1_i6_orf1	-	-	-	methionine-R-sulfoxide reductase B1 isoform X2 [Ostrinia furnacalis]	-0.984	-1.3088	0.54838	1.37063	0.3737
TRINITY_DN116972_c0_g1_i1_orf1	-	-	-	phosphofructokinase domain-containing protein [Phthorimaea operculella]	-1.3173	-1.0869	1.12452	0.63202	0.64763
TRINITY_DN6994_c0_g1_i3_orf1	-	-	-	C-type mannose receptor 2-like isoform X1 [Ostrinia furnacalis]	-0.9028	-1.0489	1.73871	0.24601	-0.033
TRINITY_DN5628_c0_g1_i3_orf1	-	-	-	muscle LIM protein Mlp84B isoform X2 [Ostrinia furnacalis]	-1.483	-0.893	0.78646	0.54815	1.04134
TRINITY_DN13602_c0_g1_i4_orf1	-	-	-	NEDD8 ultimate buster 1-like [Ostrinia furnacalis]	-0.8245	-1.4169	0.64209	1.35697	0.24241
TRINITY_DN5933_c0_g1_i1_orf1	-	-	-	peroxidase-like [Ostrinia furnacalis]	-1.5106	-0.8536	0.52624	0.81023	1.02769
TRINITY_DN10774_c0_g2_i3_orf1	-	-	-	uncharacterized protein LOC114362157, partial [Ostrinia furnacalis]	-0.9298	-1.3025	0.49185	0.28605	1.45438
TRINITY_DN51424_c0_g2_i1_orf1	-	-	-	ras suppressor protein 1 [Helicoverpa zea]	-0.9868	-1.3232	1.18492	0.90781	0.21727
TRINITY_DN554_c0_g1_i1_orf1	-	-	-	uncharacterized protein LOC114353093 isoform X1 [Ostrinia furnacalis] >XP_028160722.1 uncharacterized protein LOC114353093 isoform X1 [Ostrinia furnacalis] >XP_028160723.1 uncharacterized protein LOC114353093 isoform X1 [Ostrinia furnacalis]	-1.2348	-1.0125	0.15574	0.70045	1.39112
TRINITY_DN35763_c0_g1_i2_orf1	-	-	-	probable methylthioribulose-1-phosphate dehydratase [Helicoverpa armigera]	-1.1081	-1.2231	0.37908	0.6389	1.31316
TRINITY_DN97472_c0_g1_i5_orf1	-	-	-	microtubule-actin cross-linking factor 1 isoform X15 [Ostrinia furnacalis]	-1.3901	-0.9711	1.20786	0.55647	0.5968
TRINITY_DN59422_c0_g1_i2_orf1	-	-	-	larval cuticle protein LCP-22-like isoform X2 [Pectinophora gossypiella]	-1.8106	-0.3481	0.821	0.54295	0.79479
TRINITY_DN3374_c0_g1_i7_orf1	-	-	-	TPPP family protein CG45057 [Ostrinia furnacalis] >XP_028172578.1 TPPP family protein CG45057 [Ostrinia furnacalis]	-1.1189	-1.1384	1.29926	0.08827	0.86971
TRINITY_DN56708_c0_g3_i1_orfp1	-	-	-	TRINITY_DN56708_c0_g3_i1_m.56611 TRINITY_DN56708_c0_g3::TRINITY_DN56708_c0_g3_i1::g.56611 ORF type:internal len:69 (-),score=2.50 TRINITY_DN56708_c0_g3_i1:1-204(-)	-1.3533	-0.859	1.45736	0.27311	0.48185
TRINITY_DN4695_c0_g1_i4_orf1	-	-	-	glutathione S-transferase epsilon 3 [Ostrinia furnacalis]	-1.1137	-1.2009	0.41346	0.53865	1.3625
TRINITY_DN31225_c0_g1_i1_orf1	-	-	-	ribosome biogenesis protein BMS1 homolog [Ostrinia furnacalis]	-1.3342	-0.9015	1.13578	0.04374	1.05613
TRINITY_DN35800_c0_g1_i6_orf1	-	-	-	ommochrome-binding protein-like [Ostrinia furnacalis] >XP_028161264.1 ommochrome-binding protein-like [Ostrinia furnacalis] >XP_028161265.1 ommochrome-binding protein-like [Ostrinia furnacalis]	-1.1226	-1.1656	0.37021	1.40887	0.50912
TRINITY_DN36460_c0_g1_i2_orf1	-	-	-	N-acetylneuraminate lyase-like [Ostrinia furnacalis]	-1.1724	-1.2381	1.10292	0.55114	0.75649
TRINITY_DN1068_c0_g1_i3_orf1	-	-	-	aspartate aminotransferase, cytoplasmic [Ostrinia furnacalis]	-1.4286	-0.9827	0.69966	0.99542	0.71623
TRINITY_DN10441_c0_g1_i3_orf1	-	-	-	zonadhesin-like [Ostrinia furnacalis]	-1.4522	-0.7827	1.38412	0.40694	0.44386
TRINITY_DN38568_c0_g1_i1_orf1	-	-	-	unnamed protein product, partial [Diatraea saccharalis]	-1.728	-0.5081	0.79283	0.51439	0.92886
TRINITY_DN111488_c0_g1_i1_orf1	-	-	-	LOW QUALITY PROTEIN: formin-J-like [Chelonus insularis]	-1.7512	-0.4428	1.02693	0.55251	0.61447
TRINITY_DN57900_c0_g1_i2_orf1	-	-	-	hypothetical protein SFRURICE_000634 [Spodoptera frugiperda]	-1.408	-0.7393	0.38924	0.25696	1.50112
TRINITY_DN63030_c0_g1_i5_orf1	-	-	-	uncharacterized protein LOC114358571 [Ostrinia furnacalis]	-1.5085	-0.8569	0.88742	0.50409	0.97389
TRINITY_DN16824_c0_g1_i7_orf1	-	-	-	insulin receptor substrate 1 [Ostrinia furnacalis]	-0.9192	-1.3971	0.24814	1.07343	0.9947
TRINITY_DN1114_c0_g1_i4_orf1	-	-	-	vesicle-associated membrane protein 2 isoform X1 [Helicoverpa armigera] >XP_022834345.1 vesicle-associated membrane protein 2-like isoform X1 [Spodoptera litura] >XP_035453123.1 vesicle-associated membrane protein 2-like isoform X1 [Spodoptera frugiperda] >XP_047034881.1 vesicle-associated membrane protein 2 isoform X1 [Helicoverpa zea] >CAB3525008.1 unnamed protein product [Chilo suppressalis] >CAH0402335.1 unnamed protein product [Chilo suppressalis]	-1.2874	-0.9658	0.61554	1.4061	0.23163
TRINITY_DN60792_c0_g1_i2_orf1	-	-	-	ATP-binding cassette sub-family D member 2 [Ostrinia furnacalis] >XP_028165108.1 ATP-binding cassette sub-family D member 2 [Ostrinia furnacalis]	-1.2113	-0.7124	1.70042	-0.1215	0.34485

TRINITY_DN741_c0_g1_i10_orf1	-	-	-	talin-1 isoform X13 [Ostrinia furnacalis]	-1.2824	-1.0758	0.9068	0.32404	1.12733
TRINITY_DN62557_c0_g1_i1_orf1	-	-	-	6-phosphofructokinase [Operophtera brumata]	-1.4973	-0.795	0.60741	1.25343	0.43141
TRINITY_DN26993_c1_g1_i8_orf1	-	-	-	endocuticle structural glycoprotein ABD-4-like [Ostrinia furnacalis]	-1.2574	-0.894	1.48352	0.02083	0.64699
TRINITY_DN2722_c0_g1_i1_orf1	-	-	-	troponin C [Pieris rapae] >XP_045490973.1 troponin C-like isoform X1 [Colias croceus] >XP_049866665.1 troponin C-like [Pectinophora gossypiella]	-1.7256	-0.5359	0.83793	0.61258	0.81101
TRINITY_DN26961_c0_g1_i1_orf1	-	-	-	uncharacterized protein LOC120424957 [Culex pipiens pallens]	-1.5981	-0.6231	1.26262	0.52478	0.43382
TRINITY_DN1329_c0_g1_i5_orf1	-	-	-	neurogenic locus notch homolog protein 3 [Ostrinia furnacalis] >XP_028157678.1 neurogenic locus notch homolog protein 3 [Ostrinia furnacalis]	-1.6323	-0.6347	0.79316	0.40718	1.06669
TRINITY_DN9560_c0_g1_i5_orf1	-	-	-	uncharacterized protein LOC114357350 [Ostrinia furnacalis]	-1.7557	-0.3893	1.10851	0.51552	0.52095
TRINITY_DN12336_c0_g1_i1_orfp1	-	-	-	TRINITY_DN12336_c0_g1_i1_m.30792 TRINITY_DN12336_c0_g1::TRINITY_DN12336_c0_g1_i1::g.30792 ORF type:internal len:86 (-),score=13.56 TRINITY_DN12336_c0_g1_i1:3-257(-)	-1.2309	-0.816	1.07227	-0.2854	1.26006
TRINITY_DN1475_c0_g1_i6_orf1	-	-	-	uncharacterized protein LOC113226757 isoform X2 [Hyposmocoma kahamanoa]	-1.5383	-0.8462	0.80653	0.68408	0.89391
TRINITY_DN124171_c0_g1_i4_orf1	-	-	-	dystonin isoform X27 [Trichoplusia ni]	-1.3348	-1.0174	1.21197	0.39072	0.74949
TRINITY_DN2207_c0_g1_i4_orf1	-	-	-	methionine-R-sulfoxide reductase B1 isoform X4 [Pectinophora gossypiella] >XP_049887601.1 methionine-R-sulfoxide reductase B1 isoform X4 [Pectinophora gossypiella]	-1.4526	-0.6902	0.35132	0.31029	1.48119
TRINITY_DN47930_c0_g1_i4_orf1	-	-	-	uncharacterized protein LOC114362634 [Ostrinia furnacalis]	-1.6429	-0.676	0.89758	0.59206	0.82927
TRINITY_DN24121_c1_g1_i6_orf1	-	-	-	serine protease persephone-like [Ostrinia furnacalis]	-1.1883	-0.85	1.23011	-0.312	1.1202
TRINITY_DN120089_c0_g1_i1_orf1	-	-	-	phosphoglucomutase [Ostrinia furnacalis]	-1.5483	-0.8137	0.76377	0.59915	0.99913
TRINITY_DN15373_c0_g1_i2_orf1	-	-	-	SET domain-containing protein SmydA-8-like isoform X2 [Ostrinia furnacalis]	-1.6331	-0.6958	0.88853	0.82494	0.61543
TRINITY_DN2312_c0_g1_i4_orf1	-	-	-	endoplasmic reticulum-Golgi intermediate compartment protein 3 [Ostrinia furnacalis]	-1.74	-0.4963	0.94193	0.62577	0.66866
TRINITY_DN2956_c0_g1_i6_orf1	-	-	-	fructose-bisphosphate aldolase-like isoform X1 [Ostrinia furnacalis] >XP_028178678.1 fructose-bisphosphate aldolase-like isoform X1 [Ostrinia furnacalis]	-1.5902	-0.684	0.79378	0.36777	1.11267
TRINITY_DN105506_c0_g1_i8_orf1	-	-	-	microtubule-actin cross-linking factor 1 isoform X15 [Ostrinia furnacalis]	-1.6918	-0.5503	0.76612	0.45612	1.01982
TRINITY_DN147691_c0_g1_i1_orf1	-	-	-	WD repeat-containing protein 46 [Orussus abietinus]	-1.4423	-0.874	0.50652	0.54342	1.26643
TRINITY_DN4123_c0_g1_i1_orf1	-	-	-	uncharacterized protein LOC114355030 [Ostrinia furnacalis]	-1.1457	-1.0626	1.05329	-0.0476	1.20269
TRINITY_DN1404_c0_g1_i6_orf1	-	-	-	uncharacterized protein LOC114363065 [Ostrinia furnacalis]	-1.5995	-0.7309	1.00956	0.57081	0.74999
TRINITY_DN829_c0_g1_i8_orf1	-	-	-	cytochrome P450 6B6-like [Ostrinia furnacalis]	-1.4693	-0.6869	0.36845	0.3302	1.45756
TRINITY_DN16400_c0_g2_i1_orf1	-	-	-	superoxide dismutase [Cu-Zn]-like isoform X1 [Ostrinia furnacalis]	-1.498	-0.6059	1.48134	0.29009	0.33243
TRINITY_DN47151_c0_g1_i1_orf1	-	-	-	unnamed protein product [Danaus chrysippus]	-1.3848	-0.5394	1.03801	-0.3696	1.25586
TRINITY_DN22797_c0_g1_i5_orf1	-	-	-	phenoloxidase-activating factor 2-like isoform X1 [Ostrinia furnacalis]	-1.634	-0.4215	1.3784	0.44627	0.23085
TRINITY_DN58261_c0_g1_i2_orf1	-	-	-	15-hydroxyprostaglandin dehydrogenase [NAD(+)]-like [Ostrinia furnacalis]	-1.6742	-0.5939	0.61848	0.59972	1.04986
TRINITY_DN5628_c0_g1_i5_orf1	-	-	-	hypothetical protein O3G_MSEX015036 [Manduca sexta]	-1.7583	-0.4629	0.85726	0.56155	0.80239
TRINITY_DN10430_c0_g1_i4_orf1	-	-	-	fatty acid synthase [Ostrinia furnacalis] >XP_028160534.1 fatty acid synthase [Ostrinia furnacalis] >XP_028160535.1 fatty acid synthase [Ostrinia furnacalis] >XP_028160536.1 fatty acid synthase [Ostrinia furnacalis]	-1.3234	-0.741	0.73093	-0.132	1.46552
TRINITY_DN779_c0_g1_i3_orf1	-	-	-	uncharacterized protein LOC114351172 isoform X1 [Ostrinia furnacalis]	-1.6629	-0.5275	1.21657	0.45382	0.52004
TRINITY_DN10118_c0_g1_i4_orf1	-	-	-	glutaredoxin-C4-like [Ostrinia furnacalis]	-1.5917	-0.395	1.42158	0.53749	0.02764
TRINITY_DN2559_c0_g1_i4_orf1	-	-	-	uricase [Ostrinia furnacalis]	-1.5608	-0.5666	1.38525	0.2149	0.52718
TRINITY_DN5661_c0_g1_i5_orf1	-	-	-	cytochrome P450 6B7-like [Ostrinia furnacalis]	-1.4151	-0.7347	1.26176	-0.0414	0.92948
TRINITY_DN3231_c0_g1_i12_orf1	-	-	-	integrin-linked protein kinase [Pectinophora gossypiella]	-1.7021	-0.4761	0.71312	0.3489	1.11618
TRINITY_DN42120_c0_g1_i2_orf1	-	-	-	FAM172 family protein homolog CG10038 [Ostrinia furnacalis]	-1.2313	-0.9474	1.52644	0.47368	0.1786
TRINITY_DN15136_c0_g1_i2_orf1	-	-	-	alpha-aminoadipic semialdehyde synthase, mitochondrial isoform X3 [Ostrinia furnacalis]	-1.7206	-0.4799	1.08644	0.49171	0.6223
TRINITY_DN14922_c0_g3_i2_orf1	-	-	-	probable pseudouridine-5'-phosphatase [Ostrinia furnacalis]	-1.6839	-0.5303	1.14434	0.50938	0.56052
TRINITY_DN9820_c0_g1_i1_orf1	-	-	-	endocuticle structural glycoprotein SgAbd-2-like [Ostrinia furnacalis]	-1.8038	-0.3194	0.92167	0.40982	0.79171
TRINITY_DN19251_c0_g1_i8_orf1	-	-	-	succinate--CoA ligase [GDP-forming] subunit beta, mitochondrial [Ostrinia furnacalis]	-1.5317	-0.4039	1.55326	0.14039	0.24192
TRINITY_DN804_c0_g1_i7_orf1	-	-	-	hypothetical protein HF086_004695 [Spodoptera exigua] >CAH0695017.1 unnamed protein product [Spodoptera exigua]	-1.4215	-0.4701	1.44535	-0.3107	0.7569
TRINITY_DN1267_c0_g2_i10_orf1	-	-	-	secretory phospholipase A2 receptor-like [Ostrinia furnacalis]	-1.5298	-0.5348	1.44259	0.53347	0.08863
TRINITY_DN1993_c0_g1_i1_orf1	-	-	-	6-phosphofructo-2-kinase/fructose-2,6-bisphosphatase isoform X1 [Ostrinia furnacalis]	-1.433	-0.3865	1.65878	-0.0475	0.20822
TRINITY_DN870_c0_g1_i3_orf1	-	-	-	talin-1 isoform X12 [Ostrinia furnacalis]	-1.6363	-0.4028	1.01162	-0.0381	1.06559
TRINITY_DN120_c0_g1_i2_orf1	-	-	-	PREDICTED: myosin light chain alkali-like [Amyelois transitella]	-1.8051	-0.331	0.88496	0.44403	0.80718
TRINITY_DN3800_c0_g1_i7_orf1	-	-	-	hypothetical protein evm_004893 [Chilo suppressalis]	-1.6716	-0.4189	1.16369	0.11239	0.81445
TRINITY_DN17061_c0_g1_i1_orf1	-	-	-	uncharacterized protein LOC113511282 isoform X2 [Galleria mellonella]	-1.7599	-0.3821	0.86357	0.32817	0.95033
TRINITY_DN4793_c0_g1_i7_orf1	-	-	-	probable hydroxyacid-oxoacid transhydrogenase, mitochondrial isoform X3 [Ostrinia furnacalis] >XP_028159821.1 probable hydroxyacid-oxoacid transhydrogenase, mitochondrial isoform X4 [Ostrinia furnacalis]	-1.4163	-0.5975	0.96068	-0.2349	1.28802
TRINITY_DN14754_c0_g1_i6_orf1	-	-	-	cathepsin L [Papilio xuthus]	-1.1117	-0.8132	1.68125	-0.2292	0.4729
TRINITY_DN1333_c0_g1_i6_orf1	-	-	-	uncharacterized protein LOC114362563 [Ostrinia furnacalis]	-1.6965	-0.4758	0.9926	0.2623	0.91736
TRINITY_DN2193_c0_g1_i7_orf1	-	-	-	long-chain-fatty-acid--CoA ligase 5 isoform X1 [Ostrinia furnacalis] >XP_028176293.1 long-chain-fatty-acid--CoA ligase 5 isoform X1 [Ostrinia furnacalis] >XP_028176294.1 long-chain-fatty-acid--CoA ligase 5 isoform X1 [Ostrinia furnacalis] >XP_028176295.1 long-chain-fatty-acid--CoA ligase 5 isoform X1 [Ostrinia furnacalis] >XP_028176296.1 long-chain-fatty-acid--CoA ligase 5 isoform X1 [Ostrinia furnacalis] >XP_028176297.1 long-chain-fatty-acid--CoA ligase 5 isoform X1 [Ostrinia furnacalis] >XP_028176298.1 long-chain-fatty-acid--CoA ligase 5 isoform X2 [Ostrinia furnacalis]	-1.529	-0.5277	1.12899	-0.1188	1.04647
TRINITY_DN98995_c0_g1_i2_orf1	-	-	-	hypothetical protein HF086_008399, partial [Spodoptera exigua]	-1.6588	-0.2572	0.96638	-0.1566	1.10622
TRINITY_DN28503_c0_g1_i6_orf1	-	-	-	uncharacterized protein LOC114363584 [Ostrinia furnacalis] >AXY94663.1 seroin transcript 3 [Ostrinia nubilalis]	-1.436	-0.4692	1.62986	0.02959	0.24578
TRINITY_DN3913_c0_g1_i6_orf1	-	-	-	protein obstructor-E-like [Ostrinia furnacalis]	-1.8352	-0.2336	0.8609	0.37265	0.83523
TRINITY_DN109931_c0_g1_i1_orf1	-	-	-	hydroxymethylglutaryl-CoA lyase, mitochondrial isoform X1 [Ostrinia furnacalis]	-1.8569	-0.1798	0.93436	0.41132	0.69097
TRINITY_DN109943_c0_g1_i1_orf1	-	-	-	uncharacterized protein LOC114361588 isoform X14 [Ostrinia furnacalis]	-1.8413	-0.2089	0.87915	0.35358	0.81741

TRINITY_DN102051_c0_g1_i1_orf1	-	-	-	spectrin repeat domain-containing protein [Phthorimaea operculella]	-1.8189	-0.2968	0.97351	0.52989	0.6123
TRINITY_DN57856_c0_g2_i1_orf1	-	-	-	cytochrome P450 6B2-like [Ostrinia furnacalis]	-1.5577	-0.1669	0.94914	-0.4321	1.20756
TRINITY_DN84478_c0_g1_i8_orf1	-	-	-	uncharacterized protein LOC114359035 isoform X1 [Ostrinia furnacalis]	0.47386	-1.9622	0.50348	0.79809	0.18675
TRINITY_DN51995_c0_g3_i1_orf1	-	-	-	circadian clock-controlled protein-like [Ostrinia furnacalis]	-0.8234	-1.03	-0.2009	1.77233	0.28199
TRINITY_DN703_c0_g1_i2_orf1	-	-	-	acidic juvenile hormone-suppressible protein 1-like [Ostrinia furnacalis]	0.46724	-1.9072	0.09245	1.01136	0.33613
TRINITY_DN142657_c0_g1_i1_orf1	-	-	-	sorting and assembly machinery component 50 homolog [Diachasma alloeum]	0.27013	-1.8702	-0.007	1.06633	0.54074
TRINITY_DN106156_c1_g1_i1_orf1	-	-	-	arylphorin subunit alpha-like [Ostrinia furnacalis]	0.47645	-1.848	-0.0056	1.14116	0.23597
TRINITY_DN16091_c0_g1_i1_orfp1	-	-	-	TRINITY_DN16091_c0_g1_i1_m.64010 TRINITY_DN16091_c0_g1::TRINITY_DN16091_c0_g1_i1::g.64010 ORF type:5prime_partial len:124 (-),score=7.29,Toxin_2 PF00451.20 0.00035,Toxin_2|PF00451.20|0.00013,Toxin_2|PF00451.20|0.00037,Gamma-thionin|PF00304.21|0.37,Gamma-thionin|PF00304.21|0.052,Gamma-thionin|PF00304.21|0.022,Defensin_2|PF01097.19|0.58,Defensin_2|PF01097.19|0.12,Defensin_2|PF01097.19|0.011,Toxin_38|PF14866.7|0.18,Toxin_38|PF14866.7|0.18,Toxin_38|PF14866.7|0.4 TRINITY_DN16091_c0_g1_i1:19-390(-)	-0.2869	-1.4033	-0.0215	1.71696	-0.0053
TRINITY_DN5177_c0_g1_i2_orf1	-	-	-	hemolin-like isoform X1 [Ostrinia furnacalis]	-0.0255	-1.7366	-0.1745	0.77097	1.16566
TRINITY_DN13799_c0_g1_i1_orf1	-	-	-	uncharacterized protein LOC116345248 [Contarinia nasturtii]	0.40844	-1.6848	-0.5319	1.10038	0.70782
TRINITY_DN2049_c1_g1_i3_orf1	-	-	-	luciferin 4-monooxygenase-like [Ostrinia furnacalis]	0.57208	-1.5076	-0.2188	1.49522	-0.3409
TRINITY_DN45037_c0_g1_i1_orf1	-	-	-	trafficking protein particle complex subunit 1 [Ostrinia furnacalis]	0.51292	-1.7565	-0.1949	1.25727	0.18122
TRINITY_DN124654_c0_g1_i1_orf1	-	-	-	protein lethal(2)essential for life [Manduca sexta] >KAG6441919.1 hypothetical protein O3G_MSEX002019 [Manduca sexta]	0.39252	-1.656	-0.5998	0.86896	0.99432
TRINITY_DN17574_c0_g1_i2_orf1	-	-	-	heat shock protein Hsp-12.2-like [Ostrinia furnacalis]	0.66232	-1.4682	-0.9201	1.0496	0.67637
TRINITY_DN9733_c0_g1_i2_orf1	-	-	-	acidic juvenile hormone-suppressible protein 1-like [Ostrinia furnacalis]	0.86239	-1.4475	-0.2293	1.34974	-0.5353
TRINITY_DN295_c2_g1_i2_orf1	-	-	-	phosphoglycolate phosphatase 1A, chloroplastic [Manduca sexta]	0.39185	-1.8985	0.36483	1.04894	0.09291
TRINITY_DN10138_c0_g1_i1_orf1	-	-	-	storage protein 1 [Omphisa fuscidentalis]	0.59723	-1.7698	-0.0794	1.22648	0.02552
TRINITY_DN16234_c0_g2_i3_orf1	-	-	-	uncharacterized protein LOC114363370 [Ostrinia furnacalis]	-0.2805	-1.4257	-0.5589	1.20588	1.05928
TRINITY_DN1048_c0_g1_i6_orf1	-	-	-	uncharacterized protein LOC114360661 [Ostrinia furnacalis]	0.41615	-1.8346	0.37292	1.1455	-0.1
TRINITY_DN9090_c0_g1_i9_orf1	-	-	-	CD63 antigen [Ostrinia furnacalis]	0.2053	-1.9417	0.29934	0.89975	0.53727
TRINITY_DN276_c0_g1_i1_orf1	-	-	-	protein lethal(2)essential for life-like [Helicoverpa zea] >XP_049705426.1 protein lethal(2)essential for life [Helicoverpa armigera] >ATB54993.1 heat shock protein 20.8 [Helicoverpa armigera] >PZC74337.1 hypothetical protein B5X24_HaOG207971 [Helicoverpa armigera]	0.15398	-1.4905	-0.7438	1.17746	0.90284
TRINITY_DN2146_c0_g2_i1_orf1	-	-	-	heat shock protein 68-like [Ostrinia furnacalis]	0.01826	-1.488	-0.6286	1.35631	0.74208
TRINITY_DN6497_c0_g1_i1_orf1	-	-	-	ommochrome-binding protein-like [Ostrinia furnacalis]	0.29728	-1.8921	0.13028	1.08027	0.38426
TRINITY_DN9593_c0_g1_i2_orf1	-	-	-	uncharacterized protein LOC113518937 [Galleria mellonella]	0.57016	-1.59	-0.6368	1.25792	0.39871
TRINITY_DN2314_c0_g1_i7_orf1	-	-	-	protein dj-1beta-like isoform X1 [Ostrinia furnacalis]	0.07246	-1.8251	-0.0474	1.04376	0.75626
TRINITY_DN4235_c0_g1_i2_orf1	-	-	-	uncharacterized protein LOC114361536 [Ostrinia furnacalis]	0.25747	-1.9616	0.40485	0.84674	0.45257
TRINITY_DN71863_c0_g1_i2_orf1	-	-	-	unnamed protein product [Diatraea saccharalis]	0.45201	-1.872	-0.1037	1.00659	0.51707
TRINITY_DN2472_c0_g1_i6_orf1	-	-	-	programmed cell death protein 6 isoform X1 [Colias croceus] >XP_045492459.1 programmed cell death protein 6 isoform X1 [Colias croceus]	0.18576	-1.9091	0.36408	1.04337	0.31586
TRINITY_DN143532_c0_g1_i1_orf1	-	-	-	3-oxoacyl-[acyl-carrier-protein] reductase FabG-like [Aphidius gifuensis] >KAF7996667.1 hypothetical protein HCN44_002313 [Aphidius gifuensis]	-0.892	-0.7626	-0.6005	1.72741	0.5276
TRINITY_DN9325_c0_g1_i1_orf1	-	-	-	protein takeout-like [Ostrinia furnacalis]	0.00291	-1.7162	-0.2631	0.86146	1.11497
TRINITY_DN1534_c0_g1_i3_orf1	-	-	-	peptidoglycan recognition protein-like [Ostrinia furnacalis]	-0.625	-1.0596	-0.4714	1.76361	0.39243

TRINITY_DN142442_c0_g1_i1_orf1	-	-	-	60S ribosomal protein L38 [Homo sapiens] >NP_001002486.1 60S ribosomal protein L38 [Danio rerio] >NP_001030335.1 60S ribosomal protein L38 [Homo sapiens] >NP_001071060.1 60S ribosomal protein L38 [Rattus norvegicus] >NP_001133168.1 60S ribosomal protein L38 [Salmo salar] >NP_001187063.1 60S ribosomal protein L38 [Ictalurus punctatus] >NP_001232305.1 60S ribosomal protein L38 [Taeniopygia guttata] >NP_001264941.1 60S ribosomal protein L38 [Gallus gallus] >XP_003211558.1 60S ribosomal protein L38 [Meleagris gallopavo] >XP_003315754.1 60S ribosomal protein L38 [Pan troglodytes] >XP_003315758.1 60S ribosomal protein L38 [Pan troglodytes] >XP_003339346.1 60S ribosomal protein L38 [Pan troglodytes] >XP_003358038.1 60S ribosomal protein L38 [Sus scrofa] >XP_003417326.1 60S ribosomal protein L38 [Loxodonta africana] >XP_003453439.1 60S ribosomal protein L38 [Oreochromis niloticus] >XP_003464913.2 60S ribosomal protein L38 [Cavia porcellus] >XP_003768586.1 60S ribosomal protein L38 [Sarcophilus harrisii] >XP_003786210.1 60S ribosomal protein L38 [Otolemur garnettii] >XP_003795793.1 60S ribosomal protein L38 [Otolemur garnettii] >XP_003922345.1 60S ribosomal protein L38 [Saimiri boliviensis boliviensis] >XP_004041125.1 60S ribosomal protein L38 [Gorilla gorilla gorilla] >XP_004041126.1 60S ribosomal protein L38 [Gorilla gorilla gorilla] >XP_004041128.1 60S ribosomal protein L38 [Gorilla gorilla gorilla] >XP_004331065.1 60S ribosomal protein L38 [Tursiops truncatus] >XP_004401894.1 PREDICTED: 60S ribosomal protein L38 [Odobenus rosmarus divergens] >XP_004412345.1 PREDICTED: 60S ribosomal protein L38 [Odobenus rosmarus divergens] >XP_004469223.1 60S ribosomal protein L38 [Dasypus novemcinctus] >XP_004469224.1 60S ribosomal protein L38 [Dasypus novemcinctus] >XP_005068761.1 60S ribosomal protein L38 [Mesocricetus auratus] >XP_005070019.1 60S ribosomal protein L38 [Mesocricetus auratus] >XP_005141156.1 60S ribosomal protein L38 [Melopsittacus undulatus] >XP_005336034.1 60S ribosomal protein L38 [Ictidomys tridecemlineatus] >XP_005336035.1 60S ribosomal protein L38 [Ictidomys tridecemlineatus] >XP_005350739.1 60S ribosomal protein L38 [Microtus ochrogaster] >XP_005350740.1 60S ribosomal protein L38 [Microtus ochrogaster] >XP_005412280.1 PREDICTED: 60S ribosomal protein L38 [Chinchilla lanigera] >XP_005412281.1 PREDICTED: 60S ribosomal protein L38 [Chinchilla lanigera] >XP_005530739.1 PREDICTED: 60S ribosomal protein L38 [Pseudopodoces humilis] >XP_005584887.1 60S ribosomal protein L38 [Macaca fascicularis] >XP_005584888.1 60S ribosomal protein L38 [Macaca fascicularis] >XP_005584889.1 60S ribosomal protein L38 [Macaca fascicularis] >XP_005584890.1 60S ribosomal protein L38 [Macaca fascicularis] >XP_005584891.1 60S ribosomal protein L38 [Macaca fascicularis] >XP_005592611.1 60S ribosomal protein L38 [Macaca fascicularis] >XP_005597274.1 60S ribosomal protein L38 isoform X2 [Equus caballus] >XP_005668697.1 60S ribosomal protein L38 [Sus scrofa] >XP_005861853.1 PREDICTED: 60S ribosomal protein L38 [Myotis brandtii] >XP_005861854.1 PREDICTED: 60S ribosomal protein L38 [Myotis brandtii] >XP_005889694.1 PREDICTED: 60S ribosomal protein L38 isoform X2 [Bos mutus] >XP_006042140.1 60S ribosomal protein L38 isoform X2 [Bubalus bubalis] >XP_006042141.1 60S ribosomal protein L38 isoform X2 [Bubalus bubalis] >XP_006082202.1 60S ribosomal protein L38 [Myotis lucifugus] >XP_006106539.1 60S ribosomal protein L38 [Myotis lucifugus] >XP_006109407.1 60S ribosomal protein L38 [Myotis lucifugus] >XP_006145920.1 60S ribosomal protein L38 isoform X1 [Tupaia chinensis] >XP_006145921.1 60S ribosomal protein L38 isoform X1 [Tupaia chinensis] >XP_006754668.1 PREDICTED: 60S ribosomal protein L38 [Myotis davidii]					
osomal protein L38 [Nematolebias white	Ribosoma	Ribosoma	60S RIBO	60S RIBOSOMAL PROTEIN L38 [Oryctolagus cuniculus] >4UJD_Ak Chain Ak	60S RIBO	60S RIBO	60S ribos	60S ribos	60S ribos
TRINITY_DN69307_c0_g1_i6_orf1	-	-	-	hypothetical protein evm_010738 [Chilo suppressalis]	-0.1464	-1.7222	-0.125	1.06757	0.92597
TRINITY_DN1957_c0_g1_i4_orf1	-	-	-	NAD kinase 2, mitochondrial [Ostrinia furnacalis]	0.36452	-1.8114	-0.2614	1.02515	0.68314
TRINITY_DN6108_c0_g1_i5_orf1	-	-	-	myogenesis-regulating glycosidase-like [Ostrinia furnacalis]	0.16979	-1.7378	0.29278	1.36306	-0.0879
TRINITY_DN346_c0_g1_i7_orf1	-	-	-	CDK-activating kinase assembly factor MAT1 [Ostrinia furnacalis]	-0.2687	-1.7074	0.09995	1.28652	0.58956
TRINITY_DN36144_c0_g1_i3_orf1	-	-	-	nicotinate phosphoribosyltransferase isoform X1 [Ostrinia furnacalis] >XP_028178189.1 nicotinate phosphoribosyltransferase isoform X1 [Ostrinia furnacalis]	0.46133	-1.563	-0.4534	1.45921	0.09594
TRINITY_DN1569_c0_g1_i6_orf1	-	-	-	uncharacterized protein LOC114350603 [Ostrinia furnacalis]	0.69427	-1.1552	-0.9148	1.52461	-0.1489
TRINITY_DN8780_c0_g1_i3_orf1	-	-	-	uncharacterized protein LOC114363370 [Ostrinia furnacalis]	0.15202	-1.8012	-0.071	1.21233	0.50786
TRINITY_DN11467_c0_g1_i5_orf1	-	-	-	27 kDa hemolymph protein-like, partial [Ostrinia furnacalis]	0.35176	-1.763	-0.0622	1.31923	0.15426
TRINITY_DN47609_c0_g1_i1_orfp1	-	-	-	TRINITY_DN47609_c0_g1_i1_m.57205 TRINITY_DN47609_c0_g1::TRINITY_DN47609_c0_g1_i1::g.57205 ORF type:5prime_partial len:68 (-),score=15.96 TRINITY_DN47609_c0_g1_i1:36-239(-)	0.19633	-1.8101	0.09064	1.26998	0.25311
TRINITY_DN22375_c0_g1_i4_orf1	-	-	-	venom carboxylesterase-6-like [Ostrinia furnacalis]	0.05593	-1.6549	-0.428	1.1153	0.91165
TRINITY_DN43667_c0_g1_i1_orf1	-	-	-	carbonyl reductase [NADPH] 1-like [Ostrinia furnacalis]	-0.0304	-1.6749	-0.2594	0.669	1.29573
TRINITY_DN4822_c0_g1_i6_orf1	-	-	-	homogentisate 1,2-dioxygenase [Ostrinia furnacalis]	0.33973	-0.8878	-1.1748	1.64633	0.07652
TRINITY_DN13500_c0_g1_i1_orf1	-	-	-	phosphatidylethanolamine-binding protein homolog F40A3.3-like [Ostrinia furnacalis] >XP_028160752.1 phosphatidylethanolamine-binding protein homolog F40A3.3-like [Ostrinia furnacalis]	-0.2089	-1.6949	-0.0004	1.32013	0.58401
TRINITY_DN20347_c0_g1_i6_orf1	-	-	-	venom polypeptide precursor [Doratifera vulnerans]	-0.4397	-1.5118	-0.0145	1.52542	0.44057
TRINITY_DN2170_c0_g2_i1_orf1	-	-	-	beta-1,3-glucan-binding protein-like [Ostrinia furnacalis]	0.10304	-1.7132	-0.0115	1.41879	0.20292
TRINITY_DN987_c0_g1_i3_orf1	-	-	-	unnamed protein product [Chilo suppressalis]	-0.1544	-1.2989	-0.8215	1.02188	1.25294
TRINITY_DN7212_c0_g1_i4_orf1	-	-	-	peptidylglycine alpha-hydroxylating monooxygenase [Ostrinia furnacalis]	0.03657	-1.5934	-0.4722	1.31312	0.71588
TRINITY_DN12367_c0_g1_i8_orf1	-	-	-	aldose reductase-like isoform X2 [Ostrinia furnacalis]	-0.2754	-1.3088	-0.7296	1.18987	1.1239
TRINITY_DN15478_c0_g1_i1_orf1	-	-	-	STE20/SPS1-related proline-alanine-rich protein kinase [Vanessa tameamea] >XP_047534294.1 STE20/SPS1-related proline-alanine-rich protein kinase-like [Vanessa atalanta]	-0.1716	-1.6554	-0.2434	1.15264	0.9178
TRINITY_DN3738_c0_g1_i5_orf1	-	-	-	23 kDa integral membrane protein-like [Ostrinia furnacalis]	-0.3643	-1.5638	-0.1117	1.42541	0.61442
TRINITY_DN101_c0_g1_i4_orf1	-	-	-	disco-interacting protein 2 [Melitaea cinxia]	0.0365	-1.2436	-0.6426	1.73982	0.10995
TRINITY_DN18273_c0_g1_i4_orf1	-	-	-	venom protease-like [Ostrinia furnacalis] >XP_028156372.1 venom protease-like [Ostrinia furnacalis]	0.38956	-1.4527	-0.6086	1.53229	0.1395
TRINITY_DN1841_c0_g1_i2_orf1	-	-	-	carboxylesterase [Cnaphalocrocis medinalis]	-0.0527	-1.4363	-0.6349	1.43328	0.69058
TRINITY_DN6680_c0_g1_i1_orf1	-	-	-	hypothetical protein evm_009571 [Chilo suppressalis]	-0.4478	-1.3587	-0.5064	1.26159	1.05133
TRINITY_DN125_c0_g1_i2_orf1	-	-	-	PREDICTED: purine nucleoside phosphorylase isoform X1 [Microplitis demolitor]	-0.2222	-1.5572	-0.1666	1.52272	0.42338
TRINITY_DN26789_c0_g1_i2_orf1	-	-	-	D-2-hydroxyglutarate dehydrogenase, mitochondrial-like [Ostrinia furnacalis]	-0.3229	-1.358	-0.1156	1.74216	0.05438
TRINITY_DN7556_c0_g1_i3_orf1	-	-	-	jg27436 [Pararge aegeria aegeria]	-0.1483	-1.3883	0.16146	1.70714	-0.332

TRINITY_DN30300_c0_g2_i1_orf1	-	-	-	60S acidic ribosomal protein P2 isoform X2 [Ovis aries] >XP_017898197.1 PREDICTED: 60S acidic ribosomal protein P2 [Capra hircus] >XP_020767760.1 60S acidic ribosomal protein P2 [Odocoileus virginianus texanus] >XP_040111416.1 60S acidic ribosomal protein P2 [Oryx dammah] >XP_043307792.1 60S acidic ribosomal protein P2 [Cervus canadensis] >XP_043778683.1 60S acidic ribosomal protein P2 [Cervus elaphus] >KAB0376791.1 hypothetical protein FD755_011235 [Muntiacus reevesi] >OWK17231.1 RPLP2 [Cervus elaphus hippelaphus]	-0.1641	-1.5915	-0.2976	1.37565	0.67759
TRINITY_DN10877_c0_g1_i1_orf1	-	-	-	spodomicin-like [Ostrinia furnacalis]	-0.3942	-1.5409	1.42832	0.64179	-0.135
TRINITY_DN85290_c0_g2_i1_orf1	-	-	-	unnamed protein product, partial [Brenthis ino]	-0.2139	-1.3149	1.02085	1.26744	-0.7595
TRINITY_DN15682_c0_g1_i4_orf1	-	-	-	seroin transcript 1B [Ostrinia nubilalis]	-0.5648	-1.1209	0.68044	1.61085	-0.6056
TRINITY_DN61042_c0_g2_i2_orfp1	-	-	-	TRINITY_DN61042_c0_g2_i2_m.5292 TRINITY_DN61042_c0_g2::TRINITY_DN61042_c0_g2_i2::g.5292 ORF type:5prime_partial len:63 (-),score=21.86 TRINITY_DN61042_c0_g2_i2:77-265(-)	-0.5713	-1.3815	1.35313	0.91395	-0.3142
TRINITY_DN2407_c0_g1_i2_orf1	-	-	-	uncharacterized protein LOC114366345 isoform X2 [Ostrinia furnacalis]	-0.7624	-1.0228	1.70113	0.52949	-0.4454
TRINITY_DN27300_c0_g1_i7_orfp1	-	-	-	TRINITY_DN27300_c0_g1_i7_m.71141 TRINITY_DN27300_c0_g1::TRINITY_DN27300_c0_g1_i7::g.71141 ORF type:internal len:82 (-),score=6.59 TRINITY_DN27300_c0_g1_i7:3-245(-)	-0.4954	-1.493	1.51949	0.46551	0.00342
TRINITY_DN311_c0_g1_i8_orfp1	-	-	-	TRINITY_DN311_c0_g1_i8_m.65152 TRINITY_DN311_c0_g1::TRINITY_DN311_c0_g1_i8::g.65152 ORF type:5prime_partial len:138 (+),score=74.30 TRINITY_DN311_c0_g1_i8:2-415(+)	-0.7683	-0.6012	1.0148	1.39298	-1.0383
TRINITY_DN76633_c0_g1_i1_orfp1	-	-	-	TRINITY_DN76633_c0_g1_i1_m.53394 TRINITY_DN76633_c0_g1::TRINITY_DN76633_c0_g1_i1::g.53394 ORF type:internal len:164 (-),score=26.46,Toxin_2 PF00451.20 0.00022,Toxin_2|PF00451.20|0.00056,Toxin_2|PF00451.20|0.0002,Toxin_2|PF00451.20|5.9e-06,Gamma-thionin|PF00304.21|2,Gamma-thionin|PF00304.21|0.024,Gamma-thionin|PF00304.21|0.027,Gamma-thionin|PF00304.21|0.066,Toxin_38|PF14866.7|0.27,Toxin_38|PF14866.7|0.054,Toxin_38|PF14866.7|0.14,Defensin_2|PF01097.19|1.7,Defensin_2|PF01097.19|0.055,Defensin_2|PF01097.19|1.2,Defensin_2|PF01097.19|0.15 TRINITY_DN76633_c0_g1_i1:1-489(-)	-1.3183	-0.8921	0.97157	1.23382	0.00498
TRINITY_DN11798_c0_g2_i1_orf1	-	-	-	N-acetylglucosamine-6-sulfatase-like isoform X2 [Ostrinia furnacalis]	-0.445	-1.6011	1.08465	1.02835	-0.0669
TRINITY_DN2338_c0_g2_i1_orf1	-	-	-	prophenoloxidase PPO3 [Ostrinia furnacalis]	-0.1384	-1.0157	0.17361	1.80143	-0.8209
TRINITY_DN4794_c1_g1_i9_orf1	-	-	-	D-3-phosphoglycerate dehydrogenase [Ostrinia furnacalis]	-0.1268	-1.3417	0.55382	1.56611	-0.6515
TRINITY_DN3377_c0_g1_i1_orf1	-	-	-	calcyphosin-like protein isoform X3 [Helicoverpa armigera] >XP_047020698.1 calcyphosin-like protein isoform X2 [Helicoverpa zea]	-0.8139	-1.2692	1.30544	0.98891	-0.2113
TRINITY_DN1789_c0_g1_i5_orf1	-	-	-	uncharacterized protein LOC114365543 [Ostrinia furnacalis]	-0.5498	-1.1975	1.60329	0.65633	-0.5124
TRINITY_DN5852_c0_g1_i6_orf1	-	-	-	probable maltase isoform X6 [Ostrinia furnacalis]	-0.5155	-0.7384	1.40678	0.97196	-1.1248
TRINITY_DN21567_c0_g1_i7_orf1	-	-	-	transcription initiation factor TFIID subunit 4 isoform X1 [Ostrinia furnacalis]	-1.0373	-0.6171	1.04944	1.36522	-0.7603
TRINITY_DN7022_c0_g1_i7_orf1	-	-	-	fatty-acid amide hydrolase 2-A-like [Ostrinia furnacalis]	-1.0966	-0.8391	1.55714	0.73563	-0.3571
TRINITY_DN3126_c0_g1_i4_orf1	-	-	-	unnamed protein product, partial [Iphiclides podalirius]	-1.051	-0.0042	0.8056	1.39271	-1.1432
TRINITY_DN31390_c0_g1_i2_orf1	-	-	-	UDP-glucuronosyltransferase 2B20-like [Ostrinia furnacalis]	-1.5973	-0.357	1.09817	1.03994	-0.1839
TRINITY_DN17657_c0_g1_i1_orf1	-	-	-	alcohol dehydrogenase 18, partial [Helicoverpa assulta]	-1.6928	-0.2517	1.05774	0.97204	-0.0853
TRINITY_DN5852_c0_g1_i13_orf1	-	-	-	uncharacterized protein LOC114364714 isoform X3 [Ostrinia furnacalis] >XP_028176780.1 uncharacterized protein LOC114364714 isoform X5 [Ostrinia furnacalis]	-1.425	-0.4485	1.06017	1.21704	-0.4036
TRINITY_DN36581_c0_g1_i5_orf1	-	-	-	enoyl-CoA delta isomerase 1, mitochondrial-like isoform X1 [Ostrinia furnacalis] >XP_028158560.1 enoyl-CoA delta isomerase 1, mitochondrial-like isoform X2 [Ostrinia furnacalis]	-0.9674	-0.4728	1.78089	0.38238	-0.7231
TRINITY_DN1380_c0_g1_i5_orf1	-	-	-	ubiquitin-fold modifier-conjugating enzyme 1 [Ostrinia furnacalis]	-0.591	-0.2546	1.49966	0.70325	-1.3573
TRINITY_DN4204_c0_g1_i1_orf1	-	-	-	uncharacterized protein LOC114359352 [Ostrinia furnacalis]	-1.2259	-0.6383	-0.4705	1.4345	0.90027
TRINITY_DN18568_c0_g1_i2_orfp1	-	-	-	TRINITY_DN18568_c0_g1_i2_m.13844 TRINITY_DN18568_c0_g1::TRINITY_DN18568_c0_g1_i2::g.13844 ORF type:5prime_partial len:77 (+),score=12.07 TRINITY_DN18568_c0_g1_i2:1-231(+)	-0.7908	-0.8801	-0.769	1.3474	1.09241
TRINITY_DN1091_c0_g3_i1_orf1	-	-	-	macrophage mannose receptor 1-like [Ostrinia furnacalis]	-0.6768	-1.2184	-0.4613	0.99328	1.36315
TRINITY_DN68770_c0_g1_i1_orf1	-	-	-	seroin transcript 1A2 [Ostrinia nubilalis]	-1.2571	-0.724	-0.1714	1.60041	0.55209
TRINITY_DN1305_c0_g1_i6_orf1	-	-	-	glutathione S-transferase sigma 3 [Ostrinia furnacalis]	-0.7544	-0.9352	-0.7544	1.23288	1.2112
TRINITY_DN4242_c0_g1_i6_orf1	-	-	-	fibrohexamerin-like [Ostrinia furnacalis]	-1.5569	-0.5306	-0.0513	1.11643	1.02245
TRINITY_DN4695_c0_g1_i3_orf1	-	-	-	glutathione S-transferase epsilon 3 [Ostrinia furnacalis]	-1.5714	-0.4334	-0.1429	1.1643	0.98337
TRINITY_DN22589_c0_g1_i6_orfp1	-	-	-	TRINITY_DN22589_c0_g1_i6_m.19386 TRINITY_DN22589_c0_g1::TRINITY_DN22589_c0_g1_i6::g.19386 ORF type:internal len:183 (+),score=63.52 TRINITY_DN22589_c0_g1_i6:2-547(+)	-1.0891	-0.5513	-0.7455	0.96091	1.42504
TRINITY_DN1344_c0_g1_i1_orf1	-	-	-	ribosomal RNA small subunit methyltransferase NEP1 [Ostrinia furnacalis]	-1.6117	-0.6559	0.37478	1.08852	0.80431
TRINITY_DN5191_c0_g2_i1_orf1	-	-	-	CD151 antigen-like [Ostrinia furnacalis]	-1.4923	-0.747	0.14453	1.0556	1.03916
TRINITY_DN76307_c0_g1_i1_orf1	-	-	-	PREDICTED: quinone oxidoreductase-like protein 2 homolog [Microplitis demolitor]	-0.5703	-1.1726	-0.5629	0.75018	1.55567
TRINITY_DN55154_c0_g2_i1_orf1	-	-	-	glycosyl transferase family 8 domain-containing protein [Phthorimaea operculella]	-1.1904	-0.4107	-0.6838	1.55258	0.73229
TRINITY_DN11388_c0_g1_i4_orf1	-	-	-	limbic system-associated membrane protein-like, partial [Ostrinia furnacalis]	-1.7115	-0.4933	0.53523	1.10619	0.56335
TRINITY_DN1540_c0_g1_i9_orf1	-	-	-	alaserpin-like isoform X9 [Ostrinia furnacalis]	-0.8524	-1.0635	-0.4657	0.97874	1.40276
TRINITY_DN5126_c0_g1_i3_orf1	-	-	-	cytochrome P450 monooxygenase CYP4L47 [Ostrinia furnacalis]	-1.1557	-0.8964	-0.2827	1.33374	1.00103
TRINITY_DN18782_c0_g1_i4_orf1	-	-	-	putative riboflavin kinase [Ostrinia furnacalis] >XP_028176654.1 putative riboflavin kinase [Ostrinia furnacalis]	-1.4963	-0.6062	-0.0715	0.97421	1.19976
TRINITY_DN50743_c0_g1_i1_orf1	-	-	-	cytochrome P450 monooxygenase CYP321F7 [Ostrinia furnacalis]	-0.9464	-1.0159	-0.4319	1.09836	1.29587
TRINITY_DN8310_c0_g2_i1_orf1	-	-	-	uncharacterized protein LOC116773294 [Danaus plexippus plexippus] >OWR55545.1 hypothetical protein KGM_209260 [Danaus plexippus plexippus]	-1.7304	-0.4472	0.36911	1.03521	0.77328
TRINITY_DN72017_c0_g1_i1_orf1	-	-	-	probable peroxisomal acyl-coenzyme A oxidase 1 isoform X1 [Ostrinia furnacalis] >XP_028165840.1 probable peroxisomal acyl-coenzyme A oxidase 1 isoform X2 [Ostrinia furnacalis]	-1.5112	-0.2765	-0.3383	1.42717	0.69888
TRINITY_DN33763_c0_g1_i1_orf1	-	-	-	uncharacterized protein LOC114355186 [Ostrinia furnacalis]	-1.2902	-0.6907	-0.3421	1.01456	1.30841
TRINITY_DN133474_c0_g2_i2_orf1	-	-	-	enoyl-[acyl-carrier-protein] reductase, mitochondrial [Ostrinia furnacalis]	-1.223	-0.3014	-0.7971	1.36522	0.9562
TRINITY_DN37055_c0_g1_i1_orf1	-	-	-	ras GTPase-activating protein-binding protein 2-like, partial [Ostrinia furnacalis]	-1.1434	-0.9916	-0.1755	1.22394	1.08654
TRINITY_DN4004_c0_g1_i1_orf1	-	-	-	protein FAM114A2 isoform X1 [Ostrinia furnacalis] >XP_028175160.1 protein FAM114A2 isoform X2 [Ostrinia furnacalis]	-1.2047	-0.761	-0.3797	0.97838	1.36689
TRINITY_DN62_c1_g1_i3_orf1	-	-	-	D-2-hydroxyglutarate dehydrogenase, mitochondrial-like [Ostrinia furnacalis]	-1.6424	-0.4301	0.04312	1.18262	0.84676
TRINITY_DN18222_c0_g1_i5_orf1	-	-	-	phosphoglycerate kinase [Maniola hyperantus]	-1.5496	-0.533	0.15085	1.42759	0.50406

TRINITY_DN11649_c0_g1_i4_orf1	-	-	-	ubiquitin carboxyl-terminal hydrolase 32-like, partial [Ostrinia furnacalis]	-1.1713	-0.4288	-0.5918	0.50792	1.68402
TRINITY_DN6415_c0_g1_i1_orf1	-	-	-	D-arabinitol dehydrogenase 1-like [Ostrinia furnacalis]	-1.5894	-0.4837	0.07941	0.64603	1.34766
TRINITY_DN2098_c0_g1_i1_orf1	-	-	-	dolichyl pyrophosphate Man9GlcNAc2 alpha-1,3-glucosyltransferase [Ostrinia furnacalis]	-1.7539	-0.2161	0.09545	0.70185	1.17273
TRINITY_DN11823_c1_g1_i2_orf1	-	-	-	LOW QUALITY PROTEIN: uncharacterized protein LOC114362902 [Ostrinia furnacalis]	-1.7718	-0.4506	0.70421	0.82781	0.69038
TRINITY_DN616_c1_g1_i6_orf1	-	-	-	esterase B1-like isoform X1 [Ostrinia furnacalis] >XP_028178578.1 esterase B1-like isoform X2 [Ostrinia furnacalis]	-1.6577	-0.3794	-0.0064	1.12166	0.9219
TRINITY_DN4916_c0_g2_i1_orf1	-	-	-	uncharacterized protein LOC114357135, partial [Ostrinia furnacalis]	-1.3203	-0.6168	-0.3366	0.83819	1.43546
TRINITY_DN50074_c0_g1_i1_orf1	-	-	-	uncharacterized protein LOC114364628 [Ostrinia furnacalis]	-1.6664	-0.4551	0.37502	1.29178	0.45467
TRINITY_DN11245_c0_g1_i2_orf1	-	-	-	ITG-like peptide [Ostrinia furnacalis]	-1.5603	-0.6758	0.21517	1.11206	0.9088
TRINITY_DN90497_c0_g1_i1_orf1	-	-	-	midasin-like [Ostrinia furnacalis]	-1.9074	-0.0275	0.44582	0.58095	0.90813
TRINITY_DN2735_c0_g1_i4_orf1	-	-	-	hypothetical protein evm_008546 [Chilo suppressalis] >CAH0684158.1 unnamed protein product [Chilo suppressalis]	-1.5872	-0.4965	0.08583	0.65895	1.33892
TRINITY_DN3298_c0_g2_i4_orf1	-	-	-	macrophage mannose receptor 1-like [Ostrinia furnacalis]	-1.3868	-0.5133	0.27894	-0.0324	1.65362
TRINITY_DN4866_c0_g1_i2_orf1	-	-	-	actin-binding LIM protein 3 isoform X6 [Ostrinia furnacalis]	-1.7772	-0.1317	0.01321	1.06426	0.83144
TRINITY_DN2647_c0_g1_i3_orf1	-	-	-	DNA repair protein complementing XP-G cells homolog isoform X1 [Ostrinia furnacalis]	-1.6415	-0.0437	-0.3888	1.00936	1.06468
TRINITY_DN1989_c0_g1_i1_orf1	-	-	-	sarcoplasmic calcium-binding protein 1 isoform X1 [Ostrinia furnacalis]	-1.6933	0.21858	-0.3804	1.25886	0.59625
TRINITY_DN17907_c0_g1_i13_orf1	-	-	-	androgen-induced gene 1 protein-like isoform X1 [Galleria mellonella]	-1.5717	-0.3679	-0.2003	0.88992	1.24992
TRINITY_DN14755_c0_g1_i4_orf1	-	-	-	aldehyde dehydrogenase, dimeric NADP-preferring isoform X6 [Ostrinia furnacalis]	-1.7852	-0.3031	0.36381	1.08803	0.63647
TRINITY_DN12748_c2_g1_i1_orfp1	-	-	-	TRINITY_DN12748_c2_g1_i1_m.21305 TRINITY_DN12748_c2_g1::TRINITY_DN12748_c2_g1_i1::g.21305 ORF type:3prime_partial len:887 (+),score=-6.30 TRINITY_DN12748_c2_g1_i1:104-2761(+)	-1.7369	0.09504	-0.2443	0.68255	1.20358
TRINITY_DN143509_c0_g1_i1_orf1	-	-	-	ATP-dependent 6-phosphofructokinase isoform X3 [Diachasma alloeum]	-1.8515	0.03487	0.48585	0.19142	1.13935
TRINITY_DN5405_c1_g1_i13_orf1	-	-	-	acyl-CoA synthetase family member 3, mitochondrial [Ostrinia furnacalis]	-1.7014	-0.1766	0.34613	0.14113	1.39075
TRINITY_DN1084_c0_g2_i2_orf1	-	-	-	ATP-citrate synthase [Ostrinia furnacalis]	-1.8309	0.08043	-0.0513	0.98902	0.81273
TRINITY_DN1264_c0_g1_i2_orf1	-	-	-	L-lactate dehydrogenase isoform X1 [Ostrinia furnacalis]	-1.4161	0.10398	-0.7006	0.52282	1.48989
TRINITY_DN7861_c0_g1_i5_orf1	-	-	-	cytochrome b5-related protein-like [Ostrinia furnacalis]	-1.4007	0.08507	-0.6929	0.48752	1.52092
TRINITY_DN51776_c0_g2_i1_orf1	-	-	-	cuticle protein CP14.6-like [Ostrinia furnacalis]	-1.5536	-0.2391	-0.2632	0.61167	1.44419
TRINITY_DN128_c0_g1_i5_orf1	-	-	-	PREDICTED: muscle-specific protein 20-like [Amyelois transitella]	-1.8627	0.14185	-0.0166	0.88454	0.85291
TRINITY_DN1265_c0_g1_i4_orf1	-	-	-	fumarylacetoacetate hydrolase domain-containing protein 2 isoform X3 [Ostrinia furnacalis]	-1.5041	0.32741	-0.6726	0.44093	1.4084
TRINITY_DN9286_c0_g1_i2_orf1	-	-	-	alcohol dehydrogenase class-3 [Ostrinia furnacalis]	-1.8725	0.02269	0.22972	0.55714	1.06298
TRINITY_DN3593_c0_g1_i3_orfp1	-	-	-	TRINITY_DN3593_c0_g1_i3_m.43968 TRINITY_DN3593_c0_g1::TRINITY_DN3593_c0_g1_i3::g.43968 ORF type:5prime_partial len:72 (-),score=1.41 TRINITY_DN3593_c0_g1_i3:138-353(-)	-1.2136	-0.4815	0.37508	-0.4085	1.7285
TRINITY_DN935_c0_g1_i3_orf1	-	-	-	carboxylesterase 5A-like [Ostrinia furnacalis]	-1.3446	0.43241	-1.0165	0.72797	1.20075
TRINITY_DN41280_c0_g1_i2_orf1	-	-	-	unnamed protein product [Plutella xylostella]	-1.258	0.17401	-0.7768	0.2049	1.65585
TRINITY_DN974_c0_g2_i1_orfp1	-	-	-	TRINITY_DN974_c0_g2_i1_m.54007 TRINITY_DN974_c0_g2::TRINITY_DN974_c0_g2_i1::g.54007 ORF type:3prime_partial len:103 (-),score=5.74 TRINITY_DN974_c0_g2_i1:1-306(-)	-1.2402	0.1518	-1.0466	0.88836	1.24668
TRINITY_DN2688_c0_g2_i1_orf1	-	-	-	mitochondrial amidoxime reducing component 2 [Galleria mellonella]	-1.8272	-0.131	0.45381	0.36078	1.14367
TRINITY_DN86127_c1_g1_i2_orfp1	-	-	-	TRINITY_DN86127_c1_g1_i2_m.43062 TRINITY_DN86127_c1_g1::TRINITY_DN86127_c1_g1_i2::g.43062 ORF type:internal len:69 (-),score=14.03 TRINITY_DN86127_c1_g1_i2:2-205(-)	-1.6742	0.26686	-0.5192	0.98219	0.94429
TRINITY_DN1718_c6_g1_i4_orf1	-	-	-	adenosine kinase [Ostrinia furnacalis]	-1.8494	0.13643	0.19469	0.32865	1.18962
TRINITY_DN2350_c0_g1_i6_orf1	-	-	-	protein yellow-like isoform X2 [Ostrinia furnacalis]	-1.7126	-0.1227	0.08485	0.36888	1.38155
TRINITY_DN4279_c0_g1_i4_orf1	-	-	-	glutathione S-transferase sigma 1 [Ostrinia furnacalis]	-1.8179	0.78902	-0.3384	0.57497	0.79228
TRINITY_DN1292_c0_g1_i3_orf1	-	-	-	uncharacterized protein LOC114360660 [Ostrinia furnacalis]	-1.4564	0.18199	-0.5962	0.32682	1.54383
TRINITY_DN6482_c0_g1_i1_orf1	-	-	-	endocuticle structural glycoprotein SgAbd-5-like [Ostrinia furnacalis]	-1.913	0.06041	0.31463	0.60279	0.93516
TRINITY_DN565_c0_g2_i1_orf1	-	-	-	uncharacterized protein LOC114362323 [Ostrinia furnacalis]	-1.6028	0.06478	-0.0889	0.07332	1.5536
TRINITY_DN9242_c0_g1_i1_orf1	-	-	-	electron transfer flavoprotein subunit alpha, mitochondrial [Ostrinia furnacalis]	-1.7668	0.99173	-0.4173	0.52667	0.66576
TRINITY_DN56795_c1_g1_i1_orf1	-	-	-	uncharacterized protein LOC114365476 [Ostrinia furnacalis]	-1.4552	0.83806	-0.9108	0.46137	1.06659
TRINITY_DN3019_c0_g1_i1_orf1	-	-	-	uncharacterized protein LOC114355530 isoform X1 [Ostrinia furnacalis]	-1.6545	0.68863	-0.655	0.65908	0.96177
TRINITY_DN280_c4_g1_i5_orf1	-	-	-	fibroin light chain [Haritalodes derogata]	-1.7165	0.66141	-0.4886	1.07692	0.4667
TRINITY_DN120500_c0_g1_i1_orf1	-	-	-	cytochrome P450 6B5-like [Ostrinia furnacalis]	-1.4862	-0.1081	0.08067	-0.1454	1.65892
TRINITY_DN15000_c0_g1_i4_orf1	-	-	-	15-hydroxyprostaglandin dehydrogenase [NAD(+)]-like [Ostrinia furnacalis]	-1.2607	0.02635	-0.3524	-0.2132	1.80004
TRINITY_DN135077_c0_g1_i1_orf1	-	-	-	hypothetical protein KR038_001662 [Drosophila bunnanda]	-1.8479	0.22073	0.2027	0.22201	1.2025
TRINITY_DN779_c0_g1_i12_orf1	-	-	-	unnamed protein product [Chilo suppressalis]	-1.8823	-0.0112	0.47087	0.37718	1.04543
TRINITY_DN1585_c0_g1_i1_orf1	-	-	-	aldose reductase-like isoform X2 [Ostrinia furnacalis]	-1.8096	0.52501	-0.2463	0.43585	1.09504
TRINITY_DN4053_c0_g1_i5_orf1	-	-	-	uncharacterized protein LOC114358355 [Ostrinia furnacalis]	-1.7676	-0.031	0.41866	0.07863	1.30126
TRINITY_DN920_c0_g1_i6_orf1	-	-	-	glutathione S-transferase omega 2 [Ostrinia furnacalis]	-1.1369	0.51926	-0.9923	0.04436	1.56561
TRINITY_DN32_c0_g1_i4_orf1	-	-	-	epidermal growth factor receptor substrate 15 homolog [Ostrinia furnacalis]	-1.5061	0.53952	-0.6675	0.24255	1.39149
TRINITY_DN81258_c0_g1_i2_orf1	-	-	-	jg27820 [Pararge aegeria aegeria]	-1.9192	0.56343	0.10294	0.30735	0.94552
TRINITY_DN1215_c0_g1_i2_orf1	-	-	-	PI-stichotoxin-She2a-like [Ostrinia furnacalis]	-1.8783	0.24044	-0.0318	0.73692	0.93278
TRINITY_DN51830_c0_g1_i4_orf1	-	-	-	15-hydroxyprostaglandin dehydrogenase [NAD(+)]-like [Ostrinia furnacalis]	-1.7771	0.74458	-0.3711	0.41507	0.9886
TRINITY_DN41708_c0_g1_i1_orf1	-	-	-	facilitated trehalose transporter Tret1-like [Ostrinia furnacalis]	-1.7666	0.5142	-0.0731	0.05818	1.2673
TRINITY_DN9198_c0_g1_i4_orf1	-	-	-	4-coumarate--CoA ligase 1-like isoform X1 [Ostrinia furnacalis]	-1.5703	0.97603	-0.4375	-0.1389	1.17075
TRINITY_DN87648_c0_g1_i1_orfp1	-	-	-	TRINITY_DN87648_c0_g1_i1_m.51054 TRINITY_DN87648_c0_g1::TRINITY_DN87648_c0_g1_i1::g.51054 ORF type:internal len:180 (+),score=75.93 TRINITY_DN87648_c0_g1_i1:2-538(+)	-1.904	0.71382	0.0484	0.246	0.89577
TRINITY_DN99673_c0_g1_i1_orf1	-	-	-	PREDICTED: pistil-specific extensin-like protein isoform X2 [Microplitis demolitor]	-1.6627	-0.0734	-0.0147	0.28513	1.46574
TRINITY_DN5962_c0_g1_i1_orf1	-	-	-	tRNA (cytosine(34)-C(5))-methyltransferase [Ostrinia furnacalis]	-1.1153	0.86401	-0.7397	-0.4974	1.48833
TRINITY_DN1914_c0_g1_i4_orf1	-	-	-	loricrin-like [Ostrinia furnacalis]	-1.5262	0.81246	-0.4709	-0.1449	1.32959
TRINITY_DN17693_c0_g1_i10_orf1	-	-	-	acetylcholinesterase-like [Ostrinia furnacalis]	0.1234	-0.8678	-0.4822	1.88745	-0.6608

TRINITY_DN128231_c0_g1_i5_orf1	-	-	-	glutathione S-transferase sigma3 [Glyphodes pyloalis]	-0.1912	-0.4286	-0.3961	1.94093	-0.925
TRINITY_DN21341_c0_g1_i1_orf1	-	-	-	FAST kinase domain-containing protein 4 isoform X6 [Ostrinia furnacalis] >XP_028160336.1 FAST kinase domain-containing protein 4 isoform X7 [Ostrinia furnacalis] >XP_028160337.1 FAST kinase domain-containing protein 4 isoform X8 [Ostrinia furnacalis]	-0.3207	-0.4394	-0.6023	1.98758	-0.6252
TRINITY_DN6203_c0_g1_i1_orfp1	-	-	-	TRINITY_DN6203_c0_g1_i1_m.72736 TRINITY_DN6203_c0_g1::TRINITY_DN6203_c0_g1_i1::g.72736 ORF type:internal len:93 (+),score=12.26 TRINITY_DN6203_c0_g1_i1:3-278(+)	0.41175	-0.0773	-1.0162	1.67492	-0.9932
TRINITY_DN19866_c0_g1_i4_orf1	-	-	-	lys-63-specific deubiquitinase BRCC36-like [Ostrinia furnacalis]	-0.3493	-0.5354	-0.8024	1.967	-0.2798
TRINITY_DN8771_c0_g2_i1_orf1	-	-	-	regucalcin-like [Ostrinia furnacalis]	-0.404	0.56095	-0.6006	1.64712	-1.2035
TRINITY_DN1628_c0_g2_i3_orf1	-	-	-	uncharacterized protein LOC114363979 [Ostrinia furnacalis]	-0.0842	-0.5491	-0.2848	1.90626	-0.9881
TRINITY_DN30185_c0_g1_i3_orf1	-	-	-	organic cation transporter protein [Ostrinia furnacalis]	-0.2058	-0.3268	-0.1519	1.85773	-1.1733
TRINITY_DN2338_c0_g1_i3_orf1	-	-	-	phenoloxidase subunit 1-like [Ostrinia furnacalis]	-0.6196	-0.0718	-0.0224	1.83265	-1.1188
TRINITY_DN113327_c0_g1_i2_orf1	-	-	-	proteasome subunit beta type-6 [Helicoverpa armigera] >XP_047031479.1 proteasome subunit beta type-6 [Helicoverpa zea] >XP_049697949.1 proteasome subunit beta type-6-like [Helicoverpa armigera] >PZC87318.1 hypothetical protein B5X24_HaOG201554 [Helicoverpa armigera]	-0.3679	-0.709	-0.3105	1.97883	-0.5915
TRINITY_DN1402_c1_g1_i6_orf1	-	-	-	unnamed protein product [Parnassius apollo]	-1.102	0.13082	0.23938	1.67804	-0.9463
TRINITY_DN3092_c0_g1_i2_orf1	-	-	-	replication factor C subunit 1 isoform X1 [Ostrinia furnacalis] >XP_028157702.1 replication factor C subunit 1 isoform X2 [Ostrinia	-0.7081	0.4809	-0.6326	1.75231	-0.8925
TRINITY_DN102712_c0_g1_i1_orf1	-	-	-	transmembrane protein 177 [Ostrinia furnacalis]	-0.2559	0.53319	-0.0506	1.40683	-1.6336
TRINITY_DN52887_c0_g1_i1_orf1	-	-	-	cytochrome P450 6B5-like [Ostrinia furnacalis]	-0.5872	0.24265	0.23694	1.55952	-1.4519
TRINITY_DN9458_c0_g1_i4_orf1	-	-	-	uncharacterized protein LOC114363583 [Ostrinia furnacalis]	-0.7505	-0.2072	-0.7053	1.95256	-0.2896
TRINITY_DN109503_c0_g1_i4_orf1	-	-	-	uncharacterized protein LOC114366345 isoform X2 [Ostrinia furnacalis]	-0.8489	-0.1097	-0.9471	1.83449	0.07113
TRINITY_DN30713_c0_g1_i3_orf1	-	-	-	phosphoglucomutase [Ostrinia furnacalis]	-1.2486	-0.3786	-0.1871	1.80629	0.00795
TRINITY_DN45962_c1_g1_i2_orf1	-	-	-	protein shifted isoform X1 [Ostrinia furnacalis]	-1.5095	-0.235	0.40519	1.5655	-0.2262
TRINITY_DN34676_c1_g1_i3_orf1	-	-	-	tRNA (uracil-5-)-methyltransferase homolog A [Ostrinia furnacalis]	-1.2304	-0.7415	0.29196	1.68848	-0.0086
TRINITY_DN5564_c0_g1_i5_orf1	-	-	-	probable phosphoserine aminotransferase [Ostrinia furnacalis]	-1.4022	-0.2454	-0.2394	1.69714	0.18988
TRINITY_DN54410_c0_g2_i1_orf1	-	-	-	lysozyme-like [Ostrinia furnacalis]	-0.9193	-0.416	-0.8215	1.77878	0.37802
TRINITY_DN51342_c0_g1_i7_orf1	-	-	-	prophenoloxidase [Ostrinia furnacalis]	-1.2084	-0.0895	-0.3584	1.83601	-0.1797
TRINITY_DN2749_c4_g1_i2_orf1	-	-	-	RNA exonuclease 4-like [Ostrinia furnacalis] >QEE79882.1 REX4 [Ostrinia furnacalis]	-0.874	0.39193	-1.3738	1.41436	0.44146
TRINITY_DN1785_c0_g1_i5_orf1	-	-	-	beta-mannosidase [Ostrinia furnacalis]	-0.6828	0.17189	-1.1412	1.78449	-0.1324
TRINITY_DN11808_c0_g1_i8_orf1	-	-	-	unnamed protein product [Diatraea saccharalis]	-0.8229	-0.2515	-0.8693	1.87047	0.07315
TRINITY_DN5215_c0_g1_i1_orf1	-	-	-	glycogen [starch] synthase isoform X2 [Manduca sexta]	-1.5682	-0.1987	0.3969	1.5232	-0.1532
TRINITY_DN1083_c0_g1_i4_orf1	-	-	-	latent-transforming growth factor beta-binding protein 4-like [Ostrinia furnacalis]	-1.327	0.25484	-0.7772	1.58048	0.26884
TRINITY_DN2673_c2_g1_i2_orf1	-	-	-	aminopeptidase N3c [Ostrinia nubilalis]	-1.3995	0.51512	-0.4961	1.57823	-0.1977
TRINITY_DN14922_c0_g1_i4_orf1	-	-	-	pseudouridine-5'-phosphatase-like [Ostrinia furnacalis]	-1.8247	0.39103	0.29876	1.19362	-0.0587
TRINITY_DN16978_c0_g1_i1_orf1	-	-	-	la-related protein 7 [Helicoverpa armigera]	-1.5172	0.38497	-0.1058	1.56037	-0.3223
TRINITY_DN38482_c0_g1_i4_orf1	-	-	-	pyruvate dehydrogenase (acetyl-transferring) kinase, mitochondrial [Ostrinia furnacalis]	-1.6343	-0.0163	-0.1153	1.49782	0.26818
TRINITY_DN26195_c0_g1_i6_orf1	-	-	-	bifunctional coenzyme A synthase isoform X1 [Ostrinia furnacalis]	-1.8953	0.4166	0.18289	1.07289	0.22293
TRINITY_DN7275_c0_g1_i14_orf1	-	-	-	uncharacterized protein LOC114353817 [Ostrinia furnacalis]	-0.8047	0.40851	-1.5163	1.12579	0.78672
TRINITY_DN104597_c0_g1_i2_orf1	-	-	-	hemicentin-2-like isoform X1 [Ostrinia furnacalis]	-0.9181	0.19202	-1.2224	1.57746	0.371
TRINITY_DN3472_c0_g1_i6_orf1	-	-	-	unnamed protein product [Euphydryas editha]	-1.0363	0.76955	-1.2676	1.29157	0.24276
TRINITY_DN10398_c0_g1_i12_orf1	-	-	-	zinc finger protein ZPR1 isoform X2 [Ostrinia furnacalis]	-1.7394	0.69374	-0.1944	1.20595	0.03409
TRINITY_DN9135_c0_g1_i4_orf1	-	-	-	electron transfer flavoprotein subunit beta [Ostrinia furnacalis]	-1.8882	0.582	-0.1458	0.82745	0.62456
TRINITY_DN12397_c0_g1_i1_orf1	-	-	-	39S ribosomal protein L27, mitochondrial [Ostrinia furnacalis]	-0.9294	0.8344	-1.3788	1.2127	0.26112
TRINITY_DN16125_c0_g1_i3_orf1	-	-	-	3-ketoacyl-CoA thiolase, mitochondrial [Ostrinia furnacalis]	-1.9281	0.68284	0.09386	0.84442	0.30699
TRINITY_DN3037_c0_g1_i1_orf1	-	-	-	coiled-coil domain-containing protein 58-like [Spodoptera frugiperda] >KAF9815873.1 hypothetical protein SFRURICE_009771 [Spodoptera frugiperda] >KAG8118507.1 hypothetical protein SFRUCORN_001779 [Spodoptera frugiperda]	-1.6874	0.56132	-0.2008	1.34055	-0.0136
TRINITY_DN29_c0_g1_i4_orf1	-	-	-	sodium-dependent nutrient amino acid transporter 1-like [Ostrinia furnacalis] >XP_028167707.1 sodium-dependent nutrient amino acid transporter 1-like [Ostrinia furnacalis]	-1.6154	1.00229	-0.4256	1.09619	-0.0576
TRINITY_DN14094_c0_g1_i1_orfp1	-	-	-	TRINITY_DN14094_c0_g1_i1_m.76391 TRINITY_DN14094_c0_g1::TRINITY_DN14094_c0_g1_i1::g.76391 ORF type:5prime_partial len:120 (+),score=12.94 TRINITY_DN14094_c0_g1_i1:3-362(+)	-1.7825	0.89995	-0.1896	0.98442	0.08774
TRINITY_DN65974_c0_g1_i2_orf1	-	-	-	uncharacterized protein LOC114362364 [Ostrinia furnacalis]	-0.3233	-0.7537	-1.2172	0.8198	1.47439
TRINITY_DN1732_c0_g1_i17_orf1	-	-	-	CAD protein isoform X2 [Ostrinia furnacalis]	-0.0335	-0.8049	-1.3273	1.43225	0.73346
TRINITY_DN46715_c0_g1_i1_orf1	-	-	-	hypothetical protein evm_000885 [Chilo suppressalis] >CAH0689224.1 unnamed protein product [Chilo suppressalis]	-0.6526	-0.133	-1.4409	1.1396	1.08696
TRINITY_DN9400_c0_g1_i1_orf1	-	-	-	lysophosphatidylserine lipase ABHD12 isoform X2 [Maniola hyperantus]	-0.7784	-0.3522	-1.0976	0.57444	1.65384
TRINITY_DN2655_c0_g2_i1_orf1	-	-	-	DNA fragmentation factor subunit alpha [Ostrinia furnacalis] >XP_028176215.1 DNA fragmentation factor subunit alpha [Ostrinia	-0.2672	-0.4819	-1.4808	1.20795	1.02199
TRINITY_DN5507_c0_g1_i1_orf1	-	-	-	PREDICTED: protein mago nashi [Amyelois transitella] >XP_026764462.1 protein mago nashi [Galleria mellonella] >XP_028164484.1 protein mago nashi [Ostrinia furnacalis]	-0.7737	-0.193	-1.3161	1.02712	1.25576
TRINITY_DN7735_c0_g1_i4_orf1	-	-	-	calphotin-like [Ostrinia furnacalis]	-1.8769	-0.1974	0.73219	0.64205	0.7
TRINITY_DN1470_c0_g1_i2_orf1	-	-	-	oxidation resistance protein 1 isoform X5 [Ostrinia furnacalis]	-1.8297	-0.0886	1.16457	0.42209	0.33165
TRINITY_DN6991_c0_g1_i24_orf1	-	-	-	muscle M-line assembly protein unc-89 isoform X5 [Ostrinia furnacalis]	-1.9104	0.07125	0.69943	0.8912	0.24854
TRINITY_DN45949_c0_g1_i1_orf1	-	-	-	uncharacterized protein LOC114355167 [Ostrinia furnacalis]	-1.8697	-0.1172	0.86444	0.79938	0.32304
TRINITY_DN1134_c0_g1_i4_orf1	-	-	-	cytochrome P450 6B5-like [Ostrinia furnacalis]	-1.8473	-0.2389	0.60846	0.55384	0.92385
TRINITY_DN46372_c0_g1_i1_orf1	-	-	-	unnamed protein product [Chilo suppressalis]	-1.6909	-0.2693	1.37572	0.24345	0.34105
TRINITY_DN9468_c1_g1_i4_orf1	-	-	-	coronin-1C-A isoform X1 [Bombyx mori]	-1.8057	-0.1211	1.21056	0.3949	0.32136
TRINITY_DN841_c0_g1_i4_orf1	-	-	-	uncharacterized protein LOC114354070 isoform X3 [Ostrinia furnacalis]	-1.9559	0.40874	0.15404	0.61533	0.7778
TRINITY_DN101991_c0_g1_i5_orf1	-	-	-	hypothetical protein O3G_MSEX015044, partial [Manduca sexta]	-1.8921	-0.1364	0.65961	0.56266	0.80614

TRINITY_DN1212_c0_g1_i8_orf1	-	-	-	extensin isoform X5 [Ostrinia furnacalis] >XP_028175473.1 extensin isoform X5 [Ostrinia furnacalis] >XP_028175474.1 extensin isoform X5 [Ostrinia furnacalis]	-1.8777	-0.1557	0.76229	0.46066	0.8104
TRINITY_DN2433_c0_g1_i3_orf1	-	-	-	iron regulatory protein 1 [Manduca sexta]	-1.7338	0.57922	0.58765	1.04251	-0.4755
TRINITY_DN69707_c0_g1_i1_orf1	-	-	-	titin-like, partial [Ostrinia furnacalis]	-1.9272	-0.0253	0.62169	0.58419	0.74665
TRINITY_DN77830_c0_g2_i2_orf1	-	-	-	prostaglandin reductase 1-like [Leguminivora glycinivorella] >XP_047994907.1 prostaglandin reductase 1-like [Leguminivora	-1.4191	0.43876	1.58216	-0.0671	-0.5348
TRINITY_DN14398_c0_g1_i4_orf1	-	-	-	trimethyllysine dioxygenase, mitochondrial [Ostrinia furnacalis]	-1.8926	-0.0174	0.95143	0.31642	0.64218
TRINITY_DN1103_c0_g1_i15_orf1	-	-	-	retinal dehydrogenase 1-like [Ostrinia furnacalis]	-1.9419	0.74649	0.6235	0.53041	0.04148
TRINITY_DN1355_c0_g1_i5_orf1	-	-	-	hypothetical protein evm_006664 [Chilo suppressalis]	-1.7717	-0.0311	1.22371	-0.0225	0.60169
TRINITY_DN8258_c0_g1_i6_orf1	-	-	-	unnamed protein product [Chilo suppressalis]	-1.8797	-0.0967	0.67147	0.37384	0.93107
TRINITY_DN31118_c0_g1_i1_orf1	-	-	-	CPR9 [Ostrinia furnacalis]	-1.9267	-0.0368	0.68031	0.63153	0.65171
TRINITY_DN1029_c0_g1_i1_orfp1	-	-	-	TRINITY_DN1029_c0_g1_i1_m.64408 TRINITY_DN1029_c0_g1::TRINITY_DN1029_c0_g1_i1::g.64408 ORF type:3prime_partial len:55 (+),score=13.47 TRINITY_DN1029_c0_g1_i1:74-235(+)	-1.954	0.12389	0.79258	0.51464	0.52291
TRINITY_DN5510_c0_g1_i9_orf1	-	-	-	proteoglycan 4 [Pectinophora gossypiella]	-1.8563	-0.0563	0.64344	0.22751	1.04171
TRINITY_DN5531_c0_g3_i3_orf1	-	-	-	hypothetical protein evm_013868 [Chilo suppressalis]	-1.9564	0.28204	0.24626	0.63504	0.79308
TRINITY_DN1889_c0_g1_i1_orf1	-	-	-	titin isoform X2 [Ostrinia furnacalis]	-1.914	-0.0406	0.59488	0.51113	0.84851
TRINITY_DN2040_c0_g1_i15_orfp1	-	-	-	TRINITY_DN2040_c0_g1_i15_m.4150 TRINITY_DN2040_c0_g1::TRINITY_DN2040_c0_g1_i15::g.4150 ORF type:complete len:319 (+),score=125.43,Plasmodium_HRP PF05403.12 3.5,Plasmodium_HRP|PF05403.12|1.5 TRINITY_DN2040_c0_g1_i15:118-957(+)	-1.9192	-0.0636	0.62549	0.66567	0.69165
TRINITY_DN31118_c0_g2_i1_orf1	-	-	-	unnamed protein product [Spodoptera exigua]	-1.9488	0.06255	0.53836	0.66686	0.68102
TRINITY_DN19460_c0_g1_i1_orf1	-	-	-	cuticle protein 3-like [Ostrinia furnacalis]	-1.9179	-0.0046	0.68088	0.41174	0.82985
TRINITY_DN1211_c0_g1_i10_orf1	-	-	-	spectrin beta chain-like isoform X7 [Spodoptera frugiperda]	-1.9606	0.3986	0.73286	0.1733	0.65582
TRINITY_DN46633_c0_g1_i4_orf1	-	-	-	uncharacterized protein LOC114365425 [Ostrinia furnacalis] >QKV49448.1 fas-associated death domain protein [Ostrinia furnacalis]	-1.7215	0.88434	0.25077	1.00715	-0.4207
TRINITY_DN31645_c0_g1_i3_orf1	-	-	-	dystonin isoform X43 [Helicoverpa armigera]	-1.8506	-0.0913	1.01227	0.22986	0.69971
TRINITY_DN6621_c0_g1_i1_orf1	-	-	-	translocon-associated protein subunit delta [Ostrinia furnacalis]	-1.8663	0.07433	1.07049	0.13135	0.59012
TRINITY_DN17137_c0_g1_i2_orf1	-	-	-	unnamed protein product [Diatraea saccharalis]	-1.8383	0.01529	0.88006	0.02357	0.9194
TRINITY_DN2922_c0_g1_i1_orf1	-	-	-	uncharacterized protein LOC114354086 [Ostrinia furnacalis]	-1.9567	0.26741	0.49714	0.32946	0.86267
TRINITY_DN549_c0_g1_i7_orf1	-	-	-	titin-like, partial [Ostrinia furnacalis]	-1.9275	0.05205	0.88324	0.42667	0.56555
TRINITY_DN13350_c0_g1_i4_orf1	-	-	-	cap-specific mRNA (nucleoside-2'-O-)-methyltransferase 1 [Ostrinia furnacalis]	-1.9153	0.39374	0.96488	0.49101	0.06571
TRINITY_DN26254_c0_g1_i1_orf1	-	-	-	hypothetical protein evm_003664 [Chilo suppressalis] >CAB3521132.1 unnamed protein product [Chilo suppressalis] >CAH0398453.1 unnamed protein product [Chilo suppressalis]	-1.9329	0.02305	0.81076	0.5185	0.58061
TRINITY_DN1814_c0_g2_i1_orf1	-	-	-	titin-like, partial [Ostrinia furnacalis]	-1.9237	0.05981	0.76673	0.31808	0.77904
TRINITY_DN24322_c0_g1_i4_orf1	-	-	-	unnamed protein product, partial [Brenthis ino]	-1.8929	0.16453	1.0696	0.44924	0.20956
TRINITY_DN52395_c0_g2_i2_orf1	-	-	-	twitchin isoform X20 [Zerene cesonia]	-1.816	0.0613	1.25425	0.2495	0.25095
TRINITY_DN1262_c0_g1_i2_orf1	-	-	-	alanine aminotransferase 1 isoform X1 [Ostrinia furnacalis] >XP_028162092.1 alanine aminotransferase 1 isoform X2 [Ostrinia furnacalis] >XP_028162093.1 alanine aminotransferase 1 isoform X3 [Ostrinia furnacalis]	-1.9583	0.45028	0.46916	0.20106	0.83778
TRINITY_DN2890_c0_g1_i2_orf1	-	-	-	alanine aminotransferase 1 [Chelonus insularis]	-1.8889	0.58154	0.78969	-0.1512	0.66889
TRINITY_DN6325_c0_g1_i8_orf1	-	-	-	unnamed protein product [Pieris macdunnoughi]	-1.3883	-0.0914	1.73952	-0.0825	-0.1773
TRINITY_DN825_c0_g1_i18_orfp1	-	-	-	TRINITY_DN825_c0_g1_i18_m.8360 TRINITY_DN825_c0_g1::TRINITY_DN825_c0_g1_i18::g.8360 ORF type:complete len:415 (+),score=37.02 TRINITY_DN825_c0_g1_i18:55-1245(+)	-1.8797	0.48373	1.08594	0.20683	0.10321
TRINITY_DN11735_c0_g1_i5_orf1	-	-	-	TBC1 domain family member 22B isoform X1 [Ostrinia furnacalis] >XP_028174102.1 TBC1 domain family member 22B isoform X2 [Ostrinia furnacalis]	-1.9619	0.20865	0.40721	0.8062	0.53988
TRINITY_DN1814_c0_g2_i4_orfp1	-	-	-	TRINITY_DN1814_c0_g2_i4_m.63284 TRINITY_DN1814_c0_g2::TRINITY_DN1814_c0_g2_i4::g.63284 ORF type:internal len:258 (-),score=126.31 TRINITY_DN1814_c0_g2_i4:3-773(-)	-1.9629	0.19282	0.48149	0.48953	0.79904
TRINITY_DN46173_c0_g3_i1_orf1	-	-	-	Tropomyosin, partial [Cotesia chilonis]	-1.9194	0.14505	0.97522	0.28836	0.51071
TRINITY_DN122393_c0_g1_i1_orf1	-	-	-	microtubule-associated protein futsch isoform X4 [Ostrinia furnacalis] >XP_028162562.1 microtubule-associated protein futsch isoform X4 [Ostrinia furnacalis]	-1.7969	0.15849	1.25611	-0.027	0.40928
TRINITY_DN8651_c0_g1_i18_orf1	-	-	-	glutathione S-transferase theta 2 [Conogethes punctiferalis]	-1.7279	-0.0163	1.16951	-0.2031	0.7778
TRINITY_DN1718_c1_g1_i5_orf1	-	-	-	gelsolin-like [Ostrinia furnacalis]	-1.9429	0.45346	0.73632	0.06516	0.68792
TRINITY_DN13312_c0_g2_i1_orf1	-	-	-	von Willebrand factor A domain-containing protein 8 [Trichoplusia ni]	-1.9177	0.88228	0.59636	0.43401	0.00504
TRINITY_DN23941_c0_g1_i5_orf1	-	-	-	dystonin isoform X11 [Galleria mellonella]	-1.8745	0.36061	1.00484	-0.075	0.58398
TRINITY_DN85004_c0_g1_i1_orf1	-	-	-	uncharacterized protein LOC114357684 [Ostrinia furnacalis]	-1.9689	0.203	0.67085	0.6755	0.41952
TRINITY_DN11448_c0_g1_i11_orf1	-	-	-	hypothetical protein B5X24_HaOG201808 [Helicoverpa armigera]	-1.5012	0.10218	1.58127	-0.4221	0.23979
TRINITY_DN549_c0_g1_i14_orf1	-	-	-	titin-like [Ostrinia furnacalis]	-1.9222	0.40575	0.64714	0.01986	0.8494
TRINITY_DN1391_c1_g2_i2_orf1	-	-	-	uncharacterized protein LOC119837640 isoform X2 [Zerene cesonia]	-1.9227	0.18941	0.89427	0.17918	0.65987
TRINITY_DN12690_c0_g1_i1_orf1	-	-	-	ELAV-like protein 1 [Ostrinia furnacalis]	-1.8252	0.29718	0.92185	-0.2199	0.826
TRINITY_DN1173_c0_g1_i11_orf1	-	-	-	obscurin [Ostrinia furnacalis]	-1.9921	0.62643	0.36567	0.44805	0.55195
TRINITY_DN592_c0_g1_i6_orf1	-	-	-	PDZ and LIM domain protein Zasp isoform X4 [Pectinophora gossypiella]	-1.892	0.15657	0.71311	0.08405	0.93824
TRINITY_DN1073_c0_g1_i3_orf1	-	-	-	carboxylesterase [Loxostege sticticalis]	-1.9644	0.45805	0.21562	0.48472	0.80604
TRINITY_DN41334_c0_g1_i1_orf1	-	-	-	sarcosine dehydrogenase, mitochondrial [Chelonus insularis]	-1.9241	0.95765	0.52104	0.29435	0.15102
TRINITY_DN30208_c0_g1_i3_orf1	-	-	-	unnamed protein product [Timema cristinae]	-1.904	0.07254	0.95265	0.24468	0.63413
TRINITY_DN116_c1_g1_i8_orf1	-	-	-	uncharacterized protein LOC114350057 isoform X2 [Ostrinia furnacalis]	-1.9463	0.25522	0.91454	0.32116	0.45535
TRINITY_DN9591_c0_g1_i1_orf1	-	-	-	probable 39S ribosomal protein L49, mitochondrial [Ostrinia furnacalis]	-1.901	0.82232	0.77861	-0.0212	0.32126
TRINITY_DN101995_c0_g1_i1_orf1	-	-	-	microtubule-actin cross-linking factor 1 isoform X15 [Ostrinia furnacalis]	-1.8434	0.10655	0.71171	-0.016	1.04111
TRINITY_DN114344_c0_g1_i4_orf1	-	-	-	microtubule-actin cross-linking factor 1 isoform X15 [Ostrinia furnacalis]	-1.817	0.46892	1.13914	-0.1777	0.38664
TRINITY_DN8915_c0_g1_i3_orf1	-	-	-	filamin-A isoform X1 [Ostrinia furnacalis] >XP_028171553.1 filamin-A isoform X2 [Ostrinia furnacalis] >XP_028171561.1 filamin-A isoform X2 [Ostrinia furnacalis]	-1.9851	0.45258	0.59781	0.29391	0.64084

TRINITY_DN1895_c0_g1_i2_orf1	-	-	-	unnamed protein product [Chrysodeixis includens]	-1.8698	0.17634	0.91711	-0.0183	0.79459
TRINITY_DN13119_c0_g1_i4_orf1	-	-	-	endocuticle structural glycoprotein ABD-5-like [Bicyclus anynana]	-1.9281	0.04679	0.72368	0.37087	0.78676
TRINITY_DN6612_c0_g1_i4_orf1	-	-	-	hypothetical protein O3G_MSEX008151 [Manduca sexta]	-1.7506	0.23766	1.25007	-0.2439	0.50673
TRINITY_DN2215_c0_g2_i1_orf1	-	-	-	PREDICTED: larval cuticle protein LCP-22-like [Amyelois transitella]	-1.9489	0.10403	0.75122	0.42775	0.66596
TRINITY_DN76333_c0_g1_i2_orf1	-	-	-	larval cuticle protein 65Ag1-like [Ostrinia furnacalis]	-1.977	0.22125	0.54308	0.52779	0.68487
TRINITY_DN1982_c0_g1_i24_orf1	-	-	-	uncharacterized protein LOC114361215 isoform X5 [Ostrinia furnacalis]	-1.8801	0.43783	1.09342	0.08157	0.26725
TRINITY_DN69557_c0_g1_i1_orf1	-	-	-	hypothetical protein G9C98_005708, partial [Cotesia typhae]	-1.8584	0.50733	1.04797	-0.1174	0.42056
TRINITY_DN82628_c0_g1_i2_orf1	-	-	-	ORF type:internal len:148 hit:XP_028162129.1 TRINITY_DN82628_c0_g1_i2:3-446(-)	-1.9438	0.22348	0.86313	0.25636	0.60083
TRINITY_DN1180_c0_g1_i4_orf1	-	-	-	larval cuticle protein LCP-30-like [Ostrinia furnacalis]	-1.9629	0.16161	0.70143	0.44049	0.65939
TRINITY_DN18009_c0_g1_i1_orf1	-	-	-	pre-mRNA-splicing factor ISY1 homolog [Ostrinia furnacalis]	-1.8369	0.68825	0.98802	-0.2051	0.36577
TRINITY_DN9871_c0_g1_i11_orf1	-	-	-	PEST proteolytic signal-containing nuclear protein-like [Ostrinia furnacalis]	-1.9086	0.29687	1.03905	0.17347	0.39922
TRINITY_DN4010_c0_g2_i1_orf1	-	-	-	myophilin [Ostrinia furnacalis]	-1.8799	0.29356	1.09712	0.07692	0.41235
TRINITY_DN100_c0_g1_i13_orf1	-	-	-	hypothetical protein O3G_MSEX015273 [Manduca sexta]	-1.9572	0.88373	0.37995	0.3047	0.38883
TRINITY_DN23429_c0_g2_i1_orf1	-	-	-	muscle-specific protein 20 [Zerene cesonia]	-1.9702	0.39432	0.81288	0.30807	0.45497
TRINITY_DN11448_c0_g1_i15_orf1	-	-	-	unnamed protein product [Chilo suppressalis]	-1.9207	0.46384	0.96015	0.08926	0.40743
TRINITY_DN248_c0_g1_i1_orf1	-	-	-	unnamed protein product [Chilo suppressalis]	-1.9633	0.63613	0.70531	0.15266	0.46918
TRINITY_DN8226_c0_g1_i1_orf1	-	-	-	myosin heavy chain, muscle isoform X16 [Helicoverpa armigera]	-1.9778	0.4769	0.65135	0.22995	0.61957
TRINITY_DN29969_c0_g1_i5_orf1	-	-	-	twitchin-like [Ostrinia furnacalis]	-1.3929	-0.1888	1.72402	0.07332	-0.2156
TRINITY_DN695_c0_g1_i12_orf1	-	-	-	seroin transcript 2A, partial [Ostrinia nubilalis]	-1.978	0.47035	0.67682	0.23815	0.59273
TRINITY_DN2200_c0_g1_i4_orf1	-	-	-	uncharacterized protein LOC114363443 [Ostrinia furnacalis]	-1.8504	0.94627	0.62464	0.49428	-0.2148
TRINITY_DN3906_c0_g1_i5_orf1	-	-	-	ejaculatory bulb-specific protein 3-like [Ostrinia furnacalis]	-1.6766	0.41695	1.23185	-0.4847	0.5125
TRINITY_DN48610_c0_g1_i2_orf1	-	-	-	hypothetical protein evm_002298 [Chilo suppressalis] >CAH0682062.1 unnamed protein product [Chilo suppressalis]	-1.9159	0.3218	0.99689	0.13453	0.46265
TRINITY_DN416_c0_g1_i1_orf1	-	-	-	unnamed protein product [Diatraea saccharalis]	-1.9296	0.55715	0.87594	0.05396	0.44259
TRINITY_DN1814_c0_g1_i11_orf1	-	-	-	titin-like, partial [Ostrinia furnacalis]	-1.876	0.77793	0.72067	-0.1887	0.56609
TRINITY_DN1455_c0_g1_i8_orf1	-	-	-	troponin T, skeletal muscle isoform X1 [Galleria mellonella]	-1.9562	0.32021	0.86653	0.27585	0.49364
TRINITY_DN4724_c0_g1_i4_orf1	-	-	-	paramyosin, long form isoform X1 [Manduca sexta] >KAG6443143.1 hypothetical protein O3G_MSEX002737 [Manduca sexta]	-1.9476	0.86788	0.547	0.18741	0.34533
TRINITY_DN48097_c0_g1_i1_orf1	-	-	-	unnamed protein product [Homo sapiens]	-1.9489	0.51043	0.31759	0.2349	0.88602
TRINITY_DN100_c0_g1_i9_orf1	-	-	-	uncharacterized protein LOC114353052 [Ostrinia furnacalis]	-1.9547	0.72977	0.60298	0.09925	0.52269
TRINITY_DN7778_c0_g1_i1_orf1	-	-	-	peroxiredoxin-2 [Cotesia glomerata] >KAH0561449.1 Peroxiredoxin-4 [Cotesia glomerata]	-1.8344	0.66419	1.03895	-0.1645	0.2957
TRINITY_DN29100_c0_g1_i2_orf1	-	-	-	endocuticle structural glycoprotein ABD-5-like [Galleria mellonella]	-1.9752	0.61491	0.38656	0.26639	0.7073
TRINITY_DN928_c0_g1_i3_orf1	-	-	-	fasciclin-2-like [Ostrinia furnacalis]	-1.8923	0.8026	0.81685	0.32418	-0.0513
TRINITY_DN6881_c0_g1_i1_orf1	-	-	-	putative protein TPRXL [Ostrinia furnacalis]	-1.9888	0.46913	0.32525	0.65037	0.54402
TRINITY_DN21170_c0_g1_i5_orf1	-	-	-	twitchin-like [Ostrinia furnacalis]	-1.2331	0.10922	1.78992	-0.478	-0.1881
TRINITY_DN31001_c0_g1_i1_orf1	-	-	-	endocuticle structural glycoprotein ABD-5-like [Ostrinia furnacalis]	-1.9831	0.52299	0.30709	0.44474	0.70829
TRINITY_DN76529_c0_g1_i1_orfp1	-	-	-	TRINITY_DN76529_c0_g1_i1_m.64079 TRINITY_DN76529_c0_g1::TRINITY_DN76529_c0_g1_i1::g.64079 ORF type:internal len:70 (+),score=14.68 TRINITY_DN76529_c0_g1_i1:3-209(+)	-1.5651	0.25284	1.52508	-0.3692	0.15635
TRINITY_DN146841_c0_g1_i1_orf1	-	-	-	muscle-specific protein 20 [Temnothorax curvispinosus]	-1.9919	0.45105	0.36309	0.63086	0.54689
TRINITY_DN235_c0_g1_i2_orf1	-	-	-	unnamed protein product [Parnassius apollo]	-1.9049	0.88794	0.58814	-0.0548	0.48361
TRINITY_DN248_c0_g1_i12_orf1	-	-	-	twitchin-like [Ostrinia furnacalis]	-1.7405	0.66343	1.19718	0.15197	-0.272
TRINITY_DN1982_c0_g1_i17_orf1	-	-	-	unnamed protein product, partial [Iphiclides podalirius]	-1.9434	0.73087	0.6094	0.04109	0.56205
TRINITY_DN1123_c2_g1_i3_orf1	-	-	-	troponin I isoform X8 [Ostrinia furnacalis]	-1.9554	0.47159	0.82936	0.16581	0.48863
TRINITY_DN1123_c2_g1_i4_orf1	-	-	-	troponin I isoform X16 [Ostrinia furnacalis]	-1.9883	0.59807	0.54634	0.29117	0.55277
TRINITY_DN42461_c0_g1_i4_orf1	-	-	-	obscurin [Ostrinia furnacalis]	-1.9087	0.77504	0.7786	-0.031	0.38605
TRINITY_DN38366_c0_g1_i4_orfp1	-	-	-	TRINITY_DN38366_c0_g1_i4_m.10666 TRINITY_DN38366_c0_g1::TRINITY_DN38366_c0_g1_i4::g.10666 ORF type:internal len:143 (+),score=71.68 TRINITY_DN38366_c0_g1_i4:3-428(+)	-1.8675	0.82029	0.62004	-0.2131	0.64019
TRINITY_DN31118_c1_g1_i1_orf1	-	-	-	endocuticle structural glycoprotein ABD-4-like [Ostrinia furnacalis]	-1.9881	0.66126	0.43856	0.3356	0.55263
TRINITY_DN116951_c0_g3_i2_orf1	-	-	-	spermine oxidase-like isoform X2 [Ostrinia furnacalis]	-1.902	1.01154	0.26347	0.09775	0.52927
TRINITY_DN67231_c0_g1_i1_orf1	-	-	-	endocuticle structural glycoprotein SgAbd-8-like [Ostrinia furnacalis]	-1.9973	0.48025	0.52308	0.41698	0.57694
TRINITY_DN27500_c0_g1_i4_orf1	-	-	-	hemicentin-1-like [Ostrinia furnacalis]	-1.7358	0.50168	1.19087	-0.3761	0.4193
TRINITY_DN46216_c0_g3_i1_orf1	-	-	-	unnamed protein product, partial [Brenthis ino]	-1.8772	0.35194	0.70043	-0.0983	0.92312
TRINITY_DN115082_c0_g1_i5_orf1	-	-	-	protein dj-1beta-like isoform X2 [Ostrinia furnacalis]	-1.4333	1.01295	1.2336	-0.2235	-0.5897
TRINITY_DN2186_c0_g1_i17_orf1	-	-	-	paxillin isoform X6 [Leguminivora glycinivorella]	-1.9173	0.84617	0.69698	0.02583	0.34836
TRINITY_DN11596_c0_g1_i1_orf1	-	-	-	probable ATP-dependent RNA helicase DDX47 [Ostrinia furnacalis]	-1.085	0.89009	-0.9836	1.41652	-0.2381
TRINITY_DN33146_c0_g1_i1_orf1	-	-	-	adenylate kinase-like [Ostrinia furnacalis]	-1.39	1.12471	-0.959	0.87095	0.35329
TRINITY_DN5363_c0_g1_i1_orf1	-	-	-	cytochrome c oxidase assembly factor 6 homolog [Ostrinia furnacalis]	-1.6036	0.95023	-0.7059	0.9069	0.4524
TRINITY_DN79083_c0_g1_i2_orf1	-	-	-	unnamed protein product [Arctia plantaginis]	-1.1941	0.99699	-1.2426	0.71982	0.71982
TRINITY_DN26947_c0_g1_i1_orf1	-	-	-	probable 39S ribosomal protein L24, mitochondrial [Ostrinia furnacalis]	-1.4984	1.20902	-0.5485	0.98509	-0.1471
TRINITY_DN2338_c0_g2_i2_orf1	-	-	-	prophenoloxidase PPO3 [Ostrinia furnacalis]	-1.2977	1.43075	-0.7843	0.79556	-0.1443
TRINITY_DN4294_c0_g1_i6_orf1	-	-	-	protein vav isoform X2 [Ostrinia furnacalis]	-1.4688	1.28715	-0.7094	0.82359	0.06737
TRINITY_DN67495_c0_g1_i1_orf1	-	-	-	hypothetical protein KGM_200102A, partial [Danaus plexippus plexippus]	-1.3686	1.33382	-0.6746	0.92089	-0.2115
TRINITY_DN36928_c0_g1_i5_orf1	-	-	-	actin-interacting protein 1 isoform X2 [Ostrinia furnacalis]	-0.5651	0.98077	-1.7005	0.67108	0.61374
TRINITY_DN7908_c0_g1_i5_orf1	-	-	-	protein transport protein Sec24A [Helicoverpa zea]	-1.2332	1.64528	-0.5299	0.55113	-0.4333
TRINITY_DN6241_c0_g1_i1_orf1	-	-	-	uncharacterized protein LOC114355531 [Ostrinia furnacalis]	-1.5328	0.97035	-0.8437	0.76792	0.63825
TRINITY_DN5354_c0_g1_i4_orf1	-	-	-	NADP-dependent malic enzyme-like [Ostrinia furnacalis]	-1.2131	1.53179	-0.7871	0.70999	-0.2416

TRINITY_DN25681_c0_g1_i5_orf1	-	-	-	hypothetical protein evm_005766 [Chilo suppressalis] >CAB3520395.1 unnamed protein product [Chilo suppressalis] >CAH0397716.1 unnamed protein product [Chilo suppressalis]	-1.29	1.60363	-0.5517	0.5836	-0.3456
TRINITY_DN2749_c0_g1_i4_orf1	-	-	-	RNA exonuclease 4-like [Ostrinia furnacalis] >QEE79882.1 REX4 [Ostrinia furnacalis]	-0.9203	1.36027	-1.3529	0.62305	0.28993
TRINITY_DN4356_c0_g1_i6_orf1	-	-	-	mulatexin-like [Ostrinia furnacalis]	-0.3049	1.2879	-1.7031	0.57028	0.14983
TRINITY_DN2695_c0_g1_i8_orfp1	-	-	-	TRINITY_DN2695_c0_g1_i8_m.44478 TRINITY_DN2695_c0_g1::TRINITY_DN2695_c0_g1_i8::g.44478 ORF type:3prime_partial len:532 (+),score=80.62 TRINITY_DN2695_c0_g1_i8:101-1594(+)	-1.1632	1.22783	-0.8596	1.13387	-0.3389
TRINITY_DN99_c0_g1_i3_orf1	-	-	-	uncharacterized protein LOC126375979 [Pectinophora gossypiella] >XP_049879066.1 uncharacterized protein LOC126375979 [Pectinophora gossypiella]	-1.0207	1.53317	-0.842	0.81448	-0.4849
TRINITY_DN344_c1_g1_i1_orf1	-	-	-	chymotrypsin-like serine protease 16 [Ostrinia nubilalis]	-1.1001	1.52301	-0.6543	0.82822	-0.5969
TRINITY_DN33452_c0_g1_i1_orf1	-	-	-	lethal(2) giant larvae protein isoform X8 [Ostrinia furnacalis]	-0.7904	1.40148	-1.4046	0.14832	0.64518
TRINITY_DN39813_c0_g1_i1_orf1	-	-	-	nucleoside diphosphate kinase [Ostrinia furnacalis]	-1.4922	1.1578	-0.0191	1.00437	-0.6509
TRINITY_DN2401_c0_g2_i1_orf1	-	-	-	DNA-directed RNA polymerase I subunit RPA2 [Ostrinia furnacalis]	-1.9085	0.99242	0.05728	0.41195	0.44686
TRINITY_DN21570_c0_g1_i1_orf1	-	-	-	ceramide synthase 5-like [Ostrinia furnacalis]	-1.921	0.98884	0.15637	0.44613	0.32962
TRINITY_DN146006_c0_g1_i1_orf1	-	-	-	unnamed protein product [Chrysodeixis includens]	-1.6079	1.42795	0.5162	-0.0059	-0.3303
TRINITY_DN27885_c0_g1_i3_orf1	-	-	-	ubiquinone biosynthesis protein COQ9-B, mitochondrial-like isoform X2 [Ostrinia furnacalis]	-1.2591	1.68335	-0.6769	0.33955	-0.0869
TRINITY_DN7341_c0_g1_i8_orf1	-	-	-	LOW QUALITY PROTEIN: proteasome activator complex subunit 4-like [Ostrinia furnacalis]	-1.7085	1.30709	0.58067	0.00869	-0.188
TRINITY_DN5382_c0_g2_i1_orf1	-	-	-	protein seele [Ostrinia furnacalis]	-1.2167	1.51219	0.2824	0.41326	-0.9911
TRINITY_DN18918_c0_g1_i3_orf1	-	-	-	myrosinase 1-like isoform X2 [Ostrinia furnacalis]	-0.6908	1.59734	-0.6472	0.74175	-1.0012
TRINITY_DN26853_c0_g1_i1_orf1	-	-	-	astacin-like metalloprotease toxin 5 [Ostrinia furnacalis]	-1.6227	1.43846	-0.3461	0.12841	0.40188
TRINITY_DN9506_c0_g1_i2_orf1	-	-	-	glutathione S-transferase sigma 4 [Conogethes punctiferalis]	-1.6444	1.22118	0.28489	0.66664	-0.5283
TRINITY_DN2177_c0_g1_i1_orf1	-	-	-	uncharacterized protein LOC114349824 isoform X1 [Ostrinia furnacalis] >XP_028156186.1 uncharacterized protein LOC114349824 isoform X1 [Ostrinia furnacalis]	-1.053	1.76414	-0.7278	0.3613	-0.3446
TRINITY_DN21943_c1_g1_i1_orf1	-	-	-	myosin light chain alkali isoform X2 [Ostrinia furnacalis]	-1.8424	1.20176	0.09344	0.23505	0.31213
TRINITY_DN19080_c0_g1_i4_orf1	-	-	-	synaptic vesicle 2-related protein-like isoform X1 [Ostrinia furnacalis] >XP_028161172.1 synaptic vesicle 2-related protein-like isoform X1 [Ostrinia furnacalis]	-1.7808	1.31761	0.06853	0.12577	0.26885
TRINITY_DN146217_c0_g1_i1_orf1	-	-	-	60S acidic ribosomal protein P0 [Bombus bifarius]	-1.8807	0.77673	0.91659	0.06125	0.1261
TRINITY_DN640_c0_g1_i5_orf1	-	-	-	pancreatic triacylglycerol lipase-like [Ostrinia furnacalis]	-1.4113	1.6233	-0.524	-0.002	0.31397
TRINITY_DN35582_c0_g1_i1_orf1	-	-	-	uncharacterized protein LOC114364680 [Ostrinia furnacalis]	-1.8084	0.87728	0.97927	-0.017	-0.0312
TRINITY_DN2318_c1_g1_i1_orf1	-	-	-	transcription factor SPT20 homolog [Ostrinia furnacalis]	-1.6751	1.2347	0.78124	-0.1939	-0.1469
TRINITY_DN7335_c0_g1_i1_orf1	-	-	-	probable methylmalonate-semialdehyde dehydrogenase [acylating], mitochondrial [Bicyclus anynana]	-1.421	1.66909	0.03657	0.13425	-0.4189
TRINITY_DN23204_c0_g1_i1_orf1	-	-	-	LOW QUALITY PROTEIN: uncharacterized protein LOC114350452 [Ostrinia furnacalis]	-1.5018	1.50835	-0.5614	0.26138	0.29342
TRINITY_DN46372_c0_g2_i1_orf1	-	-	-	basic salivary proline-rich protein 1 isoform X2 [Ostrinia furnacalis]	-1.651	1.03398	1.01964	-0.4068	0.00416
TRINITY_DN5721_c0_g1_i5_orf1	-	-	-	fumarate hydratase, mitochondrial-like isoform X2 [Ostrinia furnacalis]	-1.1376	1.80896	-0.4814	0.20812	-0.3981
TRINITY_DN348_c0_g2_i1_orf1	-	-	-	pancreatic triacylglycerol lipase-like [Ostrinia furnacalis]	-1.5326	1.61119	-0.1495	-0.0881	0.15891
TRINITY_DN15160_c0_g1_i1_orf1	-	-	-	tyrosine--tRNA ligase, cytoplasmic [Ostrinia furnacalis]	-1.2071	1.44235	-0.0054	0.73244	-0.9624
TRINITY_DN8352_c0_g1_i3_orf1	-	-	-	TRPL translocation defect protein 14 isoform X1 [Ostrinia furnacalis]	-1.1973	1.77547	-0.589	0.18885	-0.1781
TRINITY_DN133228_c0_g1_i3_orf1	-	-	-	microtubule-actin cross-linking factor 1 isoform X15 [Ostrinia furnacalis]	-1.5603	1.54778	-0.1983	-0.1274	0.3382
TRINITY_DN6916_c0_g1_i4_orf1	-	-	-	isovaleryl-CoA dehydrogenase, mitochondrial [Ostrinia furnacalis]	-1.5622	1.54485	0.34377	-0.1359	-0.1905
TRINITY_DN5111_c0_g1_i2_orf1	-	-	-	uncharacterized protein LOC126368598 [Pectinophora gossypiella]	-1.2237	1.77498	-0.3941	0.22496	-0.3821
TRINITY_DN32420_c0_g1_i2_orf1	-	-	-	PREDICTED: plectin-like, partial [Papilio polytes]	-1.398	1.35326	-0.6831	-0.1274	0.85525
TRINITY_DN146126_c0_g1_i1_orf1	-	-	-	malate dehydrogenase, mitochondrial [Chelonus insularis]	-1.694	1.43713	-0.037	0.04612	0.24783
TRINITY_DN21872_c0_g1_i2_orf1	-	-	-	facilitated trehalose transporter Tret1-2 homolog [Ostrinia furnacalis] >XP_028178438.1 facilitated trehalose transporter Tret1-2 homolog [Ostrinia furnacalis] >XP_028178439.1 facilitated trehalose transporter Tret1-2 homolog [Ostrinia furnacalis]	-1.5298	1.49891	0.47672	-0.0152	-0.4306
TRINITY_DN56270_c0_g1_i1_orf1	-	-	-	PREDICTED: putative elongator complex protein 1 [Microplitis demolitor] >XP_008554512.1 PREDICTED: putative elongator complex protein 1 [Microplitis demolitor]	-1.2095	1.20812	0.73109	0.43416	-1.1638
TRINITY_DN5234_c0_g1_i2_orf1	-	-	-	proline dehydrogenase 1, mitochondrial isoform X2 [Ostrinia furnacalis]	-0.817	1.83815	-0.5801	0.28934	-0.7304
TRINITY_DN13718_c0_g1_i7_orf1	-	-	-	immulectin-4 [Ostrinia furnacalis]	-1.6406	1.4608	-0.2128	0.35763	0.03503
TRINITY_DN1125_c0_g1_i4_orf1	-	-	-	hypothetical protein evm_001907 [Chilo suppressalis] >CAH2985359.1 unnamed protein product [Chilo suppressalis]	-1.6667	1.10141	0.3905	-0.5612	0.73599
TRINITY_DN747_c0_g1_i1_orf1	-	-	-	trypsin, alkaline C-like [Ostrinia furnacalis]	-0.8449	1.62949	-0.4246	0.65242	-1.0124
TRINITY_DN38562_c0_g1_i3_orf1	-	-	-	persulfide dioxygenase ETHE1, mitochondrial isoform X1 [Ostrinia furnacalis]	-1.5107	1.44782	-0.6071	0.45489	0.21504
TRINITY_DN6365_c0_g1_i4_orf1	-	-	-	40S ribosomal protein S21 [Helicoverpa armigera] >XP_047038308.1 40S ribosomal protein S21 isoform X2 [Helicoverpa zea] >KAI5643652.1 ribosomal protein s21e domain-containing protein [Phthorimaea operculella] >PZC73652.1 hypothetical protein B5X24_HaOG209026 [Helicoverpa armigera]	-1.6359	1.31419	0.25647	-0.4816	0.54684
TRINITY_DN23740_c1_g1_i1_orf1	-	-	-	NADH dehydrogenase [ubiquinone] iron-sulfur protein 6, mitochondrial isoform X1 [Ostrinia furnacalis]	-1.4821	1.44501	0.12926	0.54315	-0.6353
TRINITY_DN72541_c0_g1_i2_orf1	-	-	-	xaa-Pro aminopeptidase ApepP-like isoform X2 [Ostrinia furnacalis]	-1.5439	1.47835	0.546	-0.147	-0.3335
TRINITY_DN6668_c0_g1_i4_orf1	-	-	-	UBX domain-containing protein 4 isoform X1 [Ostrinia furnacalis] >XP_028156702.1 UBX domain-containing protein 4 isoform X2 [Ostrinia furnacalis]	-1.0165	1.78787	-0.1819	0.23601	-0.8255
TRINITY_DN69697_c0_g1_i1_orf1	-	-	-	PREDICTED: uncharacterized protein LOC103573287 [Microplitis demolitor]	-1.0809	1.74578	0.14394	0.06254	-0.8714
TRINITY_DN6027_c0_g1_i13_orf1	-	-	-	5-demethoxyubiquinone hydroxylase, mitochondrial [Ostrinia furnacalis] >XP_028160430.1 5-demethoxyubiquinone hydroxylase, mitochondrial [Ostrinia furnacalis]	-1.6502	1.43894	0.07326	0.37829	-0.2403
TRINITY_DN2224_c0_g1_i1_orf1	-	-	-	serine--tRNA ligase, cytoplasmic [Ostrinia furnacalis]	-1.5702	1.4605	0.51197	-0.3722	-0.0301
TRINITY_DN27045_c0_g1_i1_orf1	-	-	-	cytochrome P450 6B5-like [Galleria mellonella]	-1.756	0.92164	0.17771	-0.312	0.96864
TRINITY_DN3760_c0_g1_i1_orf1	-	-	-	something about silencing protein 10 [Ostrinia furnacalis]	-1.2395	1.77445	-0.3049	0.19763	-0.4277
TRINITY_DN25492_c0_g1_i1_orf1	-	-	-	PREDICTED: myrosinase 1-like [Amyelois transitella]	-1.8119	1.20647	0.49903	0.11147	-0.0051
TRINITY_DN16673_c0_g1_i1_orf1	-	-	-	myosin heavy chain, partial [Drosophila virilis]	-1.6773	1.40382	0.4217	-0.1907	0.0425

TRINITY_DN19830_c0_g1_i1_orf1	-	-	-	macrophage migration inhibitory factor-like [Ostrinia furnacalis]	-1.6401	1.48518	-0.1096	0.30154	-0.037
TRINITY_DN13186_c0_g1_i1_orf1	-	-	-	NADH dehydrogenase [ubiquinone] iron-sulfur protein 5-like [Bicyclus anynana]	-0.7678	1.83473	-0.4855	0.27476	-0.8561
TRINITY_DN1752_c0_g1_i18_orf1	-	-	-	titin isoform X1 [Ostrinia furnacalis]	-1.5723	1.55201	0.13451	-0.2745	0.16025
TRINITY_DN863_c0_g1_i6_orf1	-	-	-	protein henna [Galleria mellonella]	-1.3493	1.48487	-0.7528	-0.0211	0.63836
TRINITY_DN1383_c0_g1_i2_orf1	-	-	-	uncharacterized protein LOC114353133 isoform X1 [Ostrinia furnacalis] >XP_028160773.1 uncharacterized protein LOC114353133 isoform X2 [Ostrinia furnacalis]	-1.107	1.64306	-0.9495	0.41655	-0.0032
TRINITY_DN8964_c0_g1_i4_orf1	-	-	-	hypothetical protein evm_010115 [Chilo suppressalis]	-1.7431	0.86215	0.80086	-0.4956	0.57566
TRINITY_DN111110_c0_g1_i1_orf1	-	-	-	NAD-dependent protein deacylase-like [Ostrinia furnacalis]	-1.6903	0.80908	0.90546	-0.59	0.56583
TRINITY_DN79673_c0_g1_i1_orf1	-	-	-	thioredoxin, mitochondrial-like [Ostrinia furnacalis]	-1.6977	1.3876	-0.1217	0.01057	0.42126
TRINITY_DN9248_c0_g1_i10_orf1	-	-	-	unnamed protein product [Arctia plantaginis]	-1.4378	1.65725	0.24101	-0.3354	-0.125
TRINITY_DN21451_c0_g1_i3_orf1	-	-	-	gelsolin-like [Ostrinia furnacalis]	-1.4443	1.61632	-0.4565	-0.0201	0.30454
TRINITY_DN7134_c0_g1_i1_orf1	-	-	-	phosphatidylglycerophosphatase and protein-tyrosine phosphatase 1 [Ostrinia furnacalis]	-1.1144	1.84737	-0.5558	0.01316	-0.1903
TRINITY_DN50676_c0_g1_i1_orf1	-	-	-	uncharacterized protein LOC114360659 [Ostrinia furnacalis]	-1.5556	1.29888	0.16687	-0.6113	0.7011
TRINITY_DN11448_c0_g1_i4_orf1	-	-	-	uncharacterized protein LOC114364760 isoform X5 [Ostrinia furnacalis]	-1.7358	1.35317	0.34225	-0.1178	0.15818
TRINITY_DN135_c0_g1_i1_orf1	-	-	-	60S ribosomal protein L11 [Nymphalis io]	-1.454	1.69197	-0.0537	-0.133	-0.0512
TRINITY_DN22956_c0_g1_i1_orf1	-	-	-	lipoamide acyltransferase component of branched-chain alpha-keto acid dehydrogenase complex, mitochondrial [Ostrinia furnacalis]	-1.5424	1.51832	-0.3338	-0.0857	0.44362
TRINITY_DN7808_c0_g1_i1_orf1	-	-	-	probable pyruvate dehydrogenase E1 component subunit alpha, mitochondrial isoform X1 [Ostrinia furnacalis] >XP_028158738.1 probable pyruvate dehydrogenase E1 component subunit alpha, mitochondrial isoform X2 [Ostrinia furnacalis] >XP_028158739.1 probable pyruvate dehydrogenase E1 component subunit alpha, mitochondrial isoform X3 [Ostrinia furnacalis] >XP_028158740.1 probable pyruvate dehydrogenase E1 component subunit alpha, mitochondrial isoform X4 [Ostrinia furnacalis]	-1.476	1.66559	-0.196	-0.0627	0.06916
TRINITY_DN105055_c0_g1_i1_orfp1	-	-	-	unnamed protein product [Euphydryas editha]	-1.0753	1.87362	-0.55	-0.1253	-0.123
TRINITY_DN5597_c0_g1_i2_orf1	-	-	-	monocarboxylate transporter 9-like [Ostrinia furnacalis] >XP_028156211.1 monocarboxylate transporter 9-like [Ostrinia furnacalis]	-1.0666	1.84346	-0.6766	-0.0255	-0.0748
TRINITY_DN38435_c0_g1_i1_orf1	-	-	-	UDP-glucuronosyltransferase 2B20-like [Ostrinia furnacalis]	-1.7565	1.26559	0.54559	0.05592	-0.1106
TRINITY_DN57918_c0_g1_i1_orf1	-	-	-	PREDICTED: serine--tRNA ligase, cytoplasmic [Fopius arisanus]	-1.6412	1.41489	0.49249	-0.2486	-0.0176
TRINITY_DN1884_c0_g2_i2_orf1	-	-	-	phosphotriesterase-related protein [Ostrinia furnacalis]	-0.8105	1.75551	-1.1157	0.05385	0.11683
TRINITY_DN4451_c0_g2_i4_orf1	-	-	-	uncharacterized protein LOC114361986 isoform X1 [Ostrinia furnacalis] >XP_028173022.1 uncharacterized protein LOC114361986 isoform X2 [Ostrinia furnacalis]	-1.4371	1.63047	0.14136	0.14913	-0.4839
TRINITY_DN1445_c0_g2_i4_orf1	-	-	-	leucine-rich PPR motif-containing protein, mitochondrial [Ostrinia furnacalis]	-1.4737	1.66909	0.01793	-0.0087	-0.2045
TRINITY_DN80245_c0_g1_i1_orf1	-	-	-	peroxisomal membrane protein 2 [Ostrinia furnacalis]	-1.5158	1.58988	-0.0325	-0.3146	0.27309
TRINITY_DN9117_c0_g1_i1_orf1	-	-	-	spherulin-2A-like [Ostrinia furnacalis]	-1.3126	1.78555	-0.0945	-0.1246	-0.2538
TRINITY_DN434_c0_g1_i4_orf1	-	-	-	uncharacterized protein LOC126367148 [Pectinophora gossypiella]	-1.6193	1.44835	-0.0994	-0.2067	0.47703
TRINITY_DN1232_c0_g1_i1_orf1	-	-	-	acanthoscurrin-2-like isoform X1 [Ostrinia furnacalis]	-1.8039	1.01414	0.74523	-0.2617	0.30617
TRINITY_DN3976_c0_g1_i6_orf1	-	-	-	grpE protein homolog, mitochondrial [Ostrinia furnacalis]	-0.9413	1.93445	-0.4665	-0.1752	-0.3515
TRINITY_DN14597_c0_g1_i5_orf1	-	-	-	UDP-glucuronosyltransferase 2B1-like isoform X3 [Ostrinia furnacalis]	-1.4093	1.57602	-0.231	-0.4549	0.51926
TRINITY_DN11194_c0_g1_i4_orf1	-	-	-	ATPase family AAA domain-containing protein 3A homolog [Ostrinia furnacalis]	-1.3619	1.70742	-0.0541	-0.4496	0.15816
TRINITY_DN55160_c0_g1_i1_orf1	-	-	-	esterase FE4-like isoform X2 [Ostrinia furnacalis]	-1.155	1.84094	-0.5001	-0.1618	-0.0241
TRINITY_DN74889_c0_g1_i1_orf1	-	-	-	probable 28S ribosomal protein S23, mitochondrial [Ostrinia furnacalis]	-1.1594	1.80761	-0.5937	-0.1582	0.10376
TRINITY_DN120439_c1_g1_i1_orf1	-	-	-	myosin heavy chain variant, partial [Bombyx mori]	-1.8054	1.15961	0.56686	-0.1495	0.22845
TRINITY_DN7580_c0_g1_i1_orf1	-	-	-	cytochrome P450 monooxygenase CYP6AB141 [Ostrinia furnacalis]	-1.6684	0.84874	0.46355	-0.6023	0.95841
TRINITY_DN107288_c0_g1_i2_orf1	-	-	-	methionine-tRNA synthetase, partial [Papilio xuthus]	-1.0387	1.68864	-0.0971	0.39716	-0.95
TRINITY_DN4920_c0_g1_i5_orf1	-	-	-	titin homolog [Ostrinia furnacalis]	-1.8415	1.08524	0.62195	-0.0659	0.20018
TRINITY_DN3461_c0_g1_i1_orf1	-	-	-	protein SCO1 homolog, mitochondrial [Ostrinia furnacalis]	-1.4052	1.71859	-0.1282	-0.2311	0.04586
TRINITY_DN4622_c0_g1_i1_orf1	-	-	-	keratin-associated protein 19-2-like [Ostrinia furnacalis]	-1.4417	1.66637	0.20873	-0.1569	-0.2764
TRINITY_DN45446_c0_g1_i2_orf1	-	-	-	peptide transporter family 1-like isoform X1 [Ostrinia furnacalis]	-0.91	1.92942	-0.5176	-0.0845	-0.4174
TRINITY_DN110460_c0_g2_i1_orf1	-	-	-	Similar to chaf1a-b: Chromatin assembly factor 1 subunit A-B (Xenopus laevis) [Cotesia congregata]	-1.7647	1.34595	0.04963	0.22627	0.1429
TRINITY_DN1173_c1_g1_i10_orf1	-	-	-	hypothetical protein evm_001011 [Chilo suppressalis]	-1.818	0.96313	0.87085	-0.0739	0.05792
TRINITY_DN1123_c2_g1_i5_orf1	-	-	-	troponin I isoform X4 [Leguminivora glycinivorella]	-1.7624	1.10226	0.81617	-0.098	-0.0581
TRINITY_DN1952_c0_g1_i2_orf1	-	-	-	uncharacterized protein LOC114354403 [Ostrinia furnacalis] >AYE20402.1 RNAi efficiency-related nuclease REase [Ostrinia furnacalis]	-1.632	1.49918	-0.0922	-0.0535	0.27855
TRINITY_DN4016_c0_g1_i1_orf1	-	-	-	60S acidic ribosomal protein P0 [Homo sapiens] >NP_444505.1 60S acidic ribosomal protein P0 [Homo sapiens] >XP_002823894.1 60S acidic ribosomal protein P0 [Pongo abelii] >XP_003280010.1 60S acidic ribosomal protein P0 [Nomascus leucogenys] >XP_004054038.1 60S acidic ribosomal protein P0 [Gorilla gorilla gorilla] >XP_004054039.1 60S acidic ribosomal protein P0 [Gorilla gorilla gorilla] >XP_008956032.1 60S acidic ribosomal protein P0 [Pan paniscus] >XP_008956033.1 60S acidic ribosomal protein P0 [Pan paniscus] >XP_012611945.1 60S acidic ribosomal protein P0 [Microcebus murinus] >XP_016802006.1 60S acidic ribosomal protein P0 [Pan troglodytes] >XP_016802007.1 60S acidic ribosomal protein P0 [Pan troglodytes] >XP_025256707.1 60S acidic ribosomal protein P0 isoform X1 [Theropithecus gelada] >XP_025256708.1 60S acidic ribosomal protein P0 isoform X1 [Theropithecus gelada] >XP_032024425.1 60S acidic ribosomal protein P0 [Hylobates moloch] >XP_032657670.1 60S acidic ribosomal protein P0 [Chelonoidis abingdonii] >XP_045390642.1 60S acidic ribosomal protein P0 [Lemur catta] >P05388.1 RecName: Full=60S acidic ribosomal protein P0; AltName: Full=60S ribosomal protein L10E; AltName: Full=Large ribosomal subunit protein uL10 [Homo sapiens] >3J92_s Structure and assembly pathway of the ribosome quality control complex [Oryctolagus cuniculus] >4V5Z_Bg Chain Bg, 60S acidic ribosomal protein P0 [Canis lupus familiaris] >4V6X_Cq Chain Cq, 60S acidic ribosomal protein P0 [Homo sapiens] >5AJ0_AK Chain AK, 60S acidic ribosomal protein P0 [Homo sapiens] >6ZM7_Ls Chain Ls, 60S acidic ribosomal protein P0 [Homo sapiens] >6ZME_Ls Chain Ls, 60S acidic ribosomal protein P0 [Homo sapiens] >6ZMI_Ls Chain Ls, 60S acidic ribosomal protein P0 [Homo sapiens] >6ZMO_Ls Chain Ls, 60S acidic ribosomal protein P0 [Homo sapiens] >ABM82739.1 ribosomal protein, large, P0 [synthetic construct] >SJX33952.1 unnamed protein product, partial [Human ORFeome Gateway entry vector] >AAA36470.1 acidic ribosomal phosphoprotein (P0) [Homo sapiens] >AAC05176.1 60S ACIDIC RIBOSOMAL PROTEIN; match to P05388 (PID:g133041) [Homo sapiens] >AAH00087.1 Ribosomal protein, large, P0 [Homo sapiens]	-1.7953	1.26996	0.29713	-0.0438	0.27196

TRINITY_DN2748_c0_g1_i6_orf1	-	-	-	uncharacterized protein LOC114352811 [Ostrinia furnacalis]	-0.9908	1.85398	0.01085	-0.1216	-0.7525
TRINITY_DN2497_c0_g1_i2_orf1	-	-	-	protein stunted-like isoform X1 [Colias croceus]	-1.0496	1.74511	0.34292	-0.206	-0.8325
TRINITY_DN36817_c0_g1_i1_orf1	-	-	-	uncharacterized protein LOC114357350 [Ostrinia furnacalis]	-1.8316	1.19413	0.44889	0.0963	0.09229
TRINITY_DN17417_c0_g1_i11_orf1	-	-	-	sodium/hydrogen exchanger 9B2-like isoform X4 [Ostrinia furnacalis]	-1.032	1.87281	-0.6061	-0.2453	0.01055
TRINITY_DN129869_c0_g4_i1_orf1	-	-	-	putative myosin heavy chain, muscle, partial [Cotesia chilonis]	-1.779	1.27825	0.39557	-0.0875	0.19268
TRINITY_DN20957_c0_g1_i1_orf1	-	-	-	adenylate kinase isoenzyme 1 isoform X2 [Ostrinia furnacalis]	-1.7615	1.26355	0.54568	0.00595	-0.0537
TRINITY_DN4501_c0_g1_i3_orf1	-	-	-	methylcrotonoyl-CoA carboxylase subunit alpha, mitochondrial [Ostrinia furnacalis]	-1.6488	1.35524	0.49637	-0.3995	0.19675
TRINITY_DN4145_c0_g1_i1_orf1	-	-	-	uncharacterized protein LOC114353175 isoform X1 [Ostrinia furnacalis]	-1.6926	1.43662	0.24454	-0.0692	0.08066
TRINITY_DN2266_c0_g1_i6_orf1	-	-	-	bilin-binding protein-like [Ostrinia furnacalis]	-0.6203	1.73985	-0.7522	0.5073	-0.8746
TRINITY_DN4408_c6_g1_i1_orf1	-	-	-	polyprotein, partial [Bemisia tabaci]	-1.6052	0.70323	0.79894	-0.7501	0.85312
TRINITY_DN15513_c0_g1_i6_orf1	-	-	-	uncharacterized protein LOC114350859 [Ostrinia furnacalis]	-1.3351	1.63254	-0.5203	-0.2468	0.46969
TRINITY_DN1666_c0_g1_i2_orf1	-	-	-	putative defense protein Hdd11 [Ostrinia furnacalis] >XP_028179344.1 putative defense protein Hdd11 [Ostrinia furnacalis] >AGV28583.1 immune-induced protein [Ostrinia furnacalis]	-0.9765	1.65913	-1.0508	-0.0628	0.43094
TRINITY_DN7512_c0_g1_i1_orf1	-	-	-	hypothetical protein evm_010529 [Chilo suppressalis] >CAB3530682.1 unnamed protein product [Chilo suppressalis] >CAH0407273.1 unnamed protein product [Chilo suppressalis]	-0.8885	1.92632	-0.6503	-0.2243	-0.1632
TRINITY_DN3355_c0_g2_i4_orf1	-	-	-	UDP-glycosyltransferase UGT33AL1 [Ostrinia furnacalis]	-1.0723	1.83471	-0.6932	-0.01	-0.0593
TRINITY_DN4514_c0_g1_i1_orf1	-	-	-	enoyl-CoA delta isomerase 1, mitochondrial-like isoform X1 [Ostrinia furnacalis] >XP_028158560.1 enoyl-CoA delta isomerase 1, mitochondrial-like isoform X2 [Ostrinia furnacalis]	-1.2947	1.55263	0.53118	0.00521	-0.7943
TRINITY_DN1578_c0_g3_i1_orf1	-	-	-	S-adenosylmethionine synthase isoform X1 [Ostrinia furnacalis]	-0.7377	1.95945	-0.5899	-0.1306	-0.5013
TRINITY_DN10458_c0_g1_i1_orf1	-	-	-	V-type proton ATPase 21 kDa proteolipid subunit [Ostrinia furnacalis]	-0.897	1.89603	-0.722	-0.2813	0.00428
TRINITY_DN2709_c0_g1_i4_orf1	-	-	-	ATP-dependent RNA helicase dbp2-like [Ostrinia furnacalis]	-1.3414	1.74613	-0.2697	0.11917	-0.2541
TRINITY_DN20682_c0_g1_i2_orf1	-	-	-	hypothetical protein B5X24_HaOG200252 [Helicoverpa armigera]	-1.282	1.77466	0.05401	-0.4386	-0.108
TRINITY_DN84357_c0_g1_i1_orf1	-	-	-	4-coumarate--CoA ligase 1-like [Ostrinia furnacalis]	-1.3008	1.39544	0.72962	0.08216	-0.9064
TRINITY_DN107261_c0_g1_i1_orf1	-	-	-	ATP synthase subunit g, mitochondrial [Ostrinia furnacalis]	-0.8575	1.93474	-0.3534	-0.1025	-0.6214
TRINITY_DN2184_c0_g1_i4_orf1	-	-	-	uncharacterized protein LOC114359356 [Ostrinia furnacalis]	-0.7069	1.82781	-1.052	-0.193	0.12413
TRINITY_DN1656_c2_g1_i5_orf1	-	-	-	15-hydroxyprostaglandin dehydrogenase [NAD(+)]-like [Ostrinia furnacalis]	-1.3283	1.45587	-0.5183	-0.4256	0.81631
TRINITY_DN2172_c0_g2_i5_orf1	-	-	-	4-hydroxyphenylpyruvate dioxygenase [Ostrinia furnacalis]	-1.3836	1.67313	-0.1642	-0.4173	0.29195
TRINITY_DN83295_c0_g1_i3_orf1	-	-	-	SSSX-APN4 [Ostrinia furnacalis]	-1.162	1.86128	-0.2126	-0.1389	-0.3477
TRINITY_DN96080_c0_g2_i1_orf1	-	-	-	ATP synthase subunit delta, mitochondrial [Ostrinia furnacalis]	-0.8875	1.94281	-0.1761	-0.3387	-0.5405
TRINITY_DN2924_c0_g1_i2_orf1	-	-	-	cuticular protein RR-2 [Spodoptera litura]	-1.5524	1.36974	-0.1202	-0.4203	0.72312
TRINITY_DN96557_c0_g1_i1_orf1	-	-	-	charged multivesicular body protein 4B [Phyllostomus discolor]	-1.8149	1.21615	0.43867	-0.0245	0.18456
TRINITY_DN28638_c0_g1_i1_orf1	-	-	-	uncharacterized protein LOC114364075 [Ostrinia furnacalis]	-1.0128	1.89526	-0.4562	-0.4171	-0.0091
TRINITY_DN3229_c0_g1_i1_orf1	-	-	-	uncharacterized protein LOC114358442 isoform X1 [Ostrinia furnacalis]	-1.1452	1.86854	-0.2124	-0.359	-0.152
TRINITY_DN105901_c0_g1_i2_orfp1	-	-	-	contactin-like [Pectinophora gossypiella]	-0.8373	1.90937	-0.5949	0.06594	-0.5431
TRINITY_DN8621_c0_g1_i5_orf1	-	-	-	aminopeptidase N-like isoform X2 [Ostrinia furnacalis]	-1.1793	1.85298	-0.243	-0.1061	-0.3246
TRINITY_DN76283_c0_g2_i1_orf1	-	-	-	fatty acid synthase-like [Ostrinia furnacalis]	-0.9308	1.90023	-0.634	-0.3473	0.0119
TRINITY_DN4929_c1_g2_i5_orf1	-	-	-	guanylate kinase isoform X2 [Ostrinia furnacalis]	-1.2004	1.65908	0.50607	-0.6886	-0.2762
TRINITY_DN23167_c0_g1_i4_orf1	-	-	-	uncharacterized protein LOC114363065 [Ostrinia furnacalis]	-1.3277	1.54021	0.64999	-0.6191	-0.2434
TRINITY_DN18396_c0_g1_i1_orf1	-	-	-	uncharacterized protein LOC114359424 [Ostrinia furnacalis]	-1.5131	1.44257	0.62547	-0.4827	-0.0722
TRINITY_DN6143_c0_g2_i1_orf1	-	-	-	uncharacterized protein LOC114365036 [Ostrinia furnacalis]	-1.3804	1.72925	0.0762	-0.2763	-0.1488
TRINITY_DN34040_c0_g2_i1_orf1	-	-	-	uncharacterized protein LOC114352849 [Ostrinia furnacalis]	-1.2874	1.76021	-0.4809	-0.077	0.08499
TRINITY_DN47_c0_g1_i2_orf1	-	-	-	uncharacterized protein LOC114356437 isoform X1 [Ostrinia furnacalis]	-0.8521	1.87847	-0.8547	-0.0785	-0.0932
TRINITY_DN3014_c0_g1_i4_orf1	-	-	-	putative inorganic phosphate cotransporter isoform X1 [Ostrinia furnacalis]	-1.2258	1.82151	-0.2654	-0.3304	4.67E-05
TRINITY_DN7336_c0_g1_i13_orf1	-	-	-	PREDICTED: calcium-transporting ATPase sarcoplasmic/endoplasmic reticulum type isoform X2 [Amyelois transitella]	-1.0784	1.87001	0.0049	-0.5051	-0.2914
TRINITY_DN19814_c0_g1_i4_orf1	-	-	-	general odorant-binding protein 28a-like [Ostrinia furnacalis]	-1.3076	1.59117	0.43731	-0.7524	0.03152
TRINITY_DN2668_c0_g1_i7_orf1	-	-	-	unnamed protein product [Chrysodeixis includens]	-1.159	1.86427	-0.2581	-0.3092	-0.138
TRINITY_DN94248_c0_g2_i3_orf1	-	-	-	uncharacterized protein LOC114357292 isoform X4 [Ostrinia furnacalis]	-0.7177	1.9633	-0.682	-0.2255	-0.3381
TRINITY_DN3135_c0_g1_i6_orf1	-	-	-	acanthoscurrin-1-like [Ostrinia furnacalis]	-1.6658	1.47513	0.01649	-0.043	0.21712
TRINITY_DN542_c0_g1_i4_orf1	-	-	-	uncharacterized protein LOC114364889 [Ostrinia furnacalis]	-0.8383	1.96751	-0.3653	-0.3609	-0.4029
TRINITY_DN42759_c0_g3_i1_orf1	-	-	-	fatty acid synthase-like [Ostrinia furnacalis]	-0.7901	1.97291	-0.4541	-0.288	-0.4407
TRINITY_DN3476_c0_g1_i5_orf1	-	-	-	maltase A1-like [Ostrinia furnacalis]	-0.8013	1.96415	-0.5534	-0.2413	-0.3682
TRINITY_DN15046_c0_g1_i8_orf1	-	-	-	epidermal retinol dehydrogenase 2-like isoform X1 [Ostrinia furnacalis] >XP_028169999.1 epidermal retinol dehydrogenase 2-like isoform X2 [Ostrinia furnacalis]	-0.7147	1.9074	-0.8621	-0.0023	-0.3283
TRINITY_DN1707_c0_g1_i1_orf1	-	-	-	inositol oxygenase-like [Ostrinia furnacalis]	-0.5994	1.91496	-0.9311	-0.064	-0.3205
TRINITY_DN72707_c0_g1_i1_orf1	-	-	-	uncharacterized protein LOC114357549 [Ostrinia furnacalis]	-0.629	1.98506	-0.5405	-0.2678	-0.5477
TRINITY_DN79319_c0_g1_i8_orfp1	-	-	-	TRINITY_DN79319_c0_g1_i8_m.49956 TRINITY_DN79319_c0_g1::TRINITY_DN79319_c0_g1_i8::g.49956 ORF type:5prime_partial len:84 (+),score=1.39 TRINITY_DN79319_c0_g1_i8:1-252(+)	-0.6544	1.95994	-0.7641	-0.2656	-0.2759
TRINITY_DN6418_c0_g1_i28_orf1	-	-	-	peritrophic membrane chitin binding protein [Loxostege sticticalis]	-0.8459	1.96479	-0.3939	-0.3072	-0.4179
TRINITY_DN7964_c0_g1_i6_orfp1	-	-	-	TRINITY_DN7964_c0_g1_i6_m.23478 TRINITY_DN7964_c0_g1::TRINITY_DN7964_c0_g1_i6::g.23478 ORF type:internal len:87 (+),score=67.94 TRINITY_DN7964_c0_g1_i6:2-259(+)	-0.9043	1.93157	-0.4321	-0.0885	-0.5066
TRINITY_DN2695_c0_g1_i14_orfp1	-	-	-	TRINITY_DN2695_c0_g1_i14_m.44485 TRINITY_DN2695_c0_g1::TRINITY_DN2695_c0_g1_i14::g.44485 ORF type:3prime_partial len:698 (+),score=187.51 TRINITY_DN2695_c0_g1_i14:101-2092(+)	-1.2164	1.6885	-0.4241	-0.5181	0.47012
TRINITY_DN55160_c0_g2_i1_orf1	-	-	-	esterase FE4-like isoform X2 [Ostrinia furnacalis]	-0.3335	1.86703	-1.0598	0.05259	-0.5263
TRINITY_DN30663_c0_g1_i1_orf1	-	-	-	surfeit locus protein 6 homolog [Ostrinia furnacalis]	-0.7084	1.95041	-0.2784	-0.2058	-0.7578
TRINITY_DN1249_c0_g1_i10_orf1	-	-	-	venom carboxylesterase-6-like [Ostrinia furnacalis]	-0.7956	1.89215	-0.8717	-0.084	-0.1408

TRINITY_DN11259_c0_g1_i1_orf1	-	-	-	uncharacterized protein LOC114357075 [Ostrinia furnacalis]	-0.6976	1.96195	-0.7059	-0.3495	-0.209
TRINITY_DN5244_c0_g1_i1_orf1	-	-	-	eukaryotic peptide chain release factor GTP-binding subunit-like [Ostrinia furnacalis]	-1.3691	1.74602	-0.1102	-0.2541	-0.0126
TRINITY_DN2825_c0_g1_i3_orf1	-	-	-	carbonic anhydrase 2-like [Ostrinia furnacalis]	-0.8123	1.96978	-0.3705	-0.3121	-0.4749
TRINITY_DN29034_c0_g1_i2_orf1	-	-	-	trypsin-like serine protease [Ostrinia nubilalis]	-0.8612	1.95549	-0.2353	-0.5006	-0.3584
TRINITY_DN69713_c0_g1_i1_orf1	-	-	-	membrane-bound alkaline phosphatase-like [Ostrinia furnacalis]	-0.925	1.92969	-0.5585	-0.291	-0.1552
TRINITY_DN29018_c0_g1_i4_orf1	-	-	-	prostaglandin reductase 1-like isoform X1 [Ostrinia furnacalis] >XP_028178925.1 prostaglandin reductase 1-like isoform X2 [Ostrinia furnacalis]	-0.7823	1.95403	-0.4845	-0.1212	-0.566
TRINITY_DN27592_c0_g1_i1_orf1	-	-	-	D-arabinitol dehydrogenase 1-like [Ostrinia furnacalis]	-0.8912	1.36271	1.05436	-0.5719	-0.954
TRINITY_DN16931_c0_g1_i1_orf1	-	-	-	pancreatic triacylglycerol lipase-like [Ostrinia furnacalis]	-0.685	1.89797	-0.8927	-0.3604	0.0402
TRINITY_DN22664_c0_g1_i1_orf1	-	-	-	larval cuticle protein LCP-14-like [Ostrinia furnacalis]	-1.2535	1.62823	0.44976	-0.7554	-0.0691
TRINITY_DN96_c0_g1_i1_orf1	-	-	-	collagenase-like [Ostrinia furnacalis]	-0.6858	1.97496	-0.6529	-0.3028	-0.3333
TRINITY_DN37699_c0_g1_i3_orfp1	-	-	-	TRINITY_DN37699_c0_g1_i3_m.58788 TRINITY_DN37699_c0_g1::TRINITY_DN37699_c0_g1_i3::g.58788 ORF type:internal len:122 (+),score=39.86 TRINITY_DN37699_c0_g1_i3:1-363(+)	-1.0826	1.76243	0.38519	-0.5884	-0.4766
TRINITY_DN3504_c0_g1_i4_orfp1	-	-	-	TRINITY_DN3504_c0_g1_i4_m.43930 TRINITY_DN3504_c0_g1::TRINITY_DN3504_c0_g1_i4::g.43930 ORF type:internal len:196 (-),score=84.82 TRINITY_DN3504_c0_g1_i4:3-587(-)	-1.5395	1.59652	-0.0099	-0.2232	0.17611
TRINITY_DN2114_c0_g1_i5_orf1	-	-	-	vegetative cell wall protein gp1-like isoform X1 [Ostrinia furnacalis]	-0.8281	1.96068	-0.5332	-0.3548	-0.2446
TRINITY_DN4612_c0_g1_i1_orf1	-	-	-	uncharacterized protein LOC114362092 [Ostrinia furnacalis]	-0.7785	1.92561	-0.7855	-0.1382	-0.2234
TRINITY_DN30704_c0_g1_i1_orf1	-	-	-	cytochrome P450 monooxygenase CYP6AE134v2 [Ostrinia furnacalis]	-1.2332	1.79163	-0.4594	-0.2129	0.11383
TRINITY_DN10940_c0_g1_i10_orfp1	-	-	-	TRINITY_DN10940_c0_g1_i10_m.52163 TRINITY_DN10940_c0_g1::TRINITY_DN10940_c0_g1_i10::g.52163 ORF type:5prime_partial len:248 (-),score=128.24 TRINITY_DN10940_c0_g1_i10:121-864(-)	-1.279	1.80827	-0.2012	-0.1611	-0.167
TRINITY_DN2986_c1_g1_i1_orf1	-	-	-	Troponin C, isoform 1 [Papilio xuthus]	-1.2486	1.64358	0.46241	-0.71	-0.1474
TRINITY_DN336_c0_g1_i6_orfp1	-	-	-	TRINITY_DN336_c0_g1_i6_m.64791 TRINITY_DN336_c0_g1::TRINITY_DN336_c0_g1_i6::g.64791 ORF type:complete len:61 (-),score=19.53 TRINITY_DN336_c0_g1_i6:236-418(-)	-1.0979	1.84736	-0.4106	0.10981	-0.4487
TRINITY_DN334_c0_g1_i4_orf1	-	-	-	collagenase-like [Ostrinia furnacalis]	-1.0204	1.89551	-0.4188	-0.4359	-0.0204
