# Supplementary material for: Comparative Proteomics and Metabonomics Analysis of Different Diapause Stages Revealed a New Regulation Mechanism of Diapause in Loxostege sticticalis (Lepidoptera: Pyralidae)
Source: Molecules. 2024 Jul 25;29(15):3472. doi: 10.3390/molecules29153472 (PMC11314584; doi:10.3390/molecules29153472)
Supplement: Supplementary file 1 [file molecules-29-03472-s001.zip › analysis process/proteomic/Cluster analysis of expression patterns/Down/RDvsD down.pdf]

Accession	Description	RD	ND	PreD	CT	D
TRINITY_DN2396_c0_g1_i9_orfp1	TRINITY_DN2396_c0_g1_i9_m.39038 TRINITY_DN2396_c0_g1::TRINITY_DN2396_c0_g1_i9::g.39038 ORF type:5prime_partial len:181 (+),score=90.33 TRINITY_DN2396_c0_g1_i9:3-545(+)	-0.04221	-1.92051	0.754172	0.677816	0.530724
TRINITY_DN971_c0_g1_i10_orfp1	TRINITY_DN971_c0_g1_i10_m.54268 TRINITY_DN971_c0_g1::TRINITY_DN971_c0_g1_i10::g.54268 ORF type:internal len:187 (+),score=141.68 TRINITY_DN971_c0_g1_i10:1-558(+)	-0.1083	-1.8854	0.891131	0.425574	0.676991
TRINITY_DN1506_c0_g1_i6_orfp1	TRINITY_DN1506_c0_g1_i6_m.57691 TRINITY_DN1506_c0_g1::TRINITY_DN1506_c0_g1_i6::g.57691 ORF type:5prime_partial len:173 (+),score=30.49 TRINITY_DN1506_c0_g1_i6:1-519(+)	-1.32434	-1.11546	0.706934	0.848064	0.884809
TRINITY_DN295_c3_g1_i1_orfp1	TRINITY_DN295_c3_g1_i1_m.18839 TRINITY_DN295_c3_g1::TRINITY_DN295_c3_g1_i1::g.18839 ORF type:5prime_partial len:118 (+),score=46.25 TRINITY_DN295_c3_g1_i1:1-354(+)	-0.12431	-1.89772	0.581712	0.781725	0.65859
TRINITY_DN971_c0_g1_i5_orfp1	TRINITY_DN971_c0_g1_i5_m.54249 TRINITY_DN971_c0_g1::TRINITY_DN971_c0_g1_i5::g.54249 ORF type:internal len:108 (+),score=66.98 TRINITY_DN971_c0_g1_i5:1-321(+)	-0.39991	-1.76323	0.48896	1.049906	0.624269
TRINITY_DN57137_c0_g1_i1_orfp1	TRINITY_DN57137_c0_g1_i1_m.46420 TRINITY_DN57137_c0_g1::TRINITY_DN57137_c0_g1_i1::g.46420 ORF type:5prime_partial len:56 (-),score=4.65 TRINITY_DN57137_c0_g1_i1:82-249(-)	-0.23025	-1.84759	0.799526	0.442793	0.835517
TRINITY_DN3166_c1_g1_i6_orf1	hypothetical protein evm_013813 [Chilo suppressalis]	-0.31599	-1.7427	0.35242	0.4768	1.229472
TRINITY_DN2044_c0_g1_i5_orfp1	TRINITY_DN2044_c0_g1_i5_m.4210 TRINITY_DN2044_c0_g1::TRINITY_DN2044_c0_g1_i5::g.4210 ORF type:complete len:151 (-),score=85.31 TRINITY_DN2044_c0_g1_i5:857-1309(-)	-0.40125	-1.76135	0.448788	1.03947	0.674341
TRINITY_DN34423_c0_g1_i3_orf1	THAP domain-containing protein 4-like [Ostrinia furnacalis]	0.072575	-1.95064	0.602757	0.718866	0.556447
TRINITY_DN7064_c0_g1_i19_orf1	unnamed protein product [Chilo suppressalis]	-1.1627	-1.23178	0.443989	0.849735	1.100758
TRINITY_DN5177_c0_g1_i2_orf1	hemolin-like isoform X1 [Ostrinia furnacalis]	-0.02554	-1.73659	-0.17449	0.770967	1.165657
TRINITY_DN36476_c1_g1_i1_orfp1	TRINITY_DN36476_c1_g1_i1_m.70910 TRINITY_DN36476_c1_g1::TRINITY_DN36476_c1_g1_i1::g.70910 ORF type:5prime_partial len:88 (-),score=0.50 TRINITY_DN36476_c1_g1_i1:49-312(-)	-0.66669	-1.62065	1.01449	0.424577	0.848274
TRINITY_DN6330_c0_g1_i1_orfp1	TRINITY_DN6330_c0_g1_i1_m.42332 TRINITY_DN6330_c0_g1::TRINITY_DN6330_c0_g1_i1::g.42332 ORF type:3prime_partial len:51 (-),score=33.30 TRINITY_DN6330_c0_g1_i1:3-152(-)	-0.47532	-1.72994	0.921904	0.409329	0.874028
TRINITY_DN15202_c0_g1_i6_orf1	uncharacterized protein LOC114364499 isoform X2 [Ostrinia furnacalis]	-0.53535	-1.67149	0.337399	0.764067	1.105382
TRINITY_DN17615_c0_g1_i3_orf1	hypothetical protein SFRUCORN_008858 [Spodoptera frugiperda]	-0.06894	-1.90641	0.525117	0.595676	0.85456
TRINITY_DN56459_c0_g1_i2_orf1	aldo-keto reductase AKR2E4-like [Ostrinia furnacalis]	-0.60488	-1.69301	0.704117	0.82862	0.765151
TRINITY_DN86772_c0_g1_i3_orfp1	x-tox [Spodoptera exigua]	-0.3059	-1.78653	0.981394	0.296282	0.814751
TRINITY_DN1597_c0_g1_i5_orfp1	TRINITY_DN1597_c0_g1_i5_m.57494 TRINITY_DN1597_c0_g1::TRINITY_DN1597_c0_g1_i5::g.57494 ORF type:complete len:86 (+),score=7.19 TRINITY_DN1597_c0_g1_i5:134-391(+)	0.103703	-1.85136	-0.00396	0.758293	0.993324
TRINITY_DN12534_c0_g1_i4_orf1	antibacterial protein [Heliothis virescens]	-0.50785	-1.74092	0.645477	0.870177	0.733112
TRINITY_DN35809_c0_g1_i1_orf1	spodomicin-like [Ostrinia furnacalis] >QKV49445.1 diapausin [Ostrinia furnacalis]	-0.59427	-1.6706	0.990783	0.814055	0.460024
TRINITY_DN124654_c0_g1_i1_orf1	protein lethal(2)essential for life [Manduca sexta] >KAG6441919.1 hypothetical protein O3G_MSEX002019 [Manduca sexta]	0.392523	-1.65603	-0.59978	0.868961	0.994321
TRINITY_DN29026_c0_g1_i4_orf1	TIL [Ostrinia furnacalis]	-0.2116	-1.71245	0.155281	0.418375	1.350398
TRINITY_DN30510_c0_g1_i6_orf1	spodomicin-like [Ostrinia furnacalis]	-1.05909	-1.29959	1.193754	0.375345	0.789588
TRINITY_DN380_c0_g2_i2_orf1	chemosensory protein 10 [Ostrinia furnacalis]	-0.34895	-1.79714	0.476404	0.952394	0.717294
TRINITY_DN4802_c0_g1_i4_orf1	uncharacterized protein LOC114366345 isoform X2 [Ostrinia furnacalis]	-0.50714	-1.48977	0.459518	0.016889	1.520508
TRINITY_DN9608_c0_g1_i3_orf1	cytochrome P450 monooxygenase CYP9G18 [Cnaphalocrocis medinalis]	-0.53724	-1.52189	0.04571	0.578782	1.43463
TRINITY_DN47784_c0_g2_i1_orfp1	arylphorin subunit alpha-like [Ostrinia furnacalis]	-0.18103	-1.66831	1.360777	-0.08201	0.570566
TRINITY_DN5439_c0_g1_i2_orf1	uncharacterized protein LOC114353087 [Ostrinia furnacalis]	-0.17705	-1.80925	0.569988	0.279551	1.136761
TRINITY_DN3439_c0_g2_i2_orf1	histone H2A.Z-specific chaperone CHZ1-like [Ostrinia furnacalis]	-0.8689	-1.46229	0.884746	0.35051	1.095938
TRINITY_DN6025_c0_g2_i1_orfp1	TRINITY_DN6025_c0_g2_i1_m.7749 TRINITY_DN6025_c0_g2::TRINITY_DN6025_c0_g2_i1::g.7749 ORF type:5prime_partial len:106 (+),score=47.10 TRINITY_DN6025_c0_g2_i1:3-320(+)	-0.29357	-1.82794	0.893104	0.51334	0.715059
TRINITY_DN8595_c0_g1_i3_orf1	aldose reductase-like isoform X4 [Trichoplusia ni]	-0.65403	-1.63749	0.590183	0.632407	1.068932
TRINITY_DN1391_c0_g1_i29_orfp1	TRINITY_DN1391_c0_g1_i29_m.70767 TRINITY_DN1391_c0_g1::TRINITY_DN1391_c0_g1_i29::g.70767 ORF type:complete len:495 (+),score=158.49 TRINITY_DN1391_c0_g1_i29:728-2212(+)	-0.10285	-1.90099	0.822202	0.56359	0.618054
TRINITY_DN1772_c1_g2_i1_orf1	aldose reductase-like isoform X2 [Ostrinia furnacalis]	-0.8172	-1.48297	0.296695	0.863851	1.139618
TRINITY_DN3275_c0_g2_i3_orf1	hypothetical protein B5X24_HaOG216046 [Helicoverpa armigera]	-0.23787	-1.74395	1.185954	0.098885	0.69698
TRINITY_DN30306_c0_g2_i1_orf1	perilipin-4-like isoform X3 [Ostrinia furnacalis]	-0.58492	-1.64237	1.14023	0.357072	0.729982
TRINITY_DN16234_c0_g2_i3_orf1	uncharacterized protein LOC114363370 [Ostrinia furnacalis]	-0.28053	-1.42573	-0.5589	1.205881	1.059279
TRINITY_DN24723_c2_g1_i1_orf1	hypothetical protein evm_001103 [Chilo suppressalis]	-0.44583	-1.76659	0.772888	0.872888	0.566651

TRINITY_DN6415_c0_g2_i1_orf1	D-arabinitol dehydrogenase 1-like [Ostrinia furnacalis]	-0.92292	-1.39165	0.407408	0.616745	1.290416
TRINITY_DN4816_c0_g2_i3_orf1	15-hydroxyprostaglandin dehydrogenase [NAD(+)]-like [Ostrinia furnacalis]	-0.14321	-1.89395	0.693786	0.73797	0.605403
TRINITY_DN581_c3_g2_i1_orf1	uncharacterized protein LOC114364499 isoform X3 [Ostrinia furnacalis]	-0.43062	-1.53007	-0.11973	0.656118	1.424298
TRINITY_DN38506_c0_g1_i4_orf1	C-1-tetrahydrofolate synthase, cytoplasmic isoform X1 [Ostrinia furnacalis] >XP_028166137.1 C-1-tetrahydrofolate synthase, cytoplasmic isoform X2 [Ostrinia furnacalis] >XP_028166140.1 C-1-tetrahydrofolate synthase, cytoplasmic isoform X4 [Ostrinia furnacalis]	-0.91799	-1.48978	0.823809	0.840481	0.743484
TRINITY_DN20344_c0_g1_i5_orf1	uncharacterized protein LOC114351483 [Ostrinia furnacalis]	-1.23363	-1.14126	0.392988	0.821767	1.160141
TRINITY_DN5337_c0_g1_i6_orf1	epoxide hydrolase 1-like [Ostrinia furnacalis]	0.151087	-1.91203	0.160323	0.714755	0.885861
TRINITY_DN135780_c0_g1_i1_orf1	flotillin-1 isoform X1 [Pectinophora gossypiella]	-0.07917	-1.87167	0.826345	0.266053	0.85845
TRINITY_DN9079_c0_g1_i5_orf1	UDP-glucuronosyltransferase-like [Ostrinia furnacalis]	-0.17022	-1.88496	0.642254	0.766763	0.646164
TRINITY_DN19731_c0_g1_i1_orf1	allergen Tha p 1-like [Ostrinia furnacalis] >XP_028174916.1 allergen Tha p 1-like [Ostrinia furnacalis] >BAV56808.1 chemosensory protein 4 [Ostrinia furnacalis]	-0.19115	-1.83232	0.274958	0.904232	0.844287
TRINITY_DN69_c0_g1_i1_orf1	glycerol-3-phosphate dehydrogenase [NAD(+)], cytoplasmic isoform X1 [Ostrinia furnacalis]	-0.19638	-1.85711	0.657815	0.467699	0.927969
TRINITY_DN14328_c0_g1_i12_orf1	larval cuticle protein LCP-30-like [Ostrinia furnacalis]	-1.19973	-1.22551	0.739662	0.629382	1.056196
TRINITY_DN31286_c0_g1_i6_orfp1	TRINITY_DN31286_c0_g1_i6_m.28438 TRINITY_DN31286_c0_g1::TRINITY_DN31286_c0_g1_i6::g.28438 ORF type:internal len:92 (-),score=3.10,Perilipin PF03036.17 2e-05 TRINITY_DN31286_c0_g1_i6:1-273(-)	-0.19318	-1.82585	0.513184	0.412498	1.093356
TRINITY_DN628_c0_g1_i1_orf1	prostamide/prostaglandin F synthase-like [Ostrinia furnacalis]	0.102876	-1.95787	0.583432	0.696311	0.575252
TRINITY_DN33346_c0_g1_i1_orf1	PREDICTED: U6 snRNA-associated Sm-like protein LSm3 [Papilio xuthus] >XP_028165558.1 U6 snRNA-associated Sm-like protein LSm3 [Ostrinia furnacalis] >KOB73597.1 LSM Sm-like protein family member [Operophtera brumata] >RVE45517.1 hypothetical protein evm_009856 [Chilo suppressalis] >CAB3523639.1 unnamed protein product [Chilo suppressalis] >CAH0400961.1 unnamed protein product [Chilo suppressalis]	0.026508	-1.9243	0.40817	0.644146	0.845478
TRINITY_DN1880_c0_g1_i4_orf1	serine protease inhibitor dipetalogastin-like [Helicoverpa zea]	-0.73259	-1.6005	0.692729	1.015692	0.624668
TRINITY_DN394_c0_g1_i2_orf1	uncharacterized protein LOC114351483 [Ostrinia furnacalis]	-1.25181	-1.07239	0.791022	0.275719	1.257461
TRINITY_DN8008_c0_g1_i6_orf1	uncharacterized protein LOC114357965 isoform X1 [Ostrinia furnacalis] >XP_028167599.1 uncharacterized protein LOC114357965 isoform X1 [Ostrinia furnacalis] >XP_028167600.1 uncharacterized protein LOC114357965 isoform X2 [Ostrinia furnacalis] >XP_028167601.1 uncharacterized protein LOC114357965 isoform X3 [Ostrinia furnacalis]	-0.84436	-1.47885	0.405145	1.178638	0.739421
TRINITY_DN59429_c0_g1_i6_orf1	uncharacterized protein LOC114366345 isoform X2 [Ostrinia furnacalis]	-0.79362	-1.52804	1.060942	0.870103	0.390616
TRINITY_DN7226_c0_g1_i2_orf1	chemosensory protein [Conogethes punctiferalis]	-0.44449	-1.7465	0.822515	0.948134	0.420342
TRINITY_DN710_c0_g1_i11_orfp1	TRINITY_DN710_c0_g1_i11_m.67699 TRINITY_DN710_c0_g1::TRINITY_DN710_c0_g1_i11::g.67699 ORF type:complete len:194 (-),score=34.99,Collagen PF01391.19 0.00029 TRINITY_DN710_c0_g1_i11:1283-1864(-)	-0.99675	-1.34384	0.985946	0.283021	1.071627
TRINITY_DN31348_c0_g1_i1_orf1	protein lethal(2)essential for life [Bombyx mori]	-0.55478	-1.64067	0.454275	1.243569	0.4976
TRINITY_DN307_c1_g1_i1_orf1	uncharacterized protein LOC114356704 [Ostrinia furnacalis]	0.400108	-1.37359	-0.9009	0.498515	1.375869
TRINITY_DN276_c0_g1_i1_orf1	protein lethal(2)essential for life-like [Helicoverpa zea] >XP_049705426.1 protein lethal(2)essential for life [Helicoverpa armigera] >ATB54993.1 heat shock protein 20.8 [Helicoverpa armigera] >PZC74337.1 hypothetical protein B5X24_HaOG207971 [Helicoverpa armigera]	0.153979	-1.49049	-0.74378	1.177455	0.902836
TRINITY_DN7226_c0_g1_i5_orf1	chemosensory protein [Dioryctria abietella]	-0.34679	-1.79643	0.944692	0.743979	0.454552
TRINITY_DN43350_c0_g3_i1_orf1	uncharacterized protein LOC114355190 [Ostrinia furnacalis]	-1.0414	-1.3572	0.501929	0.841431	1.055245
TRINITY_DN1363_c0_g1_i11_orf1	cytochrome P450 CYP12A2-like isoform X1 [Ostrinia furnacalis] >QPF77619.1 cytochrome P450 monooxygenase CYP333A20 [Ostrinia furnacalis]	-0.29938	-1.81711	0.966857	0.494753	0.654887
TRINITY_DN2314_c0_g1_i7_orf1	protein dj-1beta-like isoform X1 [Ostrinia furnacalis]	0.072462	-1.82514	-0.04735	1.043763	0.756264
TRINITY_DN3821_c1_g1_i7_orf1	mitochondrial carrier protein Rim2 isoform X1 [Ostrinia furnacalis]	-0.32247	-1.79587	0.377759	0.952021	0.788555
TRINITY_DN9325_c0_g1_i1_orf1	protein takeout-like [Ostrinia furnacalis]	0.00291	-1.71625	-0.26309	0.861463	1.114966
TRINITY_DN20658_c0_g2_i3_orf1	prostaglandin reductase 1-like [Ostrinia furnacalis]	-0.19083	-1.80774	0.555698	0.305551	1.137322
TRINITY_DN40126_c0_g1_i1_orf1	aldehyde dehydrogenase X, mitochondrial-like [Ostrinia furnacalis]	0.030099	-1.93997	0.576335	0.582282	0.751257
TRINITY_DN295_c5_g1_i2_orf1	unnamed protein product [Chilo suppressalis]	-0.38677	-1.72904	0.543831	1.191885	0.3801
TRINITY_DN20558_c0_g1_i2_orf1	Transient receptor potential channel pyrexia [Operophtera brumata]	-0.14691	-1.56759	0.41585	-0.21811	1.516762
TRINITY_DN2457_c0_g1_i8_orf1	uncharacterized protein LOC114355596 [Ostrinia furnacalis]	-0.88645	-1.35161	1.434699	0.345636	0.457731

TRINITY_DN71698_c0_g1_i1_orfp1	TRINITY_DN71698_c0_g1_i1_m.1194 TRINITY_DN71698_c0_g1::TRINITY_DN71698_c0_g1_i1::g.1194 ORF type:internal len:134 (+),score=19.66,Toxin_2 PF00451.20 4.3e-05,Toxin_2|PF00451.20|0.037,Toxin_2|PF00451.20|7.5e-05,Gamma-thionin|PF00304.21|0.017,Gamma-thionin|PF00304.21|0.05,Gamma-thionin|PF00304.21|0.021,Toxin_38|PF14866.7|0.13,Toxin_38|PF14866.7|0.15,Toxin_38|PF14866.7|0.15,Defensin_2|PF0	-0.57881	-1.67369	0.860611	0.9738	0.418091
TRINITY_DN114890_c0_g1_i4_orf1	chemosensory protein 10 [Ostrinia furnacalis]	-0.74287	-1.47498	0.568656	1.367417	0.281771
TRINITY_DN2187_c0_g1_i1_orf1	flotillin-1 [Chelonus insularis] >XP_034947202.1 flotillin-1 [Chelonus insularis]	-0.19393	-1.85825	0.814707	0.418869	0.818603
TRINITY_DN18031_c0_g1_i1_orf1	63 kDa chaperonin, mitochondrial-like [Ostrinia furnacalis]	-0.33739	-1.40851	-0.30889	1.617214	0.437574
TRINITY_DN4245_c0_g2_i1_orf1	long-chain fatty acid transport protein 4-like [Ostrinia furnacalis]	-0.15065	-1.88039	0.702725	0.483351	0.844969
TRINITY_DN13660_c0_g1_i1_orf1	Aliphatic nitrilase [Operophtera brumata]	-0.23622	-1.83886	0.546405	0.983194	0.545483
TRINITY_DN2040_c0_g1_i6_orf1	trypsin-like serine proteinase T26 protein, partial [Chilo infuscatellus]	-0.68195	-1.63528	0.555398	0.868587	0.893244
TRINITY_DN4068_c0_g2_i4_orf1	larval cuticle protein LCP-17-like precursor [Papilio polytes] >BAM18876.1 cuticular protein PpolCPR2 [Papilio	-1.46211	-0.81877	0.242558	0.852375	1.185948
TRINITY_DN6098_c1_g1_i5_orf1	unnamed protein product, partial [Iphiclides podalirius]	-0.54111	-1.72691	0.678884	0.821176	0.767962
TRINITY_DN2146_c0_g2_i1_orf1	heat shock protein 68-like [Ostrinia furnacalis]	0.018257	-1.48804	-0.62861	1.356313	0.742078
TRINITY_DN9492_c0_g1_i7_orf1	aldo-keto reductase AKR2E4-like [Ostrinia furnacalis]	0.172385	-1.9463	0.314673	0.633497	0.825747
TRINITY_DN1450_c0_g2_i1_orf1	death-associated protein 1 [Ostrinia furnacalis]	-0.69842	-1.61801	0.996718	0.537413	0.782297
TRINITY_DN5099_c0_g1_i3_orf1	trans-1,2-dihydrobenzene-1,2-diol dehydrogenase-like [Ostrinia furnacalis]	-0.16801	-1.85027	0.445186	0.549081	1.024014
TRINITY_DN122321_c0_g1_i1_orf1	uncharacterized protein LOC114356271 [Ostrinia furnacalis]	-0.32951	-1.78144	0.379501	0.672182	1.059269
TRINITY_DN9132_c0_g1_i5_orf1	ubiquitin-like-conjugating enzyme ATG3 [Spodoptera frugiperda]	-0.1254	-1.83706	0.233959	1.038761	0.689734
TRINITY_DN5153_c1_g1_i1_orf1	nose resistant to fluoxetine protein 6-like isoform X1 [Ostrinia furnacalis]	-0.57412	-1.64455	1.194551	0.427007	0.597117
TRINITY_DN8953_c0_g1_i4_orf1	gonadotropin-releasing hormone receptor [Ostrinia furnacalis] >AXF67446.1 adipokinetic hormone receptor 1 [Ostrinia furnacalis]	-0.00898	-1.87323	1.00841	0.222092	0.651704
TRINITY_DN140_c0_g1_i1_orf1	calcyphosin-like protein [Ostrinia furnacalis]	-0.13251	-1.82737	0.242369	1.092708	0.624812
TRINITY_DN130575_c0_g1_i1_orfp1	TRINITY_DN130575_c0_g1_i1_m.77798 TRINITY_DN130575_c0_g1::TRINITY_DN130575_c0_g1_i1::g.77798 ORF type:internal len:70 (+),score=15.12 TRINITY_DN130575_c0_g1_i1:3-209(+)	-0.29395	-1.80912	0.786425	0.379524	0.937115
TRINITY_DN1650_c0_g1_i5_orf1	uncharacterized protein LOC114355357 [Ostrinia furnacalis]	-0.31764	-1.61133	0.532847	-0.02451	1.420633
TRINITY_DN20658_c0_g1_i1_orf1	prostaglandin reductase 1-like [Ostrinia furnacalis]	-0.29786	-1.82141	0.834306	0.452592	0.832371
TRINITY_DN12775_c0_g1_i10_orfp1	TRINITY_DN12775_c0_g1_i10_m.21238 TRINITY_DN12775_c0_g1::TRINITY_DN12775_c0_g1_i10::g.21238 ORF type:5prime_partial len:67 (-),score=0.74 TRINITY_DN12775_c0_g1_i10:275-475(-)	-0.34124	-1.72473	0.123297	0.913684	1.028992
TRINITY_DN1149_c0_g1_i4_orf1	circadian clock-controlled protein-like [Ostrinia furnacalis]	-0.82548	-1.45568	1.263622	0.715117	0.302417
TRINITY_DN2286_c2_g1_i1_orf1	coatomer subunit zeta-1 isoform X1 [Ostrinia furnacalis]	-0.10222	-1.80723	0.17179	1.172345	0.565313
TRINITY_DN44517_c0_g1_i4_orf1	regucalcin-like [Ostrinia furnacalis]	-0.14665	-1.89074	0.742158	0.563981	0.731247
TRINITY_DN69307_c0_g1_i6_orf1	hypothetical protein evm_010738 [Chilo suppressalis]	-0.14642	-1.72216	-0.12495	1.067569	0.925965
TRINITY_DN335_c1_g1_i5_orf1	PREDICTED: perilipin-4 isoform X14 [Papilio polytes]	-0.91176	-1.39388	1.158173	0.237514	0.909953
TRINITY_DN36434_c0_g2_i3_orf1	clotting factor B isoform X1 [Ostrinia furnacalis]	-0.71747	-1.50881	0.36902	0.511599	1.345664
TRINITY_DN6325_c0_g1_i9_orf1	fructose-bisphosphate aldolase isoform X2 [Pieris brassicae]	-0.41652	-1.72079	0.276681	1.100416	0.760212
TRINITY_DN7740_c0_g1_i2_orf1	D-arabinitol dehydrogenase 1 [Eumeta japonica]	-0.76801	-1.56086	0.773002	0.489839	1.066026
TRINITY_DN43667_c0_g1_i1_orf1	carbonyl reductase [NADPH] 1-like [Ostrinia furnacalis]	-0.03045	-1.67492	-0.25936	0.668998	1.295725
TRINITY_DN2464_c0_g1_i12_orf1	uncharacterized protein LOC114362996 isoform X1 [Ostrinia furnacalis]	-1.21008	-1.23869	0.816208	0.780657	0.851903
TRINITY_DN4080_c0_g1_i8_orf1	AMP deaminase 2 isoform X3 [Ostrinia furnacalis] >XP_028163647.1 AMP deaminase 2 isoform X3 [Ostrinia furnacalis] >XP_028163648.1 AMP deaminase 2 isoform X3 [Ostrinia furnacalis]	-0.26222	-1.50535	-0.30979	0.585075	1.492277
TRINITY_DN11981_c0_g1_i7_orf1	luciferin 4-monooxygenase-like isoform X2 [Ostrinia furnacalis]	-0.55027	-1.64747	1.195498	0.661683	0.340567
TRINITY_DN8853_c0_g1_i4_orf1	uncharacterized protein LOC114351488 isoform X1 [Ostrinia furnacalis]	-1.12593	-0.99767	0.343189	0.171106	1.609308
TRINITY_DN1750_c1_g1_i5_orf1	lipid droplet localized protein-like [Ostrinia furnacalis] >XP_028161280.1 lipid droplet localized protein-like [Ostrinia furnacalis]	-0.24977	-1.84725	0.713001	0.864005	0.520009

TRINITY_DN55148_c0_g1_i1_orf1	60S ribosomal protein L11 isoform 1 [Homo sapiens] >NP_001069049.1 60S ribosomal protein L11 [Bos taurus] >NP_001240835.1 60S ribosomal protein L11 isoform 1 [Canis lupus familiaris] >NP_001269300.1 60S ribosomal protein L11 [Chinchilla lanigera] >NP_001291809.1 60S ribosomal protein L11 [Ailuropoda melanoleuca] >NP_080195.1 60S ribosomal protein L11 [Mus musculus] >XP_001504267.1 60S ribosomal protein L11 isoform X2 [Equus caballus] >XP_003471379.1 60S ribosomal protein L11 [Cavia porcellus] >XP_003810808.1 60S ribosomal protein L11 [Pan paniscus] >XP_003891370.1 60S ribosomal protein L11 [Papio anubis] >XP_003989701.1 60S ribosomal protein L11 [Felis catus] >XP_004285699.1 60S ribosomal protein L11 isoform X2 [Orcinus orca] >XP_004377186.1 60S ribosomal protein L11 [Trichechus manatus latirostris] >XP_004394821.1 PREDICTED: 60S ribosomal protein L11 isoform X1 [Odobenus rosmarus divergens] >XP_004465476.2 60S ribosomal protein L11 [Dasypus novemcinctus] >XP_004637663.1 60S ribosomal protein L11 [Octodon degus] >XP_004850617.1 60S ribosomal protein L11 isoform X2 [Heterocephalus glaber] >XP_005544522.1 60S ribosomal protein L11 isoform X1 [Macaca fascicularis] >XP_005676921.1 PREDICTED: 60S ribosomal protein L11 isoform X2 [Capra hircus] >XP_006078005.1 60S ribosomal protein L11 isoform X2 [Bubalus bubalis] >XP_006094108.1 60S ribosomal protein L11 isoform X3 [Myotis lucifugus] >XP_006239286.1 60S ribosomal protein L11 isoform X1 [Rattus norvegicus] >XP_006737645.1 60S ribosomal protein L11 [Leptonychotes weddellii] >XP_006777625.1 PREDICTED: 60S ribosomal protein L11 isoform X1 [Myotis davidii] >XP_006883588.1 PREDICTED: 60S ribosomal protein L11-like isoform X1 [Elephantulus edwardii] >XP_007121151.1 60S ribosomal protein L11 isoform X2 [Physeter catodon] >XP_007175168.1 60S ribosomal protein L11 isoform X1 [Balaenoptera acutorostrata scammoni] >XP_007459239.1 PREDICTED: 60S ribosomal protein L11 isoform X1 [Lipotes vexillifer] >XP_007524793.1 PREDICTED: 60S ribosomal protein L11 [Erinaceus europaeus] >XP_007528779.2 PREDICTED: 60S ribosomal protein L11 [Erinaceus europaeus] >XP_007933904.2 60S ribosomal protein L11 [Orycteropus afer afer] >XP_007978264.1 60S ribosomal protein L11 isoform X1 [Chlorocebus sabaeus] >XP_008059998.1 60S ribosomal protein L11 isoform X2 [Carlito syrichta] >XP_008146324.1 60S ribosomal protein L11 [Eptesicus fuscus] >XP_008263912.1 PREDICTED: 60S ribosomal protein L11 [Oryctolagus cuniculus] >XP_008518979.1 PREDICTED: 60S ribosomal protein L11 isoform X2 [Equus przewalskii] >XP_008571152.1 PREDICTED: 60S ribosomal protein L11 isoform X1 [Galeopterus variegatus] >XP_008571160.1 PREDICTED: 60S ribosomal protein L11 isoform X2 [Galeopterus variegatus] >XP_008846816.1 60S ribosomal protein L11 [Nannospalax galili] >XP_010354068.1 60S ribosomal protein L11 [Rhinopithecus roxellana] >XP_010624280.1 60S ribosomal protein L11 [Fukomys damarensis] >XP_011355886.1 60S ribosomal protein L11 [Pteropus vampyrus] >XP_011761124.1 60S ribosomal protein L11 [Macaca nemestrina] >XP_011833215.1 PREDICTED: 60S ribosomal protein L11 isoform X2 [Mandrillus leucophaeus] >XP_011935575.1	-0.25914	-1.57093	-0.30822	0.864367	1.273929
TRINITY_DN285_c0_g1_i4_orf1	catalase-like [Ostrinia furnacalis]	-0.99698	-1.39298	0.506369	0.809031	1.074564
TRINITY_DN244_c1_g1_i5_orf1	C-1-tetrahydrofolate synthase, cytoplasmic isoform X3 [Ostrinia furnacalis]	0.205647	-1.82704	-0.13075	0.692351	1.0598
TRINITY_DN1226_c0_g1_i11_orfp1	TRINITY_DN1226_c0_g1_i11_m.52385 TRINITY_DN1226_c0_g1::TRINITY_DN1226_c0_g1_i11::g.52385 ORF type:internal len:92 (-),score=5.77 TRINITY_DN1226_c0_g1_i11:2-274(-)	-1.11907	-1.19379	0.754175	0.260198	1.298486
TRINITY_DN51480_c0_g1_i1_orf1	uncharacterized protein LOC114361588 isoform X14 [Ostrinia furnacalis]	-0.32344	-1.74247	0.150788	0.921437	0.993684
TRINITY_DN15755_c0_g1_i1_orf1	cytochrome P450 monooxygenase CYP6AB141 [Ostrinia furnacalis]	-1.11964	-1.06222	0.961585	-0.07867	1.298945
TRINITY_DN1352_c0_g1_i5_orf1	uncharacterized protein LOC113491815 [Trichoplusia ni]	-0.22629	-1.84817	0.522772	0.943022	0.608669
TRINITY_DN7960_c0_g1_i2_orf1	uncharacterized protein LOC114364878 [Ostrinia furnacalis]	-0.71686	-1.55411	0.317499	1.152879	0.800584
TRINITY_DN40126_c0_g2_i1_orf1	aldehyde dehydrogenase X, mitochondrial-like [Ostrinia furnacalis]	-0.63275	-1.63685	1.095318	0.462326	0.711958
TRINITY_DN125521_c0_g2_i1_orf1	seroin transcript 1A2 [Ostrinia nubilalis]	-1.39028	-1.00326	0.501216	0.848785	1.04353
TRINITY_DN1209_c0_g1_i9_orf1	NADP-dependent malic enzyme-like isoform X1 [Ostrinia furnacalis] >XP_028161889.1 NADP-dependent malic enzyme-like isoform X1 [Ostrinia furnacalis] >XP_028161891.1 NADP-dependent malic enzyme-like isoform X3 [Ostrinia furnacalis]	-0.51542	-1.58532	0.278235	1.402641	0.419863
TRINITY_DN6580_c0_g1_i4_orf1	catalase [Ostrinia furnacalis]	-0.00777	-1.83751	0.149989	0.561295	1.133999
TRINITY_DN4497_c2_g1_i3_orf1	uncharacterized protein LOC114353086 [Ostrinia furnacalis]	-0.44873	-1.71078	0.300635	0.765472	1.093406
TRINITY_DN448_c0_g1_i20_orf1	probable cytochrome P450 9f2 isoform X1 [Ostrinia furnacalis]	-0.65214	-1.57521	0.403923	0.545765	1.277661
TRINITY_DN10222_c0_g1_i2_orf1	glutathione S-transferase sigma 3 [Ostrinia furnacalis]	-0.97388	-1.3953	0.454784	1.138026	0.776374
TRINITY_DN618_c0_g1_i3_orf1	triokinase/FMN cyclase-like isoform X1 [Ostrinia furnacalis]	-0.23513	-1.85346	0.671675	0.543444	0.873465
TRINITY_DN49530_c0_g1_i1_orf1	ommochrome-binding protein-like [Ostrinia furnacalis]	-0.33394	-1.77118	0.277032	0.956876	0.871222

TRINITY_DN142442_c0_g1_i1_orf1	60S ribosomal protein L38 [Homo sapiens] >NP_001002486.1 60S ribosomal protein L38 [Danio rerio] >NP_001030335.1 60S ribosomal protein L38 [Homo sapiens] >NP_001071060.1 60S ribosomal protein L38 [Rattus norvegicus] >NP_001133168.1 60S ribosomal protein L38 [Salmo salar] >NP_001187063.1 60S ribosomal protein L38 [Ictalurus punctatus] >NP_001232305.1 60S ribosomal protein L38 [Taeniopygia guttata] >NP_001264941.1 60S ribosomal protein L38 [Gallus gallus] >XP_003211558.1 60S ribosomal protein L38 [Meleagris gallopavo] >XP_003315754.1 60S ribosomal protein L38 [Pan troglodytes] >XP_003315758.1 60S ribosomal protein L38 [Pan troglodytes] >XP_003339346.1 60S ribosomal protein L38 [Pan troglodytes] >XP_003358038.1 60S ribosomal protein L38 [Sus scrofa] >XP_003417326.1 60S ribosomal protein L38 [Loxodonta africana] >XP_003453439.1 60S ribosomal protein L38 [Oreochromis niloticus] >XP_003464913.2 60S ribosomal protein L38 [Cavia porcellus] >XP_003768586.1 60S ribosomal protein L38 [Sarcophilus harrisii] >XP_003786210.1 60S ribosomal protein L38 [Otolemur garnettii] >XP_003795793.1 60S ribosomal protein L38 [Otolemur garnettii] >XP_003922345.1 60S ribosomal protein L38 [Saimiri boliviensis boliviensis] >XP_004041125.1 60S ribosomal protein L38 [Gorilla gorilla gorilla] >XP_004041126.1 60S ribosomal protein L38 [Gorilla gorilla gorilla] >XP_004041128.1 60S ribosomal protein L38 [Gorilla gorilla gorilla] >XP_004331065.1 60S ribosomal protein L38 [Tursiops truncatus] >XP_004401894.1 PREDICTED: 60S ribosomal protein L38 [Odobenus rosmarus divergens] >XP_004412345.1 PREDICTED: 60S ribosomal protein L38 [Odobenus rosmarus divergens] >XP_004469223.1 60S ribosomal protein L38 [Dasypus novemcinctus] >XP_004469224.1 60S ribosomal protein L38 [Dasypus novemcinctus] >XP_005068761.1 60S ribosomal protein L38 [Mesocricetus auratus] >XP_005070019.1 60S ribosomal protein L38 [Mesocricetus auratus] >XP_005141156.1 60S ribosomal protein L38 [Melopsittacus undulatus] >XP_005336034.1 60S ribosomal protein L38 [Ictidomys tridecemlineatus] >XP_005336035.1 60S ribosomal protein L38 [Ictidomys tridecemlineatus] >XP_005350739.1 60S ribosomal protein L38 [Microtus ochrogaster] >XP_005350740.1 60S ribosomal protein L38 [Microtus ochrogaster] >XP_005412280.1 PREDICTED: 60S ribosomal protein L38 [Chinchilla lanigera] >XP_005412281.1 PREDICTED: 60S ribosomal protein L38 [Chinchilla lanigera] >XP_005530739.1 PREDICTED: 60S ribosomal protein L38 [Pseudopodoces humilis] >XP_005584887.1 60S ribosomal protein L38 [Macaca fascicularis] >XP_005584888.1 60S ribosomal protein L38 [Macaca fascicularis] >XP_005584889.1 60S ribosomal protein L38 [Macaca fascicularis] >XP_005584890.1 60S ribosomal protein L38 [Macaca fascicularis] >XP_005584891.1 60S ribosomal protein L38 [Macaca fascicularis] >XP_005592611.1 60S ribosomal protein L38 [Macaca fascicularis] >XP_005597274.1 60S ribosomal protein L38 isoform X2 [Equus caballus] >XP_005668697.1 60S ribosomal protein L38 [Sus scrofa] >XP_005861853.1 PREDICTED: 60S ribosomal protein L38 [Myotis brandtii] >XP_005861854.1 PREDICTED: 60S ribosomal protein L38 [Myotis brandtii] >XP_005889694.1 PREDICTED: 60S					
osomal protein L38 [Nematolebias wh	60S RIBOSOMAL PROTEIN L38 [Oryctolagus cuniculus] >4UJD_Ak Chain Ak	60S RIBO	60S RIBO	60S ribos	60S ribos	60S ribos
TRINITY_DN74037_c0_g5_i1_orf1	hypothetical protein JYU34_010754 [Plutella xylostella]	-0.8104	-1.4078	0.253171	0.554391	1.410638
TRINITY_DN4255_c0_g1_i10_orf1	LOW QUALITY PROTEIN: lebocin-4-like [Ostrinia furnacalis]	-0.08033	-1.87367	0.876742	0.279778	0.797476
TRINITY_DN346_c0_g1_i7_orf1	CDK-activating kinase assembly factor MAT1 [Ostrinia furnacalis]	-0.26866	-1.70737	0.09995	1.286518	0.589565
TRINITY_DN1540_c0_g1_i14_orf1	alaserpin-like isoform X1 [Ostrinia furnacalis]	-0.51894	-1.59624	0.069264	0.816759	1.229158
TRINITY_DN5274_c0_g2_i2_orf1	lopap-like [Ostrinia furnacalis]	-0.37855	-1.78141	0.937039	0.780986	0.441932
TRINITY_DN66453_c0_g1_i4_orfp1	TRINITY_DN66453_c0_g1_i4_m.7345 TRINITY_DN66453_c0_g1::TRINITY_DN66453_c0_g1_i4::g.7345 ORF type:internal len:93 (+),score=24.53 TRINITY_DN66453_c0_g1_i4:3-278(+)	-1.11265	-1.14385	0.898282	0.077311	1.280909
TRINITY_DN874_c2_g1_i1_orf1	uncharacterized protein LOC114356358 [Ostrinia furnacalis]	-0.02766	-1.89379	0.54041	0.401436	0.979598
TRINITY_DN970_c0_g1_i4_orf1	spermine oxidase-like isoform X2 [Ostrinia furnacalis]	-0.40634	-1.74779	0.354301	0.767776	1.032047
TRINITY_DN5198_c0_g1_i5_orfp1	TRINITY_DN5198_c0_g1_i5_m.8637 TRINITY_DN5198_c0_g1::TRINITY_DN5198_c0_g1_i5::g.8637 ORF type:complete len:223 (-),score=54.99 TRINITY_DN5198_c0_g1_i5:319-987(-)	-0.9491	-1.36167	1.311727	0.386875	0.612172
TRINITY_DN1960_c5_g1_i3_orf1	cytochrome P450 monooxygenase CYP9G18 [Cnaphalocrocis medinalis]	-0.54873	-1.65427	0.256303	1.003029	0.943671
TRINITY_DN3175_c0_g1_i7_orf1	unnamed protein product, partial [Brenthis ino]	-0.23277	-1.86005	0.609957	0.656076	0.826779
TRINITY_DN3433_c0_g1_i15_orf1	cytosolic purine 5'-nucleotidase isoform X3 [Ostrinia furnacalis] >XP_028162965.1 cytosolic purine 5'-nucleotidase isoform X3 [Ostrinia furnacalis] >XP_028162966.1 cytosolic purine 5'-nucleotidase isoform X3 [Ostrinia furnacalis]	-0.39682	-1.69788	0.681925	0.208001	1.204768
TRINITY_DN1014_c0_g2_i8_orf1	uncharacterized protein LOC114362446 [Ostrinia furnacalis] >XP_028173663.1 uncharacterized protein LOC114362446 [Ostrinia furnacalis]	0.196412	-1.84884	0.003496	0.521394	1.127539
TRINITY_DN22375_c0_g1_i4_orf1	venom carboxylesterase-6-like [Ostrinia furnacalis]	0.055927	-1.65491	-0.42797	1.115302	0.911646
TRINITY_DN1206_c0_g1_i6_orf1	sorbitol dehydrogenase-like [Spodoptera frugiperda] >KAG8104768.1 hypothetical protein SFRUCORN_013827 [Spodoptera frugiperda]	-0.62842	-1.67648	0.728038	0.891438	0.685425
TRINITY_DN1592_c0_g1_i1_orf1	serine protease 7-like isoform X2 [Ostrinia furnacalis]	-0.08891	-1.80721	1.04569	0.057121	0.793311

TRINITY_DN54205_c0_g1_i1_orf1	aldo-keto reductase AKR2E4-like [Ostrinia furnacalis]	-0.35846	-1.76632	0.309599	0.843148	0.972038
TRINITY_DN1024_c0_g4_i1_orf1	superoxide dismutase [Cu-Zn]-like [Ostrinia furnacalis]	-0.7855	-1.57275	0.977353	0.670407	0.710487
TRINITY_DN350_c0_g1_i5_orf1	tau-like protein isoform X6 [Bombyx mori]	-0.63224	-1.62296	1.162332	0.64153	0.451335
TRINITY_DN512_c0_g1_i10_orf1	uncharacterized protein LOC114366781 [Ostrinia furnacalis]	-0.18176	-1.78009	0.112955	0.729137	1.119753
TRINITY_DN82944_c0_g1_i4_orf1	senecionine N-oxygenase isoform X2 [Ostrinia furnacalis]	-0.33426	-1.80065	0.421388	0.844683	0.868837
TRINITY_DN812_c2_g1_i1_orf1	1,4-alpha-glucan-branching enzyme [Ostrinia furnacalis]	-0.57641	-1.70553	0.867195	0.762845	0.651899
TRINITY_DN9492_c1_g1_i1_orf1	aldo-keto reductase AKR2E4-like [Galleria mellonella]	-0.30097	-1.75286	0.191697	0.748909	1.113227
TRINITY_DN574_c0_g1_i4_orf1	CD63 antigen-like [Ostrinia furnacalis]	-0.13397	-1.88994	0.787831	0.50506	0.731026
TRINITY_DN10994_c0_g1_i4_orf1	trypsin inhibitor-like [Ostrinia furnacalis]	-0.74385	-1.59247	0.541191	0.955126	0.839995
TRINITY_DN3732_c1_g1_i5_orf1	cytochrome P450 6B2-like [Ostrinia furnacalis]	-0.76765	-1.1152	1.000737	-0.50141	1.383527
TRINITY_DN4069_c0_g1_i5_orf1	putative sulfiredoxin [Ostrinia furnacalis]	-0.60588	-1.53807	1.033365	0.015513	1.095069
TRINITY_DN5748_c0_g1_i6_orf1	glycine N-methyltransferase isoform X1 [Ostrinia furnacalis] >XP_028165118.1 glycine N-methyltransferase isoform X2 [Ostrinia furnacalis] >XP_028165119.1 glycine N-methyltransferase isoform X1 [Ostrinia furnacalis] >XP_028165120.1 glycine N-methyltransferase isoform X2 [Ostrinia furnacalis]	-0.82201	-1.42537	1.382645	0.373035	0.491699
TRINITY_DN987_c0_g1_i3_orf1	unnamed protein product [Chilo suppressalis]	-0.1544	-1.2989	-0.82153	1.021883	1.252945
TRINITY_DN27300_c0_g1_i7_orfp1	TRINITY_DN27300_c0_g1_i7_m.71141 TRINITY_DN27300_c0_g1::TRINITY_DN27300_c0_g1_i7::g.71141 ORF type:internal len:82 (-),score=6.59 TRINITY_DN27300_c0_g1_i7:3-245(-)	-0.49544	-1.49298	1.519485	0.465513	0.00342
TRINITY_DN17326_c0_g1_i8_orf1	aminoacylase-1-like [Ostrinia furnacalis]	-0.46915	-1.75122	0.630969	0.633659	0.955751
TRINITY_DN5914_c1_g1_i9_orf1	unnamed protein product [Chilo suppressalis]	-0.3929	-1.75599	0.742522	0.370124	1.036245
TRINITY_DN2392_c0_g2_i1_orf1	cytochrome P450 9e2-like [Ostrinia furnacalis] >QPF77612.1 cytochrome P450 monooxygenase CYP9A185 [Ostrinia furnacalis]	-0.49679	-1.47167	-0.13139	0.622468	1.477381
TRINITY_DN14185_c0_g1_i1_orf1	uncharacterized protein LOC114358675 [Ostrinia furnacalis] >XP_028168498.1 uncharacterized protein LOC114358675 [Ostrinia furnacalis]	-0.83945	-1.4804	1.023814	0.976426	0.319605
TRINITY_DN18482_c0_g1_i3_orf1	calcyphosin-like protein isoform X3 [Helicoverpa armigera] >XP_047020698.1 calcyphosin-like protein isoform X2 [Helicoverpa zea]	-1.0723	-1.31087	0.425514	1.110234	0.847419
TRINITY_DN2461_c0_g1_i5_orf1	secretory phospholipase A2 receptor [Vanessa cardui]	-0.98072	-1.42816	0.679423	0.720841	1.008619
TRINITY_DN1216_c0_g1_i4_orf1	bifunctional purine biosynthesis protein PURH isoform X1 [Ostrinia furnacalis] >XP_028176123.1 bifunctional purine biosynthesis protein PURH isoform X2 [Ostrinia furnacalis] >XP_028176129.1 bifunctional purine biosynthesis protein PURH isoform X3 [Ostrinia furnacalis]	-0.12317	-1.82868	0.201266	0.690927	1.059656
TRINITY_DN8473_c0_g1_i6_orf1	serine/threonine-protein phosphatase 6 regulatory subunit 1 [Ostrinia furnacalis]	-0.82793	-1.40302	0.068952	1.127001	1.034988
TRINITY_DN22875_c0_g1_i6_orf1	microtubule-actin cross-linking factor 1 isoform X15 [Ostrinia furnacalis]	-0.58475	-1.63237	1.068149	0.264304	0.884669
TRINITY_DN58751_c0_g1_i2_orf1	FK506-binding protein 2 isoform X1 [Vanessa tameamea] >XP_046977568.1 FK506-binding protein 2 isoform X1 [Vanessa cardui]	-0.06837	-1.81915	1.028081	0.06933	0.790108
TRINITY_DN2255_c0_g1_i1_orf1	glutathione S-transferase sigma 3 [Ostrinia furnacalis]	-0.10372	-1.87264	0.327694	0.734861	0.913808
TRINITY_DN4062_c0_g2_i1_orf1	venom peptide BmKAPI-like isoform X2 [Ostrinia furnacalis]	-0.15121	-1.78841	1.211854	0.213328	0.514431
TRINITY_DN1230_c1_g1_i5_orf1	uncharacterized protein LOC114353440 [Ostrinia furnacalis]	-1.01605	-1.37327	0.873272	0.466477	1.049564
TRINITY_DN103511_c0_g1_i4_orf1	probable salivary secreted peptide [Ostrinia furnacalis]	-0.24027	-1.67837	-0.07876	1.248018	0.749391
TRINITY_DN11948_c0_g1_i8_orf1	cystathionine gamma-lyase [Ostrinia furnacalis]	-0.74675	-1.59051	0.845555	0.952093	0.539616
TRINITY_DN13322_c0_g1_i6_orf1	macrophage mannose receptor 1-like [Ostrinia furnacalis]	-0.46503	-1.73678	0.416879	0.875303	0.909622
TRINITY_DN14670_c0_g1_i1_orf1	heat shock protein beta-1 isoform X1 [Helicoverpa armigera] >XP_022829066.1 heat shock protein beta-1 isoform X1 [Spodoptera litura] >XP_026747148.1 heat shock protein beta-1 isoform X3 [Trichoplusia ni] >XP_026748187.1 heat shock protein beta-1 isoform X2 [Galleria mellonella] >XP_028167756.1 heat shock protein beta-1 isoform X2 [Ostrinia furnacalis] >XP_035431734.1 heat shock protein beta-1-like isoform X3 [Spodoptera frugiperda] >XP_047023072.1 heat shock protein beta-1 isoform X1 [Helicoverpa zea] >XP_049865086.1 heat shock protein beta-1 [Pectinophora gossypiella] >KAH9640995.1 hypothetical protein HF086_015091 [Spodoptera exigua] >QGZ00460.1 heat shock protein 21.4 [Glyphodes pyloalis] >QKR72095.1 heat-shock protein 21.4 [Mythimna separata] >CAB3228281.1 unnamed protein product [Arctia plantaginis] >CAH0628881.1 unnamed protein	-0.80182	-1.47876	1.299199	0.469867	0.511517
TRINITY_DN38783_c0_g1_i1_orf1	regucalcin-like [Ostrinia furnacalis]	-0.83963	-1.44952	0.262869	0.823543	1.20274

TRINITY_DN20133_c0_g1_i1_orf1	fructose-bisphosphate aldolase A isoform X2 [Microcebus murinus] >XP_012619765.1 fructose-bisphosphate aldolase A isoform X2 [Microcebus murinus] >XP_012619766.1 fructose-bisphosphate aldolase A isoform X2 [Microcebus murinus] >XP_012619767.1 fructose-bisphosphate aldolase A isoform X2 [Microcebus murinus] >XP_012619768.1 fructose-bisphosphate aldolase A isoform X2 [Microcebus murinus]	-0.59109	-1.52955	0.399365	1.439517	0.281757
TRINITY_DN2618_c0_g1_i3_orf1	CDP-diacylglycerol--inositol 3-phosphatidyltransferase [Ostrinia furnacalis]	-0.26196	-1.73619	0.075611	0.783445	1.13909
TRINITY_DN1108_c1_g2_i1_orfp1	TRINITY_DN1108_c1_g2_i1_m.5565 TRINITY_DN1108_c1_g2::TRINITY_DN1108_c1_g2_i1::g.5565 ORF type:internal len:205 (-),score=147.90 TRINITY_DN1108_c1_g2_i1:2-613(-)	-0.47359	-1.54217	1.505353	0.232024	0.278384
TRINITY_DN8685_c0_g1_i5_orf1	macrophage mannose receptor 1-like [Zerene cesonia]	-0.78348	-1.43299	1.378499	0.620658	0.217314
TRINITY_DN2076_c0_g2_i1_orf1	macrophage mannose receptor 1-like isoform X1 [Maniola jurtina]	-0.19726	-1.76747	0.147286	1.194985	0.622461
TRINITY_DN5696_c0_g1_i4_orf1	serine protease snake-like isoform X1 [Ostrinia furnacalis]	-0.66332	-1.2981	0.985512	-0.35689	1.332795
TRINITY_DN3616_c0_g2_i2_orf1	conotoxin ArMKLT2-032-like [Ostrinia furnacalis]	-1.17777	-1.1895	0.656112	0.466079	1.245082
TRINITY_DN701_c0_g1_i1_orf1	venom protease-like isoform X3 [Ostrinia furnacalis]	-1.01407	-1.31943	0.600833	0.436974	1.295692
TRINITY_DN33488_c0_g1_i2_orf1	semaphorin-1A isoform X3 [Trichoplusia ni]	-0.44616	-1.76322	0.931944	0.700102	0.577339
TRINITY_DN4621_c0_g1_i4_orf1	uncharacterized protein LOC114358242 isoform X3 [Ostrinia furnacalis]	-0.64132	-1.66414	0.590625	0.866213	0.848628
TRINITY_DN13500_c0_g1_i1_orf1	phosphatidylethanolamine-binding protein homolog F40A3.3-like [Ostrinia furnacalis] >XP_028160752.1 phosphatidylethanolamine-binding protein homolog F40A3.3-like [Ostrinia furnacalis]	-0.20892	-1.69486	-0.00036	1.320133	0.584006
TRINITY_DN3196_c0_g1_i1_orf1	organic cation transporter-like protein [Ostrinia furnacalis]	-0.41666	-1.69906	0.285306	0.612713	1.217695
TRINITY_DN26439_c0_g1_i2_orf1	uncharacterized protein LOC114351853 [Ostrinia furnacalis]	-0.90573	-1.4429	1.169831	0.458255	0.720545
TRINITY_DN4502_c0_g1_i3_orf1	kynurenine formamidase isoform X1 [Ostrinia furnacalis]	-0.53484	-1.71027	0.928377	0.484933	0.831798
TRINITY_DN11383_c0_g2_i4_orf1	aminoacylase-1A-like [Ostrinia furnacalis]	-0.59642	-1.63367	0.292008	0.891443	1.046642
TRINITY_DN12367_c0_g1_i8_orf1	aldose reductase-like isoform X2 [Ostrinia furnacalis]	-0.27538	-1.30878	-0.7296	1.18987	1.123896
TRINITY_DN91946_c0_g1_i1_orf1	protein catecholamines up [Ostrinia furnacalis]	-0.76968	-1.46719	0.91434	0.139534	1.182992
TRINITY_DN3831_c0_g1_i7_orf1	beta-ureidopropionase-like [Ostrinia furnacalis]	-0.14015	-1.86728	0.366173	0.740932	0.90032
TRINITY_DN15858_c0_g1_i2_orf1	15-hydroxyprostaglandin dehydrogenase [NAD(+)]-like [Ostrinia furnacalis]	-0.74927	-1.55418	1.127608	0.761604	0.414233
TRINITY_DN62091_c0_g1_i1_orf1	protein NipSnap [Venturia canescens]	-0.50071	-1.69178	0.345928	0.746378	1.10019
TRINITY_DN8440_c0_g1_i9_orf1	protein FAM177A1-like [Ostrinia furnacalis]	-0.05402	-1.87311	0.409628	0.46777	1.049729
TRINITY_DN5880_c0_g2_i2_orf1	macrophage mannose receptor 1 [Bombyx mori]	-0.99974	-1.1019	0.648405	-0.08269	1.535926
TRINITY_DN1493_c0_g1_i5_orf1	uncharacterized protein LOC114350869 [Ostrinia furnacalis]	-0.06347	-1.06678	-0.93853	0.38742	1.68137
TRINITY_DN11698_c0_g1_i1_orf1	hypothetical protein evm_015129 [Chilo suppressalis]	-0.80454	-1.51589	0.412082	0.775156	1.133191
TRINITY_DN34134_c0_g2_i1_orf1	THUMP domain-containing protein 1 homolog [Ostrinia furnacalis]	-0.66518	-1.52657	0.661128	0.209159	1.321467
TRINITY_DN11231_c1_g1_i1_orfp1	TRINITY_DN11231_c1_g1_i1_m.13377 TRINITY_DN11231_c1_g1::TRINITY_DN11231_c1_g1_i1::g.13377 ORF type:internal len:76 (-),score=1.43 TRINITY_DN11231_c1_g1_i1:1-225(-)	-0.82694	-1.40707	1.096039	0.075257	1.062715
TRINITY_DN3257_c0_g1_i4_orf1	N-acetylneuraminate lyase-like [Ostrinia furnacalis]	-0.14781	-1.84686	0.304222	0.693765	0.996687
TRINITY_DN11680_c0_g1_i1_orf1	uncharacterized protein LOC114355414 [Ostrinia furnacalis]	-0.26431	-1.74663	0.318613	0.43028	1.262049
TRINITY_DN8252_c0_g1_i6_orf1	scavenger receptor class B member 1 [Ostrinia furnacalis]	-0.54618	-1.5898	0.143926	1.287045	0.705006
TRINITY_DN33705_c0_g1_i1_orf1	atlastin-like isoform X4 [Ostrinia furnacalis]	-0.30778	-1.709	0.272461	0.43112	1.313201
TRINITY_DN22242_c0_g1_i1_orf1	juvenile hormone epoxide hydrolase-like [Ostrinia furnacalis] >XP_028170526.1 juvenile hormone epoxide hydrolase-like [Ostrinia furnacalis]	0.028196	-1.72365	0.555052	-0.16121	1.301605
TRINITY_DN2847_c0_g1_i20_orf1	uncharacterized protein LOC114352221 [Ostrinia furnacalis]	-0.46871	-1.47724	-0.13794	0.590426	1.493466
TRINITY_DN14611_c0_g1_i5_orf1	hsc70-interacting protein-like [Galleria mellonella]	-0.35729	-1.72201	1.236011	0.312743	0.530547
TRINITY_DN3015_c0_g1_i7_orf1	glycine-rich protein DOT1-like [Ostrinia furnacalis]	-0.21577	-1.86261	0.858558	0.570957	0.648865
TRINITY_DN5266_c0_g1_i1_orf1	malate dehydrogenase, cytoplasmic isoform X2 [Ostrinia furnacalis]	-0.68114	-1.63246	0.593191	0.719963	1.000448
TRINITY_DN6483_c0_g1_i6_orf1	transketolase-like protein 2 isoform X1 [Ostrinia furnacalis] >XP_028164795.1 transketolase-like protein 2 isoform X2 [Ostrinia furnacalis]	-0.48911	-1.73434	0.753327	0.506242	0.96388
TRINITY_DN15858_c0_g1_i1_orf1	15-hydroxyprostaglandin dehydrogenase [NAD(+)]-like [Ostrinia furnacalis]	-0.90754	-1.47824	0.5607	0.987836	0.837245
TRINITY_DN138086_c0_g1_i1_orf1	hypothetical protein evm_000614 [Chilo suppressalis]	-0.97053	-1.40586	1.125672	0.498248	0.752478
TRINITY_DN1008_c0_g1_i2_orf1	integrin beta-PS [Ostrinia furnacalis]	-0.59084	-1.55832	1.133317	0.048475	0.967367
TRINITY_DN103475_c0_g1_i4_orf1	lipid storage droplets surface-binding protein 1 isoform X3 [Ostrinia furnacalis]	-0.88567	-1.48751	1.032939	0.532597	0.807641
TRINITY_DN2514_c1_g1_i13_orf1	seroin transcript 1A2 [Ostrinia nubilalis]	-1.22862	-1.20162	0.767884	0.637208	1.025147
TRINITY_DN12873_c0_g2_i1_orf1	proteoglycan 4-like [Ostrinia furnacalis]	-0.8187	-1.55008	0.6124	0.947184	0.809194
TRINITY_DN2378_c0_g1_i5_orf1	integrin alpha-8-like isoform X1 [Ostrinia furnacalis]	-0.63927	-1.61469	0.68149	0.409852	1.162622

TRINITY_DN5497_c0_g1_i6_orf1	1,2-dihydroxy-3-keto-5-methylthiopentene dioxygenase-like [Ostrinia furnacalis]	-0.05624	-1.71331	-0.08203	0.513198	1.33839
TRINITY_DN566_c0_g1_i13_orf1	uncharacterized protein LOC114355567 isoform X2 [Ostrinia furnacalis]	-0.88611	-1.39313	0.748641	0.244626	1.285966
TRINITY_DN28299_c0_g1_i1_orf1	adenylosuccinate lyase isoform X1 [Ostrinia furnacalis]	-0.62741	-1.58328	0.208843	1.162595	0.839251
TRINITY_DN20347_c0_g1_i6_orf1	venom polypeptide precursor [Doratifera vulnerans]	-0.4397	-1.51177	-0.01452	1.52542	0.44057
TRINITY_DN65974_c0_g1_i2_orf1	uncharacterized protein LOC114362364 [Ostrinia furnacalis]	-0.32335	-0.75365	-1.21719	0.8198	1.47439
TRINITY_DN6642_c0_g1_i2_orf1	protein purity of essence [Ostrinia furnacalis]	-0.98535	-1.13845	0.12537	0.399023	1.599403
TRINITY_DN9340_c0_g1_i4_orf1	sarcosine dehydrogenase, mitochondrial [Ostrinia furnacalis]	-0.45698	-1.695	1.194102	0.608372	0.349504
TRINITY_DN89483_c0_g1_i1_orf1	mitochondrial enolase superfamily member 1-like isoform X2 [Maniola jurtina]	-0.94005	-1.1892	0.434325	0.113606	1.581318
TRINITY_DN11108_c0_g1_i4_orf1	peroxisomal leader peptide-processing protease [Ostrinia furnacalis] >XP_028165527.1 peroxisomal leader peptide-processing protease [Ostrinia furnacalis]	-0.59582	-1.4749	1.406701	-0.03569	0.699704
TRINITY_DN17031_c0_g1_i1_orf1	arginase, hepatic [Ostrinia furnacalis]	-0.237	-1.81312	0.299773	0.743442	1.006905
TRINITY_DN384_c0_g1_i8_orf1	unnamed protein product [Chilo suppressalis]	-0.83387	-1.53268	0.570163	0.806107	0.990281
TRINITY_DN7630_c0_g2_i1_orf1	flotillin-2 isoform X1 [Ostrinia furnacalis] >XP_028172931.1 flotillin-2 isoform X2 [Ostrinia furnacalis]	-0.52398	-1.70472	0.845537	0.419679	0.963486
TRINITY_DN3263_c0_g1_i2_orf1	serine hydroxymethyltransferase, cytosolic isoform X1 [Diachasma alloeum]	-0.89428	-1.49935	0.638379	0.923679	0.83157
TRINITY_DN7075_c0_g2_i1_orf1	retinal dehydrogenase 1-like [Ostrinia furnacalis]	-0.40737	-1.62161	-0.05281	0.909159	1.172632
TRINITY_DN2710_c0_g1_i4_orf1	translin-associated protein X [Ostrinia furnacalis]	-0.25979	-1.74974	0.07841	0.973568	0.957559
TRINITY_DN10403_c0_g1_i1_orf1	hypothetical protein evm_000264 [Chilo suppressalis] >CAH2987898.1 unnamed protein product [Chilo	-0.84935	-1.40298	0.304045	0.574201	1.374083
TRINITY_DN6680_c0_g1_i1_orf1	hypothetical protein evm_009571 [Chilo suppressalis]	-0.44777	-1.35872	-0.50643	1.261589	1.051333
TRINITY_DN625_c9_g1_i7_orf1	ecdysone 20-monooxygenase [Ostrinia furnacalis]	-0.71097	-1.59791	0.494065	0.748771	1.066043
TRINITY_DN21278_c0_g2_i2_orf1	mannose-1-phosphate guanyltransferase alpha-A [Ostrinia furnacalis]	-0.22394	-1.82258	0.672875	0.346345	1.0273
TRINITY_DN24970_c0_g1_i4_orf1	pyrroline-5-carboxylate reductase-like isoform X1 [Ostrinia furnacalis]	-0.87267	-1.52052	0.853041	0.690326	0.849832
TRINITY_DN67243_c0_g1_i1_orf1	39S ribosomal protein L3, mitochondrial [Ostrinia furnacalis]	-0.46474	-1.76522	0.662401	0.766465	0.801098
TRINITY_DN8651_c0_g1_i16_orf1	glutathione S-transferase theta 2 [Conogethes punctiferalis]	-0.75747	-1.31775	0.369767	0.111516	1.593933
TRINITY_DN9435_c0_g1_i7_orf1	uncharacterized protein LOC114350197 [Ostrinia furnacalis]	-0.71631	-1.42066	0.244947	1.502235	0.389781
TRINITY_DN1047_c0_g1_i6_orf1	mitochondrial genome maintenance exonuclease 1-like [Ostrinia furnacalis]	-0.53756	-1.72135	0.830136	0.576255	0.852516
TRINITY_DN104297_c0_g1_i1_orf1	tubulin-specific chaperone D [Ostrinia furnacalis]	-0.17128	-0.99677	-1.06957	0.712798	1.524817
TRINITY_DN15157_c0_g1_i1_orf1	UDP-glycosyltransferase UGT40AM2 [Ostrinia furnacalis]	-0.39708	-1.54727	0.40823	0.025839	1.510283
TRINITY_DN452_c9_g1_i1_orf1	epidermal retinol dehydrogenase 2-like [Ostrinia furnacalis]	-0.58882	-1.53889	1.358672	0.116732	0.652303
TRINITY_DN117844_c0_g1_i1_orf1	ATP-citrate synthase [Cotesia glomerata] >XP_044590631.1 ATP-citrate synthase [Cotesia glomerata] >KAH0546822.1 hypothetical protein KQX54_015428 [Cotesia glomerata]	-1.07501	-1.34835	0.593783	0.910783	0.918798
TRINITY_DN135188_c0_g1_i2_orf1	proteasome inhibitor PI31 subunit [Ostrinia furnacalis]	-0.37409	-1.80711	0.651607	0.767057	0.762532
TRINITY_DN8702_c0_g1_i1_orf1	programmed cell death protein 4 isoform X1 [Ostrinia furnacalis] >XP_028157160.1 programmed cell death protein 4 isoform X2 [Ostrinia furnacalis]	-0.70393	-1.603	0.796029	0.472799	1.038105
TRINITY_DN33_c0_g1_i14_orf1	uncharacterized protein CG45076-like isoform X1 [Ostrinia furnacalis]	-0.88362	-1.44015	1.084283	0.302463	0.937027
TRINITY_DN63536_c0_g1_i1_orf1	adenosylhomocysteinase [Chelonus insularis]	-0.55159	-1.68891	0.719685	1.05104	0.469776
TRINITY_DN23564_c0_g1_i7_orf1	cytochrome P450 6B6-like [Ostrinia furnacalis]	-0.37417	-1.61122	0.94571	-0.12403	1.163712
TRINITY_DN4245_c0_g1_i5_orf1	long-chain fatty acid transport protein 4-like [Ostrinia furnacalis]	-0.83677	-1.53122	1.016652	0.610229	0.741104
TRINITY_DN5081_c0_g1_i5_orf1	ester hydrolase C11orf54 homolog isoform X1 [Ostrinia furnacalis]	-0.45443	-1.66726	0.143704	0.853289	1.124706
TRINITY_DN44083_c0_g1_i2_orf1	putative alpha-ketoglutarate-dependent hypophosphite dioxygenase [Operophtera brumata]	-0.92689	-1.44621	0.918722	0.455454	0.998927
TRINITY_DN12873_c0_g1_i3_orf1	proteoglycan 4-like [Ostrinia furnacalis]	-0.83949	-1.4375	0.223029	1.213694	0.840267
TRINITY_DN9044_c0_g1_i1_orf1	hypothetical protein SFRURICE_005818, partial [Spodoptera frugiperda]	-0.08636	-1.4638	-0.6421	0.964703	1.227557
TRINITY_DN6813_c1_g1_i1_orf1	pantothenate kinase 3 isoform X2 [Ostrinia furnacalis] >XP_028173241.1 pantothenate kinase 3 isoform X2 [Ostrinia furnacalis]	-0.85229	-1.41261	0.17222	1.217861	0.874815
TRINITY_DN44491_c0_g1_i12_orf1	unnamed protein product [Chrysodeixis includens]	-1.06701	-1.28387	1.040874	0.28643	1.023575
TRINITY_DN8030_c0_g1_i2_orf1	chaoptin isoform X1 [Ostrinia furnacalis] >XP_028159953.1 chaoptin isoform X2 [Ostrinia furnacalis] >XP_028159954.1 chaoptin isoform X3 [Ostrinia furnacalis] >XP_028159955.1 chaoptin isoform X4 [Ostrinia furnacalis] >XP_028159956.1 chaoptin isoform X5 [Ostrinia furnacalis]	-0.9623	-1.36778	0.271464	0.956804	1.101812
TRINITY_DN36632_c0_g1_i1_orf1	hypothetical protein evm_011159, partial [Chilo suppressalis]	-0.63896	-1.19402	1.08469	-0.55039	1.298679
TRINITY_DN18196_c0_g1_i4_orf1	uncharacterized protein LOC114363471 isoform X3 [Ostrinia furnacalis]	-0.65571	-1.64965	0.98254	0.726146	0.596677
TRINITY_DN245_c0_g1_i4_orf1	ER lumen protein-retaining receptor [Ostrinia furnacalis]	-1.44148	-0.93778	0.473394	0.988049	0.91781
TRINITY_DN10742_c0_g1_i4_orf1	ethanolamine-phosphate cytidylyltransferase isoform X1 [Ostrinia furnacalis]	-0.61218	-1.54132	0.277754	0.483325	1.392417

TRINITY_DN350_c0_g1_i4_orf1	microtubule-associated protein tau-like isoform X6 [Ostrinia furnacalis]	-0.90658	-1.47686	0.923836	0.531982	0.927631
TRINITY_DN38225_c0_g2_i1_orf1	uncharacterized protein LOC114354273 [Ostrinia furnacalis]	-0.90341	-1.35795	1.389718	0.316221	0.555425
TRINITY_DN77559_c0_g1_i1_orf1	uncharacterized protein LOC114359392 isoform X2 [Ostrinia furnacalis]	-0.91765	-1.37143	0.232104	0.796489	1.260488
TRINITY_DN8527_c0_g2_i1_orfp1	TRINITY_DN8527_c0_g2_i1_m.16937 TRINITY_DN8527_c0_g2::TRINITY_DN8527_c0_g2_i1::g.16937 ORF type:5prime_partial len:54 (-),score=0.36 TRINITY_DN8527_c0_g2_i1:195-356(-)	-1.35977	-1.04698	0.528174	0.86394	1.014638
TRINITY_DN12545_c0_g1_i7_orf1	hypothetical protein evm_003491 [Chilo suppressalis]	-1.18171	-1.1449	1.218895	0.242787	0.864931
TRINITY_DN10360_c0_g1_i16_orf1	H(+)/Cl(-) exchange transporter 3 isoform X1 [Ostrinia furnacalis]	-1.23836	-1.14565	0.628953	0.547191	1.207868
TRINITY_DN19116_c0_g1_i3_orf1	UDP-glucose 4-epimerase isoform X1 [Ostrinia furnacalis]	-0.53648	-1.56725	1.166009	-0.00898	0.946707
TRINITY_DN2570_c0_g1_i1_orf1	PREDICTED: pyruvate carboxylase, mitochondrial isoform X1 [Microplitis demolitor] >XP_008556301.1 PREDICTED: pyruvate carboxylase, mitochondrial isoform X1 [Microplitis demolitor] >XP_008556302.1 PREDICTED: pyruvate carboxylase, mitochondrial isoform X1 [Microplitis demolitor]	-0.69098	-1.62725	0.53881	0.866363	0.913064
TRINITY_DN17172_c0_g1_i5_orf1	omega-amidase NIT2 isoform X1 [Zerene cesonia]	-0.29541	-1.76701	0.477051	0.401449	1.183919
TRINITY_DN4123_c0_g1_i1_orf1	uncharacterized protein LOC114355030 [Ostrinia furnacalis]	-1.14575	-1.0626	1.05329	-0.04764	1.20269
TRINITY_DN3991_c0_g1_i6_orf1	acetyl-CoA carboxylase isoform X3 [Trichoplusia ni]	-0.93951	-1.27918	0.292261	0.443587	1.482845
TRINITY_DN356_c2_g1_i3_orf1	scavenger receptor class B member 1 isoform X2 [Pectinophora gossypiella] >XP_049883835.1 scavenger receptor class B member 1 isoform X2 [Pectinophora gossypiella]	-0.75102	-1.6052	0.860982	0.738716	0.756519
TRINITY_DN51776_c0_g1_i1_orf1	unnamed protein product, partial [Iphiclides podalirius]	-0.93745	-1.33192	0.20557	0.735875	1.32793
TRINITY_DN2983_c0_g1_i6_orf1	hypothetical protein evm_002448 [Chilo suppressalis]	-0.6764	-1.63964	0.60071	0.752713	0.962618
TRINITY_DN19098_c0_g1_i4_orf1	L-xylulose reductase-like [Ostrinia furnacalis]	-0.79962	-1.17129	0.26534	-0.00272	1.708295
TRINITY_DN4314_c0_g1_i9_orf1	serine proteinase inhibitor 2 [Ostrinia furnacalis]	-1.19428	-1.19553	0.445526	1.139169	0.805114
TRINITY_DN8258_c0_g1_i3_orf1	papilin isoform X7 [Ostrinia furnacalis]	-0.72723	-1.52831	1.108705	0.220881	0.925954
TRINITY_DN30012_c1_g1_i1_orf1	ubiquitin domain-containing protein UBFD1-like [Ostrinia furnacalis]	-0.7602	-1.5659	0.663654	0.559403	1.10304
TRINITY_DN511_c0_g2_i1_orf1	pyruvate carboxylase, mitochondrial isoform X1 [Manduca sexta] >XP_037293486.1 pyruvate carboxylase, mitochondrial isoform X1 [Manduca sexta]	-0.82811	-1.54535	0.968421	0.677851	0.727188
TRINITY_DN22046_c1_g1_i5_orf1	uncharacterized protein LOC114351208 [Ostrinia furnacalis]	-0.66135	-1.25149	0.169408	0.020849	1.722576
TRINITY_DN65518_c0_g1_i1_orf1	unc-112-related protein-like, partial [Ostrinia furnacalis]	-0.9369	-1.46145	0.594467	0.956663	0.84722
TRINITY_DN15040_c0_g4_i1_orf1	hypothetical protein O3G_MSEX013320 [Manduca sexta]	-1.1747	-1.02311	0.13527	0.568657	1.493878
TRINITY_DN3433_c2_g1_i2_orf1	cytosolic purine 5'-nucleotidase isoform X4 [Ostrinia furnacalis]	-0.87901	-1.37604	1.290463	0.163446	0.801147
TRINITY_DN1622_c0_g1_i6_orf1	unnamed protein product [Parnassius apollo]	-0.87819	-1.18342	1.097754	-0.27923	1.243088
TRINITY_DN40281_c0_g1_i1_orf1	thioredoxin domain-containing protein 15 [Ostrinia furnacalis]	-1.0731	-1.21441	0.461614	1.409759	0.416133
TRINITY_DN1084_c0_g1_i2_orf1	ATP-citrate synthase [Ostrinia furnacalis]	-0.6055	-1.54292	0.104527	1.298629	0.745262
TRINITY_DN64126_c0_g1_i1_orf1	senecionine N-oxygenase isoform X2 [Galleria mellonella]	-0.94407	-1.40232	0.408152	0.748593	1.189644
TRINITY_DN8258_c0_g1_i5_orf1	papilin isoform X9 [Ostrinia furnacalis]	-0.58929	-1.64016	1.008859	0.294162	0.92643
TRINITY_DN4589_c0_g2_i1_orf1	thymidylate kinase [Ostrinia furnacalis]	-0.89228	-1.06125	0.105053	1.748285	0.100197
TRINITY_DN49038_c0_g4_i1_orf1	6-phosphogluconate dehydrogenase, decarboxylating [Ostrinia furnacalis]	-0.54053	-1.72699	0.833029	0.752845	0.68164
TRINITY_DN5748_c0_g1_i5_orf1	glycine N-methyltransferase isoform X1 [Ostrinia furnacalis] >XP_028165118.1 glycine N-methyltransferase isoform X2 [Ostrinia furnacalis] >XP_028165119.1 glycine N-methyltransferase isoform X1 [Ostrinia furnacalis] >XP_028165120.1 glycine N-methyltransferase isoform X2 [Ostrinia furnacalis]	-0.92314	-1.42587	0.601854	0.537246	1.209903
TRINITY_DN38180_c0_g1_i3_orf1	guanine deaminase [Ostrinia furnacalis]	-0.33536	-1.61687	-0.12206	0.805769	1.268512
TRINITY_DN14112_c0_g1_i3_orf1	uncharacterized protein LOC114350956 [Ostrinia furnacalis]	-0.96803	-1.36021	0.442611	0.597089	1.288538
TRINITY_DN146181_c0_g1_i1_orf1	vesicular integral-membrane protein VIP36 [Diachasma alloeum]	-0.16775	-1.83345	0.405214	0.504294	1.091697
TRINITY_DN4955_c0_g1_i2_orf1	RNA pseudouridylate synthase domain-containing protein 1-like isoform X2 [Ostrinia furnacalis]	-0.92559	-1.39704	0.326294	0.783185	1.213147
TRINITY_DN1245_c0_g1_i4_orf1	nuclear RNA export factor 1 [Ostrinia furnacalis]	-1.18626	-0.97913	0.157624	0.459048	1.548717
TRINITY_DN757_c3_g1_i2_orf1	PREDICTED: galectin-4-like [Amyelois transitella]	-0.79518	-1.55139	0.857014	0.502894	0.986663
TRINITY_DN4341_c0_g1_i4_orf1	uncharacterized protein LOC114354354 [Ostrinia furnacalis]	-0.94319	-1.06888	0.161373	0.141359	1.709343
TRINITY_DN27033_c1_g1_i3_orfp1	poly(U)-specific endoribonuclease homolog [Ostrinia furnacalis]	-0.13021	-0.91274	-1.24032	1.191099	1.092172
TRINITY_DN10774_c0_g2_i3_orf1	uncharacterized protein LOC114362157, partial [Ostrinia furnacalis]	-0.92975	-1.30253	0.491851	0.286051	1.454382
TRINITY_DN10484_c0_g1_i8_orf1	hypothetical protein evm_010330 [Chilo suppressalis]	-0.41705	-1.45395	-0.16858	0.47036	1.569218
TRINITY_DN2772_c0_g1_i3_orf1	uncharacterized protein LOC114353284 isoform X4 [Ostrinia furnacalis] >XP_028161011.1 uncharacterized protein LOC114353284 isoform X4 [Ostrinia furnacalis] >XP_028161012.1 uncharacterized protein LOC114353284 isoform X4 [Ostrinia furnacalis]	-1.05653	-1.2004	0.217572	1.417139	0.622222

TRINITY_DN33953_c0_g1_i4_orf1	leucine carboxyl methyltransferase 1 [Ostrinia furnacalis]	-0.94438	-1.25693	0.528189	0.184797	1.48833
TRINITY_DN99694_c0_g1_i1_orf1	hypothetical protein HF086_013792 [Spodoptera exigua]	-0.75442	-1.56221	0.548541	1.135435	0.632652
TRINITY_DN1328_c0_g1_i6_orf1	fungal protease inhibitor-1-like [Ostrinia furnacalis]	-1.11201	-1.22034	0.317083	1.275043	0.740223
TRINITY_DN23069_c0_g2_i3_orf1	uncharacterized protein LOC114364799 [Ostrinia furnacalis]	-1.33111	-1.06217	0.494671	0.786726	1.111888
TRINITY_DN50593_c0_g1_i1_orf1	uncharacterized protein LOC114361588 isoform X14 [Ostrinia furnacalis]	-1.52151	-0.80016	0.396222	0.832179	1.093269
TRINITY_DN10581_c0_g1_i5_orf1	facilitated trehalose transporter Tret1-like isoform X1 [Ostrinia furnacalis] >XP_028165563.1 facilitated trehalose transporter Tret1-like isoform X2 [Ostrinia furnacalis]	-1.07252	-1.24629	1.020088	0.195192	1.103531
TRINITY_DN34153_c0_g2_i2_orf1	uncharacterized protein LOC114351567 [Ostrinia furnacalis]	-0.83008	-1.22378	0.807255	-0.20875	1.455364
TRINITY_DN3374_c0_g1_i7_orf1	TPPP family protein CG45057 [Ostrinia furnacalis] >XP_028172578.1 TPPP family protein CG45057 [Ostrinia	-1.11887	-1.13836	1.299258	0.088267	0.869709
TRINITY_DN3826_c0_g1_i1_orf1	39S ribosomal protein L18, mitochondrial [Ostrinia furnacalis]	-1.09517	-1.12408	0.479514	1.499855	0.239883
TRINITY_DN7106_c0_g1_i5_orf1	TBC1 domain family member 15 isoform X5 [Helicoverpa zea]	-0.9784	-1.29831	0.473257	1.405189	0.398258
TRINITY_DN554_c0_g1_i1_orf1	uncharacterized protein LOC114353093 isoform X1 [Ostrinia furnacalis] >XP_028160722.1 uncharacterized protein LOC114353093 isoform X1 [Ostrinia furnacalis] >XP_028160723.1 uncharacterized protein LOC114353093 isoform X1 [Ostrinia furnacalis]	-1.23482	-1.01248	0.155735	0.70045	1.391119
TRINITY_DN1172_c0_g1_i1_orf1	hypothetical protein O3G_MSEX009550 [Manduca sexta]	-1.07499	-1.31364	0.506973	0.727065	1.154596
TRINITY_DN35763_c0_g1_i2_orf1	probable methylthioribulose-1-phosphate dehydratase [Helicoverpa armigera]	-1.10808	-1.22306	0.379081	0.638898	1.313161
TRINITY_DN4695_c0_g1_i4_orf1	glutathione S-transferase epsilon 3 [Ostrinia furnacalis]	-1.11368	-1.20093	0.413461	0.538652	1.362496
TRINITY_DN83622_c0_g1_i2_orf1	uncharacterized protein LOC114350344 [Ostrinia furnacalis]	-0.77637	-1.29348	0.038724	1.589986	0.441139
TRINITY_DN21285_c0_g1_i3_orf1	uncharacterized protein LOC114351683 isoform X7 [Ostrinia furnacalis]	-0.86154	-1.47903	1.143126	0.446172	0.751275
TRINITY_DN8224_c0_g1_i7_orf1	hemicentin-1-like isoform X2 [Ostrinia furnacalis]	-0.85876	-1.43353	1.246326	0.2915	0.754466
TRINITY_DN8625_c0_g1_i1_orf1	GDP-L-fucose synthase [Ostrinia furnacalis]	-0.91427	-1.18601	0.270826	1.625606	0.203845
TRINITY_DN21545_c0_g1_i2_orf1	sterile alpha and TIR motif-containing protein 1 isoform X1 [Ostrinia furnacalis]	-1.02179	-1.37428	0.665417	0.613389	1.117264
TRINITY_DN2529_c0_g1_i3_orf1	collagen alpha-1(X) chain-like [Ostrinia furnacalis]	-1.009	-1.29378	0.689293	0.291356	1.322132
TRINITY_DN11736_c0_g1_i1_orf1	uncharacterized protein LOC114352195 [Ostrinia furnacalis]	-1.0071	-1.40635	0.941659	0.59788	0.873918
TRINITY_DN52649_c0_g1_i6_orf1	twinfilin [Ostrinia furnacalis]	-0.67532	-1.52276	0.114176	0.899161	1.184743
TRINITY_DN59852_c0_g1_i1_orf1	hypothetical protein evm_008421 [Chilo suppressalis]	-0.9096	-1.3694	1.19575	0.166897	0.916351
TRINITY_DN5933_c0_g1_i1_orf1	peroxidase-like [Ostrinia furnacalis]	-1.51058	-0.85357	0.526239	0.810229	1.02769
TRINITY_DN8716_c0_g1_i3_orf1	aspartate--tRNA ligase, cytoplasmic isoform X1 [Ostrinia furnacalis] >XP_028161705.1 aspartate--tRNA ligase, cytoplasmic isoform X2 [Ostrinia furnacalis] >XP_028161706.1 aspartate--tRNA ligase, cytoplasmic isoform X1 [Ostrinia furnacalis] >XP_028161707.1 aspartate--tRNA ligase, cytoplasmic isoform X1 [Ostrinia furnacalis]	-0.85833	-0.99546	0.878019	-0.51843	1.494202
TRINITY_DN2844_c0_g1_i2_orf1	glutathione S-transferase 9 [Streltzoviella insularis]	-1.07449	-1.05596	0.851227	-0.13098	1.410206
TRINITY_DN741_c0_g1_i10_orf1	talin-1 isoform X13 [Ostrinia furnacalis]	-1.28239	-1.07578	0.9068	0.324036	1.127335
TRINITY_DN13711_c0_g1_i1_orf1	putative nuclease HARBI1 [Myzus persicae]	-1.18947	-1.12016	0.27853	1.323892	0.707206
TRINITY_DN63152_c0_g1_i7_orf1	microtubule-actin cross-linking factor 1 isoform X21 [Manduca sexta]	-1.21136	-1.12854	0.697413	0.360951	1.281536
TRINITY_DN60787_c0_g1_i5_orf1	probable transaldolase [Ostrinia furnacalis]	-1.08537	-1.34733	0.718583	0.960017	0.754103
TRINITY_DN147691_c0_g1_i1_orf1	WD repeat-containing protein 46 [Orussus abietinus]	-1.44235	-0.87402	0.506515	0.543423	1.266432
TRINITY_DN135781_c0_g1_i1_orf1	vitamin K epoxide reductase complex subunit 1-like protein 1 [Ostrinia furnacalis] >XP_028171283.1 vitamin K epoxide reductase complex subunit 1-like protein 1 [Ostrinia furnacalis]	-1.23065	-1.07818	0.58931	0.361079	1.358442
TRINITY_DN16824_c0_g1_i7_orf1	insulin receptor substrate 1 [Ostrinia furnacalis]	-0.91917	-1.39709	0.248135	1.073431	0.9947
TRINITY_DN2207_c0_g1_i6_orf1	methionine-R-sulfoxide reductase B1 isoform X2 [Ostrinia furnacalis]	-0.98395	-1.30875	0.548376	1.37063	0.373703
TRINITY_DN23164_c0_g1_i4_orf1	uncharacterized protein LOC114365928 isoform X1 [Ostrinia furnacalis]	-1.53371	-0.79884	0.528057	0.675741	1.128759
TRINITY_DN131603_c0_g1_i4_orfp1	TRINITY_DN131603_c0_g1_i4_m.86149 TRINITY_DN131603_c0_g1::TRINITY_DN131603_c0_g1_i4::g.86149 ORF type:internal len:112 (-),score=8.40 TRINITY_DN131603_c0_g1_i4:2-334(-)	-0.83367	-0.38291	-0.04622	-0.66454	1.927338
TRINITY_DN5126_c0_g2_i1_orf1	cytochrome P450 monooxygenase CYP4L47 [Ostrinia furnacalis]	-0.9471	0.349863	-0.6137	-0.59261	1.803547
TRINITY_DN17409_c0_g1_i5_orf1	integrin beta-6-like [Ostrinia furnacalis]	-0.85868	0.42408	-0.23211	-1.04626	1.712975
TRINITY_DN7291_c0_g1_i5_orf1	dynamin-1-like protein isoform X3 [Ostrinia furnacalis]	-0.75952	0.40715	0.211479	-1.37916	1.520051
TRINITY_DN20960_c0_g1_i1_orf1	aldo-keto reductase AKR2E4-like [Ostrinia furnacalis]	-1.22978	0.349996	0.530746	-1.05481	1.403856
TRINITY_DN4321_c0_g1_i1_orf1	acyl-CoA Delta(11) desaturase isoform X1 [Ostrinia furnacalis]	-0.9743	0.218589	-0.58965	-0.49998	1.845343
TRINITY_DN57749_c0_g1_i4_orf1	LOW QUALITY PROTEIN: DENN domain-containing protein Crag [Ostrinia furnacalis]	-0.43827	-0.17096	0.067081	-1.24998	1.792132
TRINITY_DN2299_c0_g1_i3_orf1	DNA-directed RNA polymerase II subunit RPB1 [Ostrinia furnacalis]	-1.30263	0.007281	0.636988	-0.82853	1.486899
TRINITY_DN56998_c0_g1_i2_orf1	glycerate kinase [Ostrinia furnacalis]	-0.90288	-0.0699	0.063276	-0.9168	1.826305

TRINITY_DN1039_c0_g1_i5_orf1	WD repeat-containing protein 26 [Ostrinia furnacalis]	-0.66535	-0.0399	-0.51453	-0.72185	1.941626
TRINITY_DN124711_c0_g1_i1_orf1	muskelin isoform X1 [Ostrinia furnacalis] >XP_028163274.1 muskelin isoform X2 [Ostrinia furnacalis]	-0.87302	0.591797	-0.67958	-0.73702	1.697819
TRINITY_DN11172_c0_g1_i4_orf1	juvenile hormone epoxide hydrolase-like isoform X1 [Ostrinia furnacalis] >XP_028170522.1 juvenile hormone epoxide hydrolase-like isoform X2 [Ostrinia furnacalis]	-0.90737	0.174469	0.345835	-1.21212	1.599188
TRINITY_DN144342_c0_g1_i1_orfp1	TRINITY_DN144342_c0_g1_i1_m.83164 TRINITY_DN144342_c0_g1::TRINITY_DN144342_c0_g1_i1::g.83164 ORF type:internal len:113 (-),score=2.22 TRINITY_DN144342_c0_g1_i1:1-336(-)	-1.03757	0.618063	-0.42328	-0.80481	1.647594
TRINITY_DN14904_c0_g1_i1_orf1	attacin [Ostrinia furnacalis]	-0.27016	-1.28866	0.175311	-0.37563	1.759132
TRINITY_DN30177_c0_g2_i1_orf1	uncharacterized protein LOC114365032 [Ostrinia furnacalis]	-0.09203	-1.21239	-0.42913	-0.09106	1.824609
TRINITY_DN26209_c0_g1_i6_orf1	uncharacterized protein LOC114352357 [Ostrinia furnacalis]	0.437306	-1.94497	0.204769	0.391515	0.911385
TRINITY_DN5310_c2_g1_i2_orf1	serine protease persephone-like [Ostrinia furnacalis]	0.518665	-1.9061	0.381913	0.030335	0.975187
TRINITY_DN955_c0_g1_i2_orf1	gloverin-like [Ostrinia furnacalis] >XP_028168251.1 gloverin-like [Ostrinia furnacalis] >AYM26645.1 gloverin [Ostrinia furnacalis]	0.625898	-1.52423	0.678908	-0.83897	1.058391
TRINITY_DN24_c0_g1_i1_orf1	hypothetical protein evm_007803 [Chilo suppressalis]	-0.03943	-1.74811	1.041612	-0.16528	0.911205
TRINITY_DN2650_c0_g1_i1_orf1	hypothetical protein HW555_002849 [Spodoptera exigua] >CAH0691914.1 unnamed protein product [Spodoptera exigua]	0.308889	-1.82894	0.04622	0.251462	1.222371
TRINITY_DN16840_c1_g1_i1_orf1	attacin-like [Ostrinia furnacalis]	0.494019	-1.58654	0.446805	-0.6339	1.279606
TRINITY_DN4343_c0_g1_i2_orf1	uncharacterized protein LOC114365231 isoform X3 [Ostrinia furnacalis]	-0.05889	-1.82167	0.807233	0.062088	1.011231
TRINITY_DN2343_c1_g1_i2_orf1	receptor expression-enhancing protein 5-like isoform X1 [Ostrinia furnacalis] >XP_028170586.1 receptor expression-enhancing protein 5-like isoform X1 [Ostrinia furnacalis]	0.225934	-1.09377	1.048108	-1.23858	1.058307
TRINITY_DN52761_c0_g1_i2_orf1	atlastin isoform X4 [Ostrinia furnacalis]	0.120247	-1.71213	0.932201	-0.36569	1.02538
TRINITY_DN2442_c0_g1_i6_orf1	cytochrome P450 6B5-like [Ostrinia furnacalis]	0.029643	-1.82068	0.593827	0.044104	1.153109
TRINITY_DN17505_c0_g1_i15_orf1	unnamed protein product [Chilo suppressalis]	-0.21987	-0.86744	-0.16714	-0.67363	1.92808
TRINITY_DN6908_c0_g1_i3_orf1	serine--pyruvate aminotransferase, mitochondrial [Ostrinia furnacalis] >XP_028157324.1 serine--pyruvate aminotransferase, mitochondrial [Ostrinia furnacalis] >XP_028157325.1 serine--pyruvate aminotransferase, mitochondrial [Ostrinia furnacalis]	0.384422	-1.68447	0.244592	-0.30833	1.363781
TRINITY_DN6698_c0_g2_i2_orf1	protein mesh isoform X1 [Ostrinia furnacalis]	0.144211	-1.86203	0.899725	-0.01985	0.837946
TRINITY_DN31609_c0_g1_i3_orf1	sorbitol dehydrogenase-like [Ostrinia furnacalis]	-0.03778	-1.52644	-0.21742	0.171739	1.609905
TRINITY_DN52761_c0_g2_i1_orf1	atlastin-like isoform X4 [Ostrinia furnacalis]	-0.20443	-1.40859	0.855559	-0.61001	1.367474
TRINITY_DN1116_c0_g1_i6_orf1	RNA exonuclease 4-like [Ostrinia furnacalis] >QEE79882.1 REX4 [Ostrinia furnacalis]	-0.16093	-0.87405	0.269393	-1.00366	1.769248
TRINITY_DN14239_c0_g1_i5_orf1	uncharacterized protein LOC114352770 [Ostrinia furnacalis] >XP_028160292.1 uncharacterized protein LOC114352770 [Ostrinia furnacalis]	-0.08755	-1.34324	-0.39226	0.083152	1.739902
TRINITY_DN1543_c0_g2_i2_orf1	regulation of enolase protein 1-like isoform X3 [Ostrinia furnacalis] >XP_028161334.1 regulation of enolase protein 1-like isoform X3 [Ostrinia furnacalis]	-0.25078	-1.12613	-0.2237	-0.28088	1.881489
TRINITY_DN27833_c0_g2_i1_orf1	uncharacterized protein LOC114359161 [Ostrinia furnacalis]	0.182443	-1.5503	-0.07612	-0.14836	1.592323
TRINITY_DN53281_c0_g1_i11_orf1	larval cuticle protein LCP-17-like [Ostrinia furnacalis]	0.020605	-1.47506	0.750751	-0.65129	1.354986
TRINITY_DN483_c0_g1_i6_orf1	uncharacterized protein LOC114354803 isoform X1 [Ostrinia furnacalis] >XP_028163172.1 uncharacterized protein LOC114354803 isoform X2 [Ostrinia furnacalis]	0.003319	-0.92616	-0.00652	-0.89736	1.826723
TRINITY_DN5174_c0_g3_i1_orf1	protein odr-4 homolog [Ostrinia furnacalis]	-0.58315	-1.10567	-0.31297	0.183617	1.818175
TRINITY_DN701_c1_g1_i4_orf1	venom serine protease Bi-VSP-like [Ostrinia furnacalis]	-0.10694	-0.95593	1.183015	-1.21506	1.094923
TRINITY_DN96801_c0_g1_i1_orf1	histone H4 isoform X2 [Gracilinanus agilis]	-0.13115	-0.94802	0.751636	-1.15256	1.480101
TRINITY_DN51498_c0_g1_i1_orf1	delta-aminolevulinic acid dehydratase isoform X3 [Ostrinia furnacalis]	0.10081	-0.80935	-0.55746	-0.63638	1.90238
TRINITY_DN4064_c0_g2_i1_orf1	disintegrin and metalloproteinase domain-containing protein 12 isoform X1 [Ostrinia furnacalis] >XP_028158112.1 disintegrin and metalloproteinase domain-containing protein 12 isoform X2 [Ostrinia furnacalis]	-0.08453	-1.28582	0.028735	-0.43343	1.775055
TRINITY_DN4246_c0_g2_i3_orf1	interferon-inducible double-stranded RNA-dependent protein kinase activator A homolog isoform X7 [Ostrinia furnacalis]	-0.60505	-0.98451	-0.54243	0.325133	1.806853
TRINITY_DN51995_c0_g3_i1_orf1	circadian clock-controlled protein-like [Ostrinia furnacalis]	-0.82341	-1.03004	-0.20087	1.772327	0.281994
TRINITY_DN18568_c0_g1_i2_orfp1	TRINITY_DN18568_c0_g1_i2_m.13844 TRINITY_DN18568_c0_g1::TRINITY_DN18568_c0_g1_i2::g.13844 ORF type:5prime_partial len:77 (+),score=12.07 TRINITY_DN18568_c0_g1_i2:1-231(+)	-0.79077	-0.88007	-0.76896	1.3474	1.092409
TRINITY_DN4204_c0_g1_i1_orf1	uncharacterized protein LOC114359352 [Ostrinia furnacalis]	-1.22593	-0.63832	-0.47051	1.434496	0.900266
TRINITY_DN1091_c0_g3_i1_orf1	macrophage mannose receptor 1-like [Ostrinia furnacalis]	-0.67679	-1.21836	-0.46129	0.993277	1.363153
TRINITY_DN975_c0_g1_i1_orf1	elongation factor 1-alpha 1 [Myotis lucifugus] >XP_008139741.1 elongation factor 1-alpha 1 [Eptesicus fuscus]	-0.73691	-1.32635	-0.23454	1.186431	1.111367

TRINITY_DN394_c0_g1_i4_orf1	uncharacterized protein LOC114351483 [Ostrinia furnacalis]	-1.15525	-1.15279	0.159437	1.03687	1.111733
TRINITY_DN3593_c0_g1_i3_orfp1	TRINITY_DN3593_c0_g1_i3_m.43968 TRINITY_DN3593_c0_g1::TRINITY_DN3593_c0_g1_i3::g.43968 ORF type:5prime_partial len:72 (-),score=1.41 TRINITY_DN3593_c0_g1_i3:138-353(-)	-1.21365	-0.48145	0.375085	-0.40849	1.728501
TRINITY_DN27247_c0_g2_i1_orfp1	TRINITY_DN27247_c0_g2_i1_m.23157 TRINITY_DN27247_c0_g2::TRINITY_DN27247_c0_g2_i1::g.23157 ORF type:5prime_partial len:70 (+),score=25.00 TRINITY_DN27247_c0_g2_i1:2-211(+)	-0.94519	-0.82413	-0.45624	0.503376	1.722177
TRINITY_DN1534_c0_g1_i3_orf1	peptidoglycan recognition protein-like [Ostrinia furnacalis]	-0.625	-1.05963	-0.47141	1.763612	0.392431
TRINITY_DN22589_c0_g1_i6_orfp1	TRINITY_DN22589_c0_g1_i6_m.19386 TRINITY_DN22589_c0_g1::TRINITY_DN22589_c0_g1_i6::g.19386 ORF type:internal len:183 (+),score=63.52 TRINITY_DN22589_c0_g1_i6:2-547(+)	-1.08912	-0.55133	-0.74549	0.960912	1.425037
TRINITY_DN1305_c0_g1_i6_orf1	glutathione S-transferase sigma 3 [Ostrinia furnacalis]	-0.75442	-0.93524	-0.75442	1.232884	1.211203
TRINITY_DN57900_c0_g1_i2_orf1	hypothetical protein SFRURICE_000634 [Spodoptera frugiperda]	-1.40804	-0.73928	0.389241	0.256963	1.501117
TRINITY_DN5001_c0_g1_i4_orf1	uncharacterized protein LOC114356665 [Ostrinia furnacalis]	-1.21607	-1.03544	0.051976	0.982825	1.216711
TRINITY_DN2207_c0_g1_i4_orf1	methionine-R-sulfoxide reductase B1 isoform X4 [Pectinophora gossypiella] >XP_049887601.1 methionine-R-sulfoxide reductase B1 isoform X4 [Pectinophora gossypiella]	-1.45256	-0.69024	0.35132	0.310294	1.48119
TRINITY_DN4242_c0_g1_i6_orf1	fibrohexamerin-like [Ostrinia furnacalis]	-1.55692	-0.53062	-0.05134	1.116429	1.022449
TRINITY_DN9991_c0_g1_i4_orf1	unnamed protein product [Parnassius apollo]	-0.99578	-1.13994	-0.12629	0.871552	1.390467
TRINITY_DN143532_c0_g1_i1_orf1	3-oxoacyl-[acyl-carrier-protein] reductase FabG-like [Aphidius gifuensis] >KAF7996667.1 hypothetical protein HCN44_002313 [Aphidius gifuensis]	-0.89196	-0.76256	-0.6005	1.727413	0.527602
TRINITY_DN4695_c0_g1_i3_orf1	glutathione S-transferase epsilon 3 [Ostrinia furnacalis]	-1.57135	-0.4334	-0.14292	1.164297	0.983368
TRINITY_DN68770_c0_g1_i1_orf1	seroin transcript 1A2 [Ostrinia nubilalis]	-1.25709	-0.72404	-0.17138	1.600414	0.552091
TRINITY_DN76307_c0_g1_i1_orf1	PREDICTED: quinone oxidoreductase-like protein 2 homolog [Microplitis demolitor]	-0.57035	-1.1726	-0.56289	0.750176	1.555666
TRINITY_DN1540_c0_g1_i9_orf1	alaserpin-like isoform X9 [Ostrinia furnacalis]	-0.85235	-1.06346	-0.46569	0.978744	1.40276
TRINITY_DN5191_c0_g2_i1_orf1	CD151 antigen-like [Ostrinia furnacalis]	-1.4923	-0.74699	0.144529	1.055602	1.039163
TRINITY_DN15000_c0_g1_i4_orf1	15-hydroxyprostaglandin dehydrogenase [NAD(+)]-like [Ostrinia furnacalis]	-1.26075	0.026352	-0.3524	-0.21325	1.800044
TRINITY_DN2848_c0_g1_i1_orf1	glyceraldehyde-3-phosphate dehydrogenase isoform 2 [Mus musculus] >XP_036021733.1 glyceraldehyde-3-phosphate dehydrogenase isoform X1 [Mus musculus] >P16858.2 RecName: Full=Glyceraldehyde-3-phosphate dehydrogenase; Short=GAPDH; AltName: Full=Peptidyl-cysteine S-nitrosylase GAPDH [Mus musculus] >6LGJ_A Crystal structure of an oxido-reductase [Mus musculus] >6LGJ_B Crystal structure of an oxido-reductase [Mus musculus] >6LGJ_C Crystal structure of an oxido-reductase [Mus musculus] >6LGJ_D Crystal structure of an oxido-reductase [Mus musculus] >AAA37659.1 glyceraldehyde-3-phosphate dehydrogenase [Mus musculus] >AAH82592.1 Glyceraldehyde-3-phosphate dehydrogenase [Mus musculus] >AAH83065.1 Glyceraldehyde-3-phosphate dehydrogenase [Mus musculus] >AAH83079.1 Glyceraldehyde-3-phosphate dehydrogenase [Mus musculus] >AAH83080.1 Glyceraldehyde-3-phosphate dehydrogenase [Mus musculus]	-0.73115	-1.06326	-0.1285	1.818655	0.10426
TRINITY_DN7064_c0_g1_i20_orfp1	TRINITY_DN7064_c0_g1_i20_m.53649 TRINITY_DN7064_c0_g1::TRINITY_DN7064_c0_g1_i20::g.53649 ORF type:internal len:256 (+),score=27.03,Kazal_1 PF00050.22 0.52,Kazal_1|PF00050.22|1.1e-10,Kazal_1|PF00050.22|6.2e-09,Kazal_1|PF00050.22|2.7e-07,Kazal_1|PF00050.22|5.1e-09,Kazal_1|PF00050.22|4.5e-11,Kazal_2|PF07648.16|2.2,Kazal_2|PF07648.16|4.8e-10,Kazal_2|PF07648.16|4.2e-10,Kazal_2|PF07648.16|1.1e-09,Kazal_2|PF07648.16|3e-10,Kazal_2|PF07648.16|1.3e-07 TRINITY_DN7064_c0_g1_i20:3-767(+)	-1.16027	-0.90264	-0.14779	1.53753	0.673176
TRINITY_DN18782_c0_g1_i4_orf1	putative riboflavin kinase [Ostrinia furnacalis] >XP_028176654.1 putative riboflavin kinase [Ostrinia furnacalis]	-1.49628	-0.60624	-0.07146	0.974214	1.199761
TRINITY_DN1344_c0_g1_i1_orf1	ribosomal RNA small subunit methyltransferase NEP1 [Ostrinia furnacalis]	-1.61167	-0.65594	0.374775	1.088523	0.804315
TRINITY_DN6415_c0_g1_i1_orf1	D-arabinitol dehydrogenase 1-like [Ostrinia furnacalis]	-1.5894	-0.4837	0.079414	0.646029	1.347657
TRINITY_DN50743_c0_g1_i1_orf1	cytochrome P450 monooxygenase CYP321F7 [Ostrinia furnacalis]	-0.9464	-1.01594	-0.43188	1.098359	1.295868
TRINITY_DN11649_c0_g1_i4_orf1	ubiquitin carboxyl-terminal hydrolase 32-like, partial [Ostrinia furnacalis]	-1.17132	-0.42884	-0.59177	0.507921	1.684017
TRINITY_DN11172_c1_g1_i1_orf1	juvenile hormone epoxide hydrolase-like isoform X1 [Ostrinia furnacalis] >XP_028170522.1 juvenile hormone epoxide hydrolase-like isoform X2 [Ostrinia furnacalis]	-0.8348	-1.14206	-0.10613	1.681075	0.401911
TRINITY_DN829_c0_g1_i8_orf1	cytochrome P450 6B6-like [Ostrinia furnacalis]	-1.46932	-0.68689	0.368453	0.330195	1.457563
TRINITY_DN5692_c0_g1_i4_orf1	E3 ubiquitin-protein ligase MARCH6 [Ostrinia furnacalis]	-1.50656	-0.6626	0.278398	0.48373	1.407033

TRINITY_DN10070_c0_g1_i1_orf1	40S ribosomal protein SA isoform 1 [Homo sapiens] >XP_002813955.1 40S ribosomal protein SA [Pongo abelii] >XP_004033937.1 40S ribosomal protein SA [Gorilla gorilla gorilla] >XP_008949773.1 40S ribosomal protein SA [Pan paniscus] >XP_009237465.1 40S ribosomal protein SA [Pongo abelii] >XP_024211184.1 40S ribosomal protein SA [Pan troglodytes] >XP_032017897.1 40S ribosomal protein SA [Hylobates moloch] >XP_032017898.1 40S ribosomal protein SA [Hylobates moloch] >XP_032615302.1 40S ribosomal protein SA [Hylobates moloch] >XP_034820112.1 40S ribosomal protein SA [Pan paniscus] >P08865.4 RecName: Full=40S ribosomal protein SA; AltName: Full=37 kDa laminin receptor precursor; Short=37LRP; AltName: Full=37/67 kDa laminin receptor; Short=LRP/LR; AltName: Full=67 kDa laminin receptor; Short=67LR; AltName: Full=Colon carcinoma laminin-binding protein; AltName: Full=Laminin receptor 1; Short=LamR; AltName: Full=Laminin-binding protein precursor p40; Short=LBP/p40; AltName: Full=Multidrug resistance-associated protein MGr1-Ag; AltName: Full=NEM/1CHD4; AltName: Full=Small ribosomal subunit protein uS2 [Homo sapiens] >4D5L_A Cryo-EM structures of ribosomal 80S complexes with termination factors and cricket paralysis virus IRES reveal the IRES in the translocated state [Oryctolagus cuniculus] >4D61_A Cryo-EM structures of ribosomal 80S complexes with termination factors and cricket paralysis virus IRES reveal the IRES in the translocated state [Oryctolagus cuniculus] >4UG0_SA Chain SA, 40S RIBOSOMAL PROTEIN SA [Homo sapiens] >4UJD_CA Chain CA, 40S RIBOSOMAL PROTEIN US2 [Oryctolagus cuniculus] >4UJE_BA Chain BA, 40S RIBOSOMAL PROTEIN SA [Oryctolagus cuniculus] >4V6X_AA Chain AA, 40S ribosomal protein SA [Homo sapiens] >5A2Q_A Structure of the HCV IRES bound to the human ribosome [Homo sapiens] >5AJ0_BA Chain BA, 40S ribosomal protein SA [Homo sapiens] >5FLX_A Mammalian 40S HCV-IRES complex [Oryctolagus cuniculus] >5LKS_SA Chain SA, 40S ribosomal protein SA [Homo sapiens] >5OA3_A Human 40S-eIF2D-re-initiation complex [Homo sapiens] >5T2C_Ao Chain Ao, 40S ribosomal protein SA [Homo sapiens] >5VYC_A1 Chain A1, 40S ribosomal protein SA [Homo sapiens] >5VYC_A2 Chain A2, 40S ribosomal protein SA [Homo sapiens] >5VYC_A3 Chain A3, 40S ribosomal protein SA [Homo sapiens] >5VYC_A4 Chain A4, 40S ribosomal protein SA [Homo sapiens] >5VYC_A5 Chain A5, 40S ribosomal protein SA [Homo sapiens] >5VYC_A6 Chain A6, 40S ribosomal protein SA [Homo sapiens] >6EK0_SA Chain SA, 40S ribosomal protein SA [Homo sapiens] >6G18_A Cryo-EM structure of a late human pre-40S ribosomal subunit - State C [Homo sapiens] >6G4S_A Cryo-EM structure of a late human pre-40S ribosomal subunit - State B [Homo sapiens] >6G51_A Cryo-EM structure of a late human pre-40S ribosomal subunit - State D [Homo sapiens] >6G53_A Cryo-EM structure of a late human pre-40S ribosomal subunit - State E [Homo sapiens] >6G5H_A Cryo-EM structure of a late human pre-40S ribosomal subunit - Mature [Homo sapiens] >6G5I_A Cryo-EM structure of a late human pre-40S ribosomal subunit - State R [Homo sapiens] >6IP5_2n Chain 2n, 40S ribosomal protein SA	-1.37402	-0.9218	0.294837	1.294999	0.705982
TRINITY_DN120500_c0_g1_i1_orf1	cytochrome P450 6B5-like [Ostrinia furnacalis]	-1.48616	-0.10807	0.080667	-0.14537	1.658922
TRINITY_DN3298_c0_g2_i4_orf1	macrophage mannose receptor 1-like [Ostrinia furnacalis]	-1.38678	-0.51335	0.278936	-0.03243	1.653621
TRINITY_DN5149_c0_g1_i14_orfp1	TRINITY_DN5149_c0_g1_i14_m.8804 TRINITY_DN5149_c0_g1::TRINITY_DN5149_c0_g1_i14::g.8804 ORF type:internal len:88 (+),score=31.42 TRINITY_DN5149_c0_g1_i14:2-262(+)	-1.34815	-0.55624	0.065102	0.152383	1.686902
TRINITY_DN5126_c0_g1_i3_orf1	cytochrome P450 monooxygenase CYP4L47 [Ostrinia furnacalis]	-1.15573	-0.89636	-0.28268	1.333742	1.001028
TRINITY_DN436_c0_g2_i5_orfp1	TRINITY_DN436_c0_g2_i5_m.4776 TRINITY_DN436_c0_g2::TRINITY_DN436_c0_g2_i5::g.4776 ORF type:complete len:158 (-),score=33.76 TRINITY_DN436_c0_g2_i5:320-772(-)	-1.14729	-0.04698	-0.41424	-0.24842	1.856936
TRINITY_DN12392_c0_g1_i3_orf1	protein KIAA0100 [Pectinophora gossypiella]	-1.11387	-1.08552	-0.07038	1.129868	1.139906
TRINITY_DN4004_c0_g1_i1_orf1	protein FAM114A2 isoform X1 [Ostrinia furnacalis] >XP_028175160.1 protein FAM114A2 isoform X2 [Ostrinia	-1.20465	-0.76096	-0.37966	0.978379	1.366892
TRINITY_DN171_c0_g1_i1_orf1	alkaline phosphatase, tissue-nonspecific isozyme-like isoform X1 [Ostrinia furnacalis]	-1.33739	-0.63134	-0.3003	0.86213	1.406897
TRINITY_DN54612_c0_g1_i3_orf1	hydroxyacid oxidase 1 isoform X1 [Ostrinia furnacalis]	-1.00289	-0.73473	0.413563	-0.43497	1.759022
TRINITY_DN33763_c0_g1_i1_orf1	uncharacterized protein LOC114355186 [Ostrinia furnacalis]	-1.29024	-0.69067	-0.34206	1.014558	1.308409
TRINITY_DN9079_c1_g1_i1_orf1	UDP-glucuronosyltransferase 2B15-like [Ostrinia furnacalis]	-1.22688	-0.29901	-0.18789	-0.11818	1.831963
TRINITY_DN20710_c0_g2_i2_orf1	acyl-CoA:lysophosphatidylglycerol acyltransferase 1-like isoform X2 [Manduca sexta]	-1.44296	-0.83042	0.195505	1.163936	0.913942
TRINITY_DN11388_c0_g1_i4_orf1	limbic system-associated membrane protein-like, partial [Ostrinia furnacalis]	-1.71148	-0.49329	0.535234	1.106193	0.563346
TRINITY_DN72017_c0_g1_i1_orf1	probable peroxisomal acyl-coenzyme A oxidase 1 isoform X1 [Ostrinia furnacalis] >XP_028165840.1 probable peroxisomal acyl-coenzyme A oxidase 1 isoform X2 [Ostrinia furnacalis]	-1.51124	-0.27651	-0.33829	1.427166	0.698883
TRINITY_DN8310_c0_g2_i1_orf1	uncharacterized protein LOC116773294 [Danaus plexippus plexippus] >OWR55545.1 hypothetical protein KGM_209260 [Danaus plexippus plexippus]	-1.73036	-0.44723	0.369109	1.035206	0.773276
TRINITY_DN55154_c0_g2_i1_orf1	glycosyl transferase family 8 domain-containing protein [Phthorimaea operculella]	-1.19037	-0.41069	-0.68381	1.552577	0.732294
TRINITY_DN2098_c0_g1_i1_orf1	dolichyl pyrophosphate Man9GlcNAc2 alpha-1,3-glucosyltransferase [Ostrinia furnacalis]	-1.75394	-0.21608	0.095448	0.701846	1.17273
TRINITY_DN6698_c0_g2_i1_orf1	protein mesh isoform X2 [Ostrinia furnacalis]	-1.60126	-0.41353	0.526146	0.080964	1.407684

TRINITY_DN19293_c0_g1_i4_orf1	carboxylesterase [Ostrinia furnacalis]	-1.29785	-0.13455	-0.33785	-0.01388	1.784135
TRINITY_DN37055_c0_g1_i1_orf1	ras GTPase-activating protein-binding protein 2-like, partial [Ostrinia furnacalis]	-1.14342	-0.99156	-0.1755	1.22394	1.08654
TRINITY_DN95530_c0_g1_i1_orf1	aldose reductase-like isoform X4 [Ostrinia furnacalis]	-0.94674	-0.64385	-0.66185	0.528581	1.723861
TRINITY_DN4916_c0_g2_i1_orf1	uncharacterized protein LOC114357135, partial [Ostrinia furnacalis]	-1.3203	-0.61675	-0.33661	0.838192	1.435465
TRINITY_DN133474_c0_g2_i2_orf1	enoyl-[acyl-carrier-protein] reductase, mitochondrial [Ostrinia furnacalis]	-1.22298	-0.30136	-0.79708	1.365225	0.956199
TRINITY_DN2350_c0_g1_i6_orf1	protein yellow-like isoform X2 [Ostrinia furnacalis]	-1.7126	-0.12269	0.08485	0.368883	1.381553
TRINITY_DN565_c0_g2_i1_orf1	uncharacterized protein LOC114362323 [Ostrinia furnacalis]	-1.60276	0.064777	-0.08893	0.073316	1.553599
TRINITY_DN3110_c0_g1_i4_orf1	hypothetical protein B5X24_HaOG214278 [Helicoverpa armigera]	-1.36649	-0.37672	-0.32093	0.41667	1.647474
TRINITY_DN2735_c0_g1_i4_orf1	hypothetical protein evm_008546 [Chilo suppressalis] >CAH0684158.1 unnamed protein product [Chilo	-1.5872	-0.4965	0.085828	0.658952	1.338917
TRINITY_DN11823_c1_g1_i2_orf1	LOW QUALITY PROTEIN: uncharacterized protein LOC114362902 [Ostrinia furnacalis]	-1.77175	-0.45065	0.704212	0.827806	0.690382
TRINITY_DN53238_c1_g1_i5_orf1	isocitrate dehydrogenase [NADP] cytoplasmic [Ostrinia furnacalis] >XP_028176272.1 isocitrate dehydrogenase [NADP] cytoplasmic [Ostrinia furnacalis]	-1.46204	-0.65195	0.298338	0.316173	1.499476
TRINITY_DN62_c1_g1_i3_orf1	D-2-hydroxyglutarate dehydrogenase, mitochondrial-like [Ostrinia furnacalis]	-1.64243	-0.43008	0.043118	1.182622	0.846764
TRINITY_DN51776_c0_g2_i1_orf1	cuticle protein CP14.6-like [Ostrinia furnacalis]	-1.55363	-0.23907	-0.26316	0.611667	1.44419
TRINITY_DN12748_c2_g1_i1_orfp1	TRINITY_DN12748_c2_g1_i1_m.21305 TRINITY_DN12748_c2_g1::TRINITY_DN12748_c2_g1_i1::g.21305 ORF type:3prime_partial len:887 (+),score=-6.30 TRINITY_DN12748_c2_g1_i1:104-2761(+)	-1.7369	0.095035	-0.24426	0.682548	1.20358
TRINITY_DN80547_c0_g1_i5_orf1	EKC/KEOPS complex subunit Tprkb-like [Ostrinia furnacalis]	-1.35769	-0.65509	0.236016	0.148985	1.627774
TRINITY_DN5405_c1_g1_i13_orf1	acyl-CoA synthetase family member 3, mitochondrial [Ostrinia furnacalis]	-1.70145	-0.17656	0.346126	0.141131	1.39075
TRINITY_DN17907_c0_g1_i13_orf1	androgen-induced gene 1 protein-like isoform X1 [Galleria mellonella]	-1.57172	-0.36785	-0.20027	0.88992	1.249923
TRINITY_DN616_c1_g1_i6_orf1	esterase B1-like isoform X1 [Ostrinia furnacalis] >XP_028178578.1 esterase B1-like isoform X2 [Ostrinia furnacalis]	-1.6577	-0.37944	-0.00642	1.121661	0.921899
TRINITY_DN18222_c0_g1_i5_orf1	phosphoglycerate kinase [Maniola hyperantus]	-1.54955	-0.53295	0.150848	1.427593	0.504061
TRINITY_DN11245_c0_g1_i2_orf1	ITG-like peptide [Ostrinia furnacalis]	-1.56027	-0.67577	0.215174	1.112061	0.908803
TRINITY_DN9904_c0_g1_i1_orf1	unnamed protein product [Chrysodeixis includens]	-1.13919	-0.57295	-0.2203	1.820118	0.112317
TRINITY_DN5564_c0_g1_i5_orf1	probable phosphoserine aminotransferase [Ostrinia furnacalis]	-1.40218	-0.24542	-0.23941	1.697138	0.189881
TRINITY_DN109503_c0_g1_i4_orf1	uncharacterized protein LOC114366345 isoform X2 [Ostrinia furnacalis]	-0.84885	-0.10967	-0.9471	1.834493	0.07113
TRINITY_DN30713_c0_g1_i3_orf1	phosphoglucomutase [Ostrinia furnacalis]	-1.24858	-0.37855	-0.18711	1.806288	0.007955
TRINITY_DN2647_c0_g1_i3_orf1	DNA repair protein complementing XP-G cells homolog isoform X1 [Ostrinia furnacalis]	-1.64152	-0.04372	-0.3888	1.009357	1.064679
TRINITY_DN13157_c0_g1_i1_orf1	TBC1 domain family member 23 [Ostrinia furnacalis]	-1.63206	0.161332	-0.05945	0.011396	1.51878
TRINITY_DN9406_c0_g1_i5_orf1	proton-coupled amino acid transporter-like protein pathetic [Ostrinia furnacalis]	-1.57797	-0.36088	-0.22039	1.076513	1.082735
TRINITY_DN4866_c0_g1_i2_orf1	actin-binding LIM protein 3 isoform X6 [Ostrinia furnacalis]	-1.77723	-0.13168	0.013207	1.06426	0.831444
TRINITY_DN2338_c3_g2_i3_orf1	charged multivesicular body protein 4b isoform X1 [Ostrinia furnacalis] >XP_028161607.1 charged multivesicular body protein 4b isoform X3 [Ostrinia furnacalis]	-1.8988	0.344506	0.028022	0.528275	0.998
TRINITY_DN4053_c0_g1_i5_orf1	uncharacterized protein LOC114358355 [Ostrinia furnacalis]	-1.76757	-0.03098	0.418659	0.078625	1.301262
TRINITY_DN1084_c0_g2_i2_orf1	ATP-citrate synthase [Ostrinia furnacalis]	-1.83091	0.080427	-0.05126	0.989017	0.812726
TRINITY_DN128_c0_g1_i5_orf1	PREDICTED: muscle-specific protein 20-like [Amyelois transitella]	-1.86272	0.141848	-0.01658	0.884544	0.852913
TRINITY_DN99673_c0_g1_i1_orf1	PREDICTED: pistil-specific extensin-like protein isoform X2 [Microplitis demolitor]	-1.66274	-0.07344	-0.01469	0.285129	1.465742
TRINITY_DN50074_c0_g1_i1_orf1	uncharacterized protein LOC114364628 [Ostrinia furnacalis]	-1.66638	-0.45509	0.375023	1.291779	0.454672
TRINITY_DN86127_c1_g1_i2_orfp1	TRINITY_DN86127_c1_g1_i2_m.43062 TRINITY_DN86127_c1_g1::TRINITY_DN86127_c1_g1_i2::g.43062 ORF type:internal len:69 (-),score=14.03 TRINITY_DN86127_c1_g1_i2:2-205(-)	-1.67417	0.266863	-0.51918	0.982193	0.944293
TRINITY_DN1215_c0_g1_i2_orf1	PI-stichotoxin-She2a-like [Ostrinia furnacalis]	-1.87831	0.240445	-0.03184	0.736923	0.932783
TRINITY_DN27300_c0_g1_i1_orfp1	TRINITY_DN27300_c0_g1_i1_m.71142 TRINITY_DN27300_c0_g1::TRINITY_DN27300_c0_g1_i1::g.71142 ORF type:internal len:82 (-),score=9.46 TRINITY_DN27300_c0_g1_i1:3-245(-)	-0.88268	-1.16404	1.670737	0.231825	0.144158
TRINITY_DN136031_c0_g1_i7_orf1	ferritin, lower subunit isoform X3 [Spodoptera litura]	-0.8095	-0.63137	1.56164	-0.92643	0.805661
TRINITY_DN15545_c0_g1_i1_orf1	larval cuticle protein LCP-14-like [Ostrinia furnacalis]	-1.35118	-1.0717	0.95578	0.599285	0.867817
TRINITY_DN609_c0_g1_i1_orf1	zonadhesin-like isoform X1 [Ostrinia furnacalis]	-1.1348	-1.14528	1.406225	0.580586	0.293272
TRINITY_DN28501_c0_g1_i2_orfp1	TRINITY_DN28501_c0_g1_i2_m.58934 TRINITY_DN28501_c0_g1::TRINITY_DN28501_c0_g1_i2::g.58934 ORF type:internal len:98 (+),score=13.70 TRINITY_DN28501_c0_g1_i2:3-293(+)	-1.27054	-0.9138	1.524181	0.260547	0.399609
TRINITY_DN84938_c0_g1_i4_orf1	vinculin-like isoform X2 [Ostrinia furnacalis]	-0.66253	-1.16778	1.505809	-0.50003	0.824533
TRINITY_DN140_c0_g1_i5_orf1	calcyphosin-like protein isoform X4 [Helicoverpa armigera]	-0.79938	-0.76048	1.310484	-0.88382	1.133202
TRINITY_DN24121_c1_g1_i6_orf1	serine protease persephone-like [Ostrinia furnacalis]	-1.18833	-0.85001	1.230111	-0.31197	1.120203
TRINITY_DN73900_c0_g1_i1_orf1	carbonic anhydrase 7 [Ostrinia furnacalis]	-0.97494	-1.41898	0.77584	1.059021	0.559055

TRINITY_DN15685_c0_g1_i5_orf1	uncharacterized protein LOC114352354 [Ostrinia furnacalis]	-1.2913	-1.12233	1.09574	0.61491	0.702974
TRINITY_DN55147_c0_g1_i1_orfp1	TRINITY_DN55147_c0_g1_i1_m.59251 TRINITY_DN55147_c0_g1::TRINITY_DN55147_c0_g1_i1::g.59251 ORF type:5prime_partial len:331 (-),score=113.97,Cuticle_3 PF11018.9 0.29,Cuticle_3|PF11018.9|2.9e-05,Cuticle_3|PF11018.9|0.00037 TRINITY_DN55147_c0_g1_i1:21-1013(-)	-1.39657	-1.02859	0.95132	0.721535	0.752307
TRINITY_DN9400_c0_g1_i8_orf1	PREDICTED: monoacylglycerol lipase ABHD12-like [Amyelois transitella]	-0.90574	-1.42476	0.800056	1.176609	0.353836
TRINITY_DN1593_c0_g1_i1_orf1	chemosensory protein csp11 [Helopeltis theivora]	-1.08597	-1.25187	1.314706	0.539952	0.48319
TRINITY_DN12586_c0_g1_i4_orf1	zonadhesin-like isoform X4 [Ostrinia furnacalis]	-1.25954	-1.11446	1.076182	0.941499	0.356314
TRINITY_DN24789_c0_g1_i9_orfp1	TRINITY_DN24789_c0_g1_i9_m.25888 TRINITY_DN24789_c0_g1::TRINITY_DN24789_c0_g1_i9::g.25888 ORF type:internal len:114 (-),score=12.65 TRINITY_DN24789_c0_g1_i9:2-340(-)	-1.02403	-1.01474	1.690654	0.211176	0.136942
TRINITY_DN15327_c2_g1_i2_orf1	protein lethal(2)essential for life-like [Ostrinia furnacalis]	-1.48605	-0.90702	0.646147	0.98654	0.760387
TRINITY_DN12336_c0_g1_i1_orfp1	TRINITY_DN12336_c0_g1_i1_m.30792 TRINITY_DN12336_c0_g1::TRINITY_DN12336_c0_g1_i1::g.30792 ORF type:internal len:86 (-),score=13.56 TRINITY_DN12336_c0_g1_i1:3-257(-)	-1.23094	-0.81597	1.072274	-0.28543	1.260059
TRINITY_DN33885_c0_g1_i1_orf1	inhibitor of nuclear factor kappa-B kinase subunit alpha [Ostrinia furnacalis]	-1.05714	-1.3368	1.10794	0.786172	0.499826
TRINITY_DN8095_c0_g1_i3_orf1	circadian clock-controlled protein-like [Ostrinia furnacalis]	-1.35996	-1.07446	0.890695	0.846609	0.697112
TRINITY_DN452_c0_g1_i4_orf1	protein CREG1 [Ostrinia furnacalis] >XP_028170592.1 protein CREG1 [Ostrinia furnacalis]	-0.39485	-1.33444	1.409919	-0.55603	0.875401
TRINITY_DN31225_c0_g1_i1_orf1	ribosome biogenesis protein BMS1 homolog [Ostrinia furnacalis]	-1.33417	-0.90148	1.135782	0.043744	1.056126
TRINITY_DN22513_c0_g1_i4_orf1	DNA-directed RNA polymerase II subunit RPB1 [Ostrinia furnacalis] >XP_028179194.1 DNA-directed RNA polymerase II subunit RPB1 [Ostrinia furnacalis] >XP_028179195.1 DNA-directed RNA polymerase II subunit RPB1	-1.45463	-0.95731	0.868044	0.666332	0.877562
TRINITY_DN29698_c0_g1_i3_orf1	15-hydroxyprostaglandin dehydrogenase [NAD(+)]-like [Ostrinia furnacalis]	-1.08779	-1.31424	1.133568	0.632146	0.636317
TRINITY_DN9694_c0_g1_i1_orf1	larval cuticle protein LCP-17 [Helicoverpa armigera] >PZC82071.1 hypothetical protein B5X24_HaOG211161 [Helicoverpa armigera] >PZC87412.1 hypothetical protein B5X24_HaOG216859 [Helicoverpa armigera]	-1.51807	-0.86956	0.730231	0.70097	0.956424
TRINITY_DN3486_c0_g1_i5_orf1	uncharacterized protein LOC114360519 [Ostrinia furnacalis]	-0.91356	-0.94961	1.282854	-0.56117	1.141494
TRINITY_DN1175_c1_g1_i2_orf1	methanethiol oxidase [Ostrinia furnacalis]	-1.04706	-1.33291	0.939056	1.042237	0.398673
TRINITY_DN14754_c0_g1_i6_orf1	cathepsin L [Papilio xuthus]	-1.11172	-0.81321	1.681249	-0.22922	0.472896
TRINITY_DN64719_c0_g1_i2_orfp1	TRINITY_DN64719_c0_g1_i2_m.37745 TRINITY_DN64719_c0_g1::TRINITY_DN64719_c0_g1_i2::g.37745 ORF type:internal len:91 (+),score=41.89 TRINITY_DN64719_c0_g1_i2:1-270(+)	-1.29225	-0.81997	1.577519	0.152843	0.381857
TRINITY_DN38230_c0_g1_i4_orf1	hypothetical protein evm_007803 [Chilo suppressalis]	-1.40544	-0.78664	1.408577	0.152044	0.631457
TRINITY_DN1038_c1_g1_i3_orf1	uncharacterized protein LOC114363583 [Ostrinia furnacalis]	-1.3507	-1.03052	1.105943	0.440957	0.834319
TRINITY_DN230_c2_g1_i5_orf1	6-pyruvoyl tetrahydrobiopterin synthase [Ostrinia furnacalis]	-0.84355	-1.0854	1.673148	-0.24505	0.500851
TRINITY_DN13236_c0_g1_i4_orf1	zonadhesin-like isoform X4 [Ostrinia furnacalis]	-1.31082	-0.9254	1.450197	0.311184	0.47484
TRINITY_DN1381_c0_g1_i5_orf1	CKLF-like MARVEL transmembrane domain-containing protein 4 isoform X1 [Ostrinia furnacalis]	-1.48066	-0.76328	1.047297	0.14432	1.052325
TRINITY_DN8621_c0_g1_i4_orf1	aminopeptidase N-like isoform X2 [Ostrinia furnacalis]	-0.64701	-1.25027	1.558796	-0.34606	0.684546
TRINITY_DN32586_c0_g2_i1_orf1	unnamed protein product [Euphydryas editha]	-1.61791	-0.71643	0.673435	0.695798	0.965108
TRINITY_DN214_c0_g1_i3_orf1	uncharacterized protein LOC114352813 [Ostrinia furnacalis]	-1.14001	-1.15132	1.369912	0.652647	0.268769
TRINITY_DN4068_c1_g2_i1_orf1	larval cuticle protein LCP-17 [Helicoverpa armigera] >PZC82071.1 hypothetical protein B5X24_HaOG211161 [Helicoverpa armigera] >PZC87412.1 hypothetical protein B5X24_HaOG216859 [Helicoverpa armigera]	-1.34265	-0.96395	1.341508	0.519628	0.445464
TRINITY_DN121802_c0_g1_i6_orfp1	TRINITY_DN121802_c0_g1_i6_m.78506 TRINITY_DN121802_c0_g1::TRINITY_DN121802_c0_g1_i6::g.78506 ORF type:3prime_partial len:123 (+),score=6.04 TRINITY_DN121802_c0_g1_i6:35-367(+)	-0.94365	-1.11741	1.353503	0.977728	-0.27017
TRINITY_DN1329_c0_g1_i5_orf1	neurogenic locus notch homolog protein 3 [Ostrinia furnacalis] >XP_028157678.1 neurogenic locus notch homolog protein 3 [Ostrinia furnacalis]	-1.63229	-0.63474	0.793158	0.40718	1.066692
TRINITY_DN214_c0_g1_i4_orf1	uncharacterized protein LOC114352813 [Ostrinia furnacalis]	-1.24034	-1.00733	1.423602	0.612854	0.211212
TRINITY_DN59422_c0_g1_i2_orf1	larval cuticle protein LCP-22-like isoform X2 [Pectinophora gossypiella]	-1.8106	-0.34815	0.821002	0.542951	0.79479
TRINITY_DN10889_c0_g1_i8_orf1	four and a half LIM domains protein 2 isoform X7 [Pectinophora gossypiella]	-1.67381	-0.61754	0.754315	0.585809	0.951228
TRINITY_DN77318_c0_g2_i1_orf1	uncharacterized protein LOC114351191 [Ostrinia furnacalis]	-0.45072	-1.0233	1.437186	-0.89906	0.935893
TRINITY_DN1455_c0_g1_i4_orf1	troponin domain-containing protein [Phthorimaea operculella]	-1.21203	-1.21648	1.025913	0.614678	0.787922
TRINITY_DN17759_c0_g1_i5_orf1	hypothetical protein evm_013530 [Chilo suppressalis]	-1.07552	-1.29768	1.224507	0.565895	0.582791
TRINITY_DN47151_c0_g1_i1_orf1	unnamed protein product [Danaus chrysippus]	-1.38484	-0.5394	1.038006	-0.36962	1.255858
TRINITY_DN13563_c0_g1_i1_orf1	Golgi resident protein GCP60 isoform X1 [Ostrinia furnacalis]	-0.84293	-0.92777	1.258267	-0.66957	1.182008
TRINITY_DN38568_c0_g1_i1_orf1	unnamed protein product, partial [Diatraea saccharalis]	-1.72797	-0.50811	0.792834	0.514386	0.928856
TRINITY_DN1470_c0_g1_i8_orf1	oxidation resistance protein 1 isoform X5 [Ostrinia furnacalis]	-1.17423	-1.20081	1.234515	0.521211	0.619315
TRINITY_DN5628_c0_g1_i3_orf1	muscle LIM protein Mlp84B isoform X2 [Ostrinia furnacalis]	-1.48296	-0.89299	0.786463	0.548149	1.041344

TRINITY_DN22597_c0_g1_i4_orf1	uncharacterized protein LOC114361588 isoform X16 [Ostrinia furnacalis]	-1.47739	-0.86723	1.115277	0.433369	0.795975
TRINITY_DN39837_c0_g1_i1_orf1	unnamed protein product [Plutella xylostella]	-1.25229	-1.17692	0.909279	0.939877	0.580056
TRINITY_DN928_c0_g2_i1_orf1	fasciclin-2-like [Ostrinia furnacalis]	-1.44215	-0.95071	0.704039	0.626929	1.061898
TRINITY_DN2302_c0_g1_i1_orf1	enoyl-CoA hydratase domain-containing protein 3, mitochondrial [Ostrinia furnacalis]	-0.86378	-1.2374	1.56134	0.006095	0.533752
TRINITY_DN135679_c0_g1_i2_orfp1	TRINITY_DN135679_c0_g1_i2_m.85525 TRINITY_DN135679_c0_g1::TRINITY_DN135679_c0_g1_i2::g.85525 ORF type:5prime_partial len:55 (+),score=5.08,Toxin_2 PF00451.20 1.9e-06 TRINITY_DN135679_c0_g1_i2:3-167(+)	-1.31445	-1.04199	0.613953	1.252961	0.489523
TRINITY_DN3859_c0_g1_i5_orf1	D-aspartate oxidase [Ostrinia furnacalis] >XP_028166452.1 D-aspartate oxidase [Ostrinia furnacalis] >XP_028166454.1 D-aspartate oxidase [Ostrinia furnacalis]	-1.22218	-1.14924	0.960593	1.071179	0.339648
TRINITY_DN5661_c0_g1_i5_orf1	cytochrome P450 6B7-like [Ostrinia furnacalis]	-1.41512	-0.73468	1.261759	-0.04145	0.929483
TRINITY_DN695_c0_g1_i5_orf1	uncharacterized protein LOC114363574 isoform X1 [Ostrinia furnacalis]	-1.24906	-1.18381	0.953219	0.872374	0.607275
TRINITY_DN610_c0_g1_i1_orf1	CAP-Gly domain-containing linker protein 1 isoform X10 [Ostrinia furnacalis]	-1.15759	-1.23202	0.966436	1.02417	0.398999
TRINITY_DN39933_c0_g1_i2_orf1	thioredoxin domain-containing protein 11 isoform X4 [Ostrinia furnacalis]	-1.26484	-1.02996	1.346638	0.669552	0.278611
TRINITY_DN61335_c0_g2_i1_orf1	uncharacterized protein LOC114352149 [Ostrinia furnacalis]	-1.21007	-1.12724	1.32051	0.461543	0.555251
TRINITY_DN56708_c0_g3_i1_orfp1	TRINITY_DN56708_c0_g3_i1_m.56611 TRINITY_DN56708_c0_g3::TRINITY_DN56708_c0_g3_i1::g.56611 ORF type:internal len:69 (-),score=2.50 TRINITY_DN56708_c0_g3_i1:1-204(-)	-1.35329	-0.85903	1.457358	0.273113	0.481854
TRINITY_DN9820_c0_g1_i1_orf1	endocuticle structural glycoprotein SgAbd-2-like [Ostrinia furnacalis]	-1.80382	-0.31938	0.921674	0.409817	0.791708
TRINITY_DN10430_c0_g1_i4_orf1	fatty acid synthase [Ostrinia furnacalis] >XP_028160534.1 fatty acid synthase [Ostrinia furnacalis] >XP_028160535.1 fatty acid synthase [Ostrinia furnacalis] >XP_028160536.1 fatty acid synthase [Ostrinia furnacalis]	-1.32343	-0.74102	0.730929	-0.132	1.465518
TRINITY_DN98995_c0_g1_i2_orf1	hypothetical protein HF086_008399, partial [Spodoptera exigua]	-1.65883	-0.25721	0.966382	-0.15656	1.106216
TRINITY_DN2956_c0_g1_i6_orf1	fructose-bisphosphate aldolase-like isoform X1 [Ostrinia furnacalis] >XP_028178678.1 fructose-bisphosphate aldolase-like isoform X1 [Ostrinia furnacalis]	-1.5902	-0.68403	0.793777	0.367775	1.112672
TRINITY_DN116972_c0_g1_i1_orf1	phosphofructokinase domain-containing protein [Phthorimaea operculella]	-1.31729	-1.08688	1.124522	0.632025	0.647628
TRINITY_DN76633_c0_g1_i1_orfp1	TRINITY_DN76633_c0_g1_i1_m.53394 TRINITY_DN76633_c0_g1::TRINITY_DN76633_c0_g1_i1::g.53394 ORF type:internal len:164 (-),score=26.46,Toxin_2 PF00451.20 0.00022,Toxin_2|PF00451.20|0.00056,Toxin_2|PF00451.20|0.0002,Toxin_2|PF00451.20|5.9e-06,Gamma-thionin|PF00304.21|2,Gamma-thionin|PF00304.21|0.024,Gamma-thionin|PF00304.21|0.027,Gamma-thionin|PF00304.21|0.066,Toxin_38|PF14866.7|0.27,Toxin_38|PF14866.7|0.054,Toxin_38|PF14866.7|0.14,Defensin_2|PF01097.19|1.7,Defensin_2|PF01097.19|0.055,Defensin_2|PF01097.19|1.2,Defensin_2|PF01097.19|0.15 TRINITY_DN76633_c0_g1_i1:1-489(-)	-1.31831	-0.89206	0.971571	1.23382	0.00498
TRINITY_DN2887_c0_g1_i1_orf1	F-box/LRR-repeat protein 4-like isoform X1 [Ostrinia furnacalis] >XP_028177606.1 F-box/LRR-repeat protein 4-like isoform X1 [Ostrinia furnacalis]	-1.31461	-0.73216	1.122779	-0.26196	1.185951
TRINITY_DN60792_c0_g1_i2_orf1	ATP-binding cassette sub-family D member 2 [Ostrinia furnacalis] >XP_028165108.1 ATP-binding cassette sub-family D member 2 [Ostrinia furnacalis]	-1.21134	-0.71242	1.700417	-0.12151	0.344854
TRINITY_DN26993_c1_g1_i8_orf1	endocuticle structural glycoprotein ABD-4-like [Ostrinia furnacalis]	-1.25735	-0.89398	1.483519	0.020832	0.646987
TRINITY_DN1607_c0_g1_i16_orf1	LOW QUALITY PROTEIN: asparagine--tRNA ligase, cytoplasmic [Ostrinia furnacalis]	-1.51342	-0.84261	1.088015	0.711028	0.556988
TRINITY_DN63030_c0_g1_i5_orf1	uncharacterized protein LOC114358571 [Ostrinia furnacalis]	-1.50853	-0.85688	0.887424	0.504088	0.973895
TRINITY_DN9569_c1_g1_i7_orf1	V-set and immunoglobulin domain-containing protein 1-like isoform X1 [Ostrinia furnacalis] >XP_028156015.1 V-set and immunoglobulin domain-containing protein 1-like isoform X2 [Ostrinia furnacalis]	-1.31084	-1.1286	0.852798	0.898092	0.688557
TRINITY_DN1034_c0_g1_i4_orf1	glycerol kinase isoform X4 [Ostrinia furnacalis]	-1.10341	-1.26569	0.881936	1.128863	0.3583
TRINITY_DN2722_c0_g1_i1_orf1	troponin C [Pieris rapae] >XP_045490973.1 troponin C-like isoform X1 [Colias croceus] >XP_049866665.1 troponin C-like [Pectinophora gossypiella]	-1.72559	-0.53593	0.83793	0.612579	0.81101
TRINITY_DN97472_c0_g1_i5_orf1	microtubule-actin cross-linking factor 1 isoform X15 [Ostrinia furnacalis]	-1.39005	-0.97108	1.207862	0.556466	0.596803
TRINITY_DN36460_c0_g1_i2_orf1	N-acetylneuraminate lyase-like [Ostrinia furnacalis]	-1.17242	-1.23813	1.10292	0.551143	0.756488
TRINITY_DN46132_c0_g2_i2_orf1	hypothetical protein evm_003834 [Chilo suppressalis]	-1.29297	-0.87124	1.526984	0.18736	0.449869
TRINITY_DN57111_c0_g1_i1_orf1	trypsin-like serine proteinase T26 protein, partial [Chilo infuscatellus]	-1.58185	-0.54962	1.383755	0.39994	0.347772
TRINITY_DN4793_c0_g1_i7_orf1	probable hydroxyacid-oxoacid transhydrogenase, mitochondrial isoform X3 [Ostrinia furnacalis] >XP_028159821.1 probable hydroxyacid-oxoacid transhydrogenase, mitochondrial isoform X4 [Ostrinia furnacalis]	-1.41631	-0.59746	0.960683	-0.23494	1.288024
TRINITY_DN268_c3_g1_i2_orf1	hypothetical protein evm_003084 [Chilo suppressalis]	-1.11548	-1.30524	1.063201	0.66837	0.689151
TRINITY_DN111488_c0_g1_i1_orf1	LOW QUALITY PROTEIN: formin-J-like [Chelonus insularis]	-1.75115	-0.44276	1.026931	0.552512	0.614468
TRINITY_DN10441_c0_g1_i3_orf1	zonadhesin-like [Ostrinia furnacalis]	-1.45224	-0.78268	1.384125	0.40694	0.443856
TRINITY_DN105506_c0_g1_i8_orf1	microtubule-actin cross-linking factor 1 isoform X15 [Ostrinia furnacalis]	-1.6918	-0.55027	0.766118	0.456121	1.019825

TRINITY_DN3231_c0_g1_i12_orf1	integrin-linked protein kinase [Pectinophora gossypiella]	-1.70213	-0.47606	0.713118	0.348895	1.116178
TRINITY_DN33995_c0_g1_i5_orf1	unnamed protein product [Spodoptera exigua]	-1.06253	-0.42753	1.67758	-0.74812	0.560607
TRINITY_DN62707_c0_g1_i1_orf1	uncharacterized protein LOC114362831 [Ostrinia furnacalis]	-1.03975	-1.15593	1.49791	0.56749	0.130277
TRINITY_DN40434_c0_g1_i2_orf1	deoxyribodipyrimidine photo-lyase [Ostrinia furnacalis]	-1.03602	-0.68309	1.69017	-0.5346	0.563538
TRINITY_DN5768_c0_g1_i2_orf1	adenosylhomocysteinase [Ostrinia furnacalis]	-1.44622	-0.91861	1.082927	0.828328	0.453579
TRINITY_DN124171_c0_g1_i4_orf1	dystonin isoform X27 [Trichoplusia ni]	-1.33478	-1.0174	1.211966	0.39072	0.749493
TRINITY_DN58261_c0_g1_i2_orf1	15-hydroxyprostaglandin dehydrogenase [NAD(+)]-like [Ostrinia furnacalis]	-1.67419	-0.59387	0.618478	0.599724	1.049863
TRINITY_DN1068_c0_g1_i3_orf1	aspartate aminotransferase, cytoplasmic [Ostrinia furnacalis]	-1.42861	-0.98271	0.699665	0.995421	0.716226
TRINITY_DN2175_c0_g1_i4_orf1	uncharacterized protein LOC114353827 [Ostrinia furnacalis]	-1.36488	-0.898	1.339078	0.697344	0.226456
TRINITY_DN120089_c0_g1_i1_orf1	phosphoglucomutase [Ostrinia furnacalis]	-1.54831	-0.81374	0.763767	0.599153	0.999125
TRINITY_DN1475_c0_g1_i6_orf1	uncharacterized protein LOC113226757 isoform X2 [Hyposmocoma kahamanoa]	-1.53829	-0.84623	0.806535	0.684079	0.893913
TRINITY_DN41952_c0_g1_i4_orf1	protein DDI1 homolog 2 [Ostrinia furnacalis]	-1.30348	-0.25921	1.207085	-0.74781	1.103403
TRINITY_DN47930_c0_g1_i4_orf1	uncharacterized protein LOC114362634 [Ostrinia furnacalis]	-1.64292	-0.67598	0.897581	0.592056	0.829266
TRINITY_DN85476_c0_g1_i1_orf1	iron-sulfur protein NUBPL-like [Ostrinia furnacalis]	-1.12704	-0.77967	1.497801	-0.42596	0.834881
TRINITY_DN24410_c0_g2_i1_orf1	spermine oxidase-like isoform X3 [Venturia canescens] >XP_043269281.1 spermine oxidase-like isoform X3 [Venturia canescens]	-1.38836	-0.99253	0.937881	1.010111	0.432897
TRINITY_DN2193_c0_g1_i7_orf1	long-chain-fatty-acid--CoA ligase 5 isoform X1 [Ostrinia furnacalis] >XP_028176293.1 long-chain-fatty-acid--CoA ligase 5 isoform X1 [Ostrinia furnacalis] >XP_028176294.1 long-chain-fatty-acid--CoA ligase 5 isoform X1 [Ostrinia furnacalis] >XP_028176295.1 long-chain-fatty-acid--CoA ligase 5 isoform X1 [Ostrinia furnacalis] >XP_028176296.1 long-chain-fatty-acid--CoA ligase 5 isoform X1 [Ostrinia furnacalis] >XP_028176297.1 long-chain-fatty-acid--CoA ligase 5 isoform X1 [Ostrinia furnacalis] >XP_028176298.1 long-chain-fatty-acid--CoA ligase 5 isoform X2 [Ostrinia furnacalis]	-1.52896	-0.52768	1.12899	-0.11883	1.046471
TRINITY_DN5628_c0_g1_i5_orf1	hypothetical protein O3G_MSEX015036 [Manduca sexta]	-1.75832	-0.46288	0.857262	0.561549	0.802387
TRINITY_DN9560_c0_g1_i5_orf1	uncharacterized protein LOC114357350 [Ostrinia furnacalis]	-1.75571	-0.38926	1.108507	0.515517	0.520954
TRINITY_DN22044_c0_g2_i1_orf1	derlin-1 [Ostrinia furnacalis]	-1.48853	-0.58352	1.153953	-0.12864	1.046731
TRINITY_DN870_c0_g1_i3_orf1	talin-1 isoform X12 [Ostrinia furnacalis]	-1.63629	-0.40283	1.011616	-0.03809	1.06559
TRINITY_DN26961_c0_g1_i1_orf1	uncharacterized protein LOC120424957 [Culex pipiens pallens]	-1.59811	-0.6231	1.262616	0.524776	0.433819
TRINITY_DN1404_c0_g1_i6_orf1	uncharacterized protein LOC114363065 [Ostrinia furnacalis]	-1.59946	-0.73089	1.009557	0.570812	0.749987
TRINITY_DN2312_c0_g1_i4_orf1	endoplasmic reticulum-Golgi intermediate compartment protein 3 [Ostrinia furnacalis]	-1.74004	-0.49633	0.941934	0.625774	0.668656
TRINITY_DN57856_c0_g2_i1_orf1	cytochrome P450 6B2-like [Ostrinia furnacalis]	-1.55768	-0.16692	0.949139	-0.4321	1.20756
TRINITY_DN2559_c0_g1_i4_orf1	uricase [Ostrinia furnacalis]	-1.56077	-0.56656	1.38525	0.214905	0.527175
TRINITY_DN3913_c0_g1_i6_orf1	protein obstructor-E-like [Ostrinia furnacalis]	-1.83515	-0.23363	0.860899	0.372654	0.83523
TRINITY_DN16400_c0_g2_i1_orf1	superoxide dismutase [Cu-Zn]-like isoform X1 [Ostrinia furnacalis]	-1.49795	-0.6059	1.481339	0.290085	0.332429
TRINITY_DN1177_c0_g1_i4_orf1	unnamed protein product [Chilo suppressalis]	-1.57777	-0.51561	1.057958	-0.02517	1.0606
TRINITY_DN120_c0_g1_i2_orf1	PREDICTED: myosin light chain alkali-like [Amyelois transitella]	-1.80515	-0.33102	0.884964	0.444031	0.807178
TRINITY_DN15373_c0_g1_i2_orf1	SET domain-containing protein SmydA-8-like isoform X2 [Ostrinia furnacalis]	-1.63314	-0.69576	0.88853	0.824938	0.615433
TRINITY_DN10512_c0_g1_i1_orf1	protein obstructor-E [Ostrinia furnacalis]	-1.53941	-0.20487	1.1508	-0.44075	1.03423
TRINITY_DN109931_c0_g1_i1_orf1	hydroxymethylglutaryl-CoA lyase, mitochondrial isoform X1 [Ostrinia furnacalis]	-1.85689	-0.17976	0.934363	0.411315	0.690968
TRINITY_DN62557_c0_g1_i1_orf1	6-phosphofructokinase [Operophtera brumata]	-1.49729	-0.79496	0.607413	1.253433	0.431406
TRINITY_DN3800_c0_g1_i7_orf1	hypothetical protein evm_004893 [Chilo suppressalis]	-1.67165	-0.41888	1.163692	0.11239	0.814447
TRINITY_DN17061_c0_g1_i1_orf1	uncharacterized protein LOC113511282 isoform X2 [Galleria mellonella]	-1.75995	-0.38212	0.863573	0.328166	0.950329
TRINITY_DN30476_c0_g1_i1_orf1	unnamed protein product [Arctia plantaginis]	-1.15971	-0.8114	1.372931	-0.38386	0.982043
TRINITY_DN779_c0_g1_i3_orf1	uncharacterized protein LOC114351172 isoform X1 [Ostrinia furnacalis]	-1.66291	-0.52753	1.216575	0.453823	0.520038
TRINITY_DN1333_c0_g1_i6_orf1	uncharacterized protein LOC114362563 [Ostrinia furnacalis]	-1.69647	-0.47579	0.992602	0.262302	0.917359
TRINITY_DN4248_c0_g1_i4_orf1	hypothetical protein SFRURICE_003580 [Spodoptera frugiperda]	-1.62233	-0.37559	1.090044	-0.10583	1.013702
TRINITY_DN15136_c0_g1_i2_orf1	alpha-aminoadipic semialdehyde synthase, mitochondrial isoform X3 [Ostrinia furnacalis]	-1.72056	-0.47989	1.086441	0.491708	0.622298
TRINITY_DN22797_c0_g1_i5_orf1	phenoloxidase-activating factor 2-like isoform X1 [Ostrinia furnacalis]	-1.63398	-0.42154	1.378395	0.44627	0.230852
TRINITY_DN14922_c0_g3_i2_orf1	probable pseudouridine-5'-phosphatase [Ostrinia furnacalis]	-1.68392	-0.53032	1.144343	0.509377	0.56052
TRINITY_DN28503_c0_g1_i6_orf1	uncharacterized protein LOC114363584 [Ostrinia furnacalis] >AXY94663.1 seroin transcript 3 [Ostrinia nubilalis]	-1.436	-0.46922	1.629859	0.029585	0.245782
TRINITY_DN1993_c0_g1_i1_orf1	6-phosphofructo-2-kinase/fructose-2,6-bisphosphatase isoform X1 [Ostrinia furnacalis]	-1.43298	-0.38654	1.658782	-0.04749	0.208224

TRINITY_DN99063_c0_g1_i1_orf1	microtubule-associated protein futsch isoform X4 [Ostrinia furnacalis] >XP_028162562.1 microtubule-associated protein futsch isoform X4 [Ostrinia furnacalis]	-1.71563	-0.40757	1.15367	0.273146	0.696381
TRINITY_DN19251_c0_g1_i8_orf1	succinate--CoA ligase [GDP-forming] subunit beta, mitochondrial [Ostrinia furnacalis]	-1.53168	-0.40389	1.553256	0.140386	0.241923
TRINITY_DN4744_c0_g1_i7_orf1	glutaryl-CoA dehydrogenase, mitochondrial [Ostrinia furnacalis]	-1.60081	0.033789	0.962448	-0.51233	1.116904
TRINITY_DN109943_c0_g1_i1_orf1	uncharacterized protein LOC114361588 isoform X14 [Ostrinia furnacalis]	-1.84127	-0.20888	0.87915	0.353582	0.817415
TRINITY_DN5513_c0_g1_i1_orf1	GDP-Man:Man(3)GlcNAc(2)-PP-Dol alpha-1,2-mannosyltransferase [Ostrinia furnacalis]	-1.68181	0.157279	0.963129	-0.44784	1.00925
TRINITY_DN5300_c0_g1_i3_orf1	valacyclovir hydrolase [Ostrinia furnacalis]	-1.70969	-0.18111	0.839446	-0.10156	1.152905
TRINITY_DN31390_c0_g1_i2_orf1	UDP-glucuronosyltransferase 2B20-like [Ostrinia furnacalis]	-1.59727	-0.35698	1.098175	1.039945	-0.18387
TRINITY_DN102051_c0_g1_i1_orf1	spectrin repeat domain-containing protein [Phthorimaea operculella]	-1.81893	-0.29677	0.973506	0.529891	0.612303
TRINITY_DN1267_c0_g2_i10_orf1	secretory phospholipase A2 receptor-like [Ostrinia furnacalis]	-1.52985	-0.53484	1.44259	0.533471	0.088629
TRINITY_DN17657_c0_g1_i1_orf1	alcohol dehydrogenase 18, partial [Helicoverpa assulta]	-1.69284	-0.25167	1.057741	0.972038	-0.08527
TRINITY_DN29190_c0_g1_i4_orf1	gloverin-like [Ostrinia furnacalis]	0.179821	-0.44145	-0.90298	-0.69818	1.862784
TRINITY_DN38392_c0_g1_i1_orf1	enoyl-CoA hydratase domain-containing protein 3 [Agrotis segetum]	0.708859	-0.84094	-1.2104	-0.1726	1.515084
TRINITY_DN48497_c0_g1_i1_orf1	unnamed protein product [Chrysodeixis includens]	0.48883	-0.0704	-1.24034	-0.78902	1.610928
TRINITY_DN34465_c0_g1_i1_orf1	putative peptidyl-tRNA hydrolase PTRHD1 [Ostrinia furnacalis]	0.169863	-0.27044	-1.23635	-0.44414	1.781064
TRINITY_DN51938_c0_g3_i1_orf1	unnamed protein product [Mus musculus]	0.070945	0.27062	-1.41058	-0.55191	1.620925
TRINITY_DN7247_c0_g1_i6_orf1	pyruvate kinase-like isoform X2 [Ostrinia furnacalis]	-0.01199	0.350256	-0.74485	-1.25269	1.659264
TRINITY_DN22_c0_g1_i3_orf1	uncharacterized protein LOC114362831 [Ostrinia furnacalis]	-0.24355	0.596595	-0.96098	-1.01446	1.62239
TRINITY_DN3732_c0_g1_i2_orf1	cytochrome P450 monooxygenase CYP6AB141 [Ostrinia furnacalis]	-0.26095	0.872404	-1.12851	-0.91698	1.43403
TRINITY_DN9400_c0_g1_i1_orf1	lysophosphatidylserine lipase ABHD12 isoform X2 [Maniola hyperantus]	-0.77844	-0.3522	-1.09764	0.574444	1.653838
TRINITY_DN41280_c0_g1_i2_orf1	unnamed protein product [Plutella xylostella]	-1.25795	0.174013	-0.77681	0.204904	1.655852
TRINITY_DN920_c0_g1_i6_orf1	glutathione S-transferase omega 2 [Ostrinia furnacalis]	-1.13695	0.519258	-0.99228	0.044358	1.565611
TRINITY_DN46715_c0_g1_i1_orf1	hypothetical protein evm_000885 [Chilo suppressalis] >CAH0689224.1 unnamed protein product [Chilo	-0.65257	-0.13305	-1.44094	1.139598	1.086956
TRINITY_DN8783_c0_g1_i9_orf1	luciferin 4-monooxygenase-like [Ostrinia furnacalis] >XP_028165580.1 luciferin 4-monooxygenase-like [Ostrinia furnacalis]	-0.7586	0.303005	-1.4089	0.380199	1.484302
TRINITY_DN30037_c0_g1_i5_orf1	cytoglobin-1-like isoform X2 [Ostrinia furnacalis]	-0.33054	0.118709	-1.59107	0.301489	1.501414
TRINITY_DN5507_c0_g1_i1_orf1	PREDICTED: protein mago nashi [Amyelois transitella] >XP_026764462.1 protein mago nashi [Galleria mellonella] >XP_028164484.1 protein mago nashi [Ostrinia furnacalis]	-0.77372	-0.19302	-1.31613	1.027117	1.255756
TRINITY_DN7861_c0_g1_i5_orf1	cytochrome b5-related protein-like [Ostrinia furnacalis]	-1.40066	0.085069	-0.69285	0.487521	1.520921
TRINITY_DN1292_c0_g1_i3_orf1	uncharacterized protein LOC114360660 [Ostrinia furnacalis]	-1.45644	0.181988	-0.59619	0.32682	1.543825
TRINITY_DN2821_c0_g1_i1_orf1	uncharacterized protein LOC114356423 [Ostrinia furnacalis]	-0.70914	-0.21699	-1.35744	1.137636	1.145937
TRINITY_DN11015_c0_g1_i8_orf1	nicotinamide riboside kinase 1 [Ostrinia furnacalis]	-1.30807	0.415684	-0.89039	0.285495	1.497281
TRINITY_DN27903_c0_g1_i1_orf1	desaturase MPVE [Helicoverpa assulta]	-1.22882	0.440031	-0.77301	-0.0791	1.640905
TRINITY_DN1264_c0_g1_i2_orf1	L-lactate dehydrogenase isoform X1 [Ostrinia furnacalis]	-1.41606	0.103976	-0.70062	0.522815	1.489888
TRINITY_DN558_c0_g1_i4_orf1	nucleoporin SEH1 isoform X1 [Ostrinia furnacalis] >XP_028169219.1 nucleoporin SEH1 isoform X2 [Ostrinia	-1.55182	0.395135	-0.34657	-0.01835	1.521606
TRINITY_DN16451_c0_g1_i7_orf1	diphthine--ammonia ligase-like [Ostrinia furnacalis]	-1.43033	0.39023	-0.65143	0.157709	1.533828
TRINITY_DN1265_c0_g1_i4_orf1	fumarylacetoacetate hydrolase domain-containing protein 2 isoform X3 [Ostrinia furnacalis]	-1.50411	0.327405	-0.67264	0.440935	1.408405
TRINITY_DN1955_c0_g1_i5_orf1	ultraviolet-B receptor UVR8-like [Ostrinia furnacalis]	-1.30366	0.413378	-1.06318	1.190329	0.763129
TRINITY_DN56877_c0_g1_i4_orf1	programmed cell death protein 2-like [Ostrinia furnacalis]	-1.18621	0.545834	-0.77792	-0.20847	1.626762
TRINITY_DN1406_c0_g2_i2_orf1	uncharacterized protein LOC114366644 [Ostrinia furnacalis]	-1.79865	0.568438	-0.14887	0.205476	1.173608
TRINITY_DN935_c0_g1_i3_orf1	carboxylesterase 5A-like [Ostrinia furnacalis]	-1.34462	0.43241	-1.01651	0.727972	1.200746
TRINITY_DN7794_c0_g1_i1_orf1	laminin subunit gamma-1-like [Ostrinia furnacalis]	-1.26112	0.419709	-0.65597	-0.16839	1.665767
TRINITY_DN49786_c0_g1_i1_orf1	UDP-glucuronosyltransferase 2B15-like isoform X1 [Ostrinia furnacalis]	-1.14294	0.800827	-1.17666	0.252202	1.266577
TRINITY_DN32_c0_g1_i4_orf1	epidermal growth factor receptor substrate 15 homolog [Ostrinia furnacalis]	-1.5061	0.53952	-0.66746	0.242553	1.391489
TRINITY_DN41708_c0_g1_i1_orf1	facilitated trehalose transporter Tret1-like [Ostrinia furnacalis]	-1.76659	0.514197	-0.07309	0.05818	1.267297
TRINITY_DN9135_c0_g1_i4_orf1	electron transfer flavoprotein subunit beta [Ostrinia furnacalis]	-1.88819	0.581998	-0.14581	0.827451	0.624556
TRINITY_DN1585_c0_g1_i1_orf1	aldose reductase-like isoform X2 [Ostrinia furnacalis]	-1.80959	0.525006	-0.2463	0.435847	1.09504
TRINITY_DN12997_c0_g2_i1_orf1	cytochrome P450 monooxygenase CYP6AE28 [Cnaphalocrocis medinalis]	-1.93074	0.833391	0.206806	0.182188	0.70835
TRINITY_DN925_c0_g1_i5_orf1	glutathione hydrolase 1 proenzyme-like [Helicoverpa zea]	-1.68983	0.720076	-0.55492	0.483289	1.041381
TRINITY_DN1989_c0_g1_i1_orf1	sarcoplasmic calcium-binding protein 1 isoform X1 [Ostrinia furnacalis]	-1.6933	0.218582	-0.38039	1.258863	0.59625
TRINITY_DN4279_c0_g1_i4_orf1	glutathione S-transferase sigma 1 [Ostrinia furnacalis]	-1.81787	0.789019	-0.33839	0.57497	0.792275

TRINITY_DN56795_c1_g1_i1_orf1	uncharacterized protein LOC114365476 [Ostrinia furnacalis]	-1.45518	0.838065	-0.91084	0.461366	1.066589
TRINITY_DN79083_c0_g1_i2_orf1	unnamed protein product [Arctia plantaginis]	-1.19407	0.996987	-1.24255	0.719818	0.719818
TRINITY_DN5363_c0_g1_i1_orf1	cytochrome c oxidase assembly factor 6 homolog [Ostrinia furnacalis]	-1.60364	0.950227	-0.70588	0.906895	0.452402
TRINITY_DN16125_c0_g1_i3_orf1	3-ketoacyl-CoA thiolase, mitochondrial [Ostrinia furnacalis]	-1.92812	0.682842	0.093859	0.844419	0.306995
TRINITY_DN5962_c0_g1_i1_orf1	tRNA (cytosine(34)-C(5))-methyltransferase [Ostrinia furnacalis]	-1.11525	0.864013	-0.73972	-0.49737	1.488331
TRINITY_DN3019_c0_g1_i1_orf1	uncharacterized protein LOC114355530 isoform X1 [Ostrinia furnacalis]	-1.6545	0.688632	-0.65499	0.659084	0.961773
TRINITY_DN10398_c0_g1_i12_orf1	zinc finger protein ZPR1 isoform X2 [Ostrinia furnacalis]	-1.73939	0.693736	-0.19439	1.205951	0.034091
TRINITY_DN81258_c0_g1_i2_orf1	jg27820 [Pararge aegeria aegeria]	-1.91924	0.56343	0.102935	0.307351	0.945522
TRINITY_DN9242_c0_g1_i1_orf1	electron transfer flavoprotein subunit alpha, mitochondrial [Ostrinia furnacalis]	-1.76683	0.991732	-0.41732	0.526669	0.665756
TRINITY_DN51830_c0_g1_i4_orf1	15-hydroxyprostaglandin dehydrogenase [NAD(+)]-like [Ostrinia furnacalis]	-1.77715	0.744583	-0.37111	0.415068	0.988603
TRINITY_DN4182_c0_g1_i6_orf1	tetratricopeptide repeat protein 19 homolog, mitochondrial [Ostrinia furnacalis]	-1.65438	1.218849	-0.49853	0.251806	0.682262
TRINITY_DN3037_c0_g1_i1_orf1	coiled-coil domain-containing protein 58-like [Spodoptera frugiperda] >KAF9815873.1 hypothetical protein SFRURICE_009771 [Spodoptera frugiperda] >KAG8118507.1 hypothetical protein SFRUCORN_001779 [Spodoptera frugiperda]	-1.6874	0.561324	-0.20085	1.340548	-0.01362
TRINITY_DN87648_c0_g1_i1_orfp1	TRINITY_DN87648_c0_g1_i1_m.51054 TRINITY_DN87648_c0_g1::TRINITY_DN87648_c0_g1_i1::g.51054 ORF type:internal len:180 (+),score=75.93 TRINITY_DN87648_c0_g1_i1:2-538(+)	-1.90399	0.713823	0.048405	0.245998	0.895769
TRINITY_DN36928_c0_g1_i5_orf1	actin-interacting protein 1 isoform X2 [Ostrinia furnacalis]	-0.56508	0.980768	-1.70051	0.671083	0.613743
TRINITY_DN2749_c4_g1_i2_orf1	RNA exonuclease 4-like [Ostrinia furnacalis] >QEE79882.1 REX4 [Ostrinia furnacalis]	-0.874	0.391931	-1.37375	1.414362	0.441464
TRINITY_DN52859_c0_g1_i4_orf1	uncharacterized protein LOC114351423 [Ostrinia furnacalis]	-1.22073	0.798189	-1.07631	1.293013	0.205845
TRINITY_DN47591_c0_g1_i2_orf1	39S ribosomal protein L30, mitochondrial [Ostrinia furnacalis]	-1.68942	1.144528	-0.45992	0.257886	0.746921
TRINITY_DN280_c4_g1_i5_orf1	fibroin light chain [Haritalodes derogata]	-1.71647	0.661415	-0.48856	1.076916	0.466705
TRINITY_DN6241_c0_g1_i1_orf1	uncharacterized protein LOC114355531 [Ostrinia furnacalis]	-1.53281	0.970352	-0.84371	0.767925	0.638245
TRINITY_DN141353_c0_g1_i1_orf1	uncharacterized protein LOC123263755 [Cotesia glomerata] >KAH0554923.1 hypothetical protein KQX54_013880 [Cotesia glomerata]	-1.85937	0.694103	-0.20767	0.880554	0.49239
TRINITY_DN29_c0_g1_i4_orf1	sodium-dependent nutrient amino acid transporter 1-like [Ostrinia furnacalis] >XP_028167707.1 sodium-dependent nutrient amino acid transporter 1-like [Ostrinia furnacalis]	-1.61535	1.002285	-0.42557	1.096194	-0.05756
TRINITY_DN14094_c0_g1_i1_orfp1	TRINITY_DN14094_c0_g1_i1_m.76391 TRINITY_DN14094_c0_g1::TRINITY_DN14094_c0_g1_i1::g.76391 ORF type:5prime_partial len:120 (+),score=12.94 TRINITY_DN14094_c0_g1_i1:3-362(+)	-1.78252	0.899952	-0.18959	0.984416	0.087736
TRINITY_DN4142_c0_g1_i5_orf1	GDP-fucose protein O-fucosyltransferase 1 [Ostrinia furnacalis]	-0.53089	-0.33269	0.965837	-1.40564	1.303385
TRINITY_DN41645_c0_g1_i1_orf1	60S acidic ribosomal protein P2 [Ostrinia furnacalis]	-0.84759	-0.30614	0.772971	-1.13588	1.516633
TRINITY_DN2072_c0_g1_i1_orf1	dnaJ homolog subfamily C member 3 [Helicoverpa zea]	-0.91359	0.068844	1.159109	-1.33355	1.019182
TRINITY_DN6358_c0_g1_i5_orf1	histone H1B-like [Ostrinia furnacalis]	-0.58123	0.242408	1.066954	-1.62897	0.90083
TRINITY_DN140613_c0_g1_i1_orf1	hypothetical protein M0804_013066 [Polistes exclamans]	-0.8668	-0.06232	1.490456	-1.24652	0.685188
TRINITY_DN2719_c1_g1_i6_orf1	unnamed protein product [Chrysodeixis includens]	-1.28481	-0.00589	1.62811	-0.73508	0.397669
TRINITY_DN920_c0_g1_i4_orf1	glutathione S-transferase omega 2 [Ostrinia furnacalis]	-1.1651	0.440012	1.479959	-1.07519	0.320324
TRINITY_DN43881_c0_g1_i2_orf1	estradiol 17-beta-dehydrogenase 8-like [Ostrinia furnacalis]	-1.27544	0.541866	1.52344	-0.86759	0.077728
TRINITY_DN146236_c0_g1_i1_orf1	vesicle-fusing ATPase 1-like [Chelonus insularis]	-1.50496	0.806648	1.299772	-0.62794	0.026478
TRINITY_DN27300_c0_g1_i6_orfp1	TRINITY_DN27300_c0_g1_i6_m.71140 TRINITY_DN27300_c0_g1::TRINITY_DN27300_c0_g1_i6::g.71140 ORF type:internal len:129 (-),score=20.75 TRINITY_DN27300_c0_g1_i6:1-384(-)	-0.98174	0.037325	1.882198	-0.63077	-0.30702
TRINITY_DN119893_c0_g2_i3_orf1	ATP-binding cassette sub-family F member 3 isoform X1 [Ostrinia furnacalis] >XP_028168051.1 ATP-binding cassette sub-family F member 3 isoform X2 [Ostrinia furnacalis]	-1.37363	0.842228	1.303791	-0.83654	0.064155
TRINITY_DN6325_c0_g1_i8_orf1	unnamed protein product [Pieris macdunnoughi]	-1.38833	-0.09141	1.739523	-0.08246	-0.17733
TRINITY_DN4822_c0_g1_i9_orf1	homogentisate 1,2-dioxygenase [Ostrinia furnacalis]	-0.75098	0.634134	1.220283	-1.53559	0.432159
TRINITY_DN21215_c0_g1_i7_orf1	phytanoyl-CoA dioxygenase, peroxisomal-like [Ostrinia furnacalis]	-0.6489	1.044676	0.920446	-1.59995	0.283735
TRINITY_DN115658_c0_g1_i1_orf1	hypothetical protein B5X24_HaOG203018 [Helicoverpa armigera]	-1.16794	1.135347	1.199317	-0.92094	-0.24578
TRINITY_DN19659_c1_g1_i1_orf1	elongation factor 1-gamma [Ostrinia furnacalis]	-1.31178	1.106814	1.201896	-0.73585	-0.26108
TRINITY_DN23474_c1_g1_i1_orf1	unnamed protein product [Chrysodeixis includens]	-1.31266	0.378942	1.583032	-0.78101	0.131704
TRINITY_DN21170_c0_g1_i5_orf1	twitchin-like [Ostrinia furnacalis]	-1.23306	0.10922	1.789915	-0.47797	-0.1881
TRINITY_DN804_c0_g1_i7_orf1	hypothetical protein HF086_004695 [Spodoptera exigua] >CAH0695017.1 unnamed protein product [Spodoptera exigua]	-1.42147	-0.47005	1.445346	-0.31072	0.756899
TRINITY_DN7735_c0_g1_i4_orf1	calphotin-like [Ostrinia furnacalis]	-1.87689	-0.19735	0.732194	0.642047	0.700003

TRINITY_DN90497_c0_g1_i1_orf1	midasin-like [Ostrinia furnacalis]	-1.90743	-0.02747	0.445823	0.580946	0.908134
TRINITY_DN143509_c0_g1_i1_orf1	ATP-dependent 6-phosphofructokinase isoform X3 [Diachasma alloeum]	-1.85148	0.034871	0.485847	0.191415	1.139348
TRINITY_DN1212_c0_g1_i8_orf1	extensin isoform X5 [Ostrinia furnacalis] >XP_028175473.1 extensin isoform X5 [Ostrinia furnacalis] >XP_028175474.1 extensin isoform X5 [Ostrinia furnacalis]	-1.87769	-0.15565	0.762292	0.460656	0.810398
TRINITY_DN1134_c0_g1_i4_orf1	cytochrome P450 6B5-like [Ostrinia furnacalis]	-1.84729	-0.23886	0.60846	0.553842	0.92385
TRINITY_DN4425_c0_g1_i4_orf1	CKLF-like MARVEL transmembrane domain-containing protein 4 [Ostrinia furnacalis] >XP_028173347.1 CKLF-like MARVEL transmembrane domain-containing protein 4 [Ostrinia furnacalis] >XP_028173348.1 CKLF-like MARVEL transmembrane domain-containing protein 4 [Ostrinia furnacalis]	-1.64484	0.116721	1.255956	-0.44115	0.713319
TRINITY_DN2688_c0_g2_i1_orf1	mitochondrial amidoxime reducing component 2 [Galleria mellonella]	-1.82722	-0.13104	0.453812	0.360783	1.143669
TRINITY_DN5510_c0_g1_i9_orf1	proteoglycan 4 [Pectinophora gossypiella]	-1.85631	-0.05635	0.643439	0.227512	1.041707
TRINITY_DN9286_c0_g1_i2_orf1	alcohol dehydrogenase class-3 [Ostrinia furnacalis]	-1.87253	0.022693	0.229715	0.557145	1.06298
TRINITY_DN2544_c1_g1_i2_orf1	hypothetical protein evm_004833 [Chilo suppressalis] >CAB3528842.1 unnamed protein product [Chilo suppressalis] >CAH0405435.1 unnamed protein product [Chilo suppressalis]	-1.79819	-0.20824	0.590647	0.276585	1.139202
TRINITY_DN5176_c0_g1_i2_orf1	uncharacterized protein LOC114361931 [Ostrinia furnacalis]	-1.77512	0.765253	0.6788	-0.44593	0.776996
TRINITY_DN8651_c0_g1_i18_orf1	glutathione S-transferase theta 2 [Conogethes punctiferalis]	-1.72793	-0.01626	1.169513	-0.20313	0.777801
TRINITY_DN11986_c0_g1_i1_orf1	DNA replication licensing factor Mcm2 [Ostrinia furnacalis]	-1.86482	0.319753	0.598463	-0.08077	1.027378
TRINITY_DN1718_c6_g1_i4_orf1	adenosine kinase [Ostrinia furnacalis]	-1.8494	0.136434	0.194691	0.32865	1.189622
TRINITY_DN1470_c0_g1_i2_orf1	oxidation resistance protein 1 isoform X5 [Ostrinia furnacalis]	-1.82969	-0.08862	1.164575	0.422086	0.331652
TRINITY_DN46372_c0_g1_i1_orf1	unnamed protein product [Chilo suppressalis]	-1.69094	-0.26928	1.375724	0.243451	0.341046
TRINITY_DN101991_c0_g1_i5_orf1	hypothetical protein O3G_MSEX015044, partial [Manduca sexta]	-1.89205	-0.13636	0.659611	0.562655	0.806144
TRINITY_DN8258_c0_g1_i6_orf1	unnamed protein product [Chilo suppressalis]	-1.87966	-0.09671	0.671472	0.373838	0.931067
TRINITY_DN1355_c0_g1_i5_orf1	hypothetical protein evm_006664 [Chilo suppressalis]	-1.77173	-0.03113	1.223707	-0.02254	0.601694
TRINITY_DN3769_c0_g1_i1_orf1	uncharacterized protein LOC114360562 [Ostrinia furnacalis] >XP_028171110.1 uncharacterized protein LOC114360562 [Ostrinia furnacalis]	-1.77125	0.318278	0.439579	-0.21947	1.232867
TRINITY_DN77480_c0_g1_i2_orf1	hypothetical protein evm_008422 [Chilo suppressalis]	-1.56245	0.100779	1.310772	-0.56451	0.715408
TRINITY_DN135077_c0_g1_i1_orf1	hypothetical protein KR038_001662 [Drosophila bunnanda]	-1.84794	0.220728	0.202703	0.222013	1.2025
TRINITY_DN69707_c0_g1_i1_orf1	titin-like, partial [Ostrinia furnacalis]	-1.9272	-0.02534	0.621694	0.584191	0.746652
TRINITY_DN1889_c0_g1_i1_orf1	titin isoform X2 [Ostrinia furnacalis]	-1.91396	-0.04056	0.594878	0.511131	0.848515
TRINITY_DN841_c0_g1_i4_orf1	uncharacterized protein LOC114354070 isoform X3 [Ostrinia furnacalis]	-1.95591	0.408745	0.154042	0.615326	0.777801
TRINITY_DN31645_c0_g1_i3_orf1	dystonin isoform X43 [Helicoverpa armigera]	-1.85056	-0.09128	1.012267	0.22986	0.69971
TRINITY_DN14398_c0_g1_i4_orf1	trimethyllysine dioxygenase, mitochondrial [Ostrinia furnacalis]	-1.89261	-0.01742	0.951431	0.316417	0.642179
TRINITY_DN9468_c1_g1_i4_orf1	coronin-1C-A isoform X1 [Bombyx mori]	-1.80573	-0.12108	1.210562	0.394898	0.321355
TRINITY_DN6991_c0_g1_i24_orf1	muscle M-line assembly protein unc-89 isoform X5 [Ostrinia furnacalis]	-1.91042	0.07125	0.699429	0.891204	0.248537
TRINITY_DN779_c0_g1_i12_orf1	unnamed protein product [Chilo suppressalis]	-1.88228	-0.01121	0.470874	0.377185	1.045425
TRINITY_DN18839_c0_g1_i4_orf1	unnamed protein product [Chilo suppressalis]	-1.34199	0.85936	0.541139	-1.06993	1.011422
TRINITY_DN5437_c0_g1_i1_orf1	unnamed protein product [Chilo suppressalis]	-1.57467	0.162861	1.339942	-0.55392	0.625788
TRINITY_DN27491_c0_g1_i1_orf1	prolactin regulatory element-binding protein [Galleria mellonella]	-1.5147	-0.31218	1.458825	-0.27003	0.638082
TRINITY_DN5149_c0_g1_i12_orfp1	TRINITY_DN5149_c0_g1_i12_m.8808 TRINITY_DN5149_c0_g1::TRINITY_DN5149_c0_g1_i12::g.8808 ORF type:internal len:254 (+),score=89.66 TRINITY_DN5149_c0_g1_i12:1-759(+)	-1.6471	0.305813	1.472122	-0.15985	0.029014
TRINITY_DN13119_c0_g1_i4_orf1	endocuticle structural glycoprotein ABD-5-like [Bicyclus anynana]	-1.9281	0.046791	0.723682	0.370866	0.786758
TRINITY_DN17137_c0_g1_i2_orf1	unnamed protein product [Diatraea saccharalis]	-1.83832	0.015292	0.88006	0.023572	0.919396
TRINITY_DN4952_c0_g1_i1_orf1	mitogen-activated protein kinase kinase kinase 4 [Ostrinia furnacalis]	-1.91678	0.295062	0.311612	0.278484	1.031621
TRINITY_DN6482_c0_g1_i1_orf1	endocuticle structural glycoprotein SgAbd-5-like [Ostrinia furnacalis]	-1.91298	0.060408	0.314625	0.602786	0.935159
TRINITY_DN69871_c0_g1_i1_orf1	translocon-associated protein subunit gamma [Venturia canescens]	-1.69659	0.082284	1.434531	-0.0529	0.232672
TRINITY_DN5531_c0_g3_i3_orf1	hypothetical protein evm_013868 [Chilo suppressalis]	-1.95642	0.282038	0.246262	0.635041	0.793075
TRINITY_DN31118_c0_g1_i1_orf1	CPR9 [Ostrinia furnacalis]	-1.92672	-0.03682	0.680307	0.631529	0.651711
TRINITY_DN592_c0_g1_i6_orf1	PDZ and LIM domain protein Zasp isoform X4 [Pectinophora gossypiella]	-1.89198	0.156572	0.713112	0.084055	0.938243
TRINITY_DN19460_c0_g1_i1_orf1	cuticle protein 3-like [Ostrinia furnacalis]	-1.91786	-0.00461	0.680883	0.411736	0.829852
TRINITY_DN26663_c0_g1_i4_orf1	phagocyte signaling-impaired protein [Ostrinia furnacalis]	-1.80251	0.030017	0.787941	-0.07541	1.059963
TRINITY_DN1814_c0_g2_i1_orf1	titin-like, partial [Ostrinia furnacalis]	-1.92366	0.059808	0.766732	0.318078	0.779042

TRINITY_DN1029_c0_g1_i1_orfp1	TRINITY_DN1029_c0_g1_i1_m.64408 TRINITY_DN1029_c0_g1::TRINITY_DN1029_c0_g1_i1::g.64408 ORF type:3prime_partial len:55 (+),score=13.47 TRINITY_DN1029_c0_g1_i1:74-235(+)	-1.95402	0.123889	0.792582	0.514635	0.52291
TRINITY_DN57636_c0_g1_i4_orf1	PREDICTED: plectin-like, partial [Papilio polytes]	-1.9853	0.524091	0.685037	0.306198	0.469975
TRINITY_DN3532_c0_g1_i12_orf1	sulfide:quinone oxidoreductase, mitochondrial-like [Ostrinia furnacalis]	-1.82127	0.791026	0.420105	-0.28426	0.894399
TRINITY_DN101995_c0_g1_i1_orf1	microtubule-actin cross-linking factor 1 isoform X15 [Ostrinia furnacalis]	-1.84335	0.106546	0.711712	-0.01601	1.041107
TRINITY_DN105157_c0_g1_i1_orf1	potential E3 ubiquitin-protein ligase ariadne-2 [Ostrinia furnacalis]	-1.84938	0.050182	0.517464	0.14685	1.134881
TRINITY_DN3010_c0_g1_i4_orf1	inositol oxygenase-like [Ostrinia furnacalis]	-1.70089	0.467179	0.986387	-0.5416	0.78893
TRINITY_DN12690_c0_g1_i1_orf1	ELAV-like protein 1 [Ostrinia furnacalis]	-1.82517	0.297182	0.921855	-0.21986	0.825996
TRINITY_DN7131_c0_g1_i2_orf1	short/branched chain specific acyl-CoA dehydrogenase, mitochondrial [Ostrinia furnacalis]	-1.75444	1.137745	0.263467	-0.32122	0.674445
TRINITY_DN350_c0_g1_i10_orf1	microtubule-associated protein tau-like isoform X5 [Ostrinia furnacalis]	-1.73903	0.79736	0.71675	-0.52038	0.745301
TRINITY_DN1814_c0_g2_i4_orfp1	TRINITY_DN1814_c0_g2_i4_m.63284 TRINITY_DN1814_c0_g2::TRINITY_DN1814_c0_g2_i4::g.63284 ORF type:internal len:258 (-),score=126.31 TRINITY_DN1814_c0_g2_i4:3-773(-)	-1.96288	0.192822	0.481488	0.489527	0.799041
TRINITY_DN1211_c0_g1_i10_orf1	spectrin beta chain-like isoform X7 [Spodoptera frugiperda]	-1.96059	0.398599	0.732861	0.173305	0.655821
TRINITY_DN2890_c0_g1_i2_orf1	alanine aminotransferase 1 [Chelonus insularis]	-1.8889	0.581536	0.789686	-0.15121	0.668887
TRINITY_DN2922_c0_g1_i1_orf1	uncharacterized protein LOC114354086 [Ostrinia furnacalis]	-1.95668	0.267405	0.497143	0.329465	0.862667
TRINITY_DN6621_c0_g1_i1_orf1	translocon-associated protein subunit delta [Ostrinia furnacalis]	-1.86629	0.074331	1.070489	0.131348	0.590121
TRINITY_DN11448_c0_g1_i11_orf1	hypothetical protein B5X24_HaOG201808 [Helicoverpa armigera]	-1.50116	0.102175	1.581274	-0.42207	0.239787
TRINITY_DN1306_c0_g1_i8_orf1	spectrin alpha chain isoform X4 [Pectinophora gossypiella]	-1.71897	0.768792	0.996358	-0.50283	0.456654
TRINITY_DN31118_c0_g2_i1_orf1	unnamed protein product [Spodoptera exigua]	-1.94879	0.062552	0.538361	0.666859	0.681016
TRINITY_DN2040_c0_g1_i15_orfp1	TRINITY_DN2040_c0_g1_i15_m.4150 TRINITY_DN2040_c0_g1::TRINITY_DN2040_c0_g1_i15::g.4150 ORF type:complete len:319 (+),score=125.43,Plasmodium_HRP PF05403.12 3.5,Plasmodium_HRP|PF05403.12|1.5 TRINITY_DN2040_c0_g1_i15:118-957(+)	-1.91917	-0.06364	0.625488	0.665669	0.691654
TRINITY_DN2822_c0_g1_i4_orf1	uncharacterized protein LOC114354271 isoform X1 [Ostrinia furnacalis]	-1.69641	0.895611	0.334884	-0.50845	0.974368
TRINITY_DN1262_c0_g1_i2_orf1	alanine aminotransferase 1 isoform X1 [Ostrinia furnacalis] >XP_028162092.1 alanine aminotransferase 1 isoform X2 [Ostrinia furnacalis] >XP_028162093.1 alanine aminotransferase 1 isoform X3 [Ostrinia furnacalis]	-1.95827	0.45028	0.469156	0.201057	0.837782
TRINITY_DN549_c0_g1_i7_orf1	titin-like, partial [Ostrinia furnacalis]	-1.92751	0.05205	0.883242	0.426669	0.565546
TRINITY_DN549_c0_g1_i14_orf1	titin-like [Ostrinia furnacalis]	-1.92216	0.405754	0.647138	0.019865	0.849403
TRINITY_DN110132_c0_g1_i1_orf1	uncharacterized protein LOC114361588 isoform X14 [Ostrinia furnacalis]	-1.4883	1.182745	0.397563	-0.82824	0.736233
TRINITY_DN2958_c0_g1_i2_orf1	uncharacterized protein LOC114356495 [Ostrinia furnacalis]	-1.63754	0.507974	1.399857	-0.31445	0.044159
TRINITY_DN2401_c0_g2_i1_orf1	DNA-directed RNA polymerase I subunit RPA2 [Ostrinia furnacalis]	-1.90852	0.99242	0.057281	0.411951	0.446863
TRINITY_DN122393_c0_g1_i1_orf1	microtubule-associated protein futsch isoform X4 [Ostrinia furnacalis] >XP_028162562.1 microtubule-associated protein futsch isoform X4 [Ostrinia furnacalis]	-1.7969	0.158489	1.256109	-0.02698	0.409276
TRINITY_DN30208_c0_g1_i3_orf1	unnamed protein product [Timema cristinae]	-1.904	0.072539	0.952649	0.244679	0.634131
TRINITY_DN1895_c0_g1_i2_orf1	unnamed protein product [Chrysodeixis includens]	-1.86979	0.17634	0.917114	-0.01825	0.794587
TRINITY_DN62_c0_g1_i7_orf1	tropomodulin-1 isoform X5 [Ostrinia furnacalis] >XP_028160021.1 tropomodulin-1 isoform X5 [Ostrinia furnacalis]	-1.70683	0.880233	0.89964	-0.53647	0.46343
TRINITY_DN12_c0_g1_i5_orf1	cAMP-dependent protein kinase type II regulatory subunit isoform X1 [Ostrinia furnacalis] >XP_028175270.1 cAMP-dependent protein kinase type II regulatory subunit isoform X1 [Ostrinia furnacalis]	-1.41245	1.06841	0.794591	-0.97714	0.526592
TRINITY_DN1391_c1_g2_i2_orf1	uncharacterized protein LOC119837640 isoform X2 [Zerene cesonia]	-1.92272	0.189405	0.894272	0.179177	0.659871
TRINITY_DN3529_c0_g1_i7_orf1	putative fatty acyl-CoA reductase CG5065 [Ostrinia furnacalis]	-1.40855	0.977934	0.708763	-1.01045	0.732305
TRINITY_DN26254_c0_g1_i1_orf1	hypothetical protein evm_003664 [Chilo suppressalis] >CAB3521132.1 unnamed protein product [Chilo suppressalis] >CAH0398453.1 unnamed protein product [Chilo suppressalis]	-1.93292	0.023048	0.810764	0.518502	0.580606
TRINITY_DN2215_c0_g2_i1_orf1	PREDICTED: larval cuticle protein LCP-22-like [Amyelois transitella]	-1.94895	0.104027	0.751216	0.427745	0.665957
TRINITY_DN3332_c0_g1_i9_orf1	glutathione S-transferase sigma3 [Glyphodes pyloalis]	-1.7714	0.636132	0.527137	-0.40116	1.009292
TRINITY_DN23941_c0_g1_i5_orf1	dystonin isoform X11 [Galleria mellonella]	-1.87446	0.360614	1.00484	-0.07497	0.58398
TRINITY_DN1073_c0_g1_i3_orf1	carboxylesterase [Loxostege sticticalis]	-1.96444	0.458052	0.215625	0.484725	0.806041
TRINITY_DN1718_c1_g1_i5_orf1	gelsolin-like [Ostrinia furnacalis]	-1.94286	0.453465	0.73632	0.065157	0.687922
TRINITY_DN6612_c0_g1_i4_orf1	hypothetical protein O3G_MSEX008151 [Manduca sexta]	-1.7506	0.237661	1.250067	-0.24386	0.506732
TRINITY_DN46173_c0_g3_i1_orf1	Tropomyosin, partial [Cotesia chilonis]	-1.91935	0.145054	0.975224	0.288364	0.510711
TRINITY_DN1104_c0_g1_i1_orfp1	TRINITY_DN1104_c0_g1_i1_m.5521 TRINITY_DN1104_c0_g1::TRINITY_DN1104_c0_g1_i1::g.5521 ORF type:5prime_partial len:204 (+),score=39.47 TRINITY_DN1104_c0_g1_i1:3-614(+)	-1.52246	-0.02673	1.34044	-0.55246	0.761199
TRINITY_DN2254_c0_g1_i4_orf1	vigilin [Ostrinia furnacalis]	-1.47744	0.743699	1.030471	-0.90935	0.612618

TRINITY_DN1180_c0_g1_i4_orf1	larval cuticle protein LCP-30-like [Ostrinia furnacalis]	-1.96292	0.161608	0.701434	0.440491	0.659386
TRINITY_DN52395_c0_g2_i2_orf1	twitchin isoform X20 [Zerene cesonia]	-1.816	0.061299	1.254249	0.249504	0.250953
TRINITY_DN15318_c0_g1_i1_orf1	hepatoma-derived growth factor-related protein 2-like [Ostrinia furnacalis]	-1.28318	0.999097	1.014599	-1.09293	0.362415
TRINITY_DN21570_c0_g1_i1_orf1	ceramide synthase 5-like [Ostrinia furnacalis]	-1.92096	0.988837	0.156372	0.446126	0.329621
TRINITY_DN8394_c1_g1_i9_orf1	uncharacterized protein LOC114364294 [Ostrinia furnacalis]	-1.57268	1.211321	0.656504	-0.68832	0.393177
TRINITY_DN45949_c0_g1_i1_orf1	uncharacterized protein LOC114355167 [Ostrinia furnacalis]	-1.86967	-0.11719	0.864443	0.799375	0.323039
TRINITY_DN47257_c0_g1_i4_orf1	PREDICTED: microtubule-actin cross-linking factor 1-like, partial [Amyelois transitella]	-1.70051	1.243263	0.474672	-0.41926	0.401835
TRINITY_DN114344_c0_g1_i4_orf1	microtubule-actin cross-linking factor 1 isoform X15 [Ostrinia furnacalis]	-1.81698	0.468916	1.139136	-0.17771	0.386638
TRINITY_DN8915_c0_g1_i3_orf1	filamin-A isoform X1 [Ostrinia furnacalis] >XP_028171553.1 filamin-A isoform X2 [Ostrinia furnacalis] >XP_028171561.1 filamin-A isoform X2 [Ostrinia furnacalis]	-1.98513	0.45258	0.597808	0.293908	0.640835
TRINITY_DN107962_c0_g1_i1_orf1	unnamed protein product [Euphydryas editha]	-1.76299	0.804782	0.437669	-0.41691	0.937449
TRINITY_DN4133_c0_g1_i2_orfp2	unnamed protein product [Spodoptera exigua]	-1.34209	0.530256	1.083385	-1.05963	0.788083
TRINITY_DN8454_c0_g1_i4_orf1	translocon-associated protein subunit alpha [Ostrinia furnacalis]	-1.67674	0.851991	1.050501	-0.52129	0.29554
TRINITY_DN46216_c0_g3_i1_orf1	unnamed protein product, partial [Brenthis ino]	-1.87715	0.351943	0.700429	-0.09834	0.923124
TRINITY_DN76333_c0_g1_i2_orf1	larval cuticle protein 65Ag1-like [Ostrinia furnacalis]	-1.97699	0.221248	0.543079	0.527794	0.684871
TRINITY_DN13350_c0_g1_i4_orf1	cap-specific mRNA (nucleoside-2'-O-)-methyltransferase 1 [Ostrinia furnacalis]	-1.91535	0.393742	0.964883	0.491012	0.065711
TRINITY_DN27045_c0_g1_i1_orf1	cytochrome P450 6B5-like [Galleria mellonella]	-1.75595	0.921642	0.177706	-0.31204	0.96864
TRINITY_DN825_c0_g1_i18_orfp1	TRINITY_DN825_c0_g1_i18_m.8360 TRINITY_DN825_c0_g1::TRINITY_DN825_c0_g1_i18::g.8360 ORF type:complete len:415 (+),score=37.02 TRINITY_DN825_c0_g1_i18:55-1245(+)	-1.87971	0.483728	1.085944	0.206826	0.10321
TRINITY_DN3906_c0_g1_i5_orf1	ejaculatory bulb-specific protein 3-like [Ostrinia furnacalis]	-1.67665	0.416946	1.231854	-0.48465	0.512499
TRINITY_DN69557_c0_g1_i1_orf1	hypothetical protein G9C98_005708, partial [Cotesia typhae]	-1.85842	0.507329	1.04797	-0.11744	0.420562
TRINITY_DN24322_c0_g1_i4_orf1	unnamed protein product, partial [Brenthis ino]	-1.89292	0.164532	1.069596	0.449241	0.209556
TRINITY_DN1173_c0_g1_i11_orf1	obscurin [Ostrinia furnacalis]	-1.9921	0.626428	0.365674	0.448052	0.551949
TRINITY_DN116_c1_g1_i8_orf1	uncharacterized protein LOC114350057 isoform X2 [Ostrinia furnacalis]	-1.94627	0.25522	0.914544	0.321161	0.455349
TRINITY_DN18009_c0_g1_i1_orf1	pre-mRNA-splicing factor ISY1 homolog [Ostrinia furnacalis]	-1.83692	0.688247	0.988016	-0.20511	0.365769
TRINITY_DN9591_c0_g1_i1_orf1	probable 39S ribosomal protein L49, mitochondrial [Ostrinia furnacalis]	-1.90103	0.822319	0.778611	-0.02116	0.321257
TRINITY_DN95971_c0_g5_i1_orf1	exportin-1 [Diachasma alloeum] >XP_015118053.1 exportin-1 [Diachasma alloeum] >XP_015118054.1 exportin-1 [Diachasma alloeum] >XP_015118055.1 exportin-1 [Diachasma alloeum] >XP_015118056.1 exportin-1 [Diachasma alloeum]	-1.79856	0.568812	1.156166	-0.18928	0.262864
TRINITY_DN82628_c0_g1_i2_orf1	ORF type:internal len:148 hit:XP_028162129.1 TRINITY_DN82628_c0_g1_i2:3-446(-)	-1.9438	0.22348	0.863126	0.256363	0.60083
TRINITY_DN11735_c0_g1_i5_orf1	TBC1 domain family member 22B isoform X1 [Ostrinia furnacalis] >XP_028174102.1 TBC1 domain family member 22B isoform X2 [Ostrinia furnacalis]	-1.96194	0.208652	0.407208	0.806204	0.539877
TRINITY_DN4010_c0_g2_i1_orf1	myophilin [Ostrinia furnacalis]	-1.87995	0.293563	1.097121	0.076915	0.412348
TRINITY_DN13312_c0_g2_i1_orf1	von Willebrand factor A domain-containing protein 8 [Trichoplusia ni]	-1.91769	0.882284	0.596361	0.434009	0.005038
TRINITY_DN1125_c0_g1_i4_orf1	hypothetical protein evm_001907 [Chilo suppressalis] >CAH2985359.1 unnamed protein product [Chilo	-1.66665	1.101408	0.390496	-0.56125	0.735994
TRINITY_DN48097_c0_g1_i1_orf1	unnamed protein product [Homo sapiens]	-1.94894	0.510428	0.317595	0.2349	0.886021
TRINITY_DN9871_c0_g1_i11_orf1	PEST proteolytic signal-containing nuclear protein-like [Ostrinia furnacalis]	-1.90861	0.296868	1.039051	0.173469	0.399217
TRINITY_DN1814_c0_g1_i11_orf1	titin-like, partial [Ostrinia furnacalis]	-1.87601	0.777926	0.720671	-0.18868	0.566089
TRINITY_DN46372_c0_g2_i1_orf1	basic salivary proline-rich protein 1 isoform X2 [Ostrinia furnacalis]	-1.65097	1.03398	1.01964	-0.40681	0.00416
TRINITY_DN8226_c0_g1_i1_orf1	myosin heavy chain, muscle isoform X16 [Helicoverpa armigera]	-1.97777	0.476896	0.651351	0.229954	0.619567
TRINITY_DN27276_c0_g1_i5_orf1	probable small nuclear ribonucleoprotein Sm D1 [Ostrinia furnacalis] >CAG9751027.1 unnamed protein product [Diatraea saccharalis] >CAG9789712.1 unnamed protein product [Diatraea saccharalis]	-1.47379	1.032556	0.32978	-0.85169	0.963143
TRINITY_DN85004_c0_g1_i1_orf1	uncharacterized protein LOC114357684 [Ostrinia furnacalis]	-1.96887	0.202999	0.670854	0.675502	0.419515
TRINITY_DN78492_c0_g1_i1_orf1	uncharacterized protein LOC114354775 [Ostrinia furnacalis]	-1.26612	1.416036	0.250968	-0.99052	0.589636
TRINITY_DN8406_c0_g1_i4_orf1	titin [Ostrinia furnacalis]	-1.60643	1.055293	1.077707	-0.3165	-0.21008
TRINITY_DN1982_c0_g1_i24_orf1	uncharacterized protein LOC114361215 isoform X5 [Ostrinia furnacalis]	-1.88007	0.437834	1.093415	0.081569	0.267252
TRINITY_DN695_c0_g1_i12_orf1	seroin transcript 2A, partial [Ostrinia nubilalis]	-1.97804	0.470347	0.676821	0.238146	0.592728
TRINITY_DN8964_c0_g1_i4_orf1	hypothetical protein evm_010115 [Chilo suppressalis]	-1.74308	0.86215	0.800862	-0.4956	0.575661
TRINITY_DN12508_c0_g1_i1_orf1	uncharacterized protein LOC114350091 [Ostrinia furnacalis]	-1.57017	1.192462	0.556246	-0.71666	0.538126
TRINITY_DN21943_c1_g1_i1_orf1	myosin light chain alkali isoform X2 [Ostrinia furnacalis]	-1.84238	1.201757	0.093444	0.235054	0.312127
TRINITY_DN111110_c0_g1_i1_orf1	NAD-dependent protein deacylase-like [Ostrinia furnacalis]	-1.69034	0.809081	0.905455	-0.59003	0.565832

TRINITY_DN19080_c0_g1_i4_orf1	synaptic vesicle 2-related protein-like isoform X1 [Ostrinia furnacalis] >XP_028161172.1 synaptic vesicle 2-related protein-like isoform X1 [Ostrinia furnacalis]	-1.78076	1.317612	0.068532	0.125767	0.268849
TRINITY_DN76529_c0_g1_i1_orfp1	TRINITY_DN76529_c0_g1_i1_m.64079 TRINITY_DN76529_c0_g1::TRINITY_DN76529_c0_g1_i1::g.64079 ORF type:internal len:70 (+),score=14.68 TRINITY_DN76529_c0_g1_i1:3-209(+)	-1.56507	0.252841	1.52508	-0.3692	0.156353
TRINITY_DN4408_c6_g1_i1_orf1	polyprotein, partial [Bemisia tabaci]	-1.60521	0.703233	0.798941	-0.75009	0.853124
TRINITY_DN11448_c0_g1_i15_orf1	unnamed protein product [Chilo suppressalis]	-1.92068	0.463841	0.960151	0.089256	0.407429
TRINITY_DN146217_c0_g1_i1_orf1	60S acidic ribosomal protein P0 [Bombus bifarius]	-1.88066	0.77673	0.916591	0.061248	0.126095
TRINITY_DN48610_c0_g1_i2_orf1	hypothetical protein evm_002298 [Chilo suppressalis] >CAH0682062.1 unnamed protein product [Chilo	-1.91586	0.321796	0.996888	0.134531	0.462646
TRINITY_DN248_c0_g1_i1_orf1	unnamed protein product [Chilo suppressalis]	-1.96327	0.636131	0.705306	0.152659	0.469179
TRINITY_DN23429_c0_g2_i1_orf1	muscle-specific protein 20 [Zerene cesonia]	-1.97024	0.394318	0.81288	0.308073	0.454967
TRINITY_DN2318_c1_g1_i1_orf1	transcription factor SPT20 homolog [Ostrinia furnacalis]	-1.67511	1.234698	0.781238	-0.19392	-0.1469
TRINITY_DN7580_c0_g1_i1_orf1	cytochrome P450 monooxygenase CYP6AB141 [Ostrinia furnacalis]	-1.66836	0.848735	0.46355	-0.60233	0.958405
TRINITY_DN35582_c0_g1_i1_orf1	uncharacterized protein LOC114364680 [Ostrinia furnacalis]	-1.80836	0.877276	0.979266	-0.01697	-0.03122
TRINITY_DN29100_c0_g1_i2_orf1	endocuticle structural glycoprotein ABD-5-like [Galleria mellonella]	-1.97515	0.614907	0.386556	0.26639	0.707299
TRINITY_DN416_c0_g1_i1_orf1	unnamed protein product [Diatraea saccharalis]	-1.92964	0.557152	0.875939	0.053963	0.442587
TRINITY_DN46633_c0_g1_i4_orf1	uncharacterized protein LOC114365425 [Ostrinia furnacalis] >QKV49448.1 fas-associated death domain protein [Ostrinia furnacalis]	-1.72154	0.884343	0.250766	1.007152	-0.42072
TRINITY_DN1455_c0_g1_i8_orf1	troponin T, skeletal muscle isoform X1 [Galleria mellonella]	-1.95623	0.32021	0.866528	0.27585	0.493638
TRINITY_DN100_c0_g1_i13_orf1	hypothetical protein O3G_MSEX015273 [Manduca sexta]	-1.9572	0.883726	0.37995	0.304696	0.38883
TRINITY_DN64_c0_g1_i4_orf1	unnamed protein product [Chilo suppressalis]	-1.70559	1.171598	0.660232	-0.43354	0.307308
TRINITY_DN100_c0_g1_i9_orf1	uncharacterized protein LOC114353052 [Ostrinia furnacalis]	-1.95469	0.72977	0.60298	0.099247	0.522688
TRINITY_DN63533_c0_g1_i2_orf1	glutathione S-transferase sigma3 [Glyphodes pyloalis]	-1.81357	0.946633	0.446812	-0.30325	0.723368
TRINITY_DN31001_c0_g1_i1_orf1	endocuticle structural glycoprotein ABD-5-like [Ostrinia furnacalis]	-1.98311	0.522988	0.307089	0.44474	0.70829
TRINITY_DN7778_c0_g1_i1_orf1	peroxiredoxin-2 [Cotesia glomerata] >KAH0561449.1 Peroxiredoxin-4 [Cotesia glomerata]	-1.83438	0.664193	1.038945	-0.16445	0.295697
TRINITY_DN1982_c0_g1_i17_orf1	unnamed protein product, partial [Iphiclides podalirius]	-1.94342	0.73087	0.609404	0.041092	0.56205
TRINITY_DN4724_c0_g1_i4_orf1	paramyosin, long form isoform X1 [Manduca sexta] >KAG6443143.1 hypothetical protein O3G_MSEX002737 [Manduca sexta]	-1.94761	0.867883	0.547	0.187405	0.345326
TRINITY_DN235_c0_g1_i2_orf1	unnamed protein product [Parnassius apollo]	-1.90493	0.887944	0.588143	-0.05477	0.48361
TRINITY_DN76815_c0_g1_i3_orf1	5-formyltetrahydrofolate cyclo-ligase [Ostrinia furnacalis]	-1.32407	0.745601	0.64253	-1.0988	1.034744
TRINITY_DN1123_c2_g1_i3_orf1	troponin I isoform X8 [Ostrinia furnacalis]	-1.95538	0.471589	0.829356	0.165813	0.488627
TRINITY_DN9506_c0_g1_i2_orf1	glutathione S-transferase sigma 4 [Conogethes punctiferalis]	-1.6444	1.221177	0.284889	0.666637	-0.5283
TRINITY_DN16673_c0_g1_i1_orf1	myosin heavy chain, partial [Drosophila virilis]	-1.67728	1.403822	0.421695	-0.19074	0.042503
TRINITY_DN3918_c0_g1_i1_orf1	odorant binding protein 3 [Ostrinia furnacalis]	-1.89299	0.775753	0.446486	0.777249	-0.1065
TRINITY_DN5281_c0_g2_i3_orf1	serine/threonine-protein kinase RIO2 isoform X2 [Ostrinia furnacalis]	-1.7295	1.284925	0.581219	-0.14128	0.00464
TRINITY_DN89613_c0_g1_i13_orf1	PREDICTED: uncharacterized protein LOC106137743 [Amyelois transitella]	-1.49752	1.314648	0.528905	-0.76018	0.41415
TRINITY_DN25492_c0_g1_i1_orf1	PREDICTED: myrosinase 1-like [Amyelois transitella]	-1.81189	1.206473	0.499033	0.111468	-0.00509
TRINITY_DN38366_c0_g1_i4_orfp1	TRINITY_DN38366_c0_g1_i4_m.10666 TRINITY_DN38366_c0_g1::TRINITY_DN38366_c0_g1_i4::g.10666 ORF type:internal len:143 (+),score=71.68 TRINITY_DN38366_c0_g1_i4:3-428(+)	-1.86747	0.82029	0.620037	-0.21305	0.640195
TRINITY_DN27500_c0_g1_i4_orf1	hemicentin-1-like [Ostrinia furnacalis]	-1.73577	0.501679	1.19087	-0.37609	0.419305
TRINITY_DN1123_c2_g1_i4_orf1	troponin I isoform X16 [Ostrinia furnacalis]	-1.98834	0.59807	0.546335	0.29117	0.552766
TRINITY_DN11448_c0_g1_i4_orf1	uncharacterized protein LOC114364760 isoform X5 [Ostrinia furnacalis]	-1.73577	1.353168	0.342247	-0.11782	0.158179
TRINITY_DN928_c0_g1_i3_orf1	fasciclin-2-like [Ostrinia furnacalis]	-1.89232	0.802601	0.81685	0.324184	-0.05132
TRINITY_DN6881_c0_g1_i1_orf1	putative protein TPRXL [Ostrinia furnacalis]	-1.98877	0.469129	0.325254	0.650368	0.544017
TRINITY_DN2200_c0_g1_i4_orf1	uncharacterized protein LOC114363443 [Ostrinia furnacalis]	-1.85039	0.946269	0.624643	0.494275	-0.21479
TRINITY_DN1232_c0_g1_i1_orf1	acanthoscurrin-2-like isoform X1 [Ostrinia furnacalis]	-1.80387	1.014137	0.745229	-0.26167	0.30617
TRINITY_DN146841_c0_g1_i1_orf1	muscle-specific protein 20 [Temnothorax curvispinosus]	-1.99189	0.451053	0.363089	0.630859	0.546894
TRINITY_DN57918_c0_g1_i1_orf1	PREDICTED: serine--tRNA ligase, cytoplasmic [Fopius arisanus]	-1.64118	1.414885	0.492491	-0.24857	-0.01763
TRINITY_DN141381_c0_g1_i1_orf1	very long-chain specific acyl-CoA dehydrogenase, mitochondrial [Chelonus insularis]	-1.87636	1.130097	0.287646	0.144853	0.313761
TRINITY_DN42461_c0_g1_i4_orf1	obscurin [Ostrinia furnacalis]	-1.90869	0.775037	0.778595	-0.03099	0.386052
TRINITY_DN67231_c0_g1_i1_orf1	endocuticle structural glycoprotein SgAbd-8-like [Ostrinia furnacalis]	-1.99725	0.480249	0.523076	0.416982	0.576945
TRINITY_DN38435_c0_g1_i1_orf1	UDP-glucuronosyltransferase 2B20-like [Ostrinia furnacalis]	-1.75649	1.265592	0.545589	0.055915	-0.11061

TRINITY_DN120439_c1_g1_i1_orf1	myosin heavy chain variant, partial [Bombyx mori]	-1.80539	1.159612	0.566858	-0.14953	0.22845
TRINITY_DN248_c0_g1_i12_orf1	twitchin-like [Ostrinia furnacalis]	-1.74055	0.663435	1.197185	0.151972	-0.27205
TRINITY_DN4920_c0_g1_i5_orf1	titin homolog [Ostrinia furnacalis]	-1.84147	1.085239	0.621955	-0.0659	0.200176
TRINITY_DN31118_c1_g1_i1_orf1	endocuticle structural glycoprotein ABD-4-like [Ostrinia furnacalis]	-1.98806	0.661257	0.438561	0.335603	0.552634
TRINITY_DN4501_c0_g1_i3_orf1	methylcrotonoyl-CoA carboxylase subunit alpha, mitochondrial [Ostrinia furnacalis]	-1.64882	1.355238	0.496372	-0.39954	0.196746
TRINITY_DN116951_c0_g3_i2_orf1	spermine oxidase-like isoform X2 [Ostrinia furnacalis]	-1.90202	1.011536	0.263472	0.097748	0.529268
TRINITY_DN4016_c0_g1_i1_orf1	60S acidic ribosomal protein P0 [Homo sapiens] >NP_444505.1 60S acidic ribosomal protein P0 [Homo sapiens] >XP_002823894.1 60S acidic ribosomal protein P0 [Pongo abelii] >XP_003280010.1 60S acidic ribosomal protein P0 [Nomascus leucogenys] >XP_004054038.1 60S acidic ribosomal protein P0 [Gorilla gorilla gorilla] >XP_004054039.1 60S acidic ribosomal protein P0 [Gorilla gorilla gorilla] >XP_008956032.1 60S acidic ribosomal protein P0 [Pan paniscus] >XP_008956033.1 60S acidic ribosomal protein P0 [Pan paniscus] >XP_012611945.1 60S acidic ribosomal protein P0 [Microcebus murinus] >XP_016802006.1 60S acidic ribosomal protein P0 [Pan troglodytes] >XP_016802007.1 60S acidic ribosomal protein P0 [Pan troglodytes] >XP_025256707.1 60S acidic ribosomal protein P0 isoform X1 [Theropithecus gelada] >XP_025256708.1 60S acidic ribosomal protein P0 isoform X1 [Theropithecus gelada] >XP_032024425.1 60S acidic ribosomal protein P0 [Hylobates moloch] >XP_032657670.1 60S acidic ribosomal protein P0 [Chelonoidis abingdonii] >XP_045390642.1 60S acidic ribosomal protein P0 [Lemur catta] >P05388.1 RecName: Full=60S acidic ribosomal protein P0; AltName: Full=60S ribosomal protein L10E; AltName: Full=Large ribosomal subunit protein uL10 [Homo sapiens] >3J92_s Structure and assembly pathway of the ribosome quality control complex [Oryctolagus cuniculus] >4V5Z_Bg Chain Bg, 60S acidic ribosomal protein P0 [Canis lupus familiaris] >4V6X_Cq Chain Cq, 60S acidic ribosomal protein P0 [Homo sapiens] >5AJ0_AK Chain AK, 60S acidic ribosomal protein P0 [Homo sapiens] >6ZM7_Ls Chain Ls, 60S acidic ribosomal protein P0 [Homo sapiens] >6ZME_Ls Chain Ls, 60S acidic ribosomal protein P0 [Homo sapiens] >6ZMI_Ls Chain Ls, 60S acidic ribosomal protein P0 [Homo sapiens] >6ZMO_Ls Chain Ls, 60S acidic ribosomal protein P0 [Homo sapiens] >ABM82739.1 ribosomal protein, large, P0 [synthetic construct] >SJX33952.1 unnamed protein product, partial [Human ORFeome Gateway entry vector] >AAA36470.1 acidic ribosomal phosphoprotein (P0) [Homo sapiens] >AAC05176.1 60S ACIDIC RIBOSOMAL PROTEIN; match to P05388 (PID:g133041) [Homo sapiens]	-1.79528	1.269962	0.297134	-0.04378	0.271962
TRINITY_DN1173_c1_g1_i10_orf1	hypothetical protein evm_001011 [Chilo suppressalis]	-1.81802	0.963132	0.870849	-0.07389	0.057925
TRINITY_DN129869_c0_g4_i1_orf1	putative myosin heavy chain, muscle, partial [Cotesia chilonis]	-1.77899	1.278248	0.39557	-0.08751	0.192682
TRINITY_DN1123_c2_g1_i5_orf1	troponin I isoform X4 [Leguminivora glycinivorella]	-1.76236	1.102257	0.816173	-0.09796	-0.05811
TRINITY_DN110460_c0_g2_i1_orf1	Similar to chaf1a-b: Chromatin assembly factor 1 subunit A-B (Xenopus laevis) [Cotesia congregata]	-1.76475	1.345948	0.04963	0.226271	0.1429
TRINITY_DN4145_c0_g1_i1_orf1	uncharacterized protein LOC114353175 isoform X1 [Ostrinia furnacalis]	-1.69264	1.436618	0.244541	-0.06918	0.08066
TRINITY_DN36817_c0_g1_i1_orf1	uncharacterized protein LOC114357350 [Ostrinia furnacalis]	-1.8316	1.194126	0.448893	0.096298	0.092285
TRINITY_DN2186_c0_g1_i17_orf1	paxillin isoform X6 [Leguminivora glycinivorella]	-1.91734	0.846168	0.69698	0.025832	0.348361
TRINITY_DN20957_c0_g1_i1_orf1	adenylate kinase isoenzyme 1 isoform X2 [Ostrinia furnacalis]	-1.76146	1.263552	0.545682	0.005954	-0.05373
TRINITY_DN21035_c0_g1_i14_orf1	mitochondrial amidoxime reducing component 2-like [Ostrinia furnacalis]	-1.16628	1.132675	0.873331	-1.20776	0.368033
TRINITY_DN96557_c0_g1_i1_orf1	charged multivesicular body protein 4B [Phyllostomus discolor]	-1.81491	1.216149	0.438673	-0.02448	0.184563
TRINITY_DN115082_c0_g1_i5_orf1	protein dj-1beta-like isoform X2 [Ostrinia furnacalis]	-1.43334	1.012946	1.233602	-0.2235	-0.58971
TRINITY_DN5991_c0_g1_i6_orf1	uncharacterized protein LOC114357071 [Ostrinia furnacalis]	-0.85134	1.045458	-0.14092	-1.28377	1.230574
TRINITY_DN1153_c1_g1_i1_orf1	gamma-butyrobetaine dioxygenase [Ostrinia furnacalis]	-0.66034	1.201221	-0.89924	-0.88104	1.23941
TRINITY_DN5011_c0_g1_i1_orf1	probable G-protein coupled receptor 158 isoform X1 [Galleria mellonella]	-1.59997	1.162136	0.02683	-0.50314	0.914146
TRINITY_DN135679_c0_g1_i1_orfp1	TRINITY_DN135679_c0_g1_i1_m.85524 TRINITY_DN135679_c0_g1::TRINITY_DN135679_c0_g1_i1::g.85524 ORF type:5prime_partial len:55 (+),score=3.74,Toxin_2 PF00451.20 1.9e-06 TRINITY_DN135679_c0_g1_i1:3-167(+)	-0.62893	0.979515	0.130123	-1.5656	1.084886
TRINITY_DN20527_c0_g1_i1_orf1	dihydrofolate reductase [Ostrinia furnacalis]	-0.59439	1.130997	-1.36929	-0.34059	1.17328
TRINITY_DN1416_c0_g2_i1_orf1	uncharacterized protein LOC114352565 [Ostrinia furnacalis]	-0.90404	1.180419	-0.70324	-0.83625	1.263114
TRINITY_DN49204_c0_g1_i1_orf1	uncharacterized protein C05D11.1-like [Chelonus insularis]	-1.24982	1.2065	-0.88852	-0.14998	1.081822
TRINITY_DN2473_c0_g1_i2_orf1	translation initiation factor eIF-2B subunit delta [Ostrinia furnacalis]	-1.08948	1.174635	0.185188	-1.22201	0.951666
TRINITY_DN5210_c0_g1_i3_orf1	uncharacterized protein LOC114358376 isoform X2 [Ostrinia furnacalis] >XP_028168126.1 uncharacterized protein LOC114358376 isoform X2 [Ostrinia furnacalis] >XP_028168127.1 uncharacterized protein LOC114358376 isoform X2 [Ostrinia furnacalis] >XP_028168128.1 uncharacterized protein LOC114358376 isoform X2 [Ostrinia furnacalis]	-0.86502	1.386367	-0.35459	-1.12982	0.963064
TRINITY_DN1309_c0_g2_i1_orf1	chymotrypsin-1-like [Ostrinia furnacalis]	-1.64851	1.253005	-0.11685	-0.27651	0.788856
TRINITY_DN9198_c0_g1_i4_orf1	4-coumarate--CoA ligase 1-like isoform X1 [Ostrinia furnacalis]	-1.57035	0.976029	-0.4375	-0.13893	1.170755

TRINITY_DN12293_c0_g1_i1_orf1	hypothetical protein evm_011848 [Chilo suppressalis]	-1.20215	1.255365	-0.27627	-0.85741	1.080466
TRINITY_DN14743_c0_g1_i4_orf1	pseudouridine-5'-phosphatase-like [Ostrinia furnacalis]	-1.50378	1.273074	-0.45426	-0.23932	0.924283
TRINITY_DN1753_c1_g1_i8_orf1	serine/threonine-protein kinase WNK1-like isoform X15 [Ostrinia furnacalis]	-1.0757	1.285562	-0.62546	-0.71757	1.133175
TRINITY_DN11746_c0_g2_i1_orf1	splicing factor 3B subunit 1 isoform X1 [Diprion similis]	-0.91969	1.636765	-0.41099	-0.94658	0.640499
TRINITY_DN5161_c0_g1_i5_orf1	glyoxylate reductase/hydroxypyruvate reductase-like isoform X1 [Ostrinia furnacalis]	-1.27299	1.279645	-0.08555	-0.89103	0.969925
TRINITY_DN14935_c0_g1_i1_orf1	kynurenine/alpha-aminoadipate aminotransferase, mitochondrial [Ostrinia furnacalis]	-1.2248	1.217387	-0.88058	-0.20722	1.095206
TRINITY_DN2146_c0_g1_i1_orf1	heat shock protein 68-like [Ostrinia furnacalis]	-1.64056	1.450834	-0.10881	-0.12207	0.420605
TRINITY_DN1914_c0_g1_i4_orf1	loricrin-like [Ostrinia furnacalis]	-1.52621	0.812463	-0.47094	-0.1449	1.329586
TRINITY_DN2120_c0_g1_i2_orf1	cullin-4A [Ostrinia furnacalis]	-1.12585	1.655468	-0.58563	-0.54091	0.596932
TRINITY_DN1351_c0_g1_i1_orf1	PREDICTED: flavin reductase (NADPH) [Microplitis demolitor] >XP_008553603.1 PREDICTED: flavin reductase (NADPH) [Microplitis demolitor]	-0.74506	1.547129	0.231079	-1.3722	0.33905
TRINITY_DN2967_c0_g1_i7_orf1	UDP-glucuronosyltransferase 1-7C-like isoform X2 [Ostrinia furnacalis]	-0.95933	1.421868	-1.29306	0.271895	0.558622
TRINITY_DN32420_c0_g1_i2_orf1	PREDICTED: plectin-like, partial [Papilio polytes]	-1.39797	1.353257	-0.68314	-0.1274	0.855255
TRINITY_DN1697_c0_g1_i1_orf1	mitogen-activated protein kinase-binding protein 1 [Ostrinia furnacalis]	-1.41098	1.57083	0.272664	-0.64854	0.216029
TRINITY_DN11050_c0_g1_i8_orf1	uncharacterized protein LOC114360965, partial [Ostrinia furnacalis]	-1.51215	1.515993	-0.12629	-0.38133	0.503782
TRINITY_DN1999_c0_g1_i9_orf1	acyl-CoA Delta(11) desaturase-like [Ostrinia furnacalis] >XP_028172986.1 acyl-CoA Delta(11) desaturase-like [Ostrinia furnacalis] >AAL27034.1 acyl-CoA delta-9 desaturase [Ostrinia furnacalis] >AAL29454.1 acyl-CoA delta-9 desaturase [Ostrinia nubilalis]	-0.32373	1.400373	-0.96787	-1.05225	0.943477
TRINITY_DN16354_c0_g1_i2_orf1	uncharacterized protein LOC114349750 isoform X1 [Ostrinia furnacalis]	-1.29884	1.549269	-0.63941	-0.26826	0.657241
TRINITY_DN3964_c1_g1_i2_orf1	phosphoinositide 3-kinase regulatory subunit 4 isoform X1 [Ostrinia furnacalis] >XP_028172384.1 phosphoinositide 3-kinase regulatory subunit 4 isoform X5 [Ostrinia furnacalis]	-0.99815	1.23654	-0.13444	-1.15904	1.055092
TRINITY_DN640_c0_g1_i5_orf1	pancreatic triacylglycerol lipase-like [Ostrinia furnacalis]	-1.4113	1.623298	-0.52399	-0.00197	0.313965
TRINITY_DN3970_c0_g1_i1_orf1	hypothetical protein evm_002369 [Chilo suppressalis]	-1.3716	1.483247	0.635601	-0.71678	-0.03047
TRINITY_DN32896_c0_g3_i1_orf1	PREDICTED: calcium-binding mitochondrial carrier protein Aralar1 isoform X1 [Microplitis demolitor]	-1.48461	1.530478	-0.20898	-0.36375	0.526862
TRINITY_DN496_c0_g1_i7_orf1	unnamed protein product [Diatraea saccharalis]	-1.40262	1.27702	0.896631	-0.77326	0.002226
TRINITY_DN61_c0_g2_i3_orf1	mitochondrial dicarboxylate carrier [Ostrinia furnacalis] >XP_028161565.1 mitochondrial dicarboxylate carrier [Ostrinia furnacalis] >XP_028161566.1 mitochondrial dicarboxylate carrier [Ostrinia furnacalis]	-1.23366	1.779669	-0.17765	-0.50935	0.140982
TRINITY_DN6365_c0_g1_i4_orf1	40S ribosomal protein S21 [Helicoverpa armigera] >XP_047038308.1 40S ribosomal protein S21 isoform X2 [Helicoverpa zea] >KAI5643652.1 ribosomal protein s21e domain-containing protein [Phthorimaea operculella] >PZC73652.1 hypothetical protein B5X24_HaOG209026 [Helicoverpa armigera]	-1.6359	1.314185	0.256471	-0.48159	0.546838
TRINITY_DN5753_c0_g1_i10_orf1	ryanodine receptor [Ostrinia furnacalis]	-1.56902	1.469286	0.058453	-0.41244	0.453717
TRINITY_DN6994_c0_g1_i4_orf1	C-type mannose receptor 2-like isoform X1 [Ostrinia furnacalis]	-0.99004	1.608339	-0.70711	-0.63718	0.725992
TRINITY_DN8584_c0_g1_i6_orf1	uncharacterized protein LOC114354070 isoform X3 [Ostrinia furnacalis]	-1.41194	1.698355	-0.17129	-0.26498	0.149852
TRINITY_DN348_c0_g2_i1_orf1	pancreatic triacylglycerol lipase-like [Ostrinia furnacalis]	-1.53256	1.611186	-0.14948	-0.08806	0.158909
TRINITY_DN8717_c0_g1_i5_orf1	hypothetical protein evm_006607 [Chilo suppressalis] >CAG9745590.1 unnamed protein product [Diatraea saccharalis] >CAG9784275.1 unnamed protein product [Diatraea saccharalis]	-0.94121	1.758362	-0.72559	-0.54158	0.450008
TRINITY_DN23204_c0_g1_i1_orf1	LOW QUALITY PROTEIN: uncharacterized protein LOC114350452 [Ostrinia furnacalis]	-1.50178	1.508347	-0.56136	0.26138	0.293415
TRINITY_DN126648_c0_g1_i1_orf1	elongation factor 1 alpha, partial [Spodoptera exigua] >QYQ52647.1 elongation factor 1 alpha, partial [Spodoptera exigua]	-0.84503	1.488137	0.377259	-1.34937	0.329006
TRINITY_DN73945_c0_g5_i3_orf1	cyclin-dependent kinase 12 isoform X1 [Diachasma alloeum] >XP_015114851.1 cyclin-dependent kinase 12 isoform X1 [Diachasma alloeum] >XP_015114852.1 cyclin-dependent kinase 12 isoform X1 [Diachasma alloeum] >XP_015114853.1 cyclin-dependent kinase 12 isoform X1 [Diachasma alloeum] >XP_015114854.1 cyclin-dependent kinase 12 isoform X1 [Diachasma alloeum]	-0.55164	1.682055	-0.26619	-1.27535	0.411119
TRINITY_DN41166_c0_g1_i1_orf1	arginine kinase isoform X1 [Ostrinia furnacalis]	-1.49214	1.336461	0.82594	-0.53588	-0.13439
TRINITY_DN50676_c0_g1_i1_orf1	uncharacterized protein LOC114360659 [Ostrinia furnacalis]	-1.55559	1.298884	0.166872	-0.61127	0.701102
TRINITY_DN133228_c0_g1_i3_orf1	microtubule-actin cross-linking factor 1 isoform X15 [Ostrinia furnacalis]	-1.56027	1.547782	-0.19828	-0.12744	0.338202
TRINITY_DN2749_c0_g2_i3_orf1	RNA exonuclease 4-like [Ostrinia furnacalis] >QEE79882.1 REX4 [Ostrinia furnacalis]	-0.69486	1.210718	-1.20897	-0.47488	1.167993
TRINITY_DN64759_c0_g1_i1_orf1	mitochondrial inner membrane protein OXA1L-like [Ostrinia furnacalis]	-1.67257	1.460837	-0.00879	-0.0382	0.258723
TRINITY_DN56250_c0_g1_i7_orf1	sex-lethal homolog isoform X3 [Ostrinia furnacalis] >XP_028172304.1 sex-lethal homolog isoform X4 [Ostrinia furnacalis]	-1.47216	1.354433	-0.71123	0.141686	0.687268

TRINITY_DN15234_c0_g1_i3_orf1	60S ribosomal protein L30 [Papilio polytes] >XP_014360326.1 60S ribosomal protein L30 [Papilio machaon] >XP_026485186.1 60S ribosomal protein L30 isoform X1 [Vanessa tameamea] >XP_028160279.1 60S ribosomal protein L30 [Ostrinia furnacalis] >XP_030027999.1 60S ribosomal protein L30 [Manduca sexta] >XP_032515151.1 60S ribosomal protein L30 [Danaus plexippus plexippus] >XP_034840952.1 60S ribosomal protein L30 [Maniola hyperantus] >XP_037301873.1 60S ribosomal protein L30 [Manduca sexta] >XP_039745408.1 60S ribosomal protein L30 [Pararge aegeria] >XP_041974708.1 60S ribosomal protein L30 [Aricia agestis] >XP_045455248.1 60S ribosomal protein L30 [Melitaea cinxia] >XP_045457914.1 60S ribosomal protein L30 [Melitaea cinxia] >XP_046969892.1 60S ribosomal protein L30 [Vanessa cardui] >XP_047539529.1 60S ribosomal protein L30 [Vanessa atalanta] >XP_049887645.1 60S ribosomal protein L30 [Pectinophora gossypiella] >XP_050360253.1 60S ribosomal protein L30 [Nymphalis io] >ADT80684.1 ribosomal protein L30 [Euphydryas aurinia] >CAG9575798.1 unnamed protein product [Danaus chrysippus] >CAH0722581.1 unnamed protein product, partial [Brenthis ino] >CAH2099946.1 unnamed protein product [Euphydryas editha] >CAH2267204.1 jg2932 [Pararge aegeria aegeria] >GBP56353.1 60S ribosomal protein L30 [Eumeta japonica]	-1.37761	1.705374	0.139958	-0.41402	-0.0537
TRINITY_DN7329_c0_g1_i6_orf1	serine hydrolase-like protein 2 isoform X2 [Ostrinia furnacalis]	-0.62332	1.697818	-1.04297	-0.58172	0.550187
TRINITY_DN34399_c0_g1_i1_orf1	cysteine synthase-like [Ostrinia furnacalis]	-1.38389	1.483563	0.590036	-0.7308	0.041089
TRINITY_DN14301_c0_g1_i1_orf1	apoptosis-inducing factor 1, mitochondrial-like [Ostrinia furnacalis]	-1.00864	1.826843	-0.74317	-0.24984	0.174813
TRINITY_DN26824_c0_g1_i1_orf1	60S ribosomal protein L6 [Hyposmocoma kahamanoa]	-0.45044	1.484164	0.053472	-1.54372	0.456531
TRINITY_DN146126_c0_g1_i1_orf1	malate dehydrogenase, mitochondrial [Chelonus insularis]	-1.69403	1.437125	-0.03705	0.046121	0.247834
TRINITY_DN863_c0_g1_i6_orf1	protein henna [Galleria mellonella]	-1.34925	1.484868	-0.75282	-0.02115	0.638355
TRINITY_DN25901_c0_g1_i2_orf1	short-chain specific acyl-CoA dehydrogenase, mitochondrial-like isoform X2 [Ostrinia furnacalis]	-1.16517	1.640975	-0.53648	-0.54409	0.604761
TRINITY_DN136906_c0_g1_i1_orf1	translational elongation factor-1alpha, partial [Ethmia eupostica]	-1.42084	1.491267	0.677518	-0.4704	-0.27754
TRINITY_DN6916_c0_g1_i4_orf1	isovaleryl-CoA dehydrogenase, mitochondrial [Ostrinia furnacalis]	-1.56221	1.544847	0.343772	-0.13592	-0.19049
TRINITY_DN2224_c0_g1_i1_orf1	serine--tRNA ligase, cytoplasmic [Ostrinia furnacalis]	-1.57016	1.4605	0.511975	-0.37218	-0.03014
TRINITY_DN130159_c0_g2_i1_orf1	lachesin-like [Chelonus insularis] >XP_034946935.1 lachesin-like [Chelonus insularis]	-1.47105	1.542543	0.443833	-0.50945	-0.00587
TRINITY_DN1752_c0_g1_i18_orf1	titin isoform X1 [Ostrinia furnacalis]	-1.57231	1.552012	0.13451	-0.27446	0.16025
TRINITY_DN147676_c0_g1_i1_orf1	PREDICTED: 60S ribosomal protein L23 [Microplitis demolitor] >XP_044591174.1 60S ribosomal protein L23 [Cotesia glomerata] >KAG8035666.1 hypothetical protein G9C98_001094 [Cotesia typhae] >KAH0547433.1 60S ribosomal protein L23A [Cotesia glomerata]	-1.29281	1.544897	0.511122	-0.82288	0.059672
TRINITY_DN7414_c0_g1_i1_orf1	uncharacterized protein LOC114357447 [Ostrinia furnacalis]	-1.28517	1.651577	0.254429	-0.73656	0.115717
TRINITY_DN95414_c0_g1_i1_orf1	protein arginine N-methyltransferase 5 [Ostrinia furnacalis]	-1.04176	1.612768	0.477902	-1.04176	-0.00715
TRINITY_DN79673_c0_g1_i1_orf1	thioredoxin, mitochondrial-like [Ostrinia furnacalis]	-1.69769	1.387605	-0.12175	0.010573	0.421261
TRINITY_DN4385_c0_g2_i1_orf1	LOW QUALITY PROTEIN: carbonic anhydrase 1-like [Ostrinia furnacalis]	-1.3891	1.509474	0.568184	-0.68487	-0.00369
TRINITY_DN21872_c0_g1_i2_orf1	facilitated trehalose transporter Tret1-2 homolog [Ostrinia furnacalis] >XP_028178438.1 facilitated trehalose transporter Tret1-2 homolog [Ostrinia furnacalis] >XP_028178439.1 facilitated trehalose transporter Tret1-2 homolog [Ostrinia furnacalis]	-1.52984	1.498906	0.476721	-0.01523	-0.43056
TRINITY_DN13718_c0_g1_i7_orf1	immulectin-4 [Ostrinia furnacalis]	-1.64062	1.4608	-0.21285	0.357633	0.035033
TRINITY_DN38562_c0_g1_i3_orf1	persulfide dioxygenase ETHE1, mitochondrial isoform X1 [Ostrinia furnacalis]	-1.51066	1.447818	-0.6071	0.454895	0.21504
TRINITY_DN12584_c0_g1_i1_orf1	carnitine O-palmitoyltransferase 1, liver isoform [Ostrinia furnacalis]	-0.9968	1.887118	-0.42235	-0.51443	0.046459
TRINITY_DN1173_c0_g1_i12_orf1	obscurin [Ostrinia furnacalis]	-1.40579	1.630416	0.076015	-0.54689	0.246245
TRINITY_DN21451_c0_g1_i3_orf1	gelsolin-like [Ostrinia furnacalis]	-1.44428	1.616321	-0.45651	-0.02007	0.304541
TRINITY_DN22956_c0_g1_i1_orf1	lipoamide acyltransferase component of branched-chain alpha-keto acid dehydrogenase complex, mitochondrial [Ostrinia furnacalis]	-1.54244	1.518321	-0.33384	-0.08567	0.443625
TRINITY_DN38835_c0_g3_i1_orf1	protein transport protein Sec61 subunit alpha [Spodoptera litura] >XP_035429226.1 protein transport protein Sec61 subunit alpha [Spodoptera frugiperda] >XP_047985890.1 protein transport protein Sec61 subunit alpha [Leguminivora glycinivorella] >KAF9413961.1 hypothetical protein HW555_007991 [Spodoptera exigua] >CAB3514725.1 unnamed protein product [Spodoptera littoralis] >KAF9810869.1 hypothetical protein SFRURICE_005295 [Spodoptera frugiperda] >KAG8115796.1 hypothetical protein SFRUCORN_012373 [Spodoptera frugiperda] >CAH0700181.1 unnamed protein product [Spodoptera exigua]	-1.60512	1.414528	0.258504	-0.45442	0.386507
TRINITY_DN7267_c1_g1_i4_orf1	probable pseudouridine-5'-phosphatase [Ostrinia furnacalis]	-1.31582	1.727576	-0.02671	-0.51599	0.130941
TRINITY_DN72541_c0_g1_i2_orf1	xaa-Pro aminopeptidase ApepP-like isoform X2 [Ostrinia furnacalis]	-1.54388	1.478351	0.545997	-0.147	-0.33348
TRINITY_DN13094_c0_g1_i1_orf1	probable ATP-dependent RNA helicase DDX56 [Ostrinia furnacalis]	-1.06868	1.515926	-1.07966	0.627851	0.004565

TRINITY_DN9248_c0_g1_i10_orf1	unnamed protein product [Arctia plantaginis]	-1.43781	1.657246	0.241015	-0.33542	-0.12502
TRINITY_DN10629_c0_g1_i1_orf1	caspase-1-like [Ostrinia furnacalis]	-1.71015	1.301042	0.083211	-0.23871	0.564605
TRINITY_DN5354_c0_g1_i4_orf1	NADP-dependent malic enzyme-like [Ostrinia furnacalis]	-1.21312	1.531794	-0.78709	0.709991	-0.24158
TRINITY_DN14597_c0_g1_i5_orf1	UDP-glucuronosyltransferase 2B1-like isoform X3 [Ostrinia furnacalis]	-1.40932	1.576022	-0.23103	-0.45492	0.519259
TRINITY_DN19830_c0_g1_i1_orf1	macrophage migration inhibitory factor-like [Ostrinia furnacalis]	-1.64011	1.485178	-0.10957	0.301538	-0.03705
TRINITY_DN6027_c0_g1_i13_orf1	5-demethoxyubiquinone hydroxylase, mitochondrial [Ostrinia furnacalis] >XP_028160430.1 5-demethoxyubiquinone hydroxylase, mitochondrial [Ostrinia furnacalis]	-1.65023	1.438938	0.073257	0.378294	-0.24026
TRINITY_DN80245_c0_g1_i1_orf1	peroxisomal membrane protein 2 [Ostrinia furnacalis]	-1.5158	1.589881	-0.03254	-0.31463	0.273091
TRINITY_DN135_c0_g1_i1_orf1	60S ribosomal protein L11 [Nymphalis io]	-1.45396	1.691973	-0.05375	-0.13305	-0.05121
TRINITY_DN7808_c0_g1_i1_orf1	probable pyruvate dehydrogenase E1 component subunit alpha, mitochondrial isoform X1 [Ostrinia furnacalis] >XP_028158738.1 probable pyruvate dehydrogenase E1 component subunit alpha, mitochondrial isoform X2 [Ostrinia furnacalis] >XP_028158739.1 probable pyruvate dehydrogenase E1 component subunit alpha, mitochondrial isoform X3 [Ostrinia furnacalis] >XP_028158740.1 probable pyruvate dehydrogenase E1 component subunit alpha, mitochondrial isoform X4 [Ostrinia furnacalis]	-1.47604	1.665586	-0.19602	-0.06268	0.069155
TRINITY_DN2265_c0_g1_i5_orf1	elongation factor G, mitochondrial [Ostrinia furnacalis]	-0.85617	1.677075	-1.17813	0.214692	0.14253
TRINITY_DN14996_c0_g1_i2_orf1	40S ribosomal protein S17 [Ostrinia furnacalis]	-1.09609	1.823644	0.016547	-0.68494	-0.05916
TRINITY_DN1383_c0_g1_i2_orf1	uncharacterized protein LOC114353133 isoform X1 [Ostrinia furnacalis] >XP_028160773.1 uncharacterized protein LOC114353133 isoform X2 [Ostrinia furnacalis]	-1.10696	1.64306	-0.94945	0.416553	-0.0032
TRINITY_DN83542_c0_g1_i1_orf1	PREDICTED: WASH complex subunit strumpellin [Microplitis demolitor]	-1.38549	1.706301	-0.29772	0.18853	-0.21162
TRINITY_DN14934_c0_g1_i17_orf1	putative tricarboxylate transport protein, mitochondrial isoform X1 [Ostrinia furnacalis] >XP_028177526.1 putative tricarboxylate transport protein, mitochondrial isoform X2 [Ostrinia furnacalis]	-1.14051	1.852509	-0.36296	-0.36791	0.018869
TRINITY_DN105055_c0_g1_i1_orfp1	unnamed protein product [Euphydryas editha]	-1.07529	1.873616	-0.54998	-0.12531	-0.12304
TRINITY_DN4497_c0_g1_i4_orf1	cytochrome P450 9e2-like [Ostrinia furnacalis] >QPF77612.1 cytochrome P450 monooxygenase CYP9A185 [Ostrinia furnacalis]	-0.55835	1.804825	-0.73395	-0.87304	0.360513
TRINITY_DN5597_c0_g1_i2_orf1	monocarboxylate transporter 9-like [Ostrinia furnacalis] >XP_028156211.1 monocarboxylate transporter 9-like [Ostrinia furnacalis]	-1.06658	1.843463	-0.67662	-0.02548	-0.07479
TRINITY_DN1884_c0_g2_i2_orf1	phosphotriesterase-related protein [Ostrinia furnacalis]	-0.81053	1.755506	-1.11566	0.053848	0.116834
TRINITY_DN3949_c1_g1_i1_orf1	probable cytochrome P450 304a1 isoform X2 [Ostrinia furnacalis]	-0.95942	1.595068	-0.74124	-0.6473	0.752889
TRINITY_DN11194_c0_g1_i4_orf1	ATPase family AAA domain-containing protein 3A homolog [Ostrinia furnacalis]	-1.36186	1.707417	-0.05414	-0.44957	0.158157
TRINITY_DN147596_c0_g1_i1_orf1	activator of basal transcription 1 [Diachasma alloeum]	-0.86284	1.7748	-0.23736	-0.97828	0.30368
TRINITY_DN2749_c0_g1_i4_orf1	RNA exonuclease 4-like [Ostrinia furnacalis] >QEE79882.1 REX4 [Ostrinia furnacalis]	-0.92033	1.360273	-1.35292	0.623052	0.289927
TRINITY_DN1791_c0_g1_i3_orf1	succinate dehydrogenase assembly factor 2-B, mitochondrial-like [Ostrinia furnacalis]	-0.89252	1.703931	0.339757	-1.08646	-0.0647
TRINITY_DN1656_c2_g1_i5_orf1	15-hydroxyprostaglandin dehydrogenase [NAD(+)]-like [Ostrinia furnacalis]	-1.32828	1.455867	-0.51829	-0.42561	0.81631
TRINITY_DN10831_c1_g1_i1_orf1	40S ribosomal protein S16 [Ostrinia furnacalis]	-1.04425	1.715194	0.416861	-0.86188	-0.22593
TRINITY_DN1081_c0_g1_i7_orf1	3-ketoacyl-CoA thiolase, mitochondrial-like [Ostrinia furnacalis]	-0.76584	1.918511	-0.72386	-0.45616	0.027349
TRINITY_DN32769_c1_g1_i5_orf1	large subunit GTPase 1 homolog [Ostrinia furnacalis]	-1.02845	1.890646	-0.22421	-0.55771	-0.08029
TRINITY_DN7464_c0_g1_i14_orf1	60S ribosomal protein L9 [Nymphalis io]	-1.05558	1.766885	0.289503	-0.79977	-0.20103
TRINITY_DN22678_c0_g1_i4_orf1	NADH-cytochrome b5 reductase 2 isoform X2 [Ostrinia furnacalis] >XP_028163866.1 NADH-cytochrome b5 reductase 2 isoform X2 [Ostrinia furnacalis]	-0.75245	1.913842	-0.56606	-0.66739	0.072058
TRINITY_DN1445_c0_g2_i4_orf1	leucine-rich PPR motif-containing protein, mitochondrial [Ostrinia furnacalis]	-1.47374	1.669088	0.01793	-0.00873	-0.20455

TRINITY_DN97589_c0_g1_i3_orf1	ribosomal protein L37A [Bombyx mori] >XP_013189707.1 PREDICTED: 60S ribosomal protein L37a [Amyelois transitella] >XP_021198447.1 60S ribosomal protein L37a [Helicoverpa armigera] >XP_022122377.1 60S ribosomal protein L37a [Pieris rapae] >XP_022822835.1 60S ribosomal protein L37a [Spodoptera litura] >XP_023937141.1 60S ribosomal protein L37a [Bicyclus anynana] >XP_026321523.1 60S ribosomal protein L37a [Hyposmocoma kahamanoa] >XP_026495655.1 60S ribosomal protein L37a [Vanessa tameamea] >XP_026746489.1 60S ribosomal protein L37a [Trichoplusia ni] >XP_026756267.1 60S ribosomal protein L37a [Galleria mellonella] >XP_028041705.1 60S ribosomal protein L37a [Bombyx mandarina] >XP_028161757.1 60S ribosomal protein L37a [Ostrinia furnacalis] >XP_030020263.1 LOW QUALITY PROTEIN: 60S ribosomal protein L37a [Manduca sexta] >XP_032518929.1 60S ribosomal protein L37a [Danaus plexippus plexippus] >XP_034834514.1 60S ribosomal protein L37a [Maniola hyperantus] >XP_035444256.1 60S ribosomal protein L37a [Spodoptera frugiperda] >XP_038222439.1 60S ribosomal protein L37a [Zerene cesonia] >XP_039756348.1 60S ribosomal protein L37a [Pararge aegeria] >XP_041981914.1 60S ribosomal protein L37a [Aricia agestis] >XP_045451710.1 60S ribosomal protein L37a [Melitaea cinxia] >XP_045500579.1 60S ribosomal protein L37a [Colias croceus] >XP_045517305.1 60S ribosomal protein L37a [Pieris brassicae] >XP_045775103.1 60S ribosomal protein L37a [Maniola jurtina] >XP_046969745.1 60S ribosomal protein L37a [Vanessa cardui] >XP_047032252.1 60S ribosomal protein L37a [Helicoverpa zea] >XP_047525321.1 60S ribosomal protein L37a [Pieris napi] >XP_047535357.1 60S ribosomal protein L37a [Vanessa atalanta] >XP_049875744.1 60S ribosomal protein L37a [Pectinophora gossypiella] >XP_050348149.1 60S ribosomal protein L37a [Nymphalis io] >ADO95156.1 ribosomal protein L37A [Antheraea yamamai] >ADT80705.1 ribosomal protein L37A [Euphydryas aurinia] >AEL28885.1 ribosomal protein L37A [Heliconius melpomene cythera] >KAF9418899.1 hypothetical protein HW555_004419 [Spodoptera exigua] >KOB75009.1 Ribosomal protein L37A [Operophtera brumata] >RVE49828.1 hypothetical protein evm_005558 [Chilo suppressalis] >CAB3234150.1 unnamed protein product [Arctia plantaginis] >CAB3509616.1 unnamed protein product [Spodoptera littoralis] >CAF4811073.1 unnamed protein product [Pieris macdunnoughi] >CAG4956733.1 unnamed protein product [Parnassius apollo] >CAG9564640.1 unnamed protein product [Danaus chrysippus] >CAG9750098.1 unnamed protein product [Diatraea saccharalis] >CAH0725676.1 unnamed protein	-1.17543	1.659064	0.310792	-0.87351	0.079085
TRINITY_DN29229_c0_g1_i4_orf1	uncharacterized protein LOC114351433 isoform X1 [Ostrinia furnacalis]	-1.17083	1.55509	0.738208	-0.69528	-0.42719
TRINITY_DN74889_c0_g1_i1_orf1	probable 28S ribosomal protein S23, mitochondrial [Ostrinia furnacalis]	-1.15941	1.807606	-0.59373	-0.15822	0.103759
TRINITY_DN15513_c0_g1_i6_orf1	uncharacterized protein LOC114350859 [Ostrinia furnacalis]	-1.33514	1.632541	-0.52026	-0.24683	0.469691
TRINITY_DN42646_c0_g2_i1_orf1	40S ribosomal protein S3 [Helicoverpa armigera] >XP_026740562.1 40S ribosomal protein S3 [Trichoplusia ni] >XP_026751545.1 40S ribosomal protein S3 [Galleria mellonella] >XP_047027704.1 40S ribosomal protein S3 [Helicoverpa zea] >CAH0591481.1 unnamed protein product [Chrysodeixis includens] >AIR07416.1 ribosomal protein S3 [Helicoverpa armigera] >AND95944.1 ribosomal protein S3 [Helicoverpa armigera] >AXY94820.1 ribosomal ribosomal protein S3 [Galleria mellonella] >PZC80336.1 hypothetical protein B5X24_HaOG214853 [Helicoverpa armigera]	-0.92257	1.809772	0.180307	-0.90206	-0.16545
TRINITY_DN2924_c0_g1_i2_orf1	cuticular protein RR-2 [Spodoptera litura]	-1.55235	1.369739	-0.12017	-0.42033	0.723117
TRINITY_DN35277_c0_g1_i1_orf1	luciferin 4-monooxygenase-like, partial [Ostrinia furnacalis]	-0.72567	1.915413	-0.82641	-0.34847	-0.01487
TRINITY_DN3461_c0_g1_i1_orf1	protein SCO1 homolog, mitochondrial [Ostrinia furnacalis]	-1.40517	1.718594	-0.12822	-0.23107	0.045863
TRINITY_DN55160_c0_g1_i1_orf1	esterase FE4-like isoform X2 [Ostrinia furnacalis]	-1.15504	1.840943	-0.50006	-0.16178	-0.02406
TRINITY_DN1952_c0_g1_i2_orf1	uncharacterized protein LOC114354403 [Ostrinia furnacalis] >AYE20402.1 RNAi efficiency-related nuclease REase [Ostrinia furnacalis]	-1.63202	1.499181	-0.0922	-0.05351	0.278549
TRINITY_DN4451_c0_g2_i4_orf1	uncharacterized protein LOC114361986 isoform X1 [Ostrinia furnacalis] >XP_028173022.1 uncharacterized protein LOC114361986 isoform X2 [Ostrinia furnacalis]	-1.43708	1.630471	0.14136	0.149126	-0.48388
TRINITY_DN1666_c0_g1_i2_orf1	putative defense protein Hdd11 [Ostrinia furnacalis] >XP_028179344.1 putative defense protein Hdd11 [Ostrinia furnacalis] >AGV28583.1 immune-induced protein [Ostrinia furnacalis]	-0.97649	1.659134	-1.05076	-0.06283	0.430937
TRINITY_DN113626_c0_g1_i3_orfp1	TRINITY_DN113626_c0_g1_i3_m.80721 TRINITY_DN113626_c0_g1::TRINITY_DN113626_c0_g1_i3::g.80721 ORF type:internal len:118 (-),score=87.34 TRINITY_DN113626_c0_g1_i3:2-352(-)	-0.99263	1.533334	-0.34335	-0.97224	0.774883
TRINITY_DN2283_c0_g2_i1_orf1	H/ACA ribonucleoprotein complex subunit 4 [Ostrinia furnacalis]	-0.94685	1.916264	-0.32917	-0.56313	-0.07712
TRINITY_DN3836_c0_g1_i4_orf1	2-oxoisovalerate dehydrogenase subunit alpha, mitochondrial [Ostrinia furnacalis]	-1.09053	1.775928	-0.20803	-0.73848	0.261117
TRINITY_DN106534_c0_g1_i1_orf1	nucleolar complex protein 2 homolog [Ostrinia furnacalis]	-1.4871	1.427802	0.505942	-0.66728	0.220636
TRINITY_DN32681_c0_g1_i3_orf1	long-chain-fatty-acid--CoA ligase ACSBG2 isoform X2 [Ostrinia furnacalis]	-1.05521	1.871884	-0.44572	-0.42538	0.054431

TRINITY_DN51934_c0_g2_i1_orf1	SCAN domain-containing protein 3-like [Pieris napi] >XP_047520696.1 SCAN domain-containing protein 3-like [Pieris napi]	-1.19098	1.766212	-0.1776	-0.61802	0.220391
TRINITY_DN4036_c0_g2_i1_orf1	microvitellogenin-like [Ostrinia furnacalis]	-0.35704	1.650419	-0.02045	-1.4544	0.181473
TRINITY_DN17417_c0_g1_i11_orf1	sodium/hydrogen exchanger 9B2-like isoform X4 [Ostrinia furnacalis]	-1.03196	1.872808	-0.60611	-0.24529	0.010553
TRINITY_DN94337_c0_g1_i1_orf1	hypothetical protein evm_006136 [Chilo suppressalis]	-0.98384	1.891657	-0.63862	-0.20394	-0.06525
TRINITY_DN1757_c0_g1_i4_orf1	F-box/LRR-repeat protein 2 isoform X1 [Ostrinia furnacalis]	-1.32652	1.755205	-0.31964	-0.21497	0.105924
TRINITY_DN4622_c0_g1_i1_orf1	keratin-associated protein 19-2-like [Ostrinia furnacalis]	-1.44174	1.666368	0.20873	-0.15691	-0.27645
TRINITY_DN48619_c0_g1_i1_orf1	PREDICTED: lysine--tRNA ligase isoform X2 [Fopius arisanus]	-1.03907	1.73373	0.311674	-0.89752	-0.10882
TRINITY_DN2172_c0_g2_i5_orf1	4-hydroxyphenylpyruvate dioxygenase [Ostrinia furnacalis]	-1.38359	1.673129	-0.16422	-0.41727	0.291948
TRINITY_DN8603_c0_g1_i1_orf1	adenosine kinase 2 isoform X2 [Cotesia glomerata] >XP_044591805.1 adenosine kinase 2 isoform X4 [Cotesia glomerata]	-1.17514	1.487799	-1.02976	0.148808	0.56829
TRINITY_DN30131_c0_g1_i1_orf1	PREDICTED: 60S ribosomal protein L44 [Amyelois transitella] >XP_021198018.1 60S ribosomal protein L44 [Helicoverpa armigera] >XP_022814294.1 60S ribosomal protein L44 [Spodoptera litura] >XP_026732397.1 60S ribosomal protein L44 [Trichoplusia ni] >XP_026752106.1 60S ribosomal protein L44 [Galleria mellonella] >XP_028158932.1 60S ribosomal protein L44 [Ostrinia furnacalis] >XP_035434364.1 60S ribosomal protein L44 [Spodoptera frugiperda] >XP_035434370.1 60S ribosomal protein L44 [Spodoptera frugiperda] >XP_047019234.1 60S ribosomal protein L44 [Helicoverpa zea] >XP_049868501.1 60S ribosomal protein L44 [Pectinophora gossypiella] >AAM53948.1 ribosomal protein L44 [Choristoneura parallela] >KAF9418375.1 hypothetical protein HW555_004805 [Spodoptera exigua] >RVE50750.1 hypothetical protein evm_004660 [Chilo suppressalis] >CAB3235328.1 unnamed protein product [Arctia plantaginis] >CAB3516516.1 unnamed protein product [Spodoptera littoralis] >CAG9747186.1 unnamed protein product [Diatraea saccharalis] >CAH0581656.1 unnamed	-0.99822	1.825588	0.062215	-0.81305	-0.07653
TRINITY_DN108200_c0_g1_i1_orf1	uncharacterized protein LOC114350842 [Ostrinia furnacalis]	-0.871	1.951196	-0.44457	-0.44924	-0.18639
TRINITY_DN27087_c0_g1_i1_orf1	2',5'-phosphodiesterase 12 [Ostrinia furnacalis]	-0.66892	1.908188	-0.31207	-0.90185	-0.02535
TRINITY_DN10458_c0_g1_i1_orf1	V-type proton ATPase 21 kDa proteolipid subunit [Ostrinia furnacalis]	-0.89704	1.896026	-0.72199	-0.28127	0.004275
TRINITY_DN7512_c0_g1_i1_orf1	hypothetical protein evm_010529 [Chilo suppressalis] >CAB3530682.1 unnamed protein product [Chilo suppressalis] >CAH0407273.1 unnamed protein product [Chilo suppressalis]	-0.88853	1.926319	-0.65029	-0.22429	-0.16322
TRINITY_DN20682_c0_g1_i2_orf1	hypothetical protein B5X24_HaOG200252 [Helicoverpa armigera]	-1.28203	1.774663	0.054011	-0.4386	-0.10804
TRINITY_DN58207_c0_g1_i1_orf1	60S ribosomal protein L6 [Ostrinia furnacalis] >XP_028170357.1 60S ribosomal protein L6 [Ostrinia furnacalis]	-0.95206	1.824443	0.192417	-0.81619	-0.24861
TRINITY_DN2184_c0_g1_i4_orf1	uncharacterized protein LOC114359356 [Ostrinia furnacalis]	-0.70694	1.82781	-1.05199	-0.19301	0.124134
TRINITY_DN3929_c0_g1_i1_orf1	glutathione S-transferase 1-1-like [Ostrinia furnacalis]	-0.83552	1.938498	-0.66623	-0.26788	-0.16887
TRINITY_DN5012_c0_g1_i6_orf1	putative serine protease K12H4.7 [Ostrinia furnacalis]	-1.2725	1.782533	-0.44481	-0.07343	0.008208
TRINITY_DN107617_c3_g1_i1_orf1	NADH dehydrogenase [ubiquinone] 1 alpha subcomplex subunit 9, mitochondrial [Ostrinia furnacalis]	-0.98831	1.919952	-0.15509	-0.46396	-0.31259
TRINITY_DN3355_c0_g2_i4_orf1	UDP-glycosyltransferase UGT33AL1 [Ostrinia furnacalis]	-1.07225	1.834711	-0.69318	-0.01	-0.05928
TRINITY_DN9647_c0_g1_i1_orf1	cytochrome P450 6B2-like [Ostrinia furnacalis]	-1.02066	1.912071	-0.2686	-0.44585	-0.17696
TRINITY_DN14063_c0_g1_i7_orf1	probable phosphorylase b kinase regulatory subunit beta isoform X1 [Ostrinia furnacalis] >XP_028175664.1 probable phosphorylase b kinase regulatory subunit beta isoform X2 [Ostrinia furnacalis] >XP_028175665.1 probable phosphorylase b kinase regulatory subunit beta isoform X3 [Ostrinia furnacalis]	-0.73777	1.938828	-0.48177	-0.68046	-0.03882
TRINITY_DN11117_c0_g1_i1_orf1	venom carboxylesterase-6-like [Ostrinia furnacalis]	-0.7702	1.958077	-0.64429	-0.34206	-0.20153
TRINITY_DN47389_c0_g1_i2_orf1	non-specific lipid-transfer protein-like [Ostrinia furnacalis]	-0.8755	1.894553	-0.56032	-0.56477	0.10604
TRINITY_DN6351_c0_g1_i4_orf1	cytochrome P450 CYP12A2-like [Ostrinia furnacalis]	-1.10519	1.889945	-0.25804	-0.23728	-0.28944
TRINITY_DN28638_c0_g1_i1_orf1	uncharacterized protein LOC114364075 [Ostrinia furnacalis]	-1.01283	1.895258	-0.45617	-0.41713	-0.00913
TRINITY_DN106730_c0_g1_i1_orf1	Photosystem I reaction center subunit II, chloroplastic, partial [Trichinella zimbabwensis]	-0.66958	1.935809	-0.77158	-0.45545	-0.0392
TRINITY_DN12683_c0_g1_i3_orf1	sulfated surface glycoprotein 185-like [Ostrinia furnacalis]	-0.95734	1.935049	-0.24797	-0.44013	-0.2896
TRINITY_DN2304_c0_g1_i4_orf1	clustered mitochondria protein homolog isoform X2 [Ostrinia furnacalis]	-0.77324	1.896802	-0.86303	-0.01739	-0.24314
TRINITY_DN2709_c0_g1_i4_orf1	ATP-dependent RNA helicase dbp2-like [Ostrinia furnacalis]	-1.34145	1.746134	-0.26973	0.119173	-0.25413
TRINITY_DN4929_c1_g2_i5_orf1	guanylate kinase isoform X2 [Ostrinia furnacalis]	-1.20038	1.659081	0.506075	-0.68858	-0.2762
TRINITY_DN76283_c0_g2_i1_orf1	fatty acid synthase-like [Ostrinia furnacalis]	-0.93082	1.900228	-0.63397	-0.34734	0.0119
TRINITY_DN23167_c0_g1_i4_orf1	uncharacterized protein LOC114363065 [Ostrinia furnacalis]	-1.32769	1.540209	0.649994	-0.61907	-0.24345
TRINITY_DN313_c0_g1_i5_orf1	collagen alpha-1(X) chain-like [Ostrinia furnacalis]	-1.31143	1.71349	0.075109	-0.57371	0.096542
TRINITY_DN18396_c0_g1_i1_orf1	uncharacterized protein LOC114359424 [Ostrinia furnacalis]	-1.51312	1.44257	0.625465	-0.48273	-0.07218
TRINITY_DN3229_c0_g1_i1_orf1	uncharacterized protein LOC114358442 isoform X1 [Ostrinia furnacalis]	-1.14521	1.868537	-0.21238	-0.35898	-0.15197

TRINITY_DN143603_c0_g1_i1_orf1	hypothetical protein KR044_005587 [Drosophila immigrans]	-0.73152	1.964445	-0.56343	-0.51402	-0.15548
TRINITY_DN3504_c0_g1_i3_orfp2	TRINITY_DN3504_c0_g1_i3_m.43947 TRINITY_DN3504_c0_g1::TRINITY_DN3504_c0_g1_i3::g.43947 ORF type:5prime_partial len:208 (-),score=77.75 TRINITY_DN3504_c0_g1_i3:185-808(-)	-1.60504	1.453067	0.306482	-0.39864	0.244129
TRINITY_DN3929_c0_g3_i3_orf1	Glutathione S-transferase 1, isoform D [Papilio machaon]	-1.05174	1.778806	-0.31473	-0.72848	0.316145
TRINITY_DN19814_c0_g1_i4_orf1	general odorant-binding protein 28a-like [Ostrinia furnacalis]	-1.30764	1.591166	0.437309	-0.75236	0.031521
TRINITY_DN39673_c0_g1_i1_orf1	uncharacterized protein LOC114359357 isoform X1 [Ostrinia furnacalis]	-0.64199	1.926672	-0.81225	-0.46473	-0.0077
TRINITY_DN24873_c0_g1_i4_orf1	uncharacterized protein LOC114365742 [Ostrinia furnacalis]	-0.74574	1.941866	-0.22662	-0.76016	-0.20934
TRINITY_DN32479_c0_g1_i8_orf1	hypothetical protein evm_009815 [Chilo suppressalis] >CAB3525305.1 unnamed protein product [Chilo suppressalis] >CAH0402632.1 unnamed protein product [Chilo suppressalis]	-1.28464	1.746604	-0.0078	-0.54004	0.085873
TRINITY_DN34040_c0_g2_i1_orf1	uncharacterized protein LOC114352849 [Ostrinia furnacalis]	-1.28735	1.760214	-0.48085	-0.07701	0.084995
TRINITY_DN6143_c0_g2_i1_orf1	uncharacterized protein LOC114365036 [Ostrinia furnacalis]	-1.38036	1.729254	0.076204	-0.27626	-0.14884
TRINITY_DN2695_c0_g1_i14_orfp1	TRINITY_DN2695_c0_g1_i14_m.44485 TRINITY_DN2695_c0_g1::TRINITY_DN2695_c0_g1_i14::g.44485 ORF type:3prime_partial len:698 (+),score=187.51 TRINITY_DN2695_c0_g1_i14:101-2092(+)	-1.21642	1.688499	-0.42407	-0.51812	0.470116
TRINITY_DN8621_c0_g1_i5_orf1	aminopeptidase N-like isoform X2 [Ostrinia furnacalis]	-1.17932	1.852984	-0.24301	-0.10607	-0.32459
TRINITY_DN33452_c0_g1_i1_orf1	lethal(2) giant larvae protein isoform X8 [Ostrinia furnacalis]	-0.79039	1.401478	-1.4046	0.148319	0.645184
TRINITY_DN868_c0_g1_i4_orf1	uncharacterized protein LOC114359357 isoform X1 [Ostrinia furnacalis]	-0.87827	1.684835	-0.50495	-0.88889	0.587267
TRINITY_DN747_c0_g1_i4_orf1	trypsin, alkaline C-like [Ostrinia furnacalis]	-0.69882	1.951857	-0.53526	-0.63978	-0.078
TRINITY_DN18909_c0_g1_i8_orf1	unnamed protein product [Euphydryas editha]	-0.62718	1.929332	-0.81361	-0.47124	-0.01731
TRINITY_DN47_c0_g1_i2_orf1	uncharacterized protein LOC114356437 isoform X1 [Ostrinia furnacalis]	-0.85208	1.878469	-0.85467	-0.07854	-0.09318
TRINITY_DN2574_c0_g1_i5_orf1	prion-like-(Q/N-rich) domain-bearing protein 25 [Ostrinia furnacalis] >XP_028158239.1 prion-like-(Q/N-rich) domain-bearing protein 25 [Ostrinia furnacalis] >XP_028158240.1 prion-like-(Q/N-rich) domain-bearing protein 25 [Ostrinia furnacalis] >XP_028158241.1 prion-like-(Q/N-rich) domain-bearing protein 25 [Ostrinia furnacalis]	-0.776	1.960801	-0.5697	-0.44782	-0.16728
TRINITY_DN7336_c0_g1_i13_orf1	PREDICTED: calcium-transporting ATPase sarcoplasmic/endoplasmic reticulum type isoform X2 [Amyelois	-1.07845	1.870006	0.004897	-0.50508	-0.29138
TRINITY_DN3135_c0_g1_i6_orf1	acanthoscurrin-1-like [Ostrinia furnacalis]	-1.66576	1.475125	0.016489	-0.04298	0.21712
TRINITY_DN96566_c0_g1_i1_orf1	NADH-ubiquinone oxidoreductase subunit 8-like [Ostrinia furnacalis]	-0.73142	1.938102	-0.7792	-0.14212	-0.28536
TRINITY_DN2668_c0_g1_i7_orf1	unnamed protein product [Chrysodeixis includens]	-1.15898	1.864269	-0.25808	-0.30924	-0.13797
TRINITY_DN48020_c0_g1_i1_orf1	aminopeptidase N4 [Cnaphalocrocis medinalis]	-0.64525	1.94389	-0.7288	-0.52096	-0.04888
TRINITY_DN18773_c0_g1_i3_orf1	keratin, type II cytoskeletal 68 kDa, component IB-like [Ostrinia furnacalis]	-1.08471	1.874936	-0.0973	-0.51747	-0.17546
TRINITY_DN38498_c0_g3_i1_orf1	unnamed protein product [Parnassius apollo]	-1.42753	1.493562	0.255041	-0.71499	0.393916
TRINITY_DN542_c0_g1_i4_orf1	uncharacterized protein LOC114364889 [Ostrinia furnacalis]	-0.83835	1.967512	-0.36532	-0.36094	-0.40291
TRINITY_DN26411_c0_g1_i2_orfp1	TRINITY_DN26411_c0_g1_i2_m.24123 TRINITY_DN26411_c0_g1::TRINITY_DN26411_c0_g1_i2::g.24123 ORF type:internal len:115 (-),score=72.83 TRINITY_DN26411_c0_g1_i2:3-344(-)	-0.71561	1.55133	-0.7419	-0.92054	0.826721
TRINITY_DN40_c0_g2_i1_orf1	trypsin CFT-1-like [Ostrinia furnacalis]	-0.81816	1.968337	-0.39909	-0.46194	-0.28915
TRINITY_DN3476_c0_g1_i5_orf1	maltase A1-like [Ostrinia furnacalis]	-0.80132	1.964154	-0.55336	-0.24131	-0.36816
TRINITY_DN1154_c0_g1_i1_orf1	calexcitin-1-like [Ostrinia furnacalis] >ADK94879.2 juvenile hormone diol kinase [Ostrinia furnacalis]	-0.90553	1.889639	-0.74629	-0.00924	-0.22857
TRINITY_DN79319_c0_g1_i8_orfp1	TRINITY_DN79319_c0_g1_i8_m.49956 TRINITY_DN79319_c0_g1::TRINITY_DN79319_c0_g1_i8::g.49956 ORF type:5prime_partial len:84 (+),score=1.39 TRINITY_DN79319_c0_g1_i8:1-252(+)	-0.65436	1.959937	-0.76406	-0.26557	-0.27594
TRINITY_DN49508_c0_g2_i8_orf1	putative fatty acyl-CoA reductase CG5065 [Ostrinia furnacalis]	-0.86536	1.93716	-0.59254	-0.36734	-0.11192
TRINITY_DN657_c0_g1_i2_orf1	cytochrome c-type heme lyase [Ostrinia furnacalis]	-0.64401	1.957341	-0.4525	-0.72942	-0.13142
TRINITY_DN117_c0_g1_i5_orf1	lipase member I-like [Ostrinia furnacalis]	-0.71122	1.977467	-0.57388	-0.4321	-0.26026
TRINITY_DN15046_c0_g1_i8_orf1	epidermal retinol dehydrogenase 2-like isoform X1 [Ostrinia furnacalis] >XP_028169999.1 epidermal retinol dehydrogenase 2-like isoform X2 [Ostrinia furnacalis]	-0.71469	1.907397	-0.86215	-0.0023	-0.32826
TRINITY_DN5244_c0_g1_i1_orf1	eukaryotic peptide chain release factor GTP-binding subunit-like [Ostrinia furnacalis]	-1.36914	1.746016	-0.11017	-0.25412	-0.01258
TRINITY_DN6418_c0_g1_i28_orf1	peritrophic membrane chitin binding protein [Loxostege sticticalis]	-0.84586	1.964791	-0.39388	-0.30718	-0.41786
TRINITY_DN22664_c0_g1_i1_orf1	larval cuticle protein LCP-14-like [Ostrinia furnacalis]	-1.2535	1.628226	0.449758	-0.75536	-0.06913
TRINITY_DN11259_c0_g1_i1_orf1	uncharacterized protein LOC114357075 [Ostrinia furnacalis]	-0.69761	1.961951	-0.70589	-0.34948	-0.20897
TRINITY_DN1249_c0_g1_i10_orf1	venom carboxylesterase-6-like [Ostrinia furnacalis]	-0.79563	1.892155	-0.87169	-0.08405	-0.14079
TRINITY_DN4731_c0_g1_i1_orf1	gelsolin-like [Ostrinia furnacalis]	-0.59518	1.980319	-0.67773	-0.43906	-0.26836
TRINITY_DN36494_c0_g1_i1_orf1	MKI67 FHA domain-interacting nucleolar phosphoprotein-like [Ostrinia furnacalis]	-0.80786	1.938281	-0.08124	-0.65389	-0.39529
TRINITY_DN64403_c0_g2_i1_orf1	carboxylesterase [Ostrinia furnacalis]	-0.80946	1.900271	-0.63727	-0.56202	0.108477
TRINITY_DN29034_c0_g1_i2_orf1	trypsin-like serine protease [Ostrinia nubilalis]	-0.86121	1.955487	-0.23532	-0.50056	-0.3584

TRINITY_DN344_c1_g1_i1_orf1	chymotrypsin-like serine protease 16 [Ostrinia nubilalis]	-1.10006	1.523007	-0.65427	0.828224	-0.5969
TRINITY_DN16931_c0_g1_i1_orf1	pancreatic triacylglycerol lipase-like [Ostrinia furnacalis]	-0.68502	1.897966	-0.89272	-0.36043	0.040201
TRINITY_DN3504_c0_g1_i4_orfp1	TRINITY_DN3504_c0_g1_i4_m.43930 TRINITY_DN3504_c0_g1::TRINITY_DN3504_c0_g1_i4::g.43930 ORF type:internal len:196 (-),score=84.82 TRINITY_DN3504_c0_g1_i4:3-587(-)	-1.53955	1.59652	-0.00991	-0.22318	0.176111
TRINITY_DN69713_c0_g1_i1_orf1	membrane-bound alkaline phosphatase-like [Ostrinia furnacalis]	-0.925	1.929689	-0.55851	-0.29096	-0.15523
TRINITY_DN311_c0_g1_i4_orfp1	TRINITY_DN311_c0_g1_i4_m.65135 TRINITY_DN311_c0_g1::TRINITY_DN311_c0_g1_i4::g.65135 ORF type:5prime_partial len:126 (+),score=71.21 TRINITY_DN311_c0_g1_i4:1-378(+)	-0.44135	1.858215	-1.14547	-0.09502	-0.17637
TRINITY_DN2343_c1_g1_i8_orf1	receptor expression-enhancing protein 5-like isoform X3 [Ostrinia furnacalis]	-0.62519	1.96638	-0.70337	-0.46784	-0.16999
TRINITY_DN2114_c0_g1_i5_orf1	vegetative cell wall protein gp1-like isoform X1 [Ostrinia furnacalis]	-0.82807	1.960682	-0.53323	-0.35476	-0.24462
TRINITY_DN30704_c0_g1_i1_orf1	cytochrome P450 monooxygenase CYP6AE134v2 [Ostrinia furnacalis]	-1.23319	1.791626	-0.4594	-0.21286	0.11383
TRINITY_DN2986_c1_g1_i1_orf1	Troponin C, isoform 1 [Papilio xuthus]	-1.24861	1.643576	0.462407	-0.70999	-0.14738
TRINITY_DN4612_c0_g1_i1_orf1	uncharacterized protein LOC114362092 [Ostrinia furnacalis]	-0.77846	1.925608	-0.78551	-0.13825	-0.2234
TRINITY_DN10940_c0_g1_i10_orfp1	TRINITY_DN10940_c0_g1_i10_m.52163 TRINITY_DN10940_c0_g1::TRINITY_DN10940_c0_g1_i10::g.52163 ORF type:5prime_partial len:248 (-),score=128.24 TRINITY_DN10940_c0_g1_i10:121-864(-)	-1.279	1.808268	-0.20121	-0.16106	-0.16699
TRINITY_DN41086_c0_g1_i4_orf1	collagenase-like [Pectinophora gossypiella]	-0.52277	1.964409	-0.78749	-0.45691	-0.19724
TRINITY_DN334_c0_g1_i4_orf1	collagenase-like [Ostrinia furnacalis]	-1.0204	1.895514	-0.41884	-0.43585	-0.02042
TRINITY_DN336_c0_g1_i6_orfp1	TRINITY_DN336_c0_g1_i6_m.64791 TRINITY_DN336_c0_g1::TRINITY_DN336_c0_g1_i6::g.64791 ORF type:complete len:61 (-),score=19.53 TRINITY_DN336_c0_g1_i6:236-418(-)	-1.09785	1.847356	-0.41063	0.109813	-0.44869
