# Supplementary material for: Comparative Proteomics and Metabonomics Analysis of Different Diapause Stages Revealed a New Regulation Mechanism of Diapause in Loxostege sticticalis (Lepidoptera: Pyralidae)
Source: Molecules. 2024 Jul 25;29(15):3472. doi: 10.3390/molecules29153472 (PMC11314584; doi:10.3390/molecules29153472)
Supplement: Supplementary file 1 [file molecules-29-03472-s001.zip › analysis process/proteomic/Cluster analysis of expression patterns/Down/RDvsPreD down.pdf]

Accession	Description	RD	ND	PreD	CT	D
TRINITY_DN2396_c0_g1_i9_orfp1	TRINITY_DN2396_c0_g1_i9_m.39038 TRINITY_DN2396_c0_g1::TRINITY_DN2396_c0_g1_i9::g.39038 ORF type:5prime_partial len:181 (+),score=90.33 TRINITY_DN2396_c0_g1_i9:3-545(+)	-0.04221	-1.92051	0.754172	0.677816	0.530724
TRINITY_DN971_c0_g1_i10_orfp1	TRINITY_DN971_c0_g1_i10_m.54268 TRINITY_DN971_c0_g1::TRINITY_DN971_c0_g1_i10::g.54268 ORF type:internal len:187 (+),score=141.68 TRINITY_DN971_c0_g1_i10:1-558(+)	-0.1083	-1.8854	0.891131	0.425574	0.676991
TRINITY_DN14242_c0_g1_i2_orfp1	TRINITY_DN14242_c0_g1_i2_m.18449 TRINITY_DN14242_c0_g1::TRINITY_DN14242_c0_g1_i2::g.18449 ORF type:internal len:148 (-),score=102.30 TRINITY_DN14242_c0_g1_i2:2-442(-)	0.087006	-1.88284	0.951973	0.72609	0.117774
TRINITY_DN10877_c0_g1_i1_orf1	spodomicin-like [Ostrinia furnacalis]	-0.39422	-1.5409	1.428318	0.641791	-0.13499
TRINITY_DN295_c3_g1_i1_orfp1	TRINITY_DN295_c3_g1_i1_m.18839 TRINITY_DN295_c3_g1::TRINITY_DN295_c3_g1_i1::g.18839 ORF type:5prime_partial len:118 (+),score=46.25 TRINITY_DN295_c3_g1_i1:1-354(+)	-0.12431	-1.89772	0.581712	0.781725	0.65859
TRINITY_DN971_c0_g1_i5_orfp1	TRINITY_DN971_c0_g1_i5_m.54249 TRINITY_DN971_c0_g1::TRINITY_DN971_c0_g1_i5::g.54249 ORF type:internal len:108 (+),score=66.98 TRINITY_DN971_c0_g1_i5:1-321(+)	-0.39991	-1.76323	0.48896	1.049906	0.624269
TRINITY_DN1506_c0_g1_i6_orfp1	TRINITY_DN1506_c0_g1_i6_m.57691 TRINITY_DN1506_c0_g1::TRINITY_DN1506_c0_g1_i6::g.57691 ORF type:5prime_partial len:173 (+),score=30.49 TRINITY_DN1506_c0_g1_i6:1-519(+)	-1.32434	-1.11546	0.706934	0.848064	0.884809
TRINITY_DN27300_c0_g1_i1_orfp1	TRINITY_DN27300_c0_g1_i1_m.71142 TRINITY_DN27300_c0_g1::TRINITY_DN27300_c0_g1_i1::g.71142 ORF type:internal len:82 (-),score=9.46 TRINITY_DN27300_c0_g1_i1:3-245(-)	-0.88268	-1.16404	1.670737	0.231825	0.144158
TRINITY_DN57137_c0_g1_i1_orfp1	TRINITY_DN57137_c0_g1_i1_m.46420 TRINITY_DN57137_c0_g1::TRINITY_DN57137_c0_g1_i1::g.46420 ORF type:5prime_partial len:56 (-),score=4.65 TRINITY_DN57137_c0_g1_i1:82-249(-)	-0.23025	-1.84759	0.799526	0.442793	0.835517
TRINITY_DN36476_c1_g1_i1_orfp1	TRINITY_DN36476_c1_g1_i1_m.70910 TRINITY_DN36476_c1_g1::TRINITY_DN36476_c1_g1_i1::g.70910 ORF type:5prime_partial len:88 (-),score=0.50 TRINITY_DN36476_c1_g1_i1:49-312(-)	-0.66669	-1.62065	1.01449	0.424577	0.848274
TRINITY_DN35809_c0_g1_i1_orf1	spodomicin-like [Ostrinia furnacalis] >QKV49445.1 diapausin [Ostrinia furnacalis]	-0.59427	-1.6706	0.990783	0.814055	0.460024
TRINITY_DN34423_c0_g1_i3_orf1	THAP domain-containing protein 4-like [Ostrinia furnacalis]	0.072575	-1.95064	0.602757	0.718866	0.556447
TRINITY_DN30510_c0_g1_i6_orf1	spodomicin-like [Ostrinia furnacalis]	-1.05909	-1.29959	1.193754	0.375345	0.789588
TRINITY_DN2044_c0_g1_i5_orfp1	TRINITY_DN2044_c0_g1_i5_m.4210 TRINITY_DN2044_c0_g1::TRINITY_DN2044_c0_g1_i5::g.4210 ORF type:complete len:151 (-),score=85.31 TRINITY_DN2044_c0_g1_i5:857-1309(-)	-0.40125	-1.76135	0.448788	1.03947	0.674341
TRINITY_DN6330_c0_g1_i1_orfp1	TRINITY_DN6330_c0_g1_i1_m.42332 TRINITY_DN6330_c0_g1::TRINITY_DN6330_c0_g1_i1::g.42332 ORF type:3prime_partial len:51 (-),score=33.30 TRINITY_DN6330_c0_g1_i1:3-152(-)	-0.47532	-1.72994	0.921904	0.409329	0.874028
TRINITY_DN47784_c0_g2_i1_orfp1	arylphorin subunit alpha-like [Ostrinia furnacalis]	-0.18103	-1.66831	1.360777	-0.08201	0.570566
TRINITY_DN28501_c0_g1_i2_orfp1	TRINITY_DN28501_c0_g1_i2_m.58934 TRINITY_DN28501_c0_g1::TRINITY_DN28501_c0_g1_i2::g.58934 ORF type:internal len:98 (+),score=13.70 TRINITY_DN28501_c0_g1_i2:3-293(+)	-1.27054	-0.9138	1.524181	0.260547	0.399609
TRINITY_DN86772_c0_g1_i3_orfp1	x-tox [Spodoptera exigua]	-0.3059	-1.78653	0.981394	0.296282	0.814751
TRINITY_DN2407_c0_g1_i2_orf1	uncharacterized protein LOC114366345 isoform X2 [Ostrinia furnacalis]	-0.7624	-1.02281	1.701134	0.529486	-0.44541
TRINITY_DN17615_c0_g1_i3_orf1	hypothetical protein SFRUCORN_008858 [Spodoptera frugiperda]	-0.06894	-1.90641	0.525117	0.595676	0.85456
TRINITY_DN56459_c0_g1_i2_orf1	aldo-keto reductase AKR2E4-like [Ostrinia furnacalis]	-0.60488	-1.69301	0.704117	0.82862	0.765151
TRINITY_DN3166_c1_g1_i6_orf1	hypothetical protein evm_013813 [Chilo suppressalis]	-0.31599	-1.7427	0.35242	0.4768	1.229472
TRINITY_DN2457_c0_g1_i8_orf1	uncharacterized protein LOC114355596 [Ostrinia furnacalis]	-0.88645	-1.35161	1.434699	0.345636	0.457731
TRINITY_DN3275_c0_g2_i3_orf1	hypothetical protein B5X24_HaOG216046 [Helicoverpa armigera]	-0.23787	-1.74395	1.185954	0.098885	0.69698
TRINITY_DN12534_c0_g1_i4_orf1	antibacterial protein [Heliothis virescens]	-0.50785	-1.74092	0.645477	0.870177	0.733112
TRINITY_DN59429_c0_g1_i6_orf1	uncharacterized protein LOC114366345 isoform X2 [Ostrinia furnacalis]	-0.79362	-1.52804	1.060942	0.870103	0.390616
TRINITY_DN30306_c0_g2_i1_orf1	perilipin-4-like isoform X3 [Ostrinia furnacalis]	-0.58492	-1.64237	1.14023	0.357072	0.729982
TRINITY_DN6025_c0_g2_i1_orfp1	TRINITY_DN6025_c0_g2_i1_m.7749 TRINITY_DN6025_c0_g2::TRINITY_DN6025_c0_g2_i1::g.7749 ORF type:5prime_partial len:106 (+),score=47.10 TRINITY_DN6025_c0_g2_i1:3-320(+)	-0.29357	-1.82794	0.893104	0.51334	0.715059
TRINITY_DN24789_c0_g1_i9_orfp1	TRINITY_DN24789_c0_g1_i9_m.25888 TRINITY_DN24789_c0_g1::TRINITY_DN24789_c0_g1_i9::g.25888 ORF type:internal len:114 (-),score=12.65 TRINITY_DN24789_c0_g1_i9:2-340(-)	-1.02403	-1.01474	1.690654	0.211176	0.136942
TRINITY_DN609_c0_g1_i1_orf1	zonadhesin-like isoform X1 [Ostrinia furnacalis]	-1.1348	-1.14528	1.406225	0.580586	0.293272
TRINITY_DN7064_c0_g1_i19_orf1	unnamed protein product [Chilo suppressalis]	-1.1627	-1.23178	0.443989	0.849735	1.100758
TRINITY_DN15202_c0_g1_i6_orf1	uncharacterized protein LOC114364499 isoform X2 [Ostrinia furnacalis]	-0.53535	-1.67149	0.337399	0.764067	1.105382
TRINITY_DN380_c0_g2_i2_orf1	chemosensory protein 10 [Ostrinia furnacalis]	-0.34895	-1.79714	0.476404	0.952394	0.717294
TRINITY_DN1391_c0_g1_i29_orfp1	TRINITY_DN1391_c0_g1_i29_m.70767 TRINITY_DN1391_c0_g1::TRINITY_DN1391_c0_g1_i29::g.70767 ORF type:complete len:495 (+),score=158.49 TRINITY_DN1391_c0_g1_i29:728-2212(+)	-0.10285	-1.90099	0.822202	0.56359	0.618054
TRINITY_DN14754_c0_g1_i6_orf1	cathepsin L [Papilio xuthus]	-1.11172	-0.81321	1.681249	-0.22922	0.472896

TRINITY_DN1149_c0_g1_i4_orf1	circadian clock-controlled protein-like [Ostrinia furnacalis]	-0.82548	-1.45568	1.263622	0.715117	0.302417
TRINITY_DN24723_c2_g1_i1_orf1	hypothetical protein evm_001103 [Chilo suppressalis]	-0.44583	-1.76659	0.772888	0.872888	0.566651
TRINITY_DN38506_c0_g1_i4_orf1	C-1-tetrahydrofolate synthase, cytoplasmic isoform X1 [Ostrinia furnacalis] >XP_028166137.1 C-1-tetrahydrofolate synthase, cytoplasmic isoform X2 [Ostrinia furnacalis] >XP_028166140.1 C-1-tetrahydrofolate synthase, cytoplasmic isoform X4 [Ostrinia furnacalis]	-0.91799	-1.48978	0.823809	0.840481	0.743484
TRINITY_DN7226_c0_g1_i2_orf1	chemosensory protein [Conogethes punctiferalis]	-0.44449	-1.7465	0.822515	0.948134	0.420342
TRINITY_DN4816_c0_g2_i3_orf1	15-hydroxyprostaglandin dehydrogenase [NAD(+)]-like [Ostrinia furnacalis]	-0.14321	-1.89395	0.693786	0.73797	0.605403
TRINITY_DN7226_c0_g1_i5_orf1	chemosensory protein [Dioryctria abietella]	-0.34679	-1.79643	0.944692	0.743979	0.454552
TRINITY_DN5439_c0_g1_i2_orf1	uncharacterized protein LOC114353087 [Ostrinia furnacalis]	-0.17705	-1.80925	0.569988	0.279551	1.136761
TRINITY_DN3439_c0_g2_i2_orf1	histone H2A.Z-specific chaperone CHZ1-like [Ostrinia furnacalis]	-0.8689	-1.46229	0.884746	0.35051	1.095938
TRINITY_DN85290_c0_g2_i1_orf1	unnamed protein product, partial [Brenthis ino]	-0.21394	-1.31486	1.020855	1.267436	-0.75949
TRINITY_DN9079_c0_g1_i5_orf1	UDP-glucuronosyltransferase-like [Ostrinia furnacalis]	-0.17022	-1.88496	0.642254	0.766763	0.646164
TRINITY_DN135780_c0_g1_i1_orf1	flotillin-1 isoform X1 [Pectinophora gossypiella]	-0.07917	-1.87167	0.826345	0.266053	0.85845
TRINITY_DN1880_c0_g1_i4_orf1	serine protease inhibitor dipetalogastin-like [Helicoverpa zea]	-0.73259	-1.6005	0.692729	1.015692	0.624668
TRINITY_DN71698_c0_g1_i1_orfp1	TRINITY_DN71698_c0_g1_i1_m.1194 TRINITY_DN71698_c0_g1::TRINITY_DN71698_c0_g1_i1::g.1194 ORF type:internal len:134 (+),score=19.66,Toxin_2 PF00451.20 4.3e-05,Toxin_2|PF00451.20|0.037,Toxin_2|PF00451.20|7.5e-05,Gamma-thionin|PF00304.21|0.017,Gamma-thionin|PF00304.21|0.05,Gamma-thionin|PF00304.21|0.021,Toxin_38|PF14866.7|0.13,Toxin_38|PF14866.7|0.15,Toxin_38|PF14866.7|0.15,Defensin_2|PF0	-0.57881	-1.67369	0.860611	0.9738	0.418091
TRINITY_DN628_c0_g1_i1_orf1	prostamide/prostaglandin F synthase-like [Ostrinia furnacalis]	0.102876	-1.95787	0.583432	0.696311	0.575252
TRINITY_DN1363_c0_g1_i11_orf1	cytochrome P450 CYP12A2-like isoform X1 [Ostrinia furnacalis] >QPF77619.1 cytochrome P450 monooxygenase CYP333A20 [Ostrinia furnacalis]	-0.29938	-1.81711	0.966857	0.494753	0.654887
TRINITY_DN5153_c1_g1_i1_orf1	nose resistant to fluoxetine protein 6-like isoform X1 [Ostrinia furnacalis]	-0.57412	-1.64455	1.194551	0.427007	0.597117
TRINITY_DN11981_c0_g1_i7_orf1	luciferin 4-monooxygenase-like isoform X2 [Ostrinia furnacalis]	-0.55027	-1.64747	1.195498	0.661683	0.340567
TRINITY_DN27300_c0_g1_i7_orfp1	TRINITY_DN27300_c0_g1_i7_m.71141 TRINITY_DN27300_c0_g1::TRINITY_DN27300_c0_g1_i7::g.71141 ORF type:internal len:82 (-),score=6.59 TRINITY_DN27300_c0_g1_i7:3-245(-)	-0.49544	-1.49298	1.519485	0.465513	0.00342
TRINITY_DN114890_c0_g1_i4_orf1	chemosensory protein 10 [Ostrinia furnacalis]	-0.74287	-1.47498	0.568656	1.367417	0.281771
TRINITY_DN69_c0_g1_i1_orf1	glycerol-3-phosphate dehydrogenase [NAD(+)], cytoplasmic isoform X1 [Ostrinia furnacalis]	-0.19638	-1.85711	0.657815	0.467699	0.927969
TRINITY_DN8595_c0_g1_i3_orf1	aldose reductase-like isoform X4 [Trichoplusia ni]	-0.65403	-1.63749	0.590183	0.632407	1.068932
TRINITY_DN5198_c0_g1_i5_orfp1	TRINITY_DN5198_c0_g1_i5_m.8637 TRINITY_DN5198_c0_g1::TRINITY_DN5198_c0_g1_i5::g.8637 ORF type:complete len:223 (-),score=54.99 TRINITY_DN5198_c0_g1_i5:319-987(-)	-0.9491	-1.36167	1.311727	0.386875	0.612172
TRINITY_DN31348_c0_g1_i1_orf1	protein lethal(2)essential for life [Bombyx mori]	-0.55478	-1.64067	0.454275	1.243569	0.4976
TRINITY_DN24_c0_g1_i1_orf1	hypothetical protein evm_007803 [Chilo suppressalis]	-0.03943	-1.74811	1.041612	-0.16528	0.911205
TRINITY_DN295_c5_g1_i2_orf1	unnamed protein product [Chilo suppressalis]	-0.38677	-1.72904	0.543831	1.191885	0.3801
TRINITY_DN710_c0_g1_i11_orfp1	TRINITY_DN710_c0_g1_i11_m.67699 TRINITY_DN710_c0_g1::TRINITY_DN710_c0_g1_i11::g.67699 ORF type:complete len:194 (-),score=34.99,Collagen PF01391.19 0.00029 TRINITY_DN710_c0_g1_i11:1283-1864(-)	-0.99675	-1.34384	0.985946	0.283021	1.071627
TRINITY_DN1201_c0_g1_i4_orf1	triosephosphate isomerase [Ostrinia furnacalis] >XP_028170843.1 triosephosphate isomerase [Ostrinia furnacalis]	0.11534	-1.95653	0.749173	0.521912	0.570108
TRINITY_DN19731_c0_g1_i1_orf1	allergen Tha p 1-like [Ostrinia furnacalis] >XP_028174916.1 allergen Tha p 1-like [Ostrinia furnacalis] >BAV56808.1 chemosensory protein 4 [Ostrinia furnacalis]	-0.19115	-1.83232	0.274958	0.904232	0.844287
TRINITY_DN8953_c0_g1_i4_orf1	gonadotropin-releasing hormone receptor [Ostrinia furnacalis] >AXF67446.1 adipokinetic hormone receptor 1 [Ostrinia furnacalis]	-0.00898	-1.87323	1.00841	0.222092	0.651704
TRINITY_DN61042_c0_g2_i2_orfp1	TRINITY_DN61042_c0_g2_i2_m.5292 TRINITY_DN61042_c0_g2::TRINITY_DN61042_c0_g2_i2::g.5292 ORF type:5prime_partial len:63 (-),score=21.86 TRINITY_DN61042_c0_g2_i2:77-265(-)	-0.57132	-1.38151	1.353132	0.913947	-0.31425
TRINITY_DN1450_c0_g2_i1_orf1	death-associated protein 1 [Ostrinia furnacalis]	-0.69842	-1.61801	0.996718	0.537413	0.782297
TRINITY_DN64719_c0_g1_i2_orfp1	TRINITY_DN64719_c0_g1_i2_m.37745 TRINITY_DN64719_c0_g1::TRINITY_DN64719_c0_g1_i2::g.37745 ORF type:internal len:91 (+),score=41.89 TRINITY_DN64719_c0_g1_i2:1-270(+)	-1.29225	-0.81997	1.577519	0.152843	0.381857
TRINITY_DN31286_c0_g1_i6_orfp1	TRINITY_DN31286_c0_g1_i6_m.28438 TRINITY_DN31286_c0_g1::TRINITY_DN31286_c0_g1_i6::g.28438 ORF type:internal len:92 (-),score=3.10,Perilipin PF03036.17 2e-05 TRINITY_DN31286_c0_g1_i6:1-273(-)	-0.19318	-1.82585	0.513184	0.412498	1.093356
TRINITY_DN4998_c0_g1_i21_orf1	phenoloxidase subunit 2-like [Ostrinia furnacalis]	0.014505	-1.92945	0.749964	0.716593	0.448389
TRINITY_DN14328_c0_g1_i12_orf1	larval cuticle protein LCP-30-like [Ostrinia furnacalis]	-1.19973	-1.22551	0.739662	0.629382	1.056196
TRINITY_DN2187_c0_g1_i1_orf1	flotillin-1 [Chelonus insularis] >XP_034947202.1 flotillin-1 [Chelonus insularis]	-0.19393	-1.85825	0.814707	0.418869	0.818603

TRINITY_DN40126_c0_g1_i1_orf1	aldehyde dehydrogenase X, mitochondrial-like [Ostrinia furnacalis]	0.030099	-1.93997	0.576335	0.582282	0.751257
TRINITY_DN13660_c0_g1_i1_orf1	Aliphatic nitrilase [Operophtera brumata]	-0.23622	-1.83886	0.546405	0.983194	0.545483
TRINITY_DN8008_c0_g1_i6_orf1	uncharacterized protein LOC114357965 isoform X1 [Ostrinia furnacalis] >XP_028167599.1 uncharacterized protein LOC114357965 isoform X1 [Ostrinia furnacalis] >XP_028167600.1 uncharacterized protein LOC114357965 isoform X2 [Ostrinia furnacalis] >XP_028167601.1 uncharacterized protein LOC114357965 isoform X3 [Ostrinia furnacalis]	-0.84436	-1.47885	0.405145	1.178638	0.739421
TRINITY_DN4802_c0_g1_i4_orf1	uncharacterized protein LOC114366345 isoform X2 [Ostrinia furnacalis]	-0.50714	-1.48977	0.459518	0.016889	1.520508
TRINITY_DN12586_c0_g1_i4_orf1	zonadhesin-like isoform X4 [Ostrinia furnacalis]	-1.25954	-1.11446	1.076182	0.941499	0.356314
TRINITY_DN8685_c0_g1_i5_orf1	macrophage mannose receptor 1-like [Zerene cesonia]	-0.78348	-1.43299	1.378499	0.620658	0.217314
TRINITY_DN29555_c0_g1_i8_orf1	fasciclin-1 [Ostrinia furnacalis]	0.271431	-1.90939	0.919619	0.061813	0.656529
TRINITY_DN21420_c0_g1_i2_orf1	glutathione peroxidase, partial [Ostrinia furnacalis]	-0.09412	-1.84002	0.445213	1.130976	0.357957
TRINITY_DN4245_c0_g2_i1_orf1	long-chain fatty acid transport protein 4-like [Ostrinia furnacalis]	-0.15065	-1.88039	0.702725	0.483351	0.844969
TRINITY_DN6098_c1_g1_i5_orf1	unnamed protein product, partial [Iphiclides podalirius]	-0.54111	-1.72691	0.678884	0.821176	0.767962
TRINITY_DN335_c1_g1_i5_orf1	PREDICTED: perilipin-4 isoform X14 [Papilio polytes]	-0.91176	-1.39388	1.158173	0.237514	0.909953
TRINITY_DN3821_c1_g1_i7_orf1	mitochondrial carrier protein Rim2 isoform X1 [Ostrinia furnacalis]	-0.32247	-1.79587	0.377759	0.952021	0.788555
TRINITY_DN5748_c0_g1_i6_orf1	glycine N-methyltransferase isoform X1 [Ostrinia furnacalis] >XP_028165118.1 glycine N-methyltransferase isoform X2 [Ostrinia furnacalis] >XP_028165119.1 glycine N-methyltransferase isoform X1 [Ostrinia furnacalis] >XP_028165120.1 glycine N-methyltransferase isoform X2 [Ostrinia furnacalis]	-0.82201	-1.42537	1.382645	0.373035	0.491699
TRINITY_DN1593_c0_g1_i1_orf1	chemosensory protein csp11 [Helopeltis theivora]	-1.08597	-1.25187	1.314706	0.539952	0.48319
TRINITY_DN20658_c0_g1_i1_orf1	prostaglandin reductase 1-like [Ostrinia furnacalis]	-0.29786	-1.82141	0.834306	0.452592	0.832371
TRINITY_DN57111_c0_g1_i1_orf1	trypsin-like serine proteinase T26 protein, partial [Chilo infuscatellus]	-1.58185	-0.54962	1.383755	0.39994	0.347772
TRINITY_DN130575_c0_g1_i1_orfp1	TRINITY_DN130575_c0_g1_i1_m.77798 TRINITY_DN130575_c0_g1::TRINITY_DN130575_c0_g1_i1::g.77798 ORF type:internal len:70 (+),score=15.12 TRINITY_DN130575_c0_g1_i1:3-209(+)	-0.29395	-1.80912	0.786425	0.379524	0.937115
TRINITY_DN1772_c1_g2_i1_orf1	aldose reductase-like isoform X2 [Ostrinia furnacalis]	-0.8172	-1.48297	0.296695	0.863851	1.139618
TRINITY_DN37307_c0_g1_i4_orf1	superoxide dismutase [Mn], mitochondrial [Ostrinia furnacalis]	-0.18805	-1.70829	1.380446	0.195959	0.319937
TRINITY_DN20658_c0_g2_i3_orf1	prostaglandin reductase 1-like [Ostrinia furnacalis]	-0.19083	-1.80774	0.555698	0.305551	1.137322
TRINITY_DN44517_c0_g1_i4_orf1	regucalcin-like [Ostrinia furnacalis]	-0.14665	-1.89074	0.742158	0.563981	0.731247
TRINITY_DN2040_c0_g1_i6_orf1	trypsin-like serine proteinase T26 protein, partial [Chilo infuscatellus]	-0.68195	-1.63528	0.555398	0.868587	0.893244
TRINITY_DN1503_c0_g1_i6_orf1	ecdysteroid-regulated 16 kDa protein [Ostrinia furnacalis]	-0.11707	-1.83672	1.086723	0.600664	0.26641
TRINITY_DN15545_c0_g1_i1_orf1	larval cuticle protein LCP-14-like [Ostrinia furnacalis]	-1.35118	-1.0717	0.95578	0.599285	0.867817
TRINITY_DN40126_c0_g2_i1_orf1	aldehyde dehydrogenase X, mitochondrial-like [Ostrinia furnacalis]	-0.63275	-1.63685	1.095318	0.462326	0.711958
TRINITY_DN1750_c1_g1_i5_orf1	lipid droplet localized protein-like [Ostrinia furnacalis] >XP_028161280.1 lipid droplet localized protein-like [Ostrinia furnacalis]	-0.24977	-1.84725	0.713001	0.864005	0.520009
TRINITY_DN11817_c0_g1_i4_orf1	glycogen phosphorylase [Heortia vitessoides]	-0.06646	-1.89425	0.743053	0.847524	0.370125
TRINITY_DN350_c0_g1_i5_orf1	tau-like protein isoform X6 [Bombyx mori]	-0.63224	-1.62296	1.162332	0.64153	0.451335
TRINITY_DN14262_c0_g1_i5_orf1	cytochrome P450 monooxygenase CYP9G18 [Cnaphalocrocis medinalis]	-0.18652	-1.78657	0.952539	0.925772	0.094782
TRINITY_DN214_c0_g1_i4_orf1	uncharacterized protein LOC114352813 [Ostrinia furnacalis]	-1.24034	-1.00733	1.423602	0.612854	0.211212
TRINITY_DN5274_c0_g2_i2_orf1	lopap-like [Ostrinia furnacalis]	-0.37855	-1.78141	0.937039	0.780986	0.441932
TRINITY_DN9608_c0_g1_i3_orf1	cytochrome P450 monooxygenase CYP9G18 [Cnaphalocrocis medinalis]	-0.53724	-1.52189	0.04571	0.578782	1.43463
TRINITY_DN4343_c0_g1_i2_orf1	uncharacterized protein LOC114365231 isoform X3 [Ostrinia furnacalis]	-0.05889	-1.82167	0.807233	0.062088	1.011231
TRINITY_DN394_c0_g1_i2_orf1	uncharacterized protein LOC114351483 [Ostrinia furnacalis]	-1.25181	-1.07239	0.791022	0.275719	1.257461
TRINITY_DN15685_c0_g1_i5_orf1	uncharacterized protein LOC114352354 [Ostrinia furnacalis]	-1.2913	-1.12233	1.09574	0.61491	0.702974
TRINITY_DN6415_c0_g2_i1_orf1	D-arabinitol dehydrogenase 1-like [Ostrinia furnacalis]	-0.92292	-1.39165	0.407408	0.616745	1.290416
TRINITY_DN1108_c1_g2_i1_orfp1	TRINITY_DN1108_c1_g2_i1_m.5565 TRINITY_DN1108_c1_g2::TRINITY_DN1108_c1_g2_i1::g.5565 ORF type:internal len:205 (-),score=147.90 TRINITY_DN1108_c1_g2_i1:2-613(-)	-0.47359	-1.54217	1.505353	0.232024	0.278384
TRINITY_DN52761_c0_g1_i2_orf1	atlastin isoform X4 [Ostrinia furnacalis]	0.120247	-1.71213	0.932201	-0.36569	1.02538
TRINITY_DN14185_c0_g1_i1_orf1	uncharacterized protein LOC114358675 [Ostrinia furnacalis] >XP_028168498.1 uncharacterized protein LOC114358675 [Ostrinia furnacalis]	-0.83945	-1.4804	1.023814	0.976426	0.319605
TRINITY_DN121802_c0_g1_i6_orfp1	TRINITY_DN121802_c0_g1_i6_m.78506 TRINITY_DN121802_c0_g1::TRINITY_DN121802_c0_g1_i6::g.78506 ORF type:3prime_partial len:123 (+),score=6.04 TRINITY_DN121802_c0_g1_i6:35-367(+)	-0.94365	-1.11741	1.353503	0.977728	-0.27017
TRINITY_DN43350_c0_g3_i1_orf1	uncharacterized protein LOC114355190 [Ostrinia furnacalis]	-1.0414	-1.3572	0.501929	0.841431	1.055245
TRINITY_DN2464_c0_g1_i12_orf1	uncharacterized protein LOC114362996 isoform X1 [Ostrinia furnacalis]	-1.21008	-1.23869	0.816208	0.780657	0.851903

TRINITY_DN14670_c0_g1_i1_orf1	heat shock protein beta-1 isoform X1 [Helicoverpa armigera] >XP_022829066.1 heat shock protein beta-1 isoform X1 [Spodoptera litura] >XP_026747148.1 heat shock protein beta-1 isoform X3 [Trichoplusia ni] >XP_026748187.1 heat shock protein beta-1 isoform X2 [Galleria mellonella] >XP_028167756.1 heat shock protein beta-1 isoform X2 [Ostrinia furnacalis] >XP_035431734.1 heat shock protein beta-1-like isoform X3 [Spodoptera frugiperda] >XP_047023072.1 heat shock protein beta-1 isoform X1 [Helicoverpa zea] >XP_049865086.1 heat shock protein beta-1 [Pectinophora gossypiella] >KAH9640995.1 hypothetical protein HF086_015091 [Spodoptera exigua] >QGZ00460.1 heat shock protein 21.4 [Glyphodes pyloalis] >QKR72095.1 heat-shock protein 21.4 [Mythimna separata] >CAB3228281.1 unnamed protein product [Arctia plantaginis] >CAH0628881.1 unnamed protein	-0.80182	-1.47876	1.299199	0.469867	0.511517
TRINITY_DN5099_c0_g1_i3_orf1	trans-1,2-dihydrobenzene-1,2-diol dehydrogenase-like [Ostrinia furnacalis]	-0.16801	-1.85027	0.445186	0.549081	1.024014
TRINITY_DN27021_c0_g1_i1_orf1	rab3 GTPase-activating protein catalytic subunit isoform X1 [Ostrinia furnacalis]	-0.66673	-1.15998	1.52958	0.791275	-0.49414
TRINITY_DN13236_c0_g1_i4_orf1	zonadhesin-like isoform X4 [Ostrinia furnacalis]	-1.31082	-0.9254	1.450197	0.311184	0.47484
TRINITY_DN1352_c0_g1_i5_orf1	uncharacterized protein LOC113491815 [Trichoplusia ni]	-0.22629	-1.84817	0.522772	0.943022	0.608669
TRINITY_DN7740_c0_g1_i2_orf1	D-arabinitol dehydrogenase 1 [Eumeta japonica]	-0.76801	-1.56086	0.773002	0.489839	1.066026
TRINITY_DN4255_c0_g1_i10_orf1	LOW QUALITY PROTEIN: lebocin-4-like [Ostrinia furnacalis]	-0.08033	-1.87367	0.876742	0.279778	0.797476
TRINITY_DN4062_c0_g2_i1_orf1	venom peptide BmKAPI-like isoform X2 [Ostrinia furnacalis]	-0.15121	-1.78841	1.211854	0.213328	0.514431
TRINITY_DN1024_c0_g4_i1_orf1	superoxide dismutase [Cu-Zn]-like [Ostrinia furnacalis]	-0.7855	-1.57275	0.977353	0.670407	0.710487
TRINITY_DN38230_c0_g1_i4_orf1	hypothetical protein evm_007803 [Chilo suppressalis]	-1.40544	-0.78664	1.408577	0.152044	0.631457
TRINITY_DN9400_c0_g1_i8_orf1	PREDICTED: monoacylglycerol lipase ABHD12-like [Amyelois transitella]	-0.90574	-1.42476	0.800056	1.176609	0.353836
TRINITY_DN73900_c0_g1_i1_orf1	carbonic anhydrase 7 [Ostrinia furnacalis]	-0.97494	-1.41898	0.77584	1.059021	0.559055
TRINITY_DN1209_c0_g1_i9_orf1	NADP-dependent malic enzyme-like isoform X1 [Ostrinia furnacalis] >XP_028161889.1 NADP-dependent malic enzyme-like isoform X1 [Ostrinia furnacalis] >XP_028161891.1 NADP-dependent malic enzyme-like isoform X3 [Ostrinia furnacalis]	-0.51542	-1.58532	0.278235	1.402641	0.419863
TRINITY_DN1592_c0_g1_i1_orf1	serine protease 7-like isoform X2 [Ostrinia furnacalis]	-0.08891	-1.80721	1.04569	0.057121	0.793311
TRINITY_DN34423_c0_g1_i2_orf1	THAP domain-containing protein 4-like [Ostrinia furnacalis]	-0.08949	-1.87707	0.582709	0.984764	0.399093
TRINITY_DN20344_c0_g1_i5_orf1	uncharacterized protein LOC114351483 [Ostrinia furnacalis]	-1.23363	-1.14126	0.392988	0.821767	1.160141
TRINITY_DN55147_c0_g1_i1_orfp1	TRINITY_DN55147_c0_g1_i1_m.59251 TRINITY_DN55147_c0_g1::TRINITY_DN55147_c0_g1_i1::g.59251 ORF type:5prime_partial len:331 (-),score=113.97,Cuticle_3 PF11018.9 0.29,Cuticle_3|PF11018.9|2.9e-05,Cuticle_3|PF11018.9|0.00037 TRINITY_DN55147_c0_g1_i1:21-1013(-)	-1.39657	-1.02859	0.95132	0.721535	0.752307
TRINITY_DN6325_c0_g1_i9_orf1	fructose-bisphosphate aldolase isoform X2 [Pieris brassicae]	-0.41652	-1.72079	0.276681	1.100416	0.760212
TRINITY_DN618_c0_g1_i3_orf1	triokinase/FMN cyclase-like isoform X1 [Ostrinia furnacalis]	-0.23513	-1.85346	0.671675	0.543444	0.873465
TRINITY_DN15858_c0_g1_i2_orf1	15-hydroxyprostaglandin dehydrogenase [NAD(+)]-like [Ostrinia furnacalis]	-0.74927	-1.55418	1.127608	0.761604	0.414233
TRINITY_DN812_c2_g1_i1_orf1	1,4-alpha-glucan-branching enzyme [Ostrinia furnacalis]	-0.57641	-1.70553	0.867195	0.762845	0.651899
TRINITY_DN33885_c0_g1_i1_orf1	inhibitor of nuclear factor kappa-B kinase subunit alpha [Ostrinia furnacalis]	-1.05714	-1.3368	1.10794	0.786172	0.499826
TRINITY_DN1206_c0_g1_i6_orf1	sorbitol dehydrogenase-like [Spodoptera frugiperda] >KAG8104768.1 hypothetical protein SFRUCORN_013827 [Spodoptera frugiperda]	-0.62842	-1.67648	0.728038	0.891438	0.685425
TRINITY_DN214_c0_g1_i3_orf1	uncharacterized protein LOC114352813 [Ostrinia furnacalis]	-1.14001	-1.15132	1.369912	0.652647	0.268769
TRINITY_DN11948_c0_g1_i8_orf1	cystathionine gamma-lyase [Ostrinia furnacalis]	-0.74675	-1.59051	0.845555	0.952093	0.539616
TRINITY_DN15755_c0_g1_i1_orf1	cytochrome P450 monooxygenase CYP6AB141 [Ostrinia furnacalis]	-1.11964	-1.06222	0.961585	-0.07867	1.298945
TRINITY_DN3175_c0_g1_i7_orf1	unnamed protein product, partial [Brenthis ino]	-0.23277	-1.86005	0.609957	0.656076	0.826779
TRINITY_DN26439_c0_g1_i2_orf1	uncharacterized protein LOC114351853 [Ostrinia furnacalis]	-0.90573	-1.4429	1.169831	0.458255	0.720545
TRINITY_DN15682_c0_g1_i4_orf1	seroin transcript 1B [Ostrinia nubilalis]	-0.56477	-1.12092	0.680438	1.610847	-0.60559
TRINITY_DN574_c0_g1_i4_orf1	CD63 antigen-like [Ostrinia furnacalis]	-0.13397	-1.88994	0.787831	0.50506	0.731026
TRINITY_DN12013_c0_g1_i6_orf1	tudor domain-containing protein 7 isoform X3 [Ostrinia furnacalis]	-0.85777	-1.00998	1.798715	0.091194	-0.02217
TRINITY_DN230_c2_g1_i5_orf1	6-pyruvoyl tetrahydrobiopterin synthase [Ostrinia furnacalis]	-0.84355	-1.0854	1.673148	-0.24505	0.500851
TRINITY_DN58751_c0_g1_i2_orf1	FK506-binding protein 2 isoform X1 [Vanessa tameamea] >XP_046977568.1 FK506-binding protein 2 isoform X1 [Vanessa cardui]	-0.06837	-1.81915	1.028081	0.06933	0.790108
TRINITY_DN4068_c1_g2_i1_orf1	larval cuticle protein LCP-17 [Helicoverpa armigera] >PZC82071.1 hypothetical protein B5X24_HaOG211161 [Helicoverpa armigera] >PZC87412.1 hypothetical protein B5X24_HaOG216859 [Helicoverpa armigera]	-1.34265	-0.96395	1.341508	0.519628	0.445464
TRINITY_DN7960_c0_g1_i2_orf1	uncharacterized protein LOC114364878 [Ostrinia furnacalis]	-0.71686	-1.55411	0.317499	1.152879	0.800584
TRINITY_DN33488_c0_g1_i2_orf1	semaphorin-1A isoform X3 [Trichoplusia ni]	-0.44616	-1.76322	0.931944	0.700102	0.577339
TRINITY_DN22875_c0_g1_i6_orf1	microtubule-actin cross-linking factor 1 isoform X15 [Ostrinia furnacalis]	-0.58475	-1.63237	1.068149	0.264304	0.884669

TRINITY_DN3029_c1_g2_i1_orf1	unnamed protein product [Plutella xylostella]	-0.39192	-1.56698	1.490967	0.404993	0.062941
TRINITY_DN10222_c0_g1_i2_orf1	glutathione S-transferase sigma 3 [Ostrinia furnacalis]	-0.97388	-1.3953	0.454784	1.138026	0.776374
TRINITY_DN14611_c0_g1_i5_orf1	hsc70-interacting protein-like [Galleria mellonella]	-0.35729	-1.72201	1.236011	0.312743	0.530547
TRINITY_DN4069_c0_g1_i5_orf1	putative sulfiredoxin [Ostrinia furnacalis]	-0.60588	-1.53807	1.033365	0.015513	1.095069
TRINITY_DN6698_c0_g2_i2_orf1	protein mesh isoform X1 [Ostrinia furnacalis]	0.144211	-1.86203	0.899725	-0.01985	0.837946
TRINITY_DN20133_c0_g1_i1_orf1	fructose-bisphosphate aldolase A isoform X2 [Microcebus murinus] >XP_012619765.1 fructose-bisphosphate aldolase A isoform X2 [Microcebus murinus] >XP_012619766.1 fructose-bisphosphate aldolase A isoform X2 [Microcebus murinus] >XP_012619767.1 fructose-bisphosphate aldolase A isoform X2 [Microcebus murinus] >XP_012619768.1 fructose-bisphosphate aldolase A isoform X2 [Microcebus murinus]	-0.59109	-1.52955	0.399365	1.439517	0.281757
TRINITY_DN49530_c0_g1_i1_orf1	ommochrome-binding protein-like [Ostrinia furnacalis]	-0.33394	-1.77118	0.277032	0.956876	0.871222
TRINITY_DN1226_c0_g1_i11_orfp1	TRINITY_DN1226_c0_g1_i11_m.52385 TRINITY_DN1226_c0_g1::TRINITY_DN1226_c0_g1_i11::g.52385 ORF type:internal len:92 (-),score=5.77 TRINITY_DN1226_c0_g1_i11:2-274(-)	-1.11907	-1.19379	0.754175	0.260198	1.298486
TRINITY_DN285_c0_g1_i4_orf1	catalase-like [Ostrinia furnacalis]	-0.99698	-1.39298	0.506369	0.809031	1.074564
TRINITY_DN9340_c0_g1_i4_orf1	sarcosine dehydrogenase, mitochondrial [Ostrinia furnacalis]	-0.45698	-1.695	1.194102	0.608372	0.349504
TRINITY_DN11108_c0_g1_i4_orf1	peroxisomal leader peptide-processing protease [Ostrinia furnacalis] >XP_028165527.1 peroxisomal leader peptide-processing protease [Ostrinia furnacalis]	-0.59582	-1.4749	1.406701	-0.03569	0.699704
TRINITY_DN28503_c0_g1_i6_orf1	uncharacterized protein LOC114363584 [Ostrinia furnacalis] >AXY94663.1 seroin transcript 3 [Ostrinia nubilalis]	-1.436	-0.46922	1.629859	0.029585	0.245782
TRINITY_DN26879_c0_g1_i1_orf1	hypothetical protein O3G_MSEX014401 [Manduca sexta]	-1.06059	-1.31954	1.085569	0.404864	0.889704
TRINITY_DN1252_c0_g1_i1_orf1	WD repeat-containing protein 47 isoform X1 [Ostrinia furnacalis]	-1.45651	-0.91999	1.048972	0.822925	0.504611
TRINITY_DN3433_c0_g1_i15_orf1	cytosolic purine 5'-nucleotidase isoform X3 [Ostrinia furnacalis] >XP_028162965.1 cytosolic purine 5'-nucleotidase isoform X3 [Ostrinia furnacalis] >XP_028162966.1 cytosolic purine 5'-nucleotidase isoform X3 [Ostrinia furnacalis]	-0.39682	-1.69788	0.681925	0.208001	1.204768
TRINITY_DN4497_c2_g1_i3_orf1	uncharacterized protein LOC114353086 [Ostrinia furnacalis]	-0.44873	-1.71078	0.300635	0.765472	1.093406
TRINITY_DN74037_c0_g5_i1_orf1	hypothetical protein JYU34_010754 [Plutella xylostella]	-0.8104	-1.4078	0.253171	0.554391	1.410638
TRINITY_DN66453_c0_g1_i4_orfp1	TRINITY_DN66453_c0_g1_i4_m.7345 TRINITY_DN66453_c0_g1::TRINITY_DN66453_c0_g1_i4::g.7345 ORF type:internal len:93 (+),score=24.53 TRINITY_DN66453_c0_g1_i4:3-278(+)	-1.11265	-1.14385	0.898282	0.077311	1.280909
TRINITY_DN10994_c0_g1_i4_orf1	trypsin inhibitor-like [Ostrinia furnacalis]	-0.74385	-1.59247	0.541191	0.955126	0.839995
TRINITY_DN970_c0_g1_i4_orf1	spermine oxidase-like isoform X2 [Ostrinia furnacalis]	-0.40634	-1.74779	0.354301	0.767776	1.032047
TRINITY_DN5914_c1_g1_i9_orf1	unnamed protein product [Chilo suppressalis]	-0.3929	-1.75599	0.742522	0.370124	1.036245
TRINITY_DN17326_c0_g1_i8_orf1	aminoacylase-1-like [Ostrinia furnacalis]	-0.46915	-1.75122	0.630969	0.633659	0.955751
TRINITY_DN82944_c0_g1_i4_orf1	senecionine N-oxygenase isoform X2 [Ostrinia furnacalis]	-0.33426	-1.80065	0.421388	0.844683	0.868837
TRINITY_DN1960_c5_g1_i3_orf1	cytochrome P450 monooxygenase CYP9G18 [Cnaphalocrocis medinalis]	-0.54873	-1.65427	0.256303	1.003029	0.943671
TRINITY_DN4502_c0_g1_i3_orf1	kynurenine formamidase isoform X1 [Ostrinia furnacalis]	-0.53484	-1.71027	0.928377	0.484933	0.831798
TRINITY_DN21435_c0_g1_i2_orf1	glycogen-binding subunit 76A isoform X1 [Ostrinia furnacalis]	-0.301	-1.81067	1.014053	0.53912	0.558495
TRINITY_DN38225_c0_g2_i1_orf1	uncharacterized protein LOC114354273 [Ostrinia furnacalis]	-0.90341	-1.35795	1.389718	0.316221	0.555425
TRINITY_DN36434_c0_g2_i3_orf1	clotting factor B isoform X1 [Ostrinia furnacalis]	-0.71747	-1.50881	0.36902	0.511599	1.345664
TRINITY_DN138086_c0_g1_i1_orf1	hypothetical protein evm_000614 [Chilo suppressalis]	-0.97053	-1.40586	1.125672	0.498248	0.752478
TRINITY_DN448_c0_g1_i20_orf1	probable cytochrome P450 9f2 isoform X1 [Ostrinia furnacalis]	-0.65214	-1.57521	0.403923	0.545765	1.277661
TRINITY_DN1230_c1_g1_i5_orf1	uncharacterized protein LOC114353440 [Ostrinia furnacalis]	-1.01605	-1.37327	0.873272	0.466477	1.049564
TRINITY_DN3015_c0_g1_i7_orf1	glycine-rich protein DOT1-like [Ostrinia furnacalis]	-0.21577	-1.86261	0.858558	0.570957	0.648865
TRINITY_DN8095_c0_g1_i3_orf1	circadian clock-controlled protein-like [Ostrinia furnacalis]	-1.35996	-1.07446	0.890695	0.846609	0.697112
TRINITY_DN1175_c1_g1_i2_orf1	methanethiol oxidase [Ostrinia furnacalis]	-1.04706	-1.33291	0.939056	1.042237	0.398673
TRINITY_DN29698_c0_g1_i3_orf1	15-hydroxyprostaglandin dehydrogenase [NAD(+)]-like [Ostrinia furnacalis]	-1.08779	-1.31424	1.133568	0.632146	0.636317
TRINITY_DN2461_c0_g1_i5_orf1	secretory phospholipase A2 receptor [Vanessa cardui]	-0.98072	-1.42816	0.679423	0.720841	1.008619
TRINITY_DN103475_c0_g1_i4_orf1	lipid storage droplets surface-binding protein 1 isoform X3 [Ostrinia furnacalis]	-0.88567	-1.48751	1.032939	0.532597	0.807641
TRINITY_DN11231_c1_g1_i1_orfp1	TRINITY_DN11231_c1_g1_i1_m.13377 TRINITY_DN11231_c1_g1::TRINITY_DN11231_c1_g1_i1::g.13377 ORF type:internal len:76 (-),score=1.43 TRINITY_DN11231_c1_g1_i1:1-225(-)	-0.82694	-1.40707	1.096039	0.075257	1.062715
TRINITY_DN4621_c0_g1_i4_orf1	uncharacterized protein LOC114358242 isoform X3 [Ostrinia furnacalis]	-0.64132	-1.66414	0.590625	0.866213	0.848628
TRINITY_DN452_c9_g1_i1_orf1	epidermal retinol dehydrogenase 2-like [Ostrinia furnacalis]	-0.58882	-1.53889	1.358672	0.116732	0.652303
TRINITY_DN8747_c0_g1_i2_orf1	lipopolysaccharide-induced tumor necrosis factor-alpha factor homolog [Ostrinia furnacalis]	-0.09526	-1.90515	0.791737	0.649148	0.559531
TRINITY_DN1008_c0_g1_i2_orf1	integrin beta-PS [Ostrinia furnacalis]	-0.59084	-1.55832	1.133317	0.048475	0.967367
TRINITY_DN13322_c0_g1_i6_orf1	macrophage mannose receptor 1-like [Ostrinia furnacalis]	-0.46503	-1.73678	0.416879	0.875303	0.909622

TRINITY_DN125521_c0_g2_i1_orf1	seroin transcript 1A2 [Ostrinia nubilalis]	-1.39028	-1.00326	0.501216	0.848785	1.04353
TRINITY_DN3766_c0_g1_i10_orf1	circadian clock-controlled protein-like [Ostrinia furnacalis]	-0.0108	-1.80718	1.140645	0.019672	0.657662
TRINITY_DN91946_c0_g1_i1_orf1	protein catecholamines up [Ostrinia furnacalis]	-0.76968	-1.46719	0.91434	0.139534	1.182992
TRINITY_DN18482_c0_g1_i3_orf1	calcyphosin-like protein isoform X3 [Helicoverpa armigera] >XP_047020698.1 calcyphosin-like protein isoform X2 [Helicoverpa zea]	-1.0723	-1.31087	0.425514	1.110234	0.847419
TRINITY_DN4394_c0_g1_i4_orf1	B-cell receptor-associated protein 31 [Ostrinia furnacalis]	-0.25193	-1.8199	1.040601	0.586132	0.445092
TRINITY_DN2706_c0_g1_i3_orf1	hypothetical protein evm_004793 [Chilo suppressalis]	-0.40812	-1.73635	1.150558	0.477069	0.516848
TRINITY_DN2624_c0_g1_i6_orf1	unnamed protein product [Danaus chrysippus]	-0.20899	-1.82551	0.95347	0.285139	0.795887
TRINITY_DN6483_c0_g1_i6_orf1	transketolase-like protein 2 isoform X1 [Ostrinia furnacalis] >XP_028164795.1 transketolase-like protein 2 isoform X2 [Ostrinia furnacalis]	-0.48911	-1.73434	0.753327	0.506242	0.96388
TRINITY_DN18196_c0_g1_i4_orf1	uncharacterized protein LOC114363471 isoform X3 [Ostrinia furnacalis]	-0.65571	-1.64965	0.98254	0.726146	0.596677
TRINITY_DN12873_c0_g2_i1_orf1	proteoglycan 4-like [Ostrinia furnacalis]	-0.8187	-1.55008	0.6124	0.947184	0.809194
TRINITY_DN1038_c1_g1_i3_orf1	uncharacterized protein LOC114363583 [Ostrinia furnacalis]	-1.3507	-1.03052	1.105943	0.440957	0.834319
TRINITY_DN4245_c0_g1_i5_orf1	long-chain fatty acid transport protein 4-like [Ostrinia furnacalis]	-0.83677	-1.53122	1.016652	0.610229	0.741104
TRINITY_DN63536_c0_g1_i1_orf1	adenosylhomocysteinase [Chelonus insularis]	-0.55159	-1.68891	0.719685	1.05104	0.469776
TRINITY_DN12545_c0_g1_i7_orf1	hypothetical protein evm_003491 [Chilo suppressalis]	-1.18171	-1.1449	1.218895	0.242787	0.864931
TRINITY_DN7630_c0_g2_i1_orf1	flotillin-2 isoform X1 [Ostrinia furnacalis] >XP_028172931.1 flotillin-2 isoform X2 [Ostrinia furnacalis]	-0.52398	-1.70472	0.845537	0.419679	0.963486
TRINITY_DN33_c0_g1_i14_orf1	uncharacterized protein CG45076-like isoform X1 [Ostrinia furnacalis]	-0.88362	-1.44015	1.084283	0.302463	0.937027
TRINITY_DN15858_c0_g1_i1_orf1	15-hydroxyprostaglandin dehydrogenase [NAD(+)]-like [Ostrinia furnacalis]	-0.90754	-1.47824	0.5607	0.987836	0.837245
TRINITY_DN5266_c0_g1_i1_orf1	malate dehydrogenase, cytoplasmic isoform X2 [Ostrinia furnacalis]	-0.68114	-1.63246	0.593191	0.719963	1.000448
TRINITY_DN24970_c0_g1_i4_orf1	pyrroline-5-carboxylate reductase-like isoform X1 [Ostrinia furnacalis]	-0.87267	-1.52052	0.853041	0.690326	0.849832
TRINITY_DN11383_c0_g2_i4_orf1	aminoacylase-1A-like [Ostrinia furnacalis]	-0.59642	-1.63367	0.292008	0.891443	1.046642
TRINITY_DN19521_c0_g1_i1_orf1	sodium/potassium-transporting ATPase subunit beta-1-like isoform X1 [Ostrinia furnacalis]	-0.01482	-1.7714	1.261493	0.004582	0.520144
TRINITY_DN2514_c1_g1_i13_orf1	seroin transcript 1A2 [Ostrinia nubilalis]	-1.22862	-1.20162	0.767884	0.637208	1.025147
TRINITY_DN34134_c0_g2_i1_orf1	THUMP domain-containing protein 1 homolog [Ostrinia furnacalis]	-0.66518	-1.52657	0.661128	0.209159	1.321467
TRINITY_DN1047_c0_g1_i6_orf1	mitochondrial genome maintenance exonuclease 1-like [Ostrinia furnacalis]	-0.53756	-1.72135	0.830136	0.576255	0.852516
TRINITY_DN3433_c2_g1_i2_orf1	cytosolic purine 5'-nucleotidase isoform X4 [Ostrinia furnacalis]	-0.87901	-1.37604	1.290463	0.163446	0.801147
TRINITY_DN2182_c0_g1_i4_orf1	growth-blocking peptide, long form-like isoform X1 [Ostrinia furnacalis] >XP_028159332.1 growth-blocking peptide, long form-like isoform X1 [Ostrinia furnacalis] >QWX20072.1 growth-blocking peptide [Ostrinia	-0.43836	-1.71093	1.025368	0.87627	0.247654
TRINITY_DN3263_c0_g1_i2_orf1	serine hydroxymethyltransferase, cytosolic isoform X1 [Diachasma alloeum]	-0.89428	-1.49935	0.638379	0.923679	0.83157
TRINITY_DN62091_c0_g1_i1_orf1	protein NipSnap [Venturia canescens]	-0.50071	-1.69178	0.345928	0.746378	1.10019
TRINITY_DN11798_c0_g2_i1_orf1	N-acetylglucosamine-6-sulfatase-like isoform X2 [Ostrinia furnacalis]	-0.44496	-1.60112	1.08465	1.028347	-0.06692
TRINITY_DN76633_c0_g1_i1_orfp1	TRINITY_DN76633_c0_g1_i1_m.53394 TRINITY_DN76633_c0_g1::TRINITY_DN76633_c0_g1_i1::g.53394 ORF type:internal len:164 (-),score=26.46,Toxin_2 PF00451.20 0.00022,Toxin_2|PF00451.20|0.00056,Toxin_2|PF00451.20|0.0002,Toxin_2|PF00451.20|5.9e-06,Gamma-thionin|PF00304.21|2,Gamma-thionin|PF00304.21|0.024,Gamma-thionin|PF00304.21|0.027,Gamma-thionin|PF00304.21|0.066,Toxin_38|PF14866.7|0.27,Toxin_38|PF14866.7|0.054,Toxin_38|PF14866.7|0.14,Defensin_2|PF01097.19|1.7,Defensin_2|PF01097.19|0.055,Defensin_2|PF01097.19|1.2,Defensin_2|PF01097.19|0.15 TRINITY_DN76633_c0_g1_i1:1-489(-)	-1.31831	-0.89206	0.971571	1.23382	0.00498
TRINITY_DN2378_c0_g1_i5_orf1	integrin alpha-8-like isoform X1 [Ostrinia furnacalis]	-0.63927	-1.61469	0.68149	0.409852	1.162622
TRINITY_DN701_c0_g1_i1_orf1	venom protease-like isoform X3 [Ostrinia furnacalis]	-1.01407	-1.31943	0.600833	0.436974	1.295692
TRINITY_DN3616_c0_g2_i2_orf1	conotoxin ArMKLT2-032-like [Ostrinia furnacalis]	-1.17777	-1.1895	0.656112	0.466079	1.245082
TRINITY_DN11698_c0_g1_i1_orf1	hypothetical protein evm_015129 [Chilo suppressalis]	-0.80454	-1.51589	0.412082	0.775156	1.133191
TRINITY_DN67243_c0_g1_i1_orf1	39S ribosomal protein L3, mitochondrial [Ostrinia furnacalis]	-0.46474	-1.76522	0.662401	0.766465	0.801098
TRINITY_DN8473_c0_g1_i6_orf1	serine/threonine-protein phosphatase 6 regulatory subunit 1 [Ostrinia furnacalis]	-0.82793	-1.40302	0.068952	1.127001	1.034988
TRINITY_DN19116_c0_g1_i3_orf1	UDP-glucose 4-epimerase isoform X1 [Ostrinia furnacalis]	-0.53648	-1.56725	1.166009	-0.00898	0.946707
TRINITY_DN135188_c0_g1_i2_orf1	proteasome inhibitor PI31 subunit [Ostrinia furnacalis]	-0.37409	-1.80711	0.651607	0.767057	0.762532
TRINITY_DN44491_c0_g1_i12_orf1	unnamed protein product [Chrysodeixis includens]	-1.06701	-1.28387	1.040874	0.28643	1.023575
TRINITY_DN394_c0_g1_i4_orf1	uncharacterized protein LOC114351483 [Ostrinia furnacalis]	-1.15525	-1.15279	0.159437	1.03687	1.111733
TRINITY_DN9435_c0_g1_i7_orf1	uncharacterized protein LOC114350197 [Ostrinia furnacalis]	-0.71631	-1.42066	0.244947	1.502235	0.389781
TRINITY_DN1926_c0_g1_i5_orf1	coiled-coil and C2 domain-containing protein 1-like [Ostrinia furnacalis]	-0.37884	-1.72935	1.077385	0.220626	0.810178

TRINITY_DN56708_c0_g3_i1_orfp1	TRINITY_DN56708_c0_g3_i1_m.56611 TRINITY_DN56708_c0_g3::TRINITY_DN56708_c0_g3_i1::g.56611 ORF type:internal len:69 (-),score=2.50 TRINITY_DN56708_c0_g3_i1:1-204(-)	-1.35329	-0.85903	1.457358	0.273113	0.481854
TRINITY_DN17845_c0_g1_i3_orf1	ataxin-10-like [Ostrinia furnacalis]	-0.39049	-1.61568	1.434271	0.195348	0.376553
TRINITY_DN44083_c0_g1_i2_orf1	putative alpha-ketoglutarate-dependent hypophosphite dioxygenase [Operophtera brumata]	-0.92689	-1.44621	0.918722	0.455454	0.998927
TRINITY_DN350_c0_g1_i4_orf1	microtubule-associated protein tau-like isoform X6 [Ostrinia furnacalis]	-0.90658	-1.47686	0.923836	0.531982	0.927631
TRINITY_DN23564_c0_g1_i7_orf1	cytochrome P450 6B6-like [Ostrinia furnacalis]	-0.37417	-1.61122	0.94571	-0.12403	1.163712
TRINITY_DN62707_c0_g1_i1_orf1	uncharacterized protein LOC114362831 [Ostrinia furnacalis]	-1.03975	-1.15593	1.49791	0.56749	0.130277
TRINITY_DN566_c0_g1_i13_orf1	uncharacterized protein LOC114355567 isoform X2 [Ostrinia furnacalis]	-0.88611	-1.39313	0.748641	0.244626	1.285966
TRINITY_DN8702_c0_g1_i1_orf1	programmed cell death protein 4 isoform X1 [Ostrinia furnacalis] >XP_028157160.1 programmed cell death protein 4 isoform X2 [Ostrinia furnacalis]	-0.70393	-1.603	0.796029	0.472799	1.038105
TRINITY_DN28299_c0_g1_i1_orf1	adenylosuccinate lyase isoform X1 [Ostrinia furnacalis]	-0.62741	-1.58328	0.208843	1.162595	0.839251
TRINITY_DN17759_c0_g1_i5_orf1	hypothetical protein evm_013530 [Chilo suppressalis]	-1.07552	-1.29768	1.224507	0.565895	0.582791
TRINITY_DN38783_c0_g1_i1_orf1	regucalcin-like [Ostrinia furnacalis]	-0.83963	-1.44952	0.262869	0.823543	1.20274
TRINITY_DN625_c9_g1_i7_orf1	ecdysone 20-monooxygenase [Ostrinia furnacalis]	-0.71097	-1.59791	0.494065	0.748771	1.066043
TRINITY_DN511_c0_g2_i1_orf1	pyruvate carboxylase, mitochondrial isoform X1 [Manduca sexta] >XP_037293486.1 pyruvate carboxylase, mitochondrial isoform X1 [Manduca sexta]	-0.82811	-1.54535	0.968421	0.677851	0.727188
TRINITY_DN8258_c0_g1_i3_orf1	papilin isoform X7 [Ostrinia furnacalis]	-0.72723	-1.52831	1.108705	0.220881	0.925954
TRINITY_DN6392_c0_g1_i9_orf1	putative mediator of RNA polymerase II transcription subunit 29 isoform X4 [Ostrinia furnacalis]	-0.84995	-1.34001	1.417141	0.682258	0.090559
TRINITY_DN1470_c0_g1_i8_orf1	oxidation resistance protein 1 isoform X5 [Ostrinia furnacalis]	-1.17423	-1.20081	1.234515	0.521211	0.619315
TRINITY_DN7152_c0_g1_i1_orf1	tissue alpha-L-fucosidase [Ostrinia furnacalis]	-0.29345	-1.81254	1.018284	0.552562	0.535138
TRINITY_DN384_c0_g1_i8_orf1	unnamed protein product [Chilo suppressalis]	-0.83387	-1.53268	0.570163	0.806107	0.990281
TRINITY_DN39933_c0_g1_i2_orf1	thioredoxin domain-containing protein 11 isoform X4 [Ostrinia furnacalis]	-1.26484	-1.02996	1.346638	0.669552	0.278611
TRINITY_DN699_c0_g1_i5_orf1	TPA_exp: putative parasitoid killing factor [Trichoplusia ni]	-0.6251	-1.42816	0.965202	1.267233	-0.17917
TRINITY_DN6994_c0_g1_i3_orf1	C-type mannose receptor 2-like isoform X1 [Ostrinia furnacalis]	-0.90284	-1.04889	1.738709	0.246007	-0.03299
TRINITY_DN4068_c0_g2_i4_orf1	larval cuticle protein LCP-17-like precursor [Papilio polytes] >BAM18876.1 cuticular protein PpolCPR2 [Papilio	-1.46211	-0.81877	0.242558	0.852375	1.185948
TRINITY_DN117844_c0_g1_i1_orf1	ATP-citrate synthase [Cotesia glomerata] >XP_044590631.1 ATP-citrate synthase [Cotesia glomerata] >KAH0546822.1 hypothetical protein KQX54_015428 [Cotesia glomerata]	-1.07501	-1.34835	0.593783	0.910783	0.918798
TRINITY_DN2688_c0_g1_i3_orf1	mitochondrial amidoxime reducing component 2-like [Ostrinia furnacalis]	-0.94582	-1.19192	1.493574	0.673163	-0.029
TRINITY_DN60792_c0_g1_i2_orf1	ATP-binding cassette sub-family D member 2 [Ostrinia furnacalis] >XP_028165108.1 ATP-binding cassette sub-family D member 2 [Ostrinia furnacalis]	-1.21134	-0.71242	1.700417	-0.12151	0.344854
TRINITY_DN12666_c0_g1_i2_orf1	sodium- and chloride-dependent GABA transporter 1 [Ostrinia furnacalis]	-0.98331	-1.31739	0.770939	1.281201	0.248552
TRINITY_DN8853_c0_g1_i4_orf1	uncharacterized protein LOC114351488 isoform X1 [Ostrinia furnacalis]	-1.12593	-0.99767	0.343189	0.171106	1.609308
TRINITY_DN1789_c0_g1_i5_orf1	uncharacterized protein LOC114365543 [Ostrinia furnacalis]	-0.54979	-1.19746	1.603289	0.65633	-0.51237
TRINITY_DN26961_c0_g1_i1_orf1	uncharacterized protein LOC120424957 [Culex pipiens pallens]	-1.59811	-0.6231	1.262616	0.524776	0.433819
TRINITY_DN10441_c0_g1_i3_orf1	zonadhesin-like [Ostrinia furnacalis]	-1.45224	-0.78268	1.384125	0.40694	0.443856
TRINITY_DN4123_c0_g1_i1_orf1	uncharacterized protein LOC114355030 [Ostrinia furnacalis]	-1.14575	-1.0626	1.05329	-0.04764	1.20269
TRINITY_DN2302_c0_g1_i1_orf1	enoyl-CoA hydratase domain-containing protein 3, mitochondrial [Ostrinia furnacalis]	-0.86378	-1.2374	1.56134	0.006095	0.533752
TRINITY_DN1455_c0_g1_i4_orf1	troponin domain-containing protein [Phthorimaea operculella]	-1.21203	-1.21648	1.025913	0.614678	0.787922
TRINITY_DN40281_c0_g1_i1_orf1	thioredoxin domain-containing protein 15 [Ostrinia furnacalis]	-1.0731	-1.21441	0.461614	1.409759	0.416133
TRINITY_DN93764_c0_g1_i1_orf1	microtubule-associated protein futsch isoform X4 [Ostrinia furnacalis] >XP_028162562.1 microtubule-associated protein futsch isoform X4 [Ostrinia furnacalis]	-0.9674	-1.25886	1.364223	0.782274	0.079759
TRINITY_DN5880_c0_g2_i2_orf1	macrophage mannose receptor 1 [Bombyx mori]	-0.99974	-1.1019	0.648405	-0.08269	1.535926
TRINITY_DN49038_c0_g4_i1_orf1	6-phosphogluconate dehydrogenase, decarboxylating [Ostrinia furnacalis]	-0.54053	-1.72699	0.833029	0.752845	0.68164
TRINITY_DN2570_c0_g1_i1_orf1	PREDICTED: pyruvate carboxylase, mitochondrial isoform X1 [Microplitis demolitor] >XP_008556301.1 PREDICTED: pyruvate carboxylase, mitochondrial isoform X1 [Microplitis demolitor] >XP_008556302.1 PREDICTED: pyruvate carboxylase, mitochondrial isoform X1 [Microplitis demolitor]	-0.69098	-1.62725	0.53881	0.866363	0.913064
TRINITY_DN8258_c0_g1_i5_orf1	papilin isoform X9 [Ostrinia furnacalis]	-0.58929	-1.64016	1.008859	0.294162	0.92643
TRINITY_DN10403_c0_g1_i1_orf1	hypothetical protein evm_000264 [Chilo suppressalis] >CAH2987898.1 unnamed protein product [Chilo	-0.84935	-1.40298	0.304045	0.574201	1.374083
TRINITY_DN2983_c0_g1_i6_orf1	hypothetical protein evm_002448 [Chilo suppressalis]	-0.6764	-1.63964	0.60071	0.752713	0.962618
TRINITY_DN12873_c0_g1_i3_orf1	proteoglycan 4-like [Ostrinia furnacalis]	-0.83949	-1.4375	0.223029	1.213694	0.840267
TRINITY_DN22597_c0_g1_i4_orf1	uncharacterized protein LOC114361588 isoform X16 [Ostrinia furnacalis]	-1.47739	-0.86723	1.115277	0.433369	0.795975

TRINITY_DN16400_c0_g2_i1_orf1	superoxide dismutase [Cu-Zn]-like isoform X1 [Ostrinia furnacalis]	-1.49795	-0.6059	1.481339	0.290085	0.332429
TRINITY_DN65518_c0_g1_i1_orf1	unc-112-related protein-like, partial [Ostrinia furnacalis]	-0.9369	-1.46145	0.594467	0.956663	0.84722
TRINITY_DN3859_c0_g1_i5_orf1	D-aspartate oxidase [Ostrinia furnacalis] >XP_028166452.1 D-aspartate oxidase [Ostrinia furnacalis] >XP_028166454.1 D-aspartate oxidase [Ostrinia furnacalis]	-1.22218	-1.14924	0.960593	1.071179	0.339648
TRINITY_DN610_c0_g1_i1_orf1	CAP-Gly domain-containing linker protein 1 isoform X10 [Ostrinia furnacalis]	-1.15759	-1.23202	0.966436	1.02417	0.398999
TRINITY_DN46132_c0_g2_i2_orf1	hypothetical protein evm_003834 [Chilo suppressalis]	-1.29297	-0.87124	1.526984	0.18736	0.449869
TRINITY_DN11263_c0_g1_i5_orf1	SET domain-containing protein SmydA-8 [Ostrinia furnacalis]	-0.24114	-1.54261	1.595085	0.10122	0.08745
TRINITY_DN3377_c0_g1_i1_orf1	calcyphosin-like protein isoform X3 [Helicoverpa armigera] >XP_047020698.1 calcyphosin-like protein isoform X2 [Helicoverpa zea]	-0.81388	-1.26918	1.305441	0.988915	-0.21129
TRINITY_DN30012_c1_g1_i1_orf1	ubiquitin domain-containing protein UBFD1-like [Ostrinia furnacalis]	-0.7602	-1.5659	0.663654	0.559403	1.10304
TRINITY_DN39837_c0_g1_i1_orf1	unnamed protein product [Plutella xylostella]	-1.25229	-1.17692	0.909279	0.939877	0.580056
TRINITY_DN8527_c0_g2_i1_orfp1	TRINITY_DN8527_c0_g2_i1_m.16937 TRINITY_DN8527_c0_g2::TRINITY_DN8527_c0_g2_i1::g.16937 ORF type:5prime_partial len:54 (-),score=0.36 TRINITY_DN8527_c0_g2_i1:195-356(-)	-1.35977	-1.04698	0.528174	0.86394	1.014638
TRINITY_DN3374_c0_g1_i7_orf1	TPPP family protein CG45057 [Ostrinia furnacalis] >XP_028172578.1 TPPP family protein CG45057 [Ostrinia	-1.11887	-1.13836	1.299258	0.088267	0.869709
TRINITY_DN2175_c0_g1_i4_orf1	uncharacterized protein LOC114353827 [Ostrinia furnacalis]	-1.36488	-0.898	1.339078	0.697344	0.226456
TRINITY_DN6813_c1_g1_i1_orf1	pantothenate kinase 3 isoform X2 [Ostrinia furnacalis] >XP_028173241.1 pantothenate kinase 3 isoform X2 [Ostrinia furnacalis]	-0.85229	-1.41261	0.17222	1.217861	0.874815
TRINITY_DN4314_c0_g1_i9_orf1	serine proteinase inhibitor 2 [Ostrinia furnacalis]	-1.19428	-1.19553	0.445526	1.139169	0.805114
TRINITY_DN245_c0_g1_i4_orf1	ER lumen protein-retaining receptor [Ostrinia furnacalis]	-1.44148	-0.93778	0.473394	0.988049	0.91781
TRINITY_DN3826_c0_g1_i1_orf1	39S ribosomal protein L18, mitochondrial [Ostrinia furnacalis]	-1.09517	-1.12408	0.479514	1.499855	0.239883
TRINITY_DN757_c3_g1_i2_orf1	PREDICTED: galectin-4-like [Amyelois transitella]	-0.79518	-1.55139	0.857014	0.502894	0.986663
TRINITY_DN10652_c0_g1_i4_orf1	uncharacterized protein LOC114364166 [Ostrinia furnacalis]	-0.88484	-0.95303	1.814154	0.10499	-0.08127
TRINITY_DN89483_c0_g1_i1_orf1	mitochondrial enolase superfamily member 1-like isoform X2 [Maniola jurtina]	-0.94005	-1.1892	0.434325	0.113606	1.581318
TRINITY_DN3433_c0_g1_i6_orf1	cytosolic purine 5'-nucleotidase isoform X2 [Ostrinia furnacalis] >XP_028162963.1 cytosolic purine 5'-nucleotidase isoform X2 [Ostrinia furnacalis]	-0.807	-1.23385	1.652493	0.09365	0.294712
TRINITY_DN1575_c0_g1_i7_orf1	adenine phosphoribosyltransferase [Ostrinia furnacalis]	-0.58185	-1.41719	1.376852	-0.21983	0.842014
TRINITY_DN61335_c0_g2_i1_orf1	uncharacterized protein LOC114352149 [Ostrinia furnacalis]	-1.21007	-1.12724	1.32051	0.461543	0.555251
TRINITY_DN8224_c0_g1_i7_orf1	hemicentin-1-like isoform X2 [Ostrinia furnacalis]	-0.85876	-1.43353	1.246326	0.2915	0.754466
TRINITY_DN8030_c0_g1_i2_orf1	chaoptin isoform X1 [Ostrinia furnacalis] >XP_028159953.1 chaoptin isoform X2 [Ostrinia furnacalis] >XP_028159954.1 chaoptin isoform X3 [Ostrinia furnacalis] >XP_028159955.1 chaoptin isoform X4 [Ostrinia furnacalis] >XP_028159956.1 chaoptin isoform X5 [Ostrinia furnacalis]	-0.9623	-1.36778	0.271464	0.956804	1.101812
TRINITY_DN21285_c0_g1_i3_orf1	uncharacterized protein LOC114351683 isoform X7 [Ostrinia furnacalis]	-0.86154	-1.47903	1.143126	0.446172	0.751275
TRINITY_DN1607_c0_g1_i16_orf1	LOW QUALITY PROTEIN: asparagine--tRNA ligase, cytoplasmic [Ostrinia furnacalis]	-1.51342	-0.84261	1.088015	0.711028	0.556988
TRINITY_DN726_c0_g1_i8_orf1	unnamed protein product [Chilo suppressalis]	-0.45814	-1.62702	1.351273	0.521346	0.212543
TRINITY_DN99694_c0_g1_i1_orf1	hypothetical protein HF086_013792 [Spodoptera exigua]	-0.75442	-1.56221	0.548541	1.135435	0.632652
TRINITY_DN26993_c1_g1_i8_orf1	endocuticle structural glycoprotein ABD-4-like [Ostrinia furnacalis]	-1.25735	-0.89398	1.483519	0.020832	0.646987
TRINITY_DN97472_c0_g1_i5_orf1	microtubule-actin cross-linking factor 1 isoform X15 [Ostrinia furnacalis]	-1.39005	-0.97108	1.207862	0.556466	0.596803
TRINITY_DN695_c0_g1_i5_orf1	uncharacterized protein LOC114363574 isoform X1 [Ostrinia furnacalis]	-1.24906	-1.18381	0.953219	0.872374	0.607275
TRINITY_DN7106_c0_g1_i5_orf1	TBC1 domain family member 15 isoform X5 [Helicoverpa zea]	-0.9784	-1.29831	0.473257	1.405189	0.398258
TRINITY_DN1267_c0_g2_i10_orf1	secretory phospholipase A2 receptor-like [Ostrinia furnacalis]	-1.52985	-0.53484	1.44259	0.533471	0.088629
TRINITY_DN5748_c0_g1_i5_orf1	glycine N-methyltransferase isoform X1 [Ostrinia furnacalis] >XP_028165118.1 glycine N-methyltransferase isoform X2 [Ostrinia furnacalis] >XP_028165119.1 glycine N-methyltransferase isoform X1 [Ostrinia furnacalis] >XP_028165120.1 glycine N-methyltransferase isoform X2 [Ostrinia furnacalis]	-0.92314	-1.42587	0.601854	0.537246	1.209903
TRINITY_DN10581_c0_g1_i5_orf1	facilitated trehalose transporter Tret1-like isoform X1 [Ostrinia furnacalis] >XP_028165563.1 facilitated trehalose transporter Tret1-like isoform X2 [Ostrinia furnacalis]	-1.07252	-1.24629	1.020088	0.195192	1.103531
TRINITY_DN5001_c0_g1_i4_orf1	uncharacterized protein LOC114356665 [Ostrinia furnacalis]	-1.21607	-1.03544	0.051976	0.982825	1.216711
TRINITY_DN7022_c0_g1_i7_orf1	fatty-acid amide hydrolase 2-A-like [Ostrinia furnacalis]	-1.09664	-0.83906	1.557141	0.735635	-0.35707
TRINITY_DN77559_c0_g1_i1_orf1	uncharacterized protein LOC114359392 isoform X2 [Ostrinia furnacalis]	-0.91765	-1.37143	0.232104	0.796489	1.260488
TRINITY_DN116972_c0_g1_i1_orf1	phosphofructokinase domain-containing protein [Phthorimaea operculella]	-1.31729	-1.08688	1.124522	0.632025	0.647628
TRINITY_DN8625_c0_g1_i1_orf1	GDP-L-fucose synthase [Ostrinia furnacalis]	-0.91427	-1.18601	0.270826	1.625606	0.203845
TRINITY_DN3991_c0_g1_i6_orf1	acetyl-CoA carboxylase isoform X3 [Trichoplusia ni]	-0.93951	-1.27918	0.292261	0.443587	1.482845

TRINITY_DN2559_c0_g1_i4_orf1	uricase [Ostrinia furnacalis]	-1.56077	-0.56656	1.38525	0.214905	0.527175
TRINITY_DN64126_c0_g1_i1_orf1	senecionine N-oxygenase isoform X2 [Galleria mellonella]	-0.94407	-1.40232	0.408152	0.748593	1.189644
TRINITY_DN59852_c0_g1_i1_orf1	hypothetical protein evm_008421 [Chilo suppressalis]	-0.9096	-1.3694	1.19575	0.166897	0.916351
TRINITY_DN5768_c0_g1_i2_orf1	adenosylhomocysteinase [Ostrinia furnacalis]	-1.44622	-0.91861	1.082927	0.828328	0.453579
TRINITY_DN14112_c0_g1_i3_orf1	uncharacterized protein LOC114350956 [Ostrinia furnacalis]	-0.96803	-1.36021	0.442611	0.597089	1.288538
TRINITY_DN1034_c0_g1_i4_orf1	glycerol kinase isoform X4 [Ostrinia furnacalis]	-1.10341	-1.26569	0.881936	1.128863	0.3583
TRINITY_DN1328_c0_g1_i6_orf1	fungal protease inhibitor-1-like [Ostrinia furnacalis]	-1.11201	-1.22034	0.317083	1.275043	0.740223
TRINITY_DN2772_c0_g1_i3_orf1	uncharacterized protein LOC114353284 isoform X4 [Ostrinia furnacalis] >XP_028161011.1 uncharacterized protein LOC114353284 isoform X4 [Ostrinia furnacalis] >XP_028161012.1 uncharacterized protein LOC114353284 isoform X4 [Ostrinia furnacalis]	-1.05653	-1.2004	0.217572	1.417139	0.622222
TRINITY_DN23069_c0_g2_i3_orf1	uncharacterized protein LOC114364799 [Ostrinia furnacalis]	-1.33111	-1.06217	0.494671	0.786726	1.111888
TRINITY_DN9569_c1_g1_i7_orf1	V-set and immunoglobulin domain-containing protein 1-like isoform X1 [Ostrinia furnacalis] >XP_028156015.1 V-set and immunoglobulin domain-containing protein 1-like isoform X2 [Ostrinia furnacalis]	-1.31084	-1.1286	0.852798	0.898092	0.688557
TRINITY_DN36460_c0_g1_i2_orf1	N-acetylneuraminate lyase-like [Ostrinia furnacalis]	-1.17242	-1.23813	1.10292	0.551143	0.756488
TRINITY_DN10774_c0_g2_i3_orf1	uncharacterized protein LOC114362157, partial [Ostrinia furnacalis]	-0.92975	-1.30253	0.491851	0.286051	1.454382
TRINITY_DN51424_c0_g2_i1_orf1	ras suppressor protein 1 [Helicoverpa zea]	-0.98677	-1.32323	1.184918	0.907808	0.217272
TRINITY_DN1172_c0_g1_i1_orf1	hypothetical protein O3G_MSEX009550 [Manduca sexta]	-1.07499	-1.31364	0.506973	0.727065	1.154596
TRINITY_DN22797_c0_g1_i5_orf1	phenoloxidase-activating factor 2-like isoform X1 [Ostrinia furnacalis]	-1.63398	-0.42154	1.378395	0.44627	0.230852
TRINITY_DN6642_c0_g1_i2_orf1	protein purity of essence [Ostrinia furnacalis]	-0.98535	-1.13845	0.12537	0.399023	1.599403
TRINITY_DN268_c3_g1_i2_orf1	hypothetical protein evm_003084 [Chilo suppressalis]	-1.11548	-1.30524	1.063201	0.66837	0.689151
TRINITY_DN741_c0_g1_i10_orf1	talin-1 isoform X13 [Ostrinia furnacalis]	-1.28239	-1.07578	0.9068	0.324036	1.127335
TRINITY_DN2596_c0_g1_i6_orf1	unnamed protein product [Arctia plantaginis]	-1.16809	-1.11274	0.117076	0.93568	1.228075
TRINITY_DN60787_c0_g1_i5_orf1	probable transaldolase [Ostrinia furnacalis]	-1.08537	-1.34733	0.718583	0.960017	0.754103
TRINITY_DN28311_c0_g1_i2_orf1	RNA polymerase II-associated protein 3-like isoform X1 [Ostrinia furnacalis] >XP_028179249.1 RNA polymerase II-associated protein 3-like isoform X2 [Ostrinia furnacalis] >XP_028179250.1 RNA polymerase II-associated protein 3-like isoform X3 [Ostrinia furnacalis]	-0.98153	-1.13374	1.584859	0.487456	0.042953
TRINITY_DN2529_c0_g1_i3_orf1	collagen alpha-1(X) chain-like [Ostrinia furnacalis]	-1.009	-1.29378	0.689293	0.291356	1.322132
TRINITY_DN57900_c0_g1_i2_orf1	hypothetical protein SFRURICE_000634 [Spodoptera frugiperda]	-1.40804	-0.73928	0.389241	0.256963	1.501117
TRINITY_DN124171_c0_g1_i4_orf1	dystonin isoform X27 [Trichoplusia ni]	-1.33478	-1.0174	1.211966	0.39072	0.749493

TRINITY_DN10070_c0_g1_i1_orf1	40S ribosomal protein SA isoform 1 [Homo sapiens] >XP_002813955.1 40S ribosomal protein SA [Pongo abelii] >XP_004033937.1 40S ribosomal protein SA [Gorilla gorilla gorilla] >XP_008949773.1 40S ribosomal protein SA [Pan paniscus] >XP_009237465.1 40S ribosomal protein SA [Pongo abelii] >XP_024211184.1 40S ribosomal protein SA [Pan troglodytes] >XP_032017897.1 40S ribosomal protein SA [Hylobates moloch] >XP_032017898.1 40S ribosomal protein SA [Hylobates moloch] >XP_032615302.1 40S ribosomal protein SA [Hylobates moloch] >XP_034820112.1 40S ribosomal protein SA [Pan paniscus] >P08865.4 RecName: Full=40S ribosomal protein SA; AltName: Full=37 kDa laminin receptor precursor; Short=37LRP; AltName: Full=37/67 kDa laminin receptor; Short=LRP/LR; AltName: Full=67 kDa laminin receptor; Short=67LR; AltName: Full=Colon carcinoma laminin-binding protein; AltName: Full=Laminin receptor 1; Short=LamR; AltName: Full=Laminin-binding protein precursor p40; Short=LBP/p40; AltName: Full=Multidrug resistance-associated protein MGr1-Ag; AltName: Full=NEM/1CHD4; AltName: Full=Small ribosomal subunit protein uS2 [Homo sapiens] >4D5L_A Cryo-EM structures of ribosomal 80S complexes with termination factors and cricket paralysis virus IRES reveal the IRES in the translocated state [Oryctolagus cuniculus] >4D61_A Cryo-EM structures of ribosomal 80S complexes with termination factors and cricket paralysis virus IRES reveal the IRES in the translocated state [Oryctolagus cuniculus] >4UG0_SA Chain SA, 40S RIBOSOMAL PROTEIN SA [Homo sapiens] >4UJD_CA Chain CA, 40S RIBOSOMAL PROTEIN US2 [Oryctolagus cuniculus] >4UJE_BA Chain BA, 40S RIBOSOMAL PROTEIN SA [Oryctolagus cuniculus] >4V6X_AA Chain AA, 40S ribosomal protein SA [Homo sapiens] >5A2Q_A Structure of the HCV IRES bound to the human ribosome [Homo sapiens] >5AJ0_BA Chain BA, 40S ribosomal protein SA [Homo sapiens] >5FLX_A Mammalian 40S HCV-IRES complex [Oryctolagus cuniculus] >5LKS_SA Chain SA, 40S ribosomal protein SA [Homo sapiens] >5OA3_A Human 40S-eIF2D-re-initiation complex [Homo sapiens] >5T2C_Ao Chain Ao, 40S ribosomal protein SA [Homo sapiens] >5VYC_A1 Chain A1, 40S ribosomal protein SA [Homo sapiens] >5VYC_A2 Chain A2, 40S ribosomal protein SA [Homo sapiens] >5VYC_A3 Chain A3, 40S ribosomal protein SA [Homo sapiens] >5VYC_A4 Chain A4, 40S ribosomal protein SA [Homo sapiens] >5VYC_A5 Chain A5, 40S ribosomal protein SA [Homo sapiens] >5VYC_A6 Chain A6, 40S ribosomal protein SA [Homo sapiens] >6EK0_SA Chain SA, 40S ribosomal protein SA [Homo sapiens] >6G18_A Cryo-EM structure of a late human pre-40S ribosomal subunit - State C [Homo sapiens] >6G4S_A Cryo-EM structure of a late human pre-40S ribosomal subunit - State B [Homo sapiens] >6G51_A Cryo-EM structure of a late human pre-40S ribosomal subunit - State D [Homo sapiens] >6G53_A Cryo-EM structure of a late human pre-40S ribosomal subunit - State E [Homo sapiens] >6G5H_A Cryo-EM structure of a late human pre-40S ribosomal subunit - Mature [Homo sapiens] >6G5I_A Cryo-EM structure of a late human pre-40S ribosomal subunit - State R [Homo sapiens] >6IP5_2n Chain 2n, 40S ribosomal protein SA	-1.37402	-0.9218	0.294837	1.294999	0.705982
TRINITY_DN50593_c0_g1_i1_orf1	uncharacterized protein LOC114361588 isoform X14 [Ostrinia furnacalis]	-1.52151	-0.80016	0.396222	0.832179	1.093269
TRINITY_DN1245_c0_g1_i4_orf1	nuclear RNA export factor 1 [Ostrinia furnacalis]	-1.18626	-0.97913	0.157624	0.459048	1.548717
TRINITY_DN24410_c0_g2_i1_orf1	spermine oxidase-like isoform X3 [Venturia canescens] >XP_043269281.1 spermine oxidase-like isoform X3 [Venturia canescens]	-1.38836	-0.99253	0.937881	1.010111	0.432897
TRINITY_DN1993_c0_g1_i1_orf1	6-phosphofructo-2-kinase/fructose-2,6-bisphosphatase isoform X1 [Ostrinia furnacalis]	-1.43298	-0.38654	1.658782	-0.04749	0.208224
TRINITY_DN5998_c0_g2_i1_orf1	protein archease-like [Ostrinia furnacalis]	-1.01076	-1.12822	1.598349	0.222143	0.318481
TRINITY_DN4695_c0_g1_i4_orf1	glutathione S-transferase epsilon 3 [Ostrinia furnacalis]	-1.11368	-1.20093	0.413461	0.538652	1.362496
TRINITY_DN13711_c0_g1_i1_orf1	putative nuclease HARBI1 [Myzus persicae]	-1.18947	-1.12016	0.27853	1.323892	0.707206
TRINITY_DN4289_c0_g1_i5_orf1	translocon-associated protein subunit beta [Ostrinia furnacalis] >XP_028157895.1 translocon-associated protein subunit beta [Ostrinia furnacalis] >XP_028157896.1 translocon-associated protein subunit beta [Ostrinia furnacalis]	-1.2145	-1.18497	1.154257	0.703951	0.541267
TRINITY_DN2844_c0_g1_i2_orf1	glutathione S-transferase 9 [Streltzoviella insularis]	-1.07449	-1.05596	0.851227	-0.13098	1.410206
TRINITY_DN5933_c0_g1_i1_orf1	peroxidase-like [Ostrinia furnacalis]	-1.51058	-0.85357	0.526239	0.810229	1.02769
TRINITY_DN35763_c0_g1_i2_orf1	probable methylthioribulose-1-phosphate dehydratase [Helicoverpa armigera]	-1.10808	-1.22306	0.379081	0.638898	1.313161
TRINITY_DN63152_c0_g1_i7_orf1	microtubule-actin cross-linking factor 1 isoform X21 [Manduca sexta]	-1.21136	-1.12854	0.697413	0.360951	1.281536
TRINITY_DN1741_c0_g1_i5_orf1	gephyrin isoform X1 [Ostrinia furnacalis]	-1.09813	-1.28475	1.160253	0.453296	0.769329
TRINITY_DN85161_c0_g1_i2_orf1	hypothetical protein evm_013813 [Chilo suppressalis]	-1.17736	-0.71667	1.663176	0.507483	-0.27664
TRINITY_DN19251_c0_g1_i8_orf1	succinate--CoA ligase [GDP-forming] subunit beta, mitochondrial [Ostrinia furnacalis]	-1.53168	-0.40389	1.553256	0.140386	0.241923
TRINITY_DN554_c0_g1_i1_orf1	uncharacterized protein LOC114353093 isoform X1 [Ostrinia furnacalis] >XP_028160722.1 uncharacterized protein LOC114353093 isoform X1 [Ostrinia furnacalis] >XP_028160723.1 uncharacterized protein LOC114353093 isoform X1 [Ostrinia furnacalis]	-1.23482	-1.01248	0.155735	0.70045	1.391119
TRINITY_DN2207_c0_g1_i6_orf1	methionine-R-sulfoxide reductase B1 isoform X2 [Ostrinia furnacalis]	-0.98395	-1.30875	0.548376	1.37063	0.373703
TRINITY_DN42120_c0_g1_i2_orf1	FAM172 family protein homolog CG10038 [Ostrinia furnacalis]	-1.2313	-0.94742	1.526442	0.473678	0.1786

TRINITY_DN2207_c0_g1_i4_orf1	methionine-R-sulfoxide reductase B1 isoform X4 [Pectinophora gossypiella] >XP_049887601.1 methionine-R-sulfoxide reductase B1 isoform X4 [Pectinophora gossypiella]	-1.45256	-0.69024	0.35132	0.310294	1.48119
TRINITY_DN17657_c0_g1_i1_orf1	alcohol dehydrogenase 18, partial [Helicoverpa assulta]	-1.69284	-0.25167	1.057741	0.972038	-0.08527
TRINITY_DN37418_c0_g1_i4_orf1	NADH dehydrogenase [ubiquinone] 1 alpha subcomplex assembly factor 3 isoform X1 [Ostrinia furnacalis] >XP_028169243.1 NADH dehydrogenase [ubiquinone] 1 alpha subcomplex assembly factor 3 isoform X1 [Ostrinia furnacalis] >XP_028169244.1 NADH dehydrogenase [ubiquinone] 1 alpha subcomplex assembly factor 3 isoform X2 [Ostrinia furnacalis] >XP_028169245.1 NADH dehydrogenase [ubiquinone] 1 alpha subcomplex assembly factor 3 isoform X2 [Ostrinia furnacalis]	-0.65466	-0.90133	1.669995	0.637131	-0.75114
TRINITY_DN135781_c0_g1_i1_orf1	vitamin K epoxide reductase complex subunit 1-like protein 1 [Ostrinia furnacalis] >XP_028171283.1 vitamin K epoxide reductase complex subunit 1-like protein 1 [Ostrinia furnacalis]	-1.23065	-1.07818	0.58931	0.361079	1.358442
TRINITY_DN31390_c0_g1_i2_orf1	UDP-glucuronosyltransferase 2B20-like [Ostrinia furnacalis]	-1.59727	-0.35698	1.098175	1.039945	-0.18387
TRINITY_DN34455_c0_g1_i1_orf1	sodium/calcium exchanger regulatory protein 1 [Manduca sexta] >AAC24317.1 cellular retinoic acid binding protein [Manduca sexta] >KAG6449763.1 hypothetical protein O3G_MSEX006229 [Manduca sexta] >KAG6449764.1 hypothetical protein O3G_MSEX006229 [Manduca sexta]	-1.35407	-0.50625	1.701395	0.117107	0.041815
TRINITY_DN10118_c0_g1_i4_orf1	glutaredoxin-C4-like [Ostrinia furnacalis]	-1.59166	-0.39504	1.421581	0.537486	0.027639
TRINITY_DN829_c0_g1_i8_orf1	cytochrome P450 6B6-like [Ostrinia furnacalis]	-1.46932	-0.68689	0.368453	0.330195	1.457563
TRINITY_DN147691_c0_g1_i1_orf1	WD repeat-containing protein 46 [Orussus abietinus]	-1.44235	-0.87402	0.506515	0.543423	1.266432
TRINITY_DN3298_c0_g2_i4_orf1	macrophage mannose receptor 1-like [Ostrinia furnacalis]	-1.38678	-0.51335	0.278936	-0.03243	1.653621
TRINITY_DN23164_c0_g1_i4_orf1	uncharacterized protein LOC114365928 isoform X1 [Ostrinia furnacalis]	-1.53371	-0.79884	0.528057	0.675741	1.128759
TRINITY_DN5852_c0_g1_i13_orf1	uncharacterized protein LOC114364714 isoform X3 [Ostrinia furnacalis] >XP_028176780.1 uncharacterized protein LOC114364714 isoform X5 [Ostrinia furnacalis]	-1.42505	-0.44853	1.060171	1.217036	-0.40363
TRINITY_DN20356_c0_g1_i5_orf1	uncharacterized protein LOC114362428 [Ostrinia furnacalis]	-0.1106	-0.50714	1.928762	-0.38067	-0.93034
TRINITY_DN103107_c0_g1_i2_orf1	superoxide dismutase [Cu-Zn] [Ostrinia furnacalis] >XP_028177872.1 superoxide dismutase [Cu-Zn] [Ostrinia	-0.31837	-0.20002	1.955549	-0.74816	-0.689
TRINITY_DN66287_c0_g1_i1_orfp1	tenascin-like isoform X4 [Trichoplusia ni]	-0.64446	-0.24384	1.980866	-0.49866	-0.5939
TRINITY_DN50725_c0_g1_i6_orf1	BTB/POZ domain-containing protein 2-like [Ostrinia furnacalis]	-0.53514	-0.21857	1.960683	-0.38764	-0.81933
TRINITY_DN52244_c1_g1_i1_orf1	triokinase/FMN cyclase-like isoform X2 [Ostrinia furnacalis]	0.391289	0.038587	1.390911	-0.11883	-1.70195
TRINITY_DN30509_c0_g1_i9_orf1	prostatic acid phosphatase [Ostrinia furnacalis]	-0.72261	-0.29101	1.980929	-0.50839	-0.45893
TRINITY_DN195_c8_g1_i1_orf1	hypothetical protein evm_009768 [Chilo suppressalis]	-0.18349	0.535507	1.640023	-1.04975	-0.94229
TRINITY_DN40911_c0_g1_i1_orf1	peroxisomal membrane protein PEX16 [Ostrinia furnacalis]	-0.38765	0.374119	1.762954	-0.68562	-1.06381
TRINITY_DN11962_c0_g1_i2_orf1	BTB/POZ domain-containing protein 2-like [Ostrinia furnacalis]	-0.70795	-0.05575	1.939193	-0.43885	-0.73664
TRINITY_DN35002_c0_g2_i2_orf1	sulfite oxidase, mitochondrial isoform X3 [Leguminivora glycinivorella]	-0.2974	0.185813	1.839445	-0.8449	-0.88295
TRINITY_DN7570_c0_g1_i18_orf1	sodium/potassium-transporting ATPase subunit alpha isoform X4 [Trichoplusia ni] >XP_026734855.1 sodium/potassium-transporting ATPase subunit alpha isoform X4 [Trichoplusia ni]	-0.28929	0.306033	1.734899	-1.24909	-0.50256
TRINITY_DN11612_c0_g2_i1_orf1	eukaryotic translation initiation factor 5B [Manduca sexta]	-0.05084	0.232174	1.728233	-1.21314	-0.69643
TRINITY_DN2694_c0_g1_i3_orf1	cubilin homolog [Ostrinia furnacalis]	-0.81317	0.214867	1.862778	-0.52454	-0.73993
TRINITY_DN1710_c0_g2_i2_orf1	relish [Ostrinia furnacalis]	0.144136	0.355177	1.543568	-1.4595	-0.58338
TRINITY_DN3307_c1_g1_i2_orf1	BTB/POZ domain-containing protein 2-like [Ostrinia furnacalis]	-0.19145	0.714513	1.48134	-0.6489	-1.3555
TRINITY_DN3797_c0_g2_i3_orf1	3'(2'),5'-bisphosphate nucleotidase 1 isoform X2 [Ostrinia furnacalis]	-0.59902	0.76343	1.570374	-1.0766	-0.65818
TRINITY_DN9510_c0_g2_i1_orf1	RNA polymerase II transcriptional coactivator [Ostrinia furnacalis]	-0.46556	0.906802	1.457009	-1.08405	-0.8142
TRINITY_DN61536_c0_g3_i1_orf1	cubilin homolog [Ostrinia furnacalis]	-0.4805	-0.12303	1.907597	-0.28819	-1.01588
TRINITY_DN28039_c0_g1_i1_orf1	translation elongation factor 2 [Athalia rosae]	-0.82294	0.849195	1.524915	-0.96691	-0.58426
TRINITY_DN19995_c0_g1_i2_orf1	E3 ubiquitin-protein ligase ZNF598 [Ostrinia furnacalis]	-0.67594	0.950549	1.425858	-0.56678	-1.13369
TRINITY_DN2813_c0_g1_i3_orf1	arylphorin subunit alpha-like [Ostrinia furnacalis]	-0.90312	0.690856	1.591946	-0.3574	-1.02229
TRINITY_DN82017_c0_g1_i5_orf1	carboxylesterase CXE17 [Ostrinia furnacalis]	-0.70859	0.899391	1.443663	-0.45042	-1.18404
TRINITY_DN32687_c0_g1_i2_orf1	protein D2-like isoform X2 [Ostrinia furnacalis] >XP_028164613.1 protein D2-like isoform X2 [Ostrinia furnacalis]	-0.95974	0.925043	1.473426	-0.62653	-0.81219
TRINITY_DN19939_c0_g1_i4_orf1	unnamed protein product [Chilo suppressalis]	-0.79461	1.13816	1.289143	-0.61828	-1.0144
TRINITY_DN69334_c0_g1_i1_orf1	PREDICTED: 15-hydroxyprostaglandin dehydrogenase [NAD(+)]-like [Papilio xuthus]	-0.65057	0.937551	1.467815	-0.91995	-0.83485
TRINITY_DN102260_c0_g1_i1_orf1	unnamed protein product [Diatraea saccharalis]	-0.94678	1.225147	1.21581	-0.81092	-0.68326
TRINITY_DN6638_c0_g1_i1_orf1	ubiquinone biosynthesis protein COQ4 homolog, mitochondrial [Ostrinia furnacalis]	-0.58307	1.291865	0.851645	-0.05623	-1.50421
TRINITY_DN33452_c0_g1_i3_orf1	lethal(2) giant larvae protein isoform X8 [Ostrinia furnacalis]	-0.17303	1.001461	1.216438	-0.57709	-1.46778

TRINITY_DN21000_c0_g1_i1_orf1	elongation factor-1 alpha, partial [Loxostege sticticalis] >QCO92153.1 elongation factor-1 alpha, partial [Sitochroa umbrosalis]	-0.99489	1.32035	1.110038	-0.76444	-0.67105
TRINITY_DN91877_c0_g1_i1_orf1	NADH dehydrogenase [ubiquinone] 1 alpha subcomplex assembly factor 2 [Ostrinia furnacalis]	-0.44823	0.975401	0.905131	0.284517	-1.71682
TRINITY_DN3370_c0_g1_i5_orf1	unnamed protein product, partial [Brenthis ino]	-0.35004	1.583704	0.702533	-0.99434	-0.94186
TRINITY_DN195_c4_g1_i1_orf1	beta-1,3-glucan-binding protein 1 [Ostrinia furnacalis]	-0.85546	1.105209	1.336887	-0.81243	-0.7742
TRINITY_DN27592_c0_g1_i1_orf1	D-arabinitol dehydrogenase 1-like [Ostrinia furnacalis]	-0.89118	1.362706	1.054356	-0.57188	-0.95399
TRINITY_DN34406_c0_g2_i9_orfp1	TRINITY_DN34406_c0_g2_i9_m.33755 TRINITY_DN34406_c0_g2::TRINITY_DN34406_c0_g2_i9::g.33755 ORF type:internal len:82 (-),score=12.88 TRINITY_DN34406_c0_g2_i9:3-245(-)	0.758426	-1.3357	1.257589	-0.98349	0.303175
TRINITY_DN2813_c0_g1_i10_orf1	arylphorin subunit alpha-like [Ostrinia furnacalis]	0.465907	-1.90318	1.035258	0.136696	0.265317
TRINITY_DN9044_c0_g1_i2_orf1	unnamed protein product [Euphydryas editha]	0.34158	-1.6119	1.420824	0.281837	-0.43234
TRINITY_DN129863_c0_g1_i1_orf1	protein PFC0760c-like isoform X2 [Ostrinia furnacalis]	0.352475	-1.88125	1.097047	0.357202	0.074526
TRINITY_DN25234_c0_g1_i1_orf1	uncharacterized protein LOC114353853 [Ostrinia furnacalis]	0.319097	-1.75627	1.289221	0.339166	-0.19121
TRINITY_DN1575_c0_g1_i10_orf1	uncharacterized protein LOC114359245 [Ostrinia furnacalis]	0.421377	-1.37917	1.181572	0.7539	-0.97768
TRINITY_DN2406_c0_g1_i6_orf1	uncharacterized protein LOC114361672 [Ostrinia furnacalis]	0.435423	-1.53018	1.51432	-0.00028	-0.41928
TRINITY_DN325_c0_g1_i15_orf1	protein draper-like [Ostrinia furnacalis]	0.679371	-0.95404	1.619198	-0.89874	-0.44579
TRINITY_DN184_c0_g1_i10_orf1	C-type mannose receptor 2-like isoform X1 [Leguminivora glycinivorella]	0.66493	-1.69794	1.276127	-0.02989	-0.21323
TRINITY_DN184_c0_g1_i1_orf1	macrophage mannose receptor 1-like isoform X1 [Maniola jurtina]	-0.04771	-0.96923	1.731471	0.277182	-0.99172
TRINITY_DN12969_c0_g1_i3_orf1	queuosine salvage protein [Ostrinia furnacalis] >XP_028167327.1 queuosine salvage protein [Ostrinia furnacalis]	-0.11583	-1.33244	1.661851	-0.56866	0.355074
TRINITY_DN12920_c0_g3_i1_orf1	zonadhesin-like isoform X4 [Ostrinia furnacalis]	-0.25238	-0.86972	1.937636	-0.6233	-0.19224
TRINITY_DN22962_c0_g1_i1_orf1	lysosomal acid glucosylceramidase-like isoform X2 [Ostrinia furnacalis]	-0.01972	-1.51127	1.625742	-0.23198	0.137228
TRINITY_DN5682_c0_g1_i6_orf1	spodomicin-like [Ostrinia furnacalis] >QKV49445.1 diapausin [Ostrinia furnacalis]	0.088899	-1.8746	1.118511	0.287093	0.380099
TRINITY_DN2343_c1_g1_i2_orf1	receptor expression-enhancing protein 5-like isoform X1 [Ostrinia furnacalis] >XP_028170586.1 receptor expression-enhancing protein 5-like isoform X1 [Ostrinia furnacalis]	0.225934	-1.09377	1.048108	-1.23858	1.058307
TRINITY_DN28741_c0_g1_i3_orf1	uncharacterized protein LOC114351652 [Ostrinia furnacalis]	0.434709	-1.1431	1.722042	-0.39464	-0.61901
TRINITY_DN3712_c0_g1_i1_orf1	ribonuclease Oy [Ostrinia furnacalis]	0.239302	-1.74258	1.324572	-0.17111	0.349815
TRINITY_DN80424_c0_g1_i1_orf1	PREDICTED: cytoplasmic FMR1-interacting protein [Dufourea novaeangliae] >KZC10094.1 Cytoplasmic FMR1-interacting protein [Dufourea novaeangliae]	0.547265	-0.55388	1.704726	-1.00044	-0.69767
TRINITY_DN1293_c1_g1_i4_orf1	putative fatty acyl-CoA reductase CG5065 [Ostrinia furnacalis]	0.694728	-0.61234	1.626979	-0.7236	-0.98576
TRINITY_DN919_c0_g1_i7_orf1	facilitated trehalose transporter Tret1-like [Ostrinia furnacalis] >XP_028161733.1 facilitated trehalose transporter Tret1-like [Ostrinia furnacalis]	0.412123	-0.70451	1.790976	-0.79017	-0.70842
TRINITY_DN14874_c0_g1_i6_orf1	uncharacterized protein LOC114358148 [Ostrinia furnacalis]	-0.25373	-1.20115	1.694046	0.425611	-0.66478
TRINITY_DN827_c1_g1_i1_orf1	peptidoglycan recognition protein 4-like isoform X1 [Ostrinia furnacalis]	0.22671	-1.66902	1.425984	-0.24621	0.262537
TRINITY_DN8644_c0_g1_i3_orf1	SEC14-like protein 2 [Ostrinia furnacalis]	0.404085	-1.31186	1.654908	-0.59484	-0.1523
TRINITY_DN8783_c0_g1_i5_orf1	luciferin 4-monooxygenase-like [Ostrinia furnacalis] >XP_028165580.1 luciferin 4-monooxygenase-like [Ostrinia furnacalis]	-0.24787	-1.1785	1.778906	-0.57817	0.225632
TRINITY_DN230_c1_g1_i3_orf1	protein artichoke-like [Ostrinia furnacalis]	0.1444	-1.62147	1.532382	-0.03927	-0.01605
TRINITY_DN3975_c0_g1_i7_orf1	low-density lipoprotein receptor domain class A domain-containing protein [Phthorimaea operculella]	-0.06657	-1.35064	1.733514	0.082927	-0.39922
TRINITY_DN2049_c1_g1_i2_orf1	luciferin 4-monooxygenase-like [Ostrinia furnacalis]	0.018609	-1.20165	1.797687	-0.0474	-0.56725
TRINITY_DN9_c0_g1_i7_orf1	heterogeneous nuclear ribonucleoprotein R isoform X1 [Ostrinia furnacalis]	0.013461	-1.17972	1.742189	-0.73909	0.163153
TRINITY_DN3627_c0_g1_i7_orf1	spermine oxidase-like isoform X2 [Ostrinia furnacalis]	0.148225	-1.00952	1.848762	-0.65719	-0.33028
TRINITY_DN2343_c1_g1_i12_orf1	receptor expression-enhancing protein 5-like isoform X3 [Ostrinia furnacalis]	-0.46255	-0.68684	1.890166	-0.85371	0.112929
TRINITY_DN22824_c0_g1_i4_orf1	LIM domain and actin-binding protein 1 [Ostrinia furnacalis]	-0.41963	-0.65832	1.71049	-1.11148	0.478931
TRINITY_DN3687_c0_g1_i1_orf1	acyl-CoA-binding domain-containing protein 5 isoform X2 [Manduca sexta]	0.010783	-0.83242	1.652266	-1.20018	0.369544
TRINITY_DN136031_c0_g1_i7_orf1	ferritin, lower subunit isoform X3 [Spodoptera litura]	-0.8095	-0.63137	1.56164	-0.92643	0.805661
TRINITY_DN84938_c0_g1_i4_orf1	vinculin-like isoform X2 [Ostrinia furnacalis]	-0.66253	-1.16778	1.505809	-0.50003	0.824533
TRINITY_DN140_c0_g1_i5_orf1	calcyphosin-like protein isoform X4 [Helicoverpa armigera]	-0.79938	-0.76048	1.310484	-0.88382	1.133202
TRINITY_DN24121_c1_g1_i6_orf1	serine protease persephone-like [Ostrinia furnacalis]	-1.18833	-0.85001	1.230111	-0.31197	1.120203
TRINITY_DN452_c0_g1_i4_orf1	protein CREG1 [Ostrinia furnacalis] >XP_028170592.1 protein CREG1 [Ostrinia furnacalis]	-0.39485	-1.33444	1.409919	-0.55603	0.875401
TRINITY_DN3732_c1_g1_i5_orf1	cytochrome P450 6B2-like [Ostrinia furnacalis]	-0.76765	-1.1152	1.000737	-0.50141	1.383527
TRINITY_DN44110_c0_g1_i4_orf1	putative phosphatidate phosphatase [Ostrinia furnacalis]	-0.40229	-1.30686	1.632233	-0.44276	0.519674
TRINITY_DN8621_c0_g1_i4_orf1	aminopeptidase N-like isoform X2 [Ostrinia furnacalis]	-0.64701	-1.25027	1.558796	-0.34606	0.684546

TRINITY_DN5696_c0_g1_i4_orf1	serine protease snake-like isoform X1 [Ostrinia furnacalis]	-0.66332	-1.2981	0.985512	-0.35689	1.332795
TRINITY_DN31225_c0_g1_i1_orf1	ribosome biogenesis protein BMS1 homolog [Ostrinia furnacalis]	-1.33417	-0.90148	1.135782	0.043744	1.056126
TRINITY_DN12336_c0_g1_i1_orfp1	TRINITY_DN12336_c0_g1_i1_m.30792 TRINITY_DN12336_c0_g1::TRINITY_DN12336_c0_g1_i1::g.30792 ORF type:internal len:86 (-),score=13.56 TRINITY_DN12336_c0_g1_i1:3-257(-)	-1.23094	-0.81597	1.072274	-0.28543	1.260059
TRINITY_DN3486_c0_g1_i5_orf1	uncharacterized protein LOC114360519 [Ostrinia furnacalis]	-0.91356	-0.94961	1.282854	-0.56117	1.141494
TRINITY_DN52761_c0_g2_i1_orf1	atlastin-like isoform X4 [Ostrinia furnacalis]	-0.20443	-1.40859	0.855559	-0.61001	1.367474
TRINITY_DN77318_c0_g2_i1_orf1	uncharacterized protein LOC114351191 [Ostrinia furnacalis]	-0.45072	-1.0233	1.437186	-0.89906	0.935893
TRINITY_DN804_c0_g1_i7_orf1	hypothetical protein HF086_004695 [Spodoptera exigua] >CAH0695017.1 unnamed protein product [Spodoptera exigua]	-1.42147	-0.47005	1.445346	-0.31072	0.756899
TRINITY_DN36632_c0_g1_i1_orf1	hypothetical protein evm_011159, partial [Chilo suppressalis]	-0.63896	-1.19402	1.08469	-0.55039	1.298679
TRINITY_DN1381_c0_g1_i5_orf1	CKLF-like MARVEL transmembrane domain-containing protein 4 isoform X1 [Ostrinia furnacalis]	-1.48066	-0.76328	1.047297	0.14432	1.052325
TRINITY_DN5558_c0_g1_i4_orf1	unnamed protein product [Parnassius apollo]	-0.34131	-1.45795	1.467167	-0.35843	0.690529
TRINITY_DN1622_c0_g1_i6_orf1	unnamed protein product [Parnassius apollo]	-0.87819	-1.18342	1.097754	-0.27923	1.243088
TRINITY_DN5661_c0_g1_i5_orf1	cytochrome P450 6B7-like [Ostrinia furnacalis]	-1.41512	-0.73468	1.261759	-0.04145	0.929483
TRINITY_DN13563_c0_g1_i1_orf1	Golgi resident protein GCP60 isoform X1 [Ostrinia furnacalis]	-0.84293	-0.92777	1.258267	-0.66957	1.182008
TRINITY_DN4991_c0_g1_i1_orf1	unnamed protein product, partial [Iphiclides podalirius]	-1.29156	-0.69028	1.506451	-0.24862	0.724004
TRINITY_DN47151_c0_g1_i1_orf1	unnamed protein product [Danaus chrysippus]	-1.38484	-0.5394	1.038006	-0.36962	1.255858
TRINITY_DN25423_c0_g1_i1_orf1	uncharacterized protein LOC114353174 [Ostrinia furnacalis]	-0.37545	-1.23763	1.622187	-0.59438	0.585267
TRINITY_DN85476_c0_g1_i1_orf1	iron-sulfur protein NUBPL-like [Ostrinia furnacalis]	-1.12704	-0.77967	1.497801	-0.42596	0.834881
TRINITY_DN2887_c0_g1_i1_orf1	F-box/LRR-repeat protein 4-like isoform X1 [Ostrinia furnacalis] >XP_028177606.1 F-box/LRR-repeat protein 4-like isoform X1 [Ostrinia furnacalis]	-1.31461	-0.73216	1.122779	-0.26196	1.185951
TRINITY_DN2468_c0_g1_i7_orf1	protein 4.1 homolog isoform X1 [Ostrinia furnacalis]	-1.0974	-0.93861	1.542719	-0.20781	0.701103
TRINITY_DN98995_c0_g1_i2_orf1	hypothetical protein HF086_008399, partial [Spodoptera exigua]	-1.65883	-0.25721	0.966382	-0.15656	1.106216
TRINITY_DN4424_c0_g1_i1_orf1	calreticulin [Ostrinia furnacalis]	-0.73784	-1.00529	1.63555	-0.56434	0.671921
TRINITY_DN2193_c0_g1_i7_orf1	long-chain-fatty-acid--CoA ligase 5 isoform X1 [Ostrinia furnacalis] >XP_028176293.1 long-chain-fatty-acid--CoA ligase 5 isoform X1 [Ostrinia furnacalis] >XP_028176294.1 long-chain-fatty-acid--CoA ligase 5 isoform X1 [Ostrinia furnacalis] >XP_028176295.1 long-chain-fatty-acid--CoA ligase 5 isoform X1 [Ostrinia furnacalis] >XP_028176296.1 long-chain-fatty-acid--CoA ligase 5 isoform X1 [Ostrinia furnacalis] >XP_028176297.1 long-chain-fatty-acid--CoA ligase 5 isoform X1 [Ostrinia furnacalis] >XP_028176298.1 long-chain-fatty-acid--CoA ligase 5 isoform X2 [Ostrinia furnacalis]	-1.52896	-0.52768	1.12899	-0.11883	1.046471
TRINITY_DN4793_c0_g1_i7_orf1	probable hydroxyacid-oxoacid transhydrogenase, mitochondrial isoform X3 [Ostrinia furnacalis] >XP_028159821.1 probable hydroxyacid-oxoacid transhydrogenase, mitochondrial isoform X4 [Ostrinia furnacalis]	-1.41631	-0.59746	0.960683	-0.23494	1.288024
TRINITY_DN41952_c0_g1_i4_orf1	protein DDI1 homolog 2 [Ostrinia furnacalis]	-1.30348	-0.25921	1.207085	-0.74781	1.103403
TRINITY_DN10430_c0_g1_i4_orf1	fatty acid synthase [Ostrinia furnacalis] >XP_028160534.1 fatty acid synthase [Ostrinia furnacalis] >XP_028160535.1 fatty acid synthase [Ostrinia furnacalis] >XP_028160536.1 fatty acid synthase [Ostrinia furnacalis]	-1.32343	-0.74102	0.730929	-0.132	1.465518
TRINITY_DN22044_c0_g2_i1_orf1	derlin-1 [Ostrinia furnacalis]	-1.48853	-0.58352	1.153953	-0.12864	1.046731
TRINITY_DN870_c0_g1_i3_orf1	talin-1 isoform X12 [Ostrinia furnacalis]	-1.63629	-0.40283	1.011616	-0.03809	1.06559
TRINITY_DN30476_c0_g1_i1_orf1	unnamed protein product [Arctia plantaginis]	-1.15971	-0.8114	1.372931	-0.38386	0.982043
TRINITY_DN10512_c0_g1_i1_orf1	protein obstructor-E [Ostrinia furnacalis]	-1.53941	-0.20487	1.1508	-0.44075	1.03423
TRINITY_DN1177_c0_g1_i4_orf1	unnamed protein product [Chilo suppressalis]	-1.57777	-0.51561	1.057958	-0.02517	1.0606
TRINITY_DN4248_c0_g1_i4_orf1	hypothetical protein SFRURICE_003580 [Spodoptera frugiperda]	-1.62233	-0.37559	1.090044	-0.10583	1.013702
TRINITY_DN57856_c0_g2_i1_orf1	cytochrome P450 6B2-like [Ostrinia furnacalis]	-1.55768	-0.16692	0.949139	-0.4321	1.20756
TRINITY_DN27491_c0_g1_i1_orf1	prolactin regulatory element-binding protein [Galleria mellonella]	-1.5147	-0.31218	1.458825	-0.27003	0.638082
TRINITY_DN41645_c0_g1_i1_orf1	60S acidic ribosomal protein P2 [Ostrinia furnacalis]	-0.84759	-0.30614	0.772971	-1.13588	1.516633
TRINITY_DN4744_c0_g1_i7_orf1	glutaryl-CoA dehydrogenase, mitochondrial [Ostrinia furnacalis]	-1.60081	0.033789	0.962448	-0.51233	1.116904
TRINITY_DN131603_c0_g1_i4_orfp1	TRINITY_DN131603_c0_g1_i4_m.86149 TRINITY_DN131603_c0_g1::TRINITY_DN131603_c0_g1_i4::g.86149 ORF type:internal len:112 (-),score=8.40 TRINITY_DN131603_c0_g1_i4:2-334(-)	-0.83367	-0.38291	-0.04622	-0.66454	1.927338
TRINITY_DN2299_c0_g1_i3_orf1	DNA-directed RNA polymerase II subunit RPB1 [Ostrinia furnacalis]	-1.30263	0.007281	0.636988	-0.82853	1.486899
TRINITY_DN5513_c0_g1_i1_orf1	GDP-Man:Man(3)GlcNAc(2)-PP-Dol alpha-1,2-mannosyltransferase [Ostrinia furnacalis]	-1.68181	0.157279	0.963129	-0.44784	1.00925
TRINITY_DN11172_c0_g1_i4_orf1	juvenile hormone epoxide hydrolase-like isoform X1 [Ostrinia furnacalis] >XP_028170522.1 juvenile hormone epoxide hydrolase-like isoform X2 [Ostrinia furnacalis]	-0.90737	0.174469	0.345835	-1.21212	1.599188

TRINITY_DN5300_c0_g1_i3_orf1	valacyclovir hydrolase [Ostrinia furnacalis]	-1.70969	-0.18111	0.839446	-0.10156	1.152905
TRINITY_DN14904_c0_g1_i1_orf1	attacin [Ostrinia furnacalis]	-0.27016	-1.28866	0.175311	-0.37563	1.759132
TRINITY_DN20558_c0_g1_i2_orf1	Transient receptor potential channel pyrexia [Operophtera brumata]	-0.14691	-1.56759	0.41585	-0.21811	1.516762
TRINITY_DN4360_c0_g1_i4_orf1	glucose-6-phosphate isomerase-like, partial [Bicyclus anynana]	-0.40961	-0.96938	1.373401	1.004362	-0.99878
TRINITY_DN5770_c0_g1_i4_orf1	rotatin-like [Ostrinia furnacalis]	-0.56363	-0.40245	1.550677	0.693824	-1.27842
TRINITY_DN311_c0_g1_i8_orfp1	TRINITY_DN311_c0_g1_i8_m.65152 TRINITY_DN311_c0_g1::TRINITY_DN311_c0_g1_i8::g.65152 ORF type:5prime_partial len:138 (+),score=74.30 TRINITY_DN311_c0_g1_i8:2-415(+)	-0.76826	-0.60118	1.014801	1.392979	-1.03834
TRINITY_DN5852_c0_g1_i6_orf1	probable maltase isoform X6 [Ostrinia furnacalis]	-0.5155	-0.7384	1.406782	0.971963	-1.12485
TRINITY_DN36581_c0_g1_i5_orf1	enoyl-CoA delta isomerase 1, mitochondrial-like isoform X1 [Ostrinia furnacalis] >XP_028158560.1 enoyl-CoA delta isomerase 1, mitochondrial-like isoform X2 [Ostrinia furnacalis]	-0.9674	-0.47281	1.780887	0.382383	-0.72306
TRINITY_DN81181_c0_g1_i6_orfp1	C-type lectin domain family 4 member E [Pieris rapae]	-0.87189	-0.13551	1.765479	0.260173	-1.01825
TRINITY_DN1380_c0_g1_i5_orf1	ubiquitin-fold modifier-conjugating enzyme 1 [Ostrinia furnacalis]	-0.59099	-0.25459	1.499664	0.703252	-1.35734
TRINITY_DN3058_c0_g1_i1_orf1	mesencephalic astrocyte-derived neurotrophic factor homolog [Ostrinia furnacalis]	-1.12276	0.099923	1.424576	0.698939	-1.10068
TRINITY_DN3126_c0_g1_i4_orf1	unnamed protein product, partial [Iphiclides podalirius]	-1.05098	-0.00418	0.805596	1.392713	-1.14315
TRINITY_DN1968_c0_g1_i3_orf1	protein ABHD4 isoform X3 [Ostrinia furnacalis]	-1.27194	0.25084	1.16861	0.911821	-1.05933
TRINITY_DN51995_c0_g3_i1_orf1	circadian clock-controlled protein-like [Ostrinia furnacalis]	-0.82341	-1.03004	-0.20087	1.772327	0.281994
TRINITY_DN68770_c0_g1_i1_orf1	seroin transcript 1A2 [Ostrinia nubilalis]	-1.25709	-0.72404	-0.17138	1.600414	0.552091
TRINITY_DN4204_c0_g1_i1_orf1	uncharacterized protein LOC114359352 [Ostrinia furnacalis]	-1.22593	-0.63832	-0.47051	1.434496	0.900266
TRINITY_DN34676_c1_g1_i3_orf1	tRNA (uracil-5-)-methyltransferase homolog A [Ostrinia furnacalis]	-1.23042	-0.74146	0.291963	1.688483	-0.00856
TRINITY_DN30713_c0_g1_i3_orf1	phosphoglucomutase [Ostrinia furnacalis]	-1.24858	-0.37855	-0.18711	1.806288	0.007955
TRINITY_DN74116_c0_g1_i2_orf1	venom protease [Galleria mellonella]	-1.34936	-0.67534	0.368551	1.607784	0.048366
TRINITY_DN33995_c0_g1_i5_orf1	unnamed protein product [Spodoptera exigua]	-1.06253	-0.42753	1.67758	-0.74812	0.560607
TRINITY_DN99900_c0_g1_i3_orf1	laminin subunit alpha-like isoform X1 [Ostrinia furnacalis] >XP_028177662.1 laminin subunit alpha-like isoform X2 [Ostrinia furnacalis]	-0.96341	-0.44105	1.860823	-0.62222	0.165851
TRINITY_DN40434_c0_g1_i2_orf1	deoxyribodipyrimidine photo-lyase [Ostrinia furnacalis]	-1.03602	-0.68309	1.69017	-0.5346	0.563538
TRINITY_DN3055_c0_g1_i9_orf1	WAP four-disulfide core domain protein 2-like [Ostrinia furnacalis]	-0.83316	-0.79956	1.752671	-0.60196	0.482011
TRINITY_DN842_c0_g1_i9_orf1	hypothetical protein evm_011651 [Chilo suppressalis]	-0.81157	-0.69645	1.877998	-0.54624	0.176256
TRINITY_DN42185_c0_g1_i7_orf1	ADP-ribosylation factor-like protein 13B isoform X2 [Ostrinia furnacalis]	-0.59783	-0.15903	1.471817	-1.40521	0.690251
TRINITY_DN14409_c0_g1_i1_orf1	endoplasmic reticulum mannosyl-oligosaccharide 1,2-alpha-mannosidase [Ostrinia furnacalis] >XP_028171972.1 endoplasmic reticulum mannosyl-oligosaccharide 1,2-alpha-mannosidase [Ostrinia furnacalis]	-0.70073	-0.29897	1.694518	-1.15575	0.460926
TRINITY_DN825_c23_g1_i5_orf1	methionine-tRNA synthetase, partial [Papilio xuthus]	-0.47551	-0.17469	1.817398	-1.19974	0.032546
TRINITY_DN2719_c1_g1_i6_orf1	unnamed protein product [Chrysodeixis includens]	-1.28481	-0.00589	1.62811	-0.73508	0.397669
TRINITY_DN9979_c0_g1_i1_orf1	ADP-dependent glucokinase [Ostrinia furnacalis]	-0.51791	0.142749	1.736877	-1.30034	-0.06138
TRINITY_DN13385_c0_g1_i4_orf1	putative aminopeptidase W07G4.4 [Ostrinia furnacalis]	-1.07983	-0.14971	1.536596	-0.99117	0.684115
TRINITY_DN4636_c0_g3_i1_orf1	microtubule-associated serine/threonine-protein kinase 4 [Ostrinia furnacalis]	-0.67237	-0.06881	1.798154	-1.14136	0.084383
TRINITY_DN17896_c0_g1_i1_orf1	probable aminopeptidase NPEPL1 isoform X1 [Ostrinia furnacalis] >XP_028174563.1 probable aminopeptidase NPEPL1 isoform X2 [Ostrinia furnacalis] >XP_028174564.1 probable aminopeptidase NPEPL1 isoform X3 [Ostrinia	-1.18253	-0.05332	1.561425	-0.90701	0.581435
TRINITY_DN3698_c0_g1_i4_orf1	neuroblastoma-amplified sequence-like [Ostrinia furnacalis]	-1.09457	0.897533	1.250836	-1.18891	0.13511
TRINITY_DN2072_c0_g1_i1_orf1	dnaJ homolog subfamily C member 3 [Helicoverpa zea]	-0.91359	0.068844	1.159109	-1.33355	1.019182
TRINITY_DN140613_c0_g1_i1_orf1	hypothetical protein M0804_013066 [Polistes exclamans]	-0.8668	-0.06232	1.490456	-1.24652	0.685188
TRINITY_DN1954_c0_g1_i4_orf1	unnamed protein product [Diatraea saccharalis]	-0.48557	0.268698	1.566663	-1.48933	0.139547
TRINITY_DN6358_c0_g1_i5_orf1	histone H1B-like [Ostrinia furnacalis]	-0.58123	0.242408	1.066954	-1.62897	0.90083
TRINITY_DN70236_c0_g1_i1_orf1	NAD-dependent protein deacetylase sirtuin-2-like [Ostrinia furnacalis]	-0.77191	0.51737	0.981484	-1.56949	0.842543
TRINITY_DN2770_c0_g2_i4_orf1	phosphatidylinositol 4-phosphate 3-kinase C2 domain-containing subunit alpha isoform X1 [Ostrinia furnacalis]	-0.4086	0.154598	1.488816	-1.57401	0.339198
TRINITY_DN2873_c0_g1_i7_orf1	transitional endoplasmic reticulum ATPase TER94 [Galleria mellonella]	-0.47368	0.675316	1.448658	-1.4806	-0.1697
TRINITY_DN35162_c0_g1_i4_orf1	mannosyl-oligosaccharide glucosidase [Ostrinia furnacalis]	-0.76082	-0.18175	1.552763	-1.25137	0.641182
TRINITY_DN14209_c0_g1_i1_orf1	unnamed protein product [Diatraea saccharalis]	-1.07661	1.071679	1.029273	-1.25661	0.232265
TRINITY_DN4822_c0_g1_i9_orf1	homogentisate 1,2-dioxygenase [Ostrinia furnacalis]	-0.75098	0.634134	1.220283	-1.53559	0.432159
TRINITY_DN15318_c0_g1_i1_orf1	hepatoma-derived growth factor-related protein 2-like [Ostrinia furnacalis]	-1.28318	0.999097	1.014599	-1.09293	0.362415
TRINITY_DN11637_c0_g1_i1_orf1	ribosome-binding protein 1 isoform X4 [Colias croceus]	-0.63544	1.080466	0.843512	-1.61564	0.327104

TRINITY_DN2031_c11_g1_i2_orfp1	TRINITY_DN2031_c11_g1_i2_m.4044 TRINITY_DN2031_c11_g1::TRINITY_DN2031_c11_g1_i2::g.4044 ORF type:3prime_partial len:149 (-),score=79.84 TRINITY_DN2031_c11_g1_i2:2-445(-)	-1.1954	0.882764	0.969779	-1.23283	0.575684
TRINITY_DN14855_c0_g1_i1_orf1	neurochondrin homolog [Ostrinia furnacalis]	-0.87211	1.324964	0.8022	-1.35306	0.098003
TRINITY_DN21215_c0_g1_i7_orf1	phytanoyl-CoA dioxygenase, peroxisomal-like [Ostrinia furnacalis]	-0.6489	1.044676	0.920446	-1.59995	0.283735
TRINITY_DN7909_c0_g2_i1_orf1	aldehyde oxidase 3 [Ostrinia furnacalis]	-0.87135	1.24356	0.972908	-1.32182	-0.02329
TRINITY_DN21035_c0_g1_i14_orf1	mitochondrial amidoxime reducing component 2-like [Ostrinia furnacalis]	-1.16628	1.132675	0.873331	-1.20776	0.368033
TRINITY_DN15327_c2_g1_i2_orf1	protein lethal(2)essential for life-like [Ostrinia furnacalis]	-1.48605	-0.90702	0.646147	0.98654	0.760387
TRINITY_DN41573_c0_g1_i1_orf1	BRISC and BRCA1-A complex member 2-like [Ostrinia furnacalis]	-1.68132	-0.62244	0.873118	0.716171	0.714479
TRINITY_DN27300_c0_g1_i6_orfp1	TRINITY_DN27300_c0_g1_i6_m.71140 TRINITY_DN27300_c0_g1::TRINITY_DN27300_c0_g1_i6::g.71140 ORF type:internal len:129 (-),score=20.75 TRINITY_DN27300_c0_g1_i6:1-384(-)	-0.98174	0.037325	1.882198	-0.63077	-0.30702
TRINITY_DN22513_c0_g1_i4_orf1	DNA-directed RNA polymerase II subunit RPB1 [Ostrinia furnacalis] >XP_028179194.1 DNA-directed RNA polymerase II subunit RPB1 [Ostrinia furnacalis] >XP_028179195.1 DNA-directed RNA polymerase II subunit RPB1	-1.45463	-0.95731	0.868044	0.666332	0.877562
TRINITY_DN9560_c0_g1_i5_orf1	uncharacterized protein LOC114357350 [Ostrinia furnacalis]	-1.75571	-0.38926	1.108507	0.515517	0.520954
TRINITY_DN9694_c0_g1_i1_orf1	larval cuticle protein LCP-17 [Helicoverpa armigera] >PZC82071.1 hypothetical protein B5X24_HaOG211161 [Helicoverpa armigera] >PZC87412.1 hypothetical protein B5X24_HaOG216859 [Helicoverpa armigera]	-1.51807	-0.86956	0.730231	0.70097	0.956424
TRINITY_DN59422_c0_g1_i2_orf1	larval cuticle protein LCP-22-like isoform X2 [Pectinophora gossypiella]	-1.8106	-0.34815	0.821002	0.542951	0.79479
TRINITY_DN111488_c0_g1_i1_orf1	LOW QUALITY PROTEIN: formin-J-like [Chelonus insularis]	-1.75115	-0.44276	1.026931	0.552512	0.614468
TRINITY_DN21170_c0_g1_i5_orf1	twitchin-like [Ostrinia furnacalis]	-1.23306	0.10922	1.789915	-0.47797	-0.1881
TRINITY_DN9820_c0_g1_i1_orf1	endocuticle structural glycoprotein SgAbd-2-like [Ostrinia furnacalis]	-1.80382	-0.31938	0.921674	0.409817	0.791708
TRINITY_DN6325_c0_g1_i8_orf1	unnamed protein product [Pieris macdunnoughi]	-1.38833	-0.09141	1.739523	-0.08246	-0.17733
TRINITY_DN135679_c0_g1_i2_orfp1	TRINITY_DN135679_c0_g1_i2_m.85525 TRINITY_DN135679_c0_g1::TRINITY_DN135679_c0_g1_i2::g.85525 ORF type:5prime_partial len:55 (+),score=5.08,Toxin_2 PF00451.20 1.9e-06 TRINITY_DN135679_c0_g1_i2:3-167(+)	-1.31445	-1.04199	0.613953	1.252961	0.489523
TRINITY_DN10889_c0_g1_i8_orf1	four and a half LIM domains protein 2 isoform X7 [Pectinophora gossypiella]	-1.67381	-0.61754	0.754315	0.585809	0.951228
TRINITY_DN32586_c0_g2_i1_orf1	unnamed protein product [Euphydryas editha]	-1.61791	-0.71643	0.673435	0.695798	0.965108
TRINITY_DN1329_c0_g1_i5_orf1	neurogenic locus notch homolog protein 3 [Ostrinia furnacalis] >XP_028157678.1 neurogenic locus notch homolog protein 3 [Ostrinia furnacalis]	-1.63229	-0.63474	0.793158	0.40718	1.066692
TRINITY_DN38568_c0_g1_i1_orf1	unnamed protein product, partial [Diatraea saccharalis]	-1.72797	-0.50811	0.792834	0.514386	0.928856
TRINITY_DN5628_c0_g1_i3_orf1	muscle LIM protein Mlp84B isoform X2 [Ostrinia furnacalis]	-1.48296	-0.89299	0.786463	0.548149	1.041344
TRINITY_DN46372_c0_g1_i1_orf1	unnamed protein product [Chilo suppressalis]	-1.69094	-0.26928	1.375724	0.243451	0.341046
TRINITY_DN7735_c0_g1_i4_orf1	calphotin-like [Ostrinia furnacalis]	-1.87689	-0.19735	0.732194	0.642047	0.700003
TRINITY_DN1344_c0_g1_i1_orf1	ribosomal RNA small subunit methyltransferase NEP1 [Ostrinia furnacalis]	-1.61167	-0.65594	0.374775	1.088523	0.804315
TRINITY_DN109931_c0_g1_i1_orf1	hydroxymethylglutaryl-CoA lyase, mitochondrial isoform X1 [Ostrinia furnacalis]	-1.85689	-0.17976	0.934363	0.411315	0.690968
TRINITY_DN2722_c0_g1_i1_orf1	troponin C [Pieris rapae] >XP_045490973.1 troponin C-like isoform X1 [Colias croceus] >XP_049866665.1 troponin C-like [Pectinophora gossypiella]	-1.72559	-0.53593	0.83793	0.612579	0.81101
TRINITY_DN11448_c0_g1_i11_orf1	hypothetical protein B5X24_HaOG201808 [Helicoverpa armigera]	-1.50116	0.102175	1.581274	-0.42207	0.239787
TRINITY_DN76529_c0_g1_i1_orfp1	TRINITY_DN76529_c0_g1_i1_m.64079 TRINITY_DN76529_c0_g1::TRINITY_DN76529_c0_g1_i1::g.64079 ORF type:internal len:70 (+),score=14.68 TRINITY_DN76529_c0_g1_i1:3-209(+)	-1.56507	0.252841	1.52508	-0.3692	0.156353
TRINITY_DN63030_c0_g1_i5_orf1	uncharacterized protein LOC114358571 [Ostrinia furnacalis]	-1.50853	-0.85688	0.887424	0.504088	0.973895
TRINITY_DN8155_c0_g1_i1_orf1	peroxisomal targeting signal 1 receptor-like [Ostrinia furnacalis] >XP_028165638.1 peroxisomal targeting signal 1 receptor-like [Ostrinia furnacalis]	-1.28987	-0.04899	1.685365	0.292227	-0.63873
TRINITY_DN2312_c0_g1_i4_orf1	endoplasmic reticulum-Golgi intermediate compartment protein 3 [Ostrinia furnacalis]	-1.74004	-0.49633	0.941934	0.625774	0.668656
TRINITY_DN11388_c0_g1_i4_orf1	limbic system-associated membrane protein-like, partial [Ostrinia furnacalis]	-1.71148	-0.49329	0.535234	1.106193	0.563346
TRINITY_DN14922_c0_g3_i2_orf1	probable pseudouridine-5'-phosphatase [Ostrinia furnacalis]	-1.68392	-0.53032	1.144343	0.509377	0.56052
TRINITY_DN2956_c0_g1_i6_orf1	fructose-bisphosphate aldolase-like isoform X1 [Ostrinia furnacalis] >XP_028178678.1 fructose-bisphosphate aldolase-like isoform X1 [Ostrinia furnacalis]	-1.5902	-0.68403	0.793777	0.367775	1.112672
TRINITY_DN9998_c0_g1_i2_orf1	GSCOCG00008521001-RA-CDS [Cotesia congregata]	-1.64079	-0.34272	1.336438	0.011346	0.635728
TRINITY_DN52395_c0_g2_i2_orf1	twitchin isoform X20 [Zerene cesonia]	-1.816	0.061299	1.254249	0.249504	0.250953
TRINITY_DN5191_c0_g2_i1_orf1	CD151 antigen-like [Ostrinia furnacalis]	-1.4923	-0.74699	0.144529	1.055602	1.039163
TRINITY_DN5628_c0_g1_i5_orf1	hypothetical protein O3G_MSEX015036 [Manduca sexta]	-1.75832	-0.46288	0.857262	0.561549	0.802387
TRINITY_DN1068_c0_g1_i3_orf1	aspartate aminotransferase, cytoplasmic [Ostrinia furnacalis]	-1.42861	-0.98271	0.699665	0.995421	0.716226
TRINITY_DN105506_c0_g1_i8_orf1	microtubule-actin cross-linking factor 1 isoform X15 [Ostrinia furnacalis]	-1.6918	-0.55027	0.766118	0.456121	1.019825

TRINITY_DN47930_c0_g1_i4_orf1	uncharacterized protein LOC114362634 [Ostrinia furnacalis]	-1.64292	-0.67598	0.897581	0.592056	0.829266
TRINITY_DN920_c0_g1_i4_orf1	glutathione S-transferase omega 2 [Ostrinia furnacalis]	-1.1651	0.440012	1.479959	-1.07519	0.320324
TRINITY_DN779_c0_g1_i3_orf1	uncharacterized protein LOC114351172 isoform X1 [Ostrinia furnacalis]	-1.66291	-0.52753	1.216575	0.453823	0.520038
TRINITY_DN1404_c0_g1_i6_orf1	uncharacterized protein LOC114363065 [Ostrinia furnacalis]	-1.59946	-0.73089	1.009557	0.570812	0.749987
TRINITY_DN1475_c0_g1_i6_orf1	uncharacterized protein LOC113226757 isoform X2 [Hyposmocoma kahamanoa]	-1.53829	-0.84623	0.806535	0.684079	0.893913
TRINITY_DN8651_c0_g1_i18_orf1	glutathione S-transferase theta 2 [Conogethes punctiferalis]	-1.72793	-0.01626	1.169513	-0.20313	0.777801
TRINITY_DN29969_c0_g1_i5_orf1	twitchin-like [Ostrinia furnacalis]	-1.39292	-0.18881	1.724023	0.073316	-0.21561
TRINITY_DN99063_c0_g1_i1_orf1	microtubule-associated protein futsch isoform X4 [Ostrinia furnacalis] >XP_028162562.1 microtubule-associated protein futsch isoform X4 [Ostrinia furnacalis]	-1.71563	-0.40757	1.15367	0.273146	0.696381
TRINITY_DN120089_c0_g1_i1_orf1	phosphoglucomutase [Ostrinia furnacalis]	-1.54831	-0.81374	0.763767	0.599153	0.999125
TRINITY_DN15373_c0_g1_i2_orf1	SET domain-containing protein SmydA-8-like isoform X2 [Ostrinia furnacalis]	-1.63314	-0.69576	0.88853	0.824938	0.615433
TRINITY_DN1459_c0_g1_i2_orf1	reticulon-1 isoform X1 [Ostrinia furnacalis]	-1.45875	-0.056	1.656673	0.168064	-0.30998
TRINITY_DN77830_c0_g2_i2_orf1	prostaglandin reductase 1-like [Leguminivora glycinivorella] >XP_047994907.1 prostaglandin reductase 1-like [Leguminivora glycinivorella]	-1.41907	0.438757	1.582159	-0.06705	-0.53479
TRINITY_DN3231_c0_g1_i12_orf1	integrin-linked protein kinase [Pectinophora gossypiella]	-1.70213	-0.47606	0.713118	0.348895	1.116178
TRINITY_DN3800_c0_g1_i7_orf1	hypothetical protein evm_004893 [Chilo suppressalis]	-1.67165	-0.41888	1.163692	0.11239	0.814447
TRINITY_DN3913_c0_g1_i6_orf1	protein obstructor-E-like [Ostrinia furnacalis]	-1.83515	-0.23363	0.860899	0.372654	0.83523
TRINITY_DN120_c0_g1_i2_orf1	PREDICTED: myosin light chain alkali-like [Amyelois transitella]	-1.80515	-0.33102	0.884964	0.444031	0.807178
TRINITY_DN11823_c1_g1_i2_orf1	LOW QUALITY PROTEIN: uncharacterized protein LOC114362902 [Ostrinia furnacalis]	-1.77175	-0.45065	0.704212	0.827806	0.690382
TRINITY_DN62557_c0_g1_i1_orf1	6-phosphofructokinase [Operophtera brumata]	-1.49729	-0.79496	0.607413	1.253433	0.431406
TRINITY_DN3732_c0_g1_i6_orf1	cytochrome P450 monooxygenase CYP6AB141 [Ostrinia furnacalis]	-1.14727	0.223477	1.690726	-0.87397	0.107036
TRINITY_DN15136_c0_g1_i2_orf1	alpha-aminoadipic semialdehyde synthase, mitochondrial isoform X3 [Ostrinia furnacalis]	-1.72056	-0.47989	1.086441	0.491708	0.622298
TRINITY_DN3492_c0_g1_i1_orf1	larval cuticle protein A2B-like [Ostrinia furnacalis]	-1.67238	-0.53551	1.166363	0.438334	0.603189
TRINITY_DN5149_c0_g1_i12_orfp1	TRINITY_DN5149_c0_g1_i12_m.8808 TRINITY_DN5149_c0_g1::TRINITY_DN5149_c0_g1_i12::g.8808 ORF type:internal len:254 (+),score=89.66 TRINITY_DN5149_c0_g1_i12:1-759(+)	-1.6471	0.305813	1.472122	-0.15985	0.029014
TRINITY_DN58261_c0_g1_i2_orf1	15-hydroxyprostaglandin dehydrogenase [NAD(+)]-like [Ostrinia furnacalis]	-1.67419	-0.59387	0.618478	0.599724	1.049863
TRINITY_DN1470_c0_g1_i2_orf1	oxidation resistance protein 1 isoform X5 [Ostrinia furnacalis]	-1.82969	-0.08862	1.164575	0.422086	0.331652
TRINITY_DN1212_c0_g1_i8_orf1	extensin isoform X5 [Ostrinia furnacalis] >XP_028175473.1 extensin isoform X5 [Ostrinia furnacalis] >XP_028175474.1 extensin isoform X5 [Ostrinia furnacalis]	-1.87769	-0.15565	0.762292	0.460656	0.810398
TRINITY_DN6698_c0_g2_i1_orf1	protein mesh isoform X2 [Ostrinia furnacalis]	-1.60126	-0.41353	0.526146	0.080964	1.407684
TRINITY_DN27332_c0_g2_i1_orf1	Alpha-D-phosphohexomutase alpha/beta/alpha domain III, partial [Trinorchestia longiramus]	-1.77915	0.519745	1.212514	-0.19244	0.239327
TRINITY_DN1333_c0_g1_i6_orf1	uncharacterized protein LOC114362563 [Ostrinia furnacalis]	-1.69647	-0.47579	0.992602	0.262302	0.917359
TRINITY_DN17061_c0_g1_i1_orf1	uncharacterized protein LOC113511282 isoform X2 [Galleria mellonella]	-1.75995	-0.38212	0.863573	0.328166	0.950329
TRINITY_DN68401_c1_g1_i1_orf1	endoplasmic reticulum chaperone BiP isoform X1 [Ostrinia furnacalis]	-1.17528	0.260429	1.708443	-0.79506	0.001466
TRINITY_DN31645_c0_g1_i3_orf1	dystonin isoform X43 [Helicoverpa armigera]	-1.85056	-0.09128	1.012267	0.22986	0.69971
TRINITY_DN5432_c1_g1_i3_orf1	electron transfer flavoprotein-ubiquinone oxidoreductase, mitochondrial [Ostrinia furnacalis]	-1.07625	0.441711	1.741587	-0.57191	-0.53514
TRINITY_DN8310_c0_g2_i1_orf1	uncharacterized protein LOC116773294 [Danaus plexippus plexippus] >OWR55545.1 hypothetical protein KGM_209260 [Danaus plexippus plexippus]	-1.73036	-0.44723	0.369109	1.035206	0.773276
TRINITY_DN77480_c0_g1_i2_orf1	hypothetical protein evm_008422 [Chilo suppressalis]	-1.56245	0.100779	1.310772	-0.56451	0.715408
TRINITY_DN1103_c0_g1_i19_orf1	retinal dehydrogenase 1-like [Ostrinia furnacalis]	-1.36605	0.205404	1.696854	-0.08285	-0.45336
TRINITY_DN122393_c0_g1_i1_orf1	microtubule-associated protein futsch isoform X4 [Ostrinia furnacalis] >XP_028162562.1 microtubule-associated protein futsch isoform X4 [Ostrinia furnacalis]	-1.7969	0.158489	1.256109	-0.02698	0.409276
TRINITY_DN9468_c1_g1_i4_orf1	coronin-1C-A isoform X1 [Bombyx mori]	-1.80573	-0.12108	1.210562	0.394898	0.321355
TRINITY_DN90497_c0_g1_i1_orf1	midasin-like [Ostrinia furnacalis]	-1.90743	-0.02747	0.445823	0.580946	0.908134
TRINITY_DN1355_c0_g1_i5_orf1	hypothetical protein evm_006664 [Chilo suppressalis]	-1.77173	-0.03113	1.223707	-0.02254	0.601694
TRINITY_DN5149_c0_g1_i1_orf1	titin [Ostrinia furnacalis]	-1.60722	0.019997	1.49285	-0.25562	0.349997
TRINITY_DN7976_c0_g1_i4_orf1	unnamed protein product [Chilo suppressalis]	-1.30231	0.470147	1.203242	-1.0705	0.69942
TRINITY_DN45949_c0_g1_i1_orf1	uncharacterized protein LOC114355167 [Ostrinia furnacalis]	-1.86967	-0.11719	0.864443	0.799375	0.323039
TRINITY_DN5176_c0_g1_i2_orf1	uncharacterized protein LOC114361931 [Ostrinia furnacalis]	-1.77512	0.765253	0.6788	-0.44593	0.776996
TRINITY_DN143509_c0_g1_i1_orf1	ATP-dependent 6-phosphofructokinase isoform X3 [Diachasma alloeum]	-1.85148	0.034871	0.485847	0.191415	1.139348
TRINITY_DN549_c0_g1_i7_orf1	titin-like, partial [Ostrinia furnacalis]	-1.92751	0.05205	0.883242	0.426669	0.565546

TRINITY_DN43881_c0_g1_i2_orf1	estradiol 17-beta-dehydrogenase 8-like [Ostrinia furnacalis]	-1.27544	0.541866	1.52344	-0.86759	0.077728
TRINITY_DN69871_c0_g1_i1_orf1	translocon-associated protein subunit gamma [Venturia canescens]	-1.69659	0.082284	1.434531	-0.0529	0.232672
TRINITY_DN5437_c0_g1_i1_orf1	unnamed protein product [Chilo suppressalis]	-1.57467	0.162861	1.339942	-0.55392	0.625788
TRINITY_DN23474_c1_g1_i1_orf1	unnamed protein product [Chrysodeixis includens]	-1.31266	0.378942	1.583032	-0.78101	0.131704
TRINITY_DN2958_c0_g1_i2_orf1	uncharacterized protein LOC114356495 [Ostrinia furnacalis]	-1.63754	0.507974	1.399857	-0.31445	0.044159
TRINITY_DN18782_c0_g1_i4_orf1	putative riboflavin kinase [Ostrinia furnacalis] >XP_028176654.1 putative riboflavin kinase [Ostrinia furnacalis]	-1.49628	-0.60624	-0.07146	0.974214	1.199761
TRINITY_DN3105_c0_g1_i4_orf1	nose resistant to fluoxetine protein 6-like [Ostrinia furnacalis]	-1.60174	0.663604	1.374712	-0.28511	-0.15147
TRINITY_DN5405_c1_g1_i13_orf1	acyl-CoA synthetase family member 3, mitochondrial [Ostrinia furnacalis]	-1.70145	-0.17656	0.346126	0.141131	1.39075
TRINITY_DN2621_c0_g1_i1_orf1	GPN-loop GTPase 1 [Ostrinia furnacalis]	-1.34119	0.651379	1.534557	-0.603	-0.24174
TRINITY_DN1029_c0_g1_i1_orfp1	TRINITY_DN1029_c0_g1_i1_m.64408 TRINITY_DN1029_c0_g1::TRINITY_DN1029_c0_g1_i1::g.64408 ORF type:3prime_partial len:55 (+),score=13.47 TRINITY_DN1029_c0_g1_i1:74-235(+)	-1.95402	0.123889	0.792582	0.514635	0.52291
TRINITY_DN1134_c0_g1_i4_orf1	cytochrome P450 6B5-like [Ostrinia furnacalis]	-1.84729	-0.23886	0.60846	0.553842	0.92385
TRINITY_DN2186_c0_g1_i13_orf1	paxillin isoform X2 [Ostrinia furnacalis]	-1.79145	0.548814	1.219857	-0.01322	0.035999
TRINITY_DN6991_c0_g1_i24_orf1	muscle M-line assembly protein unc-89 isoform X5 [Ostrinia furnacalis]	-1.91042	0.07125	0.699429	0.891204	0.248537
TRINITY_DN26254_c0_g1_i1_orf1	hypothetical protein evm_003664 [Chilo suppressalis] >CAB3521132.1 unnamed protein product [Chilo suppressalis] >CAH0398453.1 unnamed protein product [Chilo suppressalis]	-1.93292	0.023048	0.810764	0.518502	0.580606
TRINITY_DN4501_c0_g2_i1_orf1	methylcrotonoyl-CoA carboxylase subunit alpha, mitochondrial [Ostrinia furnacalis]	-1.32312	0.83942	1.438122	-0.57987	-0.37456
TRINITY_DN14398_c0_g1_i4_orf1	trimethyllysine dioxygenase, mitochondrial [Ostrinia furnacalis]	-1.89261	-0.01742	0.951431	0.316417	0.642179
TRINITY_DN62_c1_g1_i3_orf1	D-2-hydroxyglutarate dehydrogenase, mitochondrial-like [Ostrinia furnacalis]	-1.64243	-0.43008	0.043118	1.182622	0.846764
TRINITY_DN8406_c0_g1_i2_orf1	titin [Ostrinia furnacalis]	-1.56365	0.552456	1.41155	-0.49783	0.097467
TRINITY_DN6612_c0_g1_i4_orf1	hypothetical protein O3G_MSEX008151 [Manduca sexta]	-1.7506	0.237661	1.250067	-0.24386	0.506732
TRINITY_DN30208_c0_g1_i3_orf1	unnamed protein product [Timema cristinae]	-1.904	0.072539	0.952649	0.244679	0.634131
TRINITY_DN5510_c0_g1_i9_orf1	proteoglycan 4 [Pectinophora gossypiella]	-1.85631	-0.05635	0.643439	0.227512	1.041707
TRINITY_DN109943_c0_g1_i1_orf1	uncharacterized protein LOC114361588 isoform X14 [Ostrinia furnacalis]	-1.84127	-0.20888	0.87915	0.353582	0.817415
TRINITY_DN11245_c0_g1_i2_orf1	ITG-like peptide [Ostrinia furnacalis]	-1.56027	-0.67577	0.215174	1.112061	0.908803
TRINITY_DN101991_c0_g1_i5_orf1	hypothetical protein O3G_MSEX015044, partial [Manduca sexta]	-1.89205	-0.13636	0.659611	0.562655	0.806144
TRINITY_DN6653_c0_g1_i1_orf1	NEDD8-activating enzyme E1 catalytic subunit [Ostrinia furnacalis] >XP_028168359.1 NEDD8-activating enzyme E1 catalytic subunit [Ostrinia furnacalis]	-1.68331	0.449756	1.235472	-0.46881	0.4669
TRINITY_DN4242_c0_g1_i6_orf1	fibrohexamerin-like [Ostrinia furnacalis]	-1.55692	-0.53062	-0.05134	1.116429	1.022449
TRINITY_DN24322_c0_g1_i4_orf1	unnamed protein product, partial [Brenthis ino]	-1.89292	0.164532	1.069596	0.449241	0.209556
TRINITY_DN6621_c0_g1_i1_orf1	translocon-associated protein subunit delta [Ostrinia furnacalis]	-1.86629	0.074331	1.070489	0.131348	0.590121
TRINITY_DN8258_c0_g1_i6_orf1	unnamed protein product [Chilo suppressalis]	-1.87966	-0.09671	0.671472	0.373838	0.931067
TRINITY_DN6415_c0_g1_i1_orf1	D-arabinitol dehydrogenase 1-like [Ostrinia furnacalis]	-1.5894	-0.4837	0.079414	0.646029	1.347657
TRINITY_DN102051_c0_g1_i1_orf1	spectrin repeat domain-containing protein [Phthorimaea operculella]	-1.81893	-0.29677	0.973506	0.529891	0.612303
TRINITY_DN69707_c0_g1_i1_orf1	titin-like, partial [Ostrinia furnacalis]	-1.9272	-0.02534	0.621694	0.584191	0.746652
TRINITY_DN31118_c0_g1_i1_orf1	CPR9 [Ostrinia furnacalis]	-1.92672	-0.03682	0.680307	0.631529	0.651711
TRINITY_DN9871_c0_g1_i11_orf1	PEST proteolytic signal-containing nuclear protein-like [Ostrinia furnacalis]	-1.90861	0.296868	1.039051	0.173469	0.399217
TRINITY_DN11986_c0_g1_i1_orf1	DNA replication licensing factor Mcm2 [Ostrinia furnacalis]	-1.86482	0.319753	0.598463	-0.08077	1.027378
TRINITY_DN1103_c0_g1_i15_orf1	retinal dehydrogenase 1-like [Ostrinia furnacalis]	-1.94189	0.746495	0.623501	0.53041	0.041484
TRINITY_DN4010_c0_g2_i1_orf1	myophilin [Ostrinia furnacalis]	-1.87995	0.293563	1.097121	0.076915	0.412348
TRINITY_DN3010_c0_g1_i4_orf1	inositol oxygenase-like [Ostrinia furnacalis]	-1.70089	0.467179	0.986387	-0.5416	0.78893
TRINITY_DN119893_c0_g2_i3_orf1	ATP-binding cassette sub-family F member 3 isoform X1 [Ostrinia furnacalis] >XP_028168051.1 ATP-binding cassette sub-family F member 3 isoform X2 [Ostrinia furnacalis]	-1.37363	0.842228	1.303791	-0.83654	0.064155
TRINITY_DN2688_c0_g2_i1_orf1	mitochondrial amidoxime reducing component 2 [Galleria mellonella]	-1.82722	-0.13104	0.453812	0.360783	1.143669
TRINITY_DN3769_c0_g1_i1_orf1	uncharacterized protein LOC114360562 [Ostrinia furnacalis] >XP_028171110.1 uncharacterized protein LOC114360562 [Ostrinia furnacalis]	-1.77125	0.318278	0.439579	-0.21947	1.232867
TRINITY_DN17137_c0_g1_i2_orf1	unnamed protein product [Diatraea saccharalis]	-1.83832	0.015292	0.88006	0.023572	0.919396
TRINITY_DN146236_c0_g1_i1_orf1	vesicle-fusing ATPase 1-like [Chelonus insularis]	-1.50496	0.806648	1.299772	-0.62794	0.026478
TRINITY_DN57636_c0_g1_i4_orf1	PREDICTED: plectin-like, partial [Papilio polytes]	-1.9853	0.524091	0.685037	0.306198	0.469975
TRINITY_DN1104_c0_g1_i1_orfp1	TRINITY_DN1104_c0_g1_i1_m.5521 TRINITY_DN1104_c0_g1::TRINITY_DN1104_c0_g1_i1::g.5521 ORF type:5prime_partial len:204 (+),score=39.47 TRINITY_DN1104_c0_g1_i1:3-614(+)	-1.52246	-0.02673	1.34044	-0.55246	0.761199

TRINITY_DN50074_c0_g1_i1_orf1	uncharacterized protein LOC114364628 [Ostrinia furnacalis]	-1.66638	-0.45509	0.375023	1.291779	0.454672
TRINITY_DN1306_c0_g1_i8_orf1	spectrin alpha chain isoform X4 [Pectinophora gossypiella]	-1.71897	0.768792	0.996358	-0.50283	0.456654
TRINITY_DN12690_c0_g1_i1_orf1	ELAV-like protein 1 [Ostrinia furnacalis]	-1.82517	0.297182	0.921855	-0.21986	0.825996
TRINITY_DN825_c0_g1_i18_orfp1	TRINITY_DN825_c0_g1_i18_m.8360 TRINITY_DN825_c0_g1::TRINITY_DN825_c0_g1_i18::g.8360 ORF type:complete len:415 (+),score=37.02 TRINITY_DN825_c0_g1_i18:55-1245(+)	-1.87971	0.483728	1.085944	0.206826	0.10321
TRINITY_DN27500_c0_g1_i4_orf1	hemicentin-1-like [Ostrinia furnacalis]	-1.73577	0.501679	1.19087	-0.37609	0.419305
TRINITY_DN7233_c0_g2_i1_orf1	protein transport protein Sec61 subunit beta [Ostrinia furnacalis]	-1.69155	0.88777	1.040414	-0.46477	0.228139
TRINITY_DN1889_c0_g1_i1_orf1	titin isoform X2 [Ostrinia furnacalis]	-1.91396	-0.04056	0.594878	0.511131	0.848515
TRINITY_DN46173_c0_g3_i1_orf1	Tropomyosin, partial [Cotesia chilonis]	-1.91935	0.145054	0.975224	0.288364	0.510711
TRINITY_DN1814_c0_g2_i1_orf1	titin-like, partial [Ostrinia furnacalis]	-1.92366	0.059808	0.766732	0.318078	0.779042
TRINITY_DN616_c1_g1_i6_orf1	esterase B1-like isoform X1 [Ostrinia furnacalis] >XP_028178578.1 esterase B1-like isoform X2 [Ostrinia furnacalis]	-1.6577	-0.37944	-0.00642	1.121661	0.921899
TRINITY_DN46124_c0_g1_i1_orf1	protein NipSnap [Plutella xylostella] >KAG7303557.1 hypothetical protein JYU34_012086 [Plutella xylostella] >CAG9104264.1 unnamed protein product [Plutella xylostella]	-1.59076	1.485466	0.317647	-0.37022	0.157861
TRINITY_DN2890_c0_g1_i2_orf1	alanine aminotransferase 1 [Chelonus insularis]	-1.8889	0.581536	0.789686	-0.15121	0.668887
TRINITY_DN2215_c0_g2_i1_orf1	PREDICTED: larval cuticle protein LCP-22-like [Amyelois transitella]	-1.94895	0.104027	0.751216	0.427745	0.665957
TRINITY_DN19460_c0_g1_i1_orf1	cuticle protein 3-like [Ostrinia furnacalis]	-1.91786	-0.00461	0.680883	0.411736	0.829852
TRINITY_DN1237_c0_g1_i4_orf1	armadillo-like helical domain-containing protein 3 [Ostrinia furnacalis]	-1.59515	0.405	1.314981	0.464131	-0.58896
TRINITY_DN1197_c0_g1_i6_orf1	tensin-2-like isoform X6 [Ostrinia furnacalis] >XP_028159238.1 tensin-2-like isoform X6 [Ostrinia furnacalis] >XP_028159239.1 tensin-2-like isoform X6 [Ostrinia furnacalis] >XP_028159240.1 tensin-2-like isoform X6 [Ostrinia furnacalis]	-1.41881	1.013033	1.212118	-0.69164	-0.11471
TRINITY_DN26663_c0_g1_i4_orf1	phagocyte signaling-impaired protein [Ostrinia furnacalis]	-1.80251	0.030017	0.787941	-0.07541	1.059963
TRINITY_DN5563_c1_g2_i2_orf1	hypothetical protein evm_005765 [Chilo suppressalis]	-1.76927	0.936886	0.901113	-0.33231	0.263586
TRINITY_DN3906_c0_g1_i5_orf1	ejaculatory bulb-specific protein 3-like [Ostrinia furnacalis]	-1.67665	0.416946	1.231854	-0.48465	0.512499
TRINITY_DN4866_c0_g1_i2_orf1	actin-binding LIM protein 3 isoform X6 [Ostrinia furnacalis]	-1.77723	-0.13168	0.013207	1.06426	0.831444
TRINITY_DN1211_c0_g1_i10_orf1	spectrin beta chain-like isoform X7 [Spodoptera frugiperda]	-1.96059	0.398599	0.732861	0.173305	0.655821
TRINITY_DN3916_c0_g1_i6_orf1	sarcalumenin [Ostrinia furnacalis] >XP_028176617.1 sarcalumenin [Ostrinia furnacalis] >XP_028176618.1 sarcalumenin [Ostrinia furnacalis]	-1.49362	0.10978	0.178263	-0.39685	1.602427
TRINITY_DN350_c0_g1_i10_orf1	microtubule-associated protein tau-like isoform X5 [Ostrinia furnacalis]	-1.73903	0.79736	0.71675	-0.52038	0.745301
TRINITY_DN1391_c1_g2_i2_orf1	uncharacterized protein LOC119837640 isoform X2 [Zerene cesonia]	-1.92272	0.189405	0.894272	0.179177	0.659871
TRINITY_DN13119_c0_g1_i4_orf1	endocuticle structural glycoprotein ABD-5-like [Bicyclus anynana]	-1.9281	0.046791	0.723682	0.370866	0.786758
TRINITY_DN838_c0_g1_i18_orf1	hypothetical protein evm_003399, partial [Chilo suppressalis]	-1.44221	1.061484	1.138596	-0.70275	-0.05513
TRINITY_DN110400_c0_g1_i1_orf1	DNA replication factor Cdt1 [Chelonus insularis]	-1.62785	0.603061	1.021917	-0.6849	0.687769
TRINITY_DN114344_c0_g1_i4_orf1	microtubule-actin cross-linking factor 1 isoform X15 [Ostrinia furnacalis]	-1.81698	0.468916	1.139136	-0.17771	0.386638
TRINITY_DN1718_c6_g1_i4_orf1	adenosine kinase [Ostrinia furnacalis]	-1.8494	0.136434	0.194691	0.32865	1.189622
TRINITY_DN23941_c0_g1_i5_orf1	dystonin isoform X11 [Galleria mellonella]	-1.87446	0.360614	1.00484	-0.07497	0.58398
TRINITY_DN9406_c0_g1_i5_orf1	proton-coupled amino acid transporter-like protein pathetic [Ostrinia furnacalis]	-1.57797	-0.36088	-0.22039	1.076513	1.082735
TRINITY_DN42177_c0_g1_i4_orf1	androgen-dependent TFPI-regulating protein-like [Ostrinia furnacalis]	-1.34392	0.985295	1.2443	-0.81873	-0.06695
TRINITY_DN107708_c0_g1_i1_orf1	elongation factor 1-beta' [Pectinophora gossypiella]	-1.5529	1.097538	0.932225	-0.68629	0.209434
TRINITY_DN62_c0_g1_i7_orf1	tropomodulin-1 isoform X5 [Ostrinia furnacalis] >XP_028160021.1 tropomodulin-1 isoform X5 [Ostrinia furnacalis]	-1.70683	0.880233	0.89964	-0.53647	0.46343
TRINITY_DN17907_c0_g1_i13_orf1	androgen-induced gene 1 protein-like isoform X1 [Galleria mellonella]	-1.57172	-0.36785	-0.20027	0.88992	1.249923
TRINITY_DN1084_c0_g2_i2_orf1	ATP-citrate synthase [Ostrinia furnacalis]	-1.83091	0.080427	-0.05126	0.989017	0.812726
TRINITY_DN115658_c0_g1_i1_orf1	hypothetical protein B5X24_HaOG203018 [Helicoverpa armigera]	-1.16794	1.135347	1.199317	-0.92094	-0.24578
TRINITY_DN13350_c0_g1_i4_orf1	cap-specific mRNA (nucleoside-2'-O-)-methyltransferase 1 [Ostrinia furnacalis]	-1.91535	0.393742	0.964883	0.491012	0.065711
TRINITY_DN1895_c0_g1_i2_orf1	unnamed protein product [Chrysodeixis includens]	-1.86979	0.17634	0.917114	-0.01825	0.794587
TRINITY_DN9029_c0_g1_i4_orf1	venom protease-like [Ostrinia furnacalis]	-1.82737	0.853507	0.909332	0.259511	-0.19498
TRINITY_DN46372_c0_g2_i1_orf1	basic salivary proline-rich protein 1 isoform X2 [Ostrinia furnacalis]	-1.65097	1.03398	1.01964	-0.40681	0.00416
TRINITY_DN9286_c0_g1_i2_orf1	alcohol dehydrogenase class-3 [Ostrinia furnacalis]	-1.87253	0.022693	0.229715	0.557145	1.06298
TRINITY_DN21596_c0_g1_i1_orf1	peptidyl-prolyl cis-trans isomerase NIMA-interacting 4 [Zerene cesonia] >XP_038208701.1 peptidyl-prolyl cis-trans isomerase NIMA-interacting 4 [Zerene cesonia]	-1.45396	1.244499	0.682236	-0.85284	0.380064
TRINITY_DN592_c0_g1_i6_orf1	PDZ and LIM domain protein Zasp isoform X4 [Pectinophora gossypiella]	-1.89198	0.156572	0.713112	0.084055	0.938243
TRINITY_DN4695_c0_g1_i3_orf1	glutathione S-transferase epsilon 3 [Ostrinia furnacalis]	-1.57135	-0.4334	-0.14292	1.164297	0.983368

TRINITY_DN12_c0_g1_i5_orf1	cAMP-dependent protein kinase type II regulatory subunit isoform X1 [Ostrinia furnacalis] >XP_028175270.1 cAMP-dependent protein kinase type II regulatory subunit isoform X1 [Ostrinia furnacalis]	-1.41245	1.06841	0.794591	-0.97714	0.526592
TRINITY_DN8241_c0_g1_i3_orf1	transforming growth factor beta-1-induced transcript 1 protein [Ostrinia furnacalis]	-1.14956	0.970578	1.31088	-1.00039	-0.1315
TRINITY_DN128_c0_g1_i5_orf1	PREDICTED: muscle-specific protein 20-like [Amyelois transitella]	-1.86272	0.141848	-0.01658	0.884544	0.852913
TRINITY_DN2040_c0_g1_i15_orfp1	TRINITY_DN2040_c0_g1_i15_m.4150 TRINITY_DN2040_c0_g1::TRINITY_DN2040_c0_g1_i15::g.4150 ORF type:complete len:319 (+),score=125.43,Plasmodium_HRP PF05403.12 3.5,Plasmodium_HRP|PF05403.12|1.5 TRINITY_DN2040_c0_g1_i15:118-957(+)	-1.91917	-0.06364	0.625488	0.665669	0.691654
TRINITY_DN2254_c0_g1_i4_orf1	vigilin [Ostrinia furnacalis]	-1.47744	0.743699	1.030471	-0.90935	0.612618
TRINITY_DN116_c1_g1_i8_orf1	uncharacterized protein LOC114350057 isoform X2 [Ostrinia furnacalis]	-1.94627	0.25522	0.914544	0.321161	0.455349
TRINITY_DN146006_c0_g1_i1_orf1	unnamed protein product [Chrysodeixis includens]	-1.60791	1.427952	0.516196	-0.00594	-0.3303
TRINITY_DN69557_c0_g1_i1_orf1	hypothetical protein G9C98_005708, partial [Cotesia typhae]	-1.85842	0.507329	1.04797	-0.11744	0.420562
TRINITY_DN16978_c0_g1_i1_orf1	la-related protein 7 [Helicoverpa armigera]	-1.51723	0.384969	-0.10581	1.560367	-0.3223
TRINITY_DN18009_c0_g1_i1_orf1	pre-mRNA-splicing factor ISY1 homolog [Ostrinia furnacalis]	-1.83692	0.688247	0.988016	-0.20511	0.365769
TRINITY_DN48610_c0_g1_i2_orf1	hypothetical protein evm_002298 [Chilo suppressalis] >CAH0682062.1 unnamed protein product [Chilo	-1.91586	0.321796	0.996888	0.134531	0.462646
TRINITY_DN16125_c0_g1_i3_orf1	3-ketoacyl-CoA thiolase, mitochondrial [Ostrinia furnacalis]	-1.92812	0.682842	0.093859	0.844419	0.306995
TRINITY_DN5531_c0_g3_i3_orf1	hypothetical protein evm_013868 [Chilo suppressalis]	-1.95642	0.282038	0.246262	0.635041	0.793075
TRINITY_DN110132_c0_g1_i1_orf1	uncharacterized protein LOC114361588 isoform X14 [Ostrinia furnacalis]	-1.4883	1.182745	0.397563	-0.82824	0.736233
TRINITY_DN2922_c0_g1_i1_orf1	uncharacterized protein LOC114354086 [Ostrinia furnacalis]	-1.95668	0.267405	0.497143	0.329465	0.862667
TRINITY_DN8454_c0_g1_i4_orf1	translocon-associated protein subunit alpha [Ostrinia furnacalis]	-1.67674	0.851991	1.050501	-0.52129	0.29554
TRINITY_DN101995_c0_g1_i1_orf1	microtubule-actin cross-linking factor 1 isoform X15 [Ostrinia furnacalis]	-1.84335	0.106546	0.711712	-0.01601	1.041107
TRINITY_DN3529_c0_g1_i7_orf1	putative fatty acyl-CoA reductase CG5065 [Ostrinia furnacalis]	-1.40855	0.977934	0.708763	-1.01045	0.732305
TRINITY_DN549_c0_g1_i14_orf1	titin-like [Ostrinia furnacalis]	-1.92216	0.405754	0.647138	0.019865	0.849403
TRINITY_DN2822_c0_g1_i4_orf1	uncharacterized protein LOC114354271 isoform X1 [Ostrinia furnacalis]	-1.69641	0.895611	0.334884	-0.50845	0.974368
TRINITY_DN51776_c0_g2_i1_orf1	cuticle protein CP14.6-like [Ostrinia furnacalis]	-1.55363	-0.23907	-0.26316	0.611667	1.44419
TRINITY_DN4133_c0_g1_i2_orfp2	unnamed protein product [Spodoptera exigua]	-1.34209	0.530256	1.083385	-1.05963	0.788083
TRINITY_DN120500_c0_g1_i1_orf1	cytochrome P450 6B5-like [Ostrinia furnacalis]	-1.48616	-0.10807	0.080667	-0.14537	1.658922
TRINITY_DN1982_c0_g1_i24_orf1	uncharacterized protein LOC114361215 isoform X5 [Ostrinia furnacalis]	-1.88007	0.437834	1.093415	0.081569	0.267252
TRINITY_DN1718_c1_g1_i5_orf1	gelsolin-like [Ostrinia furnacalis]	-1.94286	0.453465	0.73632	0.065157	0.687922
TRINITY_DN2350_c0_g1_i6_orf1	protein yellow-like isoform X2 [Ostrinia furnacalis]	-1.7126	-0.12269	0.08485	0.368883	1.381553
TRINITY_DN1180_c0_g1_i4_orf1	larval cuticle protein LCP-30-like [Ostrinia furnacalis]	-1.96292	0.161608	0.701434	0.440491	0.659386
TRINITY_DN1262_c0_g1_i2_orf1	alanine aminotransferase 1 isoform X1 [Ostrinia furnacalis] >XP_028162092.1 alanine aminotransferase 1 isoform X2 [Ostrinia furnacalis] >XP_028162093.1 alanine aminotransferase 1 isoform X3 [Ostrinia furnacalis]	-1.95827	0.45028	0.469156	0.201057	0.837782
TRINITY_DN8406_c0_g1_i4_orf1	titin [Ostrinia furnacalis]	-1.60643	1.055293	1.077707	-0.3165	-0.21008
TRINITY_DN12748_c2_g1_i1_orfp1	TRINITY_DN12748_c2_g1_i1_m.21305 TRINITY_DN12748_c2_g1::TRINITY_DN12748_c2_g1_i1::g.21305 ORF type:3prime_partial len:887 (+),score=-6.30 TRINITY_DN12748_c2_g1_i1:104-2761(+)	-1.7369	0.095035	-0.24426	0.682548	1.20358
TRINITY_DN11448_c0_g1_i15_orf1	unnamed protein product [Chilo suppressalis]	-1.92068	0.463841	0.960151	0.089256	0.407429
TRINITY_DN19659_c1_g1_i1_orf1	elongation factor 1-gamma [Ostrinia furnacalis]	-1.31178	1.106814	1.201896	-0.73585	-0.26108
TRINITY_DN565_c0_g2_i1_orf1	uncharacterized protein LOC114362323 [Ostrinia furnacalis]	-1.60276	0.064777	-0.08893	0.073316	1.553599
TRINITY_DN95971_c0_g5_i1_orf1	exportin-1 [Diachasma alloeum] >XP_015118053.1 exportin-1 [Diachasma alloeum] >XP_015118054.1 exportin-1 [Diachasma alloeum] >XP_015118055.1 exportin-1 [Diachasma alloeum] >XP_015118056.1 exportin-1 [Diachasma alloeum]	-1.79856	0.568812	1.156166	-0.18928	0.262864
TRINITY_DN82628_c0_g1_i2_orf1	ORF type:internal len:148 hit:XP_028162129.1 TRINITY_DN82628_c0_g1_i2:3-446(-)	-1.9438	0.22348	0.863126	0.256363	0.60083
TRINITY_DN4053_c0_g1_i5_orf1	uncharacterized protein LOC114358355 [Ostrinia furnacalis]	-1.76757	-0.03098	0.418659	0.078625	1.301262
TRINITY_DN8394_c1_g1_i9_orf1	uncharacterized protein LOC114364294 [Ostrinia furnacalis]	-1.57268	1.211321	0.656504	-0.68832	0.393177
TRINITY_DN779_c0_g1_i12_orf1	unnamed protein product [Chilo suppressalis]	-1.88228	-0.01121	0.470874	0.377185	1.045425
TRINITY_DN47257_c0_g1_i4_orf1	PREDICTED: microtubule-actin cross-linking factor 1-like, partial [Amyelois transitella]	-1.70051	1.243263	0.474672	-0.41926	0.401835
TRINITY_DN1455_c0_g1_i8_orf1	troponin T, skeletal muscle isoform X1 [Galleria mellonella]	-1.95623	0.32021	0.866528	0.27585	0.493638
TRINITY_DN9591_c0_g1_i1_orf1	probable 39S ribosomal protein L49, mitochondrial [Ostrinia furnacalis]	-1.90103	0.822319	0.778611	-0.02116	0.321257
TRINITY_DN31118_c0_g2_i1_orf1	unnamed protein product [Spodoptera exigua]	-1.94879	0.062552	0.538361	0.666859	0.681016
TRINITY_DN21570_c0_g1_i1_orf1	ceramide synthase 5-like [Ostrinia furnacalis]	-1.92096	0.988837	0.156372	0.446126	0.329621
TRINITY_DN13312_c0_g2_i1_orf1	von Willebrand factor A domain-containing protein 8 [Trichoplusia ni]	-1.91769	0.882284	0.596361	0.434009	0.005038

TRINITY_DN146217_c0_g1_i1_orf1	60S acidic ribosomal protein P0 [Bombus bifarius]	-1.88066	0.77673	0.916591	0.061248	0.126095
TRINITY_DN135077_c0_g1_i1_orf1	hypothetical protein KR038_001662 [Drosophila bunnanda]	-1.84794	0.220728	0.202703	0.222013	1.2025
TRINITY_DN7341_c0_g1_i8_orf1	LOW QUALITY PROTEIN: proteasome activator complex subunit 4-like [Ostrinia furnacalis]	-1.70849	1.307088	0.580671	0.008685	-0.18795
TRINITY_DN6482_c0_g1_i1_orf1	endocuticle structural glycoprotein SgAbd-5-like [Ostrinia furnacalis]	-1.91298	0.060408	0.314625	0.602786	0.935159
TRINITY_DN1814_c0_g2_i4_orfp1	TRINITY_DN1814_c0_g2_i4_m.63284 TRINITY_DN1814_c0_g2::TRINITY_DN1814_c0_g2_i4::g.63284 ORF type:internal len:258 (-),score=126.31 TRINITY_DN1814_c0_g2_i4:3-773(-)	-1.96288	0.192822	0.481488	0.489527	0.799041
TRINITY_DN23429_c0_g2_i1_orf1	muscle-specific protein 20 [Zerene cesonia]	-1.97024	0.394318	0.81288	0.308073	0.454967
TRINITY_DN8915_c0_g1_i3_orf1	filamin-A isoform X1 [Ostrinia furnacalis] >XP_028171553.1 filamin-A isoform X2 [Ostrinia furnacalis] >XP_028171561.1 filamin-A isoform X2 [Ostrinia furnacalis]	-1.98513	0.45258	0.597808	0.293908	0.640835
TRINITY_DN248_c0_g1_i12_orf1	twitchin-like [Ostrinia furnacalis]	-1.74055	0.663435	1.197185	0.151972	-0.27205
TRINITY_DN416_c0_g1_i1_orf1	unnamed protein product [Diatraea saccharalis]	-1.92964	0.557152	0.875939	0.053963	0.442587
TRINITY_DN2318_c1_g1_i1_orf1	transcription factor SPT20 homolog [Ostrinia furnacalis]	-1.67511	1.234698	0.781238	-0.19392	-0.1469
TRINITY_DN41334_c0_g1_i1_orf1	sarcosine dehydrogenase, mitochondrial [Chelonus insularis]	-1.92405	0.95765	0.521038	0.294348	0.151018
TRINITY_DN85004_c0_g1_i1_orf1	uncharacterized protein LOC114357684 [Ostrinia furnacalis]	-1.96887	0.202999	0.670854	0.675502	0.419515
TRINITY_DN35582_c0_g1_i1_orf1	uncharacterized protein LOC114364680 [Ostrinia furnacalis]	-1.80836	0.877276	0.979266	-0.01697	-0.03122
TRINITY_DN15000_c0_g1_i4_orf1	15-hydroxyprostaglandin dehydrogenase [NAD(+)]-like [Ostrinia furnacalis]	-1.26075	0.026352	-0.3524	-0.21325	1.800044
TRINITY_DN111110_c0_g1_i1_orf1	NAD-dependent protein deacylase-like [Ostrinia furnacalis]	-1.69034	0.809081	0.905455	-0.59003	0.565832
TRINITY_DN1989_c0_g1_i1_orf1	sarcoplasmic calcium-binding protein 1 isoform X1 [Ostrinia furnacalis]	-1.6933	0.218582	-0.38039	1.258863	0.59625
TRINITY_DN1814_c0_g1_i11_orf1	titin-like, partial [Ostrinia furnacalis]	-1.87601	0.777926	0.720671	-0.18868	0.566089
TRINITY_DN8964_c0_g1_i4_orf1	hypothetical protein evm_010115 [Chilo suppressalis]	-1.74308	0.86215	0.800862	-0.4956	0.575661
TRINITY_DN7778_c0_g1_i1_orf1	peroxiredoxin-2 [Cotesia glomerata] >KAH0561449.1 Peroxiredoxin-4 [Cotesia glomerata]	-1.83438	0.664193	1.038945	-0.16445	0.295697
TRINITY_DN3332_c0_g1_i9_orf1	glutathione S-transferase sigma3 [Glyphodes pyloalis]	-1.7714	0.636132	0.527137	-0.40116	1.009292
TRINITY_DN46633_c0_g1_i4_orf1	uncharacterized protein LOC114365425 [Ostrinia furnacalis] >QKV49448.1 fas-associated death domain protein [Ostrinia furnacalis]	-1.72154	0.884343	0.250766	1.007152	-0.42072
TRINITY_DN2647_c0_g1_i3_orf1	DNA repair protein complementing XP-G cells homolog isoform X1 [Ostrinia furnacalis]	-1.64152	-0.04372	-0.3888	1.009357	1.064679
TRINITY_DN695_c0_g1_i12_orf1	seroin transcript 2A, partial [Ostrinia nubilalis]	-1.97804	0.470347	0.676821	0.238146	0.592728
TRINITY_DN1173_c0_g1_i11_orf1	obscurin [Ostrinia furnacalis]	-1.9921	0.626428	0.365674	0.448052	0.551949
TRINITY_DN248_c0_g1_i1_orf1	unnamed protein product [Chilo suppressalis]	-1.96327	0.636131	0.705306	0.152659	0.469179
TRINITY_DN64_c0_g1_i4_orf1	unnamed protein product [Chilo suppressalis]	-1.70559	1.171598	0.660232	-0.43354	0.307308
TRINITY_DN8226_c0_g1_i1_orf1	myosin heavy chain, muscle isoform X16 [Helicoverpa armigera]	-1.97777	0.476896	0.651351	0.229954	0.619567
TRINITY_DN81258_c0_g1_i2_orf1	jg27820 [Pararge aegeria aegeria]	-1.91924	0.56343	0.102935	0.307351	0.945522
TRINITY_DN1123_c2_g1_i3_orf1	troponin I isoform X8 [Ostrinia furnacalis]	-1.95538	0.471589	0.829356	0.165813	0.488627
TRINITY_DN1292_c0_g1_i3_orf1	uncharacterized protein LOC114360660 [Ostrinia furnacalis]	-1.45644	0.181988	-0.59619	0.32682	1.543825
TRINITY_DN107962_c0_g1_i1_orf1	unnamed protein product [Euphydryas editha]	-1.76299	0.804782	0.437669	-0.41691	0.937449
TRINITY_DN12508_c0_g1_i1_orf1	uncharacterized protein LOC114350091 [Ostrinia furnacalis]	-1.57017	1.192462	0.556246	-0.71666	0.538126
TRINITY_DN41708_c0_g1_i1_orf1	facilitated trehalose transporter Tret1-like [Ostrinia furnacalis]	-1.76659	0.514197	-0.07309	0.05818	1.267297
TRINITY_DN1125_c0_g1_i4_orf1	hypothetical protein evm_001907 [Chilo suppressalis] >CAH2985359.1 unnamed protein product [Chilo	-1.66665	1.101408	0.390496	-0.56125	0.735994
TRINITY_DN76333_c0_g1_i2_orf1	larval cuticle protein 65Ag1-like [Ostrinia furnacalis]	-1.97699	0.221248	0.543079	0.527794	0.684871
TRINITY_DN4408_c6_g1_i1_orf1	polyprotein, partial [Bemisia tabaci]	-1.60521	0.703233	0.798941	-0.75009	0.853124
TRINITY_DN21943_c1_g1_i1_orf1	myosin light chain alkali isoform X2 [Ostrinia furnacalis]	-1.84238	1.201757	0.093444	0.235054	0.312127
TRINITY_DN19080_c0_g1_i4_orf1	synaptic vesicle 2-related protein-like isoform X1 [Ostrinia furnacalis] >XP_028161172.1 synaptic vesicle 2-related protein-like isoform X1 [Ostrinia furnacalis]	-1.78076	1.317612	0.068532	0.125767	0.268849
TRINITY_DN100_c0_g1_i9_orf1	uncharacterized protein LOC114353052 [Ostrinia furnacalis]	-1.95469	0.72977	0.60298	0.099247	0.522688
TRINITY_DN72541_c0_g1_i2_orf1	xaa-Pro aminopeptidase ApepP-like isoform X2 [Ostrinia furnacalis]	-1.54388	1.478351	0.545997	-0.147	-0.33348
TRINITY_DN1073_c0_g1_i3_orf1	carboxylesterase [Loxostege sticticalis]	-1.96444	0.458052	0.215625	0.484725	0.806041
TRINITY_DN9198_c0_g1_i4_orf1	4-coumarate--CoA ligase 1-like isoform X1 [Ostrinia furnacalis]	-1.57035	0.976029	-0.4375	-0.13893	1.170755
TRINITY_DN6365_c0_g1_i4_orf1	40S ribosomal protein S21 [Helicoverpa armigera] >XP_047038308.1 40S ribosomal protein S21 isoform X2 [Helicoverpa zea] >KAI5643652.1 ribosomal protein s21e domain-containing protein [Phthorimaea operculella] >PZC73652.1 hypothetical protein B5X24_HaOG209026 [Helicoverpa armigera]	-1.6359	1.314185	0.256471	-0.48159	0.546838
TRINITY_DN6916_c0_g1_i4_orf1	isovaleryl-CoA dehydrogenase, mitochondrial [Ostrinia furnacalis]	-1.56221	1.544847	0.343772	-0.13592	-0.19049
TRINITY_DN100_c0_g1_i13_orf1	hypothetical protein O3G_MSEX015273 [Manduca sexta]	-1.9572	0.883726	0.37995	0.304696	0.38883

TRINITY_DN3918_c0_g1_i1_orf1	odorant binding protein 3 [Ostrinia furnacalis]	-1.89299	0.775753	0.446486	0.777249	-0.1065
TRINITY_DN21872_c0_g1_i2_orf1	facilitated trehalose transporter Tret1-2 homolog [Ostrinia furnacalis] >XP_028178438.1 facilitated trehalose transporter Tret1-2 homolog [Ostrinia furnacalis] >XP_028178439.1 facilitated trehalose transporter Tret1-2 homolog [Ostrinia furnacalis]	-1.52984	1.498906	0.476721	-0.01523	-0.43056
TRINITY_DN51830_c0_g1_i4_orf1	15-hydroxyprostaglandin dehydrogenase [NAD(+)]-like [Ostrinia furnacalis]	-1.77715	0.744583	-0.37111	0.415068	0.988603
TRINITY_DN928_c0_g1_i3_orf1	fasciclin-2-like [Ostrinia furnacalis]	-1.89232	0.802601	0.81685	0.324184	-0.05132
TRINITY_DN115082_c0_g1_i5_orf1	protein dj-1beta-like isoform X2 [Ostrinia furnacalis]	-1.43334	1.012946	1.233602	-0.2235	-0.58971
TRINITY_DN4724_c0_g1_i4_orf1	paramyosin, long form isoform X1 [Manduca sexta] >KAG6443143.1 hypothetical protein O3G_MSEX002737 [Manduca sexta]	-1.94761	0.867883	0.547	0.187405	0.345326
TRINITY_DN1585_c0_g1_i1_orf1	aldose reductase-like isoform X2 [Ostrinia furnacalis]	-1.80959	0.525006	-0.2463	0.435847	1.09504
TRINITY_DN87648_c0_g1_i1_orfp1	TRINITY_DN87648_c0_g1_i1_m.51054 TRINITY_DN87648_c0_g1::TRINITY_DN87648_c0_g1_i1::g.51054 ORF type:internal len:180 (+),score=75.93 TRINITY_DN87648_c0_g1_i1:2-538(+)	-1.90399	0.713823	0.048405	0.245998	0.895769
TRINITY_DN27045_c0_g1_i1_orf1	cytochrome P450 6B5-like [Galleria mellonella]	-1.75595	0.921642	0.177706	-0.31204	0.96864
TRINITY_DN5281_c0_g2_i3_orf1	serine/threonine-protein kinase RIO2 isoform X2 [Ostrinia furnacalis]	-1.7295	1.284925	0.581219	-0.14128	0.00464
TRINITY_DN46216_c0_g3_i1_orf1	unnamed protein product, partial [Brenthis ino]	-1.87715	0.351943	0.700429	-0.09834	0.923124
TRINITY_DN16673_c0_g1_i1_orf1	myosin heavy chain, partial [Drosophila virilis]	-1.67728	1.403822	0.421695	-0.19074	0.042503
TRINITY_DN2200_c0_g1_i4_orf1	uncharacterized protein LOC114363443 [Ostrinia furnacalis]	-1.85039	0.946269	0.624643	0.494275	-0.21479
TRINITY_DN235_c0_g1_i2_orf1	unnamed protein product [Parnassius apollo]	-1.90493	0.887944	0.588143	-0.05477	0.48361
TRINITY_DN1982_c0_g1_i17_orf1	unnamed protein product, partial [Iphiclides podalirius]	-1.94342	0.73087	0.609404	0.041092	0.56205
TRINITY_DN25492_c0_g1_i1_orf1	PREDICTED: myrosinase 1-like [Amyelois transitella]	-1.81189	1.206473	0.499033	0.111468	-0.00509
TRINITY_DN63533_c0_g1_i2_orf1	glutathione S-transferase sigma3 [Glyphodes pyloalis]	-1.81357	0.946633	0.446812	-0.30325	0.723368
TRINITY_DN146126_c0_g1_i1_orf1	malate dehydrogenase, mitochondrial [Chelonus insularis]	-1.69403	1.437125	-0.03705	0.046121	0.247834
TRINITY_DN48097_c0_g1_i1_orf1	unnamed protein product [Homo sapiens]	-1.94894	0.510428	0.317595	0.2349	0.886021
TRINITY_DN27276_c0_g1_i5_orf1	probable small nuclear ribonucleoprotein Sm D1 [Ostrinia furnacalis] >CAG9751027.1 unnamed protein product [Diatraea saccharalis] >CAG9789712.1 unnamed protein product [Diatraea saccharalis]	-1.47379	1.032556	0.32978	-0.85169	0.963143
TRINITY_DN29100_c0_g1_i2_orf1	endocuticle structural glycoprotein ABD-5-like [Galleria mellonella]	-1.97515	0.614907	0.386556	0.26639	0.707299
TRINITY_DN42461_c0_g1_i4_orf1	obscurin [Ostrinia furnacalis]	-1.90869	0.775037	0.778595	-0.03099	0.386052
TRINITY_DN50676_c0_g1_i1_orf1	uncharacterized protein LOC114360659 [Ostrinia furnacalis]	-1.55559	1.298884	0.166872	-0.61127	0.701102
TRINITY_DN89613_c0_g1_i13_orf1	PREDICTED: uncharacterized protein LOC106137743 [Amyelois transitella]	-1.49752	1.314648	0.528905	-0.76018	0.41415
TRINITY_DN1232_c0_g1_i1_orf1	acanthoscurrin-2-like isoform X1 [Ostrinia furnacalis]	-1.80387	1.014137	0.745229	-0.26167	0.30617
TRINITY_DN1173_c1_g1_i10_orf1	hypothetical protein evm_001011 [Chilo suppressalis]	-1.81802	0.963132	0.870849	-0.07389	0.057925
TRINITY_DN7580_c0_g1_i1_orf1	cytochrome P450 monooxygenase CYP6AB141 [Ostrinia furnacalis]	-1.66836	0.848735	0.46355	-0.60233	0.958405
TRINITY_DN57918_c0_g1_i1_orf1	PREDICTED: serine--tRNA ligase, cytoplasmic [Fopius arisanus]	-1.64118	1.414885	0.492491	-0.24857	-0.01763
TRINITY_DN11448_c0_g1_i4_orf1	uncharacterized protein LOC114364760 isoform X5 [Ostrinia furnacalis]	-1.73577	1.353168	0.342247	-0.11782	0.158179
TRINITY_DN1215_c0_g1_i2_orf1	PI-stichotoxin-She2a-like [Ostrinia furnacalis]	-1.87831	0.240445	-0.03184	0.736923	0.932783
TRINITY_DN86127_c1_g1_i2_orfp1	TRINITY_DN86127_c1_g1_i2_m.43062 TRINITY_DN86127_c1_g1::TRINITY_DN86127_c1_g1_i2::g.43062 ORF type:internal len:69 (-),score=14.03 TRINITY_DN86127_c1_g1_i2:2-205(-)	-1.67417	0.266863	-0.51918	0.982193	0.944293
TRINITY_DN38435_c0_g1_i1_orf1	UDP-glucuronosyltransferase 2B20-like [Ostrinia furnacalis]	-1.75649	1.265592	0.545589	0.055915	-0.11061
TRINITY_DN280_c4_g1_i5_orf1	fibroin light chain [Haritalodes derogata]	-1.71647	0.661415	-0.48856	1.076916	0.466705
TRINITY_DN6027_c0_g1_i13_orf1	5-demethoxyubiquinone hydroxylase, mitochondrial [Ostrinia furnacalis] >XP_028160430.1 5-demethoxyubiquinone hydroxylase, mitochondrial [Ostrinia furnacalis]	-1.65023	1.438938	0.073257	0.378294	-0.24026
TRINITY_DN38366_c0_g1_i4_orfp1	TRINITY_DN38366_c0_g1_i4_m.10666 TRINITY_DN38366_c0_g1::TRINITY_DN38366_c0_g1_i4::g.10666 ORF type:internal len:143 (+),score=71.68 TRINITY_DN38366_c0_g1_i4:3-428(+)	-1.86747	0.82029	0.620037	-0.21305	0.640195
TRINITY_DN13718_c0_g1_i7_orf1	immulectin-4 [Ostrinia furnacalis]	-1.64062	1.4608	-0.21285	0.357633	0.035033
TRINITY_DN1123_c2_g1_i4_orf1	troponin I isoform X16 [Ostrinia furnacalis]	-1.98834	0.59807	0.546335	0.29117	0.552766
TRINITY_DN31001_c0_g1_i1_orf1	endocuticle structural glycoprotein ABD-5-like [Ostrinia furnacalis]	-1.98311	0.522988	0.307089	0.44474	0.70829
TRINITY_DN79673_c0_g1_i1_orf1	thioredoxin, mitochondrial-like [Ostrinia furnacalis]	-1.69769	1.387605	-0.12175	0.010573	0.421261
TRINITY_DN19830_c0_g1_i1_orf1	macrophage migration inhibitory factor-like [Ostrinia furnacalis]	-1.64011	1.485178	-0.10957	0.301538	-0.03705
TRINITY_DN120439_c1_g1_i1_orf1	myosin heavy chain variant, partial [Bombyx mori]	-1.80539	1.159612	0.566858	-0.14953	0.22845
TRINITY_DN1123_c2_g1_i5_orf1	troponin I isoform X4 [Leguminivora glycinivorella]	-1.76236	1.102257	0.816173	-0.09796	-0.05811
TRINITY_DN4920_c0_g1_i5_orf1	titin homolog [Ostrinia furnacalis]	-1.84147	1.085239	0.621955	-0.0659	0.200176

TRINITY_DN141381_c0_g1_i1_orf1	very long-chain specific acyl-CoA dehydrogenase, mitochondrial [Chelonus insularis]	-1.87636	1.130097	0.287646	0.144853	0.313761
TRINITY_DN99673_c0_g1_i1_orf1	PREDICTED: pistil-specific extensin-like protein isoform X2 [Microplitis demolitor]	-1.66274	-0.07344	-0.01469	0.285129	1.465742
TRINITY_DN4501_c0_g1_i3_orf1	methylcrotonoyl-CoA carboxylase subunit alpha, mitochondrial [Ostrinia furnacalis]	-1.64882	1.355238	0.496372	-0.39954	0.196746
TRINITY_DN2186_c0_g1_i17_orf1	paxillin isoform X6 [Leguminivora glycinivorella]	-1.91734	0.846168	0.69698	0.025832	0.348361
TRINITY_DN14094_c0_g1_i1_orfp1	TRINITY_DN14094_c0_g1_i1_m.76391 TRINITY_DN14094_c0_g1::TRINITY_DN14094_c0_g1_i1::g.76391 ORF type:5prime_partial len:120 (+),score=12.94 TRINITY_DN14094_c0_g1_i1:3-362(+)	-1.78252	0.899952	-0.18959	0.984416	0.087736
TRINITY_DN141353_c0_g1_i1_orf1	uncharacterized protein LOC123263755 [Cotesia glomerata] >KAH0554923.1 hypothetical protein KQX54_013880 [Cotesia glomerata]	-1.85937	0.694103	-0.20767	0.880554	0.49239
TRINITY_DN20957_c0_g1_i1_orf1	adenylate kinase isoenzyme 1 isoform X2 [Ostrinia furnacalis]	-1.76146	1.263552	0.545682	0.005954	-0.05373
TRINITY_DN146841_c0_g1_i1_orf1	muscle-specific protein 20 [Temnothorax curvispinosus]	-1.99189	0.451053	0.363089	0.630859	0.546894
TRINITY_DN29_c0_g1_i4_orf1	sodium-dependent nutrient amino acid transporter 1-like [Ostrinia furnacalis] >XP_028167707.1 sodium-dependent nutrient amino acid transporter 1-like [Ostrinia furnacalis]	-1.61535	1.002285	-0.42557	1.096194	-0.05756
TRINITY_DN129869_c0_g4_i1_orf1	putative myosin heavy chain, muscle, partial [Cotesia chilonis]	-1.77899	1.278248	0.39557	-0.08751	0.192682
TRINITY_DN6881_c0_g1_i1_orf1	putative protein TPRXL [Ostrinia furnacalis]	-1.98877	0.469129	0.325254	0.650368	0.544017
TRINITY_DN36817_c0_g1_i1_orf1	uncharacterized protein LOC114357350 [Ostrinia furnacalis]	-1.8316	1.194126	0.448893	0.096298	0.092285
TRINITY_DN4016_c0_g1_i1_orf1	60S acidic ribosomal protein P0 [Homo sapiens] >NP_444505.1 60S acidic ribosomal protein P0 [Homo sapiens] >XP_002823894.1 60S acidic ribosomal protein P0 [Pongo abelii] >XP_003280010.1 60S acidic ribosomal protein P0 [Nomascus leucogenys] >XP_004054038.1 60S acidic ribosomal protein P0 [Gorilla gorilla gorilla] >XP_004054039.1 60S acidic ribosomal protein P0 [Gorilla gorilla gorilla] >XP_008956032.1 60S acidic ribosomal protein P0 [Pan paniscus] >XP_008956033.1 60S acidic ribosomal protein P0 [Pan paniscus] >XP_012611945.1 60S acidic ribosomal protein P0 [Microcebus murinus] >XP_016802006.1 60S acidic ribosomal protein P0 [Pan troglodytes] >XP_016802007.1 60S acidic ribosomal protein P0 [Pan troglodytes] >XP_025256707.1 60S acidic ribosomal protein P0 isoform X1 [Theropithecus gelada] >XP_025256708.1 60S acidic ribosomal protein P0 isoform X1 [Theropithecus gelada] >XP_032024425.1 60S acidic ribosomal protein P0 [Hylobates moloch] >XP_032657670.1 60S acidic ribosomal protein P0 [Chelonoidis abingdonii] >XP_045390642.1 60S acidic ribosomal protein P0 [Lemur catta] >P05388.1 RecName: Full=60S acidic ribosomal protein P0; AltName: Full=60S ribosomal protein L10E; AltName: Full=Large ribosomal subunit protein uL10 [Homo sapiens] >3J92_s Structure and assembly pathway of the ribosome quality control complex [Oryctolagus cuniculus] >4V5Z_Bg Chain Bg, 60S acidic ribosomal protein P0 [Canis lupus familiaris] >4V6X_Cq Chain Cq, 60S acidic ribosomal protein P0 [Homo sapiens] >5AJ0_AK Chain AK, 60S acidic ribosomal protein P0 [Homo sapiens] >6ZM7_Ls Chain Ls, 60S acidic ribosomal protein P0 [Homo sapiens] >6ZME_Ls Chain Ls, 60S acidic ribosomal protein P0 [Homo sapiens] >6ZMI_Ls Chain Ls, 60S acidic ribosomal protein P0 [Homo sapiens] >6ZMO_Ls Chain Ls, 60S acidic ribosomal protein P0 [Homo sapiens] >ABM82739.1 ribosomal protein, large, P0 [synthetic construct] >SJX33952.1 unnamed protein product, partial [Human ORFeome Gateway entry vector] >AAA36470.1 acidic ribosomal phosphoprotein (P0) [Homo sapiens] >AAC05176.1 60S ACIDIC RIBOSOMAL PROTEIN; match to P05388 (PID:g133041) [Homo sapiens]	-1.79528	1.269962	0.297134	-0.04378	0.271962
TRINITY_DN67231_c0_g1_i1_orf1	endocuticle structural glycoprotein SgAbd-8-like [Ostrinia furnacalis]	-1.99725	0.480249	0.523076	0.416982	0.576945
TRINITY_DN31118_c1_g1_i1_orf1	endocuticle structural glycoprotein ABD-4-like [Ostrinia furnacalis]	-1.98806	0.661257	0.438561	0.335603	0.552634
TRINITY_DN4145_c0_g1_i1_orf1	uncharacterized protein LOC114353175 isoform X1 [Ostrinia furnacalis]	-1.69264	1.436618	0.244541	-0.06918	0.08066
TRINITY_DN116951_c0_g3_i2_orf1	spermine oxidase-like isoform X2 [Ostrinia furnacalis]	-1.90202	1.011536	0.263472	0.097748	0.529268
TRINITY_DN1952_c0_g1_i2_orf1	uncharacterized protein LOC114354403 [Ostrinia furnacalis] >AYE20402.1 RNAi efficiency-related nuclease REase [Ostrinia furnacalis]	-1.63202	1.499181	-0.0922	-0.05351	0.278549
TRINITY_DN110460_c0_g2_i1_orf1	Similar to chaf1a-b: Chromatin assembly factor 1 subunit A-B (Xenopus laevis) [Cotesia congregata]	-1.76475	1.345948	0.04963	0.226271	0.1429
TRINITY_DN96557_c0_g1_i1_orf1	charged multivesicular body protein 4B [Phyllostomus discolor]	-1.81491	1.216149	0.438673	-0.02448	0.184563
TRINITY_DN76815_c0_g1_i3_orf1	5-formyltetrahydrofolate cyclo-ligase [Ostrinia furnacalis]	-1.32407	0.745601	0.64253	-1.0988	1.034744
TRINITY_DN1914_c0_g1_i4_orf1	loricrin-like [Ostrinia furnacalis]	-1.52621	0.812463	-0.47094	-0.1449	1.329586
TRINITY_DN3504_c0_g1_i3_orfp2	TRINITY_DN3504_c0_g1_i3_m.43947 TRINITY_DN3504_c0_g1::TRINITY_DN3504_c0_g1_i3::g.43947 ORF type:5prime_partial len:208 (-),score=77.75 TRINITY_DN3504_c0_g1_i3:185-808(-)	-1.60504	1.453067	0.306482	-0.39864	0.244129
TRINITY_DN3135_c0_g1_i6_orf1	acanthoscurrin-1-like [Ostrinia furnacalis]	-1.66576	1.475125	0.016489	-0.04298	0.21712
TRINITY_DN17189_c0_g1_i2_orf1	fibroin heavy chain [Haritalodes derogata]	-1.04932	1.566614	0.778086	-0.63696	-0.65842
TRINITY_DN659_c0_g2_i1_orf1	unnamed protein product [Diatraea saccharalis]	-1.34699	1.26783	0.986881	-0.76394	-0.14377
TRINITY_DN1369_c0_g2_i3_orf1	ATP-dependent Clp protease ATP-binding subunit clpX-like, mitochondrial isoform X2 [Helicoverpa zea]	-1.1275	1.360786	0.969294	-0.92869	-0.2739

TRINITY_DN14306_c0_g1_i1_orf1	prostaglandin E synthase 2 [Galleria mellonella]	-1.34979	1.523452	0.670941	-0.26368	-0.58093
TRINITY_DN3511_c0_g2_i1_orf1	eukaryotic translation initiation factor 2A [Ostrinia furnacalis]	-1.12065	1.474095	0.744836	-1.00373	-0.09455
TRINITY_DN48851_c0_g1_i2_orf1	translationally-controlled tumor protein homolog [Ostrinia furnacalis]	-1.39434	1.307892	0.938963	-0.65008	-0.20244
TRINITY_DN7686_c0_g1_i4_orf1	eIF-2-alpha kinase activator GCN1 [Ostrinia furnacalis]	-1.48459	1.480797	0.635167	-0.33127	-0.30011
TRINITY_DN129808_c0_g1_i1_orf1	uncharacterized protein LOC114354070 isoform X3 [Ostrinia furnacalis]	-1.1164	1.478566	0.688714	-1.04554	-0.00535
TRINITY_DN496_c0_g1_i7_orf1	unnamed protein product [Diatraea saccharalis]	-1.40262	1.27702	0.896631	-0.77326	0.002226
TRINITY_DN37986_c0_g1_i2_orf1	unnamed protein product [Diatraea saccharalis]	-1.13321	1.545042	0.636367	-0.95675	-0.09146
TRINITY_DN18593_c0_g1_i1_orf1	60S ribosomal protein L22-like [Ostrinia furnacalis]	-0.86303	1.605405	0.724645	-0.9293	-0.53772
TRINITY_DN3970_c0_g1_i1_orf1	hypothetical protein evm_002369 [Chilo suppressalis]	-1.3716	1.483247	0.635601	-0.71678	-0.03047
TRINITY_DN1309_c0_g2_i1_orf1	chymotrypsin-1-like [Ostrinia furnacalis]	-1.64851	1.253005	-0.11685	-0.27651	0.788856
TRINITY_DN41166_c0_g1_i1_orf1	arginine kinase isoform X1 [Ostrinia furnacalis]	-1.49214	1.336461	0.82594	-0.53588	-0.13439
TRINITY_DN15362_c0_g1_i1_orf1	probable elongation factor 1-delta isoform X1 [Ostrinia furnacalis]	-1.11953	1.474994	0.828518	-0.89505	-0.28893
TRINITY_DN817_c0_g1_i3_orf1	phenylalanine--tRNA ligase beta subunit [Ostrinia furnacalis]	-0.84789	1.589537	0.658171	-1.11314	-0.28667
TRINITY_DN14701_c0_g1_i2_orf1	staphylococcal nuclease domain-containing protein 1 [Ostrinia furnacalis]	-1.27217	1.573558	0.449487	-0.83454	0.083667
TRINITY_DN18249_c0_g1_i1_orf1	60S ribosomal protein L13 [Pectinophora gossypiella]	-0.70612	1.527846	0.713513	-1.25726	-0.27797
TRINITY_DN25960_c0_g1_i1_orf1	protein mini spindles [Ostrinia furnacalis]	-1.09175	1.577699	0.324548	-1.07052	0.260019
TRINITY_DN5382_c0_g2_i1_orf1	protein seele [Ostrinia furnacalis]	-1.21673	1.512193	0.282399	0.413255	-0.99112
TRINITY_DN1697_c0_g1_i1_orf1	mitogen-activated protein kinase-binding protein 1 [Ostrinia furnacalis]	-1.41098	1.57083	0.272664	-0.64854	0.216029
TRINITY_DN34347_c0_g1_i1_orf1	nesprin-1-like isoform X8 [Bombyx mandarina]	-1.29474	1.360564	0.905789	-0.78585	-0.18577
TRINITY_DN136906_c0_g1_i1_orf1	translational elongation factor-1alpha, partial [Ethmia eupostica]	-1.42084	1.491267	0.677518	-0.4704	-0.27754
TRINITY_DN36928_c0_g1_i2_orf1	actin-interacting protein 1 isoform X2 [Ostrinia furnacalis]	-0.53853	1.533631	0.717675	-1.29004	-0.42274
TRINITY_DN937_c0_g1_i2_orf1	protein brunelleschi [Ostrinia furnacalis]	-0.77512	1.679607	0.606188	-0.94185	-0.56882
TRINITY_DN48638_c0_g1_i5_orf1	NADH dehydrogenase [ubiquinone] flavoprotein 2, mitochondrial [Ostrinia furnacalis] >ALD03682.1 mitochondrial complex I NDUFV2 subunit [Ostrinia nubilalis]	-0.95183	1.733864	0.38225	-0.21893	-0.94535
TRINITY_DN8908_c0_g1_i1_orf1	unnamed protein product [Spodoptera littoralis] >CAH1641822.1 unnamed protein product [Spodoptera littoralis]	-1.01641	1.754678	0.439899	-0.57384	-0.60433
TRINITY_DN5218_c0_g1_i4_orf1	threonine--tRNA ligase, cytoplasmic isoform X1 [Trichoplusia ni]	-1.44284	1.685201	0.111845	-0.21635	-0.13786
TRINITY_DN34399_c0_g1_i1_orf1	cysteine synthase-like [Ostrinia furnacalis]	-1.38389	1.483563	0.590036	-0.7308	0.041089
TRINITY_DN78492_c0_g1_i1_orf1	uncharacterized protein LOC114354775 [Ostrinia furnacalis]	-1.26612	1.416036	0.250968	-0.99052	0.589636
TRINITY_DN9506_c0_g1_i2_orf1	glutathione S-transferase sigma 4 [Conogethes punctiferalis]	-1.6444	1.221177	0.284889	0.666637	-0.5283
TRINITY_DN10785_c0_g1_i4_orf1	alkylglycerol monooxygenase-like [Ostrinia furnacalis] >XP_028171363.1 alkylglycerol monooxygenase-like [Ostrinia furnacalis]	-1.16197	1.761294	0.293974	-0.62308	-0.27022
TRINITY_DN126648_c0_g1_i1_orf1	elongation factor 1 alpha, partial [Spodoptera exigua] >QYQ52647.1 elongation factor 1 alpha, partial [Spodoptera exigua]	-0.84503	1.488137	0.377259	-1.34937	0.329006
TRINITY_DN15234_c0_g1_i3_orf1	60S ribosomal protein L30 [Papilio polytes] >XP_014360326.1 60S ribosomal protein L30 [Papilio machaon] >XP_026485186.1 60S ribosomal protein L30 isoform X1 [Vanessa tameamea] >XP_028160279.1 60S ribosomal protein L30 [Ostrinia furnacalis] >XP_030027999.1 60S ribosomal protein L30 [Manduca sexta] >XP_032515151.1 60S ribosomal protein L30 [Danaus plexippus plexippus] >XP_034840952.1 60S ribosomal protein L30 [Maniola hyperantus] >XP_037301873.1 60S ribosomal protein L30 [Manduca sexta] >XP_039745408.1 60S ribosomal protein L30 [Pararge aegeria] >XP_041974708.1 60S ribosomal protein L30 [Aricia agestis] >XP_045455248.1 60S ribosomal protein L30 [Melitaea cinxia] >XP_045457914.1 60S ribosomal protein L30 [Melitaea cinxia] >XP_046969892.1 60S ribosomal protein L30 [Vanessa cardui] >XP_047539529.1 60S ribosomal protein L30 [Vanessa atalanta] >XP_049887645.1 60S ribosomal protein L30 [Pectinophora gossypiella] >XP_050360253.1 60S ribosomal protein L30 [Nymphalis io] >ADT80684.1 ribosomal protein L30 [Euphydryas aurinia] >CAG9575798.1 unnamed protein product [Danaus chrysippus] >CAH0722581.1 unnamed protein product, partial [Brenthis ino] >CAH2099946.1 unnamed protein product [Euphydryas editha] >CAH2267204.1 jg2932 [Pararge aegeria aegeria] >GBP56353.1 60S ribosomal protein L30 [Eumeta japonica]	-1.37761	1.705374	0.139958	-0.41402	-0.0537
TRINITY_DN11050_c0_g1_i8_orf1	uncharacterized protein LOC114360965, partial [Ostrinia furnacalis]	-1.51215	1.515993	-0.12629	-0.38133	0.503782
TRINITY_DN7335_c0_g1_i1_orf1	probable methylmalonate-semialdehyde dehydrogenase [acylating], mitochondrial [Bicyclus anynana]	-1.42102	1.669091	0.036568	0.134249	-0.41888
TRINITY_DN2224_c0_g1_i1_orf1	serine--tRNA ligase, cytoplasmic [Ostrinia furnacalis]	-1.57016	1.4605	0.511975	-0.37218	-0.03014
TRINITY_DN5765_c0_g2_i3_orf1	unnamed protein product [Diatraea saccharalis]	-1.35475	1.683753	0.293066	-0.46956	-0.15251

TRINITY_DN56270_c0_g1_i1_orf1	PREDICTED: putative elongator complex protein 1 [Microplitis demolitor] >XP_008554512.1 PREDICTED: putative elongator complex protein 1 [Microplitis demolitor]	-1.20952	1.208119	0.731087	0.434155	-1.16384
TRINITY_DN5753_c0_g1_i10_orf1	ryanodine receptor [Ostrinia furnacalis]	-1.56902	1.469286	0.058453	-0.41244	0.453717
TRINITY_DN15811_c0_g1_i7_orf1	mitochondrial import inner membrane translocase subunit Tim10-like [Ostrinia furnacalis] >XP_028174557.1 mitochondrial import inner membrane translocase subunit Tim10 [Ostrinia furnacalis] >XP_028174558.1 mitochondrial import inner membrane translocase subunit Tim10 [Ostrinia furnacalis] >XP_028174559.1 mitochondrial import inner membrane translocase subunit Tim10 [Ostrinia furnacalis]	-1.14466	1.645911	0.559265	-0.30047	-0.76004
TRINITY_DN81312_c0_g1_i1_orf1	atlastin isoform X4 [Ostrinia furnacalis]	-0.82951	1.614051	0.455723	-1.22426	-0.016
TRINITY_DN36893_c0_g1_i1_orf1	40S ribosomal protein S15 [Bicyclus anynana] >XP_026325996.1 40S ribosomal protein S15 [Hyposmocoma kahamanoa] >XP_028175376.1 40S ribosomal protein S15 [Ostrinia furnacalis] >XP_030034906.1 40S ribosomal protein S15 [Manduca sexta] >XP_039758445.1 40S ribosomal protein S15 [Pararge aegeria] >XP_045775675.1 40S ribosomal protein S15 [Maniola jurtina] >CAH2267288.1 jg14755 [Pararge aegeria aegeria] >ACY95351.1 ribosomal protein S15 [Manduca sexta] >KAG6461386.1 hypothetical protein O3G_MSEX012590 [Manduca sexta] >KAG6461387.1 hypothetical protein O3G_MSEX012590 [Manduca sexta]	-0.73593	1.596322	0.743881	-0.98916	-0.61511
TRINITY_DN1665_c1_g1_i2_orf1	translation elongation factor 2 [Melitaea cinxia]	-0.86346	1.643293	0.56923	-1.07454	-0.27452
TRINITY_DN348_c0_g2_i1_orf1	pancreatic triacylglycerol lipase-like [Ostrinia furnacalis]	-1.53256	1.611186	-0.14948	-0.08806	0.158909
TRINITY_DN130159_c0_g2_i1_orf1	lachesin-like [Chelonus insularis] >XP_034946935.1 lachesin-like [Chelonus insularis]	-1.47105	1.542543	0.443833	-0.50945	-0.00587
TRINITY_DN2577_c0_g1_i1_orf1	unnamed protein product [Diatraea saccharalis]	-0.76773	1.558247	0.468234	-1.32615	0.0674
TRINITY_DN143852_c0_g1_i1_orf1	60S ribosomal protein L10 [Cotesia glomerata]	-1.01306	1.492727	0.761228	-1.06548	-0.17542
TRINITY_DN5873_c0_g4_i1_orf1	hypothetical protein evm_003048 [Chilo suppressalis]	-1.03461	1.692544	0.38956	-0.95065	-0.09684
TRINITY_DN29521_c0_g1_i1_orf1	density-regulated protein homolog [Ostrinia furnacalis]	-0.77925	1.52673	0.626258	-1.2894	-0.08434
TRINITY_DN13233_c0_g1_i3_orf1	60S ribosomal protein L31 [Galleria mellonella] >XP_028158009.1 60S ribosomal protein L31 [Ostrinia furnacalis] >XP_030037192.1 60S ribosomal protein L31 [Manduca sexta] >XP_046978528.1 60S ribosomal protein L31 [Vanessa cardui] >XP_047545474.1 60S ribosomal protein L31 [Vanessa atalanta] >XP_050342244.1 60S ribosomal protein L31 [Nymphalis io] >GBP35474.1 60S ribosomal protein L31 [Eumeta japonica] >ACY95330.1 ribosomal protein L31 [Manduca sexta] >KAG6463984.1 hypothetical protein O3G_MSEX014198 [Manduca sexta] >KAG6463985.1 hypothetical protein O3G_MSEX014198 [Manduca sexta]	-1.00127	1.73542	0.465581	-0.75693	-0.4428
TRINITY_DN147676_c0_g1_i1_orf1	PREDICTED: 60S ribosomal protein L23 [Microplitis demolitor] >XP_044591174.1 60S ribosomal protein L23 [Cotesia glomerata] >KAG8035666.1 hypothetical protein G9C98_001094 [Cotesia typhae] >KAH0547433.1 60S ribosomal protein L23A [Cotesia glomerata]	-1.29281	1.544897	0.511122	-0.82288	0.059672
TRINITY_DN95414_c0_g1_i1_orf1	protein arginine N-methyltransferase 5 [Ostrinia furnacalis]	-1.04176	1.612768	0.477902	-1.04176	-0.00715
TRINITY_DN12806_c0_g2_i1_orf1	inactive pancreatic lipase-related protein 1-like isoform X2 [Ostrinia furnacalis]	-0.91151	1.598521	0.638342	-1.06772	-0.25763
TRINITY_DN42373_c0_g4_i1_orf1	unnamed protein product [Spodoptera exigua]	-0.95641	1.530804	0.719567	-1.08685	-0.20711
TRINITY_DN4385_c0_g2_i1_orf1	LOW QUALITY PROTEIN: carbonic anhydrase 1-like [Ostrinia furnacalis]	-1.3891	1.509474	0.568184	-0.68487	-0.00369
TRINITY_DN3985_c0_g2_i1_orf1	hypothetical protein evm_012077 [Chilo suppressalis] >CAB3529218.1 unnamed protein product [Chilo suppressalis] >CAH0405810.1 unnamed protein product [Chilo suppressalis]	-0.94923	1.825013	0.269368	-0.71409	-0.43107
TRINITY_DN13371_c0_g1_i4_orf1	ATP synthase mitochondrial F1 complex assembly factor 2 [Ostrinia furnacalis]	-1.24253	1.789882	0.136114	-0.35491	-0.32855
TRINITY_DN211_c1_g1_i10_orf1	protein hu-li tai shao isoform X5 [Galleria mellonella]	-1.03426	1.777981	0.251996	-0.82179	-0.17393
TRINITY_DN14498_c0_g1_i1_orf1	eukaryotic translation initiation factor 2 subunit 2 [Ostrinia furnacalis]	-1.02763	1.759721	0.380763	-0.76033	-0.35252
TRINITY_DN2682_c0_g1_i4_orf1	40S ribosomal protein S5 [Manduca sexta] >ACY95347.1 ribosomal protein S5 [Manduca sexta] >KAG6447616.1 hypothetical protein O3G_MSEX005033 [Manduca sexta] >KAG6447617.1 hypothetical protein O3G_MSEX005033 [Manduca sexta]	-0.79078	1.683287	0.526969	-1.06755	-0.35193
TRINITY_DN11065_c0_g2_i1_orf1	ribosomal protein s6e domain-containing protein [Phthorimaea operculella]	-0.97932	1.664185	0.470034	-1.01539	-0.1395
TRINITY_DN29229_c0_g1_i4_orf1	uncharacterized protein LOC114351433 isoform X1 [Ostrinia furnacalis]	-1.17083	1.55509	0.738208	-0.69528	-0.42719
TRINITY_DN7414_c0_g1_i1_orf1	uncharacterized protein LOC114357447 [Ostrinia furnacalis]	-1.28517	1.651577	0.254429	-0.73656	0.115717
TRINITY_DN7241_c0_g2_i2_orf1	40S ribosomal protein S10 [Zerene cesonia] >XP_045492164.1 40S ribosomal protein S10 [Colias croceus]	-0.74128	1.77353	0.424425	-0.90723	-0.54944
TRINITY_DN1752_c0_g1_i18_orf1	titin isoform X1 [Ostrinia furnacalis]	-1.57231	1.552012	0.13451	-0.27446	0.16025
TRINITY_DN2954_c0_g1_i1_orf1	unnamed protein product [Diatraea saccharalis]	-0.68125	1.797884	0.253421	-1.07282	-0.29724
TRINITY_DN4469_c0_g1_i2_orf1	metal transporter CNNM4-like [Ostrinia furnacalis]	-0.84112	1.777983	0.280063	-1.00368	-0.21324
TRINITY_DN9248_c0_g1_i10_orf1	unnamed protein product [Arctia plantaginis]	-1.43781	1.657246	0.241015	-0.33542	-0.12502
TRINITY_DN69697_c0_g1_i1_orf1	PREDICTED: uncharacterized protein LOC103573287 [Microplitis demolitor]	-1.08088	1.745783	0.143943	0.062537	-0.87138

TRINITY_DN24318_c0_g1_i1_orf1	60S ribosomal protein L29 [Ostrinia furnacalis]	-0.78424	1.638541	0.46623	-1.21293	-0.1076
TRINITY_DN7991_c0_g1_i9_orf1	hypothetical protein evm_006720 [Chilo suppressalis] >CAB3528247.1 unnamed protein product [Chilo suppressalis] >CAH0404834.1 unnamed protein product [Chilo suppressalis]	-0.85606	1.851699	0.152587	-0.85329	-0.29494
TRINITY_DN9965_c0_g1_i1_orf1	dihydrolipoyl dehydrogenase [Ostrinia furnacalis]	-1.26585	1.80322	-0.00908	-0.32066	-0.20763
TRINITY_DN642_c0_g1_i6_orf1	reticulon-3-B isoform X5 [Ostrinia furnacalis]	-0.81085	1.828869	0.201501	-0.93652	-0.283
TRINITY_DN15160_c0_g1_i1_orf1	tyrosine--tRNA ligase, cytoplasmic [Ostrinia furnacalis]	-1.20706	1.442352	-0.00538	0.73244	-0.96235
TRINITY_DN15737_c0_g1_i7_orf1	UPF0160 protein C27H6.8 [Ostrinia furnacalis]	-0.76283	1.659873	0.402171	-1.22284	-0.07637
TRINITY_DN23004_c0_g1_i1_orf1	uncharacterized protein LOC114365313 [Ostrinia furnacalis]	-0.84454	1.777012	0.138943	-1.05326	-0.01816
TRINITY_DN4008_c0_g1_i7_orf1	nuclear export mediator factor NEMF homolog isoform X1 [Ostrinia furnacalis]	-0.73531	1.781324	0.38607	-0.95239	-0.47969
TRINITY_DN5031_c0_g1_i1_orf1	PREDICTED: 40S ribosomal protein S12 [Trachymyrmex septentrionalis]	-0.89764	1.770552	0.305666	-0.95768	-0.22089
TRINITY_DN1173_c0_g1_i12_orf1	obscurin [Ostrinia furnacalis]	-1.40579	1.630416	0.076015	-0.54689	0.246245
TRINITY_DN7267_c1_g1_i4_orf1	probable pseudouridine-5'-phosphatase [Ostrinia furnacalis]	-1.31582	1.727576	-0.02671	-0.51599	0.130941
TRINITY_DN8824_c0_g2_i1_orf1	60S ribosomal protein L34-like [Ostrinia furnacalis]	-0.70005	1.763305	0.179784	-1.16732	-0.07572
TRINITY_DN10831_c1_g1_i1_orf1	40S ribosomal protein S16 [Ostrinia furnacalis]	-1.04425	1.715194	0.416861	-0.86188	-0.22593
TRINITY_DN82324_c0_g1_i4_orf1	hypothetical protein evm_001824 [Chilo suppressalis] >CAG9754426.1 unnamed protein product [Diatraea saccharalis] >CAG9793111.1 unnamed protein product [Diatraea saccharalis]	-0.69976	1.811481	0.314297	-0.95105	-0.47496
TRINITY_DN44261_c0_g1_i1_orf1	neural Wiskott-Aldrich syndrome protein-like [Colias croceus]	-1.28225	1.737398	0.063021	-0.57457	0.056403
TRINITY_DN5009_c0_g1_i2_orf1	GSCOCG00009487001-RA-CDS [Cotesia congregata] >CAG5088842.1 Similar to RpL18: 60S ribosomal protein L18 (Timarcha balearica) [Cotesia congregata]	-0.62917	1.708984	0.424737	-1.18314	-0.32142
TRINITY_DN23740_c1_g1_i1_orf1	NADH dehydrogenase [ubiquinone] iron-sulfur protein 6, mitochondrial isoform X1 [Ostrinia furnacalis]	-1.4821	1.445005	0.129257	0.543148	-0.63531
TRINITY_DN271_c0_g2_i6_orf1	hypothetical protein NE865_03378 [Phthorimaea operculella]	-0.71745	1.854464	0.261006	-0.71992	-0.6781
TRINITY_DN31253_c0_g1_i2_orf1	hypothetical protein evm_009655 [Chilo suppressalis]	-1.10986	1.834114	0.087934	-0.58868	-0.2235
TRINITY_DN1791_c0_g1_i3_orf1	succinate dehydrogenase assembly factor 2-B, mitochondrial-like [Ostrinia furnacalis]	-0.89252	1.703931	0.339757	-1.08646	-0.0647
TRINITY_DN3760_c0_g1_i1_orf1	something about silencing protein 10 [Ostrinia furnacalis]	-1.2395	1.774454	-0.30486	0.197633	-0.42772
TRINITY_DN11297_c0_g1_i1_orf1	ribosomal protein L13 [Conogethes punctiferalis] >QEE82690.1 ribosomal protein L13 [Conogethes pinicolalis]	-0.97759	1.834	0.046344	-0.81956	-0.08319
TRINITY_DN14996_c0_g1_i2_orf1	40S ribosomal protein S17 [Ostrinia furnacalis]	-1.09609	1.823644	0.016547	-0.68494	-0.05916
TRINITY_DN18869_c0_g1_i1_orf1	unnamed protein product [Parnassius apollo]	-1.10361	1.847531	0.057395	-0.54953	-0.25179
TRINITY_DN135_c0_g1_i1_orf1	60S ribosomal protein L11 [Nymphalis io]	-1.45396	1.691973	-0.05375	-0.13305	-0.05121
TRINITY_DN7464_c0_g1_i14_orf1	60S ribosomal protein L9 [Nymphalis io]	-1.05558	1.766885	0.289503	-0.79977	-0.20103
TRINITY_DN13496_c0_g1_i7_orf1	nucleolar protein 58 [Ostrinia furnacalis]	-0.79708	1.894731	0.119545	-0.70756	-0.50964
TRINITY_DN40650_c0_g1_i1_orf1	60S ribosomal protein L12 [Zerene cesonia]	-0.89694	1.793895	0.253497	-0.92978	-0.22068
TRINITY_DN9862_c0_g2_i1_orf1	40S ribosomal protein S4 [Manduca sexta] >ACY95325.1 ribosomal protein S4 [Manduca sexta] >KAG6465430.1 hypothetical protein O3G_MSEX015149 [Manduca sexta]	-0.83477	1.806747	0.318798	-0.87357	-0.4172

TRINITY_DN1509_c0_g1_i1_orf1	ribosomal protein S15A [Bombyx mori] >XP_011566807.1 40S ribosomal protein S15Aa [Plutella xylostella] >XP_013186470.1 PREDICTED: 40S ribosomal protein S15Aa [Amyelois transitella] >XP_021181353.1 40S ribosomal protein S15Aa [Helicoverpa armigera] >XP_022114309.1 40S ribosomal protein S15Aa [Pieris rapae] >XP_022820057.1 40S ribosomal protein S15Aa [Spodoptera litura] >XP_023947206.1 40S ribosomal protein S15Aa [Bicyclus anynana] >XP_026329660.1 40S ribosomal protein S15Aa [Hyposmocoma kahamanoa] >XP_026495126.1 40S ribosomal protein S15Aa [Vanessa tameamea] >XP_026745810.1 40S ribosomal protein S15Aa [Trichoplusia ni] >XP_026757934.1 40S ribosomal protein S15Aa [Galleria mellonella] >XP_028041686.1 40S ribosomal protein S15Aa [Bombyx mandarina] >XP_028161999.1 40S ribosomal protein S15Aa [Ostrinia furnacalis] >XP_028162000.1 40S ribosomal protein S15Aa [Ostrinia furnacalis] >XP_030024299.1 40S ribosomal protein S15Aa [Manduca sexta] >XP_032514052.1 40S ribosomal protein S15Aa [Danaus plexippus plexippus] >XP_034834075.1 40S ribosomal protein S15Aa [Maniola hyperantus] >XP_034840269.1 40S ribosomal protein S15Aa [Maniola hyperantus] >XP_035436795.1 40S ribosomal protein S15Aa [Spodoptera frugiperda] >XP_037869057.1 ribosomal protein S15A isoform X1 [Bombyx mori] >XP_039747368.1 40S ribosomal protein S15Aa [Pararge aegeria] >XP_045445511.1 40S ribosomal protein S15Aa [Melitaea cinxia] >XP_045491780.1 40S ribosomal protein S15Aa [Colias croceus] >XP_045519936.1 40S ribosomal protein S15Aa [Pieris brassicae] >XP_045785214.1 40S ribosomal protein S15Aa [Maniola jurtina] >XP_046965230.1 40S ribosomal protein S15Aa [Vanessa cardui] >XP_046965231.1 40S ribosomal protein S15Aa [Vanessa cardui] >XP_047021729.1 40S ribosomal protein S15Aa [Helicoverpa zea] >XP_047509814.1 40S ribosomal protein S15Aa [Pieris napi] >XP_047527633.1 40S ribosomal protein S15Aa [Vanessa atalanta] >XP_047527634.1 40S ribosomal protein S15Aa [Vanessa atalanta] >XP_047988443.1 40S ribosomal protein S15Aa [Leguminivora glycinivorella] >XP_050344874.1 40S ribosomal protein S15Aa [Nymphalis io] >XP_050344875.1 40S ribosomal protein S15Aa [Nymphalis io] >ADP21467.1 ribosomal protein S15A [Antheraea yamamai] >ADT80666.1 ribosomal protein S15A [Euphydryas aurinia] >AEL28847.1 ribosomal protein S15A [Heliconius melpomene cythera] >KAF9418418.1 hypothetical protein HW555_004706 [Spodoptera exigua] >KAI5633077.1 ribosomal protein s8 domain-containing protein [Phthorimaea operculella] >KOB75105.1 Ribosomal protein S15A, partial [Operophtera brumata] >CAB3516222.1 unnamed protein product [Spodoptera littoralis] >CAF4772676.1 unnamed protein product [Pieris macdunnoughi] >CAG9585503.1 unnamed protein product [Danaus chrysippus] >CAG9745029.1 unnamed protein product [Diatraea saccharalis] >CAH0604658.1 unnamed protein product [Chrysodeixis includens] >CAH2086517.1 unnamed protein product [Euphydryas editha] >CAH2990731.1 unnamed protein product [Chilo suppressalis]	-0.96957	1.867661	-0.01229	-0.74232	-0.14348
TRINITY_DN38562_c0_g1_i3_orf1	persulfide dioxygenase ETHE1, mitochondrial isoform X1 [Ostrinia furnacalis]	-1.51066	1.447818	-0.6071	0.454895	0.21504
TRINITY_DN29156_c0_g1_i1_orf1	protein FAM136A [Ostrinia furnacalis]	-0.84257	1.919504	0.007175	-0.63661	-0.4475
TRINITY_DN7808_c0_g1_i1_orf1	probable pyruvate dehydrogenase E1 component subunit alpha, mitochondrial isoform X1 [Ostrinia furnacalis] >XP_028158738.1 probable pyruvate dehydrogenase E1 component subunit alpha, mitochondrial isoform X2 [Ostrinia furnacalis] >XP_028158739.1 probable pyruvate dehydrogenase E1 component subunit alpha, mitochondrial isoform X3 [Ostrinia furnacalis] >XP_028158740.1 probable pyruvate dehydrogenase E1 component subunit alpha, mitochondrial isoform X4 [Ostrinia furnacalis]	-1.47604	1.665586	-0.19602	-0.06268	0.069155
TRINITY_DN8949_c0_g1_i2_orf1	unnamed protein product [Arctia plantaginis]	-0.61842	1.815942	0.140559	-1.119	-0.21908
TRINITY_DN5857_c0_g1_i13_orf1	uncharacterized protein LOC114353981 isoform X1 [Ostrinia furnacalis]	-0.88097	1.864154	0.068571	-0.83492	-0.21683
TRINITY_DN22956_c0_g1_i1_orf1	lipoamide acyltransferase component of branched-chain alpha-keto acid dehydrogenase complex, mitochondrial [Ostrinia furnacalis]	-1.54244	1.518321	-0.33384	-0.08567	0.443625

TRINITY_DN97589_c0_g1_i3_orf1	ribosomal protein L37A [Bombyx mori] >XP_013189707.1 PREDICTED: 60S ribosomal protein L37a [Amyelois transitella] >XP_021198447.1 60S ribosomal protein L37a [Helicoverpa armigera] >XP_022122377.1 60S ribosomal protein L37a [Pieris rapae] >XP_022822835.1 60S ribosomal protein L37a [Spodoptera litura] >XP_023937141.1 60S ribosomal protein L37a [Bicyclus anynana] >XP_026321523.1 60S ribosomal protein L37a [Hyposmocoma kahamanoa] >XP_026495655.1 60S ribosomal protein L37a [Vanessa tameamea] >XP_026746489.1 60S ribosomal protein L37a [Trichoplusia ni] >XP_026756267.1 60S ribosomal protein L37a [Galleria mellonella] >XP_028041705.1 60S ribosomal protein L37a [Bombyx mandarina] >XP_028161757.1 60S ribosomal protein L37a [Ostrinia furnacalis] >XP_030020263.1 LOW QUALITY PROTEIN: 60S ribosomal protein L37a [Manduca sexta] >XP_032518929.1 60S ribosomal protein L37a [Danaus plexippus plexippus] >XP_034834514.1 60S ribosomal protein L37a [Maniola hyperantus] >XP_035444256.1 60S ribosomal protein L37a [Spodoptera frugiperda] >XP_038222439.1 60S ribosomal protein L37a [Zerene cesonia] >XP_039756348.1 60S ribosomal protein L37a [Pararge aegeria] >XP_041981914.1 60S ribosomal protein L37a [Aricia agestis] >XP_045451710.1 60S ribosomal protein L37a [Melitaea cinxia] >XP_045500579.1 60S ribosomal protein L37a [Colias croceus] >XP_045517305.1 60S ribosomal protein L37a [Pieris brassicae] >XP_045775103.1 60S ribosomal protein L37a [Maniola jurtina] >XP_046969745.1 60S ribosomal protein L37a [Vanessa cardui] >XP_047032252.1 60S ribosomal protein L37a [Helicoverpa zea] >XP_047525321.1 60S ribosomal protein L37a [Pieris napi] >XP_047535357.1 60S ribosomal protein L37a [Vanessa atalanta] >XP_049875744.1 60S ribosomal protein L37a [Pectinophora gossypiella] >XP_050348149.1 60S ribosomal protein L37a [Nymphalis io] >ADO95156.1 ribosomal protein L37A [Antheraea yamamai] >ADT80705.1 ribosomal protein L37A [Euphydryas aurinia] >AEL28885.1 ribosomal protein L37A [Heliconius melpomene cythera] >KAF9418899.1 hypothetical protein HW555_004419 [Spodoptera exigua] >KOB75009.1 Ribosomal protein L37A [Operophtera brumata] >RVE49828.1 hypothetical protein evm_005558 [Chilo suppressalis] >CAB3234150.1 unnamed protein product [Arctia plantaginis] >CAB3509616.1 unnamed protein product [Spodoptera littoralis] >CAF4811073.1 unnamed protein product [Pieris macdunnoughi] >CAG4956733.1 unnamed protein product [Parnassius apollo] >CAG9564640.1 unnamed protein product [Danaus chrysippus] >CAG9750098.1 unnamed protein product [Diatraea saccharalis] >CAH0725676.1 unnamed protein	-1.17543	1.659064	0.310792	-0.87351	0.079085
TRINITY_DN1445_c0_g2_i4_orf1	leucine-rich PPR motif-containing protein, mitochondrial [Ostrinia furnacalis]	-1.47374	1.669088	0.01793	-0.00873	-0.20455
TRINITY_DN4451_c0_g2_i4_orf1	uncharacterized protein LOC114361986 isoform X1 [Ostrinia furnacalis] >XP_028173022.1 uncharacterized protein LOC114361986 isoform X2 [Ostrinia furnacalis]	-1.43708	1.630471	0.14136	0.149126	-0.48388
TRINITY_DN42646_c0_g2_i1_orf1	40S ribosomal protein S3 [Helicoverpa armigera] >XP_026740562.1 40S ribosomal protein S3 [Trichoplusia ni] >XP_026751545.1 40S ribosomal protein S3 [Galleria mellonella] >XP_047027704.1 40S ribosomal protein S3 [Helicoverpa zea] >CAH0591481.1 unnamed protein product [Chrysodeixis includens] >AIR07416.1 ribosomal protein S3 [Helicoverpa armigera] >AND95944.1 ribosomal protein S3 [Helicoverpa armigera] >AXY94820.1 ribosomal ribosomal protein S3 [Galleria mellonella] >PZC80336.1 hypothetical protein B5X24_HaOG214853 [Helicoverpa armigera]	-0.92257	1.809772	0.180307	-0.90206	-0.16545
TRINITY_DN137_c0_g1_i1_orf1	60S ribosomal protein L8 [Cotesia glomerata] >XP_044597650.1 60S ribosomal protein L8 [Cotesia glomerata] >KAG8034499.1 hypothetical protein G9C98_007575 [Cotesia typhae] >CAD6216378.1 GSCOCG00004534001-RA-CDS [Cotesia congregata] >KAH0544237.1 60S ribosomal protein L8 [Cotesia glomerata] >KAH0564528.1 60S ribosomal protein L8 [Cotesia glomerata] >CAG5095185.1 Similar to RpL8: 60S ribosomal protein L8 (Spodoptera frugiperda) [Cotesia congregata]	-0.58898	1.798233	0.35974	-0.95652	-0.61247
TRINITY_DN42082_c0_g2_i2_orfp1	TRINITY_DN42082_c0_g2_i2_m.7835 TRINITY_DN42082_c0_g2::TRINITY_DN42082_c0_g2_i2::g.7835 ORF type:internal len:133 (+),score=75.81 TRINITY_DN42082_c0_g2_i2:1-396(+)	-0.62243	1.803523	0.228572	-1.1011	-0.30856
TRINITY_DN11194_c0_g1_i4_orf1	ATPase family AAA domain-containing protein 3A homolog [Ostrinia furnacalis]	-1.36186	1.707417	-0.05414	-0.44957	0.158157
TRINITY_DN33_c0_g1_i1_orf1	uncharacterized protein CG45076-like isoform X2 [Ostrinia furnacalis]	-0.75248	1.806669	0.095201	-1.07475	-0.07464
TRINITY_DN3299_c0_g1_i2_orf1	metaxin-1 isoform X3 [Ostrinia furnacalis] >XP_028170907.1 metaxin-1 isoform X4 [Ostrinia furnacalis]	-0.51566	1.877829	0.106484	-0.49138	-0.97727
TRINITY_DN9117_c0_g1_i1_orf1	spherulin-2A-like [Ostrinia furnacalis]	-1.3126	1.785551	-0.09454	-0.12461	-0.2538
TRINITY_DN44557_c0_g1_i4_orf1	serine hydrolase-like protein [Ostrinia furnacalis]	-0.91526	1.809617	0.229803	-0.88059	-0.24356
TRINITY_DN14597_c0_g1_i5_orf1	UDP-glucuronosyltransferase 2B1-like isoform X3 [Ostrinia furnacalis]	-1.40932	1.576022	-0.23103	-0.45492	0.519259
TRINITY_DN106534_c0_g1_i1_orf1	nucleolar complex protein 2 homolog [Ostrinia furnacalis]	-1.4871	1.427802	0.505942	-0.66728	0.220636
TRINITY_DN21357_c0_g1_i5_orf1	40S ribosomal protein S11 isoform X2 [Ostrinia furnacalis]	-0.78524	1.826766	0.126302	-1.00132	-0.1665
TRINITY_DN4622_c0_g1_i1_orf1	keratin-associated protein 19-2-like [Ostrinia furnacalis]	-1.44174	1.666368	0.20873	-0.15691	-0.27645
TRINITY_DN2497_c0_g1_i2_orf1	protein stunted-like isoform X1 [Colias croceus]	-1.04956	1.745109	0.342922	-0.20602	-0.83246

TRINITY_DN53462_c0_g1_i1_orf1	uncharacterized protein LOC118072968 isoform X1 [Chelonus insularis] >XP_034949073.1 uncharacterized protein LOC118072968 isoform X1 [Chelonus insularis]	-1.23373	1.406599	0.888019	-0.80254	-0.25835
TRINITY_DN20_c0_g1_i11_orf1	plasma membrane calcium-transporting ATPase 2 [Ostrinia furnacalis]	-0.90548	1.922583	-0.04005	-0.5378	-0.43925
TRINITY_DN48619_c0_g1_i1_orf1	PREDICTED: lysine--tRNA ligase isoform X2 [Fopius arisanus]	-1.03907	1.73373	0.311674	-0.89752	-0.10882
TRINITY_DN84357_c0_g1_i1_orf1	4-coumarate--CoA ligase 1-like [Ostrinia furnacalis]	-1.30077	1.395442	0.729618	0.082157	-0.90645
TRINITY_DN3461_c0_g1_i1_orf1	protein SCO1 homolog, mitochondrial [Ostrinia furnacalis]	-1.40517	1.718594	-0.12822	-0.23107	0.045863
TRINITY_DN4514_c0_g1_i1_orf1	enoyl-CoA delta isomerase 1, mitochondrial-like isoform X1 [Ostrinia furnacalis] >XP_028158560.1 enoyl-CoA delta isomerase 1, mitochondrial-like isoform X2 [Ostrinia furnacalis]	-1.29469	1.552625	0.531185	0.005206	-0.79433
TRINITY_DN4938_c0_g1_i13_orf1	peroxisomal biogenesis factor 19 [Ostrinia furnacalis]	-0.97127	1.82356	0.284259	-0.5204	-0.61615
TRINITY_DN23167_c0_g1_i4_orf1	uncharacterized protein LOC114363065 [Ostrinia furnacalis]	-1.32769	1.540209	0.649994	-0.61907	-0.24345
TRINITY_DN9002_c0_g1_i1_orf1	membrane magnesium transporter 1 [Ostrinia furnacalis]	-0.53017	1.892763	0.078525	-0.93479	-0.50633
TRINITY_DN4121_c0_g1_i1_orf1	uncharacterized protein LOC114358001, partial [Ostrinia furnacalis]	-0.87462	1.909519	0.032684	-0.62825	-0.43934
TRINITY_DN2748_c0_g1_i6_orf1	uncharacterized protein LOC114352811 [Ostrinia furnacalis]	-0.99077	1.853981	0.010848	-0.12157	-0.75249
TRINITY_DN58207_c0_g1_i1_orf1	60S ribosomal protein L6 [Ostrinia furnacalis] >XP_028170357.1 60S ribosomal protein L6 [Ostrinia furnacalis]	-0.95206	1.824443	0.192417	-0.81619	-0.24861
TRINITY_DN18396_c0_g1_i1_orf1	uncharacterized protein LOC114359424 [Ostrinia furnacalis]	-1.51312	1.44257	0.625465	-0.48273	-0.07218
TRINITY_DN30131_c0_g1_i1_orf1	PREDICTED: 60S ribosomal protein L44 [Amyelois transitella] >XP_021198018.1 60S ribosomal protein L44 [Helicoverpa armigera] >XP_022814294.1 60S ribosomal protein L44 [Spodoptera litura] >XP_026732397.1 60S ribosomal protein L44 [Trichoplusia ni] >XP_026752106.1 60S ribosomal protein L44 [Galleria mellonella] >XP_028158932.1 60S ribosomal protein L44 [Ostrinia furnacalis] >XP_035434364.1 60S ribosomal protein L44 [Spodoptera frugiperda] >XP_035434370.1 60S ribosomal protein L44 [Spodoptera frugiperda] >XP_047019234.1 60S ribosomal protein L44 [Helicoverpa zea] >XP_049868501.1 60S ribosomal protein L44 [Pectinophora gossypiella] >AAM53948.1 ribosomal protein L44 [Choristoneura parallela] >KAF9418375.1 hypothetical protein HW555_004805 [Spodoptera exigua] >RVE50750.1 hypothetical protein evm_004660 [Chilo suppressalis] >CAB3235328.1 unnamed protein product [Arctia plantaginis] >CAB3516516.1 unnamed protein product [Spodoptera littoralis] >CAG9747186.1 unnamed protein product [Diatraea saccharalis] >CAH0581656.1 unnamed	-0.99822	1.825588	0.062215	-0.81305	-0.07653
TRINITY_DN4929_c1_g2_i5_orf1	guanylate kinase isoform X2 [Ostrinia furnacalis]	-1.20038	1.659081	0.506075	-0.68858	-0.2762
TRINITY_DN20682_c0_g1_i2_orf1	hypothetical protein B5X24_HaOG200252 [Helicoverpa armigera]	-1.28203	1.774663	0.054011	-0.4386	-0.10804
TRINITY_DN51934_c0_g2_i1_orf1	SCAN domain-containing protein 3-like [Pieris napi] >XP_047520696.1 SCAN domain-containing protein 3-like [Pieris napi]	-1.19098	1.766212	-0.1776	-0.61802	0.220391
TRINITY_DN3836_c0_g1_i4_orf1	2-oxoisovalerate dehydrogenase subunit alpha, mitochondrial [Ostrinia furnacalis]	-1.09053	1.775928	-0.20803	-0.73848	0.261117
TRINITY_DN107288_c0_g1_i2_orf1	methionine-tRNA synthetase, partial [Papilio xuthus]	-1.0387	1.68864	-0.09713	0.397156	-0.94996
TRINITY_DN35301_c0_g1_i3_orf1	ATP synthase subunit gamma, mitochondrial-like [Ostrinia furnacalis] >XP_028164649.1 ATP synthase subunit gamma, mitochondrial-like [Ostrinia furnacalis]	-0.90794	1.949408	-0.27897	-0.44014	-0.32236
TRINITY_DN107617_c3_g1_i1_orf1	NADH dehydrogenase [ubiquinone] 1 alpha subcomplex subunit 9, mitochondrial [Ostrinia furnacalis]	-0.98831	1.919952	-0.15509	-0.46396	-0.31259
TRINITY_DN14235_c0_g1_i1_orf1	maltase A1 [Helicoverpa armigera]	-0.64282	1.91931	0.057878	-0.73578	-0.59859
TRINITY_DN15513_c0_g1_i6_orf1	uncharacterized protein LOC114350859 [Ostrinia furnacalis]	-1.33514	1.632541	-0.52026	-0.24683	0.469691
TRINITY_DN2172_c0_g2_i5_orf1	4-hydroxyphenylpyruvate dioxygenase [Ostrinia furnacalis]	-1.38359	1.673129	-0.16422	-0.41727	0.291948
TRINITY_DN9647_c0_g1_i1_orf1	cytochrome P450 6B2-like [Ostrinia furnacalis]	-1.02066	1.912071	-0.2686	-0.44585	-0.17696
TRINITY_DN3053_c0_g1_i2_orf1	prostaglandin reductase 1-like [Ostrinia furnacalis]	-0.73149	1.948496	-0.1325	-0.71924	-0.36526
TRINITY_DN6351_c0_g1_i4_orf1	cytochrome P450 CYP12A2-like [Ostrinia furnacalis]	-1.10519	1.889945	-0.25804	-0.23728	-0.28944
TRINITY_DN19814_c0_g1_i4_orf1	general odorant-binding protein 28a-like [Ostrinia furnacalis]	-1.30764	1.591166	0.437309	-0.75236	0.031521
TRINITY_DN2924_c0_g1_i2_orf1	cuticular protein RR-2 [Spodoptera litura]	-1.55235	1.369739	-0.12017	-0.42033	0.723117
TRINITY_DN430_c0_g1_i5_orf1	hypothetical protein NE865_02252 [Phthorimaea operculella]	-0.54505	1.890125	-0.06272	-1.03118	-0.25118
TRINITY_DN5012_c0_g1_i6_orf1	putative serine protease K12H4.7 [Ostrinia furnacalis]	-1.2725	1.782533	-0.44481	-0.07343	0.008208
TRINITY_DN1656_c2_g1_i5_orf1	15-hydroxyprostaglandin dehydrogenase [NAD(+)]-like [Ostrinia furnacalis]	-1.32828	1.455867	-0.51829	-0.42561	0.81631
TRINITY_DN12683_c0_g1_i3_orf1	sulfated surface glycoprotein 185-like [Ostrinia furnacalis]	-0.95734	1.935049	-0.24797	-0.44013	-0.2896
TRINITY_DN96080_c0_g2_i1_orf1	ATP synthase subunit delta, mitochondrial [Ostrinia furnacalis]	-0.88746	1.942806	-0.17608	-0.33873	-0.54053
TRINITY_DN83295_c0_g1_i3_orf1	SSSX-APN4 [Ostrinia furnacalis]	-1.16198	1.861278	-0.21264	-0.13891	-0.34775
TRINITY_DN37699_c0_g1_i4_orfp1	TRINITY_DN37699_c0_g1_i4_m.58777 TRINITY_DN37699_c0_g1::TRINITY_DN37699_c0_g1_i4::g.58777 ORF type:internal len:122 (+),score=34.90 TRINITY_DN37699_c0_g1_i4:1-363(+)	-0.82117	1.725632	0.329749	-1.10573	-0.12849
TRINITY_DN2709_c0_g1_i4_orf1	ATP-dependent RNA helicase dbp2-like [Ostrinia furnacalis]	-1.34145	1.746134	-0.26973	0.119173	-0.25413

TRINITY_DN1557_c0_g1_i9_orf1	carboxylesterase CXE18 [Ostrinia furnacalis]	-0.86369	1.912177	0.022858	-0.64338	-0.42796
TRINITY_DN86844_c0_g2_i1_orf1	spermine oxidase-like isoform X1 [Ostrinia furnacalis]	-0.84742	1.933779	-0.05669	-0.44753	-0.58214
TRINITY_DN313_c0_g1_i5_orf1	collagen alpha-1(X) chain-like [Ostrinia furnacalis]	-1.31143	1.71349	0.075109	-0.57371	0.096542
TRINITY_DN3229_c0_g1_i1_orf1	uncharacterized protein LOC114358442 isoform X1 [Ostrinia furnacalis]	-1.14521	1.868537	-0.21238	-0.35898	-0.15197
TRINITY_DN8543_c0_g1_i1_orf1	39S ribosomal protein L38, mitochondrial [Ostrinia furnacalis]	-0.42322	1.874212	0.135835	-0.91741	-0.66942
TRINITY_DN2425_c0_g1_i1_orf1	thyroid receptor-interacting protein 11 [Ostrinia furnacalis]	-0.74073	1.693297	0.172922	-1.24127	0.115783
TRINITY_DN4550_c1_g1_i5_orfp2	TRINITY_DN4550_c1_g1_i5_m.14710 TRINITY_DN4550_c1_g1::TRINITY_DN4550_c1_g1_i5::g.14710 ORF type:5prime_partial len:168 (+),score=78.12 TRINITY_DN4550_c1_g1_i5:3-506(+)	-0.74985	1.934844	-0.06251	-0.73475	-0.38774
TRINITY_DN6143_c0_g2_i1_orf1	uncharacterized protein LOC114365036 [Ostrinia furnacalis]	-1.38036	1.729254	0.076204	-0.27626	-0.14884
TRINITY_DN28638_c0_g1_i1_orf1	uncharacterized protein LOC114364075 [Ostrinia furnacalis]	-1.01283	1.895258	-0.45617	-0.41713	-0.00913
TRINITY_DN31047_c0_g1_i4_orf1	4-coumarate--CoA ligase 1-like isoform X1 [Ostrinia furnacalis] >XP_028160248.1 4-coumarate--CoA ligase 1-like isoform X1 [Ostrinia furnacalis] >XP_028160249.1 4-coumarate--CoA ligase 1-like isoform X1 [Ostrinia furnacalis] >XP_028160250.1 4-coumarate--CoA ligase 1-like isoform X1 [Ostrinia furnacalis] >XP_028160251.1 4-coumarate--CoA ligase 1-like isoform X1 [Ostrinia furnacalis] >XP_028160253.1 4-coumarate--CoA ligase 1-like isoform X2 [Ostrinia furnacalis]	-0.73072	1.936423	-0.02886	-0.69565	-0.48119
TRINITY_DN24399_c0_g1_i1_orf1	retinol-binding protein pinta-like [Ostrinia furnacalis]	-0.76217	1.879224	0.145627	-0.4453	-0.81738
TRINITY_DN50787_c0_g2_i2_orf1	40S ribosomal protein S29 [Hyposmocoma kahamanoa] >XP_028176503.1 40S ribosomal protein S29 [Ostrinia furnacalis] >XP_049877832.1 40S ribosomal protein S29 [Pectinophora gossypiella] >ADT80654.1 ribosomal protein S29 [Euphydryas aurinia] >CAB3523209.1 unnamed protein product [Chilo suppressalis] >CAH0400531.1 unnamed protein product [Chilo suppressalis]	-0.4331	1.801742	0.090911	-1.22613	-0.23342
TRINITY_DN9003_c0_g1_i20_orf1	RNA-binding protein Nova-2 isoform X4 [Ostrinia furnacalis]	-0.47193	1.903539	0.053158	-0.89848	-0.58629
TRINITY_DN24873_c0_g1_i4_orf1	uncharacterized protein LOC114365742 [Ostrinia furnacalis]	-0.74574	1.941866	-0.22662	-0.76016	-0.20934
TRINITY_DN7073_c0_g1_i1_orf1	unnamed protein product, partial [Brenthis ino]	-0.67566	1.959352	-0.20459	-0.73997	-0.33913
TRINITY_DN25856_c0_g1_i1_orf1	myrosinase 1-like [Ostrinia furnacalis]	-0.74245	1.975167	-0.25558	-0.53735	-0.43979
TRINITY_DN8621_c0_g1_i5_orf1	aminopeptidase N-like isoform X2 [Ostrinia furnacalis]	-1.17932	1.852984	-0.24301	-0.10607	-0.32459
TRINITY_DN7336_c0_g1_i13_orf1	PREDICTED: calcium-transporting ATPase sarcoplasmic/endoplasmic reticulum type isoform X2 [Amyelois	-1.07845	1.870006	0.004897	-0.50508	-0.29138
TRINITY_DN32479_c0_g1_i8_orf1	hypothetical protein evm_009815 [Chilo suppressalis] >CAB3525305.1 unnamed protein product [Chilo suppressalis] >CAH0402632.1 unnamed protein product [Chilo suppressalis]	-1.28464	1.746604	-0.0078	-0.54004	0.085873
TRINITY_DN53167_c0_g1_i3_orf1	uncharacterized protein LOC114359219 [Ostrinia furnacalis]	-0.72651	1.979679	-0.28259	-0.51836	-0.45222
TRINITY_DN3929_c0_g3_i3_orf1	Glutathione S-transferase 1, isoform D [Papilio machaon]	-1.05174	1.778806	-0.31473	-0.72848	0.316145
TRINITY_DN3014_c0_g1_i4_orf1	putative inorganic phosphate cotransporter isoform X1 [Ostrinia furnacalis]	-1.22577	1.82151	-0.26541	-0.33038	4.67E-05
TRINITY_DN18773_c0_g1_i3_orf1	keratin, type II cytoskeletal 68 kDa, component IB-like [Ostrinia furnacalis]	-1.08471	1.874936	-0.0973	-0.51747	-0.17546
TRINITY_DN34040_c0_g2_i1_orf1	uncharacterized protein LOC114352849 [Ostrinia furnacalis]	-1.28735	1.760214	-0.48085	-0.07701	0.084995
TRINITY_DN22664_c0_g1_i1_orf1	larval cuticle protein LCP-14-like [Ostrinia furnacalis]	-1.2535	1.628226	0.449758	-0.75536	-0.06913
TRINITY_DN2668_c0_g1_i7_orf1	unnamed protein product [Chrysodeixis includens]	-1.15898	1.864269	-0.25808	-0.30924	-0.13797
TRINITY_DN542_c0_g1_i4_orf1	uncharacterized protein LOC114364889 [Ostrinia furnacalis]	-0.83835	1.967512	-0.36532	-0.36094	-0.40291
TRINITY_DN38498_c0_g3_i1_orf1	unnamed protein product [Parnassius apollo]	-1.42753	1.493562	0.255041	-0.71499	0.393916
TRINITY_DN1318_c0_g1_i5_orf1	uncharacterized protein LOC114360956 [Ostrinia furnacalis]	-0.77344	1.974851	-0.28486	-0.44121	-0.47534
TRINITY_DN2243_c0_g1_i4_orf1	WD repeat-containing protein 75 [Ostrinia furnacalis]	-0.6942	1.975764	-0.31448	-0.63833	-0.32875
TRINITY_DN37699_c0_g1_i3_orfp1	TRINITY_DN37699_c0_g1_i3_m.58788 TRINITY_DN37699_c0_g1::TRINITY_DN37699_c0_g1_i3::g.58788 ORF type:internal len:122 (+),score=39.86 TRINITY_DN37699_c0_g1_i3:1-363(+)	-1.08264	1.762435	0.385189	-0.58841	-0.47658
TRINITY_DN2695_c0_g1_i14_orfp1	TRINITY_DN2695_c0_g1_i14_m.44485 TRINITY_DN2695_c0_g1::TRINITY_DN2695_c0_g1_i14::g.44485 ORF type:3prime_partial len:698 (+),score=187.51 TRINITY_DN2695_c0_g1_i14:101-2092(+)	-1.21642	1.688499	-0.42407	-0.51812	0.470116
TRINITY_DN36494_c0_g1_i1_orf1	MKI67 FHA domain-interacting nucleolar phosphoprotein-like [Ostrinia furnacalis]	-0.80786	1.938281	-0.08124	-0.65389	-0.39529
TRINITY_DN2986_c1_g1_i1_orf1	Troponin C, isoform 1 [Papilio xuthus]	-1.24861	1.643576	0.462407	-0.70999	-0.14738
TRINITY_DN1199_c0_g1_i1_orf1	pupal cuticle protein 36a-like [Ostrinia furnacalis]	-0.67603	1.967032	-0.19955	-0.68413	-0.40732
TRINITY_DN5244_c0_g1_i1_orf1	eukaryotic peptide chain release factor GTP-binding subunit-like [Ostrinia furnacalis]	-1.36914	1.746016	-0.11017	-0.25412	-0.01258
TRINITY_DN29034_c0_g1_i2_orf1	trypsin-like serine protease [Ostrinia nubilalis]	-0.86121	1.955487	-0.23532	-0.50056	-0.3584
TRINITY_DN2825_c0_g1_i3_orf1	carbonic anhydrase 2-like [Ostrinia furnacalis]	-0.81226	1.969776	-0.37047	-0.31213	-0.47492
TRINITY_DN3504_c0_g1_i4_orfp1	TRINITY_DN3504_c0_g1_i4_m.43930 TRINITY_DN3504_c0_g1::TRINITY_DN3504_c0_g1_i4::g.43930 ORF type:internal len:196 (-),score=84.82 TRINITY_DN3504_c0_g1_i4:3-587(-)	-1.53955	1.59652	-0.00991	-0.22318	0.176111

TRINITY_DN69713_c0_g1_i1_orf1	membrane-bound alkaline phosphatase-like [Ostrinia furnacalis]	-0.925	1.929689	-0.55851	-0.29096	-0.15523
TRINITY_DN10940_c0_g1_i10_orfp1	TRINITY_DN10940_c0_g1_i10_m.52163 TRINITY_DN10940_c0_g1::TRINITY_DN10940_c0_g1_i10::g.52163 ORF type:5prime_partial len:248 (-),score=128.24 TRINITY_DN10940_c0_g1_i10:121-864(-)	-1.279	1.808268	-0.20121	-0.16106	-0.16699
TRINITY_DN94355_c0_g1_i2_orf1	uncharacterized protein LOC126369488 [Pectinophora gossypiella]	-0.51477	1.886944	0.143415	-0.70593	-0.80966
