# Supplementary material for: Comparative Proteomics and Metabonomics Analysis of Different Diapause Stages Revealed a New Regulation Mechanism of Diapause in Loxostege sticticalis (Lepidoptera: Pyralidae)
Source: Molecules. 2024 Jul 25;29(15):3472. doi: 10.3390/molecules29153472 (PMC11314584; doi:10.3390/molecules29153472)
Supplement: Supplementary file 1 [file molecules-29-03472-s001.zip › analysis process/proteomic/Cluster analysis of expression patterns/Up/CTvsD up.pdf]

<table>
<tr><th>Accession</th><th>Description</th><th>ND</th><th>D</th><th>PreD</th><th>CT</th><th>RD</th></tr>
<tr><td>TRINITY_DN971_c0_g1_i5_orfp1</td><td>TRINITY_DN971_c0_g1_i5_m.54249 TRINITY_DN971_c0_g1::TRINITY_DN971_c0_g1_i5::g.54249 ORF type:internal len:108 (+),score=66.98 TRINITY_DN971_c0_g1_i5:1-321(+)</td><td>-1.7632</td><td>0.62427</td><td>0.48896</td><td>1.04991</td><td>-0.3999</td></tr>
<tr><td>TRINITY_DN10877_c0_g1_i1_orf1</td><td>spodomicin-like [Ostrinia furnacalis]</td><td>-1.5409</td><td>-0.135</td><td>1.42832</td><td>0.64179</td><td>-0.3942</td></tr>
<tr><td>TRINITY_DN114890_c0_g1_i4_orf1</td><td>chemosensory protein 10 [Ostrinia furnacalis]</td><td>-1.475</td><td>0.28177</td><td>0.56866</td><td>1.36742</td><td>-0.7429</td></tr>
<tr><td>TRINITY_DN16091_c0_g1_i1_orfp1</td><td>TRINITY_DN16091_c0_g1_i1_m.64010 TRINITY_DN16091_c0_g1::TRINITY_DN16091_c0_g1_i1::g.64010 ORF type:5prime_partial len:124 (-),score=7.29,Toxin_2 PF00451.20 0.00035,Toxin_2|PF00451.20|0.00013,Toxin_2|PF00451.20|0.00037,Gamma-thionin|PF00304.21|0.37,Gamma-thionin|PF00304.21|0.052,Gamma-thionin|PF00304.21|0.022,Defensin_2|PF01097.19|0.58,Defensin_2|PF01097.19|0.12,Defensin_2|PF01097.19|0.011,Toxin_38|PF14866.7|0.18,Toxin_38|PF14866.7|0.18,Toxin_38|PF14866.7|0.4 TRINITY_DN16091_c0_g1_i1:19-390(-)</td><td>-1.4033</td><td>-0.0053</td><td>-0.0215</td><td>1.71696</td><td>-0.2869</td></tr>
<tr><td>TRINITY_DN59429_c0_g1_i6_orf1</td><td>uncharacterized protein LOC114366345 isoform X2 [Ostrinia furnacalis]</td><td>-1.528</td><td>0.39062</td><td>1.06094</td><td>0.8701</td><td>-0.7936</td></tr>
<tr><td>TRINITY_DN8008_c0_g1_i6_orf1</td><td>uncharacterized protein LOC114357965 isoform X1 [Ostrinia furnacalis] >XP_028167599.1 uncharacterized protein LOC114357965 isoform X1 [Ostrinia furnacalis] >XP_028167600.1 uncharacterized protein LOC114357965 isoform X2 [Ostrinia furnacalis] >XP_028167601.1 uncharacterized protein LOC114357965 isoform X3 [Ostrinia furnacalis]</td><td>-1.4788</td><td>0.73942</td><td>0.40514</td><td>1.17864</td><td>-0.8444</td></tr>
<tr><td>TRINITY_DN31348_c0_g1_i1_orf1</td><td>protein lethal(2)essential for life [Bombyx mori]</td><td>-1.6407</td><td>0.4976</td><td>0.45428</td><td>1.24357</td><td>-0.5548</td></tr>
<tr><td>TRINITY_DN1880_c0_g1_i4_orf1</td><td>serine protease inhibitor dipetalogastin-like [Helicoverpa zea]</td><td>-1.6005</td><td>0.62467</td><td>0.69273</td><td>1.01569</td><td>-0.7326</td></tr>
<tr><td>TRINITY_DN295_c5_g1_i2_orf1</td><td>unnamed protein product [Chilo suppressalis]</td><td>-1.729</td><td>0.3801</td><td>0.54383</td><td>1.19189</td><td>-0.3868</td></tr>
<tr><td>TRINITY_DN71698_c0_g1_i1_orfp1</td><td>TRINITY_DN71698_c0_g1_i1_m.1194 TRINITY_DN71698_c0_g1::TRINITY_DN71698_c0_g1_i1::g.1194 ORF type:internal len:134 (+),score=19.66,Toxin_2 PF00451.20 4.3e-05,Toxin_2|PF00451.20|0.037,Toxin_2|PF00451.20|7.5e-05,Gamma-thionin|PF00304.21|0.017,Gamma-thionin|PF00304.21|0.05,Gamma-thionin|PF00304.21|0.021,Toxin_38|PF14866.7|0.13,Toxin_38|PF14866.7|0.15,Toxin_38|PF14866.7|0.15,Defensin_2|PF01097.19|0.053,Defensin_2|PF01097.19|0.34,Defensin_2|PF01097.19|0.092 TRINITY_DN71698_c0_g1_i1:3-401(+)</td><td>-1.6737</td><td>0.41809</td><td>0.86061</td><td>0.9738</td><td>-0.5788</td></tr>
<tr><td>TRINITY_DN1149_c0_g1_i4_orf1</td><td>circadian clock-controlled protein-like [Ostrinia furnacalis]</td><td>-1.4557</td><td>0.30242</td><td>1.26362</td><td>0.71512</td><td>-0.8255</td></tr>
<tr><td>TRINITY_DN12586_c0_g1_i4_orf1</td><td>zonadhesin-like isoform X4 [Ostrinia furnacalis]</td><td>-1.1145</td><td>0.35631</td><td>1.07618</td><td>0.9415</td><td>-1.2595</td></tr>
<tr><td>TRINITY_DN1209_c0_g1_i9_orf1</td><td>NADP-dependent malic enzyme-like isoform X1 [Ostrinia furnacalis] >XP_028161889.1 NADP-dependent malic enzyme-like isoform X1 [Ostrinia furnacalis] >XP_028161891.1 NADP-dependent malic enzyme-like isoform X3 [Ostrinia furnacalis]</td><td>-1.5853</td><td>0.41986</td><td>0.27823</td><td>1.40264</td><td>-0.5154</td></tr>
<tr><td>TRINITY_DN85290_c0_g2_i1_orf1</td><td>unnamed protein product, partial [Brenthis ino]</td><td>-1.3149</td><td>-0.7595</td><td>1.02085</td><td>1.26744</td><td>-0.2139</td></tr>
<tr><td>TRINITY_DN9044_c0_g1_i2_orf1</td><td>unnamed protein product [Euphydryas editha]</td><td>-1.6119</td><td>-0.4323</td><td>1.42082</td><td>0.28184</td><td>0.34158</td></tr>
<tr><td>TRINITY_DN25234_c0_g1_i1_orf1</td><td>uncharacterized protein LOC114353853 [Ostrinia furnacalis]</td><td>-1.7563</td><td>-0.1912</td><td>1.28922</td><td>0.33917</td><td>0.3191</td></tr>
<tr><td>TRINITY_DN14185_c0_g1_i1_orf1</td><td>uncharacterized protein LOC114358675 [Ostrinia furnacalis] >XP_028168498.1 uncharacterized protein LOC114358675 [Ostrinia furnacalis]</td><td>-1.4804</td><td>0.31961</td><td>1.02381</td><td>0.97643</td><td>-0.8394</td></tr>
<tr><td>TRINITY_DN15682_c0_g1_i4_orf1</td><td>seroin transcript 1B [Ostrinia nubilalis]</td><td>-1.1209</td><td>-0.6056</td><td>0.68044</td><td>1.61085</td><td>-0.5648</td></tr>
<tr><td>TRINITY_DN2407_c0_g1_i2_orf1</td><td>uncharacterized protein LOC114366345 isoform X2 [Ostrinia furnacalis]</td><td>-1.0228</td><td>-0.4454</td><td>1.70113</td><td>0.52949</td><td>-0.7624</td></tr>
<tr><td>TRINITY_DN61042_c0_g2_i2_orfp1</td><td>TRINITY_DN61042_c0_g2_i2_m.5292 TRINITY_DN61042_c0_g2::TRINITY_DN61042_c0_g2_i2::g.5292 ORF type:5prime_partial len:63 (-),score=21.86 TRINITY_DN61042_c0_g2_i2:77-265(-)</td><td>-1.3815</td><td>-0.3142</td><td>1.35313</td><td>0.91395</td><td>-0.5713</td></tr>
<tr><td>TRINITY_DN1575_c0_g1_i10_orf1</td><td>uncharacterized protein LOC114359245 [Ostrinia furnacalis]</td><td>-1.3792</td><td>-0.9777</td><td>1.18157</td><td>0.7539</td><td>0.42138</td></tr>
<tr><td>TRINITY_DN9435_c0_g1_i7_orf1</td><td>uncharacterized protein LOC114350197 [Ostrinia furnacalis]</td><td>-1.4207</td><td>0.38978</td><td>0.24495</td><td>1.50223</td><td>-0.7163</td></tr>
<tr><td>TRINITY_DN13500_c0_g1_i1_orf1</td><td>phosphatidylethanolamine-binding protein homolog F40A3.3-like [Ostrinia furnacalis] >XP_028160752.1 phosphatidylethanolamine-binding protein homolog F40A3.3-like [Ostrinia furnacalis]</td><td>-1.6949</td><td>0.58401</td><td>-0.0004</td><td>1.32013</td><td>-0.2089</td></tr>
<tr><td>TRINITY_DN214_c0_g1_i4_orf1</td><td>uncharacterized protein LOC114352813 [Ostrinia furnacalis]</td><td>-1.0073</td><td>0.21121</td><td>1.4236</td><td>0.61285</td><td>-1.2403</td></tr>
<tr><td>TRINITY_DN1175_c1_g1_i2_orf1</td><td>methanethiol oxidase [Ostrinia furnacalis]</td><td>-1.3329</td><td>0.39867</td><td>0.93906</td><td>1.04224</td><td>-1.0471</td></tr>
<tr><td>TRINITY_DN20347_c0_g1_i6_orf1</td><td>venom polypeptide precursor [Doratifera vulnerans]</td><td>-1.5118</td><td>0.44057</td><td>-0.0145</td><td>1.52542</td><td>-0.4397</td></tr>
<tr><td>TRINITY_DN76633_c0_g1_i1_orfp1</td><td>TRINITY_DN76633_c0_g1_i1_m.53394 TRINITY_DN76633_c0_g1::TRINITY_DN76633_c0_g1_i1::g.53394 ORF type:internal len:164 (-),score=26.46,Toxin_2 PF00451.20 0.00022,Toxin_2|PF00451.20|0.00056,Toxin_2|PF00451.20|0.0002,Toxin_2|PF00451.20|5.9e-06,Gamma-thionin|PF00304.21|2,Gamma-thionin|PF00304.21|0.024,Gamma-thionin|PF00304.21|0.027,Gamma-thionin|PF00304.21|0.066,Toxin_38|PF14866.7|0.27,Toxin_38|PF14866.7|0.054,Toxin_38|PF14866.7|0.14,Defensin_2|PF01097.19|1.7,Defensin_2|PF01097.19|0.055,Defensin_2|PF01097.19|1.2,Defensin_2|PF01097.19|0.15 TRINITY_DN76633_c0_g1_i1:1-489(-)</td><td>-0.8921</td><td>0.00498</td><td>0.97157</td><td>1.23382</td><td>-1.3183</td></tr>
<tr><td>TRINITY_DN1466_c0_g1_i4_orf1</td><td>insecticyanin-A-like [Ostrinia furnacalis]</td><td>-1.656</td><td>-0.6232</td><td>0.84162</td><td>0.97152</td><td>0.46605</td></tr>
<tr><td>TRINITY_DN2170_c0_g2_i1_orf1</td><td>beta-1,3-glucan-binding protein-like [Ostrinia furnacalis]</td><td>-1.7132</td><td>0.20292</td><td>-0.0115</td><td>1.41879</td><td>0.10304</td></tr>
<tr><td>TRINITY_DN3826_c0_g1_i1_orf1</td><td>39S ribosomal protein L18, mitochondrial [Ostrinia furnacalis]</td><td>-1.1241</td><td>0.23988</td><td>0.47951</td><td>1.49986</td><td>-1.0952</td></tr>
<tr><td>TRINITY_DN135679_c0_g1_i2_orfp1</td><td>TRINITY_DN135679_c0_g1_i2_m.85525 TRINITY_DN135679_c0_g1::TRINITY_DN135679_c0_g1_i2::g.85525 ORF type:5prime_partial len:55 (+),score=5.08,Toxin_2 PF00451.20 1.9e-06 TRINITY_DN135679_c0_g1_i2:3-167(+)</td><td>-1.042</td><td>0.48952</td><td>0.61395</td><td>1.25296</td><td>-1.3145</td></tr>
<tr><td>TRINITY_DN2772_c0_g1_i3_orf1</td><td>uncharacterized protein LOC114353284 isoform X4 [Ostrinia furnacalis] >XP_028161011.1 uncharacterized protein LOC114353284 isoform X4 [Ostrinia furnacalis] >XP_028161012.1 uncharacterized protein LOC114353284 isoform X4 [Ostrinia furnacalis]</td><td>-1.2004</td><td>0.62222</td><td>0.21757</td><td>1.41714</td><td>-1.0565</td></tr>
<tr><td>TRINITY_DN80660_c0_g1_i1_orf1</td><td>probable phospholipid hydroperoxide glutathione peroxidase isoform X1 [Pieris rapae]</td><td>-1.5991</td><td>-0.0739</td><td>0.56574</td><td>1.42089</td><td>-0.3136</td></tr>
<tr><td>TRINITY_DN97883_c0_g1_i2_orf1</td><td>talin-2-like, partial [Ostrinia furnacalis]</td><td>-1.5601</td><td>-0.6613</td><td>1.25497</td><td>0.27487</td><td>0.69155</td></tr>
<tr><td>TRINITY_DN26789_c0_g1_i2_orf1</td><td>D-2-hydroxyglutarate dehydrogenase, mitochondrial-like [Ostrinia furnacalis]</td><td>-1.358</td><td>0.05438</td><td>-0.1156</td><td>1.74216</td><td>-0.3229</td></tr>
<tr><td>TRINITY_DN11798_c0_g2_i1_orf1</td><td>N-acetylglucosamine-6-sulfatase-like isoform X2 [Ostrinia furnacalis]</td><td>-1.6011</td><td>-0.0669</td><td>1.08465</td><td>1.02835</td><td>-0.445</td></tr>
<tr><td>TRINITY_DN48602_c0_g1_i6_orf1</td><td>amidophosphoribosyltransferase-like isoform X1 [Ostrinia furnacalis] >XP_028157758.1 amidophosphoribosyltransferase-like isoform X1 [Ostrinia furnacalis] >XP_028157759.1 amidophosphoribosyltransferase-like isoform X1 [Ostrinia furnacalis]</td><td>-1.6541</td><td>-0.5789</td><td>1.07896</td><td>0.79904</td><td>0.35507</td></tr>
</table>

TRINITY_DN31163_c1_g1_i4_orf1	phenoloxidase subunit 2-like [Ostrinia furnacalis]	-1.2361	0.13885	0.2996	1.6327	-0.8351
TRINITY_DN5433_c0_g1_i5_orf1	uncharacterized protein LOC114351067 [Ostrinia furnacalis]	-1.7543	-0.4226	0.88859	0.89346	0.39484
TRINITY_DN184_c0_g1_i1_orf1	macrophage mannose receptor 1-like isoform X1 [Maniola jurtina]	-0.9692	-0.9917	1.73147	0.27718	-0.0477
TRINITY_DN14874_c0_g1_i6_orf1	uncharacterized protein LOC114358148 [Ostrinia furnacalis]	-1.2011	-0.6648	1.69405	0.42561	-0.2537
TRINITY_DN4794_c1_g1_i9_orf1	D-3-phosphoglycerate dehydrogenase [Ostrinia furnacalis]	-1.3417	-0.6515	0.55382	1.56611	-0.1268
TRINITY_DN1789_c0_g1_i5_orf1	uncharacterized protein LOC114365543 [Ostrinia furnacalis]	-1.1975	-0.5124	1.60329	0.65633	-0.5498
TRINITY_DN33867_c0_g1_i8_orf1	uncharacterized protein LOC114357513 [Ostrinia furnacalis]	-1.3673	-1.0279	0.73106	1.11022	0.55393
TRINITY_DN84478_c0_g1_i8_orf1	uncharacterized protein LOC114359035 isoform X1 [Ostrinia furnacalis]	-1.9622	0.18675	0.50348	0.79809	0.47386
TRINITY_DN14242_c0_g1_i2_orfp1	TRINITY_DN14242_c0_g1_i2_m.18449 TRINITY_DN14242_c0_g1::TRINITY_DN14242_c0_g1_i2::g.18449 ORF type:internal len:148 (-),score=102.30 TRINITY_DN14242_c0_g1_i2:2-442(-)	-1.8828	0.11777	0.95197	0.72609	0.08701
TRINITY_DN1108_c3_g1_i1_orfp1	TRINITY_DN1108_c3_g1_i1_m.5561 TRINITY_DN1108_c3_g1::TRINITY_DN1108_c3_g1_i1::g.5561 ORF type:internal len:113 (+),score=89.07 TRINITY_DN1108_c3_g1_i1:1-336(+)	-1.907	0.10881	0.7938	0.83166	0.17271
TRINITY_DN5080_c0_g1_i5_orf1	storage protein [Ostrinia furnacalis]	-1.9646	0.3036	0.27671	0.61075	0.77358
TRINITY_DN143895_c0_g1_i1_orf1	cathepsin L-like [Aphidius gifuensis] >KAF7988186.1 hypothetical protein HCN44_007680 [Aphidius gifuensis]	-1.9287	0.39558	0.05375	0.85423	0.62519
TRINITY_DN703_c0_g1_i2_orf1	acidic juvenile hormone-suppressible protein 1-like [Ostrinia furnacalis]	-1.9072	0.33613	0.09245	1.01136	0.46724
TRINITY_DN142657_c0_g1_i1_orf1	sorting and assembly machinery component 50 homolog [Diachasma alloeum]	-1.8702	0.54074	-0.007	1.06633	0.27013
TRINITY_DN4767_c0_g1_i6_orf1	cysteine protease XCP2-like [Ostrinia furnacalis]	-1.8723	0.50133	-0.1558	0.9266	0.60017
TRINITY_DN4767_c0_g1_i4_orf1	cysteine protease XCP2-like [Ostrinia furnacalis]	-1.9572	0.23768	0.28587	0.67435	0.75927
TRINITY_DN64297_c0_g1_i1_orf1	vanin-like protein 2 isoform X2 [Ostrinia furnacalis]	-1.5609	-0.6142	0.25072	0.59363	1.33079
TRINITY_DN66040_c0_g1_i2_orf1	serine protease inhibitor dipetalogastin-like isoform X2 [Ostrinia furnacalis]	-1.5131	-0.361	-0.1369	0.48616	1.52484
TRINITY_DN106156_c1_g1_i1_orf1	arylphorin subunit alpha-like [Ostrinia furnacalis]	-1.848	0.23597	-0.0056	1.14116	0.47645
TRINITY_DN51813_c0_g1_i1_orf1	uncharacterized protein LOC114350216 [Ostrinia furnacalis]	-1.8579	0.02598	0.07584	0.87799	0.87806
TRINITY_DN5080_c0_g1_i1_orf1	basic juvenile hormone-suppressible protein 2-like [Ostrinia furnacalis]	-1.9059	0.1829	0.09529	0.7979	0.82977
TRINITY_DN636_c1_g1_i9_orf1	secretory phospholipase A2 receptor-like [Ostrinia furnacalis]	-1.8328	-0.1767	0.26549	0.96816	0.77582
TRINITY_DN13799_c0_g1_i1_orf1	uncharacterized protein LOC116345248 [Contarinia nasturtii]	-1.6848	0.70782	-0.5319	1.10038	0.40844
TRINITY_DN276_c0_g1_i2_orf1	protein lethal(2)essential for life-like [Ostrinia furnacalis] >UTU55753.1 small heat shock protein Hsp20.7 [Ostrinia furnacalis]	-1.3206	0.06218	-0.8035	0.5407	1.52123
TRINITY_DN19662_c0_g2_i1_orf1	storage protein [Ostrinia furnacalis]	-1.9057	0.20356	0.08663	0.89322	0.72226
TRINITY_DN6122_c0_g1_i6_orf1	deubiquitinase DESI2 isoform X1 [Helicoverpa armigera] >XP_049707835.1 deubiquitinase DESI2 isoform X1 [Helicoverpa armigera]	-1.8841	0.34394	-0.085	0.8568	0.76842
TRINITY_DN56308_c0_g1_i2_orf1	storage protein 1 [Omphisa fuscidentalis]	-1.8862	0.35573	-0.0679	0.92946	0.66885
TRINITY_DN17574_c0_g1_i2_orf1	heat shock protein Hsp-12.2-like [Ostrinia furnacalis]	-1.4682	0.67637	-0.9201	1.0496	0.66232
TRINITY_DN42719_c0_g2_i1_orf1	inter-alpha-trypsin inhibitor heavy chain H4-like isoform X11 [Ostrinia furnacalis]	-1.872	-0.1196	0.36021	0.71352	0.91789
TRINITY_DN45037_c0_g1_i1_orf1	trafficking protein particle complex subunit 1 [Ostrinia furnacalis]	-1.7565	0.18122	-0.1949	1.25727	0.51292
TRINITY_DN19662_c4_g1_i1_orf1	basic juvenile hormone-suppressible protein 1-like [Ostrinia furnacalis]	-1.9202	0.2073	0.1667	0.92615	0.62002
TRINITY_DN295_c2_g1_i2_orf1	phosphoglycolate phosphatase 1A, chloroplastic [Manduca sexta]	-1.8985	0.09291	0.36483	1.04894	0.39185
TRINITY_DN418_c1_g1_i3_orf1	hypothetical protein evm_003996 [Chilo suppressalis]	-1.7125	-0.4109	0.27099	1.15985	0.69259
TRINITY_DN2049_c1_g1_i3_orf1	luciferin 4-monooxygenase-like [Ostrinia furnacalis]	-1.5076	-0.3409	-0.2188	1.49522	0.57208
TRINITY_DN15578_c0_g2_i1_orfp1	uncharacterized protein LOC125235519 [Leguminivora glycinivorella]	-1.8883	-0.1135	0.8435	0.43946	0.71887
TRINITY_DN9538_c1_g3_i1_orf1	cilia- and flagella-associated protein 410 isoform X2 [Aphidius gifuensis]	-1.8452	0.18954	-0.0339	0.5893	1.10034
TRINITY_DN80328_c0_g1_i5_orf1	arylphorin subunit alpha-like [Ostrinia furnacalis]	-1.8607	0.32973	-0.1154	0.99701	0.64937
TRINITY_DN80328_c0_g1_i9_orf1	arylphorin subunit alpha-like [Ostrinia furnacalis]	-1.8873	0.32576	-0.0358	0.96428	0.63307
TRINITY_DN10138_c0_g1_i1_orf1	storage protein 1 [Omphisa fuscidentalis]	-1.7698	0.02552	-0.0794	1.22648	0.59723
TRINITY_DN2146_c0_g2_i1_orf1	heat shock protein 68-like [Ostrinia furnacalis]	-1.488	0.74208	-0.6286	1.35631	0.01826
TRINITY_DN21420_c0_g1_i2_orf1	glutathione peroxidase, partial [Ostrinia furnacalis]	-1.84	0.35796	0.44521	1.13098	-0.0941
TRINITY_DN9733_c0_g1_i2_orf1	acidic juvenile hormone-suppressible protein 1-like [Ostrinia furnacalis]	-1.4475	-0.5353	-0.2293	1.34974	0.86239
TRINITY_DN18128_c0_g1_i4_orf1	arylsulfatase B [Ostrinia furnacalis]	-1.8209	0.40621	-0.2345	1.05335	0.59579
TRINITY_DN6497_c0_g1_i1_orf1	ommochrome-binding protein-like [Ostrinia furnacalis]	-1.8921	0.38426	0.13028	1.08027	0.29728
TRINITY_DN3301_c0_g1_i2_orf1	hemicentin-2-like isoform X1 [Ostrinia furnacalis]	-1.8346	0.12678	-0.0534	1.05981	0.70146
TRINITY_DN13760_c1_g1_i1_orf1	pre-mRNA-processing factor 40 homolog A isoform X1 [Ostrinia furnacalis] >XP_028162665.1 pre-mRNA-processing factor 40 homolog A isoform X2 [Ostrinia furnacalis] >XP_028162667.1 pre-mRNA-processing factor 40 homolog A isoform X3 [Ostrinia furnacalis]	-1.6889	-0.3485	0.25016	0.46566	1.32162
TRINITY_DN993_c0_g1_i7_orf1	apolipophorins-like [Ostrinia furnacalis]	-1.5456	-0.1659	-0.4186	0.80089	1.32924
TRINITY_DN9593_c0_g1_i2_orf1	uncharacterized protein LOC113518937 [Galleria mellonella]	-1.59	0.39871	-0.6368	1.25792	0.57016
TRINITY_DN768_c0_g1_i7_orf1	neutral ceramidase [Leguminivora glycinivorella]	-1.9648	0.17237	0.5423	0.75656	0.49352
TRINITY_DN1048_c0_g1_i6_orf1	uncharacterized protein LOC114360661 [Ostrinia furnacalis]	-1.8346	-0.1	0.37292	1.1455	0.41615
TRINITY_DN3616_c0_g1_i4_orf1	conotoxin ArMKLT2-032-like [Ostrinia furnacalis]	-1.4273	-0.6536	-0.0476	0.69833	1.43024
TRINITY_DN5421_c0_g1_i1_orf1	arylphorin subunit alpha-like [Ostrinia furnacalis]	-1.9428	0.25159	0.46122	0.92561	0.3044
TRINITY_DN42275_c0_g1_i1_orfp1	TRINITY_DN42275_c0_g1_i1_m.44265 TRINITY_DN42275_c0_g1::TRINITY_DN42275_c0_g1_i1::g.44265 ORF type:internal len:76 (+),score=8.67 TRINITY_DN42275_c0_g1_i1:1-225(+)	-1.7028	-0.2444	-0.0228	1.20842	0.76157
TRINITY_DN2472_c0_g1_i6_orf1	programmed cell death protein 6 isoform X1 [Colias croceus] >XP_045492459.1 programmed cell death protein 6 isoform X1 [Colias croceus]	-1.9091	0.31586	0.36408	1.04337	0.18576
TRINITY_DN28711_c0_g1_i1_orf1	hypothetical protein evm_000299 [Chilo suppressalis]	-1.4105	-0.2883	-0.5329	0.83903	1.39267

TRINITY_DN81488_c0_g1_i1_orf1	apolipophorins-like [Ostrinia furnacalis]	-1.4803	-0.2184	-0.4721	0.78382	1.38699
TRINITY_DN9239_c0_g1_i1_orf1	apolipophorins-like [Ostrinia furnacalis]	-1.3268	-0.2933	-0.6047	0.71709	1.50782
TRINITY_DN85412_c0_g1_i1_orf1	unnamed protein product [Diatraea saccharalis]	-1.4122	-0.2669	-0.5218	0.75379	1.44709
TRINITY_DN578_c0_g1_i5_orf1	charged multivesicular body protein 7 [Ostrinia furnacalis]	-1.897	0.09319	0.14821	0.83508	0.82055
TRINITY_DN97042_c0_g1_i6_orf1	apolipophorins-like [Ostrinia furnacalis]	-1.4997	-0.2074	-0.466	0.83166	1.34136
TRINITY_DN699_c0_g2_i1_orf1	TPA_exp: putative parasitoid killing factor [Trichoplusia ni]	-1.4252	-0.1997	-0.5433	0.70775	1.46045
TRINITY_DN1423_c0_g1_i8_orf1	ferritin subunit-like [Ostrinia furnacalis] >XP_028168186.1 ferritin subunit-like [Ostrinia furnacalis]	-1.5296	-0.1935	-0.2715	0.4683	1.52636
TRINITY_DN14262_c0_g1_i5_orf1	cytochrome P450 monooxygenase CYP9G18 [Cnaphalocrocis medinalis]	-1.7866	0.09478	0.95254	0.92577	-0.1865
TRINITY_DN18388_c0_g1_i6_orf1	serine protease [Ostrinia furnacalis]	-1.8692	-0.0078	0.18209	0.71188	0.98296
TRINITY_DN2798_c0_g1_i5_orf1	arylsulfatase B-like isoform X1 [Ostrinia furnacalis]	-1.9403	0.07499	0.40809	0.70358	0.75365
TRINITY_DN71699_c0_g1_i1_orf1	apolipophorins-like [Ostrinia furnacalis]	-1.3854	-0.267	-0.5739	0.79524	1.43099
TRINITY_DN52944_c0_g1_i1_orf1	apolipophorins-like [Ostrinia furnacalis]	-1.4092	-0.3037	-0.4968	0.76292	1.44676
TRINITY_DN2803_c4_g1_i1_orf1	ornithine aminotransferase, mitochondrial isoform X2 [Ostrinia furnacalis]	-1.7995	-0.1428	0.34464	0.38259	1.21503
TRINITY_DN1144_c0_g1_i10_orf1	TIL [Ostrinia furnacalis]	-1.7699	0.43037	-0.2926	1.1799	0.45222
TRINITY_DN6108_c0_g1_i5_orf1	myogenesis-regulating glycosidase-like [Ostrinia furnacalis]	-1.7378	-0.0879	0.29278	1.36306	0.16979
TRINITY_DN28711_c1_g1_i1_orf1	apolipophorins-like [Ostrinia furnacalis]	-1.4115	-0.2408	-0.5321	0.71746	1.46692
TRINITY_DN745_c7_g1_i1_orf1	uncharacterized protein LOC114358822 [Ostrinia furnacalis]	-1.7551	0.1634	-0.268	1.14433	0.71535
TRINITY_DN3383_c0_g1_i5_orf1	uncharacterized protein LOC114357426 [Ostrinia furnacalis]	-1.0577	0.24695	-1.2692	1.19223	0.88771
TRINITY_DN36144_c0_g1_i3_orf1	nicotinate phosphoribosyltransferase isoform X1 [Ostrinia furnacalis] >XP_028178189.1 nicotinate phosphoribosyltransferase isoform X1 [Ostrinia furnacalis]	-1.563	0.09594	-0.4534	1.45921	0.46133
TRINITY_DN6988_c0_g1_i3_orf1	cuticle protein 1-like [Ostrinia furnacalis]	-1.8483	0.14107	-0.0593	0.87776	0.88877
TRINITY_DN14250_c0_g1_i1_orf1	apolipophorins-like [Ostrinia furnacalis]	-1.212	-0.109	-0.9575	1.19647	1.08205
TRINITY_DN1569_c0_g1_i6_orf1	uncharacterized protein LOC114350603 [Ostrinia furnacalis]	-1.1552	-0.1489	-0.9148	1.52461	0.69427
TRINITY_DN11467_c0_g1_i5_orf1	27 kDa hemolymph protein-like, partial [Ostrinia furnacalis]	-1.763	0.15426	-0.0622	1.31923	0.35176
TRINITY_DN625_c2_g2_i2_orf1	L-dopachrome tautomerase yellow-f2-like [Ostrinia furnacalis]	-1.7918	0.25406	-0.1451	1.22074	0.4621
TRINITY_DN6462_c0_g1_i5_orf1	probable histone-lysine N-methyltransferase CG1716 [Ostrinia furnacalis]	-1.8359	0.00986	0.04473	1.03365	0.74765
TRINITY_DN338_c1_g1_i9_orf1	scolexin B-like isoform X2 [Ostrinia furnacalis]	-1.9101	0.05576	0.25117	0.78856	0.81459
TRINITY_DN6205_c0_g1_i1_orf1	phenoloxidase-activating factor 2-like [Ostrinia furnacalis]	-1.6788	-0.5255	0.29831	0.94128	0.96468
TRINITY_DN21743_c0_g1_i1_orf1	uncharacterized protein LOC114357426 [Ostrinia furnacalis]	-1.4319	0.2349	-0.8565	1.18844	0.86509
TRINITY_DN9239_c0_g2_i2_orf1	apolipophorins-like [Ostrinia furnacalis]	-1.3082	-0.5911	-0.3552	0.75877	1.49575
TRINITY_DN44073_c0_g1_i3_orf1	inter-alpha-trypsin inhibitor heavy chain H4-like isoform X11 [Ostrinia furnacalis]	-1.6558	-0.3914	0.32426	0.3538	1.3692
TRINITY_DN4822_c0_g1_i6_orf1	homogentisate 1,2-dioxygenase [Ostrinia furnacalis]	-0.8878	0.07652	-1.1748	1.64633	0.33973
TRINITY_DN8703_c0_g1_i2_orf1	beta-glucuronidase-like isoform X1 [Ostrinia furnacalis] >XP_028166212.1 beta-glucuronidase-like isoform X2 [Ostrinia furnacalis]	-1.6787	-0.1631	-0.2126	1.03516	1.01919
TRINITY_DN125140_c0_g1_i1_orf1	glycogen debranching enzyme isoform X2 [Ostrinia furnacalis] >XP_028161358.1 glycogen debranching enzyme isoform X2 [Ostrinia furnacalis] >XP_028161359.1 glycogen debranching enzyme isoform X2 [Ostrinia furnacalis]	-1.9117	-0.0671	0.74014	0.73386	0.50471
TRINITY_DN23746_c0_g1_i2_orf1	protein 4.1 homolog isoform X1 [Ostrinia furnacalis]	-1.6572	-0.3168	-0.0723	0.85887	1.18754
TRINITY_DN46090_c0_g3_i1_orf1	tyrosine-protein kinase-like otk, partial [Ostrinia furnacalis]	-1.3732	-0.2057	-0.621	1.46505	0.73485
TRINITY_DN59388_c0_g1_i1_orf1	uncharacterized protein LOC114353759 [Ostrinia furnacalis]	-1.4404	-0.5556	0.48286	-0.0303	1.5435
TRINITY_DN27264_c0_g1_i1_orf1	uncharacterized protein LOC114353424 [Ostrinia furnacalis]	-0.9364	-0.6112	-0.6874	0.49325	1.74177
TRINITY_DN86833_c0_g3_i1_orf1	PREDICTED: glycerol-3-phosphate acyltransferase 1, mitochondrial isoform X1 [Microplitis demolitor]	-1.5831	-0.2993	-0.1806	0.68221	1.3807
TRINITY_DN4394_c0_g2_i1_orf1	carboxylesterase [Ostrinia furnacalis]	-1.5894	-0.4806	-0.007	1.24565	0.83133
TRINITY_DN42854_c0_g3_i2_orf1	amyloid beta (A4) precursor-like protein 2, isoform CRA_b [Homo sapiens]	-1.655	-0.0021	-0.3863	1.13084	0.9126
TRINITY_DN19110_c0_g1_i2_orf1	peroxidase [Ostrinia furnacalis]	-1.2457	-0.4603	-0.5048	0.58694	1.62388
TRINITY_DN1110_c1_g1_i9_orf1	MD-2-related lipid-recognition protein-like [Ostrinia furnacalis]	-0.9686	-0.2562	-0.8699	0.32427	1.77041
TRINITY_DN3464_c0_g1_i1_orf1	putative mitochondrial aconitate hydratase isoform X1-likeprotein, partial [Cotesia chilonis]	-0.9465	-0.5383	-0.8389	0.70861	1.61508
TRINITY_DN101_c0_g1_i4_orf1	disco-interacting protein 2 [Melitaea cinxia]	-1.2436	0.10995	-0.6426	1.73982	0.0365
TRINITY_DN15291_c0_g1_i11_orf1	uncharacterized protein LOC114353772 [Ostrinia furnacalis]	-1.275	-0.1706	-0.834	0.97899	1.30054
TRINITY_DN50237_c0_g1_i8_orf1	LOW QUALITY PROTEIN: uncharacterized protein LOC114361080 [Ostrinia furnacalis]	-1.7047	-0.1711	-0.0593	1.27487	0.66026
TRINITY_DN42719_c0_g1_i1_orf1	inter-alpha-trypsin inhibitor heavy chain H4-like isoform X11 [Ostrinia furnacalis]	-0.9083	-0.5803	-0.5474	0.16192	1.87412
TRINITY_DN12671_c0_g1_i6_orf1	hemicentin-1-like isoform X1 [Ostrinia furnacalis]	-1.0512	-0.521	-0.3467	0.04789	1.87108
TRINITY_DN5553_c0_g1_i4_orf1	uncharacterized protein LOC114353828 [Ostrinia furnacalis]	-1.2832	-0.3009	-0.558	0.498	1.64419
TRINITY_DN15175_c0_g1_i1_orf1	zinc carboxypeptidase-like [Ostrinia furnacalis]	-0.7526	-0.4163	-0.6799	-0.0977	1.94642
TRINITY_DN70_c6_g1_i1_orf1	optineurin isoform X1 [Ostrinia furnacalis] >XP_028165537.1 optineurin isoform X1 [Ostrinia furnacalis] >XP_028165538.1 optineurin isoform X1 [Ostrinia furnacalis] >XP_028165539.1 optineurin isoform X1 [Ostrinia furnacalis]	-1.8112	-0.1755	0.17523	0.99885	0.81263
TRINITY_DN2271_c0_g1_i12_orf1	plasminogen activator inhibitor 1-like [Ostrinia furnacalis]	-1.7593	-0.2962	0.17965	1.05105	0.82475
TRINITY_DN18273_c0_g1_i4_orf1	venom protease-like [Ostrinia furnacalis] >XP_028156372.1 venom protease-like [Ostrinia furnacalis]	-1.4527	0.1395	-0.6086	1.53229	0.38956
TRINITY_DN3952_c0_g1_i3_orf1	protein Skeletor, isoforms D/E-like isoform X1 [Ostrinia furnacalis] >XP_028176405.1 protein Skeletor, isoforms D/E-like isoform X2 [Ostrinia furnacalis] >XP_028176406.1 protein Skeletor, isoforms D/E-like isoform X3 [Ostrinia furnacalis] >XP_028176407.1 protein Skeletor, isoforms D/E-like isoform X4 [Ostrinia furnacalis]	-1.0343	-0.1421	-0.9681	0.49199	1.65249

TRINITY_DN12301_c0_g1_i1_orf1	ribose-phosphate pyrophosphokinase 2 [Ostrinia furnacalis]	-1.6527	0.11317	-0.449	1.19083	0.79765
TRINITY_DN2566_c0_g1_i5_orf1	uncharacterized protein LOC114349936 [Ostrinia furnacalis]	-0.9593	-0.259	-1.043	0.70157	1.55969
TRINITY_DN2897_c0_g2_i1_orf1	gem-associated protein 5-like [Ostrinia furnacalis]	-1.3491	-0.0454	-0.816	0.91897	1.29139
TRINITY_DN116874_c0_g1_i1_orfp1	TRINITY_DN116874_c0_g1_i1_m.85176 TRINITY_DN116874_c0_g1::TRINITY_DN116874_c0_g1_i1::g.85176 ORF type:5prime_partial len:95 (+),score=17.10,Baculo_p48 PF04878.14 8.5e-16 TRINITY_DN116874_c0_g1_i1:2-286(+)	-0.9332	-0.0229	-1.2568	1.33105	0.88176
TRINITY_DN32448_c0_g1_i1_orf1	unnamed protein product [Arctia plantaginis] >CAB3252297.1 unnamed protein product [Arctia plantaginis]	-1.409	-0.0767	-0.6795	1.4014	0.76372
TRINITY_DN8473_c0_g1_i5_orf1	serine/threonine-protein phosphatase 6 regulatory subunit 1 [Ostrinia furnacalis]	-1.8096	-0.0429	0.21415	1.22867	0.40969
TRINITY_DN143637_c0_g1_i1_orf1	PX domain-containing protein kinase-like protein isoform X1 [Chelonus insularis]	-1.7345	-0.3472	0.29959	0.5802	1.20193
TRINITY_DN5070_c0_g1_i1_orf1	ATP-dependent (S)-NAD(P)H-hydrate dehydratase-like [Ostrinia furnacalis]	-1.4893	-0.5874	0.07369	0.54035	1.46269
TRINITY_DN12286_c1_g1_i2_orf1	sideroflexin-1-3 [Galleria mellonella] >XP_026754161.1 sideroflexin-1-3 [Galleria mellonella]	-1.2111	-0.4446	-0.5167	0.49075	1.68161
TRINITY_DN12193_c0_g1_i6_orf1	carbonyl reductase [NADPH] 1-like [Ostrinia furnacalis]	-1.551	-0.1624	-0.426	1.2912	0.84821
TRINITY_DN875_c0_g1_i3_orf1	secernin-3 [Ostrinia furnacalis]	-1.244	-0.6161	-0.1826	0.33093	1.71172
TRINITY_DN251_c0_g1_i2_orf1	hypothetical protein evm_008466 [Chilo suppressalis]	-1.3479	-0.6763	-0.262	1.29126	0.99492
TRINITY_DN1407_c0_g1_i5_orf1	unnamed protein product [Chrysodeixis includens]	-1.2313	-0.549	-0.2163	0.24241	1.75413
TRINITY_DN18804_c0_g1_i5_orf1	zinc finger protein Xfin-like [Ostrinia furnacalis]	-0.8865	-0.7176	-0.4324	0.17022	1.86633
TRINITY_DN9412_c0_g1_i1_orf1	maspardin-like [Ostrinia furnacalis]	-1.3603	-0.6692	-0.0341	0.49746	1.5662
TRINITY_DN13856_c0_g1_i1_orf1	angiotensin-converting enzyme-like [Ostrinia furnacalis]	-1.3156	-0.6486	-0.2444	0.68637	1.52234
TRINITY_DN57998_c1_g1_i1_orf1	uncharacterized protein LOC113509309, partial [Galleria mellonella]	-0.7032	-0.6647	-0.3962	-0.202	1.96618
TRINITY_DN53294_c0_g1_i1_orf1	liver carboxylesterase 2-like [Ostrinia furnacalis]	-1.1938	-0.4802	-0.5991	1.58462	0.68859
TRINITY_DN1833_c0_g1_i5_orf1	uncharacterized protein LOC114356866 isoform X3 [Ostrinia furnacalis] >XP_028166037.1 uncharacterized protein LOC114356866 isoform X3 [Ostrinia furnacalis]	-0.9591	-1.0337	-0.3775	1.35834	1.01194
TRINITY_DN2663_c0_g1_i12_orf1	WW domain-binding protein 2 isoform X1 [Bombyx mori] >XP_028036324.1 WW domain-binding protein 2 [Bombyx mandarina]	-0.9915	-0.2477	-0.8133	1.79687	0.25561
TRINITY_DN23978_c0_g1_i2_orf1	insulin-like growth factor-binding protein complex acid labile subunit [Ostrinia furnacalis]	-0.8938	-1.2743	-0.0958	1.18271	1.08126
TRINITY_DN4550_c1_g1_i19_orf1	titin homolog [Ostrinia furnacalis]	-0.9715	-1.0242	-0.3783	1.33911	1.03487
TRINITY_DN56690_c0_g1_i4_orf1	hypothetical protein evm_002209, partial [Chilo suppressalis]	-1.2015	-1.1054	0.22576	0.79752	1.28358
TRINITY_DN17329_c0_g2_i3_orf1	uncharacterized protein LOC114354338 isoform X1 [Ostrinia furnacalis]	-0.5746	-0.8132	-0.9519	0.74256	1.59718
TRINITY_DN5028_c0_g1_i11_orf1	NTF2-related export protein [Ostrinia furnacalis]	-0.9178	-0.8496	-0.3006	0.25706	1.81091
TRINITY_DN1491_c0_g1_i4_orf1	GILT-like protein 2 isoform X1 [Ostrinia furnacalis] >XP_028156245.1 GILT-like protein 2 isoform X2 [Ostrinia furnacalis] >XP_028156247.1 GILT-like protein 2 isoform X3 [Ostrinia furnacalis]	-1.0461	-1.1382	0.74159	0.00733	1.43535
TRINITY_DN31377_c0_g2_i1_orf1	phosphatidate cytidylyltransferase, mitochondrial [Ostrinia furnacalis]	-0.2576	-0.618	-1.389	1.30289	0.9617
TRINITY_DN14391_c1_g1_i2_orf1	pre-rRNA-processing protein TSR1 homolog [Ostrinia furnacalis]	-0.5099	-0.5217	-1.3104	1.23367	1.10843
TRINITY_DN1091_c0_g2_i10_orf1	macrophage mannose receptor 1-like isoform X2 [Maniola hyperantus]	-0.951	-1.2904	0.48995	0.30271	1.44873
TRINITY_DN34426_c0_g1_i1_orf1	laminin subunit alpha-like, partial [Ostrinia furnacalis]	-0.8033	-0.5306	-1.0779	1.16655	1.24521
TRINITY_DN13221_c0_g1_i3_orf1	fasciclin-3-like [Ostrinia furnacalis]	-0.0193	-0.3721	-1.5586	0.45982	1.49017
TRINITY_DN14274_c0_g1_i3_orf1	ATP-dependent RNA helicase dbp2-like isoform X1 [Leguminivora glycinivorella]	-0.5053	-0.5934	-1.2046	0.77156	1.53174
TRINITY_DN2109_c0_g1_i4_orf1	mucin-2-like isoform X2 [Ostrinia furnacalis]	-0.3081	-0.8401	-1.0287	0.46726	1.70962
TRINITY_DN3715_c0_g1_i2_orf1	uncharacterized protein LOC114356437 isoform X1 [Ostrinia furnacalis]	-0.3023	-0.6001	-1.0332	0.07118	1.86439
TRINITY_DN1252_c0_g1_i3_orf1	unnamed protein product [Chilo suppressalis]	-0.5125	-1.0092	-0.1413	-0.2447	1.90763
TRINITY_DN2676_c0_g1_i2_orf1	probable cytochrome P450 303a1 [Ostrinia furnacalis] >XP_028178318.1 probable cytochrome P450 303a1 [Ostrinia furnacalis]	-0.6224	-1.0325	-0.6949	0.78592	1.56394
TRINITY_DN3131_c0_g1_i5_orf1	senecionine N-oxygenase-like isoform X1 [Ostrinia furnacalis] >XP_028178163.1 senecionine N-oxygenase-like isoform X2 [Ostrinia furnacalis] >XP_028178164.1 senecionine N-oxygenase-like isoform X1 [Ostrinia furnacalis]	0.09422	-0.5748	-1.5657	0.78281	1.26352
TRINITY_DN23582_c0_g1_i1_orf1	unnamed protein product [Diatraea saccharalis]	-0.1634	-0.8497	-1.0516	0.3204	1.74434
TRINITY_DN8659_c0_g2_i1_orf1	ubiquitin-like modifier-activating enzyme 1 [Ostrinia furnacalis]	-0.3919	-1.2649	0.02678	-0.164	1.79408
TRINITY_DN867_c0_g1_i1_orf1	hemicentin-2-like isoform X1 [Ostrinia furnacalis]	-0.0705	-1.0353	-0.4688	-0.3225	1.89718
TRINITY_DN51995_c0_g3_i1_orf1	circadian clock-controlled protein-like [Ostrinia furnacalis]	-1.03	0.28199	-0.2009	1.77233	-0.8234
TRINITY_DN4204_c0_g1_i1_orf1	uncharacterized protein LOC114359352 [Ostrinia furnacalis]	-0.6383	0.90027	-0.4705	1.4345	-1.2259
TRINITY_DN143532_c0_g1_i1_orf1	3-oxoacyl-[acyl-carrier-protein] reductase FabG-like [Aphidius gifuensis] >KAF7996667.1 hypothetical protein HCN44_002313 [Aphidius	-0.7626	0.5276	-0.6005	1.72741	-0.892
TRINITY_DN1534_c0_g1_i3_orf1	peptidoglycan recognition protein-like [Ostrinia furnacalis]	-1.0596	0.39243	-0.4714	1.76361	-0.625
TRINITY_DN68770_c0_g1_i1_orf1	seroin transcript 1A2 [Ostrinia nubilalis]	-0.724	0.55209	-0.1714	1.60041	-1.2571
TRINITY_DN9458_c0_g1_i4_orf1	uncharacterized protein LOC114363583 [Ostrinia furnacalis]	-0.2072	-0.2896	-0.7053	1.95256	-0.7505
TRINITY_DN55154_c0_g2_i1_orf1	glycosyl transferase family 8 domain-containing protein [Phthorimaea operculella]	-0.4107	0.73229	-0.6838	1.55258	-1.1904
TRINITY_DN109503_c0_g1_i4_orf1	uncharacterized protein LOC114366345 isoform X2 [Ostrinia furnacalis]	-0.1097	0.07113	-0.9471	1.83449	-0.8489
TRINITY_DN30713_c0_g1_i3_orf1	phosphoglucomutase [Ostrinia furnacalis]	-0.3786	0.00795	-0.1871	1.80629	-1.2486
TRINITY_DN2749_c4_g1_i2_orf1	RNA exonuclease 4-like [Ostrinia furnacalis] >QEE79882.1 REX4 [Ostrinia furnacalis]	0.39193	0.44146	-1.3738	1.41436	-0.874
TRINITY_DN5564_c0_g1_i5_orf1	probable phosphoserine aminotransferase [Ostrinia furnacalis]	-0.2454	0.18988	-0.2394	1.69714	-1.4022
TRINITY_DN1785_c0_g1_i5_orf1	beta-mannosidase [Ostrinia furnacalis]	0.17189	-0.1324	-1.1412	1.78449	-0.6828
TRINITY_DN2673_c2_g1_i2_orf1	aminopeptidase N3c [Ostrinia nubilalis]	0.51512	-0.1977	-0.4961	1.57823	-1.3995
TRINITY_DN16978_c0_g1_i1_orf1	la-related protein 7 [Helicoverpa armigera]	0.38497	-0.3223	-0.1058	1.56037	-1.5172
TRINITY_DN46633_c0_g1_i4_orf1	uncharacterized protein LOC114365425 [Ostrinia furnacalis] >QKV49448.1 fas-associated death domain protein [Ostrinia furnacalis]	0.88434	-0.4207	0.25077	1.00715	-1.7215

TRINITY_DN311_c0_g1_i8_orfp1	TRINITY_DN311_c0_g1_i8_m.65152 TRINITY_DN311_c0_g1::TRINITY_DN311_c0_g1_i8::g.65152 ORF type:5prime_partial len:138 (+),score=74.30 TRINITY_DN311_c0_g1_i8:2-415(+)	-0.6012	-1.0383	1.0148	1.39298	-0.7683
TRINITY_DN17693_c0_g1_i10_orf1	acetylcholinesterase-like [Ostrinia furnacalis]	-0.8678	-0.6608	-0.4822	1.88745	0.1234
TRINITY_DN128231_c0_g1_i5_orf1	glutathione S-transferase sigma3 [Glyphodes pyloalis]	-0.4286	-0.925	-0.3961	1.94093	-0.1912
TRINITY_DN21341_c0_g1_i1_orf1	FAST kinase domain-containing protein 4 isoform X6 [Ostrinia furnacalis] >XP_028160336.1 FAST kinase domain-containing protein 4 isoform X7 [Ostrinia furnacalis] >XP_028160337.1 FAST kinase domain-containing protein 4 isoform X8 [Ostrinia furnacalis]	-0.4394	-0.6252	-0.6023	1.98758	-0.3207
TRINITY_DN19866_c0_g1_i4_orf1	lys-63-specific deubiquitinase BRCC36-like [Ostrinia furnacalis]	-0.5354	-0.2798	-0.8024	1.967	-0.3493
TRINITY_DN2338_c0_g2_i1_orf1	prophenoloxidase PPO3 [Ostrinia furnacalis]	-1.0157	-0.8209	0.17361	1.80143	-0.1384
TRINITY_DN6203_c0_g1_i1_orfp1	TRINITY_DN6203_c0_g1_i1_m.72736 TRINITY_DN6203_c0_g1::TRINITY_DN6203_c0_g1_i1::g.72736 ORF type:internal len:93 (+),score=12.26 TRINITY_DN6203_c0_g1_i1:3-278(+)	-0.0773	-0.9932	-1.0162	1.67492	0.41175
TRINITY_DN33408_c0_g1_i1_orf1	hypothetical protein HF086_017664 [Spodoptera exigua]	-0.0013	-1.581	-0.2807	1.51723	0.3458
TRINITY_DN16123_c0_g1_i1_orf1	39S ribosomal protein L51, mitochondrial [Ostrinia furnacalis]	-0.443	-1.1693	0.20015	1.80137	-0.3892
TRINITY_DN1628_c0_g2_i3_orf1	uncharacterized protein LOC114363979 [Ostrinia furnacalis]	-0.5491	-0.9881	-0.2848	1.90626	-0.0842
TRINITY_DN8771_c0_g2_i1_orf1	regucalcin-like [Ostrinia furnacalis]	0.56095	-1.2035	-0.6006	1.64712	-0.404
TRINITY_DN5852_c0_g1_i6_orf1	probable maltase isoform X6 [Ostrinia furnacalis]	-0.7384	-1.1248	1.40678	0.97196	-0.5155
TRINITY_DN2338_c0_g1_i3_orf1	phenoloxidase subunit 1-like [Ostrinia furnacalis]	-0.0718	-1.1188	-0.0224	1.83265	-0.6196
TRINITY_DN30185_c0_g1_i3_orf1	organic cation transporter protein [Ostrinia furnacalis]	-0.3268	-1.1733	-0.1519	1.85773	-0.2058
TRINITY_DN113327_c0_g1_i2_orf1	proteasome subunit beta type-6 [Helicoverpa armigera] >XP_047031479.1 proteasome subunit beta type-6 [Helicoverpa zea] >XP_049697949.1 proteasome subunit beta type-6-like [Helicoverpa armigera] >PZC87318.1 hypothetical protein B5X24_HaOG201554 [Helicoverpa armigera]	-0.709	-0.5915	-0.3105	1.97883	-0.3679
TRINITY_DN28626_c0_g1_i5_orf1	3-ketodihydrosphingosine reductase [Ostrinia furnacalis]	-0.0088	-1.6033	0.73147	1.30823	-0.4276
TRINITY_DN3126_c0_g1_i4_orf1	unnamed protein product, partial [Iphiclides podalirius]	-0.0042	-1.1432	0.8056	1.39271	-1.051
TRINITY_DN1402_c1_g1_i6_orf1	unnamed protein product [Parnassius apollo]	0.13082	-0.9463	0.23938	1.67804	-1.102
TRINITY_DN3092_c0_g1_i2_orf1	replication factor C subunit 1 isoform X1 [Ostrinia furnacalis] >XP_028157702.1 replication factor C subunit 1 isoform X2 [Ostrinia furnacalis]	0.4809	-0.8925	-0.6326	1.75231	-0.7081
TRINITY_DN1380_c0_g1_i5_orf1	ubiquitin-fold modifier-conjugating enzyme 1 [Ostrinia furnacalis]	-0.2546	-1.3573	1.49966	0.70325	-0.591
TRINITY_DN4051_c0_g1_i1_orf1	60S acidic ribosomal protein P2 [Ostrinia furnacalis]	0.88442	-1.298	-0.6592	1.40903	-0.3362
TRINITY_DN52887_c0_g1_i1_orf1	cytochrome P450 6B5-like [Ostrinia furnacalis]	0.24265	-1.4519	0.23694	1.55952	-0.5872
TRINITY_DN3456_c0_g2_i1_orf1	protein purity of essence [Ostrinia furnacalis]	1.14726	-1.1764	-0.9056	1.18944	-0.2546
TRINITY_DN102712_c0_g1_i1_orf1	transmembrane protein 177 [Ostrinia furnacalis]	0.53319	-1.6336	-0.0506	1.40683	-0.2559
TRINITY_DN13923_c0_g2_i1_orf1	sideroflexin-2 [Zerene cesonia]	1.36103	-1.0571	-1.0605	0.9348	-0.1783
TRINITY_DN45949_c0_g1_i1_orf1	uncharacterized protein LOC114355167 [Ostrinia furnacalis]	-0.1172	0.32304	0.86444	0.79938	-1.8697
TRINITY_DN31390_c0_g1_i2_orf1	UDP-glucuronosyltransferase 2B20-like [Ostrinia furnacalis]	-0.357	-0.1839	1.09817	1.03994	-1.5973
TRINITY_DN48694_c0_g1_i1_orfp1	TRINITY_DN48694_c0_g1_i1_m.75338 TRINITY_DN48694_c0_g1::TRINITY_DN48694_c0_g1_i1::g.75338 ORF type:internal len:84 (+),score=16.02 TRINITY_DN48694_c0_g1_i1:2-250(+)	-0.5309	-1.6681	1.04725	0.27953	0.87221
TRINITY_DN526_c0_g1_i1_orf1	secretory phospholipase A2 receptor-like [Ostrinia furnacalis]	-0.2434	-1.6444	1.41687	-0.0076	0.47858
TRINITY_DN100208_c0_g1_i1_orf1	neurofilament heavy polypeptide-like isoform X2 [Ostrinia furnacalis]	-0.1176	-1.8004	0.67443	0.11339	1.13013
TRINITY_DN52244_c1_g1_i1_orf1	triokinase/FMN cyclase-like isoform X2 [Ostrinia furnacalis]	0.03859	-1.702	1.39091	-0.1188	0.39129

TRINITY_DN31584_c0_g2_i2_orf1	14-3-3 protein epsilon [Gallus gallus] >NP_001233297.1 14-3-3 protein epsilon [Pan troglodytes] >NP_006752.1 14-3-3 protein epsilon [Homo sapiens] >NP_033562.3 14-3-3 protein epsilon [Mus musculus] >NP_113791.2 14-3-3 protein epsilon [Rattus norvegicus] >NP_776916.1 14-3-3 protein epsilon [Bos taurus] >XP_001504337.1 14-3-3 protein epsilon isoform X1 [Equus caballus] >XP_002918088.2 14-3-3 protein epsilon isoform X2 [Ailuropoda melanoleuca] >XP_003416855.1 14-3-3 protein epsilon isoform X1 [Loxodonta africana] >XP_003469733.1 14-3-3 protein epsilon isoform X1 [Cavia porcellus] >XP_003816884.1 14-3-3 protein epsilon isoform X1 [Pan paniscus] >XP_003912098.1 14-3-3 protein epsilon isoform X1 [Papio anubis] >XP_003929381.1 14-3-3 protein epsilon isoform X1 [Saimiri boliviensis boliviensis] >XP_003996471.1 14-3-3 protein epsilon isoform X1 [Felis catus] >XP_004267124.1 14-3-3 protein epsilon isoform X1 [Orcinus orca] >XP_004376223.1 14-3-3 protein epsilon [Trichechus manatus latirostris] >XP_004404155.1 PREDICTED: 14-3-3 protein epsilon isoform X2 [Odobenus rosmarus divergens] >XP_004433380.1 PREDICTED: 14-3-3 protein epsilon isoform X1 [Ceratotherium simum simum] >XP_004483832.1 14-3-3 protein epsilon isoform X1 [Dasypus novemcinctus] >XP_004605045.1 PREDICTED: 14-3-3 protein epsilon [Sorex araneus] >XP_004667919.1 14-3-3 protein epsilon [Jaculus jaculus] >XP_004706944.1 14-3-3 protein epsilon [Echinops telfairi] >XP_004746947.1 14-3-3 protein epsilon isoform X1 [Mustela putorius furo] >XP_004857172.1 14-3-3 protein epsilon isoform X1 [Heterocephalus glaber] >XP_005067448.1 14-3-3 protein epsilon isoform X1 [Mesocricetus auratus] >XP_005240506.1 14-3-3 protein epsilon isoform X1 [Falco peregrinus] >XP_005327947.1 14-3-3 protein epsilon isoform X1 [Ictidomys tridecemlineatus] >XP_005349591.1 14-3-3 protein epsilon isoform X1 [Microtus ochrogaster] >XP_005402688.1 PREDICTED: 14-3-3 protein epsilon isoform X1 [Chinchilla lanigera] >XP_005525859.1 PREDICTED: 14-3-3 protein epsilon isoform X1 [Pseudopodoces humilis] >XP_005888292.1 PREDICTED: 14-3-3 protein epsilon isoform X1 [Bos mutus] >XP_006079841.1 14-3-3 protein epsilon isoform X1 [Bubalus bubalis] >XP_006099253.1 14-3-3 protein epsilon [Myotis lucifugus] >XP_006185046.1 14-3-3 protein epsilon isoform X1 [Camelus ferus] >XP_006214490.1 14-3-3 protein epsilon isoform X1 [Vicugna pacos] >XP_006259463.1 PREDICTED: 14-3-3 protein epsilon [Alligator mississippiensis] >XP_006768146.1 PREDICTED: 14-3-3 protein epsilon isoform X1 [Myotis davidii] >XP_006863283.1 PREDICTED: 14-3-3 protein epsilon [Chrysochloris asiatica] >XP_006891074.1 PREDICTED: 14-3-3 protein epsilon-like [Elephantulus edwardii] >XP_006925117.1 14-3-3 protein epsilon isoform X1 [Pteropus alecto] >XP_006977465.1 14-3-3 protein epsilon isoform X1 [Peromyscus maniculatus bairdii] >XP_007057769.1 14-3-3 protein epsilon isoform X1 [Chelonia mydas] >XP_007123613.1 14-3-3 protein epsilon isoform X1 [Physeter catodon] >XP_007183877.1 14-3-3 protein epsilon isoform X1 [Balaenoptera acutorostrata scammoni] >XP_007454293.1 PREDICTED: 14-3-3 protein epsilon [Lipotes vexillifer] >XP_007520478.1 PREDICTED: 14-3-3 protein epsilon [Erinaceus europaeus] >XP_007935626.1 14-3-3 protein epsilon [Orycteropus afer afer] >XP_008007997.1 14-3-3 protein epsilon isoform X1 [Chlorocebus sabaeus] >XP_008058985.1 14-3-3 protein epsilon isoform X2 [Carlito syrichta] >XP_008146090.1 14-3-3 protein epsilon isoform X1 [Eptesicus fuscus] >XP_008512998.1 PREDICTED: 14-3-3 protein epsilon isoform X1 [Equus przewalskii] >XP_008591162.1 PREDICTED: 14-3-3 protein epsilon [Galeopterus variegatus] >XP_008826988.1 14-3-3 protein epsilon [Nannospalax galili] >XP_009249385.2 14-3-3 protein epsilon isoform X1 [Pongo abelii] >XP_010371794.1 14-3-3 protein epsilon isoform X1 [Rhinopithecus roxellana] >XP_010571909.1 PREDICTED: 14-3-3 protein epsilon isoform X5 [Haliaeetus leucocephalus] >XP_010640470.1 14-3-3 protein epsilon [Fukomys damarensis] >XP_010859650.1 PREDICTED: 14-3-3 protein epsilon isoform X1 [Bison bison	0.41464	-0.3173	-1.7643	1.16898	0.49802
TRINITY_DN17406_c0_g1_i1_orf1	uncharacterized protein LOC114356625 [Ostrinia furnacalis]	0.06111	-1.3357	-0.6818	0.33267	1.62374
TRINITY_DN32780_c0_g1_i2_orf1	renin receptor [Ostrinia furnacalis]	0.43501	-1.5397	-0.7751	0.74984	1.13001
TRINITY_DN21380_c0_g1_i1_orf1	ankyrin repeat domain-containing protein 13C [Ostrinia furnacalis]	0.03425	-1.4295	-0.6795	0.62437	1.45041
TRINITY_DN12771_c0_g1_i1_orf1	histone acetyltransferase type B catalytic subunit [Ostrinia furnacalis]	0.73246	-1.3103	-0.826	-0.0325	1.43641
TRINITY_DN19651_c0_g1_i1_orf1	cytosolic non-specific dipeptidase [Ostrinia furnacalis]	0.86787	-1.2465	-0.8959	-0.0968	1.37145
TRINITY_DN9354_c0_g1_i7_orf1	hypothetical protein evm_012205 [Chilo suppressalis] >CAB3527181.1 unnamed protein product [Chilo suppressalis] >CAH0404510.1 unnamed protein product [Chilo suppressalis]	0.54897	-1.3367	-0.3902	-0.4274	1.60529
TRINITY_DN1313_c0_g1_i2_orf1	39S ribosomal protein L40, mitochondrial [Ostrinia furnacalis]	0.82255	-1.0424	-0.8942	-0.396	1.51007
TRINITY_DN1760_c0_g1_i4_orf1	uncharacterized protein LOC114357676 [Ostrinia furnacalis]	1.04127	-1.8661	0.46612	0.45486	-0.0961
TRINITY_DN987_c0_g1_i11_orf1	macrophage mannose receptor 1-like [Pieris napi]	1.07314	-1.821	-0.2234	0.55905	0.41221
TRINITY_DN18937_c0_g1_i1_orf1	uncharacterized protein LOC114351683 isoform X7 [Ostrinia furnacalis]	1.21932	-1.8056	-0.0299	0.13158	0.48459
TRINITY_DN3307_c1_g1_i2_orf1	BTB/POZ domain-containing protein 2-like [Ostrinia furnacalis]	0.71451	-1.3555	1.48134	-0.6489	-0.1915
TRINITY_DN6016_c0_g1_i8_orf1	hypothetical protein evm_010883 [Chilo suppressalis]	1.08612	-1.6694	0.70201	-0.5758	0.45713
TRINITY_DN33452_c0_g1_i3_orf1	lethal(2) giant larvae protein isoform X8 [Ostrinia furnacalis]	1.00146	-1.4678	1.21644	-0.5771	-0.173
TRINITY_DN48973_c0_g1_i5_orf1	uncharacterized protein LOC114351683 isoform X8 [Ostrinia furnacalis]	1.42033	-1.5416	-0.5401	0.11178	0.54958
TRINITY_DN18918_c0_g1_i3_orf1	myrosinase 1-like isoform X2 [Ostrinia furnacalis]	1.59734	-1.0012	-0.6472	0.74175	-0.6908
TRINITY_DN15160_c0_g1_i1_orf1	tyrosine--tRNA ligase, cytoplasmic [Ostrinia furnacalis]	1.44235	-0.9624	-0.0054	0.73244	-1.2071
TRINITY_DN6545_c0_g1_i6_orf1	organic cation transporter protein-like [Ostrinia furnacalis]	1.68091	-1.3157	-0.6285	0.04846	0.21481
TRINITY_DN500_c0_g1_i1_orf1	splicing factor U2AF 50 kDa subunit isoform X2 [Manduca sexta]	1.75346	-1.1926	-0.2405	0.2836	-0.604
TRINITY_DN5234_c0_g1_i2_orf1	proline dehydrogenase 1, mitochondrial isoform X2 [Ostrinia furnacalis]	1.83815	-0.7304	-0.5801	0.28934	-0.817
TRINITY_DN747_c0_g1_i1_orf1	trypsin, alkaline C-like [Ostrinia furnacalis]	1.62949	-1.0124	-0.4246	0.65242	-0.8449
TRINITY_DN13186_c0_g1_i1_orf1	NADH dehydrogenase [ubiquinone] iron-sulfur protein 5-like [Bicyclus anynana]	1.83473	-0.8561	-0.4855	0.27476	-0.7678
TRINITY_DN5593_c0_g1_i1_orf1	PREDICTED: leucine-rich repeat-containing protein 47-like [Fopius arisanus]	1.68177	-1.3723	0.31009	-0.3213	-0.2982
TRINITY_DN2630_c0_g3_i3_orf1	eukaryotic translation initiation factor 4E-binding protein 2 [Ostrinia furnacalis]	1.81823	-1.1163	-0.533	0.18898	-0.3579
TRINITY_DN2695_c0_g1_i8_orfp1	TRINITY_DN2695_c0_g1_i8_m.44478 TRINITY_DN2695_c0_g1::TRINITY_DN2695_c0_g1_i8::g.44478 ORF type:3prime_partial len:532 (+),score=80.62 TRINITY_DN2695_c0_g1_i8:101-1594(+)	1.22783	-0.3389	-0.8596	1.13387	-1.1632
TRINITY_DN14073_c0_g1_i1_orf1	cytochrome c oxidase subunit 4 isoform 1, mitochondrial-like [Ostrinia furnacalis] >XP_028164918.1 cytochrome c oxidase subunit 4 isoform 1, mitochondrial-like [Ostrinia furnacalis]	1.88216	-0.6193	-0.8953	0.13622	-0.5038
TRINITY_DN69049_c0_g2_i1_orf1	membrane alanyl aminopeptidase-like [Ostrinia furnacalis]	1.63304	-1.4731	-0.3617	0.02351	0.17825
TRINITY_DN27852_c0_g1_i1_orf1	baculoviral IAP repeat-containing protein 6-like [Ostrinia furnacalis]	1.76179	-1.1789	-0.5287	-0.3628	0.30854

TRINITY_DN99_c0_g1_i3_orf1	uncharacterized protein LOC126375979 [Pectinophora gossypiella] >XP_049879066.1 uncharacterized protein LOC126375979 [Pectinophora gossypiella]	1.53317	-0.4849	-0.842	0.81448	-1.0207
TRINITY_DN107288_c0_g1_i2_orf1	methionine-tRNA synthetase, partial [Papilio xuthus]	1.68864	-0.95	-0.0971	0.39716	-1.0387
TRINITY_DN2748_c0_g1_i6_orf1	uncharacterized protein LOC114352811 [Ostrinia furnacalis]	1.85398	-0.7525	0.01085	-0.1216	-0.9908
TRINITY_DN2497_c0_g1_i2_orf1	protein stunted-like isoform X1 [Colias croceus]	1.74511	-0.8325	0.34292	-0.206	-1.0496
TRINITY_DN4514_c0_g1_i1_orf1	enoyl-CoA delta isomerase 1, mitochondrial-like isoform X1 [Ostrinia furnacalis] >XP_028158560.1 enoyl-CoA delta isomerase 1, mitochondrial-like isoform X2 [Ostrinia furnacalis]	1.55263	-0.7943	0.53118	0.00521	-1.2947
TRINITY_DN84357_c0_g1_i1_orf1	4-coumarate--CoA ligase 1-like [Ostrinia furnacalis]	1.39544	-0.9064	0.72962	0.08216	-1.3008
TRINITY_DN2266_c0_g1_i6_orf1	bilin-binding protein-like [Ostrinia furnacalis]	1.73985	-0.8746	-0.7522	0.5073	-0.6203
TRINITY_DN344_c1_g1_i1_orf1	chymotrypsin-like serine protease 16 [Ostrinia nubilalis]	1.52301	-0.5969	-0.6543	0.82822	-1.1001
TRINITY_DN86149_c0_g1_i1_orf1	NADH dehydrogenase [ubiquinone] 1 alpha subcomplex subunit 8 [Galleria mellonella]	1.94831	-0.9156	-0.4229	-0.2826	-0.3272
TRINITY_DN478_c0_g1_i16_orf1	lipid storage droplets surface-binding protein 2 isoform X1 [Ostrinia furnacalis]	1.75638	-1.2279	0.12968	-0.6241	-0.0341
TRINITY_DN41697_c0_g1_i1_orf1	5-formyltetrahydrofolate cyclo-ligase [Ostrinia furnacalis]	1.36771	-1.4819	-0.7452	0.36306	0.49628
TRINITY_DN628_c0_g1_i7_orf1	prostamide/prostaglandin F synthase-like [Ostrinia furnacalis]	1.86953	-0.9266	-0.6395	-0.461	0.15754
TRINITY_DN46409_c0_g1_i1_orf1	unnamed protein product [Heterotrigona itama]	1.901	-0.8067	-0.8247	-0.2325	-0.0372
TRINITY_DN15624_c0_g1_i1_orf1	LOW QUALITY PROTEIN: V-type proton ATPase subunit S1-like [Ostrinia furnacalis]	1.84277	-0.9322	-0.7471	-0.3677	0.20432
TRINITY_DN8087_c0_g1_i9_orf1	cysteine-rich with EGF-like domain protein 2 isoform X1 [Ostrinia furnacalis]	1.84593	-0.8687	-0.8464	-0.3031	0.17227
TRINITY_DN30663_c0_g1_i1_orf1	surfeit locus protein 6 homolog [Ostrinia furnacalis]	1.95041	-0.7578	-0.2784	-0.2058	-0.7084
TRINITY_DN55160_c0_g2_i1_orf1	esterase FE4-like isoform X2 [Ostrinia furnacalis]	1.86703	-0.5263	-1.0598	0.05259	-0.3335
TRINITY_DN5578_c0_g1_i10_orf1	unnamed protein product [Chilo suppressalis]	1.83907	-0.6687	-1.015	-0.331	0.17567
TRINITY_DN29018_c0_g1_i4_orf1	prostaglandin reductase 1-like isoform X1 [Ostrinia furnacalis] >XP_028178925.1 prostaglandin reductase 1-like isoform X2 [Ostrinia furnacalis]	1.95403	-0.566	-0.4845	-0.1212	-0.7823
TRINITY_DN27592_c0_g1_i1_orf1	D-arabinitol dehydrogenase 1-like [Ostrinia furnacalis]	1.36271	-0.954	1.05436	-0.5719	-0.8912
TRINITY_DN336_c0_g1_i6_orfp1	TRINITY_DN336_c0_g1_i6_m.64791 TRINITY_DN336_c0_g1::TRINITY_DN336_c0_g1_i6::g.64791 ORF type:complete len:61 (-),score=19.53 TRINITY_DN336_c0_g1_i6:236-418(-)	1.84736	-0.4487	-0.4106	0.10981	-1.0979
