# Supplementary material for: Comparative Proteomics and Metabonomics Analysis of Different Diapause Stages Revealed a New Regulation Mechanism of Diapause in Loxostege sticticalis (Lepidoptera: Pyralidae)
Source: Molecules. 2024 Jul 25;29(15):3472. doi: 10.3390/molecules29153472 (PMC11314584; doi:10.3390/molecules29153472)
Supplement: Supplementary file 1 [file molecules-29-03472-s001.zip › analysis process/proteomic/Cluster analysis of expression patterns/Up/CTvsND up.pdf]

Accession	Description	ND	RD	PreD	CT	D
TRINITY_DN2396_c0_g1_i9_orfp1	TRINITY_DN2396_c0_g1_i9_m.39038 TRINITY_DN2396_c0_g1::TRINITY_DN2396_c0_g1_i9::g.39038 ORF type:5prime_partial len:181 (+),score=90.33 TRINITY_DN2396_c0_g1_i9:3-545(+)	-1.9205	-0.0422	0.75417	0.67782	0.53072
TRINITY_DN971_c0_g1_i5_orfp1	TRINITY_DN971_c0_g1_i5_m.54249 TRINITY_DN971_c0_g1::TRINITY_DN971_c0_g1_i5::g.54249 ORF type:internal len:108 (+),score=66.98 TRINITY_DN971_c0_g1_i5:1-321(+)	-1.7632	-0.3999	0.48896	1.04991	0.62427
TRINITY_DN84478_c0_g1_i8_orf1	uncharacterized protein LOC114359035 isoform X1 [Ostrinia furnacalis]	-1.9622	0.47386	0.50348	0.79809	0.18675
TRINITY_DN14242_c0_g1_i2_orfp1	TRINITY_DN14242_c0_g1_i2_m.18449 TRINITY_DN14242_c0_g1::TRINITY_DN14242_c0_g1_i2::g.18449 ORF type:internal len:148 (-),score=102.30 TRINITY_DN14242_c0_g1_i2:2-442(-)	-1.8828	0.08701	0.95197	0.72609	0.11777
TRINITY_DN971_c0_g1_i10_orfp1	TRINITY_DN971_c0_g1_i10_m.54268 TRINITY_DN971_c0_g1::TRINITY_DN971_c0_g1_i10::g.54268 ORF type:internal len:187 (+),score=141.68 TRINITY_DN971_c0_g1_i10:1-558(+)	-1.8854	-0.1083	0.89113	0.42557	0.67699
TRINITY_DN295_c3_g1_i1_orfp1	TRINITY_DN295_c3_g1_i1_m.18839 TRINITY_DN295_c3_g1::TRINITY_DN295_c3_g1_i1::g.18839 ORF type:5prime_partial len:118 (+),score=46.25 TRINITY_DN295_c3_g1_i1:1-354(+)	-1.8977	-0.1243	0.58171	0.78172	0.65859
TRINITY_DN1108_c3_g1_i1_orfp1	TRINITY_DN1108_c3_g1_i1_m.5561 TRINITY_DN1108_c3_g1::TRINITY_DN1108_c3_g1_i1::g.5561 ORF type:internal len:113 (+),score=89.07 TRINITY_DN1108_c3_g1_i1:1-336(+)	-1.907	0.17271	0.7938	0.83166	0.10881
TRINITY_DN2044_c0_g1_i5_orfp1	TRINITY_DN2044_c0_g1_i5_m.4210 TRINITY_DN2044_c0_g1::TRINITY_DN2044_c0_g1_i5::g.4210 ORF type:complete len:151 (-),score=85.31 TRINITY_DN2044_c0_g1_i5:857-1309(-)	-1.7613	-0.4013	0.44879	1.03947	0.67434
TRINITY_DN143895_c0_g1_i1_orf1	cathepsin L-like [Aphidius gifuensis] >KAF7988186.1 hypothetical protein HCN44_007680 [Aphidius gifuensis]	-1.9287	0.62519	0.05375	0.85423	0.39558
TRINITY_DN5080_c0_g1_i5_orf1	storage protein [Ostrinia furnacalis]	-1.9646	0.77358	0.27671	0.61075	0.3036
TRINITY_DN10877_c0_g1_i1_orf1	spodomicin-like [Ostrinia furnacalis]	-1.5409	-0.3942	1.42832	0.64179	-0.135
TRINITY_DN703_c0_g1_i2_orf1	acidic juvenile hormone-suppressible protein 1-like [Ostrinia furnacalis]	-1.9072	0.46724	0.09245	1.01136	0.33613
TRINITY_DN35809_c0_g1_i1_orf1	spodomicin-like [Ostrinia furnacalis] >QKV49445.1 diapausin [Ostrinia furnacalis]	-1.6706	-0.5943	0.99078	0.81406	0.46002
TRINITY_DN142657_c0_g1_i1_orf1	sorting and assembly machinery component 50 homolog [Diachasma alloeum]	-1.8702	0.27013	-0.007	1.06633	0.54074
TRINITY_DN34423_c0_g1_i3_orf1	THAP domain-containing protein 4-like [Ostrinia furnacalis]	-1.9506	0.07258	0.60276	0.71887	0.55645
TRINITY_DN57137_c0_g1_i1_orfp1	TRINITY_DN57137_c0_g1_i1_m.46420 TRINITY_DN57137_c0_g1::TRINITY_DN57137_c0_g1_i1::g.46420 ORF type:5prime_partial len:56 (-),score=4.65 TRINITY_DN57137_c0_g1_i1:82-249(-)	-1.8476	-0.2302	0.79953	0.44279	0.83552
TRINITY_DN56459_c0_g1_i2_orf1	aldo-keto reductase AKR2E4-like [Ostrinia furnacalis]	-1.693	-0.6049	0.70412	0.82862	0.76515
TRINITY_DN4767_c0_g1_i6_orf1	cysteine protease XCP2-like [Ostrinia furnacalis]	-1.8723	0.60017	-0.1558	0.9266	0.50133
TRINITY_DN106156_c1_g1_i1_orf1	arylphorin subunit alpha-like [Ostrinia furnacalis]	-1.848	0.47645	-0.0056	1.14116	0.23597
TRINITY_DN4767_c0_g1_i4_orf1	cysteine protease XCP2-like [Ostrinia furnacalis]	-1.9572	0.75927	0.28587	0.67435	0.23768
TRINITY_DN51813_c0_g1_i1_orf1	uncharacterized protein LOC114350216 [Ostrinia furnacalis]	-1.8579	0.87806	0.07584	0.87799	0.02598
TRINITY_DN703_c13_g1_i1_orf1	acidic juvenile hormone-suppressible protein 1-like [Ostrinia furnacalis]	-1.8393	0.39265	-0.0409	1.16504	0.32248
TRINITY_DN12534_c0_g1_i4_orf1	antibacterial protein [Heliothis virescens]	-1.7409	-0.5078	0.64548	0.87018	0.73311
TRINITY_DN22515_c0_g1_i10_orf1	hypothetical protein KGM_202621 [Danaus plexippus plexippus]	-1.9985	0.52323	0.42931	0.54593	0.5
TRINITY_DN15202_c0_g1_i6_orf1	uncharacterized protein LOC114364499 isoform X2 [Ostrinia furnacalis]	-1.6715	-0.5354	0.3374	0.76407	1.10538
TRINITY_DN1370_c0_g1_i2_orf1	hypothetical protein evm_000756 [Chilo suppressalis]	-1.8885	1.05558	0.09354	0.50648	0.23284
TRINITY_DN59885_c0_g1_i3_orf1	TGF-beta-activated kinase 1 and MAP3K7-binding protein 1-like [Ostrinia furnacalis]	-1.9579	0.58876	0.09603	0.65318	0.6199
TRINITY_DN5080_c0_g1_i1_orf1	basic juvenile hormone-suppressible protein 2-like [Ostrinia furnacalis]	-1.9059	0.82977	0.09529	0.7979	0.1829
TRINITY_DN3166_c1_g1_i6_orf1	hypothetical protein evm_013813 [Chilo suppressalis]	-1.7427	-0.316	0.35242	0.4768	1.22947
TRINITY_DN380_c0_g2_i2_orf1	chemosensory protein 10 [Ostrinia furnacalis]	-1.7971	-0.349	0.4764	0.95239	0.71729
TRINITY_DN67193_c0_g1_i1_orf1	A-kinase anchor protein 14-like [Ostrinia furnacalis]	-1.8754	0.88028	-0.1456	0.70511	0.4356
TRINITY_DN36476_c1_g1_i1_orfp1	TRINITY_DN36476_c1_g1_i1_m.70910 TRINITY_DN36476_c1_g1::TRINITY_DN36476_c1_g1_i1::g.70910 ORF type:5prime_partial len:88 (-),score=0.50 TRINITY_DN36476_c1_g1_i1:49-312(-)	-1.6206	-0.6667	1.01449	0.42458	0.84827
TRINITY_DN136358_c0_g1_i1_orf1	acidic juvenile hormone-suppressible protein 1-like [Ostrinia furnacalis]	-1.7189	0.10951	-0.0119	1.41028	0.21095
TRINITY_DN6330_c0_g1_i1_orfp1	TRINITY_DN6330_c0_g1_i1_m.42332 TRINITY_DN6330_c0_g1::TRINITY_DN6330_c0_g1_i1::g.42332 ORF type:3prime_partial len:51 (-),score=33.30 TRINITY_DN6330_c0_g1_i1:3-152(-)	-1.7299	-0.4753	0.9219	0.40933	0.87403
TRINITY_DN17615_c0_g1_i3_orf1	hypothetical protein SFRUCORN_008858 [Spodoptera frugiperda]	-1.9064	-0.0689	0.52512	0.59568	0.85456
TRINITY_DN31348_c0_g1_i1_orf1	protein lethal(2)essential for life [Bombyx mori]	-1.6407	-0.5548	0.45428	1.24357	0.4976
TRINITY_DN7900_c0_g1_i4_orf1	uncharacterized protein LOC114366119 [Ostrinia furnacalis]	-1.9607	0.67795	0.18363	0.72056	0.37852
TRINITY_DN52316_c0_g1_i1_orf1	arylphorin subunit alpha-like [Ostrinia furnacalis]	-1.8643	0.72891	-0.1489	0.91262	0.37161
TRINITY_DN19662_c0_g2_i1_orf1	storage protein [Ostrinia furnacalis]	-1.9057	0.72226	0.08663	0.89322	0.20356
TRINITY_DN38506_c0_g1_i4_orf1	C-1-tetrahydrofolate synthase, cytoplasmic isoform X1 [Ostrinia furnacalis] >XP_028166137.1 C-1-tetrahydrofolate synthase, cytoplasmic isoform X2 [Ostrinia furnacalis] >XP_028166140.1 C-1-tetrahydrofolate synthase, cytoplasmic isoform X4 [Ostrinia furnacalis]	-1.4898	-0.918	0.82381	0.84048	0.74348
TRINITY_DN100327_c0_g1_i1_orf1	arylphorin subunit alpha-like [Ostrinia furnacalis]	-1.9285	0.33226	0.54409	0.92673	0.1254
TRINITY_DN1880_c0_g1_i4_orf1	serine protease inhibitor dipetalogastin-like [Helicoverpa zea]	-1.6005	-0.7326	0.69273	1.01569	0.62467
TRINITY_DN13799_c0_g1_i1_orf1	uncharacterized protein LOC116345248 [Contarinia nasturtii]	-1.6848	0.40844	-0.5319	1.10038	0.70782
TRINITY_DN135449_c0_g1_i5_orf1	larval cuticle protein LCP-17-like [Galleria mellonella]	-1.9325	0.33742	0.09542	0.84503	0.6546
TRINITY_DN12582_c0_g1_i5_orf1	uncharacterized protein LOC114355527 isoform X1 [Ostrinia furnacalis]	-1.9184	0.57309	0.24067	0.95443	0.15018

TRINITY_DN6122_c0_g1_i6_orf1	deubiquitinase DESI2 isoform X1 [Helicoverpa armigera] >XP_049707835.1 deubiquitinase DESI2 isoform X1 [Helicoverpa armigera]	-1.8841	0.76842	-0.085	0.8568	0.34394
TRINITY_DN1597_c0_g1_i5_orfp1	TRINITY_DN1597_c0_g1_i5_m.57494 TRINITY_DN1597_c0_g1::TRINITY_DN1597_c0_g1_i5::g.57494 ORF type:complete len:86 (+),score=7.19 TRINITY_DN1597_c0_g1_i5:134-391(+)	-1.8514	0.1037	-0.004	0.75829	0.99332
TRINITY_DN29414_c1_g2_i1_orf1	serine protease 44-like isoform X2 [Ostrinia furnacalis]	-1.9102	0.58049	-0.0303	0.89014	0.46987
TRINITY_DN42719_c0_g2_i1_orf1	inter-alpha-trypsin inhibitor heavy chain H4-like isoform X11 [Ostrinia furnacalis]	-1.872	0.91789	0.36021	0.71352	-0.1196
TRINITY_DN24723_c2_g1_i1_orf1	hypothetical protein evm_001103 [Chilo suppressalis]	-1.7666	-0.4458	0.77289	0.87289	0.56665
TRINITY_DN7040_c0_g2_i1_orf1	uncharacterized protein LOC114353763 [Ostrinia furnacalis]	-1.9628	0.6842	0.15188	0.66746	0.45922
TRINITY_DN45037_c0_g1_i1_orf1	trafficking protein particle complex subunit 1 [Ostrinia furnacalis]	-1.7565	0.51292	-0.1949	1.25727	0.18122
TRINITY_DN7226_c0_g1_i2_orf1	chemosensory protein [Conogethes punctiferalis]	-1.7465	-0.4445	0.82251	0.94813	0.42034
TRINITY_DN56308_c0_g1_i2_orf1	storage protein 1 [Omphisa fuscidentalis]	-1.8862	0.66885	-0.0679	0.92946	0.35573
TRINITY_DN124654_c0_g1_i1_orf1	protein lethal(2)essential for life [Manduca sexta] >KAG6441919.1 hypothetical protein O3G_MSEX002019 [Manduca sexta]	-1.656	0.39252	-0.5998	0.86896	0.99432
TRINITY_DN295_c5_g1_i2_orf1	unnamed protein product [Chilo suppressalis]	-1.729	-0.3868	0.54383	1.19189	0.3801
TRINITY_DN30169_c0_g1_i1_orfp1	TRINITY_DN30169_c0_g1_i1_m.11367 TRINITY_DN30169_c0_g1::TRINITY_DN30169_c0_g1_i1::g.11367 ORF type:3prime_partial len:52 (+),score=0.98 TRINITY_DN30169_c0_g1_i1:72-224(+)	-1.8231	0.76217	-0.3079	0.86317	0.50561
TRINITY_DN295_c2_g1_i2_orf1	phosphoglycolate phosphatase 1A, chloroplastic [Manduca sexta]	-1.8985	0.39185	0.36483	1.04894	0.09291
TRINITY_DN2813_c0_g1_i10_orf1	arylphorin subunit alpha-like [Ostrinia furnacalis]	-1.9032	0.46591	1.03526	0.1367	0.26532
TRINITY_DN19662_c4_g1_i1_orf1	basic juvenile hormone-suppressible protein 1-like [Ostrinia furnacalis]	-1.9202	0.62002	0.1667	0.92615	0.2073
TRINITY_DN71698_c0_g1_i1_orfp1	TRINITY_DN71698_c0_g1_i1_m.1194 TRINITY_DN71698_c0_g1::TRINITY_DN71698_c0_g1_i1::g.1194 ORF type:internal len:134 (+),score=19.66,Toxin_2 PF00451.20 4.3e-05,Toxin_2|PF00451.20|0.037,Toxin_2|PF00451.20|7.5e-05,Gamma-thionin|PF00304.21|0.017,Gamma-thionin|PF00304.21|0.05,Gamma-thionin|PF00304.21|0.021,Toxin_38|PF14866.7|0.13,Toxin_38|PF14866.7|0.15,Toxin_38|PF14866.7|0.15,Defensin_2|PF01097.19|0.053,Defensin_2|PF01097.19 |0.34,Defensin_2|PF01097.19|0.092 TRINITY_DN71698_c0_g1_i1:3-401(+)	-1.6737	-0.5788	0.86061	0.9738	0.41809
TRINITY_DN48548_c0_g1_i1_orf1	glutathione S-transferase siama 2 [Heortia vitessoides]	-1.9616	0.24345	0.40417	0.83871	0.47532
TRINITY_DN3707_c0_g1_i1_orf1	protein FAM160B1-like isoform X1 [Ostrinia furnacalis]	-1.9349	0.85775	0.11432	0.62927	0.33359
TRINITY_DN86772_c0_g1_i3_orfp1	x-tox [Spodoptera exigua]	-1.7865	-0.3059	0.98139	0.29628	0.81475
TRINITY_DN467_c3_g1_i5_orf1	lysozyme precursor [Loxostege sticticalis]	-1.9234	0.73528	0.02681	0.37481	0.78652
TRINITY_DN15578_c0_g2_i1_orfp1	uncharacterized protein LOC125235519 [Leguminivora glycinivorella]	-1.8883	0.71887	0.8435	0.43946	-0.1135
TRINITY_DN1540_c0_g1_i7_orf1	alaserpin-like isoform X13 [Ostrinia furnacalis]	-1.9589	0.58941	0.11918	0.72358	0.52676
TRINITY_DN1093_c0_g1_i4_orf1	uncharacterized protein LOC114361723 isoform X4 [Ostrinia furnacalis]	-1.9746	0.70522	0.21267	0.51794	0.53873
TRINITY_DN4816_c0_g2_i3_orf1	15-hydroxyprostaglandin dehydrogenase [NAD(+)]-like [Ostrinia furnacalis]	-1.8939	-0.1432	0.69379	0.73797	0.6054
TRINITY_DN11826_c0_g1_i4_orf1	aldehyde dehydrogenase X, mitochondrial-like [Ostrinia furnacalis]	-1.8742	0.73404	-0.1855	0.7961	0.52956
TRINITY_DN80328_c0_g1_i5_orf1	arylphorin subunit alpha-like [Ostrinia furnacalis]	-1.8607	0.64937	-0.1154	0.99701	0.32973
TRINITY_DN6025_c0_g2_i1_orfp1	TRINITY_DN6025_c0_g2_i1_m.7749 TRINITY_DN6025_c0_g2::TRINITY_DN6025_c0_g2_i1::g.7749 ORF type:5prime_partial len:106 (+),score=47.10 TRINITY_DN6025_c0_g2_i1:3-320(+)	-1.8279	-0.2936	0.8931	0.51334	0.71506
TRINITY_DN9538_c1_g3_i1_orf1	cilia- and flagella-associated protein 410 isoform X2 [Aphidius gifuensis]	-1.8452	1.10034	-0.0339	0.5893	0.18954
TRINITY_DN19731_c0_g1_i1_orf1	allergen Tha p 1-like [Ostrinia furnacalis] >XP_028174916.1 allergen Tha p 1-like [Ostrinia furnacalis] >BAV56808.1 chemosensory protein 4 [Ostrinia furnacalis]	-1.8323	-0.1912	0.27496	0.90423	0.84429
TRINITY_DN80328_c0_g1_i9_orf1	arylphorin subunit alpha-like [Ostrinia furnacalis]	-1.8873	0.63307	-0.0358	0.96428	0.32576
TRINITY_DN9079_c0_g1_i5_orf1	UDP-glucuronosyltransferase-like [Ostrinia furnacalis]	-1.885	-0.1702	0.64225	0.76676	0.64616
TRINITY_DN1391_c0_g1_i29_orfp1	TRINITY_DN1391_c0_g1_i29_m.70767 TRINITY_DN1391_c0_g1::TRINITY_DN1391_c0_g1_i29::g.70767 ORF type:complete len:495 (+),score=158.49 TRINITY_DN1391_c0_g1_i29:728-2212(+)	-1.901	-0.1029	0.8222	0.56359	0.61805
TRINITY_DN8595_c0_g1_i3_orf1	aldose reductase-like isoform X4 [Trichoplusia ni]	-1.6375	-0.654	0.59018	0.63241	1.06893
TRINITY_DN7226_c0_g1_i5_orf1	chemosensory protein [Dioryctria abietella]	-1.7964	-0.3468	0.94469	0.74398	0.45455
TRINITY_DN1209_c0_g1_i9_orf1	NADP-dependent malic enzyme-like isoform X1 [Ostrinia furnacalis] >XP_028161889.1 NADP-dependent malic enzyme-like isoform X1 [Ostrinia furnacalis] >XP_028161891.1 NADP-dependent malic enzyme-like isoform X3 [Ostrinia furnacalis]	-1.5853	-0.5154	0.27823	1.40264	0.41986
TRINITY_DN75086_c0_g1_i5_orf1	lysosome membrane protein 2-like [Ostrinia furnacalis]	-1.9636	0.75171	0.46124	0.16937	0.58127
TRINITY_DN12526_c0_g1_i5_orf1	uncharacterized protein LOC114359035 isoform X3 [Ostrinia furnacalis]	-1.6672	1.17556	-0.4693	0.2009	0.76011
TRINITY_DN48878_c0_g2_i1_orf1	codanin-1 [Ostrinia furnacalis]	-1.8155	0.57176	-0.293	1.00344	0.53329
TRINITY_DN28221_c0_g2_i1_orf1	unnamed protein product [Chilo suppressalis]	-1.9666	0.67276	0.23764	0.70936	0.34682
TRINITY_DN30306_c0_g2_i1_orf1	perilipin-4-like isoform X3 [Ostrinia furnacalis]	-1.6424	-0.5849	1.14023	0.35707	0.72998
TRINITY_DN21420_c0_g1_i2_orf1	glutathione peroxidase, partial [Ostrinia furnacalis]	-1.84	-0.0941	0.44521	1.13098	0.35796
TRINITY_DN64772_c0_g1_i1_orf1	aldehyde dehydrogenase, partial [Mythimna separata]	-1.8838	0.77701	-0.1657	0.70898	0.56347
TRINITY_DN3821_c1_g1_i7_orf1	mitochondrial carrier protein Rim2 isoform X1 [Ostrinia furnacalis]	-1.7959	-0.3225	0.37776	0.95202	0.78856
TRINITY_DN13660_c0_g1_i1_orf1	Aliphatic nitrilase [Operophtera brumata]	-1.8389	-0.2362	0.5464	0.98319	0.54548
TRINITY_DN27035_c0_g1_i1_orf1	glucose-6-phosphate isomerase-like [Ostrinia furnacalis]	-1.8418	0.5647	-0.2544	0.92611	0.6054

TRINITY_DN129863_c0_g1_i1_orf1	protein PFC0760c-like isoform X2 [Ostrinia furnacalis]	-1.8813	0.35248	1.09705	0.3572	0.07453
TRINITY_DN628_c0_g1_i1_orf1	prostamide/prostaglandin F synthase-like [Ostrinia furnacalis]	-1.9579	0.10288	0.58343	0.69631	0.57525
TRINITY_DN9090_c0_g1_i9_orf1	CD63 antigen [Ostrinia furnacalis]	-1.9417	0.2053	0.29934	0.89975	0.53727
TRINITY_DN2314_c0_g1_i7_orf1	protein dj-1beta-like isoform X1 [Ostrinia furnacalis]	-1.8251	0.07246	-0.0474	1.04376	0.75626
TRINITY_DN1048_c0_g1_i6_orf1	uncharacterized protein LOC114360661 [Ostrinia furnacalis]	-1.8346	0.41615	0.37292	1.1455	-0.1
TRINITY_DN7040_c0_g1_i4_orf1	uncharacterized protein LOC114353763 [Ostrinia furnacalis]	-1.951	0.62077	0.06429	0.66968	0.59627
TRINITY_DN18128_c0_g1_i4_orf1	arylsulfatase B [Ostrinia furnacalis]	-1.8209	0.59579	-0.2345	1.05335	0.40621
TRINITY_DN6497_c0_g1_i1_orf1	ommochrome-binding protein-like [Ostrinia furnacalis]	-1.8921	0.29728	0.13028	1.08027	0.38426
TRINITY_DN2040_c0_g1_i6_orf1	trypsin-like serine proteinase T26 protein, partial [Chilo infuscatellus]	-1.6353	-0.6819	0.5554	0.86859	0.89324
TRINITY_DN98692_c0_g3_i1_orf1	fatty acyl-CoA hydrolase precursor, medium chain [Ostrinia furnacalis]	-1.933	0.85037	0.67659	0.21462	0.19139
TRINITY_DN5337_c0_g1_i6_orf1	epoxide hydrolase 1-like [Ostrinia furnacalis]	-1.912	0.15109	0.16032	0.71476	0.88586
TRINITY_DN140_c0_g1_i1_orf1	calcyphosin-like protein [Ostrinia furnacalis]	-1.8274	-0.1325	0.24237	1.09271	0.62481
TRINITY_DN5439_c0_g1_i2_orf1	uncharacterized protein LOC114353087 [Ostrinia furnacalis]	-1.8093	-0.177	0.56999	0.27955	1.13676
TRINITY_DN6098_c1_g1_i5_orf1	unnamed protein product, partial [Iphiclides podalirius]	-1.7269	-0.5411	0.67888	0.82118	0.76796
TRINITY_DN104663_c1_g1_i2_orf1	PREDICTED: gelsolin-like [Amyelois transitella]	-1.9257	0.74457	0.17203	0.81962	0.18949
TRINITY_DN9044_c0_g1_i2_orf1	unnamed protein product [Euphydryas editha]	-1.6119	0.34158	1.42082	0.28184	-0.4323
TRINITY_DN26209_c0_g1_i6_orf1	uncharacterized protein LOC114352357 [Ostrinia furnacalis]	-1.945	0.43731	0.20477	0.39151	0.91139
TRINITY_DN47784_c0_g2_i1_orfp1	arylphorin subunit alpha-like [Ostrinia furnacalis]	-1.6683	-0.181	1.36078	-0.082	0.57057
TRINITY_DN16905_c0_g1_i1_orf1	unnamed protein product [Leptidea sinapis]	-1.9826	0.38599	0.68762	0.58594	0.32301
TRINITY_DN4235_c0_g1_i2_orf1	uncharacterized protein LOC114361536 [Ostrinia furnacalis]	-1.9616	0.25747	0.40485	0.84674	0.45257
TRINITY_DN1575_c0_g1_i10_orf1	uncharacterized protein LOC114359245 [Ostrinia furnacalis]	-1.3792	0.42138	1.18157	0.7539	-0.9777
TRINITY_DN9132_c0_g1_i5_orf1	ubiquitin-like-conjugating enzyme ATG3 [Spodoptera frugiperda]	-1.8371	-0.1254	0.23396	1.03876	0.68973
TRINITY_DN2813_c0_g1_i7_orf1	arylphorin subunit alpha-like [Ostrinia furnacalis]	-1.8742	0.64586	0.86631	-0.1755	0.53752
TRINITY_DN73900_c0_g1_i1_orf1	carbonic anhydrase 7 [Ostrinia furnacalis]	-1.419	-0.9749	0.77584	1.05902	0.55906
TRINITY_DN33346_c0_g1_i1_orf1	PREDICTED: U6 snRNA-associated Sm-like protein LSm3 [Papilio xuthus] >XP_028165558.1 U6 snRNA-associated Sm-like protein LSm3 [Ostrinia furnacalis] >KOB73597.1 LSM Sm-like protein family member [Operophtera brumata] >RVE45517.1 hypothetical protein evm_009856 [Chilo suppressalis] >CAB3523639.1 unnamed protein product [Chilo suppressalis] >CAH0400961.1 unnamed protein product [Chilo suppressalis]	-1.9243	0.02651	0.40817	0.64415	0.84548
TRINITY_DN18323_c0_g1_i5_orf1	GILT-like protein 1 isoform X1 [Ostrinia furnacalis]	-1.9954	0.55317	0.51699	0.5546	0.37067
TRINITY_DN6325_c0_g1_i9_orf1	fructose-bisphosphate aldolase isoform X2 [Pieris brassicae]	-1.7208	-0.4165	0.27668	1.10042	0.76021
TRINITY_DN64141_c0_g1_i4_orf1	probable salivary secreted peptide [Ostrinia furnacalis]	-1.7414	0.94501	-0.3563	0.95276	0.19984
TRINITY_DN40439_c0_g1_i5_orf1	ommochrome binding protein 1, partial [Ostrinia nubilalis]	-1.8944	0.46684	0.14713	1.05978	0.22062
TRINITY_DN11981_c0_g1_i7_orf1	luciferin 4-monooxygenase-like isoform X2 [Ostrinia furnacalis]	-1.6475	-0.5503	1.1955	0.66168	0.34057
TRINITY_DN71863_c0_g1_i2_orf1	unnamed protein product [Diatraea saccharalis]	-1.872	0.45201	-0.1037	1.00659	0.51707
TRINITY_DN1363_c0_g1_i11_orf1	cytochrome P450 CYP12A2-like isoform X1 [Ostrinia furnacalis] >QPF77619.1 cytochrome P450 monooxygenase CYP333A20 [Ostrinia furnacalis]	-1.8171	-0.2994	0.96686	0.49475	0.65489
TRINITY_DN69_c0_g1_i1_orf1	glycerol-3-phosphate dehydrogenase [NAD(+)], cytoplasmic isoform X1 [Ostrinia furnacalis]	-1.8571	-0.1964	0.65782	0.4677	0.92797
TRINITY_DN768_c0_g1_i7_orf1	neutral ceramidase [Leguminivora glycinivorella]	-1.9648	0.49352	0.5423	0.75656	0.17237
TRINITY_DN5421_c0_g1_i1_orf1	arylphorin subunit alpha-like [Ostrinia furnacalis]	-1.9428	0.3044	0.46122	0.92561	0.25159
TRINITY_DN81031_c0_g1_i1_orf1	aldehyde dehydrogenase, partial [Ectropis obliqua]	-1.8861	0.77383	-0.1317	0.7842	0.45979
TRINITY_DN10539_c0_g1_i1_orf1	uncharacterized protein LOC114358962 isoform X1 [Ostrinia furnacalis]	-1.7504	0.30085	-0.1641	1.31514	0.29852
TRINITY_DN2472_c0_g1_i6_orf1	programmed cell death protein 6 isoform X1 [Colias croceus] >XP_045492459.1 programmed cell death protein 6 isoform X1 [Colias croceus]	-1.9091	0.18576	0.36408	1.04337	0.31586
TRINITY_DN1450_c0_g2_i1_orf1	death-associated protein 1 [Ostrinia furnacalis]	-1.618	-0.6984	0.99672	0.53741	0.7823
TRINITY_DN60946_c0_g2_i3_orf1	protein yellow-like [Ostrinia furnacalis]	-1.9401	0.90576	0.28682	0.20958	0.53794
TRINITY_DN578_c0_g1_i5_orf1	charged multivesicular body protein 7 [Ostrinia furnacalis]	-1.897	0.82055	0.14821	0.83508	0.09319
TRINITY_DN4998_c0_g1_i21_orf1	phenoloxidase subunit 2-like [Ostrinia furnacalis]	-1.9295	0.01451	0.74996	0.71659	0.44839
TRINITY_DN346_c0_g1_i7_orf1	CDK-activating kinase assembly factor MAT1 [Ostrinia furnacalis]	-1.7074	-0.2687	0.09995	1.28652	0.58956
TRINITY_DN31286_c0_g1_i6_orfp1	TRINITY_DN31286_c0_g1_i6_m.28438 TRINITY_DN31286_c0_g1::TRINITY_DN31286_c0_g1_i6::g.28438 ORF type:internal len:92 (-),score=3.10,Perilipin PF03036.17 2e-05 TRINITY_DN31286_c0_g1_i6:1-273(-)	-1.8259	-0.1932	0.51318	0.4125	1.09336
TRINITY_DN5153_c1_g1_i1_orf1	nose resistant to fluoxetine protein 6-like isoform X1 [Ostrinia furnacalis]	-1.6446	-0.5741	1.19455	0.42701	0.59712
TRINITY_DN3822_c0_g1_i7_orf1	hypothetical protein HF086_002539 [Spodoptera exigua] >CAH0699327.1 unnamed protein product [Spodoptera exigua]	-1.9695	0.7553	0.29464	0.60313	0.31641
TRINITY_DN25234_c0_g1_i1_orf1	uncharacterized protein LOC114353853 [Ostrinia furnacalis]	-1.7563	0.3191	1.28922	0.33917	-0.1912
TRINITY_DN40126_c0_g1_i1_orf1	aldehyde dehydrogenase X, mitochondrial-like [Ostrinia furnacalis]	-1.94	0.0301	0.57633	0.58228	0.75126
TRINITY_DN1201_c0_g1_i4_orf1	triosephosphate isomerase [Ostrinia furnacalis] >XP_028170843.1 triosephosphate isomerase [Ostrinia furnacalis]	-1.9565	0.11534	0.74917	0.52191	0.57011

TRINITY_DN1750_c1_g1_i5_orf1	lipid droplet localized protein-like [Ostrinia furnacalis] >XP_028161280.1 lipid droplet localized protein-like [Ostrinia furnacalis]	-1.8472	-0.2498	0.713	0.86401	0.52001
TRINITY_DN69307_c0_g1_i6_orf1	hypothetical protein evm_010738 [Chilo suppressalis]	-1.7222	-0.1464	-0.125	1.06757	0.92597
TRINITY_DN1161_c0_g1_i2_orf1	fructose-1,6-bisphosphatase 1 [Ostrinia furnacalis]	-1.9365	0.0716	0.61007	0.83427	0.42051
TRINITY_DN12775_c0_g1_i10_orfp1	TRINITY_DN12775_c0_g1_i10_m.21238 TRINITY_DN12775_c0_g1::TRINITY_DN12775_c0_g1_i10::g.21238 ORF type:5prime_partial len:67 (-),score=0.74 TRINITY_DN12775_c0_g1_i10:275-475(-)	-1.7247	-0.3412	0.1233	0.91368	1.02899
TRINITY_DN25976_c0_g1_i4_orf1	hypothetical protein B566_EDAN014657 [Ephemera danica]	-1.8853	1.10855	0.35248	0.24659	0.17771
TRINITY_DN135780_c0_g1_i1_orf1	flotillin-1 isoform X1 [Pectinophora gossypiella]	-1.8717	-0.0792	0.82634	0.26605	0.85845
TRINITY_DN14262_c0_g1_i5_orf1	cytochrome P450 monooxygenase CYP9G18 [Cnaphalocrocis medinalis]	-1.7866	-0.1865	0.95254	0.92577	0.09478
TRINITY_DN1352_c0_g1_i5_orf1	uncharacterized protein LOC113491815 [Trichoplusia ni]	-1.8482	-0.2263	0.52277	0.94302	0.60867
TRINITY_DN17247_c0_g1_i14_orf1	uncharacterized protein LOC114356308 [Ostrinia furnacalis]	-1.8769	0.18129	0.87831	-0.003	0.82032
TRINITY_DN805_c0_g1_i5_orf1	serine protease snake-like [Ostrinia furnacalis]	-1.9644	0.77123	0.18996	0.56145	0.44174
TRINITY_DN71308_c0_g1_i4_orf1	uncharacterized protein LOC114361536 [Ostrinia furnacalis]	-1.8757	1.13536	0.22489	0.32647	0.18895
TRINITY_DN11817_c0_g1_i4_orf1	glycogen phosphorylase [Heortia vitessoides]	-1.8942	-0.0665	0.74305	0.84752	0.37012
TRINITY_DN6108_c0_g1_i5_orf1	myogenesis-regulating glycosidase-like [Ostrinia furnacalis]	-1.7378	0.16979	0.29278	1.36306	-0.0879
TRINITY_DN122321_c0_g1_i1_orf1	uncharacterized protein LOC114356271 [Ostrinia furnacalis]	-1.7814	-0.3295	0.3795	0.67218	1.05927
TRINITY_DN39266_c0_g1_i1_orf1	PREDICTED: NECAP-like protein CG9132 [Microplitis demolitor]	-1.9542	0.69299	0.22974	0.75655	0.27496
TRINITY_DN798_c1_g1_i3_orf1	protein goliath isoform X1 [Ostrinia furnacalis] >XP_028170876.1 protein goliath isoform X1 [Ostrinia furnacalis]	-1.9941	0.57694	0.36464	0.48365	0.56891
TRINITY_DN5310_c2_g1_i2_orf1	serine protease persephone-like [Ostrinia furnacalis]	-1.9061	0.51866	0.38191	0.03033	0.97519
TRINITY_DN5132_c0_g1_i4_orf1	small heat shock protein Hsp24.2 [Ostrinia furnacalis]	-1.7392	0.72032	-0.5187	0.71778	0.81979
TRINITY_DN4245_c0_g2_i1_orf1	long-chain fatty acid transport protein 4-like [Ostrinia furnacalis]	-1.8804	-0.1507	0.70272	0.48335	0.84497
TRINITY_DN5274_c0_g2_i2_orf1	lopap-like [Ostrinia furnacalis]	-1.7814	-0.3785	0.93704	0.78099	0.44193
TRINITY_DN2187_c0_g1_i1_orf1	flotillin-1 [Chelonus insularis] >XP_034947202.1 flotillin-1 [Chelonus insularis]	-1.8582	-0.1939	0.81471	0.41887	0.8186
TRINITY_DN13098_c2_g1_i2_orf1	unnamed protein product [Arctia plantaginis] >CAB3253298.1 unnamed protein product [Arctia plantaginis]	-1.9551	0.3239	0.2367	0.8463	0.54818
TRINITY_DN51480_c0_g1_i1_orf1	uncharacterized protein LOC114361588 isoform X14 [Ostrinia furnacalis]	-1.7425	-0.3234	0.15079	0.92144	0.99368
TRINITY_DN64892_c0_g1_i1_orf1	aldehyde dehydrogenase X, mitochondrial [Manduca sexta] >KAG6450704.1 hypothetical protein O3G_MSEX006722 [Manduca sexta]	-1.7565	0.58756	-0.3139	1.17229	0.31063
TRINITY_DN27300_c0_g1_i7_orfp1	TRINITY_DN27300_c0_g1_i7_m.71141 TRINITY_DN27300_c0_g1::TRINITY_DN27300_c0_g1_i7::g.71141 ORF type:internal len:82 (-),score=6.59 TRINITY_DN27300_c0_g1_i7:3-245(-)	-1.493	-0.4954	1.51949	0.46551	0.00342
TRINITY_DN399_c3_g2_i6_orf1	proline-rich extensin-like protein EPR1 [Ostrinia furnacalis]	-1.8954	0.60869	-0.0836	0.89849	0.47187
TRINITY_DN8780_c0_g1_i3_orf1	uncharacterized protein LOC114363370 [Ostrinia furnacalis]	-1.8012	0.15202	-0.071	1.21233	0.50786
TRINITY_DN23398_c0_g1_i1_orf1	cytochrome P450 6B7-like [Ostrinia furnacalis]	-1.9096	0.81946	-0.0428	0.42792	0.70504
TRINITY_DN1206_c0_g1_i6_orf1	sorbitol dehydrogenase-like [Spodoptera frugiperda] >KAG8104768.1 hypothetical protein SFRUCORN_013827 [Spodoptera frugiperda]	-1.6765	-0.6284	0.72804	0.89144	0.68542
TRINITY_DN11948_c0_g1_i8_orf1	cystathionine gamma-lyase [Ostrinia furnacalis]	-1.5905	-0.7467	0.84555	0.95209	0.53962
TRINITY_DN1424_c0_g1_i5_orf1	insect group I lytic polysaccharide monooxygenase [Ostrinia furnacalis]	-1.9621	0.23501	0.32954	0.6286	0.76894
TRINITY_DN18388_c0_g1_i6_orf1	serine protease [Ostrinia furnacalis]	-1.8692	0.98296	0.18209	0.71188	-0.0078
TRINITY_DN1957_c0_g1_i4_orf1	NAD kinase 2, mitochondrial [Ostrinia furnacalis]	-1.8114	0.36452	-0.2614	1.02515	0.68314
TRINITY_DN1960_c5_g1_i3_orf1	cytochrome P450 monooxygenase CYP9G18 [Cnaphalocrocis medinalis]	-1.6543	-0.5487	0.2563	1.00303	0.94367
TRINITY_DN40126_c0_g2_i1_orf1	aldehyde dehydrogenase X, mitochondrial-like [Ostrinia furnacalis]	-1.6368	-0.6328	1.09532	0.46233	0.71196
TRINITY_DN1421_c0_g1_i1_orf1	uncharacterized protein LOC114352615 [Ostrinia furnacalis]	-1.9879	0.43481	0.68868	0.35769	0.50676
TRINITY_DN14460_c0_g1_i6_orf1	scavenger receptor class B member 1-like [Ostrinia furnacalis]	-1.9798	0.71023	0.27956	0.43343	0.55662
TRINITY_DN9492_c0_g1_i7_orf1	aldo-keto reductase AKR2E4-like [Ostrinia furnacalis]	-1.9463	0.17238	0.31467	0.6335	0.82575
TRINITY_DN585_c0_g1_i12_orf1	very low-density lipoprotein receptor isoform X3 [Galleria mellonella]	-1.9341	0.96795	0.28676	0.27119	0.40819
TRINITY_DN745_c7_g1_i1_orf1	uncharacterized protein LOC114358822 [Ostrinia furnacalis]	-1.7551	0.71535	-0.268	1.14433	0.1634
TRINITY_DN32532_c0_g1_i1_orf1	fatty acyl-CoA hydrolase precursor, medium chain [Ostrinia furnacalis]	-1.8865	1.07573	0.26645	0.45187	0.0925
TRINITY_DN8985_c0_g1_i4_orf1	cytochrome P450 6B6-like [Ostrinia furnacalis]	-1.6869	1.00686	-0.5577	0.79728	0.44042
TRINITY_DN5099_c0_g1_i3_orf1	trans-1,2-dihydrobenzene-1,2-diol dehydrogenase-like [Ostrinia furnacalis]	-1.8503	-0.168	0.44519	0.54908	1.02401
TRINITY_DN34423_c0_g1_i2_orf1	THAP domain-containing protein 4-like [Ostrinia furnacalis]	-1.8771	-0.0895	0.58271	0.98476	0.39909
TRINITY_DN10994_c0_g1_i4_orf1	trypsin inhibitor-like [Ostrinia furnacalis]	-1.5925	-0.7438	0.54119	0.95513	0.84
TRINITY_DN350_c0_g1_i5_orf1	tau-like protein isoform X6 [Bombyx mori]	-1.623	-0.6322	1.16233	0.64153	0.45133
TRINITY_DN49530_c0_g1_i1_orf1	ommochrome-binding protein-like [Ostrinia furnacalis]	-1.7712	-0.3339	0.27703	0.95688	0.87122
TRINITY_DN44517_c0_g1_i4_orf1	regucalcin-like [Ostrinia furnacalis]	-1.8907	-0.1466	0.74216	0.56398	0.73125
TRINITY_DN6205_c0_g1_i1_orf1	phenoloxidase-activating factor 2-like [Ostrinia furnacalis]	-1.6788	0.96468	0.29831	0.94128	-0.5255
TRINITY_DN1287_c0_g1_i5_orf1	probable chitinase 10 isoform X6 [Ostrinia furnacalis]	-1.8459	0.88095	-0.2336	0.74684	0.45177
TRINITY_DN20658_c0_g1_i1_orf1	prostaglandin reductase 1-like [Ostrinia furnacalis]	-1.8214	-0.2979	0.83431	0.45259	0.83237
TRINITY_DN2798_c0_g1_i5_orf1	arylsulfatase B-like isoform X1 [Ostrinia furnacalis]	-1.9403	0.75365	0.40809	0.70358	0.07499

TRINITY_DN95558_c0_g3_i1_orf1	cytochrome P450 monooxygenase CYP9G19 [Cnaphalocrocis medinalis]	-1.9573	0.43282	0.59371	0.15234	0.77843
TRINITY_DN1144_c0_g1_i10_orf1	TIL [Ostrinia furnacalis]	-1.7699	0.45222	-0.2926	1.1799	0.43037
TRINITY_DN7740_c0_g1_i2_orf1	D-arabinitol dehydrogenase 1 [Eumeta japonica]	-1.5609	-0.768	0.773	0.48984	1.06603
TRINITY_DN12231_c0_g1_i1_orf1	carbonyl reductase [NADPH] 3-like [Ostrinia furnacalis]	-1.9784	0.7706	0.41864	0.34293	0.44627
TRINITY_DN2614_c0_g2_i3_orf1	uncharacterized protein LOC114360661 [Ostrinia furnacalis]	-1.7553	-0.1086	0.02889	1.25097	0.58412
TRINITY_DN1024_c0_g4_i1_orf1	superoxide dismutase [Cu-Zn]-like [Ostrinia furnacalis]	-1.5727	-0.7855	0.97735	0.67041	0.71049
TRINITY_DN1466_c0_g1_i4_orf1	insecticyanin-A-like [Ostrinia furnacalis]	-1.656	0.46605	0.84162	0.97152	-0.6232
TRINITY_DN47609_c0_g1_i1_orfp1	TRINITY_DN47609_c0_g1_i1_m.57205 TRINITY_DN47609_c0_g1::TRINITY_DN47609_c0_g1_i1::g.57205 ORF type:5prime_partial len:68 (-),score=15.96 TRINITY_DN47609_c0_g1_i1:36-239(-)	-1.8101	0.19633	0.09064	1.26998	0.25311
TRINITY_DN20347_c0_g1_i6_orf1	venom polypeptide precursor [Doratifera vulnerans]	-1.5118	-0.4397	-0.0145	1.52542	0.44057
TRINITY_DN960_c1_g1_i6_orf1	hypothetical protein evm_007130 [Chilo suppressalis]	-1.9541	0.51431	0.85884	0.21719	0.36374
TRINITY_DN1710_c0_g1_i1_orf1	nuclear factor NF-kappa-B p105 subunit-like isoform X2 [Ostrinia furnacalis]	-1.9556	0.47416	0.26973	0.87427	0.33741
TRINITY_DN4076_c1_g2_i2_orf1	vacuole membrane protein 1 [Ostrinia furnacalis]	-1.8923	0.64851	-0.1421	0.78744	0.59851
TRINITY_DN130575_c0_g1_i1_orfp1	TRINITY_DN130575_c0_g1_i1_m.77798 TRINITY_DN130575_c0_g1::TRINITY_DN130575_c0_g1_i1::g.77798 ORF type:internal len:70 (+),score=15.12 TRINITY_DN130575_c0_g1_i1:3-209(+)	-1.8091	-0.2939	0.78642	0.37952	0.93712
TRINITY_DN20658_c0_g2_i3_orf1	prostaglandin reductase 1-like [Ostrinia furnacalis]	-1.8077	-0.1908	0.5557	0.30555	1.13732
TRINITY_DN812_c2_g1_i1_orf1	1,4-alpha-glucan-branching enzyme [Ostrinia furnacalis]	-1.7055	-0.5764	0.86719	0.76284	0.6519
TRINITY_DN3039_c0_g2_i1_orf1	uncharacterized protein LOC114353136 [Ostrinia furnacalis]	-1.9641	0.32785	0.27738	0.81085	0.54804
TRINITY_DN4497_c2_g1_i3_orf1	uncharacterized protein LOC114353086 [Ostrinia furnacalis]	-1.7108	-0.4487	0.30063	0.76547	1.09341
TRINITY_DN2406_c0_g1_i6_orf1	uncharacterized protein LOC114361672 [Ostrinia furnacalis]	-1.5302	0.43542	1.51432	-0.0003	-0.4193
TRINITY_DN13500_c0_g1_i1_orf1	phosphatidylethanolamine-binding protein homolog F40A3.3-like [Ostrinia furnacalis] >XP_028160752.1 phosphatidylethanolamine-binding protein homolog F40A3.3-like [Ostrinia furnacalis]	-1.6949	-0.2089	-0.0004	1.32013	0.58401
TRINITY_DN11467_c0_g1_i5_orf1	27 kDa hemolymph protein-like, partial [Ostrinia furnacalis]	-1.763	0.35176	-0.0622	1.31923	0.15426
TRINITY_DN428_c0_g1_i8_orf1	phenoloxidase-activating factor 2-like isoform X1 [Ostrinia furnacalis]	-1.9705	0.58433	0.32232	0.76222	0.30159
TRINITY_DN105574_c0_g1_i1_orf1	prolow-density lipoprotein receptor-related protein 1, partial [Ostrinia furnacalis]	-1.9447	0.93431	0.32601	0.39161	0.29274
TRINITY_DN3275_c0_g2_i3_orf1	hypothetical protein B5X24_HaOG216046 [Helicoverpa armigera]	-1.744	-0.2379	1.18595	0.09888	0.69698
TRINITY_DN29009_c0_g2_i2_orf1	juvenile hormone binding protein [Omphisa fuscidentalis]	-1.9221	-0.0056	0.53582	0.85365	0.53826
TRINITY_DN6988_c0_g1_i3_orf1	cuticle protein 1-like [Ostrinia furnacalis]	-1.8483	0.88877	-0.0593	0.87776	0.14107
TRINITY_DN1503_c0_g1_i6_orf1	ecdysteroid-regulated 16 kDa protein [Ostrinia furnacalis]	-1.8367	-0.1171	1.08672	0.60066	0.26641
TRINITY_DN2076_c0_g2_i1_orf1	macrophage mannose receptor 1-like isoform X1 [Maniola jurtina]	-1.7675	-0.1973	0.14729	1.19498	0.62246
TRINITY_DN1030_c0_g1_i6_orf1	gamma-glutamyl hydrolase A-like isoform X1 [Ostrinia furnacalis]	-1.9466	0.53576	0.16821	0.37324	0.86943
TRINITY_DN1651_c0_g2_i1_orf1	UBX domain-containing protein 6 [Ostrinia furnacalis] >XP_028162119.1 UBX domain-containing protein 6 [Ostrinia furnacalis]	-1.9123	0.85411	0.23375	0.74336	0.08103
TRINITY_DN625_c2_g2_i2_orf1	L-dopachrome tautomerase yellow-f2-like [Ostrinia furnacalis]	-1.7918	0.4621	-0.1451	1.22074	0.25406
TRINITY_DN970_c0_g1_i4_orf1	spermine oxidase-like isoform X2 [Ostrinia furnacalis]	-1.7478	-0.4063	0.3543	0.76778	1.03205
TRINITY_DN364_c1_g1_i2_orf1	talin-2-like, partial [Ostrinia furnacalis]	-1.8306	0.79969	0.99666	0.10478	-0.0705
TRINITY_DN1093_c0_g1_i6_orf1	uncharacterized protein LOC114361723 isoform X4 [Ostrinia furnacalis]	-1.9495	0.71885	0.09077	0.68166	0.45819
TRINITY_DN54205_c0_g1_i1_orf1	aldo-keto reductase AKR2E4-like [Ostrinia furnacalis]	-1.7663	-0.3585	0.3096	0.84315	0.97204
TRINITY_DN8953_c0_g1_i4_orf1	gonadotropin-releasing hormone receptor [Ostrinia furnacalis] >AXF67446.1 adipokinetic hormone receptor 1 [Ostrinia furnacalis]	-1.8732	-0.009	1.00841	0.22209	0.6517
TRINITY_DN3212_c0_g1_i7_orfp1	TRINITY_DN3212_c0_g1_i7_m.38385 TRINITY_DN3212_c0_g1::TRINITY_DN3212_c0_g1_i7::g.38385 ORF type:internal len:121 (-),score=5.33 TRINITY_DN3212_c0_g1_i7:3-362(-)	-1.7941	0.57286	-0.3232	1.05128	0.49323
TRINITY_DN15961_c0_g1_i1_orf1	uncharacterized protein LOC113522423 [Galleria mellonella]	-1.9117	0.94171	0.02293	0.54255	0.40452
TRINITY_DN4449_c0_g2_i1_orf1	calcium/calmodulin-dependent protein kinase type 1 isoform X3 [Cephus cinctus]	-1.9872	0.54699	0.28688	0.52896	0.62436
TRINITY_DN7534_c0_g1_i15_orf1	protein-glucosylgalactosylhydroxylysine glucosidase isoform X2 [Ostrinia furnacalis]	-1.8691	0.68461	0.88583	-0.1736	0.47223
TRINITY_DN1098_c1_g1_i4_orf1	lysozyme 10 [Ostrinia furnacalis]	-1.7484	0.95525	-0.4362	0.40885	0.82052
TRINITY_DN82944_c0_g1_i4_orf1	senecionine N-oxygenase isoform X2 [Ostrinia furnacalis]	-1.8006	-0.3343	0.42139	0.84468	0.86884
TRINITY_DN244_c1_g1_i5_orf1	C-1-tetrahydrofolate synthase, cytoplasmic isoform X3 [Ostrinia furnacalis]	-1.827	0.20565	-0.1308	0.69235	1.0598
TRINITY_DN618_c0_g1_i3_orf1	triokinase/FMN cyclase-like isoform X1 [Ostrinia furnacalis]	-1.8535	-0.2351	0.67167	0.54344	0.87346
TRINITY_DN74654_c0_g1_i4_orf1	limulus clotting factor C-like isoform X4 [Ostrinia furnacalis]	-1.9788	0.56655	0.34956	0.72711	0.33555
TRINITY_DN4443_c0_g1_i4_orf1	lysosome-associated membrane glycoprotein 1-like isoform X4 [Ostrinia furnacalis]	-1.9056	0.56689	0.9771	0.29729	0.06433
TRINITY_DN338_c1_g1_i9_orf1	scolexin B-like isoform X2 [Ostrinia furnacalis]	-1.9101	0.81459	0.25117	0.78856	0.05576
TRINITY_DN5667_c0_g1_i4_orf1	spodomicin-like [Ostrinia furnacalis] >QKV49445.1 diapausin [Ostrinia furnacalis]	-1.9222	0.73635	0.26706	0.82638	0.09244
TRINITY_DN18230_c1_g2_i1_orf1	hypothetical protein O3G_MSEX004459 [Manduca sexta]	-1.975	0.68332	0.2165	0.59835	0.47687
TRINITY_DN3175_c0_g1_i7_orf1	unnamed protein product, partial [Brenthis ino]	-1.86	-0.2328	0.60996	0.65608	0.82678
TRINITY_DN2170_c0_g2_i1_orf1	beta-1,3-glucan-binding protein-like [Ostrinia furnacalis]	-1.7132	0.10304	-0.0115	1.41879	0.20292
TRINITY_DN1304_c0_g1_i6_orf1	uncharacterized protein LOC114359545 [Ostrinia furnacalis]	-1.973	0.73539	0.58395	0.40343	0.25025
TRINITY_DN8245_c0_g1_i3_orf1	uncharacterized protein LOC114357622 [Ostrinia furnacalis]	-1.959	0.72716	0.28502	0.70164	0.24515

TRINITY_DN4621_c0_g1_i4_orf1	uncharacterized protein LOC114358242 isoform X3 [Ostrinia furnacalis]	-1.6641	-0.6413	0.59062	0.86621	0.84863
TRINITY_DN7102_c0_g1_i5_orf1	protein wings apart-like [Ostrinia furnacalis]	-1.8265	1.01918	-0.2491	0.57782	0.47865
TRINITY_DN2251_c0_g1_i4_orf1	serine hydroxymethyltransferase, cytosolic isoform X1 [Ostrinia furnacalis]	-1.9095	0.07408	0.81015	0.80106	0.22422
TRINITY_DN664_c0_g1_i18_orf1	chitinase-like protein EN03 isoform X2 [Ostrinia furnacalis]	-1.7578	0.83039	-0.4744	0.6197	0.78208
TRINITY_DN13322_c0_g1_i6_orf1	macrophage mannose receptor 1-like [Ostrinia furnacalis]	-1.7368	-0.465	0.41688	0.8753	0.90962
TRINITY_DN5682_c0_g1_i6_orf1	spodomicin-like [Ostrinia furnacalis] >QKV49445.1 diapausin [Ostrinia furnacalis]	-1.8746	0.0889	1.11851	0.28709	0.3801
TRINITY_DN2606_c0_g1_i5_orf1	galectin-4-like isoform X1 [Ostrinia furnacalis]	-1.9635	0.68315	0.15299	0.66156	0.46578
TRINITY_DN2650_c0_g1_i1_orf1	hypothetical protein HW555_002849 [Spodoptera exigua] >CAH0691914.1 unnamed protein product [Spodoptera exigua]	-1.8289	0.30889	0.04622	0.25146	1.22237
TRINITY_DN7633_c0_g1_i1_orf1	prolow-density lipoprotein receptor-related protein 1, partial [Ostrinia furnacalis]	-1.8974	0.93199	0.00444	0.29384	0.6671
TRINITY_DN2848_c0_g1_i2_orf1	glyceraldehyde-3-phosphate dehydrogenase isoform 1 [Homo sapiens] >NP_001276675.1 glyceraldehyde-3-phosphate dehydrogenase isoform 1 [Homo sapiens] >NP_002037.2 glyceraldehyde-3-phosphate dehydrogenase isoform 1 [Homo sapiens] >XP_003819180.1 glyceraldehyde-3-phosphate dehydrogenase [Pan paniscus] >XP_004052609.1 glyceraldehyde-3-phosphate dehydrogenase [Gorilla gorilla gorilla] >XP_008971979.1 glyceraldehyde-3-phosphate dehydrogenase [Pan paniscus] >XP_008971980.1 glyceraldehyde-3-phosphate dehydrogenase [Pan paniscus] >XP_032621678.1 glyceraldehyde-3-phosphate dehydrogenase [Chelonoidis abingdonii] >XP_508955.1 glyceraldehyde-3-phosphate dehydrogenase isoform X1 [Pan troglodytes] >P04406.3 RecName: Full=Glyceraldehyde-3-phosphate dehydrogenase; Short=GAPDH; AltName: Full=Peptidyl-cysteine S-nitrosylase GAPDH [Homo sapiens] >1U8F_O Crystal Structure Of Human Placental Glyceraldehyde-3-Phosphate Dehydrogenase At 1.75 Resolution [Homo sapiens] >1U8F_P Crystal Structure Of Human Placental Glyceraldehyde-3-Phosphate Dehydrogenase At 1.75 Resolution [Homo sapiens] >1U8F_Q Crystal Structure Of Human Placental Glyceraldehyde-3-Phosphate Dehydrogenase At 1.75 Resolution [Homo sapiens] >1U8F_R Crystal Structure Of Human Placental Glyceraldehyde-3-Phosphate Dehydrogenase At 1.75 Resolution [Homo sapiens] >4WNC_A Crystal structure of human wild-type GAPDH at 1.99 angstroms resolution [Homo sapiens] >4WNC_B Crystal structure of human wild-type GAPDH at 1.99 angstroms resolution [Homo sapiens] >4WNC_C Crystal structure of human wild-type GAPDH at 1.99 angstroms resolution [Homo sapiens] >4WNC_D Crystal structure of human wild-type GAPDH at 1.99 angstroms resolution [Homo sapiens] >4WNC_E Crystal structure of human wild-type GAPDH at 1.99 angstroms resolution [Homo sapiens] >4WNC_F Crystal structure of human wild-type GAPDH at 1.99 angstroms resolution [Homo sapiens] >4WNC_G Crystal structure of human wild-type GAPDH at 1.99 angstroms resolution [Homo sapiens] >4WNC_O Crystal structure of human wild-type GAPDH at 1.99 angstroms resolution [Homo sapiens] >6IQ6_A Crystal structure of GAPDH [Homo sapiens] >6IQ6_B Crystal structure of GAPDH [Homo sapiens] >6IQ6_C Crystal structure of GAPDH [Homo sapiens] >6IQ6_D Crystal structure of GAPDH [Homo sapiens] >6IQ6_E Crystal structure of GAPDH [Homo sapiens] >6IQ6_F Crystal structure of GAPDH [Homo sapiens] >6IQ6_G Crystal structure of GAPDH [Homo sapiens] >6IQ6_H Crystal structure of GAPDH [Homo sapiens] >6YNE_A GAPDH purified from the supernatant of HEK293F cells: crystal form 2 of 4. [Homo sapiens] >6YNE_B GAPDH purified from the supernatant of HEK293F cells: crystal form 2 of 4. [Homo sapiens] >6YNE_C GAPDH purified from the supernatant of HEK293F cells: crystal form 2 of 4. [Homo sapiens] >6YNE_D GAPDH purified from the supernatant of HEK293F cells: crystal form 2 of 4. [Homo sapiens] >AAX42270.1 glyceraldehyde-3-phosphate dehydrogenase [synthetic construct] >MXR00212.1 hypothetical protein [Bos mutus] >SJX33932.1 unnamed protein product, partial [Human ORFeome Gateway entry vector] >AAA52496.1 glyceraldehyde 3-phosphate dehydrogenase (EC 1.2.1.12) [Homo sapiens] >AAA52518.1 glyceraldehyde-3-phosphate dehydrogenase (EC 1.2.1.12) [Homo sapiens]	-1.9079	0.60011	0.06389	0.95533	0.28853
TRINITY_DN8367_c0_g2_i2_orf1	uncharacterized protein LOC114357075 [Ostrinia furnacalis]	-1.959	0.28669	0.7085	0.24351	0.7203
TRINITY_DN12873_c0_g2_i1_orf1	proteoglycan 4-like [Ostrinia furnacalis]	-1.5501	-0.8187	0.6124	0.94718	0.80919
TRINITY_DN33488_c0_g1_i2_orf1	semaphorin-1A isoform X3 [Trichoplusia ni]	-1.7632	-0.4462	0.93194	0.7001	0.57734
TRINITY_DN8037_c0_g2_i1_orf1	2-oxo-4-hydroxy-4-carboxy-5-ureidoimidazoline decarboxylase-like [Ostrinia furnacalis]	-1.9041	0.4802	0.95886	0.47375	-0.0087
TRINITY_DN1108_c1_g2_i1_orfp1	TRINITY_DN1108_c1_g2_i1_m.5565 TRINITY_DN1108_c1_g2::TRINITY_DN1108_c1_g2_i1::g.5565 ORF type:internal len:205 (-),score=147.90 TRINITY_DN1108_c1_g2_i1:2-613(-)	-1.5422	-0.4736	1.50535	0.23202	0.27838
TRINITY_DN31611_c0_g1_i2_orf1	glucose-6-phosphate isomerase-like [Ostrinia furnacalis]	-1.9161	0.28065	0.37304	1.02723	0.23521
TRINITY_DN6580_c0_g1_i4_orf1	catalase [Ostrinia furnacalis]	-1.8375	-0.0078	0.14999	0.56129	1.134
TRINITY_DN3738_c0_g1_i5_orf1	23 kDa integral membrane protein-like [Ostrinia furnacalis]	-1.5638	-0.3643	-0.1117	1.42541	0.61442
TRINITY_DN9492_c1_g1_i1_orf1	aldo-keto reductase AKR2E4-like [Galleria mellonella]	-1.7529	-0.301	0.1917	0.74891	1.11323
TRINITY_DN17326_c0_g1_i8_orf1	aminoacylase-1-like [Ostrinia furnacalis]	-1.7512	-0.4692	0.63097	0.63366	0.95575
TRINITY_DN8637_c0_g1_i1_orf1	superoxide dismutase [Cu-Zn] [Ostrinia furnacalis] >XP_028177872.1 superoxide dismutase [Cu-Zn] [Ostrinia furnacalis]	-1.8718	0.3846	-0.1377	0.74393	0.88093
TRINITY_DN5300_c0_g1_i2_orf1	valacyclovir hydrolase [Ostrinia furnacalis]	-1.9199	-0.0589	0.61565	0.64521	0.71789
TRINITY_DN4464_c0_g2_i1_orf1	glypican-6 [Pectinophora gossypiella]	-1.9247	0.98681	0.42756	0.32098	0.18936
TRINITY_DN125140_c0_g1_i1_orf1	glycogen debranching enzyme isoform X2 [Ostrinia furnacalis] >XP_028161358.1 glycogen debranching enzyme isoform X2 [Ostrinia furnacalis] >XP_028161359.1 glycogen debranching enzyme isoform X2 [Ostrinia furnacalis]	-1.9117	0.50471	0.74014	0.73386	-0.0671
TRINITY_DN28729_c0_g1_i9_orf1	serine/threonine-protein kinase mig-15 isoform X2 [Ostrinia furnacalis]	-1.703	0.9415	-0.5539	0.80175	0.51364
TRINITY_DN15774_c0_g1_i3_orf1	uncharacterized protein LOC114366599 [Ostrinia furnacalis]	-1.9475	0.80917	0.27256	0.66037	0.20538
TRINITY_DN2338_c0_g1_i5_orf1	prophenoloxidase PPO1b [Ostrinia furnacalis]	-1.8583	-0.0953	0.572	1.04866	0.33295

TRINITY_DN15478_c0_g1_i1_orf1	STE20/SPS1-related proline-alanine-rich protein kinase [Vanessa tameamea] >XP_047534294.1 STE20/SPS1-related proline-alanine-rich protein kinase-like [Vanessa atalanta]	-1.6554	-0.1716	-0.2434	1.15264	0.9178
TRINITY_DN5488_c0_g1_i5_orf1	alpha-amylase-like [Ostrinia furnacalis]	-1.9606	0.66826	0.56371	0.6156	0.11303
TRINITY_DN9542_c0_g1_i4_orf1	NAD(P)H-hydrate epimerase [Ostrinia furnacalis]	-1.9868	0.49844	0.31447	0.49669	0.67719
TRINITY_DN11383_c0_g2_i4_orf1	aminoacylase-1A-like [Ostrinia furnacalis]	-1.6337	-0.5964	0.29201	0.89144	1.04664
TRINITY_DN4343_c0_g1_i2_orf1	uncharacterized protein LOC114365231 isoform X3 [Ostrinia furnacalis]	-1.8217	-0.0589	0.80723	0.06209	1.01123
TRINITY_DN62184_c1_g1_i1_orf1	uncharacterized protein LOC114362189 [Ostrinia furnacalis]	-1.6936	0.49744	-0.5409	1.07214	0.66495
TRINITY_DN8922_c0_g1_i3_orf1	protein sidekick isoform X1 [Ostrinia furnacalis] >XP_028161979.1 protein sidekick isoform X1 [Ostrinia furnacalis] >XP_028161980.1 protein sidekick isoform X1 [Ostrinia furnacalis] >XP_028161981.1 protein sidekick isoform X1 [Ostrinia furnacalis]	-1.7033	0.28032	0.09322	1.41568	-0.0859
TRINITY_DN1694_c0_g1_i1_orf1	cuticle protein 16.5-like [Ostrinia furnacalis]	-1.9013	0.23512	0.33431	1.07287	0.25896
TRINITY_DN512_c0_g1_i10_orf1	uncharacterized protein LOC114366781 [Ostrinia furnacalis]	-1.7801	-0.1818	0.11295	0.72914	1.11975
TRINITY_DN29555_c0_g1_i8_orf1	fasciclin-1 [Ostrinia furnacalis]	-1.9094	0.27143	0.91962	0.06181	0.65653
TRINITY_DN29217_c0_g1_i3_orf1	protein yippee-like 5 [Ostrinia furnacalis]	-1.9394	0.83411	0.16419	0.66214	0.27892
TRINITY_DN2133_c0_g2_i1_orf1	glycogen debranching enzyme isoform X1 [Ostrinia furnacalis]	-1.9085	0.18858	0.84849	0.76918	0.10226
TRINITY_DN1659_c0_g1_i3_orf1	beta-catenin-like protein 1 [Ostrinia furnacalis]	-1.9883	0.56574	0.31345	0.4724	0.63667
TRINITY_DN574_c0_g1_i4_orf1	CD63 antigen-like [Ostrinia furnacalis]	-1.8899	-0.134	0.78783	0.50506	0.73103
TRINITY_DN24_c0_g1_i1_orf1	hypothetical protein evm_007803 [Chilo suppressalis]	-1.7481	-0.0394	1.04161	-0.1653	0.9112
TRINITY_DN89083_c0_g1_i1_orf1	lysine-specific demethylase 4A isoform X2 [Diachasma alloeum]	-1.8642	0.77138	-0.2257	0.72795	0.59059
TRINITY_DN1672_c0_g1_i6_orf1	cystinosin homolog isoform X1 [Ostrinia furnacalis] >XP_028162341.1 cystinosin homolog isoform X1 [Ostrinia furnacalis] >XP_028162342.1 cystinosin homolog isoform X1 [Ostrinia furnacalis] >XP_028162343.1 cystinosin homolog isoform X1 [Ostrinia furnacalis] >XP_028162344.1 cystinosin homolog isoform X1 [Ostrinia furnacalis]	-1.8624	0.60746	0.28602	-0.0684	1.03728
TRINITY_DN7868_c0_g1_i2_orf1	uncharacterized protein LOC114353432 isoform X4 [Ostrinia furnacalis]	-1.8676	0.93337	0.20381	0.773	-0.0426
TRINITY_DN37307_c0_g1_i4_orf1	superoxide dismutase [Mn], mitochondrial [Ostrinia furnacalis]	-1.7083	-0.1881	1.38045	0.19596	0.31994
TRINITY_DN104596_c0_g1_i1_orf1	unnamed protein product [Diatraea saccharalis]	-1.8252	0.53982	-0.211	1.0745	0.42193
TRINITY_DN15597_c0_g1_i1_orf1	microsomal glutathione S-transferase 1-like [Ostrinia furnacalis]	-1.9021	0.76095	0.22322	0.05162	0.86631
TRINITY_DN42854_c0_g3_i2_orf1	amyloid beta (A4) precursor-like protein 2, isoform CRA_b [Homo sapiens]	-1.655	0.9126	-0.3863	1.13084	-0.0021
TRINITY_DN97883_c0_g1_i2_orf1	talin-2-like, partial [Ostrinia furnacalis]	-1.5601	0.69155	1.25497	0.27487	-0.6613
TRINITY_DN45530_c0_g1_i1_orf1	aldose 1-epimerase isoform X1 [Ostrinia furnacalis] >XP_028178513.1 aldose 1-epimerase isoform X1 [Ostrinia furnacalis] >XP_028178514.1 aldose 1-epimerase isoform X1 [Ostrinia furnacalis]	-1.893	0.28991	0.04487	1.02025	0.538
TRINITY_DN4602_c0_g1_i4_orf1	2-iminobutanoate/2-iminopropanoate deaminase [Ostrinia furnacalis]	-1.767	1.12088	-0.333	0.36492	0.61422
TRINITY_DN2255_c0_g1_i1_orf1	glutathione S-transferase sigma 3 [Ostrinia furnacalis]	-1.8726	-0.1037	0.32769	0.73486	0.91381
TRINITY_DN2483_c0_g1_i1_orf1	UDP-glucuronosyltransferase 2B10-like [Ostrinia furnacalis]	-1.9308	0.14108	0.28512	0.89151	0.61312
TRINITY_DN63536_c0_g1_i1_orf1	adenosylhomocysteinase [Chelonus insularis]	-1.6889	-0.5516	0.71968	1.05104	0.46978
TRINITY_DN41952_c0_g1_i1_orf1	protein DDI1 homolog 2 [Ostrinia furnacalis]	-1.9222	0.05363	0.86021	0.66802	0.34038
TRINITY_DN874_c2_g1_i1_orf1	uncharacterized protein LOC114356358 [Ostrinia furnacalis]	-1.8938	-0.0277	0.54041	0.40144	0.9796
TRINITY_DN10090_c0_g1_i1_orf1	clotting factor B-like isoform X1 [Ostrinia furnacalis] >XP_028163447.1 clotting factor B-like isoform X2 [Ostrinia furnacalis] >XP_028163448.1 clotting factor B-like isoform X3 [Ostrinia furnacalis]	-1.8629	1.16435	0.17151	0.31637	0.21067
TRINITY_DN22875_c0_g1_i6_orf1	microtubule-actin cross-linking factor 1 isoform X15 [Ostrinia furnacalis]	-1.6324	-0.5848	1.06815	0.2643	0.88467
TRINITY_DN2395_c0_g1_i7_orf1	uncharacterized protein LOC114352963 [Ostrinia furnacalis]	-1.6622	1.02035	-0.572	0.86416	0.34961
TRINITY_DN4565_c0_g2_i1_orf1	acid phosphatase type 7 isoform X2 [Ostrinia furnacalis]	-1.9036	0.43712	1.03612	0.31314	0.1172
TRINITY_DN61777_c0_g1_i4_orf1	exocyst complex component 2 [Ostrinia furnacalis]	-1.9687	0.52329	0.21758	0.77086	0.45697
TRINITY_DN2264_c0_g1_i1_orf1	cytochrome P450 6B6-like [Ostrinia furnacalis]	-1.8457	0.43129	-0.1916	0.60192	1.00413
TRINITY_DN3513_c0_g1_i5_orf1	vacuolar protein sorting-associated protein 16 homolog [Ostrinia furnacalis]	-1.8949	1.01439	0.24518	0.56081	0.07457
TRINITY_DN9562_c0_g1_i3_orf1	cullin-3 isoform X1 [Ostrinia furnacalis] >XP_028159222.1 cullin-3 isoform X1 [Ostrinia furnacalis] >XP_028159223.1 cullin-3 isoform X2 [Ostrinia furnacalis] >XP_028159224.1 cullin-3 isoform X3 [Ostrinia furnacalis] >XP_028159225.1 cullin-3 isoform X1 [Ostrinia furnacalis]	-1.72	-0.1962	0.3245	1.3575	0.2342
TRINITY_DN2205_c0_g1_i3_orf1	probable chitinase 2 [Ostrinia furnacalis]	-1.8858	0.92709	-0.0334	0.28294	0.70919
TRINITY_DN53807_c0_g2_i1_orf1	aminomethyltransferase, mitochondrial [Ostrinia furnacalis]	-1.9346	0.29265	0.17363	0.91208	0.55629
TRINITY_DN2784_c0_g1_i3_orf1	toll-like receptor 6 [Ostrinia furnacalis]	-1.9242	0.80321	0.02666	0.70987	0.38444
TRINITY_DN9340_c0_g1_i4_orf1	sarcosine dehydrogenase, mitochondrial [Ostrinia furnacalis]	-1.695	-0.457	1.1941	0.60837	0.3495
TRINITY_DN1014_c0_g2_i8_orf1	uncharacterized protein LOC114362446 [Ostrinia furnacalis] >XP_028173663.1 uncharacterized protein LOC114362446 [Ostrinia furnacalis]	-1.8488	0.19641	0.0035	0.52139	1.12754
TRINITY_DN4900_c0_g1_i6_orf1	hypothetical protein evm_001963 [Chilo suppressalis]	-1.9678	0.44921	0.48915	0.79484	0.23459
TRINITY_DN3483_c0_g1_i5_orf1	phenoloxidase-activating factor 2-like isoform X1 [Ostrinia furnacalis] >XP_028178309.1 phenoloxidase-activating factor 2-like isoform X2 [Ostrinia furnacalis]	-1.713	0.80788	-0.5678	0.79082	0.68209
TRINITY_DN48250_c0_g1_i1_orf1	larval/pupal rigid cuticle protein 66-like [Hyposmocoma kahamanoa]	-1.795	0.83737	-0.3315	0.9055	0.38356
TRINITY_DN811_c0_g1_i15_orf1	uncharacterized protein LOC114364160 [Ostrinia furnacalis]	-1.8447	0.3387	1.10487	0.50145	-0.1003
TRINITY_DN41848_c0_g1_i4_orf1	vacuolar protein sorting-associated protein 28 homolog [Ostrinia furnacalis]	-1.8404	-0.1358	0.39312	1.09709	0.486

TRINITY_DN12256_c0_g1_i1_orf1	lysosome membrane protein 2-like [Ostrinia furnacalis]	-1.9243	0.85424	0.59134	0.0121	0.46658
TRINITY_DN7957_c0_g1_i5_orf1	spermidine synthase [Ostrinia furnacalis] >XP_028167892.1 spermidine synthase [Ostrinia furnacalis]	-1.7939	0.59674	-0.3824	0.91596	0.66366
TRINITY_DN2880_c0_g1_i2_orf1	sialomucin core protein 24 [Pectinophora gossypiella]	-1.8657	0.87065	0.84819	-0.0512	0.19803
TRINITY_DN22242_c0_g2_i1_orf1	juvenile hormone epoxide hydrolase-like [Ostrinia furnacalis] >XP_028170526.1 juvenile hormone epoxide hydrolase-like [Ostrinia furnacalis]	-1.9324	0.32657	0.3865	0.2447	0.97463
TRINITY_DN1216_c0_g1_i4_orf1	bifunctional purine biosynthesis protein PURH isoform X1 [Ostrinia furnacalis] >XP_028176123.1 bifunctional purine biosynthesis protein PURH isoform X2 [Ostrinia furnacalis] >XP_028176129.1 bifunctional purine biosynthesis protein PURH isoform X3 [Ostrinia furnacalis]	-1.8287	-0.1232	0.20127	0.69093	1.05966
TRINITY_DN3029_c1_g2_i1_orf1	unnamed protein product [Plutella xylostella]	-1.567	-0.3919	1.49097	0.40499	0.06294
TRINITY_DN2943_c2_g2_i1_orf1	protein phosphatase inhibitor 2-like [Ostrinia furnacalis]	-1.9964	0.55471	0.57294	0.40976	0.45896
TRINITY_DN4502_c0_g1_i3_orf1	kynurenine formamidase isoform X1 [Ostrinia furnacalis]	-1.7103	-0.5348	0.92838	0.48493	0.8318
TRINITY_DN45097_c0_g1_i5_orf1	ras-related protein Rab-24-like [Ostrinia furnacalis]	-1.9703	0.66916	0.6937	0.35016	0.2573
TRINITY_DN13997_c0_g1_i5_orf1	28 kDa heat- and acid-stable phosphoprotein [Ostrinia furnacalis]	-1.8989	0.8816	0.18111	0.76045	0.07578
TRINITY_DN3433_c0_g1_i15_orf1	cytosolic purine 5'-nucleotidase isoform X3 [Ostrinia furnacalis] >XP_028162965.1 cytosolic purine 5'-nucleotidase isoform X3 [Ostrinia furnacalis] >XP_028162966.1 cytosolic purine 5'-nucleotidase isoform X3 [Ostrinia furnacalis]	-1.6979	-0.3968	0.68193	0.208	1.20477
TRINITY_DN5266_c0_g1_i1_orf1	malate dehydrogenase, cytoplasmic isoform X2 [Ostrinia furnacalis]	-1.6325	-0.6811	0.59319	0.71996	1.00045
TRINITY_DN3831_c0_g1_i7_orf1	beta-ureidopropionase-like [Ostrinia furnacalis]	-1.8673	-0.1401	0.36617	0.74093	0.90032
TRINITY_DN913_c0_g1_i6_orf1	uncharacterized protein LOC114354578 [Ostrinia furnacalis]	-1.9674	0.74717	0.29308	0.6301	0.29703
TRINITY_DN7579_c1_g3_i1_orf1	peroxiredoxin-5, mitochondrial [Ostrinia furnacalis]	-1.948	0.8543	0.39985	0.54024	0.15364
TRINITY_DN5914_c1_g1_i9_orf1	unnamed protein product [Chilo suppressalis]	-1.756	-0.3929	0.74252	0.37012	1.03624
TRINITY_DN59028_c0_g1_i1_orf1	15-hydroxyprostaglandin dehydrogenase [NAD(+)]-like [Ostrinia furnacalis]	-1.8961	0.49496	-0.1092	0.8154	0.69498
TRINITY_DN62091_c0_g1_i1_orf1	protein NipSnap [Venturia canescens]	-1.6918	-0.5007	0.34593	0.74638	1.10019
TRINITY_DN6243_c0_g1_i5_orf1	sorting nexin-20 [Ostrinia furnacalis]	-1.9153	0.93617	0.61538	0.11011	0.25365
TRINITY_DN2618_c0_g1_i3_orf1	CDP-diacylglycerol--inositol 3-phosphatidyltransferase [Ostrinia furnacalis]	-1.7362	-0.262	0.07561	0.78345	1.13909
TRINITY_DN125_c0_g1_i2_orf1	PREDICTED: purine nucleoside phosphorylase isoform X1 [Microplitis demolitor]	-1.5572	-0.2222	-0.1666	1.52272	0.42338
TRINITY_DN7867_c0_g1_i1_orf1	putative inorganic phosphate cotransporter [Ostrinia furnacalis]	-1.8369	0.88254	-0.2803	0.56126	0.67337
TRINITY_DN2035_c0_g1_i1_orf1	uncharacterized protein LOC114356683 [Ostrinia furnacalis]	-1.9635	0.77322	0.59139	0.39307	0.20588
TRINITY_DN79657_c0_g1_i1_orf1	uncharacterized protein LOC114349955 [Ostrinia furnacalis]	-1.9682	0.45246	0.57096	0.19908	0.74565
TRINITY_DN21435_c0_g1_i2_orf1	glycogen-binding subunit 76A isoform X1 [Ostrinia furnacalis]	-1.8107	-0.301	1.01405	0.53912	0.55849
TRINITY_DN19690_c0_g1_i1_orf1	unnamed protein product [Chilo suppressalis]	-1.9602	0.55713	0.24917	0.33262	0.82127
TRINITY_DN24970_c0_g1_i4_orf1	pyrroline-5-carboxylate reductase-like isoform X1 [Ostrinia furnacalis]	-1.5205	-0.8727	0.85304	0.69033	0.84983
TRINITY_DN1592_c0_g1_i1_orf1	serine protease 7-like isoform X2 [Ostrinia furnacalis]	-1.8072	-0.0889	1.04569	0.05712	0.79331
TRINITY_DN4255_c0_g1_i10_orf1	LOW QUALITY PROTEIN: lebocin-4-like [Ostrinia furnacalis]	-1.8737	-0.0803	0.87674	0.27978	0.79748
TRINITY_DN12301_c0_g1_i1_orf1	ribose-phosphate pyrophosphokinase 2 [Ostrinia furnacalis]	-1.6527	0.79765	-0.449	1.19083	0.11317
TRINITY_DN625_c9_g1_i7_orf1	ecdysone 20-monooxygenase [Ostrinia furnacalis]	-1.5979	-0.711	0.49407	0.74877	1.06604
TRINITY_DN8580_c0_g1_i12_orf1	peroxisomal N(1)-acetyl-spermine/spermidine oxidase-like isoform X1 [Ostrinia furnacalis]	-1.5881	1.09311	-0.73	0.60665	0.61828
TRINITY_DN2710_c0_g1_i4_orf1	translin-associated protein X [Ostrinia furnacalis]	-1.7497	-0.2598	0.07841	0.97357	0.95756
TRINITY_DN11060_c0_g1_i6_orf1	extracellular matrix protein A-like isoform X3 [Ostrinia furnacalis]	-1.7923	0.93331	0.95414	-0.0756	-0.0195
TRINITY_DN3257_c0_g1_i4_orf1	N-acetylneuraminate lyase-like [Ostrinia furnacalis]	-1.8469	-0.1478	0.30422	0.69376	0.99669
TRINITY_DN16147_c0_g1_i4_orf1	aldo-keto reductase AKR2E4-like isoform X1 [Ostrinia furnacalis]	-1.7597	-0.0005	1.05503	0.87301	-0.1678
TRINITY_DN31310_c0_g1_i1_orf1	PREDICTED: multiple epidermal growth factor-like domains protein 10 isoform X3 [Polistes canadensis]	-1.9707	0.80102	0.3132	0.49913	0.3573
TRINITY_DN3251_c0_g1_i6_orf1	fatty-acid amide hydrolase 2-like [Ostrinia furnacalis] >XP_028167366.1 fatty-acid amide hydrolase 2-like [Ostrinia furnacalis] >XP_028167367.1 fatty-acid amide hydrolase 2-like [Ostrinia furnacalis] >XP_028167368.1 fatty-acid amide hydrolase 2-like [Ostrinia furnacalis] >XP_028167369.1 fatty-acid amide hydrolase 2-like [Ostrinia furnacalis] >XP_028167370.1 fatty-acid amide hydrolase 2-like [Ostrinia furnacalis] >XP_028167371.1 fatty-acid amide hydrolase 2-like [Ostrinia furnacalis] >XP_028167372.1 fatty-acid amide hydrolase 2-like [Ostrinia furnacalis]	-1.8781	1.11383	0.35537	0.30974	0.09919
TRINITY_DN4062_c0_g2_i1_orf1	venom peptide BmKAPI-like isoform X2 [Ostrinia furnacalis]	-1.7884	-0.1512	1.21185	0.21333	0.51443
TRINITY_DN98313_c0_g1_i1_orf1	tetrahydrofolate synthase, partial [Plutella xylostella]	-1.6838	1.04253	-0.4505	0.91925	0.17259
TRINITY_DN384_c0_g1_i8_orf1	unnamed protein product [Chilo suppressalis]	-1.5327	-0.8339	0.57016	0.80611	0.99028
TRINITY_DN2170_c4_g1_i2_orf1	beta-1,3-glucan-binding protein-like isoform X1 [Ostrinia furnacalis]	-1.9937	0.52217	0.41939	0.63385	0.41826
TRINITY_DN18374_c0_g1_i1_orf1	L-2-hydroxyglutarate dehydrogenase, mitochondrial [Ostrinia furnacalis]	-1.9134	0.03307	0.43095	0.95046	0.49887
TRINITY_DN1720_c0_g1_i3_orf1	monocarboxylate transporter 12 [Ostrinia furnacalis] >XP_028157531.1 monocarboxylate transporter 12 [Ostrinia furnacalis]	-1.831	0.68212	-0.3098	0.62346	0.83518
TRINITY_DN3196_c0_g1_i1_orf1	organic cation transporter-like protein [Ostrinia furnacalis]	-1.6991	-0.4167	0.28531	0.61271	1.21769
TRINITY_DN21533_c0_g1_i4_orf1	hypothetical protein evm_010931 [Chilo suppressalis]	-1.9201	0.50601	0.98082	0.19303	0.24028

TRINITY_DN9718_c0_g1_i7_orf1	uncharacterized protein LOC114365184 [Ostrinia furnacalis] >XP_028177482.1 uncharacterized protein LOC114365184 [Ostrinia furnacalis] >XP_028177483.1 uncharacterized protein LOC114365184 [Ostrinia furnacalis]	-1.9965	0.39588	0.51001	0.58058	0.51001
TRINITY_DN5029_c0_g1_i1_orf1	ribose-phosphate pyrophosphokinase 2-like [Ostrinia furnacalis]	-1.7364	0.82998	-0.4808	0.92029	0.46693
TRINITY_DN106479_c1_g1_i1_orf1	secretory phospholipase A2 receptor-like [Ostrinia furnacalis]	-1.9021	0.52408	0.8137	0.66033	-0.096
TRINITY_DN4276_c0_g1_i11_orf1	arylsulfatase B [Ostrinia furnacalis]	-1.9096	0.9702	0.35108	0.53462	0.05375
TRINITY_DN7754_c0_g1_i2_orf1	carboxypeptidase E-like isoform X1 [Ostrinia furnacalis] >XP_028174275.1 carboxypeptidase E-like isoform X2 [Ostrinia furnacalis] >XP_028174276.1 carboxypeptidase E-like isoform X2 [Ostrinia furnacalis]	-1.9665	0.63731	0.74615	0.29624	0.28679
TRINITY_DN48602_c0_g1_i6_orf1	amidophosphoribosyltransferase-like isoform X1 [Ostrinia furnacalis] >XP_028157758.1 amidophosphoribosyltransferase-like isoform X1 [Ostrinia furnacalis] >XP_028157759.1 amidophosphoribosyltransferase-like isoform X1 [Ostrinia furnacalis]	-1.6541	0.35507	1.07896	0.79904	-0.5789
TRINITY_DN13411_c0_g1_i4_orf1	facilitated trehalose transporter Tret1-like [Ostrinia furnacalis]	-1.9782	0.43877	0.65252	0.24994	0.63701
TRINITY_DN2110_c0_g1_i3_orf1	ribose-phosphate pyrophosphokinase 1 isoform X1 [Chelonus insularis]	-1.7773	0.49946	-0.403	0.9321	0.74873
TRINITY_DN2921_c1_g1_i4_orf1	uncharacterized protein LOC114352340 isoform X2 [Ostrinia furnacalis]	-1.9617	0.44564	0.62477	0.15767	0.73357
TRINITY_DN29873_c0_g1_i1_orf1	glyceraldehyde-3-phosphate dehydrogenase 2 [Pectinophora gossypiella]	-1.7002	-0.4248	0.55244	1.22621	0.34641
TRINITY_DN11798_c0_g2_i1_orf1	N-acetylglucosamine-6-sulfatase-like isoform X2 [Ostrinia furnacalis]	-1.6011	-0.445	1.08465	1.02835	-0.0669
TRINITY_DN4245_c0_g1_i5_orf1	long-chain fatty acid transport protein 4-like [Ostrinia furnacalis]	-1.5312	-0.8368	1.01665	0.61023	0.7411
TRINITY_DN1532_c0_g1_i6_orf1	mRNA (2'-O-methyladenosine-N(6)-)-methyltransferase [Ostrinia furnacalis] >XP_028179575.1 mRNA (2'-O-methyladenosine-N(6)-)-methyltransferase [Ostrinia furnacalis] >XP_028179576.1 mRNA (2'-O-methyladenosine-N(6)-)-methyltransferase [Ostrinia furnacalis]	-1.8316	0.41356	-0.0711	1.16904	0.32011
TRINITY_DN3015_c0_g1_i7_orf1	glycine-rich protein DOT1-like [Ostrinia furnacalis]	-1.8626	-0.2158	0.85856	0.57096	0.64887
TRINITY_DN2141_c0_g1_i1_orf1	low density lipoprotein receptor adapter protein 1-like [Ostrinia furnacalis]	-1.5609	0.88735	1.2249	-0.0269	-0.5245
TRINITY_DN8747_c0_g1_i2_orf1	lipopolysaccharide-induced tumor necrosis factor-alpha factor homolog [Ostrinia furnacalis]	-1.9052	-0.0953	0.79174	0.64915	0.55953
TRINITY_DN2947_c0_g1_i4_orf1	ras-related protein Rab-39B [Spodoptera litura] >XP_035450569.1 ras-related protein Rab-39B-like [Spodoptera frugiperda] >KAF9422714.1 hypothetical protein HW555_001708 [Spodoptera exigua] >CAB3507117.1 unnamed protein product [Spodoptera littoralis] >KAF9824742.1 hypothetical protein SFRURICE_016851 [Spodoptera frugiperda] >KAG8101614.1 hypothetical protein SFRUCORN_012196 [Spodoptera frugiperda] >KAH9636250.1 hypothetical protein HF086_009446 [Spodoptera exigua]	-1.9113	0.04707	0.33377	0.5886	0.94182
TRINITY_DN1827_c0_g1_i4_orf1	phosphoglycerate mutase 1 [Ostrinia furnacalis]	-1.6558	-0.5543	0.54932	1.19794	0.46286
TRINITY_DN19377_c0_g1_i4_orf1	uncharacterized protein LOC114353316 [Ostrinia furnacalis] >XP_028161061.1 uncharacterized protein LOC114353316 [Ostrinia furnacalis]	-1.8385	-0.0166	0.06405	0.82032	0.97081
TRINITY_DN12014_c0_g1_i2_orf1	unnamed protein product [Chilo suppressalis]	-1.5093	0.83407	1.33928	-0.41	-0.2541
TRINITY_DN2182_c0_g1_i4_orf1	growth-blocking peptide, long form-like isoform X1 [Ostrinia furnacalis] >XP_028159332.1 growth-blocking peptide, long form-like isoform X1 [Ostrinia furnacalis] >QWX20072.1 growth-blocking peptide [Ostrinia furnacalis]	-1.7109	-0.4384	1.02537	0.87627	0.24765
TRINITY_DN6483_c0_g1_i6_orf1	transketolase-like protein 2 isoform X1 [Ostrinia furnacalis] >XP_028164795.1 transketolase-like protein 2 isoform X2 [Ostrinia furnacalis]	-1.7343	-0.4891	0.75333	0.50624	0.96388
TRINITY_DN10264_c1_g1_i5_orf1	probable low-specificity L-threonine aldolase 2 [Ostrinia furnacalis]	-1.792	0.89194	-0.3245	0.87503	0.34956
TRINITY_DN8473_c0_g1_i5_orf1	serine/threonine-protein phosphatase 6 regulatory subunit 1 [Ostrinia furnacalis]	-1.8096	0.40969	0.21415	1.22867	-0.0429
TRINITY_DN18196_c0_g1_i4_orf1	uncharacterized protein LOC114363471 isoform X3 [Ostrinia furnacalis]	-1.6497	-0.6557	0.98254	0.72615	0.59668
TRINITY_DN10195_c0_g1_i8_orf1	charged multivesicular body protein 6-A [Ostrinia furnacalis]	-1.9105	0.30133	0.29266	1.04995	0.26658
TRINITY_DN17031_c0_g1_i1_orf1	arginase, hepatic [Ostrinia furnacalis]	-1.8131	-0.237	0.29977	0.74344	1.00691
TRINITY_DN14611_c0_g1_i5_orf1	hsc70-interacting protein-like [Galleria mellonella]	-1.722	-0.3573	1.23601	0.31274	0.53055
TRINITY_DN58751_c0_g1_i2_orf1	FK506-binding protein 2 isoform X1 [Vanessa tameamea] >XP_046977568.1 FK506-binding protein 2 isoform X1 [Vanessa cardui]	-1.8191	-0.0684	1.02808	0.06933	0.79011
TRINITY_DN3758_c0_g1_i2_orf1	S-formylglutathione hydrolase isoform X1 [Spodoptera litura]	-1.8479	-0.1422	0.51393	1.06309	0.41314
TRINITY_DN10933_c0_g2_i1_orf1	uncharacterized protein LOC114357588 [Ostrinia furnacalis]	-1.8998	0.40842	0.63101	0.90748	-0.0471
TRINITY_DN2745_c0_g1_i4_orf1	Tubulin alpha-1 chain [Harpegnathos saltator]	-1.8971	0.09405	1.00888	0.5687	0.2255
TRINITY_DN95850_c0_g4_i3_orf1	calreticulin [Cotesia glomerata] >KAH0559118.1 hypothetical protein KQX54_000875 [Cotesia glomerata]	-1.9493	0.08151	0.76388	0.56886	0.53509
TRINITY_DN1786_c0_g1_i11_orf1	ATP-binding cassette sub-family A member 1-like [Ostrinia furnacalis]	-1.9584	0.28244	0.41235	0.87332	0.39025
TRINITY_DN4394_c0_g1_i4_orf1	B-cell receptor-associated protein 31 [Ostrinia furnacalis]	-1.8199	-0.2519	1.0406	0.58613	0.44509
TRINITY_DN42856_c0_g1_i1_orf1	GSCOCG00007769001-RA-CDS [Cotesia congregata] >CAG5103393.1 Similar to GLDC: Glycine dehydrogenase (decarboxylating) [Cotesia congregata]	-1.9232	0.44652	0.90353	0.04032	0.53283
TRINITY_DN135188_c0_g1_i2_orf1	proteasome inhibitor PI31 subunit [Ostrinia furnacalis]	-1.8071	-0.3741	0.65161	0.76706	0.76253
TRINITY_DN34166_c0_g1_i1_orf1	hypothetical protein EVAR_80688_1 [Eumeta japonica]	-1.9975	0.55308	0.46223	0.42794	0.55428
TRINITY_DN17838_c0_g1_i4_orf1	mitogen-activated protein kinase kinase kinase 4 [Ostrinia furnacalis]	-1.8693	0.55131	0.02776	1.0745	0.21569
TRINITY_DN13303_c0_g1_i6_orf1	membrane-associated progesterone receptor component 1-like [Ostrinia furnacalis]	-1.9595	0.72513	0.69333	0.33178	0.20925

TRINITY_DN18538_c0_g3_i1_orf1	poly(A)-specific ribonuclease PARN-like [Ostrinia furnacalis]	-1.8149	0.79715	-0.2614	0.33594	0.9432
TRINITY_DN400_c0_g1_i1_orf1	serine/threonine-protein phosphatase 2A activator-like isoform X2 [Ostrinia furnacalis] >XP_028164470.1 serine/threonine-protein phosphatase 2A activator-like isoform X2 [Ostrinia furnacalis]	-1.8954	-0.0481	0.35409	0.8857	0.70373
TRINITY_DN50517_c0_g1_i1_orf1	cuticle protein 21-like [Ostrinia furnacalis]	-1.9501	0.86277	0.55587	0.23001	0.30144
TRINITY_DN146138_c0_g1_i1_orf1	E3 ubiquitin-protein ligase Ubr3 [Chelonus insularis] >XP_034951823.1 E3 ubiquitin-protein ligase Ubr3 [Chelonus insularis] >XP_034951824.1 E3 ubiquitin-protein ligase Ubr3 [Chelonus insularis]	-1.8931	0.73978	0.70144	0.59668	-0.1448
TRINITY_DN1047_c0_g1_i6_orf1	mitochondrial genome maintenance exonuclease 1-like [Ostrinia furnacalis]	-1.7214	-0.5376	0.83014	0.57626	0.85252
TRINITY_DN67243_c0_g1_i1_orf1	39S ribosomal protein L3, mitochondrial [Ostrinia furnacalis]	-1.7652	-0.4647	0.6624	0.76647	0.8011
TRINITY_DN2706_c0_g1_i3_orf1	hypothetical protein evm_004793 [Chilo suppressalis]	-1.7364	-0.4081	1.15056	0.47707	0.51685
TRINITY_DN3861_c0_g3_i2_orf1	glutamyl aminopeptidase-like isoform X4 [Ostrinia furnacalis]	-1.9447	0.47841	0.20498	0.35509	0.90621
TRINITY_DN6822_c0_g2_i4_orf1	gelsolin-like [Bicyclus anynana]	-1.8289	0.57086	1.15038	0.05857	0.04909
TRINITY_DN356_c2_g1_i3_orf1	scavenger receptor class B member 1 isoform X2 [Pectinophora gossypiella] >XP_049883835.1 scavenger receptor class B member 1 isoform X2 [Pectinophora gossypiella]	-1.6052	-0.751	0.86098	0.73872	0.75652
TRINITY_DN582_c0_g1_i5_orf1	hypothetical protein evm_007348 [Chilo suppressalis]	-1.9022	0.06926	0.39047	0.41779	1.02466
TRINITY_DN2378_c0_g1_i5_orf1	integrin alpha-8-like isoform X1 [Ostrinia furnacalis]	-1.6147	-0.6393	0.68149	0.40985	1.16262
TRINITY_DN511_c0_g2_i1_orf1	pyruvate carboxylase, mitochondrial isoform X1 [Manduca sexta] >XP_037293486.1 pyruvate carboxylase, mitochondrial isoform X1 [Manduca sexta]	-1.5454	-0.8281	0.96842	0.67785	0.72719
TRINITY_DN29017_c0_g1_i4_orf1	cysteine protease ATG4B [Ostrinia furnacalis]	-1.9305	0.65175	0.1847	0.8754	0.21868
TRINITY_DN8440_c0_g1_i9_orf1	protein FAM177A1-like [Ostrinia furnacalis]	-1.8731	-0.054	0.40963	0.46777	1.04973
TRINITY_DN443_c0_g1_i2_orf1	proteasome subunit beta type-4 [Ostrinia furnacalis]	-1.9388	0.35468	0.68169	0.80008	0.10233
TRINITY_DN2796_c0_g1_i28_orf1	phosphofurin acidic cluster sorting protein 2 isoform X1 [Pectinophora gossypiella]	-1.9585	0.79806	0.34301	0.21441	0.60305
TRINITY_DN6587_c0_g1_i3_orf1	phosphoribosylformylglycinamidine synthase isoform X2 [Ostrinia furnacalis]	-1.6381	-0.474	0.34297	1.33687	0.43235
TRINITY_DN2516_c0_g2_i10_orf1	unnamed protein product, partial [Chilo suppressalis]	-1.9153	0.11364	0.97887	0.30643	0.51636
TRINITY_DN2983_c0_g1_i6_orf1	hypothetical protein evm_002448 [Chilo suppressalis]	-1.6396	-0.6764	0.60071	0.75271	0.96262
TRINITY_DN108433_c0_g1_i1_orf1	alpha-2-macroglobulin receptor-associated protein [Diachasma alloeum]	-1.9043	0.98091	0.08136	0.57997	0.26203
TRINITY_DN7630_c0_g2_i1_orf1	flotillin-2 isoform X1 [Ostrinia furnacalis] >XP_028172931.1 flotillin-2 isoform X2 [Ostrinia furnacalis]	-1.7047	-0.524	0.84554	0.41968	0.96349
TRINITY_DN5433_c0_g1_i5_orf1	uncharacterized protein LOC114351067 [Ostrinia furnacalis]	-1.7543	0.39484	0.88859	0.89346	-0.4226
TRINITY_DN13515_c0_g1_i1_orf1	low-density lipoprotein receptor-related protein 4-like isoform X2 [Ostrinia furnacalis]	-1.9479	0.38831	0.17016	0.86904	0.52037
TRINITY_DN8702_c0_g1_i1_orf1	programmed cell death protein 4 isoform X1 [Ostrinia furnacalis] >XP_028157160.1 programmed cell death protein 4 isoform X2 [Ostrinia furnacalis]	-1.603	-0.7039	0.79603	0.4728	1.0381
TRINITY_DN7776_c0_g1_i1_orf1	uncharacterized protein LOC114364702 [Ostrinia furnacalis]	-1.7825	1.05961	0.17589	0.78305	-0.236
TRINITY_DN11680_c0_g1_i1_orf1	uncharacterized protein LOC114355414 [Ostrinia furnacalis]	-1.7466	-0.2643	0.31861	0.43028	1.26205
TRINITY_DN19092_c2_g1_i1_orf1	mitochondrial fission 1 protein isoform X1 [Ostrinia furnacalis] >XP_028169879.1 mitochondrial fission 1 protein isoform X2 [Ostrinia furnacalis]	-1.6755	-0.6377	0.78961	0.79152	0.73211
TRINITY_DN9420_c0_g1_i2_orf1	protein PFC0760c-like isoform X2 [Ostrinia furnacalis]	-1.7011	0.45636	1.34286	0.16283	-0.2609
TRINITY_DN2624_c0_g1_i6_orf1	unnamed protein product [Danaus chrysippus]	-1.8255	-0.209	0.95347	0.28514	0.79589
TRINITY_DN1034_c0_g2_i1_orf1	glycerol kinase isoform X7 [Ostrinia furnacalis] >XP_028168096.1 glycerol kinase isoform X8 [Ostrinia furnacalis]	-1.8283	-0.2765	0.466	0.9216	0.71719
TRINITY_DN97378_c0_g1_i2_orf1	dihydropteridine reductase [Galleria mellonella]	-1.9149	0.91987	0.00831	0.48145	0.50524
TRINITY_DN72369_c0_g1_i1_orf1	microsomal triacylglycerol transfer protein [Vanessa cardui]	-1.9669	0.81506	0.25991	0.41828	0.47367
TRINITY_DN15382_c0_g1_i3_orf1	putative aldehyde dehydrogenase family 7 member A1 homolog [Spodoptera litura]	-1.7967	0.52332	0.84206	0.80551	-0.3741
TRINITY_DN745_c5_g1_i2_orf1	facilitated trehalose transporter Tret1-like isoform X2 [Ostrinia furnacalis]	-1.6603	-0.6211	0.94847	0.47978	0.85314
TRINITY_DN73_c0_g1_i6_orf1	autophagy-related protein 16-1 isoform X1 [Vanessa cardui]	-1.8931	-0.0956	0.43588	0.70032	0.85245
TRINITY_DN8386_c0_g1_i6_orf1	F-box/WD repeat-containing protein 9-like [Ostrinia furnacalis]	-1.8011	0.85868	0.46239	0.82721	-0.3472
TRINITY_DN12686_c0_g1_i4_orf1	maltase A1-like [Ostrinia furnacalis]	-1.5748	0.11062	1.25123	0.78454	-0.5716
TRINITY_DN6472_c0_g1_i5_orf1	unnamed protein product [Chilo suppressalis]	-1.9294	0.66993	0.25128	0.14542	0.86276
TRINITY_DN5383_c0_g1_i4_orf1	vacuolar protein sorting-associated protein 35 [Ostrinia furnacalis]	-1.9008	0.77525	0.78256	-0.0679	0.41092
TRINITY_DN22257_c0_g1_i7_orf1	spatacsin isoform X1 [Ostrinia furnacalis] >XP_028159192.1 spatacsin isoform X2 [Ostrinia furnacalis]	-1.7956	-0.0729	0.01773	1.09508	0.75567
TRINITY_DN41296_c0_g1_i1_orf1	exocyst complex component 5 [Ostrinia furnacalis]	-1.7361	0.25848	-0.3985	0.9235	0.95262
TRINITY_DN3431_c0_g1_i1_orf1	3-hydroxyacyl-CoA dehydrogenase type-2-like [Ostrinia furnacalis]	-1.7076	0.16527	-0.0405	1.42487	0.15791
TRINITY_DN18230_c1_g1_i1_orf1	glycine dehydrogenase (decarboxylating), mitochondrial isoform X1 [Ostrinia furnacalis] >XP_028174269.1 glycine dehydrogenase (decarboxylating), mitochondrial isoform X3 [Ostrinia furnacalis]	-1.95	0.31575	0.91274	0.31428	0.40725
TRINITY_DN146957_c0_g1_i1_orf1	26S proteasome non-ATPase regulatory subunit 7 [Apis florea]	-1.8981	0.84811	0.61519	0.53762	-0.1029
TRINITY_DN12003_c0_g2_i1_orf1	lactoylglutathione lyase [Ostrinia furnacalis]	-1.9034	0.10543	1.02799	0.46552	0.30441
TRINITY_DN6015_c1_g1_i3_orf1	probable salivary secreted peptide [Ostrinia furnacalis]	-1.6756	1.13313	-0.5025	0.75683	0.28815
TRINITY_DN60358_c0_g1_i3_orf1	transcription termination factor 5, mitochondrial-like [Ostrinia furnacalis]	-1.7813	0.70128	0.6196	0.88035	-0.42
TRINITY_DN43505_c0_g1_i1_orf1	unnamed protein product [Chilo suppressalis]	-1.9553	0.5858	0.17883	0.81068	0.38
TRINITY_DN13615_c0_g1_i3_orf1	calcium-binding protein P-like [Ostrinia furnacalis]	-1.9891	0.69157	0.39536	0.48371	0.41843

TRINITY_DN48846_c0_g1_i1_orf1	tudor domain-containing protein 7 isoform X3 [Ostrinia furnacalis]	-1.9199	-0.0363	0.80926	0.57934	0.56762
TRINITY_DN49038_c0_g4_i1_orf1	6-phosphogluconate dehydrogenase, decarboxylating [Ostrinia furnacalis]	-1.727	-0.5405	0.83303	0.75285	0.68164
TRINITY_DN7938_c0_g1_i3_orf1	protein halfway isoform X1 [Ostrinia furnacalis] >XP_028172280.1 protein halfway isoform X2 [Ostrinia furnacalis] >XP_028172281.1 protein halfway isoform X3 [Ostrinia furnacalis]	-1.9572	0.70304	0.39875	0.15373	0.7017
TRINITY_DN1756_c0_g1_i3_orf1	UTP--glucose-1-phosphate uridylyltransferase isoform X2 [Ostrinia furnacalis]	-1.8858	0.08061	0.43918	0.28858	1.07747
TRINITY_DN938_c0_g1_i7_orf1	protein ultraspiracle homolog isoform X2 [Ostrinia furnacalis]	-1.9099	0.09907	0.93667	0.23349	0.64069
TRINITY_DN230_c1_g1_i3_orf1	protein artichoke-like [Ostrinia furnacalis]	-1.6215	0.1444	1.53238	-0.0393	-0.016
TRINITY_DN5813_c0_g1_i9_orf1	alpha-(1,6)-fucosyltransferase [Ostrinia furnacalis]	-1.8655	-0.0008	1.10813	0.4274	0.33075
TRINITY_DN21856_c0_g1_i1_orf1	anti-lipopolysaccharide factor 3 [Eriocheir sinensis]	-1.9028	0.87912	0.74598	0.06324	0.21446
TRINITY_DN1355_c0_g1_i7_orf1	larval/pupal rigid cuticle protein 66-like [Ostrinia furnacalis]	-1.7874	0.85136	-0.2542	0.98729	0.20293
TRINITY_DN364_c0_g1_i2_orf1	uncharacterized protein LOC114366657, partial [Ostrinia furnacalis]	-1.7772	0.7245	1.14092	-0.119	0.03075
TRINITY_DN19807_c0_g1_i1_orf1	DNA-directed RNA polymerase I subunit RPA1 [Ostrinia furnacalis]	-1.5116	-0.8863	0.724	0.88408	0.7898
TRINITY_DN2876_c0_g1_i1_orf1	long-chain fatty acid transport protein 4-like [Ostrinia furnacalis]	-1.8413	0.0447	1.0026	0.01803	0.77596
TRINITY_DN38685_c0_g1_i4_orf1	unnamed protein product [Euphydryas editha]	-1.8815	0.00402	0.95199	0.21261	0.71292
TRINITY_DN66671_c0_g1_i1_orf1	phosphoglucomutase-like [Ostrinia furnacalis]	-1.9576	0.59816	0.25927	0.81257	0.28756
TRINITY_DN5556_c0_g1_i3_orf1	hypothetical protein evm_006874 [Chilo suppressalis]	-1.8626	0.77596	-0.2348	0.64357	0.67782
TRINITY_DN34115_c0_g1_i1_orf1	transcription factor 23 [Ostrinia furnacalis]	-1.8888	0.08964	0.42528	1.07402	0.29988
TRINITY_DN1926_c0_g1_i5_orf1	coiled-coil and C2 domain-containing protein 1-like [Ostrinia furnacalis]	-1.7294	-0.3788	1.07738	0.22063	0.81018
TRINITY_DN1080_c0_g1_i1_orf1	bleomycin hydrolase [Ostrinia furnacalis]	-1.9083	0.58866	0.95142	0.32371	0.04449
TRINITY_DN30012_c1_g1_i1_orf1	ubiquitin domain-containing protein UBFD1-like [Ostrinia furnacalis]	-1.5659	-0.7602	0.66365	0.5594	1.10304
TRINITY_DN726_c0_g1_i2_orf1	vinculin [Ostrinia furnacalis]	-1.7885	0.02289	1.18181	0.63371	-0.0499
TRINITY_DN11492_c0_g1_i8_orf1	unnamed protein product [Chilo suppressalis]	-1.969	0.46008	0.42478	0.27447	0.80967
TRINITY_DN21715_c0_g1_i1_orf1	protein disulfide-isomerase A3 isoform X1 [Ostrinia furnacalis] >XP_028169825.1 protein disulfide-isomerase A3 isoform X2 [Ostrinia furnacalis] >XP_028169826.1 protein disulfide-isomerase A3 isoform X3 [Ostrinia furnacalis]	-1.8729	0.11684	1.03031	0.08544	0.64026
TRINITY_DN3766_c0_g1_i10_orf1	circadian clock-controlled protein-like [Ostrinia furnacalis]	-1.8072	-0.0108	1.14064	0.01967	0.65766
TRINITY_DN1054_c0_g1_i8_orf1	tubulin-specific chaperone D [Ostrinia furnacalis]	-1.8396	0.36117	1.01264	-0.1863	0.65208
TRINITY_DN24310_c0_g1_i2_orf1	glucose-6-phosphate 1-dehydrogenase [Ostrinia furnacalis]	-1.8851	0.85448	0.5354	0.63937	-0.1441
TRINITY_DN364_c0_g2_i1_orf1	unnamed protein product [Chilo suppressalis]	-1.756	0.83851	1.09362	-0.0588	-0.1172
TRINITY_DN22175_c0_g1_i1_orf1	low-density lipoprotein receptor-related protein 1B-like [Ostrinia furnacalis]	-1.9431	0.42982	0.47753	0.14675	0.88896
TRINITY_DN47260_c0_g1_i2_orf1	SET domain-containing protein SmydA-8-like [Ostrinia furnacalis]	-1.8252	0.76258	-0.3325	0.73832	0.65682
TRINITY_DN6162_c1_g1_i1_orf1	hsc70-interacting protein-like [Ostrinia furnacalis]	-1.911	0.5425	0.80655	-0.0692	0.63115
TRINITY_DN3975_c0_g1_i10_orf1	modular serine protease-like [Ostrinia furnacalis]	-1.8541	0.8115	-0.1512	0.30682	0.88702
TRINITY_DN14587_c0_g1_i7_orf1	hypothetical protein evm_001488 [Chilo suppressalis] >CAB3526337.1 unnamed protein product [Chilo suppressalis] >CAH0403665.1 unnamed protein product [Chilo suppressalis]	-1.7948	1.05331	-0.2962	0.37238	0.66537
TRINITY_DN117042_c0_g1_i2_orf1	UBX domain-containing protein 6 [Ostrinia furnacalis] >XP_028162119.1 UBX domain-containing protein 6 [Ostrinia furnacalis]	-1.8352	0.24593	-0.1571	0.7688	0.9776
TRINITY_DN7152_c0_g1_i1_orf1	tissue alpha-L-fucosidase [Ostrinia furnacalis]	-1.8125	-0.2934	1.01828	0.55256	0.53514
TRINITY_DN21278_c0_g2_i2_orf1	mannose-1-phosphate guanyltransferase alpha-A [Ostrinia furnacalis]	-1.8226	-0.2239	0.67287	0.34635	1.0273
TRINITY_DN92_c1_g2_i1_orf1	SID1 transmembrane family member 1-like isoform X3 [Ostrinia furnacalis]	-1.9533	0.7009	0.71713	0.13392	0.40137
TRINITY_DN12661_c0_g1_i3_orf1	T-complex protein 11-like protein 1 [Ostrinia furnacalis]	-1.6372	0.81103	1.24283	-0.3314	-0.0853
TRINITY_DN65299_c0_g4_i1_orf1	LOW QUALITY PROTEIN: signal transducing adapter molecule 2 [Ostrinia furnacalis]	-1.7275	0.95975	1.02117	-0.226	-0.0274
TRINITY_DN8258_c0_g1_i3_orf1	papilin isoform X7 [Ostrinia furnacalis]	-1.5283	-0.7272	1.10871	0.22088	0.92595
TRINITY_DN8555_c0_g1_i1_orf1	epoxide hydrolase 4-like [Ostrinia furnacalis]	-1.7234	0.87136	-0.5359	0.79434	0.59364
TRINITY_DN4954_c0_g1_i5_orf1	glycogen debranching enzyme [Trichoplusia ni]	-1.8089	0.52283	0.99783	0.60086	-0.3126
TRINITY_DN11370_c0_g1_i6_orf1	protein MEMO1 [Hyposmocoma kahamanoa]	-1.7672	0.54338	1.23361	-0.1782	0.16836
TRINITY_DN3712_c0_g1_i1_orf1	ribonuclease Oy [Ostrinia furnacalis]	-1.7426	0.2393	1.32457	-0.1711	0.34981
TRINITY_DN17172_c0_g1_i5_orf1	omega-amidase NIT2 isoform X1 [Zerene cesonia]	-1.767	-0.2954	0.47705	0.40145	1.18392
TRINITY_DN757_c3_g1_i2_orf1	PREDICTED: galectin-4-like [Amyelois transitella]	-1.5514	-0.7952	0.85701	0.50289	0.98666
TRINITY_DN8258_c0_g1_i5_orf1	papilin isoform X9 [Ostrinia furnacalis]	-1.6402	-0.5893	1.00886	0.29416	0.92643
TRINITY_DN1497_c0_g2_i6_orf1	unnamed protein product [Chilo suppressalis]	-1.7882	0.45609	1.22265	0.27106	-0.1616
TRINITY_DN472_c1_g1_i3_orf1	IST1 homolog isoform X1 [Ostrinia furnacalis]	-1.8696	0.1698	1.04449	0.03584	0.61943
TRINITY_DN23640_c0_g1_i5_orf1	proteasomal ATPase-associated factor 1-like [Ostrinia furnacalis]	-1.8961	0.53543	-0.1208	0.68485	0.79658
TRINITY_DN33867_c0_g1_i8_orf1	uncharacterized protein LOC114357513 [Ostrinia furnacalis]	-1.3673	0.55393	0.73106	1.11022	-1.0279
TRINITY_DN22962_c0_g1_i1_orf1	lysosomal acid glucosylceramidase-like isoform X2 [Ostrinia furnacalis]	-1.5113	-0.0197	1.62574	-0.232	0.13723
TRINITY_DN17845_c0_g1_i3_orf1	ataxin-10-like [Ostrinia furnacalis]	-1.6157	-0.3905	1.43427	0.19535	0.37655
TRINITY_DN2861_c0_g2_i1_orf1	proteasome subunit alpha type-5 [Ostrinia furnacalis]	-1.8668	1.13707	0.09748	0.39572	0.23656
TRINITY_DN827_c1_g1_i1_orf1	peptidoglycan recognition protein 4-like isoform X1 [Ostrinia furnacalis]	-1.669	0.22671	1.42598	-0.2462	0.26254
TRINITY_DN6698_c0_g2_i2_orf1	protein mesh isoform X1 [Ostrinia furnacalis]	-1.862	0.14421	0.89972	-0.0199	0.83795
TRINITY_DN19261_c0_g1_i3_orf1	UDP-glucuronic acid decarboxylase 1 isoform X1 [Papilio machaon]	-1.7699	-0.2742	0.87164	0.17074	1.00169

TRINITY_DN35991_c0_g1_i2_orf1	serine/threonine-protein kinase D1 isoform X1 [Ostrinia furnacalis] >XP_028171859.1 serine/threonine-protein kinase D1 isoform X1 [Ostrinia furnacalis]	-1.7436	-0.3688	0.30228	1.12226	0.68784
TRINITY_DN3406_c0_g1_i17_orf1	uncharacterized protein ZK1073.1-like isoform X6 [Spodoptera frugiperda]	-1.7884	-0.1685	1.04784	0.09266	0.81644
TRINITY_DN11274_c0_g1_i4_orf1	dipeptidyl peptidase 3 isoform X1 [Ostrinia furnacalis]	-1.6845	1.04519	0.37544	0.80058	-0.5367
TRINITY_DN24668_c0_g1_i8_orf1	uncharacterized protein LOC114364067 isoform X3 [Ostrinia furnacalis]	-1.3148	1.05858	1.25614	-0.3091	-0.6908
TRINITY_DN184_c0_g1_i10_orf1	C-type mannose receptor 2-like isoform X1 [Leguminivora glycinivorella]	-1.6979	0.66493	1.27613	-0.0299	-0.2132
TRINITY_DN12424_c0_g1_i2_orf1	uncharacterized protein LOC114350608 [Ostrinia furnacalis]	-1.8073	-0.1986	0.99292	0.19383	0.81908
TRINITY_DN6058_c0_g1_i3_orf1	NHL repeat-containing protein 2 isoform X2 [Ostrinia furnacalis]	-1.7127	-0.5468	0.80503	0.55113	0.90329
TRINITY_DN6771_c0_g2_i1_orf1	putative endoplasmin, partial [Cotesia chilonis]	-1.7979	0.41903	-0.2229	0.44067	1.16109
TRINITY_DN1023_c1_g1_i1_orf1	ras-related protein Rab-7a [Ostrinia furnacalis]	-1.9697	0.81238	0.37051	0.31092	0.47587
TRINITY_DN29369_c0_g1_i1_orf1	uncharacterized protein LOC114351747 [Ostrinia furnacalis]	-1.8336	0.05873	1.21465	0.24883	0.31145
TRINITY_DN21609_c0_g1_i1_orf1	translation initiation factor eIF-2B subunit epsilon [Ostrinia furnacalis]	-1.9123	0.2795	0.34636	0.24456	1.04182
TRINITY_DN28741_c0_g1_i3_orf1	uncharacterized protein LOC114351652 [Ostrinia furnacalis]	-1.1431	0.43471	1.72204	-0.3946	-0.619
TRINITY_DN1848_c0_g1_i2_orf1	uncharacterized protein LOC114351337 [Ostrinia furnacalis]	-1.8149	0.22378	-0.1535	0.63723	1.10741
TRINITY_DN8407_c0_g1_i2_orf1	dihydrolipoyllysine-residue acetyltransferase component of pyruvate dehydrogenase complex, mitochondrial isoform X1 [Ostrinia furnacalis] >XP_028161753.1 dihydrolipoyllysine-residue acetyltransferase component of pyruvate dehydrogenase complex, mitochondrial isoform X2 [Ostrinia furnacalis]	-1.6801	-0.2387	-0.0312	1.30539	0.64455
TRINITY_DN3418_c0_g1_i3_orf1	proto-oncogene tyrosine-protein kinase ROS [Ostrinia furnacalis]	-1.8578	0.69826	-0.2519	0.73454	0.67685
TRINITY_DN1063_c0_g1_i16_orf1	hypothetical protein evm_000854 [Chilo suppressalis]	-1.7788	0.29935	-0.1637	0.39178	1.25138
TRINITY_DN13901_c0_g1_i4_orf1	vesicle-associated membrane protein 7-like [Ostrinia furnacalis]	-1.9331	0.88275	0.27403	0.15614	0.62013
TRINITY_DN7992_c0_g1_i4_orf1	uncharacterized protein LOC114362579 [Ostrinia furnacalis]	-1.8937	0.26038	0.96262	0.02327	0.64738
TRINITY_DN28875_c0_g1_i1_orf1	PREDICTED: ras-related protein Rab-2 [Fopius arisanus] >XP_015110133.1 ras-related protein Rab-2 [Diachasma alloeum]	-1.9456	0.34889	0.69303	0.13206	0.77158
TRINITY_DN14831_c0_g1_i9_orf1	probable protein phosphatase 2C T23F11.1 isoform X1 [Ostrinia furnacalis]	-1.8808	0.09123	1.10098	0.29424	0.39436
TRINITY_DN582_c0_g1_i2_orf1	unnamed protein product [Chilo suppressalis]	-1.5553	0.77535	0.99089	0.59304	-0.804
TRINITY_DN10455_c0_g2_i1_orf1	ubiquitin fusion degradation protein 1 homolog [Ostrinia furnacalis]	-1.9465	0.06827	0.50685	0.75424	0.6171
TRINITY_DN21533_c0_g1_i6_orf1	annexin B9 isoform X2 [Ostrinia furnacalis]	-1.8437	0.82198	0.87886	0.33833	-0.1955
TRINITY_DN2202_c0_g1_i9_orf1	vascular endothelial growth factor receptor 1 isoform X3 [Ostrinia furnacalis]	-1.7253	1.09291	-0.4691	0.58254	0.519
TRINITY_DN1738_c0_g1_i5_orf1	baculoviral IAP repeat-containing protein 6-like [Ostrinia furnacalis]	-1.8106	0.76052	-0.3687	0.68926	0.72955
TRINITY_DN472_c0_g1_i6_orf1	hypothetical protein evm_002904 [Chilo suppressalis]	-1.8936	0.04522	0.51637	1.02535	0.30671
TRINITY_DN10396_c0_g1_i1_orf1	charged multivesicular body protein 3 isoform X1 [Ostrinia furnacalis] >XP_028178068.1 charged multivesicular body protein 3 isoform X2 [Ostrinia furnacalis]	-1.8882	0.09281	0.18178	1.02031	0.5933
TRINITY_DN29351_c0_g1_i1_orfp1	TRINITY_DN29351_c0_g1_i1_m.58077 TRINITY_DN29351_c0_g1::TRINITY_DN29351_c0_g1_i1::g.58077 ORF type:complete len:328 (+),score=-16.88 TRINITY_DN29351_c0_g1_i1:50-1033(+)	-1.661	0.82815	-0.5081	1.11648	0.22454
TRINITY_DN2374_c0_g2_i2_orf1	uncharacterized protein LOC114357127 [Ostrinia furnacalis]	-1.9722	0.36723	0.33839	0.46128	0.80526
TRINITY_DN25582_c0_g1_i3_orf1	uroporphyrinogen decarboxylase [Ostrinia furnacalis]	-1.9278	-0.0298	0.6344	0.612	0.71115
TRINITY_DN726_c0_g1_i8_orf1	unnamed protein product [Chilo suppressalis]	-1.627	-0.4581	1.35127	0.52135	0.21254
TRINITY_DN3300_c0_g2_i1_orf1	annexin B10 isoform X9 [Ostrinia furnacalis] >XP_028177766.1 annexin B10 isoform X10 [Ostrinia furnacalis]	-1.6995	0.46646	1.34882	-0.2423	0.12651
TRINITY_DN19521_c0_g1_i1_orf1	sodium/potassium-transporting ATPase subunit beta-1-like isoform X1 [Ostrinia furnacalis]	-1.7714	-0.0148	1.26149	0.00458	0.52014
TRINITY_DN9717_c0_g2_i1_orf1	proteasome subunit beta type-2 [Ostrinia furnacalis]	-1.9749	0.72411	0.43239	0.57297	0.24543
TRINITY_DN1255_c0_g1_i17_orf1	LIM and senescent cell antigen-like-containing domain protein 1 isoform X1 [Pectinophora gossypiella]	-1.7348	-0.4753	0.61988	0.5548	1.03552
TRINITY_DN65247_c1_g1_i1_orf1	beta-1,3-glucan-binding protein [Manduca sexta] >XP_030023421.1 beta-1,3-glucan-binding protein [Manduca sexta] >XP_030023422.1 beta-1,3-glucan-binding protein [Manduca sexta] >XP_030023423.1 beta-1,3-glucan-binding protein [Manduca sexta] >AEV66276.1 beta-1,3-glucanase [Manduca sexta] >KAG6448577.1 hypothetical protein O3G_MSEX005570 [Manduca sexta] >KAG6448578.1 hypothetical protein O3G_MSEX005570 [Manduca sexta] >KAG6448579.1 hypothetical protein O3G_MSEX005570 [Manduca sexta] >KAG6448580.1 hypothetical protein O3G_MSEX005570 [Manduca sexta]	-1.8756	0.55785	0.66307	0.83583	-0.1812
TRINITY_DN27771_c0_g2_i1_orf1	glycine--tRNA ligase [Ostrinia furnacalis]	-1.7689	-0.2525	0.15157	1.06886	0.80104
TRINITY_DN2013_c0_g1_i15_orf1	high mobility group protein DSP1-like isoform X1 [Ostrinia furnacalis]	-1.8667	0.08977	0.21315	1.12775	0.43604
TRINITY_DN26805_c0_g2_i3_orf1	phosphopantothenate--cysteine ligase isoform X1 [Ostrinia furnacalis] >XP_028161191.1 phosphopantothenate--cysteine ligase isoform X2 [Ostrinia furnacalis] >XP_028161192.1 phosphopantothenate--cysteine ligase isoform X1 [Ostrinia furnacalis]	-1.806	0.97684	-0.332	0.57651	0.58457
TRINITY_DN5207_c0_g2_i3_orf1	mucin-like [Galleria mellonella]	-1.7499	0.23817	-0.3265	1.12509	0.71316
TRINITY_DN146181_c0_g1_i1_orf1	vesicular integral-membrane protein VIP36 [Diachasma alloeum]	-1.8335	-0.1678	0.40521	0.50429	1.0917
TRINITY_DN143792_c0_g1_i1_orf1	ras-related GTP-binding protein C [Venturia canescens]	-1.6422	1.05972	0.50542	0.71744	-0.6404
TRINITY_DN4304_c0_g1_i3_orf1	TOM1-like protein 2 isoform X4 [Galleria mellonella]	-1.7538	-0.3085	0.50554	0.35303	1.20369

TRINITY_DN12951_c1_g2_i2_orf1	protein kinase C isoform X2 [Ostrinia furnacalis]	-1.921	0.28774	0.94441	0.12344	0.56544
TRINITY_DN10900_c0_g1_i7_orf1	fatty acid synthase-like [Ostrinia furnacalis]	-1.4691	0.54662	1.53432	-0.3344	-0.2774
TRINITY_DN22944_c0_g3_i1_orf1	transmembrane protein 115 [Ostrinia furnacalis]	-1.9177	0.48394	0.15829	0.28441	0.99108
TRINITY_DN4565_c0_g1_i3_orf1	acid phosphatase type 7 isoform X1 [Ostrinia furnacalis] >XP_028155943.1 acid phosphatase type 7 isoform X1 [Ostrinia furnacalis] >XP_028155944.1 acid phosphatase type 7 isoform X1 [Ostrinia furnacalis] >XP_028155945.1 acid phosphatase type 7 isoform X1 [Ostrinia furnacalis]	-1.958	0.84851	0.48226	0.40565	0.22158
TRINITY_DN4694_c0_g1_i6_orf1	uncharacterized protein LOC114362122 [Ostrinia furnacalis]	-1.933	0.29093	0.34135	0.32122	0.97945
TRINITY_DN5458_c1_g1_i9_orf1	histone H2A-like [Aedes aegypti] >XP_021712017.1 histone H2A-like [Aedes aegypti]	-1.8763	0.6411	0.97369	-0.0767	0.33828
TRINITY_DN22604_c0_g1_i3_orf1	cytochrome P450 6B5-like [Ostrinia furnacalis]	-1.7257	0.23345	-0.2594	0.44797	1.30368
TRINITY_DN18279_c0_g1_i1_orf1	solute carrier family 12 member 9 isoform X1 [Hyposmocoma kahamanoa]	-1.8757	0.0508	0.7749	0.92955	0.12049
TRINITY_DN16127_c0_g1_i2_orf1	transcription intermediary factor 1-alpha [Ostrinia furnacalis]	-1.8068	-0.0197	-0.0015	1.09318	0.73485
TRINITY_DN51995_c0_g3_i1_orf1	circadian clock-controlled protein-like [Ostrinia furnacalis]	-1.03	-0.8234	-0.2009	1.77233	0.28199
TRINITY_DN4204_c0_g1_i1_orf1	uncharacterized protein LOC114359352 [Ostrinia furnacalis]	-0.6383	-1.2259	-0.4705	1.4345	0.90027
TRINITY_DN143532_c0_g1_i1_orf1	3-oxoacyl-[acyl-carrier-protein] reductase FabG-like [Aphidius gifuensis] >KAF7996667.1 hypothetical protein HCN44_002313 [Aphidius gifuensis]	-0.7626	-0.892	-0.6005	1.72741	0.5276
TRINITY_DN1534_c0_g1_i3_orf1	peptidoglycan recognition protein-like [Ostrinia furnacalis]	-1.0596	-0.625	-0.4714	1.76361	0.39243
TRINITY_DN68770_c0_g1_i1_orf1	seroin transcript 1A2 [Ostrinia nubilalis]	-0.724	-1.2571	-0.1714	1.60041	0.55209
TRINITY_DN128231_c0_g1_i5_orf1	glutathione S-transferase sigma3 [Glyphodes pyloalis]	-0.4286	-0.1912	-0.3961	1.94093	-0.925
TRINITY_DN17693_c0_g1_i10_orf1	acetylcholinesterase-like [Ostrinia furnacalis]	-0.8678	0.1234	-0.4822	1.88745	-0.6608
TRINITY_DN9458_c0_g1_i4_orf1	uncharacterized protein LOC114363583 [Ostrinia furnacalis]	-0.2072	-0.7505	-0.7053	1.95256	-0.2896
TRINITY_DN8771_c0_g2_i1_orf1	regucalcin-like [Ostrinia furnacalis]	0.56095	-0.404	-0.6006	1.64712	-1.2035
TRINITY_DN21341_c0_g1_i1_orf1	FAST kinase domain-containing protein 4 isoform X6 [Ostrinia furnacalis] >XP_028160336.1 FAST kinase domain-containing protein 4 isoform X7 [Ostrinia furnacalis] >XP_028160337.1 FAST kinase domain-containing protein 4 isoform X8 [Ostrinia furnacalis]	-0.4394	-0.3207	-0.6023	1.98758	-0.6252
TRINITY_DN109503_c0_g1_i4_orf1	uncharacterized protein LOC114366345 isoform X2 [Ostrinia furnacalis]	-0.1097	-0.8489	-0.9471	1.83449	0.07113
TRINITY_DN6203_c0_g1_i1_orfp1	TRINITY_DN6203_c0_g1_i1_m.72736 TRINITY_DN6203_c0_g1::TRINITY_DN6203_c0_g1_i1::g.72736 ORF type:internal len:93 (+),score=12.26 TRINITY_DN6203_c0_g1_i1:3-278(+)	-0.0773	0.41175	-1.0162	1.67492	-0.9932
TRINITY_DN55154_c0_g2_i1_orf1	glycosyl transferase family 8 domain-containing protein [Phthorimaea operculella]	-0.4107	-1.1904	-0.6838	1.55258	0.73229
TRINITY_DN30713_c0_g1_i3_orf1	phosphoglucomutase [Ostrinia furnacalis]	-0.3786	-1.2486	-0.1871	1.80629	0.00795
TRINITY_DN2749_c4_g1_i2_orf1	RNA exonuclease 4-like [Ostrinia furnacalis] >QEE79882.1 REX4 [Ostrinia furnacalis]	0.39193	-0.874	-1.3738	1.41436	0.44146
TRINITY_DN19866_c0_g1_i4_orf1	lys-63-specific deubiquitinase BRCC36-like [Ostrinia furnacalis]	-0.5354	-0.3493	-0.8024	1.967	-0.2798
TRINITY_DN5564_c0_g1_i5_orf1	probable phosphoserine aminotransferase [Ostrinia furnacalis]	-0.2454	-1.4022	-0.2394	1.69714	0.18988
TRINITY_DN2338_c0_g1_i3_orf1	phenoloxidase subunit 1-like [Ostrinia furnacalis]	-0.0718	-0.6196	-0.0224	1.83265	-1.1188
TRINITY_DN1628_c0_g2_i3_orf1	uncharacterized protein LOC114363979 [Ostrinia furnacalis]	-0.5491	-0.0842	-0.2848	1.90626	-0.9881
TRINITY_DN2673_c2_g1_i2_orf1	aminopeptidase N3c [Ostrinia nubilalis]	0.51512	-1.3995	-0.4961	1.57823	-0.1977
TRINITY_DN30185_c0_g1_i3_orf1	organic cation transporter protein [Ostrinia furnacalis]	-0.3268	-0.2058	-0.1519	1.85773	-1.1733
TRINITY_DN54410_c0_g2_i1_orf1	lysozyme-like [Ostrinia furnacalis]	-0.416	-0.9193	-0.8215	1.77878	0.37802
TRINITY_DN1785_c0_g1_i5_orf1	beta-mannosidase [Ostrinia furnacalis]	0.17189	-0.6828	-1.1412	1.78449	-0.1324
TRINITY_DN113327_c0_g1_i2_orf1	proteasome subunit beta type-6 [Helicoverpa armigera] >XP_047031479.1 proteasome subunit beta type-6 [Helicoverpa zea] >XP_049697949.1 proteasome subunit beta type-6-like [Helicoverpa armigera] >PZC87318.1 hypothetical protein B5X24_HaOG201554 [Helicoverpa armigera]	-0.709	-0.3679	-0.3105	1.97883	-0.5915
TRINITY_DN3092_c0_g1_i2_orf1	replication factor C subunit 1 isoform X1 [Ostrinia furnacalis] >XP_028157702.1 replication factor C subunit 1 isoform X2 [Ostrinia furnacalis]	0.4809	-0.7081	-0.6326	1.75231	-0.8925
TRINITY_DN11808_c0_g1_i8_orf1	unnamed protein product [Diatraea saccharalis]	-0.2515	-0.8229	-0.8693	1.87047	0.07315
TRINITY_DN1506_c0_g1_i6_orfp1	TRINITY_DN1506_c0_g1_i6_m.57691 TRINITY_DN1506_c0_g1::TRINITY_DN1506_c0_g1_i6::g.57691 ORF type:5prime_partial len:173 (+),score=30.49 TRINITY_DN1506_c0_g1_i6:1-519(+)	-1.1155	-1.3243	0.70693	0.84806	0.88481
TRINITY_DN7064_c0_g1_i19_orf1	unnamed protein product [Chilo suppressalis]	-1.2318	-1.1627	0.44399	0.84973	1.10076
TRINITY_DN114890_c0_g1_i4_orf1	chemosensory protein 10 [Ostrinia furnacalis]	-1.475	-0.7429	0.56866	1.36742	0.28177
TRINITY_DN59429_c0_g1_i6_orf1	uncharacterized protein LOC114366345 isoform X2 [Ostrinia furnacalis]	-1.528	-0.7936	1.06094	0.8701	0.39062
TRINITY_DN8008_c0_g1_i6_orf1	uncharacterized protein LOC114357965 isoform X1 [Ostrinia furnacalis] >XP_028167599.1 uncharacterized protein LOC114357965 isoform X1 [Ostrinia furnacalis] >XP_028167600.1 uncharacterized protein LOC114357965 isoform X2 [Ostrinia furnacalis] >XP_028167601.1 uncharacterized protein LOC114357965 isoform X3 [Ostrinia furnacalis]	-1.4788	-0.8444	0.40514	1.17864	0.73942
TRINITY_DN30510_c0_g1_i6_orf1	spodomicin-like [Ostrinia furnacalis]	-1.2996	-1.0591	1.19375	0.37534	0.78959
TRINITY_DN1772_c1_g2_i1_orf1	aldose reductase-like isoform X2 [Ostrinia furnacalis]	-1.483	-0.8172	0.2967	0.86385	1.13962
TRINITY_DN20344_c0_g1_i5_orf1	uncharacterized protein LOC114351483 [Ostrinia furnacalis]	-1.1413	-1.2336	0.39299	0.82177	1.16014

TRINITY_DN21367_c0_g1_i1_orf1	[illegible] protein S15 [Macaca mulatta] >NP_001268540.1 40S ribosomal protein S15 [Mesocricetus auratus] >NP_001291817.1 40S ribosomal protein S15 [Ailuropoda melanoleuca] >NP_033117.1 40S ribosomal protein S15 isoform 1 [Mus musculus] >NP_058847.1 40S ribosomal protein S15 [Rattus norvegicus] >NP_990793.1 40S ribosomal protein S15 [Gallus gallus] >NP_999499.1 40S ribosomal protein S15 [Sus scrofa] >XP_001513459.4 40S ribosomal protein S15 [Ornithorhynchus anatinus] >XP_003277039.1 40S ribosomal protein S15 isoform X2 [Nomascus leucogenys] >XP_003461016.1 40S ribosomal protein S15 [Cavia porcellus] >XP_004378568.1 40S ribosomal protein S15 [Trichechus manatus latirostris] >XP_004395466.1 PREDICTED: 40S ribosomal protein S15 [Odobenus rosmarus divergens] >XP_004441415.1 PREDICTED: 40S ribosomal protein S15 [Ceratotherium simum simum] >XP_004462792.1 40S ribosomal protein S15 [Dasypus novemcinctus] >XP_004632738.1 40S ribosomal protein S15 [Octodon degus] >XP_004866007.1 40S ribosomal protein S15 [Heterocephalus glaber] >XP_005359120.1 40S ribosomal protein S15 isoform X2 [Microtus ochrogaster] >XP_005406026.1 PREDICTED: 40S ribosomal protein S15 [Chinchilla lanigera] >XP_005531236.1 PREDICTED: 40S ribosomal protein S15 [Pseudopodoces humilis] >XP_005587458.1 40S ribosomal protein S15 isoform X4 [Macaca fascicularis] >XP_005866755.1 PREDICTED: 40S ribosomal protein S15 [Myotis brandtii] >XP_006206493.1 40S ribosomal protein S15 [Vicugna pacos] >XP_006764872.1 PREDICTED: 40S ribosomal protein S15 [Myotis davidii] >XP_006897998.1 PREDICTED: 40S ribosomal protein S15 [Elephantulus edwardii] >XP_006904116.1 40S ribosomal protein S15 [Pteropus alecto] >XP_006928238.1 40S ribosomal protein S15 [Felis catus] >XP_006978252.1 40S ribosomal protein S15 [Peromyscus maniculatus bairdii] >XP_007992808.1 40S ribosomal protein S15 isoform X2 [Chlorocebus sabaeus] >XP_008591572.1 PREDICTED: 40S ribosomal protein S15 [Galeopterus variegatus] >XP_008835382.1 40S ribosomal protein S15 isoform X3 [Nannospalax galili] >XP_009095157.1 40S ribosomal protein S15 [Serinus canaria] >XP_010373202.1 40S ribosomal protein S15 isoform X2 [Rhinopithecus roxellana] >XP_010561484.1 PREDICTED: 40S ribosomal protein S15 [Haliaeetus leucocephalus] >XP_010633228.1 40S ribosomal protein S15 [Fukomys damarensis] >XP_010723314.1 40S ribosomal protein S15 [Meleagris gallopavo] >XP_010964943.1 40S ribosomal protein S15 [Camelus bactrianus] >XP_010996087.1 40S ribosomal protein S15 [Camelus dromedarius] >XP_011747134.1 40S ribosomal protein S15 isoform X4 [Macaca nemestrina] >XP_011819405.1 PREDICTED: 40S ribosomal protein S15 isoform X2 [Colobus angolensis palliatus] >XP_011852804.1 PREDICTED: 40S ribosomal protein S15 isoform X2 [Mandrillus leucophaeus] >XP_011928279.1 PREDICTED: 40S ribosomal protein S15 isoform X4 [Cercocebus atys] >XP_012291424.2 40S ribosomal protein S15 isoform X1 [Aotus nancymaae] >XP_012394559.1 40S ribosomal protein S15 isoform X2 [Orcinus orca] >XP_012500924.1 PREDICTED: 40S ribosomal protein S15 [Propithecus coquereli] >XP_012601179.1 40S ribosomal protein S15 [Microcebus murinus] >XP_012659873.2 40S ribosomal protein S15 [Otolemur garnettii] >XP_012878256.1 PREDICTED: 40S ribosomal protein S15 [Dipodomys ordii] >XP_013800709.1 PREDICTED: 40S ribosomal protein S15 [Apteryx mantelli mantelli] >XP_014129960.1 40S ribosomal protein S15 [Zonotrichia albicollis] >XP_014701153.1 40S ribosomal protein S15 [Equus asinus] >XP_014738550.1 PREDICTED: 40S ribosomal protein S15 [Sturnus vulgaris] >XP_015507206.1 40S ribosomal protein S15 [Parus major] >XP_015741871.1 40S ribosomal protein S15 [Coturnix japonica] >XP_015982115.1 40S ribosomal protein S15 [Rousettus aegyptiacus] >XP_016065176.1 PREDICTED: 40S ribosomal protein S15 [Miniopterus natalensis] >XP_017369931.1 40S ribosomal protein S15 isoform X1 [Cebus [illegible]	-1.3002	-0.612	0.08012	1.70668	0.12542
TRINITY_DN1149_c0_g1_i4_orf1	circadian clock-controlled protein-like [Ostrinia furnacalis]	-1.4557	-0.8255	1.26362	0.71512	0.30242
TRINITY_DN27300_c0_g1_i1_orfp1	TRINITY_DN27300_c0_g1_i1_m.71142 TRINITY_DN27300_c0_g1::TRINITY_DN27300_c0_g1_i1::g.71142 ORF type:internal len:82 (-),score=9.46 TRINITY_DN27300_c0_g1_i1:3-245(-)	-1.164	-0.8827	1.67074	0.23183	0.14416
TRINITY_DN14328_c0_g1_i12_orf1	larval cuticle protein LCP-30-like [Ostrinia furnacalis]	-1.2255	-1.1997	0.73966	0.62938	1.0562
TRINITY_DN609_c0_g1_i1_orf1	zonadhesin-like isoform X1 [Ostrinia furnacalis]	-1.1453	-1.1348	1.40622	0.58059	0.29327
TRINITY_DN43350_c0_g3_i1_orf1	uncharacterized protein LOC114355190 [Ostrinia furnacalis]	-1.3572	-1.0414	0.50193	0.84143	1.05524
TRINITY_DN12586_c0_g1_i4_orf1	zonadhesin-like isoform X4 [Ostrinia furnacalis]	-1.1145	-1.2595	1.07618	0.9415	0.35631
TRINITY_DN4068_c0_g2_i4_orf1	larval cuticle protein LCP-17-like precursor [Papilio polytes] >BAM18876.1 cuticular protein PpolCPR2 [Papilio polytes]	-0.8188	-1.4621	0.24256	0.85238	1.18595
TRINITY_DN6415_c0_g2_i1_orf1	D-arabinitol dehydrogenase 1-like [Ostrinia furnacalis]	-1.3917	-0.9229	0.40741	0.61675	1.29042
TRINITY_DN2457_c0_g1_i8_orf1	uncharacterized protein LOC114355596 [Ostrinia furnacalis]	-1.3516	-0.8865	1.4347	0.34564	0.45773
TRINITY_DN10222_c0_g1_i2_orf1	glutathione S-transferase sigma 3 [Ostrinia furnacalis]	-1.3953	-0.9739	0.45478	1.13803	0.77637
TRINITY_DN3439_c0_g2_i2_orf1	histone H2A.Z-specific chaperone CHZ1-like [Ostrinia furnacalis]	-1.4623	-0.8689	0.88475	0.35051	1.09594
TRINITY_DN2464_c0_g1_i12_orf1	uncharacterized protein LOC114362996 isoform X1 [Ostrinia furnacalis]	-1.2387	-1.2101	0.81621	0.78066	0.8519
TRINITY_DN7960_c0_g1_i2_orf1	uncharacterized protein LOC114364878 [Ostrinia furnacalis]	-1.5541	-0.7169	0.3175	1.15288	0.80058
TRINITY_DN9400_c0_g1_i8_orf1	PREDICTED: monoacylglycerol lipase ABHD12-like [Amyelois transitella]	-1.4248	-0.9057	0.80006	1.17661	0.35384
TRINITY_DN125521_c0_g2_i1_orf1	seroin transcript 1A2 [Ostrinia nubilalis]	-1.0033	-1.3903	0.50122	0.84879	1.04353
TRINITY_DN28501_c0_g1_i2_orfp1	TRINITY_DN28501_c0_g1_i2_m.58934 TRINITY_DN28501_c0_g1::TRINITY_DN28501_c0_g1_i2::g.58934 ORF type:internal len:98 (+),score=13.70 TRINITY_DN28501_c0_g1_i2:3-293(+)	-0.9138	-1.2705	1.52418	0.26055	0.39961
TRINITY_DN14185_c0_g1_i1_orf1	uncharacterized protein LOC114358675 [Ostrinia furnacalis] >XP_028168498.1 uncharacterized protein LOC114358675 [Ostrinia furnacalis]	-1.4804	-0.8394	1.02381	0.97643	0.31961
TRINITY_DN15545_c0_g1_i1_orf1	larval cuticle protein LCP-14-like [Ostrinia furnacalis]	-1.0717	-1.3512	0.95578	0.59929	0.86782
TRINITY_DN15327_c2_g1_i2_orf1	protein lethal(2)essential for life-like [Ostrinia furnacalis]	-0.907	-1.4861	0.64615	0.98654	0.76039
TRINITY_DN18482_c0_g1_i3_orf1	calcyphosin-like protein isoform X3 [Helicoverpa armigera] >XP_047020698.1 calcyphosin-like protein isoform X2 [Helicoverpa zea]	-1.3109	-1.0723	0.42551	1.11023	0.84742
TRINITY_DN55147_c0_g1_i1_orfp1	TRINITY_DN55147_c0_g1_i1_m.59251 TRINITY_DN55147_c0_g1::TRINITY_DN55147_c0_g1_i1::g.59251 ORF type:5prime_partial len:331 (-),score=113.97,Cuticle_3 PF11018.9 0.29,Cuticle_3|PF11018.9|2.9e-05,Cuticle_3|PF11018.9|0.00037 TRINITY_DN55147_c0_g1_i1:21-1013(-)	-1.0286	-1.3966	0.95132	0.72154	0.75231
TRINITY_DN394_c0_g1_i4_orf1	uncharacterized protein LOC114351483 [Ostrinia furnacalis]	-1.1528	-1.1552	0.15944	1.03687	1.11173
TRINITY_DN285_c0_g1_i4_orf1	catalase-like [Ostrinia furnacalis]	-1.393	-0.997	0.50637	0.80903	1.07456
TRINITY_DN4695_c0_g1_i3_orf1	glutathione S-transferase epsilon 3 [Ostrinia furnacalis]	-0.4334	-1.5714	-0.1429	1.1643	0.98337

TRINITY_DN4242_c0_g1_i6_orf1	fibrohexamerin-like [Ostrinia furnacalis]	-0.5306	-1.5569	-0.0513	1.11643	1.02245
TRINITY_DN15685_c0_g1_i5_orf1	uncharacterized protein LOC114352354 [Ostrinia furnacalis]	-1.1223	-1.2913	1.09574	0.61491	0.70297
TRINITY_DN710_c0_g1_i11_orfp1	TRINITY_DN710_c0_g1_i11_m.67699 TRINITY_DN710_c0_g1::TRINITY_DN710_c0_g1_i11::g.67699 ORF type:complete len:194 (-),score=34.99,Collagen PF01391.19 0.00029 TRINITY_DN710_c0_g1_i11:1283-1864(-)	-1.3438	-0.9968	0.98595	0.28302	1.07163
TRINITY_DN8685_c0_g1_i5_orf1	macrophage mannose receptor 1-like [Zerene cesonia]	-1.433	-0.7835	1.3785	0.62066	0.21731
TRINITY_DN9435_c0_g1_i7_orf1	uncharacterized protein LOC114350197 [Ostrinia furnacalis]	-1.4207	-0.7163	0.24495	1.50223	0.38978
TRINITY_DN1593_c0_g1_i1_orf1	chemosensory protein csp11 [Helopeltis theivora]	-1.2519	-1.086	1.31471	0.53995	0.48319
TRINITY_DN8095_c0_g1_i3_orf1	circadian clock-controlled protein-like [Ostrinia furnacalis]	-1.0745	-1.36	0.89069	0.84661	0.69711
TRINITY_DN394_c0_g1_i2_orf1	uncharacterized protein LOC114351483 [Ostrinia furnacalis]	-1.0724	-1.2518	0.79102	0.27572	1.25746
TRINITY_DN8473_c0_g1_i6_orf1	serine/threonine-protein phosphatase 6 regulatory subunit 1 [Ostrinia furnacalis]	-1.403	-0.8279	0.06895	1.127	1.03499
TRINITY_DN24789_c0_g1_i9_orfp1	TRINITY_DN24789_c0_g1_i9_m.25888 TRINITY_DN24789_c0_g1::TRINITY_DN24789_c0_g1_i9::g.25888 ORF type:internal len:114 (-),score=12.65 TRINITY_DN24789_c0_g1_i9:2-340(-)	-1.0147	-1.024	1.69065	0.21118	0.13694
TRINITY_DN76633_c0_g1_i1_orfp1	TRINITY_DN76633_c0_g1_i1_m.53394 TRINITY_DN76633_c0_g1::TRINITY_DN76633_c0_g1_i1::g.53394 ORF type:internal len:164 (-),score=26.46,Toxin_2 PF00451.20 0.00022,Toxin_2|PF00451.20|0.00056,Toxin_2|PF00451.20|0.0002,Toxin_2|PF00451.20|5.9e-06,Gamma-thionin|PF00304.21|2,Gamma-thionin|PF00304.21|0.024,Gamma-thionin|PF00304.21|0.027,Gamma-thionin|PF00304.21|0.066,Toxin_38|PF14866.7|0.27,Toxin_38|PF14866.7|0.054,Toxin_38|PF14866.7|0.14,Defensin_2|PF01097.19|1.7,Defensin_2|PF01097.19|0.055,Defensin_2|PF01097.19|1.2,Defensin_2|PF01097.19|0.15 TRINITY_DN76633_c0_g1_i1:1-489(-)	-0.8921	-1.3183	0.97157	1.23382	0.00498
TRINITY_DN1175_c1_g1_i2_orf1	methanethiol oxidase [Ostrinia furnacalis]	-1.3329	-1.0471	0.93906	1.04224	0.39867
TRINITY_DN335_c1_g1_i5_orf1	PREDICTED: perilipin-4 isoform X14 [Papilio polytes]	-1.3939	-0.9118	1.15817	0.23751	0.90995
TRINITY_DN214_c0_g1_i4_orf1	uncharacterized protein LOC114352813 [Ostrinia furnacalis]	-1.0073	-1.2403	1.4236	0.61285	0.21121
TRINITY_DN15858_c0_g1_i2_orf1	15-hydroxyprostaglandin dehydrogenase [NAD(+)]-like [Ostrinia furnacalis]	-1.5542	-0.7493	1.12761	0.7616	0.41423
TRINITY_DN5748_c0_g1_i6_orf1	glycine N-methyltransferase isoform X1 [Ostrinia furnacalis] >XP_028165118.1 glycine N-methyltransferase isoform X2 [Ostrinia furnacalis] >XP_028165119.1 glycine N-methyltransferase isoform X1 [Ostrinia furnacalis] >XP_028165120.1 glycine N-methyltransferase isoform X2 [Ostrinia furnacalis]	-1.4254	-0.822	1.38264	0.37303	0.4917
TRINITY_DN5198_c0_g1_i5_orfp1	TRINITY_DN5198_c0_g1_i5_m.8637 TRINITY_DN5198_c0_g1::TRINITY_DN5198_c0_g1_i5::g.8637 ORF type:complete len:223 (-),score=54.99 TRINITY_DN5198_c0_g1_i5:319-987(-)	-1.3617	-0.9491	1.31173	0.38687	0.61217
TRINITY_DN3826_c0_g1_i1_orf1	39S ribosomal protein L18, mitochondrial [Ostrinia furnacalis]	-1.1241	-1.0952	0.47951	1.49986	0.23988
TRINITY_DN14670_c0_g1_i1_orf1	heat shock protein beta-1 isoform X1 [Helicoverpa armigera] >XP_022829066.1 heat shock protein beta-1 isoform X1 [Spodoptera litura] >XP_026747148.1 heat shock protein beta-1 isoform X3 [Trichoplusia ni] >XP_026748187.1 heat shock protein beta-1 isoform X2 [Galleria mellonella] >XP_028167756.1 heat shock protein beta-1 isoform X2 [Ostrinia furnacalis] >XP_035431734.1 heat shock protein beta-1-like isoform X3 [Spodoptera frugiperda] >XP_047023072.1 heat shock protein beta-1 isoform X1 [Helicoverpa zea] >XP_049865086.1 heat shock protein beta-1 [Pectinophora gossypiella] >KAH9640995.1 hypothetical protein HF086_015091 [Spodoptera exigua] >QGZ00460.1 heat shock protein 21.4 [Glyphodes pyloalis] >QKR72095.1 heat-shock protein 21.4 [Mythimna separata] >CAB3228281.1 unnamed protein product [Arctia plantaginis] >CAH0628881.1 unnamed protein product [Chrysodeixis includens]	-1.4788	-0.8018	1.2992	0.46987	0.51152
TRINITY_DN2461_c0_g1_i5_orf1	secretory phospholipase A2 receptor [Vanessa cardui]	-1.4282	-0.9807	0.67942	0.72084	1.00862
TRINITY_DN36434_c0_g2_i3_orf1	clotting factor B isoform X1 [Ostrinia furnacalis]	-1.5088	-0.7175	0.36902	0.5116	1.34566
TRINITY_DN245_c0_g1_i4_orf1	ER lumen protein-retaining receptor [Ostrinia furnacalis]	-0.9378	-1.4415	0.47339	0.98805	0.91781
TRINITY_DN15858_c0_g1_i1_orf1	15-hydroxyprostaglandin dehydrogenase [NAD(+)]-like [Ostrinia furnacalis]	-1.4782	-0.9075	0.5607	0.98784	0.83724
TRINITY_DN448_c0_g1_i20_orf1	probable cytochrome P450 9f2 isoform X1 [Ostrinia furnacalis]	-1.5752	-0.6521	0.40392	0.54576	1.27766
TRINITY_DN214_c0_g1_i3_orf1	uncharacterized protein LOC114352813 [Ostrinia furnacalis]	-1.1513	-1.14	1.36991	0.65265	0.26877
TRINITY_DN1344_c0_g1_i1_orf1	ribosomal RNA small subunit methyltransferase NEP1 [Ostrinia furnacalis]	-0.6559	-1.6117	0.37478	1.08852	0.80431
TRINITY_DN7735_c0_g1_i4_orf1	calphotin-like [Ostrinia furnacalis]	-0.1974	-1.8769	0.73219	0.64205	0.7
TRINITY_DN59422_c0_g1_i2_orf1	larval cuticle protein LCP-22-like isoform X2 [Pectinophora gossypiella]	-0.3481	-1.8106	0.821	0.54295	0.79479
TRINITY_DN1226_c0_g1_i11_orfp1	TRINITY_DN1226_c0_g1_i11_m.52385 TRINITY_DN1226_c0_g1::TRINITY_DN1226_c0_g1_i11::g.52385 ORF type:internal len:92 (-),score=5.77 TRINITY_DN1226_c0_g1_i11:2-274(-)	-1.1938	-1.1191	0.75418	0.2602	1.29849
TRINITY_DN135679_c0_g1_i2_orfp1	TRINITY_DN135679_c0_g1_i2_m.85525 TRINITY_DN135679_c0_g1::TRINITY_DN135679_c0_g1_i2::g.85525 ORF type:5prime_partial len:55 (+),score=5.08,Toxin_2 PF00451.20 1.9e-06 TRINITY_DN135679_c0_g1_i2:3-167(+)	-1.042	-1.3145	0.61395	1.25296	0.48952
TRINITY_DN22513_c0_g1_i4_orf1	DNA-directed RNA polymerase II subunit RPB1 [Ostrinia furnacalis] >XP_028179194.1 DNA-directed RNA polymerase II subunit RPB1 [Ostrinia furnacalis] >XP_028179195.1 DNA-directed RNA polymerase II subunit RPB1 [Ostrinia furnacalis]	-0.9573	-1.4546	0.86804	0.66633	0.87756
TRINITY_DN28299_c0_g1_i1_orf1	adenylosuccinate lyase isoform X1 [Ostrinia furnacalis]	-1.5833	-0.6274	0.20884	1.1626	0.83925

TRINITY_DN5191_c0_g2_i1_orf1	CD151 antigen-like [Ostrinia furnacalis]	-0.747	-1.4923	0.14453	1.0556	1.03916
TRINITY_DN9694_c0_g1_i1_orf1	larval cuticle protein LCP-17 [Helicoverpa armigera] >PZC82071.1 hypothetical protein B5X24_HaOG211161 [Helicoverpa armigera] >PZC87412.1 hypothetical protein B5X24_HaOG216859 [Helicoverpa armigera]	-0.8696	-1.5181	0.73023	0.70097	0.95642
TRINITY_DN4068_c1_g2_i1_orf1	larval cuticle protein LCP-17 [Helicoverpa armigera] >PZC82071.1 hypothetical protein B5X24_HaOG211161 [Helicoverpa armigera] >PZC87412.1 hypothetical protein B5X24_HaOG216859 [Helicoverpa armigera]	-0.964	-1.3426	1.34151	0.51963	0.44546
TRINITY_DN26439_c0_g1_i2_orf1	uncharacterized protein LOC114351853 [Ostrinia furnacalis]	-1.4429	-0.9057	1.16983	0.45826	0.72055
TRINITY_DN4314_c0_g1_i9_orf1	serine proteinase inhibitor 2 [Ostrinia furnacalis]	-1.1955	-1.1943	0.44553	1.13917	0.80511
TRINITY_DN5001_c0_g1_i4_orf1	uncharacterized protein LOC114356665 [Ostrinia furnacalis]	-1.0354	-1.2161	0.05198	0.98282	1.21671
TRINITY_DN64719_c0_g1_i2_orfp1	TRINITY_DN64719_c0_g1_i2_m.37745 TRINITY_DN64719_c0_g1::TRINITY_DN64719_c0_g1_i2::g.37745 ORF type:internal len:91 (+),score=41.89 TRINITY_DN64719_c0_g1_i2:1-270(+)	-0.82	-1.2922	1.57752	0.15284	0.38186
TRINITY_DN57111_c0_g1_i1_orf1	trypsin-like serine proteinase T26 protein, partial [Chilo infuscatellus]	-0.5496	-1.5818	1.38376	0.39994	0.34777
TRINITY_DN138086_c0_g1_i1_orf1	hypothetical protein evm_000614 [Chilo suppressalis]	-1.4059	-0.9705	1.12567	0.49825	0.75248
TRINITY_DN2514_c1_g1_i13_orf1	seroin transcript 1A2 [Ostrinia nubilalis]	-1.2016	-1.2286	0.76788	0.63721	1.02515
TRINITY_DN6813_c1_g1_i1_orf1	pantothenate kinase 3 isoform X2 [Ostrinia furnacalis] >XP_028173241.1 pantothenate kinase 3 isoform X2 [Ostrinia furnacalis]	-1.4126	-0.8523	0.17222	1.21786	0.87482
TRINITY_DN12873_c0_g1_i3_orf1	proteoglycan 4-like [Ostrinia furnacalis]	-1.4375	-0.8395	0.22303	1.21369	0.84027
TRINITY_DN11698_c0_g1_i1_orf1	hypothetical protein evm_015129 [Chilo suppressalis]	-1.5159	-0.8045	0.41208	0.77516	1.13319
TRINITY_DN8527_c0_g2_i1_orfp1	TRINITY_DN8527_c0_g2_i1_m.16937 TRINITY_DN8527_c0_g2::TRINITY_DN8527_c0_g2_i1::g.16937 ORF type:5prime_partial len:54 (-),score=0.36 TRINITY_DN8527_c0_g2_i1:195-356(-)	-1.047	-1.3598	0.52817	0.86394	1.01464
TRINITY_DN29698_c0_g1_i3_orf1	15-hydroxyprostaglandin dehydrogenase [NAD(+)]-like [Ostrinia furnacalis]	-1.3142	-1.0878	1.13357	0.63215	0.63632
TRINITY_DN117844_c0_g1_i1_orf1	ATP-citrate synthase [Cotesia glomerata] >XP_044590631.1 ATP-citrate synthase [Cotesia glomerata] >KAH0546822.1 hypothetical protein KQX54_015428 [Cotesia glomerata]	-1.3484	-1.075	0.59378	0.91078	0.9188
TRINITY_DN11388_c0_g1_i4_orf1	limbic system-associated membrane protein-like, partial [Ostrinia furnacalis]	-0.4933	-1.7115	0.53523	1.10619	0.56335
TRINITY_DN2772_c0_g1_i3_orf1	uncharacterized protein LOC114353284 isoform X4 [Ostrinia furnacalis] >XP_028161011.1 uncharacterized protein LOC114353284 isoform X4 [Ostrinia furnacalis] >XP_028161012.1 uncharacterized protein LOC114353284 isoform X4 [Ostrinia furnacalis]	-1.2004	-1.0565	0.21757	1.41714	0.62222
TRINITY_DN32586_c0_g2_i1_orf1	unnamed protein product [Euphydryas editha]	-0.7164	-1.6179	0.67344	0.6958	0.96511
TRINITY_DN26879_c0_g1_i1_orf1	hypothetical protein O3G_MSEX014401 [Manduca sexta]	-1.3195	-1.0606	1.08557	0.40486	0.8897
TRINITY_DN8310_c0_g2_i1_orf1	uncharacterized protein LOC116773294 [Danaus plexippus plexippus] >OWR55545.1 hypothetical protein KGM_209260 [Danaus plexippus plexippus]	-0.4472	-1.7304	0.36911	1.03521	0.77328
TRINITY_DN1230_c1_g1_i5_orf1	uncharacterized protein LOC114353440 [Ostrinia furnacalis]	-1.3733	-1.016	0.87327	0.46648	1.04956
TRINITY_DN13236_c0_g1_i4_orf1	zonadhesin-like isoform X4 [Ostrinia furnacalis]	-0.9254	-1.3108	1.4502	0.31118	0.47484
TRINITY_DN12666_c0_g1_i2_orf1	sodium- and chloride-dependent GABA transporter 1 [Ostrinia furnacalis]	-1.3174	-0.9833	0.77094	1.2812	0.24855
TRINITY_DN8625_c0_g1_i1_orf1	GDP-L-fucose synthase [Ostrinia furnacalis]	-1.186	-0.9143	0.27083	1.62561	0.20384
TRINITY_DN3616_c0_g2_i2_orf1	conotoxin ArMKLT2-032-like [Ostrinia furnacalis]	-1.1895	-1.1778	0.65611	0.46608	1.24508
TRINITY_DN103475_c0_g1_i4_orf1	lipid storage droplets surface-binding protein 1 isoform X3 [Ostrinia furnacalis]	-1.4875	-0.8857	1.03294	0.5326	0.80764
TRINITY_DN1038_c1_g1_i3_orf1	uncharacterized protein LOC114363583 [Ostrinia furnacalis]	-1.0305	-1.3507	1.10594	0.44096	0.83432
TRINITY_DN18782_c0_g1_i4_orf1	putative riboflavin kinase [Ostrinia furnacalis] >XP_028176654.1 putative riboflavin kinase [Ostrinia furnacalis]	-0.6062	-1.4963	-0.0715	0.97421	1.19976
TRINITY_DN10889_c0_g1_i8_orf1	four and a half LIM domains protein 2 isoform X7 [Pectinophora gossypiella]	-0.6175	-1.6738	0.75432	0.58581	0.95123
TRINITY_DN15755_c0_g1_i1_orf1	cytochrome P450 monooxygenase CYP6AB141 [Ostrinia furnacalis]	-1.0622	-1.1196	0.96158	-0.0787	1.29894
TRINITY_DN38230_c0_g1_i4_orf1	hypothetical protein evm_007803 [Chilo suppressalis]	-0.7866	-1.4054	1.40858	0.15204	0.63146
TRINITY_DN66453_c0_g1_i4_orfp1	TRINITY_DN66453_c0_g1_i4_m.7345 TRINITY_DN66453_c0_g1::TRINITY_DN66453_c0_g1_i4::g.7345 ORF type:internal len:93 (+),score=24.53 TRINITY_DN66453_c0_g1_i4:3-278(+)	-1.1439	-1.1127	0.89828	0.07731	1.28091
TRINITY_DN701_c0_g1_i1_orf1	venom protease-like isoform X3 [Ostrinia furnacalis]	-1.3194	-1.0141	0.60083	0.43697	1.29569
TRINITY_DN9560_c0_g1_i5_orf1	uncharacterized protein LOC114357350 [Ostrinia furnacalis]	-0.3893	-1.7557	1.10851	0.51552	0.52095
TRINITY_DN8030_c0_g1_i2_orf1	chaoptin isoform X1 [Ostrinia furnacalis] >XP_028159953.1 chaoptin isoform X2 [Ostrinia furnacalis] >XP_028159954.1 chaoptin isoform X3 [Ostrinia furnacalis] >XP_028159955.1 chaoptin isoform X4 [Ostrinia furnacalis] >XP_028159956.1 chaoptin isoform X5 [Ostrinia furnacalis]	-1.3678	-0.9623	0.27146	0.9568	1.10181
TRINITY_DN111488_c0_g1_i1_orf1	LOW QUALITY PROTEIN: formin-J-like [Chelonus insularis]	-0.4428	-1.7512	1.02693	0.55251	0.61447
TRINITY_DN3859_c0_g1_i5_orf1	D-aspartate oxidase [Ostrinia furnacalis] >XP_028166452.1 D-aspartate oxidase [Ostrinia furnacalis] >XP_028166454.1 D-aspartate oxidase [Ostrinia furnacalis]	-1.1492	-1.2222	0.96059	1.07118	0.33965
TRINITY_DN10360_c0_g1_i16_orf1	H(+)/Cl(-) exchange transporter 3 isoform X1 [Ostrinia furnacalis]	-1.1457	-1.2384	0.62895	0.54719	1.20787
TRINITY_DN1328_c0_g1_i6_orf1	fungal protease inhibitor-1-like [Ostrinia furnacalis]	-1.2203	-1.112	0.31708	1.27504	0.74022
TRINITY_DN21331_c0_g1_i6_orf1	sodium-independent sulfate anion transporter-like [Ostrinia furnacalis]	-1.5348	-0.7357	0.61652	1.24836	0.40566
TRINITY_DN38568_c0_g1_i1_orf1	unnamed protein product, partial [Diatraea saccharalis]	-0.5081	-1.728	0.79283	0.51439	0.92886
TRINITY_DN1084_c0_g1_i2_orf1	ATP-citrate synthase [Ostrinia furnacalis]	-1.5429	-0.6055	0.10453	1.29863	0.74526
TRINITY_DN8853_c0_g1_i4_orf1	uncharacterized protein LOC114351488 isoform X1 [Ostrinia furnacalis]	-0.9977	-1.1259	0.34319	0.17111	1.60931

TRINITY_DN39837_c0_g1_i1_orf1	unnamed protein product [Plutella xylostella]	-1.1769	-1.2523	0.90928	0.93988	0.58006
TRINITY_DN610_c0_g1_i1_orf1	CAP-Gly domain-containing linker protein 1 isoform X10 [Ostrinia furnacalis]	-1.232	-1.1576	0.96644	1.02417	0.399
TRINITY_DN65518_c0_g1_i1_orf1	unc-112-related protein-like, partial [Ostrinia furnacalis]	-1.4615	-0.9369	0.59447	0.95666	0.84722
TRINITY_DN10403_c0_g1_i1_orf1	hypothetical protein evm_000264 [Chilo suppressalis] >CAH2987898.1 unnamed protein product [Chilo suppressalis]	-1.403	-0.8494	0.30404	0.5742	1.37408
TRINITY_DN2596_c0_g1_i6_orf1	unnamed protein product [Arctia plantaginis]	-1.1127	-1.1681	0.11708	0.93568	1.22808
TRINITY_DN12013_c0_g1_i6_orf1	tudor domain-containing protein 7 isoform X3 [Ostrinia furnacalis]	-1.01	-0.8578	1.79872	0.09119	-0.0222
TRINITY_DN4069_c0_g1_i5_orf1	putative sulfiredoxin [Ostrinia furnacalis]	-1.5381	-0.6059	1.03337	0.01551	1.09507
TRINITY_DN31163_c1_g1_i4_orf1	phenoloxidase subunit 2-like [Ostrinia furnacalis]	-1.2361	-0.8351	0.2996	1.6327	0.13885
TRINITY_DN9820_c0_g1_i1_orf1	endocuticle structural glycoprotein SgAbd-2-like [Ostrinia furnacalis]	-0.3194	-1.8038	0.92167	0.40982	0.79171
TRINITY_DN12545_c0_g1_i7_orf1	hypothetical protein evm_003491 [Chilo suppressalis]	-1.1449	-1.1817	1.2189	0.24279	0.86493
TRINITY_DN38225_c0_g2_i1_orf1	uncharacterized protein LOC114354273 [Ostrinia furnacalis]	-1.358	-0.9034	1.38972	0.31622	0.55542
TRINITY_DN44083_c0_g1_i2_orf1	putative alpha-ketoglutarate-dependent hypophosphite dioxygenase [Operophtera brumata]	-1.4462	-0.9269	0.91872	0.45545	0.99893
TRINITY_DN50593_c0_g1_i1_orf1	uncharacterized protein LOC114361588 isoform X14 [Ostrinia furnacalis]	-0.8002	-1.5215	0.39622	0.83218	1.09327
TRINITY_DN93764_c0_g1_i1_orf1	microtubule-associated protein futsch isoform X4 [Ostrinia furnacalis] >XP_028162562.1 microtubule-associated protein futsch isoform X4 [Ostrinia furnacalis]	-1.2589	-0.9674	1.36422	0.78227	0.07976
TRINITY_DN2722_c0_g1_i1_orf1	troponin C [Pieris rapae] >XP_045490973.1 troponin C-like isoform X1 [Colias croceus] >XP_049866665.1 troponin C-like [Pectinophora gossypiella]	-0.5359	-1.7256	0.83793	0.61258	0.81101
TRINITY_DN91946_c0_g1_i1_orf1	protein catecholamines up [Ostrinia furnacalis]	-1.4672	-0.7697	0.91434	0.13953	1.18299
TRINITY_DN1455_c0_g1_i4_orf1	troponin domain-containing protein [Phthorimaea operculella]	-1.2165	-1.212	1.02591	0.61468	0.78792
TRINITY_DN1607_c0_g1_i16_orf1	LOW QUALITY PROTEIN: asparagine--tRNA ligase, cytoplasmic [Ostrinia furnacalis]	-0.8426	-1.5134	1.08801	0.71103	0.55699
TRINITY_DN77559_c0_g1_i1_orf1	uncharacterized protein LOC114359392 isoform X2 [Ostrinia furnacalis]	-1.3714	-0.9177	0.2321	0.79649	1.26049
TRINITY_DN11231_c1_g1_i1_orfp1	TRINITY_DN11231_c1_g1_i1_m.13377 TRINITY_DN11231_c1_g1::TRINITY_DN11231_c1_g1_i1::g.13377 ORF type:internal len:76 (-),score=1.43 TRINITY_DN11231_c1_g1_i1:1-225(-)	-1.4071	-0.8269	1.09604	0.07526	1.06271
TRINITY_DN34134_c0_g2_i1_orf1	THUMP domain-containing protein 1 homolog [Ostrinia furnacalis]	-1.5266	-0.6652	0.66113	0.20916	1.32147
TRINITY_DN31118_c0_g2_i1_orf1	unnamed protein product [Spodoptera exigua]	0.06255	-1.9488	0.53836	0.66686	0.68102
TRINITY_DN17759_c0_g1_i5_orf1	hypothetical protein evm_013530 [Chilo suppressalis]	-1.2977	-1.0755	1.22451	0.5659	0.58279
TRINITY_DN31118_c0_g1_i1_orf1	CPR9 [Ostrinia furnacalis]	-0.0368	-1.9267	0.68031	0.63153	0.65171
TRINITY_DN1329_c0_g1_i5_orf1	neurogenic locus notch homolog protein 3 [Ostrinia furnacalis] >XP_028157678.1 neurogenic locus notch homolog protein 3 [Ostrinia furnacalis]	-0.6347	-1.6323	0.79316	0.40718	1.06669
TRINITY_DN26961_c0_g1_i1_orf1	uncharacterized protein LOC120424957 [Culex pipiens pallens]	-0.6231	-1.5981	1.26262	0.52478	0.43382
TRINITY_DN350_c0_g1_i4_orf1	microtubule-associated protein tau-like isoform X6 [Ostrinia furnacalis]	-1.4769	-0.9066	0.92384	0.53198	0.92763
TRINITY_DN22597_c0_g1_i4_orf1	uncharacterized protein LOC114361588 isoform X16 [Ostrinia furnacalis]	-0.8672	-1.4774	1.11528	0.43337	0.79598
TRINITY_DN45949_c0_g1_i1_orf1	uncharacterized protein LOC114355167 [Ostrinia furnacalis]	-0.1172	-1.8697	0.86444	0.79938	0.32304
TRINITY_DN452_c9_g1_i1_orf1	epidermal retinol dehydrogenase 2-like [Ostrinia furnacalis]	-1.5389	-0.5888	1.35867	0.11673	0.6523
TRINITY_DN566_c0_g1_i13_orf1	uncharacterized protein LOC114355567 isoform X2 [Ostrinia furnacalis]	-1.3931	-0.8861	0.74864	0.24463	1.28597
TRINITY_DN13711_c0_g1_i1_orf1	putative nuclease HARBI1 [Myzus persicae]	-1.1202	-1.1895	0.27853	1.32389	0.70721
TRINITY_DN23069_c0_g2_i3_orf1	uncharacterized protein LOC114364799 [Ostrinia furnacalis]	-1.0622	-1.3311	0.49467	0.78673	1.11189
TRINITY_DN10742_c0_g1_i4_orf1	ethanolamine-phosphate cytidylyltransferase isoform X1 [Ostrinia furnacalis]	-1.5413	-0.6122	0.27775	0.48332	1.39242
TRINITY_DN33_c0_g1_i14_orf1	uncharacterized protein CG45076-like isoform X1 [Ostrinia furnacalis]	-1.4402	-0.8836	1.08428	0.30246	0.93703
TRINITY_DN1008_c0_g1_i2_orf1	integrin beta-PS [Ostrinia furnacalis]	-1.5583	-0.5908	1.13332	0.04847	0.96737
TRINITY_DN51776_c0_g1_i1_orf1	unnamed protein product, partial [Iphiclides podalirius]	-1.3319	-0.9375	0.20557	0.73587	1.32793
TRINITY_DN695_c0_g1_i5_orf1	uncharacterized protein LOC114363574 isoform X1 [Ostrinia furnacalis]	-1.1838	-1.2491	0.95322	0.87237	0.60728
TRINITY_DN1470_c0_g1_i8_orf1	oxidation resistance protein 1 isoform X5 [Ostrinia furnacalis]	-1.2008	-1.1742	1.23451	0.52121	0.61932
TRINITY_DN5628_c0_g1_i3_orf1	muscle LIM protein Mlp84B isoform X2 [Ostrinia furnacalis]	-0.893	-1.483	0.78646	0.54815	1.04134
TRINITY_DN109931_c0_g1_i1_orf1	hydroxymethylglutaryl-CoA lyase, mitochondrial isoform X1 [Ostrinia furnacalis]	-0.1798	-1.8569	0.93436	0.41132	0.69097
TRINITY_DN5768_c0_g1_i2_orf1	adenosylhomocysteinase [Ostrinia furnacalis]	-0.9186	-1.4462	1.08293	0.82833	0.45358
TRINITY_DN44491_c0_g1_i12_orf1	unnamed protein product [Chrysodeixis includens]	-1.2839	-1.067	1.04087	0.28643	1.02358
TRINITY_DN2175_c0_g1_i4_orf1	uncharacterized protein LOC114353827 [Ostrinia furnacalis]	-0.898	-1.3649	1.33908	0.69734	0.22646
TRINITY_DN1034_c0_g1_i4_orf1	glycerol kinase isoform X4 [Ostrinia furnacalis]	-1.2657	-1.1034	0.88194	1.12886	0.3583
TRINITY_DN57900_c0_g1_i2_orf1	hypothetical protein SFRURICE_000634 [Spodoptera frugiperda]	-0.7393	-1.408	0.38924	0.25696	1.50112
TRINITY_DN928_c0_g2_i1_orf1	fasciclin-2-like [Ostrinia furnacalis]	-0.9507	-1.4422	0.70404	0.62693	1.0619
TRINITY_DN8854_c0_g1_i2_orf1	glutathione S-transferase epsilon 2 [Ostrinia furnacalis]	-1.5223	-0.6079	0.13375	1.38693	0.60955
TRINITY_DN31225_c0_g1_i1_orf1	ribosome biogenesis protein BMS1 homolog [Ostrinia furnacalis]	-0.9015	-1.3342	1.13578	0.04374	1.05613
TRINITY_DN2688_c0_g1_i3_orf1	mitochondrial amidoxime reducing component 2-like [Ostrinia furnacalis]	-1.1919	-0.9458	1.49357	0.67316	-0.029
TRINITY_DN64126_c0_g1_i1_orf1	senecionine N-oxygenase isoform X2 [Galleria mellonella]	-1.4023	-0.9441	0.40815	0.74859	1.18964
TRINITY_DN1212_c0_g1_i8_orf1	extensin isoform X5 [Ostrinia furnacalis] >XP_028175473.1 extensin isoform X5 [Ostrinia furnacalis] >XP_028175474.1 extensin isoform X5 [Ostrinia furnacalis]	-0.1557	-1.8777	0.76229	0.46066	0.8104
TRINITY_DN5933_c0_g1_i1_orf1	peroxidase-like [Ostrinia furnacalis]	-0.8536	-1.5106	0.52624	0.81023	1.02769
TRINITY_DN6415_c0_g1_i1_orf1	D-arabinitol dehydrogenase 1-like [Ostrinia furnacalis]	-0.4837	-1.5894	0.07941	0.64603	1.34766

TRINITY_DN10441_c0_g1_i3_orf1	zonadhesin-like [Ostrinia furnacalis]	-0.7827	-1.4522	1.38412	0.40694	0.44386
TRINITY_DN2312_c0_g1_i4_orf1	endoplasmic reticulum-Golgi intermediate compartment protein 3 [Ostrinia furnacalis]	-0.4963	-1.74	0.94193	0.62577	0.66866
TRINITY_DN11108_c0_g1_i4_orf1	peroxisomal leader peptide-processing protease [Ostrinia furnacalis] >XP_028165527.1 peroxisomal leader peptide-processing protease [Ostrinia furnacalis]	-1.4749	-0.5958	1.4067	-0.0357	0.6997
TRINITY_DN56708_c0_g3_i1_orfp1	TRINITY_DN56708_c0_g3_i1_m.56611 TRINITY_DN56708_c0_g3::TRINITY_DN56708_c0_g3_i1::g.56611 ORF type:internal len:69 (-),score=2.50 TRINITY_DN56708_c0_g3_i1:1-204(-)	-0.859	-1.3533	1.45736	0.27311	0.48185
TRINITY_DN6392_c0_g1_i9_orf1	putative mediator of RNA polymerase II transcription subunit 29 isoform X4 [Ostrinia furnacalis]	-1.34	-0.85	1.41714	0.68226	0.09056
TRINITY_DN71917_c0_g3_i1_orf1	leucine-rich repeat and immunoglobulin-like domain-containing nogo receptor-interacting protein 1 [Ostrinia furnacalis]	-1.5521	-0.4929	1.06974	-0.1158	1.09103
TRINITY_DN5628_c0_g1_i5_orf1	hypothetical protein O3G_MSEX015036 [Manduca sexta]	-0.4629	-1.7583	0.85726	0.56155	0.80239
TRINITY_DN3991_c0_g1_i6_orf1	acetyl-CoA carboxylase isoform X3 [Trichoplusia ni]	-1.2792	-0.9395	0.29226	0.44359	1.48284
TRINITY_DN9569_c1_g1_i7_orf1	V-set and immunoglobulin domain-containing protein 1-like isoform X1 [Ostrinia furnacalis] >XP_028156015.1 V-set and immunoglobulin domain-containing protein 1-like isoform X2 [Ostrinia furnacalis]	-1.1286	-1.3108	0.8528	0.89809	0.68856
TRINITY_DN6642_c0_g1_i2_orf1	protein purity of essence [Ostrinia furnacalis]	-1.1384	-0.9854	0.12537	0.39902	1.5994
TRINITY_DN62_c1_g1_i3_orf1	D-2-hydroxyglutarate dehydrogenase, mitochondrial-like [Ostrinia furnacalis]	-0.4301	-1.6424	0.04312	1.18262	0.84676
TRINITY_DN14112_c0_g1_i3_orf1	uncharacterized protein LOC114350956 [Ostrinia furnacalis]	-1.3602	-0.968	0.44261	0.59709	1.28854
TRINITY_DN2207_c0_g1_i4_orf1	methionine-R-sulfoxide reductase B1 isoform X4 [Pectinophora gossypiella] >XP_049887601.1 methionine-R-sulfoxide reductase B1 isoform X4 [Pectinophora gossypiella]	-0.6902	-1.4526	0.35132	0.31029	1.48119
TRINITY_DN89483_c0_g1_i1_orf1	mitochondrial enolase superfamily member 1-like isoform X2 [Maniola jurtina]	-1.1892	-0.94	0.43433	0.11361	1.58132
TRINITY_DN62707_c0_g1_i1_orf1	uncharacterized protein LOC114362831 [Ostrinia furnacalis]	-1.1559	-1.0397	1.49791	0.56749	0.13028
TRINITY_DN97472_c0_g1_i5_orf1	microtubule-actin cross-linking factor 1 isoform X15 [Ostrinia furnacalis]	-0.9711	-1.3901	1.20786	0.55647	0.5968
TRINITY_DN116972_c0_g1_i1_orf1	phosphofructokinase domain-containing protein [Phthorimaea operculella]	-1.0869	-1.3173	1.12452	0.63202	0.64763
TRINITY_DN5748_c0_g1_i5_orf1	glycine N-methyltransferase isoform X1 [Ostrinia furnacalis] >XP_028165118.1 glycine N-methyltransferase isoform X2 [Ostrinia furnacalis] >XP_028165119.1 glycine N-methyltransferase isoform X1 [Ostrinia furnacalis] >XP_028165120.1 glycine N-methyltransferase isoform X2 [Ostrinia furnacalis]	-1.4259	-0.9231	0.60185	0.53725	1.2099
TRINITY_DN26254_c0_g1_i1_orf1	hypothetical protein evm_003664 [Chilo suppressalis] >CAB3521132.1 unnamed protein product [Chilo suppressalis] >CAH0398453.1 unnamed protein product [Chilo suppressalis]	0.02305	-1.9329	0.81076	0.5185	0.58061
TRINITY_DN61335_c0_g2_i1_orf1	uncharacterized protein LOC114352149 [Ostrinia furnacalis]	-1.1272	-1.2101	1.32051	0.46154	0.55525
TRINITY_DN31390_c0_g1_i2_orf1	UDP-glucuronosyltransferase 2B20-like [Ostrinia furnacalis]	-0.357	-1.5973	1.09817	1.03994	-0.1839
TRINITY_DN2647_c0_g1_i3_orf1	DNA repair protein complementing XP-G cells homolog isoform X1 [Ostrinia furnacalis]	-0.0437	-1.6415	-0.3888	1.00936	1.06468
TRINITY_DN230_c2_g1_i5_orf1	6-pyruvoyl tetrahydrobiopterin synthase [Ostrinia furnacalis]	-1.0854	-0.8436	1.67315	-0.245	0.50085
TRINITY_DN1172_c0_g1_i1_orf1	hypothetical protein O3G_MSEX009550 [Manduca sexta]	-1.3136	-1.075	0.50697	0.72707	1.1546
TRINITY_DN616_c1_g1_i6_orf1	esterase B1-like isoform X1 [Ostrinia furnacalis] >XP_028178578.1 esterase B1-like isoform X2 [Ostrinia furnacalis]	-0.3794	-1.6577	-0.0064	1.12166	0.9219
TRINITY_DN554_c0_g1_i1_orf1	uncharacterized protein LOC114353093 isoform X1 [Ostrinia furnacalis] >XP_028160722.1 uncharacterized protein LOC114353093 isoform X1 [Ostrinia furnacalis] >XP_028160723.1 uncharacterized protein LOC114353093 isoform X1 [Ostrinia furnacalis]	-1.0125	-1.2348	0.15574	0.70045	1.39112
TRINITY_DN105506_c0_g1_i8_orf1	microtubule-actin cross-linking factor 1 isoform X15 [Ostrinia furnacalis]	-0.5503	-1.6918	0.76612	0.45612	1.01982
TRINITY_DN19116_c0_g1_i3_orf1	UDP-glucose 4-epimerase isoform X1 [Ostrinia furnacalis]	-1.5673	-0.5365	1.16601	-0.009	0.94671
TRINITY_DN11736_c0_g1_i1_orf1	uncharacterized protein LOC114352195 [Ostrinia furnacalis]	-1.4064	-1.0071	0.94166	0.59788	0.87392
TRINITY_DN5880_c0_g2_i2_orf1	macrophage mannose receptor 1 [Bombyx mori]	-1.1019	-0.9997	0.64841	-0.0827	1.53593
TRINITY_DN63030_c0_g1_i5_orf1	uncharacterized protein LOC114358571 [Ostrinia furnacalis]	-0.8569	-1.5085	0.88742	0.50409	0.97389
TRINITY_DN60787_c0_g1_i5_orf1	probable transaldolase [Ostrinia furnacalis]	-1.3473	-1.0854	0.71858	0.96002	0.7541
TRINITY_DN1068_c0_g1_i3_orf1	aspartate aminotransferase, cytoplasmic [Ostrinia furnacalis]	-0.9827	-1.4286	0.69966	0.99542	0.71623
TRINITY_DN3433_c2_g1_i2_orf1	cytosolic purine 5'-nucleotidase isoform X4 [Ostrinia furnacalis]	-1.376	-0.879	1.29046	0.16345	0.80115
TRINITY_DN16400_c0_g2_i1_orf1	superoxide dismutase [Cu-Zn]-like isoform X1 [Ostrinia furnacalis]	-0.6059	-1.498	1.48134	0.29009	0.33243
TRINITY_DN21545_c0_g1_i2_orf1	sterile alpha and TIR motif-containing protein 1 isoform X1 [Ostrinia furnacalis]	-1.3743	-1.0218	0.66542	0.61339	1.11726
TRINITY_DN86127_c1_g1_i2_orfp1	TRINITY_DN86127_c1_g1_i2_m.43062 TRINITY_DN86127_c1_g1::TRINITY_DN86127_c1_g1_i2::g.43062 ORF type:internal len:69 (-),score=14.03 TRINITY_DN86127_c1_g1_i2:2-205(-)	0.26686	-1.6742	-0.5192	0.98219	0.94429
TRINITY_DN1134_c0_g1_i4_orf1	cytochrome P450 6B5-like [Ostrinia furnacalis]	-0.2389	-1.8473	0.60846	0.55384	0.92385
TRINITY_DN12336_c0_g1_i1_orfp1	TRINITY_DN12336_c0_g1_i1_m.30792 TRINITY_DN12336_c0_g1::TRINITY_DN12336_c0_g1_i1::g.30792 ORF type:internal len:86 (-),score=13.56 TRINITY_DN12336_c0_g1_i1:3-257(-)	-0.816	-1.2309	1.07227	-0.2854	1.26006
TRINITY_DN50074_c0_g1_i1_orf1	uncharacterized protein LOC114364628 [Ostrinia furnacalis]	-0.4551	-1.6664	0.37502	1.29178	0.45467
TRINITY_DN6994_c0_g1_i3_orf1	C-type mannose receptor 2-like isoform X1 [Ostrinia furnacalis]	-1.0489	-0.9028	1.73871	0.24601	-0.033
TRINITY_DN2956_c0_g1_i6_orf1	fructose-bisphosphate aldolase-like isoform X1 [Ostrinia furnacalis] >XP_028178678.1 fructose-bisphosphate aldolase-like isoform X1 [Ostrinia furnacalis]	-0.684	-1.5902	0.79378	0.36777	1.11267
TRINITY_DN62557_c0_g1_i1_orf1	6-phosphofructokinase [Operophtera brumata]	-0.795	-1.4973	0.60741	1.25343	0.43141
TRINITY_DN2207_c0_g1_i6_orf1	methionine-R-sulfoxide reductase B1 isoform X2 [Ostrinia furnacalis]	-1.3088	-0.984	0.54838	1.37063	0.3737

TRINITY_DN58261_c0_g1_i2_orf1	15-hydroxyprostaglandin dehydrogenase [NAD(+)]-like [Ostrinia furnacalis]	-0.5939	-1.6742	0.61848	0.59972	1.04986
TRINITY_DN24121_c1_g1_i6_orf1	serine protease persephone-like [Ostrinia furnacalis]	-0.85	-1.1883	1.23011	-0.312	1.1202
TRINITY_DN3374_c0_g1_i7_orf1	TPPP family protein CG45057 [Ostrinia furnacalis] >XP_028172578.1 TPPP family protein CG45057 [Ostrinia furnacalis]	-1.1384	-1.1189	1.29926	0.08827	0.86971
TRINITY_DN47930_c0_g1_i4_orf1	uncharacterized protein LOC114362634 [Ostrinia furnacalis]	-0.676	-1.6429	0.89758	0.59206	0.82927
TRINITY_DN120_c0_g1_i2_orf1	PREDICTED: myosin light chain alkali-like [Amyelois transitella]	-0.331	-1.8051	0.88496	0.44403	0.80718
TRINITY_DN12969_c0_g1_i3_orf1	queuosine salvage protein [Ostrinia furnacalis] >XP_028167327.1 queuosine salvage protein [Ostrinia furnacalis]	-1.3324	-0.1158	1.66185	-0.5687	0.35507
TRINITY_DN8651_c0_g1_i16_orf1	glutathione S-transferase theta 2 [Conogethes punctiferalis]	-1.3177	-0.7575	0.36977	0.11152	1.59393
TRINITY_DN19098_c0_g1_i4_orf1	L-xylulose reductase-like [Ostrinia furnacalis]	-1.1713	-0.7996	0.26534	-0.0027	1.70829
TRINITY_DN35763_c0_g1_i2_orf1	probable methylthioribulose-1-phosphate dehydratase [Helicoverpa armigera]	-1.2231	-1.1081	0.37908	0.6389	1.31316
TRINITY_DN23564_c0_g1_i7_orf1	cytochrome P450 6B6-like [Ostrinia furnacalis]	-1.6112	-0.3742	0.94571	-0.124	1.16371
TRINITY_DN18222_c0_g1_i5_orf1	phosphoglycerate kinase [Maniola hyperantus]	-0.533	-1.5496	0.15085	1.42759	0.50406
TRINITY_DN3231_c0_g1_i12_orf1	integrin-linked protein kinase [Pectinophora gossypiella]	-0.4761	-1.7021	0.71312	0.3489	1.11618
TRINITY_DN4695_c0_g1_i4_orf1	glutathione S-transferase epsilon 3 [Ostrinia furnacalis]	-1.2009	-1.1137	0.41346	0.53865	1.3625
TRINITY_DN51424_c0_g2_i1_orf1	ras suppressor protein 1 [Helicoverpa zea]	-1.3232	-0.9868	1.18492	0.90781	0.21727
TRINITY_DN15373_c0_g1_i2_orf1	SET domain-containing protein SmydA-8-like isoform X2 [Ostrinia furnacalis]	-0.6958	-1.6331	0.88853	0.82494	0.61543
TRINITY_DN35800_c0_g1_i6_orf1	ommochrome-binding protein-like [Ostrinia furnacalis] >XP_028161264.1 ommochrome-binding protein-like [Ostrinia furnacalis] >XP_028161265.1 ommochrome-binding protein-like [Ostrinia furnacalis]	-1.1656	-1.1226	0.37021	1.40887	0.50912
TRINITY_DN14922_c0_g3_i2_orf1	probable pseudouridine-5'-phosphatase [Ostrinia furnacalis]	-0.5303	-1.6839	1.14434	0.50938	0.56052
TRINITY_DN2302_c0_g1_i1_orf1	enoyl-CoA hydratase domain-containing protein 3, mitochondrial [Ostrinia furnacalis]	-1.2374	-0.8638	1.56134	0.00609	0.53375
TRINITY_DN36460_c0_g1_i2_orf1	N-acetylneuraminate lyase-like [Ostrinia furnacalis]	-1.2381	-1.1724	1.10292	0.55114	0.75649
TRINITY_DN829_c0_g1_i8_orf1	cytochrome P450 6B6-like [Ostrinia furnacalis]	-0.6869	-1.4693	0.36845	0.3302	1.45756
TRINITY_DN4866_c0_g1_i2_orf1	actin-binding LIM protein 3 isoform X6 [Ostrinia furnacalis]	-0.1317	-1.7772	0.01321	1.06426	0.83144
TRINITY_DN268_c3_g1_i2_orf1	hypothetical protein evm_003084 [Chilo suppressalis]	-1.3052	-1.1155	1.0632	0.66837	0.68915
TRINITY_DN13602_c0_g1_i4_orf1	NEDD8 ultimate buster 1-like [Ostrinia furnacalis]	-1.4169	-0.8245	0.64209	1.35697	0.24241
TRINITY_DN8224_c0_g1_i7_orf1	hemicentin-1-like isoform X2 [Ostrinia furnacalis]	-1.4335	-0.8588	1.24633	0.2915	0.75447
TRINITY_DN10774_c0_g2_i3_orf1	uncharacterized protein LOC114362157, partial [Ostrinia furnacalis]	-1.3025	-0.9298	0.49185	0.28605	1.45438
TRINITY_DN2040_c0_g1_i15_orfp1	TRINITY_DN2040_c0_g1_i15_m.4150 TRINITY_DN2040_c0_g1::TRINITY_DN2040_c0_g1_i15::g.4150 ORF type:complete len:319 (+),score=125.43,Plasmodium_HRP PF05403.12 3.5,Plasmodium_HRP|PF05403.12|1.5 TRINITY_DN2040_c0_g1_i15:118-957(+)	-0.0636	-1.9192	0.62549	0.66567	0.69165
TRINITY_DN4341_c0_g1_i4_orf1	uncharacterized protein LOC114354354 [Ostrinia furnacalis]	-1.0689	-0.9432	0.16137	0.14136	1.70934
TRINITY_DN741_c0_g1_i10_orf1	talin-1 isoform X13 [Ostrinia furnacalis]	-1.0758	-1.2824	0.9068	0.32404	1.12733
TRINITY_DN1267_c0_g2_i10_orf1	secretory phospholipase A2 receptor-like [Ostrinia furnacalis]	-0.5348	-1.5298	1.44259	0.53347	0.08863
TRINITY_DN2559_c0_g1_i4_orf1	uricase [Ostrinia furnacalis]	-0.5666	-1.5608	1.38525	0.2149	0.52718
TRINITY_DN8621_c0_g1_i4_orf1	aminopeptidase N-like isoform X2 [Ostrinia furnacalis]	-1.2503	-0.647	1.5588	-0.3461	0.68455
TRINITY_DN1404_c0_g1_i6_orf1	uncharacterized protein LOC114363065 [Ostrinia furnacalis]	-0.7309	-1.5995	1.00956	0.57081	0.74999
TRINITY_DN120089_c0_g1_i1_orf1	phosphoglucomutase [Ostrinia furnacalis]	-0.8137	-1.5483	0.76377	0.59915	0.99913
TRINITY_DN2529_c0_g1_i3_orf1	collagen alpha-1(X) chain-like [Ostrinia furnacalis]	-1.2938	-1.009	0.68929	0.29136	1.32213
TRINITY_DN124171_c0_g1_i4_orf1	dystonin isoform X27 [Trichoplusia ni]	-1.0174	-1.3348	1.21197	0.39072	0.74949
TRINITY_DN13119_c0_g1_i4_orf1	endocuticle structural glycoprotein ABD-5-like [Bicyclus anynana]	0.04679	-1.9281	0.72368	0.37087	0.78676
TRINITY_DN22046_c1_g1_i5_orf1	uncharacterized protein LOC114351208 [Ostrinia furnacalis]	-1.2515	-0.6613	0.16941	0.02085	1.72258
TRINITY_DN147691_c0_g1_i1_orf1	WD repeat-containing protein 46 [Orussus abietinus]	-0.874	-1.4423	0.50652	0.54342	1.26643
TRINITY_DN5661_c0_g1_i5_orf1	cytochrome P450 6B7-like [Ostrinia furnacalis]	-0.7347	-1.4151	1.26176	-0.0414	0.92948
TRINITY_DN16824_c0_g1_i7_orf1	insulin receptor substrate 1 [Ostrinia furnacalis]	-1.3971	-0.9192	0.24814	1.07343	0.9947
TRINITY_DN101658_c0_g1_i1_orf1	uncharacterized protein LOC114349955 [Ostrinia furnacalis]	-1.3122	-1.0949	0.49038	0.9892	0.92753
TRINITY_DN3433_c0_g1_i6_orf1	cytosolic purine 5'-nucleotidase isoform X2 [Ostrinia furnacalis] >XP_028162963.1 cytosolic purine 5'-nucleotidase isoform X2 [Ostrinia furnacalis]	-1.2339	-0.807	1.65249	0.09365	0.29471
TRINITY_DN17907_c0_g1_i13_orf1	androgen-induced gene 1 protein-like isoform X1 [Galleria mellonella]	-0.3679	-1.5717	-0.2003	0.88992	1.24992
TRINITY_DN4123_c0_g1_i1_orf1	uncharacterized protein LOC114355030 [Ostrinia furnacalis]	-1.0626	-1.1457	1.05329	-0.0476	1.20269
TRINITY_DN14754_c0_g1_i6_orf1	cathepsin L [Papilio xuthus]	-0.8132	-1.1117	1.68125	-0.2292	0.4729
TRINITY_DN64297_c0_g1_i1_orf1	vanin-like protein 2 isoform X2 [Ostrinia furnacalis]	-1.5609	1.33079	0.25072	0.59363	-0.6142
TRINITY_DN66040_c0_g1_i2_orf1	serine protease inhibitor dipetalogastin-like isoform X2 [Ostrinia furnacalis]	-1.5131	1.52484	-0.1369	0.48616	-0.361
TRINITY_DN276_c0_g1_i2_orf1	protein lethal(2)essential for life-like [Ostrinia furnacalis] >UTU55753.1 small heat shock protein Hsp20.7 [Ostrinia furnacalis]	-1.3206	1.52123	-0.8035	0.5407	0.06218
TRINITY_DN38307_c0_g1_i1_orfp1	TRINITY_DN38307_c0_g1_i1_m.10661 TRINITY_DN38307_c0_g1::TRINITY_DN38307_c0_g1_i1::g.10661 ORF type:5prime_partial len:66 (+),score=5.90 TRINITY_DN38307_c0_g1_i1:2-199(+)	-1.6694	1.42198	-0.1642	0.00679	0.4049
TRINITY_DN5406_c0_g2_i1_orf1	uncharacterized protein LOC114350326 [Ostrinia furnacalis]	-1.8723	0.94781	0.7566	0.153	0.01491

TRINITY_DN5444_c0_g1_i1_orfp1	TRINITY_DN5444_c0_g1_i1_m.14077 TRINITY_DN5444_c0_g1::TRINITY_DN5444_c0_g1_i1::g.14077 ORF type:3prime_partial len:90 (-),score=25.11 TRINITY_DN5444_c0_g1_i1:2-268(-)	-1.7446	1.36434	-0.0085	0.29269	0.0961
TRINITY_DN3616_c0_g1_i4_orf1	conotoxin ArMKLT2-032-like [Ostrinia furnacalis]	-1.4273	1.43024	-0.0476	0.69833	-0.6536
TRINITY_DN64181_c0_g1_i1_orf1	uncharacterized protein LOC114356431 isoform X2 [Ostrinia furnacalis]	-1.5554	1.46639	0.54647	-0.1125	-0.3449
TRINITY_DN45220_c0_g1_i1_orf1	delta(3,5)-Delta(2,4)-dienoyl-CoA isomerase, mitochondrial isoform X1 [Ostrinia furnacalis]	-1.643	1.34972	-0.4231	0.505	0.21142
TRINITY_DN12009_c0_g1_i1_orf1	uncharacterized protein LOC114365631 [Ostrinia furnacalis]	-1.8357	0.99281	0.02278	0.01768	0.8024
TRINITY_DN993_c0_g1_i7_orf1	apolipophorins-like [Ostrinia furnacalis]	-1.5456	1.32924	-0.4186	0.80089	-0.1659
TRINITY_DN6423_c0_g1_i6_orf1	phenoloxidase-activating factor 2-like isoform X1 [Ostrinia furnacalis]	-1.7543	1.36232	0.02974	0.17188	0.19032
TRINITY_DN13760_c1_g1_i1_orf1	pre-mRNA-processing factor 40 homolog A isoform X1 [Ostrinia furnacalis] >XP_028162665.1 pre-mRNA-processing factor 40 homolog A isoform X2 [Ostrinia furnacalis] >XP_028162667.1 pre-mRNA-processing factor 40 homolog A isoform X3 [Ostrinia furnacalis]	-1.6889	1.32162	0.25016	0.46566	-0.3485
TRINITY_DN46625_c0_g1_i1_orf1	ferritin subunit isoform X1 [Belonocnema kinseyi]	-1.463	1.49897	-0.458	0.60728	-0.1853
TRINITY_DN28711_c0_g1_i1_orf1	hypothetical protein evm_000299 [Chilo suppressalis]	-1.4105	1.39267	-0.5329	0.83903	-0.2883
TRINITY_DN9239_c0_g1_i1_orf1	apolipophorins-like [Ostrinia furnacalis]	-1.3268	1.50782	-0.6047	0.71709	-0.2933
TRINITY_DN33272_c0_g1_i5_orf1	Low-density lipoprotein receptor-related protein 1 [Papilio xuthus]	-1.907	1.01785	0.43067	0.0937	0.36479
TRINITY_DN81488_c0_g1_i1_orf1	apolipophorins-like [Ostrinia furnacalis]	-1.4803	1.38699	-0.4721	0.78382	-0.2184
TRINITY_DN4255_c0_g1_i11_orf1	LOW QUALITY PROTEIN: lebocin-4-like [Ostrinia furnacalis]	-1.8751	1.11109	0.35345	0.06345	0.34713
TRINITY_DN85412_c0_g1_i1_orf1	unnamed protein product [Diatraea saccharalis]	-1.4122	1.44709	-0.5218	0.75379	-0.2669
TRINITY_DN20560_c0_g1_i6_orf1	pupal cuticle protein C1B-like [Ostrinia furnacalis]	-1.8302	1.16248	0.40775	-0.0925	0.35245
TRINITY_DN97042_c0_g1_i6_orf1	apolipophorins-like [Ostrinia furnacalis]	-1.4997	1.34136	-0.466	0.83166	-0.2074
TRINITY_DN699_c0_g2_i1_orf1	TPA_exp: putative parasitoid killing factor [Trichoplusia ni]	-1.4252	1.46045	-0.5433	0.70775	-0.1997
TRINITY_DN5655_c0_g1_i2_orf1	uncharacterized protein LOC114359603 [Ostrinia furnacalis]	-1.8202	1.22163	0.02955	0.15841	0.4106
TRINITY_DN20676_c0_g1_i6_orf1	aldo-keto reductase AKR2E4-like isoform X1 [Ostrinia furnacalis]	-1.8403	1.19071	0.05158	0.21513	0.38287
TRINITY_DN1175_c1_g1_i1_orf1	methanethiol oxidase [Ostrinia furnacalis]	-1.919	1.00209	0.44652	0.1941	0.27626
TRINITY_DN113353_c0_g1_i1_orf1	unnamed protein product [Parnassius apollo]	-1.7803	1.12965	-0.2351	0.67399	0.21173
TRINITY_DN40191_c2_g1_i1_orf1	stress-activated map kinase-interacting protein 1 [Ostrinia furnacalis]	-1.6205	1.33704	-0.3251	0.68861	-0.0801
TRINITY_DN52944_c0_g1_i1_orf1	apolipophorins-like [Ostrinia furnacalis]	-1.4092	1.44676	-0.4968	0.76292	-0.3037
TRINITY_DN4748_c0_g1_i5_orf1	unnamed protein product, partial [Brenthis ino]	-1.6662	1.46875	0.16093	0.15981	-0.1233
TRINITY_DN71699_c0_g1_i1_orf1	apolipophorins-like [Ostrinia furnacalis]	-1.3854	1.43099	-0.5739	0.79524	-0.267
TRINITY_DN1423_c0_g1_i8_orf1	ferritin subunit-like [Ostrinia furnacalis] >XP_028168186.1 ferritin subunit-like [Ostrinia furnacalis]	-1.5296	1.52636	-0.2715	0.4683	-0.1935
TRINITY_DN45948_c1_g1_i1_orf1	unnamed protein product [Leptidea sinapis]	-1.7855	1.18571	0.55625	-0.1972	0.24067
TRINITY_DN15247_c0_g1_i2_orf1	probable G-protein coupled receptor Mth-like 3 isoform X1 [Ostrinia furnacalis]	-1.8687	0.96964	0.42724	-0.1341	0.60594
TRINITY_DN121650_c0_g1_i1_orf1	carboxylesterase [Ostrinia furnacalis]	-1.6911	1.44393	-0.0336	0.0546	0.22617
TRINITY_DN28711_c1_g1_i1_orf1	apolipophorins-like [Ostrinia furnacalis]	-1.4115	1.46692	-0.5321	0.71746	-0.2408
TRINITY_DN376_c1_g1_i1_orf1	matrix metalloproteinase-25-like [Ostrinia furnacalis]	-1.777	1.19591	-0.1941	0.57975	0.19548
TRINITY_DN9239_c0_g2_i2_orf1	apolipophorins-like [Ostrinia furnacalis]	-1.3082	1.49575	-0.3552	0.75877	-0.5911
TRINITY_DN2803_c4_g1_i1_orf1	ornithine aminotransferase, mitochondrial isoform X2 [Ostrinia furnacalis]	-1.7995	1.21503	0.34464	0.38259	-0.1428
TRINITY_DN15812_c0_g1_i2_orf1	transferrin [Ostrinia furnacalis]	-1.7805	1.31766	0.15993	0.25703	0.04583
TRINITY_DN2024_c0_g1_i12_orfp1	unnamed protein product, partial [Brenthis ino]	-1.7077	1.32399	0.40147	0.28217	-0.2999
TRINITY_DN3949_c0_g1_i1_orf1	probable cytochrome P450 304a1 [Ostrinia furnacalis]	-1.7538	1.31291	-0.1414	0.36534	0.21695
TRINITY_DN801_c0_g1_i2_orf1	cathepsin L [Ostrinia furnacalis] >XP_028165920.1 cathepsin L [Ostrinia furnacalis] >UKI61015.1 cathepsin L [Ostrinia furnacalis]	-1.6549	1.39293	0.53302	-0.1484	-0.1227
TRINITY_DN41_c0_g1_i3_orf1	uncharacterized protein LOC114359035 isoform X3 [Ostrinia furnacalis]	-1.8212	1.19312	0.324	-0.081	0.38503
TRINITY_DN18338_c0_g1_i6_orf1	aquaporin AQPAn.G isoform X1 [Ostrinia furnacalis]	-1.5681	1.53526	0.18761	-0.339	0.18419
TRINITY_DN13973_c0_g1_i6_orf1	27 kDa glycoprotein-like [Ostrinia furnacalis]	-1.4116	1.38377	-0.8075	0.63146	0.20394
TRINITY_DN1423_c0_g1_i4_orf1	hypothetical protein evm_003306 [Chilo suppressalis] >CAB3526495.1 unnamed protein product [Chilo suppressalis] >CAH0403823.1 unnamed protein product [Chilo suppressalis]	-1.6014	1.4895	-0.2159	0.40544	-0.0776
TRINITY_DN44073_c0_g1_i3_orf1	inter-alpha-trypsin inhibitor heavy chain H4-like isoform X11 [Ostrinia furnacalis]	-1.6558	1.3692	0.32426	0.3538	-0.3914
TRINITY_DN391_c0_g1_i4_orf1	phenoloxidase-activating enzyme-like [Ostrinia furnacalis]	-1.4891	1.64125	-0.2772	0.10877	0.01625
TRINITY_DN3609_c0_g1_i6_orf1	leukocyte elastase inhibitor-like [Ostrinia furnacalis]	-1.8015	1.05445	0.16659	-0.1823	0.76272
TRINITY_DN49147_c0_g2_i1_orf1	glutenin, high molecular weight subunit PW212-like [Ostrinia furnacalis]	-1.1183	1.81086	-0.6812	0.04937	-0.0607
TRINITY_DN8703_c0_g1_i2_orf1	beta-glucuronidase-like isoform X1 [Ostrinia furnacalis] >XP_028166212.1 beta-glucuronidase-like isoform X2 [Ostrinia furnacalis]	-1.6787	1.01919	-0.2126	1.03516	-0.1631
TRINITY_DN19980_c0_g1_i4_orf1	hypothetical protein evm_012507 [Chilo suppressalis]	-1.8318	1.10419	0.58938	-0.1159	0.25411
TRINITY_DN2835_c0_g1_i6_orf1	probable isoaspartyl peptidase/L-asparaginase GA20639 [Ostrinia furnacalis]	-1.5731	1.32898	-0.5732	0.62849	0.18883
TRINITY_DN2097_c1_g2_i2_orf1	serine protease inhibitor 3 [Ostrinia furnacalis]	-1.6902	1.32318	-0.3501	0.43897	0.27809
TRINITY_DN23746_c0_g1_i2_orf1	protein 4.1 homolog isoform X1 [Ostrinia furnacalis]	-1.6572	1.18754	-0.0723	0.85887	-0.3168
TRINITY_DN42719_c0_g1_i1_orf1	inter-alpha-trypsin inhibitor heavy chain H4-like isoform X11 [Ostrinia furnacalis]	-0.9083	1.87412	-0.5474	0.16192	-0.5803
TRINITY_DN7618_c0_g1_i4_orf1	uncharacterized protein LOC114366712 isoform X1 [Ostrinia furnacalis]	-1.632	1.44701	-0.2805	0.06563	0.39986
TRINITY_DN5022_c0_g1_i4_orf1	syntenin-1-like [Ostrinia furnacalis]	-1.8942	0.92827	0.46804	-0.073	0.57087

TRINITY_DN33272_c0_g1_i1_orf1	unnamed protein product, partial [Iphiclides podalirius]	-1.6621	1.38353	-0.3188	0.44592	0.15137
TRINITY_DN12865_c0_g1_i1_orf1	synaptic vesicle membrane protein VAT-1 homolog-like [Ostrinia furnacalis]	-1.6522	1.48141	0.18225	0.13962	-0.151
TRINITY_DN2407_c0_g1_i6_orf1	uncharacterized protein LOC114366345 isoform X2 [Ostrinia furnacalis]	-1.7784	1.31896	0.01877	0.21652	0.2242
TRINITY_DN1407_c0_g1_i12_orf1	hypothetical protein evm_012298 [Chilo suppressalis]	-1.1238	1.56932	-0.4278	0.72978	-0.7475
TRINITY_DN138481_c0_g1_i5_orf1	hypothetical protein evm_003901 [Chilo suppressalis]	-0.869	1.93649	-0.6123	-0.1374	-0.3177
TRINITY_DN19110_c0_g1_i2_orf1	peroxidase [Ostrinia furnacalis]	-1.2457	1.62388	-0.5048	0.58694	-0.4603
TRINITY_DN5420_c0_g1_i2_orf1	DNA-directed RNA polymerase II subunit RPB1-like [Ostrinia furnacalis]	-1.5851	1.55402	0.07708	0.15793	-0.2039
TRINITY_DN7183_c0_g1_i2_orf1	seminal fluid protein CSSFP028 [Chilo suppressalis]	-1.7504	1.32529	0.38744	0.13895	-0.1013
TRINITY_DN86833_c0_g3_i1_orf1	PREDICTED: glycerol-3-phosphate acyltransferase 1, mitochondrial isoform X1 [Microplitis demolitor]	-1.5831	1.3807	-0.1806	0.68221	-0.2993
TRINITY_DN33728_c0_g2_i1_orf1	uncharacterized protein LOC114350200 [Ostrinia furnacalis]	-1.1412	1.7585	-0.6994	0.27855	-0.1965
TRINITY_DN4228_c0_g1_i5_orf1	phenoloxidase-activating enzyme-like [Ostrinia furnacalis]	-1.7664	1.32909	0.04266	0.06787	0.32681
TRINITY_DN59388_c0_g1_i1_orf1	uncharacterized protein LOC114353759 [Ostrinia furnacalis]	-1.4404	1.5435	0.48286	-0.0303	-0.5556
TRINITY_DN19043_c0_g2_i1_orf1	hypothetical protein EVAR_60653_1 [Eumeta japonica]	-1.2718	1.81138	-0.1254	-0.2038	-0.2104
TRINITY_DN19748_c0_g1_i4_orf1	PREDICTED: cysteine-rich hydrophobic domain-containing protein 2 [Amyelois transitella] >XP_028158573.1 cysteine-rich hydrophobic domain-containing protein 2 isoform X1 [Ostrinia furnacalis] >CAH0685427.1 unnamed protein product [Chilo suppressalis]	-1.9052	1.0391	0.39372	0.12705	0.34534
TRINITY_DN1110_c1_g1_i9_orf1	MD-2-related lipid-recognition protein-like [Ostrinia furnacalis]	-0.9686	1.77041	-0.8699	0.32427	-0.2562
TRINITY_DN12671_c0_g1_i6_orf1	hemicentin-1-like isoform X1 [Ostrinia furnacalis]	-1.0512	1.87108	-0.3467	0.04789	-0.521
TRINITY_DN15175_c0_g1_i1_orf1	zinc carboxypeptidase-like [Ostrinia furnacalis]	-0.7526	1.94642	-0.6799	-0.0977	-0.4163
TRINITY_DN125441_c0_g1_i5_orf1	KH domain-containing, RNA-binding, signal transduction-associated protein 2-like isoform X12 [Ostrinia furnacalis]	-1.8939	0.94752	0.69098	0.17933	0.07606
TRINITY_DN20717_c0_g1_i1_orf1	putative uncharacterized protein DDB_G0282133 isoform X1 [Ostrinia furnacalis]	-1.4066	1.5924	0.49019	-0.4303	-0.2457
TRINITY_DN4256_c0_g1_i1_orf1	chitinase-3-like protein 1 [Ostrinia furnacalis]	-1.6834	1.3046	-0.2394	0.6376	-0.0194
TRINITY_DN5553_c0_g1_i4_orf1	uncharacterized protein LOC114353828 [Ostrinia furnacalis]	-1.2832	1.64419	-0.558	0.498	-0.3009
TRINITY_DN20680_c0_g1_i5_orf1	tsukushin isoform X2 [Ostrinia furnacalis]	-1.6882	1.40701	0.10571	-0.1803	0.35582
TRINITY_DN2425_c0_g1_i3_orf1	sialic acid synthase [Ostrinia furnacalis]	-1.683	1.42428	-0.1453	0.06788	0.33623
TRINITY_DN7854_c0_g1_i4_orf1	failed axon connections [Ostrinia furnacalis]	-1.5194	1.63711	0.02109	-0.0938	-0.045
TRINITY_DN361_c0_g1_i5_orf1	hexosaminidase [Ostrinia furnacalis]	-1.5449	1.55905	-0.1804	0.34414	-0.1779
TRINITY_DN126127_c0_g1_i1_orf1	prolow-density lipoprotein receptor-related protein 1, partial [Ostrinia furnacalis]	-1.8549	1.08292	-0.0068	0.18539	0.5934
TRINITY_DN26149_c0_g1_i5_orf1	thymosin beta isoform X3 [Ostrinia furnacalis]	-1.4077	1.70773	-0.3125	-0.0399	0.05235
TRINITY_DN9615_c0_g1_i1_orf1	uncharacterized protein LOC114352730 [Ostrinia furnacalis]	-1.7911	1.24453	-0.0289	0.48371	0.09175
TRINITY_DN44709_c0_g1_i1_orf1	D-beta-hydroxybutyrate dehydrogenase, mitochondrial, partial [Chelonus insularis]	-1.5118	1.26766	-0.5698	0.88213	-0.0682
TRINITY_DN29604_c0_g2_i2_orf1	neurofilament heavy polypeptide-like isoform X2 [Ostrinia furnacalis]	-1.1205	1.86847	-0.4587	-0.1678	-0.1215
TRINITY_DN11514_c0_g1_i1_orf1	uncharacterized protein LOC114350079 [Ostrinia furnacalis]	-1.8344	1.04803	0.52924	-0.2057	0.46284
TRINITY_DN2170_c1_g1_i3_orf1	beta-1,3-glucan-binding protein-like [Ostrinia furnacalis]	-1.5408	1.58533	-0.2372	0.23363	-0.0409
TRINITY_DN8771_c0_g1_i5_orf1	regucalcin-like [Ostrinia furnacalis]	-1.8607	1.10543	0.37132	-0.0361	0.42009
TRINITY_DN3952_c0_g1_i3_orf1	protein Skeletor, isoforms D/E-like isoform X1 [Ostrinia furnacalis] >XP_028176405.1 protein Skeletor, isoforms D/E-like isoform X2 [Ostrinia furnacalis] >XP_028176406.1 protein Skeletor, isoforms D/E-like isoform X3 [Ostrinia furnacalis] >XP_028176407.1 protein Skeletor, isoforms D/E-like isoform X4 [Ostrinia furnacalis]	-1.0343	1.65249	-0.9681	0.49199	-0.1421
TRINITY_DN2684_c0_g2_i3_orf1	glutamate decarboxylase 1-like isoform X1 [Ostrinia furnacalis]	-1.1274	1.76077	-0.7601	0.2098	-0.0831
TRINITY_DN5070_c0_g1_i1_orf1	ATP-dependent (S)-NAD(P)H-hydrate dehydratase-like [Ostrinia furnacalis]	-1.4893	1.46269	0.07369	0.54035	-0.5874
TRINITY_DN10220_c1_g1_i7_orf1	uncharacterized protein LOC124645895 isoform X2 [Helicoverpa zea]	-1.8477	1.02261	0.48606	-0.1816	0.52059
TRINITY_DN5408_c0_g1_i5_orf1	uncharacterized protein LOC114359912 [Ostrinia furnacalis]	-1.4089	1.52001	-0.0488	-0.6229	0.56055
TRINITY_DN65681_c0_g1_i1_orf1	ferritin subunit-like [Ostrinia furnacalis] >XP_028168186.1 ferritin subunit-like [Ostrinia furnacalis]	-0.7607	1.68319	-1.2374	0.09588	0.21897
TRINITY_DN688_c0_g1_i8_orf1	lysosomal alpha-mannosidase-like [Ostrinia furnacalis]	-1.4246	1.70856	-0.0313	-0.0307	-0.222
TRINITY_DN2171_c0_g1_i1_orf1	probable pterin-4-alpha-carbinolamine dehydratase isoform X1 [Ostrinia furnacalis]	-1.896	1.02984	0.26072	0.08693	0.51856
TRINITY_DN23167_c0_g2_i1_orf1	hypothetical protein evm_003712 [Chilo suppressalis]	-1.5754	1.54948	-0.268	0.11528	0.17872
TRINITY_DN22053_c0_g1_i13_orf1	uncharacterized protein LOC114355104 [Ostrinia furnacalis]	-1.6744	1.43144	-0.1583	0.34498	0.05631
TRINITY_DN198_c2_g1_i2_orf1	solute carrier organic anion transporter family member 5A1-like isoform X1 [Ostrinia furnacalis]	-1.7231	1.35671	0.13983	-0.156	0.38257
TRINITY_DN38412_c0_g1_i1_orf1	translation initiation factor eIF-2B subunit alpha [Ostrinia furnacalis]	-1.657	1.40022	-0.1849	0.50553	-0.0639
TRINITY_DN23364_c0_g1_i1_orf1	PREDICTED: uncharacterized protein LOC106134920 [Amyelois transitella]	-1.4176	1.70915	-0.1917	0.06709	-0.1669
TRINITY_DN272_c0_g1_i1_orf1	vacuolar protein sorting-associated protein 11 homolog [Ostrinia furnacalis]	-1.6988	1.18205	-0.0769	0.81392	-0.2203
TRINITY_DN97097_c0_g1_i4_orf1	plectin-like, partial [Ostrinia furnacalis]	-1.4084	1.6619	-0.43	0.25249	-0.076
TRINITY_DN25273_c0_g1_i1_orf1	skin secretory protein xP2-like [Ostrinia furnacalis]	-0.9779	1.92687	-0.4592	-0.2359	-0.2539
TRINITY_DN54269_c0_g1_i3_orf1	lopap-like [Ostrinia furnacalis]	-0.838	1.72948	-1.1303	0.10137	0.13741
TRINITY_DN1664_c0_g1_i4_orf1	uncharacterized protein LOC114355246 [Ostrinia furnacalis]	-1.8103	1.09041	0.72407	0.06649	-0.0706
TRINITY_DN18804_c0_g1_i5_orf1	zinc finger protein Xfin-like [Ostrinia furnacalis]	-0.8865	1.86633	-0.4324	0.17022	-0.7176
TRINITY_DN24971_c0_g1_i3_orf1	uncharacterized protein LOC114352370 [Ostrinia furnacalis]	-1.2295	1.82537	-0.3277	-0.2146	-0.0536
TRINITY_DN125150_c0_g1_i1_orf1	aldehyde dehydrogenase, dimeric NADP-preferring isoform X5 [Ostrinia furnacalis]	-1.8976	0.88808	0.24927	0.0202	0.74008

TRINITY_DN28922_c0_g1_i2_orf1	uncharacterized protein LOC119829283 isoform X2 [Zerene cesonia]	-0.8907	1.9407	-0.3664	-0.1515	-0.5322
TRINITY_DN57998_c1_g1_i1_orf1	uncharacterized protein LOC113509309, partial [Galleria mellonella]	-0.7032	1.96618	-0.3962	-0.202	-0.6647
TRINITY_DN2515_c0_g1_i6_orf1	chitooligosaccharidolytic beta-N-acetylglucosaminidase isoform X1 [Ostrinia furnacalis]	-1.2671	1.80704	-0.1622	-0.3144	-0.0634
TRINITY_DN5467_c0_g1_i5_orf1	synaptic vesicle glycoprotein 2B-like isoform X2 [Ostrinia furnacalis] >XP_028161209.1 synaptic vesicle glycoprotein 2B-like isoform X2 [Ostrinia furnacalis] >XP_028161210.1 synaptic vesicle glycoprotein 2B-like isoform X2 [Ostrinia furnacalis]	-1.4156	1.69305	0.02883	-0.3554	0.04919
TRINITY_DN70485_c0_g1_i2_orf1	serine/threonine-protein kinase Genghis Khan-like [Ostrinia furnacalis]	-1.5568	1.56618	0.1462	0.13433	-0.2899
TRINITY_DN48765_c0_g1_i7_orf1	uncharacterized protein LOC114352307 [Ostrinia furnacalis]	-1.587	1.16355	0.74182	0.35398	-0.6724
TRINITY_DN2794_c1_g1_i8_orf1	carboxypeptidase D [Ostrinia furnacalis]	-1.5301	1.59237	0.19351	-0.2909	0.03508
TRINITY_DN2793_c0_g2_i1_orf1	PREDICTED: ras-related protein Rab-4B [Amyelois transitella]	-1.7132	1.255	-0.1687	0.67733	-0.0504
TRINITY_DN1012_c0_g1_i2_orf1	teneurin-a isoform X1 [Ostrinia furnacalis]	-1.4798	1.4208	0.19809	-0.6789	0.53979
TRINITY_DN31676_c0_g1_i4_orf1	N-acetylgalactosaminyltransferase 7 isoform X1 [Ostrinia furnacalis] >XP_028156925.1 N-acetylgalactosaminyltransferase 7 isoform X2 [Ostrinia furnacalis]	-1.7599	1.20442	-0.2199	0.61491	0.16043
TRINITY_DN479_c6_g1_i2_orf1	beta-1,3-glucan-binding protein-like [Ostrinia furnacalis]	-1.5387	1.56993	0.32255	-0.1535	-0.2003
TRINITY_DN1612_c0_g1_i3_orf1	immunoglobulin-binding protein 1b [Ostrinia furnacalis]	-1.8135	1.02892	-0.2519	0.67942	0.35709
TRINITY_DN2508_c0_g1_i2_orf1	uncharacterized protein LOC114361845 [Ostrinia furnacalis] >XP_028172853.1 uncharacterized protein LOC114361845 [Ostrinia furnacalis]	-1.7591	1.33366	0.03774	0.03523	0.35247
TRINITY_DN43431_c0_g1_i1_orf1	glycine dehydrogenase (decarboxylating), mitochondrial isoform X1 [Ostrinia furnacalis] >XP_028174269.1 glycine dehydrogenase (decarboxylating), mitochondrial isoform X3 [Ostrinia furnacalis]	-1.7688	1.20945	0.63563	-0.01	-0.0663
TRINITY_DN12286_c1_g1_i2_orf1	sideroflexin-1-3 [Galleria mellonella] >XP_026754161.1 sideroflexin-1-3 [Galleria mellonella]	-1.2111	1.68161	-0.5167	0.49075	-0.4446
TRINITY_DN2069_c1_g1_i8_orf1	lysosomal aspartic protease [Trichoplusia ni]	-1.4271	1.65635	-0.4193	-0.0187	0.20878
TRINITY_DN26337_c0_g1_i3_orf1	lysosome membrane protein 2-like [Ostrinia furnacalis]	-1.4176	1.51383	-0.576	-0.1149	0.59473
TRINITY_DN1870_c0_g1_i6_orf1	programmed cell death protein 5 [Ostrinia furnacalis]	-1.7806	1.19246	-0.1255	0.61876	0.09488
TRINITY_DN13856_c0_g1_i1_orf1	angiotensin-converting enzyme-like [Ostrinia furnacalis]	-1.3156	1.52234	-0.2444	0.68637	-0.6486
TRINITY_DN13686_c0_g2_i1_orf1	transmembrane protease serine 9-like [Ostrinia furnacalis]	-1.203	1.82352	-0.4292	-0.2077	0.01638
TRINITY_DN166_c0_g1_i4_orf1	PREDICTED: cryptochrome-1 isoform X1 [Amyelois transitella] >XP_013199861.1 PREDICTED: cryptochrome-1 isoform X1 [Amyelois transitella]	-1.601	1.33628	-0.5439	0.52374	0.28484
TRINITY_DN875_c0_g1_i3_orf1	secernin-3 [Ostrinia furnacalis]	-1.244	1.71172	-0.1826	0.33093	-0.6161
TRINITY_DN22441_c0_g1_i1_orf1	Gamma-aminobutyric acid receptor-associated protein, partial [Cotesia chilonis]	-1.8058	1.19236	-0.1335	0.2724	0.47455
TRINITY_DN143637_c0_g1_i1_orf1	PX domain-containing protein kinase-like protein isoform X1 [Chelonus insularis]	-1.7345	1.20193	0.29959	0.5802	-0.3472
TRINITY_DN45271_c0_g1_i1_orf1	double-strand break repair protein MRE11 [Ostrinia furnacalis]	-1.667	1.47481	0.21233	0.00999	-0.0302
TRINITY_DN1507_c0_g1_i5_orf1	27 kDa hemolymph protein-like, partial [Ostrinia furnacalis]	-1.7351	1.2881	0.55902	-0.1314	0.01942
TRINITY_DN9412_c0_g1_i1_orf1	maspardin-like [Ostrinia furnacalis]	-1.3603	1.5662	-0.0341	0.49746	-0.6692
TRINITY_DN1407_c0_g1_i5_orf1	unnamed protein product [Chrysodeixis includens]	-1.2313	1.75413	-0.2163	0.24241	-0.549
TRINITY_DN1630_c0_g1_i6_orf1	major facilitator superfamily domain-containing protein 1-like [Ostrinia furnacalis]	-1.3376	1.75713	-0.1486	0.04433	-0.3153
TRINITY_DN2311_c0_g3_i1_orf1	uncharacterized protein LOC114364231 isoform X1 [Ostrinia furnacalis] >XP_028176108.1 uncharacterized protein LOC114364231 isoform X2 [Ostrinia furnacalis]	-1.2125	1.75083	-0.4861	-0.3628	0.31061
TRINITY_DN4384_c0_g1_i5_orf1	chemosensory protein 5 [Conogethes punctiferalis]	-0.8811	1.9469	-0.3634	-0.1861	-0.5162
TRINITY_DN21555_c0_g1_i4_orf1	uncharacterized protein LOC114351844 [Ostrinia furnacalis] >XP_028158981.1 uncharacterized protein LOC114351844 [Ostrinia furnacalis] >XP_028158982.1 uncharacterized protein LOC114351844 [Ostrinia furnacalis] >XP_028158983.1 uncharacterized protein LOC114351844 [Ostrinia furnacalis] >XP_028158984.1 uncharacterized protein LOC114351844 [Ostrinia furnacalis] >XP_028158985.1 uncharacterized protein LOC114351844 [Ostrinia furnacalis] >5GPR_A Crystal structure of chitinase-h from Ostrinia furnacalis [Ostrinia furnacalis] >5GQB_A Crystal structure of chitinase-h from O. furnacalis in complex with chitohepatose [Ostrinia furnacalis] >6JMN_A Crystal structure of Ostrinia furnacalis Chitinase h complexed with compound 2-8-s2 [Ostrinia furnacalis] >BAE16587.1 chitinase [Ostrinia furnacalis]	-1.1256	1.85928	-0.4767	-0.2174	-0.0395
TRINITY_DN10680_c0_g1_i5_orf1	cGMP-dependent protein kinase, isozyme 2 forms cD4/T1/T3A/T3B-like isoform X3 [Ostrinia furnacalis] >XP_028158316.1 cGMP-dependent protein kinase, isozyme 2 forms cD4/T1/T3A/T3B-like isoform X3 [Ostrinia furnacalis]	-1.593	1.44791	-0.2337	0.53554	-0.1568
TRINITY_DN6423_c0_g1_i5_orf1	phenoloxidase-activating factor 2-like isoform X2 [Ostrinia furnacalis]	-1.4549	1.6612	-0.3372	0.03908	0.09183
TRINITY_DN14904_c1_g2_i2_orf1	autophagy protein 12-like [Ostrinia furnacalis]	-1.8265	1.07431	-0.1544	0.25932	0.64718
TRINITY_DN2348_c0_g1_i1_orfp1	TRINITY_DN2348_c0_g1_i1_m.39060 TRINITY_DN2348_c0_g1::TRINITY_DN2348_c0_g1_i1::g.39060 ORF type:complete len:149 (+),score=54.19 TRINITY_DN2348_c0_g1_i1:28-474(+)	-1.3293	1.73074	-0.2004	0.19699	-0.398
TRINITY_DN3647_c1_g1_i5_orf1	unnamed protein product, partial [Iphiclides podalirius]	-1.6234	1.1311	0.94416	-0.4398	-0.012
TRINITY_DN1986_c0_g1_i1_orf1	serine protease inhibitor 77Ba-like [Ostrinia furnacalis] >XP_028164032.1 serine protease inhibitor 77Ba-like [Ostrinia furnacalis]	-1.3693	1.75146	-0.1681	-0.1634	-0.0507
TRINITY_DN26301_c0_g1_i1_orf1	uncharacterized protein LOC114359193 [Ostrinia furnacalis]	-1.1117	1.88231	-0.3334	-0.3031	-0.1341
TRINITY_DN1767_c0_g2_i15_orf1	fasciclin-2 isoform X3 [Ostrinia furnacalis]	-1.6389	1.49198	0.23328	0.07922	-0.1656

TRINITY_DN12024_c0_g1_i4_orf1	pancreatic lipase-related protein 2 isoform X1 [Ostrinia furnacalis] >XP_028176200.1 pancreatic lipase-related protein 2 isoform X2 [Ostrinia furnacalis]	-1.7256	1.1905	-0.3558	0.64758	0.24332
TRINITY_DN28661_c0_g1_i1_orf1	cathepsin B [Ostrinia furnacalis]	-1.7617	1.22559	0.59612	-0.1661	0.10615
TRINITY_DN12885_c0_g1_i1_orf1	prolow-density lipoprotein receptor-related protein 1, partial [Ostrinia furnacalis]	-1.8075	1.19575	0.25047	0.47656	-0.1153
TRINITY_DN2652_c0_g2_i1_orf1	peroxidase-like isoform X1 [Ostrinia furnacalis]	-0.9509	1.93676	-0.3332	-0.2244	-0.4283
TRINITY_DN18650_c0_g1_i1_orf1	bombyxin B-9-like [Ostrinia furnacalis]	-1.3172	1.51982	-0.7953	0.56747	0.0252
TRINITY_DN3896_c0_g1_i1_orf1	glyoxalase domain-containing protein 4 [Ostrinia furnacalis]	-1.5635	1.53605	-0.3518	0.17913	0.20018
TRINITY_DN15400_c0_g1_i1_orf1	uncharacterized protein LOC114366781 [Ostrinia furnacalis]	-1.3648	1.20999	0.66042	-0.9976	0.49194
TRINITY_DN2442_c0_g1_i2_orf1	digestive cysteine proteinase 2 [Ostrinia furnacalis]	-1.2558	1.77788	0.1596	-0.387	-0.2946
TRINITY_DN2489_c0_g1_i1_orf1	uncharacterized protein LOC114354692 [Ostrinia furnacalis]	-0.9749	1.92419	-0.473	-0.1676	-0.3088
TRINITY_DN3005_c0_g1_i7_orf1	lachesin-like isoform X3 [Ostrinia furnacalis]	-1.1368	1.83031	-0.5487	0.07932	-0.2241
TRINITY_DN110402_c0_g2_i1_orf1	apolipophorins-like [Ostrinia furnacalis]	-1.2576	1.68792	-0.6595	0.34706	-0.1178
TRINITY_DN8724_c0_g1_i2_orf1	vesicle-associated membrane protein/synaptobrevin-binding protein isoform X2 [Pectinophora gossypiella]	-1.8554	1.01451	0.71257	0.14217	-0.0138
TRINITY_DN2227_c0_g1_i5_orf1	protein 60A [Ostrinia furnacalis]	-1.6524	1.37725	0.33267	-0.3895	0.33204
TRINITY_DN862_c0_g1_i4_orf1	uncharacterized protein LOC114359380 isoform X1 [Ostrinia furnacalis] >XP_028169561.1 uncharacterized protein LOC114359380 isoform X2 [Ostrinia furnacalis] >XP_028169562.1 uncharacterized protein LOC114359380 isoform X1 [Ostrinia furnacalis]	-1.3381	1.67622	-0.2905	0.3726	-0.4203
TRINITY_DN11044_c0_g1_i4_orf1	protein Skeletor, isoforms B/C isoform X3 [Ostrinia furnacalis]	-1.8075	1.20883	0.26608	0.43606	-0.1035
TRINITY_DN8766_c0_g1_i1_orf1	prolow-density lipoprotein receptor-related protein 1, partial [Ostrinia furnacalis]	-1.8444	0.90036	0.31127	-0.1789	0.81162
TRINITY_DN57998_c1_g3_i1_orf1	uncharacterized protein LOC114363305 isoform X2 [Ostrinia furnacalis]	-0.8223	1.96902	-0.4505	-0.3209	-0.3752
TRINITY_DN7590_c0_g1_i4_orf1	innexin inx1-like [Pectinophora gossypiella]	-1.7643	1.22923	-0.2236	0.24051	0.51814
TRINITY_DN9920_c0_g1_i1_orf1	uncharacterized protein LOC114351526 [Ostrinia furnacalis]	-1.239	1.70778	-0.7064	0.01599	0.22161
TRINITY_DN7128_c0_g1_i7_orf1	dystroglycan [Ostrinia furnacalis]	-1.6362	1.45888	0.39003	-0.2055	-0.0071
TRINITY_DN3109_c0_g1_i5_orf1	protein takeout isoform X2 [Ostrinia furnacalis]	-1.8045	1.07515	0.7659	-0.0372	0.00062
TRINITY_DN3616_c0_g2_i1_orf1	conotoxin ArMKLT2-032-like [Ostrinia furnacalis]	-1.1029	1.87404	-0.0539	-0.4341	-0.2831
TRINITY_DN2170_c0_g1_i2_orf1	beta-1,3-glucan-binding protein-like [Ostrinia furnacalis]	-1.5394	1.22023	0.37509	0.67881	-0.7348
TRINITY_DN8083_c0_g1_i1_orf1	solute carrier family 35 member F6 [Ostrinia furnacalis]	-1.5389	1.36715	0.75293	-0.4066	-0.1746
TRINITY_DN5122_c0_g1_i3_orf1	calumenin [Ostrinia furnacalis] >XP_028172745.1 calumenin [Ostrinia furnacalis] >XP_028172746.1 calumenin [Ostrinia furnacalis]	-1.8331	1.15217	-0.0847	0.30535	0.4603
TRINITY_DN36856_c0_g1_i1_orf1	protein enhancer of sevenless 2B isoform X2 [Formica exsecta]	-1.3952	1.63523	0.30438	-0.0089	-0.5356
TRINITY_DN5028_c0_g1_i11_orf1	NTF2-related export protein [Ostrinia furnacalis]	-0.9178	1.81091	-0.3006	0.25706	-0.8496
TRINITY_DN3292_c2_g2_i1_orf1	aldo-keto reductase AKR2E4-like [Ostrinia furnacalis]	-1.6893	1.40293	0.05954	-0.1595	0.38627
TRINITY_DN7776_c0_g1_i9_orf1	uncharacterized protein LOC114364702 [Ostrinia furnacalis]	-1.8637	1.07048	0.59691	0.14919	0.04709
TRINITY_DN4041_c0_g1_i6_orf1	tubulin-folding cofactor B isoform X3 [Ostrinia furnacalis]	-1.421	1.58499	-0.605	0.27207	0.16895
TRINITY_DN3600_c0_g1_i1_orf1	unnamed protein product [Chilo suppressalis]	-1.463	1.4593	0.33379	0.36606	-0.6961
TRINITY_DN7776_c0_g1_i5_orf1	uncharacterized protein LOC114364702 [Ostrinia furnacalis]	-1.8053	1.07891	0.13345	-0.1412	0.73415
TRINITY_DN5064_c0_g1_i4_orf1	sortilin-related receptor-like [Ostrinia furnacalis]	-1.6412	1.38948	0.42395	-0.3872	0.21493
TRINITY_DN13088_c0_g1_i5_orf1	beta-hexosaminidase subunit alpha-like isoform X2 [Ostrinia furnacalis]	-0.9931	1.89347	-0.4456	0.02415	-0.4789
TRINITY_DN537_c0_g1_i1_orf1	pupal cuticle protein C1B-like precursor [Papilio xuthus] >BAM18715.1 cuticular protein PxutCPFL6Ba [Papilio xuthus]	-1.3641	1.59296	0.539	-0.4745	-0.2934
TRINITY_DN1703_c0_g1_i6_orf1	leucine-rich repeat-containing protein 15-like [Ostrinia furnacalis] >XP_028171914.1 leucine-rich repeat-containing protein 15-like [Ostrinia furnacalis] >XP_028171921.1 leucine-rich repeat-containing protein 15-like [Ostrinia furnacalis]	-1.1532	1.84512	-0.495	-0.0749	-0.1219
TRINITY_DN15411_c0_g1_i4_orf1	uncharacterized protein LOC114362040 isoform X1 [Ostrinia furnacalis]	-1.3229	1.70426	0.01988	0.16308	-0.5643
TRINITY_DN110231_c0_g1_i1_orf1	protein singed [Ostrinia furnacalis] >XP_028161434.1 protein singed [Ostrinia furnacalis]	-1.0985	1.80145	0.27123	-0.4864	-0.4878
TRINITY_DN1465_c2_g1_i2_orf1	transcription initiation factor TFIID subunit 1-like [Ostrinia furnacalis]	-1.1896	1.7845	-0.3731	-0.4549	0.23306
TRINITY_DN6275_c0_g1_i3_orf1	CTL-like protein 1 isoform X1 [Galleria mellonella]	-1.8148	1.2096	0.1819	-0.0342	0.45747
TRINITY_DN88876_c0_g1_i1_orf1	Photosystem I reaction center subunit III, chloroplastic, partial [Trichinella zimbabwensis]	-1.2757	1.36367	-1.0326	0.45427	0.49033
TRINITY_DN6004_c0_g1_i1_orf1	endocuticle structural glycoprotein ABD-4-like [Ostrinia furnacalis]	-0.942	1.94185	-0.3786	-0.363	-0.2582
TRINITY_DN1408_c0_g1_i10_orf1	protein diaphanous [Ostrinia furnacalis]	-1.5268	1.21435	-0.3389	0.98419	-0.3328
TRINITY_DN10290_c0_g1_i7_orf1	aquaporin AQPAe.a [Ostrinia furnacalis]	-1.5041	1.46669	-0.0629	0.5875	-0.4872
TRINITY_DN19043_c0_g3_i2_orf1	hypothetical protein EVAR_60654_1 [Eumeta japonica]	-1.2933	1.5172	-0.8484	0.077	0.54751
TRINITY_DN19951_c0_g1_i5_orf1	protein croquemort-like [Ostrinia furnacalis]	-1.1742	1.85178	-0.3437	-0.0712	-0.2627
TRINITY_DN12671_c0_g1_i4_orf1	hemicentin-1-like isoform X1 [Ostrinia furnacalis]	-0.8659	1.96204	-0.3668	-0.3571	-0.3722
TRINITY_DN3545_c0_g1_i6_orf1	group XV phospholipase A2-like [Ostrinia furnacalis] >XP_028168992.1 group XV phospholipase A2-like [Ostrinia furnacalis] >XP_028168993.1 group XV phospholipase A2-like [Ostrinia furnacalis]	-1.725	1.38957	0.25706	-0.0714	0.14979
TRINITY_DN97138_c0_g1_i2_orf1	tubulin beta chain-like isoform X2 [Ostrinia furnacalis]	-1.0937	1.89179	-0.3521	-0.2513	-0.1947
TRINITY_DN906_c0_g1_i4_orf1	uncharacterized protein LOC114360441, partial [Ostrinia furnacalis]	-0.8586	1.95715	-0.2246	-0.4425	-0.4314
TRINITY_DN661_c0_g2_i2_orf1	cuticle protein 7-like [Ostrinia furnacalis]	-0.5741	1.93421	-0.9088	-0.1979	-0.2534

<table>
<tr><td>TRINITY_DN712_c0_g2_i1_orf1</td><td>serine protease inhibitor 77Ba-like [Ostrinia furnacalis]</td><td>-1.1325</td><td>1.8337</td><td>-0.5702</td><td>-0.1687</td><td>0.0377</td></tr>
<tr><td>TRINITY_DN18027_c0_g2_i1_orf1</td><td>vanin-like protein 2 isoform X2 [Ostrinia furnacalis]</td><td>-1.4336</td><td>1.66368</td><td>-0.3518</td><td>0.21206</td><td>-0.0903</td></tr>
<tr><td>TRINITY_DN125427_c0_g1_i1_orf1</td><td>heat shock protein 19.8 [Chilo suppressalis] >AGM90553.1 HSP19.8 [Chilo suppressalis] >BAE94664.1 small heat shock protein 19.7 [Chilo suppressalis]</td><td>-0.6684</td><td>1.90453</td><td>-0.9238</td><td>-0.0469</td><td>-0.2655</td></tr>
<tr><td>TRINITY_DN39404_c0_g1_i7_orf1</td><td>hypothetical protein evm_004736, partial [Chilo suppressalis]</td><td>-1.6677</td><td>1.38935</td><td>0.47197</td><td>-0.2501</td><td>0.0565</td></tr>
<tr><td>TRINITY_DN569_c0_g3_i12_orf1</td><td>prominin-like protein isoform X2 [Ostrinia furnacalis]</td><td>-1.5854</td><td>1.50013</td><td>-0.3543</td><td>0.13661</td><td>0.30303</td></tr>
<tr><td>TRINITY_DN14458_c0_g1_i2_orf1</td><td>spermatogenesis-associated protein 20 isoform X1 [Ostrinia furnacalis]</td><td>-1.2612</td><td>1.78399</td><td>-0.0471</td><td>-0.0019</td><td>-0.4737</td></tr>
<tr><td>TRINITY_DN4886_c0_g1_i6_orf1</td><td>uncharacterized protein LOC114349567 [Ostrinia furnacalis]</td><td>-1.2135</td><td>1.82588</td><td>-0.2079</td><td>-0.0172</td><td>-0.3873</td></tr>
<tr><td>TRINITY_DN13887_c0_g1_i5_orf1</td><td>transmembrane protein 184B isoform X3 [Ostrinia furnacalis]</td><td>-0.9982</td><td>1.91745</td><td>-0.4787</td><td>-0.241</td><td>-0.1995</td></tr>
<tr><td>TRINITY_DN14154_c0_g1_i1_orf1</td><td>uncharacterized protein LOC114360857, partial [Ostrinia furnacalis]</td><td>-1.5291</td><td>1.30016</td><td>-0.4698</td><td>0.85271</td><td>-0.154</td></tr>
<tr><td>TRINITY_DN13285_c0_g1_i9_orf1</td><td>E3 ubiquitin-protein ligase RNF13 isoform X1 [Ostrinia furnacalis] >XP_028158682.1 E3 ubiquitin-protein ligase RNF13 isoform X1 [Ostrinia furnacalis] >XP_028158683.1 E3 ubiquitin-protein ligase RNF13 isoform X1 [Ostrinia furnacalis]</td><td>-1.791</td><td>1.16829</td><td>0.64846</td><td>-0.0701</td><td>0.04433</td></tr>
<tr><td>TRINITY_DN26013_c0_g1_i1_orf1</td><td>E3 ubiquitin-protein ligase CHIP [Ostrinia furnacalis]</td><td>-1.7543</td><td>1.12645</td><td>0.66694</td><td>0.30295</td><td>-0.3421</td></tr>
<tr><td>TRINITY_DN10229_c0_g1_i6_orf1</td><td>autophagy protein 5 isoform X2 [Ostrinia furnacalis]</td><td>-1.8243</td><td>1.14537</td><td>0.59009</td><td>-0.0191</td><td>0.10793</td></tr>
<tr><td>TRINITY_DN61135_c0_g1_i1_orf1</td><td>uncharacterized protein LOC114362571 [Ostrinia furnacalis]</td><td>-1.7553</td><td>1.1059</td><td>-0.2687</td><td>0.77706</td><td>0.14107</td></tr>
<tr><td>TRINITY_DN1533_c0_g2_i1_orf1</td><td>unnamed protein product [Chilo suppressalis]</td><td>-1.3729</td><td>1.74635</td><td>-0.1656</td><td>-0.1943</td><td>-0.0135</td></tr>
<tr><td>TRINITY_DN364_c5_g1_i3_orf1</td><td>talin-2-like, partial [Ostrinia furnacalis]</td><td>-1.8588</td><td>1.01885</td><td>0.54405</td><td>-0.1412</td><td>0.43706</td></tr>
<tr><td>TRINITY_DN116467_c0_g1_i1_orf1</td><td>probable small nuclear ribonucleoprotein E [Ostrinia furnacalis]</td><td>-1.5376</td><td>1.56739</td><td>0.09649</td><td>0.22138</td><td>-0.3477</td></tr>
<tr><td>TRINITY_DN4367_c0_g1_i1_orf1</td><td>heat shock protein 21.7c [Chilo suppressalis] >AWT57938.1 heat shock protein 21.7c [Chilo suppressalis]</td><td>-0.8102</td><td>1.89617</td><td>-0.8468</td><td>-0.0847</td><td>-0.1545</td></tr>
<tr><td>TRINITY_DN9457_c0_g1_i9_orf1</td><td>plexin domain-containing protein 2 [Spodoptera litura]</td><td>-1.6381</td><td>1.16241</td><td>0.46415</td><td>-0.6066</td><td>0.61811</td></tr>
<tr><td>TRINITY_DN3407_c0_g1_i9_orf1</td><td>uncharacterized protein YJR142W [Ostrinia furnacalis]</td><td>-1.4534</td><td>1.26265</td><td>-0.0488</td><td>0.91423</td><td>-0.6747</td></tr>
<tr><td>TRINITY_DN291_c0_g1_i2_orf1</td><td>DNA replication licensing factor Mcm7 [Helicoverpa armigera] >XP_049698025.1 DNA replication licensing factor Mcm7-like [Helicoverpa armigera] >PZC87280.1 hypothetical protein B5X24_HaOG201516 [Helicoverpa armigera]</td><td>-1.0209</td><td>1.77544</td><td>-0.7733</td><td>0.33148</td><td>-0.3128</td></tr>
<tr><td>TRINITY_DN10747_c0_g1_i5_orf1</td><td>unnamed protein product [Plutella xylostella]</td><td>-0.6786</td><td>1.98764</td><td>-0.5397</td><td>-0.3567</td><td>-0.4126</td></tr>
<tr><td>TRINITY_DN98016_c0_g1_i1_orf1</td><td>methanethiol oxidase [Ostrinia furnacalis]</td><td>-1.4114</td><td>1.62849</td><td>-0.1443</td><td>-0.4442</td><td>0.37136</td></tr>
<tr><td>TRINITY_DN2043_c0_g1_i11_orf1</td><td>phenoloxidase-activating factor 2-like [Ostrinia furnacalis]</td><td>-1.3489</td><td>1.58366</td><td>0.5169</td><td>-0.1281</td><td>-0.6235</td></tr>
<tr><td>TRINITY_DN20796_c0_g1_i4_orf1</td><td>probable low-specificity L-threonine aldolase 2 [Ostrinia furnacalis]</td><td>-1.3023</td><td>1.72718</td><td>-0.5108</td><td>-0.1247</td><td>0.2106</td></tr>
<tr><td>TRINITY_DN17133_c0_g1_i1_orf1</td><td>unnamed protein product [Chrysodeixis includens]</td><td>-1.5187</td><td>1.49247</td><td>-0.0386</td><td>0.51331</td><td>-0.4485</td></tr>
<tr><td>TRINITY_DN8143_c0_g1_i6_orf1</td><td>gamma-soluble NSF attachment protein-like [Galleria mellonella]</td><td>-1.747</td><td>1.2495</td><td>0.36892</td><td>0.41233</td><td>-0.2837</td></tr>
<tr><td>TRINITY_DN31417_c0_g1_i3_orf1</td><td>titin-like [Ostrinia furnacalis]</td><td>-1.1043</td><td>1.88646</td><td>-0.3673</td><td>-0.188</td><td>-0.2268</td></tr>
<tr><td>TRINITY_DN4790_c0_g1_i6_orf1</td><td>ADP-ribosylation factor-like protein 8 [Ostrinia furnacalis] >CAG9746554.1 unnamed protein product [Diatraea saccharalis] >CAG9785239.1 unnamed protein product [Diatraea saccharalis] >CAH2992000.1 unnamed protein product [Chilo suppressalis]</td><td>-1.7065</td><td>1.25316</td><td>0.67652</td><td>-0.2436</td><td>0.02041</td></tr>
<tr><td>TRINITY_DN54554_c0_g1_i1_orf1</td><td>LOW QUALITY PROTEIN: signal transducing adapter molecule 2 [Ostrinia furnacalis]</td><td>-1.8262</td><td>1.10286</td><td>0.38257</td><td>0.51985</td><td>-0.1791</td></tr>
<tr><td>TRINITY_DN4273_c1_g1_i5_orf1</td><td>tetraspanin-13 isoform X1 [Ostrinia furnacalis]</td><td>-1.3045</td><td>1.77836</td><td>0.01552</td><td>-0.1558</td><td>-0.3337</td></tr>
<tr><td>TRINITY_DN1384_c0_g1_i5_orf1</td><td>vacuolar protein sorting-associated protein VTA1 homolog [Ostrinia furnacalis] >XP_028168952.1 vacuolar protein sorting-associated protein VTA1 homolog [Ostrinia furnacalis]</td><td>-1.706</td><td>1.40582</td><td>-0.1272</td><td>0.16081</td><td>0.26665</td></tr>
<tr><td>TRINITY_DN10373_c0_g1_i1_orf1</td><td>homocysteine S-methyltransferase 1-like [Ostrinia furnacalis] >XP_028162778.1 homocysteine S-methyltransferase 1-like [Ostrinia furnacalis]</td><td>-1.5007</td><td>0.81698</td><td>1.20418</td><td>-0.7577</td><td>0.23723</td></tr>
<tr><td>TRINITY_DN8812_c0_g1_i1_orf1</td><td>nuclear pore complex protein Nup160 homolog isoform X3 [Ostrinia furnacalis]</td><td>-1.8015</td><td>1.06203</td><td>0.48665</td><td>0.54936</td><td>-0.2965</td></tr>
<tr><td>TRINITY_DN4635_c0_g1_i4_orf1</td><td>putative salivary secreted peptide [Operophtera brumata]</td><td>-1.3632</td><td>1.57594</td><td>-0.6842</td><td>0.43415</td><td>0.03734</td></tr>
<tr><td>TRINITY_DN52864_c0_g1_i1_orf1</td><td>odorant binding protein 18 [Conogethes pinicolalis]</td><td>-1.1428</td><td>1.6667</td><td>-0.8285</td><td>0.45499</td><td>-0.1503</td></tr>
<tr><td>TRINITY_DN8674_c0_g2_i1_orf1</td><td>N(G),N(G)-dimethylarginine dimethylaminohydrolase 1 [Ostrinia furnacalis]</td><td>-1.5336</td><td>1.43054</td><td>-0.3484</td><td>-0.2092</td><td>0.66066</td></tr>
<tr><td>TRINITY_DN140_c1_g1_i2_orf1</td><td>modular serine protease-like isoform X1 [Ostrinia furnacalis]</td><td>-1.3605</td><td>1.57972</td><td>0.57878</td><td>-0.3909</td><td>-0.4071</td></tr>
<tr><td>TRINITY_DN246_c1_g1_i5_orf1</td><td>lachesin isoform X1 [Ostrinia furnacalis] >XP_028178464.1 lachesin isoform X2 [Ostrinia furnacalis]</td><td>-1.216</td><td>1.7594</td><td>-0.6336</td><td>0.14621</td><td>-0.0561</td></tr>
<tr><td>TRINITY_DN8726_c0_g2_i3_orf1</td><td>dnaJ homolog subfamily C member 25 homolog [Ostrinia furnacalis]</td><td>-1.828</td><td>1.11758</td><td>0.63612</td><td>0.00555</td><td>0.06875</td></tr>
<tr><td>TRINITY_DN1091_c0_g1_i1_orf1</td><td>macrophage mannose receptor 1-like [Pararge aegeria]</td><td>-1.3514</td><td>1.49356</td><td>0.73937</td><td>-0.5032</td><td>-0.3783</td></tr>
<tr><td>TRINITY_DN16091_c0_g1_i1_orfp1</td><td>TRINITY_DN16091_c0_g1_i1_m.64010 TRINITY_DN16091_c0_g1::TRINITY_DN16091_c0_g1_i1::g.64010 ORF type:5prime_partial len:124 (-),score=7.29,Toxin_2 PF00451.20 0.00035,Toxin_2|PF00451.20|0.00013,Toxin_2|PF00451.20|0.00037,Gamma-thionin|PF00304.21|0.37,Gamma-thionin|PF00304.21|0.052,Gamma-thionin|PF00304.21|0.022,Defensin_2|PF01097.19|0.58,Defensin_2|PF01097.19|0.12,Defensin_2|PF01097.19|0.011,Toxin_38|PF14866.7|0.18,Toxin_38|PF14866.7|0.18,Toxin_38|PF14866.7|0.4 TRINITY_DN16091_c0_g1_i1:19-390(-)</td><td>-1.4033</td><td>-0.2869</td><td>-0.0215</td><td>1.71696</td><td>-0.0053</td></tr>
<tr><td>TRINITY_DN85290_c0_g2_i1_orf1</td><td>unnamed protein product, partial [Brenthis ino]</td><td>-1.3149</td><td>-0.2139</td><td>1.02085</td><td>1.26744</td><td>-0.7595</td></tr>
<tr><td>TRINITY_DN15682_c0_g1_i4_orf1</td><td>seroin transcript 1B [Ostrinia nubilalis]</td><td>-1.1209</td><td>-0.5648</td><td>0.68044</td><td>1.61085</td><td>-0.6056</td></tr>
<tr><td>TRINITY_DN2407_c0_g1_i2_orf1</td><td>uncharacterized protein LOC114366345 isoform X2 [Ostrinia furnacalis]</td><td>-1.0228</td><td>-0.7624</td><td>1.70113</td><td>0.52949</td><td>-0.4454</td></tr>
</table>

TRINITY_DN61042_c0_g2_i2_orfp1	TRINITY_DN61042_c0_g2_i2_m.5292 TRINITY_DN61042_c0_g2::TRINITY_DN61042_c0_g2_i2::g.5292 ORF type:5prime_partial len:63 (-),score=21.86 TRINITY_DN61042_c0_g2_i2:77-265(-)	-1.3815	-0.5713	1.35313	0.91395	-0.3142
TRINITY_DN311_c0_g1_i8_orfp1	TRINITY_DN311_c0_g1_i8_m.65152 TRINITY_DN311_c0_g1::TRINITY_DN311_c0_g1_i8::g.65152 ORF type:5prime_partial len:138 (+),score=74.30 TRINITY_DN311_c0_g1_i8:2-415(+)	-0.6012	-0.7683	1.0148	1.39298	-1.0383
TRINITY_DN4360_c0_g1_i4_orf1	glucose-6-phosphate isomerase-like, partial [Bicyclus anynana]	-0.9694	-0.4096	1.3734	1.00436	-0.9988
TRINITY_DN80660_c0_g1_i1_orf1	probable phospholipid hydroperoxide glutathione peroxidase isoform X1 [Pieris rapae]	-1.5991	-0.3136	0.56574	1.42089	-0.0739
TRINITY_DN26789_c0_g1_i2_orf1	D-2-hydroxyglutarate dehydrogenase, mitochondrial-like [Ostrinia furnacalis]	-1.358	-0.3229	-0.1156	1.74216	0.05438
TRINITY_DN184_c0_g1_i1_orf1	macrophage mannose receptor 1-like isoform X1 [Maniola jurtina]	-0.9692	-0.0477	1.73147	0.27718	-0.9917
TRINITY_DN7556_c0_g1_i3_orf1	jg27436 [Pararge aegeria aegeria]	-1.3883	-0.1483	0.16146	1.70714	-0.332
TRINITY_DN2338_c0_g2_i1_orf1	prophenoloxidase PPO3 [Ostrinia furnacalis]	-1.0157	-0.1384	0.17361	1.80143	-0.8209
TRINITY_DN14874_c0_g1_i6_orf1	uncharacterized protein LOC114358148 [Ostrinia furnacalis]	-1.2011	-0.2537	1.69405	0.42561	-0.6648
TRINITY_DN4794_c1_g1_i9_orf1	D-3-phosphoglycerate dehydrogenase [Ostrinia furnacalis]	-1.3417	-0.1268	0.55382	1.56611	-0.6515
TRINITY_DN3377_c0_g1_i1_orf1	calcyphosin-like protein isoform X3 [Helicoverpa armigera] >XP_047020698.1 calcyphosin-like protein isoform X2 [Helicoverpa zea]	-1.2692	-0.8139	1.30544	0.98891	-0.2113
TRINITY_DN7622_c0_g2_i2_orf1	uncharacterized protein LOC114351301 [Ostrinia furnacalis]	-1.4114	0.06137	0.51543	1.51052	-0.6759
TRINITY_DN5852_c0_g1_i6_orf1	probable maltase isoform X6 [Ostrinia furnacalis]	-0.7384	-0.5155	1.40678	0.97196	-1.1248
TRINITY_DN1789_c0_g1_i5_orf1	uncharacterized protein LOC114365543 [Ostrinia furnacalis]	-1.1975	-0.5498	1.60329	0.65633	-0.5124
TRINITY_DN7022_c0_g1_i7_orf1	fatty-acid amide hydrolase 2-A-like [Ostrinia furnacalis]	-0.8391	-1.0966	1.55714	0.73563	-0.3571
TRINITY_DN8654_c0_g1_i1_orf1	argininosuccinate lyase [Ostrinia furnacalis]	-1.3958	0.16627	0.57613	1.43996	-0.7866
TRINITY_DN636_c1_g1_i9_orf1	secretory phospholipase A2 receptor-like [Ostrinia furnacalis]	-1.8328	0.77582	0.26549	0.96816	-0.1767
TRINITY_DN59965_c0_g4_i1_orf1	TKT protein, partial [Homo sapiens]	-1.5561	0.24791	-0.5895	1.37844	0.51919
TRINITY_DN2049_c1_g1_i3_orf1	luciferin 4-monooxygenase-like [Ostrinia furnacalis]	-1.5076	0.57208	-0.2188	1.49522	-0.3409
TRINITY_DN418_c1_g1_i3_orf1	hypothetical protein evm_003996 [Chilo suppressalis]	-1.7125	0.69259	0.27099	1.15985	-0.4109
TRINITY_DN9733_c0_g1_i2_orf1	acidic juvenile hormone-suppressible protein 1-like [Ostrinia furnacalis]	-1.4475	0.86239	-0.2293	1.34974	-0.5353
TRINITY_DN17574_c0_g1_i2_orf1	heat shock protein Hsp-12.2-like [Ostrinia furnacalis]	-1.4682	0.66232	-0.9201	1.0496	0.67637
TRINITY_DN10138_c0_g1_i1_orf1	storage protein 1 [Omphisa fuscidentalis]	-1.7698	0.59723	-0.0794	1.22648	0.02552
TRINITY_DN20009_c0_g1_i1_orf1	vimentin [Homo sapiens] >XP_003831224.1 vimentin [Pan paniscus] >XP_018890043.1 vimentin [Gorilla gorilla gorilla] >XP_024109584.1 vimentin [Pongo abelii] >XP_030675100.1 vimentin [Nomascus leucogenys] >XP_032020652.1 vimentin [Hylobates moloch] >P08670.4 RecName: Full=Vimentin [Homo sapiens] >AIC49963.1 VIM, partial [synthetic construct] >MXR00191.1 hypothetical protein [Bos mutus] >PNI30483.1 VIM isoform 1 [Pan troglodytes] >SJX39704.1 unnamed protein product, partial [Human ORFeome Gateway entry vector] >AAH00163.2 Vimentin [Homo sapiens]	-1.4464	0.52317	-0.6132	1.50232	0.03414
TRINITY_DN42275_c0_g1_i1_orfp1	TRINITY_DN42275_c0_g1_i1_m.44265 TRINITY_DN42275_c0_g1::TRINITY_DN42275_c0_g1_i1::g.44265 ORF type:internal len:76 (+),score=8.67 TRINITY_DN42275_c0_g1_i1:1-225(+)	-1.7028	0.76157	-0.0228	1.20842	-0.2444
TRINITY_DN3301_c0_g1_i2_orf1	hemicentin-2-like isoform X1 [Ostrinia furnacalis]	-1.8346	0.70146	-0.0534	1.05981	0.12678
TRINITY_DN9593_c0_g1_i2_orf1	uncharacterized protein LOC113518937 [Galleria mellonella]	-1.59	0.57016	-0.6368	1.25792	0.39871
TRINITY_DN1569_c0_g1_i6_orf1	uncharacterized protein LOC114350603 [Ostrinia furnacalis]	-1.1552	0.69427	-0.9148	1.52461	-0.1489
TRINITY_DN14250_c0_g1_i1_orf1	apolipophorins-like [Ostrinia furnacalis]	-1.212	1.08205	-0.9575	1.19647	-0.109
TRINITY_DN36144_c0_g1_i3_orf1	nicotinate phosphoribosyltransferase isoform X1 [Ostrinia furnacalis] >XP_028178189.1 nicotinate phosphoribosyltransferase isoform X1 [Ostrinia furnacalis]	-1.563	0.46133	-0.4534	1.45921	0.09594
TRINITY_DN3383_c0_g1_i5_orf1	uncharacterized protein LOC114357426 [Ostrinia furnacalis]	-1.0577	0.88771	-1.2692	1.19223	0.24695
TRINITY_DN30498_c0_g1_i3_orf1	lipase 3-like [Ostrinia furnacalis]	-1.7273	0.90718	0.04327	1.05545	-0.2786
TRINITY_DN4822_c0_g1_i6_orf1	homogentisate 1,2-dioxygenase [Ostrinia furnacalis]	-0.8878	0.33973	-1.1748	1.64633	0.07652
TRINITY_DN1732_c0_g1_i15_orf1	CAD protein isoform X2 [Ostrinia furnacalis]	-1.4275	0.38793	-0.8	1.40965	0.42985
TRINITY_DN46090_c0_g3_i1_orf1	tyrosine-protein kinase-like otk, partial [Ostrinia furnacalis]	-1.3732	0.73485	-0.621	1.46505	-0.2057
TRINITY_DN6462_c0_g1_i5_orf1	probable histone-lysine N-methyltransferase CG1716 [Ostrinia furnacalis]	-1.8359	0.74765	0.04473	1.03365	0.00986
TRINITY_DN21743_c0_g1_i1_orf1	uncharacterized protein LOC114357426 [Ostrinia furnacalis]	-1.4319	0.86509	-0.8565	1.18844	0.2349
TRINITY_DN4394_c0_g2_i1_orf1	carboxylesterase [Ostrinia furnacalis]	-1.5894	0.83133	-0.007	1.24565	-0.4806
TRINITY_DN17368_c0_g1_i6_orf1	protein bark beetle [Ostrinia furnacalis]	-1.5118	0.52407	-0.4703	1.48921	-0.0312
TRINITY_DN101_c0_g1_i4_orf1	disco-interacting protein 2 [Melitaea cinxia]	-1.2436	0.0365	-0.6426	1.73982	0.10995
TRINITY_DN15291_c0_g1_i11_orf1	uncharacterized protein LOC114353772 [Ostrinia furnacalis]	-1.275	1.30054	-0.834	0.97899	-0.1706
TRINITY_DN50237_c0_g1_i8_orf1	LOW QUALITY PROTEIN: uncharacterized protein LOC114361080 [Ostrinia furnacalis]	-1.7047	0.66026	-0.0593	1.27487	-0.1711
TRINITY_DN2271_c0_g1_i12_orf1	plasminogen activator inhibitor 1-like [Ostrinia furnacalis]	-1.7593	0.82475	0.17965	1.05105	-0.2962
TRINITY_DN18273_c0_g1_i4_orf1	venom protease-like [Ostrinia furnacalis] >XP_028156372.1 venom protease-like [Ostrinia furnacalis]	-1.4527	0.38956	-0.6086	1.53229	0.1395
TRINITY_DN70_c6_g1_i1_orf1	optineurin isoform X1 [Ostrinia furnacalis] >XP_028165537.1 optineurin isoform X1 [Ostrinia furnacalis] >XP_028165538.1 optineurin isoform X1 [Ostrinia furnacalis] >XP_028165539.1 optineurin isoform X1 [Ostrinia furnacalis]	-1.8112	0.81263	0.17523	0.99885	-0.1755

TRINITY_DN116874_c0_g1_i1_orfp1	TRINITY_DN116874_c0_g1_i1_m.85176 TRINITY_DN116874_c0_g1::TRINITY_DN116874_c0_g1_i1::g.85176 ORF type:5prime_partial len:95 (+),score=17.10,Baculo_p48 PF04878.14 8.5e-16 TRINITY_DN116874_c0_g1_i1:2-286(+)	-0.9332	0.88176	-1.2568	1.33105	-0.0229
TRINITY_DN32448_c0_g1_i1_orf1	unnamed protein product [Arctia plantaginis] >CAB3252297.1 unnamed protein product [Arctia plantaginis]	-1.409	0.76372	-0.6795	1.4014	-0.0767
TRINITY_DN2897_c0_g2_i1_orf1	gem-associated protein 5-like [Ostrinia furnacalis]	-1.3491	1.29139	-0.816	0.91897	-0.0454
TRINITY_DN12514_c0_g2_i1_orf1	peroxidase isoform X3 [Ostrinia furnacalis]	-1.7671	0.71375	-0.1093	1.16445	-0.0018
TRINITY_DN22674_c0_g1_i2_orf1	protein arginine N-methyltransferase 7 isoform X1 [Ostrinia furnacalis]	-1.4052	0.73343	-0.8827	1.2774	0.27701
TRINITY_DN3275_c0_g1_i4_orf1	calmodulin-like protein 4 [Ostrinia furnacalis]	-0.8927	1.09496	-1.2346	1.20433	-0.172
TRINITY_DN251_c0_g1_i2_orf1	hypothetical protein evm_008466 [Chilo suppressalis]	-1.3479	0.99492	-0.262	1.29126	-0.6763
TRINITY_DN6199_c2_g1_i3_orf1	uncharacterized protein LOC114352137 [Ostrinia furnacalis] >XP_028159411.1 uncharacterized protein LOC114352137 [Ostrinia furnacalis] >XP_028159412.1 uncharacterized protein LOC114352137 [Ostrinia furnacalis]	-1.424	1.12196	-0.9131	0.87331	0.34187
TRINITY_DN67026_c0_g1_i6_orf1	hypothetical protein O3G_MSEX011964 [Manduca sexta]	-0.8845	0.23436	-1.2652	1.56085	0.35452
TRINITY_DN8245_c0_g1_i4_orf1	uncharacterized protein LOC114357622 [Ostrinia furnacalis]	-1.4178	0.4797	-0.9423	1.16793	0.7125
TRINITY_DN781_c0_g1_i7_orf1	uncharacterized protein LOC114356786 [Ostrinia furnacalis]	-1.403	0.36678	-0.8242	1.42367	0.43682
TRINITY_DN12193_c0_g1_i6_orf1	carbonyl reductase [NADPH] 1-like [Ostrinia furnacalis]	-1.551	0.84821	-0.426	1.2912	-0.1624
TRINITY_DN53294_c0_g1_i1_orf1	liver carboxylesterase 2-like [Ostrinia furnacalis]	-1.1938	0.68859	-0.5991	1.58462	-0.4802
TRINITY_DN4270_c0_g1_i1_orf1	cytochrome b-c1 complex subunit 8-like [Ostrinia furnacalis]	-0.8978	1.31491	-1.351	0.78615	0.14771
TRINITY_DN53233_c0_g1_i1_orf1	unnamed protein product, partial [Iphiclides podalirius]	-1.2478	1.32882	-0.9273	0.90229	-0.056
TRINITY_DN6310_c0_g2_i10_orf1	BAG family molecular chaperone regulator 2 isoform X1 [Ostrinia furnacalis] >XP_028163269.1 BAG family molecular chaperone regulator 2 isoform X2 [Ostrinia furnacalis]	-1.7194	0.63682	-0.0179	1.26868	-0.1682
TRINITY_DN1004_c0_g2_i1_orf1	E3 ubiquitin-protein ligase NEDD4 isoform X6 [Ostrinia furnacalis]	-1.5886	0.318	-0.4427	1.45309	0.26023
TRINITY_DN2663_c0_g1_i12_orf1	WW domain-binding protein 2 isoform X1 [Bombyx mori] >XP_028036324.1 WW domain-binding protein 2 [Bombyx mandarina]	-0.9915	0.25561	-0.8133	1.79687	-0.2477
TRINITY_DN4558_c0_g2_i1_orf1	hypothetical protein evm_005814 [Chilo suppressalis]	-1.5983	0.71758	-0.7053	1.08928	0.49668
TRINITY_DN51429_c1_g1_i1_orf1	ER degradation-enhancing alpha-mannosidase-like protein 3 isoform X1 [Ostrinia furnacalis] >XP_028179122.1 ER degradation-enhancing alpha-mannosidase-like protein 3 isoform X2 [Ostrinia furnacalis]	-1.6932	0.69804	0.34352	1.13686	-0.4853
TRINITY_DN12024_c0_g2_i2_orf1	pancreatic lipase-related protein 2 isoform X1 [Ostrinia furnacalis] >XP_028176200.1 pancreatic lipase-related protein 2 isoform X2 [Ostrinia furnacalis]	-1.533	0.9703	-0.6758	1.11159	0.12692
TRINITY_DN6532_c2_g1_i1_orf1	nuclear receptor coactivator 5 isoform X1 [Ostrinia furnacalis] >XP_028175326.1 nuclear receptor coactivator 5 isoform X2 [Ostrinia furnacalis]	-1.6039	0.49629	-0.643	1.21835	0.53225
TRINITY_DN1783_c0_g1_i2_orf1	uncharacterized protein LOC114352783 [Ostrinia furnacalis] >XP_028160309.1 uncharacterized protein LOC114352783 [Ostrinia furnacalis]	-1.3239	0.77289	-0.4851	1.48703	-0.4509
TRINITY_DN18148_c0_g2_i1_orf1	nudC domain-containing protein 1 [Ostrinia furnacalis]	-1.1952	0.26469	-0.9145	1.61643	0.22861
TRINITY_DN213_c0_g1_i3_orf1	unnamed protein product [Chilo suppressalis]	-1.3381	0.65599	-1.0711	1.09443	0.65881
TRINITY_DN1166_c0_g3_i4_orf1	pleckstrin homology domain-containing family F member 2 isoform X1 [Ostrinia furnacalis] >XP_028157822.1 pleckstrin homology domain-containing family F member 2 isoform X2 [Ostrinia furnacalis]	-1.3751	0.87539	-0.3956	1.39253	-0.4972
TRINITY_DN16868_c0_g2_i1_orf1	gamma-glutamylcyclotransferase-like isoform X1 [Ostrinia furnacalis]	-1.1928	0.82491	-1.255	0.86021	0.7627
TRINITY_DN11159_c0_g1_i5_orf1	sphingosine-1-phosphate lyase isoform X2 [Ostrinia furnacalis]	-1.0735	0.94133	-1.2921	1.0849	0.3393
TRINITY_DN5177_c0_g1_i2_orf1	hemolin-like isoform X1 [Ostrinia furnacalis]	-1.7366	-0.0255	-0.1745	0.77097	1.16566
TRINITY_DN18568_c0_g1_i2_orfp1	TRINITY_DN18568_c0_g1_i2_m.13844 TRINITY_DN18568_c0_g1::TRINITY_DN18568_c0_g1_i2::g.13844 ORF type:5prime_partial len:77 (+),score=12.07 TRINITY_DN18568_c0_g1_i2:1-231(+)	-0.8801	-0.7908	-0.769	1.3474	1.09241
TRINITY_DN16234_c0_g2_i3_orf1	uncharacterized protein LOC114363370 [Ostrinia furnacalis]	-1.4257	-0.2805	-0.5589	1.20588	1.05928
TRINITY_DN2146_c0_g2_i1_orf1	heat shock protein 68-like [Ostrinia furnacalis]	-1.488	0.01826	-0.6286	1.35631	0.74208
TRINITY_DN29026_c0_g1_i4_orf1	TIL [Ostrinia furnacalis]	-1.7125	-0.2116	0.15528	0.41837	1.3504
TRINITY_DN276_c0_g1_i1_orf1	protein lethal(2)essential for life-like [Helicoverpa zea] >XP_049705426.1 protein lethal(2)essential for life [Helicoverpa armigera] >ATB54993.1 heat shock protein 20.8 [Helicoverpa armigera] >PZC74337.1 hypothetical protein B5X24_HaOG207971 [Helicoverpa armigera]	-1.4905	0.15398	-0.7438	1.17746	0.90284
TRINITY_DN9608_c0_g1_i3_orf1	cytochrome P450 monooxygenase CYP9G18 [Cnaphalocrocis medinalis]	-1.5219	-0.5372	0.04571	0.57878	1.43463
TRINITY_DN581_c3_g2_i1_orf1	uncharacterized protein LOC114364499 isoform X3 [Ostrinia furnacalis]	-1.5301	-0.4306	-0.1197	0.65612	1.4243

TRINITY_DN142442_c0_g1_i1_orf1	60S ribosomal protein L38 [Homo sapiens] >NP_001002486.1 60S ribosomal protein L38 [Danio rerio] >NP_001030335.1 60S ribosomal protein L38 [Homo sapiens] >NP_001071060.1 60S ribosomal protein L38 [Rattus norvegicus] >NP_001133168.1 60S ribosomal protein L38 [Salmo salar] >NP_001187063.1 60S ribosomal protein L38 [Ictalurus punctatus] >NP_001232305.1 60S ribosomal protein L38 [Taeniopygia guttata] >NP_001264941.1 60S ribosomal protein L38 [Gallus gallus] >XP_003211558.1 60S ribosomal protein L38 [Meleagris gallopavo] >XP_003315754.1 60S ribosomal protein L38 [Pan troglodytes] >XP_003315758.1 60S ribosomal protein L38 [Pan troglodytes] >XP_003339346.1 60S ribosomal protein L38 [Pan troglodytes] >XP_003358038.1 60S ribosomal protein L38 [Sus scrofa] >XP_003417326.1 60S ribosomal protein L38 [Loxodonta africana] >XP_003453439.1 60S ribosomal protein L38 [Oreochromis niloticus] >XP_003464913.2 60S ribosomal protein L38 [Cavia porcellus] >XP_003768586.1 60S ribosomal protein L38 [Sarcophilus harrisii] >XP_003786210.1 60S ribosomal protein L38 [Otolemur garnettii] >XP_003795793.1 60S ribosomal protein L38 [Otolemur garnettii] >XP_003922345.1 60S ribosomal protein L38 [Saimiri boliviensis boliviensis] >XP_004041125.1 60S ribosomal protein L38 [Gorilla gorilla gorilla] >XP_004041126.1 60S ribosomal protein L38 [Gorilla gorilla gorilla] >XP_004041128.1 60S ribosomal protein L38 [Gorilla gorilla gorilla] >XP_004331065.1 60S ribosomal protein L38 [Tursiops truncatus] >XP_004401894.1 PREDICTED: 60S ribosomal protein L38 [Odobenus rosmarus divergens] >XP_004412345.1 PREDICTED: 60S ribosomal protein L38 [Odobenus rosmarus divergens] >XP_004469223.1 60S ribosomal protein L38 [Dasypus novemcinctus] >XP_004469224.1 60S ribosomal protein L38 [Dasypus novemcinctus] >XP_005068761.1 60S ribosomal protein L38 [Mesocricetus auratus] >XP_005070019.1 60S ribosomal protein L38 [Mesocricetus auratus] >XP_005141156.1 60S ribosomal protein L38 [Melopsittacus undulatus] >XP_005336034.1 60S ribosomal protein L38 [Ictidomys tridecemlineatus] >XP_005336035.1 60S ribosomal protein L38 [Ictidomys tridecemlineatus] >XP_005350739.1 60S ribosomal protein L38 [Microtus ochrogaster] >XP_005350740.1 60S ribosomal protein L38 [Microtus ochrogaster] >XP_005412280.1 PREDICTED: 60S ribosomal protein L38 [Chinchilla lanigera] >XP_005412281.1 PREDICTED: 60S ribosomal protein L38 [Chinchilla lanigera] >XP_005530739.1 PREDICTED: 60S ribosomal protein L38 [Pseudopodoces humilis] >XP_005584887.1 60S ribosomal protein L38 [Macaca fascicularis] >XP_005584888.1 60S ribosomal protein L38 [Macaca fascicularis] >XP_005584889.1 60S ribosomal protein L38 [Macaca fascicularis] >XP_005584890.1 60S ribosomal protein L38 [Macaca fascicularis] >XP_005584891.1 60S ribosomal protein L38 [Macaca fascicularis] >XP_005592611.1 60S ribosomal protein L38 [Macaca fascicularis] >XP_005597274.1 60S ribosomal protein L38 isoform X2 [Equus caballus] >XP_005668697.1 60S ribosomal protein L38 [Sus scrofa] >XP_005861853.1 PREDICTED: 60S ribosomal protein L38 [Myotis brandtii] >XP_005861854.1 PREDICTED: 60S ribosomal protein L38 [Myotis brandtii] >XP_005889694.1 PREDICTED: 60S ribosomal protein L38 isoform X2 [Bos mutus] >XP_006042140.1 60S ribosomal protein L38 isoform X2 [Bubalus bubalis] >XP_006042141.1 60S ribosomal protein L38 isoform X2 [Bubalus bubalis] >XP_006082202.1 60S ribosomal protein L38 [Myotis lucifugus] >XP_006106539.1 60S ribosomal protein L38 [Myotis lucifugus] >XP_006109407.1 60S ribosomal protein L38 [Myotis lucifugus] >XP_006145920.1 60S ribosomal protein L38 isoform X1 [Tupaia chinensis] >XP_006145921.1 60S ribosomal protein L38 isoform X1 [Tupaia chinensis] >XP_006754668.1 PREDICTED: 60S ribosomal protein L38 [Myotis davidii] >XP_006754669.1 PREDICTED: 60S ribosomal protein L38 [Myotis davidii] >XP_006912321.1 60S ribosomal protein L38 [Pteropus alecto] >XP_006970499.1 60S ribosomal protein L38 [Peromyscus maniculatus bairdii] >XP_006970500.1 60S ribosomal protein L38 [Peromyscus maniculatus bairdii] >XP_007118971.1 60S ribosomal protein L38 [Physeter catodon] >XP_007118972.1 60S ribosomal protein L38 [Physeter catodon] >XP_007185670.1 60S ribosomal protein L38 [Balaenoptera					
osomal protein L38 [Nematolebias w	60S RIBOSOMAL PROTEIN L38 [Oryctolagus cuniculus] >4UJD_Ak Chain Ak	60S RIBO	60S RIBO	60S ribos	60S ribos	60S ribos
TRINITY_DN1091_c0_g3_i1_orf1	macrophage mannose receptor 1-like [Ostrinia furnacalis]	-1.2184	-0.6768	-0.4613	0.99328	1.36315
TRINITY_DN9325_c0_g1_i1_orf1	protein takeout-like [Ostrinia furnacalis]	-1.7162	0.00291	-0.2631	0.86146	1.11497
TRINITY_DN975_c0_g1_i1_orf1	elongation factor 1-alpha 1 [Myotis lucifugus] >XP_008139741.1 elongation factor 1-alpha 1 [Eptesicus fuscus]	-1.3263	-0.7369	-0.2345	1.18643	1.11137

TRINITY_DN55148_c0_g1_i1_orf1	60S ribosomal protein L11 isoform 1 [Homo sapiens] >NP_001069049.1 60S ribosomal protein L11 [Bos taurus] >NP_001240835.1 60S ribosomal protein L11 isoform 1 [Canis lupus familiaris] >NP_001269300.1 60S ribosomal protein L11 [Chinchilla lanigera] >NP_001291809.1 60S ribosomal protein L11 [Ailuropoda melanoleuca] >NP_080195.1 60S ribosomal protein L11 [Mus musculus] >XP_001504267.1 60S ribosomal protein L11 isoform X2 [Equus caballus] >XP_003471379.1 60S ribosomal protein L11 [Cavia porcellus] >XP_003810808.1 60S ribosomal protein L11 [Pan paniscus] >XP_003891370.1 60S ribosomal protein L11 [Papio anubis] >XP_003989701.1 60S ribosomal protein L11 [Felis catus] >XP_004285699.1 60S ribosomal protein L11 isoform X2 [Orcinus orca] >XP_004377186.1 60S ribosomal protein L11 [Trichechus manatus latirostris] >XP_004394821.1 PREDICTED: 60S ribosomal protein L11 isoform X1 [Odobenus rosmarus divergens] >XP_004465476.2 60S ribosomal protein L11 [Dasypus novemcinctus] >XP_004637663.1 60S ribosomal protein L11 [Octodon degus] >XP_004850617.1 60S ribosomal protein L11 isoform X2 [Heterocephalus glaber] >XP_005544522.1 60S ribosomal protein L11 isoform X1 [Macaca fascicularis] >XP_005676921.1 PREDICTED: 60S ribosomal protein L11 isoform X2 [Capra hircus] >XP_006078005.1 60S ribosomal protein L11 isoform X2 [Bubalus bubalis] >XP_006094108.1 60S ribosomal protein L11 isoform X3 [Myotis lucifugus] >XP_006239286.1 60S ribosomal protein L11 isoform X1 [Rattus norvegicus] >XP_006737645.1 60S ribosomal protein L11 [Leptonychotes weddellii] >XP_006777625.1 PREDICTED: 60S ribosomal protein L11 isoform X1 [Myotis davidii] >XP_006883588.1 PREDICTED: 60S ribosomal protein L11-like isoform X1 [Elephantulus edwardii] >XP_007121151.1 60S ribosomal protein L11 isoform X2 [Physeter catodon] >XP_007175168.1 60S ribosomal protein L11 isoform X1 [Balaenoptera acutorostrata scammoni] >XP_007459239.1 PREDICTED: 60S ribosomal protein L11 isoform X1 [Lipotes vexillifer] >XP_007524793.1 PREDICTED: 60S ribosomal protein L11 [Erinaceus europaeus] >XP_007528779.2 PREDICTED: 60S ribosomal protein L11 [Erinaceus europaeus] >XP_007933904.2 60S ribosomal protein L11 [Orycteropus afer afer] >XP_007978264.1 60S ribosomal protein L11 isoform X1 [Chlorocebus sabaeus] >XP_008059998.1 60S ribosomal protein L11 isoform X2 [Carlito syrichta] >XP_008146324.1 60S ribosomal protein L11 [Eptesicus fuscus] >XP_008263912.1 PREDICTED: 60S ribosomal protein L11 [Oryctolagus cuniculus] >XP_008518979.1 PREDICTED: 60S ribosomal protein L11 isoform X2 [Equus przewalskii] >XP_008571152.1 PREDICTED: 60S ribosomal protein L11 isoform X1 [Galeopterus variegatus] >XP_008571160.1 PREDICTED: 60S ribosomal protein L11 isoform X2 [Galeopterus variegatus] >XP_008846816.1 60S ribosomal protein L11 [Nannospalax galili] >XP_010354068.1 60S ribosomal protein L11 [Rhinopithecus roxellana] >XP_010624280.1 60S ribosomal protein L11 [Fukomys damarensis] >XP_011355886.1 60S ribosomal protein L11 [Pteropus vampyrus] >XP_011761124.1 60S ribosomal protein L11 [Macaca nemestrina] >XP_011833215.1 PREDICTED: 60S ribosomal protein L11 isoform X2 [Mandrillus leucophaeus] >XP_011935575.1 PREDICTED: 60S ribosomal protein L11 [Cercocebus atys] >XP_014710573.1 60S ribosomal protein L11 isoform X2 [Equus asinus] >XP_014930664.1 60S ribosomal protein L11 [Acinonyx jubatus] >XP_014949173.1 60S ribosomal protein L11 isoform X1 [Ovis aries] >XP_014986347.1 60S ribosomal protein L11 [Macaca mulatta] >XP_015341019.2 60S ribosomal protein L11 isoform X1 [Marmota marmota marmota] >XP_016811748.1 60S ribosomal protein L11 [Pan troglodytes] >XP_017390016.1 60S ribosomal protein L11 [Cebus imitator] >XP_017741964.1 PREDICTED: 60S ribosomal protein L11 [Rhinopithecus bieti] >XP_018866494.2 60S ribosomal protein L11 [Gorilla gorilla gorilla] >XP_018868111.1 60S ribosomal protein L11 [Gorilla gorilla gorilla] >XP_019317313.1 PREDICTED: 60S ribosomal protein L11 [Panthera pardus] >XP_019568603.1 PREDICTED: 60S ribosomal protein L11 [Rhinolophus sinicus] >XP_019837113.1 PREDICTED: 60S ribosomal protein L11 isoform X2 [Bos indicus] >XP_020009501.1 60S ribosomal protein L11 [Castor canadensis] >XP_020730251.1 60S ribosomal protein L11 isoform X2 [Odocoileus	-1.5709	-0.2591	-0.3082	0.86437	1.27393
TRINITY_DN22375_c0_g1_i4_orf1	venom carboxylesterase-6-like [Ostrinia furnacalis]	-1.6549	0.05593	-0.428	1.1153	0.91165
TRINITY_DN1305_c0_g1_i6_orf1	glutathione S-transferase sigma 3 [Ostrinia furnacalis]	-0.9352	-0.7544	-0.7544	1.23288	1.2112
TRINITY_DN43667_c0_g1_i1_orf1	carbonyl reductase [NADPH] 1-like [Ostrinia furnacalis]	-1.6749	-0.0304	-0.2594	0.669	1.29573
TRINITY_DN1540_c0_g1_i14_orf1	alaserpin-like isoform X1 [Ostrinia furnacalis]	-1.5962	-0.5189	0.06926	0.81676	1.22916
TRINITY_DN22589_c0_g1_i6_orfp1	TRINITY_DN22589_c0_g1_i6_m.19386 TRINITY_DN22589_c0_g1::TRINITY_DN22589_c0_g1_i6::g.19386 ORF type:internal len:183 (+),score=63.52 TRINITY_DN22589_c0_g1_i6:2-547(+)	-0.5513	-1.0891	-0.7455	0.96091	1.42504
TRINITY_DN4080_c0_g1_i8_orf1	AMP deaminase 2 isoform X3 [Ostrinia furnacalis] >XP_028163647.1 AMP deaminase 2 isoform X3 [Ostrinia furnacalis] >XP_028163648.1 AMP deaminase 2 isoform X3 [Ostrinia furnacalis]	-1.5053	-0.2622	-0.3098	0.58507	1.49228
TRINITY_DN987_c0_g1_i3_orf1	unnamed protein product [Chilo suppressalis]	-1.2989	-0.1544	-0.8215	1.02188	1.25294
TRINITY_DN12367_c0_g1_i8_orf1	aldose reductase-like isoform X2 [Ostrinia furnacalis]	-1.3088	-0.2754	-0.7296	1.18987	1.1239
TRINITY_DN7212_c0_g1_i4_orf1	peptidylglycine alpha-hydroxylating monooxygenase [Ostrinia furnacalis]	-1.5934	0.03657	-0.4722	1.31312	0.71588
TRINITY_DN1012_c0_g2_i1_orf1	teneurin-a isoform X1 [Ostrinia furnacalis]	-1.46	-0.4772	-0.1496	0.57399	1.51285
TRINITY_DN1841_c0_g1_i2_orf1	carboxylesterase [Cnaphalocrocis medinalis]	-1.4363	-0.0527	-0.6349	1.43328	0.69058
TRINITY_DN6680_c0_g1_i1_orf1	hypothetical protein evm_009571 [Chilo suppressalis]	-1.3587	-0.4478	-0.5064	1.26159	1.05133
TRINITY_DN2392_c0_g2_i1_orf1	cytochrome P450 9e2-like [Ostrinia furnacalis] >QPF77612.1 cytochrome P450 monooxygenase CYP9A185 [Ostrinia furnacalis]	-1.4717	-0.4968	-0.1314	0.62247	1.47738
TRINITY_DN7075_c0_g2_i1_orf1	retinal dehydrogenase 1-like [Ostrinia furnacalis]	-1.6216	-0.4074	-0.0528	0.90916	1.17263
TRINITY_DN65974_c0_g1_i2_orf1	uncharacterized protein LOC114362364 [Ostrinia furnacalis]	-0.7537	-0.3233	-1.2172	0.8198	1.47439
TRINITY_DN2847_c0_g1_i20_orf1	uncharacterized protein LOC114352221 [Ostrinia furnacalis]	-1.4772	-0.4687	-0.1379	0.59043	1.49347
TRINITY_DN5081_c0_g1_i5_orf1	ester hydrolase C11orf54 homolog isoform X1 [Ostrinia furnacalis]	-1.6673	-0.4544	0.1437	0.85329	1.12471
TRINITY_DN5126_c0_g1_i3_orf1	cytochrome P450 monooxygenase CYP4L47 [Ostrinia furnacalis]	-0.8964	-1.1557	-0.2827	1.33374	1.00103
TRINITY_DN1732_c0_g1_i17_orf1	CAD protein isoform X2 [Ostrinia furnacalis]	-0.8049	-0.0335	-1.3273	1.43225	0.73346
TRINITY_DN9044_c0_g1_i1_orf1	hypothetical protein SFRURICE_005818, partial [Spodoptera frugiperda]	-1.4638	-0.0864	-0.6421	0.9647	1.22756
TRINITY_DN1493_c0_g1_i5_orf1	uncharacterized protein LOC114350869 [Ostrinia furnacalis]	-1.0668	-0.0635	-0.9385	0.38742	1.68137
TRINITY_DN1103_c0_g1_i12_orf1	retinal dehydrogenase 1-like [Ostrinia furnacalis]	-1.6876	0.18779	-0.3911	0.64864	1.24223
TRINITY_DN5497_c0_g1_i6_orf1	1,2-dihydroxy-3-keto-5-methylthiopentene dioxygenase-like [Ostrinia furnacalis]	-1.7133	-0.0562	-0.082	0.5132	1.33839
TRINITY_DN1540_c0_g1_i9_orf1	alaserpin-like isoform X9 [Ostrinia furnacalis]	-1.0635	-0.8524	-0.4657	0.97874	1.40276
TRINITY_DN50743_c0_g1_i1_orf1	cytochrome P450 monooxygenase CYP321F7 [Ostrinia furnacalis]	-1.0159	-0.9464	-0.4319	1.09836	1.29587
TRINITY_DN104297_c0_g1_i1_orf1	tubulin-specific chaperone D [Ostrinia furnacalis]	-0.9968	-0.1713	-1.0696	0.7128	1.52482
TRINITY_DN76307_c0_g1_i1_orf1	PREDICTED: quinone oxidoreductase-like protein 2 homolog [Microplitis demolitor]	-1.1726	-0.5703	-0.5629	0.75018	1.55567
TRINITY_DN9400_c0_g1_i1_orf1	lysophosphatidylserine lipase ABHD12 isoform X2 [Maniola hyperantus]	-0.3522	-0.7784	-1.0976	0.57444	1.65384

TRINITY_DN512_c1_g1_i4_orf1	protein argonaute-2 [Ostrinia furnacalis] >XP_028167629.1 protein argonaute-2 [Ostrinia furnacalis]	-1.5081	0.09479	-0.6736	0.8368	1.25008
TRINITY_DN108_c0_g1_i1_orf1	cyclic GMP-AMP synthase-like [Ostrinia furnacalis]	-1.5341	-0.1822	-0.4222	0.79661	1.34186
TRINITY_DN6391_c0_g1_i1_orf1	secretory carrier-associated membrane protein 1 [Ostrinia furnacalis]	-1.4465	-0.6204	-0.1444	0.93737	1.274
TRINITY_DN38180_c0_g1_i3_orf1	guanine deaminase [Ostrinia furnacalis]	-1.6169	-0.3354	-0.1221	0.80577	1.26851
TRINITY_DN17913_c0_g1_i8_orf1	fumarylacetoacetase [Ostrinia furnacalis]	-1.4892	-0.1885	-0.3807	1.5209	0.53743
TRINITY_DN33763_c0_g1_i1_orf1	uncharacterized protein LOC114355186 [Ostrinia furnacalis]	-0.6907	-1.2902	-0.3421	1.01456	1.30841
TRINITY_DN30300_c0_g2_i1_orf1	60S acidic ribosomal protein P2 isoform X2 [Ovis aries] >XP_017898197.1 PREDICTED: 60S acidic ribosomal protein P2 [Capra hircus] >XP_020767760.1 60S acidic ribosomal protein P2 [Odocoileus virginianus texanus] >XP_040111416.1 60S acidic ribosomal protein P2 [Oryx dammah] >XP_043307792.1 60S acidic ribosomal protein P2 [Cervus canadensis] >XP_043778683.1 60S acidic ribosomal protein P2 [Cervus elaphus] >KAB0376791.1 hypothetical protein FD755_011235 [Muntiacus reevesi] >OWK17231.1 RPLP2 [Cervus elaphus hippelaphus]	-1.5915	-0.1641	-0.2976	1.37565	0.67759
TRINITY_DN62192_c0_g1_i2_orf1	ELAV-like protein 1 [Ostrinia furnacalis]	-1.3179	-0.0017	-0.7504	1.56289	0.50721
TRINITY_DN11649_c0_g1_i4_orf1	ubiquitin carboxyl-terminal hydrolase 32-like, partial [Ostrinia furnacalis]	-0.4288	-1.1713	-0.5918	0.50792	1.68402
TRINITY_DN4004_c0_g1_i1_orf1	protein FAM114A2 isoform X1 [Ostrinia furnacalis] >XP_028175160.1 protein FAM114A2 isoform X2 [Ostrinia furnacalis]	-0.761	-1.2047	-0.3797	0.97838	1.36689
TRINITY_DN52649_c0_g1_i6_orf1	twinfilin [Ostrinia furnacalis]	-1.5228	-0.6753	0.11418	0.89916	1.18474
TRINITY_DN17423_c0_g1_i2_orf1	Heterogeneous nuclear ribonucleoproteins C1/C2 variant, partial [Homo sapiens]	-1.2695	-0.5294	-0.5042	1.46918	0.83392
TRINITY_DN2655_c0_g2_i1_orf1	DNA fragmentation factor subunit alpha [Ostrinia furnacalis] >XP_028176215.1 DNA fragmentation factor subunit alpha [Ostrinia furnacalis]	-0.4819	-0.2672	-1.4808	1.20795	1.02199
TRINITY_DN37055_c0_g1_i1_orf1	ras GTPase-activating protein-binding protein 2-like, partial [Ostrinia furnacalis]	-0.9916	-1.1434	-0.1755	1.22394	1.08654
TRINITY_DN10484_c0_g1_i8_orf1	hypothetical protein evm_010330 [Chilo suppressalis]	-1.4539	-0.4171	-0.1686	0.47036	1.56922
TRINITY_DN20442_c0_g2_i1_orf1	hypothetical protein evm_008218 [Chilo suppressalis]	-1.4546	1.10153	-0.8329	0.22305	0.96294
TRINITY_DN47842_c0_g1_i1_orf1	protein lethal(2)essential for life-like [Helicoverpa armigera] >PZC74790.1 hypothetical protein B5X24_HaOG207163 [Helicoverpa armigera]	-1.5629	0.97545	-0.7147	0.29962	1.00257
TRINITY_DN4802_c0_g1_i4_orf1	uncharacterized protein LOC114366345 isoform X2 [Ostrinia furnacalis]	-1.4898	-0.5071	0.45952	0.01689	1.52051
TRINITY_DN307_c1_g1_i1_orf1	uncharacterized protein LOC114356704 [Ostrinia furnacalis]	-1.3736	0.40011	-0.9009	0.49852	1.37587
TRINITY_DN14904_c0_g1_i1_orf1	attacin [Ostrinia furnacalis]	-1.2887	-0.2702	0.17531	-0.3756	1.75913
TRINITY_DN30177_c0_g2_i1_orf1	uncharacterized protein LOC114365032 [Ostrinia furnacalis]	-1.2124	-0.092	-0.4291	-0.0911	1.82461
TRINITY_DN140212_c0_g1_i1_orf1	macrophage mannose receptor 1-like [Ostrinia furnacalis]	-1.5552	0.62509	-0.752	0.51683	1.16529
TRINITY_DN2442_c0_g1_i6_orf1	cytochrome P450 6B5-like [Ostrinia furnacalis]	-1.8207	0.02964	0.59383	0.0441	1.15311
TRINITY_DN892_c7_g1_i2_orf1	unnamed protein product [Diatraea saccharalis]	-1.8429	0.45857	0.60135	-0.2108	0.99374
TRINITY_DN20558_c0_g1_i2_orf1	Transient receptor potential channel pyrexia [Operophtera brumata]	-1.5676	-0.1469	0.41585	-0.2181	1.51676
TRINITY_DN63389_c0_g1_i4_orf1	retinol dehydrogenase 14-like [Ostrinia furnacalis]	-1.269	0.65011	-1.1363	0.61694	1.13816
TRINITY_DN13718_c0_g1_i4_orf1	immulectin-4 [Ostrinia furnacalis]	-1.78	0.10143	0.368	0.0123	1.29831
TRINITY_DN17326_c0_g1_i5_orf1	aminoacylase-1-like [Ostrinia furnacalis]	-1.5505	0.51495	-0.7569	0.621	1.17145
TRINITY_DN16840_c1_g1_i1_orf1	attacin-like [Ostrinia furnacalis]	-1.5865	0.49402	0.44681	-0.6339	1.27961
TRINITY_DN131471_c0_g1_i1_orf1	basement membrane-specific heparan sulfate proteoglycan core protein isoform X13 [Ostrinia furnacalis]	-1.6531	0.68942	-0.6196	0.51359	1.06971
TRINITY_DN52761_c0_g1_i2_orf1	atlastin isoform X4 [Ostrinia furnacalis]	-1.7121	0.12025	0.9322	-0.3657	1.02538
TRINITY_DN74020_c0_g1_i2_orf1	unnamed protein product [Euphydryas editha]	-1.4728	0.76981	-0.6815	0.05375	1.33075
TRINITY_DN1897_c0_g2_i4_orf1	phenoloxidase-activating factor 2-like [Hyposmocoma kahamanoa]	-1.352	0.87522	-0.5166	-0.4104	1.40384
TRINITY_DN1759_c0_g1_i4_orf1	protein PFC0760c-like isoform X1 [Ostrinia furnacalis]	-1.689	0.43379	0.80802	-0.553	1.0002
TRINITY_DN31609_c0_g1_i3_orf1	sorbitol dehydrogenase-like [Ostrinia furnacalis]	-1.5264	-0.0378	-0.2174	0.17174	1.6099
TRINITY_DN22242_c0_g1_i1_orf1	juvenile hormone epoxide hydrolase-like [Ostrinia furnacalis] >XP_028170526.1 juvenile hormone epoxide hydrolase-like [Ostrinia furnacalis]	-1.7236	0.0282	0.55505	-0.1612	1.30161
TRINITY_DN6908_c0_g1_i3_orf1	serine--pyruvate aminotransferase, mitochondrial [Ostrinia furnacalis] >XP_028157324.1 serine--pyruvate aminotransferase, mitochondrial [Ostrinia furnacalis] >XP_028157325.1 serine--pyruvate aminotransferase, mitochondrial [Ostrinia furnacalis]	-1.6845	0.38442	0.24459	-0.3083	1.36378
TRINITY_DN955_c0_g1_i2_orf1	gloverin-like [Ostrinia furnacalis] >XP_028168251.1 gloverin-like [Ostrinia furnacalis] >AYM26645.1 gloverin [Ostrinia furnacalis]	-1.5242	0.6259	0.67891	-0.839	1.05839
TRINITY_DN5174_c0_g3_i1_orf1	protein odr-4 homolog [Ostrinia furnacalis]	-1.1057	-0.5832	-0.313	0.18362	1.81817
TRINITY_DN810_c0_g1_i4_orf1	dicer 2 [Ostrinia nubilalis]	-1.508	0.73578	-0.448	-0.1771	1.39734
TRINITY_DN28802_c0_g1_i1_orf1	apolipoprotein D-like [Ostrinia furnacalis]	-1.6779	1.14304	-0.0293	-0.3172	0.88133
TRINITY_DN2743_c0_g1_i5_orf1	regucalcin-like [Ostrinia furnacalis]	-1.4681	0.11422	0.15847	-0.425	1.62045
TRINITY_DN35147_c0_g1_i1_orf1	collagen alpha-2(IV) chain isoform X2 [Ostrinia furnacalis]	-1.3919	0.53865	-0.4117	-0.3169	1.58191
TRINITY_DN4911_c0_g1_i6_orf1	ATP-binding cassette sub-family G member 1-like isoform X2 [Ostrinia furnacalis]	-1.6545	0.24593	-0.1369	0.06931	1.47606
TRINITY_DN14239_c0_g1_i5_orf1	uncharacterized protein LOC114352770 [Ostrinia furnacalis] >XP_028160292.1 uncharacterized protein LOC114352770 [Ostrinia furnacalis]	-1.3432	-0.0876	-0.3923	0.08315	1.7399
TRINITY_DN15157_c0_g1_i1_orf1	UDP-glycosyltransferase UGT40AM2 [Ostrinia furnacalis]	-1.5473	-0.3971	0.40823	0.02584	1.51028

TRINITY_DN3732_c1_g1_i5_orf1	cytochrome P450 6B2-like [Ostrinia furnacalis]	-1.1152	-0.7676	1.00074	-0.5014	1.38353
TRINITY_DN103_c0_g1_i1_orf1	unnamed protein product [Diatraea saccharalis]	-1.462	1.08518	-0.7002	-0.0158	1.09287
TRINITY_DN27833_c0_g2_i1_orf1	uncharacterized protein LOC114359161 [Ostrinia furnacalis]	-1.5503	0.18244	-0.0761	-0.1484	1.59232
TRINITY_DN52761_c0_g2_i1_orf1	atlastin-like isoform X4 [Ostrinia furnacalis]	-1.4086	-0.2044	0.85556	-0.61	1.36747
TRINITY_DN4125_c0_g1_i6_orf1	angiotensin-converting enzyme-like isoform X1 [Ostrinia furnacalis]	-1.5358	1.06018	-0.2884	-0.3738	1.13778
TRINITY_DN27264_c0_g1_i1_orf1	uncharacterized protein LOC114353424 [Ostrinia furnacalis]	-0.9364	1.74177	-0.6874	0.49325	-0.6112
TRINITY_DN3464_c0_g1_i1_orf1	putative mitochondrial aconitate hydratase isoform X1-likeprotein, partial [Cotesia chilonis]	-0.9465	1.61508	-0.8389	0.70861	-0.5383
TRINITY_DN2566_c0_g1_i5_orf1	uncharacterized protein LOC114349936 [Ostrinia furnacalis]	-0.9593	1.55969	-1.043	0.70157	-0.259
TRINITY_DN13221_c0_g1_i3_orf1	fasciclin-3-like [Ostrinia furnacalis]	-0.0193	1.49017	-1.5586	0.45982	-0.3721
TRINITY_DN17329_c0_g2_i3_orf1	uncharacterized protein LOC114354338 isoform X1 [Ostrinia furnacalis]	-0.5746	1.59718	-0.9519	0.74256	-0.8132
TRINITY_DN31377_c0_g2_i1_orf1	phosphatidate cytidylyltransferase, mitochondrial [Ostrinia furnacalis]	-0.2576	0.9617	-1.389	1.30289	-0.618
TRINITY_DN2109_c0_g1_i4_orf1	mucin-2-like isoform X2 [Ostrinia furnacalis]	-0.3081	1.70962	-1.0287	0.46726	-0.8401
TRINITY_DN24689_c0_g1_i1_orf1	thioredoxin domain-containing protein 17-like [Ostrinia furnacalis]	-0.4212	0.93337	-1.7364	0.91799	0.30617
TRINITY_DN14391_c1_g1_i2_orf1	pre-rRNA-processing protein TSR1 homolog [Ostrinia furnacalis]	-0.5099	1.10843	-1.3104	1.23367	-0.5217
TRINITY_DN23732_c0_g1_i1_orf1	glutathione S-transferase 1-like [Ostrinia furnacalis] >QIC35740.1 glutathione S-transferase delta 4 [Ostrinia furnacalis]	-0.8885	1.68444	-0.8093	0.60592	-0.5926
TRINITY_DN34426_c0_g1_i1_orf1	laminin subunit alpha-like, partial [Ostrinia furnacalis]	-0.8033	1.24521	-1.0779	1.16655	-0.5306
TRINITY_DN1833_c0_g1_i5_orf1	uncharacterized protein LOC114356866 isoform X3 [Ostrinia furnacalis] >XP_028166037.1 uncharacterized protein LOC114356866 isoform X3 [Ostrinia furnacalis]	-0.9591	1.01194	-0.3775	1.35834	-1.0337
TRINITY_DN23978_c0_g1_i2_orf1	insulin-like growth factor-binding protein complex acid labile subunit [Ostrinia furnacalis]	-0.8938	1.08126	-0.0958	1.18271	-1.2743
TRINITY_DN4550_c1_g1_i19_orf1	titin homolog [Ostrinia furnacalis]	-0.9715	1.03487	-0.3783	1.33911	-1.0242
TRINITY_DN5462_c0_g2_i1_orf1	acyl-protein thioesterase 1 [Ostrinia furnacalis] >XP_028165602.1 acyl-protein thioesterase 1 [Ostrinia furnacalis] >XP_028165603.1 acyl-protein thioesterase 1 [Ostrinia furnacalis] >XP_028165604.1 acyl-protein thioesterase 1 [Ostrinia furnacalis] >XP_028165606.1 acyl-protein thioesterase 1 [Ostrinia furnacalis] >XP_028165607.1 acyl-protein thioesterase 1 [Ostrinia furnacalis] >XP_028165608.1 acyl-protein thioesterase 1 [Ostrinia furnacalis] >XP_028165609.1 acyl-protein thioesterase 1 [Ostrinia furnacalis] >XP_028165610.1 acyl-protein thioesterase 1 [Ostrinia furnacalis]	-1.0578	1.01257	0.91049	0.47634	-1.3416
TRINITY_DN1491_c0_g1_i4_orf1	GILT-like protein 2 isoform X1 [Ostrinia furnacalis] >XP_028156245.1 GILT-like protein 2 isoform X2 [Ostrinia furnacalis] >XP_028156247.1 GILT-like protein 2 isoform X3 [Ostrinia furnacalis]	-1.0461	1.43535	0.74159	0.00733	-1.1382
TRINITY_DN56690_c0_g1_i4_orf1	hypothetical protein evm_002209, partial [Chilo suppressalis]	-1.2015	1.28358	0.22576	0.79752	-1.1054
TRINITY_DN1091_c0_g2_i10_orf1	macrophage mannose receptor 1-like isoform X2 [Maniola hyperantus]	-0.951	1.44873	0.48995	0.30271	-1.2904
