# Supplementary material for: Comparative Proteomics and Metabonomics Analysis of Different Diapause Stages Revealed a New Regulation Mechanism of Diapause in Loxostege sticticalis (Lepidoptera: Pyralidae)
Source: Molecules. 2024 Jul 25;29(15):3472. doi: 10.3390/molecules29153472 (PMC11314584; doi:10.3390/molecules29153472)
Supplement: Supplementary file 1 [file molecules-29-03472-s001.zip › analysis process/proteomic/Cluster analysis of expression patterns/Up/CTvsPreD up.pdf]

Accession	Description	ND	PreD	RD	CT	D
TRINITY_DN84478_c0_g1_i8_orf1	uncharacterized protein LOC114359035 isoform X1 [Ostrinia furnacalis]	-1.9622	0.50348	0.47386	0.79809	0.18675
TRINITY_DN5080_c0_g1_i5_orf1	storage protein [Ostrinia furnacalis]	-1.9646	0.27671	0.77358	0.61075	0.3036
TRINITY_DN143895_c0_g1_i1_orf1	cathepsin L-like [Aphidius gifuensis] >KAF7988186.1 hypothetical protein HCN44_007680 [Aphidius gifuensis]	-1.9287	0.05375	0.62519	0.85423	0.39558
TRINITY_DN64297_c0_g1_i1_orf1	vanin-like protein 2 isoform X2 [Ostrinia furnacalis]	-1.5609	0.25072	1.33079	0.59363	-0.6142
TRINITY_DN703_c0_g1_i2_orf1	acidic juvenile hormone-suppressible protein 1-like [Ostrinia furnacalis]	-1.9072	0.09245	0.46724	1.01136	0.33613
TRINITY_DN66040_c0_g1_i2_orf1	serine protease inhibitor dipetalogastin-like isoform X2 [Ostrinia furnacalis]	-1.5131	-0.1369	1.52484	0.48616	-0.361
TRINITY_DN4767_c0_g1_i4_orf1	cysteine protease XCP2-like [Ostrinia furnacalis]	-1.9572	0.28587	0.75927	0.67435	0.23768
TRINITY_DN142657_c0_g1_i1_orf1	sorting and assembly machinery component 50 homolog [Diachasma alloeum]	-1.8702	-0.007	0.27013	1.06633	0.54074
TRINITY_DN636_c1_g1_i9_orf1	secretory phospholipase A2 receptor-like [Ostrinia furnacalis]	-1.8328	0.26549	0.77582	0.96816	-0.1767
TRINITY_DN51813_c0_g1_i1_orf1	uncharacterized protein LOC114350216 [Ostrinia furnacalis]	-1.8579	0.07584	0.87806	0.87799	0.02598
TRINITY_DN1370_c0_g1_i2_orf1	hypothetical protein evm_000756 [Chilo suppressalis]	-1.8885	0.09354	1.05558	0.50648	0.23284
TRINITY_DN4767_c0_g1_i6_orf1	cysteine protease XCP2-like [Ostrinia furnacalis]	-1.8723	-0.1558	0.60017	0.9266	0.50133
TRINITY_DN106156_c1_g1_i1_orf1	arylphorin subunit alpha-like [Ostrinia furnacalis]	-1.848	-0.0056	0.47645	1.14116	0.23597
TRINITY_DN59885_c0_g1_i3_orf1	TGF-beta-activated kinase 1 and MAP3K7-binding protein 1-like [Ostrinia furnacalis]	-1.9579	0.09603	0.58876	0.65318	0.6199
TRINITY_DN5080_c0_g1_i1_orf1	basic juvenile hormone-suppressible protein 2-like [Ostrinia furnacalis]	-1.9059	0.09529	0.82977	0.7979	0.1829
TRINITY_DN703_c13_g1_i1_orf1	acidic juvenile hormone-suppressible protein 1-like [Ostrinia furnacalis]	-1.8393	-0.0409	0.39265	1.16504	0.32248
TRINITY_DN7900_c0_g1_i4_orf1	uncharacterized protein LOC114366119 [Ostrinia furnacalis]	-1.9607	0.18363	0.67795	0.72056	0.37852
TRINITY_DN67193_c0_g1_i1_orf1	A-kinase anchor protein 14-like [Ostrinia furnacalis]	-1.8754	-0.1456	0.88028	0.70511	0.4356
TRINITY_DN42719_c0_g2_i1_orf1	inter-alpha-trypsin inhibitor heavy chain H4-like isoform X11 [Ostrinia furnacalis]	-1.872	0.36021	0.91789	0.71352	-0.1196
TRINITY_DN19662_c0_g2_i1_orf1	storage protein [Ostrinia furnacalis]	-1.9057	0.08663	0.72226	0.89322	0.20356
TRINITY_DN7040_c0_g2_i1_orf1	uncharacterized protein LOC114353763 [Ostrinia furnacalis]	-1.9628	0.15188	0.6842	0.66746	0.45922
TRINITY_DN135449_c0_g1_i5_orf1	larval cuticle protein LCP-17-like [Galleria mellonella]	-1.9325	0.09542	0.33742	0.84503	0.6546
TRINITY_DN52316_c0_g1_i1_orf1	arylphorin subunit alpha-like [Ostrinia furnacalis]	-1.8643	-0.1489	0.72891	0.91262	0.37161
TRINITY_DN418_c1_g1_i3_orf1	hypothetical protein evm_003996 [Chilo suppressalis]	-1.7125	0.27099	0.69259	1.15985	-0.4109
TRINITY_DN6122_c0_g1_i6_orf1	deubiquitinase DESI2 isoform X1 [Helicoverpa armigera] >XP_049707835.1 deubiquitinase DESI2 isoform X1 [Helicoverpa armigera]	-1.8841	-0.085	0.76842	0.8568	0.34394
TRINITY_DN295_c2_g1_i2_orf1	phosphoglycolate phosphatase 1A, chloroplastic [Manduca sexta]	-1.8985	0.36483	0.39185	1.04894	0.09291
TRINITY_DN467_c3_g1_i5_orf1	lysozyme precursor [Loxostege sticticalis]	-1.9234	0.02681	0.73528	0.37481	0.78652
TRINITY_DN56308_c0_g1_i2_orf1	storage protein 1 [Omphisa fuscidentalis]	-1.8862	-0.0679	0.66885	0.92946	0.35573
TRINITY_DN3707_c0_g1_i1_orf1	protein FAM160B1-like isoform X1 [Ostrinia furnacalis]	-1.9349	0.11432	0.85775	0.62927	0.33359
TRINITY_DN19662_c4_g1_i1_orf1	basic juvenile hormone-suppressible protein 1-like [Ostrinia furnacalis]	-1.9202	0.1667	0.62002	0.92615	0.2073
TRINITY_DN5444_c0_g1_i1_orfp1	TRINITY_DN5444_c0_g1_i1_m.14077 TRINITY_DN5444_c0_g1::TRINITY_DN5444_c0_g1_i1::g.14077 ORF type:3prime_partial len:90 (-),score=25.11 TRINITY_DN5444_c0_g1_i1:2-268(-)	-1.7446	-0.0085	1.36434	0.29269	0.0961
TRINITY_DN2049_c1_g1_i3_orf1	luciferin 4-monooxygenase-like [Ostrinia furnacalis]	-1.5076	-0.2188	0.57208	1.49522	-0.3409
TRINITY_DN1540_c0_g1_i7_orf1	alaserpin-like isoform X13 [Ostrinia furnacalis]	-1.9589	0.11918	0.58941	0.72358	0.52676
TRINITY_DN276_c0_g1_i2_orf1	protein lethal(2)essential for life-like [Ostrinia furnacalis] >UTU55753.1 small heat shock protein Hsp20.7 [Ostrinia furnacalis]	-1.3206	-0.8035	1.52123	0.5407	0.06218
TRINITY_DN45037_c0_g1_i1_orf1	trafficking protein particle complex subunit 1 [Ostrinia furnacalis]	-1.7565	-0.1949	0.51292	1.25727	0.18122
TRINITY_DN13799_c0_g1_i1_orf1	uncharacterized protein LOC116345248 [Contarinia nasturtii]	-1.6848	-0.5319	0.40844	1.10038	0.70782
TRINITY_DN9538_c1_g3_i1_orf1	cilia- and flagella-associated protein 410 isoform X2 [Aphidius gifuensis]	-1.8452	-0.0339	1.10034	0.5893	0.18954
TRINITY_DN10138_c0_g1_i1_orf1	storage protein 1 [Omphisa fuscidentalis]	-1.7698	-0.0794	0.59723	1.22648	0.02552
TRINITY_DN11826_c0_g1_i4_orf1	aldehyde dehydrogenase X, mitochondrial-like [Ostrinia furnacalis]	-1.8742	-0.1855	0.73404	0.7961	0.52956
TRINITY_DN80328_c0_g1_i9_orf1	arylphorin subunit alpha-like [Ostrinia furnacalis]	-1.8873	-0.0358	0.63307	0.96428	0.32576
TRINITY_DN9733_c0_g1_i2_orf1	acidic juvenile hormone-suppressible protein 1-like [Ostrinia furnacalis]	-1.4475	-0.2293	0.86239	1.34974	-0.5353
TRINITY_DN80328_c0_g1_i5_orf1	arylphorin subunit alpha-like [Ostrinia furnacalis]	-1.8607	-0.1154	0.64937	0.99701	0.32973
TRINITY_DN3616_c0_g1_i4_orf1	conotoxin ArMKLT2-032-like [Ostrinia furnacalis]	-1.4273	-0.0476	1.43024	0.69833	-0.6536
TRINITY_DN9090_c0_g1_i9_orf1	CD63 antigen [Ostrinia furnacalis]	-1.9417	0.29934	0.2053	0.89975	0.53727
TRINITY_DN12526_c0_g1_i5_orf1	uncharacterized protein LOC114359035 isoform X3 [Ostrinia furnacalis]	-1.6672	-0.4693	1.17556	0.2009	0.76011
TRINITY_DN7040_c0_g1_i4_orf1	uncharacterized protein LOC114353763 [Ostrinia furnacalis]	-1.951	0.06429	0.62077	0.66968	0.59627
TRINITY_DN1048_c0_g1_i6_orf1	uncharacterized protein LOC114360661 [Ostrinia furnacalis]	-1.8346	0.37292	0.41615	1.1455	-0.1
TRINITY_DN64772_c0_g1_i1_orf1	aldehyde dehydrogenase, partial [Mythimna separata]	-1.8838	-0.1657	0.77701	0.70898	0.56347
TRINITY_DN17574_c0_g1_i2_orf1	heat shock protein Hsp-12.2-like [Ostrinia furnacalis]	-1.4682	-0.9201	0.66232	1.0496	0.67637
TRINITY_DN48878_c0_g2_i1_orf1	codanin-1 [Ostrinia furnacalis]	-1.8155	-0.293	0.57176	1.00344	0.53329
TRINITY_DN6497_c0_g1_i1_orf1	ommochrome-binding protein-like [Ostrinia furnacalis]	-1.8921	0.13028	0.29728	1.08027	0.38426
TRINITY_DN3301_c0_g1_i2_orf1	hemicentin-2-like isoform X1 [Ostrinia furnacalis]	-1.8346	-0.0534	0.70146	1.05981	0.12678
TRINITY_DN2314_c0_g1_i7_orf1	protein dj-1beta-like isoform X1 [Ostrinia furnacalis]	-1.8251	-0.0474	0.07246	1.04376	0.75626

TRINITY_DN27035_c0_g1_i1_orf1	glucose-6-phosphate isomerase-like [Ostrinia furnacalis]	-1.8418	-0.2544	0.5647	0.92611	0.6054
TRINITY_DN45220_c0_g1_i1_orf1	delta(3,5)-Delta(2,4)-dienoyl-CoA isomerase, mitochondrial isoform X1 [Ostrinia furnacalis]	-1.643	-0.4231	1.34972	0.505	0.21142
TRINITY_DN42275_c0_g1_i1_orfp1	TRINITY_DN42275_c0_g1_i1_m.44265 TRINITY_DN42275_c0_g1::TRINITY_DN42275_c0_g1_i1::g.44265 ORF type:internal len:76 (+),score=8.67 TRINITY_DN42275_c0_g1_i1:1-225(+)	-1.7028	-0.0228	0.76157	1.20842	-0.2444
TRINITY_DN2472_c0_g1_i6_orf1	programmed cell death protein 6 isoform X1 [Colias croceus] >XP_045492459.1 programmed cell death protein 6 isoform X1 [Colias croceus]	-1.9091	0.36408	0.18576	1.04337	0.31586
TRINITY_DN18128_c0_g1_i4_orf1	arylsulfatase B [Ostrinia furnacalis]	-1.8209	-0.2345	0.59579	1.05335	0.40621
TRINITY_DN993_c0_g1_i7_orf1	apolipophorins-like [Ostrinia furnacalis]	-1.5456	-0.4186	1.32924	0.80089	-0.1659
TRINITY_DN46625_c0_g1_i1_orf1	ferritin subunit isoform X1 [Belonocnema kinseyi]	-1.463	-0.458	1.49897	0.60728	-0.1853
TRINITY_DN20009_c0_g1_i1_orf1	vimentin [Homo sapiens] >XP_003831224.1 vimentin [Pan paniscus] >XP_018890043.1 vimentin [Gorilla gorilla gorilla] >XP_024109584.1 vimentin [Pongo abelii] >XP_030675100.1 vimentin [Nomascus leucogenys] >XP_032020652.1 vimentin [Hylobates moloch] >P08670.4 RecName: Full=Vimentin [Homo sapiens] >AIC49963.1 VIM, partial [synthetic construct] >MXR00191.1 hypothetical protein [Bos mutus] >PNI30483.1 VIM isoform 1 [Pan troglodytes] >SJX39704.1 unnamed protein product, partial [Human ORFeome Gateway entry vector] >AAH00163.2 Vimentin [Homo sapiens]	-1.4464	-0.6132	0.52317	1.50232	0.03414
TRINITY_DN81031_c0_g1_i1_orf1	aldehyde dehydrogenase, partial [Ectropis obliqua]	-1.8861	-0.1317	0.77383	0.7842	0.45979
TRINITY_DN20442_c0_g2_i1_orf1	hypothetical protein evm_008218 [Chilo suppressalis]	-1.4546	-0.8329	1.10153	0.22305	0.96294
TRINITY_DN71863_c0_g1_i2_orf1	unnamed protein product [Diatraea saccharalis]	-1.872	-0.1037	0.45201	1.00659	0.51707
TRINITY_DN28711_c0_g1_i1_orf1	hypothetical protein evm_000299 [Chilo suppressalis]	-1.4105	-0.5329	1.39267	0.83903	-0.2883
TRINITY_DN578_c0_g1_i5_orf1	charged multivesicular body protein 7 [Ostrinia furnacalis]	-1.897	0.14821	0.82055	0.83508	0.09319
TRINITY_DN81488_c0_g1_i1_orf1	apolipophorins-like [Ostrinia furnacalis]	-1.4803	-0.4721	1.38699	0.78382	-0.2184
TRINITY_DN85412_c0_g1_i1_orf1	unnamed protein product [Diatraea saccharalis]	-1.4122	-0.5218	1.44709	0.75379	-0.2669
TRINITY_DN9239_c0_g1_i1_orf1	apolipophorins-like [Ostrinia furnacalis]	-1.3268	-0.6047	1.50782	0.71709	-0.2933
TRINITY_DN9593_c0_g1_i2_orf1	uncharacterized protein LOC113518937 [Galleria mellonella]	-1.59	-0.6368	0.57016	1.25792	0.39871
TRINITY_DN97042_c0_g1_i6_orf1	apolipophorins-like [Ostrinia furnacalis]	-1.4997	-0.466	1.34136	0.83166	-0.2074
TRINITY_DN113353_c0_g1_i1_orf1	unnamed protein product [Parnassius apollo]	-1.7803	-0.2351	1.12965	0.67399	0.21173
TRINITY_DN1423_c0_g1_i8_orf1	ferritin subunit-like [Ostrinia furnacalis] >XP_028168186.1 ferritin subunit-like [Ostrinia furnacalis]	-1.5296	-0.2715	1.52636	0.4683	-0.1935
TRINITY_DN699_c0_g2_i1_orf1	TPA_exp: putative parasitoid killing factor [Trichoplusia ni]	-1.4252	-0.5433	1.46045	0.70775	-0.1997
TRINITY_DN6108_c0_g1_i5_orf1	myogenesis-regulating glycosidase-like [Ostrinia furnacalis]	-1.7378	0.29278	0.16979	1.36306	-0.0879
TRINITY_DN399_c3_g2_i6_orf1	proline-rich extensin-like protein EPR1 [Ostrinia furnacalis]	-1.8954	-0.0836	0.60869	0.89849	0.47187
TRINITY_DN376_c1_g1_i1_orf1	matrix metalloproteinase-25-like [Ostrinia furnacalis]	-1.777	-0.1941	1.19591	0.57975	0.19548
TRINITY_DN52944_c0_g1_i1_orf1	apolipophorins-like [Ostrinia furnacalis]	-1.4092	-0.4968	1.44676	0.76292	-0.3037
TRINITY_DN3949_c0_g1_i1_orf1	probable cytochrome P450 304a1 [Ostrinia furnacalis]	-1.7538	-0.1414	1.31291	0.36534	0.21695
TRINITY_DN8780_c0_g1_i3_orf1	uncharacterized protein LOC114363370 [Ostrinia furnacalis]	-1.8012	-0.071	0.15202	1.21233	0.50786
TRINITY_DN4076_c1_g2_i2_orf1	vacuole membrane protein 1 [Ostrinia furnacalis]	-1.8923	-0.1421	0.64851	0.78744	0.59851
TRINITY_DN5132_c0_g1_i4_orf1	small heat shock protein Hsp24.2 [Ostrinia furnacalis]	-1.7392	-0.5187	0.72032	0.71778	0.81979
TRINITY_DN1957_c0_g1_i4_orf1	NAD kinase 2, mitochondrial [Ostrinia furnacalis]	-1.8114	-0.2614	0.36452	1.02515	0.68314
TRINITY_DN1287_c0_g1_i5_orf1	probable chitinase 10 isoform X6 [Ostrinia furnacalis]	-1.8459	-0.2336	0.88095	0.74684	0.45177
TRINITY_DN71699_c0_g1_i1_orf1	apolipophorins-like [Ostrinia furnacalis]	-1.3854	-0.5739	1.43099	0.79524	-0.267
TRINITY_DN28711_c1_g1_i1_orf1	apolipophorins-like [Ostrinia furnacalis]	-1.4115	-0.5321	1.46692	0.71746	-0.2408
TRINITY_DN745_c7_g1_i1_orf1	uncharacterized protein LOC114358822 [Ostrinia furnacalis]	-1.7551	-0.268	0.71535	1.14433	0.1634
TRINITY_DN47842_c0_g1_i1_orf1	protein lethal(2)essential for life-like [Helicoverpa armigera] >PZC74790.1 hypothetical protein B5X24_HaOG207163 [Helicoverpa armigera]	-1.5629	-0.7147	0.97545	0.29962	1.00257
TRINITY_DN6988_c0_g1_i3_orf1	cuticle protein 1-like [Ostrinia furnacalis]	-1.8483	-0.0593	0.88877	0.87776	0.14107
TRINITY_DN9239_c0_g2_i2_orf1	apolipophorins-like [Ostrinia furnacalis]	-1.3082	-0.3552	1.49575	0.75877	-0.5911
TRINITY_DN1144_c0_g1_i10_orf1	TIL [Ostrinia furnacalis]	-1.7699	-0.2926	0.45222	1.1799	0.43037
TRINITY_DN1423_c0_g1_i4_orf1	hypothetical protein evm_003306 [Chilo suppressalis] >CAB3526495.1 unnamed protein product [Chilo suppressalis] >CAH0403823.1 unnamed protein product [Chilo suppressalis]	-1.6014	-0.2159	1.4895	0.40544	-0.0776
TRINITY_DN36144_c0_g1_i3_orf1	nicotinate phosphoribosyltransferase isoform X1 [Ostrinia furnacalis] >XP_028178189.1 nicotinate phosphoribosyltransferase isoform X1 [Ostrinia furnacalis]	-1.563	-0.4534	0.46133	1.45921	0.09594
TRINITY_DN6462_c0_g1_i5_orf1	probable histone-lysine N-methyltransferase CG1716 [Ostrinia furnacalis]	-1.8359	0.04473	0.74765	1.03365	0.00986
TRINITY_DN8985_c0_g1_i4_orf1	cytochrome P450 6B6-like [Ostrinia furnacalis]	-1.6869	-0.5577	1.00686	0.79728	0.44042
TRINITY_DN47609_c0_g1_i1_orfp1	TRINITY_DN47609_c0_g1_i1_m.57205 TRINITY_DN47609_c0_g1::TRINITY_DN47609_c0_g1_i1::g.57205 ORF type:5prime_partial len:68 (-),score=15.96 TRINITY_DN47609_c0_g1_i1:36-239(-)	-1.8101	0.09064	0.19633	1.26998	0.25311
TRINITY_DN11467_c0_g1_i5_orf1	27 kDa hemolymph protein-like, partial [Ostrinia furnacalis]	-1.763	-0.0622	0.35176	1.31923	0.15426
TRINITY_DN625_c2_g2_i2_orf1	L-dopachrome tautomerase yellow-f2-like [Ostrinia furnacalis]	-1.7918	-0.1451	0.4621	1.22074	0.25406

TRINITY_DN2848_c0_g1_i2_orf1	glyceraldehyde-3-phosphate dehydrogenase isoform 1 [Homo sapiens] >NP_001276675.1 glyceraldehyde-3-phosphate dehydrogenase isoform 1 [Homo sapiens] >NP_002037.2 glyceraldehyde-3-phosphate dehydrogenase isoform 1 [Homo sapiens] >XP_003819180.1 glyceraldehyde-3-phosphate dehydrogenase [Pan paniscus] >XP_004052609.1 glyceraldehyde-3-phosphate dehydrogenase [Gorilla gorilla gorilla] >XP_008971979.1 glyceraldehyde-3-phosphate dehydrogenase [Pan paniscus] >XP_008971980.1 glyceraldehyde-3-phosphate dehydrogenase [Pan paniscus] >XP_032621678.1 glyceraldehyde-3-phosphate dehydrogenase [Chelonoidis abingdonii] >XP_508955.1 glyceraldehyde-3-phosphate dehydrogenase isoform X1 [Pan troglodytes] >P04406.3 RecName: Full=Glyceraldehyde-3-phosphate dehydrogenase; Short=GAPDH; AltName: Full=Peptidyl-cysteine S-nitrosylase GAPDH [Homo sapiens] >1U8F_O Crystal Structure Of Human Placental Glyceraldehyde-3-Phosphate Dehydrogenase At 1.75 Resolution [Homo sapiens] >1U8F_P Crystal Structure Of Human Placental Glyceraldehyde-3-Phosphate Dehydrogenase At 1.75 Resolution [Homo sapiens] >1U8F_Q Crystal Structure Of Human Placental Glyceraldehyde-3-Phosphate Dehydrogenase At 1.75 Resolution [Homo sapiens] >1U8F_R Crystal Structure Of Human Placental Glyceraldehyde-3-Phosphate Dehydrogenase At 1.75 Resolution [Homo sapiens] >4WNC_A Crystal structure of human wild-type GAPDH at 1.99 angstroms resolution [Homo sapiens] >4WNC_B Crystal structure of human wild-type GAPDH at 1.99 angstroms resolution [Homo sapiens] >4WNC_C Crystal structure of human wild-type GAPDH at 1.99 angstroms resolution [Homo sapiens] >4WNC_D Crystal structure of human wild-type GAPDH at 1.99 angstroms resolution [Homo sapiens] >4WNC_E Crystal structure of human wild-type GAPDH at 1.99 angstroms resolution [Homo sapiens] >4WNC_F Crystal structure of human wild-type GAPDH at 1.99 angstroms resolution [Homo sapiens] >4WNC_G Crystal structure of human wild-type GAPDH at 1.99 angstroms resolution [Homo sapiens] >4WNC_O Crystal structure of human wild-type GAPDH at 1.99 angstroms resolution [Homo sapiens] >6IQ6_A Crystal structure of GAPDH [Homo sapiens] >6IQ6_B Crystal structure of GAPDH [Homo sapiens] >6IQ6_C Crystal structure of GAPDH [Homo sapiens] >6IQ6_D Crystal structure of GAPDH [Homo sapiens] >6IQ6_E Crystal structure of GAPDH [Homo sapiens] >6IQ6_F Crystal structure of GAPDH [Homo sapiens] >6IQ6_G Crystal structure of GAPDH [Homo sapiens] >6IQ6_H Crystal structure of GAPDH [Homo sapiens] >6YNE_A GAPDH purified from the supernatant of HEK293F cells: crystal form 2 of 4. [Homo sapiens] >6YNE_B GAPDH purified from the supernatant of HEK293F cells: crystal form 2 of 4. [Homo sapiens] >6YNE_C GAPDH purified from the supernatant of HEK293F cells: crystal form 2 of 4. [Homo sapiens] >6YNE_D GAPDH purified from the supernatant of HEK293F cells: crystal form 2 of 4. [Homo sapiens] >AAX42270.1 glyceraldehyde-3-phosphate dehydrogenase [synthetic construct] >MXR00212.1 hypothetical protein [Bos mutus] >SJX33932.1 unnamed protein product, partial [Human ORFeome Gateway entry vector] >AAA52496.1 glyceraldehyde 3-phosphate dehydrogenase (EC 1.2.1.12) [Homo sapiens] >AAA52518.1 glyceraldehyde-3-phosphate dehydrogenase (EC 1.2.1.12) [Homo sapiens]	-1.9079	0.06389	0.60011	0.95533	0.28853
TRINITY_DN1569_c0_g1_i6_orf1	uncharacterized protein LOC114350603 [Ostrinia furnacalis]	-1.1552	-0.9148	0.69427	1.52461	-0.1489
TRINITY_DN2097_c1_g2_i2_orf1	serine protease inhibitor 3 [Ostrinia furnacalis]	-1.6902	-0.3501	1.32318	0.43897	0.27809
TRINITY_DN33272_c0_g1_i1_orf1	unnamed protein product, partial [Iphiclides podalirius]	-1.6621	-0.3188	1.38353	0.44592	0.15137
TRINITY_DN23746_c0_g1_i2_orf1	protein 4.1 homolog isoform X1 [Ostrinia furnacalis]	-1.6572	-0.0723	1.18754	0.85887	-0.3168
TRINITY_DN14250_c0_g1_i1_orf1	apolipophorins-like [Ostrinia furnacalis]	-1.212	-0.9575	1.08205	1.19647	-0.109
TRINITY_DN2170_c0_g2_i1_orf1	beta-1,3-glucan-binding protein-like [Ostrinia furnacalis]	-1.7132	-0.0115	0.10304	1.41879	0.20292
TRINITY_DN8703_c0_g1_i2_orf1	beta-glucuronidase-like isoform X1 [Ostrinia furnacalis] >XP_028166212.1 beta-glucuronidase-like isoform X2 [Ostrinia furnacalis]	-1.6787	-0.2126	1.01919	1.03516	-0.1631
TRINITY_DN4394_c0_g2_i1_orf1	carboxylesterase [Ostrinia furnacalis]	-1.5894	-0.007	0.83133	1.24565	-0.4806
TRINITY_DN89083_c0_g1_i1_orf1	lysine-specific demethylase 4A isoform X2 [Diachasma alloeum]	-1.8642	-0.2257	0.77138	0.72795	0.59059
TRINITY_DN664_c0_g1_i18_orf1	chitinase-like protein EN03 isoform X2 [Ostrinia furnacalis]	-1.7578	-0.4744	0.83039	0.6197	0.78208
TRINITY_DN307_c1_g1_i1_orf1	uncharacterized protein LOC114356704 [Ostrinia furnacalis]	-1.3736	-0.9009	0.40011	0.49852	1.37587
TRINITY_DN2835_c0_g1_i6_orf1	probable isoaspartyl peptidase/L-asparaginase GA20639 [Ostrinia furnacalis]	-1.5731	-0.5732	1.32898	0.62849	0.18883
TRINITY_DN13973_c0_g1_i6_orf1	27 kDa glycoprotein-like [Ostrinia furnacalis]	-1.4116	-0.8075	1.38377	0.63146	0.20394
TRINITY_DN45530_c0_g1_i1_orf1	aldose 1-epimerase isoform X1 [Ostrinia furnacalis] >XP_028178513.1 aldose 1-epimerase isoform X1 [Ostrinia furnacalis] >XP_028178514.1 aldose 1-epimerase isoform X1 [Ostrinia furnacalis]	-1.893	0.04487	0.28991	1.02025	0.538
TRINITY_DN1732_c0_g1_i15_orf1	CAD protein isoform X2 [Ostrinia furnacalis]	-1.4275	-0.8	0.38793	1.40965	0.42985
TRINITY_DN46090_c0_g3_i1_orf1	tyrosine-protein kinase-like otk, partial [Ostrinia furnacalis]	-1.3732	-0.621	0.73485	1.46505	-0.2057
TRINITY_DN50237_c0_g1_i8_orf1	LOW QUALITY PROTEIN: uncharacterized protein LOC114361080 [Ostrinia furnacalis]	-1.7047	-0.0593	0.66026	1.27487	-0.1711
TRINITY_DN21743_c0_g1_i1_orf1	uncharacterized protein LOC114357426 [Ostrinia furnacalis]	-1.4319	-0.8565	0.86509	1.18844	0.2349
TRINITY_DN19110_c0_g1_i2_orf1	peroxidase [Ostrinia furnacalis]	-1.2457	-0.5048	1.62388	0.58694	-0.4603
TRINITY_DN104596_c0_g1_i1_orf1	unnamed protein product [Diatraea saccharalis]	-1.8252	-0.211	0.53982	1.0745	0.42193
TRINITY_DN28729_c0_g1_i9_orf1	serine/threonine-protein kinase mig-15 isoform X2 [Ostrinia furnacalis]	-1.703	-0.5539	0.9415	0.80175	0.51364
TRINITY_DN42854_c0_g3_i2_orf1	amyloid beta (A4) precursor-like protein 2, isoform CRA_b [Homo sapiens]	-1.655	-0.3863	0.9126	1.13084	-0.0021
TRINITY_DN48250_c0_g1_i1_orf1	larval/pupal rigid cuticle protein 66-like [Hyposmocoma kahamanoa]	-1.795	-0.3315	0.83737	0.9055	0.38356
TRINITY_DN140212_c0_g1_i1_orf1	macrophage mannose receptor 1-like [Ostrinia furnacalis]	-1.5552	-0.752	0.62509	0.51683	1.16529
TRINITY_DN7957_c0_g1_i5_orf1	spermidine synthase [Ostrinia furnacalis] >XP_028167892.1 spermidine synthase [Ostrinia furnacalis]	-1.7939	-0.3824	0.59674	0.91596	0.66366
TRINITY_DN5553_c0_g1_i4_orf1	uncharacterized protein LOC114353828 [Ostrinia furnacalis]	-1.2832	-0.558	1.64419	0.498	-0.3009

TRINITY_DN3483_c0_g1_i5_orf1	phenoloxidase-activating factor 2-like isoform X1 [Ostrinia furnacalis] >XP_028178309.1 phenoloxidase-activating factor 2-like isoform X2 [Ostrinia furnacalis]	-1.713	-0.5678	0.80788	0.79082	0.68209
TRINITY_DN1720_c0_g1_i3_orf1	monocarboxylate transporter 12 [Ostrinia furnacalis] >XP_028157531.1 monocarboxylate transporter 12 [Ostrinia furnacalis]	-1.831	-0.3098	0.68212	0.62346	0.83518
TRINITY_DN101_c0_g1_i4_orf1	disco-interacting protein 2 [Melitaea cinxia]	-1.2436	-0.6426	0.0365	1.73982	0.10995
TRINITY_DN15291_c0_g1_i11_orf1	uncharacterized protein LOC114353772 [Ostrinia furnacalis]	-1.275	-0.834	1.30054	0.97899	-0.1706
TRINITY_DN98313_c0_g1_i1_orf1	tetrahydrofolate synthase, partial [Plutella xylostella]	-1.6838	-0.4505	1.04253	0.91925	0.17259
TRINITY_DN44709_c0_g1_i1_orf1	D-beta-hydroxybutyrate dehydrogenase, mitochondrial, partial [Chelonus insularis]	-1.5118	-0.5698	1.26766	0.88213	-0.0682
TRINITY_DN2110_c0_g1_i3_orf1	ribose-phosphate pyrophosphokinase 1 isoform X1 [Chelonus insularis]	-1.7773	-0.403	0.49946	0.9321	0.74873
TRINITY_DN5029_c0_g1_i1_orf1	ribose-phosphate pyrophosphokinase 2-like [Ostrinia furnacalis]	-1.7364	-0.4808	0.82998	0.92029	0.46693
TRINITY_DN18273_c0_g1_i4_orf1	venom protease-like [Ostrinia furnacalis] >XP_028156372.1 venom protease-like [Ostrinia furnacalis]	-1.4527	-0.6086	0.38956	1.53229	0.1395
TRINITY_DN12301_c0_g1_i1_orf1	ribose-phosphate pyrophosphokinase 2 [Ostrinia furnacalis]	-1.6527	-0.449	0.79765	1.19083	0.11317
TRINITY_DN8580_c0_g1_i12_orf1	peroxisomal N(1)-acetyl-spermine/spermidine oxidase-like isoform X1 [Ostrinia furnacalis]	-1.5881	-0.73	1.09311	0.60665	0.61828
TRINITY_DN166_c0_g1_i4_orf1	PREDICTED: cryptochrome-1 isoform X1 [Amyelois transitella] >XP_013199861.1 PREDICTED: cryptochrome-1 isoform X1 [Amyelois transitella]	-1.601	-0.5439	1.33628	0.52374	0.28484
TRINITY_DN32448_c0_g1_i1_orf1	unnamed protein product [Arctia plantaginis] >CAB3252297.1 unnamed protein product [Arctia plantaginis]	-1.409	-0.6795	0.76372	1.4014	-0.0767
TRINITY_DN251_c0_g1_i2_orf1	hypothetical protein evm_008466 [Chilo suppressalis]	-1.3479	-0.262	0.99492	1.29126	-0.6763
TRINITY_DN2897_c0_g2_i1_orf1	gem-associated protein 5-like [Ostrinia furnacalis]	-1.3491	-0.816	1.29139	0.91897	-0.0454
TRINITY_DN131471_c0_g1_i1_orf1	basement membrane-specific heparan sulfate proteoglycan core protein isoform X13 [Ostrinia furnacalis]	-1.6531	-0.6196	0.68942	0.51359	1.06971
TRINITY_DN12286_c1_g1_i2_orf1	sideroflexin-1-3 [Galleria mellonella] >XP_026754161.1 sideroflexin-1-3 [Galleria mellonella]	-1.2111	-0.5167	1.68161	0.49075	-0.4446
TRINITY_DN17326_c0_g1_i5_orf1	aminoacylase-1-like [Ostrinia furnacalis]	-1.5505	-0.7569	0.51495	0.621	1.17145
TRINITY_DN12193_c0_g1_i6_orf1	carbonyl reductase [NADPH] 1-like [Ostrinia furnacalis]	-1.551	-0.426	0.84821	1.2912	-0.1624
TRINITY_DN22674_c0_g1_i2_orf1	protein arginine N-methyltransferase 7 isoform X1 [Ostrinia furnacalis]	-1.4052	-0.8827	0.73343	1.2774	0.27701
TRINITY_DN63389_c0_g1_i4_orf1	retinol dehydrogenase 14-like [Ostrinia furnacalis]	-1.269	-1.1363	0.65011	0.61694	1.13816
TRINITY_DN6199_c2_g1_i3_orf1	uncharacterized protein LOC114352137 [Ostrinia furnacalis] >XP_028159411.1 uncharacterized protein LOC114352137 [Ostrinia furnacalis] >XP_028159412.1 uncharacterized protein LOC114352137 [Ostrinia furnacalis]	-1.424	-0.9131	1.12196	0.87331	0.34187
TRINITY_DN23978_c0_g1_i2_orf1	insulin-like growth factor-binding protein complex acid labile subunit [Ostrinia furnacalis]	-0.8938	-0.0958	1.08126	1.18271	-1.2743
TRINITY_DN1833_c0_g1_i5_orf1	uncharacterized protein LOC114356866 isoform X3 [Ostrinia furnacalis] >XP_028166037.1 uncharacterized protein LOC114356866 isoform X3 [Ostrinia furnacalis]	-0.9591	-0.3775	1.01194	1.35834	-1.0337
TRINITY_DN8245_c0_g1_i4_orf1	uncharacterized protein LOC114357622 [Ostrinia furnacalis]	-1.4178	-0.9423	0.4797	1.16793	0.7125
TRINITY_DN781_c0_g1_i7_orf1	uncharacterized protein LOC114356786 [Ostrinia furnacalis]	-1.403	-0.8242	0.36678	1.42367	0.43682
TRINITY_DN53294_c0_g1_i1_orf1	liver carboxylesterase 2-like [Ostrinia furnacalis]	-1.1938	-0.5991	0.68859	1.58462	-0.4802
TRINITY_DN18650_c0_g1_i1_orf1	bombyxin B-9-like [Ostrinia furnacalis]	-1.3172	-0.7953	1.51982	0.56747	0.0252
TRINITY_DN1004_c0_g2_i1_orf1	E3 ubiquitin-protein ligase NEDD4 isoform X6 [Ostrinia furnacalis]	-1.5886	-0.4427	0.318	1.45309	0.26023
TRINITY_DN8555_c0_g1_i1_orf1	epoxide hydrolase 4-like [Ostrinia furnacalis]	-1.7234	-0.5359	0.87136	0.79434	0.59364
TRINITY_DN4550_c1_g1_i19_orf1	titin homolog [Ostrinia furnacalis]	-0.9715	-0.3783	1.03487	1.33911	-1.0242
TRINITY_DN12024_c0_g2_i2_orf1	pancreatic lipase-related protein 2 isoform X1 [Ostrinia furnacalis] >XP_028176200.1 pancreatic lipase-related protein 2 isoform X2 [Ostrinia furnacalis]	-1.533	-0.6758	0.9703	1.11159	0.12692
TRINITY_DN6532_c2_g1_i1_orf1	nuclear receptor coactivator 5 isoform X1 [Ostrinia furnacalis] >XP_028175326.1 nuclear receptor coactivator 5 isoform X2 [Ostrinia furnacalis]	-1.6039	-0.643	0.49629	1.21835	0.53225
TRINITY_DN2663_c0_g1_i12_orf1	WW domain-binding protein 2 isoform X1 [Bombyx mori] >XP_028036324.1 WW domain-binding protein 2 [Bombyx mandarina]	-0.9915	-0.8133	0.25561	1.79687	-0.2477
TRINITY_DN18148_c0_g2_i1_orf1	nudC domain-containing protein 1 [Ostrinia furnacalis]	-1.1952	-0.9145	0.26469	1.61643	0.22861
TRINITY_DN213_c0_g1_i3_orf1	unnamed protein product [Chilo suppressalis]	-1.3381	-1.0711	0.65599	1.09443	0.65881
TRINITY_DN16868_c0_g2_i1_orf1	gamma-glutamylcyclotransferase-like isoform X1 [Ostrinia furnacalis]	-1.1928	-1.255	0.82491	0.86021	0.7627
TRINITY_DN971_c0_g1_i5_orfp1	TRINITY_DN971_c0_g1_i5_m.54249 TRINITY_DN971_c0_g1::TRINITY_DN971_c0_g1_i5::g.54249 ORF type:internal len:108 (+),score=66.98 TRINITY_DN971_c0_g1_i5:1-321(+)	-1.7632	0.48896	-0.3999	1.04991	0.62427
TRINITY_DN2044_c0_g1_i5_orfp1	TRINITY_DN2044_c0_g1_i5_m.4210 TRINITY_DN2044_c0_g1::TRINITY_DN2044_c0_g1_i5::g.4210 ORF type:complete len:151 (-),score=85.31 TRINITY_DN2044_c0_g1_i5:857-1309(-)	-1.7613	0.44879	-0.4013	1.03947	0.67434
TRINITY_DN15202_c0_g1_i6_orf1	uncharacterized protein LOC114364499 isoform X2 [Ostrinia furnacalis]	-1.6715	0.3374	-0.5354	0.76407	1.10538
TRINITY_DN380_c0_g2_i2_orf1	chemosensory protein 10 [Ostrinia furnacalis]	-1.7971	0.4764	-0.349	0.95239	0.71729
TRINITY_DN114890_c0_g1_i4_orf1	chemosensory protein 10 [Ostrinia furnacalis]	-1.475	0.56866	-0.7429	1.36742	0.28177
TRINITY_DN1880_c0_g1_i4_orf1	serine protease inhibitor dipetalogastin-like [Helicoverpa zea]	-1.6005	0.69273	-0.7326	1.01569	0.62467
TRINITY_DN31348_c0_g1_i1_orf1	protein lethal(2)essential for life [Bombyx mori]	-1.6407	0.45428	-0.5548	1.24357	0.4976
TRINITY_DN5177_c0_g1_i2_orf1	hemolin-like isoform X1 [Ostrinia furnacalis]	-1.7366	-0.1745	-0.0255	0.77097	1.16566

TRINITY_DN8008_c0_g1_i6_orf1	uncharacterized protein LOC114357965 isoform X1 [Ostrinia furnacalis] >XP_028167599.1 uncharacterized protein LOC114357965 isoform X1 [Ostrinia furnacalis] >XP_028167600.1 uncharacterized protein LOC114357965 isoform X2 [Ostrinia furnacalis] >XP_028167601.1 uncharacterized protein LOC114357965 isoform X3 [Ostrinia furnacalis]	-1.4788	0.40514	-0.8444	1.17864	0.73942
TRINITY_DN4204_c0_g1_i1_orf1	uncharacterized protein LOC114359352 [Ostrinia furnacalis]	-0.6383	-0.4705	-1.2259	1.4345	0.90027
TRINITY_DN1597_c0_g1_i5_orfp1	TRINITY_DN1597_c0_g1_i5_m.57494 TRINITY_DN1597_c0_g1::TRINITY_DN1597_c0_g1_i5::g.57494 ORF type:complete len:86 (+),score=7.19 TRINITY_DN1597_c0_g1_i5:134-391(+)	-1.8514	-0.004	0.1037	0.75829	0.99332
TRINITY_DN295_c5_g1_i2_orf1	unnamed protein product [Chilo suppressalis]	-1.729	0.54383	-0.3868	1.19189	0.3801
TRINITY_DN1772_c1_g2_i1_orf1	aldose reductase-like isoform X2 [Ostrinia furnacalis]	-1.483	0.2967	-0.8172	0.86385	1.13962
TRINITY_DN20344_c0_g1_i5_orf1	uncharacterized protein LOC114351483 [Ostrinia furnacalis]	-1.1413	0.39299	-1.2336	0.82177	1.16014
TRINITY_DN43350_c0_g3_i1_orf1	uncharacterized protein LOC114355190 [Ostrinia furnacalis]	-1.3572	0.50193	-1.0414	0.84143	1.05524
TRINITY_DN19731_c0_g1_i1_orf1	allergen Tha p 1-like [Ostrinia furnacalis] >XP_028174916.1 allergen Tha p 1-like [Ostrinia furnacalis] >BAV56808.1 chemosensory protein 4 [Ostrinia furnacalis]	-1.8323	0.27496	-0.1912	0.90423	0.84429
TRINITY_DN124654_c0_g1_i1_orf1	protein lethal(2)essential for life [Manduca sexta] >KAG6441919.1 hypothetical protein O3G_MSEX002019 [Manduca sexta]	-1.656	-0.5998	0.39252	0.86896	0.99432
TRINITY_DN16234_c0_g2_i3_orf1	uncharacterized protein LOC114363370 [Ostrinia furnacalis]	-1.4257	-0.5589	-0.2805	1.20588	1.05928
TRINITY_DN18568_c0_g1_i2_orfp1	TRINITY_DN18568_c0_g1_i2_m.13844 TRINITY_DN18568_c0_g1::TRINITY_DN18568_c0_g1_i2::g.13844 ORF type:5prime_partial len:77 (+),score=12.07 TRINITY_DN18568_c0_g1_i2:1-231(+)	-0.8801	-0.769	-0.7908	1.3474	1.09241
TRINITY_DN21420_c0_g1_i2_orf1	glutathione peroxidase, partial [Ostrinia furnacalis]	-1.84	0.44521	-0.0941	1.13098	0.35796
TRINITY_DN3821_c1_g1_i7_orf1	mitochondrial carrier protein Rim2 isoform X1 [Ostrinia furnacalis]	-1.7959	0.37776	-0.3225	0.95202	0.78856
TRINITY_DN9608_c0_g1_i3_orf1	cytochrome P450 monooxygenase CYP9G18 [Cnaphalocrocis medinalis]	-1.5219	0.04571	-0.5372	0.57878	1.43463
TRINITY_DN1209_c0_g1_i9_orf1	NADP-dependent malic enzyme-like isoform X1 [Ostrinia furnacalis] >XP_028161889.1 NADP-dependent malic enzyme-like isoform X1 [Ostrinia furnacalis] >XP_028161891.1 NADP-dependent malic enzyme-like isoform X3 [Ostrinia furnacalis]	-1.5853	0.27823	-0.5154	1.40264	0.41986
TRINITY_DN10222_c0_g1_i2_orf1	glutathione S-transferase sigma 3 [Ostrinia furnacalis]	-1.3953	0.45478	-0.9739	1.13803	0.77637
TRINITY_DN4068_c0_g2_i4_orf1	larval cuticle protein LCP-17-like precursor [Papilio polytes] >BAM18876.1 cuticular protein PpolCPR2 [Papilio polytes]	-0.8188	0.24256	-1.4621	0.85238	1.18595
TRINITY_DN140_c0_g1_i1_orf1	calcyphosin-like protein [Ostrinia furnacalis]	-1.8274	0.24237	-0.1325	1.09271	0.62481
TRINITY_DN581_c3_g2_i1_orf1	uncharacterized protein LOC114364499 isoform X3 [Ostrinia furnacalis]	-1.5301	-0.1197	-0.4306	0.65612	1.4243
TRINITY_DN7960_c0_g1_i2_orf1	uncharacterized protein LOC114364878 [Ostrinia furnacalis]	-1.5541	0.3175	-0.7169	1.15288	0.80058
TRINITY_DN2146_c0_g2_i1_orf1	heat shock protein 68-like [Ostrinia furnacalis]	-1.488	-0.6286	0.01826	1.35631	0.74208
TRINITY_DN276_c0_g1_i1_orf1	protein lethal(2)essential for life-like [Helicoverpa zea] >XP_049705426.1 protein lethal(2)essential for life [Helicoverpa armigera] >ATB54993.1 heat shock protein 20.8 [Helicoverpa armigera] >PZC74337.1 hypothetical protein B5X24_HaOG207971 [Helicoverpa armigera]	-1.4905	-0.7438	0.15398	1.17746	0.90284
TRINITY_DN18482_c0_g1_i3_orf1	calcyphosin-like protein isoform X3 [Helicoverpa armigera] >XP_047020698.1 calcyphosin-like protein isoform X2 [Helicoverpa zea]	-1.3109	0.42551	-1.0723	1.11023	0.84742
TRINITY_DN12775_c0_g1_i10_orfp1	TRINITY_DN12775_c0_g1_i10_m.21238 TRINITY_DN12775_c0_g1::TRINITY_DN12775_c0_g1_i10::g.21238 ORF type:5prime_partial len:67 (-),score=0.74 TRINITY_DN12775_c0_g1_i10:275-475(-)	-1.7247	0.1233	-0.3412	0.91368	1.02899
TRINITY_DN9325_c0_g1_i1_orf1	protein takeout-like [Ostrinia furnacalis]	-1.7162	-0.2631	0.00291	0.86146	1.11497
TRINITY_DN346_c0_g1_i7_orf1	CDK-activating kinase assembly factor MAT1 [Ostrinia furnacalis]	-1.7074	0.09995	-0.2687	1.28652	0.58956
TRINITY_DN69307_c0_g1_i6_orf1	hypothetical protein evm_010738 [Chilo suppressalis]	-1.7222	-0.125	-0.1464	1.06757	0.92597
TRINITY_DN394_c0_g1_i4_orf1	uncharacterized protein LOC114351483 [Ostrinia furnacalis]	-1.1528	0.15944	-1.1552	1.03687	1.11173

TRINITY_DN142442_c0_g1_i1_orf1	60S ribosomal protein L38 [Homo sapiens] >NP_001002486.1 60S ribosomal protein L38 [Danio rerio] >NP_001030335.1 60S ribosomal protein L38 [Homo sapiens] >NP_001071060.1 60S ribosomal protein L38 [Rattus norvegicus] >NP_001133168.1 60S ribosomal protein L38 [Salmo salar] >NP_001187063.1 60S ribosomal protein L38 [Ictalurus punctatus] >NP_001232305.1 60S ribosomal protein L38 [Taeniopygia guttata] >NP_001264941.1 60S ribosomal protein L38 [Gallus gallus] >XP_003211558.1 60S ribosomal protein L38 [Meleagris gallopavo] >XP_003315754.1 60S ribosomal protein L38 [Pan troglodytes] >XP_003315758.1 60S ribosomal protein L38 [Pan troglodytes] >XP_003339346.1 60S ribosomal protein L38 [Pan troglodytes] >XP_003358038.1 60S ribosomal protein L38 [Sus scrofa] >XP_003417326.1 60S ribosomal protein L38 [Loxodonta africana] >XP_003453439.1 60S ribosomal protein L38 [Oreochromis niloticus] >XP_003464913.2 60S ribosomal protein L38 [Cavia porcellus] >XP_003768586.1 60S ribosomal protein L38 [Sarcophilus harrisii] >XP_003786210.1 60S ribosomal protein L38 [Otolemur garnettii] >XP_003795793.1 60S ribosomal protein L38 [Otolemur garnettii] >XP_003922345.1 60S ribosomal protein L38 [Saimiri boliviensis boliviensis] >XP_004041125.1 60S ribosomal protein L38 [Gorilla gorilla gorilla] >XP_004041126.1 60S ribosomal protein L38 [Gorilla gorilla gorilla] >XP_004041128.1 60S ribosomal protein L38 [Gorilla gorilla gorilla] >XP_004331065.1 60S ribosomal protein L38 [Tursiops truncatus] >XP_004401894.1 PREDICTED: 60S ribosomal protein L38 [Odobenus rosmarus divergens] >XP_004412345.1 PREDICTED: 60S ribosomal protein L38 [Odobenus rosmarus divergens] >XP_004469223.1 60S ribosomal protein L38 [Dasypus novemcinctus] >XP_004469224.1 60S ribosomal protein L38 [Dasypus novemcinctus] >XP_005068761.1 60S ribosomal protein L38 [Mesocricetus auratus] >XP_005070019.1 60S ribosomal protein L38 [Mesocricetus auratus] >XP_005141156.1 60S ribosomal protein L38 [Melopsittacus undulatus] >XP_005336034.1 60S ribosomal protein L38 [Ictidomys tridecemlineatus] >XP_005336035.1 60S ribosomal protein L38 [Ictidomys tridecemlineatus] >XP_005350739.1 60S ribosomal protein L38 [Microtus ochrogaster] >XP_005350740.1 60S ribosomal protein L38 [Microtus ochrogaster] >XP_005412280.1 PREDICTED: 60S ribosomal protein L38 [Chinchilla lanigera] >XP_005412281.1 PREDICTED: 60S ribosomal protein L38 [Chinchilla lanigera] >XP_005530739.1 PREDICTED: 60S ribosomal protein L38 [Pseudopodoces humilis] >XP_005584887.1 60S ribosomal protein L38 [Macaca fascicularis] >XP_005584888.1 60S ribosomal protein L38 [Macaca fascicularis] >XP_005584889.1 60S ribosomal protein L38 [Macaca fascicularis] >XP_005584890.1 60S ribosomal protein L38 [Macaca fascicularis] >XP_005584891.1 60S ribosomal protein L38 [Macaca fascicularis] >XP_005592611.1 60S ribosomal protein L38 [Macaca fascicularis] >XP_005597274.1 60S ribosomal protein L38 isoform X2 [Equus caballus] >XP_005668697.1 60S ribosomal protein L38 [Sus scrofa] >XP_005861853.1 PREDICTED: 60S ribosomal protein L38 [Myotis brandtii] >XP_005861854.1 PREDICTED: 60S ribosomal protein L38 [Myotis brandtii] >XP_005889694.1 PREDICTED: 60S ribosomal protein L38 isoform X2 [Bos mutus] >XP_006042140.1 60S ribosomal protein L38 isoform X2 [Bubalus bubalis] >XP_006042141.1 60S ribosomal protein L38 isoform X2 [Bubalus bubalis] >XP_006082202.1 60S ribosomal protein L38 [Myotis lucifugus] >XP_006106539.1 60S ribosomal protein L38 [Myotis lucifugus] >XP_006109407.1 60S ribosomal protein L38 [Myotis lucifugus] >XP_006145920.1 60S ribosomal protein L38 isoform X1 [Tupaia chinensis] >XP_006145921.1 60S ribosomal protein L38 isoform X1 [Tupaia chinensis] >XP_006754668.1 PREDICTED: 60S ribosomal protein L38 [Myotis davidii] >XP_006754669.1 PREDICTED: 60S ribosomal protein L38 [Myotis davidii] >XP_006912321.1 60S ribosomal protein L38 [Pteropus alecto] >XP_006970499.1 60S ribosomal protein L38 [Peromyscus maniculatus bairdii] >XP_006970500.1 60S ribosomal protein L38 [Peromyscus					
osomal protein L38 [Nematolebias wh	60S RIBOSOMAL PROTEIN L38 [Oryctolagus cuniculus] >4UJD_Ak Chain Ak	60S RIBO	60S RIBO	60S ribos	60S ribos	60S ribos
TRINITY_DN1091_c0_g3_i1_orf1	macrophage mannose receptor 1-like [Ostrinia furnacalis]	-1.2184	-0.4613	-0.6768	0.99328	1.36315
TRINITY_DN1960_c5_g1_i3_orf1	cytochrome P450 monooxygenase CYP9G18 [Cnaphalocrocis medinalis]	-1.6543	0.2563	-0.5487	1.00303	0.94367
TRINITY_DN9435_c0_g1_i7_orf1	uncharacterized protein LOC114350197 [Ostrinia furnacalis]	-1.4207	0.24495	-0.7163	1.50223	0.38978
TRINITY_DN68770_c0_g1_i1_orf1	seroin transcript 1A2 [Ostrinia nubilalis]	-0.724	-0.1714	-1.2571	1.60041	0.55209
TRINITY_DN4242_c0_g1_i6_orf1	fibrohexamerin-like [Ostrinia furnacalis]	-0.5306	-0.0513	-1.5569	1.11643	1.02245
TRINITY_DN13500_c0_g1_i1_orf1	phosphatidylethanolamine-binding protein homolog F40A3.3-like [Ostrinia furnacalis] >XP_028160752.1 phosphatidylethanolamine-binding protein homolog F40A3.3-like [Ostrinia furnacalis]	-1.6949	-0.0004	-0.2089	1.32013	0.58401
TRINITY_DN4695_c0_g1_i3_orf1	glutathione S-transferase epsilon 3 [Ostrinia furnacalis]	-0.4334	-0.1429	-1.5714	1.1643	0.98337
TRINITY_DN20347_c0_g1_i6_orf1	venom polypeptide precursor [Doratifera vulnerans]	-1.5118	-0.0145	-0.4397	1.52542	0.44057
TRINITY_DN3826_c0_g1_i1_orf1	39S ribosomal protein L18, mitochondrial [Ostrinia furnacalis]	-1.1241	0.47951	-1.0952	1.49986	0.23988
TRINITY_DN244_c1_g1_i5_orf1	C-1-tetrahydrofolate synthase, cytoplasmic isoform X3 [Ostrinia furnacalis]	-1.827	-0.1308	0.20565	0.69235	1.0598
TRINITY_DN43667_c0_g1_i1_orf1	carbonyl reductase [NADPH] 1-like [Ostrinia furnacalis]	-1.6749	-0.2594	-0.0304	0.669	1.29573
TRINITY_DN135679_c0_g1_i2_orfp1	TRINITY_DN135679_c0_g1_i2_m.85525 TRINITY_DN135679_c0_g1::TRINITY_DN135679_c0_g1_i2::g.85525 ORF type:5prime_partial len:55 (+),score=5.08,Toxin_2 PF00451.20 1.9e-06 TRINITY_DN135679_c0_g1_i2:3-167(+)	-1.042	0.61395	-1.3145	1.25296	0.48952
TRINITY_DN22375_c0_g1_i4_orf1	venom carboxylesterase-6-like [Ostrinia furnacalis]	-1.6549	-0.428	0.05593	1.1153	0.91165
TRINITY_DN4314_c0_g1_i9_orf1	serine proteinase inhibitor 2 [Ostrinia furnacalis]	-1.1955	0.44553	-1.1943	1.13917	0.80511
TRINITY_DN4080_c0_g1_i8_orf1	AMP deaminase 2 isoform X3 [Ostrinia furnacalis] >XP_028163647.1 AMP deaminase 2 isoform X3 [Ostrinia furnacalis] >XP_028163648.1 AMP deaminase 2 isoform X3 [Ostrinia furnacalis]	-1.5053	-0.3098	-0.2622	0.58507	1.49228
TRINITY_DN28299_c0_g1_i1_orf1	adenylosuccinate lyase isoform X1 [Ostrinia furnacalis]	-1.5833	0.20884	-0.6274	1.1626	0.83925
TRINITY_DN8637_c0_g1_i1_orf1	superoxide dismutase [Cu-Zn] [Ostrinia furnacalis] >XP_028177872.1 superoxide dismutase [Cu-Zn] [Ostrinia furnacalis]	-1.8718	-0.1377	0.3846	0.74393	0.88093
TRINITY_DN3738_c0_g1_i5_orf1	23 kDa integral membrane protein-like [Ostrinia furnacalis]	-1.5638	-0.1117	-0.3643	1.42541	0.61442
TRINITY_DN2618_c0_g1_i3_orf1	CDP-diacylglycerol--inositol 3-phosphatidyltransferase [Ostrinia furnacalis]	-1.7362	0.07561	-0.262	0.78345	1.13909
TRINITY_DN15478_c0_g1_i1_orf1	STE20/SPS1-related proline-alanine-rich protein kinase [Vanessa tameamea] >XP_047534294.1 STE20/SPS1-related proline-alanine-rich protein kinase-like [Vanessa atalanta]	-1.6554	-0.2434	-0.1716	1.15264	0.9178
TRINITY_DN7212_c0_g1_i4_orf1	peptidylglycine alpha-hydroxylating monooxygenase [Ostrinia furnacalis]	-1.5934	-0.4722	0.03657	1.31312	0.71588

TRINITY_DN2772_c0_g1_i3_orf1	uncharacterized protein LOC114353284 isoform X4 [Ostrinia furnacalis] >XP_028161011.1 uncharacterized protein LOC114353284 isoform X4 [Ostrinia furnacalis] >XP_028161012.1 uncharacterized protein LOC114353284 isoform X4 [Ostrinia furnacalis]	-1.2004	0.21757	-1.0565	1.41714	0.62222
TRINITY_DN2392_c0_g2_i1_orf1	cytochrome P450 9e2-like [Ostrinia furnacalis] >QPF77612.1 cytochrome P450 monooxygenase CYP9A185 [Ostrinia furnacalis]	-1.4717	-0.1314	-0.4968	0.62247	1.47738
TRINITY_DN8310_c0_g2_i1_orf1	uncharacterized protein LOC116773294 [Danaus plexippus plexippus] >OWR55545.1 hypothetical protein KGM_209260 [Danaus plexippus plexippus]	-0.4472	0.36911	-1.7304	1.03521	0.77328
TRINITY_DN1305_c0_g1_i6_orf1	glutathione S-transferase sigma 3 [Ostrinia furnacalis]	-0.9352	-0.7544	-0.7544	1.23288	1.2112
TRINITY_DN12367_c0_g1_i8_orf1	aldose reductase-like isoform X2 [Ostrinia furnacalis]	-1.3088	-0.7296	-0.2754	1.18987	1.1239
TRINITY_DN987_c0_g1_i3_orf1	unnamed protein product [Chilo suppressalis]	-1.2989	-0.8215	-0.1544	1.02188	1.25294
TRINITY_DN6680_c0_g1_i1_orf1	hypothetical protein evm_009571 [Chilo suppressalis]	-1.3587	-0.5064	-0.4478	1.26159	1.05133
TRINITY_DN1328_c0_g1_i6_orf1	fungal protease inhibitor-1-like [Ostrinia furnacalis]	-1.2203	0.31708	-1.112	1.27504	0.74022
TRINITY_DN1841_c0_g1_i2_orf1	carboxylesterase [Cnaphalocrocis medinalis]	-1.4363	-0.6349	-0.0527	1.43328	0.69058
TRINITY_DN22589_c0_g1_i6_orfp1	TRINITY_DN22589_c0_g1_i6_m.19386 TRINITY_DN22589_c0_g1::TRINITY_DN22589_c0_g1_i6::g.19386 ORF type:internal len:183 (+),score=63.52 TRINITY_DN22589_c0_g1_i6:2-547(+)	-0.5513	-0.7455	-1.0891	0.96091	1.42504
TRINITY_DN1084_c0_g1_i2_orf1	ATP-citrate synthase [Ostrinia furnacalis]	-1.5429	0.10453	-0.6055	1.29863	0.74526
TRINITY_DN7075_c0_g2_i1_orf1	retinal dehydrogenase 1-like [Ostrinia furnacalis]	-1.6216	-0.0528	-0.4074	0.90916	1.17263
TRINITY_DN18782_c0_g1_i4_orf1	putative riboflavin kinase [Ostrinia furnacalis] >XP_028176654.1 putative riboflavin kinase [Ostrinia furnacalis]	-0.6062	-0.0715	-1.4963	0.97421	1.19976
TRINITY_DN31163_c1_g1_i4_orf1	phenoloxidase subunit 2-like [Ostrinia furnacalis]	-1.2361	0.2996	-0.8351	1.6327	0.13885
TRINITY_DN41296_c0_g1_i1_orf1	exocyst complex component 5 [Ostrinia furnacalis]	-1.7361	-0.3985	0.25848	0.9235	0.95262
TRINITY_DN9044_c0_g1_i1_orf1	hypothetical protein SFRURICE_005818, partial [Spodoptera frugiperda]	-1.4638	-0.6421	-0.0864	0.9647	1.22756
TRINITY_DN5126_c0_g1_i3_orf1	cytochrome P450 monooxygenase CYP4L47 [Ostrinia furnacalis]	-0.8964	-0.2827	-1.1557	1.33374	1.00103
TRINITY_DN6415_c0_g1_i1_orf1	D-arabinitol dehydrogenase 1-like [Ostrinia furnacalis]	-0.4837	0.07941	-1.5894	0.64603	1.34766
TRINITY_DN55154_c0_g2_i1_orf1	glycosyl transferase family 8 domain-containing protein [Phthorimaea operculella]	-0.4107	-0.6838	-1.1904	1.55258	0.73229
TRINITY_DN50743_c0_g1_i1_orf1	cytochrome P450 monooxygenase CYP321F7 [Ostrinia furnacalis]	-1.0159	-0.4319	-0.9464	1.09836	1.29587
TRINITY_DN76307_c0_g1_i1_orf1	PREDICTED: quinone oxidoreductase-like protein 2 homolog [Microplitis demolitor]	-1.1726	-0.5629	-0.5703	0.75018	1.55567
TRINITY_DN50074_c0_g1_i1_orf1	uncharacterized protein LOC114364628 [Ostrinia furnacalis]	-0.4551	0.37502	-1.6664	1.29178	0.45467
TRINITY_DN62_c1_g1_i3_orf1	D-2-hydroxyglutarate dehydrogenase, mitochondrial-like [Ostrinia furnacalis]	-0.4301	0.04312	-1.6424	1.18262	0.84676
TRINITY_DN30300_c0_g2_i1_orf1	60S acidic ribosomal protein P2 isoform X2 [Ovis aries] >XP_017898197.1 PREDICTED: 60S acidic ribosomal protein P2 [Capra hircus] >XP_020767760.1 60S acidic ribosomal protein P2 [Odocoileus virginianus texanus] >XP_040111416.1 60S acidic ribosomal protein P2 [Oryx dammah] >XP_043307792.1 60S acidic ribosomal protein P2 [Cervus canadensis] >XP_043778683.1 60S acidic ribosomal protein P2 [Cervus elaphus] >KAB0376791.1 hypothetical protein FD755_011235 [Muntiacus reevesi] >OWK17231.1 RPLP2 [Cervus elaphus hippelaphus]	-1.5915	-0.2976	-0.1641	1.37565	0.67759
TRINITY_DN17913_c0_g1_i8_orf1	fumarylacetoacetase [Ostrinia furnacalis]	-1.4892	-0.3807	-0.1885	1.5209	0.53743
TRINITY_DN616_c1_g1_i6_orf1	esterase B1-like isoform X1 [Ostrinia furnacalis] >XP_028178578.1 esterase B1-like isoform X2 [Ostrinia furnacalis]	-0.3794	-0.0064	-1.6577	1.12166	0.9219
TRINITY_DN33763_c0_g1_i1_orf1	uncharacterized protein LOC114355186 [Ostrinia furnacalis]	-0.6907	-0.3421	-1.2902	1.01456	1.30841
TRINITY_DN4004_c0_g1_i1_orf1	protein FAM114A2 isoform X1 [Ostrinia furnacalis] >XP_028175160.1 protein FAM114A2 isoform X2 [Ostrinia furnacalis]	-0.761	-0.3797	-1.2047	0.97838	1.36689
TRINITY_DN133474_c0_g2_i2_orf1	enoyl-[acyl-carrier-protein] reductase, mitochondrial [Ostrinia furnacalis]	-0.3014	-0.7971	-1.223	1.36522	0.9562
TRINITY_DN17907_c0_g1_i13_orf1	androgen-induced gene 1 protein-like isoform X1 [Galleria mellonella]	-0.3679	-0.2003	-1.5717	0.88992	1.24992
TRINITY_DN51995_c0_g3_i1_orf1	circadian clock-controlled protein-like [Ostrinia furnacalis]	-1.03	-0.2009	-0.8234	1.77233	0.28199
TRINITY_DN16091_c0_g1_i1_orfp1	TRINITY_DN16091_c0_g1_i1_m.64010 TRINITY_DN16091_c0_g1::TRINITY_DN16091_c0_g1_i1::g.64010 ORF type:5prime_partial len:124 (-),score=7.29,Toxin_2 PF00451.20 0.00035,Toxin_2|PF00451.20|0.00013,Toxin_2|PF00451.20|0.00037,Gamma-thionin|PF00304.21|0.37,Gamma-thionin|PF00304.21|0.052,Gamma-thionin|PF00304.21|0.022,Defensin_2|PF01097.19|0.58,Defensin_2|PF01097.19|0.12,Defensin_2|PF01097.19|0.011,Toxin_38|PF14866.7|0.18,Toxin_38|PF14866.7|0.18,Toxin_38|PF14866.7|0.4 TRINITY_DN16091_c0_g1_i1:19-390(-)	-1.4033	-0.0215	-0.2869	1.71696	-0.0053
TRINITY_DN15682_c0_g1_i4_orf1	seroin transcript 1B [Ostrinia nubilalis]	-1.1209	0.68044	-0.5648	1.61085	-0.6056
TRINITY_DN143532_c0_g1_i1_orf1	3-oxoacyl-[acyl-carrier-protein] reductase FabG-like [Aphidius gifuensis] >KAF7996667.1 hypothetical protein HCN44_002313 [Aphidius gifuensis]	-0.7626	-0.6005	-0.892	1.72741	0.5276
TRINITY_DN1534_c0_g1_i3_orf1	peptidoglycan recognition protein-like [Ostrinia furnacalis]	-1.0596	-0.4714	-0.625	1.76361	0.39243
TRINITY_DN128231_c0_g1_i5_orf1	glutathione S-transferase sigma3 [Glyphodes pyloalis]	-0.4286	-0.3961	-0.1912	1.94093	-0.925

TRINITY_DN17693_c0_g1_i10_orf1	acetylcholinesterase-like [Ostrinia furnacalis]	-0.8678	-0.4822	0.1234	1.88745	-0.6608
TRINITY_DN26789_c0_g1_i2_orf1	D-2-hydroxyglutarate dehydrogenase, mitochondrial-like [Ostrinia furnacalis]	-1.358	-0.1156	-0.3229	1.74216	0.05438
TRINITY_DN21341_c0_g1_i1_orf1	FAST kinase domain-containing protein 4 isoform X6 [Ostrinia furnacalis] >XP_028160336.1 FAST kinase domain-containing protein 4 isoform X7 [Ostrinia furnacalis] >XP_028160337.1 FAST kinase domain-containing protein 4 isoform X8 [Ostrinia furnacalis]	-0.4394	-0.6023	-0.3207	1.98758	-0.6252
TRINITY_DN9458_c0_g1_i4_orf1	uncharacterized protein LOC114363583 [Ostrinia furnacalis]	-0.2072	-0.7053	-0.7505	1.95256	-0.2896
TRINITY_DN7556_c0_g1_i3_orf1	jg27436 [Pararge aegeria aegeria]	-1.3883	0.16146	-0.1483	1.70714	-0.332
TRINITY_DN2338_c0_g2_i1_orf1	prophenoloxidase PPO3 [Ostrinia furnacalis]	-1.0157	0.17361	-0.1384	1.80143	-0.8209
TRINITY_DN30713_c0_g1_i3_orf1	phosphoglucomutase [Ostrinia furnacalis]	-0.3786	-0.1871	-1.2486	1.80629	0.00795
TRINITY_DN109503_c0_g1_i4_orf1	uncharacterized protein LOC114366345 isoform X2 [Ostrinia furnacalis]	-0.1097	-0.9471	-0.8489	1.83449	0.07113
TRINITY_DN12594_c0_g1_i1_orf1	DNA-directed RNA polymerases I and III subunit RPAC1 [Ostrinia furnacalis]	-0.7571	-0.9031	-0.3328	1.86666	0.12636
TRINITY_DN19866_c0_g1_i4_orf1	lys-63-specific deubiquitinase BRCC36-like [Ostrinia furnacalis]	-0.5354	-0.8024	-0.3493	1.967	-0.2798
TRINITY_DN5564_c0_g1_i5_orf1	probable phosphoserine aminotransferase [Ostrinia furnacalis]	-0.2454	-0.2394	-1.4022	1.69714	0.18988
TRINITY_DN8771_c0_g2_i1_orf1	regucalcin-like [Ostrinia furnacalis]	0.56095	-0.6006	-0.404	1.64712	-1.2035
TRINITY_DN1628_c0_g2_i3_orf1	uncharacterized protein LOC114363979 [Ostrinia furnacalis]	-0.5491	-0.2848	-0.0842	1.90626	-0.9881
TRINITY_DN2338_c0_g1_i3_orf1	phenoloxidase subunit 1-like [Ostrinia furnacalis]	-0.0718	-0.0224	-0.6196	1.83265	-1.1188
TRINITY_DN54410_c0_g2_i1_orf1	lysozyme-like [Ostrinia furnacalis]	-0.416	-0.8215	-0.9193	1.77878	0.37802
TRINITY_DN51342_c0_g1_i7_orf1	prophenoloxidase [Ostrinia furnacalis]	-0.0895	-0.3584	-1.2084	1.83601	-0.1797
TRINITY_DN113327_c0_g1_i2_orf1	proteasome subunit beta type-6 [Helicoverpa armigera] >XP_047031479.1 proteasome subunit beta type-6 [Helicoverpa zea] >XP_049697949.1 proteasome subunit beta type-6-like [Helicoverpa armigera] >PZC87318.1 hypothetical protein B5X24_HaOG201554 [Helicoverpa armigera]	-0.709	-0.3105	-0.3679	1.97883	-0.5915
TRINITY_DN11808_c0_g1_i8_orf1	unnamed protein product [Diatraea saccharalis]	-0.2515	-0.8693	-0.8229	1.87047	0.07315
TRINITY_DN4390_c0_g1_i4_orf1	GILT-like protein 1 [Ostrinia furnacalis]	0.11944	-0.7634	-0.1625	1.8265	-1.02
TRINITY_DN1785_c0_g1_i5_orf1	beta-mannosidase [Ostrinia furnacalis]	0.17189	-1.1412	-0.6828	1.78449	-0.1324
TRINITY_DN3092_c0_g1_i2_orf1	replication factor C subunit 1 isoform X1 [Ostrinia furnacalis] >XP_028157702.1 replication factor C subunit 1 isoform X2 [Ostrinia furnacalis]	0.4809	-0.6326	-0.7081	1.75231	-0.8925
TRINITY_DN102712_c0_g1_i1_orf1	transmembrane protein 177 [Ostrinia furnacalis]	0.53319	-0.0506	-0.2559	1.40683	-1.6336
TRINITY_DN12671_c0_g1_i6_orf1	hemicentin-1-like isoform X1 [Ostrinia furnacalis]	-1.0512	-0.3467	1.87108	0.04789	-0.521
TRINITY_DN49147_c0_g2_i1_orf1	glutenin, high molecular weight subunit PW212-like [Ostrinia furnacalis]	-1.1183	-0.6812	1.81086	0.04937	-0.0607
TRINITY_DN3383_c0_g1_i5_orf1	uncharacterized protein LOC114357426 [Ostrinia furnacalis]	-1.0577	-1.2692	0.88771	1.19223	0.24695
TRINITY_DN27264_c0_g1_i1_orf1	uncharacterized protein LOC114353424 [Ostrinia furnacalis]	-0.9364	-0.6874	1.74177	0.49325	-0.6112
TRINITY_DN138481_c0_g1_i5_orf1	hypothetical protein evm_003901 [Chilo suppressalis]	-0.869	-0.6123	1.93649	-0.1374	-0.3177
TRINITY_DN4822_c0_g1_i6_orf1	homogentisate 1,2-dioxygenase [Ostrinia furnacalis]	-0.8878	-1.1748	0.33973	1.64633	0.07652
TRINITY_DN3464_c0_g1_i1_orf1	putative mitochondrial aconitate hydratase isoform X1-likeprotein, partial [Cotesia chilonis]	-0.9465	-0.8389	1.61508	0.70861	-0.5383
TRINITY_DN1110_c1_g1_i9_orf1	MD-2-related lipid-recognition protein-like [Ostrinia furnacalis]	-0.9686	-0.8699	1.77041	0.32427	-0.2562
TRINITY_DN15175_c0_g1_i1_orf1	zinc carboxypeptidase-like [Ostrinia furnacalis]	-0.7526	-0.6799	1.94642	-0.0977	-0.4163
TRINITY_DN2684_c0_g2_i3_orf1	glutamate decarboxylase 1-like isoform X1 [Ostrinia furnacalis]	-1.1274	-0.7601	1.76077	0.2098	-0.0831
TRINITY_DN2566_c0_g1_i5_orf1	uncharacterized protein LOC114349936 [Ostrinia furnacalis]	-0.9593	-1.043	1.55969	0.70157	-0.259
TRINITY_DN3952_c0_g1_i3_orf1	protein Skeletor, isoforms D/E-like isoform X1 [Ostrinia furnacalis] >XP_028176405.1 protein Skeletor, isoforms D/E-like isoform X2 [Ostrinia furnacalis] >XP_028176406.1 protein Skeletor, isoforms D/E-like isoform X3 [Ostrinia furnacalis] >XP_028176407.1 protein Skeletor, isoforms D/E-like isoform X4 [Ostrinia furnacalis]	-1.0343	-0.9681	1.65249	0.49199	-0.1421
TRINITY_DN116874_c0_g1_i1_orfp1	TRINITY_DN116874_c0_g1_i1_m.85176 TRINITY_DN116874_c0_g1::TRINITY_DN116874_c0_g1_i1::g.85176 ORF type:5prime_partial len:95 (+),score=17.10,Baculo_p48 PF04878.14 8.5e-16 TRINITY_DN116874_c0_g1_i1:2-286(+)	-0.9332	-1.2568	0.88176	1.33105	-0.0229
TRINITY_DN3227_c0_g1_i5_orf1	transmembrane emp24 domain-containing protein 5-like isoform X1 [Ostrinia furnacalis] >XP_028176732.1 transmembrane emp24 domain-containing protein 5-like isoform X2 [Ostrinia furnacalis]	-0.3242	-1.7647	1.16139	0.49221	0.43532
TRINITY_DN13435_c0_g1_i1_orf1	putative mediator of RNA polymerase II transcription subunit 26 [Ostrinia furnacalis]	-0.5256	-1.0523	0.93919	1.44415	-0.8054
TRINITY_DN9920_c0_g1_i1_orf1	uncharacterized protein LOC114351526 [Ostrinia furnacalis]	-1.239	-0.7064	1.70778	0.01599	0.22161
TRINITY_DN3275_c0_g1_i4_orf1	calmodulin-like protein 4 [Ostrinia furnacalis]	-0.8927	-1.2346	1.09496	1.20433	-0.172
TRINITY_DN54269_c0_g1_i3_orf1	lopap-like [Ostrinia furnacalis]	-0.838	-1.1303	1.72948	0.10137	0.13741
TRINITY_DN67026_c0_g1_i6_orf1	hypothetical protein O3G_MSEX011964 [Manduca sexta]	-0.8845	-1.2652	0.23436	1.56085	0.35452
TRINITY_DN6203_c0_g1_i1_orfp1	TRINITY_DN6203_c0_g1_i1_m.72736 TRINITY_DN6203_c0_g1::TRINITY_DN6203_c0_g1_i1::g.72736 ORF type:internal len:93 (+),score=12.26 TRINITY_DN6203_c0_g1_i1:3-278(+)	-0.0773	-1.0162	0.41175	1.67492	-0.9932
TRINITY_DN65681_c0_g1_i1_orf1	ferritin subunit-like [Ostrinia furnacalis] >XP_028168186.1 ferritin subunit-like [Ostrinia furnacalis]	-0.7607	-1.2374	1.68319	0.09588	0.21897
TRINITY_DN4270_c0_g1_i1_orf1	cytochrome b-c1 complex subunit 8-like [Ostrinia furnacalis]	-0.8978	-1.351	1.31491	0.78615	0.14771

TRINITY_DN88876_c0_g1_i1_orf1	Photosystem I reaction center subunit III, chloroplastic, partial [Trichinella zimbabwensis]	-1.2757	-1.0326	1.36367	0.45427	0.49033
TRINITY_DN17329_c0_g2_i3_orf1	uncharacterized protein LOC114354338 isoform X1 [Ostrinia furnacalis]	-0.5746	-0.9519	1.59718	0.74256	-0.8132
TRINITY_DN80134_c0_g1_i1_orf1	gephyrin isoform X2 [Ostrinia furnacalis] >XP_028176021.1 gephyrin isoform X3 [Ostrinia furnacalis]	-0.4062	-1.4777	0.61371	1.49183	-0.2216
TRINITY_DN23732_c0_g1_i1_orf1	glutathione S-transferase 1-like [Ostrinia furnacalis] >QIC35740.1 glutathione S-transferase delta 4 [Ostrinia furnacalis]	-0.8885	-0.8093	1.68444	0.60592	-0.5926
TRINITY_DN44285_c0_g1_i1_orf1	uncharacterized protein LOC114354962 [Ostrinia furnacalis]	-0.1855	-1.5653	-0.0811	1.5605	0.27135
TRINITY_DN4367_c0_g1_i1_orf1	heat shock protein 21.7c [Chilo suppressalis] >AWT57938.1 heat shock protein 21.7c [Chilo suppressalis]	-0.8102	-0.8468	1.89617	-0.0847	-0.1545
TRINITY_DN52864_c0_g1_i1_orf1	odorant binding protein 18 [Conogethes pinicolalis]	-1.1428	-0.8285	1.6667	0.45499	-0.1503
TRINITY_DN125427_c0_g1_i1_orf1	heat shock protein 19.8 [Chilo suppressalis] >AGM90553.1 HSP19.8 [Chilo suppressalis] >BAE94664.1 small heat shock protein 19.7 [Chilo suppressalis]	-0.6684	-0.9238	1.90453	-0.0469	-0.2655
TRINITY_DN2097_c1_g1_i1_orf1	5-oxoprolinase [Ostrinia furnacalis]	-0.9335	-1.2717	1.50541	0.36303	0.33672
TRINITY_DN11159_c0_g1_i5_orf1	sphingosine-1-phosphate lyase isoform X2 [Ostrinia furnacalis]	-1.0735	-1.2921	0.94133	1.0849	0.3393
TRINITY_DN1308_c0_g1_i4_orf1	serine proteinase stubble-like [Ostrinia furnacalis]	-0.7412	-0.81	1.92675	-0.2641	-0.1115
TRINITY_DN31377_c0_g2_i1_orf1	phosphatidate cytidylyltransferase, mitochondrial [Ostrinia furnacalis]	-0.2576	-1.389	0.9617	1.30289	-0.618
TRINITY_DN34426_c0_g1_i1_orf1	laminin subunit alpha-like, partial [Ostrinia furnacalis]	-0.8033	-1.0779	1.24521	1.16655	-0.5306
TRINITY_DN14391_c1_g1_i2_orf1	pre-rRNA-processing protein TSR1 homolog [Ostrinia furnacalis]	-0.5099	-1.3104	1.10843	1.23367	-0.5217
TRINITY_DN661_c0_g2_i2_orf1	cuticle protein 7-like [Ostrinia furnacalis]	-0.5741	-0.9088	1.93421	-0.1979	-0.2534
TRINITY_DN2109_c0_g1_i4_orf1	mucin-2-like isoform X2 [Ostrinia furnacalis]	-0.3081	-1.0287	1.70962	0.46726	-0.8401
TRINITY_DN14274_c0_g1_i3_orf1	ATP-dependent RNA helicase dbp2-like isoform X1 [Leguminivora glycinivorella]	-0.5053	-1.2046	1.53174	0.77156	-0.5934
TRINITY_DN3459_c0_g1_i4_orf1	PREDICTED: probable small nuclear ribonucleoprotein G [Papilio polytes] >XP_013168682.1 PREDICTED: probable small nuclear ribonucleoprotein G [Papilio xuthus] >XP_013200095.1 PREDICTED: probable small nuclear ribonucleoprotein G [Amyelois transitella] >XP_014365947.1 probable small nuclear ribonucleoprotein G [Papilio machaon] >XP_023949391.1 probable small nuclear ribonucleoprotein G [Bicyclus anynana] >XP_026492889.1 probable small nuclear ribonucleoprotein G [Vanessa tameamea] >XP_030032656.1 probable small nuclear ribonucleoprotein G [Manduca sexta] >XP_032524946.1 probable small nuclear ribonucleoprotein G [Danaus plexippus plexippus] >XP_032524948.1 probable small nuclear ribonucleoprotein G [Danaus plexippus plexippus] >XP_039761094.1 probable small nuclear ribonucleoprotein G [Pararge aegeria] >XP_045507396.1 probable small nuclear ribonucleoprotein G [Colias croceus] >XP_046974152.1 probable small nuclear ribonucleoprotein G [Vanessa cardui] >XP_047540860.1 probable small nuclear ribonucleoprotein G [Vanessa atalanta] >XP_050357207.1 probable small nuclear ribonucleoprotein G [Nymphalis io] >CAG5058336.1 unnamed protein product [Parnassius apollo] >CAG9570366.1 unnamed protein product [Danaus chrysippus] >CAH0731681.1 unnamed protein product, partial [Brenthis ino] >CAH2061014.1 unnamed protein product, partial [Iphiclides podalirius] >CAH2269228.1 jg1748 [Pararge aegeria aegeria]	-0.6899	-1.0563	1.72895	0.46626	-0.449
TRINITY_DN24689_c0_g1_i1_orf1	thioredoxin domain-containing protein 17-like [Ostrinia furnacalis]	-0.4212	-1.7364	0.93337	0.91799	0.30617
TRINITY_DN13067_c0_g1_i6_orf1	diphosphomevalonate decarboxylase [Ostrinia furnacalis]	-0.4742	-1.1611	1.84177	-0.1852	-0.0212
TRINITY_DN59804_c0_g1_i1_orf1	DNA methyltransferase 1-associated protein 1 [Ostrinia furnacalis]	-0.5261	-1.5932	1.06081	1.0289	0.02963
TRINITY_DN41129_c0_g1_i1_orf1	histone deacetylase 6 [Ostrinia furnacalis]	-0.1038	-1.3527	0.10935	1.73189	-0.3848
TRINITY_DN22018_c0_g1_i3_orf1	hypothetical protein evm_009110 [Chilo suppressalis] >CAH2984739.1 unnamed protein product [Chilo suppressalis]	-0.3078	-1.595	1.49813	0.33438	0.07033
TRINITY_DN3131_c0_g1_i5_orf1	senecionine N-oxygenase-like isoform X1 [Ostrinia furnacalis] >XP_028178163.1 senecionine N-oxygenase-like isoform X2 [Ostrinia furnacalis] >XP_028178164.1 senecionine N-oxygenase-like isoform X1 [Ostrinia furnacalis]	0.09422	-1.5657	1.26352	0.78281	-0.5748
TRINITY_DN11970_c0_g1_i4_orf1	myb-like protein AA [Ostrinia furnacalis]	-0.5683	-0.8333	1.95626	-0.2462	-0.3084
TRINITY_DN2971_c0_g1_i1_orf1	uncharacterized protein LOC114364864 [Ostrinia furnacalis]	-0.0592	-1.3466	1.73093	0.09647	-0.4216
TRINITY_DN3715_c0_g1_i2_orf1	uncharacterized protein LOC114356437 isoform X1 [Ostrinia furnacalis]	-0.3023	-1.0332	1.86439	0.07118	-0.6001
TRINITY_DN1604_c0_g1_i4_orf1	ubiquitin-conjugating enzyme E2 S [Ostrinia furnacalis]	-0.5025	-1.3823	1.66107	-0.0501	0.27387
TRINITY_DN106_c0_g1_i3_orf1	cytochrome b5-like heme/Steroid binding domain-containing protein [Phthorimaea operculella]	-0.4169	-1.7668	0.90276	0.45415	0.82674
TRINITY_DN23582_c0_g1_i1_orf1	unnamed protein product [Diatraea saccharalis]	-0.1634	-1.0516	1.74434	0.3204	-0.8497

TRINITY_DN31584_c0_g2_i2_orf1	14-3-3 protein epsilon [Gallus gallus] >NP_001233297.1 14-3-3 protein epsilon [Pan troglodytes] >NP_006752.1 14-3-3 protein epsilon [Homo sapiens] >NP_033562.3 14-3-3 protein epsilon [Mus musculus] >NP_113791.2 14-3-3 protein epsilon [Rattus norvegicus] >NP_776916.1 14-3-3 protein epsilon [Bos taurus] >XP_001504337.1 14-3-3 protein epsilon isoform X1 [Equus caballus] >XP_002918088.2 14-3-3 protein epsilon isoform X2 [Ailuropoda melanoleuca] >XP_003416855.1 14-3-3 protein epsilon isoform X1 [Loxodonta africana] >XP_003469733.1 14-3-3 protein epsilon isoform X1 [Cavia porcellus] >XP_003816884.1 14-3-3 protein epsilon isoform X1 [Pan paniscus] >XP_003912098.1 14-3-3 protein epsilon isoform X1 [Papio anubis] >XP_003929381.1 14-3-3 protein epsilon isoform X1 [Saimiri boliviensis boliviensis] >XP_003996471.1 14-3-3 protein epsilon isoform X1 [Felis catus] >XP_004267124.1 14-3-3 protein epsilon isoform X1 [Orcinus orca] >XP_004376223.1 14-3-3 protein epsilon [Trichechus manatus latirostris] >XP_004404155.1 PREDICTED: 14-3-3 protein epsilon isoform X2 [Odobenus rosmarus divergens] >XP_004433380.1 PREDICTED: 14-3-3 protein epsilon isoform X1 [Ceratotherium simum simum] >XP_004483832.1 14-3-3 protein epsilon isoform X1 [Dasypus novemcinctus] >XP_004605045.1 PREDICTED: 14-3-3 protein epsilon [Sorex araneus] >XP_004667919.1 14-3-3 protein epsilon [Jaculus jaculus] >XP_004706944.1 14-3-3 protein epsilon [Echinops telfairi] >XP_004746947.1 14-3-3 protein epsilon isoform X1 [Mustela putorius furo] >XP_004857172.1 14-3-3 protein epsilon isoform X1 [Heterocephalus glaber] >XP_005067448.1 14-3-3 protein epsilon isoform X1 [Mesocricetus auratus] >XP_005240506.1 14-3-3 protein epsilon isoform X1 [Falco peregrinus] >XP_005327947.1 14-3-3 protein epsilon isoform X1 [Ictidomys tridecemlineatus] >XP_005349591.1 14-3-3 protein epsilon isoform X1 [Microtus ochrogaster] >XP_005402688.1 PREDICTED: 14-3-3 protein epsilon isoform X1 [Chinchilla lanigera] >XP_005525859.1 PREDICTED: 14-3-3 protein epsilon isoform X1 [Pseudopodoces humilis] >XP_005888292.1 PREDICTED: 14-3-3 protein epsilon isoform X1 [Bos mutus] >XP_006079841.1 14-3-3 protein epsilon isoform X1 [Bubalus bubalis] >XP_006099253.1 14-3-3 protein epsilon [Myotis lucifugus] >XP_006185046.1 14-3-3 protein epsilon isoform X1 [Camelus ferus] >XP_006214490.1 14-3-3 protein epsilon isoform X1 [Vicugna pacos] >XP_006259463.1 PREDICTED: 14-3-3 protein epsilon [Alligator mississippiensis] >XP_006768146.1 PREDICTED: 14-3-3 protein epsilon isoform X1 [Myotis davidii] >XP_006863283.1 PREDICTED: 14-3-3 protein epsilon [Chrysochloris asiatica] >XP_006891074.1 PREDICTED: 14-3-3 protein epsilon-like [Elephantulus edwardii] >XP_006925117.1 14-3-3 protein epsilon isoform X1 [Pteropus alecto] >XP_006977465.1 14-3-3 protein epsilon isoform X1 [Peromyscus maniculatus bairdii] >XP_007057769.1 14-3-3 protein epsilon isoform X1 [Chelonia mydas] >XP_007123613.1 14-3-3 protein epsilon isoform X1 [Physeter catodon] >XP_007183877.1 14-3-3 protein epsilon isoform X1 [Balaenoptera acutorostrata scammoni] >XP_007454293.1 PREDICTED: 14-3-3 protein epsilon [Lipotes vexillifer] >XP_007520478.1 PREDICTED: 14-3-3 protein epsilon [Erinaceus europaeus] >XP_007935626.1 14-3-3 protein epsilon [Orycteropus afer afer] >XP_008007997.1 14-3-3 protein epsilon isoform X1 [Chlorocebus sabaeus] >XP_008058985.1 14-3-3 protein epsilon isoform X2 [Carlito syrichta] >XP_008146090.1 14-3-3 protein epsilon isoform X1 [Eptesicus fuscus] >XP_008512998.1 PREDICTED: 14-3-3 protein epsilon isoform X1 [Equus przewalskii] >XP_008591162.1 PREDICTED: 14-3-3 protein epsilon [Galeopterus variegatus] >XP_008826988.1 14-3-3 protein epsilon [Nannospalax galili] >XP_009249385.2 14-3-3 protein epsilon isoform X1 [Pongo abelii] >XP_010371794.1 14-3-3 protein epsilon isoform X1 [Rhinopithecus roxellana] >XP_010571909.1 PREDICTED: 14-3-3 protein epsilon isoform X5 [Haliaeetus leucocephalus] [illegible]	0.41464	-1.7643	0.49802	1.16898	-0.3173
TRINITY_DN13732_c0_g2_i3_orf1	60S ribosomal protein L35 [Ostrinia furnacalis]	-0.6483	-1.0354	1.6981	0.55138	-0.5658
TRINITY_DN13221_c0_g1_i3_orf1	fasciclin-3-like [Ostrinia furnacalis]	-0.0193	-1.5586	1.49017	0.45982	-0.3721
TRINITY_DN6470_c0_g3_i2_orf1	trypsin CFT-1-like [Ostrinia furnacalis]	-0.4291	-0.8548	1.96293	-0.3669	-0.3121
TRINITY_DN7565_c0_g1_i3_orf1	acylphosphatase-2-like [Ostrinia furnacalis]	-0.1334	-1.072	1.89125	-0.4453	-0.2405
TRINITY_DN1493_c0_g1_i5_orf1	uncharacterized protein LOC114350869 [Ostrinia furnacalis]	-1.0668	-0.9385	-0.0635	0.38742	1.68137
TRINITY_DN65974_c0_g1_i2_orf1	uncharacterized protein LOC114362364 [Ostrinia furnacalis]	-0.7537	-1.2172	-0.3233	0.8198	1.47439
TRINITY_DN1732_c0_g1_i17_orf1	CAD protein isoform X2 [Ostrinia furnacalis]	-0.8049	-1.3273	-0.0335	1.43225	0.73346
TRINITY_DN104297_c0_g1_i1_orf1	tubulin-specific chaperone D [Ostrinia furnacalis]	-0.9968	-1.0696	-0.1713	0.7128	1.52482
TRINITY_DN9836_c0_g1_i2_orf1	STAM-binding protein-like A isoform X2 [Ostrinia furnacalis]	-0.8416	-1.376	0.17324	0.64894	1.39543
TRINITY_DN11649_c0_g1_i4_orf1	ubiquitin carboxyl-terminal hydrolase 32-like, partial [Ostrinia furnacalis]	-0.4288	-0.5918	-1.1713	0.50792	1.68402
TRINITY_DN9400_c0_g1_i1_orf1	lysophosphatidylserine lipase ABHD12 isoform X2 [Maniola hyperantus]	-0.3522	-1.0976	-0.7784	0.57444	1.65384
TRINITY_DN46715_c0_g1_i1_orf1	hypothetical protein evm_000885 [Chilo suppressalis] >CAH0689224.1 unnamed protein product [Chilo suppressalis]	-0.133	-1.4409	-0.6526	1.1396	1.08696
TRINITY_DN2655_c0_g2_i1_orf1	DNA fragmentation factor subunit alpha [Ostrinia furnacalis] >XP_028176215.1 DNA fragmentation factor subunit alpha [Ostrinia furnacalis]	-0.4819	-1.4808	-0.2672	1.20795	1.02199
TRINITY_DN29009_c0_g2_i3_orf1	juvenile hormone binding protein [Omphisa fuscidentalis]	0.0518	-1.407	-0.7709	1.34888	0.77717
TRINITY_DN2749_c4_g1_i2_orf1	RNA exonuclease 4-like [Ostrinia furnacalis] >QEE79882.1 REX4 [Ostrinia furnacalis]	0.39193	-1.3738	-0.874	1.41436	0.44146
TRINITY_DN5507_c0_g1_i1_orf1	PREDICTED: protein mago nashi [Amyelois transitella] >XP_026764462.1 protein mago nashi [Galleria mellonella] >XP_028164484.1 protein mago nashi [Ostrinia furnacalis]	-0.193	-1.3161	-0.7737	1.02712	1.25576
TRINITY_DN2821_c0_g1_i1_orf1	uncharacterized protein LOC114356423 [Ostrinia furnacalis]	-0.217	-1.3574	-0.7091	1.13764	1.14594
TRINITY_DN22272_c0_g1_i1_orf1	28S ribosomal protein S33, mitochondrial [Galleria mellonella]	-0.1036	-1.8449	0.19198	0.93389	0.82259
TRINITY_DN104597_c0_g1_i2_orf1	hemicentin-2-like isoform X1 [Ostrinia furnacalis]	0.19202	-1.2224	-0.9181	1.57746	0.371
TRINITY_DN7275_c0_g1_i14_orf1	uncharacterized protein LOC114353817 [Ostrinia furnacalis]	0.40851	-1.5163	-0.8047	1.12579	0.78672
TRINITY_DN38392_c0_g1_i1_orf1	enoyl-CoA hydratase domain-containing protein 3 [Agrotis segetum]	-0.8409	-1.2104	0.70886	-0.1726	1.51508
TRINITY_DN42964_c0_g1_i1_orf1	protein lethal(2)essential for life-like [Galleria mellonella]	-0.4821	-1.2742	1.46893	-0.5436	0.83104
TRINITY_DN2647_c0_g1_i3_orf1	DNA repair protein complementing XP-G cells homolog isoform X1 [Ostrinia furnacalis]	-0.0437	-0.3888	-1.6415	1.00936	1.06468
TRINITY_DN2673_c2_g1_i2_orf1	aminopeptidase N3c [Ostrinia nubilalis]	0.51512	-0.4961	-1.3995	1.57823	-0.1977
TRINITY_DN1083_c0_g1_i4_orf1	latent-transforming growth factor beta-binding protein 4-like [Ostrinia furnacalis]	0.25484	-0.7772	-1.327	1.58048	0.26884

TRINITY_DN86127_c1_g1_i2_orfp1	TRINITY_DN86127_c1_g1_i2_m.43062 TRINITY_DN86127_c1_g1::TRINITY_DN86127_c1_g1_i2::g.43062 ORF type:internal len:69 (-),score=14.03 TRINITY_DN86127_c1_g1_i2:2-205(-)	0.26686	-0.5192	-1.6742	0.98219	0.94429
TRINITY_DN6881_c0_g1_i1_orf1	putative protein TPRXL [Ostrinia furnacalis]	0.46913	0.32525	-1.9888	0.65037	0.54402
TRINITY_DN7861_c0_g1_i5_orf1	cytochrome b5-related protein-like [Ostrinia furnacalis]	0.08507	-0.6929	-1.4007	0.48752	1.52092
TRINITY_DN1215_c0_g1_i2_orf1	PI-stichotoxin-She2a-like [Ostrinia furnacalis]	0.24044	-0.0318	-1.8783	0.73692	0.93278
TRINITY_DN3037_c0_g1_i1_orf1	coiled-coil domain-containing protein 58-like [Spodoptera frugiperda] >KAF9815873.1 hypothetical protein SFRURICE_009771 [Spodoptera frugiperda] >KAG8118507.1 hypothetical protein SFRUCORN_001779 [Spodoptera frugiperda]	0.56132	-0.2008	-1.6874	1.34055	-0.0136
TRINITY_DN1585_c0_g1_i1_orf1	aldose reductase-like isoform X2 [Ostrinia furnacalis]	0.52501	-0.2463	-1.8096	0.43585	1.09504
TRINITY_DN3472_c0_g1_i6_orf1	unnamed protein product [Euphydryas editha]	0.76955	-1.2676	-1.0363	1.29157	0.24276
TRINITY_DN11596_c0_g1_i1_orf1	probable ATP-dependent RNA helicase DDX47 [Ostrinia furnacalis]	0.89009	-0.9836	-1.085	1.41652	-0.2381
TRINITY_DN280_c4_g1_i5_orf1	fibroin light chain [Haritalodes derogata]	0.66141	-0.4886	-1.7165	1.07692	0.4667
TRINITY_DN3019_c0_g1_i1_orf1	uncharacterized protein LOC114355530 isoform X1 [Ostrinia furnacalis]	0.68863	-0.655	-1.6545	0.65908	0.96177
TRINITY_DN12397_c0_g1_i1_orf1	39S ribosomal protein L27, mitochondrial [Ostrinia furnacalis]	0.8344	-1.3788	-0.9294	1.2127	0.26112
TRINITY_DN79083_c0_g1_i2_orf1	unnamed protein product [Arctia plantaginis]	0.99699	-1.2426	-1.1941	0.71982	0.71982
TRINITY_DN56795_c1_g1_i1_orf1	uncharacterized protein LOC114365476 [Ostrinia furnacalis]	0.83806	-0.9108	-1.4552	0.46137	1.06659
TRINITY_DN49786_c0_g1_i1_orf1	UDP-glucuronosyltransferase 2B15-like isoform X1 [Ostrinia furnacalis]	0.80083	-1.1767	-1.1429	0.2522	1.26658
TRINITY_DN32_c0_g1_i4_orf1	epidermal growth factor receptor substrate 15 homolog [Ostrinia furnacalis]	0.53952	-0.6675	-1.5061	0.24255	1.39149
TRINITY_DN920_c0_g1_i6_orf1	glutathione S-transferase omega 2 [Ostrinia furnacalis]	0.51926	-0.9923	-1.1369	0.04436	1.56561
TRINITY_DN6241_c0_g1_i1_orf1	uncharacterized protein LOC114355531 [Ostrinia furnacalis]	0.97035	-0.8437	-1.5328	0.76792	0.63825
TRINITY_DN32780_c0_g1_i2_orf1	renin receptor [Ostrinia furnacalis]	0.43501	-0.7751	1.13001	0.74984	-1.5397
TRINITY_DN41697_c0_g1_i1_orf1	5-formyltetrahydrofolate cyclo-ligase [Ostrinia furnacalis]	1.36771	-0.7452	0.49628	0.36306	-1.4819
TRINITY_DN8646_c0_g1_i2_orf1	chromatin accessibility complex protein 1 [Ostrinia furnacalis]	1.00512	-1.8231	0.18135	0.78218	-0.1455
TRINITY_DN3456_c0_g2_i1_orf1	protein purity of essence [Ostrinia furnacalis]	1.14726	-0.9056	-0.2546	1.18944	-1.1764
TRINITY_DN3322_c0_g1_i2_orf1	myrosinase 1-like isoform X2 [Ostrinia furnacalis]	0.98843	-1.5753	0.71554	0.65023	-0.7789
TRINITY_DN2338_c0_g2_i2_orf1	prophenoloxidase PPO3 [Ostrinia furnacalis]	1.43075	-0.7843	-1.2977	0.79556	-0.1443
TRINITY_DN10234_c0_g1_i1_orf1	39S ribosomal protein L16, mitochondrial [Ostrinia furnacalis]	1.16923	-1.8123	0.36994	0.43181	-0.1587
TRINITY_DN67495_c0_g1_i1_orf1	hypothetical protein KGM_200102A, partial [Danaus plexippus plexippus]	1.33382	-0.6746	-1.3686	0.92089	-0.2115
TRINITY_DN3478_c0_g1_i10_orf1	acyl-CoA dehydrogenase family member 9, mitochondrial [Ostrinia furnacalis]	0.98292	-1.7466	0.46842	0.7477	-0.4524
TRINITY_DN23798_c0_g1_i1_orf1	arginine-glutamic acid dipeptide repeats protein [Ostrinia furnacalis]	0.92068	-1.6705	0.67226	0.71213	-0.6346
TRINITY_DN13923_c0_g2_i1_orf1	sideroflexin-2 [Zerene cesonia]	1.36103	-1.0605	-0.1783	0.9348	-1.0571
TRINITY_DN5811_c0_g1_i4_orf1	uridine-cytidine kinase-like 1 isoform X1 [Ostrinia furnacalis] >XP_028168339.1 uridine-cytidine kinase-like 1 isoform X2 [Ostrinia furnacalis]	0.93034	-1.7874	0.96576	-0.03	-0.0788
TRINITY_DN26790_c0_g1_i3_orf1	protein unc-45 homolog B [Ostrinia furnacalis]	1.43443	-1.6939	0.0611	0.25664	-0.0582
TRINITY_DN48713_c0_g1_i1_orf1	sphingomyelin phosphodiesterase 4 [Ostrinia furnacalis]	1.47871	-1.4904	0.29376	0.34173	-0.6238
TRINITY_DN23534_c0_g2_i2_orf1	armadillo repeat-containing protein 8-like [Maniola hyperantus]	1.18659	-1.6948	0.66253	0.28943	-0.4437
TRINITY_DN18918_c0_g1_i3_orf1	myrosinase 1-like isoform X2 [Ostrinia furnacalis]	1.59734	-0.6472	-0.6908	0.74175	-1.0012
TRINITY_DN1641_c0_g1_i6_orf1	rhodanese domain-containing protein CG4456-like [Ostrinia furnacalis]	1.54613	-1.5525	-0.2432	-0.1086	0.35812
TRINITY_DN38562_c0_g1_i3_orf1	persulfide dioxygenase ETHE1, mitochondrial isoform X1 [Ostrinia furnacalis]	1.44782	-0.6071	-1.5107	0.45489	0.21504
TRINITY_DN2542_c0_g2_i1_orf1	peroxiredoxin-2-like [Ostrinia furnacalis]	1.38369	-1.614	0.61003	-0.0555	-0.3242
TRINITY_DN35377_c0_g1_i3_orf1	unnamed protein product [Chilo suppressalis]	1.41846	-1.4473	0.73945	-0.1385	-0.5722
TRINITY_DN4207_c0_g1_i1_orf1	mitochondrial import inner membrane translocase subunit Tim21 [Ostrinia furnacalis]	1.45218	-1.6256	0.00876	-0.2606	0.42519
TRINITY_DN20321_c0_g1_i5_orf1	uncharacterized protein LOC114350467 isoform X3 [Ostrinia furnacalis]	1.74211	-1.1333	-0.8112	0.13548	0.06683
TRINITY_DN1383_c0_g1_i2_orf1	uncharacterized protein LOC114353133 isoform X1 [Ostrinia furnacalis] >XP_028160773.1 uncharacterized protein LOC114353133 isoform X2 [Ostrinia furnacalis]	1.64306	-0.9495	-1.107	0.41655	-0.0032
TRINITY_DN42269_c2_g1_i1_orf1	probable enoyl-CoA hydratase, mitochondrial [Ostrinia furnacalis]	1.8586	-1.0054	-0.7145	0.01612	-0.1548
TRINITY_DN2497_c0_g1_i1_orf1	protein stunted-like isoform X2 [Vanessa tameamea] >XP_046960183.1 protein stunted-like isoform X2 [Vanessa cardui] >XP_047527093.1 protein stunted-like isoform X2 [Vanessa atalanta]	1.24143	-1.7459	0.58633	-0.2191	0.13726
TRINITY_DN57765_c0_g1_i1_orf1	cytochrome P450 6B6-like [Ostrinia furnacalis]	1.70765	-1.3083	-0.5832	0.00472	0.17915
TRINITY_DN60821_c0_g1_i1_orf1	nucleolar GTP-binding protein 2 [Ostrinia furnacalis]	1.36182	-1.6372	0.44893	0.2656	-0.4391
TRINITY_DN227_c0_g1_i1_orf1	double-stranded ribonuclease 2 [Ostrinia nubilalis]	1.65806	-1.2238	0.1485	0.23838	-0.8211
TRINITY_DN28509_c0_g1_i1_orf1	39S ribosomal protein L43, mitochondrial [Ostrinia furnacalis]	1.82792	-1.1616	-0.0755	-0.0412	-0.5496
TRINITY_DN2967_c0_g1_i4_orf1	UDP-glycosyltransferase UGT41G1 [Ostrinia furnacalis]	1.87142	-0.978	-0.7238	-0.1241	-0.0455
TRINITY_DN4929_c0_g1_i1_orf1	unnamed protein product [Danaus chrysippus]	1.26549	-1.7441	0.35409	-0.2719	0.39646
TRINITY_DN32161_c0_g1_i1_orf1	uncharacterized protein LOC114352518 [Ostrinia furnacalis]	1.53445	-1.5709	0.30418	0.02329	-0.291
TRINITY_DN1884_c0_g2_i2_orf1	phosphotriesterase-related protein [Ostrinia furnacalis]	1.75551	-1.1157	-0.8105	0.05385	0.11683

TRINITY_DN14073_c0_g1_i1_orf1	cytochrome c oxidase subunit 4 isoform 1, mitochondrial-like [Ostrinia furnacalis] >XP_028164918.1 cytochrome c oxidase subunit 4 isoform 1, mitochondrial-like [Ostrinia furnacalis]	1.88216	-0.8953	-0.5038	0.13622	-0.6193
TRINITY_DN2695_c0_g1_i8_orfp1	TRINITY_DN2695_c0_g1_i8_m.44478 TRINITY_DN2695_c0_g1::TRINITY_DN2695_c0_g1_i8::g.44478 ORF type:3prime_partial len:532 (+),score=80.62 TRINITY_DN2695_c0_g1_i8:101-1594(+)	1.22783	-0.8596	-1.1632	1.13387	-0.3389
TRINITY_DN21984_c0_g1_i6_orf1	venom serine protease 34-like [Ostrinia furnacalis]	1.88274	-1.0644	-0.522	-0.0938	-0.2025
TRINITY_DN14019_c0_g1_i5_orf1	hypothetical protein evm_009768 [Chilo suppressalis]	0.73439	-1.4833	1.31237	-0.7174	0.15388
TRINITY_DN99_c0_g1_i3_orf1	uncharacterized protein LOC126375979 [Pectinophora gossypiella] >XP_049879066.1 uncharacterized protein LOC126375979 [Pectinophora gossypiella]	1.53317	-0.842	-1.0207	0.81448	-0.4849
TRINITY_DN1313_c0_g1_i2_orf1	39S ribosomal protein L40, mitochondrial [Ostrinia furnacalis]	0.82255	-0.8942	1.51007	-0.396	-1.0424
TRINITY_DN1868_c0_g1_i1_orf1	protein obstructor-E isoform X1 [Ostrinia furnacalis]	0.47972	-1.0091	1.73191	-0.4808	-0.7217
TRINITY_DN103457_c0_g1_i1_orf1	28S ribosomal protein S22, mitochondrial [Ostrinia furnacalis]	1.94612	-0.9177	-0.3134	-0.2664	-0.4486
TRINITY_DN2266_c0_g1_i6_orf1	bilin-binding protein-like [Ostrinia furnacalis]	1.73985	-0.7522	-0.6203	0.5073	-0.8746
TRINITY_DN1666_c0_g1_i2_orf1	putative defense protein Hdd11 [Ostrinia furnacalis] >XP_028179344.1 putative defense protein Hdd11 [Ostrinia furnacalis] >AGV28583.1 immune-induced protein [Ostrinia furnacalis]	1.65913	-1.0508	-0.9765	-0.0628	0.43094
TRINITY_DN6147_c0_g1_i2_orf1	uncharacterized protein LOC114352519 [Ostrinia furnacalis]	1.95109	-0.8136	-0.4867	-0.1221	-0.5286
TRINITY_DN344_c1_g1_i1_orf1	chymotrypsin-like serine protease 16 [Ostrinia nubilalis]	1.52301	-0.6543	-1.1001	0.82822	-0.5969
TRINITY_DN886_c0_g1_i1_orf1	collagenase-like isoform X1 [Ostrinia furnacalis]	1.92497	-0.9974	-0.2934	-0.2381	-0.396
TRINITY_DN2184_c0_g1_i4_orf1	uncharacterized protein LOC114359356 [Ostrinia furnacalis]	1.82781	-1.052	-0.7069	-0.193	0.12413
TRINITY_DN46778_c0_g1_i2_orf1	Deoxycytidylate deaminase [Papilio xuthus]	1.79746	-1.0052	0.33154	-0.4688	-0.6551
TRINITY_DN34821_c0_g1_i4_orf1	acetylcholine receptor subunit alpha-L1-like [Ostrinia furnacalis]	1.19076	-1.2517	1.17849	-0.5268	-0.5907
TRINITY_DN1330_c0_g1_i1_orf1	pancreatic triacylglycerol lipase-like [Ostrinia furnacalis]	1.93792	-0.9065	-0.2109	-0.266	-0.5546
TRINITY_DN3847_c1_g1_i1_orf1	ribosome production factor 2 homolog [Ostrinia furnacalis]	1.97149	-0.8085	-0.3562	-0.3434	-0.4633
TRINITY_DN47_c0_g1_i2_orf1	uncharacterized protein LOC114356437 isoform X1 [Ostrinia furnacalis]	1.87847	-0.8547	-0.8521	-0.0785	-0.0932
TRINITY_DN7920_c0_g1_i2_orf1	uncharacterized protein LOC114357268 [Ostrinia furnacalis] >XP_028166599.1 uncharacterized protein LOC114357268 [Ostrinia furnacalis]	1.88788	-1.0998	-0.344	-0.2908	-0.1532
TRINITY_DN26130_c0_g1_i1_orf1	membrane alanyl aminopeptidase-like [Ostrinia furnacalis]	1.87724	-0.9541	0.07622	-0.3258	-0.6736
TRINITY_DN4030_c0_g2_i1_orf1	putative trypsin 6 [Ostrinia nubilalis]	1.92009	-0.9526	-0.4356	-0.0728	-0.4591
TRINITY_DN94248_c0_g2_i3_orf1	uncharacterized protein LOC114357292 isoform X4 [Ostrinia furnacalis]	1.9633	-0.682	-0.7177	-0.2255	-0.3381
TRINITY_DN46409_c0_g1_i1_orf1	unnamed protein product [Heterotrigona itama]	1.901	-0.8247	-0.0372	-0.2325	-0.8067
TRINITY_DN15046_c0_g1_i8_orf1	epidermal retinol dehydrogenase 2-like isoform X1 [Ostrinia furnacalis] >XP_028169999.1 epidermal retinol dehydrogenase 2-like isoform X2 [Ostrinia furnacalis]	1.9074	-0.8621	-0.7147	-0.0023	-0.3283
TRINITY_DN79319_c0_g1_i8_orfp1	TRINITY_DN79319_c0_g1_i8_m.49956 TRINITY_DN79319_c0_g1::TRINITY_DN79319_c0_g1_i8::g.49956 ORF type:5prime_partial len:84 (+),score=1.39 TRINITY_DN79319_c0_g1_i8:1-252(+)	1.95994	-0.7641	-0.6544	-0.2656	-0.2759
TRINITY_DN98091_c0_g1_i3_orf1	UDP-glycosyltransferase UGT40AP2, partial [Ostrinia furnacalis]	1.96671	-0.788	-0.3577	-0.2586	-0.5624
TRINITY_DN1707_c0_g1_i1_orf1	inositol oxygenase-like [Ostrinia furnacalis]	1.91496	-0.9311	-0.5994	-0.064	-0.3205
TRINITY_DN108573_c0_g1_i1_orf1	uncharacterized protein LOC114366171 [Ostrinia furnacalis]	1.97779	-0.7227	-0.3539	-0.3188	-0.5824
TRINITY_DN44288_c0_g1_i2_orf1	ATP-dependent RNA helicase p62 [Ostrinia furnacalis]	1.92291	-0.9002	-0.0317	-0.5001	-0.4909
TRINITY_DN1249_c0_g1_i10_orf1	venom carboxylesterase-6-like [Ostrinia furnacalis]	1.89215	-0.8717	-0.7956	-0.084	-0.1408
TRINITY_DN29018_c0_g1_i4_orf1	prostaglandin reductase 1-like isoform X1 [Ostrinia furnacalis] >XP_028178925.1 prostaglandin reductase 1-like isoform X2 [Ostrinia furnacalis]	1.95403	-0.4845	-0.7823	-0.1212	-0.566
TRINITY_DN55160_c0_g2_i1_orf1	esterase FE4-like isoform X2 [Ostrinia furnacalis]	1.86703	-1.0598	-0.3335	0.05259	-0.5263
TRINITY_DN82320_c0_g1_i2_orf1	glutathione S-transferase sigma3 [Glyphodes pyloalis]	1.87062	-1.0779	-0.4251	0.02908	-0.3967
TRINITY_DN82801_c0_g1_i1_orf1	uncharacterized protein LOC114364712 [Ostrinia furnacalis]	1.46569	-1.0197	0.93012	-0.688	-0.688
TRINITY_DN5578_c0_g1_i10_orf1	unnamed protein product [Chilo suppressalis]	1.83907	-1.015	0.17567	-0.331	-0.6687
TRINITY_DN16931_c0_g1_i1_orf1	pancreatic triacylglycerol lipase-like [Ostrinia furnacalis]	1.89797	-0.8927	-0.685	-0.3604	0.0402
TRINITY_DN311_c0_g1_i4_orfp1	TRINITY_DN311_c0_g1_i4_m.65135 TRINITY_DN311_c0_g1::TRINITY_DN311_c0_g1_i4::g.65135 ORF type:5prime_partial len:126 (+),score=71.21 TRINITY_DN311_c0_g1_i4:1-378(+)	1.85821	-1.1455	-0.4413	-0.095	-0.1764
TRINITY_DN48410_c0_g1_i1_orf1	alpha-amylase 1-like [Ostrinia furnacalis]	1.98615	-0.7145	-0.4851	-0.3894	-0.3971
TRINITY_DN3784_c0_g1_i1_orf1	pancreatic triacylglycerol lipase-like [Ostrinia furnacalis]	1.96291	-0.8211	-0.2267	-0.4207	-0.4944
TRINITY_DN117_c0_g1_i6_orf1	lipase member I-like [Ostrinia furnacalis]	1.89133	-1.0315	-0.0346	-0.3199	-0.5054
TRINITY_DN4959_c0_g1_i1_orf1	pancreatic triacylglycerol lipase-like [Ostrinia furnacalis]	1.80133	-1.2857	-0.0878	-0.253	-0.1748
TRINITY_DN144190_c0_g1_i1_orf1	PREDICTED: uncharacterized protein LOC103572804 isoform X2 [Microplitis demolitor]	1.92351	-0.9966	-0.4019	-0.2012	-0.3237
TRINITY_DN2490_c0_g2_i1_orfp1	TRINITY_DN2490_c0_g2_i1_m.56872 TRINITY_DN2490_c0_g2::TRINITY_DN2490_c0_g2_i1::g.56872 ORF type:internal len:359 (-),score=123.59 TRINITY_DN2490_c0_g2_i1:2-1075(-)	1.96023	-0.8036	-0.2242	-0.5819	-0.3505
TRINITY_DN117_c0_g1_i4_orf1	lipase member I-like [Ostrinia furnacalis]	1.85689	-0.9577	0.1922	-0.579	-0.5123

TRINITY_DN30037_c0_g1_i5_orf1	cytoglobin-1-like isoform X2 [Ostrinia furnacalis]	0.11871	-1.5911	-0.3305	0.30149	1.50141
TRINITY_DN12367_c0_g1_i4_orf1	aldose reductase-like isoform X2 [Ostrinia furnacalis]	0.83868	-1.8073	-0.3687	0.65651	0.68076
TRINITY_DN957_c0_g1_i18_orf1	proton channel OtopLc-like isoform X6 [Ostrinia furnacalis]	0.4594	-1.9259	0.40078	0.11381	0.95193
TRINITY_DN6992_c0_g1_i6_orf1	UDP-glucuronosyltransferase 2B15-like [Ostrinia furnacalis] >XP_028166365.1 UDP-glucuronosyltransferase 2B15-like [Ostrinia furnacalis] >QNS26328.1 UDP-glycosyltransferase UGT40AN6 [Ostrinia furnacalis]	0.79443	-1.8764	0.10311	0.06612	0.91271
TRINITY_DN12087_c0_g1_i2_orf1	unnamed protein product [Euphydryas editha]	1.5221	-1.3414	-0.8223	0.35078	0.29084
TRINITY_DN36928_c0_g1_i5_orf1	actin-interacting protein 1 isoform X2 [Ostrinia furnacalis]	0.98077	-1.7005	-0.5651	0.67108	0.61374
TRINITY_DN2749_c0_g1_i4_orf1	RNA exonuclease 4-like [Ostrinia furnacalis] >QEE79882.1 REX4 [Ostrinia furnacalis]	1.36027	-1.3529	-0.9203	0.62305	0.28993
TRINITY_DN4356_c0_g1_i6_orf1	mulatexin-like [Ostrinia furnacalis]	1.2879	-1.7031	-0.3049	0.57028	0.14983
TRINITY_DN2749_c0_g2_i3_orf1	RNA exonuclease 4-like [Ostrinia furnacalis] >QEE79882.1 REX4 [Ostrinia furnacalis]	1.21072	-1.209	-0.6949	-0.4749	1.16799
TRINITY_DN33452_c0_g1_i1_orf1	lethal(2) giant larvae protein isoform X8 [Ostrinia furnacalis]	1.40148	-1.4046	-0.7904	0.14832	0.64518
