# Supplementary material for: Comparative Proteomics and Metabonomics Analysis of Different Diapause Stages Revealed a New Regulation Mechanism of Diapause in Loxostege sticticalis (Lepidoptera: Pyralidae)
Source: Molecules. 2024 Jul 25;29(15):3472. doi: 10.3390/molecules29153472 (PMC11314584; doi:10.3390/molecules29153472)
Supplement: Supplementary file 1 [file molecules-29-03472-s001.zip › analysis process/proteomic/Cluster analysis of expression patterns/Up/DvsPreD up.pdf]

Accession	Description	ND	CT	D	PreD	RD
TRINITY_DN1506_c0_g1_i6_orfp1	TRINITY_DN1506_c0_g1_i6_m.57691 TRINITY_DN1506_c0_g1::TRINITY_DN1506_c0_g1_i6::g.57691 ORF type:5prime_partial len:173 (+),score=30.49 TRINITY_DN1506_c0_g1_i6:1-519(+)	-1.11546	0.848064	0.884809	0.706934	-1.32434
TRINITY_DN7064_c0_g1_i19_orf1	unnamed protein product [Chilo suppressalis]	-1.23178	0.849735	1.100758	0.443989	-1.1627
TRINITY_DN4802_c0_g1_i4_orf1	uncharacterized protein LOC114366345 isoform X2 [Ostrinia furnacalis]	-1.48977	0.016889	1.520508	0.459518	-0.50714
TRINITY_DN14328_c0_g1_i12_orf1	larval cuticle protein LCP-30-like [Ostrinia furnacalis]	-1.22551	0.629382	1.056196	0.739662	-1.19973
TRINITY_DN8595_c0_g1_i3_orf1	aldose reductase-like isoform X4 [Trichoplusia ni]	-1.63749	0.632407	1.068932	0.590183	-0.65403
TRINITY_DN394_c0_g1_i2_orf1	uncharacterized protein LOC114351483 [Ostrinia furnacalis]	-1.07239	0.275719	1.257461	0.791022	-1.25181
TRINITY_DN20344_c0_g1_i5_orf1	uncharacterized protein LOC114351483 [Ostrinia furnacalis]	-1.14126	0.821767	1.160141	0.392988	-1.23363
TRINITY_DN1772_c1_g2_i1_orf1	aldose reductase-like isoform X2 [Ostrinia furnacalis]	-1.48297	0.863851	1.139618	0.296695	-0.8172
TRINITY_DN6415_c0_g2_i1_orf1	D-arabinitol dehydrogenase 1-like [Ostrinia furnacalis]	-1.39165	0.616745	1.290416	0.407408	-0.92292
TRINITY_DN8008_c0_g1_i6_orf1	uncharacterized protein LOC114357965 isoform X1 [Ostrinia furnacalis] >XP_028167599.1 uncharacterized protein LOC114357965 isoform X1 [Ostrinia furnacalis] >XP_028167600.1 uncharacterized protein LOC114357965 isoform X2 [Ostrinia furnacalis] >XP_028167601.1 uncharacterized protein LOC114357965 isoform X3 [Ostrinia furnacalis]	-1.47885	1.178638	0.739421	0.405145	-0.84436
TRINITY_DN43350_c0_g3_i1_orf1	uncharacterized protein LOC114355190 [Ostrinia furnacalis]	-1.3572	0.841431	1.055245	0.501929	-1.0414
TRINITY_DN51995_c0_g3_i1_orf1	circadian clock-controlled protein-like [Ostrinia furnacalis]	-1.03004	1.772327	0.281994	-0.20087	-0.82341
TRINITY_DN4204_c0_g1_i1_orf1	uncharacterized protein LOC114359352 [Ostrinia furnacalis]	-0.63832	1.434496	0.900266	-0.47051	-1.22593
TRINITY_DN1226_c0_g1_i11_orfp1	TRINITY_DN1226_c0_g1_i11_m.52385 TRINITY_DN1226_c0_g1::TRINITY_DN1226_c0_g1_i11::g.52385 ORF type:internal len:92 (-),score=5.77 TRINITY_DN1226_c0_g1_i11:2-274(-)	-1.19379	0.260198	1.298486	0.754175	-1.11907
TRINITY_DN20558_c0_g1_i2_orf1	Transient receptor potential channel pyrexia [Operophtera brumata]	-1.56759	-0.21811	1.516762	0.41585	-0.14691
TRINITY_DN285_c0_g1_i4_orf1	catalase-like [Ostrinia furnacalis]	-1.39298	0.809031	1.074564	0.506369	-0.99698
TRINITY_DN18568_c0_g1_i2_orfp1	TRINITY_DN18568_c0_g1_i2_m.13844 TRINITY_DN18568_c0_g1::TRINITY_DN18568_c0_g1_i2::g.13844 ORF type:5prime_partial len:77 (+),score=12.07 TRINITY_DN18568_c0_g1_i2:1-231(+)	-0.88007	1.3474	1.092409	-0.76896	-0.79077
TRINITY_DN8853_c0_g1_i4_orf1	uncharacterized protein LOC114351488 isoform X1 [Ostrinia furnacalis]	-0.99767	0.171106	1.609308	0.343189	-1.12593
TRINITY_DN36434_c0_g2_i3_orf1	clotting factor B isoform X1 [Ostrinia furnacalis]	-1.50881	0.511599	1.345664	0.36902	-0.71747
TRINITY_DN1091_c0_g3_i1_orf1	macrophage mannose receptor 1-like [Ostrinia furnacalis]	-1.21836	0.993277	1.363153	-0.46129	-0.67679
TRINITY_DN448_c0_g1_i20_orf1	probable cytochrome P450 9f2 isoform X1 [Ostrinia furnacalis]	-1.57521	0.545765	1.277661	0.403923	-0.65214
TRINITY_DN394_c0_g1_i4_orf1	uncharacterized protein LOC114351483 [Ostrinia furnacalis]	-1.15279	1.03687	1.111733	0.159437	-1.15525
TRINITY_DN975_c0_g1_i1_orf1	elongation factor 1-alpha 1 [Myotis lucifugus] >XP_008139741.1 elongation factor 1-alpha 1 [Eptesicus fuscus]	-1.32635	1.186431	1.111367	-0.23454	-0.73691
TRINITY_DN701_c0_g1_i1_orf1	venom protease-like isoform X3 [Ostrinia furnacalis]	-1.31943	0.436974	1.295692	0.600833	-1.01407
TRINITY_DN38783_c0_g1_i1_orf1	regucalcin-like [Ostrinia furnacalis]	-1.44952	0.823543	1.20274	0.262869	-0.83963
TRINITY_DN5880_c0_g2_i2_orf1	macrophage mannose receptor 1 [Bombyx mori]	-1.1019	-0.08269	1.535926	0.648405	-0.99974
TRINITY_DN57900_c0_g1_i2_orf1	hypothetical protein SFRURICE_000634 [Spodoptera frugiperda]	-0.73928	0.256963	1.501117	0.389241	-1.40804
TRINITY_DN2207_c0_g1_i4_orf1	methionine-R-sulfoxide reductase B1 isoform X4 [Pectinophora gossypiella] >XP_049887601.1 methionine-R-sulfoxide reductase B1 isoform X4 [Pectinophora gossypiella]	-0.69024	0.310294	1.48119	0.35132	-1.45256
TRINITY_DN89483_c0_g1_i1_orf1	mitochondrial enolase superfamily member 1-like isoform X2 [Maniola jurtina]	-1.1892	0.113606	1.581318	0.434325	-0.94005
TRINITY_DN27247_c0_g2_i1_orfp1	TRINITY_DN27247_c0_g2_i1_m.23157 TRINITY_DN27247_c0_g2::TRINITY_DN27247_c0_g2_i1::g.23157 ORF type:5prime_partial len:70 (+),score=25.00 TRINITY_DN27247_c0_g2_i1:2-211(+)	-0.82413	0.503376	1.722177	-0.45624	-0.94519
TRINITY_DN5001_c0_g1_i4_orf1	uncharacterized protein LOC114356665 [Ostrinia furnacalis]	-1.03544	0.982825	1.216711	0.051976	-1.21607
TRINITY_DN22589_c0_g1_i6_orfp1	TRINITY_DN22589_c0_g1_i6_m.19386 TRINITY_DN22589_c0_g1::TRINITY_DN22589_c0_g1_i6::g.19386 ORF type:internal len:183 (+),score=63.52 TRINITY_DN22589_c0_g1_i6:2-547(+)	-0.55133	0.960912	1.425037	-0.74549	-1.08912
TRINITY_DN1534_c0_g1_i3_orf1	peptidoglycan recognition protein-like [Ostrinia furnacalis]	-1.05963	1.763612	0.392431	-0.47141	-0.625
TRINITY_DN8651_c0_g1_i16_orf1	glutathione S-transferase theta 2 [Conogethes punctiferalis]	-1.31775	0.111516	1.593933	0.369767	-0.75747
TRINITY_DN68770_c0_g1_i1_orf1	seroin transcript 1A2 [Ostrinia nubilalis]	-0.72404	1.600414	0.552091	-0.17138	-1.25709
TRINITY_DN8030_c0_g1_i2_orf1	chaoptin isoform X1 [Ostrinia furnacalis] >XP_028159953.1 chaoptin isoform X2 [Ostrinia furnacalis] >XP_028159954.1 chaoptin isoform X3 [Ostrinia furnacalis] >XP_028159955.1 chaoptin isoform X4 [Ostrinia furnacalis] >XP_028159956.1 chaoptin isoform X5 [Ostrinia furnacalis]	-1.36778	0.956804	1.101812	0.271464	-0.9623
TRINITY_DN1305_c0_g1_i6_orf1	glutathione S-transferase sigma 3 [Ostrinia furnacalis]	-0.93524	1.232884	1.211203	-0.75442	-0.75442
TRINITY_DN77559_c0_g1_i1_orf1	uncharacterized protein LOC114359392 isoform X2 [Ostrinia furnacalis]	-1.37143	0.796489	1.260488	0.232104	-0.91765
TRINITY_DN15157_c0_g1_i1_orf1	UDP-glycosyltransferase UGT40AM2 [Ostrinia furnacalis]	-1.54727	0.025839	1.510283	0.40823	-0.39708
TRINITY_DN9991_c0_g1_i4_orf1	unnamed protein product [Parnassius apollo]	-1.13994	0.871552	1.390467	-0.12629	-0.99578
TRINITY_DN3991_c0_g1_i6_orf1	acetyl-CoA carboxylase isoform X3 [Trichoplusia ni]	-1.27918	0.443587	1.482845	0.292261	-0.93951
TRINITY_DN143532_c0_g1_i1_orf1	3-oxoacyl-[acyl-carrier-protein] reductase FabG-like [Aphidius gifuensis] >KAF7996667.1 hypothetical protein HCN44_002313 [Aphidius gifuensis]	-0.76256	1.727413	0.527602	-0.6005	-0.89196

TRINITY_DN51776_c0_g1_i1_orf1	unnamed protein product, partial [Iphiclides podalirius]	-1.33192	0.735875	1.32793	0.20557	-0.93745
TRINITY_DN7064_c0_g1_i20_orfp1	TRINITY_DN7064_c0_g1_i20_m.53649 TRINITY_DN7064_c0_g1::TRINITY_DN7064_c0_g1_i20::g.53649 ORF type:internal len:256 (+),score=27.03,Kazal_1 PF00050.22 0.52,Kazal_1|PF00050.22|1.1e-10,Kazal_1|PF00050.22|6.2e-09,Kazal_1|PF00050.22|2.7e-07,Kazal_1|PF00050.22|5.1e-09,Kazal_1|PF00050.22|4.5e-11,Kazal_2|PF07648.16|2.2,Kazal_2|PF07648.16|4.8e-10,Kazal_2|PF07648.16|4.2e-10,Kazal_2|PF07648.16|1.1e-09,Kazal_2|PF07648.16|3e-10,Kazal_2|PF07648.16|1.3e-07 TRINITY_DN7064_c0_g1_i20:3-767(+)	-0.90264	1.53753	0.673176	-0.14779	-1.16027
TRINITY_DN19098_c0_g1_i4_orf1	L-xylulose reductase-like [Ostrinia furnacalis]	-1.17129	-0.00272	1.708295	0.26534	-0.79962
TRINITY_DN829_c0_g1_i8_orf1	cytochrome P450 6B6-like [Ostrinia furnacalis]	-0.68689	0.330195	1.457563	0.368453	-1.46932
TRINITY_DN22046_c1_g1_i5_orf1	uncharacterized protein LOC114351208 [Ostrinia furnacalis]	-1.25149	0.020849	1.722576	0.169408	-0.66135
TRINITY_DN1245_c0_g1_i4_orf1	nuclear RNA export factor 1 [Ostrinia furnacalis]	-0.97913	0.459048	1.548717	0.157624	-1.18626
TRINITY_DN1540_c0_g1_i9_orf1	alaserpin-like isoform X9 [Ostrinia furnacalis]	-1.06346	0.978744	1.40276	-0.46569	-0.85235
TRINITY_DN4341_c0_g1_i4_orf1	uncharacterized protein LOC114354354 [Ostrinia furnacalis]	-1.06888	0.141359	1.709343	0.161373	-0.94319
TRINITY_DN76307_c0_g1_i1_orf1	PREDICTED: quinone oxidoreductase-like protein 2 homolog [Microplitis demolitor]	-1.1726	0.750176	1.555666	-0.56289	-0.57035
TRINITY_DN554_c0_g1_i1_orf1	uncharacterized protein LOC114353093 isoform X1 [Ostrinia furnacalis] >XP_028160722.1 uncharacterized protein LOC114353093 isoform X1 [Ostrinia furnacalis] >XP_028160723.1 uncharacterized protein LOC114353093 isoform X1 [Ostrinia furnacalis]	-1.01248	0.70045	1.391119	0.155735	-1.23482
TRINITY_DN11649_c0_g1_i4_orf1	ubiquitin carboxyl-terminal hydrolase 32-like, partial [Ostrinia furnacalis]	-0.42884	0.507921	1.684017	-0.59177	-1.17132
TRINITY_DN50743_c0_g1_i1_orf1	cytochrome P450 monooxygenase CYP321F7 [Ostrinia furnacalis]	-1.01594	1.098359	1.295868	-0.43188	-0.9464
TRINITY_DN5126_c0_g1_i3_orf1	cytochrome P450 monooxygenase CYP4L47 [Ostrinia furnacalis]	-0.89636	1.333742	1.001028	-0.28268	-1.15573
TRINITY_DN171_c0_g1_i1_orf1	alkaline phosphatase, tissue-nonspecific isozyme-like isoform X1 [Ostrinia furnacalis]	-0.63134	0.86213	1.406897	-0.3003	-1.33739
TRINITY_DN4004_c0_g1_i1_orf1	protein FAM114A2 isoform X1 [Ostrinia furnacalis] >XP_028175160.1 protein FAM114A2 isoform X2 [Ostrinia furnacalis]	-0.76096	0.978379	1.366892	-0.37966	-1.20465
TRINITY_DN33763_c0_g1_i1_orf1	uncharacterized protein LOC114355186 [Ostrinia furnacalis]	-0.69067	1.014558	1.308409	-0.34206	-1.29024
TRINITY_DN55154_c0_g2_i1_orf1	glycosyl transferase family 8 domain-containing protein [Phthorimaea operculella]	-0.41069	1.552577	0.732294	-0.68381	-1.19037
TRINITY_DN4916_c0_g2_i1_orf1	uncharacterized protein LOC114357135, partial [Ostrinia furnacalis]	-0.61675	0.838192	1.435465	-0.33661	-1.3203
TRINITY_DN133474_c0_g2_i2_orf1	enoyl-[acyl-carrier-protein] reductase, mitochondrial [Ostrinia furnacalis]	-0.30136	1.365225	0.956199	-0.79708	-1.22298
TRINITY_DN95530_c0_g1_i1_orf1	aldose reductase-like isoform X4 [Ostrinia furnacalis]	-0.64385	0.528581	1.723861	-0.66185	-0.94674
TRINITY_DN3166_c1_g1_i6_orf1	hypothetical protein evm_013813 [Chilo suppressalis]	-1.7427	0.4768	1.229472	0.35242	-0.31599
TRINITY_DN14904_c0_g1_i1_orf1	attacin [Ostrinia furnacalis]	-1.28866	-0.37563	1.759132	0.175311	-0.27016
TRINITY_DN15202_c0_g1_i6_orf1	uncharacterized protein LOC114364499 isoform X2 [Ostrinia furnacalis]	-1.67149	0.764067	1.105382	0.337399	-0.53535
TRINITY_DN143895_c0_g1_i1_orf1	cathepsin L-like [Aphidius gifuensis] >KAF7988186.1 hypothetical protein HCN44_007680 [Aphidius gifuensis]	-1.92875	0.854231	0.395579	0.05375	0.625186
TRINITY_DN17615_c0_g1_i3_orf1	hypothetical protein SFRUCORN_008858 [Spodoptera frugiperda]	-1.90641	0.595676	0.85456	0.525117	-0.06894
TRINITY_DN5177_c0_g1_i2_orf1	hemolin-like isoform X1 [Ostrinia furnacalis]	-1.73659	0.770967	1.165657	-0.17449	-0.02554
TRINITY_DN59885_c0_g1_i3_orf1	TGF-beta-activated kinase 1 and MAP3K7-binding protein 1-like [Ostrinia furnacalis]	-1.95786	0.653176	0.619902	0.096025	0.58876
TRINITY_DN12009_c0_g1_i1_orf1	uncharacterized protein LOC114365631 [Ostrinia furnacalis]	-1.83566	0.017678	0.802396	0.022779	0.99281
TRINITY_DN142657_c0_g1_i1_orf1	sorting and assembly machinery component 50 homolog [Diachasma alloeum]	-1.87016	1.066334	0.54074	-0.00704	0.270126
TRINITY_DN5439_c0_g1_i2_orf1	uncharacterized protein LOC114353087 [Ostrinia furnacalis]	-1.80925	0.279551	1.136761	0.569988	-0.17705
TRINITY_DN467_c3_g1_i5_orf1	lysozyme precursor [Loxostege sticticalis]	-1.92342	0.374809	0.786523	0.026814	0.735275
TRINITY_DN4767_c0_g1_i6_orf1	cysteine protease XCP2-like [Ostrinia furnacalis]	-1.87233	0.926598	0.501327	-0.15577	0.600171
TRINITY_DN1597_c0_g1_i5_orfp1	TRINITY_DN1597_c0_g1_i5_m.57494 TRINITY_DN1597_c0_g1::TRINITY_DN1597_c0_g1_i5::g.57494 ORF type:complete len:86 (+),score=7.19 TRINITY_DN1597_c0_g1_i5:134-391(+)	-1.85136	0.758293	0.993324	-0.00396	0.103703
TRINITY_DN29026_c0_g1_i4_orf1	TIL [Ostrinia furnacalis]	-1.71245	0.418375	1.350398	0.155281	-0.2116
TRINITY_DN9608_c0_g1_i3_orf1	cytochrome P450 monooxygenase CYP9G18 [Cnaphalocrocis medinalis]	-1.52189	0.578782	1.43463	0.04571	-0.53724
TRINITY_DN135449_c0_g1_i5_orf1	larval cuticle protein LCP-17-like [Galleria mellonella]	-1.93247	0.845035	0.6546	0.095417	0.337423
TRINITY_DN12526_c0_g1_i5_orf1	uncharacterized protein LOC114359035 isoform X3 [Ostrinia furnacalis]	-1.66723	0.200905	0.760112	-0.46935	1.175557
TRINITY_DN20442_c0_g2_i1_orf1	hypothetical protein evm_008218 [Chilo suppressalis]	-1.45463	0.223048	0.962936	-0.83288	1.101528
TRINITY_DN30177_c0_g2_i1_orf1	uncharacterized protein LOC114365032 [Ostrinia furnacalis]	-1.21239	-0.09106	1.824609	-0.42913	-0.09203
TRINITY_DN5310_c2_g1_i2_orf1	serine protease persephone-like [Ostrinia furnacalis]	-1.9061	0.030335	0.975187	0.381913	0.518665
TRINITY_DN26209_c0_g1_i6_orf1	uncharacterized protein LOC114352357 [Ostrinia furnacalis]	-1.94497	0.391515	0.911385	0.204769	0.437306
TRINITY_DN124654_c0_g1_i1_orf1	protein lethal(2)essential for life [Manduca sexta] >KAG6441919.1 hypothetical protein O3G_MSEX002019 [Manduca	-1.65603	0.868961	0.994321	-0.59978	0.392523
TRINITY_DN1540_c0_g1_i7_orf1	alaserpin-like isoform X13 [Ostrinia furnacalis]	-1.95892	0.723581	0.526755	0.119176	0.589406
TRINITY_DN31286_c0_g1_i6_orfp1	TRINITY_DN31286_c0_g1_i6_m.28438 TRINITY_DN31286_c0_g1::TRINITY_DN31286_c0_g1_i6::g.28438 ORF type:internal len:92 (-),score=3.10,Perilipin PF03036.17 2e-05 TRINITY_DN31286_c0_g1_i6:1-273(-)	-1.82585	0.412498	1.093356	0.513184	-0.19318
TRINITY_DN19731_c0_g1_i1_orf1	allergen Tha p 1-like [Ostrinia furnacalis] >XP_028174916.1 allergen Tha p 1-like [Ostrinia furnacalis] >BAV56808.1 chemosensory protein 4 [Ostrinia furnacalis]	-1.83232	0.904232	0.844287	0.274958	-0.19115

TRINITY_DN581_c3_g2_i1_orf1	uncharacterized protein LOC114364499 isoform X3 [Ostrinia furnacalis]	-1.53007	0.656118	1.424298	-0.11973	-0.43062
TRINITY_DN33346_c0_g1_i1_orf1	PREDICTED: U6 snRNA-associated Sm-like protein LSm3 [Papilio xuthus] >XP_028165558.1 U6 snRNA-associated Sm-like protein LSm3 [Ostrinia furnacalis] >KOB73597.1 LSM Sm-like protein family member [Operophtera brumata] >RVE45517.1 hypothetical protein evm_009856 [Chilo suppressalis] >CAB3523639.1 unnamed protein product [Chilo suppressalis] >CAH0400961.1 unnamed protein product [Chilo suppressalis]	-1.9243	0.644146	0.845478	0.40817	0.026508
TRINITY_DN56308_c0_g1_i2_orf1	storage protein 1 [Omphisa fuscidentalis]	-1.88616	0.929461	0.355729	-0.06788	0.668848
TRINITY_DN30169_c0_g1_i1_orfp1	TRINITY_DN30169_c0_g1_i1_m.11367 TRINITY_DN30169_c0_g1::TRINITY_DN30169_c0_g1_i1::g.11367 ORF type:3prime_partial len:52 (+),score=0.98 TRINITY_DN30169_c0_g1_i1:72-224(+)	-1.82308	0.863167	0.505605	-0.30787	0.762173
TRINITY_DN16234_c0_g2_i3_orf1	uncharacterized protein LOC114363370 [Ostrinia furnacalis]	-1.42573	1.205881	1.059279	-0.5589	-0.28053
TRINITY_DN5337_c0_g1_i6_orf1	epoxide hydrolase 1-like [Ostrinia furnacalis]	-1.91203	0.714755	0.885861	0.160323	0.151087
TRINITY_DN13799_c0_g1_i1_orf1	uncharacterized protein LOC116345248 [Contarinia nasturtii]	-1.68477	1.100378	0.70782	-0.53187	0.408441
TRINITY_DN11826_c0_g1_i4_orf1	aldehyde dehydrogenase X, mitochondrial-like [Ostrinia furnacalis]	-1.87418	0.7961	0.529556	-0.18552	0.734044
TRINITY_DN7040_c0_g1_i4_orf1	uncharacterized protein LOC114353763 [Ostrinia furnacalis]	-1.95102	0.669684	0.596271	0.064293	0.620774
TRINITY_DN20658_c0_g2_i3_orf1	prostaglandin reductase 1-like [Ostrinia furnacalis]	-1.80774	0.305551	1.137322	0.555698	-0.19083
TRINITY_DN3609_c0_g1_i6_orf1	leukocyte elastase inhibitor-like [Ostrinia furnacalis]	-1.80151	-0.18225	0.762722	0.166589	1.054449
TRINITY_DN64772_c0_g1_i1_orf1	aldehyde dehydrogenase, partial [Mythimna separata]	-1.88379	0.70898	0.563467	-0.16566	0.777007
TRINITY_DN5099_c0_g1_i3_orf1	trans-1,2-dihydrobenzene-1,2-diol dehydrogenase-like [Ostrinia furnacalis]	-1.85027	0.549081	1.024014	0.445186	-0.16801
TRINITY_DN80328_c0_g1_i5_orf1	arylphorin subunit alpha-like [Ostrinia furnacalis]	-1.86072	0.997005	0.329729	-0.11539	0.649369
TRINITY_DN2314_c0_g1_i7_orf1	protein dj-1beta-like isoform X1 [Ostrinia furnacalis]	-1.82514	1.043763	0.756264	-0.04735	0.072462
TRINITY_DN27035_c0_g1_i1_orf1	glucose-6-phosphate isomerase-like [Ostrinia furnacalis]	-1.84179	0.926109	0.605404	-0.25442	0.564701
TRINITY_DN48878_c0_g2_i1_orf1	codanin-1 [Ostrinia furnacalis]	-1.81547	1.003443	0.53329	-0.29303	0.571761
TRINITY_DN9325_c0_g1_i1_orf1	protein takeout-like [Ostrinia furnacalis]	-1.71625	0.861463	1.114966	-0.26309	0.00291
TRINITY_DN16840_c1_g1_i1_orf1	attacin-like [Ostrinia furnacalis]	-1.58654	-0.6339	1.279606	0.446805	0.494019
TRINITY_DN2650_c0_g1_i1_orf1	hypothetical protein HW555_002849 [Spodoptera exigua] >CAH0691914.1 unnamed protein product [Spodoptera exigua]	-1.82894	0.251462	1.222371	0.04622	0.308889
TRINITY_DN1897_c0_g2_i4_orf1	phenoloxidase-activating factor 2-like [Hyposmocoma kahamanoa]	-1.352	-0.41044	1.403835	-0.51662	0.875215
TRINITY_DN6325_c0_g1_i9_orf1	fructose-bisphosphate aldolase isoform X2 [Pieris brassicae]	-1.72079	1.100416	0.760212	0.276681	-0.41652
TRINITY_DN47842_c0_g1_i1_orf1	protein lethal(2)essential for life-like [Helicoverpa armigera] >PZC74790.1 hypothetical protein B5X24_HaOG207163 [Helicoverpa armigera]	-1.56292	0.299622	1.002573	-0.71473	0.975453
TRINITY_DN12775_c0_g1_i10_orfp1	TRINITY_DN12775_c0_g1_i10_m.21238 TRINITY_DN12775_c0_g1::TRINITY_DN12775_c0_g1_i10::g.21238 ORF type:5prime_partial len:67 (-),score=0.74 TRINITY_DN12775_c0_g1_i10:275-475(-)	-1.72473	0.913684	1.028992	0.123297	-0.34124
TRINITY_DN1672_c0_g1_i6_orf1	cystinosin homolog isoform X1 [Ostrinia furnacalis] >XP_028162341.1 cystinosin homolog isoform X1 [Ostrinia furnacalis] >XP_028162342.1 cystinosin homolog isoform X1 [Ostrinia furnacalis] >XP_028162343.1 cystinosin homolog isoform X1 [Ostrinia furnacalis] >XP_028162344.1 cystinosin homolog isoform X1 [Ostrinia furnacalis]	-1.86241	-0.06836	1.037283	0.286022	0.607458
TRINITY_DN81031_c0_g1_i1_orf1	aldehyde dehydrogenase, partial [Ectropis obliqua]	-1.88612	0.784203	0.459789	-0.1317	0.773829
TRINITY_DN23398_c0_g1_i1_orf1	cytochrome P450 6B7-like [Ostrinia furnacalis]	-1.90957	0.427918	0.705041	-0.04285	0.81946
TRINITY_DN6908_c0_g1_i1_orf1	serine--pyruvate aminotransferase, mitochondrial [Ostrinia furnacalis] >XP_028157324.1 serine--pyruvate aminotransferase, mitochondrial [Ostrinia furnacalis] >XP_028157325.1 serine--pyruvate aminotransferase, mitochondrial	-1.85076	0.498985	1.067925	-0.12727	0.411125
TRINITY_DN15597_c0_g1_i1_orf1	microsomal glutathione S-transferase 1-like [Ostrinia furnacalis]	-1.9021	0.05162	0.866312	0.223219	0.760946
TRINITY_DN276_c0_g1_i1_orf1	protein lethal(2)essential for life-like [Helicoverpa zea] >XP_049705426.1 protein lethal(2)essential for life [Helicoverpa armigera] >ATB54993.1 heat shock protein 20.8 [Helicoverpa armigera] >PZC74337.1 hypothetical protein B5X24_HaOG207971 [Helicoverpa armigera]	-1.49049	1.177455	0.902836	-0.74378	0.153979
TRINITY_DN1030_c0_g1_i6_orf1	gamma-glutamyl hydrolase A-like isoform X1 [Ostrinia furnacalis]	-1.94665	0.37324	0.869434	0.168213	0.535761
TRINITY_DN307_c1_g1_i1_orf1	uncharacterized protein LOC114356704 [Ostrinia furnacalis]	-1.37359	0.498515	1.375869	-0.9009	0.400108
TRINITY_DN18128_c0_g1_i4_orf1	arylsulfatase B [Ostrinia furnacalis]	-1.82085	1.05335	0.406205	-0.23449	0.595786
TRINITY_DN4497_c2_g1_i3_orf1	uncharacterized protein LOC114353086 [Ostrinia furnacalis]	-1.71078	0.765472	1.093406	0.300635	-0.44873
TRINITY_DN71863_c0_g1_i2_orf1	unnamed protein product [Diatraea saccharalis]	-1.87199	1.006589	0.517075	-0.10369	0.452013
TRINITY_DN51480_c0_g1_i1_orf1	uncharacterized protein LOC114361588 isoform X14 [Ostrinia furnacalis]	-1.74247	0.921437	0.993684	0.150788	-0.32344
TRINITY_DN69307_c0_g1_i6_orf1	hypothetical protein evm_010738 [Chilo suppressalis]	-1.72216	1.067569	0.925965	-0.12495	-0.14642
TRINITY_DN49530_c0_g1_i1_orf1	ommochrome-binding protein-like [Ostrinia furnacalis]	-1.77118	0.956876	0.871222	0.277032	-0.33394
TRINITY_DN2146_c0_g2_i1_orf1	heat shock protein 68-like [Ostrinia furnacalis]	-1.48804	1.356313	0.742078	-0.62861	0.018257
TRINITY_DN1960_c5_g1_i3_orf1	cytochrome P450 monooxygenase CYP9G18 [Cnaphalocrocis medinalis]	-1.65427	1.003029	0.943671	0.256303	-0.54873
TRINITY_DN4080_c0_g1_i8_orf1	AMP deaminase 2 isoform X3 [Ostrinia furnacalis] >XP_028163647.1 AMP deaminase 2 isoform X3 [Ostrinia furnacalis] >XP_028163648.1 AMP deaminase 2 isoform X3 [Ostrinia furnacalis]	-1.50535	0.585075	1.492277	-0.30979	-0.26222
TRINITY_DN970_c0_g1_i4_orf1	spermine oxidase-like isoform X2 [Ostrinia furnacalis]	-1.74779	0.767776	1.032047	0.354301	-0.40634

TRINITY_DN1540_c0_g1_i14_orf1	alaserpin-like isoform X1 [Ostrinia furnacalis]	-1.59624	0.816759	1.229158	0.069264	-0.51894
TRINITY_DN5132_c0_g1_i4_orf1	small heat shock protein Hsp24.2 [Ostrinia furnacalis]	-1.73921	0.717778	0.819788	-0.51867	0.720318
TRINITY_DN43667_c0_g1_i1_orf1	carbonyl reductase [NADPH] 1-like [Ostrinia furnacalis]	-1.67492	0.668998	1.295725	-0.25936	-0.03045
TRINITY_DN55148_c0_g1_i1_orf1	60S ribosomal protein L11 isoform 1 [Homo sapiens] >NP_001069049.1 60S ribosomal protein L11 [Bos taurus] >NP_001240835.1 60S ribosomal protein L11 isoform 1 [Canis lupus familiaris] >NP_001269300.1 60S ribosomal protein L11 [Chinchilla lanigera] >NP_001291809.1 60S ribosomal protein L11 [Ailuropoda melanoleuca] >NP_080195.1 60S ribosomal protein L11 [Mus musculus] >XP_001504267.1 60S ribosomal protein L11 isoform X2 [Equus caballus] >XP_003471379.1 60S ribosomal protein L11 [Cavia porcellus] >XP_003810808.1 60S ribosomal protein L11 [Pan paniscus] >XP_003891370.1 60S ribosomal protein L11 [Papio anubis] >XP_003989701.1 60S ribosomal protein L11 [Felis catus] >XP_004285699.1 60S ribosomal protein L11 isoform X2 [Orcinus orca] >XP_004377186.1 60S ribosomal protein L11 [Trichechus manatus latirostris] >XP_004394821.1 PREDICTED: 60S ribosomal protein L11 isoform X1 [Odobenus rosmarus divergens] >XP_004465476.2 60S ribosomal protein L11 [Dasypus novemcinctus] >XP_004637663.1 60S ribosomal protein L11 [Octodon degus] >XP_004850617.1 60S ribosomal protein L11 isoform X2 [Heterocephalus glaber] >XP_005544522.1 60S ribosomal protein L11 isoform X1 [Macaca fascicularis] >XP_005676921.1 PREDICTED: 60S ribosomal protein L11 isoform X2 [Capra hircus] >XP_006078005.1 60S ribosomal protein L11 isoform X2 [Bubalus bubalis] >XP_006094108.1 60S ribosomal protein L11 isoform X3 [Myotis lucifugus] >XP_006239286.1 60S ribosomal protein L11 isoform X1 [Rattus norvegicus] >XP_006737645.1 60S ribosomal protein L11 [Leptonychotes weddellii] >XP_006777625.1 PREDICTED: 60S ribosomal protein L11 isoform X1 [Myotis davidii] >XP_006883588.1 PREDICTED: 60S ribosomal protein L11-like isoform X1 [Elephantulus edwardii] >XP_007121151.1 60S ribosomal protein L11 isoform X2 [Physeter catodon] >XP_007175168.1 60S ribosomal protein L11 isoform X1 [Balaenoptera acutorostrata scammoni] >XP_007459239.1 PREDICTED: 60S ribosomal protein L11 isoform X1 [Lipotes vexillifer] >XP_007524793.1 PREDICTED: 60S ribosomal protein L11 [Erinaceus europaeus] >XP_007528779.2 PREDICTED: 60S ribosomal protein L11 [Erinaceus europaeus] >XP_007933904.2 60S ribosomal protein L11 [Orycteropus afer afer] >XP_007978264.1 60S ribosomal protein L11 isoform X1 [Chlorocebus sabaeus] >XP_008059998.1 60S ribosomal protein L11 isoform X2 [Carlito syrichta] >XP_008146324.1 60S ribosomal protein L11 [Eptesicus fuscus] >XP_008263912.1 PREDICTED: 60S ribosomal protein L11 [Oryctolagus cuniculus] >XP_008518979.1 PREDICTED: 60S ribosomal protein L11 isoform X2 [Equus przewalskii] >XP_008571152.1 PREDICTED: 60S ribosomal protein L11 isoform X1 [Galeopterus variegatus] >XP_008571160.1 PREDICTED: 60S ribosomal protein L11 isoform X2 [Galeopterus variegatus] >XP_008846816.1 60S ribosomal protein L11 [Nannospalax galili] >XP_010354068.1 60S ribosomal protein L11 [Rhinopithecus roxellana] >XP_010624280.1 60S ribosomal protein L11 [Fukomys damarensis] >XP_011355886.1 60S ribosomal protein L11 [Pteropus vampyrus] >XP_011761124.1 60S ribosomal protein L11 [Macaca nemestrina] >XP_011833215.1 PREDICTED: 60S ribosomal protein L11 isoform X2 [Mandrillus leucophaeus] >XP_011935575.1 PREDICTED: 60S ribosomal protein L11 [Cercocebus atys] >XP_014710573.1 60S ribosomal protein L11 isoform X2 [Equus asinus] >XP_014930664.1 60S ribosomal protein L11 [Acinonyx jubatus] >XP_014949173.1 60S	-1.57093	0.864367	1.273929	-0.30822	-0.25914
TRINITY_DN6580_c0_g1_i4_orf1	catalase [Ostrinia furnacalis]	-1.83751	0.561295	1.133999	0.149989	-0.00777
TRINITY_DN244_c1_g1_i5_orf1	C-1-tetrahydrofolate synthase, cytoplasmic isoform X3 [Ostrinia furnacalis]	-1.82704	0.692351	1.0598	-0.13075	0.205647
TRINITY_DN1287_c0_g1_i5_orf1	probable chitinase 10 isoform X6 [Ostrinia furnacalis]	-1.84593	0.746836	0.451772	-0.23363	0.880955
TRINITY_DN17505_c0_g1_i15_orf1	unnamed protein product [Chilo suppressalis]	-0.86744	-0.67363	1.92808	-0.16714	-0.21987
TRINITY_DN1957_c0_g1_i4_orf1	NAD kinase 2, mitochondrial [Ostrinia furnacalis]	-1.8114	1.025147	0.683143	-0.26141	0.364521
TRINITY_DN9492_c1_g1_i1_orf1	aldo-keto reductase AKR2E4-like [Galleria mellonella]	-1.75286	0.748909	1.113227	0.191697	-0.30097
TRINITY_DN1098_c1_g1_i4_orf1	lysozyme 10 [Ostrinia furnacalis]	-1.74837	0.408851	0.820515	-0.43624	0.955246

TRINITY_DN142442_c0_g1_i1_orf1	60S ribosomal protein L38 [Homo sapiens] >NP_001002486.1 60S ribosomal protein L38 [Danio rerio] >NP_001030335.1 60S ribosomal protein L38 [Homo sapiens] >NP_001071060.1 60S ribosomal protein L38 [Rattus norvegicus] >NP_001133168.1 60S ribosomal protein L38 [Salmo salar] >NP_001187063.1 60S ribosomal protein L38 [Ictalurus punctatus] >NP_001232305.1 60S ribosomal protein L38 [Taeniopygia guttata] >NP_001264941.1 60S ribosomal protein L38 [Gallus gallus] >XP_003211558.1 60S ribosomal protein L38 [Meleagris gallopavo] >XP_003315754.1 60S ribosomal protein L38 [Pan troglodytes] >XP_003315758.1 60S ribosomal protein L38 [Pan troglodytes] >XP_003339346.1 60S ribosomal protein L38 [Pan troglodytes] >XP_003358038.1 60S ribosomal protein L38 [Sus scrofa] >XP_003417326.1 60S ribosomal protein L38 [Loxodonta africana] >XP_003453439.1 60S ribosomal protein L38 [Oreochromis niloticus] >XP_003464913.2 60S ribosomal protein L38 [Cavia porcellus] >XP_003768586.1 60S ribosomal protein L38 [Sarcophilus harrisii] >XP_003786210.1 60S ribosomal protein L38 [Otolemur garnettii] >XP_003795793.1 60S ribosomal protein L38 [Otolemur garnettii] >XP_003922345.1 60S ribosomal protein L38 [Saimiri boliviensis boliviensis] >XP_004041125.1 60S ribosomal protein L38 [Gorilla gorilla gorilla] >XP_004041126.1 60S ribosomal protein L38 [Gorilla gorilla gorilla] >XP_004041128.1 60S ribosomal protein L38 [Gorilla gorilla gorilla] >XP_004331065.1 60S ribosomal protein L38 [Tursiops truncatus] >XP_004401894.1 PREDICTED: 60S ribosomal protein L38 [Odobenus rosmarus divergens] >XP_004412345.1 PREDICTED: 60S ribosomal protein L38 [Odobenus rosmarus divergens] >XP_004469223.1 60S ribosomal protein L38 [Dasypus novemcinctus] >XP_004469224.1 60S ribosomal protein L38 [Dasypus novemcinctus] >XP_005068761.1 60S ribosomal protein L38 [Mesocricetus auratus] >XP_005070019.1 60S ribosomal protein L38 [Mesocricetus auratus] >XP_005141156.1 60S ribosomal protein L38 [Melopsittacus undulatus] >XP_005336034.1 60S ribosomal protein L38 [Ictidomys tridecemlineatus] >XP_005336035.1 60S ribosomal protein L38 [Ictidomys tridecemlineatus] >XP_005350739.1 60S ribosomal protein L38 [Microtus ochrogaster] >XP_005350740.1 60S ribosomal protein L38 [Microtus ochrogaster] >XP_005412280.1 PREDICTED: 60S ribosomal protein L38 [Chinchilla lanigera] >XP_005412281.1 PREDICTED: 60S ribosomal protein L38 [Chinchilla lanigera] >XP_005530739.1 PREDICTED: 60S ribosomal protein L38 [Pseudopodoces humilis] >XP_005584887.1 60S ribosomal protein L38 [Macaca fascicularis] >XP_005584888.1 60S ribosomal protein L38 [Macaca fascicularis] >XP_005584889.1 60S ribosomal protein L38 [Macaca fascicularis] >XP_005584890.1 60S ribosomal protein L38 [Macaca fascicularis] >XP_005584891.1 60S ribosomal protein L38 [Macaca fascicularis] >XP_005592611.1 60S ribosomal protein L38 [Macaca fascicularis] >XP_005597274.1 60S ribosomal protein L38 isoform X2 [Equus caballus] >XP_005668697.1 60S ribosomal protein L38 [Sus scrofa] >XP_005861853.1 PREDICTED: 60S ribosomal protein L38 [Myotis brandtii] >XP_005861854.1 PREDICTED: 60S ribosomal protein L38 [Myotis brandtii] >XP_005889694.1 PREDICTED: 60S ribosomal protein L38 isoform X2 [Bos mutus] >XP_006042140.1 60S ribosomal protein L38 isoform X2 [Bubalus bubalis] >XP_006042141.1 60S ribosomal protein L38 isoform X2 [Bubalus bubalis] >XP_006082202.1 60S					
osomal protein L38 [Nematolebias whitei]	60S RIBOSOMAL PROTEIN L38 [Oryctolagus cuniculus] >4UJD_Ak Chain Ak	60S RIBO	60S RIBO	60S ribos	60S ribos	60S ribos
TRINITY_DN512_c0_g1_i10_orf1	uncharacterized protein LOC114366781 [Ostrinia furnacalis]	-1.78009	0.729137	1.119753	0.112955	-0.18176
TRINITY_DN2205_c0_g1_i3_orf1	probable chitinase 2 [Ostrinia furnacalis]	-1.88584	0.282945	0.709191	-0.03339	0.927093
TRINITY_DN2392_c0_g2_i1_orf1	cytochrome P450 9e2-like [Ostrinia furnacalis] >QPF77612.1 cytochrome P450 monooxygenase CYP9A185 [Ostrinia	-1.47167	0.622468	1.477381	-0.13139	-0.49679
TRINITY_DN1014_c0_g2_i8_orf1	uncharacterized protein LOC114362446 [Ostrinia furnacalis] >XP_028173663.1 uncharacterized protein LOC114362446 [Ostrinia furnacalis]	-1.84884	0.521394	1.127539	0.003496	0.196412
TRINITY_DN11383_c0_g2_i4_orf1	aminoacylase-1A-like [Ostrinia furnacalis]	-1.63367	0.891443	1.046642	0.292008	-0.59642
TRINITY_DN664_c0_g1_i18_orf1	chitinase-like protein EN03 isoform X2 [Ostrinia furnacalis]	-1.75776	0.619701	0.782076	-0.4744	0.830386
TRINITY_DN1216_c0_g1_i4_orf1	bifunctional purine biosynthesis protein PURH isoform X1 [Ostrinia furnacalis] >XP_028176123.1 bifunctional purine biosynthesis protein PURH isoform X2 [Ostrinia furnacalis] >XP_028176129.1 bifunctional purine biosynthesis protein PURH isoform X3 [Ostrinia furnacalis]	-1.82868	0.690927	1.059656	0.201266	-0.12317
TRINITY_DN6908_c0_g1_i3_orf1	serine--pyruvate aminotransferase, mitochondrial [Ostrinia furnacalis] >XP_028157324.1 serine--pyruvate aminotransferase, mitochondrial [Ostrinia furnacalis] >XP_028157325.1 serine--pyruvate aminotransferase, mitochondrial	-1.68447	-0.30833	1.363781	0.244592	0.384422
TRINITY_DN8985_c0_g1_i4_orf1	cytochrome P450 6B6-like [Ostrinia furnacalis]	-1.6869	0.797281	0.440417	-0.55766	1.00686
TRINITY_DN3196_c0_g1_i1_orf1	organic cation transporter-like protein [Ostrinia furnacalis]	-1.69906	0.612713	1.217695	0.285306	-0.41666
TRINITY_DN140212_c0_g1_i1_orf1	macrophage mannose receptor 1-like [Ostrinia furnacalis]	-1.55524	0.516832	1.165293	-0.75197	0.625087
TRINITY_DN22375_c0_g1_i4_orf1	venom carboxylesterase-6-like [Ostrinia furnacalis]	-1.65491	1.115302	0.911646	-0.42797	0.055927
TRINITY_DN8637_c0_g1_i1_orf1	superoxide dismutase [Cu-Zn] [Ostrinia furnacalis] >XP_028177872.1 superoxide dismutase [Cu-Zn] [Ostrinia furnacalis]	-1.87181	0.74393	0.880933	-0.13765	0.384597
TRINITY_DN2618_c0_g1_i3_orf1	CDP-diacylglycerol--inositol 3-phosphatidyltransferase [Ostrinia furnacalis]	-1.73619	0.783445	1.13909	0.075611	-0.26196
TRINITY_DN103511_c0_g1_i4_orf1	probable salivary secreted peptide [Ostrinia furnacalis]	-1.67837	1.248018	0.749391	-0.07876	-0.24027
TRINITY_DN4602_c0_g1_i4_orf1	2-iminobutanoate/2-iminopropanoate deaminase [Ostrinia furnacalis]	-1.76701	0.364918	0.614215	-0.33301	1.120879
TRINITY_DN2264_c0_g1_i1_orf1	cytochrome P450 6B6-like [Ostrinia furnacalis]	-1.84572	0.601916	1.004129	-0.19162	0.431293
TRINITY_DN89083_c0_g1_i1_orf1	lysine-specific demethylase 4A isoform X2 [Diachasma alloeum]	-1.86422	0.727953	0.590595	-0.22571	0.771383
TRINITY_DN64892_c0_g1_i1_orf1	aldehyde dehydrogenase X, mitochondrial [Manduca sexta] >KAG6450704.1 hypothetical protein O3G_MSEX006722 [Manduca sexta]	-1.75655	1.172294	0.31063	-0.31393	0.587556

TRINITY_DN11680_c0_g1_i1_orf1	uncharacterized protein LOC114355414 [Ostrinia furnacalis]	-1.74663	0.43028	1.262049	0.318613	-0.26431
TRINITY_DN1144_c0_g1_i10_orf1	TIL [Ostrinia furnacalis]	-1.76989	1.179899	0.430367	-0.29259	0.452216
TRINITY_DN33705_c0_g1_i1_orf1	atlastin-like isoform X4 [Ostrinia furnacalis]	-1.709	0.43112	1.313201	0.272461	-0.30778
TRINITY_DN987_c0_g1_i3_orf1	unnamed protein product [Chilo suppressalis]	-1.2989	1.021883	1.252945	-0.82153	-0.1544
TRINITY_DN2847_c0_g1_i20_orf1	uncharacterized protein LOC114352221 [Ostrinia furnacalis]	-1.47724	0.590426	1.493466	-0.13794	-0.46871
TRINITY_DN1116_c0_g1_i6_orf1	RNA exonuclease 4-like [Ostrinia furnacalis] >QEE79882.1 REX4 [Ostrinia furnacalis]	-0.87405	-1.00366	1.769248	0.269393	-0.16093
TRINITY_DN1720_c0_g1_i3_orf1	monocarboxylate transporter 12 [Ostrinia furnacalis] >XP_028157531.1 monocarboxylate transporter 12 [Ostrinia	-1.83099	0.623458	0.835175	-0.30976	0.682122
TRINITY_DN12367_c0_g1_i8_orf1	aldose reductase-like isoform X2 [Ostrinia furnacalis]	-1.30878	1.18987	1.123896	-0.7296	-0.27538
TRINITY_DN7075_c0_g2_i1_orf1	retinal dehydrogenase 1-like [Ostrinia furnacalis]	-1.62161	0.909159	1.172632	-0.05281	-0.40737
TRINITY_DN31609_c0_g1_i3_orf1	sorbitol dehydrogenase-like [Ostrinia furnacalis]	-1.52644	0.171739	1.609905	-0.21742	-0.03778
TRINITY_DN5497_c0_g1_i6_orf1	1,2-dihydroxy-3-keto-5-methylthiopentene dioxygenase-like [Ostrinia furnacalis]	-1.71331	0.513198	1.33839	-0.08203	-0.05624
TRINITY_DN38392_c0_g1_i1_orf1	enoyl-CoA hydratase domain-containing protein 3 [Agrotis segetum]	-0.84094	-0.1726	1.515084	-1.2104	0.708859
TRINITY_DN3483_c0_g1_i5_orf1	phenoloxidase-activating factor 2-like isoform X1 [Ostrinia furnacalis] >XP_028178309.1 phenoloxidase-activating factor 2-like isoform X2 [Ostrinia furnacalis]	-1.71297	0.790817	0.68209	-0.56781	0.807881
TRINITY_DN7957_c0_g1_i5_orf1	spermidine synthase [Ostrinia furnacalis] >XP_028167892.1 spermidine synthase [Ostrinia furnacalis]	-1.79395	0.915957	0.663659	-0.3824	0.596736
TRINITY_DN7212_c0_g1_i4_orf1	peptidylglycine alpha-hydroxylating monooxygenase [Ostrinia furnacalis]	-1.59341	1.313123	0.715882	-0.47216	0.036565
TRINITY_DN7590_c0_g1_i4_orf1	innexin inx1-like [Pectinophora gossypiella]	-1.76428	0.240513	0.518135	-0.2236	1.22923
TRINITY_DN1493_c0_g1_i5_orf1	uncharacterized protein LOC114350869 [Ostrinia furnacalis]	-1.06678	0.38742	1.68137	-0.93853	-0.06347
TRINITY_DN1543_c0_g2_i2_orf1	regulation of enolase protein 1-like isoform X3 [Ostrinia furnacalis] >XP_028161334.1 regulation of enolase protein 1-like isoform X3 [Ostrinia furnacalis]	-1.12613	-0.28088	1.881489	-0.2237	-0.25078
TRINITY_DN14239_c0_g1_i5_orf1	uncharacterized protein LOC114352770 [Ostrinia furnacalis] >XP_028160292.1 uncharacterized protein LOC114352770 [Ostrinia furnacalis]	-1.34324	0.083152	1.739902	-0.39226	-0.08755
TRINITY_DN6680_c0_g1_i1_orf1	hypothetical protein evm_009571 [Chilo suppressalis]	-1.35872	1.261589	1.051333	-0.50643	-0.44777
TRINITY_DN131471_c0_g1_i1_orf1	basement membrane-specific heparan sulfate proteoglycan core protein isoform X13 [Ostrinia furnacalis]	-1.6531	0.513586	1.069709	-0.61962	0.68942
TRINITY_DN35147_c0_g1_i1_orf1	collagen alpha-2(IV) chain isoform X2 [Ostrinia furnacalis]	-1.39194	-0.31689	1.581907	-0.41172	0.538648
TRINITY_DN17326_c0_g1_i5_orf1	aminoacylase-1-like [Ostrinia furnacalis]	-1.55047	0.621003	1.171447	-0.75693	0.51495
TRINITY_DN8580_c0_g1_i12_orf1	peroxisomal N(1)-acetyl-spermine/spermidine oxidase-like isoform X1 [Ostrinia furnacalis]	-1.58807	0.606648	0.618278	-0.72996	1.093108
TRINITY_DN74020_c0_g1_i2_orf1	unnamed protein product [Euphydryas editha]	-1.47281	0.053746	1.330747	-0.68149	0.769813
TRINITY_DN2110_c0_g1_i3_orf1	ribose-phosphate pyrophosphokinase 1 isoform X1 [Chelonus insularis]	-1.77728	0.932099	0.748731	-0.40301	0.499461
TRINITY_DN27833_c0_g2_i1_orf1	uncharacterized protein LOC114359161 [Ostrinia furnacalis]	-1.5503	-0.14836	1.592323	-0.07612	0.182443
TRINITY_DN6136_c0_g1_i1_orf1	uncharacterized protein LOC114354455 [Ostrinia furnacalis]	-1.57114	1.375154	0.600491	-0.51696	0.112449
TRINITY_DN1103_c0_g1_i12_orf1	retinal dehydrogenase 1-like [Ostrinia furnacalis]	-1.68758	0.648639	1.242233	-0.39108	0.187786
TRINITY_DN63389_c0_g1_i4_orf1	retinol dehydrogenase 14-like [Ostrinia furnacalis]	-1.26895	0.616936	1.138164	-1.13626	0.650112
TRINITY_DN483_c0_g1_i6_orf1	uncharacterized protein LOC114354803 isoform X1 [Ostrinia furnacalis] >XP_028163172.1 uncharacterized protein LOC114354803 isoform X2 [Ostrinia furnacalis]	-0.92616	-0.89736	1.826723	-0.00652	0.003319
TRINITY_DN5029_c0_g1_i1_orf1	ribose-phosphate pyrophosphokinase 2-like [Ostrinia furnacalis]	-1.73637	0.920287	0.466926	-0.48082	0.829981
TRINITY_DN810_c0_g1_i4_orf1	dicer 2 [Ostrinia nubilalis]	-1.50797	-0.17714	1.39734	-0.44801	0.735782
TRINITY_DN65974_c0_g1_i2_orf1	uncharacterized protein LOC114362364 [Ostrinia furnacalis]	-0.75365	0.8198	1.47439	-1.21719	-0.32335
TRINITY_DN1841_c0_g1_i2_orf1	carboxylesterase [Cnaphalocrocis medinalis]	-1.43627	1.433284	0.690584	-0.63495	-0.05265
TRINITY_DN5174_c0_g3_i1_orf1	protein odr-4 homolog [Ostrinia furnacalis]	-1.10567	0.183617	1.818175	-0.31297	-0.58315
TRINITY_DN6771_c0_g2_i1_orf1	putative endoplasmin, partial [Cotesia chilonis]	-1.79789	0.440671	1.161085	-0.2229	0.419034
TRINITY_DN9044_c0_g1_i1_orf1	hypothetical protein SFRURICE_005818, partial [Spodoptera frugiperda]	-1.4638	0.964703	1.227557	-0.6421	-0.08636
TRINITY_DN41296_c0_g1_i1_orf1	exocyst complex component 5 [Ostrinia furnacalis]	-1.7361	0.9235	0.952617	-0.39849	0.25848
TRINITY_DN104297_c0_g1_i1_orf1	tubulin-specific chaperone D [Ostrinia furnacalis]	-0.99677	0.712798	1.524817	-1.06957	-0.17128
TRINITY_DN962_c5_g1_i1_orf1	histone deacetylase 5 isoform X5 [Pectinophora gossypiella]	-0.93789	-0.67684	1.38244	-0.81233	1.044619
TRINITY_DN225_c0_g1_i6_orf1	glutathione S-transferase delta 3 [Ostrinia furnacalis]	-1.22997	-0.32005	1.621804	-0.64904	0.577263
TRINITY_DN38180_c0_g1_i3_orf1	guanine deaminase [Ostrinia furnacalis]	-1.61687	0.805769	1.268512	-0.12206	-0.33536
TRINITY_DN13330_c0_g1_i4_orf1	carboxylesterase [Cnaphalocrocis medinalis]	-1.2167	-0.42054	1.118727	-0.72989	1.2484
TRINITY_DN4064_c0_g2_i1_orf1	disintegrin and metalloproteinase domain-containing protein 12 isoform X1 [Ostrinia furnacalis] >XP_028158112.1 disintegrin and metalloproteinase domain-containing protein 12 isoform X2 [Ostrinia furnacalis]	-1.28582	-0.43343	1.775055	0.028735	-0.08453
TRINITY_DN10484_c0_g1_i8_orf1	hypothetical protein evm_010330 [Chilo suppressalis]	-1.45395	0.47036	1.569218	-0.16858	-0.41705
TRINITY_DN26089_c0_g1_i1_orf1	putative neuropeptide precursor protein isoform X1 [Ostrinia furnacalis]	-0.87148	-0.74195	1.388681	-0.81938	1.044132
TRINITY_DN35757_c0_g1_i1_orf1	ADP-ribosylation factor-like protein 6-interacting protein 1 [Ostrinia furnacalis]	-1.24391	0.032611	1.489358	-0.91194	0.633881
TRINITY_DN7388_c0_g1_i7_orf1	ras-related protein Rab-18A isoform X1 [Ostrinia furnacalis]	-1.42031	-0.56933	1.426341	-0.20078	0.764082

TRINITY_DN512_c1_g1_i4_orf1	protein argonaute-2 [Ostrinia furnacalis] >XP_028167629.1 protein argonaute-2 [Ostrinia furnacalis]	-1.5081	0.836802	1.250082	-0.67357	0.094789
TRINITY_DN54387_c0_g1_i1_orf1	catalase-like [Pectinophora gossypiella]	-1.38017	0.016037	1.178992	-0.82604	1.011187
TRINITY_DN51498_c0_g1_i1_orf1	delta-aminolevulinic acid dehydratase isoform X3 [Ostrinia furnacalis]	-0.80935	-0.63638	1.90238	-0.55746	0.10081
TRINITY_DN29956_c1_g1_i1_orf1	PREDICTED: dual specificity mitogen-activated protein kinase kinase dSOR1 isoform X1 [Fopius arisanus]	-1.38475	0.997897	1.081472	-0.92862	0.233999
TRINITY_DN115210_c0_g4_i1_orf1	PREDICTED: CAD protein [Microplitis demolitor]	-0.56204	-1.03568	1.413994	-0.80122	0.984954
TRINITY_DN3241_c0_g1_i1_orf1	cilia- and flagella-associated protein 99-like [Ostrinia furnacalis]	-1.14237	0.147279	1.427117	-1.09445	0.662427
TRINITY_DN9836_c0_g1_i2_orf1	STAM-binding protein-like A isoform X2 [Ostrinia furnacalis]	-0.84162	0.648937	1.395426	-1.37599	0.17324
TRINITY_DN2627_c0_g2_i1_orf1	phosphotriesterase-related protein [Ostrinia furnacalis]	-1.16866	-0.04075	1.005916	-1.03843	1.241923
TRINITY_DN83150_c0_g1_i1_orf1	fructose-bisphosphate aldolase-like isoform X1 [Ostrinia furnacalis] >XP_028178678.1 fructose-bisphosphate aldolase-like isoform X1 [Ostrinia furnacalis]	-0.95517	-0.36476	1.587022	-0.9703	0.7032
TRINITY_DN26994_c1_g1_i6_orf1	FGGY carbohydrate kinase domain-containing protein [Ostrinia furnacalis] >XP_028163452.1 FGGY carbohydrate kinase domain-containing protein [Ostrinia furnacalis]	-0.8484	0.068177	1.507156	-1.29616	0.569226
TRINITY_DN16868_c0_g2_i1_orf1	gamma-glutamylcyclotransferase-like isoform X1 [Ostrinia furnacalis]	-1.19277	0.86021	0.762697	-1.25505	0.824913
TRINITY_DN3209_c0_g2_i6_orf1	coatomer subunit beta, partial [Ostrinia furnacalis]	-0.6255	-0.60598	1.663382	-1.04704	0.615141
TRINITY_DN119291_c0_g1_i1_orf1	cyclophilin A, partial [Cotesia chilonis]	-0.96142	0.594412	1.457677	-1.25341	0.162743
TRINITY_DN131603_c0_g1_i4_orfp1	TRINITY_DN131603_c0_g1_i4_m.86149 TRINITY_DN131603_c0_g1::TRINITY_DN131603_c0_g1_i4::g.86149 ORF type:internal len:112 (-),score=8.40 TRINITY_DN131603_c0_g1_i4:2-334(-)	-0.38291	-0.66454	1.927338	-0.04622	-0.83367
TRINITY_DN2299_c0_g1_i3_orf1	DNA-directed RNA polymerase II subunit RPB1 [Ostrinia furnacalis]	0.007281	-0.82853	1.486899	0.636988	-1.30263
TRINITY_DN11172_c0_g1_i4_orf1	juvenile hormone epoxide hydrolase-like isoform X1 [Ostrinia furnacalis] >XP_028170522.1 juvenile hormone epoxide hydrolase-like isoform X2 [Ostrinia furnacalis]	0.174469	-1.21212	1.599188	0.345835	-0.90737
TRINITY_DN57749_c0_g1_i4_orf1	LOW QUALITY PROTEIN: DENN domain-containing protein Crag [Ostrinia furnacalis]	-0.17096	-1.24998	1.792132	0.067081	-0.43827
TRINITY_DN7247_c0_g1_i6_orf1	pyruvate kinase-like isoform X2 [Ostrinia furnacalis]	0.350256	-1.25269	1.659264	-0.74485	-0.01199
TRINITY_DN76815_c0_g1_i3_orf1	5-formyltetrahydrofolate cyclo-ligase [Ostrinia furnacalis]	0.745601	-1.0988	1.034744	0.64253	-1.32407
TRINITY_DN5991_c0_g1_i6_orf1	uncharacterized protein LOC114357071 [Ostrinia furnacalis]	1.045458	-1.28377	1.230574	-0.14092	-0.85134
TRINITY_DN32687_c0_g1_i1_orf1	protein D2-like isoform X2 [Ostrinia furnacalis] >XP_028164613.1 protein D2-like isoform X2 [Ostrinia furnacalis]	1.005629	-0.8452	1.419973	-0.85798	-0.72242
TRINITY_DN33024_c0_g1_i1_orf1	hypothetical protein evm_000959 [Chilo suppressalis]	0.706445	-1.79884	1.124533	-0.01712	-0.01501
TRINITY_DN22_c0_g1_i3_orf1	uncharacterized protein LOC114362831 [Ostrinia furnacalis]	0.596595	-1.01446	1.62239	-0.96098	-0.24355
TRINITY_DN27045_c0_g1_i1_orf1	cytochrome P450 6B5-like [Galleria mellonella]	0.921642	-0.31204	0.96864	0.177706	-1.75595
TRINITY_DN135679_c0_g1_i1_orfp1	TRINITY_DN135679_c0_g1_i1_m.85524 TRINITY_DN135679_c0_g1::TRINITY_DN135679_c0_g1_i1::g.85524 ORF type:5prime_partial len:55 (+),score=3.74,Toxin_2 PF00451.20 1.9e-06 TRINITY_DN135679_c0_g1_i1:3-167(+)	0.979515	-1.5656	1.084886	0.130123	-0.62893
TRINITY_DN12293_c0_g1_i1_orf1	hypothetical protein evm_011848 [Chilo suppressalis]	1.255365	-0.85741	1.080466	-0.27627	-1.20215
TRINITY_DN1153_c1_g1_i1_orf1	gamma-butyrobetaine dioxygenase [Ostrinia furnacalis]	1.201221	-0.88104	1.23941	-0.89924	-0.66034
TRINITY_DN5210_c0_g1_i3_orf1	uncharacterized protein LOC114358376 isoform X2 [Ostrinia furnacalis] >XP_028168126.1 uncharacterized protein LOC114358376 isoform X2 [Ostrinia furnacalis] >XP_028168127.1 uncharacterized protein LOC114358376 isoform X2 [Ostrinia furnacalis] >XP_028168128.1 uncharacterized protein LOC114358376 isoform X2 [Ostrinia furnacalis]	1.386367	-1.12982	0.963064	-0.35459	-0.86502
TRINITY_DN1416_c0_g2_i1_orf1	uncharacterized protein LOC114352565 [Ostrinia furnacalis]	1.180419	-0.83625	1.263114	-0.70324	-0.90404
TRINITY_DN3732_c0_g1_i2_orf1	cytochrome P450 monooxygenase CYP6AB141 [Ostrinia furnacalis]	0.872404	-0.91698	1.43403	-1.12851	-0.26095
TRINITY_DN1753_c1_g1_i8_orf1	serine/threonine-protein kinase WNK1-like isoform X15 [Ostrinia furnacalis]	1.285562	-0.71757	1.133175	-0.62546	-1.0757
TRINITY_DN3964_c1_g1_i2_orf1	phosphoinositide 3-kinase regulatory subunit 4 isoform X1 [Ostrinia furnacalis] >XP_028172384.1 phosphoinositide 3-kinase regulatory subunit 4 isoform X5 [Ostrinia furnacalis]	1.23654	-1.15904	1.055092	-0.13444	-0.99815
TRINITY_DN36496_c0_g1_i1_orf1	unnamed protein product [Parnassius apollo]	1.110617	-1.12772	1.143234	-1.08924	-0.0369
TRINITY_DN1999_c0_g1_i9_orf1	acyl-CoA Delta(11) desaturase-like [Ostrinia furnacalis] >XP_028172986.1 acyl-CoA Delta(11) desaturase-like [Ostrinia furnacalis] >AAL27034.1 acyl-CoA delta-9 desaturase [Ostrinia furnacalis] >AAL29454.1 acyl-CoA delta-9 desaturase [Ostrinia nubilalis]	1.400373	-1.05225	0.943477	-0.96787	-0.32373
TRINITY_DN2924_c0_g1_i2_orf1	cuticular protein RR-2 [Spodoptera litura]	1.369739	-0.42033	0.723117	-0.12017	-1.55235
TRINITY_DN5408_c0_g1_i5_orf1	uncharacterized protein LOC114359912 [Ostrinia furnacalis]	-1.40889	-0.62288	0.560553	-0.04878	1.52001
TRINITY_DN19639_c0_g2_i1_orf1	basic juvenile hormone-suppressible protein 1-like [Hyposmocoma kahamanoa]	-1.04121	-0.11238	0.602314	-1.026	1.577276
TRINITY_DN2311_c0_g3_i1_orf1	uncharacterized protein LOC114364231 isoform X1 [Ostrinia furnacalis] >XP_028176108.1 uncharacterized protein LOC114364231 isoform X2 [Ostrinia furnacalis]	-1.21249	-0.36281	0.310611	-0.48615	1.750834
TRINITY_DN29604_c0_g2_i2_orf1	neurofilament heavy polypeptide-like isoform X2 [Ostrinia furnacalis]	-1.12051	-0.16776	-0.12153	-0.45866	1.868467
TRINITY_DN49147_c0_g2_i1_orf1	glutenin, high molecular weight subunit PW212-like [Ostrinia furnacalis]	-1.11829	0.049371	-0.06071	-0.68124	1.810863
TRINITY_DN13686_c0_g2_i1_orf1	transmembrane protease serine 9-like [Ostrinia furnacalis]	-1.20297	-0.20774	0.016375	-0.42919	1.823524
TRINITY_DN1465_c2_g1_i2_orf1	transcription initiation factor TFIID subunit 1-like [Ostrinia furnacalis]	-1.18958	-0.45489	0.233062	-0.37309	1.784503
TRINITY_DN26337_c0_g1_i3_orf1	lysosome membrane protein 2-like [Ostrinia furnacalis]	-1.41761	-0.11492	0.594725	-0.57602	1.513829

TRINITY_DN2069_c1_g1_i8_orf1	lysosomal aspartic protease [Trichoplusia ni]	-1.42713	-0.01866	0.208781	-0.41934	1.656353
TRINITY_DN21555_c0_g1_i4_orf1	uncharacterized protein LOC114351844 [Ostrinia furnacalis] >XP_028158981.1 uncharacterized protein LOC114351844 [Ostrinia furnacalis] >XP_028158982.1 uncharacterized protein LOC114351844 [Ostrinia furnacalis] >XP_028158983.1 uncharacterized protein LOC114351844 [Ostrinia furnacalis] >XP_028158984.1 uncharacterized protein LOC114351844 [Ostrinia furnacalis] >XP_028158985.1 uncharacterized protein LOC114351844 [Ostrinia furnacalis] >5GPR_A Crystal structure of chitinase-h from Ostrinia furnacalis [Ostrinia furnacalis] >5GQB_A Crystal structure of chitinase-h from O. furnacalis in complex with chitohepatose [Ostrinia furnacalis] >6JMN_A Crystal structure of Ostrinia furnacalis Chitinase h complexed with compound 2-8-s2 [Ostrinia furnacalis] >BAE16587.1 chitinase [Ostrinia furnacalis]	-1.1256	-0.21744	-0.03953	-0.47671	1.859282
TRINITY_DN138481_c0_g1_i5_orf1	hypothetical protein evm_003901 [Chilo suppressalis]	-0.86904	-0.13738	-0.31774	-0.61234	1.936488
TRINITY_DN23229_c0_g1_i2_orf1	uncharacterized protein LOC114362553 [Ostrinia furnacalis]	-1.29365	-0.10522	0.402064	-0.65418	1.650993
TRINITY_DN2684_c0_g2_i3_orf1	glutamate decarboxylase 1-like isoform X1 [Ostrinia furnacalis]	-1.12739	0.2098	-0.08307	-0.76011	1.760769
TRINITY_DN9920_c0_g1_i1_orf1	uncharacterized protein LOC114351526 [Ostrinia furnacalis]	-1.23901	0.015987	0.221608	-0.70637	1.707784
TRINITY_DN98147_c0_g2_i1_orf1	hypothetical protein evm_002297 [Chilo suppressalis]	-0.48801	-0.7799	-0.20011	-0.49806	1.966086
TRINITY_DN2953_c1_g1_i10_orf1	methionine--tRNA ligase, cytoplasmic isoform X2 [Ostrinia furnacalis] >XP_028156683.1 methionine--tRNA ligase, cytoplasmic isoform X4 [Ostrinia furnacalis] >XP_028156684.1 methionine--tRNA ligase, cytoplasmic isoform X5 [Ostrinia furnacalis]	-0.98329	-0.31105	-0.05761	-0.55268	1.904631
TRINITY_DN42964_c0_g1_i1_orf1	protein lethal(2)essential for life-like [Galleria mellonella]	-0.4821	-0.54364	0.831038	-1.27423	1.468928
TRINITY_DN65681_c0_g1_i1_orf1	ferritin subunit-like [Ostrinia furnacalis] >XP_028168186.1 ferritin subunit-like [Ostrinia furnacalis]	-0.76066	0.095882	0.21897	-1.23738	1.683193
TRINITY_DN19043_c0_g3_i2_orf1	hypothetical protein EVAR_60654_1 [Eumeta japonica]	-1.29328	0.077001	0.547512	-0.84843	1.517199
TRINITY_DN54269_c0_g1_i3_orf1	lopap-like [Ostrinia furnacalis]	-0.83798	0.101371	0.137406	-1.13028	1.729481
TRINITY_DN15175_c0_g1_i1_orf1	zinc carboxypeptidase-like [Ostrinia furnacalis]	-0.7526	-0.09768	-0.41625	-0.67989	1.946417
TRINITY_DN5848_c0_g1_i6_orf1	brain tumor protein isoform X1 [Ostrinia furnacalis]	-0.9339	-0.045	0.494501	-1.12965	1.614053
TRINITY_DN1110_c1_g1_i9_orf1	MD-2-related lipid-recognition protein-like [Ostrinia furnacalis]	-0.9686	0.324272	-0.25621	-0.86986	1.770409
TRINITY_DN15222_c0_g1_i4_orf1	lysosomal alpha-mannosidase-like [Ostrinia furnacalis]	-0.52285	-0.86319	0.089934	-0.60365	1.899752
TRINITY_DN3952_c0_g1_i3_orf1	protein Skeletor, isoforms D/E-like isoform X1 [Ostrinia furnacalis] >XP_028176405.1 protein Skeletor, isoforms D/E-like isoform X2 [Ostrinia furnacalis] >XP_028176406.1 protein Skeletor, isoforms D/E-like isoform X3 [Ostrinia furnacalis] >XP_028176407.1 protein Skeletor, isoforms D/E-like isoform X4 [Ostrinia furnacalis]	-1.0343	0.491985	-0.14205	-0.96812	1.652492
TRINITY_DN29879_c0_g1_i3_orf1	uncharacterized protein LOC114350556 isoform X1 [Ostrinia furnacalis] >XP_028157201.1 uncharacterized protein LOC114350556 isoform X2 [Ostrinia furnacalis] >XP_028157202.1 uncharacterized protein LOC114350556 isoform X3 [Ostrinia furnacalis]	-0.50702	-0.91136	0.568673	-0.84557	1.695279
TRINITY_DN7539_c0_g1_i2_orf1	serine protease inhibitor 3/4 [Ostrinia furnacalis]	-0.73389	-0.35888	-0.26124	-0.61676	1.970778
TRINITY_DN31_c0_g1_i3_orfp1	TRINITY_DN31_c0_g1_i3_m.1394 TRINITY_DN31_c0_g1::TRINITY_DN31_c0_g1_i3::g.1394 ORF type:complete len:118 (+),score=43.35 TRINITY_DN31_c0_g1_i3:56-409(+)	-0.65203	-0.40295	-0.01188	-0.8528	1.919658
TRINITY_DN1308_c0_g1_i4_orf1	serine proteinase stubble-like [Ostrinia furnacalis]	-0.74116	-0.26409	-0.11148	-0.81002	1.926754
TRINITY_DN82426_c0_g1_i6_orfp1	lysosome-associated membrane glycoprotein 1-like isoform X4 [Ostrinia furnacalis]	-0.6949	-0.44854	-0.02009	-0.76705	1.930585
TRINITY_DN5581_c0_g1_i1_orf1	mucin-5AC-like [Ostrinia furnacalis]	-0.61025	-0.46086	-0.25331	-0.65574	1.980161
TRINITY_DN9109_c0_g1_i1_orf1	unnamed protein product [Chrysodeixis includens]	-0.61639	-0.91929	0.734863	-0.80671	1.607531
TRINITY_DN2097_c1_g1_i1_orf1	5-oxoprolinase [Ostrinia furnacalis]	-0.93346	0.363032	0.336719	-1.2717	1.50541
TRINITY_DN4367_c0_g1_i1_orf1	heat shock protein 21.7c [Chilo suppressalis] >AWT57938.1 heat shock protein 21.7c [Chilo suppressalis]	-0.81024	-0.08467	-0.15451	-0.84676	1.896173
TRINITY_DN9794_c0_g2_i8_orf1	acetyl-coenzyme A synthetase [Ostrinia furnacalis]	-0.82665	0.114219	0.642745	-1.35944	1.429127
TRINITY_DN13067_c0_g1_i6_orf1	diphosphomevalonate decarboxylase [Ostrinia furnacalis]	-0.47425	-0.18524	-0.02116	-1.16112	1.841773
TRINITY_DN125427_c0_g1_i1_orf1	heat shock protein 19.8 [Chilo suppressalis] >AGM90553.1 HSP19.8 [Chilo suppressalis] >BAE94664.1 small heat shock protein 19.7 [Chilo suppressalis]	-0.66839	-0.04689	-0.26548	-0.92377	1.904527
TRINITY_DN15420_c0_g3_i2_orf1	elongation factor 1-alpha 2-like [Galleria mellonella] >XP_031769625.1 elongation factor 1-alpha 2-like [Galleria	-0.66466	-0.21774	-0.17892	-0.86856	1.929873
TRINITY_DN13167_c0_g1_i1_orf1	selenoprotein M-like [Ostrinia furnacalis]	-0.16749	-1.06499	0.353394	-0.85012	1.729206
TRINITY_DN1604_c0_g1_i4_orf1	ubiquitin-conjugating enzyme E2 S [Ostrinia furnacalis]	-0.50249	-0.05011	0.27387	-1.38234	1.661065
TRINITY_DN2438_c0_g1_i1_orf1	dystrophin, isoforms A/C/F/G/H-like [Ostrinia furnacalis]	-0.15791	-1.01765	0.563756	-1.00453	1.616338
TRINITY_DN16516_c0_g1_i1_orf1	sulfotransferase 1E1 [Galleria mellonella]	-0.24369	-0.0099	-0.02911	-1.42385	1.706554
TRINITY_DN44633_c0_g1_i4_orf1	LDLR chaperone boca [Ostrinia furnacalis]	0.036838	-0.58294	0.629136	-1.50089	1.41786
TRINITY_DN10630_c0_g1_i2_orf1	J domain-containing protein [Ostrinia furnacalis]	-0.11294	-1.17141	0.628813	-0.89796	1.553486
TRINITY_DN52_c0_g1_i4_orf1	unnamed protein product [Chilo suppressalis]	0.456009	-0.58164	0.517166	-1.62923	1.237698
TRINITY_DN11566_c0_g1_i6_orf1	lens fiber major intrinsic protein-like isoform X1 [Ostrinia furnacalis]	0.326658	-0.93772	0.760958	-1.38332	1.233427
TRINITY_DN49872_c0_g1_i2_orf1	probable cytosolic iron-sulfur protein assembly protein Ciao1 [Ostrinia furnacalis]	0.136854	-0.60854	0.277939	-1.40562	1.599359
TRINITY_DN44094_c0_g1_i1_orf1	myotrophin-like [Ostrinia furnacalis]	-0.08069	-0.47359	-0.03929	-1.21839	1.811955

TRINITY_DN6362_c0_g1_i4_orf1	sodium/hydrogen exchanger 7 isoform X4 [Galleria mellonella]	0.315275	-0.98583	0.416924	-1.23751	1.491138
TRINITY_DN2201_c0_g1_i1_orf1	pleiotropic regulator 1 [Ostrinia furnacalis]	0.284058	-0.76992	0.634421	-1.47303	1.324472
TRINITY_DN7565_c0_g1_i3_orf1	acylphosphatase-2-like [Ostrinia furnacalis]	-0.13337	-0.44532	-0.24055	-1.07201	1.891251
TRINITY_DN4401_c0_g2_i1_orf1	hypothetical protein evm_003554 [Chilo suppressalis]	0.282253	-0.85935	0.644638	-1.40564	1.338091
TRINITY_DN3119_c0_g1_i7_orf1	unnamed protein product [Chilo suppressalis]	-0.03267	-0.49865	-0.10838	-1.1858	1.825493
TRINITY_DN6470_c0_g3_i2_orf1	trypsin CFT-1-like [Ostrinia furnacalis]	-0.4291	-0.3669	-0.31209	-0.85485	1.96293
TRINITY_DN4732_c0_g1_i2_orf1	reversion-inducing cysteine-rich protein with Kazal motifs [Ostrinia furnacalis]	0.676869	-0.67733	0.714139	-1.64494	0.931264
TRINITY_DN5829_c0_g2_i1_orf1	uncharacterized protein LOC114365758 isoform X2 [Ostrinia furnacalis]	0.40148	-1.20494	0.105507	-0.90247	1.600423
TRINITY_DN3715_c0_g1_i2_orf1	uncharacterized protein LOC114356437 isoform X1 [Ostrinia furnacalis]	-0.30232	0.071185	-0.60005	-1.03321	1.864393
TRINITY_DN33089_c0_g1_i1_orf1	nucleoporin NDC1 [Ostrinia furnacalis]	0.133219	-0.88664	0.103207	-1.08452	1.734731
TRINITY_DN5664_c0_g1_i1_orf1	CDGSH iron-sulfur domain-containing protein 3, mitochondrial-like [Ostrinia furnacalis]	-0.20279	-0.51408	-0.24683	-0.96199	1.925684
TRINITY_DN650_c0_g1_i3_orf1	chitinase 7 [Glyphodes pyloalis]	0.281018	-0.76033	-0.34854	-0.9787	1.806545
TRINITY_DN14532_c0_g1_i1_orf1	pupal cuticle protein-like [Trichoplusia ni]	0.095949	-0.73319	-0.45422	-0.80543	1.89689
TRINITY_DN64769_c0_g1_i3_orf1	procollagen-lysine,2-oxoglutarate 5-dioxygenase isoform X2 [Ostrinia furnacalis]	0.787371	-0.99033	-0.34964	-0.97358	1.526171
TRINITY_DN4068_c0_g2_i4_orf1	larval cuticle protein LCP-17-like precursor [Papilio polytes] >BAM18876.1 cuticular protein PpolCPR2 [Papilio polytes]	-0.81877	0.852375	1.185948	0.242558	-1.46211
TRINITY_DN3593_c0_g1_i3_orfp1	TRINITY_DN3593_c0_g1_i3_m.43968 TRINITY_DN3593_c0_g1::TRINITY_DN3593_c0_g1_i3::g.43968 ORF type:5prime_partial len:72 (-),score=1.41 TRINITY_DN3593_c0_g1_i3:138-353(-)	-0.48145	-0.40849	1.728501	0.375085	-1.21365
TRINITY_DN4242_c0_g1_i6_orf1	fibrohexamerin-like [Ostrinia furnacalis]	-0.53062	1.116429	1.022449	-0.05134	-1.55692
TRINITY_DN4695_c0_g1_i3_orf1	glutathione S-transferase epsilon 3 [Ostrinia furnacalis]	-0.4334	1.164297	0.983368	-0.14292	-1.57135
TRINITY_DN5191_c0_g2_i1_orf1	CD151 antigen-like [Ostrinia furnacalis]	-0.74699	1.055602	1.039163	0.144529	-1.4923
TRINITY_DN18782_c0_g1_i4_orf1	putative riboflavin kinase [Ostrinia furnacalis] >XP_028176654.1 putative riboflavin kinase [Ostrinia furnacalis]	-0.60624	0.974214	1.199761	-0.07146	-1.49628
TRINITY_DN6415_c0_g1_i1_orf1	D-arabinitol dehydrogenase 1-like [Ostrinia furnacalis]	-0.4837	0.646029	1.347657	0.079414	-1.5894
TRINITY_DN15000_c0_g1_i4_orf1	15-hydroxyprostaglandin dehydrogenase [NAD(+)]-like [Ostrinia furnacalis]	0.026352	-0.21325	1.800044	-0.3524	-1.26075
TRINITY_DN120500_c0_g1_i1_orf1	cytochrome P450 6B5-like [Ostrinia furnacalis]	-0.10807	-0.14537	1.658922	0.080667	-1.48616
TRINITY_DN5126_c0_g2_i1_orf1	cytochrome P450 monooxygenase CYP4L47 [Ostrinia furnacalis]	0.349863	-0.59261	1.803547	-0.6137	-0.9471
TRINITY_DN3298_c0_g2_i4_orf1	macrophage mannose receptor 1-like [Ostrinia furnacalis]	-0.51335	-0.03243	1.653621	0.278936	-1.38678
TRINITY_DN9400_c0_g1_i1_orf1	lysophosphatidylserine lipase ABHD12 isoform X2 [Maniola hyperantus]	-0.3522	0.574444	1.653838	-1.09764	-0.77844
TRINITY_DN2350_c0_g1_i6_orf1	protein yellow-like isoform X2 [Ostrinia furnacalis]	-0.12269	0.368883	1.381553	0.08485	-1.7126
TRINITY_DN41280_c0_g1_i2_orf1	unnamed protein product [Plutella xylostella]	0.174013	0.204904	1.655852	-0.77681	-1.25795
TRINITY_DN436_c0_g2_i5_orfp1	TRINITY_DN436_c0_g2_i5_m.4776 TRINITY_DN436_c0_g2::TRINITY_DN436_c0_g2_i5::g.4776 ORF type:complete len:158 (-),score=33.76 TRINITY_DN436_c0_g2_i5:320-772(-)	-0.04698	-0.24842	1.856936	-0.41424	-1.14729
TRINITY_DN779_c0_g1_i12_orf1	unnamed protein product [Chilo suppressalis]	-0.01121	0.377185	1.045425	0.470874	-1.88228
TRINITY_DN565_c0_g2_i1_orf1	uncharacterized protein LOC114362323 [Ostrinia furnacalis]	0.064777	0.073316	1.553599	-0.08893	-1.60276
TRINITY_DN9286_c0_g1_i2_orf1	alcohol dehydrogenase class-3 [Ostrinia furnacalis]	0.022693	0.557145	1.06298	0.229715	-1.87253
TRINITY_DN6482_c0_g1_i1_orf1	endocuticle structural glycoprotein SgAbd-5-like [Ostrinia furnacalis]	0.060408	0.602786	0.935159	0.314625	-1.91298
TRINITY_DN9079_c1_g1_i1_orf1	UDP-glucuronosyltransferase 2B15-like [Ostrinia furnacalis]	-0.29901	-0.11818	1.831963	-0.18789	-1.22688
TRINITY_DN12748_c2_g1_i1_orfp1	TRINITY_DN12748_c2_g1_i1_m.21305 TRINITY_DN12748_c2_g1::TRINITY_DN12748_c2_g1_i1::g.21305 ORF type:3prime_partial len:887 (+),score=-6.30 TRINITY_DN12748_c2_g1_i1:104-2761(+)	0.095035	0.682548	1.20358	-0.24426	-1.7369
TRINITY_DN616_c1_g1_i6_orf1	esterase B1-like isoform X1 [Ostrinia furnacalis] >XP_028178578.1 esterase B1-like isoform X2 [Ostrinia furnacalis]	-0.37944	1.121661	0.921899	-0.00642	-1.6577
TRINITY_DN135077_c0_g1_i1_orf1	hypothetical protein KR038_001662 [Drosophila bunnanda]	0.220728	0.222013	1.2025	0.202703	-1.84794
TRINITY_DN19293_c0_g1_i4_orf1	carboxylesterase [Ostrinia furnacalis]	-0.13455	-0.01388	1.784135	-0.33785	-1.29785
TRINITY_DN920_c0_g1_i6_orf1	glutathione S-transferase omega 2 [Ostrinia furnacalis]	0.519258	0.044358	1.565611	-0.99228	-1.13695
TRINITY_DN1292_c0_g1_i3_orf1	uncharacterized protein LOC114360660 [Ostrinia furnacalis]	0.181988	0.32682	1.543825	-0.59619	-1.45644
TRINITY_DN51776_c0_g2_i1_orf1	cuticle protein CP14.6-like [Ostrinia furnacalis]	-0.23907	0.611667	1.44419	-0.26316	-1.55363
TRINITY_DN2647_c0_g1_i3_orf1	DNA repair protein complementing XP-G cells homolog isoform X1 [Ostrinia furnacalis]	-0.04372	1.009357	1.064679	-0.3888	-1.64152
TRINITY_DN4321_c0_g1_i1_orf1	acyl-CoA Delta(11) desaturase isoform X1 [Ostrinia furnacalis]	0.218589	-0.49998	1.845343	-0.58965	-0.9743
TRINITY_DN1073_c0_g1_i3_orf1	carboxylesterase [Loxostege sticticalis]	0.458052	0.484725	0.806041	0.215625	-1.96444
TRINITY_DN7861_c0_g1_i5_orf1	cytochrome b5-related protein-like [Ostrinia furnacalis]	0.085069	0.487521	1.520921	-0.69285	-1.40066
TRINITY_DN124711_c0_g1_i1_orf1	muskelin isoform X1 [Ostrinia furnacalis] >XP_028163274.1 muskelin isoform X2 [Ostrinia furnacalis]	0.591797	-0.73702	1.697819	-0.67958	-0.87302
TRINITY_DN46715_c0_g1_i1_orf1	hypothetical protein evm_000885 [Chilo suppressalis] >CAH0689224.1 unnamed protein product [Chilo suppressalis]	-0.13305	1.139598	1.086956	-1.44094	-0.65257
TRINITY_DN1264_c0_g1_i2_orf1	L-lactate dehydrogenase isoform X1 [Ostrinia furnacalis]	0.103976	0.522815	1.489888	-0.70062	-1.41606
TRINITY_DN1585_c0_g1_i1_orf1	aldose reductase-like isoform X2 [Ostrinia furnacalis]	0.525006	0.435847	1.09504	-0.2463	-1.80959
TRINITY_DN41708_c0_g1_i1_orf1	facilitated trehalose transporter Tret1-like [Ostrinia furnacalis]	0.514197	0.05818	1.267297	-0.07309	-1.76659
TRINITY_DN27903_c0_g1_i1_orf1	desaturase MPVE [Helicoverpa assulta]	0.440031	-0.0791	1.640905	-0.77301	-1.22882

TRINITY_DN11015_c0_g1_i8_orf1	nicotinamide riboside kinase 1 [Ostrinia furnacalis]	0.415684	0.285495	1.497281	-0.89039	-1.30807
TRINITY_DN6291_c0_g1_i4_orf1	succinate-semialdehyde dehydrogenase, mitochondrial [Ostrinia furnacalis]	-0.22839	0.56118	1.598214	-1.29279	-0.63821
TRINITY_DN8783_c0_g1_i9_orf1	luciferin 4-monooxygenase-like [Ostrinia furnacalis] >XP_028165580.1 luciferin 4-monooxygenase-like [Ostrinia	0.303005	0.380199	1.484302	-1.4089	-0.7586
TRINITY_DN5507_c0_g1_i1_orf1	PREDICTED: protein mago nashi [Amyelois transitella] >XP_026764462.1 protein mago nashi [Galleria mellonella] >XP_028164484.1 protein mago nashi [Ostrinia furnacalis]	-0.19302	1.027117	1.255756	-1.31613	-0.77372
TRINITY_DN1215_c0_g1_i2_orf1	PI-stichotoxin-She2a-like [Ostrinia furnacalis]	0.240445	0.736923	0.932783	-0.03184	-1.87831
TRINITY_DN231_c1_g2_i1_orf1	G protein-activated inward rectifier potassium channel 3-like isoform X5 [Ostrinia furnacalis]	0.756923	0.380739	1.210723	-1.34566	-1.00273
TRINITY_DN86127_c1_g1_i2_orfp1	TRINITY_DN86127_c1_g1_i2_m.43062 TRINITY_DN86127_c1_g1::TRINITY_DN86127_c1_g1_i2::g.43062 ORF type:internal len:69 (-),score=14.03 TRINITY_DN86127_c1_g1_i2:2-205(-)	0.266863	0.982193	0.944293	-0.51918	-1.67417
TRINITY_DN21743_c0_g1_i2_orf1	uncharacterized protein LOC114357426 [Ostrinia furnacalis]	-0.0372	0.910821	1.28333	-1.39141	-0.76554
TRINITY_DN144342_c0_g1_i1_orfp1	TRINITY_DN144342_c0_g1_i1_m.83164 TRINITY_DN144342_c0_g1::TRINITY_DN144342_c0_g1_i1::g.83164 ORF type:internal len:113 (-),score=2.22 TRINITY_DN144342_c0_g1_i1:1-336(-)	0.618063	-0.80481	1.647594	-0.42328	-1.03757
TRINITY_DN1989_c0_g1_i1_orf1	sarcoplasmic calcium-binding protein 1 isoform X1 [Ostrinia furnacalis]	0.218582	1.258863	0.59625	-0.38039	-1.6933
TRINITY_DN2821_c0_g1_i1_orf1	uncharacterized protein LOC114356423 [Ostrinia furnacalis]	-0.21699	1.137636	1.145937	-1.35744	-0.70914
TRINITY_DN29009_c0_g2_i3_orf1	juvenile hormone binding protein [Omphisa fuscidentalis]	0.051804	1.348882	0.777167	-1.40698	-0.77087
TRINITY_DN109503_c0_g1_i4_orf1	uncharacterized protein LOC114366345 isoform X2 [Ostrinia furnacalis]	-0.10967	1.834493	0.07113	-0.9471	-0.84885
TRINITY_DN30037_c0_g1_i5_orf1	cytoglobin-1-like isoform X2 [Ostrinia furnacalis]	0.118709	0.301489	1.501414	-1.59107	-0.33054
TRINITY_DN56877_c0_g1_i4_orf1	programmed cell death protein 2-like [Ostrinia furnacalis]	0.545834	-0.20847	1.626762	-0.77792	-1.18621
TRINITY_DN87648_c0_g1_i1_orfp1	TRINITY_DN87648_c0_g1_i1_m.51054 TRINITY_DN87648_c0_g1::TRINITY_DN87648_c0_g1_i1::g.51054 ORF type:internal len:180 (+),score=75.93 TRINITY_DN87648_c0_g1_i1:2-538(+)	0.713823	0.245998	0.895769	0.048405	-1.90399
TRINITY_DN32_c0_g1_i4_orf1	epidermal growth factor receptor substrate 15 homolog [Ostrinia furnacalis]	0.53952	0.242553	1.391489	-0.66746	-1.5061
TRINITY_DN2675_c0_g1_i1_orf1	mitochondrial folate transporter/carrier [Ostrinia furnacalis]	-0.39243	0.617385	1.606953	-1.23579	-0.59612
TRINITY_DN935_c0_g1_i3_orf1	carboxylesterase 5A-like [Ostrinia furnacalis]	0.43241	0.727972	1.200746	-1.01651	-1.34462
TRINITY_DN7794_c0_g1_i1_orf1	laminin subunit gamma-1-like [Ostrinia furnacalis]	0.419709	-0.16839	1.665767	-0.65597	-1.26112
TRINITY_DN3019_c0_g1_i1_orf1	uncharacterized protein LOC114355530 isoform X1 [Ostrinia furnacalis]	0.688632	0.659084	0.961773	-0.65499	-1.6545
TRINITY_DN1865_c1_g1_i3_orf1	exosome complex exonuclease RRP44 isoform X1 [Ostrinia furnacalis] >XP_028177486.1 exosome complex exonuclease RRP44 isoform X2 [Ostrinia furnacalis]	-0.10856	0.78628	1.401828	-1.38805	-0.6915
TRINITY_DN51830_c0_g1_i4_orf1	15-hydroxyprostaglandin dehydrogenase [NAD(+)]-like [Ostrinia furnacalis]	0.744583	0.415068	0.988603	-0.37111	-1.77715
TRINITY_DN56795_c1_g1_i1_orf1	uncharacterized protein LOC114365476 [Ostrinia furnacalis]	0.838065	0.461366	1.066589	-0.91084	-1.45518
TRINITY_DN49786_c0_g1_i1_orf1	UDP-glucuronosyltransferase 2B15-like isoform X1 [Ostrinia furnacalis]	0.800827	0.252202	1.266577	-1.17666	-1.14294
TRINITY_DN5962_c0_g1_i1_orf1	tRNA (cytosine(34)-C(5))-methyltransferase [Ostrinia furnacalis]	0.864013	-0.49737	1.488331	-0.73972	-1.11525
TRINITY_DN1914_c0_g1_i4_orf1	loricrin-like [Ostrinia furnacalis]	0.812463	-0.1449	1.329586	-0.47094	-1.52621
TRINITY_DN79083_c0_g1_i2_orf1	unnamed protein product [Arctia plantaginis]	0.996987	0.719818	0.719818	-1.24255	-1.19407
TRINITY_DN280_c4_g1_i5_orf1	fibroin light chain [Haritalodes derogata]	0.661415	1.076916	0.466705	-0.48856	-1.71647
TRINITY_DN52859_c0_g1_i4_orf1	uncharacterized protein LOC114351423 [Ostrinia furnacalis]	0.798189	1.293013	0.205845	-1.07631	-1.22073
TRINITY_DN6241_c0_g1_i1_orf1	uncharacterized protein LOC114355531 [Ostrinia furnacalis]	0.970352	0.767925	0.638245	-0.84371	-1.53281
TRINITY_DN2749_c4_g1_i2_orf1	RNA exonuclease 4-like [Ostrinia furnacalis] >QEE79882.1 REX4 [Ostrinia furnacalis]	0.391931	1.414362	0.441464	-1.37375	-0.874
TRINITY_DN276_c0_g1_i2_orf1	protein lethal(2)essential for life-like [Ostrinia furnacalis] >UTU55753.1 small heat shock protein Hsp20.7 [Ostrinia	-1.32063	0.5407	0.062179	-0.80348	1.521229
TRINITY_DN45220_c0_g1_i1_orf1	delta(3,5)-Delta(2,4)-dienoyl-CoA isomerase, mitochondrial isoform X1 [Ostrinia furnacalis]	-1.64302	0.504996	0.211425	-0.42312	1.349717
TRINITY_DN17574_c0_g1_i2_orf1	heat shock protein Hsp-12.2-like [Ostrinia furnacalis]	-1.46822	1.049599	0.676373	-0.92007	0.662318
TRINITY_DN9593_c0_g1_i2_orf1	uncharacterized protein LOC113518937 [Galleria mellonella]	-1.58999	1.25792	0.398707	-0.63679	0.570161
TRINITY_DN2097_c1_g2_i2_orf1	serine protease inhibitor 3 [Ostrinia furnacalis]	-1.69015	0.438975	0.278085	-0.35009	1.323178
TRINITY_DN18218_c0_g1_i7_orf1	inhibin beta B chain [Ostrinia furnacalis]	-1.23796	0.749796	-0.30336	-0.72227	1.513792
TRINITY_DN2835_c0_g1_i6_orf1	probable isoaspartyl peptidase/L-asparaginase GA20639 [Ostrinia furnacalis]	-1.57307	0.628492	0.188826	-0.57323	1.328984
TRINITY_DN13973_c0_g1_i6_orf1	27 kDa glycoprotein-like [Ostrinia furnacalis]	-1.41164	0.631465	0.203944	-0.80754	1.383768
TRINITY_DN1732_c0_g1_i15_orf1	CAD protein isoform X2 [Ostrinia furnacalis]	-1.42747	1.409655	0.429849	-0.79996	0.38793
TRINITY_DN21743_c0_g1_i1_orf1	uncharacterized protein LOC114357426 [Ostrinia furnacalis]	-1.43193	1.188445	0.234904	-0.85651	0.865092
TRINITY_DN166_c0_g1_i4_orf1	PREDICTED: cryptochrome-1 isoform X1 [Amyelois transitella] >XP_013199861.1 PREDICTED: cryptochrome-1 isoform X1 [Amyelois transitella]	-1.60096	0.523742	0.284844	-0.54391	1.336282
TRINITY_DN3383_c0_g1_i5_orf1	uncharacterized protein LOC114357426 [Ostrinia furnacalis]	-1.0577	1.19223	0.246946	-1.26919	0.887713
TRINITY_DN6199_c2_g1_i3_orf1	uncharacterized protein LOC114352137 [Ostrinia furnacalis] >XP_028159411.1 uncharacterized protein LOC114352137 [Ostrinia furnacalis] >XP_028159412.1 uncharacterized protein LOC114352137 [Ostrinia furnacalis]	-1.42402	0.873314	0.341871	-0.91313	1.121965
TRINITY_DN14250_c0_g1_i1_orf1	apolipophorins-like [Ostrinia furnacalis]	-1.21199	1.196466	-0.109	-0.95752	1.082046
TRINITY_DN8245_c0_g1_i4_orf1	uncharacterized protein LOC114357622 [Ostrinia furnacalis]	-1.41779	1.167934	0.712502	-0.94235	0.479705

TRINITY_DN15291_c0_g1_i11_orf1	uncharacterized protein LOC114353772 [Ostrinia furnacalis]	-1.27496	0.978995	-0.17059	-0.83399	1.300542
TRINITY_DN22674_c0_g1_i2_orf1	protein arginine N-methyltransferase 7 isoform X1 [Ostrinia furnacalis]	-1.40517	1.277399	0.277006	-0.88266	0.733429
TRINITY_DN781_c0_g1_i7_orf1	uncharacterized protein LOC114356786 [Ostrinia furnacalis]	-1.40302	1.423667	0.436815	-0.82424	0.36678
TRINITY_DN18650_c0_g1_i1_orf1	bombyxin B-9-like [Ostrinia furnacalis]	-1.31721	0.567468	0.025198	-0.79528	1.519817
TRINITY_DN88876_c0_g1_i1_orf1	Photosystem I reaction center subunit III, chloroplastic, partial [Trichinella zimbabwensis]	-1.27567	0.454273	0.490334	-1.03261	1.36367
TRINITY_DN27033_c1_g1_i3_orfp1	poly(U)-specific endoribonuclease homolog [Ostrinia furnacalis]	-0.91274	1.191099	1.092172	-1.24032	-0.13021
TRINITY_DN67026_c0_g1_i6_orf1	hypothetical protein O3G_MSEX011964 [Manduca sexta]	-0.88453	1.560853	0.354523	-1.2652	0.234356
TRINITY_DN33430_c0_g1_i5_orf1	NADH dehydrogenase [ubiquinone] iron-sulfur protein 2, mitochondrial [Ostrinia furnacalis]	-0.92223	0.815111	0.373339	-1.41963	1.15341
TRINITY_DN4822_c0_g1_i6_orf1	homogentisate 1,2-dioxygenase [Ostrinia furnacalis]	-0.88778	1.646333	0.076519	-1.1748	0.339731
TRINITY_DN56164_c0_g1_i1_orf1	hypothetical protein evm_010164 [Chilo suppressalis]	-0.67602	0.59677	0.437165	-1.58104	1.223131
TRINITY_DN51523_c0_g1_i4_orf1	macrophage migration inhibitory factor homolog [Ostrinia furnacalis]	-0.80446	1.549863	0.693099	-1.18602	-0.25249
TRINITY_DN1732_c0_g1_i17_orf1	CAD protein isoform X2 [Ostrinia furnacalis]	-0.80493	1.432248	0.73346	-1.32728	-0.0335
TRINITY_DN38835_c0_g2_i1_orf1	sec61alpha [Papilio xuthus]	-1.02316	1.195425	0.994466	-1.23702	0.070282
TRINITY_DN44285_c0_g1_i1_orf1	uncharacterized protein LOC114354962 [Ostrinia furnacalis]	-0.18545	1.560498	0.271354	-1.56533	-0.08107
TRINITY_DN11159_c0_g1_i5_orf1	sphingosine-1-phosphate lyase isoform X2 [Ostrinia furnacalis]	-1.07345	1.084901	0.339304	-1.29208	0.941326
TRINITY_DN116874_c0_g1_i1_orfp1	TRINITY_DN116874_c0_g1_i1_m.85176 TRINITY_DN116874_c0_g1::TRINITY_DN116874_c0_g1_i1::g.85176 ORF type:5prime_partial len:95 (+),score=17.10,Baculo_p48 PF04878.14 8.5e-16 TRINITY_DN116874_c0_g1_i1:2-286(+)	-0.93315	1.331051	-0.02287	-1.25678	0.88176
TRINITY_DN22018_c0_g1_i3_orf1	hypothetical protein evm_009110 [Chilo suppressalis] >CAH2984739.1 unnamed protein product [Chilo suppressalis]	-0.30782	0.334377	0.070332	-1.59502	1.498131
TRINITY_DN22272_c0_g1_i1_orf1	28S ribosomal protein S33, mitochondrial [Galleria mellonella]	-0.10358	0.933894	0.822594	-1.84488	0.191976
TRINITY_DN106_c0_g1_i3_orf1	cytochrome b5-like heme/Steroid binding domain-containing protein [Phthorimaea operculella]	-0.41687	0.454148	0.826744	-1.76677	0.902756
TRINITY_DN24689_c0_g1_i1_orf1	thioredoxin domain-containing protein 17-like [Ostrinia furnacalis]	-0.42115	0.917992	0.306166	-1.73638	0.933371
TRINITY_DN13221_c0_g1_i3_orf1	fasciclin-3-like [Ostrinia furnacalis]	-0.01932	0.459815	-0.3721	-1.55856	1.490172
TRINITY_DN29190_c0_g1_i4_orf1	gloverin-like [Ostrinia furnacalis]	-0.44145	-0.69818	1.862784	-0.90298	0.179821
TRINITY_DN48497_c0_g1_i1_orf1	unnamed protein product [Chrysodeixis includens]	-0.0704	-0.78902	1.610928	-1.24034	0.48883
TRINITY_DN34465_c0_g1_i1_orf1	putative peptidyl-tRNA hydrolase PTRHD1 [Ostrinia furnacalis]	-0.27044	-0.44414	1.781064	-1.23635	0.169863
TRINITY_DN27110_c0_g1_i4_orf1	uncharacterized protein LOC114366000 [Ostrinia furnacalis]	0.151746	-0.81769	1.30934	-1.41417	0.770778
TRINITY_DN1260_c0_g2_i1_orf1	vegetative cell wall protein gp1 [Ostrinia furnacalis]	-0.10398	-0.99767	1.188636	-1.17866	1.091674
TRINITY_DN8846_c0_g1_i1_orf1	PREDICTED: synapse-associated protein of 47 kDa-like isoform X2 [Papilio xuthus]	0.63415	-1.39536	1.089622	-1.00374	0.675324
TRINITY_DN43228_c0_g1_i1_orf1	enoyl-CoA delta isomerase 2, mitochondrial [Ostrinia furnacalis]	0.220113	-0.93886	1.363236	-1.32324	0.678753
TRINITY_DN21125_c0_g1_i1_orf1	protein angel homolog 1 isoform X3 [Ostrinia furnacalis]	0.564269	-1.52884	0.629468	-0.80504	1.140143
TRINITY_DN12700_c0_g1_i7_orf1	CWF19-like protein 1 [Galleria mellonella] >XP_031765668.1 CWF19-like protein 1 [Galleria mellonella]	0.53544	-0.46927	1.42722	-1.56554	0.072155
TRINITY_DN135846_c0_g1_i1_orf1	zinc finger protein 253-like [Ostrinia furnacalis]	0.802489	0.075121	0.742969	-1.92502	0.304436
TRINITY_DN51938_c0_g3_i1_orf1	unnamed protein product [Mus musculus]	0.27062	-0.55191	1.620925	-1.41058	0.070945
TRINITY_DN27488_c0_g1_i9_orf1	hypothetical protein evm_006422 [Chilo suppressalis] >CAB3524819.1 unnamed protein product [Chilo suppressalis] >CAH0397586.1 unnamed protein product [Chilo suppressalis]	0.341005	-0.26589	1.394705	-1.68033	0.210508
TRINITY_DN323_c0_g2_i5_orf1	oxygen-dependent coproporphyrinogen-III oxidase isoform X1 [Ostrinia furnacalis]	0.317769	-0.19961	1.009659	-1.82957	0.701752
TRINITY_DN4247_c0_g1_i4_orf1	unnamed protein product [Spodoptera exigua]	0.580268	-0.07539	1.36729	-1.65567	-0.21649
TRINITY_DN3651_c0_g1_i5_orf1	probable tRNA N6-adenosine threonylcarbamoyltransferase [Ostrinia furnacalis]	1.098308	-0.61656	1.198155	-1.37238	-0.30752
TRINITY_DN957_c0_g1_i18_orf1	proton channel OtopLc-like isoform X6 [Ostrinia furnacalis]	0.459403	0.113813	0.951931	-1.92593	0.40078
TRINITY_DN45953_c0_g1_i1_orf1	UPF0587 protein GA18326-like [Ostrinia furnacalis]	0.809318	0.005994	0.904029	-1.872	0.152661
TRINITY_DN28366_c0_g1_i1_orf1	aldehyde dehydrogenase, cytosolic 2-like [Hyposmocoma kahamanoa]	1.071565	-0.32015	1.203059	-1.41991	-0.53457
TRINITY_DN12367_c0_g1_i4_orf1	aldose reductase-like isoform X2 [Ostrinia furnacalis]	0.838683	0.65651	0.68076	-1.80728	-0.36868
TRINITY_DN6153_c0_g1_i6_orf1	uncharacterized protein LOC114354884 [Ostrinia furnacalis]	1.137022	0.006691	0.957051	-1.59102	-0.50974
TRINITY_DN20527_c0_g1_i1_orf1	dihydrofolate reductase [Ostrinia furnacalis]	1.130997	-0.34059	1.17328	-1.36929	-0.59439
TRINITY_DN6992_c0_g1_i6_orf1	UDP-glucuronosyltransferase 2B15-like [Ostrinia furnacalis] >XP_028166365.1 UDP-glucuronosyltransferase 2B15-like [Ostrinia furnacalis] >QNS26328.1 UDP-glycosyltransferase UGT40AN6 [Ostrinia furnacalis]	0.794435	0.066125	0.912714	-1.87639	0.103112
TRINITY_DN64196_c0_g1_i2_orf1	ceramide-1-phosphate transfer protein [Ostrinia furnacalis]	1.46857	-0.22191	0.620871	-1.51028	-0.35724
TRINITY_DN53246_c0_g7_i1_orf1	E3 ubiquitin-protein ligase HUWE1 isoform X3 [Chelonus insularis]	1.405024	-0.31506	0.789077	-1.45846	-0.42057
TRINITY_DN1641_c0_g1_i6_orf1	rhodanese domain-containing protein CG4456-like [Ostrinia furnacalis]	1.546133	-0.10856	0.358118	-1.55252	-0.24318
TRINITY_DN1860_c0_g1_i2_orf1	26S proteasome non-ATPase regulatory subunit 5 [Ostrinia furnacalis] >XP_028175444.1 26S proteasome non-ATPase regulatory subunit 5 [Ostrinia furnacalis]	1.577422	0.03039	0.257477	-1.52631	-0.33898
TRINITY_DN4207_c0_g1_i1_orf1	mitochondrial import inner membrane translocase subunit Tim21 [Ostrinia furnacalis]	1.452175	-0.26058	0.425191	-1.62555	0.008765
TRINITY_DN36928_c0_g1_i5_orf1	actin-interacting protein 1 isoform X2 [Ostrinia furnacalis]	0.980768	0.671083	0.613743	-1.70051	-0.56508
TRINITY_DN2749_c0_g2_i3_orf1	RNA exonuclease 4-like [Ostrinia furnacalis] >QEE79882.1 REX4 [Ostrinia furnacalis]	1.210718	-0.47488	1.167993	-1.20897	-0.69486

TRINITY_DN4356_c0_g1_i6_orf1	mulatexin-like [Ostrinia furnacalis]	1.287896	0.570281	0.149829	-1.70315	-0.30486
TRINITY_DN6588_c0_g1_i4_orf1	uncharacterized protein LOC114350845 [Ostrinia furnacalis]	1.673584	-1.13067	0.266841	-0.91559	0.105831
TRINITY_DN49204_c0_g1_i1_orf1	uncharacterized protein C05D11.1-like [Chelonus insularis]	1.2065	-0.14998	1.081822	-0.88852	-1.24982
TRINITY_DN14935_c0_g1_i1_orf1	kynurenine/alpha-aminoadipate aminotransferase, mitochondrial [Ostrinia furnacalis]	1.217387	-0.20722	1.095206	-0.88058	-1.2248
TRINITY_DN5811_c0_g1_i4_orf1	uridine-cytidine kinase-like 1 isoform X1 [Ostrinia furnacalis] >XP_028168339.1 uridine-cytidine kinase-like 1 isoform X2 [Ostrinia furnacalis]	0.930336	-0.02997	-0.07876	-1.78737	0.965763
TRINITY_DN32420_c0_g1_i2_orf1	PREDICTED: plectin-like, partial [Papilio polytes]	1.353257	-0.1274	0.855255	-0.68314	-1.39797
TRINITY_DN16354_c0_g1_i2_orf1	uncharacterized protein LOC114349750 isoform X1 [Ostrinia furnacalis]	1.549269	-0.26826	0.657241	-0.63941	-1.29884
TRINITY_DN19250_c0_g2_i2_orf1	uncharacterized protein LOC114351683 isoform X8 [Ostrinia furnacalis]	1.56326	-0.6869	0.442232	-1.37325	0.054663
TRINITY_DN6994_c0_g1_i4_orf1	C-type mannose receptor 2-like isoform X1 [Ostrinia furnacalis]	1.608339	-0.63718	0.725992	-0.70711	-0.99004
TRINITY_DN2967_c0_g1_i7_orf1	UDP-glucuronosyltransferase 1-7C-like isoform X2 [Ostrinia furnacalis]	1.421868	0.271895	0.558622	-1.29306	-0.95933
TRINITY_DN56250_c0_g1_i7_orf1	sex-lethal homolog isoform X3 [Ostrinia furnacalis] >XP_028172304.1 sex-lethal homolog isoform X4 [Ostrinia furnacalis]	1.354433	0.141686	0.687268	-0.71123	-1.47216
TRINITY_DN8717_c0_g1_i5_orf1	hypothetical protein evm_006607 [Chilo suppressalis] >CAG9745590.1 unnamed protein product [Diatraea saccharalis] >CAG9784275.1 unnamed protein product [Diatraea saccharalis]	1.758362	-0.54158	0.450008	-0.72559	-0.94121
TRINITY_DN863_c0_g1_i6_orf1	protein henna [Galleria mellonella]	1.484868	-0.02115	0.638355	-0.75282	-1.34925
TRINITY_DN25901_c0_g1_i2_orf1	short-chain specific acyl-CoA dehydrogenase, mitochondrial-like isoform X2 [Ostrinia furnacalis]	1.640975	-0.54409	0.604761	-0.53648	-1.16517
TRINITY_DN2826_c0_g1_i7_orf1	ATP-binding cassette subfamily D member 1 [Chilo suppressalis] >CAB3531327.1 unnamed protein product [Chilo suppressalis] >CAH0407919.1 unnamed protein product [Chilo suppressalis]	1.638279	-0.63462	0.132077	-1.35872	0.22299
TRINITY_DN87603_c0_g2_i1_orf1	40S ribosomal protein S3-3, partial [Trichinella patagoniensis]	1.150592	-0.88121	0.403939	-1.45687	0.783555
TRINITY_DN2542_c0_g2_i1_orf1	peroxiredoxin-2-like [Ostrinia furnacalis]	1.383687	-0.05552	-0.32416	-1.61404	0.61003
TRINITY_DN11069_c0_g1_i6_orf1	ganglioside-induced differentiation-associated protein 1 [Ostrinia furnacalis]	1.839612	-0.34621	0.186322	-0.99879	-0.68093
TRINITY_DN1763_c0_g3_i2_orf1	heterogeneous nuclear ribonucleoprotein H-like isoform X2 [Ostrinia furnacalis]	1.225346	-1.38465	0.226002	-0.90741	0.840714
TRINITY_DN23783_c0_g2_i1_orf1	cytochrome b5 [Ostrinia furnacalis]	1.604444	-1.1217	0.085675	-0.99085	0.422424
TRINITY_DN18909_c0_g1_i6_orf1	unnamed protein product [Euphydryas editha]	1.574827	-0.0949	0.594778	-1.08713	-0.98757
TRINITY_DN7329_c0_g1_i6_orf1	serine hydrolase-like protein 2 isoform X2 [Ostrinia furnacalis]	1.697818	-0.58172	0.550187	-1.04297	-0.62332
TRINITY_DN4814_c0_g1_i6_orf1	vesicle transport protein GOT1B [Pectinophora gossypiella]	1.656153	-0.38782	0.071832	-1.44583	0.105667
TRINITY_DN27745_c0_g1_i6_orf1	unnamed protein product [Diatraea saccharalis]	1.495394	-0.05498	0.67258	-1.25147	-0.86153
TRINITY_DN20321_c0_g1_i5_orf1	uncharacterized protein LOC114350467 isoform X3 [Ostrinia furnacalis]	1.742105	0.135484	0.066826	-1.13325	-0.81116
TRINITY_DN3618_c0_g1_i4_orf1	WD repeat-containing protein 74-like isoform X1 [Ostrinia furnacalis] >XP_028161051.1 WD repeat-containing protein 74-like isoform X2 [Ostrinia furnacalis]	1.270906	-0.38992	0.462531	-1.70043	0.356905
TRINITY_DN2497_c0_g1_i1_orf1	protein stunted-like isoform X2 [Vanessa tameamea] >XP_046960183.1 protein stunted-like isoform X2 [Vanessa cardui] >XP_047527093.1 protein stunted-like isoform X2 [Vanessa atalanta]	1.241433	-0.21911	0.137265	-1.74591	0.586328
TRINITY_DN6556_c0_g1_i7_orf1	NFX1-type zinc finger-containing protein 1-like isoform X1 [Ostrinia furnacalis] >XP_028173496.1 NFX1-type zinc finger-containing protein 1-like isoform X1 [Ostrinia furnacalis] >XP_028173497.1 NFX1-type zinc finger-containing protein 1-like isoform X1 [Ostrinia furnacalis]	1.801966	-0.99788	0.287572	-0.74406	-0.34759
TRINITY_DN4929_c0_g1_i1_orf1	unnamed protein product [Danaus chrysippus]	1.265486	-0.27189	0.396464	-1.74415	0.354093
TRINITY_DN1824_c0_g2_i2_orf1	branched-chain-amino-acid aminotransferase, cytosolic [Manduca sexta] >KAG6453781.1 hypothetical protein O3G_MSEX008327 [Manduca sexta]	1.847051	-0.44802	0.090298	-1.11241	-0.37691
TRINITY_DN57765_c0_g1_i1_orf1	cytochrome P450 6B6-like [Ostrinia furnacalis]	1.707646	0.004719	0.179146	-1.30833	-0.58318
TRINITY_DN547_c0_g1_i1_orf1	WD repeat-containing protein 43 [Ostrinia furnacalis]	1.854891	-0.17116	-0.01508	-1.09668	-0.57197
TRINITY_DN1656_c2_g1_i5_orf1	15-hydroxyprostaglandin dehydrogenase [NAD(+)]-like [Ostrinia furnacalis]	1.455867	-0.42561	0.81631	-0.51829	-1.32828
TRINITY_DN25997_c1_g2_i4_orf1	ribokinase-like [Ostrinia furnacalis]	1.410084	-0.78946	0.466115	-1.43237	0.345633
TRINITY_DN9207_c0_g1_i1_orf1	RNA polymerases N / 8 kDa subunit domain-containing protein [Phthorimaea operculella]	1.790198	0.094451	-0.00422	-1.03573	-0.8447
TRINITY_DN13375_c0_g1_i6_orf1	thioredoxin, mitochondrial isoform X2 [Ostrinia furnacalis]	1.085717	-1.17353	-0.08173	-1.01591	1.18545
TRINITY_DN1383_c0_g1_i2_orf1	uncharacterized protein LOC114353133 isoform X1 [Ostrinia furnacalis] >XP_028160773.1 uncharacterized protein LOC114353133 isoform X2 [Ostrinia furnacalis]	1.64306	0.416553	-0.0032	-0.94945	-1.10696
TRINITY_DN15513_c0_g1_i6_orf1	uncharacterized protein LOC114350859 [Ostrinia furnacalis]	1.632541	-0.24683	0.469691	-0.52026	-1.33514
TRINITY_DN2967_c0_g1_i4_orf1	UDP-glycosyltransferase UGT41G1 [Ostrinia furnacalis]	1.871421	-0.12413	-0.04553	-0.97798	-0.72378
TRINITY_DN3949_c1_g1_i1_orf1	probable cytochrome P450 304a1 isoform X2 [Ostrinia furnacalis]	1.595068	-0.6473	0.752889	-0.74124	-0.95942
TRINITY_DN19687_c0_g1_i1_orf1	probable ribosome production factor 1 [Ostrinia furnacalis]	1.655027	0.21897	-0.26015	-1.45616	-0.15769
TRINITY_DN9156_c0_g1_i1_orf1	GTP:AMP phosphotransferase AK3, mitochondrial [Ostrinia furnacalis]	1.892352	-0.29769	-0.1688	-1.09066	-0.33521
TRINITY_DN4731_c0_g2_i1_orf1	gelsolin-like [Ostrinia furnacalis]	1.759468	0.04859	0.061125	-1.2096	-0.65959
TRINITY_DN74889_c0_g1_i1_orf1	probable 28S ribosomal protein S23, mitochondrial [Ostrinia furnacalis]	1.807606	-0.15822	0.103759	-0.59373	-1.15941
TRINITY_DN1772_c7_g1_i7_orf1	sulfotransferase family cytosolic 1B member 1-like [Ostrinia furnacalis]	1.901168	-0.39782	-0.20712	-1.06302	-0.2332

TRINITY_DN57496_c0_g1_i1_orf1	NADPH:adrenodoxin oxidoreductase, mitochondrial [Ostrinia furnacalis]	1.57794	-0.73294	0.208248	-1.35574	0.302496
TRINITY_DN2265_c0_g1_i5_orf1	elongation factor G, mitochondrial [Ostrinia furnacalis]	1.677075	0.214692	0.14253	-1.17813	-0.85617
TRINITY_DN21619_c0_g1_i1_orf1	28S ribosomal protein S15, mitochondrial [Ostrinia furnacalis]	1.859751	-0.83314	0.190593	-0.7958	-0.4214
TRINITY_DN4497_c0_g1_i4_orf1	cytochrome P450 9e2-like [Ostrinia furnacalis] >QPF77612.1 cytochrome P450 monooxygenase CYP9A185 [Ostrinia	1.804825	-0.87304	0.360513	-0.73395	-0.55835
TRINITY_DN1081_c0_g1_i7_orf1	3-ketoacyl-CoA thiolase, mitochondrial-like [Ostrinia furnacalis]	1.918511	-0.45616	0.027349	-0.72386	-0.76584
TRINITY_DN1884_c0_g2_i2_orf1	phosphotriesterase-related protein [Ostrinia furnacalis]	1.755506	0.053848	0.116834	-1.11566	-0.81053
TRINITY_DN41179_c0_g1_i1_orf1	RNA-binding protein NOB1 [Ostrinia furnacalis]	1.884408	-0.44002	-0.22648	-1.08981	-0.1281
TRINITY_DN60821_c0_g1_i1_orf1	nucleolar GTP-binding protein 2 [Ostrinia furnacalis]	1.36182	0.265595	-0.4391	-1.63724	0.448929
TRINITY_DN2749_c0_g1_i4_orf1	RNA exonuclease 4-like [Ostrinia furnacalis] >QEE79882.1 REX4 [Ostrinia furnacalis]	1.360273	0.623052	0.289927	-1.35292	-0.92033
TRINITY_DN32161_c0_g1_i1_orf1	uncharacterized protein LOC114352518 [Ostrinia furnacalis]	1.534448	0.023288	-0.29102	-1.5709	0.304177
TRINITY_DN4194_c0_g1_i1_orf1	hornerin-like [Ostrinia furnacalis]	1.065025	-1.29814	0.175754	-1.00767	1.065025
TRINITY_DN57462_c0_g1_i1_orf1	glutathione S transferase-S5 [Glyphodes pyloalis]	1.670249	-0.29127	-0.21187	-1.42006	0.252951
TRINITY_DN35277_c0_g1_i1_orf1	luciferin 4-monooxygenase-like, partial [Ostrinia furnacalis]	1.915413	-0.34847	-0.01487	-0.82641	-0.72567
TRINITY_DN2593_c0_g1_i1_orf1	midgut carboxypeptidase [Loxostege sticticalis]	1.90855	-0.21157	-0.15332	-0.99288	-0.55079
TRINITY_DN1266_c2_g1_i1_orf1	serine/threonine-protein kinase RIO3 [Ostrinia furnacalis]	1.62676	-0.94729	0.100571	-1.14565	0.365608
TRINITY_DN1666_c0_g1_i2_orf1	putative defense protein Hdd11 [Ostrinia furnacalis] >XP_028179344.1 putative defense protein Hdd11 [Ostrinia furnacalis] >AGV28583.1 immune-induced protein [Ostrinia furnacalis]	1.659134	-0.06283	0.430937	-1.05076	-0.97649
TRINITY_DN21984_c0_g1_i6_orf1	venom serine protease 34-like [Ostrinia furnacalis]	1.882736	-0.09381	-0.20251	-1.06444	-0.52199
TRINITY_DN43369_c0_g2_i1_orf1	cytochrome P450 monooxygenase 304 [Glyphodes pyloalis]	1.35978	-0.76021	0.547351	-1.47229	0.325363
TRINITY_DN14301_c0_g2_i1_orf1	unnamed protein product [Chrysodeixis includens]	1.936775	-0.41645	-0.18179	-0.94	-0.39853
TRINITY_DN30070_c0_g1_i6_orf1	uncharacterized protein LOC114361440 isoform X1 [Ostrinia furnacalis] >XP_028172261.1 uncharacterized protein LOC114361440 isoform X2 [Ostrinia furnacalis]	1.891106	-0.17777	-0.28475	-1.09548	-0.33311
TRINITY_DN8603_c0_g1_i1_orf1	adenosine kinase 2 isoform X2 [Cotesia glomerata] >XP_044591805.1 adenosine kinase 2 isoform X4 [Cotesia glomerata]	1.487799	0.148808	0.56829	-1.02976	-1.17514
TRINITY_DN14565_c0_g1_i11_orf1	4-aminobutyrate aminotransferase, mitochondrial [Galleria mellonella]	1.874486	-0.6757	-0.24294	-0.98482	0.028975
TRINITY_DN16487_c0_g1_i1_orf1	p21-activated protein kinase-interacting protein 1-like [Ostrinia furnacalis]	1.759949	0.183362	-0.08334	-1.18715	-0.67282
TRINITY_DN3355_c0_g2_i4_orf1	UDP-glycosyltransferase UGT33AL1 [Ostrinia furnacalis]	1.834711	-0.01	-0.05928	-0.69318	-1.07225
TRINITY_DN16122_c0_g1_i4_orf1	cytochrome P450 6k1-like [Ostrinia furnacalis]	1.911221	-0.21858	-0.10432	-0.91077	-0.67755
TRINITY_DN47389_c0_g1_i2_orf1	non-specific lipid-transfer protein-like [Ostrinia furnacalis]	1.894553	-0.56477	0.10604	-0.56032	-0.8755
TRINITY_DN49936_c0_g2_i1_orf1	39S ribosomal protein L20, mitochondrial [Ostrinia furnacalis]	1.878141	-0.32813	-0.30703	-1.12056	-0.12241
TRINITY_DN5678_c0_g2_i3_orf1	coiled-coil domain-containing protein 115 [Ostrinia furnacalis]	1.964543	-0.56254	-0.20858	-0.78176	-0.41166
TRINITY_DN3929_c0_g3_i3_orf1	Glutathione S-transferase 1, isoform D [Papilio machaon]	1.778806	-0.72848	0.316145	-0.31473	-1.05174
TRINITY_DN14937_c0_g1_i7_orf1	multidrug resistance protein homolog 49-like [Ostrinia furnacalis] >XP_028159925.1 multidrug resistance protein homolog 49-like [Ostrinia furnacalis]	1.893993	-0.67614	0.076818	-0.8836	-0.41107
TRINITY_DN76377_c0_g1_i1_orf1	uncharacterized protein LOC111357764, partial [Spodoptera litura]	1.196731	-1.3134	-0.26153	-0.73364	1.111839
TRINITY_DN2184_c0_g1_i4_orf1	uncharacterized protein LOC114359356 [Ostrinia furnacalis]	1.82781	-0.19301	0.124134	-1.05199	-0.70694
TRINITY_DN886_c0_g1_i1_orf1	collagenase-like isoform X1 [Ostrinia furnacalis]	1.924972	-0.23813	-0.39596	-0.99744	-0.29344
TRINITY_DN76283_c0_g2_i1_orf1	fatty acid synthase-like [Ostrinia furnacalis]	1.900228	-0.34734	0.0119	-0.63397	-0.93082
TRINITY_DN8366_c0_g1_i4_orf1	luciferin 4-monooxygenase-like [Ostrinia furnacalis]	1.934927	-0.33882	-0.10197	-0.83262	-0.66152
TRINITY_DN34040_c0_g2_i1_orf1	uncharacterized protein LOC114352849 [Ostrinia furnacalis]	1.760214	-0.07701	0.084995	-0.48085	-1.28735
TRINITY_DN3292_c2_g1_i4_orf1	ribosome biogenesis regulatory protein homolog [Ostrinia furnacalis]	1.953638	-0.3168	-0.23136	-0.84717	-0.55831
TRINITY_DN1617_c0_g1_i5_orf1	hypothetical protein evm_009822 [Chilo suppressalis] >CAB3525311.1 unnamed protein product [Chilo suppressalis] >CAH0402638.1 unnamed protein product [Chilo suppressalis]	1.943946	-0.40138	-0.12879	-0.85522	-0.55855
TRINITY_DN2695_c0_g1_i14_orfp1	TRINITY_DN2695_c0_g1_i14_m.44485 TRINITY_DN2695_c0_g1::TRINITY_DN2695_c0_g1_i14::g.44485 ORF type:3prime_partial len:698 (+),score=187.51 TRINITY_DN2695_c0_g1_i14:101-2092(+)	1.688499	-0.51812	0.470116	-0.42407	-1.21642
TRINITY_DN2304_c0_g1_i4_orf1	clustered mitochondria protein homolog isoform X2 [Ostrinia furnacalis]	1.896802	-0.01739	-0.24314	-0.86303	-0.77324
TRINITY_DN45477_c0_g1_i1_orf1	putative E3 ubiquitin-protein ligase UBR7 [Ostrinia furnacalis]	1.829175	-0.75664	-0.3198	-0.9663	0.213561
TRINITY_DN868_c0_g1_i4_orf1	uncharacterized protein LOC114359357 isoform X1 [Ostrinia furnacalis]	1.684835	-0.88889	0.587267	-0.50495	-0.87827
TRINITY_DN34821_c0_g1_i4_orf1	acetylcholine receptor subunit alpha-L1-like [Ostrinia furnacalis]	1.190755	-0.52684	-0.59068	-1.25173	1.178485
TRINITY_DN39673_c0_g1_i1_orf1	uncharacterized protein LOC114359357 isoform X1 [Ostrinia furnacalis]	1.926672	-0.46473	-0.0077	-0.81225	-0.64199
TRINITY_DN747_c0_g1_i4_orf1	trypsin, alkaline C-like [Ostrinia furnacalis]	1.951857	-0.63978	-0.078	-0.53526	-0.69882
TRINITY_DN96170_c0_g2_i1_orf1	uncharacterized protein LOC114355569 [Ostrinia furnacalis]	1.969602	-0.60538	-0.28051	-0.75195	-0.33176
TRINITY_DN7920_c0_g1_i2_orf1	uncharacterized protein LOC114357268 [Ostrinia furnacalis] >XP_028166599.1 uncharacterized protein LOC114357268 [Ostrinia furnacalis]	1.887882	-0.29084	-0.15324	-1.09976	-0.34404
TRINITY_DN42759_c0_g2_i1_orf1	fatty acid synthase-like [Ostrinia furnacalis]	1.93056	-0.53403	-0.04981	-0.8714	-0.47532

TRINITY_DN47_c0_g1_i2_orf1	uncharacterized protein LOC114356437 isoform X1 [Ostrinia furnacalis]	1.878469	-0.07854	-0.09318	-0.85467	-0.85208
TRINITY_DN18909_c0_g1_i8_orf1	unnamed protein product [Euphydryas editha]	1.929332	-0.47124	-0.01731	-0.81361	-0.62718
TRINITY_DN33452_c0_g1_i1_orf1	lethal(2) giant larvae protein isoform X8 [Ostrinia furnacalis]	1.401478	0.148319	0.645184	-1.4046	-0.79039
TRINITY_DN48020_c0_g1_i1_orf1	aminopeptidase N4 [Cnaphalocrocis medinalis]	1.94389	-0.52096	-0.04888	-0.7288	-0.64525
TRINITY_DN16900_c0_g2_i1_orf1	uncharacterized oxidoreductase dhs-27-like [Ostrinia furnacalis]	1.93946	-0.34161	-0.23859	-0.94522	-0.41404
TRINITY_DN26375_c0_g1_i1_orf1	hypothetical protein O3G_MSEX007366 [Manduca sexta]	1.9375	-0.6371	-0.37556	-0.83085	-0.09399
TRINITY_DN4030_c0_g2_i1_orf1	putative trypsin 6 [Ostrinia nubilalis]	1.920093	-0.07282	-0.45909	-0.9526	-0.43558
TRINITY_DN1154_c0_g1_i1_orf1	calexcitin-1-like [Ostrinia furnacalis] >ADK94879.2 juvenile hormone diol kinase [Ostrinia furnacalis]	1.889639	-0.00924	-0.22857	-0.74629	-0.90553
TRINITY_DN26411_c0_g1_i2_orfp1	TRINITY_DN26411_c0_g1_i2_m.24123 TRINITY_DN26411_c0_g1::TRINITY_DN26411_c0_g1_i2::g.24123 ORF type:internal len:115 (-),score=72.83 TRINITY_DN26411_c0_g1_i2:3-344(-)	1.55133	-0.92054	0.826721	-0.7419	-0.71561
TRINITY_DN79319_c0_g1_i8_orfp1	TRINITY_DN79319_c0_g1_i8_m.49956 TRINITY_DN79319_c0_g1::TRINITY_DN79319_c0_g1_i8::g.49956 ORF type:5prime_partial len:84 (+),score=1.39 TRINITY_DN79319_c0_g1_i8:1-252(+)	1.959937	-0.26557	-0.27594	-0.76406	-0.65436
TRINITY_DN66302_c0_g1_i1_orf1	carboxypeptidase B-like [Ostrinia furnacalis]	1.95823	-0.48989	-0.16229	-0.80488	-0.50118
TRINITY_DN468_c0_g1_i3_orf1	transmembrane protein 41 homolog isoform X2 [Ostrinia furnacalis]	1.94776	-0.40322	-0.21243	-0.90192	-0.4302
TRINITY_DN15046_c0_g1_i8_orf1	epidermal retinol dehydrogenase 2-like isoform X1 [Ostrinia furnacalis] >XP_028169999.1 epidermal retinol dehydrogenase 2-like isoform X2 [Ostrinia furnacalis]	1.907397	-0.0023	-0.32826	-0.86215	-0.71469
TRINITY_DN18172_c0_g1_i6_orf1	digestive cysteine proteinase 2-like [Ostrinia furnacalis]	1.958197	-0.52832	-0.45698	-0.80601	-0.16688
TRINITY_DN44288_c0_g1_i2_orf1	ATP-dependent RNA helicase p62 [Ostrinia furnacalis]	1.922906	-0.5001	-0.49093	-0.90017	-0.0317
TRINITY_DN11259_c0_g1_i1_orf1	uncharacterized protein LOC114357075 [Ostrinia furnacalis]	1.961951	-0.34948	-0.20897	-0.70589	-0.69761
TRINITY_DN64403_c0_g2_i1_orf1	carboxylesterase [Ostrinia furnacalis]	1.900271	-0.56202	0.108477	-0.63727	-0.80946
TRINITY_DN69713_c0_g1_i1_orf1	membrane-bound alkaline phosphatase-like [Ostrinia furnacalis]	1.929689	-0.29096	-0.15523	-0.55851	-0.925
TRINITY_DN1249_c0_g1_i10_orf1	venom carboxylesterase-6-like [Ostrinia furnacalis]	1.892155	-0.08405	-0.14079	-0.87169	-0.79563
TRINITY_DN4731_c0_g1_i1_orf1	gelsolin-like [Ostrinia furnacalis]	1.980319	-0.43906	-0.26836	-0.67773	-0.59518
TRINITY_DN16931_c0_g1_i1_orf1	pancreatic triacylglycerol lipase-like [Ostrinia furnacalis]	1.897966	-0.36043	0.040201	-0.89272	-0.68502
TRINITY_DN82801_c0_g1_i1_orf1	uncharacterized protein LOC114364712 [Ostrinia furnacalis]	1.465685	-0.68803	-0.68803	-1.01974	0.930118
TRINITY_DN8116_c0_g1_i2_orf1	uncharacterized protein LOC114350845 [Ostrinia furnacalis]	1.983209	-0.54943	-0.2932	-0.68984	-0.45074
TRINITY_DN542_c0_g2_i1_orf1	uncharacterized protein LOC114364889 [Ostrinia furnacalis]	1.986473	-0.47826	-0.41478	-0.71308	-0.38035
TRINITY_DN3194_c0_g1_i6_orf1	uncharacterized protein LOC114361386 [Ostrinia furnacalis]	1.974152	-0.6176	-0.33097	-0.71996	-0.30561
TRINITY_DN1310_c0_g1_i4_orf1	trypsin-like isoform X1 [Ostrinia furnacalis] >XP_028159118.1 trypsin-like isoform X2 [Ostrinia furnacalis]	1.979404	-0.35633	-0.45612	-0.76521	-0.40175
TRINITY_DN82320_c0_g1_i2_orf1	glutathione S-transferase sigma3 [Glyphodes pyloalis]	1.870615	0.029075	-0.39665	-1.07791	-0.42513
TRINITY_DN311_c0_g1_i4_orfp1	TRINITY_DN311_c0_g1_i4_m.65135 TRINITY_DN311_c0_g1::TRINITY_DN311_c0_g1_i4::g.65135 ORF type:5prime_partial len:126 (+),score=71.21 TRINITY_DN311_c0_g1_i4:1-378(+)	1.858215	-0.09502	-0.17637	-1.14547	-0.44135
TRINITY_DN48410_c0_g1_i1_orf1	alpha-amylase 1-like [Ostrinia furnacalis]	1.986149	-0.38938	-0.39711	-0.71452	-0.48513
TRINITY_DN6140_c0_g3_i3_orf1	CD63 antigen-like [Ostrinia furnacalis]	1.843749	-0.57199	0.085571	-1.09395	-0.26338
TRINITY_DN35051_c0_g1_i1_orf1	uncharacterized protein LOC114364307 [Ostrinia furnacalis]	1.982531	-0.51163	-0.27943	-0.69554	-0.49593
TRINITY_DN61674_c0_g1_i2_orf1	fatty acid-binding protein 1-like [Ostrinia furnacalis]	1.987707	-0.43691	-0.45147	-0.70785	-0.39147
TRINITY_DN1914_c0_g1_i6_orf1	loricrin-like [Ostrinia furnacalis]	1.911713	-0.61151	-0.4429	-0.88019	0.022879
TRINITY_DN14679_c0_g1_i1_orf1	hypothetical protein evm_003043 [Chilo suppressalis]	1.955962	-0.27887	-0.2885	-0.85102	-0.53757
TRINITY_DN4959_c0_g1_i1_orf1	pancreatic triacylglycerol lipase-like [Ostrinia furnacalis]	1.801331	-0.25301	-0.17483	-1.28566	-0.08783
TRINITY_DN2490_c0_g2_i1_orfp1	TRINITY_DN2490_c0_g2_i1_m.56872 TRINITY_DN2490_c0_g2::TRINITY_DN2490_c0_g2_i1::g.56872 ORF type:internal len:359 (-),score=123.59 TRINITY_DN2490_c0_g2_i1:2-1075(-)	1.960231	-0.58187	-0.35051	-0.8036	-0.22425
TRINITY_DN117_c0_g1_i4_orf1	lipase member I-like [Ostrinia furnacalis]	1.856894	-0.57903	-0.51235	-0.95772	0.192203

TRINITY_DN31584_c0_g2_i2_orf1	14-3-3 protein epsilon [Gallus gallus] >NP_001233297.1 14-3-3 protein epsilon [Pan troglodytes] >NP_006752.1 14-3-3 protein epsilon [Homo sapiens] >NP_033562.3 14-3-3 protein epsilon [Mus musculus] >NP_113791.2 14-3-3 protein epsilon [Rattus norvegicus] >NP_776916.1 14-3-3 protein epsilon [Bos taurus] >XP_001504337.1 14-3-3 protein epsilon isoform X1 [Equus caballus] >XP_002918088.2 14-3-3 protein epsilon isoform X2 [Ailuropoda melanoleuca] >XP_003416855.1 14-3-3 protein epsilon isoform X1 [Loxodonta africana] >XP_003469733.1 14-3-3 protein epsilon isoform X1 [Cavia porcellus] >XP_003816884.1 14-3-3 protein epsilon isoform X1 [Pan paniscus] >XP_003912098.1 14-3-3 protein epsilon isoform X1 [Papio anubis] >XP_003929381.1 14-3-3 protein epsilon isoform X1 [Saimiri boliviensis boliviensis] >XP_003996471.1 14-3-3 protein epsilon isoform X1 [Felis catus] >XP_004267124.1 14-3-3 protein epsilon isoform X1 [Orcinus orca] >XP_004376223.1 14-3-3 protein epsilon [Trichechus manatus latirostris] >XP_004404155.1 PREDICTED: 14-3-3 protein epsilon isoform X2 [Odobenus rosmarus divergens] >XP_004433380.1 PREDICTED: 14-3-3 protein epsilon isoform X1 [Ceratotherium simum simum] >XP_004483832.1 14-3-3 protein epsilon isoform X1 [Dasypus novemcinctus] >XP_004605045.1 PREDICTED: 14-3-3 protein epsilon [Sorex araneus] >XP_004667919.1 14-3-3 protein epsilon [Jaculus jaculus] >XP_004706944.1 14-3-3 protein epsilon [Echinops telfairi] >XP_004746947.1 14-3-3 protein epsilon isoform X1 [Mustela putorius furo] >XP_004857172.1 14-3-3 protein epsilon isoform X1 [Heterocephalus glaber] >XP_005067448.1 14-3-3 protein epsilon isoform X1 [Mesocricetus auratus] >XP_005240506.1 14-3-3 protein epsilon isoform X1 [Falco peregrinus] >XP_005327947.1 14-3-3 protein epsilon isoform X1 [Ictidomys tridecemlineatus] >XP_005349591.1 14-3-3 protein epsilon isoform X1 [Microtus ochrogaster] >XP_005402688.1 PREDICTED: 14-3-3 protein epsilon isoform X1 [Chinchilla lanigera] >XP_005525859.1 PREDICTED: 14-3-3 protein epsilon isoform X1 [Pseudopodoces humilis] >XP_005888292.1 PREDICTED: 14-3-3 protein epsilon isoform X1 [Bos mutus] >XP_006079841.1 14-3-3 protein epsilon isoform X1 [Bubalus bubalis] >XP_006099253.1 14-3-3 protein epsilon [Myotis lucifugus] >XP_006185046.1 14-3-3 protein epsilon isoform X1 [Camelus ferus] >XP_006214490.1 14-3-3 protein epsilon isoform X1 [Vicugna pacos] >XP_006259463.1 PREDICTED: 14-3-3 protein epsilon [Alligator mississippiensis] >XP_006768146.1 PREDICTED: 14-3-3 protein epsilon isoform X1 [Myotis davidii] >XP_006863283.1 PREDICTED: 14-3-3 protein epsilon [Chrysochloris asiatica] >XP_006891074.1 PREDICTED: 14-3-3 protein epsilon-like [Elephantulus edwardii] >XP_006925117.1 14-3-3 protein epsilon isoform X1 [Pteropus alecto] >XP_006977465.1 14-3-3 protein epsilon isoform X1 [Peromyscus maniculatus bairdii] >XP_007057769.1 14-3-3 protein epsilon isoform X1 [Chelonia mydas] >XP_007123613.1 14-3-3 protein epsilon isoform X1 [Physeter catodon] >XP_007183877.1 14-3-3 protein epsilon isoform X1 [Balaenoptera acutorostrata scammoni] >XP_007454293.1 PREDICTED: 14-3-3 protein epsilon [Lipotes vexillifer] >XP_007520478.1 PREDICTED: 14-3-3 protein epsilon [Erinaceus europaeus] >XP_007935626.1 14-3-3 protein epsilon [Orycteropus afer afer] >XP_008007997.1 14-3-3 protein epsilon isoform X1 [Chlorocebus sabaeus] >XP_008058985.1 14-	0.414635	1.168976	-0.31729	-1.76434	0.498016
