# Supplementary material for: Comparative Proteomics and Metabonomics Analysis of Different Diapause Stages Revealed a New Regulation Mechanism of Diapause in Loxostege sticticalis (Lepidoptera: Pyralidae)
Source: Molecules. 2024 Jul 25;29(15):3472. doi: 10.3390/molecules29153472 (PMC11314584; doi:10.3390/molecules29153472)
Supplement: Supplementary file 1 [file molecules-29-03472-s001.zip › analysis process/proteomic/Cluster analysis of expression patterns/Up/PreDvsND up.pdf]

Accession	Description	ND	RD	PreD	CT	D
TRINITY_DN2396_c0_g1_i9_orfp1	TRINITY_DN2396_c0_g1_i9_m.39038 TRINITY_DN2396_c0_g1::TRINITY_DN2396_c0_g1_i9::g.39038 ORF type:5prime_partial len:181 (+),score=90.33 TRINITY_DN2396_c0_g1_i9:3-545(+)	-1.92051	-0.04221	0.75417	0.67782	0.53072
TRINITY_DN971_c0_g1_i10_orfp1	TRINITY_DN971_c0_g1_i10_m.54268 TRINITY_DN971_c0_g1::TRINITY_DN971_c0_g1_i10::g.54268 ORF type:internal len:187 (+),score=141.68 TRINITY_DN971_c0_g1_i10:1-558(+)	-1.8854	-0.1083	0.89113	0.42557	0.67699
TRINITY_DN10877_c0_g1_i1_orf1	spodomicin-like [Ostrinia furnacalis]	-1.5409	-0.39422	1.42832	0.64179	-0.13499
TRINITY_DN1506_c0_g1_i6_orfp1	TRINITY_DN1506_c0_g1_i6_m.57691 TRINITY_DN1506_c0_g1::TRINITY_DN1506_c0_g1_i6::g.57691 ORF type:5prime_partial len:173 (+),score=30.49 TRINITY_DN1506_c0_g1_i6:1-519(+)	-1.11546	-1.32434	0.70693	0.84806	0.88481
TRINITY_DN971_c0_g1_i5_orfp1	TRINITY_DN971_c0_g1_i5_m.54249 TRINITY_DN971_c0_g1::TRINITY_DN971_c0_g1_i5::g.54249 ORF type:internal len:108 (+),score=66.98 TRINITY_DN971_c0_g1_i5:1-321(+)	-1.76323	-0.39991	0.48896	1.04991	0.62427
TRINITY_DN295_c3_g1_i1_orfp1	TRINITY_DN295_c3_g1_i1_m.18839 TRINITY_DN295_c3_g1::TRINITY_DN295_c3_g1_i1::g.18839 ORF type:5prime_partial len:118 (+),score=46.25 TRINITY_DN295_c3_g1_i1:1-354(+)	-1.89772	-0.12431	0.58171	0.78172	0.65859
TRINITY_DN30510_c0_g1_i6_orf1	spodomicin-like [Ostrinia furnacalis]	-1.29959	-1.05909	1.19375	0.37534	0.78959
TRINITY_DN57137_c0_g1_i1_orfp1	TRINITY_DN57137_c0_g1_i1_m.46420 TRINITY_DN57137_c0_g1::TRINITY_DN57137_c0_g1_i1::g.46420 ORF type:5prime_partial len:56 (-),score=4.65 TRINITY_DN57137_c0_g1_i1:82-249(-)	-1.84759	-0.23025	0.79953	0.44279	0.83552
TRINITY_DN36476_c1_g1_i1_orfp1	TRINITY_DN36476_c1_g1_i1_m.70910 TRINITY_DN36476_c1_g1::TRINITY_DN36476_c1_g1_i1::g.70910 ORF type:5prime_partial len:88 (-),score=0.50 TRINITY_DN36476_c1_g1_i1:49-312(-)	-1.62065	-0.66669	1.01449	0.42458	0.84827
TRINITY_DN35809_c0_g1_i1_orf1	spodomicin-like [Ostrinia furnacalis] >QKV49445.1 diapausin [Ostrinia furnacalis]	-1.6706	-0.59427	0.99078	0.81406	0.46002
TRINITY_DN6330_c0_g1_i1_orfp1	TRINITY_DN6330_c0_g1_i1_m.42332 TRINITY_DN6330_c0_g1::TRINITY_DN6330_c0_g1_i1::g.42332 ORF type:3prime_partial len:51 (-),score=33.30 TRINITY_DN6330_c0_g1_i1:3-152(-)	-1.72994	-0.47532	0.9219	0.40933	0.87403
TRINITY_DN47784_c0_g2_i1_orfp1	arylphorin subunit alpha-like [Ostrinia furnacalis]	-1.66831	-0.18103	1.36078	-0.08201	0.57057
TRINITY_DN2044_c0_g1_i5_orfp1	TRINITY_DN2044_c0_g1_i5_m.4210 TRINITY_DN2044_c0_g1::TRINITY_DN2044_c0_g1_i5::g.4210 ORF type:complete len:151 (-),score=85.31 TRINITY_DN2044_c0_g1_i5:857-1309(-)	-1.76135	-0.40125	0.44879	1.03947	0.67434
TRINITY_DN2457_c0_g1_i8_orf1	uncharacterized protein LOC114355596 [Ostrinia furnacalis]	-1.35161	-0.88645	1.4347	0.34564	0.45773
TRINITY_DN86772_c0_g1_i3_orfp1	x-tox [Spodoptera exigua]	-1.78653	-0.3059	0.98139	0.29628	0.81475
TRINITY_DN34423_c0_g1_i3_orf1	THAP domain-containing protein 4-like [Ostrinia furnacalis]	-1.95064	0.07258	0.60276	0.71887	0.55645
TRINITY_DN56459_c0_g1_i2_orf1	aldo-keto reductase AKR2E4-like [Ostrinia furnacalis]	-1.69301	-0.60488	0.70412	0.82862	0.76515
TRINITY_DN7064_c0_g1_i19_orf1	unnamed protein product [Chilo suppressalis]	-1.23178	-1.1627	0.44399	0.84973	1.10076
TRINITY_DN3275_c0_g2_i3_orf1	hypothetical protein B5X24_HaOG216046 [Helicoverpa armigera]	-1.74395	-0.23787	1.18595	0.09888	0.69698
TRINITY_DN30306_c0_g2_i1_orf1	perilipin-4-like isoform X3 [Ostrinia furnacalis]	-1.64237	-0.58492	1.14023	0.35707	0.72998
TRINITY_DN12534_c0_g1_i4_orf1	antibacterial protein [Heliothis virescens]	-1.74092	-0.50785	0.64548	0.87018	0.73311
TRINITY_DN3166_c1_g1_i6_orf1	hypothetical protein evm_013813 [Chilo suppressalis]	-1.7427	-0.31599	0.35242	0.4768	1.22947
TRINITY_DN17615_c0_g1_i3_orf1	hypothetical protein SFRUCORN_008858 [Spodoptera frugiperda]	-1.90641	-0.06894	0.52512	0.59568	0.85456
TRINITY_DN38506_c0_g1_i4_orf1	C-1-tetrahydrofolate synthase, cytoplasmic isoform X1 [Ostrinia furnacalis] >XP_028166137.1 C-1-tetrahydrofolate synthase, cytoplasmic isoform X2 [Ostrinia furnacalis] >XP_028166140.1 C-1-tetrahydrofolate synthase, cytoplasmic isoform X4 [Ostrinia furnacalis]	-1.48978	-0.91799	0.82381	0.84048	0.74348
TRINITY_DN6025_c0_g2_i1_orfp1	TRINITY_DN6025_c0_g2_i1_m.7749 TRINITY_DN6025_c0_g2::TRINITY_DN6025_c0_g2_i1::g.7749 ORF type:5prime_partial len:106 (+),score=47.10 TRINITY_DN6025_c0_g2_i1:3-320(+)	-1.82794	-0.29357	0.8931	0.51334	0.71506
TRINITY_DN3439_c0_g2_i2_orf1	histone H2A.Z-specific chaperone CHZ1-like [Ostrinia furnacalis]	-1.46229	-0.8689	0.88475	0.35051	1.09594
TRINITY_DN15202_c0_g1_i6_orf1	uncharacterized protein LOC114364499 isoform X2 [Ostrinia furnacalis]	-1.67149	-0.53535	0.3374	0.76407	1.10538
TRINITY_DN24723_c2_g1_i1_orf1	hypothetical protein evm_001103 [Chilo suppressalis]	-1.76659	-0.44583	0.77289	0.87289	0.56665
TRINITY_DN380_c0_g2_i2_orf1	chemosensory protein 10 [Ostrinia furnacalis]	-1.79714	-0.34895	0.4764	0.95239	0.71729
TRINITY_DN7226_c0_g1_i2_orf1	chemosensory protein [Conogethes punctiferalis]	-1.7465	-0.44449	0.82251	0.94813	0.42034
TRINITY_DN1391_c0_g1_i29_orfp1	TRINITY_DN1391_c0_g1_i29_m.70767 TRINITY_DN1391_c0_g1::TRINITY_DN1391_c0_g1_i29::g.70767 ORF type:complete len:495 (+),score=158.49 TRINITY_DN1391_c0_g1_i29:728-2212(+)	-1.90099	-0.10285	0.8222	0.56359	0.61805
TRINITY_DN5198_c0_g1_i5_orfp1	TRINITY_DN5198_c0_g1_i5_m.8637 TRINITY_DN5198_c0_g1::TRINITY_DN5198_c0_g1_i5::g.8637 ORF type:complete len:223 (-),score=54.99 TRINITY_DN5198_c0_g1_i5:319-987(-)	-1.36167	-0.9491	1.31173	0.38687	0.61217
TRINITY_DN7226_c0_g1_i5_orf1	chemosensory protein [Dioryctria abietella]	-1.79643	-0.34679	0.94469	0.74398	0.45455
TRINITY_DN1880_c0_g1_i4_orf1	serine protease inhibitor dipetalogastin-like [Helicoverpa zea]	-1.6005	-0.73259	0.69273	1.01569	0.62467
TRINITY_DN710_c0_g1_i11_orfp1	TRINITY_DN710_c0_g1_i11_m.67699 TRINITY_DN710_c0_g1::TRINITY_DN710_c0_g1_i11::g.67699 ORF type:complete len:194 (-),score=34.99,Collagen PF01391.19 0.00029 TRINITY_DN710_c0_g1_i11:1283-1864(-)	-1.34384	-0.99675	0.98595	0.28302	1.07163
TRINITY_DN27300_c0_g1_i7_orfp1	TRINITY_DN27300_c0_g1_i7_m.71141 TRINITY_DN27300_c0_g1::TRINITY_DN27300_c0_g1_i7::g.71141 ORF type:internal len:82 (-),score=6.59 TRINITY_DN27300_c0_g1_i7:3-245(-)	-1.49298	-0.49544	1.51949	0.46551	0.00342
TRINITY_DN71698_c0_g1_i1_orfp1	TRINITY_DN71698_c0_g1_i1_m.1194 TRINITY_DN71698_c0_g1::TRINITY_DN71698_c0_g1_i1::g.1194 ORF type:internal len:134 (+),score=19.66,Toxin_2 PF00451.20 4.3e-05,Toxin_2|PF00451.20|0.037,Toxin_2|PF00451.20|7.5e-05,Gamma-thionin|PF00304.21|0.017,Gamma-thionin|PF00304.21|0.05,Gamma-thionin|PF00304.21|0.021,Toxin_38|PF14866.7|0.13,Toxin_38|PF14866.7|0.15,Toxin_38|PF14866.7|0.15,Defensin_2|PF01097.19|0.053,Defensin_2|PF01097.19|0.34,Defensin_2|PF01097.19|0.092 TRINITY_DN71698_c0_g1_i1:3-401(+)	-1.67369	-0.57881	0.86061	0.9738	0.41809

TRINITY_DN14328_c0_g1_i12_orf1	larval cuticle protein LCP-30-like [Ostrinia furnacalis]	-1.22551	-1.19973	0.73966	0.62938	1.0562
TRINITY_DN5153_c1_g1_i1_orf1	nose resistant to fluoxetine protein 6-like isoform X1 [Ostrinia furnacalis]	-1.64455	-0.57412	1.19455	0.42701	0.59712
TRINITY_DN114890_c0_g1_i4_orf1	chemosensory protein 10 [Ostrinia furnacalis]	-1.47498	-0.74287	0.56866	1.36742	0.28177
TRINITY_DN11981_c0_g1_i7_orf1	luciferin 4-monooxygenase-like isoform X2 [Ostrinia furnacalis]	-1.64747	-0.55027	1.1955	0.66168	0.34057
TRINITY_DN4816_c0_g2_i3_orf1	15-hydroxyprostaglandin dehydrogenase [NAD(+)]-like [Ostrinia furnacalis]	-1.89395	-0.14321	0.69379	0.73797	0.6054
TRINITY_DN17247_c0_g1_i14_orf1	uncharacterized protein LOC114356308 [Ostrinia furnacalis]	-1.87691	0.18129	0.87831	-0.00301	0.82032
TRINITY_DN5439_c0_g1_i2_orf1	uncharacterized protein LOC114353087 [Ostrinia furnacalis]	-1.80925	-0.17705	0.56999	0.27955	1.13676
TRINITY_DN1363_c0_g1_i11_orf1	cytochrome P450 CYP12A2-like isoform X1 [Ostrinia furnacalis] >QPF77619.1 cytochrome P450 monooxygenase CYP333A20 [Ostrinia furnacalis]	-1.81711	-0.29938	0.96686	0.49475	0.65489
TRINITY_DN8595_c0_g1_i3_orf1	aldose reductase-like isoform X4 [Trichoplusia ni]	-1.63749	-0.65403	0.59018	0.63241	1.06893
TRINITY_DN15545_c0_g1_i1_orf1	larval cuticle protein LCP-14-like [Ostrinia furnacalis]	-1.0717	-1.35118	0.95578	0.59929	0.86782
TRINITY_DN135780_c0_g1_i1_orf1	flotillin-1 isoform X1 [Pectinophora gossypiella]	-1.87167	-0.07917	0.82634	0.26605	0.85845
TRINITY_DN9079_c0_g1_i5_orf1	UDP-glucuronosyltransferase-like [Ostrinia furnacalis]	-1.88496	-0.17022	0.64225	0.76676	0.64616
TRINITY_DN1450_c0_g2_i1_orf1	death-associated protein 1 [Ostrinia furnacalis]	-1.61801	-0.69842	0.99672	0.53741	0.7823
TRINITY_DN335_c1_g1_i5_orf1	PREDICTED: perilipin-4 isoform X14 [Papilio polytes]	-1.39388	-0.91176	1.15817	0.23751	0.90995
TRINITY_DN394_c0_g1_i2_orf1	uncharacterized protein LOC114351483 [Ostrinia furnacalis]	-1.07239	-1.25181	0.79102	0.27572	1.25746
TRINITY_DN31348_c0_g1_i1_orf1	protein lethal(2)essential for life [Bombyx mori]	-1.64067	-0.55478	0.45428	1.24357	0.4976
TRINITY_DN8008_c0_g1_i6_orf1	uncharacterized protein LOC114357965 isoform X1 [Ostrinia furnacalis] >XP_028167599.1 uncharacterized protein LOC114357965 isoform X1 [Ostrinia furnacalis] >XP_028167600.1 uncharacterized protein LOC114357965 isoform X2 [Ostrinia furnacalis] >XP_028167601.1 uncharacterized protein LOC114357965 isoform X3 [Ostrinia furnacalis]	-1.47885	-0.84436	0.40514	1.17864	0.73942
TRINITY_DN5748_c0_g1_i6_orf1	glycine N-methyltransferase isoform X1 [Ostrinia furnacalis] >XP_028165118.1 glycine N-methyltransferase isoform X2 [Ostrinia furnacalis] >XP_028165119.1 glycine N-methyltransferase isoform X1 [Ostrinia furnacalis] >XP_028165120.1 glycine N-methyltransferase isoform X2 [Ostrinia furnacalis]	-1.42537	-0.82201	1.38264	0.37303	0.4917
TRINITY_DN295_c5_g1_i2_orf1	unnamed protein product [Chilo suppressalis]	-1.72904	-0.38677	0.54383	1.19189	0.3801
TRINITY_DN69_c0_g1_i1_orf1	glycerol-3-phosphate dehydrogenase [NAD(+)], cytoplasmic isoform X1 [Ostrinia furnacalis]	-1.85711	-0.19638	0.65782	0.4677	0.92797
TRINITY_DN24_c0_g1_i1_orf1	hypothetical protein evm_007803 [Chilo suppressalis]	-1.74811	-0.03943	1.04161	-0.16528	0.9112
TRINITY_DN2464_c0_g1_i12_orf1	uncharacterized protein LOC114362996 isoform X1 [Ostrinia furnacalis]	-1.23869	-1.21008	0.81621	0.78066	0.8519
TRINITY_DN628_c0_g1_i1_orf1	prostamide/prostaglandin F synthase-like [Ostrinia furnacalis]	-1.95787	0.10288	0.58343	0.69631	0.57525
TRINITY_DN29026_c0_g1_i4_orf1	TIL [Ostrinia furnacalis]	-1.71245	-0.2116	0.15528	0.41837	1.3504
TRINITY_DN55147_c0_g1_i1_orfp1	TRINITY_DN55147_c0_g1_i1_m.59251 TRINITY_DN55147_c0_g1::TRINITY_DN55147_c0_g1_i1::g.59251 ORF type:5prime_partial len:331 (-),score=113.97,Cuticle_3 PF11018.9 0.29,Cuticle_3|PF11018.9|2.9e-05,Cuticle_3|PF11018.9|0.00037 TRINITY_DN55147_c0_g1_i1:21-1013(-)	-1.02859	-1.39657	0.95132	0.72154	0.75231
TRINITY_DN4802_c0_g1_i4_orf1	uncharacterized protein LOC114366345 isoform X2 [Ostrinia furnacalis]	-1.48977	-0.50714	0.45952	0.01689	1.52051
TRINITY_DN1597_c0_g1_i5_orfp1	TRINITY_DN1597_c0_g1_i5_m.57494 TRINITY_DN1597_c0_g1::TRINITY_DN1597_c0_g1_i5::g.57494 ORF type:complete len:86 (+),score=7.19 TRINITY_DN1597_c0_g1_i5:134-391(+)	-1.85136	0.1037	-0.00396	0.75829	0.99332
TRINITY_DN5177_c0_g1_i2_orf1	hemolin-like isoform X1 [Ostrinia furnacalis]	-1.73659	-0.02554	-0.17449	0.77097	1.16566
TRINITY_DN43350_c0_g3_i1_orf1	uncharacterized protein LOC114355190 [Ostrinia furnacalis]	-1.3572	-1.0414	0.50193	0.84143	1.05524
TRINITY_DN6098_c1_g1_i5_orf1	unnamed protein product, partial [Iphiclides podalirius]	-1.72691	-0.54111	0.67888	0.82118	0.76796
TRINITY_DN1201_c0_g1_i4_orf1	triosephosphate isomerase [Ostrinia furnacalis] >XP_028170843.1 triosephosphate isomerase [Ostrinia furnacalis]	-1.95653	0.11534	0.74917	0.52191	0.57011
TRINITY_DN8953_c0_g1_i4_orf1	gonadotropin-releasing hormone receptor [Ostrinia furnacalis] >AXF67446.1 adipokinetic hormone receptor 1 [Ostrinia furnacalis]	-1.87323	-0.00898	1.00841	0.22209	0.6517
TRINITY_DN1772_c1_g2_i1_orf1	aldose reductase-like isoform X2 [Ostrinia furnacalis]	-1.48297	-0.8172	0.2967	0.86385	1.13962
TRINITY_DN6415_c0_g2_i1_orf1	D-arabinitol dehydrogenase 1-like [Ostrinia furnacalis]	-1.39165	-0.92292	0.40741	0.61675	1.29042
TRINITY_DN40126_c0_g2_i1_orf1	aldehyde dehydrogenase X, mitochondrial-like [Ostrinia furnacalis]	-1.63685	-0.63275	1.09532	0.46233	0.71196
TRINITY_DN20344_c0_g1_i5_orf1	uncharacterized protein LOC114351483 [Ostrinia furnacalis]	-1.14126	-1.23363	0.39299	0.82177	1.16014
TRINITY_DN2187_c0_g1_i1_orf1	flotillin-1 [Chelonus insularis] >XP_034947202.1 flotillin-1 [Chelonus insularis]	-1.85825	-0.19393	0.81471	0.41887	0.8186
TRINITY_DN14670_c0_g1_i1_orf1	heat shock protein beta-1 isoform X1 [Helicoverpa armigera] >XP_022829066.1 heat shock protein beta-1 isoform X1 [Spodoptera litura] >XP_026747148.1 heat shock protein beta-1 isoform X3 [Trichoplusia ni] >XP_026748187.1 heat shock protein beta-1 isoform X2 [Galleria mellonella] >XP_028167756.1 heat shock protein beta-1 isoform X2 [Ostrinia furnacalis] >XP_035431734.1 heat shock protein beta-1-like isoform X3 [Spodoptera frugiperda] >XP_047023072.1 heat shock protein beta-1 isoform X1 [Helicoverpa zea] >XP_049865086.1 heat shock protein beta-1 [Pectinophora gossypiella] >KAH9640995.1 hypothetical protein HF086_015091 [Spodoptera exigua] >QGZ00460.1 heat shock protein 21.4 [Glyphodes pyloalis] >QKR72095.1 heat-shock protein 21.4 [Mythimna separata] >CAB3228281.1 unnamed protein product [Arctia plantaginis] >CAH0628881.1 unnamed protein product [Chrysodeixis includens]	-1.47876	-0.80182	1.2992	0.46987	0.51152
TRINITY_DN350_c0_g1_i5_orf1	tau-like protein isoform X6 [Bombyx mori]	-1.62296	-0.63224	1.16233	0.64153	0.45133
TRINITY_DN31286_c0_g1_i6_orfp1	TRINITY_DN31286_c0_g1_i6_m.28438 TRINITY_DN31286_c0_g1::TRINITY_DN31286_c0_g1_i6::g.28438 ORF type:internal len:92 (-),score=3.10,Perilipin PF03036.17 2e-05 TRINITY_DN31286_c0_g1_i6:1-273(-)	-1.82585	-0.19318	0.51318	0.4125	1.09336
TRINITY_DN13660_c0_g1_i1_orf1	Aliphatic nitrilase [Operophtera brumata]	-1.83886	-0.23622	0.5464	0.98319	0.54548
TRINITY_DN2040_c0_g1_i6_orf1	trypsin-like serine proteinase T26 protein, partial [Chilo infuscatellus]	-1.63528	-0.68195	0.5554	0.86859	0.89324

TRINITY_DN1108_c1_g2_i1_orfp1	TRINITY_DN1108_c1_g2_i1_m.5565 TRINITY_DN1108_c1_g2::TRINITY_DN1108_c1_g2_i1::g.5565 ORF type:internal len:205 (-),score=147.90 TRINITY_DN1108_c1_g2_i1:2-613(-)	-1.54217	-0.47359	1.50535	0.23202	0.27838
TRINITY_DN19731_c0_g1_i1_orf1	allergen Tha p 1-like [Ostrinia furnacalis] >XP_028174916.1 allergen Tha p 1-like [Ostrinia furnacalis] >BAV56808.1 chemosensory protein 4 [Ostrinia furnacalis]	-1.83232	-0.19115	0.27496	0.90423	0.84429
TRINITY_DN4998_c0_g1_i21_orf1	phenoloxidase subunit 2-like [Ostrinia furnacalis]	-1.92945	0.01451	0.74996	0.71659	0.44839
TRINITY_DN37307_c0_g1_i4_orf1	superoxide dismutase [Mn], mitochondrial [Ostrinia furnacalis]	-1.70829	-0.18805	1.38045	0.19596	0.31994
TRINITY_DN4245_c0_g2_i1_orf1	long-chain fatty acid transport protein 4-like [Ostrinia furnacalis]	-1.88039	-0.15065	0.70272	0.48335	0.84497
TRINITY_DN15755_c0_g1_i1_orf1	cytochrome P450 monooxygenase CYP6AB141 [Ostrinia furnacalis]	-1.06222	-1.11964	0.96158	-0.07867	1.29894
TRINITY_DN40126_c0_g1_i1_orf1	aldehyde dehydrogenase X, mitochondrial-like [Ostrinia furnacalis]	-1.93997	0.0301	0.57633	0.58228	0.75126
TRINITY_DN20658_c0_g1_i1_orf1	prostaglandin reductase 1-like [Ostrinia furnacalis]	-1.82141	-0.29786	0.83431	0.45259	0.83237
TRINITY_DN33346_c0_g1_i1_orf1	PREDICTED: U6 snRNA-associated Sm-like protein LSm3 [Papilio xuthus] >XP_028165558.1 U6 snRNA-associated Sm-like protein LSm3 [Ostrinia furnacalis] >KOB73597.1 LSM Sm-like protein family member [Operophtera brumata] >RVE45517.1 hypothetical protein evm_009856 [Chilo suppressalis] >CAB3523639.1 unnamed protein product [Chilo suppressalis] >CAH0400961.1 unnamed protein product [Chilo suppressalis]	-1.9243	0.02651	0.40817	0.64415	0.84548
TRINITY_DN73900_c0_g1_i1_orf1	carbonic anhydrase 7 [Ostrinia furnacalis]	-1.41898	-0.97494	0.77584	1.05902	0.55906
TRINITY_DN3821_c1_g1_i7_orf1	mitochondrial carrier protein Rim2 isoform X1 [Ostrinia furnacalis]	-1.79587	-0.32247	0.37776	0.95202	0.78856
TRINITY_DN9400_c0_g1_i8_orf1	PREDICTED: monoacylglycerol lipase ABHD12-like [Amyelois transitella]	-1.42476	-0.90574	0.80006	1.17661	0.35384
TRINITY_DN130575_c0_g1_i1_orfp1	TRINITY_DN130575_c0_g1_i1_m.77798 TRINITY_DN130575_c0_g1::TRINITY_DN130575_c0_g1_i1::g.77798 ORF type:internal len:70 (+),score=15.12 TRINITY_DN130575_c0_g1_i1:3-209(+)	-1.80912	-0.29395	0.78642	0.37952	0.93712
TRINITY_DN16091_c0_g1_i1_orfp1	TRINITY_DN16091_c0_g1_i1_m.64010 TRINITY_DN16091_c0_g1::TRINITY_DN16091_c0_g1_i1::g.64010 ORF type:5prime_partial len:124 (-),score=7.29,Toxin_2 PF00451.20 0.00035,Toxin_2|PF00451.20|0.00013,Toxin_2|PF00451.20|0.00037,Gamma-thionin|PF00304.21|0.37,Gamma-thionin|PF00304.21|0.052,Gamma-thionin|PF00304.21|0.022,Defensin_2|PF01097.19|0.58,Defensin_2|PF01097.19|0.12,Defensin_2|PF01097.19|0.011,Toxin_38|PF14866.7|0.18,Toxin_38|PF14866.7|0.18,Toxin_38|PF14866.7|0.4 TRINITY_DN16091_c0_g1_i1:19-390(-)	-1.4033	-0.28692	-0.02148	1.71696	-0.00526
TRINITY_DN5274_c0_g2_i2_orf1	lopap-like [Ostrinia furnacalis]	-1.78141	-0.37855	0.93704	0.78099	0.44193
TRINITY_DN14262_c0_g1_i5_orf1	cytochrome P450 monooxygenase CYP9G18 [Cnaphalocrocis medinalis]	-1.78657	-0.18652	0.95254	0.92577	0.09478
TRINITY_DN21420_c0_g1_i2_orf1	glutathione peroxidase, partial [Ostrinia furnacalis]	-1.84002	-0.09412	0.44521	1.13098	0.35796
TRINITY_DN29555_c0_g1_i8_orf1	fasciclin-1 [Ostrinia furnacalis]	-1.90939	0.27143	0.91962	0.06181	0.65653
TRINITY_DN7740_c0_g1_i2_orf1	D-arabinitol dehydrogenase 1 [Eumeta japonica]	-1.56086	-0.76801	0.773	0.48984	1.06603
TRINITY_DN1024_c0_g4_i1_orf1	superoxide dismutase [Cu-Zn]-like [Ostrinia furnacalis]	-1.57275	-0.7855	0.97735	0.67041	0.71049
TRINITY_DN1750_c1_g1_i5_orf1	lipid droplet localized protein-like [Ostrinia furnacalis] >XP_028161280.1 lipid droplet localized protein-like [Ostrinia furnacalis]	-1.84725	-0.24977	0.713	0.86401	0.52001
TRINITY_DN44517_c0_g1_i4_orf1	regucalcin-like [Ostrinia furnacalis]	-1.89074	-0.14665	0.74216	0.56398	0.73125
TRINITY_DN20658_c0_g2_i3_orf1	prostaglandin reductase 1-like [Ostrinia furnacalis]	-1.80774	-0.19083	0.5557	0.30555	1.13732
TRINITY_DN5337_c0_g1_i6_orf1	epoxide hydrolase 1-like [Ostrinia furnacalis]	-1.91203	0.15109	0.16032	0.71476	0.88586
TRINITY_DN9608_c0_g1_i3_orf1	cytochrome P450 monooxygenase CYP9G18 [Cnaphalocrocis medinalis]	-1.52189	-0.53724	0.04571	0.57878	1.43463
TRINITY_DN51995_c0_g3_i1_orf1	circadian clock-controlled protein-like [Ostrinia furnacalis]	-1.03004	-0.82341	-0.20087	1.77233	0.28199
TRINITY_DN1161_c0_g1_i2_orf1	fructose-1,6-bisphosphatase 1 [Ostrinia furnacalis]	-1.93645	0.0716	0.61007	0.83427	0.42051
TRINITY_DN26439_c0_g1_i2_orf1	uncharacterized protein LOC114351853 [Ostrinia furnacalis]	-1.4429	-0.90573	1.16983	0.45826	0.72055
TRINITY_DN4343_c0_g1_i2_orf1	uncharacterized protein LOC114365231 isoform X3 [Ostrinia furnacalis]	-1.82167	-0.05889	0.80723	0.06209	1.01123
TRINITY_DN11817_c0_g1_i4_orf1	glycogen phosphorylase [Heortia vitessoides]	-1.89425	-0.06646	0.74305	0.84752	0.37012
TRINITY_DN1226_c0_g1_i11_orfp1	TRINITY_DN1226_c0_g1_i11_m.52385 TRINITY_DN1226_c0_g1::TRINITY_DN1226_c0_g1_i11::g.52385 ORF type:internal len:92 (-),score=5.77 TRINITY_DN1226_c0_g1_i11:2-274(-)	-1.19379	-1.11907	0.75418	0.2602	1.29849
TRINITY_DN66453_c0_g1_i4_orfp1	TRINITY_DN66453_c0_g1_i4_m.7345 TRINITY_DN66453_c0_g1::TRINITY_DN66453_c0_g1_i4::g.7345 ORF type:internal len:93 (+),score=24.53 TRINITY_DN66453_c0_g1_i4:3-278(+)	-1.14385	-1.11265	0.89828	0.07731	1.28091
TRINITY_DN125521_c0_g2_i1_orf1	seroin transcript 1A2 [Ostrinia nubilalis]	-1.00326	-1.39028	0.50122	0.84879	1.04353
TRINITY_DN26879_c0_g1_i1_orf1	hypothetical protein O3G_MSEX014401 [Manduca sexta]	-1.31954	-1.06059	1.08557	0.40486	0.8897
TRINITY_DN11948_c0_g1_i8_orf1	cystathionine gamma-lyase [Ostrinia furnacalis]	-1.59051	-0.74675	0.84555	0.95209	0.53962
TRINITY_DN10222_c0_g1_i2_orf1	glutathione S-transferase sigma 3 [Ostrinia furnacalis]	-1.3953	-0.97388	0.45478	1.13803	0.77637
TRINITY_DN812_c2_g1_i1_orf1	1,4-alpha-glucan-branching enzyme [Ostrinia furnacalis]	-1.70553	-0.57641	0.86719	0.76284	0.6519
TRINITY_DN15327_c2_g1_i2_orf1	protein lethal(2)essential for life-like [Ostrinia furnacalis]	-0.90702	-1.48605	0.64615	0.98654	0.76039
TRINITY_DN4062_c0_g2_i1_orf1	venom peptide BmKAPI-like isoform X2 [Ostrinia furnacalis]	-1.78841	-0.15121	1.21185	0.21333	0.51443
TRINITY_DN581_c3_g2_i1_orf1	uncharacterized protein LOC114364499 isoform X3 [Ostrinia furnacalis]	-1.53007	-0.43062	-0.11973	0.65612	1.4243
TRINITY_DN1206_c0_g1_i6_orf1	sorbitol dehydrogenase-like [Spodoptera frugiperda] >KAG8104768.1 hypothetical protein SFRUCORN_013827 [Spodoptera frugiperda]	-1.67648	-0.62842	0.72804	0.89144	0.68542
TRINITY_DN5099_c0_g1_i3_orf1	trans-1,2-dihydrobenzene-1,2-diol dehydrogenase-like [Ostrinia furnacalis]	-1.85027	-0.16801	0.44519	0.54908	1.02401
TRINITY_DN285_c0_g1_i4_orf1	catalase-like [Ostrinia furnacalis]	-1.39298	-0.99698	0.50637	0.80903	1.07456
TRINITY_DN122321_c0_g1_i1_orf1	uncharacterized protein LOC114356271 [Ostrinia furnacalis]	-1.78144	-0.32951	0.3795	0.67218	1.05927
TRINITY_DN140_c0_g1_i1_orf1	calcyphosin-like protein [Ostrinia furnacalis]	-1.82737	-0.13251	0.24237	1.09271	0.62481

TRINITY_DN1209_c0_g1_i9_orf1	NADP-dependent malic enzyme-like isoform X1 [Ostrinia furnacalis] >XP_028161889.1 NADP-dependent malic enzyme-like isoform X1 [Ostrinia furnacalis] >XP_028161891.1 NADP-dependent malic enzyme-like isoform X3 [Ostrinia furnacalis]	-1.58532	-0.51542	0.27823	1.40264	0.41986
TRINITY_DN3029_c1_g2_i1_orf1	unnamed protein product [Plutella xylostella]	-1.56698	-0.39192	1.49097	0.40499	0.06294
TRINITY_DN22875_c0_g1_i6_orf1	microtubule-actin cross-linking factor 1 isoform X15 [Ostrinia furnacalis]	-1.63237	-0.58475	1.06815	0.2643	0.88467
TRINITY_DN1592_c0_g1_i1_orf1	serine protease 7-like isoform X2 [Ostrinia furnacalis]	-1.80721	-0.08891	1.04569	0.05712	0.79331
TRINITY_DN4069_c0_g1_i5_orf1	putative sulfiredoxin [Ostrinia furnacalis]	-1.53807	-0.60588	1.03337	0.01551	1.09507
TRINITY_DN1352_c0_g1_i5_orf1	uncharacterized protein LOC113491815 [Trichoplusia ni]	-1.84817	-0.22629	0.52277	0.94302	0.60867
TRINITY_DN9132_c0_g1_i5_orf1	ubiquitin-like-conjugating enzyme ATG3 [Spodoptera frugiperda]	-1.83706	-0.1254	0.23396	1.03876	0.68973
TRINITY_DN138086_c0_g1_i1_orf1	hypothetical protein evm_000614 [Chilo suppressalis]	-1.40586	-0.97053	1.12567	0.49825	0.75248
TRINITY_DN38225_c0_g2_i1_orf1	uncharacterized protein LOC114354273 [Ostrinia furnacalis]	-1.35795	-0.90341	1.38972	0.31622	0.55542
TRINITY_DN6908_c0_g1_i1_orf1	serine--pyruvate aminotransferase, mitochondrial [Ostrinia furnacalis] >XP_028157324.1 serine--pyruvate aminotransferase, mitochondrial [Ostrinia furnacalis] >XP_028157325.1 serine--pyruvate aminotransferase, mitochondrial [Ostrinia furnacalis]	-1.85076	0.41112	-0.12727	0.49898	1.06792
TRINITY_DN1424_c0_g1_i5_orf1	insect group I lytic polysaccharide monooxygenase [Ostrinia furnacalis]	-1.96208	0.23501	0.32954	0.6286	0.76894
TRINITY_DN1650_c0_g1_i5_orf1	uncharacterized protein LOC114355357 [Ostrinia furnacalis]	-1.61133	-0.31764	0.53285	-0.02451	1.42063
TRINITY_DN7960_c0_g1_i2_orf1	uncharacterized protein LOC114364878 [Ostrinia furnacalis]	-1.55411	-0.71686	0.3175	1.15288	0.80058
TRINITY_DN618_c0_g1_i3_orf1	triokinase/FMN cyclase-like isoform X1 [Ostrinia furnacalis]	-1.85346	-0.23513	0.67167	0.54344	0.87346
TRINITY_DN11108_c0_g1_i4_orf1	peroxisomal leader peptide-processing protease [Ostrinia furnacalis] >XP_028165527.1 peroxisomal leader peptide-processing protease [Ostrinia furnacalis]	-1.4749	-0.59582	1.4067	-0.03569	0.6997
TRINITY_DN4068_c0_g2_i4_orf1	larval cuticle protein LCP-17-like precursor [Papilio polytes] >BAM18876.1 cuticular protein PpolCPR2 [Papilio polytes]	-0.81877	-1.46211	0.24256	0.85238	1.18595
TRINITY_DN1230_c1_g1_i5_orf1	uncharacterized protein LOC114353440 [Ostrinia furnacalis]	-1.37327	-1.01605	0.87327	0.46648	1.04956
TRINITY_DN20558_c0_g1_i2_orf1	Transient receptor potential channel pyrexia [Operophtera brumata]	-1.56759	-0.14691	0.41585	-0.21811	1.51676
TRINITY_DN4255_c0_g1_i10_orf1	LOW QUALITY PROTEIN: lebocin-4-like [Ostrinia furnacalis]	-1.87367	-0.08033	0.87674	0.27978	0.79748
TRINITY_DN9492_c0_g1_i7_orf1	aldo-keto reductase AKR2E4-like [Ostrinia furnacalis]	-1.9463	0.17238	0.31467	0.6335	0.82575
TRINITY_DN22513_c0_g1_i4_orf1	DNA-directed RNA polymerase II subunit RPB1 [Ostrinia furnacalis] >XP_028179194.1 DNA-directed RNA polymerase II subunit RPB1 [Ostrinia furnacalis] >XP_028179195.1 DNA-directed RNA polymerase II subunit RPB1 [Ostrinia furnacalis]	-0.95731	-1.45463	0.86804	0.66633	0.87756
TRINITY_DN2286_c2_g1_i1_orf1	coatomer subunit zeta-1 isoform X1 [Ostrinia furnacalis]	-1.80723	-0.10222	0.17179	1.17235	0.56531
TRINITY_DN6325_c0_g1_i9_orf1	fructose-bisphosphate aldolase isoform X2 [Pieris brassicae]	-1.72079	-0.41652	0.27668	1.10042	0.76021
TRINITY_DN2314_c0_g1_i7_orf1	protein dj-1beta-like isoform X1 [Ostrinia furnacalis]	-1.82514	0.07246	-0.04735	1.04376	0.75626
TRINITY_DN33488_c0_g1_i2_orf1	semaphorin-1A isoform X3 [Trichoplusia ni]	-1.76322	-0.44616	0.93194	0.7001	0.57734
TRINITY_DN14611_c0_g1_i5_orf1	hsc70-interacting protein-like [Galleria mellonella]	-1.72201	-0.35729	1.23601	0.31274	0.53055
TRINITY_DN34423_c0_g1_i2_orf1	THAP domain-containing protein 4-like [Ostrinia furnacalis]	-1.87707	-0.08949	0.58271	0.98476	0.39909
TRINITY_DN9340_c0_g1_i4_orf1	sarcosine dehydrogenase, mitochondrial [Ostrinia furnacalis]	-1.695	-0.45698	1.1941	0.60837	0.3495
TRINITY_DN10994_c0_g1_i4_orf1	trypsin inhibitor-like [Ostrinia furnacalis]	-1.59247	-0.74385	0.54119	0.95513	0.84
TRINITY_DN2461_c0_g1_i5_orf1	secretory phospholipase A2 receptor [Vanessa cardui]	-1.42816	-0.98072	0.67942	0.72084	1.00862
TRINITY_DN20133_c0_g1_i1_orf1	fructose-bisphosphate aldolase A isoform X2 [Microcebus murinus] >XP_012619765.1 fructose-bisphosphate aldolase A isoform X2 [Microcebus murinus] >XP_012619766.1 fructose-bisphosphate aldolase A isoform X2 [Microcebus murinus] >XP_012619767.1 fructose-bisphosphate aldolase A isoform X2 [Microcebus murinus] >XP_012619768.1 fructose-bisphosphate aldolase A isoform X2 [Microcebus murinus]	-1.52955	-0.59109	0.39937	1.43952	0.28176
TRINITY_DN346_c0_g1_i7_orf1	CDK-activating kinase assembly factor MAT1 [Ostrinia furnacalis]	-1.70737	-0.26866	0.09995	1.28652	0.58956
TRINITY_DN11231_c1_g1_i1_orfp1	TRINITY_DN11231_c1_g1_i1_m.13377 TRINITY_DN11231_c1_g1::TRINITY_DN11231_c1_g1_i1::g.13377 ORF type:internal len:76 (-),score=1.43 TRINITY_DN11231_c1_g1_i1:1-225(-)	-1.40707	-0.82694	1.09604	0.07526	1.06271
TRINITY_DN103475_c0_g1_i4_orf1	lipid storage droplets surface-binding protein 1 isoform X3 [Ostrinia furnacalis]	-1.48751	-0.88567	1.03294	0.5326	0.80764
TRINITY_DN18482_c0_g1_i3_orf1	calcyphosin-like protein isoform X3 [Helicoverpa armigera] >XP_047020698.1 calcyphosin-like protein isoform X2 [Helicoverpa zea]	-1.31087	-1.0723	0.42551	1.11023	0.84742
TRINITY_DN58751_c0_g1_i2_orf1	FK506-binding protein 2 isoform X1 [Vanessa tameamea] >XP_046977568.1 FK506-binding protein 2 isoform X1 [Vanessa cardui]	-1.81915	-0.06837	1.02808	0.06933	0.79011
TRINITY_DN3175_c0_g1_i7_orf1	unnamed protein product, partial [Brenthis ino]	-1.86005	-0.23277	0.60996	0.65608	0.82678
TRINITY_DN574_c0_g1_i4_orf1	CD63 antigen-like [Ostrinia furnacalis]	-1.88994	-0.13397	0.78783	0.50506	0.73103
TRINITY_DN12545_c0_g1_i7_orf1	hypothetical protein evm_003491 [Chilo suppressalis]	-1.1449	-1.18171	1.2189	0.24279	0.86493
TRINITY_DN3433_c0_g1_i15_orf1	cytosolic purine 5'-nucleotidase isoform X3 [Ostrinia furnacalis] >XP_028162965.1 cytosolic purine 5'-nucleotidase isoform X3 [Ostrinia furnacalis] >XP_028162966.1 cytosolic purine 5'-nucleotidase isoform X3 [Ostrinia furnacalis]	-1.69788	-0.39682	0.68193	0.208	1.20477
TRINITY_DN29009_c0_g2_i2_orf1	juvenile hormone binding protein [Omphisa fuscidentalis]	-1.92209	-0.00564	0.53582	0.85365	0.53826
TRINITY_DN2442_c0_g1_i6_orf1	cytochrome P450 6B5-like [Ostrinia furnacalis]	-1.82068	0.02964	0.59383	0.0441	1.15311
TRINITY_DN4502_c0_g1_i3_orf1	kynurenine formamidase isoform X1 [Ostrinia furnacalis]	-1.71027	-0.53484	0.92838	0.48493	0.8318
TRINITY_DN12775_c0_g1_i10_orfp1	TRINITY_DN12775_c0_g1_i10_m.21238 TRINITY_DN12775_c0_g1::TRINITY_DN12775_c0_g1_i10::g.21238 ORF type:5prime_partial len:67 (-),score=0.74 TRINITY_DN12775_c0_g1_i10:275-475(-)	-1.72473	-0.34124	0.1233	0.91368	1.02899
TRINITY_DN9694_c0_g1_i1_orf1	larval cuticle protein LCP-17 [Helicoverpa armigera] >PZC82071.1 hypothetical protein B5X24_HaOG211161 [Helicoverpa armigera] >PZC87412.1 hypothetical protein B5X24_HaOG216859 [Helicoverpa armigera]	-0.86956	-1.51807	0.73023	0.70097	0.95642
TRINITY_DN452_c9_g1_i1_orf1	epidermal retinol dehydrogenase 2-like [Ostrinia furnacalis]	-1.53889	-0.58882	1.35867	0.11673	0.6523

TRINITY_DN36434_c0_g2_i3_orf1	clotting factor B isoform X1 [Ostrinia furnacalis]	-1.50881	-0.71747	0.36902	0.5116	1.34566
TRINITY_DN2514_c1_g1_i13_orf1	seroin transcript 1A2 [Ostrinia nubilalis]	-1.20162	-1.22862	0.76788	0.63721	1.02515
TRINITY_DN51480_c0_g1_i1_orf1	uncharacterized protein LOC114361588 isoform X14 [Ostrinia furnacalis]	-1.74247	-0.32344	0.15079	0.92144	0.99368
TRINITY_DN17326_c0_g1_i8_orf1	aminoacylase-1-like [Ostrinia furnacalis]	-1.75122	-0.46915	0.63097	0.63366	0.95575
TRINITY_DN5914_c1_g1_i9_orf1	unnamed protein product [Chilo suppressalis]	-1.75599	-0.3929	0.74252	0.37012	1.03624
TRINITY_DN1960_c5_g1_i3_orf1	cytochrome P450 monooxygenase CYP9G18 [Cnaphalocrocis medinalis]	-1.65427	-0.54873	0.2563	1.00303	0.94367
TRINITY_DN5300_c0_g1_i2_orf1	valacyclovir hydrolase [Ostrinia furnacalis]	-1.91985	-0.0589	0.61565	0.64521	0.71789
TRINITY_DN448_c0_g1_i20_orf1	probable cytochrome P450 9f2 isoform X1 [Ostrinia furnacalis]	-1.57521	-0.65214	0.40392	0.54576	1.27766
TRINITY_DN91946_c0_g1_i1_orf1	protein catecholamines up [Ostrinia furnacalis]	-1.46719	-0.76968	0.91434	0.13953	1.18299
TRINITY_DN1008_c0_g1_i2_orf1	integrin beta-PS [Ostrinia furnacalis]	-1.55832	-0.59084	1.13332	0.04847	0.96737
TRINITY_DN4497_c2_g1_i3_orf1	uncharacterized protein LOC114353086 [Ostrinia furnacalis]	-1.71078	-0.44873	0.30063	0.76547	1.09341
TRINITY_DN21435_c0_g1_i2_orf1	glycogen-binding subunit 76A isoform X1 [Ostrinia furnacalis]	-1.81067	-0.301	1.01405	0.53912	0.55849
TRINITY_DN4621_c0_g1_i4_orf1	uncharacterized protein LOC114358242 isoform X3 [Ostrinia furnacalis]	-1.66414	-0.64132	0.59062	0.86621	0.84863
TRINITY_DN874_c2_g1_i1_orf1	uncharacterized protein LOC114356358 [Ostrinia furnacalis]	-1.89379	-0.02766	0.54041	0.40144	0.9796
TRINITY_DN970_c0_g1_i4_orf1	spermine oxidase-like isoform X2 [Ostrinia furnacalis]	-1.74779	-0.40634	0.3543	0.76778	1.03205
TRINITY_DN3616_c0_g2_i2_orf1	conotoxin ArMKLT2-032-like [Ostrinia furnacalis]	-1.1895	-1.17777	0.65611	0.46608	1.24508
TRINITY_DN41952_c0_g1_i1_orf1	protein DDI1 homolog 2 [Ostrinia furnacalis]	-1.92225	0.05363	0.86021	0.66802	0.34038
TRINITY_DN49530_c0_g1_i1_orf1	ommochrome-binding protein-like [Ostrinia furnacalis]	-1.77118	-0.33394	0.27703	0.95688	0.87122
TRINITY_DN394_c0_g1_i4_orf1	uncharacterized protein LOC114351483 [Ostrinia furnacalis]	-1.15279	-1.15525	0.15944	1.03687	1.11173
TRINITY_DN12873_c0_g2_i1_orf1	proteoglycan 4-like [Ostrinia furnacalis]	-1.55008	-0.8187	0.6124	0.94718	0.80919
TRINITY_DN82944_c0_g1_i4_orf1	senecionine N-oxygenase isoform X2 [Ostrinia furnacalis]	-1.80065	-0.33426	0.42139	0.84468	0.86884
TRINITY_DN2650_c0_g1_i1_orf1	hypothetical protein HW555_002849 [Spodoptera exigua] >CAH0691914.1 unnamed protein product [Spodoptera exigua]	-1.82894	0.30889	0.04622	0.25146	1.22237
TRINITY_DN71917_c0_g3_i1_orf1	leucine-rich repeat and immunoglobulin-like domain-containing nogo receptor-interacting protein 1 [Ostrinia furnacalis]	-1.55208	-0.4929	1.06974	-0.1158	1.09103
TRINITY_DN9325_c0_g1_i1_orf1	protein takeout-like [Ostrinia furnacalis]	-1.71625	0.00291	-0.26309	0.86146	1.11497
TRINITY_DN4245_c0_g1_i5_orf1	long-chain fatty acid transport protein 4-like [Ostrinia furnacalis]	-1.53122	-0.83677	1.01665	0.61023	0.7411
TRINITY_DN33_c0_g1_i14_orf1	uncharacterized protein CG45076-like isoform X1 [Ostrinia furnacalis]	-1.44015	-0.88362	1.08428	0.30246	0.93703
TRINITY_DN54205_c0_g1_i1_orf1	aldo-keto reductase AKR2E4-like [Ostrinia furnacalis]	-1.76632	-0.35846	0.3096	0.84315	0.97204
TRINITY_DN6698_c0_g2_i2_orf1	protein mesh isoform X1 [Ostrinia furnacalis]	-1.86203	0.14421	0.89972	-0.01985	0.83795
TRINITY_DN44491_c0_g1_i12_orf1	unnamed protein product [Chrysodeixis includens]	-1.28387	-1.06701	1.04087	0.28643	1.02358
TRINITY_DN701_c0_g1_i1_orf1	venom protease-like isoform X3 [Ostrinia furnacalis]	-1.31943	-1.01407	0.60083	0.43697	1.29569
TRINITY_DN3433_c2_g1_i2_orf1	cytosolic purine 5'-nucleotidase isoform X4 [Ostrinia furnacalis]	-1.37604	-0.87901	1.29046	0.16345	0.80115
TRINITY_DN2706_c0_g1_i3_orf1	hypothetical protein evm_004793 [Chilo suppressalis]	-1.73635	-0.40812	1.15056	0.47707	0.51685
TRINITY_DN8853_c0_g1_i4_orf1	uncharacterized protein LOC114351488 isoform X1 [Ostrinia furnacalis]	-0.99767	-1.12593	0.34319	0.17111	1.60931
TRINITY_DN24970_c0_g1_i4_orf1	pyrroline-5-carboxylate reductase-like isoform X1 [Ostrinia furnacalis]	-1.52052	-0.87267	0.85304	0.69033	0.84983
TRINITY_DN15858_c0_g1_i1_orf1	15-hydroxyprostaglandin dehydrogenase [NAD(+)]-like [Ostrinia furnacalis]	-1.47824	-0.90754	0.5607	0.98784	0.83724
TRINITY_DN3015_c0_g1_i7_orf1	glycine-rich protein DOT1-like [Ostrinia furnacalis]	-1.86261	-0.21577	0.85856	0.57096	0.64887
TRINITY_DN13322_c0_g1_i6_orf1	macrophage mannose receptor 1-like [Ostrinia furnacalis]	-1.73678	-0.46503	0.41688	0.8753	0.90962
TRINITY_DN4394_c0_g1_i4_orf1	B-cell receptor-associated protein 31 [Ostrinia furnacalis]	-1.8199	-0.25193	1.0406	0.58613	0.44509
TRINITY_DN3766_c0_g1_i10_orf1	circadian clock-controlled protein-like [Ostrinia furnacalis]	-1.80718	-0.0108	1.14064	0.01967	0.65766
TRINITY_DN3263_c0_g1_i2_orf1	serine hydroxymethyltransferase, cytosolic isoform X1 [Diachasma alloeum]	-1.49935	-0.89428	0.63838	0.92368	0.83157
TRINITY_DN18196_c0_g1_i4_orf1	uncharacterized protein LOC114363471 isoform X3 [Ostrinia furnacalis]	-1.64965	-0.65571	0.98254	0.72615	0.59668
TRINITY_DN245_c0_g1_i4_orf1	ER lumen protein-retaining receptor [Ostrinia furnacalis]	-0.93778	-1.44148	0.47339	0.98805	0.91781
TRINITY_DN16147_c0_g1_i4_orf1	aldo-keto reductase AKR2E4-like isoform X1 [Ostrinia furnacalis]	-1.75971	-0.0005	1.05503	0.87301	-0.16784
TRINITY_DN6580_c0_g1_i4_orf1	catalase [Ostrinia furnacalis]	-1.83751	-0.00777	0.14999	0.56129	1.134
TRINITY_DN4123_c0_g1_i1_orf1	uncharacterized protein LOC114355030 [Ostrinia furnacalis]	-1.0626	-1.14575	1.05329	-0.04764	1.20269
TRINITY_DN2338_c0_g1_i5_orf1	prophenoloxidase PPO1b [Ostrinia furnacalis]	-1.85827	-0.09535	0.572	1.04866	0.33295
TRINITY_DN13718_c0_g1_i4_orf1	immulectin-4 [Ostrinia furnacalis]	-1.78004	0.10143	0.368	0.0123	1.29831
TRINITY_DN69307_c0_g1_i6_orf1	hypothetical protein evm_010738 [Chilo suppressalis]	-1.72216	-0.14642	-0.12495	1.06757	0.92597
TRINITY_DN44083_c0_g1_i2_orf1	putative alpha-ketoglutarate-dependent hypophosphite dioxygenase [Operophtera brumata]	-1.44621	-0.92689	0.91872	0.45545	0.99893
TRINITY_DN2516_c0_g2_i10_orf1	unnamed protein product, partial [Chilo suppressalis]	-1.9153	0.11364	0.97887	0.30643	0.51636
TRINITY_DN2876_c0_g1_i1_orf1	long-chain fatty acid transport protein 4-like [Ostrinia furnacalis]	-1.84129	0.0447	1.0026	0.01803	0.77596
TRINITY_DN6483_c0_g1_i6_orf1	transketolase-like protein 2 isoform X1 [Ostrinia furnacalis] >XP_028164795.1 transketolase-like protein 2 isoform X2 [Ostrinia furnacalis]	-1.73434	-0.48911	0.75333	0.50624	0.96388
TRINITY_DN2624_c0_g1_i6_orf1	unnamed protein product [Danaus chrysippus]	-1.82551	-0.20899	0.95347	0.28514	0.79589
TRINITY_DN5266_c0_g1_i1_orf1	malate dehydrogenase, cytoplasmic isoform X2 [Ostrinia furnacalis]	-1.63246	-0.68114	0.59319	0.71996	1.00045
TRINITY_DN34134_c0_g2_i1_orf1	THUMP domain-containing protein 1 homolog [Ostrinia furnacalis]	-1.52657	-0.66518	0.66113	0.20916	1.32147
TRINITY_DN8747_c0_g1_i2_orf1	lipopolysaccharide-induced tumor necrosis factor-alpha factor homolog [Ostrinia furnacalis]	-1.90515	-0.09526	0.79174	0.64915	0.55953
TRINITY_DN2614_c0_g2_i3_orf1	uncharacterized protein LOC114360661 [Ostrinia furnacalis]	-1.75534	-0.10865	0.02889	1.25097	0.58412

TRINITY_DN11698_c0_g1_i1_orf1	hypothetical protein evm_015129 [Chilo suppressalis]	-1.51589	-0.80454	0.41208	0.77516	1.13319
TRINITY_DN9492_c1_g1_i1_orf1	aldo-keto reductase AKR2E4-like [Galleria mellonella]	-1.75286	-0.30097	0.1917	0.74891	1.11323
TRINITY_DN566_c0_g1_i13_orf1	uncharacterized protein LOC114355567 isoform X2 [Ostrinia furnacalis]	-1.39313	-0.88611	0.74864	0.24463	1.28597
TRINITY_DN7630_c0_g2_i1_orf1	flotillin-2 isoform X1 [Ostrinia furnacalis] >XP_028172931.1 flotillin-2 isoform X2 [Ostrinia furnacalis]	-1.70472	-0.52398	0.84554	0.41968	0.96349
TRINITY_DN8527_c0_g2_i1_orfp1	TRINITY_DN8527_c0_g2_i1_m.16937 TRINITY_DN8527_c0_g2::TRINITY_DN8527_c0_g2_i1::g.16937 ORF type:5prime_partial len:54 (-),score=0.36 TRINITY_DN8527_c0_g2_i1:195-356(-)	-1.04698	-1.35977	0.52817	0.86394	1.01464
TRINITY_DN350_c0_g1_i4_orf1	microtubule-associated protein tau-like isoform X6 [Ostrinia furnacalis]	-1.47686	-0.90658	0.92384	0.53198	0.92763
TRINITY_DN63536_c0_g1_i1_orf1	adenosylhomocysteinase [Chelonus insularis]	-1.68891	-0.55159	0.71968	1.05104	0.46978
TRINITY_DN8473_c0_g1_i6_orf1	serine/threonine-protein phosphatase 6 regulatory subunit 1 [Ostrinia furnacalis]	-1.40302	-0.82793	0.06895	1.127	1.03499
TRINITY_DN117844_c0_g1_i1_orf1	ATP-citrate synthase [Cotesia glomerata] >XP_044590631.1 ATP-citrate synthase [Cotesia glomerata] >KAH0546822.1 hypothetical protein KQX54_015428 [Cotesia glomerata]	-1.34835	-1.07501	0.59378	0.91078	0.9188
TRINITY_DN12666_c0_g1_i2_orf1	sodium- and chloride-dependent GABA transporter 1 [Ostrinia furnacalis]	-1.31739	-0.98331	0.77094	1.2812	0.24855
TRINITY_DN95850_c0_g4_i3_orf1	calreticulin [Cotesia glomerata] >KAH0559118.1 hypothetical protein KQX54_000875 [Cotesia glomerata]	-1.94934	0.08151	0.76388	0.56886	0.53509
TRINITY_DN2255_c0_g1_i1_orf1	glutathione S-transferase sigma 3 [Ostrinia furnacalis]	-1.87264	-0.10372	0.32769	0.73486	0.91381
TRINITY_DN2182_c0_g1_i4_orf1	growth-blocking peptide, long form-like isoform X1 [Ostrinia furnacalis] >XP_028159332.1 growth-blocking peptide, long form-like isoform X1 [Ostrinia furnacalis] >QWX20072.1 growth-blocking peptide [Ostrinia furnacalis]	-1.71093	-0.43836	1.02537	0.87627	0.24765
TRINITY_DN40281_c0_g1_i1_orf1	thioredoxin domain-containing protein 15 [Ostrinia furnacalis]	-1.21441	-1.0731	0.46161	1.40976	0.41613
TRINITY_DN19116_c0_g1_i3_orf1	UDP-glucose 4-epimerase isoform X1 [Ostrinia furnacalis]	-1.56725	-0.53648	1.16601	-0.00898	0.94671
TRINITY_DN5880_c0_g2_i2_orf1	macrophage mannose receptor 1 [Bombyx mori]	-1.1019	-0.99974	0.64841	-0.08269	1.53593
TRINITY_DN2076_c0_g2_i1_orf1	macrophage mannose receptor 1-like isoform X1 [Maniola jurtina]	-1.76747	-0.19726	0.14729	1.19498	0.62246
TRINITY_DN244_c1_g1_i5_orf1	C-1-tetrahydrofolate synthase, cytoplasmic isoform X3 [Ostrinia furnacalis]	-1.82704	0.20565	-0.13075	0.69235	1.0598
TRINITY_DN1047_c0_g1_i6_orf1	mitochondrial genome maintenance exonuclease 1-like [Ostrinia furnacalis]	-1.72135	-0.53756	0.83014	0.57626	0.85252
TRINITY_DN2378_c0_g1_i5_orf1	integrin alpha-8-like isoform X1 [Ostrinia furnacalis]	-1.61469	-0.63927	0.68149	0.40985	1.16262
TRINITY_DN511_c0_g2_i1_orf1	pyruvate carboxylase, mitochondrial isoform X1 [Manduca sexta] >XP_037293486.1 pyruvate carboxylase, mitochondrial isoform X1 [Manduca sexta]	-1.54535	-0.82811	0.96842	0.67785	0.72719
TRINITY_DN38685_c0_g1_i4_orf1	unnamed protein product [Euphydryas editha]	-1.88155	0.00402	0.95199	0.21261	0.71292
TRINITY_DN11383_c0_g2_i4_orf1	aminoacylase-1A-like [Ostrinia furnacalis]	-1.63367	-0.59642	0.29201	0.89144	1.04664
TRINITY_DN1344_c0_g1_i1_orf1	ribosomal RNA small subunit methyltransferase NEP1 [Ostrinia furnacalis]	-0.65594	-1.61167	0.37478	1.08852	0.80431
TRINITY_DN21715_c0_g1_i1_orf1	protein disulfide-isomerase A3 isoform X1 [Ostrinia furnacalis] >XP_028169825.1 protein disulfide-isomerase A3 isoform X2 [Ostrinia furnacalis] >XP_028169826.1 protein disulfide-isomerase A3 isoform X3 [Ostrinia furnacalis]	-1.87285	0.11684	1.03031	0.08544	0.64026

TRINITY_DN55148_c0_g1_i1_orf1	60S ribosomal protein L11 isoform 1 [Homo sapiens] >NP_001069049.1 60S ribosomal protein L11 [Bos taurus] >NP_001240835.1 60S ribosomal protein L11 isoform 1 [Canis lupus familiaris] >NP_001269300.1 60S ribosomal protein L11 [Chinchilla lanigera] >NP_001291809.1 60S ribosomal protein L11 [Ailuropoda melanoleuca] >NP_080195.1 60S ribosomal protein L11 [Mus musculus] >XP_001504267.1 60S ribosomal protein L11 isoform X2 [Equus caballus] >XP_003471379.1 60S ribosomal protein L11 [Cavia porcellus] >XP_003810808.1 60S ribosomal protein L11 [Pan paniscus] >XP_003891370.1 60S ribosomal protein L11 [Papio anubis] >XP_003989701.1 60S ribosomal protein L11 [Felis catus] >XP_004285699.1 60S ribosomal protein L11 isoform X2 [Orcinus orca] >XP_004377186.1 60S ribosomal protein L11 [Trichechus manatus latirostris] >XP_004394821.1 PREDICTED: 60S ribosomal protein L11 isoform X1 [Odobenus rosmarus divergens] >XP_004465476.2 60S ribosomal protein L11 [Dasypus novemcinctus] >XP_004637663.1 60S ribosomal protein L11 [Octodon degus] >XP_004850617.1 60S ribosomal protein L11 isoform X2 [Heterocephalus glaber] >XP_005544522.1 60S ribosomal protein L11 isoform X1 [Macaca fascicularis] >XP_005676921.1 PREDICTED: 60S ribosomal protein L11 isoform X2 [Capra hircus] >XP_006078005.1 60S ribosomal protein L11 isoform X2 [Bubalus bubalis] >XP_006094108.1 60S ribosomal protein L11 isoform X3 [Myotis lucifugus] >XP_006239286.1 60S ribosomal protein L11 isoform X1 [Rattus norvegicus] >XP_006737645.1 60S ribosomal protein L11 [Leptonychotes weddellii] >XP_006777625.1 PREDICTED: 60S ribosomal protein L11 isoform X1 [Myotis davidii] >XP_006883588.1 PREDICTED: 60S ribosomal protein L11-like isoform X1 [Elephantulus edwardii] >XP_007121151.1 60S ribosomal protein L11 isoform X2 [Physeter catodon] >XP_007175168.1 60S ribosomal protein L11 isoform X1 [Balaenoptera acutorostrata scammoni] >XP_007459239.1 PREDICTED: 60S ribosomal protein L11 isoform X1 [Lipotes vexillifer] >XP_007524793.1 PREDICTED: 60S ribosomal protein L11 [Erinaceus europaeus] >XP_007528779.2 PREDICTED: 60S ribosomal protein L11 [Erinaceus europaeus] >XP_007933904.2 60S ribosomal protein L11 [Orycteropus afer afer] >XP_007978264.1 60S ribosomal protein L11 isoform X1 [Chlorocebus sabaeus] >XP_008059998.1 60S ribosomal protein L11 isoform X2 [Carlito syrichta] >XP_008146324.1 60S ribosomal protein L11 [Eptesicus fuscus] >XP_008263912.1 PREDICTED: 60S ribosomal protein L11 [Oryctolagus cuniculus] >XP_008518979.1 PREDICTED: 60S ribosomal protein L11 isoform X2 [Equus przewalskii] >XP_008571152.1 PREDICTED: 60S ribosomal protein L11 isoform X1 [Galeopterus variegatus] >XP_008571160.1 PREDICTED: 60S ribosomal protein L11 isoform X2 [Galeopterus variegatus] >XP_008846816.1 60S ribosomal protein L11 [Nannospalax galili] >XP_010354068.1 60S ribosomal protein L11 [Rhinopithecus roxellana] >XP_010624280.1 60S ribosomal protein L11 [Fukomys damarensis] >XP_011355886.1 60S ribosomal protein L11 [Pteropus vampyrus] >XP_011761124.1 60S ribosomal protein L11 [Macaca nemestrina] >XP_011833215.1 PREDICTED: 60S ribosomal protein L11 isoform X2 [Mandrillus leucophaeus] >XP_011935575.1 PREDICTED: 60S ribosomal protein L11 [Cercocebus atys] >XP_014710573.1 60S ribosomal protein L11 isoform X2 [Equus asinus] >XP_014930664.1 60S ribosomal protein L11 [Acinonyx jubatus] >XP_014949173.1 60S ribosomal protein L11 isoform X1 [Ovis aries] >XP_014986347.1 60S ribosomal protein L11 [Macaca mulatta] >XP_015341019.2 60S ribosomal protein L11 isoform X1 [Marmota marmota marmota] >XP_016811748.1 60S ribosomal protein L11 [Pan troglodytes] >XP_017390016.1 60S ribosomal protein L11 [Cebus imitator] >XP_017741964.1 PREDICTED: 60S ribosomal protein L11 [Rhinopithecus bieti] >XP_018866494.2 60S ribosomal protein L11 [Gorilla gorilla gorilla] >XP_018868111.1 60S ribosomal protein L11 [Gorilla gorilla gorilla] >XP_019317313.1 PREDICTED: 60S ribosomal protein L11 [Panthera pardus] >XP_019568603.1 PREDICTED: 60S ribosomal protein L11 [Rhinolophus sinicus] >XP_019837113.1 PREDICTED: 60S ribosomal	-1.57093	-0.25914	-0.30822	0.86437	1.27393
TRINITY_DN8702_c0_g1_i1_orf1	programmed cell death protein 4 isoform X1 [Ostrinia furnacalis] >XP_028157160.1 programmed cell death protein 4 isoform X2 [Ostrinia furnacalis]	-1.603	-0.70393	0.79603	0.4728	1.0381
TRINITY_DN1540_c0_g1_i14_orf1	alaserpin-like isoform X1 [Ostrinia furnacalis]	-1.59624	-0.51894	0.06926	0.81676	1.22916
TRINITY_DN17845_c0_g1_i3_orf1	ataxin-10-like [Ostrinia furnacalis]	-1.61568	-0.39049	1.43427	0.19535	0.37655
TRINITY_DN8258_c0_g1_i3_orf1	papilin isoform X7 [Ostrinia furnacalis]	-1.52831	-0.72723	1.10871	0.22088	0.92595
TRINITY_DN625_c9_g1_i7_orf1	ecdysone 20-monooxygenase [Ostrinia furnacalis]	-1.59791	-0.71097	0.49407	0.74877	1.06604
TRINITY_DN8252_c0_g1_i6_orf1	scavenger receptor class B member 1 [Ostrinia furnacalis]	-1.5898	-0.54618	0.14393	1.28704	0.70501
TRINITY_DN512_c0_g1_i10_orf1	uncharacterized protein LOC114366781 [Ostrinia furnacalis]	-1.78009	-0.18176	0.11295	0.72914	1.11975
TRINITY_DN1926_c0_g1_i5_orf1	coiled-coil and C2 domain-containing protein 1-like [Ostrinia furnacalis]	-1.72935	-0.37884	1.07738	0.22063	0.81018
TRINITY_DN3831_c0_g1_i7_orf1	beta-ureidopropionase-like [Ostrinia furnacalis]	-1.86728	-0.14015	0.36617	0.74093	0.90032
TRINITY_DN472_c1_g1_i3_orf1	IST1 homolog isoform X1 [Ostrinia furnacalis]	-1.86955	0.1698	1.04449	0.03584	0.61943
TRINITY_DN9435_c0_g1_i7_orf1	uncharacterized protein LOC114350197 [Ostrinia furnacalis]	-1.42066	-0.71631	0.24495	1.50223	0.38978
TRINITY_DN356_c2_g1_i3_orf1	scavenger receptor class B member 1 isoform X2 [Pectinophora gossypiella] >XP_049883835.1 scavenger receptor class B member 1 isoform X2 [Pectinophora gossypiella]	-1.6052	-0.75102	0.86098	0.73872	0.75652
TRINITY_DN38783_c0_g1_i1_orf1	regucalcin-like [Ostrinia furnacalis]	-1.44952	-0.83963	0.26287	0.82354	1.20274
TRINITY_DN6309_c0_g1_i7_orf1	apolipoprotein D-like isoform X1 [Ostrinia furnacalis]	-1.96006	0.13224	0.74048	0.51915	0.56819
TRINITY_DN1012_c0_g2_i1_orf1	teneurin-a isoform X1 [Ostrinia furnacalis]	-1.46004	-0.47718	-0.14962	0.57399	1.51285
TRINITY_DN745_c5_g1_i2_orf1	facilitated trehalose transporter Tret1-like isoform X2 [Ostrinia furnacalis]	-1.66032	-0.62106	0.94847	0.47978	0.85314
TRINITY_DN1014_c0_g2_i8_orf1	uncharacterized protein LOC114362446 [Ostrinia furnacalis] >XP_028173663.1 uncharacterized protein LOC114362446 [Ostrinia furnacalis]	-1.84884	0.19641	0.0035	0.52139	1.12754
TRINITY_DN938_c0_g1_i7_orf1	protein ultraspiracle homolog isoform X2 [Ostrinia furnacalis]	-1.90993	0.09907	0.93667	0.23349	0.64069
TRINITY_DN43667_c0_g1_i1_orf1	carbonyl reductase [NADPH] 1-like [Ostrinia furnacalis]	-1.67492	-0.03045	-0.25936	0.669	1.29573
TRINITY_DN3374_c0_g1_i7_orf1	TPPP family protein CG45057 [Ostrinia furnacalis] >XP_028172578.1 TPPP family protein CG45057 [Ostrinia furnacalis]	-1.13836	-1.11887	1.29926	0.08827	0.86971
TRINITY_DN62091_c0_g1_i1_orf1	protein NipSnap [Venturia canescens]	-1.69178	-0.50071	0.34593	0.74638	1.10019
TRINITY_DN135679_c0_g1_i2_orfp1	TRINITY_DN135679_c0_g1_i2_m.85525 TRINITY_DN135679_c0_g1::TRINITY_DN135679_c0_g1_i2::g.85525 ORF type:5prime_partial len:55 (+),score=5.08,Toxin_2 PF00451.20 1.9e-06 TRINITY_DN135679_c0_g1_i2:3-167(+)	-1.04199	-1.31445	0.61395	1.25296	0.48952
TRINITY_DN4314_c0_g1_i9_orf1	serine proteinase inhibitor 2 [Ostrinia furnacalis]	-1.19553	-1.19428	0.44553	1.13917	0.80511
TRINITY_DN19521_c0_g1_i1_orf1	sodium/potassium-transporting ATPase subunit beta-1-like isoform X1 [Ostrinia furnacalis]	-1.7714	-0.01482	1.26149	0.00458	0.52014
TRINITY_DN8637_c0_g1_i1_orf1	superoxide dismutase [Cu-Zn] [Ostrinia furnacalis] >XP_028177872.1 superoxide dismutase [Cu-Zn] [Ostrinia furnacalis]	-1.87181	0.3846	-0.13765	0.74393	0.88093

TRINITY_DN28654_c0_g1_i2_orf1	acidic juvenile hormone-suppressible protein 1-like [Ostrinia furnacalis]	-1.39002	0.08126	-0.31416	1.71855	-0.09562
TRINITY_DN1216_c0_g1_i4_orf1	bifunctional purine biosynthesis protein PURH isoform X1 [Ostrinia furnacalis] >XP_028176123.1 bifunctional purine biosynthesis protein PURH isoform X2 [Ostrinia furnacalis] >XP_028176129.1 bifunctional purine biosynthesis protein PURH isoform X3 [Ostrinia furnacalis]	-1.82868	-0.12317	0.20127	0.69093	1.05966
TRINITY_DN3257_c0_g1_i4_orf1	N-acetylneuraminate lyase-like [Ostrinia furnacalis]	-1.84686	-0.14781	0.30422	0.69376	0.99669
TRINITY_DN3826_c0_g1_i1_orf1	39S ribosomal protein L18, mitochondrial [Ostrinia furnacalis]	-1.12408	-1.09517	0.47951	1.49986	0.23988
TRINITY_DN11388_c0_g1_i4_orf1	limbic system-associated membrane protein-like, partial [Ostrinia furnacalis]	-0.49329	-1.71148	0.53523	1.10619	0.56335
TRINITY_DN23564_c0_g1_i7_orf1	cytochrome P450 6B6-like [Ostrinia furnacalis]	-1.61122	-0.37417	0.94571	-0.12403	1.16371
TRINITY_DN28299_c0_g1_i1_orf1	adenylosuccinate lyase isoform X1 [Ostrinia furnacalis]	-1.58328	-0.62741	0.20884	1.1626	0.83925
TRINITY_DN10403_c0_g1_i1_orf1	hypothetical protein evm_000264 [Chilo suppressalis] >CAH2987898.1 unnamed protein product [Chilo suppressalis]	-1.40298	-0.84935	0.30404	0.5742	1.37408
TRINITY_DN135188_c0_g1_i2_orf1	proteasome inhibitor PI31 subunit [Ostrinia furnacalis]	-1.80711	-0.37409	0.65161	0.76706	0.76253
TRINITY_DN582_c0_g1_i5_orf1	hypothetical protein evm_007348 [Chilo suppressalis]	-1.90217	0.06926	0.39047	0.41779	1.02466
TRINITY_DN22242_c0_g1_i1_orf1	juvenile hormone epoxide hydrolase-like [Ostrinia furnacalis] >XP_028170526.1 juvenile hormone epoxide hydrolase-like [Ostrinia furnacalis]	-1.72365	0.0282	0.55505	-0.16121	1.30161
TRINITY_DN20347_c0_g1_i6_orf1	venom polypeptide precursor [Doratifera vulnerans]	-1.51177	-0.4397	-0.01452	1.52542	0.44057
TRINITY_DN5628_c0_g1_i3_orf1	muscle LIM protein Mlp84B isoform X2 [Ostrinia furnacalis]	-0.89299	-1.48296	0.78646	0.54815	1.04134
TRINITY_DN13500_c0_g1_i1_orf1	phosphatidylethanolamine-binding protein homolog F40A3.3-like [Ostrinia furnacalis] >XP_028160752.1 phosphatidylethanolamine-binding protein homolog F40A3.3-like [Ostrinia furnacalis]	-1.69486	-0.20892	-0.00036	1.32013	0.58401
TRINITY_DN2947_c0_g1_i4_orf1	ras-related protein Rab-39B [Spodoptera litura] >XP_035450569.1 ras-related protein Rab-39B-like [Spodoptera frugiperda] >KAF9422714.1 hypothetical protein HW555_001708 [Spodoptera exigua] >CAB3507117.1 unnamed protein product [Spodoptera littoralis] >KAF9824742.1 hypothetical protein SFRURICE_016851 [Spodoptera frugiperda] >KAG8101614.1 hypothetical protein SFRUCORN_012196 [Spodoptera frugiperda] >KAH9636250.1 hypothetical protein HF086_009446 [Spodoptera exigua]	-1.91126	0.04707	0.33377	0.5886	0.94182
TRINITY_DN41848_c0_g1_i4_orf1	vacuolar protein sorting-associated protein 28 homolog [Ostrinia furnacalis]	-1.84044	-0.13576	0.39312	1.09709	0.486
TRINITY_DN6908_c0_g1_i3_orf1	serine--pyruvate aminotransferase, mitochondrial [Ostrinia furnacalis] >XP_028157324.1 serine--pyruvate aminotransferase, mitochondrial [Ostrinia furnacalis] >XP_028157325.1 serine--pyruvate aminotransferase, mitochondrial [Ostrinia furnacalis]	-1.68447	0.38442	0.24459	-0.30833	1.36378
TRINITY_DN3196_c0_g1_i1_orf1	organic cation transporter-like protein [Ostrinia furnacalis]	-1.69906	-0.41666	0.28531	0.61271	1.21769
TRINITY_DN3406_c0_g1_i17_orf1	uncharacterized protein ZK1073.1-like isoform X6 [Spodoptera frugiperda]	-1.78843	-0.16852	1.04784	0.09266	0.81644
TRINITY_DN125565_c1_g1_i1_orf1	uncharacterized protein LOC114352340 isoform X2 [Ostrinia furnacalis]	-1.65657	-0.24093	0.09486	1.43235	0.37028
TRINITY_DN384_c0_g1_i8_orf1	unnamed protein product [Chilo suppressalis]	-1.53268	-0.83387	0.57016	0.80611	0.99028
TRINITY_DN103511_c0_g1_i4_orf1	probable salivary secreted peptide [Ostrinia furnacalis]	-1.67837	-0.24027	-0.07876	1.24802	0.74939
TRINITY_DN12873_c0_g1_i3_orf1	proteoglycan 4-like [Ostrinia furnacalis]	-1.4375	-0.83949	0.22303	1.21369	0.84027
TRINITY_DN8440_c0_g1_i9_orf1	protein FAM177A1-like [Ostrinia furnacalis]	-1.87311	-0.05402	0.40963	0.46777	1.04973
TRINITY_DN726_c0_g1_i2_orf1	vinculin [Ostrinia furnacalis]	-1.7885	0.02289	1.18181	0.63371	-0.04991
TRINITY_DN19092_c2_g1_i1_orf1	mitochondrial fission 1 protein isoform X1 [Ostrinia furnacalis] >XP_028169879.1 mitochondrial fission 1 protein isoform X2 [Ostrinia furnacalis]	-1.6755	-0.63774	0.78961	0.79152	0.73211
TRINITY_DN67243_c0_g1_i1_orf1	39S ribosomal protein L3, mitochondrial [Ostrinia furnacalis]	-1.76522	-0.46474	0.6624	0.76647	0.8011
TRINITY_DN2264_c0_g1_i1_orf1	cytochrome P450 6B6-like [Ostrinia furnacalis]	-1.84572	0.43129	-0.19162	0.60192	1.00413
TRINITY_DN5001_c0_g1_i4_orf1	uncharacterized protein LOC114356665 [Ostrinia furnacalis]	-1.03544	-1.21607	0.05198	0.98282	1.21671
TRINITY_DN57900_c0_g1_i2_orf1	hypothetical protein SFRURICE_000634 [Spodoptera frugiperda]	-0.73928	-1.40804	0.38924	0.25696	1.50112
TRINITY_DN5531_c7_g1_i2_orf1	LOW QUALITY PROTEIN: tyrosine-protein phosphatase CDC14 homolog [Ostrinia furnacalis]	-1.43519	-0.46421	-0.10294	1.59676	0.40559
TRINITY_DN65518_c0_g1_i1_orf1	unc-112-related protein-like, partial [Ostrinia furnacalis]	-1.46145	-0.9369	0.59447	0.95666	0.84722
TRINITY_DN8258_c0_g1_i5_orf1	papilin isoform X9 [Ostrinia furnacalis]	-1.64016	-0.58929	1.00886	0.29416	0.92643
TRINITY_DN48846_c0_g1_i1_orf1	tudor domain-containing protein 7 isoform X3 [Ostrinia furnacalis]	-1.91988	-0.03634	0.80926	0.57934	0.56762
TRINITY_DN4080_c0_g1_i8_orf1	AMP deaminase 2 isoform X3 [Ostrinia furnacalis] >XP_028163647.1 AMP deaminase 2 isoform X3 [Ostrinia furnacalis] >XP_028163648.1 AMP deaminase 2 isoform X3 [Ostrinia furnacalis]	-1.50535	-0.26222	-0.30979	0.58507	1.49228
TRINITY_DN12424_c0_g1_i2_orf1	uncharacterized protein LOC114350608 [Ostrinia furnacalis]	-1.80727	-0.19856	0.99292	0.19383	0.81908
TRINITY_DN2983_c0_g1_i6_orf1	hypothetical protein evm_002448 [Chilo suppressalis]	-1.63964	-0.6764	0.60071	0.75271	0.96262
TRINITY_DN7152_c0_g1_i1_orf1	tissue alpha-L-fucosidase [Ostrinia furnacalis]	-1.81254	-0.29345	1.01828	0.55256	0.53514
TRINITY_DN89483_c0_g1_i1_orf1	mitochondrial enolase superfamily member 1-like isoform X2 [Maniola jurtina]	-1.1892	-0.94005	0.43433	0.11361	1.58132
TRINITY_DN11680_c0_g1_i1_orf1	uncharacterized protein LOC114355414 [Ostrinia furnacalis]	-1.74663	-0.26431	0.31861	0.43028	1.26205
TRINITY_DN21278_c0_g2_i2_orf1	mannose-1-phosphate guanyltransferase alpha-A [Ostrinia furnacalis]	-1.82258	-0.22394	0.67287	0.34635	1.0273
TRINITY_DN21331_c0_g1_i6_orf1	sodium-independent sulfate anion transporter-like [Ostrinia furnacalis]	-1.53482	-0.73572	0.61652	1.24836	0.40566
TRINITY_DN17031_c0_g1_i1_orf1	arginase, hepatic [Ostrinia furnacalis]	-1.81312	-0.237	0.29977	0.74344	1.00691
TRINITY_DN1756_c0_g1_i3_orf1	UTP--glucose-1-phosphate uridylyltransferase isoform X2 [Ostrinia furnacalis]	-1.88584	0.08061	0.43918	0.28858	1.07747
TRINITY_DN2570_c0_g1_i1_orf1	PREDICTED: pyruvate carboxylase, mitochondrial isoform X1 [Microplitis demolitor] >XP_008556301.1 PREDICTED: pyruvate carboxylase, mitochondrial isoform X1 [Microplitis demolitor] >XP_008556302.1 PREDICTED: pyruvate carboxylase, mitochondrial isoform X1 [Microplitis demolitor]	-1.62725	-0.69098	0.53881	0.86636	0.91306

TRINITY_DN19261_c0_g1_i3_orf1	UDP-glucuronic acid decarboxylase 1 isoform X1 [Papilio machaon]	-1.7699	-0.27418	0.87164	0.17074	1.00169
TRINITY_DN2710_c0_g1_i4_orf1	translin-associated protein X [Ostrinia furnacalis]	-1.74974	-0.25979	0.07841	0.97357	0.95756
TRINITY_DN49038_c0_g4_i1_orf1	6-phosphogluconate dehydrogenase, decarboxylating [Ostrinia furnacalis]	-1.72699	-0.54053	0.83303	0.75285	0.68164
TRINITY_DN3758_c0_g1_i2_orf1	S-formylglutathione hydrolase isoform X1 [Spodoptera litura]	-1.84792	-0.14224	0.51393	1.06309	0.41314
TRINITY_DN29873_c0_g1_i1_orf1	glyceraldehyde-3-phosphate dehydrogenase 2 [Pectinophora gossypiella]	-1.70022	-0.42484	0.55244	1.22621	0.34641
TRINITY_DN1827_c0_g1_i4_orf1	phosphoglycerate mutase 1 [Ostrinia furnacalis]	-1.6558	-0.55433	0.54932	1.19794	0.46286
TRINITY_DN33705_c0_g1_i1_orf1	atlastin-like isoform X4 [Ostrinia furnacalis]	-1.709	-0.30778	0.27246	0.43112	1.3132
TRINITY_DN5191_c0_g2_i1_orf1	CD151 antigen-like [Ostrinia furnacalis]	-0.74699	-1.4923	0.14453	1.0556	1.03916
TRINITY_DN8224_c0_g1_i7_orf1	hemicentin-1-like isoform X2 [Ostrinia furnacalis]	-1.43353	-0.85876	1.24633	0.2915	0.75447
TRINITY_DN30012_c1_g1_i1_orf1	ubiquitin domain-containing protein UBFD1-like [Ostrinia furnacalis]	-1.5659	-0.7602	0.66365	0.5594	1.10304
TRINITY_DN59028_c0_g1_i1_orf1	15-hydroxyprostaglandin dehydrogenase [NAD(+)]-like [Ostrinia furnacalis]	-1.8961	0.49496	-0.10924	0.8154	0.69498
TRINITY_DN9562_c0_g1_i3_orf1	cullin-3 isoform X1 [Ostrinia furnacalis] >XP_028159222.1 cullin-3 isoform X1 [Ostrinia furnacalis] >XP_028159223.1 cullin-3 isoform X2 [Ostrinia furnacalis] >XP_028159224.1 cullin-3 isoform X3 [Ostrinia furnacalis] >XP_028159225.1 cullin-3 isoform X1 [Ostrinia furnacalis]	-1.72005	-0.19615	0.3245	1.3575	0.2342
TRINITY_DN10581_c0_g1_i5_orf1	facilitated trehalose transporter Tret1-like isoform X1 [Ostrinia furnacalis] >XP_028165563.1 facilitated trehalose transporter Tret1-like isoform X2 [Ostrinia furnacalis]	-1.24629	-1.07252	1.02009	0.19519	1.10353
TRINITY_DN10742_c0_g1_i4_orf1	ethanolamine-phosphate cytidylyltransferase isoform X1 [Ostrinia furnacalis]	-1.54132	-0.61218	0.27775	0.48332	1.39242
TRINITY_DN15478_c0_g1_i1_orf1	STE20/SPS1-related proline-alanine-rich protein kinase [Vanessa tameamea] >XP_047534294.1 STE20/SPS1-related proline-alanine-rich protein kinase-like [Vanessa atalanta]	-1.6554	-0.17165	-0.24339	1.15264	0.9178
TRINITY_DN1575_c0_g1_i7_orf1	adenine phosphoribosyltransferase [Ostrinia furnacalis]	-1.41719	-0.58185	1.37685	-0.21983	0.84201
TRINITY_DN757_c3_g1_i2_orf1	PREDICTED: galectin-4-like [Amyelois transitella]	-1.55139	-0.79518	0.85701	0.50289	0.98666
TRINITY_DN26569_c0_g1_i4_orf1	hypothetical protein evm_009797 [Chilo suppressalis] >CAB3525291.1 unnamed protein product [Chilo suppressalis] >CAH0402618.1 unnamed protein product [Chilo suppressalis]	-1.32556	-0.16595	-0.02414	1.77423	-0.25858
TRINITY_DN50593_c0_g1_i1_orf1	uncharacterized protein LOC114361588 isoform X14 [Ostrinia furnacalis]	-0.80016	-1.52151	0.39622	0.83218	1.09327
TRINITY_DN8030_c0_g1_i2_orf1	chaoptin isoform X1 [Ostrinia furnacalis] >XP_028159953.1 chaoptin isoform X2 [Ostrinia furnacalis] >XP_028159954.1 chaoptin isoform X3 [Ostrinia furnacalis] >XP_028159955.1 chaoptin isoform X4 [Ostrinia furnacalis] >XP_028159956.1 chaoptin isoform X5 [Ostrinia furnacalis]	-1.36778	-0.9623	0.27146	0.9568	1.10181
TRINITY_DN19377_c0_g1_i4_orf1	uncharacterized protein LOC114353316 [Ostrinia furnacalis] >XP_028161061.1 uncharacterized protein LOC114353316 [Ostrinia furnacalis]	-1.83854	-0.01664	0.06405	0.82032	0.97081
TRINITY_DN6813_c1_g1_i1_orf1	pantothenate kinase 3 isoform X2 [Ostrinia furnacalis] >XP_028173241.1 pantothenate kinase 3 isoform X2 [Ostrinia furnacalis]	-1.41261	-0.85229	0.17222	1.21786	0.87482
TRINITY_DN73_c0_g1_i6_orf1	autophagy-related protein 16-1 isoform X1 [Vanessa cardui]	-1.89308	-0.09557	0.43588	0.70032	0.85245
TRINITY_DN21285_c0_g1_i3_orf1	uncharacterized protein LOC114351683 isoform X7 [Ostrinia furnacalis]	-1.47903	-0.86154	1.14313	0.44617	0.75127
TRINITY_DN23069_c0_g2_i3_orf1	uncharacterized protein LOC114364799 [Ostrinia furnacalis]	-1.06217	-1.33111	0.49467	0.78673	1.11189
TRINITY_DN7106_c0_g1_i5_orf1	TBC1 domain family member 15 isoform X5 [Helicoverpa zea]	-1.29831	-0.9784	0.47326	1.40519	0.39826
TRINITY_DN2392_c0_g2_i1_orf1	cytochrome P450 9e2-like [Ostrinia furnacalis] >QPF77612.1 cytochrome P450 monooxygenase CYP9A185 [Ostrinia furnacalis]	-1.47167	-0.49679	-0.13139	0.62247	1.47738
TRINITY_DN7992_c0_g1_i4_orf1	uncharacterized protein LOC114362579 [Ostrinia furnacalis]	-1.89365	0.26038	0.96262	0.02327	0.64738
TRINITY_DN2618_c0_g1_i3_orf1	CDP-diacylglycerol--inositol 3-phosphatidyltransferase [Ostrinia furnacalis]	-1.73619	-0.26196	0.07561	0.78345	1.13909
TRINITY_DN11263_c0_g1_i5_orf1	SET domain-containing protein SmydA-8 [Ostrinia furnacalis]	-1.54261	-0.24114	1.59509	0.10122	0.08745
TRINITY_DN9376_c1_g1_i3_orf1	RB1-inducible coiled-coil protein 1 isoform X4 [Ostrinia furnacalis]	-1.67158	-0.15858	0.69029	-0.15619	1.29606
TRINITY_DN8310_c0_g2_i1_orf1	uncharacterized protein LOC116773294 [Danaus plexippus plexippus] >OWR55545.1 hypothetical protein KGM_209260 [Danaus plexippus plexippus]	-0.44723	-1.73036	0.36911	1.03521	0.77328
TRINITY_DN19807_c0_g1_i1_orf1	DNA-directed RNA polymerase I subunit RPA1 [Ostrinia furnacalis]	-1.51161	-0.88627	0.724	0.88408	0.7898
TRINITY_DN3087_c0_g1_i1_orf1	uncharacterized protein LOC114360174 [Ostrinia furnacalis]	-1.73323	-0.39891	1.065	0.27555	0.7916
TRINITY_DN2207_c0_g1_i4_orf1	methionine-R-sulfoxide reductase B1 isoform X4 [Pectinophora gossypiella] >XP_049887601.1 methionine-R-sulfoxide reductase B1 isoform X4 [Pectinophora gossypiella]	-0.69024	-1.45256	0.35132	0.31029	1.48119

TRINITY_DN10070_c0_g1_i1_orf1	40S ribosomal protein SA isoform 1 [Homo sapiens] >XP_002813955.1 40S ribosomal protein SA [Pongo abelii] >XP_004033937.1 40S ribosomal protein SA [Gorilla gorilla gorilla] >XP_008949773.1 40S ribosomal protein SA [Pan paniscus] >XP_009237465.1 40S ribosomal protein SA [Pongo abelii] >XP_024211184.1 40S ribosomal protein SA [Pan troglodytes] >XP_032017897.1 40S ribosomal protein SA [Hylobates moloch] >XP_032017898.1 40S ribosomal protein SA [Hylobates moloch] >XP_032615302.1 40S ribosomal protein SA [Hylobates moloch] >XP_034820112.1 40S ribosomal protein SA [Pan paniscus] >P08865.4 RecName: Full=40S ribosomal protein SA; AltName: Full=37 kDa laminin receptor precursor; Short=37LRP; AltName: Full=37/67 kDa laminin receptor; Short=LRP/LR; AltName: Full=67 kDa laminin receptor; Short=67LR; AltName: Full=Colon carcinoma laminin-binding protein; AltName: Full=Laminin receptor 1; Short=LamR; AltName: Full=Laminin-binding protein precursor p40; Short=LBP/p40; AltName: Full=Multidrug resistance-associated protein MGr1-Ag; AltName: Full=NEM/1CHD4; AltName: Full=Small ribosomal subunit protein uS2 [Homo sapiens] >4D5L_A Cryo-EM structures of ribosomal 80S complexes with termination factors and cricket paralysis virus IRES reveal the IRES in the translocated state [Oryctolagus cuniculus] >4D61_A Cryo-EM structures of ribosomal 80S complexes with termination factors and cricket paralysis virus IRES reveal the IRES in the translocated state [Oryctolagus cuniculus] >4UG0_SA Chain SA, 40S RIBOSOMAL PROTEIN SA [Homo sapiens] >4UJD_CA Chain CA, 40S RIBOSOMAL PROTEIN US2 [Oryctolagus cuniculus] >4UJE_BA Chain BA, 40S RIBOSOMAL PROTEIN SA [Oryctolagus cuniculus] >4V6X_AA Chain AA, 40S ribosomal protein SA [Homo sapiens] >5A2Q_A Structure of the HCV IRES bound to the human ribosome [Homo sapiens] >5AJ0_BA Chain BA, 40S ribosomal protein SA [Homo sapiens] >5FLX_A Mammalian 40S HCV-IRES complex [Oryctolagus cuniculus] >5LKS_SA Chain SA, 40S ribosomal protein SA [Homo sapiens] >5OA3_A Human 40S-eIF2D-re-initiation complex [Homo sapiens] >5T2C_Ao Chain Ao, 40S ribosomal protein SA [Homo sapiens] >5VYC_A1 Chain A1, 40S ribosomal protein SA [Homo sapiens] >5VYC_A2 Chain A2, 40S ribosomal protein SA [Homo sapiens] >5VYC_A3 Chain A3, 40S ribosomal protein SA [Homo sapiens] >5VYC_A4 Chain A4, 40S ribosomal protein SA [Homo sapiens] >5VYC_A5 Chain A5, 40S ribosomal protein SA [Homo sapiens] >5VYC_A6 Chain A6, 40S ribosomal protein SA [Homo sapiens] >6EK0_SA Chain SA, 40S ribosomal protein SA [Homo sapiens] >6G18_A Cryo-EM structure of a late human pre-40S ribosomal subunit - State C [Homo sapiens] >6G4S_A Cryo-EM structure of a late human pre-40S ribosomal subunit - State B [Homo sapiens] >6G51_A Cryo-EM structure of a late human pre-40S ribosomal subunit - State D [Homo sapiens] >6G53_A Cryo-EM structure of a late human pre-40S ribosomal subunit - State E [Homo sapiens] >6G5H_A Cryo-EM structure of a late human pre-40S ribosomal subunit - Mature [Homo sapiens] >6G5I_A Cryo-EM structure of a late human pre-40S ribosomal subunit - State R [Homo sapiens] >6IP5_2n Chain 2n, 40S ribosomal protein SA [Homo sapiens] >6IP6_2n Chain 2n, 40S ribosomal protein SA [Homo sapiens] >6IP8_2n Chain 2n, 40S ribosomal protein SA [Homo sapiens] >6Y0G_SA Chain SA, 40S ribosomal protein SA [Homo sapiens] >6Y2L_SA Chain SA, 40S ribosomal protein SA [Homo sapiens] >6Y57_SA Chain SA, 40S ribosomal protein SA [Homo sapiens] >6YBD_N Structure of a human 48S translational initiation complex - eIF3 [Homo sapiens] >6YBW_N Structure of a human 48S translational initiation complex - 40S body [Homo sapiens] >6Z6L_SA Chain SA, 40S ribosomal protein SA [Homo sapiens] >6Z6M_SA Chain SA, 40S ribosomal protein SA [Homo sapiens] >6Z6N_SA Chain SA, 40S ribosomal protein SA [Homo sapiens] >6ZLW_B Chain B, 40S ribosomal protein SA [Homo sapiens] >6ZM7_SA Chain SA, 40S ribosomal protein SA [Homo sapiens] >6ZME_SA Chain SA, 40S ribosomal protein SA [Homo sapiens] >6ZML_SA Chain SA, 40S ribosomal protein SA [Homo sapiens] >6ZMO_SA Chain SA, 40S	-0.9218	-1.37402	0.29484	1.295	0.70598
TRINITY_DN9991_c0_g1_i4_orf1	unnamed protein product [Parnassius apollo]	-1.13994	-0.99578	-0.12629	0.87155	1.39047
TRINITY_DN63030_c0_g1_i5_orf1	uncharacterized protein LOC114358571 [Ostrinia furnacalis]	-0.85688	-1.50853	0.88742	0.50409	0.97389
TRINITY_DN1034_c0_g2_i1_orf1	glycerol kinase isoform X7 [Ostrinia furnacalis] >XP_028168096.1 glycerol kinase isoform X8 [Ostrinia furnacalis]	-1.8283	-0.27649	0.466	0.9216	0.71719
TRINITY_DN8625_c0_g1_i1_orf1	GDP-L-fucose synthase [Ostrinia furnacalis]	-1.18601	-0.91427	0.27083	1.62561	0.20384
TRINITY_DN2848_c0_g1_i1_orf1	glyceraldehyde-3-phosphate dehydrogenase isoform 2 [Mus musculus] >XP_036021733.1 glyceraldehyde-3-phosphate dehydrogenase isoform X1 [Mus musculus] >P16858.2 RecName: Full=Glyceraldehyde-3-phosphate dehydrogenase; Short=GAPDH; AltName: Full=Peptidyl-cysteine S-nitrosylase GAPDH [Mus musculus] >6LGJ_A Crystal structure of an oxido-reductase [Mus musculus] >6LGJ_B Crystal structure of an oxido-reductase [Mus musculus] >6LGJ_C Crystal structure of an oxido-reductase [Mus musculus] >6LGJ_D Crystal structure of an oxido-reductase [Mus musculus] >AAA37659.1 glyceraldehyde-3-phosphate dehydrogenase [Mus musculus] >AAH82592.1 Glyceraldehyde-3-phosphate dehydrogenase [Mus musculus] >AAH83065.1 Glyceraldehyde-3-phosphate dehydrogenase [Mus musculus] >AAH83079.1 Glyceraldehyde-3-phosphate dehydrogenase [Mus musculus] >AAH83080.1 Glyceraldehyde-3-phosphate dehydrogenase [Mus musculus]	-1.06326	-0.73115	-0.1285	1.81866	0.10426
TRINITY_DN1534_c0_g1_i3_orf1	peptidoglycan recognition protein-like [Ostrinia furnacalis]	-1.05963	-0.625	-0.47141	1.76361	0.39243
TRINITY_DN5748_c0_g1_i5_orf1	glycine N-methyltransferase isoform X1 [Ostrinia furnacalis] >XP_028165118.1 glycine N-methyltransferase isoform X2 [Ostrinia furnacalis] >XP_028165119.1 glycine N-methyltransferase isoform X1 [Ostrinia furnacalis] >XP_028165120.1 glycine N-methyltransferase isoform X2 [Ostrinia furnacalis]	-1.42587	-0.92314	0.60185	0.53725	1.2099
TRINITY_DN726_c0_g1_i8_orf1	unnamed protein product [Chilo suppressalis]	-1.62702	-0.45814	1.35127	0.52135	0.21254
TRINITY_DN3738_c0_g1_i5_orf1	23 kDa integral membrane protein-like [Ostrinia furnacalis]	-1.56381	-0.36432	-0.1117	1.42541	0.61442
TRINITY_DN14269_c0_g1_i5_orf1	probable DNA mismatch repair protein Msh6 isoform X1 [Ostrinia furnacalis]	-1.79336	0.1185	1.10709	-0.15371	0.72148
TRINITY_DN17172_c0_g1_i5_orf1	omega-amidase NIT2 isoform X1 [Zerene cesonia]	-1.76701	-0.29541	0.47705	0.40145	1.18392
TRINITY_DN1328_c0_g1_i6_orf1	fungal protease inhibitor-1-like [Ostrinia furnacalis]	-1.22034	-1.11201	0.31708	1.27504	0.74022
TRINITY_DN59852_c0_g1_i1_orf1	hypothetical protein evm_008421 [Chilo suppressalis]	-1.3694	-0.9096	1.19575	0.1669	0.91635
TRINITY_DN3991_c0_g1_i6_orf1	acetyl-CoA carboxylase isoform X3 [Trichoplusia ni]	-1.27918	-0.93951	0.29226	0.44359	1.48284
TRINITY_DN99694_c0_g1_i1_orf1	hypothetical protein HF086_013792 [Spodoptera exigua]	-1.56221	-0.75442	0.54854	1.13543	0.63265
TRINITY_DN5933_c0_g1_i1_orf1	peroxidase-like [Ostrinia furnacalis]	-0.85357	-1.51058	0.52624	0.81023	1.02769
TRINITY_DN8651_c0_g1_i16_orf1	glutathione S-transferase theta 2 [Conogethes punctiferalis]	-1.31775	-0.75747	0.36977	0.11152	1.59393
TRINITY_DN6058_c0_g1_i3_orf1	NHL repeat-containing protein 2 isoform X2 [Ostrinia furnacalis]	-1.71268	-0.54678	0.80503	0.55113	0.90329
TRINITY_DN2772_c0_g1_i3_orf1	uncharacterized protein LOC114353284 isoform X4 [Ostrinia furnacalis] >XP_028161011.1 uncharacterized protein LOC114353284 isoform X4 [Ostrinia furnacalis] >XP_028161012.1 uncharacterized protein LOC114353284 isoform X4 [Ostrinia furnacalis]	-1.2004	-1.05653	0.21757	1.41714	0.62222

TRINITY_DN5081_c0_g1_i5_orf1	ester hydrolase C11orf54 homolog isoform X1 [Ostrinia furnacalis]	-1.66726	-0.45443	0.1437	0.85329	1.12471
TRINITY_DN14112_c0_g1_i3_orf1	uncharacterized protein LOC114350956 [Ostrinia furnacalis]	-1.36021	-0.96803	0.44261	0.59709	1.28854
TRINITY_DN34153_c0_g2_i2_orf1	uncharacterized protein LOC114351567 [Ostrinia furnacalis]	-1.22378	-0.83008	0.80725	-0.20875	1.45536
TRINITY_DN741_c0_g1_i10_orf1	talin-1 isoform X13 [Ostrinia furnacalis]	-1.07578	-1.28239	0.9068	0.32404	1.12733
TRINITY_DN2847_c0_g1_i20_orf1	uncharacterized protein LOC114352221 [Ostrinia furnacalis]	-1.47724	-0.46871	-0.13794	0.59043	1.49347
TRINITY_DN77559_c0_g1_i1_orf1	uncharacterized protein LOC114359392 isoform X2 [Ostrinia furnacalis]	-1.37143	-0.91765	0.2321	0.79649	1.26049
TRINITY_DN64126_c0_g1_i1_orf1	senecionine N-oxygenase isoform X2 [Galleria mellonella]	-1.40232	-0.94407	0.40815	0.74859	1.18964
TRINITY_DN6424_c0_g1_i2_orf1	protein tweety-like [Ostrinia furnacalis]	-1.39414	0.1818	-0.45167	1.67901	-0.015
TRINITY_DN6587_c0_g1_i3_orf1	phosphoribosylformylglycinamidine synthase isoform X2 [Ostrinia furnacalis]	-1.63815	-0.47403	0.34297	1.33687	0.43235
TRINITY_DN19098_c0_g1_i4_orf1	L-xylulose reductase-like [Ostrinia furnacalis]	-1.17129	-0.79962	0.26534	-0.00272	1.70829
TRINITY_DN7075_c0_g2_i1_orf1	retinal dehydrogenase 1-like [Ostrinia furnacalis]	-1.62161	-0.40737	-0.05281	0.90916	1.17263
TRINITY_DN2596_c0_g1_i6_orf1	unnamed protein product [Arctia plantaginis]	-1.11274	-1.16809	0.11708	0.93568	1.22808
TRINITY_DN5497_c0_g1_i6_orf1	1,2-dihydroxy-3-keto-5-methylthiopentene dioxygenase-like [Ostrinia furnacalis]	-1.71331	-0.05624	-0.08203	0.5132	1.33839
TRINITY_DN4955_c0_g1_i2_orf1	RNA pseudouridylate synthase domain-containing protein 1-like isoform X2 [Ostrinia furnacalis]	-1.39704	-0.92559	0.32629	0.78318	1.21315
TRINITY_DN21545_c0_g1_i2_orf1	sterile alpha and TIR motif-containing protein 1 isoform X1 [Ostrinia furnacalis]	-1.37428	-1.02179	0.66542	0.61339	1.11726
TRINITY_DN1084_c0_g1_i2_orf1	ATP-citrate synthase [Ostrinia furnacalis]	-1.54292	-0.6055	0.10453	1.29863	0.74526
TRINITY_DN51776_c0_g1_i1_orf1	unnamed protein product, partial [Iphiclides podalirius]	-1.33192	-0.93745	0.20557	0.73587	1.32793
TRINITY_DN4242_c0_g1_i6_orf1	fibrohexamerin-like [Ostrinia furnacalis]	-0.53062	-1.55692	-0.05134	1.11643	1.02245
TRINITY_DN1172_c0_g1_i1_orf1	hypothetical protein O3G_MSEX009550 [Manduca sexta]	-1.31364	-1.07499	0.50697	0.72707	1.1546
TRINITY_DN15157_c0_g1_i1_orf1	UDP-glycosyltransferase UGT40AM2 [Ostrinia furnacalis]	-1.54727	-0.39708	0.40823	0.02584	1.51028
TRINITY_DN7064_c0_g1_i20_orfp1	TRINITY_DN7064_c0_g1_i20_m.53649 TRINITY_DN7064_c0_g1::TRINITY_DN7064_c0_g1_i20::g.53649 ORF type:internal len:256 (+),score=27.03,Kazal_1 PF00050.22 0.52,Kazal_1|PF00050.22|1.1e-10,Kazal_1|PF00050.22|6.2e-09,Kazal_1|PF00050.22|2.7e-07,Kazal_1|PF00050.22|5.1e-09,Kazal_1|PF00050.22|4.5e-11,Kazal_2|PF07648.16|2.2,Kazal_2|PF07648.16|4.8e-10,Kazal_2|PF07648.16|4.2e-10,Kazal_2|PF07648.16|1.1e-09,Kazal_2|PF07648.16|3e-10,Kazal_2|PF07648.16|1.3e-07 TRINITY_DN7064_c0_g1_i20:3-767(+)	-0.90264	-1.16027	-0.14779	1.53753	0.67318
TRINITY_DN12951_c1_g2_i2_orf1	protein kinase C isoform X2 [Ostrinia furnacalis]	-1.92102	0.28774	0.94441	0.12344	0.56544
TRINITY_DN31609_c0_g1_i3_orf1	sorbitol dehydrogenase-like [Ostrinia furnacalis]	-1.52644	-0.03778	-0.21742	0.17174	1.6099
TRINITY_DN10774_c0_g2_i3_orf1	uncharacterized protein LOC114362157, partial [Ostrinia furnacalis]	-1.30253	-0.92975	0.49185	0.28605	1.45438
TRINITY_DN6642_c0_g1_i2_orf1	protein purity of essence [Ostrinia furnacalis]	-1.13845	-0.98535	0.12537	0.39902	1.5994
TRINITY_DN7556_c0_g1_i3_orf1	jg27436 [Pararge aegeria aegeria]	-1.38829	-0.14829	0.16146	1.70714	-0.33202
TRINITY_DN11172_c1_g1_i1_orf1	juvenile hormone epoxide hydrolase-like isoform X1 [Ostrinia furnacalis] >XP_028170522.1 juvenile hormone epoxide hydrolase-like isoform X2 [Ostrinia furnacalis]	-1.14206	-0.8348	-0.10613	1.68108	0.40191
TRINITY_DN1103_c0_g1_i12_orf1	retinal dehydrogenase 1-like [Ostrinia furnacalis]	-1.68758	0.18779	-0.39108	0.64864	1.24223
TRINITY_DN976_c0_g1_i5_orf1	filaggrin-2-like [Ostrinia furnacalis]	-1.82377	0.24514	0.32807	0.02354	1.22702
TRINITY_DN60787_c0_g1_i5_orf1	probable transaldolase [Ostrinia furnacalis]	-1.34733	-1.08537	0.71858	0.96002	0.7541
TRINITY_DN31163_c1_g1_i4_orf1	phenoloxidase subunit 2-like [Ostrinia furnacalis]	-1.23608	-0.83507	0.2996	1.6327	0.13885
TRINITY_DN829_c0_g1_i8_orf1	cytochrome P450 6B6-like [Ostrinia furnacalis]	-0.68689	-1.46932	0.36845	0.3302	1.45756
TRINITY_DN2529_c0_g1_i3_orf1	collagen alpha-1(X) chain-like [Ostrinia furnacalis]	-1.29378	-1.009	0.68929	0.29136	1.32213
TRINITY_DN1068_c0_g1_i3_orf1	aspartate aminotransferase, cytoplasmic [Ostrinia furnacalis]	-0.98271	-1.42861	0.69966	0.99542	0.71623
TRINITY_DN25582_c0_g1_i3_orf1	uroporphyrinogen decarboxylase [Ostrinia furnacalis]	-1.92779	-0.02975	0.6344	0.612	0.71115
TRINITY_DN2844_c0_g1_i2_orf1	glutathione S-transferase 9 [Streltzoviella insularis]	-1.05596	-1.07449	0.85123	-0.13098	1.41021
TRINITY_DN120089_c0_g1_i1_orf1	phosphoglucomutase [Ostrinia furnacalis]	-0.81374	-1.54831	0.76377	0.59915	0.99913
TRINITY_DN1255_c0_g1_i17_orf1	LIM and senescent cell antigen-like-containing domain protein 1 isoform X1 [Pectinophora gossypiella]	-1.73485	-0.47535	0.61988	0.5548	1.03552
TRINITY_DN4695_c0_g1_i4_orf1	glutathione S-transferase epsilon 3 [Ostrinia furnacalis]	-1.20093	-1.11368	0.41346	0.53865	1.3625
TRINITY_DN63152_c0_g1_i7_orf1	microtubule-actin cross-linking factor 1 isoform X21 [Manduca sexta]	-1.12854	-1.21136	0.69741	0.36095	1.28154
TRINITY_DN23640_c0_g1_i5_orf1	proteasomal ATPase-associated factor 1-like [Ostrinia furnacalis]	-1.89609	0.53543	-0.12077	0.68485	0.79658
TRINITY_DN4341_c0_g1_i4_orf1	uncharacterized protein LOC114354354 [Ostrinia furnacalis]	-1.06888	-0.94319	0.16137	0.14136	1.70934
TRINITY_DN10455_c0_g2_i1_orf1	ubiquitin fusion degradation protein 1 homolog [Ostrinia furnacalis]	-1.94647	0.06827	0.50685	0.75424	0.6171
TRINITY_DN125_c0_g1_i2_orf1	PREDICTED: purine nucleoside phosphorylase isoform X1 [Microplitis demolitor]	-1.55722	-0.22224	-0.16664	1.52272	0.42338
TRINITY_DN83622_c0_g1_i2_orf1	uncharacterized protein LOC114350344 [Ostrinia furnacalis]	-1.29348	-0.77637	0.03872	1.58999	0.44114
TRINITY_DN22046_c1_g1_i5_orf1	uncharacterized protein LOC114351208 [Ostrinia furnacalis]	-1.25149	-0.66135	0.16941	0.02085	1.72258
TRINITY_DN117042_c0_g1_i2_orf1	UBX domain-containing protein 6 [Ostrinia furnacalis] >XP_028162119.1 UBX domain-containing protein 6 [Ostrinia furnacalis]	-1.83524	0.24593	-0.15708	0.7688	0.9776
TRINITY_DN554_c0_g1_i1_orf1	uncharacterized protein LOC114353093 isoform X1 [Ostrinia furnacalis] >XP_028160722.1 uncharacterized protein LOC114353093 isoform X1 [Ostrinia furnacalis] >XP_028160723.1 uncharacterized protein LOC114353093 isoform X1 [Ostrinia furnacalis]	-1.01248	-1.23482	0.15574	0.70045	1.39112
TRINITY_DN4304_c0_g1_i3_orf1	TOM1-like protein 2 isoform X4 [Galleria mellonella]	-1.75378	-0.30849	0.50554	0.35303	1.20369
TRINITY_DN35763_c0_g1_i2_orf1	probable methylthioribulose-1-phosphate dehydratase [Helicoverpa armigera]	-1.22306	-1.10808	0.37908	0.6389	1.31316
TRINITY_DN8854_c0_g1_i2_orf1	glutathione S-transferase epsilon 2 [Ostrinia furnacalis]	-1.5223	-0.60793	0.13375	1.38693	0.60955
TRINITY_DN1741_c0_g1_i5_orf1	gephyrin isoform X1 [Ostrinia furnacalis]	-1.28475	-1.09813	1.16025	0.4533	0.76933

TRINITY_DN146181_c0_g1_i1_orf1	vesicular integral-membrane protein VIP36 [Diachasma alloeum]	-1.83345	-0.16775	0.40521	0.50429	1.0917
TRINITY_DN62557_c0_g1_i1_orf1	6-phosphofructokinase [Operophtera brumata]	-0.79496	-1.49729	0.60741	1.25343	0.43141
TRINITY_DN147691_c0_g1_i1_orf1	WD repeat-containing protein 46 [Orussus abietinus]	-0.87402	-1.44235	0.50652	0.54342	1.26643
TRINITY_DN11569_c0_g1_i1_orf1	hypothetical protein evm_008488 [Chilo suppressalis] >CAB3522842.1 unnamed protein product [Chilo suppressalis] >CAH0400164.1 unnamed protein product [Chilo suppressalis]	-1.33847	-0.25469	-0.23827	1.7553	0.07613
TRINITY_DN6415_c0_g1_i1_orf1	D-arabinitol dehydrogenase 1-like [Ostrinia furnacalis]	-0.4837	-1.5894	0.07941	0.64603	1.34766
TRINITY_DN4911_c0_g1_i6_orf1	ATP-binding cassette sub-family G member 1-like isoform X2 [Ostrinia furnacalis]	-1.65446	0.24593	-0.13685	0.06931	1.47606
TRINITY_DN38180_c0_g1_i3_orf1	guanine deaminase [Ostrinia furnacalis]	-1.61687	-0.33536	-0.12206	0.80577	1.26851
TRINITY_DN135781_c0_g1_i1_orf1	vitamin K epoxide reductase complex subunit 1-like protein 1 [Ostrinia furnacalis] >XP_028171283.1 vitamin K epoxide reductase complex subunit 1-like protein 1 [Ostrinia furnacalis]	-1.07818	-1.23065	0.58931	0.36108	1.35844
TRINITY_DN131924_c0_g1_i2_orf1	protein FAM151A isoform X2 [Ostrinia furnacalis]	-1.62031	-0.25031	0.027	1.47487	0.36875
TRINITY_DN13602_c0_g1_i4_orf1	NEDD8 ultimate buster 1-like [Ostrinia furnacalis]	-1.41694	-0.82454	0.64209	1.35697	0.24241
TRINITY_DN2207_c0_g1_i6_orf1	methionine-R-sulfoxide reductase B1 isoform X2 [Ostrinia furnacalis]	-1.30875	-0.98395	0.54838	1.37063	0.3737
TRINITY_DN1848_c0_g1_i2_orf1	uncharacterized protein LOC114351337 [Ostrinia furnacalis]	-1.81492	0.22378	-0.15349	0.63723	1.10741
TRINITY_DN27771_c0_g2_i1_orf1	glycine--tRNA ligase [Ostrinia furnacalis]	-1.76893	-0.25254	0.15157	1.06886	0.80104
TRINITY_DN27833_c0_g2_i1_orf1	uncharacterized protein LOC114359161 [Ostrinia furnacalis]	-1.5503	0.18244	-0.07612	-0.14836	1.59232
TRINITY_DN22257_c0_g1_i7_orf1	spatacsin isoform X1 [Ostrinia furnacalis] >XP_028159192.1 spatacsin isoform X2 [Ostrinia furnacalis]	-1.79559	-0.0729	0.01773	1.09508	0.75567
TRINITY_DN50074_c0_g1_i1_orf1	uncharacterized protein LOC114364628 [Ostrinia furnacalis]	-0.45509	-1.66638	0.37502	1.29178	0.45467
TRINITY_DN9019_c0_g1_i5_orf1	exosome complex component RRP46 [Ostrinia furnacalis]	-1.66078	-0.09498	-0.31012	0.99642	1.06946
TRINITY_DN5453_c0_g1_i2_orf1	PREDICTED: MOB kinase activator-like 1 [Papilio polytes] >XP_013167887.1 PREDICTED: MOB kinase activator-like 1 isoform X1 [Papilio xuthus] >XP_014357130.2 MOB kinase activator-like 1 [Papilio machaon] >XP_026500369.1 MOB kinase activator-like 1 [Vanessa tameamea] >XP_028163142.1 MOB kinase activator-like 1 [Ostrinia furnacalis] >XP_030023123.1 MOB kinase activator-like 1 [Manduca sexta] >XP_034828352.1 MOB kinase activator-like 1 [Maniola hyperantus] >XP_039750378.1 MOB kinase activator-like 1 [Pararge aegeria] >XP_045768051.1 MOB kinase activator-like 1 isoform X1 [Maniola jurtina] >XP_046967490.1 MOB kinase activator-like 1 [Vanessa cardui] >XP_047531957.1 MOB kinase activator-like 1 [Vanessa atalanta] >XP_050349020.1 MOB kinase activator-like 1 isoform X2 [Nymphalis io] >RVE50045.1 hypothetical protein evm_005251 [Chilo suppressalis] >CAG5034166.1 unnamed protein product [Parnassius apollo] >CAH0724078.1 unnamed protein product, partial [Brenthis ino] >CAH2040061.1 unnamed protein product, partial [Iphiclides podalirius] >CAH2267987.1 jg8429 [Pararge aegeria aegeria]	-1.50488	-0.49841	1.51855	0.4205	0.06424
TRINITY_DN14242_c0_g1_i2_orfp1	TRINITY_DN14242_c0_g1_i2_m.18449 TRINITY_DN14242_c0_g1::TRINITY_DN14242_c0_g1_i2::g.18449 ORF type:internal len:148 (-),score=102.30 TRINITY_DN14242_c0_g1_i2:2-442(-)	-1.88284	0.08701	0.95197	0.72609	0.11777
TRINITY_DN84478_c0_g1_i8_orf1	uncharacterized protein LOC114359035 isoform X1 [Ostrinia furnacalis]	-1.96218	0.47386	0.50348	0.79809	0.18675
TRINITY_DN1108_c3_g1_i1_orfp1	TRINITY_DN1108_c3_g1_i1_m.5561 TRINITY_DN1108_c3_g1::TRINITY_DN1108_c3_g1_i1::g.5561 ORF type:internal len:113 (+),score=89.07 TRINITY_DN1108_c3_g1_i1:1-336(+)	-1.90698	0.17271	0.7938	0.83166	0.10881
TRINITY_DN2813_c0_g1_i10_orf1	arylphorin subunit alpha-like [Ostrinia furnacalis]	-1.90318	0.46591	1.03526	0.1367	0.26532
TRINITY_DN5080_c0_g1_i5_orf1	storage protein [Ostrinia furnacalis]	-1.96464	0.77358	0.27671	0.61075	0.3036
TRINITY_DN64297_c0_g1_i1_orf1	vanin-like protein 2 isoform X2 [Ostrinia furnacalis]	-1.56091	1.33079	0.25072	0.59363	-0.61424
TRINITY_DN2813_c0_g1_i7_orf1	arylphorin subunit alpha-like [Ostrinia furnacalis]	-1.87421	0.64586	0.86631	-0.17548	0.53752
TRINITY_DN9044_c0_g1_i2_orf1	unnamed protein product [Euphydryas editha]	-1.6119	0.34158	1.42082	0.28184	-0.43234
TRINITY_DN22515_c0_g1_i10_orf1	hypothetical protein KGM_202621 [Danaus plexippus plexippus]	-1.99847	0.52323	0.42931	0.54593	0.5
TRINITY_DN5406_c0_g2_i1_orf1	uncharacterized protein LOC114350326 [Ostrinia furnacalis]	-1.87232	0.94781	0.7566	0.153	0.01491
TRINITY_DN4767_c0_g1_i4_orf1	cysteine protease XCP2-like [Ostrinia furnacalis]	-1.95718	0.75927	0.28587	0.67435	0.23768
TRINITY_DN143895_c0_g1_i1_orf1	cathepsin L-like [Aphidius gifuensis] >KAF7988186.1 hypothetical protein HCN44_007680 [Aphidius gifuensis]	-1.92875	0.62519	0.05375	0.85423	0.39558
TRINITY_DN15578_c0_g2_i1_orfp1	uncharacterized protein LOC125235519 [Leguminivora glycinivorella]	-1.88831	0.71887	0.8435	0.43946	-0.11351
TRINITY_DN129863_c0_g1_i1_orf1	protein PFC0760c-like isoform X2 [Ostrinia furnacalis]	-1.88125	0.35248	1.09705	0.3572	0.07453
TRINITY_DN25234_c0_g1_i1_orf1	uncharacterized protein LOC114353853 [Ostrinia furnacalis]	-1.75627	0.3191	1.28922	0.33917	-0.19121
TRINITY_DN703_c0_g1_i2_orf1	acidic juvenile hormone-suppressible protein 1-like [Ostrinia furnacalis]	-1.90717	0.46724	0.09245	1.01136	0.33613
TRINITY_DN1370_c0_g1_i2_orf1	hypothetical protein evm_000756 [Chilo suppressalis]	-1.88845	1.05558	0.09354	0.50648	0.23284
TRINITY_DN66040_c0_g1_i2_orf1	serine protease inhibitor dipetalogastin-like isoform X2 [Ostrinia furnacalis]	-1.5131	1.52484	-0.13691	0.48616	-0.36099
TRINITY_DN75086_c0_g1_i5_orf1	lysosome membrane protein 2-like [Ostrinia furnacalis]	-1.96358	0.75171	0.46124	0.16937	0.58127
TRINITY_DN59885_c0_g1_i3_orf1	TGF-beta-activated kinase 1 and MAP3K7-binding protein 1-like [Ostrinia furnacalis]	-1.95786	0.58876	0.09603	0.65318	0.6199
TRINITY_DN98692_c0_g3_i1_orf1	fatty acyl-CoA hydrolase precursor, medium chain [Ostrinia furnacalis]	-1.93298	0.85037	0.67659	0.21462	0.19139
TRINITY_DN636_c1_g1_i9_orf1	secretory phospholipase A2 receptor-like [Ostrinia furnacalis]	-1.83278	0.77582	0.26549	0.96816	-0.17669
TRINITY_DN100327_c0_g1_i1_orf1	arylphorin subunit alpha-like [Ostrinia furnacalis]	-1.92848	0.33226	0.54409	0.92673	0.1254
TRINITY_DN2406_c0_g1_i6_orf1	uncharacterized protein LOC114361672 [Ostrinia furnacalis]	-1.53018	0.43542	1.51432	-0.00028	-0.41928
TRINITY_DN38307_c0_g1_i1_orfp1	TRINITY_DN38307_c0_g1_i1_m.10661 TRINITY_DN38307_c0_g1::TRINITY_DN38307_c0_g1_i1::g.10661 ORF type:5prime_partial len:66 (+),score=5.90 TRINITY_DN38307_c0_g1_i1:2-199(+)	-1.66944	1.42198	-0.16423	0.00679	0.4049
TRINITY_DN20560_c0_g1_i6_orf1	pupal cuticle protein C1B-like [Ostrinia furnacalis]	-1.83019	1.16248	0.40775	-0.09248	0.35245
TRINITY_DN1575_c0_g1_i10_orf1	uncharacterized protein LOC114359245 [Ostrinia furnacalis]	-1.37917	0.42138	1.18157	0.7539	-0.97768

TRINITY_DN45948_c1_g1_i1_orf1	unnamed protein product [Leptidea sinapis]	-1.78547	1.18571	0.55625	-0.19717	0.24067
TRINITY_DN42719_c0_g2_i1_orf1	inter-alpha-trypsin inhibitor heavy chain H4-like isoform X11 [Ostrinia furnacalis]	-1.87198	0.91789	0.36021	0.71352	-0.11964
TRINITY_DN33272_c0_g1_i5_orf1	Low-density lipoprotein receptor-related protein 1 [Papilio xuthus]	-1.907	1.01785	0.43067	0.0937	0.36479
TRINITY_DN5080_c0_g1_i1_orf1	basic juvenile hormone-suppressible protein 2-like [Ostrinia furnacalis]	-1.90586	0.82977	0.09529	0.7979	0.1829
TRINITY_DN7900_c0_g1_i4_orf1	uncharacterized protein LOC114366119 [Ostrinia furnacalis]	-1.96066	0.67795	0.18363	0.72056	0.37852
TRINITY_DN142657_c0_g1_i1_orf1	sorting and assembly machinery component 50 homolog [Diachasma alloeum]	-1.87016	0.27013	-0.00704	1.06633	0.54074
TRINITY_DN51813_c0_g1_i1_orf1	uncharacterized protein LOC114350216 [Ostrinia furnacalis]	-1.85788	0.87806	0.07584	0.87799	0.02598
TRINITY_DN4255_c0_g1_i11_orf1	LOW QUALITY PROTEIN: lebocin-4-like [Ostrinia furnacalis]	-1.87511	1.11109	0.35345	0.06345	0.34713
TRINITY_DN7040_c0_g2_i1_orf1	uncharacterized protein LOC114353763 [Ostrinia furnacalis]	-1.96276	0.6842	0.15188	0.66746	0.45922
TRINITY_DN67193_c0_g1_i1_orf1	A-kinase anchor protein 14-like [Ostrinia furnacalis]	-1.87536	0.88028	-0.14564	0.70511	0.4356
TRINITY_DN12582_c0_g1_i5_orf1	uncharacterized protein LOC114355527 isoform X1 [Ostrinia furnacalis]	-1.91837	0.57309	0.24067	0.95443	0.15018
TRINITY_DN12009_c0_g1_i1_orf1	uncharacterized protein LOC114365631 [Ostrinia furnacalis]	-1.83566	0.99281	0.02278	0.01768	0.8024
TRINITY_DN15247_c0_g1_i2_orf1	probable G-protein coupled receptor Mth-like 3 isoform X1 [Ostrinia furnacalis]	-1.86872	0.96964	0.42724	-0.1341	0.60594
TRINITY_DN1093_c0_g1_i4_orf1	uncharacterized protein LOC114361723 isoform X4 [Ostrinia furnacalis]	-1.97457	0.70522	0.21267	0.51794	0.53873
TRINITY_DN48548_c0_g1_i1_orf1	glutathione S-transferase sigma 2 [Heortia vitessoides]	-1.96164	0.24345	0.40417	0.83871	0.47532
TRINITY_DN4767_c0_g1_i6_orf1	cysteine protease XCP2-like [Ostrinia furnacalis]	-1.87233	0.60017	-0.15577	0.9266	0.50133
TRINITY_DN135449_c0_g1_i5_orf1	larval cuticle protein LCP-17-like [Galleria mellonella]	-1.93247	0.33742	0.09542	0.84503	0.6546
TRINITY_DN8158_c0_g1_i2_orf1	hypothetical protein G9C98_000136 [Cotesia typhae]	-1.73123	0.06991	-0.19615	1.27106	0.5864
TRINITY_DN467_c3_g1_i5_orf1	lysozyme precursor [Loxostege sticticalis]	-1.92342	0.73528	0.02681	0.37481	0.78652
TRINITY_DN106156_c1_g1_i1_orf1	arylphorin subunit alpha-like [Ostrinia furnacalis]	-1.84798	0.47645	-0.00559	1.14116	0.23597
TRINITY_DN801_c0_g1_i2_orf1	cathepsin L [Ostrinia furnacalis] >XP_028165920.1 cathepsin L [Ostrinia furnacalis] >UKI61015.1 cathepsin L [Ostrinia furnacalis]	-1.65486	1.39293	0.53302	-0.14842	-0.12267
TRINITY_DN19662_c0_g2_i1_orf1	storage protein [Ostrinia furnacalis]	-1.90567	0.72226	0.08663	0.89322	0.20356
TRINITY_DN16905_c0_g1_i1_orf1	unnamed protein product [Leptidea sinapis]	-1.98256	0.38599	0.68762	0.58594	0.32301
TRINITY_DN3707_c0_g1_i1_orf1	protein FAM160B1-like isoform X1 [Ostrinia furnacalis]	-1.93493	0.85775	0.11432	0.62927	0.33359
TRINITY_DN1175_c1_g1_i1_orf1	methanethiol oxidase [Ostrinia furnacalis]	-1.91897	1.00209	0.44652	0.1941	0.27626
TRINITY_DN5444_c0_g1_i1_orfp1	TRINITY_DN5444_c0_g1_i1_m.14077 TRINITY_DN5444_c0_g1::TRINITY_DN5444_c0_g1_i1::g.14077 ORF type:3prime_partial len:90 (-),score=25.11 TRINITY_DN5444_c0_g1_i1:2-268(-)	-1.74459	1.36434	-0.00854	0.29269	0.0961
TRINITY_DN295_c2_g1_i2_orf1	phosphoglycolate phosphatase 1A, chloroplastic [Manduca sexta]	-1.89854	0.39185	0.36483	1.04894	0.09291
TRINITY_DN703_c13_g1_i1_orf1	acidic juvenile hormone-suppressible protein 1-like [Ostrinia furnacalis]	-1.83925	0.39265	-0.04091	1.16504	0.32248
TRINITY_DN418_c1_g1_i3_orf1	hypothetical protein evm_003996 [Chilo suppressalis]	-1.71255	0.69259	0.27099	1.15985	-0.41088
TRINITY_DN7534_c0_g1_i15_orf1	protein-glucosylgalactosylhydroxylysine glucosidase isoform X2 [Ostrinia furnacalis]	-1.86909	0.68461	0.88583	-0.17358	0.47223
TRINITY_DN6122_c0_g1_i6_orf1	deubiquitinase DESI2 isoform X1 [Helicoverpa armigera] >XP_049707835.1 deubiquitinase DESI2 isoform X1 [Helicoverpa armigera]	-1.88412	0.76842	-0.08505	0.8568	0.34394
TRINITY_DN6423_c0_g1_i6_orf1	phenoloxidase-activating factor 2-like isoform X1 [Ostrinia furnacalis]	-1.75426	1.36232	0.02974	0.17188	0.19032
TRINITY_DN364_c1_g1_i2_orf1	talin-2-like, partial [Ostrinia furnacalis]	-1.83063	0.79969	0.99666	0.10478	-0.07049
TRINITY_DN1540_c0_g1_i7_orf1	alaserpin-like isoform X13 [Ostrinia furnacalis]	-1.95892	0.58941	0.11918	0.72358	0.52676
TRINITY_DN18338_c0_g1_i6_orf1	aquaporin AQPAn.G isoform X1 [Ostrinia furnacalis]	-1.56811	1.53526	0.18761	-0.33896	0.18419
TRINITY_DN5310_c2_g1_i2_orf1	serine protease persephone-like [Ostrinia furnacalis]	-1.9061	0.51866	0.38191	0.03033	0.97519
TRINITY_DN60946_c0_g2_i3_orf1	protein yellow-like [Ostrinia furnacalis]	-1.94011	0.90576	0.28682	0.20958	0.53794
TRINITY_DN18323_c0_g1_i5_orf1	GILT-like protein 1 isoform X1 [Ostrinia furnacalis]	-1.99543	0.55317	0.51699	0.5546	0.37067
TRINITY_DN960_c1_g1_i6_orf1	hypothetical protein evm_007130 [Chilo suppressalis]	-1.95407	0.51431	0.85884	0.21719	0.36374
TRINITY_DN19662_c4_g1_i1_orf1	basic juvenile hormone-suppressible protein 1-like [Ostrinia furnacalis]	-1.92017	0.62002	0.1667	0.92615	0.2073
TRINITY_DN13760_c1_g1_i1_orf1	pre-mRNA-processing factor 40 homolog A isoform X1 [Ostrinia furnacalis] >XP_028162665.1 pre-mRNA-processing factor 40 homolog A isoform X2 [Ostrinia furnacalis] >XP_028162667.1 pre-mRNA-processing factor 40 homolog A isoform X3 [Ostrinia furnacalis]	-1.68891	1.32162	0.25016	0.46566	-0.34853
TRINITY_DN26209_c0_g1_i6_orf1	uncharacterized protein LOC114352357 [Ostrinia furnacalis]	-1.94497	0.43731	0.20477	0.39151	0.91139
TRINITY_DN29414_c1_g2_i1_orf1	serine protease 44-like isoform X2 [Ostrinia furnacalis]	-1.91023	0.58049	-0.03026	0.89014	0.46987
TRINITY_DN9538_c1_g3_i1_orf1	cilia- and flagella-associated protein 410 isoform X2 [Aphidius gifuensis]	-1.84524	1.10034	-0.03394	0.5893	0.18954
TRINITY_DN52316_c0_g1_i1_orf1	arylphorin subunit alpha-like [Ostrinia furnacalis]	-1.86428	0.72891	-0.14886	0.91262	0.37161
TRINITY_DN28221_c0_g2_i1_orf1	unnamed protein product [Chilo suppressalis]	-1.96658	0.67276	0.23764	0.70936	0.34682
TRINITY_DN1421_c0_g1_i1_orf1	uncharacterized protein LOC114352615 [Ostrinia furnacalis]	-1.98794	0.43481	0.68868	0.35769	0.50676
TRINITY_DN95558_c0_g3_i1_orf1	cytochrome P450 monooxygenase CYP9G19 [Cnaphalocrocis medinalis]	-1.9573	0.43282	0.59371	0.15234	0.77843
TRINITY_DN25976_c0_g1_i4_orf1	hypothetical protein B566_EDAN014657 [Ephemera danica]	-1.88533	1.10855	0.35248	0.24659	0.17771
TRINITY_DN19980_c0_g1_i4_orf1	hypothetical protein evm_012507 [Chilo suppressalis]	-1.83178	1.10419	0.58938	-0.1159	0.25411
TRINITY_DN5655_c0_g1_i2_orf1	uncharacterized protein LOC114359603 [Ostrinia furnacalis]	-1.82018	1.22163	0.02955	0.15841	0.4106
TRINITY_DN768_c0_g1_i7_orf1	neutral ceramidase [Leguminivora glycinivorella]	-1.96475	0.49352	0.5423	0.75656	0.17237
TRINITY_DN56308_c0_g1_i2_orf1	storage protein 1 [Omphisa fuscidentalis]	-1.88616	0.66885	-0.06788	0.92946	0.35573
TRINITY_DN41_c0_g1_i3_orf1	uncharacterized protein LOC114359035 isoform X3 [Ostrinia furnacalis]	-1.82118	1.19312	0.324	-0.08097	0.38503

TRINITY_DN136358_c0_g1_i1_orf1	acidic juvenile hormone-suppressible protein 1-like [Ostrinia furnacalis]	-1.71886	0.10951	-0.01188	1.41028	0.21095
TRINITY_DN4443_c0_g1_i4_orf1	lysosome-associated membrane glycoprotein 1-like isoform X4 [Ostrinia furnacalis]	-1.90562	0.56689	0.9771	0.29729	0.06433
TRINITY_DN4748_c0_g1_i5_orf1	unnamed protein product, partial [Brenthis ino]	-1.66617	1.46875	0.16093	0.15981	-0.12332
TRINITY_DN20676_c0_g1_i6_orf1	aldo-keto reductase AKR2E4-like isoform X1 [Ostrinia furnacalis]	-1.84029	1.19071	0.05158	0.21513	0.38287
TRINITY_DN4235_c0_g1_i2_orf1	uncharacterized protein LOC114361536 [Ostrinia furnacalis]	-1.96163	0.25747	0.40485	0.84674	0.45257
TRINITY_DN1503_c0_g1_i6_orf1	ecdysteroid-regulated 16 kDa protein [Ostrinia furnacalis]	-1.83672	-0.11707	1.08672	0.60066	0.26641
TRINITY_DN5682_c0_g1_i6_orf1	spodomicin-like [Ostrinia furnacalis] >QKV49445.1 diapausin [Ostrinia furnacalis]	-1.8746	0.0889	1.11851	0.28709	0.3801
TRINITY_DN5408_c0_g1_i5_orf1	uncharacterized protein LOC114359912 [Ostrinia furnacalis]	-1.40889	1.52001	-0.04878	-0.62288	0.56055
TRINITY_DN11060_c0_g1_i6_orf1	extracellular matrix protein A-like isoform X3 [Ostrinia furnacalis]	-1.79233	0.93331	0.95414	-0.0756	-0.01952
TRINITY_DN9090_c0_g1_i9_orf1	CD63 antigen [Ostrinia furnacalis]	-1.94166	0.2053	0.29934	0.89975	0.53727
TRINITY_DN97883_c0_g1_i2_orf1	talin-2-like, partial [Ostrinia furnacalis]	-1.56012	0.69155	1.25497	0.27487	-0.66127
TRINITY_DN71308_c0_g1_i4_orf1	uncharacterized protein LOC114361536 [Ostrinia furnacalis]	-1.87566	1.13536	0.22489	0.32647	0.18895
TRINITY_DN5421_c0_g1_i1_orf1	arylphorin subunit alpha-like [Ostrinia furnacalis]	-1.94282	0.3044	0.46122	0.92561	0.25159
TRINITY_DN3609_c0_g1_i6_orf1	leukocyte elastase inhibitor-like [Ostrinia furnacalis]	-1.80151	1.05445	0.16659	-0.18225	0.76272
TRINITY_DN7040_c0_g1_i4_orf1	uncharacterized protein LOC114353763 [Ostrinia furnacalis]	-1.95102	0.62077	0.06429	0.66968	0.59627
TRINITY_DN104663_c1_g1_i2_orf1	PREDICTED: gelsolin-like [Amyelois transitella]	-1.92571	0.74457	0.17203	0.81962	0.18949
TRINITY_DN585_c0_g1_i12_orf1	very low-density lipoprotein receptor isoform X3 [Galleria mellonella]	-1.93408	0.96795	0.28676	0.27119	0.40819
TRINITY_DN798_c1_g1_i3_orf1	protein goliath isoform X1 [Ostrinia furnacalis] >XP_028170876.1 protein goliath isoform X1 [Ostrinia furnacalis]	-1.99414	0.57694	0.36464	0.48365	0.56891
TRINITY_DN3822_c0_g1_i7_orf1	hypothetical protein HF086_002539 [Spodoptera exigua] >CAH0699327.1 unnamed protein product [Spodoptera exigua]	-1.96948	0.7553	0.29464	0.60313	0.31641
TRINITY_DN30169_c0_g1_i1_orfp1	TRINITY_DN30169_c0_g1_i1_m.11367 TRINITY_DN30169_c0_g1::TRINITY_DN30169_c0_g1_i1::g.11367 ORF type:3prime_partial len:52 (+),score=0.98 TRINITY_DN30169_c0_g1_i1:72-224(+)	-1.82308	0.76217	-0.30787	0.86317	0.50561
TRINITY_DN121650_c0_g1_i1_orf1	carboxylesterase [Ostrinia furnacalis]	-1.69109	1.44393	-0.03362	0.0546	0.22617
TRINITY_DN2024_c0_g1_i12_orfp1	unnamed protein product, partial [Brenthis ino]	-1.70772	1.32399	0.40147	0.28217	-0.2999
TRINITY_DN2880_c0_g1_i2_orf1	sialomucin core protein 24 [Pectinophora gossypiella]	-1.86566	0.87065	0.84819	-0.0512	0.19803
TRINITY_DN184_c0_g1_i10_orf1	C-type mannose receptor 2-like isoform X1 [Leguminivora glycinivorella]	-1.69794	0.66493	1.27613	-0.02989	-0.21323
TRINITY_DN13511_c0_g1_i4_orf1	alpha-2-macroglobulin receptor-associated protein isoform X1 [Ostrinia furnacalis]	-1.60936	1.08524	0.89536	0.23963	-0.61086
TRINITY_DN12231_c0_g1_i1_orf1	carbonyl reductase [NADPH] 3-like [Ostrinia furnacalis]	-1.97843	0.7706	0.41864	0.34293	0.44627
TRINITY_DN5022_c0_g1_i4_orf1	syntenin-1-like [Ostrinia furnacalis]	-1.89421	0.92827	0.46804	-0.07298	0.57087
TRINITY_DN11826_c0_g1_i4_orf1	aldehyde dehydrogenase X, mitochondrial-like [Ostrinia furnacalis]	-1.87418	0.73404	-0.18552	0.7961	0.52956
TRINITY_DN80328_c0_g1_i9_orf1	arylphorin subunit alpha-like [Ostrinia furnacalis]	-1.88732	0.63307	-0.03579	0.96428	0.32576
TRINITY_DN578_c0_g1_i5_orf1	charged multivesicular body protein 7 [Ostrinia furnacalis]	-1.89702	0.82055	0.14821	0.83508	0.09319
TRINITY_DN1048_c0_g1_i6_orf1	uncharacterized protein LOC114360661 [Ostrinia furnacalis]	-1.83455	0.41615	0.37292	1.1455	-0.10002
TRINITY_DN6497_c0_g1_i1_orf1	ommochrome-binding protein-like [Ostrinia furnacalis]	-1.89209	0.29728	0.13028	1.08027	0.38426
TRINITY_DN64772_c0_g1_i1_orf1	aldehyde dehydrogenase, partial [Mythimna separata]	-1.88379	0.77701	-0.16566	0.70898	0.56347
TRINITY_DN14460_c0_g1_i6_orf1	scavenger receptor class B member 1-like [Ostrinia furnacalis]	-1.97983	0.71023	0.27956	0.43343	0.55662
TRINITY_DN8139_c0_g1_i2_orf1	synaptic vesicle glycoprotein 2B-like [Galleria mellonella]	-1.88173	0.36637	0.57326	-0.05452	0.99663
TRINITY_DN45220_c0_g1_i1_orf1	delta(3,5)-Delta(2,4)-dienoyl-CoA isomerase, mitochondrial isoform X1 [Ostrinia furnacalis]	-1.64302	1.34972	-0.42312	0.505	0.21142
TRINITY_DN48878_c0_g2_i1_orf1	codanin-1 [Ostrinia furnacalis]	-1.81547	0.57176	-0.29303	1.00344	0.53329
TRINITY_DN2803_c4_g1_i1_orf1	ornithine aminotransferase, mitochondrial isoform X2 [Ostrinia furnacalis]	-1.79949	1.21503	0.34464	0.38259	-0.14278
TRINITY_DN4565_c0_g2_i1_orf1	acid phosphatase type 7 isoform X2 [Ostrinia furnacalis]	-1.90357	0.43712	1.03612	0.31314	0.1172
TRINITY_DN805_c0_g1_i5_orf1	serine protease snake-like [Ostrinia furnacalis]	-1.96437	0.77123	0.18996	0.56145	0.44174
TRINITY_DN8037_c0_g2_i1_orf1	2-oxo-4-hydroxy-4-carboxy-5-ureidoimidazoline decarboxylase-like [Ostrinia furnacalis]	-1.90407	0.4802	0.95886	0.47375	-0.00874
TRINITY_DN1012_c0_g1_i2_orf1	teneurin-a isoform X1 [Ostrinia furnacalis]	-1.47982	1.4208	0.19809	-0.67885	0.53979
TRINITY_DN8367_c0_g2_i2_orf1	uncharacterized protein LOC114357075 [Ostrinia furnacalis]	-1.95901	0.28669	0.7085	0.24351	0.7203
TRINITY_DN12526_c0_g1_i5_orf1	uncharacterized protein LOC114359035 isoform X3 [Ostrinia furnacalis]	-1.66723	1.17556	-0.46935	0.2009	0.76011
TRINITY_DN80328_c0_g1_i5_orf1	arylphorin subunit alpha-like [Ostrinia furnacalis]	-1.86072	0.64937	-0.11539	0.99701	0.32973
TRINITY_DN32532_c0_g1_i1_orf1	fatty acyl-CoA hydrolase precursor, medium chain [Ostrinia furnacalis]	-1.88655	1.07573	0.26645	0.45187	0.0925
TRINITY_DN3616_c0_g1_i4_orf1	conotoxin ArMKLT2-032-like [Ostrinia furnacalis]	-1.42733	1.43024	-0.04762	0.69833	-0.65362
TRINITY_DN2472_c0_g1_i6_orf1	programmed cell death protein 6 isoform X1 [Colias croceus] >XP_045492459.1 programmed cell death protein 6 isoform X1 [Colias croceus]	-1.90907	0.18576	0.36408	1.04337	0.31586
TRINITY_DN1466_c0_g1_i4_orf1	insecticyanin-A-like [Ostrinia furnacalis]	-1.65595	0.46605	0.84162	0.97152	-0.62325
TRINITY_DN15812_c0_g1_i2_orf1	transferrin [Ostrinia furnacalis]	-1.78046	1.31766	0.15993	0.25703	0.04583
TRINITY_DN40439_c0_g1_i5_orf1	ommochrome binding protein 1, partial [Ostrinia nubilalis]	-1.89436	0.46684	0.14713	1.05978	0.22062
TRINITY_DN10138_c0_g1_i1_orf1	storage protein 1 [Omphisa fuscidentalis]	-1.76978	0.59723	-0.07945	1.22648	0.02552
TRINITY_DN105574_c0_g1_i1_orf1	prolow-density lipoprotein receptor-related protein 1, partial [Ostrinia furnacalis]	-1.94467	0.93431	0.32601	0.39161	0.29274
TRINITY_DN2141_c0_g1_i1_orf1	low density lipoprotein receptor adapter protein 1-like [Ostrinia furnacalis]	-1.56092	0.88735	1.2249	-0.02687	-0.52446
TRINITY_DN1304_c0_g1_i6_orf1	uncharacterized protein LOC114359545 [Ostrinia furnacalis]	-1.97302	0.73539	0.58395	0.40343	0.25025
TRINITY_DN10220_c1_g1_i7_orf1	uncharacterized protein LOC124645895 isoform X2 [Helicoverpa zea]	-1.84771	1.02261	0.48606	-0.18155	0.52059

TRINITY_DN892_c7_g1_i2_orf1	unnamed protein product [Diatraea saccharalis]	-1.84286	0.45857	0.60135	-0.21079	0.99374
TRINITY_DN7183_c0_g1_i2_orf1	seminal fluid protein CSSFP028 [Chilo suppressalis]	-1.75041	1.32529	0.38744	0.13895	-0.10126
TRINITY_DN811_c0_g1_i15_orf1	uncharacterized protein LOC114364160 [Ostrinia furnacalis]	-1.84474	0.3387	1.10487	0.50145	-0.10028
TRINITY_DN12256_c0_g1_i1_orf1	lysosome membrane protein 2-like [Ostrinia furnacalis]	-1.92425	0.85424	0.59134	0.0121	0.46658
TRINITY_DN125441_c0_g1_i5_orf1	KH domain-containing, RNA-binding, signal transduction-associated protein 2-like isoform X12 [Ostrinia furnacalis]	-1.89389	0.94752	0.69098	0.17933	0.07606
TRINITY_DN44073_c0_g1_i3_orf1	inter-alpha-trypsin inhibitor heavy chain H4-like isoform X11 [Ostrinia furnacalis]	-1.65582	1.3692	0.32426	0.3538	-0.39144
TRINITY_DN39266_c0_g1_i1_orf1	PREDICTED: NECAP-like protein CG9132 [Microplitis demolitor]	-1.95423	0.69299	0.22974	0.75655	0.27496
TRINITY_DN27035_c0_g1_i1_orf1	glucose-6-phosphate isomerase-like [Ostrinia furnacalis]	-1.84179	0.5647	-0.25442	0.92611	0.6054
TRINITY_DN1672_c0_g1_i6_orf1	cystinosin homolog isoform X1 [Ostrinia furnacalis] >XP_028162341.1 cystinosin homolog isoform X1 [Ostrinia furnacalis] >XP_028162342.1 cystinosin homolog isoform X1 [Ostrinia furnacalis] >XP_028162343.1 cystinosin homolog isoform X1 [Ostrinia furnacalis] >XP_028162344.1 cystinosin homolog isoform X1 [Ostrinia furnacalis]	-1.86241	0.60746	0.28602	-0.06836	1.03728
TRINITY_DN2798_c0_g1_i5_orf1	arylsulfatase B-like isoform X1 [Ostrinia furnacalis]	-1.94032	0.75365	0.40809	0.70358	0.07499
TRINITY_DN23398_c0_g1_i1_orf1	cytochrome P450 6B7-like [Ostrinia furnacalis]	-1.90957	0.81946	-0.04285	0.42792	0.70504
TRINITY_DN11514_c0_g1_i1_orf1	uncharacterized protein LOC114350079 [Ostrinia furnacalis]	-1.8344	1.04803	0.52924	-0.20571	0.46284
TRINITY_DN6243_c0_g1_i5_orf1	sorting nexin-20 [Ostrinia furnacalis]	-1.91531	0.93617	0.61538	0.11011	0.25365
TRINITY_DN2049_c1_g1_i3_orf1	luciferin 4-monooxygenase-like [Ostrinia furnacalis]	-1.50764	0.57208	-0.21881	1.49522	-0.34086
TRINITY_DN42856_c0_g1_i1_orf1	GSCOCG00007769001-RA-CDS [Cotesia congregata] >CAG5103393.1 Similar to GLDC: Glycine dehydrogenase (decarboxylating) [Cotesia congregata]	-1.9232	0.44652	0.90353	0.04032	0.53283
TRINITY_DN13098_c2_g1_i2_orf1	unnamed protein product [Arctia plantaginis] >CAB3253298.1 unnamed protein product [Arctia plantaginis]	-1.95508	0.3239	0.2367	0.8463	0.54818
TRINITY_DN6822_c0_g2_i4_orf1	gelsolin-like [Bicyclus anynana]	-1.82891	0.57086	1.15038	0.05857	0.04909
TRINITY_DN1030_c0_g1_i6_orf1	gamma-glutamyl hydrolase A-like isoform X1 [Ostrinia furnacalis]	-1.94665	0.53576	0.16821	0.37324	0.86943
TRINITY_DN2251_c0_g1_i4_orf1	serine hydroxymethyltransferase, cytosolic isoform X1 [Ostrinia furnacalis]	-1.9095	0.07408	0.81015	0.80106	0.22422
TRINITY_DN12865_c0_g1_i1_orf1	synaptic vesicle membrane protein VAT-1 homolog-like [Ostrinia furnacalis]	-1.65225	1.48141	0.18225	0.13962	-0.15102
TRINITY_DN3301_c0_g1_i2_orf1	hemicentin-2-like isoform X1 [Ostrinia furnacalis]	-1.83462	0.70146	-0.05343	1.05981	0.12678
TRINITY_DN20680_c0_g1_i5_orf1	tsukushin isoform X2 [Ostrinia furnacalis]	-1.6882	1.40701	0.10571	-0.18034	0.35582
TRINITY_DN4464_c0_g2_i1_orf1	glypican-6 [Pectinophora gossypiella]	-1.92471	0.98681	0.42756	0.32098	0.18936
TRINITY_DN18388_c0_g1_i6_orf1	serine protease [Ostrinia furnacalis]	-1.86917	0.98296	0.18209	0.71188	-0.00776
TRINITY_DN6205_c0_g1_i1_orf1	phenoloxidase-activating factor 2-like [Ostrinia furnacalis]	-1.67876	0.96468	0.29831	0.94128	-0.5255
TRINITY_DN21533_c0_g1_i4_orf1	hypothetical protein evm_010931 [Chilo suppressalis]	-1.92014	0.50601	0.98082	0.19303	0.24028
TRINITY_DN45037_c0_g1_i1_orf1	trafficking protein particle complex subunit 1 [Ostrinia furnacalis]	-1.75652	0.51292	-0.19489	1.25727	0.18122
TRINITY_DN8771_c0_g1_i5_orf1	regucalcin-like [Ostrinia furnacalis]	-1.86074	1.10543	0.37132	-0.03609	0.42009
TRINITY_DN15597_c0_g1_i1_orf1	microsomal glutathione S-transferase 1-like [Ostrinia furnacalis]	-1.9021	0.76095	0.22322	0.05162	0.86631
TRINITY_DN19043_c0_g2_i1_orf1	hypothetical protein EVAR_60653_1 [Eumeta japonica]	-1.27176	1.81138	-0.12537	-0.20385	-0.2104
TRINITY_DN19748_c0_g1_i4_orf1	PREDICTED: cysteine-rich hydrophobic domain-containing protein 2 [Amyelois transitella] >XP_028158573.1 cysteine-rich hydrophobic domain-containing protein 2 isoform X1 [Ostrinia furnacalis] >CAH0685427.1 unnamed protein product [Chilo suppressalis]	-1.90522	1.0391	0.39372	0.12705	0.34534
TRINITY_DN3039_c0_g2_i1_orf1	uncharacterized protein LOC114353136 [Ostrinia furnacalis]	-1.96412	0.32785	0.27738	0.81085	0.54804
TRINITY_DN81031_c0_g1_i1_orf1	aldehyde dehydrogenase, partial [Ectropis obliqua]	-1.88612	0.77383	-0.1317	0.7842	0.45979
TRINITY_DN71863_c0_g1_i2_orf1	unnamed protein product [Diatraea saccharalis]	-1.87199	0.45201	-0.10369	1.00659	0.51707
TRINITY_DN4449_c0_g2_i1_orf1	calcium/calmodulin-dependent protein kinase type 1 isoform X3 [Cephus cinctus]	-1.98718	0.54699	0.28688	0.52896	0.62436
TRINITY_DN428_c0_g1_i8_orf1	phenoloxidase-activating factor 2-like isoform X1 [Ostrinia furnacalis]	-1.97046	0.58433	0.32232	0.76222	0.30159
TRINITY_DN45097_c0_g1_i5_orf1	ras-related protein Rab-24-like [Ostrinia furnacalis]	-1.97033	0.66916	0.6937	0.35016	0.2573
TRINITY_DN376_c1_g1_i1_orf1	matrix metalloproteinase-25-like [Ostrinia furnacalis]	-1.77702	1.19591	-0.19412	0.57975	0.19548
TRINITY_DN9420_c0_g1_i2_orf1	protein PFC0760c-like isoform X2 [Ostrinia furnacalis]	-1.70113	0.45636	1.34286	0.16283	-0.26093
TRINITY_DN125140_c0_g1_i1_orf1	glycogen debranching enzyme isoform X2 [Ostrinia furnacalis] >XP_028161358.1 glycogen debranching enzyme isoform X2 [Ostrinia furnacalis] >XP_028161359.1 glycogen debranching enzyme isoform X2 [Ostrinia furnacalis]	-1.91165	0.50471	0.74014	0.73386	-0.06706
TRINITY_DN2133_c0_g2_i1_orf1	glycogen debranching enzyme isoform X1 [Ostrinia furnacalis]	-1.90851	0.18858	0.84849	0.76918	0.10226
TRINITY_DN1651_c0_g2_i1_orf1	UBX domain-containing protein 6 [Ostrinia furnacalis] >XP_028162119.1 UBX domain-containing protein 6 [Ostrinia furnacalis]	-1.91225	0.85411	0.23375	0.74336	0.08103
TRINITY_DN5420_c0_g1_i2_orf1	DNA-directed RNA polymerase II subunit RPB1-like [Ostrinia furnacalis]	-1.5851	1.55402	0.07708	0.15793	-0.20393
TRINITY_DN18128_c0_g1_i4_orf1	arylsulfatase B [Ostrinia furnacalis]	-1.82085	0.59579	-0.23449	1.05335	0.40621
TRINITY_DN12661_c0_g1_i3_orf1	T-complex protein 11-like protein 1 [Ostrinia furnacalis]	-1.63723	0.81103	1.24283	-0.33136	-0.08528
TRINITY_DN1710_c0_g1_i1_orf1	nuclear factor NF-kappa-B p105 subunit-like isoform X2 [Ostrinia furnacalis]	-1.95556	0.47416	0.26973	0.87427	0.33741
TRINITY_DN53358_c0_g1_i3_orf1	arylphorin subunit alpha-like [Ostrinia furnacalis]	-1.67377	0.20212	-0.30179	1.38372	0.38972
TRINITY_DN1664_c0_g1_i4_orf1	uncharacterized protein LOC114355246 [Ostrinia furnacalis]	-1.81033	1.09041	0.72407	0.06649	-0.07064
TRINITY_DN7854_c0_g1_i4_orf1	failed axon connections [Ostrinia furnacalis]	-1.51941	1.63711	0.02109	-0.09377	-0.04502
TRINITY_DN42275_c0_g1_i1_orfp1	TRINITY_DN42275_c0_g1_i1_m.44265 TRINITY_DN42275_c0_g1::TRINITY_DN42275_c0_g1_i1::g.44265 ORF type:internal len:76 (+),score=8.67 TRINITY_DN42275_c0_g1_i1:1-225(+)	-1.70278	0.76157	-0.02276	1.20842	-0.24445
TRINITY_DN79657_c0_g1_i1_orf1	uncharacterized protein LOC114349955 [Ostrinia furnacalis]	-1.96816	0.45246	0.57096	0.19908	0.74565

TRINITY_DN5488_c0_g1_i5_orf1	alpha-amylase-like [Ostrinia furnacalis]	-1.96059	0.66826	0.56371	0.6156	0.11303
TRINITY_DN74654_c0_g1_i4_orf1	limulus clotting factor C-like isoform X4 [Ostrinia furnacalis]	-1.97877	0.56655	0.34956	0.72711	0.33555
TRINITY_DN18230_c1_g2_i1_orf1	hypothetical protein O3G_MSEX004459 [Manduca sexta]	-1.97504	0.68332	0.2165	0.59835	0.47687
TRINITY_DN4228_c0_g1_i5_orf1	phenoloxidase-activating enzyme-like [Ostrinia furnacalis]	-1.76644	1.32909	0.04266	0.06787	0.32681
TRINITY_DN843_c0_g1_i2_orf1	unnamed protein product [Diatraea saccharalis]	-0.72084	1.68204	0.60207	-0.59892	-0.96434
TRINITY_DN2943_c2_g2_i1_orf1	protein phosphatase inhibitor 2-like [Ostrinia furnacalis]	-1.99637	0.55471	0.57294	0.40976	0.45896
TRINITY_DN2794_c1_g1_i8_orf1	carboxypeptidase D [Ostrinia furnacalis]	-1.53005	1.59237	0.19351	-0.2909	0.03508
TRINITY_DN2407_c0_g1_i6_orf1	uncharacterized protein LOC114366345 isoform X2 [Ostrinia furnacalis]	-1.77844	1.31896	0.01877	0.21652	0.2242
TRINITY_DN6244_c0_g1_i4_orf1	uncharacterized protein CG3556 [Ostrinia furnacalis]	-1.57853	1.56475	0.19419	-0.14418	-0.03622
TRINITY_DN6108_c0_g1_i5_orf1	myogenesis-regulating glycosidase-like [Ostrinia furnacalis]	-1.73776	0.16979	0.29278	1.36306	-0.08788
TRINITY_DN22242_c0_g2_i1_orf1	juvenile hormone epoxide hydrolase-like [Ostrinia furnacalis] >XP_028170526.1 juvenile hormone epoxide hydrolase-like [Ostrinia furnacalis]	-1.9324	0.32657	0.3865	0.2447	0.97463
TRINITY_DN3949_c0_g1_i1_orf1	probable cytochrome P450 304a1 [Ostrinia furnacalis]	-1.75378	1.31291	-0.14141	0.36534	0.21695
TRINITY_DN8480_c0_g1_i1_orf1	lysosomal Pro-X carboxypeptidase [Ostrinia furnacalis]	-1.61566	1.37775	0.52292	-0.44032	0.15532
TRINITY_DN7633_c0_g1_i1_orf1	prolow-density lipoprotein receptor-related protein 1, partial [Ostrinia furnacalis]	-1.89737	0.93199	0.00444	0.29384	0.6671
TRINITY_DN1659_c0_g1_i3_orf1	beta-catenin-like protein 1 [Ostrinia furnacalis]	-1.98826	0.56574	0.31345	0.4724	0.63667
TRINITY_DN9542_c0_g1_i4_orf1	NAD(P)H-hydrate epimerase [Ostrinia furnacalis]	-1.9868	0.49844	0.31447	0.49669	0.67719
TRINITY_DN10539_c0_g1_i1_orf1	uncharacterized protein LOC114358962 isoform X1 [Ostrinia furnacalis]	-1.75038	0.30085	-0.16413	1.31514	0.29852
TRINITY_DN47609_c0_g1_i1_orfp1	TRINITY_DN47609_c0_g1_i1_m.57205 TRINITY_DN47609_c0_g1::TRINITY_DN47609_c0_g1_i1::g.57205 ORF type:5prime_partial len:68 (-),score=15.96 TRINITY_DN47609_c0_g1_i1:36-239(-)	-1.81006	0.19633	0.09064	1.26998	0.25311
TRINITY_DN43431_c0_g1_i1_orf1	glycine dehydrogenase (decarboxylating), mitochondrial isoform X1 [Ostrinia furnacalis] >XP_028174269.1 glycine dehydrogenase (decarboxylating), mitochondrial isoform X3 [Ostrinia furnacalis]	-1.76882	1.20945	0.63563	-0.00998	-0.06628
TRINITY_DN13411_c0_g1_i4_orf1	facilitated trehalose transporter Tret1-like [Ostrinia furnacalis]	-1.97824	0.43877	0.65252	0.24994	0.63701
TRINITY_DN15961_c0_g1_i1_orf1	uncharacterized protein LOC113522423 [Galleria mellonella]	-1.91172	0.94171	0.02293	0.54255	0.40452
TRINITY_DN8245_c0_g1_i3_orf1	uncharacterized protein LOC114357622 [Ostrinia furnacalis]	-1.95898	0.72716	0.28502	0.70164	0.24515
TRINITY_DN364_c0_g1_i2_orf1	uncharacterized protein LOC114366657, partial [Ostrinia furnacalis]	-1.77715	0.7245	1.14092	-0.11902	0.03075
TRINITY_DN5467_c0_g1_i5_orf1	synaptic vesicle glycoprotein 2B-like isoform X2 [Ostrinia furnacalis] >XP_028161209.1 synaptic vesicle glycoprotein 2B-like isoform X2 [Ostrinia furnacalis] >XP_028161210.1 synaptic vesicle glycoprotein 2B-like isoform X2 [Ostrinia furnacalis]	-1.41563	1.69305	0.02883	-0.35543	0.04919
TRINITY_DN1507_c0_g1_i5_orf1	27 kDa hemolymph protein-like, partial [Ostrinia furnacalis]	-1.73512	1.2881	0.55902	-0.13143	0.01942
TRINITY_DN9733_c0_g1_i2_orf1	acidic juvenile hormone-suppressible protein 1-like [Ostrinia furnacalis]	-1.44754	0.86239	-0.22929	1.34974	-0.53529
TRINITY_DN479_c6_g1_i2_orf1	beta-1,3-glucan-binding protein-like [Ostrinia furnacalis]	-1.5387	1.56993	0.32255	-0.1535	-0.20028
TRINITY_DN2921_c1_g1_i4_orf1	uncharacterized protein LOC114352340 isoform X2 [Ostrinia furnacalis]	-1.96165	0.44564	0.62477	0.15767	0.73357
TRINITY_DN7754_c0_g1_i2_orf1	carboxypeptidase E-like isoform X1 [Ostrinia furnacalis] >XP_028174275.1 carboxypeptidase E-like isoform X2 [Ostrinia furnacalis] >XP_028174276.1 carboxypeptidase E-like isoform X2 [Ostrinia furnacalis]	-1.96649	0.63731	0.74615	0.29624	0.28679
TRINITY_DN2035_c0_g1_i1_orf1	uncharacterized protein LOC114356683 [Ostrinia furnacalis]	-1.96355	0.77322	0.59139	0.39307	0.20588
TRINITY_DN338_c1_g1_i9_orf1	scolexin B-like isoform X2 [Ostrinia furnacalis]	-1.91008	0.81459	0.25117	0.78856	0.05576
TRINITY_DN1423_c0_g1_i8_orf1	ferritin subunit-like [Ostrinia furnacalis] >XP_028168186.1 ferritin subunit-like [Ostrinia furnacalis]	-1.52962	1.52636	-0.27149	0.4683	-0.19355
TRINITY_DN113353_c0_g1_i1_orf1	unnamed protein product [Parnassius apollo]	-1.78032	1.12965	-0.23506	0.67399	0.21173
TRINITY_DN7618_c0_g1_i4_orf1	uncharacterized protein LOC114366712 isoform X1 [Ostrinia furnacalis]	-1.63196	1.44701	-0.28054	0.06563	0.39986
TRINITY_DN1093_c0_g1_i6_orf1	uncharacterized protein LOC114361723 isoform X4 [Ostrinia furnacalis]	-1.94947	0.71885	0.09077	0.68166	0.45819
TRINITY_DN44857_c0_g1_i4_orf1	UPF0183 protein CG7083 [Ostrinia furnacalis]	-1.8519	0.58841	0.36316	-0.13594	1.03627
TRINITY_DN198_c2_g1_i2_orf1	solute carrier organic anion transporter family member 5A1-like isoform X1 [Ostrinia furnacalis]	-1.7231	1.35671	0.13983	-0.15601	0.38257
TRINITY_DN2227_c0_g1_i5_orf1	protein 60A [Ostrinia furnacalis]	-1.65243	1.37725	0.33267	-0.38952	0.33204
TRINITY_DN391_c0_g1_i4_orf1	phenoloxidase-activating enzyme-like [Ostrinia furnacalis]	-1.48909	1.64125	-0.27718	0.10877	0.01625
TRINITY_DN2606_c0_g1_i5_orf1	galectin-4-like isoform X1 [Ostrinia furnacalis]	-1.96349	0.68315	0.15299	0.66156	0.46578
TRINITY_DN64141_c0_g1_i4_orf1	probable salivary secreted peptide [Ostrinia furnacalis]	-1.74136	0.94501	-0.35626	0.95276	0.19984
TRINITY_DN3109_c0_g1_i5_orf1	protein takeout isoform X2 [Ostrinia furnacalis]	-1.80446	1.07515	0.7659	-0.03722	0.00062
TRINITY_DN15774_c0_g1_i3_orf1	uncharacterized protein LOC114366599 [Ostrinia furnacalis]	-1.94748	0.80917	0.27256	0.66037	0.20538
TRINITY_DN2171_c0_g1_i1_orf1	probable pterin-4-alpha-carbinolamine dehydratase isoform X1 [Ostrinia furnacalis]	-1.89605	1.02984	0.26072	0.08693	0.51856
TRINITY_DN5667_c0_g1_i4_orf1	spodomicin-like [Ostrinia furnacalis] >QKV49445.1 diapausin [Ostrinia furnacalis]	-1.92224	0.73635	0.26706	0.82638	0.09244
TRINITY_DN661_c0_g1_i1_orf1	hypothetical protein evm_002822 [Chilo suppressalis]	-0.73025	1.92367	-0.01876	-0.78222	-0.39243
TRINITY_DN28661_c0_g1_i1_orf1	cathepsin B [Ostrinia furnacalis]	-1.76173	1.22559	0.59612	-0.16612	0.10615
TRINITY_DN993_c0_g1_i7_orf1	apolipophorins-like [Ostrinia furnacalis]	-1.54562	1.32924	-0.41864	0.80089	-0.16588
TRINITY_DN65299_c0_g4_i1_orf1	LOW QUALITY PROTEIN: signal transducing adapter molecule 2 [Ostrinia furnacalis]	-1.7275	0.95975	1.02117	-0.22598	-0.02744
TRINITY_DN125150_c0_g1_i1_orf1	aldehyde dehydrogenase, dimeric NADP-preferring isoform X5 [Ostrinia furnacalis]	-1.89764	0.88808	0.24927	0.0202	0.74008
TRINITY_DN5383_c0_g1_i4_orf1	vacuolar protein sorting-associated protein 35 [Ostrinia furnacalis]	-1.90082	0.77525	0.78256	-0.0679	0.41092
TRINITY_DN106479_c1_g1_i1_orf1	secretory phospholipase A2 receptor-like [Ostrinia furnacalis]	-1.9021	0.52408	0.8137	0.66033	-0.096
TRINITY_DN399_c3_g2_i6_orf1	proline-rich extensin-like protein EPR1 [Ostrinia furnacalis]	-1.8954	0.60869	-0.08364	0.89849	0.47187

TRINITY_DN10090_c0_g1_i1_orf1	clotting factor B-like isoform X1 [Ostrinia furnacalis] >XP_028163447.1 clotting factor B-like isoform X2 [Ostrinia furnacalis] >XP_028163448.1 clotting factor B-like isoform X3 [Ostrinia furnacalis]	-1.8629	1.16435	0.17151	0.31637	0.21067
TRINITY_DN2745_c0_g1_i4_orf1	Tubulin alpha-1 chain [Harpegnathos saltator]	-1.89712	0.09405	1.00888	0.5687	0.2255
TRINITY_DN46625_c0_g1_i1_orf1	ferritin subunit isoform X1 [Belonocnema kinseyi]	-1.46295	1.49897	-0.45797	0.60728	-0.18533
TRINITY_DN30498_c0_g1_i3_orf1	lipase 3-like [Ostrinia furnacalis]	-1.7273	0.90718	0.04327	1.05545	-0.27861
TRINITY_DN48602_c0_g1_i6_orf1	amidophosphoribosyltransferase-like isoform X1 [Ostrinia furnacalis] >XP_028157758.1 amidophosphoribosyltransferase-like isoform X1 [Ostrinia furnacalis] >XP_028157759.1 amidophosphoribosyltransferase-like isoform X1 [Ostrinia furnacalis]	-1.65412	0.35507	1.07896	0.79904	-0.57895
TRINITY_DN29217_c0_g1_i3_orf1	protein yippee-like 5 [Ostrinia furnacalis]	-1.93935	0.83411	0.16419	0.66214	0.27892
TRINITY_DN1054_c0_g1_i8_orf1	tubulin-specific chaperone D [Ostrinia furnacalis]	-1.83962	0.36117	1.01264	-0.18626	0.65208
TRINITY_DN48765_c0_g1_i7_orf1	uncharacterized protein LOC114352307 [Ostrinia furnacalis]	-1.58697	1.16355	0.74182	0.35398	-0.67238
TRINITY_DN53866_c0_g1_i1_orf1	larval/pupal cuticle protein H1C-like [Ostrinia furnacalis]	-0.88315	1.92645	-0.09137	-0.62989	-0.32205
TRINITY_DN13303_c0_g1_i6_orf1	membrane-associated progesterone receptor component 1-like [Ostrinia furnacalis]	-1.95949	0.72513	0.69333	0.33178	0.20925
TRINITY_DN4900_c0_g1_i6_orf1	hypothetical protein evm_001963 [Chilo suppressalis]	-1.96778	0.44921	0.48915	0.79484	0.23459
TRINITY_DN3251_c0_g1_i6_orf1	fatty-acid amide hydrolase 2-like [Ostrinia furnacalis] >XP_028167366.1 fatty-acid amide hydrolase 2-like [Ostrinia furnacalis] >XP_028167367.1 fatty-acid amide hydrolase 2-like [Ostrinia furnacalis] >XP_028167368.1 fatty-acid amide hydrolase 2-like [Ostrinia furnacalis] >XP_028167369.1 fatty-acid amide hydrolase 2-like [Ostrinia furnacalis] >XP_028167370.1 fatty-acid amide hydrolase 2-like [Ostrinia furnacalis] >XP_028167371.1 fatty-acid amide hydrolase 2-like [Ostrinia furnacalis] >XP_028167372.1 fatty-acid amide hydrolase 2-like [Ostrinia furnacalis]	-1.87812	1.11383	0.35537	0.30974	0.09919
TRINITY_DN1287_c0_g1_i5_orf1	probable chitinase 10 isoform X6 [Ostrinia furnacalis]	-1.84593	0.88095	-0.23363	0.74684	0.45177
TRINITY_DN2515_c0_g1_i6_orf1	chitooligosaccharidolytic beta-N-acetylglucosaminidase isoform X1 [Ostrinia furnacalis]	-1.26707	1.80704	-0.16219	-0.31439	-0.06339
TRINITY_DN7579_c1_g3_i1_orf1	peroxiredoxin-5, mitochondrial [Ostrinia furnacalis]	-1.94803	0.8543	0.39985	0.54024	0.15364
TRINITY_DN81488_c0_g1_i1_orf1	apolipophorins-like [Ostrinia furnacalis]	-1.4803	1.38699	-0.47209	0.78382	-0.21843
TRINITY_DN50517_c0_g1_i1_orf1	cuticle protein 21-like [Ostrinia furnacalis]	-1.95009	0.86277	0.55587	0.23001	0.30144
TRINITY_DN688_c0_g1_i8_orf1	lysosomal alpha-mannosidase-like [Ostrinia furnacalis]	-1.42464	1.70856	-0.03125	-0.03065	-0.22202
TRINITY_DN126127_c0_g1_i1_orf1	prolow-density lipoprotein receptor-related protein 1, partial [Ostrinia furnacalis]	-1.85492	1.08292	-0.00679	0.18539	0.5934
TRINITY_DN3513_c0_g1_i5_orf1	vacuolar protein sorting-associated protein 16 homolog [Ostrinia furnacalis]	-1.89495	1.01439	0.24518	0.56081	0.07457
TRINITY_DN1098_c1_g1_i4_orf1	lysozyme 10 [Ostrinia furnacalis]	-1.74837	0.95525	-0.43624	0.40885	0.82052
TRINITY_DN19690_c0_g1_i1_orf1	unnamed protein product [Chilo suppressalis]	-1.9602	0.55713	0.24917	0.33262	0.82127
TRINITY_DN1196_c0_g1_i4_orf1	glucosamine-6-phosphate isomerase isoform X2 [Ostrinia furnacalis]	-0.65979	1.7745	0.42066	-0.94114	-0.59424
TRINITY_DN31611_c0_g1_i2_orf1	glucose-6-phosphate isomerase-like [Ostrinia furnacalis]	-1.91613	0.28065	0.37304	1.02723	0.23521
TRINITY_DN2425_c0_g1_i3_orf1	sialic acid synthase [Ostrinia furnacalis]	-1.68305	1.42428	-0.14534	0.06788	0.33623
TRINITY_DN36899_c0_g1_i1_orf1	glucose dehydrogenase [FAD, quinone]-like [Ostrinia furnacalis]	-1.32176	1.69307	0.2017	-0.58785	0.01484
TRINITY_DN1423_c0_g1_i4_orf1	hypothetical protein evm_003306 [Chilo suppressalis] >CAB3526495.1 unnamed protein product [Chilo suppressalis] >CAH0403823.1 unnamed protein product [Chilo suppressalis]	-1.60136	1.4895	-0.21595	0.40544	-0.07763
TRINITY_DN8780_c0_g1_i3_orf1	uncharacterized protein LOC114363370 [Ostrinia furnacalis]	-1.80116	0.15202	-0.07105	1.21233	0.50786
TRINITY_DN364_c0_g2_i1_orf1	unnamed protein product [Chilo suppressalis]	-1.75605	0.83851	1.09362	-0.05883	-0.11724
TRINITY_DN3616_c0_g2_i1_orf1	conotoxin ArMKLT2-032-like [Ostrinia furnacalis]	-1.10293	1.87404	-0.05386	-0.43413	-0.28311
TRINITY_DN6162_c1_g1_i1_orf1	hsc70-interacting protein-like [Ostrinia furnacalis]	-1.91103	0.5425	0.80655	-0.06917	0.63115
TRINITY_DN2205_c0_g1_i3_orf1	probable chitinase 2 [Ostrinia furnacalis]	-1.88584	0.92709	-0.03339	0.28294	0.70919
TRINITY_DN16316_c0_g1_i7_orf1	exocyst complex component 6 isoform X1 [Ostrinia furnacalis] >XP_028166671.1 exocyst complex component 6 isoform X2 [Ostrinia furnacalis]	-1.51888	0.83452	0.81025	0.75319	-0.87908
TRINITY_DN9718_c0_g1_i7_orf1	uncharacterized protein LOC114365184 [Ostrinia furnacalis] >XP_028177482.1 uncharacterized protein LOC114365184 [Ostrinia furnacalis] >XP_028177483.1 uncharacterized protein LOC114365184 [Ostrinia furnacalis]	-1.99649	0.39588	0.51001	0.58058	0.51001
TRINITY_DN1694_c0_g1_i1_orf1	cuticle protein 16.5-like [Ostrinia furnacalis]	-1.90127	0.23512	0.33431	1.07287	0.25896
TRINITY_DN4676_c0_g1_i16_orf1	meiosis-specific nuclear structural protein 1-like isoform X2 [Ostrinia furnacalis]	-0.83685	1.89343	0.10404	-0.45735	-0.70327
TRINITY_DN1497_c0_g2_i6_orf1	unnamed protein product [Chilo suppressalis]	-1.78816	0.45609	1.22265	0.27106	-0.16164
TRINITY_DN31310_c0_g1_i1_orf1	PREDICTED: multiple epidermal growth factor-like domains protein 10 isoform X3 [Polistes canadensis]	-1.97065	0.80102	0.3132	0.49913	0.3573
TRINITY_DN1957_c0_g1_i4_orf1	NAD kinase 2, mitochondrial [Ostrinia furnacalis]	-1.8114	0.36452	-0.26141	1.02515	0.68314
TRINITY_DN913_c0_g1_i6_orf1	uncharacterized protein LOC114354578 [Ostrinia furnacalis]	-1.96738	0.74717	0.29308	0.6301	0.29703
TRINITY_DN5064_c0_g1_i4_orf1	sortilin-related receptor-like [Ostrinia furnacalis]	-1.64119	1.38948	0.42395	-0.38718	0.21493
TRINITY_DN104_c0_g1_i4_orf1	PREDICTED: heparan-alpha-glucosaminide N-acetyltransferase [Amyelois transitella]	-1.28052	1.67067	0.32396	-0.68047	-0.03364
TRINITY_DN4076_c1_g2_i2_orf1	vacuole membrane protein 1 [Ostrinia furnacalis]	-1.89235	0.64851	-0.1421	0.78744	0.59851
TRINITY_DN5813_c0_g1_i9_orf1	alpha-(1,6)-fucosyltransferase [Ostrinia furnacalis]	-1.86547	-0.00081	1.10813	0.4274	0.33075
TRINITY_DN18230_c1_g1_i1_orf1	glycine dehydrogenase (decarboxylating), mitochondrial isoform X1 [Ostrinia furnacalis] >XP_028174269.1 glycine dehydrogenase (decarboxylating), mitochondrial isoform X3 [Ostrinia furnacalis]	-1.95002	0.31575	0.91274	0.31428	0.40725
TRINITY_DN6988_c0_g1_i3_orf1	cuticle protein 1-like [Ostrinia furnacalis]	-1.8483	0.88877	-0.0593	0.87776	0.14107
TRINITY_DN4276_c0_g1_i11_orf1	arylsulfatase B [Ostrinia furnacalis]	-1.90964	0.9702	0.35108	0.53462	0.05375
TRINITY_DN23746_c0_g1_i2_orf1	protein 4.1 homolog isoform X1 [Ostrinia furnacalis]	-1.65725	1.18754	-0.07233	0.85887	-0.31684

TRINITY_DN96739_c0_g1_i1_orf1	annexin A6, isoform CRA_b [Homo sapiens]	-1.57695	1.15035	0.54816	-0.72488	0.60333
TRINITY_DN5132_c0_g1_i4_orf1	small heat shock protein Hsp24.2 [Ostrinia furnacalis]	-1.73921	0.72032	-0.51867	0.71778	0.81979
TRINITY_DN2170_c4_g1_i2_orf1	beta-1,3-glucan-binding protein-like isoform X1 [Ostrinia furnacalis]	-1.99367	0.52217	0.41939	0.63385	0.41826
TRINITY_DN10824_c0_g1_i3_orf1	endochitinase isoform X2 [Ostrinia furnacalis]	-0.69765	1.96431	-0.1412	-0.58999	-0.53547
TRINITY_DN12686_c0_g1_i4_orf1	maltase A1-like [Ostrinia furnacalis]	-1.57478	0.11062	1.25123	0.78454	-0.57161
TRINITY_DN146138_c0_g1_i1_orf1	E3 ubiquitin-protein ligase Ubr3 [Chelonus insularis] >XP_034951823.1 E3 ubiquitin-protein ligase Ubr3 [Chelonus insularis] >XP_034951824.1 E3 ubiquitin-protein ligase Ubr3 [Chelonus insularis]	-1.89307	0.73978	0.70144	0.59668	-0.14483
TRINITY_DN12003_c0_g2_i1_orf1	lactoylglutathione lyase [Ostrinia furnacalis]	-1.90335	0.10543	1.02799	0.46552	0.30441
TRINITY_DN10371_c0_g1_i2_orf1	heterogeneous nuclear ribonucleoprotein L isoform X2 [Vanessa cardui]	-1.80931	0.68237	0.14953	-0.12805	1.10545
TRINITY_DN34166_c0_g1_i1_orf1	hypothetical protein EVAR_80688_1 [Eumeta japonica]	-1.99752	0.55308	0.46223	0.42794	0.55428
TRINITY_DN97042_c0_g1_i6_orf1	apolipophorins-like [Ostrinia furnacalis]	-1.49966	1.34136	-0.46595	0.83166	-0.2074
TRINITY_DN745_c7_g1_i1_orf1	uncharacterized protein LOC114358822 [Ostrinia furnacalis]	-1.75506	0.71535	-0.26802	1.14433	0.1634
TRINITY_DN70485_c0_g1_i2_orf1	serine/threonine-protein kinase Genghis Khan-like [Ostrinia furnacalis]	-1.5568	1.56618	0.1462	0.13433	-0.2899
TRINITY_DN2483_c0_g1_i1_orf1	UDP-glucuronosyltransferase 2B10-like [Ostrinia furnacalis]	-1.93083	0.14108	0.28512	0.89151	0.61312
TRINITY_DN9615_c0_g1_i1_orf1	uncharacterized protein LOC114352730 [Ostrinia furnacalis]	-1.79107	1.24453	-0.02892	0.48371	0.09175
TRINITY_DN7128_c0_g1_i7_orf1	dystroglycan [Ostrinia furnacalis]	-1.63623	1.45888	0.39003	-0.20554	-0.00713
TRINITY_DN8766_c0_g1_i1_orf1	prolow-density lipoprotein receptor-related protein 1, partial [Ostrinia furnacalis]	-1.84438	0.90036	0.31127	-0.17886	0.81162
TRINITY_DN2097_c1_g2_i2_orf1	serine protease inhibitor 3 [Ostrinia furnacalis]	-1.69015	1.32318	-0.35009	0.43897	0.27809
TRINITY_DN2796_c0_g1_i28_orf1	phosphofurin acidic cluster sorting protein 2 isoform X1 [Pectinophora gossypiella]	-1.95852	0.79806	0.34301	0.21441	0.60305
TRINITY_DN8724_c0_g1_i2_orf1	vesicle-associated membrane protein/synaptobrevin-binding protein isoform X2 [Pectinophora gossypiella]	-1.85543	1.01451	0.71257	0.14217	-0.01382
TRINITY_DN61777_c0_g1_i4_orf1	exocyst complex component 2 [Ostrinia furnacalis]	-1.9687	0.52329	0.21758	0.77086	0.45697
TRINITY_DN45271_c0_g1_i1_orf1	double-strand break repair protein MRE11 [Ostrinia furnacalis]	-1.66698	1.47481	0.21233	0.00999	-0.03015
TRINITY_DN92_c1_g2_i1_orf1	SID1 transmembrane family member 1-like isoform X3 [Ostrinia furnacalis]	-1.95332	0.7009	0.71713	0.13392	0.40137
TRINITY_DN7868_c0_g1_i2_orf1	uncharacterized protein LOC114353432 isoform X4 [Ostrinia furnacalis]	-1.8676	0.93337	0.20381	0.773	-0.04257
TRINITY_DN21856_c0_g1_i1_orf1	anti-lipopolysaccharide factor 3 [Eriocheir sinensis]	-1.9028	0.87912	0.74598	0.06324	0.21446
TRINITY_DN85412_c0_g1_i1_orf1	unnamed protein product [Diatraea saccharalis]	-1.41217	1.44709	-0.52177	0.75379	-0.26695
TRINITY_DN63943_c0_g1_i5_orf1	uncharacterized protein LOC114357144 [Ostrinia furnacalis]	-1.79021	1.19698	0.10267	-0.09494	0.5855
TRINITY_DN2508_c0_g1_i2_orf1	uncharacterized protein LOC114361845 [Ostrinia furnacalis] >XP_028172853.1 uncharacterized protein LOC114361845 [Ostrinia furnacalis]	-1.7591	1.33366	0.03774	0.03523	0.35247
TRINITY_DN13997_c0_g1_i5_orf1	28 kDa heat- and acid-stable phosphoprotein [Ostrinia furnacalis]	-1.89894	0.8816	0.18111	0.76045	0.07578
TRINITY_DN699_c0_g2_i1_orf1	TPA_exp: putative parasitoid killing factor [Trichoplusia ni]	-1.42515	1.46045	-0.54334	0.70775	-0.19972
TRINITY_DN1080_c0_g1_i1_orf1	bleomycin hydrolase [Ostrinia furnacalis]	-1.90828	0.58866	0.95142	0.32371	0.04449
TRINITY_DN5070_c0_g1_i1_orf1	ATP-dependent (S)-NAD(P)H-hydrate dehydratase-like [Ostrinia furnacalis]	-1.48933	1.46269	0.07369	0.54035	-0.5874
TRINITY_DN26149_c0_g1_i5_orf1	thymosin beta isoform X3 [Ostrinia furnacalis]	-1.40772	1.70773	-0.3125	-0.03986	0.05235
TRINITY_DN76216_c0_g2_i3_orf1	lysosomal alpha-mannosidase isoform X1 [Pieris rapae]	-1.51791	1.50096	-0.41152	0.51584	-0.08737
TRINITY_DN8985_c0_g1_i4_orf1	cytochrome P450 6B6-like [Ostrinia furnacalis]	-1.6869	1.00686	-0.55766	0.79728	0.44042

TRINITY_DN2848_c0_g1_i2_orf1	glyceraldehyde-3-phosphate dehydrogenase isoform 1 [Homo sapiens] >NP_001276675.1 glyceraldehyde-3-phosphate dehydrogenase isoform 1 [Homo sapiens] >NP_002037.2 glyceraldehyde-3-phosphate dehydrogenase isoform 1 [Homo sapiens] >XP_003819180.1 glyceraldehyde-3-phosphate dehydrogenase [Pan paniscus] >XP_004052609.1 glyceraldehyde-3-phosphate dehydrogenase [Gorilla gorilla gorilla] >XP_008971979.1 glyceraldehyde-3-phosphate dehydrogenase [Pan paniscus] >XP_008971980.1 glyceraldehyde-3-phosphate dehydrogenase [Pan paniscus] >XP_032621678.1 glyceraldehyde-3-phosphate dehydrogenase [Chelonoidis abingdonii] >XP_508955.1 glyceraldehyde-3-phosphate dehydrogenase isoform X1 [Pan troglodytes] >P04406.3 RecName: Full=Glyceraldehyde-3-phosphate dehydrogenase; Short=GAPDH; AltName: Full=Peptidyl-cysteine S-nitrosylase GAPDH [Homo sapiens] >1U8F_O Crystal Structure Of Human Placental Glyceraldehyde-3-Phosphate Dehydrogenase At 1.75 Resolution [Homo sapiens] >1U8F_P Crystal Structure Of Human Placental Glyceraldehyde-3-Phosphate Dehydrogenase At 1.75 Resolution [Homo sapiens] >1U8F_Q Crystal Structure Of Human Placental Glyceraldehyde-3-Phosphate Dehydrogenase At 1.75 Resolution [Homo sapiens] >1U8F_R Crystal Structure Of Human Placental Glyceraldehyde-3-Phosphate Dehydrogenase At 1.75 Resolution [Homo sapiens] >4WNC_A Crystal structure of human wild-type GAPDH at 1.99 angstroms resolution [Homo sapiens] >4WNC_B Crystal structure of human wild-type GAPDH at 1.99 angstroms resolution [Homo sapiens] >4WNC_C Crystal structure of human wild-type GAPDH at 1.99 angstroms resolution [Homo sapiens] >4WNC_D Crystal structure of human wild-type GAPDH at 1.99 angstroms resolution [Homo sapiens] >4WNC_E Crystal structure of human wild-type GAPDH at 1.99 angstroms resolution [Homo sapiens] >4WNC_F Crystal structure of human wild-type GAPDH at 1.99 angstroms resolution [Homo sapiens] >4WNC_G Crystal structure of human wild-type GAPDH at 1.99 angstroms resolution [Homo sapiens] >4WNC_O Crystal structure of human wild-type GAPDH at 1.99 angstroms resolution [Homo sapiens] >6IQ6_A Crystal structure of GAPDH [Homo sapiens] >6IQ6_B Crystal structure of GAPDH [Homo sapiens] >6IQ6_C Crystal structure of GAPDH [Homo sapiens] >6IQ6_D Crystal structure of GAPDH [Homo sapiens] >6IQ6_E Crystal structure of GAPDH [Homo sapiens] >6IQ6_F Crystal structure of GAPDH [Homo sapiens] >6IQ6_G Crystal structure of GAPDH [Homo sapiens] >6IQ6_H Crystal structure of GAPDH [Homo sapiens] >6YNE_A GAPDH purified from the supernatant of HEK293F cells: crystal form 2 of 4. [Homo sapiens] >6YNE_B GAPDH purified from the supernatant of HEK293F cells: crystal form 2 of 4. [Homo sapiens] >6YNE_C GAPDH purified from the supernatant of HEK293F cells: crystal form 2 of 4. [Homo sapiens] >6YNE_D GAPDH purified from the supernatant of HEK293F cells: crystal form 2 of 4. [Homo sapiens] >AAX42270.1 glyceraldehyde-3-phosphate dehydrogenase [synthetic construct] >MXR00212.1 hypothetical protein [Bos mutus] >SJX33932.1 unnamed protein product, partial [Human ORFeome Gateway entry vector] >AAA52496.1 glyceraldehyde 3-phosphate dehydrogenase (EC 1.2.1.12) [Homo sapiens] >AAA52518.1 glyceraldehyde-3-phosphate dehydrogenase (EC 1.2.1.12) [Homo sapiens]	-1.90785	0.60011	0.06389	0.95533	0.28853
TRINITY_DN361_c0_g1_i5_orf1	hexosaminidase [Ostrinia furnacalis]	-1.54491	1.55905	-0.18039	0.34414	-0.17788
TRINITY_DN11467_c0_g1_i5_orf1	27 kDa hemolymph protein-like, partial [Ostrinia furnacalis]	-1.76302	0.35176	-0.06224	1.31923	0.15426
TRINITY_DN28711_c0_g1_i1_orf1	hypothetical protein evm_000299 [Chilo suppressalis]	-1.41045	1.39267	-0.5329	0.83903	-0.28834
TRINITY_DN74086_c0_g1_i1_orf1	UPF0489 protein C5orf22 homolog [Ostrinia furnacalis]	-0.88072	1.91781	-0.13078	-0.69662	-0.20968
TRINITY_DN6462_c0_g1_i5_orf1	probable histone-lysine N-methyltransferase CG1716 [Ostrinia furnacalis]	-1.83589	0.74765	0.04473	1.03365	0.00986
TRINITY_DN2784_c0_g1_i3_orf1	toll-like receptor 6 [Ostrinia furnacalis]	-1.92417	0.80321	0.02666	0.70987	0.38444
TRINITY_DN4602_c0_g1_i4_orf1	2-iminobutanoate/2-iminopropanoate deaminase [Ostrinia furnacalis]	-1.76701	1.12088	-0.33301	0.36492	0.61422
TRINITY_DN24971_c0_g1_i3_orf1	uncharacterized protein LOC114352370 [Ostrinia furnacalis]	-1.22952	1.82537	-0.32766	-0.21464	-0.05356
TRINITY_DN625_c2_g2_i2_orf1	L-dopachrome tautomerase yellow-f2-like [Ostrinia furnacalis]	-1.79183	0.4621	-0.14507	1.22074	0.25406
TRINITY_DN18374_c0_g1_i1_orf1	L-2-hydroxyglutarate dehydrogenase, mitochondrial [Ostrinia furnacalis]	-1.91336	0.03307	0.43095	0.95046	0.49887
TRINITY_DN52944_c0_g1_i1_orf1	apolipophorins-like [Ostrinia furnacalis]	-1.40919	1.44676	-0.49679	0.76292	-0.3037
TRINITY_DN5433_c0_g1_i5_orf1	uncharacterized protein LOC114351067 [Ostrinia furnacalis]	-1.75433	0.39484	0.88859	0.89346	-0.42256
TRINITY_DN2271_c0_g1_i12_orf1	plasminogen activator inhibitor 1-like [Ostrinia furnacalis]	-1.75928	0.82475	0.17965	1.05105	-0.29617
TRINITY_DN53807_c0_g2_i1_orf1	aminomethyltransferase, mitochondrial [Ostrinia furnacalis]	-1.93465	0.29265	0.17363	0.91208	0.55629
TRINITY_DN10933_c0_g2_i1_orf1	uncharacterized protein LOC114357588 [Ostrinia furnacalis]	-1.89981	0.40842	0.63101	0.90748	-0.0471
TRINITY_DN23364_c0_g1_i1_orf1	PREDICTED: uncharacterized protein LOC106134920 [Amyelois transitella]	-1.41764	1.70915	-0.19172	0.06709	-0.16687
TRINITY_DN3861_c0_g3_i2_orf1	glutamyl aminopeptidase-like isoform X4 [Ostrinia furnacalis]	-1.94469	0.47841	0.20498	0.35509	0.90621
TRINITY_DN15382_c0_g1_i3_orf1	putative aldehyde dehydrogenase family 7 member A1 homolog [Spodoptera litura]	-1.79673	0.52332	0.84206	0.80551	-0.37415
TRINITY_DN9239_c0_g2_i2_orf1	apolipophorins-like [Ostrinia furnacalis]	-1.30821	1.49575	-0.35523	0.75877	-0.59109
TRINITY_DN86833_c0_g3_i1_orf1	PREDICTED: glycerol-3-phosphate acyltransferase 1, mitochondrial isoform X1 [Microplitis demolitor]	-1.58306	1.3807	-0.1806	0.68221	-0.29925
TRINITY_DN23167_c0_g2_i1_orf1	hypothetical protein evm_003712 [Chilo suppressalis]	-1.57544	1.54948	-0.26804	0.11528	0.17872
TRINITY_DN443_c0_g1_i2_orf1	proteasome subunit beta type-4 [Ostrinia furnacalis]	-1.93879	0.35468	0.68169	0.80008	0.10233
TRINITY_DN6472_c0_g1_i5_orf1	unnamed protein product [Chilo suppressalis]	-1.92939	0.66993	0.25128	0.14542	0.86276
TRINITY_DN1767_c0_g2_i15_orf1	fasciclin-2 isoform X3 [Ostrinia furnacalis]	-1.63886	1.49198	0.23328	0.07922	-0.16562
TRINITY_DN3017_c0_g1_i6_orf1	uncharacterized protein LOC114364875 isoform X1 [Ostrinia furnacalis] >XP_028177022.1 uncharacterized protein LOC114364875 isoform X1 [Ostrinia furnacalis]	-1.6551	1.45098	0.2018	0.24049	-0.23817
TRINITY_DN8703_c0_g1_i2_orf1	beta-glucuronidase-like isoform X1 [Ostrinia furnacalis] >XP_028166212.1 beta-glucuronidase-like isoform X2 [Ostrinia furnacalis]	-1.67866	1.01919	-0.21258	1.03516	-0.16311
TRINITY_DN70_c6_g1_i1_orf1	optineurin isoform X1 [Ostrinia furnacalis] >XP_028165537.1 optineurin isoform X1 [Ostrinia furnacalis] >XP_028165538.1 optineurin isoform X1 [Ostrinia furnacalis] >XP_028165539.1 optineurin isoform X1 [Ostrinia furnacalis]	-1.81119	0.81263	0.17523	0.99885	-0.17553

TRINITY_DN7938_c0_g1_i3_orf1	protein halfway isoform X1 [Ostrinia furnacalis] >XP_028172280.1 protein halfway isoform X2 [Ostrinia furnacalis] >XP_028172281.1 protein halfway isoform X3 [Ostrinia furnacalis]	-1.95722	0.70304	0.39875	0.15373	0.7017
TRINITY_DN9457_c0_g1_i9_orf1	plexin domain-containing protein 2 [Spodoptera litura]	-1.6381	1.16241	0.46415	-0.60657	0.61811
TRINITY_DN33272_c0_g1_i1_orf1	unnamed protein product, partial [Iphiclides podalirius]	-1.66207	1.38353	-0.31876	0.44592	0.15137
TRINITY_DN2170_c1_g1_i3_orf1	beta-1,3-glucan-binding protein-like [Ostrinia furnacalis]	-1.54084	1.58533	-0.23724	0.23363	-0.04088
TRINITY_DN28729_c0_g1_i9_orf1	serine/threonine-protein kinase mig-15 isoform X2 [Ostrinia furnacalis]	-1.70297	0.9415	-0.55392	0.80175	0.51364
TRINITY_DN64892_c0_g1_i1_orf1	aldehyde dehydrogenase X, mitochondrial [Manduca sexta] >KAG6450704.1 hypothetical protein O3G_MSEX006722 [Manduca sexta]	-1.75655	0.58756	-0.31393	1.17229	0.31063
TRINITY_DN276_c0_g1_i2_orf1	protein lethal(2)essential for life-like [Ostrinia furnacalis] >UTU55753.1 small heat shock protein Hsp20.7 [Ostrinia furnacalis]	-1.32063	1.52123	-0.80348	0.5407	0.06218
TRINITY_DN146957_c0_g1_i1_orf1	26S proteasome non-ATPase regulatory subunit 7 [Apis florea]	-1.89806	0.84811	0.61519	0.53762	-0.10285
TRINITY_DN45530_c0_g1_i1_orf1	aldose 1-epimerase isoform X1 [Ostrinia furnacalis] >XP_028178513.1 aldose 1-epimerase isoform X1 [Ostrinia furnacalis] >XP_028178514.1 aldose 1-epimerase isoform X1 [Ostrinia furnacalis]	-1.89303	0.28991	0.04487	1.02025	0.538
TRINITY_DN7867_c0_g1_i1_orf1	putative inorganic phosphate cotransporter [Ostrinia furnacalis]	-1.83688	0.88254	-0.28029	0.56126	0.67337
TRINITY_DN2170_c0_g2_i1_orf1	beta-1,3-glucan-binding protein-like [Ostrinia furnacalis]	-1.71322	0.10304	-0.01152	1.41879	0.20292
TRINITY_DN1786_c0_g1_i11_orf1	ATP-binding cassette sub-family A member 1-like [Ostrinia furnacalis]	-1.95837	0.28244	0.41235	0.87332	0.39025
TRINITY_DN37654_c0_g1_i5_orf1	unnamed protein product [Pieris macdunnoughi]	-1.82631	1.00312	-0.26104	0.50735	0.57688
TRINITY_DN104596_c0_g1_i1_orf1	unnamed protein product [Diatraea saccharalis]	-1.82524	0.53982	-0.21101	1.0745	0.42193
TRINITY_DN72369_c0_g1_i1_orf1	microsomal triacylglycerol transfer protein [Vanessa cardui]	-1.96693	0.81506	0.25991	0.41828	0.47367
TRINITY_DN22175_c0_g1_i1_orf1	low-density lipoprotein receptor-related protein 1B-like [Ostrinia furnacalis]	-1.94305	0.42982	0.47753	0.14675	0.88896
TRINITY_DN1986_c0_g1_i1_orf1	serine protease inhibitor 77Ba-like [Ostrinia furnacalis] >XP_028164032.1 serine protease inhibitor 77Ba-like [Ostrinia furnacalis]	-1.36926	1.75146	-0.16809	-0.16344	-0.05067
TRINITY_DN25273_c0_g1_i1_orf1	skin secretory protein xP2-like [Ostrinia furnacalis]	-0.97786	1.92687	-0.45919	-0.23589	-0.25393
TRINITY_DN38412_c0_g1_i1_orf1	translation initiation factor eIF-2B subunit alpha [Ostrinia furnacalis]	-1.65697	1.40022	-0.18487	0.50553	-0.06391
TRINITY_DN22053_c0_g1_i13_orf1	uncharacterized protein LOC114355104 [Ostrinia furnacalis]	-1.67443	1.43144	-0.1583	0.34498	0.05631
TRINITY_DN2002_c0_g1_i5_orfp1	TRINITY_DN2002_c0_g1_i5_m.4230 TRINITY_DN2002_c0_g1::TRINITY_DN2002_c0_g1_i5::g.4230 ORF type:3prime_partial len:259 (+),score=29.98,Peptidase_C39 PF03412.16 1.7,Peptidase_C39|PF03412.16|0.049 TRINITY_DN2002_c0_g1_i5:65-838(+)	-0.92719	1.87811	-0.05274	-0.77071	-0.12747
TRINITY_DN906_c0_g1_i4_orf1	uncharacterized protein LOC114360441, partial [Ostrinia furnacalis]	-0.85856	1.95715	-0.22462	-0.44253	-0.43145
TRINITY_DN12671_c0_g1_i6_orf1	hemicentin-1-like isoform X1 [Ostrinia furnacalis]	-1.05123	1.87108	-0.3467	0.04789	-0.52104
TRINITY_DN71699_c0_g1_i1_orf1	apolipophorins-like [Ostrinia furnacalis]	-1.38537	1.43099	-0.57389	0.79524	-0.26696
TRINITY_DN1144_c0_g1_i10_orf1	TIL [Ostrinia furnacalis]	-1.76989	0.45222	-0.29259	1.1799	0.43037
TRINITY_DN11492_c0_g1_i8_orf1	unnamed protein product [Chilo suppressalis]	-1.96901	0.46008	0.42478	0.27447	0.80967
TRINITY_DN29604_c0_g2_i2_orf1	neurofilament heavy polypeptide-like isoform X2 [Ostrinia furnacalis]	-1.12051	1.86847	-0.45866	-0.16776	-0.12153
TRINITY_DN7739_c0_g1_i2_orf1	erlin-2-like [Ostrinia furnacalis] >XP_028161644.1 erlin-2-like [Ostrinia furnacalis]	-1.8084	1.13218	0.59362	-0.17324	0.25584
TRINITY_DN18538_c0_g3_i1_orf1	poly(A)-specific ribonuclease PARN-like [Ostrinia furnacalis]	-1.81486	0.79715	-0.26143	0.33594	0.9432
TRINITY_DN10195_c0_g1_i8_orf1	charged multivesicular body protein 6-A [Ostrinia furnacalis]	-1.91052	0.30133	0.29266	1.04995	0.26658
TRINITY_DN25987_c0_g1_i5_orf1	GILT-like protein 2 isoform X1 [Ostrinia furnacalis] >XP_028156245.1 GILT-like protein 2 isoform X2 [Ostrinia furnacalis] >XP_028156247.1 GILT-like protein 2 isoform X3 [Ostrinia furnacalis]	-0.82787	1.82718	0.29837	-0.79887	-0.49881
TRINITY_DN7062_c0_g1_i1_orf1	ribosome maturation protein SBDS [Ostrinia furnacalis]	-1.50362	1.40434	0.33179	-0.67744	0.44491
TRINITY_DN1630_c0_g1_i6_orf1	major facilitator superfamily domain-containing protein 1-like [Ostrinia furnacalis]	-1.33755	1.75713	-0.14861	0.04433	-0.31529
TRINITY_DN26301_c0_g1_i1_orf1	uncharacterized protein LOC114359193 [Ostrinia furnacalis]	-1.11169	1.88231	-0.33345	-0.30312	-0.13405
TRINITY_DN13615_c0_g1_i3_orf1	calcium-binding protein P-like [Ostrinia furnacalis]	-1.98906	0.69157	0.39536	0.48371	0.41843
TRINITY_DN57998_c1_g1_i1_orf1	uncharacterized protein LOC113509309, partial [Galleria mellonella]	-0.70318	1.96618	-0.39625	-0.20203	-0.66473
TRINITY_DN4954_c0_g1_i5_orf1	glycogen debranching enzyme [Trichoplusia ni]	-1.80894	0.52283	0.99783	0.60086	-0.31257
TRINITY_DN272_c0_g1_i1_orf1	vacuolar protein sorting-associated protein 11 homolog [Ostrinia furnacalis]	-1.69878	1.18205	-0.07685	0.81392	-0.22034
TRINITY_DN2835_c0_g1_i6_orf1	probable isoaspartyl peptidase/L-asparaginase GA20639 [Ostrinia furnacalis]	-1.57307	1.32898	-0.57323	0.62849	0.18883
TRINITY_DN4394_c0_g2_i1_orf1	carboxylesterase [Ostrinia furnacalis]	-1.58943	0.83133	-0.00699	1.24565	-0.48055
TRINITY_DN400_c0_g1_i1_orf1	serine/threonine-protein phosphatase 2A activator-like isoform X2 [Ostrinia furnacalis] >XP_028164470.1 serine/threonine-protein phosphatase 2A activator-like isoform X2 [Ostrinia furnacalis]	-1.89542	-0.0481	0.35409	0.8857	0.70373
TRINITY_DN29369_c0_g1_i1_orf1	uncharacterized protein LOC114351747 [Ostrinia furnacalis]	-1.83365	0.05873	1.21465	0.24883	0.31145
TRINITY_DN5458_c1_g1_i9_orf1	histone H2A-like [Aedes aegypti] >XP_021712017.1 histone H2A-like [Aedes aegypti]	-1.87633	0.6411	0.97369	-0.07674	0.33828
TRINITY_DN664_c0_g1_i18_orf1	chitinase-like protein EN03 isoform X2 [Ostrinia furnacalis]	-1.75776	0.83039	-0.4744	0.6197	0.78208
TRINITY_DN11670_c0_g1_i1_orf1	teneurin-m isoform X1 [Ostrinia furnacalis]	-0.93952	1.89127	-0.01638	-0.69428	-0.24108
TRINITY_DN2290_c0_g1_i2_orfp1	TRINITY_DN2290_c0_g1_i2_m.69732 TRINITY_DN2290_c0_g1::TRINITY_DN2290_c0_g1_i2::g.69732 ORF type:complete len:234 (+),score=14.43 TRINITY_DN2290_c0_g1_i2:60-761(+)	-0.57686	1.81174	0.35091	-0.74624	-0.83954
TRINITY_DN28711_c1_g1_i1_orf1	apolipophorins-like [Ostrinia furnacalis]	-1.41147	1.46692	-0.53214	0.71746	-0.24077
TRINITY_DN21533_c0_g1_i6_orf1	annexin B9 isoform X2 [Ostrinia furnacalis]	-1.84372	0.82198	0.87886	0.33833	-0.19545
TRINITY_DN9239_c0_g1_i1_orf1	apolipophorins-like [Ostrinia furnacalis]	-1.32685	1.50782	-0.60473	0.71709	-0.29332
TRINITY_DN28922_c0_g1_i2_orf1	uncharacterized protein LOC119829283 isoform X2 [Zerene cesonia]	-0.89068	1.9407	-0.36635	-0.15148	-0.53219
TRINITY_DN13515_c0_g1_i1_orf1	low-density lipoprotein receptor-related protein 4-like isoform X2 [Ostrinia furnacalis]	-1.94788	0.38831	0.17016	0.86904	0.52037

TRINITY_DN7776_c0_g1_i9_orf1	uncharacterized protein LOC114364702 [Ostrinia furnacalis]	-1.86368	1.07048	0.59691	0.14919	0.04709
TRINITY_DN143637_c0_g1_i1_orf1	PX domain-containing protein kinase-like protein isoform X1 [Chelonus insularis]	-1.73448	1.20193	0.29959	0.5802	-0.34724
TRINITY_DN29017_c0_g1_i4_orf1	cysteine protease ATG4B [Ostrinia furnacalis]	-1.93053	0.65175	0.1847	0.8754	0.21868
TRINITY_DN14774_c0_g1_i4_orf1	aminopeptidase N-like [Ostrinia furnacalis]	-0.57826	1.73297	0.51519	-0.87197	-0.79793
TRINITY_DN97378_c0_g1_i2_orf1	dihydropteridine reductase [Galleria mellonella]	-1.91487	0.91987	0.00831	0.48145	0.50524
TRINITY_DN2311_c0_g3_i1_orf1	uncharacterized protein LOC114364231 isoform X1 [Ostrinia furnacalis] >XP_028176108.1 uncharacterized protein LOC114364231 isoform X2 [Ostrinia furnacalis]	-1.21249	1.75083	-0.48615	-0.36281	0.31061
TRINITY_DN39404_c0_g1_i7_orf1	hypothetical protein evm_004736, partial [Chilo suppressalis]	-1.6677	1.38935	0.47197	-0.25011	0.0565
TRINITY_DN97097_c0_g1_i4_orf1	plectin-like, partial [Ostrinia furnacalis]	-1.40843	1.6619	-0.43	0.25249	-0.07596
TRINITY_DN8714_c0_g1_i6_orf1	stimulator of interferon genes protein isoform X1 [Nymphalis io]	-1.71548	0.85086	1.10654	-0.32027	0.07834
TRINITY_DN4256_c0_g1_i1_orf1	chitinase-3-like protein 1 [Ostrinia furnacalis]	-1.68339	1.3046	-0.2394	0.6376	-0.01941
TRINITY_DN60358_c0_g1_i3_orf1	transcription termination factor 5, mitochondrial-like [Ostrinia furnacalis]	-1.78126	0.70128	0.6196	0.88035	-0.41997
TRINITY_DN2416_c0_g1_i5_orf1	somatomedin-B and thrombospondin type-1 domain-containing protein [Ostrinia furnacalis] >XP_028177886.1 somatomedin-B and thrombospondin type-1 domain-containing protein [Ostrinia furnacalis]	-1.30679	1.61888	-0.04078	-0.69826	0.42695
TRINITY_DN49956_c0_g1_i1_orf1	UPF0585 protein CG18661 [Ostrinia furnacalis]	-1.89645	0.02115	0.37729	1.0123	0.48572
TRINITY_DN8979_c0_g1_i5_orf1	ras-related protein Rab-5B [Vanessa cardui] >XP_046961939.1 ras-related protein Rab-5B [Vanessa cardui] >XP_046961940.1 ras-related protein Rab-5B [Vanessa cardui] >XP_046961941.1 ras-related protein Rab-5B [Vanessa cardui] >XP_047545265.1 ras-related protein Rab-5B [Vanessa atalanta] >XP_047545266.1 ras-related protein Rab-5B [Vanessa atalanta] >XP_047545267.1 ras-related protein Rab-5B [Vanessa atalanta]	-1.52817	1.22049	0.48269	-0.76824	0.59323
TRINITY_DN5444_c0_g2_i1_orf1	phenoloxidase-activating factor 2-like [Ostrinia furnacalis]	-0.91223	1.93721	-0.26563	-0.54732	-0.21203
TRINITY_DN7776_c0_g1_i5_orf1	uncharacterized protein LOC114364702 [Ostrinia furnacalis]	-1.80534	1.07891	0.13345	-0.14118	0.73415
TRINITY_DN14904_c1_g2_i2_orf1	autophagy protein 12-like [Ostrinia furnacalis]	-1.82645	1.07431	-0.15436	0.25932	0.64718
TRINITY_DN8386_c0_g1_i6_orf1	F-box/WD repeat-containing protein 9-like [Ostrinia furnacalis]	-1.80113	0.85868	0.46239	0.82721	-0.34716
TRINITY_DN3292_c2_g2_i1_orf1	aldo-keto reductase AKR2E4-like [Ostrinia furnacalis]	-1.68926	1.40293	0.05954	-0.15947	0.38627
TRINITY_DN7776_c0_g1_i1_orf1	uncharacterized protein LOC114364702 [Ostrinia furnacalis]	-1.78253	1.05961	0.17589	0.78305	-0.23601
TRINITY_DN12885_c0_g1_i1_orf1	prolow-density lipoprotein receptor-related protein 1, partial [Ostrinia furnacalis]	-1.8075	1.19575	0.25047	0.47656	-0.11528
TRINITY_DN10264_c1_g1_i5_orf1	probable low-specificity L-threonine aldolase 2 [Ostrinia furnacalis]	-1.79199	0.89194	-0.32454	0.87503	0.34956
TRINITY_DN24310_c0_g1_i2_orf1	glucose-6-phosphate 1-dehydrogenase [Ostrinia furnacalis]	-1.88511	0.85448	0.5354	0.63937	-0.14415
TRINITY_DN2652_c0_g2_i1_orf1	peroxidase-like isoform X1 [Ostrinia furnacalis]	-0.95086	1.93676	-0.33325	-0.2244	-0.42826
TRINITY_DN14856_c0_g1_i1_orf1	upstream activation factor subunit spp27 [Ostrinia furnacalis]	-1.03317	1.84638	0.04922	-0.70427	-0.15816
TRINITY_DN3212_c0_g1_i7_orfp1	TRINITY_DN3212_c0_g1_i7_m.38385 TRINITY_DN3212_c0_g1::TRINITY_DN3212_c0_g1_i7::g.38385 ORF type:internal len:121 (-),score=5.33 TRINITY_DN3212_c0_g1_i7:3-362(-)	-1.79412	0.57286	-0.32325	1.05128	0.49323
TRINITY_DN7957_c0_g1_i5_orf1	spermidine synthase [Ostrinia furnacalis] >XP_028167892.1 spermidine synthase [Ostrinia furnacalis]	-1.79395	0.59674	-0.3824	0.91596	0.66366
TRINITY_DN26337_c0_g1_i3_orf1	lysosome membrane protein 2-like [Ostrinia furnacalis]	-1.41761	1.51383	-0.57602	-0.11492	0.59473
TRINITY_DN13285_c0_g1_i9_orf1	E3 ubiquitin-protein ligase RNF13 isoform X1 [Ostrinia furnacalis] >XP_028158682.1 E3 ubiquitin-protein ligase RNF13 isoform X1 [Ostrinia furnacalis] >XP_028158683.1 E3 ubiquitin-protein ligase RNF13 isoform X1 [Ostrinia furnacalis]	-1.79101	1.16829	0.64846	-0.07007	0.04433
TRINITY_DN13686_c0_g2_i1_orf1	transmembrane protease serine 9-like [Ostrinia furnacalis]	-1.20297	1.82352	-0.42919	-0.20774	0.01638
TRINITY_DN10229_c0_g1_i6_orf1	autophagy protein 5 isoform X2 [Ostrinia furnacalis]	-1.82426	1.14537	0.59009	-0.01913	0.10793
TRINITY_DN2069_c1_g1_i8_orf1	lysosomal aspartic protease [Trichoplusia ni]	-1.42713	1.65635	-0.41934	-0.01866	0.20878
TRINITY_DN364_c5_g1_i3_orf1	talin-2-like, partial [Ostrinia furnacalis]	-1.85876	1.01885	0.54405	-0.1412	0.43706
TRINITY_DN3545_c0_g1_i6_orf1	group XV phospholipase A2-like [Ostrinia furnacalis] >XP_028168992.1 group XV phospholipase A2-like [Ostrinia furnacalis] >XP_028168993.1 group XV phospholipase A2-like [Ostrinia furnacalis]	-1.72496	1.38957	0.25706	-0.07145	0.14979
TRINITY_DN2395_c0_g1_i7_orf1	uncharacterized protein LOC114352963 [Ostrinia furnacalis]	-1.66215	1.02035	-0.57196	0.86416	0.34961
TRINITY_DN6275_c0_g1_i3_orf1	CTL-like protein 1 isoform X1 [Galleria mellonella]	-1.81475	1.2096	0.1819	-0.03421	0.45747
TRINITY_DN8926_c0_g1_i4_orf1	run domain Beclin-1-interacting and cysteine-rich domain-containing protein [Ostrinia furnacalis]	-1.95527	0.79797	0.57	0.14737	0.43993
TRINITY_DN1720_c0_g1_i3_orf1	monocarboxylate transporter 12 [Ostrinia furnacalis] >XP_028157531.1 monocarboxylate transporter 12 [Ostrinia furnacalis]	-1.83099	0.68212	-0.30976	0.62346	0.83518
TRINITY_DN28875_c0_g1_i1_orf1	PREDICTED: ras-related protein Rab-2 [Fopius arisanus] >XP_015110133.1 ras-related protein Rab-2 [Diachasma alloeum]	-1.94556	0.34889	0.69303	0.13206	0.77158
TRINITY_DN3483_c0_g1_i5_orf1	phenoloxidase-activating factor 2-like isoform X1 [Ostrinia furnacalis] >XP_028178309.1 phenoloxidase-activating factor 2-like isoform X2 [Ostrinia furnacalis]	-1.71297	0.80788	-0.56781	0.79082	0.68209
TRINITY_DN14831_c0_g1_i9_orf1	probable protein phosphatase 2C T23F11.1 isoform X1 [Ostrinia furnacalis]	-1.88081	0.09123	1.10098	0.29424	0.39436
TRINITY_DN2818_c0_g1_i2_orf1	proteasome subunit alpha type-3 [Ostrinia furnacalis]	-1.7773	0.98603	0.89603	-0.22646	0.12171
TRINITY_DN18218_c0_g1_i7_orf1	inhibin beta B chain [Ostrinia furnacalis]	-1.23796	1.51379	-0.72227	0.7498	-0.30336
TRINITY_DN50237_c0_g1_i8_orf1	LOW QUALITY PROTEIN: uncharacterized protein LOC114361080 [Ostrinia furnacalis]	-1.70469	0.66026	-0.05929	1.27487	-0.17115
TRINITY_DN31676_c0_g1_i4_orf1	N-acetylgalactosaminyltransferase 7 isoform X1 [Ostrinia furnacalis] >XP_028156925.1 N-acetylgalactosaminyltransferase 7 isoform X2 [Ostrinia furnacalis]	-1.75988	1.20442	-0.21987	0.61491	0.16043
TRINITY_DN2348_c0_g1_i1_orfp1	TRINITY_DN2348_c0_g1_i1_m.39060 TRINITY_DN2348_c0_g1::TRINITY_DN2348_c0_g1_i1::g.39060 ORF type:complete len:149 (+),score=54.19 TRINITY_DN2348_c0_g1_i1:28-474(+)	-1.32934	1.73074	-0.20038	0.19699	-0.39801
TRINITY_DN8473_c0_g1_i5_orf1	serine/threonine-protein phosphatase 6 regulatory subunit 1 [Ostrinia furnacalis]	-1.80965	0.40969	0.21415	1.22867	-0.04287

TRINITY_DN9412_c0_g1_i1_orf1	maspardin-like [Ostrinia furnacalis]	-1.36034	1.5662	-0.0341	0.49746	-0.66922
TRINITY_DN1465_c2_g1_i2_orf1	transcription initiation factor TFIID subunit 1-like [Ostrinia furnacalis]	-1.18958	1.7845	-0.37309	-0.45489	0.23306
TRINITY_DN30154_c0_g1_i1_orf1	serine/threonine-protein kinase SIK2 [Ostrinia furnacalis] >XP_028174514.1 serine/threonine-protein kinase SIK2 [Ostrinia furnacalis]	-0.77251	1.97265	-0.32804	-0.54555	-0.32655
TRINITY_DN6510_c1_g1_i1_orf1	hypothetical protein evm_008080 [Chilo suppressalis]	-1.74338	1.37646	0.24018	0.05216	0.07458
TRINITY_DN4790_c0_g1_i6_orf1	ADP-ribosylation factor-like protein 8 [Ostrinia furnacalis] >CAG9746554.1 unnamed protein product [Diatraea saccharalis] >CAG9785239.1 unnamed protein product [Diatraea saccharalis] >CAH2992000.1 unnamed protein product [Chilo suppressalis]	-1.70651	1.25316	0.67652	-0.24357	0.02041
TRINITY_DN2793_c0_g2_i1_orf1	PREDICTED: ras-related protein Rab-4B [Amyelois transitella]	-1.71324	1.255	-0.16873	0.67733	-0.05036
TRINITY_DN4384_c0_g1_i5_orf1	chemosensory protein 5 [Conogethes punctiferalis]	-0.88113	1.9469	-0.36344	-0.18611	-0.51622
TRINITY_DN1612_c0_g1_i3_orf1	immunoglobulin-binding protein 1b [Ostrinia furnacalis]	-1.81348	1.02892	-0.25195	0.67942	0.35709
TRINITY_DN48250_c0_g1_i1_orf1	larval/pupal rigid cuticle protein 66-like [Hyposmocoma kahamanoa]	-1.79497	0.83737	-0.33146	0.9055	0.38356
TRINITY_DN1870_c0_g1_i6_orf1	programmed cell death protein 5 [Ostrinia furnacalis]	-1.78056	1.19246	-0.12554	0.61876	0.09488
TRINITY_DN2140_c0_g1_i1_orf1	uncharacterized protein LOC126367764 [Pectinophora gossypiella]	-0.69908	1.97988	-0.26014	-0.47268	-0.54798
TRINITY_DN108433_c0_g1_i1_orf1	alpha-2-macroglobulin receptor-associated protein [Diachasma alloeum]	-1.90425	0.98091	0.08136	0.57997	0.26203
TRINITY_DN43505_c0_g1_i1_orf1	unnamed protein product [Chilo suppressalis]	-1.95531	0.5858	0.17883	0.81068	0.38
TRINITY_DN875_c0_g1_i3_orf1	secernin-3 [Ostrinia furnacalis]	-1.24403	1.71172	-0.18255	0.33093	-0.61607
TRINITY_DN2475_c0_g1_i1_orf1	LOW QUALITY PROTEIN: uncharacterized protein LOC114363766 [Ostrinia furnacalis]	-1.77644	0.16517	0.03879	1.32435	0.24812
TRINITY_DN6423_c0_g1_i5_orf1	phenoloxidase-activating factor 2-like isoform X2 [Ostrinia furnacalis]	-1.45491	1.6612	-0.33721	0.03908	0.09183
TRINITY_DN1038_c0_g1_i4_orf1	gastric triacylglycerol lipase-like [Ostrinia furnacalis]	-1.72101	1.33143	0.46323	-0.19196	0.11831
TRINITY_DN19110_c0_g1_i2_orf1	peroxidase [Ostrinia furnacalis]	-1.24573	1.62388	-0.50481	0.58694	-0.46027
TRINITY_DN8986_c0_g1_i1_orf1	HBS1-like protein [Ostrinia furnacalis]	-1.88895	0.57894	0.98274	0.36034	-0.03307
TRINITY_DN110523_c0_g2_i1_orf1	uncharacterized protein LOC107036393 [Diachasma alloeum]	-0.35668	1.72681	0.3944	-0.58347	-1.18106
TRINITY_DN582_c0_g1_i2_orf1	unnamed protein product [Chilo suppressalis]	-1.55528	0.77535	0.99089	0.59304	-0.80399
TRINITY_DN66671_c0_g1_i1_orf1	phosphoglucomutase-like [Ostrinia furnacalis]	-1.95756	0.59816	0.25927	0.81257	0.28756
TRINITY_DN19821_c0_g2_i4_orf1	uncharacterized protein LOC114359393 isoform X1 [Ostrinia furnacalis]	-1.58849	1.00807	-0.49332	1.10285	-0.02911
TRINITY_DN89083_c0_g1_i1_orf1	lysine-specific demethylase 4A isoform X2 [Diachasma alloeum]	-1.86422	0.77138	-0.22571	0.72795	0.59059
TRINITY_DN2170_c0_g1_i2_orf1	beta-1,3-glucan-binding protein-like [Ostrinia furnacalis]	-1.53937	1.22023	0.37509	0.67881	-0.73477
TRINITY_DN1407_c0_g1_i5_orf1	unnamed protein product [Chrysodeixis includens]	-1.23131	1.75413	-0.21626	0.24241	-0.54896
TRINITY_DN10766_c0_g1_i1_orf1	hypothetical protein evm_008559 [Chilo suppressalis]	-0.89548	1.89928	-0.1069	-0.74611	-0.15079
TRINITY_DN3896_c0_g1_i1_orf1	glyoxalase domain-containing protein 4 [Ostrinia furnacalis]	-1.56353	1.53605	-0.35182	0.17913	0.20018
TRINITY_DN21555_c0_g1_i4_orf1	uncharacterized protein LOC114351844 [Ostrinia furnacalis] >XP_028158981.1 uncharacterized protein LOC114351844 [Ostrinia furnacalis] >XP_028158982.1 uncharacterized protein LOC114351844 [Ostrinia furnacalis] >XP_028158983.1 uncharacterized protein LOC114351844 [Ostrinia furnacalis] >XP_028158984.1 uncharacterized protein LOC114351844 [Ostrinia furnacalis] >XP_028158985.1 uncharacterized protein LOC114351844 [Ostrinia furnacalis] >5GPR_A Crystal structure of chitinase-h from Ostrinia furnacalis [Ostrinia furnacalis] >5GQB_A Crystal structure of chitinase-h from O. furnacalis in complex with chitohepatose [Ostrinia furnacalis] >6JMN_A Crystal structure of Ostrinia furnacalis Chitinase h complexed with compound 2-8-s2 [Ostrinia furnacalis] >BAE16587.1 chitinase [Ostrinia furnacalis]	-1.1256	1.85928	-0.47671	-0.21744	-0.03953
TRINITY_DN21609_c0_g1_i1_orf1	translation initiation factor eIF-2B subunit epsilon [Ostrinia furnacalis]	-1.91225	0.2795	0.34636	0.24456	1.04182
TRINITY_DN1023_c1_g1_i1_orf1	ras-related protein Rab-7a [Ostrinia furnacalis]	-1.96968	0.81238	0.37051	0.31092	0.47587
TRINITY_DN4217_c0_g1_i2_orf1	hypothetical protein evm_006611 [Chilo suppressalis]	-1.06386	1.89307	-0.15682	-0.46624	-0.20616
TRINITY_DN7711_c1_g1_i3_orf1	long-chain fatty acid transport protein 1-like [Ostrinia furnacalis]	-0.86408	1.76844	0.42684	-0.8355	-0.4957
TRINITY_DN26013_c0_g1_i1_orf1	E3 ubiquitin-protein ligase CHIP [Ostrinia furnacalis]	-1.75428	1.12645	0.66694	0.30295	-0.34206
TRINITY_DN28802_c0_g1_i1_orf1	apolipoprotein D-like [Ostrinia furnacalis]	-1.67787	1.14304	-0.02929	-0.3172	0.88133
TRINITY_DN98016_c0_g1_i1_orf1	methanethiol oxidase [Ostrinia furnacalis]	-1.41139	1.62849	-0.1443	-0.44416	0.37136
TRINITY_DN10680_c0_g1_i5_orf1	cGMP-dependent protein kinase, isozyme 2 forms cD4/T1/T3A/T3B-like isoform X3 [Ostrinia furnacalis] >XP_028158316.1 cGMP-dependent protein kinase, isozyme 2 forms cD4/T1/T3A/T3B-like isoform X3 [Ostrinia furnacalis]	-1.59297	1.44791	-0.23371	0.53554	-0.15677
TRINITY_DN57998_c1_g3_i1_orf1	uncharacterized protein LOC114363305 isoform X2 [Ostrinia furnacalis]	-0.82231	1.96902	-0.45052	-0.32095	-0.37524
TRINITY_DN13901_c0_g1_i4_orf1	vesicle-associated membrane protein 7-like [Ostrinia furnacalis]	-1.93306	0.88275	0.27403	0.15614	0.62013
TRINITY_DN33867_c0_g1_i8_orf1	uncharacterized protein LOC114357513 [Ostrinia furnacalis]	-1.36735	0.55393	0.73106	1.11022	-1.02786
TRINITY_DN49785_c1_g1_i3_orf1	uncharacterized protein LOC114365444, partial [Ostrinia furnacalis]	-0.6518	1.97188	-0.30342	-0.70579	-0.31087
TRINITY_DN22928_c0_g1_i6_orf1	hypothetical protein B5X24_HaOG209714 [Helicoverpa armigera]	-1.51523	1.2076	0.6141	-0.79422	0.48775
TRINITY_DN12317_c0_g1_i1_orf1	vacuolar protein sorting-associated protein 41 homolog [Ostrinia furnacalis]	-1.5828	1.30047	0.61929	-0.59464	0.25769
TRINITY_DN34115_c0_g1_i1_orf1	transcription factor 23 [Ostrinia furnacalis]	-1.88882	0.08964	0.42528	1.07402	0.29988
TRINITY_DN42854_c0_g3_i2_orf1	amyloid beta (A4) precursor-like protein 2, isoform CRA_b [Homo sapiens]	-1.65502	0.9126	-0.38635	1.13084	-0.00206
TRINITY_DN6004_c0_g1_i1_orf1	endocuticle structural glycoprotein ABD-4-like [Ostrinia furnacalis]	-0.94204	1.94185	-0.37857	-0.36302	-0.25822
TRINITY_DN44709_c0_g1_i1_orf1	D-beta-hydroxybutyrate dehydrogenase, mitochondrial, partial [Chelonus insularis]	-1.51179	1.26766	-0.56984	0.88213	-0.06816
TRINITY_DN15411_c0_g1_i4_orf1	uncharacterized protein LOC114362040 isoform X1 [Ostrinia furnacalis]	-1.32291	1.70426	0.01988	0.16308	-0.5643
TRINITY_DN2861_c0_g2_i1_orf1	proteasome subunit alpha type-5 [Ostrinia furnacalis]	-1.86682	1.13707	0.09748	0.39572	0.23656

TRINITY_DN5122_c0_g1_i3_orf1	calumenin [Ostrinia furnacalis] >XP_028172745.1 calumenin [Ostrinia furnacalis] >XP_028172746.1 calumenin [Ostrinia furnacalis]	-1.83309	1.15217	-0.08472	0.30535	0.4603
TRINITY_DN12671_c0_g1_i4_orf1	hemicentin-1-like isoform X1 [Ostrinia furnacalis]	-0.86589	1.96204	-0.36682	-0.35709	-0.37224
TRINITY_DN5556_c0_g1_i3_orf1	hypothetical protein evm_006874 [Chilo suppressalis]	-1.86256	0.77596	-0.23479	0.64357	0.67782
TRINITY_DN14587_c0_g1_i7_orf1	hypothetical protein evm_001488 [Chilo suppressalis] >CAB3526337.1 unnamed protein product [Chilo suppressalis] >CAH0403665.1 unnamed protein product [Chilo suppressalis]	-1.79483	1.05331	-0.29623	0.37238	0.66537
TRINITY_DN8922_c0_g1_i3_orf1	protein sidekick isoform X1 [Ostrinia furnacalis] >XP_028161979.1 protein sidekick isoform X1 [Ostrinia furnacalis] >XP_028161980.1 protein sidekick isoform X1 [Ostrinia furnacalis] >XP_028161981.1 protein sidekick isoform X1 [Ostrinia furnacalis]	-1.70329	0.28032	0.09322	1.41568	-0.08594
TRINITY_DN4013_c0_g1_i4_orf1	uncharacterized protein LOC114353190 isoform X1 [Ostrinia furnacalis] >XP_028160984.1 uncharacterized protein LOC114353190 isoform X2 [Ostrinia furnacalis] >XP_028161062.1 uncharacterized protein LOC114353190 isoform X1 [Ostrinia furnacalis] >XP_028161142.1 uncharacterized protein LOC114353190 isoform X1 [Ostrinia furnacalis]	-0.82999	1.85295	0.25227	-0.61603	-0.6592
TRINITY_DN11274_c0_g1_i4_orf1	dipeptidyl peptidase 3 isoform X1 [Ostrinia furnacalis]	-1.68454	1.04519	0.37544	0.80058	-0.53667
TRINITY_DN17838_c0_g1_i4_orf1	mitogen-activated protein kinase kinase kinase 4 [Ostrinia furnacalis]	-1.86928	0.55131	0.02776	1.0745	0.21569
TRINITY_DN12673_c3_g1_i2_orf1	unnamed protein product [Chilo suppressalis]	-0.917	1.85975	0.19573	-0.48536	-0.65312
TRINITY_DN12301_c0_g1_i1_orf1	ribose-phosphate pyrophosphokinase 2 [Ostrinia furnacalis]	-1.65265	0.79765	-0.449	1.19083	0.11317
TRINITY_DN41_c0_g1_i5_orf1	putative phospholipase B-like 2 [Ostrinia furnacalis]	-1.2045	1.82891	-0.02162	-0.19624	-0.40656
TRINITY_DN2489_c0_g1_i1_orf1	uncharacterized protein LOC114354692 [Ostrinia furnacalis]	-0.97486	1.92419	-0.47298	-0.16757	-0.30877
TRINITY_DN1533_c0_g2_i1_orf1	unnamed protein product [Chilo suppressalis]	-1.37291	1.74635	-0.16565	-0.19431	-0.01349
TRINITY_DN14458_c0_g1_i2_orf1	spermatogenesis-associated protein 20 isoform X1 [Ostrinia furnacalis]	-1.26124	1.78399	-0.0471	-0.0019	-0.47375
TRINITY_DN2110_c0_g1_i3_orf1	ribose-phosphate pyrophosphokinase 1 isoform X1 [Chelonus insularis]	-1.77728	0.49946	-0.40301	0.9321	0.74873
TRINITY_DN12514_c0_g2_i1_orf1	peroxidase isoform X3 [Ostrinia furnacalis]	-1.76711	0.71375	-0.1093	1.16445	-0.00178
TRINITY_DN1532_c0_g1_i6_orf1	mRNA (2'-O-methyladenosine-N(6)-)-methyltransferase [Ostrinia furnacalis] >XP_028179575.1 mRNA (2'-O-methyladenosine-N(6)-)-methyltransferase [Ostrinia furnacalis] >XP_028179576.1 mRNA (2'-O-methyladenosine-N(6)-)-methyltransferase [Ostrinia furnacalis]	-1.83161	0.41356	-0.0711	1.16904	0.32011
TRINITY_DN51429_c1_g1_i1_orf1	ER degradation-enhancing alpha-mannosidase-like protein 3 isoform X1 [Ostrinia furnacalis] >XP_028179122.1 ER degradation-enhancing alpha-mannosidase-like protein 3 isoform X2 [Ostrinia furnacalis]	-1.69316	0.69804	0.34352	1.13686	-0.48526
TRINITY_DN12106_c0_g1_i4_orf1	programmed cell death 6-interacting protein [Ostrinia furnacalis]	-1.58092	1.11795	0.5904	-0.73236	0.60493
TRINITY_DN13973_c0_g1_i6_orf1	27 kDa glycoprotein-like [Ostrinia furnacalis]	-1.41164	1.38377	-0.80754	0.63146	0.20394
TRINITY_DN5553_c0_g1_i4_orf1	uncharacterized protein LOC114353828 [Ostrinia furnacalis]	-1.28323	1.64419	-0.55803	0.498	-0.30093
TRINITY_DN97138_c0_g1_i2_orf1	tubulin beta chain-like isoform X2 [Ostrinia furnacalis]	-1.09366	1.89179	-0.35209	-0.25135	-0.19469
TRINITY_DN5029_c0_g1_i1_orf1	ribose-phosphate pyrophosphokinase 2-like [Ostrinia furnacalis]	-1.73637	0.82998	-0.48082	0.92029	0.46693
TRINITY_DN8580_c0_g1_i12_orf1	peroxisomal N(1)-acetyl-spermine/spermidine oxidase-like isoform X1 [Ostrinia furnacalis]	-1.58807	1.09311	-0.72996	0.60665	0.61828
TRINITY_DN2374_c0_g2_i2_orf1	uncharacterized protein LOC114357127 [Ostrinia furnacalis]	-1.97216	0.36723	0.33839	0.46128	0.80526
TRINITY_DN5603_c0_g1_i1_orf1	translin [Ostrinia furnacalis]	-1.88306	0.81406	0.76612	-0.13014	0.43303
TRINITY_DN12065_c0_g1_i4_orf1	protein DEK isoform X2 [Galleria mellonella]	-1.61203	1.5194	0.27381	-0.0643	-0.11688
TRINITY_DN56690_c0_g1_i4_orf1	hypothetical protein evm_002209, partial [Chilo suppressalis]	-1.20149	1.28358	0.22576	0.79752	-1.10537
TRINITY_DN4273_c1_g1_i5_orf1	tetraspanin-13 isoform X1 [Ostrinia furnacalis]	-1.30446	1.77836	0.01552	-0.15577	-0.33365
TRINITY_DN9717_c0_g2_i1_orf1	proteasome subunit beta type-2 [Ostrinia furnacalis]	-1.97489	0.72411	0.43239	0.57297	0.24543
TRINITY_DN1355_c0_g1_i7_orf1	larval/pupal rigid cuticle protein 66-like [Ostrinia furnacalis]	-1.78736	0.85136	-0.25422	0.98729	0.20293
TRINITY_DN18291_c0_g1_i1_orf1	hydroxyacylglutathione hydrolase, mitochondrial isoform X1 [Ostrinia furnacalis] >XP_028163678.1 hydroxyacylglutathione hydrolase, mitochondrial isoform X2 [Ostrinia furnacalis] >XP_028163679.1 hydroxyacylglutathione hydrolase, mitochondrial isoform X2 [Ostrinia furnacalis] >XP_028163680.1 hydroxyacylglutathione hydrolase, mitochondrial isoform X3 [Ostrinia furnacalis] >XP_028163681.1 hydroxyacylglutathione hydrolase, mitochondrial isoform X2 [Ostrinia furnacalis]	-1.9595	0.30639	0.3236	0.46727	0.86223
TRINITY_DN4688_c0_g1_i2_orf1	uncharacterized protein LOC114359411 [Ostrinia furnacalis]	-0.92553	1.92622	-0.30122	-0.57075	-0.12872
TRINITY_DN116467_c0_g1_i1_orf1	probable small nuclear ribonucleoprotein E [Ostrinia furnacalis]	-1.53755	1.56739	0.09649	0.22138	-0.3477
TRINITY_DN7590_c0_g1_i4_orf1	innexin inx1-like [Pectinophora gossypiella]	-1.76428	1.22923	-0.2236	0.24051	0.51814
TRINITY_DN65247_c1_g1_i1_orf1	beta-1,3-glucan-binding protein [Manduca sexta] >XP_030023421.1 beta-1,3-glucan-binding protein [Manduca sexta] >XP_030023422.1 beta-1,3-glucan-binding protein [Manduca sexta] >XP_030023423.1 beta-1,3-glucan-binding protein [Manduca sexta] >AEV66276.1 beta-1,3-glucanase [Manduca sexta] >KAG6448577.1 hypothetical protein O3G_MSEX005570 [Manduca sexta] >KAG6448578.1 hypothetical protein O3G_MSEX005570 [Manduca sexta] >KAG6448579.1 hypothetical protein O3G_MSEX005570 [Manduca sexta] >KAG6448580.1 hypothetical protein O3G_MSEX005570 [Manduca sexta]	-1.87555	0.55785	0.66307	0.83583	-0.18119
TRINITY_DN2107_c0_g2_i3_orf1	LIM and SH3 domain protein Lasp [Ostrinia furnacalis]	-1.17265	1.86052	-0.17419	-0.23387	-0.27981
TRINITY_DN19951_c0_g1_i5_orf1	protein croquemort-like [Ostrinia furnacalis]	-1.17419	1.85178	-0.34366	-0.07123	-0.26269
TRINITY_DN1407_c0_g1_i12_orf1	hypothetical protein evm_012298 [Chilo suppressalis]	-1.12376	1.56932	-0.4278	0.72978	-0.74753
TRINITY_DN18592_c0_g2_i1_orf1	GATOR complex protein MIOS [Ostrinia furnacalis]	-1.81691	0.30463	0.17489	1.25229	0.0851
TRINITY_DN2058_c0_g1_i2_orf1	proteasomal ubiquitin receptor ADRM1 [Ostrinia furnacalis]	-0.85566	1.81574	-0.09902	0.11281	-0.97387
TRINITY_DN3675_c0_g1_i1_orf1	unnamed protein product [Spodoptera exigua]	-1.32383	1.7693	0.00858	-0.14366	-0.31039
TRINITY_DN8726_c0_g2_i3_orf1	dnaJ homolog subfamily C member 25 homolog [Ostrinia furnacalis]	-1.82801	1.11758	0.63612	0.00555	0.06875

TRINITY_DN166_c0_g1_i4_orf1	PREDICTED: cryptochrome-1 isoform X1 [Amyelois transitella] >XP_013199861.1 PREDICTED: cryptochrome-1 isoform X1 [Amyelois transitella]	-1.60096	1.33628	-0.54391	0.52374	0.28484
TRINITY_DN8143_c0_g1_i6_orf1	gamma-soluble NSF attachment protein-like [Galleria mellonella]	-1.74703	1.2495	0.36892	0.41233	-0.28372
TRINITY_DN4065_c0_g1_i5_orf1	uncharacterized protein LOC114357176 [Ostrinia furnacalis] >XP_028166588.1 uncharacterized protein LOC114357176 [Ostrinia furnacalis]	-1.91129	0.77417	0.71142	0.48754	-0.06184
TRINITY_DN10290_c0_g1_i7_orf1	aquaporin AQPAe.a [Ostrinia furnacalis]	-1.50411	1.46669	-0.06287	0.5875	-0.48721
TRINITY_DN143792_c0_g1_i1_orf1	ras-related GTP-binding protein C [Venturia canescens]	-1.64216	1.05972	0.50542	0.71744	-0.64042
TRINITY_DN12024_c0_g1_i4_orf1	pancreatic lipase-related protein 2 isoform X1 [Ostrinia furnacalis] >XP_028176200.1 pancreatic lipase-related protein 2 isoform X2 [Ostrinia furnacalis]	-1.72556	1.1905	-0.35584	0.64758	0.24332
TRINITY_DN19328_c0_g1_i1_orf1	hypothetical protein evm_002753 [Chilo suppressalis]	-1.47152	1.43	-0.12597	-0.53262	0.70013
TRINITY_DN648_c0_g1_i5_orf1	uncharacterized protein LOC114360051 [Ostrinia furnacalis]	-1.93171	0.6994	0.83749	0.19884	0.19597
TRINITY_DN195_c0_g3_i6_orf1	E3 ubiquitin-protein ligase SIAH1-like [Ostrinia furnacalis]	-1.85216	0.84257	0.11927	-0.02868	0.91899
TRINITY_DN4565_c0_g1_i3_orf1	acid phosphatase type 7 isoform X1 [Ostrinia furnacalis] >XP_028155943.1 acid phosphatase type 7 isoform X1 [Ostrinia furnacalis] >XP_028155944.1 acid phosphatase type 7 isoform X1 [Ostrinia furnacalis] >XP_028155945.1 acid phosphatase type 7 isoform X1 [Ostrinia furnacalis]	-1.95801	0.84851	0.48226	0.40565	0.22158
TRINITY_DN22944_c0_g3_i1_orf1	transmembrane protein 115 [Ostrinia furnacalis]	-1.91771	0.48394	0.15829	0.28441	0.99108
TRINITY_DN10396_c0_g1_i1_orf1	charged multivesicular body protein 3 isoform X1 [Ostrinia furnacalis] >XP_028178068.1 charged multivesicular body protein 3 isoform X2 [Ostrinia furnacalis]	-1.8882	0.09281	0.18178	1.02031	0.5933
TRINITY_DN18027_c0_g2_i1_orf1	vanin-like protein 2 isoform X2 [Ostrinia furnacalis]	-1.43363	1.66368	-0.35178	0.21206	-0.09033
TRINITY_DN3418_c0_g1_i3_orf1	proto-oncogene tyrosine-protein kinase ROS [Ostrinia furnacalis]	-1.85777	0.69826	-0.25187	0.73454	0.67685
TRINITY_DN585_c0_g1_i5_orf1	very low-density lipoprotein receptor isoform X2 [Galleria mellonella]	-0.76484	1.93387	-0.10784	-0.30645	-0.75474
TRINITY_DN8812_c0_g1_i1_orf1	nuclear pore complex protein Nup160 homolog isoform X3 [Ostrinia furnacalis]	-1.80154	1.06203	0.48665	0.54936	-0.2965
TRINITY_DN29402_c0_g1_i1_orf1	hypothetical protein evm_000391 [Chilo suppressalis] >RVE55077.1 hypothetical protein evm_000444 [Chilo suppressalis] >CAH2982715.1 unnamed protein product [Chilo suppressalis]	-1.53303	1.42261	0.42807	-0.60171	0.28406
TRINITY_DN31417_c0_g1_i3_orf1	titin-like [Ostrinia furnacalis]	-1.10433	1.88646	-0.36732	-0.18798	-0.22684
TRINITY_DN38371_c0_g1_i7_orf1	protein smoothened isoform X2 [Ostrinia furnacalis]	-1.57356	1.47109	0.47761	-0.36268	-0.01246
TRINITY_DN47260_c0_g1_i2_orf1	SET domain-containing protein SmydA-8-like [Ostrinia furnacalis]	-1.8252	0.76258	-0.33253	0.73832	0.65682
TRINITY_DN4125_c0_g1_i6_orf1	angiotensin-converting enzyme-like isoform X1 [Ostrinia furnacalis]	-1.53577	1.06018	-0.28837	-0.37381	1.13778
TRINITY_DN18279_c0_g1_i1_orf1	solute carrier family 12 member 9 isoform X1 [Hyposmocoma kahamanoa]	-1.87573	0.0508	0.7749	0.92955	0.12049
TRINITY_DN61135_c0_g1_i1_orf1	uncharacterized protein LOC114362571 [Ostrinia furnacalis]	-1.75529	1.1059	-0.26874	0.77706	0.14107
TRINITY_DN20356_c0_g1_i5_orf1	uncharacterized protein LOC114362428 [Ostrinia furnacalis]	-0.50714	-0.1106	1.92876	-0.38067	-0.93034
TRINITY_DN103107_c0_g1_i2_orf1	superoxide dismutase [Cu-Zn] [Ostrinia furnacalis] >XP_028177872.1 superoxide dismutase [Cu-Zn] [Ostrinia furnacalis]	-0.20002	-0.31837	1.95555	-0.74816	-0.689
TRINITY_DN66287_c0_g1_i1_orfp1	tenascin-like isoform X4 [Trichoplusia ni]	-0.24384	-0.64446	1.98087	-0.49866	-0.5939
TRINITY_DN12920_c0_g3_i1_orf1	zonadhesin-like isoform X4 [Ostrinia furnacalis]	-0.86972	-0.25238	1.93764	-0.6233	-0.19224
TRINITY_DN50725_c0_g1_i6_orf1	BTB/POZ domain-containing protein 2-like [Ostrinia furnacalis]	-0.21857	-0.53514	1.96068	-0.38764	-0.81933
TRINITY_DN195_c8_g1_i1_orf1	hypothetical protein evm_009768 [Chilo suppressalis]	0.53551	-0.18349	1.64002	-1.04975	-0.94229
TRINITY_DN40911_c0_g1_i1_orf1	peroxisomal membrane protein PEX16 [Ostrinia furnacalis]	0.37412	-0.38765	1.76295	-0.68562	-1.06381
TRINITY_DN30509_c0_g1_i9_orf1	prostatic acid phosphatase [Ostrinia furnacalis]	-0.29101	-0.72261	1.98093	-0.50839	-0.45893
TRINITY_DN3627_c0_g1_i7_orf1	spermine oxidase-like isoform X2 [Ostrinia furnacalis]	-1.00952	0.14822	1.84876	-0.65719	-0.33028
TRINITY_DN29288_c0_g1_i1_orf1	uncharacterized protein LOC114355446 [Ostrinia furnacalis]	-0.83943	-0.1099	1.93797	-0.35499	-0.63366
TRINITY_DN11962_c0_g1_i2_orf1	BTB/POZ domain-containing protein 2-like [Ostrinia furnacalis]	-0.05575	-0.70795	1.93919	-0.43885	-0.73664
TRINITY_DN3307_c1_g1_i2_orf1	BTB/POZ domain-containing protein 2-like [Ostrinia furnacalis]	0.71451	-0.19145	1.48134	-0.6489	-1.3555
TRINITY_DN7570_c0_g1_i18_orf1	sodium/potassium-transporting ATPase subunit alpha isoform X4 [Trichoplusia ni] >XP_026734855.1 sodium/potassium-transporting ATPase subunit alpha isoform X4 [Trichoplusia ni]	0.30603	-0.28929	1.7349	-1.24909	-0.50256
TRINITY_DN2694_c0_g1_i3_orf1	cubilin homolog [Ostrinia furnacalis]	0.21487	-0.81317	1.86278	-0.52454	-0.73993
TRINITY_DN35002_c0_g2_i2_orf1	sulfite oxidase, mitochondrial isoform X3 [Leguminivora glycinivorella]	0.18581	-0.2974	1.83944	-0.8449	-0.88295
TRINITY_DN69334_c0_g1_i1_orf1	PREDICTED: 15-hydroxyprostaglandin dehydrogenase [NAD(+)]-like [Papilio xuthus]	0.93755	-0.65057	1.46781	-0.91995	-0.83485
TRINITY_DN11612_c0_g2_i1_orf1	eukaryotic translation initiation factor 5B [Manduca sexta]	0.23217	-0.05084	1.72823	-1.21314	-0.69643
TRINITY_DN2962_c0_g1_i7_orf1	glucose transporter type 1-like isoform X1 [Ostrinia furnacalis]	-0.63923	-0.26965	1.98396	-0.58583	-0.48926
TRINITY_DN61536_c0_g3_i1_orf1	cubilin homolog [Ostrinia furnacalis]	-0.12303	-0.4805	1.9076	-0.28819	-1.01588
TRINITY_DN27300_c0_g1_i1_orfp1	TRINITY_DN27300_c0_g1_i1_m.71142 TRINITY_DN27300_c0_g1::TRINITY_DN27300_c0_g1_i1::g.71142 ORF type:internal len:82 (-),score=9.46 TRINITY_DN27300_c0_g1_i1:3-245(-)	-1.16404	-0.88268	1.67074	0.23183	0.14416
TRINITY_DN28501_c0_g1_i2_orfp1	TRINITY_DN28501_c0_g1_i2_m.58934 TRINITY_DN28501_c0_g1::TRINITY_DN28501_c0_g1_i2::g.58934 ORF type:internal len:98 (+),score=13.70 TRINITY_DN28501_c0_g1_i2:3-293(+)	-0.9138	-1.27054	1.52418	0.26055	0.39961
TRINITY_DN2407_c0_g1_i2_orf1	uncharacterized protein LOC114366345 isoform X2 [Ostrinia furnacalis]	-1.02281	-0.7624	1.70113	0.52949	-0.44541
TRINITY_DN24789_c0_g1_i9_orfp1	TRINITY_DN24789_c0_g1_i9_m.25888 TRINITY_DN24789_c0_g1::TRINITY_DN24789_c0_g1_i9::g.25888 ORF type:internal len:114 (-),score=12.65 TRINITY_DN24789_c0_g1_i9:2-340(-)	-1.01474	-1.02403	1.69065	0.21118	0.13694
TRINITY_DN609_c0_g1_i1_orf1	zonadhesin-like isoform X1 [Ostrinia furnacalis]	-1.14528	-1.1348	1.40622	0.58059	0.29327

TRINITY_DN59429_c0_g1_i6_orf1	uncharacterized protein LOC114366345 isoform X2 [Ostrinia furnacalis]	-1.52804	-0.79362	1.06094	0.8701	0.39062
TRINITY_DN14754_c0_g1_i6_orf1	cathepsin L [Papilio xuthus]	-0.81321	-1.11172	1.68125	-0.22922	0.4729
TRINITY_DN1149_c0_g1_i4_orf1	circadian clock-controlled protein-like [Ostrinia furnacalis]	-1.45568	-0.82548	1.26362	0.71512	0.30242
TRINITY_DN64719_c0_g1_i2_orfp1	TRINITY_DN64719_c0_g1_i2_m.37745 TRINITY_DN64719_c0_g1::TRINITY_DN64719_c0_g1_i2::g.37745 ORF type:internal len:91 (+),score=41.89 TRINITY_DN64719_c0_g1_i2:1-270(+)	-0.81997	-1.29225	1.57752	0.15284	0.38186
TRINITY_DN57111_c0_g1_i1_orf1	trypsin-like serine proteinase T26 protein, partial [Chilo infuscatellus]	-0.54962	-1.58185	1.38376	0.39994	0.34777
TRINITY_DN12586_c0_g1_i4_orf1	zonadhesin-like isoform X4 [Ostrinia furnacalis]	-1.11446	-1.25954	1.07618	0.9415	0.35631
TRINITY_DN61042_c0_g2_i2_orfp1	TRINITY_DN61042_c0_g2_i2_m.5292 TRINITY_DN61042_c0_g2::TRINITY_DN61042_c0_g2_i2::g.5292 ORF type:5prime_partial len:63 (-),score=21.86 TRINITY_DN61042_c0_g2_i2:77-265(-)	-1.38151	-0.57132	1.35313	0.91395	-0.31425
TRINITY_DN1593_c0_g1_i1_orf1	chemosensory protein csp11 [Helopeltis theivora]	-1.25187	-1.08597	1.31471	0.53995	0.48319
TRINITY_DN8685_c0_g1_i5_orf1	macrophage mannose receptor 1-like [Zerene cesonia]	-1.43299	-0.78348	1.3785	0.62066	0.21731
TRINITY_DN214_c0_g1_i4_orf1	uncharacterized protein LOC114352813 [Ostrinia furnacalis]	-1.00733	-1.24034	1.4236	0.61285	0.21121
TRINITY_DN15685_c0_g1_i5_orf1	uncharacterized protein LOC114352354 [Ostrinia furnacalis]	-1.12233	-1.2913	1.09574	0.61491	0.70297
TRINITY_DN38230_c0_g1_i4_orf1	hypothetical protein evm_007803 [Chilo suppressalis]	-0.78664	-1.40544	1.40858	0.15204	0.63146
TRINITY_DN13236_c0_g1_i4_orf1	zonadhesin-like isoform X4 [Ostrinia furnacalis]	-0.9254	-1.31082	1.4502	0.31118	0.47484
TRINITY_DN121802_c0_g1_i6_orfp1	TRINITY_DN121802_c0_g1_i6_m.78506 TRINITY_DN121802_c0_g1::TRINITY_DN121802_c0_g1_i6::g.78506 ORF type:3prime_partial len:123 (+),score=6.04 TRINITY_DN121802_c0_g1_i6:35-367(+)	-1.11741	-0.94365	1.3535	0.97773	-0.27017
TRINITY_DN24121_c1_g1_i6_orf1	serine protease persephone-like [Ostrinia furnacalis]	-0.85001	-1.18833	1.23011	-0.31197	1.1202
TRINITY_DN14185_c0_g1_i1_orf1	uncharacterized protein LOC114358675 [Ostrinia furnacalis] >XP_028168498.1 uncharacterized protein LOC114358675 [Ostrinia furnacalis]	-1.4804	-0.83945	1.02381	0.97643	0.31961
TRINITY_DN28503_c0_g1_i6_orf1	uncharacterized protein LOC114363584 [Ostrinia furnacalis] >AXY94663.1 seroin transcript 3 [Ostrinia nubilalis]	-0.46922	-1.436	1.62986	0.02959	0.24578
TRINITY_DN27021_c0_g1_i1_orf1	rab3 GTPase-activating protein catalytic subunit isoform X1 [Ostrinia furnacalis]	-1.15998	-0.66673	1.52958	0.79128	-0.49414
TRINITY_DN4068_c1_g2_i1_orf1	larval cuticle protein LCP-17 [Helicoverpa armigera] >PZC82071.1 hypothetical protein B5X24_HaOG211161 [Helicoverpa armigera] >PZC87412.1 hypothetical protein B5X24_HaOG216859 [Helicoverpa armigera]	-0.96395	-1.34265	1.34151	0.51963	0.44546
TRINITY_DN214_c0_g1_i3_orf1	uncharacterized protein LOC114352813 [Ostrinia furnacalis]	-1.15132	-1.14001	1.36991	0.65265	0.26877
TRINITY_DN33885_c0_g1_i1_orf1	inhibitor of nuclear factor kappa-B kinase subunit alpha [Ostrinia furnacalis]	-1.3368	-1.05714	1.10794	0.78617	0.49983
TRINITY_DN1252_c0_g1_i1_orf1	WD repeat-containing protein 47 isoform X1 [Ostrinia furnacalis]	-0.91999	-1.45651	1.04897	0.82292	0.50461
TRINITY_DN12013_c0_g1_i6_orf1	tudor domain-containing protein 7 isoform X3 [Ostrinia furnacalis]	-1.00998	-0.85777	1.79872	0.09119	-0.02217
TRINITY_DN230_c2_g1_i5_orf1	6-pyruvoyl tetrahydrobiopterin synthase [Ostrinia furnacalis]	-1.0854	-0.84355	1.67315	-0.24505	0.50085
TRINITY_DN15858_c0_g1_i2_orf1	15-hydroxyprostaglandin dehydrogenase [NAD(+)]-like [Ostrinia furnacalis]	-1.55418	-0.74927	1.12761	0.7616	0.41423
TRINITY_DN41573_c0_g1_i1_orf1	BRISC and BRCA1-A complex member 2-like [Ostrinia furnacalis]	-0.62244	-1.68132	0.87312	0.71617	0.71448
TRINITY_DN59422_c0_g1_i2_orf1	larval cuticle protein LCP-22-like isoform X2 [Pectinophora gossypiella]	-0.34815	-1.8106	0.821	0.54295	0.79479
TRINITY_DN8095_c0_g1_i3_orf1	circadian clock-controlled protein-like [Ostrinia furnacalis]	-1.07446	-1.35996	0.89069	0.84661	0.69711
TRINITY_DN27300_c0_g1_i6_orfp1	TRINITY_DN27300_c0_g1_i6_m.71140 TRINITY_DN27300_c0_g1::TRINITY_DN27300_c0_g1_i6::g.71140 ORF type:internal len:129 (-),score=20.75 TRINITY_DN27300_c0_g1_i6:1-384(-)	0.03732	-0.98174	1.8822	-0.63077	-0.30702
TRINITY_DN9560_c0_g1_i5_orf1	uncharacterized protein LOC114357350 [Ostrinia furnacalis]	-0.38926	-1.75571	1.10851	0.51552	0.52095
TRINITY_DN31225_c0_g1_i1_orf1	ribosome biogenesis protein BMS1 homolog [Ostrinia furnacalis]	-0.90148	-1.33417	1.13578	0.04374	1.05613
TRINITY_DN12336_c0_g1_i1_orfp1	TRINITY_DN12336_c0_g1_i1_m.30792 TRINITY_DN12336_c0_g1::TRINITY_DN12336_c0_g1_i1::g.30792 ORF type:internal len:86 (-),score=13.56 TRINITY_DN12336_c0_g1_i1:3-257(-)	-0.81597	-1.23094	1.07227	-0.28543	1.26006
TRINITY_DN29698_c0_g1_i3_orf1	15-hydroxyprostaglandin dehydrogenase [NAD(+)]-like [Ostrinia furnacalis]	-1.31424	-1.08779	1.13357	0.63215	0.63632
TRINITY_DN7735_c0_g1_i4_orf1	calphotin-like [Ostrinia furnacalis]	-0.19735	-1.87689	0.73219	0.64205	0.7
TRINITY_DN1175_c1_g1_i2_orf1	methanethiol oxidase [Ostrinia furnacalis]	-1.33291	-1.04706	0.93906	1.04224	0.39867
TRINITY_DN9820_c0_g1_i1_orf1	endocuticle structural glycoprotein SgAbd-2-like [Ostrinia furnacalis]	-0.31938	-1.80382	0.92167	0.40982	0.79171
TRINITY_DN1038_c1_g1_i3_orf1	uncharacterized protein LOC114363583 [Ostrinia furnacalis]	-1.03052	-1.3507	1.10594	0.44096	0.83432
TRINITY_DN804_c0_g1_i7_orf1	hypothetical protein HF086_004695 [Spodoptera exigua] >CAH0695017.1 unnamed protein product [Spodoptera exigua]	-0.47005	-1.42147	1.44535	-0.31072	0.7569
TRINITY_DN76633_c0_g1_i1_orfp1	TRINITY_DN76633_c0_g1_i1_m.53394 TRINITY_DN76633_c0_g1::TRINITY_DN76633_c0_g1_i1::g.53394 ORF type:internal len:164 (-),score=26.46,Toxin_2 PF00451.20 0.00022,Toxin_2|PF00451.20|0.00056,Toxin_2|PF00451.20|0.0002,Toxin_2|PF00451.20|5.9e-06,Gamma-thionin|PF00304.21|2,Gamma-thionin|PF00304.21|0.024,Gamma-thionin|PF00304.21|0.027,Gamma-thionin|PF00304.21|0.066,Toxin_38|PF14866.7|0.27,Toxin_38|PF14866.7|0.054,Toxin_38|PF14866.7|0.14,Defensin_2|PF01097.19|1.7,Defensin_2|PF01097.19|0.055,Defensin_2|PF01097.19|1.2,Defensin_2|PF01097.19|0.15 TRINITY_DN76633_c0_g1_i1:1-489(-)	-0.89206	-1.31831	0.97157	1.23382	0.00498
TRINITY_DN26961_c0_g1_i1_orf1	uncharacterized protein LOC120424957 [Culex pipiens pallens]	-0.6231	-1.59811	1.26262	0.52478	0.43382
TRINITY_DN111488_c0_g1_i1_orf1	LOW QUALITY PROTEIN: formin-J-like [Chelonus insularis]	-0.44276	-1.75115	1.02693	0.55251	0.61447
TRINITY_DN56708_c0_g3_i1_orfp1	TRINITY_DN56708_c0_g3_i1_m.56611 TRINITY_DN56708_c0_g3::TRINITY_DN56708_c0_g3_i1::g.56611 ORF type:internal len:69 (-),score=2.50 TRINITY_DN56708_c0_g3_i1:1-204(-)	-0.85903	-1.35329	1.45736	0.27311	0.48185
TRINITY_DN21170_c0_g1_i5_orf1	twitchin-like [Ostrinia furnacalis]	0.10922	-1.23306	1.78992	-0.47797	-0.1881
TRINITY_DN109931_c0_g1_i1_orf1	hydroxymethylglutaryl-CoA lyase, mitochondrial isoform X1 [Ostrinia furnacalis]	-0.17976	-1.85689	0.93436	0.41132	0.69097
TRINITY_DN76529_c0_g1_i1_orfp1	TRINITY_DN76529_c0_g1_i1_m.64079 TRINITY_DN76529_c0_g1::TRINITY_DN76529_c0_g1_i1::g.64079 ORF type:internal len:70 (+),score=14.68 TRINITY_DN76529_c0_g1_i1:3-209(+)	0.25284	-1.56507	1.52508	-0.3692	0.15635

TRINITY_DN1381_c0_g1_i5_orf1	CKLF-like MARVEL transmembrane domain-containing protein 4 isoform X1 [Ostrinia furnacalis]	-0.76328	-1.48066	1.0473	0.14432	1.05232
TRINITY_DN10441_c0_g1_i3_orf1	zonadhesin-like [Ostrinia furnacalis]	-0.78268	-1.45224	1.38412	0.40694	0.44386
TRINITY_DN16400_c0_g2_i1_orf1	superoxide dismutase [Cu-Zn]-like isoform X1 [Ostrinia furnacalis]	-0.6059	-1.49795	1.48134	0.29009	0.33243
TRINITY_DN6325_c0_g1_i8_orf1	unnamed protein product [Pieris macdunnoughi]	-0.09141	-1.38833	1.73952	-0.08246	-0.17733
TRINITY_DN10889_c0_g1_i8_orf1	four and a half LIM domains protein 2 isoform X7 [Pectinophora gossypiella]	-0.61754	-1.67381	0.75432	0.58581	0.95123
TRINITY_DN38568_c0_g1_i1_orf1	unnamed protein product, partial [Diatraea saccharalis]	-0.50811	-1.72797	0.79283	0.51439	0.92886
TRINITY_DN60792_c0_g1_i2_orf1	ATP-binding cassette sub-family D member 2 [Ostrinia furnacalis] >XP_028165108.1 ATP-binding cassette sub-family D member 2 [Ostrinia furnacalis]	-0.71242	-1.21134	1.70042	-0.12151	0.34485
TRINITY_DN52395_c0_g2_i2_orf1	twitchin isoform X20 [Zerene cesonia]	0.0613	-1.816	1.25425	0.2495	0.25095
TRINITY_DN22597_c0_g1_i4_orf1	uncharacterized protein LOC114361588 isoform X16 [Ostrinia furnacalis]	-0.86723	-1.47739	1.11528	0.43337	0.79598
TRINITY_DN39933_c0_g1_i2_orf1	thioredoxin domain-containing protein 11 isoform X4 [Ostrinia furnacalis]	-1.02996	-1.26484	1.34664	0.66955	0.27861
TRINITY_DN32586_c0_g2_i1_orf1	unnamed protein product [Euphydryas editha]	-0.71643	-1.61791	0.67344	0.6958	0.96511
TRINITY_DN46372_c0_g1_i1_orf1	unnamed protein product [Chilo suppressalis]	-0.26928	-1.69094	1.37572	0.24345	0.34105
TRINITY_DN62707_c0_g1_i1_orf1	uncharacterized protein LOC114362831 [Ostrinia furnacalis]	-1.15593	-1.03975	1.49791	0.56749	0.13028
TRINITY_DN1329_c0_g1_i5_orf1	neurogenic locus notch homolog protein 3 [Ostrinia furnacalis] >XP_028157678.1 neurogenic locus notch homolog protein 3 [Ostrinia furnacalis]	-0.63474	-1.63229	0.79316	0.40718	1.06669
TRINITY_DN5661_c0_g1_i5_orf1	cytochrome P450 6B7-like [Ostrinia furnacalis]	-0.73468	-1.41512	1.26176	-0.04145	0.92948
TRINITY_DN1470_c0_g1_i8_orf1	oxidation resistance protein 1 isoform X5 [Ostrinia furnacalis]	-1.20081	-1.17423	1.23451	0.52121	0.61932
TRINITY_DN17759_c0_g1_i5_orf1	hypothetical protein evm_013530 [Chilo suppressalis]	-1.29768	-1.07552	1.22451	0.5659	0.58279
TRINITY_DN1267_c0_g2_i10_orf1	secretory phospholipase A2 receptor-like [Ostrinia furnacalis]	-0.53484	-1.52985	1.44259	0.53347	0.08863
TRINITY_DN6994_c0_g1_i3_orf1	C-type mannose receptor 2-like isoform X1 [Ostrinia furnacalis]	-1.04889	-0.90284	1.73871	0.24601	-0.03299
TRINITY_DN11448_c0_g1_i11_orf1	hypothetical protein B5X24_HaOG201808 [Helicoverpa armigera]	0.10218	-1.50116	1.58127	-0.42207	0.23979
TRINITY_DN46132_c0_g2_i2_orf1	hypothetical protein evm_003834 [Chilo suppressalis]	-0.87124	-1.29297	1.52698	0.18736	0.44987
TRINITY_DN47151_c0_g1_i1_orf1	unnamed protein product [Danaus chrysippus]	-0.5394	-1.38484	1.03801	-0.36962	1.25586
TRINITY_DN1455_c0_g1_i4_orf1	troponin domain-containing protein [Phthorimaea operculella]	-1.21648	-1.21203	1.02591	0.61468	0.78792
TRINITY_DN1607_c0_g1_i16_orf1	LOW QUALITY PROTEIN: asparagine--tRNA ligase, cytoplasmic [Ostrinia furnacalis]	-0.84261	-1.51342	1.08801	0.71103	0.55699
TRINITY_DN2688_c0_g1_i3_orf1	mitochondrial amidoxime reducing component 2-like [Ostrinia furnacalis]	-1.19192	-0.94582	1.49357	0.67316	-0.029
TRINITY_DN2175_c0_g1_i4_orf1	uncharacterized protein LOC114353827 [Ostrinia furnacalis]	-0.898	-1.36488	1.33908	0.69734	0.22646
TRINITY_DN2722_c0_g1_i1_orf1	troponin C [Pieris rapae] >XP_045490973.1 troponin C-like isoform X1 [Colias croceus] >XP_049866665.1 troponin C-like [Pectinophora gossypiella]	-0.53593	-1.72559	0.83793	0.61258	0.81101
TRINITY_DN6392_c0_g1_i9_orf1	putative mediator of RNA polymerase II transcription subunit 29 isoform X4 [Ostrinia furnacalis]	-1.34001	-0.84995	1.41714	0.68226	0.09056
TRINITY_DN2559_c0_g1_i4_orf1	uricase [Ostrinia furnacalis]	-0.56656	-1.56077	1.38525	0.2149	0.52718
TRINITY_DN11798_c0_g2_i1_orf1	N-acetylglucosamine-6-sulfatase-like isoform X2 [Ostrinia furnacalis]	-1.60112	-0.44496	1.08465	1.02835	-0.06692
TRINITY_DN920_c0_g1_i4_orf1	glutathione S-transferase omega 2 [Ostrinia furnacalis]	0.44001	-1.1651	1.47996	-1.07519	0.32032
TRINITY_DN93764_c0_g1_i1_orf1	microtubule-associated protein futsch isoform X4 [Ostrinia furnacalis] >XP_028162562.1 microtubule-associated protein futsch isoform X4 [Ostrinia furnacalis]	-1.25886	-0.9674	1.36422	0.78227	0.07976
TRINITY_DN1212_c0_g1_i8_orf1	extensin isoform X5 [Ostrinia furnacalis] >XP_028175473.1 extensin isoform X5 [Ostrinia furnacalis] >XP_028175474.1 extensin isoform X5 [Ostrinia furnacalis]	-0.15565	-1.87769	0.76229	0.46066	0.8104
TRINITY_DN3859_c0_g1_i5_orf1	D-aspartate oxidase [Ostrinia furnacalis] >XP_028166452.1 D-aspartate oxidase [Ostrinia furnacalis] >XP_028166454.1 D-aspartate oxidase [Ostrinia furnacalis]	-1.14924	-1.22218	0.96059	1.07118	0.33965
TRINITY_DN98995_c0_g1_i2_orf1	hypothetical protein HF086_008399, partial [Spodoptera exigua]	-0.25721	-1.65883	0.96638	-0.15656	1.10622
TRINITY_DN2302_c0_g1_i1_orf1	enoyl-CoA hydratase domain-containing protein 3, mitochondrial [Ostrinia furnacalis]	-1.2374	-0.86378	1.56134	0.00609	0.53375
TRINITY_DN26254_c0_g1_i1_orf1	hypothetical protein evm_003664 [Chilo suppressalis] >CAB3521132.1 unnamed protein product [Chilo suppressalis] >CAH0398453.1 unnamed protein product [Chilo suppressalis]	0.02305	-1.93292	0.81076	0.5185	0.58061
TRINITY_DN39837_c0_g1_i1_orf1	unnamed protein product [Plutella xylostella]	-1.17692	-1.25229	0.90928	0.93988	0.58006
TRINITY_DN8651_c0_g1_i18_orf1	glutathione S-transferase theta 2 [Conogethes punctiferalis]	-0.01626	-1.72793	1.16951	-0.20313	0.7778
TRINITY_DN549_c0_g1_i7_orf1	titin-like, partial [Ostrinia furnacalis]	0.05205	-1.92751	0.88324	0.42667	0.56555
TRINITY_DN610_c0_g1_i1_orf1	CAP-Gly domain-containing linker protein 1 isoform X10 [Ostrinia furnacalis]	-1.23202	-1.15759	0.96644	1.02417	0.399
TRINITY_DN1789_c0_g1_i5_orf1	uncharacterized protein LOC114365543 [Ostrinia furnacalis]	-1.19746	-0.54979	1.60329	0.65633	-0.51237
TRINITY_DN2215_c0_g2_i1_orf1	PREDICTED: larval cuticle protein LCP-22-like [Amyelois transitella]	0.10403	-1.94895	0.75122	0.42775	0.66596
TRINITY_DN22797_c0_g1_i5_orf1	phenoloxidase-activating factor 2-like isoform X1 [Ostrinia furnacalis]	-0.42154	-1.63398	1.3784	0.44627	0.23085
TRINITY_DN27500_c0_g1_i4_orf1	hemicentin-1-like [Ostrinia furnacalis]	0.50168	-1.73577	1.19087	-0.37609	0.4193
TRINITY_DN3913_c0_g1_i6_orf1	protein obstructor-E-like [Ostrinia furnacalis]	-0.23363	-1.83515	0.8609	0.37265	0.83523
TRINITY_DN699_c0_g1_i5_orf1	TPA_exp: putative parasitoid killing factor [Trichoplusia ni]	-1.42816	-0.6251	0.9652	1.26723	-0.17917
TRINITY_DN97472_c0_g1_i5_orf1	microtubule-actin cross-linking factor 1 isoform X15 [Ostrinia furnacalis]	-0.97108	-1.39005	1.20786	0.55647	0.5968
TRINITY_DN31118_c0_g1_i1_orf1	CPR9 [Ostrinia furnacalis]	-0.03682	-1.92672	0.68031	0.63153	0.65171
TRINITY_DN5628_c0_g1_i5_orf1	hypothetical protein O3G_MSEX015036 [Manduca sexta]	-0.46288	-1.75832	0.85726	0.56155	0.80239
TRINITY_DN61335_c0_g2_i1_orf1	uncharacterized protein LOC114352149 [Ostrinia furnacalis]	-1.12724	-1.21007	1.32051	0.46154	0.55525

TRINITY_DN2312_c0_g1_i4_orf1	endoplasmic reticulum-Golgi intermediate compartment protein 3 [Ostrinia furnacalis]	-0.49633	-1.74004	0.94193	0.62577	0.66866
TRINITY_DN26993_c1_g1_i8_orf1	endocuticle structural glycoprotein ABD-4-like [Ostrinia furnacalis]	-0.89398	-1.25735	1.48352	0.02083	0.64699
TRINITY_DN10652_c0_g1_i4_orf1	uncharacterized protein LOC114364166 [Ostrinia furnacalis]	-0.95303	-0.88484	1.81415	0.10499	-0.08127
TRINITY_DN45949_c0_g1_i1_orf1	uncharacterized protein LOC114355167 [Ostrinia furnacalis]	-0.11719	-1.86967	0.86444	0.79938	0.32304
TRINITY_DN5768_c0_g1_i2_orf1	adenosylhomocysteinase [Ostrinia furnacalis]	-0.91861	-1.44622	1.08293	0.82833	0.45358
TRINITY_DN3377_c0_g1_i1_orf1	calcyphosin-like protein isoform X3 [Helicoverpa armigera] >XP_047020698.1 calcyphosin-like protein isoform X2 [Helicoverpa zea]	-1.26918	-0.81388	1.30544	0.98891	-0.21129
TRINITY_DN17657_c0_g1_i1_orf1	alcohol dehydrogenase 18, partial [Helicoverpa assulta]	-0.25167	-1.69284	1.05774	0.97204	-0.08527
TRINITY_DN14922_c0_g3_i2_orf1	probable pseudouridine-5'-phosphatase [Ostrinia furnacalis]	-0.53032	-1.68392	1.14434	0.50938	0.56052
TRINITY_DN7022_c0_g1_i7_orf1	fatty-acid amide hydrolase 2-A-like [Ostrinia furnacalis]	-0.83906	-1.09664	1.55714	0.73563	-0.35707
TRINITY_DN13119_c0_g1_i4_orf1	endocuticle structural glycoprotein ABD-5-like [Bicyclus anynana]	0.04679	-1.9281	0.72368	0.37087	0.78676
TRINITY_DN3433_c0_g1_i6_orf1	cytosolic purine 5'-nucleotidase isoform X2 [Ostrinia furnacalis] >XP_028162963.1 cytosolic purine 5'-nucleotidase isoform X2 [Ostrinia furnacalis]	-1.23385	-0.807	1.65249	0.09365	0.29471
TRINITY_DN695_c0_g1_i5_orf1	uncharacterized protein LOC114363574 isoform X1 [Ostrinia furnacalis]	-1.18381	-1.24906	0.95322	0.87237	0.60728
TRINITY_DN9871_c0_g1_i11_orf1	PEST proteolytic signal-containing nuclear protein-like [Ostrinia furnacalis]	0.29687	-1.90861	1.03905	0.17347	0.39922
TRINITY_DN1180_c0_g1_i4_orf1	larval cuticle protein LCP-30-like [Ostrinia furnacalis]	0.16161	-1.96292	0.70143	0.44049	0.65939
TRINITY_DN1029_c0_g1_i1_orfp1	TRINITY_DN1029_c0_g1_i1_m.64408 TRINITY_DN1029_c0_g1::TRINITY_DN1029_c0_g1_i1::g.64408 ORF type:3prime_partial len:55 (+),score=13.47 TRINITY_DN1029_c0_g1_i1:74-235(+)	0.12389	-1.95402	0.79258	0.51464	0.52291
TRINITY_DN116972_c0_g1_i1_orf1	phosphofructokinase domain-containing protein [Phthorimaea operculella]	-1.08688	-1.31729	1.12452	0.63202	0.64763
TRINITY_DN31645_c0_g1_i3_orf1	dystonin isoform X43 [Helicoverpa armigera]	-0.09128	-1.85056	1.01227	0.22986	0.69971
TRINITY_DN30208_c0_g1_i3_orf1	unnamed protein product [Timema cristinae]	0.07254	-1.904	0.95265	0.24468	0.63413
TRINITY_DN29969_c0_g1_i5_orf1	twitchin-like [Ostrinia furnacalis]	-0.18881	-1.39292	1.72402	0.07332	-0.21561
TRINITY_DN99063_c0_g1_i1_orf1	microtubule-associated protein futsch isoform X4 [Ostrinia furnacalis] >XP_028162562.1 microtubule-associated protein futsch isoform X4 [Ostrinia furnacalis]	-0.40757	-1.71563	1.15367	0.27315	0.69638
TRINITY_DN1993_c0_g1_i1_orf1	6-phosphofructo-2-kinase/fructose-2,6-bisphosphatase isoform X1 [Ostrinia furnacalis]	-0.38654	-1.43298	1.65878	-0.04749	0.20822
TRINITY_DN122393_c0_g1_i1_orf1	microtubule-associated protein futsch isoform X4 [Ostrinia furnacalis] >XP_028162562.1 microtubule-associated protein futsch isoform X4 [Ostrinia furnacalis]	0.15849	-1.7969	1.25611	-0.02698	0.40928
TRINITY_DN120_c0_g1_i2_orf1	PREDICTED: myosin light chain alkali-like [Amyelois transitella]	-0.33102	-1.80515	0.88496	0.44403	0.80718
TRINITY_DN2956_c0_g1_i6_orf1	fructose-bisphosphate aldolase-like isoform X1 [Ostrinia furnacalis] >XP_028178678.1 fructose-bisphosphate aldolase-like isoform X1 [Ostrinia furnacalis]	-0.68403	-1.5902	0.79378	0.36777	1.11267
TRINITY_DN105506_c0_g1_i8_orf1	microtubule-actin cross-linking factor 1 isoform X15 [Ostrinia furnacalis]	-0.55027	-1.6918	0.76612	0.45612	1.01982
TRINITY_DN4010_c0_g2_i1_orf1	myophilin [Ostrinia furnacalis]	0.29356	-1.87995	1.09712	0.07692	0.41235
TRINITY_DN2887_c0_g1_i1_orf1	F-box/LRR-repeat protein 4-like isoform X1 [Ostrinia furnacalis] >XP_028177606.1 F-box/LRR-repeat protein 4-like isoform X1 [Ostrinia furnacalis]	-0.73216	-1.31461	1.12278	-0.26196	1.18595
TRINITY_DN1034_c0_g1_i4_orf1	glycerol kinase isoform X4 [Ostrinia furnacalis]	-1.26569	-1.10341	0.88194	1.12886	0.3583
TRINITY_DN31118_c0_g2_i1_orf1	unnamed protein product [Spodoptera exigua]	0.06255	-1.94879	0.53836	0.66686	0.68102
TRINITY_DN9569_c1_g1_i7_orf1	V-set and immunoglobulin domain-containing protein 1-like isoform X1 [Ostrinia furnacalis] >XP_028156015.1 V-set and immunoglobulin domain-containing protein 1-like isoform X2 [Ostrinia furnacalis]	-1.1286	-1.31084	0.8528	0.89809	0.68856
TRINITY_DN3231_c0_g1_i12_orf1	integrin-linked protein kinase [Pectinophora gossypiella]	-0.47606	-1.70213	0.71312	0.3489	1.11618
TRINITY_DN23474_c1_g1_i1_orf1	unnamed protein product [Chrysodeixis includens]	0.37894	-1.31266	1.58303	-0.78101	0.1317
TRINITY_DN779_c0_g1_i3_orf1	uncharacterized protein LOC114351172 isoform X1 [Ostrinia furnacalis]	-0.52753	-1.66291	1.21657	0.45382	0.52004
TRINITY_DN2193_c0_g1_i7_orf1	long-chain-fatty-acid--CoA ligase 5 isoform X1 [Ostrinia furnacalis] >XP_028176293.1 long-chain-fatty-acid--CoA ligase 5 isoform X1 [Ostrinia furnacalis] >XP_028176294.1 long-chain-fatty-acid--CoA ligase 5 isoform X1 [Ostrinia furnacalis] >XP_028176295.1 long-chain-fatty-acid--CoA ligase 5 isoform X1 [Ostrinia furnacalis] >XP_028176296.1 long-chain-fatty-acid--CoA ligase 5 isoform X1 [Ostrinia furnacalis] >XP_028176297.1 long-chain-fatty-acid--CoA ligase 5 isoform X1 [Ostrinia furnacalis] >XP_028176298.1 long-chain-fatty-acid--CoA ligase 5 isoform X2 [Ostrinia furnacalis]	-0.52768	-1.52896	1.12899	-0.11883	1.04647
TRINITY_DN31390_c0_g1_i2_orf1	UDP-glucuronosyltransferase 2B20-like [Ostrinia furnacalis]	-0.35698	-1.59727	1.09817	1.03994	-0.18387
TRINITY_DN24410_c0_g2_i1_orf1	spermine oxidase-like isoform X3 [Venturia canescens] >XP_043269281.1 spermine oxidase-like isoform X3 [Venturia canescens]	-0.99253	-1.38836	0.93788	1.01011	0.4329
TRINITY_DN19251_c0_g1_i8_orf1	succinate--CoA ligase [GDP-forming] subunit beta, mitochondrial [Ostrinia furnacalis]	-0.40389	-1.53168	1.55326	0.14039	0.24192
TRINITY_DN2700_c2_g1_i1_orf1	mitotic checkpoint protein BUB3 [Ostrinia furnacalis]	-1.08171	-1.00601	1.6387	0.35042	0.0986
TRINITY_DN47930_c0_g1_i4_orf1	uncharacterized protein LOC114362634 [Ostrinia furnacalis]	-0.67598	-1.64292	0.89758	0.59206	0.82927
TRINITY_DN24322_c0_g1_i4_orf1	unnamed protein product, partial [Brenthis ino]	0.16453	-1.89292	1.0696	0.44924	0.20956
TRINITY_DN57856_c0_g2_i1_orf1	cytochrome P450 6B2-like [Ostrinia furnacalis]	-0.16692	-1.55768	0.94914	-0.4321	1.20756
TRINITY_DN36460_c0_g1_i2_orf1	N-acetylneuraminate lyase-like [Ostrinia furnacalis]	-1.23813	-1.17242	1.10292	0.55114	0.75649
TRINITY_DN2719_c1_g1_i6_orf1	unnamed protein product [Chrysodeixis includens]	-0.00589	-1.28481	1.62811	-0.73508	0.39767
TRINITY_DN4793_c0_g1_i7_orf1	probable hydroxyacid-oxoacid transhydrogenase, mitochondrial isoform X3 [Ostrinia furnacalis] >XP_028159821.1 probable hydroxyacid-oxoacid transhydrogenase, mitochondrial isoform X4 [Ostrinia furnacalis]	-0.59746	-1.41631	0.96068	-0.23494	1.28802
TRINITY_DN124171_c0_g1_i4_orf1	dystonin isoform X27 [Trichoplusia ni]	-1.0174	-1.33478	1.21197	0.39072	0.74949

TRINITY_DN1134_c0_g1_i4_orf1	cytochrome P450 6B5-like [Ostrinia furnacalis]	-0.23886	-1.84729	0.60846	0.55384	0.92385
TRINITY_DN48610_c0_g1_i2_orf1	hypothetical protein evm_002298 [Chilo suppressalis] >CAH0682062.1 unnamed protein product [Chilo suppressalis]	0.3218	-1.91586	0.99689	0.13453	0.46265
TRINITY_DN6612_c0_g1_i4_orf1	hypothetical protein O3G_MSEX008151 [Manduca sexta]	0.23766	-1.7506	1.25007	-0.24386	0.50673
TRINITY_DN51424_c0_g2_i1_orf1	ras suppressor protein 1 [Helicoverpa zea]	-1.32323	-0.98677	1.18492	0.90781	0.21727
TRINITY_DN22044_c0_g2_i1_orf1	derlin-1 [Ostrinia furnacalis]	-0.58352	-1.48853	1.15395	-0.12864	1.04673
TRINITY_DN268_c3_g1_i2_orf1	hypothetical protein evm_003084 [Chilo suppressalis]	-1.30524	-1.11548	1.0632	0.66837	0.68915
TRINITY_DN28311_c0_g1_i2_orf1	RNA polymerase II-associated protein 3-like isoform X1 [Ostrinia furnacalis] >XP_028179249.1 RNA polymerase II-associated protein 3-like isoform X2 [Ostrinia furnacalis] >XP_028179250.1 RNA polymerase II-associated protein 3-like isoform X3 [Ostrinia furnacalis]	-1.13374	-0.98153	1.58486	0.48746	0.04295
TRINITY_DN1404_c0_g1_i6_orf1	uncharacterized protein LOC114363065 [Ostrinia furnacalis]	-0.73089	-1.59946	1.00956	0.57081	0.74999
TRINITY_DN870_c0_g1_i3_orf1	talin-1 isoform X12 [Ostrinia furnacalis]	-0.40283	-1.63629	1.01162	-0.03809	1.06559
TRINITY_DN58261_c0_g1_i2_orf1	15-hydroxyprostaglandin dehydrogenase [NAD(+)]-like [Ostrinia furnacalis]	-0.59387	-1.67419	0.61848	0.59972	1.04986
TRINITY_DN1455_c0_g1_i8_orf1	troponin T, skeletal muscle isoform X1 [Galleria mellonella]	0.32021	-1.95623	0.86653	0.27585	0.49364
TRINITY_DN3800_c0_g1_i7_orf1	hypothetical protein evm_004893 [Chilo suppressalis]	-0.41888	-1.67165	1.16369	0.11239	0.81445
TRINITY_DN1459_c0_g1_i2_orf1	reticulon-1 isoform X1 [Ostrinia furnacalis]	-0.056	-1.45875	1.65667	0.16806	-0.30998
TRINITY_DN15373_c0_g1_i2_orf1	SET domain-containing protein SmydA-8-like isoform X2 [Ostrinia furnacalis]	-0.69576	-1.63314	0.88853	0.82494	0.61543
TRINITY_DN10430_c0_g1_i4_orf1	fatty acid synthase [Ostrinia furnacalis] >XP_028160534.1 fatty acid synthase [Ostrinia furnacalis] >XP_028160535.1 fatty acid synthase [Ostrinia furnacalis] >XP_028160536.1 fatty acid synthase [Ostrinia furnacalis]	-0.74102	-1.32343	0.73093	-0.132	1.46552
TRINITY_DN5998_c0_g2_i1_orf1	protein archease-like [Ostrinia furnacalis]	-1.12822	-1.01076	1.59835	0.22214	0.31848
TRINITY_DN1814_c0_g2_i1_orf1	titin-like, partial [Ostrinia furnacalis]	0.05981	-1.92366	0.76673	0.31808	0.77904
TRINITY_DN77480_c0_g1_i2_orf1	hypothetical protein evm_008422 [Chilo suppressalis]	0.10078	-1.56245	1.31077	-0.56451	0.71541
TRINITY_DN85161_c0_g1_i2_orf1	hypothetical protein evm_013813 [Chilo suppressalis]	-0.71667	-1.17736	1.66318	0.50748	-0.27664
TRINITY_DN4289_c0_g1_i5_orf1	translocon-associated protein subunit beta [Ostrinia furnacalis] >XP_028157895.1 translocon-associated protein subunit beta [Ostrinia furnacalis] >XP_028157896.1 translocon-associated protein subunit beta [Ostrinia furnacalis]	-1.18497	-1.2145	1.15426	0.70395	0.54127
TRINITY_DN46173_c0_g3_i1_orf1	Tropomyosin, partial [Cotesia chilonis]	0.14505	-1.91935	0.97522	0.28836	0.51071
TRINITY_DN76333_c0_g1_i2_orf1	larval cuticle protein 65Ag1-like [Ostrinia furnacalis]	0.22125	-1.97699	0.54308	0.52779	0.68487
TRINITY_DN116_c1_g1_i8_orf1	uncharacterized protein LOC114350057 isoform X2 [Ostrinia furnacalis]	0.25522	-1.94627	0.91454	0.32116	0.45535
TRINITY_DN15136_c0_g1_i2_orf1	alpha-aminoadipic semialdehyde synthase, mitochondrial isoform X3 [Ostrinia furnacalis]	-0.47989	-1.72056	1.08644	0.49171	0.6223
TRINITY_DN2040_c0_g1_i15_orfp1	TRINITY_DN2040_c0_g1_i15_m.4150 TRINITY_DN2040_c0_g1::TRINITY_DN2040_c0_g1_i15::g.4150 ORF type:complete len:319 (+),score=125.43,Plasmodium_HRP PF05403.12 3.5,Plasmodium_HRP|PF05403.12|1.5 TRINITY_DN2040_c0_g1_i15:118-957(+)	-0.06364	-1.91917	0.62549	0.66567	0.69165
TRINITY_DN17061_c0_g1_i1_orf1	uncharacterized protein LOC113511282 isoform X2 [Galleria mellonella]	-0.38212	-1.75995	0.86357	0.32817	0.95033
TRINITY_DN1391_c1_g2_i2_orf1	uncharacterized protein LOC119837640 isoform X2 [Zerene cesonia]	0.18941	-1.92272	0.89427	0.17918	0.65987
TRINITY_DN3906_c0_g1_i5_orf1	ejaculatory bulb-specific protein 3-like [Ostrinia furnacalis]	0.41695	-1.67665	1.23185	-0.48465	0.5125
TRINITY_DN1333_c0_g1_i6_orf1	uncharacterized protein LOC114362563 [Ostrinia furnacalis]	-0.47579	-1.69647	0.9926	0.2623	0.91736
TRINITY_DN42120_c0_g1_i2_orf1	FAM172 family protein homolog CG10038 [Ostrinia furnacalis]	-0.94742	-1.2313	1.52644	0.47368	0.1786
TRINITY_DN1895_c0_g1_i2_orf1	unnamed protein product [Chrysodeixis includens]	0.17634	-1.86979	0.91711	-0.01825	0.79459
TRINITY_DN248_c0_g1_i12_orf1	twitchin-like [Ostrinia furnacalis]	0.66343	-1.74055	1.19718	0.15197	-0.27205
TRINITY_DN46216_c0_g3_i1_orf1	unnamed protein product, partial [Brenthis ino]	0.35194	-1.87715	0.70043	-0.09834	0.92312
TRINITY_DN82628_c0_g1_i2_orf1	ORF type:internal len:148 hit:XP_028162129.1 TRINITY_DN82628_c0_g1_i2:3-446(-)	0.22348	-1.9438	0.86313	0.25636	0.60083
TRINITY_DN37418_c0_g1_i4_orf1	NADH dehydrogenase [ubiquinone] 1 alpha subcomplex assembly factor 3 isoform X1 [Ostrinia furnacalis] >XP_028169243.1 NADH dehydrogenase [ubiquinone] 1 alpha subcomplex assembly factor 3 isoform X1 [Ostrinia furnacalis] >XP_028169244.1 NADH dehydrogenase [ubiquinone] 1 alpha subcomplex assembly factor 3 isoform X2 [Ostrinia furnacalis] >XP_028169245.1 NADH dehydrogenase [ubiquinone] 1 alpha subcomplex assembly factor 3 isoform X2 [Ostrinia furnacalis]	-0.90133	-0.65466	1.66999	0.63713	-0.75114
TRINITY_DN27491_c0_g1_i1_orf1	prolactin regulatory element-binding protein [Galleria mellonella]	-0.31218	-1.5147	1.45882	-0.27003	0.63808
TRINITY_DN1104_c0_g1_i1_orfp1	TRINITY_DN1104_c0_g1_i1_m.5521 TRINITY_DN1104_c0_g1::TRINITY_DN1104_c0_g1_i1::g.5521 ORF type:5prime_partial len:204 (+),score=39.47 TRINITY_DN1104_c0_g1_i1:3-614(+)	-0.02673	-1.52246	1.34044	-0.55246	0.7612
TRINITY_DN102051_c0_g1_i1_orf1	spectrin repeat domain-containing protein [Phthorimaea operculella]	-0.29677	-1.81893	0.97351	0.52989	0.6123
TRINITY_DN4567_c0_g3_i1_orf1	hypothetical protein JYU34_019391 [Plutella xylostella] >KAG7311447.1 hypothetical protein JYU34_002489 [Plutella xylostella]	-1.52234	-0.71076	1.26471	0.71755	0.25083
TRINITY_DN34406_c0_g2_i9_orfp1	TRINITY_DN34406_c0_g2_i9_m.33755 TRINITY_DN34406_c0_g2::TRINITY_DN34406_c0_g2_i9::g.33755 ORF type:internal len:82 (-),score=12.88 TRINITY_DN34406_c0_g2_i9:3-245(-)	-1.3357	0.75843	1.25759	-0.98349	0.30318
TRINITY_DN64181_c0_g1_i1_orf1	uncharacterized protein LOC114356431 isoform X2 [Ostrinia furnacalis]	-1.55545	1.46639	0.54647	-0.11249	-0.34493
TRINITY_DN15400_c0_g1_i1_orf1	uncharacterized protein LOC114366781 [Ostrinia furnacalis]	-1.36477	1.20999	0.66042	-0.99758	0.49194
TRINITY_DN325_c0_g1_i15_orf1	protein draper-like [Ostrinia furnacalis]	-0.95404	0.67937	1.6192	-0.89874	-0.44579
TRINITY_DN20717_c0_g1_i1_orf1	putative uncharacterized protein DDB_G0282133 isoform X1 [Ostrinia furnacalis]	-1.40657	1.5924	0.49019	-0.43032	-0.24569
TRINITY_DN858_c0_g1_i3_orf1	uncharacterized protein LOC114351944 [Ostrinia furnacalis]	-1.24534	1.23973	0.79527	-1.09051	0.30085
TRINITY_DN12014_c0_g1_i2_orf1	unnamed protein product [Chilo suppressalis]	-1.50932	0.83407	1.33928	-0.40995	-0.25408
TRINITY_DN59388_c0_g1_i1_orf1	uncharacterized protein LOC114353759 [Ostrinia furnacalis]	-1.44043	1.5435	0.48286	-0.03031	-0.55562
TRINITY_DN26985_c0_g1_i5_orf1	secretory phospholipase A2 receptor-like [Helicoverpa zea]	-0.57368	1.36674	1.03384	-0.73275	-1.09415

TRINITY_DN38106_c0_g1_i6_orf1	unnamed protein product [Pieris macdunnoughi]	-0.82534	0.90834	1.44438	-1.11086	-0.41652
TRINITY_DN3647_c1_g1_i5_orf1	unnamed protein product, partial [Iphiclides podalirius]	-1.62344	1.1311	0.94416	-0.43985	-0.01197
TRINITY_DN80424_c0_g1_i1_orf1	PREDICTED: cytoplasmic FMR1-interacting protein [Dufourea novaeangliae] >KZC10094.1 Cytoplasmic FMR1-interacting protein [Dufourea novaeangliae]	-0.55388	0.54726	1.70473	-1.00044	-0.69767
TRINITY_DN1293_c1_g1_i4_orf1	putative fatty acyl-CoA reductase CG5065 [Ostrinia furnacalis]	-0.61234	0.69473	1.62698	-0.7236	-0.98576
TRINITY_DN10373_c0_g1_i1_orf1	homocysteine S-methyltransferase 1-like [Ostrinia furnacalis] >XP_028162778.1 homocysteine S-methyltransferase 1-like [Ostrinia furnacalis]	-1.50072	0.81698	1.20418	-0.75767	0.23723
TRINITY_DN9383_c0_g1_i3_orf1	uncharacterized protein LOC114361502 [Ostrinia furnacalis]	-1.38247	1.4029	0.83188	-0.2453	-0.607
TRINITY_DN919_c0_g1_i7_orf1	facilitated trehalose transporter Tret1-like [Ostrinia furnacalis] >XP_028161733.1 facilitated trehalose transporter Tret1-like [Ostrinia furnacalis]	-0.70451	0.41212	1.79098	-0.79017	-0.70842
TRINITY_DN130439_c0_g1_i1_orf1	DDRGK domain-containing protein 1-like [Ostrinia furnacalis]	-1.43774	1.09269	1.14758	-0.62482	-0.17771
TRINITY_DN21533_c0_g1_i7_orf1	annexin B9 isoform X1 [Ostrinia furnacalis]	-1.46475	0.90783	1.31632	-0.44791	-0.31149
TRINITY_DN896_c0_g1_i2_orf1	uncharacterized protein LOC114356314 isoform X2 [Ostrinia furnacalis]	-1.17383	1.21433	1.06665	-0.99905	-0.1081
TRINITY_DN8083_c0_g1_i1_orf1	solute carrier family 35 member F6 [Ostrinia furnacalis]	-1.53889	1.36715	0.75293	-0.40663	-0.17457
TRINITY_DN11868_c0_g1_i2_orf1	uncharacterized protein LOC114361308 [Ostrinia furnacalis]	-1.55467	1.07345	0.98066	-0.66462	0.16518
TRINITY_DN24668_c0_g1_i8_orf1	uncharacterized protein LOC114364067 isoform X3 [Ostrinia furnacalis]	-1.31482	1.05858	1.25614	-0.30909	-0.69081
TRINITY_DN2442_c0_g1_i2_orf1	digestive cysteine proteinase 2 [Ostrinia furnacalis]	-1.25582	1.77788	0.1596	-0.38702	-0.29464
TRINITY_DN110231_c0_g1_i1_orf1	protein singed [Ostrinia furnacalis] >XP_028161434.1 protein singed [Ostrinia furnacalis]	-1.09849	1.80145	0.27123	-0.48644	-0.48775
TRINITY_DN537_c0_g1_i1_orf1	pupal cuticle protein C1B-like precursor [Papilio xuthus] >BAM18715.1 cuticular protein PxutCPFL6Ba [Papilio xuthus]	-1.3641	1.59296	0.539	-0.47446	-0.2934
TRINITY_DN132_c0_g2_i2_orf1	alpha-tocopherol transfer protein-like isoform X1 [Ostrinia furnacalis]	-1.19574	0.95664	1.40088	-0.67501	-0.48678
TRINITY_DN1194_c0_g1_i5_orf1	sequestosome-1-like isoform X4 [Ostrinia furnacalis]	-1.22234	1.48736	0.83189	-0.54737	-0.54955
TRINITY_DN1091_c0_g1_i1_orf1	macrophage mannose receptor 1-like [Pararge aegeria]	-1.3514	1.49356	0.73937	-0.50319	-0.37834
TRINITY_DN4189_c0_g2_i1_orf1	unnamed protein product [Chilo suppressalis]	-1.08714	1.81843	0.21088	-0.5786	-0.36358
TRINITY_DN4817_c0_g1_i4_orf1	palmitoyl-protein thioesterase 1 isoform X1 [Ostrinia furnacalis] >XP_028170290.1 palmitoyl-protein thioesterase 1 isoform X4 [Ostrinia furnacalis]	-1.28582	1.01208	1.16198	-0.98155	0.09331
TRINITY_DN6205_c0_g1_i4_orfp1	TRINITY_DN6205_c0_g1_i4_m.72677 TRINITY_DN6205_c0_g1::TRINITY_DN6205_c0_g1_i4::g.72677 ORF type:internal len:68 (-),score=1.69 TRINITY_DN6205_c0_g1_i4:2-202(-)	-0.61883	1.37868	1.04104	-0.97437	-0.82651
TRINITY_DN53427_c0_g1_i2_orf1	heparanase-like [Ostrinia furnacalis]	-1.10802	1.70369	0.50923	-0.55385	-0.55106
TRINITY_DN7316_c0_g2_i1_orf1	ubiquitin-protein ligase E3C [Ostrinia furnacalis]	-0.67841	1.26443	1.15401	-1.08841	-0.65163
TRINITY_DN4276_c0_g1_i6_orf1	arylsulfatase B [Ostrinia furnacalis]	-1.42492	1.53834	0.38713	0.15467	-0.65522
TRINITY_DN60949_c0_g1_i4_orf1	aldo-keto reductase AKR2E4-like [Galleria mellonella]	-0.70658	1.09138	1.29785	-1.16503	-0.51762
TRINITY_DN140_c1_g1_i2_orf1	modular serine protease-like isoform X1 [Ostrinia furnacalis]	-1.36051	1.57972	0.57878	-0.39091	-0.40708
TRINITY_DN61048_c0_g1_i2_orf1	PREDICTED: protein THEM6-like [Amyelois transitella]	-1.49946	0.90556	0.99434	-0.85698	0.45654
TRINITY_DN36856_c0_g1_i1_orf1	protein enhancer of sevenless 2B isoform X2 [Formica exsecta]	-1.39515	1.63523	0.30438	-0.00886	-0.53559
TRINITY_DN90327_c0_g1_i1_orf1	hypothetical protein HF086_000910, partial [Spodoptera exigua]	-1.07599	1.54479	0.7845	-0.79254	-0.46077
TRINITY_DN364_c2_g1_i2_orf1	vinculin-like isoform X3 [Ostrinia furnacalis]	-1.48285	1.16348	1.07447	-0.31468	-0.44041
TRINITY_DN56430_c0_g1_i1_orf1	unnamed protein product, partial [Iphiclides podalirius]	-1.13121	1.65882	0.51515	-0.8039	-0.23887
TRINITY_DN4108_c0_g1_i6_orf1	chromobox protein homolog 1-like [Ostrinia furnacalis]	-1.43671	1.19366	0.77701	-0.88534	0.35138
TRINITY_DN6205_c0_g1_i8_orf1	phenoloxidase-activating factor 2-like [Ostrinia furnacalis]	-0.38258	0.83882	1.49705	-0.95243	-1.00087
TRINITY_DN11693_c0_g1_i6_orf1	sorting nexin-32 isoform X1 [Ostrinia furnacalis] >XP_028166096.1 sorting nexin-32 isoform X2 [Ostrinia furnacalis]	-1.08412	1.30829	0.91288	-1.13122	-0.00583
TRINITY_DN855_c0_g1_i5_orf1	hypothetical protein HF086_009970 [Spodoptera exigua] >CAH0702798.1 unnamed protein product [Spodoptera exigua]	-1.09784	0.91383	1.26591	-1.16219	0.08029
TRINITY_DN1254_c0_g1_i1_orf1	lysophospholipid acyltransferase 7-like [Ostrinia furnacalis]	-0.78568	1.50035	0.81584	-1.14922	-0.38129
TRINITY_DN895_c0_g2_i1_orf1	protein N-terminal asparagine amidohydrolase [Cotesia glomerata] >XP_044583616.1 protein N-terminal asparagine amidohydrolase [Cotesia glomerata] >XP_044583617.1 protein N-terminal asparagine amidohydrolase [Cotesia glomerata] >KAH0553813.1 hypothetical protein KQX54_004640 [Cotesia glomerata]	-1.30748	1.69963	0.33988	-0.46166	-0.27037
TRINITY_DN18136_c0_g1_i1_orf1	proteoglycan 4-like [Ostrinia furnacalis]	-0.82774	1.3127	1.13269	-0.79659	-0.82105
TRINITY_DN445_c0_g1_i2_orf1	sorting nexin-17 [Ostrinia furnacalis]	-1.31795	1.71416	0.28899	-0.28616	-0.39904
TRINITY_DN2043_c0_g1_i11_orf1	phenoloxidase-activating factor 2-like [Ostrinia furnacalis]	-1.34895	1.58366	0.5169	-0.12807	-0.62353
TRINITY_DN119265_c0_g2_i1_orf1	PREDICTED: sorting nexin-12 [Fopius arisanus]	-1.45276	1.2959	0.9467	-0.36351	-0.42633
TRINITY_DN3600_c0_g1_i1_orf1	unnamed protein product [Chilo suppressalis]	-1.46301	1.4593	0.33379	0.36606	-0.69614
TRINITY_DN3637_c0_g1_i2_orf1	ATP-binding cassette sub-family G member 4 isoform X1 [Ostrinia furnacalis]	-1.53119	1.41312	0.6929	-0.20615	-0.3687
TRINITY_DN3158_c0_g1_i5_orf1	neuroglian [Ostrinia furnacalis]	-1.50228	0.87885	1.22329	-0.68361	0.08374
TRINITY_DN2326_c0_g1_i1_orf1	transmembrane protein 256 homolog isoform X1 [Ostrinia furnacalis]	-1.13511	1.25263	0.91188	-1.13964	0.11024
TRINITY_DN8652_c0_g1_i8_orf1	glucocorticoid-induced transcript 1 protein-like [Ostrinia furnacalis] >XP_028165773.1 glucocorticoid-induced transcript 1 protein-like [Ostrinia furnacalis] >XP_028165779.1 glucocorticoid-induced transcript 1 protein-like [Ostrinia furnacalis] >XP_028165785.1 glucocorticoid-induced transcript 1 protein-like [Ostrinia furnacalis]	-1.50393	1.11708	1.10261	-0.45384	-0.26192
TRINITY_DN23183_c0_g1_i2_orf1	nuclear protein localization protein 4 homolog isoform X2 [Ostrinia furnacalis]	-1.2354	1.07036	1.15467	-0.99739	0.00775
TRINITY_DN2836_c0_g1_i4_orf1	protein Diedel [Drosophila sechellia] >EDW53358.1 GM12788 [Drosophila sechellia]	-1.52702	1.23662	0.92605	-0.1186	-0.51704

TRINITY_DN7170_c0_g1_i11_orf1	sulfite oxidase isoform X1 [Ostrinia furnacalis]	-1.19255	0.62931	1.34099	-1.12409	0.34635
TRINITY_DN15114_c0_g2_i1_orf1	uncharacterized protein LOC114359552 [Ostrinia furnacalis]	-1.09499	1.63855	0.61875	-0.75118	-0.41113
TRINITY_DN34534_c0_g2_i1_orf1	hypothetical protein evm_008316 [Chilo suppressalis]	-1.50532	1.37613	0.74398	-0.52848	-0.08631
TRINITY_DN11204_c0_g1_i3_orf1	spermosin-like [Ostrinia furnacalis]	-0.6099	1.37642	1.00776	-0.61893	-1.15534
TRINITY_DN31342_c2_g2_i1_orf1	26S proteasome non-ATPase regulatory subunit 2 isoform X2 [Ostrinia furnacalis]	-0.66864	1.08326	1.34896	-0.93001	-0.83357
TRINITY_DN26488_c0_g1_i6_orf1	phosphatidate phosphatase LPIN2 isoform X1 [Ostrinia furnacalis] >XP_028176373.1 phosphatidate phosphatase LPIN2 isoform X1 [Ostrinia furnacalis] >XP_028176374.1 phosphatidate phosphatase LPIN2 isoform X2 [Ostrinia furnacalis] >XP_028176375.1 phosphatidate phosphatase LPIN2 isoform X3 [Ostrinia furnacalis] >XP_028176376.1 phosphatidate phosphatase LPIN2 isoform X4 [Ostrinia furnacalis]	-0.99485	0.81292	1.51799	-0.94425	-0.39181
TRINITY_DN2855_c0_g1_i6_orf1	uncharacterized protein LOC114359301 [Ostrinia furnacalis]	-1.07502	0.98432	0.99007	-1.31305	0.41367
TRINITY_DN7749_c1_g1_i2_orf1	uncharacterized protein LOC114364356 isoform X1 [Ostrinia furnacalis]	-1.61294	1.31664	0.73334	-0.34424	-0.0928
TRINITY_DN136031_c0_g1_i7_orf1	ferritin, lower subunit isoform X3 [Spodoptera litura]	-0.63137	-0.8095	1.56164	-0.92643	0.80566
TRINITY_DN84938_c0_g1_i4_orf1	vinculin-like isoform X2 [Ostrinia furnacalis]	-1.16778	-0.66253	1.50581	-0.50003	0.82453
TRINITY_DN184_c0_g1_i1_orf1	macrophage mannose receptor 1-like isoform X1 [Maniola jurtina]	-0.96923	-0.04771	1.73147	0.27718	-0.99172
TRINITY_DN140_c0_g1_i5_orf1	calcyphosin-like protein isoform X4 [Helicoverpa armigera]	-0.76048	-0.79938	1.31048	-0.88382	1.1332
TRINITY_DN12969_c0_g1_i3_orf1	queuosine salvage protein [Ostrinia furnacalis] >XP_028167327.1 queuosine salvage protein [Ostrinia furnacalis]	-1.33244	-0.11583	1.66185	-0.56866	0.35507
TRINITY_DN22962_c0_g1_i1_orf1	lysosomal acid glucosylceramidase-like isoform X2 [Ostrinia furnacalis]	-1.51127	-0.01972	1.62574	-0.23198	0.13723
TRINITY_DN33995_c0_g1_i5_orf1	unnamed protein product [Spodoptera exigua]	-0.42753	-1.06253	1.67758	-0.74812	0.56061
TRINITY_DN28741_c0_g1_i3_orf1	uncharacterized protein LOC114351652 [Ostrinia furnacalis]	-1.1431	0.43471	1.72204	-0.39464	-0.61901
TRINITY_DN3732_c1_g1_i5_orf1	cytochrome P450 6B2-like [Ostrinia furnacalis]	-1.1152	-0.76765	1.00074	-0.50141	1.38353
TRINITY_DN3712_c0_g1_i1_orf1	ribonuclease Oy [Ostrinia furnacalis]	-1.74258	0.2393	1.32457	-0.17111	0.34981
TRINITY_DN14874_c0_g1_i6_orf1	uncharacterized protein LOC114358148 [Ostrinia furnacalis]	-1.20115	-0.25373	1.69405	0.42561	-0.66478
TRINITY_DN452_c0_g1_i4_orf1	protein CREG1 [Ostrinia furnacalis] >XP_028170592.1 protein CREG1 [Ostrinia furnacalis]	-1.33444	-0.39485	1.40992	-0.55603	0.8754
TRINITY_DN8783_c0_g1_i5_orf1	luciferin 4-monooxygenase-like [Ostrinia furnacalis] >XP_028165580.1 luciferin 4-monooxygenase-like [Ostrinia furnacalis]	-1.1785	-0.24787	1.77891	-0.57817	0.22563
TRINITY_DN44110_c0_g1_i4_orf1	putative phosphatidate phosphatase [Ostrinia furnacalis]	-1.30686	-0.40229	1.63223	-0.44276	0.51967
TRINITY_DN3486_c0_g1_i5_orf1	uncharacterized protein LOC114360519 [Ostrinia furnacalis]	-0.94961	-0.91356	1.28285	-0.56117	1.14149
TRINITY_DN8621_c0_g1_i4_orf1	aminopeptidase N-like isoform X2 [Ostrinia furnacalis]	-1.25027	-0.64701	1.5588	-0.34606	0.68455
TRINITY_DN8644_c0_g1_i3_orf1	SEC14-like protein 2 [Ostrinia furnacalis]	-1.31186	0.40408	1.65491	-0.59484	-0.1523
TRINITY_DN827_c1_g1_i1_orf1	peptidoglycan recognition protein 4-like isoform X1 [Ostrinia furnacalis]	-1.66902	0.22671	1.42598	-0.24621	0.26254
TRINITY_DN5696_c0_g1_i4_orf1	serine protease snake-like isoform X1 [Ostrinia furnacalis]	-1.2981	-0.66332	0.98551	-0.35689	1.3328
TRINITY_DN40434_c0_g1_i2_orf1	deoxyribodipyrimidine photo-lyase [Ostrinia furnacalis]	-0.68309	-1.03602	1.69017	-0.5346	0.56354
TRINITY_DN230_c1_g1_i3_orf1	protein artichoke-like [Ostrinia furnacalis]	-1.62147	0.1444	1.53238	-0.03927	-0.01605
TRINITY_DN3975_c0_g1_i7_orf1	low-density lipoprotein receptor domain class A domain-containing protein [Phthorimaea operculella]	-1.35064	-0.06657	1.73351	0.08293	-0.39922
TRINITY_DN11370_c0_g1_i6_orf1	protein MEMO1 [Hyposmocoma kahamanoa]	-1.76716	0.54338	1.23361	-0.17819	0.16836
TRINITY_DN9_c0_g1_i7_orf1	heterogeneous nuclear ribonucleoprotein R isoform X1 [Ostrinia furnacalis]	-1.17972	0.01346	1.74219	-0.73909	0.16315
TRINITY_DN36632_c0_g1_i1_orf1	hypothetical protein evm_011159, partial [Chilo suppressalis]	-1.19402	-0.63896	1.08469	-0.55039	1.29868
TRINITY_DN77318_c0_g2_i1_orf1	uncharacterized protein LOC114351191 [Ostrinia furnacalis]	-1.0233	-0.45072	1.43719	-0.89906	0.93589
TRINITY_DN2049_c1_g1_i2_orf1	luciferin 4-monooxygenase-like [Ostrinia furnacalis]	-1.20165	0.01861	1.79769	-0.0474	-0.56725
TRINITY_DN10900_c0_g1_i7_orf1	fatty acid synthase-like [Ostrinia furnacalis]	-1.4691	0.54662	1.53432	-0.33445	-0.27739
TRINITY_DN13177_c0_g1_i9_orf1	uncharacterized protein LOC114358817 [Ostrinia furnacalis] >XP_028168676.1 uncharacterized protein LOC114358817 [Ostrinia furnacalis]	-1.2388	0.48775	1.6557	-0.25622	-0.64844
TRINITY_DN1622_c0_g1_i6_orf1	unnamed protein product [Parnassius apollo]	-1.18342	-0.87819	1.09775	-0.27923	1.24309
TRINITY_DN3300_c0_g2_i1_orf1	annexin B10 isoform X9 [Ostrinia furnacalis] >XP_028177766.1 annexin B10 isoform X10 [Ostrinia furnacalis]	-1.69953	0.46646	1.34882	-0.24225	0.12651
TRINITY_DN5558_c0_g1_i4_orf1	unnamed protein product [Parnassius apollo]	-1.45795	-0.34131	1.46717	-0.35843	0.69053
TRINITY_DN13563_c0_g1_i1_orf1	Golgi resident protein GCP60 isoform X1 [Ostrinia furnacalis]	-0.92777	-0.84293	1.25827	-0.66957	1.18201
TRINITY_DN2343_c1_g1_i12_orf1	receptor expression-enhancing protein 5-like isoform X3 [Ostrinia furnacalis]	-0.68684	-0.46255	1.89017	-0.85371	0.11293
TRINITY_DN3055_c0_g1_i9_orf1	WAP four-disulfide core domain protein 2-like [Ostrinia furnacalis]	-0.79956	-0.83316	1.75267	-0.60196	0.48201
TRINITY_DN22824_c0_g1_i4_orf1	LIM domain and actin-binding protein 1 [Ostrinia furnacalis]	-0.65832	-0.41963	1.71049	-1.11148	0.47893
TRINITY_DN10079_c0_g1_i1_orf1	PREDICTED: ubiquitin-conjugating enzyme E2 H [Amyelois transitella]	-1.46565	-0.04214	1.68343	-0.06897	-0.10667
TRINITY_DN9316_c0_g3_i1_orf1	cytochrome P450 6k1-like isoform X1 [Ostrinia furnacalis] >XP_028171821.1 cytochrome P450 6k1-like isoform X1 [Ostrinia furnacalis] >QPF77617.1 cytochrome P450 monooxygenase CYP324A34 [Ostrinia furnacalis]	-1.56479	0.31574	1.45986	-0.49162	0.2808
TRINITY_DN842_c0_g1_i9_orf1	hypothetical protein evm_011651 [Chilo suppressalis]	-0.69645	-0.81157	1.878	-0.54624	0.17626
TRINITY_DN64788_c0_g1_i1_orf1	uncharacterized protein LOC114365187 isoform X3 [Ostrinia furnacalis]	-1.50626	0.16118	1.60339	-0.35386	0.09555
TRINITY_DN3687_c0_g1_i1_orf1	acyl-CoA-binding domain-containing protein 5 isoform X2 [Manduca sexta]	-0.83242	0.01078	1.65227	-1.20018	0.36954
TRINITY_DN42185_c0_g1_i7_orf1	ADP-ribosylation factor-like protein 13B isoform X2 [Ostrinia furnacalis]	-0.15903	-0.59783	1.47182	-1.40521	0.69025
TRINITY_DN5585_c0_g1_i4_orf1	calpain-7-like isoform X3 [Ostrinia furnacalis] >XP_028163573.1 calpain-7-like isoform X4 [Ostrinia furnacalis]	-1.23092	-0.43378	1.07694	-0.69711	1.28488
TRINITY_DN85476_c0_g1_i1_orf1	iron-sulfur protein NUBPL-like [Ostrinia furnacalis]	-0.77967	-1.12704	1.4978	-0.42596	0.83488
TRINITY_DN11942_c0_g1_i1_orf1	hypothetical protein B5X24_HaOG213660 [Helicoverpa armigera]	-0.07791	0.37672	1.13675	-1.84237	0.4068

TRINITY_DN14409_c0_g1_i1_orf1	endoplasmic reticulum mannosyl-oligosaccharide 1,2-alpha-mannosidase [Ostrinia furnacalis] >XP_028171972.1 endoplasmic reticulum mannosyl-oligosaccharide 1,2-alpha-mannosidase [Ostrinia furnacalis]	-0.29897	-0.70073	1.69452	-1.15575	0.46093
TRINITY_DN10650_c0_g1_i1_orf1	protein Skeletor, isoforms B/C-like [Ostrinia furnacalis]	-1.4576	0.25467	1.46271	-0.69437	0.4346
TRINITY_DN36230_c0_g1_i1_orf1	sorting nexin-2 [Ostrinia furnacalis]	-0.995	0.19682	1.5471	-1.17984	0.43093
TRINITY_DN18216_c0_g1_i4_orf1	golgin subfamily A member 7 [Ostrinia furnacalis]	-0.64346	-0.29992	1.74534	-1.15206	0.35009
TRINITY_DN825_c23_g1_i5_orf1	methionine-tRNA synthetase, partial [Papilio xuthus]	-0.17469	-0.47551	1.8174	-1.19974	0.03255
TRINITY_DN25423_c0_g1_i1_orf1	uncharacterized protein LOC114353174 [Ostrinia furnacalis]	-1.23763	-0.37545	1.62219	-0.59438	0.58527
TRINITY_DN12003_c0_g1_i1_orf1	hypothetical protein, partial [Ostrinia furnacalis]	-1.43533	0.11923	1.62026	0.20438	-0.50854
TRINITY_DN3324_c0_g1_i3_orf1	uncharacterized protein LOC114363957 isoform X2 [Ostrinia furnacalis]	-0.56868	0.09918	1.87863	-0.97352	-0.43562
TRINITY_DN2468_c0_g1_i7_orf1	protein 4.1 homolog isoform X1 [Ostrinia furnacalis]	-0.93861	-1.0974	1.54272	-0.20781	0.7011
TRINITY_DN11133_c0_g1_i5_orf1	UHRF1-binding protein 1-like isoform X6 [Helicoverpa zea]	-1.35711	0.15249	1.5232	-0.77739	0.45881
TRINITY_DN3588_c0_g1_i4_orf1	probable peroxisomal acyl-coenzyme A oxidase 1 isoform X1 [Ostrinia furnacalis] >XP_028165840.1 probable peroxisomal acyl-coenzyme A oxidase 1 isoform X2 [Ostrinia furnacalis]	-0.52656	0.25195	1.74072	-1.25956	-0.20656
TRINITY_DN7477_c0_g1_i1_orf1	ethanolamine kinase [Ostrinia furnacalis]	-1.02364	-0.60865	1.46845	-0.75831	0.92214
TRINITY_DN2823_c0_g1_i6_orf1	proteasome-associated protein ECM29 homolog [Ostrinia furnacalis]	-1.11529	-0.43774	1.62676	-0.71341	0.63968
TRINITY_DN4424_c0_g1_i1_orf1	calreticulin [Ostrinia furnacalis]	-1.00529	-0.73784	1.63555	-0.56434	0.67192
TRINITY_DN12932_c0_g1_i1_orf1	COP9 signalosome complex subunit 3 [Ostrinia furnacalis]	-1.18464	-0.82781	1.50054	-0.2582	0.77011
TRINITY_DN6684_c0_g1_i4_orf1	26S proteasome non-ATPase regulatory subunit 6 isoform X1 [Ostrinia furnacalis]	-0.02764	0.21863	1.59817	-1.52575	-0.26341
TRINITY_DN2346_c0_g2_i1_orf1	uncharacterized protein LOC113514389 isoform X1 [Galleria mellonella]	-0.8757	-0.18634	1.69141	-1.06951	0.44013
TRINITY_DN2521_c1_g1_i2_orf1	thioredoxin-like protein 1 [Ostrinia furnacalis]	-1.32545	0.44839	1.58705	-0.72333	0.01334
TRINITY_DN9979_c0_g1_i1_orf1	ADP-dependent glucokinase [Ostrinia furnacalis]	0.14275	-0.51791	1.73688	-1.30034	-0.06138
TRINITY_DN2941_c0_g1_i1_orf1	FAS-associated factor 1 [Ostrinia furnacalis]	-1.40075	0.14162	1.54356	-0.68758	0.40315
TRINITY_DN4636_c0_g3_i1_orf1	microtubule-associated serine/threonine-protein kinase 4 [Ostrinia furnacalis]	-0.06881	-0.67237	1.79815	-1.14136	0.08438
TRINITY_DN19669_c0_g1_i1_orf1	uncharacterized protein LOC114350331 [Ostrinia furnacalis] >XP_028156889.1 uncharacterized protein LOC114350331 [Ostrinia furnacalis]	-1.44462	0.27601	1.39174	-0.77303	0.5499
TRINITY_DN30476_c0_g1_i1_orf1	unnamed protein product [Arctia plantaginis]	-0.8114	-1.15971	1.37293	-0.38386	0.98204
TRINITY_DN48694_c0_g1_i1_orfp1	TRINITY_DN48694_c0_g1_i1_m.75338 TRINITY_DN48694_c0_g1::TRINITY_DN48694_c0_g1_i1::g.75338 ORF type:internal len:84 (+),score=16.02 TRINITY_DN48694_c0_g1_i1:2-250(+)	-0.53088	0.87221	1.04725	0.27953	-1.6681
TRINITY_DN1491_c0_g1_i4_orf1	GILT-like protein 2 isoform X1 [Ostrinia furnacalis] >XP_028156245.1 GILT-like protein 2 isoform X2 [Ostrinia furnacalis] >XP_028156247.1 GILT-like protein 2 isoform X3 [Ostrinia furnacalis]	-1.04608	1.43535	0.74159	0.00733	-1.13819
TRINITY_DN52244_c1_g1_i1_orf1	triokinase/FMN cyclase-like isoform X2 [Ostrinia furnacalis]	0.03859	0.39129	1.39091	-0.11883	-1.70195
TRINITY_DN12432_c0_g1_i2_orf1	sorting nexin-8 isoform X3 [Ostrinia furnacalis]	-0.70298	0.65342	1.50509	-0.11366	-1.34187
TRINITY_DN5462_c0_g2_i1_orf1	acyl-protein thioesterase 1 [Ostrinia furnacalis] >XP_028165602.1 acyl-protein thioesterase 1 [Ostrinia furnacalis] >XP_028165603.1 acyl-protein thioesterase 1 [Ostrinia furnacalis] >XP_028165604.1 acyl-protein thioesterase 1 [Ostrinia furnacalis] >XP_028165606.1 acyl-protein thioesterase 1 [Ostrinia furnacalis] >XP_028165607.1 acyl-protein thioesterase 1 [Ostrinia furnacalis] >XP_028165608.1 acyl-protein thioesterase 1 [Ostrinia furnacalis] >XP_028165609.1 acyl-protein thioesterase 1 [Ostrinia furnacalis] >XP_028165610.1 acyl-protein thioesterase 1 [Ostrinia furnacalis]	-1.05784	1.01257	0.91049	0.47634	-1.34156
TRINITY_DN100208_c0_g1_i1_orf1	neurofilament heavy polypeptide-like isoform X2 [Ostrinia furnacalis]	-0.1176	1.13013	0.67443	0.11339	-1.80035
TRINITY_DN526_c0_g1_i1_orf1	secretory phospholipase A2 receptor-like [Ostrinia furnacalis]	-0.24343	0.47858	1.41687	-0.0076	-1.64442
TRINITY_DN1091_c0_g2_i10_orf1	macrophage mannose receptor 1-like isoform X2 [Maniola hyperantus]	-0.95099	1.44873	0.48995	0.30271	-1.2904
TRINITY_DN2499_c0_g1_i4_orf1	WD repeat-containing protein 92 isoform X1 [Ostrinia furnacalis]	-0.21588	0.3365	1.50001	-0.01127	-1.60935
TRINITY_DN85290_c0_g2_i1_orf1	unnamed protein product, partial [Brenthis ino]	-1.31486	-0.21394	1.02085	1.26744	-0.75949
TRINITY_DN4360_c0_g1_i4_orf1	glucose-6-phosphate isomerase-like, partial [Bicyclus anynana]	-0.96938	-0.40961	1.3734	1.00436	-0.99878
TRINITY_DN5770_c0_g1_i4_orf1	rotatin-like [Ostrinia furnacalis]	-0.40245	-0.56363	1.55068	0.69382	-1.27842
TRINITY_DN311_c0_g1_i8_orfp1	TRINITY_DN311_c0_g1_i8_m.65152 TRINITY_DN311_c0_g1::TRINITY_DN311_c0_g1_i8::g.65152 ORF type:5prime_partial len:138 (+),score=74.30 TRINITY_DN311_c0_g1_i8:2-415(+)	-0.60118	-0.76826	1.0148	1.39298	-1.03834
TRINITY_DN15682_c0_g1_i4_orf1	seroin transcript 1B [Ostrinia nubilalis]	-1.12092	-0.56477	0.68044	1.61085	-0.60559
TRINITY_DN80660_c0_g1_i1_orf1	probable phospholipid hydroperoxide glutathione peroxidase isoform X1 [Pieris rapae]	-1.59913	-0.31358	0.56574	1.42089	-0.07393
TRINITY_DN5852_c0_g1_i6_orf1	probable maltase isoform X6 [Ostrinia furnacalis]	-0.7384	-0.5155	1.40678	0.97196	-1.12485
TRINITY_DN81181_c0_g1_i6_orfp1	C-type lectin domain family 4 member E [Pieris rapae]	-0.13551	-0.87189	1.76548	0.26017	-1.01825
TRINITY_DN36581_c0_g1_i5_orf1	enoyl-CoA delta isomerase 1, mitochondrial-like isoform X1 [Ostrinia furnacalis] >XP_028158560.1 enoyl-CoA delta isomerase 1, mitochondrial-like isoform X2 [Ostrinia furnacalis]	-0.47281	-0.9674	1.78089	0.38238	-0.72306
TRINITY_DN4794_c1_g1_i9_orf1	D-3-phosphoglycerate dehydrogenase [Ostrinia furnacalis]	-1.34166	-0.12676	0.55382	1.56611	-0.65151
TRINITY_DN1380_c0_g1_i5_orf1	ubiquitin-fold modifier-conjugating enzyme 1 [Ostrinia furnacalis]	-0.25459	-0.59099	1.49966	0.70325	-1.35734
TRINITY_DN3058_c0_g1_i1_orf1	mesencephalic astrocyte-derived neurotrophic factor homolog [Ostrinia furnacalis]	0.09992	-1.12276	1.42458	0.69894	-1.10068
TRINITY_DN14904_c0_g1_i1_orf1	attacin [Ostrinia furnacalis]	-1.28866	-0.27016	0.17531	-0.37563	1.75913
TRINITY_DN955_c0_g1_i2_orf1	gloverin-like [Ostrinia furnacalis] >XP_028168251.1 gloverin-like [Ostrinia furnacalis] >AYM26645.1 gloverin [Ostrinia furnacalis]	-1.52423	0.6259	0.67891	-0.83897	1.05839

TRINITY_DN2343_c1_g1_i2_orf1	receptor expression-enhancing protein 5-like isoform X1 [Ostrinia furnacalis] >XP_028170586.1 receptor expression-enhancing protein 5-like isoform X1 [Ostrinia furnacalis]	-1.09377	0.22593	1.04811	-1.23858	1.05831
TRINITY_DN52761_c0_g1_i2_orf1	atlastin isoform X4 [Ostrinia furnacalis]	-1.71213	0.12025	0.9322	-0.36569	1.02538
TRINITY_DN5170_c0_g1_i5_orf1	hemolymph lipopolysaccharide-binding protein-like isoform X2 [Leguminivora glycinivorella]	-1.14401	0.60604	0.99922	-1.28399	0.82274
TRINITY_DN16840_c1_g1_i1_orf1	attacin-like [Ostrinia furnacalis]	-1.58654	0.49402	0.44681	-0.6339	1.27961
TRINITY_DN1759_c0_g1_i4_orf1	protein PFC0760c-like isoform X1 [Ostrinia furnacalis]	-1.689	0.43379	0.80802	-0.55301	1.0002
TRINITY_DN52761_c0_g2_i1_orf1	atlastin-like isoform X4 [Ostrinia furnacalis]	-1.40859	-0.20443	0.85556	-0.61001	1.36747
TRINITY_DN2743_c0_g1_i5_orf1	regucalcin-like [Ostrinia furnacalis]	-1.46811	0.11422	0.15847	-0.42503	1.62045
TRINITY_DN30177_c0_g2_i1_orf1	uncharacterized protein LOC114365032 [Ostrinia furnacalis]	-1.21239	-0.09203	-0.42913	-0.09106	1.82461
TRINITY_DN9926_c1_g1_i1_orf1	rab GTPase-activating protein 1-like isoform X6 [Ostrinia furnacalis]	-1.09858	0.58206	0.64646	-1.29541	1.16547
TRINITY_DN53281_c0_g1_i11_orf1	larval cuticle protein LCP-17-like [Ostrinia furnacalis]	-1.47506	0.02061	0.75075	-0.65129	1.35499
TRINITY_DN95850_c0_g1_i1_orf1	alpha,alpha-trehalose-phosphate synthase [UDP-forming] isoform X2 [Chelonus insularis]	-1.04684	1.08837	0.30465	-1.30597	0.95979
TRINITY_DN10231_c0_g2_i1_orf1	uncharacterized protein LOC114361472 [Ostrinia furnacalis]	-1.3533	1.29238	0.10504	-0.88413	0.84001
TRINITY_DN96801_c0_g1_i1_orf1	histone H4 isoform X2 [Gracilinanus agilis]	-0.94802	-0.13115	0.75164	-1.15256	1.4801
TRINITY_DN1116_c0_g1_i6_orf1	RNA exonuclease 4-like [Ostrinia furnacalis] >QEE79882.1 REX4 [Ostrinia furnacalis]	-0.87405	-0.16093	0.26939	-1.00366	1.76925
TRINITY_DN3219_c0_g1_i6_orf1	non-specific lipid-transfer protein [Ostrinia furnacalis]	-0.78972	0.16153	1.25176	-1.44953	0.82596
TRINITY_DN5190_c0_g3_i1_orf1	muscle LIM protein Mlp84B-like isoform X2 [Chelonus insularis]	-1.68906	0.87973	0.70185	-0.60762	0.7151
TRINITY_DN37821_c0_g1_i6_orf1	uncharacterized protein LOC114350690 [Ostrinia furnacalis]	-1.72126	0.75872	0.79699	-0.55469	0.72024
TRINITY_DN131603_c0_g1_i4_orfp1	TRINITY_DN131603_c0_g1_i4_m.86149 TRINITY_DN131603_c0_g1::TRINITY_DN131603_c0_g1_i4::g.86149 ORF type:internal len:112 (-),score=8.40 TRINITY_DN131603_c0_g1_i4:2-334(-)	-0.38291	-0.83367	-0.04622	-0.66454	1.92734
TRINITY_DN40945_c0_g1_i1_orf1	RNA exonuclease 4-like [Ostrinia furnacalis] >QEE79882.1 REX4 [Ostrinia furnacalis]	-1.19187	0.47571	0.50356	-1.13405	1.34666
TRINITY_DN8694_c1_g1_i4_orf1	sodium/potassium-transporting ATPase subunit beta-2-like isoform X2 [Ostrinia furnacalis]	-1.45209	1.15219	0.07866	-0.76495	0.98619
TRINITY_DN17505_c0_g1_i15_orf1	unnamed protein product [Chilo suppressalis]	-0.86744	-0.21987	-0.16714	-0.67363	1.92808
TRINITY_DN9544_c0_g1_i1_orf1	collagen type IV alpha-3-binding protein isoform X1 [Ostrinia furnacalis]	-1.74915	0.78786	0.55172	-0.47766	0.88723
TRINITY_DN4679_c0_g2_i13_orf1	GATOR complex protein MIOS [Ostrinia furnacalis]	-1.53499	0.36514	1.01701	-0.7793	0.93214
TRINITY_DN5055_c0_g1_i12_orf1	probable peroxisomal acyl-coenzyme A oxidase 1 [Ostrinia furnacalis]	-1.03461	0.23381	0.96302	-1.29238	1.13016
TRINITY_DN3276_c0_g1_i4_orf1	hypothetical protein B5X24_HaOG201803 [Helicoverpa armigera]	-1.37456	0.92973	0.33129	-0.97522	1.08875
TRINITY_DN2758_c0_g1_i7_orf1	hypothetical protein evm_012952 [Chilo suppressalis]	-1.43335	0.7517	0.07495	-0.74326	1.34997
TRINITY_DN1543_c0_g2_i2_orf1	regulation of enolase protein 1-like isoform X3 [Ostrinia furnacalis] >XP_028161334.1 regulation of enolase protein 1-like isoform X3 [Ostrinia furnacalis]	-1.12613	-0.25078	-0.2237	-0.28088	1.88149
TRINITY_DN1978_c0_g1_i4_orf1	RNA exonuclease 4-like [Ostrinia furnacalis]	-0.80723	0.3731	0.47497	-1.43158	1.39074
TRINITY_DN41645_c0_g1_i1_orf1	60S acidic ribosomal protein P2 [Ostrinia furnacalis]	-0.30614	-0.84759	0.77297	-1.13588	1.51663
TRINITY_DN59965_c0_g4_i1_orf1	TKT protein, partial [Homo sapiens]	-1.55609	0.24791	-0.58946	1.37844	0.51919
TRINITY_DN13799_c0_g1_i1_orf1	uncharacterized protein LOC116345248 [Contarinia nasturtii]	-1.68477	0.40844	-0.53187	1.10038	0.70782
TRINITY_DN124654_c0_g1_i1_orf1	protein lethal(2)essential for life [Manduca sexta] >KAG6441919.1 hypothetical protein O3G_MSEX002019 [Manduca sexta]	-1.65603	0.39252	-0.59978	0.86896	0.99432
TRINITY_DN16234_c0_g2_i3_orf1	uncharacterized protein LOC114363370 [Ostrinia furnacalis]	-1.42573	-0.28053	-0.5589	1.20588	1.05928
TRINITY_DN29541_c0_g1_i1_orf1	kinesin-like protein KIF23 [Ostrinia furnacalis]	-1.50449	0.81993	-0.71685	0.1676	1.23381
TRINITY_DN47842_c0_g1_i1_orf1	protein lethal(2)essential for life-like [Helicoverpa armigera] >PZC74790.1 hypothetical protein B5X24_HaOG207163 [Helicoverpa armigera]	-1.56292	0.97545	-0.71473	0.29962	1.00257
TRINITY_DN9593_c0_g1_i2_orf1	uncharacterized protein LOC113518937 [Galleria mellonella]	-1.58999	0.57016	-0.63679	1.25792	0.39871
TRINITY_DN2146_c0_g2_i1_orf1	heat shock protein 68-like [Ostrinia furnacalis]	-1.48804	0.01826	-0.62861	1.35631	0.74208
TRINITY_DN20442_c0_g2_i1_orf1	hypothetical protein evm_008218 [Chilo suppressalis]	-1.45463	1.10153	-0.83288	0.22305	0.96294
TRINITY_DN1091_c0_g3_i1_orf1	macrophage mannose receptor 1-like [Ostrinia furnacalis]	-1.21836	-0.67679	-0.46129	0.99328	1.36315
TRINITY_DN36144_c0_g1_i3_orf1	nicotinate phosphoribosyltransferase isoform X1 [Ostrinia furnacalis] >XP_028178189.1 nicotinate phosphoribosyltransferase isoform X1 [Ostrinia furnacalis]	-1.56303	0.46133	-0.45345	1.45921	0.09594
TRINITY_DN1897_c0_g2_i4_orf1	phenoloxidase-activating factor 2-like [Hyposmocoma kahamanoa]	-1.352	0.87522	-0.51662	-0.41044	1.40384
TRINITY_DN276_c0_g1_i1_orf1	protein lethal(2)essential for life-like [Helicoverpa zea] >XP_049705426.1 protein lethal(2)essential for life [Helicoverpa armigera] >ATB54993.1 heat shock protein 20.8 [Helicoverpa armigera] >PZC74337.1 hypothetical protein B5X24_HaOG207971 [Helicoverpa armigera]	-1.49049	0.15398	-0.74378	1.17746	0.90284
TRINITY_DN7212_c0_g1_i4_orf1	peptidylglycine alpha-hydroxylating monooxygenase [Ostrinia furnacalis]	-1.59341	0.03657	-0.47216	1.31312	0.71588
TRINITY_DN17574_c0_g1_i2_orf1	heat shock protein Hsp-12.2-like [Ostrinia furnacalis]	-1.46822	0.66232	-0.92007	1.0496	0.67637
TRINITY_DN307_c1_g1_i1_orf1	uncharacterized protein LOC114356704 [Ostrinia furnacalis]	-1.37359	0.40011	-0.9009	0.49852	1.37587
TRINITY_DN140212_c0_g1_i1_orf1	macrophage mannose receptor 1-like [Ostrinia furnacalis]	-1.55524	0.62509	-0.75197	0.51683	1.16529
TRINITY_DN131471_c0_g1_i1_orf1	basement membrane-specific heparan sulfate proteoglycan core protein isoform X13 [Ostrinia furnacalis]	-1.6531	0.68942	-0.61962	0.51359	1.06971
TRINITY_DN46090_c0_g3_i1_orf1	tyrosine-protein kinase-like otk, partial [Ostrinia furnacalis]	-1.37318	0.73485	-0.62105	1.46505	-0.20566
TRINITY_DN6680_c0_g1_i1_orf1	hypothetical protein evm_009571 [Chilo suppressalis]	-1.35872	-0.44777	-0.50643	1.26159	1.05133
TRINITY_DN41296_c0_g1_i1_orf1	exocyst complex component 5 [Ostrinia furnacalis]	-1.7361	0.25848	-0.39849	0.9235	0.95262
