# Supplementary material for: Comparative Proteomics and Metabonomics Analysis of Different Diapause Stages Revealed a New Regulation Mechanism of Diapause in Loxostege sticticalis (Lepidoptera: Pyralidae)
Source: Molecules. 2024 Jul 25;29(15):3472. doi: 10.3390/molecules29153472 (PMC11314584; doi:10.3390/molecules29153472)
Supplement: Supplementary file 1 [file molecules-29-03472-s001.zip › analysis process/proteomic/Cluster analysis of expression patterns/Up/RDvsCT up.pdf]

Accession	Description	CT	D	PreD	ND	RD
TRINITY_DN66040_c0_g1_i2_orf1	serine protease inhibitor dipetalogastin-like isoform X2 [Ostrinia furnacalis]	0.48616	-0.361	-0.1369	-1.5131	1.52484
TRINITY_DN32514_c0_g2_i1_orf1	mucin-5AC-like [Ostrinia furnacalis]	-0.5906	-0.5275	-0.3741	-0.5029	1.99503
TRINITY_DN64297_c0_g1_i1_orf1	vanin-like protein 2 isoform X2 [Ostrinia furnacalis]	0.59363	-0.6142	0.25072	-1.5609	1.33079
TRINITY_DN40669_c0_g2_i1_orf1	uncharacterized protein LOC114355531 [Ostrinia furnacalis]	-0.6175	-0.4538	-0.4979	-0.4265	1.99572
TRINITY_DN38307_c0_g1_i1_orfp1	TRINITY_DN38307_c0_g1_i1_m.10661 TRINITY_DN38307_c0_g1::TRINITY_DN38307_c0_g1_i1::g.10661 ORF type:5prime_partial len:66 (+),score=5.90 TRINITY_DN38307_c0_g1_i1:2-199(+)	0.00679	0.4049	-0.1642	-1.6694	1.42198
TRINITY_DN276_c0_g1_i2_orf1	protein lethal(2)essential for life-like [Ostrinia furnacalis] >UTU55753.1 small heat shock protein Hsp20.7 [Ostrinia furnacalis]	0.5407	0.06218	-0.8035	-1.3206	1.52123
TRINITY_DN56155_c0_g1_i1_orf1	hypothetical protein evm_004679 [Chilo suppressalis] >CAH2989683.1 unnamed protein product [Chilo suppressalis]	-0.5705	-0.4828	-0.5229	-0.4213	1.9976
TRINITY_DN30154_c0_g1_i1_orf1	serine/threonine-protein kinase SIK2 [Ostrinia furnacalis] >XP_028174514.1 serine/threonine-protein kinase SIK2 [Ostrinia furnacalis]	-0.5456	-0.3266	-0.328	-0.7725	1.97265
TRINITY_DN1370_c0_g1_i2_orf1	hypothetical protein evm_000756 [Chilo suppressalis]	0.50648	0.23284	0.09354	-1.8885	1.05558
TRINITY_DN1124_c0_g1_i7_orf1	PREDICTED: cuticle protein 18.6, isoform B [Amyelois transitella]	-0.84	-0.4243	-0.4181	-0.2822	1.96466
TRINITY_DN64181_c0_g1_i1_orf1	uncharacterized protein LOC114356431 isoform X2 [Ostrinia furnacalis]	-0.1125	-0.3449	0.54647	-1.5554	1.46639
TRINITY_DN87803_c0_g1_i2_orf1	hypothetical protein NE865_05903 [Phthorimaea operculella]	-0.5477	-0.5183	-0.3964	-0.5347	1.99709
TRINITY_DN18624_c0_g1_i5_orf1	uncharacterized protein LOC114353135 [Ostrinia furnacalis]	-0.4545	-0.3795	-0.5202	-0.6386	1.99275
TRINITY_DN3190_c0_g1_i1_orf1	repetitive proline-rich cell wall protein 1 precursor [Papilio polytes] >XP_013147838.1 PREDICTED: repetitive proline-rich cell wall protein 1 [Papilio polytes] >BAM19190.1 cuticular protein PpolCPG24 [Papilio polytes]	-0.5768	-0.4742	-0.4939	-0.4534	1.99824
TRINITY_DN5444_c0_g1_i1_orfp1	TRINITY_DN5444_c0_g1_i1_m.14077 TRINITY_DN5444_c0_g1::TRINITY_DN5444_c0_g1_i1::g.14077 ORF type:3prime_partial len:90 (-),score=25.11 TRINITY_DN5444_c0_g1_i1:2-268(-)	0.29269	0.0961	-0.0085	-1.7446	1.36434
TRINITY_DN15175_c0_g1_i1_orf1	zinc carboxypeptidase-like [Ostrinia furnacalis]	-0.0977	-0.4163	-0.6799	-0.7526	1.94642
TRINITY_DN52553_c0_g2_i1_orf1	hemocyte protein-glutamine gamma-glutamyltransferase-like [Ostrinia furnacalis]	-0.6098	-0.3893	-0.574	-0.4197	1.99274
TRINITY_DN12526_c0_g1_i5_orf1	uncharacterized protein LOC114359035 isoform X3 [Ostrinia furnacalis]	0.2009	0.76011	-0.4693	-1.6672	1.17556
TRINITY_DN381_c0_g1_i1_orf1	cuticle protein 8-like [Ostrinia furnacalis]	-0.6754	-0.4536	-0.4497	-0.4127	1.99137
TRINITY_DN1326_c0_g1_i1_orf1	cuticle protein 7-like [Ostrinia furnacalis]	-0.4804	-0.7953	-0.5825	-0.0884	1.94666
TRINITY_DN5406_c0_g2_i1_orf1	uncharacterized protein LOC114350326 [Ostrinia furnacalis]	0.153	0.01491	0.7566	-1.8723	0.94781
TRINITY_DN12009_c0_g1_i1_orf1	uncharacterized protein LOC114365631 [Ostrinia furnacalis]	0.01768	0.8024	0.02278	-1.8357	0.99281
TRINITY_DN138481_c0_g1_i5_orf1	hypothetical protein evm_003901 [Chilo suppressalis]	-0.1374	-0.3177	-0.6123	-0.869	1.93649
TRINITY_DN20442_c0_g2_i1_orf1	hypothetical protein evm_008218 [Chilo suppressalis]	0.22305	0.96294	-0.8329	-1.4546	1.10153
TRINITY_DN3616_c0_g1_i4_orf1	conotoxin ArMKLT2-032-like [Ostrinia furnacalis]	0.69833	-0.6536	-0.0476	-1.4273	1.43024
TRINITY_DN2813_c0_g1_i10_orf1	arylphorin subunit alpha-like [Ostrinia furnacalis]	0.1367	0.26532	1.03526	-1.9032	0.46591
TRINITY_DN6423_c0_g1_i6_orf1	phenoloxidase-activating factor 2-like isoform X1 [Ostrinia furnacalis]	0.17188	0.19032	0.02974	-1.7543	1.36232
TRINITY_DN4021_c0_g1_i1_orf1	leech-derived tryptase inhibitor C-like [Ostrinia furnacalis]	-0.8414	-0.3884	-0.3284	-0.4082	1.9664
TRINITY_DN10824_c0_g1_i3_orf1	endochitinase isoform X2 [Ostrinia furnacalis]	-0.59	-0.5355	-0.1412	-0.6977	1.96431
TRINITY_DN2101_c0_g1_i6_orf1	protein obstructor-E-like [Ostrinia furnacalis]	-0.7602	-0.5494	-0.4807	-0.1741	1.96441
TRINITY_DN129835_c0_g1_i2_orf1	alpha-tocopherol transfer protein-like [Chelonus insularis]	-0.6947	-0.8452	-0.1658	-0.2244	1.93008
TRINITY_DN74538_c0_g1_i1_orf1	tetra-peptide repeat homeobox protein 1-like [Ostrinia furnacalis]	-0.4908	-0.5482	-0.4691	-0.4912	1.99931
TRINITY_DN5408_c0_g1_i5_orf1	uncharacterized protein LOC114359912 [Ostrinia furnacalis]	-0.6229	0.56055	-0.0488	-1.4089	1.52001
TRINITY_DN644_c0_g1_i1_orf1	cuticle protein 19-like [Ostrinia furnacalis]	-0.7872	-0.4815	-0.5942	-0.0835	1.94638
TRINITY_DN4592_c0_g1_i1_orf1	hypothetical protein KGM_205563 [Danaus plexippus plexippus]	-0.5535	-0.4493	-0.4541	-0.5413	1.99815
TRINITY_DN75086_c0_g1_i5_orf1	lysosome membrane protein 2-like [Ostrinia furnacalis]	0.16937	0.58127	0.46124	-1.9636	0.75171
TRINITY_DN20560_c0_g1_i6_orf1	pupal cuticle protein C1B-like [Ostrinia furnacalis]	-0.0925	0.35245	0.40775	-1.8302	1.16248
TRINITY_DN46625_c0_g1_i1_orf1	ferritin subunit isoform X1 [Belonocnema kinseyi]	0.60728	-0.1853	-0.458	-1.463	1.49897
TRINITY_DN18338_c0_g1_i6_orf1	aquaporin AQPAn.G isoform X1 [Ostrinia furnacalis]	-0.339	0.18419	0.18761	-1.5681	1.53526
TRINITY_DN33272_c0_g1_i5_orf1	Low-density lipoprotein receptor-related protein 1 [Papilio xuthus]	0.0937	0.36479	0.43067	-1.907	1.01785
TRINITY_DN13760_c1_g1_i1_orf1	pre-mRNA-processing factor 40 homolog A isoform X1 [Ostrinia furnacalis] >XP_028162665.1 pre-mRNA-processing factor 40 homolog A isoform X2 [Ostrinia furnacalis] >XP_028162667.1 pre-mRNA-processing factor 40 homolog A isoform X3 [Ostrinia furnacalis]	0.46566	-0.3485	0.25016	-1.6889	1.32162
TRINITY_DN9538_c1_g3_i1_orf1	cilia- and flagella-associated protein 410 isoform X2 [Aphidius gifuensis]	0.5893	0.18954	-0.0339	-1.8452	1.10034
TRINITY_DN2813_c0_g1_i7_orf1	arylphorin subunit alpha-like [Ostrinia furnacalis]	-0.1755	0.53752	0.86631	-1.8742	0.64586
TRINITY_DN4255_c0_g1_i11_orf1	LOW QUALITY PROTEIN: lebocin-4-like [Ostrinia furnacalis]	0.06345	0.34713	0.35345	-1.8751	1.11109
TRINITY_DN21719_c0_g1_i2_orf1	chymotrypsin-2-like [Ostrinia furnacalis]	-0.3988	-0.5572	-0.6094	-0.4284	1.99387
TRINITY_DN18539_c0_g1_i1_orf1	pupal cuticle protein PCP52-like [Ostrinia furnacalis]	-0.4771	-0.4608	-0.6068	-0.4521	1.99685
TRINITY_DN9239_c0_g1_i1_orf1	apolipophorins-like [Ostrinia furnacalis]	0.71709	-0.2933	-0.6047	-1.3268	1.50782
TRINITY_DN5581_c0_g1_i1_orf1	mucin-5AC-like [Ostrinia furnacalis]	-0.4609	-0.2533	-0.6557	-0.6103	1.98016
TRINITY_DN45948_c1_g1_i1_orf1	unnamed protein product [Leptidea sinapis]	-0.1972	0.24067	0.55625	-1.7855	1.18571
TRINITY_DN45220_c0_g1_i1_orf1	delta(3,5)-Delta(2,4)-dienoyl-CoA isomerase, mitochondrial isoform X1 [Ostrinia furnacalis]	0.505	0.21142	-0.4231	-1.643	1.34972
TRINITY_DN9311_c0_g1_i1_orf1	cuticle protein 8-like [Ostrinia furnacalis]	-0.7592	-0.6361	-0.3658	-0.1998	1.96093
TRINITY_DN121650_c0_g1_i1_orf1	carboxylesterase [Ostrinia furnacalis]	0.0546	0.22617	-0.0336	-1.6911	1.44393
TRINITY_DN38274_c0_g1_i1_orf1	uncharacterized protein LOC114360402 [Ostrinia furnacalis]	-0.5949	-0.4612	-0.4997	-0.4414	1.99721
TRINITY_DN5829_c0_g1_i1_orf1	uncharacterized protein LOC114365758 isoform X3 [Ostrinia furnacalis]	-0.4196	-0.5035	-0.6112	-0.4616	1.99593
TRINITY_DN13576_c0_g1_i1_orf1	uncharacterized protein LOC114350099 [Ostrinia furnacalis]	-0.7288	-0.4458	-0.4226	-0.388	1.9852
TRINITY_DN29604_c0_g2_i2_orf1	neurofilament heavy polypeptide-like isoform X2 [Ostrinia furnacalis]	-0.1678	-0.1215	-0.4587	-1.1205	1.86847
TRINITY_DN98147_c0_g2_i1_orf1	hypothetical protein evm_002297 [Chilo suppressalis]	-0.7799	-0.2001	-0.4981	-0.488	1.96609
TRINITY_DN993_c0_g1_i7_orf1	apolipophorins-like [Ostrinia furnacalis]	0.80089	-0.1659	-0.4186	-1.5456	1.32924
TRINITY_DN19043_c0_g2_i1_orf1	hypothetical protein EVAR_60653_1 [Eumeta japonica]	-0.2038	-0.2104	-0.1254	-1.2718	1.81138

TRINITY_DN98692_c0_g3_i1_orf1	fatty acyl-CoA hydrolase precursor, medium chain [Ostrinia furnacalis]	0.21462	0.19139	0.67659	-1.933	0.85037
TRINITY_DN4748_c0_g1_i5_orf1	unnamed protein product, partial [Brenthis ino]	0.15981	-0.1233	0.16093	-1.6662	1.46875
TRINITY_DN661_c0_g1_i1_orf1	hypothetical protein evm_002822 [Chilo suppressalis]	-0.7822	-0.3924	-0.0188	-0.7303	1.92367
TRINITY_DN57998_c1_g1_i1_orf1	uncharacterized protein LOC113509309, partial [Galleria mellonella]	-0.202	-0.6647	-0.3962	-0.7032	1.96618
TRINITY_DN619_c0_g1_i1_orf1	putative uncharacterized protein DDB_G0271606 [Ostrinia furnacalis]	-0.8003	-0.6478	-0.4472	-0.0382	1.93351
TRINITY_DN35351_c0_g1_i3_orf1	adult-specific cuticular protein ACP-20-like [Ostrinia furnacalis]	-0.535	-0.3589	-0.5463	-0.5545	1.99475
TRINITY_DN28711_c0_g1_i1_orf1	hypothetical protein evm_000299 [Chilo suppressalis]	0.83903	-0.2883	-0.5329	-1.4105	1.39267
TRINITY_DN2400_c0_g1_i1_orf1	uncharacterized protein LOC114351021 [Ostrinia furnacalis]	-0.5394	-0.4144	-0.3292	-0.7012	1.98425
TRINITY_DN12671_c0_g1_i6_orf1	hemicentin-1-like isoform X1 [Ostrinia furnacalis]	0.04789	-0.521	-0.3467	-1.0512	1.87108
TRINITY_DN85412_c0_g1_i1_orf1	unnamed protein product [Diatraea saccharalis]	0.75379	-0.2669	-0.5218	-1.4122	1.44709
TRINITY_DN20676_c0_g1_i6_orf1	aldo-keto reductase AKR2E4-like isoform X1 [Ostrinia furnacalis]	0.21513	0.38287	0.05158	-1.8403	1.19071
TRINITY_DN2946_c0_g1_i1_orf1	histidine-rich glycoprotein [Ostrinia furnacalis]	-0.6359	-0.429	-0.6107	-0.3101	1.98559
TRINITY_DN28922_c0_g1_i2_orf1	uncharacterized protein LOC119829283 isoform X2 [Zerene cesonia]	-0.1515	-0.5322	-0.3664	-0.8907	1.9407
TRINITY_DN801_c0_g1_i2_orf1	cathepsin L [Ostrinia furnacalis] >XP_028165920.1 cathepsin L [Ostrinia furnacalis] >UKI61015.1 cathepsin L [Ostrinia furnacalis]	-0.1484	-0.1227	0.53302	-1.6549	1.39293
TRINITY_DN19639_c0_g2_i1_orf1	basic juvenile hormone-suppressible protein 1-like [Hyposmocoma kahamanoa]	-0.1124	0.60231	-1.026	-1.0412	1.57728
TRINITY_DN5655_c0_g1_i2_orf1	uncharacterized protein LOC114359603 [Ostrinia furnacalis]	0.15841	0.4106	0.02955	-1.8202	1.22163
TRINITY_DN26882_c0_g1_i1_orf1	uncharacterized protein LOC114349648 [Ostrinia furnacalis]	-0.5114	-0.5017	-0.5218	-0.4647	1.99963
TRINITY_DN81488_c0_g1_i1_orf1	apolipophorins-like [Ostrinia furnacalis]	0.78382	-0.2184	-0.4721	-1.4803	1.38699
TRINITY_DN27264_c0_g1_i1_orf1	uncharacterized protein LOC114353424 [Ostrinia furnacalis]	0.49325	-0.6112	-0.6874	-0.9364	1.74177
TRINITY_DN65681_c0_g1_i1_orf1	ferritin subunit-like [Ostrinia furnacalis] >XP_028168186.1 ferritin subunit-like [Ostrinia furnacalis]	0.09588	0.21897	-1.2374	-0.7607	1.68319
TRINITY_DN49147_c0_g2_i1_orf1	glutenin, high molecular weight subunit PW212-like [Ostrinia furnacalis]	0.04937	-0.0607	-0.6812	-1.1183	1.81086
TRINITY_DN699_c0_g2_i1_orf1	TPA_exp: putative parasitoid killing factor [Trichoplusia ni]	0.70775	-0.1997	-0.5433	-1.4252	1.46045
TRINITY_DN15247_c0_g1_i2_orf1	probable G-protein coupled receptor Mth-like 3 isoform X1 [Ostrinia furnacalis]	-0.1341	0.60594	0.42724	-1.8687	0.96964
TRINITY_DN1175_c1_g1_i1_orf1	methanethiol oxidase [Ostrinia furnacalis]	0.1941	0.27626	0.44652	-1.919	1.00209
TRINITY_DN1423_c0_g1_i8_orf1	ferritin subunit-like [Ostrinia furnacalis] >XP_028168186.1 ferritin subunit-like [Ostrinia furnacalis]	0.4683	-0.1935	-0.2715	-1.5296	1.52636
TRINITY_DN57998_c1_g3_i1_orf1	uncharacterized protein LOC114363305 isoform X2 [Ostrinia furnacalis]	-0.3209	-0.3752	-0.4505	-0.8223	1.96902
TRINITY_DN60946_c0_g2_i3_orf1	protein yellow-like [Ostrinia furnacalis]	0.20958	0.53794	0.28682	-1.9401	0.90576
TRINITY_DN25273_c0_g1_i1_orf1	skin secretory protein xP2-like [Ostrinia furnacalis]	-0.2359	-0.2539	-0.4592	-0.9779	1.92687
TRINITY_DN20717_c0_g1_i1_orf1	putative uncharacterized protein DDB_G0282133 isoform X1 [Ostrinia furnacalis]	-0.4303	-0.2457	0.49019	-1.4066	1.5924
TRINITY_DN97042_c0_g1_i6_orf1	apolipophorins-like [Ostrinia furnacalis]	0.83166	-0.2074	-0.466	-1.4997	1.34136
TRINITY_DN2908_c0_g1_i1_orf1	uncharacterized protein LOC114361337 [Ostrinia furnacalis]	-0.7403	-0.482	-0.4602	-0.2973	1.97977
TRINITY_DN3310_c0_g1_i1_orf1	hypothetical protein evm_010516 [Chilo suppressalis]	-0.6037	-0.6012	-0.6489	-0.1063	1.96004
TRINITY_DN906_c0_g1_i4_orf1	uncharacterized protein LOC114360441, partial [Ostrinia furnacalis]	-0.4425	-0.4314	-0.2246	-0.8586	1.95715
TRINITY_DN4384_c0_g1_i5_orf1	chemosensory protein 5 [Conogethes punctiferalis]	-0.1861	-0.5162	-0.3634	-0.8811	1.9469
TRINITY_DN77425_c0_g1_i2_orf1	anosmin-1 [Ostrinia furnacalis] >XP_028178811.1 anosmin-1 [Ostrinia furnacalis]	-0.4274	-0.3115	-0.553	-0.6919	1.98386
TRINITY_DN42719_c0_g1_i1_orf1	inter-alpha-trypsin inhibitor heavy chain H4-like isoform X11 [Ostrinia furnacalis]	0.16192	-0.5803	-0.5474	-0.9083	1.87412
TRINITY_DN2311_c0_g3_i1_orf1	uncharacterized protein LOC114364231 isoform X1 [Ostrinia furnacalis] >XP_028176108.1 uncharacterized protein LOC114364231 isoform X2 [Ostrinia	-0.3628	0.31061	-0.4861	-1.2125	1.75083
TRINITY_DN15222_c0_g1_i4_orf1	lysosomal alpha-mannosidase-like [Ostrinia furnacalis]	-0.8632	0.08993	-0.6036	-0.5229	1.89975
TRINITY_DN98334_c0_g1_i1_orf1	unnamed protein product [Spodoptera littoralis] >CAH1638738.1 unnamed protein product [Spodoptera littoralis]	-0.5168	-0.5237	-0.5442	-0.4133	1.99794
TRINITY_DN338_c0_g1_i1_orf1	chorion protein S36-like [Ostrinia furnacalis]	-0.5522	-0.5717	-0.3143	-0.5527	1.99096
TRINITY_DN10747_c0_g1_i5_orf1	unnamed protein product [Plutella xylostella]	-0.3567	-0.4126	-0.5397	-0.6786	1.98764
TRINITY_DN13686_c0_g2_i1_orf1	transmembrane protease serine 9-like [Ostrinia furnacalis]	-0.2077	0.01638	-0.4292	-1.203	1.82352
TRINITY_DN31_c0_g1_i3_orfp1	TRINITY_DN31_c0_g1_i3_m.1394 TRINITY_DN31_c0_g1::TRINITY_DN31_c0_g1_i3::g.1394 ORF type:complete len:118 (+),score=43.35 TRINITY_DN31_c0_g1_i3:56-	-0.403	-0.0119	-0.8528	-0.652	1.91966
TRINITY_DN391_c0_g1_i4_orf1	phenoloxidase-activating enzyme-like [Ostrinia furnacalis]	0.10877	0.01625	-0.2772	-1.4891	1.64125
TRINITY_DN41_c0_g1_i3_orf1	uncharacterized protein LOC114359035 isoform X3 [Ostrinia furnacalis]	-0.081	0.38503	0.324	-1.8212	1.19312
TRINITY_DN778_c0_g1_i1_orf1	uncharacterized protein LOC114363281 [Ostrinia furnacalis]	-0.8621	-0.4107	-0.5662	-0.0995	1.93848
TRINITY_DN138481_c0_g1_i2_orf1	cuticle protein 8-like isoform X2 [Vanessa tameamea]	-0.3871	-0.4775	-0.6857	-0.4394	1.98969
TRINITY_DN12387_c0_g1_i1_orf1	repetitive proline-rich cell wall protein 2-like [Ostrinia furnacalis]	-0.506	-0.5475	-0.4734	-0.4723	1.99925
TRINITY_DN52944_c0_g1_i1_orf1	apolipophorins-like [Ostrinia furnacalis]	0.76292	-0.3037	-0.4968	-1.4092	1.44676
TRINITY_DN7539_c0_g1_i2_orf1	serine protease inhibitor 3/4 [Ostrinia furnacalis]	-0.3589	-0.2612	-0.6168	-0.7339	1.97078
TRINITY_DN25534_c0_g1_i1_orf1	venom serine carboxypeptidase-like [Ostrinia furnacalis]	-0.3579	-0.6115	-0.713	-0.2934	1.97575
TRINITY_DN24971_c0_g1_i3_orf1	uncharacterized protein LOC114352370 [Ostrinia furnacalis]	-0.2146	-0.0536	-0.3277	-1.2295	1.82537
TRINITY_DN71699_c0_g1_i1_orf1	apolipophorins-like [Ostrinia furnacalis]	0.79524	-0.267	-0.5739	-1.3854	1.43099
TRINITY_DN28711_c1_g1_i1_orf1	apolipophorins-like [Ostrinia furnacalis]	0.71746	-0.2408	-0.5321	-1.4115	1.46692
TRINITY_DN9282_c0_g1_i2_orf1	uncharacterized protein LOC114363102 isoform X2 [Ostrinia furnacalis]	-0.8453	-0.5212	-0.5262	-0.04	1.93271
TRINITY_DN1110_c1_g1_i9_orf1	MD-2-related lipid-recognition protein-like [Ostrinia furnacalis]	0.32427	-0.2562	-0.8699	-0.9686	1.77041
TRINITY_DN2024_c0_g1_i12_orfp1	unnamed protein product, partial [Brenthis ino]	0.28217	-0.2999	0.40147	-1.7077	1.32399
TRINITY_DN3616_c0_g2_i1_orf1	conotoxin ArMKLT2-032-like [Ostrinia furnacalis]	-0.4341	-0.2831	-0.0539	-1.1029	1.87404
TRINITY_DN15812_c0_g1_i2_orf1	transferrin [Ostrinia furnacalis]	0.25703	0.04583	0.15993	-1.7805	1.31766
TRINITY_DN59388_c0_g1_i1_orf1	uncharacterized protein LOC114353759 [Ostrinia furnacalis]	-0.0303	-0.5556	0.48286	-1.4404	1.5435
TRINITY_DN25976_c0_g1_i4_orf1	hypothetical protein B566_EDAN014657 [Ephemera danica]	0.24659	0.17771	0.35248	-1.8853	1.10855
TRINITY_DN7854_c0_g1_i4_orf1	failed axon connections [Ostrinia furnacalis]	-0.0938	-0.045	0.02109	-1.5194	1.63711
TRINITY_DN2002_c0_g1_i5_orfp1	TRINITY_DN2002_c0_g1_i5_m.4230 TRINITY_DN2002_c0_g1::TRINITY_DN2002_c0_g1_i5::g.4230 ORF type:3prime_partial len:259 (+),score=29.98,Peptidase_C39 PF03412.16 1.7,Peptidase_C39|PF03412.16|0.049 TRINITY_DN2002_c0_g1_i5:65-838(+)	-0.7707	-0.1275	-0.0527	-0.9272	1.87811

TRINITY_DN12387_c1_g2_i1_orf1	skin secretory protein xP2-like [Ostrinia furnacalis]	-0.4383	-0.4958	-0.5914	-0.4719	1.99741
TRINITY_DN2803_c4_g1_i1_orf1	ornithine aminotransferase, mitochondrial isoform X2 [Ostrinia furnacalis]	0.38259	-0.1428	0.34464	-1.7995	1.21503
TRINITY_DN49785_c1_g1_i3_orf1	uncharacterized protein LOC114365444, partial [Ostrinia furnacalis]	-0.7058	-0.3109	-0.3034	-0.6518	1.97188
TRINITY_DN21555_c0_g1_i4_orf1	uncharacterized protein LOC114351844 [Ostrinia furnacalis] >XP_028158981.1 uncharacterized protein LOC114351844 [Ostrinia furnacalis] >XP_028158982.1 uncharacterized protein LOC114351844 [Ostrinia furnacalis] >XP_028158983.1 uncharacterized protein LOC114351844 [Ostrinia furnacalis] >XP_028158984.1 uncharacterized protein LOC114351844 [Ostrinia furnacalis] >XP_028158985.1 uncharacterized protein LOC114351844 [Ostrinia furnacalis] >5GPR_A Crystal structure of chitinase-h from Ostrinia furnacalis [Ostrinia furnacalis] >5GQB_A Crystal structure of chitinase-h from O. furnacalis in complex with chitohepatose [Ostrinia furnacalis] >6JMN_A Crystal structure of Ostrinia furnacalis Chitinase h complexed with compound 2-8-s2 [Ostrinia furnacalis] >BAE16587.1 chitinase [Ostrinia furnacalis]	-0.2174	-0.0395	-0.4767	-1.1256	1.85928
TRINITY_DN3609_c0_g1_i6_orf1	leukocyte elastase inhibitor-like [Ostrinia furnacalis]	-0.1823	0.76272	0.16659	-1.8015	1.05445
TRINITY_DN47842_c0_g1_i1_orf1	protein lethal(2)essential for life-like [Helicoverpa armigera] >PZC74790.1 hypothetical protein B5X24_HaOG207163 [Helicoverpa armigera]	0.29962	1.00257	-0.7147	-1.5629	0.97545
TRINITY_DN585_c0_g1_i12_orf1	very low-density lipoprotein receptor isoform X3 [Galleria mellonella]	0.27119	0.40819	0.28676	-1.9341	0.96795
TRINITY_DN12671_c0_g1_i4_orf1	hemicentin-1-like isoform X1 [Ostrinia furnacalis]	-0.3571	-0.3722	-0.3668	-0.8659	1.96204
TRINITY_DN1935_c0_g1_i1_orf1	adult-specific cuticular protein ACP-22-like [Ostrinia furnacalis]	-0.6113	-0.6333	-0.5083	-0.2259	1.97884
TRINITY_DN3949_c0_g1_i1_orf1	probable cytochrome P450 304a1 [Ostrinia furnacalis]	0.36534	0.21695	-0.1414	-1.7538	1.31291
TRINITY_DN54524_c0_g1_i6_orf1	serine protease inhibitor dipetalogastin [Ostrinia furnacalis]	-0.6461	-0.5101	-0.5214	-0.3108	1.98843
TRINITY_DN5907_c0_g1_i4_orf1	hypothetical protein evm_010265 [Chilo suppressalis] >CAB3524755.1 unnamed protein product [Chilo suppressalis] >CAH0397522.1 unnamed protein product [Chilo suppressalis]	-0.3625	-0.3875	-0.5294	-0.7056	1.98502
TRINITY_DN26149_c0_g1_i5_orf1	thymosin beta isoform X3 [Ostrinia furnacalis]	-0.0399	0.05235	-0.3125	-1.4077	1.70773
TRINITY_DN1423_c0_g1_i4_orf1	hypothetical protein evm_003306 [Chilo suppressalis] >CAB3526495.1 unnamed protein product [Chilo suppressalis] >CAH0403823.1 unnamed protein product [Chilo suppressalis]	0.40544	-0.0776	-0.2159	-1.6014	1.4895
TRINITY_DN12865_c0_g1_i1_orf1	synaptic vesicle membrane protein VAT-1 homolog-like [Ostrinia furnacalis]	0.13962	-0.151	0.18225	-1.6522	1.48141
TRINITY_DN7549_c0_g1_i1_orf1	uncharacterized protein LOC114355006 [Ostrinia furnacalis]	-0.7847	-0.4801	-0.4921	-0.2098	1.96663
TRINITY_DN2140_c0_g1_i1_orf1	uncharacterized protein LOC126367764 [Pectinophora gossypiella]	-0.4727	-0.548	-0.2601	-0.6991	1.97988
TRINITY_DN26301_c0_g1_i1_orf1	uncharacterized protein LOC114359193 [Ostrinia furnacalis]	-0.3031	-0.1341	-0.3334	-1.1117	1.88231
TRINITY_DN661_c0_g2_i2_orf1	cuticle protein 7-like [Ostrinia furnacalis]	-0.1979	-0.2534	-0.9088	-0.5741	1.93421
TRINITY_DN19990_c0_g1_i1_orf1	carboxypeptidase B-like [Ostrinia furnacalis]	-0.3138	-0.531	-0.531	-0.6143	1.99005
TRINITY_DN19980_c0_g1_i4_orf1	hypothetical protein evm_012507 [Chilo suppressalis]	-0.1159	0.25411	0.58938	-1.8318	1.10419
TRINITY_DN68397_c0_g1_i2_orf1	clavesin-1-like [Ostrinia furnacalis]	-0.353	-0.6899	-0.529	-0.4149	1.98681
TRINITY_DN91533_c0_g1_i1_orf1	uncharacterized protein LOC114361550 [Ostrinia furnacalis]	-0.5499	-0.4902	-0.4412	-0.5174	1.99873
TRINITY_DN32532_c0_g1_i1_orf1	fatty acyl-CoA hydrolase precursor, medium chain [Ostrinia furnacalis]	0.45187	0.0925	0.26645	-1.8865	1.07573
TRINITY_DN7618_c0_g1_i4_orf1	uncharacterized protein LOC114366712 isoform X1 [Ostrinia furnacalis]	0.06563	0.39986	-0.2805	-1.632	1.44701
TRINITY_DN6004_c0_g1_i1_orf1	endocuticle structural glycoprotein ABD-4-like [Ostrinia furnacalis]	-0.363	-0.2582	-0.3786	-0.942	1.94185
TRINITY_DN4676_c0_g1_i16_orf1	meiosis-specific nuclear structural protein 1-like isoform X2 [Ostrinia furnacalis]	-0.4573	-0.7033	0.10404	-0.8369	1.89343
TRINITY_DN2652_c0_g2_i1_orf1	peroxidase-like isoform X1 [Ostrinia furnacalis]	-0.2244	-0.4283	-0.3332	-0.9509	1.93676
TRINITY_DN5595_c0_g1_i1_orf1	keratin, type I cytoskeletal 10-like [Ostrinia furnacalis]	-0.7499	-0.4149	-0.3978	-0.4201	1.98264
TRINITY_DN20680_c0_g1_i5_orf1	tsukushin isoform X2 [Ostrinia furnacalis]	-0.1803	0.35582	0.10571	-1.6882	1.40701
TRINITY_DN49143_c0_g1_i1_orf1	uncharacterized protein LOC107270465 [Cephus cinctus]	-0.4524	-0.4875	-0.4875	-0.5711	1.99847
TRINITY_DN53866_c0_g1_i1_orf1	larval/pupal cuticle protein H1C-like [Ostrinia furnacalis]	-0.6299	-0.322	-0.0914	-0.8831	1.92645
TRINITY_DN2515_c0_g1_i6_orf1	chitooligosaccharidolytic beta-N-acetylglucosaminidase isoform X1 [Ostrinia furnacalis]	-0.3144	-0.0634	-0.1622	-1.2671	1.80704
TRINITY_DN2442_c0_g1_i2_orf1	digestive cysteine proteinase 2 [Ostrinia furnacalis]	-0.387	-0.2946	0.1596	-1.2558	1.77788
TRINITY_DN2489_c0_g1_i1_orf1	uncharacterized protein LOC114354692 [Ostrinia furnacalis]	-0.1676	-0.3088	-0.473	-0.9749	1.92419
TRINITY_DN31314_c0_g1_i4_orf1	uncharacterized protein LOC114355976 [Ostrinia furnacalis]	-0.4646	-0.5128	-0.5344	-0.4876	1.99945
TRINITY_DN13973_c0_g1_i6_orf1	27 kDa glycoprotein-like [Ostrinia furnacalis]	0.63146	0.20394	-0.8075	-1.4116	1.38377
TRINITY_DN3464_c0_g1_i1_orf1	putative mitochondrial aconitate hydratase isoform X1-likeprotein, partial [Cotesia chilonis]	0.70861	-0.5383	-0.8389	-0.9465	1.61508
TRINITY_DN7183_c0_g1_i2_orf1	seminal fluid protein CSSFP028 [Chilo suppressalis]	0.13895	-0.1013	0.38744	-1.7504	1.32529
TRINITY_DN44073_c0_g1_i3_orf1	inter-alpha-trypsin inhibitor heavy chain H4-like isoform X11 [Ostrinia furnacalis]	0.3538	-0.3914	0.32426	-1.6558	1.3692
TRINITY_DN1749_c0_g2_i2_orf1	putative GPI-anchored protein pfl2 isoform X1 [Ostrinia furnacalis]	-0.7738	-0.401	-0.425	-0.3791	1.97891
TRINITY_DN33272_c0_g1_i1_orf1	unnamed protein product, partial [Iphiclides podalirius]	0.44592	0.15137	-0.3188	-1.6621	1.38353
TRINITY_DN364_c1_g1_i2_orf1	talin-2-like, partial [Ostrinia furnacalis]	0.10478	-0.0705	0.99666	-1.8306	0.79969
TRINITY_DN6470_c0_g3_i2_orf1	trypsin CFT-1-like [Ostrinia furnacalis]	-0.3669	-0.3121	-0.8548	-0.4291	1.96293
TRINITY_DN5444_c0_g2_i1_orf1	phenoloxidase-activating factor 2-like [Ostrinia furnacalis]	-0.5473	-0.212	-0.2656	-0.9122	1.93721
TRINITY_DN139537_c0_g1_i1_orf1	uncharacterized protein LOC114351440 [Ostrinia furnacalis]	0.35511	-0.2022	-0.4446	-1.3696	1.66122
TRINITY_DN3056_c0_g1_i1_orf1	spidroin-2-like [Ostrinia furnacalis]	-0.7519	-0.3159	-0.3848	-0.5251	1.97764
TRINITY_DN2407_c0_g1_i6_orf1	uncharacterized protein LOC114366345 isoform X2 [Ostrinia furnacalis]	0.21652	0.2242	0.01877	-1.7784	1.31896
TRINITY_DN19110_c0_g1_i2_orf1	peroxidase [Ostrinia furnacalis]	0.58694	-0.4603	-0.5048	-1.2457	1.62388
TRINITY_DN1491_c0_g1_i4_orf1	GILT-like protein 2 isoform X1 [Ostrinia furnacalis] >XP_028156245.1 GILT-like protein 2 isoform X2 [Ostrinia furnacalis] >XP_028156247.1 GILT-like protein 2 isoform X3 [Ostrinia furnacalis]	0.00733	-1.1382	0.74159	-1.0461	1.43535
TRINITY_DN688_c0_g1_i8_orf1	lysosomal alpha-mannosidase-like [Ostrinia furnacalis]	-0.0307	-0.222	-0.0313	-1.4246	1.70856
TRINITY_DN1465_c2_g1_i2_orf1	transcription initiation factor TFIID subunit 1-like [Ostrinia furnacalis]	-0.4549	0.23306	-0.3731	-1.1896	1.7845
TRINITY_DN2097_c1_g2_i2_orf1	serine protease inhibitor 3 [Ostrinia furnacalis]	0.43897	0.27809	-0.3501	-1.6902	1.32318
TRINITY_DN7534_c0_g1_i15_orf1	protein-glucosylgalactosylhydroxylysine glucosidase isoform X2 [Ostrinia furnacalis]	-0.1736	0.47223	0.88583	-1.8691	0.68461
TRINITY_DN1749_c0_g1_i1_orf1	putative GPI-anchored protein pfl2 isoform X2 [Ostrinia furnacalis]	-0.6977	-0.3678	-0.3896	-0.531	1.98602
TRINITY_DN4228_c0_g1_i5_orf1	phenoloxidase-activating enzyme-like [Ostrinia furnacalis]	0.06787	0.32681	0.04266	-1.7664	1.32909
TRINITY_DN1326_c0_g1_i2_orf1	cuticle protein 7 [Plutella xylostella] >CAG9138501.1 unnamed protein product [Plutella xylostella]	-0.7261	0.10299	-0.6638	-0.6201	1.90707

TRINITY_DN52553_c0_g1_i1_orf1	hemocyte protein-glutamine gamma-glutamyltransferase-like [Ostrinia furnacalis]	-0.6482	-0.3947	-0.4015	-0.5465	1.991
TRINITY_DN5467_c0_g1_i5_orf1	synaptic vesicle glycoprotein 2B-like isoform X2 [Ostrinia furnacalis] >XP_028161209.1 synaptic vesicle glycoprotein 2B-like isoform X2 [Ostrinia furnacalis] >XP_028161210.1 synaptic vesicle glycoprotein 2B-like isoform X2 [Ostrinia furnacalis]	-0.3554	0.04919	0.02883	-1.4156	1.69305
TRINITY_DN13221_c0_g1_i3_orf1	fasciclin-3-like [Ostrinia furnacalis]	0.45982	-0.3721	-1.5586	-0.0193	1.49017
TRINITY_DN4324_c0_g1_i1_orf1	uncharacterized protein LOC114354985 isoform X1 [Ostrinia furnacalis]	-0.5079	-0.2788	-0.6098	-0.5896	1.98615
TRINITY_DN58125_c0_g1_i1_orf1	protein yellow-like [Ostrinia furnacalis]	-0.5842	-0.3384	-0.2791	-0.7673	1.96912
TRINITY_DN33728_c0_g2_i1_orf1	uncharacterized protein LOC114350200 [Ostrinia furnacalis]	0.27855	-0.1965	-0.6994	-1.1412	1.7585
TRINITY_DN110231_c0_g1_i1_orf1	protein singed [Ostrinia furnacalis] >XP_028161434.1 protein singed [Ostrinia furnacalis]	-0.4864	-0.4878	0.27123	-1.0985	1.80145
TRINITY_DN2896_c0_g1_i2_orf1	general odorant-binding protein 56d-like isoform X2 [Ostrinia furnacalis]	-0.6788	-0.4752	-0.3357	-0.4983	1.98805
TRINITY_DN74086_c0_g1_i1_orf1	UPF0489 protein C5orf22 homolog [Ostrinia furnacalis]	-0.6966	-0.2097	-0.1308	-0.8807	1.91781
TRINITY_DN5420_c0_g1_i2_orf1	DNA-directed RNA polymerase II subunit RPB1-like [Ostrinia furnacalis]	0.15793	-0.2039	0.07708	-1.5851	1.55402
TRINITY_DN18804_c0_g1_i5_orf1	zinc finger protein Xfin-like [Ostrinia furnacalis]	0.17022	-0.7176	-0.4324	-0.8865	1.86633
TRINITY_DN2425_c0_g1_i3_orf1	sialic acid synthase [Ostrinia furnacalis]	0.06788	0.33623	-0.1453	-1.683	1.42428
TRINITY_DN4367_c0_g1_i1_orf1	heat shock protein 21.7c [Chilo suppressalis] >AWT57938.1 heat shock protein 21.7c [Chilo suppressalis]	-0.0847	-0.1545	-0.8468	-0.8102	1.89617
TRINITY_DN27968_c0_g2_i2_orf1	nucleolin-like [Ostrinia furnacalis]	-0.487	-0.4646	-0.6176	-0.4267	1.99588
TRINITY_DN5553_c0_g1_i4_orf1	uncharacterized protein LOC114353828 [Ostrinia furnacalis]	0.498	-0.3009	-0.558	-1.2832	1.64419
TRINITY_DN2835_c0_g1_i6_orf1	probable isoaspartyl peptidase/L-asparaginase GA20639 [Ostrinia furnacalis]	0.62849	0.18883	-0.5732	-1.5731	1.32898
TRINITY_DN7785_c0_g1_i1_orf1	uncharacterized protein LOC114364098 [Ostrinia furnacalis]	-0.7398	-0.6782	-0.4829	-0.0375	1.93856
TRINITY_DN31417_c0_g1_i3_orf1	titin-like [Ostrinia furnacalis]	-0.188	-0.2268	-0.3673	-1.1043	1.88646
TRINITY_DN2684_c0_g2_i3_orf1	glutamate decarboxylase 1-like isoform X1 [Ostrinia furnacalis]	0.2098	-0.0831	-0.7601	-1.1274	1.76077
TRINITY_DN2794_c1_g1_i8_orf1	carboxypeptidase D [Ostrinia furnacalis]	-0.2909	0.03508	0.19351	-1.5301	1.59237
TRINITY_DN20793_c0_g2_i1_orf1	mucin-2-like [Ostrinia furnacalis]	-0.6181	-0.6996	-0.5752	-0.0551	1.94798
TRINITY_DN5022_c0_g1_i4_orf1	syntenin-1-like [Ostrinia furnacalis]	-0.073	0.57087	0.46804	-1.8942	0.92827
TRINITY_DN696_c1_g1_i10_orf1	titin-like [Ostrinia furnacalis]	-0.4842	-0.3397	-0.6136	-0.5541	1.99163
TRINITY_DN5664_c0_g1_i1_orf1	CDGSH iron-sulfur domain-containing protein 3, mitochondrial-like [Ostrinia furnacalis]	-0.5141	-0.2468	-0.962	-0.2028	1.92568
TRINITY_DN3952_c0_g1_i3_orf1	protein Skeletor, isoforms D/E-like isoform X1 [Ostrinia furnacalis] >XP_028176405.1 protein Skeletor, isoforms D/E-like isoform X2 [Ostrinia furnacalis] >XP_028176406.1 protein Skeletor, isoforms D/E-like isoform X3 [Ostrinia furnacalis] >XP_028176407.1 protein Skeletor, isoforms D/E-like isoform X4 [Ostrinia furnacalis]	0.49199	-0.1421	-0.9681	-1.0343	1.65249
TRINITY_DN143497_c0_g1_i1_orf1	fibroin heavy chain-like [Ostrinia furnacalis]	-0.6986	-0.5169	-0.5744	-0.1805	1.97035
TRINITY_DN2953_c1_g1_i10_orf1	methionine--tRNA ligase, cytoplasmic isoform X2 [Ostrinia furnacalis] >XP_028156683.1 methionine--tRNA ligase, cytoplasmic isoform X4 [Ostrinia furnacalis] >XP_028156684.1 methionine--tRNA ligase, cytoplasmic isoform X5 [Ostrinia furnacalis]	-0.311	-0.0576	-0.5527	-0.9833	1.90463
TRINITY_DN1902_c0_g1_i4_orf1	chemosensory protein 10 [Conogethes pinicolalis]	-0.4857	-0.6153	-0.2803	-0.6041	1.98544
TRINITY_DN2069_c1_g1_i8_orf1	lysosomal aspartic protease [Trichoplusia ni]	-0.0187	0.20878	-0.4193	-1.4271	1.65635
TRINITY_DN4464_c0_g2_i1_orf1	glypican-6 [Pectinophora gossypiella]	0.32098	0.18936	0.42756	-1.9247	0.98681
TRINITY_DN23364_c0_g1_i1_orf1	PREDICTED: uncharacterized protein LOC106134920 [Amyelois transitella]	0.06709	-0.1669	-0.1917	-1.4176	1.70915
TRINITY_DN2170_c1_g1_i3_orf1	beta-1,3-glucan-binding protein-like [Ostrinia furnacalis]	0.23363	-0.0409	-0.2372	-1.5408	1.58533
TRINITY_DN11060_c0_g1_i6_orf1	extracellular matrix protein A-like isoform X3 [Ostrinia furnacalis]	-0.0756	-0.0195	0.95414	-1.7923	0.93331
TRINITY_DN3715_c0_g1_i2_orf1	uncharacterized protein LOC114356437 isoform X1 [Ostrinia furnacalis]	0.07118	-0.6001	-1.0332	-0.3023	1.86439
TRINITY_DN15597_c0_g1_i1_orf1	microsomal glutathione S-transferase 1-like [Ostrinia furnacalis]	0.05162	0.86631	0.22322	-1.9021	0.76095
TRINITY_DN1308_c0_g1_i4_orf1	serine proteinase stubble-like [Ostrinia furnacalis]	-0.2641	-0.1115	-0.81	-0.7412	1.92675
TRINITY_DN10220_c1_g1_i7_orf1	uncharacterized protein LOC124645895 isoform X2 [Helicoverpa zea]	-0.1816	0.52059	0.48606	-1.8477	1.02261
TRINITY_DN8771_c0_g1_i5_orf1	regucalcin-like [Ostrinia furnacalis]	-0.0361	0.42009	0.37132	-1.8607	1.10543
TRINITY_DN54269_c0_g1_i3_orf1	lopap-like [Ostrinia furnacalis]	0.10137	0.13741	-1.1303	-0.838	1.72948
TRINITY_DN361_c0_g1_i5_orf1	hexosaminidase [Ostrinia furnacalis]	0.34414	-0.1779	-0.1804	-1.5449	1.55905
TRINITY_DN126127_c0_g1_i1_orf1	prolow-density lipoprotein receptor-related protein 1, partial [Ostrinia furnacalis]	0.18539	0.5934	-0.0068	-1.8549	1.08292
TRINITY_DN86833_c0_g3_i1_orf1	PREDICTED: glycerol-3-phosphate acyltransferase 1, mitochondrial isoform X1 [Microplitis demolitor]	0.68221	-0.2993	-0.1806	-1.5831	1.3807
TRINITY_DN11970_c0_g1_i4_orf1	myb-like protein AA [Ostrinia furnacalis]	-0.2462	-0.3084	-0.8333	-0.5683	1.95626
TRINITY_DN2880_c0_g1_i2_orf1	sialomucin core protein 24 [Pectinophora gossypiella]	-0.0512	0.19803	0.84819	-1.8657	0.87065
TRINITY_DN2566_c0_g1_i5_orf1	uncharacterized protein LOC114349936 [Ostrinia furnacalis]	0.70157	-0.259	-1.043	-0.9593	1.55969
TRINITY_DN479_c6_g1_i2_orf1	beta-1,3-glucan-binding protein-like [Ostrinia furnacalis]	-0.1535	-0.2003	0.32255	-1.5387	1.56993
TRINITY_DN1986_c0_g1_i1_orf1	serine protease inhibitor 77Ba-like [Ostrinia furnacalis] >XP_028164032.1 serine protease inhibitor 77Ba-like [Ostrinia furnacalis]	-0.1634	-0.0507	-0.1681	-1.3693	1.75146
TRINITY_DN10090_c0_g1_i1_orf1	clotting factor B-like isoform X1 [Ostrinia furnacalis] >XP_028163447.1 clotting factor B-like isoform X2 [Ostrinia furnacalis] >XP_028163448.1 clotting factor B-like isoform X3 [Ostrinia furnacalis]	0.31637	0.21067	0.17151	-1.8629	1.16435
TRINITY_DN6243_c0_g1_i5_orf1	sorting nexin-20 [Ostrinia furnacalis]	0.11011	0.25365	0.61538	-1.9153	0.93617
TRINITY_DN33365_c0_g1_i1_orf1	mucin-5AC isoform X1 [Ostrinia furnacalis]	-0.6088	-0.44	-0.3578	-0.5848	1.99142
TRINITY_DN125441_c0_g1_i5_orf1	KH domain-containing, RNA-binding, signal transduction-associated protein 2-like isoform X12 [Ostrinia furnacalis]	0.17933	0.07606	0.69098	-1.8939	0.94752
TRINITY_DN12256_c0_g1_i1_orf1	lysosome membrane protein 2-like [Ostrinia furnacalis]	0.0121	0.46658	0.59134	-1.9243	0.85424
TRINITY_DN7123_c0_g1_i1_orf1	activating signal cointegrator 1 complex subunit 2 homolog isoform X1 [Ostrinia furnacalis]	-0.6122	-0.5225	-0.5463	-0.3085	1.9896
TRINITY_DN267_c0_g1_i1_orf1	keratin, type I cytoskeletal 9-like [Ostrinia furnacalis]	-0.817	-0.5783	-0.293	-0.271	1.95928
TRINITY_DN268_c1_g1_i7_orf1	unnamed protein product [Spodoptera littoralis] >CAH1645252.1 unnamed protein product [Spodoptera littoralis]	-0.2939	-0.408	-0.4877	-0.7838	1.97353
TRINITY_DN23167_c0_g2_i1_orf1	hypothetical protein evm_003712 [Chilo suppressalis]	0.11528	0.17872	-0.268	-1.5754	1.54948
TRINITY_DN4602_c0_g1_i4_orf1	2-iminobutanoate/2-iminopropanoate deaminase [Ostrinia furnacalis]	0.36492	0.61422	-0.333	-1.767	1.12088
TRINITY_DN125150_c0_g1_i1_orf1	aldehyde dehydrogenase, dimeric NADP-preferring isoform X5 [Ostrinia furnacalis]	0.0202	0.74008	0.24927	-1.8976	0.88808
TRINITY_DN4189_c0_g2_i1_orf1	unnamed protein product [Chilo suppressalis]	-0.5786	-0.3636	0.21088	-1.0871	1.81843

TRINITY_DN82426_c0_g1_i6_orfp1	lysosome-associated membrane glycoprotein 1-like isoform X4 [Ostrinia furnacalis]	-0.4485	-0.0201	-0.767	-0.6949	1.93058
TRINITY_DN110519_c0_g1_i1_orf1	uncharacterized protein LOC114366601 [Ostrinia furnacalis]	-0.591	-0.3433	-0.7526	-0.2844	1.97121
TRINITY_DN11670_c0_g1_i1_orf1	teneurin-m isoform X1 [Ostrinia furnacalis]	-0.6943	-0.2411	-0.0164	-0.9395	1.89127
TRINITY_DN22053_c0_g1_i13_orf1	uncharacterized protein LOC114355104 [Ostrinia furnacalis]	0.34498	0.05631	-0.1583	-1.6744	1.43144
TRINITY_DN2227_c0_g1_i5_orf1	protein 60A [Ostrinia furnacalis]	-0.3895	0.33204	0.33267	-1.6524	1.37725
TRINITY_DN125427_c0_g1_i1_orf1	heat shock protein 19.8 [Chilo suppressalis] >AGM90553.1 HSP19.8 [Chilo suppressalis] >BAE94664.1 small heat shock protein 19.7 [Chilo suppressalis]	-0.0469	-0.2655	-0.9238	-0.6684	1.90453
TRINITY_DN198_c2_g1_i2_orf1	solute carrier organic anion transporter family member 5A1-like isoform X1 [Ostrinia furnacalis]	-0.156	0.38257	0.13983	-1.7231	1.35671
TRINITY_DN54366_c0_g1_i1_orf1	protein obstructor-E-like [Ostrinia furnacalis]	-0.735	-0.5838	-0.6847	0.09528	1.90817
TRINITY_DN9920_c0_g1_i1_orf1	uncharacterized protein LOC114351526 [Ostrinia furnacalis]	0.01599	0.22161	-0.7064	-1.239	1.70778
TRINITY_DN3255_c0_g1_i1_orf1	uncharacterized protein LOC114351042 [Ostrinia furnacalis]	-0.82	-0.5715	-0.5628	0.03529	1.91909
TRINITY_DN42337_c0_g1_i5_orf1	hypothetical protein evm_002829 [Chilo suppressalis]	-0.6177	-0.3172	-0.6686	-0.3783	1.98184
TRINITY_DN97138_c0_g1_i2_orf1	tubulin beta chain-like isoform X2 [Ostrinia furnacalis]	-0.2513	-0.1947	-0.3521	-1.0937	1.89179
TRINITY_DN1407_c0_g1_i5_orf1	unnamed protein product [Chrysodeixis includens]	0.24241	-0.549	-0.2163	-1.2313	1.75413
TRINITY_DN26337_c0_g1_i3_orf1	lysosome membrane protein 2-like [Ostrinia furnacalis]	-0.1149	0.59473	-0.576	-1.4176	1.51383
TRINITY_DN97097_c0_g1_i4_orf1	plectin-like, partial [Ostrinia furnacalis]	0.25249	-0.076	-0.43	-1.4084	1.6619
TRINITY_DN11514_c0_g1_i1_orf1	uncharacterized protein LOC114350079 [Ostrinia furnacalis]	-0.2057	0.46284	0.52924	-1.8344	1.04803
TRINITY_DN6423_c0_g1_i5_orf1	phenoloxidase-activating factor 2-like isoform X2 [Ostrinia furnacalis]	0.03908	0.09183	-0.3372	-1.4549	1.6612
TRINITY_DN4688_c0_g1_i2_orf1	uncharacterized protein LOC114359411 [Ostrinia furnacalis]	-0.5707	-0.1287	-0.3012	-0.9255	1.92622
TRINITY_DN875_c0_g1_i3_orf1	secernin-3 [Ostrinia furnacalis]	0.33093	-0.6161	-0.1826	-1.244	1.71172
TRINITY_DN843_c0_g1_i2_orf1	unnamed protein product [Diatraea saccharalis]	-0.5989	-0.9643	0.60207	-0.7208	1.68204
TRINITY_DN23229_c0_g1_i2_orf1	uncharacterized protein LOC114362553 [Ostrinia furnacalis]	-0.1052	0.40206	-0.6542	-1.2937	1.65099
TRINITY_DN261_c0_g1_i5_orfp1	TRINITY_DN261_c0_g1_i5_m.18559 TRINITY_DN261_c0_g1::TRINITY_DN261_c0_g1_i5::g.18559 ORF type:internal len:190 (+),score=69.81 TRINITY_DN261_c0_g1_i5:3-569(+)	-0.0471	-0.9402	0.07345	-0.9016	1.81538
TRINITY_DN2290_c0_g1_i2_orfp1	TRINITY_DN2290_c0_g1_i2_m.69732 TRINITY_DN2290_c0_g1::TRINITY_DN2290_c0_g1_i2::g.69732 ORF type:complete len:234 (+),score=14.43 TRINITY_DN2290_c0_g1_i2:60-761(+)	-0.7462	-0.8395	0.35091	-0.5769	1.81174
TRINITY_DN2171_c0_g1_i1_orf1	probable pterin-4-alpha-carbinolamine dehydratase isoform X1 [Ostrinia furnacalis]	0.08693	0.51856	0.26072	-1.896	1.02984
TRINITY_DN206_c0_g1_i8_orf1	A-kinase anchor protein 200-like [Ostrinia furnacalis] >XP_028173114.1 A-kinase anchor protein 200-like [Ostrinia furnacalis] >XP_028173115.1 A-kinase anchor protein 200-like [Ostrinia furnacalis]	-0.9099	-0.3558	-0.4828	-0.1944	1.94284
TRINITY_DN9383_c0_g1_i3_orf1	uncharacterized protein LOC114361502 [Ostrinia furnacalis]	-0.2453	-0.607	0.83188	-1.3825	1.4029
TRINITY_DN537_c0_g1_i1_orf1	pupal cuticle protein C1B-like precursor [Papilio xuthus] >BAM18715.1 cuticular protein PxutCPFL6Ba [Papilio xuthus]	-0.4745	-0.2934	0.539	-1.3641	1.59296
TRINITY_DN206_c0_g1_i11_orf1	A-kinase anchor protein 200-like [Ostrinia furnacalis] >XP_028173114.1 A-kinase anchor protein 200-like [Ostrinia furnacalis] >XP_028173115.1 A-kinase anchor protein 200-like [Ostrinia furnacalis]	-0.5216	-0.4167	-0.4597	-0.5984	1.99626
TRINITY_DN1563_c0_g1_i4_orf1	pupal cuticle protein 36-like [Ostrinia furnacalis]	-0.2814	-0.6023	-0.5011	-0.6014	1.98626
TRINITY_DN3251_c0_g1_i6_orf1	fatty-acid amide hydrolase 2-like [Ostrinia furnacalis] >XP_028167366.1 fatty-acid amide hydrolase 2-like [Ostrinia furnacalis] >XP_028167367.1 fatty-acid amide hydrolase 2-like [Ostrinia furnacalis] >XP_028167368.1 fatty-acid amide hydrolase 2-like [Ostrinia furnacalis] >XP_028167369.1 fatty-acid amide hydrolase 2-like [Ostrinia furnacalis] >XP_028167370.1 fatty-acid amide hydrolase 2-like [Ostrinia furnacalis] >XP_028167371.1 fatty-acid amide hydrolase 2-like [Ostrinia furnacalis] >XP_028167372.1 fatty-acid amide hydrolase 2-like [Ostrinia furnacalis]	0.30974	0.09919	0.35537	-1.8781	1.11383
TRINITY_DN5757_c0_g1_i1_orf1	ATP-dependent DNA helicase 2 subunit 1 [Ostrinia furnacalis]	-0.3079	-0.5454	-0.4574	-0.675	1.98567
TRINITY_DN36899_c0_g1_i1_orf1	glucose dehydrogenase [FAD, quinone]-like [Ostrinia furnacalis]	-0.5878	0.01484	0.2017	-1.3218	1.69307
TRINITY_DN113272_c0_g1_i1_orf1	altered inheritance of mitochondria protein 3-like [Ostrinia furnacalis]	-0.857	-0.5313	-0.6271	0.12477	1.89062
TRINITY_DN8480_c0_g1_i1_orf1	lysosomal Pro-X carboxypeptidase [Ostrinia furnacalis]	-0.4403	0.15532	0.52292	-1.6157	1.37775
TRINITY_DN1507_c0_g1_i5_orf1	27 kDa hemolymph protein-like, partial [Ostrinia furnacalis]	-0.1314	0.01942	0.55902	-1.7351	1.2881
TRINITY_DN3005_c0_g1_i7_orf1	lachesin-like isoform X3 [Ostrinia furnacalis]	0.07932	-0.2241	-0.5487	-1.1368	1.83031
TRINITY_DN4689_c0_g1_i5_orf1	pericentriolar material 1 protein-like isoform X3 [Ostrinia furnacalis]	0.07776	-0.5403	-0.8668	-0.5737	1.90303
TRINITY_DN104_c0_g1_i4_orf1	PREDICTED: heparan-alpha-glucosaminide N-acetyltransferase [Amyelois transitella]	-0.6805	-0.0336	0.32396	-1.2805	1.67067
TRINITY_DN12286_c1_g1_i2_orf1	sideroflexin-1-3 [Galleria mellonella] >XP_026754161.1 sideroflexin-1-3 [Galleria mellonella]	0.49075	-0.4446	-0.5167	-1.2111	1.68161
TRINITY_DN10304_c0_g2_i1_orf1	glycine-rich cell wall structural protein [Ostrinia furnacalis]	-0.2577	-0.6404	-0.5625	-0.5227	1.98338
TRINITY_DN1630_c0_g1_i6_orf1	major facilitator superfamily domain-containing protein 1-like [Ostrinia furnacalis]	0.04433	-0.3153	-0.1486	-1.3376	1.75713
TRINITY_DN64616_c0_g1_i1_orf1	uncharacterized protein LOC114366101 [Ostrinia furnacalis]	0.11828	-0.034	-1.0301	-0.8455	1.79134
TRINITY_DN1703_c0_g1_i6_orf1	leucine-rich repeat-containing protein 15-like [Ostrinia furnacalis] >XP_028171914.1 leucine-rich repeat-containing protein 15-like [Ostrinia furnacalis] >XP_028171921.1 leucine-rich repeat-containing protein 15-like [Ostrinia furnacalis]	-0.0749	-0.1219	-0.495	-1.1532	1.84512
TRINITY_DN37585_c0_g1_i1_orf1	cuticle protein 19.8-like [Ostrinia furnacalis]	0.1185	-0.4627	-0.672	-0.871	1.88723
TRINITY_DN1091_c0_g1_i1_orf1	macrophage mannose receptor 1-like [Pararge aegeria]	-0.5032	-0.3783	0.73937	-1.3514	1.49356
TRINITY_DN18502_c0_g1_i1_orf1	uncharacterized protein LOC114359515 [Ostrinia furnacalis]	-0.7772	-0.6549	-0.6613	0.22858	1.86481
TRINITY_DN43431_c0_g1_i1_orf1	glycine dehydrogenase (decarboxylating), mitochondrial isoform X1 [Ostrinia furnacalis] >XP_028174269.1 glycine dehydrogenase (decarboxylating), mitochondrial isoform X3 [Ostrinia furnacalis]	-0.01	-0.0663	0.63563	-1.7688	1.20945
TRINITY_DN70485_c0_g1_i2_orf1	serine/threonine-protein kinase Genghis Khan-like [Ostrinia furnacalis]	0.13433	-0.2899	0.1462	-1.5568	1.56618
TRINITY_DN38431_c0_g1_i1_orf1	neprilysin-2 isoform X1 [Ostrinia furnacalis]	-0.5199	-0.3864	-0.5575	-0.5327	1.99646
TRINITY_DN2058_c0_g1_i2_orf1	proteasomal ubiquitin receptor ADRM1 [Ostrinia furnacalis]	0.11281	-0.9739	-0.099	-0.8557	1.81574
TRINITY_DN1664_c0_g1_i4_orf1	uncharacterized protein LOC114355246 [Ostrinia furnacalis]	0.06649	-0.0706	0.72407	-1.8103	1.09041
TRINITY_DN8083_c0_g1_i1_orf1	solute carrier family 35 member F6 [Ostrinia furnacalis]	-0.4066	-0.1746	0.75293	-1.5389	1.36715
TRINITY_DN2109_c0_g1_i4_orf1	mucin-2-like isoform X2 [Ostrinia furnacalis]	0.46726	-0.8401	-1.0287	-0.3081	1.70962
TRINITY_DN7590_c0_g1_i4_orf1	innexin inx1-like [Pectinophora gossypiella]	0.24051	0.51814	-0.2236	-1.7643	1.22923
TRINITY_DN166_c0_g1_i4_orf1	PREDICTED: cryptochrome-1 isoform X1 [Amyelois transitella] >XP_013199861.1 PREDICTED: cryptochrome-1 isoform X1 [Amyelois transitella]	0.52374	0.28484	-0.5439	-1.601	1.33628

TRINITY_DN10766_c0_g1_i1_orf1	hypothetical protein evm_008559 [Chilo suppressalis]	-0.7461	-0.1508	-0.1069	-0.8955	1.89928
TRINITY_DN3109_c0_g1_i5_orf1	protein takeout isoform X2 [Ostrinia furnacalis]	-0.0372	0.00062	0.7659	-1.8045	1.07515
TRINITY_DN28661_c0_g1_i1_orf1	cathepsin B [Ostrinia furnacalis]	-0.1661	0.10615	0.59612	-1.7617	1.22559
TRINITY_DN25345_c0_g1_i1_orf1	chromodomain-helicase-DNA-binding protein 1 isoform X3 [Ostrinia furnacalis]	0.16444	-0.7785	-0.9646	-0.2572	1.83584
TRINITY_DN4125_c0_g1_i14_orf1	angiotensin-converting enzyme-like isoform X1 [Ostrinia furnacalis]	-0.8866	-0.5291	-0.5973	0.12597	1.88712
TRINITY_DN42964_c0_g1_i1_orf1	protein lethal(2)essential for life-like [Galleria mellonella]	-0.5436	0.83104	-1.2742	-0.4821	1.46893
TRINITY_DN41761_c0_g1_i4_orf1	transmembrane protease serine 9 [Ostrinia furnacalis]	-0.0317	-0.3325	-0.5152	-1.0157	1.8951
TRINITY_DN218_c0_g1_i1_orf1	altered inheritance of mitochondria protein 3-like isoform X2 [Ostrinia furnacalis]	-0.6516	-0.4519	-0.6255	-0.2501	1.97921
TRINITY_DN114834_c0_g1_i1_orf1	uncharacterized protein LOC115444227 [Manduca sexta] >XP_030025790.1 uncharacterized protein LOC115444227 [Manduca sexta] >XP_037296791.1 uncharacterized protein LOC115444227 [Manduca sexta] >XP_037296792.1 uncharacterized protein LOC115444227 [Manduca sexta] >KAG6441350.1 hypothetical protein O3G_MSEX001778 [Manduca sexta]	-0.5591	-0.5123	-0.6947	-0.2084	1.97456
TRINITY_DN15865_c0_g2_i2_orf1	carboxylesterase [Cnaphalocrocis medinalis]	-0.1067	0.04047	-0.7124	-1.0558	1.83435
TRINITY_DN110402_c0_g2_i1_orf1	apolipophorins-like [Ostrinia furnacalis]	0.34706	-0.1178	-0.6595	-1.2576	1.68792
TRINITY_DN13088_c0_g1_i5_orf1	beta-hexosaminidase subunit alpha-like isoform X2 [Ostrinia furnacalis]	0.02415	-0.4789	-0.4456	-0.9931	1.89347
TRINITY_DN36281_c0_g1_i2_orf1	putative uncharacterized protein DDB_G0282499 isoform X1 [Ostrinia furnacalis]	-0.5432	-0.2968	-0.4445	-0.6983	1.98283
TRINITY_DN18650_c0_g1_i1_orf1	bombyxin B-9-like [Ostrinia furnacalis]	0.56747	0.0252	-0.7953	-1.3172	1.51982
TRINITY_DN7128_c0_g1_i7_orf1	dystroglycan [Ostrinia furnacalis]	-0.2055	-0.0071	0.39003	-1.6362	1.45888
TRINITY_DN1767_c0_g2_i15_orf1	fasciclin-2 isoform X3 [Ostrinia furnacalis]	0.07922	-0.1656	0.23328	-1.6389	1.49198
TRINITY_DN3833_c0_g1_i4_orf1	division abnormally delayed protein [Ostrinia furnacalis]	-0.4079	-0.4736	-0.5892	-0.5258	1.99644
TRINITY_DN2348_c0_g1_i1_orfp1	TRINITY_DN2348_c0_g1_i1_m.39060 TRINITY_DN2348_c0_g1::TRINITY_DN2348_c0_g1_i1::g.39060 ORF type:complete len:149 (+),score=54.19 TRINITY_DN2348_c0_g1_i1:28-474(+)	0.19699	-0.398	-0.2004	-1.3293	1.73074
TRINITY_DN19951_c0_g1_i5_orf1	protein croquemort-like [Ostrinia furnacalis]	-0.0712	-0.2627	-0.3437	-1.1742	1.85178
TRINITY_DN5064_c0_g1_i4_orf1	sortilin-related receptor-like [Ostrinia furnacalis]	-0.3872	0.21493	0.42395	-1.6412	1.38948
TRINITY_DN14944_c0_g1_i9_orf1	casein kinase I isoform X1 [Ostrinia furnacalis] >XP_[illegible].1 casein kinase I isoform X1 [Ostrinia furnacalis] >XP_[illegible].1 casein kinase I isoform X1 [Ostrinia furnacalis] >XP_028158161.1 casein kinase I isoform X1 [Ostrinia furnacalis] >XP_028158163.1 casein kinase I isoform X1 [Ostrinia furnacalis] >XP_028158164.1 casein kinase I isoform X1 [Ostrinia furnacalis] >XP_028158165.1 casein kinase I isoform X1 [Ostrinia furnacalis] >XP_028158166.1 casein kinase I isoform X1 [Ostrinia furnacalis]	-0.717	0.14814	-0.2508	-1.0158	1.83552
TRINITY_DN806_c0_g2_i1_orf1	uncharacterized protein LOC114355167 [Ostrinia furnacalis]	-0.8625	-0.615	-0.3381	-0.1207	1.93624
TRINITY_DN2508_c0_g1_i2_orf1	uncharacterized protein LOC114361845 [Ostrinia furnacalis] >XP_028172853.1 uncharacterized protein LOC114361845 [Ostrinia furnacalis]	0.03523	0.35247	0.03774	-1.7591	1.33366
TRINITY_DN712_c0_g2_i1_orf1	serine protease inhibitor 77Ba-like [Ostrinia furnacalis]	-0.1687	0.0377	-0.5702	-1.1325	1.8337
TRINITY_DN111621_c0_g3_i1_orf1	Serine proteinase stubble [Eumeta japonica]	-0.0474	-0.6631	-0.9389	-0.2515	1.90086
TRINITY_DN1196_c0_g1_i4_orf1	glucosamine-6-phosphate isomerase isoform X2 [Ostrinia furnacalis]	-0.9411	-0.5942	0.42066	-0.6598	1.7745
TRINITY_DN585_c0_g1_i5_orf1	very low-density lipoprotein receptor isoform X2 [Galleria mellonella]	-0.3065	-0.7547	-0.1078	-0.7648	1.93387
TRINITY_DN98016_c0_g1_i1_orf1	methanethiol oxidase [Ostrinia furnacalis]	-0.4442	0.37136	-0.1443	-1.4114	1.62849
TRINITY_DN9412_c0_g1_i1_orf1	maspardin-like [Ostrinia furnacalis]	0.49746	-0.6692	-0.0341	-1.3603	1.5662
TRINITY_DN13887_c0_g1_i5_orf1	transmembrane protein 184B isoform X3 [Ostrinia furnacalis]	-0.241	-0.1995	-0.4787	-0.9982	1.91745
TRINITY_DN10364_c0_g1_i5_orf1	uncharacterized protein LOC114352615 [Ostrinia furnacalis]	-0.3554	-0.1804	-0.7427	-0.6783	1.95687
TRINITY_DN8766_c0_g1_i1_orf1	prolow-density lipoprotein receptor-related protein 1, partial [Ostrinia furnacalis]	-0.1789	0.81162	0.31127	-1.8444	0.90036
TRINITY_DN668_c0_g1_i4_orf1	fatty acid synthase-like isoform X1 [Ostrinia furnacalis]	-0.685	-0.6451	-0.5165	-0.1118	1.95841
TRINITY_DN4273_c1_g1_i5_orf1	tetraspanin-13 isoform X1 [Ostrinia furnacalis]	-0.1558	-0.3337	0.01552	-1.3045	1.77836
TRINITY_DN14856_c0_g1_i1_orf1	upstream activation factor subunit spp27 [Ostrinia furnacalis]	-0.7043	-0.1582	0.04922	-1.0332	1.84638
TRINITY_DN15420_c0_g3_i2_orf1	elongation factor 1-alpha 2-like [Galleria mellonella] >XP_031769625.1 elongation factor 1-alpha 2-like [Galleria mellonella]	-0.2177	-0.1789	-0.8686	-0.6647	1.92987
TRINITY_DN2416_c0_g1_i5_orf1	somatomedin-B and thrombospondin type-1 domain-containing protein [Ostrinia furnacalis] >XP_028177886.1 somatomedin-B and thrombospondin type-1 domain-containing protein [Ostrinia furnacalis]	-0.6983	0.42695	-0.0408	-1.3068	1.61888
TRINITY_DN45271_c0_g1_i1_orf1	double-strand break repair protein MRE11 [Ostrinia furnacalis]	0.00999	-0.0302	0.21233	-1.667	1.47481
TRINITY_DN14532_c0_g1_i1_orf1	pupal cuticle protein-like [Trichoplusia ni]	-0.7332	-0.4542	-0.8054	0.09595	1.89689
TRINITY_DN1194_c0_g1_i5_orf1	sequestosome-1-like isoform X4 [Ostrinia furnacalis]	-0.5474	-0.5495	0.83189	-1.2223	1.48736
TRINITY_DN25987_c0_g1_i5_orf1	GILT-like protein 2 isoform X1 [Ostrinia furnacalis] >XP_028156245.1 GILT-like protein 2 isoform X2 [Ostrinia furnacalis] >XP_028156247.1 GILT-like protein 2 isoform X3 [Ostrinia furnacalis]	-0.7989	-0.4988	0.29837	-0.8279	1.82718
TRINITY_DN83374_c0_g1_i1_orf1	uncharacterized protein LOC114350302, partial [Ostrinia furnacalis]	-0.5321	-0.6003	-0.6776	-0.1579	1.96785
TRINITY_DN1252_c0_g1_i3_orf1	unnamed protein product [Chilo suppressalis]	-0.2447	-1.0092	-0.1413	-0.5125	1.90763
TRINITY_DN36856_c0_g1_i1_orf1	protein enhancer of sevenless 2B isoform X2 [Formica exsecta]	-0.0089	-0.5356	0.30438	-1.3952	1.63523
TRINITY_DN11772_c0_g1_i1_orf1	conserved oligomeric Golgi complex subunit 2 [Ostrinia furnacalis]	-0.6318	-0.1514	-0.3978	-0.7737	1.95473
TRINITY_DN3896_c0_g1_i1_orf1	glyoxalase domain-containing protein 4 [Ostrinia furnacalis]	0.17913	0.20018	-0.3518	-1.5635	1.53605
TRINITY_DN7565_c0_g1_i3_orf1	acylphosphatase-2-like [Ostrinia furnacalis]	-0.4453	-0.2405	-1.072	-0.1334	1.89125
TRINITY_DN19043_c0_g3_i2_orf1	hypothetical protein EVAR_60654_1 [Eumeta japonica]	0.077	0.54751	-0.8484	-1.2933	1.5172
TRINITY_DN4886_c0_g1_i6_orf1	uncharacterized protein LOC114349567 [Ostrinia furnacalis]	-0.0172	-0.3873	-0.2079	-1.2135	1.82588
TRINITY_DN4217_c0_g1_i2_orf1	hypothetical protein evm_006611 [Chilo suppressalis]	-0.4662	-0.2062	-0.1568	-1.0639	1.89307
TRINITY_DN41_c0_g1_i5_orf1	putative phospholipase B-like 2 [Ostrinia furnacalis]	-0.1962	-0.4066	-0.0216	-1.2045	1.82891
TRINITY_DN140_c1_g1_i2_orf1	modular serine protease-like isoform X1 [Ostrinia furnacalis]	-0.3909	-0.4071	0.57878	-1.3605	1.57972
TRINITY_DN82104_c0_g1_i5_orf1	uncharacterized protein LOC114349939 [Ostrinia furnacalis] >XP_028156338.1 uncharacterized protein LOC114349939 [Ostrinia furnacalis]	-0.0627	0.17338	-0.4061	-1.3946	1.69002
TRINITY_DN321_c0_g1_i1_orf1	uncharacterized protein LOC126371336 [Pectinophora gossypiella]	-0.6877	-0.0677	-0.3948	-0.786	1.93619
TRINITY_DN661_c1_g2_i1_orf1	larval/pupal cuticle protein H1C-like [Ostrinia furnacalis]	-0.9071	-0.5386	-0.4875	0.02299	1.91021
TRINITY_DN280_c0_g1_i8_orf1	Tubulin beta-1 chain [Papilio xuthus]	-0.601	-0.5904	-0.5514	-0.2393	1.98214
TRINITY_DN53427_c0_g1_i2_orf1	heparanase-like [Ostrinia furnacalis]	-0.5538	-0.5511	0.50923	-1.108	1.70369

TRINITY_DN6275_c0_g1_i3_orf1	CTL-like protein 1 isoform X1 [Galleria mellonella]	-0.0342	0.45747	0.1819	-1.8148	1.2096
TRINITY_DN467_c0_g3_i1_orf1	histone-lysine N-methyltransferase 2B-like, partial [Ostrinia furnacalis]	-0.7922	-0.5836	-0.574	0.0258	1.92398
TRINITY_DN4013_c0_g1_i4_orf1	uncharacterized protein LOC114353190 isoform X1 [Ostrinia furnacalis] >XP_028160984.1 uncharacterized protein LOC114353190 isoform X2 [Ostrinia furnacalis] >XP_028161062.1 uncharacterized protein LOC114353190 isoform X1 [Ostrinia furnacalis] >XP_028161142.1 uncharacterized protein LOC114353190 isoform X1 [Ostrinia furnacalis]	-0.616	-0.6592	0.25227	-0.83	1.85295
TRINITY_DN31619_c0_g1_i2_orf1	endocuticle structural glycoprotein ABD-4-like [Ostrinia furnacalis]	-0.8432	-0.7035	-0.456	0.11217	1.89043
TRINITY_DN13648_c0_g1_i6_orf1	neurexin-4 [Ostrinia furnacalis]	-0.8185	-0.3519	-0.4251	-0.3754	1.97094
TRINITY_DN3545_c0_g1_i6_orf1	group XV phospholipase A2-like [Ostrinia furnacalis] >XP_028168992.1 group XV phospholipase A2-like [Ostrinia furnacalis] >XP_028168993.1 group XV phospholipase A2-like [Ostrinia furnacalis]	-0.0714	0.14979	0.25706	-1.725	1.38957
TRINITY_DN895_c0_g2_i1_orf1	protein N-terminal asparagine amidohydrolase [Cotesia glomerata] >XP_044583616.1 protein N-terminal asparagine amidohydrolase [Cotesia glomerata] >XP_044583617.1 protein N-terminal asparagine amidohydrolase [Cotesia glomerata] >KAH0553813.1 hypothetical protein KQX54_004640 [Cotesia glomerata]	-0.4617	-0.2704	0.33988	-1.3075	1.69963
TRINITY_DN5028_c0_g1_i11_orf1	NTF2-related export protein [Ostrinia furnacalis]	0.25706	-0.8496	-0.3006	-0.9178	1.81091
TRINITY_DN1196_c0_g1_i5_orf1	glucosamine-6-phosphate isomerase isoform X1 [Ostrinia furnacalis]	-0.9214	-0.3989	-0.1131	-0.4981	1.93157
TRINITY_DN661_c0_g3_i5_orf1	cuticle protein 18.6-like [Ostrinia furnacalis]	-0.6781	-0.1782	-0.6302	-0.4828	1.96931
TRINITY_DN1533_c0_g2_i1_orf1	unnamed protein product [Chilo suppressalis]	-0.1943	-0.0135	-0.1656	-1.3729	1.74635
TRINITY_DN14458_c0_g1_i2_orf1	spermatogenesis-associated protein 20 isoform X1 [Ostrinia furnacalis]	-0.0019	-0.4737	-0.0471	-1.2612	1.78399
TRINITY_DN65299_c0_g4_i1_orf1	LOW QUALITY PROTEIN: signal transducing adapter molecule 2 [Ostrinia furnacalis]	-0.226	-0.0274	1.02117	-1.7275	0.95975
TRINITY_DN37585_c0_g2_i1_orf1	cuticle protein 19.8-like [Ostrinia furnacalis]	-0.8985	-0.3197	-0.6123	-0.0947	1.92523
TRINITY_DN1491_c0_g1_i8_orf1	GILT-like protein 2 isoform X1 [Ostrinia furnacalis] >XP_028156245.1 GILT-like protein 2 isoform X2 [Ostrinia furnacalis] >XP_028156247.1 GILT-like protein 2 isoform X3 [Ostrinia furnacalis]	-0.3515	-0.2775	-0.3406	-0.9667	1.93624
TRINITY_DN18027_c0_g2_i1_orf1	vanin-like protein 2 isoform X2 [Ostrinia furnacalis]	0.21206	-0.0903	-0.3518	-1.4336	1.66368
TRINITY_DN12673_c3_g1_i2_orf1	unnamed protein product [Chilo suppressalis]	-0.4854	-0.6531	0.19573	-0.917	1.85975
TRINITY_DN501_c0_g1_i5_orf1	hypothetical protein evm_002550 [Chilo suppressalis]	-0.6224	-0.4559	-0.516	-0.4003	1.99457
TRINITY_DN2107_c0_g2_i3_orf1	LIM and SH3 domain protein Lasp [Ostrinia furnacalis]	-0.2339	-0.2798	-0.1742	-1.1727	1.86052
TRINITY_DN4041_c0_g1_i6_orf1	tubulin-folding cofactor B isoform X3 [Ostrinia furnacalis]	0.27207	0.16895	-0.605	-1.421	1.58499
TRINITY_DN3292_c2_g2_i1_orf1	aldo-keto reductase AKR2E4-like [Ostrinia furnacalis]	-0.1595	0.38627	0.05954	-1.6893	1.40293
TRINITY_DN8641_c0_g1_i1_orf1	uncharacterized protein LOC114357057 [Ostrinia furnacalis]	-0.1981	-0.2151	-0.6997	-0.823	1.93588
TRINITY_DN714_c0_g1_i3_orf1	thymosin beta isoform X4 [Ostrinia furnacalis]	-0.6771	-0.1201	-0.4391	-0.718	1.9542
TRINITY_DN5074_c0_g1_i7_orf1	zonadhesin-like [Ostrinia furnacalis]	-1.0295	-0.3991	-0.2564	-0.2288	1.91383
TRINITY_DN22443_c0_g2_i3_orf1	protein eiger [Ostrinia furnacalis] >QKV49447.1 eiger [Ostrinia furnacalis]	-0.0093	-0.274	-0.2893	-1.2429	1.81556
TRINITY_DN14009_c0_g1_i1_orf1	proline-rich extensin-like protein EPR1 [Manduca sexta]	-0.815	-0.5297	-0.4159	-0.1996	1.9602
TRINITY_DN3073_c0_g1_i7_orf1	claspin-like [Ostrinia furnacalis]	-0.6444	-0.3597	-0.4152	-0.5702	1.98945
TRINITY_DN21719_c0_g2_i4_orf1	chymotrypsin-2-like [Ostrinia furnacalis]	-0.6455	-0.4331	-0.3919	-0.522	1.99242
TRINITY_DN20796_c0_g1_i4_orf1	probable low-specificity L-threonine aldolase 2 [Ostrinia furnacalis]	-0.1247	0.2106	-0.5108	-1.3023	1.72718
TRINITY_DN20244_c0_g1_i1_orfp1	uncharacterized protein LOC125235519 [Leguminivora glycinivorella]	-0.4517	-0.8312	-0.8905	0.39736	1.77601
TRINITY_DN52788_c0_g1_i1_orf1	putative fatty acyl-CoA reductase CG5065 isoform X1 [Ostrinia furnacalis]	-0.5125	-0.5631	-0.4372	-0.4855	1.99834
TRINITY_DN34786_c0_g1_i1_orf1	small heat shock protein Hsp29.7 [Ostrinia furnacalis]	-0.4756	-0.5797	-0.765	-0.1377	1.95796
TRINITY_DN867_c0_g1_i1_orf1	hemicentin-2-like isoform X1 [Ostrinia furnacalis]	-0.3225	-1.0353	-0.4688	-0.0705	1.89718
TRINITY_DN110523_c0_g2_i1_orf1	uncharacterized protein LOC107036393 [Diachasma alloeum]	-0.5835	-1.1811	0.3944	-0.3567	1.72681
TRINITY_DN7776_c0_g1_i5_orf1	uncharacterized protein LOC114364702 [Ostrinia furnacalis]	-0.1412	0.73415	0.13345	-1.8053	1.07891
TRINITY_DN7711_c1_g1_i3_orf1	long-chain fatty acid transport protein 1-like [Ostrinia furnacalis]	-0.8355	-0.4957	0.42684	-0.8641	1.76844
TRINITY_DN48590_c0_g1_i1_orf1	acyl-CoA Delta(11) desaturase isoform X1 [Ostrinia furnacalis] >XP_028172999.1 acyl-CoA Delta(11) desaturase isoform X2 [Ostrinia furnacalis] >XP_028173000.1 acyl-CoA Delta(11) desaturase isoform X1 [Ostrinia furnacalis]	-0.7892	-0.6396	-0.6761	0.24769	1.85729
TRINITY_DN7735_c1_g1_i1_orf1	cuticular protein CPH [Spodoptera litura]	-0.8515	-0.3107	-0.5172	-0.2785	1.95789
TRINITY_DN1091_c0_g2_i10_orf1	macrophage mannose receptor 1-like isoform X2 [Maniola hyperantus]	0.30271	-1.2904	0.48995	-0.951	1.44873
TRINITY_DN23582_c0_g1_i1_orf1	unnamed protein product [Diatraea saccharalis]	0.3204	-0.8497	-1.0516	-0.1634	1.74434
TRINITY_DN15411_c0_g1_i4_orf1	uncharacterized protein LOC114362040 isoform X1 [Ostrinia furnacalis]	0.16308	-0.5643	0.01988	-1.3229	1.70426
TRINITY_DN445_c0_g1_i2_orf1	sorting nexin-17 [Ostrinia furnacalis]	-0.2862	-0.399	0.28899	-1.318	1.71416
TRINITY_DN19923_c0_g1_i1_orf1	uncharacterized protein LOC114350958 [Ostrinia furnacalis]	-0.5786	-0.6231	-0.7144	-0.0245	1.94062
TRINITY_DN14774_c0_g1_i4_orf1	aminopeptidase N-like [Ostrinia furnacalis]	-0.872	-0.7979	0.51519	-0.5783	1.73297
TRINITY_DN100208_c0_g1_i1_orf1	neurofilament heavy polypeptide-like isoform X2 [Ostrinia furnacalis]	0.11339	-1.8004	0.67443	-0.1176	1.13013
TRINITY_DN2323_c0_g1_i4_orf1	uncharacterized protein LOC114364097 isoform X2 [Ostrinia furnacalis]	-0.3288	-0.5107	-0.8006	-0.3301	1.97009
TRINITY_DN569_c0_g3_i12_orf1	prominin-like protein isoform X2 [Ostrinia furnacalis]	0.13661	0.30303	-0.3543	-1.5854	1.50013
TRINITY_DN1480_c0_g1_i5_orf1	carboxypeptidase N subunit 2-like [Ostrinia furnacalis]	-0.5145	0.35513	-0.3848	-1.1964	1.7406
TRINITY_DN13067_c0_g1_i6_orf1	diphosphomevalonate decarboxylase [Ostrinia furnacalis]	-0.1852	-0.0212	-1.1611	-0.4742	1.84177
TRINITY_DN6974_c0_g2_i1_orf1	mucolipin-3-like [Ostrinia furnacalis]	-0.8509	-0.8228	0.19967	-0.3749	1.84894
TRINITY_DN19328_c0_g1_i1_orf1	hypothetical protein evm_002753 [Chilo suppressalis]	-0.5326	0.70013	-0.126	-1.4715	1.43
TRINITY_DN3675_c0_g1_i1_orf1	unnamed protein product [Spodoptera exigua]	-0.1437	-0.3104	0.00858	-1.3238	1.7693
TRINITY_DN12464_c0_g1_i3_orf1	PH and SEC7 domain-containing protein 1 [Trichoplusia ni]	-0.263	-0.675	-0.6039	-0.4377	1.97962
TRINITY_DN56430_c0_g1_i1_orf1	unnamed protein product, partial [Iphiclides podalirius]	-0.8039	-0.2389	0.51515	-1.1312	1.65882
TRINITY_DN5893_c0_g1_i7_orf1	jupiter microtubule associated homolog 1-like [Ostrinia furnacalis] >XP_028173360.1 jupiter microtubule associated homolog 1-like [Ostrinia furnacalis]	-0.5583	-0.6639	-0.5291	-0.2276	1.97886
TRINITY_DN4635_c0_g1_i4_orf1	putative salivary secreted peptide [Operophtera brumata]	0.43415	0.03734	-0.6842	-1.3632	1.57594
TRINITY_DN31943_c0_g1_i1_orf1	proteoglycan Cow [Ostrinia furnacalis]	-0.1534	-0.3863	-1.0485	-0.3165	1.90467
TRINITY_DN2043_c0_g1_i11_orf1	phenoloxidase-activating factor 2-like [Ostrinia furnacalis]	-0.1281	-0.6235	0.5169	-1.3489	1.58366

TRINITY_DN8692_c0_g1_i2_orf1	caspase-1-like [Ostrinia furnacalis]	-0.7281	-0.1336	-0.3686	-0.7189	1.94918
TRINITY_DN58872_c0_g1_i1_orfp1	cuticle protein 64-like [Pectinophora gossypiella]	-0.1719	-0.364	-0.897	-0.5097	1.94256
TRINITY_DN14721_c0_g1_i2_orf1	protein masquerade-like isoform X2 [Ostrinia furnacalis]	-0.6418	-0.8218	-0.681	0.31441	1.8303
TRINITY_DN39404_c0_g1_i7_orf1	hypothetical protein evm_004736, partial [Chilo suppressalis]	-0.2501	0.0565	0.47197	-1.6677	1.38935
TRINITY_DN72999_c0_g1_i1_orf1	protein obstructor-E-like isoform X1 [Ostrinia furnacalis] >XP_028169319.1 protein obstructor-E-like isoform X2 [Ostrinia furnacalis]	-0.875	-0.6896	-0.5898	0.33894	1.81549
TRINITY_DN50471_c0_g1_i4_orf1	hypothetical protein evm_008982 [Chilo suppressalis]	-0.1299	-0.642	-0.7749	-0.4043	1.95116
TRINITY_DN23183_c1_g1_i2_orf1	myotubularin-related protein 9 [Ostrinia furnacalis]	-1.2249	-0.2515	0.10472	-0.4287	1.80042
TRINITY_DN1868_c0_g1_i1_orf1	protein obstructor-E isoform X1 [Ostrinia furnacalis]	-0.4808	-0.7217	-1.0091	0.47972	1.73191
TRINITY_DN12331_c0_g1_i5_orf1	septin-7 isoform X1 [Ostrinia furnacalis]	-0.4512	-0.3991	-0.5303	-0.6141	1.99469
TRINITY_DN29879_c0_g1_i3_orf1	uncharacterized protein LOC114350556 isoform X1 [Ostrinia furnacalis] >XP_028157201.1 uncharacterized protein LOC114350556 isoform X2 [Ostrinia furnacalis] >XP_028157202.1 uncharacterized protein LOC114350556 isoform X3 [Ostrinia furnacalis]	-0.9114	0.56867	-0.8456	-0.507	1.69528
TRINITY_DN8674_c0_g2_i1_orf1	N(G),N(G)-dimethylarginine dimethylaminohydrolase 1 [Ostrinia furnacalis]	-0.2092	0.66066	-0.3484	-1.5336	1.43054
TRINITY_DN978_c9_g2_i1_orf1	hypothetical protein evm_000959 [Chilo suppressalis]	-0.8889	-0.6744	-0.5439	0.26639	1.84075
TRINITY_DN4790_c0_g1_i6_orf1	ADP-ribosylation factor-like protein 8 [Ostrinia furnacalis] >CAG9746554.1 unnamed protein product [Diatraea saccharalis] >CAG9785239.1 unnamed protein product [Diatraea saccharalis] >CAH2992000.1 unnamed protein product [Chilo suppressalis]	-0.2436	0.02041	0.67652	-1.7065	1.25316
TRINITY_DN291_c0_g1_i2_orf1	DNA replication licensing factor Mcm7 [Helicoverpa armigera] >XP_049698025.1 DNA replication licensing factor Mcm7-like [Helicoverpa armigera] >PZC87280.1 hypothetical protein B5X24_HaOG201516 [Helicoverpa armigera]	0.33148	-0.3128	-0.7733	-1.0209	1.77544
TRINITY_DN19998_c0_g1_i1_orf1	ubiquitin-conjugating enzyme E2 variant 2 [Helicoverpa armigera] >XP_026325122.1 ubiquitin-conjugating enzyme E2 variant 2 [Hyposmocoma kahamanoa] >XP_026499214.1 ubiquitin-conjugating enzyme E2 variant 2 [Vanessa tameamea] >XP_026738520.1 ubiquitin-conjugating enzyme E2 variant 2 [Trichoplusia ni] >XP_026762551.1 ubiquitin-conjugating enzyme E2 variant 2 [Galleria mellonella] >XP_028172566.1 ubiquitin-conjugating enzyme E2 variant 2 [Ostrinia furnacalis] >XP_032520494.1 ubiquitin-conjugating enzyme E2 variant 2 [Danaus plexippus plexippus] >XP_034831247.1 ubiquitin-conjugating enzyme E2 variant 2 [Maniola hyperantus] >XP_039755795.1 ubiquitin-conjugating enzyme E2 variant 2 [Pararge aegeria] >XP_041987413.1 ubiquitin-conjugating enzyme E2 variant 2 [Aricia agestis] >XP_045450678.1 ubiquitin-conjugating enzyme E2 variant 2 [Melitaea cinxia] >XP_045766931.1 ubiquitin-conjugating enzyme E2 variant 2 [Maniola jurtina] >XP_046968624.1 ubiquitin-conjugating enzyme E2 variant 2 [Vanessa cardui] >XP_047027052.1 ubiquitin-conjugating enzyme E2 variant 2 [Helicoverpa zea] >XP_047534810.1 ubiquitin-conjugating enzyme E2 variant 2 [Vanessa atalanta] >KAI5645358.1 ubiquitin-conjugating enzyme domain-containing protein [Phthorimaea operculella] >RVE41109.1 hypothetical protein evm_014241 [Chilo suppressalis] >CAB3251720.1 unnamed protein product [Arctia plantaginis] >CAG9561279.1 unnamed protein product [Danaus chrysippus] >CAG9755233.1 unnamed protein product [Diatraea saccharalis] >CAH0584342.1 unnamed protein product [Chrysodeixis includens] >CAH0729826.1 unnamed protein product, partial [Brenthis ino] >CAH2039508.1 unnamed protein product, partial [Iphiclides podalirius] >CAH2239303.1 jg6875 [Pararge aegeria aegeria]	-0.3768	-0.5207	-0.0607	-0.9569	1.91508
TRINITY_DN1293_c0_g1_i4_orf1	putative fatty acyl-CoA reductase CG5065 [Ostrinia furnacalis]	-0.8903	-0.7175	-0.2584	-0.0377	1.9038
TRINITY_DN8569_c1_g2_i7_orf1	furin-like protease 1, partial [Ostrinia furnacalis]	-1.0611	-0.3704	-0.0513	-0.4064	1.8892
TRINITY_DN71832_c0_g1_i1_orf1	basement membrane-specific heparan sulfate proteoglycan core protein isoform X13 [Ostrinia furnacalis]	-1.6494	0.46383	-0.0578	-0.1808	1.42419
TRINITY_DN46090_c0_g2_i1_orf1	inactive tyrosine-protein kinase 7-like, partial [Ostrinia furnacalis]	-0.9165	-0.6278	-0.3454	-0.0198	1.90951
TRINITY_DN9455_c0_g1_i6_orf1	uncharacterized protein LOC114360866 isoform X4 [Ostrinia furnacalis]	-0.6055	-0.567	-0.9901	0.37664	1.78598
TRINITY_DN114960_c0_g1_i4_orf1	zinc finger protein on ecdysone puffs-like [Ostrinia furnacalis]	-0.6098	-0.3131	-0.1203	-0.8875	1.93077
TRINITY_DN3887_c0_g1_i1_orf1	formin-like protein isoform X3 [Ostrinia furnacalis] >XP_028170442.1 formin-like protein isoform X6 [Ostrinia furnacalis]	-0.8224	-0.6325	-0.192	-0.3015	1.94831
TRINITY_DN95850_c0_g1_i1_orf1	alpha,alpha-trehalose-phosphate synthase [UDP-forming] isoform X2 [Chelonus insularis]	-1.306	0.95979	0.30465	-1.0468	1.08837
TRINITY_DN21930_c0_g1_i1_orf1	coactosin-like protein isoform X2 [Trichoplusia ni]	-0.7003	-0.4187	-0.5391	-0.3263	1.98427
TRINITY_DN246_c1_g1_i5_orf1	lachesin isoform X1 [Ostrinia furnacalis] >XP_028178464.1 lachesin isoform X2 [Ostrinia furnacalis]	0.14621	-0.0561	-0.6336	-1.216	1.7594
TRINITY_DN3637_c0_g1_i2_orf1	ATP-binding cassette sub-family G member 4 isoform X1 [Ostrinia furnacalis]	-0.2061	-0.3687	0.6929	-1.5312	1.41312
TRINITY_DN86309_c0_g1_i4_orf1	filamin-A isoform X2 [Ostrinia furnacalis]	-0.5072	-0.4169	-0.6942	-0.3694	1.98762
TRINITY_DN3962_c0_g1_i6_orf1	follicle-stimulating hormone receptor-like [Ostrinia furnacalis]	-1.4259	0.41333	0.18937	-0.6887	1.51189
TRINITY_DN1436_c0_g1_i3_orf1	vacuolar protein sorting-associated protein 27-like [Trichoplusia ni]	-0.9711	-0.4884	-0.4288	-0.0181	1.90638
TRINITY_DN17437_c0_g1_i1_orf1	phospholipase A1 VesT1.02-like [Ostrinia furnacalis]	-0.5464	0.37483	-0.6508	-0.9671	1.78945
TRINITY_DN4320_c0_g1_i1_orf1	selenide, water dikinase [Ostrinia furnacalis] >CAG9756850.1 unnamed protein product [Diatraea saccharalis] >CAG9795535.1 unnamed protein product [Diatraea saccharalis]	-0.6031	-0.7291	-0.2895	-0.3523	1.97402
TRINITY_DN11666_c0_g1_i6_orf1	P protein-like [Ostrinia furnacalis] >XP_028167089.1 P protein-like [Ostrinia furnacalis]	-1.2893	0.41397	-0.1373	-0.6415	1.65411
TRINITY_DN4572_c0_g3_i1_orf1	putative aminopeptidase W07G4.4 isoform X2 [Ostrinia furnacalis]	-0.2935	-0.1474	-0.8309	-0.6666	1.9384
TRINITY_DN90327_c0_g1_i1_orf1	hypothetical protein HF086_000910, partial [Spodoptera exigua]	-0.7925	-0.4608	0.7845	-1.076	1.54479
TRINITY_DN1362_c0_g1_i4_orf1	heparan-alpha-glucosaminide N-acetyltransferase [Helicoverpa armigera]	-0.426	-0.9409	0.33687	-0.7674	1.79745
TRINITY_DN6870_c0_g1_i5_orf1	uncharacterized protein LOC114365835 [Ostrinia furnacalis]	-0.957	0.54686	-0.8519	-0.4327	1.69473
TRINITY_DN119265_c0_g2_i1_orf1	PREDICTED: sorting nexin-12 [Fopius arisanus]	-0.3635	-0.4263	0.9467	-1.4528	1.2959
TRINITY_DN9_c0_g1_i11_orf1	heterogeneous nuclear ribonucleoprotein Q isoform X2 [Galleria mellonella]	-0.5554	-0.8202	-0.3269	-0.2583	1.9609
TRINITY_DN34727_c0_g1_i3_orf1	tyrosine-protein kinase Src42A isoform X2 [Trichoplusia ni]	-0.9222	-0.2965	-0.3157	-0.4129	1.9472
TRINITY_DN8390_c0_g1_i2_orf1	tubulin gamma-1 chain-like isoform X1 [Ostrinia furnacalis] >XP_028160960.1 tubulin gamma-1 chain-like isoform X2 [Ostrinia furnacalis]	-0.5406	-0.5891	-0.1149	-0.7142	1.95891
TRINITY_DN19537_c0_g1_i1_orf1	disintegrin and metalloproteinase domain-containing protein 10 isoform X1 [Ostrinia furnacalis] >XP_028172845.1 disintegrin and metalloproteinase domain-containing protein 10 isoform X2 [Ostrinia furnacalis]	-1.1409	0.07783	0.06141	-0.7615	1.76315
TRINITY_DN4144_c0_g1_i7_orf1	uncharacterized protein LOC114350172 [Ostrinia furnacalis]	-0.5632	-0.84	-0.6447	0.16822	1.87969
TRINITY_DN2971_c0_g1_i1_orf1	uncharacterized protein LOC114364864 [Ostrinia furnacalis]	0.09647	-0.4216	-1.3466	-0.0592	1.73093
TRINITY_DN5495_c0_g1_i5_orf1	multiple coagulation factor deficiency protein 2 homolog isoform X1 [Ostrinia furnacalis] >XP_028177199.1 multiple coagulation factor deficiency protein 2 homolog isoform X1 [Ostrinia furnacalis]	-0.6681	0.1739	0.05116	-1.2655	1.70857
TRINITY_DN17003_c1_g1_i1_orf1	unnamed protein product [Chilo suppressalis]	-0.5845	-0.1596	-0.7054	-0.5171	1.96668
TRINITY_DN28018_c0_g6_i1_orf1	microtubule-associated protein futsch-like isoform X6 [Ostrinia furnacalis]	-0.3983	-0.6234	-0.7057	-0.246	1.97337
TRINITY_DN74069_c0_g1_i1_orf1	unnamed protein product [Parnassius apollo] >CAG5017650.1 unnamed protein product [Parnassius apollo]	-0.8181	-0.3228	-0.9765	0.34065	1.77677

TRINITY_DN57348_c0_g1_i4_orf1	facilitated trehalose transporter Tret1-like isoform X1 [Ostrinia furnacalis]	-0.7494	0.26798	-0.0045	-1.2145	1.70043
TRINITY_DN4694_c0_g2_i1_orf1	uncharacterized protein LOC114362122 [Ostrinia furnacalis]	-0.3743	-0.7986	-0.7126	-0.0414	1.92684
TRINITY_DN48023_c1_g1_i1_orf1	carboxylesterase [Ostrinia furnacalis]	-0.0881	0.34163	-0.7973	-1.1585	1.70226
TRINITY_DN15114_c0_g2_i1_orf1	uncharacterized protein LOC114359552 [Ostrinia furnacalis]	-0.7512	-0.4111	0.61875	-1.095	1.63855
TRINITY_DN2352_c0_g1_i15_orf1	polyadenylate-binding protein 1-B-like [Ostrinia furnacalis]	-0.6078	-0.3349	-0.0021	-0.9545	1.8993
TRINITY_DN139335_c0_g2_i1_orf1	unnamed protein product [Homo sapiens]	-1.3831	-0.0868	-0.0358	-0.2332	1.7389
TRINITY_DN8660_c0_g1_i1_orf1	chondroitin sulfate synthase 2 [Trichoplusia ni]	-1.3696	0.77238	0.13905	-0.8671	1.32529
TRINITY_DN16643_c0_g2_i4_orf1	heterogeneous nuclear ribonucleoprotein R isoform X6 [Danaus plexippus plexippus]	-0.6123	0.00961	-0.4987	-0.8222	1.92361
TRINITY_DN9028_c0_g1_i5_orf1	decaprenyl-diphosphate synthase subunit 2-like [Ostrinia furnacalis]	-0.9615	-0.2611	-0.5581	-0.137	1.91759
TRINITY_DN9475_c0_g1_i6_orf1	uncharacterized protein LOC114358636 [Ostrinia furnacalis]	-0.5778	0.08619	-0.8032	-0.6126	1.90743
TRINITY_DN146524_c0_g1_i1_orf1	pupal cuticle protein-like [Ostrinia furnacalis]	-0.6746	-0.3942	-0.3404	-0.5761	1.98537
TRINITY_DN1604_c0_g1_i4_orf1	ubiquitin-conjugating enzyme E2 S [Ostrinia furnacalis]	-0.0501	0.27387	-1.3823	-0.5025	1.66107
TRINITY_DN1274_c0_g1_i4_orf1	venom dipeptidyl peptidase 4-like [Ostrinia furnacalis]	-0.3309	-0.3891	-1.1455	0.01444	1.85111
TRINITY_DN2432_c0_g1_i1_orf1	unconventional myosin-XV-like [Ostrinia furnacalis]	-0.8576	-0.4542	0.92967	-1.0581	1.44025
TRINITY_DN7803_c0_g1_i2_orf1	membrane-associated protein Hem [Ostrinia furnacalis]	-0.9017	-0.2519	-0.8059	0.09822	1.86127
TRINITY_DN10057_c0_g2_i1_orf1	cell wall protein DAN4 [Ostrinia furnacalis]	-0.809	-0.6911	-0.7522	0.50736	1.74491
TRINITY_DN15865_c0_g1_i1_orf1	carboxylesterase, partial [Ostrinia furnacalis]	-0.8385	-0.3576	-0.8171	0.14796	1.86531
TRINITY_DN5954_c0_g1_i2_orf1	myosin-VIIa [Ostrinia furnacalis] >XP_028155907.1 myosin-VIIa [Ostrinia furnacalis]	-0.6794	-0.7465	-0.5828	0.10325	1.90547
TRINITY_DN1393_c0_g1_i2_orf1	uncharacterized protein LOC114357114 isoform X1 [Ostrinia furnacalis]	-0.6056	0.61069	-0.4107	-1.2131	1.61867
TRINITY_DN1254_c0_g1_i1_orf1	lysophospholipid acyltransferase 7-like [Ostrinia furnacalis]	-1.1492	-0.3813	0.81584	-0.7857	1.50035
TRINITY_DN9536_c0_g1_i4_orf1	adrenodoxin-like protein, mitochondrial isoform X1 [Ostrinia furnacalis]	-0.4643	-0.906	-0.5874	0.05631	1.90141
TRINITY_DN1768_c0_g1_i2_orf1	low-density lipoprotein receptor-related protein 1B-like [Ostrinia furnacalis]	-0.4522	0.68965	-0.9358	-0.9158	1.61415
TRINITY_DN1005_c0_g1_i5_orf1	hypothetical protein evm_008839 [Chilo suppressalis] >CAB3526474.1 unnamed protein product [Chilo suppressalis] >CAH0403802.1 unnamed protein product [Chilo suppressalis]	-1.1632	-0.0037	-0.5491	-0.1099	1.82579
TRINITY_DN650_c0_g1_i3_orf1	chitinase 7 [Glyphodes pyloalis]	-0.7603	-0.3485	-0.9787	0.28102	1.80654
TRINITY_DN5772_c0_g1_i6_orf1	uncharacterized protein LOC114364899 isoform X2 [Ostrinia furnacalis]	-0.6241	-0.4838	-0.8318	0.02036	1.91939
TRINITY_DN8659_c0_g2_i1_orf1	ubiquitin-like modifier-activating enzyme 1 [Ostrinia furnacalis]	-0.164	-1.2649	0.02678	-0.3919	1.79408
TRINITY_DN4898_c0_g1_i7_orf1	annulin-like isoform X3 [Ostrinia furnacalis]	-0.7939	-0.7138	-0.5944	0.24566	1.85648
TRINITY_DN19058_c1_g1_i1_orf1	syntaxin-7 [Helicoverpa armigera] >XP_049695901.1 syntaxin-7 [Helicoverpa armigera]	-0.6163	-0.1646	-0.2343	-0.9107	1.92585
TRINITY_DN9324_c1_g2_i2_orf1	kinesin-like protein Klp10A isoform X4 [Spodoptera frugiperda]	-0.2726	-0.9893	-0.681	0.07831	1.86466
TRINITY_DN2012_c0_g1_i3_orf1	contactin [Ostrinia furnacalis]	-0.8659	-0.183	-0.1015	-0.7561	1.90649
TRINITY_DN590_c0_g1_i4_orf1	trypsin inhibitor-like [Ostrinia furnacalis]	-0.4073	-0.2463	-0.6298	-0.6912	1.9746
TRINITY_DN41321_c1_g1_i3_orf1	uncharacterized protein LOC114357706 isoform X1 [Ostrinia furnacalis] >XP_028167261.1 uncharacterized protein LOC114357706 isoform X2 [Ostrinia	-0.5036	0.24941	-0.4741	-1.0841	1.81227
TRINITY_DN38371_c0_g1_i7_orf1	protein smoothened isoform X2 [Ostrinia furnacalis]	-0.3627	-0.0125	0.47761	-1.5736	1.47109
TRINITY_DN144_c0_g1_i4_orf1	COPII coat assembly protein sec16-like [Ostrinia furnacalis]	-0.3415	-0.4168	-0.7991	-0.4168	1.97418
TRINITY_DN6656_c0_g1_i1_orf1	sorting and assembly machinery component 50 homolog isoform X9 [Ostrinia furnacalis] >XP_028169233.1 sorting and assembly machinery component 50 homolog isoform X10 [Ostrinia furnacalis] >XP_028169234.1 sorting and assembly machinery component 50 homolog isoform X11 [Ostrinia furnacalis]	-0.9822	-0.3764	-0.6086	0.09235	1.87477
TRINITY_DN4134_c2_g1_i2_orf1	prostatic acid phosphatase-like [Ostrinia furnacalis]	-0.8382	-0.4784	-0.1775	-0.4614	1.95553
TRINITY_DN18620_c0_g1_i5_orf1	hypothetical protein evm_011254 [Chilo suppressalis]	-1.0029	0.22994	-0.5796	-0.4835	1.83616
TRINITY_DN3430_c0_g1_i1_orf1	protein HGV2-like isoform X2 [Ostrinia furnacalis]	-0.5462	-0.783	-0.6537	0.07081	1.91212
TRINITY_DN11665_c0_g1_i4_orf1	TBC1 domain family member 9 isoform X1 [Ostrinia furnacalis] >XP_028176568.1 TBC1 domain family member 9 isoform X2 [Ostrinia furnacalis]	-0.6564	-0.1699	-0.7564	-0.3739	1.95662
TRINITY_DN6586_c0_g1_i1_orf1	fatty acyl-CoA reductase wat-like isoform X1 [Ostrinia furnacalis]	-0.8028	-0.7452	-0.6965	0.49127	1.75321
TRINITY_DN20710_c0_g1_i2_orf1	plexin A3 [Ostrinia furnacalis]	-0.4848	-0.5548	-0.6995	-0.2384	1.97761
TRINITY_DN4785_c0_g2_i1_orf1	methylthioribose-1-phosphate isomerase [Ostrinia furnacalis] >XP_028172842.1 methylthioribose-1-phosphate isomerase [Ostrinia furnacalis]	-0.7025	0.54132	-0.837	-0.7287	1.72684
TRINITY_DN9109_c0_g1_i1_orf1	unnamed protein product [Chrysodeixis includens]	-0.9193	0.73486	-0.8067	-0.6164	1.60753
TRINITY_DN34830_c0_g1_i1_orf1	ubiquitin-like domain-containing CTD phosphatase 1 [Ostrinia furnacalis]	-1.0361	-0.5433	0.23935	-0.4867	1.82679
TRINITY_DN13167_c0_g1_i1_orf1	selenoprotein M-like [Ostrinia furnacalis]	-1.065	0.35339	-0.8501	-0.1675	1.72921
TRINITY_DN6014_c1_g1_i2_orf1	putative hydroxypyruvate isomerase [Ostrinia furnacalis]	-0.632	0.06343	-0.6656	-0.6852	1.91938
TRINITY_DN9872_c0_g1_i2_orf1	serine protease inhibitor 88Ea-like [Ostrinia furnacalis]	-0.5477	-0.7577	-0.7034	0.10626	1.90258
TRINITY_DN2277_c0_g1_i11_orf1	solute carrier family 12 member 4 isoform X1 [Ostrinia furnacalis]	-0.2961	-0.8611	-0.0322	-0.7211	1.91047
TRINITY_DN64222_c0_g1_i1_orf1	heat shock protein 20.2 [Glyphodes pyloalis]	-0.3292	-0.4774	-0.735	-0.4406	1.98224
TRINITY_DN9732_c0_g1_i7_orf1	CD109 antigen [Ostrinia furnacalis] >XP_028176877.1 CD109 antigen [Ostrinia furnacalis]	-0.7175	-0.4007	-0.447	-0.4215	1.98674
TRINITY_DN131264_c0_g1_i2_orf1	unnamed protein product [Parnassius apollo]	-0.5082	-1.3139	0.09463	-0.0063	1.73387
TRINITY_DN5675_c0_g1_i6_orf1	probable ATP-dependent RNA helicase DDX43 [Ostrinia furnacalis]	-0.6851	0.25845	0.1176	-1.3291	1.63813
TRINITY_DN2968_c0_g1_i3_orf1	uncharacterized protein LOC114357587 [Ostrinia furnacalis]	-0.6036	0.51642	-1.0268	-0.6036	1.71764
TRINITY_DN2043_c0_g1_i3_orf1	phenoloxidase-activating factor 2-like [Ostrinia furnacalis]	-0.9291	-0.2467	-0.3915	-0.3771	1.94434
TRINITY_DN2061_c0_g1_i3_orf1	uncharacterized protein LOC114357318 isoform X1 [Ostrinia furnacalis] >XP_028166680.1 uncharacterized protein LOC114357318 isoform X2 [Ostrinia	-0.9652	-0.1617	-0.9471	0.33181	1.74218
TRINITY_DN5568_c0_g2_i2_orf1	carboxypeptidase D isoform X5 [Ostrinia furnacalis]	-0.936	-0.4247	-0.7149	0.23837	1.83727
TRINITY_DN47219_c0_g1_i3_orf1	protein windbeutel [Ostrinia furnacalis]	-0.2263	-0.8485	-0.923	0.16783	1.82997
TRINITY_DN8306_c0_g1_i4_orf1	NAD(P) transhydrogenase, mitochondrial-like [Ostrinia furnacalis] >XP_028175067.1 NAD(P) transhydrogenase, mitochondrial-like [Ostrinia furnacalis] >XP_028175068.1 NAD(P) transhydrogenase, mitochondrial-like [Ostrinia furnacalis] >XP_028175069.1 NAD(P) transhydrogenase, mitochondrial-like [Ostrinia furnacalis]	-1.2724	0.06305	-0.0147	-0.5342	1.75824
TRINITY_DN2623_c1_g1_i3_orf1	COPII coat assembly protein sec16-like [Ostrinia furnacalis]	-0.6705	-0.8197	-0.5539	0.16098	1.88306
TRINITY_DN24631_c0_g2_i1_orf1	O-GlcNAc hydrolase [Ostrinia furnacalis]	-0.8874	-0.2713	-0.3527	-0.4426	1.9541

TRINITY_DN1716_c0_g1_i14_orf1	putative gamma-glutamylcyclotransferase CG2811 isoform X3 [Ostrinia furnacalis]	-0.2599	-0.7734	-1.0524	0.31781	1.76795
TRINITY_DN3273_c0_g1_i4_orf1	FK506-binding protein-like [Galleria mellonella]	-0.3157	-0.5443	-0.579	-0.552	1.99101
TRINITY_DN18563_c2_g1_i1_orf1	protein held out wings isoform X2 [Diachasma alloeum]	-0.1854	-0.8723	-0.7659	-0.0771	1.9006
TRINITY_DN5422_c0_g1_i1_orf1	nitrilase and fragile histidine triad fusion protein NitFhit isoform X1 [Ostrinia furnacalis]	-1.8087	0.71931	0.20846	-0.1839	1.06481
TRINITY_DN14389_c0_g1_i4_orf1	DE-cadherin [Ostrinia furnacalis]	-0.4693	-0.9363	0.15854	-0.6216	1.8686
TRINITY_DN4572_c0_g1_i2_orf1	unnamed protein product [Arctia plantaginis] >CAB3257565.1 unnamed protein product [Arctia plantaginis]	-0.5367	-0.1085	-0.7009	-0.6121	1.95815
TRINITY_DN27114_c0_g1_i1_orf1	putative inorganic phosphate cotransporter [Ostrinia furnacalis]	0.00811	-1.0182	-0.5642	-0.3096	1.88388
TRINITY_DN9302_c0_g1_i1_orf1	DEAD-box helicase Dbp80 [Ostrinia furnacalis]	-0.9019	-0.454	0.13657	-0.6589	1.87821
TRINITY_DN44094_c0_g1_i1_orf1	myotrophin-like [Ostrinia furnacalis]	-0.4736	-0.0393	-1.2184	-0.0807	1.81196
TRINITY_DN7682_c0_g1_i2_orf1	myogenesis-regulating glycosidase isoform X1 [Ostrinia furnacalis] >XP_028158488.1 myogenesis-regulating glycosidase isoform X1 [Ostrinia furnacalis] >XP_028158489.1 myogenesis-regulating glycosidase isoform X1 [Ostrinia furnacalis] >XP_028158490.1 myogenesis-regulating glycosidase isoform X1 [Ostrinia furnacalis]	-1.2353	-0.2205	0.1858	-0.5017	1.77175
TRINITY_DN5182_c0_g1_i5_orf1	rab proteins geranylgeranyltransferase component A 1 isoform X1 [Ostrinia furnacalis]	-0.5684	-0.6129	-0.9148	0.25199	1.84414
TRINITY_DN99020_c0_g1_i1_orf1	uncharacterized protein LOC114357292 isoform X4 [Ostrinia furnacalis]	-0.8778	-0.0711	-0.85	-0.0711	1.86997
TRINITY_DN2745_c0_g1_i2_orf1	PREDICTED: tubulin alpha-1A chain-like [Papilio polytes] >XP_013164648.1 PREDICTED: tubulin alpha-1A chain-like [Papilio xuthus]	-0.5528	-1.4206	0.29113	0.07417	1.60811
TRINITY_DN36324_c0_g1_i12_orf1	motile sperm domain-containing protein 1-like [Ostrinia furnacalis]	-1.559	0.4811	0.35235	-0.6248	1.35037
TRINITY_DN12222_c0_g1_i1_orf1	unnamed protein product [Chilo suppressalis]	-1.2413	-0.1786	-0.4021	0.01492	1.80703
TRINITY_DN19746_c0_g1_i5_orf1	hypothetical protein evm_004480 [Chilo suppressalis] >CAB3520922.1 unnamed protein product [Chilo suppressalis] >CAH0398243.1 unnamed protein product [Chilo suppressalis]	-0.6664	0.43896	-0.6034	-0.9363	1.76707
TRINITY_DN3177_c0_g1_i1_orf1	Pupal cuticle protein PCP52 [Papilio xuthus]	-1.1224	0.34773	-0.4813	-0.5119	1.76791
TRINITY_DN9100_c0_g1_i5_orf1	microtubule-associated protein futsch-like isoform X6 [Ostrinia furnacalis]	-0.7338	-0.5355	-0.8412	0.26785	1.84266
TRINITY_DN3119_c0_g1_i7_orf1	unnamed protein product [Chilo suppressalis]	-0.4986	-0.1084	-1.1858	-0.0327	1.82549
TRINITY_DN23194_c0_g1_i4_orf1	nucleobindin-2 isoform X2 [Ostrinia furnacalis]	-1.351	-0.0871	0.38871	-0.5856	1.635
TRINITY_DN50517_c0_g1_i5_orf1	hypothetical protein evm_004957 [Chilo suppressalis]	-0.8499	-0.4466	-0.7835	0.23342	1.84659
TRINITY_DN13898_c0_g1_i2_orf1	protein PRRC2A-like isoform X2 [Ostrinia furnacalis]	-0.5959	-1.0466	-0.1752	-0.0573	1.87497
TRINITY_DN9072_c0_g1_i1_orf1	SET and MYND domain-containing protein 4-like [Ostrinia furnacalis]	0.00769	-0.6323	-1.3411	0.32343	1.64226
TRINITY_DN9000_c0_g2_i1_orf1	uncharacterized protein LOC114356585 [Ostrinia furnacalis]	-0.7712	-0.5657	-0.811	0.32542	1.82252
TRINITY_DN35633_c0_g2_i1_orf1	uncharacterized protein LOC114353024 [Ostrinia furnacalis]	-0.6615	-0.7539	-0.8869	0.62328	1.679
TRINITY_DN13626_c0_g2_i1_orf1	charged multivesicular body protein 2b [Ostrinia furnacalis]	-0.8526	-1.0894	0.67782	-0.3238	1.58805
TRINITY_DN54925_c0_g1_i1_orf1	unnamed protein product [Arctia plantaginis]	-0.8225	-0.8541	-0.5565	0.48767	1.74542
TRINITY_DN10095_c0_g1_i5_orf1	uncharacterized protein LOC114361222 [Ostrinia furnacalis]	-1.2658	-0.0156	-0.4296	-0.0797	1.79071
TRINITY_DN13395_c0_g1_i1_orf1	cytoplasmic dynein 1 light intermediate chain 2 [Galleria mellonella]	-0.7786	-0.8862	-0.5202	0.40297	1.78199
TRINITY_DN16605_c0_g1_i3_orf1	unnamed protein product [Chrysodeixis includens]	-0.6588	-0.1136	-1.1463	0.12317	1.79553
TRINITY_DN4711_c0_g1_i2_orf1	xanthine dehydrogenase-like isoform X1 [Ostrinia furnacalis] >XP_028179066.1 xanthine dehydrogenase-like isoform X1 [Ostrinia furnacalis] >XP_028179067.1 xanthine dehydrogenase-like isoform X1 [Ostrinia furnacalis] >XP_028179068.1 xanthine dehydrogenase-like isoform X1 [Ostrinia furnacalis] >XP_028179069.1 xanthine dehydrogenase-like isoform X1 [Ostrinia furnacalis]	-1.3679	-0.2374	0.45437	-0.4742	1.62517
TRINITY_DN27456_c0_g2_i1_orf1	organic cation transporter-like protein [Ostrinia furnacalis]	-1.4812	0.24935	-0.0731	-0.3189	1.62381
TRINITY_DN19034_c0_g1_i1_orf1	tRNA-splicing ligase RtcB homolog [Ostrinia furnacalis]	-1.3336	0.06647	0.6297	-0.8253	1.4627
TRINITY_DN18756_c0_g1_i6_orf1	COP9 signalosome complex subunit 6 [Ostrinia furnacalis]	-1.2662	-0.524	0.51829	-0.3751	1.64705
TRINITY_DN23416_c1_g1_i2_orf1	ran-specific GTPase-activating protein-like [Ostrinia furnacalis]	-1.2116	0.37718	0.52164	-1.0823	1.39509
TRINITY_DN8682_c0_g1_i4_orf1	protein SYS1 homolog [Ostrinia furnacalis]	-0.2556	-0.7049	-0.9445	0.02206	1.88289
TRINITY_DN13419_c0_g1_i5_orf1	atrial natriuretic peptide-converting enzyme-like [Ostrinia furnacalis]	-1.5375	0.65088	-0.2205	-0.3271	1.4342
TRINITY_DN23042_c0_g1_i1_orf1	protoporphyrinogen oxidase [Ostrinia furnacalis]	-1.2919	-0.0438	0.35146	-0.6742	1.65857
TRINITY_DN26293_c0_g1_i4_orf1	UDP-glucose 6-dehydrogenase [Ostrinia furnacalis]	-0.3259	-0.7551	-1.0778	0.4344	1.72434
TRINITY_DN843_c0_g1_i5_orf1	fasciclin-3 isoform X4 [Helicoverpa zea]	-1.3096	0.24889	0.08081	-0.6797	1.65963
TRINITY_DN16390_c0_g1_i4_orf1	unnamed protein product, partial [Brenthis ino]	-1.029	-0.1321	-0.5754	-0.1527	1.88925
TRINITY_DN2438_c0_g1_i1_orf1	dystrophin, isoforms A/C/F/G/H-like [Ostrinia furnacalis]	-1.0176	0.56376	-1.0045	-0.1579	1.61634
TRINITY_DN28989_c0_g1_i7_orf1	26S proteasome regulatory subunit 10B [Ostrinia furnacalis]	-1.0083	-1.0041	0.5165	-0.1436	1.63945
TRINITY_DN5840_c0_g1_i6_orf1	catenin alpha isoform X2 [Ostrinia furnacalis]	-0.7722	-0.9362	-0.5009	0.45809	1.75117
TRINITY_DN2100_c0_g1_i2_orf1	uncharacterized protein LOC114362418 [Ostrinia furnacalis]	-0.4528	-0.7277	-0.7887	0.0615	1.90776
TRINITY_DN10694_c1_g2_i1_orf1	hsp70-Hsp90 organizing protein 3-like [Ostrinia furnacalis]	-0.5111	-1.0129	-0.5305	0.21463	1.8399
TRINITY_DN3521_c0_g2_i1_orf1	barrier-to-autointegration factor [Ostrinia furnacalis] >XP_045447211.1 barrier-to-autointegration factor [Melitaea cinxia] >CAG4971452.1 unnamed protein product [Parnassius apollo] >CAG9578869.1 unnamed protein product [Danaus chrysippus] >CAH0714333.1 unnamed protein product, partial [Brenthis ino] >CAH2108232.1 unnamed protein product [Euphydryas editha]	-1.0956	0.45384	-0.8029	-0.2538	1.69844
TRINITY_DN2904_c0_g1_i4_orf1	ATP-dependent DNA/RNA helicase DHX36 isoform X1 [Ostrinia furnacalis]	-1.1942	0.74742	-0.2935	-0.7821	1.52233
TRINITY_DN10630_c0_g1_i2_orf1	J domain-containing protein [Ostrinia furnacalis]	-1.1714	0.62881	-0.898	-0.1129	1.55349
TRINITY_DN19885_c0_g1_i1_orf1	transmembrane emp24 domain-containing protein 5 [Ostrinia furnacalis]	-1.0219	-0.0351	-0.6824	-0.1243	1.86371
TRINITY_DN72_c0_g1_i16_orf1	protein groucho-like [Ostrinia furnacalis]	-0.5543	-0.158	-0.4256	-0.8166	1.95446
TRINITY_DN2102_c0_g1_i11_orf1	prenylated Rab acceptor protein 1 isoform X4 [Ostrinia furnacalis]	-0.5918	0.35817	-0.942	-0.6252	1.80088
TRINITY_DN162_c0_g1_i4_orf1	ABC transporter G family member 23 isoform X1 [Ostrinia furnacalis] >XP_028178987.1 ABC transporter G family member 23 isoform X1 [Ostrinia furnacalis]	-0.7231	-0.7413	-0.5179	0.07062	1.91165
TRINITY_DN4524_c0_g1_i2_orf1	sodium/potassium-transporting ATPase subunit alpha isoform X1 [Chelonus insularis] >XP_034939982.1 sodium/potassium-transporting ATPase subunit alpha isoform X1 [Chelonus insularis] >XP_034939983.1 sodium/potassium-transporting ATPase subunit alpha isoform X1 [Chelonus insularis] >XP_034939984.1 sodium/potassium-transporting ATPase subunit alpha isoform X1 [Chelonus insularis]	-0.9216	0.07897	-0.7564	-0.2714	1.87046
TRINITY_DN4561_c0_g1_i3_orf1	host cell factor 1 [Ostrinia furnacalis]	-0.8932	-0.3079	-0.7669	0.09444	1.87358

TRINITY_DN29579_c0_g1_i1_orf1	heterogeneous nuclear ribonucleoprotein A1, A2/B1 homolog [Ostrinia furnacalis]	-0.9161	-0.8471	0.70017	-0.5611	1.6242
TRINITY_DN33967_c2_g2_i1_orf1	titin homolog [Ostrinia furnacalis]	-0.1599	-0.9713	-0.78	0.0623	1.84895
TRINITY_DN15591_c0_g1_i3_orf1	c-Jun NH2-terminal kinase [Danaus plexippus plexippus]	-1.403	0.05211	-0.4022	0.06095	1.69213
TRINITY_DN10769_c0_g1_i1_orf1	proteasome subunit beta type-5 [Ostrinia furnacalis]	-1.3118	-0.1848	0.00679	-0.2883	1.77814
TRINITY_DN5508_c0_g1_i1_orf1	tubulin beta chain [Plutella xylostella] >XP_013148991.1 PREDICTED: tubulin beta chain [Papilio polytes] >XP_013174821.1 PREDICTED: tubulin beta chain [Papilio xuthus] >XP_013197827.1 PREDICTED: tubulin beta chain [Amyelois transitella] >XP_013198295.1 PREDICTED: tubulin beta chain [Amyelois transitella] >XP_014364096.1 tubulin beta chain [Papilio machaon] >XP_021199407.1 tubulin beta chain [Helicoverpa armigera] >XP_022837146.1 tubulin beta chain [Spodoptera litura] >XP_026317095.1 tubulin beta chain [Hyposmocoma kahamanoa] >XP_026486002.1 tubulin beta chain [Vanessa tameamea] >XP_026746369.1 tubulin beta chain [Trichoplusia ni] >XP_028175399.1 tubulin beta chain isoform X1 [Ostrinia furnacalis] >XP_028175400.1 tubulin beta chain isoform X2 [Ostrinia furnacalis] >XP_032524707.1 tubulin beta chain [Danaus plexippus plexippus] >XP_035456573.1 tubulin beta chain [Spodoptera frugiperda] >XP_038219158.1 tubulin beta chain [Zerene cesonia] >XP_045456648.1 tubulin beta chain [Melitaea cinxia] >XP_045507513.1 tubulin beta chain [Colias croceus] >XP_046974148.1 tubulin beta chain [Vanessa cardui] >XP_047037076.1 tubulin beta chain [Helicoverpa zea] >XP_047540655.1 tubulin beta chain [Vanessa atalanta] >XP_048000438.1 tubulin beta chain [Leguminivora glycinivorella] >XP_049881892.1 tubulin beta chain [Pectinophora gossypiella] >XP_050357144.1 tubulin beta chain [Nymphalis io] >KAF9417727.1 hypothetical protein HW555_005234 [Spodoptera exigua] >RVE53489.1 hypothetical protein evm_001859 [Chilo suppressalis] >CAB3233188.1 unnamed protein product [Arctia plantaginis] >CAB3512351.1 unnamed protein product [Spodoptera littoralis] >CAD0206252.1 unnamed protein product [Chrysodeixis includens] >CAG9569972.1 unnamed protein product [Danaus chrysippus] >CAG9757441.1 unnamed protein product [Diatraea saccharalis] >CAH2060798.1 unnamed protein product, partial [Iphiclides podalirius] >CAH2099205.1 unnamed protein product [Euphydryas editha]	-1.0516	-0.0143	-0.5161	-0.2989	1.88099
TRINITY_DN1280_c0_g1_i1_orf1	coronin-7 isoform X1 [Ostrinia furnacalis] >XP_028164815.1 coronin-7 isoform X2 [Ostrinia furnacalis] >XP_028164817.1 coronin-7 isoform X3 [Ostrinia furnacalis] >XP_028164818.1 coronin-7 isoform X4 [Ostrinia furnacalis] >XP_028164820.1 coronin-7 isoform X6 [Ostrinia furnacalis] >XP_028164821.1 coronin-7 isoform X7 [Ostrinia furnacalis] >XP_028164822.1 coronin-7 isoform X1 [Ostrinia furnacalis] >XP_028164823.1 coronin-7 isoform X8 [Ostrinia furnacalis] >XP_028164824.1 coronin-7 isoform X9 [Ostrinia furnacalis] >XP_028164825.1 coronin-7 isoform X10 [Ostrinia furnacalis]	-0.8383	-0.7949	-0.5992	0.47808	1.75435
TRINITY_DN132857_c0_g1_i1_orf1	hypothetical protein evm_000559 [Chilo suppressalis]	-0.8837	-0.0741	-0.6476	-0.3166	1.92198
TRINITY_DN482_c0_g1_i1_orf1	fatty acyl reductase 7 [Maruca vitrata]	-0.7415	-0.766	-0.6267	0.29456	1.83957
TRINITY_DN125967_c0_g1_i1_orf1	26S proteasome non-ATPase regulatory subunit 14 [Ostrinia furnacalis]	-1.2987	-0.264	0.44296	-0.5402	1.66001
TRINITY_DN71610_c0_g1_i1_orf1	GPI transamidase component PIG-T [Ostrinia furnacalis]	-1.4086	0.3895	0.04584	-0.6062	1.57943
TRINITY_DN77005_c0_g3_i1_orf1	RNA polymerase II degradation factor 1-like [Ostrinia furnacalis]	-0.2135	-0.5621	-1.2454	0.28753	1.73343
TRINITY_DN87522_c0_g2_i1_orf1	regucalcin-like [Ostrinia furnacalis]	-1.635	0.6421	-0.0497	-0.3059	1.34848
TRINITY_DN22577_c0_g1_i2_orf1	probable beta-hexosaminidase fdl isoform X1 [Ostrinia furnacalis]	-0.989	-0.4789	-0.4196	-0.0142	1.90166
TRINITY_DN56993_c0_g1_i4_orf1	polypyrimidine tract-binding protein 1 isoform X11 [Helicoverpa zea]	-0.9313	-0.1845	-1.0351	0.47794	1.67294
TRINITY_DN62729_c0_g1_i13_orf1	G protein-coupled receptor kinase 2 isoform X3 [Manduca sexta]	-1.6444	0.49051	0.24688	-0.4362	1.3432
TRINITY_DN11084_c1_g1_i2_orf1	uncharacterized protein LOC114362953 [Ostrinia furnacalis]	-1.1336	0.53985	-0.5958	-0.4917	1.68128
TRINITY_DN33837_c0_g1_i6_orf1	bolA-like protein DDB_G0274169 [Ostrinia furnacalis] >XP_028171597.1 bolA-like protein DDB_G0274169 [Ostrinia furnacalis]	-0.9237	-0.7364	-0.3026	0.09064	1.87208
TRINITY_DN14046_c0_g1_i1_orf1	retinol dehydrogenase 14 [Ostrinia furnacalis] >XP_028165567.1 retinol dehydrogenase 14 [Ostrinia furnacalis] >XP_028165568.1 retinol dehydrogenase 14 [Ostrinia furnacalis]	-0.6707	-0.8646	-0.7336	0.54686	1.72204
TRINITY_DN5569_c0_g1_i1_orf1	PREDICTED: chromatin complexes subunit BAP18 isoform X1 [Eufriesea mexicana]	-1.1027	0.12598	0.06685	-0.8392	1.74911
TRINITY_DN467_c3_g1_i5_orf1	lysozyme precursor [Loxostege sticticalis]	0.37481	0.78652	0.02681	-1.9234	0.73528
TRINITY_DN5310_c2_g1_i2_orf1	serine protease persephone-like [Ostrinia furnacalis]	0.03033	0.97519	0.38191	-1.9061	0.51866
TRINITY_DN1672_c0_g1_i6_orf1	cystinosin homolog isoform X1 [Ostrinia furnacalis] >XP_028162341.1 cystinosin homolog isoform X1 [Ostrinia furnacalis] >XP_028162342.1 cystinosin homolog isoform X1 [Ostrinia furnacalis] >XP_028162343.1 cystinosin homolog isoform X1 [Ostrinia furnacalis] >XP_028162344.1 cystinosin homolog isoform X1 [Ostrinia furnacalis]	-0.0684	1.03728	0.28602	-1.8624	0.60746
TRINITY_DN955_c0_g1_i2_orf1	gloverin-like [Ostrinia furnacalis] >XP_028168251.1 gloverin-like [Ostrinia furnacalis] >AYM26645.1 gloverin [Ostrinia furnacalis]	-0.839	1.05839	0.67891	-1.5242	0.6259
TRINITY_DN16840_c1_g1_i1_orf1	attacin-like [Ostrinia furnacalis]	-0.6339	1.27961	0.44681	-1.5865	0.49402
TRINITY_DN10231_c0_g2_i1_orf1	uncharacterized protein LOC114361472 [Ostrinia furnacalis]	-0.8841	0.84001	0.10504	-1.3533	1.29238
TRINITY_DN1759_c0_g1_i4_orf1	protein PFC0760c-like isoform X1 [Ostrinia furnacalis]	-0.553	1.0002	0.80802	-1.689	0.43379
TRINITY_DN5170_c0_g1_i5_orf1	hemolymph lipopolysaccharide-binding protein-like isoform X2 [Leguminivora glycinivorella]	-1.284	0.82274	0.99922	-1.144	0.60604
TRINITY_DN2743_c0_g1_i5_orf1	regucalcin-like [Ostrinia furnacalis]	-0.425	1.62045	0.15847	-1.4681	0.11422
TRINITY_DN28802_c0_g1_i1_orf1	apolipoprotein D-like [Ostrinia furnacalis]	-0.3172	0.88133	-0.0293	-1.6779	1.14304
TRINITY_DN8694_c1_g1_i4_orf1	sodium/potassium-transporting ATPase subunit beta-2-like isoform X2 [Ostrinia furnacalis]	-0.765	0.98619	0.07866	-1.4521	1.15219
TRINITY_DN4125_c0_g1_i6_orf1	angiotensin-converting enzyme-like isoform X1 [Ostrinia furnacalis]	-0.3738	1.13778	-0.2884	-1.5358	1.06018
TRINITY_DN2343_c1_g1_i2_orf1	receptor expression-enhancing protein 5-like isoform X1 [Ostrinia furnacalis] >XP_028170586.1 receptor expression-enhancing protein 5-like isoform X1 [Ostrinia furnacalis]	-1.2386	1.05831	1.04811	-1.0938	0.22593
TRINITY_DN5190_c0_g3_i1_orf1	muscle LIM protein Mlp84B-like isoform X2 [Chelonus insularis]	-0.6076	0.7151	0.70185	-1.6891	0.87973
TRINITY_DN37821_c0_g1_i6_orf1	uncharacterized protein LOC114350690 [Ostrinia furnacalis]	-0.5547	0.72024	0.79699	-1.7213	0.75872
TRINITY_DN9926_c1_g1_i1_orf1	rab GTPase-activating protein 1-like isoform X6 [Ostrinia furnacalis]	-1.2954	1.16547	0.64646	-1.0986	0.58206
TRINITY_DN7388_c0_g1_i7_orf1	ras-related protein Rab-18A isoform X1 [Ostrinia furnacalis]	-0.5693	1.42634	-0.2008	-1.4203	0.76408
TRINITY_DN40945_c0_g1_i1_orf1	RNA exonuclease 4-like [Ostrinia furnacalis] >QEE79882.1 REX4 [Ostrinia furnacalis]	-1.1341	1.34666	0.50356	-1.1919	0.47571
TRINITY_DN2758_c0_g1_i7_orf1	hypothetical protein evm_012952 [Chilo suppressalis]	-0.7433	1.34997	0.07495	-1.4333	0.7517
TRINITY_DN4596_c0_g1_i14_orf1	aldehyde dehydrogenase, dimeric NADP-preferring isoform X7 [Ostrinia furnacalis]	-1.1393	1.29595	0.40628	-1.2031	0.64022
TRINITY_DN4813_c0_g1_i5_orf1	piwi-like protein Siwi [Ostrinia furnacalis]	-1.2375	1.41094	-0.0516	-0.9176	0.79576
TRINITY_DN3219_c0_g1_i6_orf1	non-specific lipid-transfer protein [Ostrinia furnacalis]	-1.4495	0.82596	1.25176	-0.7897	0.16153
TRINITY_DN5055_c0_g1_i12_orf1	probable peroxisomal acyl-coenzyme A oxidase 1 [Ostrinia furnacalis]	-1.2924	1.13016	0.96302	-1.0346	0.23381
TRINITY_DN1978_c0_g1_i4_orf1	RNA exonuclease 4-like [Ostrinia furnacalis]	-1.4316	1.39074	0.47497	-0.8072	0.3731

TRINITY_DN29144_c0_g3_i1_orf1	PREDICTED: ADP-ribosylation factor 6 [Papilio polytes] >XP_013133321.1 PREDICTED: ADP-ribosylation factor 6 [Papilio polytes] >XP_013177129.1 PREDICTED: ADP-ribosylation factor 6 [Papilio xuthus] >XP_013177130.1 PREDICTED: ADP-ribosylation factor 6 [Papilio xuthus] >XP_014356507.1 ADP-ribosylation factor 6 [Papilio machaon] >XP_021185579.1 ADP-ribosylation factor 6 [Helicoverpa armigera] >XP_021185581.1 ADP-ribosylation factor 6 [Helicoverpa armigera] >XP_022130228.1 ADP-ribosylation factor 6 [Pieris rapae] >XP_022822139.1 ADP-ribosylation factor 6 [Spodoptera litura] >XP_022822140.1 ADP-ribosylation factor 6 [Spodoptera litura] >XP_028159104.1 ADP-ribosylation factor 6 [Ostrinia furnacalis] >XP_028159105.1 ADP-ribosylation factor 6 [Ostrinia furnacalis] >XP_028159106.1 ADP-ribosylation factor 6 [Ostrinia furnacalis] >XP_028159107.1 ADP-ribosylation factor 6 [Ostrinia furnacalis] >XP_028163222.1 ADP-ribosylation factor 6 [Ostrinia furnacalis] >XP_030022165.1 ADP-ribosylation factor 6 [Manduca sexta] >XP_030022166.1 ADP-ribosylation factor 6 [Manduca sexta] >XP_030022167.1 ADP-ribosylation factor 6 [Manduca sexta] >XP_035444169.1 ADP-ribosylation factor 6 [Spodoptera frugiperda] >XP_035444175.1 ADP-ribosylation factor 6 [Spodoptera frugiperda] >XP_038207597.1 ADP-ribosylation factor 6 [Zerene cesonia] >XP_038207598.1 ADP-ribosylation factor 6 [Zerene cesonia] >XP_045510541.1 ADP-ribosylation factor 6 [Colias croceus] >XP_045510551.1 ADP-ribosylation factor 6 [Colias croceus] >XP_045527300.1 ADP-ribosylation factor 6 [Pieris brassicae] >XP_045527302.1 ADP-ribosylation factor 6 [Pieris brassicae] >XP_047029519.1 ADP-ribosylation factor 6 [Helicoverpa zea] >XP_047029551.1 ADP-ribosylation factor 6 [Helicoverpa zea] >XP_047504621.1 ADP-ribosylation factor 6 [Pieris napi] >XP_047504631.1 ADP-ribosylation factor 6 [Pieris napi] >XP_047504640.1 ADP-ribosylation factor 6 [Pieris napi] >XP_047504648.1 ADP-ribosylation factor 6 [Pieris napi] >XP_047504657.1 ADP-ribosylation factor 6 [Pieris napi] >XP_048489067.1 ADP-ribosylation factor 6 [Plutella xylostella] >XP_048489068.1 ADP-ribosylation factor 6 [Plutella xylostella] >XP_048489069.1 ADP-ribosylation factor 6 [Plutella xylostella] >XP_049883531.1 ADP-ribosylation factor 6 [Pectinophora gossypiella] >XP_049883539.1 ADP-ribosylation factor 6 [Pectinophora gossypiella] >KAG5678369.1 hypothetical protein PVAND_008051 [Polypedilum vanderplanki] >RVE51130.1 hypothetical protein evm_004273 [Chilo suppressalis] >CAB3510283.1 unnamed protein product [Spodoptera littoralis] >CAF4796780.1 unnamed protein product [Pieris macdunnoughi] >CAG4977608.1 unnamed protein product [Parnassius apollo] >CAG9757696.1 unnamed protein product [Diatraea saccharalis] >CAG9799627.1 unnamed protein product [Chironomus riparius] >CAH0695461.1 unnamed protein product [Spodoptera exigua]	-1.6304	1.44183	0.1195	-0.3162	0.38536
TRINITY_DN15400_c0_g1_i1_orf1	uncharacterized protein LOC114366781 [Ostrinia furnacalis]	-0.9976	0.49194	0.66042	-1.3648	1.20999
TRINITY_DN34406_c0_g2_i9_orfp1	TRINITY_DN34406_c0_g2_i9_m.33755 TRINITY_DN34406_c0_g2::TRINITY_DN34406_c0_g2_i9::g.33755 ORF type:internal len:82 (-),score=12.88 TRINITY_DN34406_c0_g2_i9:3-245(-)	-0.9835	0.30318	1.25759	-1.3357	0.75843
TRINITY_DN1012_c0_g1_i2_orf1	teneurin-a isoform X1 [Ostrinia furnacalis]	-0.6789	0.53979	0.19809	-1.4798	1.4208
TRINITY_DN858_c0_g1_i3_orf1	uncharacterized protein LOC114351944 [Ostrinia furnacalis]	-1.0905	0.30085	0.79527	-1.2453	1.23973
TRINITY_DN3647_c1_g1_i5_orf1	unnamed protein product, partial [Iphiclides podalirius]	-0.4398	-0.012	0.94416	-1.6234	1.1311
TRINITY_DN26985_c0_g1_i5_orf1	secretory phospholipase A2 receptor-like [Helicoverpa zea]	-0.7327	-1.0942	1.03384	-0.5737	1.36674
TRINITY_DN12014_c0_g1_i2_orf1	unnamed protein product [Chilo suppressalis]	-0.41	-0.2541	1.33928	-1.5093	0.83407
TRINITY_DN2141_c0_g1_i1_orf1	low density lipoprotein receptor adapter protein 1-like [Ostrinia furnacalis]	-0.0269	-0.5245	1.2249	-1.5609	0.88735
TRINITY_DN11868_c0_g1_i2_orf1	uncharacterized protein LOC114361308 [Ostrinia furnacalis]	-0.6646	0.16518	0.98066	-1.5547	1.07345
TRINITY_DN12661_c0_g1_i3_orf1	T-complex protein 11-like protein 1 [Ostrinia furnacalis]	-0.3314	-0.0853	1.24283	-1.6372	0.81103
TRINITY_DN96739_c0_g1_i1_orf1	annexin A6, isoform CRA_b [Homo sapiens]	-0.7249	0.60333	0.54816	-1.5769	1.15035
TRINITY_DN10373_c0_g1_i1_orf1	homocysteine S-methyltransferase 1-like [Ostrinia furnacalis] >XP_028162778.1 homocysteine S-methyltransferase 1-like [Ostrinia furnacalis]	-0.7577	0.23723	1.20418	-1.5007	0.81698
TRINITY_DN896_c0_g1_i2_orf1	uncharacterized protein LOC114356314 isoform X2 [Ostrinia furnacalis]	-0.999	-0.1081	1.06665	-1.1738	1.21433
TRINITY_DN24668_c0_g1_i8_orf1	uncharacterized protein LOC114364067 isoform X3 [Ostrinia furnacalis]	-0.3091	-0.6908	1.25614	-1.3148	1.05858
TRINITY_DN325_c0_g1_i15_orf1	protein draper-like [Ostrinia furnacalis]	-0.8987	-0.4458	1.6192	-0.954	0.67937
TRINITY_DN9457_c0_g1_i9_orf1	plexin domain-containing protein 2 [Spodoptera litura]	-0.6066	0.61811	0.46415	-1.6381	1.16241
TRINITY_DN7062_c0_g1_i1_orf1	ribosome maturation protein SBDS [Ostrinia furnacalis]	-0.6774	0.44491	0.33179	-1.5036	1.40434
TRINITY_DN38106_c0_g1_i6_orf1	unnamed protein product [Pieris macdunnoughi]	-1.1109	-0.4165	1.44438	-0.8253	0.90834
TRINITY_DN28741_c0_g1_i3_orf1	uncharacterized protein LOC114351652 [Ostrinia furnacalis]	-0.3946	-0.619	1.72204	-1.1431	0.43471
TRINITY_DN132_c0_g2_i2_orf1	alpha-tocopherol transfer protein-like isoform X1 [Ostrinia furnacalis]	-0.675	-0.4868	1.40088	-1.1957	0.95664
TRINITY_DN8979_c0_g1_i5_orf1	ras-related protein Rab-5B [Vanessa cardui] >XP_046961939.1 ras-related protein Rab-5B [Vanessa cardui] >XP_046961940.1 ras-related protein Rab-5B [Vanessa cardui] >XP_046961941.1 ras-related protein Rab-5B [Vanessa cardui] >XP_047545265.1 ras-related protein Rab-5B [Vanessa atalanta] >XP_047545266.1 ras-related protein Rab-5B [Vanessa atalanta] >XP_047545267.1 ras-related protein Rab-5B [Vanessa atalanta]	-0.7682	0.59323	0.48269	-1.5282	1.22049
TRINITY_DN6205_c0_g1_i4_orfp1	TRINITY_DN6205_c0_g1_i4_m.72677 TRINITY_DN6205_c0_g1::TRINITY_DN6205_c0_g1_i4::g.72677 ORF type:internal len:68 (-),score=1.69 TRINITY_DN6205_c0_g1_i4:2-202(-)	-0.9744	-0.8265	1.04104	-0.6188	1.37868
TRINITY_DN4817_c0_g1_i4_orf1	palmitoyl-protein thioesterase 1 isoform X1 [Ostrinia furnacalis] >XP_028170290.1 palmitoyl-protein thioesterase 1 isoform X4 [Ostrinia furnacalis]	-0.9816	0.09331	1.16198	-1.2858	1.01208
TRINITY_DN4108_c0_g1_i6_orf1	chromobox protein homolog 1-like [Ostrinia furnacalis]	-0.8853	0.35138	0.77701	-1.4367	1.19366
TRINITY_DN61048_c0_g1_i2_orf1	PREDICTED: protein THEM6-like [Amyelois transitella]	-0.857	0.45654	0.99434	-1.4995	0.90556
TRINITY_DN1293_c1_g1_i4_orf1	putative fatty acyl-CoA reductase CG5065 [Ostrinia furnacalis]	-0.7236	-0.9858	1.62698	-0.6123	0.69473
TRINITY_DN22928_c0_g1_i6_orf1	hypothetical protein B5X24_HaOG209714 [Helicoverpa armigera]	-0.7942	0.48775	0.6141	-1.5152	1.2076
TRINITY_DN7316_c0_g2_i1_orf1	ubiquitin-protein ligase E3C [Ostrinia furnacalis]	-1.0884	-0.6516	1.15401	-0.6784	1.26443
TRINITY_DN11693_c0_g1_i6_orf1	sorting nexin-32 isoform X1 [Ostrinia furnacalis] >XP_028166096.1 sorting nexin-32 isoform X2 [Ostrinia furnacalis]	-1.1312	-0.0058	0.91288	-1.0841	1.30829
TRINITY_DN28729_c0_g1_i7_orf1	serine/threonine-protein kinase mig-15 isoform X7 [Ostrinia furnacalis]	-0.8311	-1.0513	0.99398	-0.5101	1.39851
TRINITY_DN18136_c0_g1_i1_orf1	proteoglycan 4-like [Ostrinia furnacalis]	-0.7966	-0.8211	1.13269	-0.8277	1.3127
TRINITY_DN2326_c0_g1_i1_orf1	transmembrane protein 256 homolog isoform X1 [Ostrinia furnacalis]	-1.1396	0.11024	0.91188	-1.1351	1.25263
TRINITY_DN919_c0_g1_i7_orf1	facilitated trehalose transporter Tret1-like [Ostrinia furnacalis] >XP_028161733.1 facilitated trehalose transporter Tret1-like [Ostrinia furnacalis]	-0.7902	-0.7084	1.79098	-0.7045	0.41212
TRINITY_DN60949_c0_g1_i4_orf1	aldo-keto reductase AKR2E4-like [Galleria mellonella]	-1.165	-0.5176	1.29785	-0.7066	1.09138
TRINITY_DN80424_c0_g1_i1_orf1	PREDICTED: cytoplasmic FMR1-interacting protein [Dufourea novaeangliae] >KZC10094.1 Cytoplasmic FMR1-interacting protein [Dufourea novaeangliae]	-1.0004	-0.6977	1.70473	-0.5539	0.54726
TRINITY_DN11204_c0_g1_i3_orf1	spermosin-like [Ostrinia furnacalis]	-0.6189	-1.1553	1.00776	-0.6099	1.37642
TRINITY_DN855_c0_g1_i5_orf1	hypothetical protein HF086_009970 [Spodoptera exigua] >CAH0702798.1 unnamed protein product [Spodoptera exigua]	-1.1622	0.08029	1.26591	-1.0978	0.91383
TRINITY_DN2855_c0_g1_i6_orf1	uncharacterized protein LOC114359301 [Ostrinia furnacalis]	-1.313	0.41367	0.99007	-1.075	0.98432
TRINITY_DN31342_c2_g2_i1_orf1	26S proteasome non-ATPase regulatory subunit 2 isoform X2 [Ostrinia furnacalis]	-0.93	-0.8336	1.34896	-0.6686	1.08326
TRINITY_DN6205_c0_g1_i8_orf1	phenoloxidase-activating factor 2-like [Ostrinia furnacalis]	-0.9524	-1.0009	1.49705	-0.3826	0.83882

TRINITY_DN26488_c0_g1_i6_orf1	phosphatidate phosphatase LPIN2 isoform X1 [Ostrinia furnacalis] >XP_028176373.1 phosphatidate phosphatase LPIN2 isoform X1 [Ostrinia furnacalis] >XP_028176374.1 phosphatidate phosphatase LPIN2 isoform X2 [Ostrinia furnacalis] >XP_028176375.1 phosphatidate phosphatase LPIN2 isoform X3 [Ostrinia furnacalis] >XP_028176376.1 phosphatidate phosphatase LPIN2 isoform X4 [Ostrinia furnacalis]	-0.9443	-0.3918	1.51799	-0.9949	0.81292
TRINITY_DN14952_c0_g3_i1_orf1	protein DEK isoform X3 [Ostrinia furnacalis] >XP_028162408.1 protein DEK isoform X4 [Ostrinia furnacalis]	-1.3916	-0.4338	1.34707	-0.4486	0.92699
TRINITY_DN46202_c0_g1_i1_orf1	alpha-tubulin N-acetyltransferase 1-like isoform X2 [Ostrinia furnacalis]	-1.1613	-0.6424	1.1076	-0.5926	1.28871
TRINITY_DN1897_c0_g2_i4_orf1	phenoloxidase-activating factor 2-like [Hyposmocoma kahamanoa]	-0.4104	1.40384	-0.5166	-1.352	0.87522
TRINITY_DN38392_c0_g1_i1_orf1	enoyl-CoA hydratase domain-containing protein 3 [Agrotis segetum]	-0.1726	1.51508	-1.2104	-0.8409	0.70886
TRINITY_DN13330_c0_g1_i4_orf1	carboxylesterase [Cnaphalocrocis medinalis]	-0.4205	1.11873	-0.7299	-1.2167	1.2484
TRINITY_DN962_c5_g1_i1_orf1	histone deacetylase 5 isoform X5 [Pectinophora gossypiella]	-0.6768	1.38244	-0.8123	-0.9379	1.04462
TRINITY_DN26089_c0_g1_i1_orf1	putative neuropeptide precursor protein isoform X1 [Ostrinia furnacalis]	-0.742	1.38868	-0.8194	-0.8715	1.04413
TRINITY_DN115210_c0_g4_i1_orf1	PREDICTED: CAD protein [Microplitis demolitor]	-1.0357	1.41399	-0.8012	-0.562	0.98495
TRINITY_DN84631_c0_g1_i1_orf1	PREDICTED: rap guanine nucleotide exchange factor 2-like isoform X9 [Microplitis demolitor]	-0.6065	1.44155	-0.6182	-1.1449	0.928
TRINITY_DN29190_c0_g1_i4_orf1	gloverin-like [Ostrinia furnacalis]	-0.6982	1.86278	-0.903	-0.4414	0.17982
TRINITY_DN48497_c0_g1_i1_orf1	unnamed protein product [Chrysodeixis includens]	-0.789	1.61093	-1.2403	-0.0704	0.48883
TRINITY_DN1260_c0_g2_i1_orf1	vegetative cell wall protein gp1 [Ostrinia furnacalis]	-0.9977	1.18864	-1.1787	-0.104	1.09167
TRINITY_DN98242_c0_g1_i1_orf1	adenosine deaminase 2-A-like [Galleria mellonella]	-0.9611	-0.7848	-0.2907	0.21135	1.82519
TRINITY_DN147458_c0_g1_i1_orf1	60S ribosomal protein L5, partial [Cotesia chilonis]	-1.1648	-0.5694	-0.3023	0.25867	1.77783
TRINITY_DN2367_c1_g1_i20_orf1	RNA exonuclease 4-like [Ostrinia furnacalis]	-1.6044	-0.0915	1.08679	-0.4204	1.02945
TRINITY_DN86621_c0_g1_i2_orf1	protein sly1 homolog isoform X1 [Ostrinia furnacalis] >XP_028165613.1 protein sly1 homolog isoform X2 [Ostrinia furnacalis]	-1.2223	-0.3733	-0.165	-0.0656	1.82623
TRINITY_DN552_c0_g1_i3_orf1	patronin isoform X9 [Ostrinia furnacalis]	-1.2145	-0.9347	-0.0316	0.72143	1.45941
TRINITY_DN77642_c0_g1_i1_orf1	peritrophic membrane chitin binding protein [Loxostege sticticalis]	-0.9903	-1.207	0.54978	0.15185	1.49569
TRINITY_DN32956_c0_g1_i4_orf1	inositol-trisphosphate 3-kinase A isoform X1 [Vanessa tameamea] >XP_[illegible] inositol-trisphosphate 3-kinase A isoform X1 [Vanessa atalanta] >XP_047534116.1 inositol-trisphosphate 3-kinase A isoform X1 [Vanessa atalanta] >XP_047534117.1 inositol-trisphosphate 3-kinase A isoform X1 [Vanessa atalanta]	-1.0643	-0.7418	-0.3111	0.35912	1.75816
TRINITY_DN30273_c1_g1_i1_orf1	uncharacterized protein LOC114358591 isoform X2 [Ostrinia furnacalis]	-1.2877	-0.5602	-0.0802	0.20152	1.72656
TRINITY_DN29633_c0_g1_i8_orf1	transmembrane protein 87A isoform X1 [Ostrinia furnacalis] >XP_028156931.1 transmembrane protein 87A isoform X2 [Ostrinia furnacalis] >XP_028156932.1 transmembrane protein 87A isoform X3 [Ostrinia furnacalis] >XP_028156933.1 transmembrane protein 87A isoform X4 [Ostrinia furnacalis] >XP_028156934.1 transmembrane protein 87A isoform X5 [Ostrinia furnacalis] >XP_028156935.1 transmembrane protein 87A isoform X6 [Ostrinia furnacalis]	-1.1798	-0.9276	0.36902	0.12748	1.61094
TRINITY_DN1233_c0_g2_i1_orf1	unnamed protein product [Spodoptera exigua]	-0.9077	-0.8056	-0.378	0.27197	1.81938
TRINITY_DN1099_c1_g1_i2_orf1	dehydrogenase/reductase SDR family protein 7-like [Ostrinia furnacalis]	-1.7752	-0.1149	0.61882	0.06806	1.20324
TRINITY_DN210_c0_g1_i9_orf1	glycine-rich cell wall structural protein-like [Ostrinia furnacalis]	-1.6502	-0.2822	0.81109	-0.1143	1.2355
TRINITY_DN172_c8_g2_i1_orf1	sialin [Ostrinia furnacalis]	-1.0782	-1.094	0.416	0.19787	1.55834
TRINITY_DN122423_c0_g1_i1_orf1	hypothetical protein TSAR_001625, partial [Trichomalopsis sarcophagae]	-1.4884	-0.771	0.31787	0.65122	1.29035
TRINITY_DN4798_c0_g1_i3_orf1	unnamed protein product [Spodoptera exigua]	-0.7549	-1.4249	0.86152	0.03117	1.2871
TRINITY_DN1066_c0_g1_i8_orf1	hypothetical protein evm_012420 [Chilo suppressalis]	-1.2174	-1.0207	0.70251	0.13392	1.40167
TRINITY_DN5118_c0_g1_i1_orf1	AP-2 complex subunit alpha [Ostrinia furnacalis]	-1.3863	-0.5346	0.3052	-0.0269	1.64268
TRINITY_DN23020_c0_g1_i1_orf1	actin-related protein 2/3 complex subunit 1A-A [Ostrinia furnacalis] >XP_028170151.1 actin-related protein 2/3 complex subunit 1A-A [Ostrinia furnacalis]	-1.5593	-0.0557	0.74652	-0.4689	1.33731
TRINITY_DN5296_c0_g2_i1_orf1	RNA-binding protein Rsf1 [Ostrinia furnacalis]	-1.2707	-0.8883	0.75961	-0.0215	1.42087
TRINITY_DN14865_c0_g1_i2_orf1	ubiquilin-1 [Ostrinia furnacalis]	-1.3601	-0.644	0.42	-0.0156	1.59965
TRINITY_DN127056_c0_g1_i1_orf1	pantothenate kinase 4 [Ostrinia furnacalis]	-1.6842	-0.2805	0.19585	1.37526	0.39366
TRINITY_DN667_c0_g1_i5_orf1	unnamed protein product [Arctia plantaginis]	-1.6227	0.16741	-0.1533	0.08972	1.51892
TRINITY_DN2942_c0_g1_i6_orf1	microtubule-associated protein RP/EB family member 1 [Ostrinia furnacalis] >XP_028174940.1 microtubule-associated protein RP/EB family member 1 [Ostrinia furnacalis]	-1.4126	-0.4494	0.08702	0.10645	1.66848
TRINITY_DN578_c0_g1_i3_orf1	charged multivesicular body protein 7 [Ostrinia furnacalis]	-1.8142	0.50624	0.75633	-0.3277	0.87929
TRINITY_DN5666_c0_g1_i2_orf1	protein ROP isoform X2 [Ostrinia furnacalis]	-1.5937	-0.4583	0.72774	0.01256	1.31166
TRINITY_DN34703_c0_g1_i4_orf1	gamma-tubulin complex component 3 homolog [Ostrinia furnacalis]	-1.4628	-0.1778	-0.2643	0.26529	1.63962
TRINITY_DN2638_c0_g1_i7_orf1	structural maintenance of chromosomes protein 1A [Trichoplusia ni]	-1.0334	-0.9455	-0.1905	0.51516	1.65422
TRINITY_DN535_c1_g1_i2_orf1	protein tramtrack, beta isoform isoform X24 [Bicyclus anynana]	-0.9725	-0.9686	-0.1687	0.39845	1.71138
TRINITY_DN12757_c0_g1_i1_orf1	proteasome subunit alpha type-7-1-like [Ostrinia furnacalis]	-1.3744	-0.497	0.07993	0.1043	1.6872
TRINITY_DN4540_c0_g1_i9_orf1	dihydropyrimidinase isoform X2 [Manduca sexta]	-1.4941	-0.5434	0.69687	-0.0672	1.40787
TRINITY_DN19702_c0_g1_i4_orf1	AP-3 complex subunit mu-1 [Ostrinia furnacalis]	-1.4421	-0.3262	-0.2746	0.45056	1.59235
TRINITY_DN25870_c0_g2_i6_orf1	homeobox protein extradenticle isoform X3 [Ostrinia furnacalis]	-1.3043	-0.7887	0.10365	0.40838	1.58092
TRINITY_DN2054_c0_g1_i1_orf1	macrophage mannose receptor 1-like [Ostrinia furnacalis]	-0.9341	-1.1616	0.00965	0.49434	1.59174
TRINITY_DN81791_c0_g2_i2_orf1	UDP-N-acetylglucosamine--peptide N-acetylglucosaminyltransferase 110 kDa subunit isoform X2 [Diachasma alloeum]	-1.1549	-1.0702	1.03764	-0.0143	1.20168
TRINITY_DN9980_c0_g1_i1_orf1	unnamed protein product [Chrysodeixis includens]	-1.6228	-0.2192	0.48866	-0.0861	1.43949
TRINITY_DN22213_c0_g1_i3_orf1	hypothetical protein evm_003554 [Chilo suppressalis]	-1.5706	-0.1962	0.38	-0.1399	1.52671
TRINITY_DN1492_c0_g1_i4_orf1	sarcoplasmic calcium-binding protein isoform X2 [Ostrinia furnacalis]	-1.8881	0.31849	0.62545	-0.0262	0.9704
TRINITY_DN33893_c0_g1_i1_orf1	high mobility group protein I-like [Ostrinia furnacalis]	-1.4629	-0.373	-0.0217	0.22334	1.63419
TRINITY_DN9517_c0_g1_i7_orf1	transcriptional repressor p66-alpha [Ostrinia furnacalis]	-1.2439	-0.8398	-0.0186	0.53291	1.5694
TRINITY_DN9486_c1_g1_i7_orfp1	uncharacterized protein LOC114351033 isoform X1 [Ostrinia furnacalis]	-1.7423	0.09981	0.22175	0.04098	1.37974
TRINITY_DN27321_c0_g1_i1_orf1	ras-related protein Rap-2c [Bicyclus anynana] >XP_[illegible] ras-related protein Rap-2c [Vanessa tameamea] >XP_[illegible] ras-related protein Rap-2c [Maniola hyperantus] >XP_039759141.1 ras-related protein Rap-2c [Pararge aegeria] >XP_045498804.1 ras-related protein Rap-2c [Colias croceus] >XP_046959644.1 ras-related protein Rap-2c [Vanessa cardui] >XP_047530248.1 ras-related protein Rap-2c [Vanessa atalanta] >CAH2268047.1 jg10357 [Pararge aegeria aegeria]	-1.1835	-0.4248	-0.5015	0.36854	1.74117
TRINITY_DN30673_c0_g1_i5_orf1	protein phosphatase 1 regulatory subunit 21 [Ostrinia furnacalis]	-0.7031	-1.4011	0.76862	-0.0601	1.39574

TRINITY_DN359_c0_g1_i5_orf1	eukaryotic translation initiation factor 5 isoform X1 [Ostrinia furnacalis] >XP_028162054.1 eukaryotic translation initiation factor 5 isoform X1 [Ostrinia furnacalis] >XP_028162062.1 eukaryotic translation initiation factor 5 isoform X1 [Ostrinia furnacalis] >XP_028162073.1 eukaryotic translation initiation factor 5 isoform X1 [Ostrinia furnacalis] >XP_028162080.1 eukaryotic translation initiation factor 5 isoform X1 [Ostrinia furnacalis] >XP_028162091.1 eukaryotic translation initiation factor 5 isoform X1 [Ostrinia furnacalis] >XP_028162099.1 eukaryotic translation initiation factor 5 isoform X1 [Ostrinia furnacalis] >XP_028162107.1 eukaryotic translation initiation factor 5 isoform X1 [Ostrinia furnacalis]	-1.6466	0.20619	1.26184	-0.4754	0.65405
TRINITY_DN24266_c0_g2_i2_orf1	chromobox-like protein 5 [Helicoverpa armigera]	-1.3264	-1.0331	0.42261	0.72452	1.21237
TRINITY_DN5200_c0_g1_i2_orf1	uncharacterized protein LOC114351644 [Ostrinia furnacalis]	-1.4325	-0.5383	0.02647	0.35279	1.59155
TRINITY_DN29563_c0_g1_i5_orf1	N-terminal kinase-like protein [Trichoplusia ni]	-0.8525	-1.2451	0.07827	0.4273	1.59197
TRINITY_DN185_c0_g1_i6_orf1	eukaryotic translation initiation factor 4E-binding protein Mextli isoform X2 [Ostrinia furnacalis]	-1.3266	-0.9045	0.19811	0.61879	1.41419
TRINITY_DN120144_c0_g1_i1_orf1	pre-mRNA-splicing factor SPF27 [Ostrinia furnacalis] >XP_028158851.1 pre-mRNA-splicing factor SPF27 [Ostrinia furnacalis]	-1.029	-1.0654	0.14242	0.31245	1.63955
TRINITY_DN11942_c0_g1_i1_orf1	hypothetical protein B5X24_HaOG213660 [Helicoverpa armigera]	-1.8424	0.4068	1.13675	-0.0779	0.37672
TRINITY_DN123184_c0_g1_i1_orf1	double-strand break repair protein MRE11 [Ostrinia furnacalis]	-1.2518	-0.8268	0.09798	0.36647	1.61415
TRINITY_DN2345_c0_g1_i4_orf1	chromobox protein homolog 3-like [Ostrinia furnacalis] >XP_028157236.1 chromobox protein homolog 3-like [Ostrinia furnacalis]	-1.1252	-0.8462	-0.0369	0.29718	1.71118
TRINITY_DN16011_c0_g1_i3_orf1	hypothetical protein evm_002694 [Chilo suppressalis]	-1.237	-0.7044	0.03652	0.19158	1.71334
TRINITY_DN8405_c0_g1_i4_orf1	clathrin heavy chain isoform X1 [Ostrinia furnacalis] >XP_028169033.1 clathrin heavy chain isoform X2 [Ostrinia furnacalis] >XP_028169034.1 clathrin heavy chain isoform X3 [Ostrinia furnacalis] >XP_028169036.1 clathrin heavy chain isoform X5 [Ostrinia furnacalis]	-1.6654	-0.4974	0.85385	0.21104	1.09792
TRINITY_DN1853_c0_g1_i3_orf1	trans-Golgi network integral membrane protein TGN38-like isoform X1 [Ostrinia furnacalis]	-1.3018	-0.4803	-0.3019	0.40468	1.67924
TRINITY_DN24350_c0_g1_i1_orf1	zinc finger protein 771-like [Ostrinia furnacalis]	-1.2784	-0.3984	-0.3768	0.33546	1.71823
TRINITY_DN19303_c0_g1_i5_orf1	lipopolysaccharide-induced tumor necrosis factor-alpha factor-like [Ostrinia furnacalis]	-1.5891	-0.5887	0.8334	0.15729	1.18704
TRINITY_DN18922_c0_g1_i1_orf1	LOW QUALITY PROTEIN: CCR4-NOT transcription complex subunit 6 [Ostrinia furnacalis]	-1.391	-0.7759	0.51941	0.17705	1.4704
TRINITY_DN892_c0_g1_i9_orf1	calcyphosin-like protein isoform X2 [Ostrinia furnacalis]	-1.7311	-0.3674	0.45532	0.42712	1.216
TRINITY_DN30178_c0_g1_i3_orf1	LOW QUALITY PROTEIN: fibrillin-2-like [Bicyclus anynana]	-1.5203	-0.2376	-0.3188	0.60576	1.47091
TRINITY_DN486_c0_g1_i5_orf1	adaptor complexes medium subunit family domain-containing protein [Phthorimaea operculella]	-1.5421	-0.6021	0.25662	0.48981	1.39775
TRINITY_DN4782_c0_g1_i1_orf1	patched domain-containing protein 3-like [Ostrinia furnacalis]	-1.4602	-0.6795	0.40815	0.25743	1.47416
TRINITY_DN629_c0_g1_i6_orf1	annexin B9-like isoform X1 [Ostrinia furnacalis]	-0.8442	-1.5207	0.64748	0.65815	1.05934
TRINITY_DN891_c5_g1_i5_orf1	unnamed protein product, partial [Brenthis ino]	-1.8054	-0.198	0.83606	0.18316	0.98422
TRINITY_DN24218_c0_g1_i1_orf1	uncharacterized protein LOC114362624 [Ostrinia furnacalis]	-1.4585	-0.6711	0.3872	0.25686	1.48548
TRINITY_DN40176_c0_g1_i1_orf1	transcription elongation factor 1 homolog [Plutella xylostella] >XP_014365734.1 transcription elongation factor 1 homolog [Papilio machaon] >XP_021188366.1 transcription elongation factor 1 homolog [Helicoverpa armigera] >XP_022815988.1 transcription elongation factor 1 homolog [Spodoptera litura] >XP_022815989.1 transcription elongation factor 1 homolog [Spodoptera litura] >XP_026739943.1 transcription elongation factor 1 homolog [Trichoplusia ni] >XP_026739944.1 transcription elongation factor 1 homolog [Trichoplusia ni] >XP_028167273.1 transcription elongation factor 1 homolog [Ostrinia furnacalis] >XP_035429172.1 transcription elongation factor 1 homolog [Spodoptera frugiperda] >XP_038210306.1 transcription elongation factor 1 homolog [Zerene cesonia] >XP_045492874.1 transcription elongation factor 1 homolog [Colias croceus] >XP_045492875.1 transcription elongation factor 1 homolog [Colias croceus] >XP_047019949.1 transcription elongation factor 1 homolog [Helicoverpa zea] >KAF9414193.1 hypothetical protein HW555_007806 [Spodoptera exigua] >KPJ01541.1 Transcription elongation factor 1-like [Papilio xuthus] >CAB3243126.1 unnamed protein product [Arctia plantaginis] >CAB3514670.1 unnamed protein product [Spodoptera littoralis] >CAH0626924.1 unnamed protein product [Chrysodeixis includens] >CAH2075456.1 unnamed protein product, partial [Iphiclides podalirius]	-1.6551	-0.3463	1.28593	0.01773	0.69774
TRINITY_DN2004_c0_g1_i20_orf1	hypothetical protein evm_006436 [Chilo suppressalis] >CAB3522373.1 unnamed protein product [Chilo suppressalis] >CAH0399695.1 unnamed protein product [Chilo suppressalis]	-1.3209	-0.8069	0.27041	0.29388	1.56354
TRINITY_DN5162_c0_g1_i3_orf1	steroidogenic acute regulatory protein-like [Ostrinia furnacalis]	-1.3107	-1.0921	0.70765	0.57223	1.12297
TRINITY_DN8738_c0_g1_i1_orf1	unnamed protein product [Plutella xylostella]	-1.4798	-0.5197	-0.0769	0.60316	1.4732
TRINITY_DN9243_c0_g1_i4_orf1	copper-transporting ATPase 1 [Ostrinia furnacalis]	-1.5706	-0.3703	0.42462	0.02802	1.48827
TRINITY_DN14944_c0_g1_i7_orf1	alpha-tocopherol transfer protein-like isoform X1 [Ostrinia furnacalis] >XP_028158173.1 alpha-tocopherol transfer protein-like isoform X1 [Ostrinia furnacalis] >XP_028158174.1 alpha-tocopherol transfer protein-like isoform X1 [Ostrinia furnacalis]	-1.0568	-1.3366	0.66834	0.57795	1.14713
TRINITY_DN2808_c0_g1_i8_orf1	uncharacterized protein LOC114353011 isoform X2 [Ostrinia furnacalis]	-1.5968	-0.2391	-0.194	0.63079	1.39915
TRINITY_DN4766_c0_g1_i4_orf1	26S proteasome non-ATPase regulatory subunit 12 [Ostrinia furnacalis]	-1.4936	-0.8744	0.4905	0.91402	0.96354
TRINITY_DN10183_c0_g2_i3_orf1	uncharacterized protein LOC114360370 isoform X1 [Ostrinia furnacalis]	-1.8459	-0.0159	0.34699	0.35447	1.1603
TRINITY_DN2782_c0_g1_i7_orf1	CDK5 regulatory subunit-associated protein 3 [Ostrinia furnacalis]	-1.9022	0.09597	0.75343	0.17231	0.88046
TRINITY_DN3667_c0_g1_i4_orf1	coronin-2B-like isoform X2 [Ostrinia furnacalis]	-0.8368	-1.4573	1.2289	0.31086	0.7544
TRINITY_DN32359_c0_g2_i1_orf1	PREDICTED: 26S proteasome non-ATPase regulatory subunit 4 isoform X2 [Fopius arisanus]	-1.4814	-0.8745	0.55225	0.66803	1.13558
TRINITY_DN12777_c0_g1_i5_orf1	clathrin light chain isoform X2 [Ostrinia furnacalis]	-1.5815	-0.6412	0.63144	0.33592	1.25539
TRINITY_DN5475_c0_g1_i3_orf1	traB domain-containing protein-like isoform X1 [Ostrinia furnacalis] >XP_028169655.1 traB domain-containing protein-like isoform X1 [Ostrinia furnacalis]	-1.8505	0.12178	0.20784	0.3356	1.1853
TRINITY_DN8044_c0_g1_i2_orf1	protein FRG1 homolog [Pectinophora gossypiella]	-1.7598	-0.4418	0.89987	0.49112	0.81061
TRINITY_DN72816_c0_g1_i2_orf1	Golgi apparatus protein 1 [Ostrinia furnacalis]	-1.3565	-0.4945	-0.2988	0.5674	1.58243
TRINITY_DN6589_c0_g1_i2_orf1	hypothetical protein evm_004467 [Chilo suppressalis]	-1.6728	-0.6177	0.66361	0.64521	0.98163
TRINITY_DN3209_c0_g1_i1_orf1	coatomer subunit beta [Helicoverpa armigera]	-1.9336	0.16767	0.91716	0.29805	0.55068
TRINITY_DN3482_c0_g2_i1_orf1	transcription elongation factor B polypeptide 3-like isoform X2 [Ostrinia furnacalis]	-1.6658	-0.6201	0.53547	0.77094	0.97949
TRINITY_DN44658_c0_g1_i2_orf1	lipase 3-like [Ostrinia furnacalis]	-1.4609	-0.4828	-0.0625	0.44793	1.55827
TRINITY_DN1781_c0_g1_i8_orf1	transportin-1 [Pectinophora gossypiella]	-1.002	-1.3573	0.52092	0.60887	1.2295
TRINITY_DN35099_c0_g1_i1_orf1	transmembrane emp24 domain-containing protein 2 [Ostrinia furnacalis]	-1.7002	-0.5125	0.59125	0.50837	1.11302
TRINITY_DN1005_c0_g2_i1_orf1	hypothetical protein ILUMI_24512 [Ignelater luminosus]	-1.7066	-0.5389	0.83522	0.47623	0.93411
TRINITY_DN54586_c1_g1_i1_orf1	protein YIPF5 [Ostrinia furnacalis]	-1.2963	-1.0813	0.56167	0.60293	1.213
TRINITY_DN14677_c0_g2_i3_orf1	AP-3 complex subunit beta-2 [Ostrinia furnacalis]	-1.5438	-0.7718	0.6394	0.51627	1.15998
TRINITY_DN4937_c0_g1_i2_orf1	zinc finger protein 778-like [Ostrinia furnacalis]	-1.5931	-0.5393	0.28103	0.49078	1.36061
TRINITY_DN40440_c0_g1_i1_orf1	Na(+)/H(+) exchange regulatory cofactor NHE-RF1 [Helicoverpa zea] >XP_049705606.1 Na(+)/H(+) exchange regulatory cofactor NHE-RF1 [Helicoverpa	-1.4984	-0.7335	1.33714	0.56712	0.32759

TRINITY_DN1520_c0_g1_i9_orf1	adipocyte plasma membrane-associated protein-like [Ostrinia furnacalis] >XP_028176496.1 adipocyte plasma membrane-associated protein-like [Ostrinia	-1.9826	0.32577	0.48557	0.44121	0.73
TRINITY_DN53760_c0_g1_i1_orf1	unnamed protein product [Parnassius apollo]	-1.8693	-0.081	1.04056	0.49156	0.41814
TRINITY_DN4257_c0_g1_i2_orf1	dynactin subunit 1 [Ostrinia furnacalis]	-1.9598	0.77469	0.14943	0.52777	0.50795
TRINITY_DN2438_c0_g1_i4_orf1	dystrophin, isoforms A/C/F/G/H isoform X2 [Manduca sexta]	-1.6465	-0.5427	0.21692	1.01924	0.95312
TRINITY_DN10110_c1_g2_i1_orf1	venom allergen 3-like [Ostrinia furnacalis]	-1.644	-0.6571	0.83929	0.51602	0.94586
TRINITY_DN8133_c0_g1_i4_orf1	protein sel-1 homolog 1 isoform X2 [Ostrinia furnacalis]	-1.8005	-0.2195	0.19255	0.95314	0.87433
TRINITY_DN19260_c0_g1_i5_orf1	probable 26S proteasome non-ATPase regulatory subunit 3 [Ostrinia furnacalis]	-1.4761	-0.9367	0.76368	0.80779	0.84136
TRINITY_DN66442_c0_g2_i3_orf1	ubiquitin-conjugating enzyme E2 G1 [Papilio polytes] >XP_013167680.1 PREDICTED: ubiquitin-conjugating enzyme E2 G1 isoform X2 [Papilio xuthus] >XP_014357225.1 ubiquitin-conjugating enzyme E2 G1 isoform X2 [Papilio machaon] >XP_021182486.1 ubiquitin-conjugating enzyme E2 G1 isoform X2 [Helicoverpa armigera] >XP_022831375.1 ubiquitin-conjugating enzyme E2 G1 isoform X2 [Spodoptera litura] >XP_023943756.1 ubiquitin-conjugating enzyme E2 G1 isoform X2 [Bicyclus anynana] >XP_026488925.1 ubiquitin-conjugating enzyme E2 G1 isoform X2 [Vanessa tameamea] >XP_026740300.1 ubiquitin-conjugating enzyme E2 G1-like isoform X2 [Trichoplusia ni] >XP_026754924.1 ubiquitin-conjugating enzyme E2 G1 isoform X2 [Galleria mellonella] >XP_028172940.1 ubiquitin-conjugating enzyme E2 G1 isoform X2 [Ostrinia furnacalis] >XP_030034967.1 ubiquitin-conjugating enzyme E2 G1 isoform X2 [Manduca sexta] >XP_032526880.1 ubiquitin-conjugating enzyme E2 G1 isoform X2 [Danaus plexippus plexippus] >XP_034828280.1 ubiquitin-conjugating enzyme E2 G1 isoform X1 [Maniola hyperantus] >XP_035448963.1 ubiquitin-conjugating enzyme E2 G1-like isoform X2 [Spodoptera frugiperda] >XP_039752058.1 ubiquitin-conjugating enzyme E2 G1 isoform X2 [Pararge aegeria] >XP_041971665.1 ubiquitin-conjugating enzyme E2 G1 isoform X2 [Aricia agestis] >XP_045454385.1 ubiquitin-conjugating enzyme E2 G1 isoform X1 [Melitaea cinxia] >XP_045771256.1 ubiquitin-conjugating enzyme E2 G1 isoform X1 [Maniola jurtina] >XP_046964023.1 ubiquitin-conjugating enzyme E2 G1 isoform X2 [Vanessa cardui] >XP_047032842.1 ubiquitin-conjugating enzyme E2 G1 isoform X2 [Helicoverpa zea] >XP_047537963.1 ubiquitin-conjugating enzyme E2 G1 isoform X2 [Vanessa atalanta] >XP_047999421.1 ubiquitin-conjugating enzyme E2 G1 isoform X2 [Leguminivora glycinivorella] >XP_048482262.1 ubiquitin-conjugating enzyme E2 G1 isoform X2 [Plutella xylostella] >XP_049879261.1 ubiquitin-conjugating enzyme E2 G1 isoform X2 [Pectinophora gossypiella] >XP_050353097.1 ubiquitin-conjugating enzyme E2 G1 isoform X2 [Nymphalis io] >RVE50249.1 hypothetical protein evm_005084 [Chilo suppressalis] >CAB3220594.1 unnamed protein product [Arctia plantaginis] >CAH0595405.1 unnamed protein product [Chrysodeixis includens] >CAH0713838.1 unnamed protein product, partial [Brenthis ino] >KAG6461453.1 hypothetical protein O3G_MSEX012634 [Manduca sexta]	-1.6304	-0.6462	1.11315	0.64571	0.51778
TRINITY_DN95056_c0_g2_i2_orf1	40S ribosomal protein S18 [Halotydeus destructor]	-1.9836	0.32086	0.66793	0.60105	0.39375
TRINITY_DN130778_c0_g1_i1_orf1	26S proteasome non-ATPase regulatory subunit 8 [Ostrinia furnacalis]	-1.7061	-0.5699	0.64846	0.91288	0.71462
TRINITY_DN3335_c0_g1_i1_orf1	unnamed protein product [Pieris macdunnoughi]	-1.5196	-0.8372	1.02287	0.50244	0.83144
TRINITY_DN27398_c0_g1_i3_orf1	lissencephaly-1 homolog [Helicoverpa armigera] >XP_021185428.1 lissencephaly-1 homolog [Helicoverpa armigera] >XP_047030140.1 lissencephaly-1 homolog [Helicoverpa zea] >XP_047030141.1 lissencephaly-1 homolog [Helicoverpa zea]	-1.1197	-1.3126	0.65681	0.97683	0.79864
TRINITY_DN57_c0_g2_i3_orf1	transcriptional repressor CTCF-like [Ostrinia furnacalis] >XP_028163401.1 transcriptional repressor CTCF-like [Ostrinia furnacalis]	-1.7372	-0.4115	0.73154	1.07848	0.33862
TRINITY_DN41664_c0_g1_i4_orf1	uncharacterized protein LOC114356631 [Ostrinia furnacalis]	-1.409	-0.9541	0.87992	1.08079	0.40242
TRINITY_DN198_c0_g1_i2_orf1	retinol dehydrogenase 13-like [Ostrinia furnacalis]	-1.2198	-1.205	1.04043	0.59399	0.79039
TRINITY_DN5346_c0_g1_i5_orf1	syntaxin-1A isoform X2 [Pectinophora gossypiella]	-1.209	-1.2003	0.49038	1.04862	0.87033
TRINITY_DN3597_c0_g1_i10_orf1	unnamed protein product [Diatraea saccharalis]	-1.6576	-0.5	0.30892	1.23923	0.60941
TRINITY_DN24323_c0_g1_i3_orf1	ubiquitin-protein ligase E3A [Ostrinia furnacalis]	-1.7512	-0.3201	0.23779	1.13278	0.70077
TRINITY_DN34479_c0_g1_i2_orf1	PREDICTED: 26S protease regulatory subunit 4 [Amyelois transitella] >XP_021186380.1 26S proteasome regulatory subunit 4 [Helicoverpa armigera] >XP_022116536.1 26S proteasome regulatory subunit 4 [Pieris rapae] >XP_022817854.1 26S proteasome regulatory subunit 4 [Spodoptera litura] >XP_026745369.1 26S proteasome regulatory subunit 4 [Trichoplusia ni] >XP_026760570.1 26S proteasome regulatory subunit 4 [Galleria mellonella] >XP_028176505.1 26S proteasome regulatory subunit 4 [Ostrinia furnacalis] >XP_030038234.1 26S proteasome regulatory subunit 4 [Manduca sexta] >XP_035449919.1 26S proteasome regulatory subunit 4 [Spodoptera frugiperda] >XP_038206559.1 26S proteasome regulatory subunit 4 [Zerene cesonia] >XP_045502541.1 26S proteasome regulatory subunit 4 [Colias croceus] >XP_045532999.1 26S proteasome regulatory subunit 4 [Pieris brassicae] >XP_047033702.1 26S proteasome regulatory subunit 4 [Helicoverpa zea] >XP_047994509.1 26S proteasome regulatory subunit 4 [Leguminivora glycinivorella] >XP_049877826.1 26S proteasome regulatory subunit 4 [Pectinophora gossypiella] >KAH9639287.1 hypothetical protein HF086_014151 [Spodoptera exigua] >KAI5631153.1 ATPase family associated with various cellular activities (AAA) domain-containing protein [Phthorimaea operculella] >RVE50066.1 hypothetical protein evm_005272 [Chilo suppressalis] >CAB3245712.1 unnamed protein product [Arctia plantaginis] >KAF9801312.1 hypothetical protein SFRURICE_000406 [Spodoptera frugiperda]	-1.4898	-0.8697	0.73405	1.09943	0.52597
TRINITY_DN6567_c0_g1_i5_orf1	dymeclin isoform X1 [Ostrinia furnacalis] >XP_028159584.1 dymeclin isoform X2 [Ostrinia furnacalis]	-1.737	-0.2031	0.17794	1.30383	0.4583
TRINITY_DN14347_c0_g1_i1_orf1	putative nuclease HARBI1 [Ostrinia furnacalis]	-1.9671	0.15653	0.69603	0.54349	0.57108
TRINITY_DN49047_c0_g1_i2_orf1	unnamed protein product [Parnassius apollo]	-1.6794	-0.5963	0.59295	1.00968	0.67305
TRINITY_DN2224_c0_g2_i1_orf1	tumor susceptibility gene 101 protein [Ostrinia furnacalis]	-1.7206	-0.2085	0.16291	1.33572	0.43051
TRINITY_DN4741_c0_g1_i4_orf1	PREDICTED: 23 kDa integral membrane protein-like [Papilio xuthus]	-1.6083	-0.5502	1.17525	0.84247	0.14075
TRINITY_DN105359_c0_g2_i5_orf1	uncharacterized protein LOC114359499 [Ostrinia furnacalis]	-1.6942	-0.532	0.40598	0.79801	1.02222
TRINITY_DN6602_c0_g1_i4_orf1	PREDICTED: E3 ubiquitin-protein ligase RNF181-like [Amyelois transitella]	-1.7668	-0.3672	0.34035	0.9943	0.7994
TRINITY_DN152_c0_g1_i4_orf1	LOW QUALITY PROTEIN: protein tyrosine phosphatase type IVA 3 [Ostrinia furnacalis]	-1.6272	-0.5532	0.68343	1.22746	0.26945
TRINITY_DN11125_c0_g1_i1_orf1	LOW QUALITY PROTEIN: nuclear receptor-binding protein homolog [Ostrinia furnacalis]	-1.8593	0.08239	0.16139	1.12812	0.48739
TRINITY_DN31431_c0_g1_i1_orf1	carnosine N-methyltransferase [Ostrinia furnacalis]	-1.8085	-0.225	0.42392	1.12375	0.48585
TRINITY_DN10558_c0_g1_i4_orf1	unnamed protein product [Chrysodeixis includens]	-1.25	-1.1423	0.41533	0.93463	1.04237
TRINITY_DN92153_c0_g2_i2_orf1	methylenetetrahydrofolate reductase [Ostrinia furnacalis]	-1.3785	-0.9256	0.86269	1.20021	0.24122
TRINITY_DN25916_c0_g1_i1_orf1	uncharacterized protein LOC125063950 [Vanessa atalanta]	-1.4978	-0.7958	0.66733	1.23467	0.39166
TRINITY_DN38424_c0_g1_i1_orf1	glucose dehydrogenase [FAD, quinone]-like [Ostrinia furnacalis]	-1.7053	-0.518	0.41888	1.00217	0.80229
TRINITY_DN12227_c0_g2_i3_orf1	exonuclease 3'-5' domain-containing protein 2 [Ostrinia furnacalis]	-1.6725	-0.5784	0.55443	1.09503	0.60142
TRINITY_DN3702_c0_g1_i1_orf1	U4/U6.U5 tri-snRNP-associated protein 2 [Ostrinia furnacalis]	-1.7947	-0.1523	0.36501	1.22194	0.36
TRINITY_DN4571_c0_g1_i4_orf1	PREDICTED: nuclear factor NF-kappa-B p105 subunit [Microplitis demolitor] >KAG6558391.1 viral ankyrin V1 [Microplitis demolitor]	-1.2923	0.1601	-0.7996	0.33373	1.59802
TRINITY_DN36987_c0_g1_i1_orf1	basement membrane-specific heparan sulfate proteoglycan core protein isoform X13 [Ostrinia furnacalis]	-1.6387	0.60678	-0.4326	0.14623	1.31828
TRINITY_DN44633_c0_g1_i4_orf1	LDLR chaperone boca [Ostrinia furnacalis]	-0.5829	0.62914	-1.5009	0.03684	1.41786
TRINITY_DN49872_c0_g1_i2_orf1	probable cytosolic iron-sulfur protein assembly protein Ciao1 [Ostrinia furnacalis]	-0.6085	0.27794	-1.4056	0.13685	1.59936

TRINITY_DN33089_c0_g1_i1_orf1	nucleoporin NDC1 [Ostrinia furnacalis]	-0.8866	0.10321	-1.0845	0.13322	1.73473
TRINITY_DN4456_c0_g1_i1_orf1	uncharacterized protein LOC114353382 isoform X1 [Ostrinia furnacalis]	-1.4212	0.89173	-0.5947	-0.2124	1.33654
TRINITY_DN2201_c0_g1_i1_orf1	pleiotropic regulator 1 [Ostrinia furnacalis]	-0.7699	0.63442	-1.473	0.28406	1.32447
TRINITY_DN6362_c0_g1_i4_orf1	sodium/hydrogen exchanger 7 isoform X4 [Galleria mellonella]	-0.9858	0.41692	-1.2375	0.31527	1.49114
TRINITY_DN10619_c0_g5_i7_orf1	protein bunched, class 2/F/G isoform-like isoform X2 [Ostrinia furnacalis]	-1.0437	0.35846	-1.1038	0.20046	1.58859
TRINITY_DN51829_c0_g1_i1_orf1	FAS-associated factor 2 [Ostrinia furnacalis]	-1.7486	0.56182	-0.2409	0.18961	1.23807
TRINITY_DN11566_c0_g1_i6_orf1	lens fiber major intrinsic protein-like isoform X1 [Ostrinia furnacalis]	-0.9377	0.76096	-1.3833	0.32666	1.23343
TRINITY_DN5829_c0_g2_i1_orf1	uncharacterized protein LOC114365758 isoform X2 [Ostrinia furnacalis]	-1.2049	0.10551	-0.9025	0.40148	1.60042
TRINITY_DN9592_c0_g1_i2_orf1	coatomer subunit delta [Ostrinia furnacalis]	-1.3903	0.77089	-0.8746	0.20313	1.29093
TRINITY_DN9661_c0_g1_i1_orf1	tetratricopeptide repeat protein 37 [Ostrinia furnacalis]	-1.3473	1.26979	-0.9905	0.43134	0.63665
TRINITY_DN4401_c0_g2_i1_orf1	hypothetical protein evm_003554 [Chilo suppressalis]	-0.8593	0.64464	-1.4056	0.28225	1.33809
TRINITY_DN15736_c0_g1_i2_orf1	uncharacterized protein LOC114355289 isoform X3 [Ostrinia furnacalis] >XP_028163907.1 uncharacterized protein LOC114355289 isoform X3 [Ostrinia furnacalis] >XP_028163914.1 uncharacterized protein LOC114355289 isoform X3 [Ostrinia furnacalis] >XP_028163920.1 uncharacterized protein LOC114355289 isoform X3 [Ostrinia furnacalis]	-0.6159	0.37599	-1.6185	0.6716	1.18684
TRINITY_DN10415_c0_g1_i5_orf1	hypothetical protein evm_000184 [Chilo suppressalis]	-1.6694	0.75514	-0.5498	0.36515	1.09883
TRINITY_DN4732_c0_g1_i2_orf1	reversion-inducing cysteine-rich protein with Kazal motifs [Ostrinia furnacalis]	-0.6773	0.71414	-1.6449	0.67687	0.93126
TRINITY_DN14019_c0_g1_i5_orf1	hypothetical protein evm_009768 [Chilo suppressalis]	-0.7174	0.15388	-1.4833	0.73439	1.31237
TRINITY_DN21125_c0_g1_i1_orf1	protein angel homolog 1 isoform X3 [Ostrinia furnacalis]	-1.5288	0.62947	-0.805	0.56427	1.14014
TRINITY_DN7247_c0_g1_i6_orf1	pyruvate kinase-like isoform X2 [Ostrinia furnacalis]	-1.2527	1.65926	-0.7448	0.35026	-0.012
TRINITY_DN111_c0_g2_i2_orf1	hypothetical protein O3G_MSEX007696 [Manduca sexta] >KAG6452639.1 hypothetical protein O3G_MSEX007696 [Manduca sexta]	-1.6492	1.10919	-0.6035	0.69763	0.44591
TRINITY_DN11566_c2_g1_i2_orf1	transmembrane protein 18 isoform X1 [Ostrinia furnacalis]	-1.5987	0.53386	-0.7185	0.72964	1.05376
TRINITY_DN36496_c0_g1_i1_orf1	unnamed protein product [Parnassius apollo]	-1.1277	1.14323	-1.0892	1.11062	-0.0369
TRINITY_DN1999_c0_g1_i9_orf1	acyl-CoA Delta(11) desaturase-like [Ostrinia furnacalis] >XP_028172986.1 acyl-CoA Delta(11) desaturase-like [Ostrinia furnacalis] >AAL27034.1 acyl-CoA delta-9 desaturase [Ostrinia furnacalis] >AAL29454.1 acyl-CoA delta-9 desaturase [Ostrinia nubilalis]	-1.0523	0.94348	-0.9679	1.40037	-0.3237
TRINITY_DN33418_c0_g1_i1_orf1	homer protein homolog 2-like [Ostrinia furnacalis]	-1.3294	-0.8527	0.02018	1.37092	0.79102
TRINITY_DN21623_c0_g2_i1_orf1	Chlorophyll a-b binding protein, chloroplastic [Trichinella nelsoni]	-0.8884	0.05707	-1.3236	0.81913	1.33585
TRINITY_DN3499_c0_g1_i8_orf1	modular serine protease-like [Ostrinia furnacalis]	-0.4254	-1.3758	-0.3179	0.52118	1.59796
TRINITY_DN110534_c0_g1_i3_orf1	unnamed protein product [Euphydryas editha]	-0.6304	-1.1597	-0.4191	0.5383	1.67097
TRINITY_DN10745_c0_g1_i14_orf1	septin-1 [Ostrinia furnacalis]	-0.799	-0.5793	-0.9232	0.63822	1.66325
TRINITY_DN73923_c0_g1_i1_orf1	protein obstructor-E-like [Ostrinia furnacalis]	-0.7678	-0.7363	-0.8734	0.81498	1.56249
TRINITY_DN5235_c0_g1_i7_orf1	peptidoglycan-recognition protein SA-like [Ostrinia furnacalis]	-0.7522	-0.4507	-0.9978	0.46261	1.73819
TRINITY_DN286_c0_g1_i2_orf1	uncharacterized protein LOC114361329 [Ostrinia furnacalis]	-0.661	-0.5426	-1.0457	0.55637	1.69285
TRINITY_DN15256_c0_g1_i8_orf1	pre-mRNA-splicing regulator female-lethal(2)D [Ostrinia furnacalis]	-1.3245	-0.1466	-0.7996	1.07649	1.19416
TRINITY_DN5211_c0_g1_i1_orf1	elongation of very long chain fatty acids protein AAEL008004-like [Ostrinia furnacalis]	-0.9413	-0.7607	-0.6168	0.67653	1.64229
TRINITY_DN12951_c1_g1_i5_orf1	ADP-ribosylation factor-like protein 2 isoform X1 [Hyposmocoma kahamanoa]	-1.7679	0.02531	-0.1562	0.79668	1.10213
TRINITY_DN7516_c0_g2_i1_orf1	serine/threonine-protein kinase 10-like, partial [Ostrinia furnacalis]	-0.7329	-0.2382	-1.2634	0.72143	1.51307
TRINITY_DN2840_c0_g1_i5_orf1	hypothetical protein evm_002181 [Chilo suppressalis]	-0.8062	-0.656	-0.8592	0.66718	1.65421
TRINITY_DN1133_c0_g1_i6_orf1	zinc finger protein 391-like [Ostrinia furnacalis] >XP_028169193.1 zinc finger protein 391-like [Ostrinia furnacalis]	-1.0256	-0.4159	-0.6866	0.34322	1.78491
TRINITY_DN12771_c0_g1_i1_orf1	histone acetyltransferase type B catalytic subunit [Ostrinia furnacalis]	-0.0325	-1.3103	-0.826	0.73246	1.43641
TRINITY_DN17003_c0_g1_i1_orf1	mucin-5AC [Ostrinia furnacalis]	-0.9257	-0.884	-0.4724	0.62395	1.65808
TRINITY_DN2474_c0_g1_i5_orf1	glucose-6-phosphate 1-epimerase [Galleria mellonella]	-0.2361	-1.2783	-0.6091	0.47666	1.64683
TRINITY_DN2591_c0_g1_i4_orf1	26S proteasome non-ATPase regulatory subunit 13 isoform X1 [Ostrinia furnacalis]	-0.6833	-1.2085	-0.193	0.38575	1.69901
TRINITY_DN1313_c0_g1_i2_orf1	39S ribosomal protein L40, mitochondrial [Ostrinia furnacalis]	-0.396	-1.0424	-0.8942	0.82255	1.51007
TRINITY_DN6130_c0_g1_i6_orf1	tryptophan--tRNA ligase, mitochondrial [Ostrinia furnacalis]	-1.1021	-0.4752	-0.8033	0.98977	1.39087
TRINITY_DN42337_c0_g1_i6_orf1	cuticle protein 8-like [Leguminivora glycinivorella]	-1.0084	-0.4185	-0.7463	0.4195	1.75362
TRINITY_DN73224_c0_g4_i2_orf1	PREDICTED: poly(rC)-binding protein 3 isoform X2 [Vollenhovia emeryi]	-0.8232	-0.5816	-0.8913	0.62198	1.67414
TRINITY_DN27723_c0_g1_i1_orf1	putative uncharacterized protein DDB_G0282133 isoform X1 [Ostrinia furnacalis]	-1.0764	-0.313	-0.7647	0.42957	1.72456
TRINITY_DN1616_c0_g1_i3_orf1	U4/U6 small nuclear ribonucleoprotein Prp3 isoform X1 [Ostrinia furnacalis] >XP_028161035.1 U4/U6 small nuclear ribonucleoprotein Prp3 isoform X2 [Ostrinia furnacalis] >XP_028161037.1 U4/U6 small nuclear ribonucleoprotein Prp3 isoform X3 [Ostrinia furnacalis]	-0.6077	-1.127	-0.5213	0.6067	1.64941
TRINITY_DN12769_c0_g1_i5_orf1	uncharacterized protein LOC114365633 [Ostrinia furnacalis]	-1.3353	-0.3895	-0.5635	0.88953	1.39876
TRINITY_DN21124_c0_g1_i4_orf1	calsyntenin-1 [Ostrinia furnacalis]	-0.7804	-0.7098	-0.8438	0.69195	1.64206
TRINITY_DN98814_c0_g1_i2_orf1	PREDICTED: chaoptin [Amyelois transitella]	-0.3896	-1.068	-0.7509	0.51035	1.69806
TRINITY_DN17655_c0_g1_i1_orf1	BRISC and BRCA1-A complex member 1-like [Ostrinia furnacalis]	-0.6585	-1.2353	-0.172	0.3707	1.69511
TRINITY_DN782_c0_g1_i5_orf1	regulator of gene activity isoform X3 [Ostrinia furnacalis]	-0.4745	-0.86	-1.008	0.79981	1.5426
TRINITY_DN19651_c0_g1_i1_orf1	cytosolic non-specific dipeptidase [Ostrinia furnacalis]	-0.0968	-1.2465	-0.8959	0.86787	1.37145
TRINITY_DN120593_c0_g1_i1_orf1	SUMO-activating enzyme subunit 1 [Ostrinia furnacalis]	-1.1043	-0.5945	-0.5237	0.5269	1.69562
TRINITY_DN9354_c0_g1_i7_orf1	hypothetical protein evm_012205 [Chilo suppressalis] >CAB3527181.1 unnamed protein product [Chilo suppressalis] >CAH0404510.1 unnamed protein product [Chilo suppressalis]	-0.4274	-1.3367	-0.3902	0.54897	1.60529
TRINITY_DN10479_c0_g1_i6_orf1	unnamed protein product [Chrysodeixis includens]	-1.0434	-0.4979	-0.6901	0.52515	1.70627
TRINITY_DN48641_c0_g1_i4_orf1	RNA-binding protein 45-like [Galleria mellonella]	-0.9484	-1.0886	-0.27	0.85224	1.45476
TRINITY_DN20215_c0_g2_i1_orf1	unnamed protein product [Spodoptera littoralis] >CAH1638553.1 unnamed protein product [Spodoptera littoralis]	-0.467	-1.1754	-0.7266	1.0028	1.36626
TRINITY_DN2450_c0_g1_i6_orf1	oxysterol-binding protein-related protein 9 [Manduca sexta]	-0.8465	-0.7685	-0.739	0.74341	1.6106
TRINITY_DN2936_c0_g1_i1_orf1	myosin heavy chain, non-muscle isoform X1 [Hyposmocoma kahamanoa]	-0.9602	-0.6667	-0.6953	0.68481	1.6374
TRINITY_DN47914_c0_g2_i1_orf1	UBX domain-containing protein 1-A-like [Ostrinia furnacalis]	-0.9218	-1.0655	-0.2904	0.72875	1.54901

TRINITY_DN1231_c0_g1_i4_orf1	AN1-type zinc finger protein 6 isoform X1 [Galleria mellonella]	-1.0861	-0.3189	-0.8061	0.54684	1.66429
TRINITY_DN9916_c0_g1_i1_orf1	PREDICTED: dynein light chain Tctex-type [Amyelois transitella] >XP_021195381.1 dynein light chain Tctex-type [Helicoverpa armigera] >XP_022815696.1 dynein light chain Tctex-type [Spodoptera litura] >XP_028156399.1 dynein light chain Tctex-type [Ostrinia furnacalis] >XP_035458261.1 dynein light chain Tctex-type-like [Spodoptera frugiperda] >XP_047034788.1 dynein light chain Tctex-type [Helicoverpa zea] >CAB3233358.1 unnamed protein product [Arctia plantaginis] >CAB3506583.1 unnamed protein product [Spodoptera littoralis] >CAG9754627.1 unnamed protein product [Diatraea saccharalis] >CAH0596395.1 unnamed protein product [Chrysodeixis includens] >KAF9808454.1 hypothetical protein SFRURICE_008507 [Spodoptera frugiperda]	-1.1065	-0.4623	-0.659	0.54546	1.68231
TRINITY_DN6436_c0_g1_i1_orf1	serine/threonine-protein kinase PAK 3 isoform X1 [Ostrinia furnacalis] >XP_028164178.1 serine/threonine-protein kinase PAK 3 isoform X2 [Ostrinia furnacalis] >XP_028164179.1 serine/threonine-protein kinase PAK 3 isoform X3 [Ostrinia furnacalis]	-0.7112	-1.0086	-0.5783	0.64813	1.64997
TRINITY_DN2874_c0_g1_i4_orf1	ATP-binding cassette sub-family G member 1-like [Ostrinia furnacalis]	-0.8608	-0.506	-1.0152	1.45112	0.9309
TRINITY_DN1601_c0_g1_i4_orf1	cytoplasmic dynein 1 intermediate chain isoform X4 [Ostrinia furnacalis]	-0.568	-0.7299	-1.0878	0.95213	1.43349
TRINITY_DN5004_c0_g1_i2_orf1	MOXD1 homolog 1-like [Ostrinia furnacalis]	-0.5167	-0.3144	-1.3897	0.76643	1.45443
TRINITY_DN6312_c0_g1_i1_orf1	cytochrome c oxidase assembly protein COX19 [Ostrinia furnacalis]	-1.2078	-0.2053	-0.8362	0.75724	1.49211
TRINITY_DN14443_c0_g1_i1_orf1	SUMO-activating enzyme subunit 2 [Ostrinia furnacalis]	-1.1279	-0.7377	-0.4032	0.66177	1.60715
TRINITY_DN59829_c0_g1_i1_orf1	putative mediator of RNA polymerase II transcription subunit 12 [Ostrinia furnacalis]	-0.9035	-0.8859	-0.5655	0.78609	1.56881
TRINITY_DN10174_c0_g1_i4_orf1	protein D2-like isoform X2 [Aricia agestis] >XP_041972210.1 protein D2-like isoform X2 [Aricia agestis]	-0.1914	-0.7397	-1.3301	0.91783	1.34336
TRINITY_DN8659_c0_g1_i1_orf1	ubiquitin-like modifier-activating enzyme 1 [Manduca sexta]	-0.9652	-0.9082	-0.5024	0.89739	1.47845
TRINITY_DN12576_c0_g1_i2_orf1	eukaryotic translation initiation factor 4E transporter-like isoform X5 [Hyposmocoma kahamanoa]	-1.0258	-0.6342	-0.7631	1.09421	1.32888
TRINITY_DN19584_c0_g1_i2_orf1	protein NDUFAF4 homolog [Ostrinia furnacalis]	-1.103	0.33938	-1.2574	1.16079	0.86017
TRINITY_DN38644_c0_g1_i1_orf1	unnamed protein product, partial [Iphiclides podalirius]	-0.9968	-0.0714	-1.1591	0.81572	1.41155
TRINITY_DN471_c0_g1_i6_orf1	ATP-dependent RNA helicase DBP2-A-like [Ostrinia furnacalis]	-0.4577	-1.3494	-0.5139	1.08344	1.23758
TRINITY_DN4156_c0_g1_i2_orf1	calcium channel flower [Ostrinia furnacalis]	-1.3609	-0.5993	-0.3001	0.86119	1.39902
TRINITY_DN20067_c0_g1_i6_orf1	hypothetical protein evm_010712 [Chilo suppressalis] >CAB3527462.1 unnamed protein product [Chilo suppressalis] >CAH0401768.1 unnamed protein product [Chilo suppressalis]	-0.847	-0.5555	-1.0009	0.99633	1.40701
TRINITY_DN11464_c0_g1_i3_orf1	unnamed protein product [Spodoptera littoralis] >CAH1635924.1 unnamed protein product [Spodoptera littoralis]	-0.9595	-0.8136	-0.6623	1.13619	1.29919
TRINITY_DN467_c4_g1_i2_orf1	GRIP and coiled-coil domain-containing protein 1 [Ostrinia furnacalis]	-0.872	-0.0583	-1.2391	0.66761	1.50173
TRINITY_DN6588_c0_g1_i4_orf1	uncharacterized protein LOC114350845 [Ostrinia furnacalis]	-1.1307	0.26684	-0.9156	1.67358	0.10583
TRINITY_DN14987_c0_g1_i3_orf1	hypothetical protein evm_009121 [Chilo suppressalis]	-1.3684	-0.79	-0.034	0.87372	1.31866
TRINITY_DN11188_c0_g1_i2_orf1	testin [Ostrinia furnacalis] >XP_028170617.1 testin [Ostrinia furnacalis]	-1.3885	-0.8078	0.01505	1.23278	0.94847
TRINITY_DN6163_c0_g1_i4_orf1	CTD nuclear envelope phosphatase 1 homolog [Ostrinia furnacalis]	-1.3741	-0.2642	-0.6497	1.10781	1.18014
TRINITY_DN32700_c0_g1_i2_orf1	ribosomal protein S6 kinase 2 beta [Ostrinia furnacalis]	-0.5731	-0.8752	-0.9475	0.93492	1.46078
TRINITY_DN44256_c0_g1_i1_orf1	essential MCU regulator, mitochondrial [Cotesia glomerata]	-1.0918	-0.3724	-0.7849	0.6127	1.63641
TRINITY_DN67649_c0_g1_i1_orf1	proliferating cell nuclear antigen [Ostrinia furnacalis] >XP_028174842.1 proliferating cell nuclear antigen [Ostrinia furnacalis]	-0.8546	-0.9449	-0.5729	0.84402	1.52844
TRINITY_DN1405_c0_g1_i1_orf1	cyclin-dependent kinase 10 isoform X1 [Ostrinia furnacalis] >XP_028178194.1 cyclin-dependent kinase 10 isoform X2 [Ostrinia furnacalis]	-1.1636	-0.8378	-0.302	0.83562	1.46784
TRINITY_DN24539_c0_g1_i4_orf1	low molecular weight phosphotyrosine protein phosphatase 1-like isoform X2 [Ostrinia furnacalis]	-0.7428	-1.2878	-0.2885	1.06588	1.25319
TRINITY_DN13118_c0_g1_i6_orf1	AP-1 complex subunit beta-1 [Helicoverpa armigera] >XP_021189434.2 AP-1 complex subunit beta-1 [Helicoverpa armigera]	-1.5178	-0.6527	0.07436	0.86374	1.23238
TRINITY_DN8553_c0_g1_i4_orf1	coiled-coil-helix-coiled-coil-helix domain-containing protein 7 isoform X2 [Ostrinia furnacalis]	-1.0115	-0.5783	-0.8254	1.35655	1.0587
TRINITY_DN33008_c0_g1_i1_orf1	double-stranded RNA-binding protein Staufen homolog 2 isoform X3 [Helicoverpa armigera]	-0.7939	-0.5077	-0.9901	0.64299	1.6487
TRINITY_DN2596_c0_g1_i2_orf1	rho guanine nucleotide exchange factor 11-like isoform X2 [Ostrinia furnacalis]	-1.5146	-0.1717	-0.5109	1.06687	1.13024
TRINITY_DN38211_c0_g1_i1_orf1	Golgi reassembly-stacking protein 2 [Ostrinia furnacalis]	-1.1028	-0.2694	-0.9581	0.96706	1.36317
TRINITY_DN1074_c0_g1_i7_orf1	eukaryotic translation initiation factor 4E type 2 [Ostrinia furnacalis]	-1.0795	-0.5486	-0.6957	0.74375	1.58005
TRINITY_DN7603_c0_g1_i5_orf1	tetratricopeptide repeat protein 1-like [Ostrinia furnacalis]	-1.107	-0.8457	-0.387	0.87622	1.46348
TRINITY_DN41108_c0_g1_i1_orf1	coatomer subunit epsilon-like [Ostrinia furnacalis]	-1.0222	-0.0532	-1.1805	1.21245	1.04343
TRINITY_DN8701_c0_g1_i3_orf1	putative uncharacterized protein DDB_G0282133 [Ostrinia furnacalis]	-1.5552	-0.0421	-0.4973	0.78371	1.31081
TRINITY_DN64769_c0_g1_i3_orf1	procollagen-lysine,2-oxoglutarate 5-dioxygenase isoform X2 [Ostrinia furnacalis]	-0.9903	-0.3496	-0.9736	0.78737	1.52617
TRINITY_DN51737_c0_g1_i3_orf1	N-alpha-acetyltransferase 40 [Ostrinia furnacalis]	-0.8915	-0.6789	-0.8467	0.97785	1.4392
TRINITY_DN1921_c1_g1_i5_orf1	hypothetical protein evm_002627 [Chilo suppressalis] >CAB3527269.1 unnamed protein product [Chilo suppressalis]	-0.7254	-0.787	-0.9316	1.22575	1.21824
TRINITY_DN25686_c0_g1_i4_orf1	exocyst complex component 3 [Ostrinia furnacalis]	-0.8595	-0.6843	-0.8984	1.1787	1.26353
TRINITY_DN252_c0_g1_i3_orf1	probable phospholipid-transporting ATPase IM [Ostrinia furnacalis]	-0.8615	-1.0566	-0.3337	0.62882	1.6231
TRINITY_DN1552_c0_g1_i3_orf1	casein kinase II subunit alpha isoform X3 [Galleria mellonella]	-0.9361	-1.0795	-0.3622	1.19655	1.18128
TRINITY_DN13287_c0_g1_i5_orf1	dystrophin-like, partial [Ostrinia furnacalis]	-1.2892	0.35559	-1.0587	1.22639	0.76592
TRINITY_DN34745_c0_g2_i1_orf1	GSK3-beta interaction protein-like [Galleria mellonella]	-1.0273	-0.5827	-0.7347	0.77465	1.57009
TRINITY_DN24469_c0_g2_i2_orf1	unnamed protein product, partial [Brenthis ino]	-0.9513	-0.5188	-0.9071	0.89057	1.4866
TRINITY_DN4159_c1_g1_i1_orf1	F-actin-capping protein subunit beta [Ostrinia furnacalis]	-1.144	-1.0748	-0.0254	1.21434	1.02992
TRINITY_DN14298_c0_g1_i1_orf1	kinesin heavy chain [Ostrinia furnacalis]	-1.3206	-0.711	-0.2645	0.99878	1.2973

TRINITY_DN23946_c0_g1_i1_orf1	ubiquitin-conjugating enzyme E2L [Bombyx mori] >XP_013145013.1 PREDICTED: ubiquitin-conjugating enzyme E2 L3 [Papilio polytes] >XP_013145020.1 PREDICTED: ubiquitin-conjugating enzyme E2 L3 [Papilio polytes] >XP_013167448.1 PREDICTED: ubiquitin-conjugating enzyme E2 L3 [Papilio xuthus] >XP_013167449.1 PREDICTED: ubiquitin-conjugating enzyme E2 L3 [Papilio xuthus] >XP_014356324.1 ubiquitin-conjugating enzyme E2 L3 [Papilio machaon] >XP_021182538.1 ubiquitin-conjugating enzyme E2 L3 [Helicoverpa armigera] >XP_022831771.1 ubiquitin-conjugating enzyme E2 L3 [Spodoptera litura] >XP_023943862.1 ubiquitin-conjugating enzyme E2 L3 [Bicyclus anynana] >XP_026501320.1 ubiquitin-conjugating enzyme E2 L3 [Vanessa tameamea] >XP_026740672.1 ubiquitin-conjugating enzyme E2 L3 [Trichoplusia ni] >XP_030021931.1 ubiquitin-conjugating enzyme E2 L3 [Manduca sexta] >XP_035448309.1 ubiquitin-conjugating enzyme E2 L3 [Spodoptera frugiperda] >XP_038214502.1 ubiquitin-conjugating enzyme E2 L3 [Zerene cesonia] >XP_045453873.1 ubiquitin-conjugating enzyme E2 L3 [Melitaea cinxia] >XP_045503683.1 ubiquitin-conjugating enzyme E2 L3 [Colias croceus] >XP_045771134.1 ubiquitin-conjugating enzyme E2 L3 [Maniola jurtina] >XP_046963941.1 ubiquitin-conjugating enzyme E2 L3 [Vanessa cardui] >XP_047032812.1 ubiquitin-conjugating enzyme E2 L3 [Helicoverpa zea] >XP_047538650.1 ubiquitin-conjugating enzyme E2 L3 [Vanessa atalanta] >XP_050352992.1 ubiquitin-conjugating enzyme E2 L3 [Nymphalis io] >KAF9417333.1 hypothetical protein HW555_005549 [Spodoptera exigua] >CAB3511518.1 unnamed protein product [Spodoptera littoralis] >CAH0595358.1 unnamed protein product [Chrysodeixis includens] >ABB36655.1 ubiquitin-conjugating enzyme E2L [Bombyx mori] >ABF51360.1 ubiquitin-conjugating enzyme [Bombyx mori]	-0.7467	-0.8286	-0.8717	1.25849	1.18842
TRINITY_DN21559_c0_g2_i1_orf1	protein bicaudal D isoform X3 [Galleria mellonella]	-0.7024	-0.6901	-1.0375	1.18586	1.24411
TRINITY_DN3953_c0_g1_i2_orf1	TP53-binding protein 1-like [Ostrinia furnacalis]	-0.4837	-0.9792	-0.9274	0.98281	1.40753
TRINITY_DN28759_c0_g1_i1_orf1	innexin inx2 [Ostrinia furnacalis]	-1.0363	-0.4491	-0.8971	0.98902	1.3934
TRINITY_DN70409_c0_g1_i3_orf1	tyrosine-protein phosphatase non-receptor type 9 isoform X3 [Ostrinia furnacalis]	-1.5626	-0.6642	0.40246	1.29361	0.53072
TRINITY_DN12858_c0_g1_i5_orf1	unnamed protein product, partial [Iphiclides podalirius]	-1.1376	-0.8698	-0.3448	0.99682	1.3553
TRINITY_DN9146_c0_g1_i1_orf1	drebrin-like protein [Ostrinia furnacalis]	-0.9674	-0.8075	-0.6632	1.2249	1.21321
TRINITY_DN14477_c0_g1_i12_orf1	PREDICTED: phosphoribosyl pyrophosphate synthase-associated protein 2 isoform X2 [Amyelois transitella]	-0.5907	-0.6094	-1.1913	1.16886	1.22245
TRINITY_DN28428_c0_g1_i2_orf1	unnamed protein product [Chrysodeixis includens]	-1.4865	-0.4863	-0.2375	0.93955	1.2707
TRINITY_DN28938_c0_g1_i1_orf1	uncharacterized protein LOC114354070 isoform X3 [Ostrinia furnacalis]	-1.5021	-0.5908	0.35416	1.48486	0.25385
TRINITY_DN277_c1_g1_i1_orf1	uncharacterized protein LOC114363802 isoform X4 [Ostrinia furnacalis]	-1.6951	-0.0473	-0.2168	0.69836	1.26086
TRINITY_DN12134_c0_g1_i4_orf1	glutathione S-transferase 1-1 [Ostrinia furnacalis] >XP_028161942.1 glutathione S-transferase 1-1 [Ostrinia furnacalis] >XP_028161943.1 glutathione S-transferase 1-1 [Ostrinia furnacalis]	-1.3644	-0.3776	-0.4869	0.74393	1.485
TRINITY_DN2160_c0_g1_i13_orf1	unnamed protein product [Spodoptera exigua]	-0.9994	-0.4935	-0.9132	1.32306	1.08311
TRINITY_DN2172_c0_g2_i8_orf1	hypothetical protein evm_003685 [Chilo suppressalis]	-1.5516	-0.6176	0.10032	0.86022	1.2087
TRINITY_DN1554_c0_g1_i9_orf1	LOW QUALITY PROTEIN: puff-specific protein Bx42 [Ostrinia furnacalis]	-0.512	-0.8679	-1.0344	1.18985	1.22435
TRINITY_DN4808_c0_g1_i3_orf1	kinesin light chain [Ostrinia furnacalis]	-1.1353	-0.9494	-0.2482	1.0505	1.28242
TRINITY_DN5834_c0_g1_i2_orf1	CD2 antigen cytoplasmic tail-binding protein 2 homolog [Ostrinia furnacalis]	-0.7929	-0.418	-1.1231	1.49226	0.84169
TRINITY_DN21559_c0_g1_i2_orf1	protein bicaudal D [Ostrinia furnacalis]	-1.4131	-0.4604	-0.3806	1.3571	0.89701
TRINITY_DN6239_c0_g1_i1_orf1	eukaryotic translation initiation factor 6 [Ostrinia furnacalis]	-0.4174	-1.0188	-0.9579	1.14951	1.24456
TRINITY_DN28622_c0_g1_i1_orf1	actin-related protein 3 [Ostrinia furnacalis]	-1.1903	-0.6715	-0.5185	1.06613	1.31412
TRINITY_DN41736_c0_g2_i1_orf1	calcyclin-binding protein [Ostrinia furnacalis]	-0.7041	-0.9123	-0.8121	1.39246	1.03607
TRINITY_DN50225_c0_g1_i1_orf1	SRSF protein kinase 3 [Galleria mellonella]	-0.812	-1.0562	-0.5475	1.26827	1.14749
TRINITY_DN13384_c0_g1_i1_orf1	26S proteasome regulatory subunit S3 [Aphelinus abdominalis]	-1.8105	0.31719	-0.1682	1.15716	0.5043
TRINITY_DN934_c2_g1_i7_orf1	ubiquitin-40S ribosomal protein S27a [Ostrinia furnacalis]	-1.1889	-0.7396	-0.4524	1.21232	1.16847
TRINITY_DN113778_c0_g2_i1_orf1	metastasis-associated protein MTA3 [Galleria mellonella]	-1.5219	-0.6445	0.08799	0.81493	1.26352
TRINITY_DN2984_c0_g1_i3_orf1	connectin-like [Ostrinia furnacalis]	-1.1152	-0.7765	-0.5124	1.18247	1.22168
TRINITY_DN4494_c0_g1_i1_orf1	venom serine carboxypeptidase [Ostrinia furnacalis]	-1.0325	-1.1916	0.03876	0.84329	1.34211
TRINITY_DN89829_c0_g1_i1_orf1	PREDICTED: ubiquitin-conjugating enzyme E2 T [Microplitis demolitor]	-1.1785	-0.8002	-0.3785	1.33535	1.0219
TRINITY_DN36061_c0_g4_i2_orf1	putative GPI-anchored protein pfl2 [Ostrinia furnacalis] >XP_028163002.1 putative GPI-anchored protein pfl2 [Ostrinia furnacalis]	-0.7428	-0.7998	-0.8739	0.95821	1.45831
TRINITY_DN12767_c0_g1_i2_orf1	coatomer subunit alpha [Ostrinia furnacalis]	-1.4124	-0.2338	-0.5783	1.37848	0.84604
TRINITY_DN1528_c0_g1_i4_orf1	uncharacterized protein LOC114353202 [Ostrinia furnacalis]	-1.3418	-0.2234	-0.6491	0.73503	1.47924
TRINITY_DN518_c0_g1_i1_orf1	unnamed protein product [Arctia plantaginis]	-1.0733	-0.7563	-0.5892	1.25132	1.1675
TRINITY_DN9765_c0_g1_i6_orf1	hypothetical protein evm_005049 [Chilo suppressalis] >CAB3525510.1 unnamed protein product [Chilo suppressalis] >CAH0402837.1 unnamed protein product [Chilo suppressalis]	-1.2969	-0.742	-0.2675	1.0182	1.28814
TRINITY_DN42171_c0_g1_i1_orf1	amyloid-beta-like protein isoform X1 [Manduca sexta] >AAY25024.3 beta amyloid protein precursor-like protein [Manduca sexta]	-1.0483	-0.7564	-0.6016	1.01318	1.39305
TRINITY_DN14572_c0_g1_i1_orf1	ras-related protein Rab-11A [Ostrinia furnacalis]	-1.4293	-0.4573	-0.3619	1.33541	0.91308
TRINITY_DN20767_c0_g2_i1_orf1	glycosylated lysosomal membrane protein B-like [Vanessa atalanta]	-1.0848	-0.6623	-0.6009	0.80863	1.53935
TRINITY_DN4439_c0_g2_i1_orf1	unnamed protein product, partial [Brenthis ino]	-1.1544	-0.6742	-0.5577	1.02798	1.35833
TRINITY_DN103118_c0_g1_i4_orf1	hypothetical protein evm_006930 [Chilo suppressalis]	-1.7419	0.3255	-0.419	0.9054	0.92991
TRINITY_DN41259_c0_g1_i6_orf1	endocuticle structural glycoprotein SgAbd-8 [Ostrinia furnacalis]	-0.855	-0.7657	-0.8041	0.99293	1.43186
TRINITY_DN14298_c0_g3_i1_orf1	kinesin heavy chain [Ostrinia furnacalis]	-1.4649	-0.6553	-0.0534	0.91447	1.25916
TRINITY_DN12320_c0_g1_i1_orf1	brefeldin A-inhibited guanine nucleotide-exchange protein 1 [Ostrinia furnacalis]	-1.1505	-0.7013	-0.418	1.60058	0.66927
TRINITY_DN13139_c0_g1_i1_orf1	AP-1 complex subunit mu-1 [Ostrinia furnacalis]	-0.7906	-0.5406	-1.031	1.51992	0.84233
TRINITY_DN4439_c0_g1_i2_orf1	cytoplasmic FMR1-interacting protein isoform X1 [Ostrinia furnacalis] >XP_028169436.1 cytoplasmic FMR1-interacting protein isoform X2 [Ostrinia furnacalis]	-0.9859	-1.0884	-0.2753	1.23427	1.11537
TRINITY_DN802_c0_g1_i2_orf1	active breakpoint cluster region-related protein [Ostrinia furnacalis]	-0.9137	-1.1427	-0.2911	1.27153	1.07602
TRINITY_DN2812_c0_g1_i5_orf1	myotubularin-related protein 2 [Ostrinia furnacalis] >XP_028170267.1 myotubularin-related protein 2 [Ostrinia furnacalis]	-0.6807	-1.1436	-0.5577	1.39192	0.99009
TRINITY_DN397_c0_g1_i1_orf1	striatin-3 isoform X1 [Ostrinia furnacalis]	-1.1843	-0.639	-0.5696	1.16791	1.22499
TRINITY_DN10385_c0_g1_i5_orf1	unnamed protein product [Arctia plantaginis] >CAB3259747.1 unnamed protein product [Arctia plantaginis]	-1.3957	-0.4891	-0.4125	1.10536	1.19196
TRINITY_DN8596_c0_g1_i2_orf1	SWI/SNF-related matrix-associated actin-dependent regulator of chromatin subfamily E member 1-like isoform X2 [Ostrinia furnacalis]	-0.5865	-0.3579	-1.3612	1.05962	1.24594
TRINITY_DN15753_c0_g1_i1_orf1	uncharacterized protein LOC114366450 [Ostrinia furnacalis]	-0.8339	-1.2186	-0.2779	1.28076	1.04971
TRINITY_DN4403_c0_g1_i3_orf1	AP-1 complex subunit gamma-1 [Ostrinia furnacalis]	-0.7216	-0.8786	-0.8208	1.43708	0.98402
TRINITY_DN942_c0_g1_i1_orf1	probable nuclear transport factor 2 isoform X1 [Ostrinia furnacalis]	-1.1122	-0.5995	-0.6989	1.13645	1.27419

TRINITY_DN9062_c0_g2_i3_orf1	ubiquitin conjugation factor E4 B isoform X2 [Ostrinia furnacalis]	-0.9678	-0.7375	-0.7353	1.23562	1.205
TRINITY_DN14507_c0_g1_i5_orf1	PTB domain-containing adapter protein ced-6 [Ostrinia furnacalis]	-0.995	-1.267	0.09709	0.92095	1.24389
TRINITY_DN10871_c0_g1_i3_orf1	hypothetical protein HF086_013701 [Spodoptera exigua]	-1.2126	-1.0801	0.29305	1.37258	0.62705
TRINITY_DN1045_c0_g1_i6_orf1	ornithine decarboxylase 1-like isoform X1 [Ostrinia furnacalis]	-1.0703	-0.3896	-0.9024	1.40277	0.95949
TRINITY_DN15904_c0_g1_i1_orf1	papilin isoform X8 [Ostrinia furnacalis]	-1.5677	-0.0682	-0.3883	1.42334	0.60088
TRINITY_DN1661_c0_g1_i1_orf1	NAD(P) transhydrogenase, mitochondrial-like [Ostrinia furnacalis] >XP_028175067.1 NAD(P) transhydrogenase, mitochondrial-like [Ostrinia furnacalis] >XP_028175068.1 NAD(P) transhydrogenase, mitochondrial-like [Ostrinia furnacalis] >XP_028175069.1 NAD(P) transhydrogenase, mitochondrial-like [Ostrinia furnacalis]	-1.2652	-0.5545	-0.5447	1.18743	1.17694
TRINITY_DN44407_c0_g4_i2_orf1	eukaryotic translation initiation factor 5A [Antheraea pernyi]	-1.1934	-1.0374	0.22673	1.47109	0.53299
TRINITY_DN59291_c0_g1_i1_orf1	ATP-dependent RNA helicase vasa [Ostrinia furnacalis]	-1.5092	-0.5246	-0.1612	1.17592	1.01904
TRINITY_DN880_c0_g1_i6_orf1	cuticle protein 19-like [Ostrinia furnacalis]	-1.1631	-0.3266	-0.7958	0.75837	1.52714
TRINITY_DN72859_c0_g1_i1_orf1	hypothetical protein evm_010574 [Chilo suppressalis]	-1.3361	-0.4224	-0.4993	1.47401	0.78382
TRINITY_DN53311_c0_g2_i1_orf1	transcription elongation factor S-II [Chelonus insularis]	-1.0853	-0.4017	-0.8968	1.10479	1.27903
TRINITY_DN146493_c0_g1_i1_orf1	anaphase-promoting complex subunit 1 [Chelonus insularis]	-1.5013	-0.7717	0.24049	1.13254	0.9
TRINITY_DN181_c0_g1_i3_orf1	hypothetical protein evm_003589 [Chilo suppressalis]	-1.4399	-0.6663	0.12489	1.49287	0.4884
TRINITY_DN48460_c0_g1_i1_orf1	PREDICTED: signal recognition particle 54 kDa protein [Fopius arisanus]	-1.5141	-0.444	-0.1711	1.39219	0.7369
TRINITY_DN749_c0_g1_i1_orf1	serine/threonine-protein phosphatase 4 regulatory subunit 3 isoform X3 [Ostrinia furnacalis]	-1.0408	-0.5684	-0.8128	1.2095	1.2125
TRINITY_DN3472_c1_g1_i4_orf1	Krueppel homolog 2-like [Ostrinia furnacalis]	-1.4623	-0.3288	-0.4606	1.18275	1.06893
TRINITY_DN42205_c0_g1_i4_orf1	eukaryotic translation initiation factor 4H [Ostrinia furnacalis]	-0.897	-0.5834	-0.9005	1.51424	0.86669
TRINITY_DN7560_c0_g1_i4_orf1	unnamed protein product [Chilo suppressalis]	-1.1166	-1.0852	-0.0056	1.36737	0.84005
TRINITY_DN4770_c0_g1_i4_orf1	transportin-3 isoform X1 [Ostrinia furnacalis]	-1.3229	-0.6374	-0.055	1.64401	0.37129
TRINITY_DN7670_c0_g1_i1_orf1	striatin-interacting protein 1 [Ostrinia furnacalis]	-0.8642	-0.6513	-0.9214	1.29118	1.14576
TRINITY_DN1856_c0_g1_i3_orf1	hypothetical protein evm_006253 [Chilo suppressalis]	-1.1695	-0.5541	-0.5739	1.57101	0.72649
TRINITY_DN18329_c0_g1_i2_orf1	bromodomain-containing protein 3 [Ostrinia furnacalis] >XP_028169629.1 bromodomain-containing protein 3 [Ostrinia furnacalis] >XP_028169637.1 bromodomain-containing protein 3 [Ostrinia furnacalis] >XP_028169644.1 bromodomain-containing protein 3 [Ostrinia furnacalis]	-1.5734	-0.1905	-0.2803	1.42205	0.62222
TRINITY_DN43076_c0_g1_i6_orf1	protein argonaute-2 isoform X2 [Pectinophora gossypiella]	-1.2656	-0.805	-0.2318	1.30249	0.99997
TRINITY_DN87170_c0_g1_i3_orf1	uncharacterized protein LOC114360175 [Ostrinia furnacalis]	-0.9188	-1.272	-0.0602	1.10539	1.14557
TRINITY_DN1628_c0_g1_i1_orf1	uncharacterized protein LOC114363979 [Ostrinia furnacalis]	-1.215	0.09384	-1.017	0.75716	1.38104
TRINITY_DN2919_c0_g1_i5_orf1	nidogen-1 [Ostrinia furnacalis]	-1.3334	-1.0177	0.35735	1.18571	0.80799
TRINITY_DN1639_c0_g2_i2_orf1	longitudinals lacking protein-like [Plutella xylostella] >XP_021192239.1 longitudinals lacking protein-like [Helicoverpa armigera] >XP_022818289.1 longitudinals lacking protein-like [Spodoptera litura] >XP_022818290.1 longitudinals lacking protein-like [Spodoptera litura] >XP_023945541.1 longitudinals lacking protein-like [Bicyclus anynana] >XP_026320461.1 longitudinals lacking protein-like [Hyposmocoma kahamanoa] >XP_026320471.1 longitudinals lacking protein-like [Hyposmocoma kahamanoa] >XP_026499391.1 longitudinals lacking protein-like [Vanessa tameamea] >XP_026499392.1 longitudinals lacking protein-like [Vanessa tameamea] >XP_026728238.1 longitudinals lacking protein-like isoform X2 [Trichoplusia ni] >XP_026750598.1 longitudinals lacking protein-like [Galleria mellonella] >XP_028032853.1 longitudinals lacking protein-like [Bombyx mandarina] >XP_028169299.1 longitudinals lacking protein-like [Ostrinia furnacalis] >XP_030036777.1 longitudinals lacking protein-like [Manduca sexta] >XP_034826408.1 longitudinals lacking protein-like [Maniola hyperantus] >XP_034826409.1 longitudinals lacking protein-like [Maniola hyperantus] >XP_035452035.1 longitudinals lacking protein-like [Spodoptera frugiperda] >XP_035452036.1 longitudinals lacking protein-like [Spodoptera frugiperda] >XP_035452270.1 longitudinals lacking protein-like [Spodoptera frugiperda] >XP_035452271.1 longitudinals lacking protein-like [Spodoptera frugiperda] >XP_037874172.1 longitudinals lacking protein-like [Bombyx mori] >XP_038218308.1 longitudinals lacking protein-like [Zerene cesonia] >XP_039758692.1 longitudinals lacking protein-like [Pararge aegeria] >XP_041981066.1 longitudinals lacking protein-like [Aricia agestis] >XP_041981074.1 longitudinals lacking protein-like [Aricia agestis] >XP_045455814.1 longitudinals lacking protein-like [Melitaea cinxia] >XP_045505524.1 longitudinals lacking protein-like [Colias croceus] >XP_045524236.1 longitudinals lacking protein-like [Pieris brassicae] >XP_045762005.1 longitudinals lacking protein-like [Maniola jurtina] >XP_045762006.1 longitudinals lacking protein-like [Maniola jurtina] >XP_046973407.1 longitudinals lacking protein-like [Vanessa cardui] >XP_046973408.1 longitudinals lacking protein-like [Vanessa cardui] >XP_047035325.1 longitudinals lacking protein-like [Helicoverpa zea] >XP_047035326.1 longitudinals lacking protein-like [Helicoverpa zea] >XP_047520236.1 longitudinals lacking protein-like [Pieris napi] >XP_047540188.1 longitudinals lacking protein-like [Vanessa atalanta] >XP_048001034.1 longitudinals lacking protein-like [Leguminivora glycinivorella] >XP_049880222.1 longitudinals lacking protein-like isoform X2 [Pectinophora gossypiella] >XP_050356245.1 longitudinals lacking protein-like [Nymphalis io] >XP_050356247.1 longitudinals lacking protein-like [Nymphalis io] >CAB3511022.1 unnamed protein product [Spodoptera littoralis] >CAF4874356.1 unnamed protein product [Pieris macdunnoughi] >CAG9753956.1 unnamed protein product [Diatraea saccharalis] >CAH0596988.1 unnamed protein product [Chrysodeixis includens] >CAH0696275.1 unnamed protein product [Spodoptera exigua] >CAH2097688.1 unnamed protein product [Euphydryas editha] >VVC99997.1 unnamed protein product [Leptidea sinapis]	-0.9203	-0.4764	-0.8273	1.73196	0.492
TRINITY_DN19810_c1_g1_i7_orf1	RNA-binding protein spenito [Ostrinia furnacalis] >XP_028167555.1 RNA-binding protein spenito [Ostrinia furnacalis]	-1.4816	-0.5298	-0.2108	1.0377	1.18446
TRINITY_DN44335_c0_g1_i7_orf1	hypothetical protein SFRURICE_000584 [Spodoptera frugiperda]	-1.1208	-0.6229	-0.5906	1.54291	0.79142
TRINITY_DN4859_c0_g1_i5_orf1	importin-11, partial [Ostrinia furnacalis]	-1.5497	-0.2975	-0.2297	1.42603	0.65088
TRINITY_DN2019_c0_g1_i4_orf1	sushi, von Willebrand factor type A, EGF and pentraxin domain-containing protein 1 [Ostrinia furnacalis] >XP_028159318.1 sushi, von Willebrand factor type A, EGF and pentraxin domain-containing protein 1 [Ostrinia furnacalis]	-0.9413	-0.5993	-0.7946	1.61289	0.72238
TRINITY_DN57904_c0_g2_i1_orf1	cuticle protein 19 [Plutella xylostella] >CAG9138481.1 unnamed protein product [Plutella xylostella]	-0.882	-0.7657	-0.7675	0.95237	1.4628
TRINITY_DN5442_c0_g1_i4_orf1	hypothetical protein evm_004688 [Chilo suppressalis]	-0.7777	-0.9738	-0.6486	1.47048	0.92953
TRINITY_DN4757_c0_g1_i3_orf1	melanotransferrin isoform X1 [Ostrinia furnacalis] >XP_028175370.1 melanotransferrin isoform X2 [Ostrinia furnacalis] >XP_028175371.1 melanotransferrin isoform X3 [Ostrinia furnacalis]	-0.8711	-0.6736	-0.897	1.1888	1.25286
TRINITY_DN17935_c0_g1_i1_orf1	NEDD8-conjugating enzyme Ubc12 [Ostrinia furnacalis]	-0.9648	-0.4504	-0.9515	1.46637	0.90037
TRINITY_DN23790_c0_g1_i1_orf1	wiskott-Aldrich syndrome protein family member 2 [Ostrinia furnacalis]	-1.3879	-0.4741	-0.405	1.37348	0.89355
TRINITY_DN7828_c0_g1_i2_orf1	alpha-N-acetylgalactosaminidase-like isoform X1 [Ostrinia furnacalis] >XP_028171449.1 alpha-N-acetylgalactosaminidase-like isoform X2 [Ostrinia furnacalis]	-0.9675	-0.6823	-0.7875	1.28233	1.15496
TRINITY_DN13093_c0_g1_i2_orf1	enhancer of mRNA-decapping protein 4 [Ostrinia furnacalis]	-1.6046	0.12587	-0.3923	1.43816	0.43281
TRINITY_DN12820_c0_g1_i1_orf1	chromodomain-helicase-DNA-binding protein 7 [Ostrinia furnacalis] >XP_028176739.1 chromodomain-helicase-DNA-binding protein 7 [Ostrinia furnacalis]	-1.2244	-0.3064	-0.7645	1.45459	0.84067

TRINITY_DN7451_c0_g1_i10_orf1	huntingtin-interacting protein 1 isoform X4 [Pectinophora gossypiella]	-1.4419	-0.7224	0.07426	1.36859	0.72144
TRINITY_DN2802_c1_g1_i1_orf1	psi [Ostrinia furnacalis]	-0.9897	-1.1984	-0.0682	1.25928	0.99697
TRINITY_DN87603_c0_g2_i1_orf1	40S ribosomal protein S3-3, partial [Trichinella patagoniensis]	-0.8812	0.40394	-1.4569	1.15059	0.78355
TRINITY_DN1425_c0_g1_i4_orf1	fibulin-2-like [Ostrinia furnacalis]	-1.521	-0.32	-0.3709	1.11885	1.09304
TRINITY_DN122867_c1_g1_i1_orf1	nuclear migration protein nudC [Ostrinia furnacalis]	-0.9272	-0.334	-1.0781	1.42466	0.91471
TRINITY_DN4686_c0_g2_i1_orf1	lysophospholipase-like protein 1 [Ostrinia furnacalis]	-1.0964	-0.6618	-0.6593	1.24847	1.16903
TRINITY_DN18912_c1_g1_i1_orf1	engulfment and cell motility protein 1 [Ostrinia furnacalis]	-1.0502	-0.4978	-0.7783	1.57404	0.75227
TRINITY_DN4533_c0_g1_i1_orf1	neurofilament heavy polypeptide-like isoform X2 [Ostrinia furnacalis]	-1.416	-0.5561	-0.3021	1.20226	1.0719
TRINITY_DN3179_c0_g1_i1_orf1	diamine acetyltransferase 2-like [Ostrinia furnacalis]	-1.139	-0.7949	-0.344	1.56226	0.71553
TRINITY_DN4070_c0_g1_i4_orf1	alpha-N-acetylgalactosaminidase isoform X3 [Ostrinia furnacalis]	-1.0681	-0.835	-0.4759	0.96014	1.41896
TRINITY_DN4152_c0_g1_i1_orf1	importin subunit beta-1 isoform X2 [Ostrinia furnacalis]	-0.8199	-0.9972	-0.5603	1.49974	0.87763
TRINITY_DN5952_c0_g1_i6_orf1	LOW QUALITY PROTEIN: phosphoacetylglucosamine mutase [Ostrinia furnacalis]	-1.0805	-0.7265	-0.5878	1.41135	0.98347
TRINITY_DN3411_c0_g2_i1_orf1	putative U5 small nuclear ribonucleoprotein 200 kDa helicase, partial [Ostrinia furnacalis]	-1.5537	0.19622	-0.5941	1.35682	0.59473
TRINITY_DN2403_c0_g1_i3_orf1	FAD-dependent oxidoreductase domain-containing protein 1 [Ostrinia furnacalis]	-1.1286	-0.911	-0.3147	1.29116	1.06312
TRINITY_DN3801_c0_g1_i9_orf1	claspin-like isoform X2 [Ostrinia furnacalis]	-0.9955	-0.7716	-0.4534	1.71972	0.50071
TRINITY_DN21218_c0_g1_i4_orf1	leukotriene A-4 hydrolase isoform X2 [Ostrinia furnacalis]	-0.4105	-0.9735	-0.9565	1.5094	0.83107
TRINITY_DN48970_c0_g1_i1_orf1	uncharacterized protein LOC114356431 isoform X2 [Ostrinia furnacalis]	-1.2897	-0.6785	-0.3606	1.29554	1.03333
TRINITY_DN5982_c0_g1_i3_orf1	coatomer subunit gamma [Ostrinia furnacalis]	-1.1179	-0.5449	-0.5516	1.69769	0.5167
TRINITY_DN26168_c0_g1_i1_orf1	ATP-dependent RNA helicase Ddx1-like [Ostrinia furnacalis]	-1.2008	-0.6462	-0.4104	1.5892	0.66818
TRINITY_DN10131_c0_g1_i7_orf1	aldo-keto reductase AKR2E4-like [Ostrinia furnacalis]	-1.0576	-0.6712	-0.6836	1.04157	1.37075
TRINITY_DN7247_c0_g1_i7_orf1	pyruvate kinase-like isoform X3 [Ostrinia furnacalis]	-1.3649	-0.4001	-0.4864	1.44685	0.80446
TRINITY_DN31967_c0_g1_i5_orf1	N-acetylgalactosamine kinase [Ostrinia furnacalis]	-0.7734	-0.7143	-0.9451	1.35619	1.07664
TRINITY_DN11375_c0_g1_i6_orf1	uncharacterized protein LOC114363514 isoform X2 [Ostrinia furnacalis]	-0.9108	-0.3847	-1.0876	1.18958	1.19351
TRINITY_DN6380_c0_g1_i1_orf1	THAP domain-containing protein 1-like isoform X1 [Ostrinia furnacalis]	-1.0632	-0.8376	-0.4178	1.55023	0.76833
TRINITY_DN6103_c0_g1_i6_orf1	sedoheptulokinase-like [Ostrinia furnacalis]	-1.1528	-0.4743	-0.6012	1.66278	0.56554
TRINITY_DN20339_c0_g1_i3_orf1	ecto-NOX disulfide-thiol exchanger 2-like [Ostrinia furnacalis]	-1.2661	-0.9595	0.07061	1.35072	0.80429
TRINITY_DN79803_c0_g1_i7_orf1	dnaJ homolog subfamily C member 22 [Ostrinia furnacalis]	-1.4802	-0.0912	-0.5997	0.87975	1.29123
TRINITY_DN27771_c0_g1_i1_orf1	glycine--tRNA ligase [Ostrinia furnacalis]	-1.4953	-0.7274	0.43999	1.36704	0.41561
TRINITY_DN29743_c0_g1_i9_orf1	polyadenylate-binding protein 2 isoform X1 [Ostrinia furnacalis] >XP_028168980.1 polyadenylate-binding protein 2 isoform X2 [Ostrinia furnacalis]	-1.2389	-0.8889	-0.147	1.31884	0.956
TRINITY_DN20185_c0_g1_i6_orf1	zinc finger protein on ecdysone puffs [Ostrinia furnacalis]	-1.0227	-1.0106	-0.3056	1.39723	0.94178
TRINITY_DN4835_c0_g1_i2_orf1	ribonucleoside-diphosphate reductase large subunit [Ostrinia furnacalis]	-1.2435	-0.9386	-0.0832	1.12323	1.14202
TRINITY_DN5472_c0_g1_i1_orf1	serine/threonine-protein kinase 26 isoform X3 [Ostrinia furnacalis]	-1.2067	-0.2119	-0.8313	1.49695	0.75298
TRINITY_DN40197_c0_g1_i1_orf1	UDP-N-acetylglucosamine--dolichyl-phosphate N-acetylglucosaminephosphotransferase-like [Ostrinia furnacalis]	-1.0668	-0.6666	-0.4741	1.7187	0.48878
TRINITY_DN1504_c0_g1_i1_orf1	uncharacterized protein LOC114352862 [Ostrinia furnacalis] >XP_028160407.1 uncharacterized protein LOC114352862 [Ostrinia furnacalis]	-1.3897	-0.6692	-0.1957	0.9835	1.27102
TRINITY_DN78686_c0_g1_i1_orf1	myosin-2 essential light chain isoform X2 [Harpegnathos saltator] >XP_012170910.1 myosin-2 essential light chain isoform X2 [Bombus terrestris] >XP_033185931.1 myosin-2 essential light chain isoform X2 [Bombus vancouverensis nearcticus] >XP_033319091.1 myosin-2 essential light chain isoform X2 [Bombus bifarius] >XP_033349866.1 myosin-2 essential light chain isoform X2 [Bombus vosnesenskii] >XP_043597873.1 myosin-2 essential light chain isoform X2 [Bombus pyrosoma]	-1.4775	-0.2909	-0.4571	0.96003	1.26554
TRINITY_DN4908_c1_g1_i5_orf1	DNA topoisomerase 2 isoform X1 [Ostrinia furnacalis]	-0.9453	-0.7522	-0.7435	1.15561	1.28543
TRINITY_DN53115_c0_g1_i1_orf1	small glutamine-rich tetratricopeptide repeat-containing protein beta-like [Ostrinia furnacalis]	-0.8699	-0.8801	-0.5224	1.69032	0.582
TRINITY_DN3838_c0_g1_i8_orf1	ER membrane protein complex subunit 2-like isoform X1 [Ostrinia furnacalis] >XP_028161204.1 ER membrane protein complex subunit 2-like isoform X2 [Ostrinia furnacalis] >XP_028161205.1 ER membrane protein complex subunit 2-like isoform X3 [Ostrinia furnacalis]	-1.3904	-0.8388	0.11066	1.29027	0.8283
TRINITY_DN19079_c0_g1_i5_orf1	unnamed protein product [Euphydryas editha]	-1.0459	-0.9324	-0.2116	1.63999	0.54989
TRINITY_DN51050_c0_g1_i3_orf1	GRB10-interacting GYF protein 2 isoform X1 [Ostrinia furnacalis]	-1.3687	-0.3926	-0.3824	1.58586	0.55788
TRINITY_DN22156_c0_g1_i1_orf1	trafficking protein particle complex subunit 8 [Ostrinia furnacalis]	-1.1371	-0.046	-1.0311	1.4157	0.79849
TRINITY_DN4233_c0_g2_i2_orf1	actin-related protein 2/3 complex subunit 5-B [Ostrinia furnacalis]	-1.1923	-0.634	-0.5137	1.46555	0.87448
TRINITY_DN37923_c0_g1_i1_orf1	hypothetical protein NE865_05974 [Phthorimaea operculella]	-1.1651	-0.8961	-0.2704	1.32131	1.01026
TRINITY_DN288_c0_g1_i9_orf1	unnamed protein product [Chilo suppressalis]	-1.0943	-0.5357	-0.7551	1.41743	0.96763
TRINITY_DN1444_c1_g1_i5_orf1	spondin-1 isoform X1 [Ostrinia furnacalis] >XP_028167312.1 spondin-1 isoform X1 [Ostrinia furnacalis] >XP_028167313.1 spondin-1 isoform X1 [Ostrinia furnacalis] >XP_028167314.1 spondin-1 isoform X2 [Ostrinia furnacalis]	-1.1687	-0.6678	-0.5599	1.23855	1.15787
TRINITY_DN1459_c1_g1_i1_orf1	reticulon-1 isoform X2 [Ostrinia furnacalis]	-0.6651	-1.1782	-0.3615	1.65387	0.55093
TRINITY_DN9637_c0_g1_i14_orf1	zinc finger protein swm isoform X3 [Ostrinia furnacalis]	-0.8727	-0.8848	-0.6589	1.43399	0.98239
TRINITY_DN24490_c0_g1_i6_orf1	E3 ubiquitin-protein ligase Hakai [Ostrinia furnacalis]	-1.1028	-0.4342	-0.849	1.31083	1.07522
TRINITY_DN783_c0_g1_i7_orf1	microtubule-associated protein Jupiter isoform X4 [Helicoverpa armigera]	-1.0004	-0.7325	-0.701	1.14845	1.28544
TRINITY_DN46022_c0_g1_i1_orf1	mRNA-decapping enzyme 1A [Ostrinia furnacalis]	-0.9918	-0.5253	-0.7331	1.70451	0.54563
TRINITY_DN34689_c0_g1_i4_orf1	uncharacterized protein DDB_G0283357 isoform X13 [Helicoverpa armigera] >XP_049707197.1 uncharacterized protein DDB_G0283357 isoform X14 [Helicoverpa armigera] >XP_049707198.1 uncharacterized protein DDB_G0283357 isoform X15 [Helicoverpa armigera] >XP_049707199.1 uncharacterized protein DDB_G0283357 isoform X16 [Helicoverpa armigera] >XP_049707200.1 uncharacterized protein DDB_G0283357 isoform X17 [Helicoverpa armigera] >XP_049707201.1 uncharacterized protein DDB_G0283357 isoform X18 [Helicoverpa armigera] >XP_049707202.1 uncharacterized protein DDB_G0283357 isoform X19 [Helicoverpa armigera] >XP_049707203.1 uncharacterized protein DDB_G0283357 isoform X20 [Helicoverpa armigera] >XP_049707204.1 uncharacterized protein DDB_G0283357 isoform X21 [Helicoverpa armigera] >XP_049707205.1 uncharacterized protein DDB_G0283357 isoform X22 [Helicoverpa armigera] >XP_049707206.1 uncharacterized protein DDB_G0283357 isoform X23 [Helicoverpa armigera] >XP_049707207.1 uncharacterized protein DDB_G0283357 isoform X24 [Helicoverpa armigera]	-0.9152	-0.7924	-0.5979	1.6624	0.64305
TRINITY_DN63561_c1_g1_i2_orf1	unnamed protein product [Diatraea saccharalis]	-1.1236	-0.66	-0.416	1.69554	0.50402
TRINITY_DN493_c0_g1_i4_orf1	ADP-ribosylation factor GTPase-activating protein 3 [Ostrinia furnacalis]	-1.2187	-0.8012	-0.0532	1.637	0.43613
TRINITY_DN13375_c0_g1_i6_orf1	thioredoxin, mitochondrial isoform X2 [Ostrinia furnacalis]	-1.1735	-0.0817	-1.0159	1.08572	1.18545

TRINITY_DN3113_c1_g2_i1_orf1	short-chain dehydrogenase/reductase family 16C member 6-like [Ostrinia furnacalis] >XP_028174076.1 short-chain dehydrogenase/reductase family 16C member 6-like [Ostrinia furnacalis]	-1.1145	-0.7793	-0.2818	1.68137	0.49419
TRINITY_DN6185_c0_g1_i12_orf1	mitogen-activated protein kinase 1 [Ostrinia furnacalis] >AXF67444.1 mitogen-activated protein kinase 1 [Ostrinia furnacalis]	-0.4732	-0.7995	-1.0747	1.51045	0.83695
TRINITY_DN132043_c0_g1_i1_orf1	ankyrin repeat and MYND domain-containing protein 2 [Ostrinia furnacalis]	-1.0042	-0.8186	-0.4287	1.67492	0.57654
TRINITY_DN47723_c0_g1_i1_orf1	dnaJ homolog subfamily C member 21 [Ostrinia furnacalis]	-0.6962	-0.9407	-0.7626	1.49127	0.90825
TRINITY_DN18681_c0_g1_i7_orf1	fragile X mental retardation syndrome-related protein 1 isoform X3 [Ostrinia furnacalis]	-1.1499	-0.9382	-0.1542	1.48159	0.76072
TRINITY_DN11616_c0_g1_i3_orf1	coiled-coil domain-containing protein 6-like [Ostrinia furnacalis]	-1.0088	-0.3998	-0.8345	1.6739	0.56926
TRINITY_DN607_c0_g1_i16_orf1	protein muscleblind isoform X1 [Ostrinia furnacalis] >XP_028162801.1 protein muscleblind isoform X1 [Ostrinia furnacalis] >XP_028162802.1 protein muscleblind isoform X1 [Ostrinia furnacalis] >XP_028162803.1 protein muscleblind isoform X1 [Ostrinia furnacalis] >XP_028162804.1 protein muscleblind isoform X1 [Ostrinia furnacalis] >XP_028162806.1 protein muscleblind isoform X1 [Ostrinia furnacalis]	-1.1412	-0.5773	-0.4701	1.70602	0.48259
TRINITY_DN10502_c0_g1_i4_orf1	interleukin enhancer-binding factor 2 homolog [Ostrinia furnacalis]	-1.0157	-0.7219	-0.5847	1.61346	0.70871
TRINITY_DN44877_c0_g1_i2_orf1	U6 snRNA-associated Sm-like protein LSm7 [Diachasma alloeum]	-1.2132	-0.817	-0.246	1.46174	0.81448
TRINITY_DN32601_c0_g1_i2_orf1	uncharacterized protein LOC114363197 [Ostrinia furnacalis]	-1.0091	-0.6537	-0.7628	1.08832	1.33724
TRINITY_DN50875_c0_g1_i3_orf1	conserved oligomeric Golgi complex subunit 8 [Ostrinia furnacalis]	-1.2863	-0.5096	-0.5589	1.15983	1.19502
TRINITY_DN43328_c0_g1_i1_orf1	tubulin--tyrosine ligase-like protein 12 [Ostrinia furnacalis]	-1.0402	-0.6811	-0.7062	1.14772	1.27984
TRINITY_DN2879_c0_g1_i4_orf1	nucleoporin Nup35 [Ostrinia furnacalis]	-1.4411	-0.7538	0.02536	1.06596	1.10363
TRINITY_DN5562_c0_g1_i3_orf1	cell division cycle and apoptosis regulator protein 1-like [Ostrinia furnacalis]	-0.7882	-0.6909	-0.8295	1.67789	0.63079
TRINITY_DN172_c1_g1_i3_orf1	galactose oxidase, central domain-containing protein [Phthorimaea operculella]	-0.7567	-0.384	-1.1556	1.54055	0.75578
TRINITY_DN36006_c0_g1_i5_orf1	pro-resilin-like [Ostrinia furnacalis]	-1.6065	0.09725	-0.494	1.28383	0.71938
TRINITY_DN18933_c0_g1_i3_orf1	PREDICTED: protein BUD31 homolog [Papilio xuthus] >XP_014361644.1 protein BUD31 homolog [Papilio machaon] >XP_026750578.1 protein BUD31 homolog [Galleria mellonella] >XP_047995610.1 protein BUD31 homolog [Leguminivora glycinivorella] >XP_049869593.1 protein BUD31 homolog [Pectinophora gossypiella] >KAI5652084.1 g10 protein domain-containing protein [Phthorimaea operculella] >CAB3251981.1 unnamed protein product [Arctia plantaginis] >CAB3520382.1 unnamed protein product [Chilo suppressalis] >CAG9747228.1 unnamed protein product [Diatraea saccharalis] >CAH2037008.1 unnamed protein product, partial [Iphiclides podalirius]	-1.4787	-0.5058	-0.2471	1.17209	1.05956
TRINITY_DN4659_c0_g1_i2_orf1	uncharacterized protein LOC114351134 [Ostrinia furnacalis]	-0.7607	-0.735	-0.9464	1.17886	1.26323
TRINITY_DN147475_c0_g1_i1_orf1	casein kinase II subunit beta, partial [Rhincodon typus]	-1.205	-0.849	-0.2883	1.19206	1.15026
TRINITY_DN2802_c0_g1_i1_orf1	far upstream element-binding protein 1 isoform X3 [Ostrinia furnacalis]	-1.137	-0.7892	-0.3841	1.52149	0.78872
TRINITY_DN26429_c0_g1_i4_orf1	zinc transporter 9 [Ostrinia furnacalis]	-0.9608	-0.7206	-0.4799	1.78489	0.37631
TRINITY_DN48983_c0_g1_i2_orf1	E3 ubiquitin-protein ligase Su(dx) [Ostrinia furnacalis] >XP_028176753.1 E3 ubiquitin-protein ligase Su(dx) [Ostrinia furnacalis] >XP_028176754.1 E3 ubiquitin-protein ligase Su(dx) [Ostrinia furnacalis]	-1.6296	0.28094	-0.46	1.36155	0.44716
TRINITY_DN19493_c0_g1_i5_orf1	zinc finger MYM-type protein 3 isoform X1 [Ostrinia furnacalis] >XP_028159738.1 zinc finger MYM-type protein 3 isoform X2 [Ostrinia furnacalis]	-1.2551	-0.498	-0.6063	1.29326	1.06615
TRINITY_DN3176_c0_g1_i2_orf1	dnaJ homolog subfamily B member 6 isoform X2 [Ostrinia furnacalis]	-0.8475	-0.7995	-0.7051	1.61141	0.74072
TRINITY_DN7228_c0_g1_i6_orf1	neutral alpha-glucosidase AB [Ostrinia furnacalis]	-0.7908	-0.3543	-1.011	1.74987	0.4062
TRINITY_DN4085_c0_g1_i1_orf1	Protein TSSC1 [Papilio machaon]	-1.1393	-0.9864	-0.1856	1.04631	1.26488
TRINITY_DN30950_c0_g1_i13_orf1	unnamed protein product [Chilo suppressalis]	-1.1569	-0.9095	0.05061	1.64044	0.37533
TRINITY_DN37532_c0_g1_i1_orf1	transcription elongation factor S-II [Ostrinia furnacalis]	-1.3474	-0.6216	-0.326	1.3182	0.9768
TRINITY_DN17271_c0_g1_i1_orf1	uncharacterized protein LOC114350693 [Ostrinia furnacalis]	-1.1671	-0.8203	-0.3845	1.23102	1.14092
TRINITY_DN18164_c0_g1_i7_orf1	uncharacterized protein LOC114366518 isoform X5 [Ostrinia furnacalis]	-0.9433	-1.1454	-0.247	1.09892	1.23678
TRINITY_DN279_c0_g1_i10_orf1	RE1-silencing transcription factor-like isoform X1 [Ostrinia furnacalis]	-1.2477	-0.6961	-0.4128	1.10248	1.25413
TRINITY_DN2600_c0_g1_i7_orf1	mucin-5AC isoform X2 [Ostrinia furnacalis]	-1.0483	-0.6562	-0.7197	1.12898	1.29529
TRINITY_DN11402_c0_g1_i1_orf1	constitutive coactivator of PPAR-gamma-like protein 1 isoform X1 [Ostrinia furnacalis] >XP_028158538.1 constitutive coactivator of PPAR-gamma-like protein 1 isoform X2 [Ostrinia furnacalis]	-0.8613	-0.8773	-0.6545	1.51015	0.88295
TRINITY_DN1763_c0_g3_i2_orf1	heterogeneous nuclear ribonucleoprotein H-like isoform X2 [Ostrinia furnacalis]	-1.3846	0.226	-0.9074	1.22535	0.84071
TRINITY_DN972_c0_g2_i1_orf1	DNA damage-binding protein 1 [Ostrinia furnacalis]	-1.2306	-0.4084	-0.5618	1.63958	0.56121
TRINITY_DN8290_c0_g1_i3_orf1	zinc finger CCHC domain-containing protein 8 homolog [Ostrinia furnacalis]	-1.3079	-0.7028	-0.0792	1.59511	0.49477
TRINITY_DN122423_c0_g5_i1_orf1	PREDICTED: dynein heavy chain, cytoplasmic isoform X3 [Fopius arisanus]	-1.4656	0.0616	-0.4252	1.61973	0.20951
TRINITY_DN62_c0_g1_i18_orf1	hypothetical protein evm_002481 [Chilo suppressalis] >CAB3531063.1 unnamed protein product [Chilo suppressalis] >CAH0407655.1 unnamed protein product [Chilo suppressalis]	-0.9643	-0.9063	-0.379	1.66094	0.58861
TRINITY_DN2232_c1_g1_i3_orf1	protein FAM98A-like [Ostrinia furnacalis]	-1.2437	-1.0719	0.24696	1.26225	0.80632
TRINITY_DN235_c0_g3_i1_orf1	actin, muscle-type A2 [Bombyx mori] >XP_013199497.1 PREDICTED: actin, muscle-type A2 [Amyelois transitella] >XP_021196084.1 actin, muscle-type A2 [Helicoverpa armigera] >XP_022837900.1 actin, muscle-type A2 [Spodoptera litura] >XP_026314060.1 actin, muscle-type A2 [Hyposmocoma kahamanoa] >XP_026738711.1 actin, muscle-type A2 [Trichoplusia ni] >XP_028179440.1 actin, muscle-type A2 [Ostrinia furnacalis] >XP_030030527.1 actin, muscle-type A2 [Manduca sexta] >XP_035439272.1 actin, muscle-type A2 [Spodoptera frugiperda] >XP_047029939.1 actin, muscle-type A2 [Helicoverpa zea] >XP_049873365.1 actin, muscle-type A2 [Pectinophora gossypiella] >P07837.1 RecName: Full=Actin, muscle-type A2; Flags: Precursor [Bombyx mori] >KAF9423784.1 hypothetical protein HW555_000842 [Spodoptera exigua] >QLI62214.1 actin [Streltzoviella insularis] >CAB3227390.1 unnamed protein product [Arctia plantaginis] >CAB3508892.1 unnamed protein product [Spodoptera littoralis] >CAB3520808.1 unnamed protein product [Chilo suppressalis] >CAG9748331.1 unnamed protein product [Diatraea saccharalis] >CAH0585396.1 unnamed protein product [Chrysodeixis includens] >GBP21118.1 Actin, muscle-type A2 [Eumeta japonica]	-1.2914	-0.6774	-0.3021	1.45453	0.8164
TRINITY_DN46140_c0_g1_i1_orf1	protein PRRC2A-like isoform X4 [Ostrinia furnacalis]	-0.5072	-0.9366	-0.9052	1.55354	0.79543
TRINITY_DN1447_c0_g1_i5_orf1	PREDICTED: coatomer subunit beta' [Amyelois transitella]	-1.3631	-0.4299	-0.2325	1.66532	0.36019
TRINITY_DN1437_c0_g1_i6_orf1	nucleoprotein TPR isoform X1 [Ostrinia furnacalis]	-1.1463	-0.7524	-0.372	1.58465	0.68596
TRINITY_DN3856_c0_g1_i7_orf1	uncharacterized protein LOC114355702 [Ostrinia furnacalis]	-1.1724	-0.4774	-0.5522	1.67893	0.52315
TRINITY_DN48838_c0_g1_i6_orf1	merlin-like [Ostrinia furnacalis]	-1.0638	-0.6423	-0.4385	1.76654	0.37802
TRINITY_DN9938_c0_g2_i1_orf1	hypothetical protein E2986_04423 [Frieseomelitta varia]	-1.3201	-0.5514	-0.3107	1.57887	0.60334
TRINITY_DN3450_c0_g1_i3_orf1	hypothetical protein evm_008214 [Chilo suppressalis]	-1.4156	0.00435	-0.601	1.55858	0.45361

TRINITY_DN2914_c0_g1_i1_orf1	U1 small nuclear ribonucleoprotein A [Ostrinia furnacalis]	-1.1983	-0.7179	-0.2543	1.6458	0.52472
TRINITY_DN10548_c0_g2_i1_orf1	uridine 5'-monophosphate synthase-like [Ostrinia furnacalis]	-1.4345	-0.6403	0.17858	1.54083	0.35536
TRINITY_DN142588_c0_g1_i1_orf1	peptidyl-prolyl cis-trans isomerase [Cotesia flavipes]	-1.0337	-0.8832	-0.474	1.38026	1.0107
TRINITY_DN146119_c0_g1_i1_orf1	protein SEC13 homolog [Ostrinia furnacalis]	-1.3616	-0.1188	-0.7172	1.41704	0.78066
TRINITY_DN3321_c0_g1_i3_orf1	peroxidasin [Ostrinia furnacalis]	-1.304	-0.6548	0.03366	1.67555	0.24956
TRINITY_DN4950_c0_g1_i2_orf1	unnamed protein product [Diatraea saccharalis]	-1.1756	-0.5925	-0.5301	1.5643	0.73399
TRINITY_DN7037_c0_g1_i4_orf1	unnamed protein product [Chilo suppressalis]	-0.5006	-0.5421	-1.2171	1.58868	0.67107
TRINITY_DN34821_c0_g1_i4_orf1	acetylcholine receptor subunit alpha-L1-like [Ostrinia furnacalis]	-0.5268	-0.5907	-1.2517	1.19076	1.17849
TRINITY_DN14365_c0_g1_i2_orf1	leucine-rich repeat-containing protein 57-like [Colias croceus]	-0.9433	-0.0062	-1.2457	1.37043	0.82479
TRINITY_DN257_c0_g1_i7_orf1	zinc finger RNA-binding protein 2 [Ostrinia furnacalis]	-1.3049	-0.7438	-0.1459	1.49379	0.70089
TRINITY_DN2977_c0_g1_i3_orf1	transmembrane 9 superfamily member 3 [Ostrinia furnacalis]	-0.9532	-0.7554	-0.6591	1.56014	0.80761
TRINITY_DN31119_c0_g1_i1_orf1	transforming acidic coiled-coil-containing protein 3-like [Ostrinia furnacalis] >XP_028170476.1 transforming acidic coiled-coil-containing protein 3-like [Ostrinia furnacalis] >XP_028170477.1 transforming acidic coiled-coil-containing protein 3-like [Ostrinia furnacalis] >XP_028170480.1 transforming acidic coiled-coil-containing protein 3-like [Ostrinia furnacalis]	-1.6047	-0.0355	-0.0924	1.54239	0.19014
TRINITY_DN22951_c0_g1_i1_orf1	TAR DNA-binding protein 43-like [Ostrinia furnacalis]	-1.5446	0.45882	-0.4985	1.46304	0.12128
TRINITY_DN31585_c0_g1_i1_orf1	transcription elongation factor SPT5 [Ostrinia furnacalis]	-0.9288	-0.6766	-0.6332	1.74102	0.49754
TRINITY_DN91_c0_g1_i9_orf1	ribosome-binding protein 1 isoform X8 [Helicoverpa armigera]	-1.3648	-0.4203	-0.1912	1.68505	0.29122
TRINITY_DN31503_c0_g1_i4_orf1	hypothetical protein evm_001345 [Chilo suppressalis] >CAB3523265.1 unnamed protein product [Chilo suppressalis] >CAH0400587.1 unnamed protein product [Chilo suppressalis]	-0.6522	-0.8527	-0.7959	1.68292	0.61791
TRINITY_DN2738_c1_g1_i3_orf1	uridine-cytidine kinase isoform X1 [Helicoverpa zea] >XP_049697747.1 uridine-cytidine kinase-like isoform X1 [Helicoverpa armigera] >XP_049698409.1 uridine-cytidine kinase isoform X1 [Helicoverpa armigera]	-0.8628	-0.7135	-0.5899	1.80845	0.35777
TRINITY_DN29034_c0_g1_i1_orf1	trypsin-like serine protease [Ostrinia nubilalis]	-0.5563	-0.5836	-1.1527	1.58967	0.70294
TRINITY_DN23783_c0_g2_i1_orf1	cytochrome b5 [Ostrinia furnacalis]	-1.1217	0.08568	-0.9908	1.60444	0.42242
TRINITY_DN69170_c0_g2_i1_orf1	stromal membrane-associated protein 1-like [Pectinophora gossypiella]	-1.0853	-0.6814	-0.4324	1.71305	0.48607
TRINITY_DN10672_c0_g1_i3_orf1	neurofilament heavy polypeptide-like isoform X10 [Ostrinia furnacalis]	-1.5775	0.11415	-0.1709	1.57005	0.06424
TRINITY_DN3029_c4_g1_i1_orf1	proliferation marker protein Ki-67-like, partial [Ostrinia furnacalis]	-1.7367	0.32594	-0.0363	1.36743	0.07964
TRINITY_DN82311_c0_g1_i1_orf1	pleckstrin homology-like domain family B member 1 isoform X2 [Ostrinia furnacalis]	-1.4384	-0.7685	0.05088	1.16088	0.99511
TRINITY_DN30097_c0_g1_i2_orf1	unnamed protein product [Chilo suppressalis]	-1.0964	-0.1676	-0.7436	1.77888	0.22875
TRINITY_DN6572_c0_g1_i2_orf1	zinc finger protein 330 homolog [Ostrinia furnacalis]	-1.1728	-0.7916	-0.1618	1.65946	0.46676
TRINITY_DN8958_c0_g1_i1_orf1	nuclear cap-binding protein subunit 1 [Galleria mellonella]	-1.5939	-0.2301	-0.2222	1.38614	0.66007
TRINITY_DN141_c0_g1_i1_orf1	hypothetical protein evm_010402 [Chilo suppressalis]	-0.9562	-0.9811	-0.1502	1.72258	0.36495
TRINITY_DN298_c0_g1_i4_orf1	luc7-like protein 3 isoform X1 [Ostrinia furnacalis] >XP_028160033.1 luc7-like protein 3 isoform X1 [Ostrinia furnacalis]	-0.9157	-1.0282	-0.3301	1.58972	0.68426
TRINITY_DN9759_c0_g1_i1_orf1	iroquois-class homeodomain protein IRX-1-like isoform X1 [Ostrinia furnacalis]	-1.5571	-0.1734	0.20112	1.58185	-0.0525
TRINITY_DN11820_c0_g1_i1_orf1	hypothetical protein evm_000341 [Chilo suppressalis]	-1.5921	-0.398	-0.1242	1.2244	0.89
TRINITY_DN5457_c0_g1_i4_orf1	unnamed protein product [Chrysodeixis includens]	-1.1474	-0.8784	-0.2335	1.52048	0.73871
TRINITY_DN452_c1_g1_i3_orf1	ruvB-like helicase 1 [Colias croceus]	-1.076	-0.3762	-0.5363	1.84154	0.14708
TRINITY_DN1298_c0_g1_i3_orf1	ras GTPase-activating protein-binding protein 2-like, partial [Ostrinia furnacalis]	-1.5498	-0.4805	0.17597	1.48262	0.37172
TRINITY_DN170_c1_g1_i5_orf1	regulator of chromosome condensation isoform X2 [Helicoverpa zea]	-1.1308	-0.8812	0.02956	1.69088	0.29151
TRINITY_DN6189_c0_g1_i1_orf1	optic atrophy 3 protein homolog isoform X2 [Ostrinia furnacalis]	-0.6297	-0.6115	-1.1128	1.50916	0.84481
TRINITY_DN4836_c0_g1_i4_orf1	hypothetical protein O3G_MSEX014157 [Manduca sexta] >KAG6463927.1 hypothetical protein O3G_MSEX014157 [Manduca sexta]	-1.1375	-0.9629	0.33629	1.62699	0.13708
TRINITY_DN7464_c1_g1_i1_orf1	T-complex protein 1 subunit theta [Ostrinia furnacalis]	-0.8504	-0.5744	-0.6802	1.84894	0.25609
TRINITY_DN13972_c0_g1_i5_orf1	myelin expression factor 2-like [Ostrinia furnacalis] >XP_028173185.1 myelin expression factor 2-like [Ostrinia furnacalis]	-1.0354	-0.9119	-0.2259	1.67138	0.50184
TRINITY_DN3057_c0_g2_i1_orf1	chromodomain-helicase-DNA-binding protein Mi-2 homolog isoform X3 [Chelonus insularis]	-0.8677	-0.5522	-0.8319	1.72374	0.52805
TRINITY_DN14501_c0_g1_i1_orf1	28S ribosomal protein S28, mitochondrial [Ostrinia furnacalis]	-0.4558	-0.8526	-0.9075	1.73508	0.48086
TRINITY_DN45633_c0_g1_i1_orf1	ubiquitin thioesterase otubain-like [Ostrinia furnacalis]	-1.0515	-0.5145	-0.789	1.51959	0.83544
TRINITY_DN9575_c0_g1_i1_orf1	uncharacterized protein LOC114351119 [Ostrinia furnacalis]	-1.5512	-0.3619	-0.1674	1.40814	0.67233
TRINITY_DN23444_c0_g1_i10_orf1	serrate RNA effector molecule homolog isoform X2 [Ostrinia furnacalis]	-1.0477	-0.7342	-0.1449	1.82534	0.10153
TRINITY_DN3832_c0_g1_i1_orf1	serine-threonine kinase receptor-associated protein [Galleria mellonella]	-0.6151	-0.9199	-0.6994	1.74565	0.48879
TRINITY_DN79868_c0_g1_i1_orf1	lethal(2)neighbour of Tid protein [Ostrinia furnacalis]	-1.4631	-0.6632	0.11714	1.44478	0.56433
TRINITY_DN37599_c0_g1_i1_orf1	bmp-2 protein isoform X3 [Bombyx mori] >XP_028041166.1 RNA-binding protein 4.1-like isoform X2 [Bombyx mandarina]	-1.08	-0.8573	-0.265	1.65039	0.5519
TRINITY_DN140538_c0_g2_i1_orf1	peptidyl-prolyl cis-trans isomerase NIMA-interacting 1 [Urocitellus parryii]	-1.5481	-0.2911	0.42719	1.52431	-0.1122
TRINITY_DN2252_c0_g1_i4_orfp1	TRINITY_DN2252_c0_g1_i4_m.69997 TRINITY_DN2252_c0_g1::TRINITY_DN2252_c0_g1_i4::g.69997 ORF type:5prime_partial len:168 (-),score=65.67 TRINITY_DN2252_c0_g1_i4:244-747(-)	-1.2085	-0.6076	-0.4988	1.50545	0.80946
TRINITY_DN42824_c0_g1_i5_orf1	prefoldin subunit 3 [Ostrinia furnacalis]	-0.8139	-0.9515	-0.4202	1.75123	0.43445
TRINITY_DN2430_c0_g1_i1_orf1	glutathione S-transferase omega 1 [Ostrinia furnacalis]	-1.1798	-0.141	-0.5969	1.7935	0.12416
TRINITY_DN7122_c0_g1_i1_orf1	hypothetical protein evm_003965 [Chilo suppressalis]	-1.1876	0.32739	-1.1011	1.39704	0.56424
TRINITY_DN10455_c0_g1_i2_orf1	actin-related protein 2/3 complex subunit 4 [Plutella xylostella] >XP_013184242.1 PREDICTED: actin-related protein 2/3 complex subunit 4 [Amyelois transitella] >XP_026754865.1 actin-related protein 2/3 complex subunit 4 [Galleria mellonella] >XP_028168998.1 actin-related protein 2/3 complex subunit 4 [Ostrinia furnacalis] >KAI5632346.1 ARP2/3 complex 20 kDa subunit (ARPC4) domain-containing protein [Phthorimaea operculella] >KAG7303373.1 Actin- protein 2/3 complex subunit 4 [Plutella xylostella] >CAG9104981.1 unnamed protein product [Plutella xylostella]	-1.0612	-0.9467	0.08644	1.71043	0.21104
TRINITY_DN16128_c0_g1_i5_orf1	probable prefoldin subunit 4 [Ostrinia furnacalis]	-1.3429	-0.4602	0.27326	1.69768	-0.1679
TRINITY_DN4194_c0_g1_i1_orf1	hornerin-like [Ostrinia furnacalis]	-1.2981	0.17575	-1.0077	1.06503	1.06503
TRINITY_DN31637_c0_g1_i3_orf1	protein CDV3 homolog isoform X1 [Ostrinia furnacalis] >XP_028156793.1 protein CDV3 homolog isoform X2 [Ostrinia furnacalis] >XP_028156794.1 protein CDV3 homolog isoform X3 [Ostrinia furnacalis] >XP_028156795.1 protein CDV3 homolog isoform X1 [Ostrinia furnacalis]	-1.0829	-0.6833	-0.1338	1.8268	0.07321

TRINITY_DN2196_c0_g1_i2_orf1	HIRA-interacting protein 3-like [Ostrinia furnacalis]	-0.5159	-0.9575	-0.8342	1.63265	0.67497
TRINITY_DN20007_c0_g1_i1_orf1	hypothetical protein evm_011958 [Chilo suppressalis] >CAB3521085.1 unnamed protein product [Chilo suppressalis]	-0.8691	-0.7085	-0.6314	1.77689	0.43211
TRINITY_DN5046_c0_g3_i1_orf1	uncharacterized protein LOC114358520 [Ostrinia furnacalis]	-1.0212	-0.5159	-0.8406	1.46956	0.90814
TRINITY_DN4380_c0_g1_i9_orf1	hypothetical protein evm_012370 [Chilo suppressalis]	-1.0778	-0.8365	-0.0869	1.75183	0.24933
TRINITY_DN8561_c0_g4_i1_orf1	dynactin subunit 4 [Ostrinia furnacalis]	-1.1998	-0.1335	-0.735	1.69124	0.37702
TRINITY_DN57105_c0_g1_i2_orf1	transmembrane protein 161B isoform X1 [Galleria mellonella]	-0.8369	-1.0671	-0.4539	1.47487	0.88298
TRINITY_DN3343_c0_g2_i1_orf1	AFG3-like protein 2 [Ostrinia furnacalis]	-1.2682	-0.2868	-0.5778	1.65844	0.47443
TRINITY_DN3582_c0_g1_i2_orf1	uncharacterized protein LOC114357129 [Ostrinia furnacalis]	-0.9794	-0.7194	-0.5938	1.66722	0.62536
TRINITY_DN2885_c1_g1_i2_orf1	ubiquitin-like-specific protease ESD4 [Ostrinia furnacalis]	-1.2616	-0.901	-0.0536	1.37053	0.8457
TRINITY_DN2374_c0_g1_i1_orf1	uncharacterized protein LOC114357127 [Ostrinia furnacalis]	-1.1666	-0.9696	0.16727	1.58972	0.3792
TRINITY_DN5653_c0_g1_i4_orf1	hrp65 protein-like [Ostrinia furnacalis]	-0.8924	-1.0133	-0.3853	1.59492	0.69613
TRINITY_DN7251_c0_g1_i3_orf1	hypothetical protein evm_008498 [Chilo suppressalis] >CAB3527693.1 unnamed protein product [Chilo suppressalis] >CAH0401999.1 unnamed protein product [Chilo suppressalis]	-0.9628	-0.8677	-0.5158	1.5615	0.78477
TRINITY_DN3835_c0_g1_i3_orf1	protein ERGIC-53 isoform X1 [Ostrinia furnacalis] >XP_028177940.1 protein ERGIC-53 isoform X2 [Ostrinia furnacalis] >XP_028177941.1 protein ERGIC-53 isoform X3 [Ostrinia furnacalis]	-1.1016	-0.5623	-0.483	1.75628	0.39057
TRINITY_DN2718_c0_g1_i6_orf1	cleavage stimulation factor subunit 2 isoform X1 [Ostrinia furnacalis]	-1.1171	-0.8445	-0.3792	1.44914	0.89166
TRINITY_DN2356_c2_g1_i6_orf1	ER membrane protein complex subunit 3 [Ostrinia furnacalis]	-1.07	-0.9772	0.59208	1.59098	-0.1359
TRINITY_DN3649_c0_g1_i6_orf1	unnamed protein product [Chilo suppressalis]	-1.664	0.12788	-0.4378	1.18088	0.79296
TRINITY_DN41311_c0_g2_i3_orf1	ras-related protein Rab-8A isoform X2 [Ostrinia furnacalis]	-0.9724	-0.6464	-0.6643	1.68424	0.59886
TRINITY_DN1875_c0_g1_i1_orf1	uncharacterized protein LOC114366320 isoform X1 [Ostrinia furnacalis] >XP_028178963.1 uncharacterized protein LOC114366320 isoform X1 [Ostrinia furnacalis] >XP_028178964.1 uncharacterized protein LOC114366320 isoform X2 [Ostrinia furnacalis]	-1.5732	0.24249	-0.4211	1.49011	0.2617
TRINITY_DN59042_c1_g1_i1_orf1	nuclear pore complex protein Nup50 [Ostrinia furnacalis]	-1.0105	-0.2468	-0.7352	1.83065	0.16184
TRINITY_DN14487_c0_g1_i4_orf1	hypothetical protein HW555_009956 [Spodoptera exigua] >KAH9643419.1 hypothetical protein HF086_016708 [Spodoptera exigua] >CAH0702087.1 unnamed protein product [Spodoptera exigua]	-0.4858	-0.9663	-0.7307	1.76746	0.4153
TRINITY_DN25997_c1_g2_i4_orf1	ribokinase-like [Ostrinia furnacalis]	-0.7895	0.46612	-1.4324	1.41008	0.34563
TRINITY_DN49872_c0_g2_i1_orf1	NIF3-like protein 1 [Ostrinia furnacalis] >XP_028165862.1 NIF3-like protein 1 [Ostrinia furnacalis] >XP_028165864.1 NIF3-like protein 1 [Ostrinia furnacalis]	-1.5615	0.35068	-0.4012	1.50545	0.10656
TRINITY_DN104507_c0_g1_i2_orf1	replication protein A 32 kDa subunit [Ostrinia furnacalis]	-1.3638	-0.6863	-0.168	1.40976	0.80831
TRINITY_DN12964_c0_g1_i1_orf1	dnaJ homolog subfamily A member 1 [Ostrinia furnacalis]	-0.9582	-0.3383	-0.6999	1.85985	0.13648
TRINITY_DN2623_c0_g1_i3_orf1	unnamed protein product [Chilo suppressalis]	-0.9625	-0.9673	-0.3048	1.641	0.59352
TRINITY_DN26251_c0_g1_i1_orf1	serine/arginine-rich splicing factor 1A [Neodiprion lecontei] >XP_046417766.1 serine/arginine-rich splicing factor 1A [Neodiprion fabricii] >XP_046473571.1 serine/arginine-rich splicing factor 1A [Neodiprion pinetum] >XP_046610590.1 serine/arginine-rich splicing factor 1A [Neodiprion virginianus] >XP_046738887.1 serine/arginine-rich splicing factor 1A [Diprion similis]	-1.3438	-0.7146	0.06047	1.58375	0.4142
TRINITY_DN4262_c0_g1_i16_orf1	sperm-associated antigen 7 homolog [Ostrinia furnacalis]	-1.4211	-0.6211	-0.1441	1.39662	0.78959
TRINITY_DN34726_c0_g2_i1_orf1	heat shock factor-binding protein 1 [Ostrinia furnacalis]	-0.9706	-0.3594	-0.8442	1.74004	0.4341
TRINITY_DN40562_c0_g2_i1_orf1	dual specificity protein phosphatase 23-like isoform X2 [Ostrinia furnacalis]	-0.7823	-0.4315	-1.0173	1.69783	0.53325
TRINITY_DN130_c0_g1_i7_orf1	RNA-binding protein fusilli isoform X1 [Bombyx mori]	-1.3651	-0.4243	-0.0187	1.71697	0.09105
TRINITY_DN3539_c0_g1_i7_orf1	transcription elongation regulator 1-like [Ostrinia furnacalis]	-1.0791	-0.8473	-0.1925	1.70579	0.41319
TRINITY_DN9931_c0_g1_i1_orf1	syntaxin-18 [Ostrinia furnacalis]	-1.2202	-0.1258	-0.3996	1.82465	-0.0791
TRINITY_DN3893_c0_g2_i3_orf1	cleavage and polyadenylation specificity factor subunit CG7185 isoform X2 [Ostrinia furnacalis]	-1.3053	-0.7284	-0.2088	1.44358	0.79885
TRINITY_DN25210_c0_g1_i1_orf1	mitochondrial import receptor subunit TOM22 homolog [Ostrinia furnacalis]	-1.0217	-0.5785	-0.275	1.88304	-0.0079
TRINITY_DN22654_c0_g2_i4_orf1	protein EFR3 homolog cmp44E isoform X1 [Ostrinia furnacalis] >XP_028166854.1 protein EFR3 homolog cmp44E isoform X2 [Ostrinia furnacalis]	-0.9652	-0.7312	-0.5571	1.71172	0.54173
TRINITY_DN9309_c0_g1_i5_orf1	uncharacterized protein LOC114361160 [Ostrinia furnacalis]	-1.1096	-0.244	-0.7544	1.73158	0.37645
TRINITY_DN5818_c1_g1_i2_orf1	unnamed protein product [Chrysodeixis includens]	-0.8883	-0.9712	-0.1232	1.79349	0.18925
TRINITY_DN3860_c0_g1_i5_orf1	nucleoplasmin-like protein isoform X1 [Hyposmocoma kahamanoa]	-1.1636	-1.1277	0.47099	1.4081	0.41215
TRINITY_DN26355_c0_g1_i4_orf1	small integral membrane protein 12 [Ostrinia furnacalis]	-0.846	-0.7744	-0.6488	1.7215	0.54774
TRINITY_DN79000_c1_g1_i1_orf1	AT15141p, partial [Drosophila melanogaster]	-0.9003	-0.7179	-0.4361	1.85593	0.19842
TRINITY_DN4707_c0_g1_i1_orf1	PREDICTED: DNA-directed RNA polymerases I, II, and III subunit RPABC1 [Papilio xuthus] >XP_013187738.1 PREDICTED: DNA-directed RNA polymerases I, II, and III subunit RPABC1 [Amyelois transitella] >XP_028158146.1 DNA-directed RNA polymerases I, II, and III subunit RPABC1 [Ostrinia furnacalis] >XP_045537534.1 DNA-directed RNA polymerases I, II, and III subunit RPABC1 [Papilio machaon] >XP_049876738.1 DNA-directed RNA polymerases I, II, and III subunit RPABC1 [Pectinophora gossypiella] >KAG6452000.1 hypothetical protein O3G_MSEX007416 [Manduca sexta] >RVE48301.1 hypothetical protein evm_007052 [Chilo suppressalis] >CAG5049330.1 unnamed protein product [Parnassius apollo] >CAG9757053.1 unnamed protein product [Diatraea saccharalis] >CAH2042370.1 unnamed protein product, partial [Iphiclides podalirius]	-0.9588	-0.6053	-0.5392	1.82999	0.2733
TRINITY_DN2997_c0_g1_i6_orf1	titin-like [Ostrinia furnacalis]	-0.9238	-1.0573	0.18419	1.72922	0.06768
TRINITY_DN13216_c0_g1_i5_orf1	uncharacterized protein LOC114358344 isoform X1 [Ostrinia furnacalis]	-0.7701	-0.827	-0.8476	1.3129	1.13177
TRINITY_DN23502_c0_g1_i1_orf1	small nuclear ribonucleoprotein F [Ostrinia furnacalis]	-1.4141	-0.8154	0.26733	1.39261	0.56962
TRINITY_DN48413_c1_g1_i2_orf1	probable protein phosphatase 2C 11 isoform X1 [Manduca sexta] >KAG6442694.1 hypothetical protein O3G_MSEX002471 [Manduca sexta]	-0.8872	-0.9427	-0.5522	1.49262	0.88952
TRINITY_DN27556_c0_g1_i1_orf1	bystin [Ostrinia furnacalis]	-0.5723	-0.8418	-0.7078	1.83926	0.28267
TRINITY_DN1427_c0_g1_i7_orf1	SAFB-like transcription modulator isoform X1 [Ostrinia furnacalis] >XP_028158609.1 SAFB-like transcription modulator isoform X2 [Ostrinia furnacalis]	-1.4515	-0.255	0.02381	1.68155	0.00111
TRINITY_DN27_c0_g1_i1_orf1	THO complex subunit 4-A [Ostrinia furnacalis]	-1.1063	-0.744	-0.2401	1.74515	0.34532
TRINITY_DN35669_c0_g1_i1_orf1	unnamed protein product [Diatraea saccharalis]	-0.9165	-0.8673	-0.4682	1.6974	0.55456
TRINITY_DN15706_c0_g2_i5_orf1	cdc42 homolog [Galleria mellonella] >XP_028178764.1 cdc42 homolog [Ostrinia furnacalis] >XP_028178765.1 cdc42 homolog [Ostrinia furnacalis]	-0.8739	-0.4776	-0.8161	1.78845	0.37911
TRINITY_DN4132_c0_g1_i14_orf1	thyroid receptor-interacting protein 11-like isoform X1 [Ostrinia furnacalis]	-1.7324	0.24693	0.10585	1.38801	-0.0084
TRINITY_DN57496_c0_g1_i1_orf1	NADPH:adrenodoxin oxidoreductase, mitochondrial [Ostrinia furnacalis]	-0.7329	0.20825	-1.3557	1.57794	0.3025
TRINITY_DN76377_c0_g1_i1_orf1	uncharacterized protein LOC111357764, partial [Spodoptera litura]	-1.3134	-0.2615	-0.7336	1.19673	1.11184

TRINITY_DN20118_c0_g1_i4_orfp1	hypothetical protein HF086_007571 [Spodoptera exigua]	-1.4682	-0.3956	-0.3309	1.38904	0.80564
TRINITY_DN144258_c0_g1_i1_orf1	PREDICTED: enhancer of rudimentary homolog [Microplitis demolitor] >XP_044577051.1 enhancer of rudimentary homolog [Cotesia glomerata] >KAG8041963.1 hypothetical protein G9C98_007267 [Cotesia typhae] >KAH0539785.1 hypothetical protein KQX54_008036 [Cotesia glomerata] >CAD6227368.1 GSCOCG00006137001-RA-CDS [Cotesia congregata]	-1.3406	-0.9128	0.08076	1.12581	1.04677
TRINITY_DN1725_c0_g1_i7_orf1	T-complex protein 1 subunit gamma isoform X1 [Ostrinia furnacalis] >XP_028159782.1 T-complex protein 1 subunit gamma isoform X2 [Ostrinia furnacalis]	-1.1256	-0.4126	-0.469	1.81854	0.18875
TRINITY_DN5841_c0_g1_i2_orf1	hypothetical protein evm_011295 [Chilo suppressalis]	-1.3935	-0.3973	-0.427	1.46851	0.7493
TRINITY_DN120979_c0_g1_i1_orf1	la-related protein 1-like isoform X2 [Ostrinia furnacalis]	-0.8143	-0.8938	-0.2209	1.86695	0.06198
TRINITY_DN441_c0_g2_i1_orf1	guanine nucleotide-binding protein subunit beta-like protein [Diachasma alloeum]	-1.3246	-0.2336	0.41762	1.65796	-0.5174
TRINITY_DN136028_c0_g2_i1_orf1	cytochrome c oxidase subunit 5A, mitochondrial [Ostrinia furnacalis]	-0.7358	-0.3915	-0.9088	1.85666	0.17946
TRINITY_DN34432_c0_g1_i1_orf1	39S ribosomal protein L44, mitochondrial [Ostrinia furnacalis]	-0.7643	-0.6536	-0.7911	1.78389	0.42504
TRINITY_DN129226_c0_g1_i2_orf1	hypothetical protein evm_000268 [Chilo suppressalis]	-1.3617	-0.3582	-0.5597	1.36997	0.9096
TRINITY_DN10722_c0_g3_i1_orf1	inositol-3-phosphate synthase [Ostrinia furnacalis]	-1.3351	-0.7244	-0.1083	1.49096	0.67682
TRINITY_DN7868_c0_g1_i8_orf1	uncharacterized protein LOC114353432 isoform X4 [Ostrinia furnacalis]	-1.4449	-0.107	-0.0431	1.69943	-0.1044
TRINITY_DN154_c0_g1_i4_orf1	ER membrane protein complex subunit 4 [Ostrinia furnacalis]	-1.018	-0.7192	-0.4062	1.77325	0.37014
TRINITY_DN81715_c0_g1_i1_orf1	gamma-interferon-inducible lysosomal thiol reductase-like [Ostrinia furnacalis]	-1.0528	-0.7222	-0.6158	1.4531	0.93771
TRINITY_DN23264_c0_g1_i1_orf1	U5 small nuclear ribonucleoprotein 40 kDa protein [Ostrinia furnacalis]	-1.2538	-0.7846	0.36537	1.63633	0.03669
TRINITY_DN5648_c0_g1_i5_orf1	protein tumorous imaginal discs, mitochondrial-like isoform X2 [Ostrinia furnacalis]	-0.7652	-0.5897	-0.6817	1.89242	0.14415
TRINITY_DN27725_c0_g1_i2_orf1	BRISC complex subunit FAM175B-like [Ostrinia furnacalis]	-0.9973	-1.2264	-0.0127	1.12913	1.10729
TRINITY_DN5112_c0_g1_i1_orf1	unnamed protein product, partial [Iphiclides podalirius]	-1.3753	0.00116	-0.2407	1.74195	-0.1271
TRINITY_DN27960_c0_g1_i1_orf1	ATP synthase mitochondrial F1 complex assembly factor 1 [Ostrinia furnacalis]	-0.6536	-0.6706	-0.7443	1.87978	0.18868
TRINITY_DN25373_c0_g1_i1_orf1	epidermal growth factor receptor substrate 15-like 1 [Ostrinia furnacalis]	-0.7589	-0.5371	-0.792	1.85898	0.22905
TRINITY_DN9164_c0_g1_i3_orf1	unnamed protein product [Parnassius apollo]	-1.133	-0.7057	-0.1246	1.78025	0.18306
TRINITY_DN43293_c0_g1_i2_orf1	egl nine homolog 1 isoform X2 [Helicoverpa armigera]	-1.526	0.42245	-0.7058	1.32777	0.48153
TRINITY_DN5262_c0_g1_i7_orf1	T-complex protein 1 subunit beta [Ostrinia furnacalis]	-0.8931	-0.7567	-0.2774	1.88441	0.04275
TRINITY_DN19135_c0_g1_i1_orf1	ER membrane protein complex subunit 10 [Ostrinia furnacalis]	-1.2382	-0.3668	-0.3233	1.79137	0.13694
TRINITY_DN16349_c0_g1_i10_orf1	protein lingerer-like isoform X1 [Nymphalis io] >XP_050356663.1 protein lingerer-like isoform X1 [Nymphalis io] >XP_050356664.1 protein lingerer-like isoform X1 [Nymphalis io]	-1.1667	-0.6773	-0.4539	1.55748	0.74044
TRINITY_DN4116_c0_g1_i3_orf1	transmembrane protein 131 homolog [Ostrinia furnacalis]	-1.4896	-0.6864	0.2696	1.4114	0.49505
TRINITY_DN5775_c0_g1_i1_orf1	proteasome assembly chaperone 2 [Ostrinia furnacalis]	-1.131	-0.2533	-0.3792	1.87118	-0.1077
TRINITY_DN22572_c0_g1_i1_orf1	eukaryotic translation elongation factor 1 epsilon-1 [Ostrinia furnacalis]	-1.5259	-0.007	-0.2606	1.6022	0.1913
TRINITY_DN8944_c0_g1_i1_orf1	actin, clone 403 [Trichonephila clavata]	-0.9999	-0.8359	-0.4969	1.57473	0.75806
TRINITY_DN9741_c0_g1_i3_orf1	metaxin-2 isoform X4 [Manduca sexta] >KAG6447312.1 hypothetical protein O3G_MSEX004872 [Manduca sexta]	-0.9992	-0.3104	-0.7069	1.83667	0.17975
TRINITY_DN389_c0_g1_i2_orf1	uncharacterized protein LOC118068293 isoform X2 [Chelonus insularis]	-0.7755	-0.8424	-0.8106	1.41807	1.01043
TRINITY_DN3366_c0_g1_i6_orf1	eukaryotic translation initiation factor 3 subunit K [Helicoverpa zea]	-0.9596	-0.6337	-0.2105	1.90351	-0.0997
TRINITY_DN51968_c0_g1_i1_orf1	splicing factor U2af 38 kDa subunit [Aphidius gifuensis] >KAF7990547.1 hypothetical protein HCN44_000352 [Aphidius gifuensis]	-1.2234	-0.9705	0.1563	1.49887	0.53877
TRINITY_DN4779_c0_g1_i5_orf1	T-complex protein 1 subunit epsilon isoform X1 [Ostrinia furnacalis] >XP_028156782.1 T-complex protein 1 subunit epsilon isoform X2 [Ostrinia furnacalis]	-0.9634	-0.6076	-0.342	1.89353	0.01937
TRINITY_DN106038_c0_g1_i1_orf1	ankyrin-3-like isoform X1 [Galleria mellonella]	-0.8376	-0.6979	-0.6563	1.79543	0.39636
TRINITY_DN5697_c0_g1_i1_orf1	GPI ethanolamine phosphate transferase 2-like [Ostrinia furnacalis]	-0.9966	-1.0088	-0.1694	1.63305	0.54176
TRINITY_DN2769_c0_g1_i1_orf1	pseudouridylate synthase 7 homolog [Ostrinia furnacalis]	-1.0366	-0.743	-0.2498	1.80583	0.22357
TRINITY_DN36788_c0_g1_i2_orf1	isocitrate dehydrogenase [NADP] cytoplasmic-like [Bicyclus anynana]	-1.1898	-0.6941	-0.1468	1.72924	0.3014
TRINITY_DN11657_c0_g1_i2_orf1	trehalase-1 [Omphisa fuscidentalis]	-0.6532	-1.0621	-0.6632	1.48657	0.89195
TRINITY_DN5275_c0_g1_i1_orf1	paraplegin [Ostrinia furnacalis]	-1.1985	-0.7327	-0.2182	1.65307	0.49639
TRINITY_DN14018_c0_g1_i4_orf1	chitobiosyldiphosphodolichol beta-mannosyltransferase [Ostrinia furnacalis]	-0.6667	-0.8861	-0.5416	1.84839	0.24599
TRINITY_DN33801_c0_g1_i1_orf1	unnamed protein product [Diatraea saccharalis]	-1.1408	-0.5513	-0.3434	1.79402	0.24141
TRINITY_DN25341_c0_g1_i1_orf1	heat shock protein 90 [Loxostege sticticalis]	-0.9155	-0.7023	-0.5475	1.79834	0.36705
TRINITY_DN6396_c0_g1_i1_orf1	PR domain zinc finger protein 10-like [Ostrinia furnacalis]	-1.2653	-0.5429	0.34701	1.70947	-0.2483
TRINITY_DN18242_c0_g1_i3_orf1	CCHC-type zinc finger protein CG3800 [Papilio xuthus]	-1.0886	-0.921	0.1297	1.70898	0.17094
TRINITY_DN16965_c0_g2_i1_orf1	hypothetical protein evm_007405 [Chilo suppressalis]	-1.2705	-0.4179	-0.5061	1.62034	0.57414
TRINITY_DN10701_c0_g2_i2_orf1	synaptosomal-associated protein 29 [Ostrinia furnacalis]	-1.0122	-0.8964	-0.1766	1.73784	0.34735
TRINITY_DN9498_c0_g1_i3_orf1	eukaryotic translation initiation factor 4 gamma 3-like isoform X2 [Ostrinia furnacalis]	-0.9345	-0.5011	-0.442	1.91796	-0.0403
TRINITY_DN816_c0_g1_i3_orf1	calcium-binding mitochondrial carrier protein SCaMC-2 isoform X1 [Ostrinia furnacalis]	-1.0128	-0.5339	-0.3809	1.882	0.04566
TRINITY_DN7112_c0_g1_i1_orf1	heterogeneous nuclear ribonucleoprotein K isoform X2 [Ostrinia furnacalis]	-1.286	-0.9112	0.10254	1.44205	0.65261
TRINITY_DN16258_c0_g1_i2_orf1	uncharacterized protein LOC114359911 [Ostrinia furnacalis]	-0.9015	-0.9186	-0.3137	1.76277	0.37106
TRINITY_DN646_c0_g1_i5_orf1	unnamed protein product [Diatraea saccharalis]	-0.8359	-0.5247	-0.6418	1.89821	0.10418
TRINITY_DN1266_c2_g1_i1_orf1	serine/threonine-protein kinase RIO3 [Ostrinia furnacalis]	-0.9473	0.10057	-1.1456	1.62676	0.36561
TRINITY_DN24163_c0_g1_i1_orf1	luciferin 4-monooxygenase-like [Ostrinia furnacalis]	-0.9155	-0.4047	-0.7613	1.83207	0.24932
TRINITY_DN237_c1_g1_i1_orf1	PREDICTED: cytoplasmic protein NCK1 isoform X1 [Microplitis demolitor]	-0.5816	-1.0363	-0.6757	1.64735	0.6462
TRINITY_DN3647_c2_g1_i3_orf1	unnamed protein product, partial [Iphiclides podalirius]	-1.1342	-0.4066	-0.7645	1.54417	0.76116
TRINITY_DN51568_c0_g1_i1_orf1	splicing factor 3A subunit 2 [Ostrinia furnacalis]	-1.0528	-0.5702	-0.1037	1.87945	-0.1527
TRINITY_DN4056_c0_g1_i8_orf1	uncharacterized protein LOC114349672 [Ostrinia furnacalis] >XP_028155936.1 uncharacterized protein LOC114349672 [Ostrinia furnacalis] >XP_028155937.1 uncharacterized protein LOC114349672 [Ostrinia furnacalis] >XP_028155939.1 uncharacterized protein LOC114349672 [Ostrinia furnacalis]	-1.4031	-0.6528	0.42436	1.55546	0.07599
TRINITY_DN20499_c0_g3_i1_orf1	exosome RNA helicase MTR4 isoform X2 [Ostrinia furnacalis]	-1.0146	-0.7203	-0.115	1.85433	-0.0045
TRINITY_DN45449_c0_g1_i1_orf1	ATP-dependent helicase brm [Ostrinia furnacalis]	-1.2694	0.25022	-0.9234	1.5129	0.42963
TRINITY_DN27994_c0_g1_i1_orf1	uncharacterized protein LOC114364076 [Ostrinia furnacalis]	-0.5797	-0.9289	-0.5873	1.84165	0.25415

TRINITY_DN6710_c0_g1_i6_orf1	multiple C2 and transmembrane domain-containing protein-like [Ostrinia furnacalis]	-0.9766	-1.1098	-0.2121	1.40229	0.8962
TRINITY_DN23360_c0_g1_i3_orf1	protein PTCD3 homolog, mitochondrial [Ostrinia furnacalis]	-0.813	-0.6701	-0.6677	1.82748	0.32329
TRINITY_DN6671_c0_g1_i6_orf1	hypothetical protein evm_013656 [Chilo suppressalis] >CAB3521812.1 unnamed protein product [Chilo suppressalis] >CAH0399134.1 unnamed protein product [Chilo suppressalis]	-1.1425	-0.4484	-0.3977	1.81837	0.17026
TRINITY_DN7787_c0_g1_i1_orf1	trimeric intracellular cation channel type 1B.1 [Manduca sexta] >KAG6456518.1 hypothetical protein O3G_MSEX009773 [Manduca sexta]	-0.9339	-0.2127	-0.7791	1.86323	0.0625
TRINITY_DN4213_c0_g1_i4_orf1	nardilysin-like isoform X1 [Ostrinia furnacalis] >XP_028157649.1 nardilysin-like isoform X2 [Ostrinia furnacalis] >XP_028157650.1 nardilysin-like isoform X3 [Ostrinia furnacalis] >XP_028157651.1 nardilysin-like isoform X4 [Ostrinia furnacalis]	-0.7277	-0.793	-0.7689	1.70573	0.58386
TRINITY_DN19187_c0_g1_i1_orf1	fumarylacetoacetase [Chelonus insularis]	-1.2879	-0.2232	-0.1658	1.80233	-0.1256
TRINITY_DN8536_c0_g1_i2_orf1	PC4 and SFRS1-interacting protein isoform X4 [Galleria mellonella]	-1.2945	-0.6018	-0.3463	1.52606	0.71648
TRINITY_DN23926_c0_g1_i4_orf1	programmed cell death protein 10 [Ostrinia furnacalis]	-1.5383	-0.253	-0.2715	1.46054	0.6023
TRINITY_DN787_c0_g1_i7_orf1	YLP motif-containing protein 1-like isoform X1 [Ostrinia furnacalis]	-1.0159	-0.4447	-0.8073	1.65675	0.61119
TRINITY_DN108354_c0_g1_i1_orf1	WD repeat-containing protein 61-like [Ostrinia furnacalis]	-1.1597	-0.0797	-0.537	1.83227	-0.0559
TRINITY_DN2089_c0_g1_i5_orf1	eukaryotic translation initiation factor 4B [Ostrinia furnacalis]	-1.165	-0.814	0.02568	1.70868	0.24467
TRINITY_DN14920_c0_g1_i1_orf1	anamorsin homolog [Ostrinia furnacalis]	-0.797	-0.9509	-0.4453	1.75126	0.44196
TRINITY_DN220_c0_g1_i3_orf1	serine-arginine protein 55 isoform X6 [Pieris brassicae]	-1.1942	-0.8775	-0.1284	1.52846	0.67164
TRINITY_DN754_c1_g1_i8_orf1	lysophospholipid acyltransferase 5 [Ostrinia furnacalis] >XP_028169982.1 lysophospholipid acyltransferase 5 [Ostrinia furnacalis]	-0.7589	-0.51	-0.838	1.84154	0.26538
TRINITY_DN69236_c0_g1_i1_orf1	peroxiredoxin [Ostrinia furnacalis]	-1.3716	-0.2894	-0.5604	1.46662	0.75485
TRINITY_DN2065_c1_g2_i1_orf1	2-amino-3-ketobutyrate coenzyme A ligase, mitochondrial [Ostrinia furnacalis]	-0.7458	-0.733	-0.8397	1.66607	0.65242
TRINITY_DN969_c0_g1_i3_orf1	protein UBASH3A homolog isoform X3 [Ostrinia furnacalis]	-1.0576	-0.7211	-0.5019	1.64475	0.6359
TRINITY_DN14372_c0_g2_i1_orf1	12 kDa FK506-binding protein-like [Ostrinia furnacalis]	-0.9084	-0.7268	-0.2482	1.89341	-0.0101
TRINITY_DN3614_c0_g2_i1_orf1	PC4 and SFRS1-interacting protein isoform X4 [Galleria mellonella]	-1.3439	-0.9985	0.32627	1.17881	0.83727
TRINITY_DN11457_c0_g1_i5_orf1	uncharacterized protein LOC114352268 [Ostrinia furnacalis]	-0.8417	-0.6809	-0.332	1.92678	-0.0722
TRINITY_DN6439_c0_g1_i1_orf1	GPI mannosyltransferase 3 isoform X4 [Ostrinia furnacalis] >XP_028164836.1 GPI mannosyltransferase 3 isoform X5 [Ostrinia furnacalis]	-1.208	-0.6494	-0.3445	1.63769	0.56421
TRINITY_DN4237_c1_g1_i5_orf1	eukaryotic translation initiation factor 3 subunit A-like isoform X1 [Ostrinia furnacalis] >XP_028173593.1 eukaryotic translation initiation factor 3 subunit A-like isoform X2 [Ostrinia furnacalis] >XP_028173594.1 eukaryotic translation initiation factor 3 subunit A-like isoform X3 [Ostrinia furnacalis] >XP_028173595.1 eukaryotic translation initiation factor 3 subunit A-like isoform X4 [Ostrinia furnacalis]	-1.0306	-0.5733	-0.0332	1.88345	-0.2463
TRINITY_DN38540_c0_g1_i1_orf1	GSCOCG00000129001-RA-CDS [Cotesia congregata] >CAG5101050.1 Similar to LUC7L2: Putative RNA-binding protein Luc7-like 2 (Homo sapiens) [Cotesia congregata]	-1.0944	-0.55	-0.5292	1.74162	0.43194
TRINITY_DN21971_c0_g1_i4_orf1	40S ribosomal protein S26 [Nymphalis io]	-1.195	-0.5055	3.58E-06	1.81736	-0.1169
TRINITY_DN4135_c0_g1_i5_orf1	probable small nuclear ribonucleoprotein Sm D2 [Manduca sexta] >KAG6451233.1 hypothetical protein O3G_MSEX007016 [Manduca sexta]	-1.1106	-0.4903	-0.2053	1.86562	-0.0594
TRINITY_DN14429_c0_g1_i2_orf1	NADH dehydrogenase [ubiquinone] 1 beta subcomplex subunit 11, mitochondrial [Ostrinia furnacalis]	-0.4871	-0.5151	-1.2016	1.661	0.5428
TRINITY_DN10644_c0_g1_i2_orf1	carboxylesterase [Cnaphalocrocis medinalis]	-0.5631	-0.6883	-0.8947	1.81656	0.32963
TRINITY_DN21609_c0_g2_i1_orf1	translation initiation factor eIF-2B subunit epsilon [Ostrinia furnacalis]	-1.2695	-0.4683	-0.1022	1.7761	0.06388
TRINITY_DN131371_c0_g1_i1_orf1	golgin subfamily B member 1-like [Ostrinia furnacalis]	-1.6628	0.03349	0.33191	1.44941	-0.152
TRINITY_DN3127_c0_g1_i9_orf1	RNA-binding protein 1 isoform X1 [Galleria mellonella]	-0.6598	-1.037	-0.2433	1.84986	0.09022

TRINITY_DN31663_c0_g1_i2_orf1	PHD finger-like domain-containing protein 5A [Nasonia vitripennis] >XP_002427197.1 conserved hypothetical protein [Pediculus humanus corporis] >XP_003484388.1 PHD finger-like domain-containing protein 5A [Bombus impatiens] >XP_003701008.1 PREDICTED: PHD finger-like domain-containing protein 5A [Megachile rotundata] >XP_006623871.1 PHD finger-like domain-containing protein 5A [Apis dorsata] >XP_011068502.1 PREDICTED: PHD finger-like domain-containing protein 5A [Acromyrmex echinatior] >XP_011154391.1 PHD finger-like domain-containing protein 5A [Harpegnathos saltator] >XP_011164776.1 PHD finger-like domain-containing protein 5A [Solenopsis invicta] >XP_011262550.1 PHD finger-like domain-containing protein 5A [Camponotus floridanus] >XP_011297178.1 PREDICTED: PHD finger-like domain-containing protein 5A [Fopius arisanus] >XP_011334720.1 PHD finger-like domain-containing protein 5A [Ooceraea biroi] >XP_011506347.1 PREDICTED: PHD finger-like domain-containing protein 5A [Ceratosolen solmsi marchali] >XP_011506348.1 PREDICTED: PHD finger-like domain-containing protein 5A [Ceratosolen solmsi marchali] >XP_011638597.1 PHD finger-like domain-containing protein 5A isoform X2 [Pogonomyrmex barbatus] >XP_011686073.1 PREDICTED: PHD finger-like domain-containing protein 5A [Wasmannia auropunctata] >XP_011858255.1 PREDICTED: PHD finger-like domain-containing protein 5A [Vollenhovia emeryi] >XP_012058015.1 PREDICTED: PHD finger-like domain-containing protein 5A [Atta cephalotes] >XP_012135327.1 PREDICTED: PHD finger-like domain-containing protein 5A [Megachile rotundata] >XP_012135328.1 PREDICTED: PHD finger-like domain-containing protein 5A [Megachile rotundata] >XP_012222185.1 PREDICTED: PHD finger-like domain-containing protein 5A [Linepithema humile] >XP_012261946.1 PHD finger-like domain-containing protein 5A [Athalia rosae] >XP_012273120.1 PHD finger-like domain-containing protein 5A [Orussus abietinus] >XP_012526512.1 PHD finger-like domain-containing protein 5A [Monomorium pharaonis] >XP_014217558.1 PHD finger-like domain-containing protein 5A [Copidosoma floridanum] >XP_014484566.1 PREDICTED: PHD finger-like domain-containing protein 5A [Dinoponera quadriceps] >XP_014611099.1 PREDICTED: PHD finger-like domain-containing protein 5A [Polistes canadensis] >XP_015122018.1 PHD finger-like domain-containing protein 5A [Diachasma alloeum] >XP_015174163.1 PREDICTED: PHD finger-like domain-containing protein 5A [Polistes dominula] >XP_015433827.1 PREDICTED: PHD finger-like domain-containing protein 5A [Dufourea novaeangliae] >XP_015516165.1 PHD finger-like domain-containing protein 5A [Neodiprion lecontei] >XP_015586222.1 PHD finger-like domain-containing protein 5A isoform X1 [Cephus cinctus] >XP_016915535.1 PHD finger-like domain-containing protein 5A isoform X1 [Apis cerana] >XP_017786810.1 PREDICTED: PHD finger-like domain-containing protein 5A [Nicrophorus vespilloides] >XP_017793170.1 PREDICTED: PHD finger-like domain-containing protein 5A [Habropoda laboriosa] >XP_017887751.1 PHD finger-like domain-containing protein 5A [Ceratina calcarata] >XP_018054943.1 PREDICTED: PHD finger-like domain-containing protein 5A [Atta colombica] >XP_018309605.1 PREDICTED: PHD finger-like domain-containing protein 5A [Trachymyrmex zeteki] >XP_018344562.1 PREDICTED: PHD finger-like domain-containing protein 5A [Trachymyrmex septentrionalis] >XP_018378412.1 PREDICTED: PHD finger-like domain-containing protein 5A [Trachymyrmex cornetzi] >XP_018394190.1 PREDICTED: PHD finger-like domain-containing protein 5A [Cyphomyrmex costatus] >XP_018573484.1 PHD finger-like domain-containing protein 5A [Anoplophora glabripennis] >XP_018573485.1 PHD finger-like domain-containing protein 5A [Anoplophora glabripennis] >XP_019869751.1 PHD finger-like domain-containing protein 5A [Aethina tumida] >XP_019869752.1 PHD finger-like domain-containing protein 5A [Aethina tumida] >XP_019884294.1 PHD finger-like domain-containing protein 5A [Camponotus floridanus] >XP_020295914.1 PHD finger-like domain-containing protein 5A [Pseudomyrmex gracilis] >XP_021927485.1 PHD finger-like domain-containing protein 5A [Zootermopsis nevadensis] >XP_022205310.1 PHD finger-like domain-containing protein 5A [Nilaparvata lugens] >XP_022908436.1 PHD finger-like domain-containing protein 5A [Onthophagus taurus] >XP_022908437.1 PHD finger-like	-1.0421	-0.5994	-0.4223	1.82142	0.2424
TRINITY_DN1803_c0_g1_i3_orf1	translocator protein-like isoform X1 [Ostrinia furnacalis] >XP_028178947.1 translocator protein-like isoform X1 [Ostrinia furnacalis]	-0.695	-0.6924	-0.7336	1.85109	0.26992
TRINITY_DN1386_c0_g1_i6_orf1	ras-related protein Rab-36 [Ostrinia furnacalis]	-0.8193	-0.3189	-0.9021	1.83623	0.20399
TRINITY_DN32022_c0_g1_i1_orf1	striatin isoform X1 [Diachasma alloeum]	-1.22	-0.6932	0.03713	1.73489	0.14115
TRINITY_DN7574_c0_g1_i10_orf1	proteasome activator complex subunit 3 isoform X2 [Ostrinia furnacalis]	-1.1891	-0.7162	0.08871	1.74949	0.0671
TRINITY_DN26824_c0_g1_i1_orf1	60S ribosomal protein L6 [Hyposmocoma kahamanoa]	-1.5437	0.45653	0.05347	1.48416	-0.4504
TRINITY_DN50571_c1_g1_i1_orf1	WD repeat-containing protein 46 [Ostrinia furnacalis]	-0.6175	-0.5158	-1.1155	1.66286	0.58588
TRINITY_DN43369_c0_g2_i1_orf1	cytochrome P450 monooxygenase 304 [Glyphodes pyloalis]	-0.7602	0.54735	-1.4723	1.35978	0.32536
TRINITY_DN2894_c0_g2_i3_orf1	myrosinase 1-like isoform X1 [Ostrinia furnacalis]	-1.3889	-0.8434	0.08127	1.21584	0.93523
TRINITY_DN259_c0_g1_i8_orf1	hypothetical protein evm_000095 [Chilo suppressalis]	-1.044	-0.4886	-0.4138	1.86926	0.07717
TRINITY_DN124950_c0_g2_i1_orf1	TATA box-binding protein-like protein 1 [Ostrinia furnacalis] >XP_028155830.1 TATA box-binding protein-like protein 1 [Ostrinia furnacalis]	-0.9368	-1.2206	0.4144	1.55777	0.18518
TRINITY_DN37165_c0_g1_i4_orf1	pyridoxine-5'-phosphate oxidase-like [Ostrinia furnacalis]	-1.0164	-0.0451	-0.5823	1.8884	-0.2446
TRINITY_DN21539_c0_g1_i1_orf1	probable phenylalanine--tRNA ligase, mitochondrial [Ostrinia furnacalis]	-1.0135	-0.562	-0.6525	1.72611	0.50185
TRINITY_DN6563_c0_g1_i1_orf1	cytochrome c oxidase assembly protein COX15 homolog [Ostrinia furnacalis]	-0.81	-0.6396	-0.7271	1.80849	0.36817
TRINITY_DN2716_c0_g2_i1_orf1	eukaryotic translation initiation factor 1A, X-chromosomal [Ostrinia furnacalis] >XP_[illegible] eukaryotic translation initiation factor 1A, X-chromosomal [Melitaea cinxia] >XP_049867692.1 eukaryotic translation initiation factor 1A, X-chromosomal [Pectinophora gossypiella] >KOB79530.1 Eukaryotic translation initiation factor 1A [Operophtera brumata] >CAH2086435.1 unnamed protein product [Euphydryas editha] >KOB79531.1 Eukaryotic translation initiation factor 1A [Operophtera brumata]	-1.2209	-0.5956	0.00396	1.77574	0.03682
TRINITY_DN17394_c0_g1_i1_orf1	monocarboxylate transporter 14-like [Ostrinia furnacalis]	-1.0632	-0.8347	0.43498	1.70966	-0.2468
TRINITY_DN8971_c1_g1_i4_orf1	synaptosomal-associated protein 25 isoform X1 [Bombyx mori]	-1.4185	-0.2135	-0.3489	1.64573	0.33507
TRINITY_DN14565_c0_g1_i11_orf1	4-aminobutyrate aminotransferase, mitochondrial [Galleria mellonella]	-0.6757	-0.2429	-0.9848	1.87449	0.02897
TRINITY_DN29448_c0_g1_i1_orf1	28S ribosomal protein S9, mitochondrial [Ostrinia furnacalis]	-0.8406	-0.6612	-0.6011	1.8526	0.25036
TRINITY_DN4025_c0_g1_i13_orf1	tetratricopeptide repeat protein 14 homolog isoform X2 [Ostrinia furnacalis]	-1.5239	-0.2986	0.12725	1.60115	0.09401
TRINITY_DN3759_c0_g1_i1_orf1	uncharacterized protein LOC114350416 [Ostrinia furnacalis] >XP_028157016.1 uncharacterized protein LOC114350416 [Ostrinia furnacalis] >XP_028157017.1 uncharacterized protein LOC114350416 [Ostrinia furnacalis] >XP_028157018.1 uncharacterized protein LOC114350416 [Ostrinia furnacalis] >XP_028157019.1 uncharacterized protein LOC114350416 [Ostrinia furnacalis]	-1.1228	-0.5703	-0.6599	1.50072	0.8523
TRINITY_DN51252_c0_g2_i1_orf1	peroxidase-like [Ostrinia furnacalis]	-0.6792	-0.3496	-1.0921	1.75855	0.36234
TRINITY_DN50085_c0_g1_i1_orf1	hypothetical protein evm_013997 [Chilo suppressalis]	-0.9466	-0.7286	-0.2666	1.86999	0.07178
TRINITY_DN12242_c0_g1_i5_orf1	heterogeneous nuclear ribonucleoprotein 87F-like isoform X1 [Vanessa tameamea] >XP_046967652.1 heterogeneous nuclear ribonucleoprotein 87F-like isoform X1 [Vanessa cardui] >XP_047532045.1 heterogeneous nuclear ribonucleoprotein 87F-like [Vanessa atalanta]	-0.888	-0.931	-0.5033	1.60535	0.71703
TRINITY_DN17312_c0_g1_i1_orf1	mRNA cap guanine-N7 methyltransferase [Ostrinia furnacalis]	-0.9457	-0.9583	-0.2245	1.72448	0.40395
TRINITY_DN58531_c0_g1_i1_orf1	uncharacterized protein LOC114357371 [Ostrinia furnacalis] >XP_028166768.1 uncharacterized protein LOC114357371 [Ostrinia furnacalis]	-1.0856	-0.6913	-0.577	1.52328	0.83072
TRINITY_DN334_c0_g1_i3_orf1	chymotrypsin-like serine protease [Ostrinia nubilalis] >AAX62030.1 chymotrypsin-like serine protease [Ostrinia nubilalis]	-0.7195	-0.8172	-0.6967	1.76288	0.47051
TRINITY_DN886_c0_g2_i4_orf1	collagenase-like [Ostrinia furnacalis]	-0.8835	-0.5625	-0.6464	1.85128	0.24103
TRINITY_DN30_c0_g1_i6_orf1	casein kinase I-like isoform X1 [Hyposmocoma kahamanoa]	-0.9968	-0.4153	-0.7259	1.7836	0.35447
TRINITY_DN3457_c0_g1_i4_orf1	aryl hydrocarbon receptor nuclear translocator homolog [Ostrinia furnacalis]	-1.4541	-0.6077	0.18147	1.53826	0.3421

TRINITY_DN38301_c0_g1_i2_orf1	gamma-taxilin [Ostrinia furnacalis]	-0.6225	-0.5912	-0.7504	1.92305	0.04108
TRINITY_DN39532_c0_g1_i1_orf1	hypothetical protein evm_009649 [Chilo suppressalis]	-1.1996	-0.3595	-0.5425	1.73233	0.36931
TRINITY_DN3773_c0_g1_i4_orf1	peptidyl-prolyl cis-trans isomerase G isoform X2 [Ostrinia furnacalis]	-1.1799	-0.467	-0.2111	1.82875	0.02927
TRINITY_DN52893_c0_g1_i1_orf1	growth arrest and DNA damage-inducible proteins-interacting protein 1 [Galleria mellonella]	-0.8961	-0.5968	-0.3056	1.93128	-0.1328
TRINITY_DN10662_c0_g1_i4_orf1	HD domain-containing protein 2 [Ostrinia furnacalis]	-1.1347	-0.4713	-0.4947	1.77121	0.32943
TRINITY_DN46778_c0_g1_i2_orf1	Deoxycytidylate deaminase [Papilio xuthus]	-0.4688	-0.6551	-1.0052	1.79746	0.33154
TRINITY_DN44557_c0_g2_i1_orf1	serine hydrolase-like protein [Ostrinia furnacalis]	-1.1188	-0.4337	-0.3395	1.85566	0.03643
TRINITY_DN25783_c0_g1_i2_orf1	SET and MYND domain-containing protein 4 [Ostrinia furnacalis]	-1.47	-0.4323	-0.0588	1.58295	0.37817
TRINITY_DN11013_c0_g1_i3_orf1	glutamine:fructose-6-phosphate aminotransferase 1 [Heortia vitessoides]	-0.9033	-0.5946	-0.506	1.88702	0.11683
TRINITY_DN2117_c0_g1_i1_orf1	BUB3-interacting and GLEBS motif-containing protein ZNF207 [Chelonus insularis]	-1.0418	-0.8438	-0.1759	1.75429	0.30715
TRINITY_DN43412_c0_g1_i2_orf1	U1 small nuclear ribonucleoprotein C [Ostrinia furnacalis]	-1.2843	-0.813	0.00847	1.54844	0.54035
TRINITY_DN21531_c0_g1_i1_orf1	viral IAP-associated factor homolog [Ostrinia furnacalis]	-0.5588	-0.99	-0.6733	1.7378	0.48422
TRINITY_DN26963_c0_g1_i1_orf1	aminoacyl tRNA synthase complex-interacting multifunctional protein 1 isoform X2 [Ostrinia furnacalis]	-0.9424	-0.3838	-0.4913	1.92648	-0.1089
TRINITY_DN2673_c0_g3_i1_orf1	uncharacterized protein LOC114361372 [Ostrinia furnacalis]	-1.2088	-0.0201	-0.5811	1.78896	0.021
TRINITY_DN21150_c0_g1_i4_orf1	RNA-binding protein cabeza-like isoform X2 [Bicyclus anynana]	-1.1296	-0.6717	-0.2334	1.7751	0.25961
TRINITY_DN19160_c0_g1_i1_orf1	alkyldihydroxyacetonephosphate synthase [Ostrinia furnacalis]	-0.7921	-0.9248	-0.716	1.36627	1.0667
TRINITY_DN2265_c0_g2_i1_orf1	LOW QUALITY PROTEIN: elongation factor G, mitochondrial-like [Leguminivora glycinivorella]	-0.6132	-0.5064	-0.8558	1.90534	0.07001
TRINITY_DN4977_c0_g1_i2_orf1	manganese-transporting ATPase 13A1 [Ostrinia furnacalis]	-0.7282	-0.6916	-0.4645	1.94224	-0.0579
TRINITY_DN5686_c0_g1_i4_orf1	FACT complex subunit spt16 isoform X2 [Ostrinia furnacalis]	-1.0594	-0.1059	-0.4815	1.8908	-0.2441
TRINITY_DN31327_c0_g2_i1_orf1	multidrug resistance protein 1A isoform X1 [Ostrinia furnacalis]	-1.062	-0.1779	-0.3183	1.90275	-0.3446
TRINITY_DN32997_c0_g1_i8_orf1	RNA-binding protein squid isoform X1 [Ostrinia furnacalis]	-0.8212	-0.7876	-0.3703	1.88669	0.09246
TRINITY_DN478_c0_g1_i16_orf1	lipid storage droplets surface-binding protein 2 isoform X1 [Ostrinia furnacalis]	-0.6241	-1.2279	0.12968	1.75638	-0.0341
TRINITY_DN15959_c0_g1_i1_orf1	dnaJ homolog subfamily A member 2-like [Ostrinia furnacalis]	-0.7575	-0.6627	-0.4507	1.94385	-0.0729
TRINITY_DN57798_c0_g1_i1_orf1	ubiquitin carboxyl-terminal hydrolase 36 [Ostrinia furnacalis]	-0.7708	-0.5446	-0.876	1.78163	0.40968
TRINITY_DN16894_c0_g1_i5_orf1	dnaJ homolog subfamily C member 5 isoform X1 [Colias croceus]	-1.1741	-0.3088	-0.5833	1.75811	0.30809
TRINITY_DN2807_c0_g1_i4_orf1	FK506-binding protein 59 isoform X1 [Ostrinia furnacalis]	-0.9422	-0.7552	-0.2156	1.86907	0.04397
TRINITY_DN20369_c0_g1_i2_orf1	uncharacterized protein LOC114366225 [Ostrinia furnacalis]	-0.8305	-0.6323	-0.4805	1.91805	0.02527
TRINITY_DN7583_c0_g1_i1_orf1	39S ribosomal protein L21, mitochondrial [Ostrinia furnacalis]	-0.5934	-0.9478	-0.6087	1.80569	0.34421
TRINITY_DN13055_c0_g1_i5_orf1	116 kDa U5 small nuclear ribonucleoprotein component isoform X1 [Ostrinia furnacalis] >XP_028159219.1 116 kDa U5 small nuclear ribonucleoprotein component isoform X2 [Ostrinia furnacalis]	-1.143	-0.8289	-0.0618	1.70039	0.33338
TRINITY_DN698_c0_g1_i5_orf1	PREDICTED: small nuclear ribonucleoprotein Sm D3 [Amyelois transitella]	-0.849	-0.6874	-0.3976	1.90997	0.02404
TRINITY_DN123139_c0_g1_i1_orfp1	TRINITY_DN123139_c0_g1_i1_m.79879 TRINITY_DN123139_c0_g1::TRINITY_DN123139_c0_g1_i1::g.79879 ORF type:3prime_partial len:76 (+),score=3.83 TRINITY_DN123139_c0_g1_i1:25-225(+)	-1.0589	-0.5207	0.07227	1.86415	-0.3568
TRINITY_DN6248_c0_g1_i1_orf1	DNA topoisomerase I, mitochondrial [Ostrinia furnacalis]	-0.7712	-0.753	-0.6968	1.77612	0.44497
TRINITY_DN8261_c0_g1_i1_orf1	UDP-N-acetylhexosamine pyrophosphorylase-like protein 1 [Ostrinia furnacalis]	-0.703	-0.6733	-0.6512	1.90062	0.12686
TRINITY_DN3235_c0_g1_i1_orf1	SPARC [Trichoplusia ni]	-0.9764	-0.6609	-0.7754	1.42773	0.98499
TRINITY_DN27704_c0_g1_i1_orf1	PREDICTED: tRNA (guanine-N(7)-)-methyltransferase [Amyelois transitella]	-0.8247	-0.3857	-0.5605	1.95523	-0.1843
TRINITY_DN2062_c0_g1_i11_orf1	uncharacterized protein LOC114350846 [Ostrinia furnacalis]	-1.055	-0.1988	-0.4452	1.89974	-0.2008
TRINITY_DN84322_c0_g2_i1_orf1	alanyl-tRNA synthetase 1 [Homo sapiens] >KAI4055846.1 alanyl-tRNA synthetase 1 [Homo sapiens]	-1.1563	-0.535	0.10028	1.82043	-0.2294
TRINITY_DN4125_c1_g1_i5_orf1	angiotensin-converting enzyme-like isoform X2 [Ostrinia furnacalis]	-0.692	-0.7039	-0.6401	1.89647	0.13945
TRINITY_DN2082_c0_g1_i2_orf1	choline-phosphate cytidylyltransferase B-like isoform X1 [Ostrinia furnacalis]	-0.8241	-0.4227	-0.6595	1.92534	-0.0191
TRINITY_DN25779_c0_g1_i6_orf1	aldo-keto reductase AKR2E4-like [Ostrinia furnacalis]	-0.794	-0.9637	-0.5977	1.5711	0.78433
TRINITY_DN5554_c0_g1_i2_orf1	double-stranded RNA-binding protein Staufen homolog 2 isoform X5 [Pectinophora gossypiella]	-0.5573	-0.5277	-0.8627	1.91455	0.0332
TRINITY_DN5459_c0_g1_i1_orf1	protein takeout-like isoform X2 [Ostrinia furnacalis]	-0.7533	-0.8536	-0.8082	1.46689	0.94817
TRINITY_DN1706_c0_g1_i7_orf1	LOW QUALITY PROTEIN: RNA polymerase-associated protein CTR9 homolog [Ostrinia furnacalis]	-1.1753	-0.6084	-0.3393	1.72482	0.39813
TRINITY_DN5578_c0_g1_i4_orf1	chromatin modification-related protein eaf-1-like [Ostrinia furnacalis]	-1.1756	-0.6175	-0.2748	1.74919	0.31867
TRINITY_DN5087_c0_g1_i6_orf1	nascent polypeptide-associated complex subunit alpha [Ostrinia furnacalis] >XP_028156807.1 nascent polypeptide-associated complex subunit alpha [Ostrinia furnacalis] >XP_028156808.1 nascent polypeptide-associated complex subunit alpha [Ostrinia furnacalis]	-0.6973	-0.7483	-0.4722	1.93149	-0.0137
TRINITY_DN2062_c0_g1_i9_orf1	uncharacterized protein LOC114350846 [Ostrinia furnacalis]	-1.2069	-0.3636	-0.3971	1.79554	0.17204
TRINITY_DN142652_c0_g1_i1_orf1	pre-mRNA-splicing factor RBM22 [Chelonus insularis]	-1.3849	-0.4079	-0.0377	1.70224	0.12835
TRINITY_DN46173_c0_g3_i2_orf1	tropomyosin-1, isoforms 9A/A/B isoform X33 [Aedes aegypti] >EAT46020.1 AAEL002761-PB [Aedes aegypti]	-0.8766	-0.633	-0.414	1.91296	0.01061
TRINITY_DN4345_c0_g1_i9_orf1	uncharacterized protein LOC114357127 [Ostrinia furnacalis]	-1.4264	-0.7035	0.42598	1.49897	0.20499
TRINITY_DN32306_c0_g1_i3_orf1	acetyl-coenzyme A transporter 1 [Ostrinia furnacalis]	-1.3903	-0.3698	0.10981	1.70732	-0.057
TRINITY_DN47123_c0_g1_i1_orf1	WD40 repeat-containing protein SMU1 [Ostrinia furnacalis]	-0.9275	-0.7559	-0.2181	1.87622	0.02523
TRINITY_DN19361_c0_g1_i7_orf1	hydroxylysine kinase [Ostrinia furnacalis] >XP_028168144.1 hydroxylysine kinase [Ostrinia furnacalis]	-0.659	-0.765	-0.5503	1.91686	0.05746
TRINITY_DN1427_c0_g1_i9_orf1	SAFB-like transcription modulator isoform X3 [Ostrinia furnacalis]	-1.2408	-0.8711	0.15764	1.59647	0.3578
TRINITY_DN82801_c0_g1_i1_orf1	uncharacterized protein LOC114364712 [Ostrinia furnacalis]	-0.688	-0.688	-1.0197	1.46569	0.93012
TRINITY_DN45477_c0_g1_i1_orf1	putative E3 ubiquitin-protein ligase UBR7 [Ostrinia furnacalis]	-0.7566	-0.3198	-0.9663	1.82918	0.21356
TRINITY_DN1775_c0_g1_i3_orf1	ATP-dependent RNA helicase dbp2-like isoform X1 [Ostrinia furnacalis]	-0.6691	-0.7289	-0.8511	1.74393	0.50522
TRINITY_DN24317_c0_g1_i7_orf1	peptidyl-tRNA hydrolase ICT1, mitochondrial [Ostrinia furnacalis]	-0.862	-0.3203	-0.5833	1.94474	-0.1791
TRINITY_DN6231_c0_g1_i6_orf1	ran-binding protein 3 isoform X1 [Ostrinia furnacalis] >XP_028166372.1 ran-binding protein 3 isoform X2 [Ostrinia furnacalis]	-1.1339	-0.5326	-0.2317	1.83661	0.06153
TRINITY_DN5867_c0_g1_i1_orf1	NADH dehydrogenase [ubiquinone] 1 alpha subcomplex subunit 7-like [Ostrinia furnacalis]	-0.6912	-0.6229	-0.5859	1.9465	-0.0465
TRINITY_DN40704_c0_g1_i2_orf1	COX assembly mitochondrial protein homolog [Ostrinia furnacalis]	-0.9737	-0.5628	-0.5516	1.83491	0.25315
TRINITY_DN3428_c0_g1_i1_orf1	10 kDa heat shock protein, mitochondrial [Ostrinia furnacalis]	-0.8791	-0.6742	-0.2493	1.92255	-0.12

TRINITY_DN35635_c0_g1_i1_orf1	probable NADH dehydrogenase [ubiquinone] 1 alpha subcomplex subunit 12 [Ostrinia furnacalis]	-0.5404	-1.0249	-0.3006	1.88856	-0.0226
TRINITY_DN37538_c0_g4_i1_orf1	esterase FE4-like [Ostrinia furnacalis]	-0.7164	-0.544	-0.7643	1.89464	0.13013
TRINITY_DN29934_c0_g1_i6_orf1	sodium/potassium-transporting ATPase subunit beta-2-like [Ostrinia furnacalis] >XP_028176258.1 sodium/potassium-transporting ATPase subunit beta-2-like [Ostrinia furnacalis]	-1.1313	-1.0455	0.32009	1.5612	0.29549
TRINITY_DN17905_c0_g3_i1_orf1	zinc finger protein 706-like [Ostrinia furnacalis] >XP_028176219.1 zinc finger protein 706-like [Ostrinia furnacalis] >XP_028176220.1 zinc finger protein 706-like [Ostrinia furnacalis] >XP_028176221.1 zinc finger protein 706-like [Ostrinia furnacalis]	-0.65	-0.7297	-0.6989	1.87486	0.20371
TRINITY_DN100821_c0_g1_i1_orf1	putative GMP synthase, partial [Operophtera brumata]	-1.0627	-0.4233	0.07477	1.86537	-0.4541
TRINITY_DN36538_c0_g1_i2_orf1	xaa-Pro dipeptidase isoform X1 [Ostrinia furnacalis] >XP_028156507.1 xaa-Pro dipeptidase isoform X2 [Ostrinia furnacalis]	-0.7421	-0.7469	-0.353	1.93845	-0.0965
TRINITY_DN628_c0_g1_i7_orf1	prostamide/prostaglandin F synthase-like [Ostrinia furnacalis]	-0.461	-0.9266	-0.6395	1.86953	0.15754
TRINITY_DN14313_c0_g1_i1_orf1	25S rRNA (cytosine-C(5))-methyltransferase nop2 [Ostrinia furnacalis]	-0.7472	-0.6076	-0.5556	1.93983	-0.0294
TRINITY_DN63662_c0_g4_i1_orf1	polyadenylate-binding protein 1 [Ostrinia furnacalis]	-0.8612	-0.5906	-0.4768	1.91891	0.00959
TRINITY_DN1445_c0_g1_i1_orf1	leucine-rich PPR motif-containing protein, mitochondrial [Ostrinia furnacalis]	-0.6487	-0.6604	-0.5751	1.95138	-0.0672
TRINITY_DN139212_c0_g1_i4_orf1	uncharacterized protein LOC114350112 [Ostrinia furnacalis]	-1.1009	-1.0254	-0.0379	1.52551	0.63873
TRINITY_DN6308_c0_g1_i6_orf1	myc box-dependent-interacting protein 1 isoform X2 [Ostrinia furnacalis]	-0.8524	-0.6437	-0.3984	1.92338	-0.0288
TRINITY_DN44219_c0_g1_i1_orf1	mitochondrial import inner membrane translocase subunit TIM50-C-like [Ostrinia furnacalis]	-0.8574	-0.803	-0.3262	1.87094	0.11557
TRINITY_DN79804_c0_g1_i1_orf1	zinc finger protein on ecdysone puffs-like [Ostrinia furnacalis]	-0.9569	-0.5818	-0.4319	1.88466	0.08592
TRINITY_DN6685_c0_g1_i8_orf1	cleft lip and palate transmembrane protein 1 homolog [Ostrinia furnacalis]	-0.9576	-0.7784	-0.2027	1.85157	0.08722
TRINITY_DN2852_c0_g1_i9_orf1	golgin subfamily A member 4-like [Ostrinia furnacalis]	-0.8696	-0.6178	-0.2649	1.93839	-0.1861
TRINITY_DN791_c0_g1_i2_orf1	peroxiredoxin 1 isoform X1 [Maniola jurtina]	-0.839	-0.58	-0.387	1.94682	-0.1408
TRINITY_DN23343_c0_g1_i9_orf1	pre-mRNA-processing factor 6 isoform X1 [Ostrinia furnacalis] >XP_028175021.1 pre-mRNA-processing factor 6 isoform X2 [Ostrinia furnacalis]	-1.0805	-0.3928	-0.3084	1.88981	-0.1081
TRINITY_DN51045_c0_g1_i1_orf1	cell growth-regulating nucleolar protein [Ostrinia furnacalis]	-0.6324	-0.5849	-0.66	1.95359	-0.0762
TRINITY_DN2918_c0_g1_i1_orf1	28S ribosomal protein S10, mitochondrial [Ostrinia furnacalis] >XP_028175147.1 28S ribosomal protein S10, mitochondrial [Ostrinia furnacalis]	-0.655	-0.7422	-0.7104	1.85829	0.24932
TRINITY_DN1285_c0_g1_i6_orf1	bifunctional 3'-phosphoadenosine 5'-phosphosulfate synthase isoform X3 [Ostrinia furnacalis]	-0.8959	-0.4712	-0.2558	1.9501	-0.3272
TRINITY_DN14436_c0_g1_i7_orf1	V-type proton ATPase subunit C [Vanessa cardui]	-0.7087	-0.5285	-0.6322	1.95242	-0.0831
TRINITY_DN35725_c0_g1_i1_orf1	mitochondrial import inner membrane translocase subunit Tim13-like [Bicyclus anynana] >CAG9745432.1 unnamed protein product [Diatraea saccharalis] >CAG9784117.1 unnamed protein product [Diatraea saccharalis]	-0.6715	-0.632	-0.7795	1.86993	0.21306
TRINITY_DN18036_c0_g1_i7_orf1	pentatricopeptide repeat-containing protein 2, mitochondrial-like [Ostrinia furnacalis]	-0.7207	-0.5917	-0.4932	1.96512	-0.1594
TRINITY_DN4036_c0_g2_i1_orf1	microvitellogenin-like [Ostrinia furnacalis]	-1.4544	0.18147	-0.0204	1.65042	-0.357
TRINITY_DN1353_c0_g1_i1_orf1	UDP-glucose 4-epimerase-like [Ostrinia furnacalis]	-0.6118	-0.6283	-0.8934	1.82726	0.3063
TRINITY_DN30233_c0_g1_i2_orf1	39S ribosomal protein L10, mitochondrial [Ostrinia furnacalis]	-1.0175	-0.2157	-0.4707	1.91141	-0.2075
TRINITY_DN5238_c0_g1_i2_orf1	DNA-(apurinic or apyrimidinic site) lyase [Ostrinia furnacalis]	-1.1735	-0.6668	-0.2307	1.7357	0.33526
TRINITY_DN753_c0_g1_i4_orf1	venom dipeptidyl peptidase 4-like isoform X2 [Ostrinia furnacalis]	-0.954	-0.2814	-0.4113	1.93819	-0.2915
TRINITY_DN26186_c0_g1_i7_orf1	sodium- and chloride-dependent glycine transporter 1-like [Ostrinia furnacalis]	-0.779	-0.0738	-1.0439	1.81414	0.08256
TRINITY_DN107035_c0_g1_i1_orf1	splicing factor 3A subunit 3 [Ostrinia furnacalis]	-0.8058	-0.5102	-0.5509	1.94443	-0.0774
TRINITY_DN8369_c0_g1_i1_orf1	39S ribosomal protein L37, mitochondrial [Ostrinia furnacalis]	-0.5307	-0.5307	-0.8694	1.91852	0.01225
TRINITY_DN10332_c0_g1_i2_orfp1	TRINITY_DN10332_c0_g1_i2_m.42894 TRINITY_DN10332_c0_g1::TRINITY_DN10332_c0_g1_i2::g.42894 ORF type:3prime_partial len:77 (+),score=1.70 TRINITY_DN10332_c0_g1_i2:1005-1232(+)	-1.1539	-0.6293	-0.0137	1.80891	-0.012
TRINITY_DN12497_c0_g1_i1_orf1	probable N-acetyltransferase san [Ostrinia furnacalis]	-0.7645	-0.5093	-0.5137	1.96488	-0.1774
TRINITY_DN8691_c0_g1_i3_orf1	nucleolin-like [Melitaea cinxia]	-0.8562	-0.9826	-0.3777	1.70085	0.51566
TRINITY_DN25975_c0_g3_i2_orf1	V-type proton ATPase subunit D isoform X2 [Ostrinia furnacalis]	-0.6084	-0.4576	-0.7877	1.94714	-0.0935
TRINITY_DN27721_c1_g1_i2_orf1	mitochondrial import receptor subunit TOM20 homolog [Ostrinia furnacalis]	-0.806	-0.5029	-0.5055	1.95503	-0.1406
TRINITY_DN430_c0_g1_i5_orf1	hypothetical protein NE865_02252 [Phthorimaea operculella]	-1.0312	-0.2512	-0.0627	1.89012	-0.545
TRINITY_DN52296_c0_g1_i6_orf1	protein takeout-like [Ostrinia furnacalis]	-1.0236	-0.6532	0.18238	1.83707	-0.3426
TRINITY_DN4762_c0_g1_i2_orf1	ATPase family AAA domain-containing protein 1 isoform X2 [Ostrinia furnacalis]	-1.0169	-0.5365	-0.667	1.72993	0.49044
TRINITY_DN7213_c0_g1_i2_orf1	probable ATP-dependent RNA helicase CG8611 [Ostrinia furnacalis]	-0.761	-0.6377	-0.2631	1.96319	-0.3013
TRINITY_DN15900_c0_g1_i6_orf1	unnamed protein product [Diatraea saccharalis]	-0.9831	-0.4483	-0.5679	1.86899	0.13027
TRINITY_DN3062_c0_g1_i1_orf1	HEAT repeat-containing protein 1 [Ostrinia furnacalis]	-0.956	-0.5294	-0.3616	1.9158	-0.0688
TRINITY_DN23734_c0_g1_i1_orf1	histone-lysine N-methyltransferase SMYD3 [Ostrinia furnacalis]	-1.3045	-0.1929	-0.25	1.78797	-0.0405
TRINITY_DN3733_c0_g1_i1_orf1	60S ribosomal protein L37, partial [Papilio machaon]	-1.2432	-0.0482	-0.3205	1.81846	-0.2065
TRINITY_DN1249_c0_g1_i6_orf1	venom carboxylesterase-6-like [Ostrinia furnacalis]	-1.0348	-0.8416	-0.4693	1.52609	0.81961
TRINITY_DN24476_c0_g1_i1_orf1	ensconsin-like isoform X1 [Ostrinia furnacalis]	-0.7041	-0.9128	-0.3297	1.88645	0.06018
TRINITY_DN8543_c0_g1_i1_orf1	39S ribosomal protein L38, mitochondrial [Ostrinia furnacalis]	-0.9174	-0.6694	0.13584	1.87421	-0.4232
TRINITY_DN44288_c0_g1_i2_orf1	ATP-dependent RNA helicase p62 [Ostrinia furnacalis]	-0.5001	-0.4909	-0.9002	1.92291	-0.0317
TRINITY_DN28592_c0_g1_i2_orf1	UDP-glucuronosyltransferase 2B14-like isoform X1 [Ostrinia furnacalis] >XP_028167291.1 UDP-glucuronosyltransferase 2B14-like isoform X2 [Ostrinia furnacalis]	-0.9018	-0.4152	-0.4644	1.94236	-0.161
TRINITY_DN6747_c0_g1_i7_orf1	retinol dehydrogenase 12-like [Ostrinia furnacalis]	-0.8981	-0.6281	-0.0947	1.92317	-0.3023
TRINITY_DN19244_c0_g1_i7_orf1	uncharacterized protein LOC114350218 [Ostrinia furnacalis]	-1.1801	-0.6513	-0.2332	1.73855	0.32602
TRINITY_DN9003_c0_g1_i20_orf1	RNA-binding protein Nova-2 isoform X4 [Ostrinia furnacalis]	-0.8985	-0.5863	0.05316	1.90354	-0.4719
TRINITY_DN2953_c1_g1_i2_orf1	methionine--tRNA ligase, cytoplasmic isoform X6 [Ostrinia furnacalis]	-0.634	-0.5725	-0.9655	1.78577	0.38621
TRINITY_DN47731_c0_g1_i2_orf1	nucleolar GTP-binding protein 2 [Ostrinia furnacalis]	-0.6405	-0.7468	-0.8715	1.73001	0.5288
TRINITY_DN18172_c0_g1_i6_orf1	digestive cysteine proteinase 2-like [Ostrinia furnacalis]	-0.5283	-0.457	-0.806	1.9582	-0.1669
TRINITY_DN30932_c0_g1_i2_orf1	delta(24)-sterol reductase-like isoform X2 [Ostrinia furnacalis]	-0.5036	-0.5477	-0.9839	1.85642	0.17885
TRINITY_DN3082_c1_g1_i7_orf1	ribosomal RNA processing protein 1 homolog [Ostrinia furnacalis]	-0.5882	-0.4428	-0.7343	1.96907	-0.2038
TRINITY_DN35662_c0_g1_i5_orf1	hypothetical protein evm_006436 [Chilo suppressalis] >CAB3522373.1 unnamed protein product [Chilo suppressalis] >CAH0399695.1 unnamed protein product [Chilo suppressalis]	-0.5584	-0.6165	-0.6239	1.97199	-0.1731

TRINITY_DN5578_c0_g1_i10_orf1	unnamed protein product [Chilo suppressalis]	-0.331	-0.6687	-1.015	1.83907	0.17567
TRINITY_DN50787_c0_g2_i2_orf1	40S ribosomal protein S29 [Hyposmocoma kahamanoa] >XP_028176503.1 40S ribosomal protein S29 [Ostrinia furnacalis] >XP_049877832.1 40S ribosomal protein S29 [Pectinophora gossypiella] >ADT80654.1 ribosomal protein S29 [Euphydryas aurinia] >CAB3523209.1 unnamed protein product [Chilo suppressalis] >CAH0400531.1 unnamed protein product [Chilo suppressalis]	-1.2261	-0.2334	0.09091	1.80174	-0.4331
TRINITY_DN23175_c0_g1_i6_orf1	myb-binding protein 1A-like protein [Ostrinia furnacalis]	-0.6049	-0.5723	-0.6304	1.97042	-0.1627
TRINITY_DN11069_c0_g2_i1_orf1	fat storage-inducing transmembrane protein [Ostrinia furnacalis]	-1.0042	-0.3918	-0.2821	1.92312	-0.245
TRINITY_DN43942_c0_g2_i1_orf1	LOW QUALITY PROTEIN: caprin homolog [Ostrinia furnacalis]	-1.0377	-0.5226	-0.5593	1.79832	0.32132
TRINITY_DN26688_c0_g1_i2_orf1	myogenesis-regulating glycosidase-like [Ostrinia furnacalis]	-0.6997	-0.6046	-0.5238	1.96274	-0.1346
TRINITY_DN48237_c0_g1_i5_orf1	myogenesis-regulating glycosidase-like [Ostrinia furnacalis]	-0.7039	-0.3179	-0.6962	1.96421	-0.2462
TRINITY_DN8116_c0_g1_i1_orf1	uncharacterized protein LOC114350845 [Ostrinia furnacalis]	-0.6138	-0.5392	-0.6296	1.97465	-0.1921
TRINITY_DN140669_c0_g1_i1_orf1	S-methyl-5'-thioadenosine phosphorylase-like isoform X1 [Hyposmocoma kahamanoa]	-0.4874	-0.3671	-0.9141	1.93978	-0.1711
TRINITY_DN13651_c0_g1_i2_orf1	40S ribosomal protein S12, mitochondrial [Ostrinia furnacalis]	-0.9066	-0.5505	-0.4031	1.9257	-0.0656
TRINITY_DN47114_c0_g1_i5_orf1	nucleolar protein dao-5 isoform X2 [Ostrinia furnacalis]	-0.8843	-0.4082	-0.1908	1.94891	-0.4656
TRINITY_DN7047_c0_g1_i1_orf1	hypothetical protein G9C98_004728 [Cotesia typhae]	-0.7154	-0.4326	-0.6711	1.95743	-0.1383
TRINITY_DN51766_c0_g1_i2_orf1	facilitated trehalose transporter Tret1-like [Ostrinia furnacalis]	-0.7774	-0.6265	-0.6347	1.89175	0.14687
TRINITY_DN20682_c0_g2_i1_orf1	glutathione S-transferase delta3 [Glyphodes pyloalis]	-0.6929	-0.3854	-0.7389	1.95126	-0.134
TRINITY_DN29120_c0_g1_i6_orf1	putative inorganic phosphate cotransporter [Ostrinia furnacalis]	-0.7199	-0.5098	-0.8503	1.85722	0.22275
TRINITY_DN717_c0_g1_i2_orfp1	TRINITY_DN717_c0_g1_i2_m.67915 TRINITY_DN717_c0_g1::TRINITY_DN717_c0_g1_i2::g.67915 ORF type:internal len:868 (+),score=265.71,Collagen PF01391.19 0.11,Collagen|PF01391.19|0.039,Collagen|PF01391.19|0.00054,Collagen|PF01391.19|0.0019,Collagen|PF01391.19|0.0005,Collagen|PF01391.19|9.9e-05,Collagen|PF01391.19|1.7e-07 TRINITY_DN717_c0_g1_i2:3-2603(+)	-0.4451	-0.5527	-0.8451	1.94223	-0.0993
TRINITY_DN3194_c0_g1_i6_orf1	uncharacterized protein LOC114361386 [Ostrinia furnacalis]	-0.6176	-0.331	-0.72	1.97415	-0.3056
TRINITY_DN22983_c0_g1_i2_orfp1	TRINITY_DN22983_c0_g1_i2_m.10495 TRINITY_DN22983_c0_g1::TRINITY_DN22983_c0_g1_i2::g.10495 ORF type:internal len:79 (-),score=15.95,Polyhedrin PF00738.19 7.6e-40 TRINITY_DN22983_c0_g1_i2:2-235(-)	-0.6968	-0.5409	-0.3898	1.9854	-0.3579
TRINITY_DN75188_c0_g1_i1_orf1	fatty acid-binding protein 1-like [Ostrinia furnacalis]	-0.8861	-0.5152	-0.3848	1.94334	-0.1572
TRINITY_DN11587_c0_g1_i7_orf1	elongation of very long chain fatty acids protein AAEL008004-like isoform X1 [Danaus plexippus plexippus] >XP_032511006.1 elongation of very long chain fatty acids protein AAEL008004-like isoform X1 [Danaus plexippus plexippus] >XP_032511007.1 elongation of very long chain fatty acids protein AAEL008004-like isoform X1 [Danaus plexippus plexippus] >XP_032511008.1 elongation of very long chain fatty acids protein AAEL008004-like isoform X1 [Danaus plexippus plexippus] >XP_032511009.1 elongation of very long chain fatty acids protein AAEL008004-like isoform X1 [Danaus plexippus plexippus]	-0.6858	-0.5321	-0.5733	1.97115	-0.1799
TRINITY_DN64446_c0_g1_i1_orf1	uncharacterized protein LOC114364307 [Ostrinia furnacalis]	-0.8689	-0.3198	-0.5512	1.9482	-0.2083
TRINITY_DN117_c0_g1_i6_orf1	lipase member I-like [Ostrinia furnacalis]	-0.3199	-0.5054	-1.0315	1.89133	-0.0346
TRINITY_DN1914_c0_g1_i6_orf1	loricrin-like [Ostrinia furnacalis]	-0.6115	-0.4429	-0.8802	1.91171	0.02288
TRINITY_DN81803_c0_g2_i1_orf1	cathepsin K-like [Ostrinia furnacalis]	-0.4883	-0.4378	-0.8998	1.93601	-0.1101
TRINITY_DN117_c0_g1_i4_orf1	lipase member I-like [Ostrinia furnacalis]	-0.579	-0.5123	-0.9577	1.85689	0.1922
TRINITY_DN2490_c0_g2_i1_orfp1	TRINITY_DN2490_c0_g2_i1_m.56872 TRINITY_DN2490_c0_g2::TRINITY_DN2490_c0_g2_i1::g.56872 ORF type:internal len:359 (-),score=123.59 TRINITY_DN2490_c0_g2_i1:2-1075(-)	-0.5819	-0.3505	-0.8036	1.96023	-0.2242
TRINITY_DN19917_c0_g1_i1_orf1	synaptic vesicle glycoprotein 2B-like isoform X4 [Ostrinia furnacalis]	-0.5808	-0.4668	-0.5658	1.99479	-0.3814
TRINITY_DN6684_c0_g1_i4_orf1	26S proteasome non-ATPase regulatory subunit 6 isoform X1 [Ostrinia furnacalis]	-1.5257	-0.2634	1.59817	-0.0276	0.21863
TRINITY_DN1710_c0_g2_i2_orf1	relish [Ostrinia furnacalis]	-1.4595	-0.5834	1.54357	0.35518	0.14414
TRINITY_DN1272_c1_g1_i4_orf1	E3 ubiquitin-protein transferase MAEA [Ostrinia furnacalis] >XP_028157102.1 E3 ubiquitin-protein transferase MAEA [Ostrinia furnacalis]	-1.3477	-0.2774	1.75426	0.03772	-0.1668
TRINITY_DN103107_c0_g1_i2_orf1	superoxide dismutase [Cu-Zn] [Ostrinia furnacalis] >XP_028177872.1 superoxide dismutase [Cu-Zn] [Ostrinia furnacalis]	-0.7482	-0.689	1.95555	-0.2	-0.3184
TRINITY_DN7570_c0_g1_i18_orf1	sodium/potassium-transporting ATPase subunit alpha isoform X4 [Trichoplusia ni] >XP_026734855.1 sodium/potassium-transporting ATPase subunit alpha isoform X4 [Trichoplusia ni]	-1.2491	-0.5026	1.7349	0.30603	-0.2893
TRINITY_DN11612_c0_g2_i1_orf1	eukaryotic translation initiation factor 5B [Manduca sexta]	-1.2131	-0.6964	1.72823	0.23217	-0.0508
TRINITY_DN195_c8_g1_i1_orf1	hypothetical protein evm_009768 [Chilo suppressalis]	-1.0497	-0.9423	1.64002	0.53551	-0.1835
TRINITY_DN6016_c0_g1_i8_orf1	hypothetical protein evm_010883 [Chilo suppressalis]	-0.5758	-1.6694	0.70201	1.08612	0.45713
TRINITY_DN556_c0_g2_i1_orf1	serine protease inhibitor dipetalogastin-like [Ostrinia furnacalis]	-0.4111	-1.7731	0.66585	0.97254	0.54584
TRINITY_DN1013_c0_g1_i3_orf1	TELO2-interacting protein 1 homolog isoform X2 [Ostrinia furnacalis]	-0.8272	-1.5337	0.7906	1.01458	0.55579
TRINITY_DN5670_c0_g1_i2_orf1	DNA polymerase alpha subunit B [Ostrinia furnacalis]	-1.8007	0.60148	0.33308	1.10752	-0.2414
TRINITY_DN143496_c0_g1_i1_orf1	cullin-3 [Diachasma alloeum]	-1.7937	0.13984	0.55704	1.20079	-0.1039
TRINITY_DN77572_c0_g1_i1_orf1	steroid receptor RNA activator 1 [Ostrinia furnacalis]	-1.8431	0.28974	0.68008	1.01774	-0.1445
TRINITY_DN21215_c0_g1_i7_orf1	phytanoyl-CoA dioxygenase, peroxisomal-like [Ostrinia furnacalis]	-1.6	0.28373	0.92045	1.04468	-0.6489
TRINITY_DN4822_c0_g1_i9_orf1	homogentisate 1,2-dioxygenase [Ostrinia furnacalis]	-1.5356	0.43216	1.22028	0.63413	-0.751
TRINITY_DN1882_c0_g1_i4_orf1	zinc transporter ZIP13 homolog [Ostrinia furnacalis]	-1.7067	0.02889	0.64832	1.26847	-0.239
