# Supplementary material for: Comparative Proteomics and Metabonomics Analysis of Different Diapause Stages Revealed a New Regulation Mechanism of Diapause in Loxostege sticticalis (Lepidoptera: Pyralidae)
Source: Molecules. 2024 Jul 25;29(15):3472. doi: 10.3390/molecules29153472 (PMC11314584; doi:10.3390/molecules29153472)
Supplement: Supplementary file 1 [file molecules-29-03472-s001.zip › analysis process/proteomic/Cluster analysis of expression patterns/Up/RDvsD up.pdf]

Accession	Description	RD	ND	PreD	CT	D
TRINITY_DN84478_c0_g1_i8_orf1	uncharacterized protein LOC114359035 isoform X1 [Ostrinia furnacalis]	0.47386	-1.9622	0.50348	0.79809	0.18675
TRINITY_DN66040_c0_g1_i2_orf1	serine protease inhibitor dipetalogastin-like isoform X2 [Ostrinia furnacalis]	1.52484	-1.5131	-0.1369	0.48616	-0.361
TRINITY_DN38307_c0_g1_i1_orfp1	TRINITY_DN38307_c0_g1_i1_m.10661 TRINITY_DN38307_c0_g1::TRINITY_DN38307_c0_g1_i1::g.10661 ORF type:5prime_partial len:66 (+),score=5.90 TRINITY_DN38307_c0_g1_i1:2-199(+)	1.42198	-1.6694	-0.1642	0.00679	0.4049
TRINITY_DN5080_c0_g1_i5_orf1	storage protein [Ostrinia furnacalis]	0.77358	-1.9646	0.27671	0.61075	0.3036
TRINITY_DN64297_c0_g1_i1_orf1	vanin-like protein 2 isoform X2 [Ostrinia furnacalis]	1.33079	-1.5609	0.25072	0.59363	-0.6142
TRINITY_DN276_c0_g1_i2_orf1	protein lethal(2)essential for life-like [Ostrinia furnacalis] >UTU55753.1 small heat shock protein Hsp20.7 [Ostrinia furnacalis]	1.52123	-1.3206	-0.8035	0.5407	0.06218
TRINITY_DN143895_c0_g1_i1_orf1	cathepsin L-like [Aphidius gifuensis] >KAF7988186.1 hypothetical protein HCN44_007680 [Aphidius gifuensis]	0.62519	-1.9287	0.05375	0.85423	0.39558
TRINITY_DN1370_c0_g1_i2_orf1	hypothetical protein evm_000756 [Chilo suppressalis]	1.05558	-1.8885	0.09354	0.50648	0.23284
TRINITY_DN4767_c0_g1_i4_orf1	cysteine protease XCP2-like [Ostrinia furnacalis]	0.75927	-1.9572	0.28587	0.67435	0.23768
TRINITY_DN5444_c0_g1_i1_orfp1	TRINITY_DN5444_c0_g1_i1_m.14077 TRINITY_DN5444_c0_g1::TRINITY_DN5444_c0_g1_i1::g.14077 ORF type:3prime_partial len:90 (-),score=25.11 TRINITY_DN5444_c0_g1_i1:2-268(-)	1.36434	-1.7446	-0.0085	0.29269	0.0961
TRINITY_DN67193_c0_g1_i1_orf1	A-kinase anchor protein 14-like [Ostrinia furnacalis]	0.88028	-1.8754	-0.1456	0.70511	0.4356
TRINITY_DN51813_c0_g1_i1_orf1	uncharacterized protein LOC114350216 [Ostrinia furnacalis]	0.87806	-1.8579	0.07584	0.87799	0.02598
TRINITY_DN18218_c0_g1_i7_orf1	inhibin beta B chain [Ostrinia furnacalis]	1.51379	-1.238	-0.7223	0.7498	-0.3034
TRINITY_DN5080_c0_g1_i1_orf1	basic juvenile hormone-suppressible protein 2-like [Ostrinia furnacalis]	0.82977	-1.9059	0.09529	0.7979	0.1829
TRINITY_DN5406_c0_g2_i1_orf1	uncharacterized protein LOC114350326 [Ostrinia furnacalis]	0.94781	-1.8723	0.7566	0.153	0.01491
TRINITY_DN20560_c0_g1_i6_orf1	pupal cuticle protein C1B-like [Ostrinia furnacalis]	1.16248	-1.8302	0.40775	-0.0925	0.35245
TRINITY_DN6423_c0_g1_i6_orf1	phenoloxidase-activating factor 2-like isoform X1 [Ostrinia furnacalis]	1.36232	-1.7543	0.02974	0.17188	0.19032
TRINITY_DN33272_c0_g1_i5_orf1	Low-density lipoprotein receptor-related protein 1 [Papilio xuthus]	1.01785	-1.907	0.43067	0.0937	0.36479
TRINITY_DN4255_c0_g1_i11_orf1	LOW QUALITY PROTEIN: lebocin-4-like [Ostrinia furnacalis]	1.11109	-1.8751	0.35345	0.06345	0.34713
TRINITY_DN636_c1_g1_i9_orf1	secretory phospholipase A2 receptor-like [Ostrinia furnacalis]	0.77582	-1.8328	0.26549	0.96816	-0.1767
TRINITY_DN45948_c1_g1_i1_orf1	unnamed protein product [Leptidea sinapis]	1.18571	-1.7855	0.55625	-0.1972	0.24067
TRINITY_DN3707_c0_g1_i1_orf1	protein FAM160B1-like isoform X1 [Ostrinia furnacalis]	0.85775	-1.9349	0.11432	0.62927	0.33359
TRINITY_DN6122_c0_g1_i6_orf1	deubiquitinase DESI2 isoform X1 [Helicoverpa armigera] >XP_049707835.1 deubiquitinase DESI2 isoform X1 [Helicoverpa armigera]	0.76842	-1.8841	-0.085	0.8568	0.34394
TRINITY_DN19662_c0_g2_i1_orf1	storage protein [Ostrinia furnacalis]	0.72226	-1.9057	0.08663	0.89322	0.20356
TRINITY_DN121650_c0_g1_i1_orf1	carboxylesterase [Ostrinia furnacalis]	1.44393	-1.6911	-0.0336	0.0546	0.22617
TRINITY_DN42719_c0_g2_i1_orf1	inter-alpha-trypsin inhibitor heavy chain H4-like isoform X11 [Ostrinia furnacalis]	0.91789	-1.872	0.36021	0.71352	-0.1196
TRINITY_DN9538_c1_g3_i1_orf1	cilia- and flagella-associated protein 410 isoform X2 [Aphidius gifuensis]	1.10034	-1.8452	-0.0339	0.5893	0.18954
TRINITY_DN20676_c0_g1_i6_orf1	aldo-keto reductase AKR2E4-like isoform X1 [Ostrinia furnacalis]	1.19071	-1.8403	0.05158	0.21513	0.38287
TRINITY_DN98692_c0_g3_i1_orf1	fatty acyl-CoA hydrolase precursor, medium chain [Ostrinia furnacalis]	0.85037	-1.933	0.67659	0.21462	0.19139
TRINITY_DN45220_c0_g1_i1_orf1	delta(3,5)-Delta(2,4)-dienoyl-CoA isomerase, mitochondrial isoform X1 [Ostrinia furnacalis]	1.34972	-1.643	-0.4231	0.505	0.21142
TRINITY_DN5655_c0_g1_i2_orf1	uncharacterized protein LOC114359603 [Ostrinia furnacalis]	1.22163	-1.8202	0.02955	0.15841	0.4106
TRINITY_DN3616_c0_g1_i4_orf1	conotoxin ArMKLT2-032-like [Ostrinia furnacalis]	1.43024	-1.4273	-0.0476	0.69833	-0.6536
TRINITY_DN46625_c0_g1_i1_orf1	ferritin subunit isoform X1 [Belonocnema kinseyi]	1.49897	-1.463	-0.458	0.60728	-0.1853
TRINITY_DN15578_c0_g2_i1_orfp1	uncharacterized protein LOC125235519 [Leguminivora glycinivorella]	0.71887	-1.8883	0.8435	0.43946	-0.1135
TRINITY_DN13760_c1_g1_i1_orf1	pre-mRNA-processing factor 40 homolog A isoform X1 [Ostrinia furnacalis] >XP_028162665.1 pre-mRNA-processing factor 40 homolog A isoform X2 [Ostrinia furnacalis] >XP_028162667.1 pre-mRNA-processing factor 40 homolog A isoform X3 [Ostrinia furnacalis]	1.32162	-1.6889	0.25016	0.46566	-0.3485
TRINITY_DN4748_c0_g1_i5_orf1	unnamed protein product, partial [Brenthis ino]	1.46875	-1.6662	0.16093	0.15981	-0.1233
TRINITY_DN1175_c1_g1_i1_orf1	methanethiol oxidase [Ostrinia furnacalis]	1.00209	-1.919	0.44652	0.1941	0.27626
TRINITY_DN49147_c0_g2_i1_orf1	glutenin, high molecular weight subunit PW212-like [Ostrinia furnacalis]	1.81086	-1.1183	-0.6812	0.04937	-0.0607
TRINITY_DN9239_c0_g1_i1_orf1	apolipophorins-like [Ostrinia furnacalis]	1.50782	-1.3268	-0.6047	0.71709	-0.2933
TRINITY_DN41_c0_g1_i3_orf1	uncharacterized protein LOC114359035 isoform X3 [Ostrinia furnacalis]	1.19312	-1.8212	0.324	-0.081	0.38503
TRINITY_DN19662_c4_g1_i1_orf1	basic juvenile hormone-suppressible protein 1-like [Ostrinia furnacalis]	0.62002	-1.9202	0.1667	0.92615	0.2073
TRINITY_DN993_c0_g1_i7_orf1	apolipophorins-like [Ostrinia furnacalis]	1.32924	-1.5456	-0.4186	0.80089	-0.1659
TRINITY_DN1423_c0_g1_i8_orf1	ferritin subunit-like [Ostrinia furnacalis] >XP_028168186.1 ferritin subunit-like [Ostrinia furnacalis]	1.52636	-1.5296	-0.2715	0.4683	-0.1935
TRINITY_DN85412_c0_g1_i1_orf1	unnamed protein product [Diatraea saccharalis]	1.44709	-1.4122	-0.5218	0.75379	-0.2669
TRINITY_DN28711_c0_g1_i1_orf1	hypothetical protein evm_000299 [Chilo suppressalis]	1.39267	-1.4105	-0.5329	0.83903	-0.2883
TRINITY_DN81488_c0_g1_i1_orf1	apolipophorins-like [Ostrinia furnacalis]	1.38699	-1.4803	-0.4721	0.78382	-0.2184
TRINITY_DN27264_c0_g1_i1_orf1	uncharacterized protein LOC114353424 [Ostrinia furnacalis]	1.74177	-0.9364	-0.6874	0.49325	-0.6112
TRINITY_DN699_c0_g2_i1_orf1	TPA_exp: putative parasitoid killing factor [Trichoplusia ni]	1.46045	-1.4252	-0.5433	0.70775	-0.1997
TRINITY_DN418_c1_g1_i3_orf1	hypothetical protein evm_003996 [Chilo suppressalis]	0.69259	-1.7125	0.27099	1.15985	-0.4109
TRINITY_DN42719_c0_g1_i1_orf1	inter-alpha-trypsin inhibitor heavy chain H4-like isoform X11 [Ostrinia furnacalis]	1.87412	-0.9083	-0.5474	0.16192	-0.5803
TRINITY_DN76216_c0_g2_i3_orf1	lysosomal alpha-mannosidase isoform X1 [Pieris rapae]	1.50096	-1.5179	-0.4115	0.51584	-0.0874
TRINITY_DN25976_c0_g1_i4_orf1	hypothetical protein B566_EDAN014657 [Ephemera danica]	1.10855	-1.8853	0.35248	0.24659	0.17771
TRINITY_DN15812_c0_g1_i2_orf1	transferrin [Ostrinia furnacalis]	1.31766	-1.7805	0.15993	0.25703	0.04583
TRINITY_DN19980_c0_g1_i4_orf1	hypothetical protein evm_012507 [Chilo suppressalis]	1.10419	-1.8318	0.58938	-0.1159	0.25411
TRINITY_DN97042_c0_g1_i6_orf1	apolipophorins-like [Ostrinia furnacalis]	1.34136	-1.4997	-0.466	0.83166	-0.2074
TRINITY_DN20680_c0_g1_i5_orf1	tsukushin isoform X2 [Ostrinia furnacalis]	1.40701	-1.6882	0.10571	-0.1803	0.35582
TRINITY_DN7618_c0_g1_i4_orf1	uncharacterized protein LOC114366712 isoform X1 [Ostrinia furnacalis]	1.44701	-1.632	-0.2805	0.06563	0.39986
TRINITY_DN104663_c1_g1_i2_orf1	PREDICTED: gelsolin-like [Amyelois transitella]	0.74457	-1.9257	0.17203	0.81962	0.18949
TRINITY_DN113353_c0_g1_i1_orf1	unnamed protein product [Parnassius apollo]	1.12965	-1.7803	-0.2351	0.67399	0.21173

TRINITY_DN1110_c1_g1_i9_orf1	MD-2-related lipid-recognition protein-like [Ostrinia furnacalis]	1.77041	-0.9686	-0.8699	0.32427	-0.2562
TRINITY_DN10138_c0_g1_i1_orf1	storage protein 1 [Omphisa fuscidentalis]	0.59723	-1.7698	-0.0794	1.22648	0.02552
TRINITY_DN2024_c0_g1_i12_orfp1	unnamed protein product, partial [Brenthis ino]	1.32399	-1.7077	0.40147	0.28217	-0.2999
TRINITY_DN3949_c0_g1_i1_orf1	probable cytochrome P450 304a1 [Ostrinia furnacalis]	1.31291	-1.7538	-0.1414	0.36534	0.21695
TRINITY_DN71308_c0_g1_i4_orf1	uncharacterized protein LOC114361536 [Ostrinia furnacalis]	1.13536	-1.8757	0.22489	0.32647	0.18895
TRINITY_DN40191_c2_g1_i1_orf1	stress-activated map kinase-interacting protein 1 [Ostrinia furnacalis]	1.33704	-1.6205	-0.3251	0.68861	-0.0801
TRINITY_DN12865_c0_g1_i1_orf1	synaptic vesicle membrane protein VAT-1 homolog-like [Ostrinia furnacalis]	1.48141	-1.6522	0.18225	0.13962	-0.151
TRINITY_DN64141_c0_g1_i4_orf1	probable salivary secreted peptide [Ostrinia furnacalis]	0.94501	-1.7414	-0.3563	0.95276	0.19984
TRINITY_DN2803_c4_g1_i1_orf1	ornithine aminotransferase, mitochondrial isoform X2 [Ostrinia furnacalis]	1.21503	-1.7995	0.34464	0.38259	-0.1428
TRINITY_DN28711_c1_g1_i1_orf1	apolipophorins-like [Ostrinia furnacalis]	1.46692	-1.4115	-0.5321	0.71746	-0.2408
TRINITY_DN52944_c0_g1_i1_orf1	apolipophorins-like [Ostrinia furnacalis]	1.44676	-1.4092	-0.4968	0.76292	-0.3037
TRINITY_DN3301_c0_g1_i2_orf1	hemicentin-2-like isoform X1 [Ostrinia furnacalis]	0.70146	-1.8346	-0.0534	1.05981	0.12678
TRINITY_DN71699_c0_g1_i1_orf1	apolipophorins-like [Ostrinia furnacalis]	1.43099	-1.3854	-0.5739	0.79524	-0.267
TRINITY_DN1423_c0_g1_i4_orf1	hypothetical protein evm_003306 [Chilo suppressalis] >CAB3526495.1 unnamed protein product [Chilo suppressalis] >CAH0403823.1 unnamed protein product [Chilo suppressalis]	1.4895	-1.6014	-0.2159	0.40544	-0.0776
TRINITY_DN2049_c1_g1_i3_orf1	luciferin 4-monooxygenase-like [Ostrinia furnacalis]	0.57208	-1.5076	-0.2188	1.49522	-0.3409
TRINITY_DN32532_c0_g1_i1_orf1	fatty acyl-CoA hydrolase precursor, medium chain [Ostrinia furnacalis]	1.07573	-1.8865	0.26645	0.45187	0.0925
TRINITY_DN4228_c0_g1_i5_orf1	phenoloxidase-activating enzyme-like [Ostrinia furnacalis]	1.32909	-1.7664	0.04266	0.06787	0.32681
TRINITY_DN9733_c0_g1_i2_orf1	acidic juvenile hormone-suppressible protein 1-like [Ostrinia furnacalis]	0.86239	-1.4475	-0.2293	1.34974	-0.5353
TRINITY_DN2407_c0_g1_i6_orf1	uncharacterized protein LOC114366345 isoform X2 [Ostrinia furnacalis]	1.31896	-1.7784	0.01877	0.21652	0.2242
TRINITY_DN376_c1_g1_i1_orf1	matrix metalloproteinase-25-like [Ostrinia furnacalis]	1.19591	-1.777	-0.1941	0.57975	0.19548
TRINITY_DN7183_c0_g1_i2_orf1	seminal fluid protein CSSFP028 [Chilo suppressalis]	1.32529	-1.7504	0.38744	0.13895	-0.1013
TRINITY_DN364_c1_g1_i2_orf1	talin-2-like, partial [Ostrinia furnacalis]	0.79969	-1.8306	0.99666	0.10478	-0.0705
TRINITY_DN7102_c0_g1_i5_orf1	protein wings apart-like [Ostrinia furnacalis]	1.01918	-1.8265	-0.2491	0.57782	0.47865
TRINITY_DN2425_c0_g1_i3_orf1	sialic acid synthase [Ostrinia furnacalis]	1.42428	-1.683	-0.1453	0.06788	0.33623
TRINITY_DN578_c0_g1_i5_orf1	charged multivesicular body protein 7 [Ostrinia furnacalis]	0.82055	-1.897	0.14821	0.83508	0.09319
TRINITY_DN33272_c0_g1_i1_orf1	unnamed protein product, partial [Iphiclides podalirius]	1.38353	-1.6621	-0.3188	0.44592	0.15137
TRINITY_DN13973_c0_g1_i6_orf1	27 kDa glycoprotein-like [Ostrinia furnacalis]	1.38377	-1.4116	-0.8075	0.63146	0.20394
TRINITY_DN2097_c1_g2_i2_orf1	serine protease inhibitor 3 [Ostrinia furnacalis]	1.32318	-1.6902	-0.3501	0.43897	0.27809
TRINITY_DN105574_c0_g1_i1_orf1	prolow-density lipoprotein receptor-related protein 1, partial [Ostrinia furnacalis]	0.93431	-1.9447	0.32601	0.39161	0.29274
TRINITY_DN18388_c0_g1_i6_orf1	serine protease [Ostrinia furnacalis]	0.98296	-1.8692	0.18209	0.71188	-0.0078
TRINITY_DN8771_c0_g1_i5_orf1	regucalcin-like [Ostrinia furnacalis]	1.10543	-1.8607	0.37132	-0.0361	0.42009
TRINITY_DN9239_c0_g2_i2_orf1	apolipophorins-like [Ostrinia furnacalis]	1.49575	-1.3082	-0.3552	0.75877	-0.5911
TRINITY_DN42275_c0_g1_i1_orfp1	TRINITY_DN42275_c0_g1_i1_m.44265 TRINITY_DN42275_c0_g1::TRINITY_DN42275_c0_g1_i1::g.44265 ORF type:internal len:76 (+),score=8.67 TRINITY_DN42275_c0_g1_i1:1-225(+)	0.76157	-1.7028	-0.0228	1.20842	-0.2444
TRINITY_DN44073_c0_g1_i3_orf1	inter-alpha-trypsin inhibitor heavy chain H4-like isoform X11 [Ostrinia furnacalis]	1.3692	-1.6558	0.32426	0.3538	-0.3914
TRINITY_DN33728_c0_g2_i1_orf1	uncharacterized protein LOC114350200 [Ostrinia furnacalis]	1.7585	-1.1412	-0.6994	0.27855	-0.1965
TRINITY_DN5420_c0_g1_i2_orf1	DNA-directed RNA polymerase II subunit RPB1-like [Ostrinia furnacalis]	1.55402	-1.5851	0.07708	0.15793	-0.2039
TRINITY_DN11060_c0_g1_i6_orf1	extracellular matrix protein A-like isoform X3 [Ostrinia furnacalis]	0.93331	-1.7923	0.95414	-0.0756	-0.0195
TRINITY_DN1048_c0_g1_i6_orf1	uncharacterized protein LOC114360661 [Ostrinia furnacalis]	0.41615	-1.8346	0.37292	1.1455	-0.1
TRINITY_DN2684_c0_g2_i3_orf1	glutamate decarboxylase 1-like isoform X1 [Ostrinia furnacalis]	1.76077	-1.1274	-0.7601	0.2098	-0.0831
TRINITY_DN2227_c0_g1_i5_orf1	protein 60A [Ostrinia furnacalis]	1.37725	-1.6524	0.33267	-0.3895	0.33204
TRINITY_DN2798_c0_g1_i5_orf1	arylsulfatase B-like isoform X1 [Ostrinia furnacalis]	0.75365	-1.9403	0.40809	0.70358	0.07499
TRINITY_DN2880_c0_g1_i2_orf1	sialomucin core protein 24 [Pectinophora gossypiella]	0.87065	-1.8657	0.84819	-0.0512	0.19803
TRINITY_DN2835_c0_g1_i6_orf1	probable isoaspartyl peptidase/L-asparaginase GA20639 [Ostrinia furnacalis]	1.32898	-1.5731	-0.5732	0.62849	0.18883
TRINITY_DN4464_c0_g2_i1_orf1	glypican-6 [Pectinophora gossypiella]	0.98681	-1.9247	0.42756	0.32098	0.18936
TRINITY_DN2170_c1_g1_i3_orf1	beta-1,3-glucan-binding protein-like [Ostrinia furnacalis]	1.58533	-1.5408	-0.2372	0.23363	-0.0409
TRINITY_DN6243_c0_g1_i5_orf1	sorting nexin-20 [Ostrinia furnacalis]	0.93617	-1.9153	0.61538	0.11011	0.25365
TRINITY_DN19110_c0_g1_i2_orf1	peroxidase [Ostrinia furnacalis]	1.62388	-1.2457	-0.5048	0.58694	-0.4603
TRINITY_DN198_c2_g1_i2_orf1	solute carrier organic anion transporter family member 5A1-like isoform X1 [Ostrinia furnacalis]	1.35671	-1.7231	0.13983	-0.156	0.38257
TRINITY_DN3464_c0_g1_i1_orf1	putative mitochondrial aconitate hydratase isoform X1-likeprotein, partial [Cotesia chilonis]	1.61508	-0.9465	-0.8389	0.70861	-0.5383
TRINITY_DN23354_c0_g1_i7_orf1	TBC1 domain family member 20 [Ostrinia furnacalis]	1.02314	-1.2323	-0.7104	1.32786	-0.4083
TRINITY_DN18804_c0_g1_i5_orf1	zinc finger protein Xfin-like [Ostrinia furnacalis]	1.86633	-0.8865	-0.4324	0.17022	-0.7176
TRINITY_DN5553_c0_g1_i4_orf1	uncharacterized protein LOC114353828 [Ostrinia furnacalis]	1.64419	-1.2832	-0.558	0.498	-0.3009
TRINITY_DN10090_c0_g1_i1_orf1	clotting factor B-like isoform X1 [Ostrinia furnacalis] >XP_028163447.1 clotting factor B-like isoform X2 [Ostrinia furnacalis] >XP_028163448.1 clotting factor B-like isoform X3 [Ostrinia furnacalis]	1.16435	-1.8629	0.17151	0.31637	0.21067
TRINITY_DN3952_c0_g1_i3_orf1	protein Skeletor, isoforms D/E-like isoform X1 [Ostrinia furnacalis] >XP_028176405.1 protein Skeletor, isoforms D/E-like isoform X2 [Ostrinia furnacalis] >XP_028176406.1 protein Skeletor, isoforms D/E-like isoform X3 [Ostrinia furnacalis] >XP_028176407.1 protein Skeletor, isoforms D/E-like isoform X4 [Ostrinia furnacalis]	1.65249	-1.0343	-0.9681	0.49199	-0.1421
TRINITY_DN125441_c0_g1_i5_orf1	KH domain-containing, RNA-binding, signal transduction-associated protein 2-like isoform X12 [Ostrinia furnacalis]	0.94752	-1.8939	0.69098	0.17933	0.07606
TRINITY_DN6988_c0_g1_i3_orf1	cuticle protein 1-like [Ostrinia furnacalis]	0.88877	-1.8483	-0.0593	0.87776	0.14107
TRINITY_DN361_c0_g1_i5_orf1	hexosaminidase [Ostrinia furnacalis]	1.55905	-1.5449	-0.1804	0.34414	-0.1779
TRINITY_DN1407_c0_g1_i12_orf1	hypothetical protein evm_012298 [Chilo suppressalis]	1.56932	-1.1238	-0.4278	0.72978	-0.7475

TRINITY_DN8480_c0_g1_i1_orf1	lysosomal Pro-X carboxypeptidase [Ostrinia furnacalis]	1.37775	-1.6157	0.52292	-0.4403	0.15532
TRINITY_DN14250_c0_g1_i1_orf1	apolipophorins-like [Ostrinia furnacalis]	1.08205	-1.212	-0.9575	1.19647	-0.109
TRINITY_DN22053_c0_g1_i13_orf1	uncharacterized protein LOC114355104 [Ostrinia furnacalis]	1.43144	-1.6744	-0.1583	0.34498	0.05631
TRINITY_DN3383_c0_g1_i5_orf1	uncharacterized protein LOC114357426 [Ostrinia furnacalis]	0.88771	-1.0577	-1.2692	1.19223	0.24695
TRINITY_DN1507_c0_g1_i5_orf1	27 kDa hemolymph protein-like, partial [Ostrinia furnacalis]	1.2881	-1.7351	0.55902	-0.1314	0.01942
TRINITY_DN338_c1_g1_i9_orf1	scolexin B-like isoform X2 [Ostrinia furnacalis]	0.81459	-1.9101	0.25117	0.78856	0.05576
TRINITY_DN19821_c0_g2_i4_orf1	uncharacterized protein LOC114359393 isoform X1 [Ostrinia furnacalis]	1.00807	-1.5885	-0.4933	1.10285	-0.0291
TRINITY_DN4256_c0_g1_i1_orf1	chitinase-3-like protein 1 [Ostrinia furnacalis]	1.3046	-1.6834	-0.2394	0.6376	-0.0194
TRINITY_DN86833_c0_g3_i1_orf1	PREDICTED: glycerol-3-phosphate acyltransferase 1, mitochondrial isoform X1 [Microplitis demolitor]	1.3807	-1.5831	-0.1806	0.68221	-0.2993
TRINITY_DN30498_c0_g1_i3_orf1	lipase 3-like [Ostrinia furnacalis]	0.90718	-1.7273	0.04327	1.05545	-0.2786
TRINITY_DN6205_c0_g1_i1_orf1	phenoloxidase-activating factor 2-like [Ostrinia furnacalis]	0.96468	-1.6788	0.29831	0.94128	-0.5255
TRINITY_DN7590_c0_g1_i4_orf1	innexin inx1-like [Pectinophora gossypiella]	1.22923	-1.7643	-0.2236	0.24051	0.51814
TRINITY_DN3251_c0_g1_i6_orf1	fatty-acid amide hydrolase 2-like [Ostrinia furnacalis] >XP_028167366.1 fatty-acid amide hydrolase 2-like [Ostrinia furnacalis] >XP_028167367.1 fatty-acid amide hydrolase 2-like [Ostrinia furnacalis] >XP_028167368.1 fatty-acid amide hydrolase 2-like [Ostrinia furnacalis] >XP_028167369.1 fatty-acid amide hydrolase 2-like [Ostrinia furnacalis] >XP_028167370.1 fatty-acid amide hydrolase 2-like [Ostrinia furnacalis] >XP_028167371.1 fatty-acid amide hydrolase 2-like [Ostrinia furnacalis] >XP_028167372.1 fatty-acid amide hydrolase 2-like [Ostrinia furnacalis]	1.11383	-1.8781	0.35537	0.30974	0.09919
TRINITY_DN23746_c0_g1_i2_orf1	protein 4.1 homolog isoform X1 [Ostrinia furnacalis]	1.18754	-1.6572	-0.0723	0.85887	-0.3168
TRINITY_DN2566_c0_g1_i5_orf1	uncharacterized protein LOC114349936 [Ostrinia furnacalis]	1.55969	-0.9593	-1.043	0.70157	-0.259
TRINITY_DN5064_c0_g1_i4_orf1	sortilin-related receptor-like [Ostrinia furnacalis]	1.38948	-1.6412	0.42395	-0.3872	0.21493
TRINITY_DN28661_c0_g1_i1_orf1	cathepsin B [Ostrinia furnacalis]	1.22559	-1.7617	0.59612	-0.1661	0.10615
TRINITY_DN43431_c0_g1_i1_orf1	glycine dehydrogenase (decarboxylating), mitochondrial isoform X1 [Ostrinia furnacalis] >XP_028174269.1 glycine dehydrogenase (decarboxylating), mitochondrial isoform X3 [Ostrinia furnacalis]	1.20945	-1.7688	0.63563	-0.01	-0.0663
TRINITY_DN6462_c0_g1_i5_orf1	probable histone-lysine N-methyltransferase CG1716 [Ostrinia furnacalis]	0.74765	-1.8359	0.04473	1.03365	0.00986
TRINITY_DN3109_c0_g1_i5_orf1	protein takeout isoform X2 [Ostrinia furnacalis]	1.07515	-1.8045	0.7659	-0.0372	0.00062
TRINITY_DN3513_c0_g1_i5_orf1	vacuolar protein sorting-associated protein 16 homolog [Ostrinia furnacalis]	1.01439	-1.8949	0.24518	0.56081	0.07457
TRINITY_DN1664_c0_g1_i4_orf1	uncharacterized protein LOC114355246 [Ostrinia furnacalis]	1.09041	-1.8103	0.72407	0.06649	-0.0706
TRINITY_DN8703_c0_g1_i2_orf1	beta-glucuronidase-like isoform X1 [Ostrinia furnacalis] >XP_028166212.1 beta-glucuronidase-like isoform X2 [Ostrinia furnacalis]	1.01919	-1.6787	-0.2126	1.03516	-0.1631
TRINITY_DN9615_c0_g1_i1_orf1	uncharacterized protein LOC114352730 [Ostrinia furnacalis]	1.24453	-1.7911	-0.0289	0.48371	0.09175
TRINITY_DN70485_c0_g1_i2_orf1	serine/threonine-protein kinase Genghis Khan-like [Ostrinia furnacalis]	1.56618	-1.5568	0.1462	0.13433	-0.2899
TRINITY_DN261_c0_g1_i5_orfp1	TRINITY_DN261_c0_g1_i5_m.18559 TRINITY_DN261_c0_g1::TRINITY_DN261_c0_g1_i5::g.18559 ORF type:internal len:190 (+),score=69.81 TRINITY_DN261_c0_g1_i5:3-569(+)	1.81538	-0.9016	0.07345	-0.0471	-0.9402
TRINITY_DN166_c0_g1_i4_orf1	PREDICTED: cryptochrome-1 isoform X1 [Amyelois transitella] >XP_013199861.1 PREDICTED: cryptochrome-1 isoform X1 [Amyelois transitella]	1.33628	-1.601	-0.5439	0.52374	0.28484
TRINITY_DN15291_c0_g1_i11_orf1	uncharacterized protein LOC114353772 [Ostrinia furnacalis]	1.30054	-1.275	-0.834	0.97899	-0.1706
TRINITY_DN37585_c0_g1_i1_orf1	cuticle protein 19.8-like [Ostrinia furnacalis]	1.88723	-0.871	-0.672	0.1185	-0.4627
TRINITY_DN1767_c0_g2_i15_orf1	fasciclin-2 isoform X3 [Ostrinia furnacalis]	1.49198	-1.6389	0.23328	0.07922	-0.1656
TRINITY_DN12286_c1_g1_i2_orf1	sideroflexin-1-3 [Galleria mellonella] >XP_026754161.1 sideroflexin-1-3 [Galleria mellonella]	1.68161	-1.2111	-0.5167	0.49075	-0.4446
TRINITY_DN38412_c0_g1_i1_orf1	translation initiation factor eIF-2B subunit alpha [Ostrinia furnacalis]	1.40022	-1.657	-0.1849	0.50553	-0.0639
TRINITY_DN114982_c0_g1_i1_orf1	uncharacterized protein LOC114357708 [Ostrinia furnacalis]	1.73345	-0.7305	-0.4245	0.45712	-1.0355
TRINITY_DN110402_c0_g2_i1_orf1	apolipophorins-like [Ostrinia furnacalis]	1.68792	-1.2576	-0.6595	0.34706	-0.1178
TRINITY_DN45271_c0_g1_i1_orf1	double-strand break repair protein MRE11 [Ostrinia furnacalis]	1.47481	-1.667	0.21233	0.00999	-0.0302
TRINITY_DN44709_c0_g1_i1_orf1	D-beta-hydroxybutyrate dehydrogenase, mitochondrial, partial [Chelonus insularis]	1.26766	-1.5118	-0.5698	0.88213	-0.0682
TRINITY_DN143637_c0_g1_i1_orf1	PX domain-containing protein kinase-like protein isoform X1 [Chelonus insularis]	1.20193	-1.7345	0.29959	0.5802	-0.3472
TRINITY_DN5070_c0_g1_i1_orf1	ATP-dependent (S)-NAD(P)H-hydrate dehydratase-like [Ostrinia furnacalis]	1.46269	-1.4893	0.07369	0.54035	-0.5874
TRINITY_DN18650_c0_g1_i1_orf1	bombyxin B-9-like [Ostrinia furnacalis]	1.51982	-1.3172	-0.7953	0.56747	0.0252
TRINITY_DN42854_c0_g3_i2_orf1	amyloid beta (A4) precursor-like protein 2, isoform CRA_b [Homo sapiens]	0.9126	-1.655	-0.3863	1.13084	-0.0021
TRINITY_DN2058_c0_g1_i2_orf1	proteasomal ubiquitin receptor ADRM1 [Ostrinia furnacalis]	1.81574	-0.8557	-0.099	0.11281	-0.9739
TRINITY_DN1870_c0_g1_i6_orf1	programmed cell death protein 5 [Ostrinia furnacalis]	1.19246	-1.7806	-0.1255	0.61876	0.09488
TRINITY_DN3545_c0_g1_i6_orf1	group XV phospholipase A2-like [Ostrinia furnacalis] >XP_028168992.1 group XV phospholipase A2-like [Ostrinia furnacalis] >XP_028168993.1 group XV phospholipase A2-like [Ostrinia furnacalis]	1.38957	-1.725	0.25706	-0.0714	0.14979
TRINITY_DN2897_c0_g2_i1_orf1	gem-associated protein 5-like [Ostrinia furnacalis]	1.29139	-1.3491	-0.816	0.91897	-0.0454
TRINITY_DN2793_c0_g2_i1_orf1	PREDICTED: ras-related protein Rab-4B [Amyelois transitella]	1.255	-1.7132	-0.1687	0.67733	-0.0504
TRINITY_DN4394_c0_g2_i1_orf1	carboxylesterase [Ostrinia furnacalis]	0.83133	-1.5894	-0.007	1.24565	-0.4806
TRINITY_DN12024_c0_g1_i4_orf1	pancreatic lipase-related protein 2 isoform X1 [Ostrinia furnacalis] >XP_028176200.1 pancreatic lipase-related protein 2 isoform X2 [Ostrinia furnacalis]	1.1905	-1.7256	-0.3558	0.64758	0.24332
TRINITY_DN8724_c0_g1_i2_orf1	vesicle-associated membrane protein/synaptobrevin-binding protein isoform X2 [Pectinophora gossypiella]	1.01451	-1.8554	0.71257	0.14217	-0.0138
TRINITY_DN70_c6_g1_i1_orf1	optineurin isoform X1 [Ostrinia furnacalis] >XP_028165537.1 optineurin isoform X1 [Ostrinia furnacalis] >XP_028165538.1 optineurin isoform X1 [Ostrinia furnacalis] >XP_028165539.1 optineurin isoform X1 [Ostrinia furnacalis]	0.81263	-1.8112	0.17523	0.99885	-0.1755
TRINITY_DN12885_c0_g1_i1_orf1	prolow-density lipoprotein receptor-related protein 1, partial [Ostrinia furnacalis]	1.19575	-1.8075	0.25047	0.47656	-0.1153
TRINITY_DN2271_c0_g1_i12_orf1	plasminogen activator inhibitor 1-like [Ostrinia furnacalis]	0.82475	-1.7593	0.17965	1.05105	-0.2962
TRINITY_DN9412_c0_g1_i1_orf1	maspardin-like [Ostrinia furnacalis]	1.5662	-1.3603	-0.0341	0.49746	-0.6692
TRINITY_DN10680_c0_g1_i5_orf1	cGMP-dependent protein kinase, isozyme 2 forms cD4/T1/T3A/T3B-like isoform X3 [Ostrinia furnacalis] >XP_028158316.1 cGMP-dependent protein kinase, isozyme 2 forms cD4/T1/T3A/T3B-like isoform X3 [Ostrinia furnacalis]	1.44791	-1.593	-0.2337	0.53554	-0.1568
TRINITY_DN6510_c1_g1_i1_orf1	hypothetical protein evm_008080 [Chilo suppressalis]	1.37646	-1.7434	0.24018	0.05216	0.07458
TRINITY_DN12555_c0_g1_i1_orf1	uncharacterized protein LOC114350746 [Ostrinia furnacalis]	1.69736	-1.2033	-0.6031	0.44269	-0.3336

TRINITY_DN1038_c0_g1_i4_orf1	gastric triacylglycerol lipase-like [Ostrinia furnacalis]	1.33143	-1.721	0.46323	-0.192	0.11831
TRINITY_DN7776_c0_g1_i1_orf1	uncharacterized protein LOC114364702 [Ostrinia furnacalis]	1.05961	-1.7825	0.17589	0.78305	-0.236
TRINITY_DN13856_c0_g1_i1_orf1	angiotensin-converting enzyme-like [Ostrinia furnacalis]	1.52234	-1.3156	-0.2444	0.68637	-0.6486
TRINITY_DN5028_c0_g1_i11_orf1	NTF2-related export protein [Ostrinia furnacalis]	1.81091	-0.9178	-0.3006	0.25706	-0.8496
TRINITY_DN116874_c0_g1_i1_orfp1	TRINITY_DN116874_c0_g1_i1_m.85176 TRINITY_DN116874_c0_g1::TRINITY_DN116874_c0_g1_i1::g.85176 ORF type:5prime_partial len:95 (+),score=17.10,Baculo_p48 PF04878.14 8.5e-16 TRINITY_DN116874_c0_g1_i1:2-286(+)	0.88176	-0.9332	-1.2568	1.33105	-0.0229
TRINITY_DN1481_c0_g1_i4_orf1	G protein-coupled receptor kinase 1 isoform X2 [Helicoverpa armigera] >XP_047029555.1 G protein-coupled receptor kinase 1 isoform X2 [Helicoverpa zea] >ANZ22924.1 G protein-coupled receptor kinase 2 [Helicoverpa armigera armigera]	1.91824	-0.7818	-0.3904	0.0001	-0.7462
TRINITY_DN116467_c0_g1_i1_orf1	probable small nuclear ribonucleoprotein E [Ostrinia furnacalis]	1.56739	-1.5376	0.09649	0.22138	-0.3477
TRINITY_DN52864_c0_g1_i1_orf1	odorant binding protein 18 [Conogethes pinicolalis]	1.6667	-1.1428	-0.8285	0.45499	-0.1503
TRINITY_DN251_c0_g1_i2_orf1	hypothetical protein evm_008466 [Chilo suppressalis]	0.99492	-1.3479	-0.262	1.29126	-0.6763
TRINITY_DN17329_c0_g2_i3_orf1	uncharacterized protein LOC114354338 isoform X1 [Ostrinia furnacalis]	1.59718	-0.5746	-0.9519	0.74256	-0.8132
TRINITY_DN10290_c0_g1_i7_orf1	aquaporin AQPAe.a [Ostrinia furnacalis]	1.46669	-1.5041	-0.0629	0.5875	-0.4872
TRINITY_DN291_c0_g1_i2_orf1	DNA replication licensing factor Mcm7 [Helicoverpa armigera] >XP_049698025.1 DNA replication licensing factor Mcm7-like [Helicoverpa armigera] >PZC87280.1 hypothetical protein B5X24_HaOG201516 [Helicoverpa armigera]	1.77544	-1.0209	-0.7733	0.33148	-0.3128
TRINITY_DN2170_c0_g1_i2_orf1	beta-1,3-glucan-binding protein-like [Ostrinia furnacalis]	1.22023	-1.5394	0.37509	0.67881	-0.7348
TRINITY_DN246_c1_g1_i5_orf1	lachesin isoform X1 [Ostrinia furnacalis] >XP_028178464.1 lachesin isoform X2 [Ostrinia furnacalis]	1.7594	-1.216	-0.6336	0.14621	-0.0561
TRINITY_DN56690_c0_g1_i4_orf1	hypothetical protein evm_002209, partial [Chilo suppressalis]	1.28358	-1.2015	0.22576	0.79752	-1.1054
TRINITY_DN23732_c0_g1_i1_orf1	glutathione S-transferase 1-like [Ostrinia furnacalis] >QIC35740.1 glutathione S-transferase delta 4 [Ostrinia furnacalis]	1.68444	-0.8885	-0.8093	0.60592	-0.5926
TRINITY_DN17772_c0_g2_i3_orf1	uncharacterized protein LOC114351684 [Ostrinia furnacalis]	1.40731	-0.9776	-0.6629	1.01023	-0.777
TRINITY_DN8700_c9_g1_i1_orf1	uncharacterized protein LOC114351392 isoform X1 [Ostrinia furnacalis]	1.83141	-0.9098	-0.7625	0.25521	-0.4143
TRINITY_DN17133_c0_g1_i1_orf1	unnamed protein product [Chrysodeixis includens]	1.49247	-1.5187	-0.0386	0.51331	-0.4485
TRINITY_DN1833_c0_g1_i5_orf1	uncharacterized protein LOC114356866 isoform X3 [Ostrinia furnacalis] >XP_028166037.1 uncharacterized protein LOC114356866 isoform X3 [Ostrinia	1.01194	-0.9591	-0.3775	1.35834	-1.0337
TRINITY_DN11620_c0_g1_i2_orf1	serine/threonine-protein kinase PAK 3 [Ostrinia furnacalis]	1.28116	-0.8915	-0.7869	1.16447	-0.7672
TRINITY_DN23978_c0_g1_i2_orf1	insulin-like growth factor-binding protein complex acid labile subunit [Ostrinia furnacalis]	1.08126	-0.8938	-0.0958	1.18271	-1.2743
TRINITY_DN2584_c0_g1_i7_orf1	acylamino-acid-releasing enzyme-like isoform X1 [Ostrinia furnacalis] >XP_028174257.1 acylamino-acid-releasing enzyme-like isoform X2 [Ostrinia furnacalis] >XP_028174264.1 acylamino-acid-releasing enzyme-like isoform X3 [Ostrinia furnacalis] >XP_028174273.1 acylamino-acid-releasing enzyme-like isoform X4 [Ostrinia furnacalis] >XP_028174282.1 acylamino-acid-releasing enzyme-like isoform X1 [Ostrinia furnacalis]	1.82091	-0.8839	-0.8642	0.24064	-0.3134
TRINITY_DN3407_c0_g1_i9_orf1	uncharacterized protein YJR142W [Ostrinia furnacalis]	1.26265	-1.4534	-0.0488	0.91423	-0.6747
TRINITY_DN3459_c0_g1_i4_orf1	PREDICTED: probable small nuclear ribonucleoprotein G [Papilio polytes] >XP_013168682.1 PREDICTED: probable small nuclear ribonucleoprotein G [Papilio xuthus] >XP_013200095.1 PREDICTED: probable small nuclear ribonucleoprotein G [Amyelois transitella] >XP_014365947.1 probable small nuclear ribonucleoprotein G [Papilio machaon] >XP_023949391.1 probable small nuclear ribonucleoprotein G [Bicyclus anynana] >XP_026492889.1 probable small nuclear ribonucleoprotein G [Vanessa tameamea] >XP_030032656.1 probable small nuclear ribonucleoprotein G [Manduca sexta] >XP_032524946.1 probable small nuclear ribonucleoprotein G [Danaus plexippus plexippus] >XP_032524948.1 probable small nuclear ribonucleoprotein G [Danaus plexippus plexippus] >XP_039761094.1 probable small nuclear ribonucleoprotein G [Pararge aegeria] >XP_045507396.1 probable small nuclear ribonucleoprotein G [Colias croceus] >XP_046974152.1 probable small nuclear ribonucleoprotein G [Vanessa cardui] >XP_047540860.1 probable small nuclear ribonucleoprotein G [Vanessa atalanta] >XP_050357207.1 probable small nuclear ribonucleoprotein G [Nymphalis io] >CAG5058336.1 unnamed protein product [Parnassius apollo] >CAG9570366.1 unnamed protein product [Danaus chrysippus] >CAH0731681.1 unnamed protein product, partial [Brenthis ino] >CAH2061014.1 unnamed protein product, partial [Iphiclides podalirius] >CAH2269228.1 jg1748 [Pararge aegeria aegeria]	1.72895	-0.6899	-1.0563	0.46626	-0.449
TRINITY_DN4550_c1_g1_i19_orf1	titin homolog [Ostrinia furnacalis]	1.03487	-0.9715	-0.3783	1.33911	-1.0242
TRINITY_DN17995_c0_g4_i1_orf1	dynein light chain roadblock-type 2 [Bombyx mori] >XP_028159912.1 dynein light chain roadblock-type 2-like [Ostrinia furnacalis] >XP_030028262.1 dynein light chain roadblock-type 2 [Manduca sexta] >XP_038218546.1 dynein light chain roadblock-type 2-like [Zerene cesonia] >XP_045505125.1 dynein light chain roadblock-type 2-like [Colias croceus] >CAH0760167.1 unnamed protein product [Diatraea saccharalis]	1.68605	-0.7786	-0.8294	0.61675	-0.6948
TRINITY_DN4820_c0_g2_i2_orf1	tudor and KH domain-containing protein homolog isoform X1 [Ostrinia furnacalis] >XP_028179486.1 tudor and KH domain-containing protein homolog isoform X1 [Ostrinia furnacalis]	1.74561	-0.6141	-1.048	0.44501	-0.5284
TRINITY_DN14274_c0_g1_i3_orf1	ATP-dependent RNA helicase dbp2-like isoform X1 [Leguminivora glycinivorella]	1.53174	-0.5053	-1.2046	0.77156	-0.5934
TRINITY_DN4708_c0_g1_i5_orf1	GTP-binding protein 1 [Ostrinia furnacalis] >XP_028178070.1 GTP-binding protein 1 [Ostrinia furnacalis] >XP_028178072.1 GTP-binding protein 1 [Ostrinia	1.3536	-0.9863	-1.0489	0.99751	-0.3159
TRINITY_DN2676_c0_g1_i2_orf1	probable cytochrome P450 303a1 [Ostrinia furnacalis] >XP_028178318.1 probable cytochrome P450 303a1 [Ostrinia furnacalis]	1.56394	-0.6224	-0.6949	0.78592	-1.0325
TRINITY_DN50517_c0_g1_i3_orf1	PREDICTED: larval cuticle protein A2B-like [Amyelois transitella]	1.74025	-0.5932	-1.1581	0.37864	-0.3676
TRINITY_DN34426_c0_g1_i1_orf1	laminin subunit alpha-like, partial [Ostrinia furnacalis]	1.24521	-0.8033	-1.0779	1.16655	-0.5306
TRINITY_DN31980_c0_g1_i1_orf1	E3 ubiquitin-protein ligase TRIP12 isoform X2 [Ostrinia furnacalis]	1.66639	-0.3542	-0.6842	0.5323	-1.1603
TRINITY_DN77005_c0_g2_i1_orf1	RNA polymerase II degradation factor 1-like [Ostrinia furnacalis]	1.4434	-0.6625	-0.6033	0.93539	-1.113
TRINITY_DN13732_c0_g2_i3_orf1	60S ribosomal protein L35 [Ostrinia furnacalis]	1.6981	-0.6483	-1.0354	0.55138	-0.5658
TRINITY_DN32514_c0_g2_i1_orf1	mucin-5AC-like [Ostrinia furnacalis]	1.99503	-0.5029	-0.3741	-0.5906	-0.5275
TRINITY_DN40669_c0_g2_i1_orf1	uncharacterized protein LOC114355531 [Ostrinia furnacalis]	1.99572	-0.4265	-0.4979	-0.6175	-0.4538
TRINITY_DN56155_c0_g1_i1_orf1	hypothetical protein evm_004679 [Chilo suppressalis] >CAH2989683.1 unnamed protein product [Chilo suppressalis]	1.9976	-0.4213	-0.5229	-0.5705	-0.4828
TRINITY_DN30154_c0_g1_i1_orf1	serine/threonine-protein kinase SIK2 [Ostrinia furnacalis] >XP_028174514.1 serine/threonine-protein kinase SIK2 [Ostrinia furnacalis]	1.97265	-0.7725	-0.328	-0.5456	-0.3266
TRINITY_DN1124_c0_g1_i7_orf1	PREDICTED: cuticle protein 18.6, isoform B [Amyelois transitella]	1.96466	-0.2822	-0.4181	-0.84	-0.4243
TRINITY_DN87803_c0_g1_i2_orf1	hypothetical protein NE865_05903 [Phthorimaea operculella]	1.99709	-0.5347	-0.3964	-0.5477	-0.5183
TRINITY_DN64181_c0_g1_i1_orf1	uncharacterized protein LOC114356431 isoform X2 [Ostrinia furnacalis]	1.46639	-1.5554	0.54647	-0.1125	-0.3449
TRINITY_DN18624_c0_g1_i5_orf1	uncharacterized protein LOC114353135 [Ostrinia furnacalis]	1.99275	-0.6386	-0.5202	-0.4545	-0.3795
TRINITY_DN12526_c0_g1_i5_orf1	uncharacterized protein LOC114359035 isoform X3 [Ostrinia furnacalis]	1.17556	-1.6672	-0.4693	0.2009	0.76011

TRINITY_DN3190_c0_g1_i1_orf1	repetitive proline-rich cell wall protein 1 precursor [Papilio polytes] >XP_013147838.1 PREDICTED: repetitive proline-rich cell wall protein 1 [Papilio polytes] >BAM19190.1 cuticular protein PpolCPG24 [Papilio polytes]	1.99824	-0.4534	-0.4939	-0.5768	-0.4742
TRINITY_DN5408_c0_g1_i5_orf1	uncharacterized protein LOC114359912 [Ostrinia furnacalis]	1.52001	-1.4089	-0.0488	-0.6229	0.56055
TRINITY_DN52553_c0_g2_i1_orf1	hemocyte protein-glutamine gamma-glutamyltransferase-like [Ostrinia furnacalis]	1.99274	-0.4197	-0.574	-0.6098	-0.3893
TRINITY_DN15175_c0_g1_i1_orf1	zinc carboxypeptidase-like [Ostrinia furnacalis]	1.94642	-0.7526	-0.6799	-0.0977	-0.4163
TRINITY_DN381_c0_g1_i1_orf1	cuticle protein 8-like [Ostrinia furnacalis]	1.99137	-0.4127	-0.4497	-0.6754	-0.4536
TRINITY_DN4021_c0_g1_i1_orf1	leech-derived tryptase inhibitor C-like [Ostrinia furnacalis]	1.9664	-0.4082	-0.3284	-0.8414	-0.3884
TRINITY_DN138481_c0_g1_i5_orf1	hypothetical protein evm_003901 [Chilo suppressalis]	1.93649	-0.869	-0.6123	-0.1374	-0.3177
TRINITY_DN2101_c0_g1_i6_orf1	protein obstructor-E-like [Ostrinia furnacalis]	1.96441	-0.1741	-0.4807	-0.7602	-0.5494
TRINITY_DN10824_c0_g1_i3_orf1	endochitinase isoform X2 [Ostrinia furnacalis]	1.96431	-0.6977	-0.1412	-0.59	-0.5355
TRINITY_DN18338_c0_g1_i6_orf1	aquaporin AQPAn.G isoform X1 [Ostrinia furnacalis]	1.53526	-1.5681	0.18761	-0.339	0.18419
TRINITY_DN129835_c0_g1_i2_orf1	alpha-tocopherol transfer protein-like [Chelonus insularis]	1.93008	-0.2244	-0.1658	-0.6947	-0.8452
TRINITY_DN644_c0_g1_i1_orf1	cuticle protein 19-like [Ostrinia furnacalis]	1.94638	-0.0835	-0.5942	-0.7872	-0.4815
TRINITY_DN74538_c0_g1_i1_orf1	tetra-peptide repeat homeobox protein 1-like [Ostrinia furnacalis]	1.99931	-0.4912	-0.4691	-0.4908	-0.5482
TRINITY_DN4592_c0_g1_i1_orf1	hypothetical protein KGM_205563 [Danaus plexippus plexippus]	1.99815	-0.5413	-0.4541	-0.5535	-0.4493
TRINITY_DN19639_c0_g2_i1_orf1	basic juvenile hormone-suppressible protein 1-like [Hyposmocoma kahamanoa]	1.57728	-1.0412	-1.026	-0.1124	0.60231
TRINITY_DN5581_c0_g1_i1_orf1	mucin-5AC-like [Ostrinia furnacalis]	1.98016	-0.6103	-0.6557	-0.4609	-0.2533
TRINITY_DN98147_c0_g2_i1_orf1	hypothetical protein evm_002297 [Chilo suppressalis]	1.96609	-0.488	-0.4981	-0.7799	-0.2001
TRINITY_DN18539_c0_g1_i1_orf1	pupal cuticle protein PCP52-like [Ostrinia furnacalis]	1.99685	-0.4521	-0.6068	-0.4771	-0.4608
TRINITY_DN21719_c0_g1_i2_orf1	chymotrypsin-2-like [Ostrinia furnacalis]	1.99387	-0.4284	-0.6094	-0.3988	-0.5572
TRINITY_DN13576_c0_g1_i1_orf1	uncharacterized protein LOC114350099 [Ostrinia furnacalis]	1.9852	-0.388	-0.4226	-0.7288	-0.4458
TRINITY_DN661_c0_g1_i1_orf1	hypothetical protein evm_002822 [Chilo suppressalis]	1.92367	-0.7303	-0.0188	-0.7822	-0.3924
TRINITY_DN38274_c0_g1_i1_orf1	uncharacterized protein LOC114360402 [Ostrinia furnacalis]	1.99721	-0.4414	-0.4997	-0.5949	-0.4612
TRINITY_DN9311_c0_g1_i1_orf1	cuticle protein 8-like [Ostrinia furnacalis]	1.96093	-0.1998	-0.3658	-0.7592	-0.6361
TRINITY_DN29604_c0_g2_i2_orf1	neurofilament heavy polypeptide-like isoform X2 [Ostrinia furnacalis]	1.86847	-1.1205	-0.4587	-0.1678	-0.1215
TRINITY_DN5829_c0_g1_i1_orf1	uncharacterized protein LOC114365758 isoform X3 [Ostrinia furnacalis]	1.99593	-0.4616	-0.6112	-0.4196	-0.5035
TRINITY_DN35351_c0_g1_i3_orf1	adult-specific cuticular protein ACP-20-like [Ostrinia furnacalis]	1.99475	-0.5545	-0.5463	-0.535	-0.3589
TRINITY_DN619_c0_g1_i1_orf1	putative uncharacterized protein DDB_G0271606 [Ostrinia furnacalis]	1.93351	-0.0382	-0.4472	-0.8003	-0.6478
TRINITY_DN19043_c0_g2_i1_orf1	hypothetical protein EVAR_60653_1 [Eumeta japonica]	1.81138	-1.2718	-0.1254	-0.2038	-0.2104
TRINITY_DN2400_c0_g1_i1_orf1	uncharacterized protein LOC114351021 [Ostrinia furnacalis]	1.98425	-0.7012	-0.3292	-0.5394	-0.4144
TRINITY_DN2946_c0_g1_i1_orf1	histidine-rich glycoprotein [Ostrinia furnacalis]	1.98559	-0.3101	-0.6107	-0.6359	-0.429
TRINITY_DN801_c0_g1_i2_orf1	cathepsin L [Ostrinia furnacalis] >XP_028165920.1 cathepsin L [Ostrinia furnacalis] >UKI61015.1 cathepsin L [Ostrinia furnacalis]	1.39293	-1.6549	0.53302	-0.1484	-0.1227
TRINITY_DN65681_c0_g1_i1_orf1	ferritin subunit-like [Ostrinia furnacalis] >XP_028168186.1 ferritin subunit-like [Ostrinia furnacalis]	1.68319	-0.7607	-1.2374	0.09588	0.21897
TRINITY_DN26882_c0_g1_i1_orf1	uncharacterized protein LOC114349648 [Ostrinia furnacalis]	1.99963	-0.4647	-0.5218	-0.5114	-0.5017
TRINITY_DN57998_c1_g1_i1_orf1	uncharacterized protein LOC113509309, partial [Galleria mellonella]	1.96618	-0.7032	-0.3962	-0.202	-0.6647
TRINITY_DN2311_c0_g3_i1_orf1	uncharacterized protein LOC114364231 isoform X1 [Ostrinia furnacalis] >XP_028176108.1 uncharacterized protein LOC114364231 isoform X2 [Ostrinia	1.75083	-1.2125	-0.4861	-0.3628	0.31061
TRINITY_DN15222_c0_g1_i4_orf1	lysosomal alpha-mannosidase-like [Ostrinia furnacalis]	1.89975	-0.5229	-0.6036	-0.8632	0.08993
TRINITY_DN28922_c0_g1_i2_orf1	uncharacterized protein LOC119829283 isoform X2 [Zerene cesonia]	1.9407	-0.8907	-0.3664	-0.1515	-0.5322
TRINITY_DN12671_c0_g1_i6_orf1	hemicentin-1-like isoform X1 [Ostrinia furnacalis]	1.87108	-1.0512	-0.3467	0.04789	-0.521
TRINITY_DN20717_c0_g1_i1_orf1	putative uncharacterized protein DDB_G0282133 isoform X1 [Ostrinia furnacalis]	1.5924	-1.4066	0.49019	-0.4303	-0.2457
TRINITY_DN57998_c1_g3_i1_orf1	uncharacterized protein LOC114363305 isoform X2 [Ostrinia furnacalis]	1.96902	-0.8223	-0.4505	-0.3209	-0.3752
TRINITY_DN2908_c0_g1_i1_orf1	uncharacterized protein LOC114361337 [Ostrinia furnacalis]	1.97977	-0.2973	-0.4602	-0.7403	-0.482
TRINITY_DN25273_c0_g1_i1_orf1	skin secretory protein xP2-like [Ostrinia furnacalis]	1.92687	-0.9779	-0.4592	-0.2359	-0.2539
TRINITY_DN77425_c0_g1_i2_orf1	anosmin-1 [Ostrinia furnacalis] >XP_028178811.1 anosmin-1 [Ostrinia furnacalis]	1.98386	-0.6919	-0.553	-0.4274	-0.3115
TRINITY_DN906_c0_g1_i4_orf1	uncharacterized protein LOC114360441, partial [Ostrinia furnacalis]	1.95715	-0.8586	-0.2246	-0.4425	-0.4314
TRINITY_DN3310_c0_g1_i1_orf1	hypothetical protein evm_010516 [Chilo suppressalis]	1.96004	-0.1063	-0.6489	-0.6037	-0.6012
TRINITY_DN31_c0_g1_i3_orfp1	TRINITY_DN31_c0_g1_i3_m.1394 TRINITY_DN31_c0_g1::TRINITY_DN31_c0_g1_i3::g.1394 ORF type:complete len:118 (+),score=43.35 TRINITY_DN31_c0_g1_i3:56-409(+)	1.91966	-0.652	-0.8528	-0.403	-0.0119
TRINITY_DN13686_c0_g2_i1_orf1	transmembrane protease serine 9-like [Ostrinia furnacalis]	1.82352	-1.203	-0.4292	-0.2077	0.01638
TRINITY_DN778_c0_g1_i1_orf1	uncharacterized protein LOC114363281 [Ostrinia furnacalis]	1.93848	-0.0995	-0.5662	-0.8621	-0.4107
TRINITY_DN98334_c0_g1_i1_orf1	unnamed protein product [Spodoptera littoralis] >CAH1638738.1 unnamed protein product [Spodoptera littoralis]	1.99794	-0.4133	-0.5442	-0.5168	-0.5237
TRINITY_DN4384_c0_g1_i5_orf1	chemosensory protein 5 [Conogethes punctiferalis]	1.9469	-0.8811	-0.3634	-0.1861	-0.5162
TRINITY_DN338_c0_g1_i1_orf1	chorion protein S36-like [Ostrinia furnacalis]	1.99096	-0.5527	-0.3143	-0.5522	-0.5717
TRINITY_DN10747_c0_g1_i5_orf1	unnamed protein product [Plutella xylostella]	1.98764	-0.6786	-0.5397	-0.3567	-0.4126
TRINITY_DN24971_c0_g1_i3_orf1	uncharacterized protein LOC114352370 [Ostrinia furnacalis]	1.82537	-1.2295	-0.3277	-0.2146	-0.0536
TRINITY_DN1012_c0_g1_i2_orf1	teneurin-a isoform X1 [Ostrinia furnacalis]	1.4208	-1.4798	0.19809	-0.6789	0.53979
TRINITY_DN2002_c0_g1_i5_orfp1	TRINITY_DN2002_c0_g1_i5_m.4230 TRINITY_DN2002_c0_g1::TRINITY_DN2002_c0_g1_i5::g.4230 ORF type:3prime_partial len:259 (+),score=29.98,Peptidase_C39 PF03412.16 1.7,Peptidase_C39|PF03412.16|0.049 TRINITY_DN2002_c0_g1_i5:65-838(+)	1.87811	-0.9272	-0.0527	-0.7707	-0.1275
TRINITY_DN7539_c0_g1_i2_orf1	serine protease inhibitor 3/4 [Ostrinia furnacalis]	1.97078	-0.7339	-0.6168	-0.3589	-0.2612
TRINITY_DN9282_c0_g1_i2_orf1	uncharacterized protein LOC114363102 isoform X2 [Ostrinia furnacalis]	1.93271	-0.04	-0.5262	-0.8453	-0.5212
TRINITY_DN391_c0_g1_i4_orf1	phenoloxidase-activating enzyme-like [Ostrinia furnacalis]	1.64125	-1.4891	-0.2772	0.10877	0.01625
TRINITY_DN138481_c0_g1_i2_orf1	cuticle protein 8-like isoform X2 [Vanessa tameamea]	1.98969	-0.4394	-0.6857	-0.3871	-0.4775
TRINITY_DN12387_c0_g1_i1_orf1	repetitive proline-rich cell wall protein 2-like [Ostrinia furnacalis]	1.99925	-0.4723	-0.4734	-0.506	-0.5475

TRINITY_DN3616_c0_g2_i1_orf1	conotoxin ArMKLT2-032-like [Ostrinia furnacalis]	1.87404	-1.1029	-0.0539	-0.4341	-0.2831
TRINITY_DN25534_c0_g1_i1_orf1	venom serine carboxypeptidase-like [Ostrinia furnacalis]	1.97575	-0.2934	-0.713	-0.3579	-0.6115
TRINITY_DN49785_c1_g1_i3_orf1	uncharacterized protein LOC114365444, partial [Ostrinia furnacalis]	1.97188	-0.6518	-0.3034	-0.7058	-0.3109
TRINITY_DN7854_c0_g1_i4_orf1	failed axon connections [Ostrinia furnacalis]	1.63711	-1.5194	0.02109	-0.0938	-0.045
TRINITY_DN21555_c0_g1_i4_orf1	uncharacterized protein LOC114351844 [Ostrinia furnacalis] >XP_028158981.1 uncharacterized protein LOC114351844 [Ostrinia furnacalis] >XP_028158982.1 uncharacterized protein LOC114351844 [Ostrinia furnacalis] >XP_028158983.1 uncharacterized protein LOC114351844 [Ostrinia furnacalis] >XP_028158984.1 uncharacterized protein LOC114351844 [Ostrinia furnacalis] >XP_028158985.1 uncharacterized protein LOC114351844 [Ostrinia furnacalis] >5GPR_A Crystal structure of chitinase-h from Ostrinia furnacalis [Ostrinia furnacalis] >5GQB_A Crystal structure of chitinase-h from O. furnacalis in complex with chitohepatose [Ostrinia furnacalis] >6JMN_A Crystal structure of Ostrinia furnacalis Chitinase h complexed with compound 2-8-s2 [Ostrinia furnacalis] >BAE16587.1 chitinase [Ostrinia furnacalis]	1.85928	-1.1256	-0.4767	-0.2174	-0.0395
TRINITY_DN12387_c1_g2_i1_orf1	skin secretory protein xP2-like [Ostrinia furnacalis]	1.99741	-0.4719	-0.5914	-0.4383	-0.4958
TRINITY_DN7549_c0_g1_i1_orf1	uncharacterized protein LOC114355006 [Ostrinia furnacalis]	1.96663	-0.2098	-0.4921	-0.7847	-0.4801
TRINITY_DN54524_c0_g1_i6_orf1	serine protease inhibitor dipetalogastin [Ostrinia furnacalis]	1.98843	-0.3108	-0.5214	-0.6461	-0.5101
TRINITY_DN12671_c0_g1_i4_orf1	hemicentin-1-like isoform X1 [Ostrinia furnacalis]	1.96204	-0.8659	-0.3668	-0.3571	-0.3722
TRINITY_DN26149_c0_g1_i5_orf1	thymosin beta isoform X3 [Ostrinia furnacalis]	1.70773	-1.4077	-0.3125	-0.0399	0.05235
TRINITY_DN1935_c0_g1_i1_orf1	adult-specific cuticular protein ACP-22-like [Ostrinia furnacalis]	1.97884	-0.2259	-0.5083	-0.6113	-0.6333
TRINITY_DN26301_c0_g1_i1_orf1	uncharacterized protein LOC114359193 [Ostrinia furnacalis]	1.88231	-1.1117	-0.3334	-0.3031	-0.1341
TRINITY_DN5907_c0_g1_i4_orf1	hypothetical protein evm_010265 [Chilo suppressalis] >CAB3524755.1 unnamed protein product [Chilo suppressalis] >CAH0397522.1 unnamed protein product [Chilo suppressalis]	1.98502	-0.7056	-0.5294	-0.3625	-0.3875
TRINITY_DN59388_c0_g1_i1_orf1	uncharacterized protein LOC114353759 [Ostrinia furnacalis]	1.5435	-1.4404	0.48286	-0.0303	-0.5556
TRINITY_DN5595_c0_g1_i1_orf1	keratin, type I cytoskeletal 10-like [Ostrinia furnacalis]	1.98264	-0.4201	-0.3978	-0.7499	-0.4149
TRINITY_DN661_c0_g2_i2_orf1	cuticle protein 7-like [Ostrinia furnacalis]	1.93421	-0.5741	-0.9088	-0.1979	-0.2534
TRINITY_DN2140_c0_g1_i1_orf1	uncharacterized protein LOC126367764 [Pectinophora gossypiella]	1.97988	-0.6991	-0.2601	-0.4727	-0.548
TRINITY_DN1465_c2_g1_i2_orf1	transcription initiation factor TFIID subunit 1-like [Ostrinia furnacalis]	1.7845	-1.1896	-0.3731	-0.4549	0.23306
TRINITY_DN91533_c0_g1_i1_orf1	uncharacterized protein LOC114361550 [Ostrinia furnacalis]	1.99873	-0.5174	-0.4412	-0.5499	-0.4902
TRINITY_DN6004_c0_g1_i1_orf1	endocuticle structural glycoprotein ABD-4-like [Ostrinia furnacalis]	1.94185	-0.942	-0.3786	-0.363	-0.2582
TRINITY_DN1326_c0_g1_i2_orf1	cuticle protein 7 [Plutella xylostella] >CAG9138501.1 unnamed protein product [Plutella xylostella]	1.90707	-0.6201	-0.6638	-0.7261	0.10299
TRINITY_DN53866_c0_g1_i1_orf1	larval/pupal cuticle protein H1C-like [Ostrinia furnacalis]	1.92645	-0.8831	-0.0914	-0.6299	-0.322
TRINITY_DN2515_c0_g1_i6_orf1	chitooligosaccharidolytic beta-N-acetylglucosaminidase isoform X1 [Ostrinia furnacalis]	1.80704	-1.2671	-0.1622	-0.3144	-0.0634
TRINITY_DN19990_c0_g1_i1_orf1	carboxypeptidase B-like [Ostrinia furnacalis]	1.99005	-0.6143	-0.531	-0.3138	-0.531
TRINITY_DN1749_c0_g2_i2_orf1	putative GPI-anchored protein pfl2 isoform X1 [Ostrinia furnacalis]	1.97891	-0.3791	-0.425	-0.7738	-0.401
TRINITY_DN3056_c0_g1_i1_orf1	spidroin-2-like [Ostrinia furnacalis]	1.97764	-0.5251	-0.3848	-0.7519	-0.3159
TRINITY_DN5444_c0_g2_i1_orf1	phenoloxidase-activating factor 2-like [Ostrinia furnacalis]	1.93721	-0.9122	-0.2656	-0.5473	-0.212
TRINITY_DN2442_c0_g1_i2_orf1	digestive cysteine proteinase 2 [Ostrinia furnacalis]	1.77788	-1.2558	0.1596	-0.387	-0.2946
TRINITY_DN68397_c0_g1_i2_orf1	clavesin-1-like [Ostrinia furnacalis]	1.98681	-0.4149	-0.529	-0.353	-0.6899
TRINITY_DN49143_c0_g1_i1_orf1	uncharacterized protein LOC107270465 [Cephus cinctus]	1.99847	-0.5711	-0.4875	-0.4524	-0.4875
TRINITY_DN4676_c0_g1_i16_orf1	meiosis-specific nuclear structural protein 1-like isoform X2 [Ostrinia furnacalis]	1.89343	-0.8369	0.10404	-0.4573	-0.7033
TRINITY_DN2652_c0_g2_i1_orf1	peroxidase-like isoform X1 [Ostrinia furnacalis]	1.93676	-0.9509	-0.3332	-0.2244	-0.4283
TRINITY_DN31314_c0_g1_i4_orf1	uncharacterized protein LOC114355976 [Ostrinia furnacalis]	1.99945	-0.4876	-0.5344	-0.4646	-0.5128
TRINITY_DN5467_c0_g1_i5_orf1	synaptic vesicle glycoprotein 2B-like isoform X2 [Ostrinia furnacalis] >XP_028161209.1 synaptic vesicle glycoprotein 2B-like isoform X2 [Ostrinia furnacalis] >XP_028161210.1 synaptic vesicle glycoprotein 2B-like isoform X2 [Ostrinia furnacalis]	1.69305	-1.4156	0.02883	-0.3554	0.04919
TRINITY_DN6470_c0_g3_i2_orf1	trypsin CFT-1-like [Ostrinia furnacalis]	1.96293	-0.4291	-0.8548	-0.3669	-0.3121
TRINITY_DN2489_c0_g1_i1_orf1	uncharacterized protein LOC114354692 [Ostrinia furnacalis]	1.92419	-0.9749	-0.473	-0.1676	-0.3088
TRINITY_DN1749_c0_g1_i1_orf1	putative GPI-anchored protein pfl2 isoform X2 [Ostrinia furnacalis]	1.98602	-0.531	-0.3896	-0.6977	-0.3678
TRINITY_DN74086_c0_g1_i1_orf1	UPF0489 protein C5orf22 homolog [Ostrinia furnacalis]	1.91781	-0.8807	-0.1308	-0.6966	-0.2097
TRINITY_DN52553_c0_g1_i1_orf1	hemocyte protein-glutamine gamma-glutamyltransferase-like [Ostrinia furnacalis]	1.991	-0.5465	-0.4015	-0.6482	-0.3947
TRINITY_DN58125_c0_g1_i1_orf1	protein yellow-like [Ostrinia furnacalis]	1.96912	-0.7673	-0.2791	-0.5842	-0.3384
TRINITY_DN4324_c0_g1_i1_orf1	uncharacterized protein LOC114354985 isoform X1 [Ostrinia furnacalis]	1.98615	-0.5896	-0.6098	-0.5079	-0.2788
TRINITY_DN2794_c1_g1_i8_orf1	carboxypeptidase D [Ostrinia furnacalis]	1.59237	-1.5301	0.19351	-0.2909	0.03508
TRINITY_DN2896_c0_g1_i2_orf1	general odorant-binding protein 56d-like isoform X2 [Ostrinia furnacalis]	1.98805	-0.4983	-0.3357	-0.6788	-0.4752
TRINITY_DN688_c0_g1_i8_orf1	lysosomal alpha-mannosidase-like [Ostrinia furnacalis]	1.70856	-1.4246	-0.0313	-0.0307	-0.222
TRINITY_DN110231_c0_g1_i1_orf1	protein singed [Ostrinia furnacalis] >XP_028161434.1 protein singed [Ostrinia furnacalis]	1.80145	-1.0985	0.27123	-0.4864	-0.4878
TRINITY_DN27968_c0_g2_i2_orf1	nucleolin-like [Ostrinia furnacalis]	1.99588	-0.4267	-0.6176	-0.487	-0.4646
TRINITY_DN5664_c0_g1_i1_orf1	CDGSH iron-sulfur domain-containing protein 3, mitochondrial-like [Ostrinia furnacalis]	1.92568	-0.2028	-0.962	-0.5141	-0.2468
TRINITY_DN7785_c0_g1_i1_orf1	uncharacterized protein LOC114364098 [Ostrinia furnacalis]	1.93856	-0.0375	-0.4829	-0.7398	-0.6782
TRINITY_DN2953_c1_g1_i10_orf1	methionine--tRNA ligase, cytoplasmic isoform X2 [Ostrinia furnacalis] >XP_028156683.1 methionine--tRNA ligase, cytoplasmic isoform X4 [Ostrinia furnacalis] >XP_028156684.1 methionine--tRNA ligase, cytoplasmic isoform X5 [Ostrinia furnacalis]	1.90463	-0.9833	-0.5527	-0.311	-0.0576
TRINITY_DN4367_c0_g1_i1_orf1	heat shock protein 21.7c [Chilo suppressalis] >AWT57938.1 heat shock protein 21.7c [Chilo suppressalis]	1.89617	-0.8102	-0.8468	-0.0847	-0.1545
TRINITY_DN696_c1_g1_i10_orf1	titin-like [Ostrinia furnacalis]	1.99163	-0.5541	-0.6136	-0.4842	-0.3397
TRINITY_DN31417_c0_g1_i3_orf1	titin-like [Ostrinia furnacalis]	1.88646	-1.1043	-0.3673	-0.188	-0.2268
TRINITY_DN2069_c1_g1_i8_orf1	lysosomal aspartic protease [Trichoplusia ni]	1.65635	-1.4271	-0.4193	-0.0187	0.20878
TRINITY_DN143497_c0_g1_i1_orf1	fibroin heavy chain-like [Ostrinia furnacalis]	1.97035	-0.1805	-0.5744	-0.6986	-0.5169
TRINITY_DN139537_c0_g1_i1_orf1	uncharacterized protein LOC114351440 [Ostrinia furnacalis]	1.66122	-1.3696	-0.4446	0.35511	-0.2022

TRINITY_DN20793_c0_g2_i1_orf1	mucin-2-like [Ostrinia furnacalis]	1.94798	-0.0551	-0.5752	-0.6181	-0.6996
TRINITY_DN1308_c0_g1_i4_orf1	serine proteinase stubble-like [Ostrinia furnacalis]	1.92675	-0.7412	-0.81	-0.2641	-0.1115
TRINITY_DN1902_c0_g1_i4_orf1	chemosensory protein 10 [Conogethes pinicolalis]	1.98544	-0.6041	-0.2803	-0.4857	-0.6153
TRINITY_DN54269_c0_g1_i3_orf1	lopap-like [Ostrinia furnacalis]	1.72948	-0.838	-1.1303	0.10137	0.13741
TRINITY_DN23364_c0_g1_i1_orf1	PREDICTED: uncharacterized protein LOC106134920 [Amyelois transitella]	1.70915	-1.4176	-0.1917	0.06709	-0.1669
TRINITY_DN82426_c0_g1_i6_orfp1	lysosome-associated membrane glycoprotein 1-like isoform X4 [Ostrinia furnacalis]	1.93058	-0.6949	-0.767	-0.4485	-0.0201
TRINITY_DN26337_c0_g1_i3_orf1	lysosome membrane protein 2-like [Ostrinia furnacalis]	1.51383	-1.4176	-0.576	-0.1149	0.59473
TRINITY_DN11670_c0_g1_i1_orf1	teneurin-m isoform X1 [Ostrinia furnacalis]	1.89127	-0.9395	-0.0164	-0.6943	-0.2411
TRINITY_DN11970_c0_g1_i4_orf1	myb-like protein AA [Ostrinia furnacalis]	1.95626	-0.5683	-0.8333	-0.2462	-0.3084
TRINITY_DN1986_c0_g1_i1_orf1	serine protease inhibitor 77Ba-like [Ostrinia furnacalis] >XP_028164032.1 serine protease inhibitor 77Ba-like [Ostrinia furnacalis]	1.75146	-1.3693	-0.1681	-0.1634	-0.0507
TRINITY_DN33365_c0_g1_i1_orf1	mucin-5AC isoform X1 [Ostrinia furnacalis]	1.99142	-0.5848	-0.3578	-0.6088	-0.44
TRINITY_DN267_c0_g1_i1_orf1	keratin, type I cytoskeletal 9-like [Ostrinia furnacalis]	1.95928	-0.271	-0.293	-0.817	-0.5783
TRINITY_DN479_c6_g1_i2_orf1	beta-1,3-glucan-binding protein-like [Ostrinia furnacalis]	1.56993	-1.5387	0.32255	-0.1535	-0.2003
TRINITY_DN110519_c0_g1_i1_orf1	uncharacterized protein LOC114366601 [Ostrinia furnacalis]	1.97121	-0.2844	-0.7526	-0.591	-0.3433
TRINITY_DN4189_c0_g2_i1_orf1	unnamed protein product [Chilo suppressalis]	1.81843	-1.0871	0.21088	-0.5786	-0.3636
TRINITY_DN7123_c0_g1_i1_orf1	activating signal cointegrator 1 complex subunit 2 homolog isoform X1 [Ostrinia furnacalis]	1.9896	-0.3085	-0.5463	-0.6122	-0.5225
TRINITY_DN1491_c0_g1_i4_orf1	GILT-like protein 2 isoform X1 [Ostrinia furnacalis] >XP_028156245.1 GILT-like protein 2 isoform X2 [Ostrinia furnacalis] >XP_028156247.1 GILT-like protein 2 isoform X3 [Ostrinia furnacalis]	1.43535	-1.0461	0.74159	0.00733	-1.1382
TRINITY_DN42337_c0_g1_i5_orf1	hypothetical protein evm_002829 [Chilo suppressalis]	1.98184	-0.3783	-0.6686	-0.6177	-0.3172
TRINITY_DN4688_c0_g1_i2_orf1	uncharacterized protein LOC114359411 [Ostrinia furnacalis]	1.92622	-0.9255	-0.3012	-0.5707	-0.1287
TRINITY_DN23167_c0_g2_i1_orf1	hypothetical protein evm_003712 [Chilo suppressalis]	1.54948	-1.5754	-0.268	0.11528	0.17872
TRINITY_DN23229_c0_g1_i2_orf1	uncharacterized protein LOC114362553 [Ostrinia furnacalis]	1.65099	-1.2937	-0.6542	-0.1052	0.40206
TRINITY_DN206_c0_g1_i8_orf1	A-kinase anchor protein 200-like [Ostrinia furnacalis] >XP_028173114.1 A-kinase anchor protein 200-like [Ostrinia furnacalis] >XP_028173115.1 A-kinase anchor protein 200-like [Ostrinia furnacalis]	1.94284	-0.1944	-0.4828	-0.9099	-0.3558
TRINITY_DN3255_c0_g1_i1_orf1	uncharacterized protein LOC114351042 [Ostrinia furnacalis]	1.91909	0.03529	-0.5628	-0.82	-0.5715
TRINITY_DN9920_c0_g1_i1_orf1	uncharacterized protein LOC114351526 [Ostrinia furnacalis]	1.70778	-1.239	-0.7064	0.01599	0.22161
TRINITY_DN54366_c0_g1_i1_orf1	protein obstructor-E-like [Ostrinia furnacalis]	1.90817	0.09528	-0.6847	-0.735	-0.5838
TRINITY_DN268_c1_g1_i7_orf1	unnamed protein product [Spodoptera littoralis] >CAH1645252.1 unnamed protein product [Spodoptera littoralis]	1.97353	-0.7838	-0.4877	-0.2939	-0.408
TRINITY_DN104_c0_g1_i4_orf1	PREDICTED: heparan-alpha-glucosaminide N-acetyltransferase [Amyelois transitella]	1.67067	-1.2805	0.32396	-0.6805	-0.0336
TRINITY_DN36899_c0_g1_i1_orf1	glucose dehydrogenase [FAD, quinone]-like [Ostrinia furnacalis]	1.69307	-1.3218	0.2017	-0.5878	0.01484
TRINITY_DN3715_c0_g1_i2_orf1	uncharacterized protein LOC114356437 isoform X1 [Ostrinia furnacalis]	1.86439	-0.3023	-1.0332	0.07118	-0.6001
TRINITY_DN97138_c0_g1_i2_orf1	tubulin beta chain-like isoform X2 [Ostrinia furnacalis]	1.89179	-1.0937	-0.3521	-0.2513	-0.1947
TRINITY_DN6423_c0_g1_i5_orf1	phenoloxidase-activating factor 2-like isoform X2 [Ostrinia furnacalis]	1.6612	-1.4549	-0.3372	0.03908	0.09183
TRINITY_DN113272_c0_g1_i1_orf1	altered inheritance of mitochondria protein 3-like [Ostrinia furnacalis]	1.89062	0.12477	-0.6271	-0.857	-0.5313
TRINITY_DN537_c0_g1_i1_orf1	pupal cuticle protein C1B-like precursor [Papilio xuthus] >BAM18715.1 cuticular protein PxutCPFL6Ba [Papilio xuthus]	1.59296	-1.3641	0.539	-0.4745	-0.2934
TRINITY_DN125427_c0_g1_i1_orf1	heat shock protein 19.8 [Chilo suppressalis] >AGM90553.1 HSP19.8 [Chilo suppressalis] >BAE94664.1 small heat shock protein 19.7 [Chilo suppressalis]	1.90453	-0.6684	-0.9238	-0.0469	-0.2655
TRINITY_DN206_c0_g1_i11_orf1	A-kinase anchor protein 200-like [Ostrinia furnacalis] >XP_028173114.1 A-kinase anchor protein 200-like [Ostrinia furnacalis] >XP_028173115.1 A-kinase anchor protein 200-like [Ostrinia furnacalis]	1.99626	-0.5984	-0.4597	-0.5216	-0.4167
TRINITY_DN10766_c0_g1_i1_orf1	hypothetical protein evm_008559 [Chilo suppressalis]	1.89928	-0.8955	-0.1069	-0.7461	-0.1508
TRINITY_DN2290_c0_g1_i2_orfp1	TRINITY_DN2290_c0_g1_i2_m.69732 TRINITY_DN2290_c0_g1::TRINITY_DN2290_c0_g1_i2::g.69732 ORF type:complete len:234 (+),score=14.43 TRINITY_DN2290_c0_g1_i2:60-761(+)	1.81174	-0.5769	0.35091	-0.7462	-0.8395
TRINITY_DN97097_c0_g1_i4_orf1	plectin-like, partial [Ostrinia furnacalis]	1.6619	-1.4084	-0.43	0.25249	-0.076
TRINITY_DN2416_c0_g1_i5_orf1	somatomedin-B and thrombospondin type-1 domain-containing protein [Ostrinia furnacalis] >XP_028177886.1 somatomedin-B and thrombospondin type-1 domain-containing protein [Ostrinia furnacalis]	1.61888	-1.3068	-0.0408	-0.6983	0.42695
TRINITY_DN1091_c0_g1_i1_orf1	macrophage mannose receptor 1-like [Pararge aegeria]	1.49356	-1.3514	0.73937	-0.5032	-0.3783
TRINITY_DN98016_c0_g1_i1_orf1	methanethiol oxidase [Ostrinia furnacalis]	1.62849	-1.4114	-0.1443	-0.4442	0.37136
TRINITY_DN8083_c0_g1_i1_orf1	solute carrier family 35 member F6 [Ostrinia furnacalis]	1.36715	-1.5389	0.75293	-0.4066	-0.1746
TRINITY_DN14944_c0_g1_i9_orf1	casein kinase I isoform X1 [Ostrinia furnacalis] >XP_028158159.1 casein kinase I isoform X1 [Ostrinia furnacalis] >XP_028158160.1 casein kinase I isoform X1 [Ostrinia furnacalis] >XP_028158161.1 casein kinase I isoform X1 [Ostrinia furnacalis] >XP_028158163.1 casein kinase I isoform X1 [Ostrinia furnacalis] >XP_028158164.1 casein kinase I isoform X1 [Ostrinia furnacalis] >XP_028158165.1 casein kinase I isoform X1 [Ostrinia furnacalis] >XP_028158166.1 casein kinase I isoform X1 [Ostrinia furnacalis]	1.83552	-1.0158	-0.2508	-0.717	0.14814
TRINITY_DN38431_c0_g1_i1_orf1	neprilysin-2 isoform X1 [Ostrinia furnacalis]	1.99646	-0.5327	-0.5575	-0.5199	-0.3864
TRINITY_DN843_c0_g1_i2_orf1	unnamed protein product [Diatraea saccharalis]	1.68204	-0.7208	0.60207	-0.5989	-0.9643
TRINITY_DN4125_c0_g1_i14_orf1	angiotensin-converting enzyme-like isoform X1 [Ostrinia furnacalis]	1.88712	0.12597	-0.5973	-0.8866	-0.5291
TRINITY_DN1703_c0_g1_i6_orf1	leucine-rich repeat-containing protein 15-like [Ostrinia furnacalis] >XP_028171914.1 leucine-rich repeat-containing protein 15-like [Ostrinia furnacalis] >XP_028171921.1 leucine-rich repeat-containing protein 15-like [Ostrinia furnacalis]	1.84512	-1.1532	-0.495	-0.0749	-0.1219
TRINITY_DN5757_c0_g1_i1_orf1	ATP-dependent DNA helicase 2 subunit 1 [Ostrinia furnacalis]	1.98567	-0.675	-0.4574	-0.3079	-0.5454
TRINITY_DN1563_c0_g1_i4_orf1	pupal cuticle protein 36-like [Ostrinia furnacalis]	1.98626	-0.6014	-0.5011	-0.2814	-0.6023
TRINITY_DN64616_c0_g1_i1_orf1	uncharacterized protein LOC114366101 [Ostrinia furnacalis]	1.79134	-0.8455	-1.0301	0.11828	-0.034
TRINITY_DN3005_c0_g1_i7_orf1	lachesin-like isoform X3 [Ostrinia furnacalis]	1.83031	-1.1368	-0.5487	0.07932	-0.2241
TRINITY_DN218_c0_g1_i1_orf1	altered inheritance of mitochondria protein 3-like isoform X2 [Ostrinia furnacalis]	1.97921	-0.2501	-0.6255	-0.6516	-0.4519
TRINITY_DN1407_c0_g1_i5_orf1	unnamed protein product [Chrysodeixis includens]	1.75413	-1.2313	-0.2163	0.24241	-0.549
TRINITY_DN1630_c0_g1_i6_orf1	major facilitator superfamily domain-containing protein 1-like [Ostrinia furnacalis]	1.75713	-1.3376	-0.1486	0.04433	-0.3153

TRINITY_DN36281_c0_g1_i2_orf1	putative uncharacterized protein DDB_G0282499 isoform X1 [Ostrinia furnacalis]	1.98283	-0.6983	-0.4445	-0.5432	-0.2968
TRINITY_DN15865_c0_g2_i2_orf1	carboxylesterase [Cnaphalocrocis medinalis]	1.83435	-1.0558	-0.7124	-0.1067	0.04047
TRINITY_DN1196_c0_g1_i4_orf1	glucosamine-6-phosphate isomerase isoform X2 [Ostrinia furnacalis]	1.7745	-0.6598	0.42066	-0.9411	-0.5942
TRINITY_DN806_c0_g2_i1_orf1	uncharacterized protein LOC114355167 [Ostrinia furnacalis]	1.93624	-0.1207	-0.3381	-0.8625	-0.615
TRINITY_DN114834_c0_g1_i1_orf1	uncharacterized protein LOC115444227 [Manduca sexta] >XP_030025790.1 uncharacterized protein LOC115444227 [Manduca sexta] >XP_037296791.1 uncharacterized protein LOC115444227 [Manduca sexta] >XP_037296792.1 uncharacterized protein LOC115444227 [Manduca sexta] >KAG6441350.1 hypothetical protein O3G_MSEX001778 [Manduca sexta]	1.97456	-0.2084	-0.6947	-0.5591	-0.5123
TRINITY_DN10304_c0_g2_i1_orf1	glycine-rich cell wall structural protein [Ostrinia furnacalis]	1.98338	-0.5227	-0.5625	-0.2577	-0.6404
TRINITY_DN7128_c0_g1_i7_orf1	dystroglycan [Ostrinia furnacalis]	1.45888	-1.6362	0.39003	-0.2055	-0.0071
TRINITY_DN14856_c0_g1_i1_orf1	upstream activation factor subunit spp27 [Ostrinia furnacalis]	1.84638	-1.0332	0.04922	-0.7043	-0.1582
TRINITY_DN712_c0_g2_i1_orf1	serine protease inhibitor 77Ba-like [Ostrinia furnacalis]	1.8337	-1.1325	-0.5702	-0.1687	0.0377
TRINITY_DN26985_c0_g1_i5_orf1	secretory phospholipase A2 receptor-like [Helicoverpa zea]	1.36674	-0.5737	1.03384	-0.7327	-1.0942
TRINITY_DN875_c0_g1_i3_orf1	secernin-3 [Ostrinia furnacalis]	1.71172	-1.244	-0.1826	0.33093	-0.6161
TRINITY_DN4689_c0_g1_i5_orf1	pericentriolar material 1 protein-like isoform X3 [Ostrinia furnacalis]	1.90303	-0.5737	-0.8668	0.07776	-0.5403
TRINITY_DN10364_c0_g1_i5_orf1	uncharacterized protein LOC114352615 [Ostrinia furnacalis]	1.95687	-0.6783	-0.7427	-0.3554	-0.1804
TRINITY_DN3833_c0_g1_i4_orf1	division abnormally delayed protein [Ostrinia furnacalis]	1.99644	-0.5258	-0.5892	-0.4079	-0.4736
TRINITY_DN41761_c0_g1_i4_orf1	transmembrane protease serine 9 [Ostrinia furnacalis]	1.8951	-1.0157	-0.5152	-0.0317	-0.3325
TRINITY_DN13887_c0_g1_i5_orf1	transmembrane protein 184B isoform X3 [Ostrinia furnacalis]	1.91745	-0.9982	-0.4787	-0.241	-0.1995
TRINITY_DN19951_c0_g1_i5_orf1	protein croquemort-like [Ostrinia furnacalis]	1.85178	-1.1742	-0.3437	-0.0712	-0.2627
TRINITY_DN11772_c0_g1_i1_orf1	conserved oligomeric Golgi complex subunit 2 [Ostrinia furnacalis]	1.95473	-0.7737	-0.3978	-0.6318	-0.1514
TRINITY_DN25987_c0_g1_i5_orf1	GILT-like protein 2 isoform X1 [Ostrinia furnacalis] >XP_028156245.1 GILT-like protein 2 isoform X2 [Ostrinia furnacalis] >XP_028156247.1 GILT-like protein 2 isoform X3 [Ostrinia furnacalis]	1.82718	-0.8279	0.29837	-0.7989	-0.4988
TRINITY_DN321_c0_g1_i1_orf1	uncharacterized protein LOC126371336 [Pectinophora gossypiella]	1.93619	-0.786	-0.3948	-0.6877	-0.0677
TRINITY_DN668_c0_g1_i4_orf1	fatty acid synthase-like isoform X1 [Ostrinia furnacalis]	1.95841	-0.1118	-0.5165	-0.685	-0.6451
TRINITY_DN19043_c0_g3_i2_orf1	hypothetical protein EVAR_60654_1 [Eumeta japonica]	1.5172	-1.2933	-0.8484	0.077	0.54751
TRINITY_DN15420_c0_g3_i2_orf1	elongation factor 1-alpha 2-like [Galleria mellonella] >XP_031769625.1 elongation factor 1-alpha 2-like [Galleria mellonella]	1.92987	-0.6647	-0.8686	-0.2177	-0.1789
TRINITY_DN13088_c0_g1_i5_orf1	beta-hexosaminidase subunit alpha-like isoform X2 [Ostrinia furnacalis]	1.89347	-0.9931	-0.4456	0.02415	-0.4789
TRINITY_DN7565_c0_g1_i3_orf1	acylphosphatase-2-like [Ostrinia furnacalis]	1.89125	-0.1334	-1.072	-0.4453	-0.2405
TRINITY_DN661_c1_g2_i1_orf1	larval/pupal cuticle protein H1C-like [Ostrinia furnacalis]	1.91021	0.02299	-0.4875	-0.9071	-0.5386
TRINITY_DN37585_c0_g2_i1_orf1	cuticle protein 19.8-like [Ostrinia furnacalis]	1.92523	-0.0947	-0.6123	-0.8985	-0.3197
TRINITY_DN4273_c1_g1_i5_orf1	tetraspanin-13 isoform X1 [Ostrinia furnacalis]	1.77836	-1.3045	0.01552	-0.1558	-0.3337
TRINITY_DN1194_c0_g1_i5_orf1	sequestosome-1-like isoform X4 [Ostrinia furnacalis]	1.48736	-1.2223	0.83189	-0.5474	-0.5495
TRINITY_DN2348_c0_g1_i1_orfp1	TRINITY_DN2348_c0_g1_i1_m.39060 TRINITY_DN2348_c0_g1::TRINITY_DN2348_c0_g1_i1::g.39060 ORF type:complete len:149 (+),score=54.19 TRINITY_DN2348_c0_g1_i1:28-474(+)	1.73074	-1.3293	-0.2004	0.19699	-0.398
TRINITY_DN4217_c0_g1_i2_orf1	hypothetical protein evm_006611 [Chilo suppressalis]	1.89307	-1.0639	-0.1568	-0.4662	-0.2062
TRINITY_DN1196_c0_g1_i5_orf1	glucosamine-6-phosphate isomerase isoform X1 [Ostrinia furnacalis]	1.93157	-0.4981	-0.1131	-0.9214	-0.3989
TRINITY_DN82104_c0_g1_i5_orf1	uncharacterized protein LOC114349939 [Ostrinia furnacalis] >XP_028156338.1 uncharacterized protein LOC114349939 [Ostrinia furnacalis]	1.69002	-1.3946	-0.4061	-0.0627	0.17338
TRINITY_DN13648_c0_g1_i6_orf1	neurexin-4 [Ostrinia furnacalis]	1.97094	-0.3754	-0.4251	-0.8185	-0.3519
TRINITY_DN661_c0_g3_i5_orf1	cuticle protein 18.6-like [Ostrinia furnacalis]	1.96931	-0.4828	-0.6302	-0.6781	-0.1782
TRINITY_DN585_c0_g1_i5_orf1	very low-density lipoprotein receptor isoform X2 [Galleria mellonella]	1.93387	-0.7648	-0.1078	-0.3065	-0.7547
TRINITY_DN5074_c0_g1_i7_orf1	zonadhesin-like [Ostrinia furnacalis]	1.91383	-0.2288	-0.2564	-1.0295	-0.3991
TRINITY_DN83374_c0_g1_i1_orf1	uncharacterized protein LOC114350302, partial [Ostrinia furnacalis]	1.96785	-0.1579	-0.6776	-0.5321	-0.6003
TRINITY_DN3896_c0_g1_i1_orf1	glyoxalase domain-containing protein 4 [Ostrinia furnacalis]	1.53605	-1.5635	-0.3518	0.17913	0.20018
TRINITY_DN29879_c0_g1_i3_orf1	uncharacterized protein LOC114350556 isoform X1 [Ostrinia furnacalis] >XP_028157201.1 uncharacterized protein LOC114350556 isoform X2 [Ostrinia furnacalis] >XP_028157202.1 uncharacterized protein LOC114350556 isoform X3 [Ostrinia furnacalis]	1.69528	-0.507	-0.8456	-0.9114	0.56867
TRINITY_DN467_c0_g3_i1_orf1	histone-lysine N-methyltransferase 2B-like, partial [Ostrinia furnacalis]	1.92398	0.0258	-0.574	-0.7922	-0.5836
TRINITY_DN111621_c0_g3_i1_orf1	Serine proteinase stubble [Eumeta japonica]	1.90086	-0.2515	-0.9389	-0.0474	-0.6631
TRINITY_DN714_c0_g1_i3_orf1	thymosin beta isoform X4 [Ostrinia furnacalis]	1.9542	-0.718	-0.4391	-0.6771	-0.1201
TRINITY_DN140_c1_g1_i2_orf1	modular serine protease-like isoform X1 [Ostrinia furnacalis]	1.57972	-1.3605	0.57878	-0.3909	-0.4071
TRINITY_DN31619_c0_g1_i2_orf1	endocuticle structural glycoprotein ABD-4-like [Ostrinia furnacalis]	1.89043	0.11217	-0.456	-0.8432	-0.7035
TRINITY_DN895_c0_g2_i1_orf1	protein N-terminal asparagine amidohydrolase [Cotesia glomerata] >XP_044583616.1 protein N-terminal asparagine amidohydrolase [Cotesia glomerata] >XP_044583617.1 protein N-terminal asparagine amidohydrolase [Cotesia glomerata] >KAH0553813.1 hypothetical protein KQX54_004640 [Cotesia glomerata]	1.69963	-1.3075	0.33988	-0.4617	-0.2704
TRINITY_DN280_c0_g1_i8_orf1	Tubulin beta-1 chain [Papilio xuthus]	1.98214	-0.2393	-0.5514	-0.601	-0.5904
TRINITY_DN53427_c0_g1_i2_orf1	heparanase-like [Ostrinia furnacalis]	1.70369	-1.108	0.50923	-0.5538	-0.5511
TRINITY_DN1533_c0_g2_i1_orf1	unnamed protein product [Chilo suppressalis]	1.74635	-1.3729	-0.1656	-0.1943	-0.0135
TRINITY_DN41_c0_g1_i5_orf1	putative phospholipase B-like 2 [Ostrinia furnacalis]	1.82891	-1.2045	-0.0216	-0.1962	-0.4066
TRINITY_DN25345_c0_g1_i1_orf1	chromodomain-helicase-DNA-binding protein 1 isoform X3 [Ostrinia furnacalis]	1.83584	-0.2572	-0.9646	0.16444	-0.7785
TRINITY_DN4013_c0_g1_i4_orf1	uncharacterized protein LOC114353190 isoform X1 [Ostrinia furnacalis] >XP_028160984.1 uncharacterized protein LOC114353190 isoform X2 [Ostrinia furnacalis] >XP_028161062.1 uncharacterized protein LOC114353190 isoform X1 [Ostrinia furnacalis] >XP_028161142.1 uncharacterized protein LOC114353190 isoform X1 [Ostrinia furnacalis]	1.85295	-0.83	0.25227	-0.616	-0.6592
TRINITY_DN501_c0_g1_i5_orf1	hypothetical protein evm_002550 [Chilo suppressalis]	1.99457	-0.4003	-0.516	-0.6224	-0.4559
TRINITY_DN36856_c0_g1_i1_orf1	protein enhancer of sevenless 2B isoform X2 [Formica exsecta]	1.63523	-1.3952	0.30438	-0.0089	-0.5356
TRINITY_DN14009_c0_g1_i1_orf1	proline-rich extensin-like protein EPR1 [Manduca sexta]	1.9602	-0.1996	-0.4159	-0.815	-0.5297

TRINITY_DN4886_c0_g1_i6_orf1	uncharacterized protein LOC114349567 [Ostrinia furnacalis]	1.82588	-1.2135	-0.2079	-0.0172	-0.3873
TRINITY_DN1491_c0_g1_i8_orf1	GILT-like protein 2 isoform X1 [Ostrinia furnacalis] >XP_028156245.1 GILT-like protein 2 isoform X2 [Ostrinia furnacalis] >XP_028156247.1 GILT-like protein 2 isoform X3 [Ostrinia furnacalis]	1.93624	-0.9667	-0.3406	-0.3515	-0.2775
TRINITY_DN4276_c0_g1_i6_orf1	arylsulfatase B [Ostrinia furnacalis]	1.53834	-1.4249	0.38713	0.15467	-0.6552
TRINITY_DN7735_c1_g1_i1_orf1	cuticular protein CPH [Spodoptera litura]	1.95789	-0.2785	-0.5172	-0.8515	-0.3107
TRINITY_DN862_c0_g1_i4_orf1	uncharacterized protein LOC114359380 isoform X1 [Ostrinia furnacalis] >XP_028169561.1 uncharacterized protein LOC114359380 isoform X2 [Ostrinia furnacalis] >XP_028169562.1 uncharacterized protein LOC114359380 isoform X1 [Ostrinia furnacalis]	1.67622	-1.3381	-0.2905	0.3726	-0.4203
TRINITY_DN3073_c0_g1_i7_orf1	claspin-like [Ostrinia furnacalis]	1.98945	-0.5702	-0.4152	-0.6444	-0.3597
TRINITY_DN48765_c0_g1_i7_orf1	uncharacterized protein LOC114352307 [Ostrinia furnacalis]	1.16355	-1.587	0.74182	0.35398	-0.6724
TRINITY_DN20796_c0_g1_i4_orf1	probable low-specificity L-threonine aldolase 2 [Ostrinia furnacalis]	1.72718	-1.3023	-0.5108	-0.1247	0.2106
TRINITY_DN21719_c0_g2_i4_orf1	chymotrypsin-2-like [Ostrinia furnacalis]	1.99242	-0.522	-0.3919	-0.6455	-0.4331
TRINITY_DN7711_c1_g1_i3_orf1	long-chain fatty acid transport protein 1-like [Ostrinia furnacalis]	1.76844	-0.8641	0.42684	-0.8355	-0.4957
TRINITY_DN23183_c1_g1_i2_orf1	myotubularin-related protein 9 [Ostrinia furnacalis]	1.80042	-0.4287	0.10472	-1.2249	-0.2515
TRINITY_DN2107_c0_g2_i3_orf1	LIM and SH3 domain protein Lasp [Ostrinia furnacalis]	1.86052	-1.1727	-0.1742	-0.2339	-0.2798
TRINITY_DN1252_c0_g1_i3_orf1	unnamed protein product [Chilo suppressalis]	1.90763	-0.5125	-0.1413	-0.2447	-1.0092
TRINITY_DN8641_c0_g1_i1_orf1	uncharacterized protein LOC114357057 [Ostrinia furnacalis]	1.93588	-0.823	-0.6997	-0.1981	-0.2151
TRINITY_DN4041_c0_g1_i6_orf1	tubulin-folding cofactor B isoform X3 [Ostrinia furnacalis]	1.58499	-1.421	-0.605	0.27207	0.16895
TRINITY_DN12673_c3_g1_i2_orf1	unnamed protein product [Chilo suppressalis]	1.85975	-0.917	0.19573	-0.4854	-0.6531
TRINITY_DN2109_c0_g1_i4_orf1	mucin-2-like isoform X2 [Ostrinia furnacalis]	1.70962	-0.3081	-1.0287	0.46726	-0.8401
TRINITY_DN18027_c0_g2_i1_orf1	vanin-like protein 2 isoform X2 [Ostrinia furnacalis]	1.66368	-1.4336	-0.3518	0.21206	-0.0903
TRINITY_DN11666_c0_g1_i6_orf1	P protein-like [Ostrinia furnacalis] >XP_028167089.1 P protein-like [Ostrinia furnacalis]	1.65411	-0.6415	-0.1373	-1.2893	0.41397
TRINITY_DN56430_c0_g1_i1_orf1	unnamed protein product, partial [Iphiclides podalirius]	1.65882	-1.1312	0.51515	-0.8039	-0.2389
TRINITY_DN14458_c0_g1_i2_orf1	spermatogenesis-associated protein 20 isoform X1 [Ostrinia furnacalis]	1.78399	-1.2612	-0.0471	-0.0019	-0.4737
TRINITY_DN8692_c0_g1_i2_orf1	caspase-1-like [Ostrinia furnacalis]	1.94918	-0.7189	-0.3686	-0.7281	-0.1336
TRINITY_DN52788_c0_g1_i1_orf1	putative fatty acyl-CoA reductase CG5065 isoform X1 [Ostrinia furnacalis]	1.99834	-0.4855	-0.4372	-0.5125	-0.5631
TRINITY_DN22443_c0_g2_i3_orf1	protein eiger [Ostrinia furnacalis] >QKV49447.1 eiger [Ostrinia furnacalis]	1.81556	-1.2429	-0.2893	-0.0093	-0.274
TRINITY_DN34786_c0_g1_i1_orf1	small heat shock protein Hsp29.7 [Ostrinia furnacalis]	1.95796	-0.1377	-0.765	-0.4756	-0.5797
TRINITY_DN14774_c0_g1_i4_orf1	aminopeptidase N-like [Ostrinia furnacalis]	1.73297	-0.5783	0.51519	-0.872	-0.7979
TRINITY_DN13067_c0_g1_i6_orf1	diphosphomevalonate decarboxylase [Ostrinia furnacalis]	1.84177	-0.4742	-1.1611	-0.1852	-0.0212
TRINITY_DN19923_c0_g1_i1_orf1	uncharacterized protein LOC114350958 [Ostrinia furnacalis]	1.94062	-0.0245	-0.7144	-0.5786	-0.6231
TRINITY_DN445_c0_g1_i2_orf1	sorting nexin-17 [Ostrinia furnacalis]	1.71416	-1.318	0.28899	-0.2862	-0.399
TRINITY_DN42333_c0_g1_i5_orf1	regucalcin-like [Ostrinia furnacalis]	1.8641	-0.7187	-0.981	0.04558	-0.2099
TRINITY_DN6974_c0_g2_i1_orf1	mucolipin-3-like [Ostrinia furnacalis]	1.84894	-0.3749	0.19967	-0.8509	-0.8228
TRINITY_DN8569_c1_g2_i7_orf1	furin-like protease 1, partial [Ostrinia furnacalis]	1.8892	-0.4064	-0.0513	-1.0611	-0.3704
TRINITY_DN2323_c0_g1_i4_orf1	uncharacterized protein LOC114364097 isoform X2 [Ostrinia furnacalis]	1.97009	-0.3301	-0.8006	-0.3288	-0.5107
TRINITY_DN3675_c0_g1_i1_orf1	unnamed protein product [Spodoptera exigua]	1.7693	-1.3238	0.00858	-0.1437	-0.3104
TRINITY_DN5893_c0_g1_i7_orf1	jupiter microtubule associated homolog 1-like [Ostrinia furnacalis] >XP_028173360.1 jupiter microtubule associated homolog 1-like [Ostrinia furnacalis]	1.97886	-0.2276	-0.5291	-0.5583	-0.6639
TRINITY_DN12331_c0_g1_i5_orf1	septin-7 isoform X1 [Ostrinia furnacalis]	1.99469	-0.6141	-0.5303	-0.4512	-0.3991
TRINITY_DN17437_c0_g1_i1_orf1	phospholipase A1 VesT1.02-like [Ostrinia furnacalis]	1.78945	-0.9671	-0.6508	-0.5464	0.37483
TRINITY_DN110523_c0_g2_i1_orf1	uncharacterized protein LOC107036393 [Diachasma alloeum]	1.72681	-0.3567	0.3944	-0.5835	-1.1811
TRINITY_DN15411_c0_g1_i4_orf1	uncharacterized protein LOC114362040 isoform X1 [Ostrinia furnacalis]	1.70426	-1.3229	0.01988	0.16308	-0.5643
TRINITY_DN7291_c0_g1_i3_orf1	dynamin-1-like protein isoform X1 [Ostrinia furnacalis] >XP_028177409.1 dynamin-1-like protein isoform X2 [Ostrinia furnacalis]	1.77207	-1.0967	-0.2267	0.27816	-0.7268
TRINITY_DN31943_c0_g1_i1_orf1	proteoglycan Cow [Ostrinia furnacalis]	1.90467	-0.3165	-1.0485	-0.1534	-0.3863
TRINITY_DN1005_c0_g1_i5_orf1	hypothetical protein evm_008839 [Chilo suppressalis] >CAB3526474.1 unnamed protein product [Chilo suppressalis] >CAH0403802.1 unnamed protein product [Chilo suppressalis]	1.82579	-0.1099	-0.5491	-1.1632	-0.0037
TRINITY_DN19537_c0_g1_i1_orf1	disintegrin and metalloproteinase domain-containing protein 10 isoform X1 [Ostrinia furnacalis] >XP_028172845.1 disintegrin and metalloproteinase domain-containing protein 10 isoform X2 [Ostrinia furnacalis]	1.76315	-0.7615	0.06141	-1.1409	0.07783
TRINITY_DN3978_c0_g2_i1_orf1	nicastrin [Ostrinia furnacalis]	1.96209	-0.8368	-0.306	-0.3078	-0.5115
TRINITY_DN58872_c0_g1_i1_orfp1	cuticle protein 64-like [Pectinophora gossypiella]	1.94256	-0.5097	-0.897	-0.1719	-0.364
TRINITY_DN46090_c0_g2_i1_orf1	inactive tyrosine-protein kinase 7-like, partial [Ostrinia furnacalis]	1.90951	-0.0198	-0.3454	-0.9165	-0.6278
TRINITY_DN12464_c0_g1_i3_orf1	PH and SEC7 domain-containing protein 1 [Trichoplusia ni]	1.97962	-0.4377	-0.6039	-0.263	-0.675
TRINITY_DN1293_c0_g1_i4_orf1	putative fatty acyl-CoA reductase CG5065 [Ostrinia furnacalis]	1.9038	-0.0377	-0.2584	-0.8903	-0.7175
TRINITY_DN4571_c0_g1_i4_orf1	PREDICTED: nuclear factor NF-kappa-B p105 subunit [Microplitis demolitor] >KAG6558391.1 viral ankyrin V1 [Microplitis demolitor]	1.59802	0.33373	-0.7996	-1.2923	0.1601
TRINITY_DN867_c0_g1_i1_orf1	hemicentin-2-like isoform X1 [Ostrinia furnacalis]	1.89718	-0.0705	-0.4688	-0.3225	-1.0353
TRINITY_DN2043_c0_g1_i11_orf1	phenoloxidase-activating factor 2-like [Ostrinia furnacalis]	1.58366	-1.3489	0.5169	-0.1281	-0.6235
TRINITY_DN1436_c0_g1_i3_orf1	vacuolar protein sorting-associated protein 27-like [Trichoplusia ni]	1.90638	-0.0181	-0.4288	-0.9711	-0.4884
TRINITY_DN21930_c0_g1_i1_orf1	coactosin-like protein isoform X2 [Trichoplusia ni]	1.98427	-0.3263	-0.5391	-0.7003	-0.4187
TRINITY_DN57348_c0_g1_i4_orf1	facilitated trehalose transporter Tret1-like isoform X1 [Ostrinia furnacalis]	1.70043	-1.2145	-0.0045	-0.7494	0.26798
TRINITY_DN34727_c0_g1_i3_orf1	tyrosine-protein kinase Src42A isoform X2 [Trichoplusia ni]	1.9472	-0.4129	-0.3157	-0.9222	-0.2965
TRINITY_DN50471_c0_g1_i4_orf1	hypothetical protein evm_008982 [Chilo suppressalis]	1.95116	-0.4043	-0.7749	-0.1299	-0.642
TRINITY_DN6205_c0_g1_i4_orfp1	TRINITY_DN6205_c0_g1_i4_m.72677 TRINITY_DN6205_c0_g1::TRINITY_DN6205_c0_g1_i4::g.72677 ORF type:internal len:68 (-),score=1.69 TRINITY_DN6205_c0_g1_i4:2-202(-)	1.37868	-0.6188	1.04104	-0.9744	-0.8265
TRINITY_DN114960_c0_g1_i4_orf1	zinc finger protein on ecdysone puffs-like [Ostrinia furnacalis]	1.93077	-0.8875	-0.1203	-0.6098	-0.3131

TRINITY_DN16145_c0_g1_i12_orf1	Down syndrome cell adhesion molecule-like protein Dscam2 isoform X16 [Ostrinia furnacalis]	1.80249	-0.9279	-0.9379	0.09574	-0.0323
TRINITY_DN19998_c0_g1_i1_orf1	ubiquitin-conjugating enzyme E2 variant 2 [Helicoverpa armigera] >XP_026325122.1 ubiquitin-conjugating enzyme E2 variant 2 [Hyposmocoma kahamanoa] >XP_026499214.1 ubiquitin-conjugating enzyme E2 variant 2 [Vanessa tameamea] >XP_026738520.1 ubiquitin-conjugating enzyme E2 variant 2 [Trichoplusia ni] >XP_026762551.1 ubiquitin-conjugating enzyme E2 variant 2 [Galleria mellonella] >XP_028172566.1 ubiquitin-conjugating enzyme E2 variant 2 [Ostrinia furnacalis] >XP_032520494.1 ubiquitin-conjugating enzyme E2 variant 2 [Danaus plexippus plexippus] >XP_034831247.1 ubiquitin-conjugating enzyme E2 variant 2 [Maniola hyperantus] >XP_039755795.1 ubiquitin-conjugating enzyme E2 variant 2 [Pararge aegeria] >XP_041987413.1 ubiquitin-conjugating enzyme E2 variant 2 [Aricia agestis] >XP_045450678.1 ubiquitin-conjugating enzyme E2 variant 2 [Melitaea cinxia] >XP_045766931.1 ubiquitin-conjugating enzyme E2 variant 2 [Maniola jurtina] >XP_046968624.1 ubiquitin-conjugating enzyme E2 variant 2 [Vanessa cardui] >XP_047027052.1 ubiquitin-conjugating enzyme E2 variant 2 [Helicoverpa zea] >XP_047534810.1 ubiquitin-conjugating enzyme E2 variant 2 [Vanessa atalanta] >KAI5645358.1 ubiquitin-conjugating enzyme domain-containing protein [Phthorimaea operculella] >RVE41109.1 hypothetical protein evm_014241 [Chilo suppressalis] >CAB3251720.1 unnamed protein product [Arctia plantaginis] >CAG9561279.1 unnamed protein product [Danaus chrysippus] >CAG9755233.1 unnamed protein product [Diatraea saccharalis] >CAH0584342.1 unnamed protein product [Chrysodeixis includens] >CAH0729826.1 unnamed protein product, partial [Brenthis ino] >CAH2039508.1 unnamed protein product, partial [Iphiclides podalirius] >CAH2239303.1 jg6875 [Pararge aegeria aegeria]	1.91508	-0.9569	-0.0607	-0.3768	-0.5207
TRINITY_DN3887_c0_g1_i1_orf1	formin-like protein isoform X3 [Ostrinia furnacalis] >XP_028170442.1 formin-like protein isoform X6 [Ostrinia furnacalis]	1.94831	-0.3015	-0.192	-0.8224	-0.6325
TRINITY_DN9028_c0_g1_i5_orf1	decaprenyl-diphosphate synthase subunit 2-like [Ostrinia furnacalis]	1.91759	-0.137	-0.5581	-0.9615	-0.2611
TRINITY_DN13167_c0_g1_i1_orf1	selenoprotein M-like [Ostrinia furnacalis]	1.72921	-0.1675	-0.8501	-1.065	0.35339
TRINITY_DN90327_c0_g1_i1_orf1	hypothetical protein HF086_000910, partial [Spodoptera exigua]	1.54479	-1.076	0.7845	-0.7925	-0.4608
TRINITY_DN86309_c0_g1_i4_orf1	filamin-A isoform X2 [Ostrinia furnacalis]	1.98762	-0.3694	-0.6942	-0.5072	-0.4169
TRINITY_DN9475_c0_g1_i6_orf1	uncharacterized protein LOC114358636 [Ostrinia furnacalis]	1.90743	-0.6126	-0.8032	-0.5778	0.08619
TRINITY_DN17003_c1_g1_i1_orf1	unnamed protein product [Chilo suppressalis]	1.96668	-0.5171	-0.7054	-0.5845	-0.1596
TRINITY_DN3600_c0_g1_i1_orf1	unnamed protein product [Chilo suppressalis]	1.4593	-1.463	0.33379	0.36606	-0.6961
TRINITY_DN4572_c0_g3_i1_orf1	putative aminopeptidase W07G4.4 isoform X2 [Ostrinia furnacalis]	1.9384	-0.6666	-0.8309	-0.2935	-0.1474
TRINITY_DN3637_c0_g1_i2_orf1	ATP-binding cassette sub-family G member 4 isoform X1 [Ostrinia furnacalis]	1.41312	-1.5312	0.6929	-0.2061	-0.3687
TRINITY_DN16643_c0_g2_i4_orf1	heterogeneous nuclear ribonucleoprotein R isoform X6 [Danaus plexippus plexippus]	1.92361	-0.8222	-0.4987	-0.6123	0.00961
TRINITY_DN86621_c0_g1_i2_orf1	protein sly1 homolog isoform X1 [Ostrinia furnacalis] >XP_028165613.1 protein sly1 homolog isoform X2 [Ostrinia furnacalis]	1.82623	-0.0656	-0.165	-1.2223	-0.3733
TRINITY_DN1091_c0_g2_i10_orf1	macrophage mannose receptor 1-like isoform X2 [Maniola hyperantus]	1.44873	-0.951	0.48995	0.30271	-1.2904
TRINITY_DN15114_c0_g2_i1_orf1	uncharacterized protein LOC114359552 [Ostrinia furnacalis]	1.63855	-1.095	0.61875	-0.7512	-0.4111
TRINITY_DN4320_c0_g1_i1_orf1	selenide, water dikinase [Ostrinia furnacalis] >CAG9756850.1 unnamed protein product [Diatraea saccharalis] >CAG9795535.1 unnamed protein product [Diatraea saccharalis]	1.97402	-0.3523	-0.2895	-0.6031	-0.7291
TRINITY_DN8390_c0_g1_i2_orf1	tubulin gamma-1 chain-like isoform X1 [Ostrinia furnacalis] >XP_028160960.1 tubulin gamma-1 chain-like isoform X2 [Ostrinia furnacalis]	1.95891	-0.7142	-0.1149	-0.5406	-0.5891
TRINITY_DN1254_c0_g1_i1_orf1	lysophospholipid acyltransferase 7-like [Ostrinia furnacalis]	1.50035	-0.7857	0.81584	-1.1492	-0.3813
TRINITY_DN23582_c0_g1_i1_orf1	unnamed protein product [Diatraea saccharalis]	1.74434	-0.1634	-1.0516	0.3204	-0.8497
TRINITY_DN2352_c0_g1_i15_orf1	polyadenylate-binding protein 1-B-like [Ostrinia furnacalis]	1.8993	-0.9545	-0.0021	-0.6078	-0.3349
TRINITY_DN1604_c0_g1_i4_orf1	ubiquitin-conjugating enzyme E2 S [Ostrinia furnacalis]	1.66107	-0.5025	-1.3823	-0.0501	0.27387
TRINITY_DN146524_c0_g1_i1_orf1	pupal cuticle protein-like [Ostrinia furnacalis]	1.98537	-0.5761	-0.3404	-0.6746	-0.3942
TRINITY_DN10630_c0_g1_i2_orf1	J domain-containing protein [Ostrinia furnacalis]	1.55349	-0.1129	-0.898	-1.1714	0.62881
TRINITY_DN8306_c0_g1_i4_orf1	NAD(P) transhydrogenase, mitochondrial-like [Ostrinia furnacalis] >XP_028175067.1 NAD(P) transhydrogenase, mitochondrial-like [Ostrinia furnacalis] >XP_028175068.1 NAD(P) transhydrogenase, mitochondrial-like [Ostrinia furnacalis] >XP_028175069.1 NAD(P) transhydrogenase, mitochondrial-like [Ostrinia furnacalis]	1.75824	-0.5342	-0.0147	-1.2724	0.06305
TRINITY_DN9_c0_g1_i11_orf1	heterogeneous nuclear ribonucleoprotein Q isoform X2 [Galleria mellonella]	1.9609	-0.2583	-0.3269	-0.5554	-0.8202
TRINITY_DN27456_c0_g2_i1_orf1	organic cation transporter-like protein [Ostrinia furnacalis]	1.62381	-0.3189	-0.0731	-1.4812	0.24935
TRINITY_DN1362_c0_g1_i4_orf1	heparan-alpha-glucosaminide N-acetyltransferase [Helicoverpa armigera]	1.79745	-0.7674	0.33687	-0.426	-0.9409
TRINITY_DN2012_c0_g1_i3_orf1	contactin [Ostrinia furnacalis]	1.90649	-0.7561	-0.1015	-0.8659	-0.183
TRINITY_DN28018_c0_g6_i1_orf1	microtubule-associated protein futsch-like isoform X6 [Ostrinia furnacalis]	1.97337	-0.246	-0.7057	-0.3983	-0.6234
TRINITY_DN1274_c0_g1_i4_orf1	venom dipeptidyl peptidase 4-like [Ostrinia furnacalis]	1.85111	0.01444	-1.1455	-0.3309	-0.3891
TRINITY_DN19058_c1_g1_i1_orf1	syntaxin-7 [Helicoverpa armigera] >XP_049695901.1 syntaxin-7 [Helicoverpa armigera]	1.92585	-0.9107	-0.2343	-0.6163	-0.1646
TRINITY_DN12222_c0_g1_i1_orf1	unnamed protein product [Chilo suppressalis]	1.80703	0.01492	-0.4021	-1.2413	-0.1786
TRINITY_DN3177_c0_g1_i1_orf1	Pupal cuticle protein PCP52 [Papilio xuthus]	1.76791	-0.5119	-0.4813	-1.1224	0.34773
TRINITY_DN2043_c0_g1_i3_orf1	phenoloxidase-activating factor 2-like [Ostrinia furnacalis]	1.94434	-0.3771	-0.3915	-0.9291	-0.2467
TRINITY_DN6014_c1_g1_i2_orf1	putative hydroxypyruvate isomerase [Ostrinia furnacalis]	1.91938	-0.6852	-0.6656	-0.632	0.06343
TRINITY_DN11665_c0_g1_i4_orf1	TBC1 domain family member 9 isoform X1 [Ostrinia furnacalis] >XP_028176568.1 TBC1 domain family member 9 isoform X2 [Ostrinia furnacalis]	1.95662	-0.3739	-0.7564	-0.6564	-0.1699
TRINITY_DN34830_c0_g1_i1_orf1	ubiquitin-like domain-containing CTD phosphatase 1 [Ostrinia furnacalis]	1.82679	-0.4867	0.23935	-1.0361	-0.5433
TRINITY_DN4134_c2_g1_i2_orf1	prostatic acid phosphatase-like [Ostrinia furnacalis]	1.95553	-0.4614	-0.1775	-0.8382	-0.4784
TRINITY_DN16516_c0_g1_i1_orf1	sulfotransferase 1E1 [Galleria mellonella]	1.70655	-0.2437	-1.4238	-0.0099	-0.0291
TRINITY_DN99020_c0_g1_i1_orf1	uncharacterized protein LOC114357292 isoform X4 [Ostrinia furnacalis]	1.86997	-0.0711	-0.85	-0.8778	-0.0711
TRINITY_DN7682_c0_g1_i2_orf1	myogenesis-regulating glycosidase isoform X1 [Ostrinia furnacalis] >XP_028158488.1 myogenesis-regulating glycosidase isoform X1 [Ostrinia furnacalis] >XP_028158489.1 myogenesis-regulating glycosidase isoform X1 [Ostrinia furnacalis] >XP_028158490.1 myogenesis-regulating glycosidase isoform X1 [Ostrinia furnacalis]	1.77175	-0.5017	0.1858	-1.2353	-0.2205
TRINITY_DN24631_c0_g2_i1_orf1	O-GlcNAc hydrolase [Ostrinia furnacalis]	1.9541	-0.4426	-0.3527	-0.8874	-0.2713
TRINITY_DN18136_c0_g1_i1_orf1	proteoglycan 4-like [Ostrinia furnacalis]	1.3127	-0.8277	1.13269	-0.7966	-0.8211
TRINITY_DN9732_c0_g1_i7_orf1	CD109 antigen [Ostrinia furnacalis] >XP_028176877.1 CD109 antigen [Ostrinia furnacalis]	1.98674	-0.4215	-0.447	-0.7175	-0.4007
TRINITY_DN144_c0_g1_i4_orf1	COPII coat assembly protein sec16-like [Ostrinia furnacalis]	1.97418	-0.4168	-0.7991	-0.3415	-0.4168
TRINITY_DN44094_c0_g1_i1_orf1	myotrophin-like [Ostrinia furnacalis]	1.81196	-0.0807	-1.2184	-0.4736	-0.0393

TRINITY_DN14905_c0_g2_i2_orf1	hypothetical protein evm_002970 [Chilo suppressalis]	1.88434	-0.5716	0.1149	-0.5013	-0.9263
TRINITY_DN10095_c0_g1_i5_orf1	uncharacterized protein LOC114361222 [Ostrinia furnacalis]	1.79071	-0.0797	-0.4296	-1.2658	-0.0156
TRINITY_DN20710_c0_g1_i2_orf1	plexin A3 [Ostrinia furnacalis]	1.97761	-0.2384	-0.6995	-0.4848	-0.5548
TRINITY_DN4572_c0_g1_i2_orf1	unnamed protein product [Arctia plantaginis] >CAB3257565.1 unnamed protein product [Arctia plantaginis]	1.95815	-0.6121	-0.7009	-0.5367	-0.1085
TRINITY_DN77642_c0_g1_i1_orf1	peritrophic membrane chitin binding protein [Loxostege sticticalis]	1.49569	0.15185	0.54978	-0.9903	-1.207
TRINITY_DN4410_c0_g1_i1_orf1	rab GDP dissociation inhibitor alpha [Ostrinia furnacalis]	1.92595	-0.6117	-0.7947	0.0141	-0.5337
TRINITY_DN9302_c0_g1_i1_orf1	DEAD-box helicase Dbp80 [Ostrinia furnacalis]	1.87821	-0.6589	0.13657	-0.9019	-0.454
TRINITY_DN34703_c0_g1_i4_orf1	gamma-tubulin complex component 3 homolog [Ostrinia furnacalis]	1.63962	0.26529	-0.2643	-1.4628	-0.1778
TRINITY_DN210_c0_g1_i9_orf1	glycine-rich cell wall structural protein-like [Ostrinia furnacalis]	1.2355	-0.1143	0.81109	-1.6502	-0.2822
TRINITY_DN34751_c0_g1_i1_orf1	peroxisomal acyl-coenzyme A oxidase 3 isoform X3 [Ostrinia furnacalis]	1.91025	-0.223	-1.0354	-0.2346	-0.4172
TRINITY_DN64222_c0_g1_i1_orf1	heat shock protein 20.2 [Glyphodes pyloalis]	1.98224	-0.4406	-0.735	-0.3292	-0.4774
TRINITY_DN667_c0_g1_i5_orf1	unnamed protein product [Arctia plantaginis]	1.51892	0.08972	-0.1533	-1.6227	0.16741
TRINITY_DN9324_c1_g2_i2_orf1	kinesin-like protein Klp10A isoform X4 [Spodoptera frugiperda]	1.86466	0.07831	-0.681	-0.2726	-0.9893
TRINITY_DN49872_c0_g1_i2_orf1	probable cytosolic iron-sulfur protein assembly protein Ciao1 [Ostrinia furnacalis]	1.59936	0.13685	-1.4056	-0.6085	0.27794
TRINITY_DN4711_c0_g1_i2_orf1	xanthine dehydrogenase-like isoform X1 [Ostrinia furnacalis] >XP_028179066.1 xanthine dehydrogenase-like isoform X1 [Ostrinia furnacalis] >XP_028179067.1 xanthine dehydrogenase-like isoform X1 [Ostrinia furnacalis] >XP_028179068.1 xanthine dehydrogenase-like isoform X1 [Ostrinia furnacalis] >XP_028179069.1 xanthine dehydrogenase-like isoform X1 [Ostrinia furnacalis]	1.62517	-0.4742	0.45437	-1.3679	-0.2374
TRINITY_DN3273_c0_g1_i4_orf1	FK506-binding protein-like [Galleria mellonella]	1.99101	-0.552	-0.579	-0.3157	-0.5443
TRINITY_DN3119_c0_g1_i7_orf1	unnamed protein product [Chilo suppressalis]	1.82549	-0.0327	-1.1858	-0.4986	-0.1084
TRINITY_DN9465_c0_g1_i4_orf1	prisilkin-39-like [Ostrinia furnacalis]	1.77075	-0.0506	-0.9474	0.19059	-0.9633
TRINITY_DN2277_c0_g1_i11_orf1	solute carrier family 12 member 4 isoform X1 [Ostrinia furnacalis]	1.91047	-0.7211	-0.0322	-0.2961	-0.8611
TRINITY_DN16390_c0_g1_i4_orf1	unnamed protein product, partial [Brenthis ino]	1.88925	-0.1527	-0.5754	-1.029	-0.1321
TRINITY_DN101_c0_g2_i2_orf1	tyrosine-protein phosphatase non-receptor type 23 [Ostrinia furnacalis]	1.95029	-0.6346	-0.0982	-0.4622	-0.7552
TRINITY_DN29633_c0_g1_i8_orf1	transmembrane protein 87A isoform X1 [Ostrinia furnacalis] >XP_028156931.1 transmembrane protein 87A isoform X2 [Ostrinia furnacalis] >XP_028156932.1 transmembrane protein 87A isoform X3 [Ostrinia furnacalis] >XP_028156933.1 transmembrane protein 87A isoform X4 [Ostrinia furnacalis] >XP_028156934.1 transmembrane protein 87A isoform X5 [Ostrinia furnacalis] >XP_028156935.1 transmembrane protein 87A isoform X6 [Ostrinia furnacalis]	1.61094	0.12748	0.36902	-1.1798	-0.9276
TRINITY_DN16605_c0_g1_i3_orf1	unnamed protein product [Chrysodeixis includens]	1.79553	0.12317	-1.1463	-0.6588	-0.1136
TRINITY_DN44777_c0_g1_i2_orf1	probable very-long-chain enoyl-CoA reductase art-1 [Ostrinia furnacalis]	1.56303	-0.8725	0.81394	-0.7253	-0.7792
TRINITY_DN14389_c0_g1_i4_orf1	DE-cadherin [Ostrinia furnacalis]	1.8686	-0.6216	0.15854	-0.4693	-0.9363
TRINITY_DN33089_c0_g1_i1_orf1	nucleoporin NDC1 [Ostrinia furnacalis]	1.73473	0.13322	-1.0845	-0.8866	0.10321
TRINITY_DN5829_c0_g2_i1_orf1	uncharacterized protein LOC114365758 isoform X2 [Ostrinia furnacalis]	1.60042	0.40148	-0.9025	-1.2049	0.10551
TRINITY_DN18756_c0_g1_i6_orf1	COP9 signalosome complex subunit 6 [Ostrinia furnacalis]	1.64705	-0.3751	0.51829	-1.2662	-0.524
TRINITY_DN18563_c2_g1_i1_orf1	protein held out wings isoform X2 [Diachasma alloeum]	1.9006	-0.0771	-0.7659	-0.1854	-0.8723
TRINITY_DN47219_c0_g1_i3_orf1	protein windbeutel [Ostrinia furnacalis]	1.82997	0.16783	-0.923	-0.2263	-0.8485
TRINITY_DN14684_c0_g2_i1_orf1	cofilin/actin-depolymerizing factor homolog isoform X2 [Helicoverpa armigera] >XP_022828531.1 cofilin/actin-depolymerizing factor homolog [Spodoptera litura] >XP_023954560.1 cofilin/actin-depolymerizing factor homolog isoform X2 [Bicyclus anynana] >XP_023954561.1 cofilin/actin-depolymerizing factor homolog isoform X2 [Bicyclus anynana] >XP_026484174.1 cofilin/actin-depolymerizing factor homolog isoform X1 [Vanessa tameamea] >XP_026743400.1 cofilin/actin-depolymerizing factor homolog [Trichoplusia ni] >XP_035451599.1 cofilin/actin-depolymerizing factor homolog [Spodoptera frugiperda] >XP_038217031.1 cofilin/actin-depolymerizing factor homolog isoform X1 [Zerene cesonia] >XP_038217032.1 cofilin/actin-depolymerizing factor homolog isoform X2 [Zerene cesonia] >XP_039761665.1 cofilin/actin-depolymerizing factor homolog [Pararge aegeria] >XP_046974608.1 cofilin/actin-depolymerizing factor homolog [Vanessa cardui] >XP_047037753.1 cofilin/actin-depolymerizing factor homolog [Helicoverpa zea] >XP_047540992.1 cofilin/actin-depolymerizing factor homolog [Vanessa atalanta] >XP_050358240.1 cofilin/actin-depolymerizing factor homolog [Nymphalis io] >RVE53832.1 hypothetical protein evm_001494 [Chilo suppressalis] >CAH0598789.1 unnamed protein product [Chrysodeixis includens] >CAH2242550.1 jg8122 [Pararge aegeria aegeria] >AFP36378.1 cofilin [Spodoptera frugiperda] >PZC72500.1 hypothetical protein B5X24_HaOG211097 [Helicoverpa armigera]	1.96668	-0.5985	-0.2696	-0.328	-0.7705
TRINITY_DN11204_c0_g1_i3_orf1	spermosin-like [Ostrinia furnacalis]	1.37642	-0.6099	1.00776	-0.6189	-1.1553
TRINITY_DN27114_c0_g1_i1_orf1	putative inorganic phosphate cotransporter [Ostrinia furnacalis]	1.88388	-0.3096	-0.5642	0.00811	-1.0182
TRINITY_DN13626_c0_g2_i1_orf1	charged multivesicular body protein 2b [Ostrinia furnacalis]	1.58805	-0.3238	0.67782	-0.8526	-1.0894
TRINITY_DN13898_c0_g1_i2_orf1	protein PRRC2A-like isoform X2 [Ostrinia furnacalis]	1.87497	-0.0573	-0.1752	-0.5959	-1.0466
TRINITY_DN1066_c0_g1_i8_orf1	hypothetical protein evm_012420 [Chilo suppressalis]	1.40167	0.13392	0.70251	-1.2174	-1.0207
TRINITY_DN72_c0_g1_i16_orf1	protein groucho-like [Ostrinia furnacalis]	1.95446	-0.8166	-0.4256	-0.5543	-0.158
TRINITY_DN4540_c0_g1_i9_orf1	dihydropyrimidinase isoform X2 [Manduca sexta]	1.40787	-0.0672	0.69687	-1.4941	-0.5434
TRINITY_DN28989_c0_g1_i7_orf1	26S proteasome regulatory subunit 10B [Ostrinia furnacalis]	1.63945	-0.1436	0.5165	-1.0083	-1.0041
TRINITY_DN6967_c0_g1_i3_orf1	furin-like protease 2 isoform X2 [Manduca sexta] >KAG6448919.1 hypothetical protein O3G_MSEX005770 [Manduca sexta]	1.81134	-0.14	-0.8582	0.15573	-0.9688
TRINITY_DN5296_c0_g2_i1_orf1	RNA-binding protein Rsf1 [Ostrinia furnacalis]	1.42087	-0.0215	0.75961	-1.2707	-0.8883
TRINITY_DN172_c8_g2_i1_orf1	sialin [Ostrinia furnacalis]	1.55834	0.19787	0.416	-1.0782	-1.094
TRINITY_DN42753_c0_g1_i2_orf1	26S proteasome regulatory subunit 8 [Ostrinia furnacalis]	1.70738	-0.4156	0.49729	-0.7149	-1.0741
TRINITY_DN8682_c0_g1_i4_orf1	protein SYS1 homolog [Ostrinia furnacalis]	1.88289	0.02206	-0.9445	-0.2556	-0.7049
TRINITY_DN19303_c0_g1_i5_orf1	lipopolysaccharide-induced tumor necrosis factor-alpha factor-like [Ostrinia furnacalis]	1.18704	0.15729	0.8334	-1.5891	-0.5887
TRINITY_DN1675_c0_g1_i1_orf1	DNA-directed RNA polymerase II subunit Rpb4 [Trichoplusia ni] >XP_035439240.1 DNA-directed RNA polymerase II subunit Rpb4-like [Spodoptera frugiperda] >XP_035439241.1 DNA-directed RNA polymerase II subunit Rpb4-like [Spodoptera frugiperda] >XP_035439242.1 DNA-directed RNA polymerase II subunit Rpb4-like [Spodoptera frugiperda] >KAF9421095.1 hypothetical protein HW555_002807 [Spodoptera exigua] >RVE45346.1 hypothetical protein evm_009975 [Chilo suppressalis] >CAH0585697.1 unnamed protein product [Chrysodeixis includens] >KAF9801181.1 hypothetical protein SFRURICE_015021 [Spodoptera frugiperda] >KAG8120230.1 hypothetical protein SFRUCORN_011885 [Spodoptera frugiperda]	1.88854	-0.3671	0.07167	-0.6858	-0.9073

TRINITY_DN22577_c0_g1_i2_orf1	probable beta-hexosaminidase fdl isoform X1 [Ostrinia furnacalis]	1.90166	-0.0142	-0.4196	-0.989	-0.4789
TRINITY_DN29579_c0_g1_i1_orf1	heterogeneous nuclear ribonucleoprotein A1, A2/B1 homolog [Ostrinia furnacalis]	1.6242	-0.5611	0.70017	-0.9161	-0.8471
TRINITY_DN81791_c0_g2_i2_orf1	UDP-N-acetylglucosamine--peptide N-acetylglucosaminyltransferase 110 kDa subunit isoform X2 [Diachasma alloeum]	1.20168	-0.0143	1.03764	-1.1549	-1.0702
TRINITY_DN4798_c0_g1_i3_orf1	unnamed protein product [Spodoptera exigua]	1.2871	0.03117	0.86152	-0.7549	-1.4249
TRINITY_DN12777_c0_g1_i5_orf1	clathrin light chain isoform X2 [Ostrinia furnacalis]	1.25539	0.33592	0.63144	-1.5815	-0.6412
TRINITY_DN33967_c2_g2_i1_orf1	titin homolog [Ostrinia furnacalis]	1.84895	0.0623	-0.78	-0.1599	-0.9713
TRINITY_DN33837_c0_g1_i6_orf1	bolA-like protein DDB_G0274169 [Ostrinia furnacalis] >XP_028171597.1 bolA-like protein DDB_G0274169 [Ostrinia furnacalis]	1.87208	0.09064	-0.3026	-0.9237	-0.7364
TRINITY_DN30673_c0_g1_i5_orf1	protein phosphatase 1 regulatory subunit 21 [Ostrinia furnacalis]	1.39574	-0.0601	0.76862	-0.7031	-1.4011
TRINITY_DN15400_c0_g1_i1_orf1	uncharacterized protein LOC114366781 [Ostrinia furnacalis]	1.20999	-1.3648	0.66042	-0.9976	0.49194
TRINITY_DN34406_c0_g2_i9_orfp1	TRINITY_DN34406_c0_g2_i9_m.33755 TRINITY_DN34406_c0_g2::TRINITY_DN34406_c0_g2_i9::g.33755 ORF type:internal len:82 (-),score=12.88 TRINITY_DN34406_c0_g2_i9:3-245(-)	0.75843	-1.3357	1.25759	-0.9835	0.30318
TRINITY_DN9044_c0_g1_i2_orf1	unnamed protein product [Euphydryas editha]	0.34158	-1.6119	1.42082	0.28184	-0.4323
TRINITY_DN858_c0_g1_i3_orf1	uncharacterized protein LOC114351944 [Ostrinia furnacalis]	1.23973	-1.2453	0.79527	-1.0905	0.30085
TRINITY_DN25234_c0_g1_i1_orf1	uncharacterized protein LOC114353853 [Ostrinia furnacalis]	0.3191	-1.7563	1.28922	0.33917	-0.1912
TRINITY_DN2406_c0_g1_i6_orf1	uncharacterized protein LOC114361672 [Ostrinia furnacalis]	0.43542	-1.5302	1.51432	-0.0003	-0.4193
TRINITY_DN3647_c1_g1_i5_orf1	unnamed protein product, partial [Iphiclides podalirius]	1.1311	-1.6234	0.94416	-0.4398	-0.012
TRINITY_DN9383_c0_g1_i3_orf1	uncharacterized protein LOC114361502 [Ostrinia furnacalis]	1.4029	-1.3825	0.83188	-0.2453	-0.607
TRINITY_DN12014_c0_g1_i2_orf1	unnamed protein product [Chilo suppressalis]	0.83407	-1.5093	1.33928	-0.41	-0.2541
TRINITY_DN11868_c0_g1_i2_orf1	uncharacterized protein LOC114361308 [Ostrinia furnacalis]	1.07345	-1.5547	0.98066	-0.6646	0.16518
TRINITY_DN184_c0_g1_i10_orf1	C-type mannose receptor 2-like isoform X1 [Leguminivora glycinivorella]	0.66493	-1.6979	1.27613	-0.0299	-0.2132
TRINITY_DN97883_c0_g1_i2_orf1	talin-2-like, partial [Ostrinia furnacalis]	0.69155	-1.5601	1.25497	0.27487	-0.6613
TRINITY_DN896_c0_g1_i2_orf1	uncharacterized protein LOC114356314 isoform X2 [Ostrinia furnacalis]	1.21433	-1.1738	1.06665	-0.999	-0.1081
TRINITY_DN2141_c0_g1_i1_orf1	low density lipoprotein receptor adapter protein 1-like [Ostrinia furnacalis]	0.88735	-1.5609	1.2249	-0.0269	-0.5245
TRINITY_DN130439_c0_g1_i1_orf1	DDRGK domain-containing protein 1-like [Ostrinia furnacalis]	1.09269	-1.4377	1.14758	-0.6248	-0.1777
TRINITY_DN325_c0_g1_i15_orf1	protein draper-like [Ostrinia furnacalis]	0.67937	-0.954	1.6192	-0.8987	-0.4458
TRINITY_DN12661_c0_g1_i3_orf1	T-complex protein 11-like protein 1 [Ostrinia furnacalis]	0.81103	-1.6372	1.24283	-0.3314	-0.0853
TRINITY_DN2367_c1_g1_i20_orf1	RNA exonuclease 4-like [Ostrinia furnacalis]	1.02945	-0.4204	1.08679	-1.6044	-0.0915
TRINITY_DN38106_c0_g1_i6_orf1	unnamed protein product [Pieris macdunnoughi]	0.90834	-0.8253	1.44438	-1.1109	-0.4165
TRINITY_DN21533_c0_g1_i7_orf1	annexin B9 isoform X1 [Ostrinia furnacalis]	0.90783	-1.4648	1.31632	-0.4479	-0.3115
TRINITY_DN24668_c0_g1_i8_orf1	uncharacterized protein LOC114364067 isoform X3 [Ostrinia furnacalis]	1.05858	-1.3148	1.25614	-0.3091	-0.6908
TRINITY_DN28741_c0_g1_i3_orf1	uncharacterized protein LOC114351652 [Ostrinia furnacalis]	0.43471	-1.1431	1.72204	-0.3946	-0.619
TRINITY_DN132_c0_g2_i2_orf1	alpha-tocopherol transfer protein-like isoform X1 [Ostrinia furnacalis]	0.95664	-1.1957	1.40088	-0.675	-0.4868
TRINITY_DN364_c2_g1_i2_orf1	vinculin-like isoform X3 [Ostrinia furnacalis]	1.16348	-1.4829	1.07447	-0.3147	-0.4404
TRINITY_DN119265_c0_g2_i1_orf1	PREDICTED: sorting nexin-12 [Fopius arisanus]	1.2959	-1.4528	0.9467	-0.3635	-0.4263
TRINITY_DN60949_c0_g1_i4_orf1	aldo-keto reductase AKR2E4-like [Galleria mellonella]	1.09138	-0.7066	1.29785	-1.165	-0.5176
TRINITY_DN1293_c1_g1_i4_orf1	putative fatty acyl-CoA reductase CG5065 [Ostrinia furnacalis]	0.69473	-0.6123	1.62698	-0.7236	-0.9858
TRINITY_DN2836_c0_g1_i4_orf1	protein Diedel [Drosophila sechellia] >EDW53358.1 GM12788 [Drosophila sechellia]	1.23662	-1.527	0.92605	-0.1186	-0.517
TRINITY_DN919_c0_g1_i7_orf1	facilitated trehalose transporter Tret1-like [Ostrinia furnacalis] >XP_028161733.1 facilitated trehalose transporter Tret1-like [Ostrinia furnacalis]	0.41212	-0.7045	1.79098	-0.7902	-0.7084
TRINITY_DN20356_c0_g1_i5_orf1	uncharacterized protein LOC114362428 [Ostrinia furnacalis]	-0.1106	-0.5071	1.92876	-0.3807	-0.9303
TRINITY_DN6205_c0_g1_i8_orf1	phenoloxidase-activating factor 2-like [Ostrinia furnacalis]	0.83882	-0.3826	1.49705	-0.9524	-1.0009
TRINITY_DN1326_c0_g1_i1_orf1	cuticle protein 7-like [Ostrinia furnacalis]	1.94666	-0.0884	-0.5825	-0.4804	-0.7953
TRINITY_DN18502_c0_g1_i1_orf1	uncharacterized protein LOC114359515 [Ostrinia furnacalis]	1.86481	0.22858	-0.6613	-0.7772	-0.6549
TRINITY_DN14532_c0_g1_i1_orf1	pupal cuticle protein-like [Trichoplusia ni]	1.89689	0.09595	-0.8054	-0.7332	-0.4542
TRINITY_DN98242_c0_g1_i1_orf1	adenosine deaminase 2-A-like [Galleria mellonella]	1.82519	0.21135	-0.2907	-0.9611	-0.7848
TRINITY_DN147458_c0_g1_i1_orf1	60S ribosomal protein L5, partial [Cotesia chilonis]	1.77783	0.25867	-0.3023	-1.1648	-0.5694
TRINITY_DN48590_c0_g1_i1_orf1	acyl-CoA Delta(11) desaturase isoform X1 [Ostrinia furnacalis] >XP_028172999.1 acyl-CoA Delta(11) desaturase isoform X2 [Ostrinia furnacalis] >XP_028173000.1 acyl-CoA Delta(11) desaturase isoform X1 [Ostrinia furnacalis]	1.85729	0.24769	-0.6761	-0.7892	-0.6396
TRINITY_DN72999_c0_g1_i1_orf1	protein obstructor-E-like isoform X1 [Ostrinia furnacalis] >XP_028169319.1 protein obstructor-E-like isoform X2 [Ostrinia furnacalis]	1.81549	0.33894	-0.5898	-0.875	-0.6896
TRINITY_DN20244_c0_g1_i1_orfp1	uncharacterized protein LOC125235519 [Leguminivora glycinivorella]	1.77601	0.39736	-0.8905	-0.4517	-0.8312
TRINITY_DN978_c9_g2_i1_orf1	hypothetical protein evm_000959 [Chilo suppressalis]	1.84075	0.26639	-0.5439	-0.8889	-0.6744
TRINITY_DN14721_c0_g1_i2_orf1	protein masquerade-like isoform X2 [Ostrinia furnacalis]	1.8303	0.31441	-0.681	-0.6418	-0.8218
TRINITY_DN1868_c0_g1_i1_orf1	protein obstructor-E isoform X1 [Ostrinia furnacalis]	1.73191	0.47972	-1.0091	-0.4808	-0.7217
TRINITY_DN9455_c0_g1_i6_orf1	uncharacterized protein LOC114360866 isoform X4 [Ostrinia furnacalis]	1.78598	0.37664	-0.9901	-0.6055	-0.567
TRINITY_DN7803_c0_g1_i2_orf1	membrane-associated protein Hem [Ostrinia furnacalis]	1.86127	0.09822	-0.8059	-0.9017	-0.2519
TRINITY_DN74069_c0_g1_i1_orf1	unnamed protein product [Parnassius apollo] >CAG5017650.1 unnamed protein product [Parnassius apollo]	1.77677	0.34065	-0.9765	-0.8181	-0.3228
TRINITY_DN15865_c0_g1_i1_orf1	carboxylesterase, partial [Ostrinia furnacalis]	1.86531	0.14796	-0.8171	-0.8385	-0.3576
TRINITY_DN650_c0_g1_i3_orf1	chitinase 7 [Glyphodes pyloalis]	1.80654	0.28102	-0.9787	-0.7603	-0.3485
TRINITY_DN10057_c0_g2_i1_orf1	cell wall protein DAN4 [Ostrinia furnacalis]	1.74491	0.50736	-0.7522	-0.809	-0.6911
TRINITY_DN6656_c0_g1_i1_orf1	sorting and assembly machinery component 50 homolog isoform X9 [Ostrinia furnacalis] >XP_028169233.1 sorting and assembly machinery component 50 homolog isoform X10 [Ostrinia furnacalis] >XP_028169234.1 sorting and assembly machinery component 50 homolog isoform X11 [Ostrinia furnacalis]	1.87477	0.09235	-0.6086	-0.9822	-0.3764
TRINITY_DN4144_c0_g1_i7_orf1	uncharacterized protein LOC114350172 [Ostrinia furnacalis]	1.87969	0.16822	-0.6447	-0.5632	-0.84
TRINITY_DN2971_c0_g1_i1_orf1	uncharacterized protein LOC114364864 [Ostrinia furnacalis]	1.73093	-0.0592	-1.3466	0.09647	-0.4216
TRINITY_DN5772_c0_g1_i6_orf1	uncharacterized protein LOC114364899 isoform X2 [Ostrinia furnacalis]	1.91939	0.02036	-0.8318	-0.6241	-0.4838

TRINITY_DN4694_c0_g2_i1_orf1	uncharacterized protein LOC114362122 [Ostrinia furnacalis]	1.92684	-0.0414	-0.7126	-0.3743	-0.7986
TRINITY_DN5954_c0_g1_i2_orf1	myosin-VIIa [Ostrinia furnacalis] >XP_028155907.1 myosin-VIIa [Ostrinia furnacalis]	1.90547	0.10325	-0.5828	-0.6794	-0.7465
TRINITY_DN5568_c0_g2_i2_orf1	carboxypeptidase D isoform X5 [Ostrinia furnacalis]	1.83727	0.23837	-0.7149	-0.936	-0.4247
TRINITY_DN4898_c0_g1_i7_orf1	annulin-like isoform X3 [Ostrinia furnacalis]	1.85648	0.24566	-0.5944	-0.7939	-0.7138
TRINITY_DN552_c0_g1_i3_orf1	patronin isoform X9 [Ostrinia furnacalis]	1.45941	0.72143	-0.0316	-1.2145	-0.9347
TRINITY_DN32956_c0_g1_i4_orf1	inositol-trisphosphate 3-kinase A isoform X1 [Vanessa tameamea] >XP_047534115.1 inositol-trisphosphate 3-kinase A isoform X1 [Vanessa atalanta] >XP_047534116.1 inositol-trisphosphate 3-kinase A isoform X1 [Vanessa atalanta] >XP_047534117.1 inositol-trisphosphate 3-kinase A isoform X1 [Vanessa atalanta]	1.75816	0.35912	-0.3111	-1.0643	-0.7418
TRINITY_DN30273_c1_g1_i1_orf1	uncharacterized protein LOC114358591 isoform X2 [Ostrinia furnacalis]	1.72656	0.20152	-0.0802	-1.2877	-0.5602
TRINITY_DN6586_c0_g1_i1_orf1	fatty acyl-CoA reductase wat-like isoform X1 [Ostrinia furnacalis]	1.75321	0.49127	-0.6965	-0.8028	-0.7452
TRINITY_DN21623_c0_g2_i1_orf1	Chlorophyll a-b binding protein, chloroplastic [Trichinella nelsoni]	1.33585	0.81913	-1.3236	-0.8884	0.05707
TRINITY_DN9536_c0_g1_i4_orf1	adrenodoxin-like protein, mitochondrial isoform X1 [Ostrinia furnacalis]	1.90141	0.05631	-0.5874	-0.4643	-0.906
TRINITY_DN3430_c0_g1_i1_orf1	protein HGV2-like isoform X2 [Ostrinia furnacalis]	1.91212	0.07081	-0.6537	-0.5462	-0.783
TRINITY_DN15256_c0_g1_i8_orf1	pre-mRNA-splicing regulator female-lethal(2)D [Ostrinia furnacalis]	1.19416	1.07649	-0.7996	-1.3245	-0.1466
TRINITY_DN9872_c0_g1_i2_orf1	serine protease inhibitor 88Ea-like [Ostrinia furnacalis]	1.90258	0.10626	-0.7034	-0.5477	-0.7577
TRINITY_DN2623_c1_g1_i3_orf1	COPII coat assembly protein sec16-like [Ostrinia furnacalis]	1.88306	0.16098	-0.5539	-0.6705	-0.8197
TRINITY_DN50517_c0_g1_i5_orf1	hypothetical protein evm_004957 [Chilo suppressalis]	1.84659	0.23342	-0.7835	-0.8499	-0.4466
TRINITY_DN5182_c0_g1_i5_orf1	rab proteins geranylgeranyltransferase component A 1 isoform X1 [Ostrinia furnacalis]	1.84414	0.25199	-0.9148	-0.5684	-0.6129
TRINITY_DN9100_c0_g1_i5_orf1	microtubule-associated protein futsch-like isoform X6 [Ostrinia furnacalis]	1.84266	0.26785	-0.8412	-0.7338	-0.5355
TRINITY_DN8659_c0_g2_i1_orf1	ubiquitin-like modifier-activating enzyme 1 [Ostrinia furnacalis]	1.79408	-0.3919	0.02678	-0.164	-1.2649
TRINITY_DN9000_c0_g2_i1_orf1	uncharacterized protein LOC114356585 [Ostrinia furnacalis]	1.82252	0.32542	-0.811	-0.7712	-0.5657
TRINITY_DN1233_c0_g2_i1_orf1	unnamed protein product [Spodoptera exigua]	1.81938	0.27197	-0.378	-0.9077	-0.8056
TRINITY_DN1716_c0_g1_i14_orf1	putative gamma-glutamylcyclotransferase CG2811 isoform X3 [Ostrinia furnacalis]	1.76795	0.31781	-1.0524	-0.2599	-0.7734
TRINITY_DN122423_c0_g1_i1_orf1	hypothetical protein TSAR_001625, partial [Trichomalopsis sarcophagae]	1.29035	0.65122	0.31787	-1.4884	-0.771
TRINITY_DN5118_c0_g1_i1_orf1	AP-2 complex subunit alpha [Ostrinia furnacalis]	1.64268	-0.0269	0.3052	-1.3863	-0.5346
TRINITY_DN5235_c0_g1_i7_orf1	peptidoglycan-recognition protein SA-like [Ostrinia furnacalis]	1.73819	0.46261	-0.9978	-0.7522	-0.4507
TRINITY_DN10745_c0_g1_i14_orf1	septin-1 [Ostrinia furnacalis]	1.66325	0.63822	-0.9232	-0.799	-0.5793
TRINITY_DN33893_c0_g1_i1_orf1	high mobility group protein I-like [Ostrinia furnacalis]	1.63419	0.22334	-0.0217	-1.4629	-0.373
TRINITY_DN131264_c0_g1_i2_orf1	unnamed protein product [Parnassius apollo]	1.73387	-0.0063	0.09463	-0.5082	-1.3139
TRINITY_DN35633_c0_g2_i1_orf1	uncharacterized protein LOC114353024 [Ostrinia furnacalis]	1.679	0.62328	-0.8869	-0.6615	-0.7539
TRINITY_DN2942_c0_g1_i6_orf1	microtubule-associated protein RP/EB family member 1 [Ostrinia furnacalis] >XP_028174940.1 microtubule-associated protein RP/EB family member 1 [Ostrinia furnacalis]	1.66848	0.10645	0.08702	-1.4126	-0.4494
TRINITY_DN14865_c0_g1_i2_orf1	ubiquilin-1 [Ostrinia furnacalis]	1.59965	-0.0156	0.42	-1.3601	-0.644
TRINITY_DN13395_c0_g1_i1_orf1	cytoplasmic dynein 1 light intermediate chain 2 [Galleria mellonella]	1.78199	0.40297	-0.5202	-0.7786	-0.8862
TRINITY_DN42337_c0_g1_i6_orf1	cuticle protein 8-like [Leguminivora glycinivorella]	1.75362	0.4195	-0.7463	-1.0084	-0.4185
TRINITY_DN4561_c0_g1_i3_orf1	host cell factor 1 [Ostrinia furnacalis]	1.87358	0.09444	-0.7669	-0.8932	-0.3079
TRINITY_DN286_c0_g1_i2_orf1	uncharacterized protein LOC114361329 [Ostrinia furnacalis]	1.69285	0.55637	-1.0457	-0.661	-0.5426
TRINITY_DN13063_c0_g1_i1_orf1	protein unzipped [Ostrinia furnacalis]	1.65871	-0.4268	0.04035	0.15621	-1.4285
TRINITY_DN73923_c0_g1_i1_orf1	protein obstructor-E-like [Ostrinia furnacalis]	1.56249	0.81498	-0.8734	-0.7678	-0.7363
TRINITY_DN27723_c0_g1_i1_orf1	putative uncharacterized protein DDB_G0282133 isoform X1 [Ostrinia furnacalis]	1.72456	0.42957	-0.7647	-1.0764	-0.313
TRINITY_DN1133_c0_g1_i6_orf1	zinc finger protein 391-like [Ostrinia furnacalis] >XP_028169193.1 zinc finger protein 391-like [Ostrinia furnacalis]	1.78491	0.34322	-0.6866	-1.0256	-0.4159
TRINITY_DN56993_c0_g1_i4_orf1	polypyrimidine tract-binding protein 1 isoform X11 [Helicoverpa zea]	1.67294	0.47794	-1.0351	-0.9313	-0.1845
TRINITY_DN25870_c0_g2_i6_orf1	homeobox protein extradenticle isoform X3 [Ostrinia furnacalis]	1.58092	0.40838	0.10365	-1.3043	-0.7887
TRINITY_DN27321_c0_g1_i1_orf1	ras-related protein Rap-2c [Bicyclus anynana] >XP_026492616.1 ras-related protein Rap-2c [Vanessa tameamea] >XP_034838061.1 ras-related protein Rap-2c [Maniola hyperantus] >XP_039759141.1 ras-related protein Rap-2c [Pararge aegeria] >XP_045498804.1 ras-related protein Rap-2c [Colias croceus] >XP_046959644.1 ras-related protein Rap-2c [Vanessa cardui] >XP_047530248.1 ras-related protein Rap-2c [Vanessa atalanta] >CAH2268047.1 jg10357 [Pararge aegeria aegeria]	1.74117	0.36854	-0.5015	-1.1835	-0.4248
TRINITY_DN5840_c0_g1_i6_orf1	catenin alpha isoform X2 [Ostrinia furnacalis]	1.75117	0.45809	-0.5009	-0.7722	-0.9362
TRINITY_DN162_c0_g1_i4_orf1	ABC transporter G family member 23 isoform X1 [Ostrinia furnacalis] >XP_028178987.1 ABC transporter G family member 23 isoform X1 [Ostrinia furnacalis]	1.91165	0.07062	-0.5179	-0.7231	-0.7413
TRINITY_DN2745_c0_g1_i2_orf1	PREDICTED: tubulin alpha-1A chain-like [Papilio polytes] >XP_013164648.1 PREDICTED: tubulin alpha-1A chain-like [Papilio xuthus]	1.60811	0.07417	0.29113	-0.5528	-1.4206
TRINITY_DN2100_c0_g1_i2_orf1	uncharacterized protein LOC114362418 [Ostrinia furnacalis]	1.90776	0.0615	-0.7887	-0.4528	-0.7277
TRINITY_DN44070_c0_g2_i2_orf1	protein Gawky isoform X2 [Ostrinia furnacalis]	1.69246	0.17612	-1.2415	0.11464	-0.7418
TRINITY_DN5211_c0_g1_i1_orf1	elongation of very long chain fatty acids protein AAEL008004-like [Ostrinia furnacalis]	1.64229	0.67653	-0.6168	-0.9413	-0.7607
TRINITY_DN1280_c0_g1_i1_orf1	coronin-7 isoform X1 [Ostrinia furnacalis] >XP_028164815.1 coronin-7 isoform X2 [Ostrinia furnacalis] >XP_028164817.1 coronin-7 isoform X3 [Ostrinia furnacalis] >XP_028164818.1 coronin-7 isoform X4 [Ostrinia furnacalis] >XP_028164820.1 coronin-7 isoform X6 [Ostrinia furnacalis] >XP_028164821.1 coronin-7 isoform X7 [Ostrinia furnacalis] >XP_028164822.1 coronin-7 isoform X1 [Ostrinia furnacalis] >XP_028164823.1 coronin-7 isoform X8 [Ostrinia furnacalis] >XP_028164824.1 coronin-7 isoform X9 [Ostrinia furnacalis] >XP_028164825.1 coronin-7 isoform X10 [Ostrinia furnacalis]	1.75435	0.47808	-0.5992	-0.8383	-0.7949
TRINITY_DN2840_c0_g1_i5_orf1	hypothetical protein evm_002181 [Chilo suppressalis]	1.65421	0.66718	-0.8592	-0.8062	-0.656
TRINITY_DN12769_c0_g1_i5_orf1	uncharacterized protein LOC114365633 [Ostrinia furnacalis]	1.39876	0.88953	-0.5635	-1.3353	-0.3895
TRINITY_DN6130_c0_g1_i6_orf1	tryptophan--tRNA ligase, mitochondrial [Ostrinia furnacalis]	1.39087	0.98977	-0.8033	-1.1021	-0.4752
TRINITY_DN26293_c0_g1_i4_orf1	UDP-glucose 6-dehydrogenase [Ostrinia furnacalis]	1.72434	0.4344	-1.0778	-0.3259	-0.7551
TRINITY_DN9072_c0_g1_i1_orf1	SET and MYND domain-containing protein 4-like [Ostrinia furnacalis]	1.64226	0.32343	-1.3411	0.00769	-0.6323
TRINITY_DN110534_c0_g1_i3_orf1	unnamed protein product [Euphydryas editha]	1.67097	0.5383	-0.4191	-0.6304	-1.1597
TRINITY_DN5200_c0_g1_i2_orf1	uncharacterized protein LOC114351644 [Ostrinia furnacalis]	1.59155	0.35279	0.02647	-1.4325	-0.5383

TRINITY_DN3499_c0_g1_i8_orf1	modular serine protease-like [Ostrinia furnacalis]	1.59796	0.52118	-0.3179	-0.4254	-1.3758
TRINITY_DN1231_c0_g1_i4_orf1	AN1-type zinc finger protein 6 isoform X1 [Galleria mellonella]	1.66429	0.54684	-0.8061	-1.0861	-0.3189
TRINITY_DN482_c0_g1_i1_orf1	fatty acyl reductase 7 [Maruca vitrata]	1.83957	0.29456	-0.6267	-0.7415	-0.766
TRINITY_DN1853_c0_g1_i3_orf1	trans-Golgi network integral membrane protein TGN38-like isoform X1 [Ostrinia furnacalis]	1.67924	0.40468	-0.3019	-1.3018	-0.4803
TRINITY_DN30178_c0_g1_i3_orf1	LOW QUALITY PROTEIN: fibrillin-2-like [Bicyclus anynana]	1.47091	0.60576	-0.3188	-1.5203	-0.2376
TRINITY_DN2638_c0_g1_i7_orf1	structural maintenance of chromosomes protein 1A [Trichoplusia ni]	1.65422	0.51516	-0.1905	-1.0334	-0.9455
TRINITY_DN10479_c0_g1_i6_orf1	unnamed protein product [Chrysodeixis includens]	1.70627	0.52515	-0.6901	-1.0434	-0.4979
TRINITY_DN10694_c1_g2_i1_orf1	hsp70-Hsp90 organizing protein 3-like [Ostrinia furnacalis]	1.8399	0.21463	-0.5305	-0.5111	-1.0129
TRINITY_DN535_c1_g1_i2_orf1	protein tramtrack, beta isoform isoform X24 [Bicyclus anynana]	1.71138	0.39845	-0.1687	-0.9725	-0.9686
TRINITY_DN486_c0_g1_i5_orf1	adaptor complexes medium subunit family domain-containing protein [Phthorimaea operculella]	1.39775	0.48981	0.25662	-1.5421	-0.6021
TRINITY_DN9916_c0_g1_i1_orf1	PREDICTED: dynein light chain Tctex-type [Amyelois transitella] >XP_021195381.1 dynein light chain Tctex-type [Helicoverpa armigera] >XP_022815696.1 dynein light chain Tctex-type [Spodoptera litura] >XP_028156399.1 dynein light chain Tctex-type [Ostrinia furnacalis] >XP_035458261.1 dynein light chain Tctex-type-like [Spodoptera frugiperda] >XP_047034788.1 dynein light chain Tctex-type [Helicoverpa zea] >CAB3233358.1 unnamed protein product [Arctia plantaginis] >CAB3506583.1 unnamed protein product [Spodoptera littoralis] >CAG9754627.1 unnamed protein product [Diatraea saccharalis] >CAH0596395.1 unnamed protein product [Chrysodeixis includens] >KAF9808454.1 hypothetical protein SFRURICE_008507 [Spodoptera frugiperda]	1.68231	0.54546	-0.659	-1.1065	-0.4623
TRINITY_DN73224_c0_g4_i2_orf1	PREDICTED: poly(rC)-binding protein 3 isoform X2 [Vollenhovia emeryi]	1.67414	0.62198	-0.8913	-0.8232	-0.5816
TRINITY_DN8738_c0_g1_i1_orf1	unnamed protein product [Plutella xylostella]	1.4732	0.60316	-0.0769	-1.4798	-0.5197
TRINITY_DN17003_c0_g1_i1_orf1	mucin-5AC [Ostrinia furnacalis]	1.65808	0.62395	-0.4724	-0.9257	-0.884
TRINITY_DN24218_c0_g1_i1_orf1	uncharacterized protein LOC114362624 [Ostrinia furnacalis]	1.48548	0.25686	0.3872	-1.4585	-0.6711
TRINITY_DN2808_c0_g1_i8_orf1	uncharacterized protein LOC114353011 isoform X2 [Ostrinia furnacalis]	1.39915	0.63079	-0.194	-1.5968	-0.2391
TRINITY_DN6312_c0_g1_i1_orf1	cytochrome c oxidase assembly protein COX19 [Ostrinia furnacalis]	1.49211	0.75724	-0.8362	-1.2078	-0.2053
TRINITY_DN4156_c0_g1_i2_orf1	calcium channel flower [Ostrinia furnacalis]	1.39902	0.86119	-0.3001	-1.3609	-0.5993
TRINITY_DN123184_c0_g1_i1_orf1	double-strand break repair protein MRE11 [Ostrinia furnacalis]	1.61415	0.36647	0.09798	-1.2518	-0.8268
TRINITY_DN4782_c0_g1_i1_orf1	patched domain-containing protein 3-like [Ostrinia furnacalis]	1.47416	0.25743	0.40815	-1.4602	-0.6795
TRINITY_DN120593_c0_g1_i1_orf1	SUMO-activating enzyme subunit 1 [Ostrinia furnacalis]	1.69562	0.5269	-0.5237	-1.1043	-0.5945
TRINITY_DN16011_c0_g1_i3_orf1	hypothetical protein evm_002694 [Chilo suppressalis]	1.71334	0.19158	0.03652	-1.237	-0.7044
TRINITY_DN77005_c0_g3_i1_orf1	RNA polymerase II degradation factor 1-like [Ostrinia furnacalis]	1.73343	0.28753	-1.2454	-0.2135	-0.5621
TRINITY_DN277_c1_g1_i1_orf1	uncharacterized protein LOC114363802 isoform X4 [Ostrinia furnacalis]	1.26086	0.69836	-0.2168	-1.6951	-0.0473
TRINITY_DN72816_c0_g1_i2_orf1	Golgi apparatus protein 1 [Ostrinia furnacalis]	1.58243	0.5674	-0.2988	-1.3565	-0.4945
TRINITY_DN185_c0_g1_i6_orf1	eukaryotic translation initiation factor 4E-binding protein Mextli isoform X2 [Ostrinia furnacalis]	1.41419	0.61879	0.19811	-1.3266	-0.9045
TRINITY_DN21124_c0_g1_i4_orf1	calsyntenin-1 [Ostrinia furnacalis]	1.64206	0.69195	-0.8438	-0.7804	-0.7098
TRINITY_DN2054_c0_g1_i1_orf1	macrophage mannose receptor 1-like [Ostrinia furnacalis]	1.59174	0.49434	0.00965	-0.9341	-1.1616
TRINITY_DN18922_c0_g1_i1_orf1	LOW QUALITY PROTEIN: CCR4-NOT transcription complex subunit 6 [Ostrinia furnacalis]	1.4704	0.17705	0.51941	-1.391	-0.7759
TRINITY_DN18391_c0_g2_i8_orf1	protein suppressor of forked [Helicoverpa zea]	1.63927	0.63109	-0.917	-0.3958	-0.9577
TRINITY_DN2345_c0_g1_i4_orf1	chromobox protein homolog 3-like [Ostrinia furnacalis] >XP_028157236.1 chromobox protein homolog 3-like [Ostrinia furnacalis]	1.71118	0.29718	-0.0369	-1.1252	-0.8462
TRINITY_DN2004_c0_g1_i20_orf1	hypothetical protein evm_006436 [Chilo suppressalis] >CAB3522373.1 unnamed protein product [Chilo suppressalis] >CAH0399695.1 unnamed protein product [Chilo suppressalis]	1.56354	0.29388	0.27041	-1.3209	-0.8069
TRINITY_DN2591_c0_g1_i4_orf1	26S proteasome non-ATPase regulatory subunit 13 isoform X1 [Ostrinia furnacalis]	1.69901	0.38575	-0.193	-0.6833	-1.2085
TRINITY_DN2936_c0_g1_i1_orf1	myosin heavy chain, non-muscle isoform X1 [Hyposmocoma kahamanoa]	1.6374	0.68481	-0.6953	-0.9602	-0.6667
TRINITY_DN44658_c0_g1_i2_orf1	lipase 3-like [Ostrinia furnacalis]	1.55827	0.44793	-0.0625	-1.4609	-0.4828
TRINITY_DN84883_c0_g1_i1_orf1	protein yellow [Ostrinia furnacalis]	1.79129	0.34981	-0.5911	-0.5318	-1.0182
TRINITY_DN12134_c0_g1_i4_orf1	glutathione S-transferase 1-1 [Ostrinia furnacalis] >XP_028161942.1 glutathione S-transferase 1-1 [Ostrinia furnacalis] >XP_028161943.1 glutathione S-transferase 1-1 [Ostrinia furnacalis]	1.485	0.74393	-0.4869	-1.3644	-0.3776
TRINITY_DN120144_c0_g1_i1_orf1	pre-mRNA-splicing factor SPF27 [Ostrinia furnacalis] >XP_028158851.1 pre-mRNA-splicing factor SPF27 [Ostrinia furnacalis]	1.63955	0.31245	0.14242	-1.029	-1.0654
TRINITY_DN32362_c0_g1_i1_orf1	uncharacterized protein LOC114350902 [Ostrinia furnacalis]	1.60412	0.34828	0.08888	-1.3479	-0.6934
TRINITY_DN2450_c0_g1_i6_orf1	oxysterol-binding protein-related protein 9 [Manduca sexta]	1.6106	0.74341	-0.739	-0.8465	-0.7685
TRINITY_DN14443_c0_g1_i1_orf1	SUMO-activating enzyme subunit 2 [Ostrinia furnacalis]	1.60715	0.66177	-0.4032	-1.1279	-0.7377
TRINITY_DN64769_c0_g1_i3_orf1	procollagen-lysine,2-oxoglutarate 5-dioxygenase isoform X2 [Ostrinia furnacalis]	1.52617	0.78737	-0.9736	-0.9903	-0.3496
TRINITY_DN24391_c1_g1_i1_orf1	ER membrane protein complex subunit 7 [Ostrinia furnacalis]	1.56156	0.62962	-1.1383	-0.1295	-0.9234
TRINITY_DN44256_c0_g1_i1_orf1	essential MCU regulator, mitochondrial [Cotesia glomerata]	1.63641	0.6127	-0.7849	-1.0918	-0.3724
TRINITY_DN782_c0_g1_i5_orf1	regulator of gene activity isoform X3 [Ostrinia furnacalis]	1.5426	0.79981	-1.008	-0.4745	-0.86
TRINITY_DN29563_c0_g1_i5_orf1	N-terminal kinase-like protein [Trichoplusia ni]	1.59197	0.4273	0.07827	-0.8525	-1.2451
TRINITY_DN44792_c0_g1_i1_orf1	parafibromin [Ostrinia furnacalis]	1.55789	0.50034	-1.1898	0.08053	-0.949
TRINITY_DN1074_c0_g1_i7_orf1	eukaryotic translation initiation factor 4E type 2 [Ostrinia furnacalis]	1.58005	0.74375	-0.6957	-1.0795	-0.5486
TRINITY_DN13118_c0_g1_i6_orf1	AP-1 complex subunit beta-1 [Helicoverpa armigera] >XP_021189434.2 AP-1 complex subunit beta-1 [Helicoverpa armigera]	1.23238	0.86374	0.07436	-1.5178	-0.6527
TRINITY_DN47914_c0_g2_i1_orf1	UBX domain-containing protein 1-A-like [Ostrinia furnacalis]	1.54901	0.72875	-0.2904	-0.9218	-1.0655
TRINITY_DN1616_c0_g1_i3_orf1	U4/U6 small nuclear ribonucleoprotein Prp3 isoform X1 [Ostrinia furnacalis] >XP_028161035.1 U4/U6 small nuclear ribonucleoprotein Prp3 isoform X2 [Ostrinia furnacalis] >XP_028161037.1 U4/U6 small nuclear ribonucleoprotein Prp3 isoform X3 [Ostrinia furnacalis]	1.64941	0.6067	-0.5213	-0.6077	-1.127
TRINITY_DN48641_c0_g1_i4_orf1	RNA-binding protein 45-like [Galleria mellonella]	1.45476	0.85224	-0.27	-0.9484	-1.0886
TRINITY_DN38211_c0_g1_i1_orf1	Golgi reassembly-stacking protein 2 [Ostrinia furnacalis]	1.36317	0.96706	-0.9581	-1.1028	-0.2694
TRINITY_DN14987_c0_g1_i3_orf1	hypothetical protein evm_009121 [Chilo suppressalis]	1.31866	0.87372	-0.034	-1.3684	-0.79
TRINITY_DN59829_c0_g1_i1_orf1	putative mediator of RNA polymerase II transcription subunit 12 [Ostrinia furnacalis]	1.56881	0.78609	-0.5655	-0.9035	-0.8859
TRINITY_DN2474_c0_g1_i5_orf1	glucose-6-phosphate 1-epimerase [Galleria mellonella]	1.64683	0.47666	-0.6091	-0.2361	-1.2783

TRINITY_DN20067_c0_g1_i6_orf1	hypothetical protein evm_010712 [Chilo suppressalis] >CAB3527462.1 unnamed protein product [Chilo suppressalis] >CAH0401768.1 unnamed protein product [Chilo suppressalis]	1.40701	0.99633	-1.0009	-0.847	-0.5555
TRINITY_DN17655_c0_g1_i1_orf1	BRISC and BRCA1-A complex member 1-like [Ostrinia furnacalis]	1.69511	0.3707	-0.172	-0.6585	-1.2353
TRINITY_DN8659_c0_g1_i1_orf1	ubiquitin-like modifier-activating enzyme 1 [Manduca sexta]	1.47845	0.89739	-0.5024	-0.9652	-0.9082
TRINITY_DN1601_c0_g1_i4_orf1	cytoplasmic dynein 1 intermediate chain isoform X4 [Ostrinia furnacalis]	1.43349	0.95213	-1.0878	-0.568	-0.7299
TRINITY_DN14046_c0_g1_i1_orf1	retinol dehydrogenase 14 [Ostrinia furnacalis] >XP_028165567.1 retinol dehydrogenase 14 [Ostrinia furnacalis] >XP_028165568.1 retinol dehydrogenase 14 [Ostrinia furnacalis]	1.72204	0.54686	-0.7336	-0.6707	-0.8646
TRINITY_DN98814_c0_g1_i2_orf1	PREDICTED: chaoptin [Amyelois transitella]	1.69806	0.51035	-0.7509	-0.3896	-1.068
TRINITY_DN28428_c0_g1_i2_orf1	unnamed protein product [Chrysodeixis includens]	1.2707	0.93955	-0.2375	-1.4865	-0.4863
TRINITY_DN1313_c0_g1_i2_orf1	39S ribosomal protein L40, mitochondrial [Ostrinia furnacalis]	1.51007	0.82255	-0.8942	-0.396	-1.0424
TRINITY_DN12771_c0_g1_i1_orf1	histone acetyltransferase type B catalytic subunit [Ostrinia furnacalis]	1.43641	0.73246	-0.826	-0.0325	-1.3103
TRINITY_DN2172_c0_g2_i8_orf1	hypothetical protein evm_003685 [Chilo suppressalis]	1.2087	0.86022	0.10032	-1.5516	-0.6176
TRINITY_DN1405_c0_g1_i1_orf1	cyclin-dependent kinase 10 isoform X1 [Ostrinia furnacalis] >XP_028178194.1 cyclin-dependent kinase 10 isoform X2 [Ostrinia furnacalis]	1.46784	0.83562	-0.302	-1.1636	-0.8378
TRINITY_DN24469_c0_g2_i2_orf1	unnamed protein product, partial [Brenthis ino]	1.4866	0.89057	-0.9071	-0.9513	-0.5188
TRINITY_DN6436_c0_g1_i1_orf1	serine/threonine-protein kinase PAK 3 isoform X1 [Ostrinia furnacalis] >XP_028164178.1 serine/threonine-protein kinase PAK 3 isoform X2 [Ostrinia furnacalis] >XP_028164179.1 serine/threonine-protein kinase PAK 3 isoform X3 [Ostrinia furnacalis]	1.64997	0.64813	-0.5783	-0.7112	-1.0086
TRINITY_DN113778_c0_g2_i1_orf1	metastasis-associated protein MTA3 [Galleria mellonella]	1.26352	0.81493	0.08799	-1.5219	-0.6445
TRINITY_DN28759_c0_g1_i1_orf1	innexin inx2 [Ostrinia furnacalis]	1.3934	0.98902	-0.8971	-1.0363	-0.4491
TRINITY_DN34745_c0_g2_i1_orf1	GSK3-beta interaction protein-like [Galleria mellonella]	1.57009	0.77465	-0.7347	-1.0273	-0.5827
TRINITY_DN20215_c0_g2_i1_orf1	unnamed protein product [Spodoptera littoralis] >CAH1638553.1 unnamed protein product [Spodoptera littoralis]	1.36626	1.0028	-0.7266	-0.467	-1.1754
TRINITY_DN7603_c0_g1_i5_orf1	tetratricopeptide repeat protein 1-like [Ostrinia furnacalis]	1.46348	0.87622	-0.387	-1.107	-0.8457
TRINITY_DN14298_c0_g1_i1_orf1	kinesin heavy chain [Ostrinia furnacalis]	1.2973	0.99878	-0.2645	-1.3206	-0.711
TRINITY_DN1831_c0_g1_i3_orf1	hypothetical protein O3G_MSEX014886 [Manduca sexta]	1.24159	1.14198	-0.7457	-0.4605	-1.1774
TRINITY_DN51737_c0_g1_i3_orf1	N-alpha-acetyltransferase 40 [Ostrinia furnacalis]	1.4392	0.97785	-0.8467	-0.8915	-0.6789
TRINITY_DN9354_c0_g1_i7_orf1	hypothetical protein evm_012205 [Chilo suppressalis] >CAB3527181.1 unnamed protein product [Chilo suppressalis] >CAH0404510.1 unnamed protein product [Chilo suppressalis]	1.60529	0.54897	-0.3902	-0.4274	-1.3367
TRINITY_DN67649_c0_g1_i1_orf1	proliferating cell nuclear antigen [Ostrinia furnacalis] >XP_028174842.1 proliferating cell nuclear antigen [Ostrinia furnacalis]	1.52844	0.84402	-0.5729	-0.8546	-0.9449
TRINITY_DN6202_c0_g1_i2_orf1	PREDICTED: serine/threonine-protein phosphatase PP1-beta catalytic subunit [Papilio polytes] >XP_013173027.1 PREDICTED: serine/threonine-protein phosphatase PP1-beta catalytic subunit [Papilio xuthus] >XP_013196427.1 PREDICTED: serine/threonine-protein phosphatase PP1-beta catalytic subunit isoform X1 [Amyelois transitella] >XP_014358529.1 serine/threonine-protein phosphatase PP1-beta catalytic subunit isoform X2 [Papilio machaon] >XP_021196714.1 serine/threonine-protein phosphatase PP1-beta catalytic subunit isoform X3 [Helicoverpa armigera] >XP_022826582.1 serine/threonine-protein phosphatase PP1-beta catalytic subunit [Spodoptera litura] >XP_023952136.1 serine/threonine-protein phosphatase PP1-beta catalytic subunit isoform X2 [Bicyclus anynana] >XP_026730418.1 serine/threonine-protein phosphatase PP1-beta catalytic subunit [Trichoplusia ni] >XP_028156492.1 serine/threonine-protein phosphatase PP1-beta catalytic subunit [Ostrinia furnacalis] >XP_030022932.1 serine/threonine-protein phosphatase PP1-beta catalytic subunit [Manduca sexta] >XP_034832911.1 serine/threonine-protein phosphatase PP1-beta catalytic subunit isoform X2 [Maniola hyperantus] >XP_039756948.1 serine/threonine-protein phosphatase PP1-beta catalytic subunit [Pararge aegeria] >XP_045452222.1 serine/threonine-protein phosphatase PP1-beta catalytic subunit [Melitaea cinxia] >XP_045773496.1 serine/threonine-protein phosphatase PP1-beta catalytic subunit isoform X2 [Maniola jurtina] >XP_047028552.1 serine/threonine-protein phosphatase PP1-beta catalytic subunit [Helicoverpa zea] >XP_047997696.1 serine/threonine-protein phosphatase PP1-beta catalytic subunit isoform X1 [Leguminivora glycinivorella] >CAB3247342.1 unnamed protein product [Arctia plantaginis] >CAB3522774.1 unnamed protein product [Chilo suppressalis] >CAD0197791.1 unnamed protein product [Chrysodeixis includens] >CAG4945530.1 unnamed protein product [Parnassius apollo] >CAG9749509.1 unnamed protein product [Diatraea saccharalis] >CAH0686192.1 unnamed protein product [Spodoptera exigua] >CAH0727047.1 unnamed protein product, partial [Brenthis ino] >CAH2090384.1 unnamed protein product [Euphydryas editha] >CAH2243954.1 jg16796 [Pararge aegeria aegeria]	1.33273	1.05146	-0.9024	-0.4199	-1.0619
TRINITY_DN10174_c0_g1_i4_orf1	protein D2-like isoform X2 [Aricia agestis] >XP_041972210.1 protein D2-like isoform X2 [Aricia agestis]	1.34336	0.91783	-1.3301	-0.1914	-0.7397
TRINITY_DN1628_c0_g1_i1_orf1	uncharacterized protein LOC114363979 [Ostrinia furnacalis]	1.38104	0.75716	-1.017	-1.215	0.09384
TRINITY_DN14298_c0_g3_i1_orf1	kinesin heavy chain [Ostrinia furnacalis]	1.25916	0.91447	-0.0534	-1.4649	-0.6553
TRINITY_DN12858_c0_g1_i5_orf1	unnamed protein product, partial [Iphiclides podalirius]	1.3553	0.99682	-0.3448	-1.1376	-0.8698
TRINITY_DN32700_c0_g1_i2_orf1	ribosomal protein S6 kinase 2 beta [Ostrinia furnacalis]	1.46078	0.93492	-0.9475	-0.5731	-0.8752
TRINITY_DN1436_c0_g1_i5_orf1	vacuolar protein sorting-associated protein 27-like [Trichoplusia ni]	1.49643	0.12973	0.2622	-0.2763	-1.6121
TRINITY_DN53311_c0_g2_i1_orf1	transcription elongation factor S-II [Chelonus insularis]	1.27903	1.10479	-0.8968	-1.0853	-0.4017
TRINITY_DN4159_c1_g1_i1_orf1	F-actin-capping protein subunit beta [Ostrinia furnacalis]	1.02992	1.21434	-0.0254	-1.144	-1.0748
TRINITY_DN19651_c0_g1_i1_orf1	cytosolic non-specific dipeptidase [Ostrinia furnacalis]	1.37145	0.86787	-0.8959	-0.0968	-1.2465
TRINITY_DN59291_c0_g1_i1_orf1	ATP-dependent RNA helicase vasa [Ostrinia furnacalis]	1.01904	1.17592	-0.1612	-1.5092	-0.5246
TRINITY_DN252_c0_g1_i3_orf1	probable phospholipid-transporting ATPase IM [Ostrinia furnacalis]	1.6231	0.62882	-0.3337	-0.8615	-1.0566
TRINITY_DN10385_c0_g1_i5_orf1	unnamed protein product [Arctia plantaginis] >CAB3259747.1 unnamed protein product [Arctia plantaginis]	1.19196	1.10536	-0.4125	-1.3957	-0.4891
TRINITY_DN20767_c0_g2_i1_orf1	glycosylated lysosomal membrane protein B-like [Vanessa atalanta]	1.53935	0.80863	-0.6009	-1.0848	-0.6623
TRINITY_DN45400_c0_g1_i1_orf1	nuclear pore complex protein Nup107 [Ostrinia furnacalis]	1.63354	0.5823	-0.9857	-0.2516	-0.9785
TRINITY_DN3472_c1_g1_i4_orf1	Krueppel homolog 2-like [Ostrinia furnacalis]	1.06893	1.18275	-0.4606	-1.4623	-0.3288
TRINITY_DN4808_c0_g1_i3_orf1	kinesin light chain [Ostrinia furnacalis]	1.28242	1.0505	-0.2482	-1.1353	-0.9494
TRINITY_DN880_c0_g1_i6_orf1	cuticle protein 19-like [Ostrinia furnacalis]	1.52714	0.75837	-0.7958	-1.1631	-0.3266
TRINITY_DN19810_c1_g1_i7_orf1	RNA-binding protein spenito [Ostrinia furnacalis] >XP_028167555.1 RNA-binding protein spenito [Ostrinia furnacalis]	1.18446	1.0377	-0.2108	-1.4816	-0.5298
TRINITY_DN79803_c0_g1_i7_orf1	dnaJ homolog subfamily C member 22 [Ostrinia furnacalis]	1.29123	0.87975	-0.5997	-1.4802	-0.0912
TRINITY_DN9765_c0_g1_i6_orf1	hypothetical protein evm_005049 [Chilo suppressalis] >CAB3525510.1 unnamed protein product [Chilo suppressalis] >CAH0402837.1 unnamed protein product [Chilo suppressalis]	1.28814	1.0182	-0.2675	-1.2969	-0.742

TRINITY_DN1425_c0_g1_i4_orf1	fibulin-2-like [Ostrinia furnacalis]	1.09304	1.11885	-0.3709	-1.521	-0.32
TRINITY_DN1637_c0_g1_i5_orf1	ragulator complex protein LAMTOR3 homolog [Ostrinia furnacalis]	1.19498	0.86866	0.08551	-1.5749	-0.5742
TRINITY_DN78686_c0_g1_i1_orf1	myosin-2 essential light chain isoform X2 [Harpegnathos saltator] >XP_012170910.1 myosin-2 essential light chain isoform X2 [Bombus terrestris] >XP_033185931.1 myosin-2 essential light chain isoform X2 [Bombus vancouverensis nearcticus] >XP_033319091.1 myosin-2 essential light chain isoform X2 [Bombus bifarius] >XP_033349866.1 myosin-2 essential light chain isoform X2 [Bombus vosnesenskii] >XP_043597873.1 myosin-2 essential light chain isoform X2 [Bombus [illegible]	1.26554	0.96003	-0.4571	-1.4775	-0.2909
TRINITY_DN41259_c0_g1_i6_orf1	endocuticle structural glycoprotein SgAbd-8 [Ostrinia furnacalis]	1.43186	0.99293	-0.8041	-0.855	-0.7657
TRINITY_DN3953_c0_g1_i2_orf1	TP53-binding protein 1-like [Ostrinia furnacalis]	1.40753	0.98281	-0.9274	-0.4837	-0.9792
TRINITY_DN36061_c0_g4_i2_orf1	putative GPI-anchored protein pfl2 [Ostrinia furnacalis] >XP_028163002.1 putative GPI-anchored protein pfl2 [Ostrinia furnacalis]	1.45831	0.95821	-0.8739	-0.7428	-0.7998
TRINITY_DN4533_c0_g1_i1_orf1	neurofilament heavy polypeptide-like isoform X2 [Ostrinia furnacalis]	1.0719	1.20226	-0.3021	-1.416	-0.5561
TRINITY_DN1504_c0_g1_i1_orf1	uncharacterized protein LOC114352862 [Ostrinia furnacalis] >XP_028160407.1 uncharacterized protein LOC114352862 [Ostrinia furnacalis]	1.27102	0.9835	-0.1957	-1.3897	-0.6692
TRINITY_DN13375_c0_g1_i6_orf1	thioredoxin, mitochondrial isoform X2 [Ostrinia furnacalis]	1.18545	1.08572	-1.0159	-1.1735	-0.0817
TRINITY_DN6239_c0_g1_i1_orf1	eukaryotic translation initiation factor 6 [Ostrinia furnacalis]	1.24456	1.14951	-0.9579	-0.4174	-1.0188
TRINITY_DN4194_c0_g1_i1_orf1	hornerin-like [Ostrinia furnacalis]	1.06503	1.06503	-1.0077	-1.2981	0.17575
TRINITY_DN48970_c0_g1_i1_orf1	uncharacterized protein LOC114356431 isoform X2 [Ostrinia furnacalis]	1.03333	1.29554	-0.3606	-1.2897	-0.6785
TRINITY_DN57904_c0_g2_i1_orf1	cuticle protein 19 [Plutella xylostella] >CAG9138481.1 unnamed protein product [Plutella xylostella]	1.4628	0.95237	-0.7675	-0.882	-0.7657
TRINITY_DN4070_c0_g1_i4_orf1	alpha-N-acetylgalactosaminidase isoform X3 [Ostrinia furnacalis]	1.41896	0.96014	-0.4759	-1.0681	-0.835
TRINITY_DN18933_c0_g1_i3_orf1	PREDICTED: protein BUD31 homolog [Papilio xuthus] >XP_014361644.1 protein BUD31 homolog [Papilio machaon] >XP_026750578.1 protein BUD31 homolog [Galleria mellonella] >XP_047995610.1 protein BUD31 homolog [Leguminivora glycinivorella] >XP_049869593.1 protein BUD31 homolog [Pectinophora gossypiella] >KAI5652084.1 g10 protein domain-containing protein [Phthorimaea operculella] >CAB3251981.1 unnamed protein product [Arctia plantaginis] >CAB3520382.1 unnamed protein product [Chilo suppressalis] >CAG9747228.1 unnamed protein product [Diatraea saccharalis] >CAH2037008.1 unnamed protein product, partial [Iphiclides podalirius]	1.05956	1.17209	-0.2471	-1.4787	-0.5058
TRINITY_DN4835_c0_g1_i2_orf1	ribonucleoside-diphosphate reductase large subunit [Ostrinia furnacalis]	1.14202	1.12323	-0.0832	-1.2435	-0.9386
TRINITY_DN2879_c0_g1_i4_orf1	nucleoporin Nup35 [Ostrinia furnacalis]	1.10363	1.06596	0.02536	-1.4411	-0.7538
TRINITY_DN37532_c0_g1_i1_orf1	transcription elongation factor S-II [Ostrinia furnacalis]	0.9768	1.3182	-0.326	-1.3474	-0.6216
TRINITY_DN82311_c0_g1_i1_orf1	pleckstrin homology-like domain family B member 1 isoform X2 [Ostrinia furnacalis]	0.99511	1.16088	0.05088	-1.4384	-0.7685
TRINITY_DN76377_c0_g1_i1_orf1	uncharacterized protein LOC111357764, partial [Spodoptera litura]	1.11184	1.19673	-0.7336	-1.3134	-0.2615
TRINITY_DN144258_c0_g1_i1_orf1	PREDICTED: enhancer of rudimentary homolog [Microplitis demolitor] >XP_044577051.1 enhancer of rudimentary homolog [Cotesia glomerata] >KAG8041963.1 hypothetical protein G9C98_007267 [Cotesia typhae] >KAH0539785.1 hypothetical protein KQX54_008036 [Cotesia glomerata] >CAD6227368.1 GSCOCG00006137001-RA-CDS [Cotesia congregata]	1.04677	1.12581	0.08076	-1.3406	-0.9128
TRINITY_DN2894_c0_g2_i3_orf1	myrosinase 1-like isoform X1 [Ostrinia furnacalis]	0.93523	1.21584	0.08127	-1.3889	-0.8434
TRINITY_DN42964_c0_g1_i1_orf1	protein lethal(2)essential for life-like [Galleria mellonella]	1.46893	-0.4821	-1.2742	-0.5436	0.83104
TRINITY_DN13221_c0_g1_i3_orf1	fasciclin-3-like [Ostrinia furnacalis]	1.49017	-0.0193	-1.5586	0.45982	-0.3721
TRINITY_DN5256_c0_g1_i1_orf1	uncharacterized protein LOC114359599 [Ostrinia furnacalis]	1.26411	-0.1243	-1.6407	0.78389	-0.283
TRINITY_DN14391_c1_g1_i2_orf1	pre-rRNA-processing protein TSR1 homolog [Ostrinia furnacalis]	1.10843	-0.5099	-1.3104	1.23367	-0.5217
TRINITY_DN3131_c0_g1_i5_orf1	senecionine N-oxygenase-like isoform X1 [Ostrinia furnacalis] >XP_028178163.1 senecionine N-oxygenase-like isoform X2 [Ostrinia furnacalis] >XP_028178164.1 senecionine N-oxygenase-like isoform X1 [Ostrinia furnacalis]	1.26352	0.09422	-1.5657	0.78281	-0.5748
TRINITY_DN31377_c0_g2_i1_orf1	phosphatidate cytidylyltransferase, mitochondrial [Ostrinia furnacalis]	0.9617	-0.2576	-1.389	1.30289	-0.618
TRINITY_DN51836_c0_g3_i1_orf1	KRT17 isoform 1 [Pan troglodytes]	1.40267	0.1744	-0.4466	0.4749	-1.6053
TRINITY_DN10403_c0_g1_i3_orf1	hypothetical protein evm_000264 [Chilo suppressalis] >CAH2987898.1 unnamed protein product [Chilo suppressalis]	1.58158	-0.0285	-1.3219	0.48591	-0.7171
TRINITY_DN17406_c0_g1_i1_orf1	uncharacterized protein LOC114356625 [Ostrinia furnacalis]	1.62374	0.06111	-0.6818	0.33267	-1.3357
TRINITY_DN32780_c0_g1_i2_orf1	renin receptor [Ostrinia furnacalis]	1.13001	0.43501	-0.7751	0.74984	-1.5397
TRINITY_DN11856_c0_g1_i4_orf1	cartilage-associated protein-like [Ostrinia furnacalis]	0.98475	0.57867	-1.4772	0.82566	-0.9118
TRINITY_DN21380_c0_g1_i1_orf1	ankyrin repeat domain-containing protein 13C [Ostrinia furnacalis]	1.45041	0.03425	-0.6795	0.62437	-1.4295

TRINITY_DN31584_c0_g2_i2_orf1	14-3-3 protein epsilon [Gallus gallus] >NP_001233297.1 14-3-3 protein epsilon [Pan troglodytes] >NP_006752.1 14-3-3 protein epsilon [Homo sapiens] >NP_033562.3 14-3-3 protein epsilon [Mus musculus] >NP_113791.2 14-3-3 protein epsilon [Rattus norvegicus] >NP_776916.1 14-3-3 protein epsilon [Bos taurus] >XP_001504337.1 14-3-3 protein epsilon isoform X1 [Equus caballus] >XP_002918088.2 14-3-3 protein epsilon isoform X2 [Ailuropoda melanoleuca] >XP_003416855.1 14-3-3 protein epsilon isoform X1 [Loxodonta africana] >XP_003469733.1 14-3-3 protein epsilon isoform X1 [Cavia porcellus] >XP_003816884.1 14-3-3 protein epsilon isoform X1 [Pan paniscus] >XP_003912098.1 14-3-3 protein epsilon isoform X1 [Papio anubis] >XP_003929381.1 14-3-3 protein epsilon isoform X1 [Saimiri boliviensis boliviensis] >XP_003996471.1 14-3-3 protein epsilon isoform X1 [Felis catus] >XP_004267124.1 14-3-3 protein epsilon isoform X1 [Orcinus orca] >XP_004376223.1 14-3-3 protein epsilon [Trichechus manatus latirostris] >XP_004404155.1 PREDICTED: 14-3-3 protein epsilon isoform X2 [Odobenus rosmarus divergens] >XP_004433380.1 PREDICTED: 14-3-3 protein epsilon isoform X1 [Ceratotherium simum simum] >XP_004483832.1 14-3-3 protein epsilon isoform X1 [Dasypus novemcinctus] >XP_004605045.1 PREDICTED: 14-3-3 protein epsilon [Sorex araneus] >XP_004667919.1 14-3-3 protein epsilon [Jaculus jaculus] >XP_004706944.1 14-3-3 protein epsilon [Echinops telfairi] >XP_004746947.1 14-3-3 protein epsilon isoform X1 [Mustela putorius furo] >XP_004857172.1 14-3-3 protein epsilon isoform X1 [Heterocephalus glaber] >XP_005067448.1 14-3-3 protein epsilon isoform X1 [Mesocricetus auratus] >XP_005240506.1 14-3-3 protein epsilon isoform X1 [Falco peregrinus] >XP_005327947.1 14-3-3 protein epsilon isoform X1 [Ictidomys tridecemlineatus] >XP_005349591.1 14-3-3 protein epsilon isoform X1 [Microtus ochrogaster] >XP_005402688.1 PREDICTED: 14-3-3 protein epsilon isoform X1 [Chinchilla lanigera] >XP_005525859.1 PREDICTED: 14-3-3 protein epsilon isoform X1 [Pseudopodoces humilis] >XP_005888292.1 PREDICTED: 14-3-3 protein epsilon isoform X1 [Bos mutus] >XP_006079841.1 14-3-3 protein epsilon isoform X1 [Bubalus bubalis] >XP_006099253.1 14-3-3 protein epsilon [Myotis lucifugus] >XP_006185046.1 14-3-3 protein epsilon isoform X1 [Camelus ferus] >XP_006214490.1 14-3-3 protein epsilon isoform X1 [Vicugna pacos] >XP_006259463.1 PREDICTED: 14-3-3 protein epsilon [Alligator mississippiensis] >XP_006768146.1 PREDICTED: 14-3-3 protein epsilon isoform X1 [Myotis davidii] >XP_006863283.1 PREDICTED: 14-3-3 protein epsilon [Chrysochloris asiatica] >XP_006891074.1 PREDICTED: 14-3-3 protein epsilon-like [Elephantulus edwardii] >XP_006925117.1 14-3-3 protein epsilon isoform X1 [Pteropus alecto] >XP_006977465.1 14-3-3 protein epsilon isoform X1 [Peromyscus maniculatus bairdii] >XP_007057769.1 14-3-3 protein epsilon isoform X1 [Chelonia mydas] >XP_007123613.1 14-3-3 protein epsilon isoform X1 [Physeter catodon] >XP_007183877.1 14-3-3 protein epsilon isoform X1 [Balaenoptera acutorostrata scammoni] >XP_007454293.1 PREDICTED: 14-3-3 protein epsilon [Lipotes vexillifer] >XP_007520478.1 PREDICTED: 14-3-3 protein epsilon [Erinaceus europaeus] >XP_007935626.1 14-3-3 protein epsilon [Orycteropus afer afer] >XP_008007997.1 14-3-3 protein epsilon isoform X1 [Chlorocebus sabaeus] >XP_008058985.1 14-3-3 protein epsilon isoform X2 [Carlito syrichta] >XP_008146090.1 14-3-3 protein epsilon isoform X1 [Eptesicus fuscus] >XP_008512998.1 PREDICTED: 14-3-3 protein epsilon isoform X1 [Equus przewalskii] >XP_008591162.1 PREDICTED: 14-3-3 protein epsilon [Galeopterus variegatus] >XP_008826988.1 14-3-3 protein epsilon [Nannospalax galili] >XP_009249385.2 14-3-3 protein epsilon isoform X1 [Pongo abelii] >XP_010371794.1 14-3-3 protein epsilon isoform X1 [Rhinopithecus roxellana] >XP_010571909.1 PREDICTED: 14-3-3 protein epsilon isoform X5 [Haliaeetus leucocephalus] >XP_010640470.1 14-3-3 protein epsilon [Fukomys damarensis] >XP_010859650.1 PREDICTED: 14-3-3 protein epsilon isoform X1 [Bison bison bison] >XP_010956513.1 14-3-3 protein epsilon isoform X1 [Camelus bactrianus] >XP_010977216.1 14-3-3 protein epsilon isoform X1 [Camelus dromedarius] >XP_011358192.1 14-3-3 protein epsilon [Pteropus vampyrus] >XP_011722448.1 14-3-3 protein epsilon isoform X1 [Macaca nemestrina] >XP_011811232.1 PREDICTED: 14-3-3 protein epsilon isoform X2 [Colobus	0.49802	0.41464	-1.7643	1.16898	-0.3173
TRINITY_DN3322_c0_g1_i2_orf1	myrosinase 1-like isoform X2 [Ostrinia furnacalis]	0.71554	0.98843	-1.5753	0.65023	-0.7789
TRINITY_DN1322_c0_g1_i4_orf1	putative histone-binding protein Caf1 [Papilio machaon]	1.03255	0.63198	-0.7556	0.67478	-1.5837
TRINITY_DN14217_c0_g1_i1_orf1	putative serine protease K12H4.7 [Ostrinia furnacalis] >XP_028166339.1 putative serine protease K12H4.7 [Ostrinia furnacalis]	1.03265	0.82685	-1.3884	0.54269	-1.0138
TRINITY_DN23838_c0_g1_i4_orf1	ubiquitin domain-containing protein 2 isoform X1 [Ostrinia furnacalis] >XP_028177862.1 ubiquitin domain-containing protein 2 isoform X2 [Ostrinia furnacalis] >XP_028177863.1 ubiquitin domain-containing protein 2 isoform X1 [Ostrinia furnacalis]	0.77032	0.80498	-1.1379	0.86945	-1.3068
TRINITY_DN13999_c0_g1_i4_orf1	LOW QUALITY PROTEIN: succinate--hydroxymethylglutarate CoA-transferase-like [Ostrinia furnacalis]	0.58016	1.03441	-1.3303	0.80214	-1.0864
TRINITY_DN60821_c0_g1_i1_orf1	nucleolar GTP-binding protein 2 [Ostrinia furnacalis]	0.44893	1.36182	-1.6372	0.2656	-0.4391
TRINITY_DN48694_c0_g1_i1_orfp1	TRINITY_DN48694_c0_g1_i1_m.75338 TRINITY_DN48694_c0_g1::TRINITY_DN48694_c0_g1_i1::g.75338 ORF type:internal len:84 (+),score=16.02 TRINITY_DN48694_c0_g1_i1:2-250(+)	0.87221	-0.5309	1.04725	0.27953	-1.6681
TRINITY_DN100208_c0_g1_i1_orf1	neurofilament heavy polypeptide-like isoform X2 [Ostrinia furnacalis]	1.13013	-0.1176	0.67443	0.11339	-1.8004
TRINITY_DN526_c0_g1_i1_orf1	secretory phospholipase A2 receptor-like [Ostrinia furnacalis]	0.47858	-0.2434	1.41687	-0.0076	-1.6444
TRINITY_DN49221_c0_g1_i1_orf1	Similar to ND-23: NADH dehydrogenase (ubiquinone) 23 kDa subunit (Drosophila melanogaster) [Cotesia congregata]	0.19768	0.69168	1.32379	-0.7317	-1.4814
TRINITY_DN2499_c0_g1_i4_orf1	WD repeat-containing protein 92 isoform X1 [Ostrinia furnacalis]	0.3365	-0.2159	1.50001	-0.0113	-1.6094
TRINITY_DN8136_c0_g1_i1_orf1	HIG1 domain family member 2A, mitochondrial [Ostrinia furnacalis]	0.10459	0.40848	1.48866	-0.4519	-1.5498
TRINITY_DN3469_c0_g1_i4_orf1	LOW QUALITY PROTEIN: cadherin-87A-like [Ostrinia furnacalis]	0.34348	0.27493	1.41615	-0.4126	-1.6219
TRINITY_DN52244_c1_g1_i1_orf1	triokinase/FMN cyclase-like isoform X2 [Ostrinia furnacalis]	0.39129	0.03859	1.39091	-0.1188	-1.702
TRINITY_DN3307_c1_g1_i2_orf1	BTB/POZ domain-containing protein 2-like [Ostrinia furnacalis]	-0.1915	0.71451	1.48134	-0.6489	-1.3555
TRINITY_DN195_c8_g1_i1_orf1	hypothetical protein evm_009768 [Chilo suppressalis]	-0.1835	0.53551	1.64002	-1.0497	-0.9423
TRINITY_DN33452_c0_g1_i3_orf1	lethal(2) giant larvae protein isoform X8 [Ostrinia furnacalis]	-0.173	1.00146	1.21644	-0.5771	-1.4678
TRINITY_DN1575_c0_g1_i10_orf1	uncharacterized protein LOC114359245 [Ostrinia furnacalis]	0.42138	-1.3792	1.18157	0.7539	-0.9777
TRINITY_DN1466_c0_g1_i4_orf1	insecticyanin-A-like [Ostrinia furnacalis]	0.46605	-1.656	0.84162	0.97152	-0.6232
TRINITY_DN85290_c0_g2_i1_orf1	unnamed protein product, partial [Brenthis ino]	-0.2139	-1.3149	1.02085	1.26744	-0.7595
TRINITY_DN16316_c0_g1_i7_orf1	exocyst complex component 6 isoform X1 [Ostrinia furnacalis] >XP_028166671.1 exocyst complex component 6 isoform X2 [Ostrinia furnacalis]	0.83452	-1.5189	0.81025	0.75319	-0.8791
TRINITY_DN33418_c0_g1_i1_orf1	homer protein homolog 2-like [Ostrinia furnacalis]	0.79102	1.37092	0.02018	-1.3294	-0.8527
TRINITY_DN24266_c0_g2_i2_orf1	chromobox-like protein 5 [Helicoverpa armigera]	1.21237	0.72452	0.42261	-1.3264	-1.0331
TRINITY_DN7473_c0_g1_i1_orf1	zinc finger protein 664-like [Ostrinia furnacalis]	1.15069	0.95964	0.04301	-0.6104	-1.5429
TRINITY_DN32359_c0_g2_i1_orf1	PREDICTED: 26S proteasome non-ATPase regulatory subunit 4 isoform X2 [Fopius arisanus]	1.13558	0.66803	0.55225	-1.4814	-0.8745
TRINITY_DN12576_c0_g1_i2_orf1	eukaryotic translation initiation factor 4E transporter-like isoform X5 [Hyposmocoma kahamanoa]	1.32888	1.09421	-0.7631	-1.0258	-0.6342
TRINITY_DN5162_c0_g1_i3_orf1	steroidogenic acute regulatory protein-like [Ostrinia furnacalis]	1.12297	0.57223	0.70765	-1.3107	-1.0921
TRINITY_DN14677_c0_g2_i3_orf1	AP-3 complex subunit beta-2 [Ostrinia furnacalis]	1.15998	0.51627	0.6394	-1.5438	-0.7718
TRINITY_DN10110_c1_g2_i1_orf1	venom allergen 3-like [Ostrinia furnacalis]	0.94586	0.51602	0.83929	-1.644	-0.6571
TRINITY_DN14944_c0_g1_i7_orf1	alpha-tocopherol transfer protein-like isoform X1 [Ostrinia furnacalis] >XP_028158173.1 alpha-tocopherol transfer protein-like isoform X1 [Ostrinia furnacalis] >XP_028158174.1 alpha-tocopherol transfer protein-like isoform X1 [Ostrinia furnacalis]	1.14713	0.57795	0.66834	-1.0568	-1.3366

TRINITY_DN11464_c0_g1_i3_orf1	unnamed protein product [Spodoptera littoralis] >CAH1635924.1 unnamed protein product [Spodoptera littoralis]	1.29919	1.13619	-0.6623	-0.9595	-0.8136
TRINITY_DN145448_c0_g1_i1_orf1	GSCOCG00008786001-RA-CDS [Cotesia congregata] >CAG5104482.1 Similar to ubl4a: Ubiquitin-like protein 4A (Danio rerio) [Cotesia congregata]	1.41872	0.95071	-0.5219	-0.6953	-1.1522
TRINITY_DN54586_c1_g1_i1_orf1	protein YIPF5 [Ostrinia furnacalis]	1.213	0.60293	0.56167	-1.2963	-1.0813
TRINITY_DN6914_c0_g1_i2_orf1	atypical protein kinase C isoform X2 [Spodoptera litura] >XP_035444071.1 LOW QUALITY PROTEIN: atypical protein kinase C-like [Spodoptera frugiperda]	1.06406	1.34177	-1.0519	-0.528	-0.826
TRINITY_DN629_c0_g1_i6_orf1	annexin B9-like isoform X1 [Ostrinia furnacalis]	1.05934	0.65815	0.64748	-0.8442	-1.5207
TRINITY_DN3335_c0_g1_i1_orf1	unnamed protein product [Pieris macdunnoughi]	0.83144	0.50244	1.02287	-1.5196	-0.8372
TRINITY_DN25686_c0_g1_i4_orf1	exocyst complex component 3 [Ostrinia furnacalis]	1.26353	1.1787	-0.8984	-0.8595	-0.6843
TRINITY_DN2160_c0_g1_i13_orf1	unnamed protein product [Spodoptera exigua]	1.08311	1.32306	-0.9132	-0.9994	-0.4935
TRINITY_DN19260_c0_g1_i5_orf1	probable 26S proteasome non-ATPase regulatory subunit 3 [Ostrinia furnacalis]	0.84136	0.80779	0.76368	-1.4761	-0.9367
TRINITY_DN28622_c0_g1_i1_orf1	actin-related protein 3 [Ostrinia furnacalis]	1.31412	1.06613	-0.5185	-1.1903	-0.6715
TRINITY_DN1921_c1_g1_i5_orf1	hypothetical protein evm_002627 [Chilo suppressalis] >CAB3527269.1 unnamed protein product [Chilo suppressalis]	1.21824	1.22575	-0.9316	-0.7254	-0.787
TRINITY_DN1552_c0_g1_i3_orf1	casein kinase II subunit alpha isoform X3 [Galleria mellonella]	1.18128	1.19655	-0.3622	-0.9361	-1.0795
TRINITY_DN9146_c0_g1_i1_orf1	drebrin-like protein [Ostrinia furnacalis]	1.21321	1.2249	-0.6632	-0.9674	-0.8075
TRINITY_DN1781_c0_g1_i8_orf1	transportin-1 [Pectinophora gossypiella]	1.2295	0.60887	0.52092	-1.002	-1.3573
TRINITY_DN3667_c0_g1_i4_orf1	coronin-2B-like isoform X2 [Ostrinia furnacalis]	0.7544	0.31086	1.2289	-0.8368	-1.4573
TRINITY_DN471_c0_g1_i6_orf1	ATP-dependent RNA helicase DBP2-A-like [Ostrinia furnacalis]	1.23758	1.08344	-0.5139	-0.4577	-1.3494
TRINITY_DN21559_c0_g2_i1_orf1	protein bicaudal D isoform X3 [Galleria mellonella]	1.24411	1.18586	-1.0375	-0.7024	-0.6901
TRINITY_DN24539_c0_g1_i4_orf1	low molecular weight phosphotyrosine protein phosphatase 1-like isoform X2 [Ostrinia furnacalis]	1.25319	1.06588	-0.2885	-0.7428	-1.2878
TRINITY_DN23946_c0_g1_i1_orf1	ubiquitin-conjugating enzyme E2L [Bombyx mori] >XP_013145013.1 PREDICTED: ubiquitin-conjugating enzyme E2 L3 [Papilio polytes] >XP_013145026.1 PREDICTED: ubiquitin-conjugating enzyme E2 L3 [Papilio polytes] >XP_013167448.1 PREDICTED: ubiquitin-conjugating enzyme E2 L3 [Papilio xuthus] >XP_013167449.1 PREDICTED: ubiquitin-conjugating enzyme E2 L3 [Papilio xuthus] >XP_014356324.1 ubiquitin-conjugating enzyme E2 L3 [Papilio machaon] >XP_021182538.1 ubiquitin-conjugating enzyme E2 L3 [Helicoverpa armigera] >XP_022831771.1 ubiquitin-conjugating enzyme E2 L3 [Spodoptera litura] >XP_023943862.1 ubiquitin-conjugating enzyme E2 L3 [Bicyclus anynana] >XP_026501320.1 ubiquitin-conjugating enzyme E2 L3 [Vanessa tameamea] >XP_026740672.1 ubiquitin-conjugating enzyme E2 L3 [Trichoplusia ni] >XP_030021931.1 ubiquitin-conjugating enzyme E2 L3 [Manduca sexta] >XP_035448309.1 ubiquitin-conjugating enzyme E2 L3 [Spodoptera frugiperda] >XP_038214502.1 ubiquitin-conjugating enzyme E2 L3 [Zerene cesonia] >XP_045453873.1 ubiquitin-conjugating enzyme E2 L3 [Melitaea cinxia] >XP_045503683.1 ubiquitin-conjugating enzyme E2 L3 [Colias croceus] >XP_045771134.1 ubiquitin-conjugating enzyme E2 L3 [Maniola jurtina] >XP_046963941.1 ubiquitin-conjugating enzyme E2 L3 [Vanessa cardui] >XP_047032812.1 ubiquitin-conjugating enzyme E2 L3 [Helicoverpa zea] >XP_047538650.1 ubiquitin-conjugating enzyme E2 L3 [Vanessa atalanta] >XP_050352992.1 ubiquitin-conjugating enzyme E2 L3 [Nymphalis io] >KAF9417333.1 hypothetical protein HW555_005549 [Spodoptera exigua] >CAB3511518.1 unnamed protein product [Spodoptera littoralis] >CAH0595358.1 unnamed protein product [Chrysodeixis includens] >ABB36655.1 ubiquitin-conjugating enzyme E2I [Bombyx mori] >ABF51360.1 ubiquitin-conjugating enzyme [Bombyx mori]	1.18842	1.25849	-0.8717	-0.7467	-0.8286
TRINITY_DN879_c0_g1_i2_orf1	DNA-directed RNA polymerase I subunit RPA12 [Ostrinia furnacalis]	0.83239	1.42764	-1.2527	-0.1936	-0.8137
TRINITY_DN14477_c0_g1_i12_orf1	PREDICTED: phosphoribosyl pyrophosphate synthase-associated protein 2 isoform X2 [Amyelois transitella]	1.22245	1.16886	-1.1913	-0.5907	-0.6094
TRINITY_DN934_c2_g1_i7_orf1	ubiquitin-40S ribosomal protein S27a [Ostrinia furnacalis]	1.16847	1.21232	-0.4524	-1.1889	-0.7396
TRINITY_DN1045_c0_g1_i6_orf1	ornithine decarboxylase 1-like isoform X1 [Ostrinia furnacalis]	0.95949	1.40277	-0.9024	-1.0703	-0.3896
TRINITY_DN4439_c0_g2_i1_orf1	unnamed protein product, partial [Brenthis ino]	1.35833	1.02798	-0.5577	-1.1544	-0.6742
TRINITY_DN89829_c0_g1_i1_orf1	PREDICTED: ubiquitin-conjugating enzyme E2 T [Microplitis demolitor]	1.0219	1.33535	-0.3785	-1.1785	-0.8002
TRINITY_DN1691_c0_g1_i3_orf1	uncharacterized protein LOC114354192 isoform X2 [Ostrinia furnacalis]	1.14636	1.29639	-0.8531	-0.8784	-0.7112
TRINITY_DN2984_c0_g1_i3_orf1	connectin-like [Ostrinia furnacalis]	1.22168	1.18247	-0.5124	-1.1152	-0.7765
TRINITY_DN42171_c0_g1_i1_orf1	amyloid-beta-like protein isoform X1 [Manduca sexta] >AAY25024.3 beta amyloid protein precursor-like protein [Manduca sexta]	1.39305	1.01318	-0.6016	-1.0483	-0.7564
TRINITY_DN105359_c0_g2_i5_orf1	uncharacterized protein LOC114359499 [Ostrinia furnacalis]	1.02222	0.79801	0.40598	-1.6942	-0.532
TRINITY_DN397_c0_g1_i1_orf1	striatin-3 isoform X1 [Ostrinia furnacalis]	1.22499	1.16791	-0.5696	-1.1843	-0.639
TRINITY_DN942_c0_g1_i1_orf1	probable nuclear transport factor 2 isoform X1 [Ostrinia furnacalis]	1.27419	1.13645	-0.6989	-1.1122	-0.5995
TRINITY_DN33485_c0_g1_i4_orf1	luciferin 4-monooxygenase-like [Ostrinia furnacalis]	0.82107	1.28525	-0.0067	-0.5651	-1.5345
TRINITY_DN1661_c0_g1_i1_orf1	NAD(P) transhydrogenase, mitochondrial-like [Ostrinia furnacalis] >XP_028175067.1 NAD(P) transhydrogenase, mitochondrial-like [Ostrinia furnacalis] >XP_028175068.1 NAD(P) transhydrogenase, mitochondrial-like [Ostrinia furnacalis] >XP_028175069.1 NAD(P) transhydrogenase, mitochondrial-like [Ostrinia furnacalis]	1.17694	1.18743	-0.5447	-1.2652	-0.5545
TRINITY_DN518_c0_g1_i1_orf1	unnamed protein product [Arctia plantaginis]	1.1675	1.25132	-0.5892	-1.0733	-0.7563
TRINITY_DN662_c0_g1_i1_orf1	PREDICTED: actin-related protein 2 [Amyelois transitella]	0.87448	1.41755	-0.631	-0.3571	-1.3039
TRINITY_DN383_c0_g1_i1_orf1	probable Golgi SNAP receptor complex member 2 [Ostrinia furnacalis]	1.0129	1.33763	-0.7523	-1.2124	-0.3858
TRINITY_DN38667_c0_g1_i9_orf1	signal transducer and activator of transcription 5B-like [Melitaea cinxia]	0.8085	1.3957	-0.044	-0.9025	-1.2577
TRINITY_DN23790_c0_g1_i1_orf1	wiskott-Aldrich syndrome protein family member 2 [Ostrinia furnacalis]	0.89355	1.37348	-0.405	-1.3879	-0.4741
TRINITY_DN12064_c0_g2_i1_orf1	ras-related protein Rab-35 [Ostrinia furnacalis]	0.92041	1.07499	-0.1013	-0.1747	-1.7194
TRINITY_DN41664_c0_g1_i4_orf1	uncharacterized protein LOC114356631 [Ostrinia furnacalis]	0.40242	1.08079	0.87992	-1.409	-0.9541
TRINITY_DN41736_c0_g2_i1_orf1	calcyclin-binding protein [Ostrinia furnacalis]	1.03607	1.39246	-0.8121	-0.7041	-0.9123
TRINITY_DN2475_c0_g2_i1_orf1	uncharacterized protein LOC114363767 [Ostrinia furnacalis]	1.1455	1.26451	-1.0831	-0.5304	-0.7965
TRINITY_DN50225_c0_g1_i1_orf1	SRSF protein kinase 3 [Galleria mellonella]	1.14749	1.26827	-0.5475	-0.812	-1.0562
TRINITY_DN9062_c0_g2_i3_orf1	ubiquitin conjugation factor E4 B isoform X2 [Ostrinia furnacalis]	1.205	1.23562	-0.7353	-0.9678	-0.7375
TRINITY_DN1554_c0_g1_i9_orf1	LOW QUALITY PROTEIN: puff-specific protein Bx42 [Ostrinia furnacalis]	1.22435	1.18985	-1.0344	-0.512	-0.8679
TRINITY_DN8596_c0_g1_i2_orf1	SWI/SNF-related matrix-associated actin-dependent regulator of chromatin subfamily E member 1-like isoform X2 [Ostrinia furnacalis]	1.24594	1.05962	-1.3612	-0.5865	-0.3579
TRINITY_DN46_c0_g1_i2_orf1	disks large 1 tumor suppressor protein isoform X12 [Ostrinia furnacalis]	0.82754	1.48818	-0.5558	-0.5314	-1.2285
TRINITY_DN4494_c0_g1_i1_orf1	venom serine carboxypeptidase [Ostrinia furnacalis]	1.34211	0.84329	0.03876	-1.0325	-1.1916
TRINITY_DN749_c0_g1_i1_orf1	serine/threonine-protein phosphatase 4 regulatory subunit 3 isoform X3 [Ostrinia furnacalis]	1.2125	1.2095	-0.8128	-1.0408	-0.5684

TRINITY_DN43076_c0_g1_i6_orf1	protein argonaute-2 isoform X2 [Pectinophora gossypiella]	0.99997	1.30249	-0.2318	-1.2656	-0.805
TRINITY_DN7247_c0_g1_i7_orf1	pyruvate kinase-like isoform X3 [Ostrinia furnacalis]	0.80446	1.44685	-0.4864	-1.3649	-0.4001
TRINITY_DN44335_c0_g1_i7_orf1	hypothetical protein SFRURICE_000584 [Spodoptera frugiperda]	0.79142	1.54291	-0.5906	-1.1208	-0.6229
TRINITY_DN42205_c0_g1_i4_orf1	eukaryotic translation initiation factor 4H [Ostrinia furnacalis]	0.86669	1.51424	-0.9005	-0.897	-0.5834
TRINITY_DN34479_c0_g1_i2_orf1	PREDICTED: 26S protease regulatory subunit 4 [Amyelois transitella] >XP_021186380.1 26S proteasome regulatory subunit 4 [Helicoverpa armigera] >XP_022116536.1 26S proteasome regulatory subunit 4 [Pieris rapae] >XP_022817854.1 26S proteasome regulatory subunit 4 [Spodoptera litura] >XP_026745369.1 26S proteasome regulatory subunit 4 [Trichoplusia ni] >XP_026760570.1 26S proteasome regulatory subunit 4 [Galleria mellonella] >XP_028176505.1 26S proteasome regulatory subunit 4 [Ostrinia furnacalis] >XP_030038234.1 26S proteasome regulatory subunit 4 [Manduca sexta] >XP_035449919.1 26S proteasome regulatory subunit 4 [Spodoptera frugiperda] >XP_038206559.1 26S proteasome regulatory subunit 4 [Zerene cesonia] >XP_045502541.1 26S proteasome regulatory subunit 4 [Colias croceus] >XP_045532999.1 26S proteasome regulatory subunit 4 [Pieris brassicae] >XP_047033702.1 26S proteasome regulatory subunit 4 [Helicoverpa zea] >XP_047994509.1 26S proteasome regulatory subunit 4 [Leguminivora glycinivorella] >XP_049877826.1 26S proteasome regulatory subunit 4 [Pectinophora gossypiella] >KAH9639287.1 hypothetical protein HF086_014151 [Spodoptera exigua] >KAI5631153.1 ATPase family associated with various cellular activities (AAA) domain-containing protein [Phthorimaea operculella] >RVE50066.1 hypothetical protein evm_005272 [Chilo suppressalis] >CAB3245712.1 unnamed protein product [Arctia plantaginis] >KAF9801312.1 hypothetical protein SFRURICE_000406 [Spodoptera frugiperda]	0.52597	1.09943	0.73405	-1.4898	-0.8697
TRINITY_DN40211_c0_g1_i1_orf1	rho GTPase-activating protein 44-like [Ostrinia furnacalis]	0.61594	1.63112	-0.4593	-0.6191	-1.1687
TRINITY_DN49047_c0_g1_i2_orf1	unnamed protein product [Parnassius apollo]	0.67305	1.00968	0.59295	-1.6794	-0.5963
TRINITY_DN11876_c0_g1_i2_orf1	protein TAPT1 homolog [Ostrinia furnacalis]	0.80148	1.56643	-0.713	-0.713	-0.942
TRINITY_DN4439_c0_g1_i2_orf1	cytoplasmic FMR1-interacting protein isoform X1 [Ostrinia furnacalis] >XP_028169436.1 cytoplasmic FMR1-interacting protein isoform X2 [Ostrinia furnacalis]	1.11537	1.23427	-0.2753	-0.9859	-1.0884
TRINITY_DN17935_c0_g1_i1_orf1	NEDD8-conjugating enzyme Ubc12 [Ostrinia furnacalis]	0.90037	1.46637	-0.9515	-0.9648	-0.4504
TRINITY_DN122867_c1_g1_i1_orf1	nuclear migration protein nudC [Ostrinia furnacalis]	0.91471	1.42466	-1.0781	-0.9272	-0.334
TRINITY_DN7670_c0_g1_i1_orf1	striatin-interacting protein 1 [Ostrinia furnacalis]	1.14576	1.29118	-0.9214	-0.8642	-0.6513
TRINITY_DN2919_c0_g1_i5_orf1	nidogen-1 [Ostrinia furnacalis]	0.80799	1.18571	0.35735	-1.3334	-1.0177
TRINITY_DN27398_c0_g1_i3_orf1	lissencephaly-1 homolog [Helicoverpa armigera] >XP_021185428.1 lissencephaly-1 homolog [Helicoverpa armigera] >XP_047030140.1 lissencephaly-1 homolog [Helicoverpa zea] >XP_047030141.1 lissencephaly-1 homolog [Helicoverpa zea]	0.79864	0.97683	0.65681	-1.1197	-1.3126
TRINITY_DN6602_c0_g1_i4_orf1	PREDICTED: E3 ubiquitin-protein ligase RNF181-like [Amyelois transitella]	0.7994	0.9943	0.34035	-1.7668	-0.3672
TRINITY_DN198_c0_g1_i2_orf1	retinol dehydrogenase 13-like [Ostrinia furnacalis]	0.79039	0.59399	1.04043	-1.2198	-1.205
TRINITY_DN146364_c0_g1_i1_orf1	pupal cuticle protein 20-like [Ostrinia furnacalis]	0.0284	1.28234	0.63568	-0.2464	-1.7
TRINITY_DN4686_c0_g2_i1_orf1	lysophospholipase-like protein 1 [Ostrinia furnacalis]	1.16903	1.24847	-0.6593	-1.0964	-0.6618
TRINITY_DN4403_c0_g1_i3_orf1	AP-1 complex subunit gamma-1 [Ostrinia furnacalis]	0.98402	1.43708	-0.8208	-0.7216	-0.8786
TRINITY_DN7828_c0_g1_i2_orf1	alpha-N-acetylgalactosaminidase-like isoform X1 [Ostrinia furnacalis] >XP_028171449.1 alpha-N-acetylgalactosaminidase-like isoform X2 [Ostrinia furnacalis]	1.15496	1.28233	-0.7875	-0.9675	-0.6823
TRINITY_DN7560_c0_g1_i4_orf1	unnamed protein product [Chilo suppressalis]	0.84005	1.36737	-0.0056	-1.1166	-1.0852
TRINITY_DN802_c0_g1_i2_orf1	active breakpoint cluster region-related protein [Ostrinia furnacalis]	1.07602	1.27153	-0.2911	-0.9137	-1.1427
TRINITY_DN4757_c0_g1_i3_orf1	melanotransferrin isoform X1 [Ostrinia furnacalis] >XP_028175370.1 melanotransferrin isoform X2 [Ostrinia furnacalis] >XP_028175371.1 melanotransferrin isoform X3 [Ostrinia furnacalis]	1.25286	1.1888	-0.897	-0.8711	-0.6736
TRINITY_DN11375_c0_g1_i6_orf1	uncharacterized protein LOC114363514 isoform X2 [Ostrinia furnacalis]	1.19351	1.18958	-1.0876	-0.9108	-0.3847
TRINITY_DN5346_c0_g1_i5_orf1	syntaxin-1A isoform X2 [Pectinophora gossypiella]	0.87033	1.04862	0.49038	-1.209	-1.2003
TRINITY_DN2812_c0_g1_i5_orf1	myotubularin-related protein 2 [Ostrinia furnacalis] >XP_028170267.1 myotubularin-related protein 2 [Ostrinia furnacalis]	0.99009	1.39192	-0.5577	-0.6807	-1.1436
TRINITY_DN15753_c0_g1_i1_orf1	uncharacterized protein LOC114366450 [Ostrinia furnacalis]	1.04971	1.28076	-0.2779	-0.8339	-1.2186
TRINITY_DN15762_c0_g1_i2_orf1	YTH domain-containing family protein 3 isoform X3 [Maniola hyperantus]	0.49457	1.45166	0.30781	-1.2076	-1.0464
TRINITY_DN3179_c0_g1_i1_orf1	diamine acetyltransferase 2-like [Ostrinia furnacalis]	0.71553	1.56226	-0.344	-1.139	-0.7949
TRINITY_DN10131_c0_g1_i7_orf1	aldo-keto reductase AKR2E4-like [Ostrinia furnacalis]	1.37075	1.04157	-0.6836	-1.0576	-0.6712
TRINITY_DN26168_c0_g1_i1_orf1	ATP-dependent RNA helicase Ddx1-like [Ostrinia furnacalis]	0.66818	1.5892	-0.4104	-1.2008	-0.6462
TRINITY_DN5952_c0_g1_i6_orf1	LOW QUALITY PROTEIN: phosphoacetylglucosamine mutase [Ostrinia furnacalis]	0.98347	1.41135	-0.5878	-1.0805	-0.7265
TRINITY_DN14507_c0_g1_i5_orf1	PTB domain-containing adapter protein ced-6 [Ostrinia furnacalis]	1.24389	0.92095	0.09709	-0.995	-1.267
TRINITY_DN3838_c0_g1_i8_orf1	ER membrane protein complex subunit 2-like isoform X1 [Ostrinia furnacalis] >XP_028161204.1 ER membrane protein complex subunit 2-like isoform X2 [Ostrinia furnacalis] >XP_028161205.1 ER membrane protein complex subunit 2-like isoform X3 [Ostrinia furnacalis]	0.8283	1.29027	0.11066	-1.3904	-0.8388
TRINITY_DN2403_c0_g1_i3_orf1	FAD-dependent oxidoreductase domain-containing protein 1 [Ostrinia furnacalis]	1.06312	1.29116	-0.3147	-1.1286	-0.911
TRINITY_DN24490_c0_g1_i6_orf1	E3 ubiquitin-protein ligase Hakai [Ostrinia furnacalis]	1.07522	1.31083	-0.849	-1.1028	-0.4342
TRINITY_DN101358_c0_g2_i1_orf1	glycylpeptide N-tetradecanoyltransferase 2 [Ostrinia furnacalis]	0.54797	1.5537	0.01098	-0.8921	-1.2206
TRINITY_DN5525_c0_g1_i4_orf1	probable glucosamine 6-phosphate N-acetyltransferase [Ostrinia furnacalis]	0.62837	1.63912	-0.8032	-0.415	-1.0493
TRINITY_DN5442_c0_g1_i4_orf1	hypothetical protein evm_004688 [Chilo suppressalis]	0.92953	1.47048	-0.6486	-0.7777	-0.9738
TRINITY_DN1444_c1_g1_i5_orf1	spondin-1 isoform X1 [Ostrinia furnacalis] >XP_028167312.1 spondin-1 isoform X1 [Ostrinia furnacalis] >XP_028167313.1 spondin-1 isoform X1 [Ostrinia furnacalis] >XP_028167314.1 spondin-1 isoform X2 [Ostrinia furnacalis]	1.15787	1.23855	-0.5599	-1.1687	-0.6678
TRINITY_DN87170_c0_g1_i3_orf1	uncharacterized protein LOC114360175 [Ostrinia furnacalis]	1.14557	1.10539	-0.0602	-0.9188	-1.272
TRINITY_DN20339_c0_g1_i3_orf1	ecto-NOX disulfide-thiol exchanger 2-like [Ostrinia furnacalis]	0.80429	1.35072	0.07061	-1.2661	-0.9595
TRINITY_DN29743_c0_g1_i9_orf1	polyadenylate-binding protein 2 isoform X1 [Ostrinia furnacalis] >XP_028168980.1 polyadenylate-binding protein 2 isoform X2 [Ostrinia furnacalis]	0.956	1.31884	-0.147	-1.2389	-0.8889
TRINITY_DN288_c0_g1_i9_orf1	unnamed protein product [Chilo suppressalis]	0.96763	1.41743	-0.7551	-1.0943	-0.5357
TRINITY_DN6380_c0_g1_i1_orf1	THAP domain-containing protein 1-like isoform X1 [Ostrinia furnacalis]	0.76833	1.55023	-0.4178	-1.0632	-0.8376
TRINITY_DN2802_c1_g1_i1_orf1	psi [Ostrinia furnacalis]	0.99697	1.25928	-0.0682	-0.9897	-1.1984
TRINITY_DN4233_c0_g2_i2_orf1	actin-related protein 2/3 complex subunit 5-B [Ostrinia furnacalis]	0.87448	1.46555	-0.5137	-1.1923	-0.634
TRINITY_DN48973_c0_g1_i5_orf1	uncharacterized protein LOC114351683 isoform X8 [Ostrinia furnacalis]	0.54958	1.42033	-0.5401	0.11178	-1.5416
TRINITY_DN19493_c0_g1_i5_orf1	zinc finger MYM-type protein 3 isoform X1 [Ostrinia furnacalis] >XP_028159738.1 zinc finger MYM-type protein 3 isoform X2 [Ostrinia furnacalis]	1.06615	1.29326	-0.6063	-1.2551	-0.498

TRINITY_DN14734_c0_g1_i2_orf1	SET and MYND domain-containing protein 5 isoform X1 [Ostrinia furnacalis] >XP_028173610.1 SET and MYND domain-containing protein 5 isoform X2 [Ostrinia furnacalis]	0.89087	1.2421	-0.7624	0.07071	-1.4413
TRINITY_DN35377_c0_g1_i3_orf1	unnamed protein product [Chilo suppressalis]	0.73945	1.41846	-1.4473	-0.1385	-0.5722
TRINITY_DN1760_c0_g1_i4_orf1	uncharacterized protein LOC114357676 [Ostrinia furnacalis]	-0.0961	1.04127	0.46612	0.45486	-1.8661
TRINITY_DN6071_c0_g1_i1_orf1	transcription initiation factor IIB isoform X1 [Manduca sexta] >XP_038208211.1 transcription initiation factor IIB isoform X1 [Zerene cesonia] >XP_045510964.1 transcription initiation factor IIB isoform X1 [Colias croceus] >XP_049872283.1 transcription initiation factor IIB [Pectinophora gossypiella]	0.7522	1.09227	0.54597	-0.9879	-1.4025
TRINITY_DN146119_c0_g1_i1_orf1	protein SEC13 homolog [Ostrinia furnacalis]	0.78066	1.41704	-0.7172	-1.3616	-0.1188
TRINITY_DN987_c0_g1_i11_orf1	macrophage mannose receptor 1-like [Pieris napi]	0.41221	1.07314	-0.2234	0.55905	-1.821
TRINITY_DN39170_c0_g1_i4_orf1	unnamed protein product [Arctia plantaginis]	0.62901	1.37684	-1.122	0.2858	-1.1697
TRINITY_DN31967_c0_g1_i5_orf1	N-acetylgalactosamine kinase [Ostrinia furnacalis]	1.07664	1.35619	-0.9451	-0.7734	-0.7143
TRINITY_DN4908_c1_g1_i5_orf1	DNA topoisomerase 2 isoform X1 [Ostrinia furnacalis]	1.28543	1.15561	-0.7435	-0.9453	-0.7522
TRINITY_DN4152_c0_g1_i1_orf1	importin subunit beta-1 isoform X2 [Ostrinia furnacalis]	0.87763	1.49974	-0.5603	-0.8199	-0.9972
TRINITY_DN50875_c0_g1_i3_orf1	conserved oligomeric Golgi complex subunit 8 [Ostrinia furnacalis]	1.19502	1.15983	-0.5589	-1.2863	-0.5096
TRINITY_DN30150_c0_g1_i7_orf1	unnamed protein product [Chrysodeixis includens]	0.89813	1.38226	-0.9791	-0.1621	-1.1392
TRINITY_DN11820_c0_g1_i1_orf1	hypothetical protein evm_000341 [Chilo suppressalis]	0.89	1.2244	-0.1242	-1.5921	-0.398
TRINITY_DN23616_c0_g1_i4_orf1	ribosome biogenesis protein NSA2 homolog [Ostrinia furnacalis] >CAG9749295.1 unnamed protein product [Diatraea saccharalis] >CAG9787980.1 unnamed protein product [Diatraea saccharalis]	0.35293	1.62223	-1.09	0.13358	-1.0188
TRINITY_DN783_c0_g1_i7_orf1	microtubule-associated protein Jupiter isoform X4 [Helicoverpa armigera]	1.28544	1.14845	-0.701	-1.0004	-0.7325
TRINITY_DN32601_c0_g1_i2_orf1	uncharacterized protein LOC114363197 [Ostrinia furnacalis]	1.33724	1.08832	-0.7628	-1.0091	-0.6537
TRINITY_DN38424_c0_g1_i1_orf1	glucose dehydrogenase [FAD, quinone]-like [Ostrinia furnacalis]	0.80229	1.00217	0.41888	-1.7053	-0.518
TRINITY_DN20185_c0_g1_i6_orf1	zinc finger protein on ecdysone puffs [Ostrinia furnacalis]	0.94178	1.39723	-0.3056	-1.0227	-1.0106
TRINITY_DN43328_c0_g1_i1_orf1	tubulin--tyrosine ligase-like protein 12 [Ostrinia furnacalis]	1.27984	1.14772	-0.7062	-1.0402	-0.6811
TRINITY_DN37923_c0_g1_i1_orf1	hypothetical protein NE865_05974 [Phthorimaea operculella]	1.01026	1.32131	-0.2704	-1.1651	-0.8961
TRINITY_DN19079_c0_g1_i5_orf1	unnamed protein product [Euphydryas editha]	0.54989	1.63999	-0.2116	-1.0459	-0.9324
TRINITY_DN493_c0_g1_i4_orf1	ADP-ribosylation factor GTPase-activating protein 3 [Ostrinia furnacalis]	0.43613	1.637	-0.0532	-1.2187	-0.8012
TRINITY_DN2953_c1_g1_i11_orf1	methionine--tRNA ligase, cytoplasmic isoform X2 [Ostrinia furnacalis] >XP_028156683.1 methionine--tRNA ligase, cytoplasmic isoform X4 [Ostrinia furnacalis] >XP_028156684.1 methionine--tRNA ligase, cytoplasmic isoform X5 [Ostrinia furnacalis]	0.43177	1.74772	-0.7584	-0.4155	-1.0056
TRINITY_DN53115_c0_g1_i1_orf1	small glutamine-rich tetratricopeptide repeat-containing protein beta-like [Ostrinia furnacalis]	0.582	1.69032	-0.5224	-0.8699	-0.8801
TRINITY_DN279_c0_g1_i10_orf1	RE1-silencing transcription factor-like isoform X1 [Ostrinia furnacalis]	1.25413	1.10248	-0.4128	-1.2477	-0.6961
TRINITY_DN3113_c1_g2_i1_orf1	short-chain dehydrogenase/reductase family 16C member 6-like [Ostrinia furnacalis] >XP_028174076.1 short-chain dehydrogenase/reductase family 16C member 6-like [Ostrinia furnacalis]	0.49419	1.68137	-0.2818	-1.1145	-0.7793
TRINITY_DN147475_c0_g1_i1_orf1	casein kinase II subunit beta, partial [Rhincodon typus]	1.15026	1.19206	-0.2883	-1.205	-0.849
TRINITY_DN44877_c0_g1_i2_orf1	U6 snRNA-associated Sm-like protein LSm7 [Diachasma alloeum]	0.81448	1.46174	-0.246	-1.2132	-0.817
TRINITY_DN3984_c0_g1_i4_orf1	uncharacterized protein LOC114360871 isoform X1 [Ostrinia furnacalis]	0.88277	1.41005	-0.7553	-0.2788	-1.2587
TRINITY_DN10502_c0_g1_i4_orf1	interleukin enhancer-binding factor 2 homolog [Ostrinia furnacalis]	0.70871	1.61346	-0.5847	-1.0157	-0.7219
TRINITY_DN9575_c0_g1_i1_orf1	uncharacterized protein LOC114351119 [Ostrinia furnacalis]	0.67233	1.40814	-0.1674	-1.5512	-0.3619
TRINITY_DN34689_c0_g1_i4_orf1	uncharacterized protein DDB_G0283357 isoform X13 [Helicoverpa armigera] >XP_049707197.1 uncharacterized protein DDB_G0283357 isoform X14 [Helicoverpa armigera] >XP_049707198.1 uncharacterized protein DDB_G0283357 isoform X15 [Helicoverpa armigera] >XP_049707199.1 uncharacterized protein DDB_G0283357 isoform X16 [Helicoverpa armigera] >XP_049707200.1 uncharacterized protein DDB_G0283357 isoform X17 [Helicoverpa armigera] >XP_049707201.1 uncharacterized protein DDB_G0283357 isoform X18 [Helicoverpa armigera] >XP_049707202.1 uncharacterized protein DDB_G0283357 isoform X19 [Helicoverpa armigera] >XP_049707203.1 uncharacterized protein DDB_G0283357 isoform X20 [Helicoverpa armigera] >XP_049707204.1 uncharacterized protein DDB_G0283357 isoform X21 [Helicoverpa armigera] >XP_049707205.1 uncharacterized protein DDB_G0283357 isoform X22 [Helicoverpa armigera] >XP_049707206.1 uncharacterized protein DDB_G0283357 isoform X23 [Helicoverpa armigera] >XP_049707207.1 uncharacterized protein DDB_G0283357 isoform X24 [Helicoverpa armigera]	0.64305	1.6624	-0.5979	-0.9152	-0.7924
TRINITY_DN18681_c0_g1_i7_orf1	fragile X mental retardation syndrome-related protein 1 isoform X3 [Ostrinia furnacalis]	0.76072	1.48159	-0.1542	-1.1499	-0.9382
TRINITY_DN2802_c0_g1_i1_orf1	far upstream element-binding protein 1 isoform X3 [Ostrinia furnacalis]	0.78872	1.52149	-0.3841	-1.137	-0.7892
TRINITY_DN132043_c0_g1_i1_orf1	ankyrin repeat and MYND domain-containing protein 2 [Ostrinia furnacalis]	0.57654	1.67492	-0.4287	-1.0042	-0.8186
TRINITY_DN9637_c0_g1_i14_orf1	zinc finger protein swm isoform X3 [Ostrinia furnacalis]	0.98239	1.43399	-0.6589	-0.8727	-0.8848
TRINITY_DN2600_c0_g1_i7_orf1	mucin-5AC isoform X2 [Ostrinia furnacalis]	1.29529	1.12898	-0.7197	-1.0483	-0.6562
TRINITY_DN8290_c0_g1_i3_orf1	zinc finger CCHC domain-containing protein 8 homolog [Ostrinia furnacalis]	0.49477	1.59511	-0.0792	-1.3079	-0.7028
TRINITY_DN10297_c0_g1_i1_orf1	polyglutamine-binding protein 1 [Ostrinia furnacalis]	1.08533	1.27596	-0.8964	-0.334	-1.1309
TRINITY_DN8980_c0_g1_i2_orf1	putative ATP-dependent RNA helicase me31b [Ostrinia furnacalis] >XP_028162602.1 putative ATP-dependent RNA helicase me31b [Ostrinia furnacalis] >XP_028162603.1 putative ATP-dependent RNA helicase me31b [Ostrinia furnacalis]	0.39757	1.7828	-0.662	-0.5686	-0.9497
TRINITY_DN9938_c0_g2_i1_orf1	hypothetical protein E2986_04423 [Frieseomelitta varia]	0.60334	1.57887	-0.3107	-1.3201	-0.5514
TRINITY_DN2402_c1_g1_i8_orf1	interferon regulatory factor 2-binding protein 1 [Ostrinia furnacalis]	0.71919	1.19674	0.26062	-0.5342	-1.6423
TRINITY_DN18937_c0_g1_i1_orf1	uncharacterized protein LOC114351683 isoform X7 [Ostrinia furnacalis]	0.48459	1.21932	-0.0299	0.13158	-1.8056
TRINITY_DN21218_c0_g1_i4_orf1	leukotriene A-4 hydrolase isoform X2 [Ostrinia furnacalis]	0.83107	1.5094	-0.9565	-0.4105	-0.9735
TRINITY_DN17271_c0_g1_i1_orf1	uncharacterized protein LOC114350693 [Ostrinia furnacalis]	1.14092	1.23102	-0.3845	-1.1671	-0.8203
TRINITY_DN3856_c0_g1_i7_orf1	uncharacterized protein LOC114355702 [Ostrinia furnacalis]	0.52315	1.67893	-0.5522	-1.1724	-0.4774
TRINITY_DN6871_c0_g1_i3_orf1	1-acyl-sn-glycerol-3-phosphate acyltransferase gamma-like [Ostrinia furnacalis] >XP_028169689.1 1-acyl-sn-glycerol-3-phosphate acyltransferase gamma-like [Ostrinia furnacalis]	0.43051	1.61214	-0.7371	-0.0124	-1.2931

TRINITY_DN235_c0_g3_i1_orf1	actin, muscle-type A2 [Bombyx mori] >XP_013199497.1 PREDICTED: actin, muscle-type A2 [Amyelois transitella] >XP_021190684.1 actin, muscle-type A2 [Helicoverpa armigera] >XP_022837900.1 actin, muscle-type A2 [Spodoptera litura] >XP_026314060.1 actin, muscle-type A2 [Hyposmocoma kahamanoa] >XP_026738711.1 actin, muscle-type A2 [Trichoplusia ni] >XP_028179440.1 actin, muscle-type A2 [Ostrinia furnacalis] >XP_030030527.1 actin, muscle-type A2 [Manduca sexta] >XP_035439272.1 actin, muscle-type A2 [Spodoptera frugiperda] >XP_047029939.1 actin, muscle-type A2 [Helicoverpa zea] >XP_049873365.1 actin, muscle-type A2 [Pectinophora gossypiella] >P07837.1 RecName: Full=Actin, muscle-type A2; Flags: Precursor [Bombyx mori] >KAF9423784.1 hypothetical protein HW555_000842 [Spodoptera exigua] >QLI62214.1 actin [Streltzoviella insularis] >CAB3227390.1 unnamed protein product [Arctia plantaginis] >CAB3508892.1 unnamed protein product [Spodoptera littoralis] >CAB3520808.1 unnamed protein product [Chilo suppressalis] >CAG9748331.1 unnamed protein product [Diatraea saccharalis] >CAH0585396.1 unnamed protein product [Chrysodeixis includens] >GBP21118.1 Actin, muscle-type A2 [Eumeta japonica]	0.8164	1.45453	-0.3021	-1.2914	-0.6774
TRINITY_DN5562_c0_g1_i3_orf1	cell division cycle and apoptosis regulator protein 1-like [Ostrinia furnacalis]	0.63079	1.67789	-0.8295	-0.7882	-0.6909
TRINITY_DN25916_c0_g1_i1_orf1	uncharacterized protein LOC125063950 [Vanessa atalanta]	0.39166	1.23467	0.66733	-1.4978	-0.7958
TRINITY_DN4950_c0_g1_i2_orf1	unnamed protein product [Diatraea saccharalis]	0.73399	1.5643	-0.5301	-1.1756	-0.5925
TRINITY_DN30950_c0_g1_i13_orf1	unnamed protein product [Chilo suppressalis]	0.37533	1.64044	0.05061	-1.1569	-0.9095
TRINITY_DN19155_c0_g1_i1_orf1	cleavage and polyadenylation specificity factor 73 [Ostrinia furnacalis]	0.51656	1.70178	-0.4423	-0.7088	-1.0673
TRINITY_DN4659_c0_g1_i2_orf1	uncharacterized protein LOC114351134 [Ostrinia furnacalis]	1.26323	1.17886	-0.9464	-0.7607	-0.735
TRINITY_DN257_c0_g1_i7_orf1	zinc finger RNA-binding protein 2 [Ostrinia furnacalis]	0.70089	1.49379	-0.1459	-1.3049	-0.7438
TRINITY_DN21218_c0_g2_i3_orf1	leukotriene A-4 hydrolase isoform X2 [Ostrinia furnacalis]	0.39419	1.78678	-0.5507	-0.7154	-0.9149
TRINITY_DN6016_c0_g1_i8_orf1	hypothetical protein evm_010883 [Chilo suppressalis]	0.45713	1.08612	0.70201	-0.5758	-1.6694
TRINITY_DN92153_c0_g2_i2_orf1	methylenetetrahydrofolate reductase [Ostrinia furnacalis]	0.24122	1.20021	0.86269	-1.3785	-0.9256
TRINITY_DN2914_c0_g1_i1_orf1	U1 small nuclear ribonucleoprotein A [Ostrinia furnacalis]	0.52472	1.6458	-0.2543	-1.1983	-0.7179
TRINITY_DN1437_c0_g1_i6_orf1	nucleoprotein TPR isoform X1 [Ostrinia furnacalis]	0.68596	1.58465	-0.372	-1.1463	-0.7524
TRINITY_DN3176_c0_g1_i2_orf1	dnaJ homolog subfamily B member 6 isoform X2 [Ostrinia furnacalis]	0.74072	1.61141	-0.7051	-0.8475	-0.7995
TRINITY_DN6185_c0_g1_i12_orf1	mitogen-activated protein kinase 1 [Ostrinia furnacalis] >AXF67444.1 mitogen-activated protein kinase 1 [Ostrinia furnacalis]	0.83695	1.51045	-1.0747	-0.4732	-0.7995
TRINITY_DN35245_c0_g1_i1_orf1	ras GTPase-activating protein-binding protein 2 isoform X1 [Nymphalis io] >XP_050349014.1 ras GTPase-activating protein-binding protein 2 isoform X1 [Nymphalis io] >XP_050349015.1 ras GTPase-activating protein-binding protein 2 isoform X2 [Nymphalis io]	0.28353	1.67913	0.03885	-0.7821	-1.2194
TRINITY_DN1459_c1_g1_i1_orf1	reticulon-1 isoform X2 [Ostrinia furnacalis]	0.55093	1.65387	-0.3615	-0.6651	-1.1782
TRINITY_DN12227_c0_g2_i3_orf1	exonuclease 3'-5' domain-containing protein 2 [Ostrinia furnacalis]	0.60142	1.09503	0.55443	-1.6725	-0.5784
TRINITY_DN47723_c0_g1_i1_orf1	dnaJ homolog subfamily C member 21 [Ostrinia furnacalis]	0.90825	1.49127	-0.7626	-0.6962	-0.9407
TRINITY_DN79868_c0_g1_i1_orf1	lethal(2)neighbour of Tid protein [Ostrinia furnacalis]	0.56433	1.44478	0.11714	-1.4631	-0.6632
TRINITY_DN10558_c0_g1_i4_orf1	unnamed protein product [Chrysodeixis includens]	1.04237	0.93463	0.41533	-1.25	-1.1423
TRINITY_DN17828_c0_g1_i1_orf1	ER membrane protein complex subunit 8/9 homolog [Ostrinia furnacalis]	0.13446	1.47817	-0.4216	0.38173	-1.5727
TRINITY_DN2232_c1_g1_i3_orf1	protein FAM98A-like [Ostrinia furnacalis]	0.80632	1.26225	0.24696	-1.2437	-1.0719
TRINITY_DN45633_c0_g1_i1_orf1	ubiquitin thioesterase otubain-like [Ostrinia furnacalis]	0.83544	1.51959	-0.789	-1.0515	-0.5145
TRINITY_DN69170_c0_g2_i1_orf1	stromal membrane-associated protein 1-like [Pectinophora gossypiella]	0.48607	1.71305	-0.4324	-1.0853	-0.6814
TRINITY_DN6572_c0_g1_i2_orf1	zinc finger protein 330 homolog [Ostrinia furnacalis]	0.46676	1.65946	-0.1618	-1.1728	-0.7916
TRINITY_DN6545_c0_g1_i6_orf1	organic cation transporter protein-like [Ostrinia furnacalis]	0.21481	1.68091	-0.6285	0.04846	-1.3157
TRINITY_DN2977_c0_g1_i3_orf1	transmembrane 9 superfamily member 3 [Ostrinia furnacalis]	0.80761	1.56014	-0.6591	-0.9532	-0.7554
TRINITY_DN142588_c0_g1_i1_orf1	peptidyl-prolyl cis-trans isomerase [Cotesia flavipes]	1.0107	1.38026	-0.474	-1.0337	-0.8832
TRINITY_DN11402_c0_g1_i1_orf1	constitutive coactivator of PPAR-gamma-like protein 1 isoform X1 [Ostrinia furnacalis] >XP_028158538.1 constitutive coactivator of PPAR-gamma-like protein 1 isoform X2 [Ostrinia furnacalis]	0.88295	1.51015	-0.6545	-0.8613	-0.8773
TRINITY_DN31585_c0_g1_i1_orf1	transcription elongation factor SPT5 [Ostrinia furnacalis]	0.49754	1.74102	-0.6332	-0.9288	-0.6766
TRINITY_DN62_c0_g1_i18_orf1	hypothetical protein evm_002481 [Chilo suppressalis] >CAB3531063.1 unnamed protein product [Chilo suppressalis] >CAH0407655.1 unnamed protein product [Chilo suppressalis]	0.58861	1.66094	-0.379	-0.9643	-0.9063
TRINITY_DN54134_c0_g1_i1_orf1	NFU1 iron-sulfur cluster scaffold homolog, mitochondrial-like [Ostrinia furnacalis]	0.37978	1.73374	-0.3614	-0.5724	-1.1797
TRINITY_DN4262_c0_g1_i16_orf1	sperm-associated antigen 7 homolog [Ostrinia furnacalis]	0.78959	1.39662	-0.1441	-1.4211	-0.6211
TRINITY_DN2738_c1_g1_i3_orf1	uridine-cytidine kinase isoform X1 [Helicoverpa zea] >XP_049697747.1 uridine-cytidine kinase-like isoform X1 [Helicoverpa armigera] >XP_049698409.1 uridine-cytidine kinase isoform X1 [Helicoverpa armigera]	0.35777	1.80845	-0.5899	-0.8628	-0.7135
TRINITY_DN18164_c0_g1_i7_orf1	uncharacterized protein LOC114366518 isoform X5 [Ostrinia furnacalis]	1.23678	1.09892	-0.247	-0.9433	-1.1454
TRINITY_DN104507_c0_g1_i2_orf1	replication protein A 32 kDa subunit [Ostrinia furnacalis]	0.80831	1.40976	-0.168	-1.3638	-0.6863
TRINITY_DN3753_c0_g1_i7_orf1	very-long-chain 3-oxoacyl-CoA reductase isoform X2 [Ostrinia furnacalis]	0.12321	1.87177	-0.6038	-0.4226	-0.9686
TRINITY_DN5457_c0_g1_i4_orf1	unnamed protein product [Chrysodeixis includens]	0.73871	1.52048	-0.2335	-1.1474	-0.8784
TRINITY_DN5046_c0_g3_i1_orf1	uncharacterized protein LOC114358520 [Ostrinia furnacalis]	0.90814	1.46956	-0.8406	-1.0212	-0.5159
TRINITY_DN7037_c0_g1_i4_orf1	unnamed protein product [Chilo suppressalis]	0.67107	1.58868	-1.2171	-0.5006	-0.5421
TRINITY_DN170_c1_g1_i5_orf1	regulator of chromosome condensation isoform X2 [Helicoverpa zea]	0.29151	1.69088	0.02956	-1.1308	-0.8812
TRINITY_DN34821_c0_g1_i4_orf1	acetylcholine receptor subunit alpha-L1-like [Ostrinia furnacalis]	1.17849	1.19076	-1.2517	-0.5268	-0.5907
TRINITY_DN129226_c0_g1_i2_orf1	hypothetical protein evm_000268 [Chilo suppressalis]	0.9096	1.36997	-0.5597	-1.3617	-0.3582
TRINITY_DN29034_c0_g1_i1_orf1	trypsin-like serine protease [Ostrinia nubilalis]	0.70294	1.58967	-1.1527	-0.5563	-0.5836
TRINITY_DN2885_c1_g1_i2_orf1	ubiquitin-like-specific protease ESD4 [Ostrinia furnacalis]	0.8457	1.37053	-0.0536	-1.2616	-0.901
TRINITY_DN37599_c0_g1_i1_orf1	bmp-2 protein isoform X3 [Bombyx mori] >XP_028041166.1 RNA-binding protein 4.1-like isoform X2 [Bombyx mandarina]	0.5519	1.65039	-0.265	-1.08	-0.8573
TRINITY_DN26251_c0_g1_i1_orf1	serine/arginine-rich splicing factor 1A [Neodiprion lecontei] >XP_046417766.1 serine/arginine-rich splicing factor 1A [Neodiprion fabricii] >XP_046473571.1 serine/arginine-rich splicing factor 1A [Neodiprion pinetum] >XP_046610590.1 serine/arginine-rich splicing factor 1A [Neodiprion virginianus] >XP_046738887.1 serine/arginine-rich splicing factor 1A [Diprion similis]	0.4142	1.58375	0.06047	-1.3438	-0.7146
TRINITY_DN141_c0_g1_i1_orf1	hypothetical protein evm_010402 [Chilo suppressalis]	0.36495	1.72258	-0.1502	-0.9562	-0.9811
TRINITY_DN13972_c0_g1_i5_orf1	myelin expression factor 2-like [Ostrinia furnacalis] >XP_028173185.1 myelin expression factor 2-like [Ostrinia furnacalis]	0.50184	1.67138	-0.2259	-1.0354	-0.9119

TRINITY_DN18794_c0_g1_i5_orf1	hypothetical protein evm_012380 [Chilo suppressalis] >CAB3520845.1 unnamed protein product [Chilo suppressalis] >CAH0398166.1 unnamed protein product [Chilo suppressalis]	0.32501	1.74616	-0.2001	-0.77	-1.1011
TRINITY_DN31503_c0_g1_i4_orf1	hypothetical protein evm_001345 [Chilo suppressalis] >CAB3523265.1 unnamed protein product [Chilo suppressalis] >CAH0400587.1 unnamed protein product [Chilo suppressalis]	0.61791	1.68292	-0.7959	-0.6522	-0.8527
TRINITY_DN5841_c0_g1_i2_orf1	hypothetical protein evm_011295 [Chilo suppressalis]	0.7493	1.46851	-0.427	-1.3935	-0.3973
TRINITY_DN6189_c0_g1_i1_orf1	optic atrophy 3 protein homolog isoform X2 [Ostrinia furnacalis]	0.84481	1.50916	-1.1128	-0.6297	-0.6115
TRINITY_DN3893_c0_g2_i3_orf1	cleavage and polyadenylation specificity factor subunit CG7185 isoform X2 [Ostrinia furnacalis]	0.79885	1.44358	-0.2088	-1.3053	-0.7284
TRINITY_DN41311_c0_g2_i3_orf1	ras-related protein Rab-8A isoform X2 [Ostrinia furnacalis]	0.59886	1.68424	-0.6643	-0.9724	-0.6464
TRINITY_DN3582_c0_g1_i2_orf1	uncharacterized protein LOC114357129 [Ostrinia furnacalis]	0.62536	1.66722	-0.5938	-0.9794	-0.7194
TRINITY_DN2718_c0_g1_i6_orf1	cleavage stimulation factor subunit 2 isoform X1 [Ostrinia furnacalis]	0.89166	1.44914	-0.3792	-1.1171	-0.8445
TRINITY_DN4380_c0_g1_i9_orf1	hypothetical protein evm_012370 [Chilo suppressalis]	0.24933	1.75183	-0.0869	-1.0778	-0.8365
TRINITY_DN298_c0_g1_i4_orf1	luc7-like protein 3 isoform X1 [Ostrinia furnacalis] >XP_028160033.1 luc7-like protein 3 isoform X1 [Ostrinia furnacalis]	0.68426	1.58972	-0.3301	-0.9157	-1.0282
TRINITY_DN10455_c0_g1_i2_orf1	actin-related protein 2/3 complex subunit 4 [Plutella xylostella] >XP_013184242.1 PREDICTED: actin-related protein 2/3 complex subunit 4 [Amyelois transitella] >XP_026754865.1 actin-related protein 2/3 complex subunit 4 [Galleria mellonella] >XP_028168998.1 actin-related protein 2/3 complex subunit 4 [Ostrinia furnacalis] >KAI5632346.1 ARP2/3 complex 20 kDa subunit (ARPC4) domain-containing protein [Phthorimaea operculella] >KAG7303373.1 Actin-protein 2/3 complex subunit 4 [Plutella xylostella] >CAG9104981.1 unnamed protein product [Plutella xylostella]	0.21104	1.71043	0.08644	-1.0612	-0.9467
TRINITY_DN2374_c0_g1_i1_orf1	uncharacterized protein LOC114357127 [Ostrinia furnacalis]	0.3792	1.58972	0.16727	-1.1666	-0.9696
TRINITY_DN20007_c0_g1_i1_orf1	hypothetical protein evm_011958 [Chilo suppressalis] >CAB3521085.1 unnamed protein product [Chilo suppressalis]	0.43211	1.77689	-0.6314	-0.8691	-0.7085
TRINITY_DN23502_c0_g1_i1_orf1	small nuclear ribonucleoprotein F [Ostrinia furnacalis]	0.56962	1.39261	0.26733	-1.4141	-0.8154
TRINITY_DN41602_c0_g3_i1_orf1	CCR4-NOT transcription complex subunit 3 [Cotesia glomerata] >KAH0561609.1 CCR4-NOT transcription complex, subunit 3 [Cotesia glomerata]	-0.0233	1.82305	-0.5459	-0.0829	-1.1709
TRINITY_DN40562_c0_g2_i1_orf1	dual specificity protein phosphatase 23-like isoform X2 [Ostrinia furnacalis]	0.53325	1.69783	-1.0173	-0.7823	-0.4315
TRINITY_DN8432_c0_g2_i1_orf1	unnamed protein product [Chrysodeixis includens]	0.97547	1.37052	-0.6849	-0.4297	-1.2314
TRINITY_DN7251_c0_g1_i3_orf1	hypothetical protein evm_008498 [Chilo suppressalis] >CAB3527693.1 unnamed protein product [Chilo suppressalis] >CAH0401999.1 unnamed protein product [Chilo suppressalis]	0.78477	1.5615	-0.5158	-0.9628	-0.8677
TRINITY_DN42824_c0_g1_i5_orf1	prefoldin subunit 3 [Ostrinia furnacalis]	0.43445	1.75123	-0.4202	-0.8139	-0.9515
TRINITY_DN3488_c0_g1_i2_orf1	hsp70-binding protein 1 isoform X1 [Ostrinia furnacalis] >XP_028170128.1 hsp70-binding protein 1 isoform X2 [Ostrinia furnacalis]	0.13335	1.82638	-0.2651	-0.5823	-1.1123
TRINITY_DN4707_c0_g1_i1_orf1	PREDICTED: DNA-directed RNA polymerases I, II, and III subunit RPABC1 [Papilio xuthus] >XP_013187738.1 PREDICTED: DNA-directed RNA polymerases I, II, and III subunit RPABC1 [Amyelois transitella] >XP_028158146.1 DNA-directed RNA polymerases I, II, and III subunit RPABC1 [Ostrinia furnacalis] >XP_045537534.1 DNA-directed RNA polymerases I, II, and III subunit RPABC1 [Papilio machaon] >XP_049876738.1 DNA-directed RNA polymerases I, II, and III subunit RPABC1 [Pectinophora gossypiella] >KAG6452000.1 hypothetical protein O3G_MSEX007416 [Manduca sexta] >RVE48301.1 hypothetical protein evm_007052 [Chilo suppressalis] >CAG5049330.1 unnamed protein product [Parnassius apollo] >CAG9757053.1 unnamed protein product [Diatraea saccharalis] >CAH2042370.1 unnamed protein product, partial [Iphiclides podalirius]	0.2733	1.82999	-0.5392	-0.9588	-0.6053
TRINITY_DN5086_c0_g1_i1_orf1	unnamed protein product [Diatraea saccharalis]	0.12954	1.72463	0.13197	-1.0907	-0.8954
TRINITY_DN3539_c0_g1_i7_orf1	transcription elongation regulator 1-like [Ostrinia furnacalis]	0.41319	1.70579	-0.1925	-1.0791	-0.8473
TRINITY_DN3832_c0_g1_i1_orf1	serine-threonine kinase receptor-associated protein [Galleria mellonella]	0.48879	1.74565	-0.6994	-0.6151	-0.9199
TRINITY_DN5653_c0_g1_i4_orf1	hrp65 protein-like [Ostrinia furnacalis]	0.69613	1.59492	-0.3853	-0.8924	-1.0133
TRINITY_DN15706_c0_g2_i5_orf1	cdc42 homolog [Galleria mellonella] >XP_028178764.1 cdc42 homolog [Ostrinia furnacalis] >XP_028178765.1 cdc42 homolog [Ostrinia furnacalis]	0.37911	1.78845	-0.8161	-0.8739	-0.4776
TRINITY_DN4116_c0_g1_i3_orf1	transmembrane protein 131 homolog [Ostrinia furnacalis]	0.49505	1.4114	0.2696	-1.4896	-0.6864
TRINITY_DN22654_c0_g2_i4_orf1	protein EFR3 homolog cmp44E isoform X1 [Ostrinia furnacalis] >XP_028166854.1 protein EFR3 homolog cmp44E isoform X2 [Ostrinia furnacalis]	0.54173	1.71172	-0.5571	-0.9652	-0.7312
TRINITY_DN27_c0_g1_i1_orf1	THO complex subunit 4-A [Ostrinia furnacalis]	0.34532	1.74515	-0.2401	-1.1063	-0.744
TRINITY_DN14501_c0_g1_i1_orf1	28S ribosomal protein S28, mitochondrial [Ostrinia furnacalis]	0.48086	1.73508	-0.9075	-0.4558	-0.8526
TRINITY_DN57105_c0_g1_i2_orf1	transmembrane protein 161B isoform X1 [Galleria mellonella]	0.88298	1.47487	-0.4539	-0.8369	-1.0671
TRINITY_DN2623_c0_g1_i3_orf1	unnamed protein product [Chilo suppressalis]	0.59352	1.641	-0.3048	-0.9625	-0.9673
TRINITY_DN23926_c0_g1_i4_orf1	programmed cell death protein 10 [Ostrinia furnacalis]	0.6023	1.46054	-0.2715	-1.5383	-0.253
TRINITY_DN10722_c0_g3_i1_orf1	inositol-3-phosphate synthase [Ostrinia furnacalis]	0.67682	1.49096	-0.1083	-1.3351	-0.7244
TRINITY_DN79000_c1_g1_i1_orf1	AT15141p, partial [Drosophila melanogaster]	0.19842	1.85593	-0.4361	-0.9003	-0.7179
TRINITY_DN26355_c0_g1_i4_orf1	small integral membrane protein 12 [Ostrinia furnacalis]	0.54774	1.7215	-0.6488	-0.846	-0.7744
TRINITY_DN3860_c0_g1_i5_orf1	nucleoplasmin-like protein isoform X1 [Hyposmocoma kahamanoa]	0.41215	1.4081	0.47099	-1.1636	-1.1277
TRINITY_DN69236_c0_g1_i1_orf1	peroxiredoxin [Ostrinia furnacalis]	0.75485	1.46662	-0.5604	-1.3716	-0.2894
TRINITY_DN23264_c0_g1_i1_orf1	U5 small nuclear ribonucleoprotein 40 kDa protein [Ostrinia furnacalis]	0.03669	1.63633	0.36537	-1.2538	-0.7846
TRINITY_DN16349_c0_g1_i10_orf1	protein lingerer-like isoform X1 [Nymphalis io] >XP_050356663.1 protein lingerer-like isoform X1 [Nymphalis io] >XP_050356664.1 protein lingerer-like isoform X1 [Nymphalis io]	0.74044	1.55748	-0.4539	-1.1667	-0.6773
TRINITY_DN2196_c0_g1_i2_orf1	HIRA-interacting protein 3-like [Ostrinia furnacalis]	0.67497	1.63265	-0.8342	-0.5159	-0.9575
TRINITY_DN81715_c0_g1_i1_orf1	gamma-interferon-inducible lysosomal thiol reductase-like [Ostrinia furnacalis]	0.93771	1.4531	-0.6158	-1.0528	-0.7222
TRINITY_DN2876_c0_g1_i3_orf1	long-chain fatty acid transport protein 4-like isoform X1 [Ostrinia furnacalis]	0.31322	1.78276	-0.8673	-0.2852	-0.9435
TRINITY_DN16965_c0_g2_i1_orf1	hypothetical protein evm_007405 [Chilo suppressalis]	0.57414	1.62034	-0.5061	-1.2705	-0.4179
TRINITY_DN35669_c0_g1_i1_orf1	unnamed protein product [Diatraea saccharalis]	0.55456	1.6974	-0.4682	-0.9165	-0.8673
TRINITY_DN556_c0_g2_i1_orf1	serine protease inhibitor dipetalogastin-like [Ostrinia furnacalis]	0.54584	0.97254	0.66585	-0.4111	-1.7731
TRINITY_DN9164_c0_g1_i3_orf1	unnamed protein product [Parnassius apollo]	0.18306	1.78025	-0.1246	-1.133	-0.7057
TRINITY_DN48413_c1_g1_i2_orf1	probable protein phosphatase 2C 11 isoform X1 [Manduca sexta] >KAG6442694.1 hypothetical protein O3G_MSEX002471 [Manduca sexta]	0.88952	1.49262	-0.5522	-0.8872	-0.9427
TRINITY_DN13216_c0_g1_i5_orf1	uncharacterized protein LOC114358344 isoform X1 [Ostrinia furnacalis]	1.13177	1.3129	-0.8476	-0.7701	-0.827
TRINITY_DN154_c0_g1_i4_orf1	ER membrane protein complex subunit 4 [Ostrinia furnacalis]	0.37014	1.77325	-0.4062	-1.018	-0.7192
TRINITY_DN2997_c0_g1_i6_orf1	titin-like [Ostrinia furnacalis]	0.06768	1.72922	0.18419	-0.9238	-1.0573

TRINITY_DN227_c0_g1_i1_orf1	double-stranded ribonuclease 2 [Ostrinia nubilalis]	0.1485	1.65806	-1.2238	0.23838	-0.8211
TRINITY_DN14487_c0_g1_i4_orf1	hypothetical protein HW555_009956 [Spodoptera exigua] >KAH9643419.1 hypothetical protein HF086_016708 [Spodoptera exigua] >CAH0702087.1 unnamed protein product [Spodoptera exigua]	0.4153	1.76746	-0.7307	-0.4858	-0.9663
TRINITY_DN34432_c0_g1_i1_orf1	39S ribosomal protein L44, mitochondrial [Ostrinia furnacalis]	0.42504	1.78389	-0.7911	-0.7643	-0.6536
TRINITY_DN5593_c0_g1_i1_orf1	PREDICTED: leucine-rich repeat-containing protein 47-like [Fopius arisanus]	-0.2982	1.68177	0.31009	-0.3213	-1.3723
TRINITY_DN33801_c0_g1_i1_orf1	unnamed protein product [Diatraea saccharalis]	0.24141	1.79402	-0.3434	-1.1408	-0.5513
TRINITY_DN3647_c2_g1_i3_orf1	unnamed protein product, partial [Iphiclides podalirius]	0.76116	1.54417	-0.7645	-1.1342	-0.4066
TRINITY_DN36788_c0_g1_i2_orf1	isocitrate dehydrogenase [NADP] cytoplasmic-like [Bicyclus anynana]	0.3014	1.72924	-0.1468	-1.1898	-0.6941
TRINITY_DN27556_c0_g1_i1_orf1	bystin [Ostrinia furnacalis]	0.28267	1.83926	-0.7078	-0.5723	-0.8418
TRINITY_DN5275_c0_g1_i1_orf1	paraplegin [Ostrinia furnacalis]	0.49639	1.65307	-0.2182	-1.1985	-0.7327
TRINITY_DN120979_c0_g1_i1_orf1	la-related protein 1-like isoform X2 [Ostrinia furnacalis]	0.06198	1.86695	-0.2209	-0.8143	-0.8938
TRINITY_DN8944_c0_g1_i1_orf1	actin, clone 403 [Trichonephila clavata]	0.75806	1.57473	-0.4969	-0.9999	-0.8359
TRINITY_DN51968_c0_g1_i1_orf1	splicing factor U2af 38 kDa subunit [Aphidius gifuensis] >KAF7990547.1 hypothetical protein HCN44_000352 [Aphidius gifuensis]	0.53877	1.49887	0.1563	-1.2234	-0.9705
TRINITY_DN53810_c0_g1_i1_orf1	39S ribosomal protein L53, mitochondrial [Pectinophora gossypiella]	0.16597	1.87508	-0.8725	-0.5052	-0.6634
TRINITY_DN2769_c0_g1_i1_orf1	pseudouridylate synthase 7 homolog [Ostrinia furnacalis]	0.22357	1.80583	-0.2498	-1.0366	-0.743
TRINITY_DN8536_c0_g1_i2_orf1	PC4 and SFRS1-interacting protein isoform X4 [Galleria mellonella]	0.71648	1.52606	-0.3463	-1.2945	-0.6018
TRINITY_DN27960_c0_g1_i1_orf1	ATP synthase mitochondrial F1 complex assembly factor 1 [Ostrinia furnacalis]	0.18868	1.87978	-0.7443	-0.6536	-0.6706
TRINITY_DN34509_c0_g1_i1_orf1	transcription initiation factor IIA subunit 2 [Aphidius gifuensis] >KAF7997556.1 hypothetical protein HCN44_006127 [Aphidius gifuensis]	0.42004	1.77518	-0.5524	-0.7273	-0.9155
TRINITY_DN27852_c0_g1_i1_orf1	baculoviral IAP repeat-containing protein 6-like [Ostrinia furnacalis]	0.30854	1.76179	-0.5287	-0.3628	-1.1789
TRINITY_DN3673_c0_g1_i10_orf1	hypothetical protein evm_008955 [Chilo suppressalis] >CAB3526829.1 unnamed protein product [Chilo suppressalis] >CAH0404157.1 unnamed protein product [Chilo suppressalis]	0.36921	1.75951	-0.6987	-0.3564	-1.0736
TRINITY_DN106038_c0_g1_i1_orf1	ankyrin-3-like isoform X1 [Galleria mellonella]	0.39636	1.79543	-0.6563	-0.8376	-0.6979
TRINITY_DN7112_c0_g1_i1_orf1	heterogeneous nuclear ribonucleoprotein K isoform X2 [Ostrinia furnacalis]	0.65261	1.44205	0.10254	-1.286	-0.9112
TRINITY_DN787_c0_g1_i7_orf1	YLP motif-containing protein 1-like isoform X1 [Ostrinia furnacalis]	0.61119	1.65675	-0.8073	-1.0159	-0.4447
TRINITY_DN27725_c0_g1_i2_orf1	BRISC complex subunit FAM175B-like [Ostrinia furnacalis]	1.10729	1.12913	-0.0127	-0.9973	-1.2264
TRINITY_DN12101_c0_g1_i2_orf1	UPF0545 protein C22orf39 homolog [Ostrinia furnacalis]	0.28157	1.76554	-0.9986	-0.166	-0.8826
TRINITY_DN389_c0_g1_i2_orf1	uncharacterized protein LOC118068293 isoform X2 [Chelonus insularis]	1.01043	1.41807	-0.8106	-0.7755	-0.8424
TRINITY_DN25341_c0_g1_i1_orf1	heat shock protein 90 [Loxostege sticticalis]	0.36705	1.79834	-0.5475	-0.9155	-0.7023
TRINITY_DN5697_c0_g1_i1_orf1	GPI ethanolamine phosphate transferase 2-like [Ostrinia furnacalis]	0.54176	1.63305	-0.1694	-0.9966	-1.0088
TRINITY_DN18242_c0_g1_i3_orf1	CCHC-type zinc finger protein CG3800 [Papilio xuthus]	0.17094	1.70898	0.1297	-1.0886	-0.921
TRINITY_DN38540_c0_g1_i1_orf1	GSCOCG00000129001-RA-CDS [Cotesia congregata] >CAG5101050.1 Similar to LUC7L2: Putative RNA-binding protein Luc7-like 2 (Homo sapiens) [Cotesia congregata]	0.43194	1.74162	-0.5292	-1.0944	-0.55
TRINITY_DN91877_c0_g1_i1_orf1	NADH dehydrogenase [ubiquinone] 1 alpha subcomplex assembly factor 2 [Ostrinia furnacalis]	-0.4482	0.9754	0.90513	0.28452	-1.7168
TRINITY_DN6439_c0_g1_i1_orf1	GPI mannosyltransferase 3 isoform X4 [Ostrinia furnacalis] >XP_028164836.1 GPI mannosyltransferase 3 isoform X5 [Ostrinia furnacalis]	0.56421	1.63769	-0.3445	-1.208	-0.6494
TRINITY_DN10701_c0_g2_i2_orf1	synaptosomal-associated protein 29 [Ostrinia furnacalis]	0.34735	1.73784	-0.1766	-1.0122	-0.8964
TRINITY_DN3457_c0_g1_i4_orf1	aryl hydrocarbon receptor nuclear translocator homolog [Ostrinia furnacalis]	0.3421	1.53826	0.18147	-1.4541	-0.6077
TRINITY_DN2089_c0_g1_i5_orf1	eukaryotic translation initiation factor 4B [Ostrinia furnacalis]	0.24467	1.70868	0.02568	-1.165	-0.814
TRINITY_DN969_c0_g1_i3_orf1	protein UBASH3A homolog isoform X3 [Ostrinia furnacalis]	0.6359	1.64475	-0.5019	-1.0576	-0.7211
TRINITY_DN220_c0_g1_i3_orf1	serine-arginine protein 55 isoform X6 [Pieris brassicae]	0.67164	1.52846	-0.1284	-1.1942	-0.8775
TRINITY_DN3614_c0_g2_i1_orf1	PC4 and SFRS1-interacting protein isoform X4 [Galleria mellonella]	0.83727	1.17881	0.32627	-1.3439	-0.9985
TRINITY_DN14018_c0_g1_i4_orf1	chitobiosyldiphosphodolichol beta-mannosyltransferase [Ostrinia furnacalis]	0.24599	1.84839	-0.5416	-0.6667	-0.8861
TRINITY_DN754_c1_g1_i8_orf1	lysophospholipid acyltransferase 5 [Ostrinia furnacalis] >XP_028169982.1 lysophospholipid acyltransferase 5 [Ostrinia furnacalis]	0.26538	1.84154	-0.838	-0.7589	-0.51

TRINITY_DN31663_c0_g1_i2_orf1	PHD finger-like domain-containing protein 5A [Nasonia vitripennis] >XP_002427197.1 conserved hypothetical protein [Pediculus humanus corporis] >XP_003484388.1 PHD finger-like domain-containing protein 5A [Bombus impatiens] >XP_003701008.1 PREDICTED: PHD finger-like domain-containing protein 5A [Megachile rotundata] >XP_006623871.1 PHD finger-like domain-containing protein 5A [Apis dorsata] >XP_011068502.1 PREDICTED: PHD finger-like domain-containing protein 5A [Acromyrmex echinatior] >XP_011154391.1 PHD finger-like domain-containing protein 5A [Harpegnathos saltator] >XP_011164776.1 PHD finger-like domain-containing protein 5A [Solenopsis invicta] >XP_011262550.1 PHD finger-like domain-containing protein 5A [Camponotus floridanus] >XP_011297178.1 PREDICTED: PHD finger-like domain-containing protein 5A [Fopius arisanus] >XP_011334720.1 PHD finger-like domain-containing protein 5A [Ooceraea biroi] >XP_011506347.1 PREDICTED: PHD finger-like domain-containing protein 5A [Ceratosolen solmsi marchali] >XP_011506348.1 PREDICTED: PHD finger-like domain-containing protein 5A [Ceratosolen solmsi marchali] >XP_011638597.1 PHD finger-like domain-containing protein 5A isoform X2 [Pogonomyrmex barbatus] >XP_011686073.1 PREDICTED: PHD finger-like domain-containing protein 5A [Wasmannia auropunctata] >XP_011858255.1 PREDICTED: PHD finger-like domain-containing protein 5A [Vollenhovia emeryi] >XP_012058015.1 PREDICTED: PHD finger-like domain-containing protein 5A [Atta cephalotes] >XP_012135327.1 PREDICTED: PHD finger-like domain-containing protein 5A [Megachile rotundata] >XP_012135328.1 PREDICTED: PHD finger-like domain-containing protein 5A [Megachile rotundata] >XP_012222185.1 PREDICTED: PHD finger-like domain-containing protein 5A [Linepithema humile] >XP_012261946.1 PHD finger-like domain-containing protein 5A [Athalia rosae] >XP_012273120.1 PHD finger-like domain-containing protein 5A [Orussus abietinus] >XP_012526512.1 PHD finger-like domain-containing protein 5A [Monomorium pharaonis] >XP_014217558.1 PHD finger-like domain-containing protein 5A [Copidosoma floridanum] >XP_014484566.1 PREDICTED: PHD finger-like domain-containing protein 5A [Dinoponera quadriceps] >XP_014611099.1 PREDICTED: PHD finger-like domain-containing protein 5A [Polistes canadensis] >XP_015122018.1 PHD finger-like domain-containing protein 5A [Diachasma alloeum] >XP_015174163.1 PREDICTED: PHD finger-like domain-containing protein 5A [Polistes dominula] >XP_015433827.1 PREDICTED: PHD finger-like domain-containing protein 5A [Dufourea novaeangliae] >XP_015516165.1 PHD finger-like domain-containing protein 5A [Neodiprion lecontei] >XP_015586222.1 PHD finger-like domain-containing protein 5A isoform X1 [Cephus cinctus] >XP_016915535.1 PHD finger-like domain-containing protein 5A isoform X1 [Apis cerana] >XP_017786810.1 PREDICTED: PHD finger-like domain-containing protein 5A [Nicrophorus vespilloides] >XP_017793170.1 PREDICTED: PHD finger-like domain-containing protein 5A [Habropoda laboriosa] >XP_017887751.1 PHD finger-like domain-containing protein 5A [Ceratina calcarata] >XP_018054943.1 PREDICTED: PHD finger-like domain-containing protein 5A [Atta colombica] >XP_018309605.1 PREDICTED: PHD finger-like domain-containing protein 5A [Trachymyrmex zeteki] >XP_018344562.1 PREDICTED: PHD finger-like domain-containing protein 5A [Trachymyrmex septentrionalis] >XP_018378412.1 PREDICTED: PHD finger-like domain-containing protein 5A [Trachymyrmex cornetzi] >XP_018394190.1 PREDICTED: PHD finger-like domain-containing protein 5A [Cyphomyrmex costatus] >XP_018573484.1 PHD finger-like domain-containing protein 5A [Anoplophora glabripennis] >XP_018573485.1 PHD finger-like domain-containing protein 5A [Anoplophora glabripennis] >XP_019869751.1 PHD finger-like domain-containing protein 5A [Aethina tumida] >XP_019869752.1 PHD finger-like domain-containing protein 5A [Aethina tumida] >XP_019884294.1 PHD finger-like domain-containing protein 5A [Camponotus floridanus] >XP_020295914.1 PHD finger-like domain-containing protein 5A [Pseudomyrmex gracilis] >XP_021927485.1 PHD finger-like domain-containing protein 5A [Zootermopsis nevadensis] >XP_022205310.1 PHD finger-like domain-containing protein 5A [Nilaparvata lugens] >XP_022908436.1 PHD finger-like domain-containing protein 5A [Onthophagus taurus]	0.2424	1.82142	-0.4223	-1.0421	-0.5994
TRINITY_DN31851_c0_g1_i2_orf1	CDGSH iron-sulfur domain-containing protein 2 homolog [Helicoverpa armigera] >PZC85510.1 hypothetical protein B5X24_HaOG216618 [Helicoverpa	-0.0402	1.76477	-0.3377	-0.0577	-1.3291
TRINITY_DN7574_c0_g1_i10_orf1	proteasome activator complex subunit 3 isoform X2 [Ostrinia furnacalis]	0.0671	1.74949	0.08871	-1.1891	-0.7162
TRINITY_DN23360_c0_g1_i3_orf1	protein PTCD3 homolog, mitochondrial [Ostrinia furnacalis]	0.32329	1.82748	-0.6677	-0.813	-0.6701
TRINITY_DN16258_c0_g1_i2_orf1	uncharacterized protein LOC114359911 [Ostrinia furnacalis]	0.37106	1.76277	-0.3137	-0.9015	-0.9186
TRINITY_DN3759_c0_g1_i1_orf1	uncharacterized protein LOC114350416 [Ostrinia furnacalis] >XP_028157016.1 uncharacterized protein LOC114350416 [Ostrinia furnacalis] >XP_028157017.1 uncharacterized protein LOC114350416 [Ostrinia furnacalis] >XP_028157018.1 uncharacterized protein LOC114350416 [Ostrinia furnacalis] >XP_028157019.1 uncharacterized protein LOC114350416 [Ostrinia furnacalis]	0.8523	1.50072	-0.6599	-1.1228	-0.5703
TRINITY_DN69049_c0_g2_i1_orf1	membrane alanyl aminopeptidase-like [Ostrinia furnacalis]	0.17825	1.63304	-0.3617	0.02351	-1.4731
TRINITY_DN959_c0_g1_i7_orf1	Golgi to ER traffic protein 4 homolog [Ostrinia furnacalis]	0.18443	1.84772	-0.9569	-0.3671	-0.7081
TRINITY_DN14372_c0_g2_i1_orf1	12 kDa FK506-binding protein-like [Ostrinia furnacalis]	-0.0101	1.89341	-0.2482	-0.9084	-0.7268
TRINITY_DN34159_c0_g2_i1_orf1	guanine nucleotide exchange factor subunit Rich isoform X1 [Ostrinia furnacalis]	0.20861	1.67039	-0.8993	0.17258	-1.1523
TRINITY_DN10662_c0_g1_i4_orf1	HD domain-containing protein 2 [Ostrinia furnacalis]	0.32943	1.77121	-0.4947	-1.1347	-0.4713
TRINITY_DN30_c0_g1_i6_orf1	casein kinase I-like isoform X1 [Hyposmocoma kahamanoa]	0.35447	1.7836	-0.7259	-0.9968	-0.4153
TRINITY_DN21539_c0_g1_i1_orf1	probable phenylalanine--tRNA ligase, mitochondrial [Ostrinia furnacalis]	0.50185	1.72611	-0.6525	-1.0135	-0.562
TRINITY_DN11657_c0_g1_i2_orf1	trehalase-1 [Omphisa fuscidentalis]	0.89195	1.48657	-0.6632	-0.6532	-1.0621
TRINITY_DN2065_c1_g2_i1_orf1	2-amino-3-ketobutyrate coenzyme A ligase, mitochondrial [Ostrinia furnacalis]	0.65242	1.66607	-0.8397	-0.7458	-0.733
TRINITY_DN4213_c0_g1_i4_orf1	nardilysin-like isoform X1 [Ostrinia furnacalis] >XP_028157649.1 nardilysin-like isoform X2 [Ostrinia furnacalis] >XP_028157650.1 nardilysin-like isoform X3 [Ostrinia furnacalis] >XP_028157651.1 nardilysin-like isoform X4 [Ostrinia furnacalis]	0.58386	1.70573	-0.7689	-0.7277	-0.793
TRINITY_DN58531_c0_g1_i1_orf1	uncharacterized protein LOC114357371 [Ostrinia furnacalis] >XP_028166768.1 uncharacterized protein LOC114357371 [Ostrinia furnacalis]	0.83072	1.52328	-0.577	-1.0856	-0.6913
TRINITY_DN6710_c0_g1_i6_orf1	multiple C2 and transmembrane domain-containing protein-like [Ostrinia furnacalis]	0.8962	1.40229	-0.2121	-0.9766	-1.1098
TRINITY_DN14920_c0_g1_i1_orf1	anamorsin homolog [Ostrinia furnacalis]	0.44196	1.75126	-0.4453	-0.797	-0.9509
TRINITY_DN14429_c0_g1_i2_orf1	NADH dehydrogenase [ubiquinone] 1 beta subcomplex subunit 11, mitochondrial [Ostrinia furnacalis]	0.5428	1.661	-1.2016	-0.4871	-0.5151
TRINITY_DN50571_c1_g1_i1_orf1	WD repeat-containing protein 46 [Ostrinia furnacalis]	0.58588	1.66286	-1.1155	-0.6175	-0.5158
TRINITY_DN886_c0_g2_i4_orf1	collagenase-like [Ostrinia furnacalis]	0.24103	1.85128	-0.6464	-0.8835	-0.5625
TRINITY_DN6563_c0_g1_i1_orf1	cytochrome c oxidase assembly protein COX15 homolog [Ostrinia furnacalis]	0.36817	1.80849	-0.7271	-0.81	-0.6396
TRINITY_DN27994_c0_g1_i1_orf1	uncharacterized protein LOC114364076 [Ostrinia furnacalis]	0.25415	1.84165	-0.5873	-0.5797	-0.9289
TRINITY_DN1803_c0_g1_i3_orf1	translocator protein-like isoform X1 [Ostrinia furnacalis] >XP_028178947.1 translocator protein-like isoform X1 [Ostrinia furnacalis]	0.26992	1.85109	-0.7336	-0.695	-0.6924
TRINITY_DN43412_c0_g1_i2_orf1	U1 small nuclear ribonucleoprotein C [Ostrinia furnacalis]	0.54035	1.54844	0.00847	-1.2843	-0.813
TRINITY_DN338_c2_g1_i2_orf1	alpha-tocopherol transfer protein-like isoform X1 [Ostrinia furnacalis] >XP_028160444.1 alpha-tocopherol transfer protein-like isoform X1 [Ostrinia furnacalis] >XP_028160446.1 alpha-tocopherol transfer protein-like isoform X1 [Ostrinia furnacalis] >XP_028160447.1 alpha-tocopherol transfer protein-like isoform X1 [Ostrinia furnacalis] >XP_028160448.1 alpha-tocopherol transfer protein-like isoform X1 [Ostrinia furnacalis] >XP_028160449.1 alpha-tocopherol transfer protein-like isoform X1 [Ostrinia furnacalis]	-0.0695	1.93925	-0.5322	-0.5003	-0.8372
TRINITY_DN29448_c0_g1_i1_orf1	28S ribosomal protein S9, mitochondrial [Ostrinia furnacalis]	0.25036	1.8526	-0.6011	-0.8406	-0.6612

TRINITY_DN10644_c0_g1_i2_orf1	carboxylesterase [Cnaphalocrocis medinalis]	0.32963	1.81656	-0.8947	-0.5631	-0.6883
TRINITY_DN50085_c0_g1_i1_orf1	hypothetical protein evm_013997 [Chilo suppressalis]	0.07178	1.86999	-0.2666	-0.9466	-0.7286
TRINITY_DN21150_c0_g1_i4_orf1	RNA-binding protein cabeza-like isoform X2 [Bicyclus anynana]	0.25961	1.7751	-0.2334	-1.1296	-0.6717
TRINITY_DN237_c1_g1_i1_orf1	PREDICTED: cytoplasmic protein NCK1 isoform X1 [Microplitis demolitor]	0.6462	1.64735	-0.6757	-0.5816	-1.0363
TRINITY_DN11013_c0_g1_i3_orf1	glutamine:fructose-6-phosphate aminotransferase 1 [Heortia vitessoides]	0.11683	1.88702	-0.506	-0.9033	-0.5946
TRINITY_DN2117_c0_g1_i1_orf1	BUB3-interacting and GLEBS motif-containing protein ZNF207 [Chelonus insularis]	0.30715	1.75429	-0.1759	-1.0418	-0.8438
TRINITY_DN38301_c0_g1_i2_orf1	gamma-taxilin [Ostrinia furnacalis]	0.04108	1.92305	-0.7504	-0.6225	-0.5912
TRINITY_DN1013_c0_g1_i3_orf1	TELO2-interacting protein 1 homolog isoform X2 [Ostrinia furnacalis]	0.55579	1.01458	0.7906	-0.8272	-1.5337
TRINITY_DN17312_c0_g1_i1_orf1	mRNA cap guanine-N7 methyltransferase [Ostrinia furnacalis]	0.40395	1.72448	-0.2245	-0.9457	-0.9583
TRINITY_DN19115_c0_g1_i1_orf1	putative ATP synthase subunit f, mitochondrial [Ostrinia furnacalis]	-0.0443	1.88046	-0.4035	-0.3469	-1.0857
TRINITY_DN12242_c0_g1_i5_orf1	heterogeneous nuclear ribonucleoprotein 87F-like isoform X1 [Vanessa tameamea] >XP_046967652.1 heterogeneous nuclear ribonucleoprotein 87F-like isoform X1 [Vanessa cardui] >XP_047532045.1 heterogeneous nuclear ribonucleoprotein 87F-like [Vanessa atalanta]	0.71703	1.60535	-0.5033	-0.888	-0.931
TRINITY_DN3127_c0_g1_i9_orf1	RNA-binding protein 1 isoform X1 [Galleria mellonella]	0.09022	1.84986	-0.2433	-0.6598	-1.037
TRINITY_DN334_c0_g1_i3_orf1	chymotrypsin-like serine protease [Ostrinia nubilalis] >AAX62030.1 chymotrypsin-like serine protease [Ostrinia nubilalis]	0.47051	1.76288	-0.6967	-0.7195	-0.8172
TRINITY_DN4345_c0_g1_i9_orf1	uncharacterized protein LOC114357127 [Ostrinia furnacalis]	0.20499	1.49897	0.42598	-1.4264	-0.7035
TRINITY_DN57798_c0_g1_i1_orf1	ubiquitin carboxyl-terminal hydrolase 36 [Ostrinia furnacalis]	0.40968	1.78163	-0.876	-0.7708	-0.5446
TRINITY_DN43942_c0_g1_i1_orf1	LOW QUALITY PROTEIN: caprin homolog [Ostrinia furnacalis]	0.03485	1.89025	-0.5051	-0.425	-0.995
TRINITY_DN124950_c0_g2_i1_orf1	TATA box-binding protein-like protein 1 [Ostrinia furnacalis] >XP_028155830.1 TATA box-binding protein-like protein 1 [Ostrinia furnacalis]	0.18518	1.55777	0.4144	-0.9368	-1.2206
TRINITY_DN1706_c0_g1_i7_orf1	LOW QUALITY PROTEIN: RNA polymerase-associated protein CTR9 homolog [Ostrinia furnacalis]	0.39813	1.72482	-0.3393	-1.1753	-0.6084
TRINITY_DN5578_c0_g1_i4_orf1	chromatin modification-related protein eaf-1-like [Ostrinia furnacalis]	0.31867	1.74919	-0.2748	-1.1756	-0.6175
TRINITY_DN13055_c0_g1_i5_orf1	116 kDa U5 small nuclear ribonucleoprotein component isoform X1 [Ostrinia furnacalis] >XP_028159219.1 116 kDa U5 small nuclear ribonucleoprotein component isoform X2 [Ostrinia furnacalis]	0.33338	1.70039	-0.0618	-1.143	-0.8289
TRINITY_DN3235_c0_g1_i1_orf1	SPARC [Trichoplusia ni]	0.98499	1.42773	-0.7754	-0.9764	-0.6609
TRINITY_DN20369_c0_g1_i2_orf1	uncharacterized protein LOC114366225 [Ostrinia furnacalis]	0.02527	1.91805	-0.4805	-0.8305	-0.6323
TRINITY_DN2807_c0_g1_i4_orf1	FK506-binding protein 59 isoform X1 [Ostrinia furnacalis]	0.04397	1.86907	-0.2156	-0.9422	-0.7552
TRINITY_DN46778_c0_g1_i2_orf1	Deoxycytidylate deaminase [Papilio xuthus]	0.33154	1.79746	-1.0052	-0.4688	-0.6551
TRINITY_DN15667_c0_g1_i2_orf1	coiled-coil domain-containing protein 25 [Ostrinia furnacalis]	-0.0672	1.94392	-0.5357	-0.5469	-0.7941
TRINITY_DN32997_c0_g1_i8_orf1	RNA-binding protein squid isoform X1 [Ostrinia furnacalis]	0.09246	1.88669	-0.3703	-0.8212	-0.7876
TRINITY_DN1109_c0_g1_i6_orf1	1-phosphatidylinositol phosphodiesterase-like [Cotesia glomerata]	0.37742	1.7203	-1.1914	-0.2687	-0.6376
TRINITY_DN698_c0_g1_i5_orf1	PREDICTED: small nuclear ribonucleoprotein Sm D3 [Amyelois transitella]	0.02404	1.90997	-0.3976	-0.849	-0.6874
TRINITY_DN19160_c0_g1_i1_orf1	alkyldihydroxyacetonephosphate synthase [Ostrinia furnacalis]	1.0667	1.36627	-0.716	-0.7921	-0.9248
TRINITY_DN11799_c0_g1_i4_orf1	V-type proton ATPase 116 kDa subunit a1 isoform X1 [Manduca sexta]	-0.0639	1.93679	-0.7291	-0.4002	-0.7437
TRINITY_DN6248_c0_g1_i1_orf1	DNA topoisomerase I, mitochondrial [Ostrinia furnacalis]	0.44497	1.77612	-0.6968	-0.7712	-0.753
TRINITY_DN8261_c0_g1_i1_orf1	UDP-N-acetylhexosamine pyrophosphorylase-like protein 1 [Ostrinia furnacalis]	0.12686	1.90062	-0.6512	-0.703	-0.6733
TRINITY_DN45477_c0_g1_i1_orf1	putative E3 ubiquitin-protein ligase UBR7 [Ostrinia furnacalis]	0.21356	1.82918	-0.9663	-0.7566	-0.3198
TRINITY_DN46173_c0_g3_i2_orf1	tropomyosin-1, isoforms 9A/A/B isoform X33 [Aedes aegypti] >EAT46020.1 AAEL002761-PB [Aedes aegypti]	0.01061	1.91296	-0.414	-0.8766	-0.633
TRINITY_DN4125_c1_g1_i5_orf1	angiotensin-converting enzyme-like isoform X2 [Ostrinia furnacalis]	0.13945	1.89647	-0.6401	-0.692	-0.7039
TRINITY_DN6231_c0_g1_i6_orf1	ran-binding protein 3 isoform X1 [Ostrinia furnacalis] >XP_028166372.1 ran-binding protein 3 isoform X2 [Ostrinia furnacalis]	0.06153	1.83661	-0.2317	-1.1339	-0.5326
TRINITY_DN1427_c0_g1_i9_orf1	SAFB-like transcription modulator isoform X3 [Ostrinia furnacalis]	0.3578	1.59647	0.15764	-1.2408	-0.8711
TRINITY_DN21531_c0_g1_i1_orf1	viral IAP-associated factor homolog [Ostrinia furnacalis]	0.48422	1.7378	-0.6733	-0.5588	-0.99
TRINITY_DN5554_c0_g1_i2_orf1	double-stranded RNA-binding protein Staufen homolog 2 isoform X5 [Pectinophora gossypiella]	0.0332	1.91455	-0.8627	-0.5573	-0.5277
TRINITY_DN58636_c0_g1_i1_orf1	uncharacterized protein LOC114363665 [Ostrinia furnacalis]	0.03452	1.88658	-0.2827	-0.9128	-0.7257
TRINITY_DN47123_c0_g1_i1_orf1	WD40 repeat-containing protein SMU1 [Ostrinia furnacalis]	0.02523	1.87622	-0.2181	-0.9275	-0.7559
TRINITY_DN5087_c0_g1_i6_orf1	nascent polypeptide-associated complex subunit alpha [Ostrinia furnacalis] >XP_028156807.1 nascent polypeptide-associated complex subunit alpha [Ostrinia furnacalis] >XP_028156808.1 nascent polypeptide-associated complex subunit alpha [Ostrinia furnacalis]	-0.0137	1.93149	-0.4722	-0.6973	-0.7483
TRINITY_DN40704_c0_g1_i2_orf1	COX assembly mitochondrial protein homolog [Ostrinia furnacalis]	0.25315	1.83491	-0.5516	-0.9737	-0.5628
TRINITY_DN5459_c0_g1_i1_orf1	protein takeout-like isoform X2 [Ostrinia furnacalis]	0.94817	1.46689	-0.8082	-0.7533	-0.8536
TRINITY_DN25779_c0_g1_i6_orf1	aldo-keto reductase AKR2E4-like [Ostrinia furnacalis]	0.78433	1.5711	-0.5977	-0.794	-0.9637
TRINITY_DN7583_c0_g1_i1_orf1	39S ribosomal protein L21, mitochondrial [Ostrinia furnacalis]	0.34421	1.80569	-0.6087	-0.5934	-0.9478
TRINITY_DN478_c0_g1_i16_orf1	lipid storage droplets surface-binding protein 2 isoform X1 [Ostrinia furnacalis]	-0.0341	1.75638	0.12968	-0.6241	-1.2279
TRINITY_DN3428_c0_g1_i1_orf1	10 kDa heat shock protein, mitochondrial [Ostrinia furnacalis]	-0.12	1.92255	-0.2493	-0.8791	-0.6742
TRINITY_DN82801_c0_g1_i1_orf1	uncharacterized protein LOC114364712 [Ostrinia furnacalis]	0.93012	1.46569	-1.0197	-0.688	-0.688
TRINITY_DN37538_c0_g4_i1_orf1	esterase FE4-like [Ostrinia furnacalis]	0.13013	1.89464	-0.7643	-0.7164	-0.544
TRINITY_DN19361_c0_g1_i7_orf1	hydroxylysine kinase [Ostrinia furnacalis] >XP_028168144.1 hydroxylysine kinase [Ostrinia furnacalis]	0.05746	1.91686	-0.5503	-0.659	-0.765
TRINITY_DN5867_c0_g1_i1_orf1	NADH dehydrogenase [ubiquinone] 1 alpha subcomplex subunit 7-like [Ostrinia furnacalis]	-0.0465	1.9465	-0.5859	-0.6912	-0.6229
TRINITY_DN1775_c0_g1_i3_orf1	ATP-dependent RNA helicase dbp2-like isoform X1 [Ostrinia furnacalis]	0.50522	1.74393	-0.8511	-0.6691	-0.7289
TRINITY_DN29934_c0_g1_i6_orf1	sodium/potassium-transporting ATPase subunit beta-2-like [Ostrinia furnacalis] >XP_028176258.1 sodium/potassium-transporting ATPase subunit beta-2-like [Ostrinia furnacalis]	0.29549	1.5612	0.32009	-1.1313	-1.0455
TRINITY_DN63662_c0_g4_i1_orf1	polyadenylate-binding protein 1 [Ostrinia furnacalis]	0.00959	1.91891	-0.4768	-0.8612	-0.5906
TRINITY_DN6933_c1_g1_i1_orf1	Chlorophyll a-b binding protein 40, chloroplastic [Trichinella nelsoni] >KRY99282.1 Chlorophyll a-b binding protein 40, chloroplastic [Trichinella	-0.1755	1.96756	-0.5208	-0.5392	-0.7321
TRINITY_DN14313_c0_g1_i1_orf1	25S rRNA (cytosine-C(5))-methyltransferase nop2 [Ostrinia furnacalis]	-0.0294	1.93983	-0.5556	-0.7472	-0.6076
TRINITY_DN86149_c0_g1_i1_orf1	NADH dehydrogenase [ubiquinone] 1 alpha subcomplex subunit 8 [Galleria mellonella]	-0.3272	1.94831	-0.4229	-0.2826	-0.9156
TRINITY_DN6308_c0_g1_i6_orf1	myc box-dependent-interacting protein 1 isoform X2 [Ostrinia furnacalis]	-0.0288	1.92338	-0.3984	-0.8524	-0.6437

TRINITY_DN36538_c0_g1_i2_orf1	xaa-Pro dipeptidase isoform X1 [Ostrinia furnacalis] >XP_028156507.1 xaa-Pro dipeptidase isoform X2 [Ostrinia furnacalis]	-0.0965	1.93845	-0.353	-0.7421	-0.7469
TRINITY_DN17905_c0_g3_i1_orf1	zinc finger protein 706-like [Ostrinia furnacalis] >XP_028176219.1 zinc finger protein 706-like [Ostrinia furnacalis] >XP_028176220.1 zinc finger protein 706-like [Ostrinia furnacalis] >XP_028176221.1 zinc finger protein 706-like [Ostrinia furnacalis]	0.20371	1.87486	-0.6989	-0.65	-0.7297
TRINITY_DN6685_c0_g1_i8_orf1	cleft lip and palate transmembrane protein 1 homolog [Ostrinia furnacalis]	0.08722	1.85157	-0.2027	-0.9576	-0.7784
TRINITY_DN1445_c0_g1_i1_orf1	leucine-rich PPR motif-containing protein, mitochondrial [Ostrinia furnacalis]	-0.0672	1.95138	-0.5751	-0.6487	-0.6604
TRINITY_DN139212_c0_g1_i4_orf1	uncharacterized protein LOC114350112 [Ostrinia furnacalis]	0.63873	1.52551	-0.0379	-1.1009	-1.0254
TRINITY_DN4143_c0_g1_i1_orf1	zinc finger protein 530-like isoform X8 [Ostrinia furnacalis]	0.21863	1.82158	-0.8492	-0.2764	-0.9146
TRINITY_DN76036_c0_g1_i1_orf1	cytochrome c oxidase subunit 6A1, mitochondrial-like [Ostrinia furnacalis]	-0.2306	1.93779	-0.3785	-0.3765	-0.9523
TRINITY_DN905_c0_g1_i4_orf1	(11Z)-hexadec-11-enoyl-CoA conjugase-like [Ostrinia furnacalis] >XP_028172978.1 (11Z)-hexadec-11-enoyl-CoA conjugase-like [Ostrinia furnacalis]	-0.2085	1.96637	-0.4134	-0.585	-0.7595
TRINITY_DN35635_c0_g1_i1_orf1	probable NADH dehydrogenase [ubiquinone] 1 alpha subcomplex subunit 12 [Ostrinia furnacalis]	-0.0226	1.88856	-0.3006	-0.5404	-1.0249
TRINITY_DN5238_c0_g1_i2_orf1	DNA-(apurinic or apyrimidinic site) lyase [Ostrinia furnacalis]	0.33526	1.7357	-0.2307	-1.1735	-0.6668
TRINITY_DN51045_c0_g1_i1_orf1	cell growth-regulating nucleolar protein [Ostrinia furnacalis]	-0.0762	1.95359	-0.66	-0.6324	-0.5849
TRINITY_DN628_c0_g1_i7_orf1	prostamide/prostaglandin F synthase-like [Ostrinia furnacalis]	0.15754	1.86953	-0.6395	-0.461	-0.9266
TRINITY_DN2918_c0_g1_i1_orf1	28S ribosomal protein S10, mitochondrial [Ostrinia furnacalis] >XP_028175147.1 28S ribosomal protein S10, mitochondrial [Ostrinia furnacalis]	0.24932	1.85829	-0.7104	-0.655	-0.7422
TRINITY_DN10332_c0_g1_i2_orfp1	TRINITY_DN10332_c0_g1_i2_m.42894 TRINITY_DN10332_c0_g1::TRINITY_DN10332_c0_g1_i2::g.42894 ORF type:3prime_partial len:77 (+),score=1.70 TRINITY_DN10332_c0_g1_i2:1005-1232(+)	-0.012	1.80891	-0.0137	-1.1539	-0.6293
TRINITY_DN35725_c0_g1_i1_orf1	mitochondrial import inner membrane translocase subunit Tim13-like [Bicyclus anynana] >CAG9745432.1 unnamed protein product [Diatraea saccharalis] >CAG9784117.1 unnamed protein product [Diatraea saccharalis]	0.21306	1.86993	-0.7795	-0.6715	-0.632
TRINITY_DN26130_c0_g1_i1_orf1	membrane alanyl aminopeptidase-like [Ostrinia furnacalis]	0.07622	1.87724	-0.9541	-0.3258	-0.6736
TRINITY_DN41697_c0_g1_i1_orf1	5-formyltetrahydrofolate cyclo-ligase [Ostrinia furnacalis]	0.49628	1.36771	-0.7452	0.36306	-1.4819
TRINITY_DN4842_c0_g1_i5_orf1	cytochrome c oxidase assembly factor 4 homolog, mitochondrial isoform X1 [Ostrinia furnacalis] >XP_028162331.1 cytochrome c oxidase assembly factor 4 homolog, mitochondrial isoform X2 [Ostrinia furnacalis]	-0.0615	1.92861	-0.3902	-0.6086	-0.8684
TRINITY_DN107035_c0_g1_i1_orf1	splicing factor 3A subunit 3 [Ostrinia furnacalis]	-0.0774	1.94443	-0.5509	-0.8058	-0.5102
TRINITY_DN15900_c0_g1_i6_orf1	unnamed protein product [Diatraea saccharalis]	0.13027	1.86899	-0.5679	-0.9831	-0.4483
TRINITY_DN4762_c0_g1_i2_orf1	ATPase family AAA domain-containing protein 1 isoform X2 [Ostrinia furnacalis]	0.49044	1.72993	-0.667	-1.0169	-0.5365
TRINITY_DN1353_c0_g1_i1_orf1	UDP-glucose 4-epimerase-like [Ostrinia furnacalis]	0.3063	1.82726	-0.8934	-0.6118	-0.6283
TRINITY_DN3062_c0_g1_i1_orf1	HEAT repeat-containing protein 1 [Ostrinia furnacalis]	-0.0688	1.9158	-0.3616	-0.956	-0.5294
TRINITY_DN8369_c0_g1_i1_orf1	39S ribosomal protein L37, mitochondrial [Ostrinia furnacalis]	0.01225	1.91852	-0.8694	-0.5307	-0.5307
TRINITY_DN15624_c0_g1_i1_orf1	LOW QUALITY PROTEIN: V-type proton ATPase subunit S1-like [Ostrinia furnacalis]	0.20432	1.84277	-0.7471	-0.3677	-0.9322
TRINITY_DN8691_c0_g1_i3_orf1	nucleolin-like [Melitaea cinxia]	0.51566	1.70085	-0.3777	-0.8562	-0.9826
TRINITY_DN2594_c0_g2_i4_orf1	isocitrate dehydrogenase [NAD] subunit beta, mitochondrial isoform X2 [Ostrinia furnacalis]	-0.1497	1.95928	-0.4963	-0.5339	-0.7794
TRINITY_DN1249_c0_g1_i6_orf1	venom carboxylesterase-6-like [Ostrinia furnacalis]	0.81961	1.52609	-0.4693	-1.0348	-0.8416
TRINITY_DN19244_c0_g1_i7_orf1	uncharacterized protein LOC114350218 [Ostrinia furnacalis]	0.32602	1.73855	-0.2332	-1.1801	-0.6513
TRINITY_DN97680_c0_g1_i1_orf1	39S ribosomal protein L52, mitochondrial [Ostrinia furnacalis]	-0.1391	1.89732	-0.2335	-0.4759	-1.0487
TRINITY_DN8087_c0_g1_i9_orf1	cysteine-rich with EGF-like domain protein 2 isoform X1 [Ostrinia furnacalis]	0.17227	1.84593	-0.8464	-0.3031	-0.8687
TRINITY_DN46409_c0_g1_i1_orf1	unnamed protein product [Heterotrigona itama]	-0.0372	1.901	-0.8247	-0.2325	-0.8067
TRINITY_DN44288_c0_g1_i2_orf1	ATP-dependent RNA helicase p62 [Ostrinia furnacalis]	-0.0317	1.92291	-0.9002	-0.5001	-0.4909
TRINITY_DN24476_c0_g1_i1_orf1	ensconsin-like isoform X1 [Ostrinia furnacalis]	0.06018	1.88645	-0.3297	-0.7041	-0.9128
TRINITY_DN3332_c0_g1_i11_orf1	glutathione S-transferase sigma3 [Glyphodes pyloalis]	-0.2303	1.95148	-0.8231	-0.2788	-0.6193
TRINITY_DN43942_c0_g2_i1_orf1	LOW QUALITY PROTEIN: caprin homolog [Ostrinia furnacalis]	0.32132	1.79832	-0.5593	-1.0377	-0.5226
TRINITY_DN2953_c1_g1_i2_orf1	methionine--tRNA ligase, cytoplasmic isoform X6 [Ostrinia furnacalis]	0.38621	1.78577	-0.9655	-0.634	-0.5725
TRINITY_DN47731_c0_g1_i2_orf1	nucleolar GTP-binding protein 2 [Ostrinia furnacalis]	0.5288	1.73001	-0.8715	-0.6405	-0.7468
TRINITY_DN30932_c0_g1_i2_orf1	delta(24)-sterol reductase-like isoform X2 [Ostrinia furnacalis]	0.17885	1.85642	-0.9839	-0.5036	-0.5477
TRINITY_DN35662_c0_g1_i5_orf1	hypothetical protein evm_006436 [Chilo suppressalis] >CAB3522373.1 unnamed protein product [Chilo suppressalis] >CAH0399695.1 unnamed protein product [Chilo suppressalis]	-0.1731	1.97199	-0.6239	-0.5584	-0.6165
TRINITY_DN23175_c0_g1_i6_orf1	myb-binding protein 1A-like protein [Ostrinia furnacalis]	-0.1627	1.97042	-0.6304	-0.6049	-0.5723
TRINITY_DN13651_c0_g1_i2_orf1	40S ribosomal protein S12, mitochondrial [Ostrinia furnacalis]	-0.0656	1.9257	-0.4031	-0.9066	-0.5505
TRINITY_DN26688_c0_g1_i2_orf1	myogenesis-regulating glycosidase-like [Ostrinia furnacalis]	-0.1346	1.96274	-0.5238	-0.6997	-0.6046
TRINITY_DN2083_c0_g1_i4_orf1	uncharacterized protein LOC114359113 [Ostrinia furnacalis]	-0.237	1.92437	-0.9316	-0.1691	-0.5867
TRINITY_DN8116_c0_g1_i1_orf1	uncharacterized protein LOC114350845 [Ostrinia furnacalis]	-0.1921	1.97465	-0.6296	-0.6138	-0.5392
TRINITY_DN5578_c0_g1_i10_orf1	unnamed protein product [Chilo suppressalis]	0.17567	1.83907	-1.015	-0.331	-0.6687
TRINITY_DN51766_c0_g1_i2_orf1	facilitated trehalose transporter Tret1-like [Ostrinia furnacalis]	0.14687	1.89175	-0.6347	-0.7774	-0.6265
TRINITY_DN29120_c0_g1_i6_orf1	putative inorganic phosphate cotransporter [Ostrinia furnacalis]	0.22275	1.85722	-0.8503	-0.7199	-0.5098
TRINITY_DN6693_c0_g1_i1_orf1	uncharacterized protein LOC114356358 [Ostrinia furnacalis]	-0.2338	1.98013	-0.6474	-0.522	-0.5768
TRINITY_DN6074_c0_g1_i1_orf1	uncharacterized protein C1683.06c-like isoform X1 [Ostrinia furnacalis]	-0.2446	1.9738	-0.6583	-0.3986	-0.6723
TRINITY_DN75188_c0_g1_i1_orf1	fatty acid-binding protein 1-like [Ostrinia furnacalis]	-0.1572	1.94334	-0.3848	-0.8861	-0.5152
TRINITY_DN717_c0_g1_i2_orfp1	TRINITY_DN717_c0_g1_i2_m.67915 TRINITY_DN717_c0_g1::TRINITY_DN717_c0_g1_i2::g.67915 ORF type:internal len:868 (+),score=265.71,Collagen PF01391.19 0.11,Collagen|PF01391.19|0.039,Collagen|PF01391.19|0.00054,Collagen|PF01391.19|0.0019,Collagen|PF01391.19|0.0005,Collagen|PF01391.19|9.9e-05,Collagen|PF01391.19|1.7e-07 TRINITY_DN717_c0_g1_i2:3-2603(+)	-0.0993	1.94223	-0.8451	-0.4451	-0.5527
TRINITY_DN11587_c0_g1_i7_orf1	elongation of very long chain fatty acids protein AAEL008004-like isoform X1 [Danaus plexippus plexippus] >XP_032511006.1 elongation of very long chain fatty acids protein AAEL008004-like isoform X1 [Danaus plexippus plexippus] >XP_032511007.1 elongation of very long chain fatty acids protein AAEL008004-like isoform X1 [Danaus plexippus plexippus] >XP_032511008.1 elongation of very long chain fatty acids protein AAEL008004-like isoform X1 [Danaus plexippus plexippus] >XP_032511009.1 elongation of very long chain fatty acids protein AAEL008004-like isoform X1 [Danaus plexippus plexippus]	-0.1799	1.97115	-0.5733	-0.6858	-0.5321

TRINITY_DN3784_c0_g1_i1_orf1	pancreatic triacylglycerol lipase-like [Ostrinia furnacalis]	-0.2267	1.96291	-0.8211	-0.4207	-0.4944
TRINITY_DN117_c0_g1_i6_orf1	lipase member I-like [Ostrinia furnacalis]	-0.0346	1.89133	-1.0315	-0.3199	-0.5054
TRINITY_DN1914_c0_g1_i6_orf1	loricrin-like [Ostrinia furnacalis]	0.02288	1.91171	-0.8802	-0.6115	-0.4429
TRINITY_DN81803_c0_g2_i1_orf1	cathepsin K-like [Ostrinia furnacalis]	-0.1101	1.93601	-0.8998	-0.4883	-0.4378
TRINITY_DN117_c0_g1_i4_orf1	lipase member I-like [Ostrinia furnacalis]	0.1922	1.85689	-0.9577	-0.579	-0.5123
TRINITY_DN33408_c0_g1_i1_orf1	hypothetical protein HF086_017664 [Spodoptera exigua]	0.3458	-0.0013	-0.2807	1.51723	-1.581
TRINITY_DN6203_c0_g1_i1_orfp1	TRINITY_DN6203_c0_g1_i1_m.72736 TRINITY_DN6203_c0_g1::TRINITY_DN6203_c0_g1_i1::g.72736 ORF type:internal len:93 (+),score=12.26 TRINITY_DN6203_c0_g1_i1:3-278(+)	0.41175	-0.0773	-1.0162	1.67492	-0.9932
TRINITY_DN128231_c0_g1_i5_orf1	glutathione S-transferase sigma3 [Glyphodes pyloalis]	-0.1912	-0.4286	-0.3961	1.94093	-0.925
TRINITY_DN8771_c0_g2_i1_orf1	regucalcin-like [Ostrinia furnacalis]	-0.404	0.56095	-0.6006	1.64712	-1.2035
