# Supplementary material for: Comparative Proteomics and Metabonomics Analysis of Different Diapause Stages Revealed a New Regulation Mechanism of Diapause in Loxostege sticticalis (Lepidoptera: Pyralidae)
Source: Molecules. 2024 Jul 25;29(15):3472. doi: 10.3390/molecules29153472 (PMC11314584; doi:10.3390/molecules29153472)
Supplement: Supplementary file 1 [file molecules-29-03472-s001.zip › analysis process/proteomic/Cluster analysis of expression patterns/Up/RDvsND up.pdf]

Accession	Description	ND	RD	PreD	CT	D
TRINITY_DN32514_c0_g2_i1_orf1	mucin-5AC-like [Ostrinia furnacalis]	-0.5029	1.99503	-0.3741	-0.5906	-0.5275
TRINITY_DN40669_c0_g2_i1_orf1	uncharacterized protein LOC114355531 [Ostrinia furnacalis]	-0.4265	1.99572	-0.4979	-0.6175	-0.4538
TRINITY_DN56155_c0_g1_i1_orf1	hypothetical protein evm_004679 [Chilo suppressalis] >CAH2989683.1 unnamed protein product [Chilo suppressalis]	-0.4213	1.9976	-0.5229	-0.5705	-0.4828
TRINITY_DN1124_c0_g1_i7_orf1	PREDICTED: cuticle protein 18.6, isoform B [Amyelois transitella]	-0.2822	1.96466	-0.4181	-0.84	-0.4243
TRINITY_DN30154_c0_g1_i1_orf1	serine/threonine-protein kinase SIK2 [Ostrinia furnacalis] >XP_028174514.1 serine/threonine-protein kinase SIK2 [Ostrinia furnacalis]	-0.7725	1.97265	-0.328	-0.5456	-0.3266
TRINITY_DN87803_c0_g1_i2_orf1	hypothetical protein NE865_05903 [Phthorimaea operculella]	-0.5347	1.99709	-0.3964	-0.5477	-0.5183
TRINITY_DN18624_c0_g1_i5_orf1	uncharacterized protein LOC114353135 [Ostrinia furnacalis]	-0.6386	1.99275	-0.5202	-0.4545	-0.3795
TRINITY_DN3190_c0_g1_i1_orf1	repetitive proline-rich cell wall protein 1 precursor [Papilio polytes] >XP_013147838.1 PREDICTED: repetitive proline-rich cell wall protein 1 [Papilio polytes] >BAM19190.1 cuticular protein PpolCPG24 [Papilio polytes]	-0.4534	1.99824	-0.4939	-0.5768	-0.4742
TRINITY_DN52553_c0_g2_i1_orf1	hemocyte protein-glutamine gamma-glutamyltransferase-like [Ostrinia furnacalis]	-0.4197	1.99274	-0.574	-0.6098	-0.3893
TRINITY_DN381_c0_g1_i1_orf1	cuticle protein 8-like [Ostrinia furnacalis]	-0.4127	1.99137	-0.4497	-0.6754	-0.4536
TRINITY_DN1326_c0_g1_i1_orf1	cuticle protein 7-like [Ostrinia furnacalis]	-0.0884	1.94666	-0.5825	-0.4804	-0.7953
TRINITY_DN15175_c0_g1_i1_orf1	zinc carboxypeptidase-like [Ostrinia furnacalis]	-0.7526	1.94642	-0.6799	-0.0977	-0.4163
TRINITY_DN2101_c0_g1_i6_orf1	protein obstructor-E-like [Ostrinia furnacalis]	-0.1741	1.96441	-0.4807	-0.7602	-0.5494
TRINITY_DN4021_c0_g1_i1_orf1	leech-derived tryptase inhibitor C-like [Ostrinia furnacalis]	-0.4082	1.9664	-0.3284	-0.8414	-0.3884
TRINITY_DN129835_c0_g1_i2_orf1	alpha-tocopherol transfer protein-like [Chelonus insularis]	-0.2244	1.93008	-0.1658	-0.6947	-0.8452
TRINITY_DN644_c0_g1_i1_orf1	cuticle protein 19-like [Ostrinia furnacalis]	-0.0835	1.94638	-0.5942	-0.7872	-0.4815
TRINITY_DN10824_c0_g1_i3_orf1	endochitinase isoform X2 [Ostrinia furnacalis]	-0.6977	1.96431	-0.1412	-0.59	-0.5355
TRINITY_DN138481_c0_g1_i5_orf1	hypothetical protein evm_003901 [Chilo suppressalis]	-0.869	1.93649	-0.6123	-0.1374	-0.3177
TRINITY_DN74538_c0_g1_i1_orf1	tetra-peptide repeat homeobox protein 1-like [Ostrinia furnacalis]	-0.4912	1.99931	-0.4691	-0.4908	-0.5482
TRINITY_DN4592_c0_g1_i1_orf1	hypothetical protein KGM_205563 [Danaus plexippus plexippus]	-0.5413	1.99815	-0.4541	-0.5535	-0.4493
TRINITY_DN9311_c0_g1_i1_orf1	cuticle protein 8-like [Ostrinia furnacalis]	-0.1998	1.96093	-0.3658	-0.7592	-0.6361
TRINITY_DN21719_c0_g1_i2_orf1	chymotrypsin-2-like [Ostrinia furnacalis]	-0.4284	1.99387	-0.6094	-0.3988	-0.5572
TRINITY_DN5408_c0_g1_i5_orf1	uncharacterized protein LOC114359912 [Ostrinia furnacalis]	-1.4089	1.52001	-0.0488	-0.6229	0.56055
TRINITY_DN18539_c0_g1_i1_orf1	pupal cuticle protein PCP52-like [Ostrinia furnacalis]	-0.4521	1.99685	-0.6068	-0.4771	-0.4608
TRINITY_DN619_c0_g1_i1_orf1	putative uncharacterized protein DDB_G0271606 [Ostrinia furnacalis]	-0.0382	1.93351	-0.4472	-0.8003	-0.6478
TRINITY_DN13576_c0_g1_i1_orf1	uncharacterized protein LOC114350099 [Ostrinia furnacalis]	-0.388	1.9852	-0.4226	-0.7288	-0.4458
TRINITY_DN38274_c0_g1_i1_orf1	uncharacterized protein LOC114360402 [Ostrinia furnacalis]	-0.4414	1.99721	-0.4997	-0.5949	-0.4612
TRINITY_DN98147_c0_g2_i1_orf1	hypothetical protein evm_002297 [Chilo suppressalis]	-0.488	1.96609	-0.4981	-0.7799	-0.2001
TRINITY_DN5581_c0_g1_i1_orf1	mucin-5AC-like [Ostrinia furnacalis]	-0.6103	1.98016	-0.6557	-0.4609	-0.2533
TRINITY_DN5829_c0_g1_i1_orf1	uncharacterized protein LOC114365758 isoform X3 [Ostrinia furnacalis]	-0.4616	1.99593	-0.6112	-0.4196	-0.5035
TRINITY_DN2946_c0_g1_i1_orf1	histidine-rich glycoprotein [Ostrinia furnacalis]	-0.3101	1.98559	-0.6107	-0.6359	-0.429
TRINITY_DN661_c0_g1_i1_orf1	hypothetical protein evm_002822 [Chilo suppressalis]	-0.7303	1.92367	-0.0188	-0.7822	-0.3924
TRINITY_DN35351_c0_g1_i3_orf1	adult-specific cuticular protein ACP-20-like [Ostrinia furnacalis]	-0.5545	1.99475	-0.5463	-0.535	-0.3589
TRINITY_DN18338_c0_g1_i6_orf1	aquaporin AQPAn.G isoform X1 [Ostrinia furnacalis]	-1.5681	1.53526	0.18761	-0.339	0.18419
TRINITY_DN2400_c0_g1_i1_orf1	uncharacterized protein LOC114351021 [Ostrinia furnacalis]	-0.7012	1.98425	-0.3292	-0.5394	-0.4144
TRINITY_DN26882_c0_g1_i1_orf1	uncharacterized protein LOC114349648 [Ostrinia furnacalis]	-0.4647	1.99963	-0.5218	-0.5114	-0.5017
TRINITY_DN57998_c1_g1_i1_orf1	uncharacterized protein LOC113509309, partial [Galleria mellonella]	-0.7032	1.96618	-0.3962	-0.202	-0.6647
TRINITY_DN3310_c0_g1_i1_orf1	hypothetical protein evm_010516 [Chilo suppressalis]	-0.1063	1.96004	-0.6489	-0.6037	-0.6012
TRINITY_DN29604_c0_g2_i2_orf1	neurofilament heavy polypeptide-like isoform X2 [Ostrinia furnacalis]	-1.1205	1.86847	-0.4587	-0.1678	-0.1215
TRINITY_DN2908_c0_g1_i1_orf1	uncharacterized protein LOC114361337 [Ostrinia furnacalis]	-0.2973	1.97977	-0.4602	-0.7403	-0.482
TRINITY_DN19043_c0_g2_i1_orf1	hypothetical protein EVAR_60653_1 [Eumeta japonica]	-1.2718	1.81138	-0.1254	-0.2038	-0.2104
TRINITY_DN28922_c0_g1_i2_orf1	uncharacterized protein LOC119829283 isoform X2 [Zerene cesonia]	-0.8907	1.9407	-0.3664	-0.1515	-0.5322
TRINITY_DN778_c0_g1_i1_orf1	uncharacterized protein LOC114363281 [Ostrinia furnacalis]	-0.0995	1.93848	-0.5662	-0.8621	-0.4107
TRINITY_DN15222_c0_g1_i4_orf1	lysosomal alpha-mannosidase-like [Ostrinia furnacalis]	-0.5229	1.89975	-0.6036	-0.8632	0.08993
TRINITY_DN12671_c0_g1_i6_orf1	hemicentin-1-like isoform X1 [Ostrinia furnacalis]	-1.0512	1.87108	-0.3467	0.04789	-0.521
TRINITY_DN9282_c0_g1_i2_orf1	uncharacterized protein LOC114363102 isoform X2 [Ostrinia furnacalis]	-0.04	1.93271	-0.5262	-0.8453	-0.5212
TRINITY_DN57998_c1_g3_i1_orf1	uncharacterized protein LOC114363305 isoform X2 [Ostrinia furnacalis]	-0.8223	1.96902	-0.4505	-0.3209	-0.3752
TRINITY_DN98334_c0_g1_i1_orf1	unnamed protein product [Spodoptera littoralis] >CAH1638738.1 unnamed protein product [Spodoptera littoralis]	-0.4133	1.99794	-0.5442	-0.5168	-0.5237
TRINITY_DN77425_c0_g1_i2_orf1	anosmin-1 [Ostrinia furnacalis] >XP_028178811.1 anosmin-1 [Ostrinia furnacalis]	-0.6919	1.98386	-0.553	-0.4274	-0.3115
TRINITY_DN338_c0_g1_i1_orf1	chorion protein S36-like [Ostrinia furnacalis]	-0.5527	1.99096	-0.3143	-0.5522	-0.5717
TRINITY_DN906_c0_g1_i4_orf1	uncharacterized protein LOC114360441, partial [Ostrinia furnacalis]	-0.8586	1.95715	-0.2246	-0.4425	-0.4314
TRINITY_DN12387_c0_g1_i1_orf1	repetitive proline-rich cell wall protein 2-like [Ostrinia furnacalis]	-0.4723	1.99925	-0.4734	-0.506	-0.5475
TRINITY_DN25273_c0_g1_i1_orf1	skin secretory protein xP2-like [Ostrinia furnacalis]	-0.9779	1.92687	-0.4592	-0.2359	-0.2539
TRINITY_DN138481_c0_g1_i2_orf1	cuticle protein 8-like isoform X2 [Vanessa tameamea]	-0.4394	1.98969	-0.6857	-0.3871	-0.4775
TRINITY_DN10747_c0_g1_i5_orf1	unnamed protein product [Plutella xylostella]	-0.6786	1.98764	-0.5397	-0.3567	-0.4126
TRINITY_DN25534_c0_g1_i1_orf1	venom serine carboxypeptidase-like [Ostrinia furnacalis]	-0.2934	1.97575	-0.713	-0.3579	-0.6115
TRINITY_DN31_c0_g1_i3_orfp1	TRINITY_DN31_c0_g1_i3_m.1394 TRINITY_DN31_c0_g1::TRINITY_DN31_c0_g1_i3::g.1394 ORF type:complete len:118 (+),score=43.35 TRINITY_DN31_c0_g1_i3:56-409(+)	-0.652	1.91966	-0.8528	-0.403	-0.0119

TRINITY_DN4384_c0_g1_i5_orf1	chemosensory protein 5 [Conogethes punctiferalis]	-0.8811	1.9469	-0.3634	-0.1861	-0.5162
TRINITY_DN49147_c0_g2_i1_orf1	glutenin, high molecular weight subunit PW212-like [Ostrinia furnacalis]	-1.1183	1.81086	-0.6812	0.04937	-0.0607
TRINITY_DN2311_c0_g3_i1_orf1	uncharacterized protein LOC114364231 isoform X1 [Ostrinia furnacalis] >XP_028176108.1 uncharacterized protein LOC114364231 isoform X2 [Ostrinia furnacalis]	-1.2125	1.75083	-0.4861	-0.3628	0.31061
TRINITY_DN7539_c0_g1_i2_orf1	serine protease inhibitor 3/4 [Ostrinia furnacalis]	-0.7339	1.97078	-0.6168	-0.3589	-0.2612
TRINITY_DN1935_c0_g1_i1_orf1	adult-specific cuticular protein ACP-22-like [Ostrinia furnacalis]	-0.2259	1.97884	-0.5083	-0.6113	-0.6333
TRINITY_DN7549_c0_g1_i1_orf1	uncharacterized protein LOC114355006 [Ostrinia furnacalis]	-0.2098	1.96663	-0.4921	-0.7847	-0.4801
TRINITY_DN54524_c0_g1_i6_orf1	serine protease inhibitor dipetalogastin [Ostrinia furnacalis]	-0.3108	1.98843	-0.5214	-0.6461	-0.5101
TRINITY_DN42719_c0_g1_i1_orf1	inter-alpha-trypsin inhibitor heavy chain H4-like isoform X11 [Ostrinia furnacalis]	-0.9083	1.87412	-0.5474	0.16192	-0.5803
TRINITY_DN12387_c1_g2_i1_orf1	skin secretory protein xP2-like [Ostrinia furnacalis]	-0.4719	1.99741	-0.5914	-0.4383	-0.4958
TRINITY_DN13686_c0_g2_i1_orf1	transmembrane protease serine 9-like [Ostrinia furnacalis]	-1.203	1.82352	-0.4292	-0.2077	0.01638
TRINITY_DN49785_c1_g1_i3_orf1	uncharacterized protein LOC114365444, partial [Ostrinia furnacalis]	-0.6518	1.97188	-0.3034	-0.7058	-0.3109
TRINITY_DN2002_c0_g1_i5_orfp1	TRINITY_DN2002_c0_g1_i5_m.4230 TRINITY_DN2002_c0_g1::TRINITY_DN2002_c0_g1_i5::g.4230 ORF type:3prime_partial len:259 (+),score=29.98,Peptidase_C39 PF03412.16 1.7,Peptidase_C39|PF03412.16|0.049 TRINITY_DN2002_c0_g1_i5:65-838(+)	-0.9272	1.87811	-0.0527	-0.7707	-0.1275
TRINITY_DN3616_c0_g2_i1_orf1	conotoxin ArMKLT2-032-like [Ostrinia furnacalis]	-1.1029	1.87404	-0.0539	-0.4341	-0.2831
TRINITY_DN24971_c0_g1_i3_orf1	uncharacterized protein LOC114352370 [Ostrinia furnacalis]	-1.2295	1.82537	-0.3277	-0.2146	-0.0536
TRINITY_DN5595_c0_g1_i1_orf1	keratin, type I cytoskeletal 10-like [Ostrinia furnacalis]	-0.4201	1.98264	-0.3978	-0.7499	-0.4149
TRINITY_DN91533_c0_g1_i1_orf1	uncharacterized protein LOC114361550 [Ostrinia furnacalis]	-0.5174	1.99873	-0.4412	-0.5499	-0.4902
TRINITY_DN68397_c0_g1_i2_orf1	clavesin-1-like [Ostrinia furnacalis]	-0.4149	1.98681	-0.529	-0.353	-0.6899
TRINITY_DN5907_c0_g1_i4_orf1	hypothetical protein evm_010265 [Chilo suppressalis] >CAB3524755.1 unnamed protein product [Chilo suppressalis] >CAH0397522.1 unnamed protein product [Chilo suppressalis]	-0.7056	1.98502	-0.5294	-0.3625	-0.3875
TRINITY_DN2140_c0_g1_i1_orf1	uncharacterized protein LOC126367764 [Pectinophora gossypiella]	-0.6991	1.97988	-0.2601	-0.4727	-0.548
TRINITY_DN12671_c0_g1_i4_orf1	hemicentin-1-like isoform X1 [Ostrinia furnacalis]	-0.8659	1.96204	-0.3668	-0.3571	-0.3722
TRINITY_DN1749_c0_g2_i2_orf1	putative GPI-anchored protein pfl2 isoform X1 [Ostrinia furnacalis]	-0.3791	1.97891	-0.425	-0.7738	-0.401
TRINITY_DN19990_c0_g1_i1_orf1	carboxypeptidase B-like [Ostrinia furnacalis]	-0.6143	1.99005	-0.531	-0.3138	-0.531
TRINITY_DN391_c0_g1_i4_orf1	phenoloxidase-activating enzyme-like [Ostrinia furnacalis]	-1.4891	1.64125	-0.2772	0.10877	0.01625
TRINITY_DN661_c0_g2_i2_orf1	cuticle protein 7-like [Ostrinia furnacalis]	-0.5741	1.93421	-0.9088	-0.1979	-0.2534
TRINITY_DN31314_c0_g1_i4_orf1	uncharacterized protein LOC114355976 [Ostrinia furnacalis]	-0.4876	1.99945	-0.5344	-0.4646	-0.5128
TRINITY_DN21555_c0_g1_i4_orf1	uncharacterized protein LOC114351844 [Ostrinia furnacalis] >XP_028158981.1 uncharacterized protein LOC114351844 [Ostrinia furnacalis] >XP_028158982.1 uncharacterized protein LOC114351844 [Ostrinia furnacalis] >XP_028158983.1 uncharacterized protein LOC114351844 [Ostrinia furnacalis] >XP_028158984.1 uncharacterized protein LOC114351844 [Ostrinia furnacalis] >XP_028158985.1 uncharacterized protein LOC114351844 [Ostrinia furnacalis] >5GPR_A Crystal structure of chitinase-h from Ostrinia furnacalis [Ostrinia furnacalis] >5GQB_A Crystal structure of chitinase-h from O. furnacalis in complex with chitohepatose [Ostrinia furnacalis] >6JMN_A Crystal structure of Ostrinia furnacalis Chitinase h complexed with compound 2-8-s2 [Ostrinia furnacalis] >BAE16587.1 chitinase [Ostrinia furnacalis]	-1.1256	1.85928	-0.4767	-0.2174	-0.0395
TRINITY_DN49143_c0_g1_i1_orf1	uncharacterized protein LOC107270465 [Cephus cinctus]	-0.5711	1.99847	-0.4875	-0.4524	-0.4875
TRINITY_DN3056_c0_g1_i1_orf1	spidroin-2-like [Ostrinia furnacalis]	-0.5251	1.97764	-0.3848	-0.7519	-0.3159
TRINITY_DN7785_c0_g1_i1_orf1	uncharacterized protein LOC114364098 [Ostrinia furnacalis]	-0.0375	1.93856	-0.4829	-0.7398	-0.6782
TRINITY_DN4676_c0_g1_i16_orf1	meiosis-specific nuclear structural protein 1-like isoform X2 [Ostrinia furnacalis]	-0.8369	1.89343	0.10404	-0.4573	-0.7033
TRINITY_DN1110_c1_g1_i9_orf1	MD-2-related lipid-recognition protein-like [Ostrinia furnacalis]	-0.9686	1.77041	-0.8699	0.32427	-0.2562
TRINITY_DN53866_c0_g1_i1_orf1	larval/pupal cuticle protein H1C-like [Ostrinia furnacalis]	-0.8831	1.92645	-0.0914	-0.6299	-0.322
TRINITY_DN6470_c0_g3_i2_orf1	trypsin CFT-1-like [Ostrinia furnacalis]	-0.4291	1.96293	-0.8548	-0.3669	-0.3121
TRINITY_DN6004_c0_g1_i1_orf1	endocuticle structural glycoprotein ABD-4-like [Ostrinia furnacalis]	-0.942	1.94185	-0.3786	-0.363	-0.2582
TRINITY_DN26301_c0_g1_i1_orf1	uncharacterized protein LOC114359193 [Ostrinia furnacalis]	-1.1117	1.88231	-0.3334	-0.3031	-0.1341
TRINITY_DN20793_c0_g2_i1_orf1	mucin-2-like [Ostrinia furnacalis]	-0.0551	1.94798	-0.5752	-0.6181	-0.6996
TRINITY_DN1749_c0_g1_i1_orf1	putative GPI-anchored protein pfl2 isoform X2 [Ostrinia furnacalis]	-0.531	1.98602	-0.3896	-0.6977	-0.3678
TRINITY_DN1326_c0_g1_i2_orf1	cuticle protein 7 [Plutella xylostella] >CAG9138501.1 unnamed protein product [Plutella xylostella]	-0.6201	1.90707	-0.6638	-0.7261	0.10299
TRINITY_DN52553_c0_g1_i1_orf1	hemocyte protein-glutamine gamma-glutamyltransferase-like [Ostrinia furnacalis]	-0.5465	1.991	-0.4015	-0.6482	-0.3947
TRINITY_DN2652_c0_g2_i1_orf1	peroxidase-like isoform X1 [Ostrinia furnacalis]	-0.9509	1.93676	-0.3332	-0.2244	-0.4283
TRINITY_DN143497_c0_g1_i1_orf1	fibroin heavy chain-like [Ostrinia furnacalis]	-0.1805	1.97035	-0.5744	-0.6986	-0.5169
TRINITY_DN5444_c0_g2_i1_orf1	phenoloxidase-activating factor 2-like [Ostrinia furnacalis]	-0.9122	1.93721	-0.2656	-0.5473	-0.212
TRINITY_DN2896_c0_g1_i2_orf1	general odorant-binding protein 56d-like isoform X2 [Ostrinia furnacalis]	-0.4983	1.98805	-0.3357	-0.6788	-0.4752
TRINITY_DN4324_c0_g1_i1_orf1	uncharacterized protein LOC114354985 isoform X1 [Ostrinia furnacalis]	-0.5896	1.98615	-0.6098	-0.5079	-0.2788
TRINITY_DN5664_c0_g1_i1_orf1	CDGSH iron-sulfur domain-containing protein 3, mitochondrial-like [Ostrinia furnacalis]	-0.2028	1.92568	-0.962	-0.5141	-0.2468
TRINITY_DN27968_c0_g2_i2_orf1	nucleolin-like [Ostrinia furnacalis]	-0.4267	1.99588	-0.6176	-0.487	-0.4646
TRINITY_DN26149_c0_g1_i5_orf1	thymosin beta isoform X3 [Ostrinia furnacalis]	-1.4077	1.70773	-0.3125	-0.0399	0.05235
TRINITY_DN58125_c0_g1_i1_orf1	protein yellow-like [Ostrinia furnacalis]	-0.7673	1.96912	-0.2791	-0.5842	-0.3384
TRINITY_DN2489_c0_g1_i1_orf1	uncharacterized protein LOC114354692 [Ostrinia furnacalis]	-0.9749	1.92419	-0.473	-0.1676	-0.3088
TRINITY_DN2442_c0_g1_i2_orf1	digestive cysteine proteinase 2 [Ostrinia furnacalis]	-1.2558	1.77788	0.1596	-0.387	-0.2946
TRINITY_DN2515_c0_g1_i6_orf1	chitooligosaccharidolytic beta-N-acetylglucosaminidase isoform X1 [Ostrinia furnacalis]	-1.2671	1.80704	-0.1622	-0.3144	-0.0634
TRINITY_DN74086_c0_g1_i1_orf1	UPF0489 protein C5orf22 homolog [Ostrinia furnacalis]	-0.8807	1.91781	-0.1308	-0.6966	-0.2097

TRINITY_DN1465_c2_g1_i2_orf1	transcription initiation factor TFIID subunit 1-like [Ostrinia furnacalis]	-1.1896	1.7845	-0.3731	-0.4549	0.23306
TRINITY_DN696_c1_g1_i10_orf1	titin-like [Ostrinia furnacalis]	-0.5541	1.99163	-0.6136	-0.4842	-0.3397
TRINITY_DN54366_c0_g1_i1_orf1	protein obstructor-E-like [Ostrinia furnacalis]	0.09528	1.90817	-0.6847	-0.735	-0.5838
TRINITY_DN3255_c0_g1_i1_orf1	uncharacterized protein LOC114351042 [Ostrinia furnacalis]	0.03529	1.91909	-0.5628	-0.82	-0.5715
TRINITY_DN110231_c0_g1_i1_orf1	protein singed [Ostrinia furnacalis] >XP_028161434.1 protein singed [Ostrinia furnacalis]	-1.0985	1.80145	0.27123	-0.4864	-0.4878
TRINITY_DN1902_c0_g1_i4_orf1	chemosensory protein 10 [Conogethes pinicolalis]	-0.6041	1.98544	-0.2803	-0.4857	-0.6153
TRINITY_DN267_c0_g1_i1_orf1	keratin, type I cytoskeletal 9-like [Ostrinia furnacalis]	-0.271	1.95928	-0.293	-0.817	-0.5783
TRINITY_DN113272_c0_g1_i1_orf1	altered inheritance of mitochondria protein 3-like [Ostrinia furnacalis]	0.12477	1.89062	-0.6271	-0.857	-0.5313
TRINITY_DN13221_c0_g1_i3_orf1	fasciclin-3-like [Ostrinia furnacalis]	-0.0193	1.49017	-1.5586	0.45982	-0.3721
TRINITY_DN7123_c0_g1_i1_orf1	activating signal cointegrator 1 complex subunit 2 homolog isoform X1 [Ostrinia furnacalis]	-0.3085	1.9896	-0.5463	-0.6122	-0.5225
TRINITY_DN5467_c0_g1_i5_orf1	synaptic vesicle glycoprotein 2B-like isoform X2 [Ostrinia furnacalis] >XP_028161209.1 synaptic vesicle glycoprotein 2B-like isoform X2 [Ostrinia furnacalis] >XP_028161210.1 synaptic vesicle glycoprotein 2B-like isoform X2 [Ostrinia furnacalis]	-1.4156	1.69305	0.02883	-0.3554	0.04919
TRINITY_DN4367_c0_g1_i1_orf1	heat shock protein 21.7c [Chilo suppressalis] >AWT57938.1 heat shock protein 21.7c [Chilo suppressalis]	-0.8102	1.89617	-0.8468	-0.0847	-0.1545
TRINITY_DN18502_c0_g1_i1_orf1	uncharacterized protein LOC114359515 [Ostrinia furnacalis]	0.22858	1.86481	-0.6613	-0.7772	-0.6549
TRINITY_DN110519_c0_g1_i1_orf1	uncharacterized protein LOC114366601 [Ostrinia furnacalis]	-0.2844	1.97121	-0.7526	-0.591	-0.3433
TRINITY_DN206_c0_g1_i8_orf1	A-kinase anchor protein 200-like [Ostrinia furnacalis] >XP_028173114.1 A-kinase anchor protein 200-like [Ostrinia furnacalis] >XP_028173115.1 A-kinase anchor protein 200-like [Ostrinia furnacalis]	-0.1944	1.94284	-0.4828	-0.9099	-0.3558
TRINITY_DN688_c0_g1_i8_orf1	lysosomal alpha-mannosidase-like [Ostrinia furnacalis]	-1.4246	1.70856	-0.0313	-0.0307	-0.222
TRINITY_DN2953_c1_g1_i10_orf1	methionine--tRNA ligase, cytoplasmic isoform X2 [Ostrinia furnacalis] >XP_028156683.1 methionine--tRNA ligase, cytoplasmic isoform X4 [Ostrinia furnacalis] >XP_028156684.1 methionine--tRNA ligase, cytoplasmic isoform X5 [Ostrinia furnacalis]	-0.9833	1.90463	-0.5527	-0.311	-0.0576
TRINITY_DN3715_c0_g1_i2_orf1	uncharacterized protein LOC114356437 isoform X1 [Ostrinia furnacalis]	-0.3023	1.86439	-1.0332	0.07118	-0.6001
TRINITY_DN33365_c0_g1_i1_orf1	mucin-5AC isoform X1 [Ostrinia furnacalis]	-0.5848	1.99142	-0.3578	-0.6088	-0.44
TRINITY_DN31417_c0_g1_i3_orf1	titin-like [Ostrinia furnacalis]	-1.1043	1.88646	-0.3673	-0.188	-0.2268
TRINITY_DN11970_c0_g1_i4_orf1	myb-like protein AA [Ostrinia furnacalis]	-0.5683	1.95626	-0.8333	-0.2462	-0.3084
TRINITY_DN1308_c0_g1_i4_orf1	serine proteinase stubble-like [Ostrinia furnacalis]	-0.7412	1.92675	-0.81	-0.2641	-0.1115
TRINITY_DN18804_c0_g1_i5_orf1	zinc finger protein Xfin-like [Ostrinia furnacalis]	-0.8865	1.86633	-0.4324	0.17022	-0.7176
TRINITY_DN42337_c0_g1_i5_orf1	hypothetical protein evm_002829 [Chilo suppressalis]	-0.3783	1.98184	-0.6686	-0.6177	-0.3172
TRINITY_DN4125_c0_g1_i14_orf1	angiotensin-converting enzyme-like isoform X1 [Ostrinia furnacalis]	0.12597	1.88712	-0.5973	-0.8866	-0.5291
TRINITY_DN33728_c0_g2_i1_orf1	uncharacterized protein LOC114350200 [Ostrinia furnacalis]	-1.1412	1.7585	-0.6994	0.27855	-0.1965
TRINITY_DN2794_c1_g1_i8_orf1	carboxypeptidase D [Ostrinia furnacalis]	-1.5301	1.59237	0.19351	-0.2909	0.03508
TRINITY_DN82426_c0_g1_i6_orfp1	lysosome-associated membrane glycoprotein 1-like isoform X4 [Ostrinia furnacalis]	-0.6949	1.93058	-0.767	-0.4485	-0.0201
TRINITY_DN2684_c0_g2_i3_orf1	glutamate decarboxylase 1-like isoform X1 [Ostrinia furnacalis]	-1.1274	1.76077	-0.7601	0.2098	-0.0831
TRINITY_DN11670_c0_g1_i1_orf1	teneurin-m isoform X1 [Ostrinia furnacalis]	-0.9395	1.89127	-0.0164	-0.6943	-0.2411
TRINITY_DN2290_c0_g1_i2_orfp1	TRINITY_DN2290_c0_g1_i2_m.69732 TRINITY_DN2290_c0_g1::TRINITY_DN2290_c0_g1_i2::g.69732 ORF type:complete len:234 (+),score=14.43 TRINITY_DN2290_c0_g1_i2:60-761(+)	-0.5769	1.81174	0.35091	-0.7462	-0.8395
TRINITY_DN268_c1_g1_i7_orf1	unnamed protein product [Spodoptera littoralis] >CAH1645252.1 unnamed protein product [Spodoptera littoralis]	-0.7838	1.97353	-0.4877	-0.2939	-0.408
TRINITY_DN2912_c0_g1_i1_orf1	uncharacterized protein LOC114352866 [Ostrinia furnacalis] >XP_028160411.1 uncharacterized protein LOC114352866 [Ostrinia furnacalis]	-1.057	1.799	-0.6587	0.28158	-0.3648
TRINITY_DN4189_c0_g2_i1_orf1	unnamed protein product [Chilo suppressalis]	-1.0871	1.81843	0.21088	-0.5786	-0.3636
TRINITY_DN2069_c1_g1_i8_orf1	lysosomal aspartic protease [Trichoplusia ni]	-1.4271	1.65635	-0.4193	-0.0187	0.20878
TRINITY_DN806_c0_g2_i1_orf1	uncharacterized protein LOC114355167 [Ostrinia furnacalis]	-0.1207	1.93624	-0.3381	-0.8625	-0.615
TRINITY_DN23364_c0_g1_i1_orf1	PREDICTED: uncharacterized protein LOC106134920 [Amyelois transitella]	-1.4176	1.70915	-0.1917	0.06709	-0.1669
TRINITY_DN206_c0_g1_i11_orf1	A-kinase anchor protein 200-like [Ostrinia furnacalis] >XP_028173114.1 A-kinase anchor protein 200-like [Ostrinia furnacalis] >XP_028173115.1 A-kinase anchor protein 200-like [Ostrinia furnacalis]	-0.5984	1.99626	-0.4597	-0.5216	-0.4167
TRINITY_DN4688_c0_g1_i2_orf1	uncharacterized protein LOC114359411 [Ostrinia furnacalis]	-0.9255	1.92622	-0.3012	-0.5707	-0.1287
TRINITY_DN98242_c0_g1_i1_orf1	adenosine deaminase 2-A-like [Galleria mellonella]	0.21135	1.82519	-0.2907	-0.9611	-0.7848
TRINITY_DN14532_c0_g1_i1_orf1	pupal cuticle protein-like [Trichoplusia ni]	0.09595	1.89689	-0.8054	-0.7332	-0.4542
TRINITY_DN218_c0_g1_i1_orf1	altered inheritance of mitochondria protein 3-like isoform X2 [Ostrinia furnacalis]	-0.2501	1.97921	-0.6255	-0.6516	-0.4519
TRINITY_DN125427_c0_g1_i1_orf1	heat shock protein 19.8 [Chilo suppressalis] >AGM90553.1 HSP19.8 [Chilo suppressalis] >BAE94664.1 small heat shock protein 19.7 [Chilo suppressalis]	-0.6684	1.90453	-0.9238	-0.0469	-0.2655
TRINITY_DN1986_c0_g1_i1_orf1	serine protease inhibitor 77Ba-like [Ostrinia furnacalis] >XP_028164032.1 serine protease inhibitor 77Ba-like [Ostrinia furnacalis]	-1.3693	1.75146	-0.1681	-0.1634	-0.0507
TRINITY_DN114834_c0_g1_i1_orf1	uncharacterized protein LOC115444227 [Manduca sexta] >XP_030025790.1 uncharacterized protein LOC115444227 [Manduca sexta] >XP_037296791.1 uncharacterized protein LOC115444227 [Manduca sexta] >XP_037296792.1 uncharacterized protein LOC115444227 [Manduca sexta] >KAG6441350.1 hypothetical protein O3G_MSEX001778 [Manduca sexta]	-0.2084	1.97456	-0.6947	-0.5591	-0.5123
TRINITY_DN1563_c0_g1_i4_orf1	pupal cuticle protein 36-like [Ostrinia furnacalis]	-0.6014	1.98626	-0.5011	-0.2814	-0.6023
TRINITY_DN38431_c0_g1_i1_orf1	neprilysin-2 isoform X1 [Ostrinia furnacalis]	-0.5327	1.99646	-0.5575	-0.5199	-0.3864
TRINITY_DN5757_c0_g1_i1_orf1	ATP-dependent DNA helicase 2 subunit 1 [Ostrinia furnacalis]	-0.675	1.98567	-0.4574	-0.3079	-0.5454
TRINITY_DN97138_c0_g1_i2_orf1	tubulin beta chain-like isoform X2 [Ostrinia furnacalis]	-1.0937	1.89179	-0.3521	-0.2513	-0.1947
TRINITY_DN668_c0_g1_i4_orf1	fatty acid synthase-like isoform X1 [Ostrinia furnacalis]	-0.1118	1.95841	-0.5165	-0.685	-0.6451
TRINITY_DN10304_c0_g2_i1_orf1	glycine-rich cell wall structural protein [Ostrinia furnacalis]	-0.5227	1.98338	-0.5625	-0.2577	-0.6404
TRINITY_DN42964_c0_g1_i1_orf1	protein lethal(2)essential for life-like [Galleria mellonella]	-0.4821	1.46893	-1.2742	-0.5436	0.83104

TRINITY_DN661_c1_g2_i1_orf1	larval/pupal cuticle protein H1C-like [Ostrinia furnacalis]	0.02299	1.91021	-0.4875	-0.9071	-0.5386
TRINITY_DN31619_c0_g1_i2_orf1	endocuticle structural glycoprotein ABD-4-like [Ostrinia furnacalis]	0.11217	1.89043	-0.456	-0.8432	-0.7035
TRINITY_DN147458_c0_g1_i1_orf1	60S ribosomal protein L5, partial [Cotesia chilonis]	0.25867	1.77783	-0.3023	-1.1648	-0.5694
TRINITY_DN1196_c0_g1_i4_orf1	glucosamine-6-phosphate isomerase isoform X2 [Ostrinia furnacalis]	-0.6598	1.7745	0.42066	-0.9411	-0.5942
TRINITY_DN10766_c0_g1_i1_orf1	hypothetical protein evm_008559 [Chilo suppressalis]	-0.8955	1.89928	-0.1069	-0.7461	-0.1508
TRINITY_DN36899_c0_g1_i1_orf1	glucose dehydrogenase [FAD, quinone]-like [Ostrinia furnacalis]	-1.3218	1.69307	0.2017	-0.5878	0.01484
TRINITY_DN104_c0_g1_i4_orf1	PREDICTED: heparan-alpha-glucosaminide N-acetyltransferase [Amyelois transitella]	-1.2805	1.67067	0.32396	-0.6805	-0.0336
TRINITY_DN467_c0_g3_i1_orf1	histone-lysine N-methyltransferase 2B-like, partial [Ostrinia furnacalis]	0.0258	1.92398	-0.574	-0.7922	-0.5836
TRINITY_DN4689_c0_g1_i5_orf1	pericentriolar material 1 protein-like isoform X3 [Ostrinia furnacalis]	-0.5737	1.90303	-0.8668	0.07776	-0.5403
TRINITY_DN36281_c0_g1_i2_orf1	putative uncharacterized protein DDB_G0282499 isoform X1 [Ostrinia furnacalis]	-0.6983	1.98283	-0.4445	-0.5432	-0.2968
TRINITY_DN83374_c0_g1_i1_orf1	uncharacterized protein LOC114350302, partial [Ostrinia furnacalis]	-0.1579	1.96785	-0.6776	-0.5321	-0.6003
TRINITY_DN3833_c0_g1_i4_orf1	division abnormally delayed protein [Ostrinia furnacalis]	-0.5258	1.99644	-0.5892	-0.4079	-0.4736
TRINITY_DN20244_c0_g1_i1_orfp1	uncharacterized protein LOC125235519 [Leguminivora glycinivorella]	0.39736	1.77601	-0.8905	-0.4517	-0.8312
TRINITY_DN48590_c0_g1_i1_orf1	acyl-CoA Delta(11) desaturase isoform X1 [Ostrinia furnacalis] >XP_028172999.1 acyl-CoA Delta(11) desaturase isoform X2 [Ostrinia furnacalis] >XP_028173000.1 acyl-CoA Delta(11) desaturase isoform X1 [Ostrinia furnacalis]	0.24769	1.85729	-0.6761	-0.7892	-0.6396
TRINITY_DN37585_c0_g2_i1_orf1	cuticle protein 19.8-like [Ostrinia furnacalis]	-0.0947	1.92523	-0.6123	-0.8985	-0.3197
TRINITY_DN14944_c0_g1_i9_orf1	casein kinase I isoform X1 [Ostrinia furnacalis] >XP_028158159.1 casein kinase I isoform X1 [Ostrinia furnacalis] >XP_028158160.1 casein kinase I isoform X1 [Ostrinia furnacalis] >XP_028158161.1 casein kinase I isoform X1 [Ostrinia furnacalis] >XP_028158163.1 casein kinase I isoform X1 [Ostrinia furnacalis] >XP_028158164.1 casein kinase I isoform X1 [Ostrinia furnacalis] >XP_028158165.1 casein kinase I isoform X1 [Ostrinia furnacalis] >XP_028158166.1 casein kinase I isoform X1 [Ostrinia furnacalis]	-1.0158	1.83552	-0.2508	-0.717	0.14814
TRINITY_DN7565_c0_g1_i3_orf1	acylphosphatase-2-like [Ostrinia furnacalis]	-0.1334	1.89125	-1.072	-0.4453	-0.2405
TRINITY_DN111621_c0_g3_i1_orf1	Serine proteinase stubble [Eumeta japonica]	-0.2515	1.90086	-0.9389	-0.0474	-0.6631
TRINITY_DN6423_c0_g1_i5_orf1	phenoloxidase-activating factor 2-like isoform X2 [Ostrinia furnacalis]	-1.4549	1.6612	-0.3372	0.03908	0.09183
TRINITY_DN64616_c0_g1_i1_orf1	uncharacterized protein LOC114366101 [Ostrinia furnacalis]	-0.8455	1.79134	-1.0301	0.11828	-0.034
TRINITY_DN72999_c0_g1_i1_orf1	protein obstructor-E-like isoform X1 [Ostrinia furnacalis] >XP_028169319.1 protein obstructor-E-like isoform X2 [Ostrinia furnacalis]	0.33894	1.81549	-0.5898	-0.875	-0.6896
TRINITY_DN25345_c0_g1_i1_orf1	chromodomain-helicase-DNA-binding protein 1 isoform X3 [Ostrinia furnacalis]	-0.2572	1.83584	-0.9646	0.16444	-0.7785
TRINITY_DN1703_c0_g1_i6_orf1	leucine-rich repeat-containing protein 15-like [Ostrinia furnacalis] >XP_028171914.1 leucine-rich repeat-containing protein 15-like [Ostrinia furnacalis] >XP_028171921.1 leucine-rich repeat-containing protein 15-like [Ostrinia furnacalis]	-1.1532	1.84512	-0.495	-0.0749	-0.1219
TRINITY_DN5074_c0_g1_i7_orf1	zonadhesin-like [Ostrinia furnacalis]	-0.2288	1.91383	-0.2564	-1.0295	-0.3991
TRINITY_DN3005_c0_g1_i7_orf1	lachesin-like isoform X3 [Ostrinia furnacalis]	-1.1368	1.83031	-0.5487	0.07932	-0.2241
TRINITY_DN37585_c0_g1_i1_orf1	cuticle protein 19.8-like [Ostrinia furnacalis]	-0.871	1.88723	-0.672	0.1185	-0.4627
TRINITY_DN1868_c0_g1_i1_orf1	protein obstructor-E isoform X1 [Ostrinia furnacalis]	0.47972	1.73191	-1.0091	-0.4808	-0.7217
TRINITY_DN280_c0_g1_i8_orf1	Tubulin beta-1 chain [Papilio xuthus]	-0.2393	1.98214	-0.5514	-0.601	-0.5904
TRINITY_DN10364_c0_g1_i5_orf1	uncharacterized protein LOC114352615 [Ostrinia furnacalis]	-0.6783	1.95687	-0.7427	-0.3554	-0.1804
TRINITY_DN1630_c0_g1_i6_orf1	major facilitator superfamily domain-containing protein 1-like [Ostrinia furnacalis]	-1.3376	1.75713	-0.1486	0.04433	-0.3153
TRINITY_DN25987_c0_g1_i5_orf1	GILT-like protein 2 isoform X1 [Ostrinia furnacalis] >XP_028156245.1 GILT-like protein 2 isoform X2 [Ostrinia furnacalis] >XP_028156247.1 GILT-like protein 2 isoform X3 [Ostrinia furnacalis]	-0.8279	1.82718	0.29837	-0.7989	-0.4988
TRINITY_DN15865_c0_g2_i2_orf1	carboxylesterase [Cnaphalocrocis medinalis]	-1.0558	1.83435	-0.7124	-0.1067	0.04047
TRINITY_DN585_c0_g1_i5_orf1	very low-density lipoprotein receptor isoform X2 [Galleria mellonella]	-0.7648	1.93387	-0.1078	-0.3065	-0.7547
TRINITY_DN13648_c0_g1_i6_orf1	neurexin-4 [Ostrinia furnacalis]	-0.3754	1.97094	-0.4251	-0.8185	-0.3519
TRINITY_DN14856_c0_g1_i1_orf1	upstream activation factor subunit spp27 [Ostrinia furnacalis]	-1.0332	1.84638	0.04922	-0.7043	-0.1582
TRINITY_DN1196_c0_g1_i5_orf1	glucosamine-6-phosphate isomerase isoform X1 [Ostrinia furnacalis]	-0.4981	1.93157	-0.1131	-0.9214	-0.3989
TRINITY_DN14009_c0_g1_i1_orf1	proline-rich extensin-like protein EPR1 [Manduca sexta]	-0.1996	1.9602	-0.4159	-0.815	-0.5297
TRINITY_DN14721_c0_g1_i2_orf1	protein masquerade-like isoform X2 [Ostrinia furnacalis]	0.31441	1.8303	-0.681	-0.6418	-0.8218
TRINITY_DN2109_c0_g1_i4_orf1	mucin-2-like isoform X2 [Ostrinia furnacalis]	-0.3081	1.70962	-1.0287	0.46726	-0.8401
TRINITY_DN978_c9_g2_i1_orf1	hypothetical protein evm_000959 [Chilo suppressalis]	0.26639	1.84075	-0.5439	-0.8889	-0.6744
TRINITY_DN41761_c0_g1_i4_orf1	transmembrane protease serine 9 [Ostrinia furnacalis]	-1.0157	1.8951	-0.5152	-0.0317	-0.3325
TRINITY_DN11772_c0_g1_i1_orf1	conserved oligomeric Golgi complex subunit 2 [Ostrinia furnacalis]	-0.7737	1.95473	-0.3978	-0.6318	-0.1514
TRINITY_DN15420_c0_g3_i2_orf1	elongation factor 1-alpha 2-like [Galleria mellonella] >XP_031769625.1 elongation factor 1-alpha 2-like [Galleria mellonella]	-0.6647	1.92987	-0.8686	-0.2177	-0.1789
TRINITY_DN1252_c0_g1_i3_orf1	unnamed protein product [Chilo suppressalis]	-0.5125	1.90763	-0.1413	-0.2447	-1.0092
TRINITY_DN13088_c0_g1_i5_orf1	beta-hexosaminidase subunit alpha-like isoform X2 [Ostrinia furnacalis]	-0.9931	1.89347	-0.4456	0.02415	-0.4789
TRINITY_DN13887_c0_g1_i5_orf1	transmembrane protein 184B isoform X3 [Ostrinia furnacalis]	-0.9982	1.91745	-0.4787	-0.241	-0.1995
TRINITY_DN661_c0_g3_i5_orf1	cuticle protein 18.6-like [Ostrinia furnacalis]	-0.4828	1.96931	-0.6302	-0.6781	-0.1782
TRINITY_DN2416_c0_g1_i5_orf1	somatomedin-B and thrombospondin type-1 domain-containing protein [Ostrinia furnacalis] >XP_028177886.1 somatomedin-B and thrombospondin type-1 domain-containing protein [Ostrinia furnacalis]	-1.3068	1.61888	-0.0408	-0.6983	0.42695
TRINITY_DN321_c0_g1_i1_orf1	uncharacterized protein LOC126371336 [Pectinophora gossypiella]	-0.786	1.93619	-0.3948	-0.6877	-0.0677
TRINITY_DN712_c0_g2_i1_orf1	serine protease inhibitor 77Ba-like [Ostrinia furnacalis]	-1.1325	1.8337	-0.5702	-0.1687	0.0377
TRINITY_DN501_c0_g1_i5_orf1	hypothetical protein evm_002550 [Chilo suppressalis]	-0.4003	1.99457	-0.516	-0.6224	-0.4559
TRINITY_DN19951_c0_g1_i5_orf1	protein croquemort-like [Ostrinia furnacalis]	-1.1742	1.85178	-0.3437	-0.0712	-0.2627
TRINITY_DN98016_c0_g1_i1_orf1	methanethiol oxidase [Ostrinia furnacalis]	-1.4114	1.62849	-0.1443	-0.4442	0.37136

TRINITY_DN19923_c0_g1_i1_orf1	uncharacterized protein LOC114350958 [Ostrinia furnacalis]	-0.0245	1.94062	-0.7144	-0.5786	-0.6231
TRINITY_DN7735_c1_g1_i1_orf1	cuticular protein CPH [Spodoptera litura]	-0.2785	1.95789	-0.5172	-0.8515	-0.3107
TRINITY_DN34786_c0_g1_i1_orf1	small heat shock protein Hsp29.7 [Ostrinia furnacalis]	-0.1377	1.95796	-0.765	-0.4756	-0.5797
TRINITY_DN4013_c0_g1_i4_orf1	uncharacterized protein LOC114353190 isoform X1 [Ostrinia furnacalis] >XP_028160984.1 uncharacterized protein LOC114353190 isoform X2 [Ostrinia furnacalis] >XP_028161062.1 uncharacterized protein LOC114353190 isoform X1 [Ostrinia furnacalis] >XP_028161142.1 uncharacterized protein LOC114353190 isoform X1 [Ostrinia furnacalis]	-0.83	1.85295	0.25227	-0.616	-0.6592
TRINITY_DN867_c0_g1_i1_orf1	hemicentin-2-like isoform X1 [Ostrinia furnacalis]	-0.0705	1.89718	-0.4688	-0.3225	-1.0353
TRINITY_DN4217_c0_g1_i2_orf1	hypothetical protein evm_006611 [Chilo suppressalis]	-1.0639	1.89307	-0.1568	-0.4662	-0.2062
TRINITY_DN9455_c0_g1_i6_orf1	uncharacterized protein LOC114360866 isoform X4 [Ostrinia furnacalis]	0.37664	1.78598	-0.9901	-0.6055	-0.567
TRINITY_DN21719_c0_g2_i4_orf1	chymotrypsin-2-like [Ostrinia furnacalis]	-0.522	1.99242	-0.3919	-0.6455	-0.4331
TRINITY_DN3073_c0_g1_i7_orf1	claspin-like [Ostrinia furnacalis]	-0.5702	1.98945	-0.4152	-0.6444	-0.3597
TRINITY_DN714_c0_g1_i3_orf1	thymosin beta isoform X4 [Ostrinia furnacalis]	-0.718	1.9542	-0.4391	-0.6771	-0.1201
TRINITY_DN10057_c0_g2_i1_orf1	cell wall protein DAN4 [Ostrinia furnacalis]	0.50736	1.74491	-0.7522	-0.809	-0.6911
TRINITY_DN4273_c1_g1_i5_orf1	tetraspanin-13 isoform X1 [Ostrinia furnacalis]	-1.3045	1.77836	0.01552	-0.1558	-0.3337
TRINITY_DN14774_c0_g1_i4_orf1	aminopeptidase N-like [Ostrinia furnacalis]	-0.5783	1.73297	0.51519	-0.872	-0.7979
TRINITY_DN52788_c0_g1_i1_orf1	putative fatty acyl-CoA reductase CG5065 isoform X1 [Ostrinia furnacalis]	-0.4855	1.99834	-0.4372	-0.5125	-0.5631
TRINITY_DN1293_c0_g1_i4_orf1	putative fatty acyl-CoA reductase CG5065 [Ostrinia furnacalis]	-0.0377	1.9038	-0.2584	-0.8903	-0.7175
TRINITY_DN6974_c0_g2_i1_orf1	mucolipin-3-like [Ostrinia furnacalis]	-0.3749	1.84894	0.19967	-0.8509	-0.8228
TRINITY_DN12673_c3_g1_i2_orf1	unnamed protein product [Chilo suppressalis]	-0.917	1.85975	0.19573	-0.4854	-0.6531
TRINITY_DN46090_c0_g2_i1_orf1	inactive tyrosine-protein kinase 7-like, partial [Ostrinia furnacalis]	-0.0198	1.90951	-0.3454	-0.9165	-0.6278
TRINITY_DN7711_c1_g1_i3_orf1	long-chain fatty acid transport protein 1-like [Ostrinia furnacalis]	-0.8641	1.76844	0.42684	-0.8355	-0.4957
TRINITY_DN41_c0_g1_i5_orf1	putative phospholipase B-like 2 [Ostrinia furnacalis]	-1.2045	1.82891	-0.0216	-0.1962	-0.4066
TRINITY_DN5893_c0_g1_i7_orf1	jupiter microtubule associated homolog 1-like [Ostrinia furnacalis] >XP_028173360.1 jupiter microtubule associated homolog 1-like [Ostrinia furnacalis]	-0.2276	1.97886	-0.5291	-0.5583	-0.6639
TRINITY_DN1491_c0_g1_i8_orf1	GILT-like protein 2 isoform X1 [Ostrinia furnacalis] >XP_028156245.1 GILT-like protein 2 isoform X2 [Ostrinia furnacalis] >XP_028156247.1 GILT-like protein 2 isoform X3 [Ostrinia furnacalis]	-0.9667	1.93624	-0.3406	-0.3515	-0.2775
TRINITY_DN73923_c0_g1_i1_orf1	protein obstructor-E-like [Ostrinia furnacalis]	0.81498	1.56249	-0.8734	-0.7678	-0.7363
TRINITY_DN4886_c0_g1_i6_orf1	uncharacterized protein LOC114349567 [Ostrinia furnacalis]	-1.2135	1.82588	-0.2079	-0.0172	-0.3873
TRINITY_DN2323_c0_g1_i4_orf1	uncharacterized protein LOC114364097 isoform X2 [Ostrinia furnacalis]	-0.3301	1.97009	-0.8006	-0.3288	-0.5107
TRINITY_DN895_c0_g2_i1_orf1	protein N-terminal asparagine amidohydrolase [Cotesia glomerata] >XP_044583616.1 protein N-terminal asparagine amidohydrolase [Cotesia glomerata] >XP_044583617.1 protein N-terminal asparagine amidohydrolase [Cotesia glomerata] >KAH0553813.1 hypothetical protein KQX54_004640 [Cotesia glomerata]	-1.3075	1.69963	0.33988	-0.4617	-0.2704
TRINITY_DN8641_c0_g1_i1_orf1	uncharacterized protein LOC114357057 [Ostrinia furnacalis]	-0.823	1.93588	-0.6997	-0.1981	-0.2151
TRINITY_DN29879_c0_g1_i3_orf1	uncharacterized protein LOC114350556 isoform X1 [Ostrinia furnacalis] >XP_028157201.1 uncharacterized protein LOC114350556 isoform X2 [Ostrinia furnacalis] >XP_028157202.1 uncharacterized protein LOC114350556 isoform X3 [Ostrinia furnacalis]	-0.507	1.69528	-0.8456	-0.9114	0.56867
TRINITY_DN1436_c0_g1_i3_orf1	vacuolar protein sorting-associated protein 27-like [Trichoplusia ni]	-0.0181	1.90638	-0.4288	-0.9711	-0.4884
TRINITY_DN2107_c0_g2_i3_orf1	LIM and SH3 domain protein Lasp [Ostrinia furnacalis]	-1.1727	1.86052	-0.1742	-0.2339	-0.2798
TRINITY_DN8569_c1_g2_i7_orf1	furin-like protease 1, partial [Ostrinia furnacalis]	-0.4064	1.8892	-0.0513	-1.0611	-0.3704
TRINITY_DN1533_c0_g2_i1_orf1	unnamed protein product [Chilo suppressalis]	-1.3729	1.74635	-0.1656	-0.1943	-0.0135
TRINITY_DN6586_c0_g1_i1_orf1	fatty acyl-CoA reductase wat-like isoform X1 [Ostrinia furnacalis]	0.49127	1.75321	-0.6965	-0.8028	-0.7452
TRINITY_DN8692_c0_g1_i2_orf1	caspase-1-like [Ostrinia furnacalis]	-0.7189	1.94918	-0.3686	-0.7281	-0.1336
TRINITY_DN4571_c0_g1_i4_orf1	PREDICTED: nuclear factor NF-kappa-B p105 subunit [Microplitis demolitor] >KAG6558391.1 viral ankyrin V1 [Microplitis demolitor]	0.33373	1.59802	-0.7996	-1.2923	0.1601
TRINITY_DN12464_c0_g1_i3_orf1	PH and SEC7 domain-containing protein 1 [Trichoplusia ni]	-0.4377	1.97962	-0.6039	-0.263	-0.675
TRINITY_DN74069_c0_g1_i1_orf1	unnamed protein product [Parnassius apollo] >CAG5017650.1 unnamed protein product [Parnassius apollo]	0.34065	1.77677	-0.9765	-0.8181	-0.3228
TRINITY_DN13067_c0_g1_i6_orf1	diphosphomevalonate decarboxylase [Ostrinia furnacalis]	-0.4742	1.84177	-1.1611	-0.1852	-0.0212
TRINITY_DN4144_c0_g1_i7_orf1	uncharacterized protein LOC114350172 [Ostrinia furnacalis]	0.16822	1.87969	-0.6447	-0.5632	-0.84
TRINITY_DN31943_c0_g1_i1_orf1	proteoglycan Cow [Ostrinia furnacalis]	-0.3165	1.90467	-1.0485	-0.1534	-0.3863
TRINITY_DN42333_c0_g1_i5_orf1	regucalcin-like [Ostrinia furnacalis]	-0.7187	1.8641	-0.981	0.04558	-0.2099
TRINITY_DN82104_c0_g1_i5_orf1	uncharacterized protein LOC114349939 [Ostrinia furnacalis] >XP_028156338.1 uncharacterized protein LOC114349939 [Ostrinia furnacalis]	-1.3946	1.69002	-0.4061	-0.0627	0.17338
TRINITY_DN650_c0_g1_i3_orf1	chitinase 7 [Glyphodes pyloalis]	0.28102	1.80654	-0.9787	-0.7603	-0.3485
TRINITY_DN15865_c0_g1_i1_orf1	carboxylesterase, partial [Ostrinia furnacalis]	0.14796	1.86531	-0.8171	-0.8385	-0.3576
TRINITY_DN3887_c0_g1_i1_orf1	formin-like protein isoform X3 [Ostrinia furnacalis] >XP_028170442.1 formin-like protein isoform X6 [Ostrinia furnacalis]	-0.3015	1.94831	-0.192	-0.8224	-0.6325
TRINITY_DN23582_c0_g1_i1_orf1	unnamed protein product [Diatraea saccharalis]	-0.1634	1.74434	-1.0516	0.3204	-0.8497
TRINITY_DN12331_c0_g1_i5_orf1	septin-7 isoform X1 [Ostrinia furnacalis]	-0.6141	1.99469	-0.5303	-0.4512	-0.3991
TRINITY_DN7803_c0_g1_i2_orf1	membrane-associated protein Hem [Ostrinia furnacalis]	0.09822	1.86127	-0.8059	-0.9017	-0.2519
TRINITY_DN50471_c0_g1_i4_orf1	hypothetical protein evm_008982 [Chilo suppressalis]	-0.4043	1.95116	-0.7749	-0.1299	-0.642
TRINITY_DN22443_c0_g2_i3_orf1	protein eiger [Ostrinia furnacalis] >QKV49447.1 eiger [Ostrinia furnacalis]	-1.2429	1.81556	-0.2893	-0.0093	-0.274
TRINITY_DN1480_c0_g1_i5_orf1	carboxypeptidase N subunit 2-like [Ostrinia furnacalis]	-1.1964	1.7406	-0.3848	-0.5145	0.35513
TRINITY_DN21930_c0_g1_i1_orf1	coactosin-like protein isoform X2 [Trichoplusia ni]	-0.3263	1.98427	-0.5391	-0.7003	-0.4187

TRINITY_DN1005_c0_g1_i5_orf1	hypothetical protein evm_008839 [Chilo suppressalis] >CAB3526474.1 unnamed protein product [Chilo suppressalis] >CAH0403802.1 unnamed protein product [Chilo suppressalis]	-0.1099	1.82579	-0.5491	-1.1632	-0.0037
TRINITY_DN20796_c0_g1_i4_orf1	probable low-specificity L-threonine aldolase 2 [Ostrinia furnacalis]	-1.3023	1.72718	-0.5108	-0.1247	0.2106
TRINITY_DN1481_c0_g1_i4_orf1	G protein-coupled receptor kinase 1 isoform X2 [Helicoverpa armigera] >XP_047029555.1 G protein-coupled receptor kinase 1 isoform X2 [Helicoverpa zea] >ANZ22924.1 G protein-coupled receptor kinase 2 [Helicoverpa armigera armigera]	-0.7818	1.91824	-0.3904	0.0001	-0.7462
TRINITY_DN58872_c0_g1_i1_orfp1	cuticle protein 64-like [Pectinophora gossypiella]	-0.5097	1.94256	-0.897	-0.1719	-0.364
TRINITY_DN445_c0_g1_i2_orf1	sorting nexin-17 [Ostrinia furnacalis]	-1.318	1.71416	0.28899	-0.2862	-0.399
TRINITY_DN4898_c0_g1_i7_orf1	annulin-like isoform X3 [Ostrinia furnacalis]	0.24566	1.85648	-0.5944	-0.7939	-0.7138
TRINITY_DN3978_c0_g2_i1_orf1	nicastrin [Ostrinia furnacalis]	-0.8368	1.96209	-0.306	-0.3078	-0.5115
TRINITY_DN9028_c0_g1_i5_orf1	decaprenyl-diphosphate synthase subunit 2-like [Ostrinia furnacalis]	-0.137	1.91759	-0.5581	-0.9615	-0.2611
TRINITY_DN6870_c0_g1_i5_orf1	uncharacterized protein LOC114365835 [Ostrinia furnacalis]	-0.4327	1.69473	-0.8519	-0.957	0.54686
TRINITY_DN32956_c0_g1_i4_orf1	inositol-trisphosphate 3-kinase A isoform X1 [Vanessa tameamea] >XP_047534115.1 inositol-trisphosphate 3-kinase A isoform X1 [Vanessa atalanta] >XP_047534116.1 inositol-trisphosphate 3-kinase A isoform X1 [Vanessa atalanta] >XP_047534117.1 inositol-trisphosphate 3-kinase A isoform X1 [Vanessa atalanta]	0.35912	1.75816	-0.3111	-1.0643	-0.7418
TRINITY_DN5954_c0_g1_i2_orf1	myosin-VIIa [Ostrinia furnacalis] >XP_028155907.1 myosin-VIIa [Ostrinia furnacalis]	0.10325	1.90547	-0.5828	-0.6794	-0.7465
TRINITY_DN34727_c0_g1_i3_orf1	tyrosine-protein kinase Src42A isoform X2 [Trichoplusia ni]	-0.4129	1.9472	-0.3157	-0.9222	-0.2965
TRINITY_DN4320_c0_g1_i1_orf1	selenide, water dikinase [Ostrinia furnacalis] >CAG9756850.1 unnamed protein product [Diatraea saccharalis] >CAG9795535.1 unnamed protein product [Diatraea saccharalis]	-0.3523	1.97402	-0.2895	-0.6031	-0.7291
TRINITY_DN9_c0_g1_i11_orf1	heterogeneous nuclear ribonucleoprotein Q isoform X2 [Galleria mellonella]	-0.2583	1.9609	-0.3269	-0.5554	-0.8202
TRINITY_DN86621_c0_g1_i2_orf1	protein sly1 homolog isoform X1 [Ostrinia furnacalis] >XP_028165613.1 protein sly1 homolog isoform X2 [Ostrinia furnacalis]	-0.0656	1.82623	-0.165	-1.2223	-0.3733
TRINITY_DN86309_c0_g1_i4_orf1	filamin-A isoform X2 [Ostrinia furnacalis]	-0.3694	1.98762	-0.6942	-0.5072	-0.4169
TRINITY_DN48641_c0_g1_i4_orf1	RNA-binding protein 45-like [Galleria mellonella]	0.85224	1.45476	-0.27	-0.9484	-1.0886
TRINITY_DN3675_c0_g1_i1_orf1	unnamed protein product [Spodoptera exigua]	-1.3238	1.7693	0.00858	-0.1437	-0.3104
TRINITY_DN5568_c0_g2_i2_orf1	carboxypeptidase D isoform X5 [Ostrinia furnacalis]	0.23837	1.83727	-0.7149	-0.936	-0.4247
TRINITY_DN19998_c0_g1_i1_orf1	ubiquitin-conjugating enzyme E2 variant 2 [Helicoverpa armigera] >XP_026325122.1 ubiquitin-conjugating enzyme E2 variant 2 [Hyposmocoma kahamanoa] >XP_026499214.1 ubiquitin-conjugating enzyme E2 variant 2 [Vanessa tameamea] >XP_026738520.1 ubiquitin-conjugating enzyme E2 variant 2 [Trichoplusia ni] >XP_026762551.1 ubiquitin-conjugating enzyme E2 variant 2 [Galleria mellonella] >XP_028172566.1 ubiquitin-conjugating enzyme E2 variant 2 [Ostrinia furnacalis] >XP_032520494.1 ubiquitin-conjugating enzyme E2 variant 2 [Danaus plexippus plexippus] >XP_034831247.1 ubiquitin-conjugating enzyme E2 variant 2 [Maniola hyperantus] >XP_039755795.1 ubiquitin-conjugating enzyme E2 variant 2 [Pararge aegeria] >XP_041987413.1 ubiquitin-conjugating enzyme E2 variant 2 [Aricia agestis] >XP_045450678.1 ubiquitin-conjugating enzyme E2 variant 2 [Melitaea cinxia] >XP_045766931.1 ubiquitin-conjugating enzyme E2 variant 2 [Maniola jurtina] >XP_046968624.1 ubiquitin-conjugating enzyme E2 variant 2 [Vanessa cardui] >XP_047027052.1 ubiquitin-conjugating enzyme E2 variant 2 [Helicoverpa zea] >XP_047534810.1 ubiquitin-conjugating enzyme E2 variant 2 [Vanessa atalanta] >KAI5645358.1 ubiquitin-conjugating enzyme domain-containing protein [Phthorimaea operculella] >RVE41109.1 hypothetical protein evm_014241 [Chilo suppressalis] >CAB3251720.1 unnamed protein product [Arctia plantaginis] >CAG9561279.1 unnamed protein product [Danaus chrysippus] >CAG9755233.1 unnamed protein product [Diatraea saccharalis] >CAH0584342.1 unnamed protein product [Chrysodeixis includens] >CAH0729826.1 unnamed protein product, partial [Brenthis ino] >CAH2039508.1 unnamed protein product, partial [Iphiclides podalirius] >CAH2239303.1 jg6875 [Pararge aegeria aegeria]	-0.9569	1.91508	-0.0607	-0.3768	-0.5207
TRINITY_DN4694_c0_g2_i1_orf1	uncharacterized protein LOC114362122 [Ostrinia furnacalis]	-0.0414	1.92684	-0.7126	-0.3743	-0.7986
TRINITY_DN6656_c0_g1_i1_orf1	sorting and assembly machinery component 50 homolog isoform X9 [Ostrinia furnacalis] >XP_028169233.1 sorting and assembly machinery component 50 homolog isoform X10 [Ostrinia furnacalis] >XP_028169234.1 sorting and assembly machinery component 50 homolog isoform X11 [Ostrinia furnacalis]	0.09235	1.87477	-0.6086	-0.9822	-0.3764
TRINITY_DN77642_c0_g1_i1_orf1	peritrophic membrane chitin binding protein [Loxostege sticticalis]	0.15185	1.49569	0.54978	-0.9903	-1.207
TRINITY_DN114960_c0_g1_i4_orf1	zinc finger protein on ecdysone puffs-like [Ostrinia furnacalis]	-0.8875	1.93077	-0.1203	-0.6098	-0.3131
TRINITY_DN41259_c0_g1_i6_orf1	endocuticle structural glycoprotein SgAbd-8 [Ostrinia furnacalis]	0.99293	1.43186	-0.8041	-0.855	-0.7657
TRINITY_DN1274_c0_g1_i4_orf1	venom dipeptidyl peptidase 4-like [Ostrinia furnacalis]	0.01444	1.85111	-1.1455	-0.3309	-0.3891
TRINITY_DN28018_c0_g6_i1_orf1	microtubule-associated protein futsch-like isoform X6 [Ostrinia furnacalis]	-0.246	1.97337	-0.7057	-0.3983	-0.6234
TRINITY_DN2840_c0_g1_i5_orf1	hypothetical protein evm_002181 [Chilo suppressalis]	0.66718	1.65421	-0.8592	-0.8062	-0.656
TRINITY_DN9536_c0_g1_i4_orf1	adrenodoxin-like protein, mitochondrial isoform X1 [Ostrinia furnacalis]	0.05631	1.90141	-0.5874	-0.4643	-0.906
TRINITY_DN16145_c0_g1_i12_orf1	Down syndrome cell adhesion molecule-like protein Dscam2 isoform X16 [Ostrinia furnacalis]	-0.9279	1.80249	-0.9379	0.09574	-0.0323
TRINITY_DN17003_c1_g1_i1_orf1	unnamed protein product [Chilo suppressalis]	-0.5171	1.96668	-0.7054	-0.5845	-0.1596
TRINITY_DN35633_c0_g2_i1_orf1	uncharacterized protein LOC114353024 [Ostrinia furnacalis]	0.62328	1.679	-0.8869	-0.6615	-0.7539
TRINITY_DN8390_c0_g1_i2_orf1	tubulin gamma-1 chain-like isoform X1 [Ostrinia furnacalis] >XP_028160960.1 tubulin gamma-1 chain-like isoform X2 [Ostrinia furnacalis]	-0.7142	1.95891	-0.1149	-0.5406	-0.5891
TRINITY_DN30273_c1_g1_i1_orf1	uncharacterized protein LOC114358591 isoform X2 [Ostrinia furnacalis]	0.20152	1.72656	-0.0802	-1.2877	-0.5602
TRINITY_DN5772_c0_g1_i6_orf1	uncharacterized protein LOC114364899 isoform X2 [Ostrinia furnacalis]	0.02036	1.91939	-0.8318	-0.6241	-0.4838
TRINITY_DN4156_c0_g1_i2_orf1	calcium channel flower [Ostrinia furnacalis]	0.86119	1.39902	-0.3001	-1.3609	-0.5993
TRINITY_DN17437_c0_g1_i1_orf1	phospholipase A1 VesT1.02-like [Ostrinia furnacalis]	-0.9671	1.78945	-0.6508	-0.5464	0.37483
TRINITY_DN4572_c0_g3_i1_orf1	putative aminopeptidase W07G4.4 isoform X2 [Ostrinia furnacalis]	-0.6666	1.9384	-0.8309	-0.2935	-0.1474
TRINITY_DN1362_c0_g1_i4_orf1	heparan-alpha-glucosaminide N-acetyltransferase [Helicoverpa armigera]	-0.7674	1.79745	0.33687	-0.426	-0.9409
TRINITY_DN130069_c0_g6_i1_orf1	hypothetical protein HPG69_006179 [Diceros bicornis minor]	-1.312	1.55111	0.0303	-0.781	0.51162
TRINITY_DN2971_c0_g1_i1_orf1	uncharacterized protein LOC114364864 [Ostrinia furnacalis]	-0.0592	1.73093	-1.3466	0.09647	-0.4216

TRINITY_DN1313_c0_g1_i2_orf1	39S ribosomal protein L40, mitochondrial [Ostrinia furnacalis]	0.82255	1.51007	-0.8942	-0.396	-1.0424
TRINITY_DN13167_c0_g1_i1_orf1	selenoprotein M-like [Ostrinia furnacalis]	-0.1675	1.72921	-0.8501	-1.065	0.35339
TRINITY_DN5211_c0_g1_i1_orf1	elongation of very long chain fatty acids protein AAEL008004-like [Ostrinia furnacalis]	0.67653	1.64229	-0.6168	-0.9413	-0.7607
TRINITY_DN146524_c0_g1_i1_orf1	pupal cuticle protein-like [Ostrinia furnacalis]	-0.5761	1.98537	-0.3404	-0.6746	-0.3942
TRINITY_DN12222_c0_g1_i1_orf1	unnamed protein product [Chilo suppressalis]	0.01492	1.80703	-0.4021	-1.2413	-0.1786
TRINITY_DN291_c0_g1_i2_orf1	DNA replication licensing factor Mcm7 [Helicoverpa armigera] >XP_049698025.1 DNA replication licensing factor Mcm7-like [Helicoverpa armigera] >PZC87280.1 hypothetical protein B5X24_HaOG201516 [Helicoverpa armigera]	-1.0209	1.77544	-0.7733	0.33148	-0.3128
TRINITY_DN110534_c0_g1_i3_orf1	unnamed protein product [Euphydryas editha]	0.5383	1.67097	-0.4191	-0.6304	-1.1597
TRINITY_DN9475_c0_g1_i6_orf1	uncharacterized protein LOC114358636 [Ostrinia furnacalis]	-0.6126	1.90743	-0.8032	-0.5778	0.08619
TRINITY_DN36061_c0_g4_i2_orf1	putative GPI-anchored protein pfl2 [Ostrinia furnacalis] >XP_028163002.1 putative GPI-anchored protein pfl2 [Ostrinia furnacalis]	0.95821	1.45831	-0.8739	-0.7428	-0.7998
TRINITY_DN2623_c1_g1_i3_orf1	COPII coat assembly protein sec16-like [Ostrinia furnacalis]	0.16098	1.88306	-0.5539	-0.6705	-0.8197
TRINITY_DN5495_c0_g1_i5_orf1	multiple coagulation factor deficiency protein 2 homolog isoform X1 [Ostrinia furnacalis] >XP_028177199.1 multiple coagulation factor deficiency protein 2 homolog isoform X1 [Ostrinia furnacalis]	-1.2655	1.70857	0.05116	-0.6681	0.1739
TRINITY_DN57348_c0_g1_i4_orf1	facilitated trehalose transporter Tret1-like isoform X1 [Ostrinia furnacalis]	-1.2145	1.70043	-0.0045	-0.7494	0.26798
TRINITY_DN2352_c0_g1_i15_orf1	polyadenylate-binding protein 1-B-like [Ostrinia furnacalis]	-0.9545	1.8993	-0.0021	-0.6078	-0.3349
TRINITY_DN9324_c1_g2_i2_orf1	kinesin-like protein Klp10A isoform X4 [Spodoptera frugiperda]	0.07831	1.86466	-0.681	-0.2726	-0.9893
TRINITY_DN16643_c0_g2_i4_orf1	heterogeneous nuclear ribonucleoprotein R isoform X6 [Danaus plexippus plexippus]	-0.8222	1.92361	-0.4987	-0.6123	0.00961
TRINITY_DN9872_c0_g1_i2_orf1	serine protease inhibitor 88Ea-like [Ostrinia furnacalis]	0.10626	1.90258	-0.7034	-0.5477	-0.7577
TRINITY_DN3430_c0_g1_i1_orf1	protein HGV2-like isoform X2 [Ostrinia furnacalis]	0.07081	1.91212	-0.6537	-0.5462	-0.783
TRINITY_DN13395_c0_g1_i1_orf1	cytoplasmic dynein 1 light intermediate chain 2 [Galleria mellonella]	0.40297	1.78199	-0.5202	-0.7786	-0.8862
TRINITY_DN34703_c0_g1_i4_orf1	gamma-tubulin complex component 3 homolog [Ostrinia furnacalis]	0.26529	1.63962	-0.2643	-1.4628	-0.1778
TRINITY_DN286_c0_g1_i2_orf1	uncharacterized protein LOC114361329 [Ostrinia furnacalis]	0.55637	1.69285	-1.0457	-0.661	-0.5426
TRINITY_DN21124_c0_g1_i4_orf1	calsyntenin-1 [Ostrinia furnacalis]	0.69195	1.64206	-0.8438	-0.7804	-0.7098
TRINITY_DN1066_c0_g1_i8_orf1	hypothetical protein evm_012420 [Chilo suppressalis]	0.13392	1.40167	0.70251	-1.2174	-1.0207
TRINITY_DN42337_c0_g1_i6_orf1	cuticle protein 8-like [Leguminivora glycinivorella]	0.4195	1.75362	-0.7463	-1.0084	-0.4185
TRINITY_DN29633_c0_g1_i8_orf1	transmembrane protein 87A isoform X1 [Ostrinia furnacalis] >XP_028156931.1 transmembrane protein 87A isoform X2 [Ostrinia furnacalis] >XP_028156932.1 transmembrane protein 87A isoform X3 [Ostrinia furnacalis] >XP_028156933.1 transmembrane protein 87A isoform X4 [Ostrinia furnacalis] >XP_028156934.1 transmembrane protein 87A isoform X5 [Ostrinia furnacalis] >XP_028156935.1 transmembrane protein 87A isoform X6 [Ostrinia furnacalis]	0.12748	1.61094	0.36902	-1.1798	-0.9276
TRINITY_DN5235_c0_g1_i7_orf1	peptidoglycan-recognition protein SA-like [Ostrinia furnacalis]	0.46261	1.73819	-0.9978	-0.7522	-0.4507
TRINITY_DN59829_c0_g1_i1_orf1	putative mediator of RNA polymerase II transcription subunit 12 [Ostrinia furnacalis]	0.78609	1.56881	-0.5655	-0.9035	-0.8859
TRINITY_DN5182_c0_g1_i5_orf1	rab proteins geranylgeranyltransferase component A 1 isoform X1 [Ostrinia furnacalis]	0.25199	1.84414	-0.9148	-0.5684	-0.6129
TRINITY_DN1716_c0_g1_i14_orf1	putative gamma-glutamylcyclotransferase CG2811 isoform X3 [Ostrinia furnacalis]	0.31781	1.76795	-1.0524	-0.2599	-0.7734
TRINITY_DN34830_c0_g1_i1_orf1	ubiquitin-like domain-containing CTD phosphatase 1 [Ostrinia furnacalis]	-0.4867	1.82679	0.23935	-1.0361	-0.5433
TRINITY_DN10745_c0_g1_i14_orf1	septin-1 [Ostrinia furnacalis]	0.63822	1.66325	-0.9232	-0.799	-0.5793
TRINITY_DN18620_c0_g1_i5_orf1	hypothetical protein evm_011254 [Chilo suppressalis]	-0.4835	1.83616	-0.5796	-1.0029	0.22994
TRINITY_DN4134_c2_g1_i2_orf1	prostatic acid phosphatase-like [Ostrinia furnacalis]	-0.4614	1.95553	-0.1775	-0.8382	-0.4784
TRINITY_DN246_c1_g1_i5_orf1	lachesin isoform X1 [Ostrinia furnacalis] >XP_028178464.1 lachesin isoform X2 [Ostrinia furnacalis]	-1.216	1.7594	-0.6336	0.14621	-0.0561
TRINITY_DN2043_c0_g1_i3_orf1	phenoloxidase-activating factor 2-like [Ostrinia furnacalis]	-0.3771	1.94434	-0.3915	-0.9291	-0.2467
TRINITY_DN2012_c0_g1_i3_orf1	contactin [Ostrinia furnacalis]	-0.7561	1.90649	-0.1015	-0.8659	-0.183
TRINITY_DN8700_c9_g1_i1_orf1	uncharacterized protein LOC114351392 isoform X1 [Ostrinia furnacalis]	-0.9098	1.83141	-0.7625	0.25521	-0.4143
TRINITY_DN9000_c0_g2_i1_orf1	uncharacterized protein LOC114356585 [Ostrinia furnacalis]	0.32542	1.82252	-0.811	-0.7712	-0.5657
TRINITY_DN11665_c0_g1_i4_orf1	TBC1 domain family member 9 isoform X1 [Ostrinia furnacalis] >XP_028176568.1 TBC1 domain family member 9 isoform X2 [Ostrinia furnacalis]	-0.3739	1.95662	-0.7564	-0.6564	-0.1699
TRINITY_DN9100_c0_g1_i5_orf1	microtubule-associated protein futsch-like isoform X6 [Ostrinia furnacalis]	0.26785	1.84266	-0.8412	-0.7338	-0.5355
TRINITY_DN1233_c0_g2_i1_orf1	unnamed protein product [Spodoptera exigua]	0.27197	1.81938	-0.378	-0.9077	-0.8056
TRINITY_DN14905_c0_g2_i2_orf1	hypothetical protein evm_002970 [Chilo suppressalis]	-0.5716	1.88434	0.1149	-0.5013	-0.9263
TRINITY_DN99020_c0_g1_i1_orf1	uncharacterized protein LOC114357292 isoform X4 [Ostrinia furnacalis]	-0.0711	1.86997	-0.85	-0.8778	-0.0711
TRINITY_DN9109_c0_g1_i1_orf1	unnamed protein product [Chrysodeixis includens]	-0.6164	1.60753	-0.8067	-0.9193	0.73486
TRINITY_DN10479_c0_g1_i6_orf1	unnamed protein product [Chrysodeixis includens]	0.52515	1.70627	-0.6901	-1.0434	-0.4979
TRINITY_DN20710_c0_g1_i2_orf1	plexin A3 [Ostrinia furnacalis]	-0.2384	1.97761	-0.6995	-0.4848	-0.5548
TRINITY_DN57904_c0_g2_i1_orf1	cuticle protein 19 [Plutella xylostella] >CAG9138481.1 unnamed protein product [Plutella xylostella]	0.95237	1.4628	-0.7675	-0.882	-0.7657
TRINITY_DN9732_c0_g1_i7_orf1	CD109 antigen [Ostrinia furnacalis] >XP_028176877.1 CD109 antigen [Ostrinia furnacalis]	-0.4215	1.98674	-0.447	-0.7175	-0.4007
TRINITY_DN47219_c0_g1_i3_orf1	protein windbeutel [Ostrinia furnacalis]	0.16783	1.82997	-0.923	-0.2263	-0.8485
TRINITY_DN19058_c1_g1_i1_orf1	syntaxin-7 [Helicoverpa armigera] >XP_049695901.1 syntaxin-7 [Helicoverpa armigera]	-0.9107	1.92585	-0.2343	-0.6163	-0.1646
TRINITY_DN16516_c0_g1_i1_orf1	sulfotransferase 1E1 [Galleria mellonella]	-0.2437	1.70655	-1.4238	-0.0099	-0.0291
TRINITY_DN50517_c0_g1_i5_orf1	hypothetical protein evm_004957 [Chilo suppressalis]	0.23342	1.84659	-0.7835	-0.8499	-0.4466
TRINITY_DN5840_c0_g1_i6_orf1	catenin alpha isoform X2 [Ostrinia furnacalis]	0.45809	1.75117	-0.5009	-0.7722	-0.9362
TRINITY_DN24631_c0_g2_i1_orf1	O-GlcNAc hydrolase [Ostrinia furnacalis]	-0.4426	1.9541	-0.3527	-0.8874	-0.2713
TRINITY_DN144_c0_g1_i4_orf1	COPII coat assembly protein sec16-like [Ostrinia furnacalis]	-0.4168	1.97418	-0.7991	-0.3415	-0.4168
TRINITY_DN1393_c0_g1_i2_orf1	uncharacterized protein LOC114357114 isoform X1 [Ostrinia furnacalis]	-1.2131	1.61867	-0.4107	-0.6056	0.61069

TRINITY_DN73224_c0_g4_i2_orf1	PREDICTED: poly(rC)-binding protein 3 isoform X2 [Vollenhovia emeryi]	0.62198	1.67414	-0.8913	-0.8232	-0.5816
TRINITY_DN16924_c0_g1_i1_orf1	peroxiredoxin-1 [Homo sapiens] >NP_002565.1 peroxiredoxin-1 [Homo sapiens] >NP_859047.1 peroxiredoxin-1 [Homo sapiens] >NP_859048.1 peroxiredoxin-1 [Homo sapiens] >XP_001156568.1 peroxiredoxin-1 [Pan troglodytes] >XP_003308110.1 peroxiredoxin-1 [Pan troglodytes] >XP_003812829.1 peroxiredoxin-1 [Pan paniscus] >XP_016817283.1 peroxiredoxin-1 [Pan troglodytes] >XP_034508273.1 peroxiredoxin-1 [Ailuropoda melanoleuca] >XP_034819770.1 peroxiredoxin-1 [Pan paniscus] >Q06830.1 RecName: Full=Peroxiredoxin-1; AltName: Full=Natural killer cell-enhancing factor A; Short=NKEF-A; AltName: Full=Proliferation-associated gene protein; Short=PAG; AltName: Full=Thioredoxin peroxidase 2; AltName: Full=Thioredoxin-dependent peroxide reductase 2; AltName: Full=Thioredoxin-dependent peroxiredoxin 1 [Homo sapiens] >AAX41397.1 peroxiredoxin 1 [synthetic construct] >QFQ66317.1 redoxin [Phanerochaete pseudomagnoliae] >QFQ66354.1 AhpC-TSA [Xylaria sp.] >AAH07063.1 Peroxiredoxin 1 [Homo sapiens] >AAH21683.1 Peroxiredoxin 1 [Homo sapiens]	-1.1356	1.67821	-0.2718	-0.7613	0.49047
TRINITY_DN2584_c0_g1_i7_orf1	acylamino-acid-releasing enzyme-like isoform X1 [Ostrinia furnacalis] >XP_028174257.1 acylamino-acid-releasing enzyme-like isoform X2 [Ostrinia furnacalis] >XP_028174264.1 acylamino-acid-releasing enzyme-like isoform X3 [Ostrinia furnacalis] >XP_028174273.1 acylamino-acid-releasing enzyme-like isoform X4 [Ostrinia furnacalis] >XP_028174282.1 acylamino-acid-releasing enzyme-like isoform X1 [Ostrinia furnacalis]	-0.8839	1.82091	-0.8642	0.24064	-0.3134
TRINITY_DN172_c8_g2_i1_orf1	sialin [Ostrinia furnacalis]	0.19787	1.55834	0.416	-1.0782	-1.094
TRINITY_DN590_c0_g1_i4_orf1	trypsin inhibitor-like [Ostrinia furnacalis]	-0.6912	1.9746	-0.6298	-0.4073	-0.2463
TRINITY_DN535_c1_g1_i2_orf1	protein tramtrack, beta isoform isoform X24 [Bicyclus anynana]	0.39845	1.71138	-0.1687	-0.9725	-0.9686
TRINITY_DN44094_c0_g1_i1_orf1	myotrophin-like [Ostrinia furnacalis]	-0.0807	1.81196	-1.2184	-0.4736	-0.0393
TRINITY_DN4785_c0_g2_i1_orf1	methylthioribose-1-phosphate isomerase [Ostrinia furnacalis] >XP_028172842.1 methylthioribose-1-phosphate isomerase [Ostrinia furnacalis]	-0.7287	1.72684	-0.837	-0.7025	0.54132
TRINITY_DN1280_c0_g1_i1_orf1	coronin-7 isoform X1 [Ostrinia furnacalis] >XP_028164815.1 coronin-7 isoform X2 [Ostrinia furnacalis] >XP_028164817.1 coronin-7 isoform X3 [Ostrinia furnacalis] >XP_028164818.1 coronin-7 isoform X4 [Ostrinia furnacalis] >XP_028164820.1 coronin-7 isoform X6 [Ostrinia furnacalis] >XP_028164821.1 coronin-7 isoform X7 [Ostrinia furnacalis] >XP_028164822.1 coronin-7 isoform X1 [Ostrinia furnacalis] >XP_028164823.1 coronin-7 isoform X8 [Ostrinia furnacalis] >XP_028164824.1 coronin-7 isoform X9 [Ostrinia furnacalis] >XP_028164825.1 coronin-7 isoform X10 [Ostrinia furnacalis]	0.47808	1.75435	-0.5992	-0.8383	-0.7949
TRINITY_DN10630_c0_g1_i2_orf1	J domain-containing protein [Ostrinia furnacalis]	-0.1129	1.55349	-0.898	-1.1714	0.62881
TRINITY_DN10095_c0_g1_i5_orf1	uncharacterized protein LOC114361222 [Ostrinia furnacalis]	-0.0797	1.79071	-0.4296	-1.2658	-0.0156
TRINITY_DN6014_c1_g1_i2_orf1	putative hydroxypyruvate isomerase [Ostrinia furnacalis]	-0.6852	1.91938	-0.6656	-0.632	0.06343
TRINITY_DN4410_c0_g1_i1_orf1	rab GDP dissociation inhibitor alpha [Ostrinia furnacalis]	-0.6117	1.92595	-0.7947	0.0141	-0.5337
TRINITY_DN2968_c0_g1_i3_orf1	uncharacterized protein LOC114357587 [Ostrinia furnacalis]	-0.6036	1.71764	-1.0268	-0.6036	0.51642
TRINITY_DN25870_c0_g2_i6_orf1	homeobox protein extradenticle isoform X3 [Ostrinia furnacalis]	0.40838	1.58092	0.10365	-1.3043	-0.7887
TRINITY_DN41321_c1_g1_i3_orf1	uncharacterized protein LOC114357706 isoform X1 [Ostrinia furnacalis] >XP_028167261.1 uncharacterized protein LOC114357706 isoform X2 [Ostrinia furnacalis]	-1.0841	1.81227	-0.4741	-0.5036	0.24941
TRINITY_DN64222_c0_g1_i1_orf1	heat shock protein 20.2 [Glyphodes pyloalis]	-0.4406	1.98224	-0.735	-0.3292	-0.4774
TRINITY_DN22018_c0_g1_i3_orf1	hypothetical protein evm_009110 [Chilo suppressalis] >CAH2984739.1 unnamed protein product [Chilo suppressalis]	-0.3078	1.49813	-1.595	0.33438	0.07033
TRINITY_DN9302_c0_g1_i1_orf1	DEAD-box helicase Dbp80 [Ostrinia furnacalis]	-0.6589	1.87821	0.13657	-0.9019	-0.454
TRINITY_DN5829_c0_g2_i1_orf1	uncharacterized protein LOC114365758 isoform X2 [Ostrinia furnacalis]	0.40148	1.60042	-0.9025	-1.2049	0.10551
TRINITY_DN18563_c2_g1_i1_orf1	protein held out wings isoform X2 [Diachasma alloeum]	-0.0771	1.9006	-0.7659	-0.1854	-0.8723
TRINITY_DN24132_c0_g1_i2_orf1	palmitoyltransferase ZDHHC5 isoform X1 [Ostrinia furnacalis]	-0.8544	1.87851	-0.059	-0.8513	-0.1138
TRINITY_DN2054_c0_g1_i1_orf1	macrophage mannose receptor 1-like [Ostrinia furnacalis]	0.49434	1.59174	0.00965	-0.9341	-1.1616
TRINITY_DN26293_c0_g1_i4_orf1	UDP-glucose 6-dehydrogenase [Ostrinia furnacalis]	0.4344	1.72434	-1.0778	-0.3259	-0.7551
TRINITY_DN27723_c0_g1_i1_orf1	putative uncharacterized protein DDB_G0282133 isoform X1 [Ostrinia furnacalis]	0.42957	1.72456	-0.7647	-1.0764	-0.313
TRINITY_DN9916_c0_g1_i1_orf1	PREDICTED: dynein light chain Tctex-type [Amyelois transitella] >XP_021195381.1 dynein light chain Tctex-type [Helicoverpa armigera] >XP_022815696.1 dynein light chain Tctex-type [Spodoptera litura] >XP_028156399.1 dynein light chain Tctex-type [Ostrinia furnacalis] >XP_035458261.1 dynein light chain Tctex-type-like [Spodoptera frugiperda] >XP_047034788.1 dynein light chain Tctex-type [Helicoverpa zea] >CAB3233358.1 unnamed protein product [Arctia plantaginis] >CAB3506583.1 unnamed protein product [Spodoptera littoralis] >CAG9754627.1 unnamed protein product [Diatraea saccharalis] >CAH0596395.1 unnamed protein product [Chrysodeixis includens] >KAF9808454.1 hypothetical protein SFRURICE_008507 [Spodoptera frugiperda]	0.54546	1.68231	-0.659	-1.1065	-0.4623
TRINITY_DN3177_c0_g1_i1_orf1	Pupal cuticle protein PCP52 [Papilio xuthus]	-0.5119	1.76791	-0.4813	-1.1224	0.34773
TRINITY_DN16605_c0_g1_i3_orf1	unnamed protein product [Chrysodeixis includens]	0.12317	1.79553	-1.1463	-0.6588	-0.1136
TRINITY_DN3119_c0_g1_i7_orf1	unnamed protein product [Chilo suppressalis]	-0.0327	1.82549	-1.1858	-0.4986	-0.1084
TRINITY_DN2277_c0_g1_i11_orf1	solute carrier family 12 member 4 isoform X1 [Ostrinia furnacalis]	-0.7211	1.91047	-0.0322	-0.2961	-0.8611
TRINITY_DN4572_c0_g1_i2_orf1	unnamed protein product [Arctia plantaginis] >CAB3257565.1 unnamed protein product [Arctia plantaginis]	-0.6121	1.95815	-0.7009	-0.5367	-0.1085
TRINITY_DN44633_c0_g1_i4_orf1	LDLR chaperone boca [Ostrinia furnacalis]	0.03684	1.41786	-1.5009	-0.5829	0.62914
TRINITY_DN14389_c0_g1_i4_orf1	DE-cadherin [Ostrinia furnacalis]	-0.6216	1.8686	0.15854	-0.4693	-0.9363
TRINITY_DN12134_c0_g1_i4_orf1	glutathione S-transferase 1-1 [Ostrinia furnacalis] >XP_028161942.1 glutathione S-transferase 1-1 [Ostrinia furnacalis] >XP_028161943.1 glutathione S-transferase 1-1 [Ostrinia furnacalis]	0.74393	1.485	-0.4869	-1.3644	-0.3776
TRINITY_DN3273_c0_g1_i4_orf1	FK506-binding protein-like [Galleria mellonella]	-0.552	1.99101	-0.579	-0.3157	-0.5443
TRINITY_DN1231_c0_g1_i4_orf1	AN1-type zinc finger protein 6 isoform X1 [Galleria mellonella]	0.54684	1.66429	-0.8061	-1.0861	-0.3189
TRINITY_DN27321_c0_g1_i1_orf1	ras-related protein Rap-2c [Bicyclus anynana] >XP_026492616.1 ras-related protein Rap-2c [Vanessa tameamea] >XP_034838061.1 ras-related protein Rap-2c [Maniola hyperantus] >XP_039759141.1 ras-related protein Rap-2c [Pararge aegeria] >XP_045498804.1 ras-related protein Rap-2c [Colias croceus] >XP_046959644.1 ras-related protein Rap-2c [Vanessa cardui] >XP_047530248.1 ras-related protein Rap-2c [Vanessa atalanta] >CAH2268047.1 jg10357 [Pararge aegeria aegeria]	0.36854	1.74117	-0.5015	-1.1835	-0.4248

TRINITY_DN33893_c0_g1_i1_orf1	high mobility group protein I-like [Ostrinia furnacalis]	0.22334	1.63419	-0.0217	-1.4629	-0.373
TRINITY_DN49872_c0_g1_i2_orf1	probable cytosolic iron-sulfur protein assembly protein Ciao1 [Ostrinia furnacalis]	0.13685	1.59936	-1.4056	-0.6085	0.27794
TRINITY_DN101_c0_g2_i2_orf1	tyrosine-protein phosphatase non-receptor type 23 [Ostrinia furnacalis]	-0.6346	1.95029	-0.0982	-0.4622	-0.7552
TRINITY_DN2438_c0_g1_i1_orf1	dystrophin, isoforms A/C/F/G/H-like [Ostrinia furnacalis]	-0.1579	1.61634	-1.0045	-1.0176	0.56376
TRINITY_DN2201_c0_g1_i1_orf1	pleiotropic regulator 1 [Ostrinia furnacalis]	0.28406	1.32447	-1.473	-0.7699	0.63442
TRINITY_DN44070_c0_g2_i2_orf1	protein Gawky isoform X2 [Ostrinia furnacalis]	0.17612	1.69246	-1.2415	0.11464	-0.7418
TRINITY_DN16390_c0_g1_i4_orf1	unnamed protein product, partial [Brenthis ino]	-0.1527	1.88925	-0.5754	-1.029	-0.1321
TRINITY_DN5675_c0_g1_i6_orf1	probable ATP-dependent RNA helicase DDX43 [Ostrinia furnacalis]	-1.3291	1.63813	0.1176	-0.6851	0.25845
TRINITY_DN1133_c0_g1_i6_orf1	zinc finger protein 391-like [Ostrinia furnacalis] >XP_028169193.1 zinc finger protein 391-like [Ostrinia furnacalis]	0.34322	1.78491	-0.6866	-1.0256	-0.4159
TRINITY_DN5118_c0_g1_i1_orf1	AP-2 complex subunit alpha [Ostrinia furnacalis]	-0.0269	1.64268	0.3052	-1.3863	-0.5346
TRINITY_DN64769_c0_g1_i3_orf1	procollagen-lysine,2-oxoglutarate 5-dioxygenase isoform X2 [Ostrinia furnacalis]	0.78737	1.52617	-0.9736	-0.9903	-0.3496
TRINITY_DN33089_c0_g1_i1_orf1	nucleoporin NDC1 [Ostrinia furnacalis]	0.13322	1.73473	-1.0845	-0.8866	0.10321
TRINITY_DN20322_c0_g1_i1_orf1	KIF1-binding protein-like [Ostrinia furnacalis]	-0.9366	1.80485	-0.5369	-0.6766	0.34526
TRINITY_DN27114_c0_g1_i1_orf1	putative inorganic phosphate cotransporter [Ostrinia furnacalis]	-0.3096	1.88388	-0.5642	0.00811	-1.0182
TRINITY_DN9072_c0_g1_i1_orf1	SET and MYND domain-containing protein 4-like [Ostrinia furnacalis]	0.32343	1.64226	-1.3411	0.00769	-0.6323
TRINITY_DN72816_c0_g1_i2_orf1	Golgi apparatus protein 1 [Ostrinia furnacalis]	0.5674	1.58243	-0.2988	-1.3565	-0.4945
TRINITY_DN10403_c0_g1_i3_orf1	hypothetical protein evm_000264 [Chilo suppressalis] >CAH2987898.1 unnamed protein product [Chilo suppressalis]	-0.0285	1.58158	-1.3219	0.48591	-0.7171
TRINITY_DN14684_c0_g2_i1_orf1	cofilin/actin-depolymerizing factor homolog isoform X2 [Helicoverpa armigera] >XP_022828531.1 cofilin/actin-depolymerizing factor homolog [Spodoptera litura] >XP_023954560.1 cofilin/actin-depolymerizing factor homolog isoform X2 [Bicyclus anynana] >XP_023954561.1 cofilin/actin-depolymerizing factor homolog isoform X2 [Bicyclus anynana] >XP_026484174.1 cofilin/actin-depolymerizing factor homolog isoform X1 [Vanessa tameamea] >XP_026743400.1 cofilin/actin-depolymerizing factor homolog [Trichoplusia ni] >XP_035451599.1 cofilin/actin-depolymerizing factor homolog [Spodoptera frugiperda] >XP_038217031.1 cofilin/actin-depolymerizing factor homolog isoform X1 [Zerene cesonia] >XP_038217032.1 cofilin/actin-depolymerizing factor homolog isoform X2 [Zerene cesonia] >XP_039761665.1 cofilin/actin-depolymerizing factor homolog [Pararge aegeria] >XP_046974608.1 cofilin/actin-depolymerizing factor homolog [Vanessa cardui] >XP_047037753.1 cofilin/actin-depolymerizing factor homolog [Helicoverpa zea] >XP_047540992.1 cofilin/actin-depolymerizing factor homolog [Vanessa atalanta] >XP_050358240.1 cofilin/actin-depolymerizing factor homolog [Nymphalis io] >RVE53832.1 hypothetical protein evm_001494 [Chilo suppressalis] >CAH0598789.1 unnamed protein product [Chrysodeixis includens] >CAH2242550.1 jg8122 [Pararge aegeria aegeria] >AFP36378.1 cofilin [Spodoptera frugiperda] >PZC72500.1 hypothetical protein B5X24_HaOG211097 [Helicoverpa armigera]	-0.5985	1.96668	-0.2696	-0.328	-0.7705
TRINITY_DN1853_c0_g1_i3_orf1	trans-Golgi network integral membrane protein TGN38-like isoform X1 [Ostrinia furnacalis]	0.40468	1.67924	-0.3019	-1.3018	-0.4803
TRINITY_DN1074_c0_g1_i7_orf1	eukaryotic translation initiation factor 4E type 2 [Ostrinia furnacalis]	0.74375	1.58005	-0.6957	-1.0795	-0.5486
TRINITY_DN19885_c0_g1_i1_orf1	transmembrane emp24 domain-containing protein 5 [Ostrinia furnacalis]	-0.1243	1.86371	-0.6824	-1.0219	-0.0351
TRINITY_DN1103_c0_g1_i18_orf1	retinal dehydrogenase 1-like [Ostrinia furnacalis]	-1.0658	1.81637	-0.7294	0.11709	-0.1382
TRINITY_DN6436_c0_g1_i1_orf1	serine/threonine-protein kinase PAK 3 isoform X1 [Ostrinia furnacalis] >XP_028164178.1 serine/threonine-protein kinase PAK 3 isoform X2 [Ostrinia furnacalis] >XP_028164179.1 serine/threonine-protein kinase PAK 3 isoform X3 [Ostrinia furnacalis]	0.64813	1.64997	-0.5783	-0.7112	-1.0086
TRINITY_DN10694_c1_g2_i1_orf1	hsp70-Hsp90 organizing protein 3-like [Ostrinia furnacalis]	0.21463	1.8399	-0.5305	-0.5111	-1.0129
TRINITY_DN123184_c0_g1_i1_orf1	double-strand break repair protein MRE11 [Ostrinia furnacalis]	0.36647	1.61415	0.09798	-1.2518	-0.8268
TRINITY_DN3521_c0_g2_i1_orf1	barrier-to-autointegration factor [Ostrinia furnacalis] >XP_045447211.1 barrier-to-autointegration factor [Melitaea cinxia] >CAG4971452.1 unnamed protein product [Parnassius apollo] >CAG9578869.1 unnamed protein product [Danaus chrysippus] >CAH0714333.1 unnamed protein product, partial [Brenthis ino] >CAH2108232.1 unnamed protein product [Euphydryas editha]	-0.2538	1.69844	-0.8029	-1.0956	0.45384
TRINITY_DN24218_c0_g1_i1_orf1	uncharacterized protein LOC114362624 [Ostrinia furnacalis]	0.25686	1.48548	0.3872	-1.4585	-0.6711
TRINITY_DN162_c0_g1_i4_orf1	ABC transporter G family member 23 isoform X1 [Ostrinia furnacalis] >XP_028178987.1 ABC transporter G family member 23 isoform X1 [Ostrinia furnacalis]	0.07062	1.91165	-0.5179	-0.7231	-0.7413
TRINITY_DN8682_c0_g1_i4_orf1	protein SYS1 homolog [Ostrinia furnacalis]	0.02206	1.88289	-0.9445	-0.2556	-0.7049
TRINITY_DN19746_c0_g1_i5_orf1	hypothetical protein evm_004480 [Chilo suppressalis] >CAB3520922.1 unnamed protein product [Chilo suppressalis] >CAH0398243.1 unnamed protein product [Chilo suppressalis]	-0.9363	1.76707	-0.6034	-0.6664	0.43896
TRINITY_DN1616_c0_g1_i3_orf1	U4/U6 small nuclear ribonucleoprotein Prp3 isoform X1 [Ostrinia furnacalis] >XP_028161035.1 U4/U6 small nuclear ribonucleoprotein Prp3 isoform X2 [Ostrinia furnacalis] >XP_028161037.1 U4/U6 small nuclear ribonucleoprotein Prp3 isoform X3 [Ostrinia furnacalis]	0.6067	1.64941	-0.5213	-0.6077	-1.127
TRINITY_DN2100_c0_g1_i2_orf1	uncharacterized protein LOC114362418 [Ostrinia furnacalis]	0.0615	1.90776	-0.7887	-0.4528	-0.7277
TRINITY_DN14046_c0_g1_i1_orf1	retinol dehydrogenase 14 [Ostrinia furnacalis] >XP_028165567.1 retinol dehydrogenase 14 [Ostrinia furnacalis] >XP_028165568.1 retinol dehydrogenase 14 [Ostrinia furnacalis]	0.54686	1.72204	-0.7336	-0.6707	-0.8646
TRINITY_DN10619_c0_g5_i7_orf1	protein bunched, class 2/F/G isoform-like isoform X2 [Ostrinia furnacalis]	0.20046	1.58859	-1.1038	-1.0437	0.35846
TRINITY_DN467_c4_g1_i2_orf1	GRIP and coiled-coil domain-containing protein 1 [Ostrinia furnacalis]	0.66761	1.50173	-1.2391	-0.872	-0.0583
TRINITY_DN4070_c0_g1_i4_orf1	alpha-N-acetylgalactosaminidase isoform X3 [Ostrinia furnacalis]	0.96014	1.41896	-0.4759	-1.0681	-0.835
TRINITY_DN5503_c0_g1_i5_orf1	protein PRRC1-like isoform X1 [Ostrinia furnacalis] >XP_028166031.1 protein PRRC1-like isoform X1 [Ostrinia furnacalis]	-1.4243	1.58735	0.21101	-0.5977	0.2236
TRINITY_DN4524_c0_g1_i2_orf1	sodium/potassium-transporting ATPase subunit alpha isoform X1 [Chelonus insularis] >XP_034939982.1 sodium/potassium-transporting ATPase subunit alpha isoform X1 [Chelonus insularis] >XP_034939983.1 sodium/potassium-transporting ATPase subunit alpha isoform X1 [Chelonus insularis] >XP_034939984.1 sodium/potassium-transporting ATPase subunit alpha isoform X1 [Chelonus insularis]	-0.2714	1.87046	-0.7564	-0.9216	0.07897
TRINITY_DN17655_c0_g1_i1_orf1	BRISC and BRCA1-A complex member 1-like [Ostrinia furnacalis]	0.3707	1.69511	-0.172	-0.6585	-1.2353
TRINITY_DN77005_c0_g3_i1_orf1	RNA polymerase II degradation factor 1-like [Ostrinia furnacalis]	0.28753	1.73343	-1.2454	-0.2135	-0.5621
TRINITY_DN4782_c0_g1_i1_orf1	patched domain-containing protein 3-like [Ostrinia furnacalis]	0.25743	1.47416	0.40815	-1.4602	-0.6795

TRINITY_DN22577_c0_g1_i2_orf1	probable beta-hexosaminidase fdl isoform X1 [Ostrinia furnacalis]	-0.0142	1.90166	-0.4196	-0.989	-0.4789
TRINITY_DN9354_c0_g1_i7_orf1	hypothetical protein evm_012205 [Chilo suppressalis] >CAB3527181.1 unnamed protein product [Chilo suppressalis] >CAH0404510.1 unnamed protein product [Chilo suppressalis]	0.54897	1.60529	-0.3902	-0.4274	-1.3367
TRINITY_DN98814_c0_g1_i2_orf1	PREDICTED: chaoptin [Amyelois transitella]	0.51035	1.69806	-0.7509	-0.3896	-1.068
TRINITY_DN2345_c0_g1_i4_orf1	chromobox protein homolog 3-like [Ostrinia furnacalis] >XP_028157236.1 chromobox protein homolog 3-like [Ostrinia furnacalis]	0.29718	1.71118	-0.0369	-1.1252	-0.8462
TRINITY_DN33967_c2_g2_i1_orf1	titin homolog [Ostrinia furnacalis]	0.0623	1.84895	-0.78	-0.1599	-0.9713
TRINITY_DN44658_c0_g1_i2_orf1	lipase 3-like [Ostrinia furnacalis]	0.44793	1.55827	-0.0625	-1.4609	-0.4828
TRINITY_DN14019_c0_g1_i5_orf1	hypothetical protein evm_009768 [Chilo suppressalis]	0.73439	1.31237	-1.4833	-0.7174	0.15388
TRINITY_DN16011_c0_g1_i3_orf1	hypothetical protein evm_002694 [Chilo suppressalis]	0.19158	1.71334	0.03652	-1.237	-0.7044
TRINITY_DN20767_c0_g2_i1_orf1	glycosylated lysosomal membrane protein B-like [Vanessa atalanta]	0.80863	1.53935	-0.6009	-1.0848	-0.6623
TRINITY_DN2102_c0_g1_i11_orf1	prenylated Rab acceptor protein 1 isoform X4 [Ostrinia furnacalis]	-0.6252	1.80088	-0.942	-0.5918	0.35817
TRINITY_DN44256_c0_g1_i1_orf1	essential MCU regulator, mitochondrial [Cotesia glomerata]	0.6127	1.63641	-0.7849	-1.0918	-0.3724
TRINITY_DN72_c0_g1_i16_orf1	protein groucho-like [Ostrinia furnacalis]	-0.8166	1.95446	-0.4256	-0.5543	-0.158
TRINITY_DN66040_c0_g1_i2_orf1	serine protease inhibitor dipetalogastin-like isoform X2 [Ostrinia furnacalis]	-1.5131	1.52484	-0.1369	0.48616	-0.361
TRINITY_DN38307_c0_g1_i1_orfp1	TRINITY_DN38307_c0_g1_i1_m.10661 TRINITY_DN38307_c0_g1::TRINITY_DN38307_c0_g1_i1::g.10661 ORF type:5prime_partial len:66 (+),score=5.90 TRINITY_DN38307_c0_g1_i1:2-199(+)	-1.6694	1.42198	-0.1642	0.00679	0.4049
TRINITY_DN64297_c0_g1_i1_orf1	vanin-like protein 2 isoform X2 [Ostrinia furnacalis]	-1.5609	1.33079	0.25072	0.59363	-0.6142
TRINITY_DN276_c0_g1_i2_orf1	protein lethal(2)essential for life-like [Ostrinia furnacalis] >UTU55753.1 small heat shock protein Hsp20.7 [Ostrinia furnacalis]	-1.3206	1.52123	-0.8035	0.5407	0.06218
TRINITY_DN5080_c0_g1_i5_orf1	storage protein [Ostrinia furnacalis]	-1.9646	0.77358	0.27671	0.61075	0.3036
TRINITY_DN64181_c0_g1_i1_orf1	uncharacterized protein LOC114356431 isoform X2 [Ostrinia furnacalis]	-1.5554	1.46639	0.54647	-0.1125	-0.3449
TRINITY_DN1370_c0_g1_i2_orf1	hypothetical protein evm_000756 [Chilo suppressalis]	-1.8885	1.05558	0.09354	0.50648	0.23284
TRINITY_DN143895_c0_g1_i1_orf1	cathepsin L-like [Aphidius gifuensis] >KAF7988186.1 hypothetical protein HCN44_007680 [Aphidius gifuensis]	-1.9287	0.62519	0.05375	0.85423	0.39558
TRINITY_DN18218_c0_g1_i7_orf1	inhibin beta B chain [Ostrinia furnacalis]	-1.238	1.51379	-0.7223	0.7498	-0.3034
TRINITY_DN5444_c0_g1_i1_orfp1	TRINITY_DN5444_c0_g1_i1_m.14077 TRINITY_DN5444_c0_g1::TRINITY_DN5444_c0_g1_i1::g.14077 ORF type:3prime_partial len:90 (-),score=25.11 TRINITY_DN5444_c0_g1_i1:2-268(-)	-1.7446	1.36434	-0.0085	0.29269	0.0961
TRINITY_DN15400_c0_g1_i1_orf1	uncharacterized protein LOC114366781 [Ostrinia furnacalis]	-1.3648	1.20999	0.66042	-0.9976	0.49194
TRINITY_DN4767_c0_g1_i4_orf1	cysteine protease XCP2-like [Ostrinia furnacalis]	-1.9572	0.75927	0.28587	0.67435	0.23768
TRINITY_DN12526_c0_g1_i5_orf1	uncharacterized protein LOC114359035 isoform X3 [Ostrinia furnacalis]	-1.6672	1.17556	-0.4693	0.2009	0.76011
TRINITY_DN12009_c0_g1_i1_orf1	uncharacterized protein LOC114365631 [Ostrinia furnacalis]	-1.8357	0.99281	0.02278	0.01768	0.8024
TRINITY_DN51813_c0_g1_i1_orf1	uncharacterized protein LOC114350216 [Ostrinia furnacalis]	-1.8579	0.87806	0.07584	0.87799	0.02598
TRINITY_DN5406_c0_g2_i1_orf1	uncharacterized protein LOC114350326 [Ostrinia furnacalis]	-1.8723	0.94781	0.7566	0.153	0.01491
TRINITY_DN6423_c0_g1_i6_orf1	phenoloxidase-activating factor 2-like isoform X1 [Ostrinia furnacalis]	-1.7543	1.36232	0.02974	0.17188	0.19032
TRINITY_DN67193_c0_g1_i1_orf1	A-kinase anchor protein 14-like [Ostrinia furnacalis]	-1.8754	0.88028	-0.1456	0.70511	0.4356
TRINITY_DN22515_c0_g1_i10_orf1	hypothetical protein KGM_202621 [Danaus plexippus plexippus]	-1.9985	0.52323	0.42931	0.54593	0.5
TRINITY_DN703_c0_g1_i2_orf1	acidic juvenile hormone-suppressible protein 1-like [Ostrinia furnacalis]	-1.9072	0.46724	0.09245	1.01136	0.33613
TRINITY_DN5080_c0_g1_i1_orf1	basic juvenile hormone-suppressible protein 2-like [Ostrinia furnacalis]	-1.9059	0.82977	0.09529	0.7979	0.1829
TRINITY_DN20560_c0_g1_i6_orf1	pupal cuticle protein C1B-like [Ostrinia furnacalis]	-1.8302	1.16248	0.40775	-0.0925	0.35245
TRINITY_DN3616_c0_g1_i4_orf1	conotoxin ArMKLT2-032-like [Ostrinia furnacalis]	-1.4273	1.43024	-0.0476	0.69833	-0.6536
TRINITY_DN636_c1_g1_i9_orf1	secretory phospholipase A2 receptor-like [Ostrinia furnacalis]	-1.8328	0.77582	0.26549	0.96816	-0.1767
TRINITY_DN59885_c0_g1_i3_orf1	TGF-beta-activated kinase 1 and MAP3K7-binding protein 1-like [Ostrinia furnacalis]	-1.9579	0.58876	0.09603	0.65318	0.6199
TRINITY_DN4767_c0_g1_i6_orf1	cysteine protease XCP2-like [Ostrinia furnacalis]	-1.8723	0.60017	-0.1558	0.9266	0.50133
TRINITY_DN45948_c1_g1_i1_orf1	unnamed protein product [Leptidea sinapis]	-1.7855	1.18571	0.55625	-0.1972	0.24067
TRINITY_DN4255_c0_g1_i11_orf1	LOW QUALITY PROTEIN: lebocin-4-like [Ostrinia furnacalis]	-1.8751	1.11109	0.35345	0.06345	0.34713
TRINITY_DN46625_c0_g1_i1_orf1	ferritin subunit isoform X1 [Belonocnema kinseyi]	-1.463	1.49897	-0.458	0.60728	-0.1853
TRINITY_DN33272_c0_g1_i5_orf1	Low-density lipoprotein receptor-related protein 1 [Papilio xuthus]	-1.907	1.01785	0.43067	0.0937	0.36479
TRINITY_DN2813_c0_g1_i7_orf1	arylphorin subunit alpha-like [Ostrinia furnacalis]	-1.8742	0.64586	0.86631	-0.1755	0.53752
TRINITY_DN7900_c0_g1_i4_orf1	uncharacterized protein LOC114366119 [Ostrinia furnacalis]	-1.9607	0.67795	0.18363	0.72056	0.37852
TRINITY_DN121650_c0_g1_i1_orf1	carboxylesterase [Ostrinia furnacalis]	-1.6911	1.44393	-0.0336	0.0546	0.22617
TRINITY_DN13760_c1_g1_i1_orf1	pre-mRNA-processing factor 40 homolog A isoform X1 [Ostrinia furnacalis] >XP_028162665.1 pre-mRNA-processing factor 40 homolog A isoform X2 [Ostrinia furnacalis] >XP_028162667.1 pre-mRNA-processing factor 40 homolog A isoform X3 [Ostrinia furnacalis]	-1.6889	1.32162	0.25016	0.46566	-0.3485
TRINITY_DN42719_c0_g2_i1_orf1	inter-alpha-trypsin inhibitor heavy chain H4-like isoform X11 [Ostrinia furnacalis]	-1.872	0.91789	0.36021	0.71352	-0.1196
TRINITY_DN34406_c0_g2_i9_orfp1	TRINITY_DN34406_c0_g2_i9_m.33755 TRINITY_DN34406_c0_g2::TRINITY_DN34406_c0_g2_i9::g.33755 ORF type:internal len:82 (-),score=12.88 TRINITY_DN34406_c0_g2_i9:3-245(-)	-1.3357	0.75843	1.25759	-0.9835	0.30318
TRINITY_DN75086_c0_g1_i5_orf1	lysosome membrane protein 2-like [Ostrinia furnacalis]	-1.9636	0.75171	0.46124	0.16937	0.58127
TRINITY_DN467_c3_g1_i5_orf1	lysozyme precursor [Loxostege sticticalis]	-1.9234	0.73528	0.02681	0.37481	0.78652
TRINITY_DN801_c0_g1_i2_orf1	cathepsin L [Ostrinia furnacalis] >XP_028165920.1 cathepsin L [Ostrinia furnacalis] >UKI61015.1 cathepsin L [Ostrinia furnacalis]	-1.6549	1.39293	0.53302	-0.1484	-0.1227
TRINITY_DN4748_c0_g1_i5_orf1	unnamed protein product, partial [Brenthis ino]	-1.6662	1.46875	0.16093	0.15981	-0.1233
TRINITY_DN20717_c0_g1_i1_orf1	putative uncharacterized protein DDB_G0282133 isoform X1 [Ostrinia furnacalis]	-1.4066	1.5924	0.49019	-0.4303	-0.2457
TRINITY_DN45220_c0_g1_i1_orf1	delta(3,5)-Delta(2,4)-dienoyl-CoA isomerase, mitochondrial isoform X1 [Ostrinia furnacalis]	-1.643	1.34972	-0.4231	0.505	0.21142
TRINITY_DN9239_c0_g1_i1_orf1	apolipophorins-like [Ostrinia furnacalis]	-1.3268	1.50782	-0.6047	0.71709	-0.2933

TRINITY_DN9538_c1_g3_i1_orf1	cilia- and flagella-associated protein 410 isoform X2 [Aphidius gifuensis]	-1.8452	1.10034	-0.0339	0.5893	0.18954
TRINITY_DN52316_c0_g1_i1_orf1	arylphorin subunit alpha-like [Ostrinia furnacalis]	-1.8643	0.72891	-0.1489	0.91262	0.37161
TRINITY_DN7040_c0_g2_i1_orf1	uncharacterized protein LOC114353763 [Ostrinia furnacalis]	-1.9628	0.6842	0.15188	0.66746	0.45922
TRINITY_DN3707_c0_g1_i1_orf1	protein FAM160B1-like isoform X1 [Ostrinia furnacalis]	-1.9349	0.85775	0.11432	0.62927	0.33359
TRINITY_DN19662_c0_g2_i1_orf1	storage protein [Ostrinia furnacalis]	-1.9057	0.72226	0.08663	0.89322	0.20356
TRINITY_DN6122_c0_g1_i6_orf1	deubiquitinase DESI2 isoform X1 [Helicoverpa armigera] >XP_049707835.1 deubiquitinase DESI2 isoform X1 [Helicoverpa armigera]	-1.8841	0.76842	-0.085	0.8568	0.34394
TRINITY_DN20676_c0_g1_i6_orf1	aldo-keto reductase AKR2E4-like isoform X1 [Ostrinia furnacalis]	-1.8403	1.19071	0.05158	0.21513	0.38287
TRINITY_DN5655_c0_g1_i2_orf1	uncharacterized protein LOC114359603 [Ostrinia furnacalis]	-1.8202	1.22163	0.02955	0.15841	0.4106
TRINITY_DN106156_c1_g1_i1_orf1	arylphorin subunit alpha-like [Ostrinia furnacalis]	-1.848	0.47645	-0.0056	1.14116	0.23597
TRINITY_DN15247_c0_g1_i2_orf1	probable G-protein coupled receptor Mth-like 3 isoform X1 [Ostrinia furnacalis]	-1.8687	0.96964	0.42724	-0.1341	0.60594
TRINITY_DN98692_c0_g3_i1_orf1	fatty acyl-CoA hydrolase precursor, medium chain [Ostrinia furnacalis]	-1.933	0.85037	0.67659	0.21462	0.19139
TRINITY_DN1093_c0_g1_i4_orf1	uncharacterized protein LOC114361723 isoform X4 [Ostrinia furnacalis]	-1.9746	0.70522	0.21267	0.51794	0.53873
TRINITY_DN85412_c0_g1_i1_orf1	unnamed protein product [Diatraea saccharalis]	-1.4122	1.44709	-0.5218	0.75379	-0.2669
TRINITY_DN993_c0_g1_i7_orf1	apolipophorins-like [Ostrinia furnacalis]	-1.5456	1.32924	-0.4186	0.80089	-0.1659
TRINITY_DN28711_c0_g1_i1_orf1	hypothetical protein evm_000299 [Chilo suppressalis]	-1.4105	1.39267	-0.5329	0.83903	-0.2883
TRINITY_DN1423_c0_g1_i8_orf1	ferritin subunit-like [Ostrinia furnacalis] >XP_028168186.1 ferritin subunit-like [Ostrinia furnacalis]	-1.5296	1.52636	-0.2715	0.4683	-0.1935
TRINITY_DN30169_c0_g1_i1_orfp1	TRINITY_DN30169_c0_g1_i1_m.11367 TRINITY_DN30169_c0_g1::TRINITY_DN30169_c0_g1_i1::g.11367 ORF type:3prime_partial len:52 (+),score=0.98 TRINITY_DN30169_c0_g1_i1:72-224(+)	-1.8231	0.76217	-0.3079	0.86317	0.50561
TRINITY_DN699_c0_g2_i1_orf1	TPA_exp: putative parasitoid killing factor [Trichoplusia ni]	-1.4252	1.46045	-0.5433	0.70775	-0.1997
TRINITY_DN1175_c1_g1_i1_orf1	methanethiol oxidase [Ostrinia furnacalis]	-1.919	1.00209	0.44652	0.1941	0.27626
TRINITY_DN81488_c0_g1_i1_orf1	apolipophorins-like [Ostrinia furnacalis]	-1.4803	1.38699	-0.4721	0.78382	-0.2184
TRINITY_DN703_c13_g1_i1_orf1	acidic juvenile hormone-suppressible protein 1-like [Ostrinia furnacalis]	-1.8393	0.39265	-0.0409	1.16504	0.32248
TRINITY_DN12582_c0_g1_i5_orf1	uncharacterized protein LOC114355527 isoform X1 [Ostrinia furnacalis]	-1.9184	0.57309	0.24067	0.95443	0.15018
TRINITY_DN60946_c0_g2_i3_orf1	protein yellow-like [Ostrinia furnacalis]	-1.9401	0.90576	0.28682	0.20958	0.53794
TRINITY_DN29414_c1_g2_i1_orf1	serine protease 44-like isoform X2 [Ostrinia furnacalis]	-1.9102	0.58049	-0.0303	0.89014	0.46987
TRINITY_DN56308_c0_g1_i2_orf1	storage protein 1 [Omphisa fuscidentalis]	-1.8862	0.66885	-0.0679	0.92946	0.35573
TRINITY_DN41_c0_g1_i3_orf1	uncharacterized protein LOC114359035 isoform X3 [Ostrinia furnacalis]	-1.8212	1.19312	0.324	-0.081	0.38503
TRINITY_DN59388_c0_g1_i1_orf1	uncharacterized protein LOC114353759 [Ostrinia furnacalis]	-1.4404	1.5435	0.48286	-0.0303	-0.5556
TRINITY_DN76216_c0_g2_i3_orf1	lysosomal alpha-mannosidase isoform X1 [Pieris rapae]	-1.5179	1.50096	-0.4115	0.51584	-0.0874
TRINITY_DN7854_c0_g1_i4_orf1	failed axon connections [Ostrinia furnacalis]	-1.5194	1.63711	0.02109	-0.0938	-0.045
TRINITY_DN418_c1_g1_i3_orf1	hypothetical protein evm_003996 [Chilo suppressalis]	-1.7125	0.69259	0.27099	1.15985	-0.4109
TRINITY_DN11826_c0_g1_i4_orf1	aldehyde dehydrogenase X, mitochondrial-like [Ostrinia furnacalis]	-1.8742	0.73404	-0.1855	0.7961	0.52956
TRINITY_DN1540_c0_g1_i7_orf1	alaserpin-like isoform X13 [Ostrinia furnacalis]	-1.9589	0.58941	0.11918	0.72358	0.52676
TRINITY_DN1012_c0_g1_i2_orf1	teneurin-a isoform X1 [Ostrinia furnacalis]	-1.4798	1.4208	0.19809	-0.6789	0.53979
TRINITY_DN97042_c0_g1_i6_orf1	apolipophorins-like [Ostrinia furnacalis]	-1.4997	1.34136	-0.466	0.83166	-0.2074
TRINITY_DN19662_c4_g1_i1_orf1	basic juvenile hormone-suppressible protein 1-like [Ostrinia furnacalis]	-1.9202	0.62002	0.1667	0.92615	0.2073
TRINITY_DN2024_c0_g1_i12_orfp1	unnamed protein product, partial [Brenthis ino]	-1.7077	1.32399	0.40147	0.28217	-0.2999
TRINITY_DN64772_c0_g1_i1_orf1	aldehyde dehydrogenase, partial [Mythimna separata]	-1.8838	0.77701	-0.1657	0.70898	0.56347
TRINITY_DN15812_c0_g1_i2_orf1	transferrin [Ostrinia furnacalis]	-1.7805	1.31766	0.15993	0.25703	0.04583
TRINITY_DN3609_c0_g1_i6_orf1	leukocyte elastase inhibitor-like [Ostrinia furnacalis]	-1.8015	1.05445	0.16659	-0.1823	0.76272
TRINITY_DN52944_c0_g1_i1_orf1	apolipophorins-like [Ostrinia furnacalis]	-1.4092	1.44676	-0.4968	0.76292	-0.3037
TRINITY_DN25976_c0_g1_i4_orf1	hypothetical protein B566_EDAN014657 [Ephemera danica]	-1.8853	1.10855	0.35248	0.24659	0.17771
TRINITY_DN28711_c1_g1_i1_orf1	apolipophorins-like [Ostrinia furnacalis]	-1.4115	1.46692	-0.5321	0.71746	-0.2408
TRINITY_DN71699_c0_g1_i1_orf1	apolipophorins-like [Ostrinia furnacalis]	-1.3854	1.43099	-0.5739	0.79524	-0.267
TRINITY_DN28221_c0_g2_i1_orf1	unnamed protein product [Chilo suppressalis]	-1.9666	0.67276	0.23764	0.70936	0.34682
TRINITY_DN124654_c0_g1_i1_orf1	protein lethal(2)essential for life [Manduca sexta] >KAG6441919.1 hypothetical protein O3G_MSEX002019 [Manduca sexta]	-1.656	0.39252	-0.5998	0.86896	0.99432
TRINITY_DN12865_c0_g1_i1_orf1	synaptic vesicle membrane protein VAT-1 homolog-like [Ostrinia furnacalis]	-1.6522	1.48141	0.18225	0.13962	-0.151
TRINITY_DN40191_c2_g1_i1_orf1	stress-activated map kinase-interacting protein 1 [Ostrinia furnacalis]	-1.6205	1.33704	-0.3251	0.68861	-0.0801
TRINITY_DN13799_c0_g1_i1_orf1	uncharacterized protein LOC116345248 [Contarinia nasturtii]	-1.6848	0.40844	-0.5319	1.10038	0.70782
TRINITY_DN7618_c0_g1_i4_orf1	uncharacterized protein LOC114366712 isoform X1 [Ostrinia furnacalis]	-1.632	1.44701	-0.2805	0.06563	0.39986
TRINITY_DN20680_c0_g1_i5_orf1	tsukushin isoform X2 [Ostrinia furnacalis]	-1.6882	1.40701	0.10571	-0.1803	0.35582
TRINITY_DN19980_c0_g1_i4_orf1	hypothetical protein evm_012507 [Chilo suppressalis]	-1.8318	1.10419	0.58938	-0.1159	0.25411
TRINITY_DN9733_c0_g1_i2_orf1	acidic juvenile hormone-suppressible protein 1-like [Ostrinia furnacalis]	-1.4475	0.86239	-0.2293	1.34974	-0.5353
TRINITY_DN1423_c0_g1_i4_orf1	hypothetical protein evm_003306 [Chilo suppressalis] >CAB3526495.1 unnamed protein product [Chilo suppressalis] >CAH0403823.1 unnamed protein product [Chilo suppressalis]	-1.6014	1.4895	-0.2159	0.40544	-0.0776
TRINITY_DN71308_c0_g1_i4_orf1	uncharacterized protein LOC114361536 [Ostrinia furnacalis]	-1.8757	1.13536	0.22489	0.32647	0.18895
TRINITY_DN80328_c0_g1_i5_orf1	arylphorin subunit alpha-like [Ostrinia furnacalis]	-1.8607	0.64937	-0.1154	0.99701	0.32973
TRINITY_DN3949_c0_g1_i1_orf1	probable cytochrome P450 304a1 [Ostrinia furnacalis]	-1.7538	1.31291	-0.1414	0.36534	0.21695
TRINITY_DN2803_c4_g1_i1_orf1	ornithine aminotransferase, mitochondrial isoform X2 [Ostrinia furnacalis]	-1.7995	1.21503	0.34464	0.38259	-0.1428
TRINITY_DN9239_c0_g2_i2_orf1	apolipophorins-like [Ostrinia furnacalis]	-1.3082	1.49575	-0.3552	0.75877	-0.5911

TRINITY_DN80328_c0_g1_i9_orf1	arylphorin subunit alpha-like [Ostrinia furnacalis]	-1.8873	0.63307	-0.0358	0.96428	0.32576
TRINITY_DN113353_c0_g1_i1_orf1	unnamed protein product [Parnassius apollo]	-1.7803	1.12965	-0.2351	0.67399	0.21173
TRINITY_DN7040_c0_g1_i4_orf1	uncharacterized protein LOC114353763 [Ostrinia furnacalis]	-1.951	0.62077	0.06429	0.66968	0.59627
TRINITY_DN858_c0_g1_i3_orf1	uncharacterized protein LOC114351944 [Ostrinia furnacalis]	-1.2453	1.23973	0.79527	-1.0905	0.30085
TRINITY_DN585_c0_g1_i12_orf1	very low-density lipoprotein receptor isoform X3 [Galleria mellonella]	-1.9341	0.96795	0.28676	0.27119	0.40819
TRINITY_DN2049_c1_g1_i3_orf1	luciferin 4-monooxygenase-like [Ostrinia furnacalis]	-1.5076	0.57208	-0.2188	1.49522	-0.3409
TRINITY_DN48878_c0_g2_i1_orf1	codanin-1 [Ostrinia furnacalis]	-1.8155	0.57176	-0.293	1.00344	0.53329
TRINITY_DN104663_c1_g1_i2_orf1	PREDICTED: gelsolin-like [Amyelois transitella]	-1.9257	0.74457	0.17203	0.81962	0.18949
TRINITY_DN10138_c0_g1_i1_orf1	storage protein 1 [Omphisa fuscidentalis]	-1.7698	0.59723	-0.0794	1.22648	0.02552
TRINITY_DN45037_c0_g1_i1_orf1	trafficking protein particle complex subunit 1 [Ostrinia furnacalis]	-1.7565	0.51292	-0.1949	1.25727	0.18122
TRINITY_DN7183_c0_g1_i2_orf1	seminal fluid protein CSSFP028 [Chilo suppressalis]	-1.7504	1.32529	0.38744	0.13895	-0.1013
TRINITY_DN18323_c0_g1_i5_orf1	GILT-like protein 1 isoform X1 [Ostrinia furnacalis]	-1.9954	0.55317	0.51699	0.5546	0.37067
TRINITY_DN44073_c0_g1_i3_orf1	inter-alpha-trypsin inhibitor heavy chain H4-like isoform X11 [Ostrinia furnacalis]	-1.6558	1.3692	0.32426	0.3538	-0.3914
TRINITY_DN64141_c0_g1_i4_orf1	probable salivary secreted peptide [Ostrinia furnacalis]	-1.7414	0.94501	-0.3563	0.95276	0.19984
TRINITY_DN32532_c0_g1_i1_orf1	fatty acyl-CoA hydrolase precursor, medium chain [Ostrinia furnacalis]	-1.8865	1.07573	0.26645	0.45187	0.0925
TRINITY_DN139537_c0_g1_i1_orf1	uncharacterized protein LOC114351440 [Ostrinia furnacalis]	-1.3696	1.66122	-0.4446	0.35511	-0.2022
TRINITY_DN27035_c0_g1_i1_orf1	glucose-6-phosphate isomerase-like [Ostrinia furnacalis]	-1.8418	0.5647	-0.2544	0.92611	0.6054
TRINITY_DN376_c1_g1_i1_orf1	matrix metalloproteinase-25-like [Ostrinia furnacalis]	-1.777	1.19591	-0.1941	0.57975	0.19548
TRINITY_DN81031_c0_g1_i1_orf1	aldehyde dehydrogenase, partial [Ectropis obliqua]	-1.8861	0.77383	-0.1317	0.7842	0.45979
TRINITY_DN5420_c0_g1_i2_orf1	DNA-directed RNA polymerase II subunit RPB1-like [Ostrinia furnacalis]	-1.5851	1.55402	0.07708	0.15793	-0.2039
TRINITY_DN2407_c0_g1_i6_orf1	uncharacterized protein LOC114366345 isoform X2 [Ostrinia furnacalis]	-1.7784	1.31896	0.01877	0.21652	0.2242
TRINITY_DN4228_c0_g1_i5_orf1	phenoloxidase-activating enzyme-like [Ostrinia furnacalis]	-1.7664	1.32909	0.04266	0.06787	0.32681
TRINITY_DN19110_c0_g1_i2_orf1	peroxidase [Ostrinia furnacalis]	-1.2457	1.62388	-0.5048	0.58694	-0.4603
TRINITY_DN3822_c0_g1_i7_orf1	hypothetical protein HF086_002539 [Spodoptera exigua] >CAH0699327.1 unnamed protein product [Spodoptera exigua]	-1.9695	0.7553	0.29464	0.60313	0.31641
TRINITY_DN13973_c0_g1_i6_orf1	27 kDa glycoprotein-like [Ostrinia furnacalis]	-1.4116	1.38377	-0.8075	0.63146	0.20394
TRINITY_DN1407_c0_g1_i12_orf1	hypothetical protein evm_012298 [Chilo suppressalis]	-1.1238	1.56932	-0.4278	0.72978	-0.7475
TRINITY_DN16905_c0_g1_i1_orf1	unnamed protein product [Leptidea sinapis]	-1.9826	0.38599	0.68762	0.58594	0.32301
TRINITY_DN7534_c0_g1_i15_orf1	protein-glucosylgalactosylhydroxylysine glucosidase isoform X2 [Ostrinia furnacalis]	-1.8691	0.68461	0.88583	-0.1736	0.47223
TRINITY_DN33272_c0_g1_i1_orf1	unnamed protein product, partial [Iphiclides podalirius]	-1.6621	1.38353	-0.3188	0.44592	0.15137
TRINITY_DN2425_c0_g1_i3_orf1	sialic acid synthase [Ostrinia furnacalis]	-1.683	1.42428	-0.1453	0.06788	0.33623
TRINITY_DN3301_c0_g1_i2_orf1	hemicentin-2-like isoform X1 [Ostrinia furnacalis]	-1.8346	0.70146	-0.0534	1.05981	0.12678
TRINITY_DN843_c0_g1_i2_orf1	unnamed protein product [Diatraea saccharalis]	-0.7208	1.68204	0.60207	-0.5989	-0.9643
TRINITY_DN578_c0_g1_i5_orf1	charged multivesicular body protein 7 [Ostrinia furnacalis]	-1.897	0.82055	0.14821	0.83508	0.09319
TRINITY_DN364_c1_g1_i2_orf1	talin-2-like, partial [Ostrinia furnacalis]	-1.8306	0.79969	0.99666	0.10478	-0.0705
TRINITY_DN26985_c0_g1_i5_orf1	secretory phospholipase A2 receptor-like [Helicoverpa zea]	-0.5737	1.36674	1.03384	-0.7327	-1.0942
TRINITY_DN23354_c0_g1_i7_orf1	TBC1 domain family member 20 [Ostrinia furnacalis]	-1.2323	1.02314	-0.7104	1.32786	-0.4083
TRINITY_DN2097_c1_g2_i2_orf1	serine protease inhibitor 3 [Ostrinia furnacalis]	-1.6902	1.32318	-0.3501	0.43897	0.27809
TRINITY_DN805_c0_g1_i5_orf1	serine protease snake-like [Ostrinia furnacalis]	-1.9644	0.77123	0.18996	0.56145	0.44174
TRINITY_DN23398_c0_g1_i1_orf1	cytochrome P450 6B7-like [Ostrinia furnacalis]	-1.9096	0.81946	-0.0428	0.42792	0.70504
TRINITY_DN5553_c0_g1_i4_orf1	uncharacterized protein LOC114353828 [Ostrinia furnacalis]	-1.2832	1.64419	-0.558	0.498	-0.3009
TRINITY_DN18128_c0_g1_i4_orf1	arylsulfatase B [Ostrinia furnacalis]	-1.8209	0.59579	-0.2345	1.05335	0.40621
TRINITY_DN42275_c0_g1_i1_orfp1	TRINITY_DN42275_c0_g1_i1_m.44265 TRINITY_DN42275_c0_g1::TRINITY_DN42275_c0_g1_i1::g.44265 ORF type:internal len:76 (+),score=8.67 TRINITY_DN42275_c0_g1_i1:1-225(+)	-1.7028	0.76157	-0.0228	1.20842	-0.2444
TRINITY_DN5022_c0_g1_i4_orf1	syntenin-1-like [Ostrinia furnacalis]	-1.8942	0.92827	0.46804	-0.073	0.57087
TRINITY_DN955_c0_g1_i2_orf1	gloverin-like [Ostrinia furnacalis] >XP_028168251.1 gloverin-like [Ostrinia furnacalis] >AYM26645.1 gloverin [Ostrinia furnacalis]	-1.5242	0.6259	0.67891	-0.839	1.05839
TRINITY_DN18388_c0_g1_i6_orf1	serine protease [Ostrinia furnacalis]	-1.8692	0.98296	0.18209	0.71188	-0.0078
TRINITY_DN3952_c0_g1_i3_orf1	protein Skeletor, isoforms D/E-like isoform X1 [Ostrinia furnacalis] >XP_028176405.1 protein Skeletor, isoforms D/E-like isoform X2 [Ostrinia furnacalis] >XP_028176406.1 protein Skeletor, isoforms D/E-like isoform X3 [Ostrinia furnacalis] >XP_028176407.1 protein Skeletor, isoforms D/E-like isoform X4 [Ostrinia furnacalis]	-1.0343	1.65249	-0.9681	0.49199	-0.1421
TRINITY_DN12231_c0_g1_i1_orf1	carbonyl reductase [NADPH] 3-like [Ostrinia furnacalis]	-1.9784	0.7706	0.41864	0.34293	0.44627
TRINITY_DN2170_c1_g1_i3_orf1	beta-1,3-glucan-binding protein-like [Ostrinia furnacalis]	-1.5408	1.58533	-0.2372	0.23363	-0.0409
TRINITY_DN479_c6_g1_i2_orf1	beta-1,3-glucan-binding protein-like [Ostrinia furnacalis]	-1.5387	1.56993	0.32255	-0.1535	-0.2003
TRINITY_DN14460_c0_g1_i6_orf1	scavenger receptor class B member 1-like [Ostrinia furnacalis]	-1.9798	0.71023	0.27956	0.43343	0.55662
TRINITY_DN105574_c0_g1_i1_orf1	prolow-density lipoprotein receptor-related protein 1, partial [Ostrinia furnacalis]	-1.9447	0.93431	0.32601	0.39161	0.29274
TRINITY_DN798_c1_g1_i3_orf1	protein goliath isoform X1 [Ostrinia furnacalis] >XP_028170876.1 protein goliath isoform X1 [Ostrinia furnacalis]	-1.9941	0.57694	0.36464	0.48365	0.56891
TRINITY_DN10220_c1_g1_i7_orf1	uncharacterized protein LOC124645895 isoform X2 [Helicoverpa zea]	-1.8477	1.02261	0.48606	-0.1816	0.52059
TRINITY_DN2835_c0_g1_i6_orf1	probable isoaspartyl peptidase/L-asparaginase GA20639 [Ostrinia furnacalis]	-1.5731	1.32898	-0.5732	0.62849	0.18883
TRINITY_DN8985_c0_g1_i4_orf1	cytochrome P450 6B6-like [Ostrinia furnacalis]	-1.6869	1.00686	-0.5577	0.79728	0.44042
TRINITY_DN361_c0_g1_i5_orf1	hexosaminidase [Ostrinia furnacalis]	-1.5449	1.55905	-0.1804	0.34414	-0.1779
TRINITY_DN11060_c0_g1_i6_orf1	extracellular matrix protein A-like isoform X3 [Ostrinia furnacalis]	-1.7923	0.93331	0.95414	-0.0756	-0.0195

TRINITY_DN8771_c0_g1_i5_orf1	regucalcin-like [Ostrinia furnacalis]	-1.8607	1.10543	0.37132	-0.0361	0.42009
TRINITY_DN13511_c0_g1_i4_orf1	alpha-2-macroglobulin receptor-associated protein isoform X1 [Ostrinia furnacalis]	-1.6094	1.08524	0.89536	0.23963	-0.6109
TRINITY_DN2227_c0_g1_i5_orf1	protein 60A [Ostrinia furnacalis]	-1.6524	1.37725	0.33267	-0.3895	0.33204
TRINITY_DN5132_c0_g1_i4_orf1	small heat shock protein Hsp24.2 [Ostrinia furnacalis]	-1.7392	0.72032	-0.5187	0.71778	0.81979
TRINITY_DN1421_c0_g1_i1_orf1	uncharacterized protein LOC114352615 [Ostrinia furnacalis]	-1.9879	0.43481	0.68868	0.35769	0.50676
TRINITY_DN537_c0_g1_i1_orf1	pupal cuticle protein C1B-like precursor [Papilio xuthus] >BAM18715.1 cuticular protein PxutCPFL6Ba [Papilio xuthus]	-1.3641	1.59296	0.539	-0.4745	-0.2934
TRINITY_DN7102_c0_g1_i5_orf1	protein wings apart-like [Ostrinia furnacalis]	-1.8265	1.01918	-0.2491	0.57782	0.47865
TRINITY_DN39266_c0_g1_i1_orf1	PREDICTED: NECAP-like protein CG9132 [Microplitis demolitor]	-1.9542	0.69299	0.22974	0.75655	0.27496
TRINITY_DN1287_c0_g1_i5_orf1	probable chitinase 10 isoform X6 [Ostrinia furnacalis]	-1.8459	0.88095	-0.2336	0.74684	0.45177
TRINITY_DN23167_c0_g2_i1_orf1	hypothetical protein evm_003712 [Chilo suppressalis]	-1.5754	1.54948	-0.268	0.11528	0.17872
TRINITY_DN40439_c0_g1_i5_orf1	ommochrome binding protein 1, partial [Ostrinia nubilalis]	-1.8944	0.46684	0.14713	1.05978	0.22062
TRINITY_DN198_c2_g1_i2_orf1	solute carrier organic anion transporter family member 5A1-like isoform X1 [Ostrinia furnacalis]	-1.7231	1.35671	0.13983	-0.156	0.38257
TRINITY_DN1098_c1_g1_i4_orf1	lysozyme 10 [Ostrinia furnacalis]	-1.7484	0.95525	-0.4362	0.40885	0.82052
TRINITY_DN2566_c0_g1_i5_orf1	uncharacterized protein LOC114349936 [Ostrinia furnacalis]	-0.9593	1.55969	-1.043	0.70157	-0.259
TRINITY_DN71863_c0_g1_i2_orf1	unnamed protein product [Diatraea saccharalis]	-1.872	0.45201	-0.1037	1.00659	0.51707
TRINITY_DN4464_c0_g2_i1_orf1	glypican-6 [Pectinophora gossypiella]	-1.9247	0.98681	0.42756	0.32098	0.18936
TRINITY_DN2880_c0_g1_i2_orf1	sialomucin core protein 24 [Pectinophora gossypiella]	-1.8657	0.87065	0.84819	-0.0512	0.19803
TRINITY_DN126127_c0_g1_i1_orf1	prolow-density lipoprotein receptor-related protein 1, partial [Ostrinia furnacalis]	-1.8549	1.08292	-0.0068	0.18539	0.5934
TRINITY_DN7633_c0_g1_i1_orf1	prolow-density lipoprotein receptor-related protein 1, partial [Ostrinia furnacalis]	-1.8974	0.93199	0.00444	0.29384	0.6671
TRINITY_DN2798_c0_g1_i5_orf1	arylsulfatase B-like isoform X1 [Ostrinia furnacalis]	-1.9403	0.75365	0.40809	0.70358	0.07499
TRINITY_DN9383_c0_g1_i3_orf1	uncharacterized protein LOC114361502 [Ostrinia furnacalis]	-1.3825	1.4029	0.83188	-0.2453	-0.607
TRINITY_DN1407_c0_g1_i5_orf1	unnamed protein product [Chrysodeixis includens]	-1.2313	1.75413	-0.2163	0.24241	-0.549
TRINITY_DN6205_c0_g1_i1_orf1	phenoloxidase-activating factor 2-like [Ostrinia furnacalis]	-1.6788	0.96468	0.29831	0.94128	-0.5255
TRINITY_DN8480_c0_g1_i1_orf1	lysosomal Pro-X carboxypeptidase [Ostrinia furnacalis]	-1.6157	1.37775	0.52292	-0.4403	0.15532
TRINITY_DN86833_c0_g3_i1_orf1	PREDICTED: glycerol-3-phosphate acyltransferase 1, mitochondrial isoform X1 [Microplitis demolitor]	-1.5831	1.3807	-0.1806	0.68221	-0.2993
TRINITY_DN1091_c0_g1_i1_orf1	macrophage mannose receptor 1-like [Pararge aegeria]	-1.3514	1.49356	0.73937	-0.5032	-0.3783
TRINITY_DN97097_c0_g1_i4_orf1	plectin-like, partial [Ostrinia furnacalis]	-1.4084	1.6619	-0.43	0.25249	-0.076
TRINITY_DN11514_c0_g1_i1_orf1	uncharacterized protein LOC114350079 [Ostrinia furnacalis]	-1.8344	1.04803	0.52924	-0.2057	0.46284
TRINITY_DN15961_c0_g1_i1_orf1	uncharacterized protein LOC113522423 [Galleria mellonella]	-1.9117	0.94171	0.02293	0.54255	0.40452
TRINITY_DN10231_c0_g2_i1_orf1	uncharacterized protein LOC114361472 [Ostrinia furnacalis]	-1.3533	1.29238	0.10504	-0.8841	0.84001
TRINITY_DN3647_c1_g1_i5_orf1	unnamed protein product, partial [Iphiclides podalirius]	-1.6234	1.1311	0.94416	-0.4398	-0.012
TRINITY_DN12256_c0_g1_i1_orf1	lysosome membrane protein 2-like [Ostrinia furnacalis]	-1.9243	0.85424	0.59134	0.0121	0.46658
TRINITY_DN10090_c0_g1_i1_orf1	clotting factor B-like isoform X1 [Ostrinia furnacalis] >XP_028163447.1 clotting factor B-like isoform X2 [Ostrinia furnacalis] >XP_028163448.1 clotting factor B-like isoform X3 [Ostrinia furnacalis]	-1.8629	1.16435	0.17151	0.31637	0.21067
TRINITY_DN875_c0_g1_i3_orf1	secernin-3 [Ostrinia furnacalis]	-1.244	1.71172	-0.1826	0.33093	-0.6161
TRINITY_DN125441_c0_g1_i5_orf1	KH domain-containing, RNA-binding, signal transduction-associated protein 2-like isoform X12 [Ostrinia furnacalis]	-1.8939	0.94752	0.69098	0.17933	0.07606
TRINITY_DN6243_c0_g1_i5_orf1	sorting nexin-20 [Ostrinia furnacalis]	-1.9153	0.93617	0.61538	0.11011	0.25365
TRINITY_DN22053_c0_g1_i13_orf1	uncharacterized protein LOC114355104 [Ostrinia furnacalis]	-1.6744	1.43144	-0.1583	0.34498	0.05631
TRINITY_DN1304_c0_g1_i6_orf1	uncharacterized protein LOC114359545 [Ostrinia furnacalis]	-1.973	0.73539	0.58395	0.40343	0.25025
TRINITY_DN19748_c0_g1_i4_orf1	PREDICTED: cysteine-rich hydrophobic domain-containing protein 2 [Amyelois transitella] >XP_028158573.1 cysteine-rich hydrophobic domain-containing protein 2 isoform X1 [Ostrinia furnacalis] >CAH0685427.1 unnamed protein product [Chilo suppressalis]	-1.9052	1.0391	0.39372	0.12705	0.34534
TRINITY_DN6244_c0_g1_i4_orf1	uncharacterized protein CG3556 [Ostrinia furnacalis]	-1.5785	1.56475	0.19419	-0.1442	-0.0362
TRINITY_DN4602_c0_g1_i4_orf1	2-iminobutanoate/2-iminopropanoate deaminase [Ostrinia furnacalis]	-1.767	1.12088	-0.333	0.36492	0.61422
TRINITY_DN16840_c1_g1_i1_orf1	attacin-like [Ostrinia furnacalis]	-1.5865	0.49402	0.44681	-0.6339	1.27961
TRINITY_DN8083_c0_g1_i1_orf1	solute carrier family 35 member F6 [Ostrinia furnacalis]	-1.5389	1.36715	0.75293	-0.4066	-0.1746
TRINITY_DN23746_c0_g1_i2_orf1	protein 4.1 homolog isoform X1 [Ostrinia furnacalis]	-1.6572	1.18754	-0.0723	0.85887	-0.3168
TRINITY_DN30498_c0_g1_i3_orf1	lipase 3-like [Ostrinia furnacalis]	-1.7273	0.90718	0.04327	1.05545	-0.2786
TRINITY_DN1651_c0_g2_i1_orf1	UBX domain-containing protein 6 [Ostrinia furnacalis] >XP_028162119.1 UBX domain-containing protein 6 [Ostrinia furnacalis]	-1.9123	0.85411	0.23375	0.74336	0.08103
TRINITY_DN399_c3_g2_i6_orf1	proline-rich extensin-like protein EPR1 [Ostrinia furnacalis]	-1.8954	0.60869	-0.0836	0.89849	0.47187
TRINITY_DN1507_c0_g1_i5_orf1	27 kDa hemolymph protein-like, partial [Ostrinia furnacalis]	-1.7351	1.2881	0.55902	-0.1314	0.01942
TRINITY_DN4256_c0_g1_i1_orf1	chitinase-3-like protein 1 [Ostrinia furnacalis]	-1.6834	1.3046	-0.2394	0.6376	-0.0194
TRINITY_DN6988_c0_g1_i3_orf1	cuticle protein 1-like [Ostrinia furnacalis]	-1.8483	0.88877	-0.0593	0.87776	0.14107
TRINITY_DN125150_c0_g1_i1_orf1	aldehyde dehydrogenase, dimeric NADP-preferring isoform X5 [Ostrinia furnacalis]	-1.8976	0.88808	0.24927	0.0202	0.74008
TRINITY_DN12014_c0_g1_i2_orf1	unnamed protein product [Chilo suppressalis]	-1.5093	0.83407	1.33928	-0.41	-0.2541
TRINITY_DN19821_c0_g2_i4_orf1	uncharacterized protein LOC114359393 isoform X1 [Ostrinia furnacalis]	-1.5885	1.00807	-0.4933	1.10285	-0.0291
TRINITY_DN2205_c0_g1_i3_orf1	probable chitinase 2 [Ostrinia furnacalis]	-1.8858	0.92709	-0.0334	0.28294	0.70919
TRINITY_DN4076_c1_g2_i2_orf1	vacuole membrane protein 1 [Ostrinia furnacalis]	-1.8923	0.64851	-0.1421	0.78744	0.59851
TRINITY_DN70485_c0_g1_i2_orf1	serine/threonine-protein kinase Genghis Khan-like [Ostrinia furnacalis]	-1.5568	1.56618	0.1462	0.13433	-0.2899
TRINITY_DN664_c0_g1_i18_orf1	chitinase-like protein EN03 isoform X2 [Ostrinia furnacalis]	-1.7578	0.83039	-0.4744	0.6197	0.78208
TRINITY_DN1093_c0_g1_i6_orf1	uncharacterized protein LOC114361723 isoform X4 [Ostrinia furnacalis]	-1.9495	0.71885	0.09077	0.68166	0.45819

TRINITY_DN2171_c0_g1_i1_orf1	probable pterin-4-alpha-carbinolamine dehydratase isoform X1 [Ostrinia furnacalis]	-1.896	1.02984	0.26072	0.08693	0.51856
TRINITY_DN12286_c1_g1_i2_orf1	sideroflexin-1-3 [Galleria mellonella] >XP_026754161.1 sideroflexin-1-3 [Galleria mellonella]	-1.2111	1.68161	-0.5167	0.49075	-0.4446
TRINITY_DN1569_c0_g1_i6_orf1	uncharacterized protein LOC114350603 [Ostrinia furnacalis]	-1.1552	0.69427	-0.9148	1.52461	-0.1489
TRINITY_DN18230_c1_g2_i1_orf1	hypothetical protein O3G_MSEX004459 [Manduca sexta]	-1.975	0.68332	0.2165	0.59835	0.47687
TRINITY_DN10539_c0_g1_i1_orf1	uncharacterized protein LOC114358962 isoform X1 [Ostrinia furnacalis]	-1.7504	0.30085	-0.1641	1.31514	0.29852
TRINITY_DN745_c7_g1_i1_orf1	uncharacterized protein LOC114358822 [Ostrinia furnacalis]	-1.7551	0.71535	-0.268	1.14433	0.1634
TRINITY_DN4449_c0_g2_i1_orf1	calcium/calmodulin-dependent protein kinase type 1 isoform X3 [Cephus cinctus]	-1.9872	0.54699	0.28688	0.52896	0.62436
TRINITY_DN428_c0_g1_i8_orf1	phenoloxidase-activating factor 2-like isoform X1 [Ostrinia furnacalis]	-1.9705	0.58433	0.32232	0.76222	0.30159
TRINITY_DN43431_c0_g1_i1_orf1	glycine dehydrogenase (decarboxylating), mitochondrial isoform X1 [Ostrinia furnacalis] >XP_028174269.1 glycine dehydrogenase (decarboxylating), mitochondrial isoform X3 [Ostrinia furnacalis]	-1.7688	1.20945	0.63563	-0.01	-0.0663
TRINITY_DN338_c1_g1_i9_orf1	scolexin B-like isoform X2 [Ostrinia furnacalis]	-1.9101	0.81459	0.25117	0.78856	0.05576
TRINITY_DN5064_c0_g1_i4_orf1	sortilin-related receptor-like [Ostrinia furnacalis]	-1.6412	1.38948	0.42395	-0.3872	0.21493
TRINITY_DN1194_c0_g1_i5_orf1	sequestosome-1-like isoform X4 [Ostrinia furnacalis]	-1.2223	1.48736	0.83189	-0.5474	-0.5495
TRINITY_DN15291_c0_g1_i11_orf1	uncharacterized protein LOC114353772 [Ostrinia furnacalis]	-1.275	1.30054	-0.834	0.97899	-0.1706
TRINITY_DN3017_c0_g1_i6_orf1	uncharacterized protein LOC114364875 isoform X1 [Ostrinia furnacalis] >XP_028177022.1 uncharacterized protein LOC114364875 isoform X1 [Ostrinia furnacalis]	-1.6551	1.45098	0.2018	0.24049	-0.2382
TRINITY_DN8703_c0_g1_i2_orf1	beta-glucuronidase-like isoform X1 [Ostrinia furnacalis] >XP_028166212.1 beta-glucuronidase-like isoform X2 [Ostrinia furnacalis]	-1.6787	1.01919	-0.2126	1.03516	-0.1631
TRINITY_DN1664_c0_g1_i4_orf1	uncharacterized protein LOC114355246 [Ostrinia furnacalis]	-1.8103	1.09041	0.72407	0.06649	-0.0706
TRINITY_DN28661_c0_g1_i1_orf1	cathepsin B [Ostrinia furnacalis]	-1.7617	1.22559	0.59612	-0.1661	0.10615
TRINITY_DN7128_c0_g1_i7_orf1	dystroglycan [Ostrinia furnacalis]	-1.6362	1.45888	0.39003	-0.2055	-0.0071
TRINITY_DN3251_c0_g1_i6_orf1	fatty-acid amide hydrolase 2-like [Ostrinia furnacalis] >XP_028167366.1 fatty-acid amide hydrolase 2-like [Ostrinia furnacalis] >XP_028167367.1 fatty-acid amide hydrolase 2-like [Ostrinia furnacalis] >XP_028167368.1 fatty-acid amide hydrolase 2-like [Ostrinia furnacalis] >XP_028167369.1 fatty-acid amide hydrolase 2-like [Ostrinia furnacalis] >XP_028167370.1 fatty-acid amide hydrolase 2-like [Ostrinia furnacalis] >XP_028167371.1 fatty-acid amide hydrolase 2-like [Ostrinia furnacalis] >XP_028167372.1 fatty-acid amide hydrolase 2-like [Ostrinia furnacalis]	-1.8781	1.11383	0.35537	0.30974	0.09919
TRINITY_DN8245_c0_g1_i3_orf1	uncharacterized protein LOC114357622 [Ostrinia furnacalis]	-1.959	0.72716	0.28502	0.70164	0.24515
TRINITY_DN5070_c0_g1_i1_orf1	ATP-dependent (S)-NAD(P)H-hydrate dehydratase-like [Ostrinia furnacalis]	-1.4893	1.46269	0.07369	0.54035	-0.5874
TRINITY_DN2348_c0_g1_i1_orfp1	TRINITY_DN2348_c0_g1_i1_m.39060 TRINITY_DN2348_c0_g1::TRINITY_DN2348_c0_g1_i1::g.39060 ORF type:complete len:149 (+),score=54.19 TRINITY_DN2348_c0_g1_i1:28-474(+)	-1.3293	1.73074	-0.2004	0.19699	-0.398
TRINITY_DN5667_c0_g1_i4_orf1	spodomicin-like [Ostrinia furnacalis] >QKV49445.1 diapausin [Ostrinia furnacalis]	-1.9222	0.73635	0.26706	0.82638	0.09244
TRINITY_DN1710_c0_g1_i1_orf1	nuclear factor NF-kappa-B p105 subunit-like isoform X2 [Ostrinia furnacalis]	-1.9556	0.47416	0.26973	0.87427	0.33741
TRINITY_DN9615_c0_g1_i1_orf1	uncharacterized protein LOC114352730 [Ostrinia furnacalis]	-1.7911	1.24453	-0.0289	0.48371	0.09175
TRINITY_DN53427_c0_g1_i2_orf1	heparanase-like [Ostrinia furnacalis]	-1.108	1.70369	0.50923	-0.5538	-0.5511
TRINITY_DN2606_c0_g1_i5_orf1	galectin-4-like isoform X1 [Ostrinia furnacalis]	-1.9635	0.68315	0.15299	0.66156	0.46578
TRINITY_DN64892_c0_g1_i1_orf1	aldehyde dehydrogenase X, mitochondrial [Manduca sexta] >KAG6450704.1 hypothetical protein O3G_MSEX006722 [Manduca sexta]	-1.7565	0.58756	-0.3139	1.17229	0.31063
TRINITY_DN110402_c0_g2_i1_orf1	apolipophorins-like [Ostrinia furnacalis]	-1.2576	1.68792	-0.6595	0.34706	-0.1178
TRINITY_DN74654_c0_g1_i4_orf1	limulus clotting factor C-like isoform X4 [Ostrinia furnacalis]	-1.9788	0.56655	0.34956	0.72711	0.33555
TRINITY_DN3513_c0_g1_i5_orf1	vacuolar protein sorting-associated protein 16 homolog [Ostrinia furnacalis]	-1.8949	1.01439	0.24518	0.56081	0.07457
TRINITY_DN2508_c0_g1_i2_orf1	uncharacterized protein LOC114361845 [Ostrinia furnacalis] >XP_028172853.1 uncharacterized protein LOC114361845 [Ostrinia furnacalis]	-1.7591	1.33366	0.03774	0.03523	0.35247
TRINITY_DN1767_c0_g2_i15_orf1	fasciclin-2 isoform X3 [Ostrinia furnacalis]	-1.6389	1.49198	0.23328	0.07922	-0.1656
TRINITY_DN28729_c0_g1_i9_orf1	serine/threonine-protein kinase mig-15 isoform X2 [Ostrinia furnacalis]	-1.703	0.9415	-0.5539	0.80175	0.51364
TRINITY_DN896_c0_g1_i2_orf1	uncharacterized protein LOC114356314 isoform X2 [Ostrinia furnacalis]	-1.1738	1.21433	1.06665	-0.999	-0.1081
TRINITY_DN15774_c0_g1_i3_orf1	uncharacterized protein LOC114366599 [Ostrinia furnacalis]	-1.9475	0.80917	0.27256	0.66037	0.20538
TRINITY_DN1957_c0_g1_i4_orf1	NAD kinase 2, mitochondrial [Ostrinia furnacalis]	-1.8114	0.36452	-0.2614	1.02515	0.68314
TRINITY_DN38412_c0_g1_i1_orf1	translation initiation factor eIF-2B subunit alpha [Ostrinia furnacalis]	-1.657	1.40022	-0.1849	0.50553	-0.0639
TRINITY_DN5124_c0_g1_i2_orf1	glucose dehydrogenase [FAD, quinone]-like [Ostrinia furnacalis]	-1.5706	0.3187	0.05676	1.52339	-0.3283
TRINITY_DN7868_c0_g1_i2_orf1	uncharacterized protein LOC114353432 isoform X4 [Ostrinia furnacalis]	-1.8676	0.93337	0.20381	0.773	-0.0426
TRINITY_DN29217_c0_g1_i3_orf1	protein yippee-like 5 [Ostrinia furnacalis]	-1.9394	0.83411	0.16419	0.66214	0.27892
TRINITY_DN3109_c0_g1_i5_orf1	protein takeout isoform X2 [Ostrinia furnacalis]	-1.8045	1.07515	0.7659	-0.0372	0.00062
TRINITY_DN45271_c0_g1_i1_orf1	double-strand break repair protein MRE11 [Ostrinia furnacalis]	-1.667	1.47481	0.21233	0.00999	-0.0302
TRINITY_DN5488_c0_g1_i5_orf1	alpha-amylase-like [Ostrinia furnacalis]	-1.9606	0.66826	0.56371	0.6156	0.11303
TRINITY_DN45097_c0_g1_i5_orf1	ras-related protein Rab-24-like [Ostrinia furnacalis]	-1.9703	0.66916	0.6937	0.35016	0.2573
TRINITY_DN1759_c0_g1_i4_orf1	protein PFC0760c-like isoform X1 [Ostrinia furnacalis]	-1.689	0.43379	0.80802	-0.553	1.0002
TRINITY_DN1659_c0_g1_i3_orf1	beta-catenin-like protein 1 [Ostrinia furnacalis]	-1.9883	0.56574	0.31345	0.4724	0.63667
TRINITY_DN6462_c0_g1_i5_orf1	probable histone-lysine N-methyltransferase CG1716 [Ostrinia furnacalis]	-1.8359	0.74765	0.04473	1.03365	0.00986
TRINITY_DN11868_c0_g1_i2_orf1	uncharacterized protein LOC114361308 [Ostrinia furnacalis]	-1.5547	1.07345	0.98066	-0.6646	0.16518
TRINITY_DN37654_c0_g1_i5_orf1	unnamed protein product [Pieris macdunnoughi]	-1.8263	1.00312	-0.261	0.50735	0.57688
TRINITY_DN140_c1_g1_i2_orf1	modular serine protease-like isoform X1 [Ostrinia furnacalis]	-1.3605	1.57972	0.57878	-0.3909	-0.4071
TRINITY_DN9542_c0_g1_i4_orf1	NAD(P)H-hydrate epimerase [Ostrinia furnacalis]	-1.9868	0.49844	0.31447	0.49669	0.67719
TRINITY_DN4276_c0_g1_i6_orf1	arylsulfatase B [Ostrinia furnacalis]	-1.4249	1.53834	0.38713	0.15467	-0.6552
TRINITY_DN89083_c0_g1_i1_orf1	lysine-specific demethylase 4A isoform X2 [Diachasma alloeum]	-1.8642	0.77138	-0.2257	0.72795	0.59059

TRINITY_DN166_c0_g1_i4_orf1	PREDICTED: cryptochrome-1 isoform X1 [Amyelois transitella] >XP_013199861.1 PREDICTED: cryptochrome-1 isoform X1 [Amyelois transitella]	-1.601	1.33628	-0.5439	0.52374	0.28484
TRINITY_DN36856_c0_g1_i1_orf1	protein enhancer of sevenless 2B isoform X2 [Formica exsecta]	-1.3952	1.63523	0.30438	-0.0089	-0.5356
TRINITY_DN9412_c0_g1_i1_orf1	maspardin-like [Ostrinia furnacalis]	-1.3603	1.5662	-0.0341	0.49746	-0.6692
TRINITY_DN44709_c0_g1_i1_orf1	D-beta-hydroxybutyrate dehydrogenase, mitochondrial, partial [Chelonus insularis]	-1.5118	1.26766	-0.5698	0.88213	-0.0682
TRINITY_DN130439_c0_g1_i1_orf1	DDRGK domain-containing protein 1-like [Ostrinia furnacalis]	-1.4377	1.09269	1.14758	-0.6248	-0.1777
TRINITY_DN2035_c0_g1_i1_orf1	uncharacterized protein LOC114356683 [Ostrinia furnacalis]	-1.9635	0.77322	0.59139	0.39307	0.20588
TRINITY_DN7590_c0_g1_i4_orf1	innexin inx1-like [Pectinophora gossypiella]	-1.7643	1.22923	-0.2236	0.24051	0.51814
TRINITY_DN7579_c1_g3_i1_orf1	peroxiredoxin-5, mitochondrial [Ostrinia furnacalis]	-1.948	0.8543	0.39985	0.54024	0.15364
TRINITY_DN2395_c0_g1_i7_orf1	uncharacterized protein LOC114352963 [Ostrinia furnacalis]	-1.6622	1.02035	-0.572	0.86416	0.34961
TRINITY_DN18650_c0_g1_i1_orf1	bombyxin B-9-like [Ostrinia furnacalis]	-1.3172	1.51982	-0.7953	0.56747	0.0252
TRINITY_DN862_c0_g1_i4_orf1	uncharacterized protein LOC114359380 isoform X1 [Ostrinia furnacalis] >XP_028169561.1 uncharacterized protein LOC114359380 isoform X2 [Ostrinia furnacalis] >XP_028169562.1 uncharacterized protein LOC114359380 isoform X1 [Ostrinia furnacalis]	-1.3381	1.67622	-0.2905	0.3726	-0.4203
TRINITY_DN48765_c0_g1_i7_orf1	uncharacterized protein LOC114352307 [Ostrinia furnacalis]	-1.587	1.16355	0.74182	0.35398	-0.6724
TRINITY_DN96739_c0_g1_i1_orf1	annexin A6, isoform CRA_b [Homo sapiens]	-1.5769	1.15035	0.54816	-0.7249	0.60333
TRINITY_DN63943_c0_g1_i5_orf1	uncharacterized protein LOC114357144 [Ostrinia furnacalis]	-1.7902	1.19698	0.10267	-0.0949	0.5855
TRINITY_DN2943_c2_g2_i1_orf1	protein phosphatase inhibitor 2-like [Ostrinia furnacalis]	-1.9964	0.55471	0.57294	0.40976	0.45896
TRINITY_DN143637_c0_g1_i1_orf1	PX domain-containing protein kinase-like protein isoform X1 [Chelonus insularis]	-1.7345	1.20193	0.29959	0.5802	-0.3472
TRINITY_DN8766_c0_g1_i1_orf1	prolow-density lipoprotein receptor-related protein 1, partial [Ostrinia furnacalis]	-1.8444	0.90036	0.31127	-0.1789	0.81162
TRINITY_DN7867_c0_g1_i1_orf1	putative inorganic phosphate cotransporter [Ostrinia furnacalis]	-1.8369	0.88254	-0.2803	0.56126	0.67337
TRINITY_DN3896_c0_g1_i1_orf1	glyoxalase domain-containing protein 4 [Ostrinia furnacalis]	-1.5635	1.53605	-0.3518	0.17913	0.20018
TRINITY_DN2784_c0_g1_i3_orf1	toll-like receptor 6 [Ostrinia furnacalis]	-1.9242	0.80321	0.02666	0.70987	0.38444
TRINITY_DN1144_c0_g1_i10_orf1	TIL [Ostrinia furnacalis]	-1.7699	0.45222	-0.2926	1.1799	0.43037
TRINITY_DN3212_c0_g1_i7_orfp1	TRINITY_DN3212_c0_g1_i7_m.38385 TRINITY_DN3212_c0_g1::TRINITY_DN3212_c0_g1_i7::g.38385 ORF type:internal len:121 (-),score=5.33 TRINITY_DN3212_c0_g1_i7:3-362(-)	-1.7941	0.57286	-0.3232	1.05128	0.49323
TRINITY_DN50517_c0_g1_i1_orf1	cuticle protein 21-like [Ostrinia furnacalis]	-1.9501	0.86277	0.55587	0.23001	0.30144
TRINITY_DN10373_c0_g1_i1_orf1	homocysteine S-methyltransferase 1-like [Ostrinia furnacalis] >XP_028162778.1 homocysteine S-methyltransferase 1-like [Ostrinia furnacalis]	-1.5007	0.81698	1.20418	-0.7577	0.23723
TRINITY_DN13997_c0_g1_i5_orf1	28 kDa heat- and acid-stable phosphoprotein [Ostrinia furnacalis]	-1.8989	0.8816	0.18111	0.76045	0.07578
TRINITY_DN2848_c0_g1_i2_orf1	glyceraldehyde-3-phosphate dehydrogenase isoform 1 [Homo sapiens] >NP_001276675.1 glyceraldehyde-3-phosphate dehydrogenase isoform 1 [Homo sapiens] >NP_002037.2 glyceraldehyde-3-phosphate dehydrogenase isoform 1 [Homo sapiens] >XP_003819180.1 glyceraldehyde-3-phosphate dehydrogenase [Pan paniscus] >XP_004052609.1 glyceraldehyde-3-phosphate dehydrogenase [Gorilla gorilla gorilla] >XP_008971979.1 glyceraldehyde-3-phosphate dehydrogenase [Pan paniscus] >XP_008971980.1 glyceraldehyde-3-phosphate dehydrogenase [Pan paniscus] >XP_032621678.1 glyceraldehyde-3-phosphate dehydrogenase [Chelonoidis abingdonii] >XP_508955.1 glyceraldehyde-3-phosphate dehydrogenase isoform X1 [Pan troglodytes] >P04406.3 RecName: Full=Glyceraldehyde-3-phosphate dehydrogenase; Short=GAPDH; AltName: Full=Peptidyl-cysteine S-nitrosylase GAPDH [Homo sapiens] >1U8F_O Crystal Structure Of Human Placental Glyceraldehyde-3-Phosphate Dehydrogenase At 1.75 Resolution [Homo sapiens] >1U8F_P Crystal Structure Of Human Placental Glyceraldehyde-3-Phosphate Dehydrogenase At 1.75 Resolution [Homo sapiens] >1U8F_Q Crystal Structure Of Human Placental Glyceraldehyde-3-Phosphate Dehydrogenase At 1.75 Resolution [Homo sapiens] >1U8F_R Crystal Structure Of Human Placental Glyceraldehyde-3-Phosphate Dehydrogenase At 1.75 Resolution [Homo sapiens] >4WNC_A Crystal structure of human wild-type GAPDH at 1.99 angstroms resolution [Homo sapiens] >4WNC_B Crystal structure of human wild-type GAPDH at 1.99 angstroms resolution [Homo sapiens] >4WNC_C Crystal structure of human wild-type GAPDH at 1.99 angstroms resolution [Homo sapiens] >4WNC_D Crystal structure of human wild-type GAPDH at 1.99 angstroms resolution [Homo sapiens] >4WNC_E Crystal structure of human wild-type GAPDH at 1.99 angstroms resolution [Homo sapiens] >4WNC_F Crystal structure of human wild-type GAPDH at 1.99 angstroms resolution [Homo sapiens] >4WNC_G Crystal structure of human wild-type GAPDH at 1.99 angstroms resolution [Homo sapiens] >4WNC_O Crystal structure of human wild-type GAPDH at 1.99 angstroms resolution [Homo sapiens] >6IQ6_A Crystal structure of GAPDH [Homo sapiens] >6IQ6_B Crystal structure of GAPDH [Homo sapiens] >6IQ6_C Crystal structure of GAPDH [Homo sapiens] >6IQ6_D Crystal structure of GAPDH [Homo sapiens] >6IQ6_E Crystal structure of GAPDH [Homo sapiens] >6IQ6_F Crystal structure of GAPDH [Homo sapiens] >6IQ6_G Crystal structure of GAPDH [Homo sapiens] >6IQ6_H Crystal structure of GAPDH [Homo sapiens] >6YNE_A GAPDH purified from the supernatant of HEK293F cells: crystal form 2 of 4. [Homo sapiens] >6YNE_B GAPDH purified from the supernatant of HEK293F cells: crystal form 2 of 4. [Homo sapiens] >6YNE_C GAPDH purified from the supernatant of HEK293F cells: crystal form 2 of 4. [Homo sapiens] >6YNE_D GAPDH purified from the supernatant of HEK293F cells: crystal form 2 of 4. [Homo sapiens] >AAX42270.1 glyceraldehyde-3-phosphate dehydrogenase [synthetic construct] >MXR00212.1 hypothetical protein [Bos mutus] >SJX33932.1 unnamed protein product, partial [Human ORFeome Gateway entry vector] >AAA52496.1 glyceraldehyde 3-phosphate dehydrogenase (EC 1.2.1.12) [Homo sapiens] >AAA52518.1 glyceraldehyde-3-phosphate dehydrogenase (EC 1.2.1.12) [Homo sapiens]	-1.9079	0.60011	0.06389	0.95533	0.28853
TRINITY_DN2897_c0_g2_i1_orf1	gem-associated protein 5-like [Ostrinia furnacalis]	-1.3491	1.29139	-0.816	0.91897	-0.0454
TRINITY_DN184_c0_g1_i10_orf1	C-type mannose receptor 2-like isoform X1 [Leguminivora glycinivorella]	-1.6979	0.66493	1.27613	-0.0299	-0.2132
TRINITY_DN14458_c0_g1_i2_orf1	spermatogenesis-associated protein 20 isoform X1 [Ostrinia furnacalis]	-1.2612	1.78399	-0.0471	-0.0019	-0.4737
TRINITY_DN913_c0_g1_i6_orf1	uncharacterized protein LOC114354578 [Ostrinia furnacalis]	-1.9674	0.74717	0.29308	0.6301	0.29703
TRINITY_DN3483_c0_g1_i5_orf1	phenoloxidase-activating factor 2-like isoform X1 [Ostrinia furnacalis] >XP_028178309.1 phenoloxidase-activating factor 2-like isoform X2 [Ostrinia furnacalis]	-1.713	0.80788	-0.5678	0.79082	0.68209
TRINITY_DN4394_c0_g2_i1_orf1	carboxylesterase [Ostrinia furnacalis]	-1.5894	0.83133	-0.007	1.24565	-0.4806

TRINITY_DN42856_c0_g1_i1_orf1	GSCOCG00007769001-RA-CDS [Cotesia congregata] >CAG5103393.1 Similar to GLDC: Glycine dehydrogenase (decarboxylating) [Cotesia congregata]	-1.9232	0.44652	0.90353	0.04032	0.53283
TRINITY_DN52761_c0_g1_i2_orf1	atlastin isoform X4 [Ostrinia furnacalis]	-1.7121	0.12025	0.9322	-0.3657	1.02538
TRINITY_DN4276_c0_g1_i11_orf1	arylsulfatase B [Ostrinia furnacalis]	-1.9096	0.9702	0.35108	0.53462	0.05375
TRINITY_DN625_c2_g2_i2_orf1	L-dopachrome tautomerase yellow-f2-like [Ostrinia furnacalis]	-1.7918	0.4621	-0.1451	1.22074	0.25406
TRINITY_DN31310_c0_g1_i1_orf1	PREDICTED: multiple epidermal growth factor-like domains protein 10 isoform X3 [Polistes canadensis]	-1.9707	0.80102	0.3132	0.49913	0.3573
TRINITY_DN8637_c0_g1_i1_orf1	superoxide dismutase [Cu-Zn] [Ostrinia furnacalis] >XP_028177872.1 superoxide dismutase [Cu-Zn] [Ostrinia furnacalis]	-1.8718	0.3846	-0.1377	0.74393	0.88093
TRINITY_DN7754_c0_g1_i2_orf1	carboxypeptidase E-like isoform X1 [Ostrinia furnacalis] >XP_028174275.1 carboxypeptidase E-like isoform X2 [Ostrinia furnacalis] >XP_028174276.1 carboxypeptidase E-like isoform X2 [Ostrinia furnacalis]	-1.9665	0.63731	0.74615	0.29624	0.28679
TRINITY_DN6822_c0_g2_i4_orf1	gelsolin-like [Bicyclus anynana]	-1.8289	0.57086	1.15038	0.05857	0.04909
TRINITY_DN56430_c0_g1_i1_orf1	unnamed protein product, partial [Iphiclides podalirius]	-1.1312	1.65882	0.51515	-0.8039	-0.2389
TRINITY_DN13856_c0_g1_i1_orf1	angiotensin-converting enzyme-like [Ostrinia furnacalis]	-1.3156	1.52234	-0.2444	0.68637	-0.6486
TRINITY_DN48250_c0_g1_i1_orf1	larval/pupal rigid cuticle protein 66-like [Hyposmocoma kahamanoa]	-1.795	0.83737	-0.3315	0.9055	0.38356
TRINITY_DN2793_c0_g2_i1_orf1	PREDICTED: ras-related protein Rab-4B [Amyelois transitella]	-1.7132	1.255	-0.1687	0.67733	-0.0504
TRINITY_DN42854_c0_g3_i2_orf1	amyloid beta (A4) precursor-like protein 2, isoform CRA_b [Homo sapiens]	-1.655	0.9126	-0.3863	1.13084	-0.0021
TRINITY_DN12661_c0_g1_i3_orf1	T-complex protein 11-like protein 1 [Ostrinia furnacalis]	-1.6372	0.81103	1.24283	-0.3314	-0.0853
TRINITY_DN272_c0_g1_i1_orf1	vacuolar protein sorting-associated protein 11 homolog [Ostrinia furnacalis]	-1.6988	1.18205	-0.0769	0.81392	-0.2203
TRINITY_DN31676_c0_g1_i4_orf1	N-acetylgalactosaminyltransferase 7 isoform X1 [Ostrinia furnacalis] >XP_028156925.1 N-acetylgalactosaminyltransferase 7 isoform X2 [Ostrinia furnacalis]	-1.7599	1.20442	-0.2199	0.61491	0.16043
TRINITY_DN5383_c0_g1_i4_orf1	vacuolar protein sorting-associated protein 35 [Ostrinia furnacalis]	-1.9008	0.77525	0.78256	-0.0679	0.41092
TRINITY_DN12555_c0_g1_i1_orf1	uncharacterized protein LOC114350746 [Ostrinia furnacalis]	-1.2033	1.69736	-0.6031	0.44269	-0.3336
TRINITY_DN21533_c0_g1_i7_orf1	annexin B9 isoform X1 [Ostrinia furnacalis]	-1.4648	0.90783	1.31632	-0.4479	-0.3115
TRINITY_DN6205_c0_g1_i4_orfp1	TRINITY_DN6205_c0_g1_i4_m.72677 TRINITY_DN6205_c0_g1::TRINITY_DN6205_c0_g1_i4::g.72677 ORF type:internal len:68 (-),score=1.69 TRINITY_DN6205_c0_g1_i4:2-202(-)	-0.6188	1.37868	1.04104	-0.9744	-0.8265
TRINITY_DN24668_c0_g1_i8_orf1	uncharacterized protein LOC114364067 isoform X3 [Ostrinia furnacalis]	-1.3148	1.05858	1.25614	-0.3091	-0.6908
TRINITY_DN213_c0_g1_i5_orf1	protein lap4-like [Ostrinia furnacalis]	-1.5742	0.89402	0.22534	-0.6582	1.11299
TRINITY_DN1870_c0_g1_i6_orf1	programmed cell death protein 5 [Ostrinia furnacalis]	-1.7806	1.19246	-0.1255	0.61876	0.09488
TRINITY_DN46090_c0_g3_i1_orf1	tyrosine-protein kinase-like otk, partial [Ostrinia furnacalis]	-1.3732	0.73485	-0.621	1.46505	-0.2057
TRINITY_DN7062_c0_g1_i1_orf1	ribosome maturation protein SBDS [Ostrinia furnacalis]	-1.5036	1.40434	0.33179	-0.6774	0.44491
TRINITY_DN2796_c0_g1_i28_orf1	phosphofurin acidic cluster sorting protein 2 isoform X1 [Pectinophora gossypiella]	-1.9585	0.79806	0.34301	0.21441	0.60305
TRINITY_DN10680_c0_g1_i5_orf1	cGMP-dependent protein kinase, isozyme 2 forms cD4/T1/T3A/T3B-like isoform X3 [Ostrinia furnacalis] >XP_028158316.1 cGMP-dependent protein kinase, isozyme 2 forms cD4/T1/T3A/T3B-like isoform X3 [Ostrinia furnacalis]	-1.593	1.44791	-0.2337	0.53554	-0.1568
TRINITY_DN65299_c0_g4_i1_orf1	LOW QUALITY PROTEIN: signal transducing adapter molecule 2 [Ostrinia furnacalis]	-1.7275	0.95975	1.02117	-0.226	-0.0274
TRINITY_DN3292_c2_g2_i1_orf1	aldo-keto reductase AKR2E4-like [Ostrinia furnacalis]	-1.6893	1.40293	0.05954	-0.1595	0.38627
TRINITY_DN98313_c0_g1_i1_orf1	tetrahydrofolate synthase, partial [Plutella xylostella]	-1.6838	1.04253	-0.4505	0.91925	0.17259
TRINITY_DN22441_c0_g1_i1_orf1	Gamma-aminobutyric acid receptor-associated protein, partial [Cotesia chilonis]	-1.8058	1.19236	-0.1335	0.2724	0.47455
TRINITY_DN2271_c0_g1_i12_orf1	plasminogen activator inhibitor 1-like [Ostrinia furnacalis]	-1.7593	0.82475	0.17965	1.05105	-0.2962
TRINITY_DN7291_c0_g1_i3_orf1	dynamin-1-like protein isoform X1 [Ostrinia furnacalis] >XP_028177409.1 dynamin-1-like protein isoform X2 [Ostrinia furnacalis]	-1.0967	1.77207	-0.2267	0.27816	-0.7268
TRINITY_DN62184_c1_g1_i1_orf1	uncharacterized protein LOC114362189 [Ostrinia furnacalis]	-1.6936	0.49744	-0.5409	1.07214	0.66495
TRINITY_DN3545_c0_g1_i6_orf1	group XV phospholipase A2-like [Ostrinia furnacalis] >XP_028168992.1 group XV phospholipase A2-like [Ostrinia furnacalis] >XP_028168993.1 group XV phospholipase A2-like [Ostrinia furnacalis]	-1.725	1.38957	0.25706	-0.0714	0.14979
TRINITY_DN11467_c0_g1_i5_orf1	27 kDa hemolymph protein-like, partial [Ostrinia furnacalis]	-1.763	0.35176	-0.0622	1.31923	0.15426
TRINITY_DN13303_c0_g1_i6_orf1	membrane-associated progesterone receptor component 1-like [Ostrinia furnacalis]	-1.9595	0.72513	0.69333	0.33178	0.20925
TRINITY_DN1354_c5_g1_i1_orf1	PREDICTED: probable elongator complex protein 2 [Papilio xuthus]	-1.3445	1.56623	-0.4794	0.61676	-0.3591
TRINITY_DN9457_c0_g1_i9_orf1	plexin domain-containing protein 2 [Spodoptera litura]	-1.6381	1.16241	0.46415	-0.6066	0.61811
TRINITY_DN108433_c0_g1_i1_orf1	alpha-2-macroglobulin receptor-associated protein [Diachasma alloeum]	-1.9043	0.98091	0.08136	0.57997	0.26203
TRINITY_DN6275_c0_g1_i3_orf1	CTL-like protein 1 isoform X1 [Galleria mellonella]	-1.8148	1.2096	0.1819	-0.0342	0.45747
TRINITY_DN61777_c0_g1_i4_orf1	exocyst complex component 2 [Ostrinia furnacalis]	-1.9687	0.52329	0.21758	0.77086	0.45697
TRINITY_DN18027_c0_g2_i1_orf1	vanin-like protein 2 isoform X2 [Ostrinia furnacalis]	-1.4336	1.66368	-0.3518	0.21206	-0.0903
TRINITY_DN9740_c0_g1_i4_orf1	aldehyde dehydrogenase, mitochondrial [Bicyclus anynana]	-1.7793	1.20018	0.37957	-0.2392	0.43873
TRINITY_DN70_c6_g1_i1_orf1	optineurin isoform X1 [Ostrinia furnacalis] >XP_028165537.1 optineurin isoform X1 [Ostrinia furnacalis] >XP_028165538.1 optineurin isoform X1 [Ostrinia furnacalis] >XP_028165539.1 optineurin isoform X1 [Ostrinia furnacalis]	-1.8112	0.81263	0.17523	0.99885	-0.1755
TRINITY_DN1720_c0_g1_i3_orf1	monocarboxylate transporter 12 [Ostrinia furnacalis] >XP_028157531.1 monocarboxylate transporter 12 [Ostrinia furnacalis]	-1.831	0.68212	-0.3098	0.62346	0.83518
TRINITY_DN18538_c0_g3_i1_orf1	poly(A)-specific ribonuclease PARN-like [Ostrinia furnacalis]	-1.8149	0.79715	-0.2614	0.33594	0.9432
TRINITY_DN4041_c0_g1_i6_orf1	tubulin-folding cofactor B isoform X3 [Ostrinia furnacalis]	-1.421	1.58499	-0.605	0.27207	0.16895
TRINITY_DN104596_c0_g1_i1_orf1	unnamed protein product [Diatraea saccharalis]	-1.8252	0.53982	-0.211	1.0745	0.42193
TRINITY_DN364_c0_g1_i2_orf1	uncharacterized protein LOC114366657, partial [Ostrinia furnacalis]	-1.7772	0.7245	1.14092	-0.119	0.03075
TRINITY_DN12885_c0_g1_i1_orf1	prolow-density lipoprotein receptor-related protein 1, partial [Ostrinia furnacalis]	-1.8075	1.19575	0.25047	0.47656	-0.1153
TRINITY_DN14904_c1_g2_i2_orf1	autophagy protein 12-like [Ostrinia furnacalis]	-1.8265	1.07431	-0.1544	0.25932	0.64718
TRINITY_DN8724_c0_g1_i2_orf1	vesicle-associated membrane protein/synaptobrevin-binding protein isoform X2 [Pectinophora gossypiella]	-1.8554	1.01451	0.71257	0.14217	-0.0138

TRINITY_DN3515_c0_g1_i3_orf1	SCY1-like protein 2 [Ostrinia furnacalis]	-1.5764	1.30534	-0.0906	-0.4265	0.78809
TRINITY_DN1612_c0_g1_i3_orf1	immunoglobulin-binding protein 1b [Ostrinia furnacalis]	-1.8135	1.02892	-0.2519	0.67942	0.35709
TRINITY_DN7957_c0_g1_i5_orf1	spermidine synthase [Ostrinia furnacalis] >XP_028167892.1 spermidine synthase [Ostrinia furnacalis]	-1.7939	0.59674	-0.3824	0.91596	0.66366
TRINITY_DN72369_c0_g1_i1_orf1	microsomal triacylglycerol transfer protein [Vanessa cardui]	-1.9669	0.81506	0.25991	0.41828	0.47367
TRINITY_DN15411_c0_g1_i4_orf1	uncharacterized protein LOC114362040 isoform X1 [Ostrinia furnacalis]	-1.3229	1.70426	0.01988	0.16308	-0.5643
TRINITY_DN19328_c0_g1_i1_orf1	hypothetical protein evm_002753 [Chilo suppressalis]	-1.4715	1.43	-0.126	-0.5326	0.70013
TRINITY_DN8694_c1_g1_i4_orf1	sodium/potassium-transporting ATPase subunit beta-2-like isoform X2 [Ostrinia furnacalis]	-1.4521	1.15219	0.07866	-0.765	0.98619
TRINITY_DN2170_c4_g1_i2_orf1	beta-1,3-glucan-binding protein-like isoform X1 [Ostrinia furnacalis]	-1.9937	0.52217	0.41939	0.63385	0.41826
TRINITY_DN5029_c0_g1_i1_orf1	ribose-phosphate pyrophosphokinase 2-like [Ostrinia furnacalis]	-1.7364	0.82998	-0.4808	0.92029	0.46693
TRINITY_DN59028_c0_g1_i1_orf1	15-hydroxyprostaglandin dehydrogenase [NAD(+)]-like [Ostrinia furnacalis]	-1.8961	0.49496	-0.1092	0.8154	0.69498
TRINITY_DN6510_c1_g1_i1_orf1	hypothetical protein evm_008080 [Chilo suppressalis]	-1.7434	1.37646	0.24018	0.05216	0.07458
TRINITY_DN12024_c0_g1_i4_orf1	pancreatic lipase-related protein 2 isoform X1 [Ostrinia furnacalis] >XP_028176200.1 pancreatic lipase-related protein 2 isoform X2 [Ostrinia furnacalis]	-1.7256	1.1905	-0.3558	0.64758	0.24332
TRINITY_DN97378_c0_g1_i2_orf1	dihydropteridine reductase [Galleria mellonella]	-1.9149	0.91987	0.00831	0.48145	0.50524
TRINITY_DN34166_c0_g1_i1_orf1	hypothetical protein EVAR_80688_1 [Eumeta japonica]	-1.9975	0.55308	0.46223	0.42794	0.55428
TRINITY_DN7776_c0_g1_i5_orf1	uncharacterized protein LOC114364702 [Ostrinia furnacalis]	-1.8053	1.07891	0.13345	-0.1412	0.73415
TRINITY_DN10264_c1_g1_i5_orf1	probable low-specificity L-threonine aldolase 2 [Ostrinia furnacalis]	-1.792	0.89194	-0.3245	0.87503	0.34956
TRINITY_DN28802_c0_g1_i1_orf1	apolipoprotein D-like [Ostrinia furnacalis]	-1.6779	1.14304	-0.0293	-0.3172	0.88133
TRINITY_DN364_c0_g2_i1_orf1	unnamed protein product [Chilo suppressalis]	-1.756	0.83851	1.09362	-0.0588	-0.1172
TRINITY_DN50237_c0_g1_i8_orf1	LOW QUALITY PROTEIN: uncharacterized protein LOC114361080 [Ostrinia furnacalis]	-1.7047	0.66026	-0.0593	1.27487	-0.1711
TRINITY_DN21856_c0_g1_i1_orf1	anti-lipopolysaccharide factor 3 [Eriocheir sinensis]	-1.9028	0.87912	0.74598	0.06324	0.21446
TRINITY_DN9718_c0_g1_i7_orf1	uncharacterized protein LOC114365184 [Ostrinia furnacalis] >XP_028177482.1 uncharacterized protein LOC114365184 [Ostrinia furnacalis] >XP_028177483.1 uncharacterized protein LOC114365184 [Ostrinia furnacalis]	-1.9965	0.39588	0.51001	0.58058	0.51001
TRINITY_DN7776_c0_g1_i1_orf1	uncharacterized protein LOC114364702 [Ostrinia furnacalis]	-1.7825	1.05961	0.17589	0.78305	-0.236
TRINITY_DN2043_c0_g1_i11_orf1	phenoloxidase-activating factor 2-like [Ostrinia furnacalis]	-1.3489	1.58366	0.5169	-0.1281	-0.6235
TRINITY_DN460_c0_g1_i3_orf1	ribonuclease P protein subunit p30 isoform X2 [Ostrinia furnacalis]	-1.3803	1.33174	-0.6787	0.90954	-0.1822
TRINITY_DN39404_c0_g1_i7_orf1	hypothetical protein evm_004736, partial [Chilo suppressalis]	-1.6677	1.38935	0.47197	-0.2501	0.0565
TRINITY_DN7938_c0_g1_i3_orf1	protein halfway isoform X1 [Ostrinia furnacalis] >XP_028172280.1 protein halfway isoform X2 [Ostrinia furnacalis] >XP_028172281.1 protein halfway isoform X3 [Ostrinia furnacalis]	-1.9572	0.70304	0.39875	0.15373	0.7017
TRINITY_DN12301_c0_g1_i1_orf1	ribose-phosphate pyrophosphokinase 2 [Ostrinia furnacalis]	-1.6527	0.79765	-0.449	1.19083	0.11317
TRINITY_DN92_c1_g2_i1_orf1	SID1 transmembrane family member 1-like isoform X3 [Ostrinia furnacalis]	-1.9533	0.7009	0.71713	0.13392	0.40137
TRINITY_DN5538_c0_g1_i1_orf1	hypothetical protein evm_010760 [Chilo suppressalis]	-1.6033	1.01218	-0.6029	1.00241	0.1916
TRINITY_DN6162_c1_g1_i1_orf1	hsc70-interacting protein-like [Ostrinia furnacalis]	-1.911	0.5425	0.80655	-0.0692	0.63115
TRINITY_DN569_c0_g3_i12_orf1	prominin-like protein isoform X2 [Ostrinia furnacalis]	-1.5854	1.50013	-0.3543	0.13661	0.30303
TRINITY_DN4817_c0_g1_i4_orf1	palmitoyl-protein thioesterase 1 isoform X1 [Ostrinia furnacalis] >XP_028170290.1 palmitoyl-protein thioesterase 1 isoform X4 [Ostrinia furnacalis]	-1.2858	1.01208	1.16198	-0.9816	0.09331
TRINITY_DN5122_c0_g1_i3_orf1	calumenin [Ostrinia furnacalis] >XP_028172745.1 calumenin [Ostrinia furnacalis] >XP_028172746.1 calumenin [Ostrinia furnacalis]	-1.8331	1.15217	-0.0847	0.30535	0.4603
TRINITY_DN1038_c0_g1_i4_orf1	gastric triacylglycerol lipase-like [Ostrinia furnacalis]	-1.721	1.33143	0.46323	-0.192	0.11831
TRINITY_DN8979_c0_g1_i5_orf1	ras-related protein Rab-5B [Vanessa cardui] >XP_046961939.1 ras-related protein Rab-5B [Vanessa cardui] >XP_046961940.1 ras-related protein Rab-5B [Vanessa cardui] >XP_046961941.1 ras-related protein Rab-5B [Vanessa cardui] >XP_047545265.1 ras-related protein Rab-5B [Vanessa atalanta] >XP_047545266.1 ras-related protein Rab-5B [Vanessa atalanta] >XP_047545267.1 ras-related protein Rab-5B [Vanessa atalanta]	-1.5282	1.22049	0.48269	-0.7682	0.59323
TRINITY_DN90327_c0_g1_i1_orf1	hypothetical protein HF086_000910, partial [Spodoptera exigua]	-1.076	1.54479	0.7845	-0.7925	-0.4608
TRINITY_DN1054_c0_g1_i8_orf1	tubulin-specific chaperone D [Ostrinia furnacalis]	-1.8396	0.36117	1.01264	-0.1863	0.65208
TRINITY_DN14587_c0_g1_i7_orf1	hypothetical protein evm_001488 [Chilo suppressalis] >CAB3526337.1 unnamed protein product [Chilo suppressalis] >CAH0403665.1 unnamed protein product [Chilo suppressalis]	-1.7948	1.05331	-0.2962	0.37238	0.66537
TRINITY_DN7739_c0_g1_i2_orf1	erlin-2-like [Ostrinia furnacalis] >XP_028161644.1 erlin-2-like [Ostrinia furnacalis]	-1.8084	1.13218	0.59362	-0.1732	0.25584
TRINITY_DN95_c0_g1_i5_orf1	protein VAC14 homolog isoform X3 [Ostrinia furnacalis]	-1.5294	1.22187	-0.0617	0.92492	-0.5557
TRINITY_DN6015_c1_g1_i3_orf1	probable salivary secreted peptide [Ostrinia furnacalis]	-1.6756	1.13313	-0.5025	0.75683	0.28815
TRINITY_DN32448_c0_g1_i1_orf1	unnamed protein product [Arctia plantaginis] >CAB3252297.1 unnamed protein product [Arctia plantaginis]	-1.409	0.76372	-0.6795	1.4014	-0.0767
TRINITY_DN8674_c0_g2_i1_orf1	N(G),N(G)-dimethylarginine dimethylaminohydrolase 1 [Ostrinia furnacalis]	-1.5336	1.43054	-0.3484	-0.2092	0.66066
TRINITY_DN3600_c0_g1_i1_orf1	unnamed protein product [Chilo suppressalis]	-1.463	1.4593	0.33379	0.36606	-0.6961
TRINITY_DN2110_c0_g1_i3_orf1	ribose-phosphate pyrophosphokinase 1 isoform X1 [Chelonus insularis]	-1.7773	0.49946	-0.403	0.9321	0.74873
TRINITY_DN53233_c0_g1_i1_orf1	unnamed protein product, partial [Iphiclides podalirius]	-1.2478	1.32882	-0.9273	0.90229	-0.056
TRINITY_DN29017_c0_g1_i4_orf1	cysteine protease ATG4B [Ostrinia furnacalis]	-1.9305	0.65175	0.1847	0.8754	0.21868
TRINITY_DN11044_c0_g1_i4_orf1	protein Skeletor, isoforms B/C isoform X3 [Ostrinia furnacalis]	-1.8075	1.20883	0.26608	0.43606	-0.1035
TRINITY_DN10229_c0_g1_i6_orf1	autophagy protein 5 isoform X2 [Ostrinia furnacalis]	-1.8243	1.14537	0.59009	-0.0191	0.10793
TRINITY_DN2861_c0_g2_i1_orf1	proteasome subunit alpha type-5 [Ostrinia furnacalis]	-1.8668	1.13707	0.09748	0.39572	0.23656
TRINITY_DN13615_c0_g1_i3_orf1	calcium-binding protein P-like [Ostrinia furnacalis]	-1.9891	0.69157	0.39536	0.48371	0.41843
TRINITY_DN8386_c0_g1_i6_orf1	F-box/WD repeat-containing protein 9-like [Ostrinia furnacalis]	-1.8011	0.85868	0.46239	0.82721	-0.3472
TRINITY_DN7776_c0_g1_i9_orf1	uncharacterized protein LOC114364702 [Ostrinia furnacalis]	-1.8637	1.07048	0.59691	0.14919	0.04709

TRINITY_DN251_c0_g1_i2_orf1	hypothetical protein evm_008466 [Chilo suppressalis]	-1.3479	0.99492	-0.262	1.29126	-0.6763
TRINITY_DN4108_c0_g1_i6_orf1	chromobox protein homolog 1-like [Ostrinia furnacalis]	-1.4367	1.19366	0.77701	-0.8853	0.35138
TRINITY_DN47677_c0_g1_i1_orf1	unnamed protein product [Euphydryas editha]	-1.4043	1.30481	-0.087	-0.7131	0.89962
TRINITY_DN12514_c0_g2_i1_orf1	peroxidase isoform X3 [Ostrinia furnacalis]	-1.7671	0.71375	-0.1093	1.16445	-0.0018
TRINITY_DN1254_c0_g1_i1_orf1	lysophospholipid acyltransferase 7-like [Ostrinia furnacalis]	-0.7857	1.50035	0.81584	-1.1492	-0.3813
TRINITY_DN364_c5_g1_i3_orf1	talin-2-like, partial [Ostrinia furnacalis]	-1.8588	1.01885	0.54405	-0.1412	0.43706
TRINITY_DN13285_c0_g1_i9_orf1	E3 ubiquitin-protein ligase RNF13 isoform X1 [Ostrinia furnacalis] >XP_028158682.1 E3 ubiquitin-protein ligase RNF13 isoform X1 [Ostrinia furnacalis] >XP_028158683.1 E3 ubiquitin-protein ligase RNF13 isoform X1 [Ostrinia furnacalis]	-1.791	1.16829	0.64846	-0.0701	0.04433
TRINITY_DN18118_c0_g2_i10_orf1	clavesin-1-like [Ostrinia furnacalis]	-1.6365	1.42504	0.41873	0.11343	-0.3207
TRINITY_DN2170_c0_g1_i2_orf1	beta-1,3-glucan-binding protein-like [Ostrinia furnacalis]	-1.5394	1.22023	0.37509	0.67881	-0.7348
TRINITY_DN116467_c0_g1_i1_orf1	probable small nuclear ribonucleoprotein E [Ostrinia furnacalis]	-1.5376	1.56739	0.09649	0.22138	-0.3477
TRINITY_DN4790_c0_g1_i6_orf1	ADP-ribosylation factor-like protein 8 [Ostrinia furnacalis] >CAG9746554.1 unnamed protein product [Diatraea saccharalis] >CAG9785239.1 unnamed protein product [Diatraea saccharalis] >CAH2992000.1 unnamed protein product [Chilo suppressalis]	-1.7065	1.25316	0.67652	-0.2436	0.02041
TRINITY_DN15114_c0_g2_i1_orf1	uncharacterized protein LOC114359552 [Ostrinia furnacalis]	-1.095	1.63855	0.61875	-0.7512	-0.4111
TRINITY_DN10290_c0_g1_i7_orf1	aquaporin AQPAe.a [Ostrinia furnacalis]	-1.5041	1.46669	-0.0629	0.5875	-0.4872
TRINITY_DN2432_c0_g1_i1_orf1	unconventional myosin-XV-like [Ostrinia furnacalis]	-1.0581	1.44025	0.92967	-0.8576	-0.4542
TRINITY_DN4635_c0_g1_i4_orf1	putative salivary secreted peptide [Operophtera brumata]	-1.3632	1.57594	-0.6842	0.43415	0.03734
TRINITY_DN5556_c0_g1_i3_orf1	hypothetical protein evm_006874 [Chilo suppressalis]	-1.8626	0.77596	-0.2348	0.64357	0.67782
TRINITY_DN69691_c0_g2_i1_orf1	protein HGH1 homolog [Ostrinia furnacalis]	-1.8371	0.63114	-0.1986	1.01908	0.38552
TRINITY_DN52864_c0_g1_i1_orf1	odorant binding protein 18 [Conogethes pinicolalis]	-1.1428	1.6667	-0.8285	0.45499	-0.1503
TRINITY_DN4979_c0_g2_i9_orf1	arf-GAP with dual PH domain-containing protein 1-like isoform X2 [Ostrinia furnacalis]	-1.4976	1.6396	0.1487	-0.0978	-0.1929
TRINITY_DN3637_c0_g1_i2_orf1	ATP-binding cassette sub-family G member 4 isoform X1 [Ostrinia furnacalis]	-1.5312	1.41312	0.6929	-0.2061	-0.3687
TRINITY_DN4125_c0_g1_i6_orf1	angiotensin-converting enzyme-like isoform X1 [Ostrinia furnacalis]	-1.5358	1.06018	-0.2884	-0.3738	1.13778
TRINITY_DN11693_c0_g1_i6_orf1	sorting nexin-32 isoform X1 [Ostrinia furnacalis] >XP_028166096.1 sorting nexin-32 isoform X2 [Ostrinia furnacalis]	-1.0841	1.30829	0.91288	-1.1312	-0.0058
TRINITY_DN61048_c0_g1_i2_orf1	PREDICTED: protein THEM6-like [Amyelois transitella]	-1.4995	0.90556	0.99434	-0.857	0.45654
TRINITY_DN364_c2_g1_i2_orf1	vinculin-like isoform X3 [Ostrinia furnacalis]	-1.4829	1.16348	1.07447	-0.3147	-0.4404
TRINITY_DN1722_c0_g1_i2_orf1	delta(3,5)-Delta(2,4)-dienoyl-CoA isomerase, mitochondrial isoform X1 [Ostrinia furnacalis]	-1.7155	1.41823	0.13297	-0.0032	0.16745
TRINITY_DN17838_c0_g1_i4_orf1	mitogen-activated protein kinase kinase kinase 4 [Ostrinia furnacalis]	-1.8693	0.55131	0.02776	1.0745	0.21569
TRINITY_DN22928_c0_g1_i6_orf1	hypothetical protein B5X24_HaOG209714 [Helicoverpa armigera]	-1.5152	1.2076	0.6141	-0.7942	0.48775
TRINITY_DN43505_c0_g1_i1_orf1	unnamed protein product [Chilo suppressalis]	-1.9553	0.5858	0.17883	0.81068	0.38
TRINITY_DN61536_c0_g7_i1_orf1	cubilin homolog [Ostrinia furnacalis]	-1.6444	1.50358	-0.0762	0.16194	0.05516
TRINITY_DN1355_c0_g1_i7_orf1	larval/pupal rigid cuticle protein 66-like [Ostrinia furnacalis]	-1.7874	0.85136	-0.2542	0.98729	0.20293
TRINITY_DN47260_c0_g1_i2_orf1	SET domain-containing protein SmydA-8-like [Ostrinia furnacalis]	-1.8252	0.76258	-0.3325	0.73832	0.65682
TRINITY_DN8926_c0_g1_i4_orf1	run domain Beclin-1-interacting and cysteine-rich domain-containing protein [Ostrinia furnacalis]	-1.9553	0.79797	0.57	0.14737	0.43993
TRINITY_DN119265_c0_g2_i1_orf1	PREDICTED: sorting nexin-12 [Fopius arisanus]	-1.4528	1.2959	0.9467	-0.3635	-0.4263
TRINITY_DN12193_c0_g1_i6_orf1	carbonyl reductase [NADPH] 1-like [Ostrinia furnacalis]	-1.551	0.84821	-0.426	1.2912	-0.1624
TRINITY_DN12065_c0_g1_i4_orf1	protein DEK isoform X2 [Galleria mellonella]	-1.612	1.5194	0.27381	-0.0643	-0.1169
TRINITY_DN34087_c0_g1_i4_orf1	protein SMG8 [Ostrinia furnacalis]	-1.788	0.64044	-0.0475	1.17921	0.01578
TRINITY_DN1023_c1_g1_i1_orf1	ras-related protein Rab-7a [Ostrinia furnacalis]	-1.9697	0.81238	0.37051	0.31092	0.47587
TRINITY_DN1532_c0_g1_i6_orf1	mRNA (2'-O-methyladenosine-N(6)-)-methyltransferase [Ostrinia furnacalis] >XP_028179575.1 mRNA (2'-O-methyladenosine-N(6)-)-methyltransferase [Ostrinia furnacalis] >XP_028179576.1 mRNA (2'-O-methyladenosine-N(6)-)-methyltransferase [Ostrinia furnacalis]	-1.8316	0.41356	-0.0711	1.16904	0.32011
TRINITY_DN26013_c0_g1_i1_orf1	E3 ubiquitin-protein ligase CHIP [Ostrinia furnacalis]	-1.7543	1.12645	0.66694	0.30295	-0.3421
TRINITY_DN66671_c0_g1_i1_orf1	phosphoglucomutase-like [Ostrinia furnacalis]	-1.9576	0.59816	0.25927	0.81257	0.28756
TRINITY_DN18136_c0_g1_i1_orf1	proteoglycan 4-like [Ostrinia furnacalis]	-0.8277	1.3127	1.13269	-0.7966	-0.8211
TRINITY_DN2818_c0_g1_i2_orf1	proteasome subunit alpha type-3 [Ostrinia furnacalis]	-1.7773	0.98603	0.89603	-0.2265	0.12171
TRINITY_DN8555_c0_g1_i1_orf1	epoxide hydrolase 4-like [Ostrinia furnacalis]	-1.7234	0.87136	-0.5359	0.79434	0.59364
TRINITY_DN13901_c0_g1_i4_orf1	vesicle-associated membrane protein 7-like [Ostrinia furnacalis]	-1.9331	0.88275	0.27403	0.15614	0.62013
TRINITY_DN12317_c0_g1_i1_orf1	vacuolar protein sorting-associated protein 41 homolog [Ostrinia furnacalis]	-1.5828	1.30047	0.61929	-0.5946	0.25769
TRINITY_DN5190_c0_g3_i1_orf1	muscle LIM protein Mlp84B-like isoform X2 [Chelonus insularis]	-1.6891	0.87973	0.70185	-0.6076	0.7151
TRINITY_DN10646_c0_g1_i2_orf1	tyrosine-protein phosphatase non-receptor type 61F-like isoform X1 [Vanessa tameamea] >XP_047543663.1 tyrosine-protein phosphatase non-receptor type 61F-like isoform X1 [Vanessa atalanta]	-1.0903	1.58485	-1.0158	0.51724	0.00405
TRINITY_DN34816_c0_g1_i4_orf1	uncharacterized protein LOC114355576 [Ostrinia furnacalis]	-1.697	0.97353	-0.4424	0.19729	0.9685
TRINITY_DN2326_c0_g1_i1_orf1	transmembrane protein 256 homolog isoform X1 [Ostrinia furnacalis]	-1.1351	1.25263	0.91188	-1.1396	0.11024
TRINITY_DN11274_c0_g1_i4_orf1	dipeptidyl peptidase 3 isoform X1 [Ostrinia furnacalis]	-1.6845	1.04519	0.37544	0.80058	-0.5367
TRINITY_DN18222_c0_g1_i4_orf1	phosphoglycerate kinase [Manduca sexta] >KAG6457061.1 hypothetical protein O3G_MSEX010096 [Manduca sexta] >KAG6457062.1 hypothetical protein O3G_MSEX010096 [Manduca sexta]	-1.3586	0.56311	0.04493	1.50519	-0.7546
TRINITY_DN6310_c0_g2_i10_orf1	BAG family molecular chaperone regulator 2 isoform X1 [Ostrinia furnacalis] >XP_028163269.1 BAG family molecular chaperone regulator 2 isoform X2 [Ostrinia furnacalis]	-1.7194	0.63682	-0.0179	1.26868	-0.1682
TRINITY_DN2202_c0_g1_i9_orf1	vascular endothelial growth factor receptor 1 isoform X3 [Ostrinia furnacalis]	-1.7253	1.09291	-0.4691	0.58254	0.519
TRINITY_DN10336_c0_g1_i9_orf1	protein phosphatase methylesterase 1 isoform X1 [Ostrinia furnacalis]	-1.5701	1.48939	-0.1609	-0.2407	0.48228

TRINITY_DN1384_c0_g1_i5_orf1	vacuolar protein sorting-associated protein VTA1 homolog [Ostrinia furnacalis] >XP_028168952.1 vacuolar protein sorting-associated protein VTA1 homolog [Ostrinia furnacalis]	-1.706	1.40582	-0.1272	0.16081	0.26665
TRINITY_DN23640_c0_g1_i5_orf1	proteasomal ATPase-associated factor 1-like [Ostrinia furnacalis]	-1.8961	0.53543	-0.1208	0.68485	0.79658
TRINITY_DN12024_c0_g2_i2_orf1	pancreatic lipase-related protein 2 isoform X1 [Ostrinia furnacalis] >XP_028176200.1 pancreatic lipase-related protein 2 isoform X2 [Ostrinia furnacalis]	-1.533	0.9703	-0.6758	1.11159	0.12692
TRINITY_DN37821_c0_g1_i6_orf1	uncharacterized protein LOC114350690 [Ostrinia furnacalis]	-1.7213	0.75872	0.79699	-0.5547	0.72024
TRINITY_DN61135_c0_g1_i1_orf1	uncharacterized protein LOC114362571 [Ostrinia furnacalis]	-1.7553	1.1059	-0.2687	0.77706	0.14107
TRINITY_DN1408_c0_g1_i10_orf1	protein diaphanous [Ostrinia furnacalis]	-1.5268	1.21435	-0.3389	0.98419	-0.3328
TRINITY_DN17133_c0_g1_i1_orf1	unnamed protein product [Chrysodeixis includens]	-1.5187	1.49247	-0.0386	0.51331	-0.4485
TRINITY_DN5458_c1_g1_i9_orf1	histone H2A-like [Aedes aegypti] >XP_021712017.1 histone H2A-like [Aedes aegypti]	-1.8763	0.6411	0.97369	-0.0767	0.33828
TRINITY_DN14154_c0_g1_i1_orf1	uncharacterized protein LOC114360857, partial [Ostrinia furnacalis]	-1.5291	1.30016	-0.4698	0.85271	-0.154
TRINITY_DN27984_c0_g2_i1_orf1	unnamed protein product [Psylliodes chrysocephala]	-1.4836	1.52801	-0.3693	0.53326	-0.2084
TRINITY_DN42159_c0_g1_i6_orf1	regucalcin-like [Nymphalis io] >XP_050348255.1 regucalcin-like [Nymphalis io]	-1.3839	0.70599	-0.4991	1.49532	-0.3183
TRINITY_DN41296_c0_g1_i1_orf1	exocyst complex component 5 [Ostrinia furnacalis]	-1.7361	0.25848	-0.3985	0.9235	0.95262
TRINITY_DN34534_c0_g2_i1_orf1	hypothetical protein evm_008316 [Chilo suppressalis]	-1.5053	1.37613	0.74398	-0.5285	-0.0863
TRINITY_DN12106_c0_g1_i4_orf1	programmed cell death 6-interacting protein [Ostrinia furnacalis]	-1.5809	1.11795	0.5904	-0.7324	0.60493
TRINITY_DN11204_c0_g1_i3_orf1	spermosin-like [Ostrinia furnacalis]	-0.6099	1.37642	1.00776	-0.6189	-1.1553
TRINITY_DN3325_c0_g1_i1_orf1	histone H2A.V [Plutella xylostella] >XP_013137778.1 PREDICTED: histone H2A.V [Papilio polytes] >XP_013167227.1 PREDICTED: histone H2A.V [Papilio xuthus] >XP_013190422.1 PREDICTED: histone H2A.V [Amyelois transitella] >XP_013190423.1 PREDICTED: histone H2A.V [Amyelois transitella] >XP_014357142.1 histone H2A.V [Papilio machaon] >XP_021197569.1 histone H2A.V [Helicoverpa armigera] >XP_022129178.1 histone H2A.V [Pieris rapae] >XP_022826165.1 histone H2A.V [Spodoptera litura] >XP_023944430.1 histone H2A.V [Bicyclus anynana] >XP_026330284.1 histone H2A.V [Hyposmocoma kahamanoa] >XP_026500358.1 histone H2A.V [Vanessa tameamea] >XP_026727080.1 histone H2A.V [Trichoplusia ni] >XP_026759530.1 histone H2A.V [Galleria mellonella] >XP_028163156.1 histone H2A.V [Ostrinia furnacalis] >XP_028163157.1 histone H2A.V [Ostrinia furnacalis] >XP_032516010.1 histone H2A.V [Danaus plexippus plexippus] >XP_034828353.1 histone H2A.V [Maniola hyperantus] >XP_035441381.1 histone H2A.V [Spodoptera frugiperda] >XP_038218938.1 histone H2A.V [Zerene cesonia] >XP_039750757.1 histone H2A.V [Pararge aegeria] >XP_041968832.1 histone H2A.V [Aricia agestis] >XP_045449012.1 histone H2A.V [Melitaea cinxia] >XP_045496670.1 histone H2A.V [Colias croceus] >XP_045515939.1 histone H2A.V [Pieris brassicae] >XP_045768057.1 histone H2A.V [Maniola jurtina] >XP_046967022.1 histone H2A.V [Vanessa cardui] >XP_047026067.1 histone H2A.V [Helicoverpa zea] >XP_047505435.1 histone H2A.V [Pieris napi] >XP_047531958.1 histone H2A.V [Vanessa atalanta] >XP_047999605.1 histone H2A.V [Leguminivora glycinivorella] >XP_049870589.1 histone H2A.V [Pectinophora gossypiella] >XP_050349021.1 histone H2A.V [Nymphalis io] >KAF9405923.1 hypothetical protein HW555_013519 [Spodoptera exigua] >RVE50043.1 hypothetical protein evm_005249 [Chilo suppressalis] >CAD0200183.1 unnamed protein product [Chrysodeixis includens] >CAG5034067.1 unnamed protein product [Parnassius apollo] >CAG9580288.1 unnamed protein product [Danaus chrysippus] >CAH0724075.1 unnamed protein product, partial [Brenthis ino] >CAH2085910.1 unnamed protein product [Euphydryas editha] >CAH2267989.1 jg8431 [Pararge aegeria aegeria] >VVC91078.1 unnamed protein product [Leptidea sinapis] >GBP47431.1 hypothetical protein EVAR_85023_1 [Eumeta japonica]	-1.6758	1.09293	-0.0597	-0.306	0.94865
TRINITY_DN855_c0_g1_i5_orf1	hypothetical protein HF086_009970 [Spodoptera exigua] >CAH0702798.1 unnamed protein product [Spodoptera exigua]	-1.0978	0.91383	1.26591	-1.1622	0.08029
TRINITY_DN2836_c0_g1_i4_orf1	protein Diedel [Drosophila sechellia] >EDW53358.1 GM12788 [Drosophila sechellia]	-1.527	1.23662	0.92605	-0.1186	-0.517
TRINITY_DN51429_c1_g1_i1_orf1	ER degradation-enhancing alpha-mannosidase-like protein 3 isoform X1 [Ostrinia furnacalis] >XP_028179122.1 ER degradation-enhancing alpha-mannosidase-like protein 3 isoform X2 [Ostrinia furnacalis]	-1.6932	0.69804	0.34352	1.13686	-0.4853
TRINITY_DN53294_c0_g1_i1_orf1	liver carboxylesterase 2-like [Ostrinia furnacalis]	-1.1938	0.68859	-0.5991	1.58462	-0.4802
TRINITY_DN8143_c0_g1_i6_orf1	gamma-soluble NSF attachment protein-like [Galleria mellonella]	-1.747	1.2495	0.36892	0.41233	-0.2837
TRINITY_DN3418_c0_g1_i3_orf1	proto-oncogene tyrosine-protein kinase ROS [Ostrinia furnacalis]	-1.8578	0.69826	-0.2519	0.73454	0.67685
TRINITY_DN9544_c0_g1_i1_orf1	collagen type IV alpha-3-binding protein isoform X1 [Ostrinia furnacalis]	-1.7492	0.78786	0.55172	-0.4777	0.88723
TRINITY_DN57536_c0_g1_i14_orf1	hypothetical protein O3G_MSEX012216 [Manduca sexta]	-1.7868	1.0612	0.08592	-0.1646	0.80425
TRINITY_DN3276_c0_g1_i4_orf1	hypothetical protein B5X24_HaOG201803 [Helicoverpa armigera]	-1.3746	0.92973	0.33129	-0.9752	1.08875
TRINITY_DN8652_c0_g1_i8_orf1	glucocorticoid-induced transcript 1 protein-like [Ostrinia furnacalis] >XP_028165773.1 glucocorticoid-induced transcript 1 protein-like [Ostrinia furnacalis] >XP_028165779.1 glucocorticoid-induced transcript 1 protein-like [Ostrinia furnacalis] >XP_028165785.1 glucocorticoid-induced transcript 1 protein-like [Ostrinia furnacalis]	-1.5039	1.11708	1.10261	-0.4538	-0.2619
TRINITY_DN8726_c0_g2_i3_orf1	dnaJ homolog subfamily C member 25 homolog [Ostrinia furnacalis]	-1.828	1.11758	0.63612	0.00555	0.06875
TRINITY_DN1738_c0_g1_i5_orf1	baculoviral IAP repeat-containing protein 6-like [Ostrinia furnacalis]	-1.8106	0.76052	-0.3687	0.68926	0.72955
TRINITY_DN3407_c0_g1_i9_orf1	uncharacterized protein YJR142W [Ostrinia furnacalis]	-1.4534	1.26265	-0.0488	0.91423	-0.6747
TRINITY_DN23740_c0_g1_i3_orf1	ras-related protein Rab-14 [Ostrinia furnacalis] >CAB3528182.1 unnamed protein product [Chilo suppressalis] >CAH0404769.1 unnamed protein product [Chilo suppressalis]	-1.5416	1.43971	0.52256	-0.5179	0.09722
TRINITY_DN26805_c0_g2_i3_orf1	phosphopantothenate--cysteine ligase isoform X1 [Ostrinia furnacalis] >XP_028161191.1 phosphopantothenate--cysteine ligase isoform X2 [Ostrinia furnacalis] >XP_028161192.1 phosphopantothenate--cysteine ligase isoform X1 [Ostrinia furnacalis]	-1.806	0.97684	-0.332	0.57651	0.58457
TRINITY_DN3158_c0_g1_i5_orf1	neuroglian [Ostrinia furnacalis]	-1.5023	0.87885	1.22329	-0.6836	0.08374
TRINITY_DN23183_c0_g1_i2_orf1	nuclear protein localization protein 4 homolog isoform X2 [Ostrinia furnacalis]	-1.2354	1.07036	1.15467	-0.9974	0.00775
TRINITY_DN9717_c0_g2_i1_orf1	proteasome subunit beta type-2 [Ostrinia furnacalis]	-1.9749	0.72411	0.43239	0.57297	0.24543
TRINITY_DN6669_c0_g1_i3_orf1	glutamine synthetase 2 cytoplasmic [Ostrinia furnacalis]	-1.5533	1.0453	0.10506	-0.6358	1.03882
TRINITY_DN143792_c0_g1_i1_orf1	ras-related GTP-binding protein C [Venturia canescens]	-1.6422	1.05972	0.50542	0.71744	-0.6404

TRINITY_DN195_c0_g3_i6_orf1	E3 ubiquitin-protein ligase SIAH1-like [Ostrinia furnacalis]	-1.8522	0.84257	0.11927	-0.0287	0.91899
TRINITY_DN44777_c0_g1_i2_orf1	probable very-long-chain enoyl-CoA reductase art-1 [Ostrinia furnacalis]	-0.8725	1.56303	0.81394	-0.7253	-0.7792
TRINITY_DN38371_c0_g1_i7_orf1	protein smoothened isoform X2 [Ostrinia furnacalis]	-1.5736	1.47109	0.47761	-0.3627	-0.0125
TRINITY_DN5603_c0_g1_i1_orf1	translin [Ostrinia furnacalis]	-1.8831	0.81406	0.76612	-0.1301	0.43303
TRINITY_DN54554_c0_g1_i1_orf1	LOW QUALITY PROTEIN: signal transducing adapter molecule 2 [Ostrinia furnacalis]	-1.8262	1.10286	0.38257	0.51985	-0.1791
TRINITY_DN7388_c0_g1_i7_orf1	ras-related protein Rab-18A isoform X1 [Ostrinia furnacalis]	-1.4203	0.76408	-0.2008	-0.5693	1.42634
TRINITY_DN2758_c0_g1_i7_orf1	hypothetical protein evm_012952 [Chilo suppressalis]	-1.4333	0.7517	0.07495	-0.7433	1.34997
TRINITY_DN29402_c0_g1_i1_orf1	hypothetical protein evm_000391 [Chilo suppressalis] >RVE55077.1 hypothetical protein evm_000444 [Chilo suppressalis] >CAH2982715.1 unnamed protein product [Chilo suppressalis]	-1.533	1.42261	0.42807	-0.6017	0.28406
TRINITY_DN15967_c0_g1_i4_orf1	adenylyl cyclase-associated protein 1 isoform X1 [Ostrinia furnacalis] >XP_028178233.1 adenylyl cyclase-associated protein 1 isoform X1 [Ostrinia furnacalis] >XP_028178234.1 adenylyl cyclase-associated protein 1 isoform X2 [Ostrinia furnacalis]	-1.5393	1.47763	-0.5089	0.39755	0.17308
TRINITY_DN6426_c0_g1_i2_orf1	ras-related protein Rap1 [Ostrinia furnacalis]	-1.6941	1.00855	-0.4607	0.22419	0.92208
TRINITY_DN8812_c0_g1_i1_orf1	nuclear pore complex protein Nup160 homolog isoform X3 [Ostrinia furnacalis]	-1.8015	1.06203	0.48665	0.54936	-0.2965
TRINITY_DN29351_c0_g1_i1_orfp1	TRINITY_DN29351_c0_g1_i1_m.58077 TRINITY_DN29351_c0_g1::TRINITY_DN29351_c0_g1_i1::g.58077 ORF type:complete len:328 (+),score=-16.88 TRINITY_DN29351_c0_g1_i1:50-1033(+)	-1.661	0.82815	-0.5081	1.11648	0.22454
TRINITY_DN16972_c0_g1_i1_orf1	aldo-keto reductase family 1 member A1-like [Ostrinia furnacalis]	-1.339	1.7192	-0.4083	0.21942	-0.1913
TRINITY_DN1166_c0_g3_i4_orf1	pleckstrin homology domain-containing family F member 2 isoform X1 [Ostrinia furnacalis] >XP_028157822.1 pleckstrin homology domain-containing family F member 2 isoform X2 [Ostrinia furnacalis]	-1.3751	0.87539	-0.3956	1.39253	-0.4972
TRINITY_DN7749_c1_g1_i2_orf1	uncharacterized protein LOC114364356 isoform X1 [Ostrinia furnacalis]	-1.6129	1.31664	0.73334	-0.3442	-0.0928
TRINITY_DN4565_c0_g1_i3_orf1	acid phosphatase type 7 isoform X1 [Ostrinia furnacalis] >XP_028155943.1 acid phosphatase type 7 isoform X1 [Ostrinia furnacalis] >XP_028155944.1 acid phosphatase type 7 isoform X1 [Ostrinia furnacalis] >XP_028155945.1 acid phosphatase type 7 isoform X1 [Ostrinia furnacalis]	-1.958	0.84851	0.48226	0.40565	0.22158
TRINITY_DN2855_c0_g1_i6_orf1	uncharacterized protein LOC114359301 [Ostrinia furnacalis]	-1.075	0.98432	0.99007	-1.313	0.41367
TRINITY_DN23416_c1_g1_i2_orf1	ran-specific GTPase-activating protein-like [Ostrinia furnacalis]	-1.0823	1.39509	0.52164	-1.2116	0.37718
TRINITY_DN2701_c1_g1_i6_orf1	protein ELYS-like isoform X10 [Ostrinia furnacalis]	-1.3445	1.63581	-0.5981	0.38951	-0.0826
TRINITY_DN648_c0_g1_i5_orf1	uncharacterized protein LOC114360051 [Ostrinia furnacalis]	-1.9317	0.6994	0.83749	0.19884	0.19597
TRINITY_DN4679_c0_g2_i13_orf1	GATOR complex protein MIOS [Ostrinia furnacalis]	-1.535	0.36514	1.01701	-0.7793	0.93214
TRINITY_DN5207_c0_g2_i3_orf1	mucin-like [Galleria mellonella]	-1.7499	0.23817	-0.3265	1.12509	0.71316
TRINITY_DN3223_c0_g1_i4_orf1	ras-related protein Rac1 isoform X1 [Plutella xylostella] >XP_048482692.1 ras-related protein Rac1 isoform X2 [Plutella xylostella] >CAG9093870.1 unnamed protein product [Plutella xylostella]	-1.0831	1.61604	0.6478	-0.3683	-0.8124
TRINITY_DN84478_c0_g1_i8_orf1	uncharacterized protein LOC114359035 isoform X1 [Ostrinia furnacalis]	-1.9622	0.47386	0.50348	0.79809	0.18675
TRINITY_DN2396_c0_g1_i9_orfp1	TRINITY_DN2396_c0_g1_i9_m.39038 TRINITY_DN2396_c0_g1::TRINITY_DN2396_c0_g1_i9::g.39038 ORF type:5prime_partial len:181 (+),score=90.33 TRINITY_DN2396_c0_g1_i9:3-545(+)	-1.9205	-0.0422	0.75417	0.67782	0.53072
TRINITY_DN14242_c0_g1_i2_orfp1	TRINITY_DN14242_c0_g1_i2_m.18449 TRINITY_DN14242_c0_g1::TRINITY_DN14242_c0_g1_i2::g.18449 ORF type:internal len:148 (-),score=102.30 TRINITY_DN14242_c0_g1_i2:2-442(-)	-1.8828	0.08701	0.95197	0.72609	0.11777
TRINITY_DN971_c0_g1_i10_orfp1	TRINITY_DN971_c0_g1_i10_m.54268 TRINITY_DN971_c0_g1::TRINITY_DN971_c0_g1_i10::g.54268 ORF type:internal len:187 (+),score=141.68 TRINITY_DN971_c0_g1_i10:1-558(+)	-1.8854	-0.1083	0.89113	0.42557	0.67699
TRINITY_DN1108_c3_g1_i1_orfp1	TRINITY_DN1108_c3_g1_i1_m.5561 TRINITY_DN1108_c3_g1::TRINITY_DN1108_c3_g1_i1::g.5561 ORF type:internal len:113 (+),score=89.07 TRINITY_DN1108_c3_g1_i1:1-336(+)	-1.907	0.17271	0.7938	0.83166	0.10881
TRINITY_DN295_c3_g1_i1_orfp1	TRINITY_DN295_c3_g1_i1_m.18839 TRINITY_DN295_c3_g1::TRINITY_DN295_c3_g1_i1::g.18839 ORF type:5prime_partial len:118 (+),score=46.25 TRINITY_DN295_c3_g1_i1:1-354(+)	-1.8977	-0.1243	0.58171	0.78172	0.65859
TRINITY_DN2813_c0_g1_i10_orf1	arylphorin subunit alpha-like [Ostrinia furnacalis]	-1.9032	0.46591	1.03526	0.1367	0.26532
TRINITY_DN15578_c0_g2_i1_orfp1	uncharacterized protein LOC125235519 [Leguminivora glycinivorella]	-1.8883	0.71887	0.8435	0.43946	-0.1135
TRINITY_DN142657_c0_g1_i1_orf1	sorting and assembly machinery component 50 homolog [Diachasma alloeum]	-1.8702	0.27013	-0.007	1.06633	0.54074
TRINITY_DN971_c0_g1_i5_orfp1	TRINITY_DN971_c0_g1_i5_m.54249 TRINITY_DN971_c0_g1::TRINITY_DN971_c0_g1_i5::g.54249 ORF type:internal len:108 (+),score=66.98 TRINITY_DN971_c0_g1_i5:1-321(+)	-1.7632	-0.3999	0.48896	1.04991	0.62427
TRINITY_DN8158_c0_g1_i2_orf1	hypothetical protein G9C98_000136 [Cotesia typhae]	-1.7312	0.06991	-0.1962	1.27106	0.5864
TRINITY_DN34423_c0_g1_i3_orf1	THAP domain-containing protein 4-like [Ostrinia furnacalis]	-1.9506	0.07258	0.60276	0.71887	0.55645
TRINITY_DN57137_c0_g1_i1_orfp1	TRINITY_DN57137_c0_g1_i1_m.46420 TRINITY_DN57137_c0_g1::TRINITY_DN57137_c0_g1_i1::g.46420 ORF type:5prime_partial len:56 (-),score=4.65 TRINITY_DN57137_c0_g1_i1:82-249(-)	-1.8476	-0.2302	0.79953	0.44279	0.83552
TRINITY_DN135449_c0_g1_i5_orf1	larval cuticle protein LCP-17-like [Galleria mellonella]	-1.9325	0.33742	0.09542	0.84503	0.6546
TRINITY_DN2044_c0_g1_i5_orfp1	TRINITY_DN2044_c0_g1_i5_m.4210 TRINITY_DN2044_c0_g1::TRINITY_DN2044_c0_g1_i5::g.4210 ORF type:complete len:151 (-),score=85.31 TRINITY_DN2044_c0_g1_i5:857-1309(-)	-1.7613	-0.4013	0.44879	1.03947	0.67434
TRINITY_DN17615_c0_g1_i3_orf1	hypothetical protein SFRUCORN_008858 [Spodoptera frugiperda]	-1.9064	-0.0689	0.52512	0.59568	0.85456
TRINITY_DN100327_c0_g1_i1_orf1	arylphorin subunit alpha-like [Ostrinia furnacalis]	-1.9285	0.33226	0.54409	0.92673	0.1254
TRINITY_DN129863_c0_g1_i1_orf1	protein PFC0760c-like isoform X2 [Ostrinia furnacalis]	-1.8813	0.35248	1.09705	0.3572	0.07453
TRINITY_DN10877_c0_g1_i1_orf1	spodomicin-like [Ostrinia furnacalis]	-1.5409	-0.3942	1.42832	0.64179	-0.135
TRINITY_DN5177_c0_g1_i2_orf1	hemolin-like isoform X1 [Ostrinia furnacalis]	-1.7366	-0.0255	-0.1745	0.77097	1.16566
TRINITY_DN9044_c0_g1_i2_orf1	unnamed protein product [Euphydryas editha]	-1.6119	0.34158	1.42082	0.28184	-0.4323

TRINITY_DN1597_c0_g1_i5_orfp1	TRINITY_DN1597_c0_g1_i5_m.57494 TRINITY_DN1597_c0_g1::TRINITY_DN1597_c0_g1_i5::g.57494 ORF type:complete len:86 (+),score=7.19 TRINITY_DN1597_c0_g1_i5:134-391(+)	-1.8514	0.1037	-0.004	0.75829	0.99332
TRINITY_DN5310_c2_g1_i2_orf1	serine protease persephone-like [Ostrinia furnacalis]	-1.9061	0.51866	0.38191	0.03033	0.97519
TRINITY_DN295_c2_g1_i2_orf1	phosphoglycolate phosphatase 1A, chloroplastic [Manduca sexta]	-1.8985	0.39185	0.36483	1.04894	0.09291
TRINITY_DN26209_c0_g1_i6_orf1	uncharacterized protein LOC114352357 [Ostrinia furnacalis]	-1.945	0.43731	0.20477	0.39151	0.91139
TRINITY_DN3166_c1_g1_i6_orf1	hypothetical protein evm_013813 [Chilo suppressalis]	-1.7427	-0.316	0.35242	0.4768	1.22947
TRINITY_DN6330_c0_g1_i1_orfp1	TRINITY_DN6330_c0_g1_i1_m.42332 TRINITY_DN6330_c0_g1::TRINITY_DN6330_c0_g1_i1::g.42332 ORF type:3prime_partial len:51 (-),score=33.30 TRINITY_DN6330_c0_g1_i1:3-152(-)	-1.7299	-0.4753	0.9219	0.40933	0.87403
TRINITY_DN48548_c0_g1_i1_orf1	glutathione S-transferase siama 2 [Heortia vitessoides]	-1.9616	0.24345	0.40417	0.83871	0.47532
TRINITY_DN47784_c0_g2_i1_orfp1	arylphorin subunit alpha-like [Ostrinia furnacalis]	-1.6683	-0.181	1.36078	-0.082	0.57057
TRINITY_DN136358_c0_g1_i1_orf1	acidic juvenile hormone-suppressible protein 1-like [Ostrinia furnacalis]	-1.7189	0.10951	-0.0119	1.41028	0.21095
TRINITY_DN17247_c0_g1_i14_orf1	uncharacterized protein LOC114356308 [Ostrinia furnacalis]	-1.8769	0.18129	0.87831	-0.003	0.82032
TRINITY_DN25234_c0_g1_i1_orf1	uncharacterized protein LOC114353853 [Ostrinia furnacalis]	-1.7563	0.3191	1.28922	0.33917	-0.1912
TRINITY_DN1391_c0_g1_i29_orfp1	TRINITY_DN1391_c0_g1_i29_m.70767 TRINITY_DN1391_c0_g1::TRINITY_DN1391_c0_g1_i29::g.70767 ORF type:complete len:495 (+),score=158.49 TRINITY_DN1391_c0_g1_i29:728-2212(+)	-1.901	-0.1029	0.8222	0.56359	0.61805
TRINITY_DN2406_c0_g1_i6_orf1	uncharacterized protein LOC114361672 [Ostrinia furnacalis]	-1.5302	0.43542	1.51432	-0.0003	-0.4193
TRINITY_DN29026_c0_g1_i4_orf1	TIL [Ostrinia furnacalis]	-1.7125	-0.2116	0.15528	0.41837	1.3504
TRINITY_DN768_c0_g1_i7_orf1	neutral ceramidase [Leguminivora glycinivorella]	-1.9648	0.49352	0.5423	0.75656	0.17237
TRINITY_DN95558_c0_g3_i1_orf1	cytochrome P450 monooxygenase CYP9G19 [Cnaphalocrocis medinalis]	-1.9573	0.43282	0.59371	0.15234	0.77843
TRINITY_DN86772_c0_g1_i3_orfp1	x-tox [Spodoptera exigua]	-1.7865	-0.3059	0.98139	0.29628	0.81475
TRINITY_DN960_c1_g1_i6_orf1	hypothetical protein evm_007130 [Chilo suppressalis]	-1.9541	0.51431	0.85884	0.21719	0.36374
TRINITY_DN380_c0_g2_i2_orf1	chemosensory protein 10 [Ostrinia furnacalis]	-1.7971	-0.349	0.4764	0.95239	0.71729
TRINITY_DN628_c0_g1_i1_orf1	prostamide/prostaglandin F synthase-like [Ostrinia furnacalis]	-1.9579	0.10288	0.58343	0.69631	0.57525
TRINITY_DN4816_c0_g2_i3_orf1	15-hydroxyprostaglandin dehydrogenase [NAD(+)]-like [Ostrinia furnacalis]	-1.8939	-0.1432	0.69379	0.73797	0.6054
TRINITY_DN1575_c0_g1_i10_orf1	uncharacterized protein LOC114359245 [Ostrinia furnacalis]	-1.3792	0.42138	1.18157	0.7539	-0.9777
TRINITY_DN5337_c0_g1_i6_orf1	epoxide hydrolase 1-like [Ostrinia furnacalis]	-1.912	0.15109	0.16032	0.71476	0.88586
TRINITY_DN6908_c0_g1_i1_orf1	serine--pyruvate aminotransferase, mitochondrial [Ostrinia furnacalis] >XP_028157324.1 serine--pyruvate aminotransferase, mitochondrial [Ostrinia furnacalis] >XP_028157325.1 serine--pyruvate aminotransferase, mitochondrial [Ostrinia furnacalis]	-1.8508	0.41112	-0.1273	0.49898	1.06792
TRINITY_DN5439_c0_g1_i2_orf1	uncharacterized protein LOC114353087 [Ostrinia furnacalis]	-1.8093	-0.177	0.56999	0.27955	1.13676
TRINITY_DN4235_c0_g1_i2_orf1	uncharacterized protein LOC114361536 [Ostrinia furnacalis]	-1.9616	0.25747	0.40485	0.84674	0.45257
TRINITY_DN15202_c0_g1_i6_orf1	uncharacterized protein LOC114364499 isoform X2 [Ostrinia furnacalis]	-1.6715	-0.5354	0.3374	0.76407	1.10538
TRINITY_DN6497_c0_g1_i1_orf1	ommochrome-binding protein-like [Ostrinia furnacalis]	-1.8921	0.29728	0.13028	1.08027	0.38426
TRINITY_DN12534_c0_g1_i4_orf1	antibacterial protein [Heliothis virescens]	-1.7409	-0.5078	0.64548	0.87018	0.73311
TRINITY_DN9090_c0_g1_i9_orf1	CD63 antigen [Ostrinia furnacalis]	-1.9417	0.2053	0.29934	0.89975	0.53727
TRINITY_DN35809_c0_g1_i1_orf1	spodomicin-like [Ostrinia furnacalis] >QKV49445.1 diapausin [Ostrinia furnacalis]	-1.6706	-0.5943	0.99078	0.81406	0.46002
TRINITY_DN15597_c0_g1_i1_orf1	microsomal glutathione S-transferase 1-like [Ostrinia furnacalis]	-1.9021	0.76095	0.22322	0.05162	0.86631
TRINITY_DN1048_c0_g1_i6_orf1	uncharacterized protein LOC114360661 [Ostrinia furnacalis]	-1.8346	0.41615	0.37292	1.1455	-0.1
TRINITY_DN5421_c0_g1_i1_orf1	arylphorin subunit alpha-like [Ostrinia furnacalis]	-1.9428	0.3044	0.46122	0.92561	0.25159
TRINITY_DN1672_c0_g1_i6_orf1	cystinosin homolog isoform X1 [Ostrinia furnacalis] >XP_028162341.1 cystinosin homolog isoform X1 [Ostrinia furnacalis] >XP_028162342.1 cystinosin homolog isoform X1 [Ostrinia furnacalis] >XP_028162343.1 cystinosin homolog isoform X1 [Ostrinia furnacalis] >XP_028162344.1 cystinosin homolog isoform X1 [Ostrinia furnacalis]	-1.8624	0.60746	0.28602	-0.0684	1.03728
TRINITY_DN24723_c2_g1_i1_orf1	hypothetical protein evm_001103 [Chilo suppressalis]	-1.7666	-0.4458	0.77289	0.87289	0.56665
TRINITY_DN1201_c0_g1_i4_orf1	triosephosphate isomerase [Ostrinia furnacalis] >XP_028170843.1 triosephosphate isomerase [Ostrinia furnacalis]	-1.9565	0.11534	0.74917	0.52191	0.57011
TRINITY_DN4443_c0_g1_i4_orf1	lysosome-associated membrane glycoprotein 1-like isoform X4 [Ostrinia furnacalis]	-1.9056	0.56689	0.9771	0.29729	0.06433
TRINITY_DN56459_c0_g1_i2_orf1	aldo-keto reductase AKR2E4-like [Ostrinia furnacalis]	-1.693	-0.6049	0.70412	0.82862	0.76515
TRINITY_DN1030_c0_g1_i6_orf1	gamma-glutamyl hydrolase A-like isoform X1 [Ostrinia furnacalis]	-1.9466	0.53576	0.16821	0.37324	0.86943
TRINITY_DN19731_c0_g1_i1_orf1	allergen Tha p 1-like [Ostrinia furnacalis] >XP_028174916.1 allergen Tha p 1-like [Ostrinia furnacalis] >BAV56808.1 chemosensory protein 4 [Ostrinia furnacalis]	-1.8323	-0.1912	0.27496	0.90423	0.84429
TRINITY_DN33346_c0_g1_i1_orf1	PREDICTED: U6 snRNA-associated Sm-like protein LSm3 [Papilio xuthus] >XP_028165558.1 U6 snRNA-associated Sm-like protein LSm3 [Ostrinia furnacalis] >KOB73597.1 LSM Sm-like protein family member [Operophtera brumata] >RVE45517.1 hypothetical protein evm_009856 [Chilo suppressalis] >CAB3523639.1 unnamed protein product [Chilo suppressalis] >CAH0400961.1 unnamed protein product [Chilo suppressalis]	-1.9243	0.02651	0.40817	0.64415	0.84548
TRINITY_DN2314_c0_g1_i7_orf1	protein dj-1beta-like isoform X1 [Ostrinia furnacalis]	-1.8251	0.07246	-0.0474	1.04376	0.75626
TRINITY_DN9079_c0_g1_i5_orf1	UDP-glucuronosyltransferase-like [Ostrinia furnacalis]	-1.885	-0.1702	0.64225	0.76676	0.64616
TRINITY_DN135780_c0_g1_i1_orf1	flotillin-1 isoform X1 [Pectinophora gossypiella]	-1.8717	-0.0792	0.82634	0.26605	0.85845
TRINITY_DN40126_c0_g1_i1_orf1	aldehyde dehydrogenase X, mitochondrial-like [Ostrinia furnacalis]	-1.94	0.0301	0.57633	0.58228	0.75126
TRINITY_DN6025_c0_g2_i1_orfp1	TRINITY_DN6025_c0_g2_i1_m.7749 TRINITY_DN6025_c0_g2::TRINITY_DN6025_c0_g2_i1::g.7749 ORF type:5prime_partial len:106 (+),score=47.10 TRINITY_DN6025_c0_g2_i1:3-320(+)	-1.8279	-0.2936	0.8931	0.51334	0.71506
TRINITY_DN36476_c1_g1_i1_orfp1	TRINITY_DN36476_c1_g1_i1_m.70910 TRINITY_DN36476_c1_g1::TRINITY_DN36476_c1_g1_i1::g.70910 ORF type:5prime_partial len:88 (-),score=0.50 TRINITY_DN36476_c1_g1_i1:49-312(-)	-1.6206	-0.6667	1.01449	0.42458	0.84827
TRINITY_DN69_c0_g1_i1_orf1	glycerol-3-phosphate dehydrogenase [NAD(+)], cytoplasmic isoform X1 [Ostrinia furnacalis]	-1.8571	-0.1964	0.65782	0.4677	0.92797

TRINITY_DN4998_c0_g1_i21_orf1	phenoloxidase subunit 2-like [Ostrinia furnacalis]	-1.9295	0.01451	0.74996	0.71659	0.44839
TRINITY_DN97883_c0_g1_i2_orf1	talin-2-like, partial [Ostrinia furnacalis]	-1.5601	0.69155	1.25497	0.27487	-0.6613
TRINITY_DN1424_c0_g1_i5_orf1	insect group I lytic polysaccharide monooxygenase [Ostrinia furnacalis]	-1.9621	0.23501	0.32954	0.6286	0.76894
TRINITY_DN31286_c0_g1_i6_orfp1	TRINITY_DN31286_c0_g1_i6_m.28438 TRINITY_DN31286_c0_g1::TRINITY_DN31286_c0_g1_i6::g.28438 ORF type:internal len:92 (-),score=3.10,Perilipin PF03036.17 2e-05 TRINITY_DN31286_c0_g1_i6:1-273(-)	-1.8259	-0.1932	0.51318	0.4125	1.09336
TRINITY_DN13098_c2_g1_i2_orf1	unnamed protein product [Arctia plantaginis] >CAB3253298.1 unnamed protein product [Arctia plantaginis]	-1.9551	0.3239	0.2367	0.8463	0.54818
TRINITY_DN21420_c0_g1_i2_orf1	glutathione peroxidase, partial [Ostrinia furnacalis]	-1.84	-0.0941	0.44521	1.13098	0.35796
TRINITY_DN2472_c0_g1_i6_orf1	programmed cell death protein 6 isoform X1 [Colias croceus] >XP_045492459.1 programmed cell death protein 6 isoform X1 [Colias croceus]	-1.9091	0.18576	0.36408	1.04337	0.31586
TRINITY_DN7226_c0_g1_i2_orf1	chemosensory protein [Conogethes punctiferalis]	-1.7465	-0.4445	0.82251	0.94813	0.42034
TRINITY_DN892_c7_g1_i2_orf1	unnamed protein product [Diatraea saccharalis]	-1.8429	0.45857	0.60135	-0.2108	0.99374
TRINITY_DN295_c5_g1_i2_orf1	unnamed protein product [Chilo suppressalis]	-1.729	-0.3868	0.54383	1.19189	0.3801
TRINITY_DN29555_c0_g1_i8_orf1	fasciclin-1 [Ostrinia furnacalis]	-1.9094	0.27143	0.91962	0.06181	0.65653
TRINITY_DN2650_c0_g1_i1_orf1	hypothetical protein HW555_002849 [Spodoptera exigua] >CAH0691914.1 unnamed protein product [Spodoptera exigua]	-1.8289	0.30889	0.04622	0.25146	1.22237
TRINITY_DN9492_c0_g1_i7_orf1	aldo-keto reductase AKR2E4-like [Ostrinia furnacalis]	-1.9463	0.17238	0.31467	0.6335	0.82575
TRINITY_DN1363_c0_g1_i11_orf1	cytochrome P450 CYP12A2-like isoform X1 [Ostrinia furnacalis] >QPF77619.1 cytochrome P450 monooxygenase CYP333A20 [Ostrinia furnacalis]	-1.8171	-0.2994	0.96686	0.49475	0.65489
TRINITY_DN1161_c0_g1_i2_orf1	fructose-1,6-bisphosphatase 1 [Ostrinia furnacalis]	-1.9365	0.0716	0.61007	0.83427	0.42051
TRINITY_DN9325_c0_g1_i1_orf1	protein takeout-like [Ostrinia furnacalis]	-1.7162	0.00291	-0.2631	0.86146	1.11497
TRINITY_DN581_c3_g2_i1_orf1	uncharacterized protein LOC114364499 isoform X3 [Ostrinia furnacalis]	-1.5301	-0.4306	-0.1197	0.65612	1.4243
TRINITY_DN2141_c0_g1_i1_orf1	low density lipoprotein receptor adapter protein 1-like [Ostrinia furnacalis]	-1.5609	0.88735	1.2249	-0.0269	-0.5245
TRINITY_DN8367_c0_g2_i2_orf1	uncharacterized protein LOC114357075 [Ostrinia furnacalis]	-1.959	0.28669	0.7085	0.24351	0.7203
TRINITY_DN13660_c0_g1_i1_orf1	Aliphatic nitrilase [Operophtera brumata]	-1.8389	-0.2362	0.5464	0.98319	0.54548
TRINITY_DN24_c0_g1_i1_orf1	hypothetical protein evm_007803 [Chilo suppressalis]	-1.7481	-0.0394	1.04161	-0.1653	0.9112
TRINITY_DN3039_c0_g2_i1_orf1	uncharacterized protein LOC114353136 [Ostrinia furnacalis]	-1.9641	0.32785	0.27738	0.81085	0.54804
TRINITY_DN1466_c0_g1_i4_orf1	insecticyanin-A-like [Ostrinia furnacalis]	-1.656	0.46605	0.84162	0.97152	-0.6232
TRINITY_DN8037_c0_g2_i1_orf1	2-oxo-4-hydroxy-4-carboxy-5-ureidoimidazoline decarboxylase-like [Ostrinia furnacalis]	-1.9041	0.4802	0.95886	0.47375	-0.0087
TRINITY_DN30306_c0_g2_i1_orf1	perilipin-4-like isoform X3 [Ostrinia furnacalis]	-1.6424	-0.5849	1.14023	0.35707	0.72998
TRINITY_DN7493_c0_g1_i1_orf1	cofilin-1 [Homo sapiens] >XP_001494584.1 cofilin-1 [Equus caballus] >XP_002916677.1 cofilin-1 [Ailuropoda melanoleuca] >XP_004437680.1 PREDICTED: cofilin-1 [Ceratotherium simum simum] >XP_004618483.1 PREDICTED: cofilin-1 [Sorex araneus] >XP_006101632.1 cofilin-1 [Myotis lucifugus] >XP_006771640.1 PREDICTED: cofilin-1 [Myotis davidii] >XP_008531143.1 PREDICTED: cofilin-1 [Equus przewalskii] >XP_008707230.1 cofilin-1 [Ursus maritimus] >XP_008952328.1 cofilin-1 isoform X2 [Pan paniscus] >XP_009421771.1 cofilin-1 isoform X2 [Pan troglodytes] >XP_010346634.1 cofilin-1 isoform X1 [Saimiri boliviensis boliviensis] >XP_012318375.1 cofilin-1 isoform X1 [Aotus nancymaae] >XP_014700305.1 cofilin-1 [Equus asinus] >XP_015358605.1 cofilin-1 [Marmota marmota marmota] >XP_016063879.1 PREDICTED: cofilin-1 [Miniopterus natalensis] >XP_018891644.1 cofilin-1 isoform X1 [Gorilla gorilla gorilla] >XP_019500817.1 PREDICTED: cofilin-1 isoform X2 [Hipposideros armiger] >XP_021587455.1 cofilin-1 isoform X2 [Ictidomys tridecemlineatus] >XP_022349924.1 cofilin-1 [Enhydra lutris kenyoni] >XP_024110693.1 cofilin-1 isoform X2 [Pongo abelii] >XP_024430100.1 cofilin-1 [Desmodus rotundus] >XP_025302209.1 cofilin-1 [Canis lupus dingo] >XP_026338972.1 cofilin-1 [Ursus arctos] >XP_027799962.1 cofilin-1 [Marmota flaviventris] >XP_030667090.1 cofilin-1 isoform X2 [Nomascus leucogenys] >XP_032009365.1 cofilin-1 isoform X2 [Hylobates moloch] >XP_032138707.1 cofilin-1 [Sapajus apella] >XP_034507763.1 cofilin-1 [Ailuropoda melanoleuca] >XP_035119236.1 cofilin-1 isoform X2 [Callithrix jacchus] >XP_035582605.1 cofilin-1 [Zalophus californianus] >XP_036909129.1 cofilin-1 [Sturnira hondurensis] >XP_036991204.1 cofilin-1 [Artibeus jamaicensis] >XP_038280889.1 cofilin-1 [Canis lupus familiaris] >XP_041577750.1 cofilin-1 [Vulpes lagopus] >XP_045631776.1 cofilin-1 [Ursus americanus] >XP_045870967.1 cofilin-1 [Meles meles] >XP_046500850.1 cofilin-1 [Equus quagga] >XP_047374272.1 cofilin-1 [Neosciurus carolinensis] >XP_047419340.1 cofilin-1 [Neosciurus carolinensis] >XP_533231.1 cofilin-1 [Canis lupus familiaris] >P23528.3 RecName: Full=Cofilin-1; AltName: Full=18 kDa phosphoprotein; Short=p18; AltName: Full=Cofilin, non-muscle isoform [Homo sapiens] >1Q8G_A Chain A, Cofilin, non-muscle isoform [Homo sapiens] >1Q8X_A Chain A, Cofilin, non-muscle isoform [Homo sapiens] >3J0S_M Remodeling of actin filaments by ADF cofilin proteins [Homo sapiens] >3J0S_N Remodeling of actin filaments by ADF cofilin proteins [Homo sapiens] >3J0S_O Remodeling of actin filaments by ADF cofilin proteins [Homo sapiens] >3J0S_P Remodeling of actin filaments by ADF cofilin proteins [Homo sapiens] >3J0S_Q Remodeling of actin filaments by ADF cofilin proteins [Homo sapiens] >3J0S_R Remodeling of actin filaments by ADF cofilin proteins [Homo sapiens] >3J0S_S Remodeling of actin filaments by ADF cofilin proteins [Homo sapiens] >3J0S_T Remodeling of actin filaments by ADF cofilin proteins [Homo sapiens] >3J0S_U Remodeling of actin filaments by ADF cofilin proteins [Homo sapiens] >3J0S_V Remodeling of actin filaments by ADF cofilin proteins [Homo sapiens] >3J0S_W Remodeling of actin filaments by ADF cofilin proteins [Homo sapiens] >3J0S_X Remodeling of actin filaments by ADF cofilin proteins [Homo sapiens] >6UBY_I Isolated cofilin bound to an actin filament [Homo sapiens] >6UC4_I Barbed end side of a cofilactin cluster [Homo sapiens] >6UC4_M Barbed end side of a cofilactin cluster [Homo sapiens] >6UC4_N Barbed end side of a cofilactin cluster [Homo sapiens] >6UC4_O Barbed end side of a cofilactin cluster [Homo sapiens] >6UC4_P Barbed end side of a cofilactin cluster [Homo sapiens] >6VAO_F Human cofilin-1 decorated actin filament [Homo sapiens] >6VAO_G Human cofilin-1 decorated actin filament [Homo sapiens] >6VAO_H Human cofilin-1 decorated actin filament [Homo sapiens] >6VAO_I Human cofilin-1 decorated actin filament [Homo sapiens] >6VAO_J Human cofilin-1 decorated actin filament [Homo sapiens] >AAX41853.1 cofilin 1 [synthetic construct] >KAF6332759.1 cofilin 1 [Rhinolophus ferrumequinum] >KAF6437236.1 cofilin 1 [Molossus molossus] >KAF6464509.1 cofilin 1 [Rousettus aegyptiacus] >SJX27644.1 unnamed protein	-1.5598	-0.0728	-0.1561	1.57903	0.20966
TRINITY_DN3821_c1_g1_i7_orf1	mitochondrial carrier protein Rim2 isoform X1 [Ostrinia furnacalis]	-1.7959	-0.3225	0.37776	0.95202	0.78856
TRINITY_DN140_c0_g1_i1_orf1	calcyphosin-like protein [Ostrinia furnacalis]	-1.8274	-0.1325	0.24237	1.09271	0.62481
TRINITY_DN2187_c0_g1_i1_orf1	flotillin-1 [Chelonus insularis] >XP_034947202.1 flotillin-1 [Chelonus insularis]	-1.8582	-0.1939	0.81471	0.41887	0.8186
TRINITY_DN7226_c0_g1_i5_orf1	chemosensory protein [Dioryctria abietella]	-1.7964	-0.3468	0.94469	0.74398	0.45455
TRINITY_DN4245_c0_g2_i1_orf1	long-chain fatty acid transport protein 4-like [Ostrinia furnacalis]	-1.8804	-0.1507	0.70272	0.48335	0.84497

TRINITY_DN2286_c2_g1_i1_orf1	coatomer subunit zeta-1 isoform X1 [Ostrinia furnacalis]	-1.8072	-0.1022	0.17179	1.17235	0.56531
TRINITY_DN8953_c0_g1_i4_orf1	gonadotropin-releasing hormone receptor [Ostrinia furnacalis] >AXF67446.1 adipokinetic hormone receptor 1 [Ostrinia furnacalis]	-1.8732	-0.009	1.00841	0.22209	0.6517
TRINITY_DN14262_c0_g1_i5_orf1	cytochrome P450 monooxygenase CYP9G18 [Cnaphalocrocis medinalis]	-1.7866	-0.1865	0.95254	0.92577	0.09478
TRINITY_DN9132_c0_g1_i5_orf1	ubiquitin-like-conjugating enzyme ATG3 [Spodoptera frugiperda]	-1.8371	-0.1254	0.23396	1.03876	0.68973
TRINITY_DN5682_c0_g1_i6_orf1	spodomicin-like [Ostrinia furnacalis] >QKV49445.1 diapausin [Ostrinia furnacalis]	-1.8746	0.0889	1.11851	0.28709	0.3801
TRINITY_DN85290_c0_g2_i1_orf1	unnamed protein product, partial [Brenthis ino]	-1.3149	-0.2139	1.02085	1.26744	-0.7595
TRINITY_DN346_c0_g1_i7_orf1	CDK-activating kinase assembly factor MAT1 [Ostrinia furnacalis]	-1.7074	-0.2687	0.09995	1.28652	0.58956
TRINITY_DN4565_c0_g2_i1_orf1	acid phosphatase type 7 isoform X2 [Ostrinia furnacalis]	-1.9036	0.43712	1.03612	0.31314	0.1172
TRINITY_DN47609_c0_g1_i1_orfp1	TRINITY_DN47609_c0_g1_i1_m.57205 TRINITY_DN47609_c0_g1::TRINITY_DN47609_c0_g1_i1::g.57205 ORF type:5prime_partial len:68 (-),score=15.96 TRINITY_DN47609_c0_g1_i1:36-239(-)	-1.8101	0.19633	0.09064	1.26998	0.25311
TRINITY_DN10371_c0_g1_i2_orf1	heterogeneous nuclear ribonucleoprotein L isoform X2 [Vanessa cardui]	-1.8093	0.68237	0.14953	-0.1281	1.10545
TRINITY_DN20658_c0_g2_i3_orf1	prostaglandin reductase 1-like [Ostrinia furnacalis]	-1.8077	-0.1908	0.5557	0.30555	1.13732
TRINITY_DN11817_c0_g1_i4_orf1	glycogen phosphorylase [Heortia vitessoides]	-1.8942	-0.0665	0.74305	0.84752	0.37012
TRINITY_DN44857_c0_g1_i4_orf1	UPF0183 protein CG7083 [Ostrinia furnacalis]	-1.8519	0.58841	0.36316	-0.1359	1.03627
TRINITY_DN5099_c0_g1_i3_orf1	trans-1,2-dihydrobenzene-1,2-diol dehydrogenase-like [Ostrinia furnacalis]	-1.8503	-0.168	0.44519	0.54908	1.02401
TRINITY_DN4343_c0_g1_i2_orf1	uncharacterized protein LOC114365231 isoform X3 [Ostrinia furnacalis]	-1.8217	-0.0589	0.80723	0.06209	1.01123
TRINITY_DN44517_c0_g1_i4_orf1	regucalcin-like [Ostrinia furnacalis]	-1.8907	-0.1466	0.74216	0.56398	0.73125
TRINITY_DN79657_c0_g1_i1_orf1	uncharacterized protein LOC114349955 [Ostrinia furnacalis]	-1.9682	0.45246	0.57096	0.19908	0.74565
TRINITY_DN125140_c0_g1_i1_orf1	glycogen debranching enzyme isoform X2 [Ostrinia furnacalis] >XP_028161358.1 glycogen debranching enzyme isoform X2 [Ostrinia furnacalis] >XP_028161359.1 glycogen debranching enzyme isoform X2 [Ostrinia furnacalis]	-1.9117	0.50471	0.74014	0.73386	-0.0671
TRINITY_DN244_c1_g1_i5_orf1	C-1-tetrahydrofolate synthase, cytoplasmic isoform X3 [Ostrinia furnacalis]	-1.827	0.20565	-0.1308	0.69235	1.0598
TRINITY_DN22242_c0_g2_i1_orf1	juvenile hormone epoxide hydrolase-like [Ostrinia furnacalis] >XP_028170526.1 juvenile hormone epoxide hydrolase-like [Ostrinia furnacalis]	-1.9324	0.32657	0.3865	0.2447	0.97463
TRINITY_DN31348_c0_g1_i1_orf1	protein lethal(2)essential for life [Bombyx mori]	-1.6407	-0.5548	0.45428	1.24357	0.4976
TRINITY_DN71698_c0_g1_i1_orfp1	TRINITY_DN71698_c0_g1_i1_m.1194 TRINITY_DN71698_c0_g1::TRINITY_DN71698_c0_g1_i1::g.1194 ORF type:internal len:134 (+),score=19.66,Toxin_2 PF00451.20 4.3e-05,Toxin_2|PF00451.20|0.037,Toxin_2|PF00451.20|7.5e-05,Gamma-thionin|PF00304.21|0.017,Gamma-thionin|PF00304.21|0.05,Gamma-thionin|PF00304.21|0.021,Toxin_38|PF14866.7|0.13,Toxin_38|PF14866.7|0.15,Toxin_38|PF14866.7|0.15,Defensin_2|PF01097.19|0.053,Defensin_2|PF01097.19|0.34,Defensin_2|PF01097.19|0.092 TRINITY_DN71698_c0_g1_i1:3-401(+)	-1.6737	-0.5788	0.86061	0.9738	0.41809
TRINITY_DN19690_c0_g1_i1_orf1	unnamed protein product [Chilo suppressalis]	-1.9602	0.55713	0.24917	0.33262	0.82127
TRINITY_DN1750_c1_g1_i5_orf1	lipid droplet localized protein-like [Ostrinia furnacalis] >XP_028161280.1 lipid droplet localized protein-like [Ostrinia furnacalis]	-1.8472	-0.2498	0.713	0.86401	0.52001
TRINITY_DN21533_c0_g1_i4_orf1	hypothetical protein evm_010931 [Chilo suppressalis]	-1.9201	0.50601	0.98082	0.19303	0.24028
TRINITY_DN8780_c0_g1_i3_orf1	uncharacterized protein LOC114363370 [Ostrinia furnacalis]	-1.8012	0.15202	-0.071	1.21233	0.50786
TRINITY_DN6108_c0_g1_i5_orf1	myogenesis-regulating glycosidase-like [Ostrinia furnacalis]	-1.7378	0.16979	0.29278	1.36306	-0.0879
TRINITY_DN20658_c0_g1_i1_orf1	prostaglandin reductase 1-like [Ostrinia furnacalis]	-1.8214	-0.2979	0.83431	0.45259	0.83237
TRINITY_DN114890_c0_g1_i4_orf1	chemosensory protein 10 [Ostrinia furnacalis]	-1.475	-0.7429	0.56866	1.36742	0.28177
TRINITY_DN40126_c0_g2_i1_orf1	aldehyde dehydrogenase X, mitochondrial-like [Ostrinia furnacalis]	-1.6368	-0.6328	1.09532	0.46233	0.71196
TRINITY_DN2921_c1_g1_i4_orf1	uncharacterized protein LOC114352340 isoform X2 [Ostrinia furnacalis]	-1.9617	0.44564	0.62477	0.15767	0.73357
TRINITY_DN130575_c0_g1_i1_orfp1	TRINITY_DN130575_c0_g1_i1_m.77798 TRINITY_DN130575_c0_g1::TRINITY_DN130575_c0_g1_i1::g.77798 ORF type:internal len:70 (+),score=15.12 TRINITY_DN130575_c0_g1_i1:3-209(+)	-1.8091	-0.2939	0.78642	0.37952	0.93712
TRINITY_DN122321_c0_g1_i1_orf1	uncharacterized protein LOC114356271 [Ostrinia furnacalis]	-1.7814	-0.3295	0.3795	0.67218	1.05927
TRINITY_DN2264_c0_g1_i1_orf1	cytochrome P450 6B6-like [Ostrinia furnacalis]	-1.8457	0.43129	-0.1916	0.60192	1.00413
TRINITY_DN13411_c0_g1_i4_orf1	facilitated trehalose transporter Tret1-like [Ostrinia furnacalis]	-1.9782	0.43877	0.65252	0.24994	0.63701
TRINITY_DN5300_c0_g1_i2_orf1	valacyclovir hydrolase [Ostrinia furnacalis]	-1.9199	-0.0589	0.61565	0.64521	0.71789
TRINITY_DN34423_c0_g1_i2_orf1	THAP domain-containing protein 4-like [Ostrinia furnacalis]	-1.8771	-0.0895	0.58271	0.98476	0.39909
TRINITY_DN9608_c0_g1_i3_orf1	cytochrome P450 monooxygenase CYP9G18 [Cnaphalocrocis medinalis]	-1.5219	-0.5372	0.04571	0.57878	1.43463
TRINITY_DN59429_c0_g1_i6_orf1	uncharacterized protein LOC114366345 isoform X2 [Ostrinia furnacalis]	-1.528	-0.7936	1.06094	0.8701	0.39062
TRINITY_DN1450_c0_g2_i1_orf1	death-associated protein 1 [Ostrinia furnacalis]	-1.618	-0.6984	0.99672	0.53741	0.7823
TRINITY_DN1503_c0_g1_i6_orf1	ecdysteroid-regulated 16 kDa protein [Ostrinia furnacalis]	-1.8367	-0.1171	1.08672	0.60066	0.26641
TRINITY_DN2442_c0_g1_i6_orf1	cytochrome P450 6B5-like [Ostrinia furnacalis]	-1.8207	0.02964	0.59383	0.0441	1.15311
TRINITY_DN6098_c1_g1_i5_orf1	unnamed protein product, partial [Iphiclides podalirius]	-1.7269	-0.5411	0.67888	0.82118	0.76796
TRINITY_DN811_c0_g1_i15_orf1	uncharacterized protein LOC114364160 [Ostrinia furnacalis]	-1.8447	0.3387	1.10487	0.50145	-0.1003
TRINITY_DN4900_c0_g1_i6_orf1	hypothetical protein evm_001963 [Chilo suppressalis]	-1.9678	0.44921	0.48915	0.79484	0.23459
TRINITY_DN29009_c0_g2_i2_orf1	juvenile hormone binding protein [Omphisa fuscidentalis]	-1.9221	-0.0056	0.53582	0.85365	0.53826
TRINITY_DN43667_c0_g1_i1_orf1	carbonyl reductase [NADPH] 1-like [Ostrinia furnacalis]	-1.6749	-0.0304	-0.2594	0.669	1.29573
TRINITY_DN51480_c0_g1_i1_orf1	uncharacterized protein LOC114361588 isoform X14 [Ostrinia furnacalis]	-1.7425	-0.3234	0.15079	0.92144	0.99368
TRINITY_DN8595_c0_g1_i3_orf1	aldose reductase-like isoform X4 [Trichoplusia ni]	-1.6375	-0.654	0.59018	0.63241	1.06893
TRINITY_DN1880_c0_g1_i4_orf1	serine protease inhibitor dipetalogastin-like [Helicoverpa zea]	-1.6005	-0.7326	0.69273	1.01569	0.62467
TRINITY_DN2251_c0_g1_i4_orf1	serine hydroxymethyltransferase, cytosolic isoform X1 [Ostrinia furnacalis]	-1.9095	0.07408	0.81015	0.80106	0.22422
TRINITY_DN12775_c0_g1_i10_orfp1	TRINITY_DN12775_c0_g1_i10_m.21238 TRINITY_DN12775_c0_g1::TRINITY_DN12775_c0_g1_i10::g.21238 ORF type:5prime_partial len:67 (-),score=0.74 TRINITY_DN12775_c0_g1_i10:275-475(-)	-1.7247	-0.3412	0.1233	0.91368	1.02899

TRINITY_DN5153_c1_g1_i1_orf1	nose resistant to fluoxetine protein 6-like isoform X1 [Ostrinia furnacalis]	-1.6446	-0.5741	1.19455	0.42701	0.59712
TRINITY_DN1014_c0_g2_i8_orf1	uncharacterized protein LOC114362446 [Ostrinia furnacalis] >XP_028173663.1 uncharacterized protein LOC114362446 [Ostrinia furnacalis]	-1.8488	0.19641	0.0035	0.52139	1.12754
TRINITY_DN6580_c0_g1_i4_orf1	catalase [Ostrinia furnacalis]	-1.8375	-0.0078	0.14999	0.56129	1.134
TRINITY_DN1352_c0_g1_i5_orf1	uncharacterized protein LOC113491815 [Trichoplusia ni]	-1.8482	-0.2263	0.52277	0.94302	0.60867
TRINITY_DN618_c0_g1_i3_orf1	triokinase/FMN cyclase-like isoform X1 [Ostrinia furnacalis]	-1.8535	-0.2351	0.67167	0.54344	0.87346
TRINITY_DN16316_c0_g1_i7_orf1	exocyst complex component 6 isoform X1 [Ostrinia furnacalis] >XP_028166671.1 exocyst complex component 6 isoform X2 [Ostrinia furnacalis]	-1.5189	0.83452	0.81025	0.75319	-0.8791
TRINITY_DN55148_c0_g1_i1_orf1	60S ribosomal protein L11 isoform 1 [Homo sapiens] >NP_0010690[illegible] 60S ribosomal protein L11 [Bos taurus] >NP_00124[illegible] 60S ribosomal protein L11 isoform 1 [Canis lupus familiaris] >NP_001269300.1 60S ribosomal protein L11 [Chinchilla lanigera] >NP_001291809.1 60S ribosomal protein L11 [Ailuropoda melanoleuca] >NP_080195.1 60S ribosomal protein L11 [Mus musculus] >XP_001504267.1 60S ribosomal protein L11 isoform X2 [Equus caballus] >XP_003471379.1 60S ribosomal protein L11 [Cavia porcellus] >XP_003810808.1 60S ribosomal protein L11 [Pan paniscus] >XP_003891370.1 60S ribosomal protein L11 [Papio anubis] >XP_003989701.1 60S ribosomal protein L11 [Felis catus] >XP_004285699.1 60S ribosomal protein L11 isoform X2 [Orcinus orca] >XP_004377186.1 60S ribosomal protein L11 [Trichechus manatus latirostris] >XP_004394821.1 PREDICTED: 60S ribosomal protein L11 isoform X1 [Odobenus rosmarus divergens] >XP_004465476.2 60S ribosomal protein L11 [Dasypus novemcinctus] >XP_004637663.1 60S ribosomal protein L11 [Octodon degus] >XP_004850617.1 60S ribosomal protein L11 isoform X2 [Heterocephalus glaber] >XP_005544522.1 60S ribosomal protein L11 isoform X1 [Macaca fascicularis] >XP_005676921.1 PREDICTED: 60S ribosomal protein L11 isoform X2 [Capra hircus] >XP_006078005.1 60S ribosomal protein L11 isoform X2 [Bubalus bubalis] >XP_006094108.1 60S ribosomal protein L11 isoform X3 [Myotis lucifugus] >XP_006239286.1 60S ribosomal protein L11 isoform X1 [Rattus norvegicus] >XP_006737645.1 60S ribosomal protein L11 [Leptonychotes weddellii] >XP_006777625.1 PREDICTED: 60S ribosomal protein L11 isoform X1 [Myotis davidii] >XP_006883588.1 PREDICTED: 60S ribosomal protein L11-like isoform X1 [Elephantulus edwardii] >XP_007121151.1 60S ribosomal protein L11 isoform X2 [Physeter catodon] >XP_007175168.1 60S ribosomal protein L11 isoform X1 [Balaenoptera acutorostrata scammoni] >XP_007459239.1 PREDICTED: 60S ribosomal protein L11 isoform X1 [Lipotes vexillifer] >XP_007524793.1 PREDICTED: 60S ribosomal protein L11 [Erinaceus europaeus] >XP_007528779.2 PREDICTED: 60S ribosomal protein L11 [Erinaceus europaeus] >XP_007933904.2 60S ribosomal protein L11 [Orycteropus afer afer] >XP_007978264.1 60S ribosomal protein L11 isoform X1 [Chlorocebus sabaeus] >XP_008059998.1 60S ribosomal protein L11 isoform X2 [Carlito syrichta] >XP_008146324.1 60S ribosomal protein L11 [Eptesicus fuscus] >XP_008263912.1 PREDICTED: 60S ribosomal protein L11 [Oryctolagus cuniculus] >XP_008518979.1 PREDICTED: 60S ribosomal protein L11 isoform X2 [Equus przewalskii] >XP_008571152.1 PREDICTED: 60S ribosomal protein L11 isoform X1 [Galeopterus variegatus] >XP_008571160.1 PREDICTED: 60S ribosomal protein L11 isoform X2 [Galeopterus variegatus] >XP_008846816.1 60S ribosomal protein L11 [Nannospalax galili] >XP_010354068.1 60S ribosomal protein L11 [Rhinopithecus roxellana] >XP_010624280.1 60S ribosomal protein L11 [Fukomys damarensis] >XP_011355886.1 60S ribosomal protein L11 [Pteropus vampyrus] >XP_011761124.1 60S ribosomal protein L11 [Macaca nemestrina] >XP_011833215.1 PREDICTED: 60S ribosomal protein L11 isoform X2 [Mandrillus leucophaeus] >XP_011935575.1 PREDICTED: 60S ribosomal protein L11 [Cercocebus atys] >XP_014710573.1 60S ribosomal protein L11 isoform X2 [Equus asinus] >XP_014930664.1 60S ribosomal protein L11 [Acinonyx jubatus] >XP_014949173.1 60S ribosomal protein L11 isoform X1 [Ovis aries] >XP_014986347.1 60S ribosomal protein L11 [Macaca mulatta] >XP_015341019.2 60S ribosomal protein L11 isoform X1 [Marmota marmota marmota] >XP_016811748.1 60S ribosomal protein L11 [Pan troglodytes] >XP_017390016.1 60S ribosomal protein L11 [Cebus imitator] >XP_017741964.1 PREDICTED: 60S ribosomal protein L11 [Rhinopithecus bieti] >XP_018866494.2 60S ribosomal protein L11 [Gorilla gorilla gorilla] >XP_018868111.1 60S ribosomal protein L11 [Gorilla gorilla gorilla] >XP_019317313.1 PREDICTED: 60S ribosomal protein L11 [Panthera pardus] >XP_01956860[illegible] PREDICTED: 60S ribosomal protein L11 [Rhinolophus sinicus] >XP_0198371[illegible] PREDICTED: 60S ribosomal protein L11 isoform X2	-1.5709	-0.2591	-0.3082	0.86437	1.27393
TRINITY_DN2133_c0_g2_i1_orf1	glycogen debranching enzyme isoform X1 [Ostrinia furnacalis]	-1.9085	0.18858	0.84849	0.76918	0.10226
TRINITY_DN5274_c0_g2_i2_orf1	lopap-like [Ostrinia furnacalis]	-1.7814	-0.3785	0.93704	0.78099	0.44193
TRINITY_DN31611_c0_g1_i2_orf1	glucose-6-phosphate isomerase-like [Ostrinia furnacalis]	-1.9161	0.28065	0.37304	1.02723	0.23521
TRINITY_DN2040_c0_g1_i6_orf1	trypsin-like serine proteinase T26 protein, partial [Chilo infuscatellus]	-1.6353	-0.6819	0.5554	0.86859	0.89324
TRINITY_DN37307_c0_g1_i4_orf1	superoxide dismutase [Mn], mitochondrial [Ostrinia furnacalis]	-1.7083	-0.1881	1.38045	0.19596	0.31994
TRINITY_DN8008_c0_g1_i6_orf1	uncharacterized protein LOC114357965 isoform X1 [Ostrinia furnacalis] >XP_028167599.1 uncharacterized protein LOC114357965 isoform X1 [Ostrinia furnacalis] >XP_028167600.1 uncharacterized protein LOC114357965 isoform X2 [Ostrinia furnacalis] >XP_028167601.1 uncharacterized protein LOC114357965 isoform X3 [Ostrinia furnacalis]	-1.4788	-0.8444	0.40514	1.17864	0.73942
TRINITY_DN1592_c0_g1_i1_orf1	serine protease 7-like isoform X2 [Ostrinia furnacalis]	-1.8072	-0.0889	1.04569	0.05712	0.79331
TRINITY_DN2614_c0_g2_i3_orf1	uncharacterized protein LOC114360661 [Ostrinia furnacalis]	-1.7553	-0.1086	0.02889	1.25097	0.58412
TRINITY_DN41952_c0_g1_i1_orf1	protein DDI1 homolog 2 [Ostrinia furnacalis]	-1.9222	0.05363	0.86021	0.66802	0.34038
TRINITY_DN13718_c0_g1_i4_orf1	immulectin-4 [Ostrinia furnacalis]	-1.78	0.10143	0.368	0.0123	1.29831
TRINITY_DN874_c2_g1_i1_orf1	uncharacterized protein LOC114356358 [Ostrinia furnacalis]	-1.8938	-0.0277	0.54041	0.40144	0.9796
TRINITY_DN9420_c0_g1_i2_orf1	protein PFC0760c-like isoform X2 [Ostrinia furnacalis]	-1.7011	0.45636	1.34286	0.16283	-0.2609
TRINITY_DN146138_c0_g1_i1_orf1	E3 ubiquitin-protein ligase Ubr3 [Chelonus insularis] >XP_034951823.1 E3 ubiquitin-protein ligase Ubr3 [Chelonus insularis] >XP_034951824.1 E3 ubiquitin-protein ligase Ubr3 [Chelonus insularis]	-1.8931	0.73978	0.70144	0.59668	-0.1448
TRINITY_DN1694_c0_g1_i1_orf1	cuticle protein 16.5-like [Ostrinia furnacalis]	-1.9013	0.23512	0.33431	1.07287	0.25896
TRINITY_DN6472_c0_g1_i5_orf1	unnamed protein product [Chilo suppressalis]	-1.9294	0.66993	0.25128	0.14542	0.86276
TRINITY_DN28741_c0_g1_i3_orf1	uncharacterized protein LOC114351652 [Ostrinia furnacalis]	-1.1431	0.43471	1.72204	-0.3946	-0.619
TRINITY_DN3861_c0_g3_i2_orf1	glutamyl aminopeptidase-like isoform X4 [Ostrinia furnacalis]	-1.9447	0.47841	0.20498	0.35509	0.90621
TRINITY_DN1108_c1_g2_i1_orfp1	TRINITY_DN1108_c1_g2_i1_m.5565 TRINITY_DN1108_c1_g2::TRINITY_DN1108_c1_g2_i1::g.5565 ORF type:internal len:205 (-),score=147.90 TRINITY_DN1108_c1_g2_i1:2-613(-)	-1.5422	-0.4736	1.50535	0.23202	0.27838
TRINITY_DN106479_c1_g1_i1_orf1	secretory phospholipase A2 receptor-like [Ostrinia furnacalis]	-1.9021	0.52408	0.8137	0.66033	-0.096
TRINITY_DN6325_c0_g1_i9_orf1	fructose-bisphosphate aldolase isoform X2 [Pieris brassicae]	-1.7208	-0.4165	0.27668	1.10042	0.76021
TRINITY_DN574_c0_g1_i4_orf1	CD63 antigen-like [Ostrinia furnacalis]	-1.8899	-0.134	0.78783	0.50506	0.73103

TRINITY_DN3175_c0_g1_i7_orf1	unnamed protein product, partial [Brenthis ino]	-1.86	-0.2328	0.60996	0.65608	0.82678
TRINITY_DN1209_c0_g1_i9_orf1	NADP-dependent malic enzyme-like isoform X1 [Ostrinia furnacalis] >XP_028161889.1 NADP-dependent malic enzyme-like isoform X1 [Ostrinia furnacalis] >XP_028161891.1 NADP-dependent malic enzyme-like isoform X3 [Ostrinia furnacalis]	-1.5853	-0.5154	0.27823	1.40264	0.41986
TRINITY_DN11981_c0_g1_i7_orf1	luciferin 4-monooxygenase-like isoform X2 [Ostrinia furnacalis]	-1.6475	-0.5503	1.1955	0.66168	0.34057
TRINITY_DN3275_c0_g2_i3_orf1	hypothetical protein B5X24_HaOG216046 [Helicoverpa armigera]	-1.744	-0.2379	1.18595	0.09888	0.69698
TRINITY_DN27300_c0_g1_i7_orfp1	TRINITY_DN27300_c0_g1_i7_m.71141 TRINITY_DN27300_c0_g1::TRINITY_DN27300_c0_g1_i7::g.71141 ORF type:internal len:82 (-),score=6.59 TRINITY_DN27300_c0_g1_i7:3-245(-)	-1.493	-0.4954	1.51949	0.46551	0.00342
TRINITY_DN53807_c0_g2_i1_orf1	aminomethyltransferase, mitochondrial [Ostrinia furnacalis]	-1.9346	0.29265	0.17363	0.91208	0.55629
TRINITY_DN45530_c0_g1_i1_orf1	aldose 1-epimerase isoform X1 [Ostrinia furnacalis] >XP_028178513.1 aldose 1-epimerase isoform X1 [Ostrinia furnacalis] >XP_028178514.1 aldose 1-epimerase isoform X1 [Ostrinia furnacalis]	-1.893	0.28991	0.04487	1.02025	0.538
TRINITY_DN58751_c0_g1_i2_orf1	FK506-binding protein 2 isoform X1 [Vanessa tameamea] >XP_046977568.1 FK506-binding protein 2 isoform X1 [Vanessa cardui]	-1.8191	-0.0684	1.02808	0.06933	0.79011
TRINITY_DN11370_c0_g1_i6_orf1	protein MEMO1 [Hyposmocoma kahamanoa]	-1.7672	0.54338	1.23361	-0.1782	0.16836
TRINITY_DN48602_c0_g1_i6_orf1	amidophosphoribosyltransferase-like isoform X1 [Ostrinia furnacalis] >XP_028157758.1 amidophosphoribosyltransferase-like isoform X1 [Ostrinia furnacalis] >XP_028157759.1 amidophosphoribosyltransferase-like isoform X1 [Ostrinia furnacalis]	-1.6541	0.35507	1.07896	0.79904	-0.5789
TRINITY_DN2483_c0_g1_i1_orf1	UDP-glucuronosyltransferase 2B10-like [Ostrinia furnacalis]	-1.9308	0.14108	0.28512	0.89151	0.61312
TRINITY_DN970_c0_g1_i4_orf1	spermine oxidase-like isoform X2 [Ostrinia furnacalis]	-1.7478	-0.4063	0.3543	0.76778	1.03205
TRINITY_DN4080_c0_g1_i8_orf1	AMP deaminase 2 isoform X3 [Ostrinia furnacalis] >XP_028163647.1 AMP deaminase 2 isoform X3 [Ostrinia furnacalis] >XP_028163648.1 AMP deaminase 2 isoform X3 [Ostrinia furnacalis]	-1.5053	-0.2622	-0.3098	0.58507	1.49228
TRINITY_DN146957_c0_g1_i1_orf1	26S proteasome non-ATPase regulatory subunit 7 [Apis florea]	-1.8981	0.84811	0.61519	0.53762	-0.1029
TRINITY_DN1960_c5_g1_i3_orf1	cytochrome P450 monooxygenase CYP9G18 [Cnaphalocrocis medinalis]	-1.6543	-0.5487	0.2563	1.00303	0.94367
TRINITY_DN3712_c0_g1_i1_orf1	ribonuclease Oy [Ostrinia furnacalis]	-1.7426	0.2393	1.32457	-0.1711	0.34981
TRINITY_DN49530_c0_g1_i1_orf1	ommochrome-binding protein-like [Ostrinia furnacalis]	-1.7712	-0.3339	0.27703	0.95688	0.87122
TRINITY_DN2076_c0_g2_i1_orf1	macrophage mannose receptor 1-like isoform X1 [Maniola jurtina]	-1.7675	-0.1973	0.14729	1.19498	0.62246
TRINITY_DN12969_c0_g1_i3_orf1	queuosine salvage protein [Ostrinia furnacalis] >XP_028167327.1 queuosine salvage protein [Ostrinia furnacalis]	-1.3324	-0.1158	1.66185	-0.5687	0.35507
TRINITY_DN1149_c0_g1_i4_orf1	circadian clock-controlled protein-like [Ostrinia furnacalis]	-1.4557	-0.8255	1.26362	0.71512	0.30242
TRINITY_DN2170_c0_g2_i1_orf1	beta-1,3-glucan-binding protein-like [Ostrinia furnacalis]	-1.7132	0.10304	-0.0115	1.41879	0.20292
TRINITY_DN4497_c2_g1_i3_orf1	uncharacterized protein LOC114353086 [Ostrinia furnacalis]	-1.7108	-0.4487	0.30063	0.76547	1.09341
TRINITY_DN3433_c0_g1_i15_orf1	cytosolic purine 5'-nucleotidase isoform X3 [Ostrinia furnacalis] >XP_028162965.1 cytosolic purine 5'-nucleotidase isoform X3 [Ostrinia furnacalis] >XP_028162966.1 cytosolic purine 5'-nucleotidase isoform X3 [Ostrinia furnacalis]	-1.6979	-0.3968	0.68193	0.208	1.20477
TRINITY_DN4062_c0_g2_i1_orf1	venom peptide BmKAPI-like isoform X2 [Ostrinia furnacalis]	-1.7884	-0.1512	1.21185	0.21333	0.51443
TRINITY_DN54205_c0_g1_i1_orf1	aldo-keto reductase AKR2E4-like [Ostrinia furnacalis]	-1.7663	-0.3585	0.3096	0.84315	0.97204
TRINITY_DN3029_c1_g2_i1_orf1	unnamed protein product [Plutella xylostella]	-1.567	-0.3919	1.49097	0.40499	0.06294
TRINITY_DN512_c0_g1_i10_orf1	uncharacterized protein LOC114366781 [Ostrinia furnacalis]	-1.7801	-0.1818	0.11295	0.72914	1.11975
TRINITY_DN2255_c0_g1_i1_orf1	glutathione S-transferase sigma 3 [Ostrinia furnacalis]	-1.8726	-0.1037	0.32769	0.73486	0.91381
TRINITY_DN9492_c1_g1_i1_orf1	aldo-keto reductase AKR2E4-like [Galleria mellonella]	-1.7529	-0.301	0.1917	0.74891	1.11323
TRINITY_DN350_c0_g1_i5_orf1	tau-like protein isoform X6 [Bombyx mori]	-1.623	-0.6322	1.16233	0.64153	0.45133
TRINITY_DN2516_c0_g2_i10_orf1	unnamed protein product, partial [Chilo suppressalis]	-1.9153	0.11364	0.97887	0.30643	0.51636
TRINITY_DN2338_c0_g1_i5_orf1	prophenoloxidase PPO1b [Ostrinia furnacalis]	-1.8583	-0.0953	0.572	1.04866	0.33295
TRINITY_DN582_c0_g1_i5_orf1	hypothetical protein evm_007348 [Chilo suppressalis]	-1.9022	0.06926	0.39047	0.41779	1.02466
TRINITY_DN1772_c1_g2_i1_orf1	aldose reductase-like isoform X2 [Ostrinia furnacalis]	-1.483	-0.8172	0.2967	0.86385	1.13962
TRINITY_DN82944_c0_g1_i4_orf1	senecionine N-oxygenase isoform X2 [Ostrinia furnacalis]	-1.8006	-0.3343	0.42139	0.84468	0.86884
TRINITY_DN1080_c0_g1_i1_orf1	bleomycin hydrolase [Ostrinia furnacalis]	-1.9083	0.58866	0.95142	0.32371	0.04449
TRINITY_DN230_c1_g1_i3_orf1	protein artichoke-like [Ostrinia furnacalis]	-1.6215	0.1444	1.53238	-0.0393	-0.016
TRINITY_DN1012_c0_g2_i1_orf1	teneurin-a isoform X1 [Ostrinia furnacalis]	-1.46	-0.4772	-0.1496	0.57399	1.51285
TRINITY_DN4255_c0_g1_i10_orf1	LOW QUALITY PROTEIN: lebocin-4-like [Ostrinia furnacalis]	-1.8737	-0.0803	0.87674	0.27978	0.79748
TRINITY_DN10933_c0_g2_i1_orf1	uncharacterized protein LOC114357588 [Ostrinia furnacalis]	-1.8998	0.40842	0.63101	0.90748	-0.0471
TRINITY_DN2745_c0_g1_i4_orf1	Tubulin alpha-1 chain [Harpegnathos saltator]	-1.8971	0.09405	1.00888	0.5687	0.2255
TRINITY_DN18230_c1_g1_i1_orf1	glycine dehydrogenase (decarboxylating), mitochondrial isoform X1 [Ostrinia furnacalis] >XP_028174269.1 glycine dehydrogenase (decarboxylating), mitochondrial isoform X3 [Ostrinia furnacalis]	-1.95	0.31575	0.91274	0.31428	0.40725
TRINITY_DN103511_c0_g1_i4_orf1	probable salivary secreted peptide [Ostrinia furnacalis]	-1.6784	-0.2403	-0.0788	1.24802	0.74939
TRINITY_DN827_c1_g1_i1_orf1	peptidoglycan recognition protein 4-like isoform X1 [Ostrinia furnacalis]	-1.669	0.22671	1.42598	-0.2462	0.26254
TRINITY_DN22875_c0_g1_i6_orf1	microtubule-actin cross-linking factor 1 isoform X15 [Ostrinia furnacalis]	-1.6324	-0.5848	1.06815	0.2643	0.88467
TRINITY_DN13500_c0_g1_i1_orf1	phosphatidylethanolamine-binding protein homolog F40A3.3-like [Ostrinia furnacalis] >XP_028160752.1 phosphatidylethanolamine-binding protein homolog F40A3.3-like [Ostrinia furnacalis]	-1.6949	-0.2089	-0.0004	1.32013	0.58401
TRINITY_DN812_c2_g1_i1_orf1	1,4-alpha-glucan-branching enzyme [Ostrinia furnacalis]	-1.7055	-0.5764	0.86719	0.76284	0.6519
TRINITY_DN22962_c0_g1_i1_orf1	lysosomal acid glucosylceramidase-like isoform X2 [Ostrinia furnacalis]	-1.5113	-0.0197	1.62574	-0.232	0.13723
TRINITY_DN11492_c0_g1_i8_orf1	unnamed protein product [Chilo suppressalis]	-1.969	0.46008	0.42478	0.27447	0.80967
TRINITY_DN16147_c0_g1_i4_orf1	aldo-keto reductase AKR2E4-like isoform X1 [Ostrinia furnacalis]	-1.7597	-0.0005	1.05503	0.87301	-0.1678
TRINITY_DN95850_c0_g4_i3_orf1	calreticulin [Cotesia glomerata] >KAH0559118.1 hypothetical protein KQX54_000875 [Cotesia glomerata]	-1.9493	0.08151	0.76388	0.56886	0.53509
TRINITY_DN22175_c0_g1_i1_orf1	low-density lipoprotein receptor-related protein 1B-like [Ostrinia furnacalis]	-1.9431	0.42982	0.47753	0.14675	0.88896

TRINITY_DN8644_c0_g1_i3_orf1	SEC14-like protein 2 [Ostrinia furnacalis]	-1.3119	0.40408	1.65491	-0.5948	-0.1523
TRINITY_DN2947_c0_g1_i4_orf1	ras-related protein Rab-39B [Spodoptera litura] >XP_035450569.1 ras-related protein Rab-39B-like [Spodoptera frugiperda] >KAF9422714.1 hypothetical protein HW555_001708 [Spodoptera exigua] >CAB3507117.1 unnamed protein product [Spodoptera littoralis] >KAF9824742.1 hypothetical protein SFRURICE_016851 [Spodoptera frugiperda] >KAG8101614.1 hypothetical protein SFRUCORN_012196 [Spodoptera frugiperda] >KAH9636250.1 hypothetical protein HF086_009446 [Spodoptera exigua]	-1.9113	0.04707	0.33377	0.5886	0.94182
TRINITY_DN7960_c0_g1_i2_orf1	uncharacterized protein LOC114364878 [Ostrinia furnacalis]	-1.5541	-0.7169	0.3175	1.15288	0.80058
TRINITY_DN15382_c0_g1_i3_orf1	putative aldehyde dehydrogenase family 7 member A1 homolog [Spodoptera litura]	-1.7967	0.52332	0.84206	0.80551	-0.3741
TRINITY_DN10195_c0_g1_i8_orf1	charged multivesicular body protein 6-A [Ostrinia furnacalis]	-1.9105	0.30133	0.29266	1.04995	0.26658
TRINITY_DN13515_c0_g1_i1_orf1	low-density lipoprotein receptor-related protein 4-like isoform X2 [Ostrinia furnacalis]	-1.9479	0.38831	0.17016	0.86904	0.52037
TRINITY_DN1216_c0_g1_i4_orf1	bifunctional purine biosynthesis protein PURH isoform X1 [Ostrinia furnacalis] >XP_028176123.1 bifunctional purine biosynthesis protein PURH isoform X2 [Ostrinia furnacalis] >XP_028176129.1 bifunctional purine biosynthesis protein PURH isoform X3 [Ostrinia furnacalis]	-1.8287	-0.1232	0.20127	0.69093	1.05966
TRINITY_DN24310_c0_g1_i2_orf1	glucose-6-phosphate 1-dehydrogenase [Ostrinia furnacalis]	-1.8851	0.85448	0.5354	0.63937	-0.1441
TRINITY_DN3831_c0_g1_i7_orf1	beta-ureidopropionase-like [Ostrinia furnacalis]	-1.8673	-0.1401	0.36617	0.74093	0.90032
TRINITY_DN3257_c0_g1_i4_orf1	N-acetylneuraminate lyase-like [Ostrinia furnacalis]	-1.8469	-0.1478	0.30422	0.69376	0.99669
TRINITY_DN2876_c0_g1_i1_orf1	long-chain fatty acid transport protein 4-like [Ostrinia furnacalis]	-1.8413	0.0447	1.0026	0.01803	0.77596
TRINITY_DN14185_c0_g1_i1_orf1	uncharacterized protein LOC114358675 [Ostrinia furnacalis] >XP_028168498.1 uncharacterized protein LOC114358675 [Ostrinia furnacalis]	-1.4804	-0.8394	1.02381	0.97643	0.31961
TRINITY_DN18374_c0_g1_i1_orf1	L-2-hydroxyglutarate dehydrogenase, mitochondrial [Ostrinia furnacalis]	-1.9134	0.03307	0.43095	0.95046	0.49887
TRINITY_DN1786_c0_g1_i11_orf1	ATP-binding cassette sub-family A member 1-like [Ostrinia furnacalis]	-1.9584	0.28244	0.41235	0.87332	0.39025
TRINITY_DN4621_c0_g1_i4_orf1	uncharacterized protein LOC114358242 isoform X3 [Ostrinia furnacalis]	-1.6641	-0.6413	0.59062	0.86621	0.84863
TRINITY_DN17326_c0_g1_i8_orf1	aminoacylase-1-like [Ostrinia furnacalis]	-1.7512	-0.4692	0.63097	0.63366	0.95575
TRINITY_DN1206_c0_g1_i6_orf1	sorbitol dehydrogenase-like [Spodoptera frugiperda] >KAG8104768.1 hypothetical protein SFRUCORN_013827 [Spodoptera frugiperda]	-1.6765	-0.6284	0.72804	0.89144	0.68542
TRINITY_DN21715_c0_g1_i1_orf1	protein disulfide-isomerase A3 isoform X1 [Ostrinia furnacalis] >XP_028169825.1 protein disulfide-isomerase A3 isoform X2 [Ostrinia furnacalis] >XP_028169826.1 protein disulfide-isomerase A3 isoform X3 [Ostrinia furnacalis]	-1.8729	0.11684	1.03031	0.08544	0.64026
TRINITY_DN443_c0_g1_i2_orf1	proteasome subunit beta type-4 [Ostrinia furnacalis]	-1.9388	0.35468	0.68169	0.80008	0.10233
TRINITY_DN21435_c0_g1_i2_orf1	glycogen-binding subunit 76A isoform X1 [Ostrinia furnacalis]	-1.8107	-0.301	1.01405	0.53912	0.55849
TRINITY_DN5914_c1_g1_i9_orf1	unnamed protein product [Chilo suppressalis]	-1.756	-0.3929	0.74252	0.37012	1.03624
TRINITY_DN938_c0_g1_i7_orf1	protein ultraspiracle homolog isoform X2 [Ostrinia furnacalis]	-1.9099	0.09907	0.93667	0.23349	0.64069
TRINITY_DN6309_c0_g1_i7_orf1	apolipoprotein D-like isoform X1 [Ostrinia furnacalis]	-1.9601	0.13224	0.74048	0.51915	0.56819
TRINITY_DN22242_c0_g1_i1_orf1	juvenile hormone epoxide hydrolase-like [Ostrinia furnacalis] >XP_028170526.1 juvenile hormone epoxide hydrolase-like [Ostrinia furnacalis]	-1.7236	0.0282	0.55505	-0.1612	1.30161
TRINITY_DN6698_c0_g2_i2_orf1	protein mesh isoform X1 [Ostrinia furnacalis]	-1.862	0.14421	0.89972	-0.0199	0.83795
TRINITY_DN19377_c0_g1_i4_orf1	uncharacterized protein LOC114353316 [Ostrinia furnacalis] >XP_028161061.1 uncharacterized protein LOC114353316 [Ostrinia furnacalis]	-1.8385	-0.0166	0.06405	0.82032	0.97081
TRINITY_DN3766_c0_g1_i10_orf1	circadian clock-controlled protein-like [Ostrinia furnacalis]	-1.8072	-0.0108	1.14064	0.01967	0.65766
TRINITY_DN8473_c0_g1_i5_orf1	serine/threonine-protein phosphatase 6 regulatory subunit 1 [Ostrinia furnacalis]	-1.8096	0.40969	0.21415	1.22867	-0.0429
TRINITY_DN1497_c0_g2_i6_orf1	unnamed protein product [Chilo suppressalis]	-1.7882	0.45609	1.22265	0.27106	-0.1616
TRINITY_DN21533_c0_g1_i6_orf1	annexin B9 isoform X2 [Ostrinia furnacalis]	-1.8437	0.82198	0.87886	0.33833	-0.1955
TRINITY_DN1756_c0_g1_i3_orf1	UTP--glucose-1-phosphate uridylyltransferase isoform X2 [Ostrinia furnacalis]	-1.8858	0.08061	0.43918	0.28858	1.07747
TRINITY_DN5433_c0_g1_i5_orf1	uncharacterized protein LOC114351067 [Ostrinia furnacalis]	-1.7543	0.39484	0.88859	0.89346	-0.4226
TRINITY_DN2624_c0_g1_i6_orf1	unnamed protein product [Danaus chrysippus]	-1.8255	-0.209	0.95347	0.28514	0.79589
TRINITY_DN33488_c0_g1_i2_orf1	semaphorin-1A isoform X3 [Trichoplusia ni]	-1.7632	-0.4462	0.93194	0.7001	0.57734
TRINITY_DN41848_c0_g1_i4_orf1	vacuolar protein sorting-associated protein 28 homolog [Ostrinia furnacalis]	-1.8404	-0.1358	0.39312	1.09709	0.486
TRINITY_DN2710_c0_g1_i4_orf1	translin-associated protein X [Ostrinia furnacalis]	-1.7497	-0.2598	0.07841	0.97357	0.95756
TRINITY_DN1103_c0_g1_i12_orf1	retinal dehydrogenase 1-like [Ostrinia furnacalis]	-1.6876	0.18779	-0.3911	0.64864	1.24223
TRINITY_DN7740_c0_g1_i2_orf1	D-arabinitol dehydrogenase 1 [Eumeta japonica]	-1.5609	-0.768	0.773	0.48984	1.06603
TRINITY_DN20133_c0_g1_i1_orf1	fructose-bisphosphate aldolase A isoform X2 [Microcebus murinus] >XP_012619765.1 fructose-bisphosphate aldolase A isoform X2 [Microcebus murinus] >XP_012619766.1 fructose-bisphosphate aldolase A isoform X2 [Microcebus murinus] >XP_012619767.1 fructose-bisphosphate aldolase A isoform X2 [Microcebus murinus] >XP_012619768.1 fructose-bisphosphate aldolase A isoform X2 [Microcebus murinus]	-1.5296	-0.5911	0.39937	1.43952	0.28176
TRINITY_DN3300_c0_g2_i1_orf1	annexin B10 isoform X9 [Ostrinia furnacalis] >XP_028177766.1 annexin B10 isoform X10 [Ostrinia furnacalis]	-1.6995	0.46646	1.34882	-0.2423	0.12651
TRINITY_DN472_c1_g1_i3_orf1	IST1 homolog isoform X1 [Ostrinia furnacalis]	-1.8696	0.1698	1.04449	0.03584	0.61943
TRINITY_DN8440_c0_g1_i9_orf1	protein FAM177A1-like [Ostrinia furnacalis]	-1.8731	-0.054	0.40963	0.46777	1.04973
TRINITY_DN9340_c0_g1_i4_orf1	sarcosine dehydrogenase, mitochondrial [Ostrinia furnacalis]	-1.695	-0.457	1.1941	0.60837	0.3495
TRINITY_DN8747_c0_g1_i2_orf1	lipopolysaccharide-induced tumor necrosis factor-alpha factor homolog [Ostrinia furnacalis]	-1.9052	-0.0953	0.79174	0.64915	0.55953
TRINITY_DN13322_c0_g1_i6_orf1	macrophage mannose receptor 1-like [Ostrinia furnacalis]	-1.7368	-0.465	0.41688	0.8753	0.90962
TRINITY_DN10994_c0_g1_i4_orf1	trypsin inhibitor-like [Ostrinia furnacalis]	-1.5925	-0.7438	0.54119	0.95513	0.84
TRINITY_DN38685_c0_g1_i4_orf1	unnamed protein product [Euphydryas editha]	-1.8815	0.00402	0.95199	0.21261	0.71292
TRINITY_DN12003_c0_g2_i1_orf1	lactoylglutathione lyase [Ostrinia furnacalis]	-1.9034	0.10543	1.02799	0.46552	0.30441
TRINITY_DN10900_c0_g1_i7_orf1	fatty acid synthase-like [Ostrinia furnacalis]	-1.4691	0.54662	1.53432	-0.3344	-0.2774
TRINITY_DN4502_c0_g1_i3_orf1	kynurenine formamidase isoform X1 [Ostrinia furnacalis]	-1.7103	-0.5348	0.92838	0.48493	0.8318
TRINITY_DN2475_c0_g1_i1_orf1	LOW QUALITY PROTEIN: uncharacterized protein LOC114363766 [Ostrinia furnacalis]	-1.7764	0.16517	0.03879	1.32435	0.24812
TRINITY_DN2457_c0_g1_i8_orf1	uncharacterized protein LOC114355596 [Ostrinia furnacalis]	-1.3516	-0.8865	1.4347	0.34564	0.45773
TRINITY_DN6415_c0_g2_i1_orf1	D-arabinitol dehydrogenase 1-like [Ostrinia furnacalis]	-1.3917	-0.9229	0.40741	0.61675	1.29042

TRINITY_DN125565_c1_g1_i1_orf1	uncharacterized protein LOC114352340 isoform X2 [Ostrinia furnacalis]	-1.6566	-0.2409	0.09486	1.43235	0.37028
TRINITY_DN5748_c0_g1_i6_orf1	glycine N-methyltransferase isoform X1 [Ostrinia furnacalis] >XP_028165118.1 glycine N-methyltransferase isoform X2 [Ostrinia furnacalis] >XP_028165119.1 glycine N-methyltransferase isoform X1 [Ostrinia furnacalis] >XP_028165120.1 glycine N-methyltransferase isoform X2 [Ostrinia furnacalis]	-1.4254	-0.822	1.38264	0.37303	0.4917
TRINITY_DN1024_c0_g4_i1_orf1	superoxide dismutase [Cu-Zn]-like [Ostrinia furnacalis]	-1.5727	-0.7855	0.97735	0.67041	0.71049
TRINITY_DN4394_c0_g1_i4_orf1	B-cell receptor-associated protein 31 [Ostrinia furnacalis]	-1.8199	-0.2519	1.0406	0.58613	0.44509
TRINITY_DN11948_c0_g1_i8_orf1	cystathionine gamma-lyase [Ostrinia furnacalis]	-1.5905	-0.7467	0.84555	0.95209	0.53962
TRINITY_DN4954_c0_g1_i5_orf1	glycogen debranching enzyme [Trichoplusia ni]	-1.8089	0.52283	0.99783	0.60086	-0.3126
TRINITY_DN448_c0_g1_i20_orf1	probable cytochrome P450 9f2 isoform X1 [Ostrinia furnacalis]	-1.5752	-0.6521	0.40392	0.54576	1.27766
TRINITY_DN17031_c0_g1_i1_orf1	arginase, hepatic [Ostrinia furnacalis]	-1.8131	-0.237	0.29977	0.74344	1.00691
TRINITY_DN38506_c0_g1_i4_orf1	C-1-tetrahydrofolate synthase, cytoplasmic isoform X1 [Ostrinia furnacalis] >XP_028166137.1 C-1-tetrahydrofolate synthase, cytoplasmic isoform X2 [Ostrinia furnacalis] >XP_028166140.1 C-1-tetrahydrofolate synthase, cytoplasmic isoform X4 [Ostrinia furnacalis]	-1.4898	-0.918	0.82381	0.84048	0.74348
TRINITY_DN8922_c0_g1_i3_orf1	protein sidekick isoform X1 [Ostrinia furnacalis] >XP_028161979.1 protein sidekick isoform X1 [Ostrinia furnacalis] >XP_028161980.1 protein sidekick isoform X1 [Ostrinia furnacalis] >XP_028161981.1 protein sidekick isoform X1 [Ostrinia furnacalis]	-1.7033	0.28032	0.09322	1.41568	-0.0859
TRINITY_DN3015_c0_g1_i7_orf1	glycine-rich protein DOT1-like [Ostrinia furnacalis]	-1.8626	-0.2158	0.85856	0.57096	0.64887
TRINITY_DN5813_c0_g1_i9_orf1	alpha-(1,6)-fucosyltransferase [Ostrinia furnacalis]	-1.8655	-0.0008	1.10813	0.4274	0.33075
TRINITY_DN452_c0_g1_i4_orf1	protein CREG1 [Ostrinia furnacalis] >XP_028170592.1 protein CREG1 [Ostrinia furnacalis]	-1.3344	-0.3948	1.40992	-0.556	0.8754
TRINITY_DN12686_c0_g1_i4_orf1	maltase A1-like [Ostrinia furnacalis]	-1.5748	0.11062	1.25123	0.78454	-0.5716
TRINITY_DN1540_c0_g1_i14_orf1	alaserpin-like isoform X1 [Ostrinia furnacalis]	-1.5962	-0.5189	0.06926	0.81676	1.22916
TRINITY_DN8685_c0_g1_i5_orf1	macrophage mannose receptor 1-like [Zerene cesonia]	-1.433	-0.7835	1.3785	0.62066	0.21731
TRINITY_DN14611_c0_g1_i5_orf1	hsc70-interacting protein-like [Galleria mellonella]	-1.722	-0.3573	1.23601	0.31274	0.53055
TRINITY_DN5497_c0_g1_i6_orf1	1,2-dihydroxy-3-keto-5-methylthiopentene dioxygenase-like [Ostrinia furnacalis]	-1.7133	-0.0562	-0.082	0.5132	1.33839
TRINITY_DN14670_c0_g1_i1_orf1	heat shock protein beta-1 isoform X1 [Helicoverpa armigera] >XP_022829066.1 heat shock protein beta-1 isoform X1 [Spodoptera litura] >XP_026747148.1 heat shock protein beta-1 isoform X3 [Trichoplusia ni] >XP_026748187.1 heat shock protein beta-1 isoform X2 [Galleria mellonella] >XP_028167756.1 heat shock protein beta-1 isoform X2 [Ostrinia furnacalis] >XP_035431734.1 heat shock protein beta-1-like isoform X3 [Spodoptera frugiperda] >XP_047023072.1 heat shock protein beta-1 isoform X1 [Helicoverpa zea] >XP_049865086.1 heat shock protein beta-1 [Pectinophora gossypiella] >KAH9640995.1 hypothetical protein HF086_015091 [Spodoptera exigua] >QGZ00460.1 heat shock protein 21.4 [Glyphodes pyloalis] >QKR72095.1 heat-shock protein 21.4 [Mythimna separata] >CAB3228281.1 unnamed protein product [Arctia plantaginis] >CAH0628881.1 unnamed protein product [Chrysodeixis includens]	-1.4788	-0.8018	1.2992	0.46987	0.51152
TRINITY_DN2706_c0_g1_i3_orf1	hypothetical protein evm_004793 [Chilo suppressalis]	-1.7364	-0.4081	1.15056	0.47707	0.51685
TRINITY_DN3975_c0_g1_i7_orf1	low-density lipoprotein receptor domain class A domain-containing protein [Phthorimaea operculella]	-1.3506	-0.0666	1.73351	0.08293	-0.3992
TRINITY_DN400_c0_g1_i1_orf1	serine/threonine-protein phosphatase 2A activator-like isoform X2 [Ostrinia furnacalis] >XP_028164470.1 serine/threonine-protein phosphatase 2A activator-like isoform X2 [Ostrinia furnacalis]	-1.8954	-0.0481	0.35409	0.8857	0.70373
TRINITY_DN80660_c0_g1_i1_orf1	probable phospholipid hydroperoxide glutathione peroxidase isoform X1 [Pieris rapae]	-1.5991	-0.3136	0.56574	1.42089	-0.0739
TRINITY_DN4069_c0_g1_i5_orf1	putative sulfiredoxin [Ostrinia furnacalis]	-1.5381	-0.6059	1.03337	0.01551	1.09507
TRINITY_DN11680_c0_g1_i1_orf1	uncharacterized protein LOC114355414 [Ostrinia furnacalis]	-1.7466	-0.2643	0.31861	0.43028	1.26205
TRINITY_DN3758_c0_g1_i2_orf1	S-formylglutathione hydrolase isoform X1 [Spodoptera litura]	-1.8479	-0.1422	0.51393	1.06309	0.41314
TRINITY_DN15858_c0_g1_i2_orf1	15-hydroxyprostaglandin dehydrogenase [NAD(+)]-like [Ostrinia furnacalis]	-1.5542	-0.7493	1.12761	0.7616	0.41423
TRINITY_DN7992_c0_g1_i4_orf1	uncharacterized protein LOC114362579 [Ostrinia furnacalis]	-1.8937	0.26038	0.96262	0.02327	0.64738
TRINITY_DN28875_c0_g1_i1_orf1	PREDICTED: ras-related protein Rab-2 [Fopius arisanus] >XP_015110133.1 ras-related protein Rab-2 [Diachasma alloeum]	-1.9456	0.34889	0.69303	0.13206	0.77158
TRINITY_DN11383_c0_g2_i4_orf1	aminoacylase-1A-like [Ostrinia furnacalis]	-1.6337	-0.5964	0.29201	0.89144	1.04664
TRINITY_DN48846_c0_g1_i1_orf1	tudor domain-containing protein 7 isoform X3 [Ostrinia furnacalis]	-1.9199	-0.0363	0.80926	0.57934	0.56762
TRINITY_DN14874_c0_g1_i6_orf1	uncharacterized protein LOC114358148 [Ostrinia furnacalis]	-1.2011	-0.2537	1.69405	0.42561	-0.6648
TRINITY_DN21609_c0_g1_i1_orf1	translation initiation factor eIF-2B subunit epsilon [Ostrinia furnacalis]	-1.9123	0.2795	0.34636	0.24456	1.04182
TRINITY_DN3439_c0_g2_i2_orf1	histone H2A.Z-specific chaperone CHZ1-like [Ostrinia furnacalis]	-1.4623	-0.8689	0.88475	0.35051	1.09594
TRINITY_DN71917_c0_g3_i1_orf1	leucine-rich repeat and immunoglobulin-like domain-containing nogo receptor-interacting protein 1 [Ostrinia furnacalis]	-1.5521	-0.4929	1.06974	-0.1158	1.09103
TRINITY_DN6771_c0_g2_i1_orf1	putative endoplasmin, partial [Cotesia chilonis]	-1.7979	0.41903	-0.2229	0.44067	1.16109
TRINITY_DN1926_c0_g1_i5_orf1	coiled-coil and C2 domain-containing protein 1-like [Ostrinia furnacalis]	-1.7294	-0.3788	1.07738	0.22063	0.81018
TRINITY_DN726_c0_g1_i2_orf1	vinculin [Ostrinia furnacalis]	-1.7885	0.02289	1.18181	0.63371	-0.0499
TRINITY_DN3196_c0_g1_i1_orf1	organic cation transporter-like protein [Ostrinia furnacalis]	-1.6991	-0.4167	0.28531	0.61271	1.21769
TRINITY_DN73_c0_g1_i6_orf1	autophagy-related protein 16-1 isoform X1 [Vanessa cardui]	-1.8931	-0.0956	0.43588	0.70032	0.85245
TRINITY_DN9562_c0_g1_i3_orf1	cullin-3 isoform X1 [Ostrinia furnacalis] >XP_028159222.1 cullin-3 isoform X1 [Ostrinia furnacalis] >XP_028159223.1 cullin-3 isoform X2 [Ostrinia furnacalis] >XP_028159224.1 cullin-3 isoform X3 [Ostrinia furnacalis] >XP_028159225.1 cullin-3 isoform X1 [Ostrinia furnacalis]	-1.72	-0.1962	0.3245	1.3575	0.2342
TRINITY_DN9316_c0_g3_i1_orf1	cytochrome P450 6k1-like isoform X1 [Ostrinia furnacalis] >XP_028171821.1 cytochrome P450 6k1-like isoform X1 [Ostrinia furnacalis] >QPF77617.1 cytochrome P450 monooxygenase CYP324A34 [Ostrinia furnacalis]	-1.5648	0.31574	1.45986	-0.4916	0.2808
TRINITY_DN582_c0_g1_i2_orf1	unnamed protein product [Chilo suppressalis]	-1.5553	0.77535	0.99089	0.59304	-0.804
TRINITY_DN117042_c0_g1_i2_orf1	UBX domain-containing protein 6 [Ostrinia furnacalis] >XP_028162119.1 UBX domain-containing protein 6 [Ostrinia furnacalis]	-1.8352	0.24593	-0.1571	0.7688	0.9776
TRINITY_DN2182_c0_g1_i4_orf1	growth-blocking peptide, long form-like isoform X1 [Ostrinia furnacalis] >XP_028159332.1 growth-blocking peptide, long form-like isoform X1 [Ostrinia furnacalis] >QWX20072.1 growth-blocking peptide [Ostrinia furnacalis]	-1.7109	-0.4384	1.02537	0.87627	0.24765
TRINITY_DN2618_c0_g1_i3_orf1	CDP-diacylglycerol--inositol 3-phosphatidyltransferase [Ostrinia furnacalis]	-1.7362	-0.262	0.07561	0.78345	1.13909

TRINITY_DN2392_c0_g2_i1_orf1	cytochrome P450 9e2-like [Ostrinia furnacalis] >QPF77612.1 cytochrome P450 monooxygenase CYP9A185 [Ostrinia furnacalis]	-1.4717	-0.4968	-0.1314	0.62247	1.47738
TRINITY_DN28299_c0_g1_i1_orf1	adenylosuccinate lyase isoform X1 [Ostrinia furnacalis]	-1.5833	-0.6274	0.20884	1.1626	0.83925
TRINITY_DN335_c1_g1_i5_orf1	PREDICTED: perilipin-4 isoform X14 [Papilio polytes]	-1.3939	-0.9118	1.15817	0.23751	0.90995
TRINITY_DN49956_c0_g1_i1_orf1	UPF0585 protein CG18661 [Ostrinia furnacalis]	-1.8965	0.02115	0.37729	1.0123	0.48572
TRINITY_DN33705_c0_g1_i1_orf1	atlastin-like isoform X4 [Ostrinia furnacalis]	-1.709	-0.3078	0.27246	0.43112	1.3132
TRINITY_DN6483_c0_g1_i6_orf1	transketolase-like protein 2 isoform X1 [Ostrinia furnacalis] >XP_028164795.1 transketolase-like protein 2 isoform X2 [Ostrinia furnacalis]	-1.7343	-0.4891	0.75333	0.50624	0.96388
TRINITY_DN452_c9_g1_i1_orf1	epidermal retinol dehydrogenase 2-like [Ostrinia furnacalis]	-1.5389	-0.5888	1.35867	0.11673	0.6523
TRINITY_DN1063_c0_g1_i16_orf1	hypothetical protein evm_000854 [Chilo suppressalis]	-1.7788	0.29935	-0.1637	0.39178	1.25138
TRINITY_DN8986_c0_g1_i1_orf1	HBS1-like protein [Ostrinia furnacalis]	-1.8889	0.57894	0.98274	0.36034	-0.0331
TRINITY_DN3431_c0_g1_i1_orf1	3-hydroxyacyl-CoA dehydrogenase type-2-like [Ostrinia furnacalis]	-1.7076	0.16527	-0.0405	1.42487	0.15791
TRINITY_DN33867_c0_g1_i8_orf1	uncharacterized protein LOC114357513 [Ostrinia furnacalis]	-1.3673	0.55393	0.73106	1.11022	-1.0279
TRINITY_DN62091_c0_g1_i1_orf1	protein NipSnap [Venturia canescens]	-1.6918	-0.5007	0.34593	0.74638	1.10019
TRINITY_DN1091_c0_g3_i1_orf1	macrophage mannose receptor 1-like [Ostrinia furnacalis]	-1.2184	-0.6768	-0.4613	0.99328	1.36315
TRINITY_DN315_c0_g1_i1_orf1	[illegible] tropicalis] >NP_001108328.1 tubulin alpha-1B chain [Bos taurus] >NP_001182321.1 tubulin alpha-1B chain [Macaca mulatta] >NP_001182735.1 tubulin alpha-1B chain [Oryctolagus cuniculus] >NP_001230908.1 tubulin alpha-1B chain [Cricetulus griseus] >NP_006073.2 tubulin alpha-1B chain [Homo sapiens] >NP_035784.1 tubulin alpha-1B chain [Mus musculus] >XP_001509265.1 tubulin alpha-1B chain [Ornithorhynchus anatinus] >XP_002823230.1 tubulin alpha-1B chain [Pongo abelii] >XP_003476108.2 tubulin alpha-1B chain [Cavia porcellus] >XP_003988693.3 tubulin alpha-1B chain [Felis catus] >XP_004053124.1 tubulin alpha-1B chain [Gorilla gorilla gorilla] >XP_004447012.1 tubulin alpha-1B chain [Dasypus novemcinctus] >XP_004692837.1 PREDICTED: tubulin alpha-1B chain [Condylura cristata] >XP_005680059.1 PREDICTED: tubulin alpha-1B chain [Capra hircus] >XP_006091578.1 tubulin alpha-1B chain [Myotis lucifugus] >XP_006902536.1 PREDICTED: tubulin alpha-1B chain [Elephantulus edwardii] >XP_007073036.2 tubulin alpha-1B chain [Panthera tigris] >XP_007506293.1 PREDICTED: tubulin alpha-1B chain [Monodelphis domestica] >XP_007528574.2 PREDICTED: tubulin alpha-1B chain [Erinaceus europaeus] >XP_008001316.2 tubulin alpha-1B chain [Chlorocebus sabaeus] >XP_008069066.1 tubulin alpha-1B chain isoform X1 [Carlito syrichta] >XP_008702764.1 tubulin alpha-1B chain [Ursus maritimus] >XP_008949408.1 tubulin alpha-1B chain [Pan paniscus] >XP_009001972.1 tubulin alpha-1B chain [Callithrix jacchus] >XP_010348074.2 tubulin alpha-1B chain [Saimiri boliviensis boliviensis] >XP_010383711.1 tubulin alpha-1B chain [Rhinopithecus roxellana] >XP_010594685.1 tubulin alpha-1B chain [Loxodonta africana] >XP_010725238.1 tubulin alpha-1B chain [Meleagris gallopavo] >XP_010856059.1 PREDICTED: tubulin alpha-1B chain [Bison bison bison] >XP_010962544.1 tubulin alpha-1B chain isoform X1 [Camelus bactrianus] >XP_010962545.1 tubulin alpha-1B chain isoform X2 [Camelus bactrianus] >XP_011366571.1 tubulin alpha-1B chain isoform X1 [Pteropus vampyrus] >XP_011903803.1 PREDICTED: tubulin alpha-1B chain [Cercocebus atys] >XP_012811688.1 1 isoform X1 [Xenopus tropicalis] >XP_013361157.1 PREDICTED: tubulin alpha-1B chain [Chinchilla lanigera] >XP_014116068.1 PREDICTED: tubulin alpha-1B chain isoform X2 [Pseudopodoces humilis] >XP_014705495.2 tubulin alpha-1B chain [Equus asinus] >XP_014747331.1 PREDICTED: tubulin alpha-1B chain isoform X1 [Sturnus vulgaris] >XP_014747332.1 PREDICTED: tubulin alpha-1B chain isoform X2 [Sturnus vulgaris] >XP_014921273.2 tubulin alpha-1B chain isoform X1 [Acinonyx jubatus] >XP_015155803.1 tubulin alpha-1B chain [Gallus gallus] >XP_015261386.1 PREDICTED: tubulin alpha-1B chain [Gekko japonicus] >XP_016056723.1 PREDICTED: tubulin alpha-1B chain isoform X1 [Miniopterus natalensis] >XP_019274779.1 PREDICTED: tubulin alpha-1B chain [Panthera pardus] >XP_019790049.1 tubulin alpha-1B chain [Tursiops truncatus] >XP_019816220.1 PREDICTED: tubulin alpha-1B chain [Bos indicus] >XP_020742594.1 tubulin alpha-1B chain [Odocoileus virginianus texanus] >XP_020821797.1 tubulin alpha-1B chain [Phascolarctos cinereus] >XP_021014165.1 tubulin alpha-1B chain [Mus caroli] >XP_021490591.1 tubulin alpha-1B chain [Meriones unguiculatus] >XP_021560496.2 LOW QUALITY PROTEIN: tubulin alpha-1B chain-like [Neomonachus schauinslandi] >XP_022375058.1 tubulin alpha-1B chain [Enhydra lutris kenyoni] >XP_023066986.1 tubulin alpha-1B chain [Piliocolobus tephrosceles] >XP_023391180.1 tubulin alpha-1B chain isoform X2 [Pteropus vampyrus] >XP_023499251.1 tubulin alpha-1B chain [Equus caballus] >XP_024431644.1 tubulin alpha-1B chain [Desmodus rotundus] >XP_025333292.1 tubulin alpha-1B chain isoform X1 [Canis lupus dingo] >XP_025738590.1 tubulin alpha-1B chain [Callorhinus ursinus] >XP_025903158.1 tubulin alpha-1B chain [Nothoprocta perdicaria] >XP_026092992.1 [illegible]					
A Crystal structure of T2R-TTL-CF1 c	Tubulin alpha-1B chain [Sus scrofa] >6O2Q_A Acetylated Microtubules [Sus scrofa] >6O2Q_C Acetylated Microtubules [Sus scrofa] >6O2Q_E Acetylated Microtubules [Sus scrofa] >6O2Q_J Acetylated Microtubules [Sus scrofa] >6O2Q_K Acetylated Microtubules [Sus scrofa] >6O2Q_L Acetylated Microtubules [Sus scrofa] >6O2R_A Deacetylated Microtubules [Sus scrofa] >6O2R_C Deacetylated Microtubules [Sus scrofa] >6O2R_E Deacetylated Microtubules [Sus scrofa] >6O2R_J Deacetylated Microtubules [Sus scrofa] >6O2R_K Deacetylated Microtubules [Sus scrofa] >6O2R_L Deacetylated Microtubules [Sus scrofa] >6O2S_1A Chain 1A	Tubulin a	Tubulin a	Tubulin a	Tubulin a	Tubulin a
TRINITY_DN135188_c0_g1_i2_orf1	proteasome inhibitor PI31 subunit [Ostrinia furnacalis]	-1.8071	-0.3741	0.65161	0.76706	0.76253
TRINITY_DN1008_c0_g1_i2_orf1	integrin beta-PS [Ostrinia furnacalis]	-1.5583	-0.5908	1.13332	0.04847	0.96737
TRINITY_DN3406_c0_g1_i17_orf1	uncharacterized protein ZK1073.1-like isoform X6 [Spodoptera frugiperda]	-1.7884	-0.1685	1.04784	0.09266	0.81644
TRINITY_DN19261_c0_g1_i3_orf1	UDP-glucuronic acid decarboxylase 1 isoform X1 [Papilio machaon]	-1.7699	-0.2742	0.87164	0.17074	1.00169
TRINITY_DN5266_c0_g1_i1_orf1	malate dehydrogenase, cytoplasmic isoform X2 [Ostrinia furnacalis]	-1.6325	-0.6811	0.59319	0.71996	1.00045
TRINITY_DN36434_c0_g2_i3_orf1	clotting factor B isoform X1 [Ostrinia furnacalis]	-1.5088	-0.7175	0.36902	0.5116	1.34566
TRINITY_DN976_c0_g1_i5_orf1	filaggrin-2-like [Ostrinia furnacalis]	-1.8238	0.24514	0.32807	0.02354	1.22702
TRINITY_DN34134_c0_g2_i1_orf1	THUMP domain-containing protein 1 homolog [Ostrinia furnacalis]	-1.5266	-0.6652	0.66113	0.20916	1.32147
TRINITY_DN1047_c0_g1_i6_orf1	mitochondrial genome maintenance exonuclease 1-like [Ostrinia furnacalis]	-1.7214	-0.5376	0.83014	0.57626	0.85252
TRINITY_DN9_c0_g1_i7_orf1	heterogeneous nuclear ribonucleoprotein R isoform X1 [Ostrinia furnacalis]	-1.1797	0.01346	1.74219	-0.7391	0.16315
TRINITY_DN11798_c0_g2_i1_orf1	N-acetylglucosamine-6-sulfatase-like isoform X2 [Ostrinia furnacalis]	-1.6011	-0.445	1.08465	1.02835	-0.0669
TRINITY_DN2847_c0_g1_i20_orf1	uncharacterized protein LOC114352221 [Ostrinia furnacalis]	-1.4772	-0.4687	-0.1379	0.59043	1.49347
TRINITY_DN7630_c0_g2_i1_orf1	flotillin-2 isoform X1 [Ostrinia furnacalis] >XP_028172931.1 flotillin-2 isoform X2 [Ostrinia furnacalis]	-1.7047	-0.524	0.84554	0.41968	0.96349

TRINITY_DN2374_c0_g2_i2_orf1	uncharacterized protein LOC114357127 [Ostrinia furnacalis]	-1.9722	0.36723	0.33839	0.46128	0.80526
TRINITY_DN19521_c0_g1_i1_orf1	sodium/potassium-transporting ATPase subunit beta-1-like isoform X1 [Ostrinia furnacalis]	-1.7714	-0.0148	1.26149	0.00458	0.52014
TRINITY_DN12424_c0_g1_i2_orf1	uncharacterized protein LOC114350608 [Ostrinia furnacalis]	-1.8073	-0.1986	0.99292	0.19383	0.81908
TRINITY_DN7075_c0_g2_i1_orf1	retinal dehydrogenase 1-like [Ostrinia furnacalis]	-1.6216	-0.4074	-0.0528	0.90916	1.17263
TRINITY_DN2378_c0_g1_i5_orf1	integrin alpha-8-like isoform X1 [Ostrinia furnacalis]	-1.6147	-0.6393	0.68149	0.40985	1.16262
TRINITY_DN21278_c0_g2_i2_orf1	mannose-1-phosphate guanyltransferase alpha-A [Ostrinia furnacalis]	-1.8226	-0.2239	0.67287	0.34635	1.0273
TRINITY_DN29873_c0_g1_i1_orf1	glyceraldehyde-3-phosphate dehydrogenase 2 [Pectinophora gossypiella]	-1.7002	-0.4248	0.55244	1.22621	0.34641
TRINITY_DN18592_c0_g2_i1_orf1	GATOR complex protein MIOS [Ostrinia furnacalis]	-1.8169	0.30463	0.17489	1.25229	0.0851
TRINITY_DN7152_c0_g1_i1_orf1	tissue alpha-L-fucosidase [Ostrinia furnacalis]	-1.8125	-0.2934	1.01828	0.55256	0.53514
TRINITY_DN23564_c0_g1_i7_orf1	cytochrome P450 6B6-like [Ostrinia furnacalis]	-1.6112	-0.3742	0.94571	-0.124	1.16371
TRINITY_DN11108_c0_g1_i4_orf1	peroxisomal leader peptide-processing protease [Ostrinia furnacalis] >XP_028165527.1 peroxisomal leader peptide-processing protease [Ostrinia furnacalis]	-1.4749	-0.5958	1.4067	-0.0357	0.6997
TRINITY_DN18291_c0_g1_i1_orf1	hydroxyacylglutathione hydrolase, mitochondrial isoform X1 [Ostrinia furnacalis] >XP_028163678.1 hydroxyacylglutathione hydrolase, mitochondrial isoform X2 [Ostrinia furnacalis] >XP_028163679.1 hydroxyacylglutathione hydrolase, mitochondrial isoform X2 [Ostrinia furnacalis] >XP_028163680.1 hydroxyacylglutathione hydrolase, mitochondrial isoform X3 [Ostrinia furnacalis] >XP_028163681.1 hydroxyacylglutathione hydrolase, mitochondrial isoform X2 [Ostrinia furnacalis]	-1.9595	0.30639	0.3236	0.46727	0.86223
TRINITY_DN17172_c0_g1_i5_orf1	omega-amidase NIT2 isoform X1 [Zerene cesonia]	-1.767	-0.2954	0.47705	0.40145	1.18392
TRINITY_DN5081_c0_g1_i5_orf1	ester hydrolase C11orf54 homolog isoform X1 [Ostrinia furnacalis]	-1.6673	-0.4544	0.1437	0.85329	1.12471
TRINITY_DN1034_c0_g2_i1_orf1	glycerol kinase isoform X7 [Ostrinia furnacalis] >XP_028168096.1 glycerol kinase isoform X8 [Ostrinia furnacalis]	-1.8283	-0.2765	0.466	0.9216	0.71719
TRINITY_DN19116_c0_g1_i3_orf1	UDP-glucose 4-epimerase isoform X1 [Ostrinia furnacalis]	-1.5673	-0.5365	1.16601	-0.009	0.94671
TRINITY_DN1848_c0_g1_i2_orf1	uncharacterized protein LOC114351337 [Ostrinia furnacalis]	-1.8149	0.22378	-0.1535	0.63723	1.10741
TRINITY_DN30510_c0_g1_i6_orf1	spodomicin-like [Ostrinia furnacalis]	-1.2996	-1.0591	1.19375	0.37534	0.78959
TRINITY_DN22944_c0_g3_i1_orf1	transmembrane protein 115 [Ostrinia furnacalis]	-1.9177	0.48394	0.15829	0.28441	0.99108
TRINITY_DN65247_c1_g1_i1_orf1	beta-1,3-glucan-binding protein [Manduca sexta] >XP_030023421.1 beta-1,3-glucan-binding protein [Manduca sexta] >XP_030023422.1 beta-1,3-glucan-binding protein [Manduca sexta] >XP_030023423.1 beta-1,3-glucan-binding protein [Manduca sexta] >AEV66276.1 beta-1,3-glucanase [Manduca sexta] >KAG6448577.1 hypothetical protein O3G_MSEX005570 [Manduca sexta] >KAG6448578.1 hypothetical protein O3G_MSEX005570 [Manduca sexta] >KAG6448579.1 hypothetical protein O3G_MSEX005570 [Manduca sexta] >KAG6448580.1 hypothetical protein O3G_MSEX005570 [Manduca sexta]	-1.8756	0.55785	0.66307	0.83583	-0.1812
TRINITY_DN4065_c0_g1_i5_orf1	uncharacterized protein LOC114357176 [Ostrinia furnacalis] >XP_028166588.1 uncharacterized protein LOC114357176 [Ostrinia furnacalis]	-1.9113	0.77417	0.71142	0.48754	-0.0618
TRINITY_DN9435_c0_g1_i7_orf1	uncharacterized protein LOC114350197 [Ostrinia furnacalis]	-1.4207	-0.7163	0.24495	1.50223	0.38978
TRINITY_DN10650_c0_g1_i1_orf1	protein Skeletor, isoforms B/C-like [Ostrinia furnacalis]	-1.4576	0.25467	1.46271	-0.6944	0.4346
TRINITY_DN25582_c0_g1_i3_orf1	uroporphyrinogen decarboxylase [Ostrinia furnacalis]	-1.9278	-0.0298	0.6344	0.612	0.71115
TRINITY_DN49038_c0_g4_i1_orf1	6-phosphogluconate dehydrogenase, decarboxylating [Ostrinia furnacalis]	-1.727	-0.5405	0.83303	0.75285	0.68164
TRINITY_DN10222_c0_g1_i2_orf1	glutathione S-transferase sigma 3 [Ostrinia furnacalis]	-1.3953	-0.9739	0.45478	1.13803	0.77637
TRINITY_DN10396_c0_g1_i1_orf1	charged multivesicular body protein 3 isoform X1 [Ostrinia furnacalis] >XP_028178068.1 charged multivesicular body protein 3 isoform X2 [Ostrinia furnacalis]	-1.8882	0.09281	0.18178	1.02031	0.5933
TRINITY_DN4694_c0_g1_i6_orf1	uncharacterized protein LOC114362122 [Ostrinia furnacalis]	-1.933	0.29093	0.34135	0.32122	0.97945
TRINITY_DN1576_c0_g1_i4_orf1	elongator complex protein 3 [Manduca sexta]	-1.6197	-0.0181	0.03234	1.53972	0.06582
TRINITY_DN64788_c0_g1_i1_orf1	uncharacterized protein LOC114365187 isoform X3 [Ostrinia furnacalis]	-1.5063	0.16118	1.60339	-0.3539	0.09555
TRINITY_DN2570_c0_g1_i1_orf1	PREDICTED: pyruvate carboxylase, mitochondrial isoform X1 [Microplitis demolitor] >XP_008556301.1 PREDICTED: pyruvate carboxylase, mitochondrial isoform X1 [Microplitis demolitor] >XP_008556302.1 PREDICTED: pyruvate carboxylase, mitochondrial isoform X1 [Microplitis demolitor]	-1.6273	-0.691	0.53881	0.86636	0.91306
TRINITY_DN54275_c0_g1_i4_orf1	PREDICTED: aryl-hydrocarbon-interacting protein-like 1 [Amyelois transitella]	-1.812	0.29387	0.53288	1.14872	-0.1635
TRINITY_DN12920_c0_g3_i1_orf1	zonadhesin-like isoform X4 [Ostrinia furnacalis]	-0.8697	-0.2524	1.93764	-0.6233	-0.1922
TRINITY_DN67243_c0_g1_i1_orf1	39S ribosomal protein L3, mitochondrial [Ostrinia furnacalis]	-1.7652	-0.4647	0.6624	0.76647	0.8011
TRINITY_DN38783_c0_g1_i1_orf1	regucalcin-like [Ostrinia furnacalis]	-1.4495	-0.8396	0.26287	0.82354	1.20274
TRINITY_DN22604_c0_g1_i3_orf1	cytochrome P450 6B5-like [Ostrinia furnacalis]	-1.7257	0.23345	-0.2594	0.44797	1.30368
TRINITY_DN7622_c0_g2_i2_orf1	uncharacterized protein LOC114351301 [Ostrinia furnacalis]	-1.4114	0.06137	0.51543	1.51052	-0.6759
TRINITY_DN146181_c0_g1_i1_orf1	vesicular integral-membrane protein VIP36 [Diachasma alloeum]	-1.8335	-0.1678	0.40521	0.50429	1.0917
TRINITY_DN11263_c0_g1_i5_orf1	SET domain-containing protein SmydA-8 [Ostrinia furnacalis]	-1.5426	-0.2411	1.59509	0.10122	0.08745
TRINITY_DN710_c0_g1_i11_orfp1	TRINITY_DN710_c0_g1_i11_m.67699 TRINITY_DN710_c0_g1::TRINITY_DN710_c0_g1_i11::g.67699 ORF type:complete len:194 (-),score=34.99,Collagen PF01391.19 0.00029 TRINITY_DN710_c0_g1_i11:1283-1864(-)	-1.3438	-0.9968	0.98595	0.28302	1.07163
TRINITY_DN20442_c0_g2_i1_orf1	hypothetical protein evm_008218 [Chilo suppressalis]	-1.4546	1.10153	-0.8329	0.22305	0.96294
TRINITY_DN19639_c0_g2_i1_orf1	basic juvenile hormone-suppressible protein 1-like [Hyposmocoma kahamanoa]	-1.0412	1.57728	-1.026	-0.1124	0.60231
TRINITY_DN65681_c0_g1_i1_orf1	ferritin subunit-like [Ostrinia furnacalis] >XP_028168186.1 ferritin subunit-like [Ostrinia furnacalis]	-0.7607	1.68319	-1.2374	0.09588	0.21897
TRINITY_DN47842_c0_g1_i1_orf1	protein lethal(2)essential for life-like [Helicoverpa armigera] >PZC74790.1 hypothetical protein B5X24_HaOG207163 [Helicoverpa armigera]	-1.5629	0.97545	-0.7147	0.29962	1.00257
TRINITY_DN54269_c0_g1_i3_orf1	lopap-like [Ostrinia furnacalis]	-0.838	1.72948	-1.1303	0.10137	0.13741
TRINITY_DN1897_c0_g2_i4_orf1	phenoloxidase-activating factor 2-like [Hyposmocoma kahamanoa]	-1.352	0.87522	-0.5166	-0.4104	1.40384
TRINITY_DN9920_c0_g1_i1_orf1	uncharacterized protein LOC114351526 [Ostrinia furnacalis]	-1.239	1.70778	-0.7064	0.01599	0.22161
TRINITY_DN26337_c0_g1_i3_orf1	lysosome membrane protein 2-like [Ostrinia furnacalis]	-1.4176	1.51383	-0.576	-0.1149	0.59473

TRINITY_DN23229_c0_g1_i2_orf1	uncharacterized protein LOC114362553 [Ostrinia furnacalis]	-1.2937	1.65099	-0.6542	-0.1052	0.40206
TRINITY_DN5848_c0_g1_i6_orf1	brain tumor protein isoform X1 [Ostrinia furnacalis]	-0.9339	1.61405	-1.1297	-0.045	0.4945
TRINITY_DN8580_c0_g1_i12_orf1	peroxisomal N(1)-acetyl-spermine/spermidine oxidase-like isoform X1 [Ostrinia furnacalis]	-1.5881	1.09311	-0.73	0.60665	0.61828
TRINITY_DN20614_c0_g1_i1_orf1	hypothetical protein evm_003552 [Chilo suppressalis]	-1.3439	1.26543	-1.0011	0.60194	0.47762
TRINITY_DN19043_c0_g3_i2_orf1	hypothetical protein EVAR_60654_1 [Eumeta japonica]	-1.2933	1.5172	-0.8484	0.077	0.54751
TRINITY_DN38392_c0_g1_i1_orf1	enoyl-CoA hydratase domain-containing protein 3 [Agrotis segetum]	-0.8409	0.70886	-1.2104	-0.1726	1.51508
TRINITY_DN105749_c0_g1_i1_orf1	hypothetical protein evm_009828 [Chilo suppressalis] >CAH2984222.1 unnamed protein product [Chilo suppressalis]	-1.5415	0.42123	-0.4123	0.02397	1.50857
TRINITY_DN18592_c0_g1_i4_orf1	GATOR complex protein MIOS [Ostrinia furnacalis]	-1.431	1.10318	-0.951	0.55833	0.7205
TRINITY_DN88876_c0_g1_i1_orf1	Photosystem I reaction center subunit III, chloroplastic, partial [Trichinella zimbabwensis]	-1.2757	1.36367	-1.0326	0.45427	0.49033
TRINITY_DN74020_c0_g1_i2_orf1	unnamed protein product [Euphydryas editha]	-1.4728	0.76981	-0.6815	0.05375	1.33075
TRINITY_DN6199_c2_g1_i3_orf1	uncharacterized protein LOC114352137 [Ostrinia furnacalis] >XP_028159411.1 uncharacterized protein LOC114352137 [Ostrinia furnacalis] >XP_028159412.1 uncharacterized protein LOC114352137 [Ostrinia furnacalis]	-1.424	1.12196	-0.9131	0.87331	0.34187
TRINITY_DN13330_c0_g1_i4_orf1	carboxylesterase [Cnaphalocrocis medinalis]	-1.2167	1.2484	-0.7299	-0.4205	1.11873
TRINITY_DN26089_c0_g1_i1_orf1	putative neuropeptide precursor protein isoform X1 [Ostrinia furnacalis]	-0.8715	1.04413	-0.8194	-0.742	1.38868
TRINITY_DN962_c5_g1_i1_orf1	histone deacetylase 5 isoform X5 [Pectinophora gossypiella]	-0.9379	1.04462	-0.8123	-0.6768	1.38244
TRINITY_DN2097_c1_g1_i1_orf1	5-oxoprolinase [Ostrinia furnacalis]	-0.9335	1.50541	-1.2717	0.36303	0.33672
TRINITY_DN810_c0_g1_i4_orf1	dicer 2 [Ostrinia nubilalis]	-1.508	0.73578	-0.448	-0.1771	1.39734
TRINITY_DN54387_c0_g1_i1_orf1	catalase-like [Pectinophora gossypiella]	-1.3802	1.01119	-0.826	0.01604	1.17899
TRINITY_DN35147_c0_g1_i1_orf1	collagen alpha-2(IV) chain isoform X2 [Ostrinia furnacalis]	-1.3919	0.53865	-0.4117	-0.3169	1.58191
TRINITY_DN7378_c0_g1_i5_orf1	uncharacterized protein LOC114355479 isoform X2 [Ostrinia furnacalis]	-1.3322	1.45977	-0.8904	0.30818	0.45459
TRINITY_DN1604_c0_g1_i4_orf1	ubiquitin-conjugating enzyme E2 S [Ostrinia furnacalis]	-0.5025	1.66107	-1.3823	-0.0501	0.27387
TRINITY_DN15930_c0_g1_i5_orf1	ubiquitin-like modifier-activating enzyme ATG7 [Ostrinia furnacalis]	-0.7485	1.31907	-0.9358	-0.7548	1.12
TRINITY_DN1768_c0_g1_i2_orf1	low-density lipoprotein receptor-related protein 1B-like [Ostrinia furnacalis]	-0.9158	1.61415	-0.9358	-0.4522	0.68965
TRINITY_DN48023_c1_g1_i1_orf1	carboxylesterase [Ostrinia furnacalis]	-1.1585	1.70226	-0.7973	-0.0881	0.34163
TRINITY_DN2627_c0_g2_i1_orf1	phosphotriesterase-related protein [Ostrinia furnacalis]	-1.1687	1.24192	-1.0384	-0.0408	1.00592
TRINITY_DN9794_c0_g2_i8_orf1	acetyl-coenzyme A synthetase [Ostrinia furnacalis]	-0.8267	1.42913	-1.3594	0.11422	0.64275
TRINITY_DN103_c0_g1_i1_orf1	unnamed protein product [Diatraea saccharalis]	-1.462	1.08518	-0.7002	-0.0158	1.09287
TRINITY_DN225_c0_g1_i6_orf1	glutathione S-transferase delta 3 [Ostrinia furnacalis]	-1.23	0.57726	-0.649	-0.3201	1.6218
TRINITY_DN5910_c1_g1_i6_orf1	protein SAND [Ostrinia furnacalis]	-0.8339	1.56175	-1.2135	-0.128	0.61364
TRINITY_DN35757_c0_g1_i1_orf1	ADP-ribosylation factor-like protein 6-interacting protein 1 [Ostrinia furnacalis]	-1.2439	0.63388	-0.9119	0.03261	1.48936
TRINITY_DN121439_c0_g1_i1_orf1	unnamed protein product [Chilo suppressalis]	-1.2652	1.47913	-0.9638	0.3991	0.35084
TRINITY_DN84631_c0_g1_i1_orf1	PREDICTED: rap guanine nucleotide exchange factor 2-like isoform X9 [Microplitis demolitor]	-1.1449	0.928	-0.6182	-0.6065	1.44155
TRINITY_DN1260_c0_g2_i1_orf1	vegetative cell wall protein gp1 [Ostrinia furnacalis]	-0.104	1.09167	-1.1787	-0.9977	1.18864
TRINITY_DN83150_c0_g1_i1_orf1	fructose-bisphosphate aldolase-like isoform X1 [Ostrinia furnacalis] >XP_028178678.1 fructose-bisphosphate aldolase-like isoform X1 [Ostrinia furnacalis]	-0.9552	0.7032	-0.9703	-0.3648	1.58702
TRINITY_DN27264_c0_g1_i1_orf1	uncharacterized protein LOC114353424 [Ostrinia furnacalis]	-0.9364	1.74177	-0.6874	0.49325	-0.6112
TRINITY_DN3464_c0_g1_i1_orf1	putative mitochondrial aconitate hydratase isoform X1-likeprotein, partial [Cotesia chilonis]	-0.9465	1.61508	-0.8389	0.70861	-0.5383
TRINITY_DN14250_c0_g1_i1_orf1	apolipophorins-like [Ostrinia furnacalis]	-1.212	1.08205	-0.9575	1.19647	-0.109
TRINITY_DN3383_c0_g1_i5_orf1	uncharacterized protein LOC114357426 [Ostrinia furnacalis]	-1.0577	0.88771	-1.2692	1.19223	0.24695
TRINITY_DN17329_c0_g2_i3_orf1	uncharacterized protein LOC114354338 isoform X1 [Ostrinia furnacalis]	-0.5746	1.59718	-0.9519	0.74256	-0.8132
TRINITY_DN56164_c0_g1_i1_orf1	hypothetical protein evm_010164 [Chilo suppressalis]	-0.676	1.22313	-1.581	0.59677	0.43716
TRINITY_DN4270_c0_g1_i1_orf1	cytochrome b-c1 complex subunit 8-like [Ostrinia furnacalis]	-0.8978	1.31491	-1.351	0.78615	0.14771
TRINITY_DN33430_c0_g1_i5_orf1	NADH dehydrogenase [ubiquinone] iron-sulfur protein 2, mitochondrial [Ostrinia furnacalis]	-0.9222	1.15341	-1.4196	0.81511	0.37334
TRINITY_DN56690_c0_g1_i4_orf1	hypothetical protein evm_002209, partial [Chilo suppressalis]	-1.2015	1.28358	0.22576	0.79752	-1.1054
TRINITY_DN116874_c0_g1_i1_orfp1	TRINITY_DN116874_c0_g1_i1_m.85176 TRINITY_DN116874_c0_g1::TRINITY_DN116874_c0_g1_i1::g.85176 ORF type:5prime_partial len:95 (+),score=17.10,Baculo_p48 PF04878.14 8.5e-16 TRINITY_DN116874_c0_g1_i1:2-286(+)	-0.9332	0.88176	-1.2568	1.33105	-0.0229
TRINITY_DN23978_c0_g1_i2_orf1	insulin-like growth factor-binding protein complex acid labile subunit [Ostrinia furnacalis]	-0.8938	1.08126	-0.0958	1.18271	-1.2743
TRINITY_DN17772_c0_g2_i3_orf1	uncharacterized protein LOC114351684 [Ostrinia furnacalis]	-0.9776	1.40731	-0.6629	1.01023	-0.777
TRINITY_DN23732_c0_g1_i1_orf1	glutathione S-transferase 1-like [Ostrinia furnacalis] >QIC35740.1 glutathione S-transferase delta 4 [Ostrinia furnacalis]	-0.8885	1.68444	-0.8093	0.60592	-0.5926
TRINITY_DN1833_c0_g1_i5_orf1	uncharacterized protein LOC114356866 isoform X3 [Ostrinia furnacalis] >XP_028166037.1 uncharacterized protein LOC114356866 isoform X3 [Ostrinia furnacalis]	-0.9591	1.01194	-0.3775	1.35834	-1.0337
TRINITY_DN17693_c0_g1_i10_orf1	acetylcholinesterase-like [Ostrinia furnacalis]	-0.8678	0.1234	-0.4822	1.88745	-0.6608
TRINITY_DN5560_c0_g1_i5_orf1	uncharacterized protein LOC114354348 isoform X1 [Ostrinia furnacalis]	-1.1624	1.05941	-1.0559	1.18771	-0.0288
TRINITY_DN11620_c0_g1_i2_orf1	serine/threonine-protein kinase PAK 3 [Ostrinia furnacalis]	-0.8915	1.28116	-0.7869	1.16447	-0.7672
TRINITY_DN4550_c1_g1_i19_orf1	titin homolog [Ostrinia furnacalis]	-0.9715	1.03487	-0.3783	1.33911	-1.0242
TRINITY_DN2676_c0_g1_i2_orf1	probable cytochrome P450 303a1 [Ostrinia furnacalis] >XP_028178318.1 probable cytochrome P450 303a1 [Ostrinia furnacalis]	-0.6224	1.56394	-0.6949	0.78592	-1.0325
TRINITY_DN14274_c0_g1_i3_orf1	ATP-dependent RNA helicase dbp2-like isoform X1 [Leguminivora glycinivorella]	-0.5053	1.53174	-1.2046	0.77156	-0.5934

TRINITY_DN3459_c0_g1_i4_orf1	PREDICTED: probable small nuclear ribonucleoprotein G [Papilio polytes] >XP_013168682.1 PREDICTED: probable small nuclear ribonucleoprotein G [Papilio xuthus] >XP_013200095.1 PREDICTED: probable small nuclear ribonucleoprotein G [Amyelois transitella] >XP_014365947.1 probable small nuclear ribonucleoprotein G [Papilio machaon] >XP_023949391.1 probable small nuclear ribonucleoprotein G [Bicyclus anynana] >XP_026492889.1 probable small nuclear ribonucleoprotein G [Vanessa tameamea] >XP_030032656.1 probable small nuclear ribonucleoprotein G [Manduca sexta] >XP_032524946.1 probable small nuclear ribonucleoprotein G [Danaus plexippus plexippus] >XP_032524948.1 probable small nuclear ribonucleoprotein G [Danaus plexippus plexippus] >XP_039761094.1 probable small nuclear ribonucleoprotein G [Pararge aegeria] >XP_045507396.1 probable small nuclear ribonucleoprotein G [Colias croceus] >XP_046974152.1 probable small nuclear ribonucleoprotein G [Vanessa cardui] >XP_047540860.1 probable small nuclear ribonucleoprotein G [Vanessa atalanta] >XP_050357207.1 probable small nuclear ribonucleoprotein G [Nymphalis io] >CAG5058336.1 unnamed protein product [Parnassius apollo] >CAG9570366.1 unnamed protein product [Danaus chrysippus] >CAH0731681.1 unnamed protein product, partial [Brenthis ino] >CAH2061014.1 unnamed protein product, partial [Iphiclides podalirius] >CAH2269228.1 jg1748 [Pararge aegeria aegeria]	-0.6899	1.72895	-1.0563	0.46626	-0.449
TRINITY_DN17995_c0_g4_i1_orf1	dynein light chain roadblock-type 2 [Bombyx mori] >XP_028159912.1 dynein light chain roadblock-type 2-like [Ostrinia furnacalis] >XP_030028262.1 dynein light chain roadblock-type 2 [Manduca sexta] >XP_038218546.1 dynein light chain roadblock-type 2-like [Zerene cesonia] >XP_045505125.1 dynein light chain roadblock-type 2-like [Colias croceus] >CAH0760167.1 unnamed protein product [Diatraea saccharalis]	-0.7786	1.68605	-0.8294	0.61675	-0.6948
TRINITY_DN4820_c0_g2_i2_orf1	tudor and KH domain-containing protein homolog isoform X1 [Ostrinia furnacalis] >XP_028179486.1 tudor and KH domain-containing protein homolog isoform X1 [Ostrinia furnacalis]	-0.6141	1.74561	-1.048	0.44501	-0.5284
TRINITY_DN24689_c0_g1_i1_orf1	thioredoxin domain-containing protein 17-like [Ostrinia furnacalis]	-0.4212	0.93337	-1.7364	0.91799	0.30617
TRINITY_DN31377_c0_g2_i1_orf1	phosphatidate cytidylyltransferase, mitochondrial [Ostrinia furnacalis]	-0.2576	0.9617	-1.389	1.30289	-0.618
TRINITY_DN50517_c0_g1_i3_orf1	PREDICTED: larval cuticle protein A2B-like [Amyelois transitella]	-0.5932	1.74025	-1.1581	0.37864	-0.3676
TRINITY_DN4708_c0_g1_i5_orf1	GTP-binding protein 1 [Ostrinia furnacalis] >XP_028178070.1 GTP-binding protein 1 [Ostrinia furnacalis] >XP_028178072.1 GTP-binding protein 1 [Ostrinia furnacalis]	-0.9863	1.3536	-1.0489	0.99751	-0.3159
TRINITY_DN14391_c1_g1_i2_orf1	pre-rRNA-processing protein TSR1 homolog [Ostrinia furnacalis]	-0.5099	1.10843	-1.3104	1.23367	-0.5217
TRINITY_DN59804_c0_g1_i1_orf1	DNA methyltransferase 1-associated protein 1 [Ostrinia furnacalis]	-0.5261	1.06081	-1.5932	1.0289	0.02963
TRINITY_DN11159_c0_g1_i5_orf1	sphingosine-1-phosphate lyase isoform X2 [Ostrinia furnacalis]	-1.0735	0.94133	-1.2921	1.0849	0.3393
TRINITY_DN34426_c0_g1_i1_orf1	laminin subunit alpha-like, partial [Ostrinia furnacalis]	-0.8033	1.24521	-1.0779	1.16655	-0.5306
TRINITY_DN4189_c0_g1_i4_orf1	tolloid-like protein 1 [Ostrinia furnacalis]	-1.0664	1.18274	-0.7175	1.2409	-0.6397
TRINITY_DN13732_c0_g2_i3_orf1	60S ribosomal protein L35 [Ostrinia furnacalis]	-0.6483	1.6981	-1.0354	0.55138	-0.5658
TRINITY_DN59965_c0_g4_i1_orf1	TKT protein, partial [Homo sapiens]	-1.5561	0.24791	-0.5895	1.37844	0.51919
TRINITY_DN17574_c0_g1_i2_orf1	heat shock protein Hsp-12.2-like [Ostrinia furnacalis]	-1.4682	0.66232	-0.9201	1.0496	0.67637
TRINITY_DN20009_c0_g1_i1_orf1	vimentin [Homo sapiens] >XP_003831224.1 vimentin [Pan paniscus] >XP_018890043.1 vimentin [Gorilla gorilla gorilla] >XP_024109584.1 vimentin [Pongo abelii] >XP_030675100.1 vimentin [Nomascus leucogenys] >XP_032020652.1 vimentin [Hylobates moloch] >P08670.4 RecName: Full=Vimentin [Homo sapiens] >AIC49963.1 VIM, partial [synthetic construct] >MXR00191.1 hypothetical protein [Bos mutus] >PNI30483.1 VIM isoform 1 [Pan troglodytes] >SJX39704.1 unnamed protein product, partial [Human ORFeome Gateway entry vector] >AAH00163.2 Vimentin [Homo sapiens]	-1.4464	0.52317	-0.6132	1.50232	0.03414
TRINITY_DN9593_c0_g1_i2_orf1	uncharacterized protein LOC113518937 [Galleria mellonella]	-1.59	0.57016	-0.6368	1.25792	0.39871
TRINITY_DN53358_c0_g1_i3_orf1	arylphorin subunit alpha-like [Ostrinia furnacalis]	-1.6738	0.20212	-0.3018	1.38372	0.38972

TRINITY_DN111985_c0_g1_i1_orf1	Parkinson disease protein 7 [Homo sapiens] >NP_009193.2 Parkinson disease protein 7 [Homo sapiens] >XP_003891104.1 protein/nucleic acid deglycase DJ-1 [Papio anubis] >XP_003891105.1 protein/nucleic acid deglycase DJ-1 [Papio anubis] >XP_003891106.1 protein/nucleic acid deglycase DJ-1 [Papio anubis] >XP_005263481.1 Parkinson disease protein 7 isoform X1 [Homo sapiens] >XP_010355680.2 protein/nucleic acid deglycase DJ-1 [Rhinopithecus roxellana] >XP_011735530.1 protein/nucleic acid deglycase DJ-1 isoform X1 [Macaca nemestrina] >XP_011735540.1 protein/nucleic acid deglycase DJ-1 isoform X1 [Macaca nemestrina] >XP_011735547.1 protein/nucleic acid deglycase DJ-1 isoform X1 [Macaca nemestrina] >XP_011758352.1 protein/nucleic acid deglycase DJ-1 [Macaca nemestrina] >XP_011793629.1 PREDICTED: protein deglycase DJ-1 isoform X1 [Colobus angolensis palliatus] >XP_011793630.1 PREDICTED: protein deglycase DJ-1 isoform X1 [Colobus angolensis palliatus] >XP_011793631.1 PREDICTED: protein deglycase DJ-1 isoform X1 [Colobus angolensis palliatus] >XP_011793632.1 PREDICTED: protein deglycase DJ-1 isoform X1 [Colobus angolensis palliatus] >XP_011793633.1 PREDICTED: protein deglycase DJ-1 isoform X1 [Colobus angolensis palliatus] >XP_011836827.1 PREDICTED: protein deglycase DJ-1 isoform X1 [Mandrillus leucophaeus] >XP_011904889.1 PREDICTED: protein deglycase DJ-1 isoform X5 [Cercocebus atys] >XP_015000741.2 protein/nucleic acid deglycase DJ-1 isoform X1 [Macaca mulatta] >XP_015000861.2 protein/nucleic acid deglycase DJ-1 isoform X1 [Macaca mulatta] >XP_015000901.2 protein/nucleic acid deglycase DJ-1 isoform X1 [Macaca mulatta] >XP_015306634.1 Parkinson disease protein 7 [Macaca fascicularis] >XP_015306640.1 Parkinson disease protein 7 [Macaca fascicularis] >XP_015306643.1 Parkinson disease protein 7 [Macaca fascicularis] >XP_017711545.1 PREDICTED: protein deglycase DJ-1 [Rhinopithecus bieti] >XP_017711546.1 PREDICTED: protein deglycase DJ-1 [Rhinopithecus bieti] >XP_017711547.1 PREDICTED: protein deglycase DJ-1 [Rhinopithecus bieti] >XP_023071026.1 protein/nucleic acid deglycase DJ-1 [Piliocolobus tephrosceles] >XP_025220379.1 protein/nucleic acid deglycase DJ-1 isoform X1 [Theropithecus gelada] >XP_025220387.1 protein/nucleic acid deglycase DJ-1 isoform X1 [Theropithecus gelada] >XP_025220395.1 protein/nucleic acid deglycase DJ-1 isoform X1 [Theropithecus gelada] >XP_025220404.1 protein/nucleic acid deglycase DJ-1 isoform X1 [Theropithecus gelada] >XP_030661663.1 protein/nucleic acid deglycase DJ-1 [Nomascus leucogenys] >XP_030661664.1 protein/nucleic acid deglycase DJ-1 [Nomascus leucogenys] >XP_030798112.1 protein/nucleic acid deglycase DJ-1 [Rhinopithecus roxellana] >XP_032614696.1 protein/nucleic acid deglycase DJ-1 [Hylobates moloch] >XP_032614697.1 protein/nucleic acid deglycase DJ-1 [Hylobates moloch] >XP_033031677.1 protein/nucleic acid deglycase DJ-1 [Trachypithecus francoisi] >XP_033084403.1 protein/nucleic acid deglycase DJ-1 isoform X2 [Trachypithecus francoisi] >XP_033084404.1 protein/nucleic acid deglycase DJ-1 isoform X2 [Trachypithecus francoisi] >Q99497.2 RecName: Full=Parkinson disease protein 7; AltName: Full=Maillard deglycase; AltName: Full=Oncogene DJ1; AltName: Full=Parkinsonism-associated deglycase; AltName: Full=Protein DJ-1; Short=DJ-1; AltName: Full=Protein/nucleic acid deglycase DJ-1; Flags: Precursor [Homo sapiens] >1P5F_A Crystal Structure of Human DJ-1 [Homo sapiens] >1Q2U_A Crystal structure of DJ-1/RS and implication on familial Parkinson's disease [Homo sapiens] >1UCF_A The Crystal Structure of DJ-1, a Protein Related to Male Fertility and Parkinson's Disease [Homo sapiens] >1UCF_B The Crystal Structure of DJ-1, a Protein Related to Male Fertility and Parkinson's Disease [Homo sapiens] >2OR3_A Pre-oxidation Complex of Human DJ-1 [Homo sapiens] >2OR3_B Pre-oxidation Complex of Human DJ-1 [Homo sapiens] >4BTE_A DJ-1 Cu(I) complex [Homo sapiens] >4MNT_A Crystal structure of human DJ-1 in complex with Cu [Homo sapiens] >4P35_A Crystal structure of DJ-1 with Zinc(II) bound (crystal I) [Homo sapiens] >4RKW_A Crystal structure of DJ-1 [Homo sapiens] >6AF5_A	-1.4199	-0.1868	-0.3547	1.65143	0.30991
TRINITY_DN16091_c0_g1_i1_orfp1	TRINITY_DN16091_c0_g1_i1_m.64010 TRINITY_DN16091_c0_g1::TRINITY_DN16091_c0_g1_i1::g.64010 ORF type:5prime_partial len:124 (-),score=7.29,Toxin_2 PF00451.20 0.00035,Toxin_2|PF00451.20|0.00013,Toxin_2|PF00451.20|0.00037,Gamma-thionin|PF00304.21|0.37,Gamma-thionin|PF00304.21|0.052,Gamma-thionin|PF00304.21|0.022,Defensin_2|PF01097.19|0.58,Defensin_2|PF01097.19|0.12,Defensin_2|PF01097.19|0.011,Toxin_38|PF14866.7|0.18,Toxin_38|PF14866.7|0.18,Toxin_38|PF14866.7|0.4 TRINITY_DN16091_c0_g1_i1:19-390(-)	-1.4033	-0.2869	-0.0215	1.71696	-0.0053
TRINITY_DN18031_c0_g1_i1_orf1	63 kDa chaperonin, mitochondrial-like [Ostrinia furnacalis]	-1.4085	-0.3374	-0.3089	1.61721	0.43757
TRINITY_DN307_c1_g1_i1_orf1	uncharacterized protein LOC114356704 [Ostrinia furnacalis]	-1.3736	0.40011	-0.9009	0.49852	1.37587
TRINITY_DN276_c0_g1_i1_orf1	protein lethal(2)essential for life-like [Helicoverpa zea] >XP_049705426.1 protein lethal(2)essential for life [Helicoverpa armigera] >ATB54993.1 heat shock protein 20.8 [Helicoverpa armigera] >PZC74337.1 hypothetical protein B5X24_HaOG207971 [Helicoverpa armigera]	-1.4905	0.15398	-0.7438	1.17746	0.90284
TRINITY_DN16234_c0_g2_i3_orf1	uncharacterized protein LOC114363370 [Ostrinia furnacalis]	-1.4257	-0.2805	-0.5589	1.20588	1.05928
TRINITY_DN2146_c0_g2_i1_orf1	heat shock protein 68-like [Ostrinia furnacalis]	-1.488	0.01826	-0.6286	1.35631	0.74208

TRINITY_DN142442_c0_g1_i1_orf1	60S ribosomal protein L38 [Homo sapiens] >NP_001002486.1 60S ribosomal protein L38 [Danio rerio] >NP_001030333.1 60S ribosomal protein L38 [Homo sapiens] >NP_001071060.1 60S ribosomal protein L38 [Rattus norvegicus] >NP_001133168.1 60S ribosomal protein L38 [Salmo salar] >NP_001187063.1 60S ribosomal protein L38 [Ictalurus punctatus] >NP_001232305.1 60S ribosomal protein L38 [Taeniopygia guttata] >NP_001264941.1 60S ribosomal protein L38 [Gallus gallus] >XP_003211558.1 60S ribosomal protein L38 [Meleagris gallopavo] >XP_003315754.1 60S ribosomal protein L38 [Pan troglodytes] >XP_003315758.1 60S ribosomal protein L38 [Pan troglodytes] >XP_003339346.1 60S ribosomal protein L38 [Pan troglodytes] >XP_003358038.1 60S ribosomal protein L38 [Sus scrofa] >XP_003417326.1 60S ribosomal protein L38 [Loxodonta africana] >XP_003453439.1 60S ribosomal protein L38 [Oreochromis niloticus] >XP_003464913.2 60S ribosomal protein L38 [Cavia porcellus] >XP_003768586.1 60S ribosomal protein L38 [Sarcophilus harrisii] >XP_003786210.1 60S ribosomal protein L38 [Otolemur garnettii] >XP_003795793.1 60S ribosomal protein L38 [Otolemur garnettii] >XP_003922345.1 60S ribosomal protein L38 [Saimiri boliviensis boliviensis] >XP_004041125.1 60S ribosomal protein L38 [Gorilla gorilla gorilla] >XP_004041126.1 60S ribosomal protein L38 [Gorilla gorilla gorilla] >XP_004041128.1 60S ribosomal protein L38 [Gorilla gorilla gorilla] >XP_004331065.1 60S ribosomal protein L38 [Tursiops truncatus] >XP_004401894.1 PREDICTED: 60S ribosomal protein L38 [Odobenus rosmarus divergens] >XP_004412345.1 PREDICTED: 60S ribosomal protein L38 [Odobenus rosmarus divergens] >XP_004469223.1 60S ribosomal protein L38 [Dasypus novemcinctus] >XP_004469224.1 60S ribosomal protein L38 [Dasypus novemcinctus] >XP_005068761.1 60S ribosomal protein L38 [Mesocricetus auratus] >XP_005070019.1 60S ribosomal protein L38 [Mesocricetus auratus] >XP_005141156.1 60S ribosomal protein L38 [Melopsittacus undulatus] >XP_005336034.1 60S ribosomal protein L38 [Ictidomys tridecemlineatus] >XP_005336035.1 60S ribosomal protein L38 [Ictidomys tridecemlineatus] >XP_005350739.1 60S ribosomal protein L38 [Microtus ochrogaster] >XP_005350740.1 60S ribosomal protein L38 [Microtus ochrogaster] >XP_005412280.1 PREDICTED: 60S ribosomal protein L38 [Chinchilla lanigera] >XP_005412281.1 PREDICTED: 60S ribosomal protein L38 [Chinchilla lanigera] >XP_005530739.1 PREDICTED: 60S ribosomal protein L38 [Pseudopodoces humilis] >XP_005584887.1 60S ribosomal protein L38 [Macaca fascicularis] >XP_005584888.1 60S ribosomal protein L38 [Macaca fascicularis] >XP_005584889.1 60S ribosomal protein L38 [Macaca fascicularis] >XP_005584890.1 60S ribosomal protein L38 [Macaca fascicularis] >XP_005584891.1 60S ribosomal protein L38 [Macaca fascicularis] >XP_005592611.1 60S ribosomal protein L38 [Macaca fascicularis] >XP_005597274.1 60S ribosomal protein L38 isoform X2 [Equus caballus] >XP_005668697.1 60S ribosomal protein L38 [Sus scrofa] >XP_005861853.1 PREDICTED: 60S ribosomal protein L38 [Myotis brandtii] >XP_005861854.1 PREDICTED: 60S ribosomal protein L38 [Myotis brandtii] >XP_005889694.1 PREDICTED: 60S ribosomal protein L38 isoform X2 [Bos mutus] >XP_006042140.1 60S ribosomal protein L38 isoform X2 [Bubalus bubalis] >XP_006042141.1 60S ribosomal protein L38 isoform X2 [Bubalus bubalis] >XP_006082202.1 60S ribosomal protein L38 [Myotis lucifugus] >XP_006106539.1 60S ribosomal protein L38 [Myotis lucifugus] >XP_006109407.1 60S ribosomal protein L38 [Myotis lucifugus] >XP_006145920.1 60S ribosomal protein L38 isoform X1 [Tupaia chinensis] >XP_006145921.1 60S ribosomal protein L38 isoform X1 [Tupaia chinensis] >XP_006754668.1 PREDICTED: 60S ribosomal protein L38 [Myotis davidii] >XP_006754669.1 PREDICTED: 60S ribosomal protein L38 [Myotis davidii] >XP_006912321.1 60S ribosomal protein L38 [Pteropus alecto] >XP_006970499.1 60S ribosomal protein L38 [Peromyscus maniculatus bairdii] >XP_006970500.1 60S ribosomal protein L38 [Peromyscus maniculatus bairdii] >XP_007118971.1 60S ribosomal protein L38 [Physeter catodon] >XP_007118972.1 60S ribosomal protein L38 [Physeter catodon]					
osomal protein L38 [Nematolebias wl	60S RIBOSOMAL PROTEIN L38 [Oryctolagus cuniculus] >4UJD_Ak Chain Ak	60S RIBO	60S RIBO	60S ribos	60S ribos	60S ribos
TRINITY_DN140212_c0_g1_i1_orf1	macrophage mannose receptor 1-like [Ostrinia furnacalis]	-1.5552	0.62509	-0.752	0.51683	1.16529
TRINITY_DN21743_c0_g1_i1_orf1	uncharacterized protein LOC114357426 [Ostrinia furnacalis]	-1.4319	0.86509	-0.8565	1.18844	0.2349
TRINITY_DN28654_c0_g1_i2_orf1	acidic juvenile hormone-suppressible protein 1-like [Ostrinia furnacalis]	-1.39	0.08126	-0.3142	1.71855	-0.0956
TRINITY_DN36144_c0_g1_i3_orf1	nicotinate phosphoribosyltransferase isoform X1 [Ostrinia furnacalis] >XP_028178189.1 nicotinate phosphoribosyltransferase isoform X1 [Ostrinia furnacalis]	-1.563	0.46133	-0.4534	1.45921	0.09594
TRINITY_DN22375_c0_g1_i4_orf1	venom carboxylesterase-6-like [Ostrinia furnacalis]	-1.6549	0.05593	-0.428	1.1153	0.91165
TRINITY_DN69307_c0_g1_i6_orf1	hypothetical protein evm_010738 [Chilo suppressalis]	-1.7222	-0.1464	-0.125	1.06757	0.92597
TRINITY_DN63389_c0_g1_i4_orf1	retinol dehydrogenase 14-like [Ostrinia furnacalis]	-1.269	0.65011	-1.1363	0.61694	1.13816
TRINITY_DN17368_c0_g1_i6_orf1	protein bark beetle [Ostrinia furnacalis]	-1.5118	0.52407	-0.4703	1.48921	-0.0312
TRINITY_DN131471_c0_g1_i1_orf1	basement membrane-specific heparan sulfate proteoglycan core protein isoform X13 [Ostrinia furnacalis]	-1.6531	0.68942	-0.6196	0.51359	1.06971
TRINITY_DN6424_c0_g1_i2_orf1	protein tweety-like [Ostrinia furnacalis]	-1.3941	0.1818	-0.4517	1.67901	-0.015
TRINITY_DN1732_c0_g1_i15_orf1	CAD protein isoform X2 [Ostrinia furnacalis]	-1.4275	0.38793	-0.8	1.40965	0.42985
TRINITY_DN17326_c0_g1_i5_orf1	aminoacylase-1-like [Ostrinia furnacalis]	-1.5505	0.51495	-0.7569	0.621	1.17145
TRINITY_DN7212_c0_g1_i4_orf1	peptidylglycine alpha-hydroxylating monooxygenase [Ostrinia furnacalis]	-1.5934	0.03657	-0.4722	1.31312	0.71588
TRINITY_DN22674_c0_g1_i2_orf1	protein arginine N-methyltransferase 7 isoform X1 [Ostrinia furnacalis]	-1.4052	0.73343	-0.8827	1.2774	0.27701
TRINITY_DN18273_c0_g1_i4_orf1	venom protease-like [Ostrinia furnacalis] >XP_028156372.1 venom protease-like [Ostrinia furnacalis]	-1.4527	0.38956	-0.6086	1.53229	0.1395
TRINITY_DN69691_c0_g1_i1_orf1	protein HGH1 homolog [Ostrinia furnacalis]	-1.2109	0.32798	-0.5637	1.73868	-0.292
TRINITY_DN15478_c0_g1_i1_orf1	STE20/SPS1-related proline-alanine-rich protein kinase [Vanessa tameamea] >XP_047534294.1 STE20/SPS1-related proline-alanine-rich protein kinase-like [Vanessa atalanta]	-1.6554	-0.1716	-0.2434	1.15264	0.9178
TRINITY_DN4822_c0_g1_i6_orf1	homogentisate 1,2-dioxygenase [Ostrinia furnacalis]	-0.8878	0.33973	-1.1748	1.64633	0.07652
TRINITY_DN20347_c0_g1_i6_orf1	venom polypeptide precursor [Doratifera vulnerans]	-1.5118	-0.4397	-0.0145	1.52542	0.44057
TRINITY_DN5531_c7_g1_i2_orf1	LOW QUALITY PROTEIN: tyrosine-protein phosphatase CDC14 homolog [Ostrinia furnacalis]	-1.4352	-0.4642	-0.1029	1.59676	0.40559
TRINITY_DN1841_c0_g1_i2_orf1	carboxylesterase [Cnaphalocrocis medinalis]	-1.4363	-0.0527	-0.6349	1.43328	0.69058
TRINITY_DN987_c0_g1_i3_orf1	unnamed protein product [Chilo suppressalis]	-1.2989	-0.1544	-0.8215	1.02188	1.25294
TRINITY_DN101_c0_g1_i4_orf1	disco-interacting protein 2 [Melitaea cinxia]	-1.2436	0.0365	-0.6426	1.73982	0.10995
TRINITY_DN8245_c0_g1_i4_orf1	uncharacterized protein LOC114357622 [Ostrinia furnacalis]	-1.4178	0.4797	-0.9423	1.16793	0.7125
TRINITY_DN6136_c0_g1_i1_orf1	uncharacterized protein LOC114354455 [Ostrinia furnacalis]	-1.5711	0.11245	-0.517	1.37515	0.60049
TRINITY_DN781_c0_g1_i7_orf1	uncharacterized protein LOC114356786 [Ostrinia furnacalis]	-1.403	0.36678	-0.8242	1.42367	0.43682
TRINITY_DN12367_c0_g1_i8_orf1	aldose reductase-like isoform X2 [Ostrinia furnacalis]	-1.3088	-0.2754	-0.7296	1.18987	1.1239
TRINITY_DN9044_c0_g1_i1_orf1	hypothetical protein SFRURICE_005818, partial [Spodoptera frugiperda]	-1.4638	-0.0864	-0.6421	0.9647	1.22756

TRINITY_DN29956_c1_g1_i1_orf1	PREDICTED: dual specificity mitogen-activated protein kinase kinase dSOR1 isoform X1 [Fopius arisanus]	-1.3848	0.234	-0.9286	0.9979	1.08147
TRINITY_DN1004_c0_g2_i1_orf1	E3 ubiquitin-protein ligase NEDD4 isoform X6 [Ostrinia furnacalis]	-1.5886	0.318	-0.4427	1.45309	0.26023
TRINITY_DN6532_c2_g1_i1_orf1	nuclear receptor coactivator 5 isoform X1 [Ostrinia furnacalis] >XP_028175326.1 nuclear receptor coactivator 5 isoform X2 [Ostrinia furnacalis]	-1.6039	0.49629	-0.643	1.21835	0.53225
TRINITY_DN512_c1_g1_i4_orf1	protein argonaute-2 [Ostrinia furnacalis] >XP_028167629.1 protein argonaute-2 [Ostrinia furnacalis]	-1.5081	0.09479	-0.6736	0.8368	1.25008
TRINITY_DN16868_c0_g2_i1_orf1	gamma-glutamylcyclotransferase-like isoform X1 [Ostrinia furnacalis]	-1.1928	0.82491	-1.255	0.86021	0.7627
TRINITY_DN213_c0_g1_i3_orf1	unnamed protein product [Chilo suppressalis]	-1.3381	0.65599	-1.0711	1.09443	0.65881
TRINITY_DN125_c0_g1_i2_orf1	PREDICTED: purine nucleoside phosphorylase isoform X1 [Microplitis demolitor]	-1.5572	-0.2222	-0.1666	1.52272	0.42338
TRINITY_DN6680_c0_g1_i1_orf1	hypothetical protein evm_009571 [Chilo suppressalis]	-1.3587	-0.4478	-0.5064	1.26159	1.05133
TRINITY_DN9019_c0_g1_i5_orf1	exosome complex component RRP46 [Ostrinia furnacalis]	-1.6608	-0.095	-0.3101	0.99642	1.06946
TRINITY_DN48694_c0_g1_i1_orfp1	TRINITY_DN48694_c0_g1_i1_m.75338 TRINITY_DN48694_c0_g1::TRINITY_DN48694_c0_g1_i1::g.75338 ORF type:internal len:84 (+),score=16.02 TRINITY_DN48694_c0_g1_i1:2-250(+)	-0.5309	0.87221	1.04725	0.27953	-1.6681
TRINITY_DN1491_c0_g1_i4_orf1	GILT-like protein 2 isoform X1 [Ostrinia furnacalis] >XP_028156245.1 GILT-like protein 2 isoform X2 [Ostrinia furnacalis] >XP_028156247.1 GILT-like protein 2 isoform X3 [Ostrinia furnacalis]	-1.0461	1.43535	0.74159	0.00733	-1.1382
TRINITY_DN261_c0_g1_i5_orfp1	TRINITY_DN261_c0_g1_i5_m.18559 TRINITY_DN261_c0_g1::TRINITY_DN261_c0_g1_i5::g.18559 ORF type:internal len:190 (+),score=69.81 TRINITY_DN261_c0_g1_i5:3-569(+)	-0.9016	1.81538	0.07345	-0.0471	-0.9402
TRINITY_DN114982_c0_g1_i1_orf1	uncharacterized protein LOC114357708 [Ostrinia furnacalis]	-0.7305	1.73345	-0.4245	0.45712	-1.0355
TRINITY_DN2058_c0_g1_i2_orf1	proteasomal ubiquitin receptor ADRM1 [Ostrinia furnacalis]	-0.8557	1.81574	-0.099	0.11281	-0.9739
TRINITY_DN110523_c0_g2_i1_orf1	uncharacterized protein LOC107036393 [Diachasma alloeum]	-0.3567	1.72681	0.3944	-0.5835	-1.1811
TRINITY_DN5028_c0_g1_i11_orf1	NTF2-related export protein [Ostrinia furnacalis]	-0.9178	1.81091	-0.3006	0.25706	-0.8496
TRINITY_DN100208_c0_g1_i1_orf1	neurofilament heavy polypeptide-like isoform X2 [Ostrinia furnacalis]	-0.1176	1.13013	0.67443	0.11339	-1.8004
TRINITY_DN1091_c0_g2_i10_orf1	macrophage mannose receptor 1-like isoform X2 [Maniola hyperantus]	-0.951	1.44873	0.48995	0.30271	-1.2904
TRINITY_DN2455_c0_g1_i12_orf1	LOW QUALITY PROTEIN: uncharacterized protein LOC114361878 [Ostrinia furnacalis]	-0.9363	1.71993	-0.3737	0.48115	-0.8911
TRINITY_DN5462_c0_g2_i1_orf1	acyl-protein thioesterase 1 [Ostrinia furnacalis] >XP_028165602.1 acyl-protein thioesterase 1 [Ostrinia furnacalis] >XP_028165603.1 acyl-protein thioesterase 1 [Ostrinia furnacalis] >XP_028165604.1 acyl-protein thioesterase 1 [Ostrinia furnacalis] >XP_028165606.1 acyl-protein thioesterase 1 [Ostrinia furnacalis] >XP_028165607.1 acyl-protein thioesterase 1 [Ostrinia furnacalis] >XP_028165608.1 acyl-protein thioesterase 1 [Ostrinia furnacalis] >XP_028165609.1 acyl-protein thioesterase 1 [Ostrinia furnacalis] >XP_028165610.1 acyl-protein thioesterase 1 [Ostrinia furnacalis]	-1.0578	1.01257	0.91049	0.47634	-1.3416
TRINITY_DN131264_c0_g1_i2_orf1	unnamed protein product [Parnassius apollo]	-0.0063	1.73387	0.09463	-0.5082	-1.3139
TRINITY_DN8659_c0_g2_i1_orf1	ubiquitin-like modifier-activating enzyme 1 [Ostrinia furnacalis]	-0.3919	1.79408	0.02678	-0.164	-1.2649
TRINITY_DN2745_c0_g1_i2_orf1	PREDICTED: tubulin alpha-1A chain-like [Papilio polytes] >XP_013164648.1 PREDICTED: tubulin alpha-1A chain-like [Papilio xuthus]	0.07417	1.60811	0.29113	-0.5528	-1.4206
TRINITY_DN17406_c0_g1_i1_orf1	uncharacterized protein LOC114356625 [Ostrinia furnacalis]	0.06111	1.62374	-0.6818	0.33267	-1.3357
TRINITY_DN13898_c0_g1_i2_orf1	protein PRRC2A-like isoform X2 [Ostrinia furnacalis]	-0.0573	1.87497	-0.1752	-0.5959	-1.0466
TRINITY_DN13063_c0_g1_i1_orf1	protein unzipped [Ostrinia furnacalis]	-0.4268	1.65871	0.04035	0.15621	-1.4285
TRINITY_DN4798_c0_g1_i3_orf1	unnamed protein product [Spodoptera exigua]	0.03117	1.2871	0.86152	-0.7549	-1.4249
TRINITY_DN13626_c0_g2_i1_orf1	charged multivesicular body protein 2b [Ostrinia furnacalis]	-0.3238	1.58805	0.67782	-0.8526	-1.0894
TRINITY_DN14904_c0_g1_i1_orf1	attacin [Ostrinia furnacalis]	-1.2887	-0.2702	0.17531	-0.3756	1.75913
TRINITY_DN5170_c0_g1_i5_orf1	hemolymph lipopolysaccharide-binding protein-like isoform X2 [Leguminivora glycinivorella]	-1.144	0.60604	0.99922	-1.284	0.82274
TRINITY_DN30177_c0_g2_i1_orf1	uncharacterized protein LOC114365032 [Ostrinia furnacalis]	-1.2124	-0.092	-0.4291	-0.0911	1.82461
TRINITY_DN20558_c0_g1_i2_orf1	Transient receptor potential channel pyrexia [Operophtera brumata]	-1.5676	-0.1469	0.41585	-0.2181	1.51676
TRINITY_DN2343_c1_g1_i2_orf1	receptor expression-enhancing protein 5-like isoform X1 [Ostrinia furnacalis] >XP_028170586.1 receptor expression-enhancing protein 5-like isoform X1 [Ostrinia furnacalis]	-1.0938	0.22593	1.04811	-1.2386	1.05831
TRINITY_DN4802_c0_g1_i4_orf1	uncharacterized protein LOC114366345 isoform X2 [Ostrinia furnacalis]	-1.4898	-0.5071	0.45952	0.01689	1.52051
TRINITY_DN1650_c0_g1_i5_orf1	uncharacterized protein LOC114355357 [Ostrinia furnacalis]	-1.6113	-0.3176	0.53285	-0.0245	1.42063
TRINITY_DN6908_c0_g1_i3_orf1	serine--pyruvate aminotransferase, mitochondrial [Ostrinia furnacalis] >XP_028157324.1 serine--pyruvate aminotransferase, mitochondrial [Ostrinia furnacalis] >XP_028157325.1 serine--pyruvate aminotransferase, mitochondrial [Ostrinia furnacalis]	-1.6845	0.38442	0.24459	-0.3083	1.36378
TRINITY_DN2743_c0_g1_i5_orf1	regucalcin-like [Ostrinia furnacalis]	-1.4681	0.11422	0.15847	-0.425	1.62045
TRINITY_DN9926_c1_g1_i1_orf1	rab GTPase-activating protein 1-like isoform X6 [Ostrinia furnacalis]	-1.0986	0.58206	0.64646	-1.2954	1.16547
TRINITY_DN24528_c0_g1_i1_orf1	uncharacterized protein CG1161-like [Ostrinia furnacalis]	-1.468	0.17047	0.45552	-0.6406	1.4826
TRINITY_DN1493_c0_g1_i5_orf1	uncharacterized protein LOC114350869 [Ostrinia furnacalis]	-1.0668	-0.0635	-0.9385	0.38742	1.68137
TRINITY_DN31609_c0_g1_i3_orf1	sorbitol dehydrogenase-like [Ostrinia furnacalis]	-1.5264	-0.0378	-0.2174	0.17174	1.6099
TRINITY_DN53281_c0_g1_i11_orf1	larval cuticle protein LCP-17-like [Ostrinia furnacalis]	-1.4751	0.02061	0.75075	-0.6513	1.35499
TRINITY_DN40945_c0_g1_i1_orf1	RNA exonuclease 4-like [Ostrinia furnacalis] >QEE79882.1 REX4 [Ostrinia furnacalis]	-1.1919	0.47571	0.50356	-1.1341	1.34666
TRINITY_DN17505_c0_g1_i15_orf1	unnamed protein product [Chilo suppressalis]	-0.8674	-0.2199	-0.1671	-0.6736	1.92808
TRINITY_DN29190_c0_g1_i4_orf1	gloverin-like [Ostrinia furnacalis]	-0.4414	0.17982	-0.903	-0.6982	1.86278
TRINITY_DN4911_c0_g1_i6_orf1	ATP-binding cassette sub-family G member 1-like isoform X2 [Ostrinia furnacalis]	-1.6545	0.24593	-0.1369	0.06931	1.47606
TRINITY_DN4813_c0_g1_i5_orf1	piwi-like protein Siwi [Ostrinia furnacalis]	-0.9176	0.79576	-0.0516	-1.2375	1.41094
TRINITY_DN4596_c0_g1_i14_orf1	aldehyde dehydrogenase, dimeric NADP-preferring isoform X7 [Ostrinia furnacalis]	-1.2031	0.64022	0.40628	-1.1393	1.29595
TRINITY_DN52761_c0_g2_i1_orf1	atlastin-like isoform X4 [Ostrinia furnacalis]	-1.4086	-0.2044	0.85556	-0.61	1.36747
TRINITY_DN27833_c0_g2_i1_orf1	uncharacterized protein LOC114359161 [Ostrinia furnacalis]	-1.5503	0.18244	-0.0761	-0.1484	1.59232
TRINITY_DN14239_c0_g1_i5_orf1	uncharacterized protein LOC114352770 [Ostrinia furnacalis] >XP_028160292.1 uncharacterized protein LOC114352770 [Ostrinia furnacalis]	-1.3432	-0.0876	-0.3923	0.08315	1.7399

TRINITY_DN29144_c0_g3_i1_orf1	PREDICTED: ADP-ribosylation factor 6 [Papilio polytes] >XP_013133321.1 PREDICTED: ADP-ribosylation factor 6 [Papilio polytes] >XP_013177129.1 PREDICTED: ADP-ribosylation factor 6 [Papilio xuthus] >XP_013177130.1 PREDICTED: ADP-ribosylation factor 6 [Papilio xuthus] >XP_014356507.1 ADP-ribosylation factor 6 [Papilio machaon] >XP_021185579.1 ADP-ribosylation factor 6 [Helicoverpa armigera] >XP_021185581.1 ADP-ribosylation factor 6 [Helicoverpa armigera] >XP_022130228.1 ADP-ribosylation factor 6 [Pieris rapae] >XP_022822139.1 ADP-ribosylation factor 6 [Spodoptera litura] >XP_022822140.1 ADP-ribosylation factor 6 [Spodoptera litura] >XP_028159104.1 ADP-ribosylation factor 6 [Ostrinia furnacalis] >XP_028159105.1 ADP-ribosylation factor 6 [Ostrinia furnacalis] >XP_028159106.1 ADP-ribosylation factor 6 [Ostrinia furnacalis] >XP_028159107.1 ADP-ribosylation factor 6 [Ostrinia furnacalis] >XP_028163222.1 ADP-ribosylation factor 6 [Ostrinia furnacalis] >XP_030022165.1 ADP-ribosylation factor 6 [Manduca sexta] >XP_030022166.1 ADP-ribosylation factor 6 [Manduca sexta] >XP_030022167.1 ADP-ribosylation factor 6 [Manduca sexta] >XP_035444169.1 ADP-ribosylation factor 6 [Spodoptera frugiperda] >XP_035444175.1 ADP-ribosylation factor 6 [Spodoptera frugiperda] >XP_038207597.1 ADP-ribosylation factor 6 [Zerene cesonia] >XP_038207598.1 ADP-ribosylation factor 6 [Zerene cesonia] >XP_045510541.1 ADP-ribosylation factor 6 [Colias croceus] >XP_045510551.1 ADP-ribosylation factor 6 [Colias croceus] >XP_045527300.1 ADP-ribosylation factor 6 [Pieris brassicae] >XP_045527302.1 ADP-ribosylation factor 6 [Pieris brassicae] >XP_047029519.1 ADP-ribosylation factor 6 [Helicoverpa zea] >XP_047029551.1 ADP-ribosylation factor 6 [Helicoverpa zea] >XP_047504621.1 ADP-ribosylation factor 6 [Pieris napi] >XP_047504631.1 ADP-ribosylation factor 6 [Pieris napi] >XP_047504640.1 ADP-ribosylation factor 6 [Pieris napi] >XP_047504648.1 ADP-ribosylation factor 6 [Pieris napi] >XP_047504657.1 ADP-ribosylation factor 6 [Pieris napi] >XP_048489067.1 ADP-ribosylation factor 6 [Plutella xylostella] >XP_048489068.1 ADP-ribosylation factor 6 [Plutella xylostella] >XP_048489069.1 ADP-ribosylation factor 6 [Plutella xylostella] >XP_049883531.1 ADP-ribosylation factor 6 [Pectinophora gossypiella] >XP_049883539.1 ADP-ribosylation factor 6 [Pectinophora gossypiella] >KAG5678369.1 hypothetical protein PVAND_008051 [Polypedilum vanderplanki] >RVE51130.1 hypothetical protein evm_004273 [Chilo suppressalis] >CAB3510283.1 unnamed protein product [Spodoptera littoralis] >CAF4796780.1 unnamed protein product [Pieris macdunnoughi] >CAG4977608.1 unnamed protein product [Parnassius apollo] >CAG9757696.1 unnamed protein product [Diatraea saccharalis] >CAG9799627.1 unnamed protein product [Chironomus riparius] >CAH0695461.1 unnamed protein product [Spodoptera exigua]	-0.3162	0.38536	0.1195	-1.6304	1.44183
TRINITY_DN15157_c0_g1_i1_orf1	UDP-glycosyltransferase UGT40AM2 [Ostrinia furnacalis]	-1.5473	-0.3971	0.40823	0.02584	1.51028
TRINITY_DN1978_c0_g1_i4_orf1	RNA exonuclease 4-like [Ostrinia furnacalis]	-0.8072	0.3731	0.47497	-1.4316	1.39074
TRINITY_DN71832_c0_g1_i1_orf1	basement membrane-specific heparan sulfate proteoglycan core protein isoform X13 [Ostrinia furnacalis]	-0.1808	1.42419	-0.0578	-1.6494	0.46383
TRINITY_DN2367_c1_g1_i20_orf1	RNA exonuclease 4-like [Ostrinia furnacalis]	-0.4204	1.02945	1.08679	-1.6044	-0.0915
TRINITY_DN23183_c1_g1_i2_orf1	myotubularin-related protein 9 [Ostrinia furnacalis]	-0.4287	1.80042	0.10472	-1.2249	-0.2515
TRINITY_DN3962_c0_g1_i6_orf1	follicle-stimulating hormone receptor-like [Ostrinia furnacalis]	-0.6887	1.51189	0.18937	-1.4259	0.41333
TRINITY_DN11666_c0_g1_i6_orf1	P protein-like [Ostrinia furnacalis] >XP_028167089.1 P protein-like [Ostrinia furnacalis]	-0.6415	1.65411	-0.1373	-1.2893	0.41397
TRINITY_DN95850_c0_g1_i1_orf1	alpha,alpha-trehalose-phosphate synthase [UDP-forming] isoform X2 [Chelonus insularis]	-1.0468	1.08837	0.30465	-1.306	0.95979
TRINITY_DN5422_c0_g1_i1_orf1	nitrilase and fragile histidine triad fusion protein NitFhit isoform X1 [Ostrinia furnacalis]	-0.1839	1.06481	0.20846	-1.8087	0.71931
TRINITY_DN19537_c0_g1_i1_orf1	disintegrin and metalloproteinase domain-containing protein 10 isoform X1 [Ostrinia furnacalis] >XP_028172845.1 disintegrin and metalloproteinase domain-containing protein 10 isoform X2 [Ostrinia furnacalis]	-0.7615	1.76315	0.06141	-1.1409	0.07783
TRINITY_DN8660_c0_g1_i1_orf1	chondroitin sulfate synthase 2 [Trichoplusia ni]	-0.8671	1.32529	0.13905	-1.3696	0.77238
TRINITY_DN8306_c0_g1_i4_orf1	NAD(P) transhydrogenase, mitochondrial-like [Ostrinia furnacalis] >XP_028175067.1 NAD(P) transhydrogenase, mitochondrial-like [Ostrinia furnacalis] >XP_028175068.1 NAD(P) transhydrogenase, mitochondrial-like [Ostrinia furnacalis] >XP_028175069.1 NAD(P) transhydrogenase, mitochondrial-like [Ostrinia furnacalis]	-0.5342	1.75824	-0.0147	-1.2724	0.06305
TRINITY_DN27456_c0_g2_i1_orf1	organic cation transporter-like protein [Ostrinia furnacalis]	-0.3189	1.62381	-0.0731	-1.4812	0.24935
TRINITY_DN13419_c0_g1_i5_orf1	atrial natriuretic peptide-converting enzyme-like [Ostrinia furnacalis]	-0.3271	1.4342	-0.2205	-1.5375	0.65088
TRINITY_DN7682_c0_g1_i2_orf1	myogenesis-regulating glycosidase isoform X1 [Ostrinia furnacalis] >XP_028158488.1 myogenesis-regulating glycosidase isoform X1 [Ostrinia furnacalis] >XP_028158489.1 myogenesis-regulating glycosidase isoform X1 [Ostrinia furnacalis] >XP_028158490.1 myogenesis-regulating glycosidase isoform X1 [Ostrinia furnacalis]	-0.5017	1.77175	0.1858	-1.2353	-0.2205
TRINITY_DN36324_c0_g1_i12_orf1	motile sperm domain-containing protein 1-like [Ostrinia furnacalis]	-0.6248	1.35037	0.35235	-1.559	0.4811
TRINITY_DN667_c0_g1_i5_orf1	unnamed protein product [Arctia plantaginis]	0.08972	1.51892	-0.1533	-1.6227	0.16741
TRINITY_DN23194_c0_g1_i4_orf1	nucleobindin-2 isoform X2 [Ostrinia furnacalis]	-0.5856	1.635	0.38871	-1.351	-0.0871
TRINITY_DN4711_c0_g1_i2_orf1	xanthine dehydrogenase-like isoform X1 [Ostrinia furnacalis] >XP_028179066.1 xanthine dehydrogenase-like isoform X1 [Ostrinia furnacalis] >XP_028179067.1 xanthine dehydrogenase-like isoform X1 [Ostrinia furnacalis] >XP_028179068.1 xanthine dehydrogenase-like isoform X1 [Ostrinia furnacalis] >XP_028179069.1 xanthine dehydrogenase-like isoform X1 [Ostrinia furnacalis]	-0.4742	1.62517	0.45437	-1.3679	-0.2374
TRINITY_DN19303_c0_g1_i5_orf1	lipopolysaccharide-induced tumor necrosis factor-alpha factor-like [Ostrinia furnacalis]	0.15729	1.18704	0.8334	-1.5891	-0.5887
TRINITY_DN18756_c0_g1_i6_orf1	COP9 signalosome complex subunit 6 [Ostrinia furnacalis]	-0.3751	1.64705	0.51829	-1.2662	-0.524
TRINITY_DN843_c0_g1_i5_orf1	fasciclin-3 isoform X4 [Helicoverpa zea]	-0.6797	1.65963	0.08081	-1.3096	0.24889
TRINITY_DN23020_c0_g1_i1_orf1	actin-related protein 2/3 complex subunit 1A-A [Ostrinia furnacalis] >XP_028170151.1 actin-related protein 2/3 complex subunit 1A-A [Ostrinia furnacalis]	-0.4689	1.33731	0.74652	-1.5593	-0.0557
TRINITY_DN23042_c0_g1_i1_orf1	protoporphyrinogen oxidase [Ostrinia furnacalis]	-0.6742	1.65857	0.35146	-1.2919	-0.0438
TRINITY_DN2904_c0_g1_i4_orf1	ATP-dependent DNA/RNA helicase DHX36 isoform X1 [Ostrinia furnacalis]	-0.7821	1.52233	-0.2935	-1.1942	0.74742
TRINITY_DN62729_c0_g1_i13_orf1	G protein-coupled receptor kinase 2 isoform X3 [Manduca sexta]	-0.4362	1.3432	0.24688	-1.6444	0.49051
TRINITY_DN5569_c0_g1_i1_orf1	PREDICTED: chromatin complexes subunit BAP18 isoform X1 [Eufriesea mexicana]	-0.8392	1.74911	0.06685	-1.1027	0.12598
TRINITY_DN38106_c0_g1_i6_orf1	unnamed protein product [Pieris macdunnoughi]	-0.8253	0.90834	1.44438	-1.1109	-0.4165
TRINITY_DN325_c0_g1_i15_orf1	protein draper-like [Ostrinia furnacalis]	-0.954	0.67937	1.6192	-0.8987	-0.4458
TRINITY_DN132_c0_g2_i2_orf1	alpha-tocopherol transfer protein-like isoform X1 [Ostrinia furnacalis]	-1.1957	0.95664	1.40088	-0.675	-0.4868
TRINITY_DN1293_c1_g1_i4_orf1	putative fatty acyl-CoA reductase CG5065 [Ostrinia furnacalis]	-0.6123	0.69473	1.62698	-0.7236	-0.9858

TRINITY_DN20356_c0_g1_i5_orf1	uncharacterized protein LOC114362428 [Ostrinia furnacalis]	-0.5071	-0.1106	1.92876	-0.3807	-0.9303
TRINITY_DN60949_c0_g1_i4_orf1	aldo-keto reductase AKR2E4-like [Galleria mellonella]	-0.7066	1.09138	1.29785	-1.165	-0.5176
TRINITY_DN919_c0_g1_i7_orf1	facilitated trehalose transporter Tret1-like [Ostrinia furnacalis] >XP_028161733.1 facilitated trehalose transporter Tret1-like [Ostrinia furnacalis]	-0.7045	0.41212	1.79098	-0.7902	-0.7084
TRINITY_DN6205_c0_g1_i8_orf1	phenoloxidase-activating factor 2-like [Ostrinia furnacalis]	-0.3826	0.83882	1.49705	-0.9524	-1.0009
TRINITY_DN26488_c0_g1_i6_orf1	phosphatidate phosphatase LPIN2 isoform X1 [Ostrinia furnacalis] >XP_028176373.1 phosphatidate phosphatase LPIN2 isoform X1 [Ostrinia furnacalis] >XP_028176374.1 phosphatidate phosphatase LPIN2 isoform X2 [Ostrinia furnacalis] >XP_028176375.1 phosphatidate phosphatase LPIN2 isoform X3 [Ostrinia furnacalis] >XP_028176376.1 phosphatidate phosphatase LPIN2 isoform X4 [Ostrinia furnacalis]	-0.9949	0.81292	1.51799	-0.9443	-0.3918
