# Supplementary material for: Comparative Proteomics and Metabonomics Analysis of Different Diapause Stages Revealed a New Regulation Mechanism of Diapause in Loxostege sticticalis (Lepidoptera: Pyralidae)
Source: Molecules. 2024 Jul 25;29(15):3472. doi: 10.3390/molecules29153472 (PMC11314584; doi:10.3390/molecules29153472)
Supplement: Supplementary file 1 [file molecules-29-03472-s001.zip › analysis process/proteomic/Cluster analysis of expression patterns/Up/RDvsPreD up.pdf]

Accession
TRINITY_DN66040_c0_g1_i2_orf1
TRINITY_DN64297_c0_g1_i1_orf1
TRINITY_DN38307_c0_g1_i1_orfp1
TRINITY_DN5080_c0_g1_i5_orf1
TRINITY_DN64181_c0_g1_i1_orf1
TRINITY_DN276_c0_g1_i2_orf1
TRINITY_DN143895_c0_g1_i1_orf1
TRINITY_DN1370_c0_g1_i2_orf1
TRINITY_DN4767_c0_g1_i4_orf1
TRINITY_DN5444_c0_g1_i1_orfp1
TRINITY_DN703_c0_g1_i2_orf1
TRINITY_DN51813_c0_g1_i1_orf1
TRINITY_DN12009_c0_g1_i1_orf1
TRINITY_DN636_c1_g1_i9_orf1
TRINITY_DN5080_c0_g1_i1_orf1
TRINITY_DN59885_c0_g1_i3_orf1
TRINITY_DN20560_c0_g1_i6_orf1
TRINITY_DN67193_c0_g1_i1_orf1
TRINITY_DN18218_c0_g1_i7_orf1
TRINITY_DN18338_c0_g1_i6_orf1
TRINITY_DN45948_c1_g1_i1_orf1
TRINITY_DN6423_c0_g1_i6_orf1
TRINITY_DN7900_c0_g1_i4_orf1
TRINITY_DN33272_c0_g1_i5_orf1
TRINITY_DN12526_c0_g1_i5_orf1
TRINITY_DN4255_c0_g1_i11_orf1
TRINITY_DN42719_c0_g2_i1_orf1
TRINITY_DN4767_c0_g1_i6_orf1
TRINITY_DN467_c3_g1_i5_orf1
TRINITY_DN801_c0_g1_i2_orf1
TRINITY_DN3616_c0_g1_i4_orf1
TRINITY_DN13760_c1_g1_i1_orf1
TRINITY_DN7040_c0_g2_i1_orf1
TRINITY_DN19662_c0_g2_i1_orf1
TRINITY_DN15247_c0_g1_i2_orf1
TRINITY_DN106156_c1_g1_i1_orf1
TRINITY_DN19043_c0_g2_i1_orf1
TRINITY_DN1093_c0_g1_i4_orf1
TRINITY_DN20717_c0_g1_i1_orf1
TRINITY_DN3707_c0_g1_i1_orf1
TRINITY_DN121650_c0_g1_i1_orf1
TRINITY_DN4748_c0_g1_i5_orf1
TRINITY_DN52316_c0_g1_i1_orf1
TRINITY_DN9538_c1_g3_i1_orf1
TRINITY_DN29604_c0_g2_i2_orf1
TRINITY_DN6122_c0_g1_i6_orf1
TRINITY_DN1175_c1_g1_i1_orf1
TRINITY_DN20676_c0_g1_i6_orf1
TRINITY_DN703_c13_g1_i1_orf1
TRINITY_DN46625_c0_g1_i1_orf1
TRINITY_DN5655_c0_g1_i2_orf1
TRINITY_DN12671_c0_g1_i6_orf1
TRINITY_DN60946_c0_g2_i3_orf1
TRINITY_DN45220_c0_g1_i1_orf1

TRINITY_DN1540_c0_g1_i7_orf1
TRINITY_DN41_c0_g1_i3_orf1
TRINITY_DN56308_c0_g1_i2_orf1
TRINITY_DN19662_c4_g1_i1_orf1
TRINITY_DN25273_c0_g1_i1_orf1

TRINITY_DN30169_c0_g1_i1_orfp1

TRINITY_DN9239_c0_g1_i1_orf1
TRINITY_DN49147_c0_g2_i1_orf1

TRINITY_DN2311_c0_g3_i1_orf1

TRINITY_DN59388_c0_g1_i1_orf1
TRINITY_DN28221_c0_g2_i1_orf1
TRINITY_DN1423_c0_g1_i8_orf1
TRINITY_DN993_c0_g1_i7_orf1
TRINITY_DN13686_c0_g2_i1_orf1
TRINITY_DN11826_c0_g1_i4_orf1
TRINITY_DN2024_c0_g1_i12_orfp1
TRINITY_DN24971_c0_g1_i3_orf1
TRINITY_DN85412_c0_g1_i1_orf1
TRINITY_DN3609_c0_g1_i6_orf1
TRINITY_DN42719_c0_g1_i1_orf1
TRINITY_DN28711_c0_g1_i1_orf1
TRINITY_DN391_c0_g1_i4_orf1
TRINITY_DN25976_c0_g1_i4_orf1
TRINITY_DN7854_c0_g1_i4_orf1
TRINITY_DN64772_c0_g1_i1_orf1
TRINITY_DN81488_c0_g1_i1_orf1
TRINITY_DN15812_c0_g1_i2_orf1
TRINITY_DN7040_c0_g1_i4_orf1
TRINITY_DN699_c0_g2_i1_orf1
TRINITY_DN80328_c0_g1_i9_orf1
TRINITY_DN2803_c4_g1_i1_orf1
TRINITY_DN585_c0_g1_i12_orf1
TRINITY_DN80328_c0_g1_i5_orf1
TRINITY_DN76216_c0_g2_i3_orf1
TRINITY_DN12865_c0_g1_i1_orf1
TRINITY_DN2442_c0_g1_i2_orf1
TRINITY_DN104663_c1_g1_i2_orf1

TRINITY_DN21555_c0_g1_i4_orf1

TRINITY_DN71308_c0_g1_i4_orf1
TRINITY_DN97042_c0_g1_i6_orf1
TRINITY_DN26301_c0_g1_i1_orf1
TRINITY_DN20680_c0_g1_i5_orf1
TRINITY_DN124654_c0_g1_i1_orf1
TRINITY_DN45037_c0_g1_i1_orf1
TRINITY_DN13799_c0_g1_i1_orf1
TRINITY_DN10138_c0_g1_i1_orf1
TRINITY_DN26149_c0_g1_i5_orf1
TRINITY_DN6004_c0_g1_i1_orf1
TRINITY_DN110231_c0_g1_i1_orf1
TRINITY_DN7183_c0_g1_i2_orf1
TRINITY_DN9733_c0_g1_i2_orf1

TRINITY_DN2515_c0_g1_i6_orf1
TRINITY_DN2049_c1_g1_i3_orf1
TRINITY_DN32532_c0_g1_i1_orf1
TRINITY_DN3949_c0_g1_i1_orf1
TRINITY_DN113353_c0_g1_i1_orf1
TRINITY_DN40191_c2_g1_i1_orf1
TRINITY_DN1110_c1_g1_i9_orf1
TRINITY_DN7618_c0_g1_i4_orf1
TRINITY_DN48878_c0_g2_i1_orf1
TRINITY_DN52944_c0_g1_i1_orf1
TRINITY_DN44073_c0_g1_i3_orf1

TRINITY_DN5467_c0_g1_i5_orf1

TRINITY_DN81031_c0_g1_i1_orf1

TRINITY_DN1423_c0_g1_i4_orf1

TRINITY_DN3301_c0_g1_i2_orf1
TRINITY_DN28711_c1_g1_i1_orf1
TRINITY_DN27035_c0_g1_i1_orf1
TRINITY_DN1465_c2_g1_i2_orf1
TRINITY_DN71699_c0_g1_i1_orf1
TRINITY_DN2489_c0_g1_i1_orf1
TRINITY_DN2794_c1_g1_i8_orf1
TRINITY_DN688_c0_g1_i8_orf1
TRINITY_DN578_c0_g1_i5_orf1
TRINITY_DN18388_c0_g1_i6_orf1
TRINITY_DN17574_c0_g1_i2_orf1
TRINITY_DN4228_c0_g1_i5_orf1
TRINITY_DN2407_c0_g1_i6_orf1
TRINITY_DN64141_c0_g1_i4_orf1
TRINITY_DN9239_c0_g2_i2_orf1
TRINITY_DN23398_c0_g1_i1_orf1
TRINITY_DN18128_c0_g1_i4_orf1
TRINITY_DN376_c1_g1_i1_orf1
TRINITY_DN5420_c0_g1_i2_orf1
TRINITY_DN8771_c0_g1_i5_orf1
TRINITY_DN2425_c0_g1_i3_orf1
TRINITY_DN31417_c0_g1_i3_orf1
TRINITY_DN33272_c0_g1_i1_orf1
TRINITY_DN4189_c0_g2_i1_orf1
TRINITY_DN479_c6_g1_i2_orf1
TRINITY_DN1091_c0_g1_i1_orf1
TRINITY_DN2227_c0_g1_i5_orf1
TRINITY_DN139537_c0_g1_i1_orf1
TRINITY_DN71863_c0_g1_i2_orf1
TRINITY_DN2097_c1_g2_i2_orf1

TRINITY_DN2953_c1_g1_i10_orf1

TRINITY_DN18804_c0_g1_i5_orf1
TRINITY_DN23364_c0_g1_i1_orf1
TRINITY_DN7102_c0_g1_i5_orf1
TRINITY_DN33728_c0_g2_i1_orf1
TRINITY_DN2069_c1_g1_i8_orf1
TRINITY_DN8480_c0_g1_i1_orf1
TRINITY_DN19110_c0_g1_i2_orf1
TRINITY_DN104_c0_g1_i4_orf1
TRINITY_DN1407_c0_g1_i12_orf1
TRINITY_DN125150_c0_g1_i1_orf1

TRINITY_DN1986_c0_g1_i1_orf1
TRINITY_DN1287_c0_g1_i5_orf1
TRINITY_DN126127_c0_g1_i1_orf1
TRINITY_DN2170_c1_g1_i3_orf1
TRINITY_DN7633_c0_g1_i1_orf1
TRINITY_DN13973_c0_g1_i6_orf1
TRINITY_DN198_c2_g1_i2_orf1
TRINITY_DN36899_c0_g1_i1_orf1
TRINITY_DN5132_c0_g1_i4_orf1

TRINITY_DN10090_c0_g1_i1_orf1

TRINITY_DN2684_c0_g2_i3_orf1
TRINITY_DN399_c3_g2_i6_orf1
TRINITY_DN361_c0_g1_i5_orf1
TRINITY_DN5553_c0_g1_i4_orf1
TRINITY_DN23354_c0_g1_i7_orf1
TRINITY_DN8985_c0_g1_i4_orf1
TRINITY_DN4076_c1_g2_i2_orf1
TRINITY_DN2835_c0_g1_i6_orf1
TRINITY_DN30498_c0_g1_i3_orf1
TRINITY_DN9593_c0_g1_i2_orf1
TRINITY_DN6988_c0_g1_i3_orf1

TRINITY_DN20009_c0_g1_i1_orf1

TRINITY_DN2171_c0_g1_i1_orf1
TRINITY_DN1098_c1_g1_i4_orf1
TRINITY_DN23167_c0_g2_i1_orf1
TRINITY_DN97138_c0_g1_i2_orf1
TRINITY_DN2205_c0_g1_i3_orf1
TRINITY_DN86833_c0_g3_i1_orf1
TRINITY_DN23746_c0_g1_i2_orf1
TRINITY_DN22053_c0_g1_i13_orf1
TRINITY_DN5064_c0_g1_i4_orf1
TRINITY_DN4602_c0_g1_i4_orf1
TRINITY_DN6423_c0_g1_i5_orf1
TRINITY_DN1407_c0_g1_i5_orf1
TRINITY_DN26337_c0_g1_i3_orf1
TRINITY_DN4256_c0_g1_i1_orf1

TRINITY_DN3952_c0_g1_i3_orf1

TRINITY_DN7128_c0_g1_i7_orf1
TRINITY_DN745_c7_g1_i1_orf1
TRINITY_DN6462_c0_g1_i5_orf1
TRINITY_DN875_c0_g1_i3_orf1
TRINITY_DN664_c0_g1_i18_orf1
TRINITY_DN70485_c0_g1_i2_orf1
TRINITY_DN9920_c0_g1_i1_orf1
TRINITY_DN97097_c0_g1_i4_orf1
TRINITY_DN23229_c0_g1_i2_orf1
TRINITY_DN1630_c0_g1_i6_orf1
TRINITY_DN1957_c0_g1_i4_orf1
TRINITY_DN64892_c0_g1_i1_orf1

TRINITY_DN14944_c0_g1_i9_orf1

TRINITY_DN19821_c0_g2_i4_orf1

TRINITY_DN276_c0_g1_i1_orf1

TRINITY_DN89083_c0_g1_i1_orf1
TRINITY_DN8703_c0_g1_i2_orf1
TRINITY_DN143637_c0_g1_i1_orf1
TRINITY_DN140_c1_g1_i2_orf1

TRINITY_DN1703_c0_g1_i6_orf1

TRINITY_DN1767_c0_g2_i15_orf1
TRINITY_DN3005_c0_g1_i7_orf1
TRINITY_DN2146_c0_g2_i1_orf1
TRINITY_DN9615_c0_g1_i1_orf1
TRINITY_DN53427_c0_g1_i2_orf1
TRINITY_DN14250_c0_g1_i1_orf1
TRINITY_DN2508_c0_g1_i2_orf1
TRINITY_DN4276_c0_g1_i6_orf1
TRINITY_DN4273_c1_g1_i5_orf1
TRINITY_DN307_c1_g1_i1_orf1
TRINITY_DN1144_c0_g1_i10_orf1
TRINITY_DN7590_c0_g1_i4_orf1
TRINITY_DN45271_c0_g1_i1_orf1
TRINITY_DN37585_c0_g1_i1_orf1
TRINITY_DN2566_c0_g1_i5_orf1
TRINITY_DN19951_c0_g1_i5_orf1
TRINITY_DN41761_c0_g1_i4_orf1

TRINITY_DN895_c0_g2_i1_orf1

TRINITY_DN15865_c0_g2_i2_orf1
TRINITY_DN37654_c0_g1_i5_orf1
TRINITY_DN12286_c1_g1_i2_orf1
TRINITY_DN36856_c0_g1_i1_orf1

TRINITY_DN2348_c0_g1_i1_orfp1

TRINITY_DN13088_c0_g1_i5_orf1
TRINITY_DN38412_c0_g1_i1_orf1
TRINITY_DN4394_c0_g2_i1_orf1
TRINITY_DN13887_c0_g1_i5_orf1
TRINITY_DN108433_c0_g1_i1_orf1
TRINITY_DN712_c0_g2_i1_orf1
TRINITY_DN28729_c0_g1_i9_orf1
TRINITY_DN41_c0_g1_i5_orf1
TRINITY_DN9412_c0_g1_i1_orf1
TRINITY_DN36144_c0_g1_i3_orf1
TRINITY_DN7867_c0_g1_i1_orf1
TRINITY_DN140212_c0_g1_i1_orf1
TRINITY_DN1569_c0_g1_i6_orf1
TRINITY_DN6275_c0_g1_i3_orf1
TRINITY_DN15291_c0_g1_i11_orf1
TRINITY_DN166_c0_g1_i4_orf1

TRINITY_DN3545_c0_g1_i6_orf1

TRINITY_DN56430_c0_g1_i1_orf1
TRINITY_DN110402_c0_g2_i1_orf1

TRINITY_DN48250_c0_g1_i1_orf1
TRINITY_DN4886_c0_g1_i6_orf1
TRINITY_DN2395_c0_g1_i7_orf1
TRINITY_DN8580_c0_g1_i12_orf1
TRINITY_DN44709_c0_g1_i1_orf1
TRINITY_DN1870_c0_g1_i6_orf1

TRINITY_DN862_c0_g1_i4_orf1

TRINITY_DN2793_c0_g2_i1_orf1
TRINITY_DN42854_c0_g3_i2_orf1
TRINITY_DN1533_c0_g2_i1_orf1
TRINITY_DN3896_c0_g1_i1_orf1
TRINITY_DN21743_c0_g1_i1_orf1
TRINITY_DN1720_c0_g1_i3_orf1
TRINITY_DN3292_c2_g2_i1_orf1
TRINITY_DN3483_c0_g1_i5_orf1
TRINITY_DN31676_c0_g1_i4_orf1

TRINITY_DN1491_c0_g1_i8_orf1

TRINITY_DN14458_c0_g1_i2_orf1
TRINITY_DN2107_c0_g2_i3_orf1
TRINITY_DN82104_c0_g1_i5_orf1
TRINITY_DN445_c0_g1_i2_orf1
TRINITY_DN98313_c0_g1_i1_orf1
TRINITY_DN7957_c0_g1_i5_orf1

TRINITY_DN10680_c0_g1_i5_orf1

TRINITY_DN18650_c0_g1_i1_orf1
TRINITY_DN14904_c1_g2_i2_orf1
TRINITY_DN1612_c0_g1_i3_orf1
TRINITY_DN13856_c0_g1_i1_orf1
TRINITY_DN46090_c0_g3_i1_orf1
TRINITY_DN20614_c0_g1_i1_orf1
TRINITY_DN22443_c0_g2_i3_orf1
TRINITY_DN12024_c0_g1_i4_orf1
TRINITY_DN5029_c0_g1_i1_orf1
TRINITY_DN2897_c0_g2_i1_orf1
TRINITY_DN18027_c0_g2_i1_orf1
TRINITY_DN19043_c0_g3_i2_orf1
TRINITY_DN1480_c0_g1_i5_orf1
TRINITY_DN20796_c0_g1_i4_orf1
TRINITY_DN12555_c0_g1_i1_orf1
TRINITY_DN15411_c0_g1_i4_orf1
TRINITY_DN3675_c0_g1_i1_orf1
TRINITY_DN5122_c0_g1_i3_orf1
TRINITY_DN18592_c0_g1_i4_orf1
TRINITY_DN7291_c0_g1_i3_orf1
TRINITY_DN4041_c0_g1_i6_orf1
TRINITY_DN56690_c0_g1_i4_orf1
TRINITY_DN131471_c0_g1_i1_orf1
TRINITY_DN12301_c0_g1_i1_orf1

TRINITY_DN14587_c0_g1_i7_orf1

TRINITY_DN569_c0_g3_i12_orf1
TRINITY_DN1732_c0_g1_i15_orf1
TRINITY_DN5538_c0_g1_i1_orf1
TRINITY_DN88876_c0_g1_i1_orf1

TRINITY_DN6199_c2_g1_i3_orf1

TRINITY_DN251_c0_g1_i2_orf1
TRINITY_DN8674_c0_g2_i1_orf1
TRINITY_DN17326_c0_g1_i5_orf1
TRINITY_DN6015_c1_g1_i3_orf1
TRINITY_DN116467_c0_g1_i1_orf1
TRINITY_DN63389_c0_g1_i4_orf1
TRINITY_DN10290_c0_g1_i7_orf1
TRINITY_DN32448_c0_g1_i1_orf1
TRINITY_DN57348_c0_g1_i4_orf1
TRINITY_DN23978_c0_g1_i2_orf1
TRINITY_DN42159_c0_g1_i6_orf1
TRINITY_DN12193_c0_g1_i6_orf1
TRINITY_DN61135_c0_g1_i1_orf1
TRINITY_DN17437_c0_g1_i1_orf1
TRINITY_DN4635_c0_g1_i4_orf1
TRINITY_DN22674_c0_g1_i2_orf1

TRINITY_DN1833_c0_g1_i5_orf1

TRINITY_DN17693_c0_g1_i10_orf1
TRINITY_DN53233_c0_g1_i1_orf1

TRINITY_DN291_c0_g1_i2_orf1

TRINITY_DN17772_c0_g2_i3_orf1
TRINITY_DN52864_c0_g1_i1_orf1
TRINITY_DN246_c1_g1_i5_orf1
TRINITY_DN1393_c0_g1_i2_orf1
TRINITY_DN4550_c1_g1_i19_orf1
TRINITY_DN14154_c0_g1_i1_orf1
TRINITY_DN12024_c0_g2_i2_orf1
TRINITY_DN8245_c0_g1_i4_orf1
TRINITY_DN8700_c9_g1_i1_orf1
TRINITY_DN48023_c1_g1_i1_orf1
TRINITY_DN11620_c0_g1_i2_orf1
TRINITY_DN53294_c0_g1_i1_orf1

TRINITY_DN41321_c1_g1_i3_orf1

TRINITY_DN781_c0_g1_i7_orf1

TRINITY_DN10646_c0_g1_i2_orf1

TRINITY_DN2584_c0_g1_i7_orf1

TRINITY_DN19746_c0_g1_i5_orf1

TRINITY_DN16868_c0_g2_i1_orf1

TRINITY_DN4708_c0_g1_i5_orf1

TRINITY_DN34426_c0_g1_i1_orf1
TRINITY_DN4189_c0_g1_i4_orf1
TRINITY_DN32514_c0_g2_i1_orf1
TRINITY_DN40669_c0_g2_i1_orf1
TRINITY_DN56155_c0_g1_i1_orf1
TRINITY_DN30154_c0_g1_i1_orf1
TRINITY_DN1124_c0_g1_i7_orf1
TRINITY_DN87803_c0_g1_i2_orf1
TRINITY_DN18624_c0_g1_i5_orf1

TRINITY_DN3190_c0_g1_i1_orf1

TRINITY_DN52553_c0_g2_i1_orf1
TRINITY_DN381_c0_g1_i1_orf1
TRINITY_DN15175_c0_g1_i1_orf1
TRINITY_DN1326_c0_g1_i1_orf1
TRINITY_DN10824_c0_g1_i3_orf1
TRINITY_DN4021_c0_g1_i1_orf1
TRINITY_DN129835_c0_g1_i2_orf1
TRINITY_DN2101_c0_g1_i6_orf1
TRINITY_DN138481_c0_g1_i5_orf1
TRINITY_DN644_c0_g1_i1_orf1
TRINITY_DN74538_c0_g1_i1_orf1
TRINITY_DN4592_c0_g1_i1_orf1
TRINITY_DN661_c0_g1_i1_orf1
TRINITY_DN9311_c0_g1_i1_orf1
TRINITY_DN21719_c0_g1_i2_orf1
TRINITY_DN18539_c0_g1_i1_orf1
TRINITY_DN13576_c0_g1_i1_orf1
TRINITY_DN98147_c0_g2_i1_orf1
TRINITY_DN5581_c0_g1_i1_orf1
TRINITY_DN38274_c0_g1_i1_orf1
TRINITY_DN619_c0_g1_i1_orf1
TRINITY_DN2400_c0_g1_i1_orf1
TRINITY_DN5829_c0_g1_i1_orf1
TRINITY_DN35351_c0_g1_i3_orf1
TRINITY_DN57998_c1_g1_i1_orf1
TRINITY_DN2946_c0_g1_i1_orf1
TRINITY_DN26882_c0_g1_i1_orf1
TRINITY_DN28922_c0_g1_i2_orf1
TRINITY_DN906_c0_g1_i4_orf1
TRINITY_DN2908_c0_g1_i1_orf1
TRINITY_DN57998_c1_g3_i1_orf1
TRINITY_DN3310_c0_g1_i1_orf1
TRINITY_DN15222_c0_g1_i4_orf1
TRINITY_DN338_c0_g1_i1_orf1
TRINITY_DN77425_c0_g1_i2_orf1
TRINITY_DN4384_c0_g1_i5_orf1
TRINITY_DN19639_c0_g2_i1_orf1
TRINITY_DN98334_c0_g1_i1_orf1
TRINITY_DN778_c0_g1_i1_orf1

TRINITY_DN2002_c0_g1_i5_orfp1

TRINITY_DN10747_c0_g1_i5_orf1
TRINITY_DN3616_c0_g2_i1_orf1
TRINITY_DN12387_c0_g1_i1_orf1
TRINITY_DN9282_c0_g1_i2_orf1

TRINITY_DN31_c0_g1_i3_orfp1

TRINITY_DN138481_c0_g1_i2_orf1
TRINITY_DN65681_c0_g1_i1_orf1
TRINITY_DN27264_c0_g1_i1_orf1
TRINITY_DN7539_c0_g1_i2_orf1
TRINITY_DN49785_c1_g1_i3_orf1
TRINITY_DN25534_c0_g1_i1_orf1
TRINITY_DN4676_c0_g1_i16_orf1
TRINITY_DN12387_c1_g2_i1_orf1
TRINITY_DN1935_c0_g1_i1_orf1

TRINITY_DN7549_c0_g1_i1_orf1
TRINITY_DN54524_c0_g1_i6_orf1
TRINITY_DN12671_c0_g1_i4_orf1
TRINITY_DN2140_c0_g1_i1_orf1
TRINITY_DN53866_c0_g1_i1_orf1
TRINITY_DN5595_c0_g1_i1_orf1

TRINITY_DN5907_c0_g1_i4_orf1

TRINITY_DN91533_c0_g1_i1_orf1
TRINITY_DN19990_c0_g1_i1_orf1
TRINITY_DN1749_c0_g2_i2_orf1
TRINITY_DN68397_c0_g1_i2_orf1
TRINITY_DN2652_c0_g2_i1_orf1
TRINITY_DN3056_c0_g1_i1_orf1
TRINITY_DN5444_c0_g2_i1_orf1
TRINITY_DN49143_c0_g1_i1_orf1
TRINITY_DN31314_c0_g1_i4_orf1
TRINITY_DN74086_c0_g1_i1_orf1
TRINITY_DN1749_c0_g1_i1_orf1
TRINITY_DN661_c0_g2_i2_orf1
TRINITY_DN58125_c0_g1_i1_orf1
TRINITY_DN52553_c0_g1_i1_orf1
TRINITY_DN2896_c0_g1_i2_orf1
TRINITY_DN1326_c0_g1_i2_orf1
TRINITY_DN7785_c0_g1_i1_orf1
TRINITY_DN6470_c0_g3_i2_orf1
TRINITY_DN4324_c0_g1_i1_orf1
TRINITY_DN20793_c0_g2_i1_orf1
TRINITY_DN1902_c0_g1_i4_orf1
TRINITY_DN27968_c0_g2_i2_orf1
TRINITY_DN843_c0_g1_i2_orf1
TRINITY_DN143497_c0_g1_i1_orf1

TRINITY_DN2290_c0_g1_i2_orfp1

TRINITY_DN696_c1_g1_i10_orf1
TRINITY_DN11670_c0_g1_i1_orf1
TRINITY_DN267_c0_g1_i1_orf1
TRINITY_DN5664_c0_g1_i1_orf1
TRINITY_DN33365_c0_g1_i1_orf1
TRINITY_DN4367_c0_g1_i1_orf1
TRINITY_DN7123_c0_g1_i1_orf1
TRINITY_DN1308_c0_g1_i4_orf1
TRINITY_DN3255_c0_g1_i1_orf1
TRINITY_DN3464_c0_g1_i1_orf1

TRINITY_DN206_c0_g1_i8_orf1

TRINITY_DN1196_c0_g1_i4_orf1
TRINITY_DN4688_c0_g1_i2_orf1
TRINITY_DN54366_c0_g1_i1_orf1
TRINITY_DN268_c1_g1_i7_orf1
TRINITY_DN11970_c0_g1_i4_orf1
TRINITY_DN110519_c0_g1_i1_orf1
TRINITY_DN42337_c0_g1_i5_orf1

TRINITY_DN261_c0_g1_i5_orfp1

TRINITY_DN82426_c0_g1_i6_orfp1
TRINITY_DN113272_c0_g1_i1_orf1
TRINITY_DN10766_c0_g1_i1_orf1

TRINITY_DN206_c0_g1_i11_orf1

TRINITY_DN3715_c0_g1_i2_orf1
TRINITY_DN54269_c0_g1_i3_orf1
TRINITY_DN5757_c0_g1_i1_orf1
TRINITY_DN1563_c0_g1_i4_orf1

TRINITY_DN25987_c0_g1_i5_orf1

TRINITY_DN806_c0_g2_i1_orf1
TRINITY_DN4125_c0_g1_i14_orf1
TRINITY_DN38431_c0_g1_i1_orf1
TRINITY_DN114982_c0_g1_i1_orf1
TRINITY_DN14856_c0_g1_i1_orf1
TRINITY_DN125427_c0_g1_i1_orf1
TRINITY_DN10304_c0_g2_i1_orf1
TRINITY_DN2058_c0_g1_i2_orf1
TRINITY_DN218_c0_g1_i1_orf1
TRINITY_DN36281_c0_g1_i2_orf1
TRINITY_DN585_c0_g1_i5_orf1

TRINITY_DN114834_c0_g1_i1_orf1

TRINITY_DN4013_c0_g1_i4_orf1

TRINITY_DN668_c0_g1_i4_orf1
TRINITY_DN3833_c0_g1_i4_orf1
TRINITY_DN1196_c0_g1_i5_orf1
TRINITY_DN7711_c1_g1_i3_orf1
TRINITY_DN14774_c0_g1_i4_orf1
TRINITY_DN110523_c0_g2_i1_orf1
TRINITY_DN4689_c0_g1_i5_orf1
TRINITY_DN661_c1_g2_i1_orf1
TRINITY_DN11772_c0_g1_i1_orf1
TRINITY_DN5074_c0_g1_i7_orf1
TRINITY_DN1252_c0_g1_i3_orf1
TRINITY_DN4217_c0_g1_i2_orf1
TRINITY_DN12673_c3_g1_i2_orf1
TRINITY_DN64616_c0_g1_i1_orf1
TRINITY_DN31619_c0_g1_i2_orf1
TRINITY_DN321_c0_g1_i1_orf1
TRINITY_DN71832_c0_g1_i1_orf1
TRINITY_DN10364_c0_g1_i5_orf1
TRINITY_DN13648_c0_g1_i6_orf1
TRINITY_DN42964_c0_g1_i1_orf1
TRINITY_DN83374_c0_g1_i1_orf1
TRINITY_DN467_c0_g3_i1_orf1
TRINITY_DN37585_c0_g2_i1_orf1
TRINITY_DN280_c0_g1_i8_orf1
TRINITY_DN23183_c1_g1_i2_orf1
TRINITY_DN14009_c0_g1_i1_orf1
TRINITY_DN15420_c0_g3_i2_orf1
TRINITY_DN111621_c0_g3_i1_orf1
TRINITY_DN661_c0_g3_i5_orf1
TRINITY_DN714_c0_g1_i3_orf1
TRINITY_DN501_c0_g1_i5_orf1
TRINITY_DN6974_c0_g2_i1_orf1
TRINITY_DN3073_c0_g1_i7_orf1

TRINITY_DN25345_c0_g1_i1_orf1
TRINITY_DN21719_c0_g2_i4_orf1
TRINITY_DN7735_c1_g1_i1_orf1
TRINITY_DN7565_c0_g1_i3_orf1
TRINITY_DN3962_c0_g1_i6_orf1
TRINITY_DN52788_c0_g1_i1_orf1
TRINITY_DN5848_c0_g1_i6_orf1
TRINITY_DN5028_c0_g1_i11_orf1
TRINITY_DN2109_c0_g1_i4_orf1
TRINITY_DN8569_c1_g2_i7_orf1
TRINITY_DN8641_c0_g1_i1_orf1
TRINITY_DN867_c0_g1_i1_orf1
TRINITY_DN8692_c0_g1_i2_orf1
TRINITY_DN1293_c0_g1_i4_orf1
TRINITY_DN19923_c0_g1_i1_orf1
TRINITY_DN34786_c0_g1_i1_orf1
TRINITY_DN11666_c0_g1_i6_orf1
TRINITY_DN5893_c0_g1_i7_orf1
TRINITY_DN46090_c0_g2_i1_orf1
TRINITY_DN3978_c0_g2_i1_orf1

TRINITY_DN29879_c0_g1_i3_orf1

TRINITY_DN1481_c0_g1_i4_orf1

TRINITY_DN3887_c0_g1_i1_orf1
TRINITY_DN12331_c0_g1_i5_orf1
TRINITY_DN12464_c0_g1_i3_orf1
TRINITY_DN2323_c0_g1_i4_orf1

TRINITY_DN19537_c0_g1_i1_orf1

TRINITY_DN19998_c0_g1_i1_orf1

TRINITY_DN1436_c0_g1_i3_orf1
TRINITY_DN42333_c0_g1_i5_orf1
TRINITY_DN114960_c0_g1_i4_orf1
TRINITY_DN1362_c0_g1_i4_orf1
TRINITY_DN34727_c0_g1_i3_orf1
TRINITY_DN21930_c0_g1_i1_orf1

TRINITY_DN4320_c0_g1_i1_orf1

TRINITY_DN50471_c0_g1_i4_orf1
TRINITY_DN13067_c0_g1_i6_orf1
TRINITY_DN8390_c0_g1_i2_orf1
TRINITY_DN9_c0_g1_i11_orf1
TRINITY_DN31943_c0_g1_i1_orf1
TRINITY_DN86621_c0_g1_i2_orf1
TRINITY_DN58872_c0_g1_i1_orfp1
TRINITY_DN17329_c0_g2_i3_orf1

TRINITY_DN6870_c0_g1_i5_orf1

TRINITY_DN1005_c0_g1_i5_orf1

TRINITY_DN2352_c0_g1_i15_orf1
TRINITY_DN9028_c0_g1_i5_orf1
TRINITY_DN86309_c0_g1_i4_orf1
TRINITY_DN16145_c0_g1_i12_orf1
TRINITY_DN34830_c0_g1_i1_orf1
TRINITY_DN146524_c0_g1_i1_orf1
TRINITY_DN4144_c0_g1_i7_orf1
TRINITY_DN17003_c1_g1_i1_orf1

TRINITY_DN8306_c0_g1_i4_orf1

TRINITY_DN16643_c0_g2_i4_orf1
TRINITY_DN2012_c0_g1_i3_orf1
TRINITY_DN4572_c0_g3_i1_orf1
TRINITY_DN23582_c0_g1_i1_orf1
TRINITY_DN36987_c0_g1_i1_orf1
TRINITY_DN5954_c0_g1_i2_orf1
TRINITY_DN131264_c0_g1_i2_orf1
TRINITY_DN28018_c0_g6_i1_orf1
TRINITY_DN23732_c0_g1_i1_orf1
TRINITY_DN4134_c2_g1_i2_orf1
TRINITY_DN4694_c0_g2_i1_orf1
TRINITY_DN8659_c0_g2_i1_orf1

TRINITY_DN7682_c0_g1_i2_orf1

TRINITY_DN9475_c0_g1_i6_orf1
TRINITY_DN19058_c1_g1_i1_orf1
TRINITY_DN27456_c0_g2_i1_orf1
TRINITY_DN2043_c0_g1_i3_orf1
TRINITY_DN9536_c0_g1_i4_orf1
TRINITY_DN18620_c0_g1_i5_orf1
TRINITY_DN2745_c0_g1_i2_orf1
TRINITY_DN12222_c0_g1_i1_orf1
TRINITY_DN13167_c0_g1_i1_orf1
TRINITY_DN1768_c0_g1_i2_orf1
TRINITY_DN24631_c0_g2_i1_orf1
TRINITY_DN13419_c0_g1_i5_orf1
TRINITY_DN9732_c0_g1_i7_orf1
TRINITY_DN9109_c0_g1_i1_orf1
TRINITY_DN590_c0_g1_i4_orf1
TRINITY_DN11665_c0_g1_i4_orf1
TRINITY_DN3430_c0_g1_i1_orf1
TRINITY_DN9324_c1_g2_i2_orf1
TRINITY_DN2676_c0_g1_i2_orf1
TRINITY_DN6014_c1_g1_i2_orf1
TRINITY_DN843_c0_g1_i5_orf1
TRINITY_DN4785_c0_g2_i1_orf1
TRINITY_DN2623_c1_g1_i3_orf1
TRINITY_DN20710_c0_g1_i2_orf1
TRINITY_DN144_c0_g1_i4_orf1

TRINITY_DN17995_c0_g4_i1_orf1

TRINITY_DN4410_c0_g1_i1_orf1

TRINITY_DN9872_c0_g1_i2_orf1
TRINITY_DN3177_c0_g1_i1_orf1
TRINITY_DN1274_c0_g1_i4_orf1
TRINITY_DN2968_c0_g1_i3_orf1
TRINITY_DN10095_c0_g1_i5_orf1
TRINITY_DN3273_c0_g1_i4_orf1

TRINITY_DN3459_c0_g1_i4_orf1

TRINITY_DN64222_c0_g1_i1_orf1

TRINITY_DN4820_c0_g2_i2_orf1

TRINITY_DN1604_c0_g1_i4_orf1
TRINITY_DN4572_c0_g1_i2_orf1
TRINITY_DN9465_c0_g1_i4_orf1
TRINITY_DN9794_c0_g2_i8_orf1
TRINITY_DN34751_c0_g1_i1_orf1
TRINITY_DN667_c0_g1_i5_orf1
TRINITY_DN13898_c0_g1_i2_orf1
TRINITY_DN99020_c0_g1_i1_orf1
TRINITY_DN5910_c1_g1_i6_orf1
TRINITY_DN18563_c2_g1_i1_orf1
TRINITY_DN14274_c0_g1_i3_orf1
TRINITY_DN2904_c0_g1_i4_orf1
TRINITY_DN27114_c0_g1_i1_orf1
TRINITY_DN16516_c0_g1_i1_orf1
TRINITY_DN47219_c0_g1_i3_orf1
TRINITY_DN16390_c0_g1_i4_orf1
TRINITY_DN50517_c0_g1_i3_orf1
TRINITY_DN10630_c0_g1_i2_orf1
TRINITY_DN22018_c0_g1_i3_orf1
TRINITY_DN44094_c0_g1_i1_orf1
TRINITY_DN19885_c0_g1_i1_orf1
TRINITY_DN6967_c0_g1_i3_orf1
TRINITY_DN14298_c0_g1_i3_orf1

TRINITY_DN3521_c0_g2_i1_orf1

TRINITY_DN2438_c0_g1_i1_orf1
TRINITY_DN7246_c0_g1_i7_orf1
TRINITY_DN4456_c0_g1_i1_orf1
TRINITY_DN162_c0_g1_i4_orf1
TRINITY_DN72_c0_g1_i16_orf1
TRINITY_DN10694_c1_g2_i1_orf1
TRINITY_DN63568_c0_g1_i1_orf1

TRINITY_DN4524_c0_g1_i2_orf1

TRINITY_DN16605_c0_g1_i3_orf1
TRINITY_DN22577_c0_g1_i2_orf1
TRINITY_DN3119_c0_g1_i7_orf1

TRINITY_DN132857_c0_g1_i1_orf1
TRINITY_DN2100_c0_g1_i2_orf1
TRINITY_DN8682_c0_g1_i4_orf1
TRINITY_DN2102_c0_g1_i11_orf1
TRINITY_DN49872_c0_g1_i2_orf1
TRINITY_DN33089_c0_g1_i1_orf1
TRINITY_DN39975_c0_g1_i4_orf1
TRINITY_DN6161_c0_g1_i1_orf1
TRINITY_DN33967_c2_g2_i1_orf1
TRINITY_DN33837_c0_g1_i6_orf1
TRINITY_DN10415_c0_g1_i5_orf1
TRINITY_DN13732_c0_g2_i3_orf1
TRINITY_DN25360_c0_g1_i2_orf1
TRINITY_DN15400_c0_g1_i1_orf1
TRINITY_DN5408_c0_g1_i5_orf1
TRINITY_DN20442_c0_g2_i1_orf1
TRINITY_DN1012_c0_g1_i2_orf1
TRINITY_DN858_c0_g1_i3_orf1
TRINITY_DN47842_c0_g1_i1_orf1
TRINITY_DN1897_c0_g2_i4_orf1
TRINITY_DN10231_c0_g2_i1_orf1

TRINITY_DN2416_c0_g1_i5_orf1

TRINITY_DN98016_c0_g1_i1_orf1
TRINITY_DN30177_c0_g2_i1_orf1
TRINITY_DN8694_c1_g1_i4_orf1
TRINITY_DN19328_c0_g1_i1_orf1
TRINITY_DN28802_c0_g1_i1_orf1
TRINITY_DN4125_c0_g1_i6_orf1
TRINITY_DN13330_c0_g1_i4_orf1
TRINITY_DN38392_c0_g1_i1_orf1
TRINITY_DN810_c0_g1_i4_orf1
TRINITY_DN26089_c0_g1_i1_orf1
TRINITY_DN962_c5_g1_i1_orf1
TRINITY_DN54387_c0_g1_i1_orf1
TRINITY_DN103_c0_g1_i1_orf1
TRINITY_DN225_c0_g1_i6_orf1
TRINITY_DN115210_c0_g4_i1_orf1
TRINITY_DN2627_c0_g2_i1_orf1
TRINITY_DN15930_c0_g1_i5_orf1
TRINITY_DN3241_c0_g1_i1_orf1
TRINITY_DN84631_c0_g1_i1_orf1
TRINITY_DN83150_c0_g1_i1_orf1

TRINITY_DN1491_c0_g1_i4_orf1

TRINITY_DN100208_c0_g1_i1_orf1
TRINITY_DN18502_c0_g1_i1_orf1
TRINITY_DN98242_c0_g1_i1_orf1
TRINITY_DN14532_c0_g1_i1_orf1
TRINITY_DN147458_c0_g1_i1_orf1

TRINITY_DN48590_c0_g1_i1_orf1

TRINITY_DN72999_c0_g1_i1_orf1
TRINITY_DN978_c9_g2_i1_orf1
TRINITY_DN20244_c0_g1_i1_orfp1
TRINITY_DN14721_c0_g1_i2_orf1
TRINITY_DN1868_c0_g1_i1_orf1
TRINITY_DN552_c0_g1_i3_orf1

TRINITY_DN9455_c0_g1_i6_orf1

TRINITY_DN32956_c0_g1_i4_orf1

TRINITY_DN74069_c0_g1_i1_orf1
TRINITY_DN7803_c0_g1_i2_orf1
TRINITY_DN10057_c0_g2_i1_orf1

TRINITY_DN29633_c0_g1_i8_orf1

TRINITY_DN30273_c1_g1_i1_orf1
TRINITY_DN15865_c0_g1_i1_orf1
TRINITY_DN4898_c0_g1_i7_orf1
TRINITY_DN4571_c0_g1_i4_orf1

TRINITY_DN6656_c0_g1_i1_orf1

TRINITY_DN172_c8_g2_i1_orf1
TRINITY_DN5772_c0_g1_i6_orf1
TRINITY_DN6586_c0_g1_i1_orf1
TRINITY_DN24266_c0_g2_i2_orf1
TRINITY_DN650_c0_g1_i3_orf1
TRINITY_DN5568_c0_g2_i2_orf1

TRINITY_DN2061_c0_g1_i3_orf1

TRINITY_DN34703_c0_g1_i4_orf1
TRINITY_DN2971_c0_g1_i1_orf1
TRINITY_DN1233_c0_g2_i1_orf1
TRINITY_DN25870_c0_g2_i6_orf1
TRINITY_DN24218_c0_g1_i1_orf1
TRINITY_DN110534_c0_g1_i3_orf1
TRINITY_DN33893_c0_g1_i1_orf1
TRINITY_DN13395_c0_g1_i1_orf1
TRINITY_DN3499_c0_g1_i8_orf1
TRINITY_DN50517_c0_g1_i5_orf1
TRINITY_DN535_c1_g1_i2_orf1
TRINITY_DN54925_c0_g1_i1_orf1
TRINITY_DN2054_c0_g1_i1_orf1
TRINITY_DN486_c0_g1_i5_orf1
TRINITY_DN5182_c0_g1_i5_orf1
TRINITY_DN9100_c0_g1_i5_orf1
TRINITY_DN5840_c0_g1_i6_orf1
TRINITY_DN9000_c0_g2_i1_orf1
TRINITY_DN15256_c0_g1_i8_orf1
TRINITY_DN21623_c0_g2_i1_orf1
TRINITY_DN123184_c0_g1_i1_orf1
TRINITY_DN1716_c0_g1_i14_orf1
TRINITY_DN2638_c0_g1_i7_orf1
TRINITY_DN5200_c0_g1_i2_orf1
TRINITY_DN17003_c0_g1_i1_orf1
TRINITY_DN35633_c0_g2_i1_orf1
TRINITY_DN5211_c0_g1_i1_orf1
TRINITY_DN48641_c0_g1_i4_orf1
TRINITY_DN8738_c0_g1_i1_orf1
TRINITY_DN10745_c0_g1_i14_orf1

TRINITY_DN1280_c0_g1_i1_orf1

TRINITY_DN73923_c0_g1_i1_orf1
TRINITY_DN4561_c0_g1_i3_orf1
TRINITY_DN4156_c0_g1_i2_orf1

TRINITY_DN27321_c0_g1_i1_orf1

TRINITY_DN1853_c0_g1_i3_orf1
TRINITY_DN16011_c0_g1_i3_orf1
TRINITY_DN2591_c0_g1_i4_orf1
TRINITY_DN120144_c0_g1_i1_orf1
TRINITY_DN1133_c0_g1_i6_orf1
TRINITY_DN482_c0_g1_i1_orf1
TRINITY_DN2345_c0_g1_i4_orf1
TRINITY_DN5235_c0_g1_i7_orf1
TRINITY_DN42337_c0_g1_i6_orf1
TRINITY_DN12769_c0_g1_i5_orf1
TRINITY_DN30178_c0_g1_i3_orf1
TRINITY_DN17655_c0_g1_i1_orf1
TRINITY_DN27723_c0_g1_i1_orf1
TRINITY_DN286_c0_g1_i2_orf1
TRINITY_DN72816_c0_g1_i2_orf1
TRINITY_DN2840_c0_g1_i5_orf1
TRINITY_DN5829_c0_g2_i1_orf1
TRINITY_DN26293_c0_g1_i4_orf1
TRINITY_DN44658_c0_g1_i2_orf1
TRINITY_DN44070_c0_g2_i2_orf1
TRINITY_DN53847_c0_g1_i7_orf1
TRINITY_DN10479_c0_g1_i6_orf1
TRINITY_DN120593_c0_g1_i1_orf1
TRINITY_DN47914_c0_g2_i1_orf1

TRINITY_DN9916_c0_g1_i1_orf1

TRINITY_DN7516_c0_g2_i1_orf1

TRINITY_DN1616_c0_g1_i3_orf1

TRINITY_DN28660_c0_g1_i4_orf1
TRINITY_DN18391_c0_g2_i8_orf1
TRINITY_DN73224_c0_g4_i2_orf1
TRINITY_DN14443_c0_g1_i1_orf1
TRINITY_DN2474_c0_g1_i5_orf1
TRINITY_DN56993_c0_g1_i4_orf1
TRINITY_DN9072_c0_g1_i1_orf1
TRINITY_DN1231_c0_g1_i4_orf1
TRINITY_DN113778_c0_g2_i1_orf1
TRINITY_DN277_c1_g1_i1_orf1
TRINITY_DN59829_c0_g1_i1_orf1
TRINITY_DN2936_c0_g1_i1_orf1
TRINITY_DN21124_c0_g1_i4_orf1

TRINITY_DN9354_c0_g1_i7_orf1

TRINITY_DN8701_c0_g1_i3_orf1
TRINITY_DN2450_c0_g1_i6_orf1
TRINITY_DN6312_c0_g1_i1_orf1
TRINITY_DN8659_c0_g1_i1_orf1

TRINITY_DN12134_c0_g1_i4_orf1

TRINITY_DN6436_c0_g1_i1_orf1

TRINITY_DN12771_c0_g1_i1_orf1
TRINITY_DN98814_c0_g1_i2_orf1
TRINITY_DN782_c0_g1_i5_orf1
TRINITY_DN24391_c1_g1_i1_orf1
TRINITY_DN12576_c0_g1_i2_orf1
TRINITY_DN14298_c0_g3_i1_orf1

TRINITY_DN14046_c0_g1_i1_orf1

TRINITY_DN28428_c0_g1_i2_orf1
TRINITY_DN7603_c0_g1_i5_orf1
TRINITY_DN20215_c0_g2_i1_orf1
TRINITY_DN77005_c0_g3_i1_orf1
TRINITY_DN1313_c0_g1_i2_orf1
TRINITY_DN14298_c0_g1_i1_orf1
TRINITY_DN2874_c0_g1_i4_orf1
TRINITY_DN11375_c0_g1_i4_orf1
TRINITY_DN6588_c0_g1_i4_orf1
TRINITY_DN4494_c0_g1_i1_orf1
TRINITY_DN12858_c0_g1_i5_orf1
TRINITY_DN1074_c0_g1_i7_orf1
TRINITY_DN44792_c0_g1_i1_orf1
TRINITY_DN24539_c0_g1_i4_orf1
TRINITY_DN252_c0_g1_i3_orf1
TRINITY_DN11464_c0_g1_i3_orf1
TRINITY_DN44256_c0_g1_i1_orf1
TRINITY_DN67649_c0_g1_i1_orf1
TRINITY_DN471_c0_g1_i6_orf1
TRINITY_DN1831_c0_g1_i3_orf1
TRINITY_DN17255_c0_g1_i9_orf1
TRINITY_DN38644_c0_g1_i1_orf1
TRINITY_DN19584_c0_g1_i2_orf1
TRINITY_DN4808_c0_g1_i3_orf1
TRINITY_DN1601_c0_g1_i4_orf1
TRINITY_DN8553_c0_g1_i4_orf1
TRINITY_DN19651_c0_g1_i1_orf1

TRINITY_DN20067_c0_g1_i6_orf1

TRINITY_DN5004_c0_g1_i2_orf1
TRINITY_DN38211_c0_g1_i1_orf1
TRINITY_DN34745_c0_g2_i1_orf1

TRINITY_DN6202_c0_g1_i2_orf1

TRINITY_DN6914_c0_g1_i2_orf1
TRINITY_DN13287_c0_g1_i5_orf1
TRINITY_DN28622_c0_g1_i1_orf1
TRINITY_DN64769_c0_g1_i3_orf1
TRINITY_DN51737_c0_g1_i3_orf1
TRINITY_DN670_c0_g1_i3_orf1
TRINITY_DN9146_c0_g1_i1_orf1
TRINITY_DN89829_c0_g1_i1_orf1
TRINITY_DN19810_c1_g1_i7_orf1
TRINITY_DN25686_c0_g1_i4_orf1
TRINITY_DN4439_c0_g1_i2_orf1
TRINITY_DN41108_c0_g1_i1_orf1
TRINITY_DN33008_c0_g1_i1_orf1
TRINITY_DN24469_c0_g2_i2_orf1
TRINITY_DN802_c0_g1_i2_orf1
TRINITY_DN28759_c0_g1_i1_orf1
TRINITY_DN1528_c0_g1_i4_orf1
TRINITY_DN32700_c0_g1_i2_orf1
TRINITY_DN20767_c0_g2_i1_orf1
TRINITY_DN467_c4_g1_i2_orf1
TRINITY_DN1504_c0_g1_i1_orf1
TRINITY_DN10385_c0_g1_i5_orf1
TRINITY_DN1921_c1_g1_i5_orf1
TRINITY_DN4439_c0_g2_i1_orf1
TRINITY_DN2984_c0_g1_i3_orf1
TRINITY_DN42171_c0_g1_i1_orf1
TRINITY_DN87170_c0_g1_i3_orf1

TRINITY_DN23946_c0_g1_i1_orf1

TRINITY_DN2160_c0_g1_i13_orf1
TRINITY_DN50225_c0_g1_i1_orf1
TRINITY_DN3472_c1_g1_i4_orf1

TRINITY_DN518_c0_g1_i1_orf1
TRINITY_DN397_c0_g1_i1_orf1
TRINITY_DN15417_c0_g1_i6_orf1
TRINITY_DN4891_c0_g1_i4_orf1
TRINITY_DN1425_c0_g1_i4_orf1
TRINITY_DN10174_c0_g1_i4_orf1
TRINITY_DN4835_c0_g1_i2_orf1
TRINITY_DN1691_c0_g1_i3_orf1
TRINITY_DN4533_c0_g1_i1_orf1
TRINITY_DN21559_c0_g2_i1_orf1

TRINITY_DN1661_c0_g1_i1_orf1

TRINITY_DN2802_c1_g1_i1_orf1
TRINITY_DN23790_c0_g1_i1_orf1
TRINITY_DN5811_c0_g1_i4_orf1
TRINITY_DN3747_c1_g2_i1_orf1
TRINITY_DN879_c0_g1_i2_orf1
TRINITY_DN41736_c0_g2_i1_orf1
TRINITY_DN942_c0_g1_i1_orf1
TRINITY_DN4070_c0_g1_i4_orf1
TRINITY_DN3953_c0_g1_i2_orf1
TRINITY_DN2879_c0_g1_i4_orf1
TRINITY_DN5834_c0_g1_i2_orf1
TRINITY_DN9062_c0_g2_i3_orf1
TRINITY_DN880_c0_g1_i6_orf1
TRINITY_DN14477_c0_g1_i12_orf1
TRINITY_DN41259_c0_g1_i6_orf1
TRINITY_DN53311_c0_g2_i1_orf1
TRINITY_DN48970_c0_g1_i1_orf1
TRINITY_DN1045_c0_g1_i6_orf1
TRINITY_DN36061_c0_g4_i2_orf1
TRINITY_DN44335_c0_g1_i7_orf1
TRINITY_DN2475_c0_g2_i1_orf1

TRINITY_DN78686_c0_g1_i1_orf1

TRINITY_DN1554_c0_g1_i9_orf1
TRINITY_DN6239_c0_g1_i1_orf1
TRINITY_DN7247_c0_g1_i7_orf1

TRINITY_DN7391_c0_g1_i2_orf1

TRINITY_DN79803_c0_g1_i7_orf1
TRINITY_DN749_c0_g1_i1_orf1
TRINITY_DN13139_c0_g1_i1_orf1
TRINITY_DN2403_c0_g1_i3_orf1
TRINITY_DN12820_c0_g1_i1_orf1
TRINITY_DN1628_c0_g1_i1_orf1
TRINITY_DN4403_c0_g1_i3_orf1
TRINITY_DN10234_c0_g1_i1_orf1

TRINITY_DN18933_c0_g1_i3_orf1

TRINITY_DN4686_c0_g2_i1_orf1
TRINITY_DN42205_c0_g1_i4_orf1

TRINITY_DN57904_c0_g2_i1_orf1
TRINITY_DN7828_c0_g1_i2_orf1
TRINITY_DN20185_c0_g1_i6_orf1
TRINITY_DN7670_c0_g1_i1_orf1
TRINITY_DN5442_c0_g1_i4_orf1
TRINITY_DN5952_c0_g1_i6_orf1
TRINITY_DN6380_c0_g1_i1_orf1
TRINITY_DN3244_c0_g1_i4_orf1
TRINITY_DN4012_c0_g4_i2_orf1
TRINITY_DN10131_c0_g1_i7_orf1
TRINITY_DN18912_c1_g1_i1_orf1
TRINITY_DN37923_c0_g1_i1_orf1
TRINITY_DN17935_c0_g1_i1_orf1
TRINITY_DN376_c0_g1_i1_orf1
TRINITY_DN147475_c0_g1_i1_orf1

TRINITY_DN4757_c0_g1_i3_orf1

TRINITY_DN1444_c1_g1_i5_orf1

TRINITY_DN11820_c0_g1_i1_orf1
TRINITY_DN36006_c0_g1_i5_orf1
TRINITY_DN4152_c0_g1_i1_orf1
TRINITY_DN279_c0_g1_i10_orf1
TRINITY_DN14019_c0_g1_i5_orf1
TRINITY_DN4233_c0_g2_i2_orf1
TRINITY_DN8596_c0_g1_i2_orf1
TRINITY_DN18164_c0_g1_i7_orf1
TRINITY_DN45859_c0_g1_i1_orf1
TRINITY_DN122867_c1_g1_i1_orf1
TRINITY_DN17271_c0_g1_i1_orf1
TRINITY_DN8958_c0_g1_i1_orf1
TRINITY_DN23534_c0_g2_i2_orf1
TRINITY_DN39170_c0_g1_i4_orf1
TRINITY_DN37532_c0_g1_i1_orf1
TRINITY_DN4908_c1_g1_i5_orf1
TRINITY_DN2802_c0_g1_i1_orf1
TRINITY_DN783_c0_g1_i7_orf1
TRINITY_DN26790_c0_g1_i3_orf1
TRINITY_DN19493_c0_g1_i5_orf1
TRINITY_DN50875_c0_g1_i3_orf1
TRINITY_DN288_c0_g1_i9_orf1

TRINITY_DN23616_c0_g1_i4_orf1

TRINITY_DN43328_c0_g1_i1_orf1
TRINITY_DN22156_c0_g1_i1_orf1

TRINITY_DN235_c0_g3_i1_orf1

TRINITY_DN34689_c0_g1_i4_orf1

TRINITY_DN24490_c0_g1_i6_orf1
TRINITY_DN9637_c0_g1_i14_orf1
TRINITY_DN31967_c0_g1_i5_orf1
TRINITY_DN10502_c0_g1_i4_orf1
TRINITY_DN11375_c0_g1_i6_orf1
TRINITY_DN13018_c0_g1_i1_orf1
TRINITY_DN46022_c0_g1_i1_orf1
TRINITY_DN32601_c0_g1_i2_orf1
TRINITY_DN10297_c0_g1_i1_orf1
TRINITY_DN13375_c0_g1_i6_orf1
TRINITY_DN2885_c1_g1_i2_orf1
TRINITY_DN87603_c0_g2_i1_orf1
TRINITY_DN2542_c0_g2_i1_orf1
TRINITY_DN1437_c0_g1_i6_orf1
TRINITY_DN1181_c0_g1_i1_orf1
TRINITY_DN3450_c0_g1_i3_orf1
TRINITY_DN11616_c0_g1_i3_orf1
TRINITY_DN3649_c0_g1_i6_orf1
TRINITY_DN21218_c0_g1_i4_orf1
TRINITY_DN972_c0_g2_i1_orf1
TRINITY_DN104507_c0_g1_i2_orf1
TRINITY_DN142588_c0_g1_i1_orf1
TRINITY_DN4950_c0_g1_i2_orf1
TRINITY_DN5457_c0_g1_i4_orf1

TRINITY_DN144258_c0_g1_i1_orf1

TRINITY_DN19250_c0_g2_i2_orf1
TRINITY_DN4262_c0_g1_i16_orf1
TRINITY_DN1763_c0_g3_i2_orf1
TRINITY_DN2600_c0_g1_i7_orf1
TRINITY_DN146119_c0_g1_i1_orf1
TRINITY_DN1771_c0_g2_i1_orf1
TRINITY_DN3856_c0_g1_i7_orf1
TRINITY_DN47723_c0_g1_i1_orf1
TRINITY_DN5562_c0_g1_i3_orf1
TRINITY_DN3176_c0_g1_i2_orf1
TRINITY_DN133760_c0_g1_i1_orf1
TRINITY_DN35377_c0_g1_i3_orf1
TRINITY_DN3614_c0_g2_i1_orf1
TRINITY_DN298_c0_g1_i4_orf1

TRINITY_DN11402_c0_g1_i1_orf1

TRINITY_DN3893_c0_g2_i3_orf1

TRINITY_DN2826_c0_g1_i7_orf1

TRINITY_DN2783_c1_g1_i2_orf1

TRINITY_DN2977_c0_g1_i3_orf1
TRINITY_DN4659_c0_g1_i2_orf1

TRINITY_DN2718_c0_g1_i6_orf1
TRINITY_DN6185_c0_g1_i12_orf1
TRINITY_DN31585_c0_g1_i1_orf1
TRINITY_DN7228_c0_g1_i6_orf1
TRINITY_DN3343_c0_g2_i1_orf1
TRINITY_DN23783_c0_g2_i1_orf1
TRINITY_DN2623_c0_g1_i3_orf1
TRINITY_DN8432_c0_g2_i1_orf1
TRINITY_DN20118_c0_g1_i4_orfp1
TRINITY_DN5653_c0_g1_i4_orf1
TRINITY_DN45633_c0_g1_i1_orf1
TRINITY_DN10722_c0_g3_i1_orf1

TRINITY_DN4429_c0_g1_i5_orf1

TRINITY_DN7251_c0_g1_i3_orf1

TRINITY_DN4194_c0_g1_i1_orf1
TRINITY_DN57105_c0_g1_i2_orf1
TRINITY_DN14365_c0_g1_i2_orf1
TRINITY_DN3582_c0_g1_i2_orf1

TRINITY_DN31503_c0_g1_i4_orf1

TRINITY_DN7112_c0_g1_i1_orf1

TRINITY_DN2497_c0_g1_i1_orf1

TRINITY_DN5841_c0_g1_i2_orf1
TRINITY_DN3057_c0_g2_i1_orf1
TRINITY_DN41311_c0_g2_i3_orf1
TRINITY_DN20007_c0_g1_i1_orf1
TRINITY_DN5046_c0_g3_i1_orf1
TRINITY_DN12527_c0_g1_i4_orf1
TRINITY_DN46140_c0_g1_i1_orf1
TRINITY_DN129226_c0_g1_i2_orf1
TRINITY_DN2894_c0_g2_i3_orf1
TRINITY_DN7122_c0_g1_i1_orf1
TRINITY_DN4207_c0_g1_i1_orf1
TRINITY_DN76377_c0_g1_i1_orf1
TRINITY_DN2365_c0_g1_i6_orf1
TRINITY_DN3832_c0_g1_i1_orf1
TRINITY_DN4814_c0_g1_i6_orf1
TRINITY_DN129226_c0_g4_i1_orf1
TRINITY_DN43293_c0_g1_i2_orf1
TRINITY_DN22654_c0_g2_i4_orf1
TRINITY_DN9309_c0_g1_i5_orf1
TRINITY_DN48413_c1_g1_i2_orf1
TRINITY_DN35669_c0_g1_i1_orf1

TRINITY_DN16349_c0_g1_i10_orf1

TRINITY_DN7037_c0_g1_i4_orf1
TRINITY_DN34726_c0_g2_i1_orf1
TRINITY_DN5275_c0_g1_i1_orf1
TRINITY_DN5697_c0_g1_i1_orf1
TRINITY_DN26355_c0_g1_i4_orf1
TRINITY_DN29034_c0_g1_i1_orf1
TRINITY_DN23926_c0_g1_i4_orf1
TRINITY_DN14501_c0_g1_i1_orf1
TRINITY_DN82810_c0_g1_i1_orf1
TRINITY_DN6189_c0_g1_i1_orf1

TRINITY_DN81715_c0_g1_i1_orf1
TRINITY_DN2196_c0_g1_i2_orf1
TRINITY_DN8944_c0_g1_i1_orf1
TRINITY_DN3618_c0_g1_i4_orf1
TRINITY_DN27852_c0_g1_i1_orf1
TRINITY_DN34821_c0_g1_i4_orf1

TRINITY_DN14487_c0_g1_i4_orf1

TRINITY_DN220_c0_g1_i3_orf1
TRINITY_DN60821_c0_g1_i1_orf1
TRINITY_DN15706_c0_g2_i5_orf1
TRINITY_DN4929_c0_g1_i1_orf1
TRINITY_DN40562_c0_g2_i1_orf1
TRINITY_DN8012_c0_g1_i3_orf1
TRINITY_DN13216_c0_g1_i5_orf1
TRINITY_DN8536_c0_g1_i2_orf1
TRINITY_DN16965_c0_g2_i1_orf1
TRINITY_DN27556_c0_g1_i1_orf1

TRINITY_DN3673_c0_g1_i10_orf1

TRINITY_DN8971_c1_g1_i4_orf1
TRINITY_DN25997_c1_g2_i4_orf1
TRINITY_DN9741_c0_g1_i3_orf1

TRINITY_DN2257_c0_g1_i4_orf1

TRINITY_DN69236_c0_g1_i1_orf1
TRINITY_DN5648_c0_g1_i5_orf1
TRINITY_DN11396_c0_g1_i1_orf1
TRINITY_DN57454_c0_g1_i4_orf1
TRINITY_DN34432_c0_g1_i1_orf1
TRINITY_DN16258_c0_g1_i2_orf1
TRINITY_DN136028_c0_g2_i1_orf1
TRINITY_DN25373_c0_g1_i1_orf1
TRINITY_DN25341_c0_g1_i1_orf1
TRINITY_DN106038_c0_g1_i1_orf1
TRINITY_DN19687_c0_g1_i1_orf1
TRINITY_DN227_c0_g1_i1_orf1
TRINITY_DN27960_c0_g1_i1_orf1
TRINITY_DN28509_c0_g1_i1_orf1
TRINITY_DN11928_c0_g1_i3_orf1
TRINITY_DN389_c0_g1_i2_orf1
TRINITY_DN969_c0_g1_i3_orf1
TRINITY_DN3647_c2_g1_i3_orf1

TRINITY_DN38540_c0_g1_i1_orf1

TRINITY_DN45449_c0_g1_i1_orf1
TRINITY_DN12101_c0_g1_i2_orf1
TRINITY_DN32161_c0_g1_i1_orf1
TRINITY_DN57496_c0_g1_i1_orf1
TRINITY_DN11657_c0_g1_i2_orf1

TRINITY_DN31663_c0_g1_i2_orf1

TRINITY_DN14920_c0_g1_i1_orf1
TRINITY_DN646_c0_g1_i5_orf1
TRINITY_DN34159_c0_g2_i1_orf1
TRINITY_DN24163_c0_g1_i1_orf1
TRINITY_DN1772_c7_g1_i7_orf1
TRINITY_DN57462_c0_g1_i1_orf1
TRINITY_DN787_c0_g1_i7_orf1
TRINITY_DN23360_c0_g1_i3_orf1
TRINITY_DN7787_c0_g1_i1_orf1
TRINITY_DN41179_c0_g1_i1_orf1
TRINITY_DN58531_c0_g1_i1_orf1
TRINITY_DN39532_c0_g1_i1_orf1
TRINITY_DN10662_c0_g1_i4_orf1
TRINITY_DN237_c1_g1_i1_orf1
TRINITY_DN24917_c0_g2_i1_orf1

TRINITY_DN3759_c0_g1_i1_orf1

TRINITY_DN3638_c0_g1_i1_orf1
TRINITY_DN21539_c0_g1_i1_orf1

TRINITY_DN4213_c0_g1_i4_orf1

TRINITY_DN1266_c2_g1_i1_orf1

TRINITY_DN12242_c0_g1_i5_orf1

TRINITY_DN754_c1_g1_i8_orf1
TRINITY_DN959_c0_g1_i7_orf1
TRINITY_DN2065_c1_g2_i1_orf1
TRINITY_DN29448_c0_g1_i1_orf1

TRINITY_DN30_c0_g1_i6_orf1
TRINITY_DN1803_c0_g1_i3_orf1
TRINITY_DN11013_c0_g1_i3_orf1
TRINITY_DN886_c0_g2_i4_orf1
TRINITY_DN6563_c0_g1_i1_orf1
TRINITY_DN1386_c0_g1_i6_orf1
TRINITY_DN33883_c0_g1_i1_orf1
TRINITY_DN1706_c0_g1_i7_orf1
TRINITY_DN2062_c0_g1_i9_orf1
TRINITY_DN334_c0_g1_i3_orf1
TRINITY_DN10644_c0_g1_i2_orf1
TRINITY_DN8367_c0_g1_i1_orf1
TRINITY_DN15370_c0_g1_i4_orf1
TRINITY_DN14107_c0_g1_i4_orf1
TRINITY_DN38301_c0_g1_i2_orf1
TRINITY_DN19160_c0_g1_i1_orf1
TRINITY_DN139212_c0_g1_i4_orf1
TRINITY_DN14565_c0_g1_i11_orf1
TRINITY_DN19186_c0_g1_i1_orf1
TRINITY_DN50571_c1_g1_i1_orf1
TRINITY_DN21531_c0_g1_i1_orf1
TRINITY_DN3235_c0_g1_i1_orf1
TRINITY_DN51252_c0_g2_i1_orf1
TRINITY_DN11799_c0_g1_i4_orf1
TRINITY_DN14429_c0_g1_i2_orf1
TRINITY_DN25779_c0_g1_i6_orf1
TRINITY_DN2082_c0_g1_i2_orf1
TRINITY_DN6248_c0_g1_i1_orf1
TRINITY_DN7583_c0_g1_i1_orf1
TRINITY_DN8261_c0_g1_i1_orf1
TRINITY_DN43369_c0_g2_i1_orf1
TRINITY_DN4125_c1_g1_i5_orf1
TRINITY_DN2265_c0_g2_i1_orf1
TRINITY_DN57798_c0_g1_i1_orf1
TRINITY_DN49936_c0_g2_i1_orf1
TRINITY_DN46778_c0_g1_i2_orf1
TRINITY_DN5459_c0_g1_i1_orf1
TRINITY_DN40704_c0_g1_i2_orf1
TRINITY_DN28981_c0_g1_i1_orf1
TRINITY_DN42506_c0_g1_i1_orf1
TRINITY_DN19361_c0_g1_i7_orf1
TRINITY_DN1109_c0_g1_i6_orf1
TRINITY_DN44219_c0_g1_i1_orf1
TRINITY_DN5554_c0_g1_i2_orf1
TRINITY_DN5867_c0_g1_i1_orf1
TRINITY_DN5238_c0_g1_i2_orf1
TRINITY_DN886_c0_g1_i1_orf1
TRINITY_DN37538_c0_g4_i1_orf1
TRINITY_DN14313_c0_g1_i1_orf1
TRINITY_DN45477_c0_g1_i1_orf1
TRINITY_DN41697_c0_g1_i1_orf1
TRINITY_DN1775_c0_g1_i3_orf1
TRINITY_DN82801_c0_g1_i1_orf1

TRINITY_DN17905_c0_g3_i1_orf1

TRINITY_DN1445_c0_g1_i1_orf1
TRINITY_DN4143_c0_g1_i1_orf1
TRINITY_DN628_c0_g1_i7_orf1

TRINITY_DN51045_c0_g1_i1_orf1
TRINITY_DN2918_c0_g1_i1_orf1
TRINITY_DN14436_c0_g1_i7_orf1
TRINITY_DN8691_c0_g1_i3_orf1
TRINITY_DN2704_c0_g1_i5_orf1
TRINITY_DN107035_c0_g1_i1_orf1

TRINITY_DN35725_c0_g1_i1_orf1

TRINITY_DN1330_c0_g1_i1_orf1
TRINITY_DN15900_c0_g1_i6_orf1
TRINITY_DN4762_c0_g1_i2_orf1
TRINITY_DN26130_c0_g1_i1_orf1
TRINITY_DN15624_c0_g1_i1_orf1
TRINITY_DN20346_c0_g1_i1_orf1
TRINITY_DN1249_c0_g1_i6_orf1
TRINITY_DN1353_c0_g1_i1_orf1
TRINITY_DN26186_c0_g1_i7_orf1
TRINITY_DN25975_c0_g3_i2_orf1
TRINITY_DN8369_c0_g1_i1_orf1
TRINITY_DN26375_c0_g1_i1_orf1
TRINITY_DN7920_c0_g1_i2_orf1
TRINITY_DN8087_c0_g1_i9_orf1
TRINITY_DN18404_c0_g1_i5_orf1
TRINITY_DN46409_c0_g1_i1_orf1
TRINITY_DN37538_c0_g2_i1_orf1
TRINITY_DN43942_c0_g2_i1_orf1
TRINITY_DN4030_c0_g2_i1_orf1
TRINITY_DN3332_c0_g1_i11_orf1
TRINITY_DN44288_c0_g1_i2_orf1
TRINITY_DN47731_c0_g1_i2_orf1
TRINITY_DN23175_c0_g1_i6_orf1
TRINITY_DN26688_c0_g1_i2_orf1
TRINITY_DN3082_c1_g1_i7_orf1
TRINITY_DN2953_c1_g1_i2_orf1
TRINITY_DN18172_c0_g1_i6_orf1
TRINITY_DN30932_c0_g1_i2_orf1
TRINITY_DN48237_c0_g1_i5_orf1
TRINITY_DN98091_c0_g1_i3_orf1
TRINITY_DN108573_c0_g1_i1_orf1
TRINITY_DN8116_c0_g1_i1_orf1
TRINITY_DN3159_c0_g1_i4_orf1
TRINITY_DN2083_c0_g1_i4_orf1
TRINITY_DN5578_c0_g1_i10_orf1
TRINITY_DN51766_c0_g1_i2_orf1

TRINITY_DN3889_c0_g1_i7_orfp1

TRINITY_DN20682_c0_g2_i1_orf1
TRINITY_DN6074_c0_g1_i1_orf1
TRINITY_DN6693_c0_g1_i1_orf1
TRINITY_DN29120_c0_g1_i6_orf1
TRINITY_DN542_c0_g2_i1_orf1
TRINITY_DN3194_c0_g1_i6_orf1
TRINITY_DN1310_c0_g1_i4_orf1

TRINITY_DN717_c0_g1_i2_orfp1

TRINITY_DN82320_c0_g1_i2_orf1
TRINITY_DN3784_c0_g1_i1_orf1

TRINITY_DN1914_c0_g1_i6_orf1
TRINITY_DN117_c0_g1_i6_orf1
TRINITY_DN61674_c0_g1_i2_orf1
TRINITY_DN81803_c0_g2_i1_orf1
TRINITY_DN117_c0_g1_i4_orf1
TRINITY_DN4959_c0_g1_i1_orf1

TRINITY_DN2490_c0_g2_i1_orfp1

TRINITY_DN348_c0_g2_i3_orf1
TRINITY_DN13221_c0_g1_i3_orf1
TRINITY_DN3383_c0_g1_i5_orf1

TRINITY_DN3227_c0_g1_i5_orf1

TRINITY_DN4270_c0_g1_i1_orf1
TRINITY_DN33430_c0_g1_i5_orf1
TRINITY_DN56164_c0_g1_i1_orf1

TRINITY_DN116874_c0_g1_i1_orfp1

TRINITY_DN2097_c1_g1_i1_orf1
TRINITY_DN4822_c0_g1_i6_orf1
TRINITY_DN17406_c0_g1_i1_orf1
TRINITY_DN5256_c0_g1_i1_orf1
TRINITY_DN11159_c0_g1_i5_orf1
TRINITY_DN8458_c0_g2_i1_orf1
TRINITY_DN14391_c1_g1_i2_orf1
TRINITY_DN24689_c0_g1_i1_orf1

TRINITY_DN3131_c0_g1_i5_orf1

TRINITY_DN32780_c0_g1_i2_orf1
TRINITY_DN59804_c0_g1_i1_orf1
TRINITY_DN31377_c0_g2_i1_orf1
TRINITY_DN10403_c0_g1_i3_orf1

TRINITY_DN6203_c0_g1_i1_orfp1

TRINITY_DN31598_c0_g1_i1_orf1
TRINITY_DN931_c0_g1_i4_orf1
TRINITY_DN106_c0_g1_i3_orf1
TRINITY_DN1322_c0_g1_i4_orf1

TRINITY_DN5208_c0_g1_i7_orf1

TRINITY_DN3066_c0_g1_i5_orf1
TRINITY_DN7277_c0_g1_i1_orf1
TRINITY_DN11856_c0_g1_i4_orf1
TRINITY_DN3322_c0_g1_i2_orf1

TRINITY_DN23838_c0_g1_i4_orf1

TRINITY_DN14217_c0_g1_i1_orf1
TRINITY_DN23798_c0_g1_i1_orf1
TRINITY_DN3478_c0_g1_i10_orf1

TRINITY_DN31584_c0_g2_i2_orf1

TRINITY_DN13999_c0_g1_i4_orf1
TRINITY_DN987_c0_g1_i3_orf1
TRINITY_DN65974_c0_g1_i2_orf1
TRINITY_DN1732_c0_g1_i17_orf1
TRINITY_DN44633_c0_g1_i4_orf1
TRINITY_DN29190_c0_g1_i4_orf1
TRINITY_DN10619_c0_g5_i7_orf1
TRINITY_DN6362_c0_g1_i4_orf1
TRINITY_DN9661_c0_g1_i1_orf1
TRINITY_DN1260_c0_g2_i1_orf1
TRINITY_DN48497_c0_g1_i1_orf1
TRINITY_DN27110_c0_g1_i4_orf1
TRINITY_DN11566_c0_g1_i6_orf1
TRINITY_DN34465_c0_g1_i1_orf1
TRINITY_DN2201_c0_g1_i1_orf1
TRINITY_DN52_c0_g1_i4_orf1
TRINITY_DN8846_c0_g1_i1_orf1
TRINITY_DN4401_c0_g2_i1_orf1

TRINITY_DN9554_c0_g1_i1_orf1

TRINITY_DN15736_c0_g1_i2_orf1

TRINITY_DN4732_c0_g1_i2_orf1
TRINITY_DN51938_c0_g3_i1_orf1
TRINITY_DN323_c0_g2_i5_orf1
TRINITY_DN22272_c0_g1_i1_orf1
TRINITY_DN957_c0_g1_i18_orf1
TRINITY_DN30037_c0_g1_i5_orf1

TRINITY_DN6992_c0_g1_i6_orf1

TRINITY_DN36928_c0_g1_i5_orf1
TRINITY_DN4356_c0_g1_i6_orf1
TRINITY_DN36496_c0_g1_i1_orf1

TRINITY_DN1999_c0_g1_i9_orf1

TRINITY_DN2749_c0_g2_i3_orf1

Description
serine protease inhibitor dipetalogastin-like isoform X2 [Ostrinia furnacalis]
vanin-like protein 2 isoform X2 [Ostrinia furnacalis]
TRINITY_DN38307_c0_g1_i1_m.10661 TRINITY_DN38307_c0_g1::TRINITY_DN38307_c0_g1_i1::g.10661 ORF type:5prime_partial len:66 (+),score=5.90 TRINITY_DN38307_c0_g1_i1:2-199(+)
storage protein [Ostrinia furnacalis]
uncharacterized protein LOC114356431 isoform X2 [Ostrinia furnacalis]
protein lethal(2)essential for life-like [Ostrinia furnacalis] >UTU55753.1 small heat shock protein
cathepsin L-like [Aphidius gifuensis] >KAF7988186.1 hypothetical protein HCN44_007680 [Aphidius
hypothetical protein evm_000756 [Chilo suppressalis]
cysteine protease XCP2-like [Ostrinia furnacalis]
TRINITY_DN5444_c0_g1_i1_m.14077 TRINITY_DN5444_c0_g1::TRINITY_DN5444_c0_g1_i1::g.14077 ORF type:3prime_partial len:90 (-),score=25.11 TRINITY_DN5444_c0_g1_i1:2-268(-)
acidic juvenile hormone-suppressible protein 1-like [Ostrinia furnacalis]
uncharacterized protein LOC114350216 [Ostrinia furnacalis]
uncharacterized protein LOC114365631 [Ostrinia furnacalis]
secretory phospholipase A2 receptor-like [Ostrinia furnacalis]
basic juvenile hormone-suppressible protein 2-like [Ostrinia furnacalis]
TGF-beta-activated kinase 1 and MAP3K7-binding protein 1-like [Ostrinia furnacalis]
pupal cuticle protein C1B-like [Ostrinia furnacalis]
A-kinase anchor protein 14-like [Ostrinia furnacalis]
inhibin beta B chain [Ostrinia furnacalis]
aquaporin AQPAn.G isoform X1 [Ostrinia furnacalis]
unnamed protein product [Leptidea sinapis]
phenoloxidase-activating factor 2-like isoform X1 [Ostrinia furnacalis]
uncharacterized protein LOC114366119 [Ostrinia furnacalis]
Low-density lipoprotein receptor-related protein 1 [Papilio xuthus]
uncharacterized protein LOC114359035 isoform X3 [Ostrinia furnacalis]
LOW QUALITY PROTEIN: lebocin-4-like [Ostrinia furnacalis]
inter-alpha-trypsin inhibitor heavy chain H4-like isoform X11 [Ostrinia furnacalis]
cysteine protease XCP2-like [Ostrinia furnacalis]
lysozyme precursor [Loxostege sticticalis]
cathepsin L [Ostrinia furnacalis] >XP_028165920.1 cathepsin L [Ostrinia furnacalis] >UKI61015.1
conotoxin ArMKLT2-032-like [Ostrinia furnacalis]
pre-mRNA-processing factor 40 homolog A isoform X1 [Ostrinia furnacalis] >XP_028162665.1 pre-mRNA-processing factor 40 homolog A isoform X2 [Ostrinia furnacalis] >XP_028162667.1 pre-mRNA-
uncharacterized protein LOC114353763 [Ostrinia furnacalis]
storage protein [Ostrinia furnacalis]
probable G-protein coupled receptor Mth-like 3 isoform X1 [Ostrinia furnacalis]
arylphorin subunit alpha-like [Ostrinia furnacalis]
hypothetical protein EVAR_60653_1 [Eumeta japonica]
uncharacterized protein LOC114361723 isoform X4 [Ostrinia furnacalis]
putative uncharacterized protein DDB_G0282133 isoform X1 [Ostrinia furnacalis]
protein FAM160B1-like isoform X1 [Ostrinia furnacalis]
carboxylesterase [Ostrinia furnacalis]
unnamed protein product, partial [Brenthis ino]
arylphorin subunit alpha-like [Ostrinia furnacalis]
cilia- and flagella-associated protein 410 isoform X2 [Aphidius gifuensis]
neurofilament heavy polypeptide-like isoform X2 [Ostrinia furnacalis]
deubiquitinase DESI2 isoform X1 [Helicoverpa armigera] >XP_049707835.1 deubiquitinase DESI2
methanethiol oxidase [Ostrinia furnacalis]
aldo-keto reductase AKR2E4-like isoform X1 [Ostrinia furnacalis]
acidic juvenile hormone-suppressible protein 1-like [Ostrinia furnacalis]
ferritin subunit isoform X1 [Belonocnema kinseyi]
uncharacterized protein LOC114359603 [Ostrinia furnacalis]
hemicentin-1-like isoform X1 [Ostrinia furnacalis]
protein yellow-like [Ostrinia furnacalis]
delta(3,5)-Delta(2,4)-dienoyl-CoA isomerase, mitochondrial isoform X1 [Ostrinia furnacalis]

alaserpin-like isoform X13 [Ostrinia furnacalis]
uncharacterized protein LOC114359035 isoform X3 [Ostrinia furnacalis]
storage protein 1 [Omphisa fuscidentalis]
basic juvenile hormone-suppressible protein 1-like [Ostrinia furnacalis]
skin secretory protein xP2-like [Ostrinia furnacalis]
TRINITY_DN30169_c0_g1_i1_m.11367 TRINITY_DN30169_c0_g1::TRINITY_DN30169_c0_g1_i1::g.11367 ORF type:3prime_partial len:52 (+),score=0.98 TRINITY_DN30169_c0_g1_i1:72-224(+)
apolipophorins-like [Ostrinia furnacalis]
glutenin, high molecular weight subunit PW212-like [Ostrinia furnacalis]
uncharacterized protein LOC114364231 isoform X1 [Ostrinia furnacalis] >XP_028176108.1
uncharacterized protein LOC114364231 isoform X2 [Ostrinia furnacalis]
uncharacterized protein LOC114353759 [Ostrinia furnacalis]
unnamed protein product [Chilo suppressalis]
ferritin subunit-like [Ostrinia furnacalis] >XP_028168186.1 ferritin subunit-like [Ostrinia furnacalis]
apolipophorins-like [Ostrinia furnacalis]
transmembrane protease serine 9-like [Ostrinia furnacalis]
aldehyde dehydrogenase X, mitochondrial-like [Ostrinia furnacalis]
unnamed protein product, partial [Brenthis ino]
uncharacterized protein LOC114352370 [Ostrinia furnacalis]
unnamed protein product [Diatraea saccharalis]
leukocyte elastase inhibitor-like [Ostrinia furnacalis]
inter-alpha-trypsin inhibitor heavy chain H4-like isoform X11 [Ostrinia furnacalis]
hypothetical protein evm_000299 [Chilo suppressalis]
phenoloxidase-activating enzyme-like [Ostrinia furnacalis]
hypothetical protein B566_EDAN014657 [Ephemera danica]
failed axon connections [Ostrinia furnacalis]
aldehyde dehydrogenase, partial [Mythimna separata]
apolipophorins-like [Ostrinia furnacalis]
transferrin [Ostrinia furnacalis]
uncharacterized protein LOC114353763 [Ostrinia furnacalis]
TPA_exp: putative parasitoid killing factor [Trichoplusia ni]
arylphorin subunit alpha-like [Ostrinia furnacalis]
ornithine aminotransferase, mitochondrial isoform X2 [Ostrinia furnacalis]
very low-density lipoprotein receptor isoform X3 [Galleria mellonella]
arylphorin subunit alpha-like [Ostrinia furnacalis]
lysosomal alpha-mannosidase isoform X1 [Pieris rapae]
synaptic vesicle membrane protein VAT-1 homolog-like [Ostrinia furnacalis]
digestive cysteine proteinase 2 [Ostrinia furnacalis]
PREDICTED: gelsolin-like [Amyelois transitella]
uncharacterized protein LOC114351844 [Ostrinia furnacalis] >XP_028158981.1 uncharacterized protein LOC114351844 [Ostrinia furnacalis] >XP_028158982.1 uncharacterized protein LOC114351844 [Ostrinia furnacalis] >XP_028158983.1 uncharacterized protein LOC114351844 [Ostrinia furnacalis] >XP_028158984.1 uncharacterized protein LOC114351844 [Ostrinia furnacalis] >XP_028158985.1 uncharacterized protein LOC114351844 [Ostrinia furnacalis] >5GPR_A Crystal structure of chitinase-h from Ostrinia furnacalis [Ostrinia furnacalis] >5GQB_A Crystal structure of chitinase-h from O. furnacalis
uncharacterized protein LOC114361536 [Ostrinia furnacalis]
apolipophorins-like [Ostrinia furnacalis]
uncharacterized protein LOC114359193 [Ostrinia furnacalis]
tsukushin isoform X2 [Ostrinia furnacalis]
protein lethal(2)essential for life [Manduca sexta] >KAG6441919.1 hypothetical protein
trafficking protein particle complex subunit 1 [Ostrinia furnacalis]
uncharacterized protein LOC116345248 [Contarinia nasturtii]
storage protein 1 [Omphisa fuscidentalis]
thymosin beta isoform X3 [Ostrinia furnacalis]
endocuticle structural glycoprotein ABD-4-like [Ostrinia furnacalis]
protein singed [Ostrinia furnacalis] >XP_028161434.1 protein singed [Ostrinia furnacalis]
seminal fluid protein CSSFP028 [Chilo suppressalis]
acidic juvenile hormone-suppressible protein 1-like [Ostrinia furnacalis]

chitooligosaccharidolytic beta-N-acetylglucosaminidase isoform X1 [Ostrinia furnacalis]
luciferin 4-monooxygenase-like [Ostrinia furnacalis]
fatty acyl-CoA hydrolase precursor, medium chain [Ostrinia furnacalis]
probable cytochrome P450 304a1 [Ostrinia furnacalis]
unnamed protein product [Parnassius apollo]
stress-activated map kinase-interacting protein 1 [Ostrinia furnacalis]
MD-2-related lipid-recognition protein-like [Ostrinia furnacalis]
uncharacterized protein LOC114366712 isoform X1 [Ostrinia furnacalis]
codanin-1 [Ostrinia furnacalis]
apolipophorins-like [Ostrinia furnacalis]
inter-alpha-trypsin inhibitor heavy chain H4-like isoform X11 [Ostrinia furnacalis]
synaptic vesicle glycoprotein 2B-like isoform X2 [Ostrinia furnacalis] >XP_028161209.1 synaptic vesicle glycoprotein 2B-like isoform X2 [Ostrinia furnacalis] >XP_028161210.1 synaptic vesicle glycoprotein 2B-
aldehyde dehydrogenase, partial [Ectropis obliqua]
hypothetical protein evm_003306 [Chilo suppressalis] >CAB3526495.1 unnamed protein product [Chilo suppressalis] >CAH0403823.1 unnamed protein product [Chilo suppressalis]
hemicentin-2-like isoform X1 [Ostrinia furnacalis]
apolipophorins-like [Ostrinia furnacalis]
glucose-6-phosphate isomerase-like [Ostrinia furnacalis]
transcription initiation factor TFIID subunit 1-like [Ostrinia furnacalis]
apolipophorins-like [Ostrinia furnacalis]
uncharacterized protein LOC114354692 [Ostrinia furnacalis]
carboxypeptidase D [Ostrinia furnacalis]
lysosomal alpha-mannosidase-like [Ostrinia furnacalis]
charged multivesicular body protein 7 [Ostrinia furnacalis]
serine protease [Ostrinia furnacalis]
heat shock protein Hsp-12.2-like [Ostrinia furnacalis]
phenoloxidase-activating enzyme-like [Ostrinia furnacalis]
uncharacterized protein LOC114366345 isoform X2 [Ostrinia furnacalis]
probable salivary secreted peptide [Ostrinia furnacalis]
apolipophorins-like [Ostrinia furnacalis]
cytochrome P450 6B7-like [Ostrinia furnacalis]
arylsulfatase B [Ostrinia furnacalis]
matrix metalloproteinase-25-like [Ostrinia furnacalis]
DNA-directed RNA polymerase II subunit RPB1-like [Ostrinia furnacalis]
regucalcin-like [Ostrinia furnacalis]
sialic acid synthase [Ostrinia furnacalis]
titin-like [Ostrinia furnacalis]
unnamed protein product, partial [Iphiclides podalirius]
unnamed protein product [Chilo suppressalis]
beta-1,3-glucan-binding protein-like [Ostrinia furnacalis]
macrophage mannose receptor 1-like [Pararge aegeria]
protein 60A [Ostrinia furnacalis]
uncharacterized protein LOC114351440 [Ostrinia furnacalis]
unnamed protein product [Diatraea saccharalis]
serine protease inhibitor 3 [Ostrinia furnacalis]
methionine--tRNA ligase, cytoplasmic isoform X2 [Ostrinia furnacalis] >XP_028156683.1 methionine--tRNA ligase, cytoplasmic isoform X4 [Ostrinia furnacalis] >XP_028156684.1 methionine--tRNA ligase,
zinc finger protein Xfin-like [Ostrinia furnacalis]
PREDICTED: uncharacterized protein LOC106134920 [Amyelois transitella]
protein wings apart-like [Ostrinia furnacalis]
uncharacterized protein LOC114350200 [Ostrinia furnacalis]
lysosomal aspartic protease [Trichoplusia ni]
lysosomal Pro-X carboxypeptidase [Ostrinia furnacalis]
peroxidase [Ostrinia furnacalis]
PREDICTED: heparan-alpha-glucosaminide N-acetyltransferase [Amyelois transitella]
hypothetical protein evm_012298 [Chilo suppressalis]
aldehyde dehydrogenase, dimeric NADP-preferring isoform X5 [Ostrinia furnacalis]

serine protease inhibitor 77Ba-like [Ostrinia furnacalis] >XP_028164032.1 serine protease inhibitor
probable chitinase 10 isoform X6 [Ostrinia furnacalis]
prolow-density lipoprotein receptor-related protein 1, partial [Ostrinia furnacalis]
beta-1,3-glucan-binding protein-like [Ostrinia furnacalis]
prolow-density lipoprotein receptor-related protein 1, partial [Ostrinia furnacalis]
27 kDa glycoprotein-like [Ostrinia furnacalis]
solute carrier organic anion transporter family member 5A1-like isoform X1 [Ostrinia furnacalis]
glucose dehydrogenase [FAD, quinone]-like [Ostrinia furnacalis]
small heat shock protein Hsp24.2 [Ostrinia furnacalis]
clotting factor B-like isoform X1 [Ostrinia furnacalis] >XP_028163447.1 clotting factor B-like isoform X2 [Ostrinia furnacalis] >XP_028163448.1 clotting factor B-like isoform X3 [Ostrinia furnacalis]
glutamate decarboxylase 1-like isoform X1 [Ostrinia furnacalis]
proline-rich extensin-like protein EPR1 [Ostrinia furnacalis]
hexosaminidase [Ostrinia furnacalis]
uncharacterized protein LOC114353828 [Ostrinia furnacalis]
TBC1 domain family member 20 [Ostrinia furnacalis]
cytochrome P450 6B6-like [Ostrinia furnacalis]
vacuole membrane protein 1 [Ostrinia furnacalis]
probable isoaspartyl peptidase/L-asparaginase GA20639 [Ostrinia furnacalis]
lipase 3-like [Ostrinia furnacalis]
uncharacterized protein LOC113518937 [Galleria mellonella]
cuticle protein 1-like [Ostrinia furnacalis]
vimentin [Homo sapiens] >XP_003831224.1 vimentin [Pan paniscus] >XP_018890043.1 vimentin [Gorilla gorilla gorilla] >XP_024109584.1 vimentin [Pongo abelii] >XP_030675100.1 vimentin [Nomascus leucogenys] >XP_032020652.1 vimentin [Hylobates moloch] >P08670.4 RecName: Full=Vimentin [Homo sapiens] >AIC49963.1 VIM, partial [synthetic construct] >MXR00191.1 hypothetical protein [Bos
probable pterin-4-alpha-carbinolamine dehydratase isoform X1 [Ostrinia furnacalis]
lysozyme 10 [Ostrinia furnacalis]
hypothetical protein evm_003712 [Chilo suppressalis]
tubulin beta chain-like isoform X2 [Ostrinia furnacalis]
probable chitinase 2 [Ostrinia furnacalis]
PREDICTED: glycerol-3-phosphate acyltransferase 1, mitochondrial isoform X1 [Microplitis demolitor]
protein 4.1 homolog isoform X1 [Ostrinia furnacalis]
uncharacterized protein LOC114355104 [Ostrinia furnacalis]
sortilin-related receptor-like [Ostrinia furnacalis]
2-iminobutanoate/2-iminopropanoate deaminase [Ostrinia furnacalis]
phenoloxidase-activating factor 2-like isoform X2 [Ostrinia furnacalis]
unnamed protein product [Chrysodeixis includens]
lysosome membrane protein 2-like [Ostrinia furnacalis]
chitinase-3-like protein 1 [Ostrinia furnacalis]
protein Skeletor, isoforms D/E-like isoform X1 [Ostrinia furnacalis] >XP_028176405.1 protein Skeletor, isoforms D/E-like isoform X2 [Ostrinia furnacalis] >XP_028176406.1 protein Skeletor, isoforms D/E-like isoform X3 [Ostrinia furnacalis] >XP_028176407.1 protein Skeletor, isoforms D/E-like isoform X4
dystroglycan [Ostrinia furnacalis]
uncharacterized protein LOC114358822 [Ostrinia furnacalis]
probable histone-lysine N-methyltransferase CG1716 [Ostrinia furnacalis]
secernin-3 [Ostrinia furnacalis]
chitinase-like protein EN03 isoform X2 [Ostrinia furnacalis]
serine/threonine-protein kinase Genghis Khan-like [Ostrinia furnacalis]
uncharacterized protein LOC114351526 [Ostrinia furnacalis]
plectin-like, partial [Ostrinia furnacalis]
uncharacterized protein LOC114362553 [Ostrinia furnacalis]
major facilitator superfamily domain-containing protein 1-like [Ostrinia furnacalis]
NAD kinase 2, mitochondrial [Ostrinia furnacalis]
aldehyde dehydrogenase X, mitochondrial [Manduca sexta] >KAG6450704.1 hypothetical protein

casein kinase I isoform X1 [Ostrinia furnacalis] >XP_028158159.1 casein kinase I isoform X1 [Ostrinia furnacalis] >XP_028158160.1 casein kinase I isoform X1 [Ostrinia furnacalis] >XP_028158161.1 casein kinase I isoform X1 [Ostrinia furnacalis] >XP_028158163.1 casein kinase I isoform X1 [Ostrinia furnacalis] >XP_028158164.1 casein kinase I isoform X1 [Ostrinia furnacalis] >XP_028158165.1 casein kinase I
uncharacterized protein LOC114359393 isoform X1 [Ostrinia furnacalis]
protein lethal(2)essential for life-like [Helicoverpa zea] >XP_049705426.1 protein lethal(2)essential for life [Helicoverpa armigera] >ATB54993.1 heat shock protein 20.8 [Helicoverpa armigera] >PZC74337.1
lysine-specific demethylase 4A isoform X2 [Diachasma alloeum]
beta-glucuronidase-like isoform X1 [Ostrinia furnacalis] >XP_028166212.1 beta-glucuronidase-like
PX domain-containing protein kinase-like protein isoform X1 [Chelonus insularis]
modular serine protease-like isoform X1 [Ostrinia furnacalis]
leucine-rich repeat-containing protein 15-like [Ostrinia furnacalis] >XP_028171914.1 leucine-rich repeat-containing protein 15-like [Ostrinia furnacalis] >XP_028171921.1 leucine-rich repeat-containing
fasciclin-2 isoform X3 [Ostrinia furnacalis]
lachesin-like isoform X3 [Ostrinia furnacalis]
heat shock protein 68-like [Ostrinia furnacalis]
uncharacterized protein LOC114352730 [Ostrinia furnacalis]
heparanase-like [Ostrinia furnacalis]
apolipophorins-like [Ostrinia furnacalis]
uncharacterized protein LOC114361845 [Ostrinia furnacalis] >XP_028172853.1 uncharacterized protein
arylsulfatase B [Ostrinia furnacalis]
tetraspanin-13 isoform X1 [Ostrinia furnacalis]
uncharacterized protein LOC114356704 [Ostrinia furnacalis]
TIL [Ostrinia furnacalis]
innexin inx1-like [Pectinophora gossypiella]
double-strand break repair protein MRE11 [Ostrinia furnacalis]
cuticle protein 19.8-like [Ostrinia furnacalis]
uncharacterized protein LOC114349936 [Ostrinia furnacalis]
protein croquemort-like [Ostrinia furnacalis]
transmembrane protease serine 9 [Ostrinia furnacalis]
protein N-terminal asparagine amidohydrolase [Cotesia glomerata] >XP_044583616.1 protein N-terminal asparagine amidohydrolase [Cotesia glomerata] >XP_044583617.1 protein N-terminal
carboxylesterase [Cnaphalocrocis medinalis]
unnamed protein product [Pieris macdunnoughi]
sideroflexin-1-3 [Galleria mellonella] >XP_026754161.1 sideroflexin-1-3 [Galleria mellonella]
protein enhancer of sevenless 2B isoform X2 [Formica exsecta]
TRINITY_DN2348_c0_g1_i1_m.39060 TRINITY_DN2348_c0_g1::TRINITY_DN2348_c0_g1_i1::g.39060 ORF type:complete len:149 (+),score=54.19 TRINITY_DN2348_c0_g1_i1:28-474(+)
beta-hexosaminidase subunit alpha-like isoform X2 [Ostrinia furnacalis]
translation initiation factor eIF-2B subunit alpha [Ostrinia furnacalis]
carboxylesterase [Ostrinia furnacalis]
transmembrane protein 184B isoform X3 [Ostrinia furnacalis]
alpha-2-macroglobulin receptor-associated protein [Diachasma alloeum]
serine protease inhibitor 77Ba-like [Ostrinia furnacalis]
serine/threonine-protein kinase mig-15 isoform X2 [Ostrinia furnacalis]
putative phospholipase B-like 2 [Ostrinia furnacalis]
maspardin-like [Ostrinia furnacalis]
nicotinate phosphoribosyltransferase isoform X1 [Ostrinia furnacalis] >XP_028178189.1 nicotinate
putative inorganic phosphate cotransporter [Ostrinia furnacalis]
macrophage mannose receptor 1-like [Ostrinia furnacalis]
uncharacterized protein LOC114350603 [Ostrinia furnacalis]
CTL-like protein 1 isoform X1 [Galleria mellonella]
uncharacterized protein LOC114353772 [Ostrinia furnacalis]
PREDICTED: cryptochrome-1 isoform X1 [Amyelois transitella] >XP_013199861.1 PREDICTED:
group XV phospholipase A2-like [Ostrinia furnacalis] >XP_028168992.1 group XV phospholipase A2-like [Ostrinia furnacalis] >XP_028168993.1 group XV phospholipase A2-like [Ostrinia furnacalis]
unnamed protein product, partial [Iphiclides podalirius]
apolipophorins-like [Ostrinia furnacalis]

larval/pupal rigid cuticle protein 66-like [Hyposmocoma kahamanoa]
uncharacterized protein LOC114349567 [Ostrinia furnacalis]
uncharacterized protein LOC114352963 [Ostrinia furnacalis]
peroxisomal N(1)-acetyl-spermine/spermidine oxidase-like isoform X1 [Ostrinia furnacalis]
D-beta-hydroxybutyrate dehydrogenase, mitochondrial, partial [Chelonus insularis]
programmed cell death protein 5 [Ostrinia furnacalis]
uncharacterized protein LOC114359380 isoform X1 [Ostrinia furnacalis] >XP_028169561.1
uncharacterized protein LOC114359380 isoform X2 [Ostrinia furnacalis] >XP_028169562.1
PREDICTED: ras-related protein Rab-4B [Amyelois transitella]
amyloid beta (A4) precursor-like protein 2, isoform CRA_b [Homo sapiens]
unnamed protein product [Chilo suppressalis]
glyoxalase domain-containing protein 4 [Ostrinia furnacalis]
uncharacterized protein LOC114357426 [Ostrinia furnacalis]
monocarboxylate transporter 12 [Ostrinia furnacalis] >XP_028157531.1 monocarboxylate transporter 12
aldo-keto reductase AKR2E4-like [Ostrinia furnacalis]
phenoloxidase-activating factor 2-like isoform X1 [Ostrinia furnacalis] >XP_028178309.1
N-acetylgalactosaminyltransferase 7 isoform X1 [Ostrinia furnacalis] >XP_028156925.1 N-
GILT-like protein 2 isoform X1 [Ostrinia furnacalis] >XP_028156245.1 GILT-like protein 2 isoform X2
[Ostrinia furnacalis] >XP_028156247.1 GILT-like protein 2 isoform X3 [Ostrinia furnacalis]
spermatogenesis-associated protein 20 isoform X1 [Ostrinia furnacalis]
LIM and SH3 domain protein Lasp [Ostrinia furnacalis]
uncharacterized protein LOC114349939 [Ostrinia furnacalis] >XP_028156338.1 uncharacterized protein
sorting nexin-17 [Ostrinia furnacalis]
tetrahydrofolate synthase, partial [Plutella xylostella]
spermidine synthase [Ostrinia furnacalis] >XP_028167892.1 spermidine synthase [Ostrinia furnacalis]
cGMP-dependent protein kinase, isozyme 2 forms cD4/T1/T3A/T3B-like isoform X3 [Ostrinia furnacalis]
>XP_028158316.1 cGMP-dependent protein kinase, isozyme 2 forms cD4/T1/T3A/T3B-like isoform X3
bombyxin B-9-like [Ostrinia furnacalis]
autophagy protein 12-like [Ostrinia furnacalis]
immunoglobulin-binding protein 1b [Ostrinia furnacalis]
angiotensin-converting enzyme-like [Ostrinia furnacalis]
tyrosine-protein kinase-like otk, partial [Ostrinia furnacalis]
hypothetical protein evm_003552 [Chilo suppressalis]
protein eiger [Ostrinia furnacalis] >QKV49447.1 eiger [Ostrinia furnacalis]
pancreatic lipase-related protein 2 isoform X1 [Ostrinia furnacalis] >XP_028176200.1 pancreatic lipase-
ribose-phosphate pyrophosphokinase 2-like [Ostrinia furnacalis]
gem-associated protein 5-like [Ostrinia furnacalis]
vanin-like protein 2 isoform X2 [Ostrinia furnacalis]
hypothetical protein EVAR_60654_1 [Eumeta japonica]
carboxypeptidase N subunit 2-like [Ostrinia furnacalis]
probable low-specificity L-threonine aldolase 2 [Ostrinia furnacalis]
uncharacterized protein LOC114350746 [Ostrinia furnacalis]
uncharacterized protein LOC114362040 isoform X1 [Ostrinia furnacalis]
unnamed protein product [Spodoptera exigua]
calumenin [Ostrinia furnacalis] >XP_028172745.1 calumenin [Ostrinia furnacalis] >XP_028172746.1
GATOR complex protein MIOS [Ostrinia furnacalis]
dynamin-1-like protein isoform X1 [Ostrinia furnacalis] >XP_028177409.1 dynamin-1-like protein
tubulin-folding cofactor B isoform X3 [Ostrinia furnacalis]
hypothetical protein evm_002209, partial [Chilo suppressalis]
basement membrane-specific heparan sulfate proteoglycan core protein isoform X13 [Ostrinia
ribose-phosphate pyrophosphokinase 2 [Ostrinia furnacalis]
hypothetical protein evm_001488 [Chilo suppressalis] >CAB3526337.1 unnamed protein product [Chilo
suppressalis] >CAH0403665.1 unnamed protein product [Chilo suppressalis]
prominin-like protein isoform X2 [Ostrinia furnacalis]
CAD protein isoform X2 [Ostrinia furnacalis]
hypothetical protein evm_010760 [Chilo suppressalis]
Photosystem I reaction center subunit III, chloroplastic, partial [Trichinella zimbabwensis]

uncharacterized protein LOC114352137 [Ostrinia furnacalis] >XP_028159411.1 uncharacterized protein LOC114352137 [Ostrinia furnacalis] >XP_028159412.1 uncharacterized protein LOC114352137 [Ostrinia
hypothetical protein evm_008466 [Chilo suppressalis]
N(G),N(G)-dimethylarginine dimethylaminohydrolase 1 [Ostrinia furnacalis]
aminoacylase-1-like [Ostrinia furnacalis]
probable salivary secreted peptide [Ostrinia furnacalis]
probable small nuclear ribonucleoprotein E [Ostrinia furnacalis]
retinol dehydrogenase 14-like [Ostrinia furnacalis]
aquaporin AQPAe.a [Ostrinia furnacalis]
unnamed protein product [Arctia plantaginis] >CAB3252297.1 unnamed protein product [Arctia
facilitated trehalose transporter Tret1-like isoform X1 [Ostrinia furnacalis]
insulin-like growth factor-binding protein complex acid labile subunit [Ostrinia furnacalis]
regucalcin-like [Nymphalis io] >XP_050348255.1 regucalcin-like [Nymphalis io]
carbonyl reductase [NADPH] 1-like [Ostrinia furnacalis]
uncharacterized protein LOC114362571 [Ostrinia furnacalis]
phospholipase A1 VesT1.02-like [Ostrinia furnacalis]
putative salivary secreted peptide [Operophtera brumata]
protein arginine N-methyltransferase 7 isoform X1 [Ostrinia furnacalis]
uncharacterized protein LOC114356866 isoform X3 [Ostrinia furnacalis] >XP_028166037.1 uncharacterized protein LOC114356866 isoform X3 [Ostrinia furnacalis]
acetylcholinesterase-like [Ostrinia furnacalis]
unnamed protein product, partial [Iphiclides podalirius]
DNA replication licensing factor Mcm7 [Helicoverpa armigera] >XP_049698025.1 DNA replication licensing factor Mcm7-like [Helicoverpa armigera] >PZC87280.1 hypothetical protein
uncharacterized protein LOC114351684 [Ostrinia furnacalis]
odorant binding protein 18 [Conogethes pinicolalis]
lachesin isoform X1 [Ostrinia furnacalis] >XP_028178464.1 lachesin isoform X2 [Ostrinia furnacalis]
uncharacterized protein LOC114357114 isoform X1 [Ostrinia furnacalis]
titin homolog [Ostrinia furnacalis]
uncharacterized protein LOC114360857, partial [Ostrinia furnacalis]
pancreatic lipase-related protein 2 isoform X1 [Ostrinia furnacalis] >XP_028176200.1 pancreatic lipase-
uncharacterized protein LOC114357622 [Ostrinia furnacalis]
uncharacterized protein LOC114351392 isoform X1 [Ostrinia furnacalis]
carboxylesterase [Ostrinia furnacalis]
serine/threonine-protein kinase PAK 3 [Ostrinia furnacalis]
liver carboxylesterase 2-like [Ostrinia furnacalis]
uncharacterized protein LOC114357706 isoform X1 [Ostrinia furnacalis] >XP_028167261.1 uncharacterized protein LOC114357706 isoform X2 [Ostrinia furnacalis]
uncharacterized protein LOC114356786 [Ostrinia furnacalis]
tyrosine-protein phosphatase non-receptor type 61F-like isoform X1 [Vanessa tameamea] >XP_047543663.1 tyrosine-protein phosphatase non-receptor type 61F-like isoform X1 [Vanessa
acylamino-acid-releasing enzyme-like isoform X1 [Ostrinia furnacalis] >XP_028174257.1 acylamino-acid-releasing enzyme-like isoform X2 [Ostrinia furnacalis] >XP_028174264.1 acylamino-acid-releasing enzyme-like isoform X3 [Ostrinia furnacalis] >XP_028174273.1 acylamino-acid-releasing enzyme-like
hypothetical protein evm_004480 [Chilo suppressalis] >CAB3520922.1 unnamed protein product [Chilo suppressalis] >CAH0398243.1 unnamed protein product [Chilo suppressalis]
gamma-glutamylcyclotransferase-like isoform X1 [Ostrinia furnacalis]
GTP-binding protein 1 [Ostrinia furnacalis] >XP_028178070.1 GTP-binding protein 1 [Ostrinia furnacalis] >XP_028178072.1 GTP-binding protein 1 [Ostrinia furnacalis]
laminin subunit alpha-like, partial [Ostrinia furnacalis]
tolloid-like protein 1 [Ostrinia furnacalis]
mucin-5AC-like [Ostrinia furnacalis]
uncharacterized protein LOC114355531 [Ostrinia furnacalis]
hypothetical protein evm_004679 [Chilo suppressalis] >CAH2989683.1 unnamed protein product [Chilo
serine/threonine-protein kinase SIK2 [Ostrinia furnacalis] >XP_028174514.1 serine/threonine-protein
PREDICTED: cuticle protein 18.6, isoform B [Amyelois transitella]
hypothetical protein NE865_05903 [Phthorimaea operculella]
uncharacterized protein LOC114353135 [Ostrinia furnacalis]

repetitive proline-rich cell wall protein 1 precursor [Papilio polytes] >XP_013147838.1 PREDICTED: repetitive proline-rich cell wall protein 1 [Papilio polytes] >BAM19190.1 cuticular protein PpolCPG24
hemocyte protein-glutamine gamma-glutamyltransferase-like [Ostrinia furnacalis]
cuticle protein 8-like [Ostrinia furnacalis]
zinc carboxypeptidase-like [Ostrinia furnacalis]
cuticle protein 7-like [Ostrinia furnacalis]
endochitinase isoform X2 [Ostrinia furnacalis]
leech-derived tryptase inhibitor C-like [Ostrinia furnacalis]
alpha-tocopherol transfer protein-like [Chelonus insularis]
protein obstructor-E-like [Ostrinia furnacalis]
hypothetical protein evm_003901 [Chilo suppressalis]
cuticle protein 19-like [Ostrinia furnacalis]
tetra-peptide repeat homeobox protein 1-like [Ostrinia furnacalis]
hypothetical protein KGM_205563 [Danaus plexippus plexippus]
hypothetical protein evm_002822 [Chilo suppressalis]
cuticle protein 8-like [Ostrinia furnacalis]
chymotrypsin-2-like [Ostrinia furnacalis]
pupal cuticle protein PCP52-like [Ostrinia furnacalis]
uncharacterized protein LOC114350099 [Ostrinia furnacalis]
hypothetical protein evm_002297 [Chilo suppressalis]
mucin-5AC-like [Ostrinia furnacalis]
uncharacterized protein LOC114360402 [Ostrinia furnacalis]
putative uncharacterized protein DDB_G0271606 [Ostrinia furnacalis]
uncharacterized protein LOC114351021 [Ostrinia furnacalis]
uncharacterized protein LOC114365758 isoform X3 [Ostrinia furnacalis]
adult-specific cuticular protein ACP-20-like [Ostrinia furnacalis]
uncharacterized protein LOC113509309, partial [Galleria mellonella]
histidine-rich glycoprotein [Ostrinia furnacalis]
uncharacterized protein LOC114349648 [Ostrinia furnacalis]
uncharacterized protein LOC119829283 isoform X2 [Zerene cesonia]
uncharacterized protein LOC114360441, partial [Ostrinia furnacalis]
uncharacterized protein LOC114361337 [Ostrinia furnacalis]
uncharacterized protein LOC114363305 isoform X2 [Ostrinia furnacalis]
hypothetical protein evm_010516 [Chilo suppressalis]
lysosomal alpha-mannosidase-like [Ostrinia furnacalis]
chorion protein S36-like [Ostrinia furnacalis]
anosmin-1 [Ostrinia furnacalis] >XP_028178811.1 anosmin-1 [Ostrinia furnacalis]
chemosensory protein 5 [Conogethes punctiferalis]
basic juvenile hormone-suppressible protein 1-like [Hyposmocoma kahamanoa]
unnamed protein product [Spodoptera littoralis] >CAH1638738.1 unnamed protein product
uncharacterized protein LOC114363281 [Ostrinia furnacalis]
TRINITY_DN2002_c0_g1_i5_m.4230 TRINITY_DN2002_c0_g1::TRINITY_DN2002_c0_g1_i5::g.4230 ORF type:3prime_partial len:259 (+),score=29.98,Peptidase_C39 PF03412.16
unnamed protein product [Plutella xylostella]
conotoxin ArMKLT2-032-like [Ostrinia furnacalis]
repetitive proline-rich cell wall protein 2-like [Ostrinia furnacalis]
uncharacterized protein LOC114363102 isoform X2 [Ostrinia furnacalis]
TRINITY_DN31_c0_g1_i3_m.1394 TRINITY_DN31_c0_g1::TRINITY_DN31_c0_g1_i3::g.1394 ORF type:complete len:118 (+),score=43.35 TRINITY_DN31_c0_g1_i3:56-409(+)
cuticle protein 8-like isoform X2 [Vanessa tameamea]
ferritin subunit-like [Ostrinia furnacalis] >XP_028168186.1 ferritin subunit-like [Ostrinia furnacalis]
uncharacterized protein LOC114353424 [Ostrinia furnacalis]
serine protease inhibitor 3/4 [Ostrinia furnacalis]
uncharacterized protein LOC114365444, partial [Ostrinia furnacalis]
venom serine carboxypeptidase-like [Ostrinia furnacalis]
meiosis-specific nuclear structural protein 1-like isoform X2 [Ostrinia furnacalis]
skin secretory protein xP2-like [Ostrinia furnacalis]
adult-specific cuticular protein ACP-22-like [Ostrinia furnacalis]

uncharacterized protein LOC114355006 [Ostrinia furnacalis]
serine protease inhibitor dipetalogastin [Ostrinia furnacalis]
hemicentin-1-like isoform X1 [Ostrinia furnacalis]
uncharacterized protein LOC126367764 [Pectinophora gossypiella]
larval/pupal cuticle protein H1C-like [Ostrinia furnacalis]
keratin, type I cytoskeletal 10-like [Ostrinia furnacalis]
hypothetical protein evm_010265 [Chilo suppressalis] >CAB3524755.1 unnamed protein product [Chilo suppressalis] >CAH0397522.1 unnamed protein product [Chilo suppressalis]
uncharacterized protein LOC114361550 [Ostrinia furnacalis]
carboxypeptidase B-like [Ostrinia furnacalis]
putative GPI-anchored protein pfl2 isoform X1 [Ostrinia furnacalis]
clavesin-1-like [Ostrinia furnacalis]
peroxidase-like isoform X1 [Ostrinia furnacalis]
spidroin-2-like [Ostrinia furnacalis]
phenoloxidase-activating factor 2-like [Ostrinia furnacalis]
uncharacterized protein LOC107270465 [Cephus cinctus]
uncharacterized protein LOC114355976 [Ostrinia furnacalis]
UPF0489 protein C5orf22 homolog [Ostrinia furnacalis]
putative GPI-anchored protein pfl2 isoform X2 [Ostrinia furnacalis]
cuticle protein 7-like [Ostrinia furnacalis]
protein yellow-like [Ostrinia furnacalis]
hemocyte protein-glutamine gamma-glutamyltransferase-like [Ostrinia furnacalis]
general odorant-binding protein 56d-like isoform X2 [Ostrinia furnacalis]
cuticle protein 7 [Plutella xylostella] >CAG9138501.1 unnamed protein product [Plutella xylostella]
uncharacterized protein LOC114364098 [Ostrinia furnacalis]
trypsin CFT-1-like [Ostrinia furnacalis]
uncharacterized protein LOC114354985 isoform X1 [Ostrinia furnacalis]
mucin-2-like [Ostrinia furnacalis]
chemosensory protein 10 [Conogethes pinicolalis]
nucleolin-like [Ostrinia furnacalis]
unnamed protein product [Diatraea saccharalis]
fibroin heavy chain-like [Ostrinia furnacalis]
TRINITY_DN2290_c0_g1_i2_m.69732 TRINITY_DN2290_c0_g1::TRINITY_DN2290_c0_g1_i2::g.69732 ORF type:complete len:234 (+),score=14.43 TRINITY_DN2290_c0_g1_i2:60-761(+)
titin-like [Ostrinia furnacalis]
teneurin-m isoform X1 [Ostrinia furnacalis]
keratin, type I cytoskeletal 9-like [Ostrinia furnacalis]
CDGSH iron-sulfur domain-containing protein 3, mitochondrial-like [Ostrinia furnacalis]
mucin-5AC isoform X1 [Ostrinia furnacalis]
heat shock protein 21.7c [Chilo suppressalis] >AWT57938.1 heat shock protein 21.7c [Chilo
activating signal cointegrator 1 complex subunit 2 homolog isoform X1 [Ostrinia furnacalis]
serine proteinase stubble-like [Ostrinia furnacalis]
uncharacterized protein LOC114351042 [Ostrinia furnacalis]
putative mitochondrial aconitate hydratase isoform X1-likeprotein, partial [Cotesia chilonis]
A-kinase anchor protein 200-like [Ostrinia furnacalis] >XP_028173114.1 A-kinase anchor protein 200-like [Ostrinia furnacalis] >XP_028173115.1 A-kinase anchor protein 200-like [Ostrinia furnacalis]
glucosamine-6-phosphate isomerase isoform X2 [Ostrinia furnacalis]
uncharacterized protein LOC114359411 [Ostrinia furnacalis]
protein obstructor-E-like [Ostrinia furnacalis]
unnamed protein product [Spodoptera littoralis] >CAH1645252.1 unnamed protein product
myb-like protein AA [Ostrinia furnacalis]
uncharacterized protein LOC114366601 [Ostrinia furnacalis]
hypothetical protein evm_002829 [Chilo suppressalis]
TRINITY_DN261_c0_g1_i5_m.18559 TRINITY_DN261_c0_g1::TRINITY_DN261_c0_g1_i5::g.18559 ORF type:internal len:190 (+),score=69.81 TRINITY_DN261_c0_g1_i5:3-569(+)
lysosome-associated membrane glycoprotein 1-like isoform X4 [Ostrinia furnacalis]
altered inheritance of mitochondria protein 3-like [Ostrinia furnacalis]
hypothetical protein evm_008559 [Chilo suppressalis]

A-kinase anchor protein 200-like [Ostrinia furnacalis] >XP_028173114.1 A-kinase anchor protein 200-like [Ostrinia furnacalis] >XP_028173115.1 A-kinase anchor protein 200-like [Ostrinia furnacalis]
uncharacterized protein LOC114356437 isoform X1 [Ostrinia furnacalis]
lopap-like [Ostrinia furnacalis]
ATP-dependent DNA helicase 2 subunit 1 [Ostrinia furnacalis]
pupal cuticle protein 36-like [Ostrinia furnacalis]
GILT-like protein 2 isoform X1 [Ostrinia furnacalis] >XP_028156245.1 GILT-like protein 2 isoform X2 [Ostrinia furnacalis] >XP_028156247.1 GILT-like protein 2 isoform X3 [Ostrinia furnacalis]
uncharacterized protein LOC114355167 [Ostrinia furnacalis]
angiotensin-converting enzyme-like isoform X1 [Ostrinia furnacalis]
neprilysin-2 isoform X1 [Ostrinia furnacalis]
uncharacterized protein LOC114357708 [Ostrinia furnacalis]
upstream activation factor subunit spp27 [Ostrinia furnacalis]
heat shock protein 19.8 [Chilo suppressalis] >AGM90553.1 HSP19.8 [Chilo suppressalis] >BAE94664.1
glycine-rich cell wall structural protein [Ostrinia furnacalis]
proteasomal ubiquitin receptor ADRM1 [Ostrinia furnacalis]
altered inheritance of mitochondria protein 3-like isoform X2 [Ostrinia furnacalis]
putative uncharacterized protein DDB_G0282499 isoform X1 [Ostrinia furnacalis]
very low-density lipoprotein receptor isoform X2 [Galleria mellonella]
uncharacterized protein LOC115444227 [Manduca sexta] >XP_030025790.1 uncharacterized protein LOC115444227 [Manduca sexta] >XP_037296791.1 uncharacterized protein LOC115444227 [Manduca sexta] >XP_037296792.1 uncharacterized protein LOC115444227 [Manduca sexta] >KAG6441350.1
uncharacterized protein LOC114353190 isoform X1 [Ostrinia furnacalis] >XP_028160984.1
uncharacterized protein LOC114353190 isoform X2 [Ostrinia furnacalis] >XP_028161062.1
uncharacterized protein LOC114353190 isoform X1 [Ostrinia furnacalis] >XP_028161142.1
fatty acid synthase-like isoform X1 [Ostrinia furnacalis]
division abnormally delayed protein [Ostrinia furnacalis]
glucosamine-6-phosphate isomerase isoform X1 [Ostrinia furnacalis]
long-chain fatty acid transport protein 1-like [Ostrinia furnacalis]
aminopeptidase N-like [Ostrinia furnacalis]
uncharacterized protein LOC107036393 [Diachasma alloeum]
pericentriolar material 1 protein-like isoform X3 [Ostrinia furnacalis]
larval/pupal cuticle protein H1C-like [Ostrinia furnacalis]
conserved oligomeric Golgi complex subunit 2 [Ostrinia furnacalis]
zonadhesin-like [Ostrinia furnacalis]
unnamed protein product [Chilo suppressalis]
hypothetical protein evm_006611 [Chilo suppressalis]
unnamed protein product [Chilo suppressalis]
uncharacterized protein LOC114366101 [Ostrinia furnacalis]
endocuticle structural glycoprotein ABD-4-like [Ostrinia furnacalis]
uncharacterized protein LOC126371336 [Pectinophora gossypiella]
basement membrane-specific heparan sulfate proteoglycan core protein isoform X13 [Ostrinia
uncharacterized protein LOC114352615 [Ostrinia furnacalis]
neurexin-4 [Ostrinia furnacalis]
protein lethal(2)essential for life-like [Galleria mellonella]
uncharacterized protein LOC114350302, partial [Ostrinia furnacalis]
histone-lysine N-methyltransferase 2B-like, partial [Ostrinia furnacalis]
cuticle protein 19.8-like [Ostrinia furnacalis]
Tubulin beta-1 chain [Papilio xuthus]
myotubularin-related protein 9 [Ostrinia furnacalis]
proline-rich extensin-like protein EPR1 [Manduca sexta]
elongation factor 1-alpha 2-like [Galleria mellonella] >XP_031769625.1 elongation factor 1-alpha 2-like
Serine proteinase stubble [Eumeta japonica]
cuticle protein 18.6-like [Ostrinia furnacalis]
thymosin beta isoform X4 [Ostrinia furnacalis]
hypothetical protein evm_002550 [Chilo suppressalis]
mucolipin-3-like [Ostrinia furnacalis]
claspin-like [Ostrinia furnacalis]

chromodomain-helicase-DNA-binding protein 1 isoform X3 [Ostrinia furnacalis]
chymotrypsin-2-like [Ostrinia furnacalis]
cuticular protein CPH [Spodoptera litura]
acylphosphatase-2-like [Ostrinia furnacalis]
follicle-stimulating hormone receptor-like [Ostrinia furnacalis]
putative fatty acyl-CoA reductase CG5065 isoform X1 [Ostrinia furnacalis]
brain tumor protein isoform X1 [Ostrinia furnacalis]
NTF2-related export protein [Ostrinia furnacalis]
mucin-2-like isoform X2 [Ostrinia furnacalis]
furin-like protease 1, partial [Ostrinia furnacalis]
uncharacterized protein LOC114357057 [Ostrinia furnacalis]
hemicentin-2-like isoform X1 [Ostrinia furnacalis]
caspase-1-like [Ostrinia furnacalis]
putative fatty acyl-CoA reductase CG5065 [Ostrinia furnacalis]
uncharacterized protein LOC114350958 [Ostrinia furnacalis]
small heat shock protein Hsp29.7 [Ostrinia furnacalis]
P protein-like [Ostrinia furnacalis] >XP_028167089.1 P protein-like [Ostrinia furnacalis]
jupiter microtubule associated homolog 1-like [Ostrinia furnacalis] >XP_028173360.1 jupiter
inactive tyrosine-protein kinase 7-like, partial [Ostrinia furnacalis]
nicastrin [Ostrinia furnacalis]
uncharacterized protein LOC114350556 isoform X1 [Ostrinia furnacalis] >XP_028157201.1
uncharacterized protein LOC114350556 isoform X2 [Ostrinia furnacalis] >XP_028157202.1
G protein-coupled receptor kinase 1 isoform X2 [Helicoverpa armigera] >XP_047029555.1 G protein-coupled receptor kinase 1 isoform X2 [Helicoverpa zea] >ANZ22924.1 G protein-coupled receptor
formin-like protein isoform X3 [Ostrinia furnacalis] >XP_028170442.1 formin-like protein isoform X6
septin-7 isoform X1 [Ostrinia furnacalis]
PH and SEC7 domain-containing protein 1 [Trichoplusia ni]
uncharacterized protein LOC114364097 isoform X2 [Ostrinia furnacalis]
disintegrin and metalloproteinase domain-containing protein 10 isoform X1 [Ostrinia furnacalis] >XP_028172845.1 disintegrin and metalloproteinase domain-containing protein 10 isoform X2 [Ostrinia
ubiquitin-conjugating enzyme E2 variant 2 [Helicoverpa armigera] >XP_026325122.1 ubiquitin-conjugating enzyme E2 variant 2 [Hyposmocoma kahamanoa] >XP_026499214.1 ubiquitin-conjugating enzyme E2 variant 2 [Vanessa tameamea] >XP_026738520.1 ubiquitin-conjugating enzyme E2 variant 2 [Trichoplusia ni] >XP_026762551.1 ubiquitin-conjugating enzyme E2 variant 2 [Galleria mellonella] >XP_028172566.1 ubiquitin-conjugating enzyme E2 variant 2 [Ostrinia furnacalis] >XP_032520494.1 ubiquitin-conjugating enzyme E2 variant 2 [Danaus plexippus plexippus] >XP_034831247.1 ubiquitin-conjugating enzyme E2 variant 2 [Maniola hyperantus] >XP_039755795.1 ubiquitin-conjugating enzyme E2 variant 2 [Pararge aegeria] >XP_041987413.1 ubiquitin-conjugating enzyme E2 variant 2 [Aricia agestis] >XP_045450678.1 ubiquitin-conjugating enzyme E2 variant 2 [Melitaea cinxia] >XP_045766931.1 ubiquitin-conjugating enzyme E2 variant 2 [Maniola jurtina] >XP_046968624.1 ubiquitin-conjugating enzyme E2 variant 2 [Vanessa cardui] >XP_047027052.1 ubiquitin-conjugating enzyme E2 variant 2 [Helicoverpa zea] >XP_047534810.1 ubiquitin-conjugating enzyme E2 variant 2
vacuolar protein sorting-associated protein 27-like [Trichoplusia ni]
regucalcin-like [Ostrinia furnacalis]
zinc finger protein on ecdysone puffs-like [Ostrinia furnacalis]
heparan-alpha-glucosaminide N-acetyltransferase [Helicoverpa armigera]
tyrosine-protein kinase Src42A isoform X2 [Trichoplusia ni]
coactosin-like protein isoform X2 [Trichoplusia ni]
selenide, water dikinase [Ostrinia furnacalis] >CAG9756850.1 unnamed protein product [Diatraea saccharalis] >CAG9795535.1 unnamed protein product [Diatraea saccharalis]
hypothetical protein evm_008982 [Chilo suppressalis]
diphosphomevalonate decarboxylase [Ostrinia furnacalis]
tubulin gamma-1 chain-like isoform X1 [Ostrinia furnacalis] >XP_028160960.1 tubulin gamma-1 chain-
heterogeneous nuclear ribonucleoprotein Q isoform X2 [Galleria mellonella]
proteoglycan Cow [Ostrinia furnacalis]
protein sly1 homolog isoform X1 [Ostrinia furnacalis] >XP_028165613.1 protein sly1 homolog isoform
cuticle protein 64-like [Pectinophora gossypiella]
uncharacterized protein LOC114354338 isoform X1 [Ostrinia furnacalis]

uncharacterized protein LOC114365835 [Ostrinia furnacalis]
hypothetical protein evm_008839 [Chilo suppressalis] >CAB3526474.1 unnamed protein product [Chilo suppressalis] >CAH0403802.1 unnamed protein product [Chilo suppressalis]
polyadenylate-binding protein 1-B-like [Ostrinia furnacalis]
decaprenyl-diphosphate synthase subunit 2-like [Ostrinia furnacalis]
filamin-A isoform X2 [Ostrinia furnacalis]
Down syndrome cell adhesion molecule-like protein Dscam2 isoform X16 [Ostrinia furnacalis]
ubiquitin-like domain-containing CTD phosphatase 1 [Ostrinia furnacalis]
pupal cuticle protein-like [Ostrinia furnacalis]
uncharacterized protein LOC114350172 [Ostrinia furnacalis]
unnamed protein product [Chilo suppressalis]
NAD(P) transhydrogenase, mitochondrial-like [Ostrinia furnacalis] >XP_028175067.1 NAD(P) transhydrogenase, mitochondrial-like [Ostrinia furnacalis] >XP_028175068.1 NAD(P) transhydrogenase, mitochondrial-like [Ostrinia furnacalis] >XP_028175069.1 NAD(P) transhydrogenase, mitochondrial-like
heterogeneous nuclear ribonucleoprotein R isoform X6 [Danaus plexippus plexippus]
contactin [Ostrinia furnacalis]
putative aminopeptidase W07G4.4 isoform X2 [Ostrinia furnacalis]
unnamed protein product [Diatraea saccharalis]
basement membrane-specific heparan sulfate proteoglycan core protein isoform X13 [Ostrinia
myosin-VIIa [Ostrinia furnacalis] >XP_028155907.1 myosin-VIIa [Ostrinia furnacalis]
unnamed protein product [Parnassius apollo]
microtubule-associated protein futsch-like isoform X6 [Ostrinia furnacalis]
glutathione S-transferase 1-like [Ostrinia furnacalis] >QIC35740.1 glutathione S-transferase delta 4
prostatic acid phosphatase-like [Ostrinia furnacalis]
uncharacterized protein LOC114362122 [Ostrinia furnacalis]
ubiquitin-like modifier-activating enzyme 1 [Ostrinia furnacalis]
myogenesis-regulating glycosidase isoform X1 [Ostrinia furnacalis] >XP_028158488.1 myogenesis-regulating glycosidase isoform X1 [Ostrinia furnacalis] >XP_028158489.1 myogenesis-regulating glycosidase isoform X1 [Ostrinia furnacalis] >XP_028158490.1 myogenesis-regulating glycosidase
uncharacterized protein LOC114358636 [Ostrinia furnacalis]
syntaxin-7 [Helicoverpa armigera] >XP_049695901.1 syntaxin-7 [Helicoverpa armigera]
organic cation transporter-like protein [Ostrinia furnacalis]
phenoloxidase-activating factor 2-like [Ostrinia furnacalis]
adrenodoxin-like protein, mitochondrial isoform X1 [Ostrinia furnacalis]
hypothetical protein evm_011254 [Chilo suppressalis]
PREDICTED: tubulin alpha-1A chain-like [Papilio polytes] >XP_013164648.1 PREDICTED: tubulin alpha-
unnamed protein product [Chilo suppressalis]
selenoprotein M-like [Ostrinia furnacalis]
low-density lipoprotein receptor-related protein 1B-like [Ostrinia furnacalis]
O-GlcNAc hydrolase [Ostrinia furnacalis]
atrial natriuretic peptide-converting enzyme-like [Ostrinia furnacalis]
CD109 antigen [Ostrinia furnacalis] >XP_028176877.1 CD109 antigen [Ostrinia furnacalis]
unnamed protein product [Chrysodeixis includens]
trypsin inhibitor-like [Ostrinia furnacalis]
TBC1 domain family member 9 isoform X1 [Ostrinia furnacalis] >XP_028176568.1 TBC1 domain family
protein HGV2-like isoform X2 [Ostrinia furnacalis]
kinesin-like protein Klp10A isoform X4 [Spodoptera frugiperda]
probable cytochrome P450 303a1 [Ostrinia furnacalis] >XP_028178318.1 probable cytochrome P450
putative hydroxypyruvate isomerase [Ostrinia furnacalis]
fasciclin-3 isoform X4 [Helicoverpa zea]
methylthioribose-1-phosphate isomerase [Ostrinia furnacalis] >XP_028172842.1 methylthioribose-1-
COPII coat assembly protein sec16-like [Ostrinia furnacalis]
plexin A3 [Ostrinia furnacalis]
COPII coat assembly protein sec16-like [Ostrinia furnacalis]
dynein light chain roadblock-type 2 [Bombyx mori] >XP_028159912.1 dynein light chain roadblock-type 2-like [Ostrinia furnacalis] >XP_030028262.1 dynein light chain roadblock-type 2 [Manduca sexta] >XP_038218546.1 dynein light chain roadblock-type 2-like [Zerene cesonia] >XP_045505125.1 dynein
rab GDP dissociation inhibitor alpha [Ostrinia furnacalis]

serine protease inhibitor 88Ea-like [Ostrinia furnacalis]
Pupal cuticle protein PCP52 [Papilio xuthus]
venom dipeptidyl peptidase 4-like [Ostrinia furnacalis]
uncharacterized protein LOC114357587 [Ostrinia furnacalis]
uncharacterized protein LOC114361222 [Ostrinia furnacalis]
FK506-binding protein-like [Galleria mellonella]
PREDICTED: probable small nuclear ribonucleoprotein G [Papilio polytes] >XP_013168682.1 PREDICTED: probable small nuclear ribonucleoprotein G [Papilio xuthus] >XP_013200095.1 PREDICTED: probable small nuclear ribonucleoprotein G [Amyelois transitella] >XP_014365947.1 probable small nuclear ribonucleoprotein G [Papilio machaon] >XP_023949391.1 probable small nuclear ribonucleoprotein G [Bicyclus anynana] >XP_026492889.1 probable small nuclear ribonucleoprotein G [Vanessa tameamea] >XP_030032656.1 probable small nuclear ribonucleoprotein G [Manduca sexta] >XP_032524946.1 probable small nuclear ribonucleoprotein G [Danaus plexippus plexippus] >XP_032524948.1 probable small nuclear ribonucleoprotein G [Danaus plexippus plexippus] >XP_039761094.1 probable small nuclear ribonucleoprotein G [Pararge aegeria] >XP_045507396.1 probable small nuclear ribonucleoprotein G [Colias croceus] >XP_046974152.1 probable small nuclear ribonucleoprotein G
heat shock protein 20.2 [Glyphodes pyloalis]
tudor and KH domain-containing protein homolog isoform X1 [Ostrinia furnacalis] >XP_028179486.1 tudor and KH domain-containing protein homolog isoform X1 [Ostrinia furnacalis]
ubiquitin-conjugating enzyme E2 S [Ostrinia furnacalis]
unnamed protein product [Arctia plantaginis] >CAB3257565.1 unnamed protein product [Arctia
prisilkin-39-like [Ostrinia furnacalis]
acetyl-coenzyme A synthetase [Ostrinia furnacalis]
peroxisomal acyl-coenzyme A oxidase 3 isoform X3 [Ostrinia furnacalis]
unnamed protein product [Arctia plantaginis]
protein PRRC2A-like isoform X2 [Ostrinia furnacalis]
uncharacterized protein LOC114357292 isoform X4 [Ostrinia furnacalis]
protein SAND [Ostrinia furnacalis]
protein held out wings isoform X2 [Diachasma alloeum]
ATP-dependent RNA helicase dbp2-like isoform X1 [Leguminivora glycinivorella]
ATP-dependent DNA/RNA helicase DHX36 isoform X1 [Ostrinia furnacalis]
putative inorganic phosphate cotransporter [Ostrinia furnacalis]
sulfotransferase 1E1 [Galleria mellonella]
protein windbeutel [Ostrinia furnacalis]
unnamed protein product, partial [Brenthis ino]
PREDICTED: larval cuticle protein A2B-like [Amyelois transitella]
J domain-containing protein [Ostrinia furnacalis]
hypothetical protein evm_009110 [Chilo suppressalis] >CAH2984739.1 unnamed protein product [Chilo
myotrophin-like [Ostrinia furnacalis]
transmembrane emp24 domain-containing protein 5 [Ostrinia furnacalis]
furin-like protease 2 isoform X2 [Manduca sexta] >KAG6448919.1 hypothetical protein
kinesin heavy chain [Galleria mellonella]
barrier-to-autointegration factor [Ostrinia furnacalis] >XP_045447211.1 barrier-to-autointegration factor [Melitaea cinxia] >CAG4971452.1 unnamed protein product [Parnassius apollo] >CAG9578869.1 unnamed protein product [Danaus chrysippus] >CAH0714333.1 unnamed protein product, partial
dystrophin, isoforms A/C/F/G/H-like [Ostrinia furnacalis]
maternal protein tudor-like isoform X2 [Ostrinia furnacalis]
uncharacterized protein LOC114353382 isoform X1 [Ostrinia furnacalis]
ABC transporter G family member 23 isoform X1 [Ostrinia furnacalis] >XP_028178987.1 ABC transporter
protein groucho-like [Ostrinia furnacalis]
hsp70-Hsp90 organizing protein 3-like [Ostrinia furnacalis]
chromatin-remodeling complex ATPase chain Iswi isoform X1 [Ostrinia furnacalis]
sodium/potassium-transporting ATPase subunit alpha isoform X1 [Chelonus insularis] >XP_034939982.1 sodium/potassium-transporting ATPase subunit alpha isoform X1 [Chelonus insularis] >XP_034939983.1 sodium/potassium-transporting ATPase subunit alpha isoform X1
unnamed protein product [Chrysodeixis includens]
probable beta-hexosaminidase fdl isoform X1 [Ostrinia furnacalis]
unnamed protein product [Chilo suppressalis]

hypothetical protein evm_000559 [Chilo suppressalis]
uncharacterized protein LOC114362418 [Ostrinia furnacalis]
protein SYS1 homolog [Ostrinia furnacalis]
prenylated Rab acceptor protein 1 isoform X4 [Ostrinia furnacalis]
probable cytosolic iron-sulfur protein assembly protein Ciao1 [Ostrinia furnacalis]
nucleoporin NDC1 [Ostrinia furnacalis]
hsp90 co-chaperone Cdc37 [Ostrinia furnacalis]
intersectin-2 isoform X2 [Ostrinia furnacalis]
titin homolog [Ostrinia furnacalis]
bolA-like protein DDB_G0274169 [Ostrinia furnacalis] >XP_028171597.1 bolA-like protein
hypothetical protein evm_000184 [Chilo suppressalis]
60S ribosomal protein L35 [Ostrinia furnacalis]
segment polarity protein dishevelled homolog DVL-3 [Vanessa atalanta]
uncharacterized protein LOC114366781 [Ostrinia furnacalis]
uncharacterized protein LOC114359912 [Ostrinia furnacalis]
hypothetical protein evm_008218 [Chilo suppressalis]
teneurin-a isoform X1 [Ostrinia furnacalis]
uncharacterized protein LOC114351944 [Ostrinia furnacalis]
protein lethal(2)essential for life-like [Helicoverpa armigera] >PZC74790.1 hypothetical protein
phenoloxidase-activating factor 2-like [Hyposmocoma kahamanoa]
uncharacterized protein LOC114361472 [Ostrinia furnacalis]
somatomedin-B and thrombospondin type-1 domain-containing protein [Ostrinia furnacalis]
>XP_028177886.1 somatomedin-B and thrombospondin type-1 domain-containing protein [Ostrinia
methanethiol oxidase [Ostrinia furnacalis]
uncharacterized protein LOC114365032 [Ostrinia furnacalis]
sodium/potassium-transporting ATPase subunit beta-2-like isoform X2 [Ostrinia furnacalis]
hypothetical protein evm_002753 [Chilo suppressalis]
apolipoprotein D-like [Ostrinia furnacalis]
angiotensin-converting enzyme-like isoform X1 [Ostrinia furnacalis]
carboxylesterase [Cnaphalocrocis medinalis]
enoyl-CoA hydratase domain-containing protein 3 [Agrotis segetum]
dicer 2 [Ostrinia nubilalis]
putative neuropeptide precursor protein isoform X1 [Ostrinia furnacalis]
histone deacetylase 5 isoform X5 [Pectinophora gossypiella]
catalase-like [Pectinophora gossypiella]
unnamed protein product [Diatraea saccharalis]
glutathione S-transferase delta 3 [Ostrinia furnacalis]
PREDICTED: CAD protein [Microplitis demolitor]
phosphotriesterase-related protein [Ostrinia furnacalis]
ubiquitin-like modifier-activating enzyme ATG7 [Ostrinia furnacalis]
cilia- and flagella-associated protein 99-like [Ostrinia furnacalis]
PREDICTED: rap guanine nucleotide exchange factor 2-like isoform X9 [Microplitis demolitor]
fructose-bisphosphate aldolase-like isoform X1 [Ostrinia furnacalis] >XP_028178678.1 fructose-
GILT-like protein 2 isoform X1 [Ostrinia furnacalis] >XP_028156245.1 GILT-like protein 2 isoform X2
[Ostrinia furnacalis] >XP_028156247.1 GILT-like protein 2 isoform X3 [Ostrinia furnacalis]
neurofilament heavy polypeptide-like isoform X2 [Ostrinia furnacalis]
uncharacterized protein LOC114359515 [Ostrinia furnacalis]
adenosine deaminase 2-A-like [Galleria mellonella]
pupal cuticle protein-like [Trichoplusia ni]
60S ribosomal protein L5, partial [Cotesia chilonis]
acyl-CoA Delta(11) desaturase isoform X1 [Ostrinia furnacalis] >XP_028172999.1 acyl-CoA Delta(11)
desaturase isoform X2 [Ostrinia furnacalis] >XP_028173000.1 acyl-CoA Delta(11) desaturase isoform X1
protein obstructor-E-like isoform X1 [Ostrinia furnacalis] >XP_028169319.1 protein obstructor-E-like
hypothetical protein evm_000959 [Chilo suppressalis]
uncharacterized protein LOC125235519 [Leguminivora glycinivorella]
protein masquerade-like isoform X2 [Ostrinia furnacalis]
protein obstructor-E isoform X1 [Ostrinia furnacalis]
patronin isoform X9 [Ostrinia furnacalis]

uncharacterized protein LOC114360866 isoform X4 [Ostrinia furnacalis]
inositol-trisphosphate 3-kinase A isoform X1 [Vanessa tameamea] >XP_047534115.1 inositol-trisphosphate 3-kinase A isoform X1 [Vanessa atalanta] >XP_047534116.1 inositol-trisphosphate 3-kinase A isoform X1 [Vanessa atalanta] >XP_047534117.1 inositol-trisphosphate 3-kinase A isoform X1
unnamed protein product [Parnassius apollo] >CAG5017650.1 unnamed protein product [Parnassius
membrane-associated protein Hem [Ostrinia furnacalis]
cell wall protein DAN4 [Ostrinia furnacalis]
transmembrane protein 87A isoform X1 [Ostrinia furnacalis] >XP_028156931.1 transmembrane protein 87A isoform X2 [Ostrinia furnacalis] >XP_028156932.1 transmembrane protein 87A isoform X3 [Ostrinia furnacalis] >XP_028156933.1 transmembrane protein 87A isoform X4 [Ostrinia furnacalis]
uncharacterized protein LOC114358591 isoform X2 [Ostrinia furnacalis]
carboxylesterase, partial [Ostrinia furnacalis]
annulin-like isoform X3 [Ostrinia furnacalis]
PREDICTED: nuclear factor NF-kappa-B p105 subunit [Microplitis demolitor] >KAG6558391.1 viral
sorting and assembly machinery component 50 homolog isoform X9 [Ostrinia furnacalis] >XP_028169233.1 sorting and assembly machinery component 50 homolog isoform X10 [Ostrinia
sialin [Ostrinia furnacalis]
uncharacterized protein LOC114364899 isoform X2 [Ostrinia furnacalis]
fatty acyl-CoA reductase wat-like isoform X1 [Ostrinia furnacalis]
chromobox-like protein 5 [Helicoverpa armigera]
chitinase 7 [Glyphodes pyloalis]
carboxypeptidase D isoform X5 [Ostrinia furnacalis]
uncharacterized protein LOC114357318 isoform X1 [Ostrinia furnacalis] >XP_028166680.1 uncharacterized protein LOC114357318 isoform X2 [Ostrinia furnacalis]
gamma-tubulin complex component 3 homolog [Ostrinia furnacalis]
uncharacterized protein LOC114364864 [Ostrinia furnacalis]
unnamed protein product [Spodoptera exigua]
homeobox protein extradenticle isoform X3 [Ostrinia furnacalis]
uncharacterized protein LOC114362624 [Ostrinia furnacalis]
unnamed protein product [Euphydryas editha]
high mobility group protein I-like [Ostrinia furnacalis]
cytoplasmic dynein 1 light intermediate chain 2 [Galleria mellonella]
modular serine protease-like [Ostrinia furnacalis]
hypothetical protein evm_004957 [Chilo suppressalis]
protein tramtrack, beta isoform isoform X24 [Bicyclus anynana]
unnamed protein product [Arctia plantaginis]
macrophage mannose receptor 1-like [Ostrinia furnacalis]
adaptor complexes medium subunit family domain-containing protein [Phthorimaea operculella]
rab proteins geranylgeranyltransferase component A 1 isoform X1 [Ostrinia furnacalis]
microtubule-associated protein futsch-like isoform X6 [Ostrinia furnacalis]
catenin alpha isoform X2 [Ostrinia furnacalis]
uncharacterized protein LOC114356585 [Ostrinia furnacalis]
pre-mRNA-splicing regulator female-lethal(2)D [Ostrinia furnacalis]
Chlorophyll a-b binding protein, chloroplastic [Trichinella nelsoni]
double-strand break repair protein MRE11 [Ostrinia furnacalis]
putative gamma-glutamylcyclotransferase CG2811 isoform X3 [Ostrinia furnacalis]
structural maintenance of chromosomes protein 1A [Trichoplusia ni]
uncharacterized protein LOC114351644 [Ostrinia furnacalis]
mucin-5AC [Ostrinia furnacalis]
uncharacterized protein LOC114353024 [Ostrinia furnacalis]
elongation of very long chain fatty acids protein AAEL008004-like [Ostrinia furnacalis]
RNA-binding protein 45-like [Galleria mellonella]
unnamed protein product [Plutella xylostella]
septin-1 [Ostrinia furnacalis]
coronin-7 isoform X1 [Ostrinia furnacalis] >XP_028164815.1 coronin-7 isoform X2 [Ostrinia furnacalis] >XP_028164817.1 coronin-7 isoform X3 [Ostrinia furnacalis] >XP_028164818.1 coronin-7 isoform X4 [Ostrinia furnacalis] >XP_028164820.1 coronin-7 isoform X6 [Ostrinia furnacalis] >XP_028164821.1 coronin-7 isoform X7 [Ostrinia furnacalis] >XP_028164822.1 coronin-7 isoform X1 [Ostrinia furnacalis]

protein obstructor-E-like [Ostrinia furnacalis]
host cell factor 1 [Ostrinia furnacalis]
calcium channel flower [Ostrinia furnacalis]
ras-related protein Rap-2c [Bicyclus anynana] >XP_026492616.1 ras-related protein Rap-2c [Vanessa tameamea] >XP_034838061.1 ras-related protein Rap-2c [Maniola hyperantus] >XP_039759141.1 ras-related protein Rap-2c [Pararge aegeria] >XP_045498804.1 ras-related protein Rap-2c [Colias croceus] >XP_046959644.1 ras-related protein Rap-2c [Vanessa cardui] >XP_047530248.1 ras-related protein
trans-Golgi network integral membrane protein TGN38-like isoform X1 [Ostrinia furnacalis]
hypothetical protein evm_002694 [Chilo suppressalis]
26S proteasome non-ATPase regulatory subunit 13 isoform X1 [Ostrinia furnacalis]
pre-mRNA-splicing factor SPF27 [Ostrinia furnacalis] >XP_028158851.1 pre-mRNA-splicing factor
zinc finger protein 391-like [Ostrinia furnacalis] >XP_028169193.1 zinc finger protein 391-like [Ostrinia
fatty acyl reductase 7 [Maruca vitrata]
chromobox protein homolog 3-like [Ostrinia furnacalis] >XP_028157236.1 chromobox protein homolog
peptidoglycan-recognition protein SA-like [Ostrinia furnacalis]
cuticle protein 8-like [Leguminivora glycinivorella]
uncharacterized protein LOC114365633 [Ostrinia furnacalis]
LOW QUALITY PROTEIN: fibrillin-2-like [Bicyclus anynana]
BRISC and BRCA1-A complex member 1-like [Ostrinia furnacalis]
putative uncharacterized protein DDB_G0282133 isoform X1 [Ostrinia furnacalis]
uncharacterized protein LOC114361329 [Ostrinia furnacalis]
Golgi apparatus protein 1 [Ostrinia furnacalis]
hypothetical protein evm_002181 [Chilo suppressalis]
uncharacterized protein LOC114365758 isoform X2 [Ostrinia furnacalis]
UDP-glucose 6-dehydrogenase [Ostrinia furnacalis]
lipase 3-like [Ostrinia furnacalis]
protein Gawky isoform X2 [Ostrinia furnacalis]
carboxylesterase [Ostrinia furnacalis]
unnamed protein product [Chrysodeixis includens]
SUMO-activating enzyme subunit 1 [Ostrinia furnacalis]
UBX domain-containing protein 1-A-like [Ostrinia furnacalis]
PREDICTED: dynein light chain Tctex-type [Amyelois transitella] >XP_021195381.1 dynein light chain Tctex-type [Helicoverpa armigera] >XP_022815696.1 dynein light chain Tctex-type [Spodoptera litura] >XP_028156399.1 dynein light chain Tctex-type [Ostrinia furnacalis] >XP_035458261.1 dynein light chain Tctex-type-like [Spodoptera frugiperda] >XP_047034788.1 dynein light chain Tctex-type [Helicoverpa zea] >CAB3233358.1 unnamed protein product [Arctia plantaginis] >CAB3506583.1
serine/threonine-protein kinase 10-like, partial [Ostrinia furnacalis]
U4/U6 small nuclear ribonucleoprotein Prp3 isoform X1 [Ostrinia furnacalis] >XP_028161035.1 U4/U6 small nuclear ribonucleoprotein Prp3 isoform X2 [Ostrinia furnacalis] >XP_028161037.1 U4/U6 small
iron-sulfur cluster assembly 1 homolog, mitochondrial [Ostrinia furnacalis]
protein suppressor of forked [Helicoverpa zea]
PREDICTED: poly(rC)-binding protein 3 isoform X2 [Vollenhovia emeryi]
SUMO-activating enzyme subunit 2 [Ostrinia furnacalis]
glucose-6-phosphate 1-epimerase [Galleria mellonella]
polypyrimidine tract-binding protein 1 isoform X11 [Helicoverpa zea]
SET and MYND domain-containing protein 4-like [Ostrinia furnacalis]
AN1-type zinc finger protein 6 isoform X1 [Galleria mellonella]
metastasis-associated protein MTA3 [Galleria mellonella]
uncharacterized protein LOC114363802 isoform X4 [Ostrinia furnacalis]
putative mediator of RNA polymerase II transcription subunit 12 [Ostrinia furnacalis]
myosin heavy chain, non-muscle isoform X1 [Hyposmocoma kahamanoa]
calsyntenin-1 [Ostrinia furnacalis]
hypothetical protein evm_012205 [Chilo suppressalis] >CAB3527181.1 unnamed protein product [Chilo suppressalis] >CAH0404510.1 unnamed protein product [Chilo suppressalis]
putative uncharacterized protein DDB_G0282133 [Ostrinia furnacalis]
oxysterol-binding protein-related protein 9 [Manduca sexta]
cytochrome c oxidase assembly protein COX19 [Ostrinia furnacalis]
ubiquitin-like modifier-activating enzyme 1 [Manduca sexta]

glutathione S-transferase 1-1 [Ostrinia furnacalis] >XP_028161942.1 glutathione S-transferase 1-1 [Ostrinia furnacalis] >XP_028161943.1 glutathione S-transferase 1-1 [Ostrinia furnacalis]
serine/threonine-protein kinase PAK 3 isoform X1 [Ostrinia furnacalis] >XP_028164178.1
serine/threonine-protein kinase PAK 3 isoform X2 [Ostrinia furnacalis] >XP_028164179.1
histone acetyltransferase type B catalytic subunit [Ostrinia furnacalis]
PREDICTED: chaoptin [Amyelois transitella]
regulator of gene activity isoform X3 [Ostrinia furnacalis]
ER membrane protein complex subunit 7 [Ostrinia furnacalis]
eukaryotic translation initiation factor 4E transporter-like isoform X5 [Hyposmocoma kahamanoa]
kinesin heavy chain [Ostrinia furnacalis]
retinol dehydrogenase 14 [Ostrinia furnacalis] >XP_028165567.1 retinol dehydrogenase 14 [Ostrinia furnacalis] >XP_028165568.1 retinol dehydrogenase 14 [Ostrinia furnacalis]
unnamed protein product [Chrysodeixis includens]
tetratricopeptide repeat protein 1-like [Ostrinia furnacalis]
unnamed protein product [Spodoptera littoralis] >CAH1638553.1 unnamed protein product
RNA polymerase II degradation factor 1-like [Ostrinia furnacalis]
39S ribosomal protein L40, mitochondrial [Ostrinia furnacalis]
kinesin heavy chain [Ostrinia furnacalis]
ATP-binding cassette sub-family G member 1-like [Ostrinia furnacalis]
uncharacterized protein LOC114363514 isoform X3 [Ostrinia furnacalis]
uncharacterized protein LOC114350845 [Ostrinia furnacalis]
venom serine carboxypeptidase [Ostrinia furnacalis]
unnamed protein product, partial [Iphiclides podalirius]
eukaryotic translation initiation factor 4E type 2 [Ostrinia furnacalis]
parafibromin [Ostrinia furnacalis]
low molecular weight phosphotyrosine protein phosphatase 1-like isoform X2 [Ostrinia furnacalis]
probable phospholipid-transporting ATPase IM [Ostrinia furnacalis]
unnamed protein product [Spodoptera littoralis] >CAH1635924.1 unnamed protein product
essential MCU regulator, mitochondrial [Cotesia glomerata]
proliferating cell nuclear antigen [Ostrinia furnacalis] >XP_028174842.1 proliferating cell nuclear antigen
ATP-dependent RNA helicase DBP2-A-like [Ostrinia furnacalis]
hypothetical protein O3G_MSEX014886 [Manduca sexta]
focal adhesion kinase 1 isoform X2 [Galleria mellonella]
unnamed protein product, partial [Iphiclides podalirius]
protein NDUFAF4 homolog [Ostrinia furnacalis]
kinesin light chain [Ostrinia furnacalis]
cytoplasmic dynein 1 intermediate chain isoform X4 [Ostrinia furnacalis]
coiled-coil-helix-coiled-coil-helix domain-containing protein 7 isoform X2 [Ostrinia furnacalis]
cytosolic non-specific dipeptidase [Ostrinia furnacalis]
hypothetical protein evm_010712 [Chilo suppressalis] >CAB3527462.1 unnamed protein product [Chilo suppressalis] >CAH0401768.1 unnamed protein product [Chilo suppressalis]
MOXD1 homolog 1-like [Ostrinia furnacalis]
Golgi reassembly-stacking protein 2 [Ostrinia furnacalis]
GSK3-beta interaction protein-like [Galleria mellonella]

PREDICTED: serine/threonine-protein phosphatase PP1-beta catalytic subunit [Papilio polytes] >XP_013173027.1 PREDICTED: serine/threonine-protein phosphatase PP1-beta catalytic subunit [Papilio xuthus] >XP_013196427.1 PREDICTED: serine/threonine-protein phosphatase PP1-beta catalytic subunit isoform X1 [Amyelois transitella] >XP_014358529.1 serine/threonine-protein phosphatase PP1-beta catalytic subunit isoform X2 [Papilio machaon] >XP_021196714.1 serine/threonine-protein phosphatase PP1-beta catalytic subunit isoform X3 [Helicoverpa armigera] >XP_022826582.1 serine/threonine-protein phosphatase PP1-beta catalytic subunit [Spodoptera litura] >XP_023952136.1 serine/threonine-protein phosphatase PP1-beta catalytic subunit isoform X2 [Bicyclus anynana] >XP_026730418.1 serine/threonine-protein phosphatase PP1-beta catalytic subunit [Trichoplusia ni] >XP_028156492.1 serine/threonine-protein phosphatase PP1-beta catalytic subunit [Ostrinia furnacalis] >XP_030022932.1 serine/threonine-protein phosphatase PP1-beta catalytic subunit [Manduca sexta] >XP_034832911.1 serine/threonine-protein phosphatase PP1-beta catalytic subunit isoform X2 [Maniola hyperantus] >XP_039756948.1 serine/threonine-protein phosphatase PP1-beta catalytic subunit [Pararge aegeria] >XP_045452222.1 serine/threonine-protein phosphatase PP1-beta catalytic subunit [Melitaea cinxia] >XP_045773496.1 serine/threonine-protein phosphatase PP1-beta
atypical protein kinase C isoform X2 [Spodoptera litura] >XP_035444071.1 LOW QUALITY PROTEIN:
dystrophin-like, partial [Ostrinia furnacalis]
actin-related protein 3 [Ostrinia furnacalis]
procollagen-lysine,2-oxoglutarate 5-dioxygenase isoform X2 [Ostrinia furnacalis]
N-alpha-acetyltransferase 40 [Ostrinia furnacalis]
myrosinase 1-like [Ostrinia furnacalis]
drebrin-like protein [Ostrinia furnacalis]
PREDICTED: ubiquitin-conjugating enzyme E2 T [Microplitis demolitor]
RNA-binding protein spenito [Ostrinia furnacalis] >XP_028167555.1 RNA-binding protein spenito
exocyst complex component 3 [Ostrinia furnacalis]
cytoplasmic FMR1-interacting protein isoform X1 [Ostrinia furnacalis] >XP_028169436.1 cytoplasmic
coatomer subunit epsilon-like [Ostrinia furnacalis]
double-stranded RNA-binding protein Staufen homolog 2 isoform X3 [Helicoverpa armigera]
unnamed protein product, partial [Brenthis ino]
active breakpoint cluster region-related protein [Ostrinia furnacalis]
innexin inx2 [Ostrinia furnacalis]
uncharacterized protein LOC114353202 [Ostrinia furnacalis]
ribosomal protein S6 kinase 2 beta [Ostrinia furnacalis]
glycosylated lysosomal membrane protein B-like [Vanessa atalanta]
GRIP and coiled-coil domain-containing protein 1 [Ostrinia furnacalis]
uncharacterized protein LOC114352862 [Ostrinia furnacalis] >XP_028160407.1 uncharacterized protein
unnamed protein product [Arctia plantaginis] >CAB3259747.1 unnamed protein product [Arctia
hypothetical protein evm_002627 [Chilo suppressalis] >CAB3527269.1 unnamed protein product [Chilo
unnamed protein product, partial [Brenthis ino]
connectin-like [Ostrinia furnacalis]
amyloid-beta-like protein isoform X1 [Manduca sexta] >AAY25024.3 beta amyloid protein precursor-
uncharacterized protein LOC114360175 [Ostrinia furnacalis]
ubiquitin-conjugating enzyme E2L [Bombyx mori] >XP_013145013.1 PREDICTED: ubiquitin-conjugating enzyme E2 L3 [Papilio polytes] >XP_013145026.1 PREDICTED: ubiquitin-conjugating enzyme E2 L3 [Papilio polytes] >XP_013167448.1 PREDICTED: ubiquitin-conjugating enzyme E2 L3 [Papilio xuthus] >XP_013167449.1 PREDICTED: ubiquitin-conjugating enzyme E2 L3 [Papilio xuthus] >XP_014356324.1 ubiquitin-conjugating enzyme E2 L3 [Papilio machaon] >XP_021182538.1 ubiquitin-conjugating enzyme E2 L3 [Helicoverpa armigera] >XP_022831771.1 ubiquitin-conjugating enzyme E2 L3 [Spodoptera litura] >XP_023943862.1 ubiquitin-conjugating enzyme E2 L3 [Bicyclus anynana] >XP_026501320.1 ubiquitin-conjugating enzyme E2 L3 [Vanessa tameamea] >XP_026740672.1 ubiquitin-conjugating enzyme E2 L3 [Trichoplusia ni] >XP_030021931.1 ubiquitin-conjugating enzyme E2 L3 [Manduca sexta] >XP_035448309.1 ubiquitin-conjugating enzyme E2 L3 [Spodoptera frugiperda] >XP_038214502.1 ubiquitin-conjugating enzyme E2 L3 [Zerene cesonia] >XP_045453873.1 ubiquitin-conjugating enzyme E2 L3 [Melitaea cinxia] >XP_045503683.1 ubiquitin-conjugating enzyme E2 L3 [Colias croceus] >XP_045771134.1 ubiquitin-conjugating enzyme E2 L3 [Maniola jurtina]
unnamed protein product [Spodoptera exigua]
SRSF protein kinase 3 [Galleria mellonella]
Krueppel homolog 2-like [Ostrinia furnacalis]

unnamed protein product [Arctia plantaginis]
striatin-3 isoform X1 [Ostrinia furnacalis]
copper chaperone for superoxide dismutase [Ostrinia furnacalis]
xaa-Pro aminopeptidase ApepP-like isoform X1 [Ostrinia furnacalis]
fibulin-2-like [Ostrinia furnacalis]
protein D2-like isoform X2 [Aricia agestis] >XP_041972210.1 protein D2-like isoform X2 [Aricia agestis]
ribonucleoside-diphosphate reductase large subunit [Ostrinia furnacalis]
uncharacterized protein LOC114354192 isoform X2 [Ostrinia furnacalis]
neurofilament heavy polypeptide-like isoform X2 [Ostrinia furnacalis]
protein bicaudal D isoform X3 [Galleria mellonella]
NAD(P) transhydrogenase, mitochondrial-like [Ostrinia furnacalis] >XP_028175067.1 NAD(P) transhydrogenase, mitochondrial-like [Ostrinia furnacalis] >XP_028175068.1 NAD(P) transhydrogenase, mitochondrial-like [Ostrinia furnacalis] >XP_028175069.1 NAD(P) transhydrogenase, mitochondrial-like psi [Ostrinia furnacalis]
wiskott-Aldrich syndrome protein family member 2 [Ostrinia furnacalis]
uridine-cytidine kinase-like 1 isoform X1 [Ostrinia furnacalis] >XP_028168339.1 uridine-cytidine kinase-
exportin-5-like, partial [Ostrinia furnacalis]
DNA-directed RNA polymerase I subunit RPA12 [Ostrinia furnacalis]
calcyclin-binding protein [Ostrinia furnacalis]
probable nuclear transport factor 2 isoform X1 [Ostrinia furnacalis]
alpha-N-acetylgalactosaminidase isoform X3 [Ostrinia furnacalis]
TP53-binding protein 1-like [Ostrinia furnacalis]
nucleoporin Nup35 [Ostrinia furnacalis]
CD2 antigen cytoplasmic tail-binding protein 2 homolog [Ostrinia furnacalis]
ubiquitin conjugation factor E4 B isoform X2 [Ostrinia furnacalis]
cuticle protein 19-like [Ostrinia furnacalis]
PREDICTED: phosphoribosyl pyrophosphate synthase-associated protein 2 isoform X2 [Amyelois
endocuticle structural glycoprotein SgAbd-8 [Ostrinia furnacalis]
transcription elongation factor S-II [Chelonus insularis]
uncharacterized protein LOC114356431 isoform X2 [Ostrinia furnacalis]
ornithine decarboxylase 1-like isoform X1 [Ostrinia furnacalis]
putative GPI-anchored protein pfl2 [Ostrinia furnacalis] >XP_028163002.1 putative GPI-anchored
hypothetical protein SFRURICE_000584 [Spodoptera frugiperda]
uncharacterized protein LOC114363767 [Ostrinia furnacalis]
myosin-2 essential light chain isoform X2 [Harpegnathos saltator] >XP_012170910.1 myosin-2 essential light chain isoform X2 [Bombus terrestris] >XP_033185931.1 myosin-2 essential light chain isoform X2 [Bombus vancouverensis nearcticus] >XP_033319091.1 myosin-2 essential light chain isoform X2 [Bombus bifarius] >XP_033349866.1 myosin-2 essential light chain isoform X2 [Bombus vosnesenskii]
LOW QUALITY PROTEIN: puff-specific protein Bx42 [Ostrinia furnacalis]
eukaryotic translation initiation factor 6 [Ostrinia furnacalis]
pyruvate kinase-like isoform X3 [Ostrinia furnacalis]
hypothetical protein evm_000945 [Chilo suppressalis] >CAB3528924.1 unnamed protein product [Chilo suppressalis] >CAH0405517.1 unnamed protein product [Chilo suppressalis]
dnaJ homolog subfamily C member 22 [Ostrinia furnacalis]
serine/threonine-protein phosphatase 4 regulatory subunit 3 isoform X3 [Ostrinia furnacalis]
AP-1 complex subunit mu-1 [Ostrinia furnacalis]
FAD-dependent oxidoreductase domain-containing protein 1 [Ostrinia furnacalis]
chromodomain-helicase-DNA-binding protein 7 [Ostrinia furnacalis] >XP_028176739.1
uncharacterized protein LOC114363979 [Ostrinia furnacalis]
AP-1 complex subunit gamma-1 [Ostrinia furnacalis]
39S ribosomal protein L16, mitochondrial [Ostrinia furnacalis]
PREDICTED: protein BUD31 homolog [Papilio xuthus] >XP_014361644.1 protein BUD31 homolog [Papilio machaon] >XP_026750578.1 protein BUD31 homolog [Galleria mellonella] >XP_047995610.1 protein BUD31 homolog [Leguminivora glycinivorella] >XP_049869593.1 protein BUD31 homolog [Pectinophora gossypiella] >KAI5652084.1 g10 protein domain-containing protein [Phthorimaea operculella] >CAB3251981.1 unnamed protein product [Arctia plantaginis] >CAB3520382.1 unnamed
lysophospholipase-like protein 1 [Ostrinia furnacalis]
eukaryotic translation initiation factor 4H [Ostrinia furnacalis]

cuticle protein 19 [Plutella xylostella] >CAG9138481.1 unnamed protein product [Plutella xylostella]
alpha-N-acetylgalactosaminidase-like isoform X1 [Ostrinia furnacalis] >XP_028171449.1 alpha-N-
zinc finger protein on ecdysone puffs [Ostrinia furnacalis]
striatin-interacting protein 1 [Ostrinia furnacalis]
hypothetical protein evm_004688 [Chilo suppressalis]
LOW QUALITY PROTEIN: phosphoacetylglucosamine mutase [Ostrinia furnacalis]
THAP domain-containing protein 1-like isoform X1 [Ostrinia furnacalis]
protein ABHD16A isoform X1 [Ostrinia furnacalis] >XP_028156316.1 protein ABHD16A isoform X2
uncharacterized protein LOC114362418 [Ostrinia furnacalis]
aldo-keto reductase AKR2E4-like [Ostrinia furnacalis]
engulfment and cell motility protein 1 [Ostrinia furnacalis]
hypothetical protein NE865_05974 [Phthorimaea operculella]
NEDD8-conjugating enzyme Ubc12 [Ostrinia furnacalis]
thioredoxin reductase 1, mitochondrial isoform X4 [Helicoverpa zea]
casein kinase II subunit beta, partial [Rhincodon typus]
melanotransferrin isoform X1 [Ostrinia furnacalis] >XP_028175370.1 melanotransferrin isoform X2 [Ostrinia furnacalis] >XP_028175371.1 melanotransferrin isoform X3 [Ostrinia furnacalis]
spondin-1 isoform X1 [Ostrinia furnacalis] >XP_028167312.1 spondin-1 isoform X1 [Ostrinia furnacalis] >XP_028167313.1 spondin-1 isoform X1 [Ostrinia furnacalis] >XP_028167314.1 spondin-1 isoform X2
hypothetical protein evm_000341 [Chilo suppressalis]
pro-resilin-like [Ostrinia furnacalis]
importin subunit beta-1 isoform X2 [Ostrinia furnacalis]
RE1-silencing transcription factor-like isoform X1 [Ostrinia furnacalis]
hypothetical protein evm_009768 [Chilo suppressalis]
actin-related protein 2/3 complex subunit 5-B [Ostrinia furnacalis]
SWI/SNF-related matrix-associated actin-dependent regulator of chromatin subfamily E member 1-like
uncharacterized protein LOC114366518 isoform X5 [Ostrinia furnacalis]
nuclear valosin-containing protein-like [Ostrinia furnacalis]
nuclear migration protein nudC [Ostrinia furnacalis]
uncharacterized protein LOC114350693 [Ostrinia furnacalis]
nuclear cap-binding protein subunit 1 [Galleria mellonella]
armadillo repeat-containing protein 8-like [Maniola hyperantus]
unnamed protein product [Arctia plantaginis]
transcription elongation factor S-II [Ostrinia furnacalis]
DNA topoisomerase 2 isoform X1 [Ostrinia furnacalis]
far upstream element-binding protein 1 isoform X3 [Ostrinia furnacalis]
microtubule-associated protein Jupiter isoform X4 [Helicoverpa armigera]
protein unc-45 homolog B [Ostrinia furnacalis]
zinc finger MYM-type protein 3 isoform X1 [Ostrinia furnacalis] >XP_028159738.1 zinc finger MYM-
conserved oligomeric Golgi complex subunit 8 [Ostrinia furnacalis]
unnamed protein product [Chilo suppressalis]
ribosome biogenesis protein NSA2 homolog [Ostrinia furnacalis] >CAG9749295.1 unnamed protein product [Diatraea saccharalis] >CAG9787980.1 unnamed protein product [Diatraea saccharalis]
tubulin--tyrosine ligase-like protein 12 [Ostrinia furnacalis]
trafficking protein particle complex subunit 8 [Ostrinia furnacalis]
actin, muscle-type A2 [Bombyx mori] >XP_013199497.1 PREDICTED: actin, muscle-type A2 [Amyelois transitella] >XP_021196684.1 actin, muscle-type A2 [Helicoverpa armigera] >XP_022837900.1 actin, muscle-type A2 [Spodoptera litura] >XP_026314060.1 actin, muscle-type A2 [Hyposmocoma kahamanoa] >XP_026738711.1 actin, muscle-type A2 [Trichoplusia ni] >XP_028179440.1 actin, muscle-type A2 [Ostrinia furnacalis] >XP_030030527.1 actin, muscle-type A2 [Manduca sexta] >XP_035439272.1 actin, muscle-type A2 [Spodoptera frugiperda] >XP_047029939.1 actin, muscle-type A2 [Helicoverpa zea] >XP_049873365.1 actin, muscle-type A2 [Pectinophora gossypiella] >P07837.1 RecName: Full=Actin, muscle-type A2; Flags: Precursor [Bombyx mori] >KAF9423784.1 hypothetical protein HW555_000842 [Spodoptera exigua] >QLI62214.1 actin [Streltzoviella insularis] >CAB3227390.1

uncharacterized protein DDB_G0283357 isoform X13 [Helicoverpa armigera] >XP_049707197.1
uncharacterized protein DDB_G0283357 isoform X14 [Helicoverpa armigera] >XP_049707198.1
uncharacterized protein DDB_G0283357 isoform X15 [Helicoverpa armigera] >XP_049707199.1
uncharacterized protein DDB_G0283357 isoform X16 [Helicoverpa armigera] >XP_049707200.1
uncharacterized protein DDB_G0283357 isoform X17 [Helicoverpa armigera] >XP_049707201.1
uncharacterized protein DDB_G0283357 isoform X18 [Helicoverpa armigera] >XP_049707202.1
uncharacterized protein DDB_G0283357 isoform X19 [Helicoverpa armigera] >XP_049707203.1
uncharacterized protein DDB_G0283357 isoform X20 [Helicoverpa armigera] >XP_049707204.1
E3 ubiquitin-protein ligase Hakai [Ostrinia furnacalis]
zinc finger protein swm isoform X3 [Ostrinia furnacalis]
N-acetylgalactosamine kinase [Ostrinia furnacalis]
interleukin enhancer-binding factor 2 homolog [Ostrinia furnacalis]
uncharacterized protein LOC114363514 isoform X2 [Ostrinia furnacalis]
uncharacterized protein LOC114354768 isoform X2 [Ostrinia furnacalis]
mRNA-decapping enzyme 1A [Ostrinia furnacalis]
uncharacterized protein LOC114363197 [Ostrinia furnacalis]
polyglutamine-binding protein 1 [Ostrinia furnacalis]
thioredoxin, mitochondrial isoform X2 [Ostrinia furnacalis]
ubiquitin-like-specific protease ESD4 [Ostrinia furnacalis]
40S ribosomal protein S3-3, partial [Trichinella patagoniensis]
peroxiredoxin-2-like [Ostrinia furnacalis]
nucleoprotein TPR isoform X1 [Ostrinia furnacalis]
cytochrome c oxidase assembly factor 5 [Ostrinia furnacalis]
hypothetical protein evm_008214 [Chilo suppressalis]
coiled-coil domain-containing protein 6-like [Ostrinia furnacalis]
unnamed protein product [Chilo suppressalis]
leukotriene A-4 hydrolase isoform X2 [Ostrinia furnacalis]
DNA damage-binding protein 1 [Ostrinia furnacalis]
replication protein A 32 kDa subunit [Ostrinia furnacalis]
peptidyl-prolyl cis-trans isomerase [Cotesia flavipes]
unnamed protein product [Diatraea saccharalis]
unnamed protein product [Chrysodeixis includens]
PREDICTED: enhancer of rudimentary homolog [Microplitis demolitor] >XP_044577051.1 enhancer of rudimentary homolog [Cotesia glomerata] >KAG8041963.1 hypothetical protein G9C98_007267 [Cotesia typhae] >KAH0539785.1 hypothetical protein KQX54_008036 [Cotesia glomerata]
uncharacterized protein LOC114351683 isoform X8 [Ostrinia furnacalis]
sperm-associated antigen 7 homolog [Ostrinia furnacalis]
heterogeneous nuclear ribonucleoprotein H-like isoform X2 [Ostrinia furnacalis]
mucin-5AC isoform X2 [Ostrinia furnacalis]
protein SEC13 homolog [Ostrinia furnacalis]
eukaryotic peptide chain release factor subunit 1 isoform X1 [Danaus plexippus plexippus]
uncharacterized protein LOC114355702 [Ostrinia furnacalis]
dnaJ homolog subfamily C member 21 [Ostrinia furnacalis]
cell division cycle and apoptosis regulator protein 1-like [Ostrinia furnacalis]
dnaJ homolog subfamily B member 6 isoform X2 [Ostrinia furnacalis]
THO complex subunit 7 homolog [Ostrinia furnacalis]
unnamed protein product [Chilo suppressalis]
PC4 and SFRS1-interacting protein isoform X4 [Galleria mellonella]
luc7-like protein 3 isoform X1 [Ostrinia furnacalis] >XP_028160033.1 luc7-like protein 3 isoform X1
constitutive coactivator of PPAR-gamma-like protein 1 isoform X1 [Ostrinia furnacalis] >XP_028158538.1 constitutive coactivator of PPAR-gamma-like protein 1 isoform X2 [Ostrinia
cleavage and polyadenylation specificity factor subunit CG7185 isoform X2 [Ostrinia furnacalis]
ATP-binding cassette subfamily D member 1 [Chilo suppressalis] >CAB3531327.1 unnamed protein product [Chilo suppressalis] >CAH0407919.1 unnamed protein product [Chilo suppressalis]
proline-rich extensin-like protein EPR1 isoform X1 [Ostrinia furnacalis] >XP_028168549.1 proline-rich extensin-like protein EPR1 isoform X2 [Ostrinia furnacalis] >XP_028168550.1 proline-rich extensin-like
transmembrane 9 superfamily member 3 [Ostrinia furnacalis]
uncharacterized protein LOC114351134 [Ostrinia furnacalis]

cleavage stimulation factor subunit 2 isoform X1 [Ostrinia furnacalis]
mitogen-activated protein kinase 1 [Ostrinia furnacalis] >AXF67444.1 mitogen-activated protein kinase
transcription elongation factor SPT5 [Ostrinia furnacalis]
neutral alpha-glucosidase AB [Ostrinia furnacalis]
AFG3-like protein 2 [Ostrinia furnacalis]
cytochrome b5 [Ostrinia furnacalis]
unnamed protein product [Chilo suppressalis]
unnamed protein product [Chrysodeixis includens]
hypothetical protein HF086_007571 [Spodoptera exigua]
hrp65 protein-like [Ostrinia furnacalis]
ubiquitin thioesterase otubain-like [Ostrinia furnacalis]
inositol-3-phosphate synthase [Ostrinia furnacalis]
FACT complex subunit Ssrp1 isoform X1 [Ostrinia furnacalis] >XP_028173375.1 FACT complex subunit Ssrp1 isoform X2 [Ostrinia furnacalis] >XP_028173376.1 FACT complex subunit Ssrp1 isoform X3
hypothetical protein evm_008498 [Chilo suppressalis] >CAB3527693.1 unnamed protein product [Chilo suppressalis] >CAH0401999.1 unnamed protein product [Chilo suppressalis]
hornerin-like [Ostrinia furnacalis]
transmembrane protein 161B isoform X1 [Galleria mellonella]
leucine-rich repeat-containing protein 57-like [Colias croceus]
uncharacterized protein LOC114357129 [Ostrinia furnacalis]
hypothetical protein evm_001345 [Chilo suppressalis] >CAB3523265.1 unnamed protein product [Chilo suppressalis] >CAH0400587.1 unnamed protein product [Chilo suppressalis]
heterogeneous nuclear ribonucleoprotein K isoform X2 [Ostrinia furnacalis]
protein stunted-like isoform X2 [Vanessa tameamea] >XP_046960183.1 protein stunted-like isoform X2 [Vanessa cardui] >XP_047527093.1 protein stunted-like isoform X2 [Vanessa atalanta]
hypothetical protein evm_011295 [Chilo suppressalis]
chromodomain-helicase-DNA-binding protein Mi-2 homolog isoform X3 [Chelonus insularis]
ras-related protein Rab-8A isoform X2 [Ostrinia furnacalis]
hypothetical protein evm_011958 [Chilo suppressalis] >CAB3521085.1 unnamed protein product [Chilo
uncharacterized protein LOC114358520 [Ostrinia furnacalis]
DNA-directed RNA polymerase III subunit RPC4 isoform X1 [Ostrinia furnacalis]
protein PRRC2A-like isoform X4 [Ostrinia furnacalis]
hypothetical protein evm_000268 [Chilo suppressalis]
myrosinase 1-like isoform X1 [Ostrinia furnacalis]
hypothetical protein evm_003965 [Chilo suppressalis]
mitochondrial import inner membrane translocase subunit Tim21 [Ostrinia furnacalis]
uncharacterized protein LOC111357764, partial [Spodoptera litura]
carnitine O-acetyltransferase isoform X2 [Ostrinia furnacalis]
serine-threonine kinase receptor-associated protein [Galleria mellonella]
vesicle transport protein GOT1B [Pectinophora gossypiella]
hypothetical protein evm_000268 [Chilo suppressalis]
egl nine homolog 1 isoform X2 [Helicoverpa armigera]
protein EFR3 homolog cmp44E isoform X1 [Ostrinia furnacalis] >XP_028166854.1 protein EFR3
uncharacterized protein LOC114361160 [Ostrinia furnacalis]
probable protein phosphatase 2C 11 isoform X1 [Manduca sexta] >KAG6442694.1 hypothetical protein
unnamed protein product [Diatraea saccharalis]
protein lingerer-like isoform X1 [Nymphalis io] >XP_050356663.1 protein lingerer-like isoform X1 [Nymphalis io] >XP_050356664.1 protein lingerer-like isoform X1 [Nymphalis io]
unnamed protein product [Chilo suppressalis]
heat shock factor-binding protein 1 [Ostrinia furnacalis]
paraplegin [Ostrinia furnacalis]
GPI ethanolamine phosphate transferase 2-like [Ostrinia furnacalis]
small integral membrane protein 12 [Ostrinia furnacalis]
trypsin-like serine protease [Ostrinia nubilalis]
programmed cell death protein 10 [Ostrinia furnacalis]
28S ribosomal protein S28, mitochondrial [Ostrinia furnacalis]
putative carbonic anhydrase 3 [Ostrinia furnacalis]
optic atrophy 3 protein homolog isoform X2 [Ostrinia furnacalis]

gamma-interferon-inducible lysosomal thiol reductase-like [Ostrinia furnacalis]
HIRA-interacting protein 3-like [Ostrinia furnacalis]
actin, clone 403 [Trichonephila clavata]
WD repeat-containing protein 74-like isoform X1 [Ostrinia furnacalis] >XP_028161051.1 WD repeat-
baculoviral IAP repeat-containing protein 6-like [Ostrinia furnacalis]
acetylcholine receptor subunit alpha-L1-like [Ostrinia furnacalis]
hypothetical protein HW555_009956 [Spodoptera exigua] >KAH9643419.1 hypothetical protein HF086_016708 [Spodoptera exigua] >CAH0702087.1 unnamed protein product [Spodoptera exigua]
serine-arginine protein 55 isoform X6 [Pieris brassicae]
nucleolar GTP-binding protein 2 [Ostrinia furnacalis]
cdc42 homolog [Galleria mellonella] >XP_028178764.1 cdc42 homolog [Ostrinia furnacalis]
unnamed protein product [Danaus chrysippus]
dual specificity protein phosphatase 23-like isoform X2 [Ostrinia furnacalis]
uncharacterized protein LOC114354053 [Ostrinia furnacalis]
uncharacterized protein LOC114358344 isoform X1 [Ostrinia furnacalis]
PC4 and SFRS1-interacting protein isoform X4 [Galleria mellonella]
hypothetical protein evm_007405 [Chilo suppressalis]
bystin [Ostrinia furnacalis]
hypothetical protein evm_008955 [Chilo suppressalis] >CAB3526829.1 unnamed protein product [Chilo suppressalis] >CAH0404157.1 unnamed protein product [Chilo suppressalis]
synaptosomal-associated protein 25 isoform X1 [Bombyx mori]
ribokinase-like [Ostrinia furnacalis]
metaxin-2 isoform X4 [Manduca sexta] >KAG6447312.1 hypothetical protein O3G_MSEX004872
protein phosphatase 1 catalytic subunit [Bombyx mori] >NP_001296033.1 serine/threonine-protein phosphatase alpha-2 isoform [Plutella xylostella] >XP_013188351.1 PREDICTED: serine/threonine-protein phosphatase alpha-2 isoform [Amyelois transitella] >XP_021183772.1 serine/threonine-protein phosphatase alpha-2 isoform isoform X2 [Helicoverpa armigera] >XP_022831789.1 serine/threonine-protein phosphatase alpha-2 isoform [Spodoptera litura] >XP_026314622.1 serine/threonine-protein phosphatase alpha-2 isoform isoform X2 [Hyposmocoma kahamanoa] >XP_026755085.1 serine/threonine-protein phosphatase alpha-2 isoform [Galleria mellonella] >XP_028026894.1 serine/threonine-protein phosphatase alpha-2 isoform isoform X2 [Bombyx mandarina] >XP_028168459.1 serine/threonine-protein phosphatase alpha-2 isoform [Ostrinia furnacalis] >XP_035448597.1 serine/threonine-protein phosphatase alpha-2 isoform isoform X2 [Spodoptera
peroxiredoxin [Ostrinia furnacalis]
protein tumorous imaginal discs, mitochondrial-like isoform X2 [Ostrinia furnacalis]
uncharacterized protein LOC114352414 isoform X1 [Ostrinia furnacalis]
translation machinery-associated protein 7 homolog [Zerene cesonia]
39S ribosomal protein L44, mitochondrial [Ostrinia furnacalis]
uncharacterized protein LOC114359911 [Ostrinia furnacalis]
cytochrome c oxidase subunit 5A, mitochondrial [Ostrinia furnacalis]
epidermal growth factor receptor substrate 15-like 1 [Ostrinia furnacalis]
heat shock protein 90 [Loxostege sticticalis]
ankyrin-3-like isoform X1 [Galleria mellonella]
probable ribosome production factor 1 [Ostrinia furnacalis]
double-stranded ribonuclease 2 [Ostrinia nubilalis]
ATP synthase mitochondrial F1 complex assembly factor 1 [Ostrinia furnacalis]
39S ribosomal protein L43, mitochondrial [Ostrinia furnacalis]
leucyl-cystinyl aminopeptidase-like isoform X4 [Ostrinia furnacalis]
uncharacterized protein LOC118068293 isoform X2 [Chelonus insularis]
protein UBASH3A homolog isoform X3 [Ostrinia furnacalis]
unnamed protein product, partial [Iphiclides podalirius]
GSCOCG00000129001-RA-CDS [Cotesia congregata] >CAG5101050.1 Similar to LUC7L2: Putative RNA-binding protein Luc7-like 2 (Homo sapiens) [Cotesia congregata]
ATP-dependent helicase brm [Ostrinia furnacalis]
UPF0545 protein C22orf39 homolog [Ostrinia furnacalis]
uncharacterized protein LOC114352518 [Ostrinia furnacalis]
NADPH:adrenodoxin oxidoreductase, mitochondrial [Ostrinia furnacalis]
trehalase-1 [Omphisa fuscidentalis]

PHD finger-like domain-containing protein 5A [Nasonia vitripennis] >XP_002427197.1 conserved hypothetical protein [Pediculus humanus corporis] >XP_003484388.1 PHD finger-like domain-containing protein 5A [Bombus impatiens] >XP_003701008.1 PREDICTED: PHD finger-like domain-containing protein 5A [Megachile rotundata] >XP_006623871.1 PHD finger-like domain-containing protein 5A [Apis dorsata] >XP_011068502.1 PREDICTED: PHD finger-like domain-containing protein 5A [Acromyrmex echinatior] >XP_011154391.1 PHD finger-like domain-containing protein 5A [Harpegnathos saltator] >XP_011164776.1 PHD finger-like domain-containing protein 5A [Solenopsis invicta] >XP_011262550.1 PHD finger-like domain-containing protein 5A [Camponotus floridanus] >XP_011297178.1 PREDICTED: PHD finger-like domain-containing protein 5A [Fopius arisanus] >XP_011334720.1 PHD finger-like domain-containing protein 5A [Ooceraea biroi] >XP_011506347.1 PREDICTED: PHD finger-like domain-containing protein 5A [Ceratosolen solmsi marchali] >XP_011506348.1 PREDICTED: PHD finger-like domain-containing protein 5A [Ceratosolen solmsi marchali] >XP_011638597.1 PHD finger-like domain-containing protein 5A isoform X2 [Pogonomyrmex barbatus] >XP_011686073.1 PREDICTED: PHD finger-like domain-containing protein 5A [Wasmannia auropunctata] >XP_011858255.1 PREDICTED: PHD finger-like domain-containing protein 5A [Vollenhovia emeryi] >XP_012058015.1 PREDICTED: PHD finger-like domain-containing protein 5A [Atta cephalotes] >XP_012135327.1 PREDICTED: PHD finger-like domain-containing protein 5A [Megachile rotundata] >XP_012135328.1 PREDICTED: PHD finger-like domain-containing protein 5A [Megachile rotundata] >XP_012222185.1 PREDICTED: PHD finger-like domain-containing protein 5A [Linepithema humile] >XP_012261946.1 PHD finger-like domain-containing protein 5A [Athalia rosae] >XP_012273120.1 PHD finger-like domain-containing protein 5A [Orussus abietinus] >XP_012526512.1 PHD finger-like domain-containing protein 5A [Monomorium pharaonis] >XP_014217558.1 PHD finger-like domain-containing protein 5A [Copidosoma floridanum] >XP_014484566.1 PREDICTED: PHD finger-like domain-containing protein 5A [Dinoponera quadriceps] >XP_014611099.1 PREDICTED: PHD finger-like domain-containing protein 5A [Polistes canadensis] >XP_015122018.1 PHD finger-like domain-containing protein 5A [Diachasma alloeum] >XP_015174163.1 PREDICTED: PHD finger-like domain-containing protein 5A [Polistes dominula] >XP_015433827.1 PREDICTED: PHD finger-like domain-containing protein 5A [Dufourea novaeangliae] >XP_015516165.1 PHD finger-like domain-containing protein 5A [Neodiprion lecontei] >XP_015586222.1 PHD finger-like domain-containing protein 5A isoform X1 [Cephus cinctus] >XP_016915535.1 PHD finger-like domain-containing protein

anamorsin homolog [Ostrinia furnacalis]
unnamed protein product [Diatraea saccharalis]
guanine nucleotide exchange factor subunit Rich isoform X1 [Ostrinia furnacalis]
luciferin 4-monooxygenase-like [Ostrinia furnacalis]
sulfotransferase family cytosolic 1B member 1-like [Ostrinia furnacalis]
glutathione S transferase-S5 [Glyphodes pyloalis]
YLP motif-containing protein 1-like isoform X1 [Ostrinia furnacalis]
protein PTCD3 homolog, mitochondrial [Ostrinia furnacalis]
trimeric intracellular cation channel type 1B.1 [Manduca sexta] >KAG6456518.1 hypothetical protein
RNA-binding protein NOB1 [Ostrinia furnacalis]
uncharacterized protein LOC114357371 [Ostrinia furnacalis] >XP_028166768.1 uncharacterized protein
hypothetical protein evm_009649 [Chilo suppressalis]
HD domain-containing protein 2 [Ostrinia furnacalis]
PREDICTED: cytoplasmic protein NCK1 isoform X1 [Microplitis demolitor]
hypothetical protein L3Q82_022586 [Scortum barcoo]
uncharacterized protein LOC114350416 [Ostrinia furnacalis] >XP_028157016.1 uncharacterized protein LOC114350416 [Ostrinia furnacalis] >XP_028157017.1 uncharacterized protein LOC114350416 [Ostrinia furnacalis] >XP_028157018.1 uncharacterized protein LOC114350416 [Ostrinia furnacalis]
DNA replication licensing factor Mcm3 [Ostrinia furnacalis]
probable phenylalanine--tRNA ligase, mitochondrial [Ostrinia furnacalis]
nardilysin-like isoform X1 [Ostrinia furnacalis] >XP_028157649.1 nardilysin-like isoform X2 [Ostrinia furnacalis] >XP_028157650.1 nardilysin-like isoform X3 [Ostrinia furnacalis] >XP_028157651.1
serine/threonine-protein kinase RIO3 [Ostrinia furnacalis]
heterogeneous nuclear ribonucleoprotein 87F-like isoform X1 [Vanessa tameamea] >XP_046967652.1
heterogeneous nuclear ribonucleoprotein 87F-like isoform X1 [Vanessa cardui] >XP_047532045.1
lysophospholipid acyltransferase 5 [Ostrinia furnacalis] >XP_028169982.1 lysophospholipid
Golgi to ER traffic protein 4 homolog [Ostrinia furnacalis]
2-amino-3-ketobutyrate coenzyme A ligase, mitochondrial [Ostrinia furnacalis]
28S ribosomal protein S9, mitochondrial [Ostrinia furnacalis]

casein kinase I-like isoform X1 [Hyposmocoma kahamanoa]
translocator protein-like isoform X1 [Ostrinia furnacalis] >XP_028178947.1 translocator protein-like
glutamine:fructose-6-phosphate aminotransferase 1 [Heortia vitessoides]
collagenase-like [Ostrinia furnacalis]
cytochrome c oxidase assembly protein COX15 homolog [Ostrinia furnacalis]
ras-related protein Rab-36 [Ostrinia furnacalis]
probable 28S ribosomal protein S6, mitochondrial [Ostrinia furnacalis]
LOW QUALITY PROTEIN: RNA polymerase-associated protein CTR9 homolog [Ostrinia furnacalis]
uncharacterized protein LOC114350846 [Ostrinia furnacalis]
chymotrypsin-like serine protease [Ostrinia nubilalis] >AAX62030.1 chymotrypsin-like serine protease
carboxylesterase [Cnaphalocrocis medinalis]
uncharacterized protein LOC114357075 [Ostrinia furnacalis]
DNA replication licensing factor Mcm5 [Spodoptera litura]
bifunctional methylenetetrahydrofolate dehydrogenase/cyclohydrolase, mitochondrial isoform X1
gamma-taxilin [Ostrinia furnacalis]
alkyldihydroxyacetonephosphate synthase [Ostrinia furnacalis]
uncharacterized protein LOC114350112 [Ostrinia furnacalis]
4-aminobutyrate aminotransferase, mitochondrial [Galleria mellonella]
39S ribosomal protein L9, mitochondrial [Ostrinia furnacalis]
WD repeat-containing protein 46 [Ostrinia furnacalis]
viral IAP-associated factor homolog [Ostrinia furnacalis]
SPARC [Trichoplusia ni]
peroxidase-like [Ostrinia furnacalis]
V-type proton ATPase 116 kDa subunit a1 isoform X1 [Manduca sexta]
NADH dehydrogenase [ubiquinone] 1 beta subcomplex subunit 11, mitochondrial [Ostrinia furnacalis]
aldo-keto reductase AKR2E4-like [Ostrinia furnacalis]
choline-phosphate cytidylyltransferase B-like isoform X1 [Ostrinia furnacalis]
DNA topoisomerase I, mitochondrial [Ostrinia furnacalis]
39S ribosomal protein L21, mitochondrial [Ostrinia furnacalis]
UDP-N-acetylhexosamine pyrophosphorylase-like protein 1 [Ostrinia furnacalis]
cytochrome P450 monooxygenase 304 [Glyphodes pyloalis]
angiotensin-converting enzyme-like isoform X2 [Ostrinia furnacalis]
LOW QUALITY PROTEIN: elongation factor G, mitochondrial-like [Leguminivora glycinivorella]
ubiquitin carboxyl-terminal hydrolase 36 [Ostrinia furnacalis]
39S ribosomal protein L20, mitochondrial [Ostrinia furnacalis]
Deoxycytidylate deaminase [Papilio xuthus]
protein takeout-like isoform X2 [Ostrinia furnacalis]
COX assembly mitochondrial protein homolog [Ostrinia furnacalis]
uncharacterized protein C6orf203 homolog [Ostrinia furnacalis]
28S ribosomal protein S7, mitochondrial [Ostrinia furnacalis]
hydroxylysine kinase [Ostrinia furnacalis] >XP_028168144.1 hydroxylysine kinase [Ostrinia furnacalis]
1-phosphatidylinositol phosphodiesterase-like [Cotesia glomerata]
mitochondrial import inner membrane translocase subunit TIM50-C-like [Ostrinia furnacalis]
double-stranded RNA-binding protein Staufen homolog 2 isoform X5 [Pectinophora gossypiella]
NADH dehydrogenase [ubiquinone] 1 alpha subcomplex subunit 7-like [Ostrinia furnacalis]
DNA-(apurinic or apyrimidinic site) lyase [Ostrinia furnacalis]
collagenase-like isoform X1 [Ostrinia furnacalis]
esterase FE4-like [Ostrinia furnacalis]
25S rRNA (cytosine-C(5))-methyltransferase nop2 [Ostrinia furnacalis]
putative E3 ubiquitin-protein ligase UBR7 [Ostrinia furnacalis]
5-formyltetrahydrofolate cyclo-ligase [Ostrinia furnacalis]
ATP-dependent RNA helicase dbp2-like isoform X1 [Ostrinia furnacalis]
uncharacterized protein LOC114364712 [Ostrinia furnacalis]
zinc finger protein 706-like [Ostrinia furnacalis] >XP_028176219.1 zinc finger protein 706-like [Ostrinia
furnacalis] >XP_028176220.1 zinc finger protein 706-like [Ostrinia furnacalis] >XP_028176221.1 zinc
leucine-rich PPR motif-containing protein, mitochondrial [Ostrinia furnacalis]
zinc finger protein 530-like isoform X8 [Ostrinia furnacalis]
prostamide/prostaglandin F synthase-like [Ostrinia furnacalis]

cell growth-regulating nucleolar protein [Ostrinia furnacalis]
28S ribosomal protein S10, mitochondrial [Ostrinia furnacalis] >XP_028175147.1 28S ribosomal protein
V-type proton ATPase subunit C [Vanessa cardui]
nucleolin-like [Melitaea cinxia]
hypothetical protein evm_009002 [Chilo suppressalis]
splicing factor 3A subunit 3 [Ostrinia furnacalis]
mitochondrial import inner membrane translocase subunit Tim13-like [Bicyclus anynana]
>CAG9745432.1 unnamed protein product [Diatraea saccharalis] >CAG9784117.1 unnamed protein
pancreatic triacylglycerol lipase-like [Ostrinia furnacalis]
unnamed protein product [Diatraea saccharalis]
ATPase family AAA domain-containing protein 1 isoform X2 [Ostrinia furnacalis]
membrane alanyl aminopeptidase-like [Ostrinia furnacalis]
LOW QUALITY PROTEIN: V-type proton ATPase subunit S1-like [Ostrinia furnacalis]
NADH dehydrogenase [ubiquinone] 1 alpha subcomplex subunit 6 [Ostrinia furnacalis]
venom carboxylesterase-6-like [Ostrinia furnacalis]
UDP-glucose 4-epimerase-like [Ostrinia furnacalis]
sodium- and chloride-dependent glycine transporter 1-like [Ostrinia furnacalis]
V-type proton ATPase subunit D isoform X2 [Ostrinia furnacalis]
39S ribosomal protein L37, mitochondrial [Ostrinia furnacalis]
hypothetical protein O3G_MSEX007366 [Manduca sexta]
uncharacterized protein LOC114357268 [Ostrinia furnacalis] >XP_028166599.1 uncharacterized protein
cysteine-rich with EGF-like domain protein 2 isoform X1 [Ostrinia furnacalis]
periodic tryptophan protein 1 homolog isoform X1 [Ostrinia furnacalis] >XP_028157695.1 periodic
unnamed protein product [Heterotrigona itama]
esterase FE4-like [Ostrinia furnacalis]
LOW QUALITY PROTEIN: caprin homolog [Ostrinia furnacalis]
putative trypsin 6 [Ostrinia nubilalis]
glutathione S-transferase sigma3 [Glyphodes pyloalis]
ATP-dependent RNA helicase p62 [Ostrinia furnacalis]
nucleolar GTP-binding protein 2 [Ostrinia furnacalis]
myb-binding protein 1A-like protein [Ostrinia furnacalis]
myogenesis-regulating glycosidase-like [Ostrinia furnacalis]
ribosomal RNA processing protein 1 homolog [Ostrinia furnacalis]
methionine--tRNA ligase, cytoplasmic isoform X6 [Ostrinia furnacalis]
digestive cysteine proteinase 2-like [Ostrinia furnacalis]
delta(24)-sterol reductase-like isoform X2 [Ostrinia furnacalis]
myogenesis-regulating glycosidase-like [Ostrinia furnacalis]
UDP-glycosyltransferase UGT40AP2, partial [Ostrinia furnacalis]
uncharacterized protein LOC114366171 [Ostrinia furnacalis]
uncharacterized protein LOC114350845 [Ostrinia furnacalis]
uncharacterized protein LOC114362782 [Ostrinia furnacalis]
uncharacterized protein LOC114359113 [Ostrinia furnacalis]
unnamed protein product [Chilo suppressalis]
facilitated trehalose transporter Tret1-like [Ostrinia furnacalis]
TRINITY_DN3889_c0_g1_i7_m.1657 TRINITY_DN3889_c0_g1::TRINITY_DN3889_c0_g1_i7::g.1657 ORF
type:5prime_partial len:235 (+),score=70.90 TRINITY_DN3889_c0_g1_i7:1-705(+)
glutathione S-transferase delta3 [Glyphodes pyloalis]
uncharacterized protein C1683.06c-like isoform X1 [Ostrinia furnacalis]
uncharacterized protein LOC114356358 [Ostrinia furnacalis]
putative inorganic phosphate cotransporter [Ostrinia furnacalis]
uncharacterized protein LOC114364889 [Ostrinia furnacalis]
uncharacterized protein LOC114361386 [Ostrinia furnacalis]
trypsin-like isoform X1 [Ostrinia furnacalis] >XP_028159118.1 trypsin-like isoform X2 [Ostrinia
TRINITY_DN717_c0_g1_i2_m.67915 TRINITY_DN717_c0_g1::TRINITY_DN717_c0_g1_i2::g.67915 ORF
type:internal len:868 (+),score=265.71,Collagen PF01391.19
0.11,Collagen|PF01391.19|0.039,Collagen|PF01391.19|0.00054,Collagen|PF01391.19|0.0019,Collagen|PF0
glutathione S-transferase sigma3 [Glyphodes pyloalis]
pancreatic triacylglycerol lipase-like [Ostrinia furnacalis]

loricrin-like [Ostrinia furnacalis]
lipase member I-like [Ostrinia furnacalis]
fatty acid-binding protein 1-like [Ostrinia furnacalis]
cathepsin K-like [Ostrinia furnacalis]
lipase member I-like [Ostrinia furnacalis]
pancreatic triacylglycerol lipase-like [Ostrinia furnacalis]
TRINITY_DN2490_c0_g2_i1_m.56872 TRINITY_DN2490_c0_g2::TRINITY_DN2490_c0_g2_i1::g.56872 ORF type:internal len:359 (-),score=123.59 TRINITY_DN2490_c0_g2_i1:2-1075(-)
pancreatic triacylglycerol lipase-like [Ostrinia furnacalis]
fasciclin-3-like [Ostrinia furnacalis]
uncharacterized protein LOC114357426 [Ostrinia furnacalis]
transmembrane emp24 domain-containing protein 5-like isoform X1 [Ostrinia furnacalis]
>XP_028176732.1 transmembrane emp24 domain-containing protein 5-like isoform X2 [Ostrinia
cytochrome b-c1 complex subunit 8-like [Ostrinia furnacalis]
NADH dehydrogenase [ubiquinone] iron-sulfur protein 2, mitochondrial [Ostrinia furnacalis]
hypothetical protein evm_010164 [Chilo suppressalis]
TRINITY_DN116874_c0_g1_i1_m.85176
TRINITY_DN116874_c0_g1::TRINITY_DN116874_c0_g1_i1::g.85176 ORF type:5prime_partial len:95
5-oxoprolinase [Ostrinia furnacalis]
homogentisate 1,2-dioxygenase [Ostrinia furnacalis]
uncharacterized protein LOC114356625 [Ostrinia furnacalis]
uncharacterized protein LOC114359599 [Ostrinia furnacalis]
sphingosine-1-phosphate lyase isoform X2 [Ostrinia furnacalis]
poly(U)-specific endoribonuclease homolog [Ostrinia furnacalis]
pre-rRNA-processing protein TSR1 homolog [Ostrinia furnacalis]
thioredoxin domain-containing protein 17-like [Ostrinia furnacalis]
senecionine N-oxygenase-like isoform X1 [Ostrinia furnacalis] >XP_028178163.1 senecionine N-oxygenase-like isoform X2 [Ostrinia furnacalis] >XP_028178164.1 senecionine N-oxygenase-like
renin receptor [Ostrinia furnacalis]
DNA methyltransferase 1-associated protein 1 [Ostrinia furnacalis]
phosphatidate cytidylyltransferase, mitochondrial [Ostrinia furnacalis]
hypothetical protein evm_000264 [Chilo suppressalis] >CAH2987898.1 unnamed protein product [Chilo
TRINITY_DN6203_c0_g1_i1_m.72736 TRINITY_DN6203_c0_g1::TRINITY_DN6203_c0_g1_i1::g.72736 ORF type:internal len:93 (+),score=12.26 TRINITY_DN6203_c0_g1_i1:3-278(+)
39S ribosomal protein L28, mitochondrial [Ostrinia furnacalis]
plastin-2 [Galleria mellonella]
cytochrome b5-like heme/Steroid binding domain-containing protein [Phthorimaea operculella]
putative histone-binding protein Caf1 [Papilio machaon]
WD repeat-containing protein 48 homolog isoform X1 [Ostrinia furnacalis] >XP_028157984.1 WD repeat-containing protein 48 homolog isoform X2 [Ostrinia furnacalis]
thioredoxin domain-containing protein [Ostrinia furnacalis]
uncharacterized protein LOC114363024 [Ostrinia furnacalis]
cartilage-associated protein-like [Ostrinia furnacalis]
myrosinase 1-like isoform X2 [Ostrinia furnacalis]
ubiquitin domain-containing protein 2 isoform X1 [Ostrinia furnacalis] >XP_028177862.1 ubiquitin domain-containing protein 2 isoform X2 [Ostrinia furnacalis] >XP_028177863.1 ubiquitin domain-
putative serine protease K12H4.7 [Ostrinia furnacalis] >XP_028166339.1 putative serine protease
arginine-glutamic acid dipeptide repeats protein [Ostrinia furnacalis]
acyl-CoA dehydrogenase family member 9, mitochondrial [Ostrinia furnacalis]

14-3-3 protein epsilon [Gallus gallus] >NP_001233297.1 14-3-3 protein epsilon [Pan troglodytes] >NP_006752.1 14-3-3 protein epsilon [Homo sapiens] >NP_033562.3 14-3-3 protein epsilon [Mus musculus] >NP_113791.2 14-3-3 protein epsilon [Rattus norvegicus] >NP_776916.1 14-3-3 protein epsilon [Bos taurus] >XP_001504337.1 14-3-3 protein epsilon isoform X1 [Equus caballus] >XP_002918088.2 14-3-3 protein epsilon isoform X2 [Ailuropoda melanoleuca] >XP_003416855.1 14-3-3 protein epsilon isoform X1 [Loxodonta africana] >XP_003469733.1 14-3-3 protein epsilon isoform X1 [Cavia porcellus] >XP_003816884.1 14-3-3 protein epsilon isoform X1 [Pan paniscus] >XP_003912098.1 14-3-3 protein epsilon isoform X1 [Papio anubis] >XP_003929381.1 14-3-3 protein epsilon isoform X1 [Saimiri boliviensis boliviensis] >XP_003996471.1 14-3-3 protein epsilon isoform X1 [Felis catus] >XP_004267124.1 14-3-3 protein epsilon isoform X1 [Orcinus orca] >XP_004376223.1 14-3-3 protein epsilon [Trichechus manatus latirostris] >XP_004404155.1 PREDICTED: 14-3-3 protein epsilon isoform X2 [Odobenus rosmarus divergens] >XP_004433380.1 PREDICTED: 14-3-3 protein epsilon isoform X1 [Ceratotherium simum simum] >XP_004483832.1 14-3-3 protein epsilon isoform X1 [Dasypus novemcinctus] >XP_004605045.1 PREDICTED: 14-3-3 protein epsilon [Sorex araneus] >XP_004667919.1 14-3-3 protein epsilon [Jaculus jaculus] >XP_004706944.1 14-3-3 protein epsilon [Echinops telfairi] >XP_004746947.1 14-3-3 protein epsilon isoform X1 [Mustela putorius furo] >XP_004857172.1 14-3-3 protein epsilon isoform X1 [Heterocephalus glaber] >XP_005067448.1 14-3-3 protein epsilon isoform X1 [Mesocricetus auratus] >XP_005240506.1 14-3-3 protein epsilon isoform X1 [Falco peregrinus] >XP_005327947.1 14-3-3 protein epsilon isoform X1 [Ictidomys tridecemlineatus] >XP_005349591.1 14-3-3 protein epsilon isoform X1 [Microtus ochrogaster] >XP_005402688.1 PREDICTED: 14-3-3 protein epsilon isoform X1 [Chinchilla lanigera] >XP_005525859.1 PREDICTED: 14-3-3 protein epsilon isoform X1 [Pseudopodoces humilis] >XP_005888292.1 PREDICTED: 14-3-3 protein epsilon isoform X1 [Bos mutus] >XP_006079841.1 14-3-3 protein epsilon isoform X1 [Bubalus bubalis] >XP_006099253.1 14-3-3 protein epsilon [Myotis lucifugus] >XP_006185046.1 14-3-3 protein epsilon isoform X1 [Camelus ferus] >XP_006214490.1 14-3-3 protein epsilon isoform X1 [Vicugna pacos] >XP_006259463.1 PREDICTED: 14-3-3 protein epsilon [Alligator mississippiensis] >XP_006768146.1 PREDICTED: 14-3-3 protein epsilon isoform X1 [Myotis davidii] >XP_006863283.1 PREDICTED: 14-3-3 protein epsilon [Chrysochloris asiatica] >XP_006891074.1 PREDICTED: 14-3-3 protein epsilon-like [Elephantulus edwardii] >XP_006925117.1 14-3-3 protein epsilon isoform X1 [Pteropus alecto] >XP_006977465.1 14-3-3 protein epsilon isoform X1 [Peromyscus maniculatus bairdii]

LOW QUALITY PROTEIN: succinate--hydroxymethylglutarate CoA-transferase-like [Ostrinia furnacalis]
unnamed protein product [Chilo suppressalis]
uncharacterized protein LOC114362364 [Ostrinia furnacalis]
CAD protein isoform X2 [Ostrinia furnacalis]
LDLR chaperone boca [Ostrinia furnacalis]
gloverin-like [Ostrinia furnacalis]
protein bunched, class 2/F/G isoform-like isoform X2 [Ostrinia furnacalis]
sodium/hydrogen exchanger 7 isoform X4 [Galleria mellonella]
tetratricopeptide repeat protein 37 [Ostrinia furnacalis]
vegetative cell wall protein gp1 [Ostrinia furnacalis]
unnamed protein product [Chrysodeixis includens]
uncharacterized protein LOC114366000 [Ostrinia furnacalis]
lens fiber major intrinsic protein-like isoform X1 [Ostrinia furnacalis]
putative peptidyl-tRNA hydrolase PTRHD1 [Ostrinia furnacalis]
pleiotropic regulator 1 [Ostrinia furnacalis]
unnamed protein product [Chilo suppressalis]
PREDICTED: synapse-associated protein of 47 kDa-like isoform X2 [Papilio xuthus]
hypothetical protein evm_003554 [Chilo suppressalis]
zinc transporter 1 [Chelonus insularis] >XP_034950393.1 zinc transporter 1 [Chelonus insularis] >XP_034950394.1 zinc transporter 1 [Chelonus insularis] >XP_034950395.1 zinc transporter 1 [Chelonus
uncharacterized protein LOC114355289 isoform X3 [Ostrinia furnacalis] >XP_028163907.1
uncharacterized protein LOC114355289 isoform X3 [Ostrinia furnacalis] >XP_028163914.1
uncharacterized protein LOC114355289 isoform X3 [Ostrinia furnacalis] >XP_028163920.1
reversion-inducing cysteine-rich protein with Kazal motifs [Ostrinia furnacalis]
unnamed protein product [Mus musculus]
oxygen-dependent coproporphyrinogen-III oxidase isoform X1 [Ostrinia furnacalis]
28S ribosomal protein S33, mitochondrial [Galleria mellonella]
proton channel OtopLc-like isoform X6 [Ostrinia furnacalis]
cytoglobin-1-like isoform X2 [Ostrinia furnacalis]

UDP-glucuronosyltransferase 2B15-like [Ostrinia furnacalis] >XP_028166365.1 UDP-glucuronosyltransferase 2B15-like [Ostrinia furnacalis] >QNS26328.1 UDP-glycosyltransferase
actin-interacting protein 1 isoform X2 [Ostrinia furnacalis]
mulatexin-like [Ostrinia furnacalis]
unnamed protein product [Parnassius apollo]
acyl-CoA Delta(11) desaturase-like [Ostrinia furnacalis] >XP_028172986.1 acyl-CoA Delta(11) desaturase-like [Ostrinia furnacalis] >AAL27034.1 acyl-CoA delta-9 desaturase [Ostrinia furnacalis]
RNA exonuclease 4-like [Ostrinia furnacalis] >QEE79882.1 REX4 [Ostrinia furnacalis]

RD	ND	PreD	CT	D
1.524838	-1.5131	-0.13691	0.486159	-0.36099
1.330795	-1.56091	0.25072	0.593634	-0.61424
1.421976	-1.66944	-0.16423	0.006789	0.404902
0.773584	-1.96464	0.276705	0.610747	0.303605
1.466394	-1.55545	0.546475	-0.11249	-0.34493
1.521229	-1.32063	-0.80348	0.5407	0.062179
0.625186	-1.92875	0.05375	0.854231	0.395579
1.055583	-1.88845	0.09354	0.506485	0.232845
0.759273	-1.95718	0.285869	0.674351	0.237684
1.364341	-1.74459	-0.00854	0.292689	0.096097
0.467236	-1.90717	0.092446	1.011364	0.336127
0.878065	-1.85788	0.075843	0.877986	0.025985
0.99281	-1.83566	0.022779	0.017678	0.802396
0.775821	-1.83278	0.265493	0.968155	-0.17669
0.82977	-1.90586	0.095291	0.797901	0.182899
0.58876	-1.95786	0.096025	0.653176	0.619902
1.16248	-1.83019	0.40775	-0.09248	0.352447
0.880285	-1.87536	-0.14564	0.705109	0.435599
1.513792	-1.23796	-0.72227	0.749796	-0.30336
1.535257	-1.56811	0.187615	-0.33896	0.184193
1.185706	-1.78547	0.556252	-0.19717	0.240672
1.362317	-1.75426	0.029742	0.171885	0.19032
0.677952	-1.96066	0.183632	0.720555	0.37852
1.017852	-1.907	0.430668	0.093696	0.364786
1.175557	-1.66723	-0.46935	0.200905	0.760112
1.111089	-1.87511	0.353446	0.063449	0.347125
0.917893	-1.87198	0.36021	0.713515	-0.11964
0.600171	-1.87233	-0.15577	0.926598	0.501327
0.735275	-1.92342	0.026814	0.374809	0.786523
1.392926	-1.65486	0.533024	-0.14842	-0.12267
1.430237	-1.42733	-0.04762	0.698334	-0.65362
1.321623	-1.68891	0.250159	0.465658	-0.34853
0.6842	-1.96276	0.151878	0.667458	0.459223
0.722256	-1.90567	0.086632	0.893223	0.203555
0.969645	-1.86872	0.427237	-0.1341	0.605935
0.476454	-1.84798	-0.00559	1.141155	0.235969
1.811377	-1.27176	-0.12537	-0.20385	-0.2104
0.705225	-1.97457	0.212669	0.517943	0.538734
1.592397	-1.40657	0.490188	-0.43032	-0.24569
0.857751	-1.93493	0.11432	0.629269	0.333585
1.443932	-1.69109	-0.03362	0.054604	0.226173
1.468753	-1.66617	0.160931	0.159807	-0.12332
0.728911	-1.86428	-0.14886	0.912623	0.371608
1.100337	-1.84524	-0.03394	0.589301	0.189545
1.868467	-1.12051	-0.45866	-0.16776	-0.12153
0.768418	-1.88412	-0.08505	0.856802	0.343945
1.002093	-1.91897	0.44652	0.194098	0.276257
1.190708	-1.84029	0.051581	0.215131	0.382873
0.392653	-1.83925	-0.04091	1.165037	0.322476
1.498967	-1.46295	-0.45797	0.607281	-0.18533
1.22163	-1.82018	0.029547	0.158408	0.410597
1.871081	-1.05123	-0.3467	0.047886	-0.52104
0.905763	-1.94011	0.286822	0.209584	0.53794
1.349717	-1.64302	-0.42312	0.504996	0.211425

0.589406	-1.95892	0.119176	0.723581	0.526755
1.193115	-1.82118	0.324003	-0.08097	0.385027
0.668848	-1.88616	-0.06788	0.929461	0.355729
0.620018	-1.92017	0.166704	0.926153	0.207297
1.926867	-0.97786	-0.45919	-0.23589	-0.25393
0.762173	-1.82308	-0.30787	0.863167	0.505605
1.507818	-1.32685	-0.60473	0.717088	-0.29332
1.810863	-1.11829	-0.68124	0.049371	-0.06071
1.750834	-1.21249	-0.48615	-0.36281	0.310611
1.5435	-1.44043	0.482855	-0.03031	-0.55562
0.672764	-1.96658	0.237636	0.709361	0.34682
1.526364	-1.52962	-0.27149	0.468296	-0.19355
1.32924	-1.54562	-0.41864	0.800888	-0.16588
1.823524	-1.20297	-0.42919	-0.20774	0.016375
0.734044	-1.87418	-0.18552	0.7961	0.529556
1.323992	-1.70772	0.401467	0.282165	-0.2999
1.825374	-1.22952	-0.32766	-0.21464	-0.05356
1.447092	-1.41217	-0.52177	0.753793	-0.26695
1.054449	-1.80151	0.166589	-0.18225	0.762722
1.874118	-0.90834	-0.54741	0.161915	-0.58028
1.39267	-1.41045	-0.5329	0.839026	-0.28834
1.64125	-1.48909	-0.27718	0.108765	0.01625
1.108553	-1.88533	0.352482	0.246593	0.177705
1.637113	-1.51941	0.021088	-0.09377	-0.04502
0.777007	-1.88379	-0.16566	0.70898	0.563467
1.386994	-1.4803	-0.47209	0.783824	-0.21843
1.31766	-1.78046	0.159932	0.257032	0.045833
0.620774	-1.95102	0.064293	0.669684	0.596271
1.460452	-1.42515	-0.54334	0.707755	-0.19972
0.633067	-1.88732	-0.03579	0.964281	0.325761
1.215032	-1.79949	0.344644	0.382591	-0.14278
0.96795	-1.93408	0.286756	0.27119	0.408188
0.649369	-1.86072	-0.11539	0.997005	0.329729
1.500955	-1.51791	-0.41152	0.515845	-0.08737
1.481406	-1.65225	0.182247	0.139616	-0.15102
1.77788	-1.25582	0.159596	-0.38702	-0.29464
0.744567	-1.92571	0.172029	0.81962	0.18949
1.859282	-1.1256	-0.47671	-0.21744	-0.03953
1.13536	-1.87566	0.224888	0.326466	0.188947
1.341358	-1.49966	-0.46595	0.831657	-0.2074
1.882312	-1.11169	-0.33345	-0.30312	-0.13405
1.40701	-1.6882	0.105714	-0.18034	0.355816
0.392523	-1.65603	-0.59978	0.868961	0.994321
0.512918	-1.75652	-0.19489	1.257274	0.181216
0.408441	-1.68477	-0.53187	1.100378	0.70782
0.597225	-1.76978	-0.07945	1.226478	0.025524
1.707728	-1.40772	-0.3125	-0.03986	0.05235
1.941851	-0.94204	-0.37857	-0.36302	-0.25822
1.801452	-1.09849	0.271229	-0.48644	-0.48775
1.325287	-1.75041	0.387439	0.138948	-0.10126
0.862385	-1.44754	-0.22929	1.349742	-0.53529

1.807036	-1.26707	-0.16219	-0.31439	-0.06339
0.572084	-1.50764	-0.21881	1.495223	-0.34086
1.075727	-1.88655	0.266454	0.451872	0.092495
1.312905	-1.75378	-0.14141	0.365339	0.21695
1.129652	-1.78032	-0.23506	0.673994	0.211731
1.337035	-1.62052	-0.32506	0.688609	-0.08007
1.770409	-0.9686	-0.86986	0.324272	-0.25621
1.447006	-1.63196	-0.28054	0.065632	0.399855
0.571761	-1.81547	-0.29303	1.003443	0.53329
1.446757	-1.40919	-0.49679	0.76292	-0.3037
1.369201	-1.65582	0.324262	0.3538	-0.39144
1.693046	-1.41563	0.028831	-0.35543	0.049187
0.773829	-1.88612	-0.1317	0.784203	0.459789
1.489497	-1.60136	-0.21595	0.405441	-0.07763
0.701457	-1.83462	-0.05343	1.059811	0.126781
1.466924	-1.41147	-0.53214	0.717459	-0.24077
0.564701	-1.84179	-0.25442	0.926109	0.605404
1.784503	-1.18958	-0.37309	-0.45489	0.233062
1.430986	-1.38537	-0.57389	0.795238	-0.26696
1.92419	-0.97486	-0.47298	-0.16757	-0.30877
1.592369	-1.53005	0.193506	-0.2909	0.035078
1.708563	-1.42464	-0.03125	-0.03065	-0.22202
0.820546	-1.89702	0.14821	0.835075	0.093193
0.982965	-1.86917	0.182088	0.711876	-0.00776
0.662318	-1.46822	-0.92007	1.049599	0.676373
1.329088	-1.76644	0.042665	0.067873	0.32681
1.318961	-1.77844	0.018767	0.216517	0.224195
0.945012	-1.74136	-0.35626	0.952764	0.199843
1.495753	-1.30821	-0.35523	0.758773	-0.59109
0.81946	-1.90957	-0.04285	0.427918	0.705041
0.595786	-1.82085	-0.23449	1.05335	0.406205
1.195909	-1.77702	-0.19412	0.579749	0.195478
1.554021	-1.5851	0.077079	0.157933	-0.20393
1.105431	-1.86074	0.37132	-0.03609	0.420085
1.424278	-1.68305	-0.14534	0.067882	0.336226
1.886464	-1.10433	-0.36732	-0.18798	-0.22684
1.383534	-1.66207	-0.31876	0.445923	0.151367
1.818432	-1.08714	0.210881	-0.5786	-0.36358
1.569934	-1.5387	0.322551	-0.1535	-0.20028
1.493557	-1.3514	0.73937	-0.50319	-0.37834
1.377252	-1.65243	0.332666	-0.38952	0.332036
1.661218	-1.36956	-0.44462	0.355114	-0.20215
0.452013	-1.87199	-0.10369	1.006589	0.517075
1.323178	-1.69015	-0.35009	0.438975	0.278085
1.904631	-0.98329	-0.55268	-0.31105	-0.05761
1.866327	-0.88649	-0.43243	0.17022	-0.71763
1.709149	-1.41764	-0.19172	0.067088	-0.16687
1.019179	-1.82654	-0.24911	0.577821	0.478651
1.758504	-1.14121	-0.69937	0.278549	-0.19648
1.656353	-1.42713	-0.41934	-0.01866	0.208781
1.377749	-1.61566	0.522915	-0.44032	0.155316
1.623877	-1.24573	-0.50481	0.586935	-0.46027
1.670674	-1.28052	0.32396	-0.68047	-0.03364
1.569318	-1.12376	-0.4278	0.72978	-0.74753
0.888083	-1.89764	0.249272	0.020203	0.740082

1.751458	-1.36926	-0.16809	-0.16344	-0.05067
0.880955	-1.84593	-0.23363	0.746836	0.451772
1.082922	-1.85492	-0.00679	0.185386	0.593405
1.58533	-1.54084	-0.23724	0.233628	-0.04088
0.931991	-1.89737	0.004445	0.293837	0.667097
1.383768	-1.41164	-0.80754	0.631465	0.203944
1.35671	-1.7231	0.139831	-0.15601	0.382571
1.69307	-1.32176	0.201695	-0.58785	0.014841
0.720318	-1.73921	-0.51867	0.717778	0.819788
1.164348	-1.8629	0.171511	0.316369	0.210675
1.760769	-1.12739	-0.76011	0.2098	-0.08307
0.60869	-1.8954	-0.08364	0.898486	0.471872
1.559047	-1.54491	-0.18039	0.344139	-0.17788
1.644188	-1.28323	-0.55803	0.498004	-0.30093
1.023137	-1.23231	-0.71042	1.327862	-0.40827
1.00686	-1.6869	-0.55766	0.797281	0.440417
0.648505	-1.89235	-0.1421	0.787437	0.598507
1.328984	-1.57307	-0.57323	0.628492	0.188826
0.90718	-1.7273	0.043271	1.055452	-0.27861
0.570161	-1.58999	-0.63679	1.25792	0.398707
0.888768	-1.8483	-0.0593	0.877763	0.141067
0.523169	-1.44642	-0.61321	1.502322	0.034142
1.029844	-1.89605	0.260717	0.086926	0.518558
0.955246	-1.74837	-0.43624	0.408851	0.820515
1.549484	-1.57544	-0.26804	0.115282	0.178718
1.891788	-1.09366	-0.35209	-0.25135	-0.19469
0.927093	-1.88584	-0.03339	0.282945	0.709191
1.380701	-1.58306	-0.1806	0.682214	-0.29925
1.187542	-1.65725	-0.07233	0.858873	-0.31684
1.431443	-1.67443	-0.1583	0.344981	0.056312
1.38948	-1.64119	0.423954	-0.38718	0.214933
1.120879	-1.76701	-0.33301	0.364918	0.614215
1.661198	-1.45491	-0.33721	0.039082	0.091834
1.754131	-1.23131	-0.21626	0.242406	-0.54896
1.513829	-1.41761	-0.57602	-0.11492	0.594725
1.304604	-1.68339	-0.2394	0.637599	-0.01941
1.652492	-1.0343	-0.96812	0.491985	-0.14205
1.458875	-1.63623	0.390029	-0.20554	-0.00713
0.715352	-1.75506	-0.26802	1.144333	0.163396
0.747653	-1.83589	0.044731	1.033648	0.009856
1.711724	-1.24403	-0.18255	0.330931	-0.61607
0.830386	-1.75776	-0.4744	0.619701	0.782076
1.566179	-1.5568	0.146198	0.134327	-0.2899
1.707784	-1.23901	-0.70637	0.015987	0.221608
1.661897	-1.40843	-0.43	0.252493	-0.07596
1.650993	-1.29365	-0.65418	-0.10522	0.402064
1.757126	-1.33755	-0.14861	0.044331	-0.31529
0.364521	-1.8114	-0.26141	1.025147	0.683143
0.587556	-1.75655	-0.31393	1.172294	0.31063

1.835521	-1.01585	-0.25085	-0.71697	0.148143
1.008066	-1.58849	-0.49332	1.102851	-0.02911
0.153979	-1.49049	-0.74378	1.177455	0.902836
0.771383	-1.86422	-0.22571	0.727953	0.590595
1.019192	-1.67866	-0.21258	1.035158	-0.16311
1.201933	-1.73448	0.299586	0.580195	-0.34724
1.579719	-1.36051	0.578777	-0.39091	-0.40708
1.845122	-1.15325	-0.49504	-0.0749	-0.12193
1.491978	-1.63886	0.23328	0.079224	-0.16562
1.830311	-1.13684	-0.54866	0.079317	-0.22412
0.018257	-1.48804	-0.62861	1.356313	0.742078
1.244527	-1.79107	-0.02892	0.483711	0.091752
1.703691	-1.10802	0.509232	-0.55385	-0.55106
1.082046	-1.21199	-0.95752	1.196466	-0.109
1.333661	-1.7591	0.037743	0.035232	0.352468
1.538342	-1.42492	0.387131	0.15467	-0.65522
1.778357	-1.30446	0.015524	-0.15577	-0.33365
0.400108	-1.37359	-0.9009	0.498515	1.375869
0.452216	-1.76989	-0.29259	1.179899	0.430367
1.22923	-1.76428	-0.2236	0.240513	0.518135
1.474813	-1.66698	0.212334	0.009987	-0.03015
1.887228	-0.87097	-0.67202	0.118504	-0.46274
1.559693	-0.95926	-1.04303	0.701569	-0.25897
1.851779	-1.17419	-0.34366	-0.07123	-0.26269
1.895097	-1.01567	-0.51524	-0.03171	-0.33247
1.699628	-1.30748	0.339884	-0.46166	-0.27037
1.834346	-1.0558	-0.71235	-0.10667	0.040471
1.003121	-1.82631	-0.26104	0.507348	0.576879
1.681606	-1.2111	-0.51666	0.490753	-0.4446
1.635227	-1.39515	0.30438	-0.00886	-0.53559
1.730744	-1.32934	-0.20038	0.196987	-0.39801
1.893468	-0.99312	-0.44559	0.024151	-0.4789
1.400224	-1.65697	-0.18487	0.505528	-0.06391
0.831325	-1.58943	-0.00699	1.245648	-0.48055
1.917446	-0.99815	-0.47873	-0.24102	-0.19954
0.980906	-1.90425	0.081356	0.579967	0.262025
1.833697	-1.13251	-0.57016	-0.16872	0.0377
0.941501	-1.70297	-0.55392	0.801754	0.513637
1.828912	-1.2045	-0.02162	-0.19624	-0.40656
1.566203	-1.36034	-0.0341	0.497462	-0.66922
0.461332	-1.56303	-0.45345	1.459209	0.095935
0.882536	-1.83688	-0.28029	0.561257	0.673375
0.625087	-1.55524	-0.75197	0.516832	1.165293
0.694272	-1.15525	-0.91477	1.524608	-0.14886
1.2096	-1.81475	0.181902	-0.03421	0.457466
1.300542	-1.27496	-0.83399	0.978995	-0.17059
1.336282	-1.60096	-0.54391	0.523742	0.284844
1.389566	-1.72496	0.257056	-0.07145	0.149786
1.658819	-1.13121	0.515153	-0.8039	-0.23887
1.687916	-1.25762	-0.65954	0.34706	-0.11781

0.837369	-1.79497	-0.33146	0.905498	0.383557
1.82588	-1.21353	-0.20785	-0.01719	-0.3873
1.020351	-1.66215	-0.57196	0.864158	0.349606
1.093108	-1.58807	-0.72996	0.606648	0.618278
1.267662	-1.51179	-0.56984	0.882134	-0.06816
1.19246	-1.78056	-0.12554	0.618765	0.094879
1.676224	-1.33807	-0.29046	0.372602	-0.42029
1.255	-1.71324	-0.16873	0.677327	-0.05036
0.912595	-1.65502	-0.38635	1.130838	-0.00206
1.746352	-1.37291	-0.16565	-0.19431	-0.01349
1.536046	-1.56353	-0.35182	0.179126	0.200179
0.865092	-1.43193	-0.85651	1.188445	0.234904
0.682122	-1.83099	-0.30976	0.623458	0.835175
1.402927	-1.68926	0.05954	-0.15947	0.386271
0.807881	-1.71297	-0.56781	0.790817	0.68209
1.204416	-1.75988	-0.21987	0.614911	0.160427
1.93624	-0.96665	-0.34062	-0.35145	-0.27751
1.783989	-1.26124	-0.0471	-0.0019	-0.47375
1.860524	-1.17265	-0.17419	-0.23387	-0.27981
1.690022	-1.39461	-0.40608	-0.06272	0.173384
1.714165	-1.31795	0.288989	-0.28616	-0.39904
1.042535	-1.68384	-0.45054	0.919253	0.172591
0.596736	-1.79395	-0.3824	0.915957	0.663659
1.447908	-1.59297	-0.23371	0.535539	-0.15677
1.519817	-1.31721	-0.79528	0.567468	0.025198
1.074315	-1.82645	-0.15436	0.259316	0.647185
1.028922	-1.81348	-0.25195	0.679416	0.357094
1.522342	-1.31564	-0.24444	0.686365	-0.64863
0.734847	-1.37318	-0.62105	1.465049	-0.20566
1.265426	-1.34393	-1.00105	0.601938	0.477617
1.815561	-1.24293	-0.2893	-0.0093	-0.27403
1.190496	-1.72556	-0.35584	0.647576	0.243318
0.829981	-1.73637	-0.48082	0.920287	0.466926
1.291394	-1.34906	-0.81595	0.918974	-0.04536
1.663677	-1.43363	-0.35178	0.212064	-0.09033
1.517199	-1.29328	-0.84843	0.077001	0.547512
1.740601	-1.19641	-0.38478	-0.51454	0.355125
1.727179	-1.30233	-0.51079	-0.12466	0.210599
1.697356	-1.2033	-0.60314	0.442694	-0.33361
1.704256	-1.32291	0.019877	0.163077	-0.5643
1.7693	-1.32383	0.008584	-0.14366	-0.31039
1.152169	-1.83309	-0.08472	0.305345	0.460295
1.10318	-1.43097	-0.95104	0.558331	0.720497
1.77207	-1.09669	-0.22672	0.278159	-0.72681
1.584986	-1.42099	-0.60502	0.272069	0.168951
1.28358	-1.20149	0.225755	0.797523	-1.10537
0.68942	-1.6531	-0.61962	0.513586	1.069709
0.797654	-1.65265	-0.449	1.19083	0.113168
1.053311	-1.79483	-0.29623	0.372378	0.665368
1.500127	-1.58542	-0.35434	0.136614	0.303027
0.38793	-1.42747	-0.79996	1.409655	0.429849
1.012184	-1.60325	-0.60294	1.002409	0.191596
1.36367	-1.27567	-1.03261	0.454273	0.490334

1.121965	-1.42402	-0.91313	0.873314	0.341871
0.994923	-1.34787	-0.26205	1.291263	-0.67627
1.430544	-1.5336	-0.34837	-0.20924	0.660663
0.51495	-1.55047	-0.75693	0.621003	1.171447
1.133133	-1.67561	-0.5025	0.756827	0.288155
1.567389	-1.53755	0.096489	0.221381	-0.3477
0.650112	-1.26895	-1.13626	0.616936	1.138164
1.466686	-1.50411	-0.06287	0.587504	-0.48721
0.76372	-1.40898	-0.67949	1.401404	-0.07666
1.700425	-1.21455	-0.00446	-0.7494	0.26798
1.081261	-0.89383	-0.09579	1.182709	-1.27435
0.705989	-1.3839	-0.49913	1.495317	-0.31828
0.848214	-1.55095	-0.42603	1.291201	-0.16244
1.105896	-1.75529	-0.26874	0.777061	0.141073
1.789448	-0.96713	-0.65077	-0.54639	0.374834
1.57594	-1.36322	-0.68422	0.434155	0.037344
0.733429	-1.40517	-0.88266	1.277399	0.277006
1.011939	-0.9591	-0.37748	1.358339	-1.03369
0.123399	-0.86784	-0.4822	1.887445	-0.6608
1.328821	-1.24781	-0.92733	0.90229	-0.05597
1.775435	-1.02087	-0.77327	0.331485	-0.31278
1.407305	-0.97761	-0.6629	1.01023	-0.77702
1.666699	-1.14281	-0.82854	0.454987	-0.15033
1.759395	-1.21597	-0.63358	0.146208	-0.05605
1.618668	-1.21307	-0.41066	-0.60563	0.610694
1.034871	-0.97147	-0.37834	1.339111	-1.02417
1.300156	-1.52906	-0.46983	0.852708	-0.15397
0.970296	-1.53299	-0.67581	1.11159	0.126917
0.479705	-1.41779	-0.94235	1.167934	0.712502
1.831406	-0.9098	-0.76252	0.255213	-0.4143
1.702255	-1.15852	-0.79731	-0.08806	0.341626
1.281162	-0.8915	-0.78689	1.164471	-0.76724
0.688593	-1.19383	-0.59914	1.584616	-0.48024
1.812268	-1.08405	-0.47406	-0.50356	0.249408
0.36678	-1.40302	-0.82424	1.423667	0.436815
1.584853	-1.09031	-1.01583	0.517242	0.004046
1.820908	-0.88389	-0.86423	0.240636	-0.31343
1.767074	-0.93631	-0.60336	-0.66637	0.438961
0.824913	-1.19277	-1.25505	0.86021	0.762697
1.3536	-0.98627	-1.0489	0.99751	-0.31594
1.245205	-0.80333	-1.07787	1.166549	-0.53056
1.182739	-1.06643	-0.71746	1.240898	-0.63975
1.99503	-0.50286	-0.37408	-0.59056	-0.52753
1.995725	-0.42646	-0.49788	-0.61754	-0.45384
1.997603	-0.42134	-0.52292	-0.57051	-0.48283
1.972652	-0.77251	-0.32804	-0.54555	-0.32655
1.964656	-0.28224	-0.41813	-0.84002	-0.42426
1.997092	-0.53467	-0.39645	-0.54766	-0.51831
1.992747	-0.63861	-0.52015	-0.45448	-0.37951

1.998245	-0.45339	-0.49391	-0.57678	-0.47417
1.992744	-0.41972	-0.57397	-0.60977	-0.38928
1.991366	-0.41266	-0.44969	-0.67544	-0.45358
1.946417	-0.7526	-0.67989	-0.09768	-0.41625
1.946663	-0.08838	-0.58254	-0.48042	-0.79532
1.964313	-0.69765	-0.1412	-0.58999	-0.53547
1.966402	-0.40822	-0.32842	-0.84138	-0.38839
1.930081	-0.22438	-0.16582	-0.69468	-0.8452
1.964409	-0.17414	-0.48073	-0.76018	-0.54936
1.936488	-0.86904	-0.61234	-0.13738	-0.31774
1.946385	-0.08346	-0.59417	-0.78721	-0.48155
1.999313	-0.49121	-0.46912	-0.4908	-0.54818
1.99815	-0.54132	-0.4541	-0.55347	-0.44926
1.923668	-0.73025	-0.01876	-0.78222	-0.39243
1.960926	-0.19981	-0.3658	-0.75924	-0.63607
1.993873	-0.42842	-0.6094	-0.39885	-0.5572
1.996848	-0.45214	-0.60677	-0.4771	-0.46084
1.985197	-0.38803	-0.42263	-0.72876	-0.44578
1.966086	-0.48801	-0.49806	-0.7799	-0.20011
1.980161	-0.61025	-0.65574	-0.46086	-0.25331
1.997211	-0.44144	-0.49972	-0.59488	-0.46117
1.93351	-0.03825	-0.44717	-0.80031	-0.64778
1.984246	-0.70119	-0.32921	-0.53942	-0.41442
1.99593	-0.46158	-0.61125	-0.41957	-0.50354
1.994746	-0.55453	-0.54631	-0.53497	-0.35894
1.966178	-0.70318	-0.39625	-0.20203	-0.66473
1.985591	-0.31007	-0.61066	-0.63591	-0.42896
1.99963	-0.46474	-0.52177	-0.51144	-0.50168
1.940698	-0.89068	-0.36635	-0.15148	-0.53219
1.957152	-0.85856	-0.22462	-0.44253	-0.43145
1.979765	-0.29726	-0.46016	-0.74031	-0.48204
1.969016	-0.82231	-0.45052	-0.32095	-0.37524
1.960045	-0.10627	-0.64886	-0.60367	-0.60124
1.899752	-0.52285	-0.60365	-0.86319	0.089934
1.990961	-0.5527	-0.31435	-0.55217	-0.57175
1.983861	-0.69193	-0.55297	-0.42744	-0.31152
1.946896	-0.88113	-0.36344	-0.18611	-0.51622
1.577276	-1.04121	-1.026	-0.11238	0.602314
1.997937	-0.4133	-0.54419	-0.51675	-0.5237
1.938478	-0.09954	-0.56621	-0.86207	-0.41067
1.878111	-0.92719	-0.05274	-0.77071	-0.12747
1.987644	-0.67861	-0.53966	-0.35675	-0.41263
1.874036	-1.10293	-0.05386	-0.43413	-0.28311
1.999246	-0.47233	-0.47339	-0.50598	-0.54754
1.932711	-0.04004	-0.52623	-0.84528	-0.52116
1.919658	-0.65203	-0.8528	-0.40295	-0.01188
1.989693	-0.43941	-0.68574	-0.38709	-0.47745
1.683193	-0.76066	-1.23738	0.095882	0.21897
1.741769	-0.93637	-0.68744	0.493253	-0.61122
1.970778	-0.73389	-0.61676	-0.35888	-0.26124
1.97188	-0.6518	-0.30342	-0.70579	-0.31087
1.975751	-0.2934	-0.71297	-0.35791	-0.61148
1.893433	-0.83685	0.104044	-0.45735	-0.70327
1.997406	-0.47189	-0.59139	-0.43833	-0.4958
1.978837	-0.22591	-0.50828	-0.61132	-0.63334

1.966631	-0.20979	-0.49208	-0.78469	-0.48008
1.988434	-0.3108	-0.52139	-0.64609	-0.51015
1.962041	-0.86589	-0.36682	-0.35709	-0.37224
1.979876	-0.69908	-0.26014	-0.47268	-0.54798
1.926455	-0.88315	-0.09137	-0.62989	-0.32205
1.982637	-0.42006	-0.39781	-0.74987	-0.4149
1.985017	-0.70558	-0.52943	-0.36254	-0.38746
1.99873	-0.51739	-0.44119	-0.54991	-0.49023
1.99005	-0.61432	-0.53096	-0.31381	-0.53096
1.978905	-0.37907	-0.42499	-0.77383	-0.40102
1.986808	-0.4149	-0.52901	-0.35296	-0.68995
1.936764	-0.95086	-0.33325	-0.2244	-0.42826
1.977644	-0.5251	-0.38476	-0.75192	-0.31587
1.937205	-0.91223	-0.26563	-0.54732	-0.21203
1.998473	-0.57111	-0.48747	-0.45242	-0.48747
1.999449	-0.48761	-0.53443	-0.46463	-0.51277
1.917805	-0.88072	-0.13078	-0.69662	-0.20968
1.986024	-0.53096	-0.3896	-0.69765	-0.36781
1.934207	-0.5741	-0.90877	-0.19793	-0.25341
1.969117	-0.76735	-0.27911	-0.58422	-0.33844
1.991003	-0.54652	-0.40154	-0.64821	-0.39473
1.988049	-0.49834	-0.33565	-0.67884	-0.47522
1.907073	-0.62007	-0.66384	-0.72615	0.102989
1.938556	-0.03754	-0.48295	-0.73982	-0.67824
1.96293	-0.4291	-0.85485	-0.3669	-0.31209
1.986147	-0.5896	-0.60985	-0.50788	-0.27882
1.947984	-0.05511	-0.57519	-0.61807	-0.69962
1.985438	-0.60412	-0.28033	-0.48569	-0.6153
1.995875	-0.42673	-0.61755	-0.48696	-0.46464
1.682036	-0.72084	0.602066	-0.59892	-0.96434
1.970354	-0.1805	-0.57436	-0.69863	-0.51687
1.811737	-0.57686	0.350907	-0.74624	-0.83954
1.991629	-0.55411	-0.61361	-0.48423	-0.33968
1.891266	-0.93952	-0.01638	-0.69428	-0.24108
1.959285	-0.27097	-0.293	-0.81698	-0.57835
1.925684	-0.20279	-0.96199	-0.51408	-0.24683
1.991417	-0.58482	-0.3578	-0.60877	-0.44003
1.896173	-0.81024	-0.84676	-0.08467	-0.15451
1.989597	-0.30853	-0.54632	-0.61221	-0.52254
1.926754	-0.74116	-0.81002	-0.26409	-0.11148
1.919087	0.035289	-0.56282	-0.82003	-0.57152
1.615077	-0.94651	-0.83892	0.708613	-0.53826
1.942838	-0.19438	-0.48278	-0.90992	-0.35576
1.774504	-0.65979	0.420662	-0.94114	-0.59424
1.926222	-0.92553	-0.30122	-0.57075	-0.12872
1.908173	0.095282	-0.68469	-0.73496	-0.5838
1.973531	-0.78385	-0.48775	-0.29394	-0.40799
1.956255	-0.5683	-0.83326	-0.24625	-0.30844
1.971211	-0.28437	-0.75258	-0.59095	-0.34331
1.981838	-0.37833	-0.66859	-0.61768	-0.31724
1.815381	-0.90157	0.073445	-0.04707	-0.94019
1.930585	-0.6949	-0.76705	-0.44854	-0.02009
1.890625	0.124765	-0.62715	-0.85699	-0.53125
1.899281	-0.89548	-0.1069	-0.74611	-0.15079

1.996255	-0.59836	-0.45966	-0.52157	-0.41667
1.864393	-0.30232	-1.03321	0.071185	-0.60005
1.729481	-0.83798	-1.13028	0.101371	0.137406
1.985672	-0.67505	-0.45739	-0.30786	-0.54538
1.986256	-0.60141	-0.50108	-0.28143	-0.60233
1.827183	-0.82787	0.298369	-0.79887	-0.49881
1.936238	-0.12068	-0.33807	-0.86252	-0.61497
1.887121	0.125972	-0.59731	-0.88665	-0.52913
1.99646	-0.53268	-0.55753	-0.51988	-0.38637
1.733452	-0.73051	-0.42453	0.457116	-1.03553
1.846381	-1.03317	0.049222	-0.70427	-0.15816
1.904527	-0.66839	-0.92377	-0.04689	-0.26548
1.983377	-0.5227	-0.56254	-0.25772	-0.64041
1.815736	-0.85566	-0.09902	0.112812	-0.97387
1.979213	-0.25011	-0.62552	-0.65165	-0.45194
1.982831	-0.69828	-0.44451	-0.54324	-0.2968
1.933868	-0.76484	-0.10784	-0.30645	-0.75474
1.974557	-0.20842	-0.69468	-0.55912	-0.51234
1.852954	-0.82999	0.252269	-0.61603	-0.6592
1.958409	-0.11183	-0.5165	-0.68499	-0.64509
1.996438	-0.52582	-0.58916	-0.40788	-0.47358
1.931571	-0.49814	-0.11311	-0.92138	-0.39893
1.768445	-0.86408	0.426838	-0.8355	-0.4957
1.732968	-0.57826	0.515187	-0.87197	-0.79793
1.726811	-0.35668	0.394405	-0.58347	-1.18106
1.903033	-0.57365	-0.86685	0.077761	-0.54029
1.910214	0.022991	-0.48752	-0.90706	-0.53862
1.954729	-0.77375	-0.39779	-0.63182	-0.15137
1.913832	-0.22879	-0.25642	-1.02949	-0.39914
1.907635	-0.51248	-0.14127	-0.2447	-1.00919
1.893074	-1.06386	-0.15682	-0.46624	-0.20616
1.859748	-0.917	0.19573	-0.48536	-0.65312
1.791337	-0.84555	-1.03006	0.118277	-0.03401
1.890427	0.112171	-0.45596	-0.84318	-0.70345
1.936194	-0.78598	-0.39483	-0.68769	-0.0677
1.424187	-0.18084	-0.05778	-1.6494	0.463827
1.956871	-0.67833	-0.74275	-0.35542	-0.18038
1.970936	-0.37543	-0.42515	-0.81846	-0.35189
1.468928	-0.4821	-1.27423	-0.54364	0.831038
1.967852	-0.15786	-0.67764	-0.53206	-0.60029
1.923982	0.025796	-0.57397	-0.79222	-0.58359
1.925235	-0.09465	-0.61232	-0.89852	-0.31974
1.982141	-0.23931	-0.55143	-0.60101	-0.59039
1.800425	-0.42868	0.104715	-1.22494	-0.25152
1.960196	-0.1996	-0.41589	-0.81502	-0.52969
1.929873	-0.66466	-0.86856	-0.21774	-0.17892
1.900856	-0.25148	-0.93891	-0.04736	-0.6631
1.96931	-0.4828	-0.63021	-0.67809	-0.17822
1.954196	-0.71796	-0.43909	-0.67708	-0.12007
1.994568	-0.40028	-0.51595	-0.62243	-0.45591
1.848937	-0.37494	0.199673	-0.85086	-0.82282
1.989447	-0.57021	-0.41518	-0.64437	-0.35969

1.835843	-0.25722	-0.96462	0.164445	-0.77845
1.992424	-0.52201	-0.39186	-0.64548	-0.43307
1.957893	-0.27846	-0.51723	-0.8515	-0.3107
1.891251	-0.13337	-1.07201	-0.44532	-0.24055
1.511894	-0.6887	0.189366	-1.42589	0.413333
1.998343	-0.48554	-0.43724	-0.5125	-0.56306
1.614053	-0.9339	-1.12965	-0.045	0.494501
1.810906	-0.91783	-0.30055	0.257061	-0.84958
1.709622	-0.30812	-1.02869	0.467261	-0.84007
1.889197	-0.40637	-0.05131	-1.06112	-0.3704
1.935879	-0.823	-0.69966	-0.19813	-0.21509
1.897185	-0.07052	-0.46884	-0.32251	-1.03533
1.949181	-0.71895	-0.36856	-0.7281	-0.13358
1.903802	-0.03767	-0.25839	-0.89027	-0.71748
1.940621	-0.02453	-0.71441	-0.57856	-0.62312
1.957959	-0.13768	-0.76498	-0.4756	-0.5797
1.654109	-0.6415	-0.13733	-1.28925	0.413972
1.978856	-0.22764	-0.52905	-0.5583	-0.66386
1.909505	-0.01977	-0.34545	-0.91646	-0.62782
1.96209	-0.83677	-0.30603	-0.30779	-0.51151
1.695279	-0.50702	-0.84557	-0.91136	0.568673
1.918241	-0.78176	-0.3904	0.000102	-0.74618
1.948306	-0.30145	-0.19197	-0.82239	-0.6325
1.994693	-0.6141	-0.53033	-0.45118	-0.39908
1.979622	-0.43769	-0.60393	-0.263	-0.675
1.970092	-0.33006	-0.80055	-0.32877	-0.5107
1.763148	-0.76154	0.061409	-1.14085	0.077831
1.915078	-0.95691	-0.06066	-0.37679	-0.52071
1.906377	-0.01811	-0.42875	-0.97108	-0.48844
1.864103	-0.71871	-0.98103	0.045577	-0.20994
1.930771	-0.88754	-0.1203	-0.60983	-0.3131
1.797447	-0.7674	0.336871	-0.426	-0.94092
1.947202	-0.41289	-0.3157	-0.92216	-0.29646
1.984265	-0.32628	-0.53905	-0.70027	-0.41866
1.974017	-0.35228	-0.28955	-0.60305	-0.72914
1.951159	-0.40432	-0.77485	-0.12994	-0.64204
1.841773	-0.47425	-1.16112	-0.18524	-0.02116
1.958915	-0.71419	-0.11495	-0.54064	-0.58914
1.960903	-0.25833	-0.32694	-0.55544	-0.8202
1.90467	-0.31646	-1.04847	-0.1534	-0.38634
1.826233	-0.0656	-0.16503	-1.22227	-0.37333
1.942563	-0.50967	-0.89703	-0.17188	-0.36399
1.597177	-0.57461	-0.95188	0.742562	-0.81324

1.694732	-0.43269	-0.85186	-0.95705	0.546864
1.825787	-0.10991	-0.54907	-1.16316	-0.00366
1.899305	-0.9545	-0.0021	-0.60777	-0.33494
1.917591	-0.13696	-0.55807	-0.96151	-0.26105
1.987623	-0.36936	-0.69417	-0.50722	-0.41687
1.80249	-0.92795	-0.93794	0.095741	-0.03235
1.826785	-0.48672	0.239347	-1.03608	-0.54333
1.985366	-0.57614	-0.34038	-0.67462	-0.39422
1.87969	0.168221	-0.64469	-0.56318	-0.84004
1.966676	-0.51714	-0.7054	-0.58454	-0.15959
1.758238	-0.53421	-0.01467	-1.27241	0.063045
1.923608	-0.8222	-0.49871	-0.61231	0.009605
1.90649	-0.75607	-0.10145	-0.86595	-0.18302
1.938398	-0.66656	-0.83093	-0.29349	-0.14742
1.744339	-0.16339	-1.05162	0.320395	-0.84972
1.31828	0.146233	-0.43256	-1.63874	0.606782
1.905471	0.103249	-0.58283	-0.67944	-0.74645
1.733874	-0.00635	0.094632	-0.50825	-1.31391
1.973371	-0.24597	-0.70572	-0.39827	-0.62341
1.684441	-0.88849	-0.80928	0.605924	-0.5926
1.955533	-0.46137	-0.17751	-0.83823	-0.47842
1.926838	-0.0414	-0.71258	-0.37429	-0.79858
1.794082	-0.3919	0.026775	-0.16402	-1.26494
1.771752	-0.50174	0.185799	-1.23532	-0.22049
1.907427	-0.61257	-0.80323	-0.57782	0.086189
1.925849	-0.91066	-0.23429	-0.61629	-0.16461
1.623813	-0.31886	-0.07308	-1.48123	0.249354
1.944338	-0.37705	-0.39149	-0.92912	-0.24667
1.901412	0.056315	-0.58745	-0.46431	-0.90597
1.836158	-0.48355	-0.57963	-1.00293	0.229944
1.608109	0.074167	0.291133	-0.5528	-1.42061
1.807032	0.014924	-0.40209	-1.24131	-0.17856
1.729206	-0.16749	-0.85012	-1.06499	0.353394
1.614149	-0.91578	-0.93583	-0.45218	0.689648
1.954103	-0.44257	-0.35274	-0.88744	-0.27135
1.434198	-0.32709	-0.22051	-1.53747	0.650878
1.986739	-0.42151	-0.44704	-0.71749	-0.40069
1.607531	-0.61639	-0.80671	-0.91929	0.734863
1.974601	-0.69117	-0.62983	-0.40727	-0.24633
1.956615	-0.37389	-0.75642	-0.65636	-0.16994
1.912123	0.070811	-0.65374	-0.54624	-0.78295
1.864663	0.078306	-0.68103	-0.2726	-0.98934
1.563943	-0.6224	-0.69494	0.785917	-1.03251
1.91938	-0.68522	-0.66558	-0.63201	0.063428
1.65963	-0.67968	0.080808	-1.30965	0.248894
1.726835	-0.72867	-0.83696	-0.70253	0.541324
1.883056	0.160976	-0.55389	-0.67048	-0.81967
1.977607	-0.23844	-0.69953	-0.48481	-0.55482
1.974179	-0.41679	-0.79911	-0.3415	-0.41679
1.686047	-0.77864	-0.82938	0.61675	-0.69478
1.925953	-0.61168	-0.7947	0.0141	-0.53367

1.902577	0.106264	-0.70341	-0.54772	-0.75771
1.767915	-0.51192	-0.48127	-1.12244	0.347725
1.851112	0.014443	-1.14554	-0.33088	-0.38913
1.717637	-0.60363	-1.02679	-0.60363	0.516417
1.790712	-0.07974	-0.42959	-1.26578	-0.0156
1.991006	-0.55202	-0.579	-0.31567	-0.54432
1.728947	-0.68988	-1.05632	0.466255	-0.449
1.982244	-0.44065	-0.73504	-0.32917	-0.47739
1.745611	-0.61415	-1.04805	0.445006	-0.52842
1.661065	-0.50249	-1.38234	-0.05011	0.27387
1.958146	-0.61209	-0.70088	-0.53666	-0.10852
1.770751	-0.05062	-0.94739	0.190589	-0.96333
1.429127	-0.82665	-1.35944	0.114219	0.642745
1.910253	-0.22303	-1.03542	-0.2346	-0.4172
1.518923	0.089723	-0.15331	-1.62274	0.16741
1.874973	-0.05727	-0.17522	-0.59585	-1.04664
1.869974	-0.07107	-0.85002	-0.87782	-0.07107
1.561746	-0.83393	-1.2135	-0.12796	0.613642
1.900603	-0.07711	-0.76585	-0.18537	-0.87228
1.531743	-0.50532	-1.20456	0.771559	-0.59343
1.522329	-0.78208	-0.29347	-1.1942	0.747416
1.883878	-0.30957	-0.56419	0.008114	-1.01823
1.706554	-0.24369	-1.42385	-0.0099	-0.02911
1.829968	0.16783	-0.92298	-0.22633	-0.84849
1.889249	-0.15274	-0.57543	-1.029	-0.13208
1.740252	-0.59317	-1.15808	0.378637	-0.36764
1.553486	-0.11294	-0.89796	-1.17141	0.628813
1.498131	-0.30782	-1.59502	0.334377	0.070332
1.811955	-0.08069	-1.21839	-0.47359	-0.03929
1.863707	-0.1243	-0.68237	-1.0219	-0.03513
1.811338	-0.14	-0.85824	0.155725	-0.96883
1.542126	-0.22696	-1.58451	0.026516	0.242826
1.698437	-0.25381	-0.80287	-1.0956	0.453836
1.616338	-0.15791	-1.00453	-1.01765	0.563756
1.844277	-0.80028	-0.96444	-0.1513	0.071751
1.336539	-0.21239	-0.59472	-1.42116	0.891727
1.911654	0.070618	-0.51787	-0.72315	-0.74125
1.954456	-0.81656	-0.42558	-0.55431	-0.15802
1.839903	0.214635	-0.53052	-0.51109	-1.01293
1.905631	-0.64147	-0.9521	-0.1252	-0.18687
1.870457	-0.27142	-0.75644	-0.92157	0.07897
1.795529	0.123167	-1.14629	-0.6588	-0.11361
1.90166	-0.01415	-0.41965	-0.98896	-0.4789
1.825493	-0.03267	-1.1858	-0.49865	-0.10838

1.921977	-0.31662	-0.64757	-0.8837	-0.07409
1.907755	0.061501	-0.78869	-0.45283	-0.72773
1.88289	0.022057	-0.94445	-0.25556	-0.70494
1.800878	-0.62523	-0.94203	-0.59179	0.358174
1.599359	0.136854	-1.40562	-0.60854	0.277939
1.734731	0.133219	-1.08452	-0.88664	0.103207
1.693732	0.220241	-1.26833	-0.68729	0.04165
1.764462	-0.18154	-1.14572	-0.69055	0.253345
1.848952	0.062299	-0.78004	-0.15989	-0.97132
1.87208	0.090643	-0.3026	-0.92369	-0.73643
1.098826	0.365154	-0.54976	-1.66936	0.755144
1.698101	-0.64826	-1.03539	0.551378	-0.56583
1.777205	-0.67688	-1.15974	-0.1056	0.165013
1.20999	-1.36477	0.660425	-0.99758	0.491936
1.52001	-1.40889	-0.04878	-0.62288	0.560553
1.101528	-1.45463	-0.83288	0.223048	0.962936
1.420801	-1.47982	0.198088	-0.67885	0.539785
1.239735	-1.24534	0.79527	-1.09051	0.300849
0.975453	-1.56292	-0.71473	0.299622	1.002573
0.875215	-1.352	-0.51662	-0.41044	1.403835
1.292377	-1.3533	0.105042	-0.88413	0.840011
1.618882	-1.30679	-0.04078	-0.69826	0.426948
1.628488	-1.41139	-0.1443	-0.44416	0.371359
-0.09203	-1.21239	-0.42913	-0.09106	1.824609
1.152193	-1.45209	0.078657	-0.76495	0.986187
1.429995	-1.47152	-0.12597	-0.53262	0.700125
1.143035	-1.67787	-0.02929	-0.3172	0.881327
1.060178	-1.53577	-0.28837	-0.37381	1.137777
1.2484	-1.2167	-0.72989	-0.42054	1.118727
0.708859	-0.84094	-1.2104	-0.1726	1.515084
0.735782	-1.50797	-0.44801	-0.17714	1.39734
1.044132	-0.87148	-0.81938	-0.74195	1.388681
1.044619	-0.93789	-0.81233	-0.67684	1.38244
1.011187	-1.38017	-0.82604	0.016037	1.178992
1.085178	-1.46199	-0.70024	-0.01582	1.092874
0.577263	-1.22997	-0.64904	-0.32005	1.621804
0.984954	-0.56204	-0.80122	-1.03568	1.413994
1.241923	-1.16866	-1.03843	-0.04075	1.005916
1.319075	-0.7485	-0.93577	-0.7548	1.119998
0.662427	-1.14237	-1.09445	0.147279	1.427117
0.928002	-1.14488	-0.61821	-0.60647	1.441554
0.7032	-0.95517	-0.9703	-0.36476	1.587022
1.435351	-1.04608	0.741592	0.007326	-1.13819
1.130128	-0.1176	0.674427	0.113393	-1.80035
1.864808	0.228585	-0.66128	-0.7772	-0.65491
1.825191	0.21135	-0.29072	-0.96106	-0.78476
1.89689	0.095949	-0.80543	-0.73319	-0.45422
1.777832	0.258673	-0.30228	-1.16482	-0.56941
1.857285	0.247686	-0.67612	-0.78924	-0.6396
1.815492	0.338945	-0.58976	-0.87502	-0.68965
1.840747	0.266385	-0.54388	-0.88887	-0.67438
1.77601	0.397365	-0.8905	-0.45168	-0.83119
1.830295	0.314407	-0.68105	-0.64183	-0.82183
1.731908	0.47972	-1.00914	-0.48081	-0.72168
1.459413	0.721429	-0.03165	-1.21447	-0.93472

1.785979	0.376636	-0.99009	-0.60553	-0.56699
1.758158	0.359123	-0.31111	-1.06433	-0.74183
1.77677	0.340653	-0.97651	-0.81808	-0.32284
1.861274	0.098221	-0.8059	-0.90172	-0.25188
1.744915	0.507359	-0.75219	-0.80897	-0.69112
1.610937	0.127484	0.369016	-1.17983	-0.9276
1.726558	0.201521	-0.08021	-1.28769	-0.56017
1.865312	0.147958	-0.81711	-0.83855	-0.35761
1.856475	0.245659	-0.59439	-0.7939	-0.71384
1.598021	0.333735	-0.79955	-1.2923	0.160095
1.874775	0.092348	-0.60857	-0.98218	-0.37637
1.558338	0.197874	0.415998	-1.07822	-1.09399
1.91939	0.02036	-0.83183	-0.62411	-0.48381
1.753212	0.491275	-0.69654	-0.80278	-0.74516
1.212373	0.724521	0.422606	-1.32638	-1.03312
1.806545	0.281018	-0.9787	-0.76033	-0.34854
1.837271	0.238366	-0.71493	-0.93602	-0.42469
1.742176	0.331806	-0.94706	-0.96522	-0.1617
1.639616	0.265287	-0.26429	-1.46281	-0.1778
1.730926	-0.05921	-1.34661	0.096465	-0.42157
1.819382	0.271967	-0.378	-0.90774	-0.80561
1.580925	0.408383	0.103649	-1.30428	-0.78868
1.48548	0.256864	0.3872	-1.45845	-0.67109
1.670972	0.538298	-0.4191	-0.63043	-1.15975
1.634191	0.223336	-0.02168	-1.46286	-0.37299
1.781989	0.402973	-0.52018	-0.7786	-0.88618
1.597964	0.521181	-0.31791	-0.42543	-1.3758
1.846587	0.233419	-0.78351	-0.84986	-0.44664
1.711375	0.398448	-0.16871	-0.97251	-0.9686
1.745422	0.487674	-0.5565	-0.82252	-0.85408
1.591739	0.494336	0.009652	-0.93413	-1.1616
1.397755	0.489813	0.256625	-1.54206	-0.60214
1.844142	0.251994	-0.91482	-0.56843	-0.61289
1.842659	0.267849	-0.84122	-0.73382	-0.53547
1.751166	0.458091	-0.50092	-0.77218	-0.93616
1.822517	0.325424	-0.81099	-0.77121	-0.56574
1.194158	1.076491	-0.79958	-1.32452	-0.14655
1.335846	0.819127	-1.32364	-0.88841	0.057074
1.614151	0.366467	0.097984	-1.25182	-0.82679
1.767948	0.31781	-1.05244	-0.25989	-0.77343
1.654223	0.515157	-0.19049	-1.03341	-0.94549
1.591545	0.352788	0.026474	-1.4325	-0.53831
1.658083	0.623947	-0.47236	-0.92571	-0.88396
1.678998	0.623276	-0.88685	-0.66148	-0.75394
1.642294	0.676528	-0.61681	-0.94129	-0.76072
1.45476	0.85224	-0.27004	-0.94836	-1.0886
1.473201	0.603162	-0.07685	-1.47983	-0.51968
1.663254	0.638224	-0.92319	-0.79901	-0.57928
1.754352	0.478075	-0.59921	-0.83833	-0.79488

1.562491	0.81498	-0.87337	-0.76778	-0.73633
1.873579	0.094439	-0.76688	-0.89324	-0.3079
1.399018	0.861194	-0.3001	-1.36085	-0.59926
1.741174	0.368537	-0.50147	-1.18346	-0.42477
1.679238	0.404677	-0.30185	-1.30177	-0.48029
1.713338	0.191582	0.036517	-1.23703	-0.70441
1.699011	0.385749	-0.19297	-0.68333	-1.20846
1.639553	0.312445	0.142421	-1.02898	-1.06544
1.784911	0.343221	-0.68661	-1.02561	-0.41591
1.839565	0.294564	-0.62667	-0.74146	-0.76599
1.711179	0.297181	-0.03694	-1.12523	-0.8462
1.73819	0.462614	-0.99782	-0.75224	-0.45074
1.753619	0.4195	-0.74628	-1.00835	-0.41849
1.398757	0.889532	-0.56349	-1.33528	-0.38953
1.47091	0.605759	-0.31877	-1.52034	-0.23756
1.695115	0.370696	-0.17201	-0.6585	-1.2353
1.724556	0.429567	-0.76467	-1.07641	-0.31304
1.692853	0.556365	-1.04568	-0.66096	-0.54258
1.582431	0.567404	-0.2988	-1.35651	-0.49453
1.654206	0.667178	-0.85915	-0.8062	-0.65603
1.600423	0.40148	-0.90247	-1.20494	0.105507
1.724338	0.434403	-1.07776	-0.32587	-0.75512
1.558267	0.447929	-0.06249	-1.46087	-0.48284
1.692458	0.176119	-1.24145	0.114643	-0.74177
1.6999	0.330692	-1.32311	-0.36074	-0.34674
1.706272	0.525153	-0.69007	-1.04341	-0.49794
1.695624	0.526903	-0.52368	-1.10433	-0.59452
1.549012	0.728745	-0.29037	-0.92184	-1.06555
1.682307	0.545463	-0.65896	-1.10651	-0.4623
1.513074	0.721427	-1.26343	-0.73292	-0.23815
1.649413	0.606702	-0.52134	-0.60775	-1.12703
0.734262	1.018346	-1.81733	-0.21161	0.276331
1.639267	0.631094	-0.91696	-0.39575	-0.95765
1.674145	0.621982	-0.89131	-0.82322	-0.5816
1.607149	0.66177	-0.40322	-1.12795	-0.73775
1.646827	0.47666	-0.6091	-0.2361	-1.27828
1.672944	0.477937	-1.03512	-0.93131	-0.18445
1.642263	0.323434	-1.34108	0.007688	-0.63231
1.664292	0.546836	-0.80614	-1.08606	-0.31893
1.263522	0.814929	0.087992	-1.52193	-0.64452
1.260862	0.69836	-0.21681	-1.69507	-0.04734
1.568805	0.786086	-0.56548	-0.90348	-0.88593
1.6374	0.684813	-0.69533	-0.96023	-0.66665
1.642058	0.691948	-0.84379	-0.78044	-0.70977
1.605294	0.548972	-0.39016	-0.42741	-1.3367
1.310815	0.783705	-0.49726	-1.55516	-0.0421
1.6106	0.743414	-0.73898	-0.84653	-0.7685
1.492107	0.757242	-0.8362	-1.20781	-0.20534
1.478451	0.897387	-0.50245	-0.96522	-0.90817

1.484998	0.743932	-0.48685	-1.36445	-0.37763
1.649968	0.648131	-0.5783	-0.71116	-1.00864
1.436408	0.732459	-0.82605	-0.03254	-1.31027
1.698063	0.51035	-0.75089	-0.38957	-1.06795
1.542603	0.799808	-1.00798	-0.47446	-0.85997
1.561561	0.629615	-1.13832	-0.12951	-0.92335
1.328885	1.094206	-0.76307	-1.0258	-0.63422
1.25916	0.914473	-0.05341	-1.46492	-0.6553
1.722036	0.546856	-0.73361	-0.67066	-0.86463
1.270701	0.939555	-0.23749	-1.48651	-0.48625
1.463483	0.87622	-0.387	-1.10704	-0.84567
1.36626	1.002796	-0.72662	-0.46698	-1.17545
1.733429	0.287533	-1.24541	-0.21347	-0.56208
1.510073	0.822551	-0.89421	-0.39597	-1.04244
1.297301	0.998781	-0.26447	-1.32058	-0.71104
0.930903	1.45112	-1.01521	-0.86079	-0.50603
1.601427	0.255112	-1.52035	-0.13367	-0.20252
0.105831	1.673584	-0.91559	-1.13067	0.266841
1.342112	0.843291	0.038756	-1.03254	-1.19162
1.355303	0.996824	-0.34475	-1.13759	-0.86978
1.580049	0.743748	-0.69569	-1.07951	-0.5486
1.557891	0.50034	-1.18979	0.080532	-0.94898
1.253191	1.065882	-0.28849	-0.74276	-1.28782
1.623102	0.628822	-0.33375	-0.86153	-1.05664
1.299189	1.136185	-0.6623	-0.95952	-0.81355
1.636405	0.612703	-0.78491	-1.09179	-0.37241
1.528445	0.844019	-0.57293	-0.85459	-0.94494
1.237576	1.083444	-0.51387	-0.4577	-1.34944
1.241586	1.141982	-0.74571	-0.46048	-1.17738
0.530732	0.846944	-1.95726	0.256693	0.322889
1.411546	0.815724	-1.15906	-0.9968	-0.07141
0.860165	1.160789	-1.25736	-1.10297	0.339378
1.282416	1.050497	-0.24822	-1.1353	-0.94939
1.433488	0.952128	-1.08778	-0.56795	-0.72988
1.0587	1.35655	-0.82544	-1.0115	-0.57831
1.371447	0.86787	-0.89593	-0.09684	-1.24654
1.407006	0.996325	-1.00087	-0.84697	-0.55549
1.454428	0.766431	-1.38974	-0.51672	-0.3144
1.363167	0.967057	-0.95809	-1.10276	-0.26938
1.570089	0.774648	-0.73473	-1.02732	-0.58269

1.332734	1.051464	-0.90241	-0.41992	-1.06187
1.064055	1.34177	-1.0519	-0.52798	-0.82595
0.765924	1.22639	-1.05871	-1.28919	0.355588
1.314117	1.066131	-0.5185	-1.19026	-0.67149
1.526171	0.787371	-0.97358	-0.99033	-0.34964
1.439202	0.97785	-0.84666	-0.89151	-0.67888
0.865849	1.489065	-1.11543	-0.51815	-0.72133
1.213213	1.224895	-0.66325	-0.96737	-0.80749
1.021903	1.335352	-0.37852	-1.17854	-0.80019
1.184461	1.037698	-0.21075	-1.48159	-0.52981
1.263529	1.178697	-0.89839	-0.85953	-0.68431
1.115373	1.234268	-0.27527	-0.98593	-1.08844
1.043426	1.212447	-1.18046	-1.02222	-0.0532
1.648705	0.642986	-0.99012	-0.79389	-0.50768
1.486603	0.890571	-0.90711	-0.95125	-0.51881
1.076018	1.271528	-0.29113	-0.9137	-1.14272
1.393401	0.989019	-0.89707	-1.03628	-0.44907
1.479238	0.735033	-0.64907	-1.34178	-0.22342
1.460782	0.934923	-0.9475	-0.57306	-0.87515
1.539353	0.808634	-0.6009	-1.08481	-0.66228
1.50173	0.667605	-1.23911	-0.87197	-0.05826
1.271024	0.983497	-0.19569	-1.38966	-0.66917
1.191962	1.105357	-0.41251	-1.39572	-0.48909
1.218235	1.225748	-0.93164	-0.72536	-0.78699
1.358331	1.027983	-0.55773	-1.15436	-0.67422
1.221685	1.182469	-0.51243	-1.11521	-0.77651
1.393052	1.013178	-0.60158	-1.04827	-0.75638
1.145573	1.105386	-0.06018	-0.91875	-1.27203
1.188419	1.258494	-0.87165	-0.74667	-0.82859
1.083107	1.323064	-0.91318	-0.99945	-0.49354
1.147487	1.268268	-0.54749	-0.81204	-1.05623
1.068926	1.182747	-0.46063	-1.46227	-0.32878

1.167504	1.251318	-0.5892	-1.07328	-0.75634
1.224992	1.167914	-0.56959	-1.18433	-0.63898
0.777785	1.073955	-1.71716	-0.44412	0.309531
0.779417	1.350192	-1.54634	-0.22754	-0.35573
1.093038	1.118855	-0.37087	-1.52101	-0.32001
1.343357	0.917832	-1.3301	-0.19137	-0.73971
1.142023	1.123226	-0.08316	-1.24349	-0.9386
1.146355	1.29639	-0.85313	-0.87839	-0.71122
1.071895	1.202263	-0.30206	-1.41604	-0.55606
1.244107	1.185859	-1.03746	-0.70237	-0.69014
1.176937	1.187432	-0.54466	-1.26517	-0.55454
0.996972	1.259282	-0.06818	-0.98971	-1.19837
0.893548	1.373482	-0.40505	-1.38792	-0.47406
0.965763	0.930336	-1.78737	-0.02997	-0.07876
0.309961	1.227103	-1.81761	-0.02565	0.306205
0.832391	1.427637	-1.25275	-0.19356	-0.81372
1.036071	1.392462	-0.8121	-0.70411	-0.91232
1.274186	1.136454	-0.69892	-1.11222	-0.59949
1.418962	0.960137	-0.47594	-1.06811	-0.83504
1.407531	0.982807	-0.92745	-0.48369	-0.9792
1.103632	1.065957	0.025362	-1.44112	-0.75383
0.841694	1.492259	-1.12309	-0.7929	-0.41796
1.204999	1.235621	-0.73534	-0.96776	-0.73752
1.527143	0.758368	-0.79582	-1.16305	-0.32664
1.222454	1.168859	-1.19129	-0.59065	-0.60937
1.431861	0.992926	-0.80414	-0.85496	-0.76569
1.279033	1.104787	-0.89678	-1.08533	-0.40171
1.033329	1.295537	-0.36064	-1.28973	-0.6785
0.959487	1.402767	-0.90237	-1.07033	-0.38955
1.458308	0.958213	-0.87392	-0.74281	-0.79979
0.791422	1.542913	-0.59063	-1.12082	-0.62288
1.1455	1.264508	-1.0831	-0.53041	-0.79649
1.265543	0.960031	-0.45715	-1.47755	-0.29088
1.22435	1.189854	-1.03435	-0.51197	-0.86788
1.244556	1.149511	-0.95786	-0.41739	-1.01882
0.804457	1.446846	-0.48635	-1.36488	-0.40007
1.103671	1.108889	-1.46654	-0.62127	-0.12475
1.291229	0.879746	-0.59967	-1.48016	-0.09115
1.212498	1.209496	-0.81276	-1.04081	-0.56843
0.842332	1.519917	-1.03103	-0.79058	-0.54064
1.063119	1.291161	-0.31468	-1.1286	-0.91099
0.840667	1.454588	-0.76449	-1.2244	-0.30637
1.381043	0.757162	-1.017	-1.21504	0.093839
0.984021	1.437076	-0.82083	-0.72162	-0.87864
0.369944	1.169232	-1.81229	0.431814	-0.1587
1.05956	1.172089	-0.24713	-1.47873	-0.50579
1.169026	1.248467	-0.6593	-1.09636	-0.66183
0.86669	1.514237	-0.90054	-0.89702	-0.58337

1.462801	0.952365	-0.76747	-0.88196	-0.76574
1.154955	1.282328	-0.78753	-0.96747	-0.68229
0.94178	1.397228	-0.30562	-1.02274	-1.01065
1.145759	1.291184	-0.92143	-0.8642	-0.65131
0.929532	1.47048	-0.64856	-0.77766	-0.97379
0.983471	1.411353	-0.58776	-1.08055	-0.72651
0.768331	1.550227	-0.41777	-1.06316	-0.83763
0.584291	1.604509	-1.29748	-0.48728	-0.40403
0.841858	1.411589	-1.36274	-0.59526	-0.29545
1.37075	1.041565	-0.68358	-1.05755	-0.67119
0.75227	1.574041	-0.77835	-1.05017	-0.49779
1.010261	1.321309	-0.27039	-1.16509	-0.89609
0.900375	1.466368	-0.95149	-0.96481	-0.45045
0.587772	1.542361	-1.28594	-0.05763	-0.78656
1.150255	1.192061	-0.28833	-1.20496	-0.84903
1.252861	1.188798	-0.897	-0.87108	-0.67358
1.157865	1.238547	-0.55992	-1.16873	-0.66776
0.890004	1.224399	-0.12425	-1.59213	-0.39803
0.719378	1.283832	-0.49396	-1.60649	0.097246
0.877631	1.49974	-0.56026	-0.81993	-0.99718
1.254132	1.102485	-0.41282	-1.24766	-0.69614
1.312367	0.734392	-1.48325	-0.71739	0.15388
0.874477	1.46555	-0.51369	-1.19229	-0.63405
1.245943	1.05962	-1.36116	-0.58651	-0.35789
1.236784	1.098915	-0.24701	-0.9433	-1.14539
0.606929	1.554364	-1.383	-0.38532	-0.39297
0.91471	1.424663	-1.07809	-0.92724	-0.33404
1.140924	1.231016	-0.38453	-1.1671	-0.8203
0.660068	1.386137	-0.2222	-1.59393	-0.23007
0.662532	1.186591	-1.69481	0.289432	-0.44375
0.629009	1.376845	-1.12199	0.285797	-1.16966
0.976798	1.318202	-0.32598	-1.34743	-0.62159
1.285429	1.155609	-0.7435	-0.94529	-0.75225
0.788719	1.52149	-0.38408	-1.13697	-0.78916
1.285436	1.148446	-0.70103	-1.0004	-0.73245
0.061102	1.434429	-1.69394	0.256638	-0.05823
1.066145	1.293256	-0.60633	-1.25506	-0.49802
1.195023	1.159827	-0.55892	-1.28632	-0.50961
0.967632	1.417426	-0.75511	-1.09429	-0.53566
0.352933	1.622234	-1.08997	0.133584	-1.01878
1.279837	1.147724	-0.70623	-1.04024	-0.68109
0.798494	1.415701	-1.03108	-1.13708	-0.04604
0.816403	1.454532	-0.30213	-1.29136	-0.67745

0.64305	1.662405	-0.5979	-0.9152	-0.79235
1.075223	1.310834	-0.84903	-1.10283	-0.4342
0.982389	1.433987	-0.65888	-0.87272	-0.88478
1.076638	1.356187	-0.94506	-0.77344	-0.71433
0.708709	1.613465	-0.58465	-1.01565	-0.72187
1.193506	1.189579	-1.0876	-0.91083	-0.38465
0.586323	1.497021	-1.45218	-0.08412	-0.54705
0.545626	1.704514	-0.73306	-0.99176	-0.52532
1.337237	1.088315	-0.76277	-1.00905	-0.65372
1.08533	1.275961	-0.89644	-0.334	-1.13085
1.18545	1.085717	-1.01591	-1.17353	-0.08173
0.845705	1.370534	-0.05359	-1.26163	-0.90102
0.783555	1.150592	-1.45687	-0.88121	0.403939
0.61003	1.383687	-1.61404	-0.05552	-0.32416
0.685959	1.584652	-0.37198	-1.14626	-0.75237
0.517596	1.418688	-1.46781	0.242852	-0.71133
0.453609	1.558584	-0.60096	-1.41559	0.004353
0.569258	1.673902	-0.8345	-1.00883	-0.39983
0.792964	1.180877	-0.43777	-1.66395	0.12788
0.831068	1.509399	-0.95648	-0.4105	-0.97349
0.561205	1.639579	-0.56177	-1.23064	-0.40837
0.808306	1.409763	-0.16798	-1.36383	-0.68626
1.010703	1.380264	-0.47403	-1.03373	-0.8832
0.733985	1.564297	-0.53014	-1.17564	-0.59251
0.738709	1.520483	-0.23348	-1.14736	-0.87835
1.04677	1.125813	0.080763	-1.34058	-0.91277
0.054663	1.56326	-1.37325	-0.6869	0.442232
0.789592	1.396625	-0.14406	-1.4211	-0.62106
0.840714	1.225346	-0.90741	-1.38465	0.226002
1.295294	1.128976	-0.71968	-1.04835	-0.65625
0.780664	1.41704	-0.71725	-1.36161	-0.11884
0.308079	1.64349	-1.2581	0.089593	-0.78307
0.523153	1.67893	-0.55221	-1.17245	-0.47742
0.90825	1.49127	-0.76258	-0.69624	-0.9407
0.630788	1.677889	-0.82952	-0.78825	-0.69091
0.740718	1.611414	-0.70506	-0.84752	-0.79955
0.390564	1.788091	-0.89579	-0.5299	-0.75297
0.739448	1.418465	-1.44728	-0.13845	-0.57218
0.837267	1.178807	0.326272	-1.34388	-0.99847
0.684256	1.589716	-0.33011	-0.91571	-1.02816
0.882947	1.510149	-0.65451	-0.86126	-0.87733
0.798846	1.443581	-0.20876	-1.30531	-0.72835
0.22299	1.638279	-1.35872	-0.63462	0.132077
0.405432	1.791775	-0.82288	-0.64446	-0.72987
0.807614	1.560138	-0.6591	-0.95325	-0.75541
1.263232	1.178855	-0.94641	-0.7607	-0.73498

0.891663	1.449145	-0.3792	-1.11714	-0.84447
0.836945	1.510447	-1.0747	-0.47322	-0.79947
0.497535	1.741018	-0.63322	-0.92877	-0.67657
0.406197	1.749874	-1.01096	-0.7908	-0.35431
0.474428	1.658444	-0.57785	-1.26819	-0.28684
0.422424	1.604444	-0.99085	-1.1217	0.085675
0.593519	1.640999	-0.30477	-0.96248	-0.96727
0.975471	1.370521	-0.6849	-0.42965	-1.23144
0.805638	1.389037	-0.3309	-1.46817	-0.39561
0.696132	1.594916	-0.38534	-0.89244	-1.01326
0.835439	1.519589	-0.78901	-1.05148	-0.51454
0.676822	1.490964	-0.1083	-1.33509	-0.7244
0.320369	1.782725	-1.05064	-0.35075	-0.7017
0.784767	1.561498	-0.51581	-0.96277	-0.86768
1.065025	1.065025	-1.00767	-1.29814	0.175754
0.882984	1.474873	-0.45388	-0.83685	-1.06713
0.824793	1.370427	-1.24574	-0.94326	-0.00622
0.625361	1.667219	-0.59381	-0.97935	-0.71942
0.617911	1.682923	-0.79594	-0.65224	-0.85266
0.652608	1.442054	0.102538	-1.28599	-0.91121
0.586328	1.241433	-1.74591	-0.21911	0.137265
0.749305	1.468505	-0.427	-1.3935	-0.3973
0.528052	1.72374	-0.83192	-0.86769	-0.55218
0.598862	1.684236	-0.66429	-0.97244	-0.64637
0.432111	1.776887	-0.63143	-0.86906	-0.7085
0.908142	1.469563	-0.84059	-1.02125	-0.51586
0.459027	1.698712	-0.75183	-1.12136	-0.28454
0.795426	1.553541	-0.9052	-0.50719	-0.93658
0.909602	1.369967	-0.55968	-1.36172	-0.35817
0.935226	1.215839	0.081271	-1.38895	-0.84339
0.564244	1.397035	-1.1011	-1.18757	0.327388
0.008765	1.452175	-1.62555	-0.26058	0.425191
1.111839	1.196731	-0.73364	-1.3134	-0.26153
0.314885	1.819678	-0.92877	-0.59872	-0.60707
0.488789	1.745655	-0.69945	-0.61512	-0.91988
0.105667	1.656153	-1.44583	-0.38782	0.071832
0.485277	1.414494	-1.60752	0.115499	-0.40775
0.481529	1.327775	-0.70579	-1.52595	0.422445
0.541725	1.711721	-0.55707	-0.96517	-0.73121
0.376452	1.731584	-0.75441	-1.1096	-0.24402
0.889518	1.492624	-0.55217	-0.88723	-0.94275
0.554556	1.697402	-0.46823	-0.91647	-0.86726
0.740437	1.557485	-0.45391	-1.16672	-0.67729
0.671074	1.58868	-1.2171	-0.50056	-0.5421
0.434096	1.740038	-0.8442	-0.97057	-0.35936
0.496392	1.653065	-0.21822	-1.19855	-0.73269
0.541762	1.633051	-0.16938	-0.9966	-1.00883
0.54774	1.721501	-0.64881	-0.84601	-0.77441
0.702937	1.589669	-1.15274	-0.55628	-0.58359
0.602302	1.460544	-0.27154	-1.53827	-0.25303
0.480857	1.735082	-0.90747	-0.45584	-0.85263
0.24728	1.758048	-0.81609	-1.08195	-0.10729
0.844808	1.509155	-1.11282	-0.62967	-0.61147

0.937706	1.453103	-0.61581	-1.05283	-0.72217
0.674972	1.632649	-0.83422	-0.51592	-0.95749
0.758057	1.574732	-0.49693	-0.99991	-0.83594
0.356905	1.270906	-1.70043	-0.38992	0.462531
0.308545	1.761786	-0.52867	-0.36275	-1.17891
1.178485	1.190755	-1.25173	-0.52684	-0.59068
0.4153	1.767459	-0.73072	-0.48575	-0.96628
0.671645	1.528463	-0.12835	-1.19421	-0.87755
0.448929	1.36182	-1.63724	0.265595	-0.4391
0.379109	1.788446	-0.81609	-0.87386	-0.47761
0.354093	1.265486	-1.74415	-0.27189	0.396464
0.53325	1.697829	-1.01729	-0.78226	-0.43153
0.068575	1.891628	-0.92275	-0.63581	-0.40164
1.131775	1.312902	-0.84762	-0.77007	-0.82698
0.71648	1.526065	-0.34626	-1.29451	-0.60178
0.57414	1.620335	-0.50615	-1.27047	-0.41786
0.282672	1.839264	-0.70785	-0.57226	-0.84183
0.369211	1.759512	-0.69875	-0.35643	-1.07355
0.335066	1.645728	-0.34888	-1.41846	-0.21346
0.345633	1.410084	-1.43237	-0.78946	0.466115
0.179752	1.83667	-0.70686	-0.99917	-0.3104
-0.02368	1.62133	-1.1109	0.470125	-0.95687
0.754851	1.466623	-0.5604	-1.37164	-0.28943
0.144154	1.892423	-0.68171	-0.76516	-0.5897
0.170909	1.873208	-0.89446	-0.55287	-0.59679
0.160924	1.846898	-1.00168	-0.33944	-0.66671
0.425035	1.78389	-0.79108	-0.76429	-0.65356
0.371058	1.762766	-0.31371	-0.90148	-0.91864
0.179459	1.856659	-0.90882	-0.73578	-0.39152
0.229049	1.858981	-0.79201	-0.75894	-0.53708
0.367052	1.798339	-0.54753	-0.91552	-0.70233
0.396362	1.795425	-0.65632	-0.83759	-0.69788
-0.15769	1.655027	-1.45616	0.21897	-0.26015
0.148497	1.658062	-1.22381	0.238376	-0.82113
0.188684	1.87978	-0.74427	-0.6536	-0.6706
-0.07553	1.827922	-1.16158	-0.04124	-0.54957
0.066759	1.911476	-0.80785	-0.53894	-0.63145
1.010433	1.418066	-0.81064	-0.77545	-0.84241
0.635897	1.644747	-0.50194	-1.05758	-0.72112
0.761163	1.544165	-0.76453	-1.13417	-0.40664
0.43194	1.741623	-0.52923	-1.09436	-0.54998
0.42963	1.512898	-0.92336	-1.26939	0.250222
0.28157	1.76554	-0.99855	-0.16599	-0.88257
0.304177	1.534448	-1.5709	0.023288	-0.29102
0.302496	1.57794	-1.35574	-0.73294	0.208248
0.89195	1.486567	-0.66323	-0.65324	-1.06205

0.242398	1.821423	-0.42225	-1.04213	-0.59944
0.441963	1.751258	-0.44529	-0.79699	-0.95094
0.104177	1.89821	-0.64178	-0.83593	-0.52468
0.20861	1.670394	-0.89932	0.172578	-1.15226
0.24932	1.832071	-0.76126	-0.91547	-0.40466
-0.2332	1.901168	-1.06302	-0.39782	-0.20712
0.252951	1.670249	-1.42006	-0.29127	-0.21187
0.611187	1.656752	-0.80732	-1.01592	-0.4447
0.323292	1.82748	-0.66768	-0.81303	-0.67006
0.062504	1.863234	-0.77907	-0.93394	-0.21272
-0.1281	1.884408	-1.08981	-0.44002	-0.22648
0.830722	1.52328	-0.57703	-1.08564	-0.69133
0.369309	1.732328	-0.54249	-1.19963	-0.35952
0.329433	1.771212	-0.49467	-1.13465	-0.47132
0.646202	1.647346	-0.6757	-0.58156	-1.03628
-0.18108	1.731256	-1.19856	0.309783	-0.6614
0.8523	1.50072	-0.65993	-1.12281	-0.57028
-0.17122	1.947138	-0.87378	-0.38431	-0.51783
0.501852	1.726106	-0.65251	-1.01346	-0.56198
0.583862	1.705731	-0.76888	-0.72775	-0.79297
0.365608	1.62676	-1.14565	-0.94729	0.100571
0.717025	1.605351	-0.50334	-0.88803	-0.93101
0.265378	1.841542	-0.83801	-0.7589	-0.51002
0.184427	1.847719	-0.95693	-0.36708	-0.70813
0.65242	1.666066	-0.83966	-0.74583	-0.733
0.250356	1.852599	-0.60108	-0.84063	-0.66124

0.354468	1.7836	-0.72594	-0.99682	-0.41531
0.269919	1.851088	-0.73356	-0.695	-0.69244
0.116833	1.887023	-0.50599	-0.90331	-0.59456
0.241031	1.851275	-0.64635	-0.88348	-0.56248
0.36817	1.808492	-0.72712	-0.80998	-0.63957
0.203993	1.836235	-0.90205	-0.81929	-0.31888
-0.07066	1.92992	-0.88107	-0.4005	-0.5777
0.398127	1.724817	-0.33929	-1.17527	-0.60839
0.17204	1.795542	-0.39708	-1.20687	-0.36364
0.470514	1.762877	-0.69668	-0.71948	-0.81723
0.329632	1.816556	-0.89475	-0.56311	-0.68833
0.001096	1.916516	-0.89531	-0.54882	-0.47349
-0.12885	1.937023	-0.90815	-0.47814	-0.42188
-0.1423	1.936631	-0.87425	-0.31577	-0.60431
0.041084	1.923053	-0.75044	-0.62248	-0.59122
1.066699	1.366273	-0.71602	-0.79212	-0.92483
0.638732	1.525507	-0.03795	-1.10087	-1.02542
0.028975	1.874486	-0.98482	-0.6757	-0.24294
-0.09829	1.939855	-0.76734	-0.71249	-0.36174
0.585882	1.662861	-1.1155	-0.61749	-0.51575
0.484217	1.737804	-0.6733	-0.55876	-0.98996
0.984989	1.427727	-0.77542	-0.97636	-0.66093
0.362343	1.758549	-1.09211	-0.67917	-0.34961
-0.06385	1.936794	-0.7291	-0.40019	-0.74365
0.542802	1.660996	-1.20162	-0.48705	-0.51513
0.78433	1.571105	-0.5977	-0.79402	-0.96371
-0.01909	1.925345	-0.65949	-0.82407	-0.42269
0.444965	1.776118	-0.69683	-0.77124	-0.75301
0.344213	1.805694	-0.60871	-0.59338	-0.94782
0.126856	1.900616	-0.65117	-0.70302	-0.67329
0.325363	1.35978	-1.47229	-0.76021	0.547351
0.139449	1.89647	-0.64008	-0.69196	-0.70388
0.070007	1.905337	-0.85581	-0.61318	-0.50636
0.409678	1.781632	-0.87596	-0.77076	-0.54459
-0.12241	1.878141	-1.12056	-0.32813	-0.30703
0.331542	1.797458	-1.00518	-0.46876	-0.65506
0.948167	1.466891	-0.8082	-0.7533	-0.85356
0.25315	1.834907	-0.55156	-0.97368	-0.56282
-0.10348	1.947674	-0.80458	-0.5835	-0.45611
0.105638	1.886747	-0.78988	-0.80386	-0.39864
0.057463	1.916856	-0.5503	-0.65903	-0.76499
0.377422	1.720299	-1.19136	-0.26874	-0.63761
0.115566	1.870945	-0.32619	-0.85736	-0.80296
0.033204	1.914552	-0.86275	-0.55728	-0.52773
-0.04654	1.946498	-0.58591	-0.69116	-0.62288
0.33526	1.735698	-0.23066	-1.17353	-0.66677
-0.29344	1.924972	-0.99744	-0.23813	-0.39596
0.130132	1.894639	-0.76433	-0.71641	-0.54404
-0.0294	1.939825	-0.55558	-0.7472	-0.60764
0.213561	1.829175	-0.9663	-0.75664	-0.3198
0.496278	1.367713	-0.74517	0.363061	-1.48188
0.505222	1.743933	-0.85115	-0.66914	-0.72887
0.930118	1.465685	-1.01974	-0.68803	-0.68803
0.203713	1.874863	-0.69891	-0.64999	-0.72968
-0.06724	1.95138	-0.57511	-0.64867	-0.66035
0.218632	1.821578	-0.84925	-0.27639	-0.91457
0.157538	1.869528	-0.63948	-0.46096	-0.92662

-0.07625	1.953586	-0.66003	-0.63243	-0.58487
0.249322	1.858295	-0.71036	-0.65501	-0.74224
-0.08308	1.952418	-0.63219	-0.70866	-0.52848
0.515663	1.700847	-0.37775	-0.85615	-0.98261
-0.10269	1.942531	-0.84845	-0.44702	-0.54437
-0.07745	1.944426	-0.55091	-0.80583	-0.51023
0.213061	1.869933	-0.77952	-0.67147	-0.632
-0.2109	1.937923	-0.90647	-0.26599	-0.55456
0.130274	1.86899	-0.56789	-0.98307	-0.4483
0.490438	1.729932	-0.66695	-1.01694	-0.53647
0.076223	1.877242	-0.9541	-0.32579	-0.67358
0.204317	1.84277	-0.74714	-0.36775	-0.9322
-0.26927	1.974049	-0.73931	-0.57755	-0.38791
0.819609	1.526086	-0.46934	-1.0348	-0.84156
0.306303	1.827257	-0.89342	-0.61181	-0.62833
0.082557	1.814136	-1.04395	-0.77899	-0.07375
-0.09349	1.947135	-0.78765	-0.60835	-0.45764
0.012247	1.91852	-0.86943	-0.53067	-0.53067
-0.09399	1.9375	-0.83085	-0.6371	-0.37556
-0.34404	1.887882	-1.09976	-0.29084	-0.15324
0.172266	1.845929	-0.8464	-0.30312	-0.86868
-0.20859	1.967725	-0.76588	-0.44817	-0.54509
-0.03715	1.900997	-0.82466	-0.23253	-0.80666
-0.23062	1.972874	-0.74155	-0.45572	-0.54499
0.321319	1.798322	-0.55929	-1.0377	-0.52265
-0.43558	1.920093	-0.9526	-0.07282	-0.45909
-0.23028	1.951476	-0.82308	-0.27884	-0.61928
-0.0317	1.922906	-0.90017	-0.5001	-0.49093
0.5288	1.730005	-0.87146	-0.64053	-0.74682
-0.16271	1.970421	-0.63043	-0.60495	-0.57232
-0.13462	1.962743	-0.52382	-0.69973	-0.60457
-0.20377	1.969069	-0.73427	-0.58824	-0.44278
0.386209	1.785769	-0.96549	-0.63403	-0.57246
-0.16688	1.958197	-0.80601	-0.52832	-0.45698
0.178851	1.856418	-0.98391	-0.50365	-0.54771
-0.24621	1.964214	-0.69617	-0.70395	-0.31789
-0.35773	1.966706	-0.78802	-0.25859	-0.56237
-0.35394	1.977792	-0.72267	-0.31878	-0.5824
-0.19205	1.974649	-0.62961	-0.61379	-0.5392
-0.2439	1.978573	-0.69738	-0.49189	-0.54541
-0.23699	1.924366	-0.93159	-0.1691	-0.58669
0.175669	1.839069	-1.01499	-0.33103	-0.66871
0.146866	1.891749	-0.63467	-0.77741	-0.62654
-0.08969	1.93936	-0.86089	-0.51526	-0.47352
-0.13403	1.951257	-0.73886	-0.69294	-0.38543
-0.24458	1.973805	-0.65832	-0.39857	-0.67233
-0.23384	1.980128	-0.64743	-0.52201	-0.57684
0.222754	1.85722	-0.85027	-0.7199	-0.50981
-0.38035	1.986473	-0.71308	-0.47826	-0.41478
-0.30561	1.974152	-0.71996	-0.6176	-0.33097
-0.40175	1.979404	-0.76521	-0.35633	-0.45612
-0.09928	1.942234	-0.84514	-0.44507	-0.55274
-0.42513	1.870615	-1.07791	0.029075	-0.39665
-0.22673	1.96291	-0.82106	-0.42073	-0.4944

0.022879	1.911713	-0.88019	-0.61151	-0.4429
-0.03461	1.891329	-1.03148	-0.31985	-0.50539
-0.39147	1.987707	-0.70785	-0.43691	-0.45147
-0.11015	1.936008	-0.89984	-0.48826	-0.43776
0.192203	1.856894	-0.95772	-0.57903	-0.51235
-0.08783	1.801331	-1.28566	-0.25301	-0.17483
-0.22425	1.960231	-0.8036	-0.58187	-0.35051
-0.35016	1.97794	-0.77625	-0.42951	-0.42203
1.490172	-0.01932	-1.55856	0.459815	-0.3721
0.887713	-1.0577	-1.26919	1.19223	0.246946
1.161386	-0.32418	-1.76474	0.492214	0.435321
1.31491	-0.8978	-1.35097	0.78615	0.147709
1.15341	-0.92223	-1.41963	0.815111	0.373339
1.223131	-0.67602	-1.58104	0.59677	0.437165
0.88176	-0.93315	-1.25678	1.331051	-0.02287
1.50541	-0.93346	-1.2717	0.363032	0.336719
0.339731	-0.88778	-1.1748	1.646333	0.076519
1.623744	0.061114	-0.68178	0.332671	-1.33575
1.264108	-0.12431	-1.64074	0.783891	-0.28295
0.941326	-1.07345	-1.29208	1.084901	0.339304
1.281949	-0.71991	-1.50477	0.725962	0.216768
1.108428	-0.50993	-1.31042	1.233668	-0.52175
0.933371	-0.42115	-1.73638	0.917992	0.306166
1.263517	0.094219	-1.5657	0.782808	-0.57484
1.130015	0.435011	-0.77514	0.749836	-1.53972
1.060813	-0.52612	-1.59322	1.028899	0.029629
0.961696	-0.25757	-1.38901	1.302888	-0.61801
1.581585	-0.02848	-1.3219	0.485907	-0.71712
0.41175	-0.07726	-1.01625	1.674922	-0.99316
0.926276	-0.49264	-1.20137	1.423415	-0.65568
1.460908	0.478717	-1.53837	0.107177	-0.50843
0.902756	-0.41687	-1.76677	0.454148	0.826744
1.032552	0.631976	-0.75556	0.674778	-1.58374
1.527277	0.126495	-1.44696	0.414447	-0.62126
1.087479	0.77223	-1.68363	0.342625	-0.51871
1.225011	0.817028	-1.5468	0.150248	-0.64549
0.984745	0.578674	-1.47725	0.82566	-0.91183
0.715541	0.988429	-1.57527	0.650231	-0.77893
0.770324	0.804977	-1.13792	0.86945	-1.30683
1.032655	0.826851	-1.3884	0.542688	-1.01379
0.67226	0.920685	-1.67051	0.712132	-0.63457
0.46842	0.982924	-1.74662	0.747704	-0.45242

0.498016	0.414635	-1.76434	1.168976	-0.31729

0.580163	1.034411	-1.33029	0.802137	-1.08642
-0.1544	-1.2989	-0.82153	1.021883	1.252945
-0.32335	-0.75365	-1.21719	0.8198	1.47439
-0.0335	-0.80493	-1.32728	1.432248	0.73346
1.41786	0.036838	-1.50089	-0.58294	0.629136
0.179821	-0.44145	-0.90298	-0.69818	1.862784
1.58859	0.200456	-1.10383	-1.04367	0.358462
1.491138	0.315275	-1.23751	-0.98583	0.416924
0.636653	0.431339	-0.99049	-1.34729	1.269788
1.091674	-0.10398	-1.17866	-0.99767	1.188636
0.48883	-0.0704	-1.24034	-0.78902	1.610928
0.770778	0.151746	-1.41417	-0.81769	1.30934
1.233427	0.326658	-1.38332	-0.93772	0.760958
0.169863	-0.27044	-1.23635	-0.44414	1.781064
1.324472	0.284058	-1.47303	-0.76992	0.634421
1.237698	0.456009	-1.62923	-0.58164	0.517166
0.675324	0.63415	-1.00374	-1.39536	1.089622
1.338091	0.282253	-1.40564	-0.85935	0.644638
0.577397	-0.2916	-1.35294	-0.51088	1.578017
1.186841	0.671597	-1.61855	-0.61588	0.375986
0.931264	0.676869	-1.64494	-0.67733	0.714139
0.070945	0.27062	-1.41058	-0.55191	1.620925
0.701752	0.317769	-1.82957	-0.19961	1.009659
0.191976	-0.10358	-1.84488	0.933894	0.822594
0.40078	0.459403	-1.92593	0.113813	0.951931
-0.33054	0.118709	-1.59107	0.301489	1.501414

0.103112	0.794435	-1.87639	0.066125	0.912714
-0.56508	0.980768	-1.70051	0.671083	0.613743
-0.30486	1.287896	-1.70315	0.570281	0.149829
-0.0369	1.110617	-1.08924	-1.12772	1.143234
-0.32373	1.400373	-0.96787	-1.05225	0.943477
-0.69486	1.210718	-1.20897	-0.47488	1.167993
