# Supplementary material for: Comparative Proteomics and Metabonomics Analysis of Different Diapause Stages Revealed a New Regulation Mechanism of Diapause in Loxostege sticticalis (Lepidoptera: Pyralidae)
Source: Molecules. 2024 Jul 25;29(15):3472. doi: 10.3390/molecules29153472 (PMC11314584; doi:10.3390/molecules29153472)
Supplement: Supplementary file 1 [file molecules-29-03472-s001.zip › analysis process/proteomic/diffreential protein statistic table/CTvsRD.pdf]

Accession	Description	FC(CT/RD)	Log2FC(CT/RD)	P_value(CT/RD)	Regulate	Significant	CT	RD	RD1	RD2	RD3	CT1	CT2	CT3	GO Term	GO ID	KO ID	KO Name	Pathway ID	Pathway_defitior	EggNOG ID	EggNOG_Functional Categories	Pfam ID	Domain	Domain Description	Subcellular Loc	Unique Peptides	Coverage %	MW [kDa]	FDR Confidence
TRINITY_DN32514_c0_g2_i1_orf1	mucin-5AC-like [Ostrinia furnacalis]	0.046416706	-4.429212039	8.38E-05	down	yes	0.978	21.07	21.41	23.006	18.79	0.918	1.23	0.786	------	------	------	------	------	------	------	------	------	------	------	CYT	1	9	14.6	High
TRINITY_DN51995_c0_g3_i1_orf1	circadian clock-controlled protein-like [Ostrinia furnacalis]	13.05910543	3.706984168	2.36E-05	up	yes	16.35	1.252	1.003	1.365	1.389	17.669	15.542	15.853	------	------	------	------	------	------	ENOG4111G78	S:Function unknown	PF06585.14	JHBP	Haemolymph juvenile hormone binding protein	CYT	3	10	29.2	High
TRINITY_DN2396_c0_g1_i9_orfp1	TRINITY_DN2396_c0_g1_i9_m.39038 TRINITY_DN2396_c0_g1::TRINITY_DN2396_c0_g1_i9::g.39038 ORF type:5prime_partial len:181 (+),score=90.33 TRINITY_DN2396_c0_g1_i9:3-545(+)	2.495798319	1.319501357	0.004419	up	yes	29.7	11.9	10.922	13.343	11.434	29.569	24.594	34.927	------	------	------	------	------	------	------	------	------	------	------	CYT	1	6	19.3	High
TRINITY_DN971_c0_g1_i10_orfp1	TRINITY_DN971_c0_g1_i10_m.54268 TRINITY_DN971_c0_g1::TRINITY_DN971_c0_g1_i10::g.54268 ORF type:internal len:187 (+),score=141.68 TRINITY_DN971_c0_g1_i10:1-558(+)	2.051643192	1.036779849	0.001331	up	yes	17.48	8.52	8.668	8.635	8.256	17.9	15.374	19.162	------	------	------	------	------	------	------	------	------	------	------	CYT	2	20	20.5	High
TRINITY_DN971_c0_g1_i5_orfp1	TRINITY_DN971_c0_g1_i5_m.54249 TRINITY_DN971_c0_g1::TRINITY_DN971_c0_g1_i5::g.54249 ORF type:internal len:108 (+),score=66.98 TRINITY_DN971_c0_g1_i5:1-321(+)	5.072463768	2.342686655	0.001465	up	yes	21	4.14	4.683	3.422	4.314	20.606	17.52	24.873	------	------	------	------	------	------	------	------	------	------	------	CYT	1	9	11.5	High
TRINITY_DN1506_c0_g1_i6_orfp1	TRINITY_DN1506_c0_g1_i6_m.57691 TRINITY_DN1506_c0_g1::TRINITY_DN1506_c0_g1_i6::g.57691 ORF type:5prime_partial len:173 (+),score=30.49 TRINITY_DN1506_c0_g1_i6:1-519(+)	23.25350716	4.539376419	0.0009162	up	yes	16.41	0.7057	0.75	0.552	0.815	16.062	13.514	19.654	------	------	------	------	------	------	------	------	------	------	------	CYT	1	7	19	High
TRINITY_DN10877_c0_g1_i1_orf1	spodomicin-like [Ostrinia furnacalis]	2.777777778	1.473931188	0.007664	up	yes	8.7	3.132	3.104	2.969	3.322	8.217	7.054	10.829	CC:cellula	GO:00055	------	------	------	------	------	------	PF08036.14	Antimicrobial_6	Diapausin family of antimicrobial	CYT	1	19	6.9	Medium
TRINITY_DN5444_c0_g1_i1_orfp1	TRINITY_DN5444_c0_g1_i1_m.14077 TRINITY_DN5444_c0_g1::TRINITY_DN5444_c0_g1_i1::g.14077 ORF type:3prime_partial len:90 (-),score=25.11 TRINITY_DN5444_c0_g1_i1:2-268(-)	0.490705819	-1.027069715	0.000844	down	yes	3.643	7.424	6.953	8.205	7.113	3.93	3.441	3.557	------	------	------	------	------	------	------	------	------	------	------	CYT	1	8	9.9	Medium
TRINITY_DN381_c0_g1_i1_orf1	cuticle protein 8-like [Ostrinia furnacalis]	0.086062876	-3.538465138	4.72E-05	down	yes	0.7583	8.811	9.122	9.341	7.971	0.863	0.671	0.741	MF:molec	GO:0003	------	------	------	------	ENOG410ZM6M	S:Function unknown;	PF00379.26	Chitin_bind_4	Insect cuticle protein	CYT	4	22	25	High
TRINITY_DN138481_c0_g1_i5_orf1	hypothetical protein evm_003901 [Chilo suppressalis]	0.244159352	-2.034105058	6.83E-05	down	yes	1.808	7.405	7.965	7.389	6.862	1.763	1.709	1.953	MF:molec	GO:0003	------	------	------	------	ENOG410YRDB;E	S:Function unknown;	PF00379.26	Chitin_bind_4	Insect cuticle protein	CYT	2	32	22.6	High
TRINITY_DN45948_c1_g1_i1_orf1	unnamed protein product [Leptidea sinapis]	0.480535037	-1.05728647	0.0007477	down	yes	2.407	5.009	4.77	5.53	4.727	2.471	2.206	2.543	BP:metab	GO:00081	K01373	CTSF	map04210;m	Apoptosis;Lysoso	COG4870	O:Posttranslational modification, protein turnover, chaperones	PF00112.26	Peptidase_C1	Papain family cysteine protease	CYT	2	68	3.7	High
TRINITY_DN38274_c0_g1_i1_orf1	uncharacterized protein LOC114360402 [Ostrinia furnacalis]	0.14339682	-2.80191506	3.42E-06	down	yes	0.911	6.353	6.44	6.555	6.064	0.932	0.931	0.87	BP:nuclei	GO:00903	------	------	------	------	ENOG4112648;E	S:Function unknown;	PF00379.26	Chitin_bind_4	Insect cuticle protein	CYT	5	34	29.3	High
TRINITY_DN98147_c0_g2_i1_orf1	hypothetical protein evm_002297 [Chilo suppressalis]	0.157965389	-2.662319604	8.90E-06	down	yes	0.9767	6.183	6.353	6.353	5.842	1.075	0.856	0.999	MF:molec	GO:0003	------	------	------	------	ENOG4110RRZ;E	S:Function unknown;	PF00379.26	Chitin_bind_4	Insect cuticle protein	EXC	3	18	31.2	High
TRINITY_DN27264_c0_g1_i1_orf1	uncharacterized protein LOC114353424 [Ostrinia furnacalis]	0.483120948	-1.049543686	0.0005659	down	yes	2.161	4.473	4.421	4.857	4.142	2.271	1.958	2.255	MF:molec	GO:0003	------	------	------	------	ENOG4111DJS;E	S:Function unknown;	PF00379.26	Chitin_bind_4	Insect cuticle protein	CYT	2	6	29.3	High
TRINITY_DN59429_c0_g1_i6_orf1	uncharacterized protein LOC114366345 isoform X2 [Ostrinia furnacalis]	2.932038835	1.551904212	4.14E-06	up	yes	4.832	1.648	1.763	1.484	1.698	4.759	4.875	4.861	BP:respor	GO:0009	------	------	------	------	ENOG4110S6N	S:Function unknown	PF00451.22	Toxin_2	Scorpion short toxin, BmKK2	CYT	2	22	12.3	High
TRINITY_DN57998_c1_g3_i1_orf1	uncharacterized protein LOC114363305 isoform X2 [Ostrinia furnacalis]	0.269451594	-1.891901975	3.56E-06	down	yes	1.361	5.051	5.008	5.241	4.905	1.37	1.399	1.313	------	------	------	------	------	------	ENOG4111SVO	S:Function unknown	------	------	------	CYT	2	17	23.2	High
TRINITY_DN24971_c0_g1_i3_orf1	uncharacterized protein LOC114352370 [Ostrinia furnacalis]	0.389867841	-1.358942937	1.04E-05	down	yes	1.593	4.086	4.175	4.13	3.953	1.592	1.489	1.698	------	------	------	------	------	------	ENOG4111G1R	S:Function unknown	------	------	------	CYT	17	34	62.1	High
TRINITY_DN9282_c0_g1_i2_orf1	uncharacterized protein LOC114363102 isoform X2 [Ostrinia furnacalis]	0.087570184	-3.513416448	1.60E-07	down	yes	0.4523	5.165	5.158	5.07	5.268	0.484	0.419	0.454	------	------	------	------	------	------	ENOG41120R5	S:Function unknown	------	------	------	CYT	1	10	15.1	High
TRINITY_DN2140_c0_g1_i1_orf1	uncharacterized protein LOC126367764 [Pectinophora gossypiella]	0.297713845	-1.748001778	3.45E-05	down	yes	1.159	3.893	3.658	4.102	3.92	1.233	1.105	1.14	CC:intrace	GO:00432	------	------	------	------	COG2132	Q:Secondary metabolites biosynthesis, transport and catabolism	PF07732.18;PI	Cu-oxidase_3;Cu-oxidase_2;Cu-oxidase	Multicopper oxidase;Multicopper oxidase;Multicopper oxidase	CYT	4	7	84	High
TRINITY_DN68397_c0_g1_i2_orf1	clavesin-1-like [Ostrinia furnacalis]	0.254275654	-1.97553476	6.12E-06	down	yes	1.011	3.976	3.897	3.908	4.124	0.936	1.127	0.971	------	------	K19625	RLBP1	------	------	ENOG410XRSQ	S:Function unknown	PF00650.23;PI	CRAL_TRIO;CRAL_TRIO_2	CRAL/TRIO domain;Divergent CRAL/TRIO domain	CYT	2	8	34.6	High
TRINITY_DN2489_c0_g1_i1_orf1	uncharacterized protein LOC114354692 [Ostrinia furnacalis]	0.416543137	-1.263462188	9.23E-06	down	yes	1.405	3.373	3.422	3.296	3.402	1.468	1.456	1.29	------	------	------	------	------	------	ENOG41127D5	S:Function unknown	PF05335.16	DUF745	Protein of unknown function	CYT	5	24	31.8	High
TRINITY_DN15682_c0_g1_i4_orf1	seroin transcript 1B [Ostrinia nubilalis]	2.559753277	1.356004762	0.002208	up	yes	3.32	1.297	1.266	1.029	1.596	3.112	3.795	3.052	------	------	------	------	------	------	------	------	------	------	------	CYT	2	36	5.9	High
TRINITY_DN6415_c0_g2_i1_orf1	D-arabinitol dehydrogenase 1-like [Ostrinia furnacalis]	2.325493171	1.217536704	4.15E-05	up	yes	3.065	1.318	1.304	1.267	1.383	3.195	3.091	2.91	MF:oxidor	GO:00164	K17818	ARD1	map00040	Pentose and glu	COG1063	E:Amino acid transport and metabolism	PF08240.15;PI	ADH_N;ADH_zinc_N;Glu_dehyd_C	Alcohol dehydrogenase GroES-like domain;Zinc-binding dehydrogenase;Glucose dehydrogenase C-terminus	CYT	13	61	37.5	High
TRINITY_DN3715_c0_g1_i2_orf1	uncharacterized protein LOC114356437 isoform X1 [Ostrinia furnacalis]	0.421034601	-1.247989295	0.0002583	down	yes	1.229	2.919	2.845	3.143	2.77	1.384	1.175	1.128	------	------	------	------	------	------	------	------	PF00041.24	fn3	Fibronectin type III domain	CYT	3	19	19.1	High
TRINITY_DN40669_c0_g2_i1_orf1	uncharacterized protein LOC114355531 [Ostrinia furnacalis]	0.040074147	-4.641184367	4.75E-06	down	yes	0.8107	20.23	20.114	21.28	19.295	0.948	0.755	0.729	------	------	------	------	------	------	ENOG4110S60	S:Function unknown	------	------	------	CYT	6	59	9.5	High
TRINITY_DN38307_c0_g1_i1_orfp1	TRINITY_DN38307_c0_g1_i1_m.10661 TRINITY_DN38307_c0_g1::TRINITY_DN38307_c0_g1_i1::g.10661 ORF type:5prime_partial len:66 (+),score=5.90 TRINITY_DN38307_c0_g1_i1:2-199(+)	0.294536653	-1.763480917	0.0002012	down	yes	4.259	14.46	13.389	14.227	15.776	4.837	3.613	4.328	------	------	------	------	------	------	------	------	------	------	------	CYT	1	17	7.9	High
TRINITY_DN74538_c0_g1_i1_orf1	tetra-peptide repeat homeobox protein 1-like [Ostrinia furnacalis]	0.13957935	-2.840842577	5.29E-06	down	yes	1.022	7.322	7.562	6.96	7.444	1.075	1.086	0.904	------	------	------	------	------	------	------	------	------	------	------	CYT	3	32	23.1	High
TRINITY_DN114890_c0_g1_i4_orf1	chemosensory protein 10 [Ostrinia furnacalis]	3.42717855	1.777021356	0.0004728	up	yes	5.624	1.641	1.654	1.691	1.578	5.528	5.02	6.324	------	------	------	------	------	------	ENOG4111ATT;E	S:Function unknown;	PF03392.16	OS-D	Insect pheromone-binding family, A10/OS-D	CYT	1	76	6.9	High
TRINITY_DN19043_c0_g2_i1_orf1	hypothetical protein EVAR_60653_1 [Eumeta japonica]	0.33959429	-1.558115894	6.28E-05	down	yes	1.808	5.324	5.314	5.657	5.002	1.877	1.672	1.876	------	------	------	------	------	------	ENOG410YRTV	S:Function unknown	------	------	------	PLA	6	32	21	High
TRINITY_DN2400_c0_g1_i1_orf1	uncharacterized protein LOC114351021 [Ostrinia furnacalis]	0.197624376	-2.339167188	1.26E-06	down	yes	1.148	5.809	5.975	5.801	5.651	1.111	1.217	1.116	------	------	------	------	------	------	ENOG41113AM	S:Function unknown	------	------	------	PLA	1	11	12.9	High
TRINITY_DN25273_c0_g1_i1_orf1	skin secretory protein xP2-like [Ostrinia furnacalis]	0.327684813	-1.609619286	8.52E-06	down	yes	1.547	4.721	4.83	4.817	4.516	1.535	1.486	1.62	------	------	------	------	------	------	ENOG4111DG8	S:Function unknown	------	------	------	CYT	3	16	29	High
TRINITY_DN1772_c1_g2_i1_orf1	aldose reductase-like isoform X2 [Ostrinia furnacalis]	2.613013699	1.385714688	0.0001239	up	yes	3.815	1.46	1.434	1.459	1.486	3.712	4.128	3.606	------	------	K00011	AKR1B	map00051;m	Fructose and ma	COG0656	Z:Cytoskeleton	PF00248.24	Aldo_ket_red	Aldo/keto reductase family	CYT	13	77	36.1	High
TRINITY_DN5907_c0_g1_i4_orf1	hypothetical protein evm_010265 [Chilo suppressalis] >CAB3524755.1 unnamed protein product [Chilo suppressalis] >CAH0397522.1 unnamed protein product [Chilo suppressalis]	0.310600255	-1.686869079	2.37E-07	down	yes	1.216	3.915	3.893	3.869	3.983	1.244	1.215	1.19	------	------	------	------	------	------	ENOG410YTKH;E	S:Function unknown;	PF06585.14	JHBP	Haemolymph juvenile hormone binding protein	CYT	5	23	28.1	High
TRINITY_DN661_c0_g2_i2_orf1	cuticle protein 7-like [Ostrinia furnacalis]	0.326675427	-1.614070157	7.01E-06	down	yes	1.243	3.805	3.929	3.8	3.687	1.209	1.336	1.183	MF:molec	GO:0003	------	------	------	------	ENOG4110SG6;E	S:Function unknown;	PF00379.26	Chitin_bind_4	Insect cuticle protein	CYT	2	19	19.4	High
TRINITY_DN2515_c0_g1_i6_orf1	chitooligosaccharidolytic beta-N-acetylglucosaminidase isoform X1 [Ostrinia furnacalis]	0.46001221	-1.12025594	2.05E-05	down	yes	1.507	3.276	3.334	3.346	3.148	1.564	1.427	1.531	BP:amino	GO:00463	K12373	HEXA_B	map00600;m	Sphingolipid met	COG3525	G:Carbohydrate transport and metabolism	PF00728.25;PI	Glyco_hydro_20;Glycohydro_20b2	Glycosyl hydrolase family 20, catalytic domain;beta-acetyl hexosaminidase like	CYT	16	35	70.4	High
TRINITY_DN1465_c2_g1_i2_orf1	transcription initiation factor TFIID subunit 1-like [Ostrinia furnacalis]	0.403191163	-1.310464076	3.10E-05	down	yes	1.314	3.259	3.243	3.409	3.126	1.378	1.23	1.334	------	------	------	------	------	------	ENOG410YRBN;I	S:Function unknown;	PF16009.8	DUF4779	Domain of unknown function	PLA	7	26	36.9	High
TRINITY_DN5829_c0_g1_i1_orf1	uncharacterized protein LOC114365758 isoform X3 [Ostrinia furnacalis]	0.170460048	-2.552494448	1.83E-06	down	yes	1.056	6.195	6.162	6.33	6.094	0.956	1.253	0.959	------	------	------	------	------	------	ENOG410XT4C	S:Function unknown	PF00059.24	Lectin_C	Lectin C-type domain	PLA	5	39	23.4	High
TRINITY_DN26882_c0_g1_i1_orf1	uncharacterized protein LOC114349648 [Ostrinia furnacalis]	0.16315334	-2.615699571	7.48E-06	down	yes	0.9427	5.778	5.81	6.028	5.496	1.038	0.911	0.879	------	------	------	------	------	------	ENOG41120B2;E	S:Function unknown;	PF15998.8	DUF4773	Domain of unknown function	PLA	2	8	28.6	High
TRINITY_DN85290_c0_g2_i1_orf1	unnamed protein product, partial [Brenthis ino]	2.324661247	1.2170205	0.0002322	up	yes	4.289	1.845	1.771	1.858	1.907	3.913	4.42	4.533	------	------	K23615;K	SPIK5;SPINK	------	------	ENOG4111TR7;C	O:Posttranslational modification, protein turnover, chaperones;	PF00050.24;PI	Kazal_1;Kazal_2	Kazal-type serine protease inhibitor domain;Kazal-type serine protease inhibitor domain	CYT	1	56	7.6	High
TRINITY_DN64181_c0_g1_i1_orf1	uncharacterized protein LOC114356431 isoform X2 [Ostrinia furnacalis]	0.342421292	-1.546155683	0.00131	down	yes	3.165	9.243	10.081	9.715	7.934	3.356	2.457	3.683	------	------	------	------	------	------	------	------	------	------	------	PLA	1	18	7.9	High
TRINITY_DN10747_c0_g1_i5_orf1	unnamed protein product [Plutella xylostella]	0.253693542	-1.978841303	9.59E-06	down	yes	1.202	4.738	4.781	4.915	4.517	1.241	1.257	1.109	------	------	------	------	------	------	ENOG410XRSQ	S:Function unknown	PF00650.23	CRAL_TRIO	CRAL/TRIO domain	CYT	10	36	41.8	High
TRINITY_DN4021_c0_g1_i1_orf1	leech-derived tryptase inhibitor C-like [Ostrinia furnacalis]	0.089137974	-3.487816023	3.90E-05	down	yes	0.7197	8.074	7.663	8.807	7.751	0.836	0.623	0.7	------	------	K23417	SPINK1	------	------	ENOG410Z5MG;	S:Function unknown;	PF07648.18;PI	Kazal_2;Kazal_1	Kazal-type serine protease inhibitor domain;Kazal-type serine protease inhibitor domain	PLA	1	11	7.2	High
TRINITY_DN778_c0_g1_i1_orf1	uncharacterized protein LOC114363281 [Ostrinia furnacalis]	0.105240091	-3.248243687	1.03E-05	down	yes	0.5523	5.248	5.326	4.936	5.483	0.534	0.475	0.648	------	------	------	------	------	------	COG2335	O:Posttranslational modification, protein turnover, chaperones	PF02469.25	Fasciclin	Fasciclin domain	CYT	1	1	115.4	High
TRINITY_DN5581_c0_g1_i1_orf1	mucin-5AC-like [Ostrinia furnacalis]	0.170109546	-2.555463991	8.05E-06	down	yes	1.087	6.39	6.404	6.695	6.071	1.108	1.059	1.093	------	------	------	------	------	------	ENOG410YZWK	S:Function unknown	------	------	------	CYT	2	2	128.1	High
TRINITY_DN12671_c0_g1_i6_orf1	hemicentin-1-like isoform X1 [Ostrinia furnacalis]	0.354625122	-1.495633354	1.94E-05	down	yes	1.821	5.135	4.951	5.396	5.058	1.857	1.735	1.872	BP:biolog	GO:00081	------	------	------	------	ENOG410XPQS;E	S:Function unknown;U:Intracellular trafficking, secretion, and vesicular transport;U:Intracellular trafficking, secretion, and	PF07679.19;PI	I-set;Ig_3;ig;Ig_2	Immunoglobulin I-set domain;Immunoglobulin domain;Immunoglobulin domain;Immunoglobulin domain	CYT	1	13	10.3	High
TRINITY_DN49143_c0_g1_i1_orf1	uncharacterized protein LOC107270465 [Cephus cinctus]	0.286668448	-1.802544972	2.75E-06	down	yes	1.073	3.743	3.687	3.705	3.836	1.131	1.118	0.971	CC:cellula	GO:00055	------	------	------	------	ENOG410YYS2	S:Function unknown	PF00100.26	Zona_pellucida	Zona pellucida-like domain	CYT	5	7	69.1	High
TRINITY_DN53866_c0_g1_i1_orf1	larval/pupal cuticle protein H1C-like [Ostrinia furnacalis]	0.35005637	-1.514340836	1.42E-05	down	yes	1.242	3.548	3.571	3.625	3.447	1.359	1.106	1.262	------	------	------	------	------	------	ENOG4110SXG	S:Function unknown	------	------	------	CYT	2	22	21.8	High
TRINITY_DN338_c0_g1_i1_orf1	chorion protein S36-like [Ostrinia furnacalis]	0.208849198	-2.259466489	2.55E-06	down	yes	1.029	4.927	4.819	5.114	4.849	1.086	0.969	1.033	MF:molec	GO:0003	------	------	------	------	ENOG4111NR3	S:Function unknown	PF00379.26	Chitin_bind_4	Insect cuticle protein	CYT	1	3	50.9	High
TRINITY_DN4676_c0_g1_i16_orf1	meiosis-specific nuclear structural protein 1-like isoform X2 [Ostrinia furnacalis]	0.328200972	-1.607348581	3.29E-06	down	yes	1.215	3.702	3.712	3.808	3.585	1.237	1.174	1.234	BP:regula	GO:00311	K04381	STMN1	map05206;m	MicroRNAs in ca	ENOG4111K15	S:Function unknown	PF00836.22	Stathmin	Stathmin family	CYT	9	34	33.5	High
TRINITY_DN2652_c0_g2_i1_orf1	peroxidase-like isoform X1 [Ostrinia furnacalis]	0.400343643	-1.320689198	7.10E-07	down	yes	1.398	3.492	3.432	3.501	3.544	1.408	1.357	1.428	BP:respor	GO:00508	K19511	PXDN, VPO	------	------	ENOG410XPZ3	S:Function unknown	PF03098.18	An_peroxidase	Animal haem peroxidase	CYT	11	19	88.2	High
TRINITY_DN143497_c0_g1_i1_orf1	fibroin heavy chain-like [Ostrinia furnacalis]	0.207810718	-2.266658028	3.22E-07	down	yes	0.729	3.508	3.52	3.574	3.43	0.723	0.725	0.739	------	------	------	------	------	------	ENOG4111C6D	S:Function unknown	------	------	------	CYT	7	39	19.4	High
TRINITY_DN295_c3_g1_i1_orfp1	TRINITY_DN295_c3_g1_i1_m.18839 TRINITY_DN295_c3_g1::TRINITY_DN295_c3_g1_i1::g.18839 ORF type:5prime_partial len:118 (+),score=46.25 TRINITY_DN295_c3_g1_i1:1-354(+)	2.444364031	1.289459157	0.004415	up	yes	13.51	5.527	5.686	5.496	5.398	13.568	11.097	15.86	------	------	------	------	------	------	------	------	------	------	------	PLA	3	49	12.6	High
TRINITY_DN3190_c0_g1_i1_orf1	repetitive proline-rich cell wall protein 1 precursor [Papilio polytes] >XP_013147838.1 PREDICTED: repetitive proline-rich cell wall protein 1 [Papilio polytes] >BAM19190.1 cuticular protein PpolCPG24	0.087748538	-3.510481101	2.39E-06	down	yes	0.9003	10.26	10.242	10.661	9.866	0.999	0.878	0.824	------	------	------	------	------	------	ENOG4111M7J	S:Function unknown	------	------	------	CYT	17	44	44	High
TRINITY_DN13576_c0_g1_i1_orf1	uncharacterized protein LOC114350099 [Ostrinia furnacalis]	0.120213589	-3.056328104	1.65E-06	down	yes	0.7767	6.461	6.633	6.522	6.228	0.874	0.721	0.735	------	------	------	------	------	------	ENOG4111QIW	S:Function unknown	PF00379.26	Chitin_bind_4	Insect cuticle protein	CYT	9	48	29.1	High
TRINITY_DN12387_c0_g1_i1_orf1	repetitive proline-rich cell wall protein 2-like [Ostrinia furnacalis]	0.202025898	-2.307387848	2.49E-06	down	yes	0.9673	4.788	4.822	4.931	4.61	1.004	0.924	0.974	------	------	------	------	------	------	ENOG410YH2C;E	S:Function unknown;	------	------	------	CYT	18	64	40.7	High
TRINITY_DN31348_c0_g1_i1_orf1	protein lethal(2)essential for life [Bombyx mori]	2.817066369	1.494193554	0.0001347	up	yes	5.051	1.793	1.794	1.717	1.868	4.909	4.758	5.486	MF:cation	GO:00431	K09542	CRYAB	map04213;m	Longevity regula	ENOG410YERS	O:Posttranslational modification, protein turnover, chaperones	------	------	------	CYT	1	22	7.2	High
TRINITY_DN49147_c0_g2_i1_orf1	glutenin, high molecular weight subunit PW212-like [Ostrinia furnacalis]	0.413748657	-1.273173465	7.60E-05	down	yes	1.926	4.655	4.613	4.572	4.779	1.964	2.167	1.648	------	------	------	------	------	------	ENOG4110R9Z	S:Function unknown	------	------	------	PLA	1	5	31.6	High
TRINITY_DN12671_c0_g1_i4_orf1	hemicentin-1-like isoform X1 [Ostrinia furnacalis]	0.32433121	-1.624460235	1.20E-05	down	yes	1.273	3.925	4.081	3.938	3.756	1.302	1.312	1.206	BP:biolog	GO:00081	------	------	------	------	ENOG410XPQS;E	S:Function unknown;	PF07679.19;PI	I-set;Ig_3;Ig_2;ig	Immunoglobulin I-set domain;Immunoglobulin domain;Immunoglobulin domain;Immunoglobulin domain	CYT	5	9	89.1	High
TRINITY_DN3310_c0_g1_i1_orf1	hypothetical protein evm_010516 [Chilo suppressalis]	0.118587093	-3.075981096	1.54E-05	down	yes	0.6597	5.563	5.56	5.902	5.226	0.706	0.626	0.647	CC:integr	GO:0016C	------	------	------	------	ENOG4111PCZ	S:Function unknown	------	------	------	CYT	5	17	40.4	High
TRINITY_DN1935_c0_g1_i1_orf1	adult-specific cuticular protein ACP-22-like [Ostrinia furnacalis]	0.173117793	-2.53017408	1.94E-06	down	yes	0.7657	4.423	4.49	4.522	4.258	0.748	0.818	0.731	MF:molec	GO:0003	------	------	------	------	ENOG410YSFI	I:Lipid transport and metabolism	PF00379.26	Chitin_bind_4	Insect cuticle protein	CYT	6	38	19.3	High
TRINITY_DN77425_c0_g1_i2_orf1	anosmin-1 [Ostrinia furnacalis] >XP_028178811.1 anosmin-1 [Ostrinia furnacalis]	0.238592965	-2.067376591	1.07E-06	down	yes	1.187	4.975	5.038	5.061	4.827	1.208	1.141	1.212	CC:cellula	GO:00055	K23413	ANOS1, KAL	------	------	ENOG4111T0F	S:Function unknown	PF00041.24;PI	fn3;WAP	Fibronectin type III domain;WAP-type	EXC	5	16	47.5	High
TRINITY_DN5595_c0_g1_i1_orf1	keratin, type I cytoskeletal 10-like [Ostrinia furnacalis]	0.194687882	-2.360765002	1.66E-05	down	yes	0.7953	4.085	3.938	4.345	3.971	0.819	0.836	0.731	------	------	------	------	------	------	ENOG4112BJP	S:Function unknown	PF16058.8	Mucin-like	Mucin-like	EXC	1	14	16.6	High
TRINITY_DN3616_c0_g2_i1_orf1	conotoxin ArMKLT2-032-like [Ostrinia furnacalis]	0.328049952	-1.608012585	2.32E-05	down	yes	1.366	4.164	4.093	4.37	4.028	1.43	1.232	1.436	CC:cellula	GO:00055	------	------	------	------	------	------	------	------	------	CYT	2	64	8.7	High
TRINITY_DN30510_c0_g1_i6_orf1	spodomicin-like [Ostrinia furnacalis]	3.550124275	1.827869528	0.002027	up	yes	4.285	1.207	1.233	1.131	1.257	4.197	3.59	5.068	------	------	------	------	------	------	------	------	PF08036.14	Antimicrobial_6	Diapausin family of antimicrobial	CYT	3	60	6.7	High
TRINITY_DN3439_c0_g2_i2_orf1	histone H2A.Z-specific chaperone CHZ1-like [Ostrinia furnacalis]	2.24981685	1.169807561	8.67E-05	up	yes	3.071	1.365	1.32	1.384	1.392	3.173	2.864	3.175	------	------	------	------	------	------	------	------	------	------	------	PLA	4	14	31.9	High
TRINITY_DN2896_c0_g1_i2_orf1	general odorant-binding protein 56d-like isoform X2 [Ostrinia furnacalis]	0.268820306	-1.895285977	3.95E-05	down	yes	0.932	3.467	3.481	3.674	3.246	0.941	0.865	0.99	CC:integr	GO:0016C	------	------	------	------	ENOG410ZCHW	S:Function unknown;	PF01395.25	PBP_GOBP	PBP/GOBP family	CYT	7	46	15	High
TRINITY_DN91533_c0_g1_i1_orf1	uncharacterized protein LOC114361550 [Ostrinia furnacalis]	0.242288433	-2.045202566	7.61E-07	down	yes	0.9677	3.994	4.006	4.076	3.901	1.015	0.925	0.963	CC:cellula	GO:00055	------	------	------	------	ENOG410ZKIK	S:Function unknown	PF00024.29;PI	PAN_1;PAN_4	PAN domain;PAN domain	CYT	10	18	78.9	High
TRINITY_DN1149_c0_g1_i4_orf1	circadian clock-controlled protein-like [Ostrinia furnacalis]	2.376285127	1.248707953	0.0001197	up	yes	3.467	1.459	1.479	1.415	1.483	3.47	3.234	3.696	------	------	------	------	------	------	ENOG410YQCZ;I	S:Function unknown;	PF06585.14	JHBP	Haemolymph juvenile hormone binding protein	CYT	7	35	27.4	High
TRINITY_DN2442_c0_g1_i2_orf1	digestive cysteine proteinase 2 [Ostrinia furnacalis]	0.419489008	-1.253295088	1.75E-06	down	yes	1.412	3.366	3.36	3.427	3.312	1.474	1.381	1.382	BP:primar	GO:00442	------	------	------	------	COG4870	O:Posttranslational modification, protein turnover, chaperones	PF00112.26;PI	Peptidase_C1;Inhibitor_I29	Papain family cysteine protease;Cathepsin propeptide inhibitor	CYT	16	37	62.9	High
TRINITY_DN66040_c0_g1_i2_orf1	serine protease inhibitor dipetalogastin-like isoform X2 [Ostrinia furnacalis]	0.372331606	-1.425340007	2.44E-05	down	yes	7.186	19.3	19.572	19.656	18.675	7.698	6.297	7.564	------	------	------	------	------	------	------	------	PF07648.18;PI	Kazal_2;Kazal_1	Kazal-type serine protease inhibitor domain;Kazal-type serine protease inhibitor domain	CYT	1	21	6	High
TRINITY_DN6470_c0_g3_i2_orf1	trypsin CFT-1-like [Ostrinia furnacalis]	0.256608345	-1.962360009	2.78E-05	down	yes	0.9533	3.715	3.514	3.948	3.683	0.963	0.908	0.989	BP:organi	GO:00717	K01312	PRSS1_2_3	map04972;m	Pancreatic secret	COG5640	O:Posttranslational modification, protein turnover, chaperones	PF00089.29;PI	Trypsin;Trypsin_2	Trypsin;Trypsin-like peptidase domain	CYT	1	5	27.4	High
TRINITY_DN143532_c0_g1_i1_orf1	3-oxoacyl-[acyl-carrier-protein] reductase FabG-like [Aphidius gifuensis] >KAF7996667.1 hypothetical protein HCN44_002313 [Aphidius gifuensis]	4.130651045	2.046369187	0.0003645	up	yes	3.737	0.9047	0.818	0.847	1.049	4.21	3.593	3.407	MF:cataly	GO:0003	------	------	------	------	COG1028	S:Function unknown	PF13561.9;PF(	adh_short_C2;adh_short_KR	Enoyl;short chain dehydrogenase;KR domain	CYT	1	5	27.2	High
TRINITY_DN27968_c0_g2_i2_orf1	nucleolin-like [Ostrinia furnacalis]	0.248790208	-2.006998392	5.92E-06	down	yes	0.874	3.513	3.39	3.652	3.496	0.821	0.864	0.937	------	------	------	------	------	------	ENOG4111NPU	S:Function unknown	------	------	------	CYT	2	8	33.3	High
TRINITY_DN609_c0_g1_i1_orf1	zonadhesin-like isoform X1 [Ostrinia furnacalis]	3.039667897	1.603913709	0.00144	up	yes	3.295	1.084	1.117	1.048	1.088	3.268	2.821	3.796	------	------	------	------	------	------	COG4826;ENOG	O:Posttranslational modification, protein turnover, chaperones;S:Function unknown;S:Function unknown;S:Function unknown	PF01826.20	TIL	Trypsin Inhibitor like cysteine rich domain	PLA	9	18	109	High
TRINITY_DN129835_c0_g1_i2_orf1	alpha-tocopherol transfer protein-like [Chelonus insularis]	0.066465368	-3.911253364	1.38E-07	down	yes	0.5393	8.114	8.168	8.09	8.084	0.677	0.569	0.372	------	------	------	------	------	------	ENOG410XRSQ	S:Function unknown	PF00650.23;PI	CRAL_TRIO;CRAL_TRIO_2	CRAL/TRIO domain;Divergent CRAL/TRIO domain	CYT	1	2	35.4	Medium
TRINITY_DN2953_c1_g1_i10_orf1	methionine--tRNA ligase, cytoplasmic isoform X2 [Ostrinia furnacalis] >XP_028156683.1 methionine--tRNA ligase, cytoplasmic isoform X4 [Ostrinia furnacalis] >XP_028156684.1 methionine--tRNA ligase, cytoplasmic isoform X5 [Ostrinia furnacalis]	0.438179348	-1.190406606	0.0008242	down	yes	1.29	2.944	2.887	3.215	2.729	1.242	1.122	1.506	BP:methic	GO:00064	K01874	MARS, met(	map00450;m	Selenocompoun	COG0143	J:Translation, ribosomal structure and biogenesis	PF00458.23	WHEP-TRS	WHEP-TRS domain	CYT	2	57	17.6	High
TRINITY_DN4592_c0_g1_i1_orf1	hypothetical protein KGM_205563 [Danaus plexippus plexippus]	0.135555556	-2.883043854	3.62E-06	down	yes	0.976	7.2	7.378	7.365	6.858	0.962	1.032	0.934	------	------	------	------	------	------	ENOG4111M7J	S:Function unknown	------	------	------	CYT	17	56	42.8	High
TRINITY_DN121650_c0_g1_i1_orf1	carboxylesterase [Ostrinia furnacalis]	0.491382848	-1.025080595	6.00E-06	down	yes	2.395	4.874	4.906	4.919	4.798	2.531	2.342	2.312	CC:integr	GO:0016C	------	------	------	------	COG2272	I:Lipid transport and metabolism	------	------	------	CYT	1	19	7.6	High
TRINITY_DN4204_c0_g1_i1_orf1	uncharacterized protein LOC114359352 [Ostrinia furnacalis]	7.814926945	2.966232387	5.42E-05	up	yes	5.937	0.7597	0.686	0.819	0.774	6.148	5.377	6.285	CC:cellula	GO:00055	------	------	------	------	------	------	------	------	------	EXC	1	4	32.1	High
TRINITY_DN38506_c0_g1_i4_orf1	C-1-tetrahydrofolate synthase, cytoplasmic isoform X1 [Ostrinia furnacalis] >XP_028166137.1 C-1-tetrahydrofolate synthase, cytoplasmic isoform X2 [Ostrinia furnacalis] >XP_028166140.1 C-1-tetrahydrofolate synthase, cytoplasmic isoform X4 [Ostrinia furnacalis]	3.3518385	1.744952638	0.0005294	up	yes	4.649	1.387	1.12	1.376	1.665	4.298	4.446	5.202	BP:one-c	GO:00067	K00288	MTHFD	map00670;m	One carbon poo	COG0190	H:Coenzyme transport and metabolism	PF00763.26	THF_DHG_CYH	Tetrahydrofolate dehydrogenase/cyclohydrolase, catalytic domain	CYT	3	61	16.2	High
TRINITY_DN49785_c1_g1_i3_orf1	uncharacterized protein LOC114365444, partial [Ostrinia furnacalis]	0.235667293	-2.085176545	2.01E-06	down	yes	1.003	4.256	4.168	4.359	4.242	1.098	0.907	1.004	------	------	------	------	------	------	ENOG4110SB1	S:Function unknown	------	------	------	CYT	1	6	26.6	High
TRINITY_DN54524_c0_g1_i6_orf1	serine protease inhibitor dipetalogastin [Ostrinia furnacalis]	0.192693111	-2.375623103	3.11E-06	down	yes	0.8307	4.311	4.171	4.274	4.487	0.814	0.843	0.835	------	------	------	------	------	------	ENOG410ZXUD;I	S:Function unknown;	PF07648.18;PI	Kazal_2;Kazal_1	Kazal-type serine protease inhibitor domain;Kazal-type serine protease inhibitor domain	PLA	3	7	55.3	High
TRINITY_DN1326_c0_g1_i2_orf1	cuticle protein 7 [Plutella xylostella] >CAG9138501.1 unnamed protein product [Plutella xylostella]	0.253402119	-1.980499504	0.0004194	down	yes	0.9087	3.586	3.647	3.979	3.131	0.963	0.873	0.89	MF:molec	GO:0003	------	------	------	------	ENOG410ZM6M	S:Function unknown;	PF00379.26	Chitin_bind_4	Insect cuticle protein	CYT	1	24	18.5	High
TRINITY_DN13221_c0_g1_i3_orf1	fasciclin-3-like [Ostrinia furnacalis]	0.462190352	-1.11344095	9.01E-05	down	yes	1.418	3.068	3.076	3.04	3.089	1.213	1.525	1.515	------	------	------	------	------	------	ENOG4111K6M	S:Function unknown	PF08205.15;PI	C2-set_2;Ig_3	CD80-like C2-set immunoglobulin domain;Immunoglobulin domain	CYT	1	46	19	High
TRINITY_DN15400_c0_g1_i1_orf1	uncharacterized protein LOC114366781 [Ostrinia furnacalis]	0.203637448	-2.295925205	0.001001	down	yes	1.366	6.708	6.464	7.869	5.792	1.341	1.199	1.557	MF:odora	GO:00055	------	------	------	------	ENOG4111CBI	S:Function unknown	------	------	------	CYT	1	36	10.3	High
TRINITY_DN34406_c0_g2_i9_orfp1	TRINITY_DN34406_c0_g2_i9_m.33755 TRINITY_DN34406_c0_g2::TRINITY_DN34406_c0_g2_i9::g.33755 ORF type:internal len:82 (-),score=12.88 TRINITY_DN34406_c0_g2_i9:3-245(-)	0.213551119	-2.227346636	6.21E-05	down	yes	1.059	4.959	5.226	4.98	4.67	1.091	1.308	0.779	------	------	------	------	------	------	------	------	------	------	------	CYT	1	11	9.3	High
TRINITY_DN5444_c0_g2_i1_orf1	phenoloxidase-activating factor 2-like [Ostrinia furnacalis]	0.344777842	-1.536261036	6.25E-06	down	yes	1.195	3.466	3.511	3.521	3.365	1.244	1.09	1.25	BP:organi	GO:00717	------	------	------	------	COG5640	E:Amino acid transport and metabolism	PF00089.29	Trypsin	Trypsin	CYT	3	14	27.9	High
TRINITY_DN52553_c0_g1_i1_orf1	hemocyte protein-glutamine gamma-glutamyltransferase-like [Ostrinia furnacalis]	0.253964266	-1.977302579	2.09E-06	down	yes	0.9097	3.582	3.703	3.489	3.555	0.922	0.927	0.88	BP:primar	GO:00442	K05619	TGM1	------	------	ENOG410XQEZ	O:Posttranslational modification, protein turnover, chaperones	PF00927.25;PI	Transglut_C;Transglut_core;Transglut_N	Transglutaminase family, C-terminal ig like domain;Transglutaminase-like superfamily;Transglutaminase family	CYT	4	8	80	High
TRINITY_DN30154_c0_g1_i1_orf1	serine/threonine-protein kinase SIK2 [Ostrinia furnacalis] >XP_028174514.1 serine/threonine-protein kinase SIK2 [Ostrinia furnacalis]	0.090800952	-3.461148772	3.99E-07	down	yes	1.145	12.61	12.645	12.793	12.382	1.412	1.083	0.941	BP:phosp	GO:00163	K16311	SIK2	map04922	Glucagon signali	ENOG410XNQ0	T:Signal transduction mechanisms	PF00069.28;PI	Pkinase;PK_Tyr_Ser-Thr;Kinase-like	Protein kinase domain;Protein tyrosine and serine/threonine kinase;Kinase-like	CYT	1	1	136.1	Medium
TRINITY_DN7785_c0_g1_i1_orf1	uncharacterized protein LOC114364098 [Ostrinia furnacalis]	0.16355615	-2.612142089	2.01E-05	down	yes	0.6117	3.74	3.602	3.897	3.72	0.484	0.816	0.535	------	------	------	------	------	------	ENOG4110X3U;E	S:Function unknown;	PF00059.24	Lectin_C	Lectin C-type domain	CYT	2	16	29.9	High
TRINITY_DN87803_c0_g1_i2_orf1	hypothetical protein NF865_05903 [Phthorimaea operculella]	0.133396049	-2.906212159	1.05E-05	down	yes	1.418	10.63	10.94	10.257	10.691	1.937	1.029	1.289	MF:cataly	GO:0003	K10532	HGSNAT	map04142;m	Lysosome;Glycos	COG4299	S:Function unknown	------	------	------	CYT	1	23	9.7	High
TRINITY_DN36476_c1_g1_i1_orfp1	TRINITY_DN36476_c1_g1_i1_m.70910 TRINITY_DN36476_c1_g1::TRINITY_DN36476_c1_g1_i1::g.70910 ORF type:5prime_partial len:88 (-),score=0.50 TRINITY_DN36476_c1_g1_i1:49-312(-)	2.473783656	1.306719335	0.003655	up	yes	5.237	2.117	2.061	2.092	2.198	5.254	4.346	6.112	------	------	------	------	------	------	------	------	------	------	------	CYT	2	21	9.5	High
TRINITY_DN65681_c0_g1_i1_orf1	ferritin subunit-like [Ostrinia furnacalis] >XP_028168186.1 ferritin subunit-like [Ostrinia furnacalis]	0.344785526	-1.536228885	7.79E-05	down	yes	1.696	4.919	5.099	4.851	4.808	2.009	1.664	1.415	BP:ion ho	GO:00508	K00522	FTH1	map04978;m	Mineral absorpti	ENOG4111MSG;	P:Inorganic ion transport and metabolism;	PF00210.27	Ferritin	Ferritin-like domain	CYT	1	31	8.1	High
TRINITY_DN4384_c0_g1_i5_orf1	chemosensory protein 5 [Conogethes punctiferalis]	0.309790653	-1.690634477	1.77E-05	down	yes	1.465	4.729	4.778	4.807	4.602	1.51	1.239	1.646	------	------	------	------	------	------	ENOG410YU7V;E	U:Intracellular trafficking, secretion, and vesicular transport;S:Function unknown	PF03392.16	OS-D	Insect pheromone-binding family, A10/OS-D	CYT	2	14	16.9	High

TRINITY_DN295_c5_g1_i2_orf1	unnamed protein product [Chilo suppressalis]	2.100308642	1.070601349	7.59E-05	up	yes	4.083	1.944	1.949	2.04	1.844	4.008	3.932	4.309	MF:hydro	GO:00167	K21013	FPPP	map00981	Insect hormone I	COG0647	G:Carbohydrate transport and metabolism	PF13344.9	Hydrolase_6	Haloacid dehalogenase-like hydrolase	CYT	5	24	19.9	High
TRINITY_DN7539_c0_g1_i2_orf1	serine protease inhibitor 3/4 [Ostrinia furnacalis]	0.267348754	-1.90320514	1.84E-05	down	yes	1.202	4.496	4.448	4.576	4.464	1.111	1.463	1.033	CC:cellula	GO:00055	------	------	------	------	COG4826	O:Posttranslational modification, protein turnover, chaperones	PF00079.23	Serpin	Serpin	CYT	9	18	58.6	High
TRINITY_DN6004_c0_g1_i1_orf1	endocuticle structural glycoprotein ABD-4-like [Ostrinia furnacalis]	0.35365184	-1.499598329	3.73E-06	down	yes	1.288	3.642	3.515	3.705	3.706	1.267	1.325	1.271	MF:molec	GO:00036	------	------	------	------	ENOG4111PJ1;E	O:Posttranslational modification, protein turnover, chaperones;S:Function unknown	PF00379.26	Chitin_bind_4	Insect cuticle protein	CYT	2	23	16.7	High
TRINITY_DN1012_c0_g1_i2_orf1	teneurin-a isoform X1 [Ostrinia furnacalis]	0.434001179	-1.204229135	0.0003754	down	yes	1.473	3.394	3.117	3.55	3.514	1.345	1.396	1.678	CC:integr	GO:00160	K24473	TENM, ODZ	------	------	ENOG410ZZMY;	S:Function unknown;	PF07974.16;PI	EGF_2;NHL	EGF-like domain;NHL repeat	CYT	4	2	222.3	High
TRINITY_DN1491_c0_g1_i4_orf1	GILT-like protein 2 isoform X1 [Ostrinia furnacalis] >XP_028156245.1 GILT-like protein 2 isoform X2 [Ostrinia furnacalis] >XP_028156247.1 GILT-like protein 2 isoform X3 [Ostrinia furnacalis]	0.488137797	-1.034639631	0.000147	down	yes	1.502	3.077	2.968	3.015	3.247	1.462	1.404	1.64	MF:cataly	GO:00038	K08059	IFI30, GILT	map04612	Antigen processi	ENOG4111IDT	S:Function unknown	PF03227.19	GILT	Gamma interferon inducible lysosomal thiol reductase	CYT	1	16	26.5	High
TRINITY_DN110231_c0_g1_i1_orf1	protein singed [Ostrinia furnacalis] >XP_028161434.1 protein singed [Ostrinia furnacalis]	0.406200569	-1.299735833	2.80E-06	down	yes	1.284	3.161	3.206	3.135	3.142	1.359	1.208	1.286	BP:cellula	GO:00718	K23551	FSCN1	map05206	MicroRNAs in ca	ENOG410XPHV	O:Posttranslational modification, protein turnover, chaperones	PF06268.16;PI	Fascin;FRG1;FGF	Fascin domain;FRG1-like domain;Fibroblast growth factor	CYT	11	29	57	High
TRINITY_DN1902_c0_g1_i4_orf1	chemosensory protein 10 [Conogethes pinicolalis]	0.296990172	-1.751512905	1.50E-05	down	yes	0.967	3.256	3.218	3.358	3.193	0.988	1.086	0.827	------	------	------	------	------	------	ENOG4112163;E	S:Function unknown;	PF03392.16	OS-D	Insect pheromone-binding family, A10/OS-D	CYT	2	20	11.8	High
TRINITY_DN10824_c0_g1_i3_orf1	endochitinase isoform X2 [Ostrinia furnacalis]	0.143712575	-2.798741792	6.96E-08	down	yes	1.104	7.682	7.558	7.789	7.698	1.09	1.093	1.128	BP:amino	GO:00463	K01183	E3.2.1.14	map00520	Amino sugar anc	COG3325	S:Function unknown	PF00704.31;PI	Glyco_hydro_18;CBM_14	Glycosyl hydrolases family 18;Chitin binding Peritrophin-A domain	EXC	4	9	62.8	High
TRINITY_DN2101_c0_g1_i6_orf1	protein obstructor-E-like [Ostrinia furnacalis]	0.070134804	-3.83372564	1.70E-05	down	yes	0.5723	8.16	8.411	8.523	7.546	0.658	0.499	0.56	------	------	------	------	------	------	ENOG410ZKXC	S:Function unknown	PF01607.27	CBM_14	Chitin binding Peritrophin-A domain	EXC	5	20	28.1	High
TRINITY_DN16091_c0_g1_i1_orfp1	TRINITY_DN16091_c0_g1_i1_m.64010 TRINITY_DN16091_c0_g1::TRINITY_DN16091_c0_g1_i1::g.64010 ORF type:5prime_partial len:124 (-),score=7.29,Toxin_2 PF00451.20 0.00035,Toxin_2|PF00451.20|0.00013,Toxin_2|PF00451.20|0.00037,Gamma-thionin|PF00304.21|0.37,Gamma-thionin|PF00304.21|0.052,Gamma-thionin|PF00304.21|0.022,Defensin_2|PF01097.19|0.58,Defensin_2|PF01097.19|0.12,Defensin_2|PF01097.19|0.011,Toxin_38|PF14866.7|0.18,Toxin_38|PF14866.7|0.18,Toxin_38|PF14866.7|0.4 TRINITY_DN16091_c0_g1_i1:19-390(-)	3.001454193	1.58566165	0.00506	up	yes	6.192	2.063	2.228	1.806	2.154	6.238	4.908	7.431	------	------	------	------	------	------	------	------	PF00451.22;PI	Toxin_2;Gamma-thionin;Defensin_2	Scorpion short toxin, BmKK2;Gamma-thionin family;Arthropod defensin	CYT	2	43	14.3	High
TRINITY_DN12534_c0_g1_i4_orf1	antibacterial protein [Heliothis virescens]	2.549805951	1.350387457	0.0008923	up	yes	5.913	2.319	2.269	2.318	2.369	6.182	5.118	6.438	BP:respor	GO:00096	------	------	------	------	ENOG4110S6N	S:Function unknown	PF00451.22	Toxin_2	Scorpion short toxin, BmKK2	CYT	5	57	12	High
TRINITY_DN35351_c0_g1_i3_orf1	adult-specific cuticular protein ACP-20-like [Ostrinia furnacalis]	0.188797031	-2.405092021	4.72E-05	down	yes	1.119	5.927	5.556	6.377	5.849	1.132	1.265	0.961	MF:molec	GO:00036	------	------	------	------	ENOG410YWJF;E	Q:Secondary metabolites biosynthesis, transport and catabolism;S:Function unknown;S:Function unknown;S:Function unknown;S:Function unknown	PF00379.26	Chitin_bind_4	Insect cuticle protein	CYT	2	16	19.2	High
TRINITY_DN28922_c0_g1_i2_orf1	uncharacterized protein LOC119829283 isoform X2 [Zerene cesonia]	0.293300807	-1.769547055	0.0001987	down	yes	1.563	5.329	5.421	5.665	4.902	1.59	1.861	1.237	CC:cellula	GO:00055	------	------	------	------	------	------	------	------	------	CYT	1	10	12.5	High
TRINITY_DN801_c0_g1_i2_orf1	cathepsin L [Ostrinia furnacalis] >XP_028165920.1 cathepsin L [Ostrinia furnacalis] >UKI61015.1 cathepsin L [Ostrinia furnacalis]	0.455144558	-1.135603264	2.69E-05	down	yes	2.141	4.704	4.706	4.675	4.73	2.084	1.973	2.367	BP:primar	GO:00442	K01365	CTSL	map05205;m	Proteoglycans in	COG4870	S:Function unknown	PF00112.26;PI	Peptidase_C1;Inhibitor_I29	Papain family cysteine protease;Cathepsin propeptide inhibitor	CYT	16	47	38.1	High
TRINITY_DN906_c0_g1_i4_orf1	uncharacterized protein LOC114360441, partial [Ostrinia furnacalis]	0.255246166	-1.970038803	1.39E-07	down	yes	1.265	4.956	4.988	4.946	4.933	1.317	1.297	1.181	------	------	K17339	ATL	------	------	ENOG410XR6Z	S:Function unknown	PF02263.22;PI	GBP;GBP_C	Guanylate-binding protein, N-terminal domain;Guanylate-binding protein, C-terminal domain	CYT	22	38	87.8	High
TRINITY_DN15222_c0_g1_i4_orf1	lysosomal alpha-mannosidase-like [Ostrinia furnacalis]	0.143947217	-2.796388194	8.04E-06	down	yes	0.7527	5.229	5.079	5.425	5.184	0.977	0.615	0.666	BP:nuclec	GO:00091	K12311	MAN2B1, L/	map00511;m	Other glycan deg	ENOG410XQMZ	U:Intracellular trafficking, secretion, and vesicular	------	------	------	CYT	1	17	11.9	High
TRINITY_DN13686_c0_g2_i1_orf1	transmembrane protease serine 9-like [Ostrinia furnacalis]	0.361456893	-1.468104494	2.63E-06	down	yes	1.568	4.338	4.372	4.394	4.249	1.639	1.606	1.459	BP:organi	GO:00717	K20677;K	HAYAN;TMI	map05164	Influenza A	COG5640	O:Posttranslational modification, protein turnover, chaperones	PF00089.29	Trypsin	Trypsin	CYT	2	6	43.9	High
TRINITY_DN18568_c0_g1_i2_orfp1	TRINITY_DN18568_c0_g1_i2_m.13844 TRINITY_DN18568_c0_g1::TRINITY_DN18568_c0_g1_i2::g.13844 ORF type:5prime_partial len:77 (+),score=12.07 TRINITY_DN18568_c0_g1_i2:1-231(+)	5.700240082	2.511022684	0.004054	up	yes	4.986	0.8747	0.458	0.979	1.187	5.237	5.981	3.741	------	------	------	------	------	------	------	------	------	------	------	CYT	1	13	8.5	High
TRINITY_DN12387_c1_g2_i1_orf1	skin secretory protein xP2-like [Ostrinia furnacalis]	0.229226163	-2.125156377	3.15E-05	down	yes	0.9953	4.342	4.353	4.602	4.071	0.941	1.092	0.953	------	------	------	------	------	------	ENOG410ZKDO;	S:Function unknown;	------	------	------	CYT	10	50	39.7	High
TRINITY_DN21555_c0_g1_i4_orf1	uncharacterized protein LOC114351844 [Ostrinia furnacalis] >XP_028158981.1 uncharacterized protein LOC114351844 [Ostrinia furnacalis] >XP_028158982.1 uncharacterized protein LOC114351844 [Ostrinia furnacalis] >XP_028158983.1 uncharacterized protein LOC114351844 [Ostrinia furnacalis] >XP_028158984.1 uncharacterized protein LOC114351844 [Ostrinia furnacalis] >XP_028158985.1 uncharacterized protein LOC114351844 [Ostrinia furnacalis] >5GPR_A Crystal structure of chitinase-h from Ostrinia furnacalis [Ostrinia furnacalis] >5GQB_A Crystal structure of chitinase-h from O. furnacalis in complex with chitohepatose [Ostrinia furnacalis] >6JMN_A Crystal structure of Ostrinia furnacalis Chitinase h complexed with compound 2-8-s2 [Ostrinia furnacalis] >BAE16587.1 chitinase [Ostrinia	0.391419492	-1.353212495	3.37E-07	down	yes	1.478	3.776	3.826	3.778	3.724	1.515	1.472	1.448	BP:amino	GO:00463	K01183	E3.2.1.14	map00520	Amino sugar anc	COG3325	S:Function unknown	PF00704.31;PI	Glyco_hydro_18;ChitinaseA_N	Glycosyl hydrolases family 18;Chitinase A, N-terminal domain	CYT	12	28	60.8	High
TRINITY_DN56459_c0_g1_i2_orf1	aldo-keto reductase AKR2E4-like [Ostrinia furnacalis]	2.913260672	1.542634796	0.001262	up	yes	6.415	2.202	2.237	2.086	2.282	6.173	5.666	7.406	MF:cataly	GO:00038	------	------	------	------	COG0656	S:Function unknown	------	------	------	CYT	4	78	8.7	High
TRINITY_DN7064_c0_g1_i19_orf1	unnamed protein product [Chilo suppressalis]	4.777854195	2.256362828	0.001749	up	yes	6.947	1.454	1.553	1.212	1.597	6.914	5.701	8.225	------	------	------	------	------	------	------	------	PF00050.24;PI	Kazal_1;Kazal_2	Kazal-type serine protease inhibitor domain;Kazal-type serine protease inhibitor domain	EXC	1	2	73	High
TRINITY_DN20344_c0_g1_i5_orf1	uncharacterized protein LOC114351483 [Ostrinia furnacalis]	4.10400795	2.037033526	0.0003441	up	yes	3.717	0.9057	0.935	0.829	0.953	4.017	3.232	3.902	MF:molec	GO:00036	------	------	------	------	------	------	PF00379.26	Chitin_bind_4	Insect cuticle protein	CYT	4	57	13.5	High
TRINITY_DN1749_c0_g1_i1_orf1	putative GPI-anchored protein pfl2 isoform X2 [Ostrinia furnacalis]	0.250359513	-1.997926818	4.62E-05	down	yes	0.9053	3.616	3.429	3.875	3.545	0.971	0.802	0.943	------	------	------	------	------	------	COG5599	S:Function unknown	PF00102.30	Y_phosphatase	Protein-tyrosine phosphatase	CYT	2	6	59	High
TRINITY_DN2407_c0_g1_i2_orf1	uncharacterized protein LOC114366345 isoform X2 [Ostrinia furnacalis]	2.630952381	1.395585137	0.0002575	up	yes	3.094	1.176	1.32	0.99	1.219	2.859	3.274	3.148	BP:respor	GO:00096	------	------	------	------	ENOG4110S6N	S:Function unknown	PF00451.22	Toxin_2	Scorpion short toxin, BmKK2	CYT	1	36	21.5	High
TRINITY_DN71698_c0_g1_i1_orfp1	TRINITY_DN71698_c0_g1_i1_m.1194 TRINITY_DN71698_c0_g1::TRINITY_DN71698_c0_g1_i1::g.1194 ORF type:internal len:134 (+),score=19.66,Toxin_2 PF00451.20 4.3e-05,Toxin_2|PF00451.20|0.037,Toxin_2|PF00451.20|7.5e-05,Gamma-thionin|PF00304.21|0.017,Gamma-thionin|PF00304.21|0.05,Gamma-thionin|PF00304.21|0.021,Toxin_38|PF14866.7|0.13,Toxin_38|PF14866.7|0.15,Toxin_38|PF14866.7|0.15,Defensin_2|PF01097.19|0.053,Defensin_2|PF01097.19|0.34,Defensin_2|PF01097.19|0.092 TRINITY_DN71698_c0_g1_i1:3-401(+)	2.216894977	1.148540426	0.002827	up	yes	3.884	1.752	1.785	1.727	1.744	3.911	3.307	4.434	------	------	------	------	------	------	------	------	PF00451.22;PI	Toxin_2;Gamma-thionin	Scorpion short toxin, BmKK2;Gamma-thionin family	CYT	6	47	15.5	High
TRINITY_DN19990_c0_g1_i1_orf1	carboxypeptidase B-like [Ostrinia furnacalis]	0.311654527	-1.681980428	2.35E-07	down	yes	1.198	3.844	3.847	3.886	3.798	1.235	1.214	1.145	BP:nitrog	GO:00068	------	------	------	------	COG2866	U:Intracellular trafficking, secretion, and vesicular	PF00246.27;PI	Peptidase_M14;Propep_M14	Zinc carboxypeptidase;Carboxypeptidase	CYT	7	16	57.6	High
TRINITY_DN3056_c0_g1_i1_orf1	spidroin-2-like [Ostrinia furnacalis]	0.252558895	-1.98530824	4.19E-06	down	yes	0.9327	3.693	3.678	3.743	3.658	0.996	0.782	1.02	MF:molec	GO:00036	------	------	------	------	ENOG4110SY1;E	S:Function unknown;	PF00379.26	Chitin_bind_4	Insect cuticle protein	CYT	1	4	76.6	High
TRINITY_DN1308_c0_g1_i4_orf1	serine proteinase stubble-like [Ostrinia furnacalis]	0.409215017	-1.289069006	0.0001196	down	yes	1.199	2.93	3.128	2.748	2.914	1.248	1.226	1.123	BP:organi	GO:00717	------	------	------	------	COG5640	C:Energy production and conversion	PF00089.29	Trypsin	Trypsin	CYT	1	3	28.8	Medium
TRINITY_DN1124_c0_g1_i7_orf1	PREDICTED: cuticle protein 18.6, isoform B [Amyelois transitella]	0.043859649	-4.510961919	4.96E-06	down	yes	0.55	12.54	12.45	13.19	11.979	0.715	0.469	0.466	MF:molec	GO:00036	------	------	------	------	ENOG4111Y6E	S:Function unknown	PF00379.26	Chitin_bind_4	Insect cuticle protein	CYT	4	18	27.2	High
TRINITY_DN35809_c0_g1_i1_orf1	spodomicin-like [Ostrinia furnacalis] >OKV49445.1 diapausin [Ostrinia furnacalis]	3.101908066	1.633155929	0.00274	up	yes	7.153	2.306	2.392	2.294	2.233	7.27	5.828	8.361	------	------	------	------	------	------	------	------	PF08036.14	Antimicrobial_6	Diapausin family of antimicrobial	CYT	3	60	6.8	High
TRINITY_DN18338_c0_g1_i6_orf1	aquaporin AQPAn.G isoform X1 [Ostrinia furnacalis]	0.347142374	-1.526400616	0.0001248	down	yes	2.053	5.914	6.357	5.495	5.89	1.907	2.057	2.196	CC:integr	GO:00160	K09884	AQPN	------	------	COG0580	O:Posttranslational modification, protein turnover, chaperones	PF00230.23	MIP	Major intrinsic protein	CYT	1	8	29	High
TRINITY_DN380_c0_g2_i2_orf1	chemosensory protein 10 [Ostrinia furnacalis]	2.201161344	1.138264898	0.0006282	up	yes	5.307	2.411	2.252	2.512	2.469	5.487	4.745	5.689	------	------	------	------	------	------	ENOG4111ATT;E	S:Function unknown;	PF03392.16	OS-D	Insect pheromone-binding family, A10/OS-D	CYT	3	54	13.6	High
TRINITY_DN18539_c0_g1_i1_orf1	pupal cuticle protein PCP52-like [Ostrinia furnacalis]	0.14671599	-2.768901984	1.26E-05	down	yes	0.9717	6.623	6.602	7.003	6.263	1.001	0.994	0.92	MF:molec	GO:00036	------	------	------	------	ENOG4110SXG	S:Function unknown	------	------	------	CYT	13	54	28.3	High
TRINITY_DN9311_c0_g1_i1_orf1	cuticle protein 8-like [Ostrinia furnacalis]	0.093812885	-3.414070105	7.67E-05	down	yes	0.6247	6.659	6.733	7.248	5.997	0.619	0.626	0.629	MF:molec	GO:00036	------	------	------	------	ENOG410YSFI	S:Function unknown	PF00379.26	Chitin_bind_4	Insect cuticle protein	CYT	4	52	15	High
TRINITY_DN661_c0_g1_i1_orf1	hypothetical protein evm_002822 [Chilo suppressalis]	0.165432099	-2.595688907	0.000327	down	yes	1.005	6.075	6.613	6.396	5.217	1.028	0.855	1.131	MF:molec	GO:00036	------	------	------	------	ENOG4111NR3;E	S:Function unknown;	PF00379.26	Chitin_bind_4	Insect cuticle protein	CYT	1	22	17.6	High
TRINITY_DN1880_c0_g1_i4_orf1	serine protease inhibitor dipetalogastin-like [Helicoverpa zea]	2.761067314	1.465226061	0.0003121	up	yes	4.553	1.649	1.667	1.631	1.648	4.561	4.117	4.981	------	------	K23615	SPIK5	------	------	COG5640;ENOG	O:Posttranslational modification, protein turnover, chaperones;S:Function unknown	PF00050.24;PI	Kazal_1;Kazal_2	Kazal-type serine protease inhibitor domain;Kazal-type serine protease inhibitor domain	CYT	12	52	42.6	High
TRINITY_DN16234_c0_g2_i3_orf1	uncharacterized protein LOC114363370 [Ostrinia furnacalis]	2.233298539	1.159176118	0.0037	up	yes	4.279	1.916	1.907	1.945	1.896	4.371	3.565	4.901	CC:cellula	GO:00055	------	------	------	------	ENOG410Y67D	S:Function unknown	------	------	------	CYT	3	51	7.4	High
TRINITY_DN138481_c0_g1_i2_orf1	cuticle protein 8-like isoform X2 [Vanessa tameamea]	0.211715481	-2.239801328	1.28E-06	down	yes	1.012	4.78	4.769	4.874	4.696	0.887	1.066	1.082	MF:molec	GO:00036	------	------	------	------	ENOG410YRDB;E	S:Function unknown;	PF00379.26	Chitin_bind_4	Insect cuticle protein	CYT	2	27	24.2	High
TRINITY_DN7854_c0_g1_i4_orf1	failed axon connections [Ostrinia furnacalis]	0.488661504	-1.033092638	2.27E-06	down	yes	1.767	3.616	3.597	3.623	3.628	1.745	1.702	1.853	------	------	------	------	------	------	ENOG4111HSJ	S:Function unknown	PF17172.7;PF:	GST_N_4;GST_C_6;Tom37;GST_C_2;GST_N_3;GST_C_3	Glutathione S-transferase N-terminal domain;Glutathione S-transferase, C-terminal domain;Outer mitochondrial membrane transport complex protein;Glutathione S-transferase, C-terminal domain;Glutathione S-transferase, N-terminal domain;Glutathione S-transferase, C-terminal domain	CYT	22	54	42.6	High
TRINITY_DN58125_c0_g1_i1_orf1	protein yellow-like [Ostrinia furnacalis]	0.326911678	-1.613027183	2.94E-05	down	yes	1.103	3.374	3.532	3.371	3.219	0.993	1.128	1.188	BP:male r	GO:00601	------	------	------	------	COG3386	S:Function unknown	PF03022.19	MRJP	Major royal jelly protein	CYT	1	9	19.2	High
TRINITY_DN4367_c0_g1_i1_orf1	heat shock protein 21.7c [Chilo suppressalis] >AWT57938.1 heat shock protein 21.7c [Chilo suppressalis]	0.413793103	-1.273018494	1.83E-05	down	yes	1.284	3.103	3.254	3.022	3.034	1.27	1.306	1.277	MF:cation	GO:00431	K09542	CRYAB	map04213;m	Longevity regula	ENOG410YERS	S:Function unknown	PF00011.24	HSP20	Hsp20/alpha crystallin family	CYT	1	19	21.6	High
TRINITY_DN31417_c0_g1_i3_orf1	titin-like [Ostrinia furnacalis]	0.422447644	-1.243155543	0.0008489	down	yes	1.291	3.056	3.336	3.149	2.682	1.311	1.325	1.237	------	------	------	------	------	------	ENOG410ZKJ3;E	S:Function unknown;	PF00093.21	VWC	von Willebrand factor type C domain	CYT	2	17	25.3	High
TRINITY_DN11970_c0_g1_i4_orf1	myb-like protein AA [Ostrinia furnacalis]	0.373522459	-1.420733105	3.62E-05	down	yes	1.106	2.961	2.887	3.101	2.895	1.036	1.226	1.057	------	------	------	------	------	------	ENOG41117A2	S:Function unknown	------	------	------	CYT	3	5	126.8	High
TRINITY_DN73900_c0_g1_i1_orf1	carbonic anhydrase 7 [Ostrinia furnacalis]	2.42652027	1.278888912	0.001917	up	yes	2.873	1.184	1.349	1.152	1.05	3.04	2.445	3.134	MF:cation	GO:00431	K01672	CA	map00910	Nitrogen metabo	COG3338	U:Intracellular trafficking, secretion, and vesicular	PF00194.24	Carb_anhydrase	Eukaryotic-type carbonic anhydrase	CYT	1	3	36.5	High
TRINITY_DN15175_c0_g1_i1_orf1	zinc carboxypeptidase-like [Ostrinia furnacalis]	0.205240175	-2.284614936	7.12E-08	down	yes	1.739	8.473	8.516	8.539	8.363	1.675	1.72	1.822	------	------	K01298	CPA2	map04972;m	Pancreatic secret	COG2866	O:Posttranslational modification, protein turnover, chaperones	PF00246.27	Peptidase_M14	Zinc carboxypeptidase	CYT	3	7	45.8	High
TRINITY_DN15202_c0_g1_i6_orf1	uncharacterized protein LOC114364499 isoform X2 [Ostrinia furnacalis]	2.477768091	1.309041163	0.0001711	up	yes	5.684	2.294	2.318	2.266	2.297	5.555	5.331	6.166	------	------	------	------	------	------	ENOG410XU1K	S:Function unknown	------	------	------	CYT	2	41	14	High
TRINITY_DN29604_c0_g2_i2_orf1	neurofilament heavy polypeptide-like isoform X2 [Ostrinia furnacalis]	0.309549746	-1.69175682	1.36E-05	down	yes	1.705	5.508	5.473	5.714	5.338	1.741	1.518	1.855	------	------	------	------	------	------	------	------	------	------	------	CYT	22	77	40.5	High
TRINITY_DN619_c0_g1_i1_orf1	putative uncharacterized protein DDB_G0271606 [Ostrinia furnacalis]	0.073995128	-3.756425908	7.78E-06	down	yes	0.486	6.568	6.655	6.873	6.177	0.483	0.496	0.479	CC:cellula	GO:00055	------	------	------	------	ENOG4111K2A	S:Function unknown	PF01607.27	CBM_14	Chitin binding Peritrophin-A domain	CYT	3	16	39.1	High
TRINITY_DN19639_c0_g2_i1_orf1	basic juvenile hormone-suppressible protein 1-like [Hyposmocoma kahamanoa]	0.350270054	-1.513460443	0.001443	down	yes	1.751	4.999	4.955	4.538	5.504	1.807	2.252	1.193	------	------	------	------	------	------	ENOG410XR2D	S:Function unknown	PF03722.17	Hemocyanin_N	Hemocyanin, all-alpha domain	CYT	2	72	8.6	High
TRINITY_DN2908_c0_g1_i1_orf1	uncharacterized protein LOC114361337 [Ostrinia furnacalis]	0.103419561	-3.273419015	3.67E-05	down	yes	0.5837	5.644	6.058	5.658	5.216	0.58	0.705	0.466	------	------	------	------	------	------	ENOG4111MU3	S:Function unknown	PF00354.20;PI	Pentaxin;Laminin_G_3	Pentaxin family;Concanavalin A-like lectin/glucanases superfamily	CYT	1	2	61.6	High
TRINITY_DN98334_c0_g1_i1_orf1	unnamed protein product [Spodoptera littoralis] >CAH1638738.1 unnamed protein product [Spodoptera littoralis]	0.184129186	-2.441209774	1.38E-06	down	yes	0.9293	5.047	5.053	5.172	4.917	0.895	1.032	0.861	------	------	------	------	------	------	ENOG4110QDI	S:Function unknown	------	------	------	CYT	8	52	34	High
TRINITY_DN1110_c1_g1_i9_orf1	MD-2-related lipid-recognition protein-like [Ostrinia furnacalis]	0.490369181	-1.028059783	3.36E-06	down	yes	1.833	3.738	3.694	3.702	3.818	1.852	1.768	1.879	------	------	K13443	NPC2	map04979;m	Cholesterol meta	ENOG4111D1U;E	S:Function unknown;	PF02221.18	E1_DerP2_DerF2	ML domain	CYT	2	17	17.2	High
TRINITY_DN7549_c0_g1_i1_orf1	uncharacterized protein LOC114355006 [Ostrinia furnacalis]	0.145341474	-2.782481648	5.49E-06	down	yes	0.6427	4.422	4.355	4.649	4.262	0.663	0.635	0.63	------	------	------	------	------	------	ENOG4111H56;E	C:Energy production and conversion;S:Function unknown	------	------	------	CYT	2	14	20	High
TRINITY_DN31314_c0_g1_i4_orf1	uncharacterized protein LOC114355976 [Ostrinia furnacalis]	0.272097977	-1.877801867	3.46E-06	down	yes	1.022	3.756	3.774	3.82	3.675	1.132	1.018	0.917	BP:mitotic	GO:00002	K11578	ZW10, DSL1	------	------	ENOG410XQ7J;E	S:Function unknown;	------	------	------	CYT	7	9	86.8	High
TRINITY_DN10222_c0_g1_i2_orf1	glutathione S-transferase sigma 3 [Ostrinia furnacalis]	2.636595745	1.398676387	0.002956	up	yes	3.098	1.175	1.155	1.082	1.289	2.941	3.662	2.69	MF:cataly	GO:00038	K04097	HPGDS	map00590;m	Arachidonic acid	ENOG41103AU;E	O:Posttranslational modification, protein turnover, chaperones;	PF14497.9;PF(	GST_C_3;GST_N;GST_C	Glutathione S-transferase, C-terminal domain;Glutathione S-transferase, N-terminal domain;Glutathione S-transferase, C-terminal domain	CYT	14	58	22.9	High
TRINITY_DN696_c1_g1_i10_orf1	titin-like [Ostrinia furnacalis]	0.319900806	-1.644303468	2.81E-07	down	yes	1.032	3.226	3.198	3.28	3.199	1.046	0.997	1.052	------	------	------	------	------	------	ENOG410ZKJ3;E	S:Function unknown;	------	------	------	CYT	9	5	288.8	High
TRINITY_DN276_c0_g1_i2_orf1	protein lethal(2)essential for life-like [Ostrinia furnacalis] >UTU55753.1 small heat shock protein Hsp20.7 [Ostrinia furnacalis]	0.42	-1.251538767	0.0004244	down	yes	4.935	11.75	11.772	12.626	10.844	5.302	4.195	5.307	MF:cation	GO:00431	K09542	CRYAB	map04213;m	Longevity regula	ENOG410YERS	O:Posttranslational modification, protein turnover, chaperones	PF00011.24	HSP20	Hsp20/alpha crystallin family	CYT	2	43	20.8	High
TRINITY_DN56155_c0_g1_i1_orf1	hypothetical protein evm_004679 [Chilo suppressalis] >CAH2989683.1 unnamed protein product [Chilo suppressalis]	0.058468407	-4.096198915	3.70E-07	down	yes	0.8513	14.56	14.637	14.883	14.164	0.929	0.858	0.767	MF:pigme	GO:00314	K03098	APOD	------	------	COG3040	Z:Cytoskeleton	PF00061.26;PI	Lipocalin;Lipocalin_2	Lipocalin / cytosolic fatty-acid binding protein family;Lipocalin-like domain	CYT	19	58	34.1	High
TRINITY_DN1326_c0_g1_i1_orf1	cuticle protein 7-like [Ostrinia furnacalis]	0.075262557	-3.731923883	0.0007959	down	yes	0.6593	8.76	9.675	9.612	6.994	0.521	0.678	0.779	MF:molec	GO:00036	------	------	------	------	ENOG410YSFI;EI	S:Function unknown;	PF00379.26	Chitin_bind_4	Insect cuticle protein	CYT	1	27	13.9	High
TRINITY_DN21719_c0_g1_i2_orf1	chymotrypsin-2-like [Ostrinia furnacalis]	0.158648771	-2.65609175	4.98E-06	down	yes	1.052	6.631	6.673	6.89	6.331	1.13	0.976	1.049	BP:nitrog	GO:00068	K01312	PRSS1_2_3	map04972;m	Pancreatic secret	COG5640	O:Posttranslational modification, protein turnover, chaperones	PF00089.29	Trypsin	Trypsin	CYT	2	14	15.6	High
TRINITY_DN42719_c0_g1_i1_orf1	inter-alpha-trypsin inhibitor heavy chain H4-like isoform X11 [Ostrinia furnacalis]	0.439128375	-1.187285335	0.001278	down	yes	1.854	4.222	4.229	4.658	3.78	1.566	2.052	1.945	------	------	------	------	------	------	------	------	------	------	------	CYT	1	23	7.4	High
TRINITY_DN31_c0_g1_i3_orfp1	TRINITY_DN31_c0_g1_i3_m.1394 TRINITY_DN31_c0_g1::TRINITY_DN31_c0_g1_i3::g.1394 ORF type:complete len:118 (+),score=43.35 TRINITY_DN31_c0_g1_i3:56-409(+)	0.235170824	-2.08821901	2.99E-06	down	yes	1.122	4.771	4.916	4.655	4.741	1.238	1.087	1.042	------	------	------	------	------	------	------	------	------	------	------	CYT	2	15	12.6	High
TRINITY_DN2002_c0_g1_i5_orfp1	TRINITY_DN2002_c0_g1_i5_m.4230 TRINITY_DN2002_c0_g1::TRINITY_DN2002_c0_g1_i5::g.4230 ORF type:3prime_partial len:259 (+),score=29.98,Peptidase_C39 PF03412.16 1.7,Peptidase_C39|PF03412.16|0.049 TRINITY_DN2002_c0_g1_i5:65-838(+)	0.233841182	-2.096399069	9.61E-05	down	yes	1.013	4.332	3.941	4.432	4.623	0.9	1.095	1.044	------	------	------	------	------	------	------	------	------	------	------	CYT	1	5	29.4	Medium
TRINITY_DN8595_c0_g1_i3_orf1	aldose reductase-like isoform X4 [Trichoplusia ni]	2.06841166	1.048523342	2.31E-05	up	yes	3.477	1.681	1.681	1.631	1.73	3.458	3.615	3.359	MF:cataly	GO:00038	K00011	AKR1B	map00051;m	Fructose and ma	COG0656	S:Function unknown	PF00248.24	Aldo_ket_red	Aldo/keto reductase family	CYT	1	67	13.9	High
TRINITY_DN43350_c0_g3_i1_orf1	uncharacterized protein LOC114355190 [Ostrinia furnacalis]	2.770885029	1.470346851	6.13E-05	up	yes	3.35	1.209	1.143	1.202	1.283	3.431	3.124	3.496	------	------	------	------	------	------	ENOG4111DQA	S:Function unknown	------	------	------	CYT	3	25	25.8	High
TRINITY_DN1534_c0_g1_i3_orf1	peptidoglycan recognition protein-like [Ostrinia furnacalis]	2.63174859	1.396021675	3.75E-05	up	yes	3.266	1.241	1.316	1.154	1.252	3.301	3.095	3.401	BP:glycos	GO:00302	K24096;K	PGRP-SB;PC	map04624	Toll and Imd sigr	ENOG4111PAY;E	O:Posttranslational modification, protein turnover, chaperones;M:Cell wall/membrane/envelope biogenesis;M:Cell wall/membrane/envelope biogenesis	PF01510.28	Amidase_2	N-acetylmuramoyl-L-alanine amidase	CYT	2	23	11.1	High
TRINITY_DN2044_c0_g1_i5_orfp1	TRINITY_DN2044_c0_g1_i5_m.4210 TRINITY_DN2044_c0_g1::TRINITY_DN2044_c0_g1_i5::g.4210 ORF type:complete len:151 (-),score=85.31 TRINITY_DN2044_c0_g1_i5:857-1309(-)	3.208797831	1.682032896	0.005681	up	yes	10.65	3.319	3.451	3.585	2.921	10.897	8.213	12.841	------	------	------	------	------	------	------	------	------	------	------	CYT	1	5	16.2	High
TRINITY_DN52553_c0_g2_i1_orf1	hemocyte protein-glutamine gamma-glutamyltransferase-like [Ostrinia furnacalis]	0.10510883	-3.250044216	5.68E-05	down	yes	0.932	8.867	9.519	9.05	8.031	0.95	1.012	0.834	BP:primar	GO:00442	K05619	TGM1	------	------	ENOG410XQEZ	S:Function unknown	PF00868.23	Transglut_N	Transglutaminase family	CYT	2	11	25.2	High
TRINITY_DN57998_c1_g1_i1_orf1	uncharacterized protein LOC113509309, partial [Galleria mellonella]	0.265010722	-1.915877365	0.0008481	down	yes	1.483	5.596	5.526	6.395	4.866	1.558	1.257	1.635	------	------	------	------	------	------	ENOG4111SVQ	S:Function unknown	------	------	------	CYT	1	14	9.1	High
TRINITY_DN8008_c0_g1_i6_orf1	uncharacterized protein LOC114357965 isoform X1 [Ostrinia furnacalis] >XP_028167599.1 uncharacterized protein LOC114357965 isoform X1 [Ostrinia furnacalis] >XP_028167600.1 uncharacterized protein LOC114357965 isoform X2 [Ostrinia furnacalis] >XP_028167601.1 uncharacterized protein LOC114357965 isoform X3 [Ostrinia furnacalis]	3.071335079	1.618865917	2.66E-05	up	yes	4.693	1.528	1.688	1.394	1.501	4.918	4.642	4.52	MF:organ	GO:00971	------	------	------	------	ENOG410ZC30	S:Function unknown	------	------	------	CYT	1	0	506.7	Medium
TRINITY_DN2311_c0_g3_i1_orf1	uncharacterized protein LOC114364231 isoform X1 [Ostrinia furnacalis] >XP_028176108.1 uncharacterized protein LOC114364231 isoform X2 [Ostrinia furnacalis]	0.330556773	-1.597030018	0.0001081	down	yes	1.508	4.562	4.518	4.914	4.253	1.415	1.612	1.498	------	------	------	------	------	------	ENOG410Y2YK	S:Function unknown	------	------	------	CYT	1	2	41.4	High
TRINITY_DN25534_c0_g1_i1_orf1	venom serine carboxypeptidase-like [Ostrinia furnacalis]	0.213782803	-2.225782286	6.27E-05	down	yes	1.002	4.687	4.655	4.691	4.714	1.409	0.889	0.709	BP:primar	GO:00442	K09645	CPVL	------	------	ENOG410XPIW;(	S:Function unknown;E:Amino acid transport and metabolism	PF00450.25	Peptidase_S10	Serine carboxypeptidase	CYT	4	12	55.9	High
TRINITY_DN26301_c0_g1_i1_orf1	uncharacterized protein LOC114359193 [Ostrinia furnacalis]	0.393320453	-1.346222887	4.97E-06	down	yes	1.425	3.623	3.708	3.642	3.518	1.49	1.419	1.365	CC:cellula	GO:00055	------	------	------	------	ENOG410ZPH9;E	S:Function unknown;	PF01607.27	CBM_14	Chitin binding Peritrophin-A domain	CYT	3	16	28.5	High

TRINITY_DN1209_c0_g1_i9_orf1	NADP-dependent malic enzyme-like isoform X1 [Ostrinia furnacalis] >XP_028161889.1 NADP-dependent malic enzyme-like isoform X1 [Ostrinia furnacalis] >XP_028161891.1 NADP-dependent malic enzyme-like isoform X3 [Ostrinia furnacalis]	2.197809278	1.136066197	0.0002645	up	yes	3.411	1.552	1.434	1.742	1.481	3.447	3.188	3.597	MF:cation	GO:00431	K00029	E1.1.1.40, m	map00620;m	Pyruvate metabo	COG0281	S:Function unknown	PF03949.18;P	Malic_M;malic	Malic enzyme, NAD binding domain;Malic enzyme, N-terminal domain	CYT	25	41	70.7	High
TRINITY_DN4324_c0_g1_i1_orf1	uncharacterized protein LOC114354985 isoform X1 [Ostrinia furnacalis]	0.296382055	-1.754469995	9.87E-07	down	yes	1.024	3.455	3.384	3.473	3.508	1.088	0.995	0.988	CC:cellula	GO:00055	------	------	------	------	ENOG41113AM;	S:Function unknown;	------	------	------	CYT	4	20	16	High
TRINITY_DN2457_c0_g1_i8_orf1	uncharacterized protein LOC114355596 [Ostrinia furnacalis]	2.294074074	1.197911976	0.001265	up	yes	3.097	1.35	1.372	1.286	1.393	2.748	3.484	3.06	BP:biolog	GO:00081	------	------	------	------	ENOG410Y1IK	M:Cell wall/membrane/envelope biogenesis	PF12248.11	Methyltransf_FA	Farnesoic acid 0-methyl transferase	CYT	7	27	33.4	High
TRINITY_DN74086_c0_g1_i1_orf1	UPF0489 protein C5orf22 homolog [Ostrinia furnacalis]	0.355425401	-1.492381307	9.28E-05	down	yes	1.153	3.244	3.193	3.122	3.417	1.228	0.959	1.271	------	------	------	------	------	------	ENOG41109E9	S:Function unknown	PF12640.10	UPF0489	UPF0489 domain	CYT	1	4	42.6	High
TRINITY_DN12586_c0_g1_i4_orf1	zonadhesin-like isoform X4 [Ostrinia furnacalis]	4.168087698	2.059385633	0.001184	up	yes	3.422	0.821	0.682	0.853	0.928	3.797	2.813	3.656	------	------	------	------	------	------	ENOG4110RF5;C	S:Function unknown;O:Posttranslational modification, protein turnover, chaperones	PF01826.20	TIL	Trypsin Inhibitor like cysteine rich domain	PLA	1	26	14.3	High
TRINITY_DN858_c0_g1_i3_orf1	uncharacterized protein LOC114351944 [Ostrinia furnacalis]	0.365417089	-1.452383991	0.000129	down	yes	1.082	2.961	3.076	3.069	2.737	1.19	0.968	1.087	CC:cellula	GO:00055	------	------	------	------	------	------	PF16077.8	Spaetzle	Spaetzle	CYT	2	9	20.2	High
TRINITY_DN18624_c0_g1_i5_orf1	uncharacterized protein LOC114353135 [Ostrinia furnacalis]	0.112135466	-3.156685456	3.53E-06	down	yes	1.192	10.63	10.956	10.681	10.262	0.953	1.513	1.111	------	------	------	------	------	------	ENOG4110T9R	S:Function unknown	------	------	------	CYT	1	5	34.8	High
TRINITY_DN5408_c0_g1_i5_orf1	uncharacterized protein LOC114359912 [Ostrinia furnacalis]	0.250187829	-1.998916489	0.0003673	down	yes	1.665	6.655	6.785	7.3	5.879	1.919	1.353	1.722	CC:cellula	GO:00055	------	------	------	------	ENOG4110RME	S:Function unknown	------	------	------	PLA	1	5	30.4	High
TRINITY_DN644_c0_g1_i1_orf1	cuticle protein 19-like [Ostrinia furnacalis]	0.060555271	-4.04560364	3.17E-05	down	yes	0.4733	7.816	7.811	8.425	7.211	0.564	0.436	0.42	MF:molec	GO:00036	------	------	------	------	ENOG410YSFI	S:Function unknown	PF00379.26	Chitin_bind_4	Insect cuticle protein	CYT	4	34	14.8	High
TRINITY_DN2946_c0_g1_i1_orf1	histidine-rich glycoprotein [Ostrinia furnacalis]	0.128470858	-2.960486957	1.89E-05	down	yes	0.7847	6.108	6.303	6.359	5.662	0.825	0.742	0.787	------	------	------	------	------	------	ENOG410YTNT	S:Function unknown	------	------	------	PLA	9	28	23.4	High
TRINITY_DN20717_c0_g1_i1_orf1	putative uncharacterized protein DDB_G0282133 isoform X1 [Ostrinia furnacalis]	0.375137756	-1.414507623	0.000161	down	yes	1.702	4.537	4.271	4.554	4.785	1.79	1.424	1.893	------	------	------	------	------	------	ENOG410ZIVR	S:Function unknown	------	------	------	PLA	1	17	5.2	High
TRINITY_DN27300_c0_g1_i1_orfp1	TRINITY_DN27300_c0_g1_i1_m.71142 TRINITY_DN27300_c0_g1::TRINITY_DN27300_c0_g1_i1::g.71142 ORF type:internal len:82 (-),score=9.46 TRINITY_DN27300_c0_g1_i1:3-245(-)	2.549812734	1.350391295	0.0007567	up	yes	3.404	1.335	1.448	1.132	1.425	3.742	3.053	3.418	------	------	------	------	------	------	------	------	------	------	------	CYT	1	21	9.2	High
TRINITY_DN1749_c0_g2_i2_orf1	putative GPI-anchored protein pfl2 isoform X1 [Ostrinia furnacalis]	0.224194387	-2.157177938	1.13E-06	down	yes	0.8627	3.848	3.828	3.95	3.767	0.868	0.806	0.914	BP:develo	GO:00325	------	------	------	------	COG5599	T:Signal transduction	------	------	------	CYT	4	5	116.8	High
TRINITY_DN14328_c0_g1_i12_orf1	larval cuticle protein LCP-30-like [Ostrinia furnacalis]	3.184761905	1.67118552	0.0007501	up	yes	3.344	1.05	1.048	0.991	1.11	3.424	2.886	3.723	MF:molec	GO:00036	------	------	------	------	ENOG41112O7;E	S:Function unknown;	PF00379.26	Chitin_bind_4	Insect cuticle protein	CYT	10	50	24.6	High
TRINITY_DN20793_c0_g2_i1_orf1	mucin-2-like [Ostrinia furnacalis]	0.205233697	-2.284660469	3.02E-06	down	yes	0.7333	3.573	3.644	3.632	3.444	0.769	0.776	0.655	------	------	------	------	------	------	ENOG41115RA	S:Function unknown	------	------	------	EXC	4	9	52.5	High
TRINITY_DN5664_c0_g1_i1_orf1	CDGSH iron-sulfur domain-containing protein 3, mitochondrial-like [Ostrinia furnacalis]	0.205922358	-2.279827619	1.14E-05	down	yes	0.7267	3.529	3.621	3.511	3.454	0.878	0.742	0.56	CC:cytopl	GO:00057	K23886	CISD3	------	------	ENOG41122TT	S:Function unknown	PF09360.13	zf-CDGSH	Iron-binding zinc finger CDGSH type	CYT	1	12	15.7	High
TRINITY_DN7960_c0_g1_i2_orf1	uncharacterized protein LOC114364878 [Ostrinia furnacalis]	2.007851535	1.005652597	0.0001301	up	yes	2.813	1.401	1.36	1.361	1.481	2.98	2.679	2.78	------	------	------	------	------	------	COG0451	I:Lipid transport and metabolism	PF01370.24;P	Epimerase;GDP_Man_Dehyd;3Beta_HSD;NAD_binding_4;RmlD_sub_bind	NAD dependent epimerase/dehydratase family;GDP-mannose 4,6 dehydratase;3-beta hydroxysteroid dehydrogenase/isomerase family;Male sterility protein;RmlD substrate binding	CYT	13	50	34.5	High
TRINITY_DN2464_c0_g1_i12_orf1	uncharacterized protein LOC114362996 isoform X1 [Ostrinia furnacalis]	2.924151697	1.548018156	0.001626	up	yes	2.93	1.002	0.966	1.042	0.999	3.02	2.453	3.317	MF:molec	GO:00036	------	------	------	------	ENOG410YNFC	S:Function unknown	PF00379.26	Chitin_bind_4	Insect cuticle protein	CYT	8	45	37.2	High
TRINITY_DN4189_c0_g2_i1_orf1	unnamed protein product [Chilo suppressalis]	0.461625709	-1.115204522	3.30E-05	down	yes	1.221	2.645	2.743	2.573	2.618	1.23	1.135	1.297	BP:nitroge	GO:00068	K13046	tok	------	------	ENOG410ZPX7	S:Function unknown	PF00431.23;P	CUB;Astacin;FXa_inhibition;EGF_CA;cEGF;EGF	CUB domain;Astacin;Coagulation Factor Xa inhibitory site;Calcium-binding EGF domain;Complement Clr-like EGF-like;EGF-like domain	EXC	6	6	126.1	High
TRINITY_DN1091_c0_g3_i1_orf1	macrophage mannose receptor 1-like [Ostrinia furnacalis]	2.175590551	1.121407065	0.00164	up	yes	2.763	1.27	1.269	1.228	1.314	2.713	2.452	3.125	BP:respor	GO:00099	------	------	------	------	ENOG410XRS3;E	U:Intracellular trafficking, secretion, and vesicular transport;S:Function unknown;S:Function	PF00059.24	Lectin_C	Lectin C-type domain	CYT	9	34	38.3	High
TRINITY_DN125427_c0_g1_i1_orf1	heat shock protein 19.8 [Chilo suppressalis] >AGM90553.1 HSP19.8 [Chilo suppressalis] >BAE94664.1 small heat shock protein 19.7 [Chilo suppressalis]	0.4933281	-1.019380628	6.45E-05	down	yes	1.257	2.548	2.54	2.621	2.484	1.313	1.132	1.327	MF:cation	GO:00431	K09542	CRYAB	map04213;m	Longevity regula	ENOG410YERS	O:Posttranslational modification, protein turnover, chaperones	PF00011.24	HSP20	Hsp20/alpha crystallin family	CYT	3	39	21.2	High
TRINITY_DN18482_c0_g1_i3_orf1	calcyphosin-like protein isoform X3 [Helicoverpa armigera] >XP_047020698.1 calcyphosin-like protein isoform X2 [Helicoverpa zea]	2.307343608	1.206232865	0.0005374	up	yes	2.545	1.103	1.113	1.085	1.112	2.481	2.337	2.817	------	------	K23909	CAPS	------	------	ENOG410XRMJ	T:Signal transduction mechanisms	PF13405.9;P	EF-hand_6;EF-hand_7;EF-hand_1;EF-hand_5	EF-hand domain;EF-hand domain pair;EF hand;EF hand	CYT	9	56	25.6	High
TRINITY_DN285_c0_g1_i4_orf1	catalase-like [Ostrinia furnacalis]	2.158441558	1.109990031	1.94E-05	up	yes	2.493	1.155	1.176	1.105	1.184	2.555	2.392	2.533	BP:cellula	GO:00442	K03781	katE, CAT, c	map00630;m	Glyoxylate and d	COG0753	P:Inorganic ion transport and metabolism	PF00199.22;P	Catalase;Catalase-rel	Catalase;Catalase-related immune-responsive	CYT	9	23	58.6	High
TRINITY_DN10766_c0_g1_i1_orf1	hypothetical protein evm_008559 [Chilo suppressalis]	0.443006263	-1.174601	2.01E-05	down	yes	1.061	2.395	2.41	2.477	2.297	1.105	1.047	1.03	BP:primar	GO:00442	------	------	------	------	ENOG410XSGG	S:Function unknown	PF05577.15	Peptidase_S28	Serine carboxypeptidase S28	CYT	3	6	56.2	High
TRINITY_DN15685_c0_g1_i5_orf1	uncharacterized protein LOC114352354 [Ostrinia furnacalis]	2.949597017	1.560517862	1.26E-05	up	yes	2.452	0.8313	0.778	0.848	0.868	2.35	2.466	2.541	------	------	------	------	------	------	ENOG4110T90;E	S:Function unknown;	PF06585.14	JHBP	Haemolymph juvenile hormone binding protein	CYT	3	14	29.1	High
TRINITY_DN10364_c0_g1_i5_orf1	uncharacterized protein LOC114352615 [Ostrinia furnacalis]	0.498170174	-1.005289447	4.08E-05	down	yes	1.089	2.186	2.142	2.221	2.196	0.997	1.174	1.096	BP:organi	GO:00717	------	------	------	------	COG5640	I:Lipid transport and metabolism	PF00089.29;P	Trypsin;CLIP;Trypsin_2	Trypsin;Regulatory CLIP domain of proteinases;Trypsin-like peptidase	CYT	3	6	46.8	High
TRINITY_DN1563_c0_g1_i4_orf1	pupal cuticle protein 36-like [Ostrinia furnacalis]	0.428739694	-1.221826103	3.14E-05	down	yes	1.092	2.547	2.624	2.588	2.429	1.06	1.053	1.164	MF:molec	GO:00036	------	------	------	------	ENOG4111PJ1;E	S:Function unknown;	PF00379.26	Chitin_bind_4	Insect cuticle protein	CYT	5	41	30.1	High
TRINITY_DN5198_c0_g1_i5_orfp1	TRINITY_DN5198_c0_g1_i5_m.8637 TRINITY_DN5198_c0_g1::TRINITY_DN5198_c0_g1_i5::g.8637 ORF type:complete len:223 (-),score=54.99 TRINITY_DN5198_c0_g1_i5:319-987(-)	2.262536873	1.177941304	0.000313	up	yes	2.301	1.017	1.041	1.018	0.992	2.485	2.106	2.312	------	------	------	------	------	------	------	------	------	------	------	CYT	1	5	24.7	High
TRINITY_DN3255_c0_g1_i1_orf1	uncharacterized protein LOC114351042 [Ostrinia furnacalis]	0.175411363	-2.511185888	1.45E-05	down	yes	0.565	3.221	3.035	3.37	3.258	0.622	0.497	0.576	------	------	------	------	------	------	ENOG41114RO	S:Function unknown	------	------	------	CYT	1	6	13.9	High
TRINITY_DN2290_c0_g1_i2_orfp1	TRINITY_DN2290_c0_g1_i2_m.69732 TRINITY_DN2290_c0_g1::TRINITY_DN2290_c0_g1_i2::g.69732 ORF type:complete len:234 (+),score=14.43 TRINITY_DN2290_c0_g1_i2:60-761(+)	0.31509839	-1.666125712	0.001154	down	yes	0.8807	2.795	3.127	2.877	2.382	0.919	0.739	0.984	------	------	------	------	------	------	------	------	------	------	------	CYT	2	16	26.1	High
TRINITY_DN10304_c0_g2_i1_orf1	glycine-rich cell wall structural protein [Ostrinia furnacalis]	0.410763751	-1.283619221	0.0002079	down	yes	1.038	2.527	2.562	2.642	2.378	0.968	1.207	0.939	------	------	------	------	------	------	ENOG4111PWE;	S:Function unknown;	PF03103.20	DUF243	Domain of unknown function	CYT	2	16	27.6	High
TRINITY_DN15545_c0_g1_i1_orf1	larval cuticle protein LCP-14-like [Ostrinia furnacalis]	3.331265509	1.736070344	0.0003628	up	yes	2.685	0.806	0.804	0.829	0.785	2.846	2.35	2.858	MF:molec	GO:00036	------	------	------	------	ENOG4110RUZ;E	S:Function unknown;	PF00379.26	Chitin_bind_4	Insect cuticle protein	EXC	3	48	14.6	High
TRINITY_DN10231_c0_g2_i1_orf1	uncharacterized protein LOC114361472 [Ostrinia furnacalis]	0.458423093	-1.125248372	0.0005292	down	yes	1.064	2.321	2.176	2.292	2.495	1.064	1.205	0.923	CC:cellula	GO:00055	------	------	------	------	ENOG410XYE8	S:Function unknown	------	------	------	CYT	1	14	7.7	High
TRINITY_DN585_c0_g1_i5_orf1	very low-density lipoprotein receptor isoform X2 [Galleria mellonella]	0.498867241	-1.003272161	0.0002044	down	yes	1.101	2.207	2.207	2.304	2.109	1.08	1.002	1.221	CC:integr	GO:00160	K20053	VLDLR	map05417;m	Lipid and athero	ENOG410YQ6J;E	U:Intracellular trafficking, secretion, and vesicular transport;S:Function unknown	PF00057.21;P	Ldl_recept_a;Ldl_recept_b;FXa_inhibition;cEGF;EGF_CA;DUF5050	Low-density lipoprotein receptor domain class A;Low-density lipoprotein receptor repeat class B;Coagulation Factor Xa inhibitory site;Complement Clr-like EGF-like;Calcium-binding EGF domain;Domain of unknown function	PLA	2	33	94.4	High
TRINITY_DN14532_c0_g1_i1_orf1	pupal cuticle protein-like [Trichoplusia ni]	0.241525424	-2.049753035	4.65E-07	down	yes	0.627	2.596	2.547	2.647	2.595	0.62	0.604	0.657	MF:molec	GO:00036	------	------	------	------	ENOG4110QXC;E	S:Function unknown;	------	------	------	CYT	4	26	25.9	High
TRINITY_DN110519_c0_g1_i1_orf1	uncharacterized protein LOC114366601 [Ostrinia furnacalis]	0.278652058	-1.843463284	0.0001904	down	yes	0.8393	3.012	2.724	3.151	3.161	0.984	0.709	0.825	------	------	------	------	------	------	------	------	------	------	------	CYT	1	5	20	High
TRINITY_DN37585_c0_g2_i1_orf1	cuticle protein 19.8-like [Ostrinia furnacalis]	0.370574818	-1.432163249	0.002167	down	yes	0.8123	2.192	2.184	2.528	1.865	0.774	0.901	0.762	MF:molec	GO:00036	------	------	------	------	ENOG410YTXG;E	S:Function unknown;	PF00379.26	Chitin_bind_4	Insect cuticle protein	CYT	1	6	14.7	High
TRINITY_DN206_c0_g1_i11_orf1	A-kinase anchor protein 200-like [Ostrinia furnacalis] >XP_028173114.1 A-kinase anchor protein 200-like [Ostrinia furnacalis] >XP_028173115.1 A-kinase anchor protein 200-like [Ostrinia furnacalis]	0.386519421	-1.371387189	6.38E-06	down	yes	1.015	2.626	2.547	2.697	2.633	0.97	1.067	1.008	------	------	------	------	------	------	ENOG4110RAQ	S:Function unknown	------	------	------	CYT	3	12	67.6	High
TRINITY_DN1091_c0_g1_i1_orf1	macrophage mannose receptor 1-like [Pararge aegeria]	0.491368421	-1.025122952	0.0005191	down	yes	1.167	2.375	2.308	2.564	2.252	1.108	1.305	1.089	BP:respor	GO:00099	------	------	------	------	ENOG410XRS3;E	U:Intracellular trafficking, secretion, and vesicular transport;S:Function unknown;S:Function unknown;S:Function unknown;S:Function	PF00059.24	Lectin_C	Lectin C-type domain	CYT	1	6	33.2	High
TRINITY_DN661_c1_g2_i1_orf1	larval/pupal cuticle protein H1C-like [Ostrinia furnacalis]	0.273440727	-1.870699957	1.21E-05	down	yes	0.662	2.421	2.408	2.54	2.314	0.643	0.663	0.68	MF:molec	GO:00036	------	------	------	------	ENOG4111EA8;E	S:Function unknown;	PF00379.26	Chitin_bind_4	Insect cuticle protein	CYT	4	18	25.4	High
TRINITY_DN4068_c0_g2_i4_orf1	larval cuticle protein LCP-17-like precursor [Papilio polytes] >BAM18876.1 cuticular protein PpolCPR2 [Papilio polytes]	4.756004283	2.249750014	0.00029	up	yes	3.109	0.6537	0.707	0.695	0.559	3.27	2.708	3.348	MF:molec	GO:00036	------	------	------	------	ENOG4110RUZ;E	S:Function unknown;	PF00379.26	Chitin_bind_4	Insect cuticle protein	CYT	2	56	13.4	High
TRINITY_DN38431_c0_g1_i1_orf1	neprilysin-2 isoform X1 [Ostrinia furnacalis]	0.399362296	-1.324229962	1.20E-05	down	yes	1.002	2.509	2.574	2.472	2.48	0.959	1.095	0.953	BP:nitroge	GO:00068	K08635	MMEL1, NE	------	------	COG3590	O:Posttranslational modification, protein turnover, chaperones	PF05649.16;P	Peptidase_M13_N;Peptidase_M13	Peptidase family M13;Peptidase family M13	CYT	10	16	87.5	High
TRINITY_DN36281_c0_g1_i2_orf1	putative uncharacterized protein DDB_G0282499 isoform X1 [Ostrinia furnacalis]	0.495331258	-1.013534428	0.01313	down	yes	1.114	2.249	2.481	2.531	1.734	0.993	1.119	1.231	------	------	------	------	------	------	ENOG41113JC	S:Function unknown	------	------	------	EXC	1	2	81.1	High
TRINITY_DN311_c0_g1_i8_orfp1	TRINITY_DN311_c0_g1_i8_m.65152 TRINITY_DN311_c0_g1::TRINITY_DN311_c0_g1_i8::g.65152 ORF type:5prime_partial len:138 (+),score=74.30 TRINITY_DN311_c0_g1_i8:2-415(+)	2.462980319	1.3004051	0.0008439	up	yes	2.628	1.067	1.036	1.047	1.119	2.647	2.322	2.915	------	------	------	------	------	------	------	------	------	------	------	PLA	1	18	15	High
TRINITY_DN68770_c0_g1_i1_orf1	seroin transcript 1A2 [Ostrinia nubilalis]	2.704719857	1.435479174	0.0007575	up	yes	2.573	0.9513	1.039	0.967	0.848	2.898	2.468	2.352	------	------	------	------	------	------	------	------	------	------	------	CYT	1	30	8.7	High
TRINITY_DN55147_c0_g1_i1_orfp1	TRINITY_DN55147_c0_g1_i1_m.59251 TRINITY_DN55147_c0_g1::TRINITY_DN55147_c0_g1_i1::g.59251 ORF type:5prime_partial len:331 (-),score=113.97,Cuticle_3 PF11018.9 0.29,Cuticle_3|PF11018.9|2.9e-05,Cuticle_3|PF11018.9|0.00037 TRINITY_DN55147_c0_g1_i1:21-1013(-)	2.899188876	1.535649325	0.0002109	up	yes	2.502	0.863	0.86	0.847	0.882	2.477	2.296	2.734	------	------	------	------	------	------	------	------	PF11018.11	Cuticle_3	Pupal cuticle protein C1	CYT	7	50	33.7	High
TRINITY_DN1305_c0_g1_i6_orf1	glutathione S-transferase sigma 3 [Ostrinia furnacalis]	2.026264591	1.018822575	0.001749	up	yes	2.083	1.028	1.031	1.043	1.009	2.245	2.204	1.801	MF:cataly	GO:00038	K04097	HPGDS	map00590;m	Arachidonic acid	ENOG41103AU;E	O:Posttranslational modification, protein turnover, chaperones;	PF02798.23;P	GST_N;GST_C_3;GST_N_3	Glutathione S-transferase, N-terminal domain;Glutathione S-transferase, C-terminal domain;Glutathione S-transferase, N-terminal domain	CYT	2	26	19.4	High
TRINITY_DN1196_c0_g1_i5_orf1	glucosamine-6-phosphate isomerase isoform X1 [Ostrinia furnacalis]	0.423670588	-1.238985116	0.000155	down	yes	0.9003	2.125	2.226	2.108	2.041	1.031	0.794	0.876	BP:primar	GO:00442	K02564	nagB, GNPD	map00520	Amino sugar and	COG0363	G:Carbohydrate transport and metabolism	PF01182.23	Glucosamine_iso	Glucosamine-6-phosphate isomerases/6-	CYT	1	45	31.2	High
TRINITY_DN267_c0_g1_i1_orf1	keratin, type I cytoskeletal 9-like [Ostrinia furnacalis]	0.216038617	-2.210638875	1.60E-05	down	yes	0.6937	3.211	3.027	3.37	3.237	0.652	0.713	0.716	------	------	------	------	------	------	ENOG4110QOM	S:Function unknown	------	------	------	CYT	3	9	52.5	High
TRINITY_DN28501_c0_g1_i2_orfp1	TRINITY_DN28501_c0_g1_i2_m.58934 TRINITY_DN28501_c0_g1::TRINITY_DN28501_c0_g1_i2::g.58934 ORF type:internal len:98 (+),score=13.70 TRINITY_DN28501_c0_g1_i2:3-293(+)	3.814562161	1.931517473	7.32E-05	up	yes	2.74	0.7183	0.765	0.688	0.702	2.822	2.508	2.891	------	------	------	------	------	------	------	------	------	------	------	CYT	1	30	10.9	High
TRINITY_DN7565_c0_g1_i3_orf1	acylphosphatase-2-like [Ostrinia furnacalis]	0.419321267	-1.253872093	0.0007551	down	yes	0.9267	2.21	2.064	2.478	2.087	0.99	0.875	0.915	MF:hydro	GO:00167	K01512	acyP	map00620	Pyruvate metabo	ENOG41126ER	O:Posttranslational modification, protein turnover, chaperones	PF00708.21	Acylphosphatase	Acylphosphatase	CYT	1	9	13.7	High
TRINITY_DN843_c0_g1_i2_orf1	unnamed protein product [Diatraea saccharalis]	0.437451437	-1.192805226	0.001004	down	yes	1.126	2.574	2.877	2.506	2.338	1.073	1.071	1.235	------	------	------	------	------	------	ENOG4111K6M	S:Function unknown	PF08205.15;P	C2-set_2;Ig_3;I-set	CD80-like C2-set immunoglobulin domain;Immunoglobulin domain;Immunoglobulin I-set domain	CYT	2	32	48.6	High
TRINITY_DN206_c0_g1_i8_orf1	A-kinase anchor protein 200-like [Ostrinia furnacalis] >XP_028173114.1 A-kinase anchor protein 200-like [Ostrinia furnacalis] >XP_028173115.1 A-kinase anchor protein 200-like [Ostrinia furnacalis]	0.25858759	-1.951275055	0.0003057	down	yes	0.7543	2.917	2.823	3.265	2.663	0.723	0.702	0.838	------	------	------	------	------	------	ENOG4110RAQ	S:Function unknown	------	------	------	CYT	1	15	34.3	High
TRINITY_DN1196_c0_g1_i4_orf1	glucosamine-6-phosphate isomerase isoform X2 [Ostrinia furnacalis]	0.443043478	-1.17447981	0.0003185	down	yes	1.019	2.3	2.495	2.195	2.21	1.073	0.915	1.07	------	------	K02564	nagB, GNPD	map00520	Amino sugar and	COG0363	S:Function unknown	PF01182.23	Glucosamine_iso	Glucosamine-6-phosphate isomerases/6-	CYT	1	45	30.4	High
TRINITY_DN98242_c0_g1_i1_orf1	adenosine deaminase 2-A-like [Galleria mellonella]	0.214700118	-2.219605113	6.58E-05	down	yes	0.5477	2.551	2.335	2.707	2.61	0.61	0.504	0.529	BP:cellula	GO:00442	K19572	CECR1, ADA	map01232;m	Nucleotide meta	COG1816	F:Nucleotide transport and metabolism	PF00962.25;P	A_deaminase;A_deaminase_N	Adenosine deaminase;Adenosine/AMP deaminase N-terminal	CYT	1	1	57.4	High
TRINITY_DN125521_c0_g2_i1_orf1	seroin transcript 1A2 [Ostrinia nubilalis]	3.3062954	1.725215627	0.001061	up	yes	2.731	0.826	0.74	0.769	0.969	2.581	2.461	3.151	------	------	------	------	------	------	------	------	------	------	------	CYT	1	35	8.1	High
TRINITY_DN18502_c0_g1_i1_orf1	uncharacterized protein LOC114359515 [Ostrinia furnacalis]	0.158464567	-2.657767809	1.53E-05	down	yes	0.483	3.048	3.119	3.18	2.846	0.476	0.489	0.484	------	------	------	------	------	------	ENOG41117E9	S:Function unknown	------	------	------	CYT	6	15	59.7	High
TRINITY_DN33365_c0_g1_i1_orf1	mucin-5AC isoform X1 [Ostrinia furnacalis]	0.333118644	-1.585891994	2.59E-07	down	yes	0.9827	2.95	2.946	2.986	2.918	1.005	1.001	0.942	------	------	------	------	------	------	ENOG41120DH	S:Function unknown	------	------	------	CYT	3	2	204.2	High
TRINITY_DN218_c0_g1_i1_orf1	altered inheritance of mitochondria protein 3-like isoform X2 [Ostrinia furnacalis]	0.33266745	-1.587847383	0.0001225	down	yes	0.8493	2.553	2.577	2.732	2.349	0.87	0.789	0.889	MF:molec	GO:00036	------	------	------	------	ENOG4111QXK	S:Function unknown	PF00379.26	Chitin_bind_4	Insect cuticle protein	CYT	2	10	18	High
TRINITY_DN15327_c2_g1_i2_orf1	protein lethal(2)essential for life-like [Ostrinia furnacalis]	3.507273455	1.810349919	0.0002335	up	yes	2.628	0.7493	0.765	0.736	0.747	2.659	2.355	2.871	MF:cation	GO:00431	K09542	CRYAB	map04213;m	Longevity regula	ENOG410YERS	E:Amino acid transport and metabolism	PF00011.24;P	HSP20;Crystallin	Hsp20/alpha crystallin family;Alpha crystallin A chain, N terminal	CYT	7	44	22	High
TRINITY_DN3833_c0_g1_i4_orf1	division abnormally delayed protein [Ostrinia furnacalis]	0.441239776	-1.180365244	5.65E-05	down	yes	1.025	2.323	2.397	2.283	2.29	1.033	0.913	1.128	BP:regula	GO:00085	K02306	DALLY	map04310;m	Wnt signaling pa	ENOG410XST2	O:Posttranslational modification, protein turnover, chaperones	PF01153.22	Glypican	Glypican	CYT	2	3	60	High
TRINITY_DN83374_c0_g1_i1_orf1	uncharacterized protein LOC114350302, partial [Ostrinia furnacalis]	0.386899563	-1.369968994	7.25E-05	down	yes	0.886	2.29	2.415	2.282	2.173	0.967	0.811	0.88	------	------	------	------	------	------	ENOG410Y20T	S:Function unknown	------	------	------	CYT	2	7	63	High
TRINITY_DN4689_c0_g1_i5_orf1	pericentriolar material 1 protein-like isoform X3 [Ostrinia furnacalis]	0.490652264	-1.027227177	0.01706	down	yes	1.181	2.407	2.373	2.059	2.789	0.966	0.939	1.639	BP:cellula	GO:00226	K16537	PCM1	------	------	ENOG410YNFX	S:Function unknown	PF15717.8	PCM1_C	Pericentriolar material 1 C terminus	CYT	2	1	193.5	High
TRINITY_DN806_c0_g2_i1_orf1	uncharacterized protein LOC114355167 [Ostrinia furnacalis]	0.27952454	-1.83895315	1.96E-05	down	yes	0.729	2.608	2.529	2.645	2.649	0.638	0.866	0.683	MF:molec	GO:00036	------	------	------	------	ENOG410YR66;E	S:Function unknown;	PF00379.26	Chitin_bind_4	Insect cuticle protein	CYT	1	6	17.8	High
TRINITY_DN280_c0_g1_i8_orf1	Tubulin beta-1 chain [Papilio xuthus]	0.393829401	-1.344357276	2.42E-05	down	yes	0.868	2.204	2.209	2.277	2.127	0.79	0.932	0.882	BP:cellula	GO:00099	K07375	TUBB	map04540;m	Gap junction;Par	COG5023	Z:Cytoskeleton	PF00091.28;P	Tubulin;Tubulin_C	Tubulin/FtsZ family, GTPase domain;Tubulin C-terminal domain	CYT	4	53	50.6	High
TRINITY_DN11670_c0_g1_i1_orf1	teneurin-m isoform X1 [Ostrinia furnacalis]	0.397892442	-1.3295496	9.88E-07	down	yes	1.095	2.752	2.76	2.728	2.769	1.085	1.047	1.153	CC:integr	GO:00160	K24473	TENM, ODZ	------	------	ENOG410XQQD	S:Function unknown	PF15636.9;P	Tox-GHH;EGF_2;RHS_repeat	GHH signature containing HNH/Endo VII superfamily nuclease toxin;EGF-like domain;RHS Repeat	CYT	4	2	344.5	High
TRINITY_DN42337_c0_g1_i5_orf1	hypothetical protein evm_002829 [Chilo suppressalis]	0.297973901	-1.746742121	0.0004065	down	yes	0.8677	2.912	2.975	3.124	2.637	0.853	0.666	1.084	MF:molec	GO:00036	------	------	------	------	ENOG410YSFI;E	S:Function unknown;	PF00379.26	Chitin_bind_4	Insect cuticle protein	CYT	1	31	17.6	High
TRINITY_DN113272_c0_g1_i1_orf1	altered inheritance of mitochondria protein 3-like [Ostrinia furnacalis]	0.173968151	-2.523104886	5.69E-05	down	yes	0.5353	3.077	3.34	2.966	2.924	0.575	0.434	0.597	------	------	------	------	------	------	ENOG41113B8	S:Function unknown	------	------	------	PLA	1	5	33	High
TRINITY_DN54366_c0_g1_i1_orf1	protein obstructor-E-like [Ostrinia furnacalis]	0.198580889	-2.332201305	5.08E-06	down	yes	0.6297	3.171	3.04	3.29	3.184	0.682	0.594	0.613	CC:cellula	GO:00055	------	------	------	------	ENOG4111EK0	S:Function unknown	PF01607.27	CBM_14	Chitin binding Peritrophin-A domain	PLA	6	28	26.9	High
TRINITY_DN13648_c0_g1_i6_orf1	neurexin-4 [Ostrinia furnacalis]	0.409683426	-1.287418564	3.96E-05	down	yes	0.88	2.148	2.153	2.056	2.235	0.954	0.824	0.862	------	------	K07380	CNTNAP2	map04514	Cell adhesion mo	COG2091;ENOG	H:Coenzyme transport and metabolism;S:Function unknown	PF02210.27;P	Laminin_G_2;Laminin_G_1;F5_F8_type_C;Laminin_G_3;EGF	Laminin G domain;Laminin G domain;F5/8 type C domain;Concanavalin A-like lectin/glucanases superfamily;EGF-like	CYT	5	4	142.6	High
TRINITY_DN104_c0_g1_i4_orf1	PREDICTED: heparan-alpha-glucosaminide N-acetyltransferase [Amyelois transitella]	0.491878384	-1.023626439	8.27E-07	down	yes	1.181	2.401	2.437	2.366	2.401	1.196	1.158	1.188	------	------	K10532	HGSNAT	map04142;m	Lysosome;Glycos	COG4299	S:Function unknown	PF16401.8;P	DUF5009;HGSNAT_cat	Domain of unknown function;Heparan-alpha-glucosaminide N-acetyltransferase, catalytic	CYT	4	11	64.2	High
TRINITY_DN42964_c0_g1_i1_orf1	protein lethal(2)essential for life-like [Galleria mellonella]	0.414321192	-1.27117848	5.22E-05	down	yes	1.001	2.416	2.447	2.519	2.281	1.062	0.956	0.986	------	------	K09542	CRYAB	map04213;m	Longevity regula	ENOG410YERS	O:Posttranslational modification, protein turnover, chaperones	PF00011.24	HSP20	Hsp20/alpha crystallin family	CYT	1	5	23.2	High
TRINITY_DN114834_c0_g1_i1_orf1	uncharacterized protein LOC115444227 [Manduca sexta] >XP_030025790.1 uncharacterized protein LOC115444227 [Manduca sexta] >XP_037296791.1 uncharacterized protein LOC115444227 [Manduca sexta] >XP_037296792.1 uncharacterized protein LOC115444227 [Manduca sexta] >KAG6441350.1 hypothetical protein O3G_MSEX001778 [Manduca sexta]	0.343325434	-1.542351355	3.99E-06	down	yes	0.8693	2.532	2.519	2.62	2.458	0.872	0.859	0.877	CC:cellula	GO:00055	------	------	------	------	ENOG410XTAH	S:Function unknown	PF00024.29	PAN_1	PAN domain	CYT	10	19	81.4	High
TRINITY_DN668_c0_g1_i4_orf1	fatty acid synthase-like isoform X1 [Ostrinia furnacalis]	0.321422764	-1.637455986	8.98E-07	down	yes	0.7907	2.46	2.495	2.409	2.477	0.809	0.812	0.751	------	------	K00665	FASN	map04910;m	Insulin signaling	COG3321	Q:Secondary metabolites biosynthesis, transport and catabolism	PF00698.24;P	Acyl_transf_1;ketoacyl-synt;KR;Ketoacyl-synt_C;KAsynt_C_assoc;ADH_zinc_N;PS-DH;ADH_zinc_N_2;Thioesterase;PP-binding;adh_short	Acyl transferase domain;Beta-ketoacyl synthase, N-terminal domain;KR domain;Beta-ketoacyl synthase, C-terminal domain;Ketoacyl-synthetase C-terminal extension;Zinc-binding dehydrogenase;Polyketide synthase dehydratase;Zinc-binding dehydrogenase;Thioesterase domain;Phosphopantetheine attachment site;short chain	CYT	55	29	268.3	High
TRINITY_DN467_c0_g3_i1_orf1	histone-lysine N-methyltransferase 2B-like, partial [Ostrinia furnacalis]	0.280142438	-1.835767544	4.83E-06	down	yes	0.6687	2.387	2.413	2.42	2.329	0.635	0.618	0.753	MF:molec	GO:00036	------	------	------	------	ENOG4111I81	S:Function unknown	PF00379.26	Chitin_bind_4	Insect cuticle protein	CYT	7	19	58.3	High
TRINITY_DN214_c0_g1_i4_orf1	uncharacterized protein LOC114352813 [Ostrinia furnacalis]	2.543455126	1.346789641	0.0001162	up	yes	2.151	0.8457	0.869	0.738	0.93	2.277	2.052	2.124	------	------	------	------	------	------	ENOG4110S0N;E	S:Function unknown;	PF06585.14	JHBP	Haemolymph juvenile hormone binding protein	CYT	4	31	29.6	High
TRINITY_DN268_c1_g1_i7_orf1	unnamed protein product [Spodoptera littoralis] >CAH1645252.1 unnamed protein product [Spodoptera littoralis]	0.454070202	-1.139012732	1.83E-07	down	yes	1.216	2.678	2.675	2.66	2.698	1.201	1.248	1.2	CC:integr	GO:00160	K14708	SLC26A11	------	------	COG0659	S:Function unknown	PF00916.23	Sulfate_transp	Sulfate permease family	CYT	2	4	62.2	High

TRINITY_DN5757_c0_g1_i1_orf1	ATP-dependent DNA helicase 2 subunit 1 [Ostrinia furnacalis]	0.463996764	-1.107813352	0.00643	down	yes	1.147	2.472	2.133	2.329	2.953	1.101	1.261	1.079	BP:nucleic	GO:00903	K10884	XRCC6, KU7	map03450	Non-homologou	ENOG410XNXU	L:Replication, recombination and repair	PF02735.19;PI	Ku;Ku_N;Ku_C	Ku70/Ku80 beta-barrel domain;Ku70/Ku80 N-terminal alpha/beta domain;Ku70/Ku80 C-	CYT	1	2	66.8	High
TRINITY_DN25345_c0_g1_i1_orf1	chromodomain-helicase-DNA-binding protein 1 isoform X3 [Ostrinia furnacalis]	0.498466929	-1.004430302	0.006138	down	yes	1.138	2.283	2.244	2.516	2.089	0.891	1.041	1.481	CC:intrace	GO:00432	K11367	CHD1	------	------	COG0553;ENOG	U:Intracellular trafficking, secretion, and vesicular transport;L:Replication, recombination and repair	PF00176.26;PI	SNF2-rel_dom;CDH1_2_SANT_HL1;DUF4208;Chromo;Helicase_C;ResIII	SNF2-related domain;CDH1/2 SANT-Helical linker 1;Domain of unknown function;Chromo;Helicase conserved C-terminal domain;Type III restriction enzyme, res subunit	CYT	2	1	208.3	High
TRINITY_DN4125_c0_g1_i14_orf1	angiotensin-converting enzyme-like isoform X1 [Ostrinia furnacalis]	0.187938825	-2.41166496	9.35E-05	down	yes	0.5407	2.877	3.012	2.976	2.642	0.718	0.435	0.469	BP:nitrog	GO:00068	K01283	ACE, CD143	map05142;m	Chagas disease;F	ENOG410XPJ3	S:Function unknown	PF01401.21	Peptidase_M2	Angiotensin-converting enzyme	CYT	1	12	74.3	High
TRINITY_DN1593_c0_g1_i1_orf1	chemosensory protein csp11 [Helopeltis theivora]	2.134579439	1.093951854	0.001805	up	yes	2.284	1.07	1.048	1.072	1.09	2.257	2.014	2.581	------	------	------	------	------	------	ENOG4110RV8;E	S:Function unknown;	PF03392.16	OS-D	Insect pheromone-binding family, A10/OS-D	CYT	8	39	15.4	High
TRINITY_DN3826_c0_g1_i1_orf1	39S ribosomal protein L18, mitochondrial [Ostrinia furnacalis]	2.064321608	1.045667751	0.002011	up	yes	2.054	0.995	0.924	0.855	1.206	1.856	2.193	2.113	BP:cellula	GO:00442	K02881	RP-L18, MR	map03010	Ribosome	ENOG4111MCR	J:Translation, ribosomal structure and biogenesis	------	------	------	CYT	1	11	19.6	High
TRINITY_DN76633_c0_g1_i1_orfp1	TRINITY_DN76633_c0_g1_i1_m.53394 TRINITY_DN76633_c0_g1::TRINITY_DN76633_c0_g1_i1::g.53394 ORF type:internal len:164 (-),score=26.46,Toxin_2 PF00451.20 0.00022,Toxin_2|PF00451.20|0.00056,Toxin_2|PF00451.20|0.0002,Toxin_2|PF00451.20|5.9e-06,Gamma-thionin|PF00304.21|2,Gamma-thionin|PF00304.21|0.024,Gamma-thionin|PF00304.21|0.027,Gamma-thionin|PF00304.21|0.066,Toxin_38|PF14866.7|0.27,Toxin_38|PF14866.7|0.054,Toxin_38|PF14866.7|0.14,Defensin_2|PF01097.19|1.7,Defensin_2|PF01097.19|0.055,Defensin_2|PF01097.19|1.2,Defensin_2|PF01097.19|0.15 TRINITY_DN76633_c0_g1_i1:1-489(-)	2.354763749	1.235582323	0.00389	up	yes	2.128	0.9037	0.899	0.925	0.887	2.115	1.782	2.488	------	------	------	------	------	------	------	------	PF00451.22;PI	Toxin_2;Gamma-thionin	Scorpion short toxin, BmKK2;Gamma-thionin family	PLA	3	31	19	High
TRINITY_DN7123_c0_g1_i1_orf1	activating signal cointegrator 1 complex subunit 2 homolog isoform X1 [Ostrinia furnacalis]	0.276053577	-1.856979798	3.51E-06	down	yes	0.845	3.061	3.068	3.135	2.981	0.894	0.761	0.88	MF:molec	GO:00036	------	------	------	------	ENOG4111QXK	S:Function unknown	------	------	------	CYT	5	46	17.6	High
TRINITY_DN4688_c0_g1_i2_orf1	uncharacterized protein LOC114359411 [Ostrinia furnacalis]	0.423929664	-1.238103175	1.13E-05	down	yes	1.109	2.616	2.531	2.678	2.64	1.162	1.044	1.12	------	------	------	------	------	------	ENOG4110SAV	S:Function unknown	------	------	------	CYT	6	10	71.4	High
TRINITY_DN26985_c0_g1_i5_orf1	secretory phospholipase A2 receptor-like [Helicoverpa zea]	0.31842596	-1.650970135	1.73E-05	down	yes	0.8537	2.681	2.692	2.669	2.683	0.774	0.783	1.004	MF:molec	GO:00036	------	------	------	------	ENOG410XPJ1;E	S:Function unknown;U:Intracellular trafficking, secretion, and	------	------	------	CYT	2	26	9.8	High
TRINITY_DN394_c0_g1_i4_orf1	uncharacterized protein LOC114351483 [Ostrinia furnacalis]	2.327651515	1.218875081	0.0005157	up	yes	2.458	1.056	0.901	0.989	1.277	2.331	2.597	2.447	MF:molec	GO:00036	------	------	------	------	ENOG41113X7	S:Function unknown	PF00379.26	Chitin_bind_4	Insect cuticle protein	CYT	1	19	12.6	High
TRINITY_DN24789_c0_g1_i9_orfp1	TRINITY_DN24789_c0_g1_i9_m.25888 TRINITY_DN24789_c0_g1::TRINITY_DN24789_c0_g1_i9::g.25888 ORF type:internal len:114 (-),score=12.65 TRINITY_DN24789_c0_g1_i9:2-340(-)	2.220106244	1.150628719	0.001688	up	yes	2.215	0.9977	0.976	1.014	1.003	2.19	1.948	2.507	------	------	------	------	------	------	------	------	------	------	------	CYT	5	46	13.2	High
TRINITY_DN25987_c0_g1_i5_orf1	GILT-like protein 2 isoform X1 [Ostrinia furnacalis] >XP_028156245.1 GILT-like protein 2 isoform X2 [Ostrinia furnacalis] >XP_028156247.1 GILT-like protein 2 isoform X3 [Ostrinia furnacalis]	0.479534884	-1.060292327	0.002007	down	yes	1.031	2.15	2.153	2.229	2.068	0.925	1.325	0.842	MF:cataly	GO:00038	K08059	IFI30, GILT	map04612	Antigen processi	ENOG4111IDT	S:Function unknown	------	------	------	CYT	1	12	10.3	High
TRINITY_DN394_c0_g1_i2_orf1	uncharacterized protein LOC114351483 [Ostrinia furnacalis]	2.752997602	1.461003353	5.16E-05	up	yes	2.296	0.834	0.782	0.826	0.894	2.358	2.151	2.378	MF:molec	GO:00036	------	------	------	------	ENOG41113X7;E	S:Function unknown;	PF00379.26	Chitin_bind_4	Insect cuticle protein	CYT	1	28	12	High
TRINITY_DN31619_c0_g1_i2_orf1	endocuticle structural glycoprotein ABD-4-like [Ostrinia furnacalis]	0.249281314	-2.004153351	0.0004537	down	yes	0.607	2.435	2.45	2.714	2.14	0.7	0.534	0.587	MF:molec	GO:00036	------	------	------	------	ENOG4111ZVI	S:Function unknown	PF00379.26	Chitin_bind_4	Insect cuticle protein	CYT	1	7	14.7	High
TRINITY_DN4242_c0_g1_i6_orf1	fibrohexamerin-like [Ostrinia furnacalis]	4.176470588	2.062284278	4.21E-06	up	yes	2.414	0.578	0.631	0.587	0.516	2.48	2.338	2.425	CC:cellula	GO:00055	------	------	------	------	ENOG4110SS9	S:Function unknown	PF07294.14	Fibroin_P25	Fibroin P25	CYT	3	14	25.5	High
TRINITY_DN82426_c0_g1_i6_orfp1	lysosome-associated membrane glycoprotein 1-like isoform X4 [Ostrinia furnacalis]	0.388768898	-1.363015287	8.14E-05	down	yes	1.08	2.778	2.734	2.66	2.941	0.978	1.075	1.188	------	------	K06528	LAMP1_2, C	map05152;m	Tuberculosis;Pha	------	------	------	------	------	CYT	1	12	12.6	High
TRINITY_DN128231_c0_g1_i5_orf1	glutathione S-transferase sigma3 [Glyphodes pyloalis]	2.510983763	1.3282527	0.000263	up	yes	2.629	1.047	1.042	1.04	1.059	2.495	2.503	2.889	MF:cataly	GO:00038	K04097	HPGDS	map00590;m	Arachidonic acid	ENOG41103AU;E	O:Posttranslational modification, protein turnover, chaperones;	PF14497.9;PF(	GST_C_3;GST_C;GST_C_2	Glutathione S-transferase, C-terminal domain;Glutathione S-transferase, C-terminal domain;Glutathione S-transferase, C-terminal domain	CYT	1	14	9.5	High
TRINITY_DN710_c0_g1_i11_orfp1	TRINITY_DN710_c0_g1_i11_m.67699 TRINITY_DN710_c0_g1::TRINITY_DN710_c0_g1_i11::g.67699 ORF type:complete len:194 (-),score=34.99,Collagen PF01391.19 0.00029 TRINITY_DN710_c0_g1_i11:1283-1864(-)	2.220323741	1.150770048	0.0004408	up	yes	2.469	1.112	1.096	1.071	1.168	2.467	2.255	2.685	------	------	------	------	------	------	------	------	------	------	------	CYT	1	7	21.3	High
TRINITY_DN8095_c0_g1_i3_orf1	circadian clock-controlled protein-like [Ostrinia furnacalis]	2.331948439	1.22153589	0.0005954	up	yes	2.189	0.9387	1.037	0.83	0.949	2.274	2.325	1.967	------	------	------	------	------	------	ENOG41114TE;E	S:Function unknown;	PF06585.14	JHBP	Haemolymph juvenile hormone binding protein	CYT	4	18	27.1	High
TRINITY_DN4013_c0_g1_i4_orf1	uncharacterized protein LOC114353190 isoform X1 [Ostrinia furnacalis] >XP_028160984.1 uncharacterized protein LOC114353190 isoform X2 [Ostrinia furnacalis] >XP_028161062.1 uncharacterized protein LOC114353190 isoform X1 [Ostrinia furnacalis] >XP_028161142.1 uncharacterized protein LOC114353190 isoform X1 [Ostrinia furnacalis]	0.496325331	-1.010642008	0.001026	down	yes	1.013	2.041	2.156	2.111	1.856	0.929	0.946	1.164	CC:cellula	GO:00055	K12186	FAM125	map04144	Endocytosis	ENOG4110KHY	U:Intracellular trafficking, secretion, and vesicular transport	PF10240.12	DUF2464	Multivesicular body subunit 12	CYT	1	10	10.9	High
TRINITY_DN501_c0_g1_i5_orf1	hypothetical protein evm_002550 [Chilo suppressalis]	0.464933791	-1.104902811	1.76E-05	down	yes	0.948	2.039	2.024	2.082	2.01	0.988	0.869	0.987	CC:integr	GO:00160	------	------	------	------	ENOG410XSTY;E	S:Function unknown;	PF12947.10;PI	EGF_3;EGF_CA	EGF domain;Calcium-binding EGF	PLA	90	8	1895.1	High
TRINITY_DN5074_c0_g1_i7_orf1	zonadhesin-like [Ostrinia furnacalis]	0.324966383	-1.621637613	2.99E-07	down	yes	0.725	2.231	2.21	2.265	2.218	0.737	0.742	0.696	------	------	------	------	------	------	COG4826;ENOG	O:Posttranslational modification, protein turnover, chaperones;S:Function unknown;S:Function unknown;S:Function unknown;S:Function unknown	PF01826.20	TIL	Trypsin Inhibitor like cysteine rich domain	CYT	4	21	25.8	High
TRINITY_DN7711_c1_g1_i3_orf1	long-chain fatty acid transport protein 1-like [Ostrinia furnacalis]	0.495850622	-1.012022528	3.38E-06	down	yes	0.956	1.928	1.948	1.906	1.931	1.002	0.943	0.923	------	------	K08745	SLC27A1_4,	map04931;m	Insulin resistance	ENOG410XQ8T	S:Function unknown	PF00501.31;PI	AMP-binding;AMP-binding_C	AMP-binding enzyme;AMP-binding enzyme C-terminal domain	CYT	5	8	73.6	High
TRINITY_DN1436_c0_g1_i3_orf1	vacuolar protein sorting-associated protein 27-like [Trichoplusia ni]	0.485095321	-1.043659832	0.00022	down	yes	0.8397	1.731	1.835	1.758	1.6	0.823	0.861	0.835	MF:molec	GO:00036	------	------	------	------	ENOG4111O8G	S:Function unknown	PF00379.26	Chitin_bind_4	Insect cuticle protein	CYT	1	26	32.8	High
TRINITY_DN214_c0_g1_i3_orf1	uncharacterized protein LOC114352813 [Ostrinia furnacalis]	2.032327808	1.023133123	0.0003644	up	yes	1.974	0.9713	0.964	0.943	1.007	2.048	2.074	1.799	------	------	------	------	------	------	ENOG4110S0N;E	S:Function unknown;	PF06585.14	JHBP	Haemolymph juvenile hormone binding protein	CYT	3	38	19.4	High
TRINITY_DN110523_c0_g2_i1_orf1	uncharacterized protein LOC107036393 [Diachasma alloeum]	0.492875581	-1.02070459	0.0004151	down	yes	0.9547	1.937	1.996	2.028	1.787	0.929	1.052	0.883	------	------	K22628	IL16	map04060	Cytokine-cytokir	ENOG4110362;E	O:Posttranslational modification, protein turnover, chaperones;S:Function unknown	PF00595.27;PI	PDZ;PDZ_6;PDZ_2;Peptidase_M50	PDZ domain;PDZ domain;PDZ domain;Peptidase family M50	CYT	1	3	49	High
TRINITY_DN19923_c0_g1_i1_orf1	uncharacterized protein LOC114350958 [Ostrinia furnacalis]	0.40952616	-1.287972483	0.0001604	down	yes	0.8297	2.026	2.055	2.136	1.887	0.888	0.738	0.863	------	------	------	------	------	------	ENOG4111MP7	S:Function unknown	PF15430.9	SVWC	Single domain von Willebrand factor type C	CYT	7	71	16.2	High
TRINITY_DN14774_c0_g1_i4_orf1	aminopeptidase N-like [Ostrinia furnacalis]	0.494210526	-1.016802356	0.0003502	down	yes	0.939	1.9	1.98	1.759	1.961	0.871	0.916	1.03	BP:nitrog	GO:00068	K11140	ANPEP, CD1	map04640;m	Hematopoietic c	COG0308	O:Posttranslational modification, protein turnover, chaperones	PF01433.23;PI	Peptidase_M1;ERAP1_C;Peptidase_M1_N	Peptidase family M1 domain;ERAP1-like C-terminal domain;Peptidase M1 N-terminal domain	CYT	6	7	106.7	High
TRINITY_DN135679_c0_g1_i2_orfp1	TRINITY_DN135679_c0_g1_i2_m.85525 TRINITY_DN135679_c0_g1::TRINITY_DN135679_c0_g1_i2::g.85525 ORF type:5prime_partial len:55 (+),score=5.08,Toxin_2 PF00451.20 1.9e-06 TRINITY_DN135679_c0_g1_i2:3-167(+)	2.237280349	1.161746049	4.25E-05	up	yes	1.948	0.8707	0.859	0.84	0.913	1.969	1.85	2.024	------	------	------	------	------	------	------	------	PF00451.22	Toxin_2	Scorpion short toxin, BmKK2	CYT	1	46	6.1	High
TRINITY_DN978_c9_g2_i1_orf1	hypothetical protein evm_000959 [Chilo suppressalis]	0.303365385	-1.720871618	4.11E-05	down	yes	0.631	2.08	2.005	2.175	2.059	0.685	0.521	0.687	------	------	------	------	------	------	ENOG410Z4TG;E	S:Function unknown;	------	------	------	CYT	1	8	14.8	High
TRINITY_DN1344_c0_g1_i1_orf1	ribosomal RNA small subunit methyltransferase NEP1 [Ostrinia furnacalis]	2.150359967	1.104578185	0.000177	up	yes	1.822	0.8473	0.957	0.785	0.8	1.776	1.916	1.773	BP:rRNA (	GO:00311	K14568	EMG1, NEP1	map03008	Ribosome bioger	COG1756	J:Translation, ribosomal structure and biogenesis	PF03587.17	EMG1	EMG1/NEP1 methyltransferase	CYT	1	10	15.9	High
TRINITY_DN5191_c0_g2_i1_orf1	CD151 antigen-like [Ostrinia furnacalis]	2.20643141	1.1417149	0.003754	up	yes	1.832	0.8303	0.867	0.63	0.994	1.894	2.014	1.589	CC:cellula	GO:00055	K06537	CD151, TSP/	------	------	ENOG4111IRY;EI	S:Function unknown;	PF00335.23	Tetraspanin	Tetraspanin family	PLA	3	12	28.7	High
TRINITY_DN9694_c0_g1_i1_orf1	larval cuticle protein LCP-17 [Helicoverpa armigera] >PZC82071.1 hypothetical protein B5X24_HaOG211161 [Helicoverpa armigera] >PZC87412.1 hypothetical protein B5X24_HaOG216859 [Helicoverpa armigera]	2.474727583	1.307269723	1.39E-05	up	yes	1.885	0.7617	0.78	0.735	0.77	1.922	1.802	1.932	MF:molec	GO:00036	------	------	------	------	ENOG4110RUZ;E	S:Function unknown;	PF00379.26	Chitin_bind_4	Insect cuticle protein	CYT	5	82	15.7	High
TRINITY_DN13236_c0_g1_i4_orf1	zonadhesin-like isoform X4 [Ostrinia furnacalis]	2.151467186	1.105320837	0.003949	up	yes	1.767	0.8213	0.78	0.868	0.816	1.778	1.491	2.032	------	------	------	------	------	------	COG4826;ENOG	O:Posttranslational modification, protein turnover, chaperones;S:Function unknown	PF01826.20	TIL	Trypsin Inhibitor like cysteine rich domain	CYT	2	48	10.2	High
TRINITY_DN14009_c0_g1_i1_orf1	proline-rich extensin-like protein EPR1 [Manduca sexta]	0.39408424	-1.343424038	1.31E-05	down	yes	0.8327	2.113	2.069	2.113	2.157	0.916	0.804	0.778	------	------	------	------	------	------	ENOG4111PWE	S:Function unknown	PF03103.20	DUF243	Domain of unknown function	CYT	3	12	26.8	High
TRINITY_DN3073_c0_g1_i7_orf1	claspin-like [Ostrinia furnacalis]	0.498060235	-1.005607864	3.84E-05	down	yes	0.9757	1.959	2.003	1.937	1.936	1.036	0.889	1.002	BP:L-met	GO:00712	K25773	CLSPN	------	------	COG4229	U:Intracellular trafficking, secretion, and vesicular	------	------	------	CYT	17	31	81.8	High
TRINITY_DN14721_c0_g1_i2_orf1	protein masquerade-like isoform X2 [Ostrinia furnacalis]	0.321223709	-1.638349713	0.0001717	down	yes	0.672	2.092	2.27	1.973	2.033	0.587	0.661	0.768	------	------	------	------	------	------	ENOG411279V	S:Function unknown	------	------	------	CYT	1	3	38	High
TRINITY_DN4695_c0_g1_i3_orf1	glutathione S-transferase epsilon 3 [Ostrinia furnacalis]	4.46948873	2.160109809	5.62E-05	up	yes	2.439	0.5457	0.574	0.535	0.528	2.505	2.235	2.578	MF:cataly	GO:00038	K00799	GST, gst	map05207;m	Chemical carcinc	COG0625	S:Function unknown	PF13417.9;PF(	GST_N_3;GST_N;GST_C;GST_N_2;GST_C_3;GST_C_2	Glutathione S-transferase, N-terminal domain;Glutathione S-transferase, N-terminal domain;Glutathione S-transferase, C-terminal domain;Glutathione S-transferase, N-terminal domain;Glutathione S-transferase, C-terminal domain;Glutathione S-transferase, C-terminal domain	CYT	2	9	24.8	High
TRINITY_DN245_c0_g1_i4_orf1	ER lumen protein-retaining receptor [Ostrinia furnacalis]	2.142222222	1.099108145	0.01212	up	yes	1.928	0.9	0.902	1.005	0.793	1.882	2.344	1.558	BP:protei	GO:00081	K10949	KDELR	map05110	Vibrio cholerae i	COG5196	U:Intracellular trafficking, secretion, and vesicular	PF00810.21	ER_lumen_recept	ER lumen protein retaining receptor	CYT	1	9	24.4	High
TRINITY_DN21930_c0_g1_i1_orf1	coactosin-like protein isoform X2 [Trichoplusia ni]	0.460416667	-1.118988036	0.0006628	down	yes	0.8177	1.776	1.72	1.805	1.804	0.679	1.002	0.772	------	------	------	------	------	------	ENOG4111QQE	S:Function unknown	PF00241.23	Cofilin_ADF	Cofilin/tropomyosin-type actin-binding protein	CYT	1	5	29.8	High
TRINITY_DN147458_c0_g1_i1_orf1	60S ribosomal protein L5, partial [Cotesia chilonis]	0.219429058	-2.188173504	1.51E-05	down	yes	0.515	2.347	2.323	2.447	2.271	0.542	0.416	0.587	BP:cellula	GO:00346	K02932	RP-L5e, RPL	map03010;m	Ribosome;Coron	COG0256	J:Translation, ribosomal structure and biogenesis	PF17144.7;PF	Ribosomal_L5e;Ribosomal_L18_c	Ribosomal large subunit proteins 60S L5, and 50S L18;Ribosomal L18 C-terminal region	CYT	1	13	37	High
TRINITY_DN22513_c0_g1_i4_orf1	DNA-directed RNA polymerase II subunit RPB1 [Ostrinia furnacalis] >XP_028179194.1 DNA-directed RNA polymerase II subunit RPB1 [Ostrinia furnacalis] >XP_028179195.1 DNA-directed RNA polymerase II subunit RPB1 [Ostrinia furnacalis]	2.430925222	1.281505515	0.002176	up	yes	1.918	0.789	0.784	0.866	0.717	1.735	1.792	2.226	MF:molec	GO:00036	------	------	------	------	ENOG4111M49	S:Function unknown	PF00379.26	Chitin_bind_4	Insect cuticle protein	CYT	1	5	40.9	Medium
TRINITY_DN1293_c0_g1_i4_orf1	putative fatty acyl-CoA reductase CG5065 [Ostrinia furnacalis]	0.412546518	-1.277371291	1.77E-05	down	yes	0.776	1.881	1.889	1.891	1.862	0.752	0.713	0.863	BP:primar	GO:00442	K13356	FAR	map04146;m	Peroxisome;Cutir	ENOG410XS7R	S:Function unknown	PF07993.15;PI	NAD_binding_4;Sterile;Epimerase;GDP_Man_Dehyd;Polysacc_synt_2	Male sterility protein;Male sterility protein;NAD dependent epimerase/dehydratase family;GDP-mannose 4,6 dehydratase;Polysaccharide biosynthesis	CYT	3	7	59.2	High
TRINITY_DN20244_c0_g1_i1_orfp1	uncharacterized protein LOC125235519 [Leguminivora glycinivorella]	0.31025641	-1.688467076	0.00142	down	yes	0.6897	2.223	1.93	2.581	2.157	0.711	0.61	0.748	------	------	------	------	------	------	------	------	------	------	------	CYT	1	7	12.3	High
TRINITY_DN867_c0_g1_i1_orf1	hemicentin-2-like isoform X1 [Ostrinia furnacalis]	0.463245703	-1.110150502	0.0002128	down	yes	0.9163	1.978	2.101	1.95	1.884	0.818	0.935	0.996	BP:develc	GO:00325	K16680	ED	map04391	Hippo signaling	ENOG410ZJSM	S:Function unknown	PF13927.9;PF(	Ig_3;I-set;Ig_2;ig;V-set;C2-set_2;fn3	Immunoglobulin domain;Immunoglobulin I-set domain;Immunoglobulin domain;Immunoglobulin domain;Immunoglobulin V-set domain;CD80-like C2-set immunoglobulin domain;Fibronectin	CYT	1	1	138.3	High
TRINITY_DN9458_c0_g1_i4_orf1	uncharacterized protein LOC114363583 [Ostrinia furnacalis]	2.613316261	1.385881729	0.0003304	up	yes	2.041	0.781	0.801	0.792	0.75	2.054	1.846	2.222	------	------	------	------	------	------	------	------	------	------	------	CYT	1	13	9.3	High
TRINITY_DN7735_c1_g1_i1_orf1	cuticular protein CPH [Spodoptera litura]	0.397849462	-1.329705445	0.001575	down	yes	0.814	2.046	2.284	2.103	1.75	0.871	0.744	0.827	CC:cellula	GO:00055	------	------	------	------	ENOG4110SF2;E	S:Function unknown;	------	------	------	CYT	1	5	15.2	High
TRINITY_DN8771_c0_g2_i1_orf1	regucalcin-like [Ostrinia furnacalis]	3.184005309	1.670842742	0.001475	up	yes	1.919	0.6027	0.559	0.642	0.607	2.035	1.589	2.134	CC:cytopl	GO:00057	K01053	gnl, RGN	map00030;m	Pentose phospha	COG3386	G:Carbohydrate transport and metabolism	PF08450.15	SGL	SMP-30/Gluconolactonase/LRE-like region	CYT	1	11	19.2	High
TRINITY_DN109503_c0_g1_i4_orf1	uncharacterized protein LOC114366345 isoform X2 [Ostrinia furnacalis]	2.010629877	1.00764753	0.0009704	up	yes	1.778	0.8843	0.938	0.881	0.834	1.813	1.593	1.929	BP:respor	GO:00096	------	------	------	------	ENOG4110S6N	S:Function unknown	------	------	------	CYT	1	45	18.1	High
TRINITY_DN4144_c0_g1_i7_orf1	uncharacterized protein LOC114350172 [Ostrinia furnacalis]	0.431079545	-1.213973986	0.008386	down	yes	0.7587	1.76	2.053	1.377	1.849	0.836	0.779	0.661	CC:cellula	GO:00055	------	------	------	------	ENOG4110KD1	S:Function unknown	PF00100.26	Zona_pellucida	Zona pellucida-like domain	CYT	2	7	42	High
TRINITY_DN9455_c0_g1_i6_orf1	uncharacterized protein LOC114360866 isoform X4 [Ostrinia furnacalis]	0.416576964	-1.26334503	0.0007328	down	yes	0.774	1.858	1.878	1.914	1.782	0.745	0.601	0.976	CC:integr	GO:00160	------	------	------	------	ENOG410XRYE;E	S:Function unknown;	PF00086.21;PI	Thyroglobulin_1;Kunitz_BPTI;WAP;Lustrin_cystein;Antistasin	Thyroglobulin type-1 repeat;Kunitz/Bovine pancreatic trypsin inhibitor domain;WAP-type;Lustrin, cysteine-rich repeated	CYT	1	1	158.2	High
TRINITY_DN72999_c0_g1_i1_orf1	protein obstructor-E-like isoform X1 [Ostrinia furnacalis] >XP_028169319.1 protein obstructor-E-like isoform X2 [Ostrinia furnacalis]	0.233527305	-2.098336847	5.00E-06	down	yes	0.5217	2.234	2.17	2.333	2.2	0.521	0.499	0.545	CC:cellula	GO:00055	------	------	------	------	ENOG4111EXD	S:Function unknown	PF01607.27	CBM_14	Chitin binding Peritrophin-A domain	CYT	6	24	32.9	High
TRINITY_DN1868_c0_g1_i1_orf1	protein obstructor-E isoform X1 [Ostrinia furnacalis]	0.261626838	-1.934417552	5.63E-06	down	yes	0.5693	2.176	2.199	2.244	2.086	0.591	0.582	0.535	------	------	------	------	------	------	ENOG410XQ49	S:Function unknown	PF01607.27	CBM_14	Chitin binding Peritrophin-A domain	CYT	16	68	30.6	High
TRINITY_DN46090_c0_g2_i1_orf1	inactive tyrosine-protein kinase 7-like, partial [Ostrinia furnacalis]	0.42980456	-1.218247305	0.004359	down	yes	0.7917	1.842	2.187	1.76	1.58	0.805	0.759	0.811	CC:intrins	GO:00312	K05127	PTK7, CCK4	------	------	ENOG410Y8QG;	T:Signal transduction mechanisms;	PF07714.20;PI	PK_Tyr_Ser-Thr;Pkinase	Protein tyrosine and serine/threonine kinase;Protein kinase domain	CYT	1	6	16.7	High
TRINITY_DN4068_c1_g2_i1_orf1	larval cuticle protein LCP-17 [Helicoverpa armigera] >PZC82071.1 hypothetical protein B5X24_HaOG211161 [Helicoverpa armigera] >PZC87412.1 hypothetical protein B5X24_HaOG216859 [Helicoverpa armigera]	2.148894292	1.103594516	0.0005492	up	yes	1.856	0.8637	0.722	0.924	0.945	1.923	1.719	1.925	MF:molec	GO:00036	------	------	------	------	ENOG4110RUZ;E	S:Function unknown;	PF00379.26	Chitin_bind_4	Insect cuticle protein	CYT	4	64	17	High
TRINITY_DN64719_c0_g1_i2_orfp1	TRINITY_DN64719_c0_g1_i2_m.37745 TRINITY_DN64719_c0_g1::TRINITY_DN64719_c0_g1_i2::g.37745 ORF type:internal len:91 (+),score=41.89 TRINITY_DN64719_c0_g1_i2:1-270(+)	2.188614287	1.130017722	0.002858	up	yes	1.78	0.8133	0.866	0.641	0.933	1.832	1.553	1.956	------	------	------	------	------	------	------	------	------	------	------	CYT	1	14	8.4	High
TRINITY_DN34786_c0_g1_i1_orf1	small heat shock protein Hsp29.7 [Ostrinia furnacalis]	0.380028599	-1.395820104	5.00E-06	down	yes	0.7973	2.098	2.081	2.052	2.162	0.826	0.811	0.755	------	------	K09542	CRYAB	map04213;m	Longevity regula	ENOG410YERS	O:Posttranslational modification, protein turnover, chaperones	PF00011.24	HSP20	Hsp20/alpha crystallin family	CYT	2	8	28.2	High
TRINITY_DN48590_c0_g1_i1_orf1	acyl-CoA Delta(11) desaturase isoform X1 [Ostrinia furnacalis] >XP_028172999.1 acyl-CoA Delta(11) desaturase isoform X2 [Ostrinia furnacalis] >XP_028173000.1 acyl-CoA Delta(11) desaturase isoform X1 [Ostrinia furnacalis]	0.22895189	-2.12688362	6.43E-06	down	yes	0.533	2.328	2.331	2.426	2.226	0.528	0.538	0.533	BP:fatty a	GO:00066	K00507	SCD, desC	map04936;m	Alcoholic liver di:	COG1398	J:Translation, ribosomal structure and biogenesis	------	------	------	CYT	4	14	36.9	High
TRINITY_DN71832_c0_g1_i1_orf1	basement membrane-specific heparan sulfate proteoglycan core protein isoform X13 [Ostrinia	0.307734259	-1.700243033	0.003395	down	yes	0.6207	2.017	1.778	2.457	1.816	0.694	0.546	0.622	BP:develc	GO:00325	------	------	------	------	ENOG410XTD2;E	S:Function unknown;	PF07679.19	I-set	Immunoglobulin I-set domain	CYT	1	12	8.3	High
TRINITY_DN9028_c0_g1_i5_orf1	decaprenyl-diphosphate synthase subunit 2-like [Ostrinia furnacalis]	0.463236169	-1.110180193	0.001269	down	yes	0.7787	1.681	1.546	1.867	1.63	0.891	0.735	0.71	BP:organi	GO:00717	K12505	PDSS2	map00900	Terpenoid backb	COG0142	S:Function unknown	PF00348.20	polyprenyl_synt	Polyprenyl synthetase	CYT	1	2	46.4	Medium
TRINITY_DN10057_c0_g2_i1_orf1	cell wall protein DAN4 [Ostrinia furnacalis]	0.269890796	-1.889552319	9.15E-05	down	yes	0.519	1.923	1.828	1.98	1.96	0.658	0.404	0.495	CC:cellula	GO:00055	------	------	------	------	COG4826	O:Posttranslational modification, protein turnover, chaperones	PF00079.23	Serpin	Serpin	CYT	1	1	157.8	High
TRINITY_DN6586_c0_g1_i1_orf1	fatty acyl-CoA reductase wat-like isoform X1 [Ostrinia furnacalis]	0.328229937	-1.607221264	0.0001161	down	yes	0.5767	1.757	1.738	1.896	1.637	0.607	0.531	0.592	BP:primar	GO:00442	K13356	FAR	map04146;m	Peroxisome;Cutir	ENOG410XS7R	O:Posttranslational modification, protein turnover, chaperones	PF07993.15;PI	NAD_binding_4;Sterile;Epimerase	Male sterility protein;Male sterility protein;NAD dependent epimerase/dehydratase family	CYT	3	7	71.4	High
TRINITY_DN57111_c0_g1_i1_orf1	trypsin-like serine proteinase T26 protein, partial [Chilo infuscatellus]	4.073913043	2.026415187	0.0007199	up	yes	1.874	0.46	0.41	0.458	0.512	1.94	1.591	2.09	BP:organi	GO:00717	------	------	------	------	------	------	------	------	------	CYT	1	26	7.9	High
TRINITY_DN5568_c0_g2_i2_orf1	carboxypeptidase D isoform X5 [Ostrinia furnacalis]	0.442211055	-1.177193002	0.0003541	down	yes	0.704	1.592	1.708	1.551	1.518	0.642	0.809	0.661	------	------	K01296	CPM	------	------	ENOG410XX0H	S:Function unknown	PF00246.27;PI	Peptidase_M14;Carboxypep_D_reg	Zinc carboxypeptidase;Carboxypeptidase	CYT	4	8	53.7	High
TRINITY_DN35633_c0_g2_i1_orf1	uncharacterized protein LOC114353024 [Ostrinia furnacalis]	0.495727085	-1.01238201	8.45E-06	down	yes	0.7193	1.451	1.419	1.478	1.456	0.697	0.756	0.705	------	------	------	------	------	------	ENOG410YUHN	S:Function unknown	------	------	------	CYT	4	12	45.2	High

TRINITY_DN11388_c0_g1_i4_orf1	limbic system-associated membrane protein-like, partial [Ostrinia furnacalis]	2.142675159	1.099413146	0.00312	up	yes	1.682	0.785	0.828	0.801	0.726	1.649	1.462	1.935	BP:develc	GO:00325	------	------	------	------	ENOG4111UKY	S:Function unknown	PF07679.19;PI	I-set;Ig_3;ig;Ig_2;V-set	Immunoglobulin I-set domain;Immunoglobulin domain;Immunoglobulin domain;Immunoglobulin domain;Immunoglobulin V-set domain	CYT	1	2	51.3	High
TRINITY_DN5954_c0_g1_i2_orf1	myosin-VIIa [Ostrinia furnacalis] >XP_028155907.1 myosin-VIIa [Ostrinia furnacalis]	0.454856115	-1.136517846	0.0001676	down	yes	0.7587	1.668	1.789	1.561	1.653	0.77	0.744	0.762	BP:cell dif	GO:00301	K10359	MYO7A, USI	------	------	COG5022	J:Translation, ribosomal structure and biogenesis	PF00063.24;PI	Myosin_head;MyTH4;FE RM_M;IQ	Myosin head;MyTH4 domain;FERM central domain;IQ calmodulin-binding	CYT	5	2	249.6	High
TRINITY_DN7735_c0_g1_i4_orf1	calphotin-like [Ostrinia furnacalis]	7.077205882	2.823179889	0.0001344	up	yes	1.925	0.272	0.156	0.339	0.321	2.057	1.987	1.732	------	------	------	------	------	------	ENOG4110RAS;E	S:Function unknown;	------	------	------	CYT	1	3	22.5	High
TRINITY_DN2722_c0_g1_i1_orf1	troponin C [Pieris rapae] >XP_045490973.1 troponin C-like isoform X1 [Colias croceus] >XP_049866665.1 troponin C-like [Pectinophora gossypiella]	2.269172932	1.18216656	4.24E-05	up	yes	1.509	0.665	0.664	0.666	0.665	1.488	1.446	1.593	MF:cation	GO:00431	------	------	------	------	COG5126	S:Function unknown	PF13499.9;PF(	EF-hand_7;EF-hand_1;EF-hand_6;EF-hand_8;EF-hand_9;EF-hand_5	EF-hand domain pair;EF hand;EF-hand domain;EF-hand domain pair;EF-hand domain;EF hand	CYT	9	58	18.1	High
TRINITY_DN26961_c0_g1_i1_orf1	uncharacterized protein LOC120424957 [Culex pipiens pallens]	2.281391831	1.189914252	0.009895	up	yes	1.508	0.661	0.718	0.642	0.623	1.297	1.358	1.868	CC:sarcor	GO:0030(	K17751	MYH6_7	map04260;m	Cardiac muscle c	COG5022	E:Amino acid transport and metabolism	PF01576.22	Myosin_tail_1	Myosin tail	CYT	5	64	12.3	High
TRINITY_DN32586_c0_g2_i1_orf1	unnamed protein product [Euphydryas editha]	2.685894547	1.425402663	0.0001745	up	yes	1.788	0.6657	0.6	0.713	0.684	1.808	1.648	1.909	MF:molec	GO:00036	------	------	------	------	ENOG410XNMR	S:Function unknown;	PF00059.24	Lectin_C	Lectin C-type domain	CYT	4	47	20.5	High
TRINITY_DN38568_c0_g1_i1_orf1	unnamed protein product, partial [Diatraea saccharalis]	2.381097561	1.251626734	0.0001233	up	yes	1.562	0.656	0.738	0.646	0.584	1.62	1.48	1.586	------	------	K12567	TTN	map05414;m	Dilated cardiomy	ENOG410XQFD;I	T:Signal transduction mechanisms;S:Function unknown;S:Function unknown;S:Function unknown	PF07679.19;PI	I-set;Ig_3;Ig_2;ig	Immunoglobulin I-set domain;Immunoglobulin domain;Immunoglobulin domain;Immunoglobulin domain	CYT	1	4	37.5	High
TRINITY_DN33418_c0_g1_i1_orf1	homer protein homolog 2-like [Ostrinia furnacalis]	0.429677419	-1.218674133	0.003812	down	yes	0.666	1.55	1.474	1.36	1.817	0.695	0.566	0.737	BP:signal	GO:00071	K15010	HOMER	map04068;m	FoxO signaling p	ENOG410XOWT	Z:Cytoskeleton	PF00568.26	WH1	WH1 domain	CYT	1	7	20.5	High
TRINITY_DN38230_c0_g1_i4_orf1	hypothetical protein evm_007803 [Chilo suppressalis]	2.11154345	1.078297934	0.01221	up	yes	1.628	0.771	0.75	0.669	0.894	1.937	1.654	1.294	BP:organ	GO:00464	------	------	------	------	COG0737	F:Nucleotide transport and metabolism	PF02872.21	5_nucleotid_C	5'-nucleotidase, C-terminal domain	CYT	1	17	12.1	High
TRINITY_DN8310_c0_g2_i1_orf1	uncharacterized protein LOC116773294 [Danaus plexippus plexippus] >OWR55545.1 hypothetical protein KGM_209260 [Danaus plexippus plexippus]	2.735109718	1.451598707	1.57E-05	up	yes	1.745	0.638	0.571	0.677	0.666	1.687	1.78	1.769	------	------	------	------	------	------	ENOG4110R2S;C	S:Function unknown;	PF08246.15	Inhibitor_I29	Cathepsin propeptide inhibitor domain	CYT	3	46	9.3	High
TRINITY_DN111488_c0_g1_i1_orf1	LOW QUALITY PROTEIN: formin-1-like [Chelonus insularis]	2.493690852	1.318282622	0.003354	up	yes	1.581	0.634	0.694	0.518	0.69	1.859	1.473	1.411	------	------	------	------	------	------	ENOG4111I6P	S:Function unknown	------	------	------	CYT	1	3	22.4	Medium
TRINITY_DN1005_c0_g1_i5_orf1	hypothetical protein evm_008839 [Chilo suppressalis] >CAB3526474.1 unnamed protein product [Chilo suppressalis] >CAH0403802.1 unnamed protein product [Chilo suppressalis]	0.355862458	-1.490608353	0.0009046	down	yes	0.6313	1.774	1.68	1.785	1.858	0.81	0.407	0.677	BP:nuclei	GO:00903	K14406	CSTF1	map03015	mRNA surveillan	ENOG410XPWJ;I	A:RNA processing and modification;S:Function unknown	PF00400.35;PI	WD40;CSTF1_dimer;ANAPC4_WD40;NBCH_WD40;Ge1_WD40;eIF2A	WD domain, G-beta repeat;Cleavage stimulation factor subunit 1, dimerisation domain;Anaphase-promoting complex subunit 4 WD40 domain;Neurobeachin beta propeller domain;WD40 region of Ge1, enhancer of mRNA-decapping protein;Eukaryotic translation initiation factor eIF2A	CYT	1	6	41.2	High
TRINITY_DN7803_c0_g1_i2_orf1	membrane-associated protein Hem [Ostrinia furnacalis]	0.449140486	-1.154761321	1.29E-05	down	yes	0.7577	1.687	1.751	1.644	1.667	0.76	0.731	0.782	------	------	K05750	NCKAP1, N/	map05130;m	Pathogenic Esch	ENOG410XPOI	S:Function unknown	PF09735.12	Nckap1	Nck-associated protein 1	CYT	2	2	129.5	High
TRINITY_DN59422_c0_g1_i2_orf1	larval cuticle protein LCP-22-like isoform X2 [Pectinophora gossypiella]	4.616910488	2.206927762	0.0005137	up	yes	1.862	0.4033	0.393	0.386	0.431	1.87	1.612	2.103	MF:molec	GO:00036	------	------	------	------	ENOG4111CO7;I	S:Function unknown;	PF00379.26	Chitin_bind_4	Insect cuticle protein	CYT	4	38	19.5	High
TRINITY_DN12222_c0_g1_i1_orf1	unnamed protein product [Chilo suppressalis]	0.492929293	-1.020547377	0.005987	down	yes	0.732	1.485	1.32	1.741	1.393	0.693	0.842	0.661	------	------	------	------	------	------	ENOG410XRRZ	S:Function unknown	PF01607.27	CBM_14	Chitin binding Peritrophin-A domain	EXC	1	1	139.6	High
TRINITY_DN15865_c0_g1_i1_orf1	carboxylesterase, partial [Ostrinia furnacalis]	0.415214866	-1.268069996	1.92E-05	down	yes	0.715	1.722	1.759	1.733	1.674	0.778	0.708	0.659	MF:hydro	GO:00167	K01049	ACHE	map00564;m	Glycerophospho	COG2272	O:Posttranslational modification, protein turnover, chaperones	PF00135.31;PI	COesterase;BD-FAE;Abhydrolase_3	Carboxylesterase family;BD-FAE;alpha/beta hydrolase fold	PLA	4	14	38	High
TRINITY_DN650_c0_g1_i3_orf1	chitinase 7 [Glyphodes pyloalis]	0.384699769	-1.378195131	1.38E-05	down	yes	0.6663	1.732	1.759	1.77	1.666	0.64	0.717	0.642	BP:amino	GO:00463	K01183	E3.2.1.14	map00520	Amino sugar anc	COG3325	S:Function unknown	PF00704.31;PI	Glyco_hydro_18;CBM_14	Glycosyl hydrolases family 18;Chitin binding Peritrophin-A domain	CYT	7	9	111.3	High
TRINITY_DN4571_c0_g1_i4_orf1	PREDICTED: nuclear factor NF-kappa-B p105 subunit [Microplitis demolitor] >KAG6558391.1 viral ankyrin V1 [Microplitis demolitor]	0.424019608	-1.237797114	0.003215	down	yes	0.692	1.632	1.753	1.779	1.364	0.781	0.567	0.728	BP:develc	GO:00325	K04734	NFKBIA	map05140;m	Leishmaniasis;Pr	------	------	PF12796.10;PI	Ank_2;Ank_4;Ank_5	Ankyrin repeats;Ankyrin repeats;Ankyrin repeats	CYT	1	9	17	High
TRINITY_DN4898_c0_g1_i7_orf1	annulin-like isoform X3 [Ostrinia furnacalis]	0.454230067	-1.138504889	1.12E-05	down	yes	0.7463	1.643	1.643	1.696	1.589	0.739	0.77	0.73	BP:primar	GO:00442	K05619	TGM1	------	------	ENOG410XQEZ	O:Posttranslational modification, protein turnover, chaperones	PF00927.25;PI	Transglut_C;Transglut_N;Transglut_core	Transglutaminase family, C-terminal ig like domain;Transglutaminase family;Transglutaminase-like	CYT	7	9	86.1	High
TRINITY_DN18782_c0_g1_i4_orf1	putative riboflavin kinase [Ostrinia furnacalis] >XP_028176654.1 putative riboflavin kinase [Ostrinia furnacalis]	2.257589942	1.174783466	0.0005314	up	yes	1.688	0.7477	0.709	0.659	0.875	1.624	1.82	1.621	BP:small r	GO:00442	K00861	RFK, FMN1	map00740;m	Riboflavin metab	COG0196	H:Coenzyme transport and metabolism	PF01687.20	Flavokinase	Riboflavin kinase	CYT	1	7	18.2	High
TRINITY_DN10889_c0_g1_i8_orf1	four and a half LIM domains protein 2 isoform X7 [Pectinophora gossypiella]	2.469043712	1.303952378	7.14E-05	up	yes	1.655	0.6703	0.65	0.686	0.675	1.656	1.555	1.753	------	------	------	------	------	------	ENOG410XRFY;E	S:Function unknown;K:Transcription	PF00412.25	LIM	LIM domain	CYT	19	58	39.3	High
TRINITY_DN1329_c0_g1_i5_orf1	neurogenic locus notch homolog protein 3 [Ostrinia furnacalis] >XP_028157678.1 neurogenic locus notch homolog protein 3 [Ostrinia furnacalis]	2.267151295	1.18088067	0.001393	up	yes	1.497	0.6603	0.719	0.638	0.624	1.506	1.316	1.668	------	------	------	------	------	------	ENOG410XP6K	L:Replication, recombination and repair	PF12947.10	EGF_3	EGF domain	CYT	13	43	54.1	High
TRINITY_DN9560_c0_g1_i5_orf1	uncharacterized protein LOC114357350 [Ostrinia furnacalis]	3.054716981	1.611038721	0.002098	up	yes	1.619	0.53	0.629	0.538	0.423	1.673	1.351	1.833	------	------	------	------	------	------	ENOG410XV86;E	S:Function unknown;	------	------	------	CYT	1	11	27.1	High
TRINITY_DN73224_c0_g4_i2_orf1	PREDICTED: poly(rC)-binding protein 3 isoform X2 [Vollenhovia emeryi]	0.48896346	-1.032201437	0.0003136	down	yes	0.6557	1.341	1.327	1.445	1.251	0.689	0.646	0.632	MF:organ	GO:00971	K21444	PCBP3_4	------	------	ENOG410XNN8	S:Function unknown	PF00013.32;PI	KH_1;KH_2;KH_4;MOEP19	KH domain;KH domain;KH domain;KH-like RNA-binding domain	CYT	2	5	57.5	High
TRINITY_DN5235_c0_g1_i7_orf1	peptidoglycan-recognition protein SA-like [Ostrinia furnacalis]	0.495275035	-1.013698192	2.29E-05	down	yes	0.7023	1.418	1.399	1.469	1.385	0.73	0.709	0.668	BP:glycos	GO:00302	K24096;K	PGRP-SB;P(	map04624	Toll and Imd sigr	ENOG4111PAY;E	O:Posttranslational modification, protein turnover, chaperones;M:Cell wall/membrane/envelope biogenesis;M:Cell wall/membrane/envelope biogenesis	PF01510.28	Amidase_2	N-acetylmuramoyl-L-alanine amidase	CYT	2	16	20.7	High
TRINITY_DN5211_c0_g1_i1_orf1	elongation of very long chain fatty acids protein AAEL008004-like [Ostrinia furnacalis]	0.426893676	-1.228051304	0.0003567	down	yes	0.6143	1.439	1.515	1.308	1.495	0.675	0.565	0.603	BP:fatty a	GO:00066	K10250	ELOVL7	map01040;m	Biosynthesis of u	ENOG410XRWT	I:Lipid transport and metabolism	PF01151.21	ELO	GNS1/SUR4 family	PLA	1	4	32.2	High
TRINITY_DN21124_c0_g1_i4_orf1	calsyntenin-1 [Ostrinia furnacalis]	0.373870744	-1.419388514	4.46E-06	down	yes	0.538	1.439	1.411	1.481	1.425	0.526	0.569	0.519	------	------	K22659	CLSTN1	------	------	ENOG410XT2J	U:Intracellular trafficking, secretion, and vesicular	PF19699.2;PF(	CLSTN_C;Cadherin	Calsyntenin C-terminal;Cadherin domain	CYT	10	14	95.1	High
TRINITY_DN6415_c0_g1_i1_orf1	D-arabinitol dehydrogenase 1-like [Ostrinia furnacalis]	2.099219785	1.069853222	0.0001314	up	yes	1.426	0.6793	0.7	0.657	0.681	1.522	1.402	1.354	MF:oxidor	GO:00164	K17818	ARD1	map00040	Pentose and gluc	COG1063	E:Amino acid transport and metabolism	PF08240.15;PI	ADH_N;ADH_zinc_N;Glu_dehyd_C	Alcohol dehydrogenase GroES-like domain;Zinc-binding dehydrogenase;Glucose dehydrogenase C-terminus	CYT	6	42	37.8	High
TRINITY_DN1313_c0_g1_i2_orf1	39S ribosomal protein L40, mitochondrial [Ostrinia furnacalis]	0.398964088	-1.325669203	0.0001332	down	yes	0.5777	1.448	1.561	1.419	1.363	0.554	0.595	0.584	CC:large r	GO:00159	K17421	MRPL40	------	------	ENOG4111POW	S:Function unknown	PF09812.12	MRP-L28	Mitochondrial ribosomal protein L28	CYT	1	11	23.8	High
TRINITY_DN42337_c0_g1_i6_orf1	cuticle protein 8-like [Leguminivora glycinivorella]	0.282428115	-1.824044382	0.001849	down	yes	0.442	1.565	1.464	1.822	1.408	0.447	0.298	0.581	MF:molec	GO:00036	------	------	------	------	ENOG410YSFI;EI	S:Function unknown;	PF00379.26	Chitin_bind_4	Insect cuticle protein	CYT	1	36	15.3	High
TRINITY_DN1267_c0_g2_i10_orf1	secretory phospholipase A2 receptor-like [Ostrinia furnacalis]	2.063392437	1.045018234	0.003327	up	yes	1.315	0.6373	0.674	0.588	0.65	1.405	1.105	1.434	MF:molec	GO:00036	------	------	------	------	ENOG410ZPTE;E	S:Function unknown;	PF00059.24	Lectin_C	Lectin C-type domain	CYT	2	5	34.1	High
TRINITY_DN5628_c0_g1_i5_orf1	hypothetical protein O3G_MSEX015036 [Manduca sexta]	2.203683299	1.139916903	0.002236	up	yes	1.4	0.6353	0.663	0.629	0.614	1.395	1.214	1.591	MF:cation	GO:00431	------	------	------	------	ENOG410Z840;E	S:Function unknown;O:Posttranslational modification, protein turnover, chaperones	PF00412.25	LIM	LIM domain	CYT	8	55	36.3	High
TRINITY_DN9820_c0_g1_i1_orf1	endocuticle structural glycoprotein SgAbd-2-like [Ostrinia furnacalis]	3.406201205	1.768163658	0.0003784	up	yes	1.527	0.4483	0.469	0.404	0.472	1.622	1.337	1.621	MF:molec	GO:00036	------	------	------	------	ENOG4111PJ1;EI	S:Function unknown;	PF00379.26	Chitin_bind_4	Insect cuticle protein	CYT	4	56	16	High
TRINITY_DN1066_c0_g1_i8_orf1	hypothetical protein evm_012420 [Chilo suppressalis]	0.479239972	-1.061179851	0.002489	down	yes	0.681	1.421	1.542	1.51	1.211	0.731	0.63	0.682	CC:macro	GO:00329	K24990	RBMS	------	------	ENOG410XRB5	S:Function unknown	PF00076.25	RRM_1	RNA recognition motif.	EXC	1	2	47.8	High
TRINITY_DN34703_c0_g1_i4_orf1	gamma-tubulin complex component 3 homolog [Ostrinia furnacalis]	0.430474198	-1.216001328	0.003254	down	yes	0.6173	1.434	1.379	1.606	1.318	0.561	0.487	0.804	BP:cellula	GO:00718	K16570	TUBGCP3, G	------	------	ENOG410XQ5B	S:Function unknown	PF17681.4;PF(	GCP_N_terminal;GCP_C_terminal	Gamma tubulin complex component N-terminal;Gamma tubulin complex component C-terminal	CYT	1	1	93.4	High
TRINITY_DN286_c0_g1_i2_orf1	uncharacterized protein LOC114361329 [Ostrinia furnacalis]	0.490607345	-1.027359263	8.18E-06	down	yes	0.6947	1.416	1.418	1.452	1.378	0.69	0.718	0.676	MF:actin I	GO:00037	K17914	KIF13	------	------	ENOG41102XR	S:Function unknown	------	------	------	CYT	4	3	150.3	High
TRINITY_DN17003_c0_g1_i1_orf1	mucin-5AC [Ostrinia furnacalis]	0.468086643	-1.095152498	0.01095	down	yes	0.6483	1.385	1.567	1.51	1.077	0.751	0.563	0.631	CC:cellula	GO:00055	K01183	E3.2.1.14	map00520	Amino sugar anc	COG3325;ENOG	J:Translation, ribosomal structure and biogenesis;S:Function unknown	PF01607.27	CBM_14	Chitin binding Peritrophin-A domain	CYT	1	2	72.5	High
TRINITY_DN2647_c0_g1_i3_orf1	DNA repair protein complementing XP-G cells homolog isoform X1 [Ostrinia furnacalis]	2.067733416	1.048050197	0.002458	up	yes	1.331	0.6437	0.696	0.626	0.609	1.471	1.143	1.379	BP:primar	GO:00442	K10846	ERCC5, XPG	map03420	Nucleotide excisi	COG0258	L:Replication, recombination and repair	PF00752.20;PI	XPG_N;XPG_I	XPG N-terminal domain;XPG I-region	CYT	1	1	132.1	Medium
TRINITY_DN17657_c0_g1_i1_orf1	alcohol dehydrogenase 18, partial [Helicoverpa assulta]	2.23092277	1.157640571	0.007667	up	yes	1.453	0.6513	0.663	0.66	0.631	1.758	1.392	1.21	MF:cataly	GO:00038	K00069	HPGD	map00590;m	Arachidonic acid	COG1028	I:Lipid transport and metabolism	------	------	------	CYT	2	61	5.7	High
TRINITY_DN2749_c4_g1_i2_orf1	RNA exonuclease 4-like [Ostrinia furnacalis] >OEF79882.1 REX4 [Ostrinia furnacalis]	2.921900601	1.5469071	0.0006963	up	yes	1.605	0.5493	0.522	0.572	0.554	1.422	1.588	1.804	BP:nuclei	GO:00903	K18327	REXO4, REX	------	------	COG0847	S:Function unknown	PF00929.27	RNase_T	Exonuclease	CYT	3	53	9.2	High
TRINITY_DN73923_c0_g1_i1_orf1	protein obstructor-E-like [Ostrinia furnacalis]	0.159552402	-2.647897769	6.02E-07	down	yes	0.2923	1.832	1.849	1.855	1.793	0.286	0.263	0.328	CC:cellula	GO:00055	------	------	------	------	ENOG410ZSCN	S:Function unknown	PF01607.27	CBM_14	Chitin binding Peritrophin-A domain	CYT	15	65	26.5	High
TRINITY_DN2840_c0_g1_i5_orf1	hypothetical protein evm_002181 [Chilo suppressalis]	0.321913381	-1.635255549	5.45E-06	down	yes	0.498	1.547	1.553	1.578	1.509	0.462	0.485	0.547	------	------	------	------	------	------	ENOG41129M9	S:Function unknown	------	------	------	PLA	3	23	16.5	High
TRINITY_DN48641_c0_g1_i4_orf1	RNA-binding protein 45-like [Galleria mellonella]	0.302419355	-1.72537762	0.0002689	down	yes	0.45	1.488	1.367	1.652	1.444	0.431	0.449	0.47	MF:organ	GO:00971	K25081	RBM45	------	------	ENOG410XP9Z	S:Function unknown	PF00076.25;PI	RRM_1;RRM_3	RNA recognition motif.;RNA binding	CYT	1	2	53.8	High
TRINITY_DN59829_c0_g1_i1_orf1	putative mediator of RNA polymerase II transcription subunit 12 [Ostrinia furnacalis]	0.349964311	-1.514720289	4.37E-06	down	yes	0.4903	1.401	1.417	1.416	1.369	0.533	0.472	0.466	------	------	------	------	------	------	ENOG4110RMW	S:Function unknown	------	------	------	CYT	4	10	67.1	High
TRINITY_DN45949_c0_g1_i1_orf1	uncharacterized protein LOC114355167 [Ostrinia furnacalis]	3.518779343	1.815075048	0.0001248	up	yes	1.499	0.426	0.423	0.409	0.446	1.48	1.384	1.632	MF:molec	GO:00036	------	------	------	------	ENOG410ZET2;E	O:Posttranslational modification, protein turnover, chaperones;S:Function unknown;S:Function unknown;S:Function unknown	PF00379.26	Chitin_bind_4	Insect cuticle protein	CYT	3	18	22.7	High
TRINITY_DN1212_c0_g1_i8_orf1	extensin isoform X5 [Ostrinia furnacalis] >XP_028175473.1 extensin isoform X5 [Ostrinia furnacalis] >XP_028175474.1 extensin isoform X5 [Ostrinia furnacalis]	2.838201782	1.504977161	0.001138	up	yes	1.37	0.4827	0.477	0.462	0.509	1.323	1.215	1.572	------	------	------	------	------	------	ENOG4111WKY;	S:Function unknown;	PF15936.8	DUF4749	Domain of unknown function	CYT	7	26	44.1	High
TRINITY_DN3913_c0_g1_i6_orf1	protein obstructor-E-like [Ostrinia furnacalis]	2.214124588	1.146736404	0.002229	up	yes	1.276	0.5763	0.58	0.561	0.588	1.243	1.122	1.464	CC:cellula	GO:00055	------	------	------	------	ENOG4111HJZ	S:Function unknown	PF01607.27	CBM_14	Chitin binding Peritrophin-A domain	CYT	9	46	33.8	High
TRINITY_DN1074_c0_g1_i7_orf1	eukaryotic translation initiation factor 4E type 2 [Ostrinia furnacalis]	0.461594203	-1.115302989	1.18E-05	down	yes	0.5733	1.242	1.262	1.194	1.27	0.586	0.562	0.572	CC:cytopl	GO:00057	K03259	EIF4E	map04910;m	Insulin signaling	COG5053	J:Translation, ribosomal structure and biogenesis	PF01652.21	IF4E	Eukaryotic initiation factor 4E	CYT	2	11	27.8	High
TRINITY_DN109931_c0_g1_i1_orf1	hydroxymethylglutaryl-CoA lyase, mitochondrial isoform X1 [Ostrinia furnacalis]	3.589873418	1.843932974	0.001644	up	yes	1.418	0.395	0.424	0.379	0.382	1.274	1.293	1.687	MF:hydro	GO:00044	K01640	HMGCL, hm	map04146;m	Peroxisome;Valir	COG0119	E:Amino acid transport and metabolism	------	------	------	CYT	1	15	5.9	High
TRINITY_DN9916_c0_g1_i1_orf1	PREDICTED: dynein light chain Tctex-type [Amyelois transitella] >XP_021195381.1 dynein light chain Tctex-type [Helicoverpa armigera] >XP_022815696.1 dynein light chain Tctex-type [Spodoptera litura] >XP_028156399.1 dynein light chain Tctex-type [Ostrinia furnacalis] >XP_035458261.1 dynein light chain Tctex-type-like [Spodoptera frugiperda] >XP_047034788.1 dynein light chain Tctex-type [Helicoverpa zea] >CAB3233358.1 unnamed protein product [Arctia plantaginis] >CAB3506583.1 unnamed protein product [Spodoptera littoralis] >CAG9754627.1 unnamed protein product [Diatraea saccharalis] >CAH0596395.1 unnamed protein product [Chrysodeixis includens] >KAF9808454.1 hypothetical protein SFRURICE_008507 [Spodoptera frugiperda]	0.449660889	-1.153090689	0.0007824	down	yes	0.5967	1.327	1.358	1.444	1.179	0.604	0.619	0.567	------	------	K10420	DYNLT1_3	map05132	Salmonella infec	ENOG4111NHR	S:Function unknown	PF03645.16	Tctex-1	Tctex-1 family	CYT	1	16	12.9	High
TRINITY_DN1134_c0_g1_i4_orf1	cytochrome P450 6B5-like [Ostrinia furnacalis]	2.068575125	1.048637354	8.96E-06	up	yes	1.279	0.6183	0.654	0.617	0.584	1.298	1.259	1.281	CC:endor	GO:00057	K14999	CYP6	------	------	COG2124	S:Function unknown	PF00067.25	p450	Cytochrome P450	CYT	3	16	56.4	High
TRINITY_DN31118_c0_g1_i1_orf1	CPR9 [Ostrinia furnacalis]	4.92934604	2.301396262	0.0002236	up	yes	1.5	0.3043	0.309	0.285	0.319	1.467	1.357	1.677	CC:integr	GO:0016(	------	------	------	------	ENOG41125BY;E	S:Function unknown;	PF00379.26	Chitin_bind_4	Insect cuticle protein	EXC	8	78	11.4	High
TRINITY_DN4156_c0_g1_i2_orf1	calcium channel flower [Ostrinia furnacalis]	0.349092229	-1.518319851	0.0001096	down	yes	0.4807	1.377	1.277	1.425	1.429	0.455	0.543	0.444	------	------	------	------	------	------	ENOG4111G4Q	U:Intracellular trafficking, secretion, and vesicular	PF10233.12	Cg6151-P	Uncharacterized conserved protein CG6151-P	CYT	1	10	20.9	High
TRINITY_DN28503_c0_g1_i6_orf1	uncharacterized protein LOC114363584 [Ostrinia furnacalis] >AXY94663.1 seroin transcript 3 [Ostrinia nubilalis]	2.051704265	1.036822794	0.002542	up	yes	1.246	0.6073	0.656	0.583	0.583	1.203	1.113	1.422	------	------	------	------	------	------	------	------	------	------	------	PLA	5	42	12.8	High
TRINITY_DN86127_c1_g1_i2_orfp1	TRINITY_DN86127_c1_g1_i2_m.43062 TRINITY_DN86127_c1_g1::TRINITY_DN86127_c1_g1_i2::g.43062 ORF type:internal len:69 (-),score=14.03 TRINITY_DN86127_c1_g1_i2:2-205(-)	2.493697886	1.318286692	0.0001254	up	yes	1.286	0.5157	0.5	0.498	0.549	1.279	1.204	1.376	------	------	------	------	------	------	------	------	------	------	------	CYT	1	16	7.8	High
TRINITY_DN64769_c0_g1_i3_orf1	procollagen-lysine,2-oxoglutarate 5-dioxygenase isoform X2 [Ostrinia furnacalis]	0.44824	-1.157656697	1.08E-06	down	yes	0.5603	1.25	1.271	1.248	1.231	0.57	0.544	0.567	------	------	K13647	PLODN	map00310	Lysine degradati	ENOG410Y4QU	O:Posttranslational modification, protein turnover, chaperones	------	------	------	CYT	8	13	83.9	High
TRINITY_DN72816_c0_g1_i2_orf1	Golgi apparatus protein 1 [Ostrinia furnacalis]	0.479792332	-1.059517993	4.51E-05	down	yes	0.6007	1.252	1.278	1.291	1.186	0.612	0.607	0.583	CC:organ	GO:0031(	K06816	GLG1, ESL1	map04514	Cell adhesion mc	ENOG410XNWV	U:Intracellular trafficking, secretion, and vesicular	PF00839.20	Cys_rich_FGFR	Cysteine rich repeat	CYT	3	3	126.2	High
TRINITY_DN1231_c0_g1_i4_orf1	AN1-type zinc finger protein 6 isoform X1 [Galleria mellonella]	0.490564579	-1.027485028	9.03E-05	down	yes	0.6343	1.293	1.245	1.328	1.305	0.696	0.583	0.624	MF:cation	GO:00431	K24687	ZFAND5_6	------	------	ENOG4111UWC	S:Function unknown	PF01428.19	zf-AN1	AN1-like Zinc finger	CYT	2	7	28.5	High
TRINITY_DN10479_c0_g1_i6_orf1	unnamed protein product [Chrysodeixis includens]	0.33127572	-1.593895625	6.25E-05	down	yes	0.483	1.458	1.45	1.369	1.554	0.473	0.463	0.513	------	------	------	------	------	------	ENOG4111SQM;	S:Function unknown;	------	------	------	CYT	1	4	26.6	High
TRINITY_DN1029_c0_g1_i1_orfp1	TRINITY_DN1029_c0_g1_i1_m.64408 TRINITY_DN1029_c0_g1::TRINITY_DN1029_c0_g1_i1::g.64408 ORF type:3prime_partial len:55 (+),score=13.47 TRINITY_DN1029_c0_g1_i1:74-235(+)	2.222420883	1.152132061	0.009717	up	yes	1.243	0.5593	0.668	0.555	0.455	1.22	1.024	1.486	------	------	------	------	------	------	------	------	------	------	------	CYT	1	56	6.1	High
TRINITY_DN12134_c0_g1_i4_orf1	glutathione S-transferase 1-1 [Ostrinia furnacalis] >XP_028161942.1 glutathione S-transferase 1-1 [Ostrinia furnacalis] >XP_028161943.1 glutathione S-transferase 1-1 [Ostrinia furnacalis]	0.38976378	-1.359328067	5.39E-05	down	yes	0.495	1.27	1.318	1.283	1.209	0.445	0.498	0.542	MF:cataly	GO:00038	K00799	GST, gst	map05207;m	Chemical carcinc	COG0625	O:Posttranslational modification, protein turnover, chaperones	PF02798.23;PI	GST_N;GST_N_3;GST_N_2	Glutathione S-transferase, N-terminal domain;Glutathione S-transferase, N-terminal domain;Glutathione S-transferase, N-terminal domain	CYT	2	16	26.7	High
TRINITY_DN2040_c0_g1_i15_orfp1	TRINITY_DN2040_c0_g1_i15_m.4150 TRINITY_DN2040_c0_g1::TRINITY_DN2040_c0_g1_i15::g.4150 ORF type:complete len:319 (+),score=125.43,Plasmodium_HRP PF05403.12 3.5,Plasmodium_HRP|PF05403.12|1.5 TRINITY_DN2040_c0_g1_i15:118-957(+)	2.566191446	1.359628804	2.94E-05	up	yes	1.26	0.491	0.458	0.459	0.556	1.231	1.263	1.287	------	------	------	------	------	------	------	------	------	------	------	PLA	2	16	30.3	High
TRINITY_DN31118_c0_g2_i1_orf1	unnamed protein product [Spodoptera exigua]	6.427662283	2.68429413	0.000578	up	yes	1.515	0.2357	0.252	0.212	0.243	1.582	1.267	1.696	MF:molec	GO:00036	------	------	------	------	ENOG410YVGC;I	S:Function unknown;	PF00379.26	Chitin_bind_4	Insect cuticle protein	CYT	2	53	12.9	High
TRINITY_DN549_c0_g1_i7_orf1	titin-like, partial [Ostrinia furnacalis]	2.910918868	1.54147463	0.001045	up	yes	1.245	0.4277	0.395	0.436	0.452	1.285	1.065	1.385	BP:develc	GO:00325	K12567	TTN	map05414;m	Dilated cardiomy	ENOG410XQFD;I	S:Function unknown;	------	------	------	CYT	2	4	48.1	High
TRINITY_DN53311_c0_g2_i1_orf1	transcription elongation factor S-II [Chelonus insularis]	0.471932921	-1.083346279	0.0006074	down	yes	0.5347	1.133	1.103	1.233	1.063	0.582	0.471	0.551	BP:RNA b	GO:00327	K03145	TFIIS	------	------	COG1594	K:Transcription	PF07500.17;PI	TFIIS_M;TFIIS_C;Med26	Transcription factor S-II;Transcription factor S-II;TFIIS helical bundle-like	CYT	1	8	34.9	High
TRINITY_DN4070_c0_g1_i4_orf1	alpha-N-acetylgalactosaminidase isoform X3 [Ostrinia furnacalis]	0.35793045	-1.482248814	8.26E-06	down	yes	0.422	1.179	1.195	1.204	1.139	0.424	0.449	0.393	BP:primar	GO:00442	K01204	NAGA	map00603;m	Glycosphingolipi	ENOG410XPF1	O:Posttranslational modification, protein turnover, chaperones	PF16499.8;PF1	Melibiase_2;Melibiase_2_C;Melibiase_C	Alpha galactosidase A;Alpha galactosidase A C-terminal beta sandwich domain;Alpha galactosidase C-terminal beta sandwich domain	CYT	5	15	49.7	High
TRINITY_DN6482_c0_g1_i1_orf1	endocuticle structural glycoprotein SgAbd-5-like [Ostrinia furnacalis]	2.210466654	1.144350971	0.001655	up	yes	1.17	0.5293	0.564	0.486	0.538	1.192	1.019	1.3	MF:molec	GO:00036	------	------	------	------	ENOG4111D3H;I	S:Function unknown;	PF00379.26	Chitin_bind_4	Insect cuticle protein	CYT	3	46	11.3	High
TRINITY_DN26254_c0_g1_i1_orf1	hypothetical protein evm_003664 [Chilo suppressalis] >CAB3521132.1 unnamed protein product [Chilo suppressalis] >CAH0398453.1 unnamed protein product [Chilo suppressalis]	4.209864907	2.073773939	0.0005222	up	yes	1.34	0.3183	0.325	0.304	0.326	1.402	1.144	1.473	MF:molec	GO:00036	------	------	------	------	ENOG410ZNDD;	S:Function unknown;	PF00379.26	Chitin_bind_4	Insect cuticle protein	CYT	3	36	14.7	High
TRINITY_DN20767_c0_g2_i1_orf1	glycosylated lysosomal membrane protein B-like [Vanessa atalanta]	0.469475494	-1.090878241	0.0009428	down	yes	0.546	1.163	1.12	1.091	1.277	0.608	0.56	0.47	------	------	------	------	------	------	ENOG4111F22	S:Function unknown	PF15065.9	NCU-G1	Lysosomal transcription factor, NCU-G1	CYT	1	2	30	Medium
TRINITY_DN14507_c0_g1_i5_orf1	PTB domain-containing adapter protein ced-6 [Ostrinia furnacalis]	0.367508143	-1.444151877	0.02701	down	yes	0.4513	1.228	1.397	1.49	0.796	0.432	0.576	0.346	------	------	K23285	GULP1, CED	------	------	ENOG410ZHJT;C	S:Function unknown;	PF00640.26	PID	Phosphotyrosine interaction domain	CYT	1	3	57	High
TRINITY_DN198_c0_g1_i2_orf1	retinol dehydrogenase 13-like [Ostrinia furnacalis]	0.462686567	-1.11189288	0.0006682	down	yes	0.527	1.139	1.152	1.068	1.198	0.475	0.476	0.63	------	------	K11153	RDH12	map00830;m	Retinol metabolis	COG1028	V:Defense mechanisms	PF00106.28;PI	adh_short;adh_short_C2;KR	short chain dehydrogenase;Enoyl;KR domain	CYT	1	4	35.3	High

TRINITY_DN29144_c0_g3_i1_orf1	PREDICTED: ADP-ribosylation factor 6 [Papilio polytes] >XP_013133321.1 PREDICTED: ADP-ribosylation factor 6 [Papilio polytes] >XP_013177129.1 PREDICTED: ADP-ribosylation factor 6 [Papilio xuthus] >XP_013177130.1 PREDICTED: ADP-ribosylation factor 6 [Papilio xuthus] >XP_014356507.1 ADP-ribosylation factor 6 [Papilio machaon] >XP_021185579.1 ADP-ribosylation factor 6 [Helicoverpa armigera] >XP_021185581.1 ADP-ribosylation factor 6 [Helicoverpa armigera] >XP_022130228.1 ADP-ribosylation factor 6 [Pieris rapae] >XP_022822139.1 ADP-ribosylation factor 6 [Spodoptera litura] >XP_022822140.1 ADP-ribosylation factor 6 [Spodoptera litura] >XP_028159104.1 ADP-ribosylation factor 6 [Ostrinia furnacalis] >XP_028159105.1 ADP-ribosylation factor 6 [Ostrinia furnacalis] >XP_028159106.1 ADP-ribosylation factor 6 [Ostrinia furnacalis] >XP_028159107.1 ADP-ribosylation factor 6 [Ostrinia furnacalis] >XP_028163222.1 ADP-ribosylation factor 6 [Ostrinia furnacalis] >XP_030022165.1 ADP-ribosylation factor 6 [Manduca sexta] >XP_030022166.1 ADP-ribosylation factor 6 [Manduca sexta] >XP_030022167.1 ADP-ribosylation factor 6 [Manduca sexta] >XP_035444169.1 ADP-ribosylation factor 6 [Spodoptera frugiperda] >XP_035444175.1 ADP-ribosylation factor 6 [Spodoptera frugiperda] >XP_038207597.1 ADP-ribosylation factor 6 [Zerene cesonia] >XP_038207598.1 ADP-ribosylation factor 6 [Zerene cesonia] >XP_045510541.1 ADP-ribosylation factor 6 [Colias croceus] >XP_045510551.1 ADP-ribosylation factor 6 [Colias croceus] >XP_045527300.1 ADP-ribosylation factor 6 [Pieris brassicae] >XP_045527302.1 ADP-ribosylation factor 6 [Pieris brassicae] >XP_047029519.1 ADP-ribosylation factor 6 [Helicoverpa zea] >XP_047029551.1 ADP-ribosylation factor 6 [Helicoverpa zea] >XP_047504621.1 ADP-ribosylation factor 6 [Pieris napi] >XP_047504631.1 ADP-ribosylation factor 6 [Pieris napi] >XP_047504640.1 ADP-ribosylation factor 6 [Pieris napi] >XP_047504648.1 ADP-ribosylation factor 6 [Pieris napi] >XP_047504657.1 ADP-ribosylation factor 6 [Pieris napi] >XP_048489067.1 ADP-ribosylation factor 6 [Plutella xylostella] >XP_048489068.1 ADP-ribosylation factor 6 [Plutella xylostella] >XP_048489069.1 ADP-ribosylation factor 6 [Plutella xylostella] >XP_049883531.1 ADP-ribosylation factor 6 [Pectinophora gossypiella] >XP_049883539.1 ADP-ribosylation factor 6 [Pectinophora gossypiella] >KAG5678369.1 hypothetical protein PVAND_008051 [Polypedilum vanderplanki] >RVE51130.1 hypothetical protein evm_004273 [Chilo suppressalis] >CAB3510283.1 unnamed protein product [Spodoptera littoralis] >CAF4796780.1 unnamed protein product [Pieris macdunnoughi] >CAG4977608.1 unnamed protein product [Parnassius apollo] >CAG9757696.1 unnamed protein	0.481988105	-1.052930551	1.39E-05	down	yes	0.5673	1.177	1.16	1.191	1.18	0.572	0.527	0.603	BP:transp	GO:00068	K07941	ARF6	map04666;m	Fc gamma R-me	ENOG410XP1U	S:Function unknown	PF00025.24;PI	Arf;Roc;Ras;SRPRB;G-alpha;Gtr1_RagA	ADP-ribosylation factor family;Ras of Complex, Roc, domain of DAPkinase;Ras family;Signal recognition particle receptor beta subunit;G-protein alpha subunit;Gtr1/RagA G protein conserved region	CYT	1	9	20.1	High
TRINITY_DN280_c4_g1_i5_orf1	fibroin light chain [Haritalodes derogata]	2.019604217	1.014072595	5.08E-06	up	yes	1.092	0.5407	0.54	0.553	0.529	1.074	1.079	1.122	CC:cellula	GO:00055	------	------	------	------	ENOG4110RKT	S:Function unknown	PF05849.14	L-fibroin	Fibroin light chain	CYT	5	24	27.2	High
TRINITY_DN36061_c0_g4_i2_orf1	putative GPI-anchored protein pfl2 [Ostrinia furnacalis] >XP_028163002.1 putative GPI-anchored protein pfl2 [Ostrinia furnacalis]	0.175631399	-2.509377302	2.19E-05	down	yes	0.2573	1.465	1.396	1.496	1.504	0.288	0.178	0.306	CC:cellula	GO:00055	------	------	------	------	ENOG4111P12	S:Function unknown	------	------	------	CYT	1	1	91.7	High
TRINITY_DN41259_c0_g1_i6_orf1	endocuticle structural glycoprotein SgAbd-8 [Ostrinia furnacalis]	0.096649485	-3.371094152	8.64E-06	down	yes	0.15	1.552	1.547	1.639	1.471	0.149	0.156	0.145	MF:molec	GO:00036	------	------	------	------	ENOG4111PJ1	S:Function unknown	PF00379.26	Chitin_bind_4	Insect cuticle protein	CYT	7	44	24.7	High
TRINITY_DN57904_c0_g2_i1_orf1	cuticle protein 19 [Plutella xylostella] >CAG9138481.1 unnamed protein product [Plutella xylostella]	0.157943262	-2.662521699	4.77E-05	down	yes	0.2227	1.41	1.356	1.522	1.351	0.243	0.165	0.26	MF:molec	GO:00036	------	------	------	------	ENOG410YSFI	S:Function unknown	PF00379.26	Chitin_bind_4	Insect cuticle protein	CYT	1	16	14.1	High
TRINITY_DN85004_c0_g1_i1_orf1	uncharacterized protein LOC114357684 [Ostrinia furnacalis]	2.045060659	1.032143636	0.0002109	up	yes	1.062	0.5193	0.589	0.519	0.45	1.078	1.036	1.071	------	------	------	------	------	------	------	------	------	------	------	CYT	1	9	18.1	High
TRINITY_DN1814_c0_g2_i4_orfp1	TRINITY_DN1814_c0_g2_i4_m.63284 TRINITY_DN1814_c0_g2::TRINITY_DN1814_c0_g2_i4::g.63284 ORF type:internal len:258 (-),score=126.31 TRINITY_DN1814_c0_g2_i4:3-773(-)	3.012289942	1.590860641	0.0003685	up	yes	1.201	0.3987	0.415	0.382	0.399	1.26	1.059	1.284	------	------	------	------	------	------	------	------	------	------	------	CYT	3	34	30.5	High
TRINITY_DN52395_c0_g2_i2_orf1	twitchin isoform X20 [Zerene cesonia]	2.293210495	1.197368787	0.001876	up	yes	1.145	0.4993	0.414	0.579	0.505	1.292	1.052	1.09	BP:develc	GO:00325	------	------	------	------	ENOG410XP8U;E	S:Function unknown;	PF07679.19	I-set	Immunoglobulin I-set domain	CYT	1	73	10.1	High
TRINITY_DN779_c0_g1_i12_orf1	unnamed protein product [Chilo suppressalis]	2.070289994	1.049832866	0.003907	up	yes	1.078	0.5207	0.59	0.513	0.459	1.225	0.931	1.077	BP:cellula	GO:00442	------	------	------	------	ENOG410YZ5M;	S:Function unknown;	PF07004.15	SHIPPO-rpt	Sperm-tail PG-rich repeat	CYT	1	16	93.6	High
TRINITY_DN1180_c0_g1_i4_orf1	larval cuticle protein LCP-30-like [Ostrinia furnacalis]	5.081487672	2.345250927	0.0008041	up	yes	1.216	0.2393	0.247	0.225	0.246	1.159	1.066	1.423	MF:molec	GO:00036	------	------	------	------	ENOG410YZ4Z;E	S:Function unknown;	PF00379.26	Chitin_bind_4	Insect cuticle protein	CYT	13	61	31	High
TRINITY_DN11820_c0_g1_i1_orf1	hypothetical protein evm_000341 [Chilo suppressalis]	0.494448636	-1.016107434	0.001714	down	yes	0.4587	0.9277	1.035	0.857	0.891	0.416	0.441	0.519	MF:ubiqu	GO:00197	K25192	RNF157	------	------	ENOG410XRAE	S:Function unknown	PF13920.9;PF:	zf-C3HC4_3;Prok-RING_4	Zinc finger, C3HC4 type;Prokaryotic RING finger family 4	CYT	1	5	36.9	High
TRINITY_DN695_c0_g1_i12_orf1	serpin transcript 2A, partial [Ostrinia nubilalis]	2.299518072	1.201331536	0.001748	up	yes	0.9543	0.415	0.361	0.424	0.46	0.923	0.858	1.082	------	------	------	------	------	------	ENOG4110R3H;E	S:Function unknown;	------	------	------	CYT	1	30	15.1	High
TRINITY_DN1504_c0_g1_i1_orf1	uncharacterized protein LOC114352862 [Ostrinia furnacalis] >XP_028160407.1 uncharacterized protein LOC114352862 [Ostrinia furnacalis]	0.378210806	-1.402737511	0.0002135	down	yes	0.427	1.129	1.132	1.104	1.152	0.362	0.387	0.532	------	------	------	------	------	------	ENOG410Y735	S:Function unknown	------	------	------	EXC	1	1	83.4	Medium
TRINITY_DN17271_c0_g1_i1_orf1	uncharacterized protein LOC114350693 [Ostrinia furnacalis]	0.469900171	-1.089573801	2.54E-05	down	yes	0.466	0.9917	0.965	1.03	0.98	0.444	0.491	0.463	BP:nucleic	GO:00903	------	------	------	------	ENOG4111REI;EI	L:Replication, recombination and repair;	PF08661.14	Rep_fac-A_3	Replication factor A protein 3	CYT	2	14	15.3	High
TRINITY_DN18164_c0_g1_i7_orf1	uncharacterized protein LOC114366518 isoform X5 [Ostrinia furnacalis]	0.38300095	-1.384580126	0.005465	down	yes	0.4033	1.053	1.137	1.202	0.82	0.377	0.429	0.404	CC:integr	GO:00160	------	------	------	------	COG5183	O:Posttranslational modification, protein turnover, chaperones	PF12906.10;PI	RINGv;THH1_TOM1-3_dom	RING-variant domain;THH1/TOM1/TOM3 domain	CYT	1	2	64.7	High
TRINITY_DN8226_c0_g1_i1_orf1	myosin heavy chain, muscle isoform X16 [Helicoverpa armigera]	2.048935703	1.034874713	0.0003702	up	yes	0.9337	0.4557	0.456	0.452	0.459	0.941	0.856	1.004	------	------	K17751	MYH6_7	map04260;m	Cardiac muscle c	COG5022	G:Carbohydrate transport and metabolism	------	------	------	CYT	1	56	6.3	High
TRINITY_DN14094_c0_g1_i1_orfp1	TRINITY_DN14094_c0_g1_i1_m.76391 TRINITY_DN14094_c0_g1::TRINITY_DN14094_c0_g1_i1::g.76391 ORF type:5prime_partial len:120 (+),score=12.94 TRINITY_DN14094_c0_g1_i1:3-362(+)	2.368109771	1.243735957	0.008087	up	yes	1.001	0.4227	0.4	0.4	0.468	0.882	0.888	1.233	------	------	------	------	------	------	------	------	------	------	------	CYT	5	48	13.5	High
TRINITY_DN23429_c0_g2_i1_orf1	muscle-specific protein 20 [Zerene cesonia]	2.164092232	1.113761987	0.0001035	up	yes	0.9667	0.4467	0.457	0.434	0.449	0.983	0.903	1.014	------	------	K20526	TAGLN	------	------	COG5199	Z:Cytoskeleton	PF00307.34;PI	CH;Calponin	Calponin homology;Calponin family repeat	CYT	12	75	20.3	High
TRINITY_DN76333_c0_g1_i2_orf1	larval cuticle protein 65Ag1-like [Ostrinia furnacalis]	4.614503817	2.206175528	0.0002439	up	yes	1.209	0.262	0.274	0.252	0.26	1.18	1.094	1.353	MF:molec	GO:00036	------	------	------	------	ENOG410YVGC;I	S:Function unknown;	PF00379.26	Chitin_bind_4	Insect cuticle protein	CYT	4	43	15.6	High
TRINITY_DN34821_c0_g1_i4_orf1	acetylcholine receptor subunit alpha-L1-like [Ostrinia furnacalis]	0.448842191	-1.155719799	0.0002467	down	yes	0.44	0.9803	0.945	1.047	0.949	0.495	0.401	0.424	CC:cellula	GO:00055	K04803;K	CHRNA1;CH	map04080	Neuroactive liga	ENOG410XQGR	S:Function unknown	PF02931.26	Neur_chan_LBD	Neurotransmitter-gated ion-channel ligand binding domain	PLA	1	3	47.7	High
TRINITY_DN29_c0_g1_i4_orf1	sodium-dependent nutrient amino acid transporter 1-like [Ostrinia furnacalis] >XP_028167707.1 sodium-dependent nutrient amino acid transporter 1-like [Ostrinia furnacalis]	2.414102852	1.271487143	0.01259	up	yes	1.075	0.4453	0.386	0.503	0.447	0.882	0.991	1.353	CC:integr	GO:00160	K05038	SLC6A5_9, C	map04721	Synaptic vesicle c	COG0733	P:Inorganic ion transport and metabolism	PF00209.21	SNF	Sodiu	CYT	1	1	63	Medium
TRINITY_DN13119_c0_g1_i4_orf1	endocuticle structural glycoprotein ABD-5-like [Bicyclus anynana]	4.15625	2.055282436	9.83E-05	up	yes	1.197	0.288	0.266	0.289	0.309	1.222	1.088	1.28	------	------	------	------	------	------	ENOG410YVGC;I	S:Function unknown;	PF00379.26	Chitin_bind_4	Insect cuticle protein	CYT	3	49	12.3	High
TRINITY_DN2600_c0_g1_i7_orf1	mucin-5AC isoform X2 [Ostrinia furnacalis]	0.338240741	-1.563877654	8.38E-05	down	yes	0.3653	1.08	1.096	1.145	1	0.358	0.351	0.387	------	------	------	------	------	------	ENOG4112A7J	S:Function unknown	------	------	------	CYT	9	12	98	High
TRINITY_DN783_c0_g1_i7_orf1	microtubule-associated protein Jupiter isoform X4 [Helicoverpa armigera]	0.415377446	-1.26750521	0.0001093	down	yes	0.4457	1.073	1.148	1.021	1.049	0.419	0.448	0.47	------	------	------	------	------	------	ENOG410Z68G	S:Function unknown	PF17054.8	JUPITER	Microtubule-Associated protein Jupiter	EXC	2	12	24	High
TRINITY_DN279_c0_g1_i10_orf1	RE1-silencing transcription factor-like isoform X1 [Ostrinia furnacalis]	0.269912281	-1.889437476	2.76E-05	down	yes	0.3077	1.14	1.073	1.168	1.179	0.27	0.332	0.321	CC:intrace	GO:00432	------	------	------	------	COG5048	O:Posttranslational modification, protein turnover, chaperones	PF00096.29;PI	zf-C2H2;zf-C2H2_4;zf-H2C2_2;zf-AD	Zinc finger, C2H2 type;C2H2-type zinc finger;Zinc-finger double domain;Zinc-finger associated domain	CYT	1	1	120.9	High
TRINITY_DN1215_c0_g1_i2_orf1	PI-stichotoxin-She2a-like [Ostrinia furnacalis]	2.561170821	1.356803479	0.0005054	up	yes	1.12	0.4373	0.427	0.44	0.445	1.055	1.052	1.252	BP:envenc	GO:00445	K03909	TFPI	map04610	Complement anc	ENOG410XQNP	O:Posttranslational modification, protein turnover, chaperones	PF00014.26	Kunitz_BPTI	Kunitz/Bovine pancreatic trypsin inhibitor domain	CYT	2	31	8.6	High
TRINITY_DN32601_c0_g1_i2_orf1	uncharacterized protein LOC114363197 [Ostrinia furnacalis]	0.292487047	-1.773555361	4.07E-05	down	yes	0.3387	1.158	1.138	1.165	1.172	0.364	0.259	0.393	------	------	------	------	------	------	ENOG410XRFE	S:Function unknown	PF03022.19	MRJP	Major royal jelly protein	CYT	1	3	48.2	High
TRINITY_DN2215_c0_g2_i1_orf1	PREDICTED: larval cuticle protein LCP-22-like [Amyelois transitella]	5.20083682	2.378743773	0.0008714	up	yes	1.243	0.239	0.264	0.206	0.247	1.248	1.048	1.433	------	------	------	------	------	------	ENOG41119AX;E	S:Function unknown;	PF00379.26	Chitin_bind_4	Insect cuticle protein	CYT	3	33	16.5	High
TRINITY_DN30208_c0_g1_i3_orf1	unnamed protein product [Timema cristinae]	2.341304348	1.227312484	0.001691	up	yes	1.077	0.46	0.456	0.461	0.463	1.081	0.933	1.218	------	------	K10374	TPM2	map05414;m	Dilated cardiomy	ENOG410XR5K	Z:Cytoskeleton	PF00261.23;PI	Tropomyosin;Tropomyosin_1	Tropomyosin;Tropomyosin like	CYT	4	57	7.7	High
TRINITY_DN9871_c0_g1_i11_orf1	PEST proteolytic signal-containing nuclear protein-like [Ostrinia furnacalis]	2.293360901	1.197463406	0.001973	up	yes	0.9983	0.4353	0.417	0.471	0.418	1.01	0.861	1.124	BP:nitrog	GO:00068	------	------	------	------	ENOG4111S9J	S:Function unknown	PF15473.9	PCNP	PEST, proteolytic signal-containing nuclear protein family	CYT	1	6	24.7	High
TRINITY_DN105359_c0_g2_i5_orf1	uncharacterized protein LOC114359499 [Ostrinia furnacalis]	0.401546861	-1.316359734	0.001568	down	yes	0.4413	1.099	1.168	1.124	1.004	0.465	0.309	0.55	------	------	------	------	------	------	ENOG4110AQ3	S:Function unknown	PF12456.11	hSac2	Inositol phosphatase	CYT	1	4	39.7	High
TRINITY_DN10558_c0_g1_i4_orf1	unnamed protein product [Chrysodeixis includens]	0.454930857	-1.136280802	0.03282	down	yes	0.4507	0.9907	1.135	1.106	0.731	0.371	0.318	0.663	CC:macro	GO:00329	K14411	MSI	map03015	mRNA surveillan	ENOG410YA8Z	O:Posttranslational modification, protein turnover, chaperones	------	------	------	CYT	1	44	18.3	High
TRINITY_DN1073_c0_g1_i3_orf1	carboxylesterase [Loxostege sticticalis]	2.445036642	1.289856086	0.002188	up	yes	1.101	0.4503	0.44	0.393	0.518	1.103	0.952	1.248	CC:integr	GO:00160	------	------	------	------	COG2272;COG0	I:Lipid transport and metabolism;G:Carbohydrate transport and metabolism;T:Signal	PF00135.31;PI	COesterase;BD-FAE;Abhydrolase_3	Carboxylesterase family;BD-FAE;alpha/beta hydrolase fold	CYT	3	7	68.1	High
TRINITY_DN116_c1_g1_i8_orf1	uncharacterized protein LOC114350057 isoform X2 [Ostrinia furnacalis]	2.001179477	1.000850562	0.0004571	up	yes	1.018	0.5087	0.503	0.504	0.519	0.928	1.092	1.034	------	------	------	------	------	------	ENOG4111JGD	S:Function unknown	PF15929.8	Myofilin	Myofilin	CYT	18	58	37.9	High
TRINITY_DN43328_c0_g1_i1_orf1	tubulin--tyrosine ligase-like protein 12 [Ostrinia furnacalis]	0.379078341	-1.399432066	1.86E-06	down	yes	0.4113	1.085	1.081	1.068	1.107	0.429	0.391	0.414	------	------	K16609	TTLL12	------	------	ENOG410XQ0A	S:Function unknown	PF03133.18	TTL	Tubulin-tyrosine ligase family	CYT	1	2	71.9	High
TRINITY_DN38424_c0_g1_i1_orf1	glucose dehydrogenase [FAD, quinone]-like [Ostrinia furnacalis]	0.418325863	-1.257300901	0.00437	down	yes	0.4013	0.9593	0.954	1.077	0.847	0.487	0.453	0.264	MF:anion	GO:00431	------	------	------	------	COG2303	I:Lipid transport and metabolism	PF00732.22;PI	GMC_oxred_N;GMC_oxred_C	GMC oxidoreductase;GMC oxidoreductase	CYT	1	2	66.3	High
TRINITY_DN389_c0_g1_i2_orf1	uncharacterized protein LOC118068293 isoform X2 [Chelonus insularis]	0.429432624	-1.219496298	4.79E-06	down	yes	0.3633	0.846	0.846	0.838	0.854	0.338	0.385	0.367	------	------	------	------	------	------	ENOG410XX7G	S:Function unknown	PF01607.27;PI	CBM_14;Ldl_recept_a	Chitin binding Peritrophin-A domain;Low-density lipoprotein receptor domain class A	PLA	4	11	60.2	High
TRINITY_DN13216_c0_g1_i5_orf1	uncharacterized protein LOC114358344 isoform X1 [Ostrinia furnacalis]	0.34575526	-1.532176895	4.51E-05	down	yes	0.3303	0.9553	0.901	0.991	0.974	0.336	0.297	0.358	BP:cellula	GO:00099	K08451;K	ADGRG2, G	------	------	ENOG410XSD2	S:Function unknown	PF00002.27;PI	7tm_2;GPS	7 transmembrane receptor;GPCR proteolysis site, GPS, motif	CYT	1	1	148.9	Medium
TRINITY_DN1123_c2_g1_i4_orf1	troponin I isoform X16 [Ostrinia furnacalis]	2.700985065	1.433485663	8.98E-05	up	yes	0.85	0.3147	0.307	0.297	0.34	0.849	0.797	0.904	CC:cellula	GO:00055	K12044	TNNI3	map04260;m	Cardiac muscle c	ENOG410Y9IX	Z:Cytoskeleton	PF00992.23	Troponin	Troponin	CYT	8	46	24.4	High
TRINITY_DN146841_c0_g1_i1_orf1	muscle-specific protein 20 [Temnothorax curvispinosus]	5.266009852	2.396710221	0.001416	up	yes	1.069	0.203	0.211	0.236	0.162	0.906	1.027	1.273	------	------	K20526	TAGLN	------	------	COG5199	S:Function unknown	PF00307.34;PI	CH;Calponin	Calponin homology;Calponin family repeat	CYT	1	10	20.6	High
TRINITY_DN1455_c0_g1_i8_orf1	troponin T, skeletal muscle isoform X1 [Galleria mellonella]	2.685950413	1.425432671	0.001002	up	yes	0.975	0.363	0.348	0.355	0.386	0.944	0.872	1.109	BP:regula	GO:00440	K12046	TNNT3	------	------	ENOG410XS6A	Z:Cytoskeleton	------	------	------	CYT	5	41	43.1	High
TRINITY_DN1123_c2_g1_i3_orf1	troponin I isoform X8 [Ostrinia furnacalis]	2.674304919	1.419163968	0.001976	up	yes	0.8753	0.3273	0.299	0.36	0.323	0.911	0.733	0.982	CC:cellula	GO:00055	K12044	TNNI3	map04260;m	Cardiac muscle c	ENOG410Y9IX	Z:Cytoskeleton	PF00992.23	Troponin	Troponin	CYT	3	55	10.3	High
TRINITY_DN3649_c0_g1_i6_orf1	unnamed protein product [Chilo suppressalis]	0.497140294	-1.008275055	0.0002714	down	yes	0.4433	0.8917	0.889	0.874	0.912	0.487	0.373	0.47	BP:regula	GO:00800	K11649	SMARCC	map05225;m	Hepatocellular ca	COG5259;COG5	B:Chromatin structure and dynamics;K:Transcription	PF16496.8;PF	SWIRM-assoc_2;SWIRM;SWIRM-assoc_1;SWIRM-assoc_3;Myb_DNA-binding;Myb_DNA-bind_6	SWIRM-associated domain at the N-terminal;SWIRM domain;SWIRM-associated region 1;SWIRM-associated domain at the C-terminal;Myb-like DNA-binding domain;Myb-like DNA-binding domain	CYT	3	3	103.8	High
TRINITY_DN29100_c0_g1_i2_orf1	endocuticle structural glycoprotein ABD-5-like [Galleria mellonella]	2.049617436	1.035354654	0.001923	up	yes	0.884	0.4313	0.422	0.378	0.494	0.938	0.779	0.935	MF:molec	GO:00036	------	------	------	------	ENOG41113X7;E	S:Function unknown;	PF00379.26	Chitin_bind_4	Insect cuticle protein	CYT	3	61	11.2	High
TRINITY_DN99673_c0_g1_i1_orf1	PREDICTED: pistil-specific extensin-like protein isoform X2 [Microplitis demolitor]	2.318469657	1.213172846	0.002855	up	yes	0.8787	0.379	0.315	0.408	0.414	0.875	1.001	0.76	CC:intrace	GO:00432	------	------	------	------	ENOG4110YZS	S:Function unknown	PF12874.10;PI	zf-met;zf-C2H2_jaz;zf-U1	Zinc-finger of C2H2 type;Zinc-finger double-stranded RNA-binding;U1 zinc finger	PLA	1	4	27.3	Medium
TRINITY_DN76377_c0_g1_i1_orf1	uncharacterized protein LOC111357764, partial [Spodoptera litura]	0.305873852	-1.708991315	4.25E-06	down	yes	0.2963	0.9687	0.958	0.991	0.957	0.292	0.271	0.326	------	------	------	------	------	------	ENOG410Y7Q4;E	S:Function unknown;	PF03564.18	DUF1759	Protein of unknown function	CYT	1	4	27.4	Medium
TRINITY_DN144258_c0_g1_i1_orf1	PREDICTED: enhancer of rudimentary homolog [Microplitis demolitor] >XP_044577051.1 enhancer of rudimentary homolog [Cotesia glomerata] >KAG8041963.1 hypothetical protein G9C98_007267 [Cotesia typhae] >KAH0539785.1 hypothetical protein KQX54_008036 [Cotesia glomerata] >CAD6227368.1 GSCOCG00006137001-RA-CDS [Cotesia congregata]	0.434086522	-1.203945466	0.002386	down	yes	0.3823	0.8807	0.955	0.8	0.887	0.414	0.271	0.462	BP:cellula	GO:00099	------	------	------	------	ENOG4111Z48	S:Function unknown	PF01133.20	ER	Enhancer of rudimentary	CYT	1	10	12.1	Medium
TRINITY_DN31001_c0_g1_i1_orf1	endocuticle structural glycoprotein ABD-5-like [Ostrinia furnacalis]	2.808404349	1.489750667	0.0009356	up	yes	0.9557	0.3403	0.332	0.371	0.318	1.018	0.819	1.03	MF:molec	GO:00036	------	------	------	------	ENOG4111EHD;I	S:Function unknown;	PF00379.26	Chitin_bind_4	Insect cuticle protein	CYT	3	41	12.2	High
TRINITY_DN81715_c0_g1_i1_orf1	gamma-interferon-inducible lysosomal thiol reductase-like [Ostrinia furnacalis]	0.442590432	-1.175955833	0.0003461	down	yes	0.3793	0.857	0.897	0.806	0.868	0.353	0.444	0.341	MF:cataly	GO:00038	K08059	IFI30, GILT	map04612	Antigen processi	ENOG4111IDT	S:Function unknown	PF03227.19	GILT	Gamma interferon inducible lysosomal thiol reductase	CYT	1	3	26.1	High
TRINITY_DN48097_c0_g1_i1_orf1	unnamed protein product [Homo sapiens]	2.003158132	1.002276314	0.04053	up	yes	0.888	0.4433	0.419	0.443	0.468	1.164	0.844	0.656	BP:positiv	GO:00342	K03252	EIF3C	------	------	ENOG410XRU3	S:Function unknown	PF05470.15;PI	eIF-3c_N;PCI	Eukaryotic translation initiation factor 3 subunit 8 N-terminus;PCI domain	CYT	1	1	70.4	Medium
TRINITY_DN6881_c0_g1_i1_orf1	putative protein TPRXL [Ostrinia furnacalis]	6.249310535	2.643697031	0.0001687	up	yes	1.133	0.1813	0.186	0.15	0.208	1.178	1	1.222	MF:molec	GO:00036	------	------	------	------	ENOG410ZF0W;I	S:Function unknown;	PF00379.26	Chitin_bind_4	Insect cuticle protein	CYT	5	23	22.4	High
TRINITY_DN48610_c0_g1_i2_orf1	hypothetical protein evm_002298 [Chilo suppressalis] >CAH0682062.1 unnamed protein product [Chilo suppressalis]	2.229331416	1.156611106	0.0005506	up	yes	0.9303	0.4173	0.435	0.413	0.404	0.897	0.865	1.029	MF:molec	GO:00036	------	------	------	------	ENOG410XTK4;E	S:Function unknown;	PF00379.26	Chitin_bind_4	Insect cuticle protein	CYT	5	41	18.6	High
TRINITY_DN1013_c0_g1_i3_orf1	TELO2-interacting protein 1 homolog isoform X2 [Ostrinia furnacalis]	0.455468847	-1.134575715	0.002224	down	yes	0.3677	0.8073	0.702	0.858	0.862	0.369	0.307	0.427	------	------	K20403	TTI1	map04150	mTOR signaling i	ENOG410XSN6;E	S:Function unknown;	------	------	------	CYT	1	1	124.9	Medium
TRINITY_DN4194_c0_g1_i1_orf1	hornerin-like [Ostrinia furnacalis]	0.356124498	-1.489546412	0.00115	down	yes	0.3547	0.996	1.01	1.112	0.866	0.339	0.313	0.412	------	------	K19720;K	COL3A;COL	map05146;m	Amoebiasis;Prote	ENOG4111IEE;EI	S:Function unknown;	PF16058.8	Mucin-like	Mucin-like	CYT	2	1	184.6	High
TRINITY_DN129226_c0_g1_i2_orf1	hypothetical protein evm_000268 [Chilo suppressalis]	0.460660943	-1.11822281	0.0003793	down	yes	0.3917	0.8503	0.853	0.906	0.792	0.367	0.442	0.366	CC:integr	GO:00160	K10053;K	RUNX1T1, C	map05221;m	Acute myeloid le	ENOG41125M8;I	S:Function unknown;	------	------	------	CYT	1	3	28.7	High
TRINITY_DN6710_c0_g1_i6_orf1	multiple C2 and transmembrane domain-containing protein-like [Ostrinia furnacalis]	0.406222883	-1.299656583	0.004644	down	yes	0.3277	0.8067	0.666	0.953	0.801	0.332	0.347	0.304	CC:cellula	GO:00055	------	------	------	------	COG5038;ENOG	H:Coenzyme transport and metabolism;S:Function unknown	PF00168.33	C2	C2 domain	PLA	1	1	69.9	Medium
TRINITY_DN42461_c0_g1_i4_orf1	obscurin [Ostrinia furnacalis]	2.331288344	1.221127454	0.0004732	up	yes	0.722	0.3097	0.276	0.336	0.317	0.744	0.653	0.769	BP:phosp	GO:00163	------	------	------	------	ENOG410XQFD;I	S:Function unknown;	PF07679.19;PI	I-set;Ig_3	Immunoglobulin I-set domain;Immunoglobulin domain	CYT	1	37	11.1	High
TRINITY_DN2695_c0_g1_i8_orfp1	TRINITY_DN2695_c0_g1_i8_m.44478 TRINITY_DN2695_c0_g1::TRINITY_DN2695_c0_g1_i8::g.44478 ORF type:3prime_partial len:532 (+),score=80.62 TRINITY_DN2695_c0_g1_i8:101-1594(+)	4.570265871	2.192278095	0.001048	up	yes	0.8423	0.1843	0.185	0.231	0.137	0.763	0.777	0.987	------	------	------	------	------	------	------	------	------	------	------	CYT	1	2	56.2	High
TRINITY_DN36817_c0_g1_i1_orf1	uncharacterized protein LOC114357350 [Ostrinia furnacalis]	2.059752322	1.042470869	0.01207	up	yes	0.6653	0.323	0.232	0.385	0.352	0.79	0.622	0.584	MF:transl	GO:00900	------	------	------	------	ENOG410XV86;E	S:Function unknown;	------	------	------	CYT	1	29	7.6	High
TRINITY_DN3235_c0_g1_i1_orf1	SPARC [Trichoplusia ni]	0.289414563	-1.788790575	1.88E-05	down	yes	0.219	0.7567	0.758	0.795	0.717	0.218	0.224	0.215	CC:extrac	GO:00056	K24262	SPARC	------	------	ENOG41101WW	S:Function unknown	PF10591.12	SPARC_Ca_bdg	Secreted protein acidic and rich in cysteine Ca binding region	CYT	6	24	36.9	High
TRINITY_DN2186_c0_g1_i17_orf1	paxillin isoform X6 [Leguminivora glycinivorella]	3.004231312	1.586995898	0.0001242	up	yes	0.639	0.2127	0.196	0.215	0.227	0.609	0.614	0.694	BP:cell-m	GO:00071	K05760	PXN	map05205;m	Proteoglycans in	ENOG410XRD4	S:Function unknown	PF00412.25	LIM	LIM domain	CYT	2	65	26.4	High
TRINITY_DN116951_c0_g3_i2_orf1	spermine oxidase-like isoform X2 [Ostrinia furnacalis]	2.321678322	1.215168095	0.002257	up	yes	0.664	0.286	0.281	0.262	0.315	0.646	0.584	0.762	MF:cataly	GO:00038	K12259	SMOX, PAO	map00330;m	Arginine and prc	ENOG4111HZN;	S:Function unknown;E:Amino acid transport and metabolism;S:Function unknown	PF13450.9;PF	NAD_binding_8;Amino_oxidase	NA;Flavin containing amine oxidoreductase	CYT	2	38	8.6	High
TRINITY_DN46216_c0_g3_i1_orf1	unnamed protein product, partial [Brenthis ino]	3.367267414	1.751578298	0.0002239	up	yes	0.6913	0.2053	0.22	0.169	0.227	0.721	0.624	0.729	------	------	------	------	------	------	ENOG410Y7Q4;E	J:Translation, ribosomal structure and biogenesis;U:Intracellular trafficking, secretion, and vesicular transport;S:Function	PF03564.18	DUF1759	Protein of unknown function	CYT	1	11	9.6	Medium
TRINITY_DN29934_c0_g1_i6_orf1	sodium/potassium-transporting ATPase subunit beta-2-like [Ostrinia furnacalis] >XP_028176258.1 sodium/potassium-transporting ATPase subunit beta-2-like [Ostrinia furnacalis]	0.487775891	-1.035709641	0.0005338	down	yes	0.2873	0.589	0.632	0.581	0.554	0.254	0.288	0.32	BP:potass	GO:00068	K01540	ATP1B, CD2	map04918;m	Thyroid hormone	ENOG411150A	U:Intracellular trafficking, secretion, and vesicular	PF00287.21	Na_K-ATPase	Sodium / potassium ATPase beta chain	CYT	1	4	37.5	High
TRINITY_DN3759_c0_g1_i1_orf1	uncharacterized protein LOC114350416 [Ostrinia furnacalis] >XP_028157016.1 uncharacterized protein LOC114350416 [Ostrinia furnacalis] >XP_028157017.1 uncharacterized protein LOC114350416 [Ostrinia furnacalis] >XP_028157018.1 uncharacterized protein LOC114350416 [Ostrinia furnacalis] >XP_028157019.1 uncharacterized protein LOC114350416 [Ostrinia furnacalis]	0.423929099	-1.238105097	0.0006659	down	yes	0.3157	0.7447	0.714	0.83	0.69	0.326	0.292	0.329	CC:cellula	GO:00055	K22909	ZC3H13	------	------	ENOG4111GFX	S:Function unknown	PF01607.27	CBM_14	Chitin binding Peritrophin-A domain	CYT	2	2	132.4	High
TRINITY_DN3614_c0_g2_i1_orf1	PC4 and SFRS1-interacting protein isoform X4 [Galleria mellonella]	0.339227716	-1.559674049	0.0005205	down	yes	0.282	0.8313	0.779	0.805	0.91	0.257	0.236	0.353	CC:intrace	GO:00432	K25057	PSIP1, LEDC	map03250	Viral life cycle - I	ENOG4111K9I	S:Function unknown	------	------	------	CYT	1	13	11.9	High
TRINITY_DN69236_c0_g1_i1_orf1	peroxiredoxin [Ostrinia furnacalis]	0.458441558	-1.125190262	4.96E-05	down	yes	0.353	0.77	0.794	0.781	0.735	0.376	0.329	0.354	MF:antiox	GO:00162	K03386	PRDX2_4, al	map04214	Apoptosis - fly	COG0450;COG0	I:Lipid transport and metabolism;Q:Secondary metabolites biosynthesis, transport and catabolism	PF00578.24;PI	AhpC-TSA;1-cysPrx_C;Redoxin	AhpC/TSA family;C-terminal domain of 1-Cys peroxiredoxin;Redoxin	CYT	2	14	27.8	High
TRINITY_DN58531_c0_g1_i1_orf1	uncharacterized protein LOC114357371 [Ostrinia furnacalis] >XP_028166768.1 uncharacterized protein LOC114357371 [Ostrinia furnacalis]	0.358183929	-1.481227488	0.002629	down	yes	0.2777	0.7753	0.817	0.658	0.851	0.202	0.273	0.358	------	------	------	------	------	------	ENOG410Y4ER;E	S:Function unknown;	------	------	------	CYT	1	3	29.7	Medium
TRINITY_DN31118_c1_g1_i1_orf1	endocuticle structural glycoprotein ABD-4-like [Ostrinia furnacalis]	4.653192736	2.218220945	0.001154	up	yes	0.7943	0.1707	0.193	0.137	0.182	0.811	0.66	0.912	MF:molec	GO:00036	------	------	------	------	ENOG4111PJ1;EI	S:Function unknown;	PF00379.26	Chitin_bind_4	Insect cuticle protein	CYT	1	23	12	High
TRINITY_DN2894_c0_g2_i3_orf1	myrosinase 1-like isoform X1 [Ostrinia furnacalis]	0.302880559	-1.723179115	0.002532	down	yes	0.2513	0.8297	0.899	0.661	0.929	0.233	0.243	0.278	BP:primar	GO:00442	------	------	------	------	COG2723	G:Carbohydrate transport and metabolism	PF00232.21	Glyco_hydro_1	Glycosyl hydrolase family 1	CYT	1	6	50.1	High

TRINITY_DN110460_c0_g2_i1_orf1	Similar to chaf1a-b: Chromatin assembly factor 1 subunit A-B (Xenopus laevis) [Cotesia congregata]	2.006152945	1.004431598	0.005456	up	yes	0.6847	0.3413	0.323	0.335	0.366	0.681	0.58	0.793	CC:intrac	GO:00432	K10750	CHAF1A	------	------	ENOG4111K9K	K:Transcription	PF15539.9	CAF1-p150_C2	CAF1 complex subunit p150, region binding to CAF1-p60 at C-term	CYT	1	3	29.2	High
TRINITY_DN5459_c0_g1_i1_orf1	protein takeout-like isoform X2 [Ostrinia furnacalis]	0.333426223	-1.584560524	0.0004234	down	yes	0.2393	0.7177	0.755	0.749	0.649	0.265	0.183	0.27	------	------	------	------	------	------	ENOG4111E83;E	S:Function unknown;	PF06585.14	JHBP	Haemolymph juvenile hormone binding protein	CYT	1	4	21.2	Medium
TRINITY_DN38366_c0_g1_i4_orfp1	TRINITY_DN38366_c0_g1_i4_m.10666 TRINITY_DN38366_c0_g1::TRINITY_DN38366_c0_g1_i4::g.10666 ORF type:internal len:143 (+) score=71.68 TRINITY_DN38366_c0_g1_i4:3-428(+)	2.096722876	1.068136193	0.003994	up	yes	0.659	0.3143	0.28	0.335	0.328	0.652	0.567	0.758	------	------	------	------	------	------	------	------	------	------	------	CYT	1	8	13.5	High
TRINITY_DN67231_c0_g1_i1_orf1	endocuticle structural glycoprotein SgAbd-8-like [Ostrinia furnacalis]	35.16383735	5.136020612	0.0005573	up	yes	0.8907	0.02533	0.024	0.02	0.032	0.954	0.72	0.998	MF:molec	GO:00036	------	------	------	------	ENOG4111PJ1;E	S:Function unknown;	PF00379.26	Chitin_bind_4	Insect cuticle protein	CYT	1	20	13.9	High
TRINITY_DN27500_c0_g1_i4_orf1	hemicentin-1-like [Ostrinia furnacalis]	2.165452653	1.114668629	0.00472	up	yes	0.6243	0.2883	0.266	0.297	0.302	0.574	0.559	0.74	BP:develo	GO:00325	------	------	------	------	ENOG4110SA1;E	S:Function unknown;	PF13927.9;PF	Ig_3;I-set;Ig_2;ig;V-set;fn3	Immunoglobulin domain;Immunoglobulin I-set domain;Immunoglobulin domain;Immunoglobulin domain;Immunoglobulin V-set	CYT	1	2	57.4	High
TRINITY_DN19160_c0_g1_i1_orf1	alkyldihydroxyacetonephosphate synthase [Ostrinia furnacalis]	0.2139357	-2.224750848	2.43E-05	down	yes	0.179	0.8367	0.811	0.88	0.819	0.157	0.161	0.219	------	------	K00803	AGPS, agpS	map00565;m	Ether lipid metab	COG0277	C:Energy production and conversion	PF02913.22;PI	FAD-oxidase_C;FAD_binding	FAD linked oxidases, C-terminal domain;FAD binding domain	CYT	1	1	69.6	Medium
TRINITY_DN25779_c0_g1_i6_orf1	aldo-keto reductase AKR2E4-like [Ostrinia furnacalis]	0.446866075	-1.16208557	4.87E-05	down	yes	0.2973	0.6653	0.645	0.698	0.653	0.287	0.319	0.286	MF:catal	GO:00038	K00011	AKR1B	map00051;m	Fructose and ma	COG0656	S:Function unknown	PF00248.24	Aldo_ket_red	Aldo/keto reductase family	CYT	5	23	35.9	High
TRINITY_DN82801_c0_g1_i1_orf1	uncharacterized protein LOC114364712 [Ostrinia furnacalis]	0.323550777	-1.627935952	3.19E-06	down	yes	0.2227	0.6883	0.695	0.704	0.666	0.214	0.232	0.222	BP:organi	GO:00717	------	------	------	------	ENOG410XX7G	Z:Cytoskeleton	PF01607.27;PI	CBM_14;Polysacc_deac_1;Ldl_recept_a	Chitin binding Peritrophin-A domain;Polysaccharide deacetylase;Low-density lipoprotein receptor domain class A	CYT	10	27	61.3	High
TRINITY_DN139212_c0_g1_i4_orf1	uncharacterized protein LOC114350112 [Ostrinia furnacalis]	0.363784666	-1.458843364	0.0005673	down	yes	0.223	0.613	0.671	0.629	0.539	0.227	0.216	0.226	MF:catal	GO:00038	------	------	------	------	COG1028	Z:Cytoskeleton	PF13561.9;PF	adh_short_C2;adh_short:KR	Enoyl;short chain dehydrogenase;KR domain	CYT	2	8	27.3	High
TRINITY_DN344_c1_g1_i1_orf1	chymotrypsin-like serine protease 16 [Ostrinia nubilalis]	3.411111111	1.770241749	7.97E-05	up	yes	0.6447	0.189	0.2	0.172	0.195	0.647	0.598	0.689	BP:organi	GO:00717	K01312	PRSS1_2_3	map04972;m	Pancreatic secret	COG5640	I:Lipid transport and metabolism	PF00089.29	Trypsin	Trypsin	CYT	5	29	30.7	High
TRINITY_DN5238_c0_g1_i2_orf1	DNA-(apurinic or apyrimidinic site) lyase [Ostrinia furnacalis]	0.477538096	-1.066312264	4.24E-05	down	yes	0.2413	0.5053	0.517	0.512	0.487	0.235	0.261	0.228	BP:nuclei	GO:00903	K10771	APEX1	map03410	Base excision rep	COG0708	L:Replication, recombination and repair	PF03372.26	Exo_endo_phos	Endonuclease/Exonuclease/phosphatase family	CYT	5	11	73.1	High
TRINITY_DN8691_c0_g1_i3_orf1	nucleolin-like [Melitaea cinxia]	0.442619668	-1.175860537	0.0006275	down	yes	0.221	0.4993	0.54	0.496	0.462	0.255	0.196	0.212	------	------	------	------	------	------	ENOG4110RR1	S:Function unknown	------	------	------	CYT	2	15	14.4	High
TRINITY_DN47731_c0_g1_i2_orf1	nucleolar GTP-binding protein 2 [Ostrinia furnacalis]	0.413875598	-1.272730904	0.005362	down	yes	0.173	0.418	0.419	0.412	0.423	0.162	0.102	0.255	CC:organ	GO:00432	K14537	NUG2, GNL	map03008	Ribosome bioger	COG1161;ENOG	S:Function unknown;	PF07572.15	BCNT	Bucentaur or craniofacial development	CYT	1	4	30.3	Medium
TRINITY_DN30704_c0_g1_i1_orf1	cytochrome P450 monooxygenase CYP6AE134v2 [Ostrinia furnacalis]	2.298837209	1.200904306	0.01171	up	yes	0.1977	0.086	0.117	0.083	0.058	0.209	0.223	0.161	CC:integr	GO:0016C	K14999	CYP6	------	------	COG2124	Q:Secondary metabolites biosynthesis, transport and catabolism	PF00067.25	p450	Cytochrome P450	CYT	1	2	61.1	High
TRINITY_DN43942_c0_g2_i1_orf1	LOW QUALITY PROTEIN: caprin homolog [Ostrinia furnacalis]	0.408006159	-1.293337166	0.001489	down	yes	0.159	0.3897	0.402	0.419	0.348	0.126	0.197	0.154	------	------	K18743	CAPRIN1, G	------	------	ENOG4110M5O	S:Function unknown	------	------	------	CYT	1	5	22.7	High
TRINITY_DN33452_c0_g1_i1_orf1	lethal(2) giant larvae protein isoform X8 [Ostrinia furnacalis]	2.076006807	1.053811174	0.004895	up	yes	0.366	0.1763	0.211	0.129	0.189	0.327	0.364	0.407	BP:secreti	GO:00469	K06094	LLGL	map04530;m	Tight junction;Hu	ENOG410XS6Z	S:Function unknown	------	------	------	CYT	1	14	13.7	High
TRINITY_DN4762_c0_g1_i2_orf1	ATPase family AAA domain-containing protein 1 isoform X2 [Ostrinia furnacalis]	0.412951382	-1.275956156	7.63E-05	down	yes	0.2047	0.4957	0.524	0.475	0.488	0.191	0.2	0.223	MF:purine	GO:00356	K22530	ATAD1	------	------	COG0464	S:Function unknown	PF00004.32;PI	AAA;AAA_lid_3	ATPase family associated with various cellular activities;AAA+ lid domain	CYT	1	2	43.3	High
TRINITY_DN3135_c0_g1_i6_orf1	acanthoscurrin-1-like [Ostrinia furnacalis]	2.459988486	1.298651563	0.003519	up	yes	0.4273	0.1737	0.145	0.192	0.184	0.41	0.371	0.501	------	------	------	------	------	------	------	------	------	------	------	EXC	1	7	25	High
TRINITY_DN29120_c0_g1_i6_orf1	putative inorganic phosphate cotransporter [Ostrinia furnacalis]	0.49715052	-1.00824538	0.0005297	down	yes	0.1483	0.2983	0.3	0.321	0.274	0.149	0.138	0.158	CC:integr	GO:0016C	K08193	SLC17A	------	------	ENOG410XPWC;	G:Carbohydrate transport and metabolism;	PF07690.19	MFS_1	Major Facilitator Superfamily	CYT	1	4	27.3	High
TRINITY_DN10940_c0_g1_i10_orfp1	TRINITY_DN10940_c0_g1_i10_m.52163 TRINITY_DN10940_c0_g1::TRINITY_DN10940_c0_g1_i10::g.52163 ORF type:5prime_partial len:248 (-	2.546325476	1.348416839	0.0002762	up	yes	0.202	0.07933	0.085	0.085	0.068	0.193	0.194	0.219	------	------	------	------	------	------	------	------	------	------	------	CYT	1	14	22.7	High
TRINITY_DN336_c0_g1_i6_orfp1	TRINITY_DN336_c0_g1_i6_m.64791 TRINITY_DN336_c0_g1::TRINITY_DN336_c0_g1_i6::g.64791 ORF type:complete len:61 (-) score=19.53 TRINITY_DN336_c0_g1_i6:236-418(-)	3.325970918	1.733775554	0.0004638	up	yes	0.1807	0.05433	0.045	0.061	0.057	0.188	0.159	0.195	------	------	------	------	------	------	------	------	------	------	------	CYT	1	12	6.3	High
TRINITY_DN1249_c0_g1_i6_orf1	venom carboxylesterase-6-like [Ostrinia furnacalis]	0.233778446	-2.09678617	0.0004677	down	yes	0.1243	0.5317	0.543	0.519	0.533	0.159	0.166	0.048	------	------	------	------	------	------	COG2272;ENOG	I:Lipid transport and metabolism;T:Signal transduction mechanisms;G:Carbohydrate	PF00135.31;PI	COesterase;BD-FAE;Abhydrolase_3	Carboxylesterase family;BD-FAE;alpha/beta hydrolase fold	PLA	1	6	64.7	High
TRINITY_DN96557_c0_g1_i1_orf1	charged multivesicular body protein 4B [Phyllostomus discolor]	2.393239196	1.258964596	0.001546	up	yes	0.5593	0.2337	0.212	0.253	0.236	0.559	0.489	0.63	BP:protei	GO:00081	K12194	CHMP4A_B,	map03250;m	Viral life cycle - H	ENOG410YE9I	I:Lipid transport and metabolism	PF03357.24	Snf7	Snf7	CYT	1	14	8	High
TRINITY_DN19244_c0_g1_i7_orf1	uncharacterized protein LOC114350218 [Ostrinia furnacalis]	0.420966612	-1.24822228	0.00351	down	yes	0.1803	0.4283	0.491	0.434	0.36	0.177	0.159	0.205	------	------	K16830	AURKAIP1	------	------	ENOG410Y2DU;	S:Function unknown;C:Energy production and conversion	------	------	------	CYT	1	4	25.6	High
TRINITY_DN3504_c0_g1_i4_orfp1	TRINITY_DN3504_c0_g1_i4_m.43930 TRINITY_DN3504_c0_g1::TRINITY_DN3504_c0_g1_i4::g.43930 ORF type:internal len:196 (-),score=84.82 TRINITY_DN3504_c0_g1_i4:3-587(-)	2.599404089	1.378180925	0.01217	up	yes	0.253	0.09733	0.121	0.053	0.118	0.285	0.197	0.277	------	------	------	------	------	------	------	------	------	------	------	EXC	1	8	21.5	High
TRINITY_DN2953_c1_g1_i2_orf1	methionine--tRNA ligase, cytoplasmic isoform X6 [Ostrinia furnacalis]	0.48425	-1.046176046	0.000653	down	yes	0.1937	0.4	0.378	0.437	0.385	0.215	0.183	0.183	BP:methio	GO:00064	K01874	MARS, metG	map00450;m	Selenocompoun	COG0143	J:Translation, ribosomal structure and biogenesis	PF00458.23	WHEP-TRS	WHEP-TRS domain	CYT	1	43	10.3	High
TRINITY_DN117_c0_g1_i4_orf1	lipase member I-like [Ostrinia furnacalis]	0.436214242	-1.196891221	0.001427	down	yes	0.07167	0.1643	0.172	0.142	0.179	0.067	0.07	0.078	BP:organi	GO:00717	------	------	------	------	ENOG41116Y2;E	S:Function unknown;	PF00151.22	Lipase	Lipase	CYT	5	82	16.1	High
