# Supplementary material for: Comparative Proteomics and Metabonomics Analysis of Different Diapause Stages Revealed a New Regulation Mechanism of Diapause in Loxostege sticticalis (Lepidoptera: Pyralidae)
Source: Molecules. 2024 Jul 25;29(15):3472. doi: 10.3390/molecules29153472 (PMC11314584; doi:10.3390/molecules29153472)
Supplement: Supplementary file 1 [file molecules-29-03472-s001.zip › analysis process/proteomic/diffreential protein statistic table/DvsRD.pdf]

Accession	Description	FC(D/RD)	Log2FC(D/RD)	P_value(D/RD)	Regulate	Significant	D	RD	RD1	RD2	RD3	D1	D2	D3
TRINITY_DN32514_c0_g2_i1_orf1	mucin-5AC-like [Ostrinia furnacalis]	0.0500237	-4.321243542	8.68E-05	down	yes	1.054	21.07	21.41	23.006	18.79	0.797	0.961	1.404
TRINITY_DN971_c0_g1_i10_orfp1	TRINITY_DN971_c0_g1_i10_m.54268 TRINITY_DN971_c0_g1::TRINITY_DN971_c0_g1_i10::g.54268 ORF type:internal len:187 (+),score=141.68 TRINITY_DN971_c0_g1_i10:1-558(+)	2.8779343	1.525033643	1.68E-06	up	yes	24.52	8.52	8.668	8.635	8.256	25.204	24.144	24.205
TRINITY_DN276_c0_g1_i2_orf1	protein lethal(2)essential for life-like [Ostrinia furnacalis] >UTU55753.1 small heat shock protein Hsp20.7 [Ostrinia furnacalis]	0.2750638	-1.862161654	7.86E-05	down	yes	3.232	11.75	11.772	12.626	10.844	3.171	3.258	3.266
TRINITY_DN40669_c0_g2_i1_orf1	uncharacterized protein LOC114355531 [Ostrinia furnacalis]	0.0490213	-4.350448754	4.79E-06	down	yes	0.9917	20.23	20.114	21.28	19.295	0.992	1.003	0.98
TRINITY_DN295_c3_g1_i1_orfp1	TRINITY_DN295_c3_g1_i1_m.18839 TRINITY_DN295_c3_g1::TRINITY_DN295_c3_g1_i1::g.18839 ORF type:5prime_partial len:118 (+),score=46.25 TRINITY_DN295_c3_g1_i1:1-354(+)	2.1639226	1.113648872	0.0001097	up	yes	11.96	5.527	5.686	5.496	5.398	12.279	12.471	11.14
TRINITY_DN64297_c0_g1_i1_orf1	vanin-like protein 2 isoform X2 [Ostrinia furnacalis]	0.1955508	-2.354384307	1.60E-05	down	yes	2.769	14.16	13.563	14.15	14.772	2.526	3.369	2.411
TRINITY_DN66040_c0_g1_i2_orf1	serine protease inhibitor dipetalogastin-like isoform X2 [Ostrinia furnacalis]	0.1663212	-2.587955645	3.92E-06	down	yes	3.21	19.3	19.572	19.656	18.675	3.08	3.842	2.709
TRINITY_DN15175_c0_g1_i1_orf1	zinc carboxypeptidase-like [Ostrinia furnacalis]	0.1603918	-2.640327413	1.51E-07	down	yes	1.359	8.473	8.516	8.539	8.363	1.451	1.405	1.22
TRINITY_DN6330_c0_g1_i1_orfp1	TRINITY_DN6330_c0_g1_i1_m.42332 TRINITY_DN6330_c0_g1::TRINITY_DN6330_c0_g1_i1::g.42332 ORF type:3prime_partial len:51 (-),score=33.30 TRINITY_DN6330_c0_g1_i1:3-152(-)	2.5005501	1.322245485	0.002673	up	yes	6.819	2.727	2.914	2.949	2.319	5.909	7.901	6.646
TRINITY_DN56459_c0_g1_i2_orf1	aldo-keto reductase AKR2E4-like [Ostrinia furnacalis]	2.7783833	1.474245638	5.67E-05	up	yes	6.118	2.202	2.237	2.086	2.282	6.25	6.398	5.707
TRINITY_DN4592_c0_g1_i1_orf1	hypothetical protein KGM_205563 [Danaus plexippus plexippus]	0.1470833	-2.765294317	3.81E-06	down	yes	1.059	7.2	7.378	7.365	6.858	1.033	1.116	1.028
TRINITY_DN3439_c0_g2_i2_orf1	histone H2A.Z-specific chaperone CHZ1-like [Ostrinia furnacalis]	3.6923077	1.884522783	8.10E-06	up	yes	5.04	1.365	1.32	1.384	1.392	5.157	5.169	4.793
TRINITY_DN20717_c0_g1_i1_orf1	putative uncharacterized protein DDB_G0282133 isoform X1 [Ostrinia furnacalis]	0.4104034	-1.284885587	0.0001194	down	yes	1.862	4.537	4.271	4.554	4.785	1.822	1.709	2.054
TRINITY_DN38506_c0_g1_i4_orf1	C-1-tetrahydrofolate synthase, cytoplasmic isoform X1 [Ostrinia furnacalis] >XP_028166137.1 C-1-tetrahydrofolate synthase, cytoplasmic isoform X2 [Ostrinia furnacalis] >XP_028166140.1 C-1-tetrahydrofolate synthase, cytoplasmic isoform X4 [Ostrinia furnacalis]	3.1355443	1.648715921	0.0002191	up	yes	4.349	1.387	1.12	1.376	1.665	4.685	4.236	4.125
TRINITY_DN25534_c0_g1_i1_orf1	venom serine carboxypeptidase-like [Ostrinia furnacalis]	0.180862	-2.467039103	2.98E-09	down	yes	0.8477	4.687	4.655	4.691	4.714	0.847	0.858	0.838
TRINITY_DN12387_c1_g2_i1_orf1	skin secretory protein xP2-like [Ostrinia furnacalis]	0.2213957	-2.175301087	2.76E-05	down	yes	0.9613	4.342	4.353	4.602	4.071	0.894	1.007	0.983
TRINITY_DN2140_c0_g1_i1_orf1	uncharacterized protein LOC126367764 [Pectinophora gossypiella]	0.2869253	-1.801253159	2.83E-05	down	yes	1.117	3.893	3.658	4.102	3.92	1.089	1.118	1.144

Accession	GO Term	GO ID	KO ID	KO Name	Pathway ID	Pathway_defition	EggNOG ID	EggNOG_Functional Categories	Pfam ID	Domain	Domain Description	Subcellular Loc	Unique Peptides	Coverage %	MW [kDa]	FDR Confidence
TRINITY_DN32514_c0_g2_i1_orf1	------	------	------	------	------	------	------	------	------	------	------	CYT	1	9	14.6	High
TRINITY_DN971_c0_g1_i10_orfp1	------	------	------	------	------	------	------	------	------	------	------	CYT	2	20	20.5	High
TRINITY_DN276_c0_g1_i2_orf1	MF:cation binding;MF:metal ion binding;MF:molecular_function;MF:binding;MF:ion binding;	GO:0043169;GO:0046872;GO:0003674;GO:0005488;GO:0043167	K09542	CRYAB	map04213;map04141	Longevity regulating pathway - multiple species;Protein processing in endoplasmic reticulum	ENOG410YERS	O:Posttranslational modification, protein turnover, chaperones	PF00011.24	HSP20	Hsp20/alpha crystallin family	CYT	2	43	20.8	High
TRINITY_DN40669_c0_g2_i1_orf1	------	------	------	------	------	------	ENOG4110S60	S:Function unknown	------	------	------	CYT	6	59	9.5	High
TRINITY_DN295_c3_g1_i1_orfp1	------	------	------	------	------	------	------	------	------	------	------	PLA	3	49	12.6	High
TRINITY_DN64297_c0_g1_i1_orf1	BP:nitrogen compound metabolic process;BP:biological_process;BP:metabolic process;	GO:0006807;GO:0008150;GO:0008152	------	------	------	------	COG0388	O:Posttranslational modification, protein turnover, chaperones	------	------	------	CYT	1	57	7.8	High
TRINITY_DN66040_c0_g1_i2_orf1	------	------	------	------	------	------	------	------	PF07648.18;PF00050.24	Kazal_2;Kazal_1	Kazal-type serine protease inhibitor domain;Kazal-type serine protease inhibitor domain	CYT	1	21	6	High
TRINITY_DN15175_c0_g1_i1_orf1	------	------	K01298	CPA2	map04972;map04974	Pancreatic secretion;Protein digestion and absorption	COG2866	O:Posttranslational modification, protein turnover, chaperones	PF00246.27	Peptidase_M14	Zinc carboxypeptidase	CYT	3	7	45.8	High
TRINITY_DN6330_c0_g1_i1_orfp1	------	------	------	------	------	------	------	------	------	------	------	CYT	1	26	5.3	High
TRINITY_DN56459_c0_g1_i2_orf1	MF:catalytic activity;MF:molecular_function;MF:oxidoreductase activity;	GO:0003824;GO:0003674;GO:0016491	------	------	------	------	COG0656	S:Function unknown	------	------	------	CYT	4	78	8.7	High
TRINITY_DN4592_c0_g1_i1_orf1	------	------	------	------	------	------	ENOG4111M7J	S:Function unknown	------	------	------	CYT	17	56	42.8	High
TRINITY_DN3439_c0_g2_i2_orf1	------	------	------	------	------	------	------	------	------	------	------	PLA	4	14	31.9	High
TRINITY_DN20717_c0_g1_i1_orf1	------	------	------	------	------	------	ENOG410ZIVR	S:Function unknown	------	------	------	PLA	1	17	5.2	High
TRINITY_DN38506_c0_g1_i4_orf1	BP:one-carbon metabolic process;BP:nitrogen compound metabolic process;BP:cellular metabolic process;BP:tetrahydrofolate metabolic process;BP:organic cyclic compound metabolic process;BP:tetrahydrofolate interconversion;BP:pteridine-containing compound metabolic process;BP:organic substance metabolic process;BP:folic acid-containing compound metabolic process;BP:cellular process;BP:cellular aromatic compound metabolic process;BP:biological_process;BP:metabolic process;BP:heterocycle metabolic [illegible]	GO:0006730;GO:0006807;GO:0044237;GO:0046653;GO:1901360;GO:0035999;GO:0042558;GO:0071704;GO:0006760;GO:0009987;GO:0006725;GO:0008150;GO:0008152;GO:0046483;GO:1901564;GO:0006575;GO:0044281;GO:0005488;GO:0035639;GO:0016646;GO:0003674;GO:0043167;GO:1901363;GO:0016491;GO:0043168;GO:0004329;GO:0016645;GO:0000166;GO:0019238;GO:1901265;GO:0017076;GO:0005524;GO:0004486;GO:0016787;GO:0004488;GO:0036094;GO:0003824;GO:0016874;GO:0016879;GO:0097367;GO:0097159;GO:0016810;GO:0030554;GO:0004477;GO:0016814;GO:0032559;GO:0032555;GO:0032553	K00288	MTHFD	map00670;map01240	One carbon pool by folate;Biosynthesis of cofactors	COG0190	H:Coenzyme transport and metabolism	PF00763.26	THF_DHG_CYH	Tetrahydrofolate dehydrogenase/cyclohydrolase, catalytic domain	CYT	3	61	16.2	High
TRINITY_DN25534_c0_g1_i1_orf1	BP:primary metabolic process;BP:organonitrogen compound metabolic process;BP:protein metabolic process;BP:macromolecule metabolic process;BP:organic substance metabolic process;BP:nitrogen compound metabolic process;BP:biological_process;BP:metabolic process;BP:proteolysis;MF:molecular_function;MF:exopeptidase activity;MF:hydrolase activity;MF:carboxypeptidase activity;MF:catalytic activity, acting on a protein;MF:serine hydrolase activity;MF:serine-type carboxypeptidase activity;MF:serine-type exopeptidase activity;MF:peptidase [illegible]	GO:0044238;GO:1901564;GO:0019538;GO:0043170;GO:0071704;GO:0006807;GO:0008150;GO:0008152;GO:0006508;GO:0003674;GO:0008238;GO:0016787;GO:0004180;GO:0140096;GO:0017171;GO:0004185;GO:0070008;GO:0008233;GO:0003824;GO:0008236	K09645	CPVL	------	------	ENOG410XPIW;COG2939	S:Function unknown;E:Amino acid transport and metabolism	PF00450.25	Peptidase_S10	Serine carboxypeptidase	CYT	4	12	55.9	High
TRINITY_DN12387_c1_g2_i1_orf1	------	------	------	------	------	------	ENOG410ZKDQ;ENOG4111M7J;ENOG410YJ7D	S:Function unknown;	------	------	------	CYT	10	50	39.7	High
TRINITY_DN2140_c0_g1_i1_orf1	CC:intracellular membrane-bounded organelle;CC:mitochondrion;CC:organelle;CC:intracellular organelle;CC:cellular_component;CC:membrane-bounded organelle;CC:cellular anatomical entity;MF:cation binding;MF:hydroquinon;MF:transition metal ion binding;MF:metal ion binding;MF:ion binding;MF:catalytic activity;MF:molecular_function;MF:binding;MF:copper ion binding;MF:oxidoreductase activity, acting on diphenols and related substances as donors;MF:oxidoreductase activity, acting on diphenols and related substances as donors, oxygen as acceptor;MF:oxidoredu [illegible]	GO:0043231;GO:0005739;GO:0043226;GO:0043229;GO:0005575;GO:0043227;GO:0110165;GO:0043169;GO:0052716;GO:0046914;GO:0046872;GO:0043167;GO:0003824;GO:0003674;GO:0005488;GO:0005507;GO:0016679;GO:0016682;GO:0016491	------	------	------	------	COG2132	Q:Secondary metabolites biosynthesis, transport and catabolism	PF07732.18;PF07731.17;PF00394.25	Cu-oxidase_3;Cu-oxidase_2;Cu-oxidase	Multicopper oxidase;Multicopper oxidase;Multicopper oxidase	CYT	4	7	84	High

TRINITY_DN2652_c0_g2_i1_orf1	peroxidase-like isoform X1 [Ostrinia furnacalis]	0.3671249	-1.445657297	2.18E-06	down	yes	1.282	3.492	3.432	3.501	3.544	1.204	1.354	1.288	BP:response to stimulus;BP:cell development;BP:female gamete generation;BP:gamete generation;BP:cellular developmental process;BP:oogenesis;BP:developmental process;BP:multicellular organismal reproductive process;BP:cellular process;BP:germ cell development;BP:reproductive process;BP:response to stress;BP:biological_process;BP:cellular process involved in reproduction in multicellular organism;BP:response to oxidative stress;BP:developmental process involved in reproduction;BP:anatomical structure development;CC:integral component of	GO:0050896;GO:0048468;GO:0007292;GO:0007276;GO:0048869;GO:0048477;GO:0032502;GO:0048609;GO:0009987;GO:0007281;GO:0022414;GO:0006950;GO:0008150;GO:0022412;GO:0006979;GO:0003006;GO:0048856;GO:0016021;GO:0031224;GO:0110165;GO:0005575;GO:0003674;GO:0005488;GO:1901363;GO:0016491;GO:0016684;GO:0020037;GO:0016209;GO:0097159;GO:0003824;GO:0004601;GO:0046906	K19511	PXDN, VPO1	------	------	ENOG410XPZ3	S:Function unknown	PF03098.18	An_peroxidase	Animal haem peroxidase	CYT	11	19	88.2	High
TRINITY_DN6470_c0_g3_i2_orf1	trypsin CFT-1-like [Ostrinia furnacalis]	0.2649529	-1.916192211	4.46E-05	down	yes	0.9843	3.715	3.514	3.948	3.683	0.85	1.065	1.038	BP:organic substance metabolic process;BP:organonitrogen compound metabolic process;BP:protein metabolic process;BP:macromolecule metabolic process;BP:biological_process;BP:nitrogen compound metabolic process;BP:metabolic process;BP:proteolysis;BP:primary metabolic process;MF:serine-type endopeptidase activity;MF:endopeptidase activity;MF:hydrolase activity;MF:serine hydrolase activity;MF:catalytic activity, acting on a protein;MF:catalytic activity;MF:molecular_function;MF:peptidase activity;MF:serine-type	GO:0071704;GO:1901564;GO:0019538;GO:0043170;GO:0008150;GO:0006807;GO:0008152;GO:0006508;GO:0044238;GO:0004252;GO:0004175;GO:0016787;GO:0017171;GO:0140096;GO:0003824;GO:0003674;GO:0008233;GO:0008236	K01312	PRSS1_2_3	map04972;map05164;map04080;map04974	Pancreatic secretion;Influenza A;Neuroactive ligand-receptor interaction;Protein digestion and absorption	COG5640	O:Posttranslational modification, protein turnover, chaperones	PF00089.29;PF13365.9	Trypsin;Trypsin_2	Trypsin;Trypsin-like peptidase domain	CYT	1	5	27.4	High
TRINITY_DN335_c1_g1_i5_orf1	PREDICTED: perilipin-4 isoform X14 [Papilio polytes]	2.2563694	1.174003294	2.49E-05	up	yes	2.834	1.256	1.234	1.259	1.274	2.853	2.945	2.703	------	------	K20254	PLIN4	map03320	PPAR signaling pathway	ENOG4112C4W	S:Function unknown	------	------	------	CYT	13	74	15.1	High
TRINITY_DN74538_c0_g1_i1_orf1	tetra-peptide repeat homeobox protein 1-like [Ostrinia furnacalis]	0.1333379	-2.906841338	4.35E-06	down	yes	0.9763	7.322	7.562	6.96	7.444	0.972	1.016	0.941	------	------	------	------	------	------	------	------	------	------	------	CYT	3	32	23.1	High
TRINITY_DN98147_c0_g2_i1_orf1	hypothetical protein evm_002297 [Chilo suppressalis]	0.2332201	-2.100235841	1.02E-05	down	yes	1.442	6.183	6.353	6.353	5.842	1.448	1.476	1.402	MF:molecular_function;MF:structural constituent of cuticle;MF:structural molecule activity;	GO:0003674;GO:0042302;GO:0005198	------	------	------	------	ENOG4110RRZ;ENOG410XTK4;ENOG4111PJ1;ENOG4110T67	S:Function unknown;	PF00379.26	Chitin_bind_4	Insect cuticle protein	EXC	3	18	31.2	High
TRINITY_DN29604_c0_g2_i2_orf1	neurofilament heavy polypeptide-like isoform X2 [Ostrinia furnacalis]	0.3179012	-1.653349476	4.85E-06	down	yes	1.751	5.508	5.473	5.714	5.338	1.747	1.796	1.709	------	------	------	------	------	------	------	------	------	------	------	CYT	22	77	40.5	High
TRINITY_DN65681_c0_g1_i1_orf1	ferritin subunit-like [Ostrinia furnacalis] >XP_028168186.1 ferritin subunit-like [Ostrinia furnacalis]	0.3744664	-1.417091994	3.36E-05	down	yes	1.842	4.919	5.099	4.851	4.808	2.081	1.737	1.708	BP:ion homeostasis;BP:homeostatic process;BP:iron ion homeostasis;BP:transition metal ion homeostasis;BP:inorganic ion homeostasis;BP:cellular transition metal ion homeostasis;BP:transition metal ion transport;BP:biological regulation;BP:regulation of biological quality;BP:cellular homeostasis;BP:metal ion transport;BP:transport;BP:cellular iron ion homeostasis;BP:cellular metal ion homeostasis;BP:cation transport;BP:ion transport;BP:cellular process;BP:cellular ion homeostasis;BP:biological_process;BP:cellular cation	GO:0050801;GO:0042592;GO:0055072;GO:0055076;GO:0098771;GO:0046916;GO:0000041;GO:0065007;GO:0065008;GO:0019725;GO:0030001;GO:0006810;GO:0006879;GO:0006875;GO:0006812;GO:0006811;GO:0009987;GO:0006873;GO:0008150;GO:0030003;GO:0055065;GO:0055080;GO:0055082;GO:0051234;GO:0051179;GO:0048878;GO:0006826;GO:0003674;GO:0005488;GO:0016491;GO:0043169;GO:0016722;GO:0046914;GO:0004322;GO:0043167;GO:0005506;GO:0008199;GO:0016724;GO:0046872;GO:0003824	K00522	FTH1	map04978;map04217;map04216	Mineral absorption;Necroptosis;Ferroptosis	ENOG4111MSG;ENOG41124IC	P:Inorganic ion transport and metabolism;	PF00210.27	Ferritin	Ferritin-like domain	CYT	1	31	8.1	High
TRINITY_DN49147_c0_g2_i1_orf1	glutenin, high molecular weight subunit PW212-like [Ostrinia furnacalis]	0.3916219	-1.352466606	3.55E-06	down	yes	1.823	4.655	4.613	4.572	4.779	1.734	1.843	1.892	------	------	------	------	------	------	ENOG4110R9Z	S:Function unknown	------	------	------	PLA	1	5	31.6	High
TRINITY_DN12671_c0_g1_i6_orf1	hemicentin-1-like isoform X1 [Ostrinia furnacalis]	0.2566699	-1.962013906	1.00E-05	down	yes	1.318	5.135	4.951	5.396	5.058	1.258	1.355	1.341	BP:biological_process;BP:localization;	GO:0008150;GO:0051179	------	------	------	------	ENOG410XPQS;ENOG410YQ1Q;ENOG410ZN3C;ENOG410XSTY	S:Function unknown;U:Intracellular trafficking, secretion, and vesicular transport;U:Intracellular trafficking, secretion, and vesicular transport;S:Function unknown	PF07679.19;PF13927.9;PF00047.28;PF13895.9	I-set;Ig_3;ig;Ig_2	Immunoglobulin I-set domain;Immunoglobulin domain;Immunoglobulin domain;Immunoglobulin domain	CYT	1	13	10.3	High
TRINITY_DN7549_c0_g1_i1_orf1	uncharacterized protein LOC114355006 [Ostrinia furnacalis]	0.1799412	-2.474402521	1.29E-05	down	yes	0.7957	4.422	4.355	4.649	4.262	0.674	0.778	0.935	------	------	------	------	------	------	ENOG4111H56;ENOG4110SWC	C:Energy production and conversion;S:Function unknown	------	------	------	CYT	2	14	20	High
TRINITY_DN1935_c0_g1_i1_orf1	adult-specific cuticular protein ACP-22-like [Ostrinia furnacalis]	0.1705404	-2.551814911	1.66E-06	down	yes	0.7543	4.423	4.49	4.522	4.258	0.763	0.772	0.728	MF:molecular_function;MF:structural constituent of cuticle;MF:structural molecule activity;	GO:0003674;GO:0042302;GO:0005198	------	------	------	------	ENOG410YSFI	I:Lipid transport and metabolism	PF00379.26	Chitin_bind_4	Insect cuticle protein	CYT	6	38	19.3	High
TRINITY_DN5595_c0_g1_i1_orf1	keratin, type I cytoskeletal 10-like [Ostrinia furnacalis]	0.2379437	-2.071307859	4.14E-05	down	yes	0.972	4.085	3.938	4.345	3.971	0.903	1.156	0.857	------	------	------	------	------	------	ENOG4112BJP	S:Function unknown	PF16058.8	Mucin-like	Mucin-like	EXC	1	14	16.6	High

TRINITY_DN19990_c0_g1_i1_orf1	carboxypeptidase B-like [Ostrinia furnacalis]	0.2791363	-1.84095826	9.54E-08	down	yes	1.073	3.844	3.847	3.886	3.798	1.058	1.109	1.053	BP:nitrogen compound metabolic process;BP:organic substance metabolic process;BP:proteolysis;BP:biological_process;BP:metabolic process;BP:primary metabolic process;BP:organonitrogen compound metabolic process;BP:protein metabolic process;BP:macromolecule metabolic process;MF:exopeptidase activity;MF:molecular_function;MF:zinc ion binding;MF:peptidase activity;MF:metalloexopeptidase activity;MF:metallopeptidase activity;MF:cation binding;MF:transition metal ion binding;MF:metallocarboxypeptidase	GO:0006807;GO:0071704;GO:0006508;GO:0008150;GO:0008152;GO:0044238;GO:1901564;GO:0019538;GO:0043170;GO:0008238;GO:0003674;GO:0008270;GO:0008233;GO:0008235;GO:0008237;GO:0043169;GO:0046914;GO:0004181;GO:0004180;GO:0043167;GO:0046872;GO:0016787;GO:0140096;GO:0003824;GO:0005488	------	------	------	------	COG2866	U:Intracellular trafficking, secretion, and vesicular transport	PF00246.27;PF02244.19	Peptidase_M14;Propep_M14	Zinc carboxypeptidase;Carboxypeptidase activation peptide	CYT	7	16	57.6	High
TRINITY_DN3056_c0_g1_i1_orf1	spidroin-2-like [Ostrinia furnacalis]	0.3146493	-1.668183194	2.46E-05	down	yes	1.162	3.693	3.678	3.743	3.658	0.991	1.124	1.371	MF:molecular_function;MF:structural constituent of cuticle;MF:structural molecule activity;	GO:0003674;GO:0042302;GO:0005198	------	------	------	------	ENOG4110SY1;ENOG4111PJ1;ENOG410XTK4;ENOG4110T67	S:Function unknown;	PF00379.26	Chitin_bind_4	Insect cuticle protein	CYT	1	4	76.6	High
TRINITY_DN36434_c0_g2_i3_orf1	clotting factor B isoform X1 [Ostrinia furnacalis]	2.1754658	1.121324362	0.001439	up	yes	2.802	1.288	1.349	1.157	1.358	2.556	3.157	2.692	BP:organic substance metabolic process;BP:organonitrogen compound metabolic process;BP:protein metabolic process;BP:macromolecule metabolic process;BP:biological_process;BP:nitrogen compound metabolic process;BP:metabolic process;BP:proteolysis;BP:primary metabolic process;MF:serine-type endopeptidase activity;MF:endopeptidase activity;MF:hydrolase activity;MF:serine hydrolase activity;MF:catalytic activity, acting on a protein;MF:catalytic activity;MF:molecular_function;MF:peptidase activity;MF:serine-type	GO:0071704;GO:1901564;GO:0019538;GO:0043170;GO:0008150;GO:0006807;GO:0008152;GO:0006508;GO:0044238;GO:0004252;GO:0004175;GO:0016787;GO:0017171;GO:0140096;GO:0003824;GO:0003674;GO:0008233;GO:0008236	------	------	------	------	COG5640	U:Intracellular trafficking, secretion, and vesicular transport	PF00089.29	Trypsin	Trypsin	CYT	3	9	48.8	High
TRINITY_DN8008_c0_g1_i6_orf1	uncharacterized protein LOC114357965 isoform X1 [Ostrinia furnacalis] >XP_028167599.1 uncharacterized protein LOC114357965 isoform X1 [Ostrinia furnacalis] >XP_028167600.1 uncharacterized protein LOC114357965 isoform X2 [Ostrinia furnacalis] >XP_028167601.1 uncharacterized protein LOC114357965 isoform X3 [Ostrinia furnacalis]	2.4070681	1.267276936	3.96E-05	up	yes	3.678	1.528	1.688	1.394	1.501	3.789	3.691	3.555	MF:organic cyclic compound binding;MF:molecular_function;MF:binding;MF:nucleic acid binding;MF:DNA binding;MF:heterocyclic compound binding;	GO:0097159;GO:0003674;GO:0005488;GO:0003676;GO:0003677;GO:1901363	------	------	------	------	ENOG410ZC30	S:Function unknown	------	------	------	CYT	1	0	506.7	Medium
TRINITY_DN53866_c0_g1_i1_orf1	larval/pupal cuticle protein H1C-like [Ostrinia furnacalis]	0.397407	-1.331310847	1.75E-05	down	yes	1.41	3.548	3.571	3.625	3.447	1.534	1.408	1.287	------	------	------	------	------	------	ENOG4110SXG	S:Function unknown	------	------	------	CYT	2	22	21.8	High
TRINITY_DN2896_c0_g1_i2_orf1	general odorant-binding protein 56d-like isoform X2 [Ostrinia furnacalis]	0.2970868	-1.751043499	3.93E-05	down	yes	1.03	3.467	3.481	3.674	3.246	1.03	1.026	1.035	CC:integral component of membrane;CC:cellular anatomical entity;CC:cellular_component;CC:intrinsic component of membrane;MF:odorant binding;MF:molecular	GO:0016021;GO:0110165;GO:0005575;GO:0031224;GO:0005549;GO:0003674;GO:0005488	------	------	------	------	ENOG410ZCHW;ENOG4110S21	S:Function unknown;	PF01395.25	PBP_GOBP	PBP/GOBP family	CYT	7	46	15	High
TRINITY_DN27968_c0_g2_i2_orf1	nucleolin-like [Ostrinia furnacalis]	0.2519214	-1.988954216	6.68E-06	down	yes	0.885	3.513	3.39	3.652	3.496	0.866	0.829	0.96	------	------	------	------	------	------	ENOG4111NPU	S:Function unknown	------	------	------	CYT	2	8	33.3	High
TRINITY_DN11670_c0_g1_i1_orf1	teneurin-m isoform X1 [Ostrinia furnacalis]	0.4676599	-1.096468416	2.87E-06	down	yes	1.287	2.752	2.76	2.728	2.769	1.219	1.344	1.298	CC:integral component of membrane;CC:membrane;CC:cellular_component;CC:intrinsic component of membrane;CC:plasma membrane;CC:cellular anatomical entity;	GO:0016021;GO:0016020;GO:0005575;GO:0031224;GO:0005886;GO:0110165	K24473	TENM, ODZ	------	------	ENOG410XQQD	S:Function unknown	PF15636.9;PF07974.16;PF05593.17	Tox-GHH;EGF_2;RHS_repeat	GHH signature containing HNH/Endo VII superfamily nuclease toxin;EGF-like domain;RHS Repeat	CYT	4	2	344.5	High
TRINITY_DN11970_c0_g1_i4_orf1	myb-like protein AA [Ostrinia furnacalis]	0.3633907	-1.460406413	0.0002044	down	yes	1.076	2.961	2.887	3.101	2.895	0.901	1.324	1.003	------	------	------	------	------	------	ENOG41117A2	S:Function unknown	------	------	------	CYT	3	5	126.8	High
TRINITY_DN5444_c0_g1_i1_orfp1	TRINITY_DN5444_c0_g1_i1_m.14077 TRINITY_DN5444_c0_g1::TRINITY_DN5444_c0_g1_i1::g.14077 ORF type:3prime_partial len:90 (-),score=25.11 TRINITY_DN5444_c0_g1_i1:2-268(-)	0.4306304	-1.215477966	0.0005875	down	yes	3.197	7.424	6.953	8.205	7.113	3.156	3.505	2.929	------	------	------	------	------	------	------	------	------	------	------	CYT	1	8	9.9	Medium
TRINITY_DN661_c0_g1_i1_orf1	hypothetical protein evm_002822 [Chilo suppressalis]	0.214321	-2.22215496	0.0003911	down	yes	1.302	6.075	6.613	6.396	5.217	1.362	1.264	1.28	MF:molecular_function;MF:structural constituent of cuticle;MF:structural molecule activity;	GO:0003674;GO:0042302;GO:0005198	------	------	------	------	ENOG4111NR3;ENOG410YSFI;ENOG410ZM6M;ENOG4110SG6	S:Function unknown;	PF00379.26	Chitin_bind_4	Insect cuticle protein	CYT	1	22	17.6	High
TRINITY_DN30177_c0_g2_i1_orf1	uncharacterized protein LOC114365032 [Ostrinia furnacalis]	2.9323513	1.552057969	1.76E-05	up	yes	5.375	1.833	1.857	1.873	1.769	5.423	5.596	5.106	------	------	------	------	------	------	ENOG4111NJW;ENOG41113NJ	S:Function unknown;	------	------	------	CYT	1	2	63	Medium
TRINITY_DN4802_c0_g1_i4_orf1	uncharacterized protein LOC114366345 isoform X2 [Ostrinia furnacalis]	3.202524	1.6792094	8.21E-06	up	yes	5.329	1.664	1.69	1.86	1.441	5.277	5.325	5.385	BP:response to biotic stimulus;BP:response to external stimulus;BP:response to external biotic stimulus;BP:response to stimulus;BP:defense response;BP:response to stress;BP:biological_process;BP:interspecies interaction between organisms;BP:response to other organism;CC:cellular_component;CC:extracellular region;CC:cellular	GO:0009607;GO:0009605;GO:0043207;GO:0050896;GO:0006952;GO:0006950;GO:0008150;GO:0044419;GO:0051707;GO:0005575;GO:0005576;GO:0110165	------	------	------	------	------	------	------	------	------	CYT	1	12	9.9	High

TRINITY_DN801_c0_g1_i2_orf1	cathepsin L [Ostrinia furnacalis] >XP_028165920.1 cathepsin L [Ostrinia furnacalis] >UKI61015.1 cathepsin L [Ostrinia furnacalis]	0.4613095	-1.116193018	5.82E-07	down	yes	2.17	4.704	4.706	4.675	4.73	2.134	2.253	2.122	BP:primary metabolic process;BP:organonitrogen compound metabolic process;BP:protein metabolic process;BP:macromolecule metabolic process;BP:organic substance metabolic process;BP:nitrogen compound metabolic process;BP:biological_process;BP:metabolic process;BP:proteolysis;MF:molecular_function;MF:hydrolase activity;MF:catalytic activity;MF:catalytic activity, acting on a protein;MF:peptidase activity;MF:cysteine-type peptidase activity;	GO:0044238;GO:1901564;GO:0019538;GO:0043170;GO:0071704;GO:0006807;GO:0008150;GO:0008152;GO:0006508;GO:0003674;GO:0016787;GO:0003824;GO:0140096;GO:0008233;GO:0008234	K01365	CTSL	map05205;map05323;map04612;map05418;map04145;map04210;map04142;map04140	Proteoglycans in cancer;Rheumatoid arthritis;Antigen processing and presentation;Fluid shear stress and atherosclerosis;Phagosome;Apoptosis;Lysosome;Autophagy - animal	COG4870	S:Function unknown	PF00112.26;PF08246.15	Peptidase_C1;Inhibitor_I29	Papain family cysteine protease;Cathepsin propeptide inhibitor domain	CYT	16	47	38.1	High
TRINITY_DN15222_c0_g1_i4_orf1	lysosomal alpha-mannosidase-like [Ostrinia furnacalis]	0.2809333	-1.831700674	3.83E-06	down	yes	1.469	5.229	5.079	5.425	5.184	1.526	1.433	1.448	BP:nucleotide biosynthetic process;BP:small molecule metabolic process;BP:organic cyclic compound biosynthetic process;BP:organic cyclic compound metabolic process;BP:ribonucleoside triphosphate metabolic process;BP:purine ribonucleoside triphosphate biosynthetic process;BP:purine ribonucleoside triphosphate metabolic process;BP:ribonucleoside triphosphate biosynthetic process;BP:ATP metabolic process;BP:heterocycle metabolic process;BP:organonitrogen compound metabolic	GO:0009165;GO:0044281;GO:1901362;GO:1901360;GO:0009199;GO:0009206;GO:0009205;GO:0009201;GO:0046034;GO:0046483;GO:1901564;GO:1901566;GO:0006163;GO:0019637;GO:0019438;GO:0009141;GO:0009142;GO:0009144;GO:0009145;GO:0006807;GO:1901576;GO:0018130;GO:0006164;GO:0019318;GO:0009152;GO:0009150;GO:0008150;GO:0008152;GO:0034654;GO:0090407;GO:0015986;GO:0044271;GO:0009260;GO:0046390;GO:1901293;GO:0006753;GO:0006754;GO:0044249;GO:0034641;GO:0009259;GO:0006139;GO:0009987;GO:0006725;GO:0005975;GO:0055086;GO:1901137;GO:0005996;GO:1901135;GO:0044237;GO:0072521;GO:0072522;GO:0006796;GO:0006013;GO:0071704;GO:0009058;GO:0009117;GO:0044238;GO:0019693;GO:0006793;GO:0045263;GO:0033177;GO:0098800;GO:0000276;GO:0032991;GO:0005575;GO:0098796;GO:0098798;GO:0046872;GO:0004553;GO:0004559;GO:0003674;GO:0005488;GO:0015318;GO:0016787;GO:0043169;GO:0003824;GO:0022890;GO:0015075;GO:0015078;GO:0016798;GO:0043167;GO:0015923;GO:0005215;GO:0030246;GO:0008324;GO:0022857	K12311	MAN2B1, LAMAN	map00511;map04142	Other glycan degradation;Lysosome	ENOG410XQMZ	U:Intracellular trafficking, secretion, and vesicular transport	------	------	------	CYT	1	17	11.9	High
TRINITY_DN2908_c0_g1_i1_orf1	uncharacterized protein LOC114361337 [Ostrinia furnacalis]	0.1282778	-2.962656386	3.82E-05	down	yes	0.724	5.644	6.058	5.658	5.216	0.719	0.813	0.64	------	------	------	------	------	------	ENOG4111MU3	S:Function unknown	PF00354.20;PF13385.9	Pentaxin;Laminin_G_3	Pentaxin family;Concanavalin A-like lectin/glucanases superfamily	CYT	1	2	61.6	High
TRINITY_DN9239_c0_g1_i1_orf1	apolipophorins-like [Ostrinia furnacalis]	0.3699913	-1.434436789	0.0001716	down	yes	1.699	4.592	4.499	4.313	4.963	1.639	1.581	1.877	BP:lipid transport;BP:transport;BP:organic substance transport;BP:biological_process;BP:establishment of localization;BP:localization;CC:cellular_component;CC:extracellular region;CC:cellular anatomical entity;MF:lipid transporter activity;MF:transporter activity;MF:lipid binding;MF:molecular_function;MF:binding;	GO:0006869;GO:0006810;GO:0071702;GO:0008150;GO:0051234;GO:0051179;GO:0005575;GO:0005576;GO:0110165;GO:0005319;GO:0005215;GO:0008289;GO:0003674;GO:0005488	------	------	------	------	ENOG410Z6MR;ENOG41112S0	S:Function unknown;	------	------	------	CYT	10	78	18	High
TRINITY_DN7539_c0_g1_i2_orf1	serine protease inhibitor 3/4 [Ostrinia furnacalis]	0.2826957	-1.822678005	1.89E-06	down	yes	1.271	4.496	4.448	4.576	4.464	1.15	1.373	1.289	CC:cellular_component;CC:cellular anatomical entity;CC:extracellular space;MF:endopeptidase inhibitor activity;MF:endopeptidase regulator activity;MF:peptidase regulator activity;MF:molecular_function;MF:peptidase inhibitor activity;MF:serine-type endopeptidase inhibitor activity;MF:enzyme inhibitor activity;MF:molecular function regulator;MF:enzyme	GO:0005575;GO:0110165;GO:0005615;GO:0004866;GO:0061135;GO:0061134;GO:0003674;GO:0030414;GO:0004867;GO:0004857;GO:0098772;GO:0030234	------	------	------	------	COG4826	O:Posttranslational modification, protein turnover, chaperones	PF00079.23	Serpin	Serpin	CYT	9	18	58.6	High
TRINITY_DN581_c3_g2_i1_orf1	uncharacterized protein LOC114364499 isoform X3 [Ostrinia furnacalis]	2.1742257	1.120501686	0.0001568	up	yes	3.931	1.808	2.023	1.799	1.602	3.75	4.059	3.983	------	------	------	------	------	------	------	------	------	------	------	CYT	1	17	6.7	High
TRINITY_DN699_c0_g2_i1_orf1	TPA_exp: putative parasitoid killing factor [Trichoplusia ni]	0.4659864	-1.101640262	0.0002162	down	yes	1.781	3.822	3.807	4.104	3.554	1.779	1.809	1.756	------	------	------	------	------	------	------	------	------	------	------	CYT	1	3	43.9	High
TRINITY_DN1110_c1_g1_i9_orf1	MD-2-related lipid-recognition protein-like [Ostrinia furnacalis]	0.3683788	-1.44073801	2.78E-06	down	yes	1.377	3.738	3.694	3.702	3.818	1.434	1.284	1.413	------	------	K13443	NPC2	map04979;map04142	Cholesterol metabolism;Lysosome	ENOG4111D1U;ENOG41122NY	S:Function unknown;	PF02221.18	E1_DerP2_DerF2	ML domain	CYT	2	17	17.2	High
TRINITY_DN91533_c0_g1_i1_orf1	uncharacterized protein LOC114361550 [Ostrinia furnacalis]	0.2503756	-1.997834333	5.20E-07	down	yes	1	3.994	4.006	4.076	3.901	0.995	0.991	1.015	CC:cellular_component;CC:integral component of membrane;CC:cellular anatomical entity;CC:intrinsic component of membrane;	GO:0005575;GO:0016021;GO:0110165;GO:0031224	------	------	------	------	ENOG410ZKIK	S:Function unknown	PF00024.29;PF14295.9	PAN_1;PAN_4	PAN domain;PAN domain	CYT	10	18	78.9	High
TRINITY_DN31314_c0_g1_i4_orf1	uncharacterized protein LOC114355976 [Ostrinia furnacalis]	0.2653621	-1.913965829	4.33E-07	down	yes	0.9967	3.756	3.774	3.82	3.675	0.98	1.026	0.984	BP:mitotic cell cycle;BP:cellular process;BP:biological_process;BP:cell division;BP:cell cycle;CC:cytoplasm;CC:supramolecular complex;CC:chromosomal region;CC:intracellular non-membrane-bounded organelle;CC:nucleus;CC:chromosome, centromeric region;CC:cellular_component;CC:intracellular organelle;CC:non-membrane-bounded organelle;CC:cellular anatomical entity;CC:kinetochore;CC:membrane-bounded organelle;CC:organelle;CC:intracellular membrane-bounded	GO:0000278;GO:0009987;GO:0008150;GO:0051301;GO:0007049;GO:0005737;GO:0099080;GO:0098687;GO:0043232;GO:0005634;GO:0000775;GO:0005575;GO:0043229;GO:0043228;GO:0110165;GO:0000776;GO:0043227;GO:0043226;GO:0043231	K11578	ZW10, DSL1	------	------	ENOG410XQ7J;ENOG4110SRI	S:Function unknown;	------	------	------	CYT	7	9	86.8	High

TRINITY_DN3464_c0_g1_i1_orf1	putative mitochondrial aconitate hydratase isoform X1-likeprotein, partial [Cotesia chilonis]	0.4390681	-1.187483373	0.0001996	down	yes	1.225	2.79	2.905	2.59	2.875	1.354	1.183	1.138	BP:tricarboxylic acid cycle;BP:biological_process;BP:metabolic process;BP:primary metabolic process;CC:membrane-bounded organelle;CC:organelle;CC:mitochondrion;CC:cellular anatomical entity;CC:intracellular membrane-bounded organelle;CC:intracellular organelle;CC:cellular_component;MF:molecular_function;MF:binding;MF:cation binding;MF:metal cluster binding;MF:lyase activity;MF:ion binding;MF:metal ion binding;MF:catalytic activity;MF:citrate dehydratase activity;MF:4 iron, 4 sulfur cluster binding;MF:carbon-[illegible]	GO:0006099;GO:0008150;GO:0008152;GO:0044238;GO:0043227;GO:0043226;GO:0005739;GO:0110165;GO:0043231;GO:0043229;GO:0005575;GO:0003674;GO:0005488;GO:0043169;GO:0051540;GO:0016829;GO:0043167;GO:0046872;GO:0003824;GO:0047780;GO:0051539;GO:0016835;GO:0016836;GO:0003994;GO:0051536	K01681	ACO, acnA	map00020;map00630	Citrate cycl;Glyoxylate and dicarboxylate metabolism	COG1048	C:Energy production and conversion	PF00330.23;PF00694.22	Aconitase;Aconitase_C	Aconitase family;Aconitase C-terminal domain	CYT	1	6	89.4	High
TRINITY_DN971_c0_g1_i5_orfp1	TRINITY_DN971_c0_g1_i5_m.54249 TRINITY_DN971_c0_g1::TRINITY_DN971_c0_g1_i5::g.54249 ORF type:internal len:108 (+),score=66.98 TRINITY_DN971_c0_g1_i5:1-321(+)	3.1497585	1.655241197	0.000182	up	yes	13.04	4.14	4.683	3.422	4.314	13.238	13.875	11.997	------	------	------	------	------	------	------	------	------	------	------	CYT	1	9	11.5	High
TRINITY_DN30154_c0_g1_i1_orf1	serine/threonine-protein kinase SIK2 [Ostrinia furnacalis] >XP_028174514.1 serine/threonine-protein kinase SIK2 [Ostrinia furnacalis]	0.1118953	-3.159778383	1.64E-07	down	yes	1.411	12.61	12.645	12.793	12.382	1.519	1.256	1.458	BP:phosphorylation;BP:nitrogen compound metabolic process;BP:organic substance metabolic process;BP:protein phosphorylation;BP:cellular process;BP:macromolecule modification;BP:protein modification process;BP:biological_process;BP:metabolic process;BP:primary metabolic process;BP:organonitrogen compound metabolic process;BP:cellular metabolic process;BP:macromolecule metabolic process;BP:protein metabolic process;BP:phosphate-containing compound metabolic process;BP:phosphorus [illegible]	GO:0016310;GO:0006807;GO:0071704;GO:0006468;GO:0009987;GO:0043412;GO:0036211;GO:0008150;GO:0008152;GO:0044238;GO:1901564;GO:0044237;GO:0043170;GO:0019538;GO:0006796;GO:0006793;GO:0035639;GO:0003674;GO:0005488;GO:0043167;GO:1901363;GO:1901265;GO:0043168;GO:0097367;GO:0000166;GO:0004674;GO:0016740;GO:0004672;GO:0017076;GO:0005524;GO:0016301;GO:0140096;GO:0036094;GO:0003824;GO:0030554;GO:0016773;GO:0016772;GO:0032559;GO:0032555;GO:0097159;GO:0032553	K16311	SIK2	map04922	Glucagon signaling pathway	ENOG410XNQ0	T:Signal transduction mechanisms	PF00069.28;PF07714.20;PF14531.9	Pkinase;PK_Tyr_Ser-Thr;Kinase-like	Protein kinase domain;Protein tyrosine and serine/threonine kinase;Kinase-like	CYT	1	1	136.1	Medium
TRINITY_DN57137_c0_g1_i1_orfp1	TRINITY_DN57137_c0_g1_i1_m.46420 TRINITY_DN57137_c0_g1::TRINITY_DN57137_c0_g1_i1::g.46420 ORF type:5prime_partial len:56 (-),score=4.65 TRINITY_DN57137_c0_g1_i1:82-249(-)	2.4517698	1.293823524	0.0003356	up	yes	9.074	3.701	3.461	3.667	3.975	9.1	9.833	8.288	------	------	------	------	------	------	------	------	------	------	------	CYT	3	33	5.8	High
TRINITY_DN2044_c0_g1_i5_orfp1	TRINITY_DN2044_c0_g1_i5_m.4210 TRINITY_DN2044_c0_g1::TRINITY_DN2044_c0_g1_i5::g.4210 ORF type:complete len:151 (-),score=85.31 TRINITY_DN2044_c0_g1_i5:857-1309(-)	2.3880687	1.255844338	0.006592	up	yes	7.926	3.319	3.451	3.585	2.921	9.64	6.863	7.274	------	------	------	------	------	------	------	------	------	------	------	CYT	1	5	16.2	High
TRINITY_DN381_c0_g1_i1_orf1	cuticle protein 8-like [Ostrinia furnacalis]	0.1055499	-3.244003145	4.98E-05	down	yes	0.93	8.811	9.122	9.341	7.971	0.929	0.952	0.909	MF:molecular_function;MF:structural constituent of cuticle;MF:structural molecule activity;	GO:0003674;GO:0042302;GO:0005198	------	------	------	------	ENOG410ZM6M;ENOG410YSFI;ENOG4110SG6;ENOG4111NR3	S:Function unknown;	PF00379.26	Chitin_bind_4	Insect cuticle protein	CYT	4	22	25	High
TRINITY_DN5177_c0_g1_i2_orf1	hemolin-like isoform X1 [Ostrinia furnacalis]	2.1587508	1.110196721	9.90E-06	up	yes	6.636	3.074	3.065	3.132	3.026	6.835	6.665	6.408	------	------	------	------	------	------	ENOG410XSVG	S:Function unknown	PF07679.19;PF13927.9;PF00047.28;PF13895.9;PF07686.20;PF11465.11	I-set;Ig_3;ig;Ig_2;V-set;Receptor_2B4	Immunoglobulin I-set domain;Immunoglobulin domain;Immunoglobulin domain;Immunoglobulin domain;Immunoglobulin V-set domain;Natural killer cell receptor 2B4	CYT	22	58	47.3	High
TRINITY_DN138481_c0_g1_i5_orf1	hypothetical protein evm_003901 [Chilo suppressalis]	0.2160702	-2.21042783	5.79E-05	down	yes	1.6	7.405	7.965	7.389	6.862	1.529	1.731	1.539	MF:molecular_function;MF:structural constituent of cuticle;MF:structural molecule activity;	GO:0003674;GO:0042302;GO:0005198	------	------	------	------	ENOG410YRDB;ENOG410YM5B	S:Function unknown;	PF00379.26	Chitin_bind_4	Insect cuticle protein	CYT	2	32	22.6	High
TRINITY_DN12534_c0_g1_i4_orf1	antibacterial protein [Heliothis virescens]	2.322984	1.215979245	6.77E-06	up	yes	5.387	2.319	2.269	2.318	2.369	5.448	5.514	5.199	BP:response to biotic stimulus;BP:response to external stimulus;BP:response to external biotic stimulus;BP:response to stimulus;BP:defense response;BP:response to stress;BP:biological_process;BP:interspecies interaction between organisms;BP:response to other organism;CC:cellular_component;CC:extracellular region;CC:cellular	GO:0009607;GO:0009605;GO:0043207;GO:0050896;GO:0006952;GO:0006950;GO:0008150;GO:0044419;GO:0051707;GO:0005575;GO:0005576;GO:0110165	------	------	------	------	ENOG4110S6N	S:Function unknown	PF00451.22	Toxin_2	Scorpion short toxin, BmKK2	CYT	5	57	12	High
TRINITY_DN30510_c0_g1_i6_orf1	spodomicin-like [Ostrinia furnacalis]	5.1184756	2.355714195	2.74E-07	up	yes	6.178	1.207	1.233	1.131	1.257	6.132	6.3	6.102	------	------	------	------	------	------	------	------	PF08036.14	Antimicrobial_6	Diapausin family of antimicrobial peptide	CYT	3	60	6.7	High
TRINITY_DN2002_c0_g1_i5_orfp1	TRINITY_DN2002_c0_g1_i5_m.4230 TRINITY_DN2002_c0_g1::TRINITY_DN2002_c0_g1_i5::g.4230 ORF type:3prime_partial len:259 (+),score=29.98,Peptidase_C39 PF03412.16 1.7,Peptidase_C39|PF03412.16|0.049 TRINITY_DN2002_c0_g1_i5:65-838(+)	0.3328717	-1.586962078	0.0002251	down	yes	1.442	4.332	3.941	4.432	4.623	1.307	1.37	1.648	------	------	------	------	------	------	------	------	------	------	------	CYT	1	5	29.4	Medium
TRINITY_DN3616_c0_g2_i1_orf1	conotoxin ArMKLT2-032-like [Ostrinia furnacalis]	0.3527858	-1.503135673	1.47E-05	down	yes	1.469	4.164	4.093	4.37	4.028	1.444	1.453	1.511	CC:cellular_component;CC:integral component of membrane;CC:cellular anatomical entity;CC:intrinsic component of membrane;	GO:0005575;GO:0016021;GO:0110165;GO:0031224	------	------	------	------	------	------	------	------	------	CYT	2	64	8.7	High

TRINITY_DN5444_c0_g2_i1_orf1	phenoloxidase-activating factor 2-like [Ostrinia furnacalis]	0.397865	-1.329649198	9.52E-06	down	yes	1.379	3.466	3.511	3.521	3.365	1.388	1.281	1.469	BP:organic substance metabolic process;BP:organonitrogen compound metabolic process;BP:protein metabolic process;BP:macromolecule metabolic process;BP:biological_process;BP:nitrogen compound metabolic process;BP:metabolic process;BP:proteolysis;BP:primary metabolic process;MF:serine-type endopeptidase activity;MF:endopeptidase activity;MF:hydrolase activity;MF:serine hydrolase activity;MF:catalytic activity, acting on a protein;MF:catalytic activity;MF:molecular_function;MF:peptidase activity;MF:serine-type	GO:0071704;GO:1901564;GO:0019538;GO:0043170;GO:0008150;GO:0006807;GO:0008152;GO:0006508;GO:0044238;GO:0004252;GO:0004175;GO:0016787;GO:0017171;GO:0140096;GO:0003824;GO:0003674;GO:0008233;GO:0008236	------	------	------	------	COG5640	E:Amino acid transport and metabolism	PF00089.29	Trypsin	Trypsin	CYT	3	14	27.9	High
TRINITY_DN52553_c0_g1_i1_orf1	hemocyte protein-glutamine gamma-glutamyltransferase-like [Ostrinia furnacalis]	0.2897822	-1.786958894	3.23E-06	down	yes	1.038	3.582	3.703	3.489	3.555	0.986	1.047	1.08	BP:primary metabolic process;BP:peptide cross-linking;BP:organonitrogen compound metabolic process;BP:organic substance metabolic process;BP:nitrogen compound metabolic process;BP:macromolecule modification;BP:macromolecule metabolic process;BP:biological_process;BP:protein modification process;BP:metabolic process;BP:protein metabolic process;MF:molecular_function;MF:protein-glutamine gamma-glutamyltransferase activity;MF:catalytic activity, acting on a protein;MF:catalytic activity;MF:transferase activity;MF:transferase	GO:0044238;GO:0018149;GO:1901564;GO:0071704;GO:0006807;GO:0043412;GO:0043170;GO:0008150;GO:0036211;GO:0008152;GO:0019538;GO:0003674;GO:0003810;GO:0140096;GO:0003824;GO:0016740;GO:0016746;GO:0016755	K05619	TGM1	------	------	ENOG410XQEZ	O:Posttranslational modification, protein turnover, chaperones	PF00927.25;PF01841.22;PF00868.23	Transglut_C;Transglut_core;Transglut_N	Transglutaminase family, C-terminal ig like domain;Transglutaminase-like superfamily;Transglutaminase family	CYT	4	8	80	High
TRINITY_DN4324_c0_g1_i1_orf1	uncharacterized protein LOC114354985 isoform X1 [Ostrinia furnacalis]	0.3314038	-1.593338112	3.23E-06	down	yes	1.145	3.455	3.384	3.473	3.508	1.045	1.21	1.179	CC:cellular_component;CC:integral component of membrane;CC:cellular anatomical entity;CC:intrinsic component of membrane;	GO:0005575;GO:0016021;GO:0110165;GO:0031224	------	------	------	------	ENOG41113AM;ENOG4110NNY	S:Function unknown;	------	------	------	CYT	4	20	16	High
TRINITY_DN14904_c0_g1_i1_orf1	attacin [Ostrinia furnacalis]	4.0719258	2.025711256	2.78E-05	up	yes	8.775	2.155	2.209	2.181	2.076	8.823	9.278	8.223	BP:interspecies interaction between organisms;BP:response to biotic stimulus;BP:response to external stimulus;BP:response to external biotic stimulus;BP:response to stimulus;BP:response to bacterium;BP:defense response;BP:response to stress;BP:defense response to other organism;BP:defense response to bacterium;BP:biological_process;BP:response to other organism;CC:cellular_component;CC:extracell	GO:0044419;GO:0009607;GO:0009605;GO:0043207;GO:0050896;GO:0009617;GO:0006952;GO:0006950;GO:0098542;GO:0042742;GO:0008150;GO:0051707;GO:0005575;GO:0005576;GO:0110165	------	------	------	------	ENOG410ZBQ8;ENOG4110N1X	S:Function unknown;	PF03769.18	Attacin_C	Attacin, C-terminal region	CYT	3	26	17.3	High
TRINITY_DN1902_c0_g1_i4_orf1	chemosensory protein 10 [Conogethes pinicolalis]	0.2786548	-1.843449136	3.95E-06	down	yes	0.9073	3.256	3.218	3.358	3.193	0.89	0.989	0.843	------	------	------	------	------	------	ENOG4112163;ENOG410Y8R4	S:Function unknown;	PF03392.16	OS-D	Insect pheromone-binding family, A10/OS-D	CYT	2	20	11.8	High
TRINITY_DN7740_c0_g1_i2_orf1	D-arabinitol dehydrogenase 1 [Eumeta japonica]	2.0051508	1.003710774	4.58E-08	up	yes	2.725	1.359	1.371	1.338	1.368	2.711	2.729	2.735	MF:oxidoreductase activity;MF:cation binding;MF:transition metal ion binding;MF:ion binding;MF:catalytic activity;MF:molecular_function;MF:binding;MF:metal ion binding;MF:zinc ion binding;	GO:0016491;GO:0043169;GO:0046914;GO:0043167;GO:0003824;GO:0003674;GO:0005488;GO:0046872;GO:0008270	K17818	ARD1	map00040	Pentose and glucuronate interconversions	COG1063	E:Amino acid transport and metabolism	------	------	------	CYT	1	69	9.3	High
TRINITY_DN86772_c0_g1_i3_orfp1	x-tox [Spodoptera exigua]	2.2319182	1.158284178	5.82E-05	up	yes	5.678	2.544	2.561	2.63	2.441	5.964	5.684	5.386	BP:response to biotic stimulus;BP:response to external stimulus;BP:response to external biotic stimulus;BP:response to stimulus;BP:defense response;BP:response to stress;BP:biological_process;BP:interspecies interaction between organisms;BP:response to other organism;CC:cellular_component;CC:extracellular region;CC:cellular	GO:0009607;GO:0009605;GO:0043207;GO:0050896;GO:0006952;GO:0006950;GO:0008150;GO:0044419;GO:0051707;GO:0005575;GO:0005576;GO:0110165	------	------	------	------	------	------	------	------	------	CYT	1	9	8.5	High
TRINITY_DN8853_c0_g1_i4_orf1	uncharacterized protein LOC114351488 isoform X1 [Ostrinia furnacalis]	3.3967213	1.764142855	0.0001892	up	yes	3.108	0.915	0.847	0.932	0.966	3.279	2.784	3.261	MF:molecular_function;MF:structural constituent of cuticle;MF:structural molecule activity;	GO:0003674;GO:0042302;GO:0005198	------	------	------	------	------	------	------	------	------	CYT	1	9	12.9	Medium
TRINITY_DN33365_c0_g1_i1_orf1	mucin-5AC isoform X1 [Ostrinia furnacalis]	0.3576271	-1.483471956	3.47E-07	down	yes	1.055	2.95	2.946	2.986	2.918	1.014	1.088	1.064	------	------	------	------	------	------	ENOG41120DH	S:Function unknown	------	------	------	CYT	3	2	204.2	High
TRINITY_DN49785_c1_g1_i3_orf1	uncharacterized protein LOC114365444, partial [Ostrinia furnacalis]	0.2915883	-1.777995035	8.00E-07	down	yes	1.241	4.256	4.168	4.359	4.242	1.265	1.213	1.246	------	------	------	------	------	------	ENOG4110SB1	S:Function unknown	------	------	------	CYT	1	6	26.6	High
TRINITY_DN1749_c0_g1_i1_orf1	putative GPI-anchored protein pfl2 isoform X2 [Ostrinia furnacalis]	0.2967367	-1.752744602	5.32E-05	down	yes	1.073	3.616	3.429	3.875	3.545	1.14	1.078	1.002	------	------	------	------	------	------	COG5599	S:Function unknown	PF00102.30	Y_phosphatase	Protein-tyrosine phosphatase	CYT	2	6	59	High
TRINITY_DN74086_c0_g1_i1_orf1	UPF0489 protein C5orf22 homolog [Ostrinia furnacalis]	0.4309494	-1.214409459	3.50E-05	down	yes	1.398	3.244	3.193	3.122	3.417	1.414	1.358	1.421	------	------	------	------	------	------	ENOG41109F9	S:Function unknown	PF12640.10	UPF0489	UPF0489 domain	CYT	1	4	42.6	High

TRINITY_DN110231_c0_g1_i1_orf1	protein singed [Ostrinia furnacalis] >XP_028161434.1 protein singed [Ostrinia furnacalis]	0.4062006	-1.299735833	3.72E-07	down	yes	1.284	3.161	3.206	3.135	3.142	1.279	1.253	1.319	BP:cellular component organization or biogenesis;BP:fibril organization;BP:cellular component organization;BP:cell migration;BP:cellular process;BP:biological_process;BP:actin filament organization;BP:cell motility;CC:intracellular organelle;CC:non-membrane-bounded organelle;CC:organelle;CC:cytoplasm;CC:cellular anatomical entity;CC:cytoskeleton;CC:intracellular non-membrane-bounded organelle;CC:cellular_component;MF:macromolecular complex binding;MF:binding;MF:actin binding;MF:actin filament binding;MF:binding, bridging;MF:protein binding, [illegible]	GO:0071840;GO:0097435;GO:0016043;GO:0016477;GO:0009987;GO:0008150;GO:0007015;GO:0048870;GO:0043229;GO:0043228;GO:0043226;GO:0005737;GO:0110165;GO:0005856;GO:0043232;GO:0005575;GO:0044877;GO:0005488;GO:0003779;GO:0051015;GO:0060090;GO:0030674;GO:0008092;GO:0005515;GO:0003674	K23551	FSCN1	map05206	MicroRNAs in cancer	ENOG410XPHV	O:Posttranslational modification, protein turnover, chaperones	PF06268.16;PF06229.15;PF00167.21	Fascin;FRG1;FGF	Fascin domain;FRG1-like domain;Fibroblast growth factor	CYT	11	29	57	High
TRINITY_DN36476_c1_g1_i1_orfp1	TRINITY_DN36476_c1_g1_i1_m.70910 TRINITY_DN36476_c1_g1::TRINITY_DN36476_c1_g1_i1::g.70910 ORF type:5prime_partial len:88 (-),score=0.50 TRINITY_DN36476_c1_g1_i1:49-312(-)	3.516769	1.814250579	3.93E-07	up	yes	7.445	2.117	2.061	2.092	2.198	7.571	7.45	7.313	------	------	------	------	------	------	------	------	------	------	------	CYT	2	21	9.5	High
TRINITY_DN18338_c0_g1_i6_orf1	aquaporin AQPAn.G isoform X1 [Ostrinia furnacalis]	0.4665201	-1.099988787	0.0004	down	yes	2.759	5.914	6.357	5.495	5.89	2.921	2.466	2.889	CC:integral component of membrane;CC:cellular_component;CC:intrinsic component of membrane;CC:cellular anatomical entity;MF:transporter activity;MF:transmembrane transporter activity;MF:channel activity;MF:molecular_function;MF:passive transmembrane transporter activity;	GO:0016021;GO:0005575;GO:0031224;GO:0110165;GO:0005215;GO:0022857;GO:0015267;GO:0003674;GO:0022803	K09884	AQPN	------	------	COG0580	O:Posttranslational modification, protein turnover, chaperones	PF00230.23	MIP	Major intrinsic protein	CYT	1	8	29	High
TRINITY_DN129835_c0_g1_i2_orf1	alpha-tocopherol transfer protein-like [Chelonus insularis]	0.0569017	-4.135385665	8.56E-09	down	yes	0.4617	8.114	8.168	8.09	8.084	0.533	0.451	0.401	------	------	------	------	------	------	ENOG410XRSQ	S:Function unknown	PF00650.23;PF13716.9	CRAL_TRIO;CRAL_TRIO_2	CRAL/TRIO domain;Divergent CRAL/TRIO domain	CYT	1	2	35.4	Medium
TRINITY_DN13576_c0_g1_i1_orf1	uncharacterized protein LOC114350099 [Ostrinia furnacalis]	0.1499304	-2.737635628	1.53E-06	down	yes	0.9687	6.461	6.633	6.522	6.228	0.95	0.936	1.02	------	------	------	------	------	------	ENOG4111QIW	S:Function unknown	PF00379.26	Chitin_bind_4	Insect cuticle protein	CYT	9	48	29.1	High
TRINITY_DN619_c0_g1_i1_orf1	putative uncharacterized protein DDB_G0271606 [Ostrinia furnacalis]	0.0855664	-3.546812092	8.87E-06	down	yes	0.562	6.568	6.655	6.873	6.177	0.518	0.647	0.521	CC:cellular_component;CC:extracellular region;CC:cellular anatomical entity;MF:carbohydrate derivative binding;MF:molecular_function;MF:chitin binding;MF:binding;	GO:0005575;GO:0005576;GO:0110165;GO:0097367;GO:0003674;GO:0008061;GO:0005488	------	------	------	------	ENOG4111K2A	S:Function unknown	PF01607.27	CBM_14	Chitin binding Peritrophin-A domain	CYT	3	16	39.1	High
TRINITY_DN2400_c0_g1_i1_orf1	uncharacterized protein LOC114351021 [Ostrinia furnacalis]	0.2141505	-2.223303344	5.23E-06	down	yes	1.244	5.809	5.975	5.801	5.651	1.038	1.368	1.326	------	------	------	------	------	------	ENOG41113AM	S:Function unknown	------	------	------	PLA	1	11	12.9	High
TRINITY_DN57998_c1_g1_i1_orf1	uncharacterized protein LOC113509309, partial [Galleria mellonella]	0.1996069	-2.324766777	0.0005568	down	yes	1.117	5.596	5.526	6.395	4.866	1.232	1.031	1.089	------	------	------	------	------	------	ENOG4111SVO	S:Function unknown	------	------	------	CYT	1	14	9.1	High
TRINITY_DN25273_c0_g1_i1_orf1	skin secretory protein xP2-like [Ostrinia furnacalis]	0.3247193	-1.622734786	8.90E-06	down	yes	1.533	4.721	4.83	4.817	4.516	1.466	1.517	1.615	------	------	------	------	------	------	ENOG4111DG8	S:Function unknown	------	------	------	CYT	3	16	29	High
TRINITY_DN13686_c0_g2_i1_orf1	transmembrane protease serine 9-like [Ostrinia furnacalis]	0.4043338	-1.306381305	2.45E-05	down	yes	1.754	4.338	4.372	4.394	4.249	1.948	1.738	1.577	BP:organic substance metabolic process;BP:organonitrogen compound metabolic process;BP:protein metabolic process;BP:macromolecule metabolic process;BP:biological_process;BP:nitrogen compound metabolic process;BP:metabolic process;BP:proteolysis;BP:primary metabolic process;MF:serine-type endopeptidase activity;MF:endopeptidase activity;MF:hydrolase activity;MF:serine hydrolase activity;MF:catalytic activity, acting on a protein;MF:catalytic activity;MF:molecular_function;MF:peptidase activity;MF:serine-type	GO:0071704;GO:1901564;GO:0019538;GO:0043170;GO:0008150;GO:0006807;GO:0008152;GO:0006508;GO:0044238;GO:0004252;GO:0004175;GO:0016787;GO:0017171;GO:0140096;GO:0003824;GO:0003674;GO:0008233;GO:0008236	K20677;K09640;K09641	HAYAN;TMPRSS9;TMPRSS11D	map05164	Influenza A	COG5640	O:Posttranslational modification, protein turnover, chaperones	PF00089.29	Trypsin	Trypsin	CYT	2	6	43.9	High
TRINITY_DN338_c0_g1_i1_orf1	chorion protein S36-like [Ostrinia furnacalis]	0.2064136	-2.276389792	3.31E-06	down	yes	1.017	4.927	4.819	5.114	4.849	0.918	1.088	1.045	MF:molecular_function;MF:structural constituent of cuticle;MF:structural molecule activity;	GO:0003674;GO:0042302;GO:0005198	------	------	------	------	ENOG4111NR3	S:Function unknown	PF00379.26	Chitin_bind_4	Insect cuticle protein	CYT	1	3	50.9	High
TRINITY_DN1772_c1_g2_i1_orf1	aldose reductase-like isoform X2 [Ostrinia furnacalis]	3.059589	1.613337886	5.08E-07	up	yes	4.467	1.46	1.434	1.459	1.486	4.541	4.485	4.374	------	------	K00011	AKR1B	map00051;map00040;map00561;map00790;map00052	Fructose and mannose metabolism;Pentose and glucuronate interconversions;Glycerolipid metabolism;Folate biosynthesis;Galactose metabolism	COG0656	Z:Cytoskeleton	PF00248.24	Aldo_ket_red	Aldo/keto reductase family	CYT	13	77	36.1	High
TRINITY_DN28711_c0_g1_i1_orf1	hypothetical protein evm_000299 [Chilo suppressalis]	0.4312453	-1.213419292	7.02E-06	down	yes	1.728	4.007	3.926	3.962	4.132	1.736	1.793	1.655	MF:lipid transporter activity;MF:transporter activity;MF:molecular_function;	GO:0005319;GO:0005215;GO:0003674	------	------	------	------	ENOG410Z6MR;ENOG410ZRUY	S:Function unknown;	------	------	------	CYT	7	83	7.5	High
TRINITY_DN81488_c0_g1_i1_orf1	apolipophorins-like [Ostrinia furnacalis]	0.4813935	-1.05471141	0.0003216	down	yes	1.824	3.789	3.834	4.022	3.512	1.938	1.664	1.871	BP:lipid transport;BP:transport;BP:organic substance transport;BP:biological_process;BP:establishment of localization;BP:localization;CC:cellular_component;CC:extracellular region;CC:cellular anatomical entity;MF:lipid transporter activity;MF:transporter activity;MF:lipid binding;MF:molecular_function;MF:binding;	GO:0006869;GO:0006810;GO:0071702;GO:0008150;GO:0051234;GO:0051179;GO:0005575;GO:0005576;GO:0110165;GO:0005319;GO:0005215;GO:0008289;GO:0003674;GO:0005488	------	------	------	------	ENOG410Z6MR;ENOG41112S0	S:Function unknown;	------	------	------	CYT	6	75	8.8	High
TRINITY_DN27264_c0_g1_i1_orf1	uncharacterized protein LOC114353424 [Ostrinia furnacalis]	0.2539683	-1.977279924	0.0001002	down	yes	1.136	4.473	4.421	4.857	4.142	1.216	1.034	1.157	MF:molecular_function;MF:structural constituent of cuticle;MF:structural molecule activity;	GO:0003674;GO:0042302;GO:0005198	------	------	------	------	ENOG4111DJS;ENOG4111VJY	S:Function unknown;	PF00379.26	Chitin_bind_4	Insect cuticle protein	CYT	2	6	29.3	High

TRINITY_DN394_c0_g1_i2_orf1	uncharacterized protein LOC114351483 [Ostrinia furnacalis]	5.2769784	2.399712083	8.03E-05	up	yes	4.401	0.834	0.782	0.826	0.894	4.597	4.633	3.972	MF:molecular_function;MF:structural constituent of cuticle;MF:structural molecule activity;	GO:0003674;GO:0042302;GO:0005198	------	------	------	------	ENOG41113X7;ENOG410XTK4	S:Function unknown;	PF00379.26	Chitin_bind_4	Insect cuticle protein	CYT	1	28	12	High
TRINITY_DN4068_c0_g2_i4_orf1	larval cuticle protein LCP-17-like precursor [Papilio polytes] >BAM18876.1 cuticular protein PpolCPR2 [Papilio polytes]	5.9538014	2.573811108	6.36E-07	up	yes	3.892	0.6537	0.707	0.695	0.559	3.937	3.914	3.825	MF:molecular_function;MF:structural constituent of cuticle;MF:structural molecule activity;	GO:0003674;GO:0042302;GO:0005198	------	------	------	------	ENOG4110RUZ;ENOG4111PJ1;ENOG4110T0M	S:Function unknown;	PF00379.26	Chitin_bind_4	Insect cuticle protein	CYT	2	56	13.4	High
TRINITY_DN5664_c0_g1_i1_orf1	CDGSH iron-sulfur domain-containing protein 3, mitochondrial-like [Ostrinia furnacalis]	0.2448286	-2.030156213	1.27E-05	down	yes	0.864	3.529	3.621	3.511	3.454	0.848	1.026	0.718	CC:cytoplasm;CC:cellular_component;CC:intracellular organelle;CC:membrane-bounded organelle;CC:organelle;CC:cellular anatomical entity;CC:intracellular membrane-bounded organelle;MF:cation binding;MF:metal cluster binding;MF:iron-sulfur cluster binding;MF:2 iron, 2 sulfur cluster binding;MF:ion binding;MF:molecular_function;MF:binding;MF:metal ion binding;	GO:0005737;GO:0005575;GO:0043229;GO:0043227;GO:0043226;GO:0110165;GO:0043231;GO:0043169;GO:0051540;GO:0051536;GO:0051537;GO:0043167;GO:0003674;GO:0005488;GO:0046872	K23886	CISD3	------	------	ENOG41122TT	S:Function unknown	PF09360.13	zf-CDGSH	Iron-binding zinc finger CDGSH type	CYT	1	12	15.7	High
TRINITY_DN29190_c0_g1_i4_orf1	gloverin-like [Ostrinia furnacalis]	2.2355243	1.1606132	0.0001146	up	yes	2.857	1.278	1.381	1.333	1.12	2.872	2.966	2.732	BP:interspecies interaction between organisms;BP:response to biotic stimulus;BP:response to external stimulus;BP:response to external biotic stimulus;BP:response to stimulus;BP:response to bacterium;BP:defense response;BP:response to stress;BP:defense response to other organism;BP:defense response to bacterium;BP:biological_process;BP:response to other organism;CC:cellular_component;CC:extracell	GO:0044419;GO:0009607;GO:0009605;GO:0043207;GO:0050896;GO:0009617;GO:0006952;GO:0006950;GO:0098542;GO:0042742;GO:0008150;GO:0051707;GO:0005575;GO:0005576;GO:0110165	------	------	------	------	ENOG4110QR6	S:Function unknown	PF10793.12	Gloverin	Gloverin-like protein	PLA	1	28	11.5	High
TRINITY_DN38307_c0_g1_i1_orfp1	TRINITY_DN38307_c0_g1_i1_m.10661 TRINITY_DN38307_c0_g1::TRINITY_DN38307_c0_g1_i1::g.10661 ORF type:5prime_partial len:66 (+),score=5.90 TRINITY_DN38307_c0_g1_i1:2-199(+)	0.415491	-1.267110834	0.001112	down	yes	6.008	14.46	13.389	14.227	15.776	7.437	5.538	5.048	------	------	------	------	------	------	------	------	------	------	------	CYT	1	17	7.9	High
TRINITY_DN1124_c0_g1_i7_orf1	PREDICTED: cuticle protein 18.6, isoform B [Amyelois transitella]	0.0697209	-3.842265139	4.98E-06	down	yes	0.8743	12.54	12.45	13.19	11.979	0.861	0.894	0.868	MF:molecular_function;MF:structural constituent of cuticle;MF:structural molecule activity;	GO:0003674;GO:0042302;GO:0005198	------	------	------	------	ENOG4111Y6E	S:Function unknown	PF00379.26	Chitin_bind_4	Insect cuticle protein	CYT	4	18	27.2	High
TRINITY_DN7064_c0_g1_i19_orf1	unnamed protein product [Chilo suppressalis]	5.80674	2.537728445	2.87E-05	up	yes	8.443	1.454	1.553	1.212	1.597	7.966	9.01	8.353	------	------	------	------	------	------	------	------	PF00050.24;PF07648.18	Kazal_1;Kazal_2	Kazal-type serine protease inhibitor domain;Kazal-type serine protease inhibitor domain	EXC	1	2	73	High
TRINITY_DN9608_c0_g1_i3_orf1	cytochrome P450 monooxygenase CYP9G18 [Cnaphalocrocis medinalis]	3.0716383	1.619008335	2.05E-05	up	yes	5.231	1.703	1.765	1.632	1.713	5.455	4.954	5.284	CC:endoplasmic reticulum membrane;CC:intrinsic component of membrane;CC:organelle membrane;CC:integral component of membrane;CC:membrane;CC:cellular anatomical entity;CC:cellular_component;MF:monooxygenase activity;MF:oxidoreductase activity, acting on paired donors, with incorporation or reduction of molecular oxygen;MF:molecular_function;MF:binding;MF:heterocyclic compound binding;MF:oxidoreductase activity;MF:cation binding;MF:transition metal ion binding;MF:ion binding;MF:iron ion	GO:0005789;GO:0031224;GO:0031090;GO:0016021;GO:0016020;GO:0110165;GO:0005575;GO:0004497;GO:0016705;GO:0003674;GO:0005488;GO:1901363;GO:0016491;GO:0043169;GO:0046914;GO:0043167;GO:0005506;GO:0046872;GO:0020037;GO:0003824;GO:0046906;GO:0097159	K15003	CYP9	------	------	COG2124	K:Transcription	PF00067.25	p450	Cytochrome P450	CYT	7	17	58.5	High
TRINITY_DN2311_c0_g3_i1_orf1	uncharacterized protein LOC114364231 isoform X1 [Ostrinia furnacalis] >XP_028176108.1 uncharacterized protein LOC114364231 isoform X2 [Ostrinia	0.4704077	-1.088016371	0.00036	down	yes	2.146	4.562	4.518	4.914	4.253	2.267	2.218	1.953	------	------	------	------	------	------	ENOG410Y2YK	S:Function unknown	------	------	------	CYT	1	2	41.4	High
TRINITY_DN98334_c0_g1_i1_orf1	unnamed protein product [Spodoptera littoralis] >CAH1638738.1 unnamed protein product [Spodoptera littoralis]	0.1832772	-2.447900816	7.44E-07	down	yes	0.925	5.047	5.053	5.172	4.917	0.912	0.971	0.892	------	------	------	------	------	------	ENOG4110QDI	S:Function unknown	------	------	------	CYT	8	52	34	High
TRINITY_DN4384_c0_g1_i5_orf1	chemosensory protein 5 [Conogethes punctiferalis]	0.2584056	-1.952290857	1.13E-06	down	yes	1.222	4.729	4.778	4.807	4.602	1.286	1.216	1.164	------	------	------	------	------	------	ENOG410YU7V;ENOG4110RV8	U:Intracellular trafficking, secretion, and vesicular transport;S:Function unknown	PF03392.16	OS-D	Insect pheromone-binding family, A10/OS-D	CYT	2	14	16.9	High
TRINITY_DN26301_c0_g1_i1_orf1	uncharacterized protein LOC114359193 [Ostrinia furnacalis]	0.422578	-1.242710523	3.74E-06	down	yes	1.531	3.623	3.708	3.642	3.518	1.517	1.569	1.508	CC:cellular_component;CC:extracellular region;CC:cellular anatomical entity;MF:carbohydrate derivative binding;MF:molecular_function;MF:chitin binding;MF:binding;	GO:0005575;GO:0005576;GO:0110165;GO:0097367;GO:0003674;GO:0008061;GO:0005488	------	------	------	------	ENOG410ZPH9;ENOG410XQ49;ENOG4111HJZ	S:Function unknown;	PF01607.27	CBM_14	Chitin binding Peritrophin-A domain	CYT	3	16	28.5	High
TRINITY_DN59388_c0_g1_i1_orf1	uncharacterized protein LOC114353759 [Ostrinia furnacalis]	0.4004382	-1.320348357	1.74E-07	down	yes	1.462	3.651	3.681	3.653	3.62	1.472	1.495	1.419	------	------	------	------	------	------	------	------	------	------	------	CYT	5	36	20.8	High
TRINITY_DN51995_c0_g3_i1_orf1	circadian clock-controlled protein-like [Ostrinia furnacalis]	2.9880192	1.579189404	6.91E-05	up	yes	3.741	1.252	1.003	1.365	1.389	3.866	3.606	3.75	------	------	------	------	------	------	ENOG4111G78	S:Function unknown	PF06585.14	JHBP	Haemolymph juvenile hormone binding protein	CYT	3	10	29.2	High
TRINITY_DN28711_c1_g1_i1_orf1	apolipophorins-like [Ostrinia furnacalis]	0.4904733	-1.027753599	3.85E-06	down	yes	1.596	3.254	3.333	3.2	3.229	1.581	1.564	1.643	MF:lipid transporter activity;MF:transporter activity;MF:molecular_function;	GO:0005319;GO:0005215;GO:0003674	------	------	------	------	ENOG410ZRUY;ENOG410Z6MR	S:Function unknown;	PF01347.25	Vitellogenin_N	Lipoprotein amino terminal region	CYT	7	86	9.8	High

TRINITY_DN2442_c0_g1_i2_orf1	digestive cysteine proteinase 2 [Ostrinia furnacalis]	0.4355318	-1.199150073	9.22E-07	down	yes	1.466	3.366	3.36	3.427	3.312	1.431	1.487	1.479	BP:primary metabolic process;BP:organonitrogen compound metabolic process;BP:protein metabolic process;BP:macromolecule metabolic process;BP:organic substance metabolic process;BP:nitrogen compound metabolic process;BP:biological_process;BP:metabolic process;BP:proteolysis;MF:molecular_function;MF:hydrolase activity;MF:catalytic activity;MF:catalytic activity, acting on a protein;MF:peptidase activity;MF:cysteine-type peptidase activity;	GO:0044238;GO:1901564;GO:0019538;GO:0043170;GO:0071704;GO:0006807;GO:0008150;GO:0008152;GO:0006508;GO:0003674;GO:0016787;GO:0003824;GO:0140096;GO:0008233;GO:0008234	------	------	------	------	COG4870	O:Posttranslational modification, protein turnover, chaperones	PF00112.26;PF08246.15	Peptidase_C1;Inhibitor_I29	Papain family cysteine protease;Cathepsin propeptide inhibitor domain	CYT	16	37	62.9	High
TRINITY_DN2489_c0_g1_i1_orf1	uncharacterized protein LOC114354692 [Ostrinia furnacalis]	0.3925289	-1.349129196	5.92E-06	down	yes	1.324	3.373	3.422	3.296	3.402	1.276	1.269	1.427	------	------	------	------	------	------	ENOG41127D5	S:Function unknown	PF05335.16	DUF745	Protein of unknown function	CYT	5	24	31.8	High
TRINITY_DN2457_c0_g1_i8_orf1	uncharacterized protein LOC114355596 [Ostrinia furnacalis]	2.4740741	1.306888695	7.91E-05	up	yes	3.34	1.35	1.372	1.286	1.393	3.191	3.569	3.261	BP:biological_process;BP:metabolic process;BP:methylation;MF:catalytic activity;MF:transferase activity;MF:transferase activity, transferring one-carbon groups;MF:molecular_function;MF:methyltransferase activity;	GO:0008150;GO:0008152;GO:0032259;GO:0003824;GO:0016740;GO:0016741;GO:0003674;GO:0008168	------	------	------	------	ENOG410Y1IK	M:Cell wall/membrane/envelope biogenesis	PF12248.11	Methyltransf_FA	Farnesoic acid 0-methyl transferase	CYT	7	27	33.4	High
TRINITY_DN9733_c0_g1_i2_orf1	acidic juvenile hormone-suppressible protein 1-like [Ostrinia furnacalis]	0.4969285	-1.008889668	0.0002603	down	yes	1.537	3.093	3.122	3.282	2.876	1.471	1.632	1.508	------	------	------	------	------	------	ENOG410XR2D	S:Function unknown	PF00372.22	Hemocyanin_M	Hemocyanin, copper containing domain	CYT	3	54	12.1	High
TRINITY_DN1091_c0_g3_i1_orf1	macrophage mannose receptor 1-like [Ostrinia furnacalis]	2.584252	1.369746742	1.00E-04	up	yes	3.282	1.27	1.269	1.228	1.314	3.37	3.445	3.032	BP:response to extracellular stimulus;BP:nucleic acid metabolic process;BP:cellular nitrogen compound metabolic process;BP:nitrogen compound metabolic process;BP:DNA protection;BP:organic cyclic compound metabolic process;BP:nucleobase-containing compound metabolic process;BP:cellular response to stimulus;BP:cellular macromolecule metabolic process;BP:organic substance metabolic process;BP:cellular response to external stimulus;BP:cellular process;BP:cellular aromatic compound metabolic process;BP:cellular	GO:0009991;GO:0090304;GO:0034641;GO:0006807;GO:0042262;GO:1901360;GO:0006139;GO:0051716;GO:0044260;GO:0071704;GO:0071496;GO:0009987;GO:0006725;GO:0031668;GO:0006950;GO:0008150;GO:0008152;GO:0007154;GO:0046483;GO:0044238;GO:0009605;GO:0044237;GO:0043170;GO:0033554;GO:0006259;GO:0050896;GO:0003674;GO:0016787;GO:0003824;GO:0047429;GO:0016818;GO:0008413;GO:0016817;GO:0016462	------	------	------	------	ENOG410XRS3;ENOG410XPJ1;ENOG410ZPTE;ENOG410XNWT	U:Intracellular trafficking, secretion, and vesicular transport;S:Function unknown;S:Function unknown;S:Function unknown	PF00059.24	Lectin_C	Lectin C-type domain	CYT	9	34	38.3	High
TRINITY_DN143497_c0_g1_i1_orf1	fibroin heavy chain-like [Ostrinia furnacalis]	0.2312714	-2.112341354	7.70E-07	down	yes	0.8113	3.508	3.52	3.574	3.43	0.767	0.866	0.801	------	------	------	------	------	------	ENOG4111C6D	S:Function unknown	------	------	------	CYT	7	39	19.4	High
TRINITY_DN18218_c0_g1_i7_orf1	inhibin beta B chain [Ostrinia furnacalis]	0.257112	-1.959530925	0.008038	down	yes	1.907	7.417	8.375	5.186	8.689	1.828	2.12	1.773	BP:response to stimulus;BP:biological_process;BP:response to chemical;BP:response to organic substance;CC:cellular_component;CC:extracellular region;CC:cellular anatomical entity;MF:growth factor activity;MF:receptor regulator activity;MF:receptor activator activity;MF:receptor agonist activity;MF:molecular_function;MF:binding;MF:protein binding;MF:receptor binding;MF:molecular	GO:0050896;GO:0008150;GO:0042221;GO:0010033;GO:0005575;GO:0005576;GO:0110165;GO:0008083;GO:0030545;GO:0030546;GO:0048018;GO:0003674;GO:0005488;GO:0005515;GO:0005102;GO:0098772	K04669	TGFBRX	------	------	ENOG410XT8Z	O:Posttranslational modification, protein turnover, chaperones	PF00019.23;PF00688.21	TGF_beta;TGFb_propeptide	Transforming growth factor beta like domain;TGF-beta propeptide	CYT	1	4	41.4	High
TRINITY_DN29026_c0_g1_i4_orf1	TIL [Ostrinia furnacalis]	2.1445528	1.100676821	0.004718	up	yes	5.059	2.359	2.129	2.506	2.441	4.572	5.979	4.626	------	------	------	------	------	------	ENOG4110QDA;ENOG4110RUT	S:Function unknown;	------	------	------	CYT	1	14	9.9	High
TRINITY_DN6415_c0_g2_i1_orf1	D-arabinitol dehydrogenase 1-like [Ostrinia furnacalis]	3.3649469	1.750583734	1.33E-05	up	yes	4.435	1.318	1.304	1.267	1.383	4.656	4.266	4.382	MF:oxidoreductase activity;MF:cation binding;MF:transition metal ion binding;MF:ion binding;MF:catalytic activity;MF:molecular_function;MF:binding;MF:metal ion binding;MF:zinc ion binding;	GO:0016491;GO:0043169;GO:0046914;GO:0043167;GO:0003824;GO:0003674;GO:0005488;GO:0046872;GO:0008270	K17818	ARD1	map00040	Pentose and glucuronate interconversions	COG1063	E:Amino acid transport and metabolism	PF08240.15;PF00107.29;PF16912.8	ADH_N;ADH_zinc_N;Glu_dehyd_C	Alcohol dehydrogenase GroES-like domain;Zinc-binding dehydrogenase;Glucose dehydrogenase C-terminus	CYT	13	61	37.5	High
TRINITY_DN12671_c0_g1_i4_orf1	hemicentin-1-like isoform X1 [Ostrinia furnacalis]	0.3220382	-1.63469619	1.45E-05	down	yes	1.264	3.925	4.081	3.938	3.756	1.31	1.17	1.312	BP:biological_process;BP:localization;	GO:0008150;GO:0051179	------	------	------	------	ENOG410XPQS;ENOG4111FBT;ENOG410ZN3C	S:Function unknown;	PF07679.19;PF13927.9;PF13895.9;PF00047.28	I-set;Ig_3;Ig_2;ig	Immunoglobulin I-set domain;Immunoglobulin domain;Immunoglobulin domain;Immunoglobulin domain	CYT	5	9	89.1	High
TRINITY_DN20793_c0_g2_i1_orf1	mucin-2-like [Ostrinia furnacalis]	0.1951581	-2.357284527	1.65E-06	down	yes	0.6973	3.573	3.644	3.632	3.444	0.712	0.707	0.673	------	------	------	------	------	------	ENOG41115RA	S:Function unknown	------	------	------	EXC	4	9	52.5	High

TRINITY_DN1308_c0_g1_i4_orf1	serine proteinase stubble-like [Ostrinia furnacalis]	0.4354949	-1.199272336	0.0002105	down	yes	1.276	2.93	3.128	2.748	2.914	1.24	1.405	1.183	BP:organic substance metabolic process;BP:organonitrogen compound metabolic process;BP:protein metabolic process;BP:macromolecule metabolic process;BP:biological_process;BP:nitrogen compound metabolic process;BP:metabolic process;BP:proteolysis;BP:primary metabolic process;MF:serine-type endopeptidase activity;MF:endopeptidase activity;MF:hydrolase activity;MF:serine hydrolase activity;MF:catalytic activity, acting on a protein;MF:catalytic activity;MF:molecular_function;MF:peptidase activity;MF:serine-type	GO:0071704;GO:1901564;GO:0019538;GO:0043170;GO:0008150;GO:0006807;GO:0008152;GO:0006508;GO:0044238;GO:0004252;GO:0004175;GO:0016787;GO:0017171;GO:0140096;GO:0003824;GO:0003674;GO:0008233;GO:0008236	------	------	------	------	COG5640	C:Energy production and conversion	PF00089.29	Trypsin	Trypsin	CYT	1	3	28.8	Medium
TRINITY_DN136031_c0_g1_i7_orf1	ferritin, lower subunit isoform X3 [Spodoptera litura]	4.048175	2.017271659	1.16E-05	up	yes	3.294	0.8137	0.878	0.656	0.907	3.199	3.354	3.328	BP:ion homeostasis;BP:homeostatic process;BP:iron ion homeostasis;BP:transition metal ion homeostasis;BP:inorganic ion homeostasis;BP:cellular transition metal ion homeostasis;BP:transition metal ion transport;BP:biological regulation;BP:cellular homeostasis;BP:metal ion transport;BP:transport;BP:cellular iron ion homeostasis;BP:cellular metal ion homeostasis;BP:cation transport;BP:ion transport;BP:cellular process;BP:cellular ion homeostasis;BP:biological_process;BP:cellular cation homeostasis;BP:metal ion homeostasis;BP:cation	GO:0050801;GO:0042592;GO:0055072;GO:0055076;GO:0098771;GO:0046916;GO:0000041;GO:0065007;GO:0019725;GO:0030001;GO:0006810;GO:0006879;GO:0006875;GO:0006812;GO:0006811;GO:0009987;GO:0006873;GO:0008150;GO:0030003;GO:0055065;GO:0055080;GO:0055082;GO:0051234;GO:0051179;GO:0065008;GO:0048878;GO:0006826;GO:0003674;GO:0005488;GO:0016491;GO:0043169;GO:0016722;GO:0046914;GO:0004322;GO:0043167;GO:0005506;GO:0046872;GO:0003824;GO:0016724;GO:0008199	------	------	------	------	ENOG4111Q26;COG1528	P:Inorganic ion transport and metabolism;	------	------	------	EXC	1	28	11.1	High
TRINITY_DN56155_c0_g1_i1_orf1	hypothetical protein evm_004679 [Chilo suppressalis] >CAH2989683.1 unnamed protein product [Chilo suppressalis]	0.0644231	-3.956278623	3.53E-07	down	yes	0.938	14.56	14.637	14.883	14.164	0.892	0.954	0.968	MF:pigment binding;MF:molecular_function;MF:binding;	GO:0031409;GO:0003674;GO:0005488	K03098	APOD	------	------	COG3040	Z:Cytoskeleton	PF00061.26;PF08212.15	Lipocalin;Lipocalin_2	Lipocalin / cytosolic fatty-acid binding protein family;Lipocalin-like domain	CYT	19	58	34.1	High
TRINITY_DN87803_c0_g1_i2_orf1	hypothetical protein NE865_05903 [Phthorimaea operculella]	0.1365945	-2.872028238	6.90E-06	down	yes	1.452	10.63	10.94	10.257	10.691	1.037	1.506	1.812	MF:catalytic activity;MF:transferase activity;MF:molecular_function;	GO:0003824;GO:0016740;GO:0003674	K10532	HGSNAT	map04142;map00531	Lysosome;Glycosaminoglycan degradation	COG4299	S:Function unknown	------	------	------	CYT	1	23	9.7	High
TRINITY_DN15202_c0_g1_i6_orf1	uncharacterized protein LOC114364499 isoform X2 [Ostrinia furnacalis]	3.1447254	1.652934032	4.66E-06	up	yes	7.214	2.294	2.318	2.266	2.297	6.923	7.364	7.355	------	------	------	------	------	------	ENOG410XU1K	S:Function unknown	------	------	------	CYT	2	41	14	High
TRINITY_DN35809_c0_g1_i1_orf1	spodomicin-like [Ostrinia furnacalis] >QKV49445.1 diapausin [Ostrinia furnacalis]	2.3339115	1.222749878	3.26E-07	up	yes	5.382	2.306	2.392	2.294	2.233	5.366	5.391	5.388	------	------	------	------	------	------	------	------	PF08036.14	Antimicrobial_6	Diapausin family of antimicrobial peptide	CYT	3	60	6.8	High
TRINITY_DN35351_c0_g1_i3_orf1	adult-specific cuticular protein ACP-20-like [Ostrinia furnacalis]	0.2120803	-2.237317408	4.25E-05	down	yes	1.257	5.927	5.556	6.377	5.849	1.2	1.272	1.299	MF:molecular_function;MF:structural constituent of cuticle;MF:structural molecule activity;	GO:0003674;GO:0042302;GO:0005198	------	------	------	------	ENOG410YWJF;ENOG410YTXG;ENOG4110QVC;ENOG410YSFI;ENOG410ZUHH;ENOG41117GA	Q:Secondary metabolites biosynthesis, transport and catabolism;S:Function unknown;S:Function unknown;S:Function unknown;S:Function unknown;S:Function unknown	PF00379.26	Chitin_bind_4	Insect cuticle protein	CYT	2	16	19.2	High
TRINITY_DN8595_c0_g1_i3_orf1	aldose reductase-like isoform X4 [Trichoplusia ni]	2.6472338	1.404485612	1.91E-07	up	yes	4.45	1.681	1.681	1.631	1.73	4.459	4.406	4.486	MF:catalytic activity;MF:molecular_function;MF:oxidoreductase activity;	GO:0003824;GO:0003674;GO:0016491	K00011	AKR1B	map00051;map00040;map00561;map00790;map00052	Fructose and mannose metabolism;Pentose and glucuronate interconversions;Glycerolipid metabolism;Folate biosynthesis;Galactose metabolism	COG0656	S:Function unknown	PF00248.24	Aldo_ket_red	Aldo/keto reductase family	CYT	1	67	13.9	High
TRINITY_DN85412_c0_g1_i1_orf1	unnamed protein product [Diatraea saccharalis]	0.4318855	-1.211279252	2.02E-06	down	yes	1.75	4.052	4.089	4.099	3.967	1.816	1.742	1.693	MF:lipid transporter activity;MF:transporter activity;MF:molecular_function;	GO:0005319;GO:0005215;GO:0003674	------	------	------	------	ENOG410Z6MR;ENOG41112S0	S:Function unknown;	------	------	------	CYT	11	87	12	High
TRINITY_DN20344_c0_g1_i5_orf1	uncharacterized protein LOC114351483 [Ostrinia furnacalis]	5.1783151	2.37248276	7.39E-05	up	yes	4.69	0.9057	0.935	0.829	0.953	5.11	4.355	4.604	MF:molecular_function;MF:structural constituent of cuticle;MF:structural molecule activity;	GO:0003674;GO:0042302;GO:0005198	------	------	------	------	------	------	PF00379.26	Chitin_bind_4	Insect cuticle protein	CYT	4	57	13.5	High
TRINITY_DN7854_c0_g1_i4_orf1	failed axon connections [Ostrinia furnacalis]	0.4986173	-1.003995281	7.36E-07	down	yes	1.803	3.616	3.597	3.623	3.628	1.791	1.864	1.753	------	------	------	------	------	------	ENOG4111HSJ	S:Function unknown	PF17172.7;PF17171.7;PF10568.12;PF13410.9;PF13417.9; PF14497.9	GST_N_4;GST_C_6;Tom37;GST_C_2;GST_N_3;GST_C_3	Glutathione S-transferase N-terminal domain;Glutathione S-transferase, C-terminal domain;Outer mitochondrial membrane transport complex protein;Glutathione S-transferase, C-terminal domain;Glutathione S-transferase, N-terminal domain;Glutathione S-transferase, C-terminal domain	CYT	22	54	42.6	High

TRINITY_DN1749_c0_g2_i2_orf1	putative GPI-anchored protein pfl2 isoform X1 [Ostrinia furnacalis]	0.2744283	-1.865498964	1.69E-06	down	yes	1.056	3.848	3.828	3.95	3.767	1.098	1.085	0.986	BP:developmental process;BP:dephosphorylation;BP:protein dephosphorylation;BP:cell development;BP:nitrogen compound metabolic process;BP:anatomical structure morphogenesis;BP:cellular component organization or biogenesis;BP:cellular developmental process;BP:cellular component organization;BP:organic substance metabolic process;BP:neuron development;BP:cellular process;BP:macromolecule modification;BP:protein modification process;BP:biological_process;BP:metabolic process;BP:response to [illegible]	GO:0032502;GO:0016311;GO:0006470;GO:0048468;GO:0006807;GO:0009653;GO:0071840;GO:0048869;GO:0016043;GO:0071704;GO:0048666;GO:0009987;GO:0043412;GO:0036211;GO:0008150;GO:0008152;GO:0042221;GO:0040011;GO:0044238;GO:1901564;GO:0019538;GO:0048856;GO:0044237;GO:0043170;GO:0006796;GO:0006793;GO:0050896;GO:0016021;GO:0031224;GO:0110165;GO:0005575;GO:0016791;GO:0042578;GO:0003674;GO:0004721;GO:0016787;GO:0140096;GO:0016788;GO:0003824;GO:0004725	------	------	------	------	COG5599	T:Signal transduction mechanisms	------	------	------	CYT	4	5	116.8	High
TRINITY_DN68397_c0_g1_i2_orf1	clavesin-1-like [Ostrinia furnacalis]	0.208828	-2.259613154	4.55E-06	down	yes	0.8303	3.976	3.897	3.908	4.124	0.838	0.729	0.924	------	------	K19625	RLBP1	------	------	ENOG410XRSQ	S:Function unknown	PF00650.23;PF13716.9	CRAL_TRIO;CRAL_TRIO_2	CRAL/TRIO domain;Divergent CRAL/TRIO domain	CYT	2	8	34.6	High
TRINITY_DN4204_c0_g1_i1_orf1	uncharacterized protein LOC114359352 [Ostrinia furnacalis]	5.1717783	2.370660441	2.76E-05	up	yes	3.929	0.7597	0.686	0.819	0.774	4.197	3.714	3.875	CC:cellular_component;CC:extracellular region;CC:cellular anatomical entity;MF:molecular_function;MF:structural molecule activity;	GO:0005575;GO:0005576;GO:0110165;GO:0003674;GO:0005198	------	------	------	------	------	------	------	------	------	EXC	1	4	32.1	High
TRINITY_DN9239_c0_g2_i2_orf1	apolipophorins-like [Ostrinia furnacalis]	0.4478159	-1.159022217	0.0001677	down	yes	1.343	2.999	3.18	2.8	3.017	1.439	1.326	1.263	BP:lipid transport;BP:transport;BP:organic substance transport;BP:biological_process;BP:establishment of localization;BP:localization;CC:cellular_component;CC:extracellular region;CC:cellular anatomical entity;MF:lipid transporter activity;MF:transporter activity;MF:lipid binding;MF:molecular_function;MF:binding;	GO:0006869;GO:0006810;GO:0071702;GO:0008150;GO:0051234;GO:0051179;GO:0005575;GO:0005576;GO:0110165;GO:0005319;GO:0005215;GO:0008289;GO:0003674;GO:0005488	------	------	------	------	ENOG41112S0;ENOG410Z6MR	S:Function unknown;	------	------	------	CYT	5	90	7.6	High
TRINITY_DN1326_c0_g1_i1_orf1	cuticle protein 7-like [Ostrinia furnacalis]	0.0538014	-4.216213283	0.0007193	down	yes	0.4713	8.76	9.675	9.612	6.994	0.458	0.447	0.509	MF:molecular_function;MF:structural constituent of cuticle;MF:structural molecule activity;	GO:0003674;GO:0042302;GO:0005198	------	------	------	------	ENOG410YSFI;ENOG4111NR3	S:Function unknown;	PF00379.26	Chitin_bind_4	Insect cuticle protein	CYT	1	27	13.9	High
TRINITY_DN644_c0_g1_i1_orf1	cuticle protein 19-like [Ostrinia furnacalis]	0.0828685	-3.593032818	3.44E-05	down	yes	0.6477	7.816	7.811	8.425	7.211	0.606	0.716	0.621	MF:molecular_function;MF:structural constituent of cuticle;MF:structural molecule activity;	GO:0003674;GO:0042302;GO:0005198	------	------	------	------	ENOG410YSFI	S:Function unknown	PF00379.26	Chitin_bind_4	Insect cuticle protein	CYT	4	34	14.8	High
TRINITY_DN5581_c0_g1_i1_orf1	mucin-5AC-like [Ostrinia furnacalis]	0.1976526	-2.338961292	9.86E-06	down	yes	1.263	6.39	6.404	6.695	6.071	1.332	1.251	1.207	------	------	------	------	------	------	ENOG410YZWK	S:Function unknown	------	------	------	CYT	2	2	128.1	High
TRINITY_DN38274_c0_g1_i1_orf1	uncharacterized protein LOC114360402 [Ostrinia furnacalis]	0.1585078	-2.657374336	3.77E-06	down	yes	1.007	6.353	6.44	6.555	6.064	1.034	1.034	0.953	BP:nucleic acid metabolic process;BP:nitrogen compound metabolic process;BP:organic cyclic compound metabolic process;BP:nucleobase-containing compound metabolic process;BP:cellular response to stimulus;BP:cellular macromolecule metabolic process;BP:organic substance metabolic process;BP:DNA repair;BP:cellular process;BP:cellular aromatic compound metabolic process;BP:cellular response to DNA damage stimulus;BP:response to stress;BP:biological_process;BP:metabolic [illegible]	GO:0090304;GO:0006807;GO:1901360;GO:0006139;GO:0051716;GO:0044260;GO:0071704;GO:0006281;GO:0009987;GO:0006725;GO:0006974;GO:0006950;GO:0008150;GO:0008152;GO:0046483;GO:0044238;GO:0050896;GO:0034641;GO:0044237;GO:0043170;GO:0033554;GO:0006259;GO:0043229;GO:0043227;GO:0043226;GO:0005634;GO:0110165;GO:0043231;GO:0005575;GO:0042578;GO:0003674;GO:0008081;GO:0005198;GO:0016787;GO:0016788;GO:0003824;GO:0042302;GO:0004518	------	------	------	------	ENOG4112648;ENOG4111QIW	S:Function unknown;	PF00379.26	Chitin_bind_4	Insect cuticle protein	CYT	5	34	29.3	High
TRINITY_DN5829_c0_g1_i1_orf1	uncharacterized protein LOC114365758 isoform X3 [Ostrinia furnacalis]	0.1602906	-2.64123866	3.31E-07	down	yes	0.993	6.195	6.162	6.33	6.094	0.936	1.065	0.978	------	------	------	------	------	------	ENOG410XT4C	S:Function unknown	PF00059.24	Lectin_C	Lectin C-type domain	PLA	5	39	23.4	High
TRINITY_DN28922_c0_g1_i2_orf1	uncharacterized protein LOC119829283 isoform X2 [Zerene cesonia]	0.2345656	-2.091936738	5.91E-05	down	yes	1.25	5.329	5.421	5.665	4.902	1.241	1.174	1.335	CC:cellular_component;CC:cellular anatomical entity;	GO:0005575;GO:0110165	------	------	------	------	------	------	------	------	------	CYT	1	10	12.5	High
TRINITY_DN3310_c0_g1_i1_orf1	hypothetical protein evm_010516 [Chilo suppressalis]	0.1188208	-3.073140929	1.54E-05	down	yes	0.661	5.563	5.56	5.902	5.226	0.705	0.618	0.66	CC:integral component of membrane;CC:cellular_component;CC:intrinsic component of membrane;CC:cellular anatomical entity;MF:structural molecule activity;MF:molecular_function;MF:structural constituent of cuticle;	GO:0016021;GO:0005575;GO:0031224;GO:0110165;GO:0005198;GO:0003674;GO:0042302	------	------	------	------	ENOG4111PCZ	S:Function unknown	------	------	------	CYT	5	17	40.4	High
TRINITY_DN993_c0_g1_i7_orf1	apolipophorins-like [Ostrinia furnacalis]	0.4817754	-1.053567505	4.20E-08	down	yes	1.943	4.033	4.022	4.015	4.062	1.924	1.966	1.939	MF:lipid transporter activity;MF:transporter activity;MF:molecular_function;	GO:0005319;GO:0005215;GO:0003674	------	------	------	------	ENOG41112S0;ENOG410Z6MR	S:Function unknown;	PF00094.28	VWD	von Willebrand factor type D domain	CYT	27	59	66.5	High
TRINITY_DN30306_c0_g2_i1_orf1	perilipin-4-like isoform X3 [Ostrinia furnacalis]	2.1504	1.104605043	5.61E-05	up	yes	4.032	1.875	1.792	1.881	1.951	4.051	4.213	3.831	------	------	K20254	PLIN4	map03320	PPAR signaling pathway	ENOG4112C4W	S:Function unknown	------	------	------	CYT	6	64	7.2	High
TRINITY_DN10747_c0_g1_i5_orf1	unnamed protein product [Plutella xylostella]	0.2456733	-2.02518714	7.39E-06	down	yes	1.164	4.738	4.781	4.915	4.517	1.14	1.14	1.211	------	------	------	------	------	------	ENOG410XRSQ	S:Function unknown	PF00650.23	CRAL_TRIO	CRAL/TRIO domain	CYT	10	36	41.8	High
TRINITY_DN6004_c0_g1_i1_orf1	endocuticle structural glycoprotein ABD-4-like [Ostrinia furnacalis]	0.3706755	-1.431771515	5.42E-06	down	yes	1.35	3.642	3.515	3.705	3.706	1.359	1.292	1.399	MF:molecular_function;MF:structural constituent of cuticle;MF:structural molecule activity;	GO:0003674;GO:0042302;GO:0005198	------	------	------	------	ENOG4111PJ1;ENOG410YR66	O:Posttranslational modification, protein turnover, chaperones;S:Function unknown	PF00379.26	Chitin_bind_4	Insect cuticle protein	CYT	2	23	16.7	High
TRINITY_DN49143_c0_g1_i1_orf1	uncharacterized protein LOC107270465 [Cephus cinctus]	0.2815923	-1.828320181	5.88E-07	down	yes	1.054	3.743	3.687	3.705	3.836	1.06	1.064	1.039	CC:cellular_component;CC:integral component of membrane;CC:cellular anatomical entity;CC:intrinsic component of membrane;	GO:0005575;GO:0016021;GO:0110165;GO:0031224	------	------	------	------	ENOG410YYS2	S:Function unknown	PF00100.26	Zona_pellucida	Zona pellucida-like domain	CYT	5	7	69.1	High
TRINITY_DN696_c1_g1_i10_orf1	titin-like [Ostrinia furnacalis]	0.3419095	-1.548313648	3.91E-07	down	yes	1.103	3.226	3.198	3.28	3.199	1.062	1.124	1.122	------	------	------	------	------	------	ENOG410ZKJ3;ENOG4111M3T	S:Function unknown;	------	------	------	CYT	9	5	288.8	High
TRINITY_DN18624_c0_g1_i5_orf1	uncharacterized protein LOC114353135 [Ostrinia furnacalis]	0.1199436	-3.059572445	1.95E-06	down	yes	1.275	10.63	10.956	10.681	10.262	1.433	1.292	1.1	------	------	------	------	------	------	ENOG4110T9R	S:Function unknown	------	------	------	CYT	1	5	34.8	High

TRINITY_DN3190_c0_g1_i1_orf1	repetitive proline-rich cell wall protein 1 precursor [Papilio polytes] >XP_013147838.1 PREDICTED: repetitive proline-rich cell wall protein 1 [Papilio polytes] >BAM19190.1 cuticular protein PpolCPG24 [Papilio polytes]	0.0966862	-3.3705468	2.30E-06	down	yes	0.992	10.26	10.242	10.661	9.866	0.95	1.024	1.002	------	------	------	------	------	------	ENOG4111M7J	S:Function unknown	------	------	------	CYT	17	44	44	High
TRINITY_DN19110_c0_g1_i2_orf1	peroxidase [Ostrinia furnacalis]	0.4905521	-1.027521855	0.001349	down	yes	1.324	2.699	2.502	2.955	2.639	1.32	1.137	1.514	------	------	K19511	PXDN, VPO1	------	------	ENOG410XPZ3	M:Cell wall/membrane/envelope biogenesis	PF03098.18	An_peroxidase	Animal haem peroxidase	CYT	1	2	84.9	High
TRINITY_DN9311_c0_g1_i1_orf1	cuticle protein 8-like [Ostrinia furnacalis]	0.1044151	-3.259598046	8.21E-05	down	yes	0.6953	6.659	6.733	7.248	5.997	0.627	0.699	0.76	MF:molecular_function;MF:structural constituent of cuticle;MF:structural molecule activity;	GO:0003674;GO:0042302;GO:0005198	------	------	------	------	ENOG410YSFI	S:Function unknown	PF00379.26	Chitin_bind_4	Insect cuticle protein	CYT	4	52	15	High
TRINITY_DN3616_c0_g1_i4_orf1	conotoxin ArMKLT2-032-like [Ostrinia furnacalis]	0.2724806	-1.875774843	0.001262	down	yes	1.506	5.527	6.181	5.815	4.584	1.731	1.364	1.423	CC:cellular_component;CC:integral component of membrane;CC:cellular anatomical entity;CC:intrinsic component of membrane;	GO:0005575;GO:0016021;GO:0110165;GO:0031224	------	------	------	------	------	------	------	------	------	CYT	1	68	6.1	High
TRINITY_DN2946_c0_g1_i1_orf1	histidine-rich glycoprotein [Ostrinia furnacalis]	0.1510642	-2.7267665	2.13E-05	down	yes	0.9227	6.108	6.303	6.359	5.662	0.874	0.912	0.982	------	------	------	------	------	------	ENOG410YTNT	S:Function unknown	------	------	------	PLA	9	28	23.4	High
TRINITY_DN13760_c1_g1_i1_orf1	pre-mRNA-processing factor 40 homolog A isoform X1 [Ostrinia furnacalis] >XP_028162665.1 pre-mRNA-processing factor 40 homolog A isoform X2 [Ostrinia furnacalis] >XP_028162667.1 pre-mRNA-processing factor 40 homolog A isoform X3 [Ostrinia furnacalis]	0.4095964	-1.287725145	3.77E-05	down	yes	1.989	4.856	4.744	4.831	4.993	2.143	1.743	2.081	BP:nucleic acid metabolic process;BP:cellular nitrogen compound metabolic process;BP:nitrogen compound metabolic process;BP:mRNA splicing, via spliceosome;BP:organic cyclic compound metabolic process;BP:nucleobase-containing compound metabolic process;BP:organic substance metabolic process;BP:RNA splicing;BP:mRNA metabolic process;BP:cellular process;BP:cellular aromatic compound metabolic process;BP:RNA splicing, via transesterification reactions;BP:RNA splicing, via	GO:0090304;GO:0034641;GO:0006807;GO:0000398;GO:1901360;GO:0006139;GO:0071704;GO:0008380;GO:0016071;GO:0009987;GO:0006725;GO:0000375;GO:0000377;GO:0008150;GO:0008152;GO:0046483;GO:0016070;GO:0044238;GO:0045292;GO:0044237;GO:0043170;GO:0006396;GO:0006397;GO:0043227;GO:0043226;GO:0005634;GO:0110165;GO:0043231;GO:0043229;GO:0005575	K12821	PRPF40, PRP40	map03040	Spliceosome	COG5104	A:RNA processing and modification	PF01846.22;PF00397.29;PF16512.8	FF;WW;RhoGAP-FF1	FF domain;WW domain;p190-A and -B Rho GAPs FF domain	CYT	1	1	92.5	High
TRINITY_DN77425_c0_g1_i2_orf1	anosmin-1 [Ostrinia furnacalis] >XP_028178811.1 anosmin-1 [Ostrinia furnacalis]	0.2556784	-1.967597855	1.38E-06	down	yes	1.272	4.975	5.038	5.061	4.827	1.334	1.227	1.254	CC:cellular_component;CC:extracellular region;CC:cellular anatomical entity;MF:enzyme regulator activity;MF:peptidase regulator activity;MF:molecular_function;MF:peptidase inhibitor activity;MF:enzyme inhibitor activity;MF:molecular function regulator;	GO:0005575;GO:0005576;GO:0110165;GO:0030234;GO:0061134;GO:0003674;GO:0030414;GO:0004857;GO:0098772	K23413	ANOS1, KAL1, WFDC19	------	------	ENOG4111T0F	S:Function unknown	PF00041.24;PF00095.24	fn3;WAP	Fibronectin type III domain;WAP-type	EXC	5	16	47.5	High
TRINITY_DN778_c0_g1_i1_orf1	uncharacterized protein LOC114363281 [Ostrinia furnacalis]	0.1512957	-2.724556807	1.21E-05	down	yes	0.794	5.248	5.326	4.936	5.483	0.857	0.712	0.813	------	------	------	------	------	------	COG2335	O:Posttranslational modification, protein turnover, chaperones	PF02469.25	Fasciclin	Fasciclin domain	CYT	1	1	115.4	High
TRINITY_DN21555_c0_g1_i4_orf1	uncharacterized protein LOC114351844 [Ostrinia furnacalis] >XP_028158981.1 uncharacterized protein LOC114351844 [Ostrinia furnacalis] >XP_028158982.1 uncharacterized protein LOC114351844 [Ostrinia furnacalis] >XP_028158983.1 uncharacterized protein LOC114351844 [Ostrinia furnacalis] >XP_028158984.1 uncharacterized protein LOC114351844 [Ostrinia furnacalis] >XP_028158985.1 uncharacterized protein LOC114351844 [Ostrinia furnacalis] >5GPR_A Crystal structure of chitinase-h from Ostrinia furnacalis [Ostrinia furnacalis] >5GQB_A Crystal structure of chitinase-h from O. furnacalis in complex with chitohepatose [Ostrinia furnacalis] >6JMN_A Crystal structure of Ostrinia furnacalis Chitinase h complexed with compound 2-8-s2 [Ostrinia furnacalis] >BAE16587.1 chitinase [Ostrinia furnacalis]	0.4242585	-1.236984617	5.50E-07	down	yes	1.602	3.776	3.826	3.778	3.724	1.559	1.641	1.606	BP:amino sugar catabolic process;BP:nitrogen compound metabolic process;BP:aminoglycan metabolic process;BP:organic substance catabolic process;BP:organic substance metabolic process;BP:chitin metabolic process;BP:chitin catabolic process;BP:biological_process;BP:metabolic process;BP:carbohydrate metabolic process;BP:catabolic process;BP:macromolecule catabolic process;BP:primary metabolic process;BP:organonitrogen compound metabolic process;BP:organonitrogen compound catabolic	GO:0046348;GO:0006807;GO:0006022;GO:1901575;GO:0071704;GO:0006030;GO:0006032;GO:0008150;GO:0008152;GO:0005975;GO:0009056;GO:0009057;GO:0044238;GO:1901564;GO:1901565;GO:0006040;GO:1901136;GO:1901135;GO:0043170;GO:1901072;GO:1901071;GO:0006026;GO:0016798;GO:0003674;GO:0008061;GO:0016787;GO:0003824;GO:0004568;GO:0097367;GO:0004553;GO:0005488	K01183	E3.2.1.14	map00520	Amino sugar and nucleotide sugar metabolism	COG3325	S:Function unknown	PF00704.31;PF08329.13	Glyco_hydro_18;ChitinaseA_N	Glycosyl hydrolases family 18;Chitinase A, N-terminal domain	CYT	12	28	60.8	High
TRINITY_DN54524_c0_g1_i6_orf1	serine protease inhibitor dipetalogastin [Ostrinia furnacalis]	0.2097657	-2.253149194	5.08E-06	down	yes	0.9043	4.311	4.171	4.274	4.487	0.82	0.975	0.918	------	------	------	------	------	------	ENOG410ZXUD;ENOG410YKSA	S:Function unknown;	PF07648.18;PF00050.24	Kazal_2;Kazal_1	Kazal-type serine protease inhibitor domain;Kazal-type serine protease inhibitor domain	PLA	3	7	55.3	High
TRINITY_DN5907_c0_g1_i4_orf1	hypothetical protein evm_010265 [Chilo suppressalis] >CAB3524755.1 unnamed protein product [Chilo suppressalis] >CAH0397522.1 unnamed protein product [Chilo suppressalis]	0.3067688	-1.704776156	1.64E-07	down	yes	1.201	3.915	3.893	3.869	3.983	1.194	1.204	1.206	------	------	------	------	------	------	ENOG410YTKH;ENOG410ZN7P;ENOG41119GI;ENOG410ZNC7;ENOG4110RJ7;ENOG4111E83;ENOG41122YZ	S:Function unknown;	PF06585.14	JHBP	Haemolymph juvenile hormone binding protein	CYT	5	23	28.1	High
TRINITY_DN1326_c0_g1_i2_orf1	cuticle protein 7 [Plutella xylostella] >CAG9138501.1 unnamed protein product [Plutella xylostella]	0.3904071	-1.356948661	0.00254	down	yes	1.4	3.586	3.647	3.979	3.131	0.996	1.495	1.709	MF:molecular_function;MF:structural constituent of cuticle;MF:structural molecule activity;	GO:0003674;GO:0042302;GO:0005198	------	------	------	------	ENOG410ZM6M;ENOG4111NR3;ENOG410YSFI;ENOG4110SG6	S:Function unknown;	PF00379.26	Chitin_bind_4	Insect cuticle protein	CYT	1	24	18.5	High

TRINITY_DN58125_c0_g1_i1_orf1	protein yellow-like [Ostrinia furnacalis]	0.3639597	-1.458149413	8.86E-05	down	yes	1.228	3.374	3.532	3.371	3.219	1.16	1.423	1.102	BP:male mating behavior;BP:mating behavior;BP:behavior;BP:cellular biosynthetic process;BP:organic cyclic compound biosynthetic process;BP:organic cyclic compound metabolic process;BP:organic substance biosynthetic process;BP:pigment metabolic process;BP:secondary metabolite biosynthetic process;BP:organic substance metabolic process;BP:multicellular organismal process;BP:reproductive behavior;BP:phenol-containing compound biosynthetic process;BP:melanin metabolic process;BP:cellular aromatic compound	GO:0060179;GO:0007617;GO:0007610;GO:0044249;GO:1901362;GO:1901360;GO:1901576;GO:0042440;GO:0044550;GO:0071704;GO:0032501;GO:0019098;GO:0046189;GO:0006582;GO:0006725;GO:0022414;GO:0009058;GO:0008150;GO:0042438;GO:0008152;GO:0018958;GO:0046148;GO:0019748;GO:0044237;GO:0019438;GO:0009987;GO:1901617;GO:1901615	------	------	------	------	COG3386	S:Function unknown	PF03022.19	MRJP	Major royal jelly protein	CYT	1	9	19.2	High
TRINITY_DN2953_c1_g1_i10_orf1	methionine--tRNA ligase, cytoplasmic isoform X2 [Ostrinia furnacalis] >XP_028156683.1 methionine--tRNA ligase, cytoplasmic isoform X4 [Ostrinia furnacalis] >XP_028156684.1 methionine--tRNA ligase, cytoplasmic isoform X5 [Ostrinia furnacalis]	0.4816576	-1.053920139	0.0004523	down	yes	1.418	2.944	2.887	3.215	2.729	1.425	1.445	1.383	BP:methionyl-tRNA aminoacylation;BP:carboxylic acid metabolic process;BP:nucleic acid metabolic process;BP:cellular nitrogen compound metabolic process;BP:nitrogen compound metabolic process;BP:small molecule metabolic process;BP:ncRNA metabolic process;BP:organic cyclic compound metabolic process;BP:nucleobase-containing compound metabolic process;BP:cellular amino acid metabolic process;BP:organic substance metabolic process;BP:primary metabolic process;BP:cellular process;BP:cellular aromatic compound	GO:0006431;GO:0019752;GO:0090304;GO:0034641;GO:0006807;GO:0044281;GO:0034660;GO:1901360;GO:0006139;GO:0006520;GO:0071704;GO:0044238;GO:0009987;GO:0006725;GO:0008150;GO:0008152;GO:0043436;GO:1901564;GO:0046483;GO:0016070;GO:0006082;GO:0044237;GO:0043170;GO:0006399;GO:0043038;GO:0043039;GO:0006418;GO:0017101;GO:1902494;GO:0032991;GO:0005575;GO:0035639;GO:0003674;GO:0043167;GO:1901363;GO:0043168;GO:0004812;GO:0000166;GO:1901265;GO:0017076;GO:0005524;GO:0140098;GO:0036094;GO:0003824;GO:0016874;GO:0016875;GO:0004825;GO:0030554;GO:0097367;GO:0097159;GO:0032559;GO:0032555;GO:0140101;GO:0032553;GO:0005488	K01874	MARS, metG	map00450;map00970	Selenocompound metabolism;Aminoacyl-tRNA biosynthesis	COG0143	J:Translation, ribosomal structure and biogenesis	PF00458.23	WHEP-TRS	WHEP-TRS domain	CYT	2	57	17.6	High
TRINITY_DN267_c0_g1_i1_orf1	keratin, type I cytoskeletal 9-like [Ostrinia furnacalis]	0.2464341	-2.020726003	1.81E-05	down	yes	0.7913	3.211	3.027	3.37	3.237	0.761	0.818	0.795	------	------	------	------	------	------	ENOG4110QOM	S:Function unknown	------	------	------	CYT	3	9	52.5	High
TRINITY_DN2101_c0_g1_i6_orf1	protein obstructor-E-like [Ostrinia furnacalis]	0.086152	-3.536972558	1.78E-05	down	yes	0.703	8.16	8.411	8.523	7.546	0.669	0.673	0.767	------	------	------	------	------	------	ENOG410ZKXC	S:Function unknown	PF01607.27	CBM_14	Chitin binding Peritrophin-A domain	EXC	5	20	28.1	High
TRINITY_DN18539_c0_g1_i1_orf1	pupal cuticle protein PCP52-like [Ostrinia furnacalis]	0.1485732	-2.750754638	1.24E-05	down	yes	0.984	6.623	6.602	7.003	6.263	0.964	0.997	0.991	MF:molecular_function;MF:structural constituent of cuticle;MF:structural molecule activity;	GO:0003674;GO:0042302;GO:0005198	------	------	------	------	ENOG4110SXG	S:Function unknown	------	------	------	CYT	13	54	28.3	High
TRINITY_DN21719_c0_g1_i2_orf1	chymotrypsin-2-like [Ostrinia furnacalis]	0.1404011	-2.832373382	4.28E-06	down	yes	0.931	6.631	6.673	6.89	6.331	0.995	0.891	0.907	BP:nitrogen compound metabolic process;BP:organic substance metabolic process;BP:proteolysis;BP:biological_process;BP:metabolic process;BP:primary metabolic process;BP:organonitrogen compound metabolic process;BP:protein metabolic process;BP:macromolecule metabolic process;CC:intrinsic component of membrane;CC:integral component of membrane;CC:cellular anatomical entity;CC:cellular_component;MF:serine-type endopeptidase activity;MF:endopeptidase activity;MF:molecular_function;MF:peptidase	GO:0006807;GO:0071704;GO:0006508;GO:0008150;GO:0008152;GO:0044238;GO:1901564;GO:0019538;GO:0043170;GO:0031224;GO:0016021;GO:0110165;GO:0005575;GO:0004252;GO:0004175;GO:0003674;GO:0008233;GO:0008236;GO:0016787;GO:0017171;GO:0140096;GO:0003824	K01312	PRSS1_2_3	map04972;map05164;map04080;map04974	Pancreatic secretion;Influenza A;Neuroactive ligand-receptor interaction;Protein digestion and absorption	COG5640	O:Posttranslational modification, protein turnover, chaperones	PF00089.29	Trypsin	Trypsin	CYT	2	14	15.6	High
TRINITY_DN4748_c0_g1_i5_orf1	unnamed protein product, partial [Brenthis ino]	0.4644478	-1.106411616	0.0002846	down	yes	2.149	4.627	4.379	5.037	4.465	2.193	2.106	2.149	BP:response to biotic stimulus;BP:response to external stimulus;BP:response to external biotic stimulus;BP:response to other organism;BP:response to stimulus;BP:response to stress;BP:defense response;BP:biological_process;BP:interspecies interaction between organisms;CC:cellular_component;CC:extracellular region;CC:cellular anatomical entity;MF:ion channel regulator activity;MF:sodium channel inhibitor activity;MF:sodium channel regulator activity;MF:molecular_function;MF:channel inhibitor activity;MF:channel regulator	GO:0009607;GO:0009605;GO:0043207;GO:0051707;GO:0050896;GO:0006950;GO:0006952;GO:0008150;GO:0044419;GO:0005575;GO:0005576;GO:0110165;GO:0099106;GO:0019871;GO:0017080;GO:0003674;GO:0016248;GO:0016247;GO:0098772;GO:0008200	------	------	------	------	------	------	------	------	------	CYT	3	54	7.8	High
TRINITY_DN57998_c1_g3_i1_orf1	uncharacterized protein LOC114363305 isoform X2 [Ostrinia furnacalis]	0.2611364	-1.937124477	3.75E-06	down	yes	1.319	5.051	5.008	5.241	4.905	1.334	1.254	1.369	------	------	------	------	------	------	ENOG4111SVO	S:Function unknown	------	------	------	CYT	2	17	23.2	High
TRINITY_DN906_c0_g1_i4_orf1	uncharacterized protein LOC114360441, partial [Ostrinia furnacalis]	0.2568604	-1.960943768	1.20E-08	down	yes	1.273	4.956	4.988	4.946	4.933	1.241	1.274	1.304	------	------	K17339	ATL	------	------	ENOG410XR6Z	S:Function unknown	PF02263.22;PF02841.17	GBP;GBP_C	Guanylate-binding protein, N-terminal domain;Guanylate-binding protein, C-terminal domain	CYT	22	38	87.8	High
TRINITY_DN31_c0_g1_i3_orfp1	TRINITY_DN31_c0_g1_i3_m.1394 TRINITY_DN31_c0_g1::TRINITY_DN31_c0_g1_i3::g.1394 ORF type:complete len:118 (+) score=43.35 TRINITY_DN31_c0_g1_i3:56-409(+)	0.3001467	-1.736260194	5.91E-05	down	yes	1.432	4.771	4.916	4.655	4.741	1.754	1.168	1.374	------	------	------	------	------	------	------	------	------	------	------	CYT	2	15	12.6	High
TRINITY_DN9282_c0_g1_i2_orf1	uncharacterized protein LOC114363102 isoform X2 [Ostrinia furnacalis]	0.1163601	-3.103331453	4.45E-07	down	yes	0.601	5.165	5.158	5.07	5.268	0.592	0.521	0.69	------	------	------	------	------	------	ENOG41120R5	S:Function unknown	------	------	------	CYT	1	10	15.1	High
TRINITY_DN138481_c0_g1_i2_orf1	cuticle protein 8-like isoform X2 [Vanessa tameamea]	0.1995188	-2.325403196	2.87E-07	down	yes	0.9537	4.78	4.769	4.874	4.696	0.912	0.992	0.957	MF:molecular_function;MF:structural constituent of cuticle;MF:structural molecule activity;	GO:0003674;GO:0042302;GO:0005198	------	------	------	------	ENOG410YRDB;ENOG410YM5B	S:Function unknown;	PF00379.26	Chitin_bind_4	Insect cuticle protein	CYT	2	27	24.2	High

TRINITY_DN12387_c0_g1_i1_orf1	repetitive proline-rich cell wall protein 2-like [Ostrinia furnacalis]	0.1967419	-2.345624187	2.26E-06	down	yes	0.942	4.788	4.822	4.931	4.61	0.914	0.961	0.951	------	------	------	------	------	------	ENOG410YH2C;ENOG4110N1S	S:Function unknown;	------	------	------	CYT	18	64	40.7	High
TRINITY_DN661_c0_g2_i2_orf1	cuticle protein 7-like [Ostrinia furnacalis]	0.3172142	-1.656470778	0.0001203	down	yes	1.207	3.805	3.929	3.8	3.687	0.974	1.515	1.132	MF:molecular_function;MF:structural constituent of cuticle;MF:structural molecule activity;	GO:0003674;GO:0042302;GO:0005198	------	------	------	------	ENOG4110SG6;ENOG410YSFI;ENOG4110T8H;ENOG410ZM6M	S:Function unknown;	PF00379.26	Chitin_bind_4	Insect cuticle protein	CYT	2	19	19.4	High
TRINITY_DN43350_c0_g3_i1_orf1	uncharacterized protein LOC114355190 [Ostrinia furnacalis]	3.1108354	1.637302062	4.17E-05	up	yes	3.761	1.209	1.143	1.202	1.283	3.953	3.805	3.526	------	------	------	------	------	------	ENOG4111DOA	S:Function unknown	------	------	------	CYT	3	25	25.8	High
TRINITY_DN52944_c0_g1_i1_orf1	apolipophorins-like [Ostrinia furnacalis]	0.492	-1.023269779	5.85E-06	down	yes	1.599	3.25	3.317	3.283	3.15	1.601	1.581	1.616	MF:lipid transporter activity;MF:transporter activity;MF:molecular_function;	GO:0005319;GO:0005215;GO:0003674	------	------	------	------	ENOG410ZRUY;ENOG410Z6MR	S:Function unknown;	PF01347.25	Vitellogenin_N	Lipoprotein amino terminal region	CYT	5	59	11	High
TRINITY_DN4367_c0_g1_i1_orf1	heat shock protein 21.7c [Chilo suppressalis] >AWT57938.1 heat shock protein 21.7c [Chilo suppressalis]	0.4012246	-1.317517955	0.0001539	down	yes	1.245	3.103	3.254	3.022	3.034	1.134	1.136	1.465	MF:cation binding;MF:metal ion binding;MF:molecular_function;MF:binding;MF:ion binding;	GO:0043169;GO:0046872;GO:0003674;GO:0005488;GO:0043167	K09542	CRYAB	map04213;map04141	Longevity regulating pathway - multiple species;Protein processing in endoplasmic reticulum	ENOG410YERS	S:Function unknown	PF00011.24	HSP20	Hsp20/alpha crystallin family	CYT	1	19	21.6	High
TRINITY_DN2396_c0_g1_i9_orfp1	TRINITY_DN2396_c0_g1_i9_m.39038 TRINITY_DN2396_c0_g1::TRINITY_DN2396_c0_g1_i9::g.39038 ORF type:5prime_partial len:181 (+),score=90.33 TRINITY_DN2396_c0_g1_i9:3-545(+)	2.0705882	1.050040683	0.0006348	up	yes	24.64	11.9	10.922	13.343	11.434	24.391	26.632	22.885	------	------	------	------	------	------	------	------	------	------	------	CYT	1	6	19.3	High
TRINITY_DN131603_c0_g1_i4_orfp	TRINITY_DN131603_c0_g1_i4_m.86149 TRINITY_DN131603_c0_g1::TRINITY_DN131603_c0_g1_i4::g.86149 ORF type:internal len:112 (-),score=8.40 TRINITY_DN131603_c0_g1_i4:2-334(-)	22.106195	4.466378799	8.18E-05	up	yes	12.49	0.565	0.749	0.586	0.36	13.197	13.229	11.052	------	------	------	------	------	------	------	------	------	------	------	PLA	1	22	12.9	High
TRINITY_DN2464_c0_g1_i12_orf1	uncharacterized protein LOC114362996 isoform X1 [Ostrinia furnacalis]	3.0389222	1.60355972	0.0001655	up	yes	3.045	1.002	0.966	1.042	0.999	3.34	2.901	2.893	MF:molecular_function;MF:structural constituent of cuticle;MF:structural molecule activity;	GO:0003674;GO:0042302;GO:0005198	------	------	------	------	ENOG410YNFC	S:Function unknown	PF00379.26	Chitin_bind_4	Insect cuticle protein	CYT	8	45	37.2	High
TRINITY_DN19043_c0_g2_i1_orf1	hypothetical protein EVAR_60653_1 [Eumeta japonica]	0.3384673	-1.56291156	5.14E-05	down	yes	1.802	5.324	5.314	5.657	5.002	1.79	1.859	1.757	------	------	------	------	------	------	ENOG410YRTV	S:Function unknown	------	------	------	PLA	6	32	21	High
TRINITY_DN46625_c0_g1_i1_orf1	ferritin subunit isoform X1 [Belonocnema kinseyi]	0.3991537	-1.324983574	0.0001009	down	yes	1.981	4.963	5.03	4.635	5.224	1.821	2.101	2.021	BP:ion homeostasis;BP:homeostatic process;BP:iron ion homeostasis;BP:transition metal ion homeostasis;BP:inorganic ion homeostasis;BP:cellular transition metal ion homeostasis;BP:transition metal ion transport;BP:biological regulation;BP:regulation of biological quality;BP:cellular homeostasis;BP:metal ion transport;BP:transport;BP:cellular iron ion homeostasis;BP:cellular metal ion homeostasis;BP:cation transport;BP:ion transport;BP:cellular process;BP:cellular ion homeostasis;BP:biological_process;BP:cellular cation	GO:0050801;GO:0042592;GO:0055072;GO:0055076;GO:0098771;GO:0046916;GO:0000041;GO:0065007;GO:0065008;GO:0019725;GO:0030001;GO:0006810;GO:0006879;GO:0006875;GO:0006812;GO:0006811;GO:0009987;GO:0006873;GO:0008150;GO:0030003;GO:0055065;GO:0055080;GO:0055082;GO:0051234;GO:0051179;GO:0048878;GO:0006826;GO:0003674;GO:0005488;GO:0016491;GO:0043169;GO:0016722;GO:0046914;GO:0004322;GO:0043167;GO:0005506;GO:0008199;GO:0016724;GO:0046872;GO:0003824	K00522	FTH1	map04978;map04217;map04216	Mineral absorption;Necroptosis;Ferroptosis	ENOG4111MSG	P:Inorganic ion transport and metabolism	PF00210.27	Ferritin	Ferritin-like domain	CYT	1	4	24.1	High
TRINITY_DN26882_c0_g1_i1_orf1	uncharacterized protein LOC114349648 [Ostrinia furnacalis]	0.1642956	-2.605634216	6.32E-06	down	yes	0.9493	5.778	5.81	6.028	5.496	0.951	0.926	0.971	------	------	------	------	------	------	ENOG41120B2;ENOG4111JSP	S:Function unknown;	PF15998.8	DUF4773	Domain of unknown function	PLA	2	8	28.6	High
TRINITY_DN1880_c0_g1_i4_orf1	serine protease inhibitor dipetalogastin-like [Helicoverpa zea]	2.2001213	1.137583057	2.48E-05	up	yes	3.628	1.649	1.667	1.631	1.648	3.798	3.587	3.498	------	------	K23615	SPIK5	------	------	COG5640;ENOG4111TR7;ENOG410YKSA;ENOG410Z5MG	O:Posttranslational modification, protein turnover, chaperones;S:Function unknown	PF00050.24;PF07648.18	Kazal_1;Kazal_2	Kazal-type serine protease inhibitor domain;Kazal-type serine protease inhibitor domain	CYT	12	52	42.6	High
TRINITY_DN59429_c0_g1_i6_orf1	uncharacterized protein LOC114366345 isoform X2 [Ostrinia furnacalis]	2.1504854	1.104662361	0.0002642	up	yes	3.544	1.648	1.763	1.484	1.698	3.533	3.322	3.777	BP:response to biotic stimulus;BP:response to external stimulus;BP:response to external biotic stimulus;BP:response to stimulus;BP:defense response;BP:response to stress;BP:biological_process;BP:interspecies interaction between organisms;BP:response to other organism;CC:cellular_component;CC:extracellular region;CC:cellular	GO:0009607;GO:0009605;GO:0043207;GO:0050896;GO:0006952;GO:0006950;GO:0008150;GO:0044419;GO:0051707;GO:0005575;GO:0005576;GO:0110165	------	------	------	------	ENOG4110S6N	S:Function unknown	PF00451.22	Toxin_2	Scorpion short toxin, BmKK2	CYT	2	22	12.3	High
TRINITY_DN710_c0_g1_i11_orfp1	TRINITY_DN710_c0_g1_i11_m.67699 TRINITY_DN710_c0_g1::TRINITY_DN710_c0_g1_i11::g.67699 ORF type:complete len:194 (-),score=34.99,Collagen,PF01391.19,0.00029 TRINITY_DN710_c0_g1_i11:1283-1864(-)	3.6303957	1.860126799	8.48E-05	up	yes	4.037	1.112	1.096	1.071	1.168	4.342	4.044	3.725	------	------	------	------	------	------	------	------	------	------	------	CYT	1	7	21.3	High
TRINITY_DN3166_c1_g1_i6_orf1	hypothetical protein evm_013813 [Chilo suppressalis]	3.241908	1.696843153	9.63E-06	up	yes	9.515	2.935	2.99	2.731	3.083	9.892	9.487	9.167	BP:response to biotic stimulus;BP:response to external stimulus;BP:response to external biotic stimulus;BP:response to stimulus;BP:defense response;BP:response to stress;BP:biological_process;BP:interspecies interaction between organisms;BP:response to other organism;CC:cellular_component;CC:extracellular region;CC:cellular	GO:0009607;GO:0009605;GO:0043207;GO:0050896;GO:0006952;GO:0006950;GO:0008150;GO:0044419;GO:0051707;GO:0005575;GO:0005576;GO:0110165	------	------	------	------	------	------	PF00451.22	Toxin_2	Scorpion short toxin, BmKK2	CYT	1	13	10.5	High
TRINITY_DN31417_c0_g1_i3_orf1	titin-like [Ostrinia furnacalis]	0.4155759	-1.266816046	0.0008981	down	yes	1.27	3.056	3.336	3.149	2.682	1.164	1.321	1.326	------	------	------	------	------	------	ENOG410ZKJ3;ENOG4111M3T	S:Function unknown;	PF00093.21	VWC	von Willebrand factor type C domain	CYT	2	17	25.3	High

TRINITY_DN139537_c0_g1_i1_orf1	uncharacterized protein LOC114351440 [Ostrinia furnacalis]	0.438108	-1.190641478	0.007387	down	yes	1.306	2.981	2.458	3.257	3.229	1.216	1.701	1.001	BP:nucleic acid metabolic process;BP:cellular nitrogen compound metabolic process;BP:nitrogen compound metabolic process;BP:nucleobase-containing compound metabolic process;BP:cellular macromolecule metabolic process;BP:transposition;BP:organic substance metabolic process;BP:organic cyclic compound metabolic process;BP:cellular process;BP:cellular aromatic compound metabolic process;BP:biological_process;BP:metabolic process;BP:heterocycle metabolic process;BP:DNA recombination;BP:prim	GO:0090304;GO:0034641;GO:0006807;GO:0006139;GO:0044260;GO:0032196;GO:0071704;GO:1901360;GO:0009987;GO:0006725;GO:0008150;GO:0008152;GO:0046483;GO:0006310;GO:0044238;GO:0006313;GO:0044237;GO:0043170;GO:0015074;GO:0006259;GO:0031224;GO:0016021;GO:0110165;GO:0005575;GO:0003674;GO:0005488;GO:0003676;GO:0003677;GO:1901363;GO:0097159	------	------	------	------	ENOG410XQD5	S:Function unknown	------	------	------	PLA	1	5	23.3	High
TRINITY_DN52553_c0_g2_i1_orf1	hemocyte protein-glutamine gamma-glutamyltransferase-like [Ostrinia furnacalis]	0.1272133	-2.974679008	6.19E-05	down	yes	1.128	8.867	9.519	9.05	8.031	1.181	1.05	1.153	BP:primary metabolic process;BP:peptide cross-linking;BP:organonitrogen compound metabolic process;BP:organic substance metabolic process;BP:nitrogen compound metabolic process;BP:macromolecule modification;BP:macromolecule metabolic process;BP:biological_process;BP:protein modification process;BP:metabolic process;BP:protein metabolic process;MF:molecular_function;MF:protein-glutamine gamma-glutamyltransferase activity;MF:catalytic activity, acting on a protein;MF:catalytic activity;MF:transferase activity;MF:transferase	GO:0044238;GO:0018149;GO:1901564;GO:0071704;GO:0006807;GO:0043412;GO:0043170;GO:0008150;GO:0036211;GO:0008152;GO:0019538;GO:0003674;GO:0003810;GO:0140096;GO:0003824;GO:0016740;GO:0016746;GO:0016755	K05619	TGM1	------	------	ENOG410XQEZ	S:Function unknown	PF00868.23	Transglut_N	Transglutaminase family	CYT	2	11	25.2	High
TRINITY_DN4021_c0_g1_i1_orf1	leech-derived tryptase inhibitor C-like [Ostrinia furnacalis]	0.1316572	-2.92514199	5.47E-05	down	yes	1.063	8.074	7.663	8.807	7.751	0.846	1.267	1.076	------	------	K23417	SPINK1	------	------	ENOG410Z5MG;ENOG410XW7S;ENOG4112ADZ;COG1404;ENOG4111TR7	S:Function unknown;	PF07648.18;PF00050.24	Kazal_2;Kazal_1	Kazal-type serine protease inhibitor domain;Kazal-type serine protease inhibitor domain	PLA	1	11	7.2	High
TRINITY_DN82426_c0_g1_i6_orfp1	lysosome-associated membrane glycoprotein 1-like isoform X4 [Ostrinia furnacalis]	0.4611231	-1.116776124	0.0001153	down	yes	1.281	2.778	2.734	2.66	2.941	1.205	1.385	1.252	------	------	K06528	LAMP1_2, CD107	map05152;map04145;map04142;map04140	Tuberculosis;Phagosome;Lysosome;Autophagy - animal	------	------	------	------	------	CYT	1	12	12.6	High
TRINITY_DN10824_c0_g1_i3_orf1	endochitinase isoform X2 [Ostrinia furnacalis]	0.1497006	-2.739848103	1.22E-07	down	yes	1.15	7.682	7.558	7.789	7.698	1.101	1.121	1.229	BP:amino sugar catabolic process;BP:nitrogen compound metabolic process;BP:aminoglycan metabolic process;BP:organic substance catabolic process;BP:organic substance metabolic process;BP:chitin metabolic process;BP:chitin catabolic process;BP:biological_process;BP:metabolic process;BP:carbohydrate metabolic process;BP:catabolic process;BP:macromolecule catabolic process;BP:primary metabolic process;BP:organonitrogen compound metabolic process;BP:organonitrogen compound catabolic	GO:0046348;GO:0006807;GO:0006022;GO:1901575;GO:0071704;GO:0006030;GO:0006032;GO:0008150;GO:0008152;GO:0005975;GO:0009056;GO:0009057;GO:0044238;GO:1901564;GO:1901565;GO:0006040;GO:1901136;GO:1901135;GO:0043170;GO:1901072;GO:1901071;GO:0006026;GO:0110165;GO:0005575;GO:0005576;GO:0016798;GO:0003674;GO:0008061;GO:0016787;GO:0003824;GO:0004568;GO:0097367;GO:0004553;GO:0005488	K01183	E3.2.1.14	map00520	Amino sugar and nucleotide sugar metabolism	COG3325	S:Function unknown	PF00704.31;PF01607.27	Glyco_hydro_18;CBM_14	Glycosyl hydrolases family 18;Chitin binding Peritrophin-A domain	EXC	4	9	62.8	High
TRINITY_DN1506_c0_g1_i6_orfp1	TRINITY_DN1506_c0_g1_i6_m.57691 TRINITY_DN1506_c0_g1::TRINITY_DN1506_c0_g1_i6::g.57691 ORF type:5prime_partial len:173 (+),score=30.49 TRINITY_DN1506_c0_g1_i6:1-519(+)	24.528837	4.616406904	2.45E-05	up	yes	17.31	0.7057	0.75	0.552	0.815	18.374	17.674	15.873	------	------	------	------	------	------	------	------	------	------	------	CYT	1	7	19	High
TRINITY_DN64181_c0_g1_i1_orf1	uncharacterized protein LOC114356431 isoform X2 [Ostrinia furnacalis]	0.2924375	-1.773799671	0.001407	down	yes	2.703	9.243	10.081	9.715	7.934	3.462	1.759	2.889	------	------	------	------	------	------	------	------	------	------	------	PLA	1	18	7.9	High
TRINITY_DN3275_c0_g2_i3_orf1	hypothetical protein B5X24_HaOG216046 [Helicoverpa armigera]	2.0495356	1.035297052	0.01287	up	yes	3.972	1.938	1.661	1.78	2.372	4.458	4.325	3.132	MF:cation binding;MF:ion binding;MF:molecular_function;MF:binding;MF:metal ion binding;MF:calcium ion binding;	GO:0043169;GO:0043167;GO:0003674;GO:0005488;GO:0046872;GO:0005509	K02183	CALM	map05214;map05022;map04114;map05163;map04750;map05167;map04015;map04014;map04270;map04218;map04371;map04022;map04070;map05012;map04921;map05010;map04922;map04925;map04924;map05133;map04728;map05034;map04740;map04745;map05031;map04720;map05152;map04261;map05200;map04625;map04024;map04020;map05418;map05170;map05417;map04970;map04971;map04744;map04	Glioma;Pathways of neurodegeneration - multiple diseases;Oocyte meiosis;Human cytomegalovirus infection;Inflammatory mediator regulation of TRP channels;Kaposi sarcoma-associated herpesvirus infection;Rap1 signaling pathway;Ras signaling pathway;Vascular smooth muscle contraction;Cellular senescence;Apelin signaling pathway;cGMP-PKG signaling pathway;Phosphatidylinositol signaling system;Parkinson disease;Oxytocin signaling pathway;Alzheimer disease;Glucagon signaling pathway;Aldosterone synthesis and secretion;Renin secretion;Pertussis;Dopaminergic synapse;Alcoholism;Olfactory transduction;Phototransduction - fly;Amphetamine	COG5126;COG5126;COG5126;ENOG410XTJ5	D:Cell cycle control, cell division, chromosome partitioning;T:Signal transduction mechanisms;Z:Cytoskeleton;S:Function unknown	------	------	------	CYT	1	36	2.8	Medium
TRINITY_DN16234_c0_g2_i3_orf1	uncharacterized protein LOC114363370 [Ostrinia furnacalis]	2.0631524	1.044850394	0.0001236	up	yes	3.953	1.916	1.907	1.945	1.896	4.11	4.07	3.679	CC:cellular_component;CC:extracellular region;CC:cellular anatomical entity;MF:enzyme regulator activity;MF:peptidase regulator activity;MF:molecular_function;MF:peptidase inhibitor activity;MF:enzyme inhibitor activity;MF:molecular function regulator;	GO:0005575;GO:0005576;GO:0110165;GO:0030234;GO:0061134;GO:0003674;GO:0030414;GO:0004857;GO:0098772	------	------	------	------	ENOG410Y67D	S:Function unknown	------	------	------	CYT	3	51	7.4	High

TRINITY_DN1423_c0_g1_i8_orf1	ferritin subunit-like [Ostrinia furnacalis] >XP_028168186.1 ferritin subunit-like [Ostrinia furnacalis]	0.4565707	-1.131089774	6.12E-05	down	yes	1.824	3.995	3.931	4.227	3.827	1.867	1.837	1.768	BP:ion homeostasis;BP:homeostatic process;BP:iron ion homeostasis;BP:transition metal ion homeostasis;BP:inorganic ion homeostasis;BP:cellular transition metal ion homeostasis;BP:transition metal ion transport;BP:biological regulation;BP:regulation of biological quality;BP:cellular homeostasis;BP:metal ion transport;BP:transport;BP:cellular iron ion homeostasis;BP:cellular metal ion homeostasis;BP:cation transport;BP:ion transport;BP:cellular process;BP:cellular ion homeostasis;BP:biological_process;BP:cellular cation	GO:0050801;GO:0042592;GO:0055072;GO:0055076;GO:0098771;GO:0046916;GO:0000041;GO:0065007;GO:0065008;GO:0019725;GO:0030001;GO:0006810;GO:0006879;GO:0006875;GO:0006812;GO:0006811;GO:0009987;GO:0006873;GO:0008150;GO:0030003;GO:0055065;GO:0055080;GO:0055082;GO:0051234;GO:0051179;GO:0048878;GO:0006826;GO:0003674;GO:0005488;GO:0016491;GO:0043169;GO:0016722;GO:0046914;GO:0004322;GO:0043167;GO:0005506;GO:0008199;GO:0016724;GO:0046872;GO:0003824	K00522	FTH1	map04978;map04217;map04216	Mineral absorption;Necroptosis;Ferroptosis	ENOG4111MSG	P:Inorganic ion transport and metabolism	PF00210.27	Ferritin	Ferritin-like domain	CYT	6	34	23.7	High
TRINITY_DN24971_c0_g1_i3_orf1	uncharacterized protein LOC114352370 [Ostrinia furnacalis]	0.4199706	-1.251639651	5.20E-06	down	yes	1.716	4.086	4.175	4.13	3.953	1.665	1.738	1.745	------	------	------	------	------	------	ENOG4111G1R	S:Function unknown	------	------	------	CYT	17	34	62.1	High
TRINITY_DN14328_c0_g1_i12_orf1	larval cuticle protein LCP-30-like [Ostrinia furnacalis]	4.1742857	2.061529351	2.25E-05	up	yes	4.383	1.05	1.048	0.991	1.11	4.501	4.549	4.098	MF:molecular_function;MF:structural constituent of cuticle;MF:structural molecule activity;	GO:0003674;GO:0042302;GO:0005198	------	------	------	------	ENOG411127Q7;ENOG410YZ4Z	S:Function unknown;	PF00379.26	Chitin_bind_4	Insect cuticle protein	CYT	10	50	24.6	High
TRINITY_DN42719_c0_g1_i1_orf1	inter-alpha-trypsin inhibitor heavy chain H4-like isoform X11 [Ostrinia furnacalis]	0.3074372	-1.701636195	0.0003446	down	yes	1.298	4.222	4.229	4.658	3.78	1.228	1.388	1.278	------	------	------	------	------	------	------	------	------	------	------	CYT	1	23	7.4	High
TRINITY_DN76216_c0_g2_i3_orf1	lysosomal alpha-mannosidase isoform X1 [Pieris rapae]	0.4976603	-1.006766688	0.0006028	down	yes	1.808	3.633	3.404	3.862	3.633	2.055	1.611	1.758	------	------	K12311	MAN2B1, LAMAN	map00511;map04142	Other glycan degradation;Lysosome	ENOG410XQMZ;COG0564	G:Carbohydrate transport and metabolism;J:Translation, ribosomal structure and biogenesis	PF01074.25	Glyco_hydro_38N	Glycosyl hydrolases family 38 N-terminal domain	CYT	1	25	12	High
TRINITY_DN7785_c0_g1_i1_orf1	uncharacterized protein LOC114364098 [Ostrinia furnacalis]	0.170508	-2.552088484	4.38E-06	down	yes	0.6377	3.74	3.602	3.897	3.72	0.683	0.581	0.649	------	------	------	------	------	------	ENOG4110X3U;ENOG410XPJ1	S:Function unknown;	PF00059.24	Lectin_C	Lectin C-type domain	CYT	2	16	29.9	High
TRINITY_DN18568_c0_g1_i2_orfp1	TRINITY_DN18568_c0_g1_i2_m.13844 TRINITY_DN18568_c0_g1::TRINITY_DN18568_c0_g1_i2::g.13844 ORF type:5prime_partial len:77 (+),score=12.07 TRINITY_DN18568_c0_g1_i2:1-231(+)	4.6324454	2.211773975	0.0004187	up	yes	4.052	0.8747	0.458	0.979	1.187	4.39	3.7	4.065	------	------	------	------	------	------	------	------	------	------	------	CYT	1	13	8.5	High
TRINITY_DN4676_c0_g1_i16_orf1	meiosis-specific nuclear structural protein 1-like isoform X2 [Ostrinia furnacalis]	0.2920043	-1.775938372	4.37E-06	down	yes	1.081	3.702	3.712	3.808	3.585	1.018	1.159	1.067	BP:regulation of microtubule polymerization or depolymerization;BP:regulation of cellular component organization;BP:regulation of organelle organization;BP:regulation of cytoskeleton organization;BP:regulation of cellular process;BP:biological_process;BP:biological regulation;BP:regulation of microtubule-based process;BP:regulation of microtubule cytoskeleton organization;BP:regulation of biological	GO:0031110;GO:0051128;GO:0033043;GO:0051493;GO:0050794;GO:0008150;GO:0065007;GO:0032886;GO:0070507;GO:0050789	K04381	STMN1	map05206;map04010	MicroRNAs in cancer;MAPK signaling pathway	ENOG4111K15	S:Function unknown	PF00836.22	Stathmin	Stathmin family	CYT	9	34	33.5	High
TRINITY_DN27300_c0_g1_i1_orfp1	TRINITY_DN27300_c0_g1_i1_m.71142 TRINITY_DN27300_c0_g1::TRINITY_DN27300_c0_g1_i1::g.71142 ORF type:internal len:82 (-),score=9.46 TRINITY_DN27300_c0_g1_i1:3-245(-)	2.3692884	1.244453814	0.0007527	up	yes	3.163	1.335	1.448	1.132	1.425	2.949	3.043	3.496	------	------	------	------	------	------	------	------	------	------	------	CYT	1	21	9.2	High
TRINITY_DN15545_c0_g1_i1_orf1	larval cuticle protein LCP-14-like [Ostrinia furnacalis]	3.9305211	1.974720592	2.41E-05	up	yes	3.168	0.806	0.804	0.829	0.785	3.318	3.222	2.965	MF:molecular_function;MF:structural constituent of cuticle;MF:structural molecule activity;	GO:0003674;GO:0042302;GO:0005198	------	------	------	------	ENOG4110RUZ;ENOG4111PJ1;ENOG410ZNDD	S:Function unknown;	PF00379.26	Chitin_bind_4	Insect cuticle protein	EXC	3	48	14.6	High
TRINITY_DN4189_c0_g2_i1_orf1	unnamed protein product [Chilo suppressalis]	0.494518	-1.015905182	1.99E-05	down	yes	1.308	2.645	2.743	2.573	2.618	1.346	1.258	1.321	BP:nitrogen compound metabolic process;BP:organic substance metabolic process;BP:proteolysis;BP:biological_process;BP:metabolic process;BP:primary metabolic process;BP:organonitrogen compound metabolic process;BP:protein metabolic process;BP:macromolecule metabolic process;MF:endopeptidase activity;MF:molecular_function;MF:binding;MF:peptidase activity;MF:metallopeptidase activity;MF:cation binding;MF:transition metal ion binding;MF:ion binding;MF:calcium ion binding;MF:metal ion binding;MF:hydrolase	GO:0006807;GO:0071704;GO:0006508;GO:0008150;GO:0008152;GO:0044238;GO:1901564;GO:0019538;GO:0043170;GO:0004175;GO:0003674;GO:0005488;GO:0008233;GO:0008237;GO:0043169;GO:0046914;GO:0043167;GO:0005509;GO:0046872;GO:0016787;GO:0004222;GO:0140096;GO:0003824;GO:0008270	K13046	tok	------	------	ENOG410ZPX7	S:Function unknown	PF00431.23;PF01400.27;PF14670.9;PF07645.18;PF12662.10;PF00008.30	CUB;Astacin;FXa_inhibition;EGF_CA;cEGF;EGF	CUB domain;Astacin;Coagulation Factor Xa inhibitory site;Calcium-binding EGF domain;Complement Clr-like EGF-like;EGF-like domain	EXC	6	6	126.1	High
TRINITY_DN1491_c0_g1_i4_orf1	GILT-like protein 2 isoform X1 [Ostrinia furnacalis] >XP_028156245.1 GILT-like protein 2 isoform X2 [Ostrinia furnacalis] >XP_028156247.1 GILT-like protein 2 isoform X3 [Ostrinia furnacalis]	0.2746181	-1.864501197	0.0001055	down	yes	0.845	3.077	2.968	3.015	3.247	1.027	0.626	0.882	MF:catalytic activity;MF:oxidoreductase activity, acting on a sulfur group of donors, disulfide as acceptor;MF:molecular_function;MF:oxidoreductase activity, acting on a sulfur group of donors;MF:oxidoreductase activity;	GO:0003824;GO:0016671;GO:0003674;GO:0016667;GO:0016491	K08059	IFI30, GILT	map04612	Antigen processing and presentation	ENOG4111IDT	S:Function unknown	PF03227.19	GILT	Gamma interferon inducible lysosomal thiol reductase	CYT	1	16	26.5	High
TRINITY_DN268_c1_g1_i7_orf1	unnamed protein product [Spodoptera littoralis] >CAH1645252.1 unnamed protein product [Spodoptera littoralis]	0.4365198	-1.195881031	8.18E-06	down	yes	1.169	2.678	2.675	2.66	2.698	1.246	1.074	1.187	CC:integral component of membrane;CC:cellular_component;CC:intrinsic component of membrane;CC:cellular anatomical entity;MF:sulfate transmembrane transporter activity;MF:sulfur compound transmembrane transporter activity;MF:secondary active transmembrane transporter activity;MF:inorganic anion transmembrane transporter activity;MF:inorganic solute uptake transmembrane transporter activity;MF:transporter activity;MF:ion transmembrane transporter activity;MF:transmemb	GO:0016021;GO:0005575;GO:0031224;GO:0110165;GO:0015116;GO:1901682;GO:0015291;GO:0015103;GO:0015318;GO:0005215;GO:0015075;GO:0022857;GO:0008271;GO:0022853;GO:0003674;GO:0008509;GO:0022804	K14708	SLC26A11	------	------	COG0659	S:Function unknown	PF00916.23	Sulfate_transp	Sulfate permease family	CYT	2	4	62.2	High

TRINITY_DN3255_c0_g1_i1_orf1	uncharacterized protein LOC114351042 [Ostrinia furnacalis]	0.2054331	-2.283259476	1.83E-05	down	yes	0.6617	3.221	3.035	3.37	3.258	0.58	0.724	0.681	------	------	------	------	------	------	ENOG41114RO	S:Function unknown	------	------	------	CYT	1	6	13.9	High
TRINITY_DN15755_c0_g1_i1_orf1	cytochrome P450 monooxygenase CYP6AB141 [Ostrinia furnacalis]	2.7504798	1.459683332	3.60E-05	up	yes	2.866	1.042	1.052	1.048	1.025	2.72	2.847	3.031	CC:integral component of membrane;CC:cellular_component;CC:cellular anatomical entity;CC:intrinsic component of membrane;MF:cation binding;MF:monooxygenase activity;MF:heme binding;MF:transition metal ion binding;MF:tetrapyrrole binding;MF:organic cyclic compound binding;MF:oxidoreductase activity, acting on paired donors, with incorporation or reduction of molecular oxygen;MF:ion binding;MF:catalytic activity;MF:molecular_function;MF:binding;MF:iron ion binding;MF:metal ion binding;MF:heterocyclic compound	GO:0016021;GO:0005575;GO:0110165;GO:0031224;GO:0043169;GO:0004497;GO:0020037;GO:0046914;GO:0046906;GO:0097159;GO:0016705;GO:0043167;GO:0003824;GO:0003674;GO:0005488;GO:0005506;GO:0046872;GO:1901363;GO:0016491	K14999	CYP6	------	------	COG2124	Q:Secondary metabolites biosynthesis, transport and catabolism	PF00067.25	p450	Cytochrome P450	CYT	3	28	27.4	High
TRINITY_DN18804_c0_g1_i5_orf1	zinc finger protein Xfin-like [Ostrinia furnacalis]	0.3924939	-1.349258003	9.77E-06	down	yes	1.119	2.851	2.892	2.91	2.75	1.166	1.143	1.049	CC:intracellular membrane-bounded organelle;CC:nucleus;CC:cellular_component;CC:intracellular organelle;CC:membrane-bounded organelle;CC:organelle;CC:cellular anatomical entity;MF:cation binding;MF:transition metal ion binding;MF:organic cyclic compound binding;MF:heterocyclic compound binding;MF:ion binding;MF:molecular_function;MF:zinc ion binding;MF:nucleic acid binding;MF:metal ion binding;MF:binding;	GO:0043231;GO:0005634;GO:0005575;GO:0043229;GO:0043227;GO:0043226;GO:0110165;GO:0043169;GO:0046914;GO:0097159;GO:1901363;GO:0043167;GO:0003674;GO:0008270;GO:0003676;GO:0046872;GO:0005488	K09228	KRAB	map05168	Herpes simplex virus 1 infection	COG5048	I:Lipid transport and metabolism	PF13894.9;PF00096.29;PF07776.18;PF18868.4;PF13912.9;PF12874.10;PF12171.11;PF13465.9	zf-C2H2_4;zf-C2H2;zf-AD;zf-C2H2_3rep;zf-C2H2_6;zf-met;zf-C2H2_jaz;zf-H2C2_2	C2H2-type zinc finger;Zinc finger, C2H2 type;Zinc-finger associated domain;Zinc finger C2H2-type, 3 repeats;C2H2-type zinc finger;Zinc-finger of C2H2 type;Zinc-finger double-stranded RNA-binding;Zinc-finger double domain	CYT	3	2	179.5	High
TRINITY_DN285_c0_g1_i4_orf1	catalase-like [Ostrinia furnacalis]	2.417316	1.27340609	4.88E-07	up	yes	2.792	1.155	1.176	1.105	1.184	2.813	2.773	2.79	BP:cellular catabolic process;BP:hydrogen peroxide catabolic process;BP:cellular process;BP:reactive oxygen species metabolic process;BP:response to stress;BP:biological_process;BP:metabolic process;BP:catabolic process;BP:response to oxidative stress;BP:response to stimulus;BP:cellular metabolic process;BP:hydrogen peroxide metabolic process;MF:molecular_function;MF:binding;MF:heterocyclic compound binding;MF:oxidoreductase activity;MF:cation binding;MF:ion binding;MF:metal ion binding;MF:heme binding;MF:antioxidant activity;MF:catalytic	GO:0044248;GO:0042744;GO:0009987;GO:0072593;GO:0006950;GO:0008150;GO:0008152;GO:0009056;GO:0006979;GO:0050896;GO:0044237;GO:0042743;GO:0003674;GO:0005488;GO:1901363;GO:0016491;GO:0043169;GO:0043167;GO:0046872;GO:0020037;GO:0016209;GO:0003824;GO:0016684;GO:0046906;GO:0097159;GO:0004096;GO:0004601	K03781	katE, CAT, catB, srpA	map00630;map05208;map04068;map05014;map04146;map04213;map04212;map04211;map05022;map00380	Glyoxylate and dicarboxylate metabolism;Chemical carcinogenesis - reactive oxygen species;FoxO signaling pathway;Amyotrophic lateral sclerosis;Peroxisome;Longevity regulating pathway - multiple species;Longevity regulating pathway - worm;Longevity regulating pathway;Pathways of neurodegeneration - multiple diseases;Tryptophan metabolism	COG0753	P:Inorganic ion transport and metabolism	PF00199.22;PF06628.15	Catalase;Catalase-rel	Catalase;Catalase-related immune-responsive	CYT	9	23	58.6	High
TRINITY_DN13221_c0_g1_i3_orf1	fasciclin-3-like [Ostrinia furnacalis]	0.2477184	-2.013227159	5.37E-08	down	yes	0.76	3.068	3.076	3.04	3.089	0.742	0.794	0.744	------	------	------	------	------	------	ENOG4111K6M	S:Function unknown	PF08205.15;PF13927.9	C2-set_2;Ig_3	CD80-like C2-set immunoglobulin domain;Immunoglobulin domain	CYT	1	46	19	High
TRINITY_DN125427_c0_g1_i1_orf1	heat shock protein 19.8 [Chilo suppressalis] >AGM90553.1 HSP19.8 [Chilo suppressalis] >BAE94664.1 small heat shock protein 19.7 [Chilo suppressalis]	0.456044	-1.132755209	1.14E-05	down	yes	1.162	2.548	2.54	2.621	2.484	1.176	1.099	1.21	MF:cation binding;MF:structural molecule activity;MF:structural constituent of eye lens;MF:ion binding;MF:molecular_function;MF:binding;MF:metal ion binding;	GO:0043169;GO:0005198;GO:0005212;GO:0043167;GO:0003674;GO:0005488;GO:0046872	K09542	CRYAB	map04213;map04141	Longevity regulating pathway - multiple species;Protein processing in endoplasmic reticulum	ENOG410YERS	O:Posttranslational modification, protein turnover, chaperones	PF00011.24	HSP20	Hsp20/alpha crystallin family	CYT	3	39	21.2	High
TRINITY_DN2290_c0_g1_i2_orfp1	TRINITY_DN2290_c0_g1_i2_m.69732 TRINITY_DN2290_c0_g1::TRINITY_DN2290_c0_g1_i2::g.69732 ORF type:complete len:234 (+),score=14.43 TRINITY_DN2290_c0_g1_i2:60-761(+)	0.3020751	-1.727020664	0.00115	down	yes	0.8443	2.795	3.127	2.877	2.382	0.682	0.879	0.972	------	------	------	------	------	------	------	------	------	------	------	CYT	2	16	26.1	High
TRINITY_DN1575_c0_g1_i10_orf1	uncharacterized protein LOC114359245 [Ostrinia furnacalis]	0.4581641	-1.126063694	0.001991	down	yes	1.128	2.462	2.461	2.759	2.165	1.028	1.09	1.266	------	------	------	------	------	------	ENOG4111CB2;ENOG41118JD;ENOG4110T1G	S:Function unknown;	PF15868.8	MBF2	Transcription activator MBF2	CYT	1	2	45.9	High
TRINITY_DN3732_c1_g1_i5_orf1	cytochrome P450 6B2-like [Ostrinia furnacalis]	2.1686418	1.116791746	6.54E-05	up	yes	2.379	1.097	1.212	1.034	1.044	2.472	2.327	2.339	MF:cation binding;MF:monooxygenase activity;MF:heme binding;MF:transition metal ion binding;MF:tetrapyrrole binding;MF:organic cyclic compound binding;MF:oxidoreductase activity, acting on paired donors, with incorporation or reduction of molecular oxygen;MF:ion binding;MF:catalytic activity;MF:molecular_function;MF:binding;MF:iron ion binding;MF:metal ion binding;MF:heterocyclic compound binding;MF:oxidoreduc	GO:0043169;GO:0004497;GO:0020037;GO:0046914;GO:0046906;GO:0097159;GO:0016705;GO:0043167;GO:0003824;GO:0003674;GO:0005488;GO:0005506;GO:0046872;GO:1901363;GO:0016491	K14999	CYP6	------	------	COG2124	Q:Secondary metabolites biosynthesis, transport and catabolism	PF00067.25	p450	Cytochrome P450	CYT	5	23	55.1	High

TRINITY_DN114982_c0_g1_i1_orf1	uncharacterized protein LOC114357708 [Ostrinia furnacalis]	0.3594088	-1.476302257	0.002095	down	yes	0.8633	2.402	2.678	2.019	2.508	0.847	1.027	0.716	CC:cellular_component;CC:ribosome;CC:intracellular non-membrane-bounded organelle;CC:mitochondrion;CC:intracellular organelle;CC:non-membrane-bounded organelle;CC:membrane-bounded organelle;CC:organelle;CC:cellular anatomical entity;CC:intracellular membrane-bounded organelle;MF:structural molecule activity;MF:structural constituent of ribosome;MF:molecular_function;	GO:0005575;GO:0005840;GO:0043232;GO:0005739;GO:0043229;GO:0043228;GO:0043227;GO:0043226;GO:0110165;GO:0043231;GO:0005198;GO:0003735;GO:0003674	K17412	MRPS34	------	------	ENOG4111Y87	S:Function unknown	PF16053.8	MRP-S34	Mitochondrial 28S ribosomal protein S34	CYT	2	13	22.6	High
TRINITY_DN661_c1_g2_i1_orf1	larval/pupal cuticle protein H1C-like [Ostrinia furnacalis]	0.323957	-1.626125574	1.60E-05	down	yes	0.7843	2.421	2.408	2.54	2.314	0.791	0.797	0.765	MF:molecular_function;MF:structural constituent of cuticle;MF:structural molecule activity;	GO:0003674;GO:0042302;GO:0005198	------	------	------	------	ENOG4111EA8;ENOG410YSFI;ENOG4110T8H	S:Function unknown;	PF00379.26	Chitin_bind_4	Insect cuticle protein	CYT	4	18	25.4	High
TRINITY_DN15327_c2_g1_i2_orf1	protein lethal(2)essential for life-like [Ostrinia furnacalis]	3.1269185	1.644741598	4.88E-07	up	yes	2.343	0.7493	0.765	0.736	0.747	2.324	2.394	2.312	MF:cation binding;MF:structural molecule activity;MF:structural constituent of eye lens;MF:ion binding;MF:molecular_function;MF:binding;MF:metal ion binding;	GO:0043169;GO:0005198;GO:0005212;GO:0043167;GO:0003674;GO:0005488;GO:0046872	K09542	CRYAB	map04213;map04141	Longevity regulating pathway - multiple species;Protein processing in endoplasmic reticulum	ENOG410YERS	E:Amino acid transport and metabolism	PF00011.24;PF00525.21	HSP20;Crystallin	Hsp20/alpha crystallin family;Alpha crystallin A chain, N terminal	CYT	7	44	22	High
TRINITY_DN42337_c0_g1_i5_orf1	hypothetical protein evm_002829 [Chilo suppressalis]	0.3427198	-1.544898635	0.0002764	down	yes	0.998	2.912	2.975	3.124	2.637	1.023	0.869	1.102	MF:molecular_function;MF:structural constituent of cuticle;MF:structural molecule activity;	GO:0003674;GO:0042302;GO:0005198	------	------	------	------	ENOG410YSFI;ENOG4111EA8;ENOG4110T8H	S:Function unknown;	PF00379.26	Chitin_bind_4	Insect cuticle protein	CYT	1	31	17.6	High
TRINITY_DN5198_c0_g1_i5_orfp1	TRINITY_DN5198_c0_g1_i5_m.8637 TRINITY_DN5198_c0_g1::TRINITY_DN5198_c0_g1_i5::g.8637 ORF type:complete len:223 (-),score=54.99 TRINITY_DN5198_c0_g1_i5:319-987(-)	2.5968535	1.376764622	0.007285	up	yes	2.641	1.017	1.041	1.018	0.992	2.735	2.042	3.145	------	------	------	------	------	------	------	------	------	------	------	CYT	1	5	24.7	High
TRINITY_DN18482_c0_g1_i3_orf1	calcyphosin-like protein isoform X3 [Helicoverpa armigera] >XP_047020698.1 calcyphosin-like protein isoform X2 [Helicoverpa zea]	2.0861287	1.060828193	1.73E-05	up	yes	2.301	1.103	1.113	1.085	1.112	2.382	2.308	2.214	------	------	K23909	CAPS	------	------	ENOG410XRMJ	T:Signal transduction mechanisms	PF13405.9;PF13499.9;PF00036.35;PF13202.9	EF-hand_6;EF-hand_7;EF-hand_1;EF-	EF-hand domain;EF-hand domain pair;EF hand;EF hand	CYT	9	56	25.6	High
TRINITY_DN37585_c0_g2_i1_orf1	cuticle protein 19.8-like [Ostrinia furnacalis]	0.4542427	-1.138464763	0.003709	down	yes	0.9957	2.192	2.184	2.528	1.865	0.987	1.08	0.92	MF:molecular_function;MF:structural constituent of cuticle;MF:structural molecule activity;	GO:0003674;GO:0042302;GO:0005198	------	------	------	------	ENOG410YTXG;ENOG410YSFI	S:Function unknown;	PF00379.26	Chitin_bind_4	Insect cuticle protein	CYT	1	6	14.7	High
TRINITY_DN843_c0_g1_i2_orf1	unnamed protein product [Diatraea saccharalis]	0.3833333	-1.38332864	0.0006315	down	yes	0.9867	2.574	2.877	2.506	2.338	1.038	1.008	0.914	------	------	------	------	------	------	ENOG4111K6M	S:Function unknown	PF08205.15;PF13927.9;PF07679.19	C2-set_2;Ig_3;I-set	CD80-like C2-set immunoglobulin domain;Immunoglobulin domain;Immunoglobulin I-set domain	CYT	2	32	48.6	High
TRINITY_DN7565_c0_g1_i3_orf1	acylphosphatase-2-like [Ostrinia furnacalis]	0.4524887	-1.14404637	0.0009383	down	yes	1	2.21	2.064	2.478	2.087	1.009	1.051	0.94	MF:hydrolase activity;MF:hydrolase activity, acting on acid anhydrides, in phosphorus-containing anhydrides;MF:catalytic activity;MF:hydrolase activity, acting on acid anhydrides;MF:pyrophosphatase activity;MF:molecular_function;MF:acylphosph	GO:0016787;GO:0016818;GO:0003824;GO:0016817;GO:0016462;GO:0003674;GO:0003998	K01512	acyP	map00620	Pyruvate metabolism	ENOG41126ER	O:Posttranslational modification, protein turnover, chaperones	PF00708.21	Acylphosphatase	Acylphosphatase	CYT	1	9	13.7	High
TRINITY_DN110519_c0_g1_i1_orf1	uncharacterized protein LOC114366601 [Ostrinia furnacalis]	0.3153054	-1.665178011	0.0002112	down	yes	0.9497	3.012	2.724	3.151	3.161	0.875	0.883	1.091	------	------	------	------	------	------	------	------	------	------	------	CYT	1	5	20	High
TRINITY_DN609_c0_g1_i1_orf1	zonadhesin-like isoform X1 [Ostrinia furnacalis]	2.5230627	1.335176076	3.85E-05	up	yes	2.735	1.084	1.117	1.048	1.088	2.803	2.829	2.574	------	------	------	------	------	------	COG4826;ENOG4110RF5;ENOG410Y2QH;ENOG410ZQ13;ENOG410XNSK	O:Posttranslational modification, protein turnover, chaperones;S:Function unknown;S:Function unknown;S:Function unknown	PF01826.20	TIL	Trypsin Inhibitor like cysteine rich domain	PLA	9	18	109	High
TRINITY_DN24121_c1_g1_i6_orf1	serine protease persephone-like [Ostrinia furnacalis]	3.2755886	1.711754185	5.02E-06	up	yes	2.685	0.8197	0.809	0.791	0.859	2.651	2.788	2.615	BP:organic substance metabolic process;BP:organonitrogen compound metabolic process;BP:protein metabolic process;BP:macromolecule metabolic process;BP:biological_process;BP:nitrogen compound metabolic process;BP:metabolic process;BP:proteolysis;BP:primary metabolic process;MF:serine-type endopeptidase activity;MF:endopeptidase activity;MF:hydrolase activity;MF:serine hydrolase activity;MF:catalytic activity, acting on a protein;MF:catalytic activity;MF:molecular_function;MF:peptidase activity;MF:serine-type	GO:0071704;GO:1901564;GO:0019538;GO:0043170;GO:0008150;GO:0006807;GO:0008152;GO:0006508;GO:0044238;GO:0004252;GO:0004175;GO:0016787;GO:0017171;GO:0140096;GO:0003824;GO:0003674;GO:0008233;GO:0008236	K20677;K09625;K20671	HAYAN;PRSS21;PSH	map04624	Toll and Imd signaling pathway	COG5640	O:Posttranslational modification, protein turnover, chaperones	PF00089.29	Trypsin	Trypsin	CYT	7	29	28.9	High
TRINITY_DN701_c0_g1_i1_orf1	venom protease-like isoform X3 [Ostrinia furnacalis]	2.0315692	1.022594488	0.00021	up	yes	2.188	1.077	1.092	1.024	1.116	2.281	2.025	2.258	BP:organic substance metabolic process;BP:organonitrogen compound metabolic process;BP:protein metabolic process;BP:nitrogen compound metabolic process;BP:macromolecule metabolic process;BP:biological_process;BP:metabolic process;BP:proteolysis;BP:primary metabolic process;CC:cellular_component;CC:extracellular region;CC:cellular anatomical entity;MF:serine-type endopeptidase activity;MF:endopeptidase activity;MF:hydrolase activity;MF:serine hydrolase activity;MF:catalytic activity, acting on a protein;MF:catalytic	GO:0071704;GO:1901564;GO:0019538;GO:0006807;GO:0043170;GO:0008150;GO:0008152;GO:0006508;GO:0044238;GO:0005575;GO:0005576;GO:0110165;GO:0004252;GO:0004175;GO:0016787;GO:0017171;GO:0140096;GO:0003824;GO:0003674;GO:0008233;GO:0008236	------	------	------	------	COG5640	O:Posttranslational modification, protein turnover, chaperones	PF00089.29	Trypsin	Trypsin	EXC	5	30	27.1	High

TRINITY_DN27247_c0_g2_i1_orfp1	TRINITY_DN27247_c0_g2_i1_m.23157 TRINITY_DN27247_c0_g2::TRINITY_DN27247_c0_g2_i1::g.23157 ORF type:5prime_partial len:70 (+),score=25.00 TRINITY_DN27247_c0_g2_i1:2-211(+)	2.1540755	1.107068848	0.000851	up	yes	2.167	1.006	1.202	0.778	1.037	2.2	2.09	2.212	------	------	------	------	------	------	------	------	------	------	------	CYT	1	20	7	High
TRINITY_DN22589_c0_g1_i6_orfp1	TRINITY_DN22589_c0_g1_i6_m.19386 TRINITY_DN22589_c0_g1::TRINITY_DN22589_c0_g1_i6::g.19386 ORF type:internal len:183 (+),score=63.52 TRINITY_DN22589_c0_g1_i6:2-547(+)	2.1094766	1.076885107	0.0008215	up	yes	2.108	0.9993	0.981	1.083	0.934	2.078	1.927	2.319	------	------	------	------	------	------	------	------	------	------	------	CYT	1	30	17.5	High
TRINITY_DN806_c0_g2_i1_orf1	uncharacterized protein LOC114355167 [Ostrinia furnacalis]	0.3128834	-1.676302812	1.52E-06	down	yes	0.816	2.608	2.529	2.645	2.649	0.831	0.815	0.802	MF:molecular_function;MF:structural constituent of cuticle;MF:structural molecule activity;	GO:0003674;GO:0042302;GO:0005198	------	------	------	------	ENOG410YR66;ENOG4111PJ1	S:Function unknown;	PF00379.26	Chitin_bind_4	Insect cuticle protein	CYT	1	6	17.8	High
TRINITY_DN55147_c0_g1_i1_orfp1	TRINITY_DN55147_c0_g1_i1_m.59251 TRINITY_DN55147_c0_g1::TRINITY_DN55147_c0_g1_i1::g.59251 ORF type:5prime_partial len:331 (-),score=113.97,Cuticle_3 PF11018.9 0.29,Cuticle_3|PF11018.9|2.9e-05,Cuticle_3|PF11018.9|0.00037 TRINITY_DN55147_c0_g1_i1:21-1013(-)	2.9443801	1.557963911	2.39E-06	up	yes	2.541	0.863	0.86	0.847	0.882	2.621	2.485	2.518	------	------	------	------	------	------	------	------	PF11018.11	Cuticle_3	Pupal cuticle protein C1	CYT	7	50	33.7	High
TRINITY_DN14532_c0_g1_i1_orf1	pupal cuticle protein-like [Trichoplusia ni]	0.2808166	-1.832299664	1.29E-06	down	yes	0.729	2.596	2.547	2.647	2.595	0.718	0.782	0.687	MF:molecular_function;MF:structural constituent of cuticle;MF:structural molecule activity;	GO:0003674;GO:0042302;GO:0005198	------	------	------	------	ENOG4110QXC;ENOG410Z4W3	S:Function unknown;	------	------	------	CYT	4	26	25.9	High
TRINITY_DN111621_c0_g3_i1_orf1	Serine proteinase stubble [Eumeta japonica]	0.4568692	-1.130147039	1.34E-06	down	yes	0.9777	2.14	2.121	2.121	2.177	0.98	0.947	1.006	BP:organic substance metabolic process;BP:organonitrogen compound metabolic process;BP:protein metabolic process;BP:macromolecule metabolic process;BP:biological_process;BP:nitrogen compound metabolic process;BP:metabolic process;BP:proteolysis;BP:primary metabolic process;MF:serine-type endopeptidase activity;MF:endopeptidase activity;MF:hydrolase activity;MF:serine hydrolase activity;MF:catalytic activity, acting on a protein;MF:catalytic activity;MF:molecular_function;MF:peptidase activity;MF:serine-type	GO:0071704;GO:1901564;GO:0019538;GO:0043170;GO:0008150;GO:0006807;GO:0008152;GO:0006508;GO:0044238;GO:0004252;GO:0004175;GO:0016787;GO:0017171;GO:0140096;GO:0003824;GO:0003674;GO:0008233;GO:0008236	------	------	------	------	COG5640	E:Amino acid transport and metabolism	PF00089.29	Trypsin	Trypsin	CYT	1	4	18.5	Medium
TRINITY_DN1305_c0_g1_i6_orf1	glutathione S-transferase sigma 3 [Ostrinia furnacalis]	2.0107004	1.007698124	4.31E-05	up	yes	2.067	1.028	1.031	1.043	1.009	1.999	2.031	2.172	MF:catalytic activity;MF:transferase activity;MF:glutathione transferase activity;MF:molecular_function;MF:transferase activity, transferring alkyl or ary;	GO:0003824;GO:0016740;GO:0004364;GO:0003674;GO:0016765	K04097	HPGDS	map00590;map00480;map05204;map00982;map00980	Arachidonic acid metabolism;Glutathione metabolism;Chemical carcinogenesis - DNA adducts;Drug metabolism - cytochrome P450;Metabolism of xenobiotics by cytochrome P450	ENOG41103AU;ENOG4110YU0;ENOG4111I69;ENOG4111Y4Q	O:Posttranslational modification, protein turnover, chaperones;	PF02798.23;PF14497.9;PF13417.9	GST_N;GST_C_3;GST_N_3	Glutathione S-transferase, N-terminal domain;Glutathione S-transferase, C-terminal domain;Glutathione S-transferase, N-terminal domain	CYT	2	26	19.4	High
TRINITY_DN4013_c0_g1_i4_orf1	uncharacterized protein LOC114353190 isoform X1 [Ostrinia furnacalis] >XP_028160984.1 uncharacterized protein LOC114353190 isoform X2 [Ostrinia furnacalis] >XP_028161062.1 uncharacterized protein LOC114353190 isoform X1 [Ostrinia furnacalis] >XP_028161142.1 uncharacterized protein LOC114353190 isoform X1 [Ostrinia furnacalis]	0.4904459	-1.027834208	0.001453	down	yes	1.001	2.041	2.156	2.111	1.856	0.819	1.14	1.043	CC:cellular_component;CC:membrane protein complex;CC:macromolecular complex;CC:ESCRT complex;CC:ESCRT I complex;	GO:0005575;GO:0098796;GO:0032991;GO:0036452;GO:0000813	K12186	FAM125	map04144	Endocytosis	ENOG4110KHY	U:Intracellular trafficking, secretion, and vesicular transport	PF10240.12	DUF2464	Multivesicular body subunit 12	CYT	1	10	10.9	High
TRINITY_DN113272_c0_g1_i1_orf1	altered inheritance of mitochondria protein 3-like [Ostrinia furnacalis]	0.2140721	-2.223830988	5.82E-05	down	yes	0.6587	3.077	3.34	2.966	2.924	0.599	0.681	0.696	------	------	------	------	------	------	ENOG41113B8	S:Function unknown	------	------	------	PLA	1	5	33	High
TRINITY_DN4125_c0_g1_i14_orf1	angiotensin-converting enzyme-like isoform X1 [Ostrinia furnacalis]	0.2331248	-2.100825714	5.60E-05	down	yes	0.6707	2.877	3.012	2.976	2.642	0.633	0.641	0.738	BP:nitrogen compound metabolic process;BP:organic substance metabolic process;BP:proteolysis;BP:biological_process;BP:metabolic process;BP:primary metabolic process;BP:organonitrogen compound metabolic process;BP:protein metabolic process;BP:macromolecule metabolic process;CC:membrane;CC:intrinsic component of membrane;CC:integral component of membrane;CC:cellular anatomical entity;CC:cellular_component;MF:exopeptidase activity;MF:molecular_function;MF:binding;MF:peptidase	GO:0006807;GO:0071704;GO:0006508;GO:0008150;GO:0008152;GO:0044238;GO:1901564;GO:0019538;GO:0043170;GO:0016020;GO:0031224;GO:0016021;GO:0110165;GO:0005575;GO:0008238;GO:0003674;GO:0005488;GO:0008233;GO:0043169;GO:0004180;GO:0043167;GO:0008237;GO:0046872;GO:0016787;GO:0008241;GO:0140096;GO:0003824	K01283	ACE, CD143	map05142;map04614;map04924;map05171;map05415;map05410	Chagas disease;Renin-angiotensin system;Renin secretion;Coronavirus disease - COVID-19;Diabetic cardiomyopathy;Hypertrophic cardiomyopathy	ENOG410XPJ3	S:Function unknown	PF01401.21	Peptidase_M2	Angiotensin-converting enzyme	CYT	1	12	74.3	High
TRINITY_DN585_c0_g1_i5_orf1	very low-density lipoprotein receptor isoform X2 [Galleria mellonella]	0.4340734	-1.203989069	2.98E-05	down	yes	0.958	2.207	2.207	2.304	2.109	0.964	0.924	0.986	CC:integral component of membrane;CC:cellular_component;CC:intrinsic component of membrane;CC:cellular anatomical entity;MF:binding;MF:cation binding;MF:cargo receptor activity;MF:very-low-density lipoprotein particle receptor activity;MF:lipoprotein particle receptor activity;MF:ion binding;MF:molecular_function;MF:metal ion binding;MF:calcium ion binding;	GO:0016021;GO:0005575;GO:0031224;GO:0110165;GO:0005488;GO:0043169;GO:0038024;GO:0030229;GO:0030228;GO:0043167;GO:0003674;GO:0046872;GO:0005509	K20053	VLDLR	map05417;map05017	Lipid and atherosclerosis;Spinocerebellar ataxia	ENOG410YQ6J;ENOG410YEXK	U:Intracellular trafficking, secretion, and vesicular transport;S:Function unknown	PF00057.21;PF00058.20;PF14670.9;PF12662.10;PF07645.18;PF16472.8	Ldl_recept_a;Ldl_recept_b;FXa_inhibition;cEGF;EGF_CA;DUF5050	Low-density lipoprotein receptor domain class A;Low-density lipoprotein receptor repeat class B;Coagulation Factor Xa inhibitory site;Complement Clr-like EGF-like;Calcium-binding EGF domain;Domain of unknown function	PLA	2	33	94.4	High
TRINITY_DN1563_c0_g1_i4_orf1	pupal cuticle protein 36-like [Ostrinia furnacalis]	0.3804476	-1.394230389	1.42E-05	down	yes	0.969	2.547	2.624	2.588	2.429	0.937	0.982	0.988	MF:molecular_function;MF:structural constituent of cuticle;MF:structural molecule activity;	GO:0003674;GO:0042302;GO:0005198	------	------	------	------	ENOG4111PJ1;ENOG410XTK4;ENOG4110T67;ENOG4110SY1	S:Function unknown;	PF00379.26	Chitin_bind_4	Insect cuticle protein	CYT	5	41	30.1	High
TRINITY_DN114834_c0_g1_i1_orf1	uncharacterized protein LOC115444227 [Manduca sexta] >XP_030025790.1 uncharacterized protein LOC115444227 [Manduca sexta] >XP_037296791.1 uncharacterized protein LOC115444227 [Manduca sexta] >XP_037296792.1 uncharacterized protein LOC115444227 [Manduca sexta] >KAG6441350.1 hypothetical protein O3G_MSEX001778 [Manduca sexta]	0.3501975	-1.513759424	4.52E-06	down	yes	0.8867	2.532	2.519	2.62	2.458	0.87	0.908	0.882	CC:cellular_component;CC:integral component of membrane;CC:cellular anatomical entity;CC:intrinsic component of membrane;	GO:0005575;GO:0016021;GO:0110165;GO:0031224	------	------	------	------	ENOG410XTAH	S:Function unknown	PF00024.29	PAN_1	PAN domain	CYT	10	19	81.4	High

TRINITY_DN98242_c0_g1_i1_orf1	adenosine deaminase 2-A-like [Galleria mellonella]	0.2366523	-2.079159193	6.65E-05	down	yes	0.6037	2.551	2.335	2.707	2.61	0.567	0.617	0.627	BP:cellular metabolic process;BP:cellular catabolic process;BP:cellular nitrogen compound metabolic process;BP:nitrogen compound metabolic process;BP:small molecule metabolic process;BP:small molecule catabolic process;BP:purine-containing compound metabolic process;BP:purine-containing compound catabolic process;BP:purine ribonucleoside catabolic process;BP:adenosine metabolic process;BP:organic cyclic compound metabolic process;BP:organic cyclic compound catabolic [illegible]	GO:0044237;GO:0044248;GO:0034641;GO:0006807;GO:0044281;GO:0044282;GO:0072521;GO:0072523;GO:0046130;GO:0046085;GO:1901360;GO:1901361;GO:0006139;GO:1901575;GO:0042278;GO:0071704;GO:0006152;GO:1901658;GO:0042454;GO:0006154;GO:0044270;GO:0009987;GO:0006725;GO:0046700;GO:0008150;GO:0009116;GO:0008152;GO:0034655;GO:0009119;GO:0046128;GO:0009056;GO:0055086;GO:0046483;GO:0044238;GO:1901564;GO:1901565;GO:1901136;GO:0009164;GO:1901657;GO:0019439;GO:1901135;GO:0034656;GO:0005615;GO:0005575;GO:0110165;GO:0003674;GO:0019239;GO:0016787;GO:0004000;GO:0003824;GO:0016810;GO:0016814	K19572	CECR1, ADA2	map01232;map00230	Nucleotide metabolism;Purine metabolism	COG1816	F:Nucleotide transport and metabolism	PF00962.25;PF08451.14	A_deaminase;A_deaminase_N	Adenosine deaminase;Adenosine/AMP deaminase N-terminal	CYT	1	1	57.4	High
TRINITY_DN24789_c0_g1_i9_orfp1	TRINITY_DN24789_c0_g1_i9_m.25888 TRINITY_DN24789_c0_g1::TRINITY_DN24789_c0_g1_i9::g.25888 ORF type:internal len:114 (-),score=12.65 TRINITY_DN24789_c0_g1_i9:2-340(-)	2.1158665	1.081248599	4.98E-06	up	yes	2.111	0.9977	0.976	1.014	1.003	2.05	2.128	2.156	------	------	------	------	------	------	------	------	------	------	------	CYT	5	46	13.2	High
TRINITY_DN501_c0_g1_i5_orf1	hypothetical protein evm_002550 [Chilo suppressalis]	0.4881314	-1.034658427	2.45E-06	down	yes	0.9953	2.039	2.024	2.082	2.01	0.977	1.024	0.985	CC:integral component of membrane;CC:cellular_component;CC:extracellular region;CC:intrinsic component of membrane;CC:cellular anatomical entity;MF:binding;MF:cation binding;MF:molecular function regulator;MF:peptidase regulator activity;MF:ion binding;MF:molecular_function;MF:peptidase inhibitor activity;MF:enzyme inhibitor activity;MF:metal ion binding;MF:enzyme regulator activity;MF:calcium ion	GO:0016021;GO:0005575;GO:0005576;GO:0031224;GO:0110165;GO:0005488;GO:0043169;GO:0098772;GO:0061134;GO:0043167;GO:0003674;GO:0030414;GO:0004857;GO:0046872;GO:0030234;GO:0005509	------	------	------	------	ENOG410XSTY;ENOG410XP6K	S:Function unknown;	PF12947.10;PF07645.18	EGF_3;EGF_CA	EGF domain;Calcium-binding EGF domain	PLA	90	8	1895.1	High
TRINITY_DN3593_c0_g1_i3_orfp1	TRINITY_DN3593_c0_g1_i3_m.43968 TRINITY_DN3593_c0_g1::TRINITY_DN3593_c0_g1_i3::g.43968 ORF type:5prime_partial len:72 (-),score=1.41 TRINITY_DN3593_c0_g1_i3:138-353(-)	3.7902902	1.92230832	1.99E-05	up	yes	2.834	0.7477	0.806	0.793	0.644	2.693	2.935	2.874	------	------	------	------	------	------	------	------	------	------	------	CYT	1	15	7.5	High
TRINITY_DN15685_c0_g1_i5_orf1	uncharacterized protein LOC114352354 [Ostrinia furnacalis]	3.1011668	1.632811147	5.09E-07	up	yes	2.578	0.8313	0.778	0.848	0.868	2.588	2.592	2.554	------	------	------	------	------	------	ENOG4110T90;ENOG4110RJ7;ENOG41119GI;ENOG410YTKH;ENOG4110QW6;ENOG410ZNC7	S:Function unknown;	PF06585.14	JHBP	Haemolymph juvenile hormone binding protein	CYT	3	14	29.1	High
TRINITY_DN4689_c0_g1_i5_orf1	pericentriolar material 1 protein-like isoform X3 [Ostrinia furnacalis]	0.3856668	-1.374573118	0.002476	down	yes	0.9283	2.407	2.373	2.059	2.789	1.015	0.829	0.941	BP:cellular component assembly;BP:cell projection organization;BP:cell projection assembly;BP:protein localization to cytoskeleton;BP:protein localization;BP:microtubule cytoskeleton organization;BP:plasma membrane bounded cell projection organization;BP:cellular component organization or biogenesis;BP:plasma membrane bounded cell projection assembly;BP:microtubule anchoring at centrosome;BP:protein localization to centrosome;BP:microtubule anchoring at microtubule organizing center;BP:cellular [illegible]	GO:0022607;GO:0030030;GO:0030031;GO:0044380;GO:0008104;GO:0000226;GO:0120036;GO:0071840;GO:0120031;GO:0034454;GO:0071539;GO:0072393;GO:0016043;GO:1905508;GO:0033036;GO:0034453;GO:0060271;GO:0009987;GO:0008150;GO:0070925;GO:0051179;GO:0051641;GO:0006996;GO:0007017;GO:0007010;GO:0070727;GO:0072698;GO:0033365;GO:0044782;GO:0005813;GO:0005815;GO:0110165;GO:0005575	K16537	PCM1	------	------	ENOG410YNFX	S:Function unknown	PF15717.8	PCM1_C	Pericentriolar material 1 C terminus	CYT	2	1	193.5	High
TRINITY_DN5074_c0_g1_i7_orf1	zonadhesin-like [Ostrinia furnacalis]	0.4134021	-1.274382511	2.51E-06	down	yes	0.9223	2.231	2.21	2.265	2.218	0.888	0.979	0.9	------	------	------	------	------	------	COG4826;ENOG410ZAEW;ENOG410XP6K;ENOG410Y2QH;ENOG4110S7R	O:Posttranslational modification, protein turnover, chaperones;S:Function unknown;S:Function unknown;S:Function unknown;S:Function unknown	PF01826.20	TIL	Trypsin Inhibitor like cysteine rich domain	CYT	4	21	25.8	High
TRINITY_DN54366_c0_g1_i1_orf1	protein obstructor-E-like [Ostrinia furnacalis]	0.2178177	-2.198806749	5.53E-06	down	yes	0.6907	3.171	3.04	3.29	3.184	0.677	0.654	0.741	CC:cellular_component;CC:extracellular region;CC:cellular anatomical entity;MF:carbohydrate derivative binding;MF:molecular_function;MF:chitin binding;MF:binding;	GO:0005575;GO:0005576;GO:0110165;GO:0097367;GO:0003674;GO:0008061;GO:0005488	------	------	------	------	ENOG4111EK0	S:Function unknown	PF01607.27	CBM_14	Chitin binding Peritrophin-A domain	PLA	6	28	26.9	High
TRINITY_DN280_c0_g1_i8_orf1	Tubulin beta-1 chain [Papilio xuthus]	0.3953267	-1.338882776	1.06E-05	down	yes	0.8713	2.204	2.209	2.277	2.127	0.829	0.881	0.904	BP:cellular process;BP:biological_process;BP:microtubule-based process;CC:supramolecular fiber;CC:polymeric cytoskeletal fiber;CC:microtubule;CC:cytoplasm;CC:cellular_component;CC:cellular anatomical entity;CC:supramolecular complex;CC:supramolecular polymer;MF:structural constituent of cytoskeleton;MF:purine ribonucleoside triphosphate binding;MF:molecular_function;MF:binding;MF:guanyl nucleotide binding;MF:heterocyclic compound binding;MF:nucleoside phosphate binding;MF:pyrophosphatase [illegible]	GO:0009987;GO:0008150;GO:0007017;GO:0099512;GO:0099513;GO:0005874;GO:0005737;GO:0005575;GO:0110165;GO:0099080;GO:0099081;GO:0005200;GO:0035639;GO:0003674;GO:0005488;GO:0019001;GO:1901363;GO:1901265;GO:0016462;GO:0005198;GO:0032561;GO:0017076;GO:0005525;GO:0016787;GO:0000166;GO:0003924;GO:0017111;GO:0036094;GO:0003824;GO:0016818;GO:0043167;GO:0097367;GO:0097159;GO:0016817;GO:0043168;GO:0032555;GO:0032553	K07375	TUBB	map04540;map05012;map05010;map05016;map05130;map05014;map05132;map04145;map05022;map05020	Gap junction;Parkinson disease;Alzheimer disease;Huntington disease;Pathogenic Escherichia coli infection;Amyotrophic lateral sclerosis;Salmonella infection;Phagosome;Pathways of neurodegeneration - multiple diseases;Prion disease	COG5023	Z:Cytoskeleton	PF00091.28;PF03953.20	Tubulin;Tubulin_C	Tubulin/FtsZ family, GTPase domain;Tubulin C-terminal domain	CYT	4	53	50.6	High

TRINITY_DN7735_c1_g1_i1_orf1	cuticular protein CPH [Spodoptera litura]	0.4750733	-1.073777926	0.002444	down	yes	0.972	2.046	2.284	2.103	1.75	0.966	0.943	1.007	CC:cellular_component;CC:integral component of membrane;CC:cellular anatomical entity;CC:intrinsic component of membrane;	GO:0005575;GO:0016021;GO:0110165;GO:0031224	------	------	------	------	ENOG4110SF2;ENOG4110SXM	S:Function unknown;	------	------	------	CYT	1	5	15.2	High
TRINITY_DN1226_c0_g1_i11_orfp1	TRINITY_DN1226_c0_g1_i11_m.52385 TRINITY_DN1226_c0_g1::TRINITY_DN1226_c0_g1_i11::g.52385 ORF type:internal len:92 (-),score=5.77 TRINITY_DN1226_c0_g1_i11:2-274(-)	2.6936852	1.42958126	6.46E-05	up	yes	2.858	1.061	1.087	1.058	1.039	3.063	2.76	2.751	------	------	------	------	------	------	------	------	------	------	------	PLA	2	53	9.1	High
TRINITY_DN125521_c0_g2_i1_orf1	serpin transcript 1A2 [Ostrinia nubilalis]	3.6682809	1.875104107	0.001787	up	yes	3.03	0.826	0.74	0.769	0.969	3.607	2.698	2.786	------	------	------	------	------	------	------	------	------	------	------	CYT	1	35	8.1	High
TRINITY_DN3715_c0_g1_i2_orf1	uncharacterized protein LOC114356437 isoform X1 [Ostrinia furnacalis]	0.3045564	-1.715218887	5.95E-05	down	yes	0.889	2.919	2.845	3.143	2.77	0.869	0.888	0.91	------	------	------	------	------	------	------	------	PF00041.24	fn3	Fibronectin type III domain	CYT	3	19	19.1	High
TRINITY_DN18502_c0_g1_i1_orf1	uncharacterized protein LOC114359515 [Ostrinia furnacalis]	0.1725722	-2.534728198	1.73E-05	down	yes	0.526	3.048	3.119	3.18	2.846	0.504	0.562	0.512	------	------	------	------	------	------	ENOG41117F9	S:Function unknown	------	------	------	CYT	6	15	59.7	High
TRINITY_DN38431_c0_g1_i1_orf1	neprilysin-2 isoform X1 [Ostrinia furnacalis]	0.4192906	-1.253977766	1.71E-06	down	yes	1.052	2.509	2.574	2.472	2.48	1.067	1.05	1.04	BP:nitrogen compound metabolic process;BP:organic substance metabolic process;BP:proteolysis;BP:biological_process;BP:metabolic process;BP:primary metabolic process;BP:organonitrogen compound metabolic process;BP:protein metabolic process;BP:macromolecule metabolic process;CC:integral component of membrane;CC:membrane;CC:intrinsic component of membrane;CC:cellular anatomical entity;CC:plasma membrane;CC:cellular_component;MF:endopeptidase activity;MF:molecular_function;MF:binding;MF	GO:0006807;GO:0071704;GO:0006508;GO:0008150;GO:0008152;GO:0044238;GO:1901564;GO:0019538;GO:0043170;GO:0016021;GO:0016020;GO:0031224;GO:0110165;GO:0005886;GO:0005575;GO:0004175;GO:0003674;GO:0005488;GO:0008233;GO:0008237;GO:0043169;GO:0043167;GO:0046872;GO:0016787;GO:0004222;GO:0140096;GO:0003824	K08635	MMEL1, NEP	------	------	COG3590	O:Posttranslational modification, protein turnover, chaperones	PF05649.16;PF01431.24	Peptidase_M13_N;Peptidase_M13	Peptidase family M13;Peptidase family M13	CYT	10	16	87.5	High
TRINITY_DN218_c0_g1_i1_orf1	altered inheritance of mitochondria protein 3-like isoform X2 [Ostrinia furnacalis]	0.361653	-1.467322146	0.0003323	down	yes	0.9233	2.553	2.577	2.732	2.349	0.877	1.095	0.798	MF:molecular_function;MF:structural constituent of cuticle;MF:structural molecule activity;	GO:0003674;GO:0042302;GO:0005198	------	------	------	------	ENOG4111QXK	S:Function unknown	PF00379.26	Chitin_bind_4	Insect cuticle protein	CYT	2	10	18	High
TRINITY_DN1230_c1_g1_i5_orf1	uncharacterized protein LOC114353440 [Ostrinia furnacalis]	2.1944709	1.133873156	0.0001471	up	yes	2.302	1.049	1.046	0.898	1.204	2.299	2.287	2.321	------	------	------	------	------	------	ENOG4110QI3;ENOG4110SZS;ENOG4110QPA;ENOG4110T7I;ENOG41112QX	S:Function unknown;	------	------	------	CYT	1	3	35.6	High
TRINITY_DN3833_c0_g1_i4_orf1	division abnormally delayed protein [Ostrinia furnacalis]	0.4313388	-1.213106646	2.46E-05	down	yes	1.002	2.323	2.397	2.283	2.29	1.018	1.074	0.914	BP:regulation of smoothened signaling pathway;BP:positive regulation of canonical Wnt signaling pathway;BP:positive regulation of response to stimulus;BP:regulation of response to stimulus;BP:positive regulation of signaling;BP:regulation of Wnt signaling pathway;BP:positive regulation of smoothened signaling pathway;BP:regulation of signaling;BP:positive regulation of cell communication;BP:regulation of cell communication;BP:regulation of biological process;BP:regulation of cellular response to growth factor stimulus;BP:positive regulation of	GO:0008589;GO:0090263;GO:0048584;GO:0048583;GO:0023056;GO:0030111;GO:0045880;GO:0023051;GO:0010647;GO:0010646;GO:0050789;GO:0090287;GO:0090100;GO:0009966;GO:0009967;GO:0065007;GO:0048518;GO:0090092;GO:0050794;GO:0008150;GO:0030513;GO:0030510;GO:0030177;GO:0060828;GO:0048522;GO:0031012;GO:0046658;GO:0031226;GO:0031224;GO:0031225;GO:0110165;GO:0062023;GO:0030312;GO:0005575	K02306	DALLY	map04310;map04391	Wnt signaling pathway;Hippo signaling pathway - fly	ENOG410XST2	O:Posttranslational modification, protein turnover, chaperones	PF01153.22	Glypican	Glypican	CYT	2	3	60	High
TRINITY_DN147458_c0_g1_i1_orf1	60S ribosomal protein L5, partial [Cotesia chilonis]	0.2982531	-1.745391015	8.58E-06	down	yes	0.7	2.347	2.323	2.447	2.271	0.72	0.654	0.726	BP:cellular macromolecule biosynthetic process;BP:cellular amide metabolic process;BP:cellular biosynthetic process;BP:organic substance biosynthetic process;BP:cellular nitrogen compound biosynthetic process;BP:cellular macromolecule metabolic process;BP:peptide biosynthetic process;BP:organic substance metabolic process;BP:organonitrogen compound biosynthetic process;BP:macromolecule metabolic process;BP:amide biosynthetic process;BP:cellular process;BP:biosynthetic	GO:0034645;GO:0043603;GO:0044249;GO:1901576;GO:0044271;GO:0044260;GO:0043043;GO:0071704;GO:1901566;GO:0043170;GO:0043604;GO:0009987;GO:0009058;GO:0009059;GO:0008150;GO:0008152;GO:1901564;GO:0044238;GO:0019538;GO:0034641;GO:0044237;GO:0006807;GO:0006518;GO:0006412;GO:0043229;GO:0043228;GO:0043227;GO:0043226;GO:0005737;GO:0005634;GO:0110165;GO:0043231;GO:0032991;GO:0005840;GO:0043232;GO:1990904;GO:0005575;GO:0003674;GO:0005488;GO:0003676;GO:0005198;GO:1901363;GO:0003735;GO:0008097;GO:0097159;GO:0019843;GO:0003723	K02932	RP-L5e, RPL5	map03010;map05171	Ribosome;Coronavirus disease - COVID-19	COG0256	J:Translation, ribosomal structure and biogenesis	PF17144.7;PF14204.9	Ribosomal_L5e;Ribosomal_L18_c	Ribosomal large subunit proteins 60S L5, and 50S L18;Ribosomal L18 C-terminal region	CYT	1	13	37	High
TRINITY_DN4688_c0_g1_i2_orf1	uncharacterized protein LOC114359411 [Ostrinia furnacalis]	0.4935015	-1.01887354	2.17E-05	down	yes	1.291	2.616	2.531	2.678	2.64	1.234	1.362	1.276	------	------	------	------	------	------	ENOG4110SAV	S:Function unknown	------	------	------	CYT	6	10	71.4	High
TRINITY_DN83374_c0_g1_i1_orf1	uncharacterized protein LOC114350302, partial [Ostrinia furnacalis]	0.3769869	-1.407413705	4.99E-05	down	yes	0.8633	2.29	2.415	2.282	2.173	0.839	0.927	0.824	------	------	------	------	------	------	ENOG410Y20T	S:Function unknown	------	------	------	CYT	2	7	63	High
TRINITY_DN467_c0_g3_i1_orf1	histone-lysine N-methyltransferase 2B-like, partial [Ostrinia furnacalis]	0.3088814	-1.694874904	8.84E-06	down	yes	0.7373	2.387	2.413	2.42	2.329	0.727	0.828	0.657	MF:molecular_function;MF:structural constituent of cuticle;MF:structural molecule activity;	GO:0003674;GO:0042302;GO:0005198	------	------	------	------	ENOG4111I81	S:Function unknown	PF00379.26	Chitin_bind_4	Insect cuticle protein	CYT	7	19	58.3	High
TRINITY_DN206_c0_g1_i8_orf1	A-kinase anchor protein 200-like [Ostrinia furnacalis] >XP_028173114.1 A-kinase anchor protein 200-like [Ostrinia furnacalis] >XP_028173115.1 A-kinase anchor protein 200-like [Ostrinia furnacalis]	0.3363044	-1.572160345	0.0004502	down	yes	0.981	2.917	2.823	3.265	2.663	0.97	1.041	0.932	------	------	------	------	------	------	ENOG4110RAQ	S:Function unknown	------	------	------	CYT	1	15	34.3	High
TRINITY_DN66453_c0_g1_i4_orfp1	TRINITY_DN66453_c0_g1_i4_m.7345 TRINITY_DN66453_c0_g1::TRINITY_DN66453_c0_g1_i4::g.7345 ORF type:internal len:93 (+),score=24.53 TRINITY_DN66453_c0_g1_i4:3-278(+)	2.5910419	1.373532329	8.86E-05	up	yes	2.661	1.027	1.044	1.008	1.029	2.584	2.862	2.537	------	------	------	------	------	------	------	------	------	------	------	CYT	1	21	9.7	High
TRINITY_DN26985_c0_g1_i5_orf1	secretory phospholipase A2 receptor-like [Helicoverpa zea]	0.2614696	-1.93528487	3.14E-07	down	yes	0.701	2.681	2.692	2.669	2.683	0.759	0.666	0.678	MF:molecular_function;MF:carbohydrate binding;MF:binding;	GO:0003674;GO:0030246;GO:0005488	------	------	------	------	ENOG410XPJ1;ENOG410XRS3	S:Function unknown;U:Intracellular trafficking, secretion, and vesicular transport	------	------	------	CYT	2	26	9.8	High

TRINITY_DN5696_c0_g1_i4_orf1	serine protease snake-like isoform X1 [Ostrinia furnacalis]	2.1576923	1.109489148	0.001685	up	yes	2.244	1.04	1.029	1.015	1.077	1.935	2.333	2.465	BP:organic substance metabolic process;BP:organonitrogen compound metabolic process;BP:protein metabolic process;BP:macromolecule metabolic process;BP:biological_process;BP:nitrogen compound metabolic process;BP:metabolic process;BP:proteolysis;BP:primary metabolic process;MF:serine-type endopeptidase activity;MF:endopeptidase activity;MF:hydrolase activity;MF:serine hydrolase activity;MF:catalytic activity, acting on a protein;MF:catalytic activity;MF:molecular_function;MF:peptidase activity;MF:serine-type	GO:0071704;GO:1901564;GO:0019538;GO:0043170;GO:0008150;GO:0006807;GO:0008152;GO:0006508;GO:0044238;GO:0004252;GO:0004175;GO:0016787;GO:0017171;GO:0140096;GO:0003824;GO:0003674;GO:0008233;GO:0008236	K20677;K09641	HAYAN;TMPRSS11D	map05164	Influenza A	COG5640	O:Posttranslational modification, protein turnover, chaperones	PF00089.29	Trypsin	Trypsin	CYT	1	3	43.2	High
TRINITY_DN12336_c0_g1_i1_orfp1	TRINITY_DN12336_c0_g1_i1_m.30792 TRINITY_DN12336_c0_g1::TRINITY_DN12336_c0_g1_i1::g.30792 ORF type:internal len:86 (-),score=13.56 TRINITY_DN12336_c0_g1_i1:3-257(-)	2.4859076	1.313772646	6.91E-06	up	yes	2.205	0.887	0.93	0.824	0.907	2.262	2.187	2.167	------	------	------	------	------	------	------	------	------	------	------	CYT	4	51	9.3	High
TRINITY_DN7123_c0_g1_i1_orf1	activating signal cointegrator 1 complex subunit 2 homolog isoform X1 [Ostrinia furnacalis]	0.2885658	-1.793027628	1.22E-06	down	yes	0.8833	3.061	3.068	3.135	2.981	0.86	0.887	0.903	MF:molecular_function;MF:structural constituent of cuticle;MF:structural molecule activity;	GO:0003674;GO:0042302;GO:0005198	------	------	------	------	ENOG4111QXK	S:Function unknown	------	------	------	CYT	5	46	17.6	High
TRINITY_DN28501_c0_g1_i2_orfp1	TRINITY_DN28501_c0_g1_i2_m.58934 TRINITY_DN28501_c0_g1::TRINITY_DN28501_c0_g1_i2::g.58934 ORF type:internal len:98 (+),score=13.70 TRINITY_DN28501_c0_g1_i2:3-293(+)	4.3087846	2.107280989	1.80E-06	up	yes	3.095	0.7183	0.765	0.688	0.702	3.149	2.994	3.141	------	------	------	------	------	------	------	------	------	------	------	CYT	1	30	10.9	High
TRINITY_DN10222_c0_g1_i2_orf1	glutathione S-transferase sigma 3 [Ostrinia furnacalis]	2.2331915	1.159106963	2.45E-05	up	yes	2.624	1.175	1.155	1.082	1.289	2.633	2.578	2.661	MF:catalytic activity;MF:transferase activity;MF:glutathione transferase activity;MF:molecular_function;MF:transferase activity, transferring alkyl or ary;	GO:0003824;GO:0016740;GO:0004364;GO:0003674;GO:0016765	K04097	HPGDS	map00590;map00480;map05204;map00982;map00980	Arachidonic acid metabolism;Glutathione metabolism;Chemical carcinogenesis - DNA adducts;Drug metabolism - cytochrome P450;Metabolism of xenobiotics by cytochrome P450	ENOG41103AU;ENOG4110YU0;ENOG4111I69;ENOG4111VAU	O:Posttranslational modification, protein turnover, chaperones;	PF14497.9;PF02798.23;PF00043.28	GST_C_3;GST_N;GST_C	Glutathione S-transferase, C-terminal domain;Glutathione S-transferase, N-terminal domain;Glutathione S-transferase, C-terminal domain	CYT	14	58	22.9	High
TRINITY_DN37585_c0_g1_i1_orf1	cuticle protein 19.8-like [Ostrinia furnacalis]	0.4991015	-1.002594776	0.002028	down	yes	1.111	2.226	2.305	2.349	2.024	0.902	1.311	1.121	------	------	------	------	------	------	------	------	------	------	------	EXC	1	10	17.7	High
TRINITY_DN48694_c0_g1_i1_orfp1	TRINITY_DN48694_c0_g1_i1_m.75338 TRINITY_DN48694_c0_g1::TRINITY_DN48694_c0_g1_i1::g.75338 ORF type:internal len:84 (+),score=16.02 TRINITY_DN48694_c0_g1_i1:2-250(+)	0.0892226	-3.48644676	1.93E-05	down	yes	0.303	3.396	3.523	3.429	3.237	0.258	0.155	0.496	------	------	------	------	------	------	------	------	------	------	------	CYT	1	13	9.1	Medium
TRINITY_DN3616_c0_g2_i2_orf1	conotoxin ArMKLT2-032-like [Ostrinia furnacalis]	2.3231912	1.216107899	0.001827	up	yes	2.283	0.9827	1.132	0.833	0.983	2.292	2.546	2.011	CC:cellular_component;CC:integral component of membrane;CC:cellular anatomical entity;CC:intrinsic component of membrane;	GO:0005575;GO:0016021;GO:0110165;GO:0031224	------	------	------	------	------	------	------	------	------	CYT	1	64	8.7	High
TRINITY_DN206_c0_g1_i11_orf1	A-kinase anchor protein 200-like [Ostrinia furnacalis] >XP_028173114.1 A-kinase anchor protein 200-like [Ostrinia furnacalis] >XP_028173115.1 A-kinase anchor protein 200-like [Ostrinia furnacalis]	0.4021325	-1.314257082	1.16E-05	down	yes	1.056	2.626	2.547	2.697	2.633	1.007	1.134	1.027	------	------	------	------	------	------	ENOG4110RAQ	S:Function unknown	------	------	------	CYT	3	12	67.6	High
TRINITY_DN668_c0_g1_i4_orf1	fatty acid synthase-like isoform X1 [Ostrinia furnacalis]	0.3269512	-1.61285269	4.31E-07	down	yes	0.8043	2.46	2.495	2.409	2.477	0.791	0.815	0.807	------	------	K00665	FASN	map04910;map04936;map00061;map04152	Insulin signaling pathway;Alcoholic liver disease;Fatty acid biosynthesis;AMPK signaling pathway	COG3321	Q:Secondary metabolites biosynthesis, transport and catabolism	PF00698.24;PF00109.29;PF08659.13;PF02801.25;PF16197.8;PF00107.29;PF14765.9;PF13602.9;PF00975.23;PF00550.28;PF00106.28	Acyl_transf_1;ketoacyl-synt;KR;Ketoacyl-synt_C;KAsynt_C_assoc;ADH_zinc_N;PS-DH;ADH_zinc_N_2;Thioesterase;PP-binding;adh_short	Acyl transferase domain;Beta-ketoacyl synthase, N-terminal domain;KR domain;Beta-ketoacyl synthase, C-terminal domain;Ketoacyl-synthetase C-terminal extension;Zinc-binding dehydrogenase;Polyketide synthase dehydratase;Zinc-binding dehydrogenase;Thioesterase domain;Phosphopantetheine attachment site;short chain dehydrogenase	CYT	55	29	268.3	High
TRINITY_DN13648_c0_g1_i6_orf1	neurexin-4 [Ostrinia furnacalis]	0.4757914	-1.071598797	4.56E-05	down	yes	1.022	2.148	2.153	2.056	2.235	0.995	1.08	0.99	------	------	K07380	CNTNAP2	map04514	Cell adhesion molecules	COG2091;ENOG410XPHG	H:Coenzyme transport and metabolism;S:Function unknown	PF02210.27;PF00054.26;PF00754.28;PF13385.9;PF00008.30	Laminin_G_2;Laminin_G_1;F5_F8_type_C;Laminin_G_3;EGF	Laminin G domain;Laminin G domain;F5/8 type C domain;Concanavalin A-like lectin/glucanases superfamily;EGF-like domain	CYT	5	4	142.6	High
TRINITY_DN12586_c0_g1_i4_orf1	zonadhesin-like isoform X4 [Ostrinia furnacalis]	2.8514007	1.511670807	6.75E-05	up	yes	2.341	0.821	0.682	0.853	0.928	2.436	2.324	2.264	------	------	------	------	------	------	ENOG4110RF5;COG4826	S:Function unknown;O:Posttranslational modification, protein turnover, chaperones	PF01826.20	TIL	Trypsin Inhibitor like cysteine rich domain	PLA	1	26	14.3	High
TRINITY_DN261_c0_g1_i5_orfp1	TRINITY_DN261_c0_g1_i5_m.18559 TRINITY_DN261_c0_g1::TRINITY_DN261_c0_g1_i5::g.18559 ORF type:internal len:190 (+),score=69.81 TRINITY_DN261_c0_g1_i5:3-569(+)	0.4609746	-1.117240967	0.001807	down	yes	1.069	2.319	2.4	2.555	2.002	1.017	1.041	1.149	------	------	------	------	------	------	------	------	------	------	------	CYT	2	11	18.7	High
TRINITY_DN394_c0_g1_i4_orf1	uncharacterized protein LOC114351483 [Ostrinia furnacalis]	2.3958333	1.26052755	0.001357	up	yes	2.53	1.056	0.901	0.989	1.277	2.67	2.684	2.237	MF:molecular_function;MF:structural constituent of cuticle;MF:structural molecule activity;	GO:0003674;GO:0042302;GO:0005198	------	------	------	------	ENOG41113X7	S:Function unknown	PF00379.26	Chitin_bind_4	Insect cuticle protein	CYT	1	19	12.6	High
TRINITY_DN1196_c0_g1_i4_orf1	glucosamine-6-phosphate isomerase isoform X2 [Ostrinia furnacalis]	0.4917391	-1.024034932	0.00126	down	yes	1.131	2.3	2.495	2.195	2.21	1.293	0.931	1.169	------	------	K02564	nagB, GNPDA	map00520	Amino sugar and nucleotide sugar metabolism	COG0363	S:Function unknown	PF01182.23	Glucosamine_iso	Glucosamine-6-phosphate isomerases/6-phosphogluconolactonase	CYT	1	45	30.4	High
TRINITY_DN10304_c0_g2_i1_orf1	glycine-rich cell wall structural protein [Ostrinia furnacalis]	0.352869	-1.502795342	5.51E-05	down	yes	0.8917	2.527	2.562	2.642	2.378	0.825	0.979	0.871	------	------	------	------	------	------	ENOG4111PWE;ENOG4111WYB	S:Function unknown;	PF03103.20	DUF243	Domain of unknown function	CYT	2	16	27.6	High

TRINITY_DN2058_c0_g1_i2_orf1	proteasomal ubiquitin receptor ADRM1 [Ostrinia furnacalis]	0.4213152	-1.247028154	0.002858	down	yes	0.929	2.205	2.531	2.14	1.945	1.089	0.767	0.931	CC:cytoplasm;CC:macromolecular complex;CC:catalytic complex;CC:membrane-bounded organelle;CC:nucleus;CC:intracellular organelle;CC:cellular_component;CC:peptidase complex;CC:endopeptidase complex;CC:proteasome complex;CC:organelle;CC:cellular anatomical entity;CC:intracellular membrane-bounded organelle;	GO:0005737;GO:0032991;GO:1902494;GO:0043227;GO:0005634;GO:0043229;GO:0005575;GO:1905368;GO:1905369;GO:0000502;GO:0043226;GO:0110165;GO:0043231	K06691	RPN13	map03050;map05169;map05012;map05010;map05016;map05017;map05014;map05022;map05020	Proteasome;Epstein-Barr virus infection;Parkinson disease;Alzheimer disease;Huntington disease;Spinocerebellar ataxia;Amyotrophic lateral sclerosis;Pathways of neurodegeneration - multiple diseases;Prion disease	ENOG410XSJJ	O:Posttranslational modification, protein turnover, chaperones	PF16550.8	RPN13_C	UCH-binding domain	CYT	1	10	13.8	High
TRINITY_DN31619_c0_g1_i2_orf1	endocuticle structural glycoprotein ABD-4-like [Ostrinia furnacalis]	0.2676386	-1.901641872	0.0005253	down	yes	0.6517	2.435	2.45	2.714	2.14	0.539	0.702	0.714	MF:molecular_function;MF:structural constituent of cuticle;MF:structural molecule activity;	GO:0003674;GO:0042302;GO:0005198	------	------	------	------	ENOG4111ZVI	S:Function unknown	PF00379.26	Chitin_bind_4	Insect cuticle protein	CYT	1	7	14.7	High
TRINITY_DN14009_c0_g1_i1_orf1	proline-rich extensin-like protein EPR1 [Manduca sexta]	0.4336488	-1.205400843	6.51E-06	down	yes	0.9163	2.113	2.069	2.113	2.157	0.858	0.947	0.944	------	------	------	------	------	------	ENOG4111PWE	S:Function unknown	PF03103.20	DUF243	Domain of unknown function	CYT	3	12	26.8	High
TRINITY_DN1407_c0_g1_i12_orf1	hypothetical protein evm_012298 [Chilo suppressalis]	0.3972404	-1.331915821	1.56E-05	down	yes	1.094	2.754	2.674	2.861	2.728	1.02	1.134	1.129	BP:lipid transport;BP:transport;BP:organic substance transport;BP:biological_process;BP:establishment of localization;BP:localization;CC:cellular_component;CC:extracellular region;CC:cellular anatomical entity;MF:lipid binding;MF:molecular_function;MF:binding;	GO:0006869;GO:0006810;GO:0071702;GO:0008150;GO:0051234;GO:0051179;GO:0005575;GO:0005576;GO:0110165;GO:0008289;GO:0003674;GO:0005488	------	------	------	------	------	------	PF07464.14	ApoLp-III	Apolipophorin-III precursor	CYT	3	29	8	High
TRINITY_DN140_c0_g1_i5_orf1	calcyphosin-like protein isoform X4 [Helicoverpa armigera]	2.8315867	1.501610728	9.42E-07	up	yes	2.675	0.9447	0.902	0.967	0.965	2.692	2.711	2.622	MF:cation binding;MF:ion binding;MF:molecular_function;MF:binding;MF:metal ion binding;MF:calcium ion binding;	GO:0043169;GO:0043167;GO:0003674;GO:0005488;GO:0046872;GO:0005509	K23909	CAPS	------	------	ENOG410XRMJ	T:Signal transduction mechanisms	PF13405.9;PF00036.35;PF13499.9;PF13202.9;PF13833.9	EF-hand_6;EF-hand_1;EF-hand_7;EF-hand_5;EF-hand_8	EF-hand domain;EF hand;EF-hand domain pair;EF hand;EF-hand domain pair	CYT	2	73	12	High
TRINITY_DN5757_c0_g1_i1_orf1	ATP-dependent DNA helicase 2 subunit 1 [Ostrinia furnacalis]	0.4283981	-1.222976154	0.004799	down	yes	1.059	2.472	2.133	2.329	2.953	1.026	1.126	1.026	BP:nucleic acid metabolic process;BP:cellular nitrogen compound metabolic process;BP:nitrogen compound metabolic process;BP:organic cyclic compound metabolic process;BP:nucleobase-containing compound metabolic process;BP:cellular response to stimulus;BP:double-strand break repair via nonhomologous end joining;BP:double-strand break repair;BP:cellular macromolecule metabolic process;BP:cellular component organization;BP:organic substance metabolic process;BP:cellular component	GO:0090304;GO:0034641;GO:0006807;GO:1901360;GO:0006139;GO:0051716;GO:0006303;GO:0006302;GO:0044260;GO:0016043;GO:0071704;GO:0071840;GO:0000723;GO:0006281;GO:0009987;GO:0006725;GO:0006974;GO:0006950;GO:0008150;GO:0008152;GO:0006310;GO:0046483;GO:0006996;GO:0044238;GO:0051276;GO:0050896;GO:0032200;GO:0044237;GO:0043170;GO:0033554;GO:0006259;GO:0043564;GO:0032991;GO:0005575;GO:0016787;GO:0035639;GO:0036094;GO:0003674;GO:0005488;GO:0003676;GO:0003677;GO:0003678;GO:0043167;GO:1901363;GO:0043565;GO:0000166;GO:0032555;GO:0017076;GO:0008094;GO:0005524;GO:0042162;GO:0140097;GO:1901265;GO:0004386;GO:0003824;GO:0030554;GO:0097367;GO:0097159;GO:0043168;GO:0032559;GO:0003684;GO:0032553	K10884	XRCC6, KU70, G22P1	map03450	Non-homologous end-joining	ENOG410XNXU	L:Replication, recombination and repair	PF02735.19;PF03731.18;PF03730.17	Ku;Ku_N;Ku_C	Ku70/Ku80 beta-barrel domain;Ku70/Ku80 N-terminal alpha/beta domain;Ku70/Ku80 C-terminal arm	CYT	1	2	66.8	High
TRINITY_DN1593_c0_g1_i1_orf1	chemosensory protein csp11 [Helopeltis theivora]	2.0785047	1.055545991	8.13E-06	up	yes	2.224	1.07	1.048	1.072	1.09	2.2	2.298	2.175	------	------	------	------	------	------	ENOG4110RV8;ENOG410YU7V;ENOG4110R3D	S:Function unknown;	PF03392.16	OS-D	Insect pheromone-binding family, A10/OS-D	CYT	8	39	15.4	High
TRINITY_DN1196_c0_g1_i5_orf1	glucosamine-6-phosphate isomerase isoform X1 [Ostrinia furnacalis]	0.496	-1.011587974	6.18E-05	down	yes	1.054	2.125	2.226	2.108	2.041	1.021	1.03	1.11	BP:primary metabolic process;BP:carbohydrate metabolic process;BP:amino sugar metabolic process;BP:carbohydrate derivative metabolic process;BP:N-acetylglucosamine metabolic process;BP:organic substance metabolic process;BP:biological_process;BP:metabolic process;BP:glucosamine-containing compound metabolic process;CC:cytoplasm;CC:cellular_component;CC:cellular anatomical entity;MF:isomerase activity;MF:catalytic activity;MF:hydrolase activity;MF:glucosamine-6-phosphate deaminase activity;MF:deaminase activity;MF:intramolecular oxidoreductase	GO:0044238;GO:0005975;GO:0006040;GO:1901135;GO:0006044;GO:0071704;GO:0008150;GO:0008152;GO:1901071;GO:0005737;GO:0005575;GO:0110165;GO:0016853;GO:0003824;GO:0016787;GO:0004342;GO:0019239;GO:0016861;GO:0003674;GO:0016860	K02564	nagB, GNPDA	map00520	Amino sugar and nucleotide sugar metabolism	COG0363	G:Carbohydrate transport and metabolism	PF01182.23	Glucosamine_iso	Glucosamine-6-phosphate isomerases/6-phosphogluconolactonase	CYT	1	45	31.2	High
TRINITY_DN5880_c0_g2_i2_orf1	macrophage mannose receptor 1 [Bombyx mori]	2.1149653	1.080634002	0.0002624	up	yes	2.134	1.009	1.013	1.029	0.984	2.178	2.266	1.958	BP:detection of biotic stimulus;BP:detection of chemical stimulus;BP:positive regulation of response to external stimulus;BP:regulation of response to external stimulus;BP:positive regulation of response to stimulus;BP:regulation of response to biotic stimulus;BP:positive regulation of response to biotic stimulus;BP:positive regulation of defense response;BP:defense response to bacterium;BP:detection of molecule of bacterial origin;BP:regulation of biological process;BP:regulation of defense response;BP:response to bacterial	GO:0009595;GO:0009593;GO:0032103;GO:0032101;GO:0048584;GO:0002831;GO:0002833;GO:0031349;GO:0042742;GO:0032490;GO:0050789;GO:0031347;GO:0032493;GO:0043207;GO:0009617;GO:0044419;GO:0002684;GO:0065007;GO:0048518;GO:0002682;GO:0098581;GO:0045089;GO:0045088;GO:0048583;GO:0045087;GO:0006952;GO:0006950;GO:0008150;GO:0006955;GO:0042221;GO:0051707;GO:0010033;GO:0050776;GO:0009607;GO:0051606;GO:0009605;GO:0080134;GO:0042494;GO:0050778;GO:0002376;GO:0002218;GO:0002237;GO:0002253;GO:0098542;GO:0050896;GO:0140367	------	------	------	------	ENOG410XRS3;ENOG410XPJ1;ENOG410ZPTE;ENOG410ZRYS;ENOG410XNWT	U:Intracellular trafficking, secretion, and vesicular transport;S:Function unknown;S:Function unknown;S:Function unknown;S:Function unknown	PF00059.24	Lectin_C	Lectin C-type domain	CYT	3	11	38.3	High

TRINITY_DN25345_c0_g1_i1_orf1	chromodomain-helicase-DNA-binding protein 1 isoform X3 [Ostrinia furnacalis]	0.3363995	-1.571752644	0.0003121	down	yes	0.768	2.283	2.244	2.516	2.089	0.698	0.825	0.781	CC:intracellular organelle;CC:membrane-bounded organelle;CC:organelle;CC:nucleus;CC:intracellular membrane-bounded organelle;CC:cellular_component;CC:cellular anatomical entity;MF:purine ribonucleoside triphosphate binding;MF:heterocyclic compound binding;MF:molecular_function;MF:binding;MF:DNA helicase activity;MF:ion binding;MF:nucleoside phosphate binding;MF:anion binding;MF:nucleotide binding;MF:purine nucleotide binding;MF:ATP binding;MF:hydrolase activity;MF:catalytic activity, acting on	GO:0043229;GO:0043227;GO:0043226;GO:0005634;GO:0043231;GO:0005575;GO:0110165;GO:0035639;GO:1901363;GO:0003674;GO:0005488;GO:0003678;GO:0043167;GO:1901265;GO:0043168;GO:0000166;GO:0017076;GO:0005524;GO:0016787;GO:0140097;GO:0004386;GO:0036094;GO:0003824;GO:0008094;GO:0030554;GO:0097367;GO:0097159;GO:0032559;GO:0032555;GO:0003682;GO:0032553	K11367	CHD1	------	------	COG0553;ENOG410XNUT	U:Intracellular trafficking, secretion, and vesicular transport;L:Replication, recombination and repair	PF00176.26;PF18375.4;PF13907.9;PF00385.27;PF00271.34;PF04851.18	SNF2-rel_dom;CDH1_2_SANT_HL1;DUF4208;Chromo;Helicase_C;ResIII	SNF2-related domain;CDH1/2 SANT-Helical linker 1;Domain of unknown function;Chromo;Helicase conserved C-terminal domain;Type III restriction enzyme, res subunit	CYT	2	1	208.3	High
TRINITY_DN6642_c0_g1_i2_orf1	protein purity of essence [Ostrinia furnacalis]	2.0673719	1.047797925	0.01457	up	yes	2.013	0.9737	0.894	1.021	1.006	1.73	2.509	1.8	BP:nucleic acid metabolic process;BP:cellular nitrogen compound metabolic process;BP:nitrogen compound metabolic process;BP:organic cyclic compound metabolic process;BP:telomere maintenance;BP:nucleobase-containing compound metabolic process;BP:cellular response to stimulus;BP:cellular macromolecule metabolic process;BP:cellular component organization;BP:organic substance metabolic process;BP:cellular component organization or biogenesis;BP:organelle organization;BP:DNA repair;BP:cellular	GO:0090304;GO:0034641;GO:0006807;GO:1901360;GO:0000723;GO:0006139;GO:0051716;GO:0044260;GO:0016043;GO:0071704;GO:0071840;GO:0006996;GO:0006281;GO:0009987;GO:0006725;GO:0006974;GO:0006950;GO:0008150;GO:0008152;GO:0046483;GO:0006310;GO:0044238;GO:0051276;GO:0050896;GO:0032200;GO:0044237;GO:0043170;GO:0033554;GO:0006259;GO:0016787;GO:0035639;GO:0043169;GO:0008094;GO:0016817;GO:0036094;GO:0003674;GO:0008270;GO:0016887;GO:0003678;GO:0000166;GO:1901363;GO:0043167;GO:0017076;GO:0140097;GO:0046872;GO:0005524;GO:0005515;GO:0017111;GO:1901265;GO:0046914;GO:0003824;GO:0004386;GO:0032555;GO:0016818;GO:0030554;GO:0097367;GO:0097159;GO:0016462;GO:0032559;GO:0005516;GO:0043168;GO:0032553;GO:0005488	K10691	UBR4, ZUBR1	map05165;map05203	Human papillomavirus infection;Viral carcinogenesis	ENOG410XPP8	O:Posttranslational modification, protein turnover, chaperones	------	------	------	CYT	1	5	34.3	High
TRINITY_DN8095_c0_g1_i3_orf1	circadian clock-controlled protein-like [Ostrinia furnacalis]	2.2019815	1.138802324	0.000158	up	yes	2.067	0.9387	1.037	0.83	0.949	2.177	2.009	2.014	------	------	------	------	------	------	ENOG41114TE;ENOG4110QH9;ENOG410YTKH;ENOG41119GI;ENOG4110RJ7;ENOG4111E83	S:Function unknown;	PF06585.14	JHBP	Haemolymph juvenile hormone binding protein	CYT	4	18	27.1	High
TRINITY_DN13236_c0_g1_i4_orf1	zonadhesin-like isoform X4 [Ostrinia furnacalis]	2.3243638	1.2168359	0.0005295	up	yes	1.909	0.8213	0.78	0.868	0.816	2.108	1.862	1.757	------	------	------	------	------	------	COG4826;ENOG4110RF5	O:Posttranslational modification, protein turnover, chaperones;S:Function unknown	PF01826.20	TIL	Trypsin Inhibitor like cysteine rich domain	CYT	2	48	10.2	High
TRINITY_DN21930_c0_g1_i1_orf1	coactosin-like protein isoform X2 [Trichoplusia ni]	0.4994369	-1.001625572	0.0005751	down	yes	0.887	1.776	1.72	1.805	1.804	0.817	1.056	0.788	------	------	------	------	------	------	ENOG4111QQE	S:Function unknown	PF00241.23	Cofilin_ADF	Cofilin/tropomyosin-type actin-binding protein	CYT	1	5	29.8	High
TRINITY_DN15865_c0_g1_i1_orf1	carboxylesterase, partial [Ostrinia furnacalis]	0.485482	-1.042510295	8.33E-06	down	yes	0.836	1.722	1.759	1.733	1.674	0.809	0.868	0.831	MF:hydrolase activity;MF:molecular_function;MF:hydrolase activity, acting on ester bonds;MF:carboxylic ester hydrolase activity;MF:catalytic activity;	GO:0016787;GO:0003674;GO:0016788;GO:0052689;GO:0003824	K01049	ACHE	map00564;map04725	Glycerophospholipid metabolism;Cholinergic synapse	COG2272	O:Posttranslational modification, protein turnover, chaperones	PF00135.31;PF20434.1;PF07859.16	COesterase;BD-FAE;Abhydrolase_3	Carboxylesterase family;BD-FAE;alpha/beta hydrolase fold	PLA	4	14	38	High
TRINITY_DN64719_c0_g1_i2_orfp1	TRINITY_DN64719_c0_g1_i2_m.37745 TRINITY_DN64719_c0_g1::TRINITY_DN64719_c0_g1_i2::g.37745 ORF type:internal len:91 (+),score=41.89 TRINITY_DN64719_c0_g1_i2:1-270(+)	2.4787901	1.30963612	0.0009351	up	yes	2.016	0.8133	0.866	0.641	0.933	2.176	1.818	2.053	------	------	------	------	------	------	------	------	------	------	------	CYT	1	14	8.4	High
TRINITY_DN5191_c0_g2_i1_orf1	CD151 antigen-like [Ostrinia furnacalis]	2.195592	1.134609958	0.003191	up	yes	1.823	0.8303	0.867	0.63	0.994	1.939	1.593	1.937	CC:cellular_component;CC:integral component of membrane;CC:cellular anatomical entity;CC:intrinsic component of membrane;	GO:0005575;GO:0016021;GO:0110165;GO:0031224	K06537	CD151, TSPAN24	------	------	ENOG4111IRY;ENOG410ZTYS	S:Function unknown;	PF00335.23	Tetraspanin	Tetraspanin family	PLA	3	12	28.7	High
TRINITY_DN9455_c0_g1_i6_orf1	uncharacterized protein LOC114360866 isoform X4 [Ostrinia furnacalis]	0.4224973	-1.242985943	0.0002509	down	yes	0.785	1.858	1.878	1.914	1.782	0.68	0.937	0.738	CC:integral component of membrane;CC:cellular_component;CC:extracellular region;CC:intrinsic component of membrane;CC:cellular anatomical entity;MF:enzyme regulator activity;MF:peptidase inhibitor activity;MF:peptidase regulator activity;MF:molecular_function;MF:endopeptidase inhibitor activity;MF:serine-type endopeptidase inhibitor activity;MF:enzyme inhibitor activity;MF:molecular function regulator;MF:endopep	GO:0016021;GO:0005575;GO:0005576;GO:0031224;GO:0110165;GO:0030234;GO:0030414;GO:0061134;GO:0003674;GO:0004866;GO:0004867;GO:0004857;GO:0098772;GO:0061135	------	------	------	------	ENOG410XRYE;ENOG410XP6K	S:Function unknown;	PF00086.21;PF00014.26;PF00095.24;PF14625.9;PF02822.17	Thyroglobulin_1;Kunitz_BPTI;WAP;Lustrin_cystein;Antistasin	Thyroglobulin type-1 repeat;Kunitz/Bovine pancreatic trypsin inhibitor domain;WAP-type;Lustrin, cysteine-rich repeated domain;Antistasin family	CYT	1	1	158.2	High
TRINITY_DN110523_c0_g2_i1_orf1	uncharacterized protein LOC107036393 [Diachasma alloeum]	0.4104285	-1.284797188	0.0001837	down	yes	0.795	1.937	1.996	2.028	1.787	0.752	0.876	0.757	------	------	K22628	IL16	map04060	Cytokine-cytokine receptor interaction	ENOG4110362;ENOG410YQUY	O:Posttranslational modification, protein turnover, chaperones;S:Function unknown	PF00595.27;PF17820.4;PF13180.9;PF02163.25	PDZ;PDZ_6;PDZ_2;Peptidase_M50	PDZ domain;PDZ domain;PDZ domain;Peptidase family M50	CYT	1	3	49	High

TRINITY_DN18782_c0_g1_i4_orf1	putative riboflavin kinase [Ostrinia furnacalis] >XP_028176654.1 putative riboflavin kinase [Ostrinia furnacalis]	2.4327939	1.282614105	0.002089	up	yes	1.819	0.7477	0.709	0.659	0.875	1.575	2.046	1.835	[illegible] BP:small molecule biosynthetic process;BP:nucleotide metabolic process;BP:riboflavin biosynthetic process;BP:cellular biosynthetic process;BP:cellular nitrogen compound metabolic process;BP:nitrogen compound metabolic process;BP:small molecule metabolic process;BP:ribonucleoside monophosphate metabolic process;BP:nucleoside monophosphate metabolic process;BP:organic cyclic compound biosynthetic process;BP:ribonucleotide metabolic process;BP:nucleoside monophosphate biosynthetic [illegible]	GO:0044283;GO:0009117;GO:0009231;GO:0044249;GO:0034641;GO:0006807;GO:0044281;GO:0009161;GO:0009123;GO:1901362;GO:0009259;GO:0009124;GO:0006139;GO:0042727;GO:0042726;GO:0006771;GO:0009398;GO:0071704;GO:0046444;GO:1901360;GO:0018130;GO:0006767;GO:0009987;GO:0019693;GO:1901576;GO:0009156;GO:0006725;GO:0009260;GO:0009058;GO:0009110;GO:0008150;GO:0006766;GO:0008152;GO:0034654;GO:1901564;GO:0090407;GO:0055086;GO:0046483;GO:0044238;GO:0044271;GO:1901566;GO:1901135;GO:0046390;GO:0042364;GO:0044237;GO:1901137;GO:0006796;GO:0009165;GO:1901293;GO:0006793;GO:0019637;GO:0019438;GO:0006753;GO:0032553;GO:0035639;GO:0003674;GO:0043167;GO:0043168;GO:0097159;GO:0000166;GO:0016740;GO:0017076;GO:0097367;GO:0005524;GO:0016301;GO:1901265;GO:0036094;GO:0032555;GO:0030554;GO:0003824;GO:0016773;GO:0016772;GO:0008531;GO:0032559;GO:1901363;GO:0005488	K00861	RFK, FMN1	map00740;map01240	Riboflavin metabolism;Biosynthesis of cofactors	COG0196	H:Coenzyme transport and metabolism	PF01687.20	Flavokinase	Riboflavin kinase	CYT	1	7	18.2	High
TRINITY_DN867_c0_g1_i1_orf1	hemicentin-2-like isoform X1 [Ostrinia furnacalis]	0.3618301	-1.466615541	4.40E-05	down	yes	0.7157	1.978	2.101	1.95	1.884	0.742	0.716	0.689	BP:developmental process;BP:anatomical structure development;BP:biological_process;CC:integral component of membrane;CC:cellular_component;CC:intrinsic component of membrane;CC:cellular anatomical entity;	GO:0032502;GO:0048856;GO:0008150;GO:0016021;GO:0005575;GO:0031224;GO:0110165	K16680	ED	map04391	Hippo signaling pathway - fly	ENOG410ZJSM	S:Function unknown	PF13927.9;PF07679.19;PF13895.9;PF00047.28; PF07686.20;PF08205.15;PF00041.24	Ig_3;I-set;Ig_2;ig;V-set;C2-set_2;fn3	Immunoglobulin domain;Immunoglobulin I-set domain;Immunoglobulin domain;Immunoglobulin domain;Immunoglobulin V-set domain;CD80-like C2-set immunoglobulin domain;Fibronectin type III domain	CYT	1	1	138.3	High
TRINITY_DN9694_c0_g1_i1_orf1	larval cuticle protein LCP-17 [Helicoverpa armigera] >PZC82071.1 hypothetical protein B5X24_HaOG211161 [Helicoverpa armigera] >PZC87412.1 hypothetical protein B5X24_HaOG216859 [Helicoverpa armigera]	2.747801	1.458277511	1.09E-06	up	yes	2.093	0.7617	0.78	0.735	0.77	2.118	2.115	2.045	MF:molecular_function;MF:structural constituent of cuticle;MF:structural molecule activity;	GO:0003674;GO:0042302;GO:0005198	------	------	------	------	ENOG4110RUZ;ENOG4111PJ1;ENOG410ZNDD	S:Function unknown;	PF00379.26	Chitin_bind_4	Insect cuticle protein	CYT	5	82	15.7	High
TRINITY_DN38230_c0_g1_i4_orf1	hypothetical protein evm_007803 [Chilo suppressalis]	2.6588846	1.410821144	0.006937	up	yes	2.05	0.771	0.75	0.669	0.894	2.427	1.6	2.122	BP:organophosphate catabolic process;BP:nucleotide catabolic process;BP:cellular nitrogen compound metabolic process;BP:nitrogen compound metabolic process;BP:small molecule metabolic process;BP:organic cyclic compound metabolic process;BP:organic cyclic compound catabolic process;BP:nucleobase-containing compound metabolic process;BP:organic substance catabolic process;BP:organic substance metabolic process;BP:cellular catabolic process;BP:cellular process;BP:cellular aromatic compound [illegible]	GO:0046434;GO:0009166;GO:0034641;GO:0006807;GO:0044281;GO:1901360;GO:1901361;GO:0006139;GO:1901575;GO:0071704;GO:0044248;GO:0009987;GO:0006725;GO:0046700;GO:0019439;GO:0008150;GO:0008152;GO:0034655;GO:0009056;GO:0055086;GO:0046483;GO:0044238;GO:0044270;GO:0044237;GO:0006796;GO:1901292;GO:0006793;GO:0019637;GO:0009117;GO:0006753;GO:0110165;GO:0005575;GO:0016791;GO:0008252;GO:0042578;GO:0003674;GO:0000166;GO:1901363;GO:0043167;GO:1901265;GO:0046872;GO:0016787;GO:0043169;GO:0016788;GO:0036094;GO:0003824;GO:0097159;GO:0005488	------	------	------	------	COG0737	F:Nucleotide transport and metabolism	PF02872.21	5_nucleotid_C	5'-nucleotidase, C-terminal domain	CYT	1	17	12.1	High
TRINITY_DN59422_c0_g1_i2_orf1	larval cuticle protein LCP-22-like isoform X2 [Pectinophora gossypiella]	5.4376395	2.442980501	3.16E-06	up	yes	2.193	0.4033	0.393	0.386	0.431	2.267	2.203	2.108	MF:molecular_function;MF:structural constituent of cuticle;MF:structural molecule activity;	GO:0003674;GO:0042302;GO:0005198	------	------	------	------	ENOG4111CQ7;ENOG41119AX;ENOG4111DQA; ENOG410YZKX;ENOG4111PJ1;ENOG410YR66	S:Function unknown;	PF00379.26	Chitin_bind_4	Insect cuticle protein	CYT	4	38	19.5	High
TRINITY_DN10889_c0_g1_i8_orf1	four and a half LIM domains protein 2 isoform X7 [Pectinophora gossypiella]	2.8569297	1.514465553	6.61E-07	up	yes	1.915	0.6703	0.65	0.686	0.675	1.953	1.907	1.885	------	------	------	------	------	------	ENOG410XRFY;ENOG410XP0W;ENOG410YFZ1	S:Function unknown;K:Transcription	PF00412.25	LIM	LIM domain	CYT	19	58	39.3	High
TRINITY_DN4695_c0_g1_i3_orf1	glutathione S-transferase epsilon 3 [Ostrinia furnacalis]	4.0480117	2.01721347	0.0001974	up	yes	2.209	0.5457	0.574	0.535	0.528	2.436	1.999	2.193	MF:catalytic activity;MF:transferase activity;MF:glutathione transferase activity;MF:molecular_function;MF:transferase activity, transferring alkyl or ary;	GO:0003824;GO:0016740;GO:0004364;GO:0003674;GO:0016765	K00799	GST, gst	map05207;map00480;map05204;map00983;map00982;map00980;map05200;map05208;map05418;map05225;map04212;map01524	Chemical carcinogenesis - receptor activation;Glutathione metabolism;Chemical carcinogenesis - DNA adducts;Drug metabolism - other enzymes;Drug metabolism - cytochrome P450;Metabolism of xenobiotics by cytochrome P450;Pathways in cancer;Chemical carcinogenesis - reactive oxygen species;Fluid shear stress and atherosclerosis;Hepatocellular carcinoma;Longevity regulating pathway - worm;Platinum drug resistance	COG0625	S:Function unknown	PF13417.9;PF02798.23;PF00043.28;PF13409.9; PF14497.9;PF13410.9	GST_N_3;GST_N;GST_C;GST_N_2;GST_C_3;GST_C_2	Glutathione S-transferase, N-terminal domain;Glutathione S-transferase, N-terminal domain;Glutathione S-transferase, C-terminal domain;Glutathione S-transferase, N-terminal domain;Glutathione S-transferase, C-terminal domain;Glutathione S-transferase, C-terminal domain	CYT	2	9	24.8	High
TRINITY_DN57900_c0_g1_i2_orf1	hypothetical protein SFRURICE_000634 [Spodoptera frugiperda]	2.7036157	1.434890078	3.91E-06	up	yes	2.161	0.7993	0.794	0.81	0.794	2.233	2.148	2.102	MF:catalytic activity;MF:molecular_function;MF:dioxygenase activity;MF:oxidoreductase activity;	GO:0003824;GO:0003674;GO:0051213;GO:0016491	K00477	PHYH	map04146	Peroxisome	ENOG410XUSH	Q:Secondary metabolites biosynthesis, transport and catabolism	PF05721.16	PhyH	Phytanoyl-CoA dioxygenase	PLA	11	57	30.1	High

TRINITY_DN5893_c0_g1_i7_orf1	jupiter microtubule associated homolog 1-like [Ostrinia furnacalis] >XP_028173360.1 jupiter microtubule associated homolog 1-like [Ostrinia furnacalis]	0.4948454	-1.014950341	2.04E-05	down	yes	0.912	1.843	1.817	1.92	1.793	0.897	0.93	0.909	CC:cytoplasm;CC:supramolecular complex;CC:spindle;CC:supramolecular fiber;CC:intracellular membrane-bounded organelle;CC:intracellular non-membrane-bounded organelle;CC:microtubule;CC:nucleus;CC:cellular_component;CC:supramolecular polymer;CC:intracellular organelle;CC:non-membrane-bounded organelle;CC:membrane-bounded organelle;CC:organelle;CC:cellular anatomical entity;CC:polymeric cytoskeletal fiber;	GO:0005737;GO:0099080;GO:0005819;GO:0099512;GO:0043231;GO:0043232;GO:0005874;GO:0005634;GO:0005575;GO:0099081;GO:0043229;GO:0043228;GO:0043227;GO:0043226;GO:0110165;GO:0099513	------	------	------	------	ENOG410Y3IA	S:Function unknown	PF17054.8	JUPITER	Microtubule-Associated protein Jupiter	CYT	8	62	15.1	High
TRINITY_DN4123_c0_g1_i1_orf1	uncharacterized protein LOC114355030 [Ostrinia furnacalis]	2.2468466	1.167901652	0.002742	up	yes	1.906	0.8483	0.804	0.748	0.993	1.997	1.627	2.094	------	------	------	------	------	------	------	------	------	------	------	CYT	1	4	15.8	Medium
TRINITY_DN46090_c0_g2_i1_orf1	inactive tyrosine-protein kinase 7-like, partial [Ostrinia furnacalis]	0.4685125	-1.093840597	0.005659	down	yes	0.863	1.842	2.187	1.76	1.58	0.856	0.9	0.833	CC:intrinsic component of membrane;CC:integral component of membrane;CC:cellular anatomical entity;CC:cellular_component;MF:signaling receptor activity;MF:purine ribonucleoside triphosphate binding;MF:molecular transducer activity;MF:molecular_function;MF:binding;MF:ion binding;MF:heterocyclic compound binding;MF:anion binding;MF:transmembrane receptor protein tyrosine kinase activity;MF:nucleotide binding;MF:transferase activity;MF:protein tyrosine kinase activity;MF:protein kinase [illegible]	GO:0031224;GO:0016021;GO:0110165;GO:0005575;GO:0038023;GO:0035639;GO:0060089;GO:0003674;GO:0005488;GO:0043167;GO:1901363;GO:0043168;GO:0004714;GO:0000166;GO:0016740;GO:0004713;GO:0004672;GO:0017076;GO:0016301;GO:0140096;GO:1901265;GO:0036094;GO:0003824;GO:0030554;GO:0016773;GO:0016772;GO:0097367;GO:0004888;GO:0032559;GO:0005524;GO:0097159;GO:0032555;GO:0019199;GO:0032553	K05127	PTK7, CCK4	------	------	ENOG410Y8QG;ENOG410YGKQ;COG0515	T:Signal transduction mechanisms;	PF07714.20;PF00069.28	PK_Tyr_Ser-Thr;Pkinase	Protein tyrosine and serine/threonine kinase;Protein kinase domain	CYT	1	6	16.7	High
TRINITY_DN5126_c0_g2_i1_orf1	cytochrome P450 monooxygenase CYP4L47 [Ostrinia furnacalis]	4.3707934	2.127895183	9.68E-05	up	yes	2.143	0.4903	0.588	0.455	0.428	2.237	1.957	2.236	MF:cation binding;MF:monooxygenase activity;MF:heme binding;MF:transition metal ion binding;MF:tetrapyrrole binding;MF:organic cyclic compound binding;MF:oxidoreductase activity, acting on paired donors, with incorporation or reduction of molecular oxygen;MF:ion binding;MF:catalytic activity;MF:molecular_function;MF:binding;MF:iron ion binding;MF:metal ion binding;MF:heterocyclic compound binding;MF:oxidoreduc[illegible]	GO:0043169;GO:0004497;GO:0020037;GO:0046914;GO:0046906;GO:0097159;GO:0016705;GO:0043167;GO:0003824;GO:0003674;GO:0005488;GO:0005506;GO:0046872;GO:1901363;GO:0016491	K15001	CYP4	------	------	COG2124	I:Lipid transport and metabolism	PF00067.25	p450	Cytochrome P450	CYT	1	7	13	High
TRINITY_DN2109_c0_g1_i4_orf1	mucin-2-like isoform X2 [Ostrinia furnacalis]	0.3802157	-1.395110146	2.42E-06	down	yes	0.811	2.133	2.072	2.172	2.156	0.788	0.83	0.815	CC:cellular_component;CC:integral component of membrane;CC:cellular anatomical entity;CC:intrinsic component of membrane;	GO:0005575;GO:0016021;GO:0110165;GO:0031224	------	------	------	------	ENOG410Y7ID	S:Function unknown	------	------	------	EXC	3	2	236.3	High
TRINITY_DN19923_c0_g1_i1_orf1	uncharacterized protein LOC114350958 [Ostrinia furnacalis]	0.4031096	-1.310756041	8.45E-05	down	yes	0.8167	2.026	2.055	2.136	1.887	0.833	0.827	0.79	------	------	------	------	------	------	ENOG4111MP7	S:Function unknown	PF15430.9	SVWC	Single domain von Willebrand factor type C	CYT	7	71	16.2	High
TRINITY_DN1293_c0_g1_i4_orf1	putative fatty acyl-CoA reductase CG5065 [Ostrinia furnacalis]	0.4357788	-1.198331946	8.63E-07	down	yes	0.8197	1.881	1.889	1.891	1.862	0.856	0.807	0.796	BP:primary metabolic process;BP:lipid metabolic process;BP:fatty acid derivative metabolic process;BP:organic substance metabolic process;BP:biological_process;BP:metabolic process;CC:cellular_component;CC:cellular anatomical entity;CC:integral component of membrane;CC:intrinsic component of membrane;MF:fatty-acyl-CoA reductas;MF:alcohol-forming fatty acyl-CoA reductase activity;MF:catalytic activity;MF:oxidoreductase activity, acting on the aldehyde or oxo group of donors, NAD or NADP as acceptor;MF:oxidoreductase activity, acting on [illegible]	GO:0044238;GO:0006629;GO:1901568;GO:0071704;GO:0008150;GO:0008152;GO:0005575;GO:0110165;GO:0016021;GO:0031224;GO:0080019;GO:0102965;GO:0003824;GO:0016620;GO:0016903;GO:0003674;GO:0016491	K13356	FAR	map04146;map00073;map04212	Peroxisome;Cutin, suberine and wax biosynthesis;Longevity regulating pathway - worm	ENOG410XS7R	S:Function unknown	PF07993.15;PF03015.22;PF01370.24;PF16363.8;PF02719.18	NAD_binding_4;Sterile;Epimerase;GDP_Man_Dehyd;Polysacc_synt_2	Male sterility protein;Male sterility protein;NAD dependent epimerase/dehydratase family;GDP-mannose 4,6 dehydratase;Polysaccharide biosynthesis protein	CYT	3	7	59.2	High
TRINITY_DN14754_c0_g1_i6_orf1	cathepsin L [Papilio xuthus]	3.402206	1.766470496	9.57E-05	up	yes	2.19	0.6437	0.569	0.567	0.795	2.306	2.174	2.09	BP:primary metabolic process;BP:organonitrogen compound metabolic process;BP:protein metabolic process;BP:macromolecule metabolic process;BP:organic substance metabolic process;BP:nitrogen compound metabolic process;BP:biological_process;BP:metabolic process;BP:proteolysis;MF:molecular_function;MF:hydrolase activity;MF:catalytic activity;MF:catalytic activity, acting on a protein;MF:peptidase activity;MF:cysteine-type peptidase activity;	GO:0044238;GO:1901564;GO:0019538;GO:0043170;GO:0071704;GO:0006807;GO:0008150;GO:0008152;GO:0006508;GO:0003674;GO:0016787;GO:0003824;GO:0140096;GO:0008233;GO:0008234	------	------	------	------	COG4870	O:Posttranslational modification, protein turnover, chaperones	PF00112.26	Peptidase_C1	Papain family cysteine protease	CYT	1	41	12.8	High
TRINITY_DN20244_c0_g1_i1_orfp1	uncharacterized protein LOC125235519 [Leguminivora glycinivorella]	0.254161	-1.976185176	0.000993	down	yes	0.565	2.223	1.93	2.581	2.157	0.525	0.565	0.605	------	------	------	------	------	------	------	------	------	------	------	CYT	1	7	12.3	High

TRINITY_DN31225_c0_g1_i1_orf1	ribosome biogenesis protein BMS1 homolog [Ostrinia furnacalis]	2.4994041	1.321584146	2.74E-06	up	yes	2.097	0.839	0.792	0.897	0.828	2.084	2.119	2.088	BP:ribosome biogenesis;BP:cellular component organization or biogenesis;BP:ribonucleoprotein complex biogenesis;BP:cellular process;BP:biological_process;BP:cellular component biogenesis;CC:intracellular organelle;CC:non-membrane-bounded organelle;CC:organelle;CC:nucleolus;CC:cellular anatomical entity;CC:intracellular non-membrane-bounded organelle;CC:cellular_component;MF:purine ribonucleoside triphosphate binding;MF:heterocyclic compound binding;MF:molecular_function;MF:binding;MF:ion binding;MF:nucleoside [illegible]	GO:0042254;GO:0071840;GO:0022613;GO:0009987;GO:0008150;GO:0044085;GO:0043229;GO:0043228;GO:0043226;GO:0005730;GO:0110165;GO:0043232;GO:0005575;GO:0035639;GO:1901363;GO:0003674;GO:0005488;GO:0043167;GO:1901265;GO:0043168;GO:0000166;GO:0032561;GO:0017076;GO:0005525;GO:0036094;GO:0097367;GO:0097159;GO:0019001;GO:0032555;GO:0032553	K14569	BMS1	map03008	Ribosome biogenesis in eukaryotes	COG5192	J:Translation, ribosomal structure and biogenesis	PF04950.15;PF08142.15	RIBIOP_C;AARP2CN	40S ribosome biogenesis protein Tsr1 and BMS1 C-terminal;AARP2CN	CYT	2	2	124.6	High
TRINITY_DN2207_c0_g1_i4_orf1	methionine-R-sulfoxide reductase B1 isoform X4 [Pectinophora gossypiella] >XP_049887601.1 methionine-R-sulfoxide reductase B1 isoform X4 [Pectinophora gossypiella]	3.0429943	1.605491629	3.33E-05	up	yes	2.187	0.7187	0.669	0.662	0.825	2.114	2.277	2.17	------	------	K07305	msrB	------	------	COG0229	O:Posttranslational modification, protein turnover, chaperones	PF01641.21	SelR	SelR domain	CYT	1	67	5.3	High
TRINITY_DN58872_c0_g1_i1_orfp1	cuticle protein 64-like [Pectinophora gossypiella]	0.4888462	-1.032547593	0.002286	down	yes	0.8897	1.82	1.671	2.021	1.769	0.761	1.05	0.858	------	------	------	------	------	------	------	------	------	------	------	CYT	1	9	8.7	High
TRINITY_DN32586_c0_g2_i1_orf1	unnamed protein product [Euphydryas editha]	3.0133694	1.591377533	1.02E-05	up	yes	2.006	0.6657	0.6	0.713	0.684	2.057	2.022	1.94	MF:molecular_function;MF:carbohydrate binding;MF:binding;	GO:0003674;GO:0030246;GO:0005488	------	------	------	------	ENOG410XNMR;ENOG410XPJ1	S:Function unknown;	PF00059.24	Lectin_C	Lectin C-type domain	CYT	4	47	20.5	High
TRINITY_DN1868_c0_g1_i1_orf1	protein obstructor-E isoform X1 [Ostrinia furnacalis]	0.2261029	-2.144948336	3.95E-06	down	yes	0.492	2.176	2.199	2.244	2.086	0.482	0.482	0.512	------	------	------	------	------	------	ENOG410XQ49	S:Function unknown	PF01607.27	CBM_14	Chitin binding Peritrophin-A domain	CYT	16	68	30.6	High
TRINITY_DN48590_c0_g1_i1_orf1	acyl-CoA Delta(11) desaturase isoform X1 [Ostrinia furnacalis] >XP_028172999.1 acyl-CoA Delta(11) desaturase isoform X2 [Ostrinia furnacalis] >XP_028173000.1 acyl-CoA Delta(11) desaturase isoform X1 [Ostrinia furnacalis]	0.2488402	-2.006708488	7.93E-06	down	yes	0.5793	2.328	2.331	2.426	2.226	0.589	0.552	0.597	BP:fatty acid biosynthetic process;BP:fatty acid metabolic process;BP:carboxylic acid metabolic process;BP:cellular biosynthetic process;BP:small molecule metabolic process;BP:small molecule biosynthetic process;BP:monocarboxylic acid biosynthetic process;BP:organic substance biosynthetic process;BP:organic substance metabolic process;BP:lipid metabolic process;BP:cellular process;BP:monocarboxylic acid metabolic process;BP:biosynthetic process;BP:biological_process;BP:metabolic process;BP:oxoacid metabolic [illegible]	GO:0006633;GO:0006631;GO:0019752;GO:0044249;GO:0044281;GO:0044283;GO:0072330;GO:1901576;GO:0071704;GO:0006629;GO:0009987;GO:0032787;GO:0009058;GO:0008150;GO:0008152;GO:0043436;GO:0044255;GO:0008610;GO:0044238;GO:0006082;GO:0046394;GO:0016053;GO:0044237;GO:0031224;GO:0016021;GO:0110165;GO:0005575;GO:0003674;GO:0016491;GO:0016705;GO:0003824;GO:0016717	K00507	SCD, desC	map04936;map04152;map01040;map03320;map04212	Alcoholic liver disease;AMPK signaling pathway;Biosynthesis of unsaturated fatty acids;PPAR signaling pathway;Longevity regulating pathway - worm	COG1398	J:Translation, ribosomal structure and biogenesis	------	------	------	CYT	4	14	36.9	High
TRINITY_DN34786_c0_g1_i1_orf1	small heat shock protein Hsp29.7 [Ostrinia furnacalis]	0.364633	-1.455483025	3.32E-05	down	yes	0.765	2.098	2.081	2.052	2.162	0.746	0.87	0.679	------	------	K09542	CRYAB	map04213;map04141	Longevity regulating pathway - multiple species;Protein processing in endoplasmic reticulum	ENOG410YERS	O:Posttranslational modification, protein turnover, chaperones	PF00011.24	HSP20	Hsp20/alpha crystallin family	CYT	2	8	28.2	High
TRINITY_DN15000_c0_g1_i4_orf1	15-hydroxyprostaglandin dehydrogenase [NAD(+)]-like [Ostrinia furnacalis]	2.9892538	1.579785403	8.97E-05	up	yes	1.975	0.6607	0.686	0.64	0.656	1.973	1.835	2.116	------	------	K00069	HPGD	map00590;map05202	Arachidonic acid metabolism;Transcriptional misregulation in cancer	COG1028	O:Posttranslational modification, protein turnover, chaperones	PF00106.28;PF13561.9;PF08659.13	adh_short;adh_short_C2;KR	short chain dehydrogenase;Enoyl;KR domain	CYT	5	18	27.4	High
TRINITY_DN1329_c0_g1_i5_orf1	neurogenic locus notch homolog protein 3 [Ostrinia furnacalis] >XP_028157678.1 neurogenic locus notch homolog protein 3 [Ostrinia furnacalis]	2.9532031	1.562280573	1.81E-05	up	yes	1.95	0.6603	0.719	0.638	0.624	2.04	1.907	1.903	------	------	------	------	------	------	ENOG410XP6K	L:Replication, recombination and repair	PF12947.10	EGF_3	EGF domain	CYT	13	43	54.1	High
TRINITY_DN1252_c0_g1_i3_orf1	unnamed protein product [Chilo suppressalis]	0.4700893	-1.088993296	3.88E-05	down	yes	0.9477	2.016	1.943	2.068	2.037	0.901	0.917	1.025	------	------	K24746	WDR47	------	------	ENOG410Y29W;ENOG410YF9K	S:Function unknown;	PF00400.35;PF12894.10;PF20426.1	WD40;ANAPC4_WD40;NBCH_WD40	WD domain, G-beta repeat;Anaphase-promoting complex subunit 4 WD40 domain;Neurobeachin beta propeller domain	CYT	2	4	98.6	High
TRINITY_DN72999_c0_g1_i1_orf1	protein obstructor-E-like isoform X1 [Ostrinia furnacalis] >XP_028169319.1 protein obstructor-E-like isoform X2 [Ostrinia furnacalis]	0.2581468	-1.953736258	6.36E-06	down	yes	0.5767	2.234	2.17	2.333	2.2	0.545	0.608	0.577	CC:cellular_component;CC:extracellular region;CC:cellular anatomical entity;MF:carbohydrate derivative binding;MF:molecular_function;MF:chitin binding;MF:binding;	GO:0005575;GO:0005576;GO:0110165;GO:0097367;GO:0003674;GO:0008061;GO:0005488	------	------	------	------	ENOG4111EXD	S:Function unknown	PF01607.27	CBM_14	Chitin binding Peritrophin-A domain	CYT	6	24	32.9	High
TRINITY_DN245_c0_g1_i4_orf1	ER lumen protein-retaining receptor [Ostrinia furnacalis]	2.0955556	1.067332769	0.0002487	up	yes	1.886	0.9	0.902	1.005	0.793	1.96	1.911	1.787	BP:protein localization;BP:maintenance of protein localization in endoplasmic reticulum;BP:maintenance of location in cell;BP:cellular macromolecule localization;BP:nitrogen compound transport;BP:organic substance transport;BP:macromolecule localization;BP:transport;BP:maintenance of protein location in cell;BP:maintenance of protein localization in organelle;BP:cellular process;BP:protein retention in ER lumen;BP:establishment of protein localization;BP:maintenance of protein location;BP:biological_process;BP:maintenanc [illegible]	GO:0008104;GO:0035437;GO:0051651;GO:0070727;GO:0071705;GO:0071702;GO:0033036;GO:0006810;GO:0032507;GO:0072595;GO:0009987;GO:0006621;GO:0045184;GO:0045185;GO:0008150;GO:0051235;GO:0051234;GO:0051179;GO:0051641;GO:0015031;GO:0005789;GO:0031224;GO:0031090;GO:0016021;GO:0016020;GO:0110165;GO:0005575;GO:0003674;GO:0005488;GO:0042277;GO:0033218;GO:0046923;GO:0005048	K10949	KDELR	map05110	Vibrio cholerae infection	COG5196	U:Intracellular trafficking, secretion, and vesicular transport	PF00810.21	ER_lumen_recept	ER lumen protein retaining receptor	CYT	1	9	24.4	High

TRINITY_DN4242_c0_g1_i6_orf1	fibrohexamerin-like [Ostrinia furnacalis]	3.9723183	1.989981244	9.10E-06	up	yes	2.296	0.578	0.631	0.587	0.516	2.355	2.196	2.337	CC:cellular_component;CC:extracellular region;CC:cellular anatomical entity;MF:molecular_function;MF:structural molecule activity;	GO:0005575;GO:0005576;GO:0110165;GO:0003674;GO:0005198	------	------	------	------	ENOG4110SS9	S:Function unknown	PF07294.14	Fibroin_P25	Fibroin P25	CYT	3	14	25.5	High
TRINITY_DN214_c0_g1_i4_orf1	uncharacterized protein LOC114352813 [Ostrinia furnacalis]	2.0775689	1.054896308	0.0003546	up	yes	1.757	0.8457	0.869	0.738	0.93	1.838	1.788	1.645	------	------	------	------	------	------	ENOG4110S0N;ENOG4110T90;ENOG41119GI;ENOG410ZN7P;ENOG410YTKH	S:Function unknown;	PF06585.14	JHBP	Haemolymph juvenile hormone binding protein	CYT	4	31	29.6	High
TRINITY_DN978_c9_g2_i1_orf1	hypothetical protein evm_000959 [Chilo suppressalis]	0.3331731	-1.585656271	1.05E-05	down	yes	0.693	2.08	2.005	2.175	2.059	0.697	0.681	0.701	------	------	------	------	------	------	ENOG410Z4TG;ENOG410ZA8K	S:Function unknown;	------	------	------	CYT	1	8	14.8	High
TRINITY_DN22513_c0_g1_i4_orf1	DNA-directed RNA polymerase II subunit RPB1 [Ostrinia furnacalis] >XP_028179194.1 DNA-directed RNA polymerase II subunit RPB1 [Ostrinia furnacalis] >XP_028179195.1 DNA-directed RNA polymerase II subunit RPB1 [Ostrinia furnacalis]	2.6552598	1.408853039	0.0002882	up	yes	2.095	0.789	0.784	0.866	0.717	2.124	2.254	1.907	MF:molecular_function;MF:structural constituent of cuticle;MF:structural molecule activity;	GO:0003674;GO:0042302;GO:0005198	------	------	------	------	ENOG4111M49	S:Function unknown	PF00379.26	Chitin_bind_4	Insect cuticle protein	CYT	1	5	40.9	Medium
TRINITY_DN143532_c0_g1_i1_orf1	3-oxoacyl-[acyl-carrier-protein] reductase FabG-like [Aphidius gifuensis] >KAF7996667.1 hypothetical protein HCN44_002313 [Aphidius gifuensis]	2.156516	1.108702401	0.004299	up	yes	1.951	0.9047	0.818	0.847	1.049	1.63	2.169	2.055	MF:catalytic activity;MF:molecular_function;MF:oxidoreductase activity;	GO:0003824;GO:0003674;GO:0016491	------	------	------	------	COG1028	S:Function unknown	PF13561.9;PF00106.28;PF08659.13	adh_short_C2;adh_short;KR	Enoyl;short chain dehydrogenase;KR domain	CYT	1	5	27.2	High
TRINITY_DN14721_c0_g1_i2_orf1	protein masquerade-like isoform X2 [Ostrinia furnacalis]	0.2957457	-1.757570913	0.0001205	down	yes	0.6187	2.092	2.27	1.973	2.033	0.577	0.7	0.579	------	------	------	------	------	------	ENOG411279V	S:Function unknown	------	------	------	CYT	1	3	38	High
TRINITY_DN4068_c1_g2_i1_orf1	larval cuticle protein LCP-17 [Helicoverpa armigera] >PZC82071.1 hypothetical protein B5X24_HaOG211161 [Helicoverpa armigera] >PZC87412.1 hypothetical protein B5X24_HaOG216859 [Helicoverpa armigera]	2.084057	1.059394712	0.0003964	up	yes	1.8	0.8637	0.722	0.924	0.945	1.785	1.889	1.726	MF:molecular_function;MF:structural constituent of cuticle;MF:structural molecule activity;	GO:0003674;GO:0042302;GO:0005198	------	------	------	------	ENOG4110RUZ;ENOG4111PJ1;ENOG410ZNDD	S:Function unknown;	PF00379.26	Chitin_bind_4	Insect cuticle protein	CYT	4	64	17	High
TRINITY_DN23582_c0_g1_i1_orf1	unnamed protein product [Diatraea saccharalis]	0.437643	-1.192173532	3.55E-06	down	yes	0.765	1.748	1.708	1.775	1.762	0.749	0.801	0.745	------	------	------	------	------	------	ENOG4111ASG	S:Function unknown	------	------	------	CYT	1	3	26.5	Medium
TRINITY_DN38568_c0_g1_i1_orf1	unnamed protein product, partial [Diatraea saccharalis]	2.7957317	1.483225919	0.002484	up	yes	1.834	0.656	0.738	0.646	0.584	2.097	1.883	1.521	------	------	K12567	TTN	map05414;map05410	Dilated cardiomyopathy;Hypertrophic cardiomyopathy	ENOG410XQFD;ENOG410XTJ8;ENOG410ZN3C;ENOG410Y9AK	T:Signal transduction mechanisms;S:Function unknown;S:Function unknown;S:Function unknown	PF07679.19;PF13927.9;PF13895.9;PF00047.28	I-set;Ig_3;Ig_2;ig	Immunoglobulin I-set domain;Immunoglobulin domain;Immunoglobulin domain;Immunoglobulin domain	CYT	1	4	37.5	High
TRINITY_DN4144_c0_g1_i7_orf1	uncharacterized protein LOC114350172 [Ostrinia furnacalis]	0.391875	-1.351534557	0.006061	down	yes	0.6897	1.76	2.053	1.377	1.849	0.684	0.734	0.651	CC:cellular_component;CC:integral component of membrane;CC:cellular anatomical entity;CC:intrinsic component of membrane;	GO:0005575;GO:0016021;GO:0110165;GO:0031224	------	------	------	------	ENOG4110KD1	S:Function unknown	PF00100.26	Zona_pellucida	Zona pellucida-like domain	CYT	2	7	42	High
TRINITY_DN5954_c0_g1_i2_orf1	myosin-VIIa [Ostrinia furnacalis] >XP_028155907.1 myosin-VIIa [Ostrinia furnacalis]	0.4456235	-1.166102776	0.0001584	down	yes	0.7433	1.668	1.789	1.561	1.653	0.732	0.761	0.737	BP:cell differentiation;BP:anatomical structure morphogenesis;BP:cellular developmental process;BP:developmental process;BP:animal organ morphogenesis;BP:neuron differentiation;BP:cellular process;BP:biological_process;CC:cell periphery;CC:cytoplasm;CC:myosin complex;CC:cellular anatomical entity;CC:plasma membrane bounded cell projection;CC:cell projection;CC:macromolecular complex;CC:cellular_component;MF:purine ribonucleoside triphosphate binding;MF:molecular_function;MF:binding;M	GO:0030154;GO:0009653;GO:0048869;GO:0032502;GO:0009887;GO:0030182;GO:0009987;GO:0008150;GO:0071944;GO:0005737;GO:0016459;GO:0110165;GO:0120025;GO:0042995;GO:0032991;GO:0005575;GO:0035639;GO:0003674;GO:0005488;GO:1901363;GO:1901265;GO:0000166;GO:0003779;GO:0043167;GO:0017076;GO:0003774;GO:0043168;GO:0036094;GO:0008092;GO:0097367;GO:0097159;GO:0030554;GO:0032559;GO:0005524;GO:0005515;GO:0032555;GO:0032553	K10359	MYO7A, USH1B	------	------	COG5022	J:Translation, ribosomal structure and biogenesis	PF00063.24;PF00784.20;PF00373.21; PF00612.30	Myosin_head;MyTH4;FERM_M;IQ	Myosin head;MyTH4 domain;FERM central domain;IQ calmodulin-binding motif	CYT	5	2	249.6	High
TRINITY_DN650_c0_g1_i3_orf1	chitinase 7 [Glyphodes pyloalis]	0.4484411	-1.157009559	1.91E-05	down	yes	0.7767	1.732	1.759	1.77	1.666	0.735	0.816	0.779	BP:amino sugar catabolic process;BP:nitrogen compound metabolic process;BP:aminoglycan metabolic process;BP:organic substance catabolic process;BP:organic substance metabolic process;BP:chitin metabolic process;BP:chitin catabolic process;BP:biological_process;BP:metabolic process;BP:carbohydrate metabolic process;BP:catabolic process;BP:macromolecule catabolic process;BP:primary metabolic process;BP:organonitrogen compound metabolic process;BP:organonitrogen compound catabolic	GO:0046348;GO:0006807;GO:0006022;GO:1901575;GO:0071704;GO:0006030;GO:0006032;GO:0008150;GO:0008152;GO:0005975;GO:0009056;GO:0009057;GO:0044238;GO:1901564;GO:1901565;GO:0006040;GO:1901136;GO:1901135;GO:0043170;GO:1901072;GO:1901071;GO:0006026;GO:0110165;GO:0005575;GO:0005576;GO:0016798;GO:0003674;GO:0008061;GO:0016787;GO:0003824;GO:0004568;GO:0097367;GO:0004553;GO:0005488	K01183	E3.2.1.14	map00520	Amino sugar and nucleotide sugar metabolism	COG3325	S:Function unknown	PF00704.31;PF01607.27	Glyco_hydro_18;CBM_14	Glycosyl hydrolases family 18;Chitin binding Peritrophin-A domain	CYT	7	9	111.3	High
TRINITY_DN7735_c0_g1_i4_orf1	calphotin-like [Ostrinia furnacalis]	7.4044118	2.888385127	8.69E-05	up	yes	2.014	0.272	0.156	0.339	0.321	2.194	1.949	1.899	------	------	------	------	------	------	ENOG4110RAS;ENOG4110R6G	S:Function unknown;	------	------	------	CYT	1	3	22.5	High
TRINITY_DN98995_c0_g1_i2_orf1	hypothetical protein HF086_008399, partial [Spodoptera exigua]	2.0532069	1.037879015	0.001381	up	yes	1.559	0.7593	0.867	0.589	0.822	1.5	1.512	1.664	------	------	K16726	ANKRD17, MASK	------	------	COG0666	O:Posttranslational modification, protein turnover, chaperones	PF12796.10;PF13637.9;PF13606.9;PF00023.33; PF13857.9	Ank_2;Ank_4;Ank_3;Ank;Ank_5	Ankyrin repeats;Ankyrin repeats;Ankyrin repeat;Ankyrin repeat;Ankyrin repeats	CYT	1	3	74.1	High
TRINITY_DN2722_c0_g1_i1_orf1	troponin C [Pieris rapae] >XP_045490973.1 troponin C-like isoform X1 [Colias croceus] >XP_049866665.1 troponin C-like [Pectinophora gossypiella]	2.4330827	1.282785362	2.72E-06	up	yes	1.618	0.665	0.664	0.666	0.665	1.657	1.624	1.572	MF:cation binding;MF:ion binding;MF:molecular_function;MF:binding;MF:metal ion binding;MF:calcium ion binding;	GO:0043169;GO:0043167;GO:0003674;GO:0005488;GO:0046872;GO:0005509	------	------	------	------	COG5126	S:Function unknown	PF13499.9;PF00036.35;PF13405.9;PF13833.9;PF14658.9; PF13202.9	EF-hand_7;EF-hand_1;EF-hand_6;EF-hand_8;EF-hand_9;EF-hand_5	EF-hand domain pair;EF hand;EF-hand domain;EF-hand domain pair;EF-hand domain;EF hand	CYT	9	58	18.1	High
TRINITY_DN111488_c0_g1_i1_orf1	LOW QUALITY PROTEIN: formin-J-like [Chelonus insularis]	2.555205	1.353439068	0.001016	up	yes	1.62	0.634	0.694	0.518	0.69	1.447	1.791	1.623	------	------	------	------	------	------	ENOG4111I6P	S:Function unknown	------	------	------	CYT	1	3	22.4	Medium
TRINITY_DN8310_c0_g2_i1_orf1	uncharacterized protein LOC116773294 [Danaus plexippus plexippus] >OWR55545.1 hypothetical protein KGM_209260 [Danaus plexippus plexippus]	2.4874608	1.314673799	2.21E-05	up	yes	1.587	0.638	0.571	0.677	0.666	1.621	1.6	1.539	------	------	------	------	------	------	ENOG4110R2S;COG4870	S:Function unknown;	PF08246.15	Inhibitor_I29	Cathepsin propeptide inhibitor domain	CYT	3	46	9.3	High
TRINITY_DN100208_c0_g1_i1_orf1	neurofilament heavy polypeptide-like isoform X2 [Ostrinia furnacalis]	0.2888695	-1.791510389	0.0001493	down	yes	0.4957	1.716	1.776	1.548	1.824	0.461	0.517	0.509	------	------	------	------	------	------	------	------	------	------	------	CYT	1	88	8.4	High

TRINITY_DN120500_c0_g1_i1_orf1	cytochrome P450 6B5-like [Ostrinia furnacalis]	2.7755998	1.472799574	5.09E-07	up	yes	1.77	0.6377	0.634	0.616	0.663	1.778	1.788	1.743	CC:integral component of membrane;CC:cellular_component;CC:cellular anatomical entity;CC:intrinsic component of membrane;MF:cation binding;MF:monooxygenase activity;MF:heme binding;MF:transition metal ion binding;MF:tetrapyrrole binding;MF:organic cyclic compound binding;MF:oxidoreductase activity, acting on paired donors, with incorporation or reduction of molecular oxygen;MF:ion binding;MF:catalytic activity;MF:molecular_function;MF:binding;MF:iron ion binding;MF:metal ion binding;MF:heterocyclic compound	GO:0016021;GO:0005575;GO:0110165;GO:0031224;GO:0043169;GO:0004497;GO:0020037;GO:0046914;GO:0046906;GO:0097159;GO:0016705;GO:0043167;GO:0003824;GO:0003674;GO:0005488;GO:0005506;GO:0046872;GO:1901363;GO:0016491	K14999	CYP6	------	------	COG2124	Q:Secondary metabolites biosynthesis, transport and catabolism	PF00067.25	p450	Cytochrome P450	CYT	10	23	60.5	High
TRINITY_DN57111_c0_g1_i1_orf1	trypsin-like serine proteinase T26 protein, partial [Chilo infuscatellus]	3.926087	1.973092127	0.0001536	up	yes	1.806	0.46	0.41	0.458	0.512	1.788	1.973	1.656	BP:organic substance metabolic process;BP:organonitrogen compound metabolic process;BP:protein metabolic process;BP:macromolecule metabolic process;BP:biological_process;BP:nitrogen compound metabolic process;BP:metabolic process;BP:proteolysis;BP:primary metabolic process;MF:serine-type endopeptidase activity;MF:endopeptidase activity;MF:hydrolase activity;MF:serine hydrolase activity;MF:catalytic activity, acting on a protein;MF:catalytic activity;MF:molecular_function;MF:peptidase activity;MF:serine-type	GO:0071704;GO:1901564;GO:0019538;GO:0043170;GO:0008150;GO:0006807;GO:0008152;GO:0006508;GO:0044238;GO:0004252;GO:0004175;GO:0016787;GO:0017171;GO:0140096;GO:0003824;GO:0003674;GO:0008233;GO:0008236	------	------	------	------	------	------	------	------	------	CYT	1	26	7.9	High
TRINITY_DN6415_c0_g1_i1_orf1	D-arabinitol dehydrogenase 1-like [Ostrinia furnacalis]	2.6497865	1.405876147	9.21E-05	up	yes	1.8	0.6793	0.7	0.657	0.681	1.742	1.938	1.719	MF:oxidoreductase activity;MF:cation binding;MF:transition metal ion binding;MF:ion binding;MF:catalytic activity;MF:molecular_function;MF:binding;MF:metal ion binding;MF:zinc ion binding;	GO:0016491;GO:0043169;GO:0046914;GO:0043167;GO:0003824;GO:0003674;GO:0005488;GO:0046872;GO:0008270	K17818	ARD1	map00040	Pentose and glucuronate interconversions	COG1063	E:Amino acid transport and metabolism	PF08240.15;PF00107.29;PF16912.8	ADH_N;ADH_zinc_N;Glu_dehyd_C	Alcohol dehydrogenase GroES-like domain;Zinc-binding dehydrogenase;Glucose dehydrogenase C-terminus	CYT	6	42	37.8	High
TRINITY_DN10057_c0_g2_i1_orf1	cell wall protein DAN4 [Ostrinia furnacalis]	0.2866875	-1.802449256	2.50E-05	down	yes	0.5513	1.923	1.828	1.98	1.96	0.483	0.621	0.55	CC:cellular_component;CC:cellular anatomical entity;CC:extracellular space;MF:endopeptidase inhibitor activity;MF:endopeptidase regulator activity;MF:peptidase regulator activity;MF:molecular_function;MF:peptidase inhibitor activity;MF:serine-type endopeptidase inhibitor activity;MF:enzyme inhibitor activity;MF:molecular function regulator;MF:enzyme	GO:0005575;GO:0110165;GO:0005615;GO:0004866;GO:0061135;GO:0061134;GO:0003674;GO:0030414;GO:0004867;GO:0004857;GO:0098772;GO:0030234	------	------	------	------	COG4826	O:Posttranslational modification, protein turnover, chaperones	PF00079.23	Serpin	Serpin	CYT	1	1	157.8	High
TRINITY_DN920_c0_g1_i6_orf1	glutathione S-transferase omega 2 [Ostrinia furnacalis]	2.586262	1.370868423	3.69E-05	up	yes	1.619	0.626	0.65	0.591	0.637	1.69	1.635	1.532	BP:metabolic process;BP:biological_process;BP:organic substance metabolic process;CC:cytoplasm;CC:cellular_component;CC:cellular anatomical entity;MF:glutathione dehydrogenas;MF:glutathione transferase activity;MF:catalytic activity;MF:antioxidant activity;MF:oxidoreductase activity, acting on a sulfur group of donors, quinone or similar compound as acceptor;MF:glutathione disulfide oxidoreductase activity;MF:transferase activity;MF:molecular_function;MF:oxidoreductase activity, acting on a sulfur group of donors;MF:transferase activity, transferring alkyl or ary;MF:oxidoreductase	GO:0008152;GO:0008150;GO:0071704;GO:0005737;GO:0005575;GO:0110165;GO:0045174;GO:0004364;GO:0003824;GO:0016209;GO:0016672;GO:0015038;GO:0016740;GO:0003674;GO:0016667;GO:0016765;GO:0016491;GO:0015036	K00310	SE	map00480;map00790	Glutathione metabolism;Folate biosynthesis	ENOG410XSIX	O:Posttranslational modification, protein turnover, chaperones	PF13417.9;PF02798.23;PF13409.9;PF13410.9;PF14497.9;PF00043.28	GST_N_3;GST_N;GST_N_2;GST_C_2;GST_C_3;GST_C	Glutathione S-transferase, N-terminal domain;Glutathione S-transferase, N-terminal domain;Glutathione S-transferase, N-terminal domain;Glutathione S-transferase, C-terminal domain;Glutathione S-transferase, C-terminal domain;Glutathione S-transferase, C-terminal domain	CYT	1	11	32.3	High
TRINITY_DN33995_c0_g1_i5_orf1	unnamed protein product [Spodoptera exigua]	2.0483893	1.03448992	0.01414	up	yes	1.507	0.7357	0.692	0.781	0.734	1.14	1.692	1.69	CC:cellular_component;CC:integral component of membrane;CC:cellular anatomical entity;CC:intrinsic component of membrane;	GO:0005575;GO:0016021;GO:0110165;GO:0031224	------	------	------	------	ENOG410XPW0	U:Intracellular trafficking, secretion, and vesicular transport	PF03798.19	TRAM_LAG1_CLN8	TLC domain	CYT	1	4	37.8	High

TRINITY_DN4898_c0_g1_i7_orf1	annulin-like isoform X3 [Ostrinia furnacalis]	0.4651856	-1.104121545	2.41E-05	down	yes	0.7643	1.643	1.643	1.696	1.589	0.806	0.766	0.721	BP:primary metabolic process;BP:peptide cross-linking;BP:organonitrogen compound metabolic process;BP:organic substance metabolic process;BP:nitrogen compound metabolic process;BP:macromolecule modification;BP:macromolecule metabolic process;BP:biological_process;BP:protein modification process;BP:metabolic process;BP:protein metabolic process;MF:molecular_function;MF:protein-glutamine gamma-glutamyltransferase activity;MF:catalytic activity, acting on a protein;MF:catalytic activity;MF:transferase activity;MF:transferase [illegible]	GO:0044238;GO:0018149;GO:1901564;GO:0071704;GO:0006807;GO:0043412;GO:0043170;GO:0008150;GO:0036211;GO:0008152;GO:0019538;GO:0003674;GO:0003810;GO:0140096;GO:0003824;GO:0016740;GO:0016746;GO:0016755	K05619	TGM1	------	------	ENOG410XQEZ	O:Posttranslational modification, protein turnover, chaperones	PF00927.25;PF00868.23;PF01841.22	Transglut_C;Transglut_N;Transglut_core	Transglutaminase family, C-terminal ig like domain;Transglutaminase family;Transglutaminase-like superfamily	CYT	7	9	86.1	High
TRINITY_DN41280_c0_g1_i2_orf1	unnamed protein product [Plutella xylostella]	2.3023674	1.203118093	0.001411	up	yes	1.663	0.7223	0.809	0.734	0.624	1.877	1.561	1.552	MF:molecular_function;MF:structural constituent of cuticle;MF:structural molecule activity;	GO:0003674;GO:0042302;GO:0005198	------	------	------	------	ENOG4111ZRP	S:Function unknown	------	------	------	CYT	1	35	8	High
TRINITY_DN6586_c0_g1_i1_orf1	fatty acyl-CoA reductase wat-like isoform X1 [Ostrinia furnacalis]	0.3365396	-1.571152009	0.000128	down	yes	0.5913	1.757	1.738	1.896	1.637	0.563	0.567	0.644	BP:primary metabolic process;BP:lipid metabolic process;BP:fatty acid derivative metabolic process;BP:organic substance metabolic process;BP:biological_process;BP:metabolic process;CC:cellular_component;CC:cellular anatomical entity;CC:integral component of membrane;CC:intrinsic component of membrane;MF:fatty-acyl-CoA reductas;MF:alcohol-forming fatty acyl-CoA reductase activity;MF:catalytic activity;MF:oxidoreductase activity, acting on the aldehyde or oxo group of donors, NAD or NADP as acceptor;MF:oxidoreductase activity, acting on [illegible]	GO:0044238;GO:0006629;GO:1901568;GO:0071704;GO:0008150;GO:0008152;GO:0005575;GO:0110165;GO:0016021;GO:0031224;GO:0080019;GO:0102965;GO:0003824;GO:0016620;GO:0016903;GO:0003674;GO:0016491	K13356	FAR	map04146;map00073;map04212	Peroxisome;Cutin, suberine and wax biosynthesis;Longevity regulating pathway - worm	ENOG410XS7R	O:Posttranslational modification, protein turnover, chaperones	PF07993.15;PF03015.22;PF01370.24	NAD_binding_4;Sterile;Epimerase	Male sterility protein;Male sterility protein;NAD dependent epimerase/dehydratase family	CYT	3	7	71.4	High
TRINITY_DN9820_c0_g1_i1_orf1	endocuticle structural glycoprotein SgAbd-2-like [Ostrinia furnacalis]	4.2070042	2.072793272	1.69E-06	up	yes	1.886	0.4483	0.469	0.404	0.472	1.866	1.935	1.857	MF:molecular_function;MF:structural constituent of cuticle;MF:structural molecule activity;	GO:0003674;GO:0042302;GO:0005198	------	------	------	------	ENOG4111PJ1;ENOG410YXXU;ENOG410YR66	S:Function unknown;	PF00379.26	Chitin_bind_4	Insect cuticle protein	CYT	4	56	16	High
TRINITY_DN20356_c0_g1_i5_orf1	uncharacterized protein LOC114362428 [Ostrinia furnacalis]	0.4366667	-1.195395689	0.04145	down	yes	0.655	1.5	1.765	1.464	1.271	0.267	0.586	1.112	CC:cellular_component;CC:integral component of membrane;CC:cellular anatomical entity;CC:intrinsic component of membrane;	GO:0005575;GO:0016021;GO:0110165;GO:0031224	------	------	------	------	ENOG4110QHP	S:Function unknown	------	------	------	CYT	1	2	60	High
TRINITY_DN42337_c0_g1_i6_orf1	cuticle protein 8-like [Leguminivora glycinivorella]	0.3699681	-1.434527404	0.001611	down	yes	0.579	1.565	1.464	1.822	1.408	0.585	0.571	0.581	MF:molecular_function;MF:structural constituent of cuticle;MF:structural molecule activity;	GO:0003674;GO:0042302;GO:0005198	------	------	------	------	ENOG410YSFI;ENOG4111EA8;ENOG4110T8H	S:Function unknown;	PF00379.26	Chitin_bind_4	Insect cuticle protein	CYT	1	36	15.3	High
TRINITY_DN565_c0_g2_i1_orf1	uncharacterized protein LOC114362323 [Ostrinia furnacalis]	2.0778085	1.055062722	6.04E-05	up	yes	1.426	0.6863	0.685	0.671	0.703	1.433	1.493	1.352	------	------	------	------	------	------	COG1028	S:Function unknown	PF13561.9;PF00106.28;PF08659.13	adh_short_C2;adh_short;KR	Enoyl;short chain dehydrogenase;KR domain	CYT	7	34	27.4	High
TRINITY_DN1292_c0_g1_i3_orf1	uncharacterized protein LOC114360660 [Ostrinia furnacalis]	2.1327998	1.092748525	9.08E-06	up	yes	1.439	0.6747	0.688	0.692	0.644	1.435	1.479	1.403	CC:cellular_component;CC:integral component of membrane;CC:cellular anatomical entity;CC:intrinsic component of membrane;	GO:0005575;GO:0016021;GO:0110165;GO:0031224	------	------	------	------	ENOG4110R95;ENOG4110MRV	S:Function unknown;	PF15430.9	SVWC	Single domain von Willebrand factor type C	CYT	1	11	14.2	High
TRINITY_DN5628_c0_g1_i5_orf1	hypothetical protein O3G_MSEX015036 [Manduca sexta]	2.3925704	1.258561399	9.46E-05	up	yes	1.52	0.6353	0.663	0.629	0.614	1.62	1.505	1.434	MF:cation binding;MF:metal ion binding;MF:molecular_function;MF:binding;MF:ion binding;	GO:0043169;GO:0046872;GO:0003674;GO:0005488;GO:0043167	------	------	------	------	ENOG410Z840;ENOG410ZTJU;ENOG41128N9	S:Function unknown;O:Posttranslational modification, protein turnover, chaperones	PF00412.25	LIM	LIM domain	CYT	8	55	36.3	High
TRINITY_DN28503_c0_g1_i6_orf1	uncharacterized protein LOC114363584 [Ostrinia furnacalis] >AXY94663.1 seroin transcript 3 [Ostrinia nubilalis]	2.2805862	1.189404702	0.0001896	up	yes	1.385	0.6073	0.656	0.583	0.583	1.286	1.4	1.47	------	------	------	------	------	------	------	------	------	------	------	PLA	5	42	12.8	High
TRINITY_DN4156_c0_g1_i2_orf1	calcium channel flower [Ostrinia furnacalis]	0.4667393	-1.099311182	0.0001551	down	yes	0.6427	1.377	1.277	1.425	1.429	0.656	0.663	0.609	------	------	------	------	------	------	ENOG4111G4Q	U:Intracellular trafficking, secretion, and vesicular transport	PF10233.12	Cg6151-P	Uncharacterized conserved protein CG6151-P	CYT	1	10	20.9	High
TRINITY_DN73923_c0_g1_i1_orf1	protein obstructor-E-like [Ostrinia furnacalis]	0.1635917	-2.611828514	1.95E-07	down	yes	0.2997	1.832	1.849	1.855	1.793	0.296	0.311	0.292	CC:cellular_component;CC:extracellular region;CC:cellular anatomical entity;MF:carbohydrate derivative binding;MF:molecular_function;MF:chitin binding;MF:binding;	GO:0005575;GO:0005576;GO:0110165;GO:0097367;GO:0003674;GO:0008061;GO:0005488	------	------	------	------	ENOG410ZSCN	S:Function unknown	PF01607.27	CBM_14	Chitin binding Peritrophin-A domain	CYT	15	65	26.5	High
TRINITY_DN5211_c0_g1_i1_orf1	elongation of very long chain fatty acids protein AAEL008004-like [Ostrinia furnacalis]	0.4530924	-1.142122723	0.001385	down	yes	0.652	1.439	1.515	1.308	1.495	0.561	0.8	0.595	BP:fatty acid biosynthetic process;BP:fatty acid metabolic process;BP:carboxylic acid metabolic process;BP:cellular biosynthetic process;BP:small molecule metabolic process;BP:small molecule biosynthetic process;BP:monocarboxylic acid biosynthetic process;BP:organic substance biosynthetic process;BP:organic substance metabolic process;BP:lipid metabolic process;BP:cellular process;BP:monocarboxylic acid metabolic process;BP:biosynthetic process;BP:biological_process;BP:metabolic process;BP:oxoacid metabolic [illegible]	GO:0006633;GO:0006631;GO:0019752;GO:0044249;GO:0044281;GO:0044283;GO:0072330;GO:1901576;GO:0071704;GO:0006629;GO:0009987;GO:0032787;GO:0009058;GO:0008150;GO:0008152;GO:0043436;GO:0044255;GO:0008610;GO:0044238;GO:0006082;GO:0046394;GO:0016053;GO:0044237;GO:0031224;GO:0016021;GO:0110165;GO:0005575;GO:0003674;GO:0009922;GO:0016740;GO:0016746;GO:0016747;GO:0102756;GO:0003824;GO:0004312	K10250	ELOVL7	map01040;map00062	Biosynthesis of unsaturated fatty acids;Fatty acid elongation	ENOG410XRWT	I:Lipid transport and metabolism	PF01151.21	ELO	GNS1/SUR4 family	PLA	1	4	32.2	High
TRINITY_DN2840_c0_g1_i5_orf1	hypothetical protein evm_002181 [Chilo suppressalis]	0.3449903	-1.53537228	4.24E-06	down	yes	0.5337	1.547	1.553	1.578	1.509	0.552	0.491	0.558	------	------	------	------	------	------	ENOG41129M9	S:Function unknown	------	------	------	PLA	3	23	16.5	High

TRINITY_DN110534_c0_g1_i3_orf1	unnamed protein product [Euphydryas editha]	0.4700425	-1.089136909	0.00324	down	yes	0.6637	1.412	1.601	1.442	1.194	0.651	0.658	0.682	BP:nucleic acid metabolic process;BP:cellular nitrogen compound metabolic process;BP:nitrogen compound metabolic process;BP:organic cyclic compound metabolic process;BP:nucleobase-containing compound metabolic process;BP:cellular macromolecule metabolic process;BP:organic substance metabolic process;BP:cellular process;BP:cellular aromatic compound metabolic process;BP:biological_process;BP:metabolic process;BP:heterocycle metabolic process;BP:primary metabolic process;BP:cellular	GO:0090304;GO:0034641;GO:0006807;GO:1901360;GO:0006139;GO:0044260;GO:0071704;GO:0009987;GO:0006725;GO:0008150;GO:0008152;GO:0046483;GO:0044238;GO:0044237;GO:0043170;GO:0015074;GO:0006259;GO:1990234;GO:0061695;GO:1902494;GO:0032991;GO:0005575;GO:0042575;GO:0034061;GO:0003674;GO:0008270;GO:0003676;GO:1901363;GO:0043167;GO:0003887;GO:0016740;GO:0046872;GO:0043169;GO:0140097;GO:0003824;GO:0016779;GO:0046914;GO:0016772;GO:0097159;GO:0005488	------	------	------	------	ENOG410XS4D;COG2801	U:Intracellular trafficking, secretion, and vesicular transport;S:Function unknown	PF07727.17;PF00665.29;PF13976.9	RVT_2;rve;gag_pre-integrs	Reverse transcriptase;Integrase core domain;GAG-pre-integrase domain	CYT	1	1	124.4	Medium
TRINITY_DN21124_c0_g1_i4_orf1	calsyntenin-1 [Ostrinia furnacalis]	0.3847811	-1.377890165	3.71E-06	down	yes	0.5537	1.439	1.411	1.481	1.425	0.542	0.579	0.54	------	------	K22659	CLSTN1	------	------	ENOG410XT2J	U:Intracellular trafficking, secretion, and vesicular transport	PF19699.2;PF00028.20	CLSTN_C;Cadherin	Calsyntenin C-terminal;Cadherin domain	CYT	10	14	95.1	High
TRINITY_DN9560_c0_g1_i5_orf1	uncharacterized protein LOC114357350 [Ostrinia furnacalis]	3.0622642	1.614598735	0.0005993	up	yes	1.623	0.53	0.629	0.538	0.423	1.753	1.441	1.676	------	------	------	------	------	------	ENOG410XV86;ENOG4111ASB	S:Function unknown;	------	------	------	CYT	1	11	27.1	High
TRINITY_DN35633_c0_g2_i1_orf1	uncharacterized protein LOC114353024 [Ostrinia furnacalis]	0.4822192	-1.052239123	3.27E-06	down	yes	0.6997	1.451	1.419	1.478	1.456	0.678	0.707	0.714	------	------	------	------	------	------	ENOG410YUHN	S:Function unknown	------	------	------	CYT	4	12	45.2	High
TRINITY_DN57856_c0_g2_i1_orf1	cytochrome P450 6B2-like [Ostrinia furnacalis]	2.254523	1.172822204	0.0002148	up	yes	1.458	0.6467	0.676	0.588	0.676	1.568	1.383	1.423	MF:cation binding;MF:monooxygenase activity;MF:heme binding;MF:transition metal ion binding;MF:tetrapyrrole binding;MF:organic cyclic compound binding;MF:oxidoreductase activity, acting on paired donors, with incorporation or reduction of molecular oxygen;MF:ion binding;MF:catalytic activity;MF:molecular_function;MF:binding;MF:iron ion binding;MF:metal ion binding;MF:heterocyclic compound binding;MF:oxidoreduc	GO:0043169;GO:0004497;GO:0020037;GO:0046914;GO:0046906;GO:0097159;GO:0016705;GO:0043167;GO:0003824;GO:0003674;GO:0005488;GO:0005506;GO:0046872;GO:1901363;GO:0016491	K14999	CYP6	------	------	COG2124	G:Carbohydrate transport and metabolism	PF00067.25	p450	Cytochrome P450	CYT	2	38	7.2	High
TRINITY_DN17003_c0_g1_i1_orf1	mucin-5AC [Ostrinia furnacalis]	0.4738628	-1.077458638	0.01141	down	yes	0.6563	1.385	1.567	1.51	1.077	0.765	0.583	0.621	CC:cellular_component;CC:extracellular region;CC:cellular anatomical entity;MF:carbohydrate derivative binding;MF:molecular_function;MF:chitin binding;MF:binding;	GO:0005575;GO:0005576;GO:0110165;GO:0097367;GO:0003674;GO:0008061;GO:0005488	K01183	E3.2.1.14	map00520	Amino sugar and nucleotide sugar metabolism	COG3325;ENOG410YMUP	J:Translation, ribosomal structure and biogenesis;S:Function unknown	PF01607.27	CBM_14	Chitin binding Peritrophin-A domain	CYT	1	2	72.5	High
TRINITY_DN2350_c0_g1_i6_orf1	protein yellow-like isoform X2 [Ostrinia furnacalis]	2.2839029	1.191501311	1.05E-05	up	yes	1.477	0.6467	0.619	0.682	0.639	1.451	1.525	1.456	------	------	------	------	------	------	ENOG4110U30	S:Function unknown	PF03022.19	MRJP	Major royal jelly protein	CYT	4	12	55	High
TRINITY_DN109931_c0_g1_i1_orf1	hydroxymethylglutaryl-CoA lyase, mitochondrial isoform X1 [Ostrinia furnacalis]	4.2025316	2.071258683	0.0002769	up	yes	1.66	0.395	0.424	0.379	0.382	1.864	1.597	1.519	MF:hydroxymethylglutaryl-CoA lyase activity;MF:lyase activity;MF:carbon-carbon lyase activity;MF:oxo-acid-lyase activity;MF:catalytic activity;MF:molecular_function;	GO:0004419;GO:0016829;GO:0016830;GO:0016833;GO:0003824;GO:0003674	K01640	HMGCL, hmgL	map04146;map00280;map00650	Peroxisome;Valine, leucine and isoleucine degradation;Butanoate metabolism	COG0119	E:Amino acid transport and metabolism	------	------	------	CYT	1	15	5.9	High
TRINITY_DN3913_c0_g1_i6_orf1	protein obstructor-E-like [Ostrinia furnacalis]	2.6166927	1.387744504	1.08E-05	up	yes	1.508	0.5763	0.58	0.561	0.588	1.479	1.574	1.47	CC:cellular_component;CC:extracellular region;CC:cellular anatomical entity;MF:carbohydrate derivative binding;MF:molecular_function;MF:chitin binding;MF:binding;	GO:0005575;GO:0005576;GO:0110165;GO:0097367;GO:0003674;GO:0008061;GO:0005488	------	------	------	------	ENOG4111HJZ	S:Function unknown	PF01607.27	CBM_14	Chitin binding Peritrophin-A domain	CYT	9	46	33.8	High
TRINITY_DN10479_c0_g1_i6_orf1	unnamed protein product [Chrysodeixis includens]	0.4124143	-1.277833856	0.0002133	down	yes	0.6013	1.458	1.45	1.369	1.554	0.535	0.673	0.596	------	------	------	------	------	------	ENOG4111SQM;ENOG410XUIM	S:Function unknown;	------	------	------	CYT	1	4	26.6	High
TRINITY_DN131264_c0_g1_i2_orf1	unnamed protein product [Parnassius apollo]	0.4538514	-1.139708242	0.002296	down	yes	0.6717	1.48	1.323	1.592	1.525	0.563	0.838	0.614	------	------	K13718	OTUD4	------	------	ENOG4111T74	S:Function unknown	------	------	------	CYT	2	24	11.7	High
TRINITY_DN1134_c0_g1_i4_orf1	cytochrome P450 6B5-like [Ostrinia furnacalis]	2.3144105	1.210644761	3.18E-06	up	yes	1.431	0.6183	0.654	0.617	0.584	1.424	1.421	1.448	CC:endoplasmic reticulum membrane;CC:organelle membrane;CC:membrane;CC:cellular anatomical entity;CC:cellular_component;MF:monooxygenase activity;MF:oxidoreductase activity, acting on paired donors, with incorporation or reduction of molecular oxygen;MF:NADPH-hemoprotein reductase activity;MF:molecular_function;MF:binding;MF:heterocyclic compound binding;MF:oxidoreductase activity;MF:cation binding;MF:transition metal ion binding;MF:ion binding;MF:iron ion binding;MF:metal ion	GO:0005789;GO:0031090;GO:0016020;GO:0110165;GO:0005575;GO:0004497;GO:0016705;GO:0003958;GO:0003674;GO:0005488;GO:1901363;GO:0016491;GO:0043169;GO:0046914;GO:0043167;GO:0005506;GO:0046872;GO:0020037;GO:0003824;GO:0046906;GO:0097159;GO:0016651;GO:0016653	K14999	CYP6	------	------	COG2124	S:Function unknown	PF00067.25	p450	Cytochrome P450	CYT	3	16	56.4	High

TRINITY_DN26961_c0_g1_i1_orf1	uncharacterized protein LOC120424957 [Culex pipiens pallens]	2.2012103	1.138296976	0.009502	up	yes	1.455	0.661	0.718	0.642	0.623	1.515	1.711	1.14	CC:sarcomere;CC:myosin complex;CC:macromolecular complex;CC:cellular_component;CC:cellular anatomical entity;MF:purine ribonucleoside triphosphate binding;MF:macromolecular complex binding;MF:binding;MF:ion binding;MF:heterocyclic compound binding;MF:nucleoside phosphate binding;MF:anion binding;MF:nucleotide binding;MF:actin binding;MF:purine nucleotide binding;MF:motor activity;MF:ATP binding;MF:actin filament binding;MF:small molecule	GO:0030017;GO:0016459;GO:0032991;GO:0005575;GO:0110165;GO:0035639;GO:0044877;GO:0005488;GO:0043167;GO:1901363;GO:1901265;GO:0043168;GO:0000166;GO:0003779;GO:0017076;GO:0003774;GO:0005524;GO:0051015;GO:0036094;GO:0008092;GO:0097367;GO:0097159;GO:0030554;GO:0032559;GO:0005515;GO:0032555;GO:0003674;GO:0032553	K17751	MYH6_7	map04260;map04261;map04919;map04022;map05416;map05414;map05410	Cardiac muscle contraction;Adrenergic signaling in cardiomyocytes;Thyroid hormone signaling pathway;cGMP-PKG signaling pathway;Viral myocarditis;Dilated cardiomyopathy;Hypertrophic cardiomyopathy	COG5022	E:Amino acid transport and metabolism	PF01576.22	Myosin_tail_1	Myosin tail	CYT	5	64	12.3	High
TRINITY_DN124711_c0_g1_i1_orf1	muskelin isoform X1 [Ostrinia furnacalis] >XP_028163274.1 muskelin isoform X2 [Ostrinia furnacalis]	2.2370643	1.161606746	0.000849	up	yes	1.457	0.6513	0.638	0.689	0.627	1.618	1.317	1.435	------	------	K23337	MKLN1	------	------	ENOG410YJKC	S:Function unknown	PF06588.14	Muskelin_N	Muskelin N-terminus	CYT	1	4	31.3	High
TRINITY_DN1212_c0_g1_i8_orf1	extensin isoform X5 [Ostrinia furnacalis] >XP_028175473.1 extensin isoform X5 [Ostrinia furnacalis] >XP_028175474.1 extensin isoform X5 [Ostrinia furnacalis]	3.3167599	1.729774576	2.82E-05	up	yes	1.601	0.4827	0.477	0.462	0.509	1.666	1.636	1.502	------	------	------	------	------	------	ENOG4111WKY;ENOG4110SQB	S:Function unknown;	PF15936.8	DUF4749	Domain of unknown function	CYT	7	26	44.1	High
TRINITY_DN2647_c0_g1_i3_orf1	DNA repair protein complementing XP-G cells homolog isoform X1 [Ostrinia furnacalis]	2.0988038	1.069567301	7.37E-05	up	yes	1.351	0.6437	0.696	0.626	0.609	1.414	1.304	1.336	BP:primary metabolic process;BP:nucleobase-containing compound metabolic process;BP:nitrogen compound metabolic process;BP:organic cyclic compound metabolic process;BP:cellular response to stimulus;BP:cellular macromolecule metabolic process;BP:organic substance metabolic process;BP:DNA repair;BP:cellular process;BP:cellular aromatic compound metabolic process;BP:nucleotide-excision repair;BP:cellular response to DNA damage stimulus;BP:response to stress;BP:biological_pr	GO:0044238;GO:0006139;GO:0006807;GO:1901360;GO:0051716;GO:0044260;GO:0071704;GO:0006281;GO:0009987;GO:0006725;GO:0006289;GO:0006974;GO:0006950;GO:0008150;GO:0008152;GO:0046483;GO:0090304;GO:0050896;GO:0034641;GO:0044237;GO:0043170;GO:0033554;GO:0006259;GO:0043226;GO:0043229;GO:0043227;GO:0005634;GO:0110165;GO:0043231;GO:0005575;GO:0003674;GO:0005488;GO:0003676;GO:0003677;GO:1901363;GO:0043169;GO:0043167;GO:0046872;GO:0003697;GO:0016787;GO:0016788;GO:0003824;GO:0004518;GO:0004519;GO:0097159	K10846	ERCC5, XPG, RAD2	map03420	Nucleotide excision repair	COG0258	L:Replication, recombination and repair	PF00752.20;PF00867.21	XPG_N;XPG_I	XPG N-terminal domain;XPG I-region	CYT	1	1	132.1	Medium
TRINITY_DN6436_c0_g1_i1_orf1	serine/threonine-protein kinase PAK 3 isoform X1 [Ostrinia furnacalis] >XP_028164178.1 serine/threonine-protein kinase PAK 3 isoform X2 [Ostrinia furnacalis] >XP_028164179.1 serine/threonine-protein kinase PAK 3 isoform X3 [Ostrinia furnacalis]	0.4733916	-1.078894047	0.0001242	down	yes	0.596	1.259	1.198	1.264	1.314	0.565	0.567	0.656	BP:phosphorylation;BP:nitrogen compound metabolic process;BP:organic substance metabolic process;BP:protein phosphorylation;BP:cellular process;BP:macromolecule modification;BP:protein modification process;BP:biological_process;BP:metabolic process;BP:primary metabolic process;BP:organonitrogen compound metabolic process;BP:cellular metabolic process;BP:macromolecule metabolic process;BP:protein metabolic process;BP:phosphate-containing compound metabolic process;BP:phosphorus	GO:0016310;GO:0006807;GO:0071704;GO:0006468;GO:0009987;GO:0043412;GO:0036211;GO:0008150;GO:0008152;GO:0044238;GO:1901564;GO:0044237;GO:0043170;GO:0019538;GO:0006796;GO:0006793;GO:0035639;GO:0003674;GO:0005488;GO:0043167;GO:1901363;GO:1901265;GO:0043168;GO:0097367;GO:0000166;GO:0004674;GO:0016740;GO:0004672;GO:0017076;GO:0005524;GO:0016301;GO:0140096;GO:0036094;GO:0003824;GO:0030554;GO:0016773;GO:0016772;GO:0032559;GO:0032555;GO:0097159;GO:0032553	K05733	PAK3, MRX30	map04510;map04810;map04660;map04014;map04360;map04012;map05130;map05132;map05170;map05211	Focal adhesion;Regulation of actin cytoskeleton;T cell receptor signaling pathway;Ras signaling pathway;Axon guidance;ErbB signaling pathway;Pathogenic Escherichia coli infection;Salmonella infection;Human immunodeficiency virus 1 infection;Renal cell carcinoma	ENOG410XP4K	T:Signal transduction mechanisms	PF00069.28;PF07714.20;PF00786.31	Pkinase;PK_Tyr_Ser-Thr;PBD	Protein kinase domain;Protein tyrosine and serine/threonine kinase;P21-Rho-binding domain	CYT	1	2	63.7	High
TRINITY_DN99673_c0_g1_i1_orf1	PREDICTED: pistil-specific extensin-like protein isoform X2 [Microplitis demolitor]	3.8601583	1.948660016	0.03019	up	yes	1.463	0.379	0.315	0.408	0.414	0.954	2.075	1.359	CC:intracellular membrane-bounded organelle;CC:nucleus;CC:cellular_component;CC:intracellular organelle;CC:membrane-bounded organelle;CC:organelle;CC:cellular anatomical entity;MF:cation binding;MF:transition metal ion binding;MF:organic cyclic compound binding;MF:heterocyclic compound binding;MF:ion binding;MF:molecular_function;MF:zinc ion binding;MF:nucleic acid binding;MF:metal ion binding;MF:binding;	GO:0043231;GO:0005634;GO:0005575;GO:0043229;GO:0043227;GO:0043226;GO:0110165;GO:0043169;GO:0046914;GO:0097159;GO:1901363;GO:0043167;GO:0003674;GO:0008270;GO:0003676;GO:0046872;GO:0005488	------	------	------	------	ENOG4110YZS	S:Function unknown	PF12874.10;PF12171.11;PF06220.15	zf-met;zf-C2H2_jaz;zf-U1	Zinc-finger of C2H2 type;Zinc-finger double-stranded RNA-binding;U1 zinc finger	PLA	1	4	27.3	Medium
TRINITY_DN592_c0_g1_i6_orf1	PDZ and LIM domain protein Zasp isoform X4 [Pectinophora gossypiella]	2.2182912	1.149448746	2.08E-06	up	yes	1.254	0.5653	0.569	0.555	0.572	1.253	1.282	1.227	------	------	K19867	PDLIM5_6_7	------	------	ENOG410XRD4	S:Function unknown	PF00412.25;PF00595.27;PF15936.8;PF17820.4;PF02163.25;PF13180.9	LIM;PDZ;DUF4749;PDZ_6;Peptidase_M50;PDZ_2	LIM domain;PDZ domain;Domain of unknown function;PDZ domain;Peptidase family M50;PDZ domain	CYT	21	52	68.6	High
TRINITY_DN69707_c0_g1_i1_orf1	titin-like, partial [Ostrinia furnacalis]	2.048851	1.034815096	6.84E-05	up	yes	1.275	0.6223	0.595	0.671	0.601	1.328	1.269	1.227	------	------	------	------	------	------	ENOG410XQFD;ENOG410XTJ8	S:Function unknown;	------	------	------	CYT	2	49	8.4	High

TRINITY_DN779_c0_g1_i12_orf1	unnamed protein product [Chilo suppressalis]	2.5657768	1.359395696	7.79E-05	up	yes	1.336	0.5207	0.59	0.513	0.459	1.378	1.275	1.356	BP:cellular catabolic process;BP:cellular nitrogen compound metabolic process;BP:nitrogen compound metabolic process;BP:small molecule metabolic process;BP:small molecule catabolic process;BP:purine-containing compound metabolic process;BP:purine-containing compound catabolic process;BP:organic cyclic compound metabolic process;BP:organic cyclic compound catabolic process;BP:nucleobase-containing compound metabolic process;BP:organic substance catabolic process;BP:urate catabolic	GO:0044248;GO:0034641;GO:0006807;GO:0044281;GO:0044282;GO:0072521;GO:0072523;GO:1901360;GO:1901361;GO:0006139;GO:1901575;GO:0019628;GO:0071704;GO:0000255;GO:0009987;GO:0006725;GO:0046700;GO:0008150;GO:0008152;GO:0009056;GO:0055086;GO:0046483;GO:0044238;GO:1901564;GO:0044270;GO:0043603;GO:0009112;GO:0044237;GO:0019439;GO:0006144;GO:0046415;GO:1901565;GO:0003674;GO:0016829;GO:0003824;GO:0016830;GO:0016831	------	------	------	------	ENOG410YZ5M;ENOG4110RGW	S:Function unknown;	PF07004.15	SHIPPO-rpt	Sperm-tail PG-rich repeat	CYT	1	16	93.6	High
TRINITY_DN6482_c0_g1_i1_orf1	endocuticle structural glycoprotein SgAbd-5-like [Ostrinia furnacalis]	2.4560741	1.296354064	1.44E-05	up	yes	1.3	0.5293	0.564	0.486	0.538	1.316	1.323	1.26	MF:molecular_function;MF:structural constituent of cuticle;MF:structural molecule activity;	GO:0003674;GO:0042302;GO:0005198	------	------	------	------	ENOG4111D3H;ENOG410YVGC;ENOG4111EHD;ENOG41125BY	S:Function unknown;	PF00379.26	Chitin_bind_4	Insect cuticle protein	CYT	3	46	11.3	High
TRINITY_DN31118_c0_g1_i1_orf1	CPR9 [Ostrinia furnacalis]	4.9917844	2.319555631	1.69E-05	up	yes	1.519	0.3043	0.309	0.285	0.319	1.541	1.591	1.426	CC:integral component of membrane;CC:cellular_component;CC:intrinsic component of membrane;CC:cellular anatomical entity;MF:structural molecule activity;MF:molecular_function;MF:structural constituent of cuticle;	GO:0016021;GO:0005575;GO:0031224;GO:0110165;GO:0005198;GO:0003674;GO:0042302	------	------	------	------	ENOG41125BY;ENOG410YVGC	S:Function unknown;	PF00379.26	Chitin_bind_4	Insect cuticle protein	EXC	8	78	11.4	High
TRINITY_DN101995_c0_g1_i1_orf1	microtubule-actin cross-linking factor 1 isoform X15 [Ostrinia furnacalis]	2.0587736	1.041785166	1.47E-06	up	yes	1.212	0.5887	0.588	0.607	0.571	1.23	1.199	1.208	------	------	K10382	DST	------	------	COG5069;ENOG410XP41	S:Function unknown;Z:Cytoskeleton	------	------	------	CYT	1	16	8.3	High
TRINITY_DN135077_c0_g1_i1_orf1	hypothetical protein KR038_001662 [Drosophila bunnanda]	2.1845638	1.127345213	4.65E-05	up	yes	1.302	0.596	0.545	0.642	0.601	1.263	1.348	1.296	------	------	------	------	------	------	ENOG4112D9O	S:Function unknown	------	------	------	CYT	4	64	8.5	High
TRINITY_DN59829_c0_g1_i1_orf1	putative mediator of RNA polymerase II transcription subunit 12 [Ostrinia furnacalis]	0.3526053	-1.503874009	9.92E-07	down	yes	0.494	1.401	1.417	1.416	1.369	0.512	0.488	0.482	------	------	------	------	------	------	ENOG4110RMW	S:Function unknown	------	------	------	CYT	4	10	67.1	High
TRINITY_DN13119_c0_g1_i4_orf1	endocuticle structural glycoprotein ABD-5-like [Bicyclus anynana]	5.375	2.426264755	4.85E-06	up	yes	1.548	0.288	0.266	0.289	0.309	1.588	1.58	1.477	------	------	------	------	------	------	ENOG410YVGC;ENOG4110QDR;ENOG410YVKS;ENOG41125BY	S:Function unknown;	PF00379.26	Chitin_bind_4	Insect cuticle protein	CYT	3	49	12.3	High
TRINITY_DN1889_c0_g1_i1_orf1	titin isoform X2 [Ostrinia furnacalis]	2.0691895	1.049065804	2.16E-06	up	yes	1.274	0.6157	0.619	0.612	0.616	1.305	1.267	1.251	------	------	K12567	TTN	map05414;map05410	Dilated cardiomyopathy;Hypertrophic cardiomyopathy	ENOG410XTJ8	Q:Secondary metabolites biosynthesis, transport and catabolism	PF07679.19;PF13927.9;PF00047.28;PF13895.9	I-set;Ig_3;ig;Ig_2	Immunoglobulin I-set domain;Immunoglobulin domain;Immunoglobulin domain;Immunoglobulin domain	CYT	191	43	610.3	High
TRINITY_DN4053_c0_g1_i5_orf1	uncharacterized protein LOC114358355 [Ostrinia furnacalis]	2.0234463	1.016814549	0.002747	up	yes	1.26	0.6227	0.657	0.603	0.608	1.315	1.075	1.391	------	------	------	------	------	------	ENOG4110S1M	S:Function unknown	------	------	------	CYT	1	6	21.5	High
TRINITY_DN48641_c0_g1_i4_orf1	RNA-binding protein 45-like [Galleria mellonella]	0.2820565	-1.825944158	0.0003543	down	yes	0.4197	1.488	1.367	1.652	1.444	0.501	0.362	0.396	MF:organic cyclic compound binding;MF:molecular_function;MF:binding;MF:nucleic acid binding;MF:heterocyclic compound binding;MF:RNA binding;	GO:0097159;GO:0003674;GO:0005488;GO:0003676;GO:1901363;GO:0003723	K25081	RBM45	------	------	ENOG410XP9Z	S:Function unknown	PF00076.25;PF08777.14	RRM_1;RRM_3	RNA recognition motif.;RNA binding motif	CYT	1	2	53.8	High
TRINITY_DN1814_c0_g2_i1_orf1	titin-like, partial [Ostrinia furnacalis]	2.1605585	1.11140427	2.44E-06	up	yes	1.238	0.573	0.582	0.588	0.549	1.259	1.219	1.235	------	------	K12567	TTN	map05414;map05410	Dilated cardiomyopathy;Hypertrophic cardiomyopathy	ENOG410XQFD;ENOG410XTJ8;ENOG4111364	S:Function unknown;	------	------	------	CYT	32	21	230.9	High
TRINITY_DN31645_c0_g1_i3_orf1	dystonin isoform X43 [Helicoverpa armigera]	2.255939	1.173728035	0.0004029	up	yes	1.301	0.5767	0.682	0.501	0.547	1.308	1.363	1.231	------	------	K10382	DST	------	------	ENOG410XP41;ENOG410ZE6W;COG5069	Z:Cytoskeleton;S:Function unknown;Z:Cytoskeleton	------	------	------	CYT	1	6	15.8	High
TRINITY_DN1029_c0_g1_i1_orfp1	TRINITY_DN1029_c0_g1_i1_m.64408 TRINITY_DN1029_c0_g1::TRINITY_DN1029_c0_g1_i1::g.64408 ORF type:3prime_partial len:55 (+),score=13.47 TRINITY_DN1029_c0_g1_i1:74-235(+)	2.2295727	1.15676723	0.000812	up	yes	1.247	0.5593	0.668	0.555	0.455	1.296	1.159	1.285	------	------	------	------	------	------	------	------	------	------	------	CYT	1	56	6.1	High
TRINITY_DN8651_c0_g1_i18_orf1	glutathione S-transferase theta 2 [Conogethes punctiferalis]	2.2413223	1.164350131	6.06E-06	up	yes	1.356	0.605	0.594	0.602	0.619	1.347	1.322	1.399	CC:cytoplasm;CC:cellular_component;CC:cellular anatomical entity;MF:glutathione transferase activity;MF:catalytic activity;MF:transferase activity, transferring alkyl or aryl;MF:transferase activity;MF:molecular_function;	GO:0005737;GO:0005575;GO:0110165;GO:0004364;GO:0003824;GO:0016765;GO:0016740;GO:0003674	K00799	GST, gst	map05207;map00480;map05204;map00983;map00982;map00980;map05200;map05208;map05418;map05225;map04212;map01524	Chemical carcinogenesis - receptor activation;Glutathione metabolism;Chemical carcinogenesis - DNA adducts;Drug metabolism - other enzymes;Drug metabolism - cytochrome P450;Metabolism of xenobiotics by cytochrome P450;Pathways in cancer;Chemical carcinogenesis - reactive oxygen species;Fluid shear stress and atherosclerosis;Hepatocellular carcinoma;Longevity regulating pathway - worm;Platinum drug resistance	COG0625	O:Posttranslational modification, protein turnover, chaperones	PF00043.28;PF14497.9	GST_C;GST_C_3	Glutathione S-transferase, C-terminal domain;Glutathione S-transferase, C-terminal domain	CYT	2	39	16.8	High
TRINITY_DN1313_c0_g1_i2_orf1	39S ribosomal protein L40, mitochondrial [Ostrinia furnacalis]	0.2921271	-1.775332034	6.97E-05	down	yes	0.423	1.448	1.561	1.419	1.363	0.405	0.418	0.446	CC:large ribosomal subunit;CC:organellar large ribosomal subunit;CC:ribonucleoprotein complex;CC:ribosomal subunit;CC:cellular_component;CC:mitochondrial large ribosomal subunit;CC:macromolecular complex;CC:mitochondrial protein complex;	GO:0015934;GO:0000315;GO:1990904;GO:0044391;GO:0005575;GO:0005762;GO:0032991;GO:0098798	K17421	MRPL40	------	------	ENOG4111PQW	S:Function unknown	PF09812.12	MRP-L28	Mitochondrial ribosomal protein L28	CYT	1	11	23.8	High

TRINITY_DN1585_c0_g1_i1_orf1	aldose reductase-like isoform X2 [Ostrinia furnacalis]	2.2381893	1.16233208	1.34E-06	up	yes	1.246	0.5567	0.536	0.568	0.566	1.234	1.268	1.237	MF:catalytic activity;MF:molecular_function;MF:oxidoreductase activity;	GO:0003824;GO:0003674;GO:0016491	K00011	AKR1B	map00051;map00040;map00561;map00790;map00052	Fructose and mannose metabolism;Pentose and glucuronate interconversions;Glycerolipid metabolism;Folate biosynthesis;Galactose metabolism	COG0656	Z:Cytoskeleton	PF00248.24	Aldo_ket_red	Aldo/keto reductase family	CYT	2	9	35.7	High
TRINITY_DN86127_c1_g1_i2_orfp1	TRINITY_DN86127_c1_g1_i2_m.43062 TRINITY_DN86127_c1_g1::TRINITY_DN86127_c1_g1_i2::g.43062 ORF type:internal len:69 (-),score=14.03 TRINITY_DN86127_c1_g1_i2:2-205(-)	2.4626721	1.300224546	0.003625	up	yes	1.27	0.5157	0.5	0.498	0.549	1.03	1.431	1.348	------	------	------	------	------	------	------	------	------	------	------	CYT	1	16	7.8	High
TRINITY_DN14217_c0_g1_i1_orf1	putative serine protease K12H4.7 [Ostrinia furnacalis] >XP_028166339.1 putative serine protease K12H4.7 [Ostrinia furnacalis]	0.114415	-3.127651931	0.01265	down	yes	0.177	1.547	1.769	0.924	1.948	0.133	0.136	0.262	BP:primary metabolic process;BP:organonitrogen compound metabolic process;BP:protein metabolic process;BP:macromolecule metabolic process;BP:organic substance metabolic process;BP:nitrogen compound metabolic process;BP:biological_process;BP:metabolic process;BP:proteolysis;MF:molecular_function;MF:serine hydrolase activity;MF:hydrolase activity;MF:catalytic activity, acting on a protein;MF:catalytic activity;MF:peptidase activity;MF:serine-type peptidase activity;	GO:0044238;GO:1901564;GO:0019538;GO:0043170;GO:0071704;GO:0006807;GO:0008150;GO:0008152;GO:0006508;GO:0003674;GO:0017171;GO:0016787;GO:0140096;GO:0003824;GO:0008233;GO:0008236	------	------	------	------	ENOG410XSGG	S:Function unknown	PF05577.15	Peptidase_S28	Serine carboxypeptidase S28	CYT	1	3	54.9	High
TRINITY_DN5962_c0_g1_i1_orf1	tRNA (cytosine(34)-C(5))-methyltransferase [Ostrinia furnacalis]	2.6147077	1.386649693	5.97E-05	up	yes	1.248	0.4773	0.482	0.483	0.467	1.33	1.183	1.232	CC:intracellular organelle;CC:membrane-bounded organelle;CC:nucleus;CC:organelle;CC:cellular anatomical entity;CC:intracellular membrane-bounded organelle;CC:cellular_component;MF:molecular_function;MF:binding;MF:nucleic acid binding;MF:methyltransferase activity;MF:heterocyclic compound binding;MF:tRNA binding;MF:transferase activity;MF:transferase activity, transferring one-carbon groups;MF:catalytic activity, acting on RNA;MF:S-adenosylmethionine-dependent methyltransferase activity;MF:RNA methyltransferase activity;MF:tRNA	GO:0043229;GO:0043227;GO:0005634;GO:0043226;GO:0110165;GO:0043231;GO:0005575;GO:0003674;GO:0005488;GO:0003676;GO:0008168;GO:1901363;GO:0000049;GO:0016740;GO:0016741;GO:0140098;GO:0008757;GO:0008173;GO:0008175;GO:0016428;GO:0016427;GO:0097159;GO:0003824;GO:0003723;GO:0140101	K15335	NSUN2, TRM4	------	------	COG0144	J:Translation, ribosomal structure and biogenesis	PF01189.20	Methyltr_RsmB-F	16S rRNA methyltransferase RsmB/F	CYT	6	10	83.8	High
TRINITY_DN41259_c0_g1_i6_orf1	endocuticle structural glycoprotein SgAbd-8 [Ostrinia furnacalis]	0.1058634	-3.239724172	9.18E-06	down	yes	0.1643	1.552	1.547	1.639	1.471	0.154	0.174	0.165	MF:molecular_function;MF:structural constituent of cuticle;MF:structural molecule activity;	GO:0003674;GO:0042302;GO:0005198	------	------	------	------	ENOG4111PJ1	S:Function unknown	PF00379.26	Chitin_bind_4	Insect cuticle protein	CYT	7	44	24.7	High
TRINITY_DN1504_c0_g1_i1_orf1	uncharacterized protein LOC114352862 [Ostrinia furnacalis] >XP_028160407.1 uncharacterized protein LOC114352862 [Ostrinia furnacalis]	0.4922055	-1.022667341	1.36E-05	down	yes	0.5557	1.129	1.132	1.104	1.152	0.521	0.576	0.57	------	------	------	------	------	------	ENOG410Y735	S:Function unknown	------	------	------	EXC	1	1	83.4	Medium
TRINITY_DN57904_c0_g2_i1_orf1	cuticle protein 19 [Plutella xylostella] >CAG9138481.1 unnamed protein product [Plutella xylostella]	0.1730496	-2.53074211	3.72E-05	down	yes	0.244	1.41	1.356	1.522	1.351	0.217	0.272	0.243	MF:molecular_function;MF:structural constituent of cuticle;MF:structural molecule activity;	GO:0003674;GO:0042302;GO:0005198	------	------	------	------	ENOG410YSFI	S:Function unknown	PF00379.26	Chitin_bind_4	Insect cuticle protein	CYT	1	16	14.1	High
TRINITY_DN1814_c0_g2_i4_orfp1	TRINITY_DN1814_c0_g2_i4_m.63284 TRINITY_DN1814_c0_g2::TRINITY_DN1814_c0_g2_i4::g.63284 ORF type:internal len:258 (-),score=126.31 TRINITY_DN1814_c0_g2_i4:3-773(-)	3.4612491	1.791292757	1.09E-05	up	yes	1.38	0.3987	0.415	0.382	0.399	1.392	1.434	1.315	------	------	------	------	------	------	------	------	------	------	------	CYT	3	34	30.5	High
TRINITY_DN31118_c0_g2_i1_orf1	unnamed protein product [Spodoptera exigua]	6.4913025	2.698507989	6.55E-06	up	yes	1.53	0.2357	0.252	0.212	0.243	1.599	1.532	1.46	MF:molecular_function;MF:structural constituent of cuticle;MF:structural molecule activity;	GO:0003674;GO:0042302;GO:0005198	------	------	------	------	ENOG410YVGC;ENOG41125BY;ENOG410YVKS	S:Function unknown;	PF00379.26	Chitin_bind_4	Insect cuticle protein	CYT	2	53	12.9	High
TRINITY_DN1073_c0_g1_i3_orf1	carboxylesterase [Loxostege sticticalis]	2.7492783	1.459052932	0.0003973	up	yes	1.238	0.4503	0.44	0.393	0.518	1.362	1.169	1.183	CC:integral component of membrane;CC:cellular_component;CC:intrinsic component of membrane;CC:cellular anatomical entity;MF:hydrolase activity;MF:hydrolase activity, acting on ester bonds;MF:carboxylic ester hydrolase activity;MF:catalytic activity;MF:molecular_function;	GO:0016021;GO:0005575;GO:0031224;GO:0110165;GO:0016787;GO:0016788;GO:0052689;GO:0003824;GO:0003674	------	------	------	------	COG2272;COG0580;ENOG410XSWS	I:Lipid transport and metabolism;G:Carbohydrate transport and metabolism;T:Signal transduction mechanisms	PF00135.31;PF20434.1;PF07859.16	COesterase;BD-FAE;Abhydrolase_3	Carboxylesterase family;BD-FAE;alpha/beta hydrolase fold	CYT	3	7	68.1	High
TRINITY_DN2215_c0_g2_i1_orf1	PREDICTED: larval cuticle protein LCP-22-like [Amyelois transitella]	6.1338912	2.61680258	4.74E-06	up	yes	1.466	0.239	0.264	0.206	0.247	1.465	1.523	1.411	------	------	------	------	------	------	ENOG41119AX;ENOG4111CQ7;ENOG4111DQA;ENOG410YZKX;ENOG4111PJ1	S:Function unknown;	PF00379.26	Chitin_bind_4	Insect cuticle protein	CYT	3	33	16.5	High
TRINITY_DN1180_c0_g1_i4_orf1	larval cuticle protein LCP-30-like [Ostrinia furnacalis]	5.8921855	2.558802861	9.57E-06	up	yes	1.41	0.2393	0.247	0.225	0.246	1.399	1.486	1.345	MF:molecular_function;MF:structural constituent of cuticle;MF:structural molecule activity;	GO:0003674;GO:0042302;GO:0005198	------	------	------	------	ENOG410YZ4Z;ENOG4110S48	S:Function unknown;	PF00379.26	Chitin_bind_4	Insect cuticle protein	CYT	13	61	31	High
TRINITY_DN4070_c0_g1_i4_orf1	alpha-N-acetylgalactosaminidase isoform X3 [Ostrinia furnacalis]	0.3941476	-1.343192168	7.03E-06	down	yes	0.4647	1.179	1.195	1.204	1.139	0.449	0.488	0.457	BP:primary metabolic process;BP:organic substance metabolic process;BP:biological_process;BP:metabolic process;BP:carbohydrate metabolic process;BP:lipid metabolic process;CC:cellular_component;CC:lytic vacuole;CC:intracellular membrane-bounded organelle;CC:vacuole;CC:lysosome;CC:intracellular organelle;CC:membrane-bounded organelle;CC:organelle;CC:cellular anatomical entity;MF:molecular_function;MF:hydrolase activity;MF:hydrolase activity, acting on glycosyl bonds;MF:catalytic activity;MF:hydrolase activity, hydrolyzing O-glycosyl	GO:0044238;GO:0071704;GO:0008150;GO:0008152;GO:0005975;GO:0006629;GO:0005575;GO:0000323;GO:0043231;GO:0005773;GO:0005764;GO:0043229;GO:0043227;GO:0043226;GO:0110165;GO:0003674;GO:0016787;GO:0016798;GO:0003824;GO:0004553	K01204	NAGA	map00603;map04142	Glycosphingolipid biosynthesis - globo and isoglobo series;Lysosome	ENOG410XPF1	O:Posttranslational modification, protein turnover, chaperones	PF16499.8;PF17450.5;PF17801.4	Melibiase_2;Melibiase_2_C;Melibiase_C	Alpha galactosidase A;Alpha galactosidase A C-terminal beta sandwich domain;Alpha galactosidase C-terminal beta sandwich domain	CYT	5	15	49.7	High

TRINITY_DN45949_c0_g1_i1_orf1	uncharacterized protein LOC114355167 [Ostrinia furnacalis]	2.8098592	1.490497817	8.72E-07	up	yes	1.197	0.426	0.423	0.409	0.446	1.181	1.217	1.194	MF:molecular_function;MF:structural constituent of cuticle;MF:structural molecule activity;	GO:0003674;GO:0042302;GO:0005198	------	------	------	------	ENOG410ZET2;ENOG410YR66;ENOG410YXXU;ENOG4111PJ1	O:Posttranslational modification, protein turnover, chaperones;S:Function unknown;S:Function unknown;S:Function unknown	PF00379.26	Chitin_bind_4	Insect cuticle protein	CYT	3	18	22.7 High
TRINITY_DN2040_c0_g1_i15_orfp1	TRINITY_DN2040_c0_g1_i15_m.4150 TRINITY_DN2040_c0_g1::TRINITY_DN2040_c0_g1_i15::g.4150 ORF type:complete len:319 (+),score=125.43,Plasmodium_HRP PF05403.12 3.5,Plasmodium_HRP|PF05403.12|1.5 TRINITY_DN2040_c0_g1_i15:118-957(+)	2.5906314	1.373303741	0.002299	up	yes	1.272	0.491	0.458	0.459	0.556	1.268	1.462	1.087	------	------	------	------	------	------	------	------	------	------	------	PLA	2	16	30.3 High
TRINITY_DN26254_c0_g1_i1_orf1	hypothetical protein evm_003664 [Chilo suppressalis] >CAB3521132.1 unnamed protein product [Chilo suppressalis] >CAH0398453.1 unnamed protein product [Chilo suppressalis]	4.3638077	2.125587537	2.33E-08	up	yes	1.389	0.3183	0.325	0.304	0.326	1.383	1.387	1.398	MF:molecular_function;MF:structural constituent of cuticle;MF:structural molecule activity;	GO:0003674;GO:0042302;GO:0005198	------	------	------	------	ENOG410ZNDD;ENOG4110RUZ;ENOG4111PJ1;ENOG4110T0M	S:Function unknown;	PF00379.26	Chitin_bind_4	Insect cuticle protein	CYT	3	36	14.7 High
TRINITY_DN198_c0_g1_i2_orf1	retinol dehydrogenase 13-like [Ostrinia furnacalis]	0.4653205	-1.103703482	8.93E-05	down	yes	0.53	1.139	1.152	1.068	1.198	0.531	0.532	0.527	------	------	K11153	RDH12	map00830;map01240	Retinol metabolism;Biosynthesis of cofactors	COG1028	V:Defense mechanisms	PF00106.28;PF13561.9;PF08659.13	adh_short;adh_short_C2;KR	short chain dehydrogenase;Enoyl;KR domain	CYT	1	4	35.3 High
TRINITY_DN36061_c0_g4_i2_orf1	putative GPI-anchored protein pfl2 [Ostrinia furnacalis] >XP_028163002.1 putative GPI-anchored protein pfl2 [Ostrinia furnacalis]	0.1679181	-2.574170444	4.30E-06	down	yes	0.246	1.465	1.396	1.496	1.504	0.243	0.26	0.235	CC:cellular_component;CC:extracellular region;CC:cellular anatomical entity;MF:carbohydrate derivative binding;MF:molecular_function;MF:chitin binding;MF:binding;	GO:0005575;GO:0005576;GO:0110165;GO:0097367;GO:0003674;GO:0008061;GO:0005488	------	------	------	------	ENOG4111P12	S:Function unknown	------	------	------	CYT	1	1	91.7 High
TRINITY_DN3332_c0_g1_i9_orf1	glutathione S-transferase sigma3 [Glyphodes pyloalis]	2.0957427	1.06746163	0.001254	up	yes	1.147	0.5473	0.577	0.542	0.523	1.096	1.055	1.289	MF:catalytic activity;MF:transferase activity;MF:glutathione transferase activity;MF:molecular_function;MF:transferase activity, transferring alkyl or ary;	GO:0003824;GO:0016740;GO:0004364;GO:0003674;GO:0016765	K04097	HPGDS	map00590;map00480;map05204;map00982;map00980	Arachidonic acid metabolism;Glutathione metabolism;Chemical carcinogenesis - DNA adducts;Drug metabolism - cytochrome P450;Metabolism of xenobiotics by cytochrome P450	ENOG41103AU;ENOG4110YU0;ENOG4111VAU;ENOG4111I69;ENOG4111KFX	O:Posttranslational modification, protein turnover, chaperones;	PF14497.9;PF02798.23;PF00043.28	GST_C_3;GST_N;GST_C	Glutathione S-transferase, C-terminal domain;Glutathione S-transferase, N-terminal domain;Glutathione S-transferase, C-terminal domain	CYT	3	30	23.1 High
TRINITY_DN1215_c0_g1_i2_orf1	PI-stichotoxin-She2a-like [Ostrinia furnacalis]	2.7463984	1.457540898	5.69E-05	up	yes	1.201	0.4373	0.427	0.44	0.445	1.186	1.281	1.137	BP:envenomation resulting in modulation of voltage-gated potassium channel activity in other organism;BP:interspecies interaction between organisms;BP:biological_process;BP:envenomation resulting in modulation of ion channel activity in other organism;BP:envenomation resulting in negative regulation of voltage-gated potassium channel activity in other organism;BP:envenomation resulting in modification of morphology or physiology of other organism;BP:modification of morphology or physiology of other organism;MF:enzyme	GO:0044559;GO:0044419;GO:0008150;GO:0044560;GO:0044562;GO:0035738;GO:0035821;GO:0030234;GO:0061134;GO:0030414;GO:0061135;GO:0004867;GO:0004866;GO:0003674;GO:0004857;GO:0098772	K03909	TFPI	map04610	Complement and coagulation cascades	ENOG410XQNP	O:Posttranslational modification, protein turnover, chaperones	PF00014.26	Kunitz_BPTI	Kunitz/Bovine pancreatic trypsin inhibitor domain	CYT	2	31	8.6 High
TRINITY_DN52395_c0_g2_i2_orf1	twitchin isoform X20 [Zerene cesonia]	2.2932105	1.197368787	0.0003455	up	yes	1.145	0.4993	0.414	0.579	0.505	1.178	1.175	1.083	BP:developmental process;BP:cell differentiation;BP:cellular developmental process;BP:cellular process;BP:biological_process;BP:anatomical structure morphogenesis;	GO:0032502;GO:0030154;GO:0048869;GO:0009987;GO:0008150;GO:0009653	------	------	------	------	ENOG410XP8U;ENOG410XQFD;ENOG410XRMJ	S:Function unknown;	PF07679.19	I-set	Immunoglobulin I-set domain	CYT	1	73	10.1 High
TRINITY_DN14507_c0_g1_i5_orf1	PTB domain-containing adapter protein ced-6 [Ostrinia furnacalis]	0.3254886	-1.619321083	0.02261	down	yes	0.3997	1.228	1.397	1.49	0.796	0.544	0.305	0.35	------	------	K23285	GULP1, CED6	------	------	ENOG410ZHJT;COG0666	S:Function unknown;	PF00640.26	PID	Phosphotyrosine interaction domain	CYT	1	3	57 High
TRINITY_DN549_c0_g1_i7_orf1	titin-like, partial [Ostrinia furnacalis]	3.100304	1.632409663	0.0008251	up	yes	1.326	0.4277	0.395	0.436	0.452	1.513	1.183	1.282	BP:developmental process;BP:cell differentiation;BP:cellular developmental process;BP:cellular process;BP:biological_process;BP:anatomical structure morphogenesis;	GO:0032502;GO:0030154;GO:0048869;GO:0009987;GO:0008150;GO:0009653	K12567	TTN	map05414;map05410	Dilated cardiomyopathy;Hypertrophic cardiomyopathy	ENOG410XQFD;ENOG410XTJ8;ENOG410Y6GZ	S:Function unknown;	------	------	------	CYT	2	4	48.1 High
TRINITY_DN30208_c0_g1_i3_orf1	unnamed protein product [Timema cristinae]	2.7326087	1.450278883	1.86E-05	up	yes	1.257	0.46	0.456	0.461	0.463	1.269	1.308	1.194	------	------	K10374	TPM2	map05414;map04261;map04260;map05410	Dilated cardiomyopathy;Adrenergic signaling in cardiomyocytes;Cardiac muscle contraction;Hypertrophic cardiomyopathy	ENOG410XR5K	Z:Cytoskeleton	PF00261.23;PF12718.10	Tropomyosin;Tropomyosin_1	Tropomyosin;Tropomyosin like	CYT	4	57	7.7 High
TRINITY_DN8915_c0_g1_i3_orf1	filamin-A isoform X1 [Ostrinia furnacalis] >XP_028171553.1 filamin-A isoform X2 [Ostrinia furnacalis] >XP_028171561.1 filamin-A isoform X2 [Ostrinia furnacalis]	2.0544037	1.038719672	0.0001197	up	yes	1.08	0.5257	0.529	0.531	0.517	1.141	1.013	1.086	BP:organelle organization;BP:cytoskeleton organization;BP:actin filament-based process;BP:cellular process;BP:actin cytoskeleton organization;BP:biological_process;BP:cellular component organization;BP:cellular component organization or biogenesis;MF:molecular_function;MF:cytoskeletal protein binding;MF:actin filament binding;MF:actin binding;MF:protein binding;MF:macromolecular complex binding;MF:binding;	GO:0006996;GO:0007010;GO:0030029;GO:0009987;GO:0030036;GO:0008150;GO:0016043;GO:0071840;GO:0003674;GO:0008092;GO:0051015;GO:0003779;GO:0005515;GO:0044877;GO:0005488	K04437	FLNA	map05205;map04510;map04010;map05132	Proteoglycans in cancer;Focal adhesion;MAPK signaling pathway;Salmonella infection	ENOG410XNS3;COG5069	S:Function unknown;	PF00630.22	Filamin	Filamin/ABP280 repeat	CYT	37	54	100.3 High

TRINITY_DN46216_c0_g3_i1_orf1	unnamed protein product, partial [Brenthis ino]	6.7608378	2.757202035	2.64E-06	up	yes	1.388	0.2053	0.22	0.169	0.227	1.376	1.353	1.435	------	------	------	------	------	------	ENOG410Y7Q4;ENOG410XW69;ENOG4110RGQ	J:Translation, ribosomal structure and biogenesis;U:Intracellular trafficking, secretion, and vesicular transport;S:Function unknown	PF03564.18	DUF1759	Protein of unknown function	CYT	1	11	9.6	Medium
TRINITY_DN783_c0_g1_i7_orf1	microtubule-associated protein Jupiter isoform X4 [Helicoverpa armigera]	0.4603914	-1.119067129	0.0001616	down	yes	0.494	1.073	1.148	1.021	1.049	0.467	0.49	0.525	------	------	------	------	------	------	ENOG410Z68G	S:Function unknown	PF17054.8	JUPITER	Microtubule-Associated protein Jupiter	EXC	2	12	24	High
TRINITY_DN695_c0_g1_i12_orf1	seroin transcript 2A, partial [Ostrinia nubilalis]	2.626506	1.393144893	0.001203	up	yes	1.09	0.415	0.361	0.424	0.46	1.136	1.195	0.94	------	------	------	------	------	------	ENOG4110R3H;ENOG4110N4H	S:Function unknown;	------	------	------	CYT	1	30	15.1	High
TRINITY_DN76529_c0_g1_i1_orfp1	TRINITY_DN76529_c0_g1_i1_m.64079 TRINITY_DN76529_c0_g1::TRINITY_DN76529_c0_g1_i1::g.64079 ORF type:internal len:70 (+),score=14.68 TRINITY_DN76529_c0_g1_i1:3-209(+)	2.0848861	1.059968589	8.40E-05	up	yes	1.007	0.483	0.499	0.469	0.481	0.973	1.069	0.979	------	------	------	------	------	------	------	------	------	------	------	CYT	1	23	7.7	High
TRINITY_DN2749_c0_g2_i3_orf1	RNA exonuclease 4-like [Ostrinia furnacalis] >QEE79882.1 REX4 [Ostrinia furnacalis]	2.1792306	1.123818865	4.58E-05	up	yes	0.98	0.4497	0.459	0.453	0.437	0.947	1.034	0.959	BP:nucleic acid metabolic process;BP:cellular nitrogen compound metabolic process;BP:nitrogen compound metabolic process;BP:ncRNA metabolic process;BP:organic cyclic compound metabolic process;BP:nucleobase-containing compound metabolic process;BP:organic substance metabolic process;BP:rRNA processing;BP:ncRNA processing;BP:cellular process;BP:cellular aromatic compound metabolic process;BP:biological_process;BP:metabolic process;BP:heterocycle metabolic process;BP:RNA metabolic [illegible]	GO:0090304;GO:0034641;GO:0006807;GO:0034660;GO:1901360;GO:0006139;GO:0071704;GO:0006364;GO:0034470;GO:0009987;GO:0006725;GO:0008150;GO:0008152;GO:0046483;GO:0016070;GO:0044238;GO:0016072;GO:0044237;GO:0043170;GO:0006396;GO:0003674;GO:0005488;GO:0003676;GO:1901363;GO:0008408;GO:0004527;GO:0016787;GO:0016788;GO:0003824;GO:0004518;GO:0097159	K18327	REXO4, REX4	------	------	COG0847	L:Replication, recombination and repair	PF00929.27	RNase_T	Exonuclease	CYT	5	45	17.8	High
TRINITY_DN87648_c0_g1_i1_orfp1	TRINITY_DN87648_c0_g1_i1_m.51054 TRINITY_DN87648_c0_g1::TRINITY_DN87648_c0_g1_i1::g.51054 ORF type:internal len:180 (+),score=75.93 TRINITY_DN87648_c0_g1_i1:2-538(+)	2.0026154	1.001885348	1.18E-05	up	yes	1.072	0.5353	0.524	0.547	0.535	1.108	1.066	1.043	------	------	------	------	------	------	------	------	------	------	------	CYT	7	37	18	High
TRINITY_DN52244_c1_g1_i1_orf1	triokinase/FMN cyclase-like isoform X2 [Ostrinia furnacalis]	0.4412556	-1.180313489	0.005577	down	yes	0.492	1.115	1.167	0.91	1.269	0.439	0.462	0.575	BP:phosphorylation;BP:polyol metabolic process;BP:small molecule metabolic process;BP:cellular carbohydrate metabolic process;BP:organic substance metabolic process;BP:alditol metabolic process;BP:glycerol metabolic process;BP:phosphorus metabolic process;BP:cellular process;BP:primary metabolic process;BP:biological_process;BP:metabolic process;BP:carbohydrate metabolic process;BP:cellular metabolic process;BP:alcohol metabolic process;BP:phosphate-containing compound metabolic [illegible]	GO:0016310;GO:0019751;GO:0044281;GO:0044262;GO:0071704;GO:0019400;GO:0006071;GO:0006793;GO:0009987;GO:0044238;GO:0008150;GO:0008152;GO:0005975;GO:0044237;GO:0006066;GO:0006796;GO:1901615;GO:0005488;GO:0035639;GO:0003674;GO:0043167;GO:1901363;GO:1901265;GO:0043168;GO:0097367;GO:0016829;GO:0016849;GO:0000166;GO:0016740;GO:0005524;GO:0016301;GO:0036094;GO:0003824;GO:0017076;GO:0034012;GO:0004371;GO:0030554;GO:0009975;GO:0016772;GO:0016773;GO:0032559;GO:0097159;GO:0032555;GO:0032553	------	------	------	------	COG2376	G:Carbohydrate transport and metabolism	PF02733.20	Dak1	Dak1 domain	CYT	1	22	8.2	High
TRINITY_DN279_c0_g1_i10_orf1	RE1-silencing transcription factor-like isoform X1 [Ostrinia furnacalis]	0.3602632	-1.472876972	4.46E-05	down	yes	0.4107	1.14	1.073	1.168	1.179	0.38	0.443	0.409	CC:intracellular membrane-bounded organelle;CC:nucleus;CC:cellular_component;CC:intracellular organelle;CC:membrane-bounded organelle;CC:organelle;CC:cellular anatomical entity;MF:cation binding;MF:transition metal ion binding;MF:ion binding;MF:molecular_function;MF:zinc ion binding;MF:metal ion binding;MF:binding;	GO:0043231;GO:0005634;GO:0005575;GO:0043229;GO:0043227;GO:0043226;GO:0110165;GO:0043169;GO:0046914;GO:0043167;GO:0003674;GO:0008270;GO:0046872;GO:0005488	------	------	------	------	COG5048	O:Posttranslational modification, protein turnover, chaperones	PF00096.29;PF13894.9;PF13465.9;PF07776.18	zf-C2H2;zf-C2H2_4;zf-H2C2_2;zf-AD	Zinc finger, C2H2 type;C2H2-type zinc finger;Zinc-finger double domain;Zinc-finger associated domain	CYT	1	1	120.9	High
TRINITY_DN48610_c0_g1_i2_orf1	hypothetical protein evm_002298 [Chilo suppressalis] >CAH0682062.1 unnamed protein product [Chilo suppressalis]	2.5353463	1.342182802	2.04E-05	up	yes	1.058	0.4173	0.435	0.413	0.404	1.025	1.109	1.039	MF:molecular_function;MF:structural constituent of cuticle;MF:structural molecule activity;	GO:0003674;GO:0042302;GO:0005198	------	------	------	------	ENOG410XTK4;ENOG4111PJ1;ENOG4110RRZ;ENOG4110T67	S:Function unknown;	PF00379.26	Chitin_bind_4	Insect cuticle protein	CYT	5	41	18.6	High
TRINITY_DN7580_c0_g1_i1_orf1	cytochrome P450 monooxygenase CYP6AB141 [Ostrinia furnacalis]	2.3649432	1.241805531	1.66E-05	up	yes	1.02	0.4313	0.407	0.432	0.455	0.982	1.048	1.03	MF:cation binding;MF:monooxygenase activity;MF:heme binding;MF:transition metal ion binding;MF:tetrapyrrole binding;MF:organic cyclic compound binding;MF:oxidoreductase activity, acting on paired donors, with incorporation or reduction of molecular oxygen;MF:ion binding;MF:catalytic activity;MF:molecular_function;MF:binding;MF:iron ion binding;MF:metal ion binding;MF:heterocyclic compound binding;MF:oxidoreduc[illegible]	GO:0043169;GO:0004497;GO:0020037;GO:0046914;GO:0046906;GO:0097159;GO:0016705;GO:0043167;GO:0003824;GO:0003674;GO:0005488;GO:0005506;GO:0046872;GO:1901363;GO:0016491	K14999	CYP6	------	------	COG2124	S:Function unknown	PF00067.25	p450	Cytochrome P450	CYT	4	14	52.1	High
TRINITY_DN1455_c0_g1_i8_orf1	troponin T, skeletal muscle isoform X1 [Galleria mellonella]	2.9586777	1.56495254	2.20E-05	up	yes	1.074	0.363	0.348	0.355	0.386	1.057	1.13	1.034	BP:regulation of system process;BP:regulation of muscle contraction;BP:biological regulation;BP:regulation of muscle system process;BP:regulation of multicellular organismal process;BP:biological_process;BP:regulation of biological process;CC:macromolecular complex;CC:cellular_co[illegible]	GO:0044057;GO:0006937;GO:0065007;GO:0090257;GO:0051239;GO:0008150;GO:0050789;GO:0032991;GO:0005575;GO:0005861	K12046	TNNT3	------	------	ENOG410XS6A	Z:Cytoskeleton	------	------	------	CYT	5	41	43.1	High

TRINITY_DN1914_c0_g1_i4_orf1	loricrin-like [Ostrinia furnacalis]	4.0903226	2.032214625	1.14E-05	up	yes	1.268	0.31	0.366	0.268	0.296	1.261	1.306	1.236	------	------	------	------	------	------	ENOG4110QTU;ENOG4110QBC	S:Function unknown;	------	------	------	CYT	1	15	58.5	High
TRINITY_DN48097_c0_g1_i1_orf1	unnamed protein product [Homo sapiens]	2.4633431	1.300617589	6.59E-05	up	yes	1.092	0.4433	0.419	0.443	0.468	1.099	1.029	1.149	BP:positive regulation of cellular amide metabolic process;BP:positive regulation of RNA binding;BP:regulation of primary metabolic process;BP:regulation of metabolic process;BP:positive regulation of cellular biosynthetic process;BP:macromolecular complex subunit organization;BP:regulation of cellular macromolecule biosynthetic process;BP:positive regulation of cellular metabolic process;BP:regulation of cellular metabolic process;BP:positive regulation of gene expression;BP:formation of cytoplasmic translation initiation complex;BP:regulation	GO:0034250;GO:1905216;GO:0080090;GO:0019222;GO:0031328;GO:0043933;GO:2000112;GO:0031325;GO:0031323;GO:0010628;GO:0001732;GO:0050789;GO:0071840;GO:0009893;GO:0071826;GO:0010604;GO:0010608;GO:0009891;GO:0051246;GO:0051247;GO:0016043;GO:0065003;GO:0022618;GO:0065007;GO:0044093;GO:0031326;GO:0048518;GO:0065009;GO:0045727;GO:0010468;GO:0022607;GO:0060255;GO:0009987;GO:0009889;GO:0034248;GO:0008150;GO:0051171;GO:0008152;GO:0051173;GO:0050794;GO:1902415;GO:1902416;GO:0051099;GO:0051098;GO:0044237;GO:0010557;GO:0010556;GO:0006417;GO:1905214;GO:0006413;GO:0048522;GO:0070993;GO:0005852;GO:0110165;GO:0033290;GO:0032991;GO:1990904;GO:0005575;GO:0016282;GO:0005829;GO:0003674;GO:0005488;GO:0003676;GO:0031369;GO:1901363;GO:0008135;GO:0043021;GO:0090079;GO:0043022;GO:0045182;GO:0097159;GO:0003723;GO:0005515;GO:0044877;GO:0003743	K03252	EIF3C	------	------	ENOG410XRU3	S:Function unknown	PF05470.15;PF01399.30	eIF-3c_N;PCI	Eukaryotic translation initiation factor 3 subunit 8 N-terminus;PCI domain	CYT	1	1	70.4	Medium
TRINITY_DN76333_c0_g1_i2_orf1	larval cuticle protein 65Ag1-like [Ostrinia furnacalis]	5.0801527	2.344871854	1.35E-05	up	yes	1.331	0.262	0.274	0.252	0.26	1.366	1.377	1.249	MF:molecular_function;MF:structural constituent of cuticle;MF:structural molecule activity;	GO:0003674;GO:0042302;GO:0005198	------	------	------	------	ENOG410YVGC;ENOG410YVKS;ENOG4111EHD	S:Function unknown;	PF00379.26	Chitin_bind_4	Insect cuticle protein	CYT	4	43	15.6	High
TRINITY_DN416_c0_g1_i1_orf1	unnamed protein product [Diatraea saccharalis]	2.0383889	1.027429343	8.66E-07	up	yes	0.9717	0.4767	0.471	0.47	0.489	0.981	0.977	0.957	------	------	K05699	ACTN1_4	map04510;map04810;map05146;map05322;map05131;map04670;map04530;map05203;map04520	Focal adhesion;Regulation of actin cytoskeleton;Amoebiasis;Systemic lupus erythematosus;Shigellosis;Leukocyte transendothelial migration;Tight junction;Viral carcinogenesis;Adherens junction	COG5069	S:Function unknown	PF00435.24;PF00307.34;PF08726.13;PF00036.35;PF11971.11;PF13405.9;PF13499.9	Spectrin;CH;EFhand_Ca_insen;EF-hand_1;CAMSAP_CH;EF-hand_6;EF-hand_7	Spectrin repeat;Calponin homology;Ca2+ insensitive EF hand;EF hand;CAMSAP CH domain;EF-hand domain;EF-hand domain pair	CYT	42	55	103.8	High
TRINITY_DN18164_c0_g1_i7_orf1	uncharacterized protein LOC114366518 isoform X5 [Ostrinia furnacalis]	0.3504274	-1.512812715	0.007287	down	yes	0.369	1.053	1.137	1.202	0.82	0.503	0.296	0.308	CC:integral component of membrane;CC:cellular_component;CC:intrinsic component of membrane;CC:cellular anatomical entity;MF:binding;MF:cation binding;MF:transition metal ion binding;MF:ion binding;MF:molecular_function;MF:metal ion binding;MF:zinc ion binding;	GO:0016021;GO:0005575;GO:0031224;GO:0110165;GO:0005488;GO:0043169;GO:0046914;GO:0043167;GO:0003674;GO:0046872;GO:0008270	------	------	------	------	COG5183	O:Posttranslational modification, protein turnover, chaperones	PF12906.10;PF06454.14	RINGv;THH1_TOM1-3_dom	RING-variant domain;THH1/TOM1/TOM3 domain	CYT	1	2	64.7	High
TRINITY_DN32601_c0_g1_i2_orf1	uncharacterized protein LOC114363197 [Ostrinia furnacalis]	0.3523316	-1.504994196	2.17E-05	down	yes	0.408	1.158	1.138	1.165	1.172	0.47	0.382	0.372	------	------	------	------	------	------	ENOG410XRFE	S:Function unknown	PF03022.19	MRJP	Major royal jelly protein	CYT	1	3	48.2	High
TRINITY_DN4408_c6_g1_i1_orf1	polyprotein, partial [Bemisia tabaci]	2.7766497	1.473345203	1.74E-05	up	yes	1.094	0.394	0.429	0.368	0.385	1.125	1.106	1.05	BP:viral RNA genome replication;BP:RNA biosynthetic process;BP:nucleic acid-protein covalent cross-linking;BP:RNA-protein covalent cross-linking;BP:nucleic acid metabolic process;BP:cellular biosynthetic process;BP:cellular nitrogen compound metabolic process;BP:nitrogen compound metabolic process;BP:organic cyclic compound biosynthetic process;BP:organic cyclic compound metabolic process;BP:nucleobase-containing compound metabolic process;BP:cellular macromolecule metabolic process;BP:organic	GO:0039694;GO:0032774;GO:0018143;GO:0018144;GO:0090304;GO:0044249;GO:0034641;GO:0006807;GO:1901362;GO:1901360;GO:0006139;GO:0044260;GO:0071704;GO:0097659;GO:0006508;GO:0018130;GO:1901576;GO:0019079;GO:0006725;GO:0009987;GO:0043412;GO:0036211;GO:0008150;GO:0008152;GO:0019438;GO:0034654;GO:0044271;GO:0046483;GO:0016070;GO:0044238;GO:0006351;GO:1901564;GO:0019538;GO:0009058;GO:0009059;GO:0043170;GO:0016032;GO:0006259;GO:0044237;GO:0071897;GO:0034062;GO:0003674;GO:0005488;GO:0003676;GO:0008186;GO:0008233;GO:0008234;GO:1901363;GO:0016740;GO:0016787;GO:0140098;GO:0140096;GO:0004386;GO:0003824;GO:0016779;GO:0097747;GO:0016772;GO:0003724;GO:0004197;GO:0003723;GO:0097159;GO:0003968;GO:0004175	------	------	------	------	------	------	PF00680.23	RdRP_1	Viral RNA-dependent RNA polymerase	CYT	6	10	76.2	High
TRINITY_DN4010_c0_g2_i1_orf1	myophilin [Ostrinia furnacalis]	2.2180529	1.149293806	9.32E-07	up	yes	1.064	0.4797	0.481	0.478	0.48	1.071	1.079	1.041	BP:organelle organization;BP:cytoskeleton organization;BP:actomyosin structure organization;BP:actin filament-based process;BP:cellular process;BP:actin cytoskeleton organization;BP:cellular component organization;BP:biological_process;BP:cellular component organization or biogenesis;MF:cytoskeletal protein binding;MF:actin binding;MF:molecular_function;MF:calmodulin binding;MF:protein binding;MF:binding;	GO:0006996;GO:0007010;GO:0031032;GO:0030029;GO:0009987;GO:0030036;GO:0016043;GO:0008150;GO:0071840;GO:0008092;GO:0003779;GO:0003674;GO:0005516;GO:0005515;GO:0005488	K20526	TAGLN	------	------	COG5199	S:Function unknown	PF00307.34;PF00402.21	CH;Calponin	Calponin homology;Calponin family repeat	CYT	7	47	19.2	High

TRINITY_DN8226_c0_g1_i1_orf1	myosin heavy chain, muscle isoform X16 [Helicoverpa armigera]	2.3260917	1.217907989	1.18E-05	up	yes	1.06	0.4557	0.456	0.452	0.459	1.049	1.103	1.027	------	------	K17751	MYH6_7	map04260;map04261;map04919;map04022;map05416;map05414;map05410	Cardiac muscle contraction;Adrenergic signaling in cardiomyocytes;Thyroid hormone signaling pathway;cGMP-PKG signaling pathway;Viral myocarditis;Dilated cardiomyopathy;Hypertrophic cardiomyopathy	COG5022	G:Carbohydrate transport and metabolism	------	------	------	CYT	1	56	6.3	High
TRINITY_DN23429_c0_g2_i1_orf1	muscle-specific protein 20 [Zerene cesonia]	2.2744571	1.185522242	1.19E-05	up	yes	1.016	0.4467	0.457	0.434	0.449	1.02	1.049	0.979	------	------	K20526	TAGLN	------	------	COG5199	Z:Cytoskeleton	PF00307.34;PF00402.21	CH;Calponin	Calponin homology;Calponin family repeat	CYT	12	75	20.3	High
TRINITY_DN116_c1_g1_i8_orf1	uncharacterized protein LOC114350057 isoform X2 [Ostrinia furnacalis]	2.0857087	1.060537657	2.12E-06	up	yes	1.061	0.5087	0.503	0.504	0.519	1.053	1.085	1.044	------	------	------	------	------	------	ENOG4111JGD	S:Function unknown	PF15929.8	Myofilin	Myofilin	CYT	18	58	37.9	High
TRINITY_DN2600_c0_g1_i7_orf1	mucin-5AC isoform X2 [Ostrinia furnacalis]	0.4055556	-1.302028537	0.0001219	down	yes	0.438	1.08	1.096	1.145	1	0.427	0.455	0.432	------	------	------	------	------	------	ENOG4112A7I	S:Function unknown	------	------	------	CYT	9	12	98	High
TRINITY_DN11448_c0_g1_i15_orf1	unnamed protein product [Chilo suppressalis]	2.0133684	1.009611207	0.0001199	up	yes	0.994	0.4937	0.527	0.486	0.468	0.973	1.051	0.958	------	------	K23353	PDLIM1_2_3_4	------	------	ENOG4111KMK;ENOG410YX4U;ENOG4110362	S:Function unknown;	PF00595.27	PDZ	PDZ domain	CYT	2	52	27	High
TRINITY_DN82628_c0_g1_i2_orf1	ORF type:internal len:148 hit:XP_028162129.1 TRINITY_DN82628_c0_g1_i2:3-446(-)	2.1788618	1.123574685	0.0001579	up	yes	1.072	0.492	0.467	0.439	0.57	1.055	1.064	1.098	------	------	K19326	SYNE1	------	------	------	------	------	------	------	CYT	1	9	16.4	High
TRINITY_DN43328_c0_g1_i1_orf1	tubulin--tyrosine ligase-like protein 12 [Ostrinia furnacalis]	0.440553	-1.182612519	0.001311	down	yes	0.478	1.085	1.081	1.068	1.107	0.604	0.485	0.345	------	------	K16609	TTLL12	------	------	ENOG410XQ0A	S:Function unknown	PF03133.18	TTL	Tubulin-tyrosine ligase family	CYT	1	2	71.9	High
TRINITY_DN9871_c0_g1_i11_orf1	PEST proteolytic signal-containing nuclear protein-like [Ostrinia furnacalis]	2.5086147	1.326890931	0.0001726	up	yes	1.092	0.4353	0.417	0.471	0.418	1.099	1.167	1.011	BP:nitrogen compound metabolic process;BP:cell cycle;BP:protein modification by small protein conjugation;BP:protein modification by small protein conjugation or removal;BP:organic substance metabolic process;BP:cellular process;BP:macromolecule modification;BP:protein modification process;BP:biological_process;BP:metabolic process;BP:primary metabolic process;BP:organonitrogen compound metabolic process;BP:protein metabolic process;BP:macromolecule metabolic process;BP:protein ubiquitination;CC:intra	GO:0006807;GO:0007049;GO:0032446;GO:0070647;GO:0071704;GO:0009987;GO:0043412;GO:0036211;GO:0008150;GO:0008152;GO:0044238;GO:1901564;GO:0019538;GO:0043170;GO:0016567;GO:0043229;GO:0043227;GO:0043226;GO:0005634;GO:0110165;GO:0043231;GO:0005575	------	------	------	------	ENOG4111S9J	S:Function unknown	PF15473.9	PCNP	PEST, proteolytic signal-containing nuclear protein family	CYT	1	6	24.7	High
TRINITY_DN10558_c0_g1_i4_orf1	unnamed protein product [Chrysodeixis includens]	0.4720904	-1.082864823	0.02547	down	yes	0.4677	0.9907	1.135	1.106	0.731	0.487	0.328	0.588	CC:macromolecular complex;CC:ribonucleoprotein complex;CC:cellular_component;MF:organic cyclic compound binding;MF:molecular_function;MF:RNA binding;MF:nucleic acid binding;MF:heterocyclic compound	GO:0032991;GO:1990904;GO:0005575;GO:0097159;GO:0003674;GO:0003723;GO:0003676;GO:1901363;GO:0005488	K14411	MSI	map03015	mRNA surveillance pathway	ENOG410YA8Z	O:Posttranslational modification, protein turnover, chaperones	------	------	------	CYT	1	44	18.3	High
TRINITY_DN29100_c0_g1_i2_orf1	endocuticle structural glycoprotein ABD-5-like [Galleria mellonella]	2.3603061	1.23897394	0.00013	up	yes	1.018	0.4313	0.422	0.378	0.494	1.039	1.041	0.974	MF:molecular_function;MF:structural constituent of cuticle;MF:structural molecule activity;	GO:0003674;GO:0042302;GO:0005198	------	------	------	------	ENOG41113X7;ENOG41125BY;ENOG410YVGC	S:Function unknown;	PF00379.26	Chitin_bind_4	Insect cuticle protein	CYT	3	61	11.2	High
TRINITY_DN31001_c0_g1_i1_orf1	endocuticle structural glycoprotein ABD-5-like [Ostrinia furnacalis]	3.1413459	1.651382797	4.41E-05	up	yes	1.069	0.3403	0.332	0.371	0.318	1.078	1.124	1.005	MF:molecular_function;MF:structural constituent of cuticle;MF:structural molecule activity;	GO:0003674;GO:0042302;GO:0005198	------	------	------	------	ENOG4111EHD;ENOG410YVGC;ENOG41113X7	S:Function unknown;	PF00379.26	Chitin_bind_4	Insect cuticle protein	CYT	3	41	12.2	High
TRINITY_DN1982_c0_g1_i17_orf1	unnamed protein product, partial [Iphiclides podalirius]	2.2524941	1.171523303	0.0002005	up	yes	0.9483	0.421	0.441	0.395	0.427	0.881	0.951	1.013	CC:cellular_component;CC:integral component of membrane;CC:cellular anatomical entity;CC:intrinsic component of membrane;	GO:0005575;GO:0016021;GO:0110165;GO:0031224	------	------	------	------	COG0526	O:Posttranslational modification, protein turnover, chaperones	PF00085.23;PF13098.9;PF13905.9;PF07449.14	Thioredoxin;Thioredoxin_2;Thioredoxin_8;HyaE	Thioredoxin;Thioredoxin-like domain;Thioredoxin-like;Hydrogenase-1 expression protein HyaE	CYT	6	17	124.3	High
TRINITY_DN1123_c2_g1_i3_orf1	troponin I isoform X8 [Ostrinia furnacalis]	3.1072411	1.635634172	3.55E-06	up	yes	1.017	0.3273	0.299	0.36	0.323	1.009	1.031	1.01	CC:cellular_component;CC:macromolecular complex;CC:troponin complex;	GO:0005575;GO:0032991;GO:0005861	K12044	TNNI3	map04260;map04261;map04024;map05414;map05415;map05410	Cardiac muscle contraction;Adrenergic signaling in cardiomyocytes;cAMP signaling pathway;Dilated cardiomyopathy;Diabetic cardiomyopathy;Hypertrophic cardiomyopathy	ENOG410Y9IX	Z:Cytoskeleton	PF00992.23	Troponin	Troponin	CYT	3	55	10.3	High

TRINITY_DN13216_c0_g1_i5_orf1	uncharacterized protein LOC114358344 isoform X1 [Ostrinia furnacalis]	0.3349733	-1.57788196	2.11E-05	down	yes	0.32	0.9553	0.901	0.991	0.974	0.319	0.32	0.321	BP:cellular process;BP:regulation of cellular process;BP:biological regulation;BP:cell surface receptor signaling pathway;BP:biological_process;BP:regulation of biological process;BP:signal transduction;CC:integral component of membrane;CC:cellular_component;CC:cellular anatomical entity;CC:intrinsic component of membrane;MF:G-protein coupled receptor activity;MF:signaling receptor activity;MF:molecular transducer activity;MF:transmembrane signaling receptor activity;MF:molecular_f	GO:0009987;GO:0050794;GO:0065007;GO:0007166;GO:0008150;GO:0050789;GO:0007165;GO:0016021;GO:0005575;GO:0110165;GO:0031224;GO:0004930;GO:0038023;GO:0060089;GO:0004888;GO:0003674	K08451;K08455	ADGRG2, GPR64;ADGRG4, GPR112	------	------	ENOG410XSD2	S:Function unknown	PF00002.27;PF01825.24	7tm_2;GPS	7 transmembrane receptor;GPCR proteolysis site, GPS, motif	CYT	1	1	148.9	Medium
TRINITY_DN34821_c0_g1_i4_orf1	acetylcholine receptor subunit alpha-L1-like [Ostrinia furnacalis]	0.4355809	-1.198987253	9.92E-05	down	yes	0.427	0.9803	0.945	1.047	0.949	0.403	0.443	0.435	CC:cellular_component;CC:integral component of membrane;CC:cellular anatomical entity;CC:intrinsic component of membrane;MF:signaling receptor activity;MF:molecular transducer activity;MF:inorganic solute uptake transmembrane transporter activity;MF:transporter activity;MF:extracellular ligand-gated ion channel activity;MF:ligand-gated ion channel activity;MF:ion transmembrane transporter activity;MF:transmembrane signaling receptor activity;MF:transmembrane transporter [illegible]	GO:0005575;GO:0016021;GO:0110165;GO:0031224;GO:0038023;GO:0060089;GO:0015318;GO:0005215;GO:0005230;GO:0015276;GO:0015075;GO:0004888;GO:0022857;GO:0015267;GO:0003674;GO:0022836;GO:0022834;GO:0022803;GO:0005216	K04803;K04807;K04811	CHRNA1;CHRNA5;CHRNA10	map04080	Neuroactive ligand-receptor interaction	ENOG410XQGR	S:Function unknown	PF02931.26	Neur_chan_LBD	Neurotransmitter-gated ion-channel ligand binding domain	PLA	1	3	47.7	High
TRINITY_DN1123_c2_g1_i4_orf1	troponin I isoform X16 [Ostrinia furnacalis]	3.0273276	1.59804481	2.00E-05	up	yes	0.9527	0.3147	0.307	0.297	0.34	0.998	0.944	0.916	CC:cellular_component;CC:macromolecular complex;CC:troponin complex;	GO:0005575;GO:0032991;GO:0005861	K12044	TNNI3	map04260;map04261;map04024;map05414;map05415;map05410	Cardiac muscle contraction;Adrenergic signaling in cardiomyocytes;cAMP signaling pathway;Dilated cardiomyopathy;Diabetic cardiomyopathy;Hypertrophic cardiomyopathy	ENOG410Y9IX	Z:Cytoskeleton	PF00992.23	Troponin	Troponin	CYT	8	46	24.4	High
TRINITY_DN38366_c0_g1_i4_orfp1	TRINITY_DN38366_c0_g1_i4_m.10666 TRINITY_DN38366_c0_g1::TRINITY_DN38366_c0_g1_i4::g.10666 ORF type:internal len:143 (+),score=71.68 TRINITY_DN38366_c0_g1_i4:3-428(+)	3.0712695	1.618835107	1.14E-05	up	yes	0.9653	0.3143	0.28	0.335	0.328	0.96	0.997	0.939	------	------	------	------	------	------	------	------	------	------	------	CYT	1	8	13.5	High
TRINITY_DN556_c0_g2_i1_orf1	serine protease inhibitor dipetalogastin-like [Ostrinia furnacalis]	0.4412992	-1.180170965	0.01215	down	yes	0.3913	0.8867	1.036	0.806	0.818	0.558	0.344	0.272	------	------	K23615	SPIK5	------	------	ENOG410Z5MG;ENOG410YKSA	S:Function unknown;O:Posttranslational modification, protein turnover, chaperones	PF00050.24;PF07648.18	Kazal_1;Kazal_2	Kazal-type serine protease inhibitor domain;Kazal-type serine protease inhibitor domain	EXC	1	19	30.5	High
TRINITY_DN27500_c0_g1_i4_orf1	hemicentin-1-like [Ostrinia furnacalis]	3.4027055	1.7666823	3.23E-06	up	yes	0.981	0.2883	0.266	0.297	0.302	0.998	0.994	0.951	BP:developmental process;BP:anatomical structure development;BP:biological_process;CC:integral component of membrane;CC:cellular_component;CC:intrinsic component of membrane;CC:cellular anatomical entity;	GO:0032502;GO:0048856;GO:0008150;GO:0016021;GO:0005575;GO:0031224;GO:0110165	------	------	------	------	ENOG4110SA1;ENOG4111UKY	S:Function unknown;	PF13927.9;PF07679.19;PF13895.9;PF00047.28;PF07686.20;PF00041.24	Ig_3;I-set;Ig_2;ig;V-set;fn3	Immunoglobulin domain;Immunoglobulin I-set domain;Immunoglobulin domain;Immunoglobulin domain;Immunoglobulin V-set domain;Fibronectin type III domain	CYT	1	2	57.4	High
TRINITY_DN6881_c0_g1_i1_orf1	putative protein TPRXL [Ostrinia furnacalis]	5.808053	2.538054606	1.13E-06	up	yes	1.053	0.1813	0.186	0.15	0.208	1.051	1.065	1.042	MF:molecular_function;MF:structural constituent of cuticle;MF:structural molecule activity;	GO:0003674;GO:0042302;GO:0005198	------	------	------	------	ENOG410ZF0W;ENOG410YXXU;ENOG4111PJ1	S:Function unknown;	PF00379.26	Chitin_bind_4	Insect cuticle protein	CYT	5	23	22.4	High
TRINITY_DN146841_c0_g1_i1_orf1	muscle-specific protein 20 [Temnothorax curvispinosus]	4.9901478	2.319082542	0.0003498	up	yes	1.013	0.203	0.211	0.236	0.162	0.986	1.143	0.911	------	------	K20526	TAGLN	------	------	COG5199	S:Function unknown	PF00307.34;PF00402.21	CH;Calponin	Calponin homology;Calponin family repeat	CYT	1	10	20.6	High
TRINITY_DN141353_c0_g1_i1_orf1	uncharacterized protein LOC123263755 [Cotesia glomerata] >KAH0554923.1 hypothetical protein KQX54_013880 [Cotesia glomerata]	2.0945393	1.066632975	0.004891	up	yes	0.8707	0.4157	0.394	0.461	0.392	1.017	0.753	0.842	BP:nucleoside triphosphate metabolic process;BP:nucleoside triphosphate biosynthetic process;BP:purine nucleoside triphosphate metabolic process;BP:purine nucleoside triphosphate biosynthetic process;BP:cellular biosynthetic process;BP:cellular nitrogen compound metabolic process;BP:nucleotide biosynthetic process;BP:small molecule metabolic process;BP:purine-containing compound metabolic process;BP:purine-containing compound biosynthetic process;BP:organic cyclic compound [illegible]	GO:0009141;GO:0009142;GO:0009144;GO:0009145;GO:0044249;GO:0034641;GO:0009165;GO:0044281;GO:0072521;GO:0072522;GO:1901362;GO:0009259;GO:1901360;GO:0006139;GO:0009201;GO:0071704;GO:0009199;GO:0018130;GO:0009206;GO:0009205;GO:0019693;GO:1901576;GO:0046034;GO:0009987;GO:0006725;GO:0009152;GO:0009150;GO:0009260;GO:0009058;GO:0009117;GO:0008152;GO:0019438;GO:0034654;GO:0044271;GO:0090407;GO:0055086;GO:0046483;GO:0044238;GO:0015986;GO:1901564;GO:1901566;GO:1901137;GO:1901135;GO:0006164;GO:0046390;GO:0044237;GO:0006163;GO:0006796;GO:0006807;GO:1901293;GO:0006793;GO:0019637;GO:0008150;GO:0006753;GO:0006754;GO:0045263;GO:0005575;GO:0098798;GO:0098800;GO:0032991;GO:0000276;GO:0098796;GO:0033177;GO:0022890;GO:0003674;GO:0015318;GO:0022857;GO:0005215;GO:0008324;GO:0015075;GO:0015078	K02129	ATPeF0E, ATP5I	map00190;map04714	Oxidative phosphorylation;Thermogenesis	ENOG410XYSS	Z:Cytoskeleton	PF05680.15	ATP-synt_E	ATP synthase E chain	CYT	1	14	9.1	High

TRINITY_DN389_c0_g1_i2_orf1	uncharacterized protein LOC118068293 isoform X2 [Chelonus insularis]	0.4160757	-1.265082234	9.59E-07	down	yes	0.352	0.846	0.846	0.838	0.854	0.347	0.369	0.34	------	------	------	------	------	------	ENOG410XX7G	S:Function unknown	PF01607.27;PF00057.21	CBM_14;Ldl_recept_a	Chitin binding Peritrophin-A domain;Low-density lipoprotein receptor domain class A	PLA	4	11	60.2	High
TRINITY_DN76815_c0_g1_i3_orf1	5-formyltetrahydrofolate cyclo-ligase [Ostrinia furnacalis]	12.859629	3.684777157	1.48E-05	up	yes	1.256	0.09767	0.133	0.079	0.081	1.335	1.245	1.189	MF:purine ribonucleoside triphosphate binding;MF:cyclo-ligase activity;MF:molecular_function;MF:binding;MF:ion binding;MF:heterocyclic compound binding;MF:nucleoside phosphate binding;MF:anion binding;MF:cation binding;MF:nucleotide binding;MF:ligase activity, forming carbon-nitrogen bonds;MF:purine nucleotide binding;MF:metal ion binding;MF:ATP binding;MF:small molecule binding;MF:catalytic activity;MF:ligase activity;MF:5-formyltetrahydrofolate cyclo-ligase	GO:0035639;GO:0016882;GO:0003674;GO:0005488;GO:0043167;GO:1901363;GO:1901265;GO:0043168;GO:0043169;GO:0000166;GO:0016879;GO:0017076;GO:0046872;GO:0005524;GO:0036094;GO:0003824;GO:0016874;GO:0030272;GO:0030554;GO:0097367;GO:0097159;GO:0032559;GO:0032555;GO:0032553	K01934	MTHFS	map00670	One carbon pool by folate	COG0212	H:Coenzyme transport and metabolism	PF01812.23	5-FTHF_cyc-lig	5-formyltetrahydrofolate cyclo-ligase family	CYT	1	61	14.2	High
TRINITY_DN11657_c0_g1_i2_orf1	trehalase-1 [Omphisa fuscidentalis]	0.4549806	-1.136123079	3.26E-05	down	yes	0.3517	0.773	0.798	0.781	0.74	0.332	0.37	0.353	BP:primary metabolic process;BP:cellular carbohydrate metabolic process;BP:oligosaccharide metabolic process;BP:cellular process;BP:disaccharide metabolic process;BP:trehalose metabolic process;BP:biological_process;BP:cellular metabolic process;BP:metabolic process;BP:carbohydrate metabolic process;BP:organic substance metabolic process;MF:trehalase activity;MF:hydrolase activity;MF:catalytic activity;MF:hydrolase activity, acting on glycosyl bonds;MF:alpha,alpha-trehalase activity;MF:molecular_function;MF:hydrolase	GO:0044238;GO:0044262;GO:0009311;GO:0009987;GO:0005984;GO:0005991;GO:0008150;GO:0044237;GO:0008152;GO:0005975;GO:0071704;GO:0015927;GO:0016787;GO:0003824;GO:0016798;GO:0004555;GO:0003674;GO:0004553	K01194	TREH, treA, treF	map00500	Starch and sucrose metabolism	COG1626	S:Function unknown	PF01204.21	Trehalase	Trehalase	CYT	2	5	67.2	High
TRINITY_DN1013_c0_g1_i3_orf1	TELO2-interacting protein 1 homolog isoform X2 [Ostrinia furnacalis]	0.3047194	-1.714446576	0.0009937	down	yes	0.246	0.8073	0.702	0.858	0.862	0.24	0.183	0.315	------	------	K20403	TTI1	map04150	mTOR signaling pathway	ENOG410XSN6;ENOG410ZXJQ	S:Function unknown;	------	------	------	CYT	1	1	124.9	Medium
TRINITY_DN58531_c0_g1_i1_orf1	uncharacterized protein LOC114357371 [Ostrinia furnacalis] >XP_028166768.1 uncharacterized protein LOC114357371 [Ostrinia furnacalis]	0.4424094	-1.176546089	0.009794	down	yes	0.343	0.7753	0.817	0.658	0.851	0.21	0.362	0.457	------	------	------	------	------	------	ENOG410Y4ER;ENOG4110SKC	S:Function unknown;	------	------	------	CYT	1	3	29.7	Medium
TRINITY_DN31118_c1_g1_i1_orf1	endocuticle structural glycoprotein ABD-4-like [Ostrinia furnacalis]	5.3719977	2.425458675	8.35E-06	up	yes	0.917	0.1707	0.193	0.137	0.182	0.94	0.932	0.879	MF:molecular_function;MF:structural constituent of cuticle;MF:structural molecule activity;	GO:0003674;GO:0042302;GO:0005198	------	------	------	------	ENOG4111PJ1;ENOG410YR66	S:Function unknown;	PF00379.26	Chitin_bind_4	Insect cuticle protein	CYT	1	23	12	High
TRINITY_DN116951_c0_g3_i2_orf1	spermine oxidase-like isoform X2 [Ostrinia furnacalis]	2.7842657	1.477296911	6.11E-06	up	yes	0.7963	0.286	0.281	0.262	0.315	0.792	0.791	0.806	MF:catalytic activity;MF:molecular_function;MF:oxidoreductase activity;	GO:0003824;GO:0003674;GO:0016491	K12259	SMOX, PAO5	map00330;map00410	Arginine and proline metabolism;beta-Alanine metabolism	ENOG4111HZN;COG1231;ENOG410XSNC	S:Function unknown;E:Amino acid transport and metabolism;S:Function unknown	PF13450.9;PF01593.27	NAD_binding_8;Amino_oxidase	NA;Flavin containing amine oxidoreductase	CYT	2	38	8.6	High
TRINITY_DN6710_c0_g1_i6_orf1	multiple C2 and transmembrane domain-containing protein-like [Ostrinia furnacalis]	0.3809347	-1.392384489	0.005456	down	yes	0.3073	0.8067	0.666	0.953	0.801	0.254	0.382	0.286	CC:cellular_component;CC:integral component of membrane;CC:cellular anatomical entity;CC:intrinsic component of membrane;	GO:0005575;GO:0016021;GO:0110165;GO:0031224	------	------	------	------	COG5038;ENOG410XRQN	H:Coenzyme transport and metabolism;S:Function unknown	PF00168.33	C2	C2 domain	PLA	1	1	69.9	Medium
TRINITY_DN19160_c0_g1_i1_orf1	alkyldihydroxyacetonephosphate synthase [Ostrinia furnacalis]	0.191586	-2.38393601	1.16E-05	down	yes	0.1603	0.8367	0.811	0.88	0.819	0.147	0.186	0.148	------	------	K00803	AGPS, agpS	map00565;map04146	Ether lipid metabolism;Peroxisome	COG0277	C:Energy production and conversion	PF02913.22;PF01565.26	FAD-oxidase_C;FAD_binding_4	FAD linked oxidases, C-terminal domain;FAD binding domain	CYT	1	1	69.6	Medium
TRINITY_DN5459_c0_g1_i1_orf1	protein takeout-like isoform X2 [Ostrinia furnacalis]	0.3125261	-1.6779513	0.0004003	down	yes	0.2243	0.7177	0.755	0.749	0.649	0.269	0.169	0.235	------	------	------	------	------	------	ENOG4111E83;ENOG410ZNC7;ENOG4110RSP;ENOG410ZN7P;ENOG4110QH9;ENOG4110RJ7;ENOG41119GI;ENOG41114TE	S:Function unknown;	PF06585.14	JHBP	Haemolymph juvenile hormone binding protein	CYT	1	4	21.2	Medium
TRINITY_DN21035_c0_g1_i14_orf1	mitochondrial amidoxime reducing component 2-like [Ostrinia furnacalis]	2.4267667	1.279035408	3.24E-05	up	yes	0.6147	0.2533	0.248	0.244	0.268	0.595	0.603	0.646	MF:anion binding;MF:cation binding;MF:molybdenum ion binding;MF:metal ion binding;MF:transition metal ion binding;MF:organic cyclic compound binding;MF:vitamin binding;MF:vitamin B6 binding;MF:small molecule binding;MF:catalytic activity;MF:transferase activity;MF:molecular_function;MF:pyridoxal phosphate binding;MF:ion binding;MF:heterocyclic compound	GO:0043168;GO:0043169;GO:0030151;GO:0046872;GO:0046914;GO:0097159;GO:0019842;GO:0070279;GO:0036094;GO:0003824;GO:0016740;GO:0003674;GO:0030170;GO:0043167;GO:1901363;GO:0005488	------	------	------	------	COG3217	S:Function unknown	PF03476.19	MOSC_N	MOSC N-terminal beta barrel domain	CYT	1	41	15.6	High
TRINITY_DN67231_c0_g1_i1_orf1	endocuticle structural glycoprotein SgAbd-8-like [Ostrinia furnacalis]	44.492696	5.475496628	7.95E-07	up	yes	1.127	0.02533	0.024	0.02	0.032	1.118	1.167	1.097	MF:molecular_function;MF:structural constituent of cuticle;MF:structural molecule activity;	GO:0003674;GO:0042302;GO:0005198	------	------	------	------	ENOG4111PJ1;ENOG410YR66	S:Function unknown;	PF00379.26	Chitin_bind_4	Insect cuticle protein	CYT	1	20	13.9	High

TRINITY_DN25779_c0_g1_i6_orf1	aldo-keto reductase AKR2E4-like [Ostrinia furnacalis]	0.4098903	-1.286690333	1.94E-05	down	yes	0.2727	0.6653	0.645	0.698	0.653	0.273	0.277	0.268	MF:catalytic activity;MF:molecular_function;MF:oxidoreductase activity;	GO:0003824;GO:0003674;GO:0016491	K00011	AKR1B	map00051;map00040;map00561;map00790;map00052	Fructose and mannose metabolism;Pentose and glucuronate interconversions;Glycerolipid metabolism;Folate biosynthesis;Galactose metabolism	COG0656	S:Function unknown	PF00248.24	Aldo_ket_red	Aldo/keto reductase family	CYT	5	23	35.9	High
TRINITY_DN2894_c0_g2_i3_orf1	myrosinase 1-like isoform X1 [Ostrinia furnacalis]	0.4009883	-1.31836792	0.004662	down	yes	0.3327	0.8297	0.899	0.661	0.929	0.331	0.299	0.368	BP:primary metabolic process;BP:carbohydrate metabolic process;BP:organic substance metabolic process;BP:biological_process;BP:metabolic process;MF:hydrolase activity;MF:catalytic activity;MF:hydrolase activity, acting on glycosyl bonds;MF:hydrolase activity, hydrolyzing O-glycosyl compounds;MF:molecular_function;	GO:0044238;GO:0005975;GO:0071704;GO:0008150;GO:0008152;GO:0016787;GO:0003824;GO:0016798;GO:0004553;GO:0003674	------	------	------	------	COG2723	G:Carbohydrate transport and metabolism	PF00232.21	Glyco_hydro_1	Glycosyl hydrolase family 1	CYT	1	6	50.1	High
TRINITY_DN113626_c0_g1_i3_orfp	TRINITY_DN113626_c0_g1_i3_m.80721 TRINITY_DN113626_c0_g1::TRINITY_DN113626_c0_g1_i3::g.80721 ORF type:internal len:118 (-),score=87.34 TRINITY_DN113626_c0_g1_i3:2-352(-)	2.2032347	1.139623164	0.04081	up	yes	0.722	0.3277	0.391	0.36	0.232	0.478	0.815	0.873	------	------	------	------	------	------	------	------	------	------	------	PLA	1	9	11.6	High
TRINITY_DN42461_c0_g1_i4_orf1	obscurin [Ostrinia furnacalis]	2.8133678	1.49229816	8.44E-06	up	yes	0.8713	0.3097	0.276	0.336	0.317	0.859	0.886	0.869	BP:phosphorylation;BP:cell differentiation;BP:nitrogen compound metabolic process;BP:anatomical structure morphogenesis;BP:cellular developmental process;BP:organic substance metabolic process;BP:developmental process;BP:protein phosphorylation;BP:cellular process;BP:macromolecule modification;BP:protein modification process;BP:biological_process;BP:metabolic process;BP:primary metabolic process;BP:organonitrogen compound metabolic process;BP:protein metabolic process;BP:cellular	GO:0016310;GO:0030154;GO:0006807;GO:0009653;GO:0048869;GO:0071704;GO:0032502;GO:0006468;GO:0009987;GO:0043412;GO:0036211;GO:0008150;GO:0008152;GO:0044238;GO:1901564;GO:0019538;GO:0044237;GO:0043170;GO:0006796;GO:0006793;GO:0035639;GO:0005085;GO:0003674;GO:0005488;GO:0098772;GO:1901363;GO:1901265;GO:0043168;GO:0043167;GO:0000166;GO:0016740;GO:0004672;GO:0017076;GO:0097367;GO:0005524;GO:0016301;GO:0030234;GO:0140096;GO:0036094;GO:0030695;GO:0060589;GO:0003824;GO:0016772;GO:0030554;GO:0016773;GO:0032559;GO:0032555;GO:0097159;GO:0032553	------	------	------	------	ENOG410XQFD;ENOG4111QEW;ENOG410XSWY	S:Function unknown;	PF07679.19;PF13927.9	I-set;Ig_3	Immunoglobulin I-set domain;Immunoglobulin domain	CYT	1	37	11.1	High
TRINITY_DN3614_c0_g2_i1_orf1	PC4 and SFRS1-interacting protein isoform X4 [Galleria mellonella]	0.4026224	-1.312500661	0.0003457	down	yes	0.3347	0.8313	0.779	0.805	0.91	0.321	0.313	0.37	CC:intracellular membrane-bounded organelle;CC:nucleus;CC:cellular_component;CC:intracellular organelle;CC:membrane-bounded organelle;CC:organelle;CC:cellular anatomical entity;	GO:0043231;GO:0005634;GO:0005575;GO:0043229;GO:0043227;GO:0043226;GO:0110165	K25057	PSIP1, LEDGF	map03250	Viral life cycle - HIV-1	ENOG4111K9I	S:Function unknown	------	------	------	CYT	1	13	11.9	High
TRINITY_DN82801_c0_g1_i1_orf1	uncharacterized protein LOC114364712 [Ostrinia furnacalis]	0.3235508	-1.627935952	4.24E-06	down	yes	0.2227	0.6883	0.695	0.704	0.666	0.216	0.237	0.215	BP:organic substance metabolic process;BP:carbohydrate metabolic process;BP:primary metabolic process;BP:metabolic process;BP:biological_process;CC:cellular_component;CC:extracellular region;CC:cellular anatomical entity;MF:hydrolase activity;MF:deacetylase activity;MF:carbohydrate derivative binding;MF:hydrolase activity, acting on carbon-nitroge;MF:hydrolase activity, acting on carbon-nitroge;MF:catalytic activity;MF:molecular_function;MF:chitin binding;MF:chitin deacetylase activity;MF:binding;	GO:0071704;GO:0005975;GO:0044238;GO:0008152;GO:0008150;GO:0005575;GO:0005576;GO:0110165;GO:0016787;GO:0019213;GO:0097367;GO:0016810;GO:0016811;GO:0003824;GO:0003674;GO:0008061;GO:0004099;GO:0005488	------	------	------	------	ENOG410XX7G	Z:Cytoskeleton	PF01607.27;PF01522.24;PF00057.21	CBM_14;Polysacc_deac_1;Ldl_recept_a	Chitin binding Peritrophin-A domain;Polysaccharide deacetylase;Low-density lipoprotein receptor domain class A	CYT	10	27	61.3	High
TRINITY_DN1173_c1_g1_i10_orf1	hypothetical protein evm_001011 [Chilo suppressalis]	2.0609863	1.043334905	0.0006233	up	yes	0.7063	0.3427	0.32	0.352	0.356	0.647	0.77	0.702	BP:phosphorylation;BP:cell differentiation;BP:nitrogen compound metabolic process;BP:anatomical structure morphogenesis;BP:cellular developmental process;BP:organic substance metabolic process;BP:developmental process;BP:protein phosphorylation;BP:cellular process;BP:macromolecule modification;BP:protein modification process;BP:biological_process;BP:metabolic process;BP:primary metabolic process;BP:organonitrogen compound metabolic process;BP:protein metabolic process;BP:cellular	GO:0016310;GO:0030154;GO:0006807;GO:0009653;GO:0048869;GO:0071704;GO:0032502;GO:0006468;GO:0009987;GO:0043412;GO:0036211;GO:0008150;GO:0008152;GO:0044238;GO:1901564;GO:0019538;GO:0044237;GO:0043170;GO:0006796;GO:0006793;GO:0035639;GO:0005085;GO:0003674;GO:0005488;GO:0098772;GO:1901363;GO:1901265;GO:0043168;GO:0043167;GO:0000166;GO:0016740;GO:0004672;GO:0017076;GO:0097367;GO:0005524;GO:0016301;GO:0030234;GO:0140096;GO:0036094;GO:0030695;GO:0060589;GO:0003824;GO:0016772;GO:0030554;GO:0016773;GO:0032559;GO:0032555;GO:0097159;GO:0032553	------	------	------	------	ENOG410XQFD;ENOG410XSWY	S:Function unknown;T:Signal transduction mechanisms	PF07679.19;PF13927.9;PF00047.28;PF13895.9;PF00621.23;PF07686.20;PF00169.32	I-set;Ig_3;ig;Ig_2;RhoGEF;V-set;PH	Immunoglobulin I-set domain;Immunoglobulin domain;Immunoglobulin domain;Immunoglobulin domain;RhoGEF domain;Immunoglobulin V-set domain;PH domain	CYT	1	17	169.8	High
TRINITY_DN36817_c0_g1_i1_orf1	uncharacterized protein LOC114357350 [Ostrinia furnacalis]	2.0566563	1.04030075	0.001876	up	yes	0.6643	0.323	0.232	0.385	0.352	0.656	0.663	0.674	MF:translation regulator activity, nucleic acid binding;MF:nucleic acid binding;MF:organic cyclic compound binding;MF:translation factor activity, RNA binding;MF:molecular_function;MF:binding;MF:translation regulator activity;MF:heterocyclic compound	GO:0090079;GO:0003676;GO:0097159;GO:0008135;GO:0003674;GO:0005488;GO:0045182;GO:1901363;GO:0003743	------	------	------	------	ENOG410XV86;ENOG4111ASB	S:Function unknown;	------	------	------	CYT	1	29	7.6	High
TRINITY_DN139212_c0_g1_i4_orf1	uncharacterized protein LOC114350112 [Ostrinia furnacalis]	0.3800979	-1.395557119	0.001131	down	yes	0.233	0.613	0.671	0.629	0.539	0.273	0.235	0.191	MF:catalytic activity;MF:molecular_function;MF:oxidoreductase activity;	GO:0003824;GO:0003674;GO:0016491	------	------	------	------	COG1028	Z:Cytoskeleton	PF13561.9;PF00106.28;PF08659.13	adh_short_C2;adh_short;KR	Enoyl;short chain dehydrogenase;KR domain	CYT	2	8	27.3	High

TRINITY_DN2186_c0_g1_i17_orf1	paxillin isoform X6 [Leguminivora glycinivorella]	3.6060179	1.850406544	1.37E-06	up	yes	0.767	0.2127	0.196	0.215	0.227	0.753	0.781	0.767	BP:cell-matrix adhesion;BP:cell-substrate adhesion;BP:cellular process;BP:biological_process;BP:cell adhesion;CC:intracellular organelle;CC:non-membrane-bounded organelle;CC:focal adhesion;CC:organelle;CC:cell junction;CC:cell-substrate junction;CC:cytoskeleton;CC:cellular anatomical entity;CC:cytoplasm;CC:anchoring junction;CC:cell cortex;CC:intracellular non-membrane-bounded organelle;CC:cellular_component;MF:molecular_function;MF:binding;MF:cation binding;MF:ion binding;MF:metal ion	GO:0007160;GO:0031589;GO:0009987;GO:0008150;GO:0007155;GO:0043229;GO:0043228;GO:0005925;GO:0043226;GO:0030054;GO:0030055;GO:0005856;GO:0110165;GO:0005737;GO:0070161;GO:0005938;GO:0043232;GO:0005575;GO:0003674;GO:0005488;GO:0043169;GO:0043167;GO:0046872	K05760	PXN	map05205;map05165;map05203;map05163;map04510;map05135;map05131;map04370;map05170;map04670;map04062;map05100;map04810	Proteoglycans in cancer;Human papillomavirus infection;Viral carcinogenesis;Human cytomegalovirus infection;Focal adhesion;Yersinia infection;Shigellosis;VEGF signaling pathway;Human immunodeficiency virus 1 infection;Leukocyte transendothelial migration;Chemokine signaling pathway;Bacterial invasion of epithelial cells;Regulation of actin cytoskeleton	ENOG410XRD4	S:Function unknown	PF00412.25	LIM	LIM domain	CYT	2	65	26.4	High
TRINITY_DN1656_c2_g1_i5_orf1	15-hydroxyprostaglandin dehydrogenase [NAD(+)]-like [Ostrinia furnacalis]	2.1430134	1.09964087	1.50E-05	up	yes	0.7837	0.3657	0.353	0.375	0.369	0.811	0.758	0.782	MF:catalytic activity;MF:molecular_function;MF:oxidoreductase activity;	GO:0003824;GO:0003674;GO:0016491	K00069	HPGD	map00590;map05202	Arachidonic acid metabolism;Transcriptional misregulation in cancer	COG1028;COG0300	S:Function unknown;	PF00106.28;PF13561.9;PF08659.13	adh_short;adh_short_C2;KR	short chain dehydrogenase;Enoyl;KR domain	CYT	3	17	29.5	High
TRINITY_DN2924_c0_g1_i2_orf1	cuticular protein RR-2 [Spodoptera litura]	2.4281646	1.279866197	0.0005472	up	yes	0.7673	0.316	0.295	0.31	0.343	0.819	0.8	0.683	MF:molecular_function;MF:structural constituent of cuticle;MF:structural molecule activity;	GO:0003674;GO:0042302;GO:0005198	------	------	------	------	ENOG410YSFI;ENOG4111EA8;ENOG4110T8H	S:Function unknown;	------	------	------	CYT	2	14	11.6	High
TRINITY_DN96557_c0_g1_i1_orf1	charged multivesicular body protein 4B [Phyllostomus discolor]	2.6499786	1.405980712	2.73E-05	up	yes	0.6193	0.2337	0.212	0.253	0.236	0.606	0.646	0.606	BP:protein localization;BP:regulation of metabolic process;BP:anatomical structure homeostasis;BP:membrane organization;BP:plasma membrane organization;BP:establishment of organelle localization;BP:cellular component organization or biogenesis;BP:regulation of spindle assembly;BP:cellular macromolecule localization;BP:regulation of cytoskeleton organization;BP:interspecies interaction between organisms;BP:mitotic metaphase plate congression;BP:late endosome to lysosome transport;BP:negative regulation of biological	GO:0008104;GO:0019222;GO:0060249;GO:0061024;GO:0007009;GO:0051656;GO:0071840;GO:0090169;GO:0070727;GO:0051493;GO:0044419;GO:0007080;GO:1902774;GO:0048519;GO:0033036;GO:0051258;GO:0060548;GO:0006605;GO:0045184;GO:0072657;GO:0051668;GO:1902410;GO:0051701;GO:1901564;GO:0016192;GO:0019538;GO:0010506;GO:0010639;GO:0010507;GO:0090611;GO:0070507;GO:0009894;GO:1901673;GO:0009892;GO:1901215;GO:0006612;GO:0006807;GO:0050789;GO:1901575;GO:0044265;GO:0044260;GO:0006886;GO:0016043;GO:0065003;GO:0065007;GO:0071985;GO:0065008;GO:0006810;GO:0006620;GO:0060236;GO:0070972;GO:0008150;GO:0008152;GO:1901214;GO:0050794;GO:0001778;GO:0051603;GO:0009895;GO:0046907;GO:0039702;GO:0045047;GO:0006511;GO:0033043;GO:0051129;GO:0051128;GO:0044248;GO:0022607;GO:0007041;GO:0032886;GO:0051234;GO:0043162;GO:0010564;GO:0090057;GO:0071763;GO:0044000;GO:0031468;GO:0006508;GO:0072599;GO:0036438;GO:0019076;GO:0031330;GO:0072594;GO:0009987;GO:0019941;GO:0046605;GO:0051310;GO:0001894;GO:2000785;GO:0044770;GO:0061952;GO:0007034;GO:0032506;GO:1902902;GO:0043170;GO:0033365;GO:0090224;GO:0031329;GO:0043933;GO:0031324;GO:0031323;GO:1903047;GO:0042592;GO:0010824;GO:0044772;GO:0022402;GO:0046755;GO:0043632;GO:0010941;GO:0051303;GO:0046753;GO:0035891;GO:0090148;GO:0044087;GO:0071705;GO:0071704;GO:0097352;GO:1902115;GO:1902116;GO:0071702;GO:0071709;GO:0016242;GO:0016241;GO:0032984;GO:0022411;GO:0051649;GO:0009056;GO:0051179;GO:0051640;GO:0051641;GO:0010458;GO:0044238;GO:0046761	K12194	CHMP4A_B, SNF7, VPS32A_B	map03250;map04144;map04217	Viral life cycle - HIV-1;Endocytosis;Necroptosis	ENOG410YE9I	I:Lipid transport and metabolism	PF03357.24	Snf7	Snf7	CYT	1	14	8	High
TRINITY_DN3235_c0_g1_i1_orf1	SPARC [Trichoplusia ni]	0.3532444	-1.501261608	3.53E-05	down	yes	0.2673	0.7567	0.758	0.795	0.717	0.25	0.279	0.273	CC:extracellular space;CC:cellular_component;CC:cellular anatomical entity;MF:cation binding;MF:ion binding;MF:molecular_function;MF:binding;MF:metal ion binding;MF:calcium ion binding;	GO:0005615;GO:0005575;GO:0110165;GO:0043169;GO:0043167;GO:0003674;GO:0005488;GO:0046872;GO:0005509	K24262	SPARC	------	------	ENOG41101WW	S:Function unknown	PF10591.12	SPARC_Ca_bdg	Secreted protein acidic and rich in cysteine Ca binding region	CYT	6	24	36.9	High
TRINITY_DN3504_c0_g1_i3_orfp2	TRINITY_DN3504_c0_g1_i3_m.43947 TRINITY_DN3504_c0_g1::TRINITY_DN3504_c0_g1_i3::g.43947 ORF type:5prime_partial len:208 (-),score=77.75 TRINITY_DN3504_c0_g1_i3:185-808(-)	2.5806017	1.367707491	0.001022	up	yes	0.5747	0.2227	0.225	0.263	0.18	0.64	0.554	0.53	------	------	------	------	------	------	------	------	------	------	------	EXC	1	3	22.4	High
TRINITY_DN47731_c0_g1_i2_orf1	nucleolar GTP-binding protein 2 [Ostrinia furnacalis]	0.3820574	-1.38813863	0.000285	down	yes	0.1597	0.418	0.419	0.412	0.423	0.201	0.129	0.149	CC:organelle;CC:intracellular organelle;CC:non-membrane-bounded organelle;CC:nucleolus;CC:cellular anatomical entity;CC:intracellular non-membrane-bounded organelle;CC:cellular_component;MF:purine ribonucleoside triphosphate binding;MF:molecular_function;MF:binding;MF:ion binding;MF:heterocyclic compound binding;MF:nucleoside phosphate binding;MF:anion binding;MF:nucleotide binding;MF:guanyl ribonucleotide binding;MF:purine nucleotide binding;MF:GTP binding;MF:small molecule	GO:0043226;GO:0043229;GO:0043228;GO:0005730;GO:0110165;GO:0043232;GO:0005575;GO:0035639;GO:0003674;GO:0005488;GO:0043167;GO:1901363;GO:1901265;GO:0043168;GO:0000166;GO:0032561;GO:0017076;GO:0005525;GO:0036094;GO:0097367;GO:0097159;GO:0019001;GO:0032555;GO:0032553	K14537	NUG2, GNL2	map03008	Ribosome biogenesis in eukaryotes	COG1161;ENOG4111H9K	S:Function unknown;	PF07572.15	BCNT	Bucentaur or craniofacial development	CYT	1	4	30.3	Medium
TRINITY_DN38498_c0_g3_i1_orf1	unnamed protein product [Parnassius apollo]	2.9993659	1.584657523	0.004527	up	yes	0.473	0.1577	0.164	0.154	0.155	0.536	0.364	0.519	------	------	------	------	------	------	ENOG410ZSXY;ENOG410YWUP;ENOG410ZA5J;ENOG410ZHWV;ENOG410ZQZR	S:Function unknown;	------	------	------	CYT	1	3	36.4	Medium
TRINITY_DN868_c0_g1_i4_orf1	uncharacterized protein LOC114359357 isoform X1 [Ostrinia furnacalis]	2.5059701	1.32536923	0.0003114	up	yes	0.5037	0.201	0.207	0.175	0.221	0.519	0.532	0.46	CC:cellular_component;CC:extracellular region;CC:cellular anatomical entity;MF:molecular_function;MF:structural molecule activity;	GO:0005575;GO:0005576;GO:0110165;GO:0003674;GO:0005198	------	------	------	------	------	------	PF07294.14	Fibroin_P25	Fibroin P25	CYT	1	5	22.6	High
TRINITY_DN3504_c0_g1_i4_orfp1	TRINITY_DN3504_c0_g1_i4_m.43930 TRINITY_DN3504_c0_g1::TRINITY_DN3504_c0_g1_i4::g.43930 ORF type:internal len:196 (-),score=84.82 TRINITY_DN3504_c0_g1_i4:3-587(-)	3.4727217	1.796066786	0.0066	up	yes	0.338	0.09733	0.121	0.053	0.118	0.312	0.284	0.418	------	------	------	------	------	------	------	------	------	------	------	EXC	1	8	21.5	High
TRINITY_DN2986_c1_g1_i1_orf1	Troponin C, isoform 1 [Papilio xuthus]	2.407	1.267236142	9.03E-05	up	yes	0.2407	0.1	0.103	0.098	0.099	0.258	0.233	0.231	MF:cation binding;MF:ion binding;MF:molecular_function;MF:binding;MF:metal ion binding;MF:calcium ion binding;	GO:0043169;GO:0043167;GO:0003674;GO:0005488;GO:0046872;GO:0005509	------	------	------	------	COG5126	F:Nucleotide transport and metabolism	PF13499.9;PF00036.35;PF14658.9;PF13833.9;PF13405.9; PF13202.9	EF-hand_7;EF-hand_1;EF-hand_9;EF-hand_8;EF-hand_6;EF-hand_5	EF-hand domain pair;EF hand;EF-hand domain;EF-hand domain pair;EF-hand domain;EF hand	CYT	5	22	20.2	High

TRINITY_DN33452_c0_g1_i1_orf1	lethal(2) giant larvae protein isoform X8 [Ostrinia furnacalis]	3.0555871	1.611449591	0.001683	up	yes	0.5387	0.1763	0.211	0.129	0.189	0.538	0.611	0.467	BP:secretion;BP:transport;BP:vesicle-mediated transport;BP:export from cell;BP:exocytosis;BP:secretion by cell;BP:biological_process;BP:cellular process;BP:establishment of localization;BP:localization;	GO:0046903;GO:0006810;GO:0016192;GO:0140352;GO:0006887;GO:0032940;GO:0008150;GO:0009987;GO:0051234;GO:0051179	K06094	LLGL	map04530;map05165;map04390;map04391	Tight junction;Human papillomavirus infection;Hippo signaling pathway;Hippo signaling pathway - fly	ENOG410XS6Z	S:Function unknown	------	------	------	CYT	1	14	13.7	High
TRINITY_DN8691_c0_g1_i3_orf1	nucleolin-like [Melitaea cinxia]	0.4105748	-1.284282997	0.0002598	down	yes	0.205	0.4993	0.54	0.496	0.462	0.222	0.195	0.198	------	------	------	------	------	------	ENOG4110RR1	S:Function unknown	------	------	------	CYT	2	15	14.4	High
TRINITY_DN30704_c0_g1_i1_orf1	cytochrome P450 monooxygenase CYP6AE134v2 [Ostrinia furnacalis]	3	1.584962501	0.001471	up	yes	0.258	0.086	0.117	0.083	0.058	0.23	0.271	0.273	CC:integral component of membrane;CC:cellular_component;CC:cellular anatomical entity;CC:intrinsic component of membrane;MF:cation binding;MF:monooxygenase activity;MF:heme binding;MF:transition metal ion binding;MF:tetrapyrrole binding;MF:organic cyclic compound binding;MF:oxidoreductase activity, acting on paired donors, with incorporation or reduction of molecular oxygen;MF:ion binding;MF:catalytic activity;MF:molecular_function;MF:binding;MF:iron ion binding;MF:metal ion binding;MF:heterocyclic compound	GO:0016021;GO:0005575;GO:0110165;GO:0031224;GO:0043169;GO:0004497;GO:0020037;GO:0046914;GO:0046906;GO:0097159;GO:0016705;GO:0043167;GO:0003824;GO:0003674;GO:0005488;GO:0005506;GO:0046872;GO:1901363;GO:0016491	K14999	CYP6	------	------	COG2124	Q:Secondary metabolites biosynthesis, transport and catabolism	PF00067.25	p450	Cytochrome P450	CYT	1	2	61.1	High
TRINITY_DN2695_c0_g1_i14_orfp1	TRINITY_DN2695_c0_g1_i14_m.44485 TRINITY_DN2695_c0_g1::TRINITY_DN2695_c0_g1_i14::g.44485 ORF type:3prime_partial len:698 (+),score=187.51 TRINITY_DN2695_c0_g1_i14:101-2092(+)	2.5045607	1.324557594	0.0004878	up	yes	0.5217	0.2083	0.177	0.229	0.219	0.496	0.573	0.496	------	------	------	------	------	------	------	------	------	------	------	CYT	2	5	73.5	High
TRINITY_DN26411_c0_g1_i2_orfp1	TRINITY_DN26411_c0_g1_i2_m.24123 TRINITY_DN26411_c0_g1::TRINITY_DN26411_c0_g1_i2::g.24123 ORF type:internal len:115 (-),score=72.83 TRINITY_DN26411_c0_g1_i2:3-344(-)	4.8524427	2.278711169	0.004953	up	yes	0.4867	0.1003	0.103	0.118	0.08	0.377	0.472	0.611	------	------	------	------	------	------	------	------	------	------	------	CYT	1	46	10.9	High
TRINITY_DN3135_c0_g1_i6_orf1	acanthoscurrin-1-like [Ostrinia furnacalis]	2.8422568	1.507036891	2.84E-05	up	yes	0.4937	0.1737	0.145	0.192	0.184	0.501	0.49	0.49	------	------	------	------	------	------	------	------	------	------	------	EXC	1	7	25	High
TRINITY_DN5244_c0_g1_i1_orf1	eukaryotic peptide chain release factor GTP-binding subunit-like [Ostrinia furnacalis]	2.2451613	1.166819091	9.28E-07	up	yes	0.348	0.155	0.159	0.148	0.158	0.351	0.346	0.347	------	------	------	------	------	------	ENOG41116ZG;ENOG4111PWE;ENOG4111WYB	S:Function unknown;	PF03103.20	DUF243	Domain of unknown function	CYT	6	20	48.5	High
TRINITY_DN22664_c0_g1_i1_orf1	larval cuticle protein LCP-14-like [Ostrinia furnacalis]	2.162492	1.112694777	7.55E-06	up	yes	0.3367	0.1557	0.156	0.148	0.163	0.334	0.345	0.331	MF:molecular_function;MF:structural constituent of cuticle;MF:structural molecule activity;	GO:0003674;GO:0042302;GO:0005198	------	------	------	------	ENOG4111PJ1;ENOG4110T0M;ENOG4110RUZ	S:Function unknown;	PF00379.26	Chitin_bind_4	Insect cuticle protein	CYT	6	52	19.3	High
TRINITY_DN117_c0_g1_i4_orf1	lipase member I-like [Ostrinia furnacalis]	0.4686549	-1.093402129	0.001775	down	yes	0.077	0.1643	0.172	0.142	0.179	0.071	0.082	0.078	BP:organic substance metabolic process;BP:lipid metabolic process;BP:primary metabolic process;BP:metabolic process;BP:biological_process;CC:cellular_component;CC:extracellular region;CC:cellular anatomical entity;MF:hydrolase activity;MF:hydrolase activity, acting on ester bonds;MF:catalytic activity;MF:molecular_function;MF:lipase	GO:0071704;GO:0006629;GO:0044238;GO:0008152;GO:0008150;GO:0005575;GO:0005576;GO:0110165;GO:0016787;GO:0016788;GO:0003824;GO:0003674;GO:0016298	------	------	------	------	ENOG41116Y2;ENOG4110RTX;ENOG4111JY5;ENOG410YGS3;ENOG410ZKVQ;ENOG4110RNC	S:Function unknown;	PF00151.22	Lipase	Lipase	CYT	5	82	16.1	High
TRINITY_DN334_c0_g1_i4_orf1	collagenase-like [Ostrinia furnacalis]	2.8004645	1.485666133	0.02132	up	yes	0.1447	0.05167	0.042	0.07	0.043	0.101	0.182	0.151	BP:organic substance metabolic process;BP:organonitrogen compound metabolic process;BP:protein metabolic process;BP:macromolecule metabolic process;BP:biological_process;BP:nitrogen compound metabolic process;BP:metabolic process;BP:proteolysis;BP:primary metabolic process;MF:serine-type endopeptidase activity;MF:endopeptidase activity;MF:hydrolase activity;MF:serine hydrolase activity;MF:catalytic activity, acting on a protein;MF:catalytic activity;MF:molecular_function;MF:peptidase activity;MF:serine-type	GO:0071704;GO:1901564;GO:0019538;GO:0043170;GO:0008150;GO:0006807;GO:0008152;GO:0006508;GO:0044238;GO:0004252;GO:0004175;GO:0016787;GO:0017171;GO:0140096;GO:0003824;GO:0003674;GO:0008233;GO:0008236	------	------	------	------	COG5640	O:Posttranslational modification, protein turnover, chaperones	PF00089.29	Trypsin	Trypsin	EXC	1	39	29.8	High
TRINITY_DN1249_c0_g1_i6_orf1	venom carboxylesterase-6-like [Ostrinia furnacalis]	0.2721459	-1.877547545	6.95E-05	down	yes	0.1447	0.5317	0.543	0.519	0.533	0.172	0.102	0.16	------	------	------	------	------	------	COG2272;ENOG410XSWS;COG0580	I:Lipid transport and metabolism;T:Signal transduction mechanisms;G:Carbohydrate transport and metabolism	PF00135.31;PF20434.1;PF07859.16	COesterase;BD-FAE;Abhydrolase_3	Carboxylesterase family;BD-FAE;alpha/beta hydrolase fold	PLA	1	6	64.7	High
TRINITY_DN41697_c0_g1_i1_orf1	5-formyltetrahydrofolate cyclo-ligase [Ostrinia furnacalis]	0.3627125	-1.463101795	4.71E-05	down	yes	0.207	0.5707	0.563	0.557	0.592	0.221	0.175	0.225	MF:purine ribonucleoside triphosphate binding;MF:cyclo-ligase activity;MF:molecular_function;MF:binding;MF:ion binding;MF:heterocyclic compound binding;MF:nucleoside phosphate binding;MF:anion binding;MF:cation binding;MF:nucleotide binding;MF:ligase activity, forming carbon-nitrogen bonds;MF:purine nucleotide binding;MF:metal ion binding;MF:ATP binding;MF:small molecule binding;MF:catalytic activity;MF:ligase activity;MF:5-formyltetrahydrofolate cyclo-ligase	GO:0035639;GO:0016882;GO:0003674;GO:0005488;GO:0043167;GO:1901363;GO:1901265;GO:0043168;GO:0043169;GO:0000166;GO:0016879;GO:0017076;GO:0046872;GO:0005524;GO:0036094;GO:0003824;GO:0016874;GO:0030272;GO:0030554;GO:0097367;GO:0097159;GO:0032559;GO:0032555;GO:0032553	K01934	MTHFS	map00670	One carbon pool by folate	COG0212	H:Coenzyme transport and metabolism	------	------	------	CYT	1	38	4.5	High
TRINITY_DN10940_c0_g1_i10_orfp	TRINITY_DN10940_c0_g1_i10_m.52163 TRINITY_DN10940_c0_g1::TRINITY_DN10940_c0_g1_i10::g.52163 ORF type:5prime_partial len:248 (-),score=128.24 TRINITY_DN10940_c0_g1_i10:121-864(-)	2.5337199	1.341257047	0.001177	up	yes	0.201	0.07933	0.085	0.085	0.068	0.195	0.181	0.227	------	------	------	------	------	------	------	------	------	------	------	CYT	1	14	22.7	High
