# Supplementary material for: Comparative Proteomics and Metabonomics Analysis of Different Diapause Stages Revealed a New Regulation Mechanism of Diapause in Loxostege sticticalis (Lepidoptera: Pyralidae)
Source: Molecules. 2024 Jul 25;29(15):3472. doi: 10.3390/molecules29153472 (PMC11314584; doi:10.3390/molecules29153472)
Supplement: Supplementary file 1 [file molecules-29-03472-s001.zip › analysis process/proteomic/diffreential protein statistic table/NDvsCT.pdf]

Accession	Symbol	Protein Name	Entrez ID	Description	FC(ND/CT)	Log2FC(ND/CT)	P value(ND/CT)	Regulate	Significant	ND	CT	CT1	CT2	CT3	ND1	ND2	ND3	GO Term	GO ID	KO ID	KO Name	Pathway ID	Pathway defition	EggNOG ID	EggNOG Functional Categories	Pfam ID	Domain	Domain Description	Subcellular Loc	Unique Peptides	Coverage %	MW [kDa]	FDR Confidence
TRINITY_DN2396_c0_g1_i9_orfp1	-	-	-	TRINITY_DN2396_c0_g1_i9_m.39038 TRINITY_DN2396_c0_g1::TRINITY_DN2396_c0_g1_i9::g.39038 ORF type:5prime_partial len:181 (+),score=90.33 TRINITY_DN2396_c0_g1_i9:3-545(+)	0.036868687	-4.761460156	0.0006691	down	yes	1.095	29.7	29.569	24.594	34.927	1	0.759	1.526	------	------	------	------	------	------	------	------	------	------	------	CYT	1	6	19.3	High
TRINITY_DN56459_c0_g1_i2_orf1	-	-	-	aldo-keto reductase AKR2E4-like [Ostrinia furnacalis]	0.152408418	-2.713985507	0.0004617	down	yes	0.9777	6.415	6.173	5.666	7.406	1	0.951	0.982	MF:catalytic activity;	GO:0003824;GO:0003674;GO:0016491	------	------	------	------	COG0656	S:Function unknown	------	------	------	CYT	4	78	8.7	High
TRINITY_DN59965_c0_g4_i1_orf1	-	-	-	TKT protein, partial [Homo sapiens]	0.243729657	-2.036646284	0.03488	down	yes	1.273	5.223	4.655	3.405	7.609	1	1.593	1.226	BP:small molecule m	GO:0044281;GO:1901360;GO:0046483;GO:1901564;GO:0019637;GO:0006807;GO:0050789;GO:1901576;GO:0065007;GO:0008150;GO:0008152;GO:0090407;GO:0046390;GO:0019693;GO:0006753;GO:0006739;GO:0044249;GO:0034641;GO:0006139;GO:0046166;GO:0009987;GO:0006725;GO:0051156;GO:0055086;GO:0006081;GO:1901137;GO:1901135;GO:0006740;GO:0046496;GO:0044237;GO:0006098;GO:0006091;GO:0072524;GO:0040008;GO:0019682;GO:0071704;GO:0046184;GO:0009058;GO:0009117;GO:0009052;GO:0044238;GO:0006796;GO:0006793;GO:0019362;GO:0005789;GO:0031982;GO:0016020;GO:0005777;GO:0043230;GO:0043231;GO:0042579;GO:0005829;GO:0065010;GO:0043229;GO:0043227;GO:0016604;GO:0005654;GO:0110165;GO:0031090;GO:0070062;GO:0043226;GO:1903561;GO:0005575;GO:1901363;GO:0016740;GO:0016744;GO:0046872;GO:0003674;GO:0005488;GO:0030976;GO:1901681;GO:0043169;GO:0003824;GO:0097159;GO:0046983;GO:0043168;GO:0000287;GO:0043167;GO:0005509;GO:0042802;GO:0042803;GO:0005515;GO:0019842;GO:0036094;GO:0004802;GO:0050997	K00615	E2.2.1.1, tktA, t	map00030	Pentose phosphate pathway	COG0021	S:Function unknown	PF02779.27;PF02	Transket_pyr;Tr	Transketolase, pyrimidi	CYT	1	4	45.1	High
TRINITY_DN64297_c0_g1_i1_orf1	-	-	-	vanin-like protein 2 isoform X2 [Ostrinia furnacalis]	0.163979552	-2.608412173	4.83E-05	down	yes	1.251	7.629	7.733	8.118	7.036	1	1.417	1.336	BP:nitrogen compou	GO:0006807;GO:0008150;GO:0008152	------	------	------	------	COG0388	O:Posttranslational modification	------	------	------	CYT	1	57	7.8	High
TRINITY_DN1506_c0_g1_i6_orfp1	-	-	-	TRINITY_DN1506_c0_g1_i6_m.57691 TRINITY_DN1506_c0_g1::TRINITY_DN1506_c0_g1_i6::g.57691 ORF type:5prime_partial len:173 (+),score=30.49 TRINITY_DN1506_c0_g1_i6:1-519(+)	0.058196222	-4.102930695	0.0009739	down	yes	0.955	16.41	16.062	13.514	19.654	1	0.807	1.058	------	------	------	------	------	------	------	------	------	------	------	CYT	1	7	19	High
TRINITY_DN10877_c0_g1_i1_orf1	-	-	-	spodomicin-like [Ostrinia furnacalis]	0.116206897	-3.105232404	0.002408	down	yes	1.011	8.7	8.217	7.054	10.829	1	1.278	0.754	CC:cellular_compon	GO:0005575;GO:0005576;GO:0110165;GO:0003674;GO:0090729	------	------	------	------	------	------	PF08036.14	Antimicrobial_6	Diapausin family of ant	CYT	1	19	6.9	Medium
TRINITY_DN971_c0_g1_i10_orfp1	-	-	-	TRINITY_DN971_c0_g1_i10_m.54268 TRINITY_DN971_c0_g1::TRINITY_DN971_c0_g1_i10::g.54268 ORF type:internal len:187 (+),score=141.68 TRINITY_DN971_c0_g1_i10:1-558(+)	0.044565217	-4.487938046	0.0001191	down	yes	0.779	17.48	17.9	15.374	19.162	1	0.834	0.503	------	------	------	------	------	------	------	------	------	------	------	CYT	2	20	20.5	High
TRINITY_DN295_c2_g1_i2_orf1	-	-	-	phosphoglycolate phosphatase 1A, chloroplastic [Manduca sexta]	0.249378419	-2.003591476	0.0002569	down	yes	1.003	4.022	4.436	3.582	4.048	1	1.002	1.008	MF:hydrolase activity	GO:0016787;GO:0003674;GO:0003824	K21013	FPPP	map00981	Insect hormone biosynthesis	COG0647	G:Carbohydrate transport and m	PF13242.9	Hydrolase_like	HAD-hyrolase-like	PLA	4	28	13.9	High
TRINITY_DN7064_c0_g1_i19_orf1	-	-	-	unnamed protein product [Chilo suppressalis]	0.198359004	-2.333814209	0.002029	down	yes	1.378	6.947	6.914	5.701	8.225	1	1.915	1.219	------	------	------	------	------	------	------	------	PF00050.24;PF07	Kazal_1;Kazal_2	Kazal-type serine prote	EXC	1	2	73	High
TRINITY_DN2044_c0_g1_i5_orfp1	-	-	-	TRINITY_DN2044_c0_g1_i5_m.4210 TRINITY_DN2044_c0_g1::TRINITY_DN2044_c0_g1_i5::g.4210 ORF type:complete len:151 (-),score=85.31 TRINITY_DN2044_c0_g1_i5:857-1309(-)	0.103661972	-3.270041353	0.002131	down	yes	1.104	10.65	10.897	8.213	12.841	1	1.451	0.861	------	------	------	------	------	------	------	------	------	------	------	CYT	1	5	16.2	High
TRINITY_DN143895_c0_g1_i1_orf1	-	-	-	cathepsin L-like [Aphidius gifuensis] >KAF7988186.1 hypothetical protein HCN44_007680 [Aphidius gifuensis]	0.105350799	-3.246726835	1.92E-05	down	yes	0.949	9.008	9.535	8.381	9.108	1	0.834	1.013	BP:primary metaboli	GO:0044238;GO:1901564;GO:0019538;GO:0043170;GO:0071704;GO:0006807;GO:0008150;GO:0008152;GO:0006508;GO:0003674;GO:0016787;GO:0003824;GO:0140096;GO:0008233;GO:0008234	K01365	CTSL	map05205;map05	Proteoglycans in cancer;Rhe	COG4870	O:Posttranslational modification	PF00112.26;PF08	Peptidase_C1;I	Papain family cysteine p	CYT	1	2	38.3	High
TRINITY_DN971_c0_g1_i5_orfp1	-	-	-	TRINITY_DN971_c0_g1_i5_m.54249 TRINITY_DN971_c0_g1::TRINITY_DN971_c0_g1_i5::g.54249 ORF type:internal len:108 (+),score=66.98 TRINITY_DN971_c0_g1_i5:1-321(+)	0.042809524	-4.545924402	0.000708	down	yes	0.899	21	20.606	17.52	24.873	1	0.708	0.989	------	------	------	------	------	------	------	------	------	------	------	CYT	1	9	11.5	High
TRINITY_DN84478_c0_g1_i8_orf1	-	-	-	uncharacterized protein LOC114359035 isoform X1 [Ostrinia furnacalis]	0.051459293	-4.280424543	0.000215	down	yes	1.005	19.53	20.042	16.81	21.746	1	0.936	1.078	BP:nitrogen compou	GO:0006807;GO:0006643;GO:0071704;GO:0006665;GO:0006629;GO:0009987;GO:0008150;GO:0008152;GO:0044255;GO:0044238;GO:1901564;GO:0044237;GO:0000323;GO:0043229;GO:0043227;GO:0043226;GO:0005764;GO:0005773;GO:0110165;GO:0043231;GO:0005575;GO:0005576	K12382	PSAP, SGP1	map00600;map04	Sphingolipid metabolism;Lys	ENOG410XSI5	O:Posttranslational modification	PF02199.18	SapA	Saposin A-type domain	CYT	1	52	5.9	High
TRINITY_DN51995_c0_g3_i1_orf1	-	-	-	circadian clock-controlled protein-like [Ostrinia furnacalis]	0.062446483	-4.001235864	2.08E-05	down	yes	1.021	16.35	17.669	15.542	15.853	1	1.02	1.042	------	------	------	------	------	------	ENOG4111G78	S:Function unknown	PF06585.14	JHBP	Haemolymph juvenile h	CYT	3	10	29.2	High
TRINITY_DN1108_c3_g1_i1_orfp1	-	-	-	TRINITY_DN1108_c3_g1_i1_m.5561 TRINITY_DN1108_c3_g1::TRINITY_DN1108_c3_g1_i1::g.5561 ORF type:internal len:113 (+),score=89.07 TRINITY_DN1108_c3_g1_i1:1-336(+)	0.100846154	-3.309772033	0.0007305	down	yes	1.311	13	12.943	11.131	14.914	1	2.486	0.447	------	------	------	------	------	------	------	------	------	------	------	PLA	1	11	12.3	High
TRINITY_DN31348_c0_g1_i1_orf1	-	-	-	protein lethal(2)essential for life [Bombyx mori]	0.189922788	-2.39651508	5.22E-05	down	yes	0.9593	5.051	4.909	4.758	5.486	1	0.911	0.967	MF:cation binding;M	GO:0043169;GO:0046872;GO:0003674;GO:0005488;GO:0043167	K09542	CRYAB	map04213;map04	Longevity regulating pathwa	ENOG410YERS	O:Posttranslational modification	------	------	------	CYT	1	22	7.2	High
TRINITY_DN38506_c0_g1_i4_orf1	-	-	-	C-1-tetrahydrofolate synthase, cytoplasmic isoform X1 [Ostrinia furnacalis] >XP_028166137.1 C-1-tetrahydrofolate synthase, cytoplasmic isoform X2 [Ostrinia furnacalis] >XP_028166140.1 C-1-tetrahydrofolate synthase, cytoplasmic isoform X4 [Ostrinia furnacalis]	0.20133362	-2.312339991	0.0001942	down	yes	0.936	4.649	4.298	4.446	5.202	1	0.864	0.944	BP:one-carbon meta	GO:0006730;GO:0006807;GO:0044237;GO:0046653;GO:1901360;GO:0035999;GO:0042558;GO:0071704;GO:0006760;GO:0009987;GO:0006725;GO:0008150;GO:0008152;GO:0046483;GO:1901564;GO:0006575;GO:0044281;GO:0005488;GO:0035639;GO:0016646;GO:0003674;GO:0043167;GO:1901363;GO:0016491;GO:0043168;GO:0004329;GO:0016645;GO:0000166;GO:0019238;GO:1901265;GO:0017076;GO:0005524;GO:0004486;GO:0016787;GO:0004488;GO:0036094;GO:0003824;GO:0016874;GO:0016879;GO:0097367;GO:0097159;GO:0016810;GO:0030554;GO:0004477;GO:0016814;GO:0032559;GO:0032555;GO:0032553	K00288	MTHFD	map00670;map01	One carbon pool by folate;B	COG0190	H:Coenzyme transport and meta	PF00763.26	THF_DHG_CYH	Tetrahydrofolate dehyd	CYT	3	61	16.2	High
TRINITY_DN7226_c0_g1_i2_orf1	-	-	-	chemosensory protein [Conogethes punctiferalis]	0.23395646	-2.09568803	0.01812	down	yes	0.9887	4.226	3.278	5.897	3.502	1	0.963	1.003	------	------	------	------	------	------	ENOG4111U5H;I	S:Function unknown;	PF03392.16	OS-D	Insect pheromone-binc	CYT	5	54	9	High
TRINITY_DN1772_c1_g2_i1_orf1	-	-	-	aldose reductase-like isoform X2 [Ostrinia furnacalis]	0.261520315	-1.935005077	6.05E-05	down	yes	0.9977	3.815	3.712	4.128	3.606	1	0.979	1.014	------	------	K00011	AKR1B	map00051;map00	Fructose and mannose meta	COG0656	Z:Cytoskeleton	PF00248.24	Aldo_ket_red	Aldo/keto reductase fa	CYT	13	77	36.1	High
TRINITY_DN1391_c0_g1_i29_orfp1	-	-	-	TRINITY_DN1391_c0_g1_i29_m.70767 TRINITY_DN1391_c0_g1::TRINITY_DN1391_c0_g1_i29::g.70767 ORF type:complete len:495 (+),score=158.49 TRINITY_DN1391_c0_g1_i29:728-2212(+)	0.307533843	-1.70118291	0.002065	down	yes	1.045	3.398	3.497	2.784	3.912	1	1.035	1.101	------	------	------	------	------	------	------	------	------	------	------	CYT	5	14	52.3	High
TRINITY_DN14328_c0_g1_i12_orf1	-	-	-	larval cuticle protein LCP-30-like [Ostrinia furnacalis]	0.308911483	-1.694734593	0.0007186	down	yes	1.033	3.344	3.424	2.886	3.723	1	1.012	1.086	MF:molecular_functi	GO:0003674;GO:0042302;GO:0005198	------	------	------	------	ENOG41112O7;I	S:Function unknown;	PF00379.26	Chitin_bind_4	Insect cuticle protein	CYT	10	50	24.6	High
TRINITY_DN15682_c0_g1_i4_orf1	-	-	-	seroin transcript 1B [Ostrinia nubilalis]	0.307228916	-1.702614089	0.00069	down	yes	1.02	3.32	3.112	3.795	3.052	1	1.106	0.954	------	------	------	------	------	------	------	------	------	------	------	CYT	2	36	5.9	High
TRINITY_DN28221_c0_g2_i1_orf1	-	-	-	unnamed protein product [Chilo suppressalis]	0.290735695	-1.782219887	0.0005845	down	yes	0.9603	3.303	3.376	2.863	3.67	1	0.935	0.946	BP:small molecule bi	GO:0044283;GO:0019752;GO:0044249;GO:0034641;GO:0006807;GO:0044281;GO:0071941;GO:0009064;GO:1901576;GO:0006520;GO:0006526;GO:0006525;GO:0071704;GO:0006082;GO:0008652;GO:1901605;GO:1901607;GO:0019627;GO:0044238;GO:0009987;GO:0009058;GO:0008150;GO:0008152;GO:0043436;GO:0044271;GO:0043604;GO:1901564;GO:1901566;GO:0043603;GO:0046394;GO:0000050;GO:0016053;GO:0044237;GO:0009084;GO:0004055;GO:0035639;GO:0003674;GO:0043167;GO:1901363;GO:1901265;GO:0043168;GO:0000166;GO:0017076;GO:0036094;GO:0003824;GO:0016874;GO:0016879;GO:0030554;GO:0097367;GO:0032559;GO:0005524;GO:0097159;GO:0032555;GO:0032553;GO:0005488	K01940	argG, ASS1	map00220;map00	Arginine biosynthesis;Alanin	COG0137	E:Amino acid transport and meta	PF00764.22	Arginosuc_synt	Arginosuccinate syntha	CYT	1	9	10.1	High
TRINITY_DN64772_c0_g1_i1_orf1	-	-	-	aldehyde dehydrogenase, partial [Mythimna separata]	0.319774718	-1.644872212	0.000118	down	yes	1.022	3.196	3.415	2.921	3.251	1	1.023	1.044	MF:catalytic activity;	GO:0003824;GO:0016903;GO:0003674;GO:0016620;GO:0016491	K00128	ALDH	map00310;map00	Lysine degradation;Glyceroli	COG1012	C:Energy production and conver	PF00171.25	Aldedh	Aldehyde dehydrogena	CYT	2	42	5.9	High
TRINITY_DN12009_c0_g1_i1_orf1	-	-	-	uncharacterized protein LOC114365631 [Ostrinia furnacalis]	0.298657089	-1.743438127	0.000108	down	yes	0.9563	3.202	3.352	3.343	2.911	1	0.946	0.923	------	------	------	------	------	------	------	------	------	------	------	CYT	2	17	8.2	High
TRINITY_DN18128_c0_g1_i4_orf1	-	-	-	arylsulfatase B [Ostrinia furnacalis]	0.333003953	-1.586388794	6.23E-05	down	yes	1.011	3.036	2.807	3.134	3.167	1	0.996	1.036	MF:hydrolase activity	GO:0016787;GO:0003674;GO:0016788;GO:0008484;GO:0003824	K01135	ARSB	map04142;map00	Lysosome;Glycosaminoglyca	COG3119	P:Inorganic ion transport and me	------	------	------	CYT	1	14	9.5	High
TRINITY_DN104663_c1_g1_i2_orf1	-	-	-	PREDICTED: gelsolin-like [Amyelois transitella]	0.348940533	-1.518946903	0.003087	down	yes	1.021	2.926	3.394	3.011	2.372	1	1.027	1.036	MF:cytoskeletal prot	GO:0008092;GO:0051015;GO:0003779;GO:0044877;GO:0005488;GO:0005515;GO:0003674	K05768	GSN	map04666;map05	Fc gamma R-mediated pha	ENOG410XR0A	S:Function unknown	------	------	------	CYT	1	53	12.4	High
TRINITY_DN71863_c0_g1_i2_orf1	-	-	-	unnamed protein product [Diatraea saccharalis]	0.369549419	-1.436160791	0.0002988	down	yes	1.017	2.752	2.787	2.987	2.481	1	1.02	1.032	BP:organic substanc	GO:0071704;GO:1901564;GO:0019538;GO:0043170;GO:0008150;GO:0006807;GO:0008152;GO:0006508;GO:0044238;GO:0004252;GO:0004175;GO:0016787;GO:0017171;GO:0140096;GO:0003824;GO:0003674;GO:0008233;GO:0008236	------	------	------	------	COG5640	O:Posttranslational modification	PF16030.8	GD_N	Serine protease gd N-t	CYT	3	25	17.8	High
TRINITY_DN15545_c0_g1_i1_orf1	-	-	-	larval cuticle protein LCP-14-like [Ostrinia furnacalis]	0.356685289	-1.487276381	0.0005133	down	yes	0.9577	2.685	2.846	2.35	2.858	1	0.934	0.939	MF:molecular_functi	GO:0003674;GO:0042302;GO:0005198	------	------	------	------	ENOG4110RUZ;I	S:Function unknown;	PF00379.26	Chitin_bind_4	Insect cuticle protein	EXC	3	48	14.6	High
TRINITY_DN36144_c0_g1_i3_orf1	-	-	-	nicotinate phosphoribosyltransferase isoform X1 [Ostrinia furnacalis] >XP_028178189.1 nicotinate phosphoribosyltransferase isoform X1 [Ostrinia furnacalis]	0.4375	-1.192645078	0.0001998	down	yes	1.015	2.32	2.121	2.414	2.426	1	1.014	1.031	BP:NAD biosynthetic	GO:0009435;GO:0034641;GO:0009165;GO:0046496;GO:1901362;GO:0072525;GO:1901360;GO:0006139;GO:0019362;GO:0072524;GO:0071704;GO:0019359;GO:0018130;GO:1901576;GO:0009987;GO:0006725;GO:0009058;GO:0008150;GO:0008152;GO:0019438;GO:0034654;GO:1901564;GO:0090407;GO:0055086;GO:0046483;GO:0044238;GO:0044271;GO:1901566;GO:0044249;GO:0044237;GO:0006796;GO:0006807;GO:1901293;GO:0006793;GO:0019637;GO:0009117;GO:0006753;GO:0019363;GO:0044281;GO:0003674;GO:0016879;GO:0004516;GO:0003824;GO:0016874	K00763	pncB, NAPRT1	map00760;map01	Nicotinate and nicotinamide	COG1488	H:Coenzyme transport and meta	PF17767.4;PF17	NAPRTase_N;N	Nicotinate phosphoribc	CYT	8	16	66.9	High
TRINITY_DN1593_c0_g1_i1_orf1	-	-	-	chemosensory protein csp11 [Helopeltis theivora]	0.433581436	-1.205625106	0.001417	down	yes	0.9903	2.284	2.257	2.014	2.581	1	1.003	0.968	------	------	------	------	------	------	ENOG4110RV8;I	S:Function unknown;	PF03392.16	OS-D	Insect pheromone-binc	CYT	8	39	15.4	High
TRINITY_DN350_c0_g1_i5_orf1	-	-	-	tau-like protein isoform X6 [Bombyx mori]	0.458333333	-1.125530882	0.001381	down	yes	1.023	2.232	2.108	2.066	2.523	1	0.957	1.111	CC:cytoplasm;CC:su	GO:0005737;GO:0099080;GO:0099512;GO:0099513;GO:0005575;GO:0099081;GO:0005874;GO:0110165;GO:0015631;GO:0008092;GO:0003674;GO:0005488;GO:0005515	K04380	MAPT, TAU	map05012;map05	Parkinson disease;Alzheimer	ENOG4111J07	O:Posttranslational modification	PF00418.22	Tubulin-binding	Tau and MAP protein, t	CYT	1	16	26.7	High
TRINITY_DN20658_c0_g1_i1_orf1	-	-	-	prostaglandin reductase 1-like [Ostrinia furnacalis]	0.460045147	-1.120152647	0.0002801	down	yes	1.019	2.215	2.276	2.021	2.348	1	1.014	1.043	------	------	K13948	PTGR1, LTB4D	map00590	Arachidonic acid metabolism	COG2130	S:Function unknown	PF16884.8	ADH_N_2	N-terminal domain of c	CYT	8	49	17.6	High
TRINITY_DN12231_c0_g1_i1_orf1	-	-	-	carbonyl reductase [NADPH] 3-like [Ostrinia furnacalis]	0.462242563	-1.113277987	0.0002449	down	yes	1.01	2.185	2.371	2.114	2.069	1	0.995	1.035	------	------	K00079	CBR1	map00590;map05	Arachidonic acid metabolism	COG1028	K:Transcription	PF00106.28;PF13	adh_short;adh	short chain dehydroger	CYT	10	39	32.2	High
TRINITY_DN18338_c0_g1_i6_orf1	-	-	-	aquaporin AQPAn.G isoform X1 [Ostrinia furnacalis]	0.499756454	-1.000702897	0.0004088	down	yes	1.026	2.053	1.907	2.057	2.196	1	1.113	0.965	CC:integral compon	GO:0016021;GO:0005575;GO:0031224;GO:0110165;GO:0005215;GO:0022857;GO:0015267;GO:0003674;GO:0022803	K09884	AQPN	------	------	COG0580	O:Posttranslational modification	PF00230.23	MIP	Major intrinsic protein	CYT	1	8	29	High
TRINITY_DN1423_c0_g1_i4_orf1	-	-	-	hypothetical protein evm_003306 [Chilo suppressalis] >CAB3526495.1 unnamed protein product [Chilo suppressalis] >CAH0403823.1 unnamed protein product [Chilo suppressalis]	0.483317121	-1.048957994	0.0003595	down	yes	0.9937	2.056	2.015	1.916	2.236	1	0.987	0.994	BP:ion homeostasis;	GO:0050801;GO:0042592;GO:0055072;GO:0055076;GO:0098771;GO:0046916;GO:0000041;GO:0065007;GO:0019725;GO:0030001;GO:0006810;GO:0006879;GO:0006875;GO:0006812;GO:0006811;GO:0009987;GO:0006873;GO:0008150;GO:0030003;GO:0055065;GO:0055080;GO:0055082;GO:0051234;GO:0051179;GO:0065008;GO:0048878;GO:0006826;GO:0003674;GO:0005488;GO:0016491;GO:0043169;GO:0016722;GO:0046914;GO:0004322;GO:0043167;GO:0005506;GO:0046872;GO:0003824;GO:0016724;GO:0008199	------	------	------	------	ENOG4111Q26	P:Inorganic ion transport and me	PF00210.27	Ferritin	Ferritin-like domain	CYT	9	44	26.4	High
TRINITY_DN100327_c0_g1_i1_orf1	-	-	-	arylphorin subunit alpha-like [Ostrinia furnacalis]	0.213378437	-2.228513701	0.003676	down	yes	0.9777	4.582	4.999	5.332	3.414	1	0.967	0.966	------	------	------	------	------	------	ENOG410XR2D	S:Function unknown	PF00372.22;PF03	Hemocyanin_M	Hemocyanin, copper cc	PLA	35	56	83.5	High
TRINITY_DN29414_c1_g2_i1_orf1	-	-	-	serine protease 44-like isoform X2 [Ostrinia furnacalis]	0.219528772	-2.187518059	0.01131	down	yes	0.969	4.414	5.225	5.152	2.865	1	0.911	0.996	BP:organic substanc	GO:0071704;GO:1901564;GO:0019538;GO:0043170;GO:0008150;GO:0006807;GO:0008152;GO:0006508;GO:0044238;GO:0004252;GO:0004175;GO:0016787;GO:0017171;GO:0140096;GO:0003824;GO:0003674;GO:0008233;GO:0008236	------	------	------	------	COG5640	S:Function unknown	------	------	------	CYT	1	15	8.8	High
TRINITY_DN30169_c0_g1_i1_orfp1	-	-	-	TRINITY_DN30169_c0_g1_i1_m.11367 TRINITY_DN30169_c0_g1::TRINITY_DN30169_c0_g1_i1::g.11367 ORF type:3prime_partial len:52 (+),score=0.98 TRINITY_DN30169_c0_g1_i1:72-224(+)	0.257992565	-1.954598605	0.01025	down	yes	1.041	4.035	4.8	4.572	2.733	1	1.034	1.09	------	------	------	------	------	------	------	------	------	------	------	CYT	1	12	6.1	High
TRINITY_DN80328_c0_g1_i5_orf1	-	-	-	arylphorin subunit alpha-like [Ostrinia furnacalis]	0.275049116	-1.862238829	2.96E-05	down	yes	0.98	3.563	3.643	3.721	3.324	1	0.955	0.985	------	------	------	------	------	------	ENOG410XR2D	S:Function unknown	PF00372.22	Hemocyanin_M	Hemocyanin, copper cc	CYT	7	74	12.5	High
TRINITY_DN128231_c0_g1_i5_orf1	-	-	-	glutathione S-transferase sigma3 [Glyphodes pyloalis]	0.359452263	-1.476127908	0.0002243	down	yes	0.945	2.629	2.495	2.503	2.889	1	0.907	0.928	MF:catalytic activity;	GO:0003824;GO:0016740;GO:0004364;GO:0003674;GO:0016765	K04097	HPGDS	map00590;map00	Arachidonic acid metabolism	ENOG41103AU;I	O:Posttranslational modification	PF14497.9;PF00	GST_C_3;GST_C	Glutathione S-transfera	CYT	1	14	9.5	High
TRINITY_DN27300_c0_g1_i7_orfp1	-	-	-	TRINITY_DN27300_c0_g1_i7_m.71141 TRINITY_DN27300_c0_g1::TRINITY_DN27300_c0_g1_i7::g.71141 ORF type:internal len:82 (-),score=6.59 TRINITY_DN27300_c0_g1_i7:3-245(-)	0.429184549	-1.220329955	0.002736	down	yes	1	2.33	2.397	1.954	2.64	1	0.968	1.032	------	------	------	------	------	------	------	------	------	------	------	CYT	2	35	9.2	High
TRINITY_DN44517_c0_g1_i4_orf1	-	-	-	regucalcin-like [Ostrinia furnacalis]	0.442128603	-1.177462024	0.00344	down	yes	0.997	2.255	2.177	2.625	1.964	1	0.897	1.094	CC:cytoplasm;CC:ce	GO:0005737;GO:0005575;GO:0110165;GO:0016787;GO:0046872;GO:0043169;GO:0030234;GO:0004341;GO:0052689;GO:0043167;GO:0003824;GO:0003674;GO:0016788;GO:0005488;GO:0098772;GO:0005509	K01053	gnl, RGN	map00030;map00	Pentose phosphate pathway	COG3386;COG3	I:Lipid transport and metabolism	PF08450.15;PF0	SGL;Reg_prop	SMP-30/Gluconolactor	CYT	1	4	36.9	High
TRINITY_DN394_c0_g1_i2_orf1	-	-	-	uncharacterized protein LOC114351483 [Ostrinia furnacalis]	0.409102787	-1.289464728	7.12E-05	down	yes	0.9393	2.296	2.358	2.151	2.378	1	0.883	0.935	MF:molecular_functi	GO:0003674;GO:0042302;GO:0005198	------	------	------	------	ENOG41113X7;I	S:Function unknown;	PF00379.26	Chitin_bind_4	Insect cuticle protein	CYT	1	28	12	High
TRINITY_DN15858_c0_g1_i2_orf1	-	-	-	15-hydroxyprostaglandin dehydrogenase [NAD(+)]-like [Ostrinia furnacalis]	0.478053435	-1.064756208	0.0001106	down	yes	1.002	2.096	2.121	2.203	1.963	1	0.975	1.03	MF:catalytic activity;	GO:0003824;GO:0003674;GO:0016491	K00069	HPGD	map00590;map05	Arachidonic acid metabolism	COG1028;COG0	S:Function unknown;	PF00106.28;PF13	adh_short;adh	short chain dehydroger	CYT	3	30	30.1	High
TRINITY_DN13973_c0_g1_i6_orf1	-	-	-	27 kDa glycoprotein-like [Ostrinia furnacalis]	0.48160697	-1.054071823	1.12E-05	down	yes	0.995	2.066	2.017	2.137	2.045	1	0.965	1.02	------	------	------	------	------	------	ENOG4111POK	S:Function unknown	PF07165.14	DUF1397	Protein of unknown fun	CYT	6	35	29.1	High
TRINITY_DN1305_c0_g1_i6_orf1	-	-	-	glutathione S-transferase sigma 3 [Ostrinia furnacalis]	0.462650024	-1.112006829	0.001471	down	yes	0.9637	2.083	2.245	2.204	1.801	1	0.915	0.976	MF:catalytic activity;	GO:0003824;GO:0016740;GO:0004364;GO:0003674;GO:0016765	K04097	HPGDS	map00590;map00	Arachidonic acid metabolism	ENOG41103AU;I	O:Posttranslational modification	PF02798.23;PF14	GST_N;GST_C	Glutathione S-transfera	CYT	2	26	19.4	High
TRINITY_DN12367_c0_g1_i8_orf1	-	-	-	aldose reductase-like isoform X2 [Ostrinia furnacalis]	0.496302003	-1.010709818	0.0007022	down	yes	0.9663	1.947	1.783	2.135	1.922	1	0.941	0.958	MF:catalytic activity;	GO:0003824;GO:0003674;GO:0016491	K00011	AKR1B	map00051;map00	Fructose and mannose meta	COG0656	S:Function unknown	PF00248.24	Aldo_ket_red	Aldo/keto reductase fa	CYT	1	46	25.1	High
TRINITY_DN66040_c0_g1_i2_orf1	-	-	-	serine protease inhibitor dipetalogastin-like isoform X2 [Ostrinia furnacalis]	0.149318119	-2.743538859	0.0001704	down	yes	1.073	7.186	7.698	6.297	7.564	1	1.191	1.028	------	------	------	------	------	------	------	------	PF07648.18;PF0	Kazal_2;Kazal_	Kazal-type serine prote	CYT	1	21	6	High
TRINITY_DN57137_c0_g1_i1_orfp1	-	-	-	TRINITY_DN57137_c0_g1_i1_m.46420 TRINITY_DN57137_c0_g1::TRINITY_DN57137_c0_g1_i1::g.46420 ORF type:5prime_partial len:56 (-),score=4.65 TRINITY_DN57137_c0_g1_i1:82-249(-)	0.145552147	-2.780391972	0.002611	down	yes	0.949	6.52	6.167	5.286	8.108	1	0.936	0.911	------	------	------	------	------	------	------	------	------	------	------	CYT	3	33	5.8	High
TRINITY_DN703_c13_g1_i1_orf1	-	-	-	acidic juvenile hormone-suppressible protein 1-like [Ostrinia furnacalis]	0.164532266	-2.603557559	0.007442	down	yes	0.9867	5.997	6.059	7.698	4.235	1	0.952	1.008	------	------	------	------	------	------	ENOG410XR2D	S:Function unknown	PF00372.22;PF03	Hemocyanin_M	Hemocyanin, copper cc	CYT	15	47	57.7	High
TRINITY_DN6122_c0_g1_i6_orf1	-	-	-	deubiquitinase DESI2 isoform X1 [Helicoverpa armigera] >XP_049707835.1 deubiquitinase DESI2 isoform X1 [Helicoverpa armigera]	0.259113982	-1.948341226	0.0005991	down	yes	1.123	4.334	4.963	4.129	3.911	1	1.182	1.188	MF:hydrolase activity	GO:0016787;GO:0008233;GO:0140096;GO:0003674;GO:0003824	K22763	DESI2, PPPDE1	------	------	ENOG4111H3Z	S:Function unknown	PF05903.17	Peptidase_C97	PPPDE putative peptida	CYT	1	8	23.8	High
TRINITY_DN1597_c0_g1_i5_orfp1	-	-	-	TRINITY_DN1597_c0_g1_i5_m.57494 TRINITY_DN1597_c0_g1::TRINITY_DN1597_c0_g1_i5::g.57494 ORF type:complete len:86 (+),score=7.19 TRINITY_DN1597_c0_g1_i5:134-391(+)	0.223413418	-2.16221226	0.0009783	down	yes	0.9857	4.412	4.186	3.869	5.182	1	0.991	0.966	------	------	------	------	------	------	------	------	------	------	------	CYT	2	27	9.2	High
TRINITY_DN295_c5_g1_i2_orf1	-	-	-	unnamed protein product [Chilo suppressalis]	0.253490081	-1.9799988	1.46E-05	down	yes	1.035	4.083	4.008	3.932	4.309	1	0.997	1.107	MF:hydrolase activity	GO:0016787;GO:0003674;GO:0003824	K21013	FPPP	map00981	Insect hormone biosynthesis	COG0647	G:Carbohydrate transport and m	PF13344.9	Hydrolase_6	Haloacid dehalogenase	CYT	5	24	19.9	High
TRINITY_DN1880_c0_g1_i4_orf1	-	-	-	serine protease inhibitor dipetalogastin-like [Helicoverpa zea]	0.218690973	-2.193034424	0.0001405	down	yes	0.9957	4.553	4.561	4.117	4.981	1	0.998	0.989	------	------	K23615	SPIK5	------	------	COG5640;ENOG	O:Posttranslational modification	PF00050.24;PF07	Kazal_1;Kazal_2	Kazal-type serine prote	CYT	12	52	42.6	High
TRINITY_DN1540_c0_g1_i7_orf1	-	-	-	alaserpin-like isoform X13 [Ostrinia furnacalis]	0.280315389	-1.834877147	7.76E-05	down	yes	1.031	3.678	3.712	3.391	3.932	1	1.089	1.005	CC:cellular_compon	GO:0005575;GO:0110165;GO:0005615;GO:0004866;GO:0061135;GO:0061134;GO:0003674;GO:0030414;GO:0004867;GO:0004857;GO:0098772;GO:0030234	------	------	------	------	COG4826	O:Posttranslational modification	PF00079.23	Serpin	Serpin	CYT	1	35	42.9	High
TRINITY_DN418_c1_g1_i3_orf1	-	-	-	hypothetical protein evm_003996 [Chilo suppressalis]	0.231867887	-2.108625072	0.0004619	down	yes	1.039	4.481	4.661	3.851	4.931	1	0.995	1.123	MF:molecular_functi	GO:0003674;GO:0030246;GO:0005488	------	------	------	------	------	------	------	------	------	PLA	1	46	8.8	High
TRINITY_DN21367_c0_g1_i1_orf1	-	-	-	40S ribosomal protein S15 isoform 2 [Homo sapiens] >NP_001125055.1 40S ribosomal protein S15 [Pongo abelii] >NP_001252851.1 40S ribosomal protein S15 [Macaca mulatta] >NP_001268540.1 40S ribosomal protein S15 [Mesocricetus auratus] >NP_001291817.1 40S ribosomal protein S15 [Ailuropoda melanoleuca] >NP_033117.1 40S ribosomal protein S15 isoform 1 [Mus musculus] >NP_058847.1 40S ribosomal protein S15 [Rattus norvegicus] >NP_990793.1 40S ribosomal protein S15 [Gallus gallus] >NP_999499.1 40S ribosomal protein S15 [Sus scrofa] >XP_001513459.4 40S ribosomal protein S15 [Ornithorhynchus anatinus] >XP_003277039.1 40S ribosomal protein S15 isoform X2 [Nomascus leucogenys] >XP_003461016.1 40S ribosomal protein S15 [Cavia porcellus] >XP_004378568.1 40S ribosomal protein S15 [Trichechus manatus latirostris] >XP_004395466.1 PREDICTED: 40S ribosomal protein S15 [Odobenus rosmarus divergens] >XP_004441415.1 PREDICTED: 40S ribosomal protein S15 [Ceratotherium simum simum] >XP_004462792.1 40S ribosomal protein S15 [Dasypus novemcinctus] >XP_004632738.1 40S ribosomal protein S15 [Octodon degus] >XP_004866007.1 40S ribosomal protein S15 [Heterocephalus glaber] >XP_005359120.1 40S ribosomal protein S15 isoform X2 [Microtus ochrogaster] >XP_005406026.1 PREDICTED: 40S ribosomal protein S15 [Chinchilla lanigera] >XP_005531236.1 PREDICTED: 40S ribosomal protein S15 [Pseudopodoces humilis] >XP_005587458.1 40S ribosomal protein S15 isoform X4 [Macaca fascicularis] >XP_005866755.1 PREDICTED: 40S ribosomal protein S15 [Myotis brandtii] >XP_006206493.1 40S ribosomal protein S15 [Vicugna pacos] >XP_006764872.1 PREDICTED: 40S ribosomal protein S15 [Myotis davidii] >XP_006897998.1 PREDICTED: 40S ribosomal protein S15 [Elephantulus edwardii] >XP_006904116.1 40S ribosomal protein S15 [Pteropus alecto] >XP_006928238.1 40S ribosomal protein S15 [Felis catus] >XP_006978252.1 40S ribosomal protein S15 [Peromyscus maniculatus bairdii] >XP_007992808.1 40S ribosomal protein S15 isoform X2 [Chlorocebus sabaeus] >XP_008591572.1 PREDICTED: 40S ribosomal protein S15 [Galeopterus variegatus] >XP_008835382.1 40S ribosomal protein S15 isoform X3 [Nannospalax galili] >XP_009095157.1 40S ribosomal protein S15 [Serinus canaria] >XP_010373202.1 40S ribosomal protein S15 isoform X2 [Rhinopithecus roxellana] >XP_010561484.1	0.440331844	-1.183336913	0.02681	down	yes	1.38	3.134	2.657	2.691	4.055	1	1.358	1.783	BP:nuclear transport	GO:0051169;GO:0051168;GO:0048584;GO:0048583;GO:0071840;GO:0065003;GO:0009966;GO:0009967;GO:0043043;GO:1901798;GO:0048518;GO:1901796;GO:0006364;GO:0034470;GO:0046483;GO:0031503;GO:1901564;GO:0019538;GO:0022607;GO:0000028;GO:0006807;GO:0034660;GO:0050789;GO:1901576;GO:0051656;GO:0044260;GO:0016043;GO:0065007;GO:0006810;GO:0050794;GO:0008150;GO:0008152;GO:1902533;GO:1902531;GO:0016070;GO:0016072;GO:0044271;GO:0046907;GO:0048869;GO:0006518;GO:0030154;GO:0033750;GO:0023056;GO:0044249;GO:0034641;GO:0034645;GO:0023051;GO:0010647;GO:0010646;GO:1901566;GO:0006139;GO:0051234;GO:0042274;GO:0022618;GO:0022613;GO:0032502;GO:0009987;GO:0006725;GO:0002181;GO:0043604;GO:0043603;GO:0000054;GO:0043170;GO:0043933;GO:0090304;GO:0000056;GO:0001649;GO:0071826;GO:1901360;GO:0071704;GO:0006913;GO:0009058;GO:0009059;GO:0051649;GO:0051179;GO:0051640;GO:0051641;GO:0044238;GO:0044237;GO:0044085;GO:0006412;GO:0006396;GO:0048522;GO:0022626;GO:0022627;GO:0030055;GO:0016020;GO:0043231;GO:0043232;GO:0005829;GO:0043229;GO:0005925;GO:0043227;GO:0005654;GO:0030054;GO:0110165;GO:0070161;GO:0005840;GO:0015935;GO:0005737;GO:0005634;GO:0044391;GO:1990904;GO:0043228;GO:0043226;GO:0032991;GO:0005575;GO:0005198;GO:1901363;GO:1990948;GO:0003674;GO:0005488;GO:0003676;GO:0003677;GO:0055106;GO:0097159;GO:0098772;GO:0097371;GO:0004857;GO:0003723;GO:0005515;GO:0055105;GO:0003735;GO:0030234	K02958	RP-S15e, RPS1	map03010;map05	Ribosome;Coronavirus disea	COG0185	J:Translation, ribosomal structure	PF00203.24	Ribosomal_S19	Ribosomal protein S19	EXC	1	19	17	High
TRINITY_DN30510_c0_g1_i6_orf1	-	-	-	spodomicin-like [Ostrinia furnacalis]	0.227771295	-2.134342151	0.001522	down	yes	0.976	4.285	4.197	3.59	5.068	1	0.961	0.967	------	------	------	------	------	------	------	------	PF08036.14	Antimicrobial_6	Diapausin family of ant	CYT	3	60	6.7	High
TRINITY_DN43350_c0_g3_i1_orf1	-	-	-	uncharacterized protein LOC114355190 [Ostrinia furnacalis]	0.304179104	-1.717007044	3.57E-05	down	yes	1.019	3.35	3.431	3.124	3.496	1	1.011	1.047	------	------	------	------	------	------	ENOG4111DOA	S:Function unknown	------	------	------	CYT	3	25	25.8	High
TRINITY_DN48878_c0_g2_i1_orf1	-	-	-	codanin-1 [Ostrinia furnacalis]	0.373459716	-1.420975464	0.00797	down	yes	1.182	3.165	3.927	2.951	2.617	1	1.291	1.256	------	------	K19531	CDAN1	------	------	ENOG410XXWY	S:Function unknown	PF15296.9	Codanin-1_C	Codanin-1 C-terminus	CYT	1	1	83.1	Medium
TRINITY_DN20344_c0_g1_i5_orf1	-	-	-	uncharacterized protein LOC114351483 [Ostrinia furnacalis]	0.259617971	-1.945537841	0.0003729	down	yes	0.965	3.717	4.017	3.232	3.902	1	0.89	1.005	MF:molecular_functi	GO:0003674;GO:0042302;GO:0005198	------	------	------	------	------	------	PF00379.26	Chitin_bind_4	Insect cuticle protein	CYT	4	57	13.5	High
TRINITY_DN13660_c0_g1_i1_orf1	-	-	-	Aliphatic nitrilase [Operophtera brumata]	0.315590551	-1.663874083	3.08E-06	down	yes	1.002	3.175	3.095	3.144	3.287	1	0.989	1.018	BP:nitrogen compou	GO:0006807;GO:0008152;GO:0008150;GO:0016787;GO:0003824;GO:0016810;GO:0016811;GO:0003837;GO:0003674	K01431	UPB1, pydC	map00240;map00	Pyrimidine metabolism;Drug	COG0388	S:Function unknown	PF00795.25	CN_hydrolase	Carbon-nitrogen hydro	CYT	17	50	44.5	High
TRINITY_DN21420_c0_g1_i2_orf1	-	-	-	glutathione peroxidase, partial [Ostrinia furnacalis]	0.306652294	-1.705324349	4.87E-05	down	yes	0.9957	3.247	3.269	3.035	3.437	1	0.939	1.048	BP:response to stimu	GO:0050896;GO:0006950;GO:0008150;GO:0006979;GO:0016209;GO:0004601;GO:0003824;GO:0003674;GO:0016684;GO:0004602;GO:0016491	K05361;K00432	GPX4;gpx, btu	map00480;map00	Glutathione metabolism;Ara	COG0386	U:Intracellular trafficking, secreti	------	------	------	CYT	5	76	8.3	High
TRINITY_DN6415_c0_g2_i1_orf1	-	-	-	D-arabinitol dehydrogenase 1-like [Ostrinia furnacalis]	0.332463295	-1.588733022	1.86E-05	down	yes	1.019	3.065	3.195	3.091	2.91	1	0.995	1.063	MF:oxidoreductase a	GO:0016491;GO:0043169;GO:0046914;GO:0043167;GO:0003824;GO:0003674;GO:0005488;GO:0046872;GO:0008270	K17818	ARD1	map00040	Pentose and glucuronate int	COG1063	E:Amino acid transport and meta	PF08240.15;PF00	ADH_N;ADH_zi	Alcohol dehydrogenase	CYT	13	61	37.5	High
TRINITY_DN42275_c0_g1_i1_orfp1	-	-	-	TRINITY_DN42275_c0_g1_i1_m.44265 TRINITY_DN42275_c0_g1::TRINITY_DN42275_c0_g1_i1::g.44265 ORF type:internal len:76 (+),score=8.67 TRINITY_DN42275_c0_g1_i1:1-225(+)	0.300222859	-1.735894267	0.006428	down	yes	0.943	3.141	3.073	2.476	3.874	1	1.108	0.721	------	------	------	------	------	------	------	------	------	------	------	CYT	1	16	7.9	Medium
TRINITY_DN2314_c0_g1_i7_orf1	-	-	-	protein dj-1beta-like isoform X1 [Ostrinia furnacalis]	0.333006536	-1.586377601	7.44E-05	down	yes	1.019	3.06	3.214	2.824	3.143	1	1.062	0.995	------	------	K05687	PARK7	map05012;map05	Parkinson disease;Pathways	COG0693	S:Function unknown	PF01965.27	DJ-1_PfpI	DJ-1/PfpI family	CYT	4	45	24.5	High
TRINITY_DN73900_c0_g1_i1_orf1	-	-	-	carbonic anhydrase 7 [Ostrinia furnacalis]	0.339470936	-1.558640031	0.0009575	down	yes	0.9753	2.873	3.04	2.445	3.134	1	0.914	1.012	MF:cation binding;M	GO:0043169;GO:0046872;GO:0046914;GO:0016835;GO:0016836;GO:0043167;GO:0003824;GO:0003674;GO:0008270;GO:0004089;GO:0016829;GO:0005488	K01672	CA	map00910	Nitrogen metabolism	COG3338	U:Intracellular trafficking, secreti	PF00194.24	Carb_anhydras	Eukaryotic-type carbor	CYT	1	3	36.5	High

TRINITY_DN2407_c0_g1_i2_orf1	-	-	-	uncharacterized protein LOC114366345 isoform X2 [Ostrinia furnacalis]	0.312863607	-1.676394244	7.85E-05	down	yes	0.968	3.094	2.859	3.274	3.148	1	0.892	1.012	BP:response to bioti	GO:0009607;GO:0009605;GO:0043207;GO:0050896;GO:0006952;GO:0006950;GO:0008150;GO:0044419;GO:0051707;GO:0005575;GO:0005576;GO:0110165	------	------	------	------	ENOG4110S6N	S:Function unknown	PF00451.22	Toxin_2	Scorpion short toxin, Br	CYT	1	36	21.5	High
TRINITY_DN768_c0_g1_i7_orf1	-	-	-	neutral ceramidase [Leguminivora glycinivorella]	0.339978602	-1.556484148	9.82E-05	down	yes	0.9533	2.804	2.591	2.83	2.991	1	0.932	0.928	BP:sphingolipid cata	GO:0030149;GO:0044248;GO:0034641;GO:0006807;GO:0044242;GO:0006629;GO:1901575;GO:0016042;GO:0071704;GO:0046466;GO:0006665;GO:0006643;GO:0009987;GO:0008150;GO:0008152;GO:0044255;GO:0009056;GO:0044238;GO:1901564;GO:1901565;GO:0043603;GO:0044237;GO:0046514;GO:0006672;GO:0017040;GO:0003674;GO:0102121;GO:0016787;GO:0003824;GO:0016810;GO:0016811	K12349	ASAH2	map00600;map04	Sphingolipid metabolism;Sp	ENOG410XQWE	S:Function unknown	PF04734.16;PF17	Ceramidase_all	Neutral/alkaline non-ly	CYT	10	20	77.2	High
TRINITY_DN2040_c0_g1_i6_orf1	-	-	-	trypsin-like serine proteinase T26 protein, partial [Chilo infuscatellus]	0.352505886	-1.504280746	0.0009684	down	yes	1.048	2.973	3.374	2.625	2.919	1	1.016	1.129	BP:organic substanc	GO:0071704;GO:1901564;GO:0019538;GO:0043170;GO:0008150;GO:0006807;GO:0008152;GO:0006508;GO:0044238;GO:0004252;GO:0004175;GO:0016787;GO:0017171;GO:0140096;GO:0003824;GO:0003674;GO:0008233;GO:0008236	------	------	------	------	------	------	------	------	------	PLA	2	17	37.1	High
TRINITY_DN81031_c0_g1_i1_orf1	-	-	-	aldehyde dehydrogenase, partial [Ectropis obliqua]	0.348232323	-1.521877973	0.0002417	down	yes	0.9653	2.772	2.491	2.971	2.853	1	0.95	0.946	MF:catalytic activity;I	GO:0003824;GO:0016903;GO:0003674;GO:0016620;GO:0016491	K00128	ALDH	map00310;map00	Lysine degradation;Glyceroli	COG1012	C:Energy production and conver	PF00171.25	Aldedh	Aldehyde dehydrogena	CYT	5	57	8.6	High
TRINITY_DN98692_c0_g3_i1_orf1	-	-	-	fatty acyl-CoA hydrolase precursor, medium chain [Ostrinia furnacalis]	0.33533557	-1.576322571	0.0004344	down	yes	0.9993	2.98	3.119	2.613	3.207	1	0.983	1.015	------	------	------	------	------	------	------	------	------	------	------	CYT	1	13	7.2	High
TRINITY_DN20676_c0_g1_i6_orf1	-	-	-	aldo-keto reductase AKR2E4-like isoform X1 [Ostrinia furnacalis]	0.375	-1.415037499	3.87E-06	down	yes	0.999	2.664	2.674	2.578	2.74	1	0.991	1.006	MF:catalytic activity;I	GO:0003824;GO:0003674;GO:0016491	------	------	------	------	COG0656	S:Function unknown	PF00248.24	Aldo_ket_red	Aldo/keto reductase fa	CYT	13	47	40.6	High
TRINITY_DN9325_c0_g1_i1_orf1	-	-	-	protein takeout-like [Ostrinia furnacalis]	0.386097469	-1.372962999	0.0008718	down	yes	1.022	2.647	3.001	2.534	2.407	1	1.069	0.998	------	------	------	------	------	------	ENOG4110SYW;	S:Function unknown;	PF06585.14	JHBP	Haemolymph juvenile h	CYT	4	25	26	High
TRINITY_DN5153_c1_g1_i1_orf1	-	-	-	nose resistant to fluoxetine protein 6-like isoform X1 [Ostrinia furnacalis]	0.392410016	-1.34956623	0.001782	down	yes	1.003	2.556	2.91	2.466	2.292	1	0.83	1.18	CC:integral compon	GO:0016021;GO:0005575;GO:0031224;GO:0110165;GO:0003824;GO:0016740;GO:0016746;GO:0003674;GO:0016747	------	------	------	------	ENOG410YD1T	S:Function unknown	------	------	------	CYT	1	1	71.6	High
TRINITY_DN18482_c0_g1_i3_orf1	-	-	-	calcyphosin-like protein isoform X3 [Helicoverpa armigera] >XP_047020698.1 calcyphosin-like protein isoform X2 [Helicoverpa zea]	0.3956778	-1.337601973	0.0004169	down	yes	1.007	2.545	2.481	2.337	2.817	1	1.022	0.999	------	------	K23909	CAPS	------	------	ENOG410XRMJ	T:Signal transduction mechanism	PF13405.9;PF134	EF-hand_6;EF-	EF-hand domain;EF-ha	CYT	9	56	25.6	High
TRINITY_DN69307_c0_g1_i6_orf1	-	-	-	hypothetical protein evm_010738 [Chilo suppressalis]	0.386580087	-1.371160771	0.0001943	down	yes	0.9823	2.541	2.504	2.357	2.762	1	0.97	0.977	------	------	------	------	------	------	ENOG410XU1K	S:Function unknown	------	------	------	CYT	1	29	9.5	High
TRINITY_DN1161_c0_g1_i2_orf1	-	-	-	fructose-1,6-bisphosphatase 1 [Ostrinia furnacalis]	0.398487261	-1.32739449	8.30E-08	down	yes	1.001	2.512	2.487	2.519	2.531	1	0.984	1.018	BP:monosaccharide	GO:0046364;GO:0044283;GO:0006094;GO:0071704;GO:1901576;GO:0019318;GO:0019319;GO:0009058;GO:0008150;GO:0008152;GO:0044238;GO:0005975;GO:0005996;GO:0016051;GO:0006006;GO:0044281;GO:0019203;GO:0042132;GO:0016791;GO:0042578;GO:0003674;GO:0005488;GO:0043169;GO:0043167;GO:0046872;GO:0016787;GO:0016788;GO:0003824;GO:0050308	K03841	FBP, fbp	map00051;map00	Fructose and mannose meta	COG0158	G:Carbohydrate transport and m	PF00316.23;PF18	FBPase;FBPase_	Fructose-1-6-bisphosp	CYT	12	41	36.3	High
TRINITY_DN4748_c0_g1_i5_orf1	-	-	-	unnamed protein product, partial [Brenthis ino]	0.414941129	-1.269021432	0.0005159	down	yes	1.022	2.463	2.644	2.19	2.556	1	0.998	1.069	BP:response to bioti	GO:0009607;GO:0009605;GO:0043207;GO:0051707;GO:0050896;GO:0006950;GO:0006952;GO:0008150;GO:0044419;GO:0005575;GO:0005576;GO:0110165;GO:0099106;GO:0019871;GO:0017080;GO:0003674;GO:0016248;GO:0016247;GO:0098772;GO:0008200	------	------	------	------	------	------	------	------	------	CYT	3	54	7.8	High
TRINITY_DN11817_c0_g1_i4_orf1	-	-	-	glycogen phosphorylase [Heortia vitessoides]	0.418265221	-1.257510053	5.74E-05	down	yes	1.003	2.398	2.352	2.55	2.292	1	1.002	1.007	BP:cellular metabolic	GO:0044237;GO:0006091;GO:0044264;GO:0044262;GO:0044260;GO:0071704;GO:0006112;GO:0006073;GO:0009987;GO:0008150;GO:0008152;GO:0044042;GO:0044238;GO:0005975;GO:0015980;GO:0005977;GO:0005976;GO:0043170;GO:0070279;GO:0003674;GO:0005488;GO:0008184;GO:1901363;GO:0043168;GO:0043167;GO:0016740;GO:0036094;GO:0003824;GO:0102499;GO:0097159;GO:0004645;GO:0019842;GO:0102250;GO:0016757;GO:0016758;GO:0030170	K00688	PYG, glgP	map04910;map04	Insulin signaling pathway;Gl	COG0058	G:Carbohydrate transport and m	PF00343.23	Phosphorylase	Carbohydrate phospho	CYT	48	62	96.7	High
TRINITY_DN15247_c0_g1_i2_orf1	-	-	-	probable G-protein coupled receptor Mth-like 3 isoform X1 [Ostrinia furnacalis]	0.423544198	-1.239415569	0.0009989	down	yes	1.011	2.387	2.64	2.428	2.093	1	1.037	0.996	BP:cellular process;B	GO:0009987;GO:0050794;GO:0065007;GO:0007166;GO:0008150;GO:0050789;GO:0007165;GO:0016021;GO:0005575;GO:0110165;GO:0031224;GO:0004930;GO:0038023;GO:0060089;GO:0004888;GO:0003674	K04599	MTH	------	------	ENOG410XSJF;E	S:Function unknown;	PF00002.27	7tm_2	7 transmembrane rece	CYT	1	2	46.6	High
TRINITY_DN13098_c2_g1_i2_orf1	-	-	-	unnamed protein product [Arctia plantaginis] >CAB3253298.1 unnamed protein product [Arctia plantaginis]	0.444108761	-1.171015062	9.84E-05	down	yes	1.029	2.317	2.433	2.171	2.347	1	1.088	1	MF:cation binding;M	GO:0043169;GO:0043167;GO:0003674;GO:0005488;GO:0046872;GO:0005509	K23922	EFHD2	------	------	ENOG4111JG9	S:Function unknown	PF00036.35;PF13	EF-hand_1;EF-	EF hand;EF-hand doma	CYT	6	41	22.9	High
TRINITY_DN47842_c0_g1_i1_orf1	-	-	-	protein lethal(2)essential for life-like [Helicoverpa armigera] >PZC74790.1 hypothetical protein B5X24_HaOG207163 [Helicoverpa armigera]	0.475286849	-1.073129611	0.0001229	down	yes	1.077	2.266	2.137	2.282	2.38	1	1.105	1.126	MF:cation binding;M	GO:0043169;GO:0046872;GO:0003674;GO:0005488;GO:0043167	K09542	CRYAB	map04213;map04	Longevity regulating pathwa	ENOG410YERS	O:Posttranslational modification	PF00011.24	HSP20	Hsp20/alpha crystallin f	CYT	6	43	20.7	High
TRINITY_DN23398_c0_g1_i1_orf1	-	-	-	cytochrome P450 6B7-like [Ostrinia furnacalis]	0.489228007	-1.031421098	0.0007187	down	yes	1.09	2.228	2.347	2.333	2.003	1	1.146	1.124	CC:integral compon	GO:0016021;GO:0005575;GO:0110165;GO:0031224;GO:0043169;GO:0004497;GO:0020037;GO:0046914;GO:0046906;GO:0097159;GO:0016705;GO:0043167;GO:0003824;GO:0003674;GO:0005488;GO:0005506;GO:0046872;GO:1901363;GO:0016491	K14999	CYP6	------	------	COG2124	Q:Secondary metabolites biosyr	PF00067.25	p450	Cytochrome P450	CYT	6	12	57.2	High
TRINITY_DN8473_c0_g1_i6_orf1	-	-	-	serine/threonine-protein phosphatase 6 regulatory subunit 1 [Ostrinia furnacalis]	0.469545455	-1.09066327	0.01236	down	yes	1.033	2.2	1.958	2.732	1.909	1	0.985	1.113	BP:regulation of pho	GO:0019220;GO:0080090;GO:0019222;GO:0010921;GO:0031323;GO:0050789;GO:0051246;GO:0065007;GO:0031399;GO:0065009;GO:0060255;GO:0050790;GO:0050794;GO:0051174;GO:0008150;GO:0051171;GO:0035303;GO:0035304;GO:0051336;GO:0043666;GO:0019899;GO:0003674;GO:0005488;GO:0019903;GO:0019902;GO:0005515	------	------	------	------	ENOG410XRM1	T:Signal transduction mechanism	PF04499.18	SAPS	SIT4 phosphatase-asso	CYT	1	3	110.3	High
TRINITY_DN745_c7_g1_i1_orf1	-	-	-	uncharacterized protein LOC114358822 [Ostrinia furnacalis]	0.457702582	-1.127517663	0.0002854	down	yes	1.028	2.246	2.349	2.345	2.045	1	1.021	1.063	------	------	------	------	------	------	ENOG4110RMS;	S:Function unknown;	------	------	------	CYT	4	17	33.1	High
TRINITY_DN76633_c0_g1_i1_orfp1	-	-	-	TRINITY_DN76633_c0_g1_i1_m.53394 TRINITY_DN76633_c0_g1::TRINITY_DN76633_c0_g1_i1::g.53394 ORF type:internal len:164 (-),score=26.46,Toxin_2 PF00451.20 0.00022,Toxin_2|PF00451.20|0.00056,Toxin_2|PF00451.20|0.0002,Toxin_2|PF00451.20|5.9e-06,Gamma-thionin|PF00304.21|2,Gamma-thionin|PF00304.21|0.024,Gamma-thionin|PF00304.21|0.027,Gamma-thionin|PF00304.21|0.066,Toxin_38|PF14866.7|0.27,Toxin_38|PF14866.7|0.054,Toxin_38|PF14866.7|0.14,Defensin_2|PF01097.19|1.7,Defensin_2|PF01097.19|0.055,Defensin_2|PF01097.19|1.2,Defensin_2|PF01097.19|0.15 TRINITY_DN76633_c0_g1_i1:1-489(-)	0.490131579	-1.028758993	0.00622	down	yes	1.043	2.128	2.115	1.782	2.488	1	1.028	1.1	------	------	------	------	------	------	------	------	PF00451.22;PF0(	Toxin_2;Gamm	Scorpion short toxin, Br	PLA	3	31	19	High
TRINITY_DN55148_c0_g1_i1_orf1	-	-	-	60S ribosomal protein L11 isoform 1 [Homo sapiens] >NP_001069049.1 60S ribosomal protein L11 [Bos taurus] >NP_001240835.1 60S ribosomal protein L11 isoform 1 [Canis lupus familiaris] >NP_001269300.1 60S ribosomal protein L11 [Chinchilla lanigera] >NP_001291809.1 60S ribosomal protein L11 [Ailuropoda melanoleuca] >NP_080195.1 60S ribosomal protein L11 [Mus musculus] >XP_001504267.1 60S ribosomal protein L11 isoform X2 [Equus caballus] >XP_003471379.1 60S ribosomal protein L11 [Cavia porcellus] >XP_003810808.1 60S ribosomal protein L11 [Pan paniscus] >XP_003891370.1 60S ribosomal protein L11 [Papio anubis] >XP_003989701.1 60S ribosomal protein L11 [Felis catus] >XP_004285699.1 60S ribosomal protein L11 isoform X2 [Orcinus orca] >XP_004377186.1 60S ribosomal protein L11 [Trichechus manatus latirostris] >XP_004394821.1 PREDICTED: 60S ribosomal protein L11 isoform X1 [Odobenus rosmarus divergens] >XP_004465476.2 60S ribosomal protein L11 [Dasypus novemcinctus] >XP_004637663.1 60S ribosomal protein L11 [Octodon degus] >XP_004850617.1 60S ribosomal protein L11 isoform X2 [Heterocephalus glaber] >XP_005544522.1 60S ribosomal protein L11 isoform X1 [Macaca fascicularis] >XP_005676921.1 PREDICTED: 60S ribosomal protein L11 isoform X2 [Capra hircus] >XP_006078005.1 60S ribosomal protein L11 isoform X2 [Bubalus bubalis] >XP_006094108.1 60S ribosomal protein L11 isoform X3 [Myotis lucifugus] >XP_006239286.1 60S ribosomal protein L11 isoform X1 [Rattus norvegicus] >XP_006737645.1 60S ribosomal protein L11 [Leptonychotes weddellii] >XP_006777625.1 PREDICTED: 60S ribosomal protein L11 isoform X1 [Myotis davidii] >XP_006883588.1 PREDICTED: 60S ribosomal protein L11-like isoform X1 [Elephantulus edwardii] >XP_007121151.1 60S ribosomal protein L11 isoform X2 [Physeter catodon] >XP_007175168.1 60S ribosomal protein L11 isoform X1 [Balaenoptera acutorostrata scammoni] >XP_007459239.1 PREDICTED: 60S ribosomal protein L11 isoform X1 [Lipotes vexillifer] >XP_007524793.1 PREDICTED: 60S ribosomal protein L11 [Erinaceus europaeus] >XP_007528779.2 PREDICTED: 60S ribosomal protein L11 [Erinaceus europaeus] >XP_007933904.2 60S ribosomal protein L11 [Orycteropus afer afer] >XP_007978264.1 60S ribosomal protein L11 isoform X1 [Chlorocebus sabaeus] >XP_008059998.1 60S	0.497428705	-1.007438332	0.01126	down	yes	1.064	2.139	1.805	2.009	2.603	1	1.076	1.115	BP:positive regulatio	GO:2001235;GO:0008104;GO:0080090;GO:0032434;GO:0032435;GO:0048584;GO:0048583;GO:0071705;GO:2000058;GO:0071840;GO:0065003;GO:0010605;GO:0010604;GO:0070727;GO:0009966;GO:0009967;GO:0043043;GO:0002181;GO:0042981;GO:1901799;GO:0044092;GO:0048518;GO:0048519;GO:0033036;GO:0034470;GO:0060255;GO:0071702;GO:0006605;GO:0045184;GO:0030162;GO:1901564;GO:0046483;GO:1901566;GO:0019538;GO:0034641;GO:0042177;GO:2000435;GO:2000434;GO:1902255;GO:0009894;GO:0022607;GO:0009892;GO:0009893;GO:1901798;GO:0050821;GO:0034660;GO:0050789;GO:0000027;GO:2001244;GO:1901576;GO:0032092;GO:0044260;GO:0006886;GO:0051348;GO:0016043;GO:0065007;GO:0006364;GO:0065009;GO:0065008;GO:0006810;GO:0050790;GO:2001233;GO:0050794;GO:0043393;GO:0008152;GO:1902533;GO:1902531;GO:0016070;GO:1902253;GO:0016072;GO:0044271;GO:0046907;GO:0031400;GO:0031647;GO:0051338;GO:0008150;GO:0006518;GO:0023056;GO:0044249;GO:0042176;GO:0009895;GO:0034645;GO:0023051;GO:0010647;GO:0010646;GO:0043086;GO:0042273;GO:0006139;GO:0051248;GO:0051234;GO:0051246;GO:1903320;GO:1903321;GO:0022618;GO:0043068;GO:0031399;GO:0022613;GO:0031397;GO:2001242;GO:0051444;GO:0044093;GO:0031330;GO:1903050;GO:1903051;GO:0006725;GO:0031396;GO:1901796;GO:0043604;GO:0043603;GO:0051099;GO:0051098;GO:0043170;GO:0010628;GO:0033365;GO:0045861;GO:0034504;GO:0031329;GO:0043933;GO:0031324;GO:0031323;GO:0006807;GO:0090304;GO:0051438;GO:0043065;GO:0010942;GO:0010941;GO:0071826;GO:1901360;GO:2000059;GO:0071704;GO:0043067;GO:1904666;GO:1904667;GO:0010468;GO:0009987;GO:0061136;GO:0009058;GO:0009059;GO:0051171;GO:0051172;GO:0051649;GO:0019222;GO:0051179;GO:0051641;GO:0044238;GO:0044237;GO:0044085;GO:0006396;GO:0015031;GO:0006412;GO:0048523;GO:0048522;GO:0022626;GO:0022625;GO:0043230;GO:0043232;GO:0005829;GO:0065010;GO:0043229;GO:0043228;GO:0043227;GO:0005654;GO:0110165;GO:0005737;GO:0031982;GO:0005840;GO:0016020;GO:0015934;GO:0005730;GO:0044391;GO:1990904;GO:0070062;GO:0043226;GO:0042788;GO:1903561;GO:0032991;GO:0005575;GO:1901363;GO:0005198;GO:1990948;GO:00	K02868	RP-L11e, RPL1	map03010;map05	Ribosome;Coronavirus disea	COG0094	J:Translation, ribosomal structure	PF00281.22;PF0(	Ribosomal_L5;R	Ribosomal protein L5;ri	CYT	1	12	20.3	High
TRINITY_DN4822_c0_g1_i6_orf1	-	-	-	homogentisate 1,2-dioxygenase [Ostrinia furnacalis]	0.437979251	-1.191065569	2.90E-05	down	yes	0.971	2.217	2.32	2.204	2.126	1	0.969	0.944	BP:carboxylic acid m	GO:0019752;GO:0009063;GO:0009074;GO:0006807;GO:0044282;GO:1901360;GO:1901575;GO:1901361;GO:0006520;GO:0071704;GO:0044248;GO:1901605;GO:1901606;GO:1902221;GO:1902222;GO:0009987;GO:0006725;GO:0008150;GO:0008152;GO:0043436;GO:0006558;GO:0009056;GO:0009072;GO:1901565;GO:0006570;GO:0044238;GO:1901564;GO:0006082;GO:0006559;GO:0046395;GO:0016054;GO:0044237;GO:0019439;GO:0044281;GO:0003674;GO:0005488;GO:0016701;GO:0016491;GO:0043169;GO:0016702;GO:0043167;GO:0046872;GO:0003824;GO:0051213;GO:0004411	K00451	HGD, hmgA	map00350	Tyrosine metabolism	COG3508	Q:Secondary metabolites biosyr	PF20510.1;PF04;	HgmA_N;Hgm	Homogentisate 1,2-dic	CYT	1	43	48.9	High
TRINITY_DN13500_c0_g1_i1_orf1	-	-	-	phosphatidylethanolamine-binding protein homolog F40A3.3-like [Ostrinia furnacalis] >XP_028160752.1 phosphatidylethanolamine-binding protein homolog F40A3.3-like [Ostrinia furnacalis]	0.461147388	-1.116700168	0.0001342	down	yes	0.9887	2.144	2.064	2.304	2.065	1	0.977	0.989	------	------	K06910	PEBP, TFS1	------	------	COG1881	U:Intracellular trafficking, secreti	PF01161.23	PBP	Phosphatidylethanolam	CYT	10	64	20.5	High
TRINITY_DN3949_c0_g1_i1_orf1	-	-	-	probable cytochrome P450 304a1 [Ostrinia furnacalis]	0.465332713	-1.103665483	5.65E-07	down	yes	1	2.149	2.122	2.136	2.188	1	0.998	1.003	CC:integral compon	GO:0016021;GO:0005575;GO:0110165;GO:0031224;GO:0043169;GO:0004497;GO:0020037;GO:0046914;GO:0046906;GO:0097159;GO:0016705;GO:0043167;GO:0003824;GO:0003674;GO:0005488;GO:0005506;GO:0046872;GO:1901363;GO:0016491	------	------	------	------	COG2124	Q:Secondary metabolites biosyr	PF00067.25	p450	Cytochrome P450	CYT	3	5	59	High
TRINITY_DN214_c0_g1_i4_orf1	-	-	-	uncharacterized protein LOC114352813 [Ostrinia furnacalis]	0.442119944	-1.177490279	8.44E-05	down	yes	0.951	2.151	2.277	2.052	2.124	1	0.889	0.964	------	------	------	------	------	------	ENOG4110S0N;E	S:Function unknown;	PF06585.14	JHBP	Haemolymph juvenile h	CYT	4	31	29.6	High
TRINITY_DN4497_c2_g1_i3_orf1	-	-	-	uncharacterized protein LOC114353086 [Ostrinia furnacalis]	0.471830986	-1.083657929	1.54E-06	down	yes	1.005	2.13	2.166	2.092	2.132	1	1.031	0.985	CC:integral compon	GO:0016021;GO:0005575;GO:0110165;GO:0031224;GO:0043169;GO:0004497;GO:0020037;GO:0046914;GO:0046906;GO:0097159;GO:0016705;GO:0043167;GO:0003824;GO:0003674;GO:0005488;GO:0005506;GO:0046872;GO:1901363;GO:0016491	K15003	CYP9	------	------	COG2124	Q:Secondary metabolites biosyr	PF00067.25	p450	Cytochrome P450	CYT	9	20	59.8	High
TRINITY_DN3826_c0_g1_i1_orf1	-	-	-	39S ribosomal protein L18, mitochondrial [Ostrinia furnacalis]	0.480525803	-1.057314192	0.0004734	down	yes	0.987	2.054	1.856	2.193	2.113	1	0.969	0.992	BP:cellular biosynthe	GO:0044249;GO:0034641;GO:0006807;GO:0034645;GO:1901576;GO:0044271;GO:0044260;GO:0043043;GO:0071704;GO:0043604;GO:0009987;GO:0009058;GO:0009059;GO:0008150;GO:0008152;GO:1901564;GO:0044238;GO:0043603;GO:1901566;GO:0019538;GO:0044237;GO:0043170;GO:0006518;GO:0006412;GO:0043232;GO:0043226;GO:0005737;GO:0110165;GO:0005840;GO:0032991;GO:1990904;GO:0043229;GO:0005575;GO:0043228;GO:0003674;GO:0005198;GO:0003735	K02881	RP-L18, MRPL	map03010	Ribosome	ENOG4111MCR	J:Translation, ribosomal structure	------	------	------	CYT	1	11	19.6	High
TRINITY_DN1732_c0_g1_i15_orf1	-	-	-	CAD protein isoform X2 [Ostrinia furnacalis]	0.453339649	-1.141335749	0.003507	down	yes	0.957	2.111	1.781	2.424	2.128	1	0.941	0.93	BP:primary metaboli	GO:0044238;GO:0005975;GO:0071704;GO:0008150;GO:0008152;GO:0015925;GO:0016787;GO:0003824;GO:0003674;GO:0016798;GO:0004553;GO:0004565	K12309;K11540	GLB1, ELNR1;(	map01240;map00	Biosynthesis of cofactors;Sp	COG1874;COG0!	G:Carbohydrate transport and m	PF01301.22;PF02	Glyco_hydro_3!	Glycosyl hydrolases fan	CYT	1	23	72.9	High
TRINITY_DN6462_c0_g1_i5_orf1	-	-	-	probable histone-lysine N-methyltransferase CG1716 [Ostrinia furnacalis]	0.441551642	-1.179345915	0.002111	down	yes	0.9277	2.101	2.187	1.792	2.323	1	0.942	0.841	BP:protein methylati	GO:0006479;GO:0097198;GO:0080090;GO:0019222;GO:0018022;GO:0031326;GO:0031323;GO:0008213;GO:0006807;GO:0050789;GO:0018205;GO:0018023;GO:0044260;GO:0071704;GO:0018193;GO:0060255;GO:0034968;GO:0065007;GO:0016570;GO:0016571;GO:0032259;GO:0010468;GO:0019219;GO:0009987;GO:0009889;GO:1903506;GO:0050794;GO:0043412;GO:0036211;GO:0008150;GO:0051171;GO:0008152;GO:2001141;GO:0044238;GO:1901564;GO:0019538;GO:0010452;GO:0051252;GO:0044237;GO:0043170;GO:0006355;GO:0010556;GO:0043414;GO:0043229;GO:0043228;GO:0043227;GO:0005634;GO:0043226;GO:0005694;GO:0110165;GO:0043231;GO:0043232;GO:0005575;GO:0018024;GO:0003674;GO:0042054;GO:0016278;GO:0016279;GO:0046975;GO:0008168;GO:0016740;GO:0016741;GO:0008757;GO:0140096;GO:0003824;GO:0008170;GO:0008276	K11423	SETD2	map00310	Lysine degradation	COG2940	S:Function unknown	PF08236.14;PF0(	SRI;SET;AWS;W	SRI;SET domain;AWS do	CYT	1	1	177	Medium
TRINITY_DN3212_c0_g1_i7_orfp1	-	-	-	TRINITY_DN3212_c0_g1_i7_m.38385 TRINITY_DN3212_c0_g1::TRINITY_DN3212_c0_g1_i7::g.38385 ORF type:internal len:121 (-),score=5.33 TRINITY_DN3212_c0_g1_i7:3-362(-)	0.45460241	-1.137322764	0.02805	down	yes	0.9433	2.075	2.62	2.138	1.468	1	0.877	0.953	------	------	------	------	------	------	------	------	------	------	------	CYT	1	9	13.8	Medium
TRINITY_DN2170_c0_g2_i1_orf1	-	-	-	beta-1,3-glucan-binding protein-like [Ostrinia furnacalis]	0.498895028	-1.003191804	3.37E-06	down	yes	0.9933	1.991	2.04	1.982	1.951	1	1.003	0.977	BP:positive regulatio	GO:0032103;GO:0032101;GO:0048584;GO:0002831;GO:0002833;GO:0031349;GO:0007165;GO:0007166;GO:0050789;GO:0031347;GO:0002764;GO:0043207;GO:0002768;GO:0044419;GO:0071704;GO:0002684;GO:0002682;GO:0048518;GO:0065007;GO:0002220;GO:0002221;GO:0045089;GO:0045088;GO:0002757;GO:0048583;GO:0045087;GO:0009987;GO:0050794;GO:0006952;GO:0006950;GO:0002429;GO:0008150;GO:0008152;GO:0006955;GO:0009607;GO:0051707;GO:0044238;GO:0050776;GO:0005975;GO:0009605;GO:0002752;GO:0031663;GO:0080134;GO:0002758;GO:0050778;GO:0002376;GO:0002218;GO:0002253;GO:0098542;GO:0050896;GO:0005575;GO:0005576;GO:0110165;GO:0038023;GO:0030246;GO:0060089;GO:0003674;GO:0001875;GO:0038187;GO:0001873;GO:0030247;GO:0016787;GO:0008289;GO:0003824;GO:0001530;GO:0097367;GO:0070891;GO:0004553;GO:0016798;GO:0001872;GO:0005488	K20692	GNBP3	map04624	Toll and Imd signaling pathw	ENOG410Z83Q;(	S:Function unknown;	PF00722.24	Glyco_hydro_1(	Glycosyl hydrolases fan	CYT	1	8	19.1	High
TRINITY_DN2650_c0_g1_i1_orf1	-	-	-	hypothetical protein HW555_002849 [Spodoptera exigua] >CAH0691914.1 unnamed protein product [Spodoptera exigua]	0.480921053	-1.056128012	1.34E-05	down	yes	0.9503	1.976	1.937	1.979	2.012	1	0.887	0.964	------	------	------	------	------	------	ENOG4111T57	S:Function unknown	PF12974.10	Phosphonate-t	ABC transporter, phosp	CYT	5	16	31.9	High
TRINITY_DN9608_c0_g1_i3_orf1	-	-	-	cytochrome P450 monooxygenase CYP9G18 [Cnaphalocrocis medinalis]	0.30264468	-1.724303106	4.86E-07	down	yes	0.9727	3.214	3.184	3.282	3.177	1	0.942	0.976	CC:endoplasmic reti	GO:0005789;GO:0031224;GO:0031090;GO:0016021;GO:0016020;GO:0110165;GO:0005575;GO:0004497;GO:0016705;GO:0003674;GO:0005488;GO:1901363;GO:0016491;GO:0043169;GO:0046914;GO:0043167;GO:0005506;GO:0046872;GO:0020037;GO:0003824;GO:0046906;GO:0097159	K15003	CYP9	------	------	COG2124	K:Transcription	PF00067.25	p450	Cytochrome P450	CYT	7	17	58.5	High
TRINITY_DN7040_c0_g1_i4_orf1	-	-	-	uncharacterized protein LOC114353763 [Ostrinia furnacalis]	0.303828829	-1.718669328	0.0006835	down	yes	0.9443	3.108	3.263	2.665	3.396	1	0.878	0.955	------	------	------	------	------	------	------	------	------	------	------	CYT	2	39	7.4	High
TRINITY_DN3439_c0_g2_i2_orf1	-	-	-	histone H2A.Z-specific chaperone CHZ1-like [Ostrinia furnacalis]	0.299674373	-1.738532379	4.15E-05	down	yes	0.9203	3.071	3.173	2.864	3.175	1	0.884	0.877	------	------	------	------	------	------	------	------	------	------	------	PLA	4	14	31.9	High
TRINITY_DN9593_c0_g1_i2_orf1	-	-	-	uncharacterized protein LOC113518937 [Galleria mellonella]	0.370600414	-1.432063602	9.29E-06	down	yes	1.074	2.898	2.828	2.865	3.002	1	1.113	1.108	------	------	------	------	------	------	------	------	------	------	------	CYT	3	32	15	High
TRINITY_DN4255_c0_g1_i11_orf1	-	-	-	LOW QUALITY PROTEIN: lebocin-4-like [Ostrinia furnacalis]	0.362683438	-1.463217228	0.0004363	down	yes	1.038	2.862	2.711	2.676	3.2	1	1.03	1.084	------	------	------	------	------	------	ENOG4111ASS	S:Function unknown	------	------	------	CYT	1	13	22.2	High
TRINITY_DN9132_c0_g1_i5_orf1	-	-	-	ubiquitin-like-conjugating enzyme ATG3 [Spodoptera frugiperda]	0.339237435	-1.559632714	0.005654	down	yes	0.9787	2.885	2.489	3.587	2.578	1	0.959	0.977	MF:ubiquitin-like pro	GO:0019787;GO:0003824;GO:0016740;GO:0140096;GO:0003674	K08343	ATG3	map04136;map05	Autophagy - other;Kaposi sa	ENOG410Y3BC	U:Intracellular trafficking, secreti	PF03987.18	Autophagy_act	Autophagocytosis asso	CYT	5	18	36.1	High
TRINITY_DN2472_c0_g1_i6_orf1	-	-	-	programmed cell death protein 6 isoform X1 [Colias croceus] >XP_045492459.1 programmed cell death protein 6 isoform X1 [Colias croceus]	0.352831567	-1.502948452	1.23E-05	down	yes	0.9657	2.737	2.84	2.618	2.753	1	0.936	0.961	MF:cation binding;M	GO:0043169;GO:0043167;GO:0003674;GO:0005488;GO:0046872;GO:0005509	K23902	PDCD6	------	------	ENOG410YKQK	S:Function unknown	PF00036.35;PF13	EF-hand_1;EF-	EF hand;EF-hand doma	CYT	3	22	21	High
TRINITY_DN31286_c0_g1_i6_orfp1	-	-	-	TRINITY_DN31286_c0_g1_i6_m.28438 TRINITY_DN31286_c0_g1::TRINITY_DN31286_c0_g1_i6::g.28438 ORF type:internal len:92 (-),score=3.10,Perilipin PF03036.17 2e-05 TRINITY_DN31286_c0_g1_i6:1-273(-)	0.400860383	-1.318828251	0.0001367	down	yes	1.025	2.557	2.554	2.375	2.742	1	1.037	1.038	------	------	------	------	------	------	------	------	PF03036.19	Perilipin	Perilipin family	CYT	5	71	9.4	High
TRINITY_DN1750_c1_g1_i5_orf1	-	-	-	lipid droplet localized protein-like [Ostrinia furnacalis] >XP_028161280.1 lipid droplet localized protein-like [Ostrinia furnacalis]	0.402385686	-1.31334911	0.0001824	down	yes	1.012	2.515	2.309	2.695	2.541	1	1.004	1.032	BP:nucleic acid meta	GO:0090304;GO:0044249;GO:0034641;GO:0006807;GO:1901362;GO:0006139;GO:0044260;GO:0071704;GO:1901360;GO:0018130;GO:1901576;GO:0009987;GO:0006725;GO:0009058;GO:0009059;GO:0008150;GO:0008152;GO:0034654;GO:0046483;GO:0044238;GO:0044271;GO:0044237;GO:0043170;GO:0006259;GO:0019438;GO:0071897;GO:0031224;GO:0016021;GO:0110165;GO:0005575;GO:0003674;GO:0016491;GO:0003824	------	------	------	------	COG3268	S:Function unknown	PF03435.21	Sacchrp_dh_NA	Saccharopine dehydrog	CYT	15	35	47.8	High
TRINITY_DN4242_c0_g1_i6_orf1	-	-	-	fibrohexamerin-like [Ostrinia furnacalis]	0.414664457	-1.269983702	6.69E-06	down	yes	1.001	2.414	2.48	2.338	2.425	1	0.966	1.036	CC:cellular compon	GO:0005575;GO:0005576;GO:0110165;GO:0003674;GO:0005198	------	------	------	------	ENOG4110SS9	S:Function unknown	PF07294.14	Fibroin_P25	Fibroin P25	CYT	3	14	25.5	High
TRINITY_DN32532_c0_g1_i1_orf1	-	-	-	fatty acyl-CoA hydrolase precursor, medium chain [Ostrinia furnacalis]	0.46720575	-1.097870064	0.0004295	down	yes	1.04	2.226	2.411	2.054	2.214	1	1.001	1.12	------	------	------	------	------	------	ENOG410Y6DN;	S:Function unknown;I:Lipid trans	------	------	------	CYT	2	33	8.6	High
TRINITY_DN24789_c0_g1_i9_orfp1	-	-	-	TRINITY_DN24789_c0_g1_i9_m.25888 TRINITY_DN24789_c0_g1::TRINITY_DN24789_c0_g1_i9::g.25888 ORF type:internal len:114 (-),score=12.65 TRINITY_DN24789_c0_g1_i9:2-340(-)	0.453273138	-1.141547429	0.002105	down	yes	1.004	2.215	2.19	1.948	2.507	1	1.102	0.909	------	------	------	------	------	------	------	------	------	------	------	CYT	5	46	13.2	High
TRINITY_DN1144_c0_g1_i10_orf1	-	-	-	TIL [Ostrinia furnacalis]	0.429593205	-1.21895692	0.0008838	down	yes	0.961	2.237	2.195	2.018	2.499	1	0.903	0.98	------	------	------	------	------	------	ENOG4110ODA	S:Function unknown	------	------	------	CYT	2	55	10.1	High
TRINITY_DN7740_c0_g1_i2_orf1	-	-	-	D-arabinitol dehydrogenase 1 [Eumeta japonica]	0.459360731	-1.122300565	0.0005202	down	yes	1.006	2.19	2.163	2.007	2.4	1	0.974	1.044	MF:oxidoreductase a	GO:0016491;GO:0043169;GO:0046914;GO:0043167;GO:0003824;GO:0003674;GO:0005488;GO:0046872;GO:0008270	K17818	ARD1	map00040	Pentose and glucuronate int	COG1063	E:Amino acid transport and met	------	------	------	CYT	1	69	9.3	High
TRINITY_DN1024_c0_g4_i1_orf1	-	-	-	superoxide dismutase [Cu-Zn]-like [Ostrinia furnacalis]	0.462207971	-1.113385956	0.0003646	down	yes	1.009	2.183	2.079	2.078	2.393	1	1.023	1.003	MF:oxidoreductase a	GO:0016721;GO:0043169;GO:0016209;GO:0043167;GO:0003824;GO:0003674;GO:0005488;GO:0004784;GO:0046872;GO:0016491	K04565	SOD1	map05208;map05	Chemical carcinogenesis - r	COG2032	S:Function unknown	PF00080.23	Sod_Cu	Copper/zinc superoxide	PLA	8	58	18.2	High
TRINITY_DN11467_c0_g1_i5_orf1	-	-	-	27 kDa hemolymph protein-like, partial [Ostrinia furnacalis]	0.469534556	-1.090696757	0.001875	down	yes	0.9987	2.127	2.145	1.851	2.385	1	1.012	0.984	------	------	------	------	------	------	ENOG4111POK	S:Function unknown	PF07165.14	DUF1397	Protein of unknown fun	CYT	20	76	25.3	High
TRINITY_DN1503_c0_g1_i6_orf1	-	-	-	ecdysteroid-regulated 16 kDa protein [Ostrinia furnacalis]	0.45933082	-1.122394507	0.0002099	down	yes	0.9747	2.122	2.106	1.978	2.281	1	0.976	0.948	------	------	K13443	NPC2	map04979;map04	Cholesterol metabolism;Lyso	ENOG4111Q8S	O:Posttranslational modification	PF02221.18	E1_DerP2_DerF	ML domain	CYT	6	38	17.1	High
TRINITY_DN43667_c0_g1_i1_orf1	-	-	-	carbonyl reductase [NADPH] 1-like [Ostrinia furnacalis]	0.498517787	-1.004283116	2.43E-05	down	yes	1.009	2.024	2.056	2.08	1.935	1	1.002	1.026	------	------	K00079	CBR1	map00590;map05	Arachidonic acid metabolisr	COG1028	S:Function unknown	PF00106.28;PF13	adh_short;adh	short chain dehydroger	CYT	8	32	32.5	High
TRINITY_DN8953_c0_g1_i4_orf1	-	-	-	gonadotropin-releasing hormone receptor [Ostrinia furnacalis] >AXF67446.1 adipokinetic hormone receptor 1 [Ostrinia furnacalis]	0.447011	-1.161617763	0.004822	down	yes	0.9347	2.091	1.845	1.937	2.491	1	0.889	0.915	CC:integral compon	GO:0016021;GO:0005575;GO:0110165;GO:0031224;GO:0004930;GO:0038023;GO:0060089;GO:0004888;GO:0003674	K04280	GNRHR	map04080;map04	Neuroactive ligand-recepto	ENOG410XRW9	U:Intracellular trafficking, secreti	PF00001.24	7tm_1	7 transmembrane rece	CYT	2	6	45	High
TRINITY_DN35809_c0_g1_i1_orf1	-	-	-	spodomicin-like [Ostrinia furnacalis] >QKV49445.1 diapausin [Ostrinia furnacalis]	0.135747239	-2.881005241	0.001087	down	yes	0.971	7.153	7.27	5.828	8.361	1	0.978	0.935	------	------	------	------	------	------	------	------	PF08036.14	Antimicrobial_6	Diapausin family of ant	CYT	3	60	6.8	High
TRINITY_DN4767_c0_g1_i6_orf1	-	-	-	cysteine protease XCP2-like [Ostrinia furnacalis]	0.155909091	-2.681223042	0.0003381	down	yes	0.9947	6.38	6.573	5.483	7.085	1	1.013	0.971	BP:primary metaboli	GO:0044238;GO:1901564;GO:0019538;GO:0043170;GO:0071704;GO:0006807;GO:0008150;GO:0008152;GO:0006508;GO:0003674;GO:0016787;GO:0003824;GO:0140096;GO:0008233	------	------	------	------	ENOG4110R2S	S:Function unknown	PF08246.15	Inhibitor_I29	Cathepsin propeptide i	CYT	2	44	5.3	High

TRINITY_DN3166_c1_g1_i6_orf1	-	-	-	hypothetical protein evm_013813 [Chilo suppressalis]	0.184625419	-2.437326897	0.0007443	down	yes	0.9907	5.366	5.488	4.497	6.112	1	0.958	1.014	BP:response to bioti	GO:0009607;GO:0009605;GO:0043207;GO:0050896;GO:0006952;GO:0006950;GO:0008150;GO:0044419;GO:0051707;GO:0005575;GO:0005576;GO:0110165	------	------	------	------	------	------	PF00451.22	Toxin_2	Scorpion short toxin, Br	CYT	1	13	10.5	High
TRINITY_DN8008_c0_g1_i6_orf1	-	-	-	uncharacterized protein LOC114357965 isoform X1 [Ostrinia furnacalis] >XP_028167599.1 uncharacterized protein LOC114357965 isoform X1 [Ostrinia furnacalis] >XP_028167600.1 uncharacterized protein LOC114357965 isoform X2 [Ostrinia furnacalis] >XP_028167601.1 uncharacterized protein LOC114357965 isoform X3 [Ostrinia furnacalis]	0.228851481	-2.127516467	8.37E-06	down	yes	1.074	4.693	4.918	4.642	4.52	1	1.141	1.082	MF:organic cyclic co	GO:0097159;GO:0003674;GO:0005488;GO:0003676;GO:0003677;GO:1901363	------	------	------	------	ENOG410ZC30	S:Function unknown	------	------	------	CYT	1	0	506.7	Medium
TRINITY_DN19662_c0_g2_i1_orf1	-	-	-	storage protein [Ostrinia furnacalis]	0.211599745	-2.240590206	0.002118	down	yes	0.996	4.707	4.398	5.731	3.992	1	0.985	1.003	------	------	------	------	------	------	ENOG410XR2D	S:Function unknown	PF00372.22;PF0	Hemocyanin_M	Hemocyanin, copper co	CYT	43	69	59	High
TRINITY_DN86772_c0_g1_i3_orfp1	-	-	-	x-tox [Spodoptera exigua]	0.224897855	-2.152658194	0.001286	down	yes	0.8807	3.916	4.197	3.183	4.369	1	0.884	0.758	BP:response to bioti	GO:0009607;GO:0009605;GO:0043207;GO:0050896;GO:0006952;GO:0006950;GO:0008150;GO:0044419;GO:0051707;GO:0005575;GO:0005576;GO:0110165	------	------	------	------	------	------	------	------	------	CYT	1	9	8.5	High
TRINITY_DN467_c3_g1_i5_orf1	-	-	-	lysozyme precursor [Loxostege sticticalis]	0.25656963	-1.962577684	0.0001964	down	yes	0.9783	3.813	3.945	3.391	4.102	1	0.994	0.941	BP:metabolic proces	GO:0008152;GO:0008150;GO:0016787;GO:0003824;GO:0016798;GO:0003796;GO:0003674;GO:0004553;GO:0061783	K13915	LYZ	map04970	Salivary secretion	ENOG4111QHM	G:Carbohydrate transport and m	PF00062.23	Lys	C-type lysozyme/alpha	CYT	7	52	16	High
TRINITY_DN609_c0_g1_i1_orf1	-	-	-	zonadhesin-like isoform X1 [Ostrinia furnacalis]	0.326858877	-1.613260215	0.001463	down	yes	1.077	3.295	3.268	2.821	3.796	1	1.135	1.096	------	------	------	------	------	------	COG4826;ENOG	O:Posttranslational modification	PF01826.20	TIL	Trypsin Inhibitor like cys	PLA	9	18	109	High
TRINITY_DN46625_c0_g1_i1_orf1	-	-	-	ferritin subunit isoform X1 [Belonocnema kinseyi]	0.323394495	-1.628632972	0.009103	down	yes	0.987	3.052	3.703	3.229	2.224	1	0.945	1.016	BP:ion homeostasis;	GO:0050801;GO:0042592;GO:0055072;GO:0055076;GO:0098771;GO:0046916;GO:0000041;GO:0065007;GO:0065008;GO:0019725;GO:0030001;GO:0006810;GO:0006879;GO:0006875;GO:0006812;GO:0006811;GO:0009987;GO:0006873;GO:0008150;GO:0030003;GO:0055065;GO:0055080;GO:0055082;GO:0051234;GO:0051179;GO:0048878;GO:0006826;GO:0003674;GO:0005488;GO:0016491;GO:0043169;GO:0016722;GO:0046914;GO:0004322;GO:0043167;GO:0005506;GO:0008199;GO:0016724;GO:0046872;GO:0003824	K00522	FTH1	map04978;map04	Mineral absorption;Necropt	ENOG4111MSG	P:Inorganic ion transport and me	PF00210.27	Ferritin	Ferritin-like domain	CYT	1	4	24.1	High
TRINITY_DN20560_c0_g1_i6_orf1	-	-	-	pupal cuticle protein C1B-like [Ostrinia furnacalis]	0.394612304	-1.341492154	0.0002383	down	yes	1.084	2.747	2.755	2.53	2.957	1	1.077	1.176	------	------	------	------	------	------	ENOG410Y08D	S:Function unknown	------	------	------	CYT	3	30	22.6	High
TRINITY_DN1091_c0_g3_i1_orf1	-	-	-	macrophage mannose receptor 1-like [Ostrinia furnacalis]	0.35732899	-1.48467513	0.0008315	down	yes	0.9873	2.763	2.713	2.452	3.125	1	0.955	1.007	BP:response to extra	GO:0009991;GO:0090304;GO:0034641;GO:0006807;GO:0042262;GO:1901360;GO:0006139;GO:0051716;GO:0044260;GO:0071704;GO:0071496;GO:0009987;GO:0006725;GO:0031668;GO:0006950;GO:0008150;GO:0008152;GO:0007154;GO:0046483;GO:0044238;GO:0009605;GO:0044237;GO:0043170;GO:0033554;GO:0006259;GO:0050896;GO:0003674;GO:0016787;GO:0003824;GO:0047429;GO:0016818;GO:0008413;GO:0016817;GO:0016462	------	------	------	------	ENOG410XRS3;E	U:Intracellular trafficking, secreti	PF00059.24	Lectin_C	Lectin C-type domain	CYT	9	34	38.3	High
TRINITY_DN28501_c0_g1_i2_orfp1	-	-	-	TRINITY_DN28501_c0_g1_i2_m.58934 TRINITY_DN28501_c0_g1::TRINITY_DN28501_c0_g1_i2::g.58934 ORF type:internal len:98 (+) score=13.70 TRINITY_DN28501_c0_g1_i2:3-293(+)	0.358138686	-1.481409728	0.0001405	down	yes	0.9813	2.74	2.822	2.508	2.891	1	0.911	1.033	------	------	------	------	------	------	------	------	------	------	------	CYT	1	30	10.9	High
TRINITY_DN10539_c0_g1_i1_orf1	-	-	-	uncharacterized protein LOC114358962 isoform X1 [Ostrinia furnacalis]	0.408209806	-1.292617255	0.04772	down	yes	1.074	2.631	3.597	2.562	1.735	1	0.912	1.309	------	------	------	------	------	------	ENOG41115U6	S:Function unknown	------	------	------	CYT	1	2	40.4	Medium
TRINITY_DN25976_c0_g1_i4_orf1	-	-	-	hypothetical protein B566_EDAN014657 [Ephemera danica]	0.494006849	-1.01739705	0.0007047	down	yes	1.154	2.336	2.341	2.42	2.248	1	1.082	1.379	BP:developmental p	GO:0032502;GO:0009987;GO:0048869;GO:0048856;GO:0048468;GO:0016043;GO:0048513;GO:0008150;GO:0071840;GO:0016021;GO:0005575;GO:0031224;GO:0110165	K24068	MEGF10_11	------	------	ENOG410XQWV	S:Function unknown	PF00053.27;PF07	Laminin_EGF;E(	Laminin EGF domain;E(	CYT	1	30	8.4	High
TRINITY_DN380_c0_g2_i2_orf1	-	-	-	chemosensory protein 10 [Ostrinia furnacalis]	0.188807236	-2.40501404	0.0001151	down	yes	1.002	5.307	5.487	4.745	5.689	1	1.005	1.001	------	------	------	------	------	------	ENOG4111ATT;E	S:Function unknown;	PF03392.16	OS-D	Insect pheromone-binc	CYT	3	54	13.6	High
TRINITY_DN15685_c0_g1_i5_orf1	-	-	-	uncharacterized protein LOC114352354 [Ostrinia furnacalis]	0.373164763	-1.422115331	2.91E-05	down	yes	0.915	2.452	2.35	2.466	2.541	1	0.905	0.84	------	------	------	------	------	------	ENOG4110T90;E	S:Function unknown;	PF06585.14	JHBP	Haemolymph juvenile h	CYT	3	14	29.1	High
TRINITY_DN136358_c0_g1_i1_orf1	-	-	-	acidic juvenile hormone-suppressible protein 1-like [Ostrinia furnacalis]	0.199532801	-2.325302164	0.01944	down	yes	1.025	5.137	3.728	7.277	4.406	1	1.026	1.049	------	------	------	------	------	------	ENOG410XR2D	S:Function unknown	PF03723.17	Hemocyanin_C	Hemocyanin, ig-like do	CYT	2	55	9.7	High
TRINITY_DN5274_c0_g2_i2_orf1	-	-	-	lopap-like [Ostrinia furnacalis]	0.42838929	-1.223005682	8.14E-05	down	yes	1.008	2.353	2.511	2.253	2.295	1	0.98	1.044	------	------	K03098	APOD	------	------	ENOG4110TA7;E	S:Function unknown;	PF00061.26	Lipocalin	Lipocalin / cytosolic fat	CYT	2	14	24.4	High
TRINITY_DN1209_c0_g1_i9_orf1	-	-	-	NADP-dependent malic enzyme-like isoform X1 [Ostrinia furnacalis] >XP_028161889.1 NADP-dependent malic enzyme-like isoform X1 [Ostrinia furnacalis] >XP_028161891.1 NADP-dependent malic enzyme-like isoform X3 [Ostrinia furnacalis]	0.293462328	-1.768752781	3.69E-05	down	yes	1.001	3.411	3.447	3.188	3.597	1	0.974	1.028	MF:cation binding;M	GO:0043169;GO:0016614;GO:0016615;GO:0016616;GO:0043167;GO:0051287;GO:0004471;GO:0004470;GO:0000166;GO:0036094;GO:0003824;GO:0003674;GO:0005488;GO:0097159;GO:0046872;GO:1901363;GO:1901265;GO:0016491	K00029	E1.1.1.40, mae	map00620;map03	Pyruvate metabolism;PPAR s	COG0281	S:Function unknown	PF03949.18;PF0(	Malic_M;malic	Malic enzyme, NAD bin	CYT	25	41	70.7	High
TRINITY_DN64892_c0_g1_i1_orf1	-	-	-	aldehyde dehydrogenase X, mitochondrial [Manduca sexta] >KAG6450704.1 hypothetical protein O3G_MSEX006722 [Manduca sexta]	0.402734539	-1.312098889	0.03046	down	yes	0.9573	2.377	1.854	3.234	2.043	1	0.917	0.955	MF:catalytic activity;	GO:0003824;GO:0016903;GO:0003674;GO:0016620;GO:0016491	K00128	ALDH	map00310;map00	Lysine degradation;Glyceroli	COG1012	U:Intracellular trafficking, secreti	PF00171.25	Aldedh	Aldehyde dehydrogena	CYT	3	59	5.7	High
TRINITY_DN75086_c0_g1_i5_orf1	-	-	-	lysosome membrane protein 2-like [Ostrinia furnacalis]	0.285878877	-1.806524067	0.0004798	down	yes	0.9677	3.385	3.71	3.508	2.937	1	0.959	0.944	BP:multicellular orga	GO:0032501;GO:0007608;GO:0050877;GO:0050896;GO:0007606;GO:0007600;GO:0008150;GO:0003008;GO:0016020;GO:0005575;GO:0110165;GO:0005886;GO:0016021;GO:0031224	K13885	SCARB1	map04979;map05	Cholesterol metabolism;Hep	ENOG410XS17	U:Intracellular trafficking, secreti	------	------	------	CYT	1	11	7.7	High
TRINITY_DN1206_c0_g1_i6_orf1	-	-	-	sorbitol dehydrogenase-like [Spodoptera frugiperda] >KAG8104768.1 hypothetical protein SFRUCORN_013827 [Spodoptera frugiperda]	0.42887931	-1.221356375	2.15E-05	down	yes	0.995	2.32	2.42	2.221	2.319	1	0.982	1.003	MF:oxidoreductase a	GO:0016491;GO:0043169;GO:0016614;GO:0016616;GO:0046914;GO:0043167;GO:0003824;GO:0003674;GO:0005488;GO:0046872;GO:0008270	K00008	SORD, gutB	map00040;map00	Pentose and glucuronate int	COG1063	E:Amino acid transport and met	PF08240.15;PF0(	ADH_N;ADH_zi	Alcohol dehydrogenase	CYT	12	46	38.7	High
TRINITY_DN20009_c0_g1_i1_orf1	-	-	-	vimentin [Homo sapiens] >XP_003831224.1 vimentin [Pan paniscus] >XP_018890043.1 vimentin [Gorilla gorilla gorilla] >XP_024109584.1 vimentin [Pongo abelii] >XP_030675100.1 vimentin [Nomascus leucogenys] >XP_032020652.1 vimentin [Hylobates moloch] >P08670.4 RecName: Full=Vimentin [Homo sapiens] >AIC49963.1 VIM, partial [synthetic construct] >MXR00191.1 hypothetical protein [Bos mutus] >PNI30483.1 VIM isoform 1 [Pan troglodytes] >SJX39704.1 unnamed protein product, partial [Human ORFeome Gateway entry vector] >AAH00163.2 Vimentin [Homo sapiens]	0.33558697	-1.575241395	0.02804	down	yes	1.092	3.254	3.281	2.156	4.325	1	0.912	1.365	BP:regulation of prin	GO:0080090;GO:0019222;GO:0007165;GO:0031345;GO:0071840;GO:0051716;GO:0043207;GO:0010608;GO:0048518;GO:0048519;GO:0002064;GO:0060255;GO:0048468;GO:0061013;GO:0010977;GO:0010975;GO:0010033;GO:0045727;GO:0009607;GO:0009605;GO:0045104;GO:0060395;GO:0010243;GO:0045103;GO:0032967;GO:0009894;GO:0070887;GO:0021782;GO:0009891;GO:0031175;GO:0120036;GO:0032495;GO:0032496;GO:0016043;GO:0031344;GO:0065007;GO:0071219;GO:0071216;GO:0065008;GO:0034097;GO:0009889;GO:0048708;GO:0050794;GO:0008150;GO:0014002;GO:0010604;GO:0071345;GO:1901698;GO:0010714;GO:0010712;GO:0010557;GO:0010556;GO:0048869;GO:1903311;GO:0034250;GO:0030154;GO:0051129;GO:0051128;GO:0032965;GO:0071225;GO:0010001;GO:0009893;GO:0009719;GO:0043488;GO:0043487;GO:0097435;GO:0051246;GO:0051247;GO:0045109;GO:0071495;GO:0006996;GO:0071346;GO:1901699;GO:0009987;GO:0071396;GO:0070307;GO:0034248;GO:0007010;GO:0051252;GO:0002237;GO:0050896;GO:0032502;GO:0071222;GO:0031329;GO:0031328;GO:0030030;GO:0031326;GO:0031325;GO:0031323;GO:0060020;GO:0010628;GO:0120035;GO:0033993;GO:0071417;GO:2000112;GO:0050789;GO:0071310;GO:0010468;GO:0019219;GO:0034341;GO:0051171;GO:0051173;GO:0042221;GO:1901700;GO:1901701;GO:0048856;GO:1901652;GO:1901653;GO:0006417;GO:0048523;GO:0048522;GO:0099512;GO:0016363;GO:0030424;GO:0030055;GO:0031982;GO:0016020;GO:0005777;GO:0043230;GO:0042995;GO:0043231;GO:0043232;GO:0042579;GO:0065010;GO:0005829;GO:0043229;GO:0043228;GO:0005925;GO:0043227;GO:0043226;GO:0005856;GO:0045111;GO:0030054;GO:0110165;GO:0005737;GO:0070161;GO:0045335;GO:0097708;GO:0120025;GO:0005844;GO:0031252;GO:0031410;GO:0043005;GO:0005886;GO:0099513;GO:0005815;GO:1990904;GO:0099080;GO:0099081;GO:0030139;GO:0070062;GO:0005882;GO:1903561;GO:0032991;GO:0005575;GO:0019215;GO:0005198;GO:1901363;GO:0060090;GO:0019904;GO:0003674;GO:0005488;GO:0003676;GO:0097159;GO:0042802;GO:0005212;GO:0003725;GO:0003723;GO:0005515;GO:0044877;GO:0005200;GO:0097110;GO:1990254	K07606	VIM	map05206;map05	MicroRNAs in cancer;Epstei	ENOG410XRBS	Z:Cytoskeleton	PF00038.24;PF0	Filament;Filame	Intermediate filament p	CYT	1	5	52.1	High
TRINITY_DN18388_c0_g1_i6_orf1	-	-	-	serine protease [Ostrinia furnacalis]	0.415207176	-1.268096716	0.000755	down	yes	0.972	2.341	2.427	2.054	2.541	1	0.955	0.961	BP:organic substanc	GO:0071704;GO:1901564;GO:0019538;GO:0006807;GO:0043170;GO:0008150;GO:0008152;GO:0006508;GO:0044238;GO:0005575;GO:0005576;GO:0110165;GO:0004252;GO:0004175;GO:0016787;GO:0017171;GO:0140096;GO:0003824;GO:0003674;GO:0008233;GO:0008236	------	------	------	------	COG5640	O:Posttranslational modification	PF00089.29;PF1	Trypsin;CLIP;Tr	Trypsin;Regulatory CLIF	CYT	4	13	47	High
TRINITY_DN29026_c0_g1_i4_orf1	-	-	-	TIL [Ostrinia furnacalis]	0.353069492	-1.501975928	0.0002936	down	yes	1.133	3.209	3.107	3.441	3.078	1	1.003	1.396	------	------	------	------	------	------	ENOG4110ODA;	S:Function unknown;	------	------	------	CYT	1	14	9.9	High
TRINITY_DN69_c0_g1_i1_orf1	-	-	-	glycerol-3-phosphate dehydrogenase [NAD(+)], cytoplasmic isoform X1 [Ostrinia	0.352391226	-1.50475009	1.95E-05	down	yes	0.98	2.781	2.789	2.909	2.646	1	0.966	0.974	------	------	K00006	GPD1	map00564	Glycerophospholipid metab	COG0240	C:Energy production and conver	PF01210.26;PF0	NAD_Gly3P_dh	NAD-dependent glycer	CYT	21	63	39.8	High
TRINITY_DN15858_c0_g1_i1_orf1	-	-	-	15-hydroxyprostaglandin dehydrogenase [NAD(+)]-like [Ostrinia furnacalis]	0.468296296	-1.094506467	2.96E-05	down	yes	0.9483	2.025	1.967	2.016	2.092	1	0.88	0.965	MF:catalytic activity;	GO:0003824;GO:0003674;GO:0016491	K00069	HPGD	map00590;map05	Arachidonic acid metabolis	COG1028;COG0	S:Function unknown;	PF00106.28;PF1	adh_short;adh	short chain dehydroger	CYT	1	21	29.6	High
TRINITY_DN125521_c0_g2_i1_orf1	-	-	-	serpin transcript 1A2 [Ostrinia nubilalis]	0.372024899	-1.426528912	0.001507	down	yes	1.016	2.731	2.581	2.461	3.151	1	0.916	1.131	------	------	------	------	------	------	------	------	------	------	------	CYT	1	35	8.1	High
TRINITY_DN3822_c0_g1_i7_orf1	-	-	-	hypothetical protein HF086_002539 [Spodoptera exigua] >CAH0699327.1 unnamed protein product [Spodoptera exigua]	0.389301054	-1.361041843	2.65E-05	down	yes	0.997	2.561	2.624	2.418	2.642	1	0.99	1.001	BP:nucleoside mono	GO:0009124;GO:0046390;GO:0009161;GO:0044249;GO:0034641;GO:0009165;GO:0008150;GO:0009123;GO:0072521;GO:0072522;GO:1901362;GO:0009259;GO:0009168;GO:0006139;GO:0071704;GO:0090407;GO:0009127;GO:1901360;GO:0046040;GO:0018130;GO:0009987;GO:0009167;GO:0019693;GO:1901576;GO:0009156;GO:0006793;GO:0006725;GO:0009152;GO:0009150;GO:0009260;GO:0009058;GO:0006189;GO:0006188;GO:0008152;GO:0019438;GO:0034654;GO:0044271;GO:0055086;GO:0046483;GO:0044238;GO:1901564;GO:0009126;GO:1901566;GO:1901137;GO:0044237;GO:0006163;GO:0006796;GO:0006807;GO:1901293;GO:0006164;GO:0019637;GO:0009117;GO:0006753;GO:1901135;GO:0044281;GO:0005488;GO:0035639;GO:1901363;GO:0043727;GO:0016881;GO:0003674;GO:0000166;GO:0043168;GO:0016829;GO:0004639;GO:0004638;GO:0017076;GO:0005524;GO:1901265;GO:0036094;GO:0016874;GO:0032555;GO:0016879;GO:0097159;GO:0003824;GO:0030554;GO:0016830;GO:0016831;GO:0043167;GO:0097367;GO:0032559;GO:0032553	K01587	PAICS	map00230	Purine metabolism	COG0152	F:Nucleotide transport and met	PF01259.21;PF0	SAICAR_synt;AI	SAICAR synthetase;AIR	CYT	28	60	47.1	High
TRINITY_DN214_c0_g1_i3_orf1	-	-	-	uncharacterized protein LOC114352813 [Ostrinia furnacalis]	0.489868288	-1.029534195	0.0003518	down	yes	0.967	1.974	2.048	2.074	1.799	1	0.946	0.955	------	------	------	------	------	------	ENOG4110S0N;E	S:Function unknown;	PF06585.14	JHBP	Haemolymph juvenile h	CYT	3	38	19.4	High
TRINITY_DN25234_c0_g1_i1_orf1	-	-	-	uncharacterized protein LOC114353853 [Ostrinia furnacalis]	0.325261194	-1.620329386	0.0002211	down	yes	0.8717	2.68	2.661	2.883	2.495	1	0.705	0.91	------	------	------	------	------	------	------	------	------	------	------	CYT	1	15	10	High
TRINITY_DN59429_c0_g1_i6_orf1	-	-	-	uncharacterized protein LOC114366345 isoform X2 [Ostrinia furnacalis]	0.212127483	-2.236996545	1.06E-07	down	yes	1.025	4.832	4.759	4.875	4.861	1	1.074	1.002	BP:response to bioti	GO:0009607;GO:0009605;GO:0043207;GO:0050896;GO:0006952;GO:0006950;GO:0008150;GO:0044419;GO:0051707;GO:0005575;GO:0005576;GO:0110165	------	------	------	------	ENOG4110S6N	S:Function unknown	PF00451.22	Toxin_2	Scorpion short toxin, Br	CYT	2	22	12.3	High
TRINITY_DN33272_c0_g1_i1_orf1	-	-	-	unnamed protein product, partial [Iphiclides podalirius]	0.482945344	-1.050068169	3.09E-06	down	yes	0.9543	1.976	1.947	1.983	1.997	1	0.927	0.936	------	------	K04550	LRP1, CD91	map05010;map04	Alzheimer disease;Cholester	ENOG410YM97;I	S:Function unknown;	PF00057.21	Ldl_recept_a	Low-density lipoprotei	CYT	1	4	47.8	High
TRINITY_DN52316_c0_g1_i1_orf1	-	-	-	arylphorin subunit alpha-like [Ostrinia furnacalis]	0.206425452	-2.276307229	0.003564	down	yes	0.9927	4.809	5.461	5.399	3.567	1	0.967	1.011	------	------	------	------	------	------	ENOG410XR2D	J:Translation, ribosomal structure	PF03722.17	Hemocyanin_N	Hemocyanin, all-alpha	PLA	4	55	13.9	High
TRINITY_DN8685_c0_g1_i5_orf1	-	-	-	macrophage mannose receptor 1-like [Zerene cesonia]	0.427595045	-1.22568296	0.0003247	down	yes	1.001	2.341	2.255	2.202	2.567	1	1.043	0.959	BP:detection of bioti	GO:0009595;GO:0009593;GO:0032103;GO:0032101;GO:0048584;GO:0002831;GO:0002833;GO:0031349;GO:0042742;GO:0032490;GO:0050789;GO:0031347;GO:0032493;GO:0043207;GO:0009617;GO:0044419;GO:0002684;GO:0065007;GO:0048518;GO:0002682;GO:0098581;GO:0045089;GO:0045088;GO:0048583;GO:0045087;GO:0006952;GO:0006950;GO:0008150;GO:0006955;GO:0042221;GO:0051707;GO:0010033;GO:0050776;GO:0009607;GO:0051606;GO:0009605;GO:0080134;GO:0042494;GO:0050778;GO:0002376;GO:0002218;GO:0002237;GO:0002253;GO:0098542;GO:0050896;GO:0140367	------	------	------	------	ENOG410XRS3;E	U:Intracellular trafficking, secreti	PF00059.24	Lectin_C	Lectin C-type domain	PLA	2	6	40.6	High
TRINITY_DN13799_c0_g1_i1_orf1	-	-	-	uncharacterized protein LOC116345248 [Contarinia nasturtii]	0.220220507	-2.182979274	6.79E-05	down	yes	0.9987	4.535	4.597	4.151	4.857	1	1.008	0.988	------	------	------	------	------	------	------	------	PF06355.16	Aegerolysin	Aegerolysin	EXC	6	44	15.8	High
TRINITY_DN12582_c0_g1_i5_orf1	-	-	-	uncharacterized protein LOC114355527 isoform X1 [Ostrinia furnacalis]	0.238202247	-2.069741071	0.003415	down	yes	1.06	4.45	4.757	5.199	3.393	1	1.025	1.154	MF:hydrolase activity	GO:0016787;GO:0016818;GO:0003824;GO:0016817;GO:0016462;GO:0003674;GO:0047429	------	------	------	------	COG0424	D:Cell cycle control, cell division,	PF02545.17	Maf	Maf-like protein	CYT	1	5	22.9	High
TRINITY_DN11948_c0_g1_i8_orf1	-	-	-	cystathionine gamma-lyase [Ostrinia furnacalis]	0.434443964	-1.202757991	7.33E-06	down	yes	1.004	2.311	2.386	2.236	2.311	1	0.999	1.013	BP:carboxylic acid m	GO:0019752;GO:0044249;GO:0006807;GO:0044281;GO:0044283;GO:0009069;GO:1901576;GO:0050667;GO:0006520;GO:0071704;GO:0009092;GO:0000097;GO:1901607;GO:0000096;GO:0008652;GO:0009987;GO:0009058;GO:0008150;GO:0008152;GO:0043436;GO:0009070;GO:0044238;GO:1901564;GO:1901566;GO:0006082;GO:0044272;GO:0046394;GO:1901605;GO:0016053;GO:0044237;GO:0006534;GO:0019346;GO:0019344;GO:0006790;GO:0005488;GO:0070279;GO:0003674;GO:1901363;GO:0043168;GO:0016846;GO:0016829;GO:0080146;GO:0043167;GO:0004123;GO:0036094;GO:0003824;GO:0019842;GO:0044540;GO:0097159;GO:0030170	K01758	CTH	map00450;map00	Selenocompound metabolis	COG0626	E:Amino acid transport and met	PF01053.23;PF0	Cys_Met_Meta_	Cys/Met metabolism Pl	CYT	16	56	43	High
TRINITY_DN38307_c0_g1_i1_orfp1	-	-	-	TRINITY_DN38307_c0_g1_i1_m.10661 TRINITY_DN38307_c0_g1::TRINITY_DN38307_c0_g1_i1::g.10661 ORF type:5prime_partial len:66 (+) score=5.90 TRINITY_DN38307_c0_g1_i1:2-199(+)	0.235031698	-2.089072756	0.001074	down	yes	1.001	4.259	4.837	3.613	4.328	1	0.741	1.262	------	------	------	------	------	------	------	------	------	------	------	CYT	1	17	7.9	High
TRINITY_DN56308_c0_g1_i2_orf1	-	-	-	storage protein 1 [Omphisa fuscidentalis]	0.23764566	-2.073116041	7.54E-06	down	yes	0.9993	4.205	4	4.357	4.258	1	0.972	1.026	CC:extracellular spac	GO:0005615;GO:0005575;GO:0005576;GO:0110165;GO:0003674;GO:0045735	------	------	------	------	ENOG410XR2D	S:Function unknown	PF03722.17	Hemocyanin_N	Hemocyanin, all-alpha	CYT	9	60	11.3	High
TRINITY_DN1421_c0_g1_i1_orf1	-	-	-	uncharacterized protein LOC114352615 [Ostrinia furnacalis]	0.435596171	-1.198936824	0.0002545	down	yes	1.001	2.298	2.338	2.099	2.458	1	0.987	1.016	BP:nitrogen compou	GO:0006807;GO:0071704;GO:0006508;GO:0008150;GO:0008152;GO:0044238;GO:1901564;GO:0019538;GO:0043170;GO:0005737;GO:0110165;GO:0005575;GO:0005576;GO:0004252;GO:0004175;GO:0003674;GO:0005488;GO:0008233;GO:0008236;GO:0043169;GO:0043167;GO:0046872;GO:0016787;GO:0017171;GO:0140096;GO:0003824	------	------	------	------	COG5640	U:Intracellular trafficking, secreti	PF00089.29;PF1	Trypsin;CLIP	Trypsin;Regulatory CLIF	CYT	7	22	44.4	High
TRINITY_DN9733_c0_g1_i2_orf1	-	-	-	acidic juvenile hormone-suppressible protein 1-like [Ostrinia furnacalis]	0.246631206	-2.019572743	2.58E-06	down	yes	0.9737	3.948	3.948	3.823	4.072	1	0.923	0.998	------	------	------	------	------	------	ENOG410XR2D	S:Function unknown	PF00372.22	Hemocyanin_M	Hemocyanin, copper co	CYT	3	54	12.1	High
TRINITY_DN4802_c0_g1_i4_orf1	-	-	-	uncharacterized protein LOC114366345 isoform X2 [Ostrinia furnacalis]	0.420951957	-1.248272505	0.0005989	down	yes	0.9463	2.248	2.145	2.093	2.505	1	0.921	0.918	BP:response to bioti	GO:0009607;GO:0009605;GO:0043207;GO:0050896;GO:0006952;GO:0006950;GO:0008150;GO:0044419;GO:0051707;GO:0005575;GO:0005576;GO:0110165	------	------	------	------	------	------	------	------	------	CYT	1	12	9.9	High
TRINITY_DN6025_c0_g2_i1_orfp1	-	-	-	TRINITY_DN6025_c0_g2_i1_m.7749 TRINITY_DN6025_c0_g2::TRINITY_DN6025_c0_g2_i1::g.7749 ORF type:5prime_partial len:106 (+) score=47.10 TRINITY_DN6025_c0_g2_i1:3-320(+)	0.235005453	-2.089233865	0.003705	down	yes	0.862	3.668	3.441	3.037	4.525	1	0.611	0.975	------	------	------	------	------	------	------	------	------	------	------	PLA	1	10	11.6	Medium
TRINITY_DN993_c0_g1_i7_orf1	-	-	-	apolipophorins-like [Ostrinia furnacalis]	0.317811297	-1.653757689	4.79E-06	down	yes	0.9903	3.116	3.037	3.241	3.069	1	0.98	0.991	MF:lipid transporter	GO:0005319;GO:0005215;GO:0003674	------	------	------	------	ENOG41112S0;E	S:Function unknown;	PF00094.28	VWD	von Willebrand factor t	CYT	27	59	66.5	High
TRINITY_DN15812_c0_g1_i2_orf1	-	-	-	transferrin [Ostrinia furnacalis]	0.455114155	-1.135699636	0.0003857	down	yes	0.9967	2.19	2.173	2.012	2.386	1	0.992	0.998	BP:ion homeostasis;	GO:0050801;GO:0042592;GO:0055072;GO:0055076;GO:0098771;GO:0065007;GO:0006811;GO:0006810;GO:0008150;GO:0055065;GO:0055080;GO:0051234;GO:0051179;GO:0065008;GO:0048878;GO:0005615;GO:0110165;GO:0005575;GO:0003674;GO:0005488;GO:0043169;GO:0043167;GO:0046872	K06569	MFI2, CD228	------	------	ENOG410XQ36	O:Posttranslational modification	PF00405.20;PF1	Transferrin;Pho	Transferrin;ABC transpo	CYT	30	52	75.6	High
TRINITY_DN628_c0_g1_i1_orf1	-	-	-	prostamide/prostaglandin F synthase-like [Ostrinia furnacalis]	0.321410174	-1.637512498	9.00E-06	down	yes	0.9983	3.106	3.041	3.253	3.023	1	0.993	1.002	------	------	K15717	PRXL2B, FAM2	map00590	Arachidonic acid metabolis	ENOG410YFY9	O:Posttranslational modification	PF13911.9	AhpC-TSA_2	AhpC/TSA antioxidant e	CYT	9	46	26.8	High
TRINITY_DN307_c1_g1_i1_orf1	-	-	-	uncharacterized protein LOC114356704 [Ostrinia furnacalis]	0.482871891	-1.050287611	0.002236	down	yes	1.029	2.131	2.225	1.825	2.344	1	1.068	1.02	------	------	------	------	------	------	ENOG410Y4WH	S:Function unknown	PF07648.18	Kazal_2	Kazal-type serine prote	CYT	3	35	10.4	High
TRINITY_DN581_c3_g2_i1_orf1	-	-	-	uncharacterized protein LOC114364499 isoform X3 [Ostrinia furnacalis]	0.400350877	-1.320663128	0.0001538	down	yes	1.141	2.85	2.969	2.678	2.902	1	1.128	1.295	------	------	------	------	------	------	------	------	------	------	------	CYT	1	17	6.7	High
TRINITY_DN27264_c0_g1_i1_orf1	-	-	-	uncharacterized protein LOC114353424 [Ostrinia furnacalis]	0.434844979	-1.201426918	0.0003262	down	yes	0.9397	2.161	2.271	1.958	2.255	1	0.916	0.903	MF:molecular functi	GO:0003674;GO:0042302;GO:0005198	------	------	------	------	ENOG4111DJS;E	S:Function unknown;	PF00379.26	Chitin_bind_4	Insect cuticle protein	CYT	2	6	29.3	High
TRINITY_DN2464_c0_g1_i12_orf1	-	-	-	uncharacterized protein LOC114362996 isoform X1 [Ostrinia furnacalis]	0.336860068	-1.569778675	0.001558	down	yes	0.987	2.93	3.02	2.453	3.317	1	0.981	0.98	MF:molecular functi	GO:0003674;GO:0042302;GO:0005198	------	------	------	------	ENOG410YNFC	S:Function unknown	PF00379.26	Chitin_bind_4	Insect cuticle protein	CYT	8	45	37.2	High
TRINITY_DN41_c0_g1_i3_orf1	-	-	-	uncharacterized protein LOC114359035 isoform X3 [Ostrinia furnacalis]	0.466524621	-1.099974872	0.0002644	down	yes	0.9853	2.112	2.127	1.946	2.263	1	0.958	0.998	BP:nitrogen compou	GO:0006807;GO:0006643;GO:0071704;GO:0006665;GO:0006629;GO:0009987;GO:0008150;GO:0008152;GO:0044255;GO:0044238;GO:1901564;GO:0044237;GO:0000323;GO:0043229;GO:0043227;GO:0043226;GO:0005764;GO:0005773;GO:0110165;GO:0043231;GO:0005575;GO:0005576	K12382	PSAP, SGP1	map00600;map04	Sphingolipid metabolism;Lys	ENOG410XSI5	J:Translation, ribosomal structure	PF03489.20;PF0	SapB_2;SapB_1	Saposin-like type B, reg	CYT	40	46	125.9	High
TRINITY_DN625_c2_g2_i2_orf1	-	-	-	L-dopachrome tautomerase yellow-f2-like [Ostrinia furnacalis]	0.490785645	-1.026835043	0.001591	down	yes	1.012	2.062	1.882	2.33	1.975	1	0.988	1.049	------	------	K22203	DCE	map00350	Tyrosine metabolism	ENOG410YB1P;E	S:Function unknown;	PF03022.19	MRJP	Major royal jelly protein	CYT	6	23	45.4	High
TRINITY_DN47784_c0_g2_i1_orfp1	-	-	-	arylphorin subunit alpha-like [Ostrinia furnacalis]	0.372618207	-1.424229924	0.002606	down	yes	1.056	2.834	2.818	2.386	3.297	1	1.037	1.131	------	------	------	------	------	------	------	------	------	------	------	CYT	2	27	10.1	High
TRINITY_DN7960_c0_g1_i2_orf1	-	-	-	uncharacterized protein LOC114364878 [Ostrinia furnacalis]	0.364379666	-1.456485643	3.68E-05	down	yes	1.025	2.813	2.98	2.679	2.78	1	1.038	1.037	------	------	------	------	------	------	COG0451	I:Lipid transport and metabolism	PF01370.24;PF1	Epimerase;GDF	NAD dependent epime	CYT	13	50	34.5	High
TRINITY_DN46090_c0_g3_i1_orf1	-	-	-	tyrosine-protein kinase-like otk, partial [Ostrinia furnacalis]	0.487426614	-1.036743068	2.81E-05	down	yes	0.9963	2.044	1.952	2.063	2.117	1	0.985	1.004	CC:intrinsic compon	GO:0031224;GO:0016021;GO:0110165;GO:0005575;GO:0038023;GO:0035639;GO:0060089;GO:0003674;GO:0005488;GO:0043167;GO:1901363;GO:0043168;GO:0004714;GO:0000166;GO:0016740;GO:0004713;GO:0004672;GO:0017076;GO:0016301;GO:0140096;GO:1901265;GO:0036094;GO:0003824;GO:0030554;GO:0016773;GO:0016772;GO:0097367;GO:0004888;GO:0032559;GO:0005524;GO:0097159;GO:0032555;GO:0019199;GO:0032553	K05127	PTK7, CCK4	------	------	ENOG410YGKQ;	I:Lipid transport and metabolism	PF13927.9;PF07	Ig_3;I-set;Ig_2;I	Immunoglobulin domai	CYT	1	1	55	Medium
TRINITY_DN33346_c0_g1_i1_orf1	-	-	-	PREDICTED: U6 snRNA-associated Sm-like protein LSm3 [Papilio xuthus] >XP_028165558.1 U6 snRNA-associated Sm-like protein LSm3 [Ostrinia furnacalis] >KOB73597.1 LSM Sm-like protein family member [Operophtera brumata] >RVE45517.1 hypothetical protein evm_009856 [Chilo suppressalis] >CAB3523639.1 unnamed protein product [Chilo suppressalis] >CAH0400961.1 unnamed protein product [Chilo suppressalis]	0.324315899	-1.624528344	0.0001972	down	yes	0.9363	2.887	2.772	2.717	3.171	1	0.952	0.857	BP:primary metaboli	GO:0044238;GO:0044237;GO:0090304;GO:0034641;GO:0006807;GO:1901360;GO:0016071;GO:0071704;GO:0008380;GO:0006139;GO:0009987;GO:0006725;GO:0000375;GO:0000377;GO:0008150;GO:0008152;GO:0046483;GO:0016070;GO:0000398;GO:0043170;GO:0006396;GO:0006397;GO:0120114;GO:0005681;GO:0046540;GO:0097525;GO:0032991;GO:0030532;GO:1990904;GO:0005575;GO:0097526;GO:0005688;GO:0003674;GO:0005488;GO:0003676;GO:1901363;GO:0097159;GO:0003723	K12622	LSM3	map03018;map03	RNA degradation;Spliceoso	ENOG4111PB8	K:Transcription	PF01423.25	LSM	LSM domain	CYT	1	14	11.7	Medium
TRINITY_DN21743_c0_g1_i1_orf1	-	-	-	uncharacterized protein LOC114357426 [Ostrinia furnacalis]	0.474890297	-1.074333814	0.0002135	down	yes	0.974	2.051	2.211	1.937	2.004	1	0.977	0.945	------	------	------	------	------	------	ENOG4110OG2;	S:Function unknown;	------	------	------	CYT	1	36	11.7	High
TRINITY_DN97042_c0_g1_i6_orf1	-	-	-	apolipophorins-like [Ostrinia furnacalis]	0.359566787	-1.475668329	1.64E-06	down	yes	0.996	2.77	2.728	2.85	2.733	1	0.981	1.007	MF:lipid transporter	GO:0005319;GO:0005215;GO:0003674	------	------	------	------	ENOG410Z6MR;	S:Function unknown;	PF09172.14	Vit_open_b-sh	Vitellinogen, open beta	CYT	2	29	12.7	High
TRINITY_DN5421_c0_g1_i1_orf1	-	-	-	arylphorin subunit alpha-like [Ostrinia furnacalis]	0.379639838	-1.397296702	0.0009668	down	yes	1.033	2.721	2.667	2.417	3.079	1	1.021	1.077	------	------	------	------	------	------	------	------	------	------	------	EXC	1	21	17.2	High
TRINITY_DN36434_c0_g2_i3_orf1	-	-	-	clotting factor B isoform X1 [Ostrinia furnacalis]	0.467253177	-1.097723622	0.0003906	down	yes	0.956	2.046	2.234	1.993	1.912	1	0.926	0.942	BP:organic substanc	GO:0071704;GO:1901564;GO:0019538;GO:0043170;GO:0008150;GO:0006807;GO:0008152;GO:0006508;GO:0044238;GO:0004252;GO:0004175;GO:0016787;GO:0017171;GO:0140096;GO:0003824;GO:0003674;GO:0008233;GO:0008236	------	------	------	------	COG5640	U:Intracellular trafficking, secreti	PF00089.29	Trypsin	Trypsin	CYT	3	9	48.8	High
TRINITY_DN578_c0_g1_i5_orf1	-	-	-	charged multivesicular body protein 7 [Ostrinia furnacalis]	0.45988024	-1.120669887	0.0007336	down	yes	1.152	2.505	2.592	2.269	2.655	1	1.173	1.282	BP:cellular process;B	GO:0009987;GO:0007034;GO:0046907;GO:0006810;GO:0008150;GO:0051649;GO:0051234;GO:0051179;GO:0051641	K15053	CHMP7	map04144;map04	Endocytosis;Necroptosis	ENOG4110IXB	U:Intracellular trafficking, secreti	PF03357.24	Snf7	Snf7	CYT	1	10	43.1	High
TRINITY_DN1407_c0_g1_i12_orf1	-	-	-	hypothetical protein evm_012298 [Chilo suppressalis]	0.477929985	-1.065128811	0.0407	down	yes	0.942	1.971	1.579	2.656	1.679	1	0.946	0.88	BP:lipid transport;BP	GO:0006869;GO:0006810;GO:0071702;GO:0008150;GO:0051234;GO:0051179;GO:0005575;GO:0005576;GO:0110165;GO:0008289;GO:0003674;GO:0005488	------	------	------	------	------	------	PF07464.14	ApoLp-III	Apolipophorin-III precu	CYT	3	29	8	High

TRINITY_DN346_c0_g1_i7_orf1	-	-	-	CDK-activating kinase assembly factor MAT1 [Ostrinia furnacalis]	0.47826087	-1.064130337	0.002591	down	yes	1.166	2.438	2.247	2.352	2.714	1	1.415	1.082	BP:nucleic acid meta	GO:0090304;GO:0006807;GO:1901360;GO:0051716;GO:0044260;GO:0071704;GO:0006725;GO:0009987;GO:0006289;GO:0006974;GO:0006950;GO:0008150;GO:0006281;GO:0008152;GO:0046483;GO:0044238;GO:0050896;GO:0034641;GO:0044237;GO:0043170;GO:0006139;GO:0006259;GO:0033554;GO:0032806;GO:0090575;GO:1902911;GO:0005675;GO:1902494;GO:0061695;GO:1902554;GO:1990234;GO:0032991;GO:0005575;GO:0005667;GO:0061575;GO:0019207;GO:0019209;GO:0043169;GO:0003674;GO:0008047;GO:0098772;GO:0019887;GO:0043167;GO:0016538;GO:0046872;GO:0030234;GO:0030295;GO:0043539;GO:0005488	K10842	MNAT1	map03022;map03	Basal transcription factors;N	COG5220	K:Transcription	PF06391.16;PF1	MAT1;zf-C3HC	CDK-activating kinase	CYT	1	5	35.7	High
TRINITY_DN5132_c0_g1_i4_orf1	-	-	-	small heat shock protein Hsp24.2 [Ostrinia furnacalis]	0.438139932	-1.190536388	0.002302	down	yes	1.027	2.344	2.577	2.487	1.968	1	1.059	1.023	------	------	K09541	CRYAA	map04141	Protein processing in endop	ENOG410YERS	J:Translation, ribosomal structure	PF00011.24	HSP20	Hsp20/alpha crystallin f	CYT	5	21	24.7	High
TRINITY_DN1957_c0_g1_i4_orf1	-	-	-	NAD kinase 2, mitochondrial [Ostrinia furnacalis]	0.437065972	-1.194077033	8.36E-06	down	yes	1.007	2.304	2.376	2.309	2.226	1	0.994	1.028	BP:NADP metabolic	GO:0006739;GO:0019674;GO:0016310;GO:0044249;GO:0034641;GO:0006807;GO:1901576;GO:0072524;GO:0072525;GO:1901360;GO:1901362;GO:0071704;GO:0019359;GO:0044281;GO:0006741;GO:0018130;GO:0006139;GO:0009987;GO:0006725;GO:0009058;GO:0008150;GO:0008152;GO:0019438;GO:0034654;GO:0044271;GO:0090407;GO:0055086;GO:0046483;GO:0044238;GO:0006753;GO:1901564;GO:1901566;GO:0044237;GO:0006796;GO:0009165;GO:1901293;GO:0006793;GO:0019637;GO:0009117;GO:0019362;GO:0019363;GO:0046496;GO:0003674;GO:0005488;GO:0003951;GO:0043169;GO:0043167;GO:0016740;GO:0046872;GO:0016301;GO:0003824;GO:0016773;GO:0016772	K00858	ppnK, NADK	map00760;map01	Nicotinate and nicotinamide	ENOG410XQA4	S:Function unknown	PF01513.24	NAD_kinase	ATP-NAD kinase N-ter	CYT	7	21	49.1	High
TRINITY_DN4076_c1_g2_i2_orf1	-	-	-	vacuole membrane protein 1 [Ostrinia furnacalis]	0.414120267	-1.271878284	4.28E-05	down	yes	0.9297	2.245	2.335	2.265	2.136	1	0.904	0.885	CC:cellular_compone	GO:0005575;GO:0016021;GO:0110165;GO:0031224	K21248	VMP1	map04140	Autophagy - animal	ENOG410XOHA	S:Function unknown	PF09335.14	SNARE_assoc	SNARE associated Golc	CYT	1	4	44.2	High
TRINITY_DN335_c1_g1_i5_orf1	-	-	-	PREDICTED: perilipin-4 isoform X14 [Papilio polytes]	0.482364156	-1.051805388	0.001055	down	yes	1.012	2.098	2.072	1.892	2.331	1	1.035	1.002	------	------	K20254	PLIN4	map03320	PPAR signaling pathway	ENOG4112C4W	S:Function unknown	------	------	------	CYT	13	74	15.1	High
TRINITY_DN1540_c0_g1_i14_orf1	-	-	-	alaserpin-like isoform X1 [Ostrinia furnacalis]	0.46503632	-1.104584699	0.0001317	down	yes	0.9603	2.065	1.967	2.019	2.209	1	0.944	0.937	CC:cellular_compone	GO:0005575;GO:0110165;GO:0005615;GO:0004866;GO:0061135;GO:0061134;GO:0003674;GO:0030414;GO:0004867;GO:0004857;GO:0098772;GO:0030234	------	------	------	------	COG4826	O:Posttranslational modification	PF00079.23	Serpin	Serpin	CYT	1	33	43.8	High
TRINITY_DN135679_c0_g1_i2_orfp1	-	-	-	TRINITY_DN135679_c0_g1_i2_m.85525 TRINITY_DN135679_c0_g1::TRINITY_DN135679_c0_g1_i2::g.85525 ORF type:5prime_partial len:55 (+),score=5.08,Toxin_2 PF00451.20 1.9e-06	0.486806982	-1.038578236	8.58E-05	down	yes	0.9483	1.948	1.969	1.85	2.024	1	0.883	0.962	------	------	------	------	------	------	------	------	PF00451.22	Toxin_2	Scorpion short toxin, Br	CYT	1	46	6.1	High
TRINITY_DN34423_c0_g1_i3_orf1	-	-	-	THAP domain-containing protein 4-like [Ostrinia furnacalis]	0.100474342	-3.31510096	0.0008176	down	yes	0.699	6.957	6.697	5.946	8.228	1	0.628	0.469	------	------	K23205	THAP4	------	------	ENOG4111TBC	S:Function unknown	PF08768.14	THAP4_heme-l	THAP4-like, heme-binc	CYT	1	23	12.2	High
TRINITY_DN636_c1_g1_i9_orf1	-	-	-	secretory phospholipase A2 receptor-like [Ostrinia furnacalis]	0.154512695	-2.694202722	0.0002425	down	yes	0.9433	6.105	6.816	6.119	5.379	1	0.931	0.899	MF:molecular_functi	GO:0003674;GO:0030246;GO:0005488	------	------	------	------	ENOG410ZRYS;E	S:Function unknown;U:Intracellul	PF00059.24	Lectin_C	Lectin C-type domain	PLA	2	18	31	High
TRINITY_DN114890_c0_g1_i4_orf1	-	-	-	chemosensory protein 10 [Ostrinia furnacalis]	0.190256046	-2.393985798	0.0002821	down	yes	1.07	5.624	5.528	5.02	6.324	1	1.077	1.134	------	------	------	------	------	------	ENOG4111ATT;E	S:Function unknown;	PF03392.16	OS-D	Insect pheromone-binc	CYT	1	76	6.9	High
TRINITY_DN1370_c0_g1_i2_orf1	-	-	-	hypothetical protein evm_000756 [Chilo suppressalis]	0.201873622	-2.308475681	0.0003455	down	yes	1.099	5.444	5.736	5.899	4.696	1	1.06	1.236	------	------	------	------	------	------	------	------	------	------	------	CYT	2	35	23	High
TRINITY_DN2049_c1_g1_i3_orf1	-	-	-	luciferin 4-monooxygenase-like [Ostrinia furnacalis]	0.226502311	-2.142402323	0.0004443	down	yes	1.029	4.543	4.291	5.127	4.211	1	0.778	1.308	------	------	K01904	4CL	map00130	Ubiquinone and other terpe	COG0318;COG0	I:Lipid transport and metabolism	PF00501.31;PF13	AMP-binding;A	AMP-binding enzyme;A	CYT	1	33	59.6	High
TRINITY_DN19662_c4_g1_i1_orf1	-	-	-	basic juvenile hormone-suppressible protein 1-like [Ostrinia furnacalis]	0.25509165	-1.970912421	0.0001634	down	yes	1.002	3.928	3.72	4.354	3.709	1	0.999	1.007	CC:cellular_compone	GO:0005575;GO:0016020;GO:0005576;GO:0110165;GO:0038023;GO:0003824;GO:0016773;GO:0016772;GO:0016301;GO:0140096;GO:0004888;GO:0004675;GO:0004674;GO:0016740;GO:0003674;GO:0004672;GO:0019199;GO:0060089	------	------	------	------	ENOG410XR2D	S:Function unknown	PF03723.17	Hemocyanin_C	Hemocyanin, ig-like do	CYT	31	82	30.7	High
TRINITY_DN11826_c0_g1_i4_orf1	-	-	-	aldehyde dehydrogenase X, mitochondrial-like [Ostrinia furnacalis]	0.275395723	-1.860421939	0.002318	down	yes	0.9917	3.601	4.064	3.888	2.852	1	1.014	0.961	MF:catalytic activity;I	GO:0003824;GO:0016903;GO:0003674;GO:0016620;GO:0016491	K00128	ALDH	map00310;map00	Lysine degradation;Glyceroli	COG1012	C:Energy production and conver	PF00171.25	Aldedh	Aldehyde dehydrogena	CYT	3	48	11.9	High
TRINITY_DN1149_c0_g1_i4_orf1	-	-	-	circadian clock-controlled protein-like [Ostrinia furnacalis]	0.295356216	-1.759472121	6.20E-05	down	yes	1.024	3.467	3.47	3.234	3.696	1	0.97	1.102	------	------	------	------	------	------	ENOG410YOCZ;	S:Function unknown;	PF06585.14	JHBP	Haemolymph juvenile h	CYT	7	35	27.4	High
TRINITY_DN7226_c0_g1_i5_orf1	-	-	-	chemosensory protein [Dioryctria abietella]	0.256871096	-1.960883533	1.87E-06	down	yes	0.9047	3.522	3.458	3.515	3.594	1	0.854	0.86	------	------	------	------	------	------	ENOG4111U5H;I	S:Function unknown;	PF03392.16	OS-D	Insect pheromone-binc	CYT	4	28	8.8	High
TRINITY_DN2146_c0_g2_i1_orf1	-	-	-	heat shock protein 68-like [Ostrinia furnacalis]	0.299584323	-1.738965964	0.0007175	down	yes	1.009	3.368	3.313	2.963	3.828	1	1.012	1.016	MF:binding;MF:carbo	GO:0005488;GO:0097367;GO:0043168;GO:0030554;GO:0035639;GO:0097159;GO:0044183;GO:0000166;GO:0036094;GO:0032553;GO:0003674;GO:1901265;GO:0017076;GO:0032555;GO:0032559;GO:0043167;GO:1901363;GO:0005524	K03283	HSPA1s	map05162;map05	Measles;Toxoplasmosis;Estr	COG0443	O:Posttranslational modification	PF00012.23;PF06	HSP70;MreB_M	Hsp70 protein;MreB/MI	CYT	10	33	70.4	High
TRINITY_DN64181_c0_g1_i1_orf1	-	-	-	uncharacterized protein LOC114356431 isoform X2 [Ostrinia furnacalis]	0.375671406	-1.412456785	0.006421	down	yes	1.189	3.165	3.356	2.457	3.683	1	1.297	1.269	------	------	------	------	------	------	------	------	------	------	------	PLA	1	18	7.9	High
TRINITY_DN45220_c0_g1_i1_orf1	-	-	-	delta(3,5)-Delta(2,4)-dienoyl-CoA isomerase, mitochondrial isoform X1 [Ostrinia furnacalis]	0.389696169	-1.359578346	0.003744	down	yes	1.18	3.028	3.525	2.517	3.043	1	1.245	1.295	BP:lipid oxidation;BP	GO:0034440;GO:0006631;GO:0006635;GO:0019752;GO:0044248;GO:0009062;GO:0044281;GO:0044282;GO:0044242;GO:1901575;GO:0030258;GO:0016042;GO:0071704;GO:0006629;GO:0009987;GO:0032787;GO:0008150;GO:0008152;GO:0072329;GO:0043436;GO:0019395;GO:0044255;GO:0009056;GO:0044238;GO:0006082;GO:0046395;GO:0016054;GO:0044237;GO:0003674;GO:0003824;GO:0016853	K12663	ECH1	map04146	Peroxisome	ENOG410XTHX	S:Function unknown	PF00378.23	ECH_1	Enoyl-CoA hydratase/is	CYT	1	59	7.4	High
TRINITY_DN26209_c0_g1_i6_orf1	-	-	-	uncharacterized protein LOC114352357 [Ostrinia furnacalis]	0.344728655	-1.536466868	3.40E-05	down	yes	0.9973	2.893	2.921	2.72	3.037	1	0.985	1.007	------	------	------	------	------	------	COG1028	S:Function unknown	PF13561.9;PF00	adh_short_C2;a	Enoyl;short chain dehyc	CYT	11	54	27.3	High
TRINITY_DN2813_c0_g1_i7_orf1	-	-	-	arylphorin subunit alpha-like [Ostrinia furnacalis]	0.329533679	-1.601502177	0.001043	down	yes	0.954	2.895	2.949	2.478	3.257	1	0.948	0.914	------	------	------	------	------	------	ENOG4110QFU;I	S:Function unknown;	PF03723.17;PF0	Hemocyanin_C	Hemocyanin, ig-like do	CYT	6	36	139.8	High
TRINITY_DN6325_c0_g1_i9_orf1	-	-	-	fructose-bisphosphate aldolase isoform X2 [Pieris brassicae]	0.342751323	-1.544765861	0.02213	down	yes	0.9717	2.835	2.254	3.857	2.393	1	0.969	0.946	BP:ribonucleoside di	GO:0009185;GO:0016310;GO:0009144;GO:0019752;GO:0034641;GO:0006807;GO:0044281;GO:0006725;GO:0072521;GO:0006091;GO:0006090;GO:0006096;GO:1901360;GO:0006139;GO:1901575;GO:0009135;GO:0046939;GO:0009259;GO:0071704;GO:0009199;GO:0009141;GO:0009150;GO:0009205;GO:0019693;GO:0046034;GO:0009987;GO:0046031;GO:0032787;GO:0008150;GO:0008152;GO:0043436;GO:0005975;GO:0009132;GO:0009179;GO:0055086;GO:0046483;GO:0044238;GO:1901564;GO:0006082;GO:1901135;GO:0016052;GO:0044237;GO:0006163;GO:0006796;GO:0006165;GO:0006793;GO:0019637;GO:0009117;GO:0006753;GO:0009056;GO:0006757;GO:0003674;GO:0016829;GO:0016830;GO:0004332;GO:0016832;GO:0003824	K01623	ALDO	map00010;map00	Glycolysis / Gluconeogenesi	COG3588	G:Carbohydrate transport and m	PF00274.22	Glycolytic	Fructose-bisphosphate	CYT	5	82	42.3	High
TRINITY_DN14262_c0_g1_i5_orf1	-	-	-	cytochrome P450 monooxygenase CYP9G18 [Cnaphalocrocis medinalis]	0.474446337	-1.075683176	0.000115	down	yes	1.114	2.348	2.247	2.346	2.451	1	1.18	1.163	CC:endoplasmic reti	GO:0005789;GO:0031224;GO:0031090;GO:0016021;GO:0016020;GO:0110165;GO:0005575;GO:0004497;GO:0016705;GO:0003674;GO:0005488;GO:1901363;GO:0016491;GO:0043169;GO:0046914;GO:0043167;GO:0005506;GO:0046872;GO:0020037;GO:0003824;GO:0046906;GO:0097159	K15003	CYP9	------	------	COG2124	Q:Secondary metabolites biosyr	PF00067.25	p450	Cytochrome P450	CYT	1	39	12.9	High
TRINITY_DN17247_c0_g1_i14_orf1	-	-	-	uncharacterized protein LOC114356308 [Ostrinia furnacalis]	0.451476793	-1.147276263	0.0005929	down	yes	1.07	2.37	2.518	2.443	2.148	1	1.206	1.005	------	------	------	------	------	------	------	------	------	------	------	CYT	1	5	24.8	Medium
TRINITY_DN805_c0_g1_i5_orf1	-	-	-	serine protease snake-like [Ostrinia furnacalis]	0.44346364	-1.173112273	5.28E-05	down	yes	1.055	2.379	2.41	2.261	2.467	1	1.035	1.129	BP:organic substanc	GO:0071704;GO:1901564;GO:0019538;GO:0043170;GO:0008150;GO:0006807;GO:0008152;GO:0006508;GO:0044238;GO:0004252;GO:0004175;GO:0016787;GO:0017171;GO:0140096;GO:0003824;GO:0003674;GO:0008233;GO:0008236	------	------	------	------	COG5640	O:Posttranslational modification	PF00089.29	Trypsin	Trypsin	EXC	6	23	43.9	High
TRINITY_DN710_c0_g1_i11_orfp1	-	-	-	TRINITY_DN710_c0_g1_i11_m.67699 TRINITY_DN710_c0_g1::TRINITY_DN710_c0_g1_i11::g.67699 ORF type:complete len:194 (-),score=34.99,Collagen PF01391.19 0.00029 TRINITY_DN710_c0_g1_i11:1283-1864(-)	0.362616444	-1.463483745	0.0003177	down	yes	0.8953	2.469	2.467	2.255	2.685	1	0.874	0.812	------	------	------	------	------	------	------	------	------	------	------	CYT	1	7	21.3	High
TRINITY_DN14250_c0_g1_i1_orf1	-	-	-	apolipophorins-like [Ostrinia furnacalis]	0.393094842	-1.347050662	0.0008875	down	yes	0.945	2.404	2.305	2.188	2.72	1	0.941	0.894	MF:lipid transporter	GO:0005319;GO:0005215;GO:0003674	------	------	------	------	ENOG41112S0;E	S:Function unknown;	------	------	------	CYT	2	22	9.7	High
TRINITY_DN9239_c0_g2_i2_orf1	-	-	-	apolipophorins-like [Ostrinia furnacalis]	0.451284322	-1.147891435	0.008693	down	yes	1.019	2.258	2.042	1.963	2.769	1	0.974	1.082	BP:lipid transport;BP	GO:0006869;GO:0006810;GO:0071702;GO:0008150;GO:0051234;GO:0051179;GO:0005575;GO:0005576;GO:0110165;GO:0005319;GO:0005215;GO:0008289;GO:0003674;GO:0005488	------	------	------	------	ENOG41112S0;E	S:Function unknown;	------	------	------	CYT	5	90	7.6	High
TRINITY_DN34423_c0_g1_i2_orf1	-	-	-	THAP domain-containing protein 4-like [Ostrinia furnacalis]	0.449133718	-1.15478306	4.11E-06	down	yes	1.011	2.251	2.25	2.307	2.195	1	0.992	1.041	------	------	K23205	THAP4	------	------	ENOG4111TBC	S:Function unknown	PF08768.14	THAP4_heme-l	THAP4-like, heme-binc	CYT	1	24	9.9	High
TRINITY_DN2614_c0_g2_i3_orf1	-	-	-	uncharacterized protein LOC114360661 [Ostrinia furnacalis]	0.48880597	-1.032666189	0.007364	down	yes	1.048	2.144	1.954	2.555	1.924	1	0.951	1.192	------	------	------	------	------	------	ENOG4111BN3;I	S:Function unknown;	PF15430.9	SVWC	Single domain von Wille	CYT	1	18	12.7	High
TRINITY_DN1466_c0_g1_i4_orf1	-	-	-	insecticyanin-A-like [Ostrinia furnacalis]	0.480742459	-1.056663866	0.0003714	down	yes	1.036	2.155	2.325	1.984	2.156	1	1.035	1.072	MF:pigment binding	GO:0031409;GO:0003674;GO:0005488	K03098	APOD	------	------	ENOG4110R73;E	S:Function unknown;M:Cell wall/	PF00061.26	Lipocalin	Lipocalin / cytosolic fat	CYT	5	23	25.4	High
TRINITY_DN20347_c0_g1_i6_orf1	-	-	-	venom polypeptide precursor [Doratifera vulnerans]	0.484593838	-1.045152036	0.0002548	down	yes	1.038	2.142	2.142	2.008	2.275	1	1.131	0.984	CC:cellular_compone	GO:0005575;GO:0005576;GO:0110165	------	------	------	------	ENOG4111POK	S:Function unknown	PF07165.14	DUF1397	Protein of unknown fun	EXC	5	28	24.4	High
TRINITY_DN1710_c0_g1_i1_orf1	-	-	-	nuclear factor NF-kappa-B p105 subunit-like isoform X2 [Ostrinia furnacalis]	0.462246777	-1.113264834	0.001643	down	yes	1.004	2.172	2.476	2.043	1.998	1	0.96	1.051	BP:positive regulatio	GO:1903508;GO:0051252;GO:0080090;GO:0019222;GO:0031328;GO:0031326;GO:0031325;GO:0051173;GO:0048468;GO:0050789;GO:0009893;GO:0010604;GO:0048869;GO:0009891;GO:0019219;GO:0010556;GO:0065007;GO:0048518;GO:0010468;GO:0032502;GO:1902680;GO:0045935;GO:0060255;GO:0031323;GO:0009987;GO:0009889;GO:1903506;GO:0050794;GO:0008150;GO:0051171;GO:2001141;GO:0009605;GO:0007399;GO:0050896;GO:0048856;GO:0051254;GO:0006355;GO:0010557;GO:0006357;GO:0048731;GO:0048522;GO:0045893;GO:0005737;GO:0110165;GO:0005575;GO:0003674;GO:0005488;GO:0003676;GO:0003677;GO:0043565;GO:1990837;GO:1901363;GO:0003690;GO:0140110;GO:0001067;GO:0003700;GO:0097159;GO:0000976;GO:0000977	K02580;K09258	NFKB1;BCL3	map05140;map05	Leishmaniasis;Prostate canc	COG0666	S:Function unknown	------	------	------	CYT	2	13	20.8	High
TRINITY_DN29009_c0_g2_i2_orf1	-	-	-	juvenile hormone binding protein [Omphisa fuscidentalis]	0.458724203	-1.124301068	0.004009	down	yes	0.978	2.132	2.141	1.793	2.462	1	0.942	0.992	------	------	------	------	------	------	ENOG41117DM;	S:Function unknown;	PF06585.14	JHBP	Haemolymph juvenile h	CYT	1	23	22	High
TRINITY_DN5080_c0_g1_i5_orf1	-	-	-	storage protein [Ostrinia furnacalis]	0.112735101	-3.148991317	0.0001782	down	yes	0.995	8.826	9.982	8.38	8.115	1	0.967	1.018	------	------	------	------	------	------	ENOG410XR2D	J:Translation, ribosomal structure	PF00372.22;PF03	Hemocyanin_M	Hemocyanin, copper cc	CYT	3	69	49.2	High
TRINITY_DN16091_c0_g1_i1_orfp1	-	-	-	TRINITY_DN16091_c0_g1_i1_m.64010 TRINITY_DN16091_c0_g1::TRINITY_DN16091_c0_g1_i1::g.64010 ORF type:5prime_partial len:124 (-),score=7.29,Toxin_2 PF00451.20 0.00035,Toxin_2|PF00451.20|0.00013,Toxin_2|PF00451.20|0.00037,Gamma-thionin|PF00304.21|0.37,Gamma-thionin|PF00304.21|0.052,Gamma-thionin|PF00304.21|0.022,Defensin_2|PF01097.19|0.58,Defensin_2|PF01097.19|0.12,Defensin_2|PF01097.19|0.011,Toxin_38|PF14866.7|0.18,Toxin_38|PF14866.7|0.18,Toxin_38|PF14866.7|0.4 TRINITY_DN16091_c0_g1_i1:19-390(-)	0.180555556	-2.469485283	0.002263	down	yes	1.118	6.192	6.238	4.908	7.431	1	1.171	1.183	------	------	------	------	------	------	------	------	PF00451.22;PF0	Toxin_2;Gamma	Scorpion short toxin, Br	CYT	2	43	14.3	High
TRINITY_DN106156_c1_g1_i1_orf1	-	-	-	arylphorin subunit alpha-like [Ostrinia furnacalis]	0.156933609	-2.671773738	0.0004016	down	yes	0.9857	6.281	6.414	7.048	5.382	1	0.96	0.997	------	------	------	------	------	------	ENOG410XR2D	S:Function unknown	PF03723.17	Hemocyanin_C	Hemocyanin, ig-like do	CYT	12	59	13.5	High
TRINITY_DN51813_c0_g1_i1_orf1	-	-	-	uncharacterized protein LOC114350216 [Ostrinia furnacalis]	0.147262295	-2.763540004	0.002402	down	yes	0.8983	6.1	4.837	5.999	7.463	1	0.85	0.845	BP:'de novo' NAD bi	GO:0034354;GO:0009074;GO:0009165;GO:0044281;GO:0044282;GO:0044283;GO:1901362;GO:1901360;GO:1901361;GO:0043650;GO:0042537;GO:0019359;GO:0046496;GO:0032787;GO:0043436;GO:0009072;GO:0046483;GO:0043648;GO:1901564;GO:1901565;GO:1901566;GO:0016054;GO:0016053;GO:0019439;GO:0019438;GO:0009063;GO:0006807;GO:0043420;GO:1901576;GO:1901575;GO:0070189;GO:0006569;GO:0006568;GO:0019637;GO:0018130;GO:0009308;GO:0006139;GO:0006586;GO:0008150;GO:0008152;GO:0034654;GO:0042436;GO:0090407;GO:0042430;GO:0044271;GO:0044270;GO:0046394;GO:0046395;GO:1901293;GO:0006753;GO:0006576;GO:0044248;GO:0044249;GO:0034641;GO:1901605;GO:1901606;GO:0009987;GO:0006725;GO:0044106;GO:0055086;GO:0006082;GO:0046700;GO:0046874;GO:0019752;GO:0009435;GO:0034627;GO:0044237;GO:0072524;GO:0072525;GO:0019805;GO:0006520;GO:0071704;GO:0009058;GO:0009117;GO:0009056;GO:0044238;GO:0042180;GO:0042182;GO:0097053;GO:0097052;GO:0006796;GO:0006793;GO:0019363;GO:0019362;GO:0110165;GO:0005737;GO:0005575;GO:0030429;GO:1901363;GO:0061981;GO:0016787;GO:0003674;GO:0030170;GO:0016823;GO:0003824;GO:0070279;GO:0043168;GO:0016822;GO:0043167;GO:0019842;GO:0097159;GO:0005488;GO:0036094	K01556	KYNU, kynU	map00380;map01	Tryptophan metabolism;Bio	COG3844	E:Amino acid transport and meta	PF00266.22	Aminotran_5	Aminotransferase class	CYT	1	1	48.1	High
TRINITY_DN12534_c0_g1_i4_orf1	-	-	-	antibacterial protein [Heliothis virescens]	0.169626247	-2.559568671	0.0002643	down	yes	1.003	5.913	6.182	5.118	6.438	1	1.032	0.978	BP:response to bioti	GO:0009607;GO:0009605;GO:0043207;GO:0050896;GO:0006952;GO:0006950;GO:0008150;GO:0044419;GO:0051707;GO:0005575;GO:0005576;GO:0110165	------	------	------	------	ENOG4110S6N	S:Function unknown	PF00451.22	Toxin_2	Scorpion short toxin, Br	CYT	5	57	12	High
TRINITY_DN22515_c0_g1_i10_orf1	-	-	-	hypothetical protein KGM_202621 [Danaus plexippus plexippus]	0.176634699	-2.501159312	0.0008951	down	yes	1.013	5.735	6.216	4.671	6.317	1	1.004	1.035	------	------	------	------	------	------	ENOG41119TW	S:Function unknown	------	------	------	CYT	2	29	7.7	High
TRINITY_DN5177_c0_g1_i2_orf1	-	-	-	hemolin-like isoform X1 [Ostrinia furnacalis]	0.197938946	-2.336872592	6.56E-05	down	yes	1.018	5.143	5.201	4.704	5.523	1	1.013	1.041	------	------	------	------	------	------	ENOG410XSVG	S:Function unknown	PF07679.19;PF13	I-set;Ig_3;ig;Ig	Immunoglobulin I-set d	CYT	22	58	47.3	High
TRINITY_DN18568_c0_g1_i2_orfp1	-	-	-	TRINITY_DN18568_c0_g1_i2_m.13844 TRINITY_DN18568_c0_g1::TRINITY_DN18568_c0_g1_i2::g.13844 ORF type:5prime_partial len:77 (+),score=12.07 TRINITY_DN18568_c0_g1_i2:1-231(+)	0.163116727	-2.616023364	0.003314	down	yes	0.8133	4.986	5.237	5.981	3.741	1	0.659	0.781	------	------	------	------	------	------	------	------	------	------	------	CYT	1	13	8.5	High
TRINITY_DN135449_c0_g1_i5_orf1	-	-	-	larval cuticle protein LCP-17-like [Galleria mellonella]	0.240611648	-2.055221608	0.0003679	down	yes	1.07	4.447	5.005	4.36	3.975	1	1.086	1.125	MF:molecular_functi	GO:0003674;GO:0042302;GO:0005198	------	------	------	------	ENOG4110RUZ;I	S:Function unknown;	------	------	------	CYT	1	68	8.6	High
TRINITY_DN42719_c0_g2_i1_orf1	-	-	-	inter-alpha-trypsin inhibitor heavy chain H4-like isoform X11 [Ostrinia furnacalis]	0.25176139	-1.989871044	4.77E-07	down	yes	1.072	4.258	4.255	4.284	4.236	1	1.043	1.172	------	------	------	------	------	------	COG2304	S:Function unknown	------	------	------	CYT	1	12	12.1	High
TRINITY_DN7040_c0_g2_i1_orf1	-	-	-	uncharacterized protein LOC114353763 [Ostrinia furnacalis]	0.23240028	-2.105316288	4.50E-05	down	yes	0.9963	4.287	4.352	4.548	3.96	1	1.003	0.986	------	------	------	------	------	------	ENOG4110RZK	O:Posttranslational modification	------	------	------	CYT	8	32	22	High
TRINITY_DN124654_c0_g1_i1_orf1	-	-	-	protein lethal(2)essential for life [Manduca sexta] >KAG6441919.1 hypothetical protein O3G_MSEX002019 [Manduca sexta]	0.242774566	-2.042310805	6.20E-05	down	yes	1.008	4.152	4.061	3.898	4.496	1	0.984	1.039	------	------	K09542	CRYAB	map04213;map04	Longevity regulating pathwa	ENOG410YERS	P:Inorganic ion transport and me	PF00011.24	HSP20	Hsp20/alpha crystallin f	CYT	6	44	22.4	High
TRINITY_DN5444_c0_g1_i1_orfp1	-	-	-	TRINITY_DN5444_c0_g1_i1_m.14077 TRINITY_DN5444_c0_g1::TRINITY_DN5444_c0_g1_i1::g.14077 ORF type:3prime_partial len:90 (-),score=25.11 TRINITY_DN5444_c0_g1_i1:2-268(-)	0.258303596	-1.952860367	5.69E-05	down	yes	0.941	3.643	3.93	3.441	3.557	1	0.896	0.927	------	------	------	------	------	------	------	------	------	------	------	CYT	1	8	9.9	Medium
TRINITY_DN2457_c0_g1_i8_orf1	-	-	-	uncharacterized protein LOC114355596 [Ostrinia furnacalis]	0.318695512	-1.649749393	0.0006003	down	yes	0.987	3.097	2.748	3.484	3.06	1	0.94	1.021	BP:biological_proces	GO:0008150;GO:0008152;GO:0032259;GO:0003824;GO:0016740;GO:0016741;GO:0003674;GO:0008168	------	------	------	------	ENOG410Y1IK	M:Cell wall/membrane/envelope	PF12248.11	Methyltransf_F	Farnesoic acid 0-methy	CYT	7	27	33.4	High
TRINITY_DN85412_c0_g1_i1_orf1	-	-	-	unnamed protein product [Diatraea saccharalis]	0.34627383	-1.530014736	0.0001201	down	yes	0.999	2.885	2.86	2.679	3.117	1	0.993	1.004	MF:lipid transporter	GO:0005319;GO:0005215;GO:0003674	------	------	------	------	ENOG4110Z6MR;	S:Function unknown;	------	------	------	CYT	11	87	12	High
TRINITY_DN9400_c0_g1_i8_orf1	-	-	-	PREDICTED: monoacylglycerol lipase ABHD12-like [Amyelois transitella]	0.416543575	-1.263460671	0.0317	down	yes	1.128	2.708	2.206	3.672	2.245	1	1.134	1.25	------	------	K13704	ABHD12	------	------	ENOG4111FHA;(	S:Function unknown;	PF12146.11;PF0(	Hydrolase_4;Ab	Serine aminopeptidase	PLA	1	5	24.6	High
TRINITY_DN311_c0_g1_i8_orfp1	-	-	-	TRINITY_DN311_c0_g1_i8_m.65152 TRINITY_DN311_c0_g1::TRINITY_DN311_c0_g1_i8::g.65152 ORF type:5prime_partial len:138 (+),score=74.30 TRINITY_DN311_c0_g1_i8:2-415(+)	0.435312024	-1.199878224	0.001339	down	yes	1.144	2.628	2.647	2.322	2.915	1	1.215	1.218	------	------	------	------	------	------	------	------	------	------	------	PLA	1	18	15	High
TRINITY_DN1175_c1_g1_i1_orf1	-	-	-	methanethiol oxidase [Ostrinia furnacalis]	0.378203184	-1.402766585	6.63E-05	down	yes	0.9977	2.638	2.827	2.529	2.559	1	0.982	1.011	MF:selenium bindinc	GO:0008430;GO:0003674;GO:0005488	K17285	SELENBP1	map00920	Sulfur metabolism	ENOG410XP4F	S:Function unknown	PF05694.14	SBP56	56kDa selenium bindin	CYT	11	46	53.7	High
TRINITY_DN40126_c0_g1_i1_orf1	-	-	-	aldehyde dehydrogenase X, mitochondrial-like [Ostrinia furnacalis]	0.407613006	-1.294728011	0.000242	down	yes	1.028	2.522	2.472	2.749	2.345	1	1.027	1.056	MF:catalytic activity;I	GO:0003824;GO:0016903;GO:0003674;GO:0016620;GO:0016491	K00128	ALDH	map00310;map00	Lysine degradation;Glyceroli	COG1012	C:Energy production and conver	PF00171.25	Aldedh	Aldehyde dehydrogena	CYT	19	65	45.5	High
TRINITY_DN135780_c0_g1_i1_orf1	-	-	-	flotillin-1 isoform X1 [Pectinophora gossypiella]	0.366914935	-1.446482464	0.0005778	down	yes	0.936	2.551	2.521	2.291	2.841	1	0.906	0.902	CC:cellular_compone	GO:0005575;GO:0016020;GO:0110165	K07192	FLOT	map04910	Insulin signaling pathway	COG2268	S:Function unknown	PF01145.28	Band_7	SPFH domain / Band 7	CYT	2	27	12.8	High
TRINITY_DN285_c0_g1_i4_orf1	-	-	-	catalase-like [Ostrinia furnacalis]	0.391375852	-1.353373349	1.96E-05	down	yes	0.9757	2.493	2.555	2.392	2.533	1	0.898	1.029	BP:cellular catabolic	GO:0044248;GO:0042744;GO:0009987;GO:0072593;GO:0006950;GO:0008150;GO:0008152;GO:0009056;GO:0006979;GO:0050896;GO:0044237;GO:0042743;GO:0003674;GO:0005488;GO:1901363;GO:0016491;GO:0043169;GO:0043167;GO:0046872;GO:0020037;GO:0016209;GO:0003824;GO:0016684;GO:0046906;GO:0097159;GO:0004096;GO:0004601	K03781	katE, CAT, catl	map00630;map05	Glyoxylate and dicarboxylat	COG0753	P:Inorganic ion transport and me	PF00199.22;PF06	Catalase;Catal	Catalase;Catalase-relat	CYT	9	23	58.6	High
TRINITY_DN121650_c0_g1_i1_orf1	-	-	-	carboxylesterase [Ostrinia furnacalis]	0.409478079	-1.288141873	3.56E-05	down	yes	0.9807	2.395	2.531	2.342	2.312	1	0.952	0.99	CC:integral compone	GO:0016021;GO:0005575;GO:0031224;GO:0110165;GO:0016787;GO:0016788;GO:0052689;GO:0003824;GO:0003674	------	------	------	------	COG2272	I:Lipid transport and metabolism	------	------	------	CYT	1	19	7.6	High
TRINITY_DN3383_c0_g1_i5_orf1	-	-	-	uncharacterized protein LOC114357426 [Ostrinia furnacalis]	0.431515672	-1.212515138	0.0001535	down	yes	1.005	2.329	2.419	2.141	2.426	1	0.983	1.032	------	------	------	------	------	------	ENOG4110OG2;I	S:Function unknown;	------	------	------	CYT	4	14	35.5	High
TRINITY_DN1424_c0_g1_i5_orf1	-	-	-	insect group I lytic polysaccharide monooxygenase [Ostrinia furnacalis]	0.417450813	-1.260321877	6.37E-06	down	yes	0.976	2.338	2.404	2.271	2.34	1	0.934	0.994	------	------	------	------	------	------	ENOG410Y8ZO	S:Function unknown	PF03067.18	LPMO_10	Lytic polysaccharide m	CYT	6	21	39	High
TRINITY_DN1960_c5_g1_i3_orf1	-	-	-	cytochrome P450 monooxygenase CYP9G18 [Cnaphalocrocis medinalis]	0.466903598	-1.098803387	5.08E-05	down	yes	1.051	2.251	2.365	2.197	2.192	1	1.043	1.109	CC:endoplasmic reti	GO:0005789;GO:0031224;GO:0031090;GO:0016021;GO:0016020;GO:0110165;GO:0005575;GO:0004497;GO:0016705;GO:0003674;GO:0005488;GO:1901363;GO:0016491;GO:0043169;GO:0046914;GO:0043167;GO:0005506;GO:0046872;GO:0020037;GO:0003824;GO:0046906;GO:0097159	K15003	CYP9	------	------	COG2124	Q:Secondary metabolites biosyr	PF00067.25	p450	Cytochrome P450	CYT	3	21	43.4	High
TRINITY_DN1287_c0_g1_i5_orf1	-	-	-	probable chitinase 10 isoform X6 [Ostrinia furnacalis]	0.451815329	-1.146194875	1.24E-05	down	yes	1.008	2.231	2.186	2.185	2.323	1	1	1.023	BP:amino sugar cata	GO:0046348;GO:0006807;GO:0006022;GO:1901575;GO:0071704;GO:0006030;GO:0006032;GO:0008150;GO:0008152;GO:0005975;GO:0009056;GO:0009057;GO:0044238;GO:1901564;GO:1901565;GO:0006040;GO:1901136;GO:1901135;GO:0043170;GO:1901072;GO:1901071;GO:0006026;GO:0110165;GO:0005575;GO:0005576;GO:0016798;GO:0003674;GO:0008061;GO:0016787;GO:0003824;GO:0004568;GO:0097367;GO:0004553;GO:0005488	K01183	E3.2.1.14	map00520	Amino sugar and nucleotide	COG3325	S:Function unknown	PF00704.31;PF0	Glyco_hydro_18	Glycosyl hydrolases fan	CYT	51	24	327.2	High
TRINITY_DN2024_c0_g1_i12_orfp1	-	-	-	unnamed protein product, partial [Brenthis ino]	0.44430727	-1.170370346	0.002654	down	yes	0.9717	2.187	2.258	1.842	2.461	1	0.954	0.961	------	------	------	------	------	------	------	------	PF13930.9	Endonuclea_NS	DNA/RNA non-specific	CYT	2	6	46	High
TRINITY_DN1175_c1_g1_i2_orf1	-	-	-	methanethiol oxidase [Ostrinia furnacalis]	0.467837079	-1.095921887	0.0002336	down	yes	0.9993	2.136	2.2	1.957	2.251	1	1.003	0.995	MF:selenium bindinc	GO:0008430;GO:0003674;GO:0005488	K17285	SELENBP1	map00920	Sulfur metabolism	ENOG410XP4F	S:Function unknown	PF05694.14	SBP56	56kDa selenium bindin	CYT	10	45	53.7	High
TRINITY_DN3275_c0_g2_i3_orf1	-	-	-	hypothetical protein B5X24_HaOG216046 [Helicoverpa armigera]	0.243005181	-2.04094102	0.02013	down	yes	0.6097	2.509	2.48	1.718	3.33	1	0.513	0.316	MF:cation binding;M	GO:0043169;GO:0043167;GO:0003674;GO:0005488;GO:0046872;GO:0005509	K02183	CALM	map05214;map05	Glioma;Pathways of neurod	COG5126;COG5	D:Cell cycle control, cell division,	------	------	------	CYT	1	36	2.8	Medium
TRINITY_DN5748_c0_g1_i6_orf1	-	-	-	glycine N-methyltransferase isoform X1 [Ostrinia furnacalis] >XP_028165118.1 glycine N-methyltransferase isoform X2 [Ostrinia furnacalis] >XP_028165119.1 glycine N-methyltransferase isoform X1 [Ostrinia furnacalis] >XP_028165120.1 glycine N-methyltransferase isoform X2 [Ostrinia furnacalis]	0.499757869	-1.000698811	0.002998	down	yes	1.032	2.065	2.067	1.787	2.34	1	1.045	1.051	BP:organic substanc	GO:0071704;GO:0008150;GO:0008152;GO:0032259;GO:0017174;GO:0003824;GO:0008757;GO:0003674;GO:0016740;GO:0008170;GO:0016741;GO:0008168	K00552	GNMT	map00260	Glycine, serine and threonin	ENOG410ZWXN	S:Function unknown	PF13649.9;PF08	Methyltransf_2	Methyltransferase dom	CYT	3	48	27.4	High
TRINITY_DN54205_c0_g1_i1_orf1	-	-	-	aldo-keto reductase AKR2E4-like [Ostrinia furnacalis]	0.498514851	-1.00429161	1.39E-06	down	yes	1.007	2.02	2.01	1.997	2.052	1	0.985	1.035	MF:catalytic activity;I	GO:0003824;GO:0003674;GO:0016491	------	------	------	------	COG0656	S:Function unknown	PF00248.24	Aldo_ket_red	Aldo/keto reductase fa	CYT	7	32	34.5	High
TRINITY_DN5667_c0_g1_i4_orf1	-	-	-	spodomicin-like [Ostrinia furnacalis] >QKV49445.1 diapausin [Ostrinia furnacalis]	0.467595308	-1.096667642	0.005514	down	yes	0.9567	2.046	1.983	1.77	2.386	1	0.791	1.079	BP:antifungal humor	GO:0019732;GO:0019730;GO:0002376;GO:0043207;GO:0044419;GO:0001906;GO:0009987;GO:0050832;GO:0006950;GO:0008150;GO:0006955;GO:0006959;GO:0051707;GO:0006952;GO:0009607;GO:0009605;GO:0009620;GO:0050896;GO:0031640;GO:0098542;GO:0110165;GO:0005575;GO:0005576;GO:0099106;GO:0090729;GO:0003674;GO:0098772;GO:0008200;GO:0005246;GO:0016248;GO:0016247;GO:0019855	------	------	------	------	------	------	PF08036.14	Antimicrobial_6	Diapausin family of ant	CYT	1	16	7.1	High
TRINITY_DN4394_c0_g2_i1_orf1	-	-	-	carboxylesterase [Ostrinia furnacalis]	0.48512355	-1.043575878	0.004486	down	yes	0.962	1.983	1.902	2.318	1.73	1	0.903	0.983	CC:integral compone	GO:0016021;GO:0005575;GO:0031224;GO:0110165;GO:0016787;GO:0016788;GO:0052689;GO:0003824;GO:0003674	------	------	------	------	COG2272	I:Lipid transport and metabolism	PF00135.31;PF20	COesterase;BD	Carboxylesterase family	CYT	4	20	48.8	High
TRINITY_DN36476_c1_g1_i1_orfp1	-	-	-	TRINITY_DN36476_c1_g1_i1_m.70910 TRINITY_DN36476_c1_g1::TRINITY_DN36476_c1_g1_i1::g.70910 ORF type:5prime_partial len:88 (-),score=0.50 TRINITY_DN36476_c1_g1_i1:49-312(-)	0.183120107	-2.449137884	0.001107	down	yes	0.959	5.237	5.254	4.346	6.112	1	0.942	0.935	------	------	------	------	------	------	------	------	------	------	------	CYT	2	21	9.5	High
TRINITY_DN3707_c0_g1_i1_orf1	-	-	-	protein FAM160B1-like isoform X1 [Ostrinia furnacalis]	0.272296764	-1.876748257	0.0001714	down	yes	1.035	3.801	3.998	3.395	4.01	1	1.069	1.037	------	------	------	------	------	------	ENOG410XTDG;I	S:Function unknown;	PF10257.12;PF19	RAI16-like;DUF	Retinoic acid induced 1	CYT	1	1	93.1	High
TRINITY_DN4816_c0_g2_i3_orf1	-	-	-	15-hydroxyprostaglandin dehydrogenase [NAD(+)]-like [Ostrinia furnacalis]	0.283930058	-1.816392507	6.78E-06	down	yes	1.023	3.603	3.47	3.637	3.702	1	0.954	1.116	MF:catalytic activity;I	GO:0003824;GO:0003674;GO:0016491	K00069	HPGD	map00590;map05	Arachidonic acid metabolism	COG1028;COG0	S:Function unknown;	PF00106.28;PF13	adh_short;adh	short chain dehydroger	CYT	7	38	29.1	High
TRINITY_DN80328_c0_g1_i9_orf1	-	-	-	arylphorin subunit alpha-like [Ostrinia furnacalis]	0.280325528	-1.834824963	0.0002244	down	yes	0.9817	3.502	3.768	3.626	3.113	1	0.969	0.976	------	------	------	------	------	------	ENOG410XR2D	S:Function unknown	PF00372.22	Hemocyanin_M	Hemocyanin, copper cc	CYT	4	84	9.1	High
TRINITY_DN27300_c0_g1_i1_orfp1	-	-	-	TRINITY_DN27300_c0_g1_i1_m.71142 TRINITY_DN27300_c0_g1::TRINITY_DN27300_c0_g1_i1::g.71142 ORF type:internal len:82 (-),score=9.46 TRINITY_DN27300_c0_g1_i1:3-245(-)	0.309635723	-1.69135617	0.0003397	down	yes	1.054	3.404	3.742	3.053	3.418	1	0.998	1.164	------	------	------	------	------	------	------	------	------	------	------	CYT	1	21	9.2	High
TRINITY_DN3616_c0_g1_i4_orf1	-	-	-	conotoxin ArMKLT2-032-like [Ostrinia furnacalis]	0.265438446	-1.913550749	0.003889	down	yes	0.9293	3.501	3.646	2.701	4.155	1	0.952	0.836	CC:cellular_compone	GO:0005575;GO:0016021;GO:0110165;GO:0031224	------	------	------	------	------	------	------	------	------	CYT	1	68	6.1	High

TRINITY_DN12526_c0_g1_i5_orf1	-	-	-	uncharacterized protein LOC114359035 isoform X3 [Ostrinia furnacalis]	0.292543078	-1.773279013	0.001708	down	yes	0.9847	3.366	3.032	3.071	3.995	1	0.895	1.059	BP:nitrogen compou	GO:0006807;GO:0006643;GO:0071704;GO:0006665;GO:0006629;GO:0009987;GO:0008150;GO:0008152;GO:0044255;GO:0044238;GO:1901564;GO:0044237;GO:0000323;GO:0043229;GO:0043227;GO:0043226;GO:0005764;GO:0005773;GO:0110165;GO:0043231;GO:0005575;GO:0005576	K12382	PSAP, SGP1	map00600;map04	Sphingolipid metabolism;Lys	ENOG410XSI5	O:Posttranslational modification	PF05184.18;PF03	SapB_1;SapB_2	Saposin-like type B, reg	CYT	2	37	15.2	High
TRINITY_DN12586_c0_g1_i4_orf1	-	-	-	zonadhesin-like isoform X4 [Ostrinia furnacalis]	0.263588545	-1.923640421	0.001266	down	yes	0.902	3.422	3.797	2.813	3.656	1	0.873	0.833	------	------	------	------	------	------	ENOG4110RF5;C	S:Function unknown;O:Posttransl	PF01826.20	TIL	Trypsin Inhibitor like cys	PLA	1	26	14.3	High
TRINITY_DN30306_c0_g2_i1_orf1	-	-	-	perilipin-4-like isoform X3 [Ostrinia furnacalis]	0.312172573	-1.679584304	0.00112	down	yes	1.013	3.245	3.228	2.791	3.715	1	1.032	1.006	------	------	K20254	PLIN4	map03320	PPAR signaling pathway	ENOG4112C4W	S:Function unknown	------	------	------	CYT	6	64	7.2	High
TRINITY_DN20442_c0_g2_i1_orf1	-	-	-	hypothetical protein evm_008218 [Chilo suppressalis]	0.300313578	-1.735458391	0.0004606	down	yes	0.9577	3.189	3.591	3.004	2.972	1	0.827	1.046	BP:nucleosome orga	GO:0034728;GO:0022607;GO:0019222;GO:0043933;GO:0031326;GO:0031323;GO:0071840;GO:0080090;GO:0071824;GO:0006325;GO:0016043;GO:0050789;GO:0065003;GO:0019219;GO:0065004;GO:0065007;GO:0010468;GO:0060255;GO:0009889;GO:0009987;GO:1903506;GO:0050794;GO:0008150;GO:0051171;GO:2001141;GO:0006338;GO:0006334;GO:0051252;GO:0006355;GO:0010556;GO:0000786;GO:0044815;GO:0032991;GO:0032993;GO:0005575;GO:0003676;GO:0061733;GO:0003674;GO:0005488;GO:0034212;GO:0003677;GO:0043167;GO:1901363;GO:0043169;GO:0008080;GO:0004402;GO:0016740;GO:0016746;GO:0016747;GO:0016410;GO:0046872;GO:0140096;GO:0003824;GO:0097159;GO:0016407	K11306	MYST4, KAT6E	------	------	COG5027	B:Chromatin structure and dynai	------	------	------	CYT	1	7	13.7	Medium
TRINITY_DN13760_c1_g1_i1_orf1	-	-	-	pre-mRNA-processing factor 40 homolog A isoform X1 [Ostrinia furnacalis] >XP_028162665.1 pre-mRNA-processing factor 40 homolog A isoform X2 [Ostrinia furnacalis] >XP_028162667.1 pre-mRNA-processing factor 40 homolog A isoform X3 [Ostrinia furnacalis]	0.316205662	-1.661064893	6.36E-05	down	yes	0.9717	3.073	3.206	2.987	3.027	1	1.129	0.786	BP:nucleic acid meta	GO:0090304;GO:0034641;GO:0006807;GO:0000398;GO:1901360;GO:0006139;GO:0071704;GO:0008380;GO:0016071;GO:0009987;GO:0006725;GO:0000375;GO:0000377;GO:0008150;GO:0008152;GO:0046483;GO:0016070;GO:0044238;GO:0045292;GO:0044237;GO:0043170;GO:0006396;GO:0006397;GO:0043227;GO:0043226;GO:0005634;GO:0110165;GO:0043231;GO:0043229;GO:0005575	K12821	PRPF40, PRP40	map03040	Spliceosome	COG5104	A:RNA processing and modificat	PF01846.22;PF0(	FF;WW;RhoGAI	FF domain;WW domain	CYT	1	1	92.5	High
TRINITY_DN28711_c0_g1_i1_orf1	-	-	-	hypothetical protein evm_000299 [Chilo suppressalis]	0.324563714	-1.623426377	0.00126	down	yes	0.9857	3.037	2.636	3.504	2.972	1	0.956	1.001	MF:lipid transporter	GO:0005319;GO:0005215;GO:0003674	------	------	------	------	ENOG410Z6MR;	S:Function unknown;	------	------	------	CYT	7	83	7.5	High
TRINITY_DN140_c0_g1_i1_orf1	-	-	-	calcyphosin-like protein [Ostrinia furnacalis]	0.337288709	-1.567944073	5.42E-06	down	yes	0.9977	2.958	2.907	2.888	3.078	1	0.991	1.002	------	------	K23909	CAPS	------	------	ENOG410XRMJ	T:Signal transduction mechanism	PF13405.9;PF134	EF-hand_6;EF-	EF-hand domain;EF-ha	CYT	7	55	27.1	High
TRINITY_DN81488_c0_g1_i1_orf1	-	-	-	apolipophorins-like [Ostrinia furnacalis]	0.356721084	-1.487131607	0.001047	down	yes	1.027	2.879	3.275	2.837	2.526	1	1.031	1.05	BP:lipid transport;BP	GO:0006869;GO:0006810;GO:0071702;GO:0008150;GO:0051234;GO:0051179;GO:0005575;GO:0005576;GO:0110165;GO:0005319;GO:0005215;GO:0008289;GO:0003674;GO:0005488	------	------	------	------	ENOG410Z6MR;	S:Function unknown;	------	------	------	CYT	6	75	8.8	High
TRINITY_DN16905_c0_g1_i1_orf1	-	-	-	unnamed protein product [Leptidea sinapis]	0.421961072	-1.244818184	0.001029	down	yes	1.149	2.723	2.946	2.69	2.534	1	1.428	1.018	BP:phosphorylation;I	GO:0016310;GO:0006807;GO:0071704;GO:0006796;GO:0006468;GO:0009987;GO:0043412;GO:0036211;GO:0008150;GO:0008152;GO:0044238;GO:1901564;GO:0044237;GO:0043170;GO:0019538;GO:0006793;GO:0031224;GO:0016021;GO:0110165;GO:0005575;GO:0038023;GO:0035639;GO:0060089;GO:0003674;GO:0005488;GO:0000166;GO:1901363;GO:0043168;GO:0043169;GO:0097159;GO:0043167;GO:0004675;GO:0004674;GO:0016740;GO:1901265;GO:0004672;GO:0017076;GO:0046872;GO:0097367;GO:0016301;GO:0140096;GO:0036094;GO:0003824;GO:0030554;GO:0016773;GO:0016772;GO:0004888;GO:0032559;GO:0005524;GO:0032555;GO:0019199;GO:0032553	K13578	BMPR1B, ALK6	map04390;map04	Hippo signaling pathway;Sig	ENOG410XQT0	T:Signal transduction mechanism	PF00069.28;PF07	Pkinase;PK_Tyr_	Protein kinase domain;I	CYT	1	2	40.3	High
TRINITY_DN4235_c0_g1_i2_orf1	-	-	-	uncharacterized protein LOC114361536 [Ostrinia furnacalis]	0.368234878	-1.441301815	1.77E-05	down	yes	1.041	2.827	2.908	2.887	2.685	1	1.053	1.071	CC:cellular_compon	GO:0005575;GO:0110165;GO:0005615;GO:0004866;GO:0061135;GO:0061134;GO:0003674;GO:0030414;GO:0004867;GO:0004857;GO:0098772;GO:0030234	------	------	------	------	COG4826	P:Inorganic ion transport and me	PF00079.23	Serpin	Serpin	CYT	5	16	43.7	High
TRINITY_DN15327_c2_g1_i2_orf1	-	-	-	protein lethal(2)essential for life-like [Ostrinia furnacalis]	0.382420091	-1.386769774	0.0004183	down	yes	1.005	2.628	2.659	2.355	2.871	1	1.032	0.984	MF:cation binding;M	GO:0043169;GO:0005198;GO:0005212;GO:0043167;GO:0003674;GO:0005488;GO:0046872	K09542	CRYAB	map04213;map04	Longevity regulating pathwa	ENOG410YERS	E:Amino acid transport and meta	PF00011.24;PF0(	HSP20;Crystalli	Hsp20/alpha crystallin f	CYT	7	44	22	High
TRINITY_DN394_c0_g1_i4_orf1	-	-	-	uncharacterized protein LOC114351483 [Ostrinia furnacalis]	0.43002441	-1.217509539	7.59E-05	down	yes	1.057	2.458	2.331	2.597	2.447	1	1.117	1.053	MF:molecular_functi	GO:0003674;GO:0042302;GO:0005198	------	------	------	------	ENOG41113X7	S:Function unknown	PF00379.26	Chitin_bind_4	Insect cuticle protein	CYT	1	19	12.6	High
TRINITY_DN8780_c0_g1_i3_orf1	-	-	-	uncharacterized protein LOC114363370 [Ostrinia furnacalis]	0.435797665	-1.198269627	0.0003421	down	yes	1.008	2.313	2.302	2.12	2.517	1	1.004	1.019	CC:cellular_compon	GO:0005575;GO:0005576;GO:0110165;GO:0030234;GO:0061134;GO:0003674;GO:0030414;GO:0004857;GO:0098772	------	------	------	------	ENOG410Y67D	S:Function unknown	------	------	------	CYT	2	32	12.2	High
TRINITY_DN9492_c0_g1_i7_orf1	-	-	-	aldo-keto reductase AKR2E4-like [Ostrinia furnacalis]	0.417146541	-1.261373814	8.86E-06	down	yes	0.9707	2.327	2.281	2.29	2.41	1	0.926	0.986	MF:catalytic activity;I	GO:0003824;GO:0003674;GO:0016491	------	------	------	------	COG0656	S:Function unknown	PF00248.24	Aldo_ket_red	Aldo/keto reductase fa	CYT	7	30	38.3	High
TRINITY_DN8095_c0_g1_i3_orf1	-	-	-	circadian clock-controlled protein-like [Ostrinia furnacalis]	0.478300594	-1.064010512	0.0006059	down	yes	1.047	2.189	2.274	2.325	1.967	1	1.109	1.033	------	------	------	------	------	------	ENOG41114TE;E	S:Function unknown;	PF06585.14	JHBP	Haemolymph juvenile h	CYT	4	18	27.1	High
TRINITY_DN95558_c0_g3_i1_orf1	-	-	-	cytochrome P450 monooxygenase CYP9G19 [Cnaphalocrocis medinalis]	0.48180562	-1.053476874	0.0002731	down	yes	1.046	2.171	2.295	2.011	2.207	1	1.01	1.128	CC:membrane;CC:er	GO:0016020;GO:0005789;GO:0031090;GO:0005575;GO:0110165;GO:0043169;GO:0004497;GO:0020037;GO:0046914;GO:0046906;GO:0097159;GO:0016705;GO:0043167;GO:0003824;GO:0003674;GO:0005488;GO:0005506;GO:0046872;GO:1901363;GO:0016491	K15003	CYP9	------	------	COG2124	J:Translation, ribosomal structure	PF00067.25	p450	Cytochrome P450	CYT	1	16	8	High
TRINITY_DN130575_c0_g1_i1_orfp1	-	-	-	TRINITY_DN130575_c0_g1_i1_m.77798 TRINITY_DN130575_c0_g1::TRINITY_DN130575_c0_g1_i1::g.77798 ORF type:internal len:70 (+),score=15.12 TRINITY_DN130575_c0_g1_i1:3-209(+)	0.457504604	-1.128141833	0.0001232	down	yes	0.9937	2.172	2.148	2.048	2.321	1	0.982	0.999	------	------	------	------	------	------	------	------	------	------	------	CYT	2	74	6.7	High
TRINITY_DN20658_c0_g2_i3_orf1	-	-	-	prostaglandin reductase 1-like [Ostrinia furnacalis]	0.455939227	-1.133086559	0.001615	down	yes	0.9903	2.172	2.24	2.401	1.876	1	0.968	1.003	CC:cytoplasm;CC:ce	GO:0005737;GO:0005575;GO:0110165;GO:0036132;GO:0016614;GO:0016628;GO:0016616;GO:0016627;GO:0003824;GO:0003674;GO:0047522;GO:0032440;GO:0016491	K13948	PTGR1, LTB4D	map00590	Arachidonic acid metabolism	COG2130	S:Function unknown	PF00107.29;PF1:	ADH_zinc_N;AD	Zinc-binding dehydrog	CYT	7	27	25.7	High
TRINITY_DN9458_c0_g1_i4_orf1	-	-	-	uncharacterized protein LOC114363583 [Ostrinia furnacalis]	0.464135228	-1.107382893	0.0007252	down	yes	0.9473	2.041	2.054	1.846	2.222	1	0.863	0.979	------	------	------	------	------	------	------	------	------	------	------	CYT	1	13	9.3	High
TRINITY_DN7102_c0_g1_i5_orf1	-	-	-	protein wings apart-like [Ostrinia furnacalis]	0.395238095	-1.339206086	0.001094	down	yes	0.8383	2.121	2.236	1.864	2.263	1	0.736	0.779	BP:regulation of chro	GO:0033044;GO:0051726;GO:0033043;GO:0010564;GO:0050794;GO:0065007;GO:0008150;GO:0051128;GO:0050789;GO:0007063	K25163	WAPL	------	------	ENOG410XSDS	S:Function unknown	PF07814.16	WAPL	Wings apart-like protei	CYT	1	1	119.6	Medium
TRINITY_DN703_c0_g1_i2_orf1	-	-	-	acidic juvenile hormone-suppressible protein 1-like [Ostrinia furnacalis]	0.116589753	-3.100487102	6.77E-06	down	yes	0.9853	8.451	8.693	7.964	8.697	1	0.982	0.974	------	------	------	------	------	------	ENOG410XR2D	S:Function unknown	PF03723.17	Hemocyanin_C	Hemocyanin, ig-like do	CYT	6	97	10.1	High
TRINITY_DN4767_c0_g1_i4_orf1	-	-	-	cysteine protease XCP2-like [Ostrinia furnacalis]	0.15457015	-2.693666355	0.0005063	down	yes	0.9673	6.258	6.492	5.274	7.009	1	0.912	0.99	BP:primary metaboli	GO:0044238;GO:1901564;GO:0019538;GO:0043170;GO:0071704;GO:0006807;GO:0008150;GO:0008152;GO:0006508;GO:0003674;GO:0016787;GO:0003824;GO:0140096;GO:0008233	------	------	------	------	ENOG4110R2S;E	S:Function unknown;	PF08246.15	Inhibitor_I29	Cathepsin propeptide ii	CYT	6	38	11.7	High
TRINITY_DN17615_c0_g1_i3_orf1	-	-	-	hypothetical protein SFRUCORN_008858 [Spodoptera frugiperda]	0.218630797	-2.193431457	0.0004898	down	yes	1.089	4.981	4.501	4.73	5.712	1	1.076	1.192	------	------	K20674	MODSP	map04624	Toll and Imd signaling pathw	COG5640	O:Posttranslational modification	------	------	------	CYT	1	20	8.4	High
TRINITY_DN276_c0_g1_i2_orf1	-	-	-	protein lethal(2)essential for life-like [Ostrinia furnacalis] >UTU55753.1 small heat shock protein Hsp20.7 [Ostrinia furnacalis]	0.192705167	-2.375532838	0.0004332	down	yes	0.951	4.935	5.302	4.195	5.307	1	0.991	0.862	MF:cation binding;M	GO:0043169;GO:0046872;GO:0003674;GO:0005488;GO:0043167	K09542	CRYAB	map04213;map04	Longevity regulating pathwa	ENOG410YERS	O:Posttranslational modification	PF00011.24	HSP20	Hsp20/alpha crystallin f	CYT	2	43	20.8	High
TRINITY_DN17574_c0_g1_i2_orf1	-	-	-	heat shock protein Hsp-12.2-like [Ostrinia furnacalis]	0.249860015	-2.000808047	1.81E-05	down	yes	0.9817	3.929	3.965	3.7	4.121	1	0.975	0.97	------	------	K09542	CRYAB	map04213;map04	Longevity regulating pathwa	ENOG410YERS	O:Secondary metabolites biosyr	PF00011.24	HSP20	Hsp20/alpha crystallin f	CYT	7	51	19.4	High
TRINITY_DN71698_c0_g1_i1_orfp1	-	-	-	TRINITY_DN71698_c0_g1_i1_m.1194 TRINITY_DN71698_c0_g1::TRINITY_DN71698_c0_g1_i1::g.1194 ORF type:internal len:134 (+),score=19.66,Toxin_2 PF00451.20 4.3e-05,Toxin_2|PF00451.20|0.037,Toxin_2|PF00451.20|7.5e-05,Gamma-thionin|PF00304.21|0.017,Gamma-thionin|PF00304.21|0.05,Gamma-thionin|PF00304.21|0.021,Toxin_38|PF14866.7|0.13,Toxin_38|PF14866.7|0.15,Toxin_38|PF14866.7|0.15,Defensin_2|PF01097.19|0.053,Defensin_2|PF01097.19|0.34,Defensin_2|PF01097.19|0.092 TRINITY_DN71698_c0_g1_i1:3-401(+)	0.257286303	-1.958553441	0.0008999	down	yes	0.9993	3.884	3.911	3.307	4.434	1	1.024	0.974	------	------	------	------	------	------	------	------	PF00451.22;PF0(	Toxin_2;Gamma	Scorpion short toxin, Br	CYT	6	47	15.5	High
TRINITY_DN10138_c0_g1_i1_orf1	-	-	-	storage protein 1 [Omphisa fuscidentalis]	0.261292876	-1.936260304	0.0001244	down	yes	0.9903	3.79	3.411	4.007	3.953	1	0.979	0.992	------	------	------	------	------	------	ENOG410XR2D	S:Function unknown	PF03723.17	Hemocyanin_C	Hemocyanin, ig-like do	PLA	6	91	10.1	High
TRINITY_DN1093_c0_g1_i4_orf1	-	-	-	uncharacterized protein LOC114361723 isoform X4 [Ostrinia furnacalis]	0.270982628	-1.88372773	4.07E-06	down	yes	0.9983	3.684	3.613	3.833	3.605	1	1.031	0.964	------	------	------	------	------	------	ENOG410XRJB	S:Function unknown	------	------	------	CYT	1	32	26.1	High
TRINITY_DN9079_c0_g1_i5_orf1	-	-	-	UDP-glucuronosyltransferase-like [Ostrinia furnacalis]	0.25740062	-1.957912565	3.53E-05	down	yes	0.913	3.547	3.61	3.719	3.313	1	0.902	0.837	CC:integral compon	GO:0016021;GO:0005575;GO:0110165;GO:0031224;GO:0003824;GO:0016740;GO:0003674;GO:0016757;GO:0016758;GO:0015020;GO:0008194	K00699	UGT	map05207;map05	Chemical carcinogenesis - r	COG1819	S:Function unknown	PF00201.21	UDPGT	UDP-glucoronosyl and	CYT	5	14	48.5	High
TRINITY_DN9090_c0_g1_i9_orf1	-	-	-	CD63 antigen [Ostrinia furnacalis]	0.31443299	-1.669175505	0.0002985	down	yes	0.976	3.104	3.214	2.752	3.347	1	0.95	0.978	CC:cellular_compon	GO:0005575;GO:0016021;GO:0110165;GO:0031224	K06497	CD63, MLA1, T	map05205;map04	Proteoglycans in cancer;Lys	ENOG4111IRY	S:Function unknown	PF00335.23	Tetraspanin	Tetraspanin family	EXC	3	13	25.6	High
TRINITY_DN6423_c0_g1_i6_orf1	-	-	-	phenoloxidase-activating factor 2-like isoform X1 [Ostrinia furnacalis]	0.347898047	-1.523263515	0.000454	down	yes	1.051	3.021	2.926	2.759	3.377	1	1.07	1.084	BP:organic substanc	GO:0071704;GO:1901564;GO:0019538;GO:0043170;GO:0008150;GO:0006807;GO:0008152;GO:0006508;GO:0044238;GO:0004252;GO:0004175;GO:0016787;GO:0017171;GO:0140096;GO:0003824;GO:0003674;GO:0008233;GO:0008236	------	------	------	------	COG5640	O:Posttranslational modification	PF00089.29;PF18	Trypsin;CLIP_1	Trypsin;Serine protease	CYT	3	13	55.7	High
TRINITY_DN10222_c0_g1_i2_orf1	-	-	-	glutathione S-transferase sigma 3 [Ostrinia furnacalis]	0.312685604	-1.677215297	0.001879	down	yes	0.9687	3.098	2.941	3.662	2.69	1	0.971	0.935	MF:catalytic activity;I	GO:0003824;GO:0016740;GO:0004364;GO:0003674;GO:0016765	K04097	HPGDS	map00590;map00	Arachidonic acid metabolism	ENOG41103AU;I	O:Posttranslational modification	PF14497.9;PF02	GST_C_3;GST_N	Glutathione S-transfera	CYT	14	58	22.9	High
TRINITY_DN1048_c0_g1_i6_orf1	-	-	-	uncharacterized protein LOC114360661 [Ostrinia furnacalis]	0.293309971	-1.769501977	0.0009064	down	yes	0.9207	3.139	3.084	2.74	3.593	1	0.898	0.864	------	------	------	------	------	------	ENOG4111BN3;I	S:Function unknown;	PF15430.9	SVWC	Single domain von Will	CYT	4	39	12.1	High
TRINITY_DN5337_c0_g1_i6_orf1	-	-	-	epoxide hydrolase 1-like [Ostrinia furnacalis]	0.337933356	-1.565189336	0.0007138	down	yes	1.004	2.971	3.304	3.025	2.585	1	0.999	1.012	CC:integral compon	GO:0016021;GO:0005575;GO:0031224;GO:0110165;GO:0016787;GO:0003824;GO:0003674	K22368;K22369	EPHX3;EPHX4	map05207;map05	Chemical carcinogenesis - r	ENOG410XRPK;(	S:Function unknown;	PF00561.23;PF12	Abhydrolase_1;	alpha/beta hydrolase fo	CYT	11	39	39.7	High
TRINITY_DN1575_c0_g1_i10_orf1	-	-	-	uncharacterized protein LOC114359245 [Ostrinia furnacalis]	0.304319946	-1.7163392	0.001624	down	yes	0.9017	2.963	3.025	2.493	3.372	1	0.716	0.989	------	------	------	------	------	------	ENOG4111CB2;E	S:Function unknown;	PF15868.8	MBF2	Transcription activator I	CYT	1	2	45.9	High
TRINITY_DN1363_c0_g1_i11_orf1	-	-	-	cytochrome P450 CYP12A2-like isoform X1 [Ostrinia furnacalis] >QPF77619.1 cytochrome P450 monooxygenase CYP333A20 [Ostrinia furnacalis]	0.36637618	-1.448602385	2.27E-05	down	yes	1.009	2.754	2.676	2.679	2.908	1	1.004	1.024	MF:cation binding;M	GO:0043169;GO:0004497;GO:0020037;GO:0046914;GO:0046906;GO:0097159;GO:0016705;GO:0043167;GO:0003824;GO:0003674;GO:0005488;GO:0005506;GO:0046872;GO:1901363;GO:0016491	K15004	CYP12	------	------	COG2124	Q:Secondary metabolites biosyr	PF00067.25	p450	Cytochrome P450	CYT	8	17	57.7	High
TRINITY_DN12775_c0_g1_i10_orfp1	-	-	-	TRINITY_DN12775_c0_g1_i10_m.21238 TRINITY_DN12775_c0_g1::TRINITY_DN12775_c0_g1_i10::g.21238 ORF type:5prime_partial len:67 (-),score=0.74 TRINITY_DN12775_c0_g1_i10:275-475(-)	0.403761505	-1.308424727	0.0005227	down	yes	1.009	2.499	2.755	2.479	2.263	1	1.071	0.957	------	------	------	------	------	------	------	------	------	------	------	CYT	2	14	7.7	High
TRINITY_DN1352_c0_g1_i5_orf1	-	-	-	uncharacterized protein LOC113491815 [Trichoplusia ni]	0.38	-1.395928676	0.001701	down	yes	0.95	2.5	2.443	2.177	2.881	1	0.906	0.944	BP:positive regulatio	GO:0032414;GO:0032411;GO:0032412;GO:0051049;GO:0050804;GO:0043266;GO:0023051;GO:0034764;GO:0034765;GO:0034767;GO:0050789;GO:0034762;GO:0043268;GO:0010646;GO:0048511;GO:0032409;GO:0065007;GO:0043269;GO:0044093;GO:0099177;GO:0048518;GO:0065009;GO:1901381;GO:0032501;GO:0051050;GO:0050794;GO:0043270;GO:0008150;GO:0030431;GO:1903818;GO:2001257;GO:0032879;GO:1901018;GO:0010959;GO:0032222;GO:0022898;GO:1904064;GO:1904062;GO:1901016;GO:2001259;GO:1901379;GO:0048522;GO:0005886;GO:0031224;GO:0016020;GO:0031225;GO:0110165;GO:0005575	------	------	------	------	ENOG410Z7FK	S:Function unknown	PF17064.8	QVR	Sleepless protein	CYT	3	28	16.5	High
TRINITY_DN6108_c0_g1_i5_orf1	-	-	-	myogenesis-regulating glycosidase-like [Ostrinia furnacalis]	0.426772975	-1.228459272	3.63E-05	down	yes	1.017	2.383	2.511	2.282	2.357	1	1.019	1.032	BP:primary metaboli	GO:0044238;GO:0005975;GO:0071704;GO:0008150;GO:0008152;GO:0016787;GO:0003824;GO:0016798;GO:0004553;GO:0003674	K24727	MYORG	------	------	COG1501	E:Amino acid transport and meta	PF01055.29	Glyco_hydro_31	Glycosyl hydrolases fan	CYT	11	20	66.3	High
TRINITY_DN39266_c0_g1_i1_orf1	-	-	-	PREDICTED: NECAP-like protein CG9132 [Microplitis demolitor]	0.424548129	-1.235999979	0.0003916	down	yes	1.01	2.379	2.621	2.206	2.31	1	1.013	1.016	BP:endocytosis;BP:ve	GO:0006897;GO:0016192;GO:0006810;GO:0008150;GO:0051234;GO:0051179;GO:0016020;GO:0005575;GO:0110165	K20069	NECAP1_2	------	------	ENOG41113PS	S:Function unknown	PF07933.17	DUF1681	Protein of unknown fun	CYT	1	6	28.7	High
TRINITY_DN2187_c0_g1_i1_orf1	-	-	-	flotillin-1 [Chelonus insularis] >XP_034947202.1 flotillin-1 [Chelonus insularis]	0.425053079	-1.234285086	0.0003209	down	yes	1.001	2.355	2.406	2.131	2.527	1	1.003	1.001	------	------	K07192	FLOT	map04910	Insulin signaling pathway	COG2268	S:Function unknown	PF01145.28;PF15	Band_7;Flot	SPFH domain / Band 7	CYT	3	10	46.9	High
TRINITY_DN51480_c0_g1_i1_orf1	-	-	-	uncharacterized protein LOC114361588 isoform X14 [Ostrinia furnacalis]	0.463799912	-1.108425548	0.001653	down	yes	1.057	2.279	2.551	2.287	2	1	1.067	1.104	------	------	K10382	DST	------	------	COG5069;ENOG	Z:Cytoskeleton;	PF00681.23	Plectin	Plectin repeat	CYT	3	27	12.6	High
TRINITY_DN585_c0_g1_i12_orf1	-	-	-	very low-density lipoprotein receptor isoform X3 [Galleria mellonella]	0.44591029	-1.165174602	1.16E-05	down	yes	1.014	2.274	2.246	2.36	2.216	1	1.048	0.994	CC:integral compon	GO:0016021;GO:0005575;GO:0031224;GO:0110165;GO:0005488;GO:0043169;GO:0038024;GO:0030229;GO:0030228;GO:0043167;GO:0003674;GO:0046872;GO:0005509	K20053	VLDLR	map05417;map05	Lipid and atherosclerosis;Sp	ENOG410YQ6J	U:Intracellular trafficking, secreti	PF00057.21;PF0(	Ldl_recept_a;Lc	Low-density lipoprotein	PLA	3	31	100.3	High
TRINITY_DN2798_c0_g1_i5_orf1	-	-	-	arylsulfatase B-like isoform X1 [Ostrinia furnacalis]	0.415317287	-1.267714173	3.54E-05	down	yes	0.949	2.285	2.231	2.217	2.406	1	0.915	0.932	MF:hydrolase activity	GO:0016787;GO:0003674;GO:0016788;GO:0008484;GO:0003824	K01135	ARSB	map04142;map00	Lysosome;Glycosaminoglyca	COG3119	S:Function unknown	PF00884.26;PF0:	Sulfatase;Phos	Sulfatase;Type I phosph	CYT	3	7	66.8	High
TRINITY_DN30498_c0_g1_i3_orf1	-	-	-	lipase 3-like [Ostrinia furnacalis]	0.464399093	-1.10656294	0.009364	down	yes	1.024	2.205	2.161	2.634	1.82	1	0.885	1.186	------	------	K01052	LIPA	map04979;map04	Cholesterol metabolism;Lys	COG0596	S:Function unknown	PF00561.23;PF04	Abhydrolase_1;	alpha/beta hydrolase fo	CYT	1	3	56.9	High
TRINITY_DN2076_c0_g2_i1_orf1	-	-	-	macrophage mannose receptor 1-like isoform X1 [Maniola jurtina]	0.485563041	-1.04226948	0.01263	down	yes	1.009	2.078	2.562	1.925	1.747	1	0.976	1.052	------	------	------	------	------	------	ENOG410XRS3;E	U:Intracellular trafficking, secreti	PF00059.24	Lectin_C	Lectin C-type domain	CYT	2	8	35.6	High
TRINITY_DN2461_c0_g1_i5_orf1	-	-	-	secretory phospholipase A2 receptor [Vanessa cardui]	0.496774194	-1.009337865	5.83E-06	down	yes	1.001	2.015	1.957	2.067	2.021	1	1.001	1.001	------	------	------	------	------	------	ENOG410XRS3;E	U:Intracellular trafficking, secreti	PF00059.24	Lectin_C	Lectin C-type domain	CYT	7	30	31.9	High
TRINITY_DN14242_c0_g1_i2_orfp1	-	-	-	TRINITY_DN14242_c0_g1_i2_m.18449 TRINITY_DN14242_c0_g1::TRINITY_DN14242_c0_g1_i2::g.18449 ORF type:internal len:148 (-),score=102.30 TRINITY_DN14242_c0_g1_i2:2-442(-)	0.071658615	-3.802716027	0.001119	down	yes	1.335	18.63	15.959	17.239	22.679	1	1.386	1.618	------	------	------	------	------	------	------	------	------	------	------	CYT	1	8	16	High
TRINITY_DN295_c3_g1_i1_orfp1	-	-	-	TRINITY_DN295_c3_g1_i1_m.18839 TRINITY_DN295_c3_g1::TRINITY_DN295_c3_g1_i1::g.18839 ORF type:5prime_partial len:118 (+),score=46.25 TRINITY_DN295_c3_g1_i1:1-354(+)	0.071132494	-3.813347433	0.0008014	down	yes	0.961	13.51	13.568	11.097	15.86	1	0.975	0.908	------	------	------	------	------	------	------	------	------	------	------	PLA	3	49	12.6	High
TRINITY_DN59885_c0_g1_i3_orf1	-	-	-	TGF-beta-activated kinase 1 and MAP3K7-binding protein 1-like [Ostrinia furnacalis]	0.184920198	-2.435025281	0.0005413	down	yes	1.008	5.451	5.411	4.712	6.231	1	1.065	0.959	BP:dephosphorylatio	GO:0016311;GO:0006470;GO:0006807;GO:0071704;GO:0009987;GO:0043412;GO:0036211;GO:0008150;GO:0008152;GO:0044238;GO:1901564;GO:0019538;GO:0044237;GO:0043170;GO:0006796;GO:0006793;GO:0016791;GO:0042578;GO:0003674;GO:0004721;GO:0016787;GO:0140096;GO:0016788;GO:0003824;GO:0004722	K04403	MAP3K7IP1, T/	map05140;map05	Leishmaniasis;Human immu	COG0631	S:Function unknown	PF00481.24	PP2C	Protein phosphatase 2C	CYT	1	3	51	High
TRINITY_DN7900_c0_g1_i4_orf1	-	-	-	uncharacterized protein LOC114366119 [Ostrinia furnacalis]	0.170595122	-2.551351701	0.001116	down	yes	0.8743	5.125	5.919	5.255	4.201	1	0.92	0.703	------	------	K20701	PIRK	map04624	Toll and Imd signaling pathw	ENOG410Y5Y5	S:Function unknown	------	------	------	CYT	1	9	13.5	Medium
TRINITY_DN24723_c2_g1_i1_orf1	-	-	-	hypothetical protein evm_001103 [Chilo suppressalis]	0.249823902	-2.001016579	0.0002746	down	yes	1.064	4.259	4.555	3.785	4.438	1	0.905	1.288	BP:small molecule bi	GO:0044283;GO:0019752;GO:0044249;GO:0034641;GO:0006807;GO:0044281;GO:0071941;GO:0009064;GO:1901576;GO:0006520;GO:0006526;GO:0006525;GO:0071704;GO:0006082;GO:0008652;GO:1901605;GO:1901607;GO:0019627;GO:0044238;GO:0009987;GO:0009058;GO:0008150;GO:0008152;GO:0043436;GO:0044271;GO:0043604;GO:1901564;GO:1901566;GO:0043603;GO:0046394;GO:0000050;GO:0016053;GO:0044237;GO:0009084;GO:0004055;GO:0035639;GO:0003674;GO:0043167;GO:1901363;GO:1901265;GO:0043168;GO:0000166;GO:0017076;GO:0036094;GO:0003824;GO:0016874;GO:0016879;GO:0030554;GO:0097367;GO:0032559;GO:0005524;GO:0097159;GO:0032555;GO:0032553;GO:0005488	K01940	argG, ASS1	map00220;map00	Arginine biosynthesis;Alanin	COG0137	E:Amino acid transport and meta	PF00764.22	Arginosuc_synt	Arginosuccinate syntha	CYT	1	9	11.6	High
TRINITY_DN48548_c0_g1_i1_orf1	-	-	-	glutathione S-transferase sigma 2 [Heortia vitessoides]	0.249499101	-2.002893478	0.01194	down	yes	0.9713	3.893	3.156	5.226	3.296	1	0.942	0.972	MF:catalytic activity;I	GO:0003824;GO:0016740;GO:0004364;GO:0003674;GO:0016765	K04097	HPGDS	map00590;map00	Arachidonic acid metabolism	ENOG41103AU;I	O:Posttranslational modification	PF14497.9;PF02	GST_C_3;GST_N	Glutathione S-transfera	CYT	18	60	23.2	High
TRINITY_DN5406_c0_g2_i1_orf1	-	-	-	uncharacterized protein LOC114350326 [Ostrinia furnacalis]	0.312362031	-1.678708997	0.0002127	down	yes	1.132	3.624	3.927	3.624	3.321	1	1.108	1.289	BP:positive regulatio	GO:0032414;GO:0032411;GO:0032412;GO:0051049;GO:0050804;GO:0043266;GO:0023051;GO:0034764;GO:0034765;GO:0034767;GO:0050789;GO:0034762;GO:0043268;GO:0010646;GO:0048511;GO:0032409;GO:0065007;GO:0043269;GO:0044093;GO:0099177;GO:0048518;GO:0065009;GO:1901381;GO:0032501;GO:0051050;GO:0050794;GO:0043270;GO:0008150;GO:0030431;GO:1903818;GO:2001257;GO:0032879;GO:1901018;GO:0010959;GO:0032222;GO:0022898;GO:1904064;GO:1904062;GO:1901016;GO:2001259;GO:1901379;GO:0048522;GO:0005886;GO:0031224;GO:0016020;GO:0031225;GO:0110165;GO:0005575	------	------	------	------	ENOG410ZEPJ;E	S:Function unknown;	PF17064.8	QVR	Sleepless protein	CYT	1	9	15.6	High
TRINITY_DN19731_c0_g1_i1_orf1	-	-	-	allergen Tha p 1-like [Ostrinia furnacalis] >XP_028174916.1 allergen Tha p 1-like [Ostrinia furnacalis] >BAV56808.1 chemosensory protein 4 [Ostrinia furnacalis]	0.291523482	-1.778315998	0.0002712	down	yes	1.018	3.492	3.473	3.147	3.856	1	1.02	1.035	------	------	------	------	------	------	ENOG4111U5H;I	S:Function unknown;	PF03392.16	OS-D	Insect pheromone-binc	CYT	3	23	14.9	High
TRINITY_DN3821_c1_g1_i7_orf1	-	-	-	mitochondrial carrier protein Rim2 isoform X1 [Ostrinia furnacalis]	0.315261673	-1.665378303	0.000434	down	yes	1.006	3.191	3.03	2.954	3.588	1	0.936	1.083	BP:cellular process;B	GO:0009987;GO:0046907;GO:0006810;GO:0055085;GO:0008150;GO:0051649;GO:0006839;GO:0051234;GO:0051179;GO:0051641;GO:0016020;GO:0031090;GO:0016021;GO:0005575;GO:0110165;GO:0031966;GO:0031224	K15116	SLC25A33_36,	------	------	ENOG410XS20	S:Function unknown	PF00153.30	Mito_carr	Mitochondrial carrier pr	CYT	1	4	40.2	High
TRINITY_DN4068_c0_g2_i4_orf1	-	-	-	larval cuticle protein LCP-17-like precursor [Papilio polytes] >BAM18876.1 cuticular protein PpolCPR2 [Papilio polytes]	0.324220006	-1.624954978	0.0004844	down	yes	1.008	3.109	3.27	2.708	3.348	1	0.988	1.037	MF:molecular_functi	GO:0003674;GO:0042302;GO:0005198	------	------	------	------	ENOG4110RUZ;I	S:Function unknown;	PF00379.26	Chitin_bind_4	Insect cuticle protein	CYT	2	56	13.4	High
TRINITY_DN3301_c0_g1_i2_orf1	-	-	-	hemicentin-2-like isoform X1 [Ostrinia furnacalis]	0.307689843	-1.700451275	0.0001221	down	yes	0.9603	3.121	2.854	3.352	3.156	1	0.943	0.938	MF:hydrolase activity	GO:0016787;GO:0043169;GO:0097159;GO:0043167;GO:0003824;GO:0003674;GO:0005488;GO:0003676;GO:0046872;GO:1901363	------	------	------	------	ENOG410ZN3C;	S:Function unknown;	PF07679.19;PF13	I-set;Ig_3;Ig_2;I	Immunoglobulin I-set c	CYT	3	3	129.9	High
TRINITY_DN61042_c0_g2_i2_orfp1	-	-	-	TRINITY_DN61042_c0_g2_i2_m.5292 TRINITY_DN61042_c0_g2::TRINITY_DN61042_c0_g2_i2::g.5292 ORF type:5prime_partial len:63 (-),score=21.86 TRINITY_DN61042_c0_g2_i2:77-265(-)	0.483858859	-1.047341819	0.001557	down	yes	1.289	2.664	2.818	2.463	2.71	1	1.457	1.411	------	------	------	------	------	------	------	------	------	------	------	CYT	1	10	7.1	Medium
TRINITY_DN6098_c1_g1_i5_orf1	-	-	-	unnamed protein product, partial [Iphiclides podalirius]	0.340230665	-1.555414918	0.001229	down	yes	1.003	2.948	2.962	2.529	3.354	1	1.023	0.985	BP:immune system p	GO:0002376;GO:0043207;GO:0051707;GO:0044419;GO:0045087;GO:0009987;GO:0006952;GO:0006950;GO:0098542;GO:0006955;GO:0001906;GO:0050832;GO:0009607;GO:0009605;GO:0009620;GO:0050896;GO:0031640;GO:0008150;GO:0005615;GO:0110165;GO:0005575;GO:0005576;GO:0099106;GO:0003674;GO:0098772;GO:0019871;GO:0016248;GO:0016247;GO:0008200;GO:0017080	------	------	------	------	------	------	------	------	------	CYT	2	28	7.7	High
TRINITY_DN9239_c0_g1_i1_orf1	-	-	-	apolipophorins-like [Ostrinia furnacalis]	0.323551213	-1.627934009	0.0005556	down	yes	0.9603	2.968	3.226	3.1	2.577	1	0.917	0.964	BP:lipid transport;BP	GO:0006869;GO:0006810;GO:0071702;GO:0008150;GO:0051234;GO:0051179;GO:0005575;GO:0005576;GO:0110165;GO:0005319;GO:0005215;GO:0008289;GO:0003674;GO:0005488	------	------	------	------	ENOG410Z6MR;	S:Function unknown;	------	------	------	CYT	10	78	18	High
TRINITY_DN18323_c0_g1_i5_orf1	-	-	-	GILT-like protein 1 isoform X1 [Ostrinia furnacalis]	0.350548673	-1.512313323	0.0006531	down	yes	0.9903	2.825	2.82	2.497	3.157	1	0.996	0.975	------	------	K08059	IFI30, GILT	map04612	Antigen processing and pres	ENOG4111IDT	U:Intracellular trafficking, secreti	PF03227.19	GILT	Gamma interferon indu	CYT	15	62	25	High
TRINITY_DN64141_c0_g1_i4_orf1	-	-	-	probable salivary secreted peptide [Ostrinia furnacalis]	0.366810655	-1.446892548	0.01773	down	yes	1.019	2.778	2.078	3.623	2.632	1	1.056	1.002	------	------	------	------	------	------	ENOG410ZS6V;E	S:Function unknown;	PF15868.8	MBF2	Transcription activator I	CYT	3	33	13.3	High
TRINITY_DN11981_c0_g1_i7_orf1	-	-	-	luciferin 4-monooxygenase-like isoform X2 [Ostrinia furnacalis]	0.337331918	-1.567759264	0.0001122	down	yes	0.9533	2.826	2.977	2.588	2.913	1	0.898	0.962	------	------	K01904	4CL	map00130	Ubiquinone and other terpe	COG0365;COG0:	I:Lipid transport and metabolism	PF00501.31;PF13	AMP-binding;A	AMP-binding enzyme;A	CYT	3	7	62.4	High
TRINITY_DN699_c0_g2_i1_orf1	-	-	-	TPA_exp: putative parasitoid killing factor [Trichoplusia ni]	0.375	-1.415037499	4.85E-05	down	yes	1.014	2.704	2.695	2.552	2.864	1	1.014	1.028	------	------	------	------	------	------	------	------	------	------	------	CYT	1	3	43.9	High
TRINITY_DN14185_c0_g1_i1_orf1	-	-	-	uncharacterized protein LOC114358675 [Ostrinia furnacalis] >XP_028168498.1 uncharacterized protein LOC114358675 [Ostrinia furnacalis]	0.406023637	-1.300364377	0.0003685	down	yes	1.065	2.623	2.565	2.423	2.881	1	1.076	1.119	------	------	------	------	------	------	------	------	------	------	------	CYT	2	19	18.4	High
TRINITY_DN60946_c0_g2_i3_orf1	-	-	-	protein yellow-like [Ostrinia furnacalis]	0.369073316	-1.438020661	1.30E-05	down	yes	0.9917	2.687	2.638	2.608	2.816	1	0.981	0.994	------	------	------	------	------	------	COG3386	S:Function unknown	PF03022.19	MRJP	Major royal jelly proteir	CYT	13	36	49.2	High
TRINITY_DN52944_c0_g1_i1_orf1	-	-	-	apolipophorins-like [Ostrinia furnacalis]	0.414772727	-1.26960706	2.97E-05	down	yes	1.022	2.464	2.444	2.576	2.371	1	0.98	1.086	MF:lipid transporter	GO:0005319;GO:0005215;GO:0003674	------	------	------	------	ENOG410ZRUY;I	S:Function unknown;	PF01347.25	Vitellogenin_N	Lipoprotein amino term	CYT	5	59	11	High
TRINITY_DN122321_c0_g1_i1_orf1	-	-	-	uncharacterized protein LOC114356271 [Ostrinia furnacalis]	0.427429533	-1.226241501	0.003364	down	yes	1.016	2.377	2.681	2.47	1.981	1	1.141	0.906	CC:cellular_compon	GO:0005575;GO:0110165;GO:0005615;GO:0004866;GO:0061135;GO:0061134;GO:0003674;GO:0030414;GO:0004867;GO:0004857;GO:0098772;GO:0030234	------	------	------	------	COG4826	J:Translation, ribosomal structure	PF00079.23	Serpin	Serpin	CYT	1	32	7.6	High
TRINITY_DN9435_c0_g1_i7_orf1	-	-	-	uncharacterized protein LOC114350197 [Ostrinia furnacalis]	0.4056	-1.301870443	0.00123	down	yes	0.9633	2.375	2.164	2.245	2.716	1	0.941	0.949	------	------	K02437	gcvH, GCSH	map00630;map00	Glyoxylate and dicarboxylate	COG0509	E:Amino acid transport and meta	PF01597.22	GCV_H	Glycine cleavage H-prc	CYT	5	34	16.8	High
TRINITY_DN5099_c0_g1_i3_orf1	-	-	-	trans-1,2-dihydrobenzene-1,2-diol dehydrogenase-like [Ostrinia furnacalis]	0.446563193	-1.16306375	7.69E-06	down	yes	1.007	2.255	2.325	2.18	2.259	1	1.01	1.011	MF:organic cyclic co	GO:0097159;GO:0000166;GO:0036094;GO:0003674;GO:0005488;GO:1901363;GO:1901265	K00078	DHDH	map00040;map00	Pentose and glucuronate int	COG0673	S:Function unknown	PF01408.25;PF02	GFO_IDH_Moc	Oxidoreductase family,	CYT	17	55	38.1	High
TRINITY_DN960_c1_g1_i6_orf1	-	-	-	hypothetical protein evm_007130 [Chilo suppressalis]	0.462068966	-1.1138199	0.000252	down	yes	1.005	2.175	2.231	1.995	2.3	1	1.048	0.968	BP:transport;BP:intra	GO:0006810;GO:0046907;GO:0015918;GO:0006869;GO:0032366;GO:0032367;GO:0032365;GO:0015850;GO:0030301;GO:0008150;GO:0071702;GO:0009987;GO:0051234;GO:0051179;GO:0051649;GO:0051641;GO:0005575;GO:0005576;GO:0110165	K13443	NPC2	map04979;map04	Cholesterol metabolism;Lys	ENOG4111Q8S	S:Function unknown	PF02221.18	E1_DerP2_DerF	ML domain	PLA	5	38	15.5	High
TRINITY_DN364_c1_g1_i2_orf1	-	-	-	talin-2-like, partial [Ostrinia furnacalis]	0.491691105	-1.02417584	0.000271	down	yes	1.006	2.046	2.17	1.885	2.083	1	1.04	0.979	CC:cytoskeleton;CC:	GO:0005856;GO:0005575;GO:0001726;GO:0005925;GO:0043232;GO:0030054;GO:0070161;GO:0043229;GO:0043228;GO:0120025;GO:0042995;GO:0043226;GO:0110165;GO:0030055;GO:0005200;GO:0008092;GO:0005198;GO:0003779;GO:0003674;GO:0005488;GO:0005515	K06271	TLN	map05166;map04	Human T-cell leukemia virus	ENOG410XQ0V;	S:Function unknown;	PF09141.13	Talin_middle	Talin, middle domain	CYT	7	9	81.6	High
TRINITY_DN1093_c0_g1_i6_orf1	-	-	-	uncharacterized protein LOC114361723 isoform X4 [Ostrinia furnacalis]	0.495088409	-1.014241923	0.00106	down	yes	1.008	2.036	2.219	2.082	1.808	1	1.028	0.997	------	------	------	------	------	------	ENOG410XRJB	S:Function unknown	PF16061.8	DUF4803	Domain of unknown fu	CYT	4	20	47.1	High

TRINITY_DN5080_c0_g1_i1_orf1	-	-	-	basic juvenile hormone-suppressible protein 2-like [Ostrinia furnacalis]	0.185089974	-2.433701344	0.0008585	down	yes	1.008	5.446	5.219	6.394	4.724	1	1.007	1.018	------	------	------	------	------	------	ENOG410XR2D	S:Function unknown	PF00372.22;PF03	Hemocyanin_M	Hemocyanin, copper cc	CYT	46	75	89.5	High
TRINITY_DN85290_c0_g2_i1_orf1	-	-	-	unnamed protein product, partial [Brenthis ino]	0.229890417	-2.120981764	6.79E-05	down	yes	0.986	4.289	3.913	4.42	4.533	1	1.025	0.933	------	------	K23615;K23420	SPIK5;SPINK5	------	------	ENOG4111TR7;C	O:Posttranslational modification	PF00050.24;PF07	Kazal_1;Kazal_2	Kazal-type serine prote	CYT	1	56	7.6	High
TRINITY_DN45037_c0_g1_i1_orf1	-	-	-	trafficking protein particle complex subunit 1 [Ostrinia furnacalis]	0.202717515	-2.302457347	3.56E-05	down	yes	0.8877	4.379	4.667	4.32	4.151	1	0.726	0.937	BP:ER to Golgi vesicl	GO:0006888;GO:0051179;GO:0051641;GO:0006810;GO:0048193;GO:0009987;GO:0008150;GO:0051649;GO:0051234;GO:0016192;GO:0046907;GO:0005783;GO:0043229;GO:0099023;GO:0043227;GO:0043226;GO:0110165;GO:0005794;GO:0030008;GO:0043231;GO:0032991;GO:0005575	K20300	TRAPPC1, BET	------	------	ENOG4111X41	U:Intracellular trafficking, secreti	PF04099.15;PF04	Sybindin;Sedlin	Sybindin-like family;Sec	CYT	1	9	16.8	High
TRINITY_DN15578_c0_g2_i1_orfp1	-	-	-	uncharacterized protein LOC125235519 [Leguminivora glycinivorella]	0.301381215	-1.730338596	0.0001393	down	yes	1.091	3.62	3.919	3.397	3.544	1	1.012	1.26	------	------	------	------	------	------	------	------	------	------	------	CYT	1	9	10.9	High
TRINITY_DN8595_c0_g1_i3_orf1	-	-	-	aldose reductase-like isoform X4 [Trichoplusia ni]	0.277250503	-1.850738016	5.18E-06	down	yes	0.964	3.477	3.458	3.615	3.359	1	0.951	0.941	MF:catalytic activity;I	GO:0003824;GO:0003674;GO:0016491	K00011	AKR1B	map00051;map00	Fructose and mannose meta	COG0656	S:Function unknown	PF00248.24	Aldo_ket_red	Aldo/keto reductase fa	CYT	1	67	13.9	High
TRINITY_DN129863_c0_g1_i1_orf1	-	-	-	protein PFC0760c-like isoform X2 [Ostrinia furnacalis]	0.329178651	-1.603057321	0.01424	down	yes	1.03	3.129	2.69	2.561	4.135	1	0.995	1.094	------	------	------	------	------	------	------	------	------	------	------	CYT	1	21	19.3	High
TRINITY_DN5439_c0_g1_i2_orf1	-	-	-	uncharacterized protein LOC114353087 [Ostrinia furnacalis]	0.342042755	-1.547751422	5.54E-07	down	yes	1.008	2.947	2.901	3.012	2.929	1	1.004	1.019	CC:membrane;CC:er	GO:0016020;GO:0005789;GO:0031090;GO:0005575;GO:0110165;GO:0043169;GO:0004497;GO:0020037;GO:0046914;GO:0046906;GO:0097159;GO:0016705;GO:0043167;GO:0003824;GO:0003674;GO:0005488;GO:0005506;GO:0046872;GO:1901363;GO:0016491	K15003	CYP9	------	------	COG2124	S:Function unknown	PF00067.25	p450	Cytochrome P450	CYT	16	34	61.8	High
TRINITY_DN9044_c0_g1_i2_orf1	-	-	-	unnamed protein product [Euphydryas editha]	0.36294504	-1.462176995	0.000142	down	yes	1.05	2.893	2.961	2.658	3.059	1	1.005	1.144	BP:immune system p	GO:0002376;GO:0043207;GO:0044419;GO:0071704;GO:0045087;GO:0006952;GO:0006950;GO:0008150;GO:0008152;GO:0006955;GO:0005975;GO:0051707;GO:0044238;GO:0009607;GO:0009605;GO:0050896;GO:0098542;GO:0110165;GO:0005575;GO:0005576;GO:0030246;GO:0003674;GO:0005488;GO:0016787;GO:0016798;GO:0003824;GO:0004553	K20692	GNBP3	map04624	Toll and Imd signaling pathw	ENOG410Z83Q;I	S:Function unknown;	PF15886.8	CBM39	Carbohydrate binding c	CYT	1	10	11.4	High
TRINITY_DN33272_c0_g1_i5_orf1	-	-	-	Low-density lipoprotein receptor-related protein 1 [Papilio xuthus]	0.323183508	-1.629574516	0.01363	down	yes	0.9563	2.959	2.337	2.648	3.892	1	0.892	0.977	BP:endocytosis;BP:ve	GO:0006897;GO:0016192;GO:0006810;GO:0008150;GO:0051234;GO:0051179;GO:0016021;GO:0005575;GO:0031224;GO:0110165;GO:0005488;GO:0043169;GO:0046872;GO:0043167;GO:0003674;GO:0005509	K04550;K24498	LRP1, CD91;SC	map05010;map04	Alzheimer disease;Cholester	ENOG410XP34	U:Intracellular trafficking, secreti	PF00057.21	Ldl_recept_a	Low-density lipoprotei	CYT	1	13	9.2	High
TRINITY_DN40439_c0_g1_i5_orf1	-	-	-	ommochrome binding protein 1, partial [Ostrinia nubilalis]	0.394174041	-1.343095325	0.009457	down	yes	1.069	2.712	2.962	3.152	2.023	1	1.07	1.137	------	------	------	------	------	------	ENOG4110SZS;E	S:Function unknown;	------	------	------	CYT	1	8	15.4	High
TRINITY_DN5655_c0_g1_i2_orf1	-	-	-	uncharacterized protein LOC114359603 [Ostrinia furnacalis]	0.419653179	-1.252730585	0.0001571	down	yes	1.089	2.595	2.702	2.398	2.686	1	1.147	1.121	------	------	------	------	------	------	ENOG4111C52	S:Function unknown	------	------	------	CYT	1	8	10.8	High
TRINITY_DN975_c0_g1_i1_orf1	-	-	-	elongation factor 1-alpha 1 [Myotis lucifugus] >XP_008139741.1 elongation factor 1-alpha 1 [Eptesicus fuscus]	0.471996753	-1.083151159	0.04256	down	yes	1.163	2.464	2.052	2.005	3.335	1	1.251	1.237	BP:regulation of pho	GO:0019220;GO:0080090;GO:0019222;GO:0043043;GO:0051716;GO:0070849;GO:0070848;GO:0044419;GO:0044794;GO:0060255;GO:0051702;GO:0010033;GO:0042325;GO:0044788;GO:0009894;GO:0034645;GO:0050789;GO:1901576;GO:0044260;GO:0043549;GO:0009719;GO:0065007;GO:0065009;GO:0050790;GO:0050794;GO:0008150;GO:0008152;GO:0034641;GO:0044271;GO:0050896;GO:0051338;GO:1904714;GO:0051246;GO:0044249;GO:0042176;GO:0070887;GO:1901566;GO:1900022;GO:0051851;GO:0071495;GO:0044829;GO:0009987;GO:0044827;GO:0043604;GO:0071364;GO:0071363;GO:0043603;GO:0043170;GO:0006807;GO:0019538;GO:0031329;GO:0031323;GO:1901564;GO:0071704;GO:0071310;GO:0010506;GO:0051174;GO:0009058;GO:0009059;GO:0051171;GO:0042221;GO:0006518;GO:0044238;GO:0044237;GO:0044403;GO:0006414;GO:0006412;GO:0098574;GO:0031974;GO:0031983;GO:0031982;GO:0016020;GO:0034774;GO:0043230;GO:0043231;GO:0043232;GO:0043233;GO:0005829;GO:0065010;GO:0098590;GO:0043229;GO:0043228;GO:0043227;GO:0005856;GO:0005853;GO:0110165;GO:0031256;GO:0031253;GO:0030863;GO:0030864;GO:1904813;GO:0060205;GO:0005737;GO:0005730;GO:0005634;GO:0032587;GO:0098552;GO:0005615;GO:0043226;GO:0098562;GO:0005886;GO:1903561;GO:0070062;GO:0032991;GO:0005575;GO:0070013;GO:0005576;GO:1901363;GO:0000166;GO:0003924;GO:0019901;GO:0019900;GO:0045182;GO:0016818;GO:0090079;GO:0097367;GO:0016817;GO:0003674;GO:0005488;GO:0003676;GO:1901265;GO:0017076;GO:0005525;GO:0016787;GO:0003824;GO:0036094;GO:0097159;GO:0019001;GO:0032555;GO:0003746;GO:0032553;GO:0035639;GO:0019899;GO:0000049;GO:0043167;GO:0032561;GO:0005516;GO:0003723;GO:0005515;GO:0016462;GO:0008135;GO:0017111;GO:0043168 [illegible]	K03231	EEF1A	map05140;map05	Leishmaniasis;Legionellosis;I	COG5256	S:Function unknown	PF00009.30;PF03	GTP_EFTU;GTP_	Elongation factor Tu GT	CYT	2	20	50.1	High
TRINITY_DN4998_c0_g1_i21_orf1	-	-	-	phenoloxidase subunit 2-like [Ostrinia furnacalis]	0.387480799	-1.367803274	6.02E-06	down	yes	1.009	2.604	2.623	2.509	2.68	1	1.007	1.019	CC:cellular_compone	GO:0005575;GO:0005576;GO:0110165;GO:0043169;GO:0004497;GO:0016705;GO:0004503;GO:0016716;GO:0043167;GO:0003824;GO:0003674;GO:0005488;GO:0046872;GO:0016491	------	------	------	------	ENOG4111KTW	S:Function unknown	PF03723.17;PF0(	Hemocyanin_C	Hemocyanin, ig-like do	PLA	16	38	80	High
TRINITY_DN71699_c0_g1_i1_orf1	-	-	-	apolipophorins-like [Ostrinia furnacalis]	0.41109761	-1.282447109	3.43E-06	down	yes	1.015	2.469	2.454	2.411	2.543	1	1.033	1.013	MF:lipid transporter	GO:0005319;GO:0005215;GO:0003674	------	------	------	------	ENOG410Z6MR;	S:Function unknown;	------	------	------	CYT	6	63	8.2	High
TRINITY_DN1423_c0_g1_i8_orf1	-	-	-	ferritin subunit-like [Ostrinia furnacalis] >XP_028168186.1 ferritin subunit-like [Ostrinia furnacalis]	0.402270884	-1.313760774	5.63E-05	down	yes	0.992	2.466	2.63	2.379	2.39	1	0.99	0.986	BP:ion homeostasis;I	GO:0050801;GO:0042592;GO:0055072;GO:0055076;GO:0098771;GO:0046916;GO:0000041;GO:0065007;GO:0065008;GO:0019725;GO:0030001;GO:0006810;GO:0006879;GO:0006875;GO:0006812;GO:0006811;GO:0009987;GO:0006873;GO:0008150;GO:0030003;GO:0055065;GO:0055080;GO:0055082;GO:0051234;GO:0051179;GO:0048878;GO:0006826;GO:0003674;GO:0005488;GO:0016491;GO:0043169;GO:0016722;GO:0046914;GO:0004322;GO:0043167;GO:0005506;GO:0008199;GO:0016724;GO:0046872;GO:0003824	K00522	FTH1	map04978;map04	Mineral absorption;Necropto	ENOG4111MSG	P:Inorganic ion transport and me	PF00210.27	Ferritin	Ferritin-like domain	CYT	6	34	23.7	High
TRINITY_DN17693_c0_g1_i10_orf1	-	-	-	acetylcholinesterase-like [Ostrinia furnacalis]	0.422305764	-1.243640155	0.0008388	down	yes	1.011	2.394	2.561	2.088	2.534	1	1.017	1.016	BP:neurotransmitter	GO:0042133;GO:0042135;GO:0044248;GO:0065007;GO:0065008;GO:0009987;GO:0008150;GO:0008152;GO:0009056;GO:0044237;GO:0001505;GO:0045202;GO:0030054;GO:0110165;GO:0070161;GO:0005575;GO:0003674;GO:0016787;GO:0004104;GO:0016788;GO:0003824;GO:0052689;GO:0003990	K01050;K01049	BCHE;ACHE	map00564;map04	Glycerophospholipid metab	COG2272	U:Intracellular trafficking, secreti	PF00135.31;PF2(	COesterase;BD	Carboxylesterase family	CYT	1	2	74	High
TRINITY_DN798_c1_g1_i3_orf1	-	-	-	protein goliath isoform X1 [Ostrinia furnacalis] >XP_028170876.1 protein goliath isoform X1 [Ostrinia furnacalis]	0.475774771	-1.071649324	0.001984	down	yes	1.09	2.291	2.105	2.161	2.607	1	1.18	1.089	CC:integral compone	GO:0016021;GO:0005575;GO:0031224;GO:0110165;GO:0043169;GO:0043167;GO:0003674;GO:0046872;GO:0005488	------	------	------	------	ENOG41121N2	O:Posttranslational modification	PF13639.9;PF171	zf-RING_2;zf-F	Ring finger domain;RIN	CYT	1	3	47	High
TRINITY_DN4245_c0_g2_i1_orf1	-	-	-	long-chain fatty acid transport protein 4-like [Ostrinia furnacalis]	0.416715789	-1.26286433	1.62E-05	down	yes	0.9897	2.375	2.485	2.323	2.317	1	0.966	1.003	------	------	K08745	SLC27A1_4, FA	map04931;map03	Insulin resistance;PPAR signa	ENOG410XQ8T	I:Lipid transport and metabolism	PF00501.31	AMP-binding	AMP-binding enzyme	CYT	2	21	19.7	High
TRINITY_DN399_c3_g2_i6_orf1	-	-	-	proline-rich extensin-like protein EPR1 [Ostrinia furnacalis]	0.415368242	-1.26753718	0.0007841	down	yes	0.9757	2.349	2.318	2.109	2.621	1	0.94	0.987	CC:cellular_compone	GO:0005575;GO:0005576;GO:0110165;GO:0004866;GO:0061135;GO:0061134;GO:0003674;GO:0030414;GO:0004867;GO:0004857;GO:0098772;GO:0030234	------	------	------	------	ENOG410ZDQ9;	S:Function unknown;	PF00050.24	Kazal_1	Kazal-type serine prote	PLA	3	33	18.8	High
TRINITY_DN801_c0_g1_i2_orf1	-	-	-	cathepsin L [Ostrinia furnacalis] >XP_028165920.1 cathepsin L [Ostrinia furnacalis] >UKI61015.1 cathepsin L [Ostrinia furnacalis]	0.463475012	-1.109436537	0.0006245	down	yes	0.9923	2.141	2.084	1.973	2.367	1	0.966	1.011	BP:primary metaboli	GO:0044238;GO:1901564;GO:0019538;GO:0043170;GO:0071704;GO:0006807;GO:0008150;GO:0008152;GO:0006508;GO:0003674;GO:0016787;GO:0003824;GO:0140096;GO:0008233;GO:0008234	K01365	CTSL	map05205;map05	Proteoglycans in cancer;Rhe	COG4870	S:Function unknown	PF00112.26;PF08	Peptidase_C1;I	Papain family cysteine p	CYT	16	47	38.1	High
TRINITY_DN428_c0_g1_i8_orf1	-	-	-	phenoloxidase-activating factor 2-like isoform X1 [Ostrinia furnacalis]	0.482202183	-1.052289912	0.0001785	down	yes	1.016	2.107	2.128	1.963	2.229	1	0.984	1.064	BP:organic substanc	GO:0071704;GO:1901564;GO:0019538;GO:0043170;GO:0008150;GO:0006807;GO:0008152;GO:0006508;GO:0044238;GO:0004252;GO:0004175;GO:0016787;GO:0017171;GO:0140096;GO:0003824;GO:0003674;GO:0008233;GO:0008236	------	------	------	------	COG5640	O:Posttranslational modification	PF00089.29	Trypsin	Trypsin	CYT	4	13	46.8	High
TRINITY_DN142657_c0_g1_i1_orf1	-	-	-	sorting and assembly machinery component 50 homolog [Diachasma alloeum]	0.145296671	-2.782926441	0.0008029	down	yes	1.004	6.91	8.167	6.551	6.011	1	1.007	1.006	CC:cellular_compone	GO:0005575;GO:0016020;GO:0019867;GO:0110165	K07277	SAM50, TOB55	------	------	COG4775	M:Cell wall/membrane/envelope	PF01103.26	Omp85	Omp85 superfamily do	CYT	1	2	53.8	High
TRINITY_DN6330_c0_g1_i1_orfp1	-	-	-	TRINITY_DN6330_c0_g1_i1_m.42332 TRINITY_DN6330_c0_g1::TRINITY_DN6330_c0_g1_i1::g.42332 ORF type:3prime_partial len:51 (-),score=33.30 TRINITY_DN6330_c0_g1_i1:3-152(-)	0.233862859	-2.096265334	0.000227	down	yes	1.163	4.973	5.385	4.417	5.118	1	1.304	1.186	------	------	------	------	------	------	------	------	------	------	------	CYT	1	26	5.3	High
TRINITY_DN9538_c1_g3_i1_orf1	-	-	-	cilia- and flagella-associated protein 410 isoform X2 [Aphidius gifuensis]	0.310795125	-1.685964221	0.0002969	down	yes	1.071	3.446	3.689	3.056	3.593	1	1.065	1.148	------	------	K23456	CFAP410, C21(	------	------	ENOG410Y9AE	S:Function unknown	PF14580.9;PF127	LRR_9;LRR_4	Leucine-rich repeat;Leu	CYT	1	2	47.7	Medium
TRINITY_DN1534_c0_g1_i3_orf1	-	-	-	peptidoglycan recognition protein-like [Ostrinia furnacalis]	0.318432333	-1.650941263	2.26E-05	down	yes	1.04	3.266	3.301	3.095	3.401	1	1.002	1.119	BP:glycosaminoglyca	GO:0030203;GO:0006807;GO:0009253;GO:0043170;GO:1901575;GO:0043207;GO:0009056;GO:0044419;GO:0071704;GO:1901135;GO:0045087;GO:0000270;GO:0006952;GO:0006950;GO:0008150;GO:0008152;GO:0006955;GO:1901564;GO:0051707;GO:0009057;GO:0009607;GO:1901565;GO:0009605;GO:1901136;GO:0050896;GO:0006027;GO:0002376;GO:0006022;GO:0098542;GO:0006026;GO:0042834;GO:0008745;GO:0003674;GO:0005488;GO:0005539;GO:0043167;GO:0046872;GO:0016787;GO:0043169;GO:0046914;GO:0003824;GO:0061783;GO:0097367;GO:0016811;GO:0016810;GO:0008270	K24096;K01446	PGRP-SB;PGRI	map04624	Toll and Imd signaling pathw	ENOG4111PAY;E	O:Posttranslational modification	PF01510.28	Amidase_2	N-acetylmuramoyl-L-a	CYT	2	23	11.1	High
TRINITY_DN276_c0_g1_i1_orf1	-	-	-	protein lethal(2)essential for life-like [Helicoverpa zea] >XP_049705426.1 protein lethal(2)essential for life [Helicoverpa armigera] >ATB54993.1 heat shock protein 20.8 [Helicoverpa armigera] >PZC74337.1 hypothetical protein B5X24_HaOG207971 [Helicoverpa armigera]	0.295466908	-1.758931534	0.0005085	down	yes	0.9777	3.309	3.246	2.952	3.73	1	0.953	0.98	MF:cation binding;M	GO:0043169;GO:0046872;GO:0003674;GO:0005488;GO:0043167	K09542	CRYAB	map04213;map04	Longevity regulating pathwa	ENOG410YERS	O:Posttranslational modification	PF00011.24	HSP20	Hsp20/alpha crystallin f	CYT	5	65	21.3	High
TRINITY_DN6497_c0_g1_i1_orf1	-	-	-	ommochrome-binding protein-like [Ostrinia furnacalis]	0.35560779	-1.491641165	7.52E-05	down	yes	1.059	2.978	3.015	2.771	3.148	1	1.109	1.068	------	------	------	------	------	------	ENOG4110SZS;E	S:Function unknown;G:Carbohyd	------	------	------	CYT	2	15	17	High
TRINITY_DN68770_c0_g1_i1_orf1	-	-	-	seroin transcript 1A2 [Ostrinia nubilalis]	0.44500583	-1.168103859	0.001478	down	yes	1.145	2.573	2.898	2.468	2.352	1	1.167	1.269	------	------	------	------	------	------	------	------	------	------	------	CYT	1	30	8.7	High
TRINITY_DN1450_c0_g2_i1_orf1	-	-	-	death-associated protein 1 [Ostrinia furnacalis]	0.418908532	-1.255292827	0.006469	down	yes	1.09	2.602	2.599	2.113	3.095	1	1.209	1.062	------	------	------	------	------	------	ENOG41122SE	S:Function unknown	PF15228.9	DAP	Death-associated prote	CYT	2	37	10.7	High
TRINITY_DN1201_c0_g1_i4_orf1	-	-	-	triosephosphate isomerase [Ostrinia furnacalis] >XP_028170843.1 triosephosphate isomerase [Ostrinia furnacalis]	0.400948992	-1.318509385	2.39E-05	down	yes	1.014	2.529	2.604	2.394	2.588	1	1.019	1.023	BP:ribonucleoside di	GO:0009185;GO:0016310;GO:0009144;GO:0019752;GO:0034641;GO:0006807;GO:0044281;GO:0044283;GO:0006725;GO:0072521;GO:0006091;GO:0006090;GO:0006096;GO:1901360;GO:0006139;GO:1901575;GO:0009135;GO:0046939;GO:0009259;GO:0071704;GO:0009199;GO:0009141;GO:0006094;GO:0009150;GO:0046364;GO:0009205;GO:0019318;GO:0019693;GO:1901576;GO:0046034;GO:0009987;GO:0046031;GO:0019319;GO:0032787;GO:0009058;GO:0008150;GO:0008152;GO:0043436;GO:0005975;GO:0009132;GO:0009179;GO:0055086;GO:0046483;GO:0044238;GO:1901564;GO:0006082;GO:0005996;GO:1901135;GO:0016052;GO:0044237;GO:0016051;GO:0006163;GO:0006796;GO:0006165;GO:0006793;GO:0019637;GO:0009117;GO:0006006;GO:0006753;GO:0009056;GO:0006757;GO:0003674;GO:0016860;GO:0016861;GO:0003824;GO:0004807;GO:0016853	K01803	TPI, tpiA	map00010;map00	Glycolysis / Gluconeogenesi	COG0149	G:Carbohydrate transport and m	PF00121.21	TIM	Triosephosphate isome	CYT	16	71	26.4	High
TRINITY_DN113353_c0_g1_i1_orf1	-	-	-	unnamed protein product [Parnassius apollo]	0.386110012	-1.372916131	3.39E-05	down	yes	0.9757	2.527	2.484	2.67	2.427	1	0.99	0.937	BP:cellular process;B	GO:0009987;GO:0006810;GO:0006869;GO:0006914;GO:0044237;GO:0051179;GO:0061919;GO:0008152;GO:0071702;GO:0008150;GO:0051234;GO:0009056;GO:0044248;GO:0005575;GO:0016020;GO:0034045;GO:0031224;GO:0110165;GO:0016021	K17907	ATG9	map04136;map04	Autophagy - other;Mitopha	ENOG410XQBE	U:Intracellular trafficking, secreti	------	------	------	CYT	1	19	7.5	High
TRINITY_DN40191_c2_g1_i1_orf1	-	-	-	stress-activated map kinase-interacting protein 1 [Ostrinia furnacalis]	0.436445907	-1.196125237	0.003218	down	yes	1.061	2.431	2.773	2.428	2.091	1	0.942	1.24	CC:cellular_compone	GO:0005575;GO:0031932;GO:0032991;GO:0038201	K20410	MAPKAP1, SIN	map04150	mTOR signaling pathway	ENOG410XR57	S:Function unknown	PF05422.15;PF16	SIN1;SIN1_PH;(	Stress-activated map k	CYT	1	1	60.4	Medium
TRINITY_DN4695_c0_g1_i3_orf1	-	-	-	glutathione S-transferase epsilon 3 [Ostrinia furnacalis]	0.41697417	-1.261970079	0.0001827	down	yes	1.017	2.439	2.505	2.235	2.578	1	0.991	1.061	MF:catalytic activity;I	GO:0003824;GO:0016740;GO:0004364;GO:0003674;GO:0016765	K00799	GST, gst	map05207;map00	Chemical carcinogenesis - r	COG0625	S:Function unknown	PF13417.9;PF027	GST_N_3;GST_I	Glutathione S-transfera	CYT	2	9	24.8	High
TRINITY_DN5310_c2_g1_i2_orf1	-	-	-	serine protease persephone-like [Ostrinia furnacalis]	0.418713204	-1.25596568	7.53E-06	down	yes	0.9957	2.378	2.43	2.417	2.288	1	0.977	1.01	BP:organic substanc	GO:0071704;GO:1901564;GO:0019538;GO:0043170;GO:0008150;GO:0006807;GO:0008152;GO:0006508;GO:0044238;GO:0004252;GO:0004175;GO:0016787;GO:0017171;GO:0140096;GO:0003824;GO:0003674;GO:0008233;GO:0008236	K20677;K20671	HAYAN;PSH	map04624	Toll and Imd signaling pathw	COG5640	O:Posttranslational modification	PF00089.29	Trypsin	Trypsin	CYT	9	34	34.8	High
TRINITY_DN28711_c1_g1_i1_orf1	-	-	-	apolipophorins-like [Ostrinia furnacalis]	0.411470588	-1.281138784	8.80E-06	down	yes	0.9793	2.38	2.296	2.453	2.392	1	0.945	0.993	MF:lipid transporter	GO:0005319;GO:0005215;GO:0003674	------	------	------	------	ENOG410ZRUY;I	S:Function unknown;	PF01347.25	Vitellogenin_N	Lipoprotein amino term	CYT	7	86	9.8	High
TRINITY_DN14460_c0_g1_i6_orf1	-	-	-	scavenger receptor class B member 1-like [Ostrinia furnacalis]	0.47606264	-1.070776681	8.55E-05	down	yes	1.064	2.235	2.282	2.113	2.311	1	1.061	1.131	BP:multicellular orga	GO:0032501;GO:0007608;GO:0050877;GO:0050896;GO:0007606;GO:0007600;GO:0008150;GO:0003008;GO:0016020;GO:0005575;GO:0110165;GO:0005886;GO:0016021;GO:0031224	K13885	SCARB1	map04979;map05	Cholesterol metabolism;Hep	ENOG410XS17	C:Energy production and conver	PF01130.24	CD36	CD36 family	CYT	3	6	62.9	High
TRINITY_DN10994_c0_g1_i4_orf1	-	-	-	trypsin inhibitor-like [Ostrinia furnacalis]	0.459641256	-1.1214198	0.0003362	down	yes	1.025	2.23	2.419	2.074	2.198	1	0.988	1.087	BP:nitrogen compou	GO:0006807;GO:0008152;GO:0071704;GO:0006508;GO:0008150;GO:0044238;GO:1901564;GO:0019538;GO:0043170;GO:0043229;GO:0043228;GO:0043226;GO:0005576;GO:0110165;GO:0043232;GO:0005575;GO:0042151;GO:0004866;GO:0099106;GO:0090729;GO:0003674;GO:0004857;GO:0098772;GO:0015459;GO:0016247;GO:0061135;GO:0016787;GO:0030234;GO:0061134;GO:0140096;GO:0003824;GO:0030414;GO:0004867;GO:0008233	K23622	EPPIN	------	------	ENOG410XQNP	U:Intracellular trafficking, secreti	PF00014.26	Kunitz_BPTI	Kunitz/Bovine pancreat	EXC	2	49	8.1	High
TRINITY_DN6988_c0_g1_i3_orf1	-	-	-	cuticle protein 1-like [Ostrinia furnacalis]	0.446153846	-1.164386818	8.46E-05	down	yes	0.957	2.145	2.184	2.009	2.242	1	0.937	0.934	MF:molecular_functi	GO:0003674;GO:0042302;GO:0005198	------	------	------	------	ENOG4110SHP	S:Function unknown	PF17223.6	CPCFC	Cuticle protein CPCFC	CYT	3	59	10.1	High
TRINITY_DN5198_c0_g1_i5_orfp1	-	-	-	TRINITY_DN5198_c0_g1_i5_m.8637 TRINITY_DN5198_c0_g1::TRINITY_DN5198_c0_g1_i5::g.8637 ORF type:complete len:223 (-),score=54.99 TRINITY_DN5198_c0_g1_i5:319-987(-)	0.343459365	-1.54178867	0.0006067	down	yes	0.7903	2.301	2.485	2.106	2.312	1	0.638	0.733	------	------	------	------	------	------	------	------	------	------	------	CYT	1	5	24.7	High
TRINITY_DN664_c0_g1_i18_orf1	-	-	-	chitinase-like protein EN03 isoform X2 [Ostrinia furnacalis]	0.495526182	-1.012966808	0.0004438	down	yes	0.9747	1.967	2.018	1.788	2.096	1	0.969	0.955	BP:primary metaboli	GO:0044238;GO:0005975;GO:0071704;GO:0008150;GO:0008152;GO:0005575;GO:0005576;GO:0110165;GO:0097367;GO:0008061;GO:0003674;GO:0005488	------	------	------	------	COG3325	S:Function unknown	PF00704.31	Glyco_hydro_18	Glycosyl hydrolases fan	CYT	19	48	48	High
TRINITY_DN2848_c0_g1_i2_orf1	-	-	-	[illegible] glyceraldehyde-3-phosphate dehydrogenase isoform 1 [Homo sapiens] >NP_001276675.1 glyceraldehyde-3-phosphate dehydrogenase isoform 1 [Homo sapiens] >NP_002037.2 glyceraldehyde-3-phosphate dehydrogenase isoform 1 [Homo sapiens] >XP_003819180.1 glyceraldehyde-3-phosphate dehydrogenase [Pan paniscus] >XP_004052609.1 glyceraldehyde-3-phosphate dehydrogenase [Gorilla gorilla gorilla] >XP_008971979.1 glyceraldehyde-3-phosphate dehydrogenase [Pan paniscus] >XP_008971980.1 glyceraldehyde-3-phosphate dehydrogenase [Pan paniscus] >XP_032621678.1 glyceraldehyde-3-phosphate dehydrogenase [Chelonoidis abingdonii] >XP_508955.1 glyceraldehyde-3-phosphate dehydrogenase isoform X1 [Pan troglodytes] >P04406.3 RecName: Full=Glyceraldehyde-3-phosphate dehydrogenase; Short=GAPDH; AltName: Full=Peptidyl-cysteine S-nitrosylase GAPDH [Homo sapiens] >1U8F_O Crystal Structure Of Human Placental Glyceraldehyde-3-Phosphate Dehydrogenase At 1.75 Resolution [Homo sapiens] >1U8F_P Crystal Structure Of Human Placental Glyceraldehyde-3-Phosphate Dehydrogenase At 1.75 Resolution [Homo sapiens] >1U8F_Q Crystal Structure Of Human Placental Glyceraldehyde-3-Phosphate Dehydrogenase At 1.75 Resolution [Homo sapiens] >1U8F_R Crystal Structure Of Human Placental Glyceraldehyde-3-Phosphate Dehydrogenase At 1.75 Resolution [Homo sapiens] >4WNC_A Crystal structure of human wild-type GAPDH at 1.99 angstroms resolution [Homo sapiens] >4WNC_B Crystal structure of human wild-type GAPDH at 1.99 angstroms resolution [Homo sapiens] >4WNC_C Crystal structure of human wild-type GAPDH at 1.99 angstroms resolution [Homo sapiens] >4WNC_D Crystal structure of human wild-type GAPDH at 1.99 angstroms resolution [Homo sapiens] >4WNC_E Crystal structure of human wild-type GAPDH at 1.99 angstroms resolution [Homo sapiens] >4WNC_F Crystal structure of human wild-type GAPDH at 1.99 angstroms resolution [Homo sapiens] >4WNC_G Crystal structure of human wild-type GAPDH at 1.99 angstroms resolution [Homo sapiens] >4WNC_O Crystal structure of human wild-type GAPDH at 1.99 angstroms resolution [Homo sapiens] >6IQ6_A Crystal structure of GAPDH [Homo sapiens] >6IQ6_B Crystal structure of GAPDH [Homo sapiens] >6IQ6_C Crystal structure of GAPDH [Homo sapiens] >6IQ6_D Crystal structure of GAPDH [Homo [illegible]	0.487888041	-1.035377975	2.11E-05	down	yes	0.9587	1.965	1.969	1.896	2.029	1	0.942	0.934	BP:regulation of prin	[illegible]:0071840;GO:0010605;GO:0043207;GO:0009966;GO:0009179;GO:0018193;GO:0044419;GO:0009199;GO:0044092;GO:0048518;GO:0048519;GO:0001906;GO:0043122;GO:0043123;GO:0009205;GO:0060255;GO:0046034;GO:0032481;GO:0046031;GO:0032787;GO:0050832;GO:0030162;GO:0043436;GO:0009607;GO:0051707;GO:0065009;GO:0050794;GO:0046483;GO:1901564;GO:0009605;GO:0019538;GO:0016052;GO:0006163;GO:0016241;GO:0006165;GO:0019637;GO:0009894;GO:0051716;GO:0009892;GO:0009893;GO:0009890;GO:0009141;GO:0009144;GO:0050821;GO:0006807;GO:0000226;GO:0008219;GO:0061844;GO:0002376;GO:1901575;GO:0051346;GO:0070997;GO:0016043;GO:0043086;GO:0065007;GO:1901360;GO:0051873;GO:0010608;GO:0065008;GO:0034097;GO:0018119;GO:0050790;GO:0009889;GO:0017014;GO:0009150;GO:0006952;GO:0006950;GO:0036211;GO:0008150;GO:0008152;GO:0006955;GO:1902533;GO:0006959;GO:0006006;GO:0018198;GO:0051336;GO:0071345;GO:0050896;GO:0031647;GO:0031640;GO:0043412;GO:0009967;GO:0006753;GO:0051240;GO:0006757;GO:0051239;GO:0016310;GO:0023056;GO:0034641;GO:0070887;GO:0023051;GO:0010647;GO:0010646;GO:0009259;GO:0006139;GO:0051248;GO:1902531;GO:0051246;GO:0046939;GO:0017148;GO:0035606;GO:0010556;GO:0009987;GO:0006725;GO:0034249;GO:0034248;GO:0098542;GO:0005975;GO:0009132;GO:0009135;GO:0055086;GO:0006082;GO:0005996;GO:1901135;GO:0010629;GO:0043170;GO:0001817;GO:0032479;GO:0045861;GO:0001819;GO:0009185;GO:0071346;GO:0031329;GO:0031327;GO:0031326;GO:0031324;GO:0031323;GO:0010604;GO:0019752;GO:0044237;GO:0072521;GO:0006091;GO:0006090;GO:0006096;GO:0010628;GO:0012501;GO:2000112;GO:2000113;GO:0050789;GO:0071704;GO:0071310;GO:0010466;GO:0010506;GO:0010468;GO:0010033;GO:0019318;GO:0052547;GO:0052548;GO:0045087;GO:0006915;GO:0034341;GO:0051402;GO:0009117;GO:0051171;GO:0051172;GO:0010951;GO:0042221;GO:0019222;GO:0009056;GO:0006996;GO:0044238;GO:0007017;GO:0007010;GO:0009620;GO:0019693;GO:0010558;GO:0006796;GO:0006793;GO:0006417;GO:0044403;GO:0048523;GO:0048522;GO:0031982;GO:0016020;GO:00 [illegible]	K00134	GAPDH, gapA	map00010;map05	Glycolysis / Gluconeogenesi	COG0057	G:Carbohydrate transport and m	PF02800.23;PF00	Gp_dh_C;Gp_dh	Glyceraldehyde 3-phos	CYT	2	16	36	High
TRINITY_DN4204_c0_g1_i1_orf1	-	-	-	uncharacterized protein LOC114359352 [Ostrinia furnacalis]	0.201448543	-2.311516724	9.45E-05	down	yes	1.196	5.937	6.148	5.377	6.285	1	1.345	1.244	CC:cellular_compone	GO:0005575;GO:0005576;GO:0110165;GO:0003674;GO:0005198	------	------	------	------	------	------	------	------	------	EXC	1	4	32.1	High
TRINITY_DN15202_c0_g1_i6_orf1	-	-	-	uncharacterized protein LOC114364499 isoform X2 [Ostrinia furnacalis]	0.182441942	-2.45449066	5.26E-05	down	yes	1.037	5.684	5.555	5.331	6.166	1	1.132	0.98	------	------	------	------	------	------	ENOG410XU1K	S:Function unknown	------	------	------	CYT	2	41	14	High
TRINITY_DN67193_c0_g1_i1_orf1	-	-	-	A-kinase anchor protein 14-like [Ostrinia furnacalis]	0.210842207	-2.245764395	0.0003636	down	yes	1.089	5.165	5.528	5.501	4.466	1	0.973	1.294	------	------	------	------	------	------	------	------	------	------	------	CYT	1	6	15.2	High
TRINITY_DN16234_c0_g2_i3_orf1	-	-	-	uncharacterized protein LOC114363370 [Ostrinia furnacalis]	0.241177845	-2.051830708	0.001124	down	yes	1.032	4.279	4.371	3.565	4.901	1	1.031	1.064	CC:cellular_compone	GO:0005575;GO:0005576;GO:0110165;GO:0030234;GO:0061134;GO:0003674;GO:0030414;GO:0004857;GO:0098772	------	------	------	------	ENOG410Y67D	S:Function unknown	------	------	------	CYT	3	51	7.4	High
TRINITY_DN2813_c0_g1_i10_orf1	-	-	-	arylphorin subunit alpha-like [Ostrinia furnacalis]	0.245855247	-2.024118952	0.0001084	down	yes	0.9817	3.993	4.086	3.616	4.277	1	0.936	1.009	------	------	------	------	------	------	ENOG4110OFU;I	S:Function unknown;	PF03723.17;PF0(	Hemocyanin_C	Hemocyanin, ig-like do	CYT	1	33	139.5	High
TRINITY_DN143532_c0_g1_i1_orf1	-	-	-	3-oxoacyl-[acyl-carrier-protein] reductase FabG-like [Aphidius gifuensis] >KAF7996667.1 hypothetical protein HCN44_002313 [Aphidius gifuensis]	0.259646775	-1.945377786	0.0003405	down	yes	0.9703	3.737	4.21	3.593	3.407	1	0.962	0.949	MF:catalytic activity;I	GO:0003824;GO:0003674;GO:0016491	------	------	------	------	COG1028	S:Function unknown	PF13561.9;PF001	adh_short_C2;a	Enoyl;short chain dehyc	CYT	1	5	27.2	High
TRINITY_DN27035_c0_g1_i1_orf1	-	-	-	glucose-6-phosphate isomerase-like [Ostrinia furnacalis]	0.341062802	-1.551890679	0.003116	down	yes	1.059	3.105	2.988	2.619	3.707	1	1.065	1.113	BP:ribonucleoside di	GO:0009185;GO:0016310;GO:0009144;GO:0019752;GO:0034641;GO:0006807;GO:0044283;GO:0006725;GO:0072521;GO:0006091;GO:0006090;GO:0006096;GO:0006139;GO:1901575;GO:0009135;GO:0046939;GO:0009259;GO:0071704;GO:0009199;GO:1901360;GO:0009141;GO:0006094;GO:0044281;GO:0009150;GO:0046364;GO:0009205;GO:0019318;GO:0019693;GO:1901576;GO:0046034;GO:0009987;GO:0046031;GO:0019319;GO:0032787;GO:0009058;GO:0008150;GO:0008152;GO:0043436;GO:0005975;GO:0009132;GO:0009179;GO:0055086;GO:0046483;GO:0044238;GO:1901564;GO:0006082;GO:0005996;GO:1901135;GO:0016052;GO:0044237;GO:0016051;GO:0006163;GO:0006796;GO:0006165;GO:0006793;GO:0019637;GO:0009117;GO:0006006;GO:0006753;GO:0009056;GO:0006757;GO:0003674;GO:0005488;GO:0016861;GO:0016860;GO:0004347;GO:0003824;GO:0016853;GO:0097367	K01810	GPI, pgi	map00520;map00	Amino sugar and nucleotide	COG0166	S:Function unknown	PF00342.22	PGI	Phosphoglucose isome	CYT	3	39	13.3	High

TRINITY_DN142442_c0_g1_i1_orf1	-	-	-	60S ribosomal protein L38 [Homo sapiens] >NP_001002486.1 60S ribosomal protein L38 [Danio rerio] >NP_001030335.1 60S ribosomal protein L38 [Homo sapiens] >NP_001071060.1 60S ribosomal protein L38 [Rattus norvegicus] >NP_001133168.1 60S ribosomal protein L38 [Salmo salar] >NP_001187063.1 60S ribosomal protein L38 [Ictalurus punctatus] >NP_001232305.1 60S ribosomal protein L38 [Taeniopygia guttata] >NP_001264941.1 60S ribosomal protein L38 [Gallus gallus] >XP_003211558.1 60S ribosomal protein L38 [Meleagris gallopavo] >XP_003315754.1 60S ribosomal protein L38 [Pan troglodytes] >XP_003315758.1 60S ribosomal protein L38 [Pan troglodytes] >XP_003339346.1 60S ribosomal protein L38 [Pan troglodytes] >XP_003358038.1 60S ribosomal protein L38 [Sus scrofa] >XP_003417326.1 60S ribosomal protein L38 [Loxodonta africana] >XP_003453439.1 60S ribosomal protein L38 [Oreochromis niloticus] >XP_003464913.2 60S ribosomal protein L38 [Cavia porcellus] >XP_003768586.1 60S ribosomal protein L38 [Sarcophilus harrisii] >XP_003786210.1 60S ribosomal protein L38 [Otolemur garnettii] >XP_003795793.1 60S ribosomal protein L38 [Otolemur garnettii] >XP_003922345.1 60S ribosomal protein L38 [Saimiri boliviensis boliviensis] >XP_004041125.1 60S ribosomal protein L38 [Gorilla gorilla gorilla] >XP_004041126.1 60S ribosomal protein L38 [Gorilla gorilla gorilla] >XP_004041128.1 60S ribosomal protein L38 [Gorilla gorilla gorilla] >XP_004331065.1 60S ribosomal protein L38 [Tursiops truncatus] >XP_004401894.1 PREDICTED: 60S ribosomal protein L38 [Odobenus rosmarus divergens] >XP_004412345.1 PREDICTED: 60S ribosomal protein L38 [Odobenus rosmarus divergens] >XP_004469223.1 60S ribosomal protein L38 [Dasypus novemcinctus] >XP_004469224.1 60S ribosomal protein L38 [Dasypus novemcinctus] >XP_005068761.1 60S ribosomal protein L38 [Mesocricetus auratus] >XP_005070019.1 60S ribosomal protein L38 [Mesocricetus auratus] >XP_005141156.1 60S ribosomal protein L38 [Melopsittacus undulatus] >XP_005336034.1 60S ribosomal protein L38 [Ictidomys tridecemlineatus] >XP_005336035.1 60S ribosomal protein L38 [Ictidomys tridecemlineatus] >XP_005350739.1 60S ribosomal protein L38 [Microtus ochrogaster] >XP_005350740.1 60S ribosomal protein L38 [Microtus ochrogaster] >XP_005412280.1 PREDICTED: 60S ribosomal protein L38 [Chinchilla lanigera] >XP_005412281.1																													
osomal protein L38 [Nematolebias w	Ribosom	Ribosomal prc	60S RIBC	60S RIBOSOMAL PROTEIN L38 [Oryctolagus cuniculus] >4UJD_Ak Chain Ak	60S RIBOSOM/	60S RIBOSOMAL	60S ribosomal prc	60S ribosoı	60S ribosoma	eL38 [Or	eL38 [Or	eL38 [Or	eL38 [Or	eL38 [Or	eL38 [Or	eL38 [Oryctola	eL38 [Oryctolagus	60S ribosomal prote	eL38 [Oryctolagus cuniculus] >6FK0_Lk Chain Lk	60S ribosomal p	eL38 [Oryctol	eL38 [Oryctolagus	eL38 [Oryctolagus cuniculus	eL38 [Oryctolag	60S ribosomal protein L38 [Hor	60S ribosomal r	60S ribosomal	eL38 [Oryctolagus cun	60S ribosomal r	eL38 [Oryctolagus c	60S ribosomal prc	60S ribosomal	60S ribosomal protei
TRINITY_DN55147_c0_g1_i1_orfp1	-	-	-	TRINITY_DN55147_c0_g1_i1_m.59251 TRINITY_DN55147_c0_g1::TRINITY_DN55147_c0_g1_i1::g.59251 ORF type:5prime_partial len:331 (-),score=113.97,Cuticle_3 PF11018.9 0.29,Cuticle_3|PF11018.9|2.9e-05 Cuticle_3|PF11018.9|0.00037 TRINITY_DN55147_c0_g1_i1:21-1013(-)	0.414868106	-1.269275346	0.000352	down	yes	1.038	2.502	2.477	2.296	2.734	1	1.026	1.089	------	------	------	------	------	------	------	------	PF11018.11	Cuticle_3	Pupal cuticle protein C1	CYT	7	50	33.7	High
TRINITY_DN45948_c1_g1_i1_orf1	-	-	-	unnamed protein product [Leptidea sinapis]	0.430826755	-1.214820248	0.0001994	down	yes	1.037	2.407	2.471	2.206	2.543	1	1.032	1.079	BP:metabolic proces	GO:0008152;GO:0006807;GO:0071704;GO:0006508;GO:0008150;GO:0044238;GO:1901564;GO:0019538;GO:0043170;GO:0004175;GO:0030414;GO:0003674;GO:0004857;GO:0098772;GO:0008233;GO:0008234;GO:0030234;GO:0016787;GO:0061135;GO:0061134;GO:0140096;GO:0003824;GO:0004866;GO:0004869;GO:0004197	K01373	CTSF	map04210;map04	Apoptosis;Lysosome	COG4870	O:Posttranslational modification	PF00112.26	Peptidase_C1	Papain family cysteine p	CYT	2	68	3.7	High
TRINITY_DN8985_c0_g1_i4_orf1	-	-	-	cytochrome P450 6B6-like [Ostrinia furnacalis]	0.477807971	-1.065497173	3.29E-05	down	yes	1.055	2.208	2.182	2.154	2.289	1	1.037	1.128	MF:cation binding;M	GO:0043169;GO:0004497;GO:0020037;GO:0046914;GO:0046906;GO:0097159;GO:0016705;GO:0043167;GO:0003824;GO:0003674;GO:0005488;GO:0005506;GO:0046872;GO:1901363;GO:0016491	K14999	CYP6	------	------	COG2124	Q:Secondary metabolites biosyr	PF00067.25	p450	Cytochrome P450	CYT	3	8	59.5	High
TRINITY_DN49530_c0_g1_i1_orf1	-	-	-	ommochrome-binding protein-like [Ostrinia furnacalis]	0.422824661	-1.241868571	0.001021	down	yes	0.967	2.287	2.302	2.544	2.016	1	0.925	0.976	------	------	------	------	------	------	ENOG4110SZS	S:Function unknown	------	------	------	CYT	3	50	13.6	High
TRINITY_DN2803_c4_g1_i1_orf1	-	-	-	ornithine aminotransferase, mitochondrial isoform X2 [Ostrinia furnacalis]	0.442382022	-1.176635336	5.17E-07	down	yes	0.9843	2.225	2.214	2.262	2.199	1	0.967	0.986	BP:small molecule bi	GO:0044283;GO:0019752;GO:0006807;GO:0044281;GO:0009064;GO:0055129;GO:1901362;GO:1901360;GO:1901576;GO:0006561;GO:0006560;GO:0006520;GO:0071704;GO:1901605;GO:1901607;GO:0018130;GO:0008652;GO:0009987;GO:0009058;GO:0008150;GO:0008152;GO:0043436;GO:0046483;GO:0044238;GO:1901564;GO:1901566;GO:0006082;GO:0044249;GO:0046394;GO:0016053;GO:0044237;GO:0009084;GO:0005488;GO:0070279;GO:0008483;GO:0003674;GO:1901363;GO:0043168;GO:0043167;GO:0050155;GO:0016740;GO:0004587;GO:0016769;GO:0036094;GO:0003824;GO:0019842;GO:0097159;GO:0030170	K00819	rocD, OAT	map00330	Arginine and proline metabo	COG4992	E:Amino acid transport and meta	PF00202.24	Aminotran_3	Aminotransferase class	CYT	25	68	44.7	High
TRINITY_DN812_c2_g1_i1_orf1	-	-	-	1,4-alpha-glucan-branching enzyme [Ostrinia furnacalis]	0.472209248	-1.082501798	2.08E-05	down	yes	1.011	2.141	2.228	2.132	2.063	1	1.002	1.031	BP:cellular biosynthe	GO:0044249;GO:0034645;GO:1901576;GO:0006091;GO:0033692;GO:0044264;GO:0044262;GO:0044260;GO:0016051;GO:0071704;GO:0006112;GO:0006073;GO:0009250;GO:0009987;GO:0000271;GO:0034637;GO:0009058;GO:0009059;GO:0008150;GO:0008152;GO:0044042;GO:0044238;GO:0005975;GO:0015980;GO:0005977;GO:0005976;GO:0005978;GO:0044237;GO:0043170;GO:0016798;GO:0003674;GO:0005488;GO:0043169;GO:0043167;GO:0016740;GO:0102752;GO:0016787;GO:0003824;GO:0003844;GO:0016757;GO:0004553;GO:0016758	K00700	GBE1, glgB	map00500	Starch and sucrose metabol	COG0296	G:Carbohydrate transport and m	PF02806.21;PF0(	Alpha-amylase	Alpha amylase, C-term	CYT	35	57	79.1	High
TRINITY_DN82944_c0_g1_i4_orf1	-	-	-	senecionine N-oxygenase isoform X2 [Ostrinia furnacalis]	0.487302372	-1.037110851	9.15E-06	down	yes	0.9863	2.024	1.991	2.063	2.019	1	0.929	1.03	MF:anion binding;MF	GO:0043168;GO:0050661;GO:0050660;GO:0016709;GO:0097159;GO:0016705;GO:0004499;GO:0004497;GO:0000166;GO:0036094;GO:0003824;GO:0003674;GO:0005488;GO:0043167;GO:1901363;GO:1901265;GO:0016491	------	------	------	------	COG2072	P:Inorganic ion transport and me	PF00743.22;PF13	FMO-like;Pyr_r	Flavin-binding monoox	CYT	2	20	17.9	High
TRINITY_DN4443_c0_g1_i4_orf1	-	-	-	lysosome-associated membrane glycoprotein 1-like isoform X4 [Ostrinia furnacalis]	0.496764559	-1.009365843	0.001024	down	yes	0.998	2.009	2.049	1.789	2.19	1	0.974	1.02	CC:organelle membr	GO:0031090;GO:0016021;GO:0016020;GO:0005575;GO:0030659;GO:0031224;GO:0012506;GO:0098588;GO:0110165;GO:0010008	K06528	LAMP1_2, CD1	map05152;map04	Tuberculosis;Phagosome;Lys	ENOG410XQ96	U:Intracellular trafficking, secreti	PF01299.20	Lamp	Lysosome-associated r	CYT	1	3	24.7	High
TRINITY_DN1230_c1_g1_i5_orf1	-	-	-	uncharacterized protein LOC114353440 [Ostrinia furnacalis]	0.496746204	-1.009419152	0.0001352	down	yes	0.916	1.844	1.875	1.786	1.872	1	0.942	0.806	------	------	------	------	------	------	ENOG4110OI3;E	S:Function unknown;	------	------	------	CYT	1	3	35.6	High
TRINITY_DN2943_c2_g2_i1_orf1	-	-	-	protein phosphatase inhibitor 2-like [Ostrinia furnacalis]	0.499174463	-1.002383964	0.007576	down	yes	0.907	1.817	1.725	1.581	2.145	1	0.773	0.948	BP:regulation of pho	GO:0019220;GO:0080090;GO:0019222;GO:0010921;GO:0048583;GO:0023051;GO:0050789;GO:0010646;GO:0009966;GO:0051246;GO:0060255;GO:0065007;GO:0031399;GO:0065009;GO:0031323;GO:0050790;GO:0050794;GO:0051174;GO:0008150;GO:0051171;GO:0035303;GO:0035304;GO:0051336;GO:0043666;GO:0019208;GO:0003674;GO:0004857;GO:0098772;GO:0019212;GO:0019888;GO:0030234;GO:0004864	K16833	PPP1R2, IPP2	------	------	ENOG4111SYV	T:Signal transduction mechanisn	PF04979.17	IPP-2	Protein phosphatase inl	CYT	1	9	18.8	High
TRINITY_DN89083_c0_g1_i1_orf1	-	-	-	lysine-specific demethylase 4A isoform X2 [Diachasma alloeum]	0.437684855	-1.192035632	0.003334	down	yes	0.8583	1.961	1.697	1.932	2.254	1	0.792	0.783	BP:cellular compone	GO:0071840;GO:0016043;GO:0009987;GO:0008150;GO:0006325;GO:0043229;GO:0043227;GO:0043226;GO:0005634;GO:0110165;GO:0043231;GO:0005575;GO:0003674;GO:0005488;GO:0016491;GO:0043169;GO:0032451;GO:0016706;GO:0016705;GO:0043167;GO:0046872;GO:0140096;GO:0003824;GO:0051213;GO:0032452;GO:0032454;GO:0140457	K06709	KDM4, JMJD2,	------	------	COG5141;ENOG	S:Function unknown;	PF18104.4	Tudor_2	Jumonji domain-contai	PLA	1	6	12.5	Medium
TRINITY_DN384_c0_g1_i8_orf1	-	-	-	unnamed protein product [Chilo suppressalis]	0.46409545	-1.107506543	0.002051	down	yes	0.8363	1.802	1.772	1.983	1.652	1	0.838	0.671	------	------	K23028	SPECC1	------	------	COG5069;ENOG	E:Amino acid transport and meta	------	------	------	CYT	1	1	216.4	High
TRINITY_DN8922_c0_g1_i3_orf1	-	-	-	protein sidekick isoform X1 [Ostrinia furnacalis] >XP_028161979.1 protein sidekick isoform X1 [Ostrinia furnacalis] >XP_028161980.1 protein sidekick isoform X1 [Ostrinia furnacalis] >XP_028161981.1 protein sidekick isoform X1 [Ostrinia furnacalis]	0.4328125	-1.208185929	0.02443	down	yes	0.8587	1.984	1.747	2.587	1.618	1	0.909	0.667	------	------	K16353	SDK	------	------	ENOG410YMNG	U:Intracellular trafficking, secreti	PF00041.24;PF07	fn3;I-set;Ig_3;Ig	Fibronectin type III dom	CYT	1	1	245.2	High
TRINITY_DN7868_c0_g1_i2_orf1	-	-	-	uncharacterized protein LOC114353432 isoform X4 [Ostrinia furnacalis]	0.489835892	-1.029629606	0.00143	down	yes	0.9253	1.889	1.819	2.106	1.742	1	0.954	0.822	------	------	------	------	------	------	ENOG410ZHS3	S:Function unknown	PF02995.20;PF0(	DUF229;Sulfata	Protein of unknown fun	EXC	1	5	36.1	High
TRINITY_DN4255_c0_g1_i10_orf1	-	-	-	LOW QUALITY PROTEIN: lebocin-4-like [Ostrinia furnacalis]	0.47816156	-1.064429941	0.0004616	down	yes	0.8583	1.795	1.831	1.727	1.827	1	0.715	0.86	------	------	------	------	------	------	ENOG4111ASS	S:Function unknown	------	------	------	CYT	2	21	22.7	High
TRINITY_DN30097_c0_g1_i2_orf1	-	-	-	unnamed protein product [Chilo suppressalis]	2.005110733	1.003681912	0.004869	up	yes	1.177	0.587	0.623	0.611	0.527	1	1.185	1.347	BP:nucleic acid meta	GO:0090304;GO:0034641;GO:0006807;GO:0044237;GO:1901360;GO:0006139;GO:0071704;GO:0008380;GO:0016071;GO:0009987;GO:0006725;GO:0000375;GO:0000377;GO:0008150;GO:0008152;GO:0046483;GO:0016070;GO:0044238;GO:0000398;GO:0043170;GO:0006396;GO:0006397;GO:1902494;GO:0071013;GO:0032991;GO:1990904;GO:0005575;GO:0005681	K12816	CDC40, PRP17	map03040	Spliceosome	ENOG410XP87	A:RNA processing and modificat	PF00400.35;PF12	WD40;ANAPC4	WD domain, G-beta re	CYT	1	1	65.3	High
TRINITY_DN3638_c0_g1_i1_orf1	-	-	-	DNA replication licensing factor Mcm3 [Ostrinia furnacalis]	2.011945392	1.008591149	0.007366	up	yes	1.179	0.586	0.64	0.568	0.55	1	1.144	1.393	------	------	K02541	MCM3	map04110;map03	Cell cycle;DNA replication	COG1241	L:Replication, recombination anc	PF00493.26;PF17	MCM;MCM_OE	MCM P-loop domain;M	CYT	1	1	90.7	High
TRINITY_DN7647_c0_g1_i4_orf1	-	-	-	E3 ubiquitin-protein ligase Bre1 isoform X6 [Ostrinia furnacalis]	2.099944883	1.070351462	0.002991	up	yes	1.143	0.5443	0.591	0.457	0.585	1	1.144	1.284	BP:histone monoubi	GO:0010390;GO:0006807;GO:0071840;GO:0016567;GO:0006325;GO:0070647;GO:0016043;GO:0016574;GO:0016570;GO:0009987;GO:0032446;GO:0043412;GO:0036211;GO:0008150;GO:0008152;GO:0071704;GO:0044238;GO:1901564;GO:0019538;GO:0043170;GO:0006513;GO:0043229;GO:0043227;GO:0043226;GO:0005634;GO:0110165;GO:0043231;GO:0005575;GO:0003674;GO:0005488;GO:0019787;GO:0043169;GO:0043167;GO:0016740;GO:0046872;GO:0140096;GO:0003824;GO:0004842	K10696	BRE1	------	------	ENOG410ZW9A;	O:Posttranslational modification	PF13920.9;PF16	zf-C3HC4_3;Tc	Zinc finger, C3HC4 type	CYT	1	1	105.9	High
TRINITY_DN441_c0_g2_i1_orf1	-	-	-	guanine nucleotide-binding protein subunit beta-like protein [Diachasma alloeum]	2.086523055	1.061100862	0.002449	up	yes	1.172	0.5617	0.56	0.563	0.562	1	1.212	1.303	CC:macromolecular	GO:0032991;GO:0015935;GO:1990904;GO:0044391;GO:0005575;GO:0003674;GO:0005488;GO:0043021;GO:0044877;GO:0043022	K14753	RACK1	map05162	Measles	ENOG410XQGZ	S:Function unknown	PF00400.35;PF2(	WD40;NBCH_W	WD domain, G-beta re	PLA	1	38	35.8	High
TRINITY_DN6572_c0_g1_i2_orf1	-	-	-	zinc finger protein 330 homolog [Ostrinia furnacalis]	2.008944544	1.00643774	0.001436	up	yes	1.123	0.559	0.537	0.537	0.603	1	1.237	1.131	------	------	------	------	------	------	ENOG410XNTY	S:Function unknown	PF06524.15	NOA36	NOA36 protein	CYT	1	3	34.4	High
TRINITY_DN18696_c0_g1_i1_orf1	-	-	-	rap guanine nucleotide exchange factor 2 [Ostrinia furnacalis] >XP_028159576.1 rap guanine nucleotide exchange factor 2 [Ostrinia furnacalis]	2.146775109	1.102171066	0.01515	up	yes	1.135	0.5287	0.46	0.535	0.591	1	0.983	1.423	BP:small GTPase me	GO:0007264;GO:0009987;GO:0050794;GO:0065007;GO:0007165;GO:0008150;GO:0035556;GO:0050789;GO:0005096;GO:0030234;GO:0005085;GO:0030695;GO:0003674;GO:0098772;GO:0060589;GO:0008047	K08018	RAPGEF2, PDZ	map04015;map04	Rap1 signaling pathway;Tigh	ENOG410XS6B	T:Signal transduction mechanisn	PF00617.22;PF0(	RasGEF;PDZ;Ra	RasGEF domain;PDZ dc	CYT	1	1	132.4	High
TRINITY_DN3299_c0_g1_i2_orf1	-	-	-	metaxin-1 isoform X3 [Ostrinia furnacalis] >XP_028170907.1 metaxin-1 isoform X4 [Ostrinia furnacalis]	2.314229249	1.210531786	0.004043	up	yes	1.171	0.506	0.49	0.435	0.593	1	1.353	1.16	BP:nitrogen compou	GO:0071705;GO:0033036;GO:0008104;GO:0070727;GO:0009987;GO:0045184;GO:0008150;GO:0006810;GO:0071702;GO:0015031;GO:0051234;GO:0051179;GO:0051641;GO:0032991;GO:0098796;GO:0001401;GO:0005575;GO:0005742;GO:0098798;GO:0098799	K17776	MTX	------	------	ENOG41100SS	U:Intracellular trafficking, secreti	PF10568.12;PF17	Tom37;GST_C_	Outer mitochondrial me	CYT	1	4	36.5	High
TRINITY_DN659_c0_g1_i3_orf1	-	-	-	probable malonyl-CoA-acyl carrier protein transacylase, mitochondrial [Ostrinia furnacalis]	2.024876532	1.017833941	0.000635	up	yes	1.107	0.5467	0.562	0.503	0.575	1	1.156	1.164	BP:fatty acid biosynt	GO:0006633;GO:0006631;GO:0019752;GO:0044249;GO:0044281;GO:0044283;GO:0072330;GO:1901576;GO:0071704;GO:0006629;GO:0009987;GO:0032787;GO:0009058;GO:0008150;GO:0008152;GO:0043436;GO:0044255;GO:0008610;GO:0044238;GO:0006082;GO:0046394;GO:0016053;GO:0044237;GO:0003674;GO:0016740;GO:0003824	K00645	fabD, MCAT, M	map00061	Fatty acid biosynthesis	COG0331	I:Lipid transport and metabolism	PF00698.24	Acyl_transf_1	Acyl transferase domai	CYT	1	3	33	High
TRINITY_DN1791_c0_g1_i3_orf1	-	-	-	succinate dehydrogenase assembly factor 2-B, mitochondrial-like [Ostrinia furnacalis]	2.192235831	1.132403006	0.0005888	up	yes	1.056	0.4817	0.516	0.382	0.547	1	1.094	1.073	BP:nitrogen compou	GO:0006807;GO:0044237;GO:0006091;GO:0071704;GO:0022900;GO:0022904;GO:0018293;GO:0009987;GO:0019646;GO:0043412;GO:0036211;GO:0008150;GO:0008152;GO:0044238;GO:1901564;GO:0015980;GO:0019538;GO:0006121;GO:0043170;GO:0009060;GO:0045333;GO:0031974;GO:0005759;GO:0110165;GO:0043233;GO:0005575;GO:0070013	K18168	SDHAF2, SDH5	------	------	COG2938	S:Function unknown	PF03937.19	Sdh5	Flavinator of succinate	CYT	1	4	16.8	Medium
TRINITY_DN57798_c0_g1_i1_orf1	-	-	-	ubiquitin carboxyl-terminal hydrolase 36 [Ostrinia furnacalis]	3.512755817	1.812603294	0.002318	up	yes	1.253	0.3567	0.403	0.326	0.341	1	1.346	1.412	BP:cellular catabolic	GO:0044248;GO:0006807;GO:0043632;GO:1901575;GO:0044265;GO:0044260;GO:0070647;GO:0070646;GO:0071704;GO:0006508;GO:0016579;GO:0044238;GO:0009987;GO:0019941;GO:0043412;GO:0036211;GO:0008150;GO:0008152;GO:0009056;GO:0009057;GO:0051603;GO:1901564;GO:0019538;GO:0044237;GO:0043170;GO:0006511;GO:0101005;GO:0003674;GO:0008234;GO:0019783;GO:0004843;GO:0016787;GO:0140096;GO:0003824;GO:0008233	K11855	USP36_42	------	------	ENOG410XQ92	O:Posttranslational modification	PF00443.32;PF1	UCH;UCH_1	Ubiquitin carboxyl-term	CYT	1	2	79	High
TRINITY_DN48641_c0_g1_i4_orf1	-	-	-	RNA-binding protein 45-like [Galleria mellonella]	2.448888889	1.292127317	0.0005308	up	yes	1.102	0.45	0.431	0.449	0.47	1	1.218	1.089	MF:organic cyclic co	GO:0097159;GO:0003674;GO:0005488;GO:0003676;GO:1901363;GO:0003723	K25081	RBM45	------	------	ENOG410XP9Z	S:Function unknown	PF00076.25;PF08	RRM_1;RRM_3	RNA recognition motif.	CYT	1	2	53.8	High
TRINITY_DN2168_c0_g1_i2_orf1	-	-	-	protein arginine methyltransferase NDUFAF7 homolog, mitochondrial [Ostrinia furnacalis]	2.112262859	1.078789381	0.0002769	up	yes	1.08	0.5113	0.518	0.515	0.501	1	1.076	1.163	BP:methylation;BP:bi	GO:0032259;GO:0008150;GO:0008152;GO:0043229;GO:0043227;GO:0043226;GO:0005739;GO:0110165;GO:0043231;GO:0005575;GO:0016274;GO:0016273;GO:0008276;GO:0003674;GO:0035243;GO:0008168;GO:0016740;GO:0016741;GO:0008757;GO:0140096;GO:0003824;GO:0008170	K18164	NDUFAF7	map04714	Thermogenesis	COG1565	S:Function unknown	PF02636.20	Methyltransf_2(	Putative S-adenosyl-L-	PLA	1	5	49.6	High
TRINITY_DN3814_c1_g1_i1_orf1	-	-	-	39S ribosomal protein L11, mitochondrial [Ostrinia furnacalis]	2.763990268	1.466752536	0.003708	up	yes	1.136	0.411	0.417	0.392	0.424	1	1.373	1.035	BP:cellular biosynthe	GO:0044249;GO:0034641;GO:0006807;GO:0034645;GO:1901576;GO:0044271;GO:0044260;GO:0043043;GO:0071704;GO:0043604;GO:0009987;GO:0009058;GO:0009059;GO:0008150;GO:0008152;GO:1901564;GO:0044238;GO:0043603;GO:1901566;GO:0019538;GO:0044237;GO:0043170;GO:0006518;GO:0006412;GO:0043232;GO:0043226;GO:0110165;GO:0005840;GO:0032991;GO:1990904;GO:0043229;GO:0005575;GO:0043228;GO:0003674;GO:0005198;GO:0003735	K02867	RP-L11, MRPL	map03010	Ribosome	COG0080	J:Translation, ribosomal structure	PF00298.22;PF03	Ribosomal_L11	Ribosomal protein L11,	CYT	1	5	22	High
TRINITY_DN21181_c0_g1_i6_orf1	-	-	-	unnamed protein product, partial [Brenthis ino]	2.21930002	1.150104714	0.0007774	up	yes	1.097	0.4943	0.544	0.523	0.416	1	1.111	1.179	BP:phosphorylation;I	GO:0016310;GO:0006807;GO:0071704;GO:0006468;GO:0009987;GO:0043412;GO:0036211;GO:0008150;GO:0008152;GO:0044238;GO:1901564;GO:0019538;GO:0044237;GO:0043170;GO:0006796;GO:0006793;GO:0035639;GO:0003674;GO:0005488;GO:0043167;GO:1901363;GO:1901265;GO:0043168;GO:0097367;GO:0000166;GO:0004674;GO:0016740;GO:0004672;GO:0017076;GO:0004679;GO:0005524;GO:0016301;GO:0140096;GO:0036094;GO:0003824;GO:0030554;GO:0016772;GO:0016773;GO:0032559;GO:0032555;GO:0097159;GO:0032553	K07198	PRKAA, AMPK	map04152;map04	AMPK signaling pathway;Ox	ENOG410XNQ0	O:Posttranslational modification	PF00069.28;PF07	Pkinase;PK_Tyr_	Protein kinase domain;I	CYT	1	3	58.3	High
TRINITY_DN3027_c0_g1_i4_orf1	-	-	-	ribosome biogenesis protein BOP1 homolog [Ostrinia furnacalis]	2.090044634	1.063533752	0.0002551	up	yes	1.077	0.5153	0.518	0.507	0.521	1	1.158	1.073	------	------	K14824	ERB1, BOP1	------	------	ENOG410XRBX	J:Translation, ribosomal structure	PF08145.15;PF0(	BOP1NT;WD40	BOP1NT;WD domain, G	CYT	4	6	95.8	High
TRINITY_DN71465_c0_g1_i1_orf1	-	-	-	adenylate kinase isoenzyme 6 [Ostrinia furnacalis]	2.23632203	1.161127951	0.0005654	up	yes	1.075	0.4807	0.487	0.475	0.48	1	1.192	1.032	BP:phosphorylation;I	GO:0016310;GO:0009987;GO:0006793;GO:0008150;GO:0008152;GO:0044237;GO:0006796;GO:0043229;GO:0043227;GO:0043226;GO:0005634;GO:0043231;GO:0005575;GO:0110165;GO:0019205;GO:0035639;GO:1901363;GO:0004017;GO:0016776;GO:0003674;GO:0005488;GO:0016887;GO:0043167;GO:1901265;GO:0043168;GO:0000166;GO:0016740;GO:0017076;GO:0005524;GO:0016787;GO:0016301;GO:0017111;GO:0036094;GO:0003824;GO:0016818;GO:0030554;GO:0097367;GO:0097159;GO:0016817;GO:0016462;GO:0050145;GO:0032559;GO:0016772;GO:0032555;GO:0032553	K18532	AK6, FAP7	map03008;map01	Ribosome biogenesis in euk	COG1936	F:Nucleotide transport and meta	PF13238.9	AAA_18	AAA domain	CYT	1	9	20.6	High
TRINITY_DN147458_c0_g1_i1_orf1	-	-	-	60S ribosomal protein L5, partial [Cotesia chilonis]	2.083495146	1.059005739	0.00106	up	yes	1.073	0.515	0.542	0.416	0.587	1	1.075	1.143	BP:cellular macromo	GO:0034645;GO:0043603;GO:0044249;GO:1901576;GO:0044271;GO:0044260;GO:0043043;GO:0071704;GO:1901566;GO:0043170;GO:0043604;GO:0009987;GO:0009058;GO:0009059;GO:0008150;GO:0008152;GO:1901564;GO:0044238;GO:0019538;GO:0034641;GO:0044237;GO:0006807;GO:0006518;GO:0006412;GO:0043229;GO:0043228;GO:0043227;GO:0043226;GO:0005737;GO:0005634;GO:0110165;GO:0043231;GO:0032991;GO:0005840;GO:0043232;GO:1990904;GO:0005575;GO:0003674;GO:0005488;GO:0003676;GO:0005198;GO:1901363;GO:0003735;GO:0008097;GO:0097159;GO:0019843;GO:0003723	K02932	RP-L5e, RPL5	map03010;map05	Ribosome;Coronavirus disea	COG0256	J:Translation, ribosomal structure	PF17144.7;PF14	Ribosomal_L5e	Ribosomal large subuni	CYT	1	13	37	High
TRINITY_DN198_c0_g1_i2_orf1	-	-	-	retinol dehydrogenase 13-like [Ostrinia furnacalis]	2.0056926	1.00410051	0.001917	up	yes	1.057	0.527	0.475	0.476	0.63	1	1.01	1.16	------	------	K11153	RDH12	map00830;map01	Retinol metabolism;Biosynth	COG1028	V:Defense mechanisms	PF00106.28;PF13	adh_short;adh	short chain dehydroger	CYT	1	4	35.3	High
TRINITY_DN7289_c0_g1_i1_orf1	-	-	-	nuclear cap-binding protein subunit 2 [Ostrinia furnacalis]	2.078307297	1.055408986	0.0005509	up	yes	1.051	0.5057	0.508	0.572	0.437	1	1.029	1.125	BP:negative regulati	GO:0009892;GO:0019222;GO:0044237;GO:0090304;GO:0050789;GO:0006807;GO:1901360;GO:0006139;GO:0010605;GO:0010468;GO:0071704;GO:0065007;GO:0048519;GO:0008380;GO:0016071;GO:0060255;GO:0009987;GO:0006725;GO:0000375;GO:0000377;GO:0008150;GO:0031047;GO:0008152;GO:0046483;GO:0016070;GO:0044238;GO:0045292;GO:0034641;GO:0000398;GO:0043170;GO:0010629;GO:0006396;GO:0006397;GO:0043229;GO:0043227;GO:0005634;GO:0043226;GO:0034518;GO:0110165;GO:0005846;GO:0032991;GO:0043231;GO:0005575;GO:0003674;GO:0005488;GO:0003676;GO:1901363;GO:0000339;GO:0097159;GO:0003723	K12883	NCBP2, CBP2(	map03013;map03	Nucleocytoplasmic transpor	ENOG4111FJQ	A:RNA processing and modificat	PF00076.25	RRM_1	RNA recognition motif.	CYT	1	9	18.8	High
TRINITY_DN3889_c0_g1_i7_orfp1	-	-	-	TRINITY_DN3889_c0_g1_i7_m.1657 TRINITY_DN3889_c0_g1::TRINITY_DN3889_c0_g1_i7::g.1657 ORF type:5prime_partial len:235 (+),score=70.90 TRINITY_DN3889_c0_g1_i7:1-705(+)	6.207207207	2.633944306	0.006302	up	yes	1.378	0.222	0.313	0.157	0.196	1	1.745	1.388	------	------	------	------	------	------	------	------	------	------	------	EXC	1	38	24.3	High
TRINITY_DN6396_c0_g1_i1_orf1	-	-	-	PR domain zinc finger protein 10-like [Ostrinia furnacalis]	2.071526285	1.050694126	0.000332	up	yes	1.06	0.5117	0.514	0.574	0.447	1	1.078	1.102	------	------	K24643	PRDM10	------	------	ENOG4111EA0;(	S:Function unknown;	PF13894.9;PF00(	zf-C2H2_4;zf-(	C2H2-type zinc finger;:	CYT	1	1	79.4	High
TRINITY_DN4905_c0_g1_i6_orf1	-	-	-	uncharacterized protein LOC114351759 [Ostrinia furnacalis]	2.434298441	1.283506051	0.001444	up	yes	1.093	0.449	0.521	0.511	0.315	1	1.159	1.119	------	------	------	------	------	------	------	------	------	------	------	EXC	1	4	62.4	High
TRINITY_DN24318_c0_g1_i1_orf1	-	-	-	60S ribosomal protein L29 [Ostrinia furnacalis]	2.09349184	1.065911294	0.0001065	up	yes	1.039	0.4963	0.532	0.438	0.519	1	1.064	1.054	BP:cellular biosynthe	GO:0044249;GO:0034641;GO:0006807;GO:0034645;GO:1901576;GO:0044271;GO:0044260;GO:0043043;GO:0071704;GO:0043604;GO:0009987;GO:0009058;GO:0009059;GO:0008150;GO:0008152;GO:1901564;GO:0044238;GO:0043603;GO:1901566;GO:0019538;GO:0044237;GO:0043170;GO:0006518;GO:0006412;GO:0043232;GO:0043226;GO:0110165;GO:0005840;GO:0032991;GO:1990904;GO:0043229;GO:0005575;GO:0043228;GO:0003674;GO:0005198;GO:0003735	K02905	RP-L29e, RPL2	map03010;map05	Ribosome;Coronavirus disea	ENOG4112428	J:Translation, ribosomal structure	PF01779.20	Ribosomal_L29	Ribosomal L29e proteir	CYT	4	38	8.5	High
TRINITY_DN19493_c0_g1_i5_orf1	-	-	-	zinc finger MYM-type protein 3 isoform X1 [Ostrinia furnacalis] >XP_028159738.1 zinc finger MYM-type protein 3 isoform X2 [Ostrinia furnacalis]	2.044867977	1.032007701	7.48E-05	up	yes	1.03	0.5037	0.544	0.451	0.516	1	1.048	1.043	------	------	K24675	ZMYM2_3_4	------	------	ENOG410XQR6	S:Function unknown	------	------	------	CYT	2	1	142.6	High
TRINITY_DN4497_c0_g1_i4_orf1	-	-	-	cytochrome P450 9e2-like [Ostrinia furnacalis] >QPF77612.1 cytochrome P450 monooxygenase CYP9A185 [Ostrinia furnacalis]	2.53258427	1.340610274	0.0008292	up	yes	1.127	0.445	0.404	0.405	0.526	1	1.2	1.18	CC:intrinsic compone	GO:0031224;GO:0016021;GO:0110165;GO:0005575;GO:0004497;GO:0003958;GO:0003674;GO:0005488;GO:1901363;GO:0016491;GO:0043169;GO:0046914;GO:0016705;GO:0043167;GO:0005506;GO:0046872;GO:0020037;GO:0003824;GO:0046906;GO:0097159;GO:0016651;GO:0016653	K15003	CYP9	------	------	COG2124	Q:Secondary metabolites biosyr	PF00067.25	p450	Cytochrome P450	CYT	1	15	31.2	High
TRINITY_DN19135_c0_g1_i1_orf1	-	-	-	ER membrane protein complex subunit 10 [Ostrinia furnacalis]	2.00776699	1.005591848	5.07E-05	up	yes	1.034	0.515	0.531	0.474	0.54	1	1.066	1.036	CC:intracellular mem	GO:0043231;GO:0016021;GO:0005575;GO:0043229;GO:0031224;GO:0043227;GO:0043226;GO:0110165;GO:0005783	K23570	EMC10	------	------	ENOG4111PEN	S:Function unknown	------	------	------	CYT	1	4	25.1	High

TRINITY_DN2954_c0_g1_i1_orf1	-	-	-	unnamed protein product [Diatraea saccharalis]	2.033457249	1.02393466	0.001076	up	yes	1.094	0.538	0.563	0.46	0.591	1	1.1	1.181	BP:organic substanc	GO:0071704;GO:0044238;GO:1901564;GO:0032446;GO:0019538;GO:0006807;GO:0070647;GO:0043170;GO:0016567;GO:0043412;GO:0036211;GO:0008150;GO:0008152;GO:0043231;GO:0043229;GO:0043226;GO:0005575;GO:0043227;GO:0005634;GO:0110165	K11789	DCAF1, VPRBP	map05170	Human immunodeficiency v	ENOG410XR8C;	S:Function unknown;	------	------	------	CYT	2	1	162.1	High
TRINITY_DN53311_c0_g2_i1_orf1	-	-	-	transcription elongation factor S-II [Chelonus insularis]	2.00486254	1.003503324	0.0004371	up	yes	1.072	0.5347	0.582	0.471	0.551	1	1.129	1.087	BP:RNA biosynthetic	GO:0032774;GO:0090304;GO:0044249;GO:0034641;GO:0006807;GO:1901362;GO:1901360;GO:1901576;GO:0071704;GO:0006368;GO:0018130;GO:0006139;GO:0009987;GO:0006725;GO:0009058;GO:0009059;GO:0008150;GO:0008152;GO:0034654;GO:0046483;GO:0016070;GO:0044238;GO:0044271;GO:0044237;GO:0043170;GO:0006354;GO:0019438;GO:0043227;GO:0005634;GO:0043226;GO:0005575;GO:0110165;GO:0043231;GO:0043229;GO:0043169;GO:0003674;GO:0005488;GO:0003676;GO:0003677;GO:0043167;GO:1901363;GO:0046914;GO:0046872;GO:0008135;GO:0090079;GO:0097159;GO:0003746;GO:0045182;GO:0008270	K03145	TFIIS	------	------	COG1594	K:Transcription	PF07500.17;PF0	TFIIS_M;TFIIS_C	Transcription factor S-I	CYT	1	8	34.9	High
TRINITY_DN14730_c0_g1_i7_orf1	-	-	-	titin homolog [Ostrinia furnacalis]	2.406576981	1.266982572	0.0005752	up	yes	1.127	0.4683	0.443	0.459	0.503	1	1.18	1.201	------	------	------	------	------	------	ENOG410ZVTG:	S:Function unknown;	PF07679.19;PF1	I-set;Ig_3;Ig_2	Immunoglobulin I-set c	CYT	1	0	324.7	High
TRINITY_DN8971_c1_g1_i4_orf1	-	-	-	synaptosomal-associated protein 25 isoform X1 [Bombyx mori]	3.327735796	1.734540896	0.02422	up	yes	1.189	0.3573	0.32	0.351	0.401	1	1.655	0.912	------	------	K18211	SNAP25	map04911;map04	Insulin secretion;Synaptic ve	ENOG410Y3Y0	U:Intracellular trafficking, secreti	PF00835.22	SNAP-25	SNAP-25 family	CYT	1	6	23.6	High
TRINITY_DN1578_c0_g3_i1_orf1	-	-	-	S-adenosylmethionine synthase isoform X1 [Ostrinia furnacalis]	2.046667985	1.033277084	0.0001374	up	yes	1.035	0.5057	0.456	0.559	0.502	1	1.075	1.03	BP:one-carbon meta	GO:0006730;GO:0044249;GO:0044281;GO:1901576;GO:0071704;GO:0006790;GO:0046500;GO:0009987;GO:0009058;GO:0008150;GO:0008152;GO:0044272;GO:0006556;GO:0044237;GO:0035639;GO:0003674;GO:0000166;GO:1901363;GO:1901265;GO:0043168;GO:0016765;GO:0043167;GO:0016740;GO:0046872;GO:0005524;GO:0043169;GO:0036094;GO:0003824;GO:0017076;GO:0004478;GO:0097159;GO:0030554;GO:0097367;GO:0032559;GO:0032555;GO:0032553;GO:0005488	K00789	metK, MAT	map00270;map01	Cysteine and methionine me	COG0192	H:Coenzyme transport and meta	PF02772.19;PF0	S-AdoMet_syn	S-adenosylmethionine	CYT	7	42	28.2	High
TRINITY_DN4156_c0_g1_i2_orf1	-	-	-	calcium channel flower [Ostrinia furnacalis]	2.33409611	1.222863967	0.001329	up	yes	1.122	0.4807	0.455	0.543	0.444	1	1.11	1.255	------	------	------	------	------	------	ENOG4111G4O	U:Intracellular trafficking, secreti	PF10233.12	Cg6151-P	Uncharacterized conse	CYT	1	10	20.9	High
TRINITY_DN52893_c0_g1_i1_orf1	-	-	-	growth arrest and DNA damage-inducible proteins-interacting protein 1 [Galleria mellonella]	2.308852035	1.20717572	0.0001696	up	yes	1.072	0.4643	0.471	0.446	0.476	1	1.151	1.065	BP:cellular process;B	GO:0009987;GO:0008150;GO:0007049;GO:0043231;GO:0005634;GO:0005739;GO:0005575;GO:0043229;GO:0043227;GO:0043226;GO:0110165	------	------	------	------	ENOG410XVD1	S:Function unknown	PF10147.12	CR6_interact	Growth arrest and DNA	CYT	3	13	29.1	High
TRINITY_DN82324_c0_g1_i4_orf1	-	-	-	hypothetical protein evm_001824 [Chilo suppressalis] >CAG9754426.1 unnamed protein product [Diatraea saccharalis] >CAG9793111.1 unnamed protein product [Diatraea saccharalis]	2.045855379	1.032704165	3.89E-05	up	yes	1.044	0.5103	0.485	0.517	0.529	1	1.081	1.051	BP:cellular biosynthe	GO:0044249;GO:0034641;GO:0006807;GO:0034645;GO:1901576;GO:0044260;GO:0043043;GO:0071704;GO:0043604;GO:0009987;GO:0009058;GO:0009059;GO:0008150;GO:0008152;GO:0044271;GO:0044238;GO:1901564;GO:0043603;GO:1901566;GO:0019538;GO:0044237;GO:0043170;GO:0006518;GO:0006412;GO:0015935;GO:0032991;GO:0044391;GO:1990904;GO:0005575;GO:0003674;GO:0005198;GO:0003735	K02969	RP-S20e, RPS2	map03010;map05	Ribosome;Coronavirus disea	COG0051	U:Intracellular trafficking, secreti	PF00338.25	Ribosomal_S10	Ribosomal protein S10	CYT	4	37	13.7	High
TRINITY_DN2252_c0_g1_i4_orfp1	-	-	-	TRINITY_DN2252_c0_g1_i4_m.69997 TRINITY_DN2252_c0_g1::TRINITY_DN2252_c0_g1_i4::g.69997 ORF type:5prime_partial len:168 (-) score=65.67 TRINITY_DN2252_c0_g1_i4:244-747(-)	2.093442952	1.065877604	0.0002328	up	yes	1.044	0.4987	0.44	0.54	0.516	1	1.028	1.105	------	------	------	------	------	------	------	------	------	------	------	CYT	1	5	19.1	High
TRINITY_DN49742_c0_g1_i4_orf1	-	-	-	neuroglobin-like [Ostrinia furnacalis]	2.37143452	1.245760033	0.002836	up	yes	1.139	0.4803	0.524	0.367	0.55	1	1.287	1.129	MF:oxygen binding;M	GO:0019825;GO:0020037;GO:0046906;GO:0097159;GO:0036094;GO:0003674;GO:0005488;GO:1901363	K21893	NGB	------	------	ENOG4111QDK	S:Function unknown	PF00042.25	Globin	Globin	CYT	1	11	21.5	High
TRINITY_DN11457_c0_g1_i5_orf1	-	-	-	uncharacterized protein LOC114352268 [Ostrinia furnacalis]	2.238866397	1.162768439	0.001184	up	yes	1.106	0.494	0.476	0.445	0.561	1	1.226	1.091	------	------	------	------	------	------	ENOG41121MH	S:Function unknown	------	------	------	PLA	1	13	14.3	High
TRINITY_DN8958_c0_g1_i1_orf1	-	-	-	nuclear cap-binding protein subunit 1 [Galleria mellonella]	2.033832394	1.024200793	7.78E-05	up	yes	1.046	0.5143	0.559	0.497	0.487	1	1.067	1.07	------	------	K12882	NCBP1, CBP8	map03013;map03	Nucleocytoplasmic transpor	ENOG410XR7H	A:RNA processing and modificat	PF09090.14;PF0	MIF4G_like_2;M	MIF4G like;MIF4G like;M	CYT	2	3	92.5	High
TRINITY_DN10458_c0_g1_i1_orf1	-	-	-	V-type proton ATPase 21 kDa proteolipid subunit [Ostrinia furnacalis]	2.108952534	1.076526625	0.0001736	up	yes	1.053	0.4993	0.519	0.499	0.48	1	1.029	1.13	CC:intrinsic compone	GO:0031224;GO:0005575;GO:0033179;GO:0016021;GO:0110165;GO:0032991;GO:0098796;GO:0033177;GO:0003674;GO:0042626;GO:0015318;GO:0009678;GO:0015399;GO:0042625;GO:0005215;GO:0008324;GO:0046961;GO:0044769;GO:0022890;GO:0019829;GO:0015075;GO:0022857;GO:0022804;GO:0022853;GO:0015078	K03661	ATPeV0B, ATP	map05152;map05	Tuberculosis;Human papillo	COG0636	C:Energy production and conver	PF00137.24	ATP-synt_C	ATP synthase subunit C	CYT	1	5	21.6	High
TRINITY_DN72934_c0_g1_i1_orf1	-	-	-	carboxylesterase 8 [Streltzoviella insularis]	2.484978541	1.313233393	0.001962	up	yes	1.158	0.466	0.429	0.555	0.414	1	1.291	1.183	MF:hydrolase activity	GO:0016787;GO:0003674;GO:0016788;GO:0052689;GO:0003824	------	------	------	------	COG2272	I:Lipid transport and metabolism	PF00135.31;PF2	COesterase;BD	Carboxylesterase famil	CYT	1	5	61.5	High
TRINITY_DN83327_c0_g1_i1_orf1	-	-	-	trypsin-like serine proteinase T22 [Ostrinia nubilalis]	2.695924765	1.430780236	0.0008754	up	yes	1.118	0.4147	0.387	0.343	0.514	1	1.16	1.195	BP:organic substanc	GO:0071704;GO:1901564;GO:0019538;GO:0043170;GO:0008150;GO:0006807;GO:0008152;GO:0006508;GO:0044238;GO:0004252;GO:0004175;GO:0016787;GO:0017171;GO:0140096;GO:0003824;GO:0003674;GO:0008233;GO:0008236	K09640;K01312	TMPRSS9;PRS	map04972;map05	Pancreatic secretion;Influenz	COG5640	O:Posttranslational modification	PF00089.29	Trypsin	Trypsin	PLA	1	4	28.4	Medium
TRINITY_DN8367_c0_g1_i1_orf1	-	-	-	uncharacterized protein LOC114357075 [Ostrinia furnacalis]	2.01852947	1.01330465	4.64E-05	up	yes	1.024	0.5073	0.514	0.471	0.537	1	1.062	1.009	------	------	------	------	------	------	COG1028	S:Function unknown	PF00106.28;PF1	adh_short;adh	short chain dehydroger	CYT	3	36	23.4	High
TRINITY_DN1999_c0_g1_i9_orf1	-	-	-	acyl-CoA Delta(11) desaturase-like [Ostrinia furnacalis] >XP_028172986.1 acyl-CoA Delta(11) desaturase-like [Ostrinia furnacalis] >AAL27034.1 acyl-CoA delta-9 desaturase [Ostrinia furnacalis] >AAL29454.1 acyl-CoA delta-9 desaturase [Ostrinia nubilalis]	2.021868787	1.015689374	4.11E-05	up	yes	1.017	0.503	0.536	0.464	0.509	1	1.002	1.049	BP:fatty acid biosynt	GO:0006633;GO:0006631;GO:0019752;GO:0044249;GO:0044281;GO:0044283;GO:0072330;GO:1901576;GO:0071704;GO:0006629;GO:0009987;GO:0032787;GO:0009058;GO:0008150;GO:0008152;GO:0043436;GO:0044255;GO:0008610;GO:0044238;GO:0006082;GO:0046394;GO:0016053;GO:0044237;GO:0031224;GO:0016021;GO:0110165;GO:0005575;GO:0003674;GO:0016491;GO:0016705;GO:0043167;GO:0046872;GO:0043169;GO:0003824;GO:0016717;GO:0005488	K00507	SCD, desC	map04936;map04	Alcoholic liver disease;AMPK	COG1398	S:Function unknown	------	------	------	CYT	1	3	40.8	High
TRINITY_DN21619_c0_g1_i1_orf1	-	-	-	28S ribosomal protein S15, mitochondrial [Ostrinia furnacalis]	2.064646465	1.045894766	6.67E-06	up	yes	1.022	0.495	0.485	0.479	0.521	1	1.029	1.036	BP:cellular biosynthe	GO:0044249;GO:0034641;GO:0006807;GO:0034645;GO:1901576;GO:0044271;GO:0044260;GO:0043043;GO:0071704;GO:0043604;GO:0009987;GO:0009058;GO:0009059;GO:0008150;GO:0008152;GO:1901564;GO:0044238;GO:0043603;GO:1901566;GO:0019538;GO:0044237;GO:0043170;GO:0006518;GO:0006412;GO:0043232;GO:0043226;GO:0110165;GO:0005840;GO:0032991;GO:1990904;GO:0043229;GO:0005575;GO:0043228;GO:0003674;GO:0005198;GO:0003735	K02956	RP-S15, MRPS	map03010	Ribosome	COG0184	S:Function unknown	PF00312.25	Ribosomal_S15	Ribosomal protein S15	CYT	3	14	32.8	High
TRINITY_DN14507_c0_g1_i5_orf1	-	-	-	PTB domain-containing adapter protein ced-6 [Ostrinia furnacalis]	2.355417682	1.235982913	0.001327	up	yes	1.063	0.4513	0.432	0.576	0.346	1	1.127	1.061	------	------	K23285	GULP1, CED6	------	------	ENOG410ZHJT;C	S:Function unknown;	PF00640.26	PID	Phosphotyrosine intera	CYT	1	3	57	High
TRINITY_DN1422_c0_g1_i4_orf1	-	-	-	unnamed protein product [Chilo suppressalis]	2.098075729	1.069066752	8.44E-07	up	yes	1.014	0.4833	0.484	0.472	0.494	1	1.028	1.014	BP:generation of pre	GO:0006091;GO:0022900;GO:0009987;GO:0022904;GO:0008150;GO:0008152;GO:0042773;GO:0044237;GO:0016020;GO:0110165;GO:0005575;GO:0003674;GO:0005488;GO:0003955;GO:0003954;GO:0016491;GO:0051540;GO:0015399;GO:0050136;GO:0005215;GO:0022804;GO:0015453;GO:0008137;GO:0003824;GO:0009055;GO:0051539;GO:0051536;GO:0016655;GO:0022857;GO:0016651	K03934	NDUFS1	map04714;map00	Thermogenesis;Oxidative ph	COG1034	C:Energy production and conver	PF00384.25;PF1	Molybdopterin;	Molybdopterin oxidore	CYT	23	40	79.7	High
TRINITY_DN38301_c0_g1_i2_orf1	-	-	-	gamma-taxilin [Ostrinia furnacalis]	2.097195868	1.068461608	2.96E-07	up	yes	0.9947	0.4743	0.485	0.464	0.474	1	0.999	0.985	MF:molecular_functi	GO:0003674;GO:0000149;GO:0019905;GO:0005488;GO:0005515	------	------	------	------	ENOG410XOH1	S:Function unknown	PF09728.12	Taxilin	Myosin-like coiled-coil	CYT	7	12	71.1	High
TRINITY_DN3836_c0_g1_i4_orf1	-	-	-	2-oxoisovalerate dehydrogenase subunit alpha, mitochondrial [Ostrinia furnacalis]	2.143010984	1.099639245	5.08E-06	up	yes	0.995	0.4643	0.464	0.445	0.484	1	1.012	0.973	BP:carboxylic acid m	GO:0019752;GO:0009063;GO:0006807;GO:0044281;GO:0044282;GO:1901575;GO:0006520;GO:0071704;GO:0009987;GO:0008150;GO:0008152;GO:0043436;GO:0009056;GO:0044248;GO:0044238;GO:1901564;GO:1901565;GO:0006082;GO:0046395;GO:0016054;GO:0044237;GO:0009081;GO:0009083;GO:0003674;GO:0016491;GO:0016624;GO:0003824;GO:0016903;GO:0003863	K00166	BCKDHA, bkdA	map00280;map00	Valine, leucine and isoleucin	COG1071	S:Function unknown	PF00676.23	E1_dh	Dehydrogenase E1 con	CYT	8	21	48.9	High
TRINITY_DN2780_c0_g1_i5_orf1	-	-	-	probable ATP-dependent RNA helicase DDX27 [Ostrinia furnacalis]	2.271194063	1.183450983	9.57E-07	up	yes	1.01	0.4447	0.453	0.428	0.453	1	1.005	1.025	------	------	K13181	DDX27, DRS1	------	------	COG0513	O:Posttranslational modification	PF00270.32;PF0	DEAD;Helicase	DEAD/DEAH box helica	CYT	4	7	90.9	High
TRINITY_DN1386_c0_g1_i6_orf1	-	-	-	ras-related protein Rab-36 [Ostrinia furnacalis]	2.093023256	1.065588342	1.95E-05	up	yes	0.981	0.4687	0.478	0.497	0.431	1	0.973	0.97	------	------	K07918	RAB32	------	------	ENOG410YITC	S:Function unknown	PF00071.25;PF08	Ras;Roc;Arf	Ras family;Ras of Comp	CYT	7	13	54.1	High
TRINITY_DN83005_c0_g1_i1_orf1	-	-	-	ATP synthase subunit O, mitochondrial [Danaus plexippus plexippus] >OWR53927.1 H+ transporting ATP synthase O subunit [Danaus plexippus plexippus]	2.223458714	1.152805616	2.84E-06	up	yes	0.999	0.4493	0.475	0.432	0.441	1	1.009	0.988	CC:cellular_compone	GO:0005575;GO:0031090;GO:0016020;GO:0110165;GO:0031966;GO:0019866;GO:0005743;GO:0005261;GO:0003674;GO:0046933;GO:0015318;GO:0015252;GO:0022803;GO:0005215;GO:0005216;GO:0008324;GO:0003824;GO:0016874;GO:0022890;GO:0015075;GO:0022857;GO:0015267;GO:0015078	K02137	ATPeF0O, ATP	map04714;map05	Thermogenesis;Chemical ca	COG0712;COG0	C:Energy production and conver	PF00213.21	OSCP	ATP synthase delta	CYT	13	55	22.7	High
TRINITY_DN8173_c0_g1_i3_orf1	-	-	-	dihydroceramide fatty acyl 2-hydroxylase FAH1 [Ostrinia furnacalis]	2.912937347	1.542474672	0.001285	up	yes	1.074	0.3687	0.31	0.414	0.382	1	0.985	1.238	BP:fatty acid biosynt	GO:0006633;GO:0006631;GO:0019752;GO:0044249;GO:0044281;GO:0044283;GO:0072330;GO:1901576;GO:0071704;GO:0006629;GO:0009987;GO:0032787;GO:0009058;GO:0008150;GO:0008152;GO:0043436;GO:0044255;GO:0008610;GO:0044238;GO:0006082;GO:0046394;GO:0016053;GO:0044237;GO:0005789;GO:0031224;GO:0031090;GO:0016021;GO:0016020;GO:0110165;GO:0005575;GO:0003674;GO:0043167;GO:0016491;GO:0046914;GO:1901363;GO:0005506;GO:0046872;GO:0020037;GO:0043169;GO:0003824;GO:0046906;GO:0097159;GO:0080132;GO:0005488	K19703	FA2H, SCS7	------	------	COG5274;COG5	C:Energy production and conver	PF04116.16;PF0	FA_hydroxylase	Fatty acid hydroxylase;	CYT	1	4	39.3	High
TRINITY_DN24142_c0_g1_i1_orf1	-	-	-	arylalkylamine N-acetyltransferase [Chilo suppressalis]	2.339438906	1.226162553	1.73E-05	up	yes	1.009	0.4313	0.464	0.444	0.386	1	1.012	1.016	MF:N-acetyltransfer	GO:0008080;GO:0004059;GO:0016740;GO:0003824;GO:0016407;GO:0003674;GO:0016747;GO:0016410;GO:0016746	------	------	------	------	ENOG4111REY	S:Function unknown	------	------	------	CYT	1	3	29.3	High
TRINITY_DN33_c0_g1_i1_orf1	-	-	-	uncharacterized protein CG45076-like isoform X2 [Ostrinia furnacalis]	2.110151188	1.077346369	0.0001192	up	yes	0.977	0.463	0.435	0.426	0.528	1	0.966	0.965	------	------	------	------	------	------	ENOG4112AMP:	S:Function unknown;	------	------	------	CYT	5	32	54.2	High
TRINITY_DN19186_c0_g1_i1_orf1	-	-	-	39S ribosomal protein L9, mitochondrial [Ostrinia furnacalis]	2.068869936	1.04884295	2.92E-05	up	yes	0.9703	0.469	0.457	0.495	0.455	1	0.933	0.978	CC:macromolecular	GO:0032991;GO:0005840;GO:0043232;GO:1990904;GO:0005575;GO:0043229;GO:0043228;GO:0043226;GO:0110165	K02939	RP-L9, MRPL9	map03010	Ribosome	ENOG4111HFB	J:Translation, ribosomal structure	PF01281.22	Ribosomal_L9_	Ribosomal protein L9, N	CYT	2	10	29.2	High
TRINITY_DN131371_c0_g1_i1_orf1	-	-	-	golgin subfamily B member 1-like [Ostrinia furnacalis]	2.262309961	1.177796608	0.000224	up	yes	0.997	0.4407	0.501	0.369	0.452	1	1.032	0.959	------	------	K20478	GOLGB1	------	------	------	------	------	------	------	CYT	1	8	11.9	High
TRINITY_DN45924_c0_g1_i14_orf1	-	-	-	adenylosuccinate synthetase isoform X1 [Ostrinia furnacalis] >XP_028166048.1 adenylosuccinate synthetase isoform X2 [Ostrinia furnacalis]	2.394799054	1.259904606	4.70E-07	up	yes	1.013	0.423	0.427	0.417	0.425	1	1.031	1.007	BP:nucleoside mono	GO:0009124;GO:0009260;GO:0009161;GO:0009127;GO:0044249;GO:0034641;GO:0009165;GO:0072521;GO:0006163;GO:0072522;GO:1901362;GO:0009259;GO:0009168;GO:0044208;GO:0090407;GO:0071704;GO:1901360;GO:0018130;GO:0009987;GO:0009167;GO:0019693;GO:0006139;GO:0009156;GO:0006793;GO:0006725;GO:0009152;GO:0046033;GO:0009150;GO:0009058;GO:0009117;GO:0008152;GO:0019438;GO:0034654;GO:0044271;GO:0055086;GO:0046483;GO:0044238;GO:0009123;GO:1901564;GO:0009126;GO:1901566;GO:1901576;GO:1901135;GO:0046390;GO:0044237;GO:1901137;GO:0006796;GO:0006807;GO:0006167;GO:1901293;GO:0006164;GO:0019637;GO:0008150;GO:0006753;GO:0044281;GO:0005737;GO:0110165;GO:0005575;GO:0004019;GO:0000166;GO:0035639;GO:0003674;GO:0005488;GO:0019001;GO:0043169;GO:0032561;GO:0000287;GO:0005525;GO:0043167;GO:0032553;GO:0017076;GO:0046872;GO:0097367;GO:0043168;GO:1901265;GO:0036094;GO:0003824;GO:0016874;GO:0032555;GO:0016879;GO:0097159;GO:1901363	K01939	purA, ADSS	map00250;map01	Alanine, aspartate and gluta	COG0104	F:Nucleotide transport and meta	PF00709.24	Adenylsucc_syn	Adenylosuccinate synth	CYT	10	19	49.7	High
TRINITY_DN38424_c0_g1_i1_orf1	-	-	-	glucose dehydrogenase [FAD, quinone]-like [Ostrinia furnacalis]	2.561674558	1.357087203	0.001075	up	yes	1.028	0.4013	0.487	0.453	0.264	1	1.004	1.081	MF:anion binding;MF	GO:0043168;GO:0050660;GO:0016614;GO:0097159;GO:0000166;GO:0036094;GO:0003824;GO:0003674;GO:0005488;GO:0043167;GO:1901363;GO:1901265;GO:0016491	------	------	------	------	COG2303	I:Lipid transport and metabolism	PF00732.22;PF05	GMC_oxred_N;	GMC oxidoreductase;G	CYT	1	2	66.3	High
TRINITY_DN76283_c0_g2_i1_orf1	-	-	-	fatty acid synthase-like [Ostrinia furnacalis]	2.391923991	1.258171545	4.15E-05	up	yes	1.007	0.421	0.426	0.467	0.37	1	1.029	0.993	BP:fatty acid biosynt	GO:0006633;GO:0006631;GO:0019752;GO:0044249;GO:0044281;GO:0044283;GO:0072330;GO:1901576;GO:0071704;GO:0006629;GO:0009987;GO:0032787;GO:0009058;GO:0008150;GO:0008152;GO:0043436;GO:0044255;GO:0008610;GO:0044238;GO:0006082;GO:0046394;GO:0016053;GO:0044237;GO:0003674;GO:0016491;GO:0016740;GO:0016746;GO:0003824	K00665	FASN	map04910;map04	Insulin signaling pathway;Alc	COG3321	Q:Secondary metabolites biosyn	PF00698.24	Acyl_transf_1	Acyl transferase domai	CYT	3	8	32	High
TRINITY_DN32022_c0_g1_i1_orf1	-	-	-	striatin isoform X1 [Diachasma alloeum]	2.126948775	1.088785288	0.0003987	up	yes	0.955	0.449	0.46	0.398	0.489	1	0.88	0.985	CC:macromolecular	GO:0032991;GO:0016020;GO:0005575;GO:0110165;GO:0044877;GO:0005488;GO:0005515;GO:0003674;GO:0005516	K17608	STRN1_3_4	map04013	MAPK signaling pathway - f	ENOG410XR31	S:Function unknown	PF08232.15;PF0	Striatin;WD40	Striatin family;WD dom	CYT	1	1	80	High
TRINITY_DN20749_c0_g1_i3_orf1	-	-	-	protein arginine N-methyltransferase 1-like [Ostrinia furnacalis]	2.172816869	1.119566585	0.0003485	up	yes	0.948	0.4363	0.505	0.403	0.401	1	0.946	0.898	BP:protein methylati	GO:0006479;GO:0006807;GO:0008213;GO:0035246;GO:0035247;GO:0044260;GO:0018216;GO:0018193;GO:0071704;GO:0032259;GO:0009987;GO:0043412;GO:0036211;GO:0043414;GO:0008152;GO:0044238;GO:1901564;GO:0019538;GO:0044237;GO:0043170;GO:0018195;GO:0008150;GO:0016274;GO:0016273;GO:0003674;GO:0008168;GO:0016740;GO:0016741;GO:0008757;GO:0140096;GO:0003824;GO:0008170;GO:0008276	K11436	PRMT3	------	------	ENOG410XQYH	O:Posttranslational modification	PF06325.16;PF03	PrmA;TehB;Met	Ribosomal protein L11	CYT	2	4	59.5	High
TRINITY_DN8944_c0_g1_i1_orf1	-	-	-	actin, clone 403 [Trichonephila clavata]	2.755555556	1.462343214	0.0007706	up	yes	1.116	0.405	0.472	0.376	0.367	1	1.24	1.107	------	------	K05692	ACTB_G1	map05164;map04	Influenza A;Hippo signaling	COG5277	O:Posttranslational modification	PF00022.22	Actin	Actin	CYT	1	64	42.8	High
TRINITY_DN86090_c0_g1_i1_orf1	-	-	-	ATP synthase subunit b, mitochondrial [Ostrinia furnacalis]	2.105479171	1.074148603	3.30E-05	up	yes	1.026	0.4873	0.504	0.479	0.479	1	1.076	1.003	BP:nucleoside tripho	GO:0009141;GO:0009142;GO:0009144;GO:0009145;GO:0044249;GO:0034641;GO:0009165;GO:0044281;GO:0072521;GO:0072522;GO:1901362;GO:0009259;GO:1901360;GO:0006139;GO:0009201;GO:0071704;GO:0009199;GO:0018130;GO:0009206;GO:0009205;GO:0019693;GO:1901576;GO:0046034;GO:0009987;GO:0006725;GO:0009152;GO:0009150;GO:0009260;GO:0009058;GO:0009117;GO:0008152;GO:0019438;GO:0034654;GO:0044271;GO:0090407;GO:0055086;GO:0046483;GO:0044238;GO:0015986;GO:1901564;GO:1901566;GO:1901137;GO:1901135;GO:0006164;GO:0046390;GO:0044237;GO:0006163;GO:0006796;GO:0006807;GO:1901293;GO:0006793;GO:0019637;GO:0008150;GO:0006753;GO:0006754;GO:0045263;GO:0005575;GO:0098798;GO:0098800;GO:0032991;GO:0000276;GO:0098796;GO:0033177;GO:0022890;GO:0003674;GO:0015318;GO:0022857;GO:0005215;GO:0008324;GO:0015075;GO:0015078	K02127	ATPeF0B, ATP	map04714;map05	Thermogenesis;Chemical ca	ENOG410ZUQH	I:Lipid transport and metabolism	PF05405.17	Mt_ATP-synt_B	Mitochondrial ATP synt	CYT	12	46	27.4	High
TRINITY_DN15737_c0_g1_i7_orf1	-	-	-	UPF0160 protein C27H6.8 [Ostrinia furnacalis]	2.031470648	1.022524519	8.93E-06	up	yes	1.007	0.4957	0.516	0.506	0.465	1	0.996	1.024	------	------	K25877	MYG1	------	------	COG4286	S:Function unknown	PF03690.16	UPF0160	Uncharacterised protei	CYT	4	14	42	High
TRINITY_DN37165_c0_g1_i4_orf1	-	-	-	pyridoxine-5'-phosphate oxidase-like [Ostrinia furnacalis]	2.074074074	1.05246742	5.22E-08	up	yes	1.008	0.486	0.486	0.488	0.484	1	1.008	1.017	BP:cellular biosynthe	GO:0044249;GO:0034641;GO:0006807;GO:0044281;GO:0044283;GO:0072524;GO:0072525;GO:1901360;GO:1901576;GO:1901362;GO:0071704;GO:0018130;GO:0006767;GO:0006766;GO:0009987;GO:0006725;GO:0009058;GO:0009110;GO:0008150;GO:0008152;GO:0044271;GO:0008615;GO:0008614;GO:0046483;GO:1901564;GO:1901566;GO:0042364;GO:0044237;GO:0042816;GO:0019438;GO:0042819;GO:1901617;GO:1901615;GO:0005488;GO:1901363;GO:0003674;GO:0043167;GO:0016491;GO:0043168;GO:0016641;GO:0004733;GO:0000166;GO:1901265;GO:0010181;GO:0036094;GO:0003824;GO:0016638;GO:0097367;GO:0097159;GO:0032553	K00275	pdxH, PNPO	map00750;map01	Vitamin B6 metabolism;Bios	COG0259;ENOG	H:Coenzyme transport and meta	PF01243.23;PF1	Putative_PNPO	Pyridoxamine 5'-phosp	CYT	2	9	29.5	High
TRINITY_DN48413_c1_g1_i2_orf1	-	-	-	probable protein phosphatase 2C 11 isoform X1 [Manduca sexta] >KAG6442694.1 hypothetical protein O3G_MSEX002471 [Manduca sexta]	2.193899782	1.133497625	5.18E-06	up	yes	1.007	0.459	0.469	0.455	0.453	1	1.037	0.983	------	------	K17499	PPM1G, PP2C	------	------	COG0631	T:Signal transduction mechanism	PF00481.24	PP2C	Protein phosphatase 2C	CYT	6	13	70.5	High
TRINITY_DN48619_c0_g1_i1_orf1	-	-	-	PREDICTED: lysine--tRNA ligase isoform X2 [Fopius arisanus]	2.237341066	1.161785201	5.33E-06	up	yes	1.003	0.4483	0.475	0.453	0.417	1	1.001	1.007	BP:lysyl-tRNA aminc	GO:0006430;GO:0019752;GO:0090304;GO:0034641;GO:0006807;GO:0034660;GO:0006139;GO:0006520;GO:0071704;GO:1901360;GO:0016070;GO:0009987;GO:0006725;GO:0008150;GO:0008152;GO:0043436;GO:0046483;GO:0044238;GO:1901564;GO:0006082;GO:0044237;GO:0043170;GO:0006399;GO:0043038;GO:0043039;GO:0006418;GO:0044281;GO:0035639;GO:0003674;GO:0005488;GO:0003676;GO:0043167;GO:1901363;GO:1901265;GO:0043168;GO:0004812;GO:0000166;GO:0017076;GO:0005524;GO:0140098;GO:0036094;GO:0003824;GO:0016874;GO:0016875;GO:0004824;GO:0030554;GO:0097367;GO:0097159;GO:0032559;GO:0032555;GO:0140101;GO:0032553	K04567	KARS, lysS	map00970	Aminoacyl-tRNA biosynthes	COG1190	J:Translation, ribosomal structure	PF00152.23;PF01	tRNA-synt_2;tR	tRNA synthetases class	CYT	1	5	66.7	High
TRINITY_DN30131_c0_g1_i1_orf1	-	-	-	PREDICTED: 60S ribosomal protein L44 [Amyelois transitella] >XP_021198018.1 60S ribosomal protein L44 [Helicoverpa armigera] >XP_022814294.1 60S ribosomal protein L44 [Spodoptera litura] >XP_026732397.1 60S ribosomal protein L44 [Trichoplusia ni] >XP_026752106.1 60S ribosomal protein L44 [Galleria mellonella] >XP_028158932.1 60S ribosomal protein L44 [Ostrinia furnacalis] >XP_035434364.1 60S ribosomal protein L44 [Spodoptera frugiperda] >XP_035434370.1 60S ribosomal protein L44 [Spodoptera frugiperda] >XP_047019234.1 60S ribosomal protein L44 [Helicoverpa zea] >XP_049868501.1 60S ribosomal protein L44 [Pectinophora gossypiella] >AAM53948.1 ribosomal protein L44 [Choristoneura parallela] >KAF9418375.1 hypothetical protein HW555_004805 [Spodoptera exigua] >RVE50750.1 hypothetical protein evm_004660 [Chilo suppressalis] >CAB3235328.1 unnamed protein product [Arctia plantaginis] >CAB3516516.1 unnamed protein product [Spodoptera littoralis] >CAG9747186.1 unnamed protein product [Diatraea saccharalis] >CAH0581656.1 unnamed protein product [Chrysodeixis includens]	2.24201947	1.164798807	0.0001722	up	yes	0.9903	0.4417	0.395	0.519	0.411	1	1.003	0.968	BP:cellular biosynthe	GO:0044249;GO:0034641;GO:0006807;GO:0034645;GO:1901576;GO:0044271;GO:0044260;GO:0043043;GO:0071704;GO:0043604;GO:0009987;GO:0009058;GO:0009059;GO:0008150;GO:0008152;GO:1901564;GO:0044238;GO:0043603;GO:1901566;GO:0019538;GO:0044237;GO:0043170;GO:0006518;GO:0006412;GO:0043232;GO:0043226;GO:0110165;GO:0005840;GO:0032991;GO:1990904;GO:0043229;GO:0005575;GO:0043228;GO:0003674;GO:0005198;GO:0003735	K02929	RP-L44e, RPL4	map03010;map05	Ribosome;Coronavirus disea	COG1631	J:Translation, ribosomal structure	PF00935.22	Ribosomal_L44	Ribosomal protein L44	CYT	3	28	12.3	High
TRINITY_DN124950_c0_g2_i1_orf1	-	-	-	TATA box-binding protein-like protein 1 [Ostrinia furnacalis] >XP_028155830.1 TATA box-binding protein-like protein 1 [Ostrinia furnacalis]	2.591112156	1.373571464	0.0004277	up	yes	1.102	0.4253	0.402	0.377	0.497	1	1.164	1.142	BP:RNA biosynthetic	GO:0032774;GO:0090304;GO:0044249;GO:0034641;GO:0006807;GO:1901362;GO:1901360;GO:1901576;GO:0071704;GO:0018130;GO:0006139;GO:0009987;GO:0006725;GO:0009058;GO:0009059;GO:0008150;GO:0008152;GO:0034654;GO:0046483;GO:0016070;GO:0044238;GO:0044271;GO:0044237;GO:0043170;GO:0006352;GO:0019438;GO:0003674;GO:0005488;GO:0003676;GO:0003677;GO:1901363;GO:0097159	K03120	TBP, tbp	map05166;map05	Human T-cell leukemia virus	COG2101	K:Transcription	PF00352.24	TBP	Transcription factor TFI	CYT	1	4	31.8	High
TRINITY_DN5087_c0_g1_i6_orf1	-	-	-	nascent polypeptide-associated complex subunit alpha [Ostrinia furnacalis] >XP_028156807.1 nascent polypeptide-associated complex subunit alpha [Ostrinia furnacalis] >XP_028156808.1 nascent polypeptide-associated complex subunit alpha [Ostrinia furnacalis]	2.361682022	1.239814733	8.84E-06	up	yes	0.9997	0.4233	0.453	0.407	0.41	1	0.976	1.023	CC:cellular_compone	GO:0005575;GO:0005854;GO:0032991	K03626	EGD2, NACA	map04928	Parathyroid hormone synthe	COG1308	K:Transcription	PF01849.21;PF19	NAC;HYPK_UBA	NAC domain;HYPK UBA	CYT	7	34	22.8	High
TRINITY_DN106730_c0_g1_i1_orf1	-	-	-	Photosystem I reaction center subunit II, chloroplastic, partial [Trichinella zimbabwensis]	2.524875622	1.336212321	2.42E-05	up	yes	1.015	0.402	0.412	0.355	0.439	1	1.039	1.006	------	------	K02692	psaD	------	------	ENOG4111HAV	C:Energy production and conver	PF02531.19	PsaD	PsaD	CYT	2	11	22.6	High
TRINITY_DN12133_c0_g2_i1_orf1	-	-	-	O-acyltransferase like protein-like [Ostrinia furnacalis]	2.229066849	1.156439883	0.0004122	up	yes	0.977	0.4383	0.372	0.518	0.425	1	1.005	0.926	CC:integral compone	GO:0016021;GO:0005575;GO:0031224;GO:0110165;GO:0003824;GO:0016740;GO:0016746;GO:0003674;GO:0016747	------	------	------	------	ENOG410YD1T;E	S:Function unknown;	PF01757.25;PF2	Acyl_transf_3;N	Acyltransferase family;N	CYT	1	1	84.1	High
TRINITY_DN4589_c0_g1_i1_orf1	-	-	-	protein penguin [Ostrinia furnacalis]	2.118348058	1.082939653	0.0002906	up	yes	1.031	0.4867	0.514	0.543	0.403	1	1.059	1.035	MF:cation binding;M	GO:0043169;GO:0097159;GO:0043167;GO:0003674;GO:0003723;GO:0003676;GO:0003677;GO:0046872;GO:1901363;GO:0005488	K14844	PUF6	------	------	ENOG410XSHY	S:Function unknown	PF08144.14;PF0	CPL;PUF	CPL;Pumilio-family RN	CYT	2	3	70.9	High

TRINITY_DN6535_c0_g2_i1_orf1	-	-	-	NADH dehydrogenase [ubiquinone] 1 beta subcomplex subunit 3 [Ostrinia furnacalis] >XP_028166399.1 NADH dehydrogenase [ubiquinone] 1 beta subcomplex subunit 3 [Ostrinia furnacalis]	2.300700935	1.202073462	1.20E-05	up	yes	0.9847	0.428	0.424	0.413	0.447	1	1.006	0.948	BP:cellular process;B	GO:0009987;GO:0044237;GO:0022900;GO:0008150;GO:0008152;GO:0006091;GO:0031090;GO:0016021;GO:0016020;GO:0019866;GO:0031966;GO:0005575;GO:0005743;GO:0031224;GO:0070469;GO:0110165	K03959	NDUFB3	map04714;map00	Thermogenesis;Oxidative ph	ENOG411294X	I:Lipid transport and metabolism	PF08122.15	NDUF_B12	NADH-ubiquinone oxic	CYT	4	39	11	High
TRINITY_DN14168_c0_g1_i1_orf1	-	-	-	transmembrane 7 superfamily member 3-like [Ostrinia furnacalis]	2.515081207	1.330604982	0.001328	up	yes	1.084	0.431	0.466	0.494	0.333	1	1.041	1.212	CC:cellular_compone	GO:0005575;GO:0016021;GO:0110165;GO:0031224	------	------	------	------	ENOG41117R0	S:Function unknown	PF13886.9	DUF4203	Domain of unknown fur	CYT	1	3	62.2	High
TRINITY_DN3749_c0_g1_i1_orf1	-	-	-	cytochrome c oxidase subunit 6B1 [Ostrinia furnacalis]	2.572151899	1.362975844	2.94E-05	up	yes	1.016	0.395	0.397	0.358	0.43	1	0.991	1.057	CC:macromolecular	GO:0032991;GO:0045277;GO:0098803;GO:0070069;GO:0043231;GO:0005575;GO:0098796;GO:0043229;GO:0005739;GO:0043227;GO:0043226;GO:0110165;GO:1902494	K02267	COX6B	map04714;map00	Thermogenesis;Oxidative ph	ENOG41121WM	S:Function unknown	PF02297.20	COX6B	Cytochrome oxidase c	CYT	5	43	9.9	High
TRINITY_DN3028_c0_g1_i1_orf1	-	-	-	pre-rRNA processing protein FTSJ3 [Ostrinia furnacalis]	2.222912353	1.152451066	0.0001632	up	yes	0.9663	0.4347	0.499	0.418	0.387	1	0.932	0.967	BP:metabolic proces	GO:0008152;GO:0009451;GO:0090304;GO:0034641;GO:0006807;GO:0034660;GO:0000451;GO:0000453;GO:0006139;GO:0044260;GO:1901360;GO:0071704;GO:0006364;GO:0032259;GO:0034470;GO:0009987;GO:0006725;GO:0043412;GO:0008150;GO:0031167;GO:0001510;GO:0046483;GO:0016070;GO:0044238;GO:0016072;GO:0000154;GO:0044237;GO:0043170;GO:0043414;GO:0006396;GO:0043226;GO:0005730;GO:0110165;GO:0043232;GO:0043229;GO:0005575;GO:0043228;GO:0003674;GO:0008168;GO:0008649;GO:0016740;GO:0016741;GO:0140098;GO:0008757;GO:0003824;GO:0008173;GO:0140102	K14857	SPB1, FTSJ3	------	------	COG0293	J:Translation, ribosomal structure	PF07780.15;PF0	Spb1_C;FtsJ;DU	Spb1 C-terminal doma	CYT	2	3	99	High
TRINITY_DN2070_c1_g1_i1_orf1	-	-	-	mitochondrial carrier homolog 2-like [Helicoverpa zea] >PZC82360.1 hypothetical protein B5X24_HaOG210663 [Helicoverpa armigera]	2.06657137	1.047239189	0.0006799	up	yes	1.012	0.4897	0.465	0.564	0.44	1	0.95	1.086	CC:cellular_compone	GO:0005575;GO:0016021;GO:0110165;GO:0031224	K17885	MTCH	------	------	ENOG410XSXF	O:Posttranslational modification	PF00153.30	Mito_carr	Mitochondrial carrier pr	CYT	3	12	33.1	High
TRINITY_DN620_c0_g1_i4_orf1	-	-	-	lysine--tRNA ligase isoform X1 [Ostrinia furnacalis]	2.471177945	1.3051989	1.26E-06	up	yes	0.986	0.399	0.401	0.383	0.413	1	0.989	0.969	BP:lysyl-tRNA aminc	GO:0006430;GO:0016070;GO:0019752;GO:0090304;GO:0034641;GO:0006807;GO:0034660;GO:0006139;GO:0006520;GO:0071704;GO:1901360;GO:0009987;GO:0006725;GO:0008150;GO:0008152;GO:0043436;GO:0046483;GO:0044238;GO:1901564;GO:0006082;GO:0044237;GO:0043170;GO:0006399;GO:0043038;GO:0043039;GO:0006418;GO:0044281;GO:0005737;GO:0005575;GO:0110165;GO:0035639;GO:0003674;GO:0005488;GO:0003676;GO:0043167;GO:1901363;GO:1901265;GO:0004812;GO:0000166;GO:0017076;GO:0005524;GO:0043168;GO:0140098;GO:0036094;GO:0003824;GO:0016874;GO:0016875;GO:0004824;GO:0030554;GO:0097367;GO:0097159;GO:0032559;GO:0032555;GO:0140101;GO:0032553	K04567	KARS, lysS	map00970	Aminoacyl-tRNA biosynthes	COG1190	J:Translation, ribosomal structure	PF00152.23;PF0	tRNA-synt_2;tR	tRNA synthetases class	CYT	13	42	50.6	High
TRINITY_DN17049_c0_g1_i6_orf1	-	-	-	unnamed protein product [Arctia plantaginis] >CAB3248215.1 unnamed protein product [Arctia plantaginis]	2.229437229	1.156679581	2.36E-05	up	yes	1.03	0.462	0.485	0.429	0.472	1	1.065	1.025	BP:cellular compone	GO:0022607;GO:0043933;GO:0071826;GO:0016043;GO:0065003;GO:0022618;GO:0071840;GO:0009987;GO:0008150;GO:0001732;GO:0033290;GO:0005852;GO:0016282;GO:0070993;GO:0032991;GO:1990904;GO:0005575;GO:0003674;GO:0005488;GO:0003676;GO:0008233;GO:1901363;GO:0008237;GO:0016787;GO:0008135;GO:0140096;GO:0003824;GO:0045182;GO:0090079;GO:0097159;GO:0003743	K03247	EIF3H	map05162	Measles	ENOG410XSR9	J:Translation, ribosomal structure	PF19445.2;PF01	eIF3h_C;JAB	C-terminal region of el	CYT	5	16	38.7	High
TRINITY_DN7112_c0_g1_i1_orf1	-	-	-	heterogeneous nuclear ribonucleoprotein K isoform X2 [Ostrinia furnacalis]	2.494949495	1.319010612	1.35E-05	up	yes	0.988	0.396	0.377	0.392	0.419	1	1.014	0.95	MF:organic cyclic co	GO:0097159;GO:0003674;GO:0005488;GO:0003676;GO:1901363;GO:0003723	K12886	HNRNPK	map05206;map05	MicroRNAs in cancer;Viral ca	ENOG4111GSB;E	K:Transcription;S:Function unkno	PF00013.32;PF07	KH_1;KH_2	KH domain;KH domain	CYT	7	48	22.2	High
TRINITY_DN25341_c0_g1_i1_orf1	-	-	-	heat shock protein 90 [Loxostege sticticalis]	2.036322594	1.025966131	1.29E-05	up	yes	0.9923	0.4873	0.489	0.455	0.518	1	0.998	0.979	MF:purine ribonucle	GO:0035639;GO:0003674;GO:0005488;GO:0016887;GO:0043167;GO:1901363;GO:1901265;GO:0043168;GO:0051082;GO:0000166;GO:0017076;GO:0005524;GO:0016787;GO:0017111;GO:0036094;GO:0003824;GO:0016818;GO:0030554;GO:0097367;GO:0097159;GO:0044183;GO:0016817;GO:0016462;GO:0032559;GO:0005515;GO:0032555;GO:0032553	K04079	HSP90A, htpG	map05207;map05	Chemical carcinogenesis - r	COG0326	S:Function unknown	PF00183.21;PF02	HSP90;HATPase	Hsp90 protein;Histidine	CYT	32	49	82.7	High
TRINITY_DN69334_c0_g1_i1_orf1	-	-	-	PREDICTED: 15-hydroxyprostaglandin dehydrogenase [NAD(+)]-like [Papilio xuthus]	2.209884607	1.143971039	0.0001351	up	yes	1.015	0.4593	0.43	0.531	0.417	1	1.043	1.003	MF:catalytic activity;I	GO:0003824;GO:0003674;GO:0016491	K00069	HPGD	map00590;map05	Arachidonic acid metabolism	COG1028	S:Function unknown	PF00106.28;PF1	adh_short;adh	short chain dehydroger	CYT	3	11	29.1	High
TRINITY_DN5009_c0_g1_i2_orf1	-	-	-	GSCOCG00009487001-RA-CDS [Cotesia congregata] >CAG5088842.1 Similar to RpL18: 60S ribosomal protein L18 (Timarcha balearica) [Cotesia congregata]	2.228070175	1.155794673	0.0005453	up	yes	1.016	0.456	0.516	0.355	0.497	1	1.061	0.988	BP:cellular biosynthe	GO:0044249;GO:0034641;GO:0006807;GO:0034645;GO:1901576;GO:0044260;GO:0043043;GO:0071704;GO:0043604;GO:0009987;GO:0009058;GO:0009059;GO:0008150;GO:0008152;GO:0044271;GO:0044238;GO:1901564;GO:0043603;GO:1901566;GO:0019538;GO:0044237;GO:0043170;GO:0006518;GO:0006412;GO:0043232;GO:0043226;GO:0110165;GO:0005840;GO:0043229;GO:0005575;GO:0043228;GO:0003674;GO:0005198;GO:0003735	K02883	RP-L18e, RPL1	map03010;map05	Ribosome;Coronavirus disea	COG1727	J:Translation, ribosomal structure	PF17135.7	Ribosomal_L18	Ribosomal protein 60S	CYT	1	4	22	High
TRINITY_DN147596_c0_g1_i1_orf1	-	-	-	activator of basal transcription 1 [Diachasma alloeum]	2.058034779	1.041267363	0.0009523	up	yes	0.9823	0.4773	0.556	0.51	0.366	1	0.973	0.974	CC:intracellular non-	GO:0043232;GO:0005730;GO:0043226;GO:0005575;GO:0043229;GO:0043228;GO:0110165;GO:0097159;GO:0003674;GO:0005488;GO:0003676;GO:1901363	K14785	ESF2, ABT1	------	------	ENOG4111JJV	K:Transcription	------	------	------	CYT	1	3	25.4	Medium
TRINITY_DN27641_c0_g1_i1_orf1	-	-	-	succinate dehydrogenase [ubiquinone] iron-sulfur subunit, mitochondrial [Ostrinia furnacalis]	2.211453744	1.144995067	1.02E-06	up	yes	1.004	0.454	0.453	0.471	0.438	1	1.015	0.996	BP:tricarboxylic acid	GO:0006099;GO:0008150;GO:0008152;GO:0044238;GO:0031090;GO:0016020;GO:0110165;GO:0031966;GO:0019866;GO:0005575;GO:0005743;GO:0016635;GO:0003674;GO:0005488;GO:0000104;GO:0016491;GO:0043169;GO:0016627;GO:0043167;GO:0046872;GO:0003824;GO:0008177;GO:0051540;GO:0009055;GO:0051538;GO:0051539;GO:0051536;GO:0051537	K00235	SDHB, SDH2	map04714;map00	Thermogenesis;Oxidative ph	COG0479	C:Energy production and conver	PF13085.9;PF13	Fer2_3;Fer4_17	2Fe-2S iron-sulfur clust	CYT	6	30	31.8	High
TRINITY_DN4056_c0_g1_i8_orf1	-	-	-	uncharacterized protein LOC114349672 [Ostrinia furnacalis] >XP_028155936.1 uncharacterized protein LOC114349672 [Ostrinia furnacalis] >XP_028155937.1 uncharacterized protein LOC114349672 [Ostrinia furnacalis] >XP_028155939.1 uncharacterized protein LOC114349672 [Ostrinia furnacalis]	2.070194154	1.049766078	0.001651	up	yes	0.9703	0.4687	0.45	0.383	0.573	1	1.013	0.898	CC:organelle;CC:intr	GO:0043226;GO:0043229;GO:0043228;GO:0005730;GO:0043232;GO:0110165;GO:0005575;GO:0035639;GO:0003674;GO:0005488;GO:0003676;GO:0043167;GO:1901363;GO:1901265;GO:0043168;GO:0043169;GO:0000166;GO:0016740;GO:0017076;GO:0046872;GO:0005524;GO:0036094;GO:0003824;GO:0016779;GO:0030554;GO:0097367;GO:0016772;GO:0032559;GO:0003723;GO:0097159;GO:0032555;GO:0032553	K18709	TUT1	------	------	COG5260	L:Replication, recombination anc	PF03828.22	PAP_assoc	Cid1 family poly A poly	CYT	1	1	82.7	High
TRINITY_DN11013_c0_g1_i3_orf1	-	-	-	glutamine:fructose-6-phosphate aminotransferase 1 [Heortia vitessoides]	2.267926246	1.181373724	3.35E-05	up	yes	0.9963	0.4393	0.456	0.467	0.395	1	0.968	1.021	BP:amino sugar bios	GO:0046349;GO:1901137;GO:0034641;GO:0006807;GO:0009064;GO:1901362;GO:1901360;GO:0006139;GO:0006520;GO:0006541;GO:0071704;GO:1901605;GO:0018130;GO:0009226;GO:1901576;GO:0009225;GO:0009987;GO:0006725;GO:0009058;GO:0008150;GO:0019752;GO:0008152;GO:0034654;GO:1901564;GO:0055086;GO:0046483;GO:0006048;GO:0044238;GO:0044271;GO:0006082;GO:0044249;GO:1901135;GO:0044237;GO:0006047;GO:0006040;GO:0006793;GO:0043436;GO:0019438;GO:0044281;GO:0003674;GO:0005488;GO:0008483;GO:0004360;GO:0016740;GO:0016769;GO:0070548;GO:0003824;GO:0097367	K00820	glmS, GFPT	map00250;map00	Alanine, aspartate and gluta	COG0449	M:Cell wall/membrane/envelope	PF01380.25;PF1	SIS;GATase_6;G	SIS domain;Glutamine	CYT	15	29	75.4	High
TRINITY_DN886_c0_g2_i4_orf1	-	-	-	collagenase-like [Ostrinia furnacalis]	2.274845785	1.185768746	2.27E-05	up	yes	0.9957	0.4377	0.414	0.416	0.483	1	0.977	1.01	------	------	------	------	------	------	COG5640	S:Function unknown	PF00089.29	Trypsin	Trypsin	EXC	2	8	30.5	High
TRINITY_DN16900_c0_g2_i1_orf1	-	-	-	uncharacterized oxidoreductase dhs-27-like [Ostrinia furnacalis]	3.133121571	1.64760075	0.0002598	up	yes	1.085	0.3463	0.391	0.332	0.316	1	1.191	1.064	------	------	------	------	------	------	ENOG410XT8F;E	S:Function unknown;	PF02958.23;PF07	EcKL;DUF1679	Ecdysteroid kinase-like	CYT	1	3	32.1	High
TRINITY_DN10637_c0_g1_i4_orf1	-	-	-	V-type proton ATPase subunit d [Bombyx mandarina]	2.665809769	1.414573834	1.18E-05	up	yes	1.037	0.389	0.385	0.415	0.367	1	1.043	1.068	CC:cellular_compone	GO:0005575;GO:0033179;GO:0032991;GO:0098796;GO:0033177;GO:0003674;GO:0042626;GO:0015318;GO:0009678;GO:0015399;GO:0022804;GO:0042625;GO:0005215;GO:0008324;GO:0046961;GO:0044769;GO:0022890;GO:0019829;GO:0015075;GO:0022857;GO:0022853;GO:0015078	K02146	ATPeV0D, ATF	map05152;map05	Tuberculosis;Human papillor	COG1527	S:Function unknown	PF01992.19	vATP-synt_AC3	ATP synthase	CYT	7	20	39.6	High
TRINITY_DN8621_c0_g1_i5_orf1	-	-	-	aminopeptidase N-like isoform X2 [Ostrinia furnacalis]	2.112882096	1.079212264	7.21E-05	up	yes	0.9677	0.458	0.421	0.493	0.46	1	0.926	0.977	MF:exopeptidase ac	GO:0008238;GO:0004177;GO:0046914;GO:0016787;GO:0140096;GO:0043169;GO:0043167;GO:0003824;GO:0003674;GO:0008270;GO:0008233;GO:0046872;GO:0008237;GO:0005488	K11140;K11141	ANPEP, CD13;	map04640;map00	Hematopoietic cell lineage;C	COG0308	S:Function unknown	PF11838.11;PF0	ERAP1_C;Peptic	ERAP1-like C-terminal	CYT	1	2	69.5	High
TRINITY_DN698_c0_g1_i5_orf1	-	-	-	PREDICTED: small nuclear ribonucleoprotein Sm D3 [Amyelois transitella]	2.340325242	1.226709039	1.79E-05	up	yes	0.993	0.4243	0.429	0.461	0.383	1	0.979	1	BP:spliceosomal snR	GO:0000387;GO:0022607;GO:0043933;GO:0009987;GO:0071826;GO:0016043;GO:0065003;GO:0022618;GO:0008150;GO:0071840;GO:0005575;GO:0032991;GO:0005829;GO:1990904;GO:0110165;GO:0005681	K11088	SNRPD3, SMD	map05322;map03	Systemic lupus erythematos	COG1958	S:Function unknown	PF01423.25	LSM	LSM domain	CYT	3	31	14.1	High
TRINITY_DN5019_c0_g1_i2_orf1	-	-	-	RRP12-like protein isoform X4 [Ostrinia furnacalis] >XP_028175539.1 RRP12-like protein isoform X5 [Ostrinia furnacalis]	2.553838358	1.352667214	1.91E-06	up	yes	1.008	0.3947	0.419	0.372	0.393	1	1.018	1.006	CC:intracellular mem	GO:0043231;GO:0005634;GO:0005575;GO:0043229;GO:0043227;GO:0043226;GO:0110165	K14794	RRP12	------	------	ENOG410XQ3D	S:Function unknown	PF08161.15	NUC173	NUC173 domain	CYT	8	6	153.7	High
TRINITY_DN32479_c0_g1_i8_orf1	-	-	-	hypothetical protein evm_009815 [Chilo suppressalis] >CAB3525305.1 unnamed protein product [Chilo suppressalis] >CAH0402632.1 unnamed protein product [Chilo	2.85399449	1.51298255	1.53E-05	up	yes	1.036	0.363	0.372	0.325	0.392	1	1.047	1.06	CC:integral compone	GO:0016021;GO:0005575;GO:0110165;GO:0031224;GO:0005215;GO:0022857;GO:0003674	------	------	------	------	COG0477	Q:Secondary metabolites biosyr	PF07690.19;PF0	MFS_1;Sugar_tr	Major Facilitator Superf	CYT	1	2	56.4	High
TRINITY_DN4944_c0_g1_i2_orf1	-	-	-	bifunctional glutamate/proline--tRNA ligase [Ostrinia furnacalis]	2.401275448	1.263800903	1.15E-06	up	yes	0.979	0.4077	0.405	0.4	0.418	1	0.965	0.972	BP:prolyl-tRNA amir	GO:0006433;GO:0019752;GO:0090304;GO:0034641;GO:0006807;GO:0044281;GO:0034660;GO:1901360;GO:0006139;GO:0006520;GO:0071704;GO:0009987;GO:0006725;GO:0008150;GO:0008152;GO:0043436;GO:0046483;GO:0016070;GO:0044238;GO:1901564;GO:0006082;GO:0044237;GO:0043170;GO:0006399;GO:0043038;GO:0043039;GO:0006418;GO:0005737;GO:0110165;GO:0005575;GO:0035639;GO:0003674;GO:0043167;GO:1901363;GO:0004812;GO:0000166;GO:1901265;GO:0017076;GO:0043168;GO:0140098;GO:0036094;GO:0003824;GO:0016874;GO:0016875;GO:0004827;GO:0030554;GO:0097367;GO:0097159;GO:0032559;GO:0005524;GO:0032555;GO:0140101;GO:0032553;GO:0005488	K14163	EPRS	map00860;map00	Porphyrin metabolism;Amin	COG0442	J:Translation, ribosomal structure	PF09180.14;PF0	ProRS-C_1;tRN	Prolyl-tRNA synthetase	CYT	6	28	51	High
TRINITY_DN6248_c0_g1_i1_orf1	-	-	-	DNA topoisomerase I, mitochondrial [Ostrinia furnacalis]	2.681371246	1.42297098	1.14E-05	up	yes	1.009	0.3763	0.372	0.416	0.341	1	1.027	1	BP:DNA conformatic	GO:0071103;GO:0090304;GO:0034641;GO:0006807;GO:0071840;GO:0006139;GO:0044260;GO:0006265;GO:0016043;GO:0071704;GO:1901360;GO:0009987;GO:0006725;GO:0008150;GO:0008152;GO:0046483;GO:0006996;GO:0044238;GO:0051276;GO:0044237;GO:0043170;GO:0006259;GO:0043227;GO:0043226;GO:0005634;GO:0005694;GO:0110165;GO:0043229;GO:0043231;GO:0043228;GO:0043232;GO:0005575;GO:0003674;GO:0005488;GO:0003676;GO:0003677;GO:1901363;GO:0140097;GO:0003824;GO:0016853;GO:0097159;GO:0003917;GO:0003916	K03163	TOP1	------	------	COG3569	L:Replication, recombination anc	PF01028.23;PF0	Topoisom_I;Top	Eukaryotic DNA topois	CYT	8	9	110.1	High
TRINITY_DN3401_c0_g1_i1_orf1	-	-	-	28S ribosomal protein S5, mitochondrial [Ostrinia furnacalis]	2.269458245	1.182347945	9.38E-05	up	yes	0.9593	0.4227	0.419	0.471	0.378	1	0.946	0.932	BP:cellular biosynthe	GO:0044249;GO:0034645;GO:1901576;GO:0044271;GO:0044260;GO:0043603;GO:0043043;GO:0071704;GO:1901566;GO:0043604;GO:0009987;GO:0009058;GO:0009059;GO:0008150;GO:0008152;GO:1901564;GO:0044238;GO:0019538;GO:0034641;GO:0044237;GO:0043170;GO:0006807;GO:0006518;GO:0006412;GO:0043226;GO:0043229;GO:0043228;GO:0110165;GO:0032991;GO:0005840;GO:0043232;GO:1990904;GO:0005575;GO:0003676;GO:0003674;GO:0005488;GO:1901363;GO:0005198;GO:0003735;GO:0097159;GO:0003723	K02988	RP-S5, MRPS5	map03010	Ribosome	COG0098	J:Translation, ribosomal structure	PF03719.18;PF0	Ribosomal_S5_	Ribosomal protein S5, (	CYT	2	6	49	High
TRINITY_DN29038_c0_g2_i1_orf1	-	-	-	PREDICTED: ATP synthase lipid-binding protein, mitochondrial [Fopius arisanus] >XP_011314178.1 PREDICTED: ATP synthase lipid-binding protein, mitochondrial [Fopius arisanus] >XP_011314186.1 PREDICTED: ATP synthase lipid-binding protein, mitochondrial [Fopius arisanus]	2.018940157	1.013598149	1.89E-05	up	yes	1.002	0.4963	0.49	0.531	0.468	1	0.984	1.021	BP:nucleoside tripho	GO:0009141;GO:0009142;GO:0009144;GO:0009145;GO:0044249;GO:0034641;GO:0009165;GO:0044281;GO:1901137;GO:0072521;GO:0072522;GO:1901362;GO:0009259;GO:1901360;GO:0006163;GO:0044271;GO:0009201;GO:0071704;GO:0009199;GO:0018130;GO:0009206;GO:0009205;GO:0009987;GO:0019693;GO:0006139;GO:0046034;GO:0006793;GO:0006725;GO:0009152;GO:0009150;GO:0009260;GO:0009058;GO:0009117;GO:0008152;GO:0019438;GO:0034654;GO:1901564;GO:0090407;GO:0055086;GO:0046483;GO:0044238;GO:0015986;GO:1901566;GO:1901576;GO:1901135;GO:0046390;GO:0044237;GO:0006796;GO:0006807;GO:0019693;GO:0006164;GO:0019637;GO:0008150;GO:0006753;GO:0006754;GO:0031090;GO:0045263;GO:0016021;GO:0016020;GO:0031224;GO:0110165;GO:0031966;GO:0032991;GO:0005575;GO:0098796;GO:0033177;GO:0005488;GO:0022890;GO:0003674;GO:0008289;GO:0015318;GO:0022857;GO:0005215;GO:0008324;GO:0015075;GO:0015078	K02128	ATPeF0C, ATP	map04714;map05	Thermogenesis;Chemical ca	COG0636	G:Carbohydrate transport and m	PF00137.24	ATP-synt_C	ATP synthase subunit C	EXC	1	5	14.4	Medium
TRINITY_DN10476_c0_g1_i1_orf1	-	-	-	prohibitin-2 [Ostrinia furnacalis]	2.187299551	1.129150811	5.13E-06	up	yes	1.023	0.4677	0.481	0.447	0.475	1	1.023	1.046	CC:organelle membr	GO:0031090;GO:0016020;GO:0019866;GO:0031966;GO:0005575;GO:0005743;GO:0110165	K17081	PHB2	------	------	COG0330	O:Posttranslational modification	PF01145.28	Band_7	SPFH domain / Band 7	CYT	10	36	32.9	High
TRINITY_DN17271_c0_g1_i1_orf1	-	-	-	uncharacterized protein LOC114350693 [Ostrinia furnacalis]	2.190987124	1.131581006	1.84E-05	up	yes	1.021	0.466	0.444	0.491	0.463	1	1.059	1.005	BP:nucleic acid meta	GO:0090304;GO:0034641;GO:0006807;GO:1901360;GO:0006139;GO:0051716;GO:0044260;GO:0071704;GO:0006260;GO:0006281;GO:0009987;GO:0006725;GO:0006974;GO:0006950;GO:0008150;GO:0008152;GO:0046483;GO:0006310;GO:0044238;GO:0050896;GO:0044237;GO:0043170;GO:0033554;GO:0006259;GO:0031974;GO:0110165;GO:0031981;GO:0043233;GO:0005575;GO:0070013;GO:0003674;GO:0005488;GO:0003676;GO:0003677;GO:1901363;GO:0097159	------	------	------	------	ENOG4111REI;EI	L:Replication, recombination anc	PF08661.14	Rep_fac-A_3	Replication factor A prc	CYT	2	14	15.3	High
TRINITY_DN23502_c0_g1_i1_orf1	-	-	-	small nuclear ribonucleoprotein F [Ostrinia furnacalis]	2.093168999	1.065688797	1.01E-06	up	yes	1.002	0.4787	0.464	0.478	0.494	1	0.992	1.013	BP:cellular compone	GO:0022607;GO:0043933;GO:0071840;GO:0000387;GO:0016043;GO:0065003;GO:0022618;GO:0009987;GO:0071826;GO:0008150;GO:0120114;GO:0032991;GO:1990904;GO:0005575;GO:0005681;GO:0003674;GO:0005488;GO:0003676;GO:1901363;GO:0097159;GO:0003723	K11098	SNRPF, SMF	map03040	Spliceosome	ENOG4111W47	K:Transcription	PF01423.25	LSM	LSM domain	CYT	4	31	9.7	High
TRINITY_DN11820_c0_g1_i1_orf1	-	-	-	hypothetical protein evm_000341 [Chilo suppressalis]	2.223675605	1.15294634	7.18E-05	up	yes	1.02	0.4587	0.416	0.441	0.519	1	1.019	1.041	MF:ubiquitin-like pro	GO:0019787;GO:0043169;GO:0004842;GO:0140096;GO:0043167;GO:0003824;GO:0016740;GO:0003674;GO:0005488;GO:0046872;GO:0061630;GO:0061659	K25192	RNF157	------	------	ENOG410XRAE	S:Function unknown	PF13920.9;PF14	zf-C3HC4_3;Pr	Zinc finger, C3HC4 type	CYT	1	5	36.9	High
TRINITY_DN123139_c0_g1_i1_orfp1	-	-	-	TRINITY_DN123139_c0_g1_i1_m.79879 TRINITY_DN123139_c0_g1::TRINITY_DN123139_c0_g1_i1::g.79879 ORF type:3prime_partial len:76 (+),score=3.83 TRINITY_DN123139_c0_g1_i1:25-225(+)	2.307692308	1.206450877	0.0001234	up	yes	1.017	0.4407	0.387	0.47	0.465	1	0.978	1.072	------	------	------	------	------	------	------	------	------	------	------	CYT	1	9	7.6	Medium
TRINITY_DN44557_c0_g1_i4_orf1	-	-	-	serine hydrolase-like protein [Ostrinia furnacalis]	2.015145228	1.010883815	3.59E-05	up	yes	0.9713	0.482	0.45	0.516	0.48	1	0.965	0.949	MF:catalytic activity;I	GO:0003824;GO:0016740;GO:0003674	------	------	------	------	COG0596;ENOG	S:Function unknown;	------	------	------	PLA	2	16	10.4	High
TRINITY_DN96080_c0_g2_i1_orf1	-	-	-	ATP synthase subunit delta, mitochondrial [Ostrinia furnacalis]	2.262921348	1.178186442	8.17E-06	up	yes	1.007	0.445	0.452	0.42	0.463	1	1.034	0.986	CC:proton-transport	GO:0045261;GO:0005575;GO:0032991;GO:0033178;GO:0098796;GO:0008324;GO:0046933;GO:0015318;GO:0005215;GO:0005216;GO:0022803;GO:0015075;GO:0003824;GO:0022857;GO:0015252;GO:0003674;GO:0015267;GO:0005261;GO:0022890;GO:0016874;GO:0015078	K02134	ATPeF1D, ATP	map04714;map05	Thermogenesis;Chemical ca	COG0355	C:Energy production and conver	PF02823.19	ATP-synt_DE_N	ATP synthase, Delta/Ep	CYT	6	53	17.2	High
TRINITY_DN12683_c0_g1_i3_orf1	-	-	-	sulfated surface glycoprotein 185-like [Ostrinia furnacalis]	2.239775281	1.163353992	9.37E-07	up	yes	0.9967	0.445	0.457	0.425	0.453	1	1.002	0.988	------	------	------	------	------	------	ENOG4110OPT;E	S:Function unknown;	PF11901.11	DUF3421	Protein of unknown fun	CYT	2	9	27.1	High
TRINITY_DN34040_c0_g2_i1_orf1	-	-	-	uncharacterized protein LOC114352849 [Ostrinia furnacalis]	2.240857788	1.164051094	2.35E-05	up	yes	0.9927	0.443	0.449	0.419	0.461	1	1.025	0.953	------	------	------	------	------	------	------	------	------	------	------	CYT	1	6	24	High
TRINITY_DN10662_c0_g1_i4_orf1	-	-	-	HD domain-containing protein 2 [Ostrinia furnacalis]	2.507425743	1.326206976	4.49E-06	up	yes	1.013	0.404	0.396	0.386	0.43	1	1.037	1.002	MF:hydrolase activity	GO:0016787;GO:0016791;GO:0016788;GO:0042578;GO:0003824;GO:0003674;GO:0002953	K07023	YGK1, HDDC2	map00240;map01	Pyrimidine metabolism;Nucl	COG1896	S:Function unknown	PF13023.9;PF12	HD_3;YfbR-like	HD domain;5'-deoxynu	CYT	2	10	24.1	High
TRINITY_DN19361_c0_g1_i7_orf1	-	-	-	hydroxylysine kinase [Ostrinia furnacalis] >XP_028168144.1 hydroxylysine kinase [Ostrinia furnacalis]	2.538847118	1.344173523	8.72E-07	up	yes	1.013	0.399	0.406	0.381	0.41	1	1.027	1.011	------	------	K18201	AGPHD1	map00310	Lysine degradation	COG2334	S:Function unknown	PF01636.26	APH	Phosphotransferase en	CYT	9	31	42.1	High
TRINITY_DN2082_c0_g1_i2_orf1	-	-	-	choline-phosphate cytidylyltransferase B-like isoform X1 [Ostrinia furnacalis]	2.324699788	1.217044419	1.02E-06	up	yes	0.9873	0.4247	0.441	0.409	0.424	1	0.985	0.977	MF:nucleotidyltransf	GO:0016779;GO:0004105;GO:0003824;GO:0016772;GO:0070567;GO:0016740;GO:0003674	K00968	PCYT1	map00564;map05	Glycerophospholipid metab	COG0615;COG0	I:Lipid transport and metabolism	PF01467.29	CTP_transf_like	Cytidylyltransferase-like	CYT	5	17	45.3	High
TRINITY_DN42082_c0_g2_i2_orfp1	-	-	-	TRINITY_DN42082_c0_g2_i2_m.7835 TRINITY_DN42082_c0_g2::TRINITY_DN42082_c0_g2_i2::g.7835 ORF type:internal len:133 (+),score=75.81 TRINITY_DN42082_c0_g2_i2:1-396(+)	2.154382022	1.107274096	3.52E-05	up	yes	0.9587	0.445	0.464	0.45	0.421	1	0.951	0.925	------	------	------	------	------	------	------	------	------	------	------	CYT	1	7	13.9	High
TRINITY_DN46173_c0_g3_i2_orf1	-	-	-	tropomyosin-1, isoforms 9A/A/B isoform X33 [Aedes aegypti] >EAT46020.1 AAEL002761-PB [Aedes aegypti]	2.490049751	1.316174568	1.46E-06	up	yes	1.001	0.402	0.403	0.391	0.412	1	1.022	0.981	------	------	K10373	TPM1	map05206;map05	MicroRNAs in cancer;Dilatec	ENOG410XR5K	S:Function unknown	PF00261.23;PF1	Tropomyosin;T	Tropomyosin;Tropomy	CYT	2	24	29	High
TRINITY_DN3457_c0_g1_i4_orf1	-	-	-	aryl hydrocarbon receptor nuclear translocator homolog [Ostrinia furnacalis]	2.81974366	1.495564014	0.008976	up	yes	1.034	0.3667	0.529	0.181	0.39	1	1.218	0.885	BP:positive regulatio	GO:1903508;GO:0051252;GO:0080090;GO:0019222;GO:0031328;GO:0031326;GO:0031325;GO:0051173;GO:0031323;GO:0009893;GO:1902680;GO:0010604;GO:0009891;GO:0050789;GO:0019219;GO:0010556;GO:0065007;GO:0048518;GO:0010468;GO:0032502;GO:0045935;GO:0060255;GO:0009889;GO:1903506;GO:0050794;GO:0008150;GO:0045893;GO:2001141;GO:0048856;GO:0051254;GO:0006355;GO:0010557;GO:0006357;GO:0045944;GO:0048522;GO:0051171;GO:0043229;GO:0043227;GO:0043226;GO:0005737;GO:0005634;GO:0110165;GO:0032991;GO:0043231;GO:0005575;GO:0005667;GO:0003674;GO:0005488;GO:0003676;GO:0003677;GO:0140110;GO:0005515;GO:0003700;GO:0097159;GO:0046983;GO:1901363	K09097	ARNT	map05207;map05	Chemical carcinogenesis - r	ENOG410XVHF;E	K:Transcription;	PF14598.9;PF08	PAS_11;PAS_3;I	PAS domain;PAS fold;P	CYT	1	2	58.6	High
TRINITY_DN969_c0_g1_i3_orf1	-	-	-	protein UBASH3A homolog isoform X3 [Ostrinia furnacalis]	2.423716381	1.277220887	0.000342	up	yes	0.9913	0.409	0.388	0.373	0.466	1	0.914	1.06	------	------	K18993	UBASH3, STS	------	------	ENOG410YGUZ	O:Posttranslational modification	PF00300.25;PF1	His_Phos_1;SH3	Histidine phosphatase	CYT	2	3	77	High
TRINITY_DN5867_c0_g1_i1_orf1	-	-	-	NADH dehydrogenase [ubiquinone] 1 alpha subcomplex subunit 7-like [Ostrinia furnacalis]	2.557251908	1.354594284	4.25E-06	up	yes	1.005	0.393	0.395	0.364	0.42	1	1.019	0.995	BP:cellular process;B	GO:0009987;GO:0044237;GO:0022900;GO:0008150;GO:0008152;GO:0022904;GO:0006091;GO:0042773;GO:0031090;GO:0016020;GO:0019866;GO:0031966;GO:0005575;GO:0005743;GO:0110165	K03951	NDUFA7	map04714;map00	Thermogenesis;Oxidative ph	ENOG410XW5P	C:Energy production and conver	PF07347.15	CI-B14_5a	NAD	CYT	6	60	12.3	High
TRINITY_DN43369_c0_g2_i1_orf1	-	-	-	cytochrome P450 monooxygenase 304 [Glyphodes pyloalis]	2.195065714	1.13426413	0.0002723	up	yes	0.952	0.4337	0.455	0.481	0.365	1	0.917	0.939	CC:integral compone	GO:0016021;GO:0005575;GO:0110165;GO:0031224;GO:0043169;GO:0004497;GO:0020037;GO:0046914;GO:0046906;GO:0097159;GO:0016705;GO:0043167;GO:0003824;GO:0003674;GO:0005488;GO:0005506;GO:0046872;GO:1901363;GO:0016491	------	------	------	------	COG2124	S:Function unknown	------	------	------	CYT	1	8	13	High
TRINITY_DN14967_c0_g2_i1_orf1	-	-	-	glyceraldehyde-3-phosphate dehydrogenase 2 [Holotrichia oblita]	2.722371968	1.444864201	1.56E-05	up	yes	1.01	0.371	0.389	0.386	0.338	1	0.982	1.048	BP:small molecule m	GO:0044281;GO:1901360;GO:0009205;GO:0046034;GO:0046031;GO:0032787;GO:0043436;GO:0009179;GO:0046483;GO:1901564;GO:0016052;GO:0006163;GO:0006165;GO:0019637;GO:0009141;GO:0009144;GO:1901575;GO:0009199;GO:0019318;GO:0009150;GO:0012501;GO:0008150;GO:0008152;GO:0006753;GO:0006757;GO:0016310;GO:0034641;GO:0009259;GO:0006139;GO:0046939;GO:0009987;GO:0006725;GO:0009132;GO:0009135;GO:0055086;GO:0006082;GO:0005996;GO:1901135;GO:0006807;GO:0006006;GO:0009185;GO:0019752;GO:0019693;GO:0072521;GO:0008219;GO:0006090;GO:0006096;GO:0071704;GO:0006915;GO:0009117;GO:0009056;GO:0006091;GO:0044238;GO:0005975;GO:0044237;GO:0006796;GO:0006793;GO:0043231;GO:0043232;GO:0005829;GO:0043229;GO:0043228;GO:0043227;GO:0043226;GO:0005856;GO:0110165;GO:0005634;GO:0005575;GO:0051287;GO:1901363;GO:0004365;GO:0043891;GO:0000166;GO:0016740;GO:0016744;GO:0046872;GO:0016209;GO:0004784;GO:0003674;GO:0005488;GO:1901265;GO:0043169;GO:0004743;GO:0003824;GO:0016773;GO:0097159;GO:0016620;GO:0016491;GO:0030955;GO:0000287;GO:0043167;GO:0031420;GO:0016772;GO:0016721;GO:0050661;GO:0036094;GO:0016903;GO:0004802	K05298	GAPA	------	------	COG0057	C:Energy production and conver	PF02800.23;PF0	Gp_dh_C;Gp_dl	Glyceraldehyde 3-phos	CYT	2	6	35.5	High

TRINITY_DN2803_c2_g1_i8_orf1	-	-	-	trans-1,2-dihydrobenzene-1,2-diol dehydrogenase-like isoform X1 [Ostrinia furnacalis]	2.024588539	1.017628736	0.0001684	up	yes	1.021	0.5043	0.508	0.445	0.56	1	1.057	1.005	MF:organic cyclic co	GO:0097159;GO:0000166;GO:0036094;GO:0003674;GO:0005488;GO:1901363;GO:1901265	K00078	DHDH	map00040;map00	Pentose and glucuronate int	COG0673	S:Function unknown	PF01408.25;PF0	GFO_IDH_Moc	Oxidoreductase family,	CYT	1	3	38.6	High
TRINITY_DN29291_c0_g1_i1_orf1	-	-	-	carboxylesterase [Ostrinia furnacalis]	2.00907835	1.006533827	0.001143	up	yes	1.018	0.5067	0.438	0.624	0.458	1	1.001	1.053	MF:hydrolase activity	GO:0016787;GO:0003674;GO:0016788;GO:0052689;GO:0003824	K03927	CES2	map00983	Drug metabolism - other en	COG2272	I:Lipid transport and metabolism	PF00135.31	COesterase	Carboxylesterase family	CYT	1	8	26.3	High
TRINITY_DN106038_c0_g1_i1_orf1	-	-	-	ankyrin-3-like isoform X1 [Galleria mellonella]	2.084091369	1.059418528	0.0001471	up	yes	1.031	0.4947	0.549	0.438	0.497	1	1.07	1.024	------	------	K10380	ANK	map05205;map04	Proteoglycans in cancer;Toll	COG0666	F:Nucleotide transport and meta	PF12796.10;PF1	Ank_2;Ank_4;A	Ankyrin repeats;Ankyrin	CYT	3	8	47.2	High
TRINITY_DN51934_c0_g2_i1_orf1	-	-	-	SCAN domain-containing protein 3-like [Pieris napi] >XP_047520696.1 SCAN domain-containing protein 3-like [Pieris napi]	2.087368638	1.061685409	0.0001649	up	yes	1.013	0.4853	0.482	0.426	0.548	1	0.995	1.044	BP:nucleic acid meta	GO:0090304;GO:0034641;GO:0006807;GO:1901360;GO:0006139;GO:0044260;GO:0071704;GO:0009987;GO:0006725;GO:0008150;GO:0008152;GO:0046483;GO:0044238;GO:0044237;GO:0043170;GO:0015074;GO:0006259;GO:0003674;GO:0005488;GO:0003676;GO:1901363;GO:0097159	------	------	------	------	COG2801;ENOG	E:Amino acid transport and meta	------	------	------	CYT	1	4	17.4	Medium
TRINITY_DN101682_c0_g1_i1_orf1	-	-	-	cysteine-rich with EGF-like domain protein 2 [Ostrinia furnacalis]	2.576555024	1.365443402	0.003334	up	yes	1.077	0.418	0.47	0.26	0.524	1	1.213	1.019	BP:developmental p	GO:0032502;GO:0009987;GO:0048869;GO:0048856;GO:0048468;GO:0016043;GO:0048513;GO:0008150;GO:0071840;GO:0016021;GO:0005575;GO:0031224;GO:0110165;GO:0043169;GO:0003674;GO:0043167;GO:0005488;GO:0046872;GO:0005509	K24335	CRELD	------	------	ENOG41107X2	U:Intracellular trafficking, secreti	PF11938.11	DUF3456	TLR4 regulator and MIF	CYT	1	2	40.4	High
TRINITY_DN16343_c0_g1_i6_orf1	-	-	-	aminopeptidase N6 [Ostrinia nubilalis]	2.156070593	1.108404415	3.59E-06	up	yes	1.014	0.4703	0.446	0.474	0.491	1	1.026	1.015	BP:nitrogen compou	GO:0006807;GO:0071704;GO:0006508;GO:0008150;GO:0008152;GO:0044238;GO:1901564;GO:0019538;GO:0043170;GO:0016021;GO:0031224;GO:0110165;GO:0005575;GO:0004177;GO:0008238;GO:0003674;GO:0005488;GO:0008233;GO:0008237;GO:0043169;GO:0046914;GO:0043167;GO:0046872;GO:0016787;GO:0140096;GO:0003824;GO:0008270	K11140	ANPEP, CD13	map04640;map00	Hematopoietic cell lineage;(	COG0308	E:Amino acid transport and meta	PF11838.11;PF0	ERAP1_C;Pepti	ERAP1-like C-terminal	CYT	5	7	100.2	High
TRINITY_DN26824_c0_g1_i1_orf1	-	-	-	60S ribosomal protein L6 [Hyposmocoma kahamanoa]	2.183809319	1.126846892	0.0003453	up	yes	1.017	0.4657	0.456	0.388	0.553	1	1.027	1.023	BP:cellular biosynthe	GO:0044249;GO:0034641;GO:0006807;GO:0034645;GO:1901576;GO:0044271;GO:0044260;GO:0043043;GO:0071704;GO:0043604;GO:0009987;GO:0009058;GO:0009059;GO:0008150;GO:0008152;GO:1901564;GO:0044238;GO:0043603;GO:1901566;GO:0019538;GO:0044237;GO:0043170;GO:0006518;GO:0006412;GO:0043232;GO:0043226;GO:0110165;GO:0005840;GO:0032991;GO:1990904;GO:0043229;GO:0005575;GO:0043228;GO:0003674;GO:0005198;GO:0003735	K02934	RP-L6e, RPL6	map03010;map05	Ribosome;Coronavirus disea	COG2163	S:Function unknown	PF03868.18	Ribosomal_L6e	Ribosomal protein L6, N	CYT	2	38	8.6	High
TRINITY_DN2885_c1_g1_i2_orf1	-	-	-	ubiquitin-like-specific protease ESD4 [Ostrinia furnacalis]	2.006948702	1.005003742	0.0002453	up	yes	0.982	0.4893	0.553	0.452	0.463	1	1.011	0.935	BP:primary metaboli	GO:0044238;GO:1901564;GO:0019538;GO:0043170;GO:0071704;GO:0006807;GO:0008150;GO:0008152;GO:0006508;GO:0003674;GO:0016787;GO:0003824;GO:0140096;GO:0008233;GO:0008234	K08592	SENP1	------	------	COG5160	E:Amino acid transport and meta	PF02902.22	Peptidase_C48	Ulp1 protease family, C	CYT	2	3	79.7	High
TRINITY_DN3504_c0_g1_i3_orfp2	-	-	-	TRINITY_DN3504_c0_g1_i3_m.43947 TRINITY_DN3504_c0_g1::TRINITY_DN3504_c0_g1_i3::g.43947 ORF type:5prime_partial len:208 (-) score=77.75 TRINITY_DN3504_c0_g1_i3:185-808(-)	2.584079361	1.369650378	0.001303	up	yes	1.068	0.4133	0.51	0.291	0.439	1	1.165	1.039	------	------	------	------	------	------	------	------	------	------	------	EXC	1	3	22.4	High
TRINITY_DN53136_c0_g1_i1_orf1	-	-	-	glutathione S transferase-E4 [Glyphodes pyloalis]	2.183982684	1.126961418	2.14E-05	up	yes	1.009	0.462	0.461	0.503	0.422	1	1.015	1.013	MF:catalytic activity;I	GO:0003824;GO:0016740;GO:0004364;GO:0003674;GO:0016765	K00799	GST, gst	map05207;map00	Chemical carcinogenesis - r	COG0625	U:Intracellular trafficking, secreti	PF13417.9;PF00	GST_N_3;GST_(	Glutathione S-transfera	CYT	3	15	25.1	High
TRINITY_DN4025_c0_g1_i13_orf1	-	-	-	tetratricopeptide repeat protein 14 homolog isoform X2 [Ostrinia furnacalis]	2.682926829	1.423807709	0.0006317	up	yes	1.067	0.3977	0.503	0.326	0.364	1	1.054	1.148	------	------	------	------	------	------	ENOG410XQJ7	S:Function unknown	------	------	------	CYT	2	8	35.8	High
TRINITY_DN107617_c3_g1_i1_orf1	-	-	-	NADH dehydrogenase [ubiquinone] 1 alpha subcomplex subunit 9, mitochondrial [Ostrinia furnacalis]	2.03853325	1.027531489	5.59E-07	up	yes	0.984	0.4827	0.488	0.481	0.479	1	0.972	0.98	------	------	K03953	NDUFA9	map04714;map00	Thermogenesis;Oxidative ph	COG0702;COG0	G:Carbohydrate transport and m	PF01073.22;PF0	3Beta_HSD;Epi	3-beta hydroxysteroid	CYT	8	16	45.2	High
TRINITY_DN6671_c0_g1_i6_orf1	-	-	-	hypothetical protein evm_013656 [Chilo suppressalis] >CAB3521812.1 unnamed protein product [Chilo suppressalis] >CAH0399134.1 unnamed protein product [Chilo	2.056722689	1.040347286	1.69E-05	up	yes	0.979	0.476	0.501	0.447	0.48	1	0.983	0.954	BP:cellular process;B	GO:0009987;GO:0008150;GO:0006457;GO:0016272;GO:0032991;GO:0005575;GO:0003674;GO:0051082;GO:0005488;GO:0005515	K04797	pfdA, PFDN5	------	------	COG1730	O:Posttranslational modification	PF02996.20	Prefoldin	Prefoldin subunit	CYT	6	35	17.9	High
TRINITY_DN32997_c0_g1_i8_orf1	-	-	-	RNA-binding protein squid isoform X1 [Ostrinia furnacalis]	2.390949382	1.257583587	1.55E-06	up	yes	1.025	0.4287	0.433	0.423	0.43	1	1.032	1.044	------	------	K03102	SOD	map04320	Dorso-ventral axis formatio	COG0724	G:Carbohydrate transport and m	PF00076.25;PF1	RRM_1;RRM_7	RNA recognition motif.	CYT	8	21	30	High
TRINITY_DN19829_c0_g1_i1_orf1	-	-	-	28S ribosomal protein S35, mitochondrial [Ostrinia furnacalis]	2.113592441	1.079697212	0.0001124	up	yes	0.9843	0.4657	0.514	0.409	0.474	1	1	0.953	BP:cellular biosynthe	GO:0044249;GO:0034641;GO:0006807;GO:0034645;GO:1901576;GO:0044260;GO:0043043;GO:0071704;GO:0032543;GO:0043604;GO:0009987;GO:0009058;GO:0009059;GO:0008150;GO:0008152;GO:0044271;GO:0044238;GO:1901564;GO:0043603;GO:1901566;GO:0019538;GO:0044237;GO:0043170;GO:0006518;GO:0006412;GO:0043227;GO:0043226;GO:0005739;GO:0110165;GO:0043231;GO:0043229;GO:0005575;GO:0003674;GO:0005198;GO:0003735	K17413	MRPS35	------	------	ENOG410ZX0Y	S:Function unknown	PF10213.12	MRP-S28	Mitochondrial ribosoma	CYT	2	8	27.2	High
TRINITY_DN783_c0_g1_i7_orf1	-	-	-	microtubule-associated protein Jupiter isoform X4 [Helicoverpa armigera]	2.284047566	1.191592695	5.34E-05	up	yes	1.018	0.4457	0.419	0.448	0.47	1	0.981	1.072	------	------	------	------	------	------	ENOG410Z68G	S:Function unknown	PF17054.8	JUPITER	Microtubule-Associate	EXC	2	12	24	High
TRINITY_DN644_c0_g1_i1_orf1	-	-	-	cuticle protein 19-like [Ostrinia furnacalis]	2.058525248	1.041611144	0.0005547	up	yes	0.9743	0.4733	0.564	0.436	0.42	1	0.989	0.934	MF:molecular functi	GO:0003674;GO:0042302;GO:0005198	------	------	------	------	ENOG410YSFI	S:Function unknown	PF00379.26	Chitin_bind_4	Insect cuticle protein	CYT	4	34	14.8	High
TRINITY_DN1465_c0_g2_i1_orf1	-	-	-	unnamed protein product, partial [Iphiclides podalirius]	2.358974359	1.238159737	0.0001434	up	yes	1.012	0.429	0.406	0.506	0.375	1	1.001	1.034	MF:catalytic activity;I	GO:0003824;GO:0004497;GO:0003674;GO:0016491	K01904	4CL	map00130	Ubiquinone and other terpe	COG0318;COG0	I:Lipid transport and metabolism	PF00501.31;PF1	AMP-binding;A	AMP-binding enzyme;A	CYT	8	23	57.9	High
TRINITY_DN1109_c0_g1_i6_orf1	-	-	-	1-phosphatidylinositol phosphodiesterase-like [Cotesia glomerata]	2.120759548	1.084581057	0.001118	up	yes	0.994	0.4687	0.433	0.59	0.383	1	0.979	1.003	BP:primary metaboli	GO:0044238;GO:0006629;GO:0071704;GO:0008150;GO:0008152;GO:0008081;GO:0016787;GO:0016788;GO:0042578;GO:0003674;GO:0003824	K01771	plc	map00562	Inositol phosphate metaboli	------	------	------	------	------	EXC	2	8	34.7	High
TRINITY_DN15959_c0_g1_i1_orf1	-	-	-	dnaJ homolog subfamily A member 2-like [Ostrinia furnacalis]	2.213473121	1.146311854	9.88E-07	up	yes	0.9923	0.4483	0.434	0.446	0.465	1	0.997	0.98	BP:protein folding;BI	GO:0006457;GO:0009987;GO:0006950;GO:0008150;GO:0009266;GO:0009408;GO:0009628;GO:0050896;GO:0016020;GO:0005575;GO:0110165;GO:0035639;GO:0031072;GO:0003674;GO:0005488;GO:0000166;GO:1901363;GO:1901265;GO:0043168;GO:0043169;GO:0030544;GO:0051082;GO:0043167;GO:0017076;GO:0046872;GO:0005524;GO:0036094;GO:0030554;GO:0097367;GO:0097159;GO:0032559;GO:0005515;GO:0032555;GO:0032553	K09503	DNAJA2	map04141	Protein processing in endop	COG0484	O:Posttranslational modification	PF01556.21;PF0	DnaJ_C;DnaJ;D	DnaJ C terminal domai	CYT	10	33	44.9	High
TRINITY_DN2062_c0_g1_i11_orf1	-	-	-	uncharacterized protein LOC114350846 [Ostrinia furnacalis]	2.435294118	1.284096021	3.01E-05	up	yes	1.035	0.425	0.454	0.393	0.428	1	1.078	1.026	------	------	------	------	------	------	COG1028	V:Defense mechanisms	PF13561.9;PF00	adh_short_C2;a	Enoyl:short chain dehyd	CYT	1	61	15.4	High
TRINITY_DN478_c0_g1_i16_orf1	-	-	-	lipid storage droplets surface-binding protein 2 isoform X1 [Ostrinia furnacalis]	2.185840708	1.128188269	1.59E-06	up	yes	0.988	0.452	0.449	0.456	0.451	1	1	0.964	------	------	K17284	PLIN2, ADRP	map03320	PPAR signaling pathway	ENOG410XYYG;E	S:Function unknown;U:Intracellul	PF03036.19	Perilipin	Perilipin family	CYT	3	9	33.9	High
TRINITY_DN21357_c0_g1_i5_orf1	-	-	-	40S ribosomal protein S11 isoform X2 [Ostrinia furnacalis]	2.09864019	1.069454839	3.75E-05	up	yes	0.9723	0.4633	0.455	0.431	0.504	1	0.958	0.959	BP:cellular biosynthe	GO:0044249;GO:0034641;GO:0006807;GO:0034645;GO:1901576;GO:0044271;GO:0044260;GO:0043043;GO:0071704;GO:0043604;GO:0009987;GO:0009058;GO:0009059;GO:0008150;GO:0008152;GO:1901564;GO:0044238;GO:0043603;GO:1901566;GO:0019538;GO:0044237;GO:0043170;GO:0006518;GO:0006412;GO:0043232;GO:0043226;GO:0110165;GO:0005840;GO:0032991;GO:1990904;GO:0043229;GO:0005575;GO:0043228;GO:0003674;GO:0005198;GO:0003735	K02949	RP-S11e, RPS1	map03010;map05	Ribosome;Coronavirus disea	COG0186	J:Translation, ribosomal structure	PF16205.8;PF00	Ribosomal_S17	Ribosomal_S17 N-term	CYT	9	54	17.6	High
TRINITY_DN14235_c0_g1_i1_orf1	-	-	-	maltase A1 [Helicoverpa armigera]	2.629722922	1.394910799	2.31E-05	up	yes	1.044	0.397	0.378	0.379	0.434	1	1.07	1.061	BP:primary metaboli	GO:0044238;GO:0005975;GO:0071704;GO:0008150;GO:0008152;GO:0016787;GO:0003824;GO:0016798;GO:0003674	K01187	malZ	map00500;map00	Starch and sucrose metabol	COG0366	G:Carbohydrate transport and m	PF00128.27	Alpha-amylase	Alpha amylase, catalyti	CYT	10	29	66.7	High
TRINITY_DN1266_c2_g1_i1_orf1	-	-	-	serine/threonine-protein kinase RIO3 [Ostrinia furnacalis]	2.408482143	1.268124227	0.002946	up	yes	1.079	0.448	0.37	0.49	0.484	1	1.257	0.979	BP:phosphorylation;I	GO:0016310;GO:0006807;GO:0071704;GO:0006468;GO:0009987;GO:0043412;GO:0036211;GO:0008150;GO:0008152;GO:0044238;GO:1901564;GO:0044237;GO:0043170;GO:0019538;GO:0006796;GO:0006793;GO:0035639;GO:0003674;GO:0005488;GO:0000166;GO:1901363;GO:1901265;GO:0043168;GO:0043169;GO:0097367;GO:0043167;GO:0004674;GO:0016740;GO:0004672;GO:0017076;GO:0046872;GO:0005524;GO:0016301;GO:0140096;GO:0036094;GO:0003824;GO:0030554;GO:0016773;GO:0016772;GO:0032559;GO:0032555;GO:0097159;GO:0032553	K08872	RIOK3, SUDD	------	------	COG1718	S:Function unknown	PF01163.25	RIO1	RIO1 family	CYT	1	2	57.3	Medium
TRINITY_DN27704_c0_g1_i1_orf1	-	-	-	PREDICTED: tRNA (guanine-N(7)-)-methyltransferase [Amyelois transitella]	2.225733634	1.154280948	9.40E-06	up	yes	0.986	0.443	0.441	0.443	0.445	1	1.01	0.948	------	------	K03439	trmB, METTL1	------	------	COG0220	S:Function unknown	PF02390.20	Methyltransf_4	Putative methyltransfer	CYT	2	7	28	High
TRINITY_DN14372_c0_g2_i1_orf1	-	-	-	12 kDa FK506-binding protein-like [Ostrinia furnacalis]	2.081897426	1.057898989	4.85E-05	up	yes	1.027	0.4933	0.518	0.455	0.507	1	1.068	1.012	MF:isomerase activit	GO:0016853;GO:0140096;GO:0016859;GO:0003824;GO:0003674;GO:0003755	K09568	FKBP1	------	------	COG0545	O:Posttranslational modification	PF00254.31	FKBP_C	FKBP-type peptidyl-pr	PLA	2	19	11.7	High
TRINITY_DN3665_c0_g1_i2_orf1	-	-	-	regucalcin-like isoform X2 [Ostrinia furnacalis] >XP_028175354.1 regucalcin-like isoform X2 [Ostrinia furnacalis]	3.209439528	1.682321378	0.0006291	up	yes	1.088	0.339	0.289	0.342	0.386	1	1.23	1.033	------	------	K01053	gnl, RGN	map00030;map00	Pentose phosphate pathway	COG3386	G:Carbohydrate transport and m	PF08450.15	SGL	SMP-30/Gluconolactor	CYT	1	16	6.9	High
TRINITY_DN3598_c0_g1_i1_orf1	-	-	-	esterase FE4-like [Ostrinia furnacalis]	2.370283019	1.245059332	3.36E-06	up	yes	1.005	0.424	0.404	0.455	0.413	1	1.007	1.008	MF:hydrolase activity	GO:0016787;GO:0003674;GO:0016788;GO:0052689;GO:0003824	------	------	------	------	COG2272	T:Signal transduction mechanism	PF00135.31;PF0	COesterase;BD	Carboxylesterase family	CYT	4	10	61.6	High
TRINITY_DN13530_c0_g1_i1_orf1	-	-	-	unnamed protein product [Euphydryas editha]	2.30281844	1.20340067	9.37E-05	up	yes	1.054	0.4577	0.506	0.452	0.415	1	1.077	1.084	------	------	K16815	PNPLA8	------	------	ENOG410XOXJ	S:Function unknown	PF01734.25	Patatin	Patatin-like phospholip	CYT	1	2	63.4	High
TRINITY_DN86956_c0_g5_i1_orf1	-	-	-	PREDICTED: protein sly1 homolog [Microplitis demolitor]	3.267326733	1.708110731	0.0003315	up	yes	1.089	0.3333	0.393	0.275	0.332	1	1.074	1.194	BP:transport;BP:biolo	GO:0006810;GO:0008150;GO:0016192;GO:0051234;GO:0051179	K19998	SCFD1, SLY1	------	------	COG5158	U:Intracellular trafficking, secreti	PF00995.26	Sec1	Sec1 family	CYT	1	1	71.3	High
TRINITY_DN2993_c0_g1_i4_orf1	-	-	-	heat shock 70 kDa protein cognate 5 [Ostrinia furnacalis]	2.389697114	1.256827773	1.27E-06	up	yes	1.002	0.4193	0.416	0.434	0.408	1	1.02	0.986	MF:purine ribonucle	GO:0035639;GO:0003674;GO:0005488;GO:0043167;GO:1901363;GO:1901265;GO:0043168;GO:0051082;GO:0000166;GO:0017076;GO:0005524;GO:0036094;GO:0030554;GO:0097367;GO:0097159;GO:0044183;GO:0032559;GO:0005515;GO:0032555;GO:0032553	K04043	dnaK, HSPA9	map03018;map05	RNA degradation;Tuberculo	COG0443	O:Posttranslational modification	PF00012.23;PF0	HSP70;MreB_M	Hsp70 protein;MreB/Ml	CYT	24	34	74.7	High
TRINITY_DN1617_c0_g1_i5_orf1	-	-	-	hypothetical protein evm_009822 [Chilo suppressalis] >CAB3525311.1 unnamed protein product [Chilo suppressalis] >CAH0402638.1 unnamed protein product [Chilo suppressalis]	2.28845709	1.194375241	1.39E-07	up	yes	0.9893	0.4323	0.425	0.436	0.436	1	0.98	0.988	CC:integral compon	GO:0016021;GO:0031224;GO:0005575;GO:0110165;GO:0035639;GO:0003674;GO:0005488;GO:0043167;GO:1901363;GO:1901265;GO:0043168;GO:0000166;GO:0017076;GO:0005524;GO:0016787;GO:0036094;GO:0003824;GO:0030554;GO:0097367;GO:0097159;GO:0032559;GO:0032555;GO:0032553	K01511	ENTPD5_6	map00240;map01	Pyrimidine metabolism;Nucl	COG5371;COG5	G:Carbohydrate transport and m	PF01150.20	GDA1_CD39	GDA1/CD39	CYT	3	7	51.5	High
TRINITY_DN3135_c0_g1_i6_orf1	-	-	-	acanthoscurrin-1-like [Ostrinia furnacalis]	2.321553943	1.215090804	0.000559	up	yes	0.992	0.4273	0.41	0.371	0.501	1	1.059	0.917	------	------	------	------	------	------	------	------	------	------	------	EXC	1	7	25	High
TRINITY_DN75746_c0_g1_i1_orfp1	-	-	-	TRINITY_DN75746_c0_g1_i1_m.54871 TRINITY_DN75746_c0_g1::TRINITY_DN75746_c0_g1_i1::g.54871 ORF type:internal len:83 (+),score=-1.09,Polyhedrin,PF00738.19,1.7e-25 TRINITY_DN75746_c0_g1_i1:2-247(+)	4.383769435	2.132171922	0.0004918	up	yes	1.156	0.2637	0.286	0.244	0.261	1	1.294	1.174	------	------	------	------	------	------	------	------	PF00738.21	Polyhedrin	Polyhedrin	CYT	2	29	9.6	High
TRINITY_DN334_c0_g1_i3_orf1	-	-	-	chymotrypsin-like serine protease [Ostrinia nubilalis] >AAX62030.1 chymotrypsin-like serine protease [Ostrinia nubilalis]	2.369928401	1.244843474	5.74E-06	up	yes	0.993	0.419	0.4	0.406	0.451	1	1.002	0.977	BP:organic substanc	GO:0071704;GO:1901564;GO:0019538;GO:0043170;GO:0008150;GO:0006807;GO:0008152;GO:0006508;GO:0044238;GO:0004252;GO:0004175;GO:0016787;GO:0017171;GO:0140096;GO:0003824;GO:0003674;GO:0008233;GO:0008236	------	------	------	------	COG5640	O:Posttranslational modification	PF00089.29	Trypsin	Trypsin	PLA	1	35	29.6	High
TRINITY_DN18242_c0_g1_i3_orf1	-	-	-	CCHC-type zinc finger protein CG3800 [Papilio xuthus]	2.089062179	1.062855434	0.0003131	up	yes	1.018	0.4873	0.488	0.42	0.554	1	0.988	1.066	MF:cation binding;M	GO:0043169;GO:0046914;GO:0097159;GO:0043167;GO:0003674;GO:0005488;GO:0003676;GO:0046872;GO:1901363;GO:0008270	K09250	CNBP	------	------	COG5082	O:Posttranslational modification	PF00098.26;PF1	zf-CCHC;zf-CC	Zinc knuckle;GAG-poly	PLA	4	27	18.3	High
TRINITY_DN4116_c0_g1_i3_orf1	-	-	-	transmembrane protein 131 homolog [Ostrinia furnacalis]	2.056803456	1.040403939	7.69E-05	up	yes	0.9523	0.463	0.475	0.485	0.429	1	0.926	0.931	CC:cellular compon	GO:0005575;GO:0016021;GO:0110165;GO:0031224	------	------	------	------	ENOG410Z7NH	S:Function unknown	PF12371.11;PF1	TMEM131_like	Transmembrane protei	CYT	1	1	66.6	High
TRINITY_DN13216_c0_g1_i5_orf1	-	-	-	uncharacterized protein LOC114358344 isoform X1 [Ostrinia furnacalis]	3.200121102	1.678126502	0.0002166	up	yes	1.057	0.3303	0.336	0.297	0.358	1	1.165	1.006	BP:cellular process;B	GO:0009987;GO:0050794;GO:0065007;GO:0007166;GO:0008150;GO:0050789;GO:0007165;GO:0016021;GO:0005575;GO:0110165;GO:0031224;GO:0004930;GO:0038023;GO:0060089;GO:0004888;GO:0003674	K08451;K08455	ADGRG2, GPR	------	------	ENOG410XSD2	S:Function unknown	PF00002.27;PF0	7tm_2;GPS	7 transmembrane rece	CYT	1	1	148.9	Medium
TRINITY_DN3949_c1_g1_i1_orf1	-	-	-	probable cytochrome P450 304a1 isoform X2 [Ostrinia furnacalis]	2.314304607	1.210578763	0.0003534	up	yes	1.05	0.4537	0.375	0.49	0.496	1	1.031	1.118	CC:integral compon	GO:0016021;GO:0005575;GO:0110165;GO:0031224;GO:0043169;GO:0004497;GO:0020037;GO:0046914;GO:0046906;GO:0097159;GO:0016705;GO:0043167;GO:0003824;GO:0003674;GO:0005488;GO:0005506;GO:0046872;GO:1901363;GO:0016491	------	------	------	------	COG2124	Q:Secondary metabolites biosyr	PF00067.25	p450	Cytochrome P450	CYT	2	17	16.8	High
TRINITY_DN7964_c0_g1_i1_orfp1	-	-	-	TRINITY_DN7964_c0_g1_i1_m.23483 TRINITY_DN7964_c0_g1::TRINITY_DN7964_c0_g1_i1::g.23483 ORF type:internal len:79 (+),score=44.25 TRINITY_DN7964_c0_g1_i1:3-236(+)	2.711286089	1.438977351	3.69E-05	up	yes	1.033	0.381	0.431	0.342	0.37	1	1.067	1.033	------	------	------	------	------	------	------	------	------	------	------	CYT	2	17	8.7	High
TRINITY_DN22815_c0_g1_i2_orf1	-	-	-	acyl carrier protein, mitochondrial isoform X1 [Ostrinia furnacalis]	2.155580866	1.108076686	0.0001826	up	yes	0.9463	0.439	0.397	0.432	0.488	1	0.928	0.911	------	------	K03955	NDUFAB1	map04714;map00	Thermogenesis;Oxidative ph	COG0236	I:Lipid transport and metabolism	PF00550.28	PP-binding	Phosphopantetheine at	CYT	2	15	17.3	High
TRINITY_DN21150_c0_g1_i4_orf1	-	-	-	RNA-binding protein cabeza-like isoform X2 [Bicyclus anynana]	2.493130152	1.3179582	4.46E-06	up	yes	0.998	0.4003	0.42	0.367	0.414	1	1.006	0.988	BP:regulation of prin	GO:0080090;GO:0019222;GO:0031326;GO:0031323;GO:0050789;GO:0060255;GO:0065007;GO:0010468;GO:0019219;GO:0009889;GO:1903506;GO:0050794;GO:0008150;GO:0051171;GO:2001141;GO:0051252;GO:0006355;GO:0010556;GO:0003674;GO:0005488;GO:0003676;GO:1901363;GO:0097159;GO:0003723	K13098	TLS, FUS	map03015;map03	mRNA surveillance pathway	ENOG4111Q2F;E	S:Function unknown;	PF00076.25	RRM_1	RNA recognition motif.	CYT	3	31	13.2	High
TRINITY_DN2718_c0_g1_i6_orf1	-	-	-	cleavage stimulation factor subunit 2 isoform X1 [Ostrinia furnacalis]	2.181856809	1.125556424	2.13E-05	up	yes	1.027	0.4707	0.496	0.445	0.471	1	1.016	1.064	BP:nucleic acid meta	GO:0090304;GO:0034641;GO:0006807;GO:0043631;GO:1901360;GO:0006139;GO:0071704;GO:0016071;GO:0031124;GO:0009987;GO:0006725;GO:0031123;GO:0008150;GO:0008152;GO:0046483;GO:0016070;GO:0044238;GO:0044237;GO:0043170;GO:0006378;GO:0006396;GO:0006397;GO:0005847;GO:0032991;GO:0005849;GO:0005575;GO:0003674;GO:0005488;GO:0003676;GO:1901363;GO:0097159;GO:0003723	K14407	CSTF2, RNA15	map03015	mRNA surveillance pathway	ENOG410XQBV	S:Function unknown	PF14327.9;PF00	CSTF2_hinge;R	Hinge domain of cleav	CYT	3	8	42.8	High
TRINITY_DN50571_c1_g1_i1_orf1	-	-	-	WD repeat-containing protein 46 [Ostrinia furnacalis]	2.289411765	1.194976964	0.0001932	up	yes	0.973	0.425	0.464	0.457	0.354	1	0.989	0.93	CC:intracellular non-	GO:0043232;GO:0005730;GO:0005575;GO:0043229;GO:0043228;GO:0043226;GO:0110165	K14768	UTP7, WDR46	------	------	ENOG410XP5D	S:Function unknown	PF08149.14	BING4CT	BING4CT	CYT	1	2	65.3	High
TRINITY_DN26130_c0_g1_i1_orf1	-	-	-	membrane alanyl aminopeptidase-like [Ostrinia furnacalis]	2.661776264	1.41238931	1.62E-06	up	yes	1.016	0.3817	0.382	0.397	0.366	1	1.038	1.01	BP:nitrogen compou	GO:0006807;GO:0071704;GO:0006508;GO:0008150;GO:0008152;GO:0044238;GO:1901564;GO:0019538;GO:0043170;GO:0016021;GO:0031224;GO:0110165;GO:0005575;GO:0004177;GO:0008238;GO:0003674;GO:0005488;GO:0008233;GO:0008237;GO:0043169;GO:0046914;GO:0043167;GO:0046872;GO:0016787;GO:0140096;GO:0003824;GO:0008270	K11140	ANPEP, CD13	map04640;map00	Hematopoietic cell lineage;(	COG0308	E:Amino acid transport and meta	PF01433.23;PF1	Peptidase_M1;E	Peptidase family M1 dc	CYT	9	13	104.2	High
TRINITY_DN10796_c0_g2_i1_orf1	-	-	-	F-BAR domain only protein 2 [Ostrinia furnacalis]	2.032304527	1.023116596	2.37E-05	up	yes	0.9877	0.486	0.508	0.444	0.506	1	0.973	0.99	CC:cellular compon	GO:0005575;GO:0098590;GO:0016020;GO:0005905;GO:0110165	K20042	FCHO	------	------	ENOG410Y7RY	S:Function unknown	PF10291.12;PF0	muHD;FCH	Muniscin C-terminal mu	CYT	3	3	99.2	High
TRINITY_DN38540_c0_g1_i1_orf1	-	-	-	GSCOCG00000129001-RA-CDS [Cotesia congregata] >CAG5101050.1 Similar to LUC7L2: Putative RNA-binding protein Luc7-like 2 (Homo sapiens) [Cotesia congregata]	2.475059382	1.307463139	0.000108	up	yes	1.042	0.421	0.405	0.419	0.439	1	1.121	1.005	BP:cellular compone	GO:0022607;GO:0043933;GO:0071840;GO:0071826;GO:0016043;GO:0065003;GO:0022618;GO:0009987;GO:0008150;GO:0006376;GO:0120114;GO:0005575;GO:0097525;GO:0032991;GO:0030532;GO:1990904;GO:0005685;GO:0003674;GO:0005488;GO:0003676;GO:1901363;GO:0097159;GO:0003723;GO:0003729	K13212	LUC7L2	------	------	COG5200	A:RNA processing and modificat	PF03194.18	LUC7	LUC7 N_terminus	CYT	2	9	38.6	High
TRINITY_DN4070_c0_g1_i4_orf1	-	-	-	alpha-N-acetylgalactosaminidase isoform X3 [Ostrinia furnacalis]	2.312085308	1.209194629	1.19E-05	up	yes	0.9757	0.422	0.424	0.449	0.393	1	0.972	0.955	BP:primary metaboli	GO:0044238;GO:0071704;GO:0008150;GO:0008152;GO:0005975;GO:0006629;GO:0005575;GO:0000323;GO:0043231;GO:0005773;GO:0005764;GO:0043229;GO:0043227;GO:0043226;GO:0110165;GO:0003674;GO:0016787;GO:0016798;GO:0003824;GO:0004553	K01204	NAGA	map00603;map04	Glycosphingolipid biosynthe	ENOG410XPF1	O:Posttranslational modification	PF16499.8;PF17	Melibiase_2;Me	Alpha galactosidase A	CYT	5	15	49.7	High
TRINITY_DN391_c5_g1_i1_orf1	-	-	-	hypothetical protein B5X24_HaOG210395 [Helicoverpa armigera]	2.208791209	1.143257051	2.57E-07	up	yes	1.005	0.455	0.468	0.454	0.443	1	1.011	1.004	MF:oxidoreductase a	GO:0016491;GO:0051287;GO:0000166;GO:0036094;GO:0003824;GO:0003674;GO:0005488;GO:0097159;GO:0048038;GO:1901363;GO:1901265;GO:0016651	K03935	NDUFS2	map04714;map00	Thermogenesis;Oxidative ph	COG0649	C:Energy production and conver	PF00346.22	Complex1_49kl	Respiratory-chain NAD	CYT	8	26	52.6	High
TRINITY_DN11657_c0_g1_i2_orf1	-	-	-	trehalase-1 [Omphisa fuscidentalis]	2.368700265	1.244095652	1.37E-05	up	yes	0.9823	0.4147	0.384	0.447	0.413	1	0.958	0.989	BP:primary metaboli	GO:0044238;GO:0044262;GO:0009311;GO:0009987;GO:0005984;GO:0005991;GO:0008150;GO:0044237;GO:0008152;GO:0005975;GO:0071704;GO:0015927;GO:0016787;GO:0003824;GO:0016798;GO:0004555;GO:0003674;GO:0004553	K01194	TREH, treA, tre	map00500	Starch and sucrose metabol	COG1626	S:Function unknown	PF01204.21	Trehalase	Trehalase	CYT	2	5	67.2	High
TRINITY_DN18502_c0_g1_i1_orf1	-	-	-	uncharacterized protein LOC114359515 [Ostrinia furnacalis]	2.016563147	1.011898583	4.83E-05	up	yes	0.974	0.483	0.476	0.489	0.484	1	1	0.922	------	------	------	------	------	------	ENOG41117E9	S:Function unknown	------	------	------	CYT	6	15	59.7	High
TRINITY_DN905_c0_g1_i4_orf1	-	-	-	(11Z)-hexadec-11-enoyl-CoA conjugase-like [Ostrinia furnacalis] >XP_028172978.1 (11Z)-hexadec-11-enoyl-CoA conjugase-like [Ostrinia furnacalis]	2.693387797	1.429421966	0.0002886	up	yes	1.055	0.3917	0.426	0.367	0.382	1	1.161	1.004	BP:fatty acid biosynt	GO:0006633;GO:0006631;GO:0019752;GO:0044249;GO:0044281;GO:0044283;GO:0072330;GO:1901576;GO:0071704;GO:0006629;GO:0009987;GO:0032787;GO:0009058;GO:0008150;GO:0008152;GO:0043436;GO:0044255;GO:0008610;GO:0044238;GO:0006082;GO:0046394;GO:0016053;GO:0044237;GO:0031224;GO:0016021;GO:0110165;GO:0005575;GO:0003674;GO:0016491;GO:0016705;GO:0003824;GO:0016717	K00507	SCD, desC	map04936;map04	Alcoholic liver disease;AMPK	COG1398	J:Translation, ribosomal structure	PF00487.27	FA_desaturase	Fatty acid desaturase	CYT	2	8	41.9	High
TRINITY_DN47123_c0_g1_i1_orf1	-	-	-	WD40 repeat-containing protein SMU1 [Ostrinia furnacalis]	2.599948414	1.378482999	9.29E-06	up	yes	1.008	0.3877	0.42	0.368	0.375	1	0.987	1.036	BP:nucleic acid meta	GO:0090304;GO:0034641;GO:0006807;GO:0000398;GO:1901360;GO:0006139;GO:0071704;GO:0008380;GO:0016071;GO:0009987;GO:0006725;GO:0000375;GO:0000377;GO:0008150;GO:0008152;GO:0046483;GO:0016070;GO:0044238;GO:0044237;GO:0043170;GO:0006396;GO:0006397;GO:0016607;GO:0016604;GO:0110165;GO:0005575	K13111	SMU1	------	------	ENOG410XP39	S:Function unknown	PF00400.35;PF1	WD40;ANAPC4	WD domain, G-beta re	CYT	2	4	56.4	High
TRINITY_DN45477_c0_g1_i1_orf1	-	-	-	putative E3 ubiquitin-protein ligase UBR7 [Ostrinia furnacalis]	2.986437863	1.578425705	0.0002849	up	yes	1.079	0.3613	0.42	0.324	0.34	1	1.058	1.178	------	------	K11979	UBR7	------	------	ENOG410XSV6	S:Function unknown	PF02207.23	zf-UBR	Putative zinc finger in N	CYT	2	4	80.9	High
TRINITY_DN12442_c0_g1_i4_orf1	-	-	-	midasin-like [Ostrinia furnacalis]	2.529875413	1.339066339	2.72E-05	up	yes	0.995	0.3933	0.444	0.366	0.37	1	0.973	1.012	BP:cellular compone	GO:0022607;GO:0000027;GO:0065003;GO:0071826;GO:0016043;GO:0043933;GO:0022618;GO:0071840;GO:0009987;GO:0008150;GO:0043229;GO:0043228;GO:0043226;GO:0005730;GO:0005654;GO:0043232;GO:0005575;GO:0110165;GO:0035639;GO:1901363;GO:0003674;GO:0005488;GO:0016887;GO:0043167;GO:1901265;GO:0043168;GO:0000166;GO:0017076;GO:0005524;GO:0016787;GO:0017111;GO:0036094;GO:0003824;GO:0016818;GO:0030554;GO:0097367;GO:0097159;GO:0016817;GO:0016462;GO:0032559;GO:0032555;GO:0032553	K14572	MDN1, REA1	map03008	Ribosome biogenesis in euk	COG5271	S:Function unknown	PF13519.9;PF00	VWA_2;VWA	von Willebrand factor t	CYT	2	1	270.7	High
TRINITY_DN9647_c0_g1_i1_orf1	-	-	-	cytochrome P450 6B2-like [Ostrinia furnacalis]	2.041551834	1.029666197	3.27E-05	up	yes	0.963	0.4717	0.462	0.474	0.479	1	0.92	0.969	MF:cation binding;M	GO:0043169;GO:0004497;GO:0020037;GO:0046914;GO:0046906;GO:0097159;GO:0016705;GO:0043167;GO:0003824;GO:0003674;GO:0005488;GO:0005506;GO:0046872;GO:1901363;GO:0016491	K14999	CYP6	------	------	COG2124	Q:Secondary metabolites biosyr	PF00067.25	p450	Cytochrome P450	CYT	5	11	59.3	High
TRINITY_DN3292_c2_g1_i4_orf1	-	-	-	ribosome biogenesis regulatory protein homolog [Ostrinia furnacalis]	2.157929515	1.109647743	5.68E-06	up	yes	0.9797	0.454	0.467	0.453	0.442	1	0.951	0.988	BP:cellular process;B	GO:0009987;GO:0042254;GO:0044085;GO:0008150;GO:0071840;GO:0022613;GO:0043231;GO:0005634;GO:0005575;GO:0043229;GO:0043227;GO:0043226;GO:0110165	K14852	RRS1	------	------	COG5225	J:Translation, ribosomal structure	PF04939.15	RRS1	Ribosome biogenesis re	CYT	4	12	40	High
TRINITY_DN100821_c0_g1_i1_orf1	-	-	-	putative GMP synthase, partial [Operophtera brumata]	2.526127963	1.336927722	1.24E-06	up	yes	0.991	0.3923	0.403	0.369	0.405	1	0.982	0.991	BP:carboxylic acid m	GO:0019752;GO:0006807;GO:0009064;GO:0006520;GO:0006541;GO:0071704;GO:1901605;GO:0009987;GO:0008150;GO:0008152;GO:0043436;GO:0044238;GO:1901564;GO:0006082;GO:0044237;GO:0044281;GO:0005488;GO:0035639;GO:0003674;GO:0016884;GO:0043167;GO:1901363;GO:1901265;GO:0043168;GO:0000166;GO:0017076;GO:0005524;GO:0016787;GO:0003922;GO:0036094;GO:0003824;GO:0016874;GO:0016818;GO:0016879;GO:0097367;GO:0097159;GO:0030554;GO:0016817;GO:0016462;GO:0032559;GO:0032555;GO:0032553	K01951	guaA, GMPS	map01232;map00	Nucleotide metabolism;Drug	COG0519	F:Nucleotide transport and meta	------	------	------	CYT	2	32	7.3	High
TRINITY_DN15624_c0_g1_i1_orf1	-	-	-	LOW QUALITY PROTEIN: V-type proton ATPase subunit S1-like [Ostrinia furnacalis]	3.152492669	1.656493015	7.13E-05	up	yes	1.075	0.341	0.318	0.321	0.384	1	1.118	1.107	------	------	K03662	ATPeVS1, ATP	map05152;map05	Tuberculosis;Human papillo	ENOG410XTHY	U:Intracellular trafficking, secreti	PF20520.1;PF05	Ac45-VOA1_TI	V0 complex accessory	CYT	3	9	45.2	High
TRINITY_DN37699_c0_g1_i3_orfp1	-	-	-	TRINITY_DN37699_c0_g1_i3_m.58788 TRINITY_DN37699_c0_g1::TRINITY_DN37699_c0_g1_i3::g.58788 ORF type:internal len:122 (+),score=39.86 TRINITY_DN37699_c0_g1_i3:1-363(+)	5.633333333	2.493988841	0.0005696	up	yes	1.183	0.21	0.209	0.219	0.202	1	1.333	1.215	------	------	------	------	------	------	------	------	------	------	------	CYT	1	12	12.6	High
TRINITY_DN4820_c0_g1_i1_orf1	-	-	-	tudor and KH domain-containing protein homolog [Galleria mellonella]	2.094966375	1.066927088	0.0003154	up	yes	1.028	0.4907	0.468	0.466	0.538	1	1.107	0.978	CC:cytoplasm;CC:int	GO:0005737;GO:0016021;GO:0005575;GO:0110165;GO:0031224;GO:0097159;GO:0003674;GO:0003676;GO:0005488;GO:1901363;GO:0003723	K18406	TDRKH, TDRD	------	------	ENOG410Z0DP	S:Function unknown	------	------	------	CYT	1	10	16.2	High
TRINITY_DN42646_c0_g2_i1_orf1	-	-	-	40S ribosomal protein S3 [Helicoverpa armigera] >XP_026740562.1 40S ribosomal protein S3 [Trichoplusia ni] >XP_026751545.1 40S ribosomal protein S3 [Galleria mellonella] >XP_047027704.1 40S ribosomal protein S3 [Helicoverpa zea] >CAH0591481.1 unnamed protein product [Chrysodeixis includens] >AIR07416.1 ribosomal protein S3 [Helicoverpa armigera] >AND95944.1 ribosomal protein S3 [Helicoverpa armigera] >AXY94820.1 ribosomal ribosomal protein S3 [Galleria mellonella] >PZC80336.1 hypothetical protein B5X24_HaOG214853 [Helicoverpa armigera]	2.047435638	1.033818101	1.65E-05	up	yes	1.01	0.4933	0.511	0.485	0.484	1	0.983	1.047	BP:cellular biosynthe	GO:0044249;GO:0034641;GO:0006807;GO:0034645;GO:1901576;GO:0044271;GO:0044260;GO:0043043;GO:0071704;GO:0043604;GO:0009987;GO:0009058;GO:0009059;GO:0008150;GO:0008152;GO:1901564;GO:0044238;GO:0043603;GO:1901566;GO:0019538;GO:0044237;GO:0043170;GO:0006518;GO:0006412;GO:0015935;GO:0005737;GO:0110165;GO:0044391;GO:0032991;GO:1990904;GO:0005575;GO:0003674;GO:0005488;GO:1901363;GO:0005198;GO:0003735;GO:0003676;GO:0097159;GO:0003723	K02985	RP-S3e, RPS3	map03010;map05	Ribosome;Coronavirus disea	COG0092	J:Translation, ribosomal structure	PF00189.23;PF0	Ribosomal_S3_	Ribosomal protein S3, C	CYT	13	56	26.8	High

TRINITY_DN33801_c0_g1_i1_orf1	-	-	-	unnamed protein product [Diatraea saccharalis]	2.051699573	1.036819495	7.15E-06	up	yes	1.008	0.4913	0.493	0.462	0.519	1	1.009	1.016	CC:cytoplasm;CC:ce	GO:0005737;GO:0005575;GO:0110165;GO:0035639;GO:0003674;GO:0005488;GO:0016887;GO:0043167;GO:1901363;GO:1901265;GO:0051082;GO:0000166;GO:0017076;GO:0005524;GO:0016787;GO:0017111;GO:0036094;GO:0003824;GO:0016818;GO:0030554;GO:0097367;GO:0097159;GO:0044183;GO:0016817;GO:0043168;GO:0016462;GO:0032559;GO:0005515;GO:0032555;GO:0032553	K09496	CCT4	------	------	COG0459	O:Posttranslational modification	PF00118.27	Cpn60_TCP1	TCP-1/cpn60 chaperor	CYT	15	46	42.9	High
TRINITY_DN79734_c0_g2_i3_orf1	-	-	-	60S ribosomal protein L27a [Ostrinia furnacalis]	2.007277138	1.005239818	4.01E-05	up	yes	0.993	0.4947	0.535	0.457	0.492	1	1.009	0.97	BP:cellular biosynthe	GO:0044249;GO:0034641;GO:0006807;GO:0034645;GO:1901576;GO:0044271;GO:0044260;GO:0043043;GO:0071704;GO:0043604;GO:0009987;GO:0009058;GO:0009059;GO:0008150;GO:0008152;GO:0044238;GO:1901564;GO:0043603;GO:1901566;GO:0019538;GO:0044237;GO:0043170;GO:0006518;GO:0006412;GO:0015934;GO:0044391;GO:0032991;GO:1990904;GO:0005575;GO:0003674;GO:0005198;GO:0003735	K02900	RP-L27Ae, RPI	map03010;map05	Ribosome;Coronavirus disea	COG0200	J:Translation, ribosomal structure	PF00828.22	Ribosomal_L27	Ribosomal proteins 50S	CYT	7	39	17.1	High
TRINITY_DN2924_c0_g1_i2_orf1	-	-	-	cuticular protein RR-2 [Spodoptera litura]	2.009566456	1.006884288	9.61E-05	up	yes	0.9873	0.4913	0.478	0.446	0.55	1	0.975	0.987	MF:molecular_functio	GO:0003674;GO:0042302;GO:0005198	------	------	------	------	ENOG410YSFI;EI	S:Function unknown;	------	------	------	CYT	2	14	11.6	High
TRINITY_DN8949_c0_g1_i2_orf1	-	-	-	unnamed protein product [Arctia plantaginis]	2.151305849	1.105212646	0.0001113	up	yes	0.9967	0.4633	0.451	0.42	0.519	1	1.029	0.961	BP:cellular biosynthe	GO:0044249;GO:0034641;GO:0006807;GO:0034645;GO:1901576;GO:0044271;GO:0044260;GO:0043043;GO:0071704;GO:0043604;GO:0009987;GO:0009058;GO:0009059;GO:0008150;GO:0008152;GO:0044238;GO:1901564;GO:0043603;GO:1901566;GO:0019538;GO:0044237;GO:0043170;GO:0006518;GO:0006412;GO:0015934;GO:0044391;GO:0032991;GO:1990904;GO:0005575;GO:0003674;GO:0005198;GO:0003735	K02880	RP-L17e, RPL1	map03010;map05	Ribosome;Coronavirus disea	COG0091	J:Translation, ribosomal structure	PF00237.22	Ribosomal_L22	Ribosomal protein L22p	CYT	7	29	24	High
TRINITY_DN3773_c0_g1_i4_orf1	-	-	-	peptidyl-prolyl cis-trans isomerase G isoform X2 [Ostrinia furnacalis]	2.360119733	1.238860052	0.0004693	up	yes	1.025	0.4343	0.456	0.338	0.509	1	1.075	1	BP:primary metaboli	GO:0044238;GO:0000413;GO:1901564;GO:0018208;GO:0019538;GO:0006807;GO:0018193;GO:0043170;GO:0071704;GO:0043412;GO:0036211;GO:0008150;GO:0008152;GO:0003755;GO:0003824;GO:0140096;GO:0003674;GO:0016859;GO:0016853	K12740;K09566	NKTR;PPIG	------	------	COG0652	Q:Secondary metabolites biosyr	PF00160.24	Pro_isomerase	Cyclophilin type peptid	CYT	2	3	93.9	High
TRINITY_DN81926_c0_g1_i1_orf1	-	-	-	membrane-bound alkaline phosphatase-like isoform X3 [Ostrinia furnacalis]	2.227147984	1.155197422	0.0001029	up	yes	1.055	0.4737	0.494	0.5	0.427	1	1.065	1.101	MF:hydrolase activity	GO:0016787;GO:0016791;GO:0004035;GO:0016788;GO:0042578;GO:0003824;GO:0003674	K01077	E3.1.3.1, phoA	map00730;map00	Thiamine metabolism;Folate	COG1785	P:Inorganic ion transport and me	PF00245.23	Alk_phosphatas	Alkaline phosphatase	CYT	1	4	59.1	High
TRINITY_DN51252_c0_g2_i1_orf1	-	-	-	peroxidase-like [Ostrinia furnacalis]	2.347103623	1.230881537	3.21E-06	up	yes	1.017	0.4333	0.41	0.451	0.439	1	1.017	1.035	BP:response to stres	GO:0006950;GO:0006979;GO:0050896;GO:0008150;GO:0042600;GO:0030312;GO:0005575;GO:0110165;GO:0020037;GO:0046906;GO:0097159;GO:0016209;GO:1901363;GO:0003824;GO:0003674;GO:0005488;GO:0016684;GO:0004601;GO:0016491	K19511	PXDN, VPO1	------	------	ENOG410XPZ3	U:Intracellular trafficking, secreti	PF03098.18	An_peroxidase	Animal haem peroxidas	CYT	1	13	18.9	High
TRINITY_DN16349_c0_g1_i10_orf1	-	-	-	protein lingerer-like isoform X1 [Nymphalis io] >XP_050356663.1 protein lingerer-like isoform X1 [Nymphalis io] >XP_050356664.1 protein lingerer-like isoform X1 [Nymphalis	2.327130714	1.218552249	1.30E-05	up	yes	1.013	0.4353	0.434	0.4	0.472	1	1.012	1.027	------	------	------	------	------	------	ENOG4110RI8	O:Posttranslational modification	PF12478.11	DUF3697	Ubiquitin-associated pr	CYT	5	14	69.3	High
TRINITY_DN15667_c0_g1_i2_orf1	-	-	-	coiled-coil domain-containing protein 25 [Ostrinia furnacalis]	2.143593976	1.100031667	7.95E-05	up	yes	1.039	0.4847	0.494	0.491	0.469	1	1.104	1.013	CC:cellular_compone	GO:0005575;GO:0012505;GO:0110165	------	------	------	------	ENOG410Z5JK	S:Function unknown	PF05670.16	NFACT-R_1	NFACT protein RNA bir	CYT	2	10	24.3	High
TRINITY_DN106476_c0_g1_i3_orf1	-	-	-	mitochondrial import inner membrane translocase subunit TIM44 [Ostrinia furnacalis]	2.322580645	1.215728691	2.88E-05	up	yes	1.008	0.434	0.485	0.405	0.412	1	0.999	1.025	BP:establishment of	GO:0051649;GO:0030150;GO:0008104;GO:0071806;GO:0070727;GO:0006886;GO:0055085;GO:0071705;GO:0065002;GO:1990542;GO:0071702;GO:0033036;GO:0009987;GO:0072594;GO:0006810;GO:0045184;GO:0033365;GO:0008150;GO:0072655;GO:0006839;GO:0051234;GO:0051179;GO:0044743;GO:0051641;GO:0070585;GO:0046907;GO:0015031;GO:0016020;GO:0031090;GO:0110165;GO:0031966;GO:0019866;GO:0005575;GO:0005743;GO:0003674;GO:0005488;GO:0051087;GO:0005515	K17804	TIM44	------	------	ENOG410Y5IK	O:Posttranslational modification	PF04280.18	Tim44	Tim44-like domain	CYT	7	18	49.7	High
TRINITY_DN5275_c0_g1_i1_orf1	-	-	-	paraplegin [Ostrinia furnacalis]	2.081707056	1.057767062	0.0001345	up	yes	0.9707	0.4663	0.472	0.439	0.488	1	1.005	0.907	------	------	K09552	SPG7	------	------	COG0465	O:Posttranslational modification	PF01434.21;PF0(	Peptidase_M41	Peptidase family M41;A	CYT	1	1	87.8	High
TRINITY_DN63914_c0_g1_i1_orf1	-	-	-	myophilin-like [Ostrinia furnacalis]	2.426488166	1.278869824	2.63E-06	up	yes	1.015	0.4183	0.403	0.409	0.443	1	1.031	1.013	------	------	K20526	TAGLN	------	------	COG5199	Z:Cytoskeleton	PF00307.34	CH	Calponin homology	CYT	9	51	20.7	High
TRINITY_DN1803_c0_g1_i3_orf1	-	-	-	translocator protein-like isoform X1 [Ostrinia furnacalis] >XP_028178947.1 translocator protein-like isoform X1 [Ostrinia furnacalis]	2.011796924	1.008484683	0.0005112	up	yes	0.955	0.4747	0.485	0.535	0.404	1	0.959	0.906	CC:cellular_compone	GO:0005575;GO:0016021;GO:0110165;GO:0031224	K05770	TSPO, BZRP	map05166;map04	Human T-cell leukemia virus	COG3476;ENOG	T:Signal transduction mechanism	PF03073.18	TspO_MBR	TspO/MBR family	CYT	3	12	21.3	High
TRINITY_DN2716_c0_g2_i1_orf1	-	-	-	eukaryotic translation initiation factor 1A, X-chromosomal [Ostrinia furnacalis] >XP_045445466.1 eukaryotic translation initiation factor 1A, X-chromosomal [Melitaea cinxia] >XP_049867692.1 eukaryotic translation initiation factor 1A, X-chromosomal [Pectinophora gossypiella] >KOB79530.1 Eukaryotic translation initiation factor 1A [Operophtera brumata] >CAH2086435.1 unnamed protein product [Euphydryas editha] >KOB79531.1 Eukaryotic translation initiation factor 1A [Operophtera brumata]	2.191869556	1.132161942	0.0001498	up	yes	0.9813	0.4477	0.44	0.394	0.509	1	0.999	0.945	MF:translation regula	GO:0090079;GO:0045182;GO:0097159;GO:0008135;GO:0003674;GO:0005488;GO:0003676;GO:0003743;GO:1901363;GO:0003723	K03236	EIF1A	------	------	COG0361	J:Translation, ribosomal structure	PF01176.22	eIF-1a	Translation initiation fac	CYT	3	21	16.9	High
TRINITY_DN58207_c0_g1_i1_orf1	-	-	-	60S ribosomal protein L6 [Ostrinia furnacalis] >XP_028170357.1 60S ribosomal protein L6 [Ostrinia furnacalis]	2.276536956	1.186840879	2.02E-05	up	yes	0.9887	0.4343	0.426	0.417	0.46	1	1.016	0.95	BP:cellular biosynthe	GO:0044249;GO:0034641;GO:0006807;GO:0034645;GO:1901576;GO:0044271;GO:0044260;GO:0043043;GO:0071704;GO:0043604;GO:0009987;GO:0009058;GO:0009059;GO:0008150;GO:0008152;GO:1901564;GO:0044238;GO:0043603;GO:1901566;GO:0019538;GO:0044237;GO:0043170;GO:0006518;GO:0006412;GO:0043232;GO:0043226;GO:0110165;GO:0005840;GO:0032991;GO:1990904;GO:0043229;GO:0005575;GO:0043228;GO:0003674;GO:0005198;GO:0003735	K02934	RP-L6e, RPL6	map03010;map05	Ribosome;Coronavirus disea	COG2163	J:Translation, ribosomal structure	PF01159.22;PF0	Ribosomal_L6e	Ribosomal protein L6e;	CYT	9	32	28.2	High
TRINITY_DN2975_c0_g1_i4_orf1	-	-	-	ubiquitin-like protein 4A [Ostrinia furnacalis]	2.221276596	1.15138905	0.0004291	up	yes	1.044	0.47	0.526	0.373	0.511	1	1.072	1.06	------	------	K23388	UBL4	------	------	COG5272	S:Function unknown	PF00240.26;PF1	ubiquitin;Rad6(	Ubiquitin family;Ubiquit	CYT	2	15	17	High
TRINITY_DN142652_c0_g1_i1_orf1	-	-	-	pre-mRNA-splicing factor RBM22 [Chelonus insularis]	3.521383076	1.81614218	0.0008605	up	yes	1.161	0.3297	0.336	0.289	0.364	1	1.17	1.312	CC:macromolecular	GO:0032991;GO:0005575;GO:1990904;GO:0005681;GO:0043169;GO:0097159;GO:0043167;GO:0003674;GO:0003723;GO:0003676;GO:0046872;GO:1901363;GO:0005488	K12872	RBM22, SLT11	map03040	Spliceosome	ENOG410XSFK	S:Function unknown	PF00076.25;PF1	RRM_1;zf_CCCI	RNA recognition motif.;	CYT	1	2	45.3	High
TRINITY_DN2668_c0_g1_i7_orf1	-	-	-	unnamed protein product [Chrysodeixis includens]	2.788487646	1.479482879	1.75E-05	up	yes	1.027	0.3683	0.379	0.327	0.399	1	1.023	1.058	BP:organic substanc	GO:0071704;GO:0006629;GO:0044238;GO:0008152;GO:0008150;GO:0005575;GO:0005576;GO:0110165;GO:0016787;GO:0016788;GO:0003824;GO:0003674;GO:0016298	------	------	------	------	ENOG410YGS3;E	S:Function unknown;	PF00151.22	Lipase	Lipase	CYT	6	26	38.5	High
TRINITY_DN94625_c0_g1_i1_orf1	-	-	-	uncharacterized protein LOC114354112 [Ostrinia furnacalis]	2.165957447	1.1150049	3.92E-06	up	yes	1.018	0.47	0.472	0.447	0.491	1	1.027	1.027	CC:cytoplasm;CC:ce	GO:0005737;GO:0110165;GO:0005575;GO:0035639;GO:0003674;GO:0005488;GO:0003676;GO:0043167;GO:1901363;GO:1901265;GO:0019001;GO:0000166;GO:0032561;GO:0017076;GO:0005525;GO:0016787;GO:0008135;GO:0003924;GO:0017111;GO:0036094;GO:0003824;GO:0045182;GO:0016818;GO:0090079;GO:0097367;GO:0097159;GO:0016817;GO:0043168;GO:0016462;GO:0032555;GO:0003746;GO:0032553	K02358	tuf, TUFM	------	------	COG0050	J:Translation, ribosomal structure	PF00009.30;PF0	GTP_EFTU;GTP_	Elongation factor Tu GT	CYT	12	36	50.9	High
TRINITY_DN129226_c0_g1_i2_orf1	-	-	-	hypothetical protein evm_000268 [Chilo suppressalis]	2.540209344	1.344947397	0.0002481	up	yes	0.995	0.3917	0.367	0.442	0.366	1	1.065	0.92	CC:integral compone	GO:0016021;GO:0005575;GO:0031224;GO:0110165;GO:0043169;GO:0097159;GO:0043167;GO:0003674;GO:0003676;GO:0003723;GO:0046872;GO:1901363;GO:0005488	K10053;K18415	RUNX1T1, CBI	map05221;map05	Acute myeloid leukemia;Tra	ENOG41125M8;I	S:Function unknown;	------	------	------	CYT	1	3	28.7	High
TRINITY_DN10644_c0_g1_i2_orf1	-	-	-	carboxylesterase [Cnaphalocrocis medinalis]	2.183591266	1.126702831	0.0001402	up	yes	1.03	0.4717	0.426	0.542	0.447	1	1.036	1.055	MF:hydrolase activity	GO:0016787;GO:0003674;GO:0016788;GO:0052689;GO:0003824	------	------	------	------	COG2272	I:Lipid transport and metabolism	PF00135.31;PF2	COesterase;BD	Carboxylesterase family	CYT	6	17	55	High
TRINITY_DN76036_c0_g1_i1_orf1	-	-	-	cytochrome c oxidase subunit 6A1, mitochondrial-like [Ostrinia furnacalis]	2.325842697	1.217753527	0.0002349	up	yes	1.035	0.445	0.526	0.383	0.426	1	1.034	1.072	BP:aerobic respiratic	GO:0009060;GO:0006091;GO:0006119;GO:0009987;GO:0045333;GO:0008152;GO:0044237;GO:0015980;GO:0008150;GO:0031224;GO:0005575;GO:0016021;GO:0098803;GO:0070069;GO:0098800;GO:0005751;GO:1902494;GO:0032991;GO:0045277;GO:0098796;GO:0110165;GO:0098798;GO:0015453;GO:0003674;GO:0016491;GO:0015318;GO:0015399;GO:0022804;GO:0004129;GO:0005215;GO:0008324;GO:0003824;GO:0009055;GO:0022890;GO:0015075;GO:0022857;GO:0022853;GO:0016675;GO:0015078	K02266	COX6A	map04714;map00	Thermogenesis;Oxidative ph	ENOG411253X	O:Posttranslational modification	PF02046.18	COX6A	Cytochrome c oxidase s	CYT	2	24	12	High
TRINITY_DN35301_c0_g1_i3_orf1	-	-	-	ATP synthase subunit gamma, mitochondrial-like [Ostrinia furnacalis] >XP_028164649.1 ATP synthase subunit gamma, mitochondrial-like [Ostrinia furnacalis]	2.045500711	1.032454039	1.36E-05	up	yes	1.007	0.4923	0.527	0.472	0.478	1	0.994	1.026	------	------	K02136	ATPeF1G, ATP	map04714;map05	Thermogenesis;Chemical ca	COG0224	C:Energy production and conver	PF00231.22	ATP-synt	ATP synthase	CYT	10	32	32.5	High
TRINITY_DN21909_c0_g1_i1_orf1	-	-	-	complement component 1 Q subcomponent-binding protein, mitochondrial [Ostrinia furnacalis]	2.750879091	1.459892731	3.22E-06	up	yes	1.017	0.3697	0.395	0.351	0.363	1	1.039	1.011	CC:organelle lumen;	GO:0043233;GO:0031974;GO:0005575;GO:0070013;GO:0005759;GO:0110165	K15414	C1QBP	------	------	ENOG4111G4Z	S:Function unknown	PF02330.19	MAM33	Mitochondrial glycopro	CYT	6	24	31.9	High
TRINITY_DN825_c8_g1_i5_orf1	-	-	-	ATP-binding cassette sub-family F member 2 [Ostrinia furnacalis] >XP_028169527.1 ATP-binding cassette sub-family F member 2 [Ostrinia furnacalis]	2.144957983	1.100949388	4.18E-06	up	yes	1.021	0.476	0.493	0.474	0.461	1	1.019	1.044	MF:anion binding;MF	GO:0043168;GO:0097367;GO:0003674;GO:0030554;GO:0035639;GO:0097159;GO:0000166;GO:0036094;GO:0032553;GO:0032559;GO:1901265;GO:0032555;GO:0017076;GO:0043167;GO:1901363;GO:0005524;GO:0005488	K06185	ABCF2	map05130	Pathogenic Escherichia coli i	COG0488	S:Function unknown	PF00005.30;PF1	ABC_tran;ABC_	ABC transporter;ABC tra	CYT	15	26	70.8	High
TRINITY_DN2265_c0_g2_i1_orf1	-	-	-	LOW QUALITY PROTEIN: elongation factor G, mitochondrial-like [Leguminivora glycinivorella]	2.278481013	1.188072348	9.44E-05	up	yes	1.026	0.4503	0.503	0.443	0.405	1	1.071	1.006	BP:cellular macromo	GO:0034645;GO:0044249;GO:1901576;GO:0044260;GO:0009987;GO:0071704;GO:0070125;GO:0009058;GO:0009059;GO:0008150;GO:0008152;GO:0044237;GO:0043170;GO:0006414;GO:0043229;GO:0043227;GO:0005739;GO:0043226;GO:0110165;GO:0043231;GO:0005575;GO:0035639;GO:0003674;GO:0005488;GO:0003676;GO:0019001;GO:1901363;GO:1901265;GO:0043168;GO:0032561;GO:0017076;GO:0005525;GO:0016787;GO:0000166;GO:0008135;GO:0003924;GO:0017111;GO:0036094;GO:0003824;GO:0016818;GO:0090079;GO:0043167;GO:0097367;GO:0097159;GO:0016817;GO:0016462;GO:0032555;GO:0003746;GO:0045182;GO:0032553	K02355	fusA, GFM, EF(	------	------	COG0480	U:Intracellular trafficking, secreti	PF00679.27;PF0	EFG_C;EFG_IV	Elongation factor G C-t	CYT	1	7	18.1	High
TRINITY_DN44557_c0_g2_i1_orf1	-	-	-	serine hydrolase-like protein [Ostrinia furnacalis]	2.11161015	1.078343506	0.0007621	up	yes	0.9403	0.4453	0.435	0.393	0.508	1	0.961	0.86	MF:hydrolase activity	GO:0016787;GO:0016740;GO:0003674;GO:0003824	------	------	------	------	ENOG4111NQ7;	S:Function unknown;	PF00561.23;PF1	Abhydrolase_1;	alpha/beta hydrolase fo	CYT	1	5	32.3	High
TRINITY_DN24163_c0_g1_i1_orf1	-	-	-	luciferin 4-monooxygenase-like [Ostrinia furnacalis]	2.076131687	1.053897956	4.38E-06	up	yes	1.009	0.486	0.507	0.493	0.458	1	1.016	1.01	------	------	------	------	------	------	COG0318	S:Function unknown	PF00501.31;PF1	AMP-binding;A	AMP-binding enzyme;A	CYT	6	11	62.7	High
TRINITY_DN631_c0_g1_i6_orf1	-	-	-	cytosolic 10-formyltetrahydrofolate dehydrogenase isoform X1 [Ostrinia furnacalis] >XP_028172896.1 cytosolic 10-formyltetrahydrofolate dehydrogenase isoform X2 [Ostrinia furnacalis]	2.128156164	1.089604019	3.38E-06	up	yes	1.003	0.4713	0.449	0.467	0.498	1	1.009	1.001	BP:one-carbon meta	GO:0006730;GO:0019752;GO:0044248;GO:0009256;GO:0006807;GO:0044281;GO:0044282;GO:0046653;GO:0009258;GO:1901360;GO:0009397;GO:0042219;GO:0071704;GO:0042560;GO:0044270;GO:0006760;GO:1901361;GO:0009987;GO:0006725;GO:1901575;GO:0046700;GO:0009058;GO:0008150;GO:0008152;GO:0043436;GO:0009056;GO:0046483;GO:0043649;GO:0043648;GO:1901564;GO:0006575;GO:0006082;GO:0046395;GO:0016054;GO:0034641;GO:0044237;GO:0042558;GO:0019439;GO:1901565;GO:0005737;GO:0110165;GO:0005575;GO:0016155;GO:0016620;GO:0003674;GO:0016491;GO:0016646;GO:0016645;GO:0016742;GO:0016740;GO:0016741;GO:0003824;GO:0016903	K00289	ALDH1L	map00670	One carbon pool by folate	COG1012	S:Function unknown	PF00171.25;PF0	Aldedh;Formyl_	Aldehyde dehydrogena	CYT	17	25	101.7	High
TRINITY_DN24399_c0_g1_i1_orf1	-	-	-	retinol-binding protein pinta-like [Ostrinia furnacalis]	2.931818182	1.551795637	1.62E-05	up	yes	1.032	0.352	0.366	0.316	0.374	1	1.071	1.024	------	------	------	------	------	------	ENOG410XRSO	S:Function unknown	PF00650.23	CRAL_TRIO	CRAL/TRIO domain	CYT	4	15	35.4	High
TRINITY_DN86844_c0_g2_i1_orf1	-	-	-	spermine oxidase-like isoform X1 [Ostrinia furnacalis]	2.41937082	1.27463191	7.16E-06	up	yes	0.9767	0.4037	0.376	0.409	0.426	1	0.97	0.96	MF:catalytic activity;I	GO:0003824;GO:0003674;GO:0016491	K12259	SMOX, PAO5	map00330;map00	Arginine and proline metabo	ENOG410YT60;E	F:Nucleotide transport and meta	PF01593.27	Amino_oxidase	Flavin containing amine	CYT	1	5	46.8	High
TRINITY_DN14565_c0_g1_i11_orf1	-	-	-	4-aminobutyrate aminotransferase, mitochondrial [Galleria mellonella]	2.075625	1.053545818	1.54E-06	up	yes	0.9963	0.48	0.476	0.484	0.48	1	0.975	1.014	BP:neurotransmitter	GO:0042133;GO:0042135;GO:0019752;GO:0044248;GO:0006807;GO:0001505;GO:0006520;GO:0071704;GO:0065007;GO:0065008;GO:0044281;GO:0009448;GO:0009987;GO:0032787;GO:0008150;GO:0008152;GO:0043436;GO:0009056;GO:0044238;GO:1901564;GO:0006082;GO:0044237;GO:0005488;GO:0070279;GO:0008483;GO:0003674;GO:1901363;GO:0043168;GO:0043167;GO:0016740;GO:0047298;GO:0016769;GO:0036094;GO:0003824;GO:0019842;GO:0003867;GO:0097159;GO:0034386;GO:0030170	K13524	ABAT	map00640;map00	Propanoate metabolism;But	COG0160	S:Function unknown	PF00202.24	Aminotran_3	Aminotransferase class	CYT	6	18	52.2	High
TRINITY_DN2184_c0_g1_i4_orf1	-	-	-	uncharacterized protein LOC114359356 [Ostrinia furnacalis]	2.086637298	1.061179851	1.71E-05	up	yes	0.9947	0.4767	0.504	0.486	0.44	1	0.976	1.008	CC:cellular_compone	GO:0005575;GO:0005576;GO:0110165;GO:0003674;GO:0005198	------	------	------	------	------	------	PF07294.14	Fibroin_P25	Fibroin P25	CYT	2	8	28	High
TRINITY_DN33248_c0_g1_i1_orf1	-	-	-	elongation factor Ts, mitochondrial isoform X3 [Ostrinia furnacalis] >XP_028155866.1 elongation factor Ts, mitochondrial isoform X3 [Ostrinia furnacalis]	2.128156164	1.089604019	5.51E-07	up	yes	1.003	0.4713	0.454	0.475	0.485	1	1.004	1.005	CC:mitochondrion;C	GO:0005739;GO:0043226;GO:0005575;GO:0043229;GO:0043227;GO:0110165;GO:0043231;GO:0090079;GO:0003676;GO:0097159;GO:0008135;GO:0003674;GO:0005488;GO:0045182;GO:0003746;GO:1901363	K02357	tsf, TSFM	------	------	COG0264	J:Translation, ribosomal structure	PF00889.22	EF_TS	Elongation factor TS	CYT	2	7	35.6	High
TRINITY_DN5697_c0_g1_i1_orf1	-	-	-	GPI ethanolamine phosphate transferase 2-like [Ostrinia furnacalis]	2.174194943	1.120481301	8.51E-05	up	yes	1.006	0.4627	0.422	0.447	0.519	1	1.037	0.98	BP:glycerophospholi	GO:0006650;GO:0044249;GO:0006807;GO:0046486;GO:0006629;GO:0045017;GO:0071704;GO:0006505;GO:0006506;GO:0006661;GO:0006664;GO:1903509;GO:0006644;GO:0006643;GO:1901576;GO:0009987;GO:0009247;GO:0009058;GO:0043412;GO:0036211;GO:0008150;GO:0008152;GO:0046474;GO:0044255;GO:0090407;GO:0008610;GO:0006497;GO:0044238;GO:0046467;GO:1901564;GO:0008654;GO:0019538;GO:1901135;GO:0044237;GO:0043170;GO:1901137;GO:0006796;GO:0006793;GO:0019637;GO:0046488;GO:0005789;GO:0031224;GO:0031090;GO:0016021;GO:0016020;GO:0110165;GO:0005575;GO:0003674;GO:0016740;GO:0016780;GO:0003824;GO:0016772;GO:0051377	K05310	PIGG, GPI7	map00563	Glycosylphosphatidylinosito	COG1524	S:Function unknown	PF01663.25	Phosphodiest	Type I phosphodiestera	CYT	1	1	89.9	Medium
TRINITY_DN4121_c0_g1_i1_orf1	-	-	-	uncharacterized protein LOC114358001, partial [Ostrinia furnacalis]	2.077407174	1.054784014	5.88E-05	up	yes	0.9903	0.4767	0.422	0.515	0.493	1	0.994	0.977	BP:cellular catabolic	GO:0044248;GO:0006807;GO:0043632;GO:1901575;GO:0044265;GO:0044260;GO:0070647;GO:0070646;GO:0071704;GO:0006508;GO:0016579;GO:0044238;GO:0009987;GO:0019941;GO:0043412;GO:0036211;GO:0008150;GO:0008152;GO:0009056;GO:0009057;GO:0051603;GO:1901564;GO:0019538;GO:0044237;GO:0043170;GO:0006511;GO:0101005;GO:0003674;GO:0008234;GO:0019783;GO:0004843;GO:0016787;GO:0140096;GO:0003824;GO:0008233	K11833	USP2	------	------	ENOG410XP8T	S:Function unknown	PF00443.32;PF1	UCH;UCH_1	Ubiquitin carboxyl-term	CYT	3	4	104.2	High
TRINITY_DN4040_c0_g1_i10_orf1	-	-	-	hypothetical protein evm_007488 [Chilo suppressalis]	2.056548245	1.040224917	4.30E-06	up	yes	0.9783	0.4757	0.495	0.463	0.469	1	0.969	0.966	CC:cellular anatomic	GO:0110165;GO:0031224;GO:0005575;GO:0016021;GO:0015297;GO:0015291;GO:0015103;GO:0015318;GO:0005215;GO:0008509;GO:0140323;GO:0015075;GO:0022857;GO:0003674;GO:0022853;GO:0005452;GO:0022804	K13855	SLC4A2, AE2	map04972;map04	Pancreatic secretion;Salivary	ENOG410XPHD	U:Intracellular trafficking, secreti	PF00955.24;PF0	HCO3_cotransp	HCO3- transporter fam	CYT	5	6	128.9	High
TRINITY_DN2089_c0_g1_i5_orf1	-	-	-	eukaryotic translation initiation factor 4B [Ostrinia furnacalis]	2.183431953	1.12659757	5.46E-05	up	yes	0.9963	0.4563	0.462	0.404	0.503	1	0.981	1.008	------	------	K03258	EIF4B	map05205;map04	Proteoglycans in cancer;mT(	ENOG4111NS2;(	S:Function unknown;	PF00076.25	RRM_1	RNA recognition motif.	CYT	10	17	65.7	High
TRINITY_DN42738_c0_g1_i1_orf1	-	-	-	arf-GAP domain and FG repeat-containing protein 1 [Ostrinia furnacalis]	2.442440066	1.288323161	0.0001481	up	yes	1.029	0.4213	0.5	0.364	0.4	1	1.046	1.04	BP:carboxylic acid m	GO:0019752;GO:0044249;GO:0006807;GO:0009067;GO:0009066;GO:0044283;GO:1901576;GO:0006529;GO:0006528;GO:0006520;GO:0071704;GO:1901605;GO:1901607;GO:0008652;GO:0009987;GO:0009058;GO:0008150;GO:0008152;GO:0043436;GO:0044238;GO:1901564;GO:0006082;GO:1901566;GO:0046394;GO:0016053;GO:0044237;GO:0044281;GO:0008047;GO:0098772;GO:0016874;GO:0030234;GO:0005096;GO:0030695;GO:0060589;GO:0016884;GO:0016879;GO:0003824;GO:0004066;GO:0003674	K15044	AGFG1	------	------	ENOG4111QGH;	U:Intracellular trafficking, secreti	PF01412.21	ArfGap	Putative GTPase activat	EXC	1	5	38.3	High
TRINITY_DN2274_c0_g1_i6_orf1	-	-	-	membrane alanyl aminopeptidase-like [Ostrinia furnacalis]	2.207422134	1.142362548	1.33E-05	up	yes	0.9993	0.4527	0.432	0.49	0.436	1	0.982	1.016	BP:nitrogen compou	GO:0006807;GO:0071704;GO:0006508;GO:0008150;GO:0008152;GO:0044238;GO:1901564;GO:0019538;GO:0043170;GO:0004177;GO:0008238;GO:0003674;GO:0008270;GO:0008233;GO:0008237;GO:0043169;GO:0046914;GO:0043167;GO:0046872;GO:0016787;GO:0140096;GO:0003824;GO:0005488	------	------	------	------	COG0308	E:Amino acid transport and meta	PF01433.23;PF1	Peptidase_M1;I	Peptidase family M1 do	CYT	11	15	114.7	High
TRINITY_DN4143_c0_g1_i1_orf1	-	-	-	zinc finger protein 530-like isoform X8 [Ostrinia furnacalis]	2.447368421	1.291231298	0.002559	up	yes	1.023	0.418	0.26	0.558	0.436	1	0.996	1.074	------	------	K09228	KRAB	map05168	Herpes simplex virus 1 infect	COG5048;ENOG	S:Function unknown;	PF07776.18	zf-AD	Zinc-finger associated	CYT	1	5	20.3	High
TRINITY_DN43293_c0_g1_i2_orf1	-	-	-	egl nine homolog 1 isoform X2 [Helicoverpa armigera]	2.32408893	1.216665274	4.91E-05	up	yes	1.014	0.4363	0.487	0.394	0.428	1	1.044	0.998	MF:anion binding;MF	GO:0043168;GO:0031418;GO:0051213;GO:0046914;GO:0043169;GO:0043177;GO:0097159;GO:0016705;GO:0031406;GO:0030246;GO:0019842;GO:0043167;GO:0036094;GO:0003824;GO:0016491;GO:0003674;GO:0048029;GO:0005506;GO:0046872;GO:1901363;GO:0005488	K09592	EGLN, HPH	map05211;map04	Renal cell carcinoma;HIF-1 s	ENOG410ZHZN	O:Posttranslational modification	PF13640.9;PF01	2OG-FeII_Oxy_	2OG-F;MYND finger;2C	CYT	2	5	54	High
TRINITY_DN2065_c1_g2_i1_orf1	-	-	-	2-amino-3-ketobutyrate coenzyme A ligase, mitochondrial [Ostrinia furnacalis]	2.450394454	1.293014007	4.15E-06	up	yes	1.025	0.4183	0.431	0.401	0.423	1	1.052	1.023	BP:carboxylic acid m	GO:0019752;GO:0009063;GO:0006807;GO:0009066;GO:0044282;GO:0009068;GO:0006567;GO:0006566;GO:1901575;GO:0006520;GO:0071704;GO:1901605;GO:1901606;GO:0009987;GO:0009058;GO:0008150;GO:0008152;GO:0043436;GO:0009056;GO:0044248;GO:0044238;GO:1901564;GO:1901565;GO:0006082;GO:0046395;GO:0016054;GO:0044237;GO:0044281;GO:0030170;GO:0070279;GO:0003674;GO:1901363;GO:0016874;GO:0008890;GO:0043167;GO:0016740;GO:0016746;GO:0016747;GO:0016453;GO:0043168;GO:0036094;GO:0003824;GO:0019842;GO:0016407;GO:0097159;GO:0005488;GO:0016408	K00639	kbl, GCAT	map00260	Glycine, serine and threonine	COG0156;COG0	J:Translation, ribosomal structure	PF00155.24	Aminotran_1_2	Aminotransferase class	CYT	4	13	46.2	High
TRINITY_DN1066_c0_g1_i4_orf1	-	-	-	ribosome biogenesis protein WDR12 homolog [Ostrinia furnacalis]	2.198444444	1.136483076	3.99E-07	up	yes	0.9893	0.45	0.445	0.452	0.453	1	0.973	0.995	BP:maturation of 5.8	GO:0000466;GO:0090304;GO:0000470;GO:0034660;GO:1901360;GO:0006139;GO:0071704;GO:0006364;GO:0034470;GO:0009987;GO:0006725;GO:0000460;GO:0000463;GO:0008150;GO:0008152;GO:0046483;GO:0016070;GO:0044238;GO:0016072;GO:0034641;GO:0044237;GO:0043170;GO:0006807;GO:0006396;GO:0030687;GO:0030684;GO:0043229;GO:0043228;GO:0043226;GO:0005730;GO:0005654;GO:0110165;GO:0032991;GO:0043232;GO:1990904;GO:0005575;GO:0044877;GO:0005488;GO:0043021;GO:0003674	K14863	WDR12, YTM1	------	------	ENOG410XPW5	J:Translation, ribosomal structure	PF00400.35;PF0	WD40;NLE;NBC	WD domain, G-beta re	CYT	3	6	46.5	High
TRINITY_DN536_c0_g1_i7_orf1	-	-	-	polymerase delta-interacting protein 2 isoform X3 [Ostrinia furnacalis]	2.016537576	1.011880289	8.36E-05	up	yes	0.9633	0.4777	0.454	0.468	0.511	1	0.973	0.917	MF:organic cyclic co	GO:0097159;GO:0003674;GO:0005488;GO:0003676;GO:0003677;GO:1901363	K17809	POLDIP2	------	------	COG2967	P:Inorganic ion transport and me	PF04379.17;PF0	DUF525;YccV-l	ApaG domain;Hemimet	CYT	2	12	39.3	High
TRINITY_DN9853_c0_g3_i1_orf1	-	-	-	importin-7 isoform X1 [Ostrinia furnacalis]	2.248394495	1.168895188	6.14E-06	up	yes	0.9803	0.436	0.444	0.444	0.42	1	0.991	0.95	------	------	K20223	IPO7, RANBP7	map03013;map04	Nucleocytoplasmic transpor	COG5656	O:Posttranslational modification	PF03810.22	IBN_N	Importin-beta N-termi	CYT	12	13	120.6	High
TRINITY_DN9002_c0_g1_i1_orf1	-	-	-	membrane magnesium transporter 1 [Ostrinia furnacalis]	2.556409621	1.354119022	0.0005874	up	yes	1.031	0.4033	0.38	0.403	0.427	1	0.943	1.151	BP:localization;BP:bic	GO:0051179;GO:0008150;GO:0051234;GO:0000139;GO:0032991;GO:0031090;GO:0030659;GO:0098588;GO:0016020;GO:0072546;GO:0005575;GO:0098796;GO:0110165;GO:0012506;GO:0031901;GO:0010008	K23566	MMGT1, EMG5	------	------	ENOG4111TZW	U:Intracellular trafficking, secreti	PF10270.12	MMgT	Membrane magnesium	CYT	1	8	13.1	High
TRINITY_DN19000_c0_g1_i4_orf1	-	-	-	NADH dehydrogenase [ubiquinone] 1 beta subcomplex subunit 9 [Ostrinia furnacalis]	2.183231913	1.126465389	6.53E-06	up	yes	0.9687	0.4437	0.44	0.435	0.456	1	0.956	0.95	BP:electron transpor	GO:0022900;GO:0009987;GO:0019646;GO:0044237;GO:0009060;GO:0045333;GO:0008152;GO:0022904;GO:0008150;GO:0006120;GO:0006091;GO:0015980;GO:0031090;GO:0016020;GO:0019866;GO:0031966;GO:0005575;GO:0005743;GO:0070469;GO:0110165	K03965	NDUFB9	map04714;map00	Thermogenesis;Oxidative ph	ENOG4111PES	C:Energy production and conver	------	------	------	CYT	6	43	17.6	High
TRINITY_DN4929_c1_g2_i5_orf1	-	-	-	guanylate kinase isoform X2 [Ostrinia furnacalis]	2.424462171	1.277664743	2.04E-06	up	yes	1.003	0.4137	0.441	0.402	0.398	1	1.011	0.999	BP:phosphorylation;I	GO:0016310;GO:0009987;GO:0008150;GO:0008152;GO:0044237;GO:0006796;GO:0006793;GO:0019205;GO:0035639;GO:0003674;GO:0005488;GO:0043167;GO:1901363;GO:1901265;GO:0043168;GO:0000166;GO:0016740;GO:0017076;GO:0005524;GO:0016301;GO:0004385;GO:0036094;GO:0003824;GO:0030554;GO:0097367;GO:0016772;GO:0016776;GO:0050145;GO:0032559;GO:0032555;GO:0097159;GO:0032553	K00942	gmk, GUK1	map01232;map00	Nucleotide metabolism;Purir	COG0194	U:Intracellular trafficking, secreti	PF00625.24;PF1	Guanylate_kin;A	Guanylate kinase;AAA (	CYT	2	12	22.7	High

TRINITY_DN7583_c0_g1_i1_orf1	-	-	-	39S ribosomal protein L21, mitochondrial [Ostrinia furnacalis]	2.449229264	1.292327826	8.55E-06	up	yes	1.001	0.4087	0.402	0.377	0.447	1	1	1.003	BP:cellular biosynthe	GO:0044249;GO:0034645;GO:1901576;GO:0044271;GO:0044260;GO:0043603;GO:0043043;GO:0071704;GO:1901566;GO:0043604;GO:0009987;GO:0009058;GO:0009059;GO:0008150;GO:0008152;GO:1901564;GO:0044238;GO:0019538;GO:0034641;GO:0044237;GO:0043170;GO:0006807;GO:0006518;GO:0006412;GO:0043226;GO:0043229;GO:0043228;GO:0005737;GO:0110165;GO:0032991;GO:0005840;GO:0043232;GO:1990904;GO:0005575;GO:0003676;GO:0003674;GO:0005488;GO:1901363;GO:0005198;GO:0003735;GO:0097159;GO:0003723	K02888	RP-L21, MRPL	map03010	Ribosome	ENOG4111TX4	J:Translation, ribosomal structure	PF00829.24	Ribosomal_L21	Ribosomal prokaryotic	CYT	1	5	19.8	High
TRINITY_DN16965_c0_g2_i1_orf1	-	-	-	hypothetical protein evm_007405 [Chilo suppressalis]	2.277083333	1.18718709	3.87E-05	up	yes	0.9837	0.432	0.415	0.435	0.446	1	0.932	1.019	MF:organic cyclic co	GO:0097159;GO:0003674;GO:0005488;GO:0003676;GO:1901363;GO:0003723	K23625	ATXN2_2L	map04711;map05	Circadian rhythm - fly;Spino	ENOG4110S1X;C	S:Function unknown;	PF07145.18	PAM2	Ataxin-2 C-terminal re	CYT	1	3	27	High
TRINITY_DN105901_c0_g1_i2_orfp1	-	-	-	contactin-like [Pectinophora gossypiella]	2.100506943	1.070737554	0.000907	up	yes	0.953	0.4537	0.413	0.535	0.413	1	0.875	0.984	------	------	------	------	------	------	------	------	------	------	------	CYT	2	16	16.7	High
TRINITY_DN12973_c0_g1_i1_orf1	-	-	-	mitochondrial-processing peptidase subunit alpha [Ostrinia furnacalis]	2.159614954	1.110774112	0.0006719	up	yes	0.9647	0.4467	0.394	0.549	0.397	1	0.953	0.941	BP:mitochondrial pro	GO:0034982;GO:0006807;GO:0071704;GO:0006508;GO:0006627;GO:0008150;GO:0008152;GO:0016485;GO:0044238;GO:1901564;GO:0051604;GO:0019538;GO:0043170;GO:0031974;GO:0005759;GO:0110165;GO:0043233;GO:0005575;GO:0070013;GO:0003674;GO:0005488;GO:0043169;GO:0043167;GO:0046872	K01412	PMPCA, MAS2	------	------	COG0612	O:Posttranslational modification	PF05193.24;PF0	Peptidase_M16	Peptidase M16 inactive	CYT	5	10	59.2	High
TRINITY_DN29934_c0_g1_i6_orf1	-	-	-	sodium/potassium-transporting ATPase subunit beta-2-like [Ostrinia furnacalis] >XP_028176258.1 sodium/potassium-transporting ATPase subunit beta-2-like [Ostrinia furnacalis]	3.873999304	1.953823695	0.0002511	up	yes	1.113	0.2873	0.254	0.288	0.32	1	1.223	1.117	BP:potassium ion tra	GO:0006813;GO:0006812;GO:0006811;GO:0006810;GO:0006814;GO:0030001;GO:0008150;GO:0051234;GO:0051179;GO:0098533;GO:1902495;GO:0005575;GO:1990351;GO:0098797;GO:0098796;GO:0005890;GO:0032991;GO:0090533	K01540	ATP1B, CD298	map04918;map04	Thyroid hormone synthesis;I	ENOG411150A	U:Intracellular trafficking, secreti	PF00287.21	Na_K-ATPase	Sodium / potassium AT	CYT	1	4	37.5	High
TRINITY_DN35635_c0_g1_i1_orf1	-	-	-	probable NADH dehydrogenase [ubiquinone] 1 alpha subcomplex subunit 12 [Ostrinia furnacalis]	2.46925	1.30407291	1.17E-06	up	yes	0.9877	0.4	0.397	0.384	0.419	1	0.987	0.976	BP:mitochondrial res	GO:0033108;GO:0022607;GO:0032981;GO:0043933;GO:0010257;GO:0016043;GO:0009987;GO:0008150;GO:0071840;GO:0065003;GO:0031090;GO:0016020;GO:0019866;GO:0031966;GO:0005575;GO:0005743;GO:0070469;GO:0110165	K11352	NDUFA12	map04714;map00	Thermogenesis;Oxidative ph	ENOG4111XVR	I:Lipid transport and metabolism	PF05071.19	NDUFA12	NADH ubiquinone oxid	CYT	5	44	16.9	High
TRINITY_DN2704_c0_g1_i5_orf1	-	-	-	hypothetical protein evm_009002 [Chilo suppressalis]	2.579395817	1.367033177	2.78E-05	up	yes	0.999	0.3873	0.347	0.374	0.441	1	1.008	0.989	------	------	K06569	MFI2, CD228	------	------	ENOG410ZEJE	S:Function unknown	PF00405.20	Transferrin	Transferrin	CYT	9	16	84.2	High
TRINITY_DN51045_c0_g1_i1_orf1	-	-	-	cell growth-regulating nucleolar protein [Ostrinia furnacalis]	2.871536524	1.521822912	2.20E-06	up	yes	1.026	0.3573	0.362	0.342	0.368	1	1.028	1.05	CC:intracellular mem	GO:0043231;GO:0005634;GO:0005575;GO:0043229;GO:0043227;GO:0043226;GO:0110165;GO:0043169;GO:0097159;GO:1901363;GO:0043167;GO:0003674;GO:0005488;GO:0003676;GO:0003677;GO:0046872	K15263	LYER	------	------	ENOG411081U	S:Function unknown	PF08790.14	zf-LYAR	LYAR-type C2HC zinc f	CYT	4	13	48.3	High
TRINITY_DN2749_c0_g2_i3_orf1	-	-	-	RNA exonuclease 4-like [Ostrinia furnacalis] >QEE79882.1 REX4 [Ostrinia furnacalis]	2.023732252	1.017018428	9.94E-05	up	yes	0.9977	0.493	0.469	0.453	0.557	1	1	0.993	BP:nucleic acid meta	GO:0090304;GO:0034641;GO:0006807;GO:0034660;GO:1901360;GO:0006139;GO:0071704;GO:0006364;GO:0034470;GO:0009987;GO:0006725;GO:0008150;GO:0008152;GO:0046483;GO:0016070;GO:0044238;GO:0016072;GO:0044237;GO:0043170;GO:0006396;GO:0003674;GO:0005488;GO:0003676;GO:1901363;GO:0008408;GO:0004527;GO:0016787;GO:0016788;GO:0003824;GO:0004518;GO:0097159	K18327	REXO4, REX4	------	------	COG0847	L:Replication, recombination and	PF00929.27	RNase_T	Exonuclease	CYT	5	45	17.8	High
TRINITY_DN86149_c0_g1_i1_orf1	-	-	-	NADH dehydrogenase [ubiquinone] 1 alpha subcomplex subunit 8 [Galleria mellonella]	2.079783919	1.056433646	5.14E-07	up	yes	1.001	0.4813	0.49	0.483	0.471	1	1.014	0.99	BP:electron transpor	GO:0022900;GO:0009987;GO:0019646;GO:0006120;GO:0009060;GO:0045333;GO:0044237;GO:0008152;GO:0022904;GO:0008150;GO:0006091;GO:0015980;GO:0043231;GO:0005739;GO:0005575;GO:0043229;GO:0043227;GO:0043226;GO:0110165	K03952	NDUFA8	map04714;map00	Thermogenesis;Oxidative ph	ENOG4111IST	I:Lipid transport and metabolism	------	------	------	EXC	6	42	20.1	High
TRINITY_DN58636_c0_g1_i1_orf1	-	-	-	uncharacterized protein LOC114363665 [Ostrinia furnacalis]	2.813875227	1.492558358	0.0003426	up	yes	1.087	0.3863	0.449	0.306	0.404	1	1.112	1.15	BP:nucleic acid meta	GO:0090304;GO:0034641;GO:0006807;GO:0034660;GO:1901360;GO:0006139;GO:0071704;GO:0006364;GO:0034470;GO:0009987;GO:0006725;GO:0008150;GO:0008152;GO:0046483;GO:0016070;GO:0044238;GO:0016072;GO:0030490;GO:0044237;GO:0043170;GO:0006396;GO:0043226;GO:0005730;GO:0110165;GO:0043232;GO:0043229;GO:0005575;GO:0043228	------	------	------	------	ENOG41118C1	S:Function unknown	------	------	------	CYT	1	1	66.5	Medium
TRINITY_DN4262_c0_g1_i16_orf1	-	-	-	sperm-associated antigen 7 homolog [Ostrinia furnacalis]	2.158087443	1.109753323	4.68E-06	up	yes	1.002	0.4643	0.478	0.458	0.457	1	0.978	1.028	MF:organic cyclic co	GO:0097159;GO:0003674;GO:1901363;GO:0003676;GO:0005488	------	------	------	------	ENOG4111H0W	S:Function unknown	PF01424.25	R3H	R3H domain	CYT	3	13	27	High
TRINITY_DN17394_c0_g1_i1_orf1	-	-	-	monocarboxylate transporter 14-like [Ostrinia furnacalis]	2.060041841	1.04267364	5.09E-05	up	yes	0.9847	0.478	0.496	0.427	0.511	1	0.986	0.968	CC:integral compon	GO:0016021;GO:0005575;GO:0110165;GO:0031224;GO:0005215;GO:0022857;GO:0003674	------	------	------	------	ENOG410ZW1J;E	G:Carbohydrate transport and m	PF07690.19	MFS_1	Major Facilitator Superf	CYT	2	2	89.3	High
TRINITY_DN20582_c0_g1_i1_orf1	-	-	-	2-oxoglutarate dehydrogenase, mitochondrial isoform X3 [Ostrinia furnacalis] >XP_028167081.1 2-oxoglutarate dehydrogenase, mitochondrial isoform X3 [Ostrinia furnacalis]	2.384490365	1.253680953	4.48E-06	up	yes	1.027	0.4307	0.443	0.429	0.42	1	1.056	1.024	------	------	K00164	OGDH, sucA	map00020	Citrate cycl	COG0567	Z:Cytoskeleton	PF16870.8;PF02	OxoGdeHyase_	2-oxoglutarate dehydr	CYT	7	32	25.6	High
TRINITY_DN21609_c0_g2_i1_orf1	-	-	-	translation initiation factor eIF-2B subunit epsilon [Ostrinia furnacalis]	2.136304348	1.095117195	2.73E-05	up	yes	0.9827	0.46	0.453	0.431	0.496	1	0.995	0.953	MF:translation regula	GO:0090079;GO:0003676;GO:0097159;GO:0030234;GO:0008135;GO:0005085;GO:1901363;GO:0030695;GO:0003674;GO:0005488;GO:0005515;GO:0031369;GO:0098772;GO:0060589;GO:0045182;GO:0003743	K03240	EIF2B5	map05168	Herpes simplex virus 1 infect	COG1208	M:Cell wall/membrane/envelope	PF02020.21;PF0	W2;Hexapep	eIF4-gamma/eIF5/eIF2	CYT	3	8	40.1	High
TRINITY_DN78873_c0_g1_i4_orf1	-	-	-	hypothetical protein evm_008224 [Chilo suppressalis]	2.426907535	1.279119143	0.0007736	up	yes	1.021	0.4207	0.302	0.468	0.492	1	1.073	0.99	BP:developmental p	GO:0032502;GO:0048856;GO:0008150;GO:0016021;GO:0005575;GO:0031224;GO:0110165	------	------	------	------	ENOG410XQIR;C	S:Function unknown;	PF00041.24	fn3	Fibronectin type III dom	CYT	1	6	14.6	High
TRINITY_DN4434_c0_g1_i7_orf1	-	-	-	V-type proton ATPase catalytic subunit A [Ostrinia furnacalis] >XP_028155921.1 V-type proton ATPase catalytic subunit A [Ostrinia furnacalis] >XP_028155922.1 V-type proton ATPase catalytic subunit A [Ostrinia furnacalis] >ADP23923.1 V-ATPase subunit A [Ostrinia furnacalis] >ADT80587.1 V-type proton ATPase catalytic subunit A [Ostrinia furnacalis] >CBY05457.1 V-type proton ATPase catalytic subunit A [Ostrinia furnacalis]	2.260504202	1.176644599	2.04E-07	up	yes	0.9953	0.4403	0.443	0.446	0.432	1	1.003	0.983	CC:proton-transport	GO:0033180;GO:0033178;GO:0032991;GO:0005575;GO:0098796;GO:0005261;GO:0035639;GO:0003674;GO:0005488;GO:0043167;GO:1901363;GO:1901265;GO:0043168;GO:0015075;GO:0042626;GO:0015318;GO:0009678;GO:0000166;GO:0015399;GO:0017076;GO:0022804;GO:0015252;GO:0042625;GO:0005215;GO:0005216;GO:0008324;GO:0036094;GO:0003824;GO:0046961;GO:0022803;GO:0016874;GO:0044769;GO:0022890;GO:0030554;GO:0097367;GO:0097159;GO:0046933;GO:0019829;GO:0022857;GO:0015267;GO:0032559;GO:0005524;GO:0022853;GO:0032555;GO:0015078;GO:0032553	K02145	ATPeV1A, ATF	map05323;map00	Rheumatoid arthritis;Oxidati	COG1155	C:Energy production and conver	PF00006.28;PF16	ATP-synt_ab;A	ATP synthase alpha/bet	CYT	29	62	68.1	High
TRINITY_DN55160_c0_g2_i1_orf1	-	-	-	esterase FE4-like isoform X2 [Ostrinia furnacalis]	3.955017301	1.983684006	0.0002915	up	yes	1.143	0.289	0.304	0.275	0.288	1	1.217	1.213	------	------	------	------	------	------	COG2272	I:Lipid transport and metabolism	PF00135.31	COesterase	Carboxylesterase family	CYT	1	4	34.3	High
TRINITY_DN23167_c0_g1_i4_orf1	-	-	-	uncharacterized protein LOC114363065 [Ostrinia furnacalis]	2.345101707	1.229650494	5.24E-05	up	yes	1.003	0.4277	0.45	0.369	0.464	1	1.022	0.986	BP:organic substanc	GO:0071704;GO:1901564;GO:0019538;GO:0043170;GO:0008150;GO:0006807;GO:0008152;GO:0006508;GO:0044238;GO:0004252;GO:0004175;GO:0016787;GO:0017171;GO:0140096;GO:0003824;GO:0003674;GO:0008233;GO:0008236	------	------	------	------	COG5640	O:Posttranslational modification	PF18322.4	CLIP_1	Serine protease Clip do	CYT	7	14	69.4	High
TRINITY_DN29448_c0_g1_i1_orf1	-	-	-	28S ribosomal protein S9, mitochondrial [Ostrinia furnacalis]	2.1890625	1.130313146	2.20E-05	up	yes	0.9807	0.448	0.485	0.415	0.444	1	0.982	0.96	BP:cellular biosynthe	GO:0044249;GO:0034641;GO:0006807;GO:0034645;GO:1901576;GO:0044271;GO:0044260;GO:0043043;GO:0071704;GO:0043604;GO:0009987;GO:0009058;GO:0009059;GO:0008150;GO:0008152;GO:1901564;GO:0044238;GO:0043603;GO:1901566;GO:0019538;GO:0044237;GO:0043170;GO:0006518;GO:0006412;GO:0043232;GO:0043226;GO:0110165;GO:0005840;GO:0032991;GO:1990904;GO:0043229;GO:0005575;GO:0043228;GO:0003674;GO:0005198;GO:0003735	K02996	RP-S9, MRPS9	map03010	Ribosome	COG0103	J:Translation, ribosomal structure	PF00380.22	Ribosomal_S9	Ribosomal protein S9/S	CYT	2	5	45.9	High
TRINITY_DN4977_c0_g1_i2_orf1	-	-	-	manganese-transporting ATPase 13A1 [Ostrinia furnacalis]	2.091345106	1.06443115	4.99E-05	up	yes	0.9593	0.4587	0.437	0.486	0.453	1	0.921	0.957	CC:intrinsic compon	GO:0031224;GO:0016021;GO:0110165;GO:0005575;GO:0035639;GO:0003674;GO:0005488;GO:0016887;GO:0043167;GO:1901363;GO:0043168;GO:0015075;GO:0042626;GO:0015318;GO:0000166;GO:0015399;GO:1901265;GO:0017076;GO:0022804;GO:0016787;GO:0005215;GO:0008324;GO:0140358;GO:0017111;GO:0036094;GO:0003824;GO:0022890;GO:0016818;GO:0030554;GO:0097367;GO:0097159;GO:0016817;GO:0019829;GO:0016462;GO:0022857;GO:0032559;GO:0005524;GO:0022853;GO:0032555;GO:0032553	K14950	ATP13A1, SPF	------	------	COG0474	J:Translation, ribosomal structure	PF00122.23;PF0	E1-E2_ATPase;	E1-E2 ATPase;haloacid	CYT	6	6	130.6	High
TRINITY_DN84322_c0_g2_i1_orf1	-	-	-	alanyl-tRNA synthetase 1 [Homo sapiens] >KAI4055846.1 alanyl-tRNA synthetase 1 [Homo sapiens]	2.341232227	1.227268043	2.28E-06	up	yes	0.988	0.422	0.439	0.405	0.422	1	0.996	0.968	BP:carboxylic acid m	GO:0019752;GO:0090304;GO:0034641;GO:0006807;GO:0034660;GO:1901360;GO:0006520;GO:0071704;GO:0044281;GO:0006139;GO:0009987;GO:0006725;GO:0008150;GO:0008152;GO:0043436;GO:0046483;GO:0016070;GO:0044238;GO:1901564;GO:0006082;GO:0044237;GO:0043170;GO:0006399;GO:0043038;GO:0043039;GO:0006419;GO:0006418;GO:0005737;GO:0110165;GO:0005575;GO:0035639;GO:0003674;GO:0008270;GO:0003676;GO:0005524;GO:0000166;GO:1901363;GO:0043169;GO:0004813;GO:0004812;GO:0046914;GO:0043167;GO:1901265;GO:0017076;GO:0046872;GO:0043168;GO:0140098;GO:0036094;GO:0003824;GO:0000049;GO:0016874;GO:0016875;GO:0030554;GO:0097367;GO:0097159;GO:0032559;GO:0003723;GO:0032555;GO:0140101;GO:0032553;GO:0005488	K01872	AARS, alaS	map00970	Aminoacyl-tRNA biosynthes	COG0013	J:Translation, ribosomal structure	PF01411.22	tRNA-synt_2c	tRNA synthetases class	CYT	1	14	12.3	High
TRINITY_DN31327_c0_g2_i1_orf1	-	-	-	multidrug resistance protein 1A isoform X1 [Ostrinia furnacalis]	2.392461197	1.258495526	0.000164	up	yes	1.079	0.451	0.417	0.442	0.494	1	1.11	1.127	CC:integral compon	GO:0016021;GO:0031224;GO:0005575;GO:0110165;GO:0035639;GO:0003674;GO:0005488;GO:0043167;GO:1901363;GO:1901265;GO:0043168;GO:0042626;GO:0000166;GO:0015399;GO:0017076;GO:0005524;GO:0022804;GO:0005215;GO:0140359;GO:0036094;GO:0030554;GO:0097367;GO:0097159;GO:0022857;GO:0032559;GO:0032555;GO:0032553	K05658	ABCB1, CD243	map05206;map02	MicroRNAs in cancer;ABC tr	COG1132	S:Function unknown	PF00664.26;PF0	ABC_membran	ABC transporter transm	CYT	2	2	137.8	High
TRINITY_DN4030_c0_g2_i1_orf1	-	-	-	putative trypsin 6 [Ostrinia nubilalis]	2.543483741	1.346805872	8.04E-06	up	yes	1.009	0.3967	0.396	0.425	0.369	1	0.992	1.035	BP:organic substanc	GO:0071704;GO:1901564;GO:0019538;GO:0043170;GO:0008150;GO:0006807;GO:0008152;GO:0006508;GO:0044238;GO:0004252;GO:0004175;GO:0016787;GO:0017171;GO:0140096;GO:0003824;GO:0003674;GO:0008233;GO:0008236	K09628;K09640	PRSS27;TMPRS	------	------	COG5640	O:Posttranslational modification	PF00089.29	Trypsin	Trypsin	CYT	3	20	28.7	High
TRINITY_DN58531_c0_g1_i1_orf1	-	-	-	uncharacterized protein LOC114357371 [Ostrinia furnacalis] >XP_028166768.1 uncharacterized protein LOC114357371 [Ostrinia furnacalis]	4.047533309	2.017042953	0.0004158	up	yes	1.124	0.2777	0.202	0.273	0.358	1	1.157	1.214	------	------	------	------	------	------	ENOG410Y4ER;E	S:Function unknown;	------	------	------	CYT	1	3	29.7	Medium
TRINITY_DN18164_c0_g1_i7_orf1	-	-	-	uncharacterized protein LOC114366518 isoform X5 [Ostrinia furnacalis]	2.45722787	1.297031652	4.91E-05	up	yes	0.991	0.4033	0.377	0.429	0.404	1	0.939	1.034	CC:integral compon	GO:0016021;GO:0005575;GO:0031224;GO:0110165;GO:0005488;GO:0043169;GO:0046914;GO:0043167;GO:0003674;GO:0046872;GO:0008270	------	------	------	------	COG5183	O:Posttranslational modification	PF12906.10;PF0	RINGv;THH1_T	RING-variant domain;T	CYT	1	2	64.7	High
TRINITY_DN8261_c0_g1_i1_orf1	-	-	-	UDP-N-acetylhexosamine pyrophosphorylase-like protein 1 [Ostrinia furnacalis]	2.272192136	1.184084834	8.25E-06	up	yes	0.965	0.4247	0.429	0.43	0.415	1	0.954	0.941	MF:nucleotidyltransf	GO:0016779;GO:0016772;GO:0003824;GO:0016740;GO:0003977;GO:0003674;GO:0070569	K00972	UAP1	map00520;map01	Amino sugar and nucleotide	COG4284	O:Posttranslational modification	PF01704.21	UDPGP	UTP--glucose-1-phos	CYT	5	11	54.6	High
TRINITY_DN27848_c0_g1_i2_orf1	-	-	-	cystathionine beta-synthase-like [Ostrinia furnacalis] >XP_028159011.1 cystathionine beta-synthase-like [Ostrinia furnacalis]	2.533180778	1.340950037	5.31E-08	up	yes	0.9963	0.3933	0.399	0.387	0.394	1	1.002	0.987	BP:carboxylic acid m	GO:0019752;GO:0044249;GO:0006807;GO:0044281;GO:0044283;GO:0009069;GO:0006534;GO:0006563;GO:0006520;GO:0071704;GO:0019343;GO:0008652;GO:1901605;GO:0000096;GO:1901576;GO:0000097;GO:0009987;GO:0009058;GO:0008150;GO:0008152;GO:0043436;GO:0009070;GO:0044238;GO:1901564;GO:1901566;GO:0006082;GO:0044272;GO:0046394;GO:0016053;GO:0044237;GO:0006535;GO:0019344;GO:0006790;GO:1901607;GO:0005737;GO:0110165;GO:0005575;GO:0003674;GO:0016829;GO:0004122;GO:0003824;GO:0016835;GO:0016836	K01697	CBS	map00270;map00	Cysteine and methionine me	COG0031	E:Amino acid transport and met	PF00291.28	PALP	Pyridoxal-phosphate de	CYT	12	31	54.7	High
TRINITY_DN24751_c0_g1_i1_orf1	-	-	-	NADH dehydrogenase [ubiquinone] 1 beta subcomplex subunit 7-like [Ostrinia furnacalis]	2.211270624	1.144875598	3.72E-05	up	yes	1.032	0.4667	0.496	0.438	0.466	1	1.076	1.019	CC:organelle membr	GO:0031090;GO:0070013;GO:0031970;GO:0016020;GO:0019866;GO:0031974;GO:0031966;GO:0005575;GO:0005743;GO:0043233;GO:0005758;GO:0070469;GO:0110165	K03963	NDUFB7	map04714;map00	Thermogenesis;Oxidative ph	ENOG411230N	I:Lipid transport and metabolism	PF05676.16	NDUF_B7	NADH-ubiquinone oxic	CYT	3	30	14.3	High
TRINITY_DN3053_c0_g1_i2_orf1	-	-	-	prostaglandin reductase 1-like [Ostrinia furnacalis]	2.509486466	1.327392165	8.66E-07	up	yes	0.992	0.3953	0.398	0.386	0.402	1	1.005	0.971	CC:cytoplasm;CC:cel	GO:0005737;GO:0005575;GO:0110165;GO:0036132;GO:0016614;GO:0016628;GO:0016616;GO:0016627;GO:0003824;GO:0003674;GO:0047522;GO:0032440;GO:0016491	K13948	PTGR1, LTB4D	map00590	Arachidonic acid metabolism	COG2130	S:Function unknown	PF16884.8;PF00	ADH_N_2;ADH_	N-terminal domain of	CYT	9	28	39.1	High
TRINITY_DN12323_c0_g2_i2_orf1	-	-	-	polyribonucleotide nucleotidyltransferase 1, mitochondrial [Ostrinia furnacalis]	2.306487696	1.205697596	0.0002671	up	yes	1.031	0.447	0.433	0.409	0.499	1	1.11	0.982	BP:negative regulati	GO:0009892;GO:0019222;GO:0090304;GO:0034641;GO:0006807;GO:0050789;GO:1901360;GO:1901361;GO:0006139;GO:1901575;GO:0044265;GO:0010605;GO:0044260;GO:0071704;GO:0065007;GO:0006401;GO:0006402;GO:0010468;GO:0044238;GO:0060255;GO:0009987;GO:0006725;GO:0046700;GO:0008150;GO:0008152;GO:0048519;GO:0034655;GO:0009056;GO:0009057;GO:0044248;GO:0046483;GO:0016070;GO:0016071;GO:0044270;GO:0010629;GO:0044237;GO:0043170;GO:0019439;GO:0006396;GO:0003674;GO:0005488;GO:0003676;GO:0004654;GO:1901363;GO:0016740;GO:0003824;GO:0016779;GO:0016772;GO:0003723;GO:0097159	K00962	pnp, PNPT1	map03018	RNA degradation	COG1185	J:Translation, ribosomal structure	PF01138.24;PF0	RNase_PH;RNa	3' exoribonuclease fam	CYT	1	1	82.8	Medium
TRINITY_DN108200_c0_g1_i1_orf1	-	-	-	uncharacterized protein LOC114350842 [Ostrinia furnacalis]	2.011625535	1.008361772	0.0001439	up	yes	0.9863	0.4903	0.478	0.467	0.526	1	0.929	1.03	MF:catalytic activity;	GO:0003824;GO:0003674;GO:0016491	------	------	------	------	COG1028	S:Function unknown	PF13561.9;PF00	adh_short_C2;a	Enoyl:short chain dehyc	CYT	3	16	26	High
TRINITY_DN5678_c0_g2_i3_orf1	-	-	-	coiled-coil domain-containing protein 115 [Ostrinia furnacalis]	2.289291303	1.194901052	8.38E-05	up	yes	1.024	0.4473	0.447	0.428	0.467	1	1.09	0.981	BP:proton-transport	GO:0070070;GO:0070071;GO:0070072;GO:0022607;GO:0043933;GO:0009987;GO:0016043;GO:0065003;GO:0008150;GO:0071840	K23543	CCDC115	------	------	ENOG41124X6	S:Function unknown	------	------	------	CYT	1	9	17.6	High
TRINITY_DN754_c1_g1_i8_orf1	-	-	-	lysophospholipid acyltransferase 5 [Ostrinia furnacalis] >XP_028169982.1 lysophospholipid acyltransferase 5 [Ostrinia furnacalis]	2.031798472	1.022757313	0.0001726	up	yes	0.984	0.4843	0.51	0.512	0.431	1	0.934	1.018	CC:integral compon	GO:0016021;GO:0005575;GO:0110165;GO:0031224;GO:0003824;GO:0016740;GO:0003674	K13515	LPCAT3, MBO	map00564;map04	Glycerophospholipid metab	COG5202	I:Lipid transport and metabolism	PF03062.22	MBOAT	MBOAT, membrane-bo	CYT	2	3	55.3	High
TRINITY_DN3534_c0_g1_i2_orf1	-	-	-	guanine nucleotide-binding protein subunit beta-like protein [Ostrinia furnacalis]	2.064185658	1.045572736	1.24E-05	up	yes	0.9873	0.4783	0.472	0.473	0.49	1	0.951	1.011	BP:phosphorylation;	GO:0016310;GO:0009987;GO:0044237;GO:0006796;GO:0008150;GO:0008152;GO:0006793;GO:0032991;GO:0015935;GO:1990904;GO:0044391;GO:0005575;GO:0044877;GO:0016301;GO:0016740;GO:0003824;GO:0043022;GO:0043021;GO:0003674;GO:0016772;GO:0005488	K14753	RACK1	map05162	Measles	ENOG410XQGZ	S:Function unknown	PF00400.35;PF2	WD40;NBCH_V	WD domain, G-beta re	PLA	8	66	35.9	High
TRINITY_DN3893_c0_g2_i3_orf1	-	-	-	cleavage and polyadenylation specificity factor subunit CG7185 isoform X2 [Ostrinia furnacalis]	2.118625987	1.083128923	8.16E-05	up	yes	0.993	0.4687	0.475	0.415	0.516	1	0.968	1.011	------	------	K14398	CPSF6_7	map03250;map03	Viral life cycle - HIV-1;mRN	ENOG410Y0H0	A:RNA processing and modificat	------	------	------	CYT	4	8	69.6	High
TRINITY_DN3702_c0_g1_i1_orf1	-	-	-	U4/U6.U5 tri-snRNP-associated protein 2 [Ostrinia furnacalis]	2.124062567	1.086826263	3.39E-05	up	yes	0.9913	0.4667	0.465	0.444	0.491	1	1.024	0.95	BP:cellular compone	GO:0022607;GO:0000245;GO:0043933;GO:0006807;GO:0071840;GO:0071826;GO:0070647;GO:0070646;GO:0016043;GO:0065003;GO:0071704;GO:0022618;GO:0006508;GO:0016579;GO:0009987;GO:0043412;GO:0036211;GO:0008150;GO:0008152;GO:0044238;GO:1901564;GO:0019538;GO:0043170;GO:0043229;GO:0043227;GO:0043226;GO:0005634;GO:0110165;GO:0043231;GO:0005575;GO:0101005;GO:0003674;GO:0008270;GO:0008233;GO:0043167;GO:0043169;GO:0019783;GO:0046914;GO:0008234;GO:0046872;GO:0016787;GO:0140096;GO:0003824;GO:0004843;GO:0005488	K12847	USP39, SAD1	map03040	Spliceosome	ENOG410XS41	O:Posttranslational modification	PF00443.32;PF0	UCH;zf-UBP	Ubiquitin carboxyl-term	EXC	1	2	56	High
TRINITY_DN50085_c0_g1_i1_orf1	-	-	-	hypothetical protein evm_013997 [Chilo suppressalis]	2.186466828	1.128601461	1.16E-06	up	yes	0.992	0.4537	0.47	0.456	0.435	1	0.983	0.993	BP:cellular compone	GO:0022607;GO:0080090;GO:0019222;GO:0043933;GO:0031323;GO:0001732;GO:0050789;GO:0071840;GO:0071826;GO:0010608;GO:0051246;GO:0016043;GO:0065003;GO:0022618;GO:0065007;GO:0031326;GO:2000112;GO:0010468;GO:0060255;GO:0006446;GO:0009987;GO:0009889;GO:0034248;GO:0008150;GO:0051171;GO:0050794;GO:0010556;GO:0006417;GO:0070993;GO:0005852;GO:0033290;GO:0032991;GO:1990904;GO:0005575;GO:0016282;GO:0003674;GO:0005488;GO:0003676;GO:0031369;GO:1901363;GO:0008135;GO:0045182;GO:0090079;GO:0097159;GO:0003723;GO:0005515;GO:0003743	K03253	EIF3B	------	------	COG5354	S:Function unknown	PF08662.14;PF0	eIF2A;RRM_1	Eukaryotic translation in	CYT	13	22	82.4	High
TRINITY_DN3649_c0_g1_i6_orf1	-	-	-	unnamed protein product [Chilo suppressalis]	2.24610873	1.167427768	0.0001151	up	yes	0.9957	0.4433	0.487	0.373	0.47	1	1.01	0.977	BP:regulation of prin	GO:0080090;GO:0019222;GO:0031326;GO:0031323;GO:0050789;GO:0060255;GO:0065007;GO:0010468;GO:0019219;GO:0009889;GO:1903506;GO:0050794;GO:0008150;GO:0051171;GO:2001141;GO:0051252;GO:0006355;GO:0010556;GO:0005575;GO:0070603;GO:0016514;GO:1902494;GO:1904949;GO:0032991;GO:0071565;GO:0071564	K11649	SMARCC	map05225;map04	Hepatocellular carcinoma;Th	COG5259;COG5	B:Chromatin structure and dyna	PF16496.8;PF04	SWIRM-assoc_	SWIRM-associated dor	CYT	3	3	103.8	High
TRINITY_DN1330_c0_g1_i1_orf1	-	-	-	pancreatic triacylglycerol lipase-like [Ostrinia furnacalis]	2.416925651	1.273173094	9.82E-06	up	yes	1.011	0.4183	0.425	0.414	0.416	1	0.982	1.052	CC:cellular_compone	GO:0005575;GO:0005576;GO:0110165;GO:0016787;GO:0016788;GO:0003824;GO:0003674;GO:0016298	------	------	------	------	ENOG41116Y2;E	S:Function unknown;	PF00151.22	Lipase	Lipase	CYT	7	15	65	High
TRINITY_DN137_c0_g1_i1_orf1	-	-	-	60S ribosomal protein L8 [Cotesia glomerata] >XP_044597650.1 60S ribosomal protein L8 [Cotesia glomerata] >KAG8034499.1 hypothetical protein G9C98_007575 [Cotesia typhae] >CAD6216378.1 GSCOCG00004534001-RA-CDS [Cotesia congregata] >KAH0544237.1 60S ribosomal protein L8 [Cotesia glomerata] >KAH0564528.1 60S ribosomal protein L8 [Cotesia glomerata] >CAG5095185.1 Similar to RpL8: 60S ribosomal protein L8 (Spodoptera frugiperda) [Cotesia congregata]	2.209072535	1.143440791	8.50E-06	up	yes	0.9837	0.4453	0.441	0.419	0.476	1	0.979	0.972	BP:cellular biosynthe	GO:0044249;GO:0034645;GO:1901576;GO:0044271;GO:0044260;GO:0043603;GO:0043043;GO:0071704;GO:1901566;GO:0043604;GO:0009987;GO:0009058;GO:0009059;GO:0008150;GO:0008152;GO:1901564;GO:0044238;GO:0019538;GO:0034641;GO:0044237;GO:0043170;GO:0006807;GO:0006518;GO:0006412;GO:0015934;GO:0043226;GO:0043229;GO:0043228;GO:0110165;GO:0044391;GO:0032991;GO:0005840;GO:0043232;GO:1990904;GO:0005575;GO:0003676;GO:0003674;GO:0005488;GO:1901363;GO:0005198;GO:0003735;GO:0097159;GO:0003723	K02938	RP-L8e, RPL8	map03010;map05	Ribosome;Coronavirus disea	COG0090	J:Translation, ribosomal structure	PF03947.21;PF0	Ribosomal_L2_	Ribosomal Proteins L2,	CYT	3	9	28.1	High
TRINITY_DN44219_c0_g1_i1_orf1	-	-	-	mitochondrial import inner membrane translocase subunit TIM50-C-like [Ostrinia furnacalis]	3.34450198	1.741791399	0.0005667	up	yes	1.098	0.3283	0.4	0.221	0.364	1	1.188	1.106	BP:protein localizatic	GO:0008104;GO:0070727;GO:0071705;GO:0071702;GO:0033036;GO:0006810;GO:0009987;GO:0045184;GO:0008150;GO:0051234;GO:0051179;GO:0051641;GO:0015031;GO:0031224;GO:0016021;GO:0098800;GO:0110165;GO:0032991;GO:0005575;GO:0098796;GO:0005744;GO:0098798	K17496	TIM50	------	------	COG5190	K:Transcription	PF03031.21	NIF	NLI interacting factor-li	CYT	1	3	44.9	High
TRINITY_DN34821_c0_g1_i4_orf1	-	-	-	acetylcholine receptor subunit alpha-L1-like [Ostrinia furnacalis]	2.240909091	1.164084123	0.0002236	up	yes	0.986	0.44	0.495	0.401	0.424	1	0.924	1.034	CC:cellular_compone	GO:0005575;GO:0016021;GO:0110165;GO:0031224;GO:0038023;GO:0060089;GO:0015318;GO:0005215;GO:0005230;GO:0015276;GO:0015075;GO:0004888;GO:0022857;GO:0015267;GO:0003674;GO:0022836;GO:0022834;GO:0022803;GO:0005216	K04803;K04807;K	CHRNA1;CHRI	map04080	Neuroactive ligand-recepto	ENOG410XQGR	S:Function unknown	PF02931.26	Neur_chan_LBD	Neurotransmitter-gate	PLA	1	3	47.7	High
TRINITY_DN6933_c1_g1_i1_orf1	-	-	-	Chlorophyll a-b binding protein 40, chloroplastic [Trichinella nelsoni] >KRY99282.1 Chlorophyll a-b binding protein 40, chloroplastic [Trichinella zimbabwensis]	2.349752534	1.232508826	0.0001228	up	yes	0.997	0.4243	0.421	0.417	0.435	1	0.929	1.062	CC:cellular_compone	GO:0005575;GO:0016020;GO:0110165	K08913	LHCB2	------	------	ENOG410ZHBU	S:Function unknown	PF00504.24	Chloroa_b-bind	Chlorophyll A-B binding	CYT	2	8	24	High
TRINITY_DN195_c8_g1_i1_orf1	-	-	-	hypothetical protein evm_009768 [Chilo suppressalis]	2.309129115	1.207348844	1.24E-05	up	yes	0.989	0.4283	0.397	0.462	0.426	1	0.969	0.998	BP:interspecies intera	GO:0044419;GO:0009607;GO:0009605;GO:0043207;GO:0050896;GO:0009617;GO:0006952;GO:0006950;GO:0098542;GO:0042742;GO:0008150;GO:0051707;GO:0005575;GO:0005576;GO:0110165	------	------	------	------	ENOG410ZBQ8	S:Function unknown	PF03769.18	Attacin_C	Attacin, C-terminal regi	CYT	1	4	21.6	Medium
TRINITY_DN1013_c0_g1_i3_orf1	-	-	-	TELO2-interacting protein 1 homolog isoform X2 [Ostrinia furnacalis]	2.850149578	1.511037635	0.0003021	up	yes	1.048	0.3677	0.369	0.307	0.427	1	1.141	1.003	------	------	K20403	TTI1	map04150	mTOR signaling pathway	ENOG410XSN6;E	S:Function unknown;	------	------	------	CYT	1	1	124.9	Medium
TRINITY_DN14826_c0_g1_i1_orf1	-	-	-	uncharacterized protein LOC114350939 [Ostrinia furnacalis]	2.228904642	1.156334896	1.30E-05	up	yes	0.9747	0.4373	0.445	0.406	0.461	1	0.962	0.962	CC:cellular_compone	GO:0005575;GO:0016021;GO:0110165;GO:0031224	------	------	------	------	ENOG410Z38Q	S:Function unknown	------	------	------	CYT	1	16	8.6	High
TRINITY_DN20118_c0_g1_i4_orfp1	-	-	-	hypothetical protein HF086_007571 [Spodoptera exigua]	2.083719903	1.059161361	0.001458	up	yes	0.9433	0.4527	0.368	0.438	0.552	1	0.944	0.886	------	------	------	------	------	------	------	------	------	------	------	CYT	2	9	29.7	High
TRINITY_DN36538_c0_g1_i2_orf1	-	-	-	xaa-Pro dipeptidase isoform X1 [Ostrinia furnacalis] >XP_028156507.1 xaa-Pro dipeptidase isoform X2 [Ostrinia furnacalis]	2.662628391	1.412851093	1.23E-05	up	yes	1.011	0.3797	0.362	0.424	0.353	1	1.028	1.005	MF:cation binding;M	GO:0043169;GO:0046914;GO:0004177;GO:0016787;GO:0140096;GO:0030145;GO:0003824;GO:0043167;GO:0008238;GO:0003674;GO:0005488;GO:0070006;GO:0008233;GO:0046872;GO:0008235;GO:0008237	K14213	PEPD	------	------	COG0006	E:Amino acid transport and met	PF00557.27;PF0	Peptidase_M24	Metallopeptidase famil	CYT	9	18	60.8	High
TRINITY_DN389_c0_g1_i2_orf1	-	-	-	uncharacterized protein LOC118068293 isoform X2 [Chelonus insularis]	2.824112304	1.49779746	5.27E-06	up	yes	1.026	0.3633	0.338	0.385	0.367	1	1.052	1.026	------	------	------	------	------	------	ENOG410XX7G	S:Function unknown	PF01607.27;PF0	CBM_14;Ldl_re	Chitin binding Peritroph	PLA	4	11	60.2	High
TRINITY_DN1445_c0_g1_i1_orf1	-	-	-	leucine-rich PPR motif-containing protein, mitochondrial [Ostrinia furnacalis]	2.676727562	1.420470308	3.80E-08	up	yes	1.011	0.3777	0.381	0.377	0.375	1	1.017	1.015	------	------	K17964	LRPPRC	------	------	ENOG410XSG9	S:Function unknown	PF01535.23;PF1	PPR;PPR_2;PPR	PPR repeat;PPR repeat	CYT	9	14	87.6	High
TRINITY_DN1239_c0_g1_i3_orf1	-	-	-	uncharacterized protein LOC114355269 [Ostrinia furnacalis] >XP_028163822.1 uncharacterized protein LOC114355269 [Ostrinia furnacalis]	2.844036697	1.507940081	7.83E-05	up	yes	1.023	0.3597	0.36	0.362	0.357	1	0.968	1.101	CC:integral compon	GO:0016021;GO:0005575;GO:0110165;GO:0031224;GO:0005215;GO:0022857;GO:0003674	K08190	SLC16A14	------	------	ENOG41116NW	P:Inorganic ion transport and me	PF07690.19	MFS_1	Major Facilitator Superf	CYT	1	2	63.4	High
TRINITY_DN23586_c0_g1_i3_orf1	-	-	-	myrosinase 1-like isoform X1 [Ostrinia furnacalis]	2.109196595	1.076693573	0.0001111	up	yes	1.016	0.4817	0.415	0.525	0.505	1	1.014	1.035	BP:primary metaboli	GO:0044238;GO:0005975;GO:0071704;GO:0008150;GO:0008152;GO:0016787;GO:0003824;GO:0016798;GO:0004553;GO:0003674	------	------	------	------	COG2723	G:Carbohydrate transport and m	PF00232.21	Glyco_hydro_1	Glycosyl hydrolase fam	CYT	5	29	39.2	High

TRINITY_DN108354_c0_g1_i1_orf1	-	-	-	WD repeat-containing protein 61-like [Ostrinia furnacalis]	2.066352652	1.047086491	0.0001053	up	yes	1.009	0.4883	0.539	0.426	0.5	1	1.024	1.004	------	------	K12602	WDR61, REC1	map03018	RNA degradation	ENOG410XP1Z	O:Posttranslational modification	PF00400.35;PF1	WD40;ANAPC4	WD domain, G-beta re	CYT	1	2	35.8	High
TRINITY_DN46778_c0_g1_i2_orf1	-	-	-	Deoxycytidylate deaminase [Papilio xuthus]	2.343926076	1.22892707	3.21E-05	up	yes	1.04	0.4437	0.468	0.414	0.449	1	1.083	1.036	MF:hydrolase activity	GO:0016787;GO:0046914;GO:0043169;GO:0043167;GO:0003824;GO:0003674;GO:0008270;GO:0046872;GO:0005488	K01493	comEB	map00240;map01	Pyrimidine metabolism;Nucl	COG2131	F:Nucleotide transport and meta	PF00383.26;PF1	dCMP_cyt_dea	Cytidine and deoxycytic	CYT	1	5	21.4	Medium
TRINITY_DN11117_c0_g1_i1_orf1	-	-	-	venom carboxylesterase-6-like [Ostrinia furnacalis]	2.133757962	1.093396536	2.89E-05	up	yes	1.005	0.471	0.451	0.514	0.448	1	1.03	0.986	MF:hydrolase activity	GO:0016787;GO:0003674;GO:0016788;GO:0052689;GO:0003824	------	------	------	------	COG2272;COG0	S:Function unknown;G:Carbohyd	PF00135.31	COesterase	Carboxylesterase family	CYT	4	11	63.8	High
TRINITY_DN51968_c0_g1_i1_orf1	-	-	-	splicing factor U2af 38 kDa subunit [Aphidius gifuensis] >KAF7990547.1 hypothetical protein HCN44_000352 [Aphidius gifuensis]	2.386859637	1.255113729	0.0001019	up	yes	1.039	0.4353	0.458	0.406	0.442	1	1.111	1.007	BP:cellular metabolic	GO:0044237;GO:0090304;GO:0034641;GO:0006807;GO:1901360;GO:0016071;GO:0071704;GO:0008380;GO:0006139;GO:0009987;GO:0006725;GO:0000375;GO:0000377;GO:0008150;GO:0008152;GO:0046483;GO:0016070;GO:0044238;GO:0000398;GO:0043170;GO:0006396;GO:0006397;GO:0089701;GO:0032991;GO:0005575;GO:0003674;GO:0005488;GO:0003676;GO:1901363;GO:0043169;GO:0043167;GO:0046872;GO:0097159;GO:0003723	K12836	U2AF1	map05131;map03	Shigellosis;Spliceosome	ENOG410Z5PX	S:Function unknown	PF00642.27;PF0	zf-CCCH;RRM_	Zinc finger C-x8-C-x5-	CYT	1	6	28.6	High
TRINITY_DN108051_c0_g1_i2_orf1	-	-	-	uncharacterized protein LOC114351921 [Ostrinia furnacalis]	2.126439232	1.088439627	1.02E-05	up	yes	0.9973	0.469	0.46	0.504	0.443	1	1.006	0.986	BP:aerobic respiratio	GO:0009060;GO:0006091;GO:0022900;GO:0022904;GO:0009987;GO:0019646;GO:0045333;GO:0008152;GO:0015980;GO:0044237;GO:0006120;GO:0008150;GO:1990204;GO:0098803;GO:0098800;GO:1902495;GO:1902494;GO:1990351;GO:0045271;GO:0032991;GO:0030964;GO:0005575;GO:0098796;GO:0005747;GO:0098798	K03962	NDUFB6	map04714;map00	Thermogenesis;Oxidative ph	ENOG4111RT5	U:Intracellular trafficking, secreti	PF09782.12	NDUF_B6	NAD	CYT	4	24	18.7	High
TRINITY_DN8754_c0_g1_i2_orf1	-	-	-	dnaJ homolog subfamily C member 11 [Ostrinia furnacalis]	2.351592941	1.233638352	7.57E-06	up	yes	1.026	0.4363	0.43	0.44	0.439	1	1.064	1.013	------	------	K09531	DNAJC11	------	------	COG0484	J:Translation, ribosomal structure	PF11875.11;PF0	DnaJ-like_C11	DnaJ-like protein C11,	CYT	2	3	65.1	High
TRINITY_DN26963_c0_g1_i1_orf1	-	-	-	aminoacyl tRNA synthase complex-interacting multifunctional protein 1 isoform X2 [Ostrinia furnacalis]	2.190455245	1.131230738	3.11E-05	up	yes	0.996	0.4547	0.486	0.417	0.461	1	1.022	0.966	------	------	K15437	AIMP1, ARC1	------	------	COG0073	J:Translation, ribosomal structure	PF01588.23	tRNA_bind	Putative tRNA binding c	CYT	7	25	32.8	High
TRINITY_DN81715_c0_g1_i1_orf1	-	-	-	gamma-interferon-inducible lysosomal thiol reductase-like [Ostrinia furnacalis]	2.7893488	1.479928351	0.0001031	up	yes	1.058	0.3793	0.353	0.444	0.341	1	1.078	1.097	MF:catalytic activity;	GO:0003824;GO:0016671;GO:0003674;GO:0016667;GO:0016491	K08059	IFI30, GILT	map04612	Antigen processing and pre	ENOG4111IDT	S:Function unknown	PF03227.19	GILT	Gamma interferon indu	CYT	1	3	26.1	High
TRINITY_DN17559_c0_g1_i4_orf1	-	-	-	GDP-mannose 4,6 dehydratase isoform X2 [Ostrinia furnacalis] >XP_028166204.1 GDP-mannose 4,6 dehydratase isoform X2 [Ostrinia furnacalis]	2.394799054	1.259904606	6.36E-05	up	yes	1.013	0.423	0.395	0.479	0.395	1	1.05	0.988	BP:GDP-mannose m	GO:0019673;GO:0046368;GO:0044249;GO:0034641;GO:0006807;GO:0044281;GO:1901362;GO:1901360;GO:0006139;GO:0042350;GO:0042351;GO:0071704;GO:0018130;GO:0009226;GO:1901576;GO:0009225;GO:0009987;GO:0006725;GO:0009058;GO:0008150;GO:0008152;GO:0034654;GO:0055086;GO:0046483;GO:0044238;GO:0044271;GO:1901137;GO:0044237;GO:0006793;GO:0019438;GO:1901135;GO:0003674;GO:0008446;GO:0016829;GO:0003824;GO:0016835;GO:0016836	K01711	gmd, GMDS	map00520;map01	Amino sugar and nucleotide	COG1089	M:Cell wall/membrane/envelope	PF16363.8;PF01	GDP_Man_Deh	GDP-mannose 4,6 deh	CYT	4	16	40.5	High
TRINITY_DN19092_c0_g1_i2_orf1	-	-	-	eukaryotic translation initiation factor 3 subunit L [Ostrinia furnacalis]	2.099934966	1.070344649	0.0003789	up	yes	0.9687	0.4613	0.452	0.54	0.392	1	0.948	0.958	BP:cellular compone	GO:0022607;GO:0043933;GO:0001732;GO:0071840;GO:0071826;GO:0016043;GO:0065003;GO:0022618;GO:0009987;GO:0008150;GO:0005852;GO:0016282;GO:0070993;GO:0033290;GO:0032991;GO:1990904;GO:0005575;GO:0003674;GO:0005488;GO:0003676;GO:1901363;GO:0008135;GO:0045182;GO:0090079;GO:0097159;GO:0003743	K15029	EIF3L	------	------	ENOG410XPJT	J:Translation, ribosomal structure	PF10255.12	Paf67	RNA polymerase I-asso	CYT	10	19	63.7	High
TRINITY_DN15965_c0_g1_i1_orf1	-	-	-	U3 small nucleolar RNA-associated protein 15 homolog [Ostrinia furnacalis]	2.278122843	1.187845544	1.40E-05	up	yes	0.9903	0.4347	0.446	0.464	0.394	1	0.991	0.98	BP:nucleic acid meta	GO:0090304;GO:0034641;GO:0006807;GO:0034660;GO:1901360;GO:0006139;GO:0071704;GO:0006364;GO:0034470;GO:0009987;GO:0006725;GO:0008150;GO:0008152;GO:0046483;GO:0016070;GO:0044238;GO:0016072;GO:0044237;GO:0043170;GO:0006396;GO:0043226;GO:0005730;GO:0110165;GO:0043232;GO:0043229;GO:0005575;GO:0043228	K14549	UTP15	map03008	Ribosome biogenesis in euk	ENOG410XNZ0	I:Lipid transport and metabolism	PF09384.13;PF0	UTP15_C;WD4	UTP15 C terminal;WD c	CYT	2	4	57.9	High
TRINITY_DN47389_c0_g1_i2_orf1	-	-	-	non-specific lipid-transfer protein-like [Ostrinia furnacalis]	2.434131013	1.283406821	5.92E-06	up	yes	1.007	0.4137	0.385	0.447	0.409	1	1.017	1.005	BP:lipid transport;BP	GO:0006869;GO:0071702;GO:0006810;GO:0008150;GO:0051234;GO:0051179;GO:0042579;GO:0043229;GO:0043227;GO:0043226;GO:0005777;GO:0005739;GO:0110165;GO:0043231;GO:0005575;GO:0003674;GO:0005488;GO:0033814;GO:0050633;GO:0016740;GO:0016746;GO:0016747;GO:0003988;GO:0008289;GO:0019107;GO:0003824;GO:0050632;GO:0016408	K08764	SCP2, SCPX	map04146;map00	Peroxisome;Primary bile acid	ENOG410XPRW	I:Lipid transport and metabolism	PF00108.26;PF0	Thiolase_N;Thi	Thiolase, N-terminal dc	CYT	10	38	43.9	High
TRINITY_DN1294_c0_g1_i3_orf1	-	-	-	46 kDa FK506-binding nuclear protein-like isoform X1 [Ostrinia furnacalis] >XP_028157904.1 46 kDa FK506-binding nuclear protein-like isoform X2 [Ostrinia furnacalis]	2.570877283	1.362260747	2.19E-07	up	yes	0.9993	0.3887	0.384	0.392	0.39	1	1.013	0.985	CC:intracellular mem	GO:0043231;GO:0005634;GO:0005575;GO:0043229;GO:0043227;GO:0043226;GO:0110165;GO:0097159;GO:0140096;GO:1901363;GO:0016859;GO:0003824;GO:0003674;GO:0005488;GO:0003755;GO:0003677;GO:0003676;GO:0016853	K14826	FPR3_4	------	------	COG0545	C:Energy production and conver	PF00254.31;PF1	FKBP_C;NPL	FKBP-type peptidyl-pr	CYT	12	32	44.7	High
TRINITY_DN755_c0_g1_i3_orf1	-	-	-	uncharacterized protein LOC114358844 [Ostrinia furnacalis]	2.036704568	1.026236727	3.01E-05	up	yes	1.021	0.5013	0.478	0.541	0.485	1	1.049	1.014	CC:cellular compone	GO:0005575;GO:0016021;GO:0110165;GO:0031224	------	------	------	------	ENOG411170P	S:Function unknown	------	------	------	CYT	2	37	7	High
TRINITY_DN1477_c0_g1_i5_orf1	-	-	-	mitochondrial import inner membrane translocase subunit Tim9 [Ostrinia furnacalis]	2.113584356	1.079691694	0.0001085	up	yes	1.016	0.4807	0.516	0.418	0.508	1	1.048	1.001	------	------	K17777	TIM9	------	------	ENOG4111TUA	O:Posttranslational modification	PF02953.18	zf-Tim10_DDP	Tim10/DDP family zinc	PLA	3	35	10.7	High
TRINITY_DN59829_c0_g1_i1_orf1	-	-	-	putative mediator of RNA polymerase II transcription subunit 12 [Ostrinia furnacalis]	2.04976545	1.035458835	0.0001468	up	yes	1.005	0.4903	0.533	0.472	0.466	1	0.956	1.058	------	------	------	------	------	------	ENOG4110RMW	S:Function unknown	------	------	------	CYT	4	10	67.1	High
TRINITY_DN3229_c0_g1_i1_orf1	-	-	-	uncharacterized protein LOC114358442 isoform X1 [Ostrinia furnacalis]	2.33557047	1.223774975	5.18E-05	up	yes	1.044	0.447	0.462	0.426	0.453	1	1.029	1.103	MF:catalytic activity;	GO:0003824;GO:0016740;GO:0003674	------	------	------	------	ENOG41129KS	S:Function unknown	PF11901.11	DUF3421	Protein of unknown fun	CYT	6	37	23.3	High
TRINITY_DN4956_c0_g1_i6_orf1	-	-	-	nucleolar GTP-binding protein 1 [Ostrinia furnacalis]	2.158487359	1.110020644	3.42E-06	up	yes	1.016	0.4707	0.488	0.457	0.467	1	1.039	1.008	BP:ribosome biogen	GO:0042254;GO:0071840;GO:0022613;GO:0009987;GO:0008150;GO:0044085;GO:0043229;GO:0043228;GO:0043226;GO:0005730;GO:0110165;GO:0043232;GO:0005575;GO:0035639;GO:1901363;GO:0003674;GO:0005488;GO:0043167;GO:1901265;GO:0043168;GO:0000166;GO:0032561;GO:0017076;GO:0005525;GO:0036094;GO:0097367;GO:0097159;GO:0019001;GO:0032555;GO:0032553	K06943	NOG1	map03008	Ribosome biogenesis in euk	COG1084	S:Function unknown	PF17835.4;PF06	NOG1_N;NOG	NOG1 N-terminal helic	CYT	6	10	75.1	High
TRINITY_DN8676_c0_g1_i1_orf1	-	-	-	probable rRNA-processing protein EBP2 homolog [Ostrinia furnacalis]	2.033724039	1.024123929	7.07E-05	up	yes	0.989	0.4863	0.488	0.447	0.524	1	1.016	0.951	BP:cellular process;B	GO:0009987;GO:0042254;GO:0044085;GO:0008150;GO:0022613;GO:0071840;GO:0043228;GO:0043232;GO:0005730;GO:0005575;GO:0043229;GO:0110165;GO:0043226	K14823	EBP2, EBNA1B	------	------	ENOG410YYHN	S:Function unknown	PF05890.15	Ebp2	Eukaryotic rRNA proces	CYT	3	10	36	High
TRINITY_DN58413_c0_g1_i4_orf1	-	-	-	cysteine and histidine-rich protein 1 isoform X1 [Ostrinia furnacalis]	2.532374101	1.340490546	7.58E-05	up	yes	1.056	0.417	0.382	0.45	0.419	1	1.055	1.114	BP:cellular catabolic	GO:0044248;GO:0006807;GO:0043632;GO:1901575;GO:0044265;GO:0044260;GO:0071704;GO:0070647;GO:0032446;GO:0006508;GO:0051603;GO:0009987;GO:0019941;GO:0043412;GO:0036211;GO:0008150;GO:0008152;GO:0009056;GO:0009057;GO:0044238;GO:1901564;GO:0019538;GO:0044237;GO:0043170;GO:0016567;GO:0006511;GO:0005737;GO:0110165;GO:0005575;GO:0003674;GO:0005488;GO:0043169;GO:0043167;GO:0046872	K04506	SIAH1	map04310;map04	Wnt signaling pathway;Ubiq	ENOG410XVP0	O:Posttranslational modification	PF03145.19	Sina	Seven in absentia prote	CYT	2	7	34.8	High
TRINITY_DN4213_c0_g1_i4_orf1	-	-	-	nardilysin-like isoform X1 [Ostrinia furnacalis] >XP_028157649.1 nardilysin-like isoform X2 [Ostrinia furnacalis] >XP_028157650.1 nardilysin-like isoform X3 [Ostrinia furnacalis] >XP_028157651.1 nardilysin-like isoform X4 [Ostrinia furnacalis]	2.379967838	1.250942078	6.57E-06	up	yes	1.036	0.4353	0.431	0.44	0.435	1	1.042	1.066	------	------	K01411	NRD1	------	------	COG1025	O:Posttranslational modification	PF16187.8;PF00	Peptidase_M16	Middle or third domain	CYT	3	3	131.4	High
TRINITY_DN36788_c0_g1_i2_orf1	-	-	-	isocitrate dehydrogenase [NADP] cytoplasmic-like [Bicyclus anynana]	2.003267307	1.00235494	1.80E-06	up	yes	0.981	0.4897	0.496	0.477	0.496	1	0.969	0.974	BP:carboxylic acid m	GO:0019752;GO:0006099;GO:0006097;GO:0072350;GO:0044262;GO:0071704;GO:0009987;GO:0032787;GO:0008150;GO:0008152;GO:0043436;GO:0005975;GO:0046487;GO:0006081;GO:0044238;GO:0006082;GO:1902652;GO:0044237;GO:0006066;GO:0006102;GO:1901615;GO:0044281;GO:0051287;GO:0003674;GO:0005488;GO:0043167;GO:1901363;GO:1901265;GO:0016491;GO:0043169;GO:0000287;GO:0000166;GO:0004448;GO:0046872;GO:0036094;GO:0003824;GO:0016614;GO:0016616;GO:0097159;GO:0004450	K00031	IDH1, IDH2, ic	map00020;map04	Citrate cycl;Peroxisome;Glut	COG0538	S:Function unknown	PF00180.23	Iso_dh	Isocitrate/isopropylmal	CYT	18	49	49.2	High
TRINITY_DN26649_c0_g1_i2_orf1	-	-	-	ATP synthase-coupling factor 6, mitochondrial [Ostrinia furnacalis]	2.062330623	1.044275637	5.62E-07	up	yes	0.9893	0.4797	0.468	0.484	0.487	1	0.991	0.977	BP:nucleoside tripho	GO:0009141;GO:0009142;GO:0009144;GO:0009145;GO:0044249;GO:0034641;GO:0009165;GO:0044281;GO:0072521;GO:0072522;GO:1901362;GO:0009259;GO:1901360;GO:0006139;GO:0009201;GO:0071704;GO:0009199;GO:0018130;GO:0009206;GO:0009205;GO:0019693;GO:1901576;GO:0046034;GO:0009987;GO:0006725;GO:0009152;GO:0009150;GO:0009260;GO:0009058;GO:0009117;GO:0008152;GO:0019438;GO:0034654;GO:0044271;GO:0090407;GO:0055086;GO:0046483;GO:0044238;GO:0015986;GO:1901564;GO:1901566;GO:1901137;GO:1901135;GO:0006164;GO:0046390;GO:0044237;GO:0006163;GO:0006796;GO:0006807;GO:1901293;GO:0006793;GO:0019637;GO:0008150;GO:0006753;GO:0006754;GO:0045263;GO:0005575;GO:0098798;GO:0098800;GO:0032991;GO:0000276;GO:0098796;GO:0033177;GO:0022890;GO:0003674;GO:0015318;GO:0022857;GO:0005215;GO:0008324;GO:0015075;GO:0015078	K02131	ATPeF0F6, ATI	map04714;map05	Thermogenesis;Chemical ca	ENOG41122G1	C:Energy production and conver	PF05511.14	ATP-synt_F6	Mitochondrial ATP synt	CYT	5	62	10.5	High
TRINITY_DN4135_c0_g1_i5_orf1	-	-	-	probable small nuclear ribonucleoprotein Sm D2 [Manduca sexta] >KAG6451233.1 hypothetical protein O3G_MSEX007016 [Manduca sexta]	2.016494845	1.011849718	6.44E-05	up	yes	0.978	0.485	0.484	0.458	0.513	1	1.003	0.931	BP:nucleic acid meta	GO:0090304;GO:0034641;GO:0006807;GO:1901360;GO:0006139;GO:0071704;GO:0008380;GO:0016071;GO:0009987;GO:0006725;GO:0008150;GO:0008152;GO:0046483;GO:0016070;GO:0044238;GO:0044237;GO:0043170;GO:0006396;GO:0006397;GO:0120114;GO:0110165;GO:0032991;GO:0005575;GO:0030532;GO:0005829;GO:1990904;GO:0005681	K11096	SNRPD2, SMD	map03040	Spliceosome	COG1958	S:Function unknown	PF01423.25	LSM	LSM domain	CYT	3	24	13.3	High
TRINITY_DN2238_c0_g2_i1_orf1	-	-	-	mitochondrial import inner membrane translocase subunit Tim8 [Ostrinia furnacalis]	2.044486612	1.031738616	0.0006032	up	yes	0.9697	0.4743	0.435	0.546	0.442	1	1.01	0.899	BP:protein localizatic	GO:0008104;GO:0070727;GO:0071705;GO:0071702;GO:0033036;GO:0006810;GO:0009987;GO:0045184;GO:0008150;GO:0051234;GO:0051179;GO:0051641;GO:0015031;GO:0031970;GO:0031974;GO:0031090;GO:0016020;GO:0005758;GO:0110165;GO:0031966;GO:0070013;GO:0043233;GO:0019866;GO:0005575;GO:0005743	K17780	TIM8	------	------	ENOG41126D5	O:Posttranslational modification	PF02953.18	zf-Tim10_DDP	Tim10/DDP family zinc	CYT	4	44	10.2	High
TRINITY_DN7626_c0_g1_i1_orf1	-	-	-	NADH dehydrogenase [ubiquinone] flavoprotein 1, mitochondrial isoform X1 [Ostrinia furnacalis]	2.25706534	1.174448184	3.64E-06	up	yes	0.9983	0.4423	0.429	0.436	0.462	1	1.018	0.977	CC:oxidoreductase c	GO:1990204;GO:0005743;GO:0016020;GO:0098803;GO:0110165;GO:1902495;GO:1902494;GO:1990351;GO:0031966;GO:0031090;GO:0045271;GO:0032991;GO:0030964;GO:0019866;GO:0005575;GO:0098796;GO:0051287;GO:0046872;GO:0003674;GO:0005488;GO:0043167;GO:0003955;GO:1901363;GO:1901265;GO:0016491;GO:0043168;GO:0043169;GO:0051540;GO:0005215;GO:0000166;GO:0015399;GO:0050136;GO:0010181;GO:0009055;GO:0016651;GO:0015453;GO:0008137;GO:0036094;GO:0003824;GO:0003954;GO:0016655;GO:0051539;GO:0097367;GO:0097159;GO:0022857;GO:0022804;GO:0051536;GO:0032553	K03942	NDUFV1	map04714;map00	Thermogenesis;Oxidative ph	COG1894	C:Energy production and conver	PF01512.20;PF1	Complex1_51K	Respiratory-chain NAD	CYT	9	24	50.5	High
TRINITY_DN18860_c0_g1_i1_orf1	-	-	-	DDB1- and CUL4-associated factor 13 [Ostrinia furnacalis]	2.183727614	1.126792914	5.25E-06	up	yes	0.9877	0.4523	0.476	0.454	0.427	1	0.972	0.991	BP:nucleic acid meta	GO:0090304;GO:0034641;GO:0006807;GO:0034660;GO:1901360;GO:0006139;GO:0032446;GO:0070647;GO:0071704;GO:0006364;GO:0034470;GO:0009987;GO:0006725;GO:0043412;GO:0036211;GO:0008150;GO:0008152;GO:0046483;GO:0016070;GO:0044238;GO:0016072;GO:1901564;GO:0019538;GO:0044237;GO:0043170;GO:0016567;GO:0006396;GO:0043226;GO:0110165;GO:0043232;GO:0005730;GO:0043229;GO:0005575;GO:0043228	K11806	DCAF13, WDS	------	------	ENOG410XNX6	S:Function unknown	PF04158.17;PF0	Sof1;WD40;AN	Sof1-like domain;WD c	CYT	2	3	51.5	High
TRINITY_DN27979_c0_g1_i2_orf1	-	-	-	zinc finger CCCH domain-containing protein 15 homolog [Ostrinia furnacalis]	2.340227748	1.226648938	9.34E-07	up	yes	1.007	0.4303	0.436	0.444	0.411	1	1.018	1.002	MF:cation binding;M	GO:0043169;GO:0046872;GO:0003674;GO:0005488;GO:0043167	------	------	------	------	COG5252	S:Function unknown	PF16543.8;PF16	DFRP_C;Torus	DRG Family Regulatory	CYT	5	13	43.1	High
TRINITY_DN5417_c0_g1_i1_orf1	-	-	-	NADH dehydrogenase [ubiquinone] 1 alpha subcomplex subunit 10, mitochondrial isoform X1 [Ostrinia furnacalis] >XP_028175885.1 NADH dehydrogenase [ubiquinone] 1 alpha subcomplex subunit 10, mitochondrial isoform X2 [Ostrinia furnacalis]	2.237998648	1.162209165	6.46E-07	up	yes	0.993	0.4437	0.43	0.445	0.456	1	0.98	0.999	BP:aerobic respiratic	GO:0009060;GO:0006091;GO:0022900;GO:0022904;GO:0009987;GO:0019646;GO:0045333;GO:0008152;GO:0015980;GO:0044237;GO:0006120;GO:0008150;GO:0031974;GO:1990204;GO:0070013;GO:0098803;GO:0098800;GO:0005759;GO:0110165;GO:1902495;GO:1902494;GO:1990351;GO:0045271;GO:0032991;GO:0043233;GO:0030964;GO:0005575;GO:0098796;GO:0005747;GO:0098798	K03954	NDUFA10	map04714;map00	Thermogenesis;Oxidative ph	ENOG41101CN	F:Nucleotide transport and meta	PF01712.22	dNK	Deoxynucleoside kinase	CYT	9	22	45.9	High
TRINITY_DN6143_c0_g2_i1_orf1	-	-	-	uncharacterized protein LOC114365036 [Ostrinia furnacalis]	2.310177706	1.208003832	8.03E-06	up	yes	1.001	0.4333	0.422	0.408	0.47	1	1.009	0.993	------	------	------	------	------	------	ENOG41123CP	S:Function unknown	------	------	------	CYT	8	41	28.8	High
TRINITY_DN42506_c0_g1_i1_orf1	-	-	-	28S ribosomal protein S7, mitochondrial [Ostrinia furnacalis]	2.678983834	1.421685875	0.0002963	up	yes	1.044	0.3897	0.486	0.363	0.32	1	1.085	1.046	BP:cellular biosynthe	GO:0044249;GO:0034645;GO:1901576;GO:0044271;GO:0044260;GO:0043603;GO:0043043;GO:0071704;GO:1901566;GO:0043604;GO:0009987;GO:0009058;GO:0009059;GO:0008150;GO:0008152;GO:1901564;GO:0044238;GO:0019538;GO:0034641;GO:0044237;GO:0043170;GO:0006807;GO:0006518;GO:0006412;GO:0043226;GO:0043229;GO:0043228;GO:0110165;GO:0032991;GO:0005840;GO:0043232;GO:1990904;GO:0005575;GO:0003676;GO:0003674;GO:0005488;GO:1901363;GO:0005198;GO:0003735;GO:0097159;GO:0003723	K02992	RP-S7, MRPS7	map03010	Ribosome	COG0049	S:Function unknown	PF00177.24	Ribosomal_S7	Ribosomal protein S7p/	CYT	1	4	27.3	High
TRINITY_DN53167_c0_g1_i2_orf1	-	-	-	uncharacterized protein LOC114359219 [Ostrinia furnacalis]	2.187847763	1.129512355	0.0001328	up	yes	0.983	0.4493	0.506	0.458	0.384	1	0.984	0.965	BP:primary metaboli	GO:0044238;GO:0005975;GO:0071704;GO:0008150;GO:0008152;GO:0005737;GO:0005575;GO:0110165;GO:0016787;GO:0003824;GO:0016798;GO:0004564;GO:0004553;GO:0003674	------	------	------	------	COG1621	G:Carbohydrate transport and m	PF00251.23;PF0	Glyco_hydro_3	Glycosyl hydrolases fan	CYT	2	5	59.8	High
TRINITY_DN28638_c0_g1_i1_orf1	-	-	-	uncharacterized protein LOC114364075 [Ostrinia furnacalis]	2.293967517	1.197844963	4.28E-06	up	yes	0.9887	0.431	0.411	0.456	0.426	1	0.996	0.97	------	------	------	------	------	------	ENOG410XRSO	G:Carbohydrate transport and m	PF00650.23	CRAL_TRIO	CRAL/TRIO domain	CYT	7	25	34.8	High
TRINITY_DN45449_c0_g1_i1_orf1	-	-	-	ATP-dependent helicase brm [Ostrinia furnacalis]	2.334272962	1.222973275	3.00E-07	up	yes	0.9937	0.4257	0.411	0.428	0.438	1	0.99	0.991	BP:regulation of prin	GO:0080090;GO:0019222;GO:0031326;GO:0031323;GO:0050789;GO:0019219;GO:0065007;GO:0010468;GO:0060255;GO:0009889;GO:1903506;GO:0008150;GO:0051171;GO:2001141;GO:0050794;GO:0006355;GO:0010556;GO:0051252;GO:0005575;GO:0070603;GO:0016514;GO:1902494;GO:1904949;GO:0032991;GO:0042393;GO:0035639;GO:0003674;GO:0005488;GO:0016887;GO:0043167;GO:1901265;GO:0043168;GO:0016462;GO:0036094;GO:0000166;GO:0017076;GO:0140097;GO:0005524;GO:0016787;GO:0017111;GO:0004386;GO:0003824;GO:0008094;GO:0016818;GO:0030554;GO:0097367;GO:0097159;GO:0016817;GO:0032559;GO:0032553;GO:0005515;GO:0032555;GO:1901363	K11647	SMARCA2_4	map05225;map04	Hepatocellular carcinoma;Th	COG5076;ENOG	I:Lipid transport and metabolism	PF08880.14	QLQ	QLQ	CYT	3	20	15.3	High
TRINITY_DN14601_c0_g1_i2_orf1	-	-	-	unnamed protein product [Chilo suppressalis]	2.416123582	1.272694249	0.0001617	up	yes	1.001	0.4143	0.393	0.496	0.354	1	0.993	1.01	CC:cytoplasm;CC:int	GO:0005737;GO:0016021;GO:0005575;GO:0110165;GO:0031224;GO:0043169;GO:0043167;GO:0003674;GO:0046872;GO:0005488	K26089	ANKZF1, VMS	------	------	ENOG410XQAG	S:Function unknown	PF18826.4;PF18	bVLRF1;VATC	Bacteroidetes VLRF1 re	CYT	2	2	76.4	High
TRINITY_DN14063_c0_g1_i7_orf1	-	-	-	probable phosphorylase b kinase regulatory subunit beta isoform X1 [Ostrinia furnacalis] >XP_028175664.1 probable phosphorylase b kinase regulatory subunit beta isoform X2 [Ostrinia furnacalis] >XP_028175665.1 probable phosphorylase b kinase regulatory subunit beta isoform X3 [Ostrinia furnacalis]	2.484594528	1.313010431	0.0001192	up	yes	1.008	0.4057	0.466	0.37	0.381	1	0.966	1.058	------	------	K07190	PHKA_B	map04910;map04	Insulin signaling pathway;Gl	ENOG410XPJZ	S:Function unknown	PF00723.24;PF1	Glyco_hydro_1	Glycosyl hydrolases fan	CYT	1	1	134.3	High
TRINITY_DN21539_c0_g1_i1_orf1	-	-	-	probable phenylalanine--tRNA ligase, mitochondrial [Ostrinia furnacalis]	2.434814633	1.283811941	0.001158	up	yes	0.9917	0.4073	0.386	0.42	0.416	1	0.867	1.108	BP:phenylalanyl-tRN	GO:0006432;GO:0019752;GO:0090304;GO:0034641;GO:0006807;GO:0034660;GO:1901360;GO:0006139;GO:0006520;GO:0071704;GO:0009987;GO:0006725;GO:0008150;GO:0008152;GO:0043436;GO:0046483;GO:0016070;GO:0044238;GO:1901564;GO:0006082;GO:0044237;GO:0043170;GO:0006399;GO:0043038;GO:0043039;GO:0006418;GO:0044281;GO:0005737;GO:0070013;GO:0005759;GO:0110165;GO:0031974;GO:0043233;GO:0005575;GO:0035639;GO:0003674;GO:0005488;GO:0003676;GO:0000166;GO:1901363;GO:0004812;GO:0005524;GO:1901265;GO:0017076;GO:0043168;GO:0140098;GO:0030554;GO:0036094;GO:0003824;GO:0000049;GO:0016874;GO:0016875;GO:0004826;GO:0097367;GO:0097159;GO:0032559;GO:0003723;GO:0032555;GO:0140101;GO:0043167;GO:0032553	K01889	FARSA, pheS	map00970	Aminoacyl-tRNA biosynthes	COG0072	S:Function unknown	PF01409.23;PF0	tRNA-synt_2d;	tRNA synthetases class	CYT	1	3	51	High
TRINITY_DN109540_c0_g1_i3_orf1	-	-	-	4-coumarate--CoA ligase 1-like isoform X4 [Ostrinia furnacalis]	2.604651163	1.381090167	5.76E-07	up	yes	1.008	0.387	0.388	0.404	0.369	1	1.013	1.012	CC:integral compone	GO:0016021;GO:0005575;GO:0031224;GO:0110165;GO:0004497;GO:0003824;GO:0003674;GO:0016491	K01904	4CL	map00130	Ubiquinone and other terpe	COG0318;COG0	I:Lipid transport and metabolism	PF00501.31;PF1	AMP-binding;A	AMP-binding enzyme;A	CYT	6	14	58.4	High
TRINITY_DN5925_c0_g1_i5_orf1	-	-	-	isocitrate dehydrogenase [NAD] subunit gamma, mitochondrial-like isoform X1 [Ostrinia furnacalis]	2.634464752	1.397509877	4.88E-05	up	yes	1.009	0.383	0.392	0.329	0.428	1	1.042	0.985	BP:primary metaboli	GO:0044238;GO:0006099;GO:0008150;GO:0008152;GO:0043231;GO:0043229;GO:0005739;GO:0005575;GO:0043227;GO:0043226;GO:0110165	K00030	IDH3	map00020	Citrate cycl	COG0473	E:Amino acid transport and met	PF00180.23	Iso_dh	Isocitrate/isopropylmal	CYT	2	6	42.4	High
TRINITY_DN7574_c0_g1_i10_orf1	-	-	-	proteasome activator complex subunit 3 isoform X2 [Ostrinia furnacalis]	2.48173516	1.311349165	0.0001871	up	yes	1.087	0.438	0.462	0.408	0.444	1	1.158	1.103	------	------	K06698	PSME3	map03050;map04	Proteasome;Antigen proces	ENOG410XQVX	O:Posttranslational modification	PF02252.21;PF0	PA28_beta;PA2	Proteasome activator p	CYT	3	14	28.3	High
TRINITY_DN11799_c0_g1_i4_orf1	-	-	-	V-type proton ATPase 116 kDa subunit a1 isoform X1 [Manduca sexta]	2.052525253	1.037399972	3.99E-07	up	yes	1.016	0.495	0.497	0.493	0.495	1	1.027	1.022	------	------	K02154	ATPeV0A, ATP	map05152;map05	Tuberculosis;Rheumatoid ar	COG1269	F:Nucleotide transport and meta	PF01496.22	V_ATPase_I	V-type ATPase 116kDa	CYT	8	12	97.5	High
TRINITY_DN2172_c0_g2_i5_orf1	-	-	-	4-hydroxyphenylpyruvate dioxygenase [Ostrinia furnacalis]	2.007204323	1.005187483	7.32E-06	up	yes	1.003	0.4997	0.517	0.512	0.47	1	0.992	1.018	------	------	K00457	HPD, hppD	map00130;map00	Ubiquinone and other terpe	COG3185	E:Amino acid transport and met	PF00903.28;PF1	Glyoxalase;Gly	Glyoxalase/Bleomycin r	CYT	9	26	43.6	High
TRINITY_DN16258_c0_g1_i2_orf1	-	-	-	uncharacterized protein LOC114359911 [Ostrinia furnacalis]	2.160271359	1.111212546	0.0003136	up	yes	1.019	0.4717	0.492	0.388	0.535	1	1.054	1.002	BP:organonitrogen c	GO:1901564;GO:0019538;GO:0006807;GO:0043170;GO:0008150;GO:0008152;GO:0006508;GO:0044238;GO:0071704;GO:0004176;GO:0016787;GO:0004175;GO:0004252;GO:0017171;GO:0140096;GO:0003824;GO:0003674;GO:0008233;GO:0008236	K01358	clpP, CLPP	map04212	Longevity regulating pathwa	COG0740	O:Posttranslational modification	PF00574.26	CLP_protease	Clp protease	CYT	3	15	27.3	High
TRINITY_DN12134_c0_g1_i4_orf1	-	-	-	glutathione S-transferase 1-1 [Ostrinia furnacalis] >XP_028161942.1 glutathione S-transferase 1-1 [Ostrinia furnacalis] >XP_028161943.1 glutathione S-transferase 1-1 [Ostrinia furnacalis]	2.008080808	1.005817327	0.0001169	up	yes	0.994	0.495	0.445	0.498	0.542	1	1.022	0.96	MF:catalytic activity;	GO:0003824;GO:0016740;GO:0004364;GO:0003674;GO:0016765	K00799	GST, gst	map05207;map00	Chemical carcinogenesis - r	COG0625	O:Posttranslational modification	PF02798.23;PF1	GST_N;GST_N_	Glutathione S-transfera	CYT	2	16	26.7	High

TRINITY_DN31663_c0_g1_i2_orf1	-	-	-	PHD finger-like domain-containing protein 5A [Nasonia vitripennis] >XP_002427197.1 conserved hypothetical protein [Pediculus humanus corporis] >XP_003484388.1 PHD finger-like domain-containing protein 5A [Bombus impatiens] >XP_003701008.1 PREDICTED: PHD finger-like domain-containing protein 5A [Megachile rotundata] >XP_006623871.1 PHD finger-like domain-containing protein 5A [Apis dorsata] >XP_011068502.1 PREDICTED: PHD finger-like domain-containing protein 5A [Acromyrmex echinatior] >XP_011154391.1 PHD finger-like domain-containing protein 5A [Harpegnathos saltator] >XP_011164776.1 PHD finger-like domain-containing protein 5A [Solenopsis invicta] >XP_011262550.1 PHD finger-like domain-containing protein 5A [Camponotus floridanus] >XP_011297178.1 PREDICTED: PHD finger-like domain-containing protein 5A [Fopius arisanus] >XP_011334720.1 PHD finger-like domain-containing protein 5A [Ooceraea biroi] >XP_011506347.1 PREDICTED: PHD finger-like domain-containing protein 5A [Ceratosolen solmsi marchali] >XP_011506348.1 PREDICTED: PHD finger-like domain-containing protein 5A [Ceratosolen solmsi marchali] >XP_011638597.1 PHD finger-like domain-containing protein 5A isoform X2 [Pogonomyrmex barbatus] >XP_011686073.1 PREDICTED: PHD finger-like domain-containing protein 5A [Wasmannia auropunctata] >XP_011858255.1 PREDICTED: PHD finger-like domain-containing protein 5A [Vollenhovia emeryi] >XP_012058015.1 PREDICTED: PHD finger-like domain-containing protein 5A [Atta cephalotes] >XP_012135327.1 PREDICTED: PHD finger-like domain-containing protein 5A [Megachile rotundata] >XP_012135328.1 PREDICTED: PHD finger-like domain-containing protein 5A [Megachile rotundata] >XP_012222185.1 PREDICTED: PHD finger-like domain-containing protein 5A [Linepithema humile] >XP_012261946.1 PHD finger-like domain-containing protein 5A [Athalia rosae] >XP_012273120.1 PHD finger-like domain-containing protein 5A [Orussus abietinus] >XP_012526512.1 PHD finger-like domain-containing protein 5A [Monomorium pharaonis] >XP_014217558.1 PHD finger-like domain-containing protein 5A [Copidosoma floridanum] >XP_014484566.1 PREDICTED: PHD finger-like domain-containing protein 5A [Dinoponera quadriceps] >XP_014611099.1 PREDICTED: PHD finger-like domain-containing protein 5A [Polistes canadensis] >XP_015122018.1 PHD [illegible]	2.338510016	1.225589608	1.87E-05	up	yes	1.039	0.4443	0.452	0.447	0.434	1	1.032	1.084	BP:nucleic acid meta	GO:0090304;GO:0034641;GO:0006807;GO:0044237;GO:1901360;GO:0006139;GO:0016071;GO:0071704;GO:0008380;GO:0044238;GO:0009987;GO:0006725;GO:0000375;GO:0000377;GO:0008150;GO:0008152;GO:0046483;GO:0016070;GO:0000398;GO:0043170;GO:0006396;GO:0006397;GO:0120114;GO:0071011;GO:0097525;GO:0032991;GO:0030532;GO:1990904;GO:0005575;GO:0005681;GO:0005689;GO:0005686	K12834	PHF5A	map03040	Spliceosome	ENOG4111JIM	A:RNA processing and modificat	PF03660.17	PHF5	PHF5-like protein	CYT	2	18	12.5	High
TRINITY_DN28981_c0_g1_i1_orf1	-	-	-	uncharacterized protein C6orf203 homolog [Ostrinia furnacalis]	2.322869955	1.215908388	0.0003575	up	yes	1.036	0.446	0.491	0.375	0.472	1	0.996	1.113	BP:regulation of tran	GO:1903108;GO:0080090;GO:0019222;GO:0031326;GO:0031323;GO:0050789;GO:0019219;GO:0065007;GO:0010468;GO:0060255;GO:0009889;GO:1903506;GO:0050794;GO:0008150;GO:0051171;GO:2001141;GO:0062125;GO:0051252;GO:0006355;GO:0010556;GO:0043226;GO:0043229;GO:0043227;GO:0005739;GO:0110165;GO:0043231;GO:0005575;GO:0003674;GO:0005488;GO:0003676;GO:1901363;GO:0097159;GO:0003723	------	------	------	------	ENOG4111QEK	S:Function unknown	------	------	------	CYT	1	5	22.6	High
TRINITY_DN23360_c0_g1_i3_orf1	-	-	-	protein PTCD3 homolog, mitochondrial [Ostrinia furnacalis]	2.111344538	1.078162023	4.89E-06	up	yes	1.005	0.476	0.498	0.454	0.476	1	0.992	1.024	BP:regulation of prin	GO:0080090;GO:0019222;GO:2000112;GO:0031323;GO:0006807;GO:0044249;GO:0043043;GO:1901576;GO:0044271;GO:0044260;GO:0010608;GO:0043603;GO:0051246;GO:0050789;GO:0071704;GO:0043604;GO:0065007;GO:0031326;GO:1901566;GO:0043170;GO:0044238;GO:0060255;GO:0009987;GO:0009889;GO:0034248;GO:0034645;GO:0009059;GO:0008150;GO:0051171;GO:0008152;GO:1901564;GO:0034641;GO:0050794;GO:0032543;GO:0019538;GO:0009058;GO:0044237;GO:0010468;GO:0010556;GO:0006417;GO:0006518;GO:0006412;GO:0043229;GO:0043228;GO:0043227;GO:0043226;GO:0005739;GO:0110165;GO:0043231;GO:0032991;GO:0005840;GO:0043232;GO:1990904;GO:0005575;GO:0044877;GO:0005488;GO:0003676;GO:0019843;GO:0043021;GO:0043024;GO:0097159;GO:0003723;GO:0003674;GO:1901363	K17659	PTCD3	------	------	ENOG410XNQS	S:Function unknown	PF17177.7	PPR_long	Pentacotripeptide-repe	CYT	5	8	78.8	High
TRINITY_DN14920_c0_g1_i1_orf1	-	-	-	anamorsin homolog [Ostrinia furnacalis]	2.250165453	1.170031086	1.61E-05	up	yes	1.02	0.4533	0.474	0.412	0.474	1	1.033	1.026	BP:cellular compone	GO:0022607;GO:0071840;GO:0016043;GO:0009987;GO:0016226;GO:0031163;GO:0008150;GO:0008152;GO:0044237;GO:0006790;GO:0031970;GO:0031974;GO:0005758;GO:0110165;GO:0043233;GO:0005575;GO:0070013;GO:0003674;GO:0005488;GO:0016491;GO:0043169;GO:0051540;GO:0043167;GO:0046872;GO:0003824;GO:0009055;GO:0051539;GO:0051536;GO:0051537	K22746	CIAPIN1, DRE2	------	------	COG5636	S:Function unknown	PF05093.16	CIAPIN1	Cytokine-induced anti-	CYT	2	8	27.1	High
TRINITY_DN237_c1_g1_i1_orf1	-	-	-	PREDICTED: cytoplasmic protein NCK1 isoform X1 [Microplitis demolitor]	2.294382022	1.198105625	0.001313	up	yes	1.021	0.445	0.458	0.561	0.316	1	1.035	1.027	BP:developmental p	GO:0032502;GO:0002009;GO:0009987;GO:0048869;GO:0048856;GO:0048468;GO:0048513;GO:0008150;GO:0048729;GO:0009653;GO:0051179	K19862	NCK2, GRB4	map04360;map04	Axon guidance;Axon regene	ENOG410XRPF	T:Signal transduction mechanism	PF00018.31;PF14	SH3_1;SH3_9;SH	SH3 domain;Variant SH	PLA	2	3	44.1	High
TRINITY_DN105359_c0_g2_i5_orf1	-	-	-	uncharacterized protein LOC114359499 [Ostrinia furnacalis]	2.309086789	1.207322399	0.001935	up	yes	1.019	0.4413	0.465	0.309	0.55	1	1.091	0.966	------	------	------	------	------	------	ENOG4110AO3	S:Function unknown	PF12456.11	hSac2	Inositol phosphatase	CYT	1	4	39.7	High
TRINITY_DN80560_c0_g1_i1_orf1	-	-	-	ATP synthase subunit alpha, mitochondrial [Ostrinia furnacalis]	2.227100513	1.155166671	1.94E-08	up	yes	0.9993	0.4487	0.453	0.45	0.443	1	1.004	0.994	CC:proton-transport	GO:0045261;GO:0033178;GO:0032991;GO:0005575;GO:0098796;GO:0005261;GO:0035639;GO:0003674;GO:0005488;GO:0043167;GO:1901363;GO:1901265;GO:0046933;GO:0015318;GO:0000166;GO:0015252;GO:0017076;GO:0005524;GO:0043168;GO:0005215;GO:0005216;GO:0008324;GO:0036094;GO:0003824;GO:0022803;GO:0016874;GO:0022890;GO:0030554;GO:0097367;GO:0097159;GO:0015075;GO:0022857;GO:0015267;GO:0032559;GO:0032555;GO:0015078;GO:0032553	K02132	ATPeF1A, ATP	map04714;map05	Thermogenesis;Chemical ca	COG0056	C:Energy production and conver	PF00006.28;PF0	ATP-synt_ab;AT	ATP synthase alpha/be	CYT	23	45	59.4	High
TRINITY_DN1504_c0_g1_i1_orf1	-	-	-	uncharacterized protein LOC114352862 [Ostrinia furnacalis] >XP_028160407.1 uncharacterized protein LOC114352862 [Ostrinia furnacalis]	2.381733021	1.252011704	0.002128	up	yes	1.017	0.427	0.362	0.387	0.532	1	1.136	0.914	------	------	------	------	------	------	ENOG410Y735	S:Function unknown	------	------	------	EXC	1	1	83.4	Medium
TRINITY_DN754_c1_g1_i6_orf1	-	-	-	28S ribosomal protein S2, mitochondrial [Ostrinia furnacalis]	3.360606061	1.748721436	0.0005571	up	yes	1.109	0.33	0.322	0.239	0.429	1	1.174	1.154	BP:cellular biosynthe	GO:0044249;GO:0034641;GO:0006807;GO:0034645;GO:1901576;GO:0044260;GO:0043043;GO:0071704;GO:0043604;GO:0009987;GO:0009058;GO:0009059;GO:0008150;GO:0008152;GO:0044271;GO:0044238;GO:1901564;GO:0043603;GO:1901566;GO:0019538;GO:0044237;GO:0043170;GO:0006518;GO:0006412;GO:0015935;GO:0032991;GO:0044391;GO:1990904;GO:0005575;GO:0003674;GO:0005198;GO:0003735	K02967	RP-S2, MRPS2	map03010	Ribosome	COG0052	S:Function unknown	PF00318.23	Ribosomal_S2	Ribosomal protein S2	CYT	1	5	28.4	High
TRINITY_DN2807_c0_g1_i4_orf1	-	-	-	FK506-binding protein 59 isoform X1 [Ostrinia furnacalis]	2.415931314	1.272579439	7.78E-07	up	yes	1.013	0.4193	0.433	0.408	0.417	1	1.029	1.009	MF:isomerase activit	GO:0016853;GO:0140096;GO:0016859;GO:0003824;GO:0003674;GO:0003755	K09571	FKBP4_5	map04915	Estrogen signaling pathway	COG0545	S:Function unknown	PF00254.31;PF18	FKBP_C;FKBP_N	FKBP-type peptidyl-pro	CYT	17	47	52.8	High
TRINITY_DN5554_c0_g1_i2_orf1	-	-	-	double-stranded RNA-binding protein Staufen homolog 2 isoform X5 [Pectinophora gossypiella]	2.306978901	1.20600481	3.31E-06	up	yes	0.995	0.4313	0.417	0.433	0.444	1	0.97	1.015	------	------	K17597	STAU	------	------	ENOG410XSCK	K:Transcription	PF00035.29;PF16	dsrm;Staufen_C	Double-stranded RNA	CYT	1	2	56.8	High
TRINITY_DN4125_c1_g1_i5_orf1	-	-	-	angiotensin-converting enzyme-like isoform X2 [Ostrinia furnacalis]	2.378074994	1.249794212	1.12E-06	up	yes	0.9957	0.4187	0.41	0.442	0.404	1	0.994	0.993	BP:nitrogen compou	GO:0006807;GO:0071704;GO:0006508;GO:0008150;GO:0008152;GO:0044238;GO:1901564;GO:0019538;GO:0043170;GO:0016020;GO:0110165;GO:0005575;GO:0004180;GO:0008238;GO:0003674;GO:0005488;GO:0008233;GO:0008237;GO:0043169;GO:0043167;GO:0046872;GO:0016787;GO:0008241;GO:0140096;GO:0003824	K01283	ACE, CD143	map05142;map04	Chagas disease;Renin-angic	ENOG410XPJ3	S:Function unknown	PF01401.21	Peptidase_M2	Angiotensin-converting	CYT	10	21	74.4	High
TRINITY_DN8019_c0_g1_i4_orf1	-	-	-	deoxyhypusine hydroxylase [Ostrinia furnacalis]	2.397608006	1.261595806	1.87E-05	up	yes	0.9823	0.4097	0.405	0.387	0.437	1	1.003	0.944	BP:nitrogen compou	GO:0006807;GO:0018193;GO:0071704;GO:0043412;GO:0036211;GO:0008150;GO:0008152;GO:0008612;GO:0044238;GO:1901564;GO:0051604;GO:0018205;GO:0009058;GO:0043170;GO:0019538;GO:0004497;GO:0019135;GO:0003674;GO:0005488;GO:0016491;GO:0043169;GO:0016705;GO:0043167;GO:0046872;GO:0140096;GO:0003824	K06072	DOHH	------	------	COG1413	C:Energy production and conver	PF13646.9;PF03	HEAT_2;HEAT_	HEAT repeats;PBS lyase	CYT	5	19	34.1	High
TRINITY_DN17312_c0_g1_i1_orf1	-	-	-	mRNA cap guanine-N7 methyltransferase [Ostrinia furnacalis]	2.347700458	1.231248347	0.0004254	up	yes	0.975	0.4153	0.508	0.392	0.346	1	0.989	0.936	CC:intracellular orga	GO:0043229;GO:0043227;GO:0043226;GO:0005634;GO:0110165;GO:0043231;GO:0005575;GO:0003674;GO:0005488;GO:0003676;GO:0008168;GO:1901363;GO:0016740;GO:0016741;GO:0140098;GO:0004482;GO:0008757;GO:0008173;GO:0008170;GO:0008174;GO:0003824;GO:0097159;GO:0003723	K00565	RNMT	map03015	mRNA surveillance pathway	ENOG410Y7HG	A:RNA processing and modificat	PF03291.19;PF13	Pox_MCEL;Meth	mRNA capping enzyme	CYT	2	5	47.5	High
TRINITY_DN1079_c0_g1_i4_orf1	-	-	-	CD109 antigen-like [Ostrinia furnacalis]	2.75291565	1.460960406	1.80E-05	up	yes	1.015	0.3687	0.395	0.318	0.393	1	1.014	1.032	CC:cellular_compone	GO:0005575;GO:0110165;GO:0005615;GO:0004866;GO:0061135;GO:0061134;GO:0003674;GO:0030414;GO:0004857;GO:0098772;GO:0030234	K06530	CD109	------	------	ENOG410XRED;I	S:Function unknown;	PF07678.17;PF01	TED_compleme	A-macroglobulin TED c	CYT	3	2	146.3	High
TRINITY_DN3503_c0_g1_i1_orfp1	-	-	-	uncharacterized protein LOC114356429 [Ostrinia furnacalis]	2.180414747	1.124602583	0.0002825	up	yes	0.9463	0.434	0.486	0.405	0.411	1	0.883	0.956	------	------	------	------	------	------	------	------	------	------	------	CYT	1	9	14.4	High
TRINITY_DN106534_c0_g1_i1_orf1	-	-	-	nucleolar complex protein 2 homolog [Ostrinia furnacalis]	2.070083046	1.049688646	0.001644	up	yes	1.022	0.4937	0.482	0.417	0.582	1	1.119	0.947	CC:intracellular mem	GO:0043231;GO:0005634;GO:0005575;GO:0043229;GO:0043227;GO:0043226;GO:0110165	K14833	NOC2	------	------	COG5604	S:Function unknown	PF03715.16	Noc2	Noc2p family	CYT	1	2	78.2	High
TRINITY_DN10520_c0_g1_i2_orf1	-	-	-	probable 39S ribosomal protein L45, mitochondrial [Ostrinia furnacalis]	2.292351523	1.196828293	0.0003486	up	yes	1.046	0.4563	0.454	0.499	0.416	1	1	1.139	CC:ribosome;CC:intr	GO:0005840;GO:0043232;GO:0005575;GO:0043229;GO:0043228;GO:0043226;GO:0110165	K17426	MRPL45	------	------	ENOG411019B	S:Function unknown	PF04280.18	Tim44	Tim44-like domain	CYT	4	16	30	High
TRINITY_DN14429_c0_g1_i2_orf1	-	-	-	NADH dehydrogenase [ubiquinone] 1 beta subcomplex subunit 11, mitochondrial [Ostrinia furnacalis]	2.404745608	1.265884283	0.0001682	up	yes	1.054	0.4383	0.487	0.447	0.381	1	1.114	1.049	CC:cellular_compone	GO:0005575;GO:0016021;GO:0110165;GO:0031224	K11351	NDUFB11	map04714;map00	Thermogenesis;Oxidative ph	ENOG41120A5	U:Intracellular trafficking, secreti	PF10183.12	ESSS	ESSS subunit of NAD	PLA	1	8	16.4	High
TRINITY_DN143603_c0_g1_i1_orf1	-	-	-	hypothetical protein KR044_005587 [Drosophila immigrans]	2.847921508	1.509909384	0.0003675	up	yes	1.103	0.3873	0.337	0.397	0.428	1	1.108	1.202	BP:response to extra	GO:0009991;GO:0015976;GO:0071704;GO:0008150;GO:0008152;GO:0044238;GO:0005975;GO:0009605;GO:0050896;GO:0031667;GO:0016020;GO:0110165;GO:0005575;GO:0016787;GO:0030246;GO:0016829;GO:0003674;GO:0005488;GO:0043167;GO:1901363;GO:1901265;GO:0016491;GO:0043168;GO:0043169;GO:0050660;GO:0046914;GO:0016627;GO:0000166;GO:0090599;GO:0016835;GO:0017150;GO:0046872;GO:0015926;GO:0140098;GO:0016798;GO:0036094;GO:0003824;GO:0004089;GO:0097159;GO:0004553;GO:0140101;GO:0016836;GO:0008270	K01673	cynT, can	map00910	Nitrogen metabolism	COG0288	P:Inorganic ion transport and me	PF00484.22	Pro_CA	Carbonic anhydrase	CYT	1	5	26.9	High
TRINITY_DN619_c0_g1_i1_orf1	-	-	-	putative uncharacterized protein DDB_G0271606 [Ostrinia furnacalis]	2.065843621	1.04673105	2.84E-06	up	yes	1.004	0.486	0.483	0.496	0.479	1	0.985	1.028	CC:cellular_compone	GO:0005575;GO:0005576;GO:0110165;GO:0097367;GO:0003674;GO:0008061;GO:0005488	------	------	------	------	ENOG4111K2A	S:Function unknown	PF01607.27	CBM_14	Chitin binding Peritroph	CYT	3	16	39.1	High
TRINITY_DN104507_c0_g1_i2_orf1	-	-	-	replication protein A 32 kDa subunit [Ostrinia furnacalis]	2.246012672	1.167366067	0.0003747	up	yes	1.028	0.4577	0.538	0.37	0.465	1	1.026	1.057	BP:nucleic acid meta	GO:0090304;GO:0006807;GO:1901360;GO:0006139;GO:0051716;GO:0044260;GO:0071704;GO:0006260;GO:0006281;GO:0009987;GO:0006725;GO:0006974;GO:0006950;GO:0008150;GO:0008152;GO:0046483;GO:0006310;GO:0044238;GO:0050896;GO:0034641;GO:0044237;GO:0043170;GO:0033554;GO:0006259;GO:0043229;GO:0043227;GO:0043226;GO:0005634;GO:0110165;GO:0043231;GO:0005575;GO:0003674;GO:0005488;GO:0003676;GO:0003677;GO:1901363;GO:0097159	K10739	RFA2, RPA2	map03460;map03	Fanconi anemia pathway;Mi	COG5235	L:Replication, recombination and	PF08784.14;PF01	RPA_C;tRNA_an	Replication protein A C	EXC	1	4	29.8	High
TRINITY_DN18396_c0_g1_i1_orf1	-	-	-	uncharacterized protein LOC114359424 [Ostrinia furnacalis]	2.293495386	1.197548005	5.25E-06	up	yes	1.019	0.4443	0.427	0.462	0.444	1	1.047	1.009	MF:molecular_functi	GO:0003674;GO:0042302;GO:0005198	------	------	------	------	ENOG410YSFI;EI	K:Transcription;S:Function unkno	PF00379.26	Chitin_bind_4	Insect cuticle protein	CYT	1	4	39.7	High
TRINITY_DN20369_c0_g1_i2_orf1	-	-	-	uncharacterized protein LOC114366225 [Ostrinia furnacalis]	2.410484437	1.269323115	1.39E-05	up	yes	1.03	0.4273	0.453	0.398	0.431	1	1.06	1.03	------	------	K17406	MRPS27	------	------	ENOG4110AQE	S:Function unknown	PF10037.12	MRP-S27	Mitochondrial 28S ribo	CYT	4	11	51.4	High
TRINITY_DN45227_c0_g1_i3_orf1	-	-	-	uncharacterized protein LOC114359191 [Ostrinia furnacalis]	2.733794517	1.450904808	0.0001636	up	yes	1.067	0.3903	0.389	0.338	0.444	1	1.134	1.067	CC:respiratory chain	GO:0045271;GO:1902495;GO:1902494;GO:1990351;GO:0030964;GO:0098803;GO:0098800;GO:0005575;GO:0098796;GO:0005747;GO:0032991;GO:1990204;GO:0098798	------	------	------	------	ENOG410XXY6	S:Function unknown	PF15880.8	NDUFV3	NADH dehydrogenase	CYT	1	8	11.2	High
TRINITY_DN5686_c0_g1_i4_orf1	-	-	-	FACT complex subunit spt16 isoform X2 [Ostrinia furnacalis]	2.247767857	1.168493046	4.82E-05	up	yes	1.007	0.448	0.458	0.424	0.462	1	0.963	1.057	BP:nucleic acid meta	GO:0090304;GO:0034641;GO:0006807;GO:1901360;GO:0006139;GO:0051716;GO:0044260;GO:0071704;GO:0006260;GO:0006281;GO:0009987;GO:0006725;GO:0006974;GO:0006950;GO:0008150;GO:0008152;GO:0046483;GO:0044238;GO:0050896;GO:0044237;GO:0043170;GO:0033554;GO:0006259;GO:0008023;GO:0035101;GO:0032991;GO:0005575	K25639	SUPT16H, SPT	------	------	COG5406	K:Transcription	PF08644.14;PF14	SPT16;FACT-Sp	FACT complex subunit;I	CYT	3	3	127	High
TRINITY_DN19829_c0_g2_i1_orf1	-	-	-	28S ribosomal protein S35, mitochondrial [Ostrinia furnacalis]	2.03611691	1.025820401	0.0001154	up	yes	0.9753	0.479	0.527	0.486	0.424	1	0.975	0.951	BP:cellular biosynthe	GO:0044249;GO:0034641;GO:0006807;GO:0034645;GO:1901576;GO:0044260;GO:0043043;GO:0071704;GO:0032543;GO:0043604;GO:0009987;GO:0009058;GO:0009059;GO:0008150;GO:0008152;GO:0044271;GO:0044238;GO:1901564;GO:0043603;GO:1901566;GO:0019538;GO:0044237;GO:0043170;GO:0006518;GO:0006412;GO:0043227;GO:0043226;GO:0005739;GO:0110165;GO:0043231;GO:0043229;GO:0005575;GO:0003674;GO:0005198;GO:0003735	K17413	MRPS35	------	------	ENOG410ZX0Y	S:Function unknown	PF10213.12	MRP-S28	Mitochondrial ribosoma	CYT	1	6	18.6	High
TRINITY_DN30_c0_g1_i6_orf1	-	-	-	casein kinase I-like isoform X1 [Hyposmocoma kahamanoa]	2.551470588	1.351329011	4.45E-05	up	yes	1.041	0.408	0.411	0.439	0.374	1	1.031	1.093	BP:phosphorylation;I	GO:0016310;GO:0006807;GO:0071704;GO:0006468;GO:0009987;GO:0043412;GO:0036211;GO:0008150;GO:0008152;GO:0044238;GO:1901564;GO:0044237;GO:0043170;GO:0019538;GO:0006796;GO:0006793;GO:0035639;GO:0003674;GO:0005488;GO:0043167;GO:1901363;GO:1901265;GO:0043168;GO:0097367;GO:0000166;GO:0004674;GO:0016740;GO:0004672;GO:0017076;GO:0005524;GO:0016301;GO:0140096;GO:0036094;GO:0003824;GO:0030554;GO:0016773;GO:0016772;GO:0032559;GO:0032555;GO:0097159;GO:0032553	K08957	CSNK1A	map05165;map04	Human papillomavirus infec	ENOG410XPGP	T:Signal transduction mechanism	PF00069.28;PF07	Pkinase;PK_Tyr_	Protein kinase domain;I	CYT	1	11	43.4	High
TRINITY_DN6563_c0_g1_i1_orf1	-	-	-	cytochrome c oxidase assembly protein COX15 homolog [Ostrinia furnacalis]	2.302752294	1.203359229	2.47E-05	up	yes	1.004	0.436	0.478	0.437	0.393	1	0.994	1.019	BP:porphyrin-contai	GO:0006778;GO:0006779;GO:0044249;GO:0034641;GO:0006807;GO:1901362;GO:1901360;GO:1901576;GO:0042440;GO:0006783;GO:0006784;GO:0071704;GO:0033014;GO:0033013;GO:0018130;GO:0042168;GO:0009987;GO:0006725;GO:0046160;GO:0009058;GO:0008150;GO:0008152;GO:0044271;GO:0046148;GO:0046483;GO:1901564;GO:1901566;GO:0044237;GO:0019438;GO:0016021;GO:0031224;GO:0110165;GO:0005575;GO:0003674;GO:0016491;GO:0003824;GO:0016651;GO:0016653	K02259	COX15, ctaA	map00190;map00	Oxidative phosphorylation;P	COG5023;COG1	T:Signal transduction mechanism	PF02628.18	COX15-CtaA	Cytochrome oxidase as	CYT	3	7	47.4	High
TRINITY_DN43328_c0_g1_i1_orf1	-	-	-	tubulin--tyrosine ligase-like protein 12 [Ostrinia furnacalis]	2.496960856	1.320173205	0.0001627	up	yes	1.027	0.4113	0.429	0.391	0.414	1	1.112	0.969	------	------	K16609	TTLL12	------	------	ENOG410XO0A	S:Function unknown	PF03133.18	TTL	Tubulin-tyrosine ligase	CYT	1	2	71.9	High
TRINITY_DN11894_c1_g1_i5_orf1	-	-	-	39S ribosomal protein L33, mitochondrial [Ostrinia furnacalis]	2.417399572	1.273455956	0.0007094	up	yes	1.017	0.4207	0.336	0.449	0.477	1	1.104	0.946	------	------	K02913	RP-L33, MRPL	map03010	Ribosome	ENOG4112B1M	J:Translation, ribosomal structure	------	------	------	CYT	1	18	7.9	High
TRINITY_DN10722_c0_g3_i1_orf1	-	-	-	inositol-3-phosphate synthase [Ostrinia furnacalis]	2.032119914	1.022985537	0.003778	up	yes	0.949	0.467	0.39	0.393	0.618	1	0.919	0.928	BP:polyol biosynthet	GO:0046173;GO:0019751;GO:0044249;GO:0044281;GO:0044283;GO:0044255;GO:1901576;GO:0044262;GO:0071704;GO:0006644;GO:0006629;GO:0046165;GO:0009987;GO:0034637;GO:0009058;GO:0008150;GO:0008152;GO:0090407;GO:0008610;GO:0044238;GO:0005975;GO:0008654;GO:0044237;GO:0016051;GO:0006066;GO:0006796;GO:0006793;GO:0019637;GO:0006020;GO:0006021;GO:1901617;GO:1901615;GO:0003674;GO:0016872;GO:0004512;GO:0016853;GO:0003824	K01858	INO1, ISYNA1	map00562	Inositol phosphate metaboli	COG1260	I:Lipid transport and metabolism	PF07994.15;PF01	NAD_binding_5	Myo-inositol-1-phospl	EXC	1	2	55.4	High
TRINITY_DN10558_c0_g1_i4_orf1	-	-	-	unnamed protein product [Chrysodeixis includens]	2.118260484	1.082880009	0.01033	up	yes	0.9547	0.4507	0.371	0.318	0.663	1	0.956	0.908	CC:macromolecular	GO:0032991;GO:1990904;GO:0005575;GO:0097159;GO:0003674;GO:0003723;GO:0003676;GO:1901363;GO:0005488	K14411	MSI	map03015	mRNA surveillance pathway	ENOG410YA8Z	O:Posttranslational modification	------	------	------	CYT	1	44	18.3	High
TRINITY_DN21531_c0_g1_i1_orf1	-	-	-	viral IAP-associated factor homolog [Ostrinia furnacalis]	2.406015038	1.266645659	1.94E-05	up	yes	0.992	0.4123	0.396	0.383	0.458	1	1.001	0.975	------	------	------	------	------	------	ENOG4111H24	S:Function unknown	PF02114.19	Phosducin	Phosducin	CYT	4	21	26.4	High
TRINITY_DN16482_c0_g1_i6_orf1	-	-	-	transmembrane protein 120 homolog isoform X2 [Ostrinia furnacalis]	2.32853717	1.219423912	0.0002533	up	yes	0.971	0.417	0.445	0.349	0.457	1	0.912	1.001	CC:cellular_compone	GO:0005575;GO:0016021;GO:0110165;GO:0031224	------	------	------	------	ENOG410XT6G	S:Function unknown	PF07851.16	TMPIT	TMPIT-like protein	CYT	1	2	47.7	High
TRINITY_DN47_c0_g1_i2_orf1	-	-	-	uncharacterized protein LOC114356437 isoform X1 [Ostrinia furnacalis]	2.418024691	1.273828977	2.83E-06	up	yes	0.9793	0.405	0.389	0.401	0.425	1	0.964	0.974	------	------	------	------	------	------	------	------	PF00041.24	fn3	Fibronectin type III dom	CYT	1	8	17.9	High
TRINITY_DN11153_c0_g1_i1_orf1	-	-	-	unnamed protein product [Chilo suppressalis]	2.467235752	1.302895576	3.70E-06	up	yes	0.9827	0.3983	0.397	0.415	0.383	1	0.992	0.956	------	------	K20792	NAA15_16	------	------	ENOG410XR7D	S:Function unknown	PF12569.11;PF07	NatA_aux_su;TF	N-terminal acetyltransf	CYT	16	20	100.1	High
TRINITY_DN92232_c0_g1_i1_orf1	-	-	-	protein SDA1 homolog [Ostrinia furnacalis]	2.873595506	1.522856999	4.26E-05	up	yes	1.023	0.356	0.387	0.39	0.291	1	1.03	1.039	BP:nuclear transport	GO:0051169;GO:0008104;GO:0033750;GO:0030036;GO:0051168;GO:0051656;GO:0071840;GO:0042273;GO:0070727;GO:0016043;GO:0071705;GO:0071702;GO:0022613;GO:0033036;GO:0006996;GO:0006810;GO:0030029;GO:0009987;GO:0006913;GO:0045184;GO:0008150;GO:0051649;GO:0051234;GO:0051179;GO:0051640;GO:0051641;GO:0031503;GO:0007010;GO:0046907;GO:0000055;GO:0000054;GO:0044085;GO:0015031;GO:0043229;GO:0043228;GO:0043226;GO:0110165;GO:0043232;GO:0005730;GO:0005575	K14856	SDA1, SDAD1	------	------	ENOG410XPHI	S:Function unknown	PF05285.15;PF08	SDA1;NUC130_	SDA1;NUC130/3NT dor	CYT	1	1	84.7	Medium
TRINITY_DN53462_c0_g1_i1_orf1	-	-	-	uncharacterized protein LOC118072968 isoform X1 [Chelonus insularis] >XP_034949073.1 uncharacterized protein LOC118072968 isoform X1 [Chelonus insularis]	2.80154568	1.486223017	2.06E-05	up	yes	1.015	0.3623	0.396	0.309	0.382	1	1.017	1.028	------	------	------	------	------	------	ENOG41125KI	S:Function unknown	PF15376.9	DUF4603	Domain of unknown fu	CYT	1	1	69.1	Medium
TRINITY_DN3428_c0_g1_i1_orf1	-	-	-	10 kDa heat shock protein, mitochondrial [Ostrinia furnacalis]	2.542635659	1.346324749	8.31E-05	up	yes	0.984	0.387	0.401	0.347	0.413	1	1.027	0.925	MF:binding;MF:carbo	GO:0005488;GO:0097367;GO:0043168;GO:0030554;GO:0035639;GO:0097159;GO:0044183;GO:0000166;GO:0036094;GO:0032553;GO:0003674;GO:1901265;GO:0017076;GO:0032555;GO:0032559;GO:0043167;GO:1901363;GO:0005524	K04078	groES, HSPE1	------	------	COG0234	O:Posttranslational modification	PF00166.24	Cpn10	Chaperonin 10 Kd subu	CYT	8	64	11.1	High
TRINITY_DN4895_c0_g1_i2_orf1	-	-	-	coiled-coil domain-containing protein 86 [Ostrinia furnacalis]	2.786703601	1.478559563	4.60E-06	up	yes	1.006	0.361	0.358	0.394	0.331	1	1.017	1	CC:intracellular mem	GO:0043231;GO:0005634;GO:0005575;GO:0043229;GO:0043227;GO:0043226;GO:0110165	K14822	CGR1	------	------	ENOG41129IK	S:Function unknown	PF03879.17	Cgr1	Cgr1 family	CYT	1	8	19.7	High
TRINITY_DN121893_c0_g1_i1_orf1	-	-	-	hypothetical protein, partial [Ostrinia furnacalis]	2.134120917	1.09364192	0.0003119	up	yes	0.9213	0.4317	0.45	0.418	0.427	1	0.902	0.862	BP:cellular biosynthe	GO:0044249;GO:0034641;GO:0006807;GO:0034645;GO:1901576;GO:0044271;GO:0044260;GO:0043043;GO:0071704;GO:0043604;GO:0009987;GO:0009058;GO:0009059;GO:0008150;GO:0008152;GO:1901564;GO:0044238;GO:0043603;GO:1901566;GO:0019538;GO:0044237;GO:0043170;GO:0006518;GO:0006412;GO:0015935;GO:0043232;GO:0043226;GO:0022627;GO:0110165;GO:0044391;GO:0005840;GO:0032991;GO:1990904;GO:0043229;GO:0005575;GO:0043228;GO:0003674;GO:0005198;GO:0003735	K02984	RP-S3Ae, RPS	map03010;map05	Ribosome;Coronavirus disea	COG1890	J:Translation, ribosomal structure	PF01015.21	Ribosomal_S3A	Ribosomal S3Ae family	CYT	1	24	8.5	High
TRINITY_DN39532_c0_g1_i1_orf1	-	-	-	hypothetical protein evm_009649 [Chilo suppressalis]	2.434652726	1.283716004	0.0005752	up	yes	0.978	0.4017	0.483	0.361	0.361	1	1.036	0.898	------	------	K12587	MTR3, EXOSC	map03018	RNA degradation	COG0689	S:Function unknown	PF01138.24	RNase_PH	3' exoribonuclease fam	CYT	1	2	35.1	High
TRINITY_DN3647_c2_g1_i3_orf1	-	-	-	unnamed protein product, partial [Iphiclides podalirius]	2.474829244	1.307328987	4.70E-05	up	yes	0.9783	0.3953	0.382	0.354	0.45	1	0.958	0.977	CC:cellular_compone	GO:0005575;GO:0016021;GO:0110165;GO:0031224	------	------	------	------	ENOG410ZUNN	S:Function unknown	PF10961.11	SelK_SelG	Selenoprotein SelK_Sel	EXC	1	13	9.5	High
TRINITY_DN27087_c0_g1_i1_orf1	-	-	-	2',5'-phosphodiesterase 12 [Ostrinia furnacalis]	2.420300752	1.275186331	1.81E-05	up	yes	0.9657	0.399	0.41	0.406	0.381	1	0.972	0.925	MF:catalytic activity;I	GO:0003824;GO:0003674	K19612	PDE12	------	------	COG5239	O:Posttranslational modification	PF03372.26	Exo_endo_phos	Endonuclease/Exonucle	CYT	2	3	67.3	High
TRINITY_DN1882_c0_g1_i4_orf1	-	-	-	zinc transporter ZIP13 homolog [Ostrinia furnacalis]	2.075892857	1.053731984	0.001209	up	yes	0.93	0.448	0.449	0.367	0.528	1	0.88	0.91	CC:cellular_compone	GO:0005575;GO:0016021;GO:0110165;GO:0031224;GO:0022890;GO:0015318;GO:0005215;GO:0008324;GO:0015075;GO:0022857;GO:0003674;GO:0046873	K14719	SLC39A13, ZIP	map05012;map05	Parkinson disease;Alzheimer	COG0428	P:Inorganic ion transport and me	PF02535.25	Zip	ZIP Zinc transporter	CYT	2	7	42.2	High
TRINITY_DN20346_c0_g1_i1_orf1	-	-	-	NADH dehydrogenase [ubiquinone] 1 alpha subcomplex subunit 6 [Ostrinia furnacalis]	2.87362792	1.522873272	4.10E-06	up	yes	1.021	0.3553	0.349	0.368	0.349	1	1.057	1.006	CC:respiratory chain	GO:0045271;GO:1902495;GO:1902494;GO:1990351;GO:0030964;GO:0098803;GO:0098800;GO:0005575;GO:0098796;GO:0005747;GO:0032991;GO:1990204;GO:0098798	K03950	NDUFA6	map04714;map00	Thermogenesis;Oxidative ph	ENOG4111S2F	I:Lipid transport and metabolism	PF05347.18	Complex1_LYR	Complex 1 protein	CYT	2	12	14.8	High
TRINITY_DN2117_c0_g1_i1_orf1	-	-	-	BUB3-interacting and GLEBS motif-containing protein ZNF207 [Chelonus insularis]	2.353786219	1.234983294	6.74E-06	up	yes	0.9667	0.4107	0.414	0.417	0.401	1	0.941	0.959	MF:cation binding;M	GO:0043169;GO:0097159;GO:0043167;GO:0003674;GO:0005488;GO:0003676;GO:0003677;GO:0046872;GO:1901363	------	------	------	------	ENOG410ZJGQ	S:Function unknown	PF03153.16	TFIIA	Transcription factor IIA,	CYT	2	4	52.5	High
TRINITY_DN1557_c0_g1_i9_orf1	-	-	-	carboxylesterase CXE18 [Ostrinia furnacalis]	2.729239924	1.448499226	1.97E-05	up	yes	1.009	0.3697	0.416	0.368	0.325	1	1.024	1.004	MF:hydrolase activity	GO:0016787;GO:0003674;GO:0016788;GO:0052689;GO:0003824	------	------	------	------	COG2272	S:Function unknown	PF00135.31;PF20	COesterase;BD	Carboxylesterase family	CYT	4	7	63.7	High

TRINITY_DN760_c1_g2_i6_orf1	-	-	-	ADP,ATP carrier protein [Pieris napi]	2.252358491	1.171436468	0.0009346	up	yes	0.955	0.424	0.369	0.534	0.369	1	0.912	0.953	BP:nucleobase-cont	GO:0015931;GO:0006862;GO:0006820;GO:1901264;GO:0051503;GO:0055085;GO:0071705;GO:1990542;GO:1990544;GO:0071702;GO:0015865;GO:0015868;GO:0015866;GO:0015867;GO:0006811;GO:0006810;GO:0015711;GO:0034220;GO:0008150;GO:0051234;GO:0051179;GO:0072530;GO:1901679;GO:0140021;GO:0015748;GO:0098656;GO:0009987;GO:0031224;GO:0031090;GO:0016020;GO:0110165;GO:0031966;GO:0019866;GO:0005575;GO:0005743;GO:0016021;GO:0005346;GO:0015932;GO:0015216;GO:0005347;GO:0140323;GO:0015605;GO:0008509;GO:0003674;GO:0015075;GO:0015215;GO:0005215;GO:1901505;GO:0015297;GO:0015291;GO:0000295;GO:0008514;GO:0005471;GO:0022857;GO:0022804;GO:0022853;GO:0015217	K05863	SLC25A4S, AN	map05166;map05	Human T-cell leukemia virus	ENOG410XNW0	U:Intracellular trafficking, secreti	PF00153.30	Mito_carr	Mitochondrial carrier pr	CYT	21	57	33	High
TRINITY_DN10636_c0_g1_i1_orf1	-	-	-	unnamed protein product [Arctia plantaginis] >CAB3253774.1 unnamed protein product [Arctia plantaginis]	2.012582781	1.009048125	0.004637	up	yes	0.9117	0.453	0.388	0.584	0.387	1	0.843	0.892	BP:regulation of prin	GO:0080090;GO:0019222;GO:0031326;GO:0031323;GO:0050789;GO:0071840;GO:0006325;GO:0016043;GO:0060255;GO:0065007;GO:0010468;GO:0019219;GO:0009987;GO:0009889;GO:1903506;GO:0050794;GO:0008150;GO:0051171;GO:2001141;GO:0051252;GO:0006355;GO:0010556;GO:0031248;GO:1902562;GO:0070822;GO:0000123;GO:0043189;GO:1902493;GO:1902494;GO:0000118;GO:1990234;GO:0032991;GO:0016580;GO:0005575;GO:0035267;GO:0003674;GO:0005488;GO:0003682	K11339	MORF4L1, MR	------	------	ENOG410XR9F	K:Transcription	PF05712.16;PF11	MRG;Tudor-kn	MRG;RNA binding activ	CYT	3	9	38.3	High
TRINITY_DN12495_c0_g1_i2_orf1	-	-	-	probable ATP-dependent RNA helicase pitchoune [Manduca sexta] >KAG6441249.1 hypothetical protein O3G_MSEX001749 [Manduca sexta]	2.576044129	1.365157308	8.57E-07	up	yes	0.9807	0.3807	0.389	0.385	0.368	1	0.973	0.969	MF:purine ribonucle	GO:0035639;GO:0003674;GO:0005488;GO:0003676;GO:0008186;GO:0043167;GO:1901363;GO:1901265;GO:0043168;GO:0000166;GO:0017076;GO:0005524;GO:0016787;GO:0140098;GO:0004386;GO:0036094;GO:0003824;GO:0030554;GO:0097367;GO:0097159;GO:0003724;GO:0032559;GO:0003723;GO:0032555;GO:0032553	K13179	DDX18, HAS1	------	------	COG0513	S:Function unknown	PF00270.32;PF00	DEAD;Helicase_	DEAD/DEAH box helica	CYT	2	3	69	High
TRINITY_DN279_c0_g1_i10_orf1	-	-	-	RE1-silencing transcription factor-like isoform X1 [Ostrinia furnacalis]	3.422164446	1.774909088	2.24E-05	up	yes	1.053	0.3077	0.27	0.332	0.321	1	1.086	1.073	CC:intracellular mem	GO:0043231;GO:0005634;GO:0005575;GO:0043229;GO:0043227;GO:0043226;GO:0110165;GO:0043169;GO:0046914;GO:0043167;GO:0003674;GO:0008270;GO:0046872;GO:0005488	------	------	------	------	COG5048	O:Posttranslational modification	PF00096.29;PF13	zf-C2H2;zf-C2	Zinc finger, C2H2 type;(	CYT	1	1	120.9	High
TRINITY_DN11347_c0_g1_i1_orf1	-	-	-	N(4)-(Beta-N-acetylglucosaminyl)-L-asparaginase-like [Ostrinia furnacalis]	3.247700603	1.69941864	1.74E-05	up	yes	1.024	0.3153	0.341	0.305	0.3	1	1.077	0.996	MF:hydrolase activity	GO:0016787;GO:0003674;GO:0003824	K01444	AGA, aspG	map00511;map04	Other glycan degradation;Ly	COG1446	E:Amino acid transport and meta	PF01112.21	Asparaginase_2	Asparaginase	CYT	4	13	37	High
TRINITY_DN3134_c0_g1_i1_orf1	-	-	-	cytochrome c oxidase subunit 6C-1 isoform X1 [Hyposmocoma kahamanoa]	3.291626564	1.718800671	3.91E-06	up	yes	1.026	0.3117	0.339	0.299	0.297	1	1.025	1.052	CC:organelle membr	GO:0031090;GO:0016021;GO:0016020;GO:0019866;GO:0031966;GO:0005575;GO:0005743;GO:0031224;GO:0110165	K02268	COX6C	map04714;map00	Thermogenesis;Oxidative ph	ENOG41124VH	O:Posttranslational modification	PF02937.18	COX6C	Cytochrome c oxidase s	CYT	3	21	11.6	High
TRINITY_DN6231_c0_g1_i6_orf1	-	-	-	ran-binding protein 3 isoform X1 [Ostrinia furnacalis] >XP_028166372.1 ran-binding protein 3 isoform X2 [Ostrinia furnacalis]	2.749295775	1.459062123	1.13E-05	up	yes	0.976	0.355	0.342	0.344	0.379	1	0.937	0.991	BP:cellular process;B	GO:0009987;GO:0046907;GO:0006810;GO:0008150;GO:0051649;GO:0051234;GO:0051179;GO:0051641	K15304	RANBP3	map05166	Human T-cell leukemia virus	ENOG4111NCK	U:Intracellular trafficking, secreti	PF00638.21	Ran_BP1	RanBP1 domain	CYT	4	11	48.6	High
TRINITY_DN2852_c0_g1_i9_orf1	-	-	-	golgin subfamily A member 4-like [Ostrinia furnacalis]	2.629700796	1.394898661	1.03E-05	up	yes	0.958	0.3643	0.372	0.364	0.357	1	0.94	0.934	------	------	K20283	GOLGA4	------	------	ENOG410XQQG	U:Intracellular trafficking, secreti	PF01465.23	GRIP	GRIP domain	CYT	4	3	155.2	High
TRINITY_DN4036_c0_g2_i1_orf1	-	-	-	microvitellogenin-like [Ostrinia furnacalis]	3.294681203	1.720138875	4.78E-06	up	yes	0.9973	0.3027	0.317	0.267	0.324	1	1.014	0.978	CC:cellular_compone	GO:0005575;GO:0005576;GO:0110165	------	------	------	------	ENOG410Z5ZD	S:Function unknown	PF03260.16	Lipoprotein_11	Lepidopteran low mole	CYT	5	20	33.5	High
TRINITY_DN30233_c0_g1_i2_orf1	-	-	-	39S ribosomal protein L10, mitochondrial [Ostrinia furnacalis]	3.010377358	1.589944344	2.62E-05	up	yes	0.9573	0.318	0.284	0.317	0.353	1	0.931	0.941	CC:ribosome;CC:intr	GO:0005840;GO:0043232;GO:0005575;GO:0043229;GO:0043228;GO:0043226;GO:0110165	K02864	RP-L10, MRPL	map03010	Ribosome	ENOG4111GA4	J:Translation, ribosomal structure	------	------	------	CYT	1	2	27.9	High
TRINITY_DN26375_c0_g1_i1_orf1	-	-	-	hypothetical protein O3G_MSEX007366 [Manduca sexta]	3.127302632	1.644918837	4.72E-05	up	yes	0.9507	0.304	0.301	0.271	0.34	1	0.949	0.903	------	------	------	------	------	------	ENOG410Y78S;E	S:Function unknown;	------	------	------	CYT	1	2	129.6	High
TRINITY_DN19160_c0_g1_i1_orf1	-	-	-	alkyldihydroxyacetonephosphate synthase [Ostrinia furnacalis]	5.994413408	2.583618583	4.19E-05	up	yes	1.073	0.179	0.157	0.161	0.219	1	1.074	1.144	------	------	K00803	AGPS, agpS	map00565;map04	Ether lipid metabolism;Perox	COG0277	C:Energy production and conver	PF02913.22;PF01	FAD-oxidase_C	FAD linked oxidases, C-	CYT	1	1	69.6	Medium
TRINITY_DN36061_c0_g4_i2_orf1	-	-	-	putative GPI-anchored protein pfl2 [Ostrinia furnacalis] >XP_028163002.1 putative GPI-anchored protein pfl2 [Ostrinia furnacalis]	3.835989118	1.939598628	0.000131	up	yes	0.987	0.2573	0.288	0.178	0.306	1	0.929	1.032	CC:cellular_compone	GO:0005575;GO:0005576;GO:0110165;GO:0097367;GO:0003674;GO:0008061;GO:0005488	------	------	------	------	ENOG4111P12	S:Function unknown	------	------	------	CYT	1	1	91.7	High
TRINITY_DN9003_c0_g1_i20_orf1	-	-	-	RNA-binding protein Nova-2 isoform X4 [Ostrinia furnacalis]	3.563857195	1.833439529	1.79E-05	up	yes	0.9683	0.2717	0.269	0.256	0.29	1	0.914	0.991	------	------	K14944	NOVA	------	------	ENOG410XRZD	S:Function unknown	PF00013.32;PF07	KH_1;KH_2;KH	KH domain;KH domain;	CYT	4	11	42.2	High
TRINITY_DN3159_c0_g1_i4_orf1	-	-	-	uncharacterized protein LOC114362782 [Ostrinia furnacalis]	3.980699638	1.993022018	1.73E-07	up	yes	0.99	0.2487	0.243	0.24	0.263	1	0.992	0.978	BP:response to host;	GO:0075136;GO:0052173;GO:0009607;GO:0009605;GO:0043207;GO:0030682;GO:0050896;GO:0044419;GO:0008150;GO:0044403;GO:0051701;GO:0052200;GO:0051707;GO:0031409;GO:0003674;GO:0005488	K18271	RBP4	------	------	COG3040	M:Cell wall/membrane/envelope	PF00061.26;PF08	Lipocalin;Lipoc	Lipocalin / cytosolic fat	CYT	22	10	323.1	High
TRINITY_DN657_c0_g1_i2_orf1	-	-	-	cytochrome c-type heme lyase [Ostrinia furnacalis]	4.517857143	2.175638653	8.38E-07	up	yes	1.012	0.224	0.238	0.238	0.196	1	1.02	1.015	------	------	K01764	HCCS	map00860	Porphyrin metabolism	ENOG410YAUW	O:Posttranslational modification	PF01265.20	Cyto_heme_lya	Cytochrome c/c1 heme	CYT	2	7	31.6	High
TRINITY_DN2343_c1_g1_i8_orf1	-	-	-	receptor expression-enhancing protein 5-like isoform X3 [Ostrinia furnacalis]	5.434897206	2.442252749	1.34E-05	up	yes	1.031	0.1897	0.197	0.177	0.195	1	1.095	0.998	CC:cellular_compone	GO:0005575;GO:0016021;GO:0110165;GO:0031224	K17279	REEP5_6	------	------	COG5052	U:Intracellular trafficking, secreti	PF03134.22	TB2_DP1_HVA2	TB2/DP1, HVA22 family	CYT	1	21	22.4	High
TRINITY_DN16931_c0_g1_i1_orf1	-	-	-	pancreatic triacylglycerol lipase-like [Ostrinia furnacalis]	4.76076555	2.251193583	1.64E-08	up	yes	0.995	0.209	0.209	0.215	0.203	1	0.986	0.999	------	------	------	------	------	------	ENOG4110RTX;E	S:Function unknown;	PF00151.22	Lipase	Lipase	PLA	5	26	32.3	High
TRINITY_DN10792_c0_g2_i5_orf1	-	-	-	uncharacterized protein LOC114366171 [Ostrinia furnacalis]	4.851001466	2.278282615	5.31E-07	up	yes	0.993	0.2047	0.209	0.209	0.196	1	1.011	0.968	------	------	------	------	------	------	ENOG410XT8F;E	S:Function unknown;	PF02958.23;PF07	EcKL;DUF1679;	Ecdysteroid kinase-like	CYT	9	20	48	High
TRINITY_DN30932_c0_g1_i2_orf1	-	-	-	delta(24)-sterol reductase-like isoform X2 [Ostrinia furnacalis]	4.042918455	2.015397105	3.53E-05	up	yes	0.942	0.233	0.267	0.219	0.213	1	0.93	0.896	CC:microbody;CC:int	GO:0042579;GO:0043229;GO:0043227;GO:0043226;GO:0016021;GO:0005777;GO:0031224;GO:0110165;GO:0043231;GO:0005575;GO:1901363;GO:0003674;GO:0005488;GO:0071949;GO:0043167;GO:1901265;GO:0016491;GO:0043168;GO:0050660;GO:0016628;GO:0000166;GO:0036094;GO:0003824;GO:0050614;GO:0097159;GO:0016627	K09828	DHCR24, DWF	map00100	Steroid biosynthesis	COG0277	O:Posttranslational modification	PF01565.26	FAD_binding_4	FAD binding domain	CYT	1	2	59.2	High
TRINITY_DN23734_c0_g1_i1_orf1	-	-	-	histone-lysine N-methyltransferase SMYD3 [Ostrinia furnacalis]	3.539929879	1.823720783	0.001088	up	yes	0.9087	0.2567	0.328	0.195	0.247	1	0.778	0.948	CC:cellular_compone	GO:0005575;GO:0043229;GO:0043226;GO:0110165;GO:0043169;GO:0043167;GO:0003674;GO:0005488;GO:0046872	K11426	SMYD	map00310	Lysine degradation	COG2940	S:Function unknown	PF01753.21	zf-MYND	MYND finger	CYT	1	1	56.4	Medium
TRINITY_DN94755_c0_g1_i5_orfp1	-	-	-	TRINITY_DN94755_c0_g1_i5_m.62794 TRINITY_DN94755_c0_g1::TRINITY_DN94755_c0_g1_i5::g.62794 ORF type:internal len:70 (+),score=5.17 TRINITY_DN94755_c0_g1_i5:2-208(+)	11.40422552	3.511496569	1.38E-05	up	yes	0.977	0.08567	0.089	0.078	0.09	1	1.022	0.909	------	------	------	------	------	------	------	------	------	------	------	CYT	1	16	8	High
TRINITY_DN10530_c0_g1_i1_orf1	-	-	-	cytochrome c oxidase subunit NDUFA4 [Ostrinia furnacalis]	2.652968037	1.407607294	8.93E-08	up	yes	0.9877	0.3723	0.37	0.373	0.374	1	0.986	0.977	CC:cellular_compone	GO:0005575;GO:0016021;GO:0110165;GO:0031224	K03948	NDUFA4	map04714;map00	Thermogenesis;Oxidative ph	ENOG41124SW	U:Intracellular trafficking, secreti	PF06522.14	B12D	NADH-ubiquinone redu	CYT	5	48	9.2	High
TRINITY_DN24317_c0_g1_i7_orf1	-	-	-	peptidyl-tRNA hydrolase ICT1, mitochondrial [Ostrinia furnacalis]	2.407072987	1.267279888	2.03E-05	up	yes	0.9597	0.3987	0.391	0.388	0.417	1	0.956	0.923	MF:translation termi	GO:0008079;GO:0090079;GO:0003676;GO:0097159;GO:0008135;GO:0003674;GO:0005488;GO:0045182;GO:0003747;GO:1901363	K15033	ICT1, MRPL58	------	------	COG1186	J:Translation, ribosomal structure	PF00472.23	RF-1	RF-1 domain	CYT	3	16	22.9	High
TRINITY_DN28152_c0_g1_i1_orf1	-	-	-	mitochondrial import inner membrane translocase subunit Tim29 [Ostrinia furnacalis]	2.947674419	1.559577182	5.32E-06	up	yes	1.014	0.344	0.363	0.353	0.316	1	1.044	0.999	CC:mitochondrial inr	GO:0042721;GO:0032991;GO:0098800;GO:0005575;GO:0098796;GO:0098798	------	------	------	------	ENOG4111K8C	S:Function unknown	PF10171.12	Tim29	Translocase of the Inne	CYT	1	4	21.8	High
TRINITY_DN18036_c0_g1_i7_orf1	-	-	-	pentatricopeptide repeat-containing protein 2, mitochondrial-like [Ostrinia furnacalis]	2.996752288	1.583399835	6.05E-07	up	yes	1.015	0.3387	0.348	0.32	0.348	1	1.025	1.02	BP:regulation of prin	GO:0080090;GO:0019222;GO:0019219;GO:0050684;GO:0051252;GO:0031323;GO:0050794;GO:0060255;GO:0065007;GO:0051171;GO:0008150;GO:1903311;GO:0050789;GO:0005575;GO:0043229;GO:0005739;GO:0043226;GO:0043227;GO:0110165;GO:0043231	K17711	PTCD2	------	------	ENOG4111GFQ	S:Function unknown	------	------	------	CYT	6	16	44.3	High
TRINITY_DN1173_c1_g1_i9_orf1	-	-	-	hypothetical protein evm_001011 [Chilo suppressalis]	2.383084577	1.252830155	2.18E-05	up	yes	0.958	0.402	0.398	0.385	0.423	1	0.946	0.928	BP:phosphorylation;I	GO:0016310;GO:0030154;GO:0006807;GO:0009653;GO:0048869;GO:0071704;GO:0032502;GO:0006468;GO:0009987;GO:0043412;GO:0036211;GO:0008150;GO:0008152;GO:0044238;GO:1901564;GO:0019538;GO:0044237;GO:0043170;GO:0006796;GO:0006793;GO:0035639;GO:0005085;GO:0003674;GO:0005488;GO:0098772;GO:1901363;GO:1901265;GO:0043168;GO:0043167;GO:0000166;GO:0016740;GO:0004672;GO:0017076;GO:0097367;GO:0005524;GO:0016301;GO:0030234;GO:0140096;GO:0036094;GO:0030695;GO:0060589;GO:0003824;GO:0016772;GO:0030554;GO:0016773;GO:0032559;GO:0032555;GO:0097159;GO:0032553	------	------	------	------	ENOG410XQFD;I	S:Function unknown;T:Signal trar	PF07679.19;PF13	I-set;Ig_3;ig;Ig_	Immunoglobulin I-set c	CYT	1	18	160.4	High
TRINITY_DN34536_c0_g1_i6_orf1	-	-	-	clustered mitochondria protein homolog isoform X2 [Ostrinia furnacalis]	3.303225806	1.723875595	1.15E-06	up	yes	1.024	0.31	0.306	0.297	0.327	1	1.035	1.037	BP:organelle organiz	GO:0006996;GO:0071840;GO:0009987;GO:0016043;GO:0008150;GO:0007005;GO:0005737;GO:0005575;GO:0110165;GO:0097159;GO:0003674;GO:0003723;GO:0003676;GO:1901363;GO:0005488	K03255	TIF31, CLU1	------	------	ENOG410XQUQ	S:Function unknown	PF13236.9;PF12	CLU;eIF3_p135	Clustered mitochondria	CYT	9	8	124.7	High
TRINITY_DN63662_c0_g4_i1_orf1	-	-	-	polyadenylate-binding protein 1 [Ostrinia furnacalis]	2.803476311	1.487216879	6.23E-08	up	yes	1	0.3567	0.351	0.363	0.356	1	1.01	0.991	CC:cytoplasm;CC:cel	GO:0005737;GO:0005575;GO:0110165;GO:0097159;GO:0003674;GO:0003723;GO:0003676;GO:1901363;GO:0005488	K13126	PABPC	map03018;map03	RNA degradation;mRNA sur	ENOG410XR5X	S:Function unknown	PF00076.25;PF0(	RRM_1;PABP;N	RNA recognition motif.;	CYT	21	40	68.2	High
TRINITY_DN1277_c4_g1_i5_orf1	-	-	-	GTP cyclohydrolase 1 isoform X1 [Ostrinia furnacalis] >XP_028166842.1 GTP cyclohydrolase 1 isoform X1 [Ostrinia furnacalis]	3.277419355	1.712560282	3.24E-07	up	yes	1.016	0.31	0.301	0.309	0.32	1	1.032	1.015	BP:cellular modified	GO:0042398;GO:0051066;GO:0046173;GO:0019751;GO:0034311;GO:0044249;GO:0034641;GO:0034312;GO:0044281;GO:0044283;GO:0046653;GO:1901362;GO:0046654;GO:1901360;GO:1901576;GO:0009396;GO:0035998;GO:0042558;GO:0042559;GO:0071704;GO:0018130;GO:0006729;GO:0006760;GO:0046165;GO:0009987;GO:0006725;GO:0009058;GO:0046146;GO:0008150;GO:0008152;GO:1901564;GO:0090407;GO:0046483;GO:0044271;GO:0006575;GO:1901566;GO:0044237;GO:0006066;GO:0006796;GO:0006807;GO:0006793;GO:0019637;GO:0019438;GO:1901617;GO:1901615;GO:0003933;GO:0003674;GO:0016814;GO:0019238;GO:0016787;GO:0003934;GO:0003824;GO:0016810	K01495	GCH1, folE	map00790;map01	Folate biosynthesis;Biosynth	COG0302	H:Coenzyme transport and meta	PF01227.25	GTP_cyclohydr	GTP cyclohydrolase I	CYT	5	19	32.6	High
TRINITY_DN1707_c0_g1_i1_orf1	-	-	-	inositol oxygenase-like [Ostrinia furnacalis]	3.090414273	1.627800246	1.51E-05	up	yes	1.022	0.3307	0.299	0.363	0.33	1	1.063	1.003	BP:polyol catabolic p	GO:0046174;GO:0019751;GO:0044248;GO:0044281;GO:0044282;GO:0006066;GO:1901575;GO:0044262;GO:0071704;GO:0046164;GO:0019310;GO:0009987;GO:0008150;GO:0008152;GO:0009056;GO:0044275;GO:0044238;GO:0005975;GO:0016052;GO:0044237;GO:0006020;GO:1901616;GO:1901615;GO:0005737;GO:0110165;GO:0005575;GO:0005488;GO:0004497;GO:0003674;GO:0043167;GO:0016491;GO:0046914;GO:0016701;GO:0050113;GO:0046872;GO:0043169;GO:0003824;GO:0005506	K00469	MIOX	map01250;map00	Biosynthesis of nucleotide su	ENOG410XQ4J	S:Function unknown	PF05153.18	MIOX	Myo-inositol oxygenase	CYT	4	21	34.5	High
TRINITY_DN1901_c0_g1_i6_orf1	-	-	-	mitochondrial intermembrane space import and assembly protein 40 [Ostrinia furnacalis] >XP_028171079.1 mitochondrial intermembrane space import and assembly protein 40 [Ostrinia furnacalis] >XP_028171080.1 mitochondrial intermembrane space import and assembly protein 40 [Ostrinia furnacalis]	3.334425156	1.737438067	3.63E-06	up	yes	1.018	0.3053	0.334	0.273	0.309	1	1.029	1.025	BP:protein localizatio	GO:0008104;GO:0051649;GO:0071806;GO:0070727;GO:0006886;GO:0051179;GO:0071705;GO:0065002;GO:1990542;GO:0071702;GO:0033036;GO:0006810;GO:0072594;GO:0009987;GO:0045184;GO:0033365;GO:0008150;GO:0072655;GO:0006839;GO:0051234;GO:0055085;GO:0044743;GO:0051641;GO:0070585;GO:0046907;GO:0045041;GO:0015031;GO:0003674;GO:0016491;GO:0016667;GO:0140096;GO:0003824;GO:0015035;GO:0015036	K17782	MIA40, CHCHD	------	------	ENOG4111V0I	O:Posttranslational modification	PF06747.16	CHCH	CHCH domain	CYT	2	13	15	High
TRINITY_DN56110_c0_g1_i1_orf1	-	-	-	pescadillo homolog [Ostrinia furnacalis]	3.168224299	1.663674477	7.51E-06	up	yes	1.017	0.321	0.33	0.35	0.283	1	1.041	1.011	BP:maturation of 5.8	GO:0000466;GO:0090304;GO:0000470;GO:0034660;GO:1901360;GO:0006139;GO:0071704;GO:0006364;GO:0034470;GO:0009987;GO:0006725;GO:0000460;GO:0000463;GO:0008150;GO:0008152;GO:0046483;GO:0016070;GO:0044238;GO:0016072;GO:0034641;GO:0044237;GO:0043170;GO:0006807;GO:0006396;GO:0030687;GO:0030684;GO:0043229;GO:0043228;GO:0043226;GO:0005730;GO:0005654;GO:0110165;GO:0032991;GO:0043232;GO:1990904;GO:0005575;GO:0044877;GO:0005488;GO:0043021;GO:0003674	K14843	PES1, NOP7	------	------	COG5163	J:Translation, ribosomal structure	PF06732.14;PF16	Pescadillo_N;BI	Pescadillo N-terminus;E	CYT	2	3	71.4	High
TRINITY_DN40704_c0_g1_i2_orf1	-	-	-	COX assembly mitochondrial protein homolog [Ostrinia furnacalis]	2.817310485	1.494318566	4.33E-06	up	yes	0.97	0.3443	0.351	0.357	0.325	1	0.961	0.949	CC:intracellular mem	GO:0043231;GO:0005739;GO:0005575;GO:0043229;GO:0043227;GO:0043226;GO:0110165	K18171	CMC1	------	------	ENOG4111YK0	S:Function unknown	PF08583.13	Cmc1	Cytochrome c oxidase I	CYT	1	9	13.6	High
TRINITY_DN2574_c0_g1_i5_orf1	-	-	-	prion-like-(Q/N-rich) domain-bearing protein 25 [Ostrinia furnacalis] >XP_028158239.1 prion-like-(Q/N-rich) domain-bearing protein 25 [Ostrinia furnacalis] >XP_028158240.1 prion-like-(Q/N-rich) domain-bearing protein 25 [Ostrinia furnacalis] >XP_028158241.1 prion-like-(Q/N-rich) domain-bearing protein 25 [Ostrinia furnacalis]	2.917374744	1.544670716	3.69E-05	up	yes	0.9957	0.3413	0.357	0.286	0.381	1	1.021	0.966	------	------	------	------	------	------	ENOG410XTK6;E	S:Function unknown;	PF01683.21	EB	EB module	CYT	4	20	34.9	High
TRINITY_DN96566_c0_g1_i1_orf1	-	-	-	NADH-ubiquinone oxidoreductase subunit 8-like [Ostrinia furnacalis]	2.429244049	1.280507435	0.0006406	up	yes	0.9287	0.3823	0.45	0.395	0.302	1	0.906	0.88	CC:membrane;CC:ce	GO:0016020;GO:0005575;GO:0110165;GO:0051539;GO:0016651;GO:0051536;GO:0003824;GO:0003674;GO:0005488;GO:0016491;GO:0051540	K03941	NDUFS8	map04714;map00	Thermogenesis;Oxidative ph	COG1143	C:Energy production and conver	PF12838.10;PF0(	Fer4_7;Fer4;Fer	4Fe-4S dicluster domai	CYT	1	4	28.6	High
TRINITY_DN6087_c0_g1_i7_orf1	-	-	-	uncharacterized protein LOC114355564 [Ostrinia furnacalis]	2.716285714	1.441635238	3.59E-05	up	yes	0.9507	0.35	0.32	0.36	0.37	1	0.914	0.938	MF:hydrolase activity	GO:0016787;GO:0080030;GO:0016788;GO:0003824;GO:0003674;GO:0052689	K03927	CES2	map00983	Drug metabolism - other en	COG2272;ENOG	I:Lipid transport and metabolism	PF00135.31;PF2(	COesterase;BD	Carboxylesterase family	CYT	5	9	61.5	High
TRINITY_DN4923_c0_g1_i4_orf1	-	-	-	O-acyltransferase like protein-like [Ostrinia furnacalis]	2.820417501	1.495908738	2.22E-05	up	yes	0.9863	0.3497	0.351	0.342	0.356	1	0.933	1.026	------	------	------	------	------	------	ENOG410YD1T;E	O:Posttranslational modification	------	------	------	PLA	1	1	70.2	Medium
TRINITY_DN9464_c0_g1_i1_orf1	-	-	-	angio-associated migratory cell protein [Ostrinia furnacalis] >XP_028162594.1 angio-associated migratory cell protein [Ostrinia furnacalis]	3.538246594	1.823034599	2.14E-06	up	yes	1.013	0.2863	0.309	0.261	0.289	1	1.035	1.004	------	------	K24725	AAMP	------	------	ENOG410XR6A	S:Function unknown	PF00400.35;PF12	WD40;ANAPC4	WD domain, G-beta re	CYT	6	20	44.7	High
TRINITY_DN5578_c0_g1_i4_orf1	-	-	-	chromatin modification-related protein eaf-1-like [Ostrinia furnacalis]	2.689396048	1.427282226	0.0002513	up	yes	0.9663	0.3593	0.4	0.316	0.362	1	1.018	0.881	CC:intracellular mem	GO:0043231;GO:0005634;GO:0005575;GO:0043229;GO:0043227;GO:0043226;GO:0110165	K23390	BAG6	------	------	ENOG410XS9P;E	O:Posttranslational modification	PF12057.11;PF0(	BAG6;ubiquitin	BCL2-associated athan	CYT	5	6	150.2	High
TRINITY_DN1706_c0_g1_i7_orf1	-	-	-	LOW QUALITY PROTEIN: RNA polymerase-associated protein CTR9 homolog [Ostrinia furnacalis]	2.629463031	1.394768213	6.18E-05	up	yes	0.9353	0.3557	0.356	0.352	0.359	1	0.914	0.892	BP:regulation of prin	GO:0080090;GO:0019222;GO:0031326;GO:0031323;GO:0006807;GO:0050789;GO:0071704;GO:0060255;GO:0065007;GO:0016570;GO:0010468;GO:0019219;GO:0009889;GO:1903506;GO:0050794;GO:0043412;GO:0036211;GO:0008150;GO:0051171;GO:0008152;GO:2001141;GO:0044238;GO:1901564;GO:0019538;GO:0051252;GO:0043170;GO:0006355;GO:0010556;GO:0043229;GO:0043227;GO:0043226;GO:0110165;GO:0043231;GO:0005575	K15176	CTR9	------	------	COG0457	S:Function unknown	PF00515.31;PF07	TPR_1;TPR_2;TF	Tetratricopeptide repea	CYT	1	1	136.5	High
TRINITY_DN43391_c0_g1_i5_orf1	-	-	-	myrosinase 1-like isoform X2 [Ostrinia furnacalis]	3.04587156	1.606855107	5.42E-06	up	yes	0.996	0.327	0.346	0.33	0.305	1	0.965	1.023	BP:primary metaboli	GO:0044238;GO:0005975;GO:0071704;GO:0008150;GO:0008152;GO:0016787;GO:0003824;GO:0016798;GO:0004553;GO:0003674	------	------	------	------	COG2723	G:Carbohydrate transport and m	PF00232.21	Glyco_hydro_1	Glycosyl hydrolase fam	CYT	3	6	59.3	High
TRINITY_DN130051_c0_g1_i1_orf1	-	-	-	5-methyltetrahydropteroyltriglutamate--homocysteine S-methyltransferase-like protein [Leptotrombidium deliense]	2.962264151	1.566700294	0.0002393	up	yes	0.9577	0.3233	0.335	0.238	0.397	1	0.932	0.941	BP:one-carbon meta	GO:0006730;GO:0019752;GO:0044249;GO:0006807;GO:0009067;GO:0044281;GO:0044283;GO:0046653;GO:0043170;GO:1901576;GO:0006520;GO:0035999;GO:0042558;GO:0071704;GO:1901360;GO:0006508;GO:0032259;GO:1901605;GO:1901607;GO:0000096;GO:0006760;GO:0044238;GO:0000097;GO:0009987;GO:0006725;GO:0009058;GO:0008150;GO:0008152;GO:0043436;GO:0006575;GO:0008652;GO:1901564;GO:1901566;GO:0006082;GO:0044272;GO:0046394;GO:0006555;GO:0016053;GO:0044237;GO:0046483;GO:0019538;GO:0006790;GO:0009086;GO:0009066;GO:0031224;GO:0016021;GO:0005575;GO:0110165;GO:0005488;GO:0043169;GO:0016646;GO:0008236;GO:0008168;GO:0008233;GO:0016491;GO:0016645;GO:0046914;GO:0003871;GO:0008270;GO:0043167;GO:0016740;GO:0016741;GO:0016787;GO:0140096;GO:0004489;GO:0008172;GO:0003824;GO:0017171;GO:0046872;GO:0042085;GO:0003674	K00549	metE	map00450;map00	Selenocompound metabolis	------	------	PF08267.15	Meth_synt_1	Cobalamin-independer	CYT	1	10	13	High
TRINITY_DN3332_c0_g1_i11_orf1	-	-	-	glutathione S-transferase sigma3 [Glyphodes pyloalis]	3.014285714	1.591816172	0.0002891	up	yes	0.9917	0.329	0.28	0.436	0.271	1	0.961	1.014	MF:catalytic activity;I	GO:0003824;GO:0016740;GO:0004364;GO:0003674;GO:0016765	K04097	HPGDS	map00590;map00	Arachidonic acid metabolisr	ENOG41103AU;I	O:Posttranslational modification	PF14497.9;PF02	GST_C_3;GST_N	Glutathione S-transfera	CYT	2	45	23.1	High
TRINITY_DN76283_c0_g6_i1_orf1	-	-	-	fatty acid synthase-like [Ostrinia furnacalis]	3.840332955	1.941231397	1.69E-05	up	yes	1.015	0.2643	0.251	0.27	0.272	1	0.972	1.073	BP:fatty acid biosynt	GO:0006633;GO:0006631;GO:0019752;GO:0044249;GO:0044281;GO:0044283;GO:0072330;GO:1901576;GO:0071704;GO:0006629;GO:0009987;GO:0032787;GO:0009058;GO:0008150;GO:0008152;GO:0043436;GO:0044255;GO:0008610;GO:0044238;GO:0006082;GO:0046394;GO:0016053;GO:0044237;GO:0016790;GO:0003674;GO:0016491;GO:0004320;GO:0016740;GO:0016746;GO:0016747;GO:0016787;GO:0016788;GO:0003824;GO:0004312;GO:0016295;GO:0016296;GO:0016297	K00665	FASN	map04910;map04	Insulin signaling pathway;Alc	COG3321;ENOG	Q:Secondary metabolites biosyr	PF13602.9;PF00	ADH_zinc_N_2;	Zinc-binding dehydrog	CYT	1	2	95	High
TRINITY_DN679_c0_g1_i2_orf1	-	-	-	cytochrome b-c1 complex subunit 7-like [Ostrinia furnacalis]	3.049492776	1.608569298	2.74E-06	up	yes	0.992	0.3253	0.328	0.335	0.313	1	1.015	0.961	BP:aerobic respiratio	GO:0009060;GO:0006091;GO:0022900;GO:0022904;GO:0009987;GO:0019646;GO:0045333;GO:0008152;GO:0015980;GO:0006122;GO:0044237;GO:0008150;GO:1990204;GO:0098803;GO:0070069;GO:0098800;GO:0005750;GO:1902495;GO:1902494;GO:1990351;GO:0032991;GO:0045275;GO:0005575;GO:0098796;GO:0098798	K00417	QCR7, UQCRB	map04714;map00	Thermogenesis;Oxidative ph	ENOG4111V1G	C:Energy production and conver	PF02271.19	UCR_14kD	Ubiquinol-cytochrome	CYT	6	44	13.2	High
TRINITY_DN1249_c0_g1_i10_orf1	-	-	-	venom carboxylesterase-6-like [Ostrinia furnacalis]	3.305096186	1.724692258	3.70E-06	up	yes	0.9793	0.2963	0.299	0.305	0.285	1	0.995	0.943	MF:hydrolase activity	GO:0016787;GO:0003674;GO:0016788;GO:0052689;GO:0003824	------	------	------	------	COG2272;ENOG	I:Lipid transport and metabolism	PF00135.31;PF2(	COesterase;BD	Carboxylesterase family	PLA	1	6	55.8	High
TRINITY_DN12474_c0_g1_i6_orf1	-	-	-	aromatic-L-amino-acid decarboxylase [Ostrinia furnacalis]	3.354283824	1.746004768	1.16E-06	up	yes	1.014	0.3023	0.317	0.314	0.276	1	1.018	1.023	BP:carboxylic acid m	GO:0019752;GO:0006807;GO:0006520;GO:0071704;GO:0009987;GO:0008150;GO:0008152;GO:0043436;GO:0044238;GO:1901564;GO:0006082;GO:0044237;GO:0044281;GO:0005488;GO:0004058;GO:0070279;GO:0003674;GO:0030170;GO:1901363;GO:0043168;GO:0016829;GO:0043167;GO:0036094;GO:0003824;GO:0019842;GO:0097159;GO:0016830;GO:0016831	K01593	DDC, TDC	map00350;map00	Tyrosine metabolism;Trypto	COG0076	S:Function unknown	PF00282.22	Pyridoxal_deC	Pyridoxal-dependent d	CYT	10	20	54.3	High
TRINITY_DN29229_c0_g1_i5_orfp1	-	-	-	TRINITY_DN29229_c0_g1_i5_m.11187 TRINITY_DN29229_c0_g1::TRINITY_DN29229_c0_g1_i5::g.11187 ORF type:internal len:133 (+),score=48.64 TRINITY_DN29229_c0_g1_i5:3-398(+)	2.632478632	1.396421821	0.0002276	up	yes	0.924	0.351	0.387	0.31	0.356	1	0.867	0.905	------	------	------	------	------	------	------	------	------	------	------	CYT	1	10	14.2	High
TRINITY_DN2894_c0_g3_i1_orf1	-	-	-	lactase-phlorizin hydrolase-like [Ostrinia furnacalis]	3.288442542	1.717404463	1.72E-06	up	yes	0.9987	0.3037	0.291	0.33	0.29	1	1.014	0.982	BP:primary metaboli	GO:0044238;GO:0005975;GO:0071704;GO:0008150;GO:0008152;GO:0016787;GO:0003824;GO:0016798;GO:0004553;GO:0003674	------	------	------	------	COG2723	G:Carbohydrate transport and m	PF00232.21	Glyco_hydro_1	Glycosyl hydrolase fam	CYT	10	28	57.5	High
TRINITY_DN6221_c0_g1_i5_orf1	-	-	-	unnamed protein product [Diatraea saccharalis]	3.39109752	1.761752273	8.93E-05	up	yes	0.998	0.2943	0.3	0.346	0.237	1	0.944	1.05	CC:intrinsic compone	GO:0031224;GO:0005575;GO:0033179;GO:0016021;GO:0110165;GO:0032991;GO:0098796;GO:0033177;GO:0003674;GO:0042626;GO:0015318;GO:0009678;GO:0015399;GO:0042625;GO:0005215;GO:0008324;GO:0046961;GO:0044769;GO:0022890;GO:0019829;GO:0015075;GO:0022857;GO:0022804;GO:0022853;GO:0015078	K02153	ATPeV0E, ATP	map05165;map04	Human papillomavirus infec	ENOG41121M0	C:Energy production and conver	PF05493.16	ATP_synt_H	ATP synthase subunit H	CYT	1	9	9.6	High
TRINITY_DN6074_c0_g1_i1_orf1	-	-	-	uncharacterized protein C1683.06c-like isoform X1 [Ostrinia furnacalis]	4.340538156	2.117873924	4.16E-07	up	yes	0.984	0.2267	0.237	0.225	0.218	1	0.989	0.963	MF:hydrolase activity	GO:0016787;GO:0003674;GO:0016799;GO:0016798;GO:0003824	------	------	------	------	COG1957;COG1	F:Nucleotide transport and meta	PF01156.22	IU_nuc_hydro	Inosine-uridine preferri	CYT	2	5	38.9	High
TRINITY_DN3062_c0_g1_i1_orf1	-	-	-	HEAT repeat-containing protein 1 [Ostrinia furnacalis]	3.638658493	1.863406653	2.74E-07	up	yes	1.009	0.2773	0.262	0.277	0.293	1	1.007	1.02	BP:nucleic acid meta	GO:0090304;GO:0034641;GO:0006807;GO:0034660;GO:1901360;GO:0006139;GO:0071704;GO:0006364;GO:0034470;GO:0009987;GO:0006725;GO:0008150;GO:0008152;GO:0046483;GO:0016070;GO:0044238;GO:0016072;GO:0044237;GO:0043170;GO:0006396;GO:0043226;GO:0005730;GO:0110165;GO:0032991;GO:0043232;GO:1990904;GO:0043229;GO:0005575;GO:0043228	K14550	UTP10, HEATR	map03008	Ribosome biogenesis in euk	ENOG410XNQQ	J:Translation, ribosomal structure	PF08146.15;PF12	BP28CT;U3snol	BP28CT;U3 small nucle	CYT	4	2	240.8	High
TRINITY_DN717_c0_g1_i2_orfp1	-	-	-	TRINITY_DN717_c0_g1_i2_m.67915 TRINITY_DN717_c0_g1::TRINITY_DN717_c0_g1_i2::g.67915 ORF type:internal len:868 (+),score=265.71,Collagen PF01391.19 0.11,Collagen|PF01391.19|0.039,Collagen|PF01391.19|0.00054,Collagen|PF01391.19|0.0019 ,Collagen|PF01391.19|0.0005,Collagen|PF01391.19|9.9e-05,Collagen|PF01391.19|1.7e-07 TRINITY_DN717_c0_g1_i2:3-2603(+)	5.732954545	2.51927884	6.98E-06	up	yes	1.009	0.176	0.185	0.146	0.197	1	1.052	0.975	------	------	------	------	------	------	------	------	PF01391.21	Collagen	Collagen triple helix rep	PLA	8	20	90.1	High
TRINITY_DN7966_c0_g1_i4_orf1	-	-	-	leucine-rich repeat neuronal protein 1-like [Ostrinia furnacalis]	3.328818151	1.73501006	9.47E-06	up	yes	0.983	0.2953	0.317	0.29	0.279	1	0.94	1.009	BP:locomotion;BP:bi	GO:0040011;GO:0008150;GO:0016021;GO:0005575;GO:0071944;GO:0031224;GO:0110165	------	------	------	------	COG4886	P:Inorganic ion transport and me	PF13855.9;PF12	LRR_8;LRR_4	Leucine rich repeat;Leu	PLA	2	4	57.2	High

TRINITY_DN542_c0_g2_i1_orf1	-	-	-	uncharacterized protein LOC114364889 [Ostrinia furnacalis]	4.982260517	2.316800461	7.25E-06	up	yes	0.983	0.1973	0.197	0.218	0.177	1	1.012	0.937	BP:amino sugar cata	GO:0046348;GO:0006807;GO:0006022;GO:1901575;GO:1901072;GO:0071704;GO:0006030;GO:0000272;GO:0008150;GO:0008152;GO:1901564;GO:0009056;GO:0009057;GO:0044238;GO:0006032;GO:0005975;GO:1901565;GO:0005976;GO:0006040;GO:1901136;GO:0016052;GO:0043170;GO:1901071;GO:0006026;GO:1901135;GO:0005615;GO:0110165;GO:0005575;GO:0003674;GO:0008270;GO:0043169;GO:0046914;GO:0043167;GO:0046872;GO:0016787;GO:0019213;GO:0003824;GO:0016810;GO:0016811;GO:0004099;GO:0005488	------	------	------	------	ENOG410ZWU3	S:Function unknown	PF01522.24	Polysacc_deac_	Polysaccharide deacety	PLA	4	16	44.5	High
TRINITY_DN15900_c0_g1_i6_orf1	-	-	-	unnamed protein product [Diatraea saccharalis]	3.996078431	1.998584899	5.65E-05	up	yes	1.019	0.255	0.259	0.243	0.263	1	1.1	0.958	BP:RNA metabolic p	GO:0016070;GO:0016071;GO:0044238;GO:0006139;GO:0009987;GO:0006725;GO:0044237;GO:0043170;GO:0090304;GO:0071704;GO:0034641;GO:0006807;GO:0008150;GO:1901360;GO:0008152;GO:0046483;GO:0006396;GO:0006397	K13171	SRRM1, SRM1	map03013;map03	Nucleocytoplasmic transpor	ENOG4111IMU	S:Function unknown	PF01480.20	PWI	PWI domain	CYT	2	2	111.5	High
TRINITY_DN7688_c0_g1_i10_orf1	-	-	-	uncharacterized protein LOC114352518 [Ostrinia furnacalis]	6.079854809	2.604036872	1.66E-05	up	yes	1.005	0.1653	0.134	0.172	0.19	1	1.059	0.955	BP:phosphorylation;	GO:0016310;GO:0009987;GO:0044237;GO:0006796;GO:0008150;GO:0008152;GO:0006793;GO:0016301;GO:0016740;GO:0003824;GO:0003674;GO:0016772	------	------	------	------	ENOG410YKCN;	S:Function unknown;	PF02958.23;PF0	EcKL;DUF1679	Ecdysteroid kinase-like	CYT	1	10	47.9	High
TRINITY_DN79210_c0_g1_i1_orf1	-	-	-	V-type proton ATPase 16 kDa proteolipid subunit [Frieseomelitta varia]	3.048997773	1.608335097	3.57E-05	up	yes	0.9583	0.3143	0.302	0.361	0.28	1	0.939	0.936	CC:intrinsic compon	GO:0031224;GO:0005575;GO:0031090;GO:0033179;GO:0016021;GO:0016020;GO:0005774;GO:0110165;GO:0098588;GO:0032991;GO:0098796;GO:0033177;GO:0003674;GO:0042626;GO:0015318;GO:0009678;GO:0015399;GO:0042625;GO:0005215;GO:0008324;GO:0046961;GO:0044769;GO:0022890;GO:0019829;GO:0015075;GO:0022857;GO:0022804;GO:0022853;GO:0015078	K02155	ATPeV0C, ATP	map05152;map05	Tuberculosis;Rheumatoid ar	COG0636	J:Translation, ribosomal structure	PF00137.24	ATP-synt_C	ATP synthase subunit C	CYT	1	11	16	High
TRINITY_DN26411_c0_g1_i2_orfp1	-	-	-	TRINITY_DN26411_c0_g1_i2_m.24123 TRINITY_DN26411_c0_g1::TRINITY_DN26411_c0_g1_i2::g.24123 ORF type:internal len:115 (-) score=72.83 TRINITY_DN26411_c0_g1_i2:3-344(-)	12.56608877	3.651463772	1.33E-05	up	yes	1.022	0.08133	0.102	0.05	0.092	1	1.086	0.979	------	------	------	------	------	------	------	------	------	------	------	CYT	1	46	10.9	High
TRINITY_DN5107_c0_g1_i4_orf1	-	-	-	peptide methionine sulfoxide reductase [Ostrinia furnacalis]	4.093495935	2.033333463	1.14E-05	up	yes	1.007	0.246	0.226	0.227	0.285	1	1.046	0.976	MF:peptide-methior	GO:0008113;GO:0003824;GO:0016671;GO:0003674;GO:0016667;GO:0016491	K07304	msrA	------	------	COG0225	O:Posttranslational modification	PF01625.24	PMSR	Peptide methionine sulf	CYT	1	4	26.1	Medium
TRINITY_DN37538_c0_g1_i1_orf1	-	-	-	esterase FE4-like [Ostrinia furnacalis]	7.696	2.944108799	2.77E-06	up	yes	0.962	0.125	0.12	0.116	0.139	1	0.957	0.929	------	------	K07378	NLGN	map04514	Cell adhesion molecules	COG2272;ENOG	I:Lipid transport and metabolism	PF00135.31;PF2	COesterase;BD	Carboxylesterase family	CYT	1	4	22.5	High
TRINITY_DN13783_c0_g4_i2_orf1	-	-	-	hypothetical protein evm_010131 [Chilo suppressalis]	3.937226857	1.977179841	4.41E-06	up	yes	0.991	0.2517	0.268	0.231	0.256	1	1.018	0.955	BP:protein localizatio	GO:0008104;GO:0035437;GO:0051651;GO:0070727;GO:0071705;GO:0071702;GO:0033036;GO:0006810;GO:0032507;GO:0072595;GO:0009987;GO:0006621;GO:0045184;GO:0045185;GO:0008150;GO:0051235;GO:0051234;GO:0051179;GO:0051641;GO:0015031;GO:0005789;GO:0031224;GO:0031090;GO:0016021;GO:0016020;GO:0110165;GO:0005575;GO:0003674;GO:0005488;GO:0042277;GO:0033218;GO:0046923;GO:0005048	------	------	------	------	ENOG410Z4PE	S:Function unknown	PF14880.9	COX14	Cytochrome oxidase c	CYT	1	10	11.2	High
TRINITY_DN69713_c0_g1_i1_orf1	-	-	-	membrane-bound alkaline phosphatase-like [Ostrinia furnacalis]	4.0283135	2.010175964	2.04E-06	up	yes	0.9817	0.2437	0.262	0.25	0.219	1	0.987	0.958	MF:hydrolase activity	GO:0016787;GO:0016791;GO:0004035;GO:0016788;GO:0042578;GO:0003824;GO:0003674	K01077	E3.1.3.1, phoA	map00730;map00	Thiamine metabolism;Folate	COG1785	P:Inorganic ion transport and me	PF00245.23	Alk_phosphata	Alkaline phosphatase	CYT	3	7	57.6	High
TRINITY_DN82801_c0_g1_i1_orf1	-	-	-	uncharacterized protein LOC114364712 [Ostrinia furnacalis]	4.490345757	2.166826537	1.36E-06	up	yes	1	0.2227	0.214	0.232	0.222	1	1.028	0.972	BP:organic substanc	GO:0071704;GO:0005975;GO:0044238;GO:0008152;GO:0008150;GO:0005575;GO:0005576;GO:0110165;GO:0016787;GO:0019213;GO:0097367;GO:0016810;GO:0016811;GO:0003824;GO:0003674;GO:0008061;GO:0004099;GO:0005488	------	------	------	------	ENOG410XX7G	Z:Cytoskeleton	PF01607.27;PF0	CBM_14;Polysa	Chitin binding Peritroph	CYT	10	27	61.3	High
TRINITY_DN96_c0_g1_i1_orf1	-	-	-	collagenase-like [Ostrinia furnacalis]	4.84804631	2.27740348	7.58E-05	up	yes	1.005	0.2073	0.2	0.202	0.22	1	0.925	1.089	BP:organic substanc	GO:0071704;GO:1901564;GO:0019538;GO:0043170;GO:0008150;GO:0006807;GO:0008152;GO:0006508;GO:0044238;GO:0004252;GO:0004175;GO:0016787;GO:0017171;GO:0140096;GO:0003824;GO:0003674;GO:0008233;GO:0008236	------	------	------	------	COG5640	O:Posttranslational modification	PF00089.29	Trypsin	Trypsin	CYT	1	3	44.4	High
TRINITY_DN69049_c0_g1_i2_orf1	-	-	-	membrane alanyl aminopeptidase-like [Ostrinia furnacalis]	4.909852217	2.295679601	2.92E-06	up	yes	0.9967	0.203	0.218	0.188	0.203	1	1.028	0.962	CC:integral compon	GO:0016021;GO:0005575;GO:0110165;GO:0031224;GO:0008238;GO:0004177;GO:0046914;GO:0016787;GO:0140096;GO:0043169;GO:0043167;GO:0003824;GO:0008237;GO:0003674;GO:0005488;GO:0008233;GO:0046872;GO:0008270	K11140	ANPEP, CD13	map04640;map00	Hematopoietic cell lineage;	COG0308	E:Amino acid transport and meta	PF11838.11	ERAP1_C	ERAP1-like C-terminal	CYT	1	2	51.3	High
TRINITY_DN27721_c1_g1_i2_orf1	-	-	-	mitochondrial import receptor subunit TOM20 homolog [Ostrinia furnacalis]	2.756283805	1.462724445	0.001499	up	yes	0.8663	0.3143	0.31	0.277	0.356	1	0.783	0.816	BP:protein localizatio	GO:0008104;GO:0070727;GO:0071705;GO:0071702;GO:0033036;GO:0006810;GO:0009987;GO:0006605;GO:0045184;GO:0008150;GO:0051649;GO:0051234;GO:0051179;GO:0051641;GO:0046907;GO:0015031;GO:0006886;GO:0031224;GO:0005742;GO:0016021;GO:0110165;GO:0032991;GO:0005575;GO:0098796;GO:0098799;GO:0098798	K17770	TOM20	------	------	ENOG4111NAH	U:Intracellular trafficking, secreti	PF02064.18	MAS20	MAS20 protein import	CYT	2	15	17.2	High
TRINITY_DN20682_c0_g2_i1_orf1	-	-	-	glutathione S-transferase delta3 [Glyphodes pyloalis]	5.323134729	2.412276081	1.42E-06	up	yes	0.9917	0.1863	0.209	0.173	0.177	1	0.965	1.01	MF:catalytic activity;	GO:0003824;GO:0016740;GO:0004364;GO:0003674;GO:0016765	K00799	GST, gst	map05207;map00	Chemical carcinogenesis - r	COG0625	O:Posttranslational modification	PF13417.9;PF02	GST_N_3;GST_I	Glutathione S-transfera	PLA	1	8	14.1	Medium
TRINITY_DN29120_c0_g1_i6_orf1	-	-	-	putative inorganic phosphate cotransporter [Ostrinia furnacalis]	6.756574511	2.756292006	1.88E-06	up	yes	1.002	0.1483	0.149	0.138	0.158	1	1.036	0.969	CC:integral compon	GO:0016021;GO:0005575;GO:0110165;GO:0031224;GO:0005215;GO:0022857;GO:0003674	K08193	SLC17A	------	------	ENOG410XPWC;	G:Carbohydrate transport and m	PF07690.19	MFS_1	Major Facilitator Superf	CYT	1	4	27.3	High
TRINITY_DN41259_c0_g1_i6_orf1	-	-	-	endocuticle structural glycoprotein SgAbd-8 [Ostrinia furnacalis]	6.608666667	2.72435923	3.05E-08	up	yes	0.9913	0.15	0.149	0.156	0.145	1	0.995	0.979	MF:molecular_functi	GO:0003674;GO:0042302;GO:0005198	------	------	------	------	ENOG4111PJ1	S:Function unknown	PF00379.26	Chitin_bind_4	Insect cuticle protein	CYT	7	44	24.7	High
TRINITY_DN348_c0_g2_i3_orf1	-	-	-	pancreatic triacylglycerol lipase-like [Ostrinia furnacalis]	11.22721612	3.488928339	3.12E-08	up	yes	0.9917	0.08833	0.091	0.084	0.09	1	0.998	0.977	------	------	------	------	------	------	ENOG4110T03;E	S:Function unknown;	PF00151.22	Lipase	Lipase	CYT	4	45	36.1	High
TRINITY_DN6933_c0_g1_i2_orf1	-	-	-	Chlorophyll a-b binding protein 40, chloroplastic [Trichinella nelsoni] >KRY99282.1 Chlorophyll a-b binding protein 40, chloroplastic [Trichinella zimbabwensis]	7.699193548	2.944707339	9.91E-06	up	yes	0.9547	0.124	0.117	0.116	0.139	1	0.901	0.963	CC:cellular_compon	GO:0005575;GO:0016020;GO:0110165	K08912	LHCB1	------	------	ENOG410ZHBU;	J:Translation, ribosomal structure	PF00504.24	Chloroa_b-bin	Chlorophyll A-B binding	CYT	2	7	28.3	High
TRINITY_DN39200_c0_g1_i5_orf1	-	-	-	juvenile hormone epoxide hydrolase-like [Ostrinia furnacalis]	11.29394252	3.497477288	2.28E-07	up	yes	0.9863	0.08733	0.086	0.093	0.083	1	0.962	0.997	CC:integral compon	GO:0016021;GO:0005575;GO:0031224;GO:0110165;GO:0016787;GO:0033961;GO:0016801;GO:0003824;GO:0003674;GO:0016803	K10719	JHEH	map00981	Insect hormone biosynthesis	COG0596;COG0	U:Intracellular trafficking, secreti	PF06441.15;PF0	EHN;Abhydrola	Epoxide hydrolase N te	PLA	1	4	56.8	High
TRINITY_DN21494_c0_g1_i2_orf1	-	-	-	pancreatic triacylglycerol lipase-like [Ostrinia furnacalis]	2.921380911	1.54665048	3.39E-05	up	yes	1.007	0.3447	0.354	0.372	0.308	1	1.055	0.965	BP:organic substanc	GO:0071704;GO:0006629;GO:0044238;GO:0008152;GO:0008150;GO:0005575;GO:0005576;GO:0110165;GO:0016787;GO:0016788;GO:0003824;GO:0003674;GO:0016298	------	------	------	------	ENOG41116Y2;E	S:Function unknown;	PF00151.22	Lipase	Lipase	CYT	1	4	35.8	High
TRINITY_DN7073_c0_g1_i1_orf1	-	-	-	unnamed protein product, partial [Brenthis ino]	3.150600554	1.655626855	1.51E-05	up	yes	1.023	0.3247	0.318	0.295	0.361	1	1.063	1.006	CC:cellular_compon	GO:0005575;GO:0032991;GO:1990904	K11129	NHP2, NOLA2	map03008	Ribosome biogenesis in euk	COG1358	L:Replication, recombination and	PF01248.29	Ribosomal_L7A	Ribosomal protein L7Ae	CYT	2	10	17.7	High
TRINITY_DN1505_c0_g1_i1_orf1	-	-	-	uncharacterized protein LOC114362816 isoform X1 [Ostrinia furnacalis] >XP_028174154.1 uncharacterized protein LOC114362816 isoform X2 [Ostrinia furnacalis]	2.933235294	1.552492804	0.0001476	up	yes	0.9973	0.34	0.354	0.254	0.412	1	1.008	0.984	------	------	K16913	PELP1, MNAR	------	------	ENOG4111B29;E	S:Function unknown;	------	------	------	CYT	2	4	56	High
TRINITY_DN33452_c0_g1_i1_orf1	-	-	-	lethal(2) giant larvae protein isoform X8 [Ostrinia furnacalis]	2.650273224	1.406141099	2.56E-05	up	yes	0.97	0.366	0.327	0.364	0.407	1	0.955	0.955	BP:secretion;BP:trans	GO:0046903;GO:0006810;GO:0016192;GO:0140352;GO:0006887;GO:0032940;GO:0008150;GO:0009987;GO:0051234;GO:0051179	K06094	LLGL	map04530;map05	Tight junction;Human papillo	ENOG410XS6Z	S:Function unknown	------	------	------	CYT	1	14	13.7	High
TRINITY_DN787_c0_g1_i7_orf1	-	-	-	YLP motif-containing protein 1-like isoform X1 [Ostrinia furnacalis]	2.169397646	1.11729452	0.0006446	up	yes	0.94	0.4333	0.492	0.435	0.373	1	0.955	0.865	CC:intracellular mem	GO:0043231;GO:0005634;GO:0005575;GO:0043229;GO:0043227;GO:0043226;GO:0110165	K17602	YLPM1	------	------	ENOG410YIR5	S:Function unknown	------	------	------	CYT	1	1	137.9	Medium
TRINITY_DN23266_c0_g2_i1_orf1	-	-	-	medium-chain acyl-CoA ligase ACSF2, mitochondrial [Chelonus insularis]	3.54772494	1.82689416	5.62E-06	up	yes	1.037	0.2923	0.296	0.267	0.314	1	1.059	1.053	------	------	K23617	ACSF2	------	------	COG0318	S:Function unknown	PF00501.31;PF1	AMP-binding;A	AMP-binding enzyme;A	CYT	1	1	65	Medium
TRINITY_DN37538_c0_g4_i1_orf1	-	-	-	esterase FE4-like [Ostrinia furnacalis]	2.678225806	1.421277602	0.0001086	up	yes	0.9963	0.372	0.417	0.292	0.407	1	1.009	0.98	------	------	------	------	------	------	COG2272;COG0	I:Lipid transport and metabolism	PF00135.31	COesterase	Carboxylesterase family	CYT	1	12	12.7	High
TRINITY_DN1427_c0_g1_i9_orf1	-	-	-	SAFB-like transcription modulator isoform X3 [Ostrinia furnacalis]	3.210745123	1.682908145	3.63E-05	up	yes	1.004	0.3127	0.249	0.324	0.365	1	0.997	1.015	------	------	------	------	------	------	ENOG4111F1G;E	K:Transcription;S:Function unkno	PF00076.25;PF0	RRM_1;SAP	RNA recognition motif.	CYT	1	2	71.9	High
TRINITY_DN7336_c0_g1_i13_orf1	-	-	-	PREDICTED: calcium-transporting ATPase sarcoplasmic/endoplasmic reticulum type isoform X2 [Amyelois transitella]	2.78551532	1.477944251	2.07E-07	up	yes	1	0.359	0.357	0.375	0.345	1	1.001	0.999	CC:endoplasmic reti	GO:0005789;GO:0031224;GO:0031090;GO:0016021;GO:0016020;GO:0033017;GO:0098588;GO:0005575;GO:0110165;GO:0035639;GO:0003674;GO:0005488;GO:0016887;GO:0000166;GO:1901363;GO:0043168;GO:0015085;GO:0042626;GO:0043167;GO:0015318;GO:0005524;GO:0015399;GO:1901265;GO:0017076;GO:0046873;GO:0022804;GO:0016787;GO:0005215;GO:0008324;GO:0140358;GO:0017111;GO:0036094;GO:0003824;GO:0015662;GO:0022890;GO:0016818;GO:0030554;GO:0097367;GO:0097159;GO:0015075;GO:0016817;GO:0019829;GO:0016462;GO:0022857;GO:0032559;GO:0022853;GO:0032555;GO:0005388;GO:0032553	K05853	ATP2A	map04972;map04	Pancreatic secretion;Cardiac	COG0474	G:Carbohydrate transport and m	PF00122.23;PF0	E1-E2_ATPase;	E1-E2 ATPase;Cation tr	CYT	37	37	109.8	High
TRINITY_DN16894_c0_g1_i5_orf1	-	-	-	dnaJ homolog subfamily C member 5 isoform X1 [Colias croceus]	2.364985923	1.241831596	0.0006675	up	yes	0.924	0.3907	0.377	0.328	0.467	1	0.891	0.881	------	------	K09525	DNAJC5	map04141	Protein processing in endop	COG0484;ENOG	S:Function unknown;U:Intracellul	PF00226.34	DnaJ	DnaJ domain	PLA	1	10	22.2	High
TRINITY_DN20294_c0_g2_i1_orf1	-	-	-	cytochrome b-c1 complex subunit 2, mitochondrial isoform X1 [Ostrinia furnacalis] >XP_028170208.1 cytochrome b-c1 complex subunit 2, mitochondrial isoform X2 [Ostrinia furnacalis]	2.598118631	1.377467306	6.64E-07	up	yes	0.9943	0.3827	0.388	0.366	0.394	1	1.003	0.98	MF:cation binding;M	GO:0043169;GO:0046872;GO:0003674;GO:0005488;GO:0043167	K00415	QCR2, UQCRC	map04714;map00	Thermogenesis;Oxidative ph	COG0612	T:Signal transduction mechanism	PF00675.23;PF0	Peptidase_M16	Insulinase;Peptidase M	CYT	17	43	45.7	High
TRINITY_DN4550_c1_g1_i5_orfp2	-	-	-	TRINITY_DN4550_c1_g1_i5_m.14710 TRINITY_DN4550_c1_g1::TRINITY_DN4550_c1_g1_i5::g.14710 ORF type:5prime_partial len:168 (+) score=78.12 TRINITY_DN4550_c1_g1_i5:3-506(+)	2.813831339	1.492535856	7.08E-05	up	yes	0.9643	0.3427	0.356	0.283	0.389	1	0.935	0.958	------	------	------	------	------	------	------	------	------	------	------	CYT	1	7	20.9	High
TRINITY_DN46409_c0_g1_i1_orf1	-	-	-	unnamed protein product [Heterotrigona itama]	2.919075145	1.54551135	0.0002453	up	yes	1.01	0.346	0.344	0.357	0.337	1	1.107	0.923	BP:regulation of T ce	GO:0002709;GO:0051234;GO:0080090;GO:0019222;GO:0048584;GO:0048583;GO:0002706;GO:0002705;GO:0071705;GO:0065009;GO:0007165;GO:0042110;GO:1901360;GO:0002708;GO:0070661;GO:0051716;GO:0006986;GO:0045785;GO:0071310;GO:0010950;GO:0001817;GO:0044419;GO:0002366;GO:0048291;GO:0032729;GO:0048518;GO:0032727;GO:0033036;GO:0009266;GO:0034514;GO:0032655;GO:0032653;GO:0070585;GO:0060255;GO:0060548;GO:0002824;GO:0071702;GO:0002822;GO:0008637;GO:0002821;GO:0045184;GO:0045041;GO:0050865;GO:0046649;GO:0030162;GO:0002819;GO:0072655;GO:0006839;GO:0051702;GO:0009628;GO:0010033;GO:0046483;GO:0046907;GO:0002833;GO:0010468;GO:0002376;GO:0019538;GO:0002204;GO:0033554;GO:0032735;GO:0002703;GO:0043030;GO:0016445;GO:0016444;GO:0016447;GO:0032647;GO:0022607;GO:0006457;GO:0009893;GO:0051131;GO:0002834;GO:0002837;GO:0006458;GO:0032649;GO:0050867;GO:0008104;GO:0050821;GO:0045321;GO:0006807;GO:0046651;GO:0051179;GO:0042981;GO:0043280;GO:0044260;GO:0002285;GO:0002684;GO:0006886;GO:0052547;GO:0016043;GO:1990542;GO:0065003;GO:0065002;GO:0002208;GO:0035966;GO:0065007;GO:0071840;GO:0002221;GO:0032943;GO:0002200;GO:0065008;GO:0006810;GO:0006139;GO:0050790;GO:0050794;GO:0006950;GO:0051249;GO:0008150;GO:0008152;GO:0045862;GO:0032755;GO:0002694;GO:0010604;GO:0051336;GO:0002755;GO:0051604;GO:0050896;GO:0031647;GO:0001775;GO:0002697;GO:0002696;GO:0032733;GO:0032481;GO:0002840;GO:0002699;GO:0002263;GO:0051239;GO:0030155;GO:0034641;GO:0070887;GO:0002839;GO:0007005;GO:0043085;GO:0071806;GO:0051649;GO:0051240;GO:0051246;GO:0051247;GO:1903039;GO:0002224;GO:0051641;GO:1903037;GO:0006996;GO:0045190;GO:0044093;GO:0002764;GO:0044238;GO:0002831;GO:0009987;GO:0006725;GO:0050870;GO:0043281;GO:0050863;GO:0048519;GO:0009409;GO:0055085;GO:0044743;GO:0050776;GO:0051251;GO:0050778;GO:0032675;GO:0043170;GO:0035967;GO:0032479;GO:0033365;GO:0015031;GO:0010952;GO:0001819;GO:0043933;GO:0042100;GO:0042113;GO:2001056;GO:1901564;GO:0090304;GO:0022407;GO:0034620;GO:0043032;GO:0010942;GO:0010628;GO:0002682;GO:00	K04077	groEL, HSPD1	map05152;map04	Tuberculosis;Type I diabetes	COG0459	G:Carbohydrate transport and m	PF00118.27	Cpn60_TCP1	TCP-1/cpn60 chaperon	CYT	2	7	54.5	High
TRINITY_DN35725_c0_g1_i1_orf1	-	-	-	mitochondrial import inner membrane translocase subunit Tim13-like [Bicyclus anynana] >CAG9745432.1 unnamed protein product [Diatraea saccharalis] >CAG9784117.1 unnamed protein product [Diatraea saccharalis]	3.185897436	1.671699823	8.58E-06	up	yes	0.994	0.312	0.309	0.29	0.337	1	1.024	0.958	------	------	K17781	TIM13	------	------	ENOG41124YI	O:Posttranslational modification	PF02953.18	zf-Tim10_DDP	Tim10/DDP family zinc	CYT	3	39	10.9	High
TRINITY_DN14436_c0_g1_i7_orf1	-	-	-	V-type proton ATPase subunit C [Vanessa cardui]	2.905456668	1.538764938	2.80E-07	up	yes	0.9957	0.3427	0.359	0.341	0.328	1	0.998	0.989	------	------	K02148	ATPeV1C, ATP	map05323;map00	Rheumatoid arthritis;Oxidati	COG5127	C:Energy production and conver	PF03223.18	V-ATPase_C	V-ATPase subunit C	CYT	20	51	44.1	High
TRINITY_DN18909_c0_g1_i8_orf1	-	-	-	unnamed protein product [Euphydryas editha]	3.041835779	1.604942268	0.0002252	up	yes	0.9743	0.3203	0.267	0.376	0.318	1	1.029	0.894	MF:hydrolase activity	GO:0016787;GO:0003674;GO:0016788;GO:0052689;GO:0003824	------	------	------	------	COG2272;COG0	I:Lipid transport and metabolism	PF00135.31;PF2	COesterase;BD	Carboxylesterase family	CYT	1	4	60.6	High
TRINITY_DN3332_c0_g1_i2_orf1	-	-	-	glutathione S-transferase sigma3 [Glyphodes pyloalis]	3.277419355	1.712560282	4.04E-06	up	yes	1.016	0.31	0.291	0.346	0.293	1	1.032	1.015	MF:catalytic activity;	GO:0003824;GO:0016740;GO:0004364;GO:0003674;GO:0016765	K04097	HPGDS	map00590;map00	Arachidonic acid metabolism	ENOG41103AU;	O:Posttranslational modification	PF02798.23;PF1	GST_N;GST_C	Glutathione S-transfera	CYT	3	50	20.6	High
TRINITY_DN25779_c0_g1_i6_orf1	-	-	-	aldo-keto reductase AKR2E4-like [Ostrinia furnacalis]	3.343424151	1.741326389	4.61E-06	up	yes	0.994	0.2973	0.287	0.319	0.286	1	1.021	0.961	MF:catalytic activity;	GO:0003824;GO:0003674;GO:0016491	K00011	AKR1B	map00051;map00	Fructose and mannose meta	COG0656	S:Function unknown	PF00248.24	Aldo_ket_red	Aldo/keto reductase fa	CYT	5	23	35.9	High
TRINITY_DN32306_c0_g1_i3_orf1	-	-	-	acetyl-coenzyme A transporter 1 [Ostrinia furnacalis]	2.540112691	1.344892503	0.0007916	up	yes	0.9467	0.3727	0.282	0.361	0.475	1	0.936	0.904	------	------	K03372	SLC33A1, ACA	map00604	Glycosphingolipid biosynthe	COG0477	S:Function unknown	PF13000.10	Acatn	Acetyl-coenzyme A tra	CYT	1	4	57	High
TRINITY_DN1073_c0_g1_i4_orf1	-	-	-	carboxylesterase [Loxostege sticticalis]	3.094963106	1.629922211	1.07E-05	up	yes	0.9647	0.3117	0.306	0.331	0.298	1	0.969	0.925	CC:integral compon	GO:0016021;GO:0005575;GO:0031224;GO:0110165;GO:0016787;GO:0016788;GO:0052689;GO:0003824;GO:0003674	------	------	------	------	COG2272;COG0	I:Lipid transport and metabolism	PF00135.31;PF2	COesterase;BD	Carboxylesterase family	EXC	5	14	65.7	High
TRINITY_DN43412_c0_g1_i2_orf1	-	-	-	U1 small nuclear ribonucleoprotein C [Ostrinia furnacalis]	2.685938816	1.425426441	5.64E-05	up	yes	0.957	0.3563	0.362	0.314	0.393	1	0.955	0.916	BP:macromolecular	GO:0043933;GO:0022607;GO:0071840;GO:0000387;GO:0016043;GO:0065003;GO:0022618;GO:0009987;GO:0071826;GO:0008150;GO:0000395;GO:0006376;GO:0000243;GO:0071004;GO:0120114;GO:0005575;GO:0071010;GO:0097525;GO:0032991;GO:0030532;GO:1990904;GO:0005685;GO:0005684;GO:0005681;GO:0003674;GO:0005488;GO:0003676;GO:1901363;GO:0003729;GO:0046914;GO:0043167;GO:0046872;GO:0043169;GO:0030619;GO:0097159;GO:0017069;GO:0003723;GO:0008270	K11095	SNRPC	map03040	Spliceosome	COG5136	A:RNA processing and modificat	PF06220.15	zf-U1	U1 zinc finger	CYT	1	6	17.6	High
TRINITY_DN7024_c0_g1_i1_orf1	-	-	-	uncharacterized protein LOC114363116 [Ostrinia furnacalis]	20.34523211	4.346618834	0.0004746	up	yes	1.214	0.05967	0.063	0.054	0.062	1	1.369	1.272	MF:molecular_functi	GO:0003674;GO:0008289;GO:0005488	K08753;K08752	FABP4, aP2;FA	map04923;map03	Regulation of lipolysis in adi	ENOG4111US8	I:Lipid transport and metabolism	------	------	------	CYT	7	68	15.1	High
TRINITY_DN4345_c0_g1_i9_orf1	-	-	-	uncharacterized protein LOC114357127 [Ostrinia furnacalis]	2.973636364	1.572228236	2.82E-06	up	yes	0.9813	0.33	0.325	0.322	0.343	1	0.95	0.994	------	------	K13172;K22909	SRRM2, SRM3	------	------	ENOG4110R6X	S:Function unknown	------	------	------	CYT	1	2	92.7	High
TRINITY_DN122170_c0_g1_i2_orfp1	-	-	-	TRINITY_DN122170_c0_g1_i2_m.81408 TRINITY_DN122170_c0_g1::TRINITY_DN122170_c0_g1_i2::g.81408 ORF type:internal len:87 (+),score=9.58,Baculo_E25,PF05274.12,1.7e-07 TRINITY_DN122170_c0_g1_i2:2-	6.668674699	2.737400075	9.90E-05	up	yes	1.107	0.166	0.135	0.155	0.208	1	1.13	1.191	------	------	------	------	------	------	------	------	PF05274.14	Baculo_E25	Occlusion-derived virus	CYT	1	12	9	Medium
TRINITY_DN3759_c0_g1_i1_orf1	-	-	-	uncharacterized protein LOC114350416 [Ostrinia furnacalis] >XP_028157016.1 uncharacterized protein LOC114350416 [Ostrinia furnacalis] >XP_028157017.1 uncharacterized protein LOC114350416 [Ostrinia furnacalis] >XP_028157018.1 uncharacterized protein LOC114350416 [Ostrinia furnacalis] >XP_028157019.1 uncharacterized protein LOC114350416 [Ostrinia furnacalis]	3.126385809	1.644495824	4.31E-06	up	yes	0.987	0.3157	0.326	0.292	0.329	1	1.005	0.956	CC:cellular_compon	GO:0005575;GO:0005576;GO:0110165;GO:0097367;GO:0003674;GO:0008061;GO:0005488	K22909	ZC3H13	------	------	ENOG4111GFX	S:Function unknown	PF01607.27	CBM_14	Chitin binding Peritroph	CYT	2	2	132.4	High
TRINITY_DN117_c0_g1_i5_orf1	-	-	-	lipase member I-like [Ostrinia furnacalis]	3.561397191	1.832443344	5.09E-07	up	yes	0.989	0.2777	0.269	0.265	0.299	1	0.986	0.981	BP:organic substanc	GO:0071704;GO:0006629;GO:0044238;GO:0008152;GO:0008150;GO:0005575;GO:0005576;GO:0110165;GO:0016787;GO:0016788;GO:0003824;GO:0003674;GO:0016298	------	------	------	------	ENOG41116Y2;E	S:Function unknown;	PF00151.22	Lipase	Lipase	CYT	6	21	46.9	High
TRINITY_DN109733_c0_g1_i1_orf1	-	-	-	uncharacterized protein LOC112452128 [Temnothorax curvispinosus]	4.42348907	2.145184758	0.0001895	up	yes	1.032	0.2333	0.24	0.222	0.238	1	1.148	0.947	BP:nucleic acid meta	GO:0090304;GO:0034641;GO:0006807;GO:1901360;GO:0000723;GO:0006139;GO:0051716;GO:0044260;GO:0006281;GO:0016043;GO:0071704;GO:0071840;GO:0044238;GO:0009987;GO:0006725;GO:0006974;GO:0006950;GO:0008150;GO:0008152;GO:0046483;GO:0006996;GO:0051276;GO:0050896;GO:0032200;GO:0044237;GO:0043170;GO:0033554;GO:0006259;GO:0003674;GO:0003678;GO:0140097;GO:0004386;GO:0003824;GO:0008094	------	------	------	------	COG0507;ENOG	S:Function unknown;	------	------	------	CYT	1	2	37.9	High
TRINITY_DN140669_c0_g1_i1_orf1	-	-	-	S-methyl-5'-thioadenosine phosphorylase-like isoform X1 [Hyposmocoma kahamanoa]	4.512931034	2.174064732	1.45E-05	up	yes	1.047	0.232	0.245	0.233	0.218	1	1.034	1.106	BP:nucleobase-cont	GO:0034404;GO:0044249;GO:0034641;GO:0006807;GO:0009163;GO:0044283;GO:0072521;GO:0072522;GO:1901362;GO:0042451;GO:1901360;GO:0006139;GO:0042278;GO:0071704;GO:0042455;GO:0043101;GO:0044281;GO:0018130;GO:0009119;GO:1901576;GO:0009987;GO:0006725;GO:0009058;GO:0008150;GO:0009116;GO:0008152;GO:0034654;GO:1901564;GO:0046129;GO:0046128;GO:0055086;GO:0046483;GO:0043094;GO:0044238;GO:0044271;GO:1901566;GO:1901137;GO:1901135;GO:0043174;GO:0044237;GO:1901657;GO:0006166;GO:0019438;GO:1901659;GO:0003674;GO:0004731;GO:0016740;GO:0016763;GO:0003824;GO:0017061;GO:0016757	K00772	mtaP, MTAP	map00270	Cysteine and methionine me	COG0005	F:Nucleotide transport and meta	PF01048.23	PNP_UDP_1	Phosphorylase superfan	CYT	1	10	8.1	Medium
TRINITY_DN97680_c0_g1_i1_orf1	-	-	-	39S ribosomal protein L52, mitochondrial [Ostrinia furnacalis]	2.742967131	1.455737334	0.0007135	up	yes	0.9263	0.3377	0.394	0.363	0.256	1	0.939	0.84	BP:cellular biosynthe	GO:0044249;GO:0034641;GO:0006807;GO:0034645;GO:1901576;GO:0044260;GO:0043043;GO:0071704;GO:0032543;GO:0043604;GO:0009987;GO:0009058;GO:0009059;GO:0008150;GO:0008152;GO:0044271;GO:0044238;GO:1901564;GO:0043603;GO:1901566;GO:0019538;GO:0044237;GO:0043170;GO:0006518;GO:0006412;GO:0015934;GO:0000315;GO:0032991;GO:0044391;GO:1990904;GO:0005575;GO:0005762;GO:0098798;GO:0003674;GO:0005198;GO:0003735	K17433	MRPL52	------	------	ENOG4111XV4;(	J:Translation, ribosomal structure	PF18699.4	MRPL52	Mitoribosomal protein	CYT	1	20	14.8	Medium
TRINITY_DN18172_c0_g1_i6_orf1	-	-	-	digestive cysteine proteinase 2-like [Ostrinia furnacalis]	3.788164342	1.921498921	4.81E-07	up	yes	1.005	0.2653	0.263	0.286	0.247	1	0.999	1.015	BP:primary metaboli	GO:0044238;GO:1901564;GO:0019538;GO:0043170;GO:0071704;GO:0006807;GO:0008150;GO:0008152;GO:0006508;GO:0003674;GO:0016787;GO:0003824;GO:0140096;GO:0008233;GO:0008234	------	------	------	------	COG4870	O:Posttranslational modification	PF00112.26;PF0	Peptidase_C1;I	Papain family cysteine	EXC	3	7	57.6	High
TRINITY_DN49508_c0_g2_i8_orf1	-	-	-	putative fatty acyl-CoA reductase CG5065 [Ostrinia furnacalis]	3.397212544	1.764351482	0.0001023	up	yes	0.975	0.287	0.258	0.365	0.238	1	0.991	0.934	BP:primary metaboli	GO:0044238;GO:0006629;GO:1901568;GO:0071704;GO:0008150;GO:0008152;GO:0005575;GO:0110165;GO:0016021;GO:0031224;GO:0080019;GO:0102965;GO:0003824;GO:0016620;GO:0016903;GO:0003674;GO:0016491	K13356	FAR	map04146;map00	Peroxisome;Cutin, suberine a	ENOG410XS7R	S:Function unknown	PF07993.15;PF0	NAD_binding_4	Male sterility protein;M	CYT	1	2	60.6	High
TRINITY_DN3733_c0_g1_i1_orf1	-	-	-	60S ribosomal protein L37, partial [Papilio machaon]	3.944596176	1.979877614	8.31E-05	up	yes	1.011	0.2563	0.248	0.211	0.31	1	1.079	0.955	BP:organonitrogen c	GO:1901566;GO:0034645;GO:0044249;GO:0044271;GO:0044260;GO:0043603;GO:0043043;GO:0071704;GO:0043170;GO:1901576;GO:0009987;GO:0009058;GO:0009059;GO:0008150;GO:0008152;GO:1901564;GO:0043604;GO:0044238;GO:0019538;GO:0034641;GO:0044237;GO:0006807;GO:0006518;GO:0006412;GO:0043226;GO:0043229;GO:0043228;GO:0110165;GO:0032991;GO:0005840;GO:0043232;GO:1990904;GO:0005575;GO:0003674;GO:0005488;GO:0003676;GO:0005198;GO:1901363;GO:0043169;GO:0043167;GO:0003735;GO:0046872;GO:0097159;GO:0019843;GO:0003723	K02922	RP-L37e, RPL3	map03010;map05	Ribosome;Coronavirus disea	COG2126	J:Translation, ribosomal structure	PF01907.22	Ribosomal_L37	Ribosomal protein L37e	PLA	4	30	11.4	High
TRINITY_DN5238_c0_g1_i2_orf1	-	-	-	DNA-(apurinic or apyrimidinic site) lyase [Ostrinia furnacalis]	4.156651471	2.055421785	9.96E-07	up	yes	1.003	0.2413	0.235	0.261	0.228	1	1.025	0.985	BP:nucleic acid meta	GO:0090304;GO:0034641;GO:0006807;GO:1901360;GO:0006139;GO:0051716;GO:0044260;GO:0071704;GO:0006281;GO:0009987;GO:0006725;GO:0006974;GO:0006950;GO:0008150;GO:0008152;GO:0046483;GO:0044238;GO:0050896;GO:0044237;GO:0043170;GO:0033554;GO:0006259;GO:0043169;GO:0003674;GO:0005488;GO:0003676;GO:0003677;GO:1901363;GO:0043167;GO:0046872;GO:0016787;GO:0016788;GO:0003824;GO:0004518;GO:0004519;GO:0097159	K10771	APEX1	map03410	Base excision repair	COG0708	L:Replication, recombination and	PF03372.26	Exo_endo_phos	Endonuclease/Exonucle	CYT	5	11	73.1	High
TRINITY_DN8838_c0_g1_i1_orf1	-	-	-	mannose-P-dolichol utilization defect 1 protein homolog [Ostrinia furnacalis]	2.590239677	1.373085598	0.002367	up	yes	0.897	0.3463	0.432	0.379	0.228	1	0.832	0.859	CC:cellular_compon	GO:0005575;GO:0016021;GO:0110165;GO:0031224	K09660	MPDU1	------	------	ENOG4111FE3	S:Function unknown	PF04193.17	PQ-loop	PQ loop repeat	CYT	1	4	26.8	High
TRINITY_DN5459_c0_g1_i1_orf1	-	-	-	protein takeout-like isoform X2 [Ostrinia furnacalis]	4.191391559	2.067429304	2.14E-05	up	yes	1.003	0.2393	0.265	0.183	0.27	1	1.035	0.974	------	------	------	------	------	------	ENOG4111E83;E	S:Function unknown;	PF06585.14	JHBP	Haemolymph juvenile h	CYT	1	4	21.2	Medium

TRINITY_DN4762_c0_g1_i2_orf1	-	-	-	ATPase family AAA domain-containing protein 1 isoform X2 [Ostrinia furnacalis]	5.012212995	2.325447723	0.0001098	up	yes	1.026	0.2047	0.191	0.2	0.223	1	1.128	0.949	MF:purine ribonucle	GO:0035639;GO:0003674;GO:0005488;GO:0016887;GO:0043167;GO:1901363;GO:1901265;GO:0043168;GO:0000166;GO:0017076;GO:0005524;GO:0016787;GO:0017111;GO:0036094;GO:0003824;GO:0016818;GO:0030554;GO:0097367;GO:0097159;GO:0016817;GO:0016462;GO:0032559;GO:0032555;GO:0032553	K22530	ATAD1	------	------	COG0464	S:Function unknown	PF00004.32;PF1	AAA;AAA_lid_3	ATPase family associat	CYT	1	2	43.3	High
TRINITY_DN4731_c0_g1_i1_orf1	-	-	-	gelsolin-like [Ostrinia furnacalis]	4.285104567	2.09933041	3.91E-07	up	yes	1.004	0.2343	0.217	0.249	0.237	1	1.019	0.992	------	------	K05768	GSN	map04666;map05	Fc gamma R-mediated pha	ENOG410XR0A	O:Posttranslational modification	PF00626.25	Gelsolin	Gelsolin repeat	CYT	7	15	72	High
TRINITY_DN48020_c0_g1_i1_orf1	-	-	-	aminopeptidase N4 [Cnaphalocrocis medinalis]	3.197813461	1.677085784	6.01E-05	up	yes	0.936	0.2927	0.297	0.303	0.278	1	0.877	0.931	BP:nitrogen compou	GO:0006807;GO:0071704;GO:0006508;GO:0008150;GO:0008152;GO:0044238;GO:1901564;GO:0019538;GO:0043170;GO:0016021;GO:0031224;GO:0110165;GO:0005575;GO:0004177;GO:0008238;GO:0003674;GO:0005488;GO:0008233;GO:0008237;GO:0043169;GO:0046914;GO:0043167;GO:0046872;GO:0016787;GO:0140096;GO:0003824;GO:0008270	K11140	ANPEP, CD13	map04640;map00	Hematopoietic cell lineage;(	COG0308	E:Amino acid transport and met	PF17900.4	Peptidase_M1_	Peptidase M1 N-termir	CYT	1	21	15.7	High
TRINITY_DN11259_c0_g1_i1_orf1	-	-	-	uncharacterized protein LOC114357075 [Ostrinia furnacalis]	3.810324599	1.929913905	1.56E-06	up	yes	0.9743	0.2557	0.263	0.268	0.236	1	0.962	0.961	------	------	------	------	------	------	COG1028	S:Function unknown	PF13561.9;PF00	adh_short_C2;a	Enoyl:short chain dehyc	CYT	6	46	26.7	High
TRINITY_DN1199_c0_g1_i1_orf1	-	-	-	pupal cuticle protein 36a-like [Ostrinia furnacalis]	4.740104912	2.24491899	2.99E-07	up	yes	0.994	0.2097	0.196	0.221	0.212	1	1.006	0.976	------	------	------	------	------	------	ENOG4110OGB	S:Function unknown	------	------	------	CYT	1	2	23.9	High
TRINITY_DN5578_c0_g1_i10_orf1	-	-	-	unnamed protein product [Chilo suppressalis]	4.091561181	2.032651425	1.54E-06	up	yes	0.9697	0.237	0.227	0.239	0.245	1	0.961	0.948	------	------	K23390	BAG6	------	------	ENOG410XS9P	O:Posttranslational modification	PF00240.26;PF1	ubiquitin;BAG6	Ubiquitin family;BCL2-a	CYT	1	4	62.9	High
TRINITY_DN4959_c0_g1_i1_orf1	-	-	-	pancreatic triacylglycerol lipase-like [Ostrinia furnacalis]	8.765466297	3.13183084	9.33E-06	up	yes	0.9493	0.1083	0.128	0.115	0.082	1	0.937	0.911	------	------	------	------	------	------	ENOG410YGS3;E	S:Function unknown;	PF00151.22	Lipase	Lipase	CYT	1	7	37.1	High
TRINITY_DN51766_c0_g1_i2_orf1	-	-	-	facilitated trehalose transporter Tret1-like [Ostrinia furnacalis]	6.399499061	2.677958978	3.93E-07	up	yes	1.022	0.1597	0.164	0.146	0.169	1	1.041	1.024	BP:transport;BP:carb	GO:0006810;GO:0008643;GO:0071702;GO:0008150;GO:0051234;GO:0051179;GO:0016021;GO:0005575;GO:0110165;GO:0031224;GO:0005215;GO:0022857;GO:0003674	------	------	------	------	ENOG410XNQK	E:Amino acid transport and met	PF00083.27;PF0	Sugar_tr;MFS_1	Sugar;Major Facilitator	CYT	1	3	49.7	High
TRINITY_DN9282_c0_g1_i2_orf1	-	-	-	uncharacterized protein LOC114363102 isoform X2 [Ostrinia furnacalis]	2.025867787	1.018540023	0.001235	up	yes	0.9163	0.4523	0.484	0.419	0.454	1	0.816	0.933	------	------	------	------	------	------	ENOG41120R5	S:Function unknown	------	------	------	CYT	1	10	15.1	High
TRINITY_DN3476_c0_g1_i5_orf1	-	-	-	maltase A1-like [Ostrinia furnacalis]	3.059347181	1.613223836	3.67E-06	up	yes	1.031	0.337	0.337	0.356	0.318	1	1.053	1.041	BP:primary metaboli	GO:0044238;GO:0005975;GO:0071704;GO:0008150;GO:0008152;GO:0016787;GO:0003824;GO:0016798;GO:0003674	K01187	malZ	map00500;map00	Starch and sucrose metabol	COG0366	E:Amino acid transport and met	PF00128.27	Alpha-amylase	Alpha amylase, catalytic	CYT	8	22	66.3	High
TRINITY_DN1249_c0_g1_i6_orf1	-	-	-	venom carboxylesterase-6-like [Ostrinia furnacalis]	7.439259855	2.895159092	0.0001298	up	yes	0.9247	0.1243	0.159	0.166	0.048	1	0.908	0.866	------	------	------	------	------	------	COG2272;ENOG	I:Lipid transport and metabolism	PF00135.31;PF2	COesterase;BD	Carboxylesterase family	PLA	1	6	64.7	High
TRINITY_DN7688_c0_g1_i2_orf1	-	-	-	uncharacterized protein LOC114352518 [Ostrinia furnacalis]	7.769230769	2.957771765	6.25E-06	up	yes	1.01	0.13	0.12	0.127	0.143	1	1.062	0.969	BP:phosphorylation;I	GO:0016310;GO:0009987;GO:0044237;GO:0006796;GO:0008150;GO:0008152;GO:0006793;GO:0016301;GO:0016740;GO:0003824;GO:0003674;GO:0016772	------	------	------	------	ENOG410YKCN;	S:Function unknown;	PF02958.23;PF0	EcKL;DUF1679	Ecdysteroid kinase-like	CYT	1	10	47.9	High
TRINITY_DN3082_c1_g1_i7_orf1	-	-	-	ribosomal RNA processing protein 1 homolog [Ostrinia furnacalis]	4.266718811	2.093127036	7.20E-05	up	yes	1.091	0.2557	0.232	0.241	0.294	1	1.138	1.135	BP:nucleic acid meta	GO:0090304;GO:0034641;GO:0006807;GO:0034660;GO:1901360;GO:0006139;GO:0071704;GO:0006364;GO:0034470;GO:0009987;GO:0006725;GO:0008150;GO:0008152;GO:0046483;GO:0016070;GO:0044238;GO:0016072;GO:0044237;GO:0043170;GO:0006396;GO:0030688;GO:0030684;GO:0032991;GO:1990904;GO:0005575	K14849	RRP1	------	------	ENOG4111GJ6	J:Translation, ribosomal structure	PF05997.15	Nop52	Nucleolar protein,Nop5	CYT	1	1	84.5	Medium
TRINITY_DN2815_c0_g1_i3_orf1	-	-	-	uncharacterized protein LOC114364075 [Ostrinia furnacalis]	5.188888889	2.375425643	0.0001059	up	yes	0.934	0.18	0.181	0.174	0.185	1	0.964	0.838	------	------	------	------	------	------	ENOG410XRSO	S:Function unknown	PF00650.23	CRAL_TRIO	CRAL/TRIO domain	CYT	1	3	35.8	High
TRINITY_DN753_c0_g1_i4_orf1	-	-	-	venom dipeptidyl peptidase 4-like isoform X2 [Ostrinia furnacalis]	3.136462543	1.649138333	2.22E-06	up	yes	1.009	0.3217	0.319	0.296	0.35	1	1.006	1.022	BP:primary metaboli	GO:0044238;GO:1901564;GO:0019538;GO:0043170;GO:0071704;GO:0006807;GO:0008150;GO:0008152;GO:0006508;GO:0016021;GO:0005575;GO:0110165;GO:0031224;GO:0003674;GO:0017171;GO:0016787;GO:0140096;GO:0003824;GO:0008233;GO:0008236	K01278	DPP4, CD26	map04974	Protein digestion and absor	COG1506;COG1	J:Translation, ribosomal structure	PF00930.24;PF0	DPPIV_N;Peptic	Dipeptidyl peptidase IV	CYT	3	5	92.6	High
TRINITY_DN144190_c0_g1_i1_orf1	-	-	-	PREDICTED: uncharacterized protein LOC103572804 isoform X2 [Microplitis demolitor]	10.29459372	3.363814989	1.42E-05	up	yes	0.954	0.09267	0.109	0.077	0.092	1	0.971	0.891	------	------	------	------	------	------	ENOG4112B12	S:Function unknown	------	------	------	CYT	1	3	26.8	Medium
TRINITY_DN16830_c0_g1_i5_orf1	-	-	-	adrenodoxin [Ostrinia furnacalis]	3.326790972	1.734131222	9.65E-05	up	yes	1.017	0.3057	0.256	0.319	0.342	1	1.089	0.963	BP:cellular process;B	GO:0009987;GO:0044237;GO:0022900;GO:0008152;GO:0008150;GO:0006091;GO:0051540;GO:0051536;GO:0051537;GO:0003674;GO:0005488	K22070	FDX1, ADX	------	------	COG0633	C:Energy production and conver	PF00111.30	Fer2	2Fe-2S iron-sulfur clust	CYT	1	6	17.6	High
TRINITY_DN13055_c0_g1_i5_orf1	-	-	-	116 kDa U5 small nuclear ribonucleoprotein component isoform X1 [Ostrinia furnacalis] >XP_028159219.1 116 kDa U5 small nuclear ribonucleoprotein component isoform X2 [Ostrinia furnacalis]	2.478249407	1.309321385	0.00343	up	yes	0.94	0.3793	0.368	0.376	0.394	1	0.763	1.057	BP:nucleic acid meta	GO:0090304;GO:0034641;GO:0006807;GO:1901360;GO:0006139;GO:0071704;GO:0008380;GO:0044238;GO:0006725;GO:0009987;GO:0008150;GO:0008152;GO:0046483;GO:0016070;GO:0016071;GO:0044237;GO:0043170;GO:0006396;GO:0006397;GO:0032991;GO:0005575;GO:1990904;GO:0005681;GO:0035639;GO:0032553;GO:0003674;GO:0005488;GO:0019001;GO:1901363;GO:0043168;GO:0032561;GO:0000166;GO:1901265;GO:0017076;GO:0005525;GO:0016787;GO:0003924;GO:0017111;GO:0036094;GO:0003824;GO:0016818;GO:0043167;GO:0097367;GO:0097159;GO:0016817;GO:0016462;GO:0032555	K12852	EFTUD2	map03040	Spliceosome	COG0480	J:Translation, ribosomal structure	PF03764.21;PF0	EFG_IV;EFG_C;(	Elongation factor G, do	CYT	6	8	79.1	High
TRINITY_DN2695_c0_g1_i14_orfp1	-	-	-	TRINITY_DN2695_c0_g1_i14_m.44485 TRINITY_DN2695_c0_g1::TRINITY_DN2695_c0_g1_i14::g.44485 ORF type:3prime_partial len:698 (+) score=187.51 TRINITY_DN2695_c0_g1_i14:101-2092(+)	3.321299639	1.731747885	8.14E-05	up	yes	1.012	0.3047	0.301	0.284	0.329	1	1.089	0.948	------	------	------	------	------	------	------	------	------	------	------	CYT	2	5	73.5	High
TRINITY_DN73923_c0_g1_i1_orf1	-	-	-	protein obstructor-E-like [Ostrinia furnacalis]	3.479302087	1.798797945	4.95E-06	up	yes	1.017	0.2923	0.286	0.263	0.328	1	1.037	1.014	CC:cellular_compon	GO:0005575;GO:0005576;GO:0110165;GO:0097367;GO:0003674;GO:0008061;GO:0005488	------	------	------	------	ENOG410ZSCN	S:Function unknown	PF01607.27	CBM_14	Chitin binding Peritroph	CYT	15	65	26.5	High
TRINITY_DN76377_c0_g1_i1_orf1	-	-	-	uncharacterized protein LOC111357764, partial [Spodoptera litura]	3.408707391	1.769224762	3.37E-06	up	yes	1.01	0.2963	0.292	0.271	0.326	1	1.032	0.997	------	------	------	------	------	------	ENOG410Y7Q4;E	S:Function unknown;	PF03564.18	DUF1759	Protein of unknown fun	CYT	1	4	27.4	Medium
TRINITY_DN2267_c0_g1_i1_orf1	-	-	-	hypothetical protein evm_006312 [Chilo suppressalis]	3.320958488	1.731599689	2.89E-06	up	yes	0.984	0.2963	0.315	0.309	0.265	1	0.983	0.969	BP:phosphate ion tra	GO:0035435;GO:0006820;GO:0055085;GO:1990542;GO:0098661;GO:1990547;GO:0015698;GO:0009987;GO:0006811;GO:0006810;GO:0006817;GO:0034220;GO:0008150;GO:0051234;GO:0051179;GO:0098660;GO:0098656;GO:0031224;GO:0031090;GO:0016021;GO:0016020;GO:0110165;GO:0031966;GO:0019866;GO:0005575;GO:0005743;GO:0003674;GO:0015318;GO:0005215;GO:0015291;GO:0005315;GO:0022857;GO:0022804	K15102	SLC25A3, PHC	------	------	ENOG410XPST	S:Function unknown	PF00153.30	Mito_carr	Mitochondrial carrier pr	CYT	9	19	41.3	High
TRINITY_DN139212_c0_g1_i4_orf1	-	-	-	uncharacterized protein LOC114350112 [Ostrinia furnacalis]	4.600896861	2.201915116	4.14E-06	up	yes	1.026	0.223	0.227	0.216	0.226	1	1.072	1.007	MF:catalytic activity;I	GO:0003824;GO:0003674;GO:0016491	------	------	------	------	COG1028	Z:Cytoskeleton	PF13561.9;PF00	adh_short_C2;a	Enoyl;short chain dehyc	CYT	2	8	27.3	High
TRINITY_DN17825_c1_g1_i1_orf1	-	-	-	39S ribosomal protein L1, mitochondrial [Ostrinia furnacalis]	3.533941606	1.821278201	3.93E-05	up	yes	0.9683	0.274	0.268	0.229	0.325	1	0.978	0.927	CC:ribosome;CC:intr	GO:0005840;GO:0043232;GO:0005575;GO:0043229;GO:0043228;GO:0043226;GO:0110165	K02863	RP-L1, MRPL1	map03010	Ribosome	COG0081	J:Translation, ribosomal structure	PF00687.24	Ribosomal_L1	Ribosomal protein L1p/	CYT	1	3	39.6	High
TRINITY_DN22664_c0_g1_i1_orf1	-	-	-	larval cuticle protein LCP-14-like [Ostrinia furnacalis]	4.723641431	2.239899455	3.16E-07	up	yes	1.017	0.2153	0.215	0.206	0.225	1	1.013	1.037	MF:molecular_functi	GO:0003674;GO:0042302;GO:0005198	------	------	------	------	ENOG4111PJ1;E	S:Function unknown;	PF00379.26	Chitin_bind_4	Insect cuticle protein	CYT	6	52	19.3	High
TRINITY_DN21722_c0_g1_i3_orf1	-	-	-	V-type proton ATPase 116 kDa subunit a isoform X1 [Ostrinia furnacalis] >XP_028177509.1 V-type proton ATPase 116 kDa subunit a isoform X1 [Ostrinia furnacalis]	3.961739831	1.986134141	5.82E-07	up	yes	0.9837	0.2483	0.245	0.255	0.245	1	0.992	0.959	CC:vacuolar proton-	GO:0000220;GO:0031224;GO:0005575;GO:0033179;GO:0016021;GO:0110165;GO:0032991;GO:0098796;GO:0033177;GO:0003674;GO:0042626;GO:0015318;GO:0009678;GO:0015399;GO:0042625;GO:0005215;GO:0008324;GO:0046961;GO:0044769;GO:0022890;GO:0019829;GO:0015075;GO:0022857;GO:0022804;GO:0022853;GO:0015078	K02154	ATPeV0A, ATF	map05152;map05	Tuberculosis;Rheumatoid ar	COG1269	C:Energy production and conver	PF01496.22	V_ATPase_I	V-type ATPase 116kDa	CYT	11	20	95.5	High
TRINITY_DN23175_c0_g1_i6_orf1	-	-	-	myb-binding protein 1A-like protein [Ostrinia furnacalis]	3.946153846	1.980447203	2.33E-05	up	yes	0.9747	0.247	0.27	0.248	0.223	1	0.916	1.008	------	------	K02331	POL5, MYBBP1	------	------	ENOG410YFVF;E	S:Function unknown;L:Replicatio	PF04931.16	DNA_pol_phi	DNA polymerase phi	CYT	1	1	130.2	High
TRINITY_DN30704_c0_g1_i1_orf1	-	-	-	cytochrome P450 monooxygenase CYP6AE134v2 [Ostrinia furnacalis]	5.123925139	2.357249398	1.67E-05	up	yes	1.013	0.1977	0.209	0.223	0.161	1	0.973	1.066	CC:integral compone	GO:0016021;GO:0005575;GO:0110165;GO:0031224;GO:0043169;GO:0004497;GO:0020037;GO:0046914;GO:0046906;GO:0097159;GO:0016705;GO:0043167;GO:0003824;GO:0003674;GO:0005488;GO:0005506;GO:0046872;GO:1901363;GO:0016491	K14999	CYP6	------	------	COG2124	Q:Secondary metabolites biosyr	PF00067.25	p450	Cytochrome P450	CYT	1	2	61.1	High
TRINITY_DN57202_c0_g1_i1_orf1	-	-	-	PREDICTED: U4/U6 small nuclear ribonucleoprotein Prp31 [Amyelois transitella]	3.497571909	1.806353719	3.46E-05	up	yes	0.9363	0.2677	0.282	0.267	0.254	1	0.908	0.901	BP:cellular compone	GO:0022607;GO:0000244;GO:0043933;GO:0071840;GO:0000387;GO:0016043;GO:0065003;GO:0022618;GO:0009987;GO:0071826;GO:0008150;GO:0120114;GO:0046540;GO:0097525;GO:0032991;GO:0030532;GO:1990904;GO:0005575;GO:0097526;GO:0005681;GO:0003674;GO:0005488;GO:0003676;GO:1901363;GO:0097159;GO:0003723	K12844	PRPF31	map03040	Spliceosome	COG1498	S:Function unknown	PF01798.21;PF0	Nop;Prp31_C	snoRNA binding domai	CYT	1	3	55.5	High
TRINITY_DN713_c0_g1_i4_orf1	-	-	-	periodic tryptophan protein 2 homolog isoform X1 [Ostrinia furnacalis] >XP_028176443.1 periodic tryptophan protein 2 homolog isoform X2 [Ostrinia furnacalis] >XP_028176445.1 periodic tryptophan protein 2 homolog isoform X3 [Ostrinia furnacalis]	5.443243243	2.444466507	3.80E-06	up	yes	1.007	0.185	0.165	0.16	0.23	1	1.003	1.018	------	------	K14558	PWP2, UTP1	map03008	Ribosome biogenesis in euk	ENOG410XP2P	S:Function unknown	PF00400.35;PF1	WD40;ANAPC4	WD domain, G-beta re	CYT	1	1	102.3	High
TRINITY_DN26010_c0_g1_i2_orf1	-	-	-	cytochrome b-c1 complex subunit 10-like [Ostrinia furnacalis]	4.267857143	2.093511886	2.49E-05	up	yes	0.956	0.224	0.245	0.19	0.237	1	0.965	0.903	BP:aerobic respiratic	GO:0009060;GO:0006091;GO:0022900;GO:0022904;GO:0009987;GO:0019646;GO:0045333;GO:0008152;GO:0015980;GO:0006122;GO:0044237;GO:0008150;GO:1990204;GO:0098803;GO:0070069;GO:0098800;GO:0005750;GO:1902495;GO:1902494;GO:1990351;GO:0032991;GO:0045275;GO:0005575;GO:0098796;GO:0098798	K00420	QCR10, UQCR	map04714;map00	Thermogenesis;Oxidative ph	ENOG410XVMI	C:Energy production and conver	PF08997.13	UCR_6-4kD	Ubiquinol-cytochrome	PLA	1	14	6.3	High
TRINITY_DN2673_c0_g3_i1_orf1	-	-	-	uncharacterized protein LOC114361372 [Ostrinia furnacalis]	2.185614849	1.128039191	0.001252	up	yes	0.942	0.431	0.465	0.503	0.325	1	0.937	0.889	BP:nitrogen compou	GO:0006807;GO:0071704;GO:0006508;GO:0008150;GO:0008152;GO:0044238;GO:1901564;GO:0019538;GO:0043170;GO:0004177;GO:0008238;GO:0003674;GO:0008270;GO:0008233;GO:0008237;GO:0043169;GO:0046914;GO:0043167;GO:0046872;GO:0016787;GO:0140096;GO:0003824;GO:0005488	K11140	ANPEP, CD13	map04640;map00	Hematopoietic cell lineage;(	COG0308	E:Amino acid transport and met	PF11838.11;PF0	ERAP1_C;Peptic	ERAP1-like C-terminal	CYT	1	2	115.7	High
TRINITY_DN6439_c0_g1_i1_orf1	-	-	-	GPI mannosyltransferase 3 isoform X4 [Ostrinia furnacalis] >XP_028164836.1 GPI mannosyltransferase 3 isoform X5 [Ostrinia furnacalis]	2.340632603	1.2268985	0.0003103	up	yes	0.962	0.411	0.484	0.412	0.337	1	0.958	0.928	------	------	K05286	PIGB	map00563	Glycosylphosphatidylinosito	ENOG410XYK9	S:Function unknown	PF03901.20	Glyco_transf_2	Alg9-like mannosyltran	CYT	1	3	57.2	High
TRINITY_DN7920_c0_g1_i2_orf1	-	-	-	uncharacterized protein LOC114357268 [Ostrinia furnacalis] >XP_028166599.1 uncharacterized protein LOC114357268 [Ostrinia furnacalis]	2.493761141	1.318323287	8.96E-06	up	yes	0.9793	0.3927	0.377	0.423	0.378	1	0.953	0.985	------	------	K03098	APOD	------	------	COG3040	K:Transcription	PF08212.15;PF0	Lipocalin_2;Lip	Lipocalin-like domain;L	CYT	3	14	22.3	High
TRINITY_DN3014_c0_g1_i4_orf1	-	-	-	putative inorganic phosphate cotransporter isoform X1 [Ostrinia furnacalis]	2.56222164	1.357395279	0.000131	up	yes	0.978	0.3817	0.385	0.342	0.418	1	0.91	1.024	CC:integral compone	GO:0016021;GO:0005575;GO:0110165;GO:0031224;GO:0005215;GO:0022857;GO:0003674	K12301	SLC17A5	map04142	Lysosome	ENOG410XPWC	S:Function unknown	PF07690.19;PF0	MFS_1;Sugar_tr	Major Facilitator Superf	CYT	1	1	56.6	Medium
TRINITY_DN948_c0_g1_i1_orf1	-	-	-	mitochondrial-processing peptidase subunit beta [Ostrinia furnacalis]	2.729945055	1.448871915	1.11E-06	up	yes	0.9937	0.364	0.373	0.34	0.379	1	0.997	0.984	BP:primary metaboli	GO:0044238;GO:1901564;GO:0019538;GO:0043170;GO:0071704;GO:0006807;GO:0008150;GO:0008152;GO:0006508;GO:0003674;GO:0004175;GO:0043169;GO:0016787;GO:0043167;GO:0140096;GO:0003824;GO:0004222;GO:0005488;GO:0008233;GO:0046872;GO:0008237	K17732	PMPCB, MAS1	------	------	COG0612	O:Posttranslational modification	PF05193.24;PF0	Peptidase_M16	Peptidase M16 inactive	CYT	13	44	37.5	High
TRINITY_DN49265_c0_g3_i2_orf1	-	-	-	cytochrome c [Ostrinia furnacalis] >XP_028160278.1 cytochrome c [Ostrinia furnacalis]	2.648233073	1.4050301	5.77E-07	up	yes	0.9817	0.3707	0.374	0.36	0.378	1	0.975	0.97	CC:organelle lumen;	GO:0043233;GO:0031970;GO:0031974;GO:0005575;GO:0070013;GO:0005758;GO:0070469;GO:0110165;GO:0016491;GO:0043169;GO:0020037;GO:0097159;GO:0046906;GO:0043167;GO:0003674;GO:0005488;GO:0003824;GO:0046872;GO:1901363;GO:0009055	K08738	CYC	map05167;map05	Kaposi sarcoma-associated	COG3474	C:Energy production and conver	PF00034.24;PF1	Cytochrom_C;C	Cytochrome c;Cytochrc	PLA	7	50	11.8	High
TRINITY_DN64510_c0_g1_i1_orf1	-	-	-	39S ribosomal protein L15, mitochondrial [Ostrinia furnacalis]	2.697796143	1.431781336	4.06E-05	up	yes	0.9793	0.363	0.346	0.419	0.324	1	0.983	0.955	BP:cellular biosynthe	GO:0044249;GO:0034641;GO:0006807;GO:0034645;GO:1901576;GO:0044271;GO:0044260;GO:0043043;GO:0071704;GO:0043604;GO:0009987;GO:0009058;GO:0009059;GO:0008150;GO:0008152;GO:0044238;GO:1901564;GO:0043603;GO:1901566;GO:0019538;GO:0044237;GO:0043170;GO:0006518;GO:0006412;GO:0015934;GO:0044391;GO:0032991;GO:1990904;GO:0005575;GO:0003674;GO:0005198;GO:0003735	K02876	RP-L15, MRPL	map03010	Ribosome	COG0200	U:Intracellular trafficking, secreti	PF00828.22	Ribosomal_L27	Ribosomal proteins 50S	CYT	2	7	33.9	High
TRINITY_DN1285_c0_g1_i6_orf1	-	-	-	bifunctional 3'-phosphoadenosine 5'-phosphosulfate synthase isoform X3 [Ostrinia furnacalis]	2.72838106	1.448045153	9.79E-06	up	yes	0.9623	0.3527	0.353	0.367	0.338	1	0.931	0.956	BP:sulfate assimilatic	GO:0000103;GO:0006790;GO:0009987;GO:0008150;GO:0008152;GO:0044237;GO:0035639;GO:0003674;GO:0005488;GO:0043167;GO:1901363;GO:1901265;GO:0043168;GO:0000166;GO:0016740;GO:0004779;GO:0005524;GO:0016301;GO:0004020;GO:0036094;GO:0003824;GO:0017076;GO:0004781;GO:0016779;GO:0030554;GO:0016773;GO:0016772;GO:0070566;GO:0097367;GO:0032559;GO:0097159;GO:0032555;GO:0032553	K13811	PAPSS	map00450;map00	Selenocompound metabolis	COG2046	P:Inorganic ion transport and me	PF01747.20;PF1	ATP-sulfurylase	ATP-sulfurylase;PUA-lik	CYT	5	16	43.5	High
TRINITY_DN96170_c0_g2_i1_orf1	-	-	-	uncharacterized protein LOC114355569 [Ostrinia furnacalis]	2.845430503	1.508646943	1.23E-05	up	yes	0.9683	0.3403	0.36	0.333	0.328	1	0.927	0.978	BP:phosphorylation;I	GO:0016310;GO:0009987;GO:0008150;GO:0008152;GO:0044237;GO:0006796;GO:0006793;GO:0031224;GO:0016021;GO:0005575;GO:0110165;GO:0035639;GO:0003674;GO:0005488;GO:0043167;GO:1901363;GO:0043168;GO:0000166;GO:0016740;GO:1901265;GO:0017076;GO:0005524;GO:0016301;GO:0036094;GO:0003824;GO:0030554;GO:0097367;GO:0016772;GO:0032559;GO:0032555;GO:0097159;GO:0032553	------	------	------	------	COG0574	G:Carbohydrate transport and m	PF00391.26	PEP-utilizers	PEP-utilising enzyme, n	CYT	1	4	31.7	High
TRINITY_DN1285_c0_g2_i1_orf1	-	-	-	bifunctional 3'-phosphoadenosine 5'-phosphosulfate synthase isoform X3 [Ostrinia furnacalis]	2.781213873	1.475714691	4.64E-05	up	yes	0.9623	0.346	0.327	0.363	0.348	1	0.901	0.986	------	------	K13811	PAPSS	map00450;map00	Selenocompound metabolis	COG0529;COG2	P:Inorganic ion transport and me	PF01583.23;PF1	APS_kinase;PU	Adenylylsulphate kinase	CYT	3	14	32.9	High
TRINITY_DN39673_c0_g1_i1_orf1	-	-	-	uncharacterized protein LOC114359357 isoform X1 [Ostrinia furnacalis]	2.711719418	1.439207911	4.64E-05	up	yes	0.951	0.3507	0.321	0.355	0.376	1	0.948	0.905	------	------	------	------	------	------	------	------	PF07294.14	Fibroin_P25	Fibroin P25	PLA	1	4	27.9	High
TRINITY_DN5244_c0_g1_i1_orf1	-	-	-	eukaryotic peptide chain release factor GTP-binding subunit-like [Ostrinia furnacalis]	3.294722868	1.720157119	1.14E-06	up	yes	0.9927	0.3013	0.302	0.28	0.322	1	1.001	0.977	------	------	------	------	------	------	ENOG41116ZG;E	S:Function unknown;	PF03103.20	DUF243	Domain of unknown fu	CYT	6	20	48.5	High
TRINITY_DN7213_c0_g1_i2_orf1	-	-	-	probable ATP-dependent RNA helicase CG8611 [Ostrinia furnacalis]	3.038522261	1.603369861	1.28E-05	up	yes	0.9623	0.3167	0.297	0.311	0.342	1	0.928	0.959	MF:purine ribonucle	GO:0035639;GO:0003674;GO:0005488;GO:0003676;GO:0008186;GO:0043167;GO:1901363;GO:1901265;GO:0043168;GO:0000166;GO:0017076;GO:0005524;GO:0140098;GO:0004386;GO:0036094;GO:0003824;GO:0030554;GO:0097367;GO:0097159;GO:0003724;GO:0032559;GO:0003723;GO:0032555;GO:0032553	K14806	DDX31, DBP7	------	------	ENOG410XNT7	L:Replication, recombination anc	PF00270.32;PF0	DEAD;Helicase_	DEAD/DEAH box helica	CYT	1	1	106.1	Medium
TRINITY_DN30663_c0_g1_i1_orf1	-	-	-	surfeit locus protein 6 homolog [Ostrinia furnacalis]	3.429616725	1.778047358	5.87E-06	up	yes	0.9843	0.287	0.296	0.259	0.306	1	1.002	0.951	CC:intracellular mem	GO:0043231;GO:0005634;GO:0005575;GO:0043229;GO:0043227;GO:0043226;GO:0110165	------	------	------	------	ENOG4111G0Y	S:Function unknown	PF04935.15	SURF6	Surfeit locus protein 6	CYT	2	6	33.5	High
TRINITY_DN8116_c0_g1_i2_orf1	-	-	-	uncharacterized protein LOC114350845 [Ostrinia furnacalis]	5.268357105	2.397353139	6.94E-08	up	yes	0.9973	0.1893	0.189	0.177	0.202	1	1.003	0.989	------	------	------	------	------	------	COG1028	S:Function unknown	PF13561.9;PF00	adh_short_C2;a	Enoyl:short chain dehyc	CYT	6	37	26.1	High
TRINITY_DN61674_c0_g1_i2_orf1	-	-	-	fatty acid-binding protein 1-like [Ostrinia furnacalis]	7.241134752	2.856215798	8.47E-06	up	yes	1.021	0.141	0.13	0.135	0.158	1	0.985	1.079	MF:molecular_functi	GO:0003674;GO:0008289;GO:0005488	K08753;K08752	FABP4, aP2;FA	map04923;map03	Regulation of lipolysis in adi	ENOG4111US8	I:Lipid transport and metabolism	------	------	------	CYT	2	12	14.2	High
TRINITY_DN1914_c0_g1_i6_orf1	-	-	-	loricrin-like [Ostrinia furnacalis]	9.961649089	3.316384591	1.97E-05	up	yes	1.039	0.1043	0.107	0.106	0.1	1	1.119	0.999	------	------	------	------	------	------	ENOG4110OTU;E	S:Function unknown;	------	------	------	CYT	1	15	58.9	High
TRINITY_DN64446_c0_g1_i1_orf1	-	-	-	uncharacterized protein LOC114364307 [Ostrinia furnacalis]	7.784530387	2.960610009	2.20E-07	up	yes	0.9863	0.1267	0.124	0.141	0.115	1	0.99	0.969	------	------	------	------	------	------	ENOG410Y27O	S:Function unknown	PF06757.16	Ins_allergen_rp	Insect allergen related	CYT	3	15	23.7	High
TRINITY_DN9119_c0_g1_i3_orf1	-	-	-	actin-binding Rho-activating protein [Helicoverpa armigera] >XP_047029227.1 actin-binding Rho-activating protein-like [Helicoverpa zea] >PZC85229.1 hypothetical protein B5X24_HaOG202414 [Helicoverpa armigera]	2.278502415	1.1880859	0.00022	up	yes	0.9433	0.414	0.387	0.422	0.433	1	0.962	0.868	BP:regulation of Rho	GO:0035023;GO:0048583;GO:0046579;GO:0023056;GO:0035025;GO:0023051;GO:0010647;GO:0010646;GO:0050789;GO:0009966;GO:0009967;GO:0065007;GO:0048518;GO:0051057;GO:0051056;GO:0048584;GO:0046578;GO:0050794;GO:0008150;GO:1902533;GO:1902531;GO:0048522;GO:0003674;GO:0005488;GO:0003779;GO:0008092;GO:0005515	------	------	------	------	ENOG410XR7V	S:Function unknown	PF14705.9	Costars	Costars	CYT	1	4	21.7	High
TRINITY_DN8833_c0_g1_i1_orf1	-	-	-	nucleolar protein 16 [Ostrinia furnacalis]	3.066306631	1.616501974	1.15E-05	up	yes	1.022	0.3333	0.371	0.337	0.292	1	1.04	1.026	------	------	------	------	------	------	ENOG4111OB6	S:Function unknown	PF09420.13	Nop16	Ribosome biogenesis p	CYT	2	8	24.1	High
TRINITY_DN5956_c1_g1_i5_orf1	-	-	-	uncharacterized protein DDB_G0286299-like [Ostrinia furnacalis] >XP_028170990.1 uncharacterized protein DDB_G0286299-like [Ostrinia furnacalis] >XP_028170991.1 uncharacterized protein DDB_G0286299-like [Ostrinia furnacalis]	3.199502023	1.677847379	1.07E-05	up	yes	1.028	0.3213	0.329	0.285	0.35	1	1.06	1.025	MF:organic cyclic co	GO:0097159;GO:0003674;GO:0005488;GO:0003676;GO:1901363;GO:0003723	------	------	------	------	ENOG4111E9J;E	S:Function unknown;	PF00076.25	RRM_1	RNA recognition motif.	CYT	3	7	66.4	High
TRINITY_DN3929_c0_g3_i3_orf1	-	-	-	Glutathione S-transferase 1, isoform D [Papilio machaon]	3.191222571	1.674109232	7.91E-07	up	yes	1.018	0.319	0.303	0.32	0.334	1	1.034	1.02	MF:catalytic activity;I	GO:0003824;GO:0016740;GO:0004364;GO:0003674;GO:0016765	K00799	GST, gst	map05207;map00	Chemical carcinogenesis - r	COG0625	O:Posttranslational modification	PF13417.9;PF02	GST_N_3;GST_I	Glutathione S-transfera	CYT	1	23	12.3	High
TRINITY_DN2270_c0_g2_i1_orf1	-	-	-	integrin beta-nu [Ostrinia furnacalis]	3.055708601	1.611506971	1.11E-05	up	yes	0.9983	0.3267	0.375	0.302	0.303	1	0.988	1.007	BP:cell-cell adhesion	GO:0098609;GO:0007229;GO:0009987;GO:0050794;GO:0008150;GO:0007165;GO:0007166;GO:0007155;GO:0065007;GO:0007157;GO:0050789;GO:0098742;GO:0110165;GO:0016021;GO:0005575;GO:0031224	------	------	------	------	ENOG410XP60	J:Translation, ribosomal structure	PF00362.21;PF1	Integrin_beta;P	Integrin beta chain VW	CYT	2	3	89	High
TRINITY_DN23343_c0_g1_i9_orf1	-	-	-	pre-mRNA-processing factor 6 isoform X1 [Ostrinia furnacalis] >XP_028175021.1 pre-mRNA-processing factor 6 isoform X2 [Ostrinia furnacalis]	2.948602344	1.56003127	0.000126	up	yes	0.981	0.3327	0.338	0.275	0.385	1	1.022	0.921	BP:nucleic acid meta	GO:0090304;GO:0034641;GO:0006807;GO:0000398;GO:1901360;GO:0006139;GO:0071704;GO:0008380;GO:0016071;GO:0009987;GO:0006725;GO:0000375;GO:0000377;GO:0008150;GO:0008152;GO:0046483;GO:0016070;GO:0044238;GO:0044237;GO:0043170;GO:0006396;GO:0006397	K12855	PRPF6, PRP6	map03040	Spliceosome	ENOG410XRKD	A:RNA processing and modificat	PF06424.15;PF1	PRP1_N;TPR_14	PRP1 splicing factor, N-	CYT	1	1	104.7	High
TRINITY_DN28577_c0_g1_i6_orf1	-	-	-	delta-1-pyrroline-5-carboxylate dehydrogenase, mitochondrial [Nymphalis io]	3.238504797	1.695327881	6.04E-07	up	yes	0.979	0.3023	0.309	0.295	0.303	1	0.961	0.976	BP:small molecule ca	GO:0044282;GO:0019752;GO:0009063;GO:0006807;GO:0044281;GO:0009065;GO:0009064;GO:1901575;GO:0006562;GO:0006560;GO:0006520;GO:0071704;GO:1901605;GO:1901606;GO:0044238;GO:0009987;GO:0010133;GO:0008152;GO:0043436;GO:0009056;GO:0044248;GO:0043648;GO:1901564;GO:1901565;GO:0006082;GO:0046395;GO:0016054;GO:0044237;GO:0006536;GO:0008150;GO:0003674;GO:0016491;GO:0016620;GO:0003824;GO:0016903;GO:0003842	K00294	E1.2.1.88	map00250;map00	Alanine, aspartate and gluta	COG1012	S:Function unknown	PF00171.25	Aldedh	Aldehyde dehydrogena	CYT	3	34	11.3	High
TRINITY_DN35662_c0_g1_i5_orf1	-	-	-	hypothetical protein evm_006436 [Chilo suppressalis] >CAB3522373.1 unnamed protein product [Chilo suppressalis] >CAH0399695.1 unnamed protein product [Chilo	3.979711276	1.992663768	1.40E-06	up	yes	1.02	0.2563	0.277	0.251	0.241	1	1.044	1.015	MF:hydrolase activity	GO:0016787;GO:0003674;GO:0003824	K21013	FPPP	map00981	Insect hormone biosynthesis	COG0647	G:Carbohydrate transport and m	PF13344.9;PF13	Hydrolase_6;Hy	Haloacid dehalogenase	CYT	2	6	32.9	High
TRINITY_DN4476_c0_g1_i5_orf1	-	-	-	trypsin, alkaline C-like isoform X1 [Ostrinia furnacalis]	2.834385332	1.503035905	0.0001073	up	yes	0.943	0.3327	0.285	0.331	0.382	1	0.913	0.916	BP:organic substanc	GO:0071704;GO:1901564;GO:0019538;GO:0043170;GO:0008150;GO:0006807;GO:0008152;GO:0006508;GO:0044238;GO:0004252;GO:0004175;GO:0016787;GO:0017171;GO:0140096;GO:0003824;GO:0003674;GO:0008233;GO:0008236	K01312	PRSS1_2_3	map04972;map05	Pancreatic secretion;Influenz	COG5640	O:Posttranslational modification	PF00089.29	Trypsin	Trypsin	PLA	1	22	26.3	High
TRINITY_DN5740_c0_g1_i4_orf1	-	-	-	unconventional myosin IC isoform X1 [Ostrinia furnacalis]	4.050733254	2.018183085	8.87E-07	up	yes	1.022	0.2523	0.243	0.273	0.241	1	1.033	1.033	CC:cytoplasm;CC:my	GO:0005737;GO:0016459;GO:0032991;GO:0005575;GO:0110165;GO:0035639;GO:0003674;GO:0005488;GO:0043167;GO:1901363;GO:1901265;GO:0000166;GO:0003779;GO:0017076;GO:0003774;GO:0005524;GO:0043168;GO:0036094;GO:0008092;GO:0097367;GO:0097159;GO:0030554;GO:0032559;GO:0005515;GO:0032555;GO:0032553	K10356	MYO1	map05130	Pathogenic Escherichia coli i	COG5022	S:Function unknown	PF00063.24;PF0	Myosin_head;M	Myosin head;Unconven	CYT	15	15	119.7	High
TRINITY_DN700_c0_g1_i3_orf1	-	-	-	V-type proton ATPase subunit H isoform X1 [Chelonus insularis]	4.075411151	2.026945614	9.01E-07	up	yes	1.016	0.2493	0.257	0.266	0.225	1	1.029	1.02	CC:vacuolar proton-	GO:0000221;GO:0033180;GO:0033178;GO:0032991;GO:0005575;GO:0098796;GO:0003674;GO:0015075;GO:0042626;GO:0015318;GO:0009678;GO:0015399;GO:0022804;GO:0042625;GO:0005215;GO:0008324;GO:0046961;GO:0044769;GO:0022890;GO:0019829;GO:0022857;GO:0022853;GO:0015078	K02144	ATPeV1H	map05152;map05	Tuberculosis;Human papillor	COG5231	C:Energy production and conver	PF03224.17;PF1	V-ATPase_H_N	V-ATPase subunit H;V-	CYT	1	3	59.4	High
TRINITY_DN44288_c0_g1_i2_orf1	-	-	-	ATP-dependent RNA helicase p62 [Ostrinia furnacalis]	3.566051393	1.834327495	3.88E-07	up	yes	0.9853	0.2763	0.275	0.287	0.267	1	0.967	0.989	CC:intracellular orga	GO:0043229;GO:0043227;GO:0005634;GO:0043226;GO:0043231;GO:0005575;GO:0110165;GO:0035639;GO:0003674;GO:0005488;GO:0003676;GO:0003677;GO:0008186;GO:0000166;GO:1901363;GO:1901265;GO:0043168;GO:0043167;GO:0017076;GO:0005524;GO:0016787;GO:0140098;GO:0004386;GO:0036094;GO:0003824;GO:0030554;GO:0097367;GO:0097159;GO:0003724;GO:0032559;GO:0032555;GO:0032553	K12823	DDX5, DBP2	map05205;map05	Proteoglycans in cancer;Trar	COG0513	I:Lipid transport and metabolism	PF00270.32;PF0	DEAD;Helicase_	DEAD/DEAH box helica	CYT	8	25	60.3	High
TRINITY_DN141462_c0_g1_i1_orf1	-	-	-	mitochondrial-processing peptidase subunit beta [Diachasma alloeum] >THK33262.1 core protein 1, ubiquinol-cytochrome c reductase [Diachasma alloeum]	4.205020921	2.072112978	6.64E-08	up	yes	1.005	0.239	0.247	0.231	0.239	1	1.018	0.998	BP:mitochondrial pro	GO:0034982;GO:0006807;GO:0071704;GO:0006508;GO:0006627;GO:0008150;GO:0008152;GO:0016485;GO:0044238;GO:1901564;GO:0051604;GO:0019538;GO:0043170;GO:1902494;GO:0032991;GO:0005575;GO:0017087;GO:0098798;GO:0004175;GO:0003674;GO:0005488;GO:0008233;GO:0008237;GO:0043169;GO:0043167;GO:0046872;GO:0016787;GO:0004222;GO:0140096;GO:0003824	K17732	PMPCB, MAS1	------	------	COG0612	S:Function unknown	PF00675.23;PF0	Peptidase_M16	Insulinase;Peptidase M	CYT	1	2	53.3	High

TRINITY_DN15380_c0_g1_i1_orf1	-	-	-	39S ribosomal protein L32, mitochondrial [Ostrinia furnacalis]	4.015428339	2.005553893	5.30E-05	up	yes	0.989	0.2463	0.266	0.242	0.231	1	0.916	1.051	BP:cellular biosynthe	GO:0044249;GO:0034641;GO:0006807;GO:0034645;GO:1901576;GO:0044271;GO:0044260;GO:0043043;GO:0071704;GO:0043604;GO:0009987;GO:0009058;GO:0009059;GO:0008150;GO:0008152;GO:0044238;GO:1901564;GO:0043603;GO:1901566;GO:0019538;GO:0044237;GO:0043170;GO:0006518;GO:0006412;GO:0015934;GO:0043232;GO:0043226;GO:0110165;GO:0044391;GO:0032991;GO:0005840;GO:1990904;GO:0043229;GO:0005575;GO:0043228;GO:0003674;GO:0005198;GO:0003735	K02911	RP-L32, MRPL	map03010	Ribosome	COG0333	J:Translation, ribosomal structure	------	------	------	CYT	1	5	22.6	High
TRINITY_DN3235_c0_g1_i1_orf1	-	-	-	SPARC [Trichoplusia ni]	4.570776256	2.192439199	3.62E-06	up	yes	1.001	0.219	0.218	0.224	0.215	1	1.039	0.964	CC:extracellular spac	GO:0005615;GO:0005575;GO:0110165;GO:0043169;GO:0043167;GO:0003674;GO:0005488;GO:0046872;GO:0005509	K24262	SPARC	------	------	ENOG41101WW	S:Function unknown	PF10591.12	SPARC_Ca_bdg	Secreted protein acidic	CYT	6	24	36.9	High
TRINITY_DN1310_c0_g1_i4_orf1	-	-	-	trypsin-like isoform X1 [Ostrinia furnacalis] >XP_028159118.1 trypsin-like isoform X2 [Ostrinia furnacalis]	4.7432878	2.245887407	2.60E-06	up	yes	1.007	0.2123	0.208	0.242	0.187	1	1.032	0.99	BP:organic substanc	GO:0071704;GO:1901564;GO:0019538;GO:0043170;GO:0008150;GO:0006807;GO:0008152;GO:0006508;GO:0044238;GO:0004252;GO:0004175;GO:0016787;GO:0017171;GO:0140096;GO:0003824;GO:0003674;GO:0008233;GO:0008236	------	------	------	------	ENOG411152E;C	O:Posttranslational modification	PF00089.29	Trypsin	Trypsin	CYT	3	26	26.7	High
TRINITY_DN336_c0_g1_i6_orfp1	-	-	-	TRINITY_DN336_c0_g1_i6_m.64791 TRINITY_DN336_c0_g1::TRINITY_DN336_c0_g1_i6::g.64791 ORF type:complete len:61 (-) score=19.53 TRINITY_DN336_c0_g1_i6:236-418(-)	5.62257886	2.491231991	5.26E-07	up	yes	1.016	0.1807	0.188	0.159	0.195	1	1.017	1.032	------	------	------	------	------	------	------	------	------	------	------	CYT	1	12	6.3	High
TRINITY_DN27592_c0_g1_i1_orf1	-	-	-	D-arabinitol dehydrogenase 1-like [Ostrinia furnacalis]	4.534297713	2.180879119	1.65E-06	up	yes	0.9717	0.2143	0.223	0.204	0.216	1	0.972	0.943	MF:oxidoreductase a	GO:0016491;GO:0043169;GO:0046914;GO:0043167;GO:0003824;GO:0003674;GO:0005488;GO:0046872;GO:0008270	K17818	ARD1	map00040	Pentose and glucuronate int	COG1063	E:Amino acid transport and met	PF00107.29	ADH_zinc_N	Zinc-binding dehydrog	CYT	1	59	15.1	High
TRINITY_DN2114_c0_g1_i5_orf1	-	-	-	vegetative cell wall protein gp1-like isoform X1 [Ostrinia furnacalis]	4.905669844	2.294450143	9.57E-07	up	yes	0.9777	0.1993	0.189	0.195	0.214	1	0.98	0.953	BP:biological_proces	GO:0008150;GO:0008152;GO:0032259;GO:0003824;GO:0016740;GO:0016741;GO:0003674;GO:0008168	------	------	------	------	ENOG410Y1IK;El	S:Function unknown;	PF11901.11	DUF3421	Protein of unknown fun	CYT	4	16	25.5	High
TRINITY_DN57904_c0_g2_i1_orf1	-	-	-	cuticle protein 19 [Plutella xylostella] >CAG9138481.1 unnamed protein product [Plutella xylostella]	4.235743152	2.08261511	0.0002127	up	yes	0.9433	0.2227	0.243	0.165	0.26	1	0.982	0.848	MF:molecular_functi	GO:0003674;GO:0042302;GO:0005198	------	------	------	------	ENOG410YSFI	S:Function unknown	PF00379.26	Chitin_bind_4	Insect cuticle protein	CYT	1	16	14.1	High
TRINITY_DN334_c0_g1_i4_orf1	-	-	-	collagenase-like [Ostrinia furnacalis]	11.0039224	3.459945966	1.33E-06	up	yes	1.038	0.09433	0.102	0.089	0.092	1	1.068	1.047	BP:organic substanc	GO:0071704;GO:1901564;GO:0019538;GO:0043170;GO:0008150;GO:0006807;GO:0008152;GO:0006508;GO:0044238;GO:0004252;GO:0004175;GO:0016787;GO:0017171;GO:0140096;GO:0003824;GO:0003674;GO:0008233;GO:0008236	------	------	------	------	COG5640	O:Posttranslational modification	PF00089.29	Trypsin	Trypsin	EXC	1	39	29.8	High
TRINITY_DN81803_c0_g2_i1_orf1	-	-	-	cathepsin K-like [Ostrinia furnacalis]	8.442455243	3.077662626	3.83E-08	up	yes	0.9903	0.1173	0.116	0.119	0.117	1	0.975	0.996	BP:primary metaboli	GO:0044238;GO:1901564;GO:0019538;GO:0043170;GO:0071704;GO:0006807;GO:0008150;GO:0008152;GO:0006508;GO:0003674;GO:0016787;GO:0003824;GO:0140096;GO:0008233;GO:0008234	------	------	------	------	COG4870	O:Posttranslational modification	------	------	------	CYT	1	8	13.3	High
TRINITY_DN17738_c0_g1_i2_orf1	-	-	-	unnamed protein product [Diatraea saccharalis]	2.722023646	1.444679599	3.07E-06	up	yes	0.99	0.3637	0.374	0.355	0.362	1	1.011	0.959	BP:nucleic acid meta	GO:0090304;GO:0034641;GO:0006807;GO:1901360;GO:0006139;GO:0071704;GO:0009987;GO:0006725;GO:0008150;GO:0008152;GO:0046483;GO:0016070;GO:0044238;GO:0044237;GO:0043170;GO:0006396;GO:0043227;GO:0005634;GO:0043226;GO:0110165;GO:0032991;GO:0043231;GO:1990904;GO:0043229;GO:0005575;GO:0003674;GO:0005488;GO:0003676;GO:1901363;GO:0097159;GO:0003723	K11090	LA, SSB	map05322	Systemic lupus erythematos	COG5193	G:Carbohydrate transport and m	PF05383.20;PF0	La;RRM_3;RRM	La domain;RNA binding	CYT	10	28	43.9	High
TRINITY_DN17905_c0_g3_i1_orf1	-	-	-	zinc finger protein 706-like [Ostrinia furnacalis] >XP_028176219.1 zinc finger protein 706-like [Ostrinia furnacalis] >XP_028176220.1 zinc finger protein 706-like [Ostrinia furnacalis] >XP_028176221.1 zinc finger protein 706-like [Ostrinia furnacalis]	2.741774952	1.455110158	2.35E-05	up	yes	0.9917	0.3617	0.348	0.336	0.401	1	1.021	0.954	CC:cytoplasm;CC:int	GO:0005737;GO:0043231;GO:0005634;GO:0005575;GO:0043229;GO:0043227;GO:0043226;GO:0110165	------	------	------	------	ENOG411234V	S:Function unknown	PF04419.17	4F5	4F5 protein related disc	CYT	2	18	8.7	High
TRINITY_DN108122_c0_g1_i9_orf1	-	-	-	hypothetical protein SFRUCORN_003152 [Spodoptera frugiperda]	3.003003003	1.586405918	1.08E-05	up	yes	1	0.333	0.368	0.286	0.345	1	1.004	0.997	BP:cellular compone	GO:0016043;GO:0008219;GO:0007005;GO:0071840;GO:0009987;GO:0006915;GO:0008150;GO:0006996;GO:0012501;GO:0043229;GO:0043227;GO:0043226;GO:0005739;GO:0110165;GO:0043231;GO:0005575;GO:0003674;GO:0016787;GO:0003924;GO:0017111;GO:0003824;GO:0016818;GO:0016817;GO:0016462	K17079	OPA1	map05017	Spinocerebellar ataxia	COG0699	S:Function unknown	PF19434.2	OPA1_C	Dynamin-like GTPase C	CYT	1	20	10.8	High
TRINITY_DN628_c0_g1_i7_orf1	-	-	-	prostamide/prostaglandin F synthase-like [Ostrinia furnacalis]	2.512078577	1.328881592	3.80E-05	up	yes	0.9463	0.3767	0.37	0.392	0.368	1	0.908	0.931	------	------	K15717	PRXL2B, FAM2	map00590	Arachidonic acid metabolisr	ENOG410YFY9	O:Posttranslational modification	PF13911.9	AhpC-TSA_2	AhpC/TSA antioxidant e	CYT	2	8	26.6	High
TRINITY_DN2243_c0_g1_i4_orf1	-	-	-	WD repeat-containing protein 75 [Ostrinia furnacalis]	3.791208791	1.922657911	4.05E-06	up	yes	1.035	0.273	0.267	0.268	0.284	1	1.073	1.031	------	------	K14552	NAN1, UTP17,	map03008	Ribosome biogenesis in euk	ENOG410YY9G	S:Function unknown	------	------	------	CYT	1	1	99.1	Medium
TRINITY_DN43420_c0_g2_i1_orf1	-	-	-	collagenase-like [Ostrinia furnacalis]	3.055989991	1.611639818	1.24E-05	up	yes	0.977	0.3197	0.343	0.305	0.311	1	0.998	0.933	BP:organic substanc	GO:0071704;GO:1901564;GO:0019538;GO:0043170;GO:0008150;GO:0006807;GO:0008152;GO:0006508;GO:0044238;GO:0004252;GO:0004175;GO:0016787;GO:0017171;GO:0140096;GO:0003824;GO:0003674;GO:0008233;GO:0008236	------	------	------	------	COG5640	O:Posttranslational modification	PF00089.29	Trypsin	Trypsin	CYT	4	24	29.8	High
TRINITY_DN48237_c0_g1_i5_orf1	-	-	-	myogenesis-regulating glycosidase-like [Ostrinia furnacalis]	4.505059393	2.171546126	7.28E-05	up	yes	1.024	0.2273	0.188	0.246	0.248	1	1.108	0.965	BP:primary metaboli	GO:0044238;GO:0005975;GO:0071704;GO:0008150;GO:0008152;GO:0016021;GO:0005575;GO:0031224;GO:0110165;GO:0016787;GO:0016798;GO:0003824;GO:0004553;GO:0003674	K24727	MYORG	------	------	COG1501	G:Carbohydrate transport and m	PF01055.29	Glyco_hydro_3	Glycosyl hydrolases fan	CYT	1	1	76.4	High
TRINITY_DN17864_c0_g1_i1_orf1	-	-	-	PREDICTED: erlin-2-B [Microplitis demolitor]	3.130231001	1.646269127	0.0004137	up	yes	0.935	0.2987	0.274	0.375	0.247	1	0.954	0.851	CC:endoplasmic reti	GO:0005783;GO:0016021;GO:0043231;GO:0005575;GO:0043229;GO:0031224;GO:0043227;GO:0043226;GO:0110165;GO:0031625;GO:0044389;GO:0019899;GO:0003674;GO:0005515;GO:0005488	K23341	ERLIN	------	------	ENOG410XQSH	O:Posttranslational modification	PF01145.28	Band_7	SPFH domain / Band 7	CYT	1	2	37.3	Medium
TRINITY_DN195_c4_g1_i1_orf1	-	-	-	beta-1,3-glucan-binding protein 1 [Ostrinia furnacalis]	2.93713355	1.554408866	0.0005701	up	yes	0.9017	0.307	0.372	0.26	0.289	1	0.845	0.86	BP:immune system p	GO:0002376;GO:0043207;GO:0044419;GO:0071704;GO:0045087;GO:0006952;GO:0006950;GO:0008150;GO:0008152;GO:0006955;GO:0005975;GO:0051707;GO:0044238;GO:0009607;GO:0009605;GO:0050896;GO:0098542;GO:0110165;GO:0005575;GO:0005576;GO:0030246;GO:0003674;GO:0005488;GO:0016787;GO:0016798;GO:0003824;GO:0004553	K20692	GNBP3	map04624	Toll and Imd signaling pathw	ENOG410Z83Q;I	S:Function unknown;	PF15886.8	CBM39	Carbohydrate binding c	CYT	1	19	14.1	High
TRINITY_DN6693_c0_g1_i1_orf1	-	-	-	uncharacterized protein LOC114356358 [Ostrinia furnacalis]	4.767788462	2.253320227	5.60E-07	up	yes	0.9917	0.208	0.209	0.217	0.198	1	1.008	0.967	MF:catalytic activity;I	GO:0003824;GO:0016740;GO:0016782;GO:0008146;GO:0003674	------	------	------	------	ENOG4111H56	S:Function unknown	PF00685.30;PF13	Sulfotransfer_1	Sulfotransferase domai	CYT	6	24	40.4	High
TRINITY_DN8116_c0_g1_i1_orf1	-	-	-	uncharacterized protein LOC114350845 [Ostrinia furnacalis]	4.223684211	2.078501974	6.84E-06	up	yes	0.963	0.228	0.23	0.225	0.229	1	0.971	0.918	------	------	------	------	------	------	COG1028	S:Function unknown	PF13561.9;PF00	adh_short_C2;a	Enoyl:short chain dehyc	CYT	4	26	25.5	High
TRINITY_DN3194_c0_g1_i6_orf1	-	-	-	uncharacterized protein LOC114361386 [Ostrinia furnacalis]	5.814622913	2.539685634	3.47E-06	up	yes	1.01	0.1737	0.181	0.161	0.179	1	0.977	1.052	BP:nitrogen compou	GO:0006807;GO:0071704;GO:0006508;GO:0008150;GO:0008152;GO:0044238;GO:1901564;GO:0019538;GO:0043170;GO:0016021;GO:0031224;GO:0110165;GO:0005575;GO:0004177;GO:0008238;GO:0003674;GO:0005488;GO:0008233;GO:0008237;GO:0043169;GO:0046914;GO:0043167;GO:0046872;GO:0016787;GO:0140096;GO:0003824;GO:0008270	K11140	ANPEP, CD13	map04640;map00	Hematopoietic cell lineage;(	COG0308	E:Amino acid transport and met	PF01433.23;PF11	Peptidase_M1;l	Peptidase family M1 dc	CYT	4	7	109.4	High
TRINITY_DN26408_c0_g1_i7_orf1	-	-	-	venom carboxylesterase-6-like [Ostrinia furnacalis]	5.527666857	2.46667067	6.30E-05	up	yes	0.969	0.1753	0.169	0.242	0.115	1	0.916	0.991	CC:integral compon	GO:0016021;GO:0005575;GO:0031224;GO:0110165;GO:0016787;GO:0016788;GO:0052689;GO:0003824;GO:0003674	------	------	------	------	COG2272;ENOG	I:Lipid transport and metabolism	PF00135.31;PF2(	COesterase;BD	Carboxylesterase family	PLA	1	2	64.2	High
TRINITY_DN35051_c0_g1_i1_orf1	-	-	-	uncharacterized protein LOC114364307 [Ostrinia furnacalis]	7.84651527	2.972052078	2.05E-07	up	yes	1.002	0.1277	0.127	0.127	0.129	1	1.023	0.982	------	------	------	------	------	------	ENOG410Y27O	S:Function unknown	PF06757.16	Ins_allergen_rp	Insect allergen related r	CYT	5	30	14.8	High
TRINITY_DN14679_c0_g1_i1_orf1	-	-	-	hypothetical protein evm_003043 [Chilo suppressalis]	10.19940179	3.350412634	6.49E-07	up	yes	1.023	0.1003	0.098	0.081	0.122	1	1.032	1.038	CC:cellular compon	GO:0005575;GO:0016021;GO:0110165;GO:0031224	------	------	------	------	ENOG410ZMUH	S:Function unknown;	PF13927.9;PF07(	Ig_3;I-set;Ig_2;I	Immunoglobulin domai	CYT	1	1	90	High
TRINITY_DN313_c0_g1_i5_orf1	-	-	-	collagen alpha-1(X) chain-like [Ostrinia furnacalis]	2.452261307	1.294112717	7.33E-05	up	yes	0.976	0.398	0.436	0.334	0.424	1	0.966	0.962	CC:macromolecular	GO:0032991;GO:0005581;GO:0005575;GO:0005198;GO:0003674;GO:0042302	K06237	COL4A	map05165;map04	Human papillomavirus infec	ENOG410XNMV	S:Function unknown;	PF01391.21	Collagen	Collagen triple helix rep	PLA	1	2	36.1	High
TRINITY_DN15607_c0_g1_i6_orf1	-	-	-	protein artichoke-like [Ostrinia furnacalis]	2.661831856	1.412419441	1.26E-06	up	yes	0.991	0.3723	0.373	0.374	0.37	1	1.008	0.965	CC:cellular compon	GO:0005575;GO:0016021;GO:0110165;GO:0031224	------	------	------	------	COG4886	I:Lipid transport and metabolism	PF13855.9;PF127	LRR_8;LRR_4;LR	Leucine rich repeat;Leu	CYT	5	6	109	High
TRINITY_DN19814_c0_g1_i4_orf1	-	-	-	general odorant-binding protein 28a-like [Ostrinia furnacalis]	2.899799024	1.535952915	1.13E-05	up	yes	1.01	0.3483	0.363	0.348	0.334	1	1.054	0.976	MF:odorant binding;	GO:0005549;GO:0003674;GO:0005488	------	------	------	------	ENOG4110ONN	S:Function unknown;	PF01395.25	PBP_GOBP	PBP/GOBP family	CYT	1	5	16.7	High
TRINITY_DN7964_c0_g1_i6_orfp1	-	-	-	TRINITY_DN7964_c0_g1_i6_m.23478 TRINITY_DN7964_c0_g1::TRINITY_DN7964_c0_g1_i6::g.23478 ORF type:internal len:87 (+) score=67.94 TRINITY_DN7964_c0_g1_i6:2-259(+)	3.216981132	1.685707474	4.28E-05	up	yes	1.023	0.318	0.364	0.268	0.322	1	1.071	0.999	------	------	------	------	------	------	------	------	------	------	------	CYT	2	30	9	High
TRINITY_DN1154_c0_g1_i1_orf1	-	-	-	calexcitin-1-like [Ostrinia furnacalis] >ADK94879.2 juvenile hormone diol kinase [Ostrinia furnacalis]	2.6	1.378511623	0.0005542	up	yes	0.962	0.37	0.481	0.325	0.304	1	0.945	0.941	BP:phosphorylation;I	GO:0016310;GO:0009987;GO:0044237;GO:0006796;GO:0008150;GO:0008152;GO:0006793;GO:0043169;GO:0016301;GO:0046872;GO:0016740;GO:0043167;GO:0003824;GO:0005509;GO:0003674;GO:0016772;GO:0005488	------	------	------	------	ENOG4111I7F	S:Function unknown	PF00036.35;PF13	EF-hand_1;EF-	EF hand;EF hand;EF-ha	CYT	5	35	20.6	High
TRINITY_DN3959_c1_g2_i1_orf1	-	-	-	PREDICTED: probable isocitrate dehydrogenase [NAD] subunit alpha, mitochondrial isoform X3 [Papilio xuthus] >XP_014363280.1 probable isocitrate dehydrogenase [NAD] subunit alpha, mitochondrial isoform X3 [Papilio machaon]	3.070094888	1.618283246	8.37E-07	up	yes	1.003	0.3267	0.324	0.348	0.308	1	1.015	0.995	BP:tricarboxylic acid	GO:0006099;GO:0008150;GO:0008152;GO:0044238;GO:1990204;GO:0045242;GO:0005962;GO:1902494;GO:0030062;GO:0045239;GO:0032991;GO:0005575;GO:0098798;GO:0051287;GO:0003674;GO:0005488;GO:0000166;GO:1901363;GO:1901265;GO:0016491;GO:0043169;GO:0000287;GO:0043167;GO:0004448;GO:0046872;GO:0036094;GO:0016614;GO:0004449;GO:0016616;GO:0003824;GO:0097159	K00030	IDH3	map00020	Citrate cycl	COG0473	S:Function unknown	PF00180.23	Iso_dh	Isocitrate/isopropylmal	CYT	16	55	38.7	High
TRINITY_DN18404_c0_g1_i5_orf1	-	-	-	periodic tryptophan protein 1 homolog isoform X1 [Ostrinia furnacalis] >XP_028157695.1 periodic tryptophan protein 1 homolog isoform X2 [Ostrinia furnacalis]	3.079765938	1.62282071	7.31E-07	up	yes	1	0.3247	0.323	0.305	0.346	1	1.008	0.993	BP:RNA metabolic p	GO:0016070;GO:0006139;GO:0016072;GO:0034470;GO:0044238;GO:0006396;GO:0009987;GO:0006725;GO:0044237;GO:0043170;GO:0090304;GO:0071704;GO:0034641;GO:0006807;GO:0008150;GO:0006364;GO:0008152;GO:0034660;GO:1901360;GO:0046483	K14791	PWP1	------	------	ENOG410XT1V	S:Function unknown	PF00400.35	WD40	WD domain, G-beta re	CYT	1	2	51.1	High
TRINITY_DN40_c0_g2_i1_orf1	-	-	-	trypsin CFT-1-like [Ostrinia furnacalis]	3.424840978	1.776037002	2.68E-06	up	yes	1.023	0.2987	0.295	0.297	0.304	1	1.01	1.06	BP:organic substanc	GO:0071704;GO:1901564;GO:0019538;GO:0043170;GO:0008150;GO:0006807;GO:0008152;GO:0006508;GO:0044238;GO:0004252;GO:0004175;GO:0016787;GO:0017171;GO:0140096;GO:0003824;GO:0003674;GO:0008233;GO:0008236	K01312	PRSS1_2_3	map04972;map05	Pancreatic secretion;Influenz	COG5640	O:Posttranslational modification	PF00089.29	Trypsin	Trypsin	CYT	1	3	28.5	High
TRINITY_DN72707_c0_g1_i1_orf1	-	-	-	uncharacterized protein LOC114357549 [Ostrinia furnacalis]	3.247385621	1.699278712	1.28E-07	up	yes	0.9937	0.306	0.301	0.296	0.321	1	0.989	0.992	MF:hydrolase activity	GO:0016787;GO:0003674;GO:0016788;GO:0052689;GO:0003824	K03927;K01049	CES2;ACHE	map00564;map04	Glycerophospholipid metab	COG2272	I:Lipid transport and metabolism	PF00135.31;PF2(	COesterase;BD	Carboxylesterase family	CYT	3	7	60.3	High
TRINITY_DN8087_c0_g1_i9_orf1	-	-	-	cysteine-rich with EGF-like domain protein 2 isoform X1 [Ostrinia furnacalis]	2.889865077	1.531002137	0.0001982	up	yes	0.921	0.3187	0.303	0.365	0.288	1	0.887	0.876	BP:developmental p	GO:0032502;GO:0009987;GO:0048869;GO:0048856;GO:0048468;GO:0016043;GO:0048513;GO:0008150;GO:0071840;GO:0043169;GO:0043167;GO:0005509;GO:0003674;GO:0046872;GO:0005488	K24335	CRELD	------	------	ENOG41107X2	O:Posttranslational modification	PF11938.11	DUF3456	TLR4 regulator and MIR	CYT	1	2	42.5	High
TRINITY_DN47114_c0_g1_i5_orf1	-	-	-	nucleolar protein dao-5 isoform X2 [Ostrinia furnacalis]	4.6	2.201633861	9.88E-07	up	yes	1.012	0.22	0.22	0.221	0.219	1	1.044	0.993	CC:intracellular non-	GO:0043232;GO:0005730;GO:0005575;GO:0043229;GO:0043228;GO:0043226;GO:0110165	K25818	NOLC1	------	------	ENOG4111U5P;E	S:Function unknown;	PF05022.15	SRP40_C	SRP40, C-terminal dom	CYT	7	10	72.7	High
TRINITY_DN311_c0_g1_i4_orfp1	-	-	-	TRINITY_DN311_c0_g1_i4_m.65135 TRINITY_DN311_c0_g1::TRINITY_DN311_c0_g1_i4::g.65135 ORF type:5prime_partial len:126 (+) score=71.21 TRINITY_DN311_c0_g1_i4:1-378(+)	4.102381947	2.036461818	1.12E-05	up	yes	0.9817	0.2393	0.264	0.188	0.266	1	0.966	0.979	------	------	------	------	------	------	------	------	------	------	------	EXC	1	6	13.8	High
TRINITY_DN11376_c0_g2_i1_orf1	-	-	-	cathepsin K-like [Ostrinia furnacalis]	3.79501385	1.924105151	3.65E-05	up	yes	0.959	0.2527	0.223	0.298	0.237	1	0.968	0.909	BP:primary metaboli	GO:0044238;GO:1901564;GO:0019538;GO:0043170;GO:0071704;GO:0006807;GO:0008150;GO:0008152;GO:0006508;GO:0003674;GO:0016787;GO:0003824;GO:0140096;GO:0008233;GO:0008234	------	------	------	------	COG4870	O:Posttranslational modification	PF00112.26;PF08	Peptidase_C1;I	Papain family cysteine	CYT	2	6	53.7	High
TRINITY_DN2894_c0_g2_i3_orf1	-	-	-	myrosinase 1-like isoform X1 [Ostrinia furnacalis]	3.813370474	1.931066697	9.88E-06	up	yes	0.9583	0.2513	0.233	0.243	0.278	1	0.946	0.929	BP:primary metaboli	GO:0044238;GO:0005975;GO:0071704;GO:0008150;GO:0008152;GO:0016787;GO:0003824;GO:0016798;GO:0004553;GO:0003674	------	------	------	------	COG2723	G:Carbohydrate transport and m	PF00232.21	Glyco_hydro_1	Glycosyl hydrolase fam	CYT	1	6	50.1	High
TRINITY_DN2894_c0_g1_i2_orf1	-	-	-	myrosinase 1-like isoform X1 [Ostrinia furnacalis]	5.040201005	2.33348127	3.06E-06	up	yes	1.003	0.199	0.202	0.2	0.195	1	1.042	0.968	BP:primary metaboli	GO:0044238;GO:0005975;GO:0071704;GO:0008150;GO:0008152;GO:0016787;GO:0003824;GO:0016798;GO:0004553;GO:0003674	------	------	------	------	COG2723	G:Carbohydrate transport and m	PF00232.21	Glyco_hydro_1	Glycosyl hydrolase fam	CYT	6	17	59.3	High
TRINITY_DN104586_c0_g1_i1_orf1	-	-	-	Chlorophyll a-b binding protein 37, chloroplastic, partial [Trichinella patagoniensis]	3.468976018	1.794509867	0.0001317	up	yes	0.9113	0.2627	0.257	0.274	0.257	1	0.863	0.871	CC:cellular compon	GO:0005575;GO:0016020;GO:0110165	K08913	LHCB2	------	------	ENOG410ZHBU	L:Replication, recombination anc	------	------	------	CYT	1	15	10.4	High
TRINITY_DN38498_c0_g3_i1_orf1	-	-	-	unnamed protein product [Parnassius apollo]	3.788691705	1.921699749	0.008057	up	yes	0.918	0.2423	0.398	0.169	0.16	1	0.693	1.061	------	------	------	------	------	------	ENOG410ZSXY;E	S:Function unknown;	------	------	------	CYT	1	3	36.4	Medium
TRINITY_DN19244_c0_g1_i7_orf1	-	-	-	uncharacterized protein LOC114350218 [Ostrinia furnacalis]	5.346644481	2.41863375	4.01E-06	up	yes	0.964	0.1803	0.177	0.159	0.205	1	0.946	0.946	------	------	K16830	AURKAIP1	------	------	ENOG410Y2DU;(	S:Function unknown;C:Energy pr	------	------	------	CYT	1	4	25.6	High
TRINITY_DN78546_c0_g5_i1_orf1	-	-	-	kinesin-like protein KIF13A isoform X9 [Cephus cinctus]	2.773648649	1.471785046	0.006317	up	yes	0.821	0.296	0.344	0.321	0.223	1	0.688	0.775	------	------	K17914	KIF13	------	------	COG5059	O:Secondary metabolites biosyr	PF16183.8;PF004	Kinesin_assoc;F	Kinesin-associated;FHA	CYT	1	2	34.4	Medium
TRINITY_DN6140_c0_g3_i3_orf1	-	-	-	CD63 antigen-like [Ostrinia furnacalis]	7.591129032	2.924314474	2.85E-05	up	yes	0.9413	0.124	0.103	0.108	0.161	1	0.884	0.94	CC:cellular compon	GO:0005575;GO:0016021;GO:0110165;GO:0031224	K06497	CD63, MLA1, T	map05205;map04	Proteoglycans in cancer;Lysc	ENOG4111IRY	O:Posttranslational modification	PF00335.23	Tetraspanin	Tetraspanin family	CYT	1	4	32.2	High
TRINITY_DN117_c0_g1_i4_orf1	-	-	-	lipase member I-like [Ostrinia furnacalis]	13.73377982	3.779656835	1.17E-07	up	yes	0.9843	0.07167	0.067	0.07	0.078	1	0.988	0.965	BP:organic substanc	GO:0071704;GO:0006629;GO:0044238;GO:0008152;GO:0008150;GO:0005575;GO:0005576;GO:0110165;GO:0016787;GO:0016788;GO:0003824;GO:0003674;GO:0016298	------	------	------	------	ENOG41116Y2;E	S:Function unknown;	PF00151.22	Lipase	Lipase	CYT	5	82	16.1	High
TRINITY_DN19917_c0_g1_i1_orf1	-	-	-	synaptic vesicle glycoprotein 2B-like isoform X4 [Ostrinia furnacalis]	15.69158424	3.971919111	6.11E-07	up	yes	0.9677	0.06167	0.06	0.063	0.062	1	0.951	0.952	CC:integral compon	GO:0016021;GO:0005575;GO:0110165;GO:0031224;GO:0005215;GO:0022857;GO:0003674	K06258	SV2	map04512	ECM-receptor interaction	ENOG410ZYFU;E	G:Carbohydrate transport and m	PF07690.19;PF0(	MFS_1;Sugar_tr	Major Facilitator Superf	CYT	1	2	57.3	High
TRINITY_DN14937_c0_g1_i7_orf1	-	-	-	multidrug resistance protein homolog 49-like [Ostrinia furnacalis] >XP_028159925.1 multidrug resistance protein homolog 49-like [Ostrinia furnacalis]	2.411852219	1.270141512	2.22E-05	up	yes	0.9727	0.4033	0.441	0.385	0.384	1	0.943	0.975	CC:integral compon	GO:0016021;GO:0031224;GO:0005575;GO:0110165;GO:0035639;GO:0003674;GO:0005488;GO:0043167;GO:1901363;GO:1901265;GO:0043168;GO:0042626;GO:0000166;GO:0015399;GO:0017076;GO:0005524;GO:0022804;GO:0005215;GO:0140359;GO:0036094;GO:0030554;GO:0097367;GO:0097159;GO:0022857;GO:0032559;GO:0032555;GO:0032553	K05658	ABCB1, CD24	map05206;map02	MicroRNAs in cancer;ABC tr	COG1132	S:Function unknown	PF00664.26;PF0(	ABC_membran	ABC transporter transm	CYT	3	2	143.2	High
TRINITY_DN37538_c0_g2_i1_orf1	-	-	-	esterase FE4-like [Ostrinia furnacalis]	3.42394822	1.775660884	4.27E-05	up	yes	1.058	0.309	0.317	0.34	0.27	1	1.059	1.114	------	------	K07378	NLGN	map04514	Cell adhesion molecules	COG2272	I:Lipid transport and metabolism	PF00135.31	COesterase	Carboxylesterase family	CYT	3	31	15.9	High
TRINITY_DN12242_c0_g1_i5_orf1	-	-	-	heterogeneous nuclear ribonucleoprotein 87F-like isoform X1 [Vanessa tameamea] >XP_046967652.1 heterogeneous nuclear ribonucleoprotein 87F-like isoform X1 [Vanessa cardui] >XP_047532045.1 heterogeneous nuclear ribonucleoprotein 87F-like [Vanessa atalanta]	2.813284957	1.49225569	1.25E-06	up	yes	1.008	0.3583	0.384	0.346	0.345	1	1.018	1.006	MF:organic cyclic co	GO:0097159;GO:0003674;GO:0005488;GO:0003676;GO:1901363;GO:0003723	K12741	HNRNPA1_3	map05014;map03	Amyotrophic lateral sclerosi	COG0724	S:Function unknown	PF00076.25;PF16	RRM_1;RRM_7	RNA recognition motif.;	CYT	8	41	34	High
TRINITY_DN2600_c0_g1_i7_orf1	-	-	-	mucin-5AC isoform X2 [Ostrinia furnacalis]	2.737476047	1.452846341	5.44E-07	up	yes	1	0.3653	0.358	0.351	0.387	1	1.001	1	------	------	------	------	------	------	ENOG4112A7J	S:Function unknown	------	------	------	CYT	9	12	98	High
TRINITY_DN6685_c0_g1_i8_orf1	-	-	-	cleft lip and palate transmembrane protein 1 homolog [Ostrinia furnacalis]	3.051515152	1.609525754	5.09E-07	up	yes	1.007	0.33	0.351	0.319	0.32	1	1.004	1.016	CC:cellular compon	GO:0005575;GO:0016021;GO:0110165;GO:0031224	------	------	------	------	ENOG410XPEV	S:Function unknown	PF05602.15	CLPTM1	Cleft lip and palate trar	CYT	3	7	72.4	High
TRINITY_DN79319_c0_g1_i8_orfp1	-	-	-	TRINITY_DN79319_c0_g1_i8_m.49956 TRINITY_DN79319_c0_g1::TRINITY_DN79319_c0_g1_i8::g.49956 ORF type:5prime_partial len:84 (+) score=1.39 TRINITY_DN79319_c0_g1_i8:1-252(+)	3.22875817	1.690979389	2.51E-06	up	yes	0.988	0.306	0.306	0.332	0.28	1	0.993	0.971	------	------	------	------	------	------	------	------	------	------	------	CYT	2	20	9.6	High
TRINITY_DN8543_c0_g1_i1_orf1	-	-	-	39S ribosomal protein L38, mitochondrial [Ostrinia furnacalis]	3.531316726	1.820206223	2.27E-06	up	yes	0.9923	0.281	0.296	0.254	0.293	1	1.007	0.97	------	------	K17419	MRPL38	------	------	COG1881	S:Function unknown	PF01161.23	PBP	Phosphatidylethanolam	CYT	1	4	46.4	High
TRINITY_DN3614_c0_g2_i1_orf1	-	-	-	PC4 and SFRS1-interacting protein isoform X4 [Galleria mellonella]	3.491843972	1.803989096	9.32E-05	up	yes	0.9847	0.282	0.257	0.236	0.353	1	0.934	1.02	CC:intracellular mem	GO:0043231;GO:0005634;GO:0005575;GO:0043229;GO:0043227;GO:0043226;GO:0110165	K25057	PSIP1, LEDGF	map03250	Viral life cycle - HIV-1	ENOG4111K9I	S:Function unknown	------	------	------	CYT	1	13	11.9	High
TRINITY_DN98091_c0_g1_i3_orf1	-	-	-	UDP-glycosyltransferase UGT40AP2, partial [Ostrinia furnacalis]	3.104693141	1.634450684	2.65E-05	up	yes	0.946	0.3047	0.321	0.311	0.282	1	0.923	0.915	CC:integral compon	GO:0016021;GO:0005575;GO:0110165;GO:0031224;GO:0003824;GO:0016740;GO:0003674;GO:0016757;GO:0016758;GO:0015020;GO:0008194	K00699	UGT	map05207;map05	Chemical carcinogenesis - r	COG1819	S:Function unknown	PF00201.21;PF04	UDPGT;Glyco_t	UDP-glucoronosyl and	CYT	4	11	57.4	High
TRINITY_DN29034_c0_g1_i2_orf1	-	-	-	trypsin-like serine protease [Ostrinia nubilalis]	4.204783886	2.072031652	1.45E-07	up	yes	1.002	0.2383	0.255	0.228	0.232	1	1.011	0.996	BP:organic substanc	GO:0071704;GO:1901564;GO:0019538;GO:0043170;GO:0008150;GO:0006807;GO:0008152;GO:0006508;GO:0044238;GO:0004252;GO:0004175;GO:0016787;GO:0017171;GO:0140096;GO:0003824;GO:0003674;GO:0008233;GO:0008236	K01312	PRSS1_2_3	map04972;map05	Pancreatic secretion;Influenz	COG5640	O:Posttranslational modification	PF00089.29	Trypsin	Trypsin	CYT	1	8	15.3	High
TRINITY_DN48410_c0_g2_i1_orf1	-	-	-	alpha-amylase 2-like isoform X3 [Ostrinia furnacalis]	4.051503914	2.018457534	3.15E-06	up	yes	0.9833	0.2427	0.248	0.242	0.238	1	1.006	0.944	BP:organic substanc	GO:0071704;GO:0044238;GO:0005975;GO:0008152;GO:0008150;GO:0016787;GO:0043169;GO:0016160;GO:0016798;GO:0043167;GO:0003824;GO:0004556;GO:0003674;GO:0005488;GO:0004553;GO:0046872	K01176	AMY, amyA, m	map04972;map04	Pancreatic secretion;Carboh	COG0366	G:Carbohydrate transport and m	PF00128.27;PF02	Alpha-amylase	Alpha amylase, catalytic	EXC	8	15	56.5	High
TRINITY_DN21035_c0_g1_i14_orf1	-	-	-	mitochondrial amidoxime reducing component 2-like [Ostrinia furnacalis]	3.865750101	1.950748378	1.65E-05	up	yes	0.956	0.2473	0.277	0.226	0.239	1	0.953	0.915	MF:anion binding;MF	GO:0043168;GO:0043169;GO:0030151;GO:0046872;GO:0046914;GO:0097159;GO:0019842;GO:0070279;GO:0036094;GO:0003824;GO:0016740;GO:0003674;GO:0030170;GO:0043167;GO:1901363;GO:0005488	------	------	------	------	COG3217	S:Function unknown	PF03476.19	MOSC_N	MOSC N-terminal beta	CYT	1	41	15.6	High
TRINITY_DN868_c0_g1_i4_orf1	-	-	-	uncharacterized protein LOC114359357 isoform X1 [Ostrinia furnacalis]	5.017526289	2.326976271	3.58E-07	up	yes	1.002	0.1997	0.211	0.187	0.201	1	1.021	0.985	CC:cellular compon	GO:0005575;GO:0005576;GO:0110165;GO:0003674;GO:0005198	------	------	------	------	------	------	PF07294.14	Fibroin_P25	Fibroin P25	CYT	1	5	22.6	High
TRINITY_DN61112_c0_g1_i4_orfp1	-	-	-	TRINITY_DN61112_c0_g1_i4_m.53012 TRINITY_DN61112_c0_g1::TRINITY_DN61112_c0_g1_i4::g.53012 ORF type:3prime_partial len:87 (-) score=12.29 HMMR_N PF15905.6 0.0011 TRINITY_DN61112_c0_g1_i4:2-232(-)	4.960466295	2.310475744	4.92E-07	up	yes	0.9787	0.1973	0.209	0.2	0.183	1	0.971	0.965	------	------	------	------	------	------	------	------	------	------	------	CYT	3	39	9.2	High
TRINITY_DN4612_c0_g1_i1_orf1	-	-	-	uncharacterized protein LOC114362092 [Ostrinia furnacalis]	4.738831615	2.244531399	4.13E-05	up	yes	0.9653	0.2037	0.222	0.185	0.204	1	1.006	0.89	CC:cellular compon	GO:0005575;GO:0016021;GO:0110165;GO:0031224	------	------	------	------	ENOG4110SII	S:Function unknown	------	------	------	CYT	1	1	70.8	Medium
TRINITY_DN22430_c0_g3_i1_orf1	-	-	-	V-type proton ATPase 16 kDa proteolipid subunit c [Homo sapiens] >NP_001685.1 V-type proton ATPase 16 kDa proteolipid subunit c [Homo sapiens] >P27449.1 RecName: Full=V-type proton ATPase 16 kDa proteolipid subunit c; Short=V-ATPase 16 kDa proteolipid subunit c; AltName: Full=Vacuolar proton pump 16 kDa proteolipid subunit c [Homo sapiens] >6WLW_1 The Vo region of human V-ATPase in state 1 (focused refinement) [Homo sapiens] >6WLW_2 The Vo region of human V-ATPase in state 1 (focused refinement) [Homo sapiens] >6WLW_3 The Vo region of human V-ATPase in state 1 (focused refinement) [Homo sapiens] >6WLW_4 The Vo region of human V-ATPase in state 1 (focused refinement) [Homo sapiens] >6WLW_5 The Vo region of human V-ATPase in state 1 (focused refinement) [Homo sapiens] >6WLW_6 The Vo region of human V-ATPase in state 1 (focused refinement) [Homo sapiens] >6WLW_7 The Vo region of human V-ATPase in state 1 (focused refinement) [Homo sapiens] >6WLW_8 The Vo region of human V-ATPase in state 1 (focused refinement) [Homo sapiens] >6WLW_9 The Vo region of human V-ATPase in state 1 (focused refinement) [Homo sapiens] >6WM2_1 Human V-ATPase in state 1 with SidK and ADP [Homo sapiens] >6WM2_2 Human V-ATPase in state 1 with SidK and ADP [Homo sapiens] >6WM2_3 Human V-ATPase in state 1 with SidK and ADP [Homo sapiens] >6WM2_4 Human V-ATPase in state 1 with SidK and ADP [Homo sapiens] >6WM2_5 Human V-ATPase in state 1 with SidK and ADP [Homo sapiens] >6WM2_6 Human V-ATPase in state 1 with SidK and ADP [Homo sapiens] >6WM2_7 Human V-ATPase in state 1 with SidK and ADP [Homo sapiens] >6WM2_8 Human V-ATPase in state 1 with SidK and ADP [Homo sapiens] >6WM2_9 Human V-ATPase in state 1 with SidK and ADP [Homo sapiens] >6WM3_1 Human V-ATPase in state 2 with SidK and ADP [Homo sapiens] >6WM3_2 Human V-ATPase in state 2 with SidK and ADP [Homo sapiens] >6WM3_3 Human V-ATPase in state 2 with SidK and ADP [Homo sapiens] >6WM3_4 Human V-ATPase in state 2 with SidK and ADP [Homo sapiens] >6WM3_5 Human V-ATPase in state 2 with SidK and ADP [Homo sapiens] >6WM3_6 Human V-ATPase in state 2 with SidK and ADP [Homo sapiens] >6WM3_7 Human V-ATPase in state 2 with SidK and ADP [Homo sapiens] >6WM3_8 Human V-ATPase in state 2 with SidK and ADP [Homo	9.039704524	3.176275617	2.45E-06	up	yes	0.979	0.1083	0.095	0.115	0.115	1	1	0.937	BP:regulation of met	GO:0019222;GO:0048584;GO:0048583;GO:0030111;GO:0098771;GO:0009966;GO:0009967;GO:0048518;GO:0019725;GO:0045851;GO:0048878;GO:0009894;GO:0030177;GO:0061795;GO:0050789;GO:0006885;GO:0065007;GO:0098662;GO:0098660;GO:0065008;GO:0006810;GO:0006812;GO:0006811;GO:0050794;GO:0008150;GO:0051234;GO:1902600;GO:0050801;GO:0023056;GO:0048388;GO:0007042;GO:0023051;GO:0051453;GO:0051452;GO:0010647;GO:0010646;GO:0009987;GO:0006873;GO:0030004;GO:0030003;GO:0055080;GO:0055082;GO:0055085;GO:0007035;GO:0035751;GO:0031329;GO:0031323;GO:0042592;GO:0030641;GO:0010506;GO:0016241;GO:0034220;GO:0055067;GO:0051179;GO:0098655;GO:0048522;GO:0035577;GO:0030054;GO:0030055;GO:0031982;GO:0016021;GO:0016020;GO:0005774;GO:0099501;GO:0098588;GO:0043230;GO:0065010;GO:0098852;GO:0005925;GO:0043227;GO:0110165;GO:0070161;GO:0012506;GO:0030672;GO:0030670;GO:0000139;GO:0033179;GO:0016469;GO:0033176;GO:0033177;GO:0101003;GO:0031224;GO:0010008;GO:0030665;GO:0030667;GO:0030666;GO:0031090;GO:0030662;GO:0030658;GO:0000220;GO:0070821;GO:0070062;GO:0098796;GO:0043226;GO:0005886;GO:1903561;GO:0032991;GO:0030659;GO:0005575;GO:0005765;GO:0005261;GO:0046961;GO:0022804;GO:0003674;GO:0005488;GO:0042625;GO:0042626;GO:0015318;GO:0009678;GO:0015399;GO:0015252;GO:0022803;GO:0016787;GO:0003824;GO:0022890;GO:0046933;GO:0015075;GO:0015267;GO:0015078;GO:0019899;GO:0019829;GO:0005215;GO:0005216;GO:0031625;GO:0005515;GO:0044389;GO:0008324;GO:0016874;GO:0044769;GO:0022857;GO:0022853	K02155	ATPeV0C, ATP	map05152;map05	Tuberculosis;Rheumatoid ar	COG0636	S:Function unknown	PF00137.24	ATP-synt_C	ATP synthase subunit C	CYT	1	12	15.7	Medium
TRINITY_DN76815_c0_g1_i3_orf1	-	-	-	5-formyltetrahydrofolate cyclo-ligase [Ostrinia furnacalis]	7.367281476	2.881132364	9.60E-05	up	yes	0.9187	0.1247	0.176	0.082	0.116	1	0.857	0.899	MF:purine ribonuclec	GO:0035639;GO:0016882;GO:0003674;GO:0005488;GO:0043167;GO:1901363;GO:1901265;GO:0043168;GO:0043169;GO:0000166;GO:0016879;GO:0017076;GO:0046872;GO:0005524;GO:0036094;GO:0003824;GO:0016874;GO:0030272;GO:0030554;GO:0097367;GO:0097159;GO:0032559;GO:0032555;GO:0032553	K01934	MTHFS	map00670	One carbon pool by folate	COG0212	H:Coenzyme transport and meta	PF01812.23	5-FTHF_cyc-lig	5-formyltetrahydrofola	CYT	1	61	14.2	High

TRINITY_DN327_c1_g1_i4_orf1	-	-	-	mitochondrial import receptor subunit TOM40 homolog 1-like [Ostrinia furnacalis]	2.734340659	1.451192993	5.62E-07	up	yes	0.9953	0.364	0.381	0.359	0.352	1	0.982	1.004	BP:protein localizatic	GO:0008104;GO:0051649;GO:0030150;GO:0071806;GO:0070727;GO:0051179;GO:0071705;GO:0065002;GO:1990542;GO:0071702;GO:0033036;GO:0006810;GO:0072594;GO:0009987;GO:0045184;GO:0033365;GO:0008150;GO:0072655;GO:0006839;GO:0051234;GO:0055085;GO:0044743;GO:0051641;GO:0070585;GO:0046907;GO:0015031;GO:0006886;GO:0031224;GO:0031090;GO:0016021;GO:0016020;GO:0110165;GO:0031968;GO:0098588;GO:0031966;GO:0019867;GO:0005575;GO:0005741;GO:0140318;GO:0003674;GO:0022884;GO:0005215;GO:0008320;GO:0022857	K11518	TOM40	map05022;map05	Pathways of neurodegenera	ENOG410XS2U	O:Posttranslational modification	PF01459.25	Porin_3	Eukaryotic porin	CYT	6	26	34	High
TRINITY_DN6710_c0_g1_i6_orf1	-	-	-	multiple C2 and transmembrane domain-containing protein-like [Ostrinia furnacalis]	3.140067135	1.650795404	2.90E-05	up	yes	1.029	0.3277	0.332	0.347	0.304	1	0.997	1.09	CC:cellular_compon	GO:0005575;GO:0016021;GO:0110165;GO:0031224	------		------	------	COG5038;ENOG	H:Coenzyme transport and meta	PF00168.33	C2	C2 domain	PLA	1	1	69.9	Medium
TRINITY_DN32601_c0_g1_i2_orf1	-	-	-	uncharacterized protein LOC114363197 [Ostrinia furnacalis]	3.002657219	1.586239787	9.19E-05	up	yes	1.017	0.3387	0.364	0.259	0.393	1	1.042	1.008	------	------	------	------	------	------	ENOG410XRFE	S:Function unknown	PF03022.19	MRJP	Major royal jelly protein	CYT	1	3	48.2	High
TRINITY_DN17351_c0_g1_i3_orf1	-	-	-	V-type proton ATPase subunit F [Ostrinia furnacalis]	3.04822566	1.607969709	1.22E-06	up	yes	1.005	0.3297	0.324	0.313	0.352	1	1.021	0.993	CC:proton-transport	GO:0033180;GO:0033178;GO:0032991;GO:0005575;GO:0098796;GO:0003674;GO:0015075;GO:0042626;GO:0015318;GO:0009678;GO:0015399;GO:0022804;GO:0042625;GO:0005215;GO:0008324;GO:0046961;GO:0044769;GO:0022890;GO:0019829;GO:0022857;GO:0022853;GO:0015078	K02151	ATPeV1F, ATP	map05323;map00	Rheumatoid arthritis;Oxidati	COG1436	C:Energy production and conver	PF01990.20	ATP-synt_F	ATP synthase	CYT	6	40	13.8	High
TRINITY_DN430_c0_g1_i5_orf1	-	-	-	hypothetical protein NE865_02252 [Phthorimaea operculella]	3.120743034	1.641889569	8.42E-07	up	yes	1.008	0.323	0.319	0.308	0.342	1	0.998	1.025	MF:cation binding;M	GO:0043169;GO:0046914;GO:0097159;GO:0043167;GO:0003674;GO:0005488;GO:0003676;GO:0046872;GO:1901363;GO:0008270	------	------	------	------	ENOG4110QHB	S:Function unknown	------	------	------	CYT	5	8	83.6	High
TRINITY_DN94248_c0_g2_i3_orf1	-	-	-	uncharacterized protein LOC114357292 isoform X4 [Ostrinia furnacalis]	2.850437318	1.511183276	1.06E-06	up	yes	0.9777	0.343	0.332	0.342	0.355	1	0.968	0.965	------	------	------	------	------	------	COG1028;ENOG	C:Energy production and conver	PF13561.9;PF001	adh_short_C2;a	Enoyl:short chain dehyc	CYT	7	29	28.7	High
TRINITY_DN26186_c0_g1_i7_orf1	-	-	-	sodium- and chloride-dependent glycine transporter 1-like [Ostrinia furnacalis]	3.462717058	1.791904508	1.49E-05	up	yes	1.017	0.2937	0.303	0.288	0.29	1	0.978	1.072	CC:integral compon	GO:0016021;GO:0005575;GO:0031224;GO:0110165;GO:0015291;GO:0015293;GO:0005215;GO:0022857;GO:0003674;GO:0022804	K05038	SLC6A5_9, GL	map04721	Synaptic vesicle cycle	COG0733	U:Intracellular trafficking, secreti	PF00209.21	SNF	Sodiu	CYT	1	2	71.2	High
TRINITY_DN42759_c0_g2_i1_orf1	-	-	-	fatty acid synthase-like [Ostrinia furnacalis]	2.971489233	1.571186153	2.31E-05	up	yes	0.9797	0.3297	0.337	0.335	0.317	1	1.015	0.924	BP:fatty acid biosynt	GO:0006633;GO:0006631;GO:0019752;GO:0044249;GO:0044281;GO:0044283;GO:0072330;GO:1901576;GO:0071704;GO:0006629;GO:0009987;GO:0032787;GO:0009058;GO:0008150;GO:0008152;GO:0043436;GO:0044255;GO:0008610;GO:0044238;GO:0006082;GO:0046394;GO:0016053;GO:0044237;GO:0047451;GO:0016790;GO:0016417;GO:0047117;GO:0016627;GO:0003674;GO:0016491;GO:0016628;GO:0004313;GO:0016829;GO:0004320;GO:0016407;GO:0016740;GO:0016746;GO:0016747;GO:0016418;GO:0016787;GO:0008693;GO:0016788;GO:0003824;GO:0016614;GO:0016616;GO:0016835;GO:0016836;GO:0004312;GO:0016295;GO:0016296;GO:0016297;GO:0004316;GO:0004317;GO:0004315;GO:0019171;GO:0008659	K00665	FASN	map04910;map04	Insulin signaling pathway;Alc	COG3321	Q:Secondary metabolites biosyn	PF00975.23;PF14	Thioesterase;PS	Thioesterase domain;Pc	CYT	5	3	166.4	High
TRINITY_DN52768_c0_g1_i1_orf1	-	-	-	carboxypeptidase Q-like isoform X2 [Ostrinia furnacalis]	3.714182082	1.893044543	1.14E-05	up	yes	1.024	0.2757	0.256	0.247	0.324	1	1.026	1.047	CC:lytic vacuole;CC:a	GO:0000323;GO:0005783;GO:0043229;GO:0043226;GO:0005764;GO:0005773;GO:0043227;GO:0110165;GO:0005794;GO:0043231;GO:0005575;GO:0005576;GO:0016787;GO:0008238;GO:0003674;GO:0008233;GO:0008235;GO:0008237;GO:0016805;GO:0070573;GO:0140096;GO:0003824	K01302	CPQ	------	------	COG2234	S:Function unknown	PF04389.20;PF02	Peptidase_M28	Peptidase family M28;P	CYT	1	2	52	High
TRINITY_DN2825_c0_g1_i3_orf1	-	-	-	carbonic anhydrase 2-like [Ostrinia furnacalis]	4.039735099	2.014260693	1.02E-05	up	yes	1.037	0.2567	0.216	0.276	0.278	1	1.067	1.043	MF:cation binding;M	GO:0043169;GO:0046872;GO:0046914;GO:0016835;GO:0016836;GO:0043167;GO:0003824;GO:0003674;GO:0008270;GO:0004089;GO:0016829;GO:0005488	K01672	CA	map00910	Nitrogen metabolism	COG3338	I:Lipid transport and metabolism	PF00194.24	Carb_anhydras	Eukaryotic-type carbor	CYT	2	8	30.6	High
TRINITY_DN9715_c0_g1_i1_orf1	-	-	-	V-type proton ATPase subunit E [Manduca sexta] >P31402.1 RecName: Full=V-type proton ATPase subunit E; Short=V-ATPase subunit E; AltName: Full=V-ATPase 26 kDa subunit; AltName: Full=Vacuolar proton pump subunit E [Manduca sexta] >KAG6457535.1 hypothetical protein O3G_MSEX010354 [Manduca sexta] >CAA47610.1 H(+)-transporting ATPase [Manduca sexta]	3.39680888	1.764180048	9.01E-07	up	yes	0.9793	0.2883	0.3	0.286	0.279	1	0.98	0.958	CC:proton-transport	GO:0033178;GO:0032991;GO:0005575;GO:0098796;GO:0003674;GO:0015075;GO:0042626;GO:0015318;GO:0009678;GO:0015399;GO:0022804;GO:0042625;GO:0005215;GO:0008324;GO:0046961;GO:0044769;GO:0022890;GO:0019829;GO:0022857;GO:0022853;GO:0015078	K02150	ATPeV1E, ATP	map05323;map00	Rheumatoid arthritis;Oxidati	COG1390	C:Energy production and conver	PF01991.21	vATP-synt_E	ATP synthase	CYT	12	44	26	High
TRINITY_DN8366_c0_g1_i4_orf1	-	-	-	luciferin 4-monooxygenase-like [Ostrinia furnacalis]	2.11219623	1.078743872	0.0002142	up	yes	0.93	0.4403	0.45	0.423	0.448	1	0.873	0.917	------	------	------	------	------	------	COG0318;COG0	I:Lipid transport and metabolism	PF00501.31;PF13	AMP-binding;A	AMP-binding enzyme;A	CYT	3	5	61.5	High
TRINITY_DN468_c0_g1_i3_orf1	-	-	-	transmembrane protein 41 homolog isoform X2 [Ostrinia furnacalis]	3.534892086	1.821666173	2.60E-07	up	yes	0.9827	0.278	0.284	0.282	0.268	1	0.977	0.971	------	------	------	------	------	------	COG0398	S:Function unknown	PF09335.14	SNARE_assoc	SNARE associated Golc	CYT	1	7	30.6	High
TRINITY_DN15870_c0_g1_i3_orf1	-	-	-	PREDICTED: mitochondrial import inner membrane translocase subunit Tim23 isoform X1 [Fopius arisanus]	3.699245283	1.887230963	4.64E-06	up	yes	0.9803	0.265	0.248	0.283	0.264	1	0.998	0.943	CC:integral compon	GO:0016021;GO:0005575;GO:0110165;GO:0031224;GO:0005215;GO:0022857;GO:0003674	K17794	TIM23	map04212	Longevity regulating pathwa	COG5596	O:Posttranslational modification	PF02466.22	Tim17	Tim17/Tim22/Tim23/Pr	CYT	1	5	22.1	High
TRINITY_DN145647_c0_g1_i1_orf1	-	-	-	PREDICTED: U6 snRNA-associated Sm-like protein LSm5 isoform X1 [Fopius arisanus]	2.874964559	1.523544171	5.79E-07	up	yes	1.014	0.3527	0.364	0.36	0.334	1	1.022	1.019	BP:nucleic acid meta	GO:0090304;GO:0034641;GO:0006807;GO:1901360;GO:0006139;GO:0071704;GO:0008380;GO:0016071;GO:0009987;GO:0006725;GO:0008150;GO:0008152;GO:0046483;GO:0016070;GO:0044238;GO:0044237;GO:0043170;GO:0006396;GO:0006397;GO:0005681;GO:0032991;GO:0005575;GO:1990904;GO:0003674;GO:0005488;GO:0003676;GO:1901363;GO:0097159;GO:0003723	K12624	LSM5	map03018;map03	RNA degradation;Spliceosor	COG1958	O:Posttranslational modification	PF01423.25	LSM	LSM domain	CYT	1	6	10.5	High
TRINITY_DN6747_c0_g1_i7_orf1	-	-	-	retinol dehydrogenase 12-like [Ostrinia furnacalis]	3.715909091	1.893715207	7.75E-07	up	yes	0.981	0.264	0.25	0.262	0.28	1	0.964	0.979	------	------	K11153	RDH12	map00830;map01	Retinol metabolism;Biosynth	COG1028	S:Function unknown	PF00106.28;PF13	adh_short;adh	short chain dehydroger	CYT	3	8	34.2	High
TRINITY_DN23926_c0_g1_i4_orf1	-	-	-	programmed cell death protein 10 [Ostrinia furnacalis]	2.582368421	1.368694842	9.59E-07	up	yes	0.9813	0.38	0.382	0.368	0.39	1	0.979	0.965	CC:cytoplasm;CC:cel	GO:0005737;GO:0005575;GO:0016020;GO:0110165;GO:0005886	K18269	PDCD10	------	------	ENOG410XTA6	S:Function unknown	PF06840.14	DUF1241	Protein of unknown fun	CYT	1	5	23.5	High
TRINITY_DN10332_c0_g1_i2_orfp1	-	-	-	TRINITY_DN10332_c0_g1_i2_m.42894 TRINITY_DN10332_c0_g1::TRINITY_DN10332_c0_g1_i2::g.42894 ORF type:3prime_partial len:77 (+) score=1.70 TRINITY_DN10332_c0_g1_i2:1005-1232(+)	3.418253683	1.773259469	1.51E-05	up	yes	0.9513	0.2783	0.284	0.257	0.294	1	0.932	0.922	------	------	------	------	------	------	------	------	------	------	------	CYT	1	8	9	Medium
TRINITY_DN2579_c0_g1_i7_orf1	-	-	-	aminopeptidase N5 [Ostrinia nubilalis]	2.626100881	1.392922338	2.91E-06	up	yes	0.984	0.3747	0.357	0.365	0.402	1	0.973	0.979	BP:nitrogen compou	GO:0006807;GO:0071704;GO:0006508;GO:0008150;GO:0008152;GO:0044238;GO:1901564;GO:0019538;GO:0043170;GO:0016021;GO:0031224;GO:0110165;GO:0005575;GO:0004177;GO:0008238;GO:0003674;GO:0005488;GO:0008233;GO:0008237;GO:0043169;GO:0046914;GO:0043167;GO:0046872;GO:0016787;GO:0140096;GO:0003824;GO:0008270	K11140	ANPEP, CD13	map04640;map00	Hematopoietic cell lineage;(	COG0308	L:Replication, recombination anc	PF01433.23;PF11	Peptidase_M1;E	Peptidase family M1 dc	CYT	3	3	108	High
TRINITY_DN43611_c0_g1_i1_orf1	-	-	-	39S ribosomal protein L41, mitochondrial [Ostrinia furnacalis]	3.794681267	1.923978712	2.69E-06	up	yes	0.9703	0.2557	0.271	0.242	0.254	1	0.943	0.968	CC:cellular_compon	GO:0005575;GO:0015934;GO:0000315;GO:1990904;GO:0005762;GO:0032991;GO:0044391;GO:0098798;GO:0005198;GO:0003735;GO:0003674	K17422	MRPL41	------	------	ENOG4111ND6	S:Function unknown	PF09809.12	MRP-L27	Mitochondrial ribosoma	CYT	2	25	18.2	High
TRINITY_DN53167_c0_g1_i3_orf1	-	-	-	uncharacterized protein LOC114359219 [Ostrinia furnacalis]	2.953156823	1.562257971	1.04E-05	up	yes	1.015	0.3437	0.319	0.39	0.322	1	1.023	1.023	BP:primary metaboli	GO:0044238;GO:0005975;GO:0071704;GO:0008150;GO:0008152;GO:0005737;GO:0005575;GO:0110165;GO:0016787;GO:0003824;GO:0016798;GO:0004564;GO:0004553;GO:0003674	------	------	------	------	COG1621	G:Carbohydrate transport and m	PF00251.23;PF08	Glyco_hydro_3	Glycosyl hydrolases fan	CYT	6	16	55.9	High
TRINITY_DN29018_c0_g1_i4_orf1	-	-	-	prostaglandin reductase 1-like isoform X1 [Ostrinia furnacalis] >XP_028178925.1 prostaglandin reductase 1-like isoform X2 [Ostrinia furnacalis]	4.115189873	2.040958998	1.55E-06	up	yes	0.9753	0.237	0.241	0.248	0.222	1	0.977	0.949	CC:cytoplasm;CC:cel	GO:0005737;GO:0005575;GO:0110165;GO:0036132;GO:0016614;GO:0016628;GO:0016616;GO:0016627;GO:0003824;GO:0003674;GO:0047522;GO:0032440;GO:0016491	K13948	PTGR1, LTB4D	map00590	Arachidonic acid metabolism	COG2130	S:Function unknown	PF16884.8;PF00	ADH_N_2;ADH	N-terminal domain of (	CYT	7	24	36.5	High
TRINITY_DN8536_c0_g1_i2_orf1	-	-	-	PC4 and SFRS1-interacting protein isoform X4 [Galleria mellonella]	2.553753597	1.352619331	8.02E-05	up	yes	0.9763	0.3823	0.373	0.361	0.413	1	1.017	0.912	------	------	K25057	PSIP1, LEDGF	map03250	Viral life cycle - HIV-1	ENOG4111K9I	S:Function unknown	PF11467.11	LEDGF	Lens epithelium-derive	CYT	2	6	31.1	High
TRINITY_DN334_c0_g1_i1_orf1	-	-	-	putative chymotrypsin 12 [Ostrinia nubilalis]	4.668694431	2.223019167	8.30E-07	up	yes	0.9977	0.2137	0.22	0.212	0.209	1	0.971	1.022	BP:organic substanc	GO:0071704;GO:1901564;GO:0019538;GO:0043170;GO:0008150;GO:0006807;GO:0008152;GO:0006508;GO:0044238;GO:0004252;GO:0004175;GO:0016787;GO:0017171;GO:0140096;GO:0003824;GO:0003674;GO:0008233;GO:0008236	------	------	------	------	COG5640	O:Posttranslational modification	PF00089.29	Trypsin	Trypsin	CYT	5	53	21.6	High
TRINITY_DN4194_c0_g1_i1_orf1	-	-	-	hornerin-like [Ostrinia furnacalis]	2.808006766	1.489546412	0.0009648	up	yes	0.996	0.3547	0.339	0.313	0.412	1	1.111	0.877	------	------	K19720;K19721	COL3A;COL5A	map05146;map04	Amoebiasis;Protein digestio	ENOG4111IEE;EI	S:Function unknown;	PF16058.8	Mucin-like	Mucin-like	CYT	2	1	184.6	High
TRINITY_DN47731_c0_g1_i2_orf1	-	-	-	nucleolar GTP-binding protein 2 [Ostrinia furnacalis]	5.976878613	2.579392243	0.0008071	up	yes	1.034	0.173	0.162	0.102	0.255	1	1.193	0.91	CC:organelle;CC:intr	GO:0043226;GO:0043229;GO:0043228;GO:0005730;GO:0110165;GO:0043232;GO:0005575;GO:0035639;GO:0003674;GO:0005488;GO:0043167;GO:1901363;GO:1901265;GO:0043168;GO:0000166;GO:0032561;GO:0017076;GO:0005525;GO:0036094;GO:0097367;GO:0097159;GO:0019001;GO:0032555;GO:0032553	K14537	NUG2, GNL2	map03008	Ribosome biogenesis in euk	COG1161;ENOG	S:Function unknown;	PF07572.15	BCNT	Bucentaur or craniofaci	CYT	1	4	30.3	Medium
TRINITY_DN501_c1_g1_i1_orf1	-	-	-	sodium- and chloride-dependent GABA transporter ine isoform X1 [Ostrinia furnacalis]	2.851862464	1.511904407	1.89E-05	up	yes	0.9953	0.349	0.337	0.322	0.388	1	1.025	0.961	BP:transmission of n	GO:0019226;GO:0035637;GO:0048588;GO:0048589;GO:0016049;GO:0042065;GO:0032501;GO:0050877;GO:0008150;GO:0040007;GO:0003008;GO:0023052;GO:0009987;GO:0031224;GO:0005575;GO:0016021;GO:0110165;GO:0005328;GO:0005326;GO:0060089;GO:0003674;GO:0038023;GO:0015081;GO:0015318;GO:0004888;GO:0005034;GO:0015370;GO:0005215;GO:0046873;GO:0140299;GO:0022804;GO:0015294;GO:0015291;GO:0015293;GO:0008324;GO:0022890;GO:0015075;GO:0022857;GO:0022853	K05039	SLC6A6, TAUT	------	------	COG0733	S:Function unknown	PF00209.21	SNF	Sodiu	CYT	1	1	80.8	High
TRINITY_DN50787_c0_g2_i2_orf1	-	-	-	40S ribosomal protein S29 [Hyposmocoma kahamanoa] >XP_028176503.1 40S ribosomal protein S29 [Ostrinia furnacalis] >XP_049877832.1 40S ribosomal protein S29 [Pectinophora gossypiella] >ADT80654.1 ribosomal protein S29 [Euphydryas aurinia] >CAB3523209.1 unnamed protein product [Chilo suppressalis] >CAH0400531.1 unnamed protein product [Chilo suppressalis]	4.155613351	2.055061428	1.37E-05	up	yes	0.9587	0.2307	0.243	0.206	0.243	1	0.964	0.912	BP:organonitrogen c	GO:1901566;GO:0044249;GO:0034645;GO:0044271;GO:0043043;GO:0071704;GO:0043604;GO:0002181;GO:1901576;GO:0044260;GO:0009987;GO:0009058;GO:0009059;GO:0008152;GO:1901564;GO:0044238;GO:0019538;GO:0034641;GO:0044237;GO:0043170;GO:0006807;GO:0043603;GO:0008150;GO:0006518;GO:0006412;GO:0005783;GO:0043229;GO:0043228;GO:0043227;GO:0043226;GO:0022627;GO:0110165;GO:0005791;GO:0044391;GO:0015935;GO:0043231;GO:0042788;GO:0032991;GO:0005840;GO:0043232;GO:0005829;GO:1990904;GO:0005575;GO:1901363;GO:0003674;GO:0008270;GO:0003676;GO:0043167;GO:0043169;GO:0046914;GO:0005198;GO:0003735;GO:0046872;GO:0003723;GO:0097159;GO:0005488	K02980	RP-S29e, RPS2	map03010;map05	Ribosome;Coronavirus disea	COG0199	J:Translation, ribosomal structure	PF00253.24	Ribosomal_S14	Ribosomal protein S14p	CYT	2	38	6.6	High
TRINITY_DN79804_c0_g1_i1_orf1	-	-	-	zinc finger protein on ecdysone puffs-like [Ostrinia furnacalis]	3.057651675	1.612424066	0.001439	up	yes	1.013	0.3313	0.41	0.305	0.279	1	1.153	0.886	------	------	------	------	------	------	ENOG4111SKG	S:Function unknown	------	------	------	CYT	1	21	8.9	High
TRINITY_DN3784_c0_g1_i1_orf1	-	-	-	pancreatic triacylglycerol lipase-like [Ostrinia furnacalis]	6.327575176	2.661652743	2.36E-08	up	yes	0.989	0.1563	0.157	0.159	0.153	1	0.989	0.978	BP:organic substanc	GO:0071704;GO:0006629;GO:0044238;GO:0008152;GO:0008150;GO:0005575;GO:0005576;GO:0110165;GO:0016787;GO:0016788;GO:0003824;GO:0003674;GO:0016298	------	------	------	------	ENOG41116Y2;E	S:Function unknown;	PF00151.22	Lipase	Lipase	PLA	13	30	61.9	High
TRINITY_DN113626_c0_g1_i3_orfp1	-	-	-	TRINITY_DN113626_c0_g1_i3_m.80721 TRINITY_DN113626_c0_g1::TRINITY_DN113626_c0_g1_i3::g.80721 ORF type:internal len:118 (-) score=87.34 TRINITY_DN113626_c0_g1_i3:2-352(-)	3.063199274	1.615039224	0.0001533	up	yes	1.013	0.3307	0.392	0.297	0.303	1	0.955	1.085	------	------	------	------	------	------	------	------	------	------	------	PLA	1	9	11.6	High
TRINITY_DN6044_c0_g1_i4_orf1	-	-	-	acyl-CoA-binding protein-like [Ostrinia furnacalis]	7.596081387	2.925255363	2.71E-06	up	yes	1.008	0.1327	0.136	0.124	0.138	1	1.05	0.974	MF:sulfur compound	GO:1901681;GO:0003674;GO:1901567;GO:0097367;GO:0033218;GO:0030554;GO:0032553;GO:0008289;GO:0000062;GO:0000166;GO:0036094;GO:0032555;GO:0032559;GO:0005488;GO:0097159;GO:0120227;GO:0017076;GO:1901363;GO:1901265	K08762	DBI, ACBP	map03320	PPAR signaling pathway	COG4281	I:Lipid transport and metabolism	PF00887.22	ACBP	Acyl CoA binding prote	CYT	5	54	9.5	High
TRINITY_DN14313_c0_g1_i1_orf1	-	-	-	25S rRNA (cytosine-C(5))-methyltransferase nop2 [Ostrinia furnacalis]	2.617480442	1.388178758	8.25E-06	up	yes	0.9703	0.3707	0.376	0.374	0.362	1	0.979	0.932	BP:RNA modification	GO:0009451;GO:0090304;GO:0071840;GO:0006139;GO:0044260;GO:0042254;GO:0071704;GO:1901360;GO:0022613;GO:0032259;GO:0009987;GO:0006725;GO:0043412;GO:0008150;GO:0008152;GO:0001510;GO:0046483;GO:0016070;GO:0044238;GO:0006807;GO:0034641;GO:0044237;GO:0043170;GO:0044085;GO:0043414;GO:0006396;GO:0043229;GO:0043228;GO:0043226;GO:0005730;GO:0110165;GO:0043232;GO:0005575;GO:0003674;GO:0005488;GO:0003676;GO:0008168;GO:1901363;GO:0016740;GO:0016741;GO:0008757;GO:0003824;GO:0097159;GO:0003723	K14835	NOP2	------	------	COG0144	O:Posttranslational modification	PF01189.20;PF17	Methyltr_RsmB	16S rRNA methyltransfe	CYT	9	12	95.6	High
TRINITY_DN75188_c0_g1_i1_orf1	-	-	-	fatty acid-binding protein 1-like [Ostrinia furnacalis]	7.260583942	2.860085583	9.16E-06	up	yes	0.9947	0.137	0.133	0.139	0.139	1	0.94	1.044	MF:molecular_functi	GO:0003674;GO:0008289;GO:0005488	K08753;K08752	FABP4, aP2;FA	map04923;map03	Regulation of lipolysis in adi	ENOG4111US8	I:Lipid transport and metabolism	------	------	------	CYT	1	7	14.7	High
TRINITY_DN747_c0_g1_i4_orf1	-	-	-	trypsin, alkaline C-like [Ostrinia furnacalis]	3.373770492	1.754361834	7.45E-05	up	yes	1.029	0.305	0.318	0.303	0.294	1	0.974	1.113	BP:organic substanc	GO:0071704;GO:1901564;GO:0019538;GO:0043170;GO:0008150;GO:0006807;GO:0008152;GO:0006508;GO:0044238;GO:0004252;GO:0004175;GO:0016787;GO:0017171;GO:0140096;GO:0003824;GO:0003674;GO:0008233;GO:0008236	K09628;K09640	PRSS27;TMPRS	------	------	COG5640	O:Posttranslational modification	PF00089.29	Trypsin	Trypsin	EXC	1	5	28	High
TRINITY_DN9790_c0_g1_i4_orf1	-	-	-	protein IWS1 homolog [Ostrinia furnacalis]	2.497239022	1.320333915	0.004245	up	yes	0.9497	0.3803	0.219	0.494	0.428	1	0.848	1.001	CC:intracellular mem	GO:0043231;GO:0005634;GO:0005575;GO:0043229;GO:0043227;GO:0043226;GO:0110165	K17498	SPN1, IWS1	------	------	COG5139	S:Function unknown	------	------	------	CYT	1	9	12.2	Medium
TRINITY_DN42759_c0_g3_i1_orf1	-	-	-	fatty acid synthase-like [Ostrinia furnacalis]	2.996377905	1.583219589	2.03E-07	up	yes	0.9927	0.3313	0.321	0.336	0.337	1	0.978	1	BP:fatty acid biosynt	GO:0006633;GO:0006631;GO:0019752;GO:0044249;GO:0044281;GO:0044283;GO:0072330;GO:1901576;GO:0071704;GO:0006629;GO:0009987;GO:0032787;GO:0009058;GO:0008150;GO:0008152;GO:0043436;GO:0044255;GO:0008610;GO:0044238;GO:0006082;GO:0046394;GO:0016053;GO:0044237;GO:0047451;GO:0016790;GO:0016417;GO:0047117;GO:0016627;GO:0003674;GO:0016491;GO:0016628;GO:0004313;GO:0016829;GO:0004320;GO:0016407;GO:0016740;GO:0016746;GO:0016747;GO:0016418;GO:0016787;GO:0008693;GO:0016788;GO:0003824;GO:0016614;GO:0016616;GO:0016835;GO:0016836;GO:0004312;GO:0016295;GO:0016296;GO:0016297;GO:0004316;GO:0004317;GO:0004315;GO:0019171;GO:0008659	K00665	FASN	map04910;map04	Insulin signaling pathway;Alc	COG3321	Q:Secondary metabolites biosyn	PF00698.24;PF00	Acyl_transf_1;k	Acyl transferase domai	CYT	2	3	84.3	High
TRINITY_DN25975_c0_g3_i2_orf1	-	-	-	V-type proton ATPase subunit D isoform X2 [Ostrinia furnacalis]	3.167349512	1.663276075	4.63E-07	up	yes	1.005	0.3173	0.324	0.31	0.318	1	0.99	1.026	MF:inorganic cation	GO:0022890;GO:0042626;GO:0022853;GO:0042625;GO:0015318;GO:0005215;GO:0008324;GO:0009678;GO:0019829;GO:0015075;GO:0015399;GO:0022857;GO:0003674;GO:0022804;GO:0046961;GO:0015078;GO:0044769	K02149	ATPeV1D, ATF	map05323;map00	Rheumatoid arthritis;Oxidati	COG1394	C:Energy production and conver	PF01813.20	ATP-synt_D	ATP synthase subunit D	CYT	7	20	27.4	High
TRINITY_DN64472_c0_g2_i1_orf1	-	-	-	repressed by EFG1 protein 1-like isoform X3 [Ostrinia furnacalis]	2.334847338	1.223328224	0.001698	up	yes	0.9253	0.3963	0.397	0.372	0.42	1	0.787	0.989	CC:cellular_compon	GO:0005575;GO:0005576;GO:0110165	K24835	GLIPR2	------	------	COG2340	G:Carbohydrate transport and m	PF00188.29	CAP	Cysteine-rich secretory	PLA	1	3	21.3	High
TRINITY_DN40_c0_g1_i3_orf1	-	-	-	trypsin CFT-1-like [Ostrinia furnacalis]	3.190946764	1.67398454	3.79E-06	up	yes	1.001	0.3137	0.332	0.328	0.281	1	0.983	1.019	BP:organic substanc	GO:0071704;GO:1901564;GO:0019538;GO:0043170;GO:0008150;GO:0006807;GO:0008152;GO:0006508;GO:0044238;GO:0004252;GO:0004175;GO:0016787;GO:0017171;GO:0140096;GO:0003824;GO:0003674;GO:0008233;GO:0008236	K01312	PRSS1_2_3	map04972;map05	Pancreatic secretion;Influenz	COG5640	O:Posttranslational modification	PF00089.29	Trypsin	Trypsin	CYT	4	18	28.1	High
TRINITY_DN31047_c0_g1_i4_orf1	-	-	-	4-coumarate--CoA ligase 1-like isoform X1 [Ostrinia furnacalis] >XP_028160248.1 4-coumarate--CoA ligase 1-like isoform X1 [Ostrinia furnacalis] >XP_028160249.1 4-coumarate--CoA ligase 1-like isoform X1 [Ostrinia furnacalis] >XP_028160250.1 4-coumarate--CoA ligase 1-like isoform X1 [Ostrinia furnacalis] >XP_028160251.1 4-coumarate--CoA ligase 1-like isoform X1 [Ostrinia furnacalis] >XP_028160253.1 4-coumarate--CoA ligase 1-like isoform X2 [Ostrinia furnacalis]	2.853702052	1.512834714	1.98E-05	up	yes	0.9597	0.3363	0.343	0.341	0.325	1	0.968	0.911	------	------	K01904	4CL	map00130	Ubiquinone and other terpe	COG0318;COG0	I:Lipid transport and metabolism	PF00501.31;PF13	AMP-binding;A	AMP-binding enzyme;A	CYT	4	8	59.4	High
TRINITY_DN37699_c0_g1_i4_orfp1	-	-	-	TRINITY_DN37699_c0_g1_i4_m.58777 TRINITY_DN37699_c0_g1::TRINITY_DN37699_c0_g1_i4::g.58777 ORF type:internal len:122 (+),score=34.90 TRINITY_DN37699_c0_g1_i4:1-363(+)	3.871089333	1.952739601	0.0003031	up	yes	1.027	0.2653	0.184	0.369	0.243	1	1.097	0.984	------	------	------	------	------	------	------	------	------	------	------	PLA	1	18	12.7	High
TRINITY_DN2425_c0_g1_i1_orf1	-	-	-	thyroid receptor-interacting protein 11 [Ostrinia furnacalis]	4.511310286	2.173546517	1.89E-05	up	yes	1.057	0.2343	0.196	0.247	0.26	1	1.092	1.079	------	------	------	------	------	------	ENOG410XUHJ	S:Function unknown	PF11365.11	SOGA	Protein SOGA	CYT	1	1	262.5	Medium
TRINITY_DN36262_c0_g1_i1_orf1	-	-	-	trypsin, alkaline C-like [Maniola jurtina]	4.0796216	2.028435343	7.13E-05	up	yes	1.035	0.2537	0.283	0.178	0.3	1	1.086	1.02	BP:organic substanc	GO:0071704;GO:1901564;GO:0019538;GO:0043170;GO:0008150;GO:0006807;GO:0008152;GO:0006508;GO:0044238;GO:0004252;GO:0004175;GO:0016787;GO:0017171;GO:0140096;GO:0003824;GO:0003674;GO:0008233;GO:0008236	K01312	PRSS1_2_3	map04972;map05	Pancreatic secretion;Influenz	COG5640	O:Posttranslational modification	PF00089.29	Trypsin	Trypsin	CYT	1	9	11.8	High
TRINITY_DN24476_c0_g1_i1_orf1	-	-	-	ensconsin-like isoform X1 [Ostrinia furnacalis]	3.822512486	1.934521214	4.74E-06	up	yes	0.995	0.2603	0.278	0.252	0.251	1	1.027	0.958	------	------	K24203;K16806	MAP7D2;MAP	------	------	ENOG4111S2A;E	S:Function unknown;				CYT	4	6	92	High
TRINITY_DN82320_c0_g1_i2_orf1	-	-	-	glutathione S-transferase sigma3 [Glyphodes pyloalis]	3.493720847	1.80476434	0.0003289	up	yes	0.9737	0.2787	0.242	0.27	0.324	1	0.867	1.054	MF:catalytic activity;I	GO:0003824;GO:0016740;GO:0004364;GO:0003674;GO:0016765	K04097	HPGDS	map00590;map00	Arachidonic acid metabolism	ENOG4110YU0;E	O:Posttranslational modification	PF14497.9	GST_C_3	Glutathione S-transfera	CYT	1	13	7.2	High
TRINITY_DN52296_c0_g1_i6_orf1	-	-	-	protein takeout-like [Ostrinia furnacalis]	3.070684039	1.618560072	0.0001073	up	yes	0.9427	0.307	0.344	0.265	0.312	1	0.948	0.88	------	------	------	------	------	------	ENOG4110RSP;E	S:Function unknown;	PF06585.14	JHBP	Haemolymph juvenile h	CYT	1	3	19.5	High
TRINITY_DN18773_c0_g1_i3_orf1	-	-	-	keratin, type II cytoskeletal 68 kDa, component IB-like [Ostrinia furnacalis]	2.80733945	1.489203518	0.0009856	up	yes	0.918	0.327	0.272	0.292	0.417	1	0.824	0.93	------	------	------	------	------	------	------	------	------	------	------	CYT	3	47	13.9	High
TRINITY_DN2394_c0_g1_i4_orf1	-	-	-	uncharacterized protein LOC114363116 [Ostrinia furnacalis]	10.5	3.392317423	0.0001339	up	yes	1.134	0.108	0.109	0.098	0.117	1	1.241	1.161	MF:molecular_functi	GO:0003674;GO:0008289;GO:0005488	K08752	FABP3	map03320	PPAR signaling pathway	ENOG4111US8	I:Lipid transport and metabolism	PF14651.9	Lipocalin_7	Lipocalin / cytosolic fat	CYT	6	33	18.8	High
TRINITY_DN28592_c0_g1_i2_orf1	-	-	-	UDP-glucuronosyltransferase 2B14-like isoform X1 [Ostrinia furnacalis] >XP_028167291.1 UDP-glucuronosyltransferase 2B14-like isoform X2 [Ostrinia furnacalis]	3.745173745	1.905032649	7.17E-05	up	yes	0.97	0.259	0.271	0.227	0.279	1	1.017	0.893	CC:integral compon	GO:0016021;GO:0005575;GO:0110165;GO:0031224;GO:0003824;GO:0016740;GO:0003674;GO:0016757;GO:0016758;GO:0015020;GO:0008194	K00699	UGT	map05207;map05	Chemical carcinogenesis - r	COG1819	S:Function unknown	PF00201.21	UDPGT	UDP-glucoronosyl and	CYT	1	4	58.6	High
TRINITY_DN3504_c0_g1_i4_orfp1	-	-	-	TRINITY_DN3504_c0_g1_i4_m.43930 TRINITY_DN3504_c0_g1::TRINITY_DN3504_c0_g1_i4::g.43930 ORF type:internal len:196 (-),score=84.82 TRINITY_DN3504_c0_g1_i4:3-587(-)	3.743083004	1.904227041	8.57E-05	up	yes	0.947	0.253	0.285	0.197	0.277	1	0.888	0.953	------	------	------	------	------	------	------	------	------	------	------	EXC	1	8	21.5	High
TRINITY_DN2986_c1_g1_i1_orf1	-	-	-	Troponin C, isoform 1 [Papilio xuthus]	6.525699414	2.706132536	4.88E-06	up	yes	1.003	0.1537	0.16	0.142	0.159	1	1.048	0.962	MF:cation binding;M	GO:0043169;GO:0043167;GO:0003674;GO:0005488;GO:0046872;GO:0005509	------	------	------	------	COG5126	F:Nucleotide transport and meta	PF13499.9;PF00	EF-hand_7;EF-	EF-hand domain pair;E	CYT	5	22	20.2	High
TRINITY_DN117_c0_g1_i6_orf1	-	-	-	lipase member I-like [Ostrinia furnacalis]	6.943866944	2.795739304	3.71E-07	up	yes	1.002	0.1443	0.155	0.149	0.129	1	1.022	0.984	BP:organic substanc	GO:0071704;GO:0006629;GO:0044238;GO:0008152;GO:0008150;GO:0005575;GO:0005576;GO:0110165;GO:0016787;GO:0016788;GO:0003824;GO:0003674;GO:0016298	------	------	------	------	ENOG41116Y2;E	S:Function unknown;	PF00151.22	Lipase	Lipase	CYT	1	11	42.3	High
TRINITY_DN4869_c0_g1_i10_orf1	-	-	-	estrogen sulfotransferase-like isoform X1 [Ostrinia furnacalis]	6.789115646	2.763223661	6.70E-07	up	yes	0.998	0.147	0.151	0.151	0.139	1	1.023	0.971	------	------	------	------	------	------	ENOG4111H56	S:Function unknown	PF00685.30;PF13	Sulfotransfer_1	Sulfotransferase domai	CYT	9	34	39.9	High
TRINITY_DN94355_c0_g1_i2_orf1	-	-	-	uncharacterized protein LOC126369488 [Pectinophora gossypiella]	13.2181599	3.724449449	2.53E-06	up	yes	0.987	0.07467	0.073	0.079	0.072	1	1.019	0.942	------	------	------	------	------	------	ENOG410Z1OR;I	S:Function unknown;L:Replicatio	PF05380.16;PF18	Peptidase_A17	Pao retrotransposon pe	CYT	1	0	179.7	High
TRINITY_DN96170_c0_g1_i1_orf1	-	-	-	uncharacterized protein LOC114355569 [Ostrinia furnacalis]	2.72850556	1.448110984	0.0002948	up	yes	1.006	0.3687	0.328	0.335	0.443	1	0.941	1.076	BP:phosphorylation;I	GO:0016310;GO:0009987;GO:0008150;GO:0008152;GO:0044237;GO:0006796;GO:0006793;GO:0031224;GO:0016021;GO:0005575;GO:0110165;GO:0035639;GO:0003674;GO:0005488;GO:0043167;GO:1901363;GO:0043168;GO:0000166;GO:0016740;GO:1901265;GO:0017076;GO:0005524;GO:0016301;GO:0036094;GO:0003824;GO:0030554;GO:0097367;GO:0016772;GO:0032559;GO:0032555;GO:0097159;GO:0032553	------	------	------	------	COG0574	G:Carbohydrate transport and m	PF01326.22	PPDK_N	Pyruvate phosphate dik	CYT	3	4	87.6	High
TRINITY_DN2062_c0_g1_i9_orf1	-	-	-	uncharacterized protein LOC114350846 [Ostrinia furnacalis]	2.702702703	1.434402824	1.02E-06	up	yes	0.99	0.3663	0.35	0.388	0.361	1	0.99	0.98	------	------	------	------	------	------	COG1028	S:Function unknown	PF13561.9;PF00	adh_short_C2;a	Enoyl:short chain dehyc	CYT	6	61	25.6	High
TRINITY_DN2594_c0_g2_i4_orf1	-	-	-	isocitrate dehydrogenase [NAD] subunit beta, mitochondrial isoform X2 [Ostrinia furnacalis]	3.112602991	1.638121574	6.85E-07	up	yes	1.02	0.3277	0.32	0.338	0.325	1	1.02	1.04	BP:tricarboxylic acid	GO:0006099;GO:0008150;GO:0008152;GO:0044238;GO:1990204;GO:0045242;GO:0005962;GO:1902494;GO:0030062;GO:0045239;GO:0032991;GO:0005575;GO:0098798;GO:0051287;GO:0003674;GO:0005488;GO:0000166;GO:1901363;GO:1901265;GO:0016491;GO:0043169;GO:0000287;GO:0043167;GO:0004448;GO:0046872;GO:0036094;GO:0016614;GO:0004449;GO:0016616;GO:0003824;GO:0097159	K00030	IDH3	map00020	Citrate cycl	COG0473	E:Amino acid transport and meta	PF00180.23	Iso_dh	Isocitrate/isopropylmal	CYT	3	13	41.2	High
TRINITY_DN29440_c1_g1_i4_orf1	-	-	-	neutral lipase [Helicoverpa armigera]	2.435897436	1.284453389	0.0001624	up	yes	0.95	0.39	0.361	0.355	0.454	1	0.93	0.92	BP:organic substanc	GO:0071704;GO:0006629;GO:0044238;GO:0008152;GO:0008150;GO:0005575;GO:0005576;GO:0110165;GO:0016787;GO:0016788;GO:0003824;GO:0003674;GO:0016298	------	------	------	------	ENOG410YGS3;E	S:Function unknown;	PF00151.22	Lipase	Lipase	PLA	1	10	11.7	High
TRINITY_DN2083_c0_g1_i4_orf1	-	-	-	uncharacterized protein LOC114359113 [Ostrinia furnacalis]	3.370860927	1.753117107	0.000219	up	yes	1.018	0.302	0.265	0.403	0.238	1	1.064	0.989	------	------	------	------	------	------	ENOG410ZGJA	S:Function unknown	PF06585.14	JHBP	Haemolymph juvenile h	CYT	3	17	24.2	High

TRINITY_DN1366_c0_g1_i5_orf1	-	-	-	unnamed protein product, partial [Iphiclides podalirius]	3.089635854	1.627436812	2.26E-07	up	yes	0.9927	0.3213	0.312	0.337	0.315	1	0.995	0.983	BP:nucleoside tripho	GO:0009141;GO:0009144;GO:0034641;GO:0006807;GO:0044281;GO:0072521;GO:0009259;GO:1901360;GO:0006139;GO:0071704;GO:0009199;GO:0009205;GO:0019693;GO:0046034;GO:0009987;GO:0006725;GO:0009150;GO:0009117;GO:0008152;GO:0055086;GO:0046483;GO:0044238;GO:1901564;GO:1901135;GO:0044237;GO:0006163;GO:0006796;GO:0006793;GO:0019637;GO:0008150;GO:0006753;GO:0033178;GO:0032991;GO:0005575;GO:0033180;GO:0098796;GO:0046961;GO:0035639;GO:0032553;GO:0003674;GO:0043167;GO:0043168;GO:0042626;GO:0015399;GO:0015318;GO:0022857;GO:0009678;GO:0000166;GO:0044769;GO:0005215;GO:0017076;GO:0022804;GO:0008324;GO:0022890;GO:1901265;GO:0036094;GO:0032555;GO:0042625;GO:0030554;GO:0097367;GO:0097159;GO:0015075;GO:0019829;GO:0032559;GO:0005524;GO:0022853;GO:0015078;GO:1901363;GO:0005488	K02147	ATPeV1B, ATP	map05323;map00	Rheumatoid arthritis;Oxidati	COG1156	C:Energy production and conver	PF00006.28;PF02	ATP-synt_ab;AT	ATP synthase alpha/bet	CYT	16	45	54.8	High
TRINITY_DN107035_c0_g1_i1_orf1	-	-	-	splicing factor 3A subunit 3 [Ostrinia furnacalis]	3.207297385	1.681358128	3.50E-05	up	yes	0.9933	0.3097	0.322	0.267	0.34	1	1.034	0.946	BP:cellular metabolic	GO:0044237;GO:0090304;GO:0034641;GO:0006807;GO:1901360;GO:0006139;GO:0071704;GO:0008380;GO:0016071;GO:0009987;GO:0006725;GO:0000375;GO:0000377;GO:0008150;GO:0008152;GO:0046483;GO:0016070;GO:0044238;GO:0000398;GO:0043170;GO:0006396;GO:0006397;GO:0005681;GO:0032991;GO:1990904;GO:0005575;GO:0003674;GO:0005488;GO:0003676;GO:1901363;GO:0046914;GO:0008270;GO:0043167;GO:0043169;GO:0046872;GO:0097159;GO:0003723	K12827	SF3A3, SAP61,	map03040	Spliceosome	COG5188	A:RNA processing and modificat	PF11931.11;PF1	SF3a60_Prp9_C	SF3a60/Prp9 C-termina	CYT	1	1	57.7	High
TRINITY_DN7556_c0_g1_i1_orf1	-	-	-	venom carboxylesterase-6-like [Ostrinia furnacalis]	3.737226277	1.901967917	6.38E-06	up	yes	1.024	0.274	0.235	0.296	0.291	1	1.049	1.024	------	------	------	------	------	------	COG2272;COG0	I:Lipid transport and metabolism	PF00135.31;PF0	COesterase;Ab	Carboxylesterase family	CYT	1	1	54.8	Medium
TRINITY_DN1318_c0_g1_i5_orf1	-	-	-	uncharacterized protein LOC114360956 [Ostrinia furnacalis]	3.231350641	1.692137309	9.09E-06	up	yes	0.9833	0.3043	0.293	0.304	0.316	1	1.012	0.938	CC:cellular_compone	GO:0005575;GO:0016021;GO:0110165;GO:0031224	------	------	------	------	ENOG4110SBJ;C	S:Function unknown;	PF13855.9;PF12	LRR_8;LRR_4	Leucine rich repeat;Leu	CYT	5	21	47.9	High
TRINITY_DN334_c0_g1_i2_orf1	-	-	-	chymotrypsin-like serine protease, partial [Ostrinia nubilalis]	5.004807692	2.323314635	1.14E-05	up	yes	1.041	0.208	0.191	0.251	0.182	1	1.076	1.047	BP:organic substanc	GO:0071704;GO:1901564;GO:0019538;GO:0043170;GO:0008150;GO:0006807;GO:0008152;GO:0006508;GO:0044238;GO:0004252;GO:0004175;GO:0016787;GO:0017171;GO:0140096;GO:0003824;GO:0003674;GO:0008233;GO:0008236	------	------	------	------	COG5640	O:Posttranslational modification	PF00089.29	Trypsin	Trypsin	CYT	1	18	13.2	High
TRINITY_DN5891_c0_g2_i4_orf1	-	-	-	amino acid transporter AVT1A-like [Ostrinia furnacalis] >XP_028156666.1 amino acid transporter AVT1A-like [Ostrinia furnacalis]	4.672469079	2.224185117	1.13E-05	up	yes	1.02	0.2183	0.232	0.244	0.179	1	0.996	1.064	CC:cellular_compone	GO:0005575;GO:0016021;GO:0110165;GO:0031224	K15015	SLC32A, VGAT	map04727;map04	GABAergic synapse;Retrogr	COG0814	E:Amino acid transport and met	PF01490.21;PF0	Aa_trans;Trp_Ty	Transmembrane amino	CYT	1	2	48.1	High
TRINITY_DN8691_c0_g1_i3_orf1	-	-	-	nucleolin-like [Melitaea cinxia]	4.570135747	2.192237018	3.67E-06	up	yes	1.01	0.221	0.255	0.196	0.212	1	1.036	0.993	------	------	------	------	------	------	ENOG4110RR1	S:Function unknown	------	------	------	CYT	2	15	14.4	High
TRINITY_DN123396_c0_g1_i1_orf1	-	-	-	PREDICTED: delta-1-pyrroline-5-carboxylate dehydrogenase, mitochondrial isoform X1 [Megachile rotundata]	3.920883534	1.971178789	1.66E-05	up	yes	0.9763	0.249	0.255	0.231	0.261	1	1.009	0.92	BP:small molecule ca	GO:0044282;GO:0019752;GO:0009063;GO:0006807;GO:0044281;GO:0009065;GO:0009064;GO:1901575;GO:0006562;GO:0006560;GO:0006520;GO:0071704;GO:1901605;GO:1901606;GO:0044238;GO:0009987;GO:0010133;GO:0008152;GO:0043436;GO:0009056;GO:0044248;GO:0043648;GO:1901564;GO:1901565;GO:0006082;GO:0046395;GO:0016054;GO:0044237;GO:0006536;GO:0008150;GO:0003674;GO:0016491;GO:0016620;GO:0003824;GO:0016903;GO:0003842	K00294	E1.2.1.88	map00250;map00	Alanine, aspartate and gluta	COG1012	Z:Cytoskeleton	PF00171.25	Aldedh	Aldehyde dehydrogena	CYT	1	1	63.5	High
TRINITY_DN36494_c0_g1_i1_orf1	-	-	-	MKI67 FHA domain-interacting nucleolar phosphoprotein-like [Ostrinia furnacalis]	4.51962111	2.176201833	5.22E-07	up	yes	1.002	0.2217	0.231	0.214	0.22	1	1.024	0.981	MF:organic cyclic co	GO:0097159;GO:0003674;GO:0005488;GO:0003676;GO:1901363;GO:0003723	K14838	NOP15	------	------	ENOG4111NF9	S:Function unknown	PF00076.25	RRM_1	RNA recognition motif.	CYT	2	6	30.6	High
TRINITY_DN16939_c0_g1_i4_orf1	-	-	-	39S ribosomal protein L17, mitochondrial [Ostrinia furnacalis]	3.766024381	1.91304234	1.16E-05	up	yes	0.9577	0.2543	0.259	0.227	0.277	1	0.946	0.927	BP:cellular biosynthe	GO:0044249;GO:0034641;GO:0006807;GO:0034645;GO:1901576;GO:0044271;GO:0044260;GO:0043043;GO:0071704;GO:0043604;GO:0009987;GO:0009058;GO:0009059;GO:0008150;GO:0008152;GO:1901564;GO:0044238;GO:0043603;GO:1901566;GO:0019538;GO:0044237;GO:0043170;GO:0006518;GO:0006412;GO:0043232;GO:0043226;GO:0110165;GO:0005840;GO:0032991;GO:1990904;GO:0043229;GO:0005575;GO:0043228;GO:0003674;GO:0005198;GO:0003735	K02879	RP-L17, MRPL	map03010	Ribosome	COG0203	J:Translation, ribosomal structure	PF01196.22	Ribosomal_L17	Ribosomal protein L17	CYT	2	7	24	High
TRINITY_DN43942_c0_g2_i1_orf1	-	-	-	LOW QUALITY PROTEIN: caprin homolog [Ostrinia furnacalis]	6.490566038	2.6983443	9.49E-06	up	yes	1.032	0.159	0.126	0.197	0.154	1	1.077	1.019	------	------	K18743	CAPRIN1, GPI	------	------	ENOG4110M5Q	S:Function unknown	------	------	------	CYT	1	5	22.7	High
TRINITY_DN2490_c0_g2_i1_orfp1	-	-	-	TRINITY_DN2490_c0_g2_i1_m.56872 TRINITY_DN2490_c0_g2::TRINITY_DN2490_c0_g2_i1::g.56872 ORF type:internal len:359 (-) score=123.59 TRINITY_DN2490_c0_g2_i1:2-1075(-)	13.76726664	3.783170249	1.08E-05	up	yes	0.9867	0.07167	0.071	0.074	0.07	1	0.923	1.037	------	------	------	------	------	------	------	------	------	------	------	EXC	7	34	36.3	High
TRINITY_DN4842_c0_g1_i5_orf1	-	-	-	cytochrome c oxidase assembly factor 4 homolog, mitochondrial isoform X1 [Ostrinia furnacalis] >XP_028162331.1 cytochrome c oxidase assembly factor 4 homolog, mitochondrial isoform X2 [Ostrinia furnacalis]	2.811722422	1.491454176	1.89E-05	up	yes	1.017	0.3617	0.335	0.345	0.405	1	1	1.051	BP:mitochondrial res	GO:0033108;GO:0033617;GO:0022607;GO:0043933;GO:0009987;GO:0008535;GO:0008150;GO:0017004;GO:0071840;GO:0065003;GO:0016043;GO:0043233;GO:0031970;GO:0031974;GO:0005575;GO:0070013;GO:0005758;GO:0110165	K18177	COA4	map04714	Thermogenesis	ENOG410XXMU	O:Posttranslational modification	------	------	------	CYT	1	13	8.2	High
TRINITY_DN52861_c0_g1_i1_orf1	-	-	-	protein MAK16 homolog A [Ostrinia furnacalis]	2.840782123	1.506288187	6.95E-06	up	yes	1.017	0.358	0.388	0.322	0.364	1	1.034	1.016	CC:intracellular non-	GO:0043232;GO:0005730;GO:0005575;GO:0043229;GO:0043228;GO:0043226;GO:0110165	K14831	MAK16	------	------	COG5129	S:Function unknown	PF01778.20;PF0	Ribosomal_L28	Ribosomal L28e protein	CYT	1	2	35.4	Medium
TRINITY_DN3847_c1_g1_i1_orf1	-	-	-	ribosome production factor 2 homolog [Ostrinia furnacalis]	2.602952808	1.380149156	2.17E-05	up	yes	0.9873	0.3793	0.378	0.372	0.388	1	0.937	1.025	BP:cellular compone	GO:0022607;GO:0043933;GO:0090304;GO:0000470;GO:0034660;GO:1901360;GO:0000027;GO:0006139;GO:0071826;GO:0071840;GO:0016043;GO:0065003;GO:0071704;GO:0022618;GO:0006364;GO:0034470;GO:0009987;GO:0006725;GO:0008150;GO:0008152;GO:0046483;GO:0016070;GO:0044238;GO:0016072;GO:0034641;GO:0044237;GO:0043170;GO:0006807;GO:0006396;GO:0043229;GO:0043228;GO:0043226;GO:0005730;GO:0110165;GO:0043232;GO:0005575;GO:0003674;GO:0005488;GO:0003676;GO:0019843;GO:1901363;GO:0097159;GO:0003723	K14847	RPF2	------	------	COG5106	S:Function unknown	PF04427.21	Brix	Brix domain	CYT	2	7	35.9	High
TRINITY_DN54543_c0_g5_i2_orf1	-	-	-	reactive oxygen species modulator 1 [Papilio machaon] >XP_022123076.1 reactive oxygen species modulator 1 [Pieris rapae] >XP_022820703.1 reactive oxygen species modulator 1 [Spodoptera litura] >XP_028176790.1 reactive oxygen species modulator 1 [Ostrinia furnacalis] >XP_030021418.1 reactive oxygen species modulator 1 [Manduca sexta] >XP_034836896.1 reactive oxygen species modulator 1 [Maniola hyperantus] >XP_035446679.1 reactive oxygen species modulator 1-like [Spodoptera frugiperda] >XP_045458971.1 reactive oxygen species modulator 1 [Melitaea cinxia] >XP_045521377.1 reactive oxygen species modulator 1 [Pieris brassicae] >XP_045765649.1 reactive oxygen species modulator 1 [Maniola jurtina] >XP_047042173.1 reactive oxygen species modulator 1 [Helicoverpa zea] >XP_049691668.1 reactive oxygen species modulator 1 [Helicoverpa armigera] >KAF9414699.1 hypothetical protein HW555_007477 [Spodoptera exigua] >KPI93862.1 Reactive oxygen species modulator 1 [Papilio xuthus] >CAB3515149.1 unnamed protein product [Spodoptera littoralis] >CAG5021695.1 unnamed protein product [Parnassius apollo] >KAF9823017.1 hypothetical protein SFRURICE_018191 [Spodoptera frugiperda]	2.941518766	1.55656124	8.69E-05	up	yes	1.011	0.3437	0.37	0.317	0.344	1	0.95	1.082	CC:cellular_compone	GO:0005575;GO:0016021;GO:0110165;GO:0031224	------	------	------	------	ENOG411279J	S:Function unknown	PF10247.12	Romo1	Reactive mitochondrial	CYT	1	23	8.2	High
TRINITY_DN24873_c0_g1_i4_orf1	-	-	-	uncharacterized protein LOC114365742 [Ostrinia furnacalis]	3.10962648	1.636741298	1.51E-06	up	yes	1.024	0.3293	0.335	0.311	0.342	1	1.029	1.042	CC:endoplasmic reti	GO:0005789;GO:0031224;GO:0031090;GO:0016021;GO:0016020;GO:0110165;GO:0005575;GO:0004497;GO:0016705;GO:0003674;GO:0005488;GO:1901363;GO:0016491;GO:0043169;GO:0046914;GO:0043167;GO:0005506;GO:0046872;GO:0020037;GO:0003824;GO:0046906;GO:0097159	K15001	CYP4	------	------	COG2124	S:Function unknown	PF00067.25	p450	Cytochrome P450	CYT	2	3	59.3	High
TRINITY_DN15046_c0_g1_i8_orf1	-	-	-	epidermal retinol dehydrogenase 2-like isoform X1 [Ostrinia furnacalis] >XP_028169999.1 epidermal retinol dehydrogenase 2-like isoform X2 [Ostrinia furnacalis]	2.835859308	1.50378596	1.22E-05	up	yes	0.9917	0.3497	0.326	0.394	0.329	1	1.003	0.972	MF:catalytic activity;I	GO:0003824;GO:0003674;GO:0016491	K15734	SDR16C5	map00830;map01	Retinol metabolism;Biosynth	COG1028;COG4	S:Function unknown;	PF00106.28;PF1	adh_short;adh_	short chain dehydroger	CYT	3	13	34.7	High
TRINITY_DN25783_c0_g1_i2_orf1	-	-	-	SET and MYND domain-containing protein 4 [Ostrinia furnacalis]	2.570476952	1.362036076	0.0001226	up	yes	0.9647	0.3753	0.339	0.354	0.433	1	0.911	0.983	CC:integral compone	GO:0016021;GO:0005575;GO:0043229;GO:0031224;GO:0043226;GO:0110165	------	------	------	------	COG2940	U:Intracellular trafficking, secreti	PF00856.31	SET	SET domain	CYT	1	1	84	High
TRINITY_DN85319_c0_g1_i1_orf1	-	-	-	cholinesterase 2-like [Ostrinia furnacalis]	3.054623131	1.610994396	1.84E-06	up	yes	1.001	0.3277	0.343	0.319	0.321	1	1.025	0.977	MF:hydrolase activity	GO:0016787;GO:0003674;GO:0016788;GO:0052689;GO:0003824	K12298	CEL	map04972;map00	Pancreatic secretion;Steroid	COG2272	I:Lipid transport and metabolism	PF00135.31;PF2	COesterase;BD	Carboxylesterase family	CYT	9	18	61.4	High
TRINITY_DN37538_c0_g3_i1_orf1	-	-	-	esterase FE4-like [Ostrinia furnacalis]	2.295992034	1.199117636	0.001208	up	yes	0.9223	0.4017	0.371	0.485	0.349	1	0.836	0.931	------	------	K07378	NLGN	map04514	Cell adhesion molecules	COG2272;COG0	I:Lipid transport and metabolism	PF00135.31	COesterase	Carboxylesterase family	CYT	1	13	13.4	High
TRINITY_DN791_c0_g1_i2_orf1	-	-	-	peroxiredoxin 1 isoform X1 [Maniola jurtina]	2.718583076	1.442854915	4.85E-05	up	yes	0.967	0.3557	0.386	0.36	0.321	1	0.987	0.914	MF:antioxidant activi	GO:0016209;GO:0051920;GO:0003824;GO:0003674;GO:0016684;GO:0004601;GO:0016491	K13279	PRDX1	map04146;map05	Peroxisome;Amoebiasis	COG0450	L:Replication, recombination and	PF00578.24;PF1	AhpC-TSA;1-cy	AhpC/TSA family;C-ter	CYT	7	32	26.5	High
TRINITY_DN146544_c0_g1_i1_orf1	-	-	-	UPF0047 protein YjbQ [Aphidius gifuensis] >KAF7996225.1 hypothetical protein HCN44_001857 [Aphidius gifuensis]	2.374456104	1.247597086	0.0003257	up	yes	0.9277	0.3907	0.416	0.332	0.424	1	0.888	0.895	------	------	------	------	------	------	COG0432	S:Function unknown	PF01894.20	UPF0047	Uncharacterised protei	CYT	1	9	16.6	High
TRINITY_DN6418_c0_g1_i28_orf1	-	-	-	peritrophic membrane chitin binding protein [Loxostege sticticalis]	3.449324324	1.786313785	1.40E-05	up	yes	1.021	0.296	0.312	0.271	0.305	1	1.072	0.992	CC:cellular_compone	GO:0005575;GO:0005576;GO:0110165;GO:0097367;GO:0003674;GO:0008061;GO:0005488	------	------	------	------	ENOG41121EJ;E	S:Function unknown;	PF01607.27	CBM_14	Chitin binding Peritroph	CYT	1	3	48	High
TRINITY_DN542_c0_g1_i4_orf1	-	-	-	uncharacterized protein LOC114364889 [Ostrinia furnacalis]	2.900900901	1.536501012	8.63E-06	up	yes	0.966	0.333	0.331	0.335	0.333	1	0.925	0.973	BP:amino sugar cata	GO:0046348;GO:0006807;GO:0006022;GO:1901575;GO:1901072;GO:0071704;GO:0006030;GO:0000272;GO:0008150;GO:0008152;GO:1901564;GO:0009056;GO:0009057;GO:0044238;GO:0006032;GO:0005975;GO:1901565;GO:0005976;GO:0006040;GO:1901136;GO:0016052;GO:0043170;GO:1901071;GO:0006026;GO:1901135;GO:0005615;GO:0110165;GO:0005575;GO:0016798;GO:0051060;GO:0003674;GO:0008270;GO:0043169;GO:0046914;GO:0043167;GO:0046872;GO:0016787;GO:0019213;GO:0003824;GO:0016810;GO:0016811;GO:0004553;GO:0004099;GO:0005488	------	------	------	------	ENOG410ZWU3	S:Function unknown	PF01522.24	Polysacc_deac_	Polysaccharide deacety	CYT	5	14	44.8	High
TRINITY_DN2918_c0_g1_i1_orf1	-	-	-	28S ribosomal protein S10, mitochondrial [Ostrinia furnacalis] >XP_028175147.1 28S ribosomal protein S10, mitochondrial [Ostrinia furnacalis]	2.953273901	1.562315165	1.53E-05	up	yes	0.9607	0.3253	0.309	0.341	0.326	1	0.964	0.918	------	------	K02946	RP-S10, MRPS	map03010	Ribosome	COG0051	J:Translation, ribosomal structure	PF00338.25	Ribosomal_S10	Ribosomal protein S10p	CYT	1	5	19.9	High
TRINITY_DN6059_c0_g1_i1_orf1	-	-	-	brachyurin-like [Ostrinia furnacalis]	4.36039099	2.124457505	7.10E-05	up	yes	1.026	0.2353	0.247	0.221	0.238	1	0.962	1.115	BP:organic substanc	GO:0071704;GO:1901564;GO:0019538;GO:0043170;GO:0008150;GO:0006807;GO:0008152;GO:0006508;GO:0044238;GO:0004252;GO:0004175;GO:0016787;GO:0017171;GO:0140096;GO:0003824;GO:0003674;GO:0008233;GO:0008236	K09634	TMPRSS3	------	------	COG5640	S:Function unknown	PF00089.29	Trypsin	Trypsin	CYT	1	4	30.1	High
TRINITY_DN1353_c0_g1_i1_orf1	-	-	-	UDP-glucose 4-epimerase-like [Ostrinia furnacalis]	3.237172953	1.694734447	3.55E-05	up	yes	0.9527	0.2943	0.318	0.283	0.282	1	0.962	0.896	BP:primary metaboli	GO:0044238;GO:0005975;GO:0071704;GO:0005996;GO:0019318;GO:0006012;GO:0008150;GO:0044281;GO:0008152;GO:0016854;GO:0016853;GO:0003824;GO:0016857;GO:0003974;GO:0003674;GO:0003978	K01784	galE, GALE	map00520;map01	Amino sugar and nucleotide	COG1087	M:Cell wall/membrane/envelope	PF16363.8;PF01	GDP_Man_Deh	GDP-mannose 4,6 deh	CYT	2	8	40.3	High
TRINITY_DN11069_c0_g2_i1_orf1	-	-	-	fat storage-inducing transmembrane protein [Ostrinia furnacalis]	4.564148413	2.190345705	5.02E-05	up	yes	1.021	0.2237	0.211	0.205	0.255	1	0.965	1.099	BP:lipid storage;BP:c	GO:0019915;GO:0016043;GO:0071840;GO:0009987;GO:0008150;GO:0051235;GO:0051179;GO:0006996;GO:0034389;GO:0005789;GO:0031224;GO:0031090;GO:0016021;GO:0016020;GO:0110165;GO:0005575;GO:0003674;GO:0016787;GO:0003824;GO:0016818;GO:0016817;GO:0016462	------	------	------	------	ENOG4111CNJ	S:Function unknown	PF10261.12	Scs3p	Inositol phospholipid sy	CYT	2	6	42.2	High
TRINITY_DN26688_c0_g1_i2_orf1	-	-	-	myogenesis-regulating glycosidase-like [Ostrinia furnacalis]	4.506726457	2.172079886	6.11E-08	up	yes	1.005	0.223	0.231	0.223	0.215	1	1.018	0.998	BP:primary metaboli	GO:0044238;GO:0005975;GO:0071704;GO:0008150;GO:0008152;GO:0016787;GO:0003824;GO:0016798;GO:0004553;GO:0003674	K24727	MYORG	------	------	COG1501	G:Carbohydrate transport and m	PF01055.29	Glyco_hydro_31	Glycosyl hydrolases fam	CYT	11	21	71.1	High
TRINITY_DN7047_c0_g1_i1_orf1	-	-	-	hypothetical protein G9C98_004728 [Cotesia typhae]	4.94321608	2.305449972	3.94E-06	up	yes	0.9837	0.199	0.228	0.18	0.189	1	1.001	0.95	CC:macromolecular	GO:0032991;GO:0005840;GO:0043232;GO:1990904;GO:0005739;GO:0005575;GO:0043229;GO:0043228;GO:0043227;GO:0043226;GO:0110165;GO:0043231	K17431	MRPL50	------	------	ENOG4111QY3	S:Function unknown	------	------	------	CYT	1	4	24	High
TRINITY_DN13651_c0_g1_i2_orf1	-	-	-	40S ribosomal protein S12, mitochondrial [Ostrinia furnacalis]	5.067936181	2.341398357	3.99E-07	up	yes	0.9847	0.1943	0.193	0.186	0.204	1	0.992	0.962	BP:cellular biosynthe	GO:0044249;GO:0034641;GO:0006807;GO:0034645;GO:1901576;GO:0044260;GO:0043043;GO:0071704;GO:0043604;GO:0009987;GO:0009058;GO:0009059;GO:0008150;GO:0008152;GO:0044271;GO:0044238;GO:1901564;GO:0043603;GO:1901566;GO:0019538;GO:0044237;GO:0043170;GO:0006518;GO:0006412;GO:0015935;GO:0032991;GO:0044391;GO:1990904;GO:0005575;GO:0003674;GO:0005198;GO:0003735	K02950	RP-S12, MRPS	map03010	Ribosome	COG0048	S:Function unknown	PF00164.28	Ribosom_S12_S	Ribosomal protein S12/	CYT	1	6	17.4	High
TRINITY_DN144807_c0_g1_i1_orf1	-	-	-	hypothetical protein G9C98_004245 [Cotesia typhae]	4.981218274	2.31649863	3.55E-06	up	yes	0.9813	0.197	0.164	0.218	0.209	1	0.954	0.99	BP:L-methionine sal	GO:0071267;GO:0019752;GO:0071265;GO:0044249;GO:0006807;GO:0009067;GO:0044281;GO:0044283;GO:0019284;GO:1901576;GO:0019509;GO:0006520;GO:0071704;GO:0043102;GO:1901605;GO:0000096;GO:0044238;GO:0000097;GO:0009987;GO:0009058;GO:0008150;GO:0008152;GO:0043436;GO:0043094;GO:0008652;GO:1901564;GO:1901566;GO:0006082;GO:0044272;GO:0046394;GO:0006555;GO:0016053;GO:0044237;GO:0006790;GO:1901607;GO:0009086;GO:0009066;GO:0043229;GO:0043227;GO:0005737;GO:0005634;GO:0110165;GO:0043231;GO:0005575;GO:0043226;GO:0005488;GO:0000287;GO:0016791;GO:0043874;GO:0042578;GO:0016862;GO:0016860;GO:0043716;GO:0043715;GO:0043167;GO:0043169;GO:0046872;GO:0016787;GO:0016788;GO:0003824;GO:0016853;GO:0003674	K09880	mtnC, ENOPH	map00270	Cysteine and methionine me	COG4229	C:Energy production and conver	------	------	------	CYT	1	1	75.4	Medium
TRINITY_DN22983_c0_g1_i2_orfp1	-	-	-	TRINITY_DN22983_c0_g1_i2_m.10495 TRINITY_DN22983_c0_g1::TRINITY_DN22983_c0_g1_i2::g.10495 ORF type:internal len:79 (-) score=15.95 Polyhedrin PF00738.19 7.6e-40 TRINITY_DN22983_c0_g1_i2:2-235(-)	5.995731707	2.583935828	1.76E-07	up	yes	0.9833	0.164	0.16	0.155	0.177	1	0.973	0.977	------	------	------	------	------	------	------	------	PF00738.21	Polyhedrin	Polyhedrin	CYT	3	21	9.2	High
TRINITY_DN11587_c0_g1_i7_orf1	-	-	-	elongation of very long chain fatty acids protein AAEL008004-like isoform X1 [Danaus plexippus plexippus] >XP_032511006.1 elongation of very long chain fatty acids protein AAEL008004-like isoform X1 [Danaus plexippus plexippus] >XP_032511007.1 elongation of very long chain fatty acids protein AAEL008004-like isoform X1 [Danaus plexippus plexippus] >XP_032511008.1 elongation of very long chain fatty acids protein AAEL008004-like isoform X1 [Danaus plexippus plexippus] >XP_032511009.1 elongation of very long chain fatty acids protein AAEL008004-like isoform X1 [Danaus plexippus	7.466666667	2.900464326	3.77E-08	up	yes	1.008	0.135	0.122	0.141	0.142	1	1.011	1.014	------	------	K10250	ELOVL7	map01040;map00	Biosynthesis of unsaturated	ENOG410XRWT	C:Energy production and conver	PF01151.21	ELO	GNS1/SUR4 family	CYT	1	4	36.4	High
TRINITY_DN3862_c0_g1_i7_orf1	-	-	-	venom acid phosphatase Acph-1-like [Ostrinia furnacalis]	8.318219291	3.056274719	2.30E-07	up	yes	1.009	0.1213	0.133	0.101	0.13	1	1.023	1.004	BP:nucleic acid meta	GO:0090304;GO:0034641;GO:0006807;GO:1901360;GO:0006139;GO:0044260;GO:0071704;GO:0009987;GO:0006725;GO:0008150;GO:0008152;GO:0046483;GO:0044238;GO:0044237;GO:0043170;GO:0015074;GO:0006259;GO:0016791;GO:0042578;GO:0003674;GO:0005488;GO:0003676;GO:1901363;GO:0016787;GO:0016788;GO:0003824;GO:0097159;GO:0003993	K19283	ACPP	------	------	ENOG410ZVBQ	C:Energy production and conver	PF00328.25	His_Phos_2	Histidine phosphatase s	CYT	1	2	43.7	Medium
TRINITY_DN41086_c0_g1_i4_orf1	-	-	-	collagenase-like [Pectinophora gossypiella]	9.942424242	3.313597664	2.75E-07	up	yes	0.9843	0.099	0.096	0.103	0.098	1	0.994	0.959	BP:organic substanc	GO:0071704;GO:1901564;GO:0019538;GO:0043170;GO:0008150;GO:0006807;GO:0008152;GO:0006508;GO:0044238;GO:0004252;GO:0004175;GO:0016787;GO:0017171;GO:0140096;GO:0003824;GO:0003674;GO:0008233;GO:0008236	------	------	------	------	COG5640	O:Posttranslational modification	PF00089.29;PF1	Trypsin;Trypsin_	Trypsin;Trypsin-like pep	CYT	3	10	36.1	High
TRINITY_DN36199_c0_g1_i1_orf1	-	-	-	fatty acid-binding protein 1-like [Ostrinia furnacalis]	14.95425229	3.902483872	1.75E-07	up	yes	0.997	0.06667	0.059	0.074	0.067	1	1.015	0.976	MF:molecular_functi	GO:0003674;GO:0008289;GO:0005488	K08753;K08752	FABP4, aP2;FA	map04923;map03	Regulation of lipolysis in adi	ENOG4111US8	I:Lipid transport and metabolism	------	------	------	CYT	5	48	15.2	High
TRINITY_DN25856_c0_g1_i1_orf1	-	-	-	myrosinase 1-like [Ostrinia furnacalis]	2.781767956	1.476002081	4.04E-06	up	yes	1.007	0.362	0.384	0.361	0.341	1	1.033	0.987	------	------	------	------	------	------	COG2723;ENOG	G:Carbohydrate transport and m	PF00232.21	Glyco_hydro_1	Glycosyl hydrolase fam	CYT	3	9	57.7	High
TRINITY_DN220_c0_g1_i3_orf1	-	-	-	serine-arginine protein 55 isoform X6 [Pieris brassicae]	2.395238095	1.260169072	1.94E-05	up	yes	0.9557	0.399	0.4	0.387	0.41	1	0.925	0.942	------	------	K12893	SRSF4_5_6, SF	map05168;map03	Herpes simplex virus 1 infect	COG0724	S:Function unknown	PF00076.25;PF0	RRM_1;RRM_3	RNA recognition motif.;	CYT	6	28	26.5	High
TRINITY_DN69236_c0_g1_i1_orf1	-	-	-	peroxiredoxin [Ostrinia furnacalis]	2.832011331	1.501827038	1.22E-06	up	yes	0.9997	0.353	0.376	0.329	0.354	1	0.996	1.003	MF:antioxidant activi	GO:0016209;GO:0051920;GO:0003824;GO:0003674;GO:0016684;GO:0004601;GO:0016491	K03386	PRDX2_4, ahpl	map04214	Apoptosis - fly	COG0450;COG0	I:Lipid transport and metabolism	PF00578.24;PF1	AhpC-TSA;1-cy	AhpC/TSA family;C-ter	CYT	2	14	27.8	High
TRINITY_DN6308_c0_g1_i6_orf1	-	-	-	myc box-dependent-interacting protein 1 isoform X2 [Ostrinia furnacalis]	2.816384181	1.493844144	3.66E-06	up	yes	0.997	0.354	0.324	0.362	0.376	1	1.011	0.98	CC:cytoplasm;CC:ce	GO:0005737;GO:0005575;GO:0110165	K12562	AMPH	map04666;map04	Fc gamma R-mediated pha	ENOG410ZBW1	S:Function unknown	PF03114.21;PF1	BAR;SH3_9;SH3	BAR domain;Variant SH	CYT	3	33	52.4	High
TRINITY_DN1775_c0_g1_i3_orf1	-	-	-	ATP-dependent RNA helicase dbp2-like isoform X1 [Ostrinia furnacalis]	3.054545455	1.610957709	5.63E-07	up	yes	1.008	0.33	0.345	0.309	0.336	1	1.017	1.008	------	------	K12823	DDX5, DBP2	map05205;map05	Proteoglycans in cancer;Tran	COG0513	L:Replication, recombination and	PF00270.32;PF0	DEAD;Helicase	DEAD/DEAH box helica	CYT	13	24	92.9	High
TRINITY_DN12497_c0_g1_i1_orf1	-	-	-	probable N-acetyltransferase san [Ostrinia furnacalis]	3.164717041	1.662076513	4.11E-07	up	yes	1.001	0.3163	0.326	0.328	0.295	1	1.007	0.997	MF:transferase activi	GO:0016740;GO:0003824;GO:0016407;GO:0016746;GO:0016747;GO:0003674	K20793	NAA50, NAT5	------	------	COG0456	S:Function unknown	PF00583.28;PF1	Acetyltransf_1;	Acetyltransferase;Acetv	CYT	5	36	20.2	High
TRINITY_DN66302_c0_g1_i1_orf1	-	-	-	carboxypeptidase B-like [Ostrinia furnacalis]	4.058755315	2.021037369	3.24E-05	up	yes	1.05	0.2587	0.237	0.314	0.225	1	1.089	1.06	BP:nitrogen compou	GO:0006807;GO:0071704;GO:0006508;GO:0008150;GO:0008152;GO:0044238;GO:1901564;GO:0019538;GO:0043170;GO:0031224;GO:0110165;GO:0005575;GO:0016021;GO:0008238;GO:0003674;GO:0008270;GO:0008233;GO:0008235;GO:0043169;GO:0046914;GO:0004181;GO:0004180;GO:0043167;GO:0008237;GO:0046872;GO:0016787;GO:0140096;GO:0003824;GO:0005488	K01298	CPA2	map04972;map04	Pancreatic secretion;Protein	COG2866	E:Amino acid transport and met	PF00246.27	Peptidase_M14	Zinc carboxypeptidase	CYT	1	3	47.1	High
TRINITY_DN8369_c0_g1_i1_orf1	-	-	-	39S ribosomal protein L37, mitochondrial [Ostrinia furnacalis]	3.066812363	1.616739901	1.63E-06	up	yes	0.9823	0.3203	0.299	0.34	0.322	1	0.968	0.979	------	------	K17418	MRPL37	------	------	ENOG410ZT8U	J:Translation, ribosomal structure	PF07147.15	PDCD9	Mitochondrial 28S ribos	CYT	3	6	47.1	High
TRINITY_DN144258_c0_g1_i1_orf1	-	-	-	PREDICTED: enhancer of rudimentary homolog [Microplitis demolitor] >XP_044577051.1 enhancer of rudimentary homolog [Cotesia glomerata] >KAG8041963.1 hypothetical protein G9C98_007267 [Cotesia typhae] >KAH0539785.1 hypothetical protein KQX54_008036 [Cotesia glomerata] >CAD6227368.1 GSCOCG00006137001-RA-CDS [Cotesia congregata]	2.368035574	1.243690754	0.005071	up	yes	0.9053	0.3823	0.414	0.271	0.462	1	0.759	0.957	BP:cellular process;B	GO:0009987;GO:0008150;GO:0007049	------	------	------	------	ENOG4111Z48	S:Function unknown	PF01133.20	ER	Enhancer of rudimentar	CYT	1	10	12.1	Medium
TRINITY_DN1044_c0_g1_i2_orf1	-	-	-	V-type proton ATPase subunit H isoform X3 [Ostrinia furnacalis] >QRR19186.1 V-type proton ATPase subunit H [Ostrinia nubilalis]	4.18115649	2.063902041	4.24E-06	up	yes	1.034	0.2473	0.259	0.259	0.224	1	1.068	1.034	CC:vacuolar proton-	GO:0000221;GO:0033180;GO:0033178;GO:0032991;GO:0005575;GO:0098796;GO:0003674;GO:0015075;GO:0042626;GO:0015318;GO:0009678;GO:0015399;GO:0022804;GO:0042625;GO:0005215;GO:0008324;GO:0046961;GO:0044769;GO:0022890;GO:0019829;GO:0022857;GO:0022853;GO:0015078	K02144	ATPeV1H	map05152;map05	Tuberculosis;Human papillor	COG5231	C:Energy production and conver	PF03224.17;PF1	V-ATPase_H_N	V-ATPase subunit H;V-	CYT	15	38	55.2	High
TRINITY_DN2953_c1_g1_i2_orf1	-	-	-	methionine--tRNA ligase, cytoplasmic isoform X6 [Ostrinia furnacalis]	5.585957666	2.48180464	8.48E-05	up	yes	1.082	0.1937	0.215	0.183	0.183	1	1.183	1.062	BP:methionyl-tRNA	GO:0006431;GO:0019752;GO:0090304;GO:0034641;GO:0006807;GO:0044281;GO:0034660;GO:1901360;GO:0006139;GO:0006520;GO:0071704;GO:0044238;GO:0009987;GO:0006725;GO:0008150;GO:0008152;GO:0043436;GO:1901564;GO:0046483;GO:0016070;GO:0006082;GO:0044237;GO:0043170;GO:0006399;GO:0043038;GO:0043039;GO:0006418;GO:0017101;GO:1902494;GO:0032991;GO:0005575;GO:0035639;GO:0003674;GO:0043167;GO:1901363;GO:0043168;GO:0004812;GO:0000166;GO:1901265;GO:0017076;GO:0005524;GO:0140098;GO:0036094;GO:0003824;GO:0016874;GO:0016875;GO:0004825;GO:0030554;GO:0097367;GO:0097159;GO:0032559;GO:0032555;GO:0140101;GO:0032553;GO:0005488	K01874	MARS, metG	map00450;map00	Selenocompound metabolis	COG0143	J:Translation, ribosomal structure	PF00458.23	WHEP-TRS	WHEP-TRS domain	CYT	1	43	10.3	High
TRINITY_DN108573_c0_g1_i1_orf1	-	-	-	uncharacterized protein LOC114366171 [Ostrinia furnacalis]	3.374740484	1.754776564	1.19E-06	up	yes	0.9753	0.289	0.297	0.275	0.295	1	0.958	0.968	------	------	------	------	------	------	ENOG410XT8F;E	G:Carbohydrate transport and m	PF02958.23;PF0	EcKL;DUF1679	Ecdysteroid kinase-like	CYT	3	7	47.8	High
TRINITY_DN10940_c0_g1_i10_orfp1	-	-	-	TRINITY_DN10940_c0_g1_i10_m.52163 TRINITY_DN10940_c0_g1::TRINITY_DN10940_c0_g1_i10::g.52163 ORF type:5prime_partial len:248 (-) score=128.24 TRINITY_DN10940_c0_g1_i10:121-864(-)	5.183168317	2.373834244	6.83E-06	up	yes	1.047	0.202	0.193	0.194	0.219	1	1.091	1.05	------	------	------	------	------	------	------	------	------	------	------	CYT	1	14	22.7	High
TRINITY_DN2178_c0_g1_i1_orf1	-	-	-	carboxypeptidase B-like [Ostrinia furnacalis]	4.380201489	2.130997235	0.0001239	up	yes	1	0.2283	0.18	0.33	0.175	1	1.022	0.978	BP:nitrogen compou	GO:0006807;GO:0071704;GO:0006508;GO:0008150;GO:0008152;GO:0044238;GO:1901564;GO:0019538;GO:0043170;GO:0008238;GO:0003674;GO:0008270;GO:0008233;GO:0008235;GO:0008237;GO:0043169;GO:0046914;GO:0004181;GO:0004180;GO:0043167;GO:0046872;GO:0016787;GO:0140096;GO:0003824;GO:0005488	K01291;K01298	CPB1;CPA2	map04972;map04	Pancreatic secretion;Protein	COG2866	E:Amino acid transport and met	PF00246.27;PF0	Peptidase_M14	Zinc carboxypeptidase;	CYT	1	2	48.1	High

TRINITY_DN67623_c0_g1_i1_orf1	-	-	-	maltase A1-like [Ostrinia furnacalis]	5.349206349	2.419324858	2.75E-07	up	yes	1.011	0.189	0.189	0.172	0.206	1	1.024	1.01
TRINITY_DN23570_c0_g1_i2_orf1	-	-	-	putative trypsin 6 [Ostrinia nubilalis]	4.158415842	2.056034035	6.45E-05	up	yes	0.966	0.2323	0.256	0.194	0.247	1	0.891	1.007
TRINITY_DN64403_c0_g2_i1_orf1	-	-	-	carboxylesterase [Ostrinia furnacalis]	5.209804955	2.381229362	5.94E-07	up	yes	0.9883	0.1897	0.167	0.206	0.196	1	0.973	0.992
TRINITY_DN48410_c0_g1_i1_orf1	-	-	-	alpha-amylase 1-like [Ostrinia furnacalis]	5.310363538	2.408810629	4.33E-07	up	yes	0.9787	0.1843	0.179	0.192	0.182	1	0.979	0.957

TRINITY_DN67623_c0_g1_i1_orf1	BP:primary metaboli	GO:0044238;GO:0005975;GO:0071704;GO:0008150;GO:0008152;GO:0016021;GO:0005575;GO:0031224;GO:0110165;GO:0016787;GO:0016798;GO:0003824;GO:0003674	------	------	------	------	COG0366	G:Carbohydrate transport and m	PF00128.27	Alpha-amylase	Alpha amylase, catalyti	CYT	4	9	68.6	High
TRINITY_DN23570_c0_g1_i2_orf1	BP:organic substanc	GO:0071704;GO:1901564;GO:0019538;GO:0043170;GO:0006807;GO:0008150;GO:0008152;GO:0006508;GO:0044238;GO:0005615;GO:0005575;GO:0110165;GO:0004252;GO:0004175;GO:0016787;GO:0017171;GO:0140096;GO:0003824;GO:0003674;GO:0008233;GO:0008236	K09628;K09640;I	PRSS27;TMPR	map04972;map05	Pancreatic secretion;Influenz	COG5640	O:Posttranslational modification	PF00089.29	Trypsin	Trypsin	CYT	1	4	26.1	High
TRINITY_DN64403_c0_g2_i1_orf1	MF:hydrolase activity	GO:0016787;GO:0003674;GO:0016788;GO:0052689;GO:0003824	K03927	CES2	map00983	Drug metabolism - other en	COG2272	K:Transcription	PF00135.31	COesterase	Carboxylesterase famil	CYT	1	14	10.6	High
TRINITY_DN48410_c0_g1_i1_orf1	BP:organic substanc	GO:0071704;GO:0044238;GO:0005975;GO:0008152;GO:0008150;GO:0016787;GO:0043169;GO:0016160;GO:0016798;GO:0043167;GO:0003824;GO:0004556;GO:0003674;GO:0005488;GO:0004553;GO:0046872	K01176	AMY, amyA, m	map04972;map04	Pancreatic secretion;Carboh	COG0366	G:Carbohydrate transport and m	PF00128.27;PF0	Alpha-amylase	Alpha amylase, catalyti	EXC	11	29	56	High
