# Supplementary material for: Comparative Proteomics and Metabonomics Analysis of Different Diapause Stages Revealed a New Regulation Mechanism of Diapause in Loxostege sticticalis (Lepidoptera: Pyralidae)
Source: Molecules. 2024 Jul 25;29(15):3472. doi: 10.3390/molecules29153472 (PMC11314584; doi:10.3390/molecules29153472)
Supplement: Supplementary file 1 [file molecules-29-03472-s001.zip › analysis process/proteomic/diffreential protein statistic table/NDvsD.pdf]

Accession	Description	FC(ND/D)	Log2FC(ND/D)	P value(ND/D)	Regulate	Significant	ND	D	D1	D2	D3	ND1	ND2	ND3	GO Term	GO ID	KO ID	KO Name	Pathway ID	Pathway defition	EggNOG ID	EggNOG Functional Categories	Pfam ID	Domain	Domain Description	Subcellular Loc	Unique Peptides	Coverage %	MW [kDa]	FDR Confidence
TRINITY_DN30510_c0_g1_i6_orf1	spodomicin-like [Ostrinia furnacalis]	0.157979929	-2.662186818	1.27E-07	down	yes	0.976	6.178	6.132	6.3	6.102	1	0.961	0.967	------	------	------	------	------	------	------	------	PF08036.14	Antimicrobial_6	Diapausin family of antimicrobial peptide	CYT	3	60	6.7	High
TRINITY_DN971_c0_g1_i5_orfp1	TRINITY_DN971_c0_g1_i5_m.54249 TRINITY_DN971_c0_g1::TRINITY_DN971_c0_g1_i5::g.54249 ORF type:internal len:108 (+),score=66.98 TRINITY_DN971_c0_g1_i5:1-321(+)	0.068941718	-3.858478944	2.67E-05	down	yes	0.899	13.04	13.238	13.875	11.997	1	0.708	0.989	------	------	------	------	------	------	------	------	------	------	------	CYT	1	9	11.5	High
TRINITY_DN15202_c0_g1_i6_orf1	uncharacterized protein LOC114364499 isoform X2 [Ostrinia furnacalis]	0.143748267	-2.798383529	2.26E-06	down	yes	1.037	7.214	6.923	7.364	7.355	1	1.132	0.98	------	------	------	------	------	------	ENOG410XU1K	S:Function unknown	------	------	------	CYT	2	41	14	High
TRINITY_DN971_c0_g1_i10_orfp1	TRINITY_DN971_c0_g1_i10_m.54268 TRINITY_DN971_c0_g1::TRINITY_DN971_c0_g1_i10::g.54268 ORF type:internal len:187 (+),score=141.68 TRINITY_DN971_c0_g1_i10:1-558(+)	0.031769984	-4.976191841	3.67E-07	down	yes	0.779	24.52	25.204	24.144	24.205	1	0.834	0.503	------	------	------	------	------	------	------	------	------	------	------	CYT	2	20	20.5	High
TRINITY_DN20442_c0_g2_i1_orf1	hypothetical protein evm_008218 [Chilo suppressalis]	0.176664822	-2.5009133	1.53E-05	down	yes	0.9577	5.421	5.673	5.108	5.481	1	0.827	1.046	BP:nucleosome organization;BP:cellular component assembly;BP:regulation of metabolic process;BP:macromolecular complex subunit organization;BP:regulation of cellular biosynthetic process;BP:regulation of cellular metabolic process;BP:cellular component organization or biogenesis;BP:regulation of primary metabolic process;BP:protein-DNA complex subunit organization;BP:chromatin organization;BP:cellular component organization;BP:regulation of biological process;BP:macromolecular complex assembly;BP:regulation of nucleobase-containing compound metabolic process;BP:protein-DNA complex assembly;BP:biological regulation;BP:regulation of gene expression;BP:regulation of macromolecule metabolic process;BP:regulation of biosynthetic process;BP:cellular process;BP:regulation of nucleic acid-templated transcription;BP:regulation of cellular process;BP:biological_process;BP:regulation of nitrogen compound metabolic process;BP:regulation of RNA biosynthetic process;BP:chromatin remodeling;BP:nucleosome assembly;BP:regulation of RNA metabolic process;BP:regulation of transcription, DNA-templated;BP:regulation of macromolecule biosynthetic process;CC:nucleosome;CC:DNA packaging complex;CC:macromolecular complex;CC:protein-DNA complex;CC:cellular_component;MF:nucleic acid binding;MF:peptide-lysine-N-acetyltransferase activity;MF:molecular_function;MF:binding;MF:peptide N-acetyltransferase activity;MF:DNA binding;MF:ion binding;MF:heterocyclic compound binding;MF:cation binding;MF:N-acetyltransferase activity;MF:histone acetyltransferase activity;MF:transferase activity;MF:transferase activity, transferring acyl groups;MF:transferase activity, transferring acyl groups other than amino-acyl groups;MF:N-acyltransferase activity;MF:metal ion binding;MF:catalytic activity, acting on a protein;MF:catalytic activity;MF:organic cyclic compound	GO:0034728;GO:0022607;GO:0019222;GO:0043933;GO:0031326;GO:0031323;GO:0071840;GO:0080090;GO:0071824;GO:0006325;GO:0016043;GO:0050789;GO:0065003;GO:0019219;GO:0065004;GO:0065007;GO:0010468;GO:0060255;GO:0009889;GO:0009987;GO:1903506;GO:0050794;GO:0008150;GO:0051171;GO:2001141;GO:0006338;GO:0006334;GO:0051252;GO:0006355;GO:0010556;GO:0000786;GO:0044815;GO:0032991;GO:0032993;GO:0005575;GO:0003676;GO:0061733;GO:0003674;GO:0005488;GO:0034212;GO:0003677;GO:0043167;GO:1901363;GO:0043169;GO:0008080;GO:0004402;GO:0016740;GO:0016746;GO:0016747;GO:0016410;GO:0046872;GO:0140096;GO:0003824;GO:0097159;GO:0016407	K11306	MYST4, KAT6	------	------	COG5027	B:Chromatin structure and dynamics	------	------	------	CYT	1	7	13.7	Medium
TRINITY_DN38307_c0_g1_i1_orfp1	TRINITY_DN38307_c0_g1_i1_m.10661 TRINITY_DN38307_c0_g1::TRINITY_DN38307_c0_g1_i1::g.10661 ORF type:5prime_partial len:66 (+),score=5.90 TRINITY_DN38307_c0_g1_i1:2-199(+)	0.166611185	-2.585442839	0.002539	down	yes	1.001	6.008	7.437	5.538	5.048	1	0.741	1.262	------	------	------	------	------	------	------	------	------	------	------	CYT	1	17	7.9	High
TRINITY_DN295_c3_g1_i1_orfp1	TRINITY_DN295_c3_g1_i1_m.18839 TRINITY_DN295_c3_g1::TRINITY_DN295_c3_g1_i1::g.18839 ORF type:5prime_partial len:118 (+),score=46.25 TRINITY_DN295_c3_g1_i1:1-354(+)	0.080351171	-3.637537148	1.22E-05	down	yes	0.961	11.96	12.279	12.471	11.14	1	0.975	0.908	------	------	------	------	------	------	------	------	------	------	------	PLA	3	49	12.6	High
TRINITY_DN3166_c1_g1_i6_orf1	hypothetical protein evm_013813 [Chilo suppressalis]	0.104119811	-3.263683499	2.22E-06	down	yes	0.9907	9.515	9.892	9.487	9.167	1	0.958	1.014	BP:response to biotic stimulus;BP:response to external stimulus;BP:response to external biotic stimulus;BP:response to stimulus;BP:defense response;BP:response to stress;BP:biological_process;BP:interspecies interaction between organisms;BP:response to other organism;CC:cellular_component;CC:extracellular region;CC:cellular anatomical entity	GO:0009607;GO:0009605;GO:0043207;GO:0050896;GO:0006952;GO:0006950;GO:0008150;GO:0044419;GO:0051707;GO:0005575;GO:0005576;GO:0110165	------	------	------	------	------	------	PF00451.22	Toxin_2	Scorpion short toxin, BmKK2	CYT	1	13	10.5	High
TRINITY_DN380_c0_g2_i2_orf1	chemosensory protein 10 [Ostrinia furnacalis]	0.217731421	-2.199378475	6.74E-07	down	yes	1.002	4.602	4.518	4.732	4.556	1	1.005	1.001	------	------	------	------	------	------	ENOG4111ATT;ENO	S:Function unknown;	PF03392.16	OS-D	Insect pheromone-binding family, A10/OS-D	CYT	3	54	13.6	High
TRINITY_DN14904_c0_g1_i1_orf1	attacin [Ostrinia furnacalis]	0.121367521	-3.042545695	1.50E-05	down	yes	1.065	8.775	8.823	9.278	8.223	1	1.089	1.107	BP:interspecies interaction between organisms;BP:response to biotic stimulus;BP:response to external stimulus;BP:response to external biotic stimulus;BP:response to stimulus;BP:response to bacterium;BP:defense response;BP:response to stress;BP:defense response to other organism;BP:defense response to bacterium;BP:biological_process;BP:response to other organism;CC:cellular_component;CC:extracellular region;CC:cellular anatomical entity	GO:0044419;GO:0009607;GO:0009605;GO:0043207;GO:0050896;GO:0009617;GO:0006952;GO:0006950;GO:0098542;GO:0042742;GO:0008150;GO:0051707;GO:0005575;GO:0005576;GO:0110165	------	------	------	------	ENOG410ZBQ8;ENO	S:Function unknown;	PF03769.18	Attacin_C	Attacin, C-terminal region	CYT	3	26	17.3	High
TRINITY_DN143895_c0_g1_i1_orf1	cathepsin L-like [Aphidius gifuensis] >KAF7988186.1 hypothetical protein HCN44_007680 [Aphidius gifuensis]	0.152645971	-2.711738587	2.88E-05	down	yes	0.949	6.217	6.675	5.86	6.115	1	0.834	1.013	BP:primary metabolic process;BP:organonitrogen compound metabolic process;BP:protein metabolic process;BP:macromolecule metabolic process;BP:organic substance metabolic process;BP:nitrogen compound metabolic process;BP:biological_process;BP:metabolic process;BP:proteolysis;MF:molecular_function;MF:hydrolase activity;MF:catalytic activity;MF:catalytic activity, acting on a protein;MF:peptidase activity;MF:cysteine-type peptidase activity;	GO:0044238;GO:1901564;GO:0019538;GO:0043170;GO:0071704;GO:0006807;GO:0008150;GO:0008152;GO:0006508;GO:0003674;GO:0016787;GO:0003824;GO:0140096;GO:0008233;GO:0008234	K01365	CTSL	map05205;map	Proteoglycans in car	COG4870	O:Posttranslational modification, pro	PF00112.26;PF082	Peptidase_C1;Inhibitor_	Papain family cysteine protease;Cathepsin propeptide inhibitor domain;Peptidase C1-like family	CYT	1	2	38.3	High
TRINITY_DN2396_c0_g1_i9_orfp1	TRINITY_DN2396_c0_g1_i9_m.39038 TRINITY_DN2396_c0_g1::TRINITY_DN2396_c0_g1_i9::g.39038 ORF type:5prime_partial len:181 (+),score=90.33 TRINITY_DN2396_c0_g1_i9:3-545(+)	0.044439935	-4.491999481	2.94E-05	down	yes	1.095	24.64	24.391	26.632	22.885	1	0.759	1.526	------	------	------	------	------	------	------	------	------	------	------	CYT	1	6	19.3	High
TRINITY_DN14242_c0_g1_i2_orfp1	TRINITY_DN14242_c0_g1_i2_m.18449 TRINITY_DN14242_c0_g1::TRINITY_DN14242_c0_g1_i2::g.18449 ORF type:internal len:148 (-),score=102.30 TRINITY_DN14242_c0_g1_i2:2-442(-)	0.132571996	-2.915152036	0.002107	down	yes	1.335	10.07	12.447	8.38	9.395	1	1.386	1.618	------	------	------	------	------	------	------	------	------	------	------	CYT	1	8	16	High
TRINITY_DN14328_c0_g1_i12_orf1	larval cuticle protein LCP-30-like [Ostrinia furnacalis]	0.235683322	-2.085078425	2.11E-05	down	yes	1.033	4.383	4.501	4.549	4.098	1	1.012	1.086	MF:molecular_function;MF:structural constituent of cuticle;MF:structural molecule activity;	GO:0003674;GO:0042302;GO:0005198	------	------	------	------	ENOG41112O7;ENO	S:Function unknown;	PF00379.26	Chitin_bind_4	Insect cuticle protein	CYT	10	50	24.6	High
TRINITY_DN1108_c3_g1_i1_orfp1	TRINITY_DN1108_c3_g1_i1_m.5561 TRINITY_DN1108_c3_g1::TRINITY_DN1108_c3_g1_i1::g.5561 ORF type:internal len:113 (+),score=89.07 TRINITY_DN1108_c3_g1_i1:1-336(+)	0.18480406	-2.435931645	0.00722	down	yes	1.311	7.094	8.19	5.161	7.93	1	2.486	0.447	------	------	------	------	------	------	------	------	------	------	------	PLA	1	11	12.3	High
TRINITY_DN9608_c0_g1_i3_orf1	cytochrome P450 monooxygenase CYP9G18 [Cnaphalocrocis medinalis]	0.185949149	-2.427019947	8.68E-06	down	yes	0.9727	5.231	5.455	4.954	5.284	1	0.942	0.976	CC:endoplasmic reticulum membrane;CC:intrinsic component of membrane;CC:organelle membrane;CC:integral component of membrane;CC:membrane;CC:cellular anatomical entity;CC:cellular_component;MF:monooxygenase activity;MF:oxidoreductase activity, acting on paired donors, with incorporation or reduction of molecular oxygen;MF:molecular_function;MF:binding;MF:heterocyclic compound binding;MF:oxidoreductase activity;MF:cation binding;MF:transition metal ion binding;MF:ion binding;MF:iron ion binding;MF:metal ion binding;MF:heme binding;MF:catalytic activity;MF:tetrapyrrole	GO:0005789;GO:0031224;GO:0031090;GO:0016021;GO:0016020;GO:0110165;GO:0005575;GO:0004497;GO:0016705;GO:0003674;GO:0005488;GO:1901363;GO:0016491;GO:0043169;GO:0046914;GO:0043167;GO:0005506;GO:0046872;GO:0020037;GO:0003824;GO:0046906;GO:0097159	K15003	CYP9	------	------	COG2124	K:Transcription	PF00067.25	p450	Cytochrome P450	CYT	7	17	58.5	High
TRINITY_DN5406_c0_g2_i1_orf1	uncharacterized protein LOC114350326 [Ostrinia furnacalis]	0.338112306	-1.56442557	0.0008322	down	yes	1.132	3.348	3.659	3.486	2.898	1	1.108	1.289	BP:positive regulation of ion transmembrane transporter activity;BP:positive regulation of transporter activity;BP:regulation of ion transmembrane transporter activity;BP:regulation of transport;BP:modulation of synaptic transmission;BP:regulation of potassium ion transport;BP:regulation of signaling;BP:positive regulation of transmembrane transport;BP:regulation of ion transmembrane transport;BP:positive regulation of ion transmembrane transport;BP:regulation of biological process;BP:regulation of transmembrane transport;BP:positive regulation of potassium ion transport;BP:regulation of cell communication;BP:rhythmic process;BP:regulation of transporter activity;BP:biological regulation;BP:regulation of ion transport;BP:positive regulation of molecular function;BP:regulation of trans-synaptic signaling;BP:positive regulation of biological process;BP:regulation of molecular function;BP:positive regulation of potassium ion transmembrane transport;BP:multicellular organismal process;BP:positive regulation of transport;BP:regulation of cellular process;BP:positive regulation of ion transport;BP:biological_process;BP:sleep;BP:positive regulation of voltage-gated potassium channel activity;BP:regulation of cation channel activity;BP:regulation of localization;BP:positive regulation of potassium ion transmembrane transporter activity;BP:regulation of metal ion transport;BP:regulation of synaptic transmission, cholinergic;BP:regulation of transmembrane transporter activity;BP:positive regulation of cation transmembrane transport;BP:regulation of cation transmembrane transport;BP:regulation of potassium ion transmembrane transporter activity;BP:positive regulation of cation channel activity;BP:regulation of potassium ion transmembrane transport;BP:positive regulation of cellular process;CC:plasma membrane;CC:intrinsic component of membrane;CC:membrane;CC:anchored component of membrane;CC:cellular anatomical entity;CC:cellular_component;	GO:0032414;GO:0032411;GO:0032412;GO:0051049;GO:0050804;GO:0043266;GO:0023051;GO:0034764;GO:0034765;GO:0034767;GO:0050789;GO:0034762;GO:0043268;GO:0010646;GO:0048511;GO:0032409;GO:0065007;GO:0043269;GO:0044093;GO:0099177;GO:0048518;GO:0065009;GO:1901381;GO:0032501;GO:0051050;GO:0050794;GO:0043270;GO:0008150;GO:0030431;GO:1903818;GO:2001257;GO:0032879;GO:1901018;GO:0010959;GO:0032222;GO:0022898;GO:1904064;GO:1904062;GO:1901016;GO:2001259;GO:1901379;GO:0048522;GO:0005886;GO:0031224;GO:0016020;GO:0031225;GO:0110165;GO:0005575	------	------	------	------	ENOG410ZEPJ;ENOG	S:Function unknown;	PF17064.8	QVR	Sleepless protein	CYT	1	9	15.6	High
TRINITY_DN15247_c0_g1_i2_orf1	probable G-protein coupled receptor Mth-like 3 isoform X1 [Ostrinia furnacalis]	0.293554007	-1.768302145	8.80E-06	down	yes	1.011	3.444	3.494	3.28	3.557	1	1.037	0.996	BP:cellular process;BP:regulation of cellular process;BP:biological regulation;BP:cell surface receptor signaling pathway;BP:biological_process;BP:regulation of biological process;BP:signal transduction;CC:integral component of membrane;CC:cellular_component;CC:cellular anatomical entity;CC:intrinsic component of membrane;MF:G-protein coupled receptor activity;MF:signaling receptor activity;MF:molecular transducer activity;MF:transmembrane signaling receptor activity;MF:molecular_function;	GO:0009987;GO:0050794;GO:0065007;GO:0007166;GO:0008150;GO:0050789;GO:0007165;GO:0016021;GO:0005575;GO:0110165;GO:0031224;GO:0004930;GO:0038023;GO:0060089;GO:0004888;GO:0003674	K04599	MTH	------	------	ENOG410XSJF;ENOG	S:Function unknown;	PF00002.27	7tm_2	7 transmembrane receptor	CYT	1	2	46.6	High
TRINITY_DN31286_c0_g1_i6_orfp1	TRINITY_DN31286_c0_g1_i6_m.28438 TRINITY_DN31286_c0_g1::TRINITY_DN31286_c0_g1_i6::g.28438 ORF type:internal len:92 (-),score=3.10,Perilipin PF03036.17 2e-05 TRINITY_DN31286_c0_g1_i6:1-273(-)	0.303523838	-1.72011827	4.49E-06	down	yes	1.025	3.377	3.504	3.355	3.271	1	1.037	1.038	------	------	------	------	------	------	------	------	PF03036.19	Perilipin	Perilipin family	CYT	5	71	9.4	High
TRINITY_DN2457_c0_g1_i8_orf1	uncharacterized protein LOC114355596 [Ostrinia furnacalis]	0.295508982	-1.758726113	3.81E-05	down	yes	0.987	3.34	3.191	3.569	3.261	1	0.94	1.021	BP:biological_process;BP:metabolic process;BP:methylation;MF:catalytic activity;MF:transferase activity;MF:transferase activity, transferring one-carbon groups;MF:molecular_function;MF:methyltransferase	GO:0008150;GO:0008152;GO:0032259;GO:0003824;GO:0016740;GO:0016741;GO:0003674;GO:0008168	------	------	------	------	ENOG410Y1IK	M:Cell wall/membrane/envelope bio	PF12248.11	Methyltransf_FA	Farnesoic acid 0-methyl transferase	CYT	7	27	33.4	High
TRINITY_DN60946_c0_g2_i3_orf1	protein yellow-like [Ostrinia furnacalis]	0.316938319	-1.657725998	1.28E-06	down	yes	0.9917	3.129	3.186	3.163	3.039	1	0.981	0.994	------	------	------	------	------	------	COG3386	S:Function unknown	PF03022.19	MRJP	Major royal jelly protein	CYT	13	36	49.2	High
TRINITY_DN2464_c0_g1_i12_orf1	uncharacterized protein LOC114362996 isoform X1 [Ostrinia furnacalis]	0.324137931	-1.625320238	0.0001545	down	yes	0.987	3.045	3.34	2.901	2.893	1	0.981	0.98	MF:molecular_function;MF:structural constituent of cuticle;MF:structural molecule activity;	GO:0003674;GO:0042302;GO:0005198	------	------	------	------	ENOG410YNFC	S:Function unknown	PF00379.26	Chitin_bind_4	Insect cuticle protein	CYT	8	45	37.2	High
TRINITY_DN7040_c0_g1_i4_orf1	uncharacterized protein LOC114353763 [Ostrinia furnacalis]	0.31413839	-1.670527834	7.38E-05	down	yes	0.9443	3.006	3.213	2.999	2.806	1	0.878	0.955	------	------	------	------	------	------	------	------	------	------	------	CYT	2	39	7.4	High
TRINITY_DN276_c0_g1_i1_orf1	protein lethal(2)essential for life-like [Helicoverpa zea] >XP_049705426.1 protein lethal(2)essential for life [Helicoverpa armigera] >ATB54993.1 heat shock protein 20.8 [Helicoverpa armigera] >PZC74337.1 hypothetical protein B5X24_HaOG207971 [Helicoverpa armigera]	0.334943474	-1.578010453	2.44E-06	down	yes	0.9777	2.919	2.998	2.924	2.835	1	0.953	0.98	MF:cation binding;MF:metal ion binding;MF:molecular_function;MF:binding;MF:ion binding;	GO:0043169;GO:0046872;GO:0003674;GO:0005488;GO:0043167	K09542	CRYAB	map04213;map	Longevity regulating	ENOG410YERS	O:Posttranslational modification, pro	PF00011.24	HSP20	Hsp20/alpha crystallin family	CYT	5	65	21.3	High
TRINITY_DN295_c5_g1_i2_orf1	unnamed protein product [Chilo suppressalis]	0.371233859	-1.429599793	6.01E-05	down	yes	1.035	2.788	2.729	2.969	2.666	1	0.997	1.107	MF:hydrolase activity;MF:molecular_function;MF:catalytic activity;	GO:0016787;GO:0003674;GO:0003824	K21013	FPPP	map00981	Insect hormone bios	COG0647	G:Carbohydrate transport and metab	PF13344.9	Hydrolase_6	Haloacid dehalogenase-like hydrolase	CYT	5	24	19.9	High
TRINITY_DN122321_c0_g1_i1_orf1	uncharacterized protein LOC114356271 [Ostrinia furnacalis]	0.373666789	-1.420175749	0.003554	down	yes	1.016	2.719	2.592	2.33	3.234	1	1.141	0.906	CC:cellular_component;CC:cellular anatomical entity;CC:extracellular space;MF:endopeptidase inhibitor activity;MF:endopeptidase regulator activity;MF:peptidase regulator activity;MF:molecular_function;MF:peptidase inhibitor activity;MF:serine-type endopeptidase inhibitor activity;MF:enzyme inhibitor activity;MF:molecular function regulator;MF:enzyme regulator activity;	GO:0005575;GO:0110165;GO:0005615;GO:0004866;GO:0061135;GO:0061134;GO:0030414;GO:0004867;GO:0004857;GO:0098772;GO:0030234	------	------	------	------	COG4826	J:Translation, ribosomal structure and	PF00079.23	Serpin	Serpin	CYT	1	32	7.6	High
TRINITY_DN69307_c0_g1_i6_orf1	hypothetical protein evm_010738 [Chilo suppressalis]	0.405741429	-1.301367475	1.89E-06	down	yes	0.9823	2.421	2.356	2.447	2.461	1	0.97	0.977	------	------	------	------	------	------	ENOG410XU1K	S:Function unknown	------	------	------	CYT	1	29	9.5	High
TRINITY_DN51480_c0_g1_i1_orf1	uncharacterized protein LOC114361588 isoform X14 [Ostrinia furnacalis]	0.454232918	-1.138495834	3.63E-05	down	yes	1.057	2.327	2.229	2.332	2.421	1	1.067	1.104	------	------	K10382	DST	------	------	COG5069;ENOG410	Z:Cytoskeleton;	PF00681.23	Plectin	Plectin repeat	CYT	3	27	12.6	High
TRINITY_DN21420_c0_g1_i2_orf1	glutathione peroxidase, partial [Ostrinia furnacalis]	0.417134478	-1.261415531	8.13E-06	down	yes	0.9957	2.387	2.321	2.443	2.398	1	0.939	1.048	BP:response to stimulus;BP:response to stress;BP:biological_process;BP:response to oxidative stress;MF:antioxidant activity;MF:peroxidase activity;MF:catalytic activity;MF:molecular_function;MF:oxidoreductase activity, acting on peroxide as acceptor;MF:glutathione	GO:0050896;GO:0006950;GO:0008150;GO:0006979;GO:0016209;GO:0004601;GO:0003824;GO:0003674;GO:0016684;GO:0004602;GO:0016491	K05361;K00432	GPX4;gpx, btu	map00480;map	Glutathione metabo	COG0386	U:Intracellular trafficking, secretion, a	------	------	------	CYT	5	76	8.3	High
TRINITY_DN44517_c0_g1_i4_orf1	regucalcin-like [Ostrinia furnacalis]	0.418204698	-1.257718826	0.0001309	down	yes	0.997	2.384	2.243	2.403	2.507	1	0.897	1.094	CC:cytoplasm;CC:cellular_component;CC:cellular anatomical entity;MF:hydrolase activity;MF:metal ion binding;MF:cation binding;MF:enzyme regulator activity;MF:gluconolactonase activity;MF:carboxylic ester hydrolase activity;MF:ion binding;MF:catalytic activity;MF:molecular_function;MF:hydrolase activity, acting on ester bonds;MF:binding;MF:molecular function regulator;MF:calcium ion binding;	GO:0005737;GO:0005575;GO:0110165;GO:0016787;GO:0046872;GO:0043169;GO:0030234;GO:0004341;GO:0052689;GO:0043167;GO:0003824;GO:0003674;GO:0016788;GO:0005488;GO:0098772;GO:0005509	K01053	gnl, RGN	map00030;map	Pentose phosphate	COG3386;COG3386;	I:Lipid transport and metabolism;Q:S	PF08450.15;PF074	SGL;Reg_prop	SMP-30/Gluconolactonase/LRE-like region;Two component regulator propeller	CYT	1	4	36.9	High
TRINITY_DN71863_c0_g1_i2_orf1	unnamed protein product [Diatraea saccharalis]	0.437795954	-1.191669475	3.63E-06	down	yes	1.017	2.323	2.383	2.326	2.261	1	1.02	1.032	BP:organic substance metabolic process;BP:organonitrogen compound metabolic process;BP:protein metabolic process;BP:macromolecule metabolic process;BP:biological_process;BP:nitrogen compound metabolic process;BP:metabolic process;BP:proteolysis;BP:primary metabolic process;MF:serine-type endopeptidase activity;MF:endopeptidase activity;MF:hydrolase activity;MF:serine hydrolase activity;MF:catalytic activity, acting on a protein;MF:catalytic activity;MF:molecular_function;MF:peptidase activity;MF:serine-type peptidase activity;	GO:0071704;GO:1901564;GO:0019538;GO:0043170;GO:0008150;GO:0006807;GO:0008152;GO:0006508;GO:0044238;GO:0004252;GO:0004175;GO:0016787;GO:0017171;GO:0140096;GO:0003824;GO:0003674;GO:0008233;GO:0008236	------	------	------	------	COG5640	O:Posttranslational modification, pro	PF16030.8	GD_N	Serine protease gd N-terminus	CYT	3	25	17.8	High
TRINITY_DN960_c1_g1_i6_orf1	hypothetical protein evm_007130 [Chilo suppressalis]	0.438481675	-1.189411543	1.36E-06	down	yes	1.005	2.292	2.279	2.273	2.323	1	1.048	0.968	BP:transport;BP:intracellular transport;BP:sterol transport;BP:lipid transport;BP:intracellular sterol transport;BP:intracellular cholesterol transport;BP:intracellular lipid transport;BP:organic hydroxy compound transport;BP:cholesterol transport;BP:biological_process;BP:organic substance transport;BP:cellular process;BP:establishment of localization;BP:localization;BP:establishment of localization in cell;BP:cellular localization;CC:cellular_component;CC:extracellular region;CC:cellular anatomical entity;	GO:0006810;GO:0046907;GO:0015918;GO:0006869;GO:0032366;GO:0032367;GO:0032365;GO:0015850;GO:0030301;GO:0008150;GO:0071702;GO:0009987;GO:0051234;GO:0051179;GO:0051649;GO:0051641;GO:0005575;GO:0005576;GO:0110165	K13443	NPC2	map04979;map	Cholesterol metabol	ENOG4111Q8S	S:Function unknown	PF02221.18	E1_DerP2_DerF2	ML domain	PLA	5	38	15.5	High
TRINITY_DN1750_c1_g1_i5_orf1	lipid droplet localized protein-like [Ostrinia furnacalis] >XP_028161280.1 lipid droplet localized protein-like [Ostrinia furnacalis]	0.451584114	-1.146933359	1.21E-05	down	yes	1.012	2.241	2.239	2.32	2.163	1	1.004	1.032	BP:nucleic acid metabolic process;BP:cellular biosynthetic process;BP:cellular nitrogen compound metabolic process;BP:nitrogen compound metabolic process;BP:organic cyclic compound biosynthetic process;BP:nucleobase-containing compound metabolic process;BP:cellular macromolecule metabolic process;BP:organic substance metabolic process;BP:organic cyclic compound metabolic process;BP:heterocycle biosynthetic process;BP:organic substance biosynthetic process;BP:cellular process;BP:cellular aromatic compound metabolic process;BP:biosynthetic process;BP:macromolecule biosynthetic process;BP:biological_process;BP:metabolic process;BP:nucleobase-containing compound biosynthetic process;BP:heterocycle metabolic process;BP:primary metabolic process;BP:cellular nitrogen compound biosynthetic process;BP:cellular metabolic process;BP:macromolecule metabolic process;BP:DNA metabolic process;BP:aromatic compound biosynthetic process;BP:DNA biosynthetic process;CC:intrinsic component of membrane;CC:integral component of membrane;CC:cellular anatomical entity;CC:cellular_component;MF:molecular_function;MF:oxidoreductase activity;MF:catalytic activity;	GO:0090304;GO:0044249;GO:0034641;GO:0006807;GO:1901362;GO:0006139;GO:0044260;GO:0071704;GO:1901360;GO:0018130;GO:1901576;GO:0009987;GO:0006725;GO:0009058;GO:0009059;GO:0008150;GO:0008152;GO:0034654;GO:0046483;GO:0044238;GO:0044271;GO:0044237;GO:0043170;GO:0006259;GO:0019438;GO:0071897;GO:0031224;GO:0016021;GO:0110165;GO:0005575;GO:0003674;GO:0016491;GO:0003824	------	------	------	------	COG3268	S:Function unknown	PF03435.21	Sacchrp_dh_NADP	Saccharopine dehydrogenase NADP binding domain	CYT	15	35	47.8	High
TRINITY_DN1230_c1_g1_i5_orf1	uncharacterized protein LOC114353440 [Ostrinia furnacalis]	0.397914857	-1.32946833	1.86E-05	down	yes	0.916	2.302	2.299	2.287	2.321	1	0.942	0.806	------	------	------	------	------	------	ENOG4110OI3;ENO	S:Function unknown;	------	------	------	CYT	1	3	35.6	High
TRINITY_DN13098_c2_g1_i2_orf1	unnamed protein product [Arctia plantaginis] >CAB3253298.1 unnamed protein product [Arctia plantaginis]	0.484235294	-1.046219859	5.33E-06	down	yes	1.029	2.125	2.135	2.093	2.148	1	1.088	1	MF:cation binding;MF:ion binding;MF:molecular_function;MF:binding;MF:metal ion binding;MF:calcium ion binding;	GO:0043169;GO:0043167;GO:0003674;GO:0005488;GO:0046872;GO:0005509	K23922	EFHD2	------	------	ENOG4111JG9	S:Function unknown	PF00036.35;PF134	EF-hand_1;EF-hand_7;	EF hand;EF-hand domain pair;EF-hand domain;EF	CYT	6	41	22.9	High
TRINITY_DN2207_c0_g1_i4_orf1	methionine-R-sulfoxide reductase B1 isoform X4 [Pectinophora gossypiella] >XP_049887601.1 methionine-R-sulfoxide reductase B1 isoform X4 [Pectinophora gossypiella]	0.438820302	-1.188297822	3.09E-05	down	yes	0.9597	2.187	2.114	2.277	2.17	1	0.987	0.892	------	------	K07305	msrB	------	------	COG0229	O:Posttranslational modification, pro	PF01641.21	SelR	SelR domain	CYT	1	67	5.3	High
TRINITY_DN2392_c0_g2_i1_orf1	cytochrome P450 9e2-like [Ostrinia furnacalis] >QPF77612.1 cytochrome P450 monooxygenase CYP9A185 [Ostrinia furnacalis]	0.462867819	-1.111327833	2.08E-06	down	yes	0.991	2.141	2.185	2.106	2.131	1	0.961	1.012	CC:integral component of membrane;CC:cellular_component;CC:cellular anatomical entity;CC:intrinsic component of membrane;MF:cation binding;MF:monooxygenase activity;MF:heme binding;MF:transition metal ion binding;MF:tetrapyrrole binding;MF:organic cyclic compound binding;MF:oxidoreductase activity, acting on paired donors, with incorporation or reduction of molecular oxygen;MF:ion binding;MF:catalytic activity;MF:molecular_function;MF:binding;MF:iron ion binding;MF:metal ion binding;MF:heterocyclic compound binding;MF:oxidoreductase activity;	GO:0016021;GO:0005575;GO:0110165;GO:0031224;GO:0043169;GO:0004497;GO:0020037;GO:0046914;GO:0046906;GO:0097159;GO:0016705;GO:0043167;GO:0003824;GO:0003674;GO:0005488;GO:0005506;GO:0046872;GO:1901363;GO:0016491	K15003	CYP9	------	------	COG2124	Q:Secondary metabolites biosynthesi	PF00067.25	p450	Cytochrome P450	CYT	6	17	61.5	High
TRINITY_DN6330_c0_g1_i1_orfp1	TRINITY_DN6330_c0_g1_i1_m.42332 TRINITY_DN6330_c0_g1::TRINITY_DN6330_c0_g1_i1::g.42332 ORF type:3prime_partial len:51 (-),score=33.30 TRINITY_DN6330_c0_g1_i1:3-152(-)	0.170552867	-2.551709088	0.0006541	down	yes	1.163	6.819	5.909	7.901	6.646	1	1.304	1.186	------	------	------	------	------	------	------	------	------	------	------	CYT	1	26	5.3	High
TRINITY_DN12009_c0_g1_i1_orf1	uncharacterized protein LOC114365631 [Ostrinia furnacalis]	0.179048867	-2.481574703	3.93E-05	down	yes	0.9563	5.341	5.649	4.911	5.463	1	0.946	0.923	------	------	------	------	------	------	------	------	------	------	------	CYT	2	17	8.2	High
TRINITY_DN2813_c0_g1_i7_orf1	arylphorin subunit alpha-like [Ostrinia furnacalis]	0.206851691	-2.273331342	9.13E-06	down	yes	0.954	4.612	4.702	4.772	4.363	1	0.948	0.914	------	------	------	------	------	------	ENOG4110QFU;ENO	S:Function unknown;	PF03723.17;PF003	Hemocyanin_C;Hemoc	Hemocyanin, ig-like domain;Hemocyanin, copper containing domain;Hemocyanin, all-alpha domain	CYT	6	36	139.8	High
TRINITY_DN394_c0_g1_i2_orf1	uncharacterized protein LOC114351483 [Ostrinia furnacalis]	0.213428766	-2.228173457	9.07E-05	down	yes	0.9393	4.401	4.597	4.633	3.972	1	0.883	0.935	MF:molecular_function;MF:structural constituent of cuticle;MF:structural molecule activity;	GO:0003674;GO:0042302;GO:0005198	------	------	------	------	ENOG41113X7;ENO	S:Function unknown;	PF00379.26	Chitin_bind_4	Insect cuticle protein	CYT	1	28	12	High
TRINITY_DN4068_c0_g2_i4_orf1	larval cuticle protein LCP-17-like precursor [Papilio polytes] >BAM18876.1 cuticular protein PcolCPR2 [Papilio polytes]	0.258992806	-1.949016071	1.66E-07	down	yes	1.008	3.892	3.937	3.914	3.825	1	0.988	1.037	MF:molecular_function;MF:structural constituent of cuticle;MF:structural molecule activity;	GO:0003674;GO:0042302;GO:0005198	------	------	------	------	ENOG4110RUZ;ENO	S:Function unknown;	PF00379.26	Chitin_bind_4	Insect cuticle protein	CYT	2	56	13.4	High
TRINITY_DN51813_c0_g1_i1_orf1	uncharacterized protein LOC114350216 [Ostrinia furnacalis]	0.267430783	-1.902762557	0.00196	down	yes	0.8983	3.359	3.097	4.029	2.952	1	0.85	0.845	BP:'de novo' NAD biosynthetic process from tryptophan;BP:aromatic amino acid family catabolic process;BP:nucleotide biosynthetic process;BP:small molecule metabolic process;BP:small molecule catabolic process;BP:small molecule biosynthetic process;BP:organic cyclic compound biosynthetic process;BP:organic cyclic compound metabolic process;BP:organic cyclic compound catabolic process;BP:dicarboxylic acid biosynthetic process;BP:benzene-containing compound metabolic process;BP:nicotinamide nucleotide biosynthetic process;BP:nicotinamide nucleotide metabolic process;BP:monocarboxylic acid metabolic process;BP:oxoacid metabolic process;BP:aromatic amino acid family metabolic process;BP:heterocycle metabolic process;BP:dicarboxylic acid metabolic process;BP:organonitrogen compound metabolic process;BP:organonitrogen compound catabolic process;BP:organonitrogen compound biosynthetic process;BP:organic acid catabolic process;BP:organic acid biosynthetic process;BP:aromatic compound catabolic process;BP:aromatic compound biosynthetic process;BP:cellular amino acid catabolic process;BP:nitrogen compound metabolic process;BP:anthranilate metabolic process;BP:organic substance biosynthetic process;BP:organic substance catabolic process;BP:kynurenine metabolic process;BP:tryptophan catabolic process;BP:tryptophan metabolic process;BP:organophosphate metabolic process;BP:heterocycle biosynthetic process;BP:amine metabolic process;BP:nucleobase-containing compound metabolic process;BP:indolalkylamine metabolic process;BP:biological_process;BP:metabolic process;BP:nucleobase-containing compound biosynthetic process;BP:indole-containing compound catabolic process;BP:organophosphate biosynthetic process;BP:indole-containing compound metabolic process;BP:cellular nitrogen compound biosynthetic process;BP:cellular nitrogen compound catabolic process;BP:carboxylic acid biosynthetic process;BP:carboxylic acid catabolic process;BP:nucleoside phosphate biosynthetic process;BP:nucleoside phosphate metabolic process;BP:cellular biogenic amine metabolic process;BP:cellular catabolic process;BP:cellular biosynthetic process;BP:cellular nitrogen compound metabolic process;BP:alpha-amino acid metabolic process;BP:alpha-amino acid catabolic process;BP:cellular process;BP:cellular aromatic compound metabolic process;BP:cellular amine metabolic process;BP:nucleobase-containing small molecule metabolic process;BP:organic acid metabolic process;BP:heterocycle catabolic process;BP:quinolinate metabolic process;BP:carboxylic acid metabolic process;BP:NAD biosynthetic process;BP:'de novo' NAD biosynthetic process;BP:cellular metabolic process;BP:pyridine-containing compound metabolic process;BP:pyridine-containing compound biosynthetic process;BP:quinolinate biosynthetic process;BP:cellular amino acid metabolic process;BP:organic substance metabolic process;BP:biosynthetic process;BP:nucleotide metabolic process;BP:catabolic process;BP:primary	GO:0034354;GO:0009074;GO:0009165;GO:0044281;GO:0044282;GO:0044283;GO:1901362;GO:1901360;GO:1901361;GO:0043650;GO:0042537;GO:0019359;GO:0046496;GO:0032787;GO:0043436;GO:0009072;GO:0046483;GO:0043648;GO:1901564;GO:1901565;GO:1901566;GO:0016054;GO:0016053;GO:0019439;GO:0019438;GO:0009063;GO:0006807;GO:0043420;GO:1901576;GO:1901575;GO:0070189;GO:0006569;GO:0006568;GO:0019637;GO:0018130;GO:0009308;GO:0006139;GO:0006586;GO:0008150;GO:0008152;GO:0034654;GO:0042436;GO:0090407;GO:0042430;GO:0044271;GO:0044270;GO:0046394;GO:0046395;GO:1901293;GO:0006753;GO:0006576;GO:0044248;GO:0044249;GO:0034641;GO:1901605;GO:1901606;GO:0009987;GO:0006725;GO:0044106;GO:0055086;GO:0006082;GO:0046700;GO:0046874;GO:0019752;GO:0009435;GO:0034627;GO:0044237;GO:0072524;GO:0072525;GO:0019805;GO:0006520;GO:0071704;GO:0009058;GO:0009117;GO:0009056;GO:0044238;GO:0042180;GO:0042182;GO:0097053;GO:0097052;GO:0006796;GO:0006793;GO:0019363;GO:0019362;GO:0110165;GO:0005737;GO:0005575;GO:0030429;GO:1901363;GO:0061981;GO:0016787;GO:0003674;GO:0030170;GO:0016823;GO:0003824;GO:0070279;GO:0043168;GO:0016822;GO:0043167;GO:0019842;GO:0097159;GO:0005488;GO:0036094	K01556	KYNU, kynU	map00380;map	Tryptophan metabo	COG3844	E:Amino acid transport and metaboli	PF00266.22	Aminotran_5	Aminotransferase class-V	CYT	1	1	48.1	High
TRINITY_DN5337_c0_g1_i6_orf1	epoxide hydrolase 1-like [Ostrinia furnacalis]	0.314832236	-1.667344828	0.0001118	down	yes	1.004	3.189	3.205	2.931	3.431	1	0.999	1.012	CC:integral component of membrane;CC:cellular_component;CC:intrinsic component of membrane;CC:cellular anatomical entity;MF:hydrolase activity;MF:catalytic activity;MF:molecular_function;	GO:0016021;GO:0005575;GO:0031224;GO:0110165;GO:0016787;GO:0003824;GO:0003674	K22368;K22369	EPHX3;EPHX4	map05207;map	Chemical carcinoger	ENOG410XRPK;COG	S:Function unknown;	PF00561.23;PF126	Abhydrolase_1;Abhydro	alpha/beta hydrolase fold;Alpha/beta hydrolase family;Serine aminopeptidase, S33	CYT	11	39	39.7	High
TRINITY_DN1897_c0_g2_i4_orf1	phenoloxidase-activating factor 2-like [Hyposmocoma kahamanoa]	0.301242622	-1.731002188	0.004053	down	yes	0.9697	3.219	3.95	3.015	2.691	1	1.013	0.896	------	------	------	------	------	------	------	------	PF18322.4	CLIP_1	Serine protease Clip domain PPAF-2	CYT	1	7	13.9	Medium
TRINITY_DN7226_c0_g1_i2_orf1	chemosensory protein [Conogethes punctiferalis]	0.311009751	-1.684968279	2.27E-06	down	yes	0.9887	3.179	3.074	3.239	3.225	1	0.963	1.003	------	------	------	------	------	------	ENOG4111U5H;ENO	S:Function unknown;	PF03392.16	OS-D	Insect pheromone-binding family, A10/OS-D	CYT	5	54	9	High

TRINITY_DN114890_c0_g1_i4_orf1	chemosensory protein 10 [Ostrinia furnacalis]	0.358579088	-1.479636739	1.55E-05	down	yes	1.07	2.984	3.117	2.914	2.922	1	1.077	1.134	------	------	------	------	------	------	ENOG4111ATT;ENO(	S:Function unknown;	PF03392.16	OS-D	Insect pheromone-binding family, A10/OS-D	CYT	1	76	6.9	High
TRINITY_DN1363_c0_g1_i11_orf1	cytochrome P450 CYP12A2-like isoform X1 [Ostrinia furnacalis] >QPF77619.1 cytochrome P450 monooxygenase CYP333A20 [Ostrinia furnacalis]	0.341686421	-1.549255182	1.23E-06	down	yes	1.009	2.953	2.962	3.018	2.878	1	1.004	1.024	[illegible]	[illegible]	K15004	CYP12	------	------	COG2124	Q:Secondary metabolites biosynthesi	PF00067.25	p450	Cytochrome P450	CYT	8	17	57.7	High
TRINITY_DN45220_c0_g1_i1_orf1	delta(3,5)-Delta(2,4)-dienoyl-CoA isomerase, mitochondrial isoform X1 [Ostrinia furnacalis]	0.443275733	-1.173723712	0.0001634	down	yes	1.18	2.662	2.555	2.754	2.678	1	1.245	1.295	[illegible]	[illegible]	K12663	ECH1	map04146	Peroxisome	ENOG410XTHX	S:Function unknown	PF00378.23	ECH_1	Enoyl-CoA hydratase/isomerase	CYT	1	59	7.4	High
TRINITY_DN27035_c0_g1_i1_orf1	glucose-6-phosphate isomerase-like [Ostrinia furnacalis]	0.386355345	-1.371999739	3.10E-05	down	yes	1.059	2.741	2.88	2.713	2.63	1	1.065	1.113	[illegible]	[illegible]	K01810	GPI, pgi	map00520;map	Amino sugar and nu	COG0166	S:Function unknown	PF00342.22	PGI	Phosphoglucose isomerase	CYT	3	39	13.3	High
TRINITY_DN7740_c0_g1_i2_orf1	D-arabinitol dehydrogenase 1 [Eumeta japonica]	0.369174312	-1.437625925	1.51E-07	down	yes	1.006	2.725	2.711	2.729	2.735	1	0.974	1.044	[illegible]	[illegible]	K17818	ARD1	map00040	Pentose and glucuro	COG1063	E:Amino acid transport and metaboli	------	------	------	CYT	1	69	9.3	High
TRINITY_DN66453_c0_g1_i4_orfp1	TRINITY_DN66453_c0_g1_i4_m.7345 TRINITY_DN66453_c0_g1::TRINITY_DN66453_c0_g1_i4::g.7345 ORF type:internal len:93 (+),score=24.53 TRINITY_DN66453_c0_g1_i4:3-278(+)	0.381059752	-1.391910858	9.08E-05	down	yes	1.014	2.661	2.584	2.862	2.537	1	0.989	1.054	------	------	------	------	------	------	------	------	------	------	------	CYT	1	21	9.7	High
TRINITY_DN16905_c0_g1_i1_orf1	unnamed protein product [Leptidea sinapis]	0.460890493	-1.117504085	0.008129	down	yes	1.149	2.493	2.723	2.019	2.737	1	1.428	1.018	[illegible]	[illegible]	K13578	BMPR1B, ALK	map04390;map	Hippo signaling path	ENOG410XQT0	T:Signal transduction mechanisms	PF00069.28;PF077	Pkinase;PK_Tyr_Ser-Thr	Protein kinase domain;Protein tyrosine and serine/threonine kinase;Transforming growth factor beta type I GS-motif	CYT	1	2	40.3	High
TRINITY_DN41_c0_g1_i3_orf1	uncharacterized protein LOC114359035 isoform X3 [Ostrinia furnacalis]	0.38042471	-1.394317136	8.45E-08	down	yes	0.9853	2.59	2.611	2.586	2.574	1	0.958	0.998	[illegible]	[illegible]	K12382	PSAP, SGP1	map00600;map	Sphingolipid metabo	ENOG410XSI5	J:Translation, ribosomal structure and	PF03489.20;PF051	SapB_2;SapB_1;SapA	Saposin-like type B, region 2;Saposin-like type B, region 1;Saposin A-type domain	CYT	40	46	125.9	High
TRINITY_DN7534_c0_g1_i15_orf1	protein-glucosylgalactosylhydroxylysine glucosidase isoform X2 [Ostrinia furnacalis]	0.443528442	-1.172901473	3.84E-05	down	yes	1.076	2.426	2.536	2.348	2.394	1	1.11	1.118	[illegible]	[illegible]	K22078	PGGHG, ATHL	------	------	COG1554	G:Carbohydrate transport and metab	PF03632.18	Glyco_hydro_65m	Glycosyl hydrolase family 65 central catalytic domain	CYT	2	5	48.7	High
TRINITY_DN6497_c0_g1_i1_orf1	ommochrome-binding protein-like [Ostrinia furnacalis]	0.45295124	-1.142572341	4.47E-05	down	yes	1.059	2.338	2.42	2.37	2.223	1	1.109	1.068	------	------	------	------	------	------	ENOG4110SZS;ENO(	S:Function unknown;G:Carbohydrate	------	------	------	CYT	2	15	17	High
TRINITY_DN1030_c0_g1_i6_orf1	gamma-glutamyl hydrolase A-like isoform X1 [Ostrinia furnacalis]	0.448099103	-1.158110257	0.000557	down	yes	1.049	2.341	2.129	2.554	2.339	1	1.127	1.02	[illegible]	[illegible]	K01307	GGH	map01523;map	Antifolate resistance	ENOG410XQKI	S:Function unknown	PF07722.16;PF001	Peptidase_C26;GATase	Peptidase C26;Glutamine amidotransferase class-I	CYT	2	6	48.4	High
TRINITY_DN3822_c0_g1_i7_orf1	hypothetical protein HF086_002539 [Spodoptera exigua] >CAH0699327.1 unnamed protein product [Spodoptera exigua]	0.43235039	-1.209727103	1.69E-06	down	yes	0.997	2.306	2.34	2.246	2.331	1	0.99	1.001	[illegible]	[illegible]	K01587	PAICS	map00230	Purine metabolism	COG0152	F:Nucleotide transport and metabolis	PF01259.21;PF007	SAICAR_synt;AIRC	SAICAR synthetase;AIR carboxylase	CYT	28	60	47.1	High
TRINITY_DN24789_c0_g1_i9_orfp1	TRINITY_DN24789_c0_g1_i9_m.25888 TRINITY_DN24789_c0_g1::TRINITY_DN24789_c0_g1_i9::g.25888 ORF type:internal len:114 (-),score=12.65 TRINITY_DN24789_c0_g1_i9:2-340(-)	0.475603979	-1.072167309	6.60E-05	down	yes	1.004	2.111	2.05	2.128	2.156	1	1.102	0.909	------	------	------	------	------	------	------	------	------	------	------	CYT	5	46	13.2	High
TRINITY_DN5274_c0_g2_i2_orf1	lopap-like [Ostrinia furnacalis]	0.479315264	-1.060953211	0.0007662	down	yes	1.008	2.103	2.294	1.89	2.126	1	0.98	1.044	------	------	K03098	APOD	------	------	ENOG4110TA7;ENO(	S:Function unknown;	PF00061.26	Lipocalin	Lipocalin / cytosolic fatty-acid binding protein family	CYT	2	14	24.4	High
TRINITY_DN22513_c0_g1_i4_orf1	DNA-directed RNA polymerase II subunit RPB1 [Ostrinia furnacalis] >XP_028179194.1 DNA-directed RNA polymerase II subunit RPB1 [Ostrinia furnacalis] >XP_028179195.1 DNA-directed RNA polymerase II subunit RPB1 [Ostrinia furnacalis]	0.463818616	-1.10836737	0.0006275	down	yes	0.9717	2.095	2.124	2.254	1.907	1	1.051	0.864	MF:molecular_function;MF:structural constituent of cuticle;MF:structural molecule activity;	GO:0003674;GO:0042302;GO:0005198	------	------	------	------	ENOG4111M49	S:Function unknown	PF00379.26	Chitin_bind_4	Insect cuticle protein	CYT	1	5	40.9	Medium
TRINITY_DN17326_c0_g1_i8_orf1	aminoacylase-1-like [Ostrinia furnacalis]	0.489236791	-1.031395196	5.16E-05	down	yes	1	2.044	2.048	1.949	2.136	1	1.031	0.97	[illegible]	[illegible]	K14677	ACY1	map00220	Arginine biosynthesi	COG0624	E:Amino acid transport and metaboli	PF01546.31	Peptidase_M20	Peptidase family M20/M25/M40	CYT	1	33	20.8	High
TRINITY_DN33885_c0_g1_i1_orf1	inhibitor of nuclear factor kappa-B kinase subunit alpha [Ostrinia furnacalis]	0.468444666	-1.094049452	0.0153	down	yes	0.9397	2.006	1.866	2.509	1.642	1	0.876	0.943	------	------	K07209	IKBKB, IKKB	map05166;map	Human T-cell leuker	ENOG410XRMU	T:Signal transduction mechanisms	PF00069.28;PF077	Pkinase;PK_Tyr_Ser-Thr	Protein kinase domain;Protein tyrosine and serine/threonine	CYT	1	2	89.6	High
TRINITY_DN25234_c0_g1_i1_orf1	uncharacterized protein LOC114353853 [Ostrinia furnacalis]	0.4321765	-1.210307469	0.001036	down	yes	0.8717	2.017	1.88	1.954	2.216	1	0.705	0.91	------	------	------	------	------	------	------	------	------	------	------	CYT	1	15	10	High
TRINITY_DN4767_c0_g1_i6_orf1	cysteine protease XCP2-like [Ostrinia furnacalis]	0.206755352	-2.274003418	1.22E-05	down	yes	0.9947	4.811	4.81	5.06	4.562	1	1.013	0.971	[illegible]	[illegible]	------	------	------	------	ENOG4110R2S	S:Function unknown	PF08246.15	Inhibitor_I29	Cathepsin propeptide inhibitor domain	CYT	2	44	5.3	High
TRINITY_DN51995_c0_g3_i1_orf1	circadian clock-controlled protein-like [Ostrinia furnacalis]	0.272921679	-1.8734411	3.67E-06	down	yes	1.021	3.741	3.866	3.606	3.75	1	1.02	1.042	------	------	------	------	------	------	ENOG4111G78	S:Function unknown	PF06585.14	JHBP	Haemolymph juvenile hormone binding protein	CYT	3	10	29.2	High
TRINITY_DN29414_c1_g2_i1_orf1	serine protease 44-like isoform X2 [Ostrinia furnacalis]	0.27559727	-1.8593665	0.001111	down	yes	0.969	3.516	3.612	2.95	3.985	1	0.911	0.996	[illegible]	[illegible]	------	------	------	------	COG5640	S:Function unknown	------	------	------	CYT	1	15	8.8	High
TRINITY_DN19731_c0_g1_i1_orf1	allergen Tha p 1-like [Ostrinia furnacalis] >XP_028174916.1 allergen Tha p 1-like [Ostrinia furnacalis] >BAV56808.1 chemosensory protein 4 [Ostrinia furnacalis]	0.299499853	-1.7393728	9.18E-07	down	yes	1.018	3.399	3.491	3.355	3.351	1	1.02	1.035	------	------	------	------	------	------	ENOG4111U5H;ENO	S:Function unknown;	PF03392.16	OS-D	Insect pheromone-binding family, A10/OS-D	CYT	3	23	14.9	High
TRINITY_DN69_c0_g1_i1_orf1	glycerol-3-phosphate dehydrogenase [NAD(+)], cytoplasmic isoform X1 [Ostrinia furnacalis]	0.286633519	-1.802720768	4.62E-07	down	yes	0.98	3.419	3.441	3.343	3.474	1	0.966	0.974	------	------	K00006	GPD1	map00564	Glycerophospholipic	COG0240	C:Energy production and conversion	PF01210.26;PF074	NAD_Gly3P_dh_N;NAD	NAD-dependent glycerol-3-phosphate dehydrogenase N-terminus;NAD-dependent glycerol-3-phosphate dehydrogenase C-terminus	CYT	21	63	39.8	High
TRINITY_DN33346_c0_g1_i1_orf1	PREDICTED: U6 snRNA-associated Sm-like protein LSm3 [Papilio xuthus] >XP_028165558.1 U6 snRNA-associated Sm-like protein LSm3 [Ostrinia furnacalis] >KOB73597.1 LSM Sm-like protein family member [Operophtera brumata] >RVE45517.1 hypothetical protein evm_009856 [Chilo suppressalis] >CAB3523639.1 unnamed protein product [Chilo suppressalis] >CAH0400961.1 unnamed protein product [Chilo suppressalis]	0.296955281	-1.751682407	3.08E-06	down	yes	0.9363	3.153	3.089	3.232	3.138	1	0.952	0.857	[illegible]	[illegible]	K12622	LSM3	map03018;map	RNA degradation;Sp	ENOG4111PB8	K:Transcription	PF01423.25	LSM	LSM domain	CYT	1	14	11.7	Medium
TRINITY_DN636_c1_g1_i9_orf1	secretory phospholipase A2 receptor-like [Ostrinia furnacalis]	0.331447646	-1.593147089	0.0009995	down	yes	0.9433	2.846	2.668	2.588	3.281	1	0.931	0.899	[illegible]	[illegible]	------	------	------	------	ENOG410ZRYS;ENO(	S:Function unknown;U:Intracellular tr	PF00059.24	Lectin_C	Lectin C-type domain	PLA	2	18	31	High
TRINITY_DN295_c2_g1_i2_orf1	phosphoglycolate phosphatase 1A, chloroplastic [Manduca sexta]	0.391185647	-1.354074656	2.38E-05	down	yes	1.003	2.564	2.435	2.675	2.581	1	1.002	1.008	[illegible]	[illegible]	K21013	FPPP	map00981	Insect hormone bios	COG0647	G:Carbohydrate transport and metab	PF13242.9	Hydrolase_like	HAD-hyrolase-like	PLA	4	28	13.9	High
TRINITY_DN16091_c0_g1_i1_orfp1	TRINITY_DN16091_c0_g1_i1_m.64010 TRINITY_DN16091_c0_g1::TRINITY_DN16091_c0_g1_i1::g.64010 ORF type:5prime_partial len:124 (-),score=7.29,Toxin_2 PF00451.20 0.00035,Toxin_2|PF00451.20|0.00013,Toxin_2|PF00451.20|0.00037,Gamma-thionin|PF00304.21|0.37,Gamma-thionin|PF00304.21|0.052,Gamma-thionin|PF00304.21|0.022,Defensin_2|PF01097.19|0.58,Defensin_2|PF01097.19|0.12,Defensin_2|PF01097.19|0.011,Toxin_38|PF14866.7|0.18,Toxin_38|PF14866.7|0.18,Toxin_38|PF14866.7|0.4 TRINITY_DN16091_c0_g1_i1:19-390(-)	0.464478604	-1.106315954	0.02701	down	yes	1.118	2.407	2.868	1.668	2.686	1	1.171	1.183	------	------	------	------	------	------	------	------	PF00451.22;PF003	Toxin_2;Gamma-thioni	Scorpion short toxin, BmKK2;Gamma-thionin family;Arthropod defensin	CYT	2	43	14.3	High
TRINITY_DN448_c0_g1_i20_orf1	probable cytochrome P450 9f2 isoform X1 [Ostrinia furnacalis]	0.401740186	-1.315665313	4.86E-05	down	yes	0.9927	2.471	2.517	2.317	2.578	1	0.977	1.001	[illegible]	[illegible]	K15003	CYP9	------	------	COG2124	Q:Secondary metabolites biosynthesi	PF00067.25	p450	Cytochrome P450	CYT	8	21	60.7	High
TRINITY_DN11981_c0_g1_i7_orf1	luciferin 4-monooxygenase-like isoform X2 [Ostrinia furnacalis]	0.392304527	-1.349954112	9.16E-05	down	yes	0.9533	2.43	2.284	2.417	2.588	1	0.898	0.962	------	------	K01904	4CL	map00130	Ubiquinone and oth	COG0365;COG0318;(	I:Lipid transport and metabolism;Q:S	PF00501.31;PF131	AMP-binding;AMP-bin	AMP-binding enzyme;AMP-binding enzyme C-terminal domain	CYT	3	7	62.4	High
TRINITY_DN1209_c0_g1_i9_orf1	NADP-dependent malic enzyme-like isoform X1 [Ostrinia furnacalis] >XP_028161889.1 NADP-dependent malic enzyme-like isoform X1 [Ostrinia furnacalis] >XP_028161891.1 NADP-dependent malic enzyme-like isoform X3 [Ostrinia furnacalis]	0.439227731	-1.18695895	2.19E-07	down	yes	1.001	2.279	2.295	2.268	2.273	1	0.974	1.028	[illegible]	[illegible]	K00029	E1.1.1.40, mae	map00620;map	Pyruvate metabolism	COG0281	S:Function unknown	PF03949.18;PF003	Malic_M;malic	Malic enzyme, NAD binding domain;Malic enzyme, N-terminal domain	CYT	25	41	70.7	High
TRINITY_DN19980_c0_g1_i4_orf1	hypothetical protein evm_012507 [Chilo suppressalis]	0.466427932	-1.100273908	0.009538	down	yes	1.042	2.234	1.733	2.43	2.539	1	1.007	1.119	[illegible]	[illegible]	------	------	------	------	ENOG410Y7WV	S:Function unknown	PF07974.16;PF000	EGF_2;EGF;Laminin_EG	EGF-like domain;EGF-like domain;Laminin EGF domain;Delta serrate ligand	PLA	1	4	37.3	High
TRINITY_DN1960_c5_g1_i3_orf1	cytochrome P450 monooxygenase CYP9G18 [Cnaphalocrocis medinalis]	0.474920922	-1.074240782	3.35E-05	down	yes	1.051	2.213	2.211	2.133	2.296	1	1.043	1.109	[illegible]	[illegible]	K15003	CYP9	------	------	COG2124	Q:Secondary metabolites biosynthesi	PF00067.25	p450	Cytochrome P450	CYT	3	21	43.4	High
TRINITY_DN12586_c0_g1_i4_orf1	zonadhesin-like isoform X4 [Ostrinia furnacalis]	0.385305425	-1.375925596	3.54E-05	down	yes	0.902	2.341	2.436	2.324	2.264	1	0.873	0.833	------	------	------	------	------	------	ENOG4110RF5;COG4	S:Function unknown;O:Posttranslatic	PF01826.20	TIL	Trypsin Inhibitor like cysteine rich domain	PLA	1	26	14.3	High
TRINITY_DN3433_c0_g1_i15_orf1	cytosolic purine 5'-nucleotidase isoform X3 [Ostrinia furnacalis] >XP_028162965.1 cytosolic purine 5'-nucleotidase isoform X3 [Ostrinia furnacalis] >XP_028162966.1 cytosolic purine 5'-nucleotidase isoform X3 [Ostrinia furnacalis]	0.479185939	-1.06134252	9.59E-07	down	yes	1.036	2.162	2.188	2.155	2.142	1	1.056	1.051	------	------	K01081	E3.1.3.5	map00240;map	Pyrimidine metaboli	ENOG410XQAV	S:Function unknown	PF05761.17	5_nucleotid	5' nucleotidase family	CYT	1	36	66.4	High
TRINITY_DN39266_c0_g1_i1_orf1	PREDICTED: NECAP-like protein CG9132 [Microplitis demolitor]	0.494371023	-1.016333911	6.23E-07	down	yes	1.01	2.043	2.008	2.067	2.054	1	1.013	1.016	[illegible]	[illegible]	K20069	NECAP1_2	------	------	ENOG41113PS	S:Function unknown	PF07933.17	DUF1681	Protein of unknown function	CYT	1	6	28.7	High

TRINITY_DN12534_c0_g1_i4_orf1	antibacterial protein [Heliothis virescens]	0.186188973	-2.425160459	1.45E-06	down	yes	1.003	5.387	5.448	5.514	5.199	1	1.032	0.978	BP:response to biotic stimulus;BP:response to external stimulus;BP:response to external biotic stimulus;BP:response to stimulus;BP:defense response;BP:response to stress;BP:biological_process;BP:interspecies interaction between organisms;BP:response to other organism;CC:cellular_component;CC:extracellular region;CC:cellular anatomical entity;	GO:0009607;GO:0009605;GO:0043207;GO:0050896;GO:0006952;GO:0006950;GO:0008150;GO:0044419;GO:0051707;GO:0005575;GO:0005576;GO:0110165	------	------	------	------	ENOG4110S6N	S:Function unknown	PF00451.22	Toxin_2	Scorpion short toxin, BmKK2	CYT	5	57	12	High
TRINITY_DN4204_c0_g1_i1_orf1	uncharacterized protein LOC114359352 [Ostrinia furnacalis]	0.304403156	-1.715944778	9.84E-05	down	yes	1.196	3.929	4.197	3.714	3.875	1	1.345	1.244	CC:cellular_component;CC:extracellular region;CC:cellular anatomical entity;MF:molecular_function;MF:structural molecule activity;	GO:0005575;GO:0005576;GO:0110165;GO:0003674;GO:0005198	------	------	------	------	------	------	------	------	------	EXC	1	4	32.1	High
TRINITY_DN24723_c2_g1_i1_orf1	hypothetical protein evm_001103 [Chilo suppressalis]	0.293436293	-1.768880774	0.005727	down	yes	1.064	3.626	2.766	3.769	4.344	1	0.905	1.288	BP:small molecule biosynthetic process;BP:carboxylic acid metabolic process;BP:cellular biosynthetic process;BP:cellular nitrogen compound metabolic process;BP:nitrogen compound metabolic process;BP:small molecule metabolic process;BP:nitrogen cycle metabolic process;BP:glutamine family amino acid metabolic process;BP:organic substance biosynthetic process;BP:cellular amino acid metabolic process;BP:arginine biosynthetic process;BP:arginine metabolic process;BP:organic substance metabolic process;BP:organic acid metabolic process;BP:cellular amino acid biosynthetic process;BP:alpha-amino acid metabolic process;BP:alpha-amino acid biosynthetic process;BP:urea metabolic process;BP:primary metabolic process;BP:cellular process;BP:biosynthetic process;BP:biological_process;BP:metabolic process;BP:oxoacid metabolic process;BP:cellular nitrogen compound biosynthetic process;BP:amide biosynthetic process;BP:organonitrogen compound metabolic process;BP:organonitrogen compound biosynthetic process;BP:cellular amide metabolic process;BP:carboxylic acid biosynthetic process;BP:urea cycle;BP:organic acid biosynthetic process;BP:cellular metabolic process;BP:glutamine family amino acid biosynthetic process;MF:argininosuccinate synthase activity;MF:purine ribonucleoside triphosphate binding;MF:molecular_function;MF:ion binding;MF:heterocyclic compound binding;MF:nucleoside phosphate binding;MF:anion binding;MF:nucleotide binding;MF:purine nucleotide binding;MF:small molecule binding;MF:catalytic activity;MF:ligase activity;MF:ligase activity, forming carbon-nitrogen bonds;MF:adenyl nucleotide binding;MF:carbohydrate derivative binding;MF:adenyl ribonucleotide binding;MF:ATP binding;MF:organic cyclic compound binding;MF:purine ribonucleotide binding;MF:ribonucleotide binding;MF:binding;	GO:0044283;GO:0019752;GO:0044249;GO:0034641;GO:0006807;GO:0044281;GO:0071941;GO:0009064;GO:1901576;GO:0006520;GO:0006526;GO:0006525;GO:0071704;GO:0006082;GO:0008652;GO:1901605;GO:1901607;GO:0019627;GO:0044238;GO:0009987;GO:0009058;GO:0008150;GO:0008152;GO:0043436;GO:0044271;GO:0043604;GO:1901564;GO:1901566;GO:0043603;GO:0046394;GO:0000050;GO:0016053;GO:0044237;GO:0009084;GO:0004055;GO:0035639;GO:0003674;GO:0043167;GO:1901363;GO:1901265;GO:0043168;GO:0000166;GO:0017076;GO:0036094;GO:0003824;GO:0016874;GO:0016879;GO:0030554;GO:0097367;GO:0032559;GO:0005524;GO:0097159;GO:0032555;GO:0032553;GO:0005488	K01940	argG, ASS1	map00220;map	Arginine biosynthesi	COG0137	E:Amino acid transport and metaboli	PF00764.22	Arginosuc_synth	Arginosuccinate synthase	CYT	1	9	11.6	High
TRINITY_DN17574_c0_g1_i2_orf1	heat shock protein Hsp-12.2-like [Ostrinia furnacalis]	0.306877149	-1.704266872	8.63E-06	down	yes	0.9817	3.199	3.271	3.279	3.046	1	0.975	0.97	------	------	K09542	CRYAB	map04213;map	Longevity regulatinc	ENOG410YERS	O:Secondary metabolites biosynthesi	PF00011.24	HSP20	Hsp20/alpha crystallin family	CYT	7	51	19.4	High
TRINITY_DN11826_c0_g1_i4_orf1	aldehyde dehydrogenase X, mitochondrial-like [Ostrinia furnacalis]	0.313234365	-1.674685595	0.0009485	down	yes	0.9917	3.166	3.176	2.731	3.592	1	1.014	0.961	MF:catalytic activity;MF:oxidoreductase activity, acting on the aldehyde or oxo group of donors;MF:molecular_function;MF:oxidoreductase activity, acting on the aldehyde or oxo group of donors, NAD or NADP as acceptor;MF:oxidoreductase activity;	GO:0003824;GO:0016903;GO:0003674;GO:0016620;GO:0016491	K00128	ALDH	map00310;map	Lysine degradation;(	COG1012	C:Energy production and conversion	PF00171.25	Aldedh	Aldehyde dehydrogenase family	CYT	3	48	11.9	High
TRINITY_DN56308_c0_g1_i2_orf1	storage protein 1 [Omphisa fuscidentalis]	0.318451243	-1.650855592	2.42E-07	down	yes	0.9993	3.138	3.13	3.186	3.097	1	0.972	1.026	CC:extracellular space;CC:cellular_component;CC:extracellular region;CC:cellular anatomical entity;MF:molecular_function;MF:nutrient reservoir activity;	GO:0005615;GO:0005575;GO:0005576;GO:0110165;GO:0003674;GO:0045735	------	------	------	------	ENOG410XR2D	S:Function unknown	PF03722.17	Hemocyanin_N	Hemocyanin, all-alpha domain	CYT	9	60	11.3	High
TRINITY_DN34406_c0_g2_i9_orfp1	TRINITY_DN34406_c0_g2_i9_m.33755 TRINITY_DN34406_c0_g2::TRINITY_DN34406_c0_g2_i9::g.33755 ORF type:internal len:82 (-),score=12.88 TRINITY_DN34406_c0_g2_i9:3-245(-)	0.234017507	-2.095311633	0.0001248	down	yes	0.7753	3.313	3.269	3.114	3.555	1	0.702	0.624	------	------	------	------	------	------	------	------	------	------	------	CYT	1	11	9.3	High
TRINITY_DN28501_c0_g1_i2_orfp1	TRINITY_DN28501_c0_g1_i2_m.58934 TRINITY_DN28501_c0_g1::TRINITY_DN28501_c0_g1_i2::g.58934 ORF type:internal len:98 (+),score=13.70 TRINITY_DN28501_c0_g1_i2:3-293(+)	0.317059774	-1.657173244	4.47E-06	down	yes	0.9813	3.095	3.149	2.994	3.141	1	0.911	1.033	------	------	------	------	------	------	------	------	------	------	------	CYT	1	30	10.9	High
TRINITY_DN12582_c0_g1_i5_orf1	uncharacterized protein LOC114355527 isoform X1 [Ostrinia furnacalis]	0.355943586	-1.490279489	0.02879	down	yes	1.06	2.978	2.684	2.167	4.082	1	1.025	1.154	MF:hydrolase activity;MF:hydrolase activity, acting on acid anhydrides, in phosphorus-containing anhydrides;MF:catalytic activity;MF:hydrolase activity, acting on acid anhydrides;MF:pyrophosphatase activity;MF:molecular_function;MF:nucleoside-triphosphate diphosphatase activity;	GO:0016787;GO:0016818;GO:0003824;GO:0016817;GO:0016462;GO:0003674;GO:0047429	------	------	------	------	COG0424	D:Cell cycle control, cell division, chrc	PF02545.17	Maf	Maf-like protein	CYT	1	5	22.9	High
TRINITY_DN5655_c0_g1_i2_orf1	uncharacterized protein LOC114359603 [Ostrinia furnacalis]	0.375646775	-1.412551379	0.0001132	down	yes	1.089	2.899	3.086	2.701	2.91	1	1.147	1.121	------	------	------	------	------	------	ENOG4111C52	S:Function unknown	------	------	------	CYT	1	8	10.8	High
TRINITY_DN71698_c0_g1_i1_orfp1	TRINITY_DN71698_c0_g1_i1_m.1194 TRINITY_DN71698_c0_g1::TRINITY_DN71698_c0_g1_i1::g.1194 ORF type:internal len:134 (+),score=19.66,Toxin_2 PF00451.20 4.3e-05,Toxin_2|PF00451.20|0.037,Toxin_2|PF00451.20|7.5e-05,Gamma-thionin|PF00304.21|0.017,Gamma-thionin|PF00304.21|0.05,Gamma-thionin|PF00304.21|0.021,Toxin_38|PF14866.7|0.13,Toxin_38|PF14866.7|0.15,Toxin_38|PF14866.7|0.15,Defensin_2|PF01097.19|0.053,Defensin_2|PF01097.19|0.34,Defensin_2|PF01097.19|0.092 TRINITY_DN71698_c0_g1_i1:3-401(+)	0.342108867	-1.547472598	7.63E-08	down	yes	0.9993	2.921	2.941	2.929	2.893	1	1.024	0.974	------	------	------	------	------	------	------	------	PF00451.22;PF003	Toxin_2;Gamma-thioni	Scorpion short toxin, BmKK2;Gamma-thionin family	CYT	6	47	15.5	High
TRINITY_DN3593_c0_g1_i3_orfp1	TRINITY_DN3593_c0_g1_i3_m.43968 TRINITY_DN3593_c0_g1::TRINITY_DN3593_c0_g1_i3::g.43968 ORF type:5prime_partial len:72 (-),score=1.41 TRINITY_DN3593_c0_g1_i3:138-353(-)	0.367678193	-1.443484481	2.01E-05	down	yes	1.042	2.834	2.693	2.935	2.874	1	1.038	1.087	------	------	------	------	------	------	------	------	------	------	------	CYT	1	15	7.5	High
TRINITY_DN136358_c0_g1_i1_orf1	acidic juvenile hormone-suppressible protein 1-like [Ostrinia furnacalis]	0.370036101	-1.434262067	4.91E-06	down	yes	1.025	2.77	2.786	2.848	2.675	1	1.026	1.049	------	------	------	------	------	------	ENOG410XR2D	S:Function unknown	PF03723.17	Hemocyanin_C	Hemocyanin, ig-like domain	CYT	2	55	9.7	High
TRINITY_DN2187_c0_g1_i1_orf1	flotillin-1 [Chelonus insularis] >XP_034947202.1 flotillin-1 [Chelonus insularis]	0.365862573	-1.450626256	2.60E-06	down	yes	1.001	2.736	2.825	2.694	2.689	1	1.003	1.001	------	------	K07192	FLOT	map04910	Insulin signaling pat	COG2268	S:Function unknown	PF01145.28;PF159	Band_7;Flot	SPFH domain / Band 7 family;Flotillin	CYT	3	10	46.9	High
TRINITY_DN15685_c0_g1_i5_orf1	uncharacterized protein LOC114352354 [Ostrinia furnacalis]	0.354926299	-1.494408615	4.14E-06	down	yes	0.915	2.578	2.588	2.592	2.554	1	0.905	0.84	------	------	------	------	------	------	ENOG4110T90;ENOC	S:Function unknown;	PF06585.14	JHBP	Haemolymph juvenile hormone binding protein	CYT	3	14	29.1	High
TRINITY_DN81031_c0_g1_i1_orf1	aldehyde dehydrogenase, partial [Ectropis obliqua]	0.395939295	-1.336648842	1.27E-05	down	yes	0.9653	2.438	2.446	2.527	2.342	1	0.95	0.946	MF:catalytic activity;MF:oxidoreductase activity, acting on the aldehyde or oxo group of donors;MF:molecular_function;MF:oxidoreductase activity, acting on the aldehyde or oxo group of donors, NAD or NADP as acceptor;MF:oxidoreductase activity;	GO:0003824;GO:0016903;GO:0003674;GO:0016620;GO:0016491	K00128	ALDH	map00310;map	Lysine degradation;(	COG1012	C:Energy production and conversion	PF00171.25	Aldedh	Aldehyde dehydrogenase family	CYT	5	57	8.6	High
TRINITY_DN140212_c0_g1_i1_orf1	macrophage mannose receptor 1-like [Ostrinia furnacalis]	0.453674121	-1.140271727	0.0001258	down	yes	0.994	2.191	2.295	2.238	2.041	1	0.944	1.038	BP:response to extracellular stimulus;BP:nucleic acid metabolic process;BP:cellular nitrogen compound metabolic process;BP:nitrogen compound metabolic process;BP:DNA protection;BP:organic cyclic compound metabolic process;BP:nucleobase-containing compound metabolic process;BP:cellular response to stimulus;BP:cellular macromolecule metabolic process;BP:organic substance metabolic process;BP:cellular response to external stimulus;BP:cellular process;BP:cellular aromatic compound metabolic process;BP:cellular response to extracellular stimulus;BP:response to stress;BP:biological_process;BP:metabolic process;BP:cell communication;BP:heterocycle metabolic process;BP:primary metabolic process;BP:response to external stimulus;BP:cellular metabolic process;BP:macromolecule metabolic process;BP:cellular response to stress;BP:DNA metabolic process;BP:response to stimulus;MF:carbohydrate binding;MF:molecular_function;MF:hydrolase activity;MF:catalytic activity;MF:nucleoside-triphosphate diphosphatase activity;MF:hydrolase activity, acting on acid anhydrides, in phosphorus-containing anhydrides;MF:8-oxo-7,8-dihydroguanosine triphosphate pyrophosphatase activity;MF:hydrolase activity, acting on acid anhydrides;MF:pyrophosphatase	GO:0009991;GO:0090304;GO:0034641;GO:0006807;GO:0042262;GO:1901360;GO:0006139;GO:0051716;GO:0044260;GO:0071704;GO:0071496;GO:0009987;GO:0006725;GO:0031668;GO:0006950;GO:0008150;GO:0008152;GO:0007154;GO:0046483;GO:0044238;GO:0009605;GO:0044237;GO:0043170;GO:0033554;GO:0006259;GO:0050896;GO:0030246;GO:0003674;GO:0016787;GO:0003824;GO:0047429;GO:0016818;GO:0008413;GO:0016817;GO:0016462;GO:0005488	------	------	------	------	ENOG410XPJ1;ENOC	S:Function unknown;U:Intracellular tr	------	------	------	CYT	2	59	10.3	High
TRINITY_DN1305_c0_g1_i6_orf1	glutathione S-transferase sigma 3 [Ostrinia furnacalis]	0.466231253	-1.100882379	4.76E-05	down	yes	0.9637	2.067	1.999	2.031	2.172	1	0.915	0.976	MF:catalytic activity;MF:transferase activity;MF:glutathione transferase activity;MF:molecular_function;MF:transferase activity, transferring alkyl or ary;	GO:0003824;GO:0016740;GO:0004364;GO:0003674;GO:0016765	K04097	HPGDS	map00590;map	Arachidonic acid me	ENOG41103AU;ENOI	O:Posttranslational modification, pro	PF02798.23;PF144	GST_N;GST_C_3;GST_N	Glutathione S-transferase, N-terminal domain;Glutathione S-transferase, C-terminal domain;Glutathione S-transferase, N-terminal	CYT	2	26	19.4	High
TRINITY_DN11948_c0_g1_i8_orf1	cystathionine gamma-lyase [Ostrinia furnacalis]	0.497275879	-1.007881641	4.94E-07	down	yes	1.004	2.019	2.023	1.988	2.045	1	0.999	1.013	BP:carboxylic acid metabolic process;BP:cellular biosynthetic process;BP:nitrogen compound metabolic process;BP:small molecule metabolic process;BP:small molecule biosynthetic process;BP:serine family amino acid metabolic process;BP:organic substance biosynthetic process;BP:homocysteine metabolic process;BP:cellular amino acid metabolic process;BP:organic substance metabolic process;BP:homoserine metabolic process;BP:sulfur amino acid biosynthetic process;BP:alpha-amino acid biosynthetic process;BP:sulfur amino acid metabolic process;BP:cellular amino acid biosynthetic process;BP:cellular process;BP:biosynthetic process;BP:biological_process;BP:metabolic process;BP:oxoacid metabolic process;BP:serine family amino acid biosynthetic process;BP:primary metabolic process;BP:organonitrogen compound metabolic process;BP:organonitrogen compound biosynthetic process;BP:organic acid metabolic process;BP:sulfur compound biosynthetic process;BP:carboxylic acid biosynthetic process;BP:alpha-amino acid metabolic process;BP:organic acid biosynthetic process;BP:cellular metabolic process;BP:cysteine metabolic process;BP:transsulfuration;BP:cysteine biosynthetic process;BP:sulfur compound metabolic process;MF:binding;MF:vitamin B6 binding;MF:molecular_function;MF:heterocyclic compound binding;MF:anion binding;MF:carbon-sulfur lyase activity;MF:lyase activity;MF:L-cysteine desulfhydrase activity;MF:ion binding;MF:cystathionine gamma-lyase activity;MF:small molecule binding;MF:catalytic activity;MF:vitamin binding;MF:L-cystine L-cysteine-lyas;MF:organic cyclic compound binding;MF:pyridoxal phosphate binding;	GO:0019752;GO:0044249;GO:0006807;GO:0044281;GO:0044283;GO:0009069;GO:1901576;GO:0050667;GO:0006520;GO:0071704;GO:0009092;GO:0000097;GO:1901607;GO:0000096;GO:0008652;GO:0009987;GO:0009058;GO:0008150;GO:0008152;GO:0043436;GO:0009070;GO:0044238;GO:1901564;GO:1901566;GO:0006082;GO:0044272;GO:0046394;GO:1901605;GO:0016053;GO:0044237;GO:0006534;GO:0019346;GO:0019344;GO:0006790;GO:0005488;GO:0070279;GO:0003674;GO:1901363;GO:0043168;GO:0016846;GO:0016829;GO:0080146;GO:0043167;GO:0004123;GO:0036094;GO:0003824;GO:0019842;GO:0044540;GO:0097159;GO:0030170	K01758	CTH	map00450;map	Selenocompound m	COG0626	E:Amino acid transport and metaboli	PF01053.23;PF001	Cys_Met_Meta_PP;Ami	Cys/Met metabolism PLP-dependent enzyme;Aminotransferase class I and II;Methionine gamma-lyase	CYT	16	56	43	High
TRINITY_DN7735_c0_g1_i4_orf1	calphotin-like [Ostrinia furnacalis]	0.498013903	-1.005742077	0.0009574	down	yes	1.003	2.014	2.194	1.949	1.899	1	0.88	1.129	------	------	------	------	------	------	ENOG4110RAS;ENO(	S:Function unknown;	------	------	------	CYT	1	3	22.5	High
TRINITY_DN15858_c0_g1_i1_orf1	15-hydroxyprostaglandin dehydrogenase [NAD(+)]-like [Ostrinia furnacalis]	0.490584584	-1.027426196	1.67E-05	down	yes	0.9483	1.933	1.898	1.963	1.939	1	0.88	0.965	MF:catalytic activity;MF:molecular_function;MF:oxidoreductase activity;	GO:0003824;GO:0003674;GO:0016491	K00069	HPGD	map00590;map	Arachidonic acid me	COG1028;COG0300	S:Function unknown;	PF00106.28;PF135	adh_short;adh_short_C	short chain dehydrogenase;Enoyl;KR domain	CYT	1	21	29.6	High
TRINITY_DN12526_c0_g1_i5_orf1	uncharacterized protein LOC114359035 isoform X3 [Ostrinia furnacalis]	0.202488176	-2.304090428	2.54E-05	down	yes	0.9847	4.863	4.838	5.169	4.582	1	0.895	1.059	BP:nitrogen compound metabolic process;BP:membrane lipid metabolic process;BP:organic substance metabolic process;BP:sphingolipid metabolic process;BP:lipid metabolic process;BP:cellular process;BP:biological_process;BP:metabolic process;BP:cellular lipid metabolic process;BP:primary metabolic process;BP:organonitrogen compound metabolic process;BP:cellular metabolic process;CC:lytic vacuole;CC:intracellular organelle;CC:membrane-bounded organelle;CC:organelle;CC:lysosome;CC:vacuole;CC:cellular anatomical entity;CC:intracellular membrane-bounded organelle;CC:cellular_component;CC:extracellular region;	GO:0006807;GO:0006643;GO:0071704;GO:0006665;GO:0006629;GO:0009987;GO:0008150;GO:0008152;GO:0044255;GO:0044238;GO:1901564;GO:0044237;GO:0000323;GO:0043229;GO:0043227;GO:0043226;GO:0005764;GO:0005773;GO:0110165;GO:0043231;GO:0005575;GO:0005576	K12382	PSAP, SGP1	map00600;map	Sphingolipid metab(	ENOG410XSI5	O:Posttranslational modification, pro	PF05184.18;PF034	SapB_1;SapB_2	Saposin-like type B, region 1;Saposin-like type B, region 2	CYT	2	37	15.2	High
TRINITY_DN8595_c0_g1_i3_orf1	aldose reductase-like isoform X4 [Trichoplusia ni]	0.216629213	-2.206700285	3.18E-08	down	yes	0.964	4.45	4.459	4.406	4.486	1	0.951	0.941	MF:catalytic activity;MF:molecular_function;MF:oxidoreductase activity;	GO:0003824;GO:0003674;GO:0016491	K00011	AKR1B	map00051;map	Fructose and mann(	COG0656	S:Function unknown	PF00248.24	Aldo_ket_red	Aldo/keto reductase family	CYT	1	67	13.9	High
TRINITY_DN135449_c0_g1_i5_orf1	larval cuticle protein LCP-17-like [Galleria mellonella]	0.265311183	-1.914242609	6.63E-05	down	yes	1.07	4.033	4.004	3.758	4.337	1	1.086	1.125	MF:molecular_function;MF:structural constituent of cuticle;MF:structural molecule activity;	GO:0003674;GO:0042302;GO:0005198	------	------	------	------	ENOG4110RUZ;ENO(	S:Function unknown;	------	------	------	CYT	1	68	8.6	High
TRINITY_DN7900_c0_g1_i4_orf1	uncharacterized protein LOC114366119 [Ostrinia furnacalis]	0.213765281	-2.225900539	0.00128	down	yes	0.8743	4.09	4.16	3.385	4.725	1	0.92	0.703	------	------	K20701	PIRK	map04624	Toll and Imd signalir	ENOG410Y5Y5	S:Function unknown	------	------	------	CYT	1	9	13.5	Medium
TRINITY_DN52316_c0_g1_i1_orf1	arylphorin subunit alpha-like [Ostrinia furnacalis]	0.28074095	-1.832688577	0.001349	down	yes	0.9927	3.536	3.324	3.121	4.164	1	0.967	1.011	------	------	------	------	------	------	ENOG410XR2D	J:Translation, ribosomal structure anc	PF03722.17	Hemocyanin_N	Hemocyanin, all-alpha domain	PLA	4	55	13.9	High
TRINITY_DN136031_c0_g1_i7_orf1	ferritin, lower subunit isoform X3 [Spodoptera litura]	0.28819065	-1.794904567	1.76E-06	down	yes	0.9493	3.294	3.199	3.354	3.328	1	0.913	0.935	BP:ion homeostasis;BP:homeostatic process;BP:iron ion homeostasis;BP:transition metal ion homeostasis;BP:inorganic ion homeostasis;BP:cellular transition metal ion homeostasis;BP:transition metal ion transport;BP:biological regulation;BP:cellular homeostasis;BP:metal ion transport;BP:transport;BP:cellular iron ion homeostasis;BP:cellular metal ion homeostasis;BP:cation transport;BP:ion transport;BP:cellular process;BP:cellular ion homeostasis;BP:biological_process;BP:cellular cation homeostasis;BP:metal ion homeostasis;BP:cation homeostasis;BP:cellular chemical homeostasis;BP:establishment of localization;BP:localization;BP:regulation of biological quality;BP:chemical homeostasis;BP:iron ion transport;MF:molecular_function;MF:binding;MF:oxidoreductase activity;MF:cation binding;MF:oxidoreductase activity, oxidizing metal ions;MF:transition metal ion binding;MF:ferroxidase activity;MF:ion binding;MF:iron ion binding;MF:metal ion binding;MF:catalytic activity;MF:oxidoreductase activity, oxidizing metal ions, oxygen as acceptor;MF:ferric iron binding;	GO:0050801;GO:0042592;GO:0055072;GO:0055076;GO:0098771;GO:0046916;GO:0000041;GO:0065007;GO:0019725;GO:0030001;GO:0006810;GO:0006879;GO:0006875;GO:0006812;GO:0006811;GO:0009987;GO:0006873;GO:0008150;GO:0030003;GO:0055065;GO:0055080;GO:0055082;GO:0051234;GO:0051179;GO:0065008;GO:0048878;GO:0006826;GO:0003674;GO:0005488;GO:0016491;GO:0043169;GO:0016722;GO:0046914;GO:0004322;GO:0043167;GO:0005506;GO:0046872;GO:0003824;GO:0016724;GO:0008199	------	------	------	------	ENOG4111Q26;COG	P:Inorganic ion transport and metab(	------	------	------	EXC	1	28	11.1	High
TRINITY_DN15755_c0_g1_i1_orf1	cytochrome P450 monooxygenase CYP6AB141 [Ostrinia furnacalis]	0.372295883	-1.425478433	5.12E-05	down	yes	1.067	2.866	2.72	2.847	3.031	1	1.129	1.072	CC:integral component of membrane;CC:cellular_component;CC:cellular anatomical entity;CC:intrinsic component of membrane;MF:cation binding;MF:monooxygenase activity;MF:heme binding;MF:transition metal ion binding;MF:tetrapyrrole binding;MF:organic cyclic compound binding;MF:oxidoreductase activity, acting on paired donors, with incorporation or reduction of molecular oxygen;MF:ion binding;MF:catalytic activity;MF:molecular_function;MF:binding;MF:iron ion binding;MF:metal ion binding;MF:heterocyclic compound binding;MF:oxidoreductase activity;	GO:0016021;GO:0005575;GO:0110165;GO:0031224;GO:0043169;GO:0004497;GO:0020037;GO:0046914;GO:0046906;GO:0097159;GO:0016705;GO:0043167;GO:0003824;GO:0003674;GO:0005488;GO:0005506;GO:0046872;GO:1901363;GO:0016491	K14999	CYP6	------	------	COG2124	Q:Secondary metabolites biosynthesi	PF00067.25	p450	Cytochrome P450	CYT	3	28	27.4	High
TRINITY_DN20676_c0_g1_i6_orf1	aldo-keto reductase AKR2E4-like isoform X1 [Ostrinia furnacalis]	0.346153846	-1.530514717	5.29E-07	down	yes	0.999	2.886	2.879	2.945	2.834	1	0.991	1.006	MF:catalytic activity;MF:molecular_function;MF:oxidoreductase activity;	GO:0003824;GO:0003674;GO:0016491	------	------	------	------	COG0656	S:Function unknown	PF00248.24	Aldo_ket_red	Aldo/keto reductase family	CYT	13	47	40.6	High
TRINITY_DN48878_c0_g2_i1_orf1	codanin-1 [Ostrinia furnacalis]	0.440059568	-1.184229269	0.0001299	down	yes	1.182	2.686	2.742	2.591	2.724	1	1.291	1.256	------	------	K19531	CDAN1	------	------	ENOG410XXWY	S:Function unknown	PF15296.9	Codanin-1_C	Codanin-1 C-terminus	CYT	1	1	83.1	Medium
TRINITY_DN13660_c0_g1_i1_orf1	Aliphatic nitrilase [Operophtera brumata]	0.37740113	-1.405829353	9.96E-08	down	yes	1.002	2.655	2.685	2.654	2.627	1	0.989	1.018	BP:nitrogen compound metabolic process;BP:metabolic process;BP:biological_process;MF:hydrolase activity;MF:catalytic activity;MF:hydrolase activity, acting on carbon-nitroge;MF:hydrolase activity, acting on carbon-nitroge;MF:beta-ureidopropionase activity;MF:molecular_function;	GO:0006807;GO:0008152;GO:0008150;GO:0016787;GO:0003824;GO:0016810;GO:0016811;GO:0003837;GO:0003674	K01431	UPB1, pydC	map00240;map	Pyrimidine metaboli	COG0388	S:Function unknown	PF00795.25	CN_hydrolase	Carbon-nitrogen hydrolase	CYT	17	50	44.5	High
TRINITY_DN140_c0_g1_i5_orf1	calcyphosin-like protein isoform X4 [Helicoverpa armigera]	0.360635514	-1.47138662	8.23E-07	down	yes	0.9647	2.675	2.692	2.711	2.622	1	0.936	0.958	MF:cation binding;MF:ion binding;MF:molecular_function;MF:binding;MF:metal ion binding;MF:calcium ion binding;	GO:0043169;GO:0043167;GO:0003674;GO:0005488;GO:0046872;GO:0005509	K23909	CAPS	------	------	ENOG410XRMJ	T:Signal transduction mechanisms	PF13405.9;PF0003	EF-hand_6;EF-hand_1;	EF-hand domain;EF hand;EF-hand domain pair;EF hand;EF-hand	CYT	2	73	12	High
TRINITY_DN80328_c0_g1_i5_orf1	arylphorin subunit alpha-like [Ostrinia furnacalis]	0.371775417	-1.427496716	7.91E-07	down	yes	0.98	2.636	2.621	2.595	2.691	1	0.955	0.985	------	------	------	------	------	------	ENOG410XR2D	S:Function unknown	PF00372.22	Hemocyanin_M	Hemocyanin, copper containing domain	CYT	7	74	12.5	High
TRINITY_DN55147_c0_g1_i1_orfp1	TRINITY_DN55147_c0_g1_i1_m.59251 TRINITY_DN55147_c0_g1::TRINITY_DN55147_c0_g1_i1::g.59251 ORF type:5prime_partial len:331 (-),score=113.97,Cuticle_3 PF11018.9 0.29,Cuticle_3|PF11018.9|2.9e-05,Cuticle_3|PF11018.9|0.00037 TRINITY_DN55147_c0_g1_i1:21-1013(-)	0.40850059	-1.291589932	6.59E-06	down	yes	1.038	2.541	2.621	2.485	2.518	1	1.026	1.089	------	------	------	------	------	------	------	------	PF11018.11	Cuticle_3	Pupal cuticle protein C1	CYT	7	50	33.7	High
TRINITY_DN9132_c0_g1_i5_orf1	ubiquitin-like-conjugating enzyme ATG3 [Spodoptera frugiperda]	0.386837945	-1.37019878	6.71E-06	down	yes	0.9787	2.53	2.628	2.487	2.475	1	0.959	0.977	MF:ubiquitin-like protein transferase activity;MF:catalytic activity;MF:transferase activity;MF:catalytic activity, acting on a protein;MF:molecular_function;	GO:0019787;GO:0003824;GO:0016740;GO:0140096;GO:0003674	K08343	ATG3	map04136;map	Autophagy - other;I	ENOG410Y3BC	U:Intracellular trafficking, secretion, a	PF03987.18	Autophagy_act_C	Autophagocytosis associated protein, active-site domain	CYT	5	18	36.1	High
TRINITY_DN5132_c0_g1_i4_orf1	small heat shock protein Hsp24.2 [Ostrinia furnacalis]	0.423330585	-1.240143369	2.91E-05	down	yes	1.027	2.426	2.458	2.303	2.516	1	1.059	1.023	------	------	K09541	CRYAA	map04141	Protein processing ii	ENOG410YERS	J:Translation, ribosomal structure anc	PF00011.24	HSP20	Hsp20/alpha crystallin family	CYT	5	21	24.7	High
TRINITY_DN17505_c0_g1_i15_orf1	unnamed protein product [Chilo suppressalis]	0.41426216	-1.271384049	0.002452	down	yes	1.005	2.426	2.787	2.429	2.063	1	1.025	0.989	CC:cellular_component;CC:integral component of membrane;CC:cellular anatomical entity;CC:intrinsic component of membrane;	GO:0005575;GO:0016021;GO:0110165;GO:0031224	------	------	------	------	ENOG410ZWQR	S:Function unknown	PF07782.16	DC_STAMP	DC-STAMP-like protein	CYT	1	0	297.6	Medium
TRINITY_DN585_c0_g1_i12_orf1	very low-density lipoprotein receptor isoform X3 [Galleria mellonella]	0.424090339	-1.237556478	0.004659	down	yes	1.014	2.391	1.99	2.822	2.361	1	1.048	0.994	CC:integral component of membrane;CC:cellular_component;CC:intrinsic component of membrane;CC:cellular anatomical entity;MF:binding;MF:cation binding;MF:cargo receptor activity;MF:very-low-density lipoprotein particle receptor activity;MF:lipoprotein particle receptor activity;MF:ion binding;MF:molecular_function;MF:metal ion binding;MF:calcium ion binding;	GO:0016021;GO:0005575;GO:0031224;GO:0110165;GO:0005488;GO:0043169;GO:0038024;GO:0030229;GO:0030228;GO:0043167;GO:0003674;GO:0046872;GO:0005509	K20053	VLDLR	map05417;map	Lipid and atheroscle	ENOG410YQ6J	U:Intracellular trafficking, secretion, a	PF00057.21;PF000	Ldl_recept_a;Ldl_recept	Low-density lipoprotein receptor domain class A;Low-density lipoprotein receptor repeat class B;Coagulation Factor Xa inhibitory site;Complement Clr-like EGF-like;Calcium-binding EGF domain;Domain of unknown function	PLA	3	31	100.3	High
TRINITY_DN4998_c0_g1_i21_orf1	phenoloxidase subunit 2-like [Ostrinia furnacalis]	0.426638478	-1.228914009	4.56E-06	down	yes	1.009	2.365	2.401	2.409	2.286	1	1.007	1.019	CC:cellular_component;CC:extracellular region;CC:cellular anatomical entity;MF:cation binding;MF:monooxygenase activity;MF:oxidoreductase activity, acting on paired donors, with incorporation or reduction of molecular oxygen;MF:monophenol monooxygenase activity;MF:oxidoreductase activity, acting on paired donors, with incorporation or reduction of molecular oxygen, another compound as one donor, and incorporation of one atom of oxygen;MF:ion binding;MF:catalytic activity;MF:molecular_function;MF:binding;MF:metal ion binding;MF:oxidoreductase activity;	GO:0005575;GO:0005576;GO:0110165;GO:0043169;GO:0004497;GO:0016705;GO:0004503;GO:0016716;GO:0043167;GO:0003824;GO:0003674;GO:0005488;GO:0046872;GO:0016491	------	------	------	------	ENOG4111KTW	S:Function unknown	PF03723.17;PF003	Hemocyanin_C;Hemoc	Hemocyanin, ig-like domain;Hemocyanin, copper containing domain;Hemocyanin, all-alpha domain;Common central domain of tyrosinase	PLA	16	38	80	High
TRINITY_DN45037_c0_g1_i1_orf1	trafficking protein particle complex subunit 1 [Ostrinia furnacalis]	0.358377069	-1.480449766	0.005802	down	yes	0.8877	2.477	2.124	2.268	3.039	1	0.726	0.937	BP:ER to Golgi vesicle-mediated transport;BP:localization;BP:cellular localization;BP:transport;BP:Golgi vesicle transport;BP:cellular process;BP:biological_process;BP:establishment of localization in cell;BP:establishment of localization;BP:vesicle-mediated transport;BP:intracellular transport;CC:endoplasmic reticulum;CC:intracellular organelle;CC:tethering complex;CC:membrane-bounded organelle;CC:organelle;CC:cellular anatomical entity;CC:Golgi apparatus;CC:TRAPP complex;CC:intracellular membrane-bounded organelle;CC:macromolecular complex;CC:cellular_component;	GO:0006888;GO:0051179;GO:0051641;GO:0006810;GO:0048193;GO:0009987;GO:0008150;GO:0051649;GO:0051234;GO:0016192;GO:0046907;GO:0005783;GO:0043229;GO:0099023;GO:0043227;GO:0043226;GO:0110165;GO:0005794;GO:0030008;GO:0043231;GO:0032991;GO:0005575	K20300	TRAPPC1, BE1	------	------	ENOG4111X41	U:Intracellular trafficking, secretion, a	PF04099.15;PF046	Sybindin;Sedlin_N	Sybindin-like family;Sedlin, N-terminal conserved region	CYT	1	9	16.8	High
TRINITY_DN59422_c0_g1_i2_orf1	larval cuticle protein LCP-22-like isoform X2 [Pectinophora gossypiella]	0.475604195	-1.072166654	3.70E-05	down	yes	1.043	2.193	2.267	2.203	2.108	1	1.019	1.111	MF:molecular_function;MF:structural constituent of cuticle;MF:structural molecule activity;	GO:0003674;GO:0042302;GO:0005198	------	------	------	------	ENOG4111CO7;ENO	S:Function unknown;	PF00379.26	Chitin_bind_4	Insect cuticle protein	CYT	4	38	19.5	High
TRINITY_DN970_c0_g1_i4_orf1	spermine oxidase-like isoform X2 [Ostrinia furnacalis]	0.46776406	-1.096147075	7.57E-07	down	yes	1.023	2.187	2.205	2.15	2.206	1	1.038	1.032	------	------	K12259	SMOX, PAO5	map00330;map	Arginine and proline	ENOG4111HZN;ENO	S:Function unknown;	PF01593.27;PF134	Amino_oxidase;NAD_b	Flavin containing amine oxidoreductase;NA	CYT	16	32	55.5	High
TRINITY_DN10994_c0_g1_i4_orf1	trypsin inhibitor-like [Ostrinia furnacalis]	0.476079889	-1.07072441	0.0001818	down	yes	1.025	2.153	2.289	2.017	2.154	1	0.988	1.087	BP:nitrogen compound metabolic process;BP:metabolic process;BP:organic substance metabolic process;BP:proteolysis;BP:biological_process;BP:primary metabolic process;BP:organonitrogen compound metabolic process;BP:protein metabolic process;BP:macromolecule metabolic process;CC:intracellular organelle;CC:non-membrane-bounded organelle;CC:organelle;CC:extracellular region;CC:cellular anatomical entity;CC:intracellular non-membrane-bounded organelle;CC:cellular_component;CC:nematocyst;MF:endopeptidase inhibitor activity;MF:ion channel regulator activity;MF:toxin activity;MF:molecular_function;MF:enzyme inhibitor activity;MF:molecular function regulator;MF:potassium channel regulator activity;MF:channel regulator activity;MF:endopeptidase regulator activity;MF:hydrolase activity;MF:enzyme regulator activity;MF:peptidase regulator activity;MF:catalytic activity, acting on a protein;MF:catalytic activity;MF:peptidase inhibitor activity;MF:serine-type endopeptidase inhibitor activity;MF:peptidase activity;	GO:0006807;GO:0008152;GO:0071704;GO:0006508;GO:0008150;GO:0044238;GO:1901564;GO:0019538;GO:0043170;GO:0043229;GO:0043228;GO:0043226;GO:0005576;GO:0110165;GO:0043232;GO:0005575;GO:0042151;GO:0004866;GO:0099106;GO:0090729;GO:0003674;GO:0004857;GO:0098772;GO:0015459;GO:0016247;GO:0061135;GO:0016787;GO:0030234;GO:0061134;GO:0140096;GO:0003824;GO:0030414;GO:0004867;GO:0008233	K23622	EPPIN	------	------	ENOG410XQNP	U:Intracellular trafficking, secretion, a	PF00014.26	Kunitz_BPTI	Kunitz/Bovine pancreatic trypsin inhibitor domain	EXC	2	49	8.1	High
TRINITY_DN874_c2_g1_i1_orf1	uncharacterized protein LOC114356358 [Ostrinia furnacalis]	0.472993753	-1.080106965	3.45E-05	down	yes	0.9843	2.081	2.181	2.004	2.058	1	0.994	0.959	MF:catalytic activity;MF:transferase activity;MF:transferase activity, transferring sulfur-containing groups;MF:sulfotransferase activity;MF:molecular_function;	GO:0003824;GO:0016740;GO:0016782;GO:0008146;GO:0003674	K01025	SULT1	------	------	ENOG4111H56	S:Function unknown	PF00685.30;PF134	Sulfotransfer_1;Sulfotra	Sulfotransferase domain;Sulfotransferase family	CYT	6	19	46.2	High
TRINITY_DN131603_c0_g1_i4_orfp1	TRINITY_DN131603_c0_g1_i4_m.86149 TRINITY_DN131603_c0_g1::TRINITY_DN131603_c0_g1_i4::g.86149 ORF type:internal len:112 (-),score=8.40 TRINITY_DN131603_c0_g1_i4:2-334(-)	0.074995997	-3.737042602	8.89E-05	down	yes	0.9367	12.49	13.197	13.229	11.052	1	0.855	0.955	------	------	------	------	------	------	------	------	------	------	------	PLA	1	22	12.9	High
TRINITY_DN86772_c0_g1_i3_orfp1	x-tox [Spodoptera exigua]	0.155107432	-2.688660278	1.20E-05	down	yes	0.8807	5.678	5.964	5.684	5.386	1	0.884	0.758	BP:response to biotic stimulus;BP:response to external stimulus;BP:response to external biotic stimulus;BP:response to stimulus;BP:defense response;BP:response to stress;BP:biological_process;BP:interspecies interaction between organisms;BP:response to other organism;CC:cellular_component;CC:extracellular region;CC:cellular anatomical entity;	GO:0009607;GO:0009605;GO:0043207;GO:0050896;GO:0006952;GO:0006950;GO:0008150;GO:0044419;GO:0051707;GO:0005575;GO:0005576;GO:0110165	------	------	------	------	------	------	------	------	------	CYT	1	9	8.5	High

TRINITY_DN59885_c0_g1_i3_orf1	TGF-beta-activated kinase 1 and MAP3K7-binding protein 1-like [Ostrinia furnacalis]	0.188940956	-2.403992632	2.38E-06	down	yes	1.008	5.335	5.379	5.49	5.137	1	1.065	0.959	[illegible]	[illegible]	K04403	MAP3K7IP1, T	map05140;map	Leishmaniasis;Huma	COG0631	S:Function unknown	PF00481.24	PP2C	Protein phosphatase 2C	CYT	1	3	51	High
TRINITY_DN3439_c0_g2_i2_orf1	histone H2A.Z-specific chaperone CHZ1-like [Ostrinia furnacalis]	0.182599206	-2.4532476	5.85E-06	down	yes	0.9203	5.04	5.157	5.169	4.793	1	0.884	0.877	------	------	------	------	------	------	------	------	------	------	------	PLA	4	14	31.9	High
TRINITY_DN67193_c0_g1_i1_orf1	A-kinase anchor protein 14-like [Ostrinia furnacalis]	0.248063781	-2.011216986	9.10E-05	down	yes	1.089	4.39	4.345	4.103	4.722	1	0.973	1.294	------	------	------	------	------	------	------	------	------	------	------	CYT	1	6	15.2	High
TRINITY_DN18568_c0_g1_i2_orfp1	TRINITY_DN18568_c0_g1_i2_m.13844 TRINITY_DN18568_c0_g1::TRINITY_DN18568_c0_g1_i2::g.13844 ORF type:5prime_partial len:77 (+),score=12.07 TRINITY_DN18568_c0_g1_i2:1-231(+)	0.200715696	-2.316774655	0.0001305	down	yes	0.8133	4.052	4.39	3.7	4.065	1	0.659	0.781	------	------	------	------	------	------	------	------	------	------	------	CYT	1	13	8.5	High
TRINITY_DN8008_c0_g1_i6_orf1	uncharacterized protein LOC114357965 isoform X1 [Ostrinia furnacalis] >XP_028167599.1 uncharacterized protein LOC114357965 isoform X1 [Ostrinia furnacalis] >XP_028167600.1 uncharacterized protein LOC114357965 isoform X2 [Ostrinia furnacalis] >XP_028167601.1 uncharacterized protein LOC114357965 isoform X3 [Ostrinia furnacalis]	0.292006525	-1.775927486	5.11E-06	down	yes	1.074	3.678	3.789	3.691	3.555	1	1.141	1.082	MF:organic cyclic compound binding;MF:molecular_function;MF:binding;MF:nucleic acid binding;MF:DNA binding;MF:heterocyclic compound binding;	GO:0097159;GO:0003674;GO:0005488;GO:0003676;GO:0003677;GO:1901363	------	------	------	------	ENOG410ZC30	S:Function unknown	------	------	------	CYT	1	0	506.7	Medium
TRINITY_DN5444_c0_g1_i1_orfp1	TRINITY_DN5444_c0_g1_i1_m.14077 TRINITY_DN5444_c0_g1::TRINITY_DN5444_c0_g1_i1::g.14077 ORF type:3prime_partial len:90 (-),score=25.11 TRINITY_DN5444_c0_g1_i1:2-268(-)	0.294338442	-1.764452116	0.0001879	down	yes	0.941	3.197	3.156	3.505	2.929	1	0.896	0.927	------	------	------	------	------	------	------	------	------	------	------	CYT	1	8	9.9	Medium
TRINITY_DN8853_c0_g1_i4_orf1	uncharacterized protein LOC114351488 isoform X1 [Ostrinia furnacalis]	0.311776062	-1.681417933	0.0002286	down	yes	0.969	3.108	3.279	2.784	3.261	1	1.037	0.87	MF:molecular_function;MF:structural constituent of cuticle;MF:structural molecule activity	GO:0003674;GO:0042302;GO:0005198	------	------	------	------	------	------	------	------	------	CYT	1	9	12.9	Medium
TRINITY_DN98692_c0_g3_i1_orf1	fatty acyl-CoA hydrolase precursor, medium chain [Ostrinia furnacalis]	0.339320883	-1.559277874	0.0001029	down	yes	0.9993	2.945	3.053	3.087	2.694	1	0.983	1.015	------	------	------	------	------	------	------	------	------	------	------	CYT	1	13	7.2	High
TRINITY_DN7226_c0_g1_i5_orf1	chemosensory protein [Dioryctria abietella]	0.299867418	-1.73760332	3.80E-05	down	yes	0.9047	3.017	2.87	3.195	2.986	1	0.854	0.86	------	------	------	------	------	------	ENOG4111U5H;ENO	S:Function unknown;	PF03392.16	OS-D	Insect pheromone-binding family, A10/OS-D	CYT	4	28	8.8	High
TRINITY_DN2650_c0_g1_i1_orf1	hypothetical protein HW555_002849 [Spodoptera exigua] >CAH0691914.1 unnamed protein product [Spodoptera exigua]	0.341711615	-1.54914881	2.19E-05	down	yes	0.9503	2.781	2.681	2.738	2.923	1	0.887	0.964	------	------	------	------	------	------	ENOG4111T57	S:Function unknown	PF12974.10	Phosphonate-bd	ABC transporter, phosphonate, periplasmic substrate-binding protein	CYT	5	16	31.9	High
TRINITY_DN7960_c0_g1_i2_orf1	uncharacterized protein LOC114364878 [Ostrinia furnacalis]	0.415484394	-1.267133806	4.78E-05	down	yes	1.025	2.467	2.591	2.329	2.48	1	1.038	1.037	------	------	------	------	------	------	COG0451	I:Lipid transport and metabolism	PF01370.24;PF163	Epimerase;GDP_Man_D	NAD dependent epimerase/dehydratase family;GDP-mannose 4,6 dehydratase;3-beta hydroxysteroid dehydrogenase/isomerase family;Male sterility protein;RmlD substrate binding domain	CYT	13	50	34.5	High
TRINITY_DN1424_c0_g1_i5_orf1	insect group I lytic polysaccharide monooxygenase [Ostrinia furnacalis]	0.398042414	-1.329005926	8.46E-06	down	yes	0.976	2.452	2.48	2.361	2.514	1	0.934	0.994	------	------	------	------	------	------	ENOG410Y8ZQ	S:Function unknown	PF03067.18	LPMO_10	Lytic polysaccharide mono-oxygenase, cellulose-degrading	CYT	6	21	39	High
TRINITY_DN1421_c0_g1_i1_orf1	uncharacterized protein LOC114352615 [Ostrinia furnacalis]	0.413124226	-1.275352431	3.06E-06	down	yes	1.001	2.423	2.448	2.35	2.471	1	0.987	1.016	[illegible]	[illegible]	------	------	------	------	COG5640	U:Intracellular trafficking, secretion, a	PF00089.29;PF120	Trypsin;CLIP	Trypsin;Regulatory CLIP domain of proteinases	CYT	7	22	44.4	High
TRINITY_DN2343_c1_g1_i2_orf1	receptor expression-enhancing protein 5-like isoform X1 [Ostrinia furnacalis] >XP_028170586.1 receptor expression-enhancing protein 5-like isoform X1 [Ostrinia furnacalis]	0.404185661	-1.306909954	9.67E-05	down	yes	0.9753	2.413	2.505	2.496	2.237	1	0.923	1.003	CC:cellular_component;CC:integral component of membrane;CC:cellular anatomical entity;CC:intrinsic component of membrane;	GO:0005575;GO:0016021;GO:0110165;GO:0031224	K17279	REEP5_6	------	------	COG5052	U:Intracellular trafficking, secretion, a	PF03134.22	TB2_DP1_HVA22	TB2/DP1, HVA22 family	CYT	2	26	22.8	High
TRINITY_DN1540_c0_g1_i14_orf1	alaserpin-like isoform X1 [Ostrinia furnacalis]	0.407943925	-1.293557238	3.77E-05	down	yes	0.9603	2.354	2.409	2.432	2.22	1	0.944	0.937	[illegible]	[illegible]	------	------	------	------	COG4826	O:Posttranslational modification, pro	PF00079.23	Serpin	Serpin	CYT	1	33	43.8	High
TRINITY_DN618_c0_g1_i3_orf1	triokinase/FMN cyclase-like isoform X1 [Ostrinia furnacalis]	0.465201465	-1.104072454	1.87E-05	down	yes	1.016	2.184	2.247	2.09	2.216	1	1.01	1.037	[illegible]	[illegible]	K00863	DAK, TKFC	map04622;map	RIG-I-like receptor s	COG2376	G:Carbohydrate transport and metab	PF02733.20;PF027	Dak1;Dak2	Dak1 domain;DAK2 domain	CYT	12	25	62.6	High
TRINITY_DN768_c0_g1_i7_orf1	neutral ceramidase [Leguminivora glycinivorella]	0.428642086	-1.222154587	1.29E-06	down	yes	0.9533	2.224	2.242	2.235	2.196	1	0.932	0.928	[illegible]	[illegible]	K12349	ASAH2	map00600;map	Sphingolipid metab	ENOG410XQWE	S:Function unknown	PF04734.16;PF170	Ceramidase_alk;Ceram	Neutral/alkaline non-lysosomal ceramidase, N-terminal;Neutral/alkaline non-lysosomal ceramidase, C-terminal	CYT	10	20	77.2	High
TRINITY_DN5880_c0_g2_i2_orf1	macrophage mannose receptor 1 [Bombyx mori]	0.458622306	-1.124621571	0.0002559	down	yes	0.9787	2.134	2.178	2.266	1.958	1	0.933	1.003	[illegible]	[illegible]	------	------	------	------	ENOG410XRS3;ENO	U:Intracellular trafficking, secretion, a	PF00059.24	Lectin_C	Lectin C-type domain	CYT	3	11	38.3	High
TRINITY_DN987_c0_g1_i3_orf1	unnamed protein product [Chilo suppressalis]	0.470591062	-1.087454175	1.39E-05	down	yes	0.9793	2.081	2.065	2.159	2.02	1	0.984	0.954	[illegible]	[illegible]	K01934	MTHFS	map00670	One carbon pool by	COG0212	H:Coenzyme transport and metabolis	PF01812.23	5-FTHF_cyc-lig	5-formyltetrahydrofolate cyclo-ligase family	CYT	3	49	21.6	High
TRINITY_DN399_c3_g2_i6_orf1	proline-rich extensin-like protein EPR1 [Ostrinia furnacalis]	0.475024343	-1.073926648	1.94E-05	down	yes	0.9757	2.054	2.08	2.111	1.972	1	0.94	0.987	[illegible]	[illegible]	------	------	------	------	ENOG410ZDQ9;ENO	S:Function unknown;	PF00050.24	Kazal_1	Kazal-type serine protease inhibitor domain	PLA	3	33	18.8	High
TRINITY_DN82944_c0_g1_i4_orf1	senecionine N-oxygenase isoform X2 [Ostrinia furnacalis]	0.483954858	-1.047055613	1.76E-05	down	yes	0.9863	2.038	2.101	2.002	2.01	1	0.929	1.03	[illegible]	[illegible]	------	------	------	------	COG2072	P:Inorganic ion transport and metab	PF00743.22;PF137	FMO-like;Pyr_redox_3;	Flavin-binding monooxygenase-like;Pyridine nucleotide-disulphide oxidoreductase;Pyridine nucleotide-disulphide oxidoreductase;L-lysine 6-monooxygenase/L-ornithine 5-monooxygenase	CYT	2	20	17.9	High
TRINITY_DN4255_c0_g1_i10_orf1	LOW QUALITY PROTEIN: lebocin-4-like [Ostrinia furnacalis]	0.400513299	-1.320077947	0.0002801	down	yes	0.8583	2.143	2.281	2.085	2.064	1	0.715	0.86	------	------	------	------	------	------	ENOG4111ASS	S:Function unknown	------	------	------	CYT	2	21	22.7	High
TRINITY_DN17615_c0_g1_i3_orf1	hypothetical protein SFRICORN_008858 [Spodoptera frugiperda]	0.186824498	-2.420244448	4.03E-05	down	yes	1.089	5.829	5.854	6.225	5.409	1	1.076	1.192	------	------	K20674	MODSP	map04624	Toll and Imd signalir	COG5640	O:Posttranslational modification, pro	------	------	------	CYT	1	20	8.4	High
TRINITY_DN15400_c0_g1_i1_orf1	uncharacterized protein LOC114366781 [Ostrinia furnacalis]	0.262196647	-1.931278856	3.81E-06	down	yes	1.048	3.997	4.133	3.865	3.994	1	1.106	1.038	MF:odorant binding;MF:molecular_function;MF:binding	GO:0005549;GO:0003674;GO:0005488	------	------	------	------	ENOG4111CBI	S:Function unknown	------	------	------	CYT	1	36	10.3	High
TRINITY_DN710_c0_g1_i11_orfp1	TRINITY_DN710_c0_g1_i11_m.67699 TRINITY_DN710_c0_g1::TRINITY_DN710_c0_g1_i11::g.67699 ORF type:complete len:194 (-),score=34.99,Collagen PF01391.19 0.00029 TRINITY_DN710_c0_g1_i11:1283-1864(-)	0.221773594	-2.172840495	7.29E-05	down	yes	0.8953	4.037	4.342	4.044	3.725	1	0.874	0.812	------	------	------	------	------	------	------	------	------	------	------	CYT	1	7	21.3	High
TRINITY_DN13799_c0_g1_i1_orf1	uncharacterized protein LOC116345248 [Contarinia nasturtii]	0.272570961	-1.875296227	2.08E-06	down	yes	0.9987	3.664	3.639	3.786	3.567	1	1.008	0.988	------	------	------	------	------	------	------	------	PF06355.16	Aegerolysin	Aegerolysin	EXC	6	44	15.8	High
TRINITY_DN3275_c0_g2_i3_orf1	hypothetical protein B5X24_HaOG216046 [Helicoverpa armigera]	0.153499496	-2.703694172	0.00199	down	yes	0.6097	3.972	4.458	4.325	3.132	1	0.513	0.316	MF:cation binding;MF:ion binding;MF:molecular_function;MF:binding;MF:metal ion binding;MF:calcium ion binding;	GO:0043169;GO:0043167;GO:0003674;GO:0005488;GO:0046872;GO:0005509	K02183	CALM	map05214;map	Glioma;Pathways of	COG5126;COG5126;(	D:Cell cycle control, cell division, chrc	------	------	------	CYT	1	36	2.8	Medium
TRINITY_DN5408_c0_g1_i5_orf1	uncharacterized protein LOC114359912 [Ostrinia furnacalis]	0.279765232	-1.837711413	3.36E-05	down	yes	1.001	3.578	3.671	3.353	3.711	1	0.907	1.097	CC:cellular_component;CC:integral component of membrane;CC:cellular anatomical entity;CC:intrinsic component of membrane;	GO:0005575;GO:0016021;GO:0110165;GO:0031224	------	------	------	------	ENOG4110RME	S:Function unknown	------	------	------	PLA	1	5	30.4	High
TRINITY_DN9079_c0_g1_i5_orf1	UDP-glucuronosyltransferase-like [Ostrinia furnacalis]	0.273763118	-1.868999996	1.87E-06	down	yes	0.913	3.335	3.272	3.355	3.378	1	0.902	0.837	[illegible]	[illegible]	K00699	UGT	map05207;map	Chemical carcinoger	COG1819	S:Function unknown	PF00201.21	UDPGT	UDP-glucoronosyl and UDP-glucosyl transferase	CYT	5	14	48.5	High
TRINITY_DN6423_c0_g1_i6_orf1	phenoloxidase-activating factor 2-like isoform X1 [Ostrinia furnacalis]	0.344477221	-1.537519511	9.83E-06	down	yes	1.051	3.051	2.999	3.184	2.971	1	1.07	1.084	[illegible]	[illegible]	------	------	------	------	COG5640	O:Posttranslational modification, pro	PF00089.29;PF183	Trypsin;CLIP_1	Trypsin;Serine protease Clip domain PPAF-2	CYT	3	13	55.7	High
TRINITY_DN955_c0_g1_i2_orf1	gloverin-like [Ostrinia furnacalis] >XP_028168251.1 gloverin-like [Ostrinia furnacalis] >AYM26645.1 gloverin [Ostrinia furnacalis]	0.323479675	-1.628253029	5.06E-05	down	yes	0.9947	3.075	3.106	3.242	2.878	1	1.057	0.927	------	------	------	------	------	------	ENOG4110QR6	S:Function unknown	PF10793.12	Gloverin	Gloverin-like protein	PLA	2	20	21.1	High
TRINITY_DN24121_c1_g1_i6_orf1	serine protease persephone-like [Ostrinia furnacalis]	0.363240223	-1.461004126	6.16E-06	down	yes	0.9753	2.685	2.651	2.788	2.615	1	0.952	0.974	[illegible]	[illegible]	K20677;K09625	HAYAN;PRSS2	map04624	Toll and Imd signalir	COG5640	O:Posttranslational modification, pro	PF00089.29	Trypsin	Trypsin	CYT	7	29	28.9	High
TRINITY_DN975_c0_g1_i1_orf1	elongation factor 1-alpha 1 [Myotis lucifugus] >XP_008139741.1 elongation factor 1-alpha 1 [Eptesicus fuscus]	0.482772935	-1.050583297	0.001065	down	yes	1.163	2.409	2.614	2.424	2.19	1	1.251	1.237	[illegible]	[illegible]	K03231	EEF1A	map05140;map	Leishmaniasis;Legior	COG5256	S:Function unknown	PF00009.30;PF031	GTP_EFTU;GTP_EFTU_D	Elongation factor Tu GTP binding domain;Elongation factor Tu C-terminal domain;Elongation factor Tu domain 2	CYT	2	20	50.1	High
TRINITY_DN2743_c0_g1_i5_orf1	regucalcin-like [Ostrinia furnacalis]	0.346750285	-1.528031026	0.0231	down	yes	0.9123	2.631	1.961	3.553	2.379	1	0.813	0.924	------	------	K01053	gnl, RGN	map00030;map	Pentose phosphate	COG3386	G:Carbohydrate transport and metab	PF08450.15	SGL	SMP-30/Gluconolactonase/LRE-like region	CYT	2	29	10	High
TRINITY_DN23398_c0_g1_i1_orf1	cytochrome P450 6B7-like [Ostrinia furnacalis]	0.449484536	-1.153656612	1.16E-05	down	yes	1.09	2.425	2.387	2.429	2.458	1	1.146	1.124	[illegible]	[illegible]	K14999	CYP6	------	------	COG2124	Q:Secondary metabolites biosynthesi	PF00067.25	p450	Cytochrome P450	CYT	6	12	57.2	High
TRINITY_DN1012_c0_g1_i2_orf1	teneurin-a isoform X1 [Ostrinia furnacalis]	0.447929737	-1.15865565	0.0007493	down	yes	1.071	2.391	2.519	2.531	2.123	1	1.161	1.053	[illegible]	[illegible]	K24473	TENM, ODZ	------	------	ENOG410ZZMY;ENC	S:Function unknown;	PF07974.16;PF014	EGF_2;NHL	EGF-like domain;NHL repeat	CYT	4	2	222.3	High
TRINITY_DN798_c1_g1_i3_orf1	protein goliath isoform X1 [Ostrinia furnacalis] >XP_028170876.1 protein goliath isoform X1 [Ostrinia furnacalis]	0.463829787	-1.108332622	0.001127	down	yes	1.09	2.35	2.388	2.088	2.575	1	1.18	1.089	[illegible]	[illegible]	------	------	------	------	ENOG41121N2	O:Posttranslational modification, pro	PF13639.9;PF1712	zf-RING_2;zf-RING_11;	Ring finger domain;RING-like zinc finger;Zinc finger, C3HC4 type;Zinc finger, C3HC4 type;RING-H2 zinc finger domain;PA domain	CYT	1	3	47	High
TRINITY_DN8953_c0_g1_i4_orf1	gonadotropin-releasing hormone receptor [Ostrinia furnacalis] >AXF67446.1 adipokinetic hormone receptor 1 [Ostrinia furnacalis]	0.379034874	-1.399597501	6.92E-05	down	yes	0.9347	2.466	2.307	2.504	2.588	1	0.889	0.915	[illegible]	[illegible]	K04280	GNRHR	map04080;map	Neuroactive ligand-	ENOG410XRW9	U:Intracellular trafficking, secretion, a	PF00001.24	7tm_1	7 transmembrane receptor	CYT	2	6	45	High
TRINITY_DN73900_c0_g1_i1_orf1	carbonic anhydrase 7 [Ostrinia furnacalis]	0.422207792	-1.24397489	0.0005943	down	yes	0.9753	2.31	2.503	2.058	2.37	1	0.914	1.012	[illegible]	[illegible]	K01672	CA	map00910	Nitrogen metabolisr	COG3338	U:Intracellular trafficking, secretion, a	PF00194.24	Carb_anhydrase	Eukaryotic-type carbonic anhydrase	CYT	1	3	36.5	High
TRINITY_DN1024_c0_g4_i1_orf1	superoxide dismutase [Cu-Zn]-like [Ostrinia furnacalis]	0.455736224	-1.133729048	2.03E-05	down	yes	1.009	2.214	2.316	2.169	2.156	1	1.023	1.003	[illegible]	[illegible]	K04565	SOD1	map05208;map	Chemical carcinoger	COG2032	S:Function unknown	PF00080.23	Sod_Cu	Copper/zinc superoxide dismutase	PLA	8	58	18.2	High
TRINITY_DN20680_c0_g1_i5_orf1	tsukushin isoform X2 [Ostrinia furnacalis]	0.477993374	-1.064937474	3.59E-05	down	yes	1.01	2.113	2.198	2.012	2.128	1	1.006	1.025	CC:cellular_component;CC:integral component of membrane;CC:cellular anatomical entity;CC:intrinsic component of membrane;	GO:0005575;GO:0016021;GO:0110165;GO:0031224	------	------	------	------	COG4886	S:Function unknown	PF13855.9;PF1279	LRR_8;LRR_4	Leucine rich repeat;Leucine Rich repeats	CYT	5	15	40.6	High

TRINITY_DN2311_c0_g3_i1_orf1	uncharacterized protein LOC114364231 isoform X1 [Ostrinia furnacalis] >XP_028176108.1 uncharacterized protein LOC114364231 isoform X2 [Ostrinia furnacalis]	0.450465983	-1.150509929	0.0002999	down	yes	0.9667	2.146	2.267	2.218	1.953	1	0.979	0.921	------	------	------	------	------	------	ENOG410Y2YK	S:Function unknown	------	------	------	CYT	1	2	41.4	High
TRINITY_DN3301_c0_g1_i2_orf1	hemicentin-2-like isoform X1 [Ostrinia furnacalis]	0.45	-1.152003093	4.64E-05	down	yes	0.9603	2.134	2.209	2.176	2.018	1	0.943	0.938	MF:hydrolase activity;MF:cation binding;MF:organic cyclic compound binding;MF:ion binding;MF:catalytic activity;MF:molecular_function;MF:binding;MF:nucleic acid binding;MF:metal ion binding;MF:heterocyclic compound binding;	GO:0016787;GO:0043169;GO:0097159;GO:0043167;GO:0003824;GO:0003674;GO:0005488;GO:0003676;GO:0046872;GO:1901363	------	------	------	------	ENOG410ZN3C;ENO	S:Function unknown;	PF07679.19;PF139	I-set;Ig_3;Ig_2;ig;VWA;V	Immunoglobulin I-set domain;Immunoglobulin domain;Immunoglobulin domain;Immunoglobulin domain;von Willebrand factor type A domain;von Willebrand factor type A domain	CYT	3	3	129.9	High
TRINITY_DN5696_c0_g1_i4_orf1	serine protease snake-like isoform X1 [Ostrinia furnacalis]	0.363057041	-1.461731863	0.001563	down	yes	0.8147	2.244	1.935	2.333	2.465	1	0.67	0.774	BP:organic substance metabolic process;BP:organonitrogen compound metabolic process;BP:protein metabolic process;BP:macromolecule metabolic process;BP:biological_process;BP:nitrogen compound metabolic process;BP:metabolic process;BP:proteolysis;BP:primary metabolic process;MF:serine-type endopeptidase activity;MF:endopeptidase activity;MF:hydrolase activity;MF:serine hydrolase activity;MF:catalytic activity, acting on a protein;MF:catalytic activity;MF:molecular_function;MF:peptidase activity;MF:serine-type peptidase activity;	GO:0071704;GO:1901564;GO:0019538;GO:0043170;GO:0008150;GO:0006807;GO:0008152;GO:0006508;GO:0044238;GO:0004252;GO:0004175;GO:0016787;GO:0017171;GO:0140096;GO:0003824;GO:0003674;GO:0008233;GO:0008236	K20677;K09641	HAYAN;TMPR	map05164	Influenza A	COG5640	O:Posttranslational modification, pro	PF00089.29	Trypsin	Trypsin	CYT	1	3	43.2	High
TRINITY_DN4076_c1_g2_i2_orf1	vacuole membrane protein 1 [Ostrinia furnacalis]	0.440616114	-1.182405838	0.002195	down	yes	0.9297	2.11	2.019	2.43	1.881	1	0.904	0.885	CC:cellular_component;CC:integral component of membrane;CC:cellular anatomical entity;CC:intrinsic component of membrane;	GO:0005575;GO:0016021;GO:0110165;GO:0031224	K21248	VMP1	map04140	Autophagy - animal	ENOG410XQHA	S:Function unknown	PF09335.14	SNARE_assoc	SNARE associated Golgi protein	CYT	1	4	44.2	High
TRINITY_DN664_c0_g1_i18_orf1	chitinase-like protein EN03 isoform X2 [Ostrinia furnacalis]	0.472238372	-1.082412821	5.77E-06	down	yes	0.9747	2.064	2.108	2.081	2.003	1	0.969	0.955	BP:primary metabolic process;BP:carbohydrate metabolic process;BP:organic substance metabolic process;BP:biological_process;BP:metabolic process;CC:cellular_component;CC:extracellular region;CC:cellular anatomical entity;MF:carbohydrate derivative binding;MF:chitin binding;MF:molecular_function;MF:binding;	GO:0044238;GO:0005975;GO:0071704;GO:0008150;GO:0008152;GO:0005575;GO:0005576;GO:0110165;GO:0097367;GO:0008061;GO:0003674;GO:0005488	------	------	------	------	COG3325	S:Function unknown	PF00704.31	Glyco_hydro_18	Glycosyl hydrolases family 18	CYT	19	48	48	High
TRINITY_DN14754_c0_g1_i6_orf1	cathepsin L [Papilio xuthus]	0.370182648	-1.433690822	0.0002773	down	yes	0.8107	2.19	2.306	2.174	2.09	1	0.685	0.747	BP:primary metabolic process;BP:organonitrogen compound metabolic process;BP:protein metabolic process;BP:macromolecule metabolic process;BP:organic substance metabolic process;BP:nitrogen compound metabolic process;BP:biological_process;BP:metabolic process;BP:proteolysis;MF:molecular_function;MF:hydrolase activity;MF:catalytic activity;MF:catalytic activity, acting on a protein;MF:peptidase activity;MF:cysteine-type peptidase activity;	GO:0044238;GO:1901564;GO:0019538;GO:0043170;GO:0071704;GO:0006807;GO:0008150;GO:0008152;GO:0006508;GO:0003674;GO:0016787;GO:0003824;GO:0140096;GO:0008233;GO:0008234	------	------	------	------	COG4870	O:Posttranslational modification, pro	PF00112.26	Peptidase_C1	Papain family cysteine protease	CYT	1	41	12.8	High
TRINITY_DN143532_c0_g1_i1_orf1	3-oxoacyl-[acyl-carrier-protein] reductase FabG-like [Aphidius gifuensis] >KAF7996667.1 hypothetical protein HCN44_002313 [Aphidius gifuensis]	0.4973347	-1.007711	0.003989	down	yes	0.9703	1.951	1.63	2.169	2.055	1	0.962	0.949	MF:catalytic activity;MF:molecular_function;MF:oxidoreductase activity;	GO:0003824;GO:0003674;GO:0016491	------	------	------	------	COG1028	S:Function unknown	PF13561.9;PF0010	adh_short_C2;adh_shor	Enoyl;short chain dehydrogenase;KR domain	CYT	1	5	27.2	High
TRINITY_DN5080_c0_g1_i5_orf1	storage protein [Ostrinia furnacalis]	0.146259003	-2.773402658	9.40E-06	down	yes	0.995	6.803	6.829	6.435	7.145	1	0.967	1.018	------	------	------	------	------	------	ENOG410XR2D	J:Translation, ribosomal structure and	PF00372.22;PF037	Hemocyanin_M;Hemoc	Hemocyanin, copper containing domain;Hemocyanin, all-alpha domain	CYT	3	69	49.2	High
TRINITY_DN56459_c0_g1_i2_orf1	aldo-keto reductase AKR2E4-like [Ostrinia furnacalis]	0.159807127	-2.645596349	1.67E-05	down	yes	0.9777	6.118	6.25	6.398	5.707	1	0.951	0.982	MF:catalytic activity;MF:molecular_function;MF:oxidoreductase activity;	GO:0003824;GO:0003674;GO:0016491	------	------	------	------	COG0656	S:Function unknown	------	------	------	CYT	4	78	8.7	High
TRINITY_DN29026_c0_g1_i4_orf1	TIL [Ostrinia furnacalis]	0.223957304	-2.158704378	0.001204	down	yes	1.133	5.059	4.572	5.979	4.626	1	1.003	1.396	------	------	------	------	------	------	ENOG4110ODA;ENC	S:Function unknown;	------	------	------	CYT	1	14	9.9	High
TRINITY_DN1597_c0_g1_i5_orfp1	TRINITY_DN1597_c0_g1_i5_m.57494 TRINITY_DN1597_c0_g1::TRINITY_DN1597_c0_g1_i5::g.57494 ORF type:complete len:86 (+),score=7.19 TRINITY_DN1597_c0_g1_i5:134-391(+)	0.195188119	-2.357062857	9.77E-05	down	yes	0.9857	5.05	5.528	4.987	4.635	1	0.991	0.966	------	------	------	------	------	------	------	------	------	------	------	CYT	2	27	9.2	High
TRINITY_DN16234_c0_g2_i3_orf1	uncharacterized protein LOC114363370 [Ostrinia furnacalis]	0.261067544	-1.937504984	3.01E-05	down	yes	1.032	3.953	4.11	4.07	3.679	1	1.031	1.064	CC:cellular_component;CC:extracellular region;CC:cellular anatomical entity;MF:enzyme regulator activity;MF:peptidase regulator activity;MF:molecular_function;MF:peptidase inhibitor activity;MF:enzyme inhibitor activity;MF:molecular function regulator;	GO:0005575;GO:0005576;GO:0110165;GO:0030234;GO:0061134;GO:0003674;GO:0030414;GO:0004857;GO:0098772	------	------	------	------	ENOG410Y67D	S:Function unknown	------	------	------	CYT	3	51	7.4	High
TRINITY_DN5310_c2_g1_i2_orf1	serine protease persephone-like [Ostrinia furnacalis]	0.27376959	-1.868965891	4.00E-07	down	yes	0.9957	3.637	3.711	3.568	3.633	1	0.977	1.01	BP:organic substance metabolic process;BP:organonitrogen compound metabolic process;BP:protein metabolic process;BP:macromolecule metabolic process;BP:biological_process;BP:nitrogen compound metabolic process;BP:metabolic process;BP:proteolysis;BP:primary metabolic process;MF:serine-type endopeptidase activity;MF:endopeptidase activity;MF:hydrolase activity;MF:serine hydrolase activity;MF:catalytic activity, acting on a protein;MF:catalytic activity;MF:molecular_function;MF:peptidase activity;MF:serine-type peptidase activity;	GO:0071704;GO:1901564;GO:0019538;GO:0043170;GO:0008150;GO:0006807;GO:0008152;GO:0006508;GO:0044238;GO:0004252;GO:0004175;GO:0016787;GO:0017171;GO:0140096;GO:0003824;GO:0003674;GO:0008233;GO:0008236	K20677;K20671	HAYAN;PSH	map04624	Toll and Imd signalir	COG5640	O:Posttranslational modification, pro	PF00089.29	Trypsin	Trypsin	CYT	9	34	34.8	High
TRINITY_DN66040_c0_g1_i2_orf1	serine protease inhibitor dipetalogastin-like isoform X2 [Ostrinia furnacalis]	0.334267913	-1.580923221	0.003227	down	yes	1.073	3.21	3.08	3.842	2.709	1	1.191	1.028	------	------	------	------	------	------	------	------	PF07648.18;PF000	Kazal_2;Kazal_1	Kazal-type serine protease inhibitor domain;Kazal-type serine protease inhibitor domain	CYT	1	21	6	High
TRINITY_DN48548_c0_g1_i1_orf1	glutathione S-transferase siama 2 [Heortia vitessoides]	0.298769609	-1.742894689	5.30E-08	down	yes	0.9713	3.251	3.223	3.271	3.259	1	0.942	0.972	MF:catalytic activity;MF:transferase activity;MF:glutathione transferase activity;MF:molecular_function;MF:transferase activity, transferring alkyl or ary;	GO:0003824;GO:0016740;GO:0004364;GO:0003674;GO:0016765	K04097	HPGDS	map00590;map	Arachidonic acid me	ENOG41103AU;ENO	O:Posttranslational modification, pro	PF14497.9;PF0279	GST_C_3;GST_N;GST_C	Glutathione S-transferase, C-terminal domain;Glutathione S-transferase, N-terminal domain;Glutathione S-transferase, C-terminal domain	CYT	18	60	23.2	High
TRINITY_DN20658_c0_g2_i3_orf1	prostaglandin reductase 1-like [Ostrinia furnacalis]	0.334673876	-1.579172152	3.40E-05	down	yes	0.9903	2.959	3.029	2.77	3.079	1	0.968	1.003	CC:cytoplasm;CC:cellular_component;CC:cellular anatomical entity;MF:13-prostaglandin reductase activity;MF:oxidoreductase activity, acting on CH-OH group of donors;MF:oxidoreductase activity, acting on the CH-CH group of donors, NAD or NADP as acceptor;MF:oxidoreductase activity, acting on the CH-OH group of donors, NAD or NADP as acceptor;MF:oxidoreductase activity, acting on the CH-CH group of donors;MF:catalytic activity;MF:molecular_function;MF:15-oxoprostaglandin 13-oxidase activity;MF:2-alkenal reductase [NA;MF:oxidoreductase activity;	GO:0005737;GO:0005575;GO:0110165;GO:0036132;GO:0016614;GO:0016628;GO:0016616;GO:0016627;GO:0003824;GO:0003674;GO:0047522;GO:0032440;GO:0016491	K13948	PTGR1, LTB4D	map00590	Arachidonic acid me	COG2130	S:Function unknown	PF00107.29;PF136	ADH_zinc_N;ADH_zinc_	Zinc-binding dehydrogenase;Zinc-binding dehydrogenase	CYT	7	27	25.7	High
TRINITY_DN3609_c0_g1_i6_orf1	leukocyte elastase inhibitor-like [Ostrinia furnacalis]	0.355272226	-1.493003187	2.65E-07	down	yes	1.031	2.902	2.944	2.895	2.868	1	1.048	1.045	CC:cellular_component;CC:cellular anatomical entity;CC:extracellular space;MF:endopeptidase inhibitor activity;MF:endopeptidase regulator activity;MF:peptidase regulator activity;MF:molecular_function;MF:peptidase inhibitor activity;MF:serine-type endopeptidase inhibitor activity;MF:enzyme inhibitor activity;MF:molecular function regulator;MF:enzyme regulator activity;	GO:0005575;GO:0110165;GO:0005615;GO:0004866;GO:0061135;GO:0061134;GO:0003674;GO:0030414;GO:0004867;GO:0004857;GO:0098772;GO:0030234	------	------	------	------	COG4826	S:Function unknown	PF00079.23	Serpin	Serpin	CYT	8	17	56.7	High
TRINITY_DN9325_c0_g1_i1_orf1	protein takeout-like [Ostrinia furnacalis]	0.351565187	-1.508135875	0.0004297	down	yes	1.022	2.907	3.177	2.581	2.963	1	1.069	0.998	------	------	------	------	------	------	ENOG4110SYW;ENO	S:Function unknown;	PF06585.14	JHBP	Haemolymph juvenile hormone binding protein	CYT	4	25	26	High
TRINITY_DN6098_c1_g1_i5_orf1	unnamed protein product, partial [Iphiclides podalirius]	0.347901492	-1.523249231	0.002599	down	yes	1.003	2.883	3.381	2.409	2.858	1	1.023	0.985	BP:immune system process;BP:response to external biotic stimulus;BP:response to other organism;BP:interspecies interaction between organisms;BP:innate immune response;BP:cellular process;BP:defense response;BP:response to stress;BP:defense response to other organism;BP:immune response;BP:cell killing;BP:defense response to fungus;BP:response to biotic stimulus;BP:response to external stimulus;BP:response to fungus;BP:response to stimulus;BP:killing of cells of other organism;BP:biological_process;CC:extracellular space;CC:cellular anatomical entity;CC:cellular_component;CC:extracellular region;MF:ion channel regulator activity;MF:molecular_function;MF:molecular function regulator;MF:sodium channel inhibitor activity;MF:channel inhibitor activity;MF:channel regulator activity;MF:ion channel inhibitor activity;MF:sodium channel regulator activity;	GO:0002376;GO:0043207;GO:0051707;GO:0044419;GO:0045087;GO:0009987;GO:0006952;GO:0006950;GO:0098542;GO:0006955;GO:0001906;GO:0050832;GO:0009607;GO:0009605;GO:0009620;GO:0050896;GO:0031640;GO:0008150;GO:0005615;GO:0110165;GO:0005575;GO:0005576;GO:0099106;GO:0003674;GO:0098772;GO:0019871;GO:0016248;GO:0016247;GO:0008200;GO:0017080	------	------	------	------	------	------	------	------	------	CYT	2	28	7.7	High
TRINITY_DN18338_c0_g1_i6_orf1	aquaporin AQPAn.G isoform X1 [Ostrinia furnacalis]	0.371873867	-1.427114726	0.000349	down	yes	1.026	2.759	2.921	2.466	2.889	1	1.113	0.965	CC:integral component of membrane;CC:cellular_component;CC:intrinsic component of membrane;CC:cellular anatomical entity;MF:transporter activity;MF:transmembrane transporter activity;MF:channel activity;MF:molecular_function;MF:passive transmembrane transporter activity;	GO:0016021;GO:0005575;GO:0031224;GO:0110165;GO:0005215;GO:0022857;GO:0015267;GO:0003674;GO:0022803	K09884	AQPN	------	------	COG0580	O:Posttranslational modification, pro	PF00230.23	MIP	Major intrinsic protein	CYT	1	8	29	High
TRINITY_DN19662_c4_g1_i1_orf1	basic juvenile hormone-suppressible protein 1-like [Ostrinia furnacalis]	0.360172538	-1.473239911	1.62E-07	down	yes	1.002	2.782	2.76	2.758	2.827	1	0.999	1.007	CC:cellular_component;CC:membrane;CC:extracellular region;CC:cellular anatomical entity;MF:signaling receptor activity;MF:catalytic activity;MF:phosphotransferase activity, alcohol group as acceptor;MF:transferase activity, transferring phosphorus-containing groups;MF:kinase activity;MF:catalytic activity, acting on a protein;MF:transmembrane signaling receptor activity;MF:transmembrane receptor protein serine/threonine kinase activity;MF:protein serine/threonine kinase activity;MF:transferase activity;MF:molecular_function;MF:protein kinase activity;MF:transmembrane receptor protein kinase	GO:0005575;GO:0016020;GO:0005576;GO:0110165;GO:0038023;GO:0003824;GO:0016773;GO:0016772;GO:0016301;GO:0140096;GO:0004888;GO:0004675;GO:0004674;GO:0016740;GO:0003674;GO:0004672;GO:0019199;GO:0060089	------	------	------	------	ENOG410XR2D	S:Function unknown	PF03723.17	Hemocyanin_C	Hemocyanin, ig-like domain	CYT	31	82	30.7	High
TRINITY_DN1175_c1_g1_i1_orf1	methanethiol oxidase [Ostrinia furnacalis]	0.364124088	-1.457497914	2.63E-06	down	yes	0.9977	2.74	2.809	2.658	2.753	1	0.982	1.011	MF:selenium binding;MF:molecular_function;MF:binding;	GO:0008430;GO:0003674;GO:0005488	K17285	SELENBP1	map00920	Sulfur metabolism	ENOG410XP4F	S:Function unknown	PF05694.14	SBP56	56kDa selenium binding protein	CYT	11	46	53.7	High
TRINITY_DN55148_c0_g1_i1_orf1	60S ribosomal protein L11 isoform 1 [Homo sapiens] >NP_001069049.1 60S ribosomal protein L11 [Bos taurus] >NP_001240835.1 60S ribosomal protein L11 isoform 1 [Canis lupus familiaris] >NP_001269300.1 60S ribosomal protein L11 [Chinchilla lanigera] >NP_001291809.1 60S ribosomal protein L11 [Ailuropoda melanoleuca] >NP_080195.1 60S ribosomal protein L11 [Mus musculus] >XP_001504267.1 60S ribosomal protein L11 isoform X2 [Equus caballus] >XP_003471379.1 60S ribosomal protein L11 [Cavia porcellus] >XP_003810808.1 60S ribosomal protein L11 [Pan paniscus] >XP_003891370.1 60S ribosomal protein L11 [Papio anubis] >XP_003989701.1 60S ribosomal protein L11 [Felis catus] >XP_004285699.1 60S ribosomal protein L11 isoform X2 [Orcinus orca] >XP_004377186.1 60S ribosomal protein L11 [Trichechus manatus latirostris] >XP_004394821.1 PREDICTED: 60S ribosomal protein L11 isoform X1 [Odobenus rosmarus divergens] >XP_004465476.2 60S ribosomal protein L11 [Dasypus novemcinctus] >XP_004637663.1 60S ribosomal protein L11 [Octodon degus] >XP_004850617.1 60S ribosomal protein L11 isoform X2 [Heterocephalus glaber] >XP_005544522.1 60S ribosomal protein L11 isoform X1 [Macaca fascicularis] >XP_005676921.1 PREDICTED: 60S ribosomal protein L11 isoform X2 [Capra hircus] >XP_006078005.1 60S ribosomal protein L11 isoform X2 [Bubalus bubalis] >XP_006094108.1 60S ribosomal protein L11 isoform X3 [Myotis lucifugus] >XP_006239286.1 60S ribosomal protein L11 isoform X1 [Rattus norvegicus] >XP_006737645.1 60S ribosomal protein L11 [Leptonychotes weddellii] >XP_006777625.1 PREDICTED: 60S ribosomal protein L11 isoform X1 [Myotis davidii] >XP_006883588.1 PREDICTED: 60S ribosomal protein L11-like isoform X1 [Elephantulus edwardii] >XP_007121151.1 60S ribosomal protein L11 isoform X2 [Physeter catodon] >XP_007175168.1 60S ribosomal protein L11 isoform X1 [Balaenoptera acutorostrata scammoni] >XP_007459239.1 PREDICTED: 60S ribosomal protein L11 isoform X1 [Lipotes vexillifer] >XP_007524793.1 PREDICTED: 60S ribosomal protein L11 [Erinaceus europaeus] >XP_007528779.2 PREDICTED: 60S ribosomal protein L11 [Erinaceus europaeus] >XP_007933904.2 60S ribosomal protein L11 [Orycteropus afer afer] >XP_007978264.1 60S ribosomal protein L11 isoform X1 [Chlorocebus sabaeus] >XP_008059998.1 60S ribosomal protein L11 isoform X2 [Carlito syrichta] >XP_008146324.1 60S ribosomal protein L11 [Eptesicus fuscus] >XP_008263912.1 PREDICTED: 60S ribosomal protein L11 [Oryctolagus cuniculus] >XP_008518979.1 PREDICTED: 60S ribosomal protein L11 isoform X2 [Equus przewalskii] >XP_008571152.1 PREDICTED: 60S ribosomal protein L11 isoform X1 [Galeopterus	0.442227764	-1.177138492	0.0002261	down	yes	1.064	2.406	2.451	2.553	2.213	1	1.076	1.115	BP:positive regulation of apoptotic signaling pathway;BP:protein localization;BP:regulation of primary metabolic process;BP:regulation of proteasomal ubiquitin-dependent protein catabolic process;BP:negative regulation of proteasomal ubiquitin-dependent protein catabolic process;BP:positive regulation of response to stimulus;BP:regulation of response to stimulus;BP:nitrogen compound transport;BP:regulation of protein ubiquitination involved in ubiquitin-dependent protein catabolic process;BP:cellular component organization or biogenesis;BP:macromolecular complex assembly;BP:negative regulation of macromolecule metabolic process;BP:positive regulation of macromolecule metabolic process;BP:cellular macromolecule localization;BP:regulation of signal transduction;BP:positive regulation of signal transduction;BP:peptide biosynthetic process;BP:cytoplasmic translation;BP:regulation of apoptotic process;BP:negative regulation of proteasomal protein catabolic process;BP:negative regulation of molecular function;BP:positive regulation of biological process;BP:negative regulation of biological process;BP:macromolecule localization;BP:ncRNA processing;BP:regulation of macromolecule metabolic process;BP:organic substance transport;BP:protein targeting;BP:establishment of protein localization;BP:regulation of proteolysis;BP:organonitrogen compound metabolic process;BP:heterocycle metabolic process;BP:organonitrogen compound biosynthetic process;BP:protein metabolic process;BP:cellular nitrogen compound metabolic process;BP:negative regulation of protein catabolic process;BP:negative regulation of protein neddylation;BP:regulation of protein neddylation;BP:positive regulation of intrinsic apoptotic signaling pathway by p53 class mediator;BP:regulation of catabolic process;BP:cellular component assembly;BP:negative regulation of metabolic process;BP:positive regulation of metabolic process;BP:positive regulation of signal transduction by p53 class mediator;BP:protein stabilization;BP:ncRNA metabolic process;BP:regulation of biological process;BP:ribosomal large subunit assembly;BP:positive regulation of intrinsic apoptotic signaling pathway;BP:organic substance biosynthetic process;BP:positive regulation of protein binding;BP:cellular macromolecule metabolic process;BP:intracellular protein transport;BP:negative regulation of transferase activity;BP:cellular component organization;BP:biological regulation;BP:rRNA processing;BP:regulation of molecular function;BP:regulation of biological quality;BP:transport;BP:regulation of catalytic activity;BP:regulation of apoptotic signaling pathway;BP:regulation of cellular process;BP:regulation of protein binding;BP:metabolic process;BP:positive regulation of intracellular signal transduction;BP:regulation of intracellular signal transduction;BP:RNA metabolic process;BP:regulation of intrinsic apoptotic signaling pathway by p53 class mediator;BP:rRNA metabolic process;BP:cellular nitrogen compound biosynthetic process;BP:intracellular transport;BP:negative regulation of protein modification	GO:2001235;GO:0008104;GO:0080090;GO:0032434;GO:0032435;GO:0048584;GO:0048583;GO:0071705;GO:2000058;GO:0071840;GO:0065003;GO:0010605;GO:0010604;GO:0070727;GO:0009966;GO:0009967;GO:0043043;GO:0002181;GO:0042981;GO:1901799;GO:0044092;GO:0048518;GO:0048519;GO:0033036;GO:0034470;GO:0060255;GO:0071702;GO:0006605;GO:0045184;GO:0030162;GO:1901564;GO:0046483;GO:1901566;GO:0019538;GO:0034641;GO:0042177;GO:2000435;GO:2000434;GO:1902255;GO:0009894;GO:0022607;GO:0009892;GO:0009893;GO:1901798;GO:0050821;GO:0034660;GO:0050789;GO:0000027;GO:2001244;GO:1901576;GO:0032092;GO:0044260;GO:0006886;GO:0051348;GO:0016043;GO:0065007;GO:0006364;GO:0065009;GO:0065008;GO:0006810;GO:0050790;GO:2001233;GO:0050794;GO:0043393;GO:0008152;GO:1902533;GO:1902531;GO:0016070;GO:1902253;GO:0016072;GO:0044271;GO:0046907;GO:0031400;GO:0031647;GO:0051338;GO:0008150;GO:0006518;GO:0023056;GO:0044249;GO:0042176;GO:0009895;GO:0034645;GO:0023051;GO:0010647;GO:0010646;GO:0043086;GO:0042273;GO:0006139;GO:0051248;GO:0051234;GO:0051246;GO:1903320;GO:1903321;GO:0022618;GO:0043068;GO:0031399;GO:0022613;GO:0031397;GO:2001242;GO:0051444;GO:0044093;GO:0031330;GO:1903050;GO:1903051;GO:0006725;GO:0031396;GO:1901796;GO:0043604;GO:0043603;GO:0051099;GO:0051098;GO:0043170;GO:0010628;GO:0033365;GO:0045861;GO:0034504;GO:0031329;GO:0043933;GO:0031324;GO:0031323;GO:0006807;GO:0090304;GO:0051438;GO:0043065;GO:0010942;GO:0010941;GO:0071826;GO:1901360;GO:2000059;GO:0071704;GO:0043067;GO:1904666;GO:1904667;GO:0010468;GO:0009987;GO:0061136;GO:0009058;GO:0009059;GO:0051171;GO:0051172;GO:0051649;GO:0019222;GO:0051179;GO:0051641;GO:0044238;GO:0044237;GO:0044085;GO:0006396;GO:0015031;GO:0006412;GO:0048523;GO:0048522;GO:0022626;GO:0022625;GO:0043	K02868	RP-L11e, RPL:	map03010;map	Ribosome;Coronavir	COG0094	J:Translation, ribosomal structure and	PF00281.22;PF006	Ribosomal_L5;Ribosom	Ribosomal protein L5;ribosomal L5P family C-terminus	CYT	1	12	20.3	High
TRINITY_DN43667_c0_g1_i1_orf1	carbonyl reductase [NADPH] 1-like [Ostrinia furnacalis]	0.414033648	-1.272180077	2.14E-05	down	yes	1.009	2.437	2.447	2.326	2.539	1	1.002	1.026	------	------	K00079	CBR1	map00590;map	Arachidonic acid me	COG1028	S:Function unknown	PF00106.28;PF135	adh_short;adh_short_C	short chain dehydrogenase;Enoyl;KR domain	CYT	8	32	32.5	High
TRINITY_DN104663_c1_g1_i2_orf1	PREDICTED: gelsolin-like [Amyelois transitella]	0.444299391	-1.170395932	2.36E-05	down	yes	1.021	2.298	2.186	2.346	2.361	1	1.027	1.036	MF:cytoskeletal protein binding;MF:actin filament binding;MF:actin binding;MF:macromolecular complex binding;MF:binding;MF:protein binding;MF:molecular_function;	GO:0008092;GO:0051015;GO:0003779;GO:0044877;GO:0005488;GO:0005515;GO:0003674	K05768	GSN	map04666;map	Fc gamma R-mediat	ENOG410XR0A	S:Function unknown	------	------	------	CYT	1	53	12.4	High
TRINITY_DN52761_c0_g1_i2_orf1	atlastin isoform X4 [Ostrinia furnacalis]	0.424087591	-1.237565824	7.03E-05	down	yes	0.9877	2.329	2.438	2.372	2.176	1	0.982	0.981	CC:integral component of membrane;CC:intrinsic component of membrane;CC:cellular anatomical entity;CC:cellular_component;MF:purine ribonucleoside triphosphate binding;MF:molecular_function;MF:binding;MF:ion binding;MF:heterocyclic compound binding;MF:nucleoside phosphate binding;MF:anion binding;MF:guanyl nucleotide binding;MF:nucleotide binding;MF:guanyl ribonucleotide binding;MF:purine nucleotide binding;MF:GTP binding;MF:hydrolase activity;MF:GTPase activity;MF:nucleoside-triphosphatase activity;MF:small molecule binding;MF:catalytic activity;MF:hydrolase activity, acting on acid anhydrides, in phosphorus-containing anhydrides;MF:carbohydrate derivative binding;MF:organic cyclic compound binding;MF:hydrolase activity, acting on acid anhydrides;MF:pyrophosphatase activity;MF:purine ribonucleotide binding;MF:ribonucleotide binding;	GO:0016021;GO:0031224;GO:0110165;GO:0005575;GO:0035639;GO:0003674;GO:0005488;GO:0043167;GO:1901363;GO:1901265;GO:0043168;GO:0019001;GO:0000166;GO:0032561;GO:0017076;GO:0005525;GO:0016787;GO:0003924;GO:0017111;GO:0036094;GO:0003824;GO:0016818;GO:0097367;GO:0097159;GO:0016817;GO:0016462;GO:0032555;GO:0032553	K17339	ATL	------	------	ENOG410XR6Z	S:Function unknown	PF02263.22	GBP	Guanylate-binding protein, N-terminal domain	CYT	2	31	12.4	High
TRINITY_DN892_c7_g1_i2_orf1	unnamed protein product [Diatraea saccharalis]	0.463218903	-1.110233966	2.05E-06	down	yes	1.039	2.243	2.276	2.251	2.203	1	1.051	1.065	BP:primary metabolic process;BP:organonitrogen compound metabolic process;BP:protein metabolic process;BP:macromolecule metabolic process;BP:organic substance metabolic process;BP:nitrogen compound metabolic process;BP:biological_process;BP:metabolic process;BP:proteolysis;MF:molecular_function;MF:hydrolase activity;MF:catalytic activity;MF:catalytic activity, acting on a protein;MF:peptidase activity;MF:cysteine-type peptidase activity;	GO:0044238;GO:1901564;GO:0019538;GO:0043170;GO:0071704;GO:0006807;GO:0008150;GO:0008152;GO:0006508;GO:0003674;GO:0016787;GO:0003824;GO:0140096;GO:0008233;GO:0008234	------	------	------	------	ENOG410YN9K	O:Posttranslational modification, pro	PF00112.26	Peptidase_C1	Papain family cysteine protease	PLA	11	29	48.7	High
TRINITY_DN29555_c0_g1_i8_orf1	fasciclin-1 [Ostrinia furnacalis]	0.42145979	-1.246533099	9.10E-06	down	yes	0.9643	2.288	2.274	2.364	2.227	1	0.92	0.973	------	------	K19897	FAS1	------	------	COG2335	M:Cell wall/membrane/envelope bio	PF02469.25	Fasciclin	Fasciclin domain	CYT	9	15	76	High
TRINITY_DN6580_c0_g1_i4_orf1	catalase [Ostrinia furnacalis]	0.452018141	-1.145547422	9.84E-06	down	yes	0.9967	2.205	2.291	2.169	2.156	1	1.004	0.986	BP:cellular catabolic process;BP:hydrogen peroxide catabolic process;BP:cellular process;BP:reactive oxygen species metabolic process;BP:response to stress;BP:biological_process;BP:metabolic process;BP:catabolic process;BP:response to oxidative stress;BP:response to stimulus;BP:cellular metabolic process;BP:hydrogen peroxide metabolic process;MF:molecular_function;MF:binding;MF:heterocyclic compound binding;MF:oxidoreductase activity;MF:cation binding;MF:ion binding;MF:metal ion binding;MF:heme binding;MF:antioxidant activity;MF:catalytic activity;MF:oxidoreductase activity, acting on peroxide as acceptor;MF:tetrapyrrole binding;MF:organic cyclic compound binding;MF:catalase activity;MF:peroxidase	GO:0044248;GO:0042744;GO:0009987;GO:0072593;GO:0006950;GO:0008150;GO:0008152;GO:0009056;GO:0006979;GO:0050896;GO:0044237;GO:0042743;GO:0003674;GO:0005488;GO:1901363;GO:0016491;GO:0043169;GO:0043167;GO:0046872;GO:0020037;GO:0016209;GO:0003824;GO:0016684;GO:0046906;GO:0097159;GO:0004096;GO:0004601	K03781	katE, CAT, cat	map00630;map	Glyoxylate and dicar	COG0753	P:Inorganic ion transport and metab	PF00199.22;PF066	Catalase;Catalase-rel	Catalase;Catalase-related immune-responsive	PLA	32	63	61.4	High
TRINITY_DN7618_c0_g1_i4_orf1	uncharacterized protein LOC114366712 isoform X1 [Ostrinia furnacalis]	0.49882574	-1.003392184	0.005151	down	yes	1.062	2.129	1.909	2.482	1.997	1	1.206	0.979	------	------	------	------	------	------	ENOG4111M89;COG	S:Function unknown;	------	------	------	CYT	1	4	36.6	High
TRINITY_DN4748_c0_g1_i5_orf1	unnamed protein product, partial [Brenthis ino]	0.475570033	-1.072270286	5.11E-06	down	yes	1.022	2.149	2.193	2.106	2.149	1	0.998	1.069	BP:response to biotic stimulus;BP:response to external stimulus;BP:response to external biotic stimulus;BP:response to other organism;BP:response to stimulus;BP:response to stress;BP:defense response;BP:biological_process;BP:interspecies interaction between organisms;CC:cellular_component;CC:extracellular region;CC:cellular anatomical entity;MF:ion channel regulator activity;MF:sodium channel inhibitor activity;MF:sodium channel regulator activity;MF:molecular_function;MF:channel inhibitor activity;MF:channel regulator activity;MF:molecular function regulator;MF:ion channel inhibitor activity;	GO:0009607;GO:0009605;GO:0043207;GO:0051707;GO:0050896;GO:0006950;GO:0006952;GO:0008150;GO:0044419;GO:0005575;GO:0005576;GO:0110165;GO:0099106;GO:0019871;GO:0017080;GO:0003674;GO:0016248;GO:0016247;GO:0098772;GO:0008200	------	------	------	------	------	------	------	------	------	CYT	3	54	7.8	High
TRINITY_DN2472_c0_g1_i6_orf1	programmed cell death protein 6 isoform X1 [Colias croceus] >XP_045492459.1 programmed cell death protein 6 isoform X1 [Colias croceus]	0.456164384	-1.132374287	1.28E-06	down	yes	0.9657	2.117	2.143	2.122	2.087	1	0.936	0.961	MF:cation binding;MF:ion binding;MF:molecular_function;MF:binding;MF:metal ion binding;MF:calcium ion binding;	GO:0043169;GO:0043167;GO:0003674;GO:0005488;GO:0046872;GO:0005509	K23902	PDCD6	------	------	ENOG410YKQK	S:Function unknown	PF00036.35;PF134	EF-hand_1;EF-hand_6;	EF hand;EF-hand domain;EF-hand domain pair;EF-hand domain pair;EF	CYT	3	22	21	High
TRINITY_DN14670_c0_g1_i1_orf1	heat shock protein beta-1 isoform X1 [Helicoverpa armigera] >XP_022829066.1 heat shock protein beta-1 isoform X1 [Spodoptera litura] >XP_026747148.1 heat shock protein beta-1 isoform X3 [Trichoplusia ni] >XP_026748187.1 heat shock protein beta-1 isoform X2 [Galleria mellonella] >XP_028167756.1 heat shock protein beta-1 isoform X2 [Ostrinia furnacalis] >XP_035431734.1 heat shock protein beta-1-like isoform X3 [Spodoptera frugiperda] >XP_047023072.1 heat shock protein beta-1 isoform X1 [Helicoverpa zea] >XP_049865086.1 heat shock protein beta-1 [Pectinophora gossypiella] >KAH9640995.1 hypothetical protein HF086_015091 [Spodoptera exigua] >QGZ00460.1 heat shock protein 21.4 [Glyphodes pyloalis] >QKR72095.1 heat-shock protein 21.4 [Mythimna separata] >CAB3228281.1 unnamed protein product [Arctia plantaginis] >CAH0628881.1 unnamed protein product [Chrysodeixis includens]	0.497553816	-1.007075517	2.56E-06	down	yes	1.017	2.044	2.059	2.078	1.996	1	1.03	1.02	MF:cation binding;MF:metal ion binding;MF:molecular_function;MF:binding;MF:ion binding;	GO:0043169;GO:0046872;GO:0003674;GO:0005488;GO:0043167	------	------	------	------	ENOG410YERS	K:Transcription	PF00011.24	HSP20	Hsp20/alpha crystallin family	CYT	13	72	21.4	High
TRINITY_DN15812_c0_g1_i2_orf1	transferrin [Ostrinia furnacalis]	0.493660228	-1.018409677	2.53E-05	down	yes	0.9967	2.019	2.01	2.103	1.943	1	0.992	0.998	BP:ion homeostasis;BP:homeostatic process;BP:iron ion homeostasis;BP:transition metal ion homeostasis;BP:inorganic ion homeostasis;BP:biological regulation;BP:ion transport;BP:transport;BP:biological_process;BP:metal ion homeostasis;BP:cation homeostasis;BP:establishment of localization;BP:localization;BP:regulation of biological quality;BP:chemical homeostasis;CC:extracellular space;CC:cellular anatomical entity;CC:cellular_component;MF:molecular_function;MF:binding;MF:cation binding;MF:ion binding;MF:metal ion binding;	GO:0050801;GO:0042592;GO:0055072;GO:0055076;GO:0098771;GO:0065007;GO:0006811;GO:0006810;GO:0008150;GO:0055065;GO:0055080;GO:0051234;GO:0051179;GO:0065008;GO:0048878;GO:0005615;GO:0110165;GO:0005575;GO:0003674;GO:0005488;GO:0043169;GO:0043167;GO:0046872	K06569	MFI2, CD228	------	------	ENOG410XQ36	O:Posttranslational modification, pro	PF00405.20;PF129	Transferrin;Phosphona	Transferrin;ABC transporter, phosphonate, periplasmic substrate-binding protein	CYT	30	52	75.6	High
TRINITY_DN13322_c0_g1_i6_orf1	macrophage mannose receptor 1-like [Ostrinia furnacalis]	0.49908583	-1.00264015	6.34E-05	down	yes	0.9827	1.969	2.069	1.951	1.887	1	0.945	1.003	------	------	------	------	------	------	ENOG410ZPTE;ENO	S:Function unknown;U:Intracellular tr	PF00059.24	Lectin_C	Lectin C-type domain	CYT	6	25	36.3	High
TRINITY_DN84478_c0_g1_i8_orf1	uncharacterized protein LOC114359035 isoform X1 [Ostrinia furnacalis]	0.0993083	-3.331941884	1.98E-08	down	yes	1.005	10.12	10.233	10.051	10.087	1	0.936	1.078	BP:nitrogen compound metabolic process;BP:membrane lipid metabolic process;BP:organic substance metabolic process;BP:sphingolipid metabolic process;BP:lipid metabolic process;BP:cellular process;BP:biological_process;BP:metabolic process;BP:cellular lipid metabolic process;BP:primary metabolic process;BP:organonitrogen compound metabolic process;BP:cellular metabolic process;CC:lytic vacuole;CC:intracellular organelle;CC:membrane-bounded organelle;CC:organelle;CC:lysosome;CC:vacuole;CC:cellular anatomical entity;CC:intracellular membrane-bounded organelle;CC:cellular_component;CC:extracellular region;	GO:0006807;GO:0006643;GO:0071704;GO:0006665;GO:0006629;GO:0009987;GO:0008150;GO:0008152;GO:0044255;GO:0044238;GO:1901564;GO:0044237;GO:0000323;GO:0043229;GO:0043227;GO:0043226;GO:0005764;GO:0005773;GO:0110165;GO:0043231;GO:0005575;GO:0005576	K12382	PSAP, SGP1	map00600;map	Sphingolipid metabo	ENOG410XSI5	O:Posttranslational modification, pro	PF02199.18	SapA	Saposin A-type domain	CYT	1	52	5.9	High
TRINITY_DN57137_c0_g1_i1_orfp1	TRINITY_DN57137_c0_g1_i1_m.46420 TRINITY_DN57137_c0_g1::TRINITY_DN57137_c0_g1_i1::g.46420 ORF type:5prime_partial len:56 (-),score=4.65 TRINITY_DN57137_c0_g1_i1:82-249(-)	0.104584527	-3.257258667	5.39E-05	down	yes	0.949	9.074	9.1	9.833	8.288	1	0.936	0.911	------	------	------	------	------	------	------	------	------	------	------	CYT	3	33	5.8	High
TRINITY_DN8158_c0_g1_i2_orf1	hypothetical protein G9C98_000136 [Cotesia typhae]	0.190104167	-2.395137942	0.01055	down	yes	1.022	5.376	5.546	3.634	6.947	1	1.036	1.03	------	------	------	------	------	------	ENOG410XR2D	S:Function unknown	PF00372.22;PF037	Hemocyanin_M;Hemoc	Hemocyanin, copper containing domain;Hemocyanin, ig-like domain;Hemocyanin, all-alpha domain	CYT	2	1	88	High
TRINITY_DN5439_c0_g1_i2_orf1	uncharacterized protein LOC114353087 [Ostrinia furnacalis]	0.220183486	-2.183221824	7.57E-10	down	yes	1.008	4.578	4.599	4.565	4.571	1	1.004	1.019	CC:membrane;CC:endoplasmic reticulum membrane;CC:organelle membrane;CC:cellular_component;CC:cellular anatomical entity;MF:cation binding;MF:monooxygenase activity;MF:heme binding;MF:transition metal ion binding;MF:tetrapyrrole binding;MF:organic cyclic compound binding;MF:oxidoreductase activity, acting on paired donors, with incorporation or reduction of molecular oxygen;MF:ion binding;MF:catalytic activity;MF:molecular_function;MF:binding;MF:iron ion binding;MF:metal ion binding;MF:heterocyclic compound binding;MF:oxidoreductase activity;	GO:0016020;GO:0005789;GO:0031090;GO:0005575;GO:0110165;GO:0043169;GO:0004497;GO:0020037;GO:0046914;GO:0046906;GO:0097159;GO:0016705;GO:0043167;GO:0003824;GO:0003674;GO:0005488;GO:0005506;GO:0046872;GO:1901363;GO:0016491	K15003	CYP9	------	------	COG2124	S:Function unknown	PF00067.25	p450	Cytochrome P450	CYT	16	34	61.8	High

TRINITY_DN6415_c0_g2_i1_orf1	D-arabinitol dehydrogenase 1-like [Ostrinia furnacalis]	0.229763247	-2.121780053	8.39E-06	down	yes	1.019	4.435	4.656	4.266	4.382	1	0.995	1.063	MF:oxidoreductase activity;MF:cation binding;MF:transition metal ion binding;MF:ion binding;MF:catalytic activity;MF:molecular_function;MF:binding;MF:metal ion binding;MF:zinc ion binding;	GO:0016491;GO:0043169;GO:0046914;GO:0043167;GO:0003824;GO:0003674;GO:0005488;GO:0046872;GO:0008270	K17818	ARD1	map00040	Pentose and glucur	COG1063	E:Amino acid transport and metaboli	PF08240.15;PF001	ADH_N;ADH_zinc_N;Gl	Alcohol dehydrogenase GroES-like domain;Zinc-binding dehydrogenase;Glucose dehydrogenase C-terminus	CYT	13	61	37.5	High
TRINITY_DN38506_c0_g1_i4_orf1	C-1-tetrahydrofolate synthase, cytoplasmic isoform X1 [Ostrinia furnacalis] >XP_028166137.1 C-1-tetrahydrofolate synthase, cytoplasmic isoform X2 [Ostrinia furnacalis] >XP_028166140.1 C-1-tetrahydrofolate synthase, cytoplasmic isoform X4 [Ostrinia furnacalis]	0.21522189	-2.216103274	4.14E-05	down	yes	0.936	4.349	4.685	4.236	4.125	1	0.864	0.944	BP:one-carbon metabolic process;BP:nitrogen compound metabolic process;BP:cellular metabolic process;BP:tetrahydrofolate metabolic process;BP:organic cyclic compound metabolic process;BP:tetrahydrofolate interconversion;BP:pteridine-containing compound metabolic process;BP:organic substance metabolic process;BP:folic acid-containing compound metabolic process;BP:cellular process;BP:cellular aromatic compound metabolic process;BP:biological_process;BP:metabolic process;BP:heterocycle metabolic process;BP:organonitrogen compound metabolic process;BP:cellular modified amino acid metabolic process;BP:small molecule metabolic process;MF:binding;MF:purine ribonucleoside triphosphate binding;MF:oxidoreductase activity, acting on the CH-NH group of donors, NAD or NADP as acceptor;MF:molecular_function;MF:ion binding;MF:heterocyclic compound binding;MF:oxidoreductase activity;MF:anion binding;MF:formate-tetrahydrofolate ligase activity;MF:oxidoreductase activity, acting on the CH-NH group of donors;MF:nucleotide binding;MF:cyclohydrolase activity;MF:nucleoside phosphate binding;MF:purine nucleotide binding;MF:ATP binding;MF:methylenetetrahydrofolate dehydrogenase [NA;MF:hydrolase activity;MF:methylenetetrahydrofolate dehydrogenas;MF:small molecule binding;MF:catalytic activity;MF:ligase activity;MF:ligase activity, forming carbon-nitrogen bonds;MF:carbohydrate derivative binding;MF:organic cyclic compound binding;MF:hydrolase activity, acting on carbon-nitroge;MF:adenyl nucleotide binding;MF:methenyltetrahydrofolate cyclohydrolase activity;MF:hydrolase activity, acting on carbon-nitroge;MF:adenyl ribonucleotide binding;MF:purine ribonucleotide binding;MF:ribonucleotide binding;	GO:0006730;GO:0006807;GO:0044237;GO:0046653;GO:1901360;GO:0035999;GO:0042558;GO:0071704;GO:0006760;GO:0009987;GO:0006725;GO:0008150;GO:0008152;GO:0046483;GO:1901564;GO:0006575;GO:0044281;GO:0005488;GO:0035639;GO:0016646;GO:0003674;GO:0043167;GO:1901363;GO:0016491;GO:0043168;GO:0004329;GO:0016645;GO:0000166;GO:0019238;GO:1901265;GO:0017076;GO:0005524;GO:0004486;GO:0016787;GO:0004488;GO:0036094;GO:0003824;GO:0016874;GO:0016879;GO:0097367;GO:0097159;GO:0016810;GO:0030554;GO:0004477;GO:0016814;GO:0032559;GO:0032555;GO:0032553	K00288	MTHFD	map00670;map	One carbon pool by	COG0190	H:Coenzyme transport and metabolis	PF00763.26	THF_DHG_CYH	Tetrahydrofolate dehydrogenase/cyclohydrolase, catalytic domain	CYT	3	61	16.2	High
TRINITY_DN17247_c0_g1_i14_orf1	uncharacterized protein LOC114356308 [Ostrinia furnacalis]	0.318452381	-1.650850436	0.0005151	down	yes	1.07	3.36	2.988	3.727	3.365	1	1.206	1.005	------	------	------	------	------	------	------	------	------	------	------	CYT	1	5	24.8	Medium
TRINITY_DN4816_c0_g2_i3_orf1	15-hydroxyprostaglandin dehydrogenase [NAD(+)]-like [Ostrinia furnacalis]	0.302483737	-1.725070515	1.45E-06	down	yes	1.023	3.382	3.341	3.404	3.4	1	0.954	1.116	MF:catalytic activity;MF:molecular_function;MF:oxidoreductase activity;	GO:0003824;GO:0003674;GO:0016491	K00069	HPGD	map00590;map	Arachidonic acid me	COG1028;COG0300	S:Function unknown;	PF00106.28;PF135	adh_short;adh_short_C	short chain dehydrogenase;Enoyl;KR domain;NAD dependent epimerase/dehydratase family;3-beta hydroxysteroid dehydrogenase/isomerase family	CYT	7	38	29.1	High
TRINITY_DN47842_c0_g1_i1_orf1	protein lethal(2)essential for life-like [Helicoverpa armigera] >PZC74790.1 hypothetical protein B5X24_HaOG207163 [Helicoverpa armigera]	0.358880373	-1.478425069	3.03E-05	down	yes	1.077	3.001	3.165	2.939	2.899	1	1.105	1.126	MF:cation binding;MF:metal ion binding;MF:molecular_function;MF:binding;MF:ion binding;	GO:0043169;GO:0046872;GO:0003674;GO:0005488;GO:0043167	K09542	CRYAB	map04213;map	Longevity regulating	ENOG410YERS	O:Posttranslational modification, pro	PF00011.24	HSP20	Hsp20/alpha crystallin family	CYT	6	43	20.7	High
TRINITY_DN45948_c1_g1_i1_orf1	unnamed protein product [Leptidea sinapis]	0.341680395	-1.549280622	9.82E-05	down	yes	1.037	3.035	3.07	2.802	3.234	1	1.032	1.079	BP:metabolic process;BP:nitrogen compound metabolic process;BP:organic substance metabolic process;BP:proteolysis;BP:biological_process;BP:primary metabolic process;BP:organonitrogen compound metabolic process;BP:protein metabolic process;BP:macromolecule metabolic process;MF:endopeptidase activity;MF:peptidase inhibitor activity;MF:molecular_function;MF:enzyme inhibitor activity;MF:molecular function regulator;MF:peptidase activity;MF:cysteine-type peptidase activity;MF:enzyme regulator activity;MF:hydrolase activity;MF:endopeptidase regulator activity;MF:peptidase regulator activity;MF:catalytic activity, acting on a protein;MF:catalytic activity;MF:endopeptidase inhibitor activity;MF:cysteine-type endopeptidase inhibitor activity;MF:cysteine-type endopeptidase activity;	GO:0008152;GO:0006807;GO:0071704;GO:0006508;GO:0008150;GO:0044238;GO:1901564;GO:0019538;GO:0043170;GO:0004175;GO:0030414;GO:0003674;GO:0004857;GO:0098772;GO:0008233;GO:0008234;GO:0030234;GO:0016787;GO:0061135;GO:0061134;GO:0140096;GO:0003824;GO:0004866;GO:0004869;GO:0004197	K01373	CTSF	map04210;map	Apoptosis;Lysosome	COG4870	O:Posttranslational modification, pro	PF00112.26	Peptidase_C1	Papain family cysteine protease	CYT	2	68	3.7	High
TRINITY_DN335_c1_g1_i5_orf1	PREDICTED: perilipin-4 isoform X14 [Papilio polytes]	0.357092449	-1.485630468	1.41E-05	down	yes	1.012	2.834	2.853	2.945	2.703	1	1.035	1.002	------	------	K20254	PLIN4	map03320	PPAR signaling path	ENOG4112C4W	S:Function unknown	------	------	------	CYT	13	74	15.1	High
TRINITY_DN1650_c0_g1_i5_orf1	uncharacterized protein LOC114355357 [Ostrinia furnacalis]	0.37641475	-1.409604933	0.003979	down	yes	1.031	2.739	2.861	2.194	3.162	1	1.027	1.065	CC:cellular_component;CC:integral component of membrane;CC:cellular anatomical entity;CC:intrinsic component of membrane;	GO:0005575;GO:0016021;GO:0110165;GO:0031224	------	------	------	------	ENOG4111DXB	S:Function unknown	PF13273.9	DUF4064	Protein of unknown function	CYT	1	4	19.4	High
TRINITY_DN121650_c0_g1_i1_orf1	carboxylesterase [Ostrinia furnacalis]	0.375028681	-1.414927164	6.16E-08	down	yes	0.9807	2.615	2.619	2.6	2.625	1	0.952	0.99	CC:integral component of membrane;CC:cellular_component;CC:intrinsic component of membrane;CC:cellular anatomical entity;MF:hydrolase activity;MF:hydrolase activity, acting on ester bonds;MF:carboxylic ester hydrolase activity;MF:catalytic activity;MF:molecular_function;	GO:0016021;GO:0005575;GO:0031224;GO:0110165;GO:0016787;GO:0016788;GO:0052689;GO:0003824;GO:0003674	------	------	------	------	COG2272	I:Lipid transport and metabolism	------	------	------	CYT	1	19	7.6	High
TRINITY_DN20658_c0_g1_i1_orf1	prostaglandin reductase 1-like [Ostrinia furnacalis]	0.404044409	-1.307414224	9.63E-08	down	yes	1.019	2.522	2.505	2.543	2.517	1	1.014	1.043	------	------	K13948	PTGR1, LTB4D	map00590	Arachidonic acid me	COG2130	S:Function unknown	PF16884.8	ADH_N_2	N-terminal domain of oxidoreductase	CYT	8	49	17.6	High
TRINITY_DN53358_c0_g1_i3_orf1	arylphorin subunit alpha-like [Ostrinia furnacalis]	0.385395458	-1.375588525	0.002308	down	yes	0.9843	2.554	2.356	2.3	3.005	1	0.941	1.012	CC:cellular_component;CC:extracellular space;CC:cellular anatomical entity;MF:nutrient reservoir activity;MF:molecular_function;	GO:0005575;GO:0005615;GO:0110165;GO:0045735;GO:0003674	------	------	------	------	ENOG410XR2D	S:Function unknown	PF00372.22	Hemocyanin_M	Hemocyanin, copper containing domain	CYT	4	39	13.3	High
TRINITY_DN18128_c0_g1_i4_orf1	arylsulfatase B [Ostrinia furnacalis]	0.426582278	-1.229104062	0.000205	down	yes	1.011	2.37	2.577	2.287	2.246	1	0.996	1.036	MF:hydrolase activity;MF:molecular_function;MF:hydrolase activity, acting on ester bonds;MF:sulfuric ester hydrolase activity;MF:catalytic activity;	GO:0016787;GO:0003674;GO:0016788;GO:0008484;GO:0003824	K01135	ARSB	map04142;map	Lysosome;Glycosami	COG3119	P:Inorganic ion transport and metabo	------	------	------	CYT	1	14	9.5	High
TRINITY_DN3732_c1_g1_i5_orf1	cytochrome P450 6B2-like [Ostrinia furnacalis]	0.406767549	-1.297723505	1.17E-05	down	yes	0.9677	2.379	2.472	2.327	2.339	1	0.918	0.985	MF:cation binding;MF:monooxygenase activity;MF:heme binding;MF:transition metal ion binding;MF:tetrapyrrole binding;MF:organic cyclic compound binding;MF:oxidoreductase activity, acting on paired donors, with incorporation or reduction of molecular oxygen;MF:ion binding;MF:catalytic activity;MF:molecular_function;MF:binding;MF:iron ion binding;MF:metal ion binding;MF:heterocyclic compound binding;MF:oxidoreductase activity;	GO:0043169;GO:0004497;GO:0020037;GO:0046914;GO:0046906;GO:0097159;GO:0016705;GO:0043167;GO:0003824;GO:0003674;GO:0005488;GO:0005506;GO:0046872;GO:1901363;GO:0016491	K14999	CYP6	------	------	COG2124	Q:Secondary metabolites biosynthesi	PF00067.25	p450	Cytochrome P450	CYT	5	23	55.1	High
TRINITY_DN18482_c0_g1_i3_orf1	calcyphosin-like protein isoform X3 [Helicoverpa armigera] >XP_047020698.1 calcyphosin-like protein isoform X2 [Helicoverpa zea]	0.437635811	-1.1921973	1.24E-05	down	yes	1.007	2.301	2.382	2.308	2.214	1	1.022	0.999	------	------	K23909	CAPS	------	------	ENOG410XRMJ	T:Signal transduction mechanisms	PF13405.9;PF1349	EF-hand_6;EF-hand_7;	EF-hand domain;EF-hand domain pair;EF hand;EF hand	CYT	9	56	25.6	High
TRINITY_DN4242_c0_g1_i6_orf1	fibrohexamerin-like [Ostrinia furnacalis]	0.43597561	-1.197680668	1.82E-05	down	yes	1.001	2.296	2.355	2.196	2.337	1	0.966	1.036	CC:cellular_component;CC:extracellular region;CC:cellular anatomical entity;MF:molecular_function;MF:structural molecule activity;	GO:0005575;GO:0005576;GO:0110165;GO:0003674;GO:0005198	------	------	------	------	ENOG4110SS9	S:Function unknown	PF07294.14	Fibroin_P25	Fibroin P25	CYT	3	14	25.5	High
TRINITY_DN12231_c0_g1_i1_orf1	carbonyl reductase [NADPH] 3-like [Ostrinia furnacalis]	0.446704998	-1.162605699	8.77E-05	down	yes	1.01	2.261	2.265	2.126	2.392	1	0.995	1.035	------	------	K00079	CBR1	map00590;map	Arachidonic acid me	COG1028	K:Transcription	PF00106.28;PF135	adh_short;adh_short_C	short chain dehydrogenase;Enoyl;KR domain	CYT	10	39	32.2	High
TRINITY_DN5748_c0_g1_i6_orf1	glycine N-methyltransferase isoform X1 [Ostrinia furnacalis] >XP_028165118.1 glycine N-methyltransferase isoform X2 [Ostrinia furnacalis] >XP_028165119.1 glycine N-methyltransferase isoform X1 [Ostrinia furnacalis] >XP_028165120.1 glycine N-methyltransferase isoform X2 [Ostrinia furnacalis]	0.477556687	-1.066256101	1.12E-06	down	yes	1.032	2.161	2.139	2.195	2.15	1	1.045	1.051	BP:organic substance metabolic process;BP:biological_process;BP:metabolic process;BP:methylation;MF:glycine N-methyltransferase activity;MF:catalytic activity;MF:S-adenosylmethionine-dependent methyltransferase activity;MF:molecular_function;MF:transferase activity;MF:N-methyltransferase activity;MF:transferase activity, transferring one-carbon groups;MF:methyltransferase activity;	GO:0071704;GO:0008150;GO:0008152;GO:0032259;GO:0017174;GO:0003824;GO:0008757;GO:0003674;GO:0016740;GO:0008170;GO:0016741;GO:0008168	K00552	GNMT	map00260	Glycine, serine and t	ENOG410ZWXN	S:Function unknown	PF13649.9;PF0824	Methyltransf_25;Methy	Methyltransferase domain;Methyltransferase domain;Methyltransferase domain;Methyltransferase domain;Methyltransferase domain	CYT	3	48	27.4	High
TRINITY_DN1161_c0_g1_i2_orf1	fructose-1,6-bisphosphatase 1 [Ostrinia furnacalis]	0.457077626	-1.129488896	6.61E-07	down	yes	1.001	2.19	2.152	2.201	2.216	1	0.984	1.018	BP:monosaccharide biosynthetic process;BP:small molecule biosynthetic process;BP:gluconeogenesis;BP:organic substance metabolic process;BP:organic substance biosynthetic process;BP:hexose metabolic process;BP:hexose biosynthetic process;BP:biosynthetic process;BP:biological_process;BP:metabolic process;BP:primary metabolic process;BP:carbohydrate metabolic process;BP:monosaccharide metabolic process;BP:carbohydrate biosynthetic process;BP:glucose metabolic process;BP:small molecule metabolic process;MF:carbohydrate phosphatase activity;MF:fructose 1,6-bisphosphate 1-phosphatase activity;MF:phosphatase activity;MF:phosphoric ester hydrolase activity;MF:molecular_function;MF:binding;MF:cation binding;MF:ion binding;MF:metal ion binding;MF:hydrolase activity;MF:hydrolase activity, acting on ester bonds;MF:catalytic activity;MF:sugar-phosphatase activity;	GO:0046364;GO:0044283;GO:0006094;GO:0071704;GO:1901576;GO:0019318;GO:0019319;GO:0009058;GO:0008150;GO:0008152;GO:0044238;GO:0005975;GO:0005996;GO:0016051;GO:0006006;GO:0044281;GO:0019203;GO:0042132;GO:0016791;GO:0042578;GO:0003674;GO:0005488;GO:0043169;GO:0043167;GO:0046872;GO:0016787;GO:0016788;GO:0003824;GO:0050308	K03841	FBP, fbp	map00051;map	Fructose and mann	COG0158	G:Carbohydrate transport and metab	PF00316.23;PF189	FBPase;FBPase_C;Inosit	Fructose-1-6-bisphosphatase, N-terminal domain;Fructose-1-6-bisphosphatase, C-terminal domain;Inositol monophosphatase family	CYT	12	41	36.3	High
TRINITY_DN126127_c0_g1_i1_orf1	prolow-density lipoprotein receptor-related protein 1, partial [Ostrinia furnacalis]	0.487568922	-1.036321924	6.81E-05	down	yes	0.9727	1.995	1.987	2.098	1.899	1	0.972	0.946	------	------	K04550	LRP1, CD91	map05010;map	Alzheimer disease;C	ENOG410XP34	S:Function unknown	PF00058.20;PF146	Ldl_recept_b;FXa_inhib	Low-density lipoprotein receptor repeat class B;Coagulation Factor Xa inhibitory site;Low-density lipoprotein receptor domain class A	CYT	9	25	56.4	High
TRINITY_DN6642_c0_g1_i2_orf1	protein purity of essence [Ostrinia furnacalis]	0.463338301	-1.10986215	0.01328	down	yes	0.9327	2.013	1.73	2.509	1.8	1	0.975	0.823	BP:nucleic acid metabolic process;BP:cellular nitrogen compound metabolic process;BP:nitrogen compound metabolic process;BP:organic cyclic compound metabolic process;BP:telomere maintenance;BP:nucleobase-containing compound metabolic process;BP:cellular response to stimulus;BP:cellular macromolecule metabolic process;BP:cellular component organization;BP:organic substance metabolic process;BP:cellular component organization or biogenesis;BP:organelle organization;BP:DNA repair;BP:cellular process;BP:cellular aromatic compound metabolic process;BP:cellular response to DNA damage stimulus;BP:response to stress;BP:biological_process;BP:metabolic process;BP:heterocycle metabolic process;BP:DNA recombination;BP:primary metabolic process;BP:chromosome organization;BP:response to stimulus;BP:telomere organization;BP:cellular metabolic process;BP:macromolecule metabolic process;BP:cellular response to stress;BP:DNA metabolic process;MF:hydrolase activity;MF:purine ribonucleoside triphosphate binding;MF:cation binding;MF:DNA-dependent ATPase activity;MF:hydrolase activity, acting on acid anhydrides;MF:small molecule binding;MF:molecular_function;MF:zinc ion binding;MF:ATPase activity;MF:DNA helicase activity;MF:nucleotide binding;MF:heterocyclic compound binding;MF:ion binding;MF:purine nucleotide binding;MF:catalytic activity, acting on DNA;MF:metal ion binding;MF:ATP binding;MF:protein binding;MF:nucleoside-triphosphatase activity;MF:nucleoside phosphate binding;MF:transition metal ion binding;MF:catalytic activity;MF:helicase activity;MF:purine ribonucleotide binding;MF:hydrolase activity, acting on acid anhydrides, in phosphorus-containing anhydrides;MF:adenyl nucleotide binding;MF:carbohydrate derivative binding;MF:organic cyclic compound binding;MF:pyrophosphatase activity;MF:adenyl ribonucleotide binding;MF:calmodulin binding;MF:anion binding;MF:ribonucleotide binding;MF:binding;	GO:0090304;GO:0034641;GO:0006807;GO:1901360;GO:0000723;GO:0006139;GO:0051716;GO:0044260;GO:0016043;GO:0071704;GO:0071840;GO:0006996;GO:0006281;GO:0009987;GO:0006725;GO:0006974;GO:0006950;GO:0008150;GO:0008152;GO:0046483;GO:0006310;GO:0044238;GO:0051276;GO:0050896;GO:0032200;GO:0044237;GO:0043170;GO:0033554;GO:0006259;GO:0016787;GO:0035639;GO:0043169;GO:0008094;GO:0016817;GO:0036094;GO:0003674;GO:0008270;GO:0016887;GO:0003678;GO:0000166;GO:1901363;GO:0043167;GO:0017076;GO:0140097;GO:0046872;GO:0005524;GO:0005515;GO:0017111;GO:1901265;GO:0046914;GO:0003824;GO:0004386;GO:0032555;GO:0016818;GO:0030554;GO:0097367;GO:0097159;GO:0016462;GO:0032559;GO:0005516;GO:0043168;GO:0032553;GO:0005488	K10691	UBR4, ZUBR1	map05165;map	Human papillomavir	ENOG410XPP8	O:Posttranslational modification, pro	------	------	------	CYT	1	5	34.3	High
TRINITY_DN43350_c0_g3_i1_orf1	uncharacterized protein LOC114355190 [Ostrinia furnacalis]	0.27093858	-1.883962255	2.64E-05	down	yes	1.019	3.761	3.953	3.805	3.526	1	1.011	1.047	------	------	------	------	------	------	ENOG4111DOA	S:Function unknown	------	------	------	CYT	3	25	25.8	High
TRINITY_DN26209_c0_g1_i6_orf1	uncharacterized protein LOC114352357 [Ostrinia furnacalis]	0.272040371	-1.878107331	9.39E-08	down	yes	0.9973	3.666	3.723	3.648	3.627	1	0.985	1.007	------	------	------	------	------	------	COG1028	S:Function unknown	PF13561.9;PF0010	adh_short_C2;adh_shor	Enoyl;short chain dehydrogenase;KR domain	CYT	11	54	27.3	High
TRINITY_DN20560_c0_g1_i6_orf1	pupal cuticle protein C1B-like [Ostrinia furnacalis]	0.310958118	-1.685207813	2.48E-05	down	yes	1.084	3.486	3.575	3.588	3.294	1	1.077	1.176	------	------	------	------	------	------	ENOG410Y08D	S:Function unknown	------	------	------	CYT	3	30	22.6	High
TRINITY_DN6122_c0_g1_i6_orf1	deubiquitinase DESI2 isoform X1 [Helicoverpa armigera] >XP_049707835.1 deubiquitinase DESI2 isoform X1 [Helicoverpa armigera]	0.333531334	-1.584105793	7.29E-05	down	yes	1.123	3.367	3.579	3.35	3.171	1	1.182	1.188	MF:hydrolase activity;MF:peptidase activity;MF:catalytic activity, acting on a protein;MF:molecular_function;MF:catalytic activity;	GO:0016787;GO:0008233;GO:0140096;GO:0003674;GO:0003824	K22763	DESI2, PPPDE	------	------	ENOG4111H3Z	S:Function unknown	PF05903.17	Peptidase_C97	PPPDE putative peptidase domain	CYT	1	8	23.8	High
TRINITY_DN30169_c0_g1_i1_orfp1	TRINITY_DN30169_c0_g1_i1_m.11367 TRINITY_DN30169_c0_g1::TRINITY_DN30169_c0_g1_i1::g.11367 ORF type:3prime_partial len:52 (+),score=0.98 TRINITY_DN30169_c0_g1_i1:72-224(+)	0.308993767	-1.69435036	0.0001726	down	yes	1.041	3.369	3.045	3.443	3.62	1	1.034	1.09	------	------	------	------	------	------	------	------	------	------	------	CYT	1	12	6.1	High
TRINITY_DN15545_c0_g1_i1_orf1	larval cuticle protein LCP-14-like [Ostrinia furnacalis]	0.302304293	-1.725926629	3.30E-05	down	yes	0.9577	3.168	3.318	3.222	2.965	1	0.934	0.939	MF:molecular_function;MF:structural constituent of cuticle;MF:structural molecule activity;	GO:0003674;GO:0042302;GO:0005198	------	------	------	------	ENOG4110RUZ;ENO	S:Function unknown;	PF00379.26	Chitin_bind_4	Insect cuticle protein	EXC	3	48	14.6	High
TRINITY_DN3821_c1_g1_i7_orf1	mitochondrial carrier protein Rim2 isoform X1 [Ostrinia furnacalis]	0.337697214	-1.566197818	0.0002776	down	yes	1.006	2.979	3.291	2.876	2.77	1	0.936	1.083	BP:cellular process;BP:intracellular transport;BP:transport;BP:transmembrane transport;BP:biological_process;BP:establishment of localization in cell;BP:mitochondrial transport;BP:establishment of localization;BP:localization;BP:cellular localization;CC:membrane;CC:organelle membrane;CC:integral component of membrane;CC:cellular_component;CC:cellular anatomical entity;CC:mitochondrial membrane;CC:intrinsic component of membrane;	GO:0009987;GO:0046907;GO:0006810;GO:0055085;GO:0008150;GO:0051649;GO:0006839;GO:0051234;GO:0051179;GO:0051641;GO:0016020;GO:0031090;GO:0016021;GO:0005575;GO:0110165;GO:0031966;GO:0031224	K15116	SLC25A33_36,	------	------	ENOG410XS20	S:Function unknown	PF00153.30	Mito_carr	Mitochondrial carrier protein	CYT	1	4	40.2	High
TRINITY_DN18031_c0_g1_i1_orf1	63 kDa chaperonin, mitochondrial-like [Ostrinia furnacalis]	0.452443118	-1.144191669	0.000719	down	yes	1.213	2.681	2.611	2.589	2.842	1	1.46	1.179	BP:protein folding;BP:cellular process;BP:biological_process;BP:protein refolding;CC:intracellular organelle;CC:membrane-bounded organelle;CC:organelle;CC:mitochondrion;CC:intracellular membrane-bounded organelle;CC:cellular_component;CC:cellular anatomical entity;MF:purine ribonucleoside triphosphate binding;MF:heterocyclic compound binding;MF:molecular_function;MF:binding;MF:ion binding;MF:nucleoside phosphate binding;MF:anion binding;MF:nucleotide binding;MF:purine nucleotide binding;MF:ATP binding;MF:small molecule binding;MF:adenyl nucleotide binding;MF:carbohydrate derivative binding;MF:organic cyclic compound binding;MF:protein binding involved in protein folding;MF:adenyl ribonucleotide binding;MF:purine ribonucleotide binding;MF:ribonucleotide binding;	GO:0006457;GO:0009987;GO:0008150;GO:0042026;GO:0043229;GO:0043227;GO:0043226;GO:0005739;GO:0043231;GO:0005575;GO:0110165;GO:0035639;GO:1901363;GO:0003674;GO:0005488;GO:0043167;GO:1901265;GO:0043168;GO:0000166;GO:0017076;GO:0005524;GO:0036094;GO:0030554;GO:0097367;GO:0097159;GO:0044183;GO:0032559;GO:0032555;GO:0032553	K04077	groEL, HSPD1	map05152;map	Tuberculosis;Type I c	COG0459	C:Energy production and conversion	PF00118.27	Cpn60_TCP1	TCP-1/cpn60 chaperonin family	CYT	1	5	60.7	High
TRINITY_DN15578_c0_g2_i1_orfp1	uncharacterized protein LOC125235519 [Leguminivora glycinivorella]	0.400808229	-1.319015965	0.0001785	down	yes	1.091	2.722	2.717	2.574	2.876	1	1.012	1.26	------	------	------	------	------	------	------	------	------	------	------	CYT	1	9	10.9	High
TRINITY_DN1149_c0_g1_i4_orf1	circadian clock-controlled protein-like [Ostrinia furnacalis]	0.372499091	-1.424691192	1.28E-05	down	yes	1.024	2.749	2.682	2.853	2.713	1	0.97	1.102	------	------	------	------	------	------	ENOG410YQCZ;ENO	S:Function unknown;	PF06585.14	JHBP	Haemolymph juvenile hormone binding protein	CYT	7	35	27.4	High
TRINITY_DN19639_c0_g2_i1_orf1	basic juvenile hormone-suppressible protein 1-like [Hyposmocoma kahamanoa]	0.360315134	-1.472668847	0.003623	down	yes	0.9833	2.729	2.838	2.194	3.154	1	1.045	0.905	------	------	------	------	------	------	ENOG410XR2D	S:Function unknown	PF03722.17	Hemocyanin_N	Hemocyanin, all-alpha domain	CYT	2	72	8.6	High
TRINITY_DN2146_c0_g2_i1_orf1	heat shock protein 68-like [Ostrinia furnacalis]	0.388674884	-1.363364209	4.59E-05	down	yes	1.009	2.596	2.599	2.74	2.45	1	1.012	1.016	MF:binding;MF:carbohydrate derivative binding;MF:anion binding;MF:adenyl nucleotide binding;MF:purine ribonucleoside triphosphate binding;MF:organic cyclic compound binding;MF:protein binding involved in protein folding;MF:nucleotide binding;MF:small molecule binding;MF:ribonucleotide binding;MF:molecular_function;MF:nucleoside phosphate binding;MF:purine nucleotide binding;MF:purine ribonucleotide binding;MF:adenyl ribonucleotide binding;MF:ion binding;MF:heterocyclic compound binding;MF:ATP binding;	GO:0005488;GO:0097367;GO:0043168;GO:0030554;GO:0035639;GO:0097159;GO:0044183;GO:0000166;GO:0036094;GO:0032553;GO:0003674;GO:1901265;GO:0017076;GO:0032555;GO:0032559;GO:0043167;GO:1901363;GO:0005524	K03283	HSPA1s	map05162;map	Measles;Toxoplasmo	COG0443	O:Posttranslational modification, pro	PF00012.23;PF067	HSP70;MreB_Mbl	Hsp70 protein;MreB/Mbl protein	CYT	10	33	70.4	High
TRINITY_DN394_c0_g1_i4_orf1	uncharacterized protein LOC114351483 [Ostrinia furnacalis]	0.417786561	-1.259162008	0.0006108	down	yes	1.057	2.53	2.67	2.684	2.237	1	1.117	1.053	MF:molecular_function;MF:structural constituent of cuticle;MF:structural molecule activity;	GO:0003674;GO:0042302;GO:0005198	------	------	------	------	ENOG41113X7	S:Function unknown	PF00379.26	Chitin_bind_4	Insect cuticle protein	CYT	1	19	12.6	High
TRINITY_DN6325_c0_g1_i9_orf1	fructose-bisphosphate aldolase isoform X2 [Pieris brassicae]	0.390084303	-1.358142148	3.91E-06	down	yes	0.9717	2.491	2.462	2.571	2.441	1	0.969	0.946	BP:ribonucleoside diphosphate metabolic process;BP:phosphorylation;BP:purine nucleoside triphosphate metabolic process;BP:carboxylic acid metabolic process;BP:cellular nitrogen compound metabolic process;BP:nitrogen compound metabolic process;BP:small molecule metabolic process;BP:cellular aromatic compound metabolic process;BP:purine-containing compound metabolic process;BP:generation of precursor metabolites and energy;BP:pyruvate metabolic process;BP:glycolytic process;BP:organic cyclic compound metabolic process;BP:nucleobase-containing compound metabolic process;BP:organic substance catabolic process;BP:purine nucleoside diphosphate metabolic process;BP:nucleotide phosphorylation;BP:ribonucleotide metabolic process;BP:organic substance metabolic process;BP:ribonucleoside triphosphate metabolic process;BP:nucleoside triphosphate metabolic process;BP:purine ribonucleotide metabolic process;BP:purine ribonucleoside triphosphate metabolic process;BP:ribose phosphate metabolic process;BP:ATP metabolic process;BP:cellular process;BP:ADP metabolic process;BP:monocarboxylic acid metabolic process;BP:biological_process;BP:metabolic process;BP:oxoacid metabolic process;BP:carbohydrate metabolic process;BP:nucleoside diphosphate metabolic process;BP:purine ribonucleoside diphosphate metabolic process;BP:nucleobase-containing small molecule metabolic process;BP:heterocycle metabolic process;BP:primary metabolic process;BP:organonitrogen compound metabolic process;BP:organic acid metabolic process;BP:carbohydrate derivative metabolic process;BP:carbohydrate catabolic process;BP:cellular metabolic process;BP:purine nucleotide metabolic process;BP:phosphate-containing compound metabolic process;BP:nucleoside diphosphate phosphorylation;BP:phosphorus metabolic process;BP:organophosphate metabolic process;BP:nucleotide metabolic process;BP:nucleoside phosphate metabolic process;BP:catabolic process;BP:ATP generation from ADP;MF:molecular_function;MF:lyase activity;MF:carbon-carbon lyase activity;MF:fructose-bisphosphate aldolase activity;MF:aldehyde-lyase activity;MF:catalytic activity;	GO:0009185;GO:0016310;GO:0009144;GO:0019752;GO:0034641;GO:0006807;GO:0044281;GO:0006725;GO:0072521;GO:0006091;GO:0006090;GO:0006096;GO:1901360;GO:0006139;GO:1901575;GO:0009135;GO:0046939;GO:0009259;GO:0071704;GO:0009199;GO:0009141;GO:0009150;GO:0009205;GO:0019693;GO:0046034;GO:0009987;GO:0046031;GO:0032787;GO:0008150;GO:0008152;GO:0043436;GO:0005975;GO:0009132;GO:0009179;GO:0055086;GO:0046483;GO:0044238;GO:1901564;GO:0006082;GO:1901135;GO:0016052;GO:0044237;GO:0006163;GO:0006796;GO:0006165;GO:0006793;GO:0019637;GO:0009117;GO:0006753;GO:0009056;GO:0006757;GO:0003674;GO:0016829;GO:0016830;GO:0004332;GO:0016832;GO:0003824	K01623	ALDO	map00010;map	Glycolysis / Glucone	COG3588	G:Carbohydrate transport and metab	PF00274.22	Glycolytic	Fructose-bisphosphate aldolase class-I	CYT	5	82	42.3	High
TRINITY_DN15327_c2_g1_i2_orf1	protein lethal(2)essential for life-like [Ostrinia furnacalis]	0.42893726	-1.221161453	1.36E-06	down	yes	1.005	2.343	2.324	2.394	2.312	1	1.032	0.984	MF:cation binding;MF:structural molecule activity;MF:structural constituent of eye lens;MF:ion binding;MF:molecular_function;MF:binding;MF:metal ion binding;	GO:0043169;GO:0005198;GO:0005212;GO:0043167;GO:0003674;GO:0005488;GO:0046872	K09542	CRYAB	map04213;map	Longevity regulating	ENOG410YERS	E:Amino acid transport and metaboli	PF00011.24;PF005	HSP20;Crystallin	Hsp20/alpha crystallin family;Alpha crystallin A chain, N terminal	CYT	7	44	22	High
TRINITY_DN244_c1_g1_i5_orf1	C-1-tetrahydrofolate synthase, cytoplasmic isoform X3 [Ostrinia furnacalis]	0.464236902	-1.107066888	5.34E-06	down	yes	1.019	2.195	2.263	2.163	2.159	1	1.042	1.014	BP:one-carbon metabolic process;BP:nitrogen compound metabolic process;BP:cellular metabolic process;BP:tetrahydrofolate metabolic process;BP:organic cyclic compound metabolic process;BP:tetrahydrofolate interconversion;BP:pteridine-containing compound metabolic process;BP:organic substance metabolic process;BP:folic acid-containing compound metabolic process;BP:cellular process;BP:cellular aromatic compound metabolic process;BP:biological_process;BP:metabolic process;BP:heterocycle metabolic process;BP:organonitrogen compound metabolic process;BP:cellular modified amino acid metabolic process;BP:small molecule metabolic process;MF:binding;MF:purine ribonucleoside triphosphate binding;MF:oxidoreductase activity, acting on the CH-NH group of donors, NAD or NADP as acceptor;MF:molecular_function;MF:ion binding;MF:heterocyclic compound binding;MF:oxidoreductase activity;MF:anion binding;MF:formate-tetrahydrofolate ligase activity;MF:oxidoreductase activity, acting on the CH-NH group of donors;MF:nucleotide binding;MF:cyclohydrolase activity;MF:nucleoside phosphate binding;MF:purine nucleotide binding;MF:ATP binding;MF:methylenetetrahydrofolate dehydrogenase [NA;MF:hydrolase activity;MF:methylenetetrahydrofolate dehydrogenas;MF:small molecule binding;MF:catalytic activity;MF:ligase activity;MF:ligase activity, forming carbon-nitrogen bonds;MF:carbohydrate derivative binding;MF:organic cyclic compound binding;MF:hydrolase activity, acting on carbon-nitroge;MF:adenyl nucleotide binding;MF:methenyltetrahydrofolate cyclohydrolase activity;MF:hydrolase activity, acting on carbon-nitroge;MF:adenyl ribonucleotide binding;MF:purine ribonucleotide binding;MF:ribonucleotide binding;	GO:0006730;GO:0006807;GO:0044237;GO:0046653;GO:1901360;GO:0035999;GO:0042558;GO:0071704;GO:0006760;GO:0009987;GO:0006725;GO:0008150;GO:0008152;GO:0046483;GO:1901564;GO:0006575;GO:0044281;GO:0005488;GO:0035639;GO:0016646;GO:0003674;GO:0043167;GO:1901363;GO:0016491;GO:0043168;GO:0004329;GO:0016645;GO:0000166;GO:0019238;GO:1901265;GO:0017076;GO:0005524;GO:0004486;GO:0016787;GO:0004488;GO:0036094;GO:0003824;GO:0016874;GO:0016879;GO:0097367;GO:0097159;GO:0016810;GO:0030554;GO:0004477;GO:0016814;GO:0032559;GO:0032555;GO:0032553	K00288	MTHFD	map00670;map	One carbon pool by	COG0190	H:Coenzyme transport and metabolis	PF01268.22;PF028	FTHFS;THF_DHG_CYH_	Formate--tetrahydrofolate ligase;Tetrahydrofolate dehydrogenase/cyclohydrolase, NA;Tetrahydrofolate dehydrogenase/cyclohydrolase, catalytic domain	CYT	27	42	99.9	High
TRINITY_DN27247_c0_g2_i1_orfp1	TRINITY_DN27247_c0_g2_i1_m.23157 TRINITY_DN27247_c0_g2::TRINITY_DN27247_c0_g2_i1::g.23157 ORF type:5prime_partial len:70 (+),score=25.00 TRINITY_DN27247_c0_g2_i1:2-211(+)	0.480387633	-1.057729085	0.0007263	down	yes	1.041	2.167	2.2	2.09	2.212	1	0.868	1.256	------	------	------	------	------	------	------	------	------	------	------	CYT	1	20	7	High
TRINITY_DN10138_c0_g1_i1_orf1	storage protein 1 [Omphisa fuscidentalis]	0.447492092	-1.160065907	8.98E-05	down	yes	0.9903	2.213	2.189	2.356	2.095	1	0.979	0.992	------	------	------	------	------	------	ENOG410XR2D	S:Function unknown	PF03723.17	Hemocyanin_C	Hemocyanin, ig-like domain	PLA	6	91	10.1	High

TRINITY_DN1352_c0_g1_i5_orf1	uncharacterized protein LOC113491815 [Trichoplusia ni]	0.426582847	-1.22910214	0.0001558	down	yes	0.95	2.227	2.299	2.329	2.052	1	0.906	0.944	BP:positive regulation of ion transmembrane transporter activity;BP:positive regulation of transporter activity;BP:regulation of ion transmembrane transporter activity;BP:regulation of transport;BP:modulation of synaptic transmission;BP:regulation of potassium ion transport;BP:regulation of signaling;BP:positive regulation of transmembrane transport;BP:regulation of ion transmembrane transport;BP:positive regulation of ion transmembrane transport;BP:regulation of biological process;BP:regulation of transmembrane transport;BP:positive regulation of potassium ion transport;BP:regulation of cell communication;BP:rhythmic process;BP:regulation of transporter activity;BP:biological regulation;BP:regulation of ion transport;BP:positive regulation of molecular function;BP:regulation of trans-synaptic signaling;BP:positive regulation of biological process;BP:regulation of molecular function;BP:positive regulation of potassium ion transmembrane transport;BP:multicellular organismal process;BP:positive regulation of transport;BP:regulation of cellular process;BP:positive regulation of ion transport;BP:biological_process;BP:sleep;BP:positive regulation of voltage-gated potassium channel activity;BP:regulation of cation channel activity;BP:regulation of localization;BP:positive regulation of potassium ion transmembrane transporter activity;BP:regulation of metal ion transport;BP:regulation of synaptic transmission, cholinergic;BP:regulation of transmembrane transporter activity;BP:positive regulation of cation transmembrane transport;BP:regulation of cation transmembrane transport;BP:regulation of potassium ion transmembrane transporter activity;BP:positive regulation of cation channel activity;BP:regulation of potassium ion transmembrane transport;BP:positive regulation of cellular process;CC:plasma membrane;CC:intrinsic component of membrane;CC:membrane;CC:anchored component of membrane;CC:cellular anatomical entity;CC:cellular_component;	GO:0032414;GO:0032411;GO:0032412;GO:0051049;GO:0050804;GO:0043266;GO:0023051;GO:0034764;GO:0034765;GO:0034767;GO:0050789;GO:0034762;GO:0043268;GO:0010646;GO:0048511;GO:0032409;GO:0065007;GO:0043269;GO:0044093;GO:0099177;GO:0048518;GO:0065009;GO:1901381;GO:0032501;GO:0051050;GO:0050794;GO:0043270;GO:0008150;GO:0030431;GO:1903818;GO:2001257;GO:0032879;GO:1901018;GO:0010959;GO:0032222;GO:0022898;GO:1904064;GO:1904062;GO:1901016;GO:2001259;GO:1901379;GO:0048522;GO:0005886;GO:0031224;GO:0016020;GO:0031225;GO:0110165;GO:0005575	------	------	------	------	ENOG410Z7FK	S:Function unknown	PF17064.8	QVR	Sleepless protein	CYT	3	28	16.5	High
TRINITY_DN1206_c0_g1_i6_orf1	sorbitol dehydrogenase-like [Spodoptera frugiperda] >KAG8104768.1 hypothetical protein SFRUCORN_013827 [Spodoptera frugiperda]	0.458948339	-1.123596326	0.001093	down	yes	0.995	2.168	2.288	1.89	2.325	1	0.982	1.003	MF:oxidoreductase activity;MF:cation binding;MF:oxidoreductase activity, acting on CH-OH group of donors;MF:oxidoreductase activity, acting on the CH-OH group of donors, NAD or NADP as acceptor;MF:transition metal ion binding;MF:ion binding;MF:catalytic activity;MF:molecular_function;MF:binding;MF:metal ion binding;MF:zinc ion binding;	GO:0016491;GO:0043169;GO:0016614;GO:0016616;GO:0046914;GO:0043167;GO:0003824;GO:0003674;GO:0005488;GO:0046872;GO:0008270	K00008	SORD, gutB	map00040;map	Pentose and glucuro	COG1063	E:Amino acid transport and metaboli	PF08240.15;PF001	ADH_N;ADH_zinc_N;Gl	Alcohol dehydrogenase GroES-like domain;Zinc-binding dehydrogenase;Glucose dehydrogenase C-terminus	CYT	12	46	38.7	High
TRINITY_DN113353_c0_g1_i1_orf1	unnamed protein product [Parnassius apollo]	0.461979167	-1.114100301	0.00252	down	yes	0.9757	2.112	2.378	1.803	2.156	1	0.99	0.937	BP:cellular process;BP:transport;BP:lipid transport;BP:autophagy;BP:cellular metabolic process;BP:localization;BP:process utilizing autophagic mechanism;BP:metabolic process;BP:organic substance transport;BP:biological_process;BP:establishment of localization;BP:catabolic process;BP:cellular catabolic process;CC:cellular_component;CC:membrane;CC:pre-autophagosomal structure membrane;CC:intrinsic component of membrane;CC:cellular anatomical entity;CC:integral component of membrane;	GO:0009987;GO:0006810;GO:0006869;GO:0006914;GO:0044237;GO:0051179;GO:0061919;GO:0008152;GO:0071702;GO:0008150;GO:0051234;GO:0009056;GO:0044248;GO:0005575;GO:0016020;GO:0034045;GO:0031224;GO:0110165;GO:0016021	K17907	ATG9	map04136;map	Autophagy - other;I	ENOG410XQBE	U:Intracellular trafficking, secretion, a	------	------	------	CYT	1	19	7.5	High
TRINITY_DN32586_c0_g2_i1_orf1	unnamed protein product [Euphydryas editha]	0.487686939	-1.035972759	9.62E-06	down	yes	0.9783	2.006	2.057	2.022	1.94	1	0.976	0.959	MF:molecular_function;MF:carbohydrate binding;MF:binding;	GO:0003674;GO:0030246;GO:0005488	------	------	------	------	ENOG410XNMR;ENC	S:Function unknown;	PF00059.24	Lectin_C	Lectin C-type domain	CYT	4	47	20.5	High
TRINITY_DN1772_c1_g2_i1_orf1	aldose reductase-like isoform X2 [Ostrinia furnacalis]	0.223349004	-2.162628275	2.61E-07	down	yes	0.9977	4.467	4.541	4.485	4.374	1	0.979	1.014	------	------	K00011	AKR1B	map00051;map	Fructose and manno	COG0656	Z:Cytoskeleton	PF00248.24	Aldo_ket_red	Aldo/keto reductase family	CYT	13	77	36.1	High
TRINITY_DN124654_c0_g1_i1_orf1	protein lethal(2)essential for life [Manduca sexta] >KAG6441919.1 hypothetical protein O3G_MSEX002019 [Manduca sexta]	0.226313426	-2.143605919	0.0003832	down	yes	1.008	4.454	4.577	4.922	3.863	1	0.984	1.039	------	------	K09542	CRYAB	map04213;map	Longevity regulating	ENOG410YERS	P:Inorganic ion transport and metabo	PF00011.24	HSP20	Hsp20/alpha crystallin family	CYT	6	44	22.4	High
TRINITY_DN47784_c0_g2_i1_orfp1	arylphorin subunit alpha-like [Ostrinia furnacalis]	0.248295321	-2.00987102	5.18E-06	down	yes	1.056	4.253	4.255	4.407	4.097	1	1.037	1.131	------	------	------	------	------	------	------	------	------	------	------	CYT	2	27	10.1	High
TRINITY_DN75086_c0_g1_i5_orf1	lysosome membrane protein 2-like [Ostrinia furnacalis]	0.22447228	-2.155390795	7.80E-06	down	yes	0.9677	4.311	4.446	4.398	4.089	1	0.959	0.944	BP:multicellular organismal process;BP:sensory perception of smell;BP:neurological system process;BP:response to stimulus;BP:sensory perception of chemical stimulus;BP:sensory perception;BP:biological_process;BP:system process;CC:membrane;CC:cellular_component;CC:cellular anatomical entity;CC:plasma membrane;CC:integral component of membrane;CC:intrinsic component of membrane;	GO:0032501;GO:0007608;GO:0050877;GO:0050896;GO:0007606;GO:0007600;GO:0008150;GO:0003008;GO:0016020;GO:0005575;GO:0110165;GO:0005886;GO:0016021;GO:0031224	K13885	SCARB1	map04979;map	Cholesterol metabol	ENOG410XS17	U:Intracellular trafficking, secretion, a	------	------	------	CYT	1	11	7.7	High
TRINITY_DN10877_c0_g1_i1_orf1	spodomicin-like [Ostrinia furnacalis]	0.25	-2	0.001589	down	yes	1.011	4.044	3.708	3.645	4.779	1	1.278	0.754	CC:cellular_component;CC:extracellular region;CC:cellular anatomical entity;MF:molecular_function;MF:toxin activity;	GO:0005575;GO:0005576;GO:0110165;GO:0003674;GO:0090729	------	------	------	------	------	------	PF08036.14	Antimicrobial_6	Diapausin family of antimicrobial peptide	CYT	1	19	6.9	Medium
TRINITY_DN6025_c0_g2_i1_orfp1	TRINITY_DN6025_c0_g2_i1_m.7749 TRINITY_DN6025_c0_g2::TRINITY_DN6025_c0_g2_i1::g.7749 ORF type:5prime_partial len:106 (+),score=47.10 TRINITY_DN6025_c0_g2_i1:3-320(+)	0.20746089	-2.269088703	0.002345	down	yes	0.862	4.155	5.022	3.999	3.444	1	0.611	0.975	------	------	------	------	------	------	------	------	------	------	------	PLA	1	10	11.6	Medium
TRINITY_DN59429_c0_g1_i6_orf1	uncharacterized protein LOC114366345 isoform X2 [Ostrinia furnacalis]	0.289221219	-1.789754694	4.68E-05	down	yes	1.025	3.544	3.533	3.322	3.777	1	1.074	1.002	BP:response to biotic stimulus;BP:response to external stimulus;BP:response to external biotic stimulus;BP:response to stimulus;BP:defense response;BP:response to stress;BP:biological_process;BP:interspecies interaction between organisms;BP:response to other organism;CC:cellular_component;CC:extracellular region;CC:cellular anatomical entity;	GO:0009607;GO:0009605;GO:0043207;GO:0050896;GO:0006952;GO:0006950;GO:0008150;GO:0044419;GO:0051707;GO:0005575;GO:0005576;GO:0110165	------	------	------	------	ENOG4110S6N	S:Function unknown	PF00451.22	Toxin_2	Scorpion short toxin, BmKK2	CYT	2	22	12.3	High
TRINITY_DN1391_c0_g1_i29_orfp1	TRINITY_DN1391_c0_g1_i29_m.70767 TRINITY_DN1391_c0_g1::TRINITY_DN1391_c0_g1_i29::g.70767 ORF type:complete len:495 (+),score=158.49 TRINITY_DN1391_c0_g1_i29:728-2212(+)	0.299684543	-1.738483422	0.001609	down	yes	1.045	3.487	4.05	2.942	3.47	1	1.035	1.101	------	------	------	------	------	------	------	------	------	------	------	CYT	5	14	52.3	High
TRINITY_DN33272_c0_g1_i5_orf1	Low-density lipoprotein receptor-related protein 1 [Papilio xuthus]	0.277349188	-1.850224594	4.48E-07	down	yes	0.9563	3.448	3.437	3.412	3.496	1	0.892	0.977	BP:endocytosis;BP:vesicle-mediated transport;BP:transport;BP:biological_process;BP:establishment of localization;BP:localization;CC:integral component of membrane;CC:cellular_component;CC:intrinsic component of membrane;CC:cellular anatomical entity;MF:binding;MF:cation binding;MF:metal ion binding;MF:ion binding;MF:molecular_function;MF:calcium ion binding;	GO:0006897;GO:0016192;GO:0006810;GO:0008150;GO:0051234;GO:0051179;GO:0016021;GO:0005575;GO:0031224;GO:0110165;GO:0005488;GO:0043169;GO:0046872;GO:0043167;GO:0003674;GO:0005509	K04550;K24498	LRP1, CD91;S(	map05010;map	Alzheimer disease;C	ENOG410XP34	U:Intracellular trafficking, secretion, a	PF00057.21	Ldl_recept_a	Low-density lipoprotein receptor domain class A	CYT	1	13	9.2	High
TRINITY_DN307_c1_g1_i1_orf1	uncharacterized protein LOC114356704 [Ostrinia furnacalis]	0.343228819	-1.542757401	5.33E-05	down	yes	1.029	2.998	3.168	3.022	2.803	1	1.068	1.02	------	------	------	------	------	------	ENOG410Y4WH	S:Function unknown	PF07648.18	Kazal_2	Kazal-type serine protease inhibitor domain	CYT	3	35	10.4	High
TRINITY_DN100327_c0_g1_i1_orf1	arylphorin subunit alpha-like [Ostrinia furnacalis]	0.329191919	-1.602999173	0.0005231	down	yes	0.9777	2.97	3.009	2.614	3.287	1	0.967	0.966	------	------	------	------	------	------	ENOG410XR2D	S:Function unknown	PF00372.22;PF037	Hemocyanin_M;Hemoc	Hemocyanin, copper containing domain;Hemocyanin, ig-like domain;Hemocyanin, all-alpha domain	PLA	35	56	83.5	High
TRINITY_DN30306_c0_g2_i1_orf1	perilipin-4-like isoform X3 [Ostrinia furnacalis]	0.251240079	-1.992861465	1.09E-05	down	yes	1.013	4.032	4.051	4.213	3.831	1	1.032	1.006	------	------	K20254	PLIN4	map03320	PPAR signaling path	ENOG4112C4W	S:Function unknown	------	------	------	CYT	6	64	7.2	High
TRINITY_DN36434_c0_g2_i3_orf1	clotting factor B isoform X1 [Ostrinia furnacalis]	0.341184868	-1.551374432	0.0005476	down	yes	0.956	2.802	2.556	3.157	2.692	1	0.926	0.942	BP:organic substance metabolic process;BP:organonitrogen compound metabolic process;BP:protein metabolic process;BP:macromolecule metabolic process;BP:biological_process;BP:nitrogen compound metabolic process;BP:metabolic process;BP:proteolysis;BP:primary metabolic process;MF:serine-type endopeptidase activity;MF:endopeptidase activity;MF:hydrolase activity;MF:serine hydrolase activity;MF:catalytic activity, acting on a protein;MF:catalytic activity;MF:molecular_function;MF:peptidase activity;MF:serine-type peptidase activity;	GO:0071704;GO:1901564;GO:0019538;GO:0043170;GO:0008150;GO:0006807;GO:0008152;GO:0006508;GO:0044238;GO:0004252;GO:0004175;GO:0016787;GO:0017171;GO:0140096;GO:0003824;GO:0003674;GO:0008233;GO:0008236	------	------	------	------	COG5640	U:Intracellular trafficking, secretion, a	PF00089.29	Trypsin	Trypsin	CYT	3	9	48.8	High
TRINITY_DN129863_c0_g1_i1_orf1	protein PFC0760c-like isoform X2 [Ostrinia furnacalis]	0.378815741	-1.400431814	0.001176	down	yes	1.03	2.719	2.382	3.081	2.694	1	0.995	1.094	------	------	------	------	------	------	------	------	------	------	------	CYT	1	21	19.3	High
TRINITY_DN3707_c0_g1_i1_orf1	protein FAM160B1-like isoform X1 [Ostrinia furnacalis]	0.316320293	-1.660541981	0.000262	down	yes	1.035	3.272	3.251	2.966	3.598	1	1.069	1.037	------	------	------	------	------	------	ENOG410XTDG;ENO	S:Function unknown;	PF10257.12;PF193	RAI16-like;DUF5917;KE	Retinoic acid induced 16-like protein;Family of unknown function;KELAA motif	CYT	1	1	93.1	High
TRINITY_DN130575_c0_g1_i1_orfp1	TRINITY_DN130575_c0_g1_i1_m.77798 TRINITY_DN130575_c0_g1::TRINITY_DN130575_c0_g1_i1::g.77798 ORF type:internal len:70 (+),score=15.12 TRINITY_DN130575_c0_g1_i1:3-209(+)	0.374839683	-1.4156544	5.38E-06	down	yes	0.9937	2.651	2.666	2.731	2.557	1	0.982	0.999	------	------	------	------	------	------	------	------	------	------	------	CYT	2	74	6.7	High
TRINITY_DN64772_c0_g1_i1_orf1	aldehyde dehydrogenase, partial [Mythimna separata]	0.340893929	-1.552605187	2.89E-06	down	yes	1.022	2.998	2.979	3.093	2.921	1	1.023	1.044	MF:catalytic activity;MF:oxidoreductase activity, acting on the aldehyde or oxo group of donors;MF:molecular_function;MF:oxidoreductase activity, acting on the aldehyde or oxo group of donors, NAD or NADP as acceptor;MF:oxidoreductase activity;	GO:0003824;GO:0016903;GO:0003674;GO:0016620;GO:0016491	K00128	ALDH	map00310;map	Lysine degradation;(	COG1012	C:Energy production and conversion	PF00171.25	Aldedh	Aldehyde dehydrogenase family	CYT	2	42	5.9	High
TRINITY_DN12775_c0_g1_i10_orfp1	TRINITY_DN12775_c0_g1_i10_m.21238 TRINITY_DN12775_c0_g1::TRINITY_DN12775_c0_g1_i10::g.21238 ORF type:5prime_partial len:67 (-),score=0.74 TRINITY_DN12775_c0_g1_i10:275-475(-)	0.388076923	-1.365585449	9.27E-06	down	yes	1.009	2.6	2.61	2.517	2.673	1	1.071	0.957	------	------	------	------	------	------	------	------	------	------	------	CYT	2	14	7.7	High
TRINITY_DN1450_c0_g2_i1_orf1	death-associated protein 1 [Ostrinia furnacalis]	0.379394361	-1.398229858	0.0001875	down	yes	1.09	2.873	3.101	2.819	2.698	1	1.209	1.062	------	------	------	------	------	------	ENOG41122SE	S:Function unknown	PF15228.9	DAP	Death-associated protein	CYT	2	37	10.7	High
TRINITY_DN9538_c1_g3_i1_orf1	cilia- and flagella-associated protein 410 isoform X2 [Aphidius gifuensis]	0.376582278	-1.408962985	1.50E-05	down	yes	1.071	2.844	2.897	2.904	2.732	1	1.065	1.148	------	------	K23456	CFAP410, C21	------	------	ENOG410Y9AE	S:Function unknown	PF14580.9;PF1279	LRR_9;LRR_4	Leucine-rich repeat;Leucine Rich repeats	CYT	1	2	47.7	Medium
TRINITY_DN4080_c0_g1_i8_orf1	AMP deaminase 2 isoform X3 [Ostrinia furnacalis] >XP_028163647.1 AMP deaminase 2 isoform X3 [Ostrinia furnacalis] >XP_028163648.1 AMP deaminase 2 isoform X3 [Ostrinia furnacalis]	0.39624119	-1.335549235	8.22E-06	down	yes	1.012	2.554	2.653	2.474	2.536	1	1.016	1.021	------	------	K01490	AMPD	map01232;map	Nucleotide metaboli	COG1816	S:Function unknown	PF19326.2;PF0096	AMP_deaminase;A_dea	AMP deaminase;Adenosine deaminase	CYT	21	35	87.3	High
TRINITY_DN64181_c0_g1_i1_orf1	uncharacterized protein LOC114356431 isoform X2 [Ostrinia furnacalis]	0.439881613	-1.184812797	0.04095	down	yes	1.189	2.703	3.462	1.759	2.889	1	1.297	1.269	------	------	------	------	------	------	------	------	------	------	------	PLA	1	18	7.9	High
TRINITY_DN1226_c0_g1_i11_orfp1	TRINITY_DN1226_c0_g1_i11_m.52385 TRINITY_DN1226_c0_g1::TRINITY_DN1226_c0_g1_i11::g.52385 ORF type:internal len:92 (-),score=5.77 TRINITY_DN1226_c0_g1_i11:2-274(-)	0.360041987	-1.473762934	6.46E-05	down	yes	1.029	2.858	3.063	2.76	2.751	1	1.077	1.011	------	------	------	------	------	------	------	------	------	------	------	PLA	2	53	9.1	High
TRINITY_DN36476_c1_g1_i1_orfp1	TRINITY_DN36476_c1_g1_i1_m.70910 TRINITY_DN36476_c1_g1::TRINITY_DN36476_c1_g1_i1::g.70910 ORF type:5prime_partial len:88 (-),score=0.50 TRINITY_DN36476_c1_g1_i1:49-312(-)	0.128811283	-2.956669128	1.21E-07	down	yes	0.959	7.445	7.571	7.45	7.313	1	0.942	0.935	------	------	------	------	------	------	------	------	------	------	------	CYT	2	21	9.5	High
TRINITY_DN9593_c0_g1_i2_orf1	uncharacterized protein LOC113518937 [Galleria mellonella]	0.5	-1	2.42E-05	down	yes	1.074	2.148	2.096	2.204	2.144	1	1.113	1.108	------	------	------	------	------	------	------	------	------	------	------	CYT	3	32	15	High
TRINITY_DN4245_c0_g2_i1_orf1	long-chain fatty acid transport protein 4-like [Ostrinia furnacalis]	0.364530387	-1.455889014	5.51E-05	down	yes	0.9897	2.715	2.708	2.555	2.883	1	0.966	1.003	------	------	K08745	SLC27A1_4, F/	map04931;map	Insulin resistance;PP	ENOG410XQ8T	I:Lipid transport and metabolism	PF00501.31	AMP-binding	AMP-binding enzyme	CYT	2	21	19.7	High
TRINITY_DN1759_c0_g1_i4_orf1	protein PFC0760c-like isoform X1 [Ostrinia furnacalis]	0.457025921	-1.129652103	5.58E-07	down	yes	1.005	2.199	2.202	2.232	2.163	1	0.998	1.017	------	------	------	------	------	------	COG3752;ENOG411(	S:Function unknown;	------	------	------	CYT	9	12	100.6	High
TRINITY_DN5177_c0_g1_i2_orf1	hemolin-like isoform X1 [Ostrinia furnacalis]	0.153405666	-2.704576325	1.45E-06	down	yes	1.018	6.636	6.835	6.665	6.408	1	1.013	1.041	------	------	------	------	------	------	ENOG410XSVG	S:Function unknown	PF07679.19;PF139	I-set;Ig_3;ig;Ig_2;V-set;	Immunoglobulin I-set domain;Immunoglobulin domain;Immunoglobulin domain;Immunoglobulin domain;Immunoglobulin V-set domain;Natural killer cell receptor 2B4	CYT	22	58	47.3	High
TRINITY_DN10222_c0_g1_i2_orf1	glutathione S-transferase sigma 3 [Ostrinia furnacalis]	0.369169207	-1.437645873	7.16E-07	down	yes	0.9687	2.624	2.633	2.578	2.661	1	0.971	0.935	MF:catalytic activity;MF:transferase activity;MF:glutathione transferase activity;MF:molecular_function;MF:transferase activity, transferring alkyl or ary;	GO:0003824;GO:0016740;GO:0004364;GO:0003674;GO:0016765	K04097	HPGDS	map00590;map	Arachidonic acid me	ENOG41103AU;ENO(	O:Posttranslational modification, pro	PF14497.9;PF0279	GST_C_3;GST_N;GST_C	Glutathione S-transferase, C-terminal domain;Glutathione S-transferase, N-terminal domain;Glutathione S-transferase, C-terminal domain	CYT	14	58	22.9	High
TRINITY_DN5126_c0_g2_i1_orf1	cytochrome P450 monooxygenase CYP4L47 [Ostrinia furnacalis]	0.458702753	-1.124368528	0.0002479	down	yes	0.983	2.143	2.237	1.957	2.236	1	0.958	0.991	MF:cation binding;MF:monooxygenase activity;MF:heme binding;MF:transition metal ion binding;MF:tetrapyrrole binding;MF:organic cyclic compound binding;MF:oxidoreductase activity, acting on paired donors, with incorporation or reduction of molecular oxygen;MF:ion binding;MF:catalytic activity;MF:molecular_function;MF:binding;MF:iron ion binding;MF:metal ion binding;MF:heterocyclic compound binding;MF:oxidoreductase activity;	GO:0043169;GO:0004497;GO:0020037;GO:0046914;GO:0046906;GO:0097159;GO:0016705;GO:0043167;GO:0003824;GO:0003674;GO:0005488;GO:0005506;GO:0046872;GO:1901363;GO:0016491	K15001	CYP4	------	------	COG2124	I:Lipid transport and metabolism	PF00067.25	p450	Cytochrome P450	CYT	1	7	13	High
TRINITY_DN35809_c0_g1_i1_orf1	spodomicin-like [Ostrinia furnacalis] >QKV49445.1 diapausin [Ostrinia furnacalis]	0.180416202	-2.47059919	2.88E-09	down	yes	0.971	5.382	5.366	5.391	5.388	1	0.978	0.935	------	------	------	------	------	------	------	------	PF08036.14	Antimicrobial_6	Diapausin family of antimicrobial peptide	CYT	3	60	6.8	High
TRINITY_DN9492_c0_g1_i7_orf1	aldo-keto reductase AKR2E4-like [Ostrinia furnacalis]	0.390780998	-1.355567776	1.27E-06	down	yes	0.9707	2.484	2.479	2.526	2.446	1	0.926	0.986	MF:catalytic activity;MF:molecular_function;MF:oxidoreductase activity;	GO:0003824;GO:0003674;GO:0016491	------	------	------	------	COG0656	S:Function unknown	PF00248.24	Aldo_ket_red	Aldo/keto reductase family	CYT	7	30	38.3	High
TRINITY_DN581_c3_g2_i1_orf1	uncharacterized protein LOC114364499 isoform X3 [Ostrinia furnacalis]	0.290256932	-1.784597572	2.48E-05	down	yes	1.141	3.931	3.75	4.059	3.983	1	1.128	1.295	------	------	------	------	------	------	------	------	------	------	------	CYT	1	17	6.7	High
TRINITY_DN14460_c0_g1_i6_orf1	scavenger receptor class B member 1-like [Ostrinia furnacalis]	0.458225668	-1.125869821	6.42E-06	down	yes	1.064	2.322	2.323	2.296	2.346	1	1.061	1.131	BP:multicellular organismal process;BP:sensory perception of smell;BP:neurological system process;BP:response to stimulus;BP:sensory perception of chemical stimulus;BP:sensory perception;BP:biological_process;BP:system process;CC:membrane;CC:cellular_component;CC:cellular anatomical entity;CC:plasma membrane;CC:integral component of membrane;CC:intrinsic component of membrane;	GO:0032501;GO:0007608;GO:0050877;GO:0050896;GO:0007606;GO:0007600;GO:0008150;GO:0003008;GO:0016020;GO:0005575;GO:0110165;GO:0005886;GO:0016021;GO:0031224	K13885	SCARB1	map04979;map	Cholesterol metabol	ENOG410XS17	C:Energy production and conversion	PF01130.24	CD36	CD36 family	CYT	3	6	62.9	High
TRINITY_DN7040_c0_g2_i1_orf1	uncharacterized protein LOC114353763 [Ostrinia furnacalis]	0.260879811	-1.938542791	1.55E-06	down	yes	0.9963	3.819	3.77	3.742	3.945	1	1.003	0.986	------	------	------	------	------	------	ENOG4110RZK	O:Posttranslational modification, pro	------	------	------	CYT	8	32	22	High
TRINITY_DN27300_c0_g1_i1_orfp1	TRINITY_DN27300_c0_g1_i1_m.71142 TRINITY_DN27300_c0_g1::TRINITY_DN27300_c0_g1_i1::g.71142 ORF type:internal len:82 (-),score=9.46 TRINITY_DN27300_c0_g1_i1:3-245(-)	0.333227948	-1.585418689	0.0002881	down	yes	1.054	3.163	2.949	3.043	3.496	1	0.998	1.164	------	------	------	------	------	------	------	------	------	------	------	CYT	1	21	9.2	High
TRINITY_DN1593_c0_g1_i1_orf1	chemosensory protein csp11 [Helopeltis theivora]	0.445278777	-1.167219244	6.05E-06	down	yes	0.9903	2.224	2.2	2.298	2.175	1	1.003	0.968	------	------	------	------	------	------	ENOG4110RV8;ENO(	S:Function unknown;	PF03392.16	OS-D	Insect pheromone-binding family, A10/OS-D	CYT	8	39	15.4	High
TRINITY_DN3949_c0_g1_i1_orf1	probable cytochrome P450 304a1 [Ostrinia furnacalis]	0.490918017	-1.02644598	5.37E-05	down	yes	1	2.037	1.925	2.073	2.112	1	0.998	1.003	CC:integral component of membrane;CC:cellular_component;CC:cellular anatomical entity;CC:intrinsic component of membrane;MF:cation binding;MF:monooxygenase activity;MF:heme binding;MF:transition metal ion binding;MF:tetrapyrrole binding;MF:organic cyclic compound binding;MF:oxidoreductase activity, acting on paired donors, with incorporation or reduction of molecular oxygen;MF:ion binding;MF:catalytic activity;MF:molecular_function;MF:binding;MF:iron ion binding;MF:metal ion binding;MF:heterocyclic compound binding;MF:oxidoreductase activity;	GO:0016021;GO:0005575;GO:0110165;GO:0031224;GO:0043169;GO:0004497;GO:0020037;GO:0046914;GO:0046906;GO:0097159;GO:0016705;GO:0043167;GO:0003824;GO:0003674;GO:0005488;GO:0005506;GO:0046872;GO:1901363;GO:0016491	------	------	------	------	COG2124	Q:Secondary metabolites biosynthesi	PF00067.25	p450	Cytochrome P450	CYT	3	5	59	High
TRINITY_DN2461_c0_g1_i5_orf1	secretory phospholipase A2 receptor [Vanessa cardui]	0.452327158	-1.144561477	1.51E-05	down	yes	1.001	2.213	2.119	2.28	2.24	1	1.001	1.001	------	------	------	------	------	------	ENOG410XRS3;ENO(	U:Intracellular trafficking, secretion, a	PF00059.24	Lectin_C	Lectin C-type domain	CYT	7	30	31.9	High
TRINITY_DN64297_c0_g1_i1_orf1	vanin-like protein 2 isoform X2 [Ostrinia furnacalis]	0.451787649	-1.146283264	0.009816	down	yes	1.251	2.769	2.526	3.369	2.411	1	1.417	1.336	BP:nitrogen compound metabolic process;BP:biological_process;BP:metabolic process;	GO:0006807;GO:0008150;GO:0008152	------	------	------	------	COG0388	O:Posttranslational modification, pro	------	------	------	CYT	1	57	7.8	High
TRINITY_DN628_c0_g1_i1_orf1	prostamide/prostaglandin F synthase-like [Ostrinia furnacalis]	0.338521533	-1.562680491	6.36E-08	down	yes	0.9983	2.949	2.926	2.988	2.933	1	0.993	1.002	------	------	K15717	PRXL2B, FAM:	map00590	Arachidonic acid me	ENOG410YFY9	O:Posttranslational modification, pro	PF13911.9	AhpC-TSA_2	AhpC/TSA antioxidant enzyme	CYT	9	46	26.8	High
TRINITY_DN8473_c0_g1_i6_orf1	serine/threonine-protein phosphatase 6 regulatory subunit 1 [Ostrinia furnacalis]	0.48271028	-1.050770542	0.006128	down	yes	1.033	2.14	1.84	2.533	2.047	1	0.985	1.113	BP:regulation of phosphate metabolic process;BP:regulation of primary metabolic process;BP:regulation of metabolic process;BP:regulation of phosphatase activity;BP:regulation of cellular metabolic process;BP:regulation of biological process;BP:regulation of protein metabolic process;BP:biological regulation;BP:regulation of protein modification process;BP:regulation of molecular function;BP:regulation of macromolecule metabolic process;BP:regulation of catalytic activity;BP:regulation of cellular process;BP:regulation of phosphorus metabolic process;BP:biological_process;BP:regulation of nitrogen compound metabolic process;BP:regulation of dephosphorylation;BP:regulation of protein dephosphorylation;BP:regulation of hydrolase activity;BP:regulation of phosphoprotein phosphatase activity;MF:enzyme binding;MF:molecular_function;MF:binding;MF:protein phosphatase binding;MF:phosphatase binding;MF:protein binding;	GO:0019220;GO:0080090;GO:0019222;GO:0010921;GO:0031323;GO:0050789;GO:0051246;GO:0065007;GO:0031399;GO:0065009;GO:0060255;GO:0050790;GO:0050794;GO:0051174;GO:0008150;GO:0051171;GO:0035303;GO:0035304;GO:0051336;GO:0043666;GO:0019899;GO:0003674;GO:0005488;GO:0019903;GO:0019902;GO:0005515	------	------	------	------	ENOG410XRM1	T:Signal transduction mechanisms	PF04499.18	SAPS	SIT4 phosphatase-associated protein	CYT	1	3	110.3	High
TRINITY_DN29190_c0_g1_i4_orf1	gloverin-like [Ostrinia furnacalis]	0.332411621	-1.588957278	2.98E-05	down	yes	0.9497	2.857	2.872	2.966	2.732	1	0.831	1.018	BP:interspecies interaction between organisms;BP:response to biotic stimulus;BP:response to external stimulus;BP:response to external biotic stimulus;BP:response to stimulus;BP:response to bacterium;BP:defense response;BP:response to stress;BP:defense response to other organism;BP:defense response to bacterium;BP:biological_process;BP:response to other organism;CC:cellular_component;CC:extracellular region;CC:cellular anatomical entity;	GO:0044419;GO:0009607;GO:0009605;GO:0043207;GO:0050896;GO:0009617;GO:0006952;GO:0006950;GO:0098542;GO:0042742;GO:0008150;GO:0051707;GO:0005575;GO:0005576;GO:0110165	------	------	------	------	ENOG4110QR6	S:Function unknown	PF10793.12	Gloverin	Gloverin-like protein	PLA	1	28	11.5	High
TRINITY_DN512_c0_g1_i10_orf1	uncharacterized protein LOC114366781 [Ostrinia furnacalis]	0.488839725	-1.032566567	2.11E-06	down	yes	0.9943	2.034	2.076	2.027	2	1	0.971	1.012	MF:odorant binding;MF:molecular_function;MF:binding;	GO:0005549;GO:0003674;GO:0005488	------	------	------	------	ENOG4111CBI	S:Function unknown	------	------	------	CYT	4	43	16.8	High
TRINITY_DN5914_c1_g1_i9_orf1	unnamed protein product [Chilo suppressalis]	0.485503686	-1.042445848	5.14E-06	down	yes	0.988	2.035	2.045	2.083	1.978	1	0.971	0.993	------	------	K00477	PHYH	map04146	Peroxisome	ENOG410XUSH	S:Function unknown	PF05721.16	PhyH	Phytanoyl-CoA dioxygenase	CYT	9	43	32.7	High
TRINITY_DN28221_c0_g2_i1_orf1	unnamed protein product [Chilo suppressalis]	0.343700787	-1.540774938	3.70E-05	down	yes	0.9603	2.794	2.79	2.951	2.641	1	0.935	0.946	BP:small molecule biosynthetic process;BP:carboxylic acid metabolic process;BP:cellular biosynthetic process;BP:cellular nitrogen compound metabolic process;BP:nitrogen compound metabolic process;BP:small molecule metabolic process;BP:nitrogen cycle metabolic process;BP:glutamine family amino acid metabolic process;BP:organic substance biosynthetic process;BP:cellular amino acid metabolic process;BP:arginine biosynthetic process;BP:arginine metabolic process;BP:organic substance metabolic process;BP:organic acid metabolic process;BP:cellular amino acid biosynthetic process;BP:alpha-amino acid metabolic process;BP:alpha-amino acid biosynthetic process;BP:urea metabolic process;BP:primary metabolic process;BP:cellular process;BP:biosynthetic process;BP:biological_process;BP:metabolic process;BP:oxoacid metabolic process;BP:cellular nitrogen compound biosynthetic process;BP:amide biosynthetic process;BP:organonitrogen compound metabolic process;BP:organonitrogen compound biosynthetic process;BP:cellular amide metabolic process;BP:carboxylic acid biosynthetic process;BP:urea cycle;BP:organic acid biosynthetic process;BP:cellular metabolic process;BP:glutamine family amino acid biosynthetic process;MF:argininosuccinate synthase activity;MF:purine ribonucleoside triphosphate binding;MF:molecular_function;MF:ion binding;MF:heterocyclic compound binding;MF:nucleoside phosphate binding;MF:anion binding;MF:nucleotide binding;MF:purine nucleotide binding;MF:small molecule binding;MF:catalytic activity;MF:ligase activity;MF:ligase activity, forming carbon-nitrogen bonds;MF:adenyl nucleotide binding;MF:carbohydrate derivative binding;MF:adenyl ribonucleotide binding;MF:ATP binding;MF:organic cyclic compound binding;MF:purine ribonucleotide binding;MF:ribonucleotide binding;MF:binding;	GO:0044283;GO:0019752;GO:0044249;GO:0034641;GO:0006807;GO:0044281;GO:0071941;GO:0009064;GO:1901576;GO:0006520;GO:0006526;GO:0006525;GO:0071704;GO:0006082;GO:0008652;GO:1901605;GO:1901607;GO:0019627;GO:0044238;GO:0009987;GO:0009058;GO:0008150;GO:0008152;GO:0043436;GO:0044271;GO:0043604;GO:1901564;GO:1901566;GO:0043603;GO:0046394;GO:0000050;GO:0016053;GO:0044237;GO:0009084;GO:0004055;GO:0035639;GO:0003674;GO:0043167;GO:1901363;GO:1901265;GO:0043168;GO:0000166;GO:0017076;GO:0036094;GO:0003824;GO:0016874;GO:0016879;GO:0030554;GO:0097367;GO:0032559;GO:0005524;GO:0097159;GO:0032555;GO:0032553;GO:0005488	K01940	argG, ASS1	map00220;map	Arginine biosynthesi	COG0137	E:Amino acid transport and metaboli	PF00764.22	Arginosuc_synth	Arginosuccinate synthase	CYT	1	9	10.1	High
TRINITY_DN701_c0_g1_i1_orf1	venom protease-like isoform X3 [Ostrinia furnacalis]	0.448354662	-1.157287697	0.0001266	down	yes	0.981	2.188	2.281	2.025	2.258	1	0.979	0.964	BP:organic substance metabolic process;BP:organonitrogen compound metabolic process;BP:protein metabolic process;BP:nitrogen compound metabolic process;BP:macromolecule metabolic process;BP:biological_process;BP:metabolic process;BP:proteolysis;BP:primary metabolic process;CC:cellular_component;CC:extracellular region;CC:cellular anatomical entity;MF:serine-type endopeptidase activity;MF:endopeptidase activity;MF:hydrolase activity;MF:serine hydrolase activity;MF:catalytic activity, acting on a protein;MF:catalytic activity;MF:molecular_function;MF:peptidase activity;MF:serine-type	GO:0071704;GO:1901564;GO:0019538;GO:0006807;GO:0043170;GO:0008150;GO:0008152;GO:0006508;GO:0044238;GO:0005575;GO:0005576;GO:0110165;GO:0004252;GO:0004175;GO:0016787;GO:0017171;GO:0140096;GO:0003824;GO:0003674;GO:0008233;GO:0008236	------	------	------	------	COG5640	O:Posttranslational modification, pro	PF00089.29	Trypsin	Trypsin	EXC	5	30	27.1	High
TRINITY_DN95558_c0_g3_i1_orf1	cytochrome P450 monooxygenase CYP9G19 [Cnaphalocrocis medinalis]	0.387982196	-1.365937645	0.0001881	down	yes	1.046	2.696	2.761	2.468	2.86	1	1.01	1.128	CC:membrane;CC:endoplasmic reticulum membrane;CC:organelle membrane;CC:cellular_component;CC:cellular anatomical entity;MF:cation binding;MF:monooxygenase activity;MF:heme binding;MF:transition metal ion binding;MF:tetrapyrrole binding;MF:organic cyclic compound binding;MF:oxidoreductase activity, acting on paired donors, with incorporation or reduction of molecular oxygen;MF:ion binding;MF:catalytic activity;MF:molecular_function;MF:binding;MF:iron ion binding;MF:metal ion binding;MF:heterocyclic compound binding;MF:oxidoreductase activity;	GO:0016020;GO:0005789;GO:0031090;GO:0005575;GO:0110165;GO:0043169;GO:0004497;GO:0020037;GO:0046914;GO:0046906;GO:0097159;GO:0016705;GO:0043167;GO:0003824;GO:0003674;GO:0005488;GO:0005506;GO:0046872;GO:1901363;GO:0016491	K15003	CYP9	------	------	COG2124	J:Translation, ribosomal structure anc	PF00067.25	p450	Cytochrome P450	CYT	1	16	8	High
TRINITY_DN4235_c0_g1_i2_orf1	uncharacterized protein LOC114361536 [Ostrinia furnacalis]	0.423687424	-1.238927789	1.07E-05	down	yes	1.041	2.457	2.474	2.368	2.529	1	1.053	1.071	CC:cellular_component;CC:cellular anatomical entity;CC:extracellular space;MF:endopeptidase inhibitor activity;MF:endopeptidase regulator activity;MF:peptidase regulator activity;MF:molecular_function;MF:peptidase inhibitor activity;MF:serine-type endopeptidase inhibitor activity;MF:enzyme inhibitor activity;MF:molecular function regulator;MF:enzyme regulator activity;	GO:0005575;GO:0110165;GO:0005615;GO:0004866;GO:0061135;GO:0061134;GO:0003674;GO:0030414;GO:0004867;GO:0004857;GO:0098772;GO:0030234	------	------	------	------	COG4826	P:Inorganic ion transport and metab	PF00079.23	Serpin	Serpin	CYT	5	16	43.7	High
TRINITY_DN31225_c0_g1_i1_orf1	ribosome biogenesis protein BMS1 homolog [Ostrinia furnacalis]	0.472246066	-1.082389317	1.63E-06	down	yes	0.9903	2.097	2.084	2.119	2.088	1	1.024	0.947	BP:ribosome biogenesis;BP:cellular component organization or biogenesis;BP:ribonucleoprotein complex biogenesis;BP:cellular process;BP:biological_process;BP:cellular component biogenesis;CC:intracellular organelle;CC:non-membrane-bounded organelle;CC:organelle;CC:nucleolus;CC:cellular anatomical entity;CC:intracellular non-membrane-bounded organelle;CC:cellular_component;MF:purine ribonucleoside triphosphate binding;MF:heterocyclic compound binding;MF:molecular_function;MF:binding;MF:ion binding;MF:nucleoside phosphate binding;MF:anion binding;MF:nucleotide binding;MF:guanyl ribonucleotide binding;MF:purine nucleotide binding;MF:GTP binding;MF:small molecule binding;MF:carbohydrate derivative binding;MF:organic cyclic compound binding;MF:guanyl nucleotide binding;MF:purine ribonucleotide binding;MF:ribonucleotide binding;	GO:0042254;GO:0071840;GO:0022613;GO:0009987;GO:0008150;GO:0044085;GO:0043229;GO:0043228;GO:0043226;GO:0005730;GO:0110165;GO:0043232;GO:0005575;GO:0035639;GO:1901363;GO:0003674;GO:0005488;GO:0043167;GO:1901265;GO:0043168;GO:0000166;GO:0032561;GO:0017076;GO:0005525;GO:0036094;GO:0097367;GO:0097159;GO:0019001;GO:0032555;GO:0032553	K14569	BMS1	map03008	Ribosome biogenesi	COG5192	J:Translation, ribosomal structure anc	PF04950.15;PF081	RIBIOP_C;AARP2CN	40S ribosome biogenesis protein Tsr1 and BMS1 C-terminal;AARP2CN	CYT	2	2	124.6	High

TRINITY_DN125150_c0_g1_i1_orf1	aldehyde dehydrogenase, dimeric NADP-preferring isoform X5 [Ostrinia furnacalis]	0.433746425	-1.205076227	0.0003847	down	yes	0.91	2.098	2.261	2.052	1.981	1	0.952	0.778	BP:cellular aldehyde metabolic process;BP:cellular process;BP:cellular metabolic process;BP:organic substance metabolic process;BP:biological_process;BP:metabolic process;MF:catalytic activity;MF:molecular_function;MF:oxidoreductase activity;	GO:0006081;GO:0009987;GO:0044237;GO:0071704;GO:0008150;GO:0008152;GO:0003824;GO:0003674;GO:0016491	K00129	ALDH3	map00982;map	Drug metabolism -	------	------	------	------	------	CYT	2	22	8.3	High
TRINITY_DN2442_c0_g1_i6_orf1	cytochrome P450 6B5-like [Ostrinia furnacalis]	0.454545455	-1.137503524	0.0001649	down	yes	1.01	2.222	2.236	2.064	2.365	1	0.991	1.04	MF:cation binding;MF:monooxygenase activity;MF:heme binding;MF:transition metal ion binding;MF:tetrapyrrole binding;MF:organic cyclic compound binding;MF:oxidoreductase activity, acting on paired donors, with incorporation or reduction of molecular oxygen;MF:ion binding;MF:catalytic activity;MF:molecular_function;MF:binding;MF:iron ion binding;MF:metal ion binding;MF:heterocyclic compound binding;MF:oxidoreductase activity;	GO:0043169;GO:0004497;GO:0020037;GO:0046914;GO:0046906;GO:0097159;GO:0016705;GO:0043167;GO:0003824;GO:0003674;GO:0005488;GO:0005506;GO:0046872;GO:1901363;GO:0016491	K14999	CYP6	------	------	COG2124	Q:Secondary metabolites biosynthesi	PF00067.25	p450	Cytochrome P450	CYT	7	20	56.7	High
TRINITY_DN49530_c0_g1_i1_orf1	ommochrome-binding protein-like [Ostrinia furnacalis]	0.4344115	-1.202865798	0.000215	down	yes	0.967	2.226	2.038	2.289	2.352	1	0.925	0.976	------	------	------	------	------	------	ENOG4110SZS	S:Function unknown	------	------	------	CYT	3	50	13.6	High
TRINITY_DN64141_c0_g1_i4_orf1	probable salivary secreted peptide [Ostrinia furnacalis]	0.485469271	-1.042548115	0.0001958	down	yes	1.019	2.099	2.068	2.251	1.978	1	1.056	1.002	------	------	------	------	------	------	ENOG410ZS6V;ENO	S:Function unknown;	PF15868.8	MBF2	Transcription activator MBF2	CYT	3	33	13.3	High
TRINITY_DN3175_c0_g1_i7_orf1	unnamed protein product, partial [Brenthis ino]	0.478763183	-1.062615882	1.67E-05	down	yes	0.9987	2.086	2.053	2.173	2.031	1	0.988	1.008	BP:fatty acid metabolic process;BP:carboxylic acid metabolic process;BP:small molecule metabolic process;BP:organic substance metabolic process;BP:lipid metabolic process;BP:cellular process;BP:monocarboxylic acid metabolic process;BP:biological_process;BP:metabolic process;BP:oxoacid metabolic process;BP:cellular lipid metabolic process;BP:primary metabolic process;BP:organic acid metabolic process;BP:cellular metabolic process;MF:NAD binding;MF:molecular_function;MF:nucleotide binding;MF:heterocyclic compound binding;MF:nucleoside phosphate binding;MF:oxidoreductase activity;MF:anion binding;MF:ion binding;MF:small molecule binding;MF:catalytic activity;MF:oxidoreductase activity, acting on CH-OH group of donors;MF:oxidoreductase activity, acting on the CH-OH group of donors, NAD or NADP as acceptor;MF:organic cyclic compound binding;MF:NAD+ binding;MF:binding;	GO:0006631;GO:0019752;GO:0044281;GO:0071704;GO:0006629;GO:0009987;GO:0032787;GO:0008150;GO:0008152;GO:0043436;GO:0044255;GO:0044238;GO:0006082;GO:0044237;GO:0051287;GO:0003674;GO:0000166;GO:1901363;GO:1901265;GO:0016491;GO:0043168;GO:0043167;GO:0036094;GO:0003824;GO:0016614;GO:0016616;GO:0097159;GO:0070403;GO:0005488	K13247	CRYL1	map00040	Pentose and glucur	COG1250	S:Function unknown	PF02737.21;PF007	3HCDH_N;3HCDH	3-hydroxyacyl-CoA dehydrogenase, NAD binding domain;3-hydroxyacyl-CoA dehydrogenase, C-terminal domain	CYT	18	70	36.2	High
TRINITY_DN11817_c0_g1_i4_orf1	glycogen phosphorylase [Heortia vitessoides]	0.486893204	-1.038322731	1.40E-07	down	yes	1.003	2.06	2.05	2.045	2.086	1	1.002	1.007	BP:cellular metabolic process;BP:generation of precursor metabolites and energy;BP:cellular polysaccharide metabolic process;BP:cellular carbohydrate metabolic process;BP:cellular macromolecule metabolic process;BP:organic substance metabolic process;BP:energy reserve metabolic process;BP:cellular glucan metabolic process;BP:cellular process;BP:biological_process;BP:metabolic process;BP:glucan metabolic process;BP:primary metabolic process;BP:carbohydrate metabolic process;BP:energy derivation by oxidation of organic compounds;BP:glycogen metabolic process;BP:polysaccharide metabolic process;BP:macromolecule metabolic process;MF:vitamin B6 binding;MF:molecular_function;MF:binding;MF:glycogen phosphorylase activity;MF:heterocyclic compound binding;MF:anion binding;MF:ion binding;MF:transferase activity;MF:small molecule binding;MF:catalytic activity;MF:SHG alpha-glucan phosphorylase activity;MF:organic cyclic compound binding;MF:phosphorylase activity;MF:vitamin binding;MF:linear malto-oligosaccharide phosphorylase activity;MF:transferase activity, transferring glycosyl groups;MF:transferase activity, transferring hexosyl groups;MF:pyridoxal phosphate binding;	GO:0044237;GO:0006091;GO:0044264;GO:0044262;GO:0044260;GO:0071704;GO:0006112;GO:0006073;GO:0009987;GO:0008150;GO:0008152;GO:0044042;GO:0044238;GO:0005975;GO:0015980;GO:0005977;GO:0005976;GO:0043170;GO:0070279;GO:0003674;GO:0005488;GO:0008184;GO:1901363;GO:0043168;GO:0043167;GO:0016740;GO:0036094;GO:0003824;GO:0102499;GO:0097159;GO:0004645;GO:0019842;GO:0102250;GO:0016757;GO:0016758;GO:0030170	K00688	PYG, glgP	map04910;map	Insulin signaling pat	COG0058	G:Carbohydrate transport and metab	PF00343.23	Phosphorylase	Carbohydrate phosphorylase	CYT	48	62	96.7	High
TRINITY_DN46625_c0_g1_i1_orf1	ferritin subunit isoform X1 [Belonocnema kinseyi]	0.498233216	-1.00510689	0.00032	down	yes	0.987	1.981	1.821	2.101	2.021	1	0.945	1.016	BP:ion homeostasis;BP:homeostatic process;BP:iron ion homeostasis;BP:transition metal ion homeostasis;BP:inorganic ion homeostasis;BP:cellular transition metal ion homeostasis;BP:transition metal ion transport;BP:biological regulation;BP:regulation of biological quality;BP:cellular homeostasis;BP:metal ion transport;BP:transport;BP:cellular iron ion homeostasis;BP:cellular metal ion homeostasis;BP:cation transport;BP:ion transport;BP:cellular process;BP:cellular ion homeostasis;BP:biological_process;BP:cellular cation homeostasis;BP:metal ion homeostasis;BP:cation homeostasis;BP:cellular chemical homeostasis;BP:establishment of localization;BP:localization;BP:chemical homeostasis;BP:iron ion transport;MF:molecular_function;MF:binding;MF:oxidoreductase activity;MF:cation binding;MF:oxidoreductase activity, oxidizing metal ions;MF:transition metal ion binding;MF:ferroxidase activity;MF:ion binding;MF:iron ion binding;MF:ferric iron binding;MF:oxidoreductase activity, oxidizing metal ions, oxygen as acceptor;MF:metal ion binding;MF:catalytic activity;	GO:0050801;GO:0042592;GO:0055072;GO:0055076;GO:0098771;GO:0046916;GO:0000041;GO:0065007;GO:0065008;GO:0019725;GO:0030001;GO:0006810;GO:0006879;GO:0006875;GO:0006812;GO:0006811;GO:0009987;GO:0006873;GO:0008150;GO:0030003;GO:0055065;GO:0055080;GO:0055082;GO:0051234;GO:0051179;GO:0048878;GO:0006826;GO:0003674;GO:0005488;GO:0016491;GO:0043169;GO:0016722;GO:0046914;GO:0004322;GO:0043167;GO:0005506;GO:0008199;GO:0016724;GO:0046872;GO:0003824	K00522	FTH1	map04978;map	Mineral absorption;I	ENOG4111MSG	P:Inorganic ion transport and metab	PF00210.27	Ferritin	Ferritin-like domain	CYT	1	4	24.1	High
TRINITY_DN13760_c1_g1_i1_orf1	pre-mRNA-processing factor 40 homolog A isoform X1 [Ostrinia furnacalis] >XP_028162665.1 pre-mRNA-processing factor 40 homolog A isoform X2 [Ostrinia furnacalis] >XP_028162667.1 pre-mRNA-processing factor 40 homolog A isoform X3 [Ostrinia furnacalis]	0.488536953	-1.033460402	0.003103	down	yes	0.9717	1.989	2.143	1.743	2.081	1	1.129	0.786	BP:nucleic acid metabolic process;BP:cellular nitrogen compound metabolic process;BP:nitrogen compound metabolic process;BP:mRNA splicing, via spliceosome;BP:organic cyclic compound metabolic process;BP:nucleobase-containing compound metabolic process;BP:organic substance metabolic process;BP:RNA splicing;BP:mRNA metabolic process;BP:cellular process;BP:cellular aromatic compound metabolic process;BP:RNA splicing, via transesterification reactions;BP:RNA splicing, via transesterification reactions with bulged adenosine as nucleophile;BP:biological_process;BP:metabolic process;BP:heterocycle metabolic process;BP:RNA metabolic process;BP:primary metabolic process;BP:mRNA cis splicing, via spliceosome;BP:cellular metabolic process;BP:macromolecule metabolic process;BP:RNA processing;BP:mRNA processing;CC:membrane-bounded organelle;CC:organelle;CC:nucleus;CC:cellular anatomical entity;CC:intracellular membrane-bounded organelle;CC:intracellular organelle;CC:cellular_component;	GO:0090304;GO:0034641;GO:0006807;GO:0000398;GO:1901360;GO:0006139;GO:0071704;GO:0008380;GO:0016071;GO:0009987;GO:0006725;GO:0000375;GO:0000377;GO:0008150;GO:0008152;GO:0046483;GO:0016070;GO:0044238;GO:0045292;GO:0044237;GO:0043170;GO:0006396;GO:0006397;GO:0043227;GO:0043226;GO:0005634;GO:0110165;GO:0043231;GO:0043229;GO:0005575	K12821	PRPF40, PRP4	map03040	Spliceosome	COG5104	A:RNA processing and modification	PF01846.22;PF003	FF;WW;RhoGAP-FF1	FF domain;WW domain;p190-A and -B Rho GAPs FF domain	CYT	1	1	92.5	High
TRINITY_DN7102_c0_g1_i5_orf1	protein wings apart-like [Ostrinia furnacalis]	0.410730034	-1.283737648	0.0006051	down	yes	0.8383	2.041	1.946	2.224	1.954	1	0.736	0.779	BP:regulation of chromosome organization;BP:regulation of cell cycle;BP:regulation of organelle organization;BP:regulation of cell cycle process;BP:regulation of cellular process;BP:biological regulation;BP:biological_process;BP:regulation of cellular component organization;BP:regulation of biological process;BP:regulation of sister chromatid cohesion;	GO:0033044;GO:0051726;GO:0033043;GO:0010564;GO:0050794;GO:0065007;GO:0008150;GO:0051128;GO:0050789;GO:0007063	K25163	WAPL	------	------	ENOG410XSDS	S:Function unknown	PF07814.16	WAPL	Wings apart-like protein regulation of heterochromatin	CYT	1	1	119.6	Medium
TRINITY_DN1506_c0_g1_i6_orfp1	TRINITY_DN1506_c0_g1_i6_m.57691 TRINITY_DN1506_c0_g1::TRINITY_DN1506_c0_g1_i6::g.57691 ORF type:5prime_partial len:173 (+),score=30.49 TRINITY_DN1506_c0_g1_i6:1-519(+)	0.055170422	-4.179961181	2.60E-05	down	yes	0.955	17.31	18.374	17.674	15.873	1	0.807	1.058	------	------	------	------	------	------	------	------	------	------	------	CYT	1	7	19	High
TRINITY_DN2044_c0_g1_i5_orfp1	TRINITY_DN2044_c0_g1_i5_m.4210 TRINITY_DN2044_c0_g1::TRINITY_DN2044_c0_g1_i5::g.4210 ORF type:complete len:151 (-),score=85.31 TRINITY_DN2044_c0_g1_i5:857-1309(-)	0.139288418	-2.843852795	0.001515	down	yes	1.104	7.926	9.64	6.863	7.274	1	1.451	0.861	------	------	------	------	------	------	------	------	------	------	------	CYT	1	5	16.2	High
TRINITY_DN34423_c0_g1_i3_orf1	THAP domain-containing protein 4-like [Ostrinia furnacalis]	0.11555629	-3.1133323	0.0005251	down	yes	0.699	6.049	7.049	5.623	5.476	1	0.628	0.469	------	------	K23205	THAP4	------	------	ENOG4111TBC	S:Function unknown	PF08768.14	THAP4_heme-bd	THAP4-like, heme-binding beta-barrel domain	CYT	1	23	12.2	High
TRINITY_DN703_c0_g1_i2_orf1	acidic juvenile hormone-suppressible protein 1-like [Ostrinia furnacalis]	0.191692607	-2.383133397	2.64E-08	down	yes	0.9853	5.14	5.19	5.153	5.078	1	0.982	0.974	------	------	------	------	------	------	ENOG410XR2D	S:Function unknown	PF03723.17	Hemocyanin_C	Hemocyanin, ig-like domain	CYT	6	97	10.1	High
TRINITY_DN1370_c0_g1_i2_orf1	hypothetical protein evm_000756 [Chilo suppressalis]	0.242390825	-2.044593005	5.45E-05	down	yes	1.099	4.534	4.533	4.23	4.839	1	1.06	1.236	------	------	------	------	------	------	------	------	------	------	------	CYT	2	35	23	High
TRINITY_DN59965_c0_g4_i1_orf1	TKT protein, partial [Homo sapiens]	0.36845152	-1.440453291	0.02452	down	yes	1.273	3.455	3.936	2.27	4.158	1	1.593	1.226	BP:small molecule metabolic process;BP:organic cyclic compound metabolic process;BP:heterocycle metabolic process;BP:organonitrogen compound metabolic process;BP:organophosphate metabolic process;BP:nitrogen compound metabolic process;BP:regulation of biological process;BP:organic substance biosynthetic process;BP:biological regulation;BP:biological_process;BP:metabolic process;BP:organophosphate biosynthetic process;BP:ribose phosphate biosynthetic process;BP:ribose phosphate metabolic process;BP:nucleoside phosphate metabolic process;BP:NADP metabolic process;BP:cellular biosynthetic process;BP:cellular nitrogen compound metabolic process;BP:nucleobase-containing compound metabolic process;BP:glyceraldehyde-3-phosphate biosynthetic process;BP:cellular process;BP:cellular aromatic compound metabolic process;BP:glucose 6-phosphate metabolic process;BP:nucleobase-containing small molecule metabolic process;BP:cellular aldehyde metabolic process;BP:carbohydrate derivative biosynthetic process;BP:carbohydrate derivative metabolic process;BP:NADPH regeneration;BP:nicotinamide nucleotide metabolic process;BP:cellular metabolic process;BP:pentose-phosphate shunt;BP:generation of precursor metabolites and energy;BP:pyridine-containing compound metabolic process;BP:regulation of growth;BP:glyceraldehyde-3-phosphate metabolic process;BP:organic substance metabolic process;BP:aldehyde biosynthetic process;BP:biosynthetic process;BP:nucleotide metabolic process;BP:pentose-phosphate shunt, non-oxidative branch;BP:primary metabolic process;BP:phosphate-containing compound metabolic process;BP:phosphorus metabolic process;BP:pyridine nucleotide metabolic process;CC:endoplasmic reticulum membrane;CC:vesicle;CC:membrane;CC:peroxisome;CC:extracellular organelle;CC:intracellular membrane-bounded organelle;CC:microbody;CC:cytosol;CC:extracellular membrane-bounded organelle;CC:intracellular organelle;CC:membrane-bounded organelle;CC:nuclear body;CC:nucleoplasm;CC:cellular anatomical entity;CC:organelle membrane;CC:extracellular exosome;CC:organelle;CC:extracellular vesicle;CC:cellular_component;MF:heterocyclic compound binding;MF:transferase activity;MF:transferase activity, transferring aldehyde or ketonic groups;MF:metal ion binding;MF:molecular_function;MF:binding;MF:thiamine pyrophosphate binding;MF:sulfur compound binding;MF:cation binding;MF:catalytic activity;MF:organic cyclic compound binding;MF:protein dimerization activity;MF:anion binding;MF:magnesium ion binding;MF:ion binding;MF:calcium ion binding;MF:identical protein binding;MF:protein homodimerization activity;MF:protein binding;MF:vitamin binding;MF:small molecule binding;MF:transketolase activity;MF:quaternary ammonium group binding;	GO:0044281;GO:1901360;GO:0046483;GO:1901564;GO:0019637;GO:0006807;GO:0050789;GO:1901576;GO:0065007;GO:0008150;GO:0008152;GO:0090407;GO:0046390;GO:0019693;GO:0006753;GO:0006739;GO:0044249;GO:0034641;GO:0006139;GO:0046166;GO:0009987;GO:0006725;GO:0051156;GO:0055086;GO:0006081;GO:1901137;GO:1901135;GO:0006740;GO:0046496;GO:0044237;GO:0006098;GO:0006091;GO:0072524;GO:0040008;GO:0019682;GO:0071704;GO:0046184;GO:0009058;GO:0009117;GO:0009052;GO:0044238;GO:0006796;GO:0006793;GO:0019362;GO:0005789;GO:0031982;GO:0016020;GO:0005777;GO:0043230;GO:0043231;GO:0042579;GO:0005829;GO:0065010;GO:0043229;GO:0043227;GO:0016604;GO:0005654;GO:0110165;GO:0031090;GO:0070062;GO:0043226;GO:1903561;GO:0005575;GO:1901363;GO:0016740;GO:0016744;GO:0046872;GO:0003674;GO:0005488;GO:0030976;GO:1901681;GO:0043169;GO:0003824;GO:0097159;GO:0046983;GO:0043168;GO:0000287;GO:0043167;GO:0005509;GO:0042802;GO:0042803;GO:0005515;GO:0019842;GO:0036094;GO:0004802;GO:0050997	K00615	E2.2.1.1, tktA,	map00030	Pentose phosphate	COG0021	S:Function unknown	PF02779.27;PF027	Transket_pyr;Transketo	Transketolase, pyrimidine binding domain;Transketolase, C-terminal domain;Transketolase, thiamine diphosphate binding domain	CYT	1	4	45.1	High
TRINITY_DN1093_c0_g1_i4_orf1	uncharacterized protein LOC114361723 isoform X4 [Ostrinia furnacalis]	0.268071966	-1.899307742	4.77E-06	down	yes	0.9983	3.724	3.636	3.654	3.882	1	1.031	0.964	------	------	------	------	------	------	ENOG410XRJB	S:Function unknown	------	------	------	CYT	1	32	26.1	High
TRINITY_DN5080_c0_g1_i1_orf1	basic juvenile hormone-suppressible protein 2-like [Ostrinia furnacalis]	0.271624899	-1.880312362	2.18E-06	down	yes	1.008	3.711	3.717	3.593	3.822	1	1.007	1.018	------	------	------	------	------	------	ENOG410XR2D	S:Function unknown	PF00372.22;PF037	Hemocyanin_M;Hemoc	Hemocyanin, copper containing domain;Hemocyanin, ig-like domain;Hemocyanin, all-alpha domain	CYT	46	75	89.5	High
TRINITY_DN703_c13_g1_i1_orf1	acidic juvenile hormone-suppressible protein 1-like [Ostrinia furnacalis]	0.272946058	-1.873312233	0.000966	down	yes	0.9867	3.615	3.63	3.085	4.131	1	0.952	1.008	------	------	------	------	------	------	ENOG410XR2D	S:Function unknown	PF00372.22;PF037	Hemocyanin_M;Hemoc	Hemocyanin, copper containing domain;Hemocyanin, all-alpha domain;Hemocyanin, ig-like domain	CYT	15	47	57.7	High
TRINITY_DN106156_c1_g1_i1_orf1	arylphorin subunit alpha-like [Ostrinia furnacalis]	0.274951185	-1.862752588	7.09E-05	down	yes	0.9857	3.585	3.592	3.317	3.846	1	0.96	0.997	------	------	------	------	------	------	ENOG410XR2D	S:Function unknown	PF03723.17	Hemocyanin_C	Hemocyanin, ig-like domain	CYT	12	59	13.5	High
TRINITY_DN1540_c0_g1_i7_orf1	alaserpin-like isoform X13 [Ostrinia furnacalis]	0.307669352	-1.700547354	4.65E-05	down	yes	1.031	3.351	3.112	3.458	3.482	1	1.089	1.005	CC:cellular_component;CC:cellular anatomical entity;CC:extracellular space;MF:endopeptidase inhibitor activity;MF:endopeptidase regulator activity;MF:peptidase regulator activity;MF:molecular_function;MF:peptidase inhibitor activity;MF:serine-type endopeptidase inhibitor activity;MF:enzyme inhibitor activity;MF:molecular function regulator;MF:enzyme regulator activity;	GO:0005575;GO:0110165;GO:0005615;GO:0004866;GO:0061135;GO:0061134;GO:0003674;GO:0030414;GO:0004867;GO:0004857;GO:0098772;GO:0030234	------	------	------	------	COG4826	O:Posttranslational modification, pro	PF00079.23	Serpin	Serpin	CYT	1	35	42.9	High
TRINITY_DN4255_c0_g1_i11_orf1	LOW QUALITY PROTEIN: lebocin-4-like [Ostrinia furnacalis]	0.312650602	-1.677376798	1.58E-05	down	yes	1.038	3.32	3.493	3.27	3.198	1	1.03	1.084	------	------	------	------	------	------	ENOG4111ASS	S:Function unknown	------	------	------	CYT	1	13	22.2	High
TRINITY_DN19662_c0_g2_i1_orf1	storage protein [Ostrinia furnacalis]	0.310280374	-1.68835565	3.11E-07	down	yes	0.996	3.21	3.189	3.167	3.275	1	0.985	1.003	------	------	------	------	------	------	ENOG410XR2D	S:Function unknown	PF00372.22;PF037	Hemocyanin_M;Hemoc	Hemocyanin, copper containing domain;Hemocyanin, all-alpha domain;Hemocyanin, ig-like domain	CYT	43	69	59	High
TRINITY_DN20558_c0_g1_i2_orf1	Transient receptor potential channel pyrexia [Operophtera brumata]	0.336204041	-1.572591029	3.73E-06	down	yes	1.015	3.019	3.091	2.94	3.027	1	0.963	1.083	BP:response to stimulus;BP:biological_process;CC:transporter complex;CC:transmembrane transporter complex;CC:ion channel complex;CC:cellular_component;CC:membrane protein complex;CC:cellular anatomical entity;CC:macromolecular complex;CC:integral component of membrane;CC:intrinsic component of membrane;CC:cation channel complex;MF:ion transmembrane transporter activity;MF:inorganic solute uptake transmembrane transporter activity;MF:transporter activity;MF:ion channel activity;MF:transmembrane transporter activity;MF:channel activity;MF:passive transmembrane transporter activity;MF:molecular_function;	GO:0050896;GO:0008150;GO:1990351;GO:1902495;GO:0034702;GO:0005575;GO:0098796;GO:0110165;GO:0032991;GO:0016021;GO:0031224;GO:0034703;GO:0015075;GO:0015318;GO:0005215;GO:0005216;GO:0022857;GO:0015267;GO:0022803;GO:0003674	K04984	TRPA1, ANKTI	map04750	Inflammatory media	COG0666	Q:Secondary metabolites biosynthesi	------	------	------	CYT	1	14	8.5	High
TRINITY_DN2314_c0_g1_i7_orf1	protein dj-1beta-like isoform X1 [Ostrinia furnacalis]	0.371762131	-1.427548277	1.45E-05	down	yes	1.019	2.741	2.844	2.757	2.622	1	1.062	0.995	------	------	K05687	PARK7	map05012;map	Parkinson disease;Pa	COG0693	S:Function unknown	PF01965.27	DJ-1_PfpI	DJ-1/PfpI family	CYT	4	45	24.5	High
TRINITY_DN4343_c0_g1_i2_orf1	uncharacterized protein LOC114365231 isoform X3 [Ostrinia furnacalis]	0.412337662	-1.278101854	3.80E-06	down	yes	1.016	2.464	2.434	2.478	2.48	1	0.959	1.088	------	------	------	------	------	------	ENOG410YXRP	S:Function unknown	PF16091.8	DUF4820	Domain of unknown function	CYT	9	28	43.4	High
TRINITY_DN5198_c0_g1_i5_orfp1	TRINITY_DN5198_c0_g1_i5_m.8637 TRINITY_DN5198_c0_g1::TRINITY_DN5198_c0_g1_i5::g.8637 ORF type:complete len:223 (-),score=54.99 TRINITY_DN5198_c0_g1_i5:319-987(-)	0.299242711	-1.740611988	0.005514	down	yes	0.7903	2.641	2.735	2.042	3.145	1	0.638	0.733	------	------	------	------	------	------	------	------	------	------	------	CYT	1	5	24.7	High
TRINITY_DN2286_c2_g1_i1_orf1	coatomer subunit zeta-1 isoform X1 [Ostrinia furnacalis]	0.431188752	-1.213608552	9.51E-05	down	yes	1.012	2.347	2.229	2.316	2.495	1	0.962	1.073	BP:protein localization;BP:cellular macromolecule localization;BP:nitrogen compound transport;BP:organic substance transport;BP:macromolecule localization;BP:transport;BP:Golgi vesicle transport;BP:cellular process;BP:establishment of protein localization;BP:biological_process;BP:establishment of localization;BP:localization;BP:cellular localization;BP:retrograde vesicle-mediated transport, Golgi to ER;BP:vesicle-mediated transport;BP:protein transport;CC:membrane coat;CC:organelle membrane;CC:membrane;CC:cellular anatomical entity;CC:bounding membrane of organelle;CC:vesicle coat;CC:COPI vesicle coat;CC:macromolecular complex;CC:Golgi	GO:0008104;GO:0070727;GO:0071705;GO:0071702;GO:0033036;GO:0006810;GO:0048193;GO:0009987;GO:0045184;GO:0008150;GO:0051234;GO:0051179;GO:0051641;GO:0006890;GO:0016192;GO:0015031;GO:0030117;GO:0031090;GO:0016020;GO:0110165;GO:0098588;GO:0030120;GO:0030126;GO:0032991;GO:0000139;GO:0005575;GO:0098796	K20472	COPZ, RET3	------	------	COG5541	U:Intracellular trafficking, secretion, a	PF01217.23	Clat_adaptor_s	Clathrin adaptor complex small chain	CYT	2	21	20.6	High
TRINITY_DN12336_c0_g1_i1_orfp1	TRINITY_DN12336_c0_g1_i1_m.30792 TRINITY_DN12336_c0_g1::TRINITY_DN12336_c0_g1_i1::g.30792 ORF type:internal len:86 (-),score=13.56 TRINITY_DN12336_c0_g1_i1:3-257(-)	0.468027211	-1.095335685	3.80E-06	down	yes	1.032	2.205	2.262	2.187	2.167	1	1.05	1.047	------	------	------	------	------	------	------	------	------	------	------	CYT	4	51	9.3	High
TRINITY_DN4695_c0_g1_i3_orf1	glutathione S-transferase epsilon 3 [Ostrinia furnacalis]	0.460389316	-1.11907374	0.000747	down	yes	1.017	2.209	2.436	1.999	2.193	1	0.991	1.061	MF:catalytic activity;MF:transferase activity;MF:glutathione transferase activity;MF:molecular_function;MF:transferase activity, transferring alkyl or ary;	GO:0003824;GO:0016740;GO:0004364;GO:0003674;GO:0016765	K00799	GST, gst	map05207;map	Chemical carcinoger	COG0625	S:Function unknown	PF13417.9;PF0279	GST_N_3;GST_N;GST_C	Glutathione S-transferase, N-terminal domain;Glutathione S-transferase, N-terminal domain;Glutathione S-transferase, C-terminal domain;Glutathione S-transferase, N-terminal domain;Glutathione S-transferase, C-terminal domain;Glutathione S-transferase, C-terminal	CYT	2	9	24.8	High
TRINITY_DN38783_c0_g1_i1_orf1	regucalcin-like [Ostrinia furnacalis]	0.433884298	-1.20461772	0.0002338	down	yes	0.945	2.178	2.013	2.34	2.18	1	0.925	0.91	CC:cytoplasm;CC:cellular_component;CC:cellular anatomical entity;MF:hydrolase activity;MF:metal ion binding;MF:cation binding;MF:enzyme regulator activity;MF:gluconolactonase activity;MF:carboxylic ester hydrolase activity;MF:ion binding;MF:catalytic activity;MF:molecular_function;MF:hydrolase activity, acting on ester bonds;MF:binding;MF:molecular function regulator;MF:calcium ion binding;	GO:0005737;GO:0005575;GO:0110165;GO:0016787;GO:0046872;GO:0043169;GO:0030234;GO:0004341;GO:0052689;GO:0043167;GO:0003824;GO:0003674;GO:0016788;GO:0005488;GO:0098772;GO:0005509	K01053	gnl, RGN	map00030;map	Pentose phosphate	COG3386	G:Carbohydrate transport and metab	PF08450.15	SGL	SMP-30/Gluconolactonase/LRE-like region	CYT	2	34	18.3	High
TRINITY_DN10371_c0_g1_i2_orf1	heterogeneous nuclear ribonucleoprotein L isoform X2 [Vanessa cardui]	0.476805897	-1.068526017	0.01644	down	yes	0.9703	2.035	1.766	1.906	2.432	1	0.654	1.257	------	------	K13159	HNRNPL	------	------	ENOG410XQHN	S:Function unknown	PF13893.9;PF0007	RRM_5;RRM_1;RRM_8	RNA recognition motif.;RNA recognition motif.;RRM-like domain	PLA	2	5	52.6	High
TRINITY_DN22515_c0_g1_i10_orf1	hypothetical protein KGM_202621 [Danaus plexippus plexippus]	0.182259806	-2.45593166	0.0003419	down	yes	1.013	5.558	6.182	5.679	4.813	1	1.004	1.035	------	------	------	------	------	------	ENOG41119TW	S:Function unknown	------	------	------	CYT	2	29	7.7	High
TRINITY_DN467_c3_g1_i5_orf1	lysozyme precursor [Loxostege sticticalis]	0.201089414	-2.314090958	1.08E-05	down	yes	0.9783	4.865	5.068	4.933	4.593	1	0.994	0.941	BP:metabolic process;BP:biological_process;MF:hydrolase activity;MF:catalytic activity;MF:hydrolase activity, acting on glycosyl bonds;MF:lysozyme activity;MF:molecular_function;MF:hydrolase activity, hydrolyzing O-glycosyl compounds;MF:peptidoglycan muralytic activity;	GO:0008152;GO:0008150;GO:0016787;GO:0003824;GO:0016798;GO:0003796;GO:0003674;GO:0004553;GO:0061783	K13915	LYZ	map04970	Salivary secretion	ENOG4111QHM	G:Carbohydrate transport and metab	PF00062.23	Lys	C-type lysozyme/alpha-lactalbumin family	CYT	7	52	16	High
TRINITY_DN2813_c0_g1_i10_orf1	arylphorin subunit alpha-like [Ostrinia furnacalis]	0.225057313	-2.15163565	2.99E-06	down	yes	0.9817	4.362	4.228	4.525	4.334	1	0.936	1.009	------	------	------	------	------	------	ENOG4110QFU;ENO	S:Function unknown;	PF03723.17;PF003	Hemocyanin_C;Hemoc	Hemocyanin, ig-like domain;Hemocyanin, copper containing domain;Hemocyanin, all-alpha domain	CYT	1	33	139.5	High
TRINITY_DN1880_c0_g1_i4_orf1	serine protease inhibitor dipetalogastin-like [Helicoverpa zea]	0.274448732	-1.865391421	7.79E-06	down	yes	0.9957	3.628	3.798	3.587	3.498	1	0.998	0.989	------	------	K23615	SPIK5	------	------	COG5640;ENOG4111	O:Posttranslational modification, pro	PF00050.24;PF076	Kazal_1;Kazal_2	Kazal-type serine protease inhibitor domain;Kazal-type serine protease inhibitor domain	CYT	12	52	42.6	High
TRINITY_DN135780_c0_g1_i1_orf1	flotillin-1 isoform X1 [Pectinophora gossypiella]	0.277909739	-1.847311703	2.78E-05	down	yes	0.936	3.368	3.418	3.526	3.16	1	0.906	0.902	CC:cellular_component;CC:membrane;CC:cellular anatomical entity;	GO:0005575;GO:0016020;GO:0110165	K07192	FLOT	map04910	Insulin signaling pat	COG2268	S:Function unknown	PF01145.28	Band_7	SPFH domain / Band 7 family	CYT	2	27	12.8	High
TRINITY_DN31348_c0_g1_i1_orf1	protein lethal(2)essential for life [Bombyx mori]	0.291846669	-1.776717494	1.27E-06	down	yes	0.9593	3.287	3.287	3.361	3.213	1	0.911	0.967	MF:cation binding;MF:metal ion binding;MF:molecular_function;MF:binding;MF:ion binding;	GO:0043169;GO:0046872;GO:0003674;GO:0005488;GO:0043167	K09542	CRYAB	map04213;map	Longevity regulating	ENOG410YERS	O:Posttranslational modification, pro	------	------	------	CYT	1	22	7.2	High
TRINITY_DN2040_c0_g1_i6_orf1	trypsin-like serine proteinase T26 protein, partial [Chilo infuscatellus]	0.348984349	-1.518765758	3.33E-06	down	yes	1.048	3.003	3.027	2.935	3.048	1	1.016	1.129	BP:organic substance metabolic process;BP:organonitrogen compound metabolic process;BP:protein metabolic process;BP:macromolecule metabolic process;BP:biological_process;BP:nitrogen compound metabolic process;BP:metabolic process;BP:proteolysis;BP:primary metabolic process;MF:serine-type endopeptidase activity;MF:endopeptidase activity;MF:hydrolase activity;MF:serine hydrolase activity;MF:catalytic activity, acting on a protein;MF:catalytic activity;MF:molecular_function;MF:peptidase activity;MF:serine-type peptidase activity;	GO:0071704;GO:1901564;GO:0019538;GO:0043170;GO:0008150;GO:0006807;GO:0008152;GO:0006508;GO:0044238;GO:0004252;GO:0004175;GO:0016787;GO:0017171;GO:0140096;GO:0003824;GO:0003674;GO:0008233;GO:0008236	------	------	------	------	------	------	------	------	------	PLA	2	17	37.1	High
TRINITY_DN125521_c0_g2_i1_orf1	serpin transcript 1A2 [Ostrinia nubilalis]	0.335313531	-1.576417392	0.002439	down	yes	1.016	3.03	3.607	2.698	2.786	1	0.916	1.131	------	------	------	------	------	------	------	------	------	------	------	CYT	1	35	8.1	High
TRINITY_DN24_c0_g1_i1_orf1	hypothetical protein evm_007803 [Chilo suppressalis]	0.340181374	-1.555623943	6.27E-07	down	yes	0.9753	2.867	2.856	2.835	2.91	1	1.002	0.924	BP:organophosphate catabolic process;BP:nucleotide catabolic process;BP:cellular nitrogen compound metabolic process;BP:nitrogen compound metabolic process;BP:small molecule metabolic process;BP:organic cyclic compound metabolic process;BP:organic cyclic compound catabolic process;BP:nucleobase-containing compound metabolic process;BP:organic substance catabolic process;BP:organic substance metabolic process;BP:cellular catabolic process;BP:cellular process;BP:cellular aromatic compound metabolic process;BP:heterocycle catabolic process;BP:aromatic compound catabolic process;BP:biological_process;BP:metabolic process;BP:nucleobase-containing compound catabolic process;BP:catabolic process;BP:nucleobase-containing small molecule metabolic process;BP:heterocycle metabolic process;BP:primary metabolic process;BP:cellular nitrogen compound catabolic process;BP:cellular metabolic process;BP:phosphate-containing compound metabolic process;BP:nucleoside phosphate catabolic process;BP:phosphorus metabolic process;BP:organophosphate metabolic process;BP:nucleotide metabolic process;BP:nucleoside phosphate metabolic process;CC:cellular anatomical entity;CC:cellular_component;MF:phosphatase activity;MF:nucleotidase activity;MF:phosphoric ester hydrolase activity;MF:molecular_function;MF:nucleotide binding;MF:heterocyclic compound binding;MF:ion binding;MF:nucleoside phosphate binding;MF:metal ion binding;MF:hydrolase activity;MF:cation binding;MF:hydrolase activity, acting on ester bonds;MF:small molecule binding;MF:catalytic activity;MF:organic cyclic compound binding;MF:binding;	GO:0046434;GO:0009166;GO:0034641;GO:0006807;GO:0044281;GO:1901360;GO:1901361;GO:0006139;GO:1901575;GO:0071704;GO:0044248;GO:0009987;GO:0006725;GO:0046700;GO:0019439;GO:0008150;GO:0008152;GO:0034655;GO:0009056;GO:0055086;GO:0046483;GO:0044238;GO:0044270;GO:0044237;GO:0006796;GO:1901292;GO:0006793;GO:0019637;GO:0009117;GO:0006753;GO:0110165;GO:0005575;GO:0016791;GO:0008252;GO:0042578;GO:0003674;GO:0000166;GO:1901363;GO:0043167;GO:1901265;GO:0046872;GO:0016787;GO:0043169;GO:0016788;GO:0036094;GO:0003824;GO:0097159;GO:0005488	------	------	------	------	COG0737	S:Function unknown	PF02872.21;PF001	5_nucleotid_C;Metallop	5'-nucleotidase, C-terminal domain;Calcineurin-like phosphoesterase	CYT	9	19	60.2	High
TRINITY_DN609_c0_g1_i1_orf1	zonadhesin-like isoform X1 [Ostrinia furnacalis]	0.393784278	-1.344522583	5.20E-05	down	yes	1.077	2.735	2.803	2.829	2.574	1	1.135	1.096	------	------	------	------	------	------	COG4826;ENOG411	O:Posttranslational modification, pro	PF01826.20	TIL	Trypsin Inhibitor like cysteine rich domain	PLA	9	18	109	High
TRINITY_DN42719_c0_g2_i1_orf1	inter-alpha-trypsin inhibitor heavy chain H4-like isoform X11 [Ostrinia furnacalis]	0.392673993	-1.348596045	0.001068	down	yes	1.072	2.73	2.444	3.087	2.659	1	1.043	1.172	------	------	------	------	------	------	COG2304	S:Function unknown	------	------	------	CYT	1	12	12.1	High

TRINITY_DN9090_c0_g1_i9_orf1	CD63 antigen [Ostrinia furnacalis]	0.364451083	-1.456202908	5.02E-05	down	yes	0.976	2.678	2.829	2.691	2.515	1	0.95	0.978	CC:cellular_component;CC:integral component of membrane;CC:cellular anatomical entity;CC:intrinsic component of membrane;	GO:0005575;GO:0016021;GO:0110165;GO:0031224	K06497	CD63, MLA1,	map05205;map	Proteoglycans in car	ENOG4111IRY	S:Function unknown	PF00335.23	Tetraspanin	Tetraspanin family	EXC	3	13	25.6	High
TRINITY_DN80328_c0_g1_i9_orf1	arylphorin subunit alpha-like [Ostrinia furnacalis]	0.372703113	-1.423901225	2.27E-05	down	yes	0.9817	2.634	2.611	2.522	2.77	1	0.969	0.976	------	------	------	------	------	------	ENOG410XR2D	S:Function unknown	PF00372.22	Hemocyanin_M	Hemocyanin, copper containing domain	CYT	4	84	9.1	High
TRINITY_DN4497_c2_g1_i3_orf1	uncharacterized protein LOC114353086 [Ostrinia furnacalis]	0.427114322	-1.227305819	1.30E-06	down	yes	1.005	2.353	2.386	2.302	2.37	1	1.031	0.985	CC:integral component of membrane;CC:cellular_component;CC:cellular anatomical entity;CC:intrinsic component of membrane;MF:cation binding;MF:monooxygenase activity;MF:heme binding;MF:transition metal ion binding;MF:tetrapyrrole binding;MF:organic cyclic compound binding;MF:oxidoreductase activity, acting on paired donors, with incorporation or reduction of molecular oxygen;MF:ion binding;MF:catalytic activity;MF:molecular_function;MF:binding;MF:iron ion binding;MF:metal ion binding;MF:heterocyclic compound binding;MF:oxidoreductase activity;	GO:0016021;GO:0005575;GO:0110165;GO:0031224;GO:0043169;GO:0004497;GO:0020037;GO:0046914;GO:0046906;GO:0097159;GO:0016705;GO:0043167;GO:0003824;GO:0003674;GO:0005488;GO:0005506;GO:0046872;GO:1901363;GO:0016491	K15003	CYP9	------	------	COG2124	Q:Secondary metabolites biosynthesi	PF00067.25	p450	Cytochrome P450	CYT	9	20	59.8	High
TRINITY_DN805_c0_g1_i5_orf1	serine protease snake-like [Ostrinia furnacalis]	0.460899956	-1.117474464	5.72E-05	down	yes	1.055	2.289	2.401	2.212	2.255	1	1.035	1.129	BP:organic substance metabolic process;BP:organonitrogen compound metabolic process;BP:protein metabolic process;BP:macromolecule metabolic process;BP:biological_process;BP:nitrogen compound metabolic process;BP:metabolic process;BP:proteolysis;BP:primary metabolic process;MF:serine-type endopeptidase activity;MF:endopeptidase activity;MF:hydrolase activity;MF:serine hydrolase activity;MF:catalytic activity, acting on a protein;MF:catalytic activity;MF:molecular_function;MF:peptidase activity;MF:serine-type peptidase activity;	GO:0071704;GO:1901564;GO:0019538;GO:0043170;GO:0008150;GO:0006807;GO:0008152;GO:0006508;GO:0044238;GO:0004252;GO:0004175;GO:0016787;GO:0017171;GO:0140096;GO:0003824;GO:0003674;GO:0008233;GO:0008236	------	------	------	------	COG5640	O:Posttranslational modification, pro	PF00089.29	Trypsin	Trypsin	EXC	6	23	43.9	High
TRINITY_DN5421_c0_g1_i1_orf1	arylphorin subunit alpha-like [Ostrinia furnacalis]	0.476695893	-1.068858899	1.52E-05	down	yes	1.033	2.167	2.174	2.231	2.096	1	1.021	1.077	------	------	------	------	------	------	------	------	------	------	------	EXC	1	21	17.2	High
TRINITY_DN350_c0_g1_i5_orf1	tau-like protein isoform X6 [Bombyx mori]	0.489239598	-1.031386917	0.0009658	down	yes	1.023	2.091	2.051	2.305	1.916	1	0.957	1.111	CC:cytoplasm;CC:supramolecular complex;CC:supramolecular fiber;CC:polymeric cytoskeletal fiber;CC:cellular_component;CC:supramolecular polymer;CC:microtubule;CC:cellular anatomical entity;MF:tubulin binding;MF:cytoskeletal protein binding;MF:molecular_function;MF:binding;MF:protein binding;	GO:0005737;GO:0099080;GO:0099512;GO:0099513;GO:0005575;GO:0099081;GO:0005874;GO:0110165;GO:0015631;GO:0008092;GO:0003674;GO:0005488;GO:0005515	K04380	MAPT, TAU	map05012;map	Parkinson disease;Al	ENOG4111J07	O:Posttranslational modification, pro	PF00418.22	Tubulin-binding	Tau and MAP protein, tubulin-binding repeat	CYT	1	16	26.7	High
TRINITY_DN54205_c0_g1_i1_orf1	aldo-keto reductase AKR2E4-like [Ostrinia furnacalis]	0.481818182	-1.053439259	2.88E-05	down	yes	1.007	2.09	2.185	2.063	2.023	1	0.985	1.035	MF:catalytic activity;MF:molecular_function;MF:oxidoreductase activity;	GO:0003824;GO:0003674;GO:0016491	------	------	------	------	COG0656	S:Function unknown	PF00248.24	Aldo_ket_red	Aldo/keto reductase family	CYT	7	32	34.5	High
TRINITY_DN1957_c0_g1_i4_orf1	NAD kinase 2, mitochondrial [Ostrinia furnacalis]	0.482973621	-1.0499837	8.21E-07	down	yes	1.007	2.085	2.096	2.05	2.109	1	0.994	1.028	BP:NADP metabolic process;BP:NAD metabolic process;BP:phosphorylation;BP:cellular biosynthetic process;BP:cellular nitrogen compound metabolic process;BP:nitrogen compound metabolic process;BP:organic substance biosynthetic process;BP:pyridine-containing compound metabolic process;BP:pyridine-containing compound biosynthetic process;BP:organic cyclic compound metabolic process;BP:organic cyclic compound biosynthetic process;BP:organic substance metabolic process;BP:nicotinamide nucleotide biosynthetic process;BP:small molecule metabolic process;BP:NADP biosynthetic process;BP:heterocycle biosynthetic process;BP:nucleobase-containing compound metabolic process;BP:cellular process;BP:cellular aromatic compound metabolic process;BP:biosynthetic process;BP:biological_process;BP:metabolic process;BP:aromatic compound biosynthetic process;BP:nucleobase-containing compound biosynthetic process;BP:cellular nitrogen compound biosynthetic process;BP:organophosphate biosynthetic process;BP:nucleobase-containing small molecule metabolic process;BP:heterocycle metabolic process;BP:primary metabolic process;BP:nucleoside phosphate metabolic process;BP:organonitrogen compound metabolic process;BP:organonitrogen compound biosynthetic process;BP:cellular metabolic process;BP:phosphate-containing compound metabolic process;BP:nucleotide biosynthetic process;BP:nucleoside phosphate biosynthetic process;BP:phosphorus metabolic process;BP:organophosphate metabolic process;BP:nucleotide metabolic process;BP:pyridine nucleotide metabolic process;BP:pyridine nucleotide biosynthetic process;BP:nicotinamide nucleotide metabolic process;MF:molecular_function;MF:binding;MF:NAD+ kinase activity;MF:cation binding;MF:ion binding;MF:transferase activity;MF:metal ion binding;MF:kinase activity;MF:catalytic activity;MF:phosphotransferase activity, alcohol group as acceptor;MF:transferase activity, transferring	GO:0006739;GO:0019674;GO:0016310;GO:0044249;GO:0034641;GO:0006807;GO:1901576;GO:0072524;GO:0072525;GO:1901360;GO:1901362;GO:0071704;GO:0019359;GO:0044281;GO:0006741;GO:0018130;GO:0006139;GO:0009987;GO:0006725;GO:0009058;GO:0008150;GO:0008152;GO:0019438;GO:0034654;GO:0044271;GO:0090407;GO:0055086;GO:0046483;GO:0044238;GO:0006753;GO:1901564;GO:1901566;GO:0044237;GO:0006796;GO:0009165;GO:1901293;GO:0006793;GO:0019637;GO:0009117;GO:0019362;GO:0019363;GO:0046496;GO:0003674;GO:0005488;GO:0003951;GO:0043169;GO:0043167;GO:0016740;GO:0046872;GO:0016301;GO:0003824;GO:0016773;GO:0016772	K00858	ppnK, NADK	map00760;map	Nicotinate and nicot	ENOG410XQA4	S:Function unknown	PF01513.24	NAD_kinase	ATP-NAD kinase N-terminal domain	CYT	7	21	49.1	High
TRINITY_DN22242_c0_g2_i1_orf1	juvenile hormone epoxide hydrolase-like [Ostrinia furnacalis] >XP_028170526.1 juvenile hormone epoxide hydrolase-like [Ostrinia furnacalis]	0.475682145	-1.071930222	1.05E-06	down	yes	0.9937	2.089	2.123	2.093	2.052	1	1.005	0.976	BP:cellular process;BP:cellular aromatic compound metabolic process;BP:cellular metabolic process;BP:cellular catabolic process;BP:biological_process;BP:metabolic process;BP:aromatic compound catabolic process;BP:catabolic process;CC:membrane;CC:organelle membrane;CC:endoplasmic reticulum membrane;CC:cellular_component;CC:cellular anatomical entity;MF:hydrolase activity;MF:cis-stilbene-oxide hydrolase activity;MF:hydrolase activity, acting on ether bonds;MF:ether hydrolase activity;MF:catalytic activity;MF:molecular_function;	GO:0009987;GO:0006725;GO:0044237;GO:0044248;GO:0008150;GO:0008152;GO:0019439;GO:0009056;GO:0016020;GO:0031090;GO:0005789;GO:0005575;GO:0110165;GO:0016787;GO:0033961;GO:0016801;GO:0016803;GO:0003824;GO:0003674	K10719	JHEH	map00981	Insect hormone bios	COG0596;COG0386	S:Function unknown;O:Posttranslatic	PF06441.15;PF005	EHN;Abhydrolase_1	Epoxide hydrolase N terminus;alpha/beta hydrolase fold	CYT	5	16	38.1	High
TRINITY_DN1287_c0_g1_i5_orf1	probable chitinase 10 isoform X6 [Ostrinia furnacalis]	0.494602552	-1.015658413	3.57E-06	down	yes	1.008	2.038	2.057	2.074	1.984	1	1	1.023	BP:amino sugar catabolic process;BP:nitrogen compound metabolic process;BP:aminoglycan metabolic process;BP:organic substance catabolic process;BP:organic substance metabolic process;BP:chitin metabolic process;BP:chitin catabolic process;BP:biological_process;BP:metabolic process;BP:carbohydrate metabolic process;BP:catabolic process;BP:macromolecule catabolic process;BP:primary metabolic process;BP:organonitrogen compound metabolic process;BP:organonitrogen compound catabolic process;BP:amino sugar metabolic process;BP:carbohydrate derivative catabolic process;BP:carbohydrate derivative metabolic process;BP:macromolecule metabolic process;BP:glucosamine-containing compound catabolic process;BP:glucosamine-containing compound metabolic process;BP:aminoglycan catabolic process;CC:cellular anatomical entity;CC:cellular_component;CC:extracellular region;MF:hydrolase activity, acting on glycosyl bonds;MF:molecular_function;MF:chitin binding;MF:hydrolase activity;MF:catalytic activity;MF:chitinase activity;MF:carbohydrate derivative binding;MF:hydrolase activity, hydrolyzing O-glycosyl	GO:0046348;GO:0006807;GO:0006022;GO:1901575;GO:0071704;GO:0006030;GO:0006032;GO:0008150;GO:0008152;GO:0005975;GO:0009056;GO:0009057;GO:0044238;GO:1901564;GO:1901565;GO:0006040;GO:1901136;GO:1901135;GO:0043170;GO:1901072;GO:1901071;GO:0006026;GO:0110165;GO:0005575;GO:0005576;GO:0016798;GO:0003674;GO:0008061;GO:0016787;GO:0003824;GO:0004568;GO:0097367;GO:0004553;GO:0005488	K01183	E3.2.1.14	map00520	Amino sugar and nu	COG3325	S:Function unknown	PF00704.31;PF016	Glyco_hydro_18;CBM_1	Glycosyl hydrolases family 18;Chitin binding Peritrophin-A domain	CYT	51	24	327.2	High
TRINITY_DN7064_c0_g1_i19_orf1	unnamed protein product [Chilo suppressalis]	0.163212128	-2.615179826	6.72E-05	down	yes	1.378	8.443	7.966	9.01	8.353	1	1.915	1.219	------	------	------	------	------	------	------	------	PF00050.24;PF076	Kazal_1;Kazal_2	Kazal-type serine protease inhibitor domain;Kazal-type serine protease inhibitor domain	EXC	1	2	73	High
TRINITY_DN30177_c0_g2_i1_orf1	uncharacterized protein LOC114365032 [Ostrinia furnacalis]	0.181823256	-2.459391358	7.23E-06	down	yes	0.9773	5.375	5.423	5.596	5.106	1	1.008	0.924	------	------	------	------	------	------	ENOG4111NJW;ENO	S:Function unknown;	------	------	------	CYT	1	2	63	Medium
TRINITY_DN4802_c0_g1_i4_orf1	uncharacterized protein LOC114366345 isoform X2 [Ostrinia furnacalis]	0.17757553	-2.493495303	4.68E-08	down	yes	0.9463	5.329	5.277	5.325	5.385	1	0.921	0.918	BP:response to biotic stimulus;BP:response to external stimulus;BP:response to external biotic stimulus;BP:response to stimulus;BP:defense response;BP:response to stress;BP:biological_process;BP:interspecies interaction between organisms;BP:response to other organism;CC:cellular_component;CC:extracellular region;CC:cellular anatomical entity;	GO:0009607;GO:0009605;GO:0043207;GO:0050896;GO:0006952;GO:0006950;GO:0008150;GO:0044419;GO:0051707;GO:0005575;GO:0005576;GO:0110165	------	------	------	------	------	------	------	------	------	CYT	1	12	9.9	High
TRINITY_DN142657_c0_g1_i1_orf1	sorting and assembly machinery component 50 homolog [Diachasma alloeum]	0.205191089	-2.284960013	0.000124	down	yes	1.004	4.893	4.866	4.449	5.363	1	1.007	1.006	CC:cellular_component;CC:membrane;CC:outer membrane;CC:cellular anatomical entity;	GO:0005575;GO:0016020;GO:0019867;GO:0110165	K07277	SAM50, TOB5	------	------	COG4775	M:Cell wall/membrane/envelope bio	PF01103.26	Omp85	Omp85 superfamily domain	CYT	1	2	53.8	High
TRINITY_DN20344_c0_g1_i5_orf1	uncharacterized protein LOC114351483 [Ostrinia furnacalis]	0.20575693	-2.280987075	7.84E-05	down	yes	0.965	4.69	5.11	4.355	4.604	1	0.89	1.005	MF:molecular_function;MF:structural constituent of cuticle;MF:structural molecule activity;	GO:0003674;GO:0042302;GO:0005198	------	------	------	------	------	------	PF00379.26	Chitin_bind_4	Insect cuticle protein	CYT	4	57	13.5	High
TRINITY_DN4767_c0_g1_i4_orf1	cysteine protease XCP2-like [Ostrinia furnacalis]	0.210694838	-2.246773128	1.88E-06	down	yes	0.9673	4.591	4.682	4.662	4.429	1	0.912	0.99	BP:primary metabolic process;BP:organonitrogen compound metabolic process;BP:protein metabolic process;BP:macromolecule metabolic process;BP:organic substance metabolic process;BP:nitrogen compound metabolic process;BP:biological_process;BP:metabolic process;BP:proteolysis;MF:molecular_function;MF:hydrolase activity;MF:catalytic activity;MF:catalytic activity, acting on a protein;MF:peptidase activity;	GO:0044238;GO:1901564;GO:0019538;GO:0043170;GO:0071704;GO:0006807;GO:0008150;GO:0008152;GO:0006508;GO:0003674;GO:0016787;GO:0003824;GO:0140096;GO:0008233	------	------	------	------	ENOG4110R2S;ENO(	S:Function unknown;	PF08246.15	Inhibitor_I29	Cathepsin propeptide inhibitor domain	CYT	6	38	11.7	High
TRINITY_DN1091_c0_g3_i1_orf1	macrophage mannose receptor 1-like [Ostrinia furnacalis]	0.300822669	-1.733014807	5.70E-05	down	yes	0.9873	3.282	3.37	3.445	3.032	1	0.955	1.007	BP:response to extracellular stimulus;BP:nucleic acid metabolic process;BP:cellular nitrogen compound metabolic process;BP:nitrogen compound metabolic process;BP:DNA protection;BP:organic cyclic compound metabolic process;BP:nucleobase-containing compound metabolic process;BP:cellular response to stimulus;BP:cellular macromolecule metabolic process;BP:organic substance metabolic process;BP:cellular response to external stimulus;BP:cellular process;BP:cellular aromatic compound metabolic process;BP:cellular response to extracellular stimulus;BP:response to stress;BP:biological_process;BP:metabolic process;BP:cell communication;BP:heterocycle metabolic process;BP:primary metabolic process;BP:response to external stimulus;BP:cellular metabolic process;BP:macromolecule metabolic process;BP:cellular response to stress;BP:DNA metabolic process;BP:response to stimulus;MF:molecular_function;MF:hydrolase activity;MF:catalytic activity;MF:nucleoside-triphosphate diphosphatase activity;MF:hydrolase activity, acting on acid anhydrides, in phosphorus-containing anhydrides;MF:8-oxo-7,8-dihydroguanosine triphosphate pyrophosphatase activity;MF:hydrolase activity, acting on acid anhydrides;MF:pyrophosphatase activity;	GO:0009991;GO:0090304;GO:0034641;GO:0006807;GO:0042262;GO:1901360;GO:0006139;GO:0051716;GO:0044260;GO:0071704;GO:0071496;GO:0009987;GO:0006725;GO:0031668;GO:0006950;GO:0008150;GO:0008152;GO:0007154;GO:0046483;GO:0044238;GO:0009605;GO:0044237;GO:0043170;GO:0033554;GO:0006259;GO:0050896;GO:0003674;GO:0016787;GO:0003824;GO:0047429;GO:0016818;GO:0008413;GO:0016817;GO:0016462	------	------	------	------	ENOG410XRS3;ENO(	U:Intracellular trafficking, secretion, a	PF00059.24	Lectin_C	Lectin C-type domain	CYT	9	34	38.3	High
TRINITY_DN276_c0_g1_i2_orf1	protein lethal(2)essential for life-like [Ostrinia furnacalis] >UTU55753.1 small heat shock protein Hsp20.7 [Ostrinia furnacalis]	0.29424505	-1.764909952	1.87E-06	down	yes	0.951	3.232	3.171	3.258	3.266	1	0.991	0.862	MF:cation binding;MF:metal ion binding;MF:molecular_function;MF:binding;MF:ion binding;	GO:0043169;GO:0046872;GO:0003674;GO:0005488;GO:0043167	K09542	CRYAB	map04213;map	Longevity regulating	ENOG410YERS	O:Posttranslational modification, pro	PF00011.24	HSP20	Hsp20/alpha crystallin family	CYT	2	43	20.8	High
TRINITY_DN285_c0_g1_i4_orf1	catalase-like [Ostrinia furnacalis]	0.349462751	-1.516789408	1.61E-06	down	yes	0.9757	2.792	2.813	2.773	2.79	1	0.898	1.029	BP:cellular catabolic process;BP:hydrogen peroxide catabolic process;BP:cellular process;BP:reactive oxygen species metabolic process;BP:response to stress;BP:biological_process;BP:metabolic process;BP:catabolic process;BP:response to oxidative stress;BP:response to stimulus;BP:cellular metabolic process;BP:hydrogen peroxide metabolic process;MF:molecular_function;MF:binding;MF:heterocyclic compound binding;MF:oxidoreductase activity;MF:cation binding;MF:ion binding;MF:metal ion binding;MF:heme binding;MF:antioxidant activity;MF:catalytic activity;MF:oxidoreductase activity, acting on peroxide as acceptor;MF:tetrapyrrole binding;MF:organic cyclic compound binding;MF:catalase activity;MF:peroxidase	GO:0044248;GO:0042744;GO:0009987;GO:0072593;GO:0006950;GO:0008150;GO:0008152;GO:0009056;GO:0006979;GO:0050896;GO:0044237;GO:0042743;GO:0003674;GO:0005488;GO:1901363;GO:0016491;GO:0043169;GO:0043167;GO:0046872;GO:0020037;GO:0016209;GO:0003824;GO:0016684;GO:0046906;GO:0097159;GO:0004096;GO:0004601	K03781	katE, CAT, cat	map00630;map	Glyoxylate and dicar	COG0753	P:Inorganic ion transport and metab	PF00199.22;PF066	Catalase;Catalase-rel	Catalase;Catalase-related immune-responsive	CYT	9	23	58.6	High
TRINITY_DN5153_c1_g1_i1_orf1	nose resistant to fluoxetine protein 6-like isoform X1 [Ostrinia furnacalis]	0.363405797	-1.460346661	0.0006979	down	yes	1.003	2.76	2.84	2.459	2.981	1	0.83	1.18	CC:integral component of membrane;CC:cellular_component;CC:intrinsic component of membrane;CC:cellular anatomical entity;MF:catalytic activity;MF:transferase activity;MF:transferase activity, transferring acyl groups;MF:molecular_function;MF:transferase activity, transferring acyl groups other than amino-acyl groups;	GO:0016021;GO:0005575;GO:0031224;GO:0110165;GO:0003824;GO:0016740;GO:0016746;GO:0003674;GO:0016747	------	------	------	------	ENOG410YD1T	S:Function unknown	------	------	------	CYT	1	1	71.6	High
TRINITY_DN16840_c1_g1_i1_orf1	attacin-like [Ostrinia furnacalis]	0.41441098	-1.270865867	0.000162	down	yes	1.087	2.623	2.815	2.573	2.48	1	1.173	1.089	BP:interspecies interaction between organisms;BP:response to biotic stimulus;BP:response to external stimulus;BP:response to external biotic stimulus;BP:response to stimulus;BP:response to bacterium;BP:defense response;BP:response to stress;BP:defense response to other organism;BP:defense response to bacterium;BP:biological_process;BP:response to other organism;CC:cellular_component;CC:extracellular region;CC:cellular anatomical entity;	GO:0044419;GO:0009607;GO:0009605;GO:0043207;GO:0050896;GO:0009617;GO:0006952;GO:0006950;GO:0098542;GO:0042742;GO:0008150;GO:0051707;GO:0005575;GO:0005576;GO:0110165	------	------	------	------	ENOG410ZBQ8;ENO	S:Function unknown;	PF03769.18;PF037	Attacin_C;Attacin_N	Attacin, C-terminal region;Attacin, N-terminal region	EXC	1	6	22.2	High
TRINITY_DN40126_c0_g1_i1_orf1	aldehyde dehydrogenase X, mitochondrial-like [Ostrinia furnacalis]	0.383868559	-1.381315696	5.08E-07	down	yes	1.028	2.678	2.646	2.666	2.723	1	1.027	1.056	MF:catalytic activity;MF:oxidoreductase activity, acting on the aldehyde or oxo group of donors;MF:molecular_function;MF:oxidoreductase activity, acting on the aldehyde or oxo group of donors, NAD or NADP as acceptor;MF:oxidoreductase activity;	GO:0003824;GO:0016903;GO:0003674;GO:0016620;GO:0016491	K00128	ALDH	map00310;map	Lysine degradation;(	COG1012	C:Energy production and conversion	PF00171.25	Aldedh	Aldehyde dehydrogenase family	CYT	19	65	45.5	High
TRINITY_DN5099_c0_g1_i3_orf1	trans-1,2-dihydrobenzene-1,2-diol dehydrogenase-like [Ostrinia furnacalis]	0.380718336	-1.393204039	2.77E-05	down	yes	1.007	2.645	2.739	2.494	2.701	1	1.01	1.011	MF:organic cyclic compound binding;MF:nucleotide binding;MF:small molecule binding;MF:molecular_function;MF:binding;MF:heterocyclic compound binding;MF:nucleoside phosphate binding;	GO:0097159;GO:0000166;GO:0036094;GO:0003674;GO:0005488;GO:1901363;GO:1901265	K00078	DHDH	map00040;map	Pentose and glucuro	COG0673	S:Function unknown	PF01408.25;PF028	GFO_IDH_MocA;GFO_I	Oxidoreductase family, NAD-binding Rossmann fold;Oxidoreductase family, C-terminal alpha/beta domain	CYT	17	55	38.1	High
TRINITY_DN18323_c0_g1_i5_orf1	GILT-like protein 1 isoform X1 [Ostrinia furnacalis]	0.37812142	-1.403078515	2.71E-06	down	yes	0.9903	2.619	2.664	2.657	2.536	1	0.996	0.975	------	------	K08059	IFI30, GILT	map04612	Antigen processing	ENOG4111IDT	U:Intracellular trafficking, secretion, a	PF03227.19	GILT	Gamma interferon inducible lysosomal thiol reductase	CYT	15	62	25	High
TRINITY_DN1201_c0_g1_i4_orf1	triosephosphate isomerase [Ostrinia furnacalis] >XP_028170843.1 triosephosphate isomerase [Ostrinia furnacalis]	0.393939394	-1.343954401	1.52E-08	down	yes	1.014	2.574	2.577	2.587	2.558	1	1.019	1.023	BP:ribonucleoside diphosphate metabolic process;BP:phosphorylation;BP:purine nucleoside triphosphate metabolic process;BP:carboxylic acid metabolic process;BP:cellular nitrogen compound metabolic process;BP:nitrogen compound metabolic process;BP:small molecule metabolic process;BP:small molecule biosynthetic process;BP:cellular aromatic compound metabolic process;BP:purine-containing compound metabolic process;BP:generation of precursor metabolites and energy;BP:pyruvate metabolic process;BP:glycolytic process;BP:organic cyclic compound metabolic process;BP:nucleobase-containing compound metabolic process;BP:organic substance catabolic process;BP:purine nucleoside diphosphate metabolic process;BP:nucleotide phosphorylation;BP:ribonucleotide metabolic process;BP:organic substance metabolic process;BP:ribonucleoside triphosphate metabolic process;BP:nucleoside triphosphate metabolic process;BP:gluconeogenesis;BP:purine ribonucleotide metabolic process;BP:monosaccharide biosynthetic process;BP:purine ribonucleoside triphosphate metabolic process;BP:hexose metabolic process;BP:ribose phosphate metabolic process;BP:organic substance biosynthetic process;BP:ATP metabolic process;BP:cellular [illegible] process;BP:ADP metabolic process;BP:hexose biosynthetic process;BP:monocarboxylic acid metabolic process;BP:biosynthetic process;BP:biological_process;BP:metabolic process;BP:oxoacid metabolic process;BP:carbohydrate metabolic process;BP:nucleoside diphosphate metabolic process;BP:purine ribonucleoside diphosphate metabolic process;BP:nucleobase-containing small molecule metabolic process;BP:heterocycle metabolic process;BP:primary metabolic process;BP:organonitrogen compound metabolic process;BP:organic acid metabolic process;BP:monosaccharide metabolic process;BP:carbohydrate derivative metabolic process;BP:carbohydrate catabolic process;BP:cellular metabolic process;BP:carbohydrate biosynthetic process;BP:purine nucleotide metabolic process;BP:phosphate-containing compound metabolic process;BP:nucleoside diphosphate phosphorylation;BP:phosphorus metabolic process;BP:organophosphate metabolic process;BP:nucleotide metabolic process;BP:glucose metabolic process;BP:nucleoside phosphate metabolic process;BP:catabolic process;BP:ATP generation from ADP;MF:molecular_function;MF:intramolecular oxidoreductase activity;MF:intramolecular oxidoreductase activity, interconverting aldoses and ketoses;MF:catalytic activity;MF:triose-phosphate isomerase activity;MF:isomerase activity;	GO:0009185;GO:0016310;GO:0009144;GO:0019752;GO:0034641;GO:0006807;GO:0044281;GO:0044283;GO:0006725;GO:0072521;GO:0006091;GO:0006090;GO:0006096;GO:1901360;GO:0006139;GO:1901575;GO:0009135;GO:0046939;GO:0009259;GO:0071704;GO:0009199;GO:0009141;GO:0006094;GO:0009150;GO:0046364;GO:0009205;GO:0019318;GO:0019693;GO:1901576;GO:0046034;GO:0009987;GO:0046031;GO:0019319;GO:0032787;GO:0009058;GO:0008150;GO:0008152;GO:0043436;GO:0005975;GO:0009132;GO:0009179;GO:0055086;GO:0046483;GO:0044238;GO:1901564;GO:0006082;GO:0005996;GO:1901135;GO:0016052;GO:0044237;GO:0016051;GO:0006163;GO:0006796;GO:0006165;GO:0006793;GO:0019637;GO:0009117;GO:0006006;GO:0006753;GO:0009056;GO:0006757;GO:0003674;GO:0016860;GO:0016861;GO:0003824;GO:0004807;GO:0016853	K01803	TPI, tpiA	map00010;map	Glycolysis / Glucone	COG0149	G:Carbohydrate transport and metab	PF00121.21	TIM	Triosephosphate isomerase	CYT	16	71	26.4	High
TRINITY_DN1672_c0_g1_i6_orf1	cystinosin homolog isoform X1 [Ostrinia furnacalis] >XP_028162341.1 cystinosin homolog isoform X1 [Ostrinia furnacalis] >XP_028162342.1 cystinosin homolog isoform X1 [Ostrinia furnacalis] >XP_028162343.1 cystinosin homolog isoform X1 [Ostrinia furnacalis] >XP_028162344.1 cystinosin homolog isoform X1 [Ostrinia furnacalis]	0.414663462	-1.269987166	1.38E-05	down	yes	1.035	2.496	2.437	2.497	2.554	1	0.979	1.126	CC:cellular_component;CC:integral component of membrane;CC:cellular anatomical entity;CC:intrinsic component of membrane;	GO:0005575;GO:0016021;GO:0110165;GO:0031224	K12386	CTNS	map04142	Lysosome	ENOG410XQSD	U:Intracellular trafficking, secretion, a	PF04193.17	PQ-loop	PQ loop repeat	PLA	1	2	48.7	High
TRINITY_DN140_c0_g1_i1_orf1	calcyphosin-like protein [Ostrinia furnacalis]	0.401488934	-1.316567872	2.28E-05	down	yes	0.9977	2.485	2.485	2.599	2.371	1	0.991	1.002	------	------	K23909	CAPS	------	------	ENOG410XRMJ	T:Signal transduction mechanisms	PF13405.9;PF1349	EF-hand_6;EF-hand_7;	EF-hand domain;EF-hand domain pair;EF hand;EF hand;EF-hand domain pair	CYT	7	55	27.1	High
TRINITY_DN15597_c0_g1_i1_orf1	microsomal glutathione S-transferase 1-like [Ostrinia furnacalis]	0.40784396	-1.293910809	7.40E-06	down	yes	0.9723	2.384	2.452	2.38	2.32	1	0.917	1	CC:integral component of membrane;CC:cellular_component;CC:intrinsic component of membrane;CC:cellular anatomical entity;MF:glutathione transferase activity;MF:catalytic activity;MF:transferase activity, transferring alkyl or aryl;MF:transferase activity;MF:molecular_function;	GO:0016021;GO:0005575;GO:0031224;GO:0110165;GO:0004364;GO:0003824;GO:0016765;GO:0016740;GO:0003674	K00799	GST, gst	map05207;map	Chemical carcinoger	ENOG4111VJG	S:Function unknown	PF01124.21	MAPEG	MAPEG family	CYT	1	7	16.4	High
TRINITY_DN5022_c0_g1_i4_orf1	syntenin-1-like [Ostrinia furnacalis]	0.496383363	-1.010473331	0.0002243	down	yes	1.098	2.212	2.225	2.079	2.331	1	1.15	1.144	------	------	K17254	SDCBP	------	------	ENOG410YNPQ	U:Intracellular trafficking, secretion, a	PF00595.27;PF178	PDZ;PDZ_6;Peptidase_I	PDZ domain;PDZ domain;Peptidase family M50;PDZ domain	CYT	4	20	30.8	High
TRINITY_DN3616_c0_g2_i2_orf1	conotoxin ArMKLT2-032-like [Ostrinia furnacalis]	0.42869032	-1.221992255	0.001148	down	yes	0.9787	2.283	2.292	2.546	2.011	1	1.013	0.923	CC:cellular_component;CC:integral component of membrane;CC:cellular anatomical entity;CC:intrinsic component of membrane;	GO:0005575;GO:0016021;GO:0110165;GO:0031224	------	------	------	------	------	------	------	------	------	CYT	1	64	8.7	High
TRINITY_DN57900_c0_g1_i2_orf1	hypothetical protein SFRURICE_000634 [Spodoptera frugiperda]	0.465062471	-1.104503571	7.44E-06	down	yes	1.005	2.161	2.233	2.148	2.102	1	1.014	1	MF:catalytic activity;MF:molecular_function;MF:dioxygenase activity;MF:oxidoreductase activity;	GO:0003824;GO:0003674;GO:0051213;GO:0016491	K00477	PHYH	map04146	Peroxisome	ENOG410XUSH	O:Secondary metabolites biosynthesi	PF05721.16	PhyH	Phytanoyl-CoA dioxygenase	PLA	11	57	30.1	High
TRINITY_DN801_c0_g1_i2_orf1	cathepsin L [Ostrinia furnacalis] >XP_028165920.1 cathepsin L [Ostrinia furnacalis] >UKI61015.1 cathepsin L [Ostrinia furnacalis]	0.457281106	-1.128846784	1.15E-05	down	yes	0.9923	2.17	2.134	2.253	2.122	1	0.966	1.011	BP:primary metabolic process;BP:organonitrogen compound metabolic process;BP:protein metabolic process;BP:macromolecule metabolic process;BP:organic substance metabolic process;BP:nitrogen compound metabolic process;BP:biological_process;BP:metabolic process;BP:proteolysis;MF:molecular_function;MF:hydrolase activity;MF:catalytic activity;MF:catalytic activity, acting on a protein;MF:peptidase activity;MF:cysteine-type peptidase activity;	GO:0044238;GO:1901564;GO:0019538;GO:0043170;GO:0071704;GO:0006807;GO:0008150;GO:0008152;GO:0006508;GO:0003674;GO:0016787;GO:0003824;GO:0140096;GO:0008233;GO:0008234	K01365	CTSL	map05205;map	Proteoglycans in car	COG4870	S:Function unknown	PF00112.26;PF082	Peptidase_C1;Inhibitor_	Papain family cysteine protease;Cathepsin propeptide inhibitor domain	CYT	16	47	38.1	High
TRINITY_DN4069_c0_g1_i5_orf1	putative sulfiredoxin [Ostrinia furnacalis]	0.45983341	-1.120816802	7.88E-05	down	yes	0.9937	2.161	2.243	2.022	2.219	1	0.974	1.007	------	------	K12260	SRX1	------	------	COG5119	S:Function unknown	PF02195.21	ParBc	ParB/Sulfiredoxin domain	CYT	4	47	14.6	High
TRINITY_DN9694_c0_g1_i1_orf1	larval cuticle protein LCP-17 [Helicoverpa armigera] >PZC82071.1 hypothetical protein B5X24_HaOG211161 [Helicoverpa armigera] >PZC87412.1 hypothetical protein B5X24_HaOG216859 [Helicoverpa armigera]	0.47429527	-1.076142614	1.55E-06	down	yes	0.9927	2.093	2.118	2.115	2.045	1	0.979	0.999	MF:molecular_function;MF:structural constituent of cuticle;MF:structural molecule activity;	GO:0003674;GO:0042302;GO:0005198	------	------	------	------	ENOG4110RUZ;ENO(	S:Function unknown;	PF00379.26	Chitin_bind_4	Insect cuticle protein	CYT	5	82	15.7	High
TRINITY_DN10220_c1_g1_i7_orf1	uncharacterized protein LOC124645895 isoform X2 [Helicoverpa zea]	0.473709369	-1.077925889	7.86E-05	down	yes	0.991	2.092	2.059	1.997	2.22	1	0.99	0.983	CC:cellular_component;CC:integral component of membrane;CC:cellular anatomical entity;CC:intrinsic component of membrane;	GO:0005575;GO:0016021;GO:0110165;GO:0031224	K14788	NOL10, ENP2	------	------	ENOG410ZV5F	S:Function unknown	------	------	------	CYT	1	2	52.5	Medium
TRINITY_DN812_c2_g1_i1_orf1	1,4-alpha-glucan-branching enzyme [Ostrinia furnacalis]	0.488405797	-1.03384777	4.47E-06	down	yes	1.011	2.07	2.1	2.011	2.099	1	1.002	1.031	BP:cellular biosynthetic process;BP:cellular macromolecule biosynthetic process;BP:organic substance biosynthetic process;BP:generation of precursor metabolites and energy;BP:cellular polysaccharide biosynthetic process;BP:cellular polysaccharide metabolic process;BP:cellular carbohydrate metabolic process;BP:cellular macromolecule metabolic process;BP:carbohydrate biosynthetic process;BP:organic substance metabolic process;BP:energy reserve metabolic process;BP:cellular glucan metabolic process;BP:glucan biosynthetic process;BP:cellular process;BP:polysaccharide biosynthetic process;BP:cellular carbohydrate biosynthetic process;BP:biosynthetic process;BP:macromolecule biosynthetic process;BP:biological_process;BP:metabolic process;BP:glucan metabolic process;BP:primary metabolic process;BP:carbohydrate metabolic process;BP:energy derivation by oxidation of organic compounds;BP:glycogen metabolic process;BP:polysaccharide metabolic process;BP:glycogen biosynthetic process;BP:cellular metabolic process;BP:macromolecule metabolic process;MF:hydrolase activity, acting on glycosyl bonds;MF:molecular_function;MF:binding;MF:cation binding;MF:ion binding;MF:transferase activity;MF:1,4-alpha-glucan branching enzyme activit;MF:hydrolase activity;MF:catalytic activity;MF:1,4-alpha-glucan branching enzyme activity;MF:transferase activity, transferring glycosyl groups;MF:hydrolase activity, hydrolyzing O-glycosyl compounds;MF:transferase activity, transferring hexosyl groups;	GO:0044249;GO:0034645;GO:1901576;GO:0006091;GO:0033692;GO:0044264;GO:0044262;GO:0044260;GO:0016051;GO:0071704;GO:0006112;GO:0006073;GO:0009250;GO:0009987;GO:0000271;GO:0034637;GO:0009058;GO:0009059;GO:0008150;GO:0008152;GO:0044042;GO:0044238;GO:0005975;GO:0015980;GO:0005977;GO:0005976;GO:0005978;GO:0044237;GO:0043170;GO:0016798;GO:0003674;GO:0005488;GO:0043169;GO:0043167;GO:0016740;GO:0102752;GO:0016787;GO:0003824;GO:0003844;GO:0016757;GO:0004553;GO:0016758	K00700	GBE1, glgB	map00500	Starch and sucrose r	COG0296	G:Carbohydrate transport and metab	PF02806.21;PF001	Alpha-amylase_C;Alph	Alpha amylase, C-terminal all-beta domain;Alpha amylase, catalytic domain;Carbohydrate-binding module 48	CYT	35	57	79.1	High
TRINITY_DN4449_c0_g2_i1_orf1	calcium/calmodulin-dependent protein kinase type 1 isoform X3 [Cephus cinctus]	0.497315764	-1.007765933	0.0002984	down	yes	1.019	2.049	1.967	2.22	1.961	1	1.058	0.998	BP:phosphorylation;BP:nitrogen compound metabolic process;BP:organic substance metabolic process;BP:protein phosphorylation;BP:cellular process;BP:macromolecule modification;BP:protein modification process;BP:biological_process;BP:metabolic process;BP:primary metabolic process;BP:organonitrogen compound metabolic process;BP:cellular metabolic process;BP:macromolecule metabolic process;BP:protein metabolic process;BP:phosphate-containing compound metabolic process;BP:phosphorus metabolic process;MF:purine ribonucleoside triphosphate binding;MF:molecular_function;MF:binding;MF:ion binding;MF:heterocyclic compound binding;MF:nucleoside phosphate binding;MF:anion binding;MF:carbohydrate derivative binding;MF:nucleotide binding;MF:protein serine/threonine kinase activity;MF:transferase activity;MF:protein kinase activity;MF:purine nucleotide binding;MF:ATP binding;MF:kinase activity;MF:catalytic activity, acting on a protein;MF:small molecule binding;MF:catalytic activity;MF:adenyl nucleotide binding;MF:phosphotransferase activity, alcohol group as acceptor;MF:transferase activity, transferring phosphorus-containing groups;MF:adenyl ribonucleotide binding;MF:purine ribonucleotide binding;MF:organic cyclic compound binding;MF:ribonucleotide	GO:0016310;GO:0006807;GO:0071704;GO:0006468;GO:0009987;GO:0043412;GO:0036211;GO:0008150;GO:0008152;GO:0044238;GO:1901564;GO:0044237;GO:0043170;GO:0019538;GO:0006796;GO:0006793;GO:0035639;GO:0003674;GO:0005488;GO:0043167;GO:1901363;GO:1901265;GO:0043168;GO:0097367;GO:0000166;GO:0004674;GO:0016740;GO:0004672;GO:0017076;GO:0005524;GO:0016301;GO:0140096;GO:0036094;GO:0003824;GO:0030554;GO:0016773;GO:0016772;GO:0032559;GO:0032555;GO:0097159;GO:0032553	K08794	CAMK1	map05214;map	Glioma;Oxytocin sig	ENOG410XRMJ	S:Function unknown	PF00069.28;PF077	Pkinase;PK_Tyr_Ser-Thr	Protein kinase domain;Protein tyrosine and serine/threonine kinase	PLA	3	10	49	High
TRINITY_DN91946_c0_g1_i1_orf1	protein catecholamines up [Ostrinia furnacalis]	0.493130911	-1.019957407	0.0001653	down	yes	0.9907	2.009	2.081	1.903	2.044	1	0.898	1.074	CC:cellular_component;CC:integral component of membrane;CC:cellular anatomical entity;CC:intrinsic component of membrane;MF:inorganic cation transmembrane transporter activity;MF:inorganic solute uptake transmembrane transporter activity;MF:transporter activity;MF:cation transmembrane transporter activity;MF:ion transmembrane transporter activity;MF:transmembrane transporter activity;MF:molecular_function;MF:metal ion transmembrane transporter activity;	GO:0005575;GO:0016021;GO:0110165;GO:0031224;GO:0022890;GO:0015318;GO:0005215;GO:0008324;GO:0015075;GO:0022857;GO:0003674;GO:0046873	K14713	SLC39A7, KE4	map05012;map	Parkinson disease;Al	COG0428	O:Posttranslational modification, pro	PF02535.25	Zip	ZIP Zinc transporter	CYT	1	4	38	High

TRINITY_DN10231_c0_g2_i1_orf1	uncharacterized protein LOC114361472 [Ostrinia furnacalis]	0.455572442	-1.134247616	0.001045	down	yes	0.8993	1.974	2.012	2.152	1.757	1	0.858	0.84	CC:cellular_component;CC:integral component of membrane;CC:cellular anatomical entity;CC:intrinsic component of membrane;	GO:0005575;GO:0016021;GO:0110165;GO:0031224	------	------	------	------	ENOG410XYE8	S:Function unknown	------	------	------	CYT	1	14	7.7	High
TRINITY_DN384_c0_g1_i8_orf1	unnamed protein product [Chilo suppressalis]	0.436710183	-1.195251924	0.001005	down	yes	0.8363	1.915	2.077	1.815	1.852	1	0.838	0.671	------	------	K23028	SPECC1	------	------	COG5069;ENOG410Y	E:Amino acid transport and metaboli	------	------	------	CYT	1	1	216.4	High
TRINITY_DN315_c0_g1_i1_orf1	tubulin alpha-1B chain [Rattus norvegicus] >NP_001038009.1 tubulin alpha-1B chain [Sus scrofa] >NP_001107705.1 1 [Xenopus tropicalis] [Xenopus tropicalis] >NP_001108328.1 tubulin alpha-1B chain [Bos taurus] >NP_001182321.1 tubulin alpha-1B chain [Macaca mulatta] >NP_001182735.1 tubulin alpha-1B chain [Oryctolagus cuniculus] >NP_001230908.1 tubulin alpha-1B chain [Cricetulus griseus] >NP_006073.2 tubulin alpha-1B chain [Homo sapiens] >NP_035784.1 tubulin alpha-1B chain [Mus musculus] >XP_001509265.1 tubulin alpha-1B chain [Ornithorhynchus anatinus] >XP_002823230.1 tubulin alpha-1B chain [Pongo abelii] >XP_003476108.2 tubulin alpha-1B chain [Cavia porcellus] >XP_003988693.3 tubulin alpha-1B chain [Felis catus] >XP_004053124.1 tubulin alpha-1B chain [Gorilla gorilla gorilla] >XP_004447012.1 tubulin alpha-1B chain [Dasypus novemcinctus] >XP_004692837.1 PREDICTED: tubulin alpha-1B chain [Condylura cristata] >XP_005680059.1 PREDICTED: tubulin alpha-1B chain [Capra hircus] >XP_006091578.1 tubulin alpha-1B chain [Myotis lucifugus] >XP_006902536.1 PREDICTED: tubulin alpha-1B chain [Elephantulus edwardii] >XP_007073036.2 tubulin alpha-1B chain [Panthera tigris] >XP_007506293.1 PREDICTED: tubulin alpha-1B chain [Monodelphis domestica] >XP_007528574.2 PREDICTED: tubulin alpha-1B chain [Erinaceus europaeus] >XP_008001316.2 tubulin alpha-1B chain [Chlorocebus sabaeus] >XP_008069066.1 tubulin alpha-1B chain isoform X1 [Carlito syrichta] >XP_008702764.1 tubulin alpha-1B chain [Ursus maritimus] >XP_008949408.1 tubulin alpha-1B chain [Pan paniscus] >XP_009001972.1 tubulin alpha-1B chain [Callithrix jacchus] >XP_010348074.2 tubulin alpha-1B chain [Saimiri boliviensis boliviensis] >XP_010383711.1 tubulin alpha-1B chain [Rhinopithecus roxellana] >XP_010594685.1 tubulin alpha-1B chain [Loxodonta africana] >XP_010725238.1 tubulin alpha-1B chain [Meleagris gallopavo] >XP_010856059.1 PREDICTED: tubulin alpha-1B chain [Bison bison bison] >XP_010962544.1 tubulin alpha-1B chain isoform X1 [Camelus bactrianus] >XP_010962545.1 tubulin alpha-1B chain isoform X2 [Camelus bactrianus] >XP_011366571.1 tubulin alpha-1B chain isoform X1 [Pteropus vampyrus] >XP_011903803.1 PREDICTED: tubulin alpha-1B chain [Cercocebus atys] >XP_012811688.1 1 isoform X1 [Xenopus tropicalis] >XP_013361157.1 PREDICTED: tubulin alpha-1B chain [Chinchilla lanigera] >XP_014116068.1 PREDICTED: tubulin alpha-1B chain isoform X2 [Pseudopodoces humilis] >XP_014705495.2 tubulin alpha-1B chain [Equus asinus] >XP_014747331.1 PREDICTED: tubulin alpha-1B chain isoform X1 [Sturnus vulgaris] >XP_014747332.1 PREDICTED: tubulin alpha-1B chain isoform X2 [Sturnus vulgaris]																												
A Crystal structure of T2R-TTL-CF1 c	Tubulin alpha-1B chain [Sus scrofa] >6O2Q_A Acetylated Microtubules [Sus scrofa] >6O2Q_C Acetylated Microtubules [Sus scrofa] >6O2Q_E Acetylated Microtubules [Sus scrofa] >6O2Q_J Acetylated Microtubules [Sus scrofa] >6O2Q_K Acetylated Microtubules [Sus scrofa] >6O2Q_L Acetylated Microtubules [Sus scrofa] >6O2R_A Deacetylated Microtubules [Sus scrofa] >6O2R_C Deacetylated Microtubules [Sus scrofa] >6O2R_E Deacetylated Microtubules [Sus scrofa] >6O2R_J Deacetylated Microtubules [Sus scrofa] >6O2R_K Deacetylated Microtubules [Sus scrofa] >6O2R_L Deacetylated Microtubules [Sus scrofa] >6O2S_1A Chain 1A	Tubulin alpha-1B	Tubulin alpha-1B c	Tubulin alpha-1	Tubulin alph	Tubulin alph	Tubulin a	Tubulin a	Tubulin a	Tubulin a	Tubulin a	Tubulin a	Tubulin a	Tubulin a	Tubulin alpha-1B chain [Sus scrofa] >6O2S_2B Chain 2B	Tubulin alpha-1B chain [Sus scrofa] >6O2S_2C Chain 2C	Tubulin alpha	Tubulin alpha	Tubulin alpha-	Tubulin alpha-1B cl	Tubulin alpha-1B ch	Tubulin alpha-1B chain [Sus scrofa]	Tubulin alpha-1B	Tubulin alpha-1B chai	Tubulin alpha-1B chain [Sus scrofa] >6O2S_2M Chain 2M	Tubulin alpha-1B	Tubulin alpha-1B c	Tubulin alpha-	Tubulin alpha	Tubulin alpha-1E
TRINITY_DN18218_c0_g1_i7_orf1	inhibin beta B chain [Ostrinia furnacalis]	0.497273204	-1.007889403	0.001298	down	yes	0.9483	1.907	1.828	2.12	1.773	1	0.846	0.999	BP:response to stimulus;BP:biological_process;BP:response to chemical;BP:response to organic substance;CC:cellular_component;CC:extracellular region;CC:cellular anatomical entity;MF:growth factor activity;MF:receptor regulator activity;MF:receptor activator activity;MF:receptor agonist activity;MF:molecular_function;MF:binding;MF:protein binding;MF:receptor binding;MF:molecular function regulator;	GO:0050896;GO:0008150;GO:0042221;GO:0010033;GO:0005575;GO:0005576;GO:0110165;GO:0008083;GO:0030545;GO:0030546;GO:0048018;GO:0003674;GO:0005488;GO:0005515;GO:0005102;GO:0098772	K04669	TGFBRX	------	------	ENOG410XT8Z	O:Posttranslational modification, pro	PF00019.23;PF006	TGF_beta;TGFb_propep	Transforming growth factor beta like domain;TGF-beta propeptide	CYT	1	4	41.4	High
TRINITY_DN1048_c0_g1_i6_orf1	uncharacterized protein LOC114360661 [Ostrinia furnacalis]	0.489734043	-1.02992961	8.15E-05	down	yes	0.9207	1.88	1.873	1.956	1.811	1	0.898	0.864	------	------	------	------	------	------	ENOG4111BN3;ENO	S:Function unknown;	PF15430.9	SVWC	Single domain von Willebrand factor type C	CYT	4	39	12.1	High
TRINITY_DN6698_c0_g2_i2_orf1	protein mesh isoform X1 [Ostrinia furnacalis]	0.463817204	-1.10837176	0.0008742	down	yes	0.8627	1.86	1.958	1.707	1.916	1	0.867	0.721	------	------	K23820	SUSD2	------	------	ENOG410YZ7Z	S:Function unknown	PF03782.20;PF000	AMOP;VWD;NIDO	AMOP domain;von Willebrand factor type D domain;Nidogen-like	PLA	1	18	155	High
TRINITY_DN4123_c0_g1_i1_orf1	uncharacterized protein LOC114355030 [Ostrinia furnacalis]	0.458027282	-1.12649456	0.00295	down	yes	0.873	1.906	1.997	1.627	2.094	1	0.748	0.871	------	------	------	------	------	------	------	------	------	------	------	CYT	1	4	15.8	Medium
TRINITY_DN2943_c2_g2_i1_orf1	protein phosphatase inhibitor 2-like [Ostrinia furnacalis]	0.492132393	-1.022881615	0.002206	down	yes	0.907	1.843	1.727	2.073	1.729	1	0.773	0.948	BP:regulation of phosphate metabolic process;BP:regulation of primary metabolic process;BP:regulation of metabolic process;BP:regulation of phosphatase activity;BP:regulation of response to stimulus;BP:regulation of signaling;BP:regulation of biological process;BP:regulation of cell communication;BP:regulation of signal transduction;BP:regulation of protein metabolic process;BP:regulation of macromolecule metabolic process;BP:biological regulation;BP:regulation of protein modification process;BP:regulation of molecular function;BP:regulation of cellular metabolic process;BP:regulation of catalytic activity;BP:regulation of cellular process;BP:regulation of phosphorus metabolic process;BP:biological_process;BP:regulation of nitrogen compound metabolic process;BP:regulation of dephosphorylation;BP:regulation of protein dephosphorylation;BP:regulation of hydrolase activity;BP:regulation of phosphoprotein phosphatase activity;MF:phosphatase regulator activity;MF:molecular_function;MF:enzyme inhibitor activity;MF:molecular function regulator;MF:phosphatase inhibitor activity;MF:protein phosphatase regulator activity;MF:enzyme regulator activity;MF:protein phosphatase inhibitor activity;	GO:0019220;GO:0080090;GO:0019222;GO:0010921;GO:0048583;GO:0023051;GO:0050789;GO:0010646;GO:0009966;GO:0051246;GO:0060255;GO:0065007;GO:0031399;GO:0065009;GO:0031323;GO:0050790;GO:0050794;GO:0051174;GO:0008150;GO:0051171;GO:0035303;GO:0035304;GO:0051336;GO:0043666;GO:0019208;GO:0003674;GO:0004857;GO:0098772;GO:0019212;GO:0019888;GO:0030234;GO:0004864	K16833	PPP1R2, IPP2	------	------	ENOG4111SYV	T:Signal transduction mechanisms	PF04979.17	IPP-2	Protein phosphatase inhibitor 2	CYT	1	9	18.8	High
TRINITY_DN2618_c0_g1_i3_orf1	CDP-diacylglycerol--inositol 3-phosphatidyltransferase [Ostrinia furnacalis]	0.473892894	-1.077367067	3.62E-05	down	yes	0.9203	1.942	1.988	1.882	1.956	1	0.874	0.887	BP:cellular biosynthetic process;BP:organic substance biosynthetic process;BP:organic substance metabolic process;BP:phospholipid metabolic process;BP:lipid metabolic process;BP:cellular process;BP:biosynthetic process;BP:biological_process;BP:metabolic process;BP:cellular lipid metabolic process;BP:organophosphate biosynthetic process;BP:lipid biosynthetic process;BP:primary metabolic process;BP:phospholipid biosynthetic process;BP:cellular metabolic process;BP:phosphate-containing compound metabolic process;BP:phosphorus metabolic process;BP:organophosphate metabolic process;CC:intrinsic component of membrane;CC:integral component of membrane;CC:cellular anatomical entity;CC:cellular_component;MF:CDP-alcohol phosphatidyltransferase activity;MF:molecular_function;MF:transferase activity;MF:CDP-diacylglycerol-inositol 3-phosphatidyltransferase activity;MF:phosphotransferase activity, for other substituted phosphate groups;MF:catalytic activity;MF:transferase activity, transferring phosphorus-containing groups;	GO:0044249;GO:1901576;GO:0071704;GO:0006644;GO:0006629;GO:0009987;GO:0009058;GO:0008150;GO:0008152;GO:0044255;GO:0090407;GO:0008610;GO:0044238;GO:0008654;GO:0044237;GO:0006796;GO:0006793;GO:0019637;GO:0031224;GO:0016021;GO:0110165;GO:0005575;GO:0017169;GO:0003674;GO:0016740;GO:0003881;GO:0016780;GO:0003824;GO:0016772	K00999	CDIPT	map00564;map	Glycerophospholipic	COG0558	I:Lipid transport and metabolism	PF01066.24	CDP-OH_P_transf	CDP-alcohol phosphatidyltransferase	CYT	2	8	24.6	High
TRINITY_DN89083_c0_g1_i1_orf1	lysine-specific demethylase 4A isoform X2 [Diachasma alloeum]	0.457272243	-1.128874747	0.0008235	down	yes	0.8583	1.877	2.051	1.777	1.803	1	0.792	0.783	BP:cellular component organization or biogenesis;BP:cellular component organization;BP:cellular process;BP:biological_process;BP:chromatin organization;CC:intracellular organelle;CC:membrane-bounded organelle;CC:organelle;CC:nucleus;CC:cellular anatomical entity;CC:intracellular membrane-bounded organelle;CC:cellular_component;MF:molecular_function;MF:binding;MF:oxidoreductase activity;MF:cation binding;MF:demethylase activity;MF:oxidoreductase activity, acting on paired donors, with incorporation or reduction of molecular oxygen, 2-oxoglutarate as one donor, and incorporation of one atom each of oxygen into both donors;MF:oxidoreductase activity, acting on paired donors, with incorporation or reduction of molecular oxygen;MF:ion binding;MF:metal ion binding;MF:catalytic activity, acting on a protein;MF:catalytic activity;MF:dioxygenase activity;MF:histone demethylase activity;MF:histone demethylase activit;MF:protein demethylase activity;	GO:0071840;GO:0016043;GO:0009987;GO:0008150;GO:0006325;GO:0043229;GO:0043227;GO:0043226;GO:0005634;GO:0110165;GO:0043231;GO:0005575;GO:0003674;GO:0005488;GO:0016491;GO:0043169;GO:0032451;GO:0016706;GO:0016705;GO:0043167;GO:0046872;GO:0140096;GO:0003824;GO:0051213;GO:0032452;GO:0032454;GO:0140457	K06709	KDM4, JMJD2	------	------	COG5141;ENOG410X	S:Function unknown;	PF18104.4	Tudor_2	Jumonji domain-containing protein 2A Tudor domain	PLA	1	6	12.5	Medium
TRINITY_DN7647_c0_g1_i4_orf1	E3 ubiquitin-protein ligase Bre1 isoform X6 [Ostrinia furnacalis]	2.00772879	1.005564399	0.00431	up	yes	1.143	0.5693	0.609	0.637	0.462	1	1.144	1.284	BP:histone monoubiquitination;BP:nitrogen compound metabolic process;BP:cellular component organization or biogenesis;BP:protein ubiquitination;BP:chromatin organization;BP:protein modification by small protein conjugation or removal;BP:cellular component organization;BP:histone ubiquitination;BP:histone modification;BP:cellular process;BP:protein modification by small protein conjugation;BP:macromolecule modification;BP:protein modification process;BP:biological_process;BP:metabolic process;BP:organic substance metabolic process;BP:primary metabolic process;BP:organonitrogen compound metabolic process;BP:protein metabolic process;BP:macromolecule metabolic process;BP:protein monoubiquitination;CC:intracellular organelle;CC:membrane-bounded organelle;CC:organelle;CC:nucleus;CC:cellular anatomical entity;CC:intracellular membrane-bounded organelle;CC:cellular_component;MF:molecular_function;MF:binding;MF:ubiquitin-like protein transferase activity;MF:cation binding;MF:ion binding;MF:transferase activity;MF:metal ion binding;MF:catalytic activity	GO:0010390;GO:0006807;GO:0071840;GO:0016567;GO:0006325;GO:0070647;GO:0016043;GO:0016574;GO:0016570;GO:0009987;GO:0032446;GO:0043412;GO:0036211;GO:0008150;GO:0008152;GO:0071704;GO:0044238;GO:1901564;GO:0019538;GO:0043170;GO:0006513;GO:0043229;GO:0043227;GO:0043226;GO:0005634;GO:0110165;GO:0043231;GO:0005575;GO:0003674;GO:0005488;GO:0019787;GO:0043169;GO:0043167;GO:0016740;GO:0046872;GO:0140096;GO:0003824;GO:0004842	K10696	BRE1	------	------	ENOG410ZW9A;ENC	O:Posttranslational modification, pro	PF13920.9;PF1633	zf-C3HC4_3;TolA_bind_	Zinc finger, C3HC4 type;TolA binding protein trimerisation;Zinc finger, C3HC4 type	CYT	1	1	105.9	High
TRINITY_DN24917_c0_g2_i1_orf1	hypothetical protein L3Q82_022586 [Scortum barcoo]	2.007092199	1.00510689	0.002235	up	yes	1.132	0.564	0.564	0.493	0.635	1	1.241	1.155	CC:DNA packaging complex;CC:intracellular organelle;CC:nucleosome;CC:membrane-bounded organelle;CC:nucleus;CC:nucleoplasm;CC:cellular anatomical entity;CC:macromolecular complex;CC:intracellular membrane-bounded organelle;CC:protein-DNA complex;CC:cellular_component;CC:organelle;MF:DNA binding;MF:molecular_function;MF:binding;MF:nucleic acid binding;MF:heterocyclic compound binding;MF:structural constituent of chromatin;MF:structural molecule activity;MF:protein binding;MF:organic cyclic compound binding;MF:protein dimerization activity;MF:protein heterodimerization activity;	GO:0044815;GO:0043229;GO:0000786;GO:0043227;GO:0005634;GO:0005654;GO:0110165;GO:0032991;GO:0043231;GO:0032993;GO:0005575;GO:0043226;GO:0003677;GO:0003674;GO:0005488;GO:0003676;GO:1901363;GO:0030527;GO:0005198;GO:0005515;GO:0097159;GO:0046983;GO:0046982	K11254	H4	map05034;map	Alcoholism;Neutropl	COG2036	B:Chromatin structure and dynamics	PF15511.9	CENP-T_C	Centromere kinetochore component CENP-T histone fold	CYT	1	44	13.1	High
TRINITY_DN3638_c0_g1_i1_orf1	DNA replication licensing factor Mcm3 [Ostrinia furnacalis]	2.094138544	1.066356891	0.006144	up	yes	1.179	0.563	0.581	0.585	0.523	1	1.144	1.393	------	------	K02541	MCM3	map04110;map	Cell cycle;DNA replic	COG1241	L:Replication, recombination and rep	PF00493.26;PF172	MCM;MCM_OB;MCM_	MCM P-loop domain;MCM OB domain;MCM AAA-lid domain;MCM N-terminal domain;Magnesium chelatase, subunit ChlI	CYT	1	1	90.7	High
TRINITY_DN18696_c0_g1_i1_orf1	rap guanine nucleotide exchange factor 2 [Ostrinia furnacalis] >XP_028159576.1 rap guanine nucleotide exchange factor 2 [Ostrinia furnacalis]	2.275917385	1.186448189	0.01194	up	yes	1.135	0.4987	0.484	0.47	0.542	1	0.983	1.423	BP:small GTPase mediated signal transduction;BP:cellular process;BP:regulation of cellular process;BP:biological regulation;BP:signal transduction;BP:biological_process;BP:intracellular signal transduction;BP:regulation of biological process;MF:GTPase activator activity;MF:enzyme regulator activity;MF:guanyl-nucleotide exchange factor activity;MF:GTPase regulator activity;MF:molecular_function;MF:molecular function regulator;MF:nucleoside-triphosphatase regulator activity;MF:enzyme activator activity;	GO:0007264;GO:0009987;GO:0050794;GO:0065007;GO:0007165;GO:0008150;GO:0035556;GO:0050789;GO:0005096;GO:0030234;GO:0005085;GO:0030695;GO:0003674;GO:0098772;GO:0060589;GO:0008047	K08018	RAPGEF2, PDZ	map04015;map	Rap1 signaling path	ENOG410XS6B	T:Signal transduction mechanisms	PF00617.22;PF005	RasGEF;PDZ;RasGEF_N	RasGEF domain;PDZ domain;RasGEF N-terminal motif;Ras association;PDZ domain;Peptidase family M50	CYT	1	1	132.4	High
TRINITY_DN7574_c0_g1_i10_orf1	proteasome activator complex subunit 3 isoform X2 [Ostrinia furnacalis]	2.143984221	1.100294288	0.0002742	up	yes	1.087	0.507	0.52	0.48	0.521	1	1.158	1.103	------	------	K06698	PSME3	map03050;map	Proteasome;Antigen	ENOG410XQVX	O:Posttranslational modification, pro	PF02252.21;PF022	PA28_beta;PA28_alpha	Proteasome activator pa28 beta subunit;Proteasome activator pa28 alpha subunit	CYT	3	14	28.3	High
TRINITY_DN33883_c0_g1_i1_orf1	probable 28S ribosomal protein S6, mitochondrial [Ostrinia furnacalis]	2.030017318	1.021492035	0.0001417	up	yes	1.055	0.5197	0.509	0.525	0.525	1	1.039	1.126	BP:cellular biosynthetic process;BP:cellular macromolecule biosynthetic process;BP:organic substance biosynthetic process;BP:cellular nitrogen compound biosynthetic process;BP:cellular macromolecule metabolic process;BP:peptide biosynthetic process;BP:organic substance metabolic process;BP:organonitrogen compound biosynthetic process;BP:amide biosynthetic process;BP:cellular process;BP:biosynthetic process;BP:macromolecule biosynthetic process;BP:biological_process;BP:metabolic process;BP:organonitrogen compound metabolic process;BP:primary metabolic process;BP:protein metabolic process;BP:cellular nitrogen compound metabolic process;BP:cellular metabolic process;BP:macromolecule metabolic process;BP:nitrogen compound metabolic process;BP:cellular amide metabolic process;BP:peptide metabolic process;BP:translation;CC:intracellular organelle;CC:non-membrane-bounded organelle;CC:organelle;CC:cellular anatomical entity;CC:macromolecular complex;CC:ribosome;CC:intracellular non-membrane-bounded organelle;CC:ribonucleoprotein complex;CC:cellular_component;MF:nucleic acid binding;MF:heterocyclic compound binding;MF:molecular_function;MF:binding;MF:rRNA binding;MF:structural molecule activity;MF:structural constituent of ribosome;MF:RNA binding;MF:organic cyclic compound binding;	GO:0044249;GO:0034645;GO:1901576;GO:0044271;GO:0044260;GO:0043043;GO:0071704;GO:1901566;GO:0043604;GO:0009987;GO:0009058;GO:0009059;GO:0008150;GO:0008152;GO:1901564;GO:0044238;GO:0019538;GO:0034641;GO:0044237;GO:0043170;GO:0006807;GO:0043603;GO:0006518;GO:0006412;GO:0043229;GO:0043228;GO:0043226;GO:0110165;GO:0032991;GO:0005840;GO:0043232;GO:1990904;GO:0005575;GO:0003676;GO:1901363;GO:0003674;GO:0005488;GO:0019843;GO:0005198;GO:0003735;GO:0003723;GO:0097159	K02990	RP-S6, MRPS(	map03010	Ribosome	COG0360	J:Translation, ribosomal structure anc	PF01250.20	Ribosomal_S6	Ribosomal protein S6	CYT	2	17	17.7	High
TRINITY_DN57798_c0_g1_i1_orf1	ubiquitin carboxyl-terminal hydrolase 36 [Ostrinia furnacalis]	3.14271382	1.652010904	0.002882	up	yes	1.253	0.3987	0.459	0.372	0.365	1	1.346	1.412	BP:cellular catabolic process;BP:nitrogen compound metabolic process;BP:modification-dependent macromolecule catabolic process;BP:organic substance catabolic process;BP:cellular macromolecule catabolic process;BP:cellular macromolecule metabolic process;BP:protein modification by small protein conjugation or removal;BP:protein modification by small protein removal;BP:organic substance metabolic process;BP:proteolysis;BP:protein deubiquitination;BP:primary metabolic process;BP:cellular process;BP:modification-dependent protein catabolic process;BP:macromolecule modification;BP:protein modification process;BP:biological_process;BP:metabolic process;BP:catabolic process;BP:macromolecule catabolic process;BP:proteolysis involved in cellular protein catabolic process;BP:organonitrogen compound metabolic process;BP:protein metabolic process;BP:cellular metabolic process;BP:macromolecule metabolic process;BP:ubiquitin-dependent protein catabolic process;MF:ubiquitinyl hydrolase activity;MF:molecular_function;MF:cysteine-type peptidase activity;MF:ubiquitin-like protein-specific protease activity;MF:thiol-dependent ubiquitin-specific protease activity;MF:hydrolase activity;MF:catalytic activity, acting on a protein;MF:catalytic activity;MF:peptidase activity;	GO:0044248;GO:0006807;GO:0043632;GO:1901575;GO:0044265;GO:0044260;GO:0070647;GO:0070646;GO:0071704;GO:0006508;GO:0016579;GO:0044238;GO:0009987;GO:0019941;GO:0043412;GO:0036211;GO:0008150;GO:0008152;GO:0009056;GO:0009057;GO:0051603;GO:1901564;GO:0019538;GO:0044237;GO:0043170;GO:0006511;GO:0101005;GO:0003674;GO:0008234;GO:0019783;GO:0004843;GO:0016787;GO:0140096;GO:0003824;GO:0008233	K11855	USP36_42	------	------	ENOG410XQ92	O:Posttranslational modification, pro	PF00443.32;PF134	UCH;UCH_1	Ubiquitin carboxyl-terminal hydrolase;Ubiquitin carboxyl-terminal hydrolase	CYT	1	2	79	High
TRINITY_DN14730_c0_g1_i7_orf1	titin homolog [Ostrinia furnacalis]	2.276767677	1.186987085	0.001244	up	yes	1.127	0.495	0.408	0.554	0.523	1	1.18	1.201	------	------	------	------	------	------	ENOG410ZVTG;ENO	S:Function unknown;	PF07679.19;PF139	I-set;Ig_3;Ig_2	Immunoglobulin I-set domain;Immunoglobulin domain;Immunoglobulin domain	CYT	1	0	324.7	High
TRINITY_DN3299_c0_g1_i2_orf1	metaxin-1 isoform X3 [Ostrinia furnacalis] >XP_028170907.1 metaxin-1 isoform X4 [Ostrinia furnacalis]	2.748826291	1.45881574	0.002783	up	yes	1.171	0.426	0.516	0.345	0.417	1	1.353	1.16	BP:nitrogen compound transport;BP:macromolecule localization;BP:protein localization;BP:cellular macromolecule localization;BP:cellular process;BP:establishment of protein localization;BP:biological_process;BP:transport;BP:organic substance transport;BP:protein transport;BP:establishment of localization;BP:localization;BP:cellular localization;CC:macromolecular complex;CC:membrane protein complex;CC:mitochondrial sorting and assembly machinery complex;CC:cellular_component;CC:mitochondrial outer membrane translocase complex;CC:mitochondrial protein complex;CC:outer mitochondrial membrane protein complex;	GO:0071705;GO:0033036;GO:0008104;GO:0070727;GO:0009987;GO:0045184;GO:0008150;GO:0006810;GO:0071702;GO:0015031;GO:0051234;GO:0051179;GO:0051641;GO:0032991;GO:0098796;GO:0001401;GO:0005575;GO:0005742;GO:0098798;GO:0098799	K17776	MTX	------	------	ENOG41100SS	U:Intracellular trafficking, secretion, a	PF10568.12;PF171	Tom37;GST_C_6;GST_N	Outer mitochondrial membrane transport complex protein;Glutathione S-transferase, C-terminal domain;Glutathione S-transferase N-terminal domain;Glutathione S-transferase, C-terminal domain	CYT	1	4	36.5	High
TRINITY_DN3814_c1_g1_i1_orf1	39S ribosomal protein L11, mitochondrial [Ostrinia furnacalis]	2.319787625	1.213992734	0.006891	up	yes	1.136	0.4897	0.528	0.536	0.405	1	1.373	1.035	BP:cellular biosynthetic process;BP:cellular nitrogen compound metabolic process;BP:nitrogen compound metabolic process;BP:cellular macromolecule biosynthetic process;BP:organic substance biosynthetic process;BP:cellular nitrogen compound biosynthetic process;BP:cellular macromolecule metabolic process;BP:peptide biosynthetic process;BP:organic substance metabolic process;BP:amide biosynthetic process;BP:cellular process;BP:biosynthetic process;BP:macromolecule biosynthetic process;BP:biological_process;BP:metabolic process;BP:organonitrogen compound metabolic process;BP:primary metabolic process;BP:cellular amide metabolic process;BP:organonitrogen compound biosynthetic process;BP:protein metabolic process;BP:cellular metabolic process;BP:macromolecule metabolic process;BP:peptide metabolic process;BP:translation;CC:intracellular non-membrane-bounded organelle;CC:organelle;CC:cellular anatomical entity;CC:ribosome;CC:macromolecular complex;CC:ribonucleoprotein complex;CC:intracellular organelle;CC:cellular_component;CC:non-membrane-bounded organelle;MF:molecular_function;MF:structural molecule activity;MF:structural constituent of ribosome;	GO:0044249;GO:0034641;GO:0006807;GO:0034645;GO:1901576;GO:0044271;GO:0044260;GO:0043043;GO:0071704;GO:0043604;GO:0009987;GO:0009058;GO:0009059;GO:0008150;GO:0008152;GO:1901564;GO:0044238;GO:0043603;GO:1901566;GO:0019538;GO:0044237;GO:0043170;GO:0006518;GO:0006412;GO:0043232;GO:0043226;GO:0110165;GO:0005840;GO:0032991;GO:1990904;GO:0043229;GO:0005575;GO:0043228;GO:0003674;GO:0005198;GO:0003735	K02867	RP-L11, MRPL	map03010	Ribosome	COG0080	J:Translation, ribosomal structure anc	PF00298.22;PF039	Ribosomal_L11;Ribosor	Ribosomal protein L11, RNA binding domain;Ribosomal protein L11, N-terminal domain	CYT	1	5	22	High
TRINITY_DN142652_c0_g1_i1_orf1	pre-mRNA-splicing factor RBM22 [Chelonus insularis]	2.364562118	1.241573043	0.003352	up	yes	1.161	0.491	0.43	0.607	0.436	1	1.17	1.312	CC:macromolecular complex;CC:cellular_component;CC:ribonucleoprotein complex;CC:spliceosomal complex;MF:cation binding;MF:organic cyclic compound binding;MF:ion binding;MF:molecular_function;MF:RNA binding;MF:nucleic acid binding;MF:metal ion binding;MF:heterocyclic compound binding;MF:binding;	GO:0032991;GO:0005575;GO:1990904;GO:0005681;GO:0043169;GO:0097159;GO:0043167;GO:0003674;GO:0003723;GO:0003676;GO:0046872;GO:1901363;GO:0005488	K12872	RBM22, SLT11	map03040	Spliceosome	ENOG410XSFK	S:Function unknown	PF00076.25;PF183	RRM_1;zf_CCCH_4	RNA recognition motif.;Zinc finger domain	CYT	1	2	45.3	High
TRINITY_DN52893_c0_g1_i1_orf1	growth arrest and DNA damage-inducible proteins-interacting protein 1 [Galleria mellonella]	2.113148039	1.07939384	0.000537	up	yes	1.072	0.5073	0.543	0.541	0.438	1	1.151	1.065	BP:cellular process;BP:biological_process;BP:cell cycle;CC:intracellular membrane-bounded organelle;CC:nucleus;CC:mitochondrion;CC:cellular_component;CC:intracellular organelle;CC:membrane-bounded organelle;CC:organelle;CC:cellular anatomical entity;	GO:0009987;GO:0008150;GO:0007049;GO:0043231;GO:0005634;GO:0005739;GO:0005575;GO:0043229;GO:0043227;GO:0043226;GO:0110165	------	------	------	------	ENOG410XVD1	S:Function unknown	PF10147.12	CR6_interact	Growth arrest and DNA-damage-inducible proteins-interacting protein 1	CYT	3	13	29.1	High
TRINITY_DN49742_c0_g1_i4_orf1	neuroglobin-like [Ostrinia furnacalis]	2.135758485	1.094748514	0.002067	up	yes	1.139	0.5333	0.539	0.498	0.563	1	1.287	1.129	MF:oxygen binding;MF:heme binding;MF:tetrapyrrole binding;MF:organic cyclic compound binding;MF:small molecule binding;MF:molecular_function;MF:binding;MF:heterocyclic compound binding;	GO:0019825;GO:0020037;GO:0046906;GO:0097159;GO:0036094;GO:0003674;GO:0005488;GO:1901363	K21893	NGB	------	------	ENOG4111QDK	S:Function unknown	PF00042.25	Globin	Globin	CYT	1	11	21.5	High
TRINITY_DN747_c0_g1_i1_orf1	trypsin, alkaline C-like [Ostrinia furnacalis]	2.204408818	1.140391803	0.001348	up	yes	1.1	0.499	0.516	0.454	0.527	1	1.061	1.24	BP:organic substance metabolic process;BP:organonitrogen compound metabolic process;BP:protein metabolic process;BP:macromolecule metabolic process;BP:biological_process;BP:nitrogen compound metabolic process;BP:metabolic process;BP:proteolysis;BP:primary metabolic process;MF:serine-type endopeptidase activity;MF:endopeptidase activity;MF:hydrolase activity;MF:serine hydrolase activity;MF:catalytic activity, acting on a protein;MF:catalytic activity;MF:molecular_function;MF:peptidase activity;MF:serine-type peptidase activity;	GO:0071704;GO:1901564;GO:0019538;GO:0043170;GO:0008150;GO:0006807;GO:0008152;GO:0006508;GO:0044238;GO:0004252;GO:0004175;GO:0016787;GO:0017171;GO:0140096;GO:0003824;GO:0003674;GO:0008233;GO:0008236	K09628;K09640	PRSS27;TMPR	------	------	COG5640	O:Posttranslational modification, pro	PF00089.29	Trypsin	Trypsin	CYT	1	6	30.5	High
TRINITY_DN72934_c0_g1_i1_orf1	carboxylesterase 8 [Streltzoviella insularis]	2.655963303	1.409235213	0.001102	up	yes	1.158	0.436	0.463	0.42	0.425	1	1.291	1.183	MF:hydrolase activity;MF:molecular_function;MF:hydrolase activity, acting on ester bonds;MF:carboxylic ester hydrolase activity;MF:catalytic activity;	GO:0016787;GO:0003674;GO:0016788;GO:0052689;GO:0003824	------	------	------	------	COG2272	I:Lipid transport and metabolism	PF00135.31;PF204	COesterase;BD-FAE;Ab	Carboxylesterase family;BD-FAE;alpha/beta hydrolase	CYT	1	5	61.5	High
TRINITY_DN47723_c0_g1_i1_orf1	dnaJ homolog subfamily C member 21 [Ostrinia furnacalis]	2.0760727	1.053856965	0.004065	up	yes	1.108	0.5337	0.518	0.527	0.556	1	1.3	1.024	MF:cation binding;MF:transition metal ion binding;MF:organic cyclic compound binding;MF:ion binding;MF:molecular_function;MF:binding;MF:nucleic acid binding;MF:metal ion binding;MF:heterocyclic compound binding;MF:zinc ion binding;	GO:0043169;GO:0046914;GO:0097159;GO:0043167;GO:0003674;GO:0005488;GO:0003676;GO:0046872;GO:1901363;GO:0008270	K09506	DNAJA5	------	------	ENOG410XNV5	O:Posttranslational modification, pro	PF00226.34;PF128	DnaJ;zf-met;zf-C2H2_j	DnaJ domain;Zinc-finger of C2H2 type;Zinc-finger double-stranded RNA-binding;C2H2 type zinc-	CYT	1	1	69.6	High
TRINITY_DN11457_c0_g1_i5_orf1	uncharacterized protein LOC114352268 [Ostrinia furnacalis]	2.136372416	1.095163162	0.001933	up	yes	1.106	0.5177	0.565	0.566	0.422	1	1.226	1.091	------	------	------	------	------	------	ENOG41121MH	S:Function unknown	------	------	------	PLA	1	13	14.3	High

TRINITY_DN21181_c0_g1_i6_orf1	unnamed protein product, partial [Brenthis ino]	2.125968992	1.088120555	0.0006467	up	yes	1.097	0.516	0.469	0.572	0.507	1	1.111	1.179	BP:phosphorylation;BP:nitrogen compound metabolic process;BP:organic substance metabolic process;BP:protein phosphorylation;BP:cellular process;BP:macromolecule modification;BP:protein modification process;BP:biological_process;BP:metabolic process;BP:primary metabolic process;BP:organonitrogen compound metabolic process;BP:protein metabolic process;BP:cellular metabolic process;BP:macromolecule metabolic process;BP:phosphate-containing compound metabolic process;BP:phosphorus metabolic process;MF:purine ribonucleoside triphosphate binding;MF:molecular_function;MF:binding;MF:ion binding;MF:heterocyclic compound binding;MF:nucleoside phosphate binding;MF:anion binding;MF:carbohydrate derivative binding;MF:nucleotide binding;MF:protein serine/threonine kinase activity;MF:transferase activity;MF:protein kinase activity;MF:purine nucleotide binding;MF:AMP-activated protein kinase activity;MF:ATP binding;MF:kinase activity;MF:catalytic activity, acting on a protein;MF:small molecule binding;MF:catalytic activity;MF:adenyl nucleotide binding;MF:transferase activity, transferring phosphorus-containing groups;MF:phosphotransferase activity, alcohol group as acceptor;MF:adenyl ribonucleotide binding;MF:purine ribonucleotide binding;MF:organic cyclic compound binding;MF:ribonucleotide binding;	GO:0016310;GO:0006807;GO:0071704;GO:0006468;GO:0009987;GO:0043412;GO:0036211;GO:0008150;GO:0008152;GO:0044238;GO:1901564;GO:0019538;GO:0044237;GO:0043170;GO:0006796;GO:0006793;GO:0035639;GO:0003674;GO:0005488;GO:0043167;GO:1901363;GO:1901265;GO:0043168;GO:0097367;GO:0000166;GO:0004674;GO:0016740;GO:0004672;GO:0017076;GO:0004679;GO:0005524;GO:0016301;GO:0140096;GO:0036094;GO:0003824;GO:0030554;GO:0016772;GO:0016773;GO:0032559;GO:0032555;GO:0097159;GO:0032553	K07198	PRKAA, AMPK	map04152;map	AMPK signaling patl	ENOG410XNQ0	O:Posttranslational modification, pro	PF00069.28;PF077	Pkinase;PK_Tyr_Ser-Thr	Protein kinase domain;Protein tyrosine and serine/threonine kinase;Adenylate sensor of SNF1-like protein kinase;Kinase-like	CYT	1	3	58.3	High
TRINITY_DN71465_c0_g1_i1_orf1	adenylate kinase isoenzyme 6 [Ostrinia furnacalis]	2.005597015	1.004031754	0.001081	up	yes	1.075	0.536	0.578	0.533	0.497	1	1.192	1.032	BP:phosphorylation;BP:cellular process;BP:phosphorus metabolic process;BP:biological_process;BP:metabolic process;BP:cellular metabolic process;BP:phosphate-containing compound metabolic process;CC:intracellular organelle;CC:membrane-bounded organelle;CC:organelle;CC:nucleus;CC:intracellular membrane-bounded organelle;CC:cellular_component;CC:cellular anatomical entity;MF:nucleobase-containing compound kinase activity;MF:purine ribonucleoside triphosphate binding;MF:heterocyclic compound binding;MF:adenylate kinase activity;MF:phosphotransferase activity, phosphate group as acceptor;MF:molecular_function;MF:binding;MF:ATPase activity;MF:ion binding;MF:nucleoside phosphate binding;MF:anion binding;MF:nucleotide binding;MF:transferase activity;MF:purine nucleotide binding;MF:ATP binding;MF:hydrolase activity;MF:kinase activity;MF:nucleoside-triphosphatase activity;MF:small molecule binding;MF:catalytic activity;MF:hydrolase activity, acting on acid anhydrides, in phosphorus-containing anhydrides;MF:adenyl nucleotide binding;MF:carbohydrate derivative binding;MF:organic cyclic compound binding;MF:hydrolase activity, acting on acid anhydrides;MF:pyrophosphatase activity;MF:nucleoside phosphate kinase activity;MF:adenyl ribonucleotide binding;MF:transferase activity, transferring phosphorus-containing groups;MF:purine ribonucleotide binding;MF:ribonucleotide binding;	GO:0016310;GO:0009987;GO:0006793;GO:0008150;GO:0008152;GO:0044237;GO:0006796;GO:0043229;GO:0043227;GO:0043226;GO:0005634;GO:0043231;GO:0005575;GO:0110165;GO:0019205;GO:0035639;GO:1901363;GO:0004017;GO:0016776;GO:0003674;GO:0005488;GO:0016887;GO:0043167;GO:1901265;GO:0043168;GO:0000166;GO:0016740;GO:0017076;GO:0005524;GO:0016787;GO:0016301;GO:0017111;GO:0036094;GO:0003824;GO:0016818;GO:0030554;GO:0097367;GO:0097159;GO:0016817;GO:0016462;GO:0050145;GO:0032559;GO:0016772;GO:0032555;GO:0032553	K18532	AK6, FAP7	map03008;map	Ribosome biogenesi	COG1936	F:Nucleotide transport and metabolis	PF13238.9	AAA_18	AAA domain	CYT	1	9	20.6	High
TRINITY_DN3889_c0_g1_i7_orfp1	TRINITY_DN3889_c0_g1_i7_m.1657 TRINITY_DN3889_c0_g1::TRINITY_DN3889_c0_g1_i7::g.1657 ORF type:5prime_partial len:235 (+),score=70.90 TRINITY_DN3889_c0_g1_i7:1-705(+)	6.017467249	2.589156385	0.006631	up	yes	1.378	0.229	0.139	0.22	0.328	1	1.745	1.388	------	------	------	------	------	------	------	------	------	------	------	EXC	1	38	24.3	High
TRINITY_DN3027_c0_g1_i4_orf1	ribosome biogenesis protein BOP1 homolog [Ostrinia furnacalis]	2.088423502	1.062414299	0.0002845	up	yes	1.077	0.5157	0.505	0.539	0.503	1	1.158	1.073	------	------	K14824	ERB1, BOP1	------	------	ENOG410XRBX	J:Translation, ribosomal structure anc	PF08145.15;PF004	BOP1NT;WD40;ANAPC	BOP1NT;WD domain, G-beta repeat;Anaphase-promoting complex subunit 4 WD40 domain	CYT	4	6	95.8	High
TRINITY_DN1868_c0_g1_i1_orf1	protein obstructor-E isoform X1 [Ostrinia furnacalis]	2.071138211	1.050423831	6.60E-06	up	yes	1.019	0.492	0.482	0.482	0.512	1	1.046	1.011	------	------	------	------	------	------	ENOG410XQ49	S:Function unknown	PF01607.27	CBM_14	Chitin binding Peritrophin-A domain	CYT	16	68	30.6	High
TRINITY_DN43328_c0_g1_i1_orf1	tubulin--tyrosine ligase-like protein 12 [Ostrinia furnacalis]	2.148535565	1.103353658	0.003162	up	yes	1.027	0.478	0.604	0.485	0.345	1	1.112	0.969	------	------	K16609	TTLL12	------	------	ENOG410XO0A	S:Function unknown	PF03133.18	TTL	Tubulin-tyrosine ligase family	CYT	1	2	71.9	High
TRINITY_DN31851_c0_g1_i2_orf1	CDGSH iron-sulfur domain-containing protein 2 homolog [Helicoverpa armigera] >PZC85510.1 hypothetical protein B5X24_HaOG216618 [Helicoverpa armigera]	2.040567951	1.028970753	4.87E-07	up	yes	1.006	0.493	0.486	0.495	0.498	1	1.022	0.997	BP:regulation of catabolic process;BP:regulation of cellular catabolic process;BP:regulation of cellular metabolic process;BP:regulation of biological process;BP:biological regulation;BP:regulation of autophagy;BP:regulation of cellular process;BP:biological_process;BP:regulation of metabolic process;CC:endoplasmic reticulum membrane;CC:intracellular organelle;CC:membrane-bounded organelle;CC:organelle;CC:intrinsic component of membrane;CC:organelle membrane;CC:integral component of membrane;CC:membrane;CC:cellular anatomical entity;CC:intracellular membrane-bounded organelle;CC:cellular_component;MF:molecular_function;MF:binding;MF:cation binding;MF:ion binding;MF:metal ion binding;MF:metal cluster binding;MF:iron-sulfur cluster binding;MF:2 iron, 2 sulfur cluster binding;	GO:0009894;GO:0031329;GO:0031323;GO:0050789;GO:0065007;GO:0010506;GO:0050794;GO:0008150;GO:0019222;GO:0005789;GO:0043229;GO:0043227;GO:0043226;GO:0031224;GO:0031090;GO:0016021;GO:0016020;GO:0110165;GO:0043231;GO:0005575;GO:0003674;GO:0005488;GO:0043169;GO:0043167;GO:0046872;GO:0051540;GO:0051536;GO:0051537	K23882	CISD2	------	------	ENOG41122II	S:Function unknown	PF10660.12;PF093	MitoNEET_N;zf-CDGSH	Iron-containing outer mitochondrial membrane protein N-terminus;Iron-binding zinc finger CDGSH type	CYT	5	38	14.7	High
TRINITY_DN4451_c0_g1_i1_orf1	uncharacterized protein LOC114361986 isoform X1 [Ostrinia furnacalis] >XP_028173022.1 uncharacterized protein LOC114361986 isoform X2 [Ostrinia furnacalis]	2.324433475	1.216879137	1.29E-05	up	yes	1.036	0.4457	0.453	0.44	0.444	1	1.032	1.077	BP:carboxylic acid metabolic process;BP:organonitrogen compound metabolic process;BP:cellular amino acid catabolic process;BP:nitrogen compound metabolic process;BP:small molecule catabolic process;BP:cellular amino acid metabolic process;BP:organic substance metabolic process;BP:alpha-amino acid metabolic process;BP:alpha-amino acid catabolic process;BP:cellular process;BP:organic substance catabolic process;BP:biological_process;BP:metabolic process;BP:oxoacid metabolic process;BP:catabolic process;BP:cellular catabolic process;BP:organonitrogen compound catabolic process;BP:primary metabolic process;BP:valine metabolic process;BP:valine catabolic process;BP:organic acid metabolic process;BP:organic acid catabolic process;BP:cellular metabolic process;BP:carboxylic acid catabolic process;BP:branched-chain amino acid metabolic process;BP:branched-chain amino acid catabolic process;BP:small molecule metabolic process;MF:NAD binding;MF:molecular_function;MF:binding;MF:heterocyclic compound binding;MF:nucleoside phosphate binding;MF:oxidoreductase activity;MF:NADP binding;MF:3-hydroxyisobutyrate dehydrogenase activity;MF:nucleotide binding;MF:small molecule binding;MF:catalytic activity;MF:oxidoreductase activity, acting on CH-OH group of donors;MF:oxidoreductase activity, acting on the CH-OH group of donors, NAD or NADP as acceptor;MF:organic cyclic compound binding;	GO:0019752;GO:1901564;GO:0009063;GO:0006807;GO:0044282;GO:0006520;GO:0071704;GO:1901605;GO:1901606;GO:0009987;GO:1901575;GO:0008150;GO:0008152;GO:0043436;GO:0009056;GO:0044248;GO:1901565;GO:0044238;GO:0006573;GO:0006574;GO:0006082;GO:0016054;GO:0044237;GO:0046395;GO:0009081;GO:0009083;GO:0044281;GO:0051287;GO:0003674;GO:0005488;GO:1901363;GO:1901265;GO:0016491;GO:0050661;GO:0008442;GO:0000166;GO:0036094;GO:0003824;GO:0016614;GO:0016616;GO:0097159	K23146	HPD1	map00280;map	Valine, leucine and i	COG2084	I:Lipid transport and metabolism	PF14833.9;PF0344	NAD_binding_11;NAD_	NAD-binding of NADP-dependent 3-hydroxyisobutyrate dehydrogenase;NAD binding domain of 6-phosphogluconate dehydrogenase	CYT	3	39	20.6	High
TRINITY_DN1578_c0_g3_i1_orf1	S-adenosylmethionine synthase isoform X1 [Ostrinia furnacalis]	2.324275769	1.216781251	1.70E-05	up	yes	1.035	0.4453	0.424	0.455	0.457	1	1.075	1.03	BP:one-carbon metabolic process;BP:cellular biosynthetic process;BP:small molecule metabolic process;BP:organic substance biosynthetic process;BP:organic substance metabolic process;BP:sulfur compound metabolic process;BP:S-adenosylmethionine metabolic process;BP:cellular process;BP:biosynthetic process;BP:biological_process;BP:metabolic process;BP:sulfur compound biosynthetic process;BP:S-adenosylmethionine biosynthetic process;BP:cellular metabolic process;MF:purine ribonucleoside triphosphate binding;MF:molecular_function;MF:nucleotide binding;MF:heterocyclic compound binding;MF:nucleoside phosphate binding;MF:anion binding;MF:transferase activity, transferring alkyl or ary;MF:ion binding;MF:transferase activity;MF:metal ion binding;MF:ATP binding;MF:cation binding;MF:small molecule binding;MF:catalytic activity;MF:purine nucleotide binding;MF:methionine adenosyltransferase activity;MF:organic cyclic compound binding;MF:adenyl nucleotide binding;MF:carbohydrate derivative binding;MF:adenyl ribonucleotide binding;MF:purine ribonucleotide binding;MF:ribonucleotide binding;MF:binding;	GO:0006730;GO:0044249;GO:0044281;GO:1901576;GO:0071704;GO:0006790;GO:0046500;GO:0009987;GO:0009058;GO:0008150;GO:0008152;GO:0044272;GO:0006556;GO:0044237;GO:0035639;GO:0003674;GO:0000166;GO:1901363;GO:1901265;GO:0043168;GO:0016765;GO:0043167;GO:0016740;GO:0046872;GO:0005524;GO:0043169;GO:0036094;GO:0003824;GO:0017076;GO:0004478;GO:0097159;GO:0030554;GO:0097367;GO:0032559;GO:0032555;GO:0032553;GO:0005488	K00789	metK, MAT	map00270;map	Cysteine and methic	COG0192	H:Coenzyme transport and metabolis	PF02772.19;PF004	S-AdoMet_synt_M;S-A	S-adenosylmethionine synthetase, central domain;S-adenosylmethionine synthetase, N-terminal domain	CYT	7	42	28.2	High
TRINITY_DN12683_c0_g1_i3_orf1	sulfated surface glycoprotein 185-like [Ostrinia furnacalis]	2.128336536	1.08972629	1.36E-06	up	yes	0.9967	0.4683	0.479	0.479	0.447	1	1.002	0.988	------	------	------	------	------	------	ENOG4110OPT;ENO	S:Function unknown;	PF11901.11	DUF3421	Protein of unknown function	CYT	2	9	27.1	High
TRINITY_DN21150_c0_g1_i4_orf1	RNA-binding protein cabeza-like isoform X2 [Bicyclus anynana]	2.158771361	1.110210453	1.13E-07	up	yes	0.998	0.4623	0.469	0.458	0.46	1	1.006	0.988	BP:regulation of primary metabolic process;BP:regulation of metabolic process;BP:regulation of cellular biosynthetic process;BP:regulation of cellular metabolic process;BP:regulation of biological process;BP:regulation of macromolecule metabolic process;BP:biological regulation;BP:regulation of gene expression;BP:regulation of nucleobase-containing compound metabolic process;BP:regulation of biosynthetic process;BP:regulation of nucleic acid-templated transcription;BP:regulation of cellular process;BP:biological_process;BP:regulation of nitrogen compound metabolic process;BP:regulation of RNA biosynthetic process;BP:regulation of RNA metabolic process;BP:regulation of transcription, DNA-templated;BP:regulation of macromolecule biosynthetic process;MF:molecular_function;MF:binding;MF:nucleic acid binding;MF:heterocyclic compound binding;MF:organic cyclic compound binding;MF:RNA binding;	GO:0080090;GO:0019222;GO:0031326;GO:0031323;GO:0050789;GO:0060255;GO:0065007;GO:0010468;GO:0019219;GO:0009889;GO:1903506;GO:0050794;GO:0008150;GO:0051171;GO:2001141;GO:0051252;GO:0006355;GO:0010556;GO:0003674;GO:0005488;GO:0003676;GO:1901363;GO:0097159;GO:0003723	K13098	TLS, FUS	map03015;map	mRNA surveillance p	ENOG4111Q2F;ENO	S:Function unknown;	PF00076.25	RRM_1	RNA recognition motif.	CYT	3	31	13.2	High
TRINITY_DN8536_c0_g1_i2_orf1	PC4 and SFRS1-interacting protein isoform X4 [Galleria mellonella]	2.028464575	1.020388108	0.0002191	up	yes	0.9763	0.4813	0.515	0.442	0.487	1	1.017	0.912	------	------	K25057	PSIP1, LEDGF	map03250	Viral life cycle - HIV-	ENOG4111K9I	S:Function unknown	PF11467.11	LEDGF	Lens epithelium-derived growth factor	CYT	2	6	31.1	High
TRINITY_DN80560_c0_g1_i1_orf1	ATP synthase subunit alpha, mitochondrial [Ostrinia furnacalis]	2.252197431	1.171333302	3.39E-08	up	yes	0.9993	0.4437	0.442	0.438	0.451	1	1.004	0.994	CC:proton-transporting ATP synthase complex, catalytic core;CC:proton-transporting two-sector ATPase complex, catalytic domain;CC:macromolecular complex;CC:cellular_component;CC:membrane protein complex;MF:cation channel activity;MF:purine ribonucleoside triphosphate binding;MF:molecular_function;MF:binding;MF:ion binding;MF:heterocyclic compound binding;MF:nucleoside phosphate binding;MF:proton-transporting ATP synthase activity, rotational mechanism;MF:inorganic solute uptake transmembrane transporter activity;MF:nucleotide binding;MF:hydrogen ion channel activity;MF:purine nucleotide binding;MF:ATP binding;MF:anion binding;MF:transporter activity;MF:ion channel activity;MF:cation transmembrane transporter activity;MF:small molecule binding;MF:catalytic activity;MF:passive transmembrane transporter activity;MF:ligase activity;MF:inorganic cation transmembrane transporter activity;MF:adenyl nucleotide binding;MF:carbohydrate derivative binding;MF:organic cyclic compound binding;MF:ion transmembrane transporter activity;MF:transmembrane transporter activity;MF:channel activity;MF:adenyl ribonucleotide binding;MF:purine ribonucleotide binding;MF:hydrogen ion transmembrane transporter	GO:0045261;GO:0033178;GO:0032991;GO:0005575;GO:0098796;GO:0005261;GO:0035639;GO:0003674;GO:0005488;GO:0043167;GO:1901363;GO:1901265;GO:0046933;GO:0015318;GO:0000166;GO:0015252;GO:0017076;GO:0005524;GO:0043168;GO:0005215;GO:0005216;GO:0008324;GO:0036094;GO:0003824;GO:0022803;GO:0016874;GO:0022890;GO:0030554;GO:0097367;GO:0097159;GO:0015075;GO:0022857;GO:0015267;GO:0032559;GO:0032555;GO:0015078;GO:0032553	K02132	ATPeF1A, ATF	map04714;map	Thermogenesis;Cher	COG0056	C:Energy production and conversion	PF00006.28;PF003	ATP-synt_ab;ATP-synt_	ATP synthase alpha/beta family, nucleotide-binding domain;ATP synthase alpha/beta chain, C terminal domain;ATP synthase alpha/beta family, beta-barrel domain	CYT	23	45	59.4	High
TRINITY_DN24873_c0_g1_i4_orf1	uncharacterized protein LOC114365742 [Ostrinia furnacalis]	2.46746988	1.303032474	2.99E-06	up	yes	1.024	0.415	0.433	0.397	0.415	1	1.029	1.042	CC:endoplasmic reticulum membrane;CC:intrinsic component of membrane;CC:organelle membrane;CC:integral component of membrane;CC:membrane;CC:cellular anatomical entity;CC:cellular_component;MF:monooxygenase activity;MF:oxidoreductase activity, acting on paired donors, with incorporation or reduction of molecular oxygen;MF:molecular_function;MF:binding;MF:heterocyclic compound binding;MF:oxidoreductase activity;MF:cation binding;MF:transition metal ion binding;MF:ion binding;MF:iron ion binding;MF:metal ion binding;MF:heme binding;MF:catalytic activity;MF:tetrapyrrole	GO:0005789;GO:0031224;GO:0031090;GO:0016021;GO:0016020;GO:0110165;GO:0005575;GO:0004497;GO:0016705;GO:0003674;GO:0005488;GO:1901363;GO:0016491;GO:0043169;GO:0046914;GO:0043167;GO:0005506;GO:0046872;GO:0020037;GO:0003824;GO:0046906;GO:0097159	K15001	CYP4	------	------	COG2124	S:Function unknown	PF00067.25	p450	Cytochrome P450	CYT	2	3	59.3	High
TRINITY_DN75746_c0_g1_i1_orfp1	TRINITY_DN75746_c0_g1_i1_m.54871 TRINITY_DN75746_c0_g1::TRINITY_DN75746_c0_g1_i1::g.54871 ORF type:internal len:83 (+),score=-1.09,Polyhedrin PF00738.19 1.7e-25 TRINITY_DN75746_c0_g1_i1:2-247(+)	4.124152694	2.044097748	0.0005314	up	yes	1.156	0.2803	0.266	0.306	0.269	1	1.294	1.174	------	------	------	------	------	------	------	------	PF00738.21	Polyhedrin	Polyhedrin	CYT	2	29	9.6	High
TRINITY_DN1803_c0_g1_i3_orf1	translocator protein-like isoform X1 [Ostrinia furnacalis] >XP_028178947.1 translocator protein-like isoform X1 [Ostrinia furnacalis]	2.010526316	1.00757322	8.29E-05	up	yes	0.955	0.475	0.494	0.455	0.476	1	0.959	0.906	CC:cellular_component;CC:integral component of membrane;CC:cellular anatomical entity;CC:intrinsic component of membrane;	GO:0005575;GO:0016021;GO:0110165;GO:0031224	K05770	TSPO, BZRP	map05166;map	Human T-cell leuker	COG3476;ENOG410X	T:Signal transduction mechanisms;S:f	PF03073.18	TspO_MBR	TspO/MBR family	CYT	3	12	21.3	High
TRINITY_DN26186_c0_g1_i7_orf1	sodium- and chloride-dependent glycine transporter 1-like [Ostrinia furnacalis]	2.470245324	1.304654325	0.001243	up	yes	1.017	0.4117	0.349	0.337	0.549	1	0.978	1.072	CC:integral component of membrane;CC:cellular_component;CC:intrinsic component of membrane;CC:cellular anatomical entity;MF:secondary active transmembrane transporter activity;MF:symporter activity;MF:transporter activity;MF:transmembrane transporter activity;MF:molecular_function;MF:active transmembrane transporter activity;	GO:0016021;GO:0005575;GO:0031224;GO:0110165;GO:0015291;GO:0015293;GO:0005215;GO:0022857;GO:0003674;GO:0022804	K05038	SLC6A5_9, GL'	map04721	Synaptic vesicle cycl	COG0733	U:Intracellular trafficking, secretion, a	PF00209.21	SNF	Sodiu	CYT	1	2	71.2	High
TRINITY_DN8771_c0_g2_i1_orf1	regucalcin-like [Ostrinia furnacalis]	2.70784467	1.437144984	0.00318	up	yes	1.039	0.3837	0.264	0.499	0.388	1	1.19	0.928	CC:cytoplasm;CC:cellular_component;CC:cellular anatomical entity;MF:hydrolase activity;MF:metal ion binding;MF:cation binding;MF:enzyme regulator activity;MF:gluconolactonase activity;MF:carboxylic ester hydrolase activity;MF:ion binding;MF:catalytic activity;MF:molecular_function;MF:hydrolase activity, acting on ester bonds;MF:binding;MF:molecular_function_regulator;MF:calcium ion binding;	GO:0005737;GO:0005575;GO:0110165;GO:0016787;GO:0046872;GO:0043169;GO:0030234;GO:0004341;GO:0052689;GO:0043167;GO:0003824;GO:0003674;GO:0016788;GO:0005488;GO:0098772;GO:0005509	K01053	gnl, RGN	map00030;map	Pentose phosphate	COG3386	G:Carbohydrate transport and metab	PF08450.15	SGL	SMP-30/Gluconolactonase/LRE-like region	CYT	1	11	19.2	High
TRINITY_DN4977_c0_g1_i2_orf1	manganese-transporting ATPase 13A1 [Ostrinia furnacalis]	2.070580617	1.050035375	4.15E-05	up	yes	0.9593	0.4633	0.459	0.446	0.485	1	0.921	0.957	CC:intrinsic component of membrane;CC:integral component of membrane;CC:cellular anatomical entity;CC:cellular_component;MF:purine ribonucleoside triphosphate binding;MF:molecular_function;MF:binding;MF:ATPase activity;MF:ion binding;MF:heterocyclic compound binding;MF:anion binding;MF:ion transmembrane transporter activity;MF:ATPase activity, coupled to transmembrane movement of substances;MF:inorganic solute uptake transmembrane transporter activity;MF:nucleotide binding;MF:primary active transmembrane transporter activity;MF:nucleoside phosphate binding;MF:purine nucleotide binding;MF:active transmembrane transporter activity;MF:hydrolase activity;MF:transporter activity;MF:cation transmembrane transporter activity;MF:P-type transmembrane transporter activity;MF:nucleoside-triphosphatase activity;MF:small molecule binding;MF:catalytic activity;MF:inorganic cation transmembrane transporter activity;MF:hydrolase activity, acting on acid anhydrides, in phosphorus-containing anhydrides;MF:adenyl nucleotide binding;MF:carbohydrate derivative binding;MF:organic cyclic compound binding;MF:hydrolase activity, acting on acid anhydrides;MF:cation-transporting ATPase activity;MF:pyrophosphatase activity;MF:transmembrane transporter activity;MF:adenyl ribonucleotide binding;MF:ATP binding;MF:active ion transmembrane transporter activity;MF:purine ribonucleotide binding;MF:ribonucleotide binding;	GO:0031224;GO:0016021;GO:0110165;GO:0005575;GO:0035639;GO:0003674;GO:0005488;GO:0016887;GO:0043167;GO:1901363;GO:0043168;GO:0015075;GO:0042626;GO:0015318;GO:0000166;GO:0015399;GO:1901265;GO:0017076;GO:0022804;GO:0016787;GO:0005215;GO:0008324;GO:0140358;GO:0017111;GO:0036094;GO:0003824;GO:0022890;GO:0016818;GO:0030554;GO:0097367;GO:0097159;GO:0016817;GO:0019829;GO:0016462;GO:0022857;GO:0032559;GO:0005524;GO:0022853;GO:0032555;GO:0032553	K14950	ATP13A1, SPF	------	------	COG0474	J:Translation, ribosomal structure anc	PF00122.23;PF007	E1-E2_ATPase;Hydrolas	E1-E2 ATPase;haloacid dehalogenase-like hydrolase	CYT	6	6	130.6	High
TRINITY_DN1557_c0_g1_i9_orf1	carboxylesterase CXE18 [Ostrinia furnacalis]	2.508078548	1.326582531	2.00E-06	up	yes	1.009	0.4023	0.378	0.409	0.42	1	1.024	1.004	MF:hydrolase activity;MF:molecular_function;MF:hydrolase activity, acting on ester bonds;MF:carboxylic ester hydrolase activity;MF:catalytic activity;	GO:0016787;GO:0003674;GO:0016788;GO:0052689;GO:0003824	------	------	------	------	COG2272	S:Function unknown	PF00135.31;PF204	COesterase;BD-FAE;Ab	Carboxylesterase family;BD-FAE;alpha/beta hydrolase	CYT	4	7	63.7	High
TRINITY_DN37699_c0_g1_i3_orfp1	TRINITY_DN37699_c0_g1_i3_m.58788 TRINITY_DN37699_c0_g1::TRINITY_DN37699_c0_g1_i3::g.58788 ORF type:internal len:122 (+),score=39.86 TRINITY_DN37699_c0_g1_i3:1-363(+)	5.188596491	2.375344344	0.0007681	up	yes	1.183	0.228	0.181	0.207	0.296	1	1.333	1.215	------	------	------	------	------	------	------	------	------	------	------	CYT	1	12	12.6	High
TRINITY_DN20346_c0_g1_i1_orf1	NADH dehydrogenase [ubiquinone] 1 alpha subcomplex subunit 6 [Ostrinia furnacalis]	2.656778558	1.409677985	1.48E-05	up	yes	1.021	0.3843	0.361	0.419	0.373	1	1.057	1.006	CC:respiratory chain complex I;CC:transmembrane transporter complex;CC:catalytic complex;CC:transporter complex;CC:NADH dehydrogenase complex;CC:respiratory chain complex;CC:inner mitochondrial membrane protein complex;CC:cellular_component;CC:membrane protein complex;CC:mitochondrial respiratory chain complex I;CC:macromolecular complex;CC:oxidoreductase complex;CC:mitochondrial protein complex;	GO:0045271;GO:1902495;GO:1902494;GO:1990351;GO:0030964;GO:0098803;GO:0098800;GO:0005575;GO:0098796;GO:0005747;GO:0032991;GO:1990204;GO:0098798	K03950	NDUFA6	map04714;map	Thermogenesis;Oxic	ENOG4111S2F	I:Lipid transport and metabolism	PF05347.18	Complex1_LYR	Complex 1 protein	CYT	2	12	14.8	High
TRINITY_DN31232_c1_g1_i9_orf1	PREDICTED: elongation factor 1-alpha 1, partial [Haliaeetus albicilla]	2.277625185	1.187530351	0.001608	up	yes	1.078	0.4733	0.474	0.574	0.372	1	1.182	1.052	CC:cytoplasm;CC:cellular anatomical entity;CC:cellular_component;MF:purine ribonucleoside triphosphate binding;MF:molecular_function;MF:binding;MF:nucleic acid binding;MF:ion binding;MF:heterocyclic compound binding;MF:nucleoside phosphate binding;MF:guanyl nucleotide binding;MF:nucleotide binding;MF:guanyl ribonucleotide binding;MF:purine nucleotide binding;MF:GTP binding;MF:hydrolase activity;MF:translation factor activity, RNA binding;MF:GTPase activity;MF:nucleoside-triphosphatase activity;MF:small molecule binding;MF:catalytic activity;MF:translation regulator activity;MF:hydrolase activity, acting on acid anhydrides, in phosphorus-containing anhydrides;MF:translation regulator activity, nucleic acid binding;MF:carbohydrate derivative binding;MF:organic cyclic compound binding;MF:hydrolase activity, acting on acid anhydrides;MF:anion binding;MF:pyrophosphatase activity;MF:purine ribonucleotide binding;MF:translation elongation factor activity;MF:ribonucleotide binding;	GO:0005737;GO:0110165;GO:0005575;GO:0035639;GO:0003674;GO:0005488;GO:0003676;GO:0043167;GO:1901363;GO:1901265;GO:0019001;GO:0000166;GO:0032561;GO:0017076;GO:0005525;GO:0016787;GO:0008135;GO:0003924;GO:0017111;GO:0036094;GO:0003824;GO:0045182;GO:0016818;GO:0090079;GO:0097367;GO:0097159;GO:0016817;GO:0043168;GO:0016462;GO:0032555;GO:0003746;GO:0032553	K03231	EEF1A	map05140;map	Leishmaniasis;Legior	COG5256	U:Intracellular trafficking, secretion, a	PF00009.30;PF031	GTP_EFTU;GTP_EFTU_D	Elongation factor Tu GTP binding domain;Elongation factor Tu C-terminal domain;Elongation factor Tu domain 2	CYT	1	14	45.4	High
TRINITY_DN2425_c0_g1_i1_orf1	thyroid receptor-interacting protein 11 [Ostrinia furnacalis]	2.247501595	1.168322139	0.001604	up	yes	1.057	0.4703	0.611	0.423	0.377	1	1.092	1.079	------	------	------	------	------	------	ENOG410XUHJ	S:Function unknown	PF11365.11	SOGA	Protein SOGA	CYT	1	1	262.5	Medium
TRINITY_DN28981_c0_g1_i1_orf1	uncharacterized protein C6orf203 homolog [Ostrinia furnacalis]	2.226520524	1.154790911	0.0001641	up	yes	1.036	0.4653	0.456	0.443	0.497	1	0.996	1.113	BP:regulation of transcription from mitochondrial promoter;BP:regulation of primary metabolic process;BP:regulation of metabolic process;BP:regulation of cellular biosynthetic process;BP:regulation of cellular metabolic process;BP:regulation of biological process;BP:regulation of nucleobase-containing compound metabolic process;BP:biological regulation;BP:regulation of gene expression;BP:regulation of macromolecule metabolic process;BP:regulation of biosynthetic process;BP:regulation of nucleic acid-templated transcription;BP:regulation of cellular process;BP:biological_process;BP:regulation of nitrogen compound metabolic process;BP:regulation of RNA biosynthetic process;BP:regulation of mitochondrial gene expression;BP:regulation of RNA metabolic process;BP:regulation of transcription, DNA-templated;BP:regulation of macromolecule biosynthetic process;CC:organelle;CC:intracellular organelle;CC:membrane-bounded organelle;CC:mitochondrion;CC:cellular anatomical entity;CC:intracellular membrane-bounded organelle;CC:cellular_component;MF:molecular_function;MF:binding;MF:nucleic acid binding;MF:heterocyclic compound binding;MF:organic cyclic compound binding;MF:RNA binding;	GO:1903108;GO:0080090;GO:0019222;GO:0031326;GO:0031323;GO:0050789;GO:0019219;GO:0065007;GO:0010468;GO:0060255;GO:0009889;GO:1903506;GO:0050794;GO:0008150;GO:0051171;GO:2001141;GO:0062125;GO:0051252;GO:0006355;GO:0010556;GO:0043226;GO:0043229;GO:0043227;GO:0005739;GO:0110165;GO:0043231;GO:0005575;GO:0003674;GO:0005488;GO:0003676;GO:1901363;GO:0097159;GO:0003723	------	------	------	------	ENOG4111QEK	S:Function unknown	------	------	------	CYT	1	5	22.6	High
TRINITY_DN23586_c0_g1_i3_orf1	myrosinase 1-like isoform X1 [Ostrinia furnacalis]	2.134453782	1.093866923	1.81E-06	up	yes	1.016	0.476	0.478	0.488	0.462	1	1.014	1.035	BP:primary metabolic process;BP:carbohydrate metabolic process;BP:organic substance metabolic process;BP:biological_process;BP:metabolic process;MF:hydrolase activity;MF:catalytic activity;MF:hydrolase activity, acting on glycosyl bonds;MF:hydrolase activity, hydrolyzing O-glycosyl	GO:0044238;GO:0005975;GO:0071704;GO:0008150;GO:0008152;GO:0016787;GO:0003824;GO:0016798;GO:0004553;GO:0003674	------	------	------	------	COG2723	G:Carbohydrate transport and metab	PF00232.21	Glyco_hydro_1	Glycosyl hydrolase family 1	CYT	5	29	39.2	High
TRINITY_DN2748_c0_g1_i6_orf1	uncharacterized protein LOC114352811 [Ostrinia furnacalis]	2.229437229	1.156679581	6.91E-05	up	yes	1.03	0.462	0.426	0.449	0.511	1	1.072	1.019	------	------	------	------	------	------	ENOG41117PX	S:Function unknown	------	------	------	CYT	3	8	48	High
TRINITY_DN76283_c0_g2_i1_orf1	fatty acid synthase-like [Ostrinia furnacalis]	2.080578512	1.056984731	7.69E-05	up	yes	1.007	0.484	0.425	0.515	0.512	1	1.029	0.993	BP:fatty acid biosynthetic process;BP:fatty acid metabolic process;BP:carboxylic acid metabolic process;BP:cellular biosynthetic process;BP:small molecule metabolic process;BP:small molecule biosynthetic process;BP:monocarboxylic acid biosynthetic process;BP:organic substance biosynthetic process;BP:organic substance metabolic process;BP:lipid metabolic process;BP:cellular process;BP:monocarboxylic acid metabolic process;BP:biosynthetic process;BP:biological_process;BP:metabolic process;BP:oxoacid metabolic process;BP:cellular lipid metabolic process;BP:lipid biosynthetic process;BP:primary metabolic process;BP:organic acid metabolic process;BP:carboxylic acid biosynthetic process;BP:organic acid biosynthetic process;BP:cellular metabolic process;MF:molecular_function;MF:oxidoreductase activity;MF:transferase activity;MF:transferase activity, transferring acyl groups;MF:catalytic activity;	GO:0006633;GO:0006631;GO:0019752;GO:0044249;GO:0044281;GO:0044283;GO:0072330;GO:1901576;GO:0071704;GO:0006629;GO:0009987;GO:0032787;GO:0009058;GO:0008150;GO:0008152;GO:0043436;GO:0044255;GO:0008610;GO:0044238;GO:0006082;GO:0046394;GO:0016053;GO:0044237;GO:0003674;GO:0016491;GO:0016740;GO:0016746;GO:0003824	K00665	FASN	map04910;map	Insulin signaling pat	COG3321	Q:Secondary metabolites biosynthesi	PF00698.24	Acyl_transf_1	Acyl transferase domain	CYT	3	8	32	High
TRINITY_DN106730_c0_g1_i1_orf1	Photosystem I reaction center subunit II, chloroplastic, partial [Trichinella zimbabwensis]	2.149057802	1.103704287	6.88E-05	up	yes	1.015	0.4723	0.441	0.445	0.531	1	1.039	1.006	------	------	K02692	psaD	------	------	ENOG4111HAV	C:Energy production and conversion	PF02531.19	PsaD	PsaD	CYT	2	11	22.6	High
TRINITY_DN46140_c0_g1_i1_orf1	protein PRRC2A-like isoform X4 [Ostrinia furnacalis]	2.046601942	1.03323053	0.0138	up	yes	1.054	0.515	0.739	0.328	0.478	1	1.145	1.016	------	------	------	------	------	------	ENOG410XORI;ENO(	S:Function unknown;	PF07001.14	BAT2_N	BAT2 N-terminus	CYT	1	10	24.1	High
TRINITY_DN6351_c0_g1_i4_orf1	cytochrome P450 CYP12A2-like [Ostrinia furnacalis]	2.016453382	1.011820053	7.83E-05	up	yes	0.9927	0.4923	0.545	0.451	0.481	1	1.009	0.969	MF:cation binding;MF:monooxygenase activity;MF:heme binding;MF:transition metal ion binding;MF:tetrapyrrole binding;MF:organic cyclic compound binding;MF:oxidoreductase activity, acting on paired donors, with incorporation or reduction of molecular oxygen;MF:ion binding;MF:catalytic activity;MF:molecular_function;MF:binding;MF:iron ion binding;MF:metal ion binding;MF:heterocyclic compound binding;MF:oxidoreductase activity;	GO:0043169;GO:0004497;GO:0020037;GO:0046914;GO:0046906;GO:0097159;GO:0016705;GO:0043167;GO:0003824;GO:0003674;GO:0005488;GO:0005506;GO:0046872;GO:1901363;GO:0016491	K15004	CYP12	------	------	COG2124;ENOG410X	G:Carbohydrate transport and metab	PF00067.25	p450	Cytochrome P450	CYT	2	4	57.5	High

TRINITY_DN5697_c0_g1_i1_orf1	GPI ethanolamine phosphate transferase 2-like [Ostrinia furnacalis]	2.182212581	1.125791649	6.66E-05	up	yes	1.006	0.461	0.414	0.507	0.462	1	1.037	0.98	BP:glycerophospholipid metabolic process;BP:cellular biosynthetic process;BP:nitrogen compound metabolic process;BP:glycerolipid metabolic process;BP:lipid metabolic process;BP:glycerolipid biosynthetic process;BP:organic substance metabolic process;BP:GPI anchor metabolic process;BP:GPI anchor biosynthetic process;BP:phosphatidylinositol biosynthetic process;BP:glycolipid metabolic process;BP:liposaccharide metabolic process;BP:phospholipid metabolic process;BP:membrane lipid metabolic process;BP:organic substance biosynthetic process;BP:cellular process;BP:glycolipid biosynthetic process;BP:biosynthetic process;BP:macromolecule modification;BP:protein modification process;BP:biological_process;BP:metabolic process;BP:glycerophospholipid biosynthetic process;BP:cellular lipid metabolic process;BP:organophosphate biosynthetic process;BP:lipid biosynthetic process;BP:protein lipidation;BP:primary metabolic process;BP:membrane lipid biosynthetic process;BP:organonitrogen compound metabolic process;BP:phospholipid biosynthetic process;BP:protein metabolic process;BP:carbohydrate derivative metabolic process;BP:cellular metabolic process;BP:macromolecule metabolic process;BP:carbohydrate derivative biosynthetic process;BP:phosphate-containing compound metabolic process;BP:phosphorus metabolic process;BP:organophosphate metabolic process;BP:phosphatidylinositol metabolic process;CC:endoplasmic reticulum membrane;CC:intrinsic component of membrane;CC:organelle membrane;CC:integral component of membrane;CC:membrane;CC:cellular anatomical entity;CC:cellular_component;MF:molecular_function;MF:transferase activity;MF:phosphotransferase activity, for [illegible]	GO:0006650;GO:0044249;GO:0006807;GO:0046486;GO:0006629;GO:0045017;GO:0071704;GO:0006505;GO:0006506;GO:0006661;GO:0006664;GO:1903509;GO:0006644;GO:0006643;GO:1901576;GO:0009987;GO:0009247;GO:0009058;GO:0043412;GO:0036211;GO:0008150;GO:0008152;GO:0046474;GO:0044255;GO:0090407;GO:0008610;GO:0006497;GO:0044238;GO:0046467;GO:1901564;GO:0008654;GO:0019538;GO:1901135;GO:0044237;GO:0043170;GO:1901137;GO:0006796;GO:0006793;GO:0019637;GO:0046488;GO:0005789;GO:0031224;GO:0031090;GO:0016021;GO:0016020;GO:0110165;GO:0005575;GO:0003674;GO:0016740;GO:0016780;GO:0003824;GO:0016772;GO:0051377	K05310	PIGG, GPI7	map00563	Glycosylphosphatidy	COG1524	S:Function unknown	PF01663.25	Phosphodiest	Type I phosphodiesterase / nucleotide pyrophosphatase	CYT	1	1	89.9	Medium
TRINITY_DN92232_c0_g1_i1_orf1	protein SDA1 homolog [Ostrinia furnacalis]	2.405360922	1.266253385	0.0003826	up	yes	1.023	0.4253	0.327	0.441	0.508	1	1.03	1.039	BP:nuclear transport;BP:protein localization;BP:ribosome localization;BP:actin cytoskeleton organization;BP:nuclear export;BP:establishment of organelle localization;BP:cellular component organization or biogenesis;BP:ribosomal large subunit biogenesis;BP:cellular macromolecule localization;BP:cellular component organization;BP:nitrogen compound transport;BP:organic substance transport;BP:ribonucleoprotein complex biogenesis;BP:macromolecule localization;BP:organelle organization;BP:transport;BP:actin filament-based process;BP:cellular process;BP:nucleocytoplasmic transport;BP:establishment of protein localization;BP:biological_process;BP:establishment of localization in cell;BP:establishment of localization;BP:localization;BP:organelle localization;BP:cellular localization;BP:protein complex localization;BP:cytoskeleton organization;BP:intracellular transport;BP:ribosomal large subunit export from nucleus;BP:ribosomal subunit export from nucleus;BP:cellular component biogenesis;BP:protein transport;CC:intracellular organelle;CC:non-membrane-bounded organelle;CC:organelle;CC:cellular anatomical entity;CC:intracellular non-membrane-bounded organelle;CC:nucleolus;CC:cellular_component;	GO:0051169;GO:0008104;GO:0033750;GO:0030036;GO:0051168;GO:0051656;GO:0071840;GO:0042273;GO:0070727;GO:0016043;GO:0071705;GO:0071702;GO:0022613;GO:0033036;GO:0006996;GO:0006810;GO:0030029;GO:0009987;GO:0006913;GO:0045184;GO:0008150;GO:0051649;GO:0051234;GO:0051179;GO:0051640;GO:0051641;GO:0031503;GO:0007010;GO:0046907;GO:0000055;GO:0000054;GO:0044085;GO:0015031;GO:0043229;GO:0043228;GO:0043226;GO:0110165;GO:0043232;GO:0005730;GO:0005575	K14856	SDA1, SDAD1	------	------	ENOG410XPHI	S:Function unknown	PF05285.15;PF081	SDA1;NUC130_3NT	SDA1;NUC130/3NT domain	CYT	1	1	84.7	Medium
TRINITY_DN2065_c1_g2_i1_orf1	2-amino-3-ketobutyrate coenzyme A ligase, mitochondrial [Ostrinia furnacalis]	2.438734237	1.286132548	4.77E-06	up	yes	1.025	0.4203	0.414	0.407	0.44	1	1.052	1.023	BP:carboxylic acid metabolic process;BP:cellular amino acid catabolic process;BP:nitrogen compound metabolic process;BP:aspartate family amino acid metabolic process;BP:small molecule catabolic process;BP:aspartate family amino acid catabolic process;BP:threonine catabolic process;BP:threonine metabolic process;BP:organic substance catabolic process;BP:cellular amino acid metabolic process;BP:organic substance metabolic process;BP:alpha-amino acid metabolic process;BP:alpha-amino acid catabolic process;BP:cellular process;BP:biosynthetic process;BP:biological_process;BP:metabolic process;BP:oxoacid metabolic process;BP:catabolic process;BP:cellular catabolic process;BP:primary metabolic process;BP:organonitrogen compound metabolic process;BP:organonitrogen compound catabolic process;BP:organic acid metabolic process;BP:carboxylic acid catabolic process;BP:organic acid catabolic process;BP:cellular metabolic process;BP:small molecule metabolic process;MF:pyridoxal phosphate binding;MF:vitamin B6 binding;MF:molecular_function;MF:heterocyclic compound binding;MF:ligase activity;MF:glycine C-acetyltransferase activity;MF:ion binding;MF:transferase activity;MF:transferase activity, transferring acyl groups;MF:transferase activity, transferring acyl groups other than amino-acyl groups;MF:C-acetyltransferase activity;MF:anion binding;MF:small molecule binding;MF:catalytic activity;MF:vitamin binding;MF:acetyltransferase activity;MF:organic cyclic compound binding;MF:binding;MF:C-acyltransferase	GO:0019752;GO:0009063;GO:0006807;GO:0009066;GO:0044282;GO:0009068;GO:0006567;GO:0006566;GO:1901575;GO:0006520;GO:0071704;GO:1901605;GO:1901606;GO:0009987;GO:0009058;GO:0008150;GO:0008152;GO:0043436;GO:0009056;GO:0044248;GO:0044238;GO:1901564;GO:1901565;GO:0006082;GO:0046395;GO:0016054;GO:0044237;GO:0044281;GO:0030170;GO:0070279;GO:0003674;GO:1901363;GO:0016874;GO:0008890;GO:0043167;GO:0016740;GO:0016746;GO:0016747;GO:0016453;GO:0043168;GO:0036094;GO:0003824;GO:0019842;GO:0016407;GO:0097159;GO:0005488;GO:0016408	K00639	kbl, GCAT	map00260	Glycine, serine and t	COG0156;COG0366	J:Translation, ribosomal structure anc	PF00155.24	Aminotran_1_2	Aminotransferase class I and II	CYT	4	13	46.2	High
TRINITY_DN24317_c0_g1_i7_orf1	peptidyl-tRNA hydrolase ICT1, mitochondrial [Ostrinia furnacalis]	2.031971205	1.022879958	0.0001569	up	yes	0.9597	0.4723	0.43	0.523	0.464	1	0.956	0.923	MF:translation termination factor activity;MF:translation regulator activity, nucleic acid binding;MF:nucleic acid binding;MF:organic cyclic compound binding;MF:translation factor activity, RNA binding;MF:molecular_function;MF:binding;MF:translation regulator activity;MF:translation release factor activity;MF:heterocyclic compound binding;	GO:0008079;GO:0090079;GO:0003676;GO:0097159;GO:0008135;GO:0003674;GO:0005488;GO:0045182;GO:0003747;GO:1901363	K15033	ICT1, MRPL58	------	------	COG1186	J:Translation, ribosomal structure anc	PF00472.23	RF-1	RF-1 domain	CYT	3	16	22.9	High
TRINITY_DN96080_c0_g2_i1_orf1	ATP synthase subunit delta, mitochondrial [Ostrinia furnacalis]	2.43236715	1.282361011	2.43E-06	up	yes	1.007	0.414	0.423	0.408	0.411	1	1.034	0.986	CC:proton-transporting ATP synthase complex, catalytic core;CC:cellular_component;CC:macromolecular complex;CC:proton-transporting two-sector ATPase complex, catalytic domain;CC:membrane protein complex;MF:cation transmembrane transporter activity;MF:proton-transporting ATP synthase activity, rotational mechanism;MF:inorganic solute uptake transmembrane transporter activity;MF:transporter activity;MF:ion channel activity;MF:passive transmembrane transporter activity;MF:ion transmembrane transporter activity;MF:catalytic activity;MF:transmembrane transporter activity;MF:hydrogen ion channel activity;MF:molecular_function;MF:channel activity;MF:cation channel activity;MF:inorganic cation transmembrane transporter activity;MF:ligase activity;MF:hydrogen ion transmembrane transporter activity;	GO:0045261;GO:0005575;GO:0032991;GO:0033178;GO:0098796;GO:0008324;GO:0046933;GO:0015318;GO:0005215;GO:0005216;GO:0022803;GO:0015075;GO:0003824;GO:0022857;GO:0015252;GO:0003674;GO:0015267;GO:0005261;GO:0022890;GO:0016874;GO:0015078	K02134	ATPeF1D, ATF	map04714;map	Thermogenesis;Cher	COG0355	C:Energy production and conversion	PF02823.19	ATP-synt_DE_N	ATP synthase, Delta/Epsilon chain, beta-sandwich domain	CYT	6	53	17.2	High
TRINITY_DN3127_c0_g1_i9_orf1	RNA-binding protein 1 isoform X1 [Galleria mellonella]	2.151691687	1.105471371	8.37E-05	up	yes	0.9603	0.4463	0.485	0.415	0.439	1	0.917	0.964	MF:organic cyclic compound binding;MF:molecular_function;MF:binding;MF:nucleic acid binding;MF:heterocyclic compound binding;MF:RNA binding;	GO:0097159;GO:0003674;GO:0005488;GO:0003676;GO:1901363;GO:0003723	K12896	SRSF7, SFRS7	map05168;map	Herpes simplex virus	ENOG4111N8Y	I:Lipid transport and metabolism	PF00076.25;PF138	RRM_1;RRM_5	RNA recognition motif.;RNA recognition motif	CYT	2	18	18	High
TRINITY_DN83295_c0_g1_i3_orf1	SSSX-APN4 [Ostrinia furnacalis]	2.054466231	1.038763617	0.0002967	up	yes	0.943	0.459	0.517	0.44	0.42	1	0.915	0.914	BP:nitrogen compound metabolic process;BP:organic substance metabolic process;BP:proteolysis;BP:biological_process;BP:metabolic process;BP:primary metabolic process;BP:organonitrogen compound metabolic process;BP:protein metabolic process;BP:macromolecule metabolic process;CC:integral component of membrane;CC:intrinsic component of membrane;CC:cellular anatomical entity;CC:cellular_component;MF:aminopeptidase activity;MF:exopeptidase activity;MF:molecular_function;MF:binding;MF:peptidase activity;MF:metallopeptidase activity;MF:cation binding;MF:transition metal ion binding;MF:ion binding;MF:metal ion binding;MF:hydrolase activity;MF:catalytic activity, acting on a protein;MF:catalytic activity;MF:zinc ion binding;	GO:0006807;GO:0071704;GO:0006508;GO:0008150;GO:0008152;GO:0044238;GO:1901564;GO:0019538;GO:0043170;GO:0016021;GO:0031224;GO:0110165;GO:0005575;GO:0004177;GO:0008238;GO:0003674;GO:0005488;GO:0008233;GO:0008237;GO:0043169;GO:0046914;GO:0043167;GO:0046872;GO:0016787;GO:0140096;GO:0003824;GO:0008270	K11140	ANPEP, CD13	map04640;map	Hematopoietic cell l	COG0308	E:Amino acid transport and metaboli	PF01433.23	Peptidase_M1	Peptidase family M1 domain	CYT	1	3	35.8	High
TRINITY_DN8173_c0_g1_i3_orf1	dihydroceramide fatty acyl 2-hydroxylase FAH1 [Ostrinia furnacalis]	2.334782609	1.223288227	0.003235	up	yes	1.074	0.46	0.393	0.423	0.564	1	0.985	1.238	BP:fatty acid biosynthetic process;BP:fatty acid metabolic process;BP:carboxylic acid metabolic process;BP:cellular biosynthetic process;BP:small molecule metabolic process;BP:small molecule biosynthetic process;BP:monocarboxylic acid biosynthetic process;BP:organic substance biosynthetic process;BP:organic substance metabolic process;BP:lipid metabolic process;BP:cellular process;BP:monocarboxylic acid metabolic process;BP:biosynthetic process;BP:biological_process;BP:metabolic process;BP:oxoacid metabolic process;BP:cellular lipid metabolic process;BP:lipid biosynthetic process;BP:primary metabolic process;BP:organic acid metabolic process;BP:carboxylic acid biosynthetic process;BP:organic acid biosynthetic process;BP:cellular metabolic process;CC:endoplasmic reticulum membrane;CC:intrinsic component of membrane;CC:organelle membrane;CC:integral component of membrane;CC:membrane;CC:cellular anatomical entity;CC:cellular_component;MF:molecular_function;MF:ion binding;MF:oxidoreductase activity;MF:transition metal ion binding;MF:heterocyclic compound binding;MF:iron ion binding;MF:metal ion binding;MF:heme binding;MF:cation binding;MF:catalytic activity;MF:tetrapyrrole binding;MF:organic cyclic compound binding;MF:fatty acid alpha-hydroxylase activity;MF:binding;	GO:0006633;GO:0006631;GO:0019752;GO:0044249;GO:0044281;GO:0044283;GO:0072330;GO:1901576;GO:0071704;GO:0006629;GO:0009987;GO:0032787;GO:0009058;GO:0008150;GO:0008152;GO:0043436;GO:0044255;GO:0008610;GO:0044238;GO:0006082;GO:0046394;GO:0016053;GO:0044237;GO:0005789;GO:0031224;GO:0031090;GO:0016021;GO:0016020;GO:0110165;GO:0005575;GO:0003674;GO:0043167;GO:0016491;GO:0046914;GO:1901363;GO:0005506;GO:0046872;GO:0020037;GO:0043169;GO:0003824;GO:0046906;GO:0097159;GO:0080132;GO:0005488	K19703	FA2H, SCS7	------	------	COG5274;COG5274;(	C:Energy production and conversion;	PF04116.16;PF001	FA_hydroxylase;Cyt-b5	Fatty acid hydroxylase;Cytochrome b5-like Heme/Steroid binding domain	CYT	1	4	39.3	High
TRINITY_DN755_c0_g1_i3_orf1	uncharacterized protein LOC114358844 [Ostrinia furnacalis]	2.000783853	1.000565319	3.75E-05	up	yes	1.021	0.5103	0.512	0.473	0.546	1	1.049	1.014	CC:cellular_component;CC:integral component of membrane;CC:cellular anatomical entity;CC:intrinsic component of membrane;	GO:0005575;GO:0016021;GO:0110165;GO:0031224	------	------	------	------	ENOG411170P	S:Function unknown	------	------	------	CYT	2	37	7	High
TRINITY_DN21214_c0_g2_i1_orf1	heat shock protein family A (Hsp70) member 1A [Homo sapiens] >KAI4017664.1 heat shock protein family A (Hsp70) member 1A [Homo sapiens] >PNI76655.1 HSPA1A isoform 2 [Pan troglodytes]	2.078185133	1.055324181	1.84E-05	up	yes	1.026	0.4937	0.488	0.499	0.494	1	1.07	1.008	BP:regulation of microtubule polymerization or depolymerization;BP:positive regulation of microtubule polymerization or depolymerization;BP:regulation of microtubule polymerization;BP:positive regulation of proteasomal ubiquitin-dependent protein catabolic process;BP:regulation of primary metabolic process;BP:regulation of metabolic process;BP:regulation of microtubule nucleation;BP:regulation of spindle assembly;BP:cellular response to stimulus;BP:response to unfolded protein;BP:negative regulation of macromolecule metabolic process;BP:regulation of proteasomal ubiquitin-dependent protein catabolic process;BP:positive regulation of cytoskeleton organization;BP:regulation of cytoskeleton organization;BP:positive regulation of biological process;BP:negative regulation of biological process;BP:regulation of macromolecule metabolic process;BP:regulation of proteolysis;BP:regulation of RNA biosynthetic process;BP:response to organic substance;BP:vesicle-mediated transport;BP:regulation of mitotic spindle assembly;BP:positive regulation of organelle organization;BP:cellular response to stress;BP:regulation of microtubule cytoskeleton organization;BP:positive regulation of fibril organization;BP:positive regulation of catabolic process;BP:regulation [illegible]	GO:0031110;GO:0031112;GO:0031113;GO:0032436;GO:0080090;GO:0019222;GO:0010968;GO:0090169;GO:0051716;GO:0006986;GO:0010605;GO:0032434;GO:0051495;GO:0051493;GO:0048518;GO:0048519;GO:0060255;GO:0030162;GO:2001141;GO:0010033;GO:0016192;GO:1901673;GO:0010638;GO:0033554;GO:0070507;GO:1902905;GO:0009896;GO:0009894;GO:0009892;GO:0070887;GO:0009890;GO:0006458;GO:0050789;GO:0090063;GO:0065007;GO:1901800;GO:0051130;GO:0006810;GO:0009889;GO:0050794;GO:0006950;GO:0008150;GO:0043254;GO:0051234;GO:0061077;GO:0010604;GO:0046907;GO:0050896;GO:2000060;GO:0006357;GO:0010558;GO:0006457;GO:0033043;GO:0010564;GO:0051173;GO:0051128;GO:0042176;GO:0007041;GO:0032886;GO:0009893;GO:0000122;GO:0051084;GO:0051085;GO:0051246; [illegible]	K03283	HSPA1s	map05162;map	Measles;Toxoplasmc	COG0443	A:RNA processing and modification	PF00012.23	HSP70	Hsp70 protein	CYT	1	5	45.4	High
TRINITY_DN11013_c0_g1_i3_orf1	glutamine:fructose-6-phosphate aminotransferase 1 [Heortia vitessoides]	2.071309771	1.05054333	6.57E-06	up	yes	0.9963	0.481	0.479	0.471	0.493	1	0.968	1.021	[illegible] process;BP:aromatic compound biosynthetic process;BP:small molecule metabolic process;MF:molecular_function;MF:binding;MF:transaminase activity;MF:glutamine-fructose-6-phosphate transaminas;MF:transferase activity;MF:transferase activity, transferring nitrogenous groups;MF:L-glutamine aminotransferase activity;MF:catalytic activity;MF:carbohydrate derivative binding;	[illegible]	K00820	glmS, GFPT	map00250;map	Alanine, aspartate a	COG0449	M:Cell wall/membrane/envelope bio;	PF01380.25;PF135	SIS;GATase_6;GATase_7	SIS domain;Glutamine amidotransferase domain;Glutamine amidotransferase domain;Glutamine amidotransferases class-II	CYT	15	29	75.4	High
TRINITY_DN1617_c0_g1_i5_orf1	hypothetical protein evm_009822 [Chilo suppressalis] >CAB3525311.1 unnamed protein product [Chilo suppressalis] >CAH0402638.1 unnamed protein product [Chilo suppressalis]	2.078361345	1.055446503	8.03E-06	up	yes	0.9893	0.476	0.493	0.443	0.492	1	0.98	0.988	CC:integral component of membrane;CC:intrinsic component of membrane;CC:cellular_component;CC:cellular anatomical entity;MF:purine ribonucleoside triphosphate binding;MF:molecular_function;MF:binding;MF:ion binding;MF:heterocyclic compound binding;MF:nucleoside phosphate binding;MF:anion binding;MF:nucleotide binding;MF:purine nucleotide binding;MF:ATP binding;MF:hydrolase activity;MF:small molecule binding;MF:catalytic activity;MF:adenyl nucleotide binding;MF:carbohydrate derivative binding;MF:organic cyclic compound binding;MF:adenyl ribonucleotide binding;MF:purine ribonucleotide binding;MF:ribonucleotide binding;	GO:0016021;GO:0031224;GO:0005575;GO:0110165;GO:0035639;GO:0003674;GO:0005488;GO:0043167;GO:1901363;GO:1901265;GO:0043168;GO:0000166;GO:0017076;GO:0005524;GO:0016787;GO:0036094;GO:0003824;GO:0030554;GO:0097367;GO:0097159;GO:0032559;GO:0032555;GO:0032553	K01511	ENTPD5_6	map00240;map	Pyrimidine metaboli	COG5371;COG5371	G:Carbohydrate transport and metab	PF01150.20	GDA1_CD39	GDA1/CD39	CYT	3	7	51.5	High
TRINITY_DN14235_c0_g1_i1_orf1	maltase A1 [Helicoverpa armigera]	2.501797268	1.322964886	1.56E-05	up	yes	1.044	0.4173	0.433	0.426	0.393	1	1.07	1.061	BP:primary metabolic process;BP:carbohydrate metabolic process;BP:organic substance metabolic process;BP:biological_process;BP:metabolic process;MF:hydrolase activity;MF:catalytic activity;MF:hydrolase activity, acting on glycosyl bonds;MF:molecular_function;	GO:0044238;GO:0005975;GO:0071704;GO:0008150;GO:0008152;GO:0016787;GO:0003824;GO:0016798;GO:0003674	K01187	malZ	map00500;map	Starch and sucrose r	COG0366	G:Carbohydrate transport and metab	PF00128.27	Alpha-amylase	Alpha amylase, catalytic domain	CYT	10	29	66.7	High
TRINITY_DN11799_c0_g1_i4_orf1	V-type proton ATPase 116 kDa subunit a1 isoform X1 [Manduca sexta]	2.281607905	1.190050885	5.77E-06	up	yes	1.016	0.4453	0.418	0.445	0.473	1	1.027	1.022	------	------	K02154	ATPeV0A, ATF	map05152;map	Tuberculosis;Rheum	COG1269	F:Nucleotide transport and metabolis	PF01496.22	V_ATPase_I	V-type ATPase 116kDa subunit family	CYT	8	12	97.5	High
TRINITY_DN9853_c0_g3_i1_orf1	importin-7 isoform X1 [Ostrinia furnacalis]	2.047837894	1.034101517	3.12E-05	up	yes	0.9803	0.4787	0.501	0.442	0.493	1	0.991	0.95	------	------	K20223	IPO7, RANBP7	map03013;map	Nucleocytoplasmic t	COG5656	O:Posttranslational modification, pro	PF03810.22	IBN_N	Importin-beta N-terminal domain	CYT	12	13	120.6	High
TRINITY_DN2807_c0_g1_i4_orf1	FK506-binding protein 59 isoform X1 [Ostrinia furnacalis]	2.277940184	1.187729864	5.48E-07	up	yes	1.013	0.4447	0.448	0.435	0.451	1	1.029	1.009	MF:isomerase activity;MF:catalytic activity, acting on a protein;MF:cis-trans isomerase activity;MF:catalytic activity;MF:molecular_function;MF:peptidyl-prolyl cis-trans isomerase activity;	GO:0016853;GO:0140096;GO:0016859;GO:0003824;GO:0003674;GO:0003755	K09571	FKBP4_5	map04915	Estrogen signaling p	COG0545	S:Function unknown	PF00254.31;PF180	FKBP_C;FKBP_N_2;TPR_	FKBP-type peptidyl-prolyl cis-trans isomerase;BDBT FKBP like N-terminal;Tetratricopeptide repeat;Tetratricopeptide repeat	CYT	17	47	52.8	High
TRINITY_DN15959_c0_g1_i1_orf1	dnaJ homolog subfamily A member 2-like [Ostrinia furnacalis]	2.152494577	1.106009603	2.87E-06	up	yes	0.9923	0.461	0.436	0.475	0.472	1	0.997	0.98	BP:protein folding;BP:cellular process;BP:response to stress;BP:biological_process;BP:response to temperature stimulus;BP:response to heat;BP:response to abiotic stimulus;BP:response to stimulus;CC:membrane;CC:cellular_component;CC:cellular anatomical entity;MF:purine ribonucleoside triphosphate binding;MF:heat shock protein binding;MF:molecular_function;MF:binding;MF:nucleotide binding;MF:heterocyclic compound binding;MF:nucleoside phosphate binding;MF:anion binding;MF:cation binding;MF:Hsp70 protein binding;MF:unfolded protein binding;MF:ion binding;MF:purine nucleotide binding;MF:metal ion binding;MF:ATP binding;MF:small molecule binding;MF:adenyl nucleotide binding;MF:carbohydrate derivative binding;MF:organic cyclic compound binding;MF:adenyl ribonucleotide binding;MF:protein binding;MF:purine ribonucleotide binding;MF:ribonucleotide binding;	GO:0006457;GO:0009987;GO:0006950;GO:0008150;GO:0009266;GO:0009408;GO:0009628;GO:0050896;GO:0016020;GO:0005575;GO:0110165;GO:0035639;GO:0031072;GO:0003674;GO:0005488;GO:0000166;GO:1901363;GO:1901265;GO:0043168;GO:0043169;GO:0030544;GO:0051082;GO:0043167;GO:0017076;GO:0046872;GO:0005524;GO:0036094;GO:0030554;GO:0097367;GO:0097159;GO:0032559;GO:0005515;GO:0032555;GO:0032553	K09503	DNAJA2	map04141	Protein processing ii	COG0484	O:Posttranslational modification, pro	PF01556.21;PF002	DnaJ_C;DnaJ;DnaJ_CXX	DnaJ C terminal domain;DnaJ domain;DnaJ central domain	CYT	10	33	44.9	High
TRINITY_DN2304_c0_g1_i4_orf1	clustered mitochondria protein homolog isoform X2 [Ostrinia furnacalis]	2.162938596	1.112992709	1.74E-05	up	yes	0.9863	0.456	0.422	0.489	0.457	1	0.993	0.966	BP:organelle organization;BP:cellular component organization or biogenesis;BP:cellular process;BP:cellular component organization;BP:biological_process;BP:mitochondrion organization;CC:cytoplasm;CC:cellular_component;CC:cellular anatomical entity;MF:organic cyclic compound binding;MF:molecular_function;MF:RNA binding;MF:nucleic acid binding;MF:heterocyclic compound binding;MF:binding;	GO:0006996;GO:0071840;GO:0009987;GO:0016043;GO:0008150;GO:0007005;GO:0005737;GO:0005575;GO:0110165;GO:0097159;GO:0003674;GO:0003723;GO:0003676;GO:1901363;GO:0005488	K03255	TIF31, CLU1	------	------	ENOG410XQUQ	S:Function unknown	PF13424.9;PF1337	TPR_12;TPR_10;eIF3_p1	Tetratricopeptide repeat;Tetratricopeptide repeat;Translation initiation factor eIF3 subunit 135	CYT	4	11	48	High
TRINITY_DN195_c8_g1_i1_orf1	hypothetical protein evm_009768 [Chilo suppressalis]	2.181778072	1.12550436	5.55E-05	up	yes	0.989	0.4533	0.505	0.409	0.446	1	0.969	0.998	BP:interspecies interaction between organisms;BP:response to biotic stimulus;BP:response to external stimulus;BP:response to external biotic stimulus;BP:response to stimulus;BP:response to bacterium;BP:defense response;BP:response to stress;BP:defense response to other organism;BP:defense response to bacterium;BP:biological_process;BP:response to other organism;CC:cellular_component;CC:extracellular region;CC:cellular anatomical entity;	GO:0044419;GO:0009607;GO:0009605;GO:0043207;GO:0050896;GO:0009617;GO:0006952;GO:0006950;GO:0098542;GO:0042742;GO:0008150;GO:0051707;GO:0005575;GO:0005576;GO:0110165	------	------	------	------	ENOG410ZBQ8	S:Function unknown	PF03769.18	Attacin_C	Attacin, C-terminal region	CYT	1	4	21.6	Medium
TRINITY_DN4434_c0_g1_i7_orf1	V-type proton ATPase catalytic subunit A [Ostrinia furnacalis] >XP_028155921.1 V-type proton ATPase catalytic subunit A [Ostrinia furnacalis] >XP_028155922.1 V-type proton ATPase catalytic subunit A [Ostrinia furnacalis] >ADP23923.1 V-ATPase subunit A [Ostrinia furnacalis] >ADT80587.1 V-type proton ATPase catalytic subunit A [Ostrinia furnacalis] >CBY05457.1 V-type proton ATPase catalytic subunit A [Ostrinia furnacalis]	2.298614319	1.200764419	2.00E-07	up	yes	0.9953	0.433	0.425	0.434	0.44	1	1.003	0.983	CC:proton-transporting V-type ATPase, V1 domain;CC:proton-transporting two-sector ATPase complex, catalytic domain;CC:macromolecular complex;CC:cellular_component;CC:membrane protein complex;MF:cation channel activity;MF:purine ribonucleoside triphosphate binding;MF:molecular_function;MF:binding;MF:ion binding;MF:heterocyclic compound binding;MF:nucleoside phosphate binding;MF:anion binding;MF:ion transmembrane transporter activity;MF:ATPase activity, coupled to transmembrane movement of substances;MF:inorganic solute uptake transmembrane transporter activity;MF:hydrogen-translocating pyrophosphatase activity;MF:nucleotide binding;MF:primary active transmembrane transporter activity;MF:purine nucleotide binding;MF:active transmembrane transporter activity;MF:hydrogen ion channel activity;MF:ATPase coupled ion transmembrane transporter activity;MF:transporter activity;MF:ion channel activity;MF:cation transmembrane transporter activity;MF:small molecule binding;MF:catalytic activity;MF:proton-transporting ATPase activity, rotational mechanism;MF:passive transmembrane transporter activity;MF:ligase activity;MF:ATPase activity, coupled to transmembrane movement of ions, rotational mechanism;MF:inorganic cation transmembrane transporter activity;MF:adenyl nucleotide binding;MF:carbohydrate derivative binding;MF:organic cyclic compound binding;MF:proton-transporting ATP synthase activity, rotational mechanism;MF:cation-transporting ATPase activity;MF:transmembrane transporter activity;MF:channel activity;MF:adenyl ribonucleotide binding;MF:ATP binding;MF:active ion transmembrane transporter activity;MF:purine ribonucleotide binding;MF:hydrogen ion transmembrane transporter	GO:0033180;GO:0033178;GO:0032991;GO:0005575;GO:0098796;GO:0005261;GO:0035639;GO:0003674;GO:0005488;GO:0043167;GO:1901363;GO:1901265;GO:0043168;GO:0015075;GO:0042626;GO:0015318;GO:0009678;GO:0000166;GO:0015399;GO:0017076;GO:0022804;GO:0015252;GO:0042625;GO:0005215;GO:0005216;GO:0008324;GO:0036094;GO:0003824;GO:0046961;GO:0022803;GO:0016874;GO:0044769;GO:0022890;GO:0030554;GO:0097367;GO:0097159;GO:0046933;GO:0019829;GO:0022857;GO:0015267;GO:0032559;GO:0005524;GO:0022853;GO:0032555;GO:0015078;GO:0032553	K02145	ATPeV1A, ATF	map05323;map	Rheumatoid arthritis	COG1155	C:Energy production and conversion	PF00006.28;PF168	ATP-synt_ab;ATP-synt_	ATP synthase alpha/beta family, nucleotide-binding domain;ATPsynthase alpha/beta subunit N-term extension;ATP synthase alpha/beta family, beta-barrel domain	CYT	29	62	68.1	High
TRINITY_DN34821_c0_g1_i4_orf1	acetylcholine receptor subunit alpha-L1-like [Ostrinia furnacalis]	2.309133489	1.207351577	8.72E-05	up	yes	0.986	0.427	0.403	0.443	0.435	1	0.924	1.034	CC:cellular_component;CC:integral component of membrane;CC:cellular anatomical entity;CC:intrinsic component of membrane;MF:signaling receptor activity;MF:molecular transducer activity;MF:inorganic solute uptake transmembrane transporter activity;MF:transporter activity;MF:extracellular ligand-gated ion channel activity;MF:ligand-gated ion channel activity;MF:ion transmembrane transporter activity;MF:transmembrane signaling receptor activity;MF:transmembrane transporter activity;MF:channel activity;MF:molecular_function;MF:gated channel activity;MF:ligand-gated channel activity;MF:passive transmembrane transporter activity;MF:ion channel activity;	GO:0005575;GO:0016021;GO:0110165;GO:0031224;GO:0038023;GO:0060089;GO:0015318;GO:0005215;GO:0005230;GO:0015276;GO:0015075;GO:0004888;GO:0022857;GO:0015267;GO:0003674;GO:0022836;GO:0022834;GO:0022803;GO:0005216	K04803;K04807	CHRNA1;CHR	map04080	Neuroactive ligand-	ENOG410XQGR	S:Function unknown	PF02931.26	Neur_chan_LBD	Neurotransmitter-gated ion-channel ligand binding domain	PLA	1	3	47.7	High
TRINITY_DN14168_c0_g1_i1_orf1	transmembrane 7 superfamily member 3-like [Ostrinia furnacalis]	2.277310924	1.187331278	0.001209	up	yes	1.084	0.476	0.442	0.438	0.548	1	1.041	1.212	CC:cellular_component;CC:integral component of membrane;CC:cellular anatomical entity;CC:intrinsic component of membrane;	GO:0005575;GO:0016021;GO:0110165;GO:0031224	------	------	------	------	ENOG41117R0	S:Function unknown	PF13886.9	DUF4203	Domain of unknown function	CYT	1	3	62.2	High

TRINITY_DN143603_c0_g1_i1_orf1	hypothetical protein KR044_005587 [Drosophila immigrans]	2.447304193	1.291193436	0.0004549	up	yes	1.103	0.4507	0.47	0.472	0.41	1	1.108	1.202	BP:response to extracellular stimulus;BP:carbon utilization;BP:organic substance metabolic process;BP:biological_process;BP:metabolic process;BP:primary metabolic process;BP:carbohydrate metabolic process;BP:response to external stimulus;BP:response to stimulus;BP:response to nutrient levels;CC:membrane;CC:cellular anatomical entity;CC:cellular_component;MF:hydrolase activity;MF:carbohydrate binding;MF:lyase activity;MF:molecular_function;MF:binding;MF:ion binding;MF:heterocyclic compound binding;MF:nucleoside phosphate binding;MF:oxidoreductase activity;MF:anion binding;MF:cation binding;MF:flavin adenine dinucleotide binding;MF:transition metal ion binding;MF:oxidoreductase activity, acting on the CH-CH group of donors;MF:nucleotide binding;MF:alpha-glucosidase activity;MF:carbon-oxygen lyase activity;MF:tRNA dihydrouridine synthase activity;MF:metal ion binding;MF:glucosidase activity;MF:catalytic activity, acting on RNA;MF:hydrolase activity, acting on glycosyl bonds;MF:small molecule binding;MF:catalytic activity;MF:carbonate dehydratase activity;MF:organic cyclic compound binding;MF:hydrolase activity, hydrolyzing O-glycosyl compounds;MF:catalytic activity, acting on a tRNA;MF:hydro-lyase activity;MF:zinc ion	GO:0009991;GO:0015976;GO:0071704;GO:0008150;GO:0008152;GO:0044238;GO:0005975;GO:0009605;GO:0050896;GO:0031667;GO:0016020;GO:0110165;GO:0005575;GO:0016787;GO:0030246;GO:0016829;GO:0003674;GO:0005488;GO:0043167;GO:1901363;GO:1901265;GO:0016491;GO:0043168;GO:0043169;GO:0050660;GO:0046914;GO:0016627;GO:0000166;GO:0090599;GO:0016835;GO:0017150;GO:0046872;GO:0015926;GO:0140098;GO:0016798;GO:0036094;GO:0003824;GO:0004089;GO:0097159;GO:0004553;GO:0140101;GO:0016836;GO:0008270	K01673	cynT, can	map00910	Nitrogen metabolisr	COG0288	P:Inorganic ion transport and metabo	PF00484.22	Pro_CA	Carbonic anhydrase	CYT	1	5	26.9	High
TRINITY_DN94625_c0_g1_i1_orf1	uncharacterized protein LOC114354112 [Ostrinia furnacalis]	2.106352162	1.074746662	1.11E-06	up	yes	1.018	0.4833	0.493	0.471	0.486	1	1.027	1.027	CC:cytoplasm;CC:cellular anatomical entity;CC:cellular_component;MF:purine ribonucleoside triphosphate binding;MF:molecular_function;MF:binding;MF:nucleic acid binding;MF:ion binding;MF:heterocyclic compound binding;MF:nucleoside phosphate binding;MF:guanyl nucleotide binding;MF:nucleotide binding;MF:guanyl ribonucleotide binding;MF:purine nucleotide binding;MF:GTP binding;MF:hydrolase activity;MF:translation factor activity, RNA binding;MF:GTPase activity;MF:nucleoside-triphosphatase activity;MF:small molecule binding;MF:catalytic activity;MF:translation regulator activity;MF:hydrolase activity, acting on acid anhydrides, in phosphorus-containing anhydrides;MF:translation regulator activity, nucleic acid binding;MF:carbohydrate derivative binding;MF:organic cyclic compound binding;MF:hydrolase activity, acting on acid anhydrides;MF:anion binding;MF:pyrophosphatase activity;MF:purine ribonucleotide binding;MF:translation elongation factor activity;MF:ribonucleotide binding;	GO:0005737;GO:0110165;GO:0005575;GO:0035639;GO:0003674;GO:0005488;GO:0003676;GO:0043167;GO:1901363;GO:1901265;GO:0019001;GO:0000166;GO:0032561;GO:0017076;GO:0005525;GO:0016787;GO:0008135;GO:0003924;GO:0017111;GO:0036094;GO:0003824;GO:0045182;GO:0016818;GO:0090079;GO:0097367;GO:0097159;GO:0016817;GO:0043168;GO:0016462;GO:0032555;GO:0003746;GO:0032553	K02358	tuf, TUFM	------	------	COG0050	J:Translation, ribosomal structure anc	PF00009.30;PF031	GTP_EFTU;GTP_EFTU_D	Elongation factor Tu GTP binding domain;Elongation factor Tu C-terminal domain;Elongation factor Tu domain 2	CYT	12	36	50.9	High
TRINITY_DN10520_c0_g1_i2_orf1	probable 39S ribosomal protein L45, mitochondrial [Ostrinia furnacalis]	2.377272727	1.249307423	0.0002233	up	yes	1.046	0.44	0.417	0.446	0.457	1	1	1.139	CC:ribosome;CC:intracellular non-membrane-bounded organelle;CC:cellular_component;CC:intracellular organelle;CC:non-membrane-bounded organelle;CC:organelle;CC:cellular anatomical entity;	GO:0005840;GO:0043232;GO:0005575;GO:0043229;GO:0043228;GO:0043226;GO:0110165	K17426	MRPL45	------	------	ENOG411019B	S:Function unknown	PF04280.18	Tim44	Tim44-like domain	CYT	4	16	30	High
TRINITY_DN137_c0_g1_i1_orf1	60S ribosomal protein L8 [Cotesia glomerata] >XP_044597650.1 60S ribosomal protein L8 [Cotesia glomerata] >KAG8034499.1 hypothetical protein G9C98_007575 [Cotesia typhae] >CAD6216378.1 GSCOCG00004534001-RA-CDS [Cotesia congregata] >KAH0544237.1 60S ribosomal protein L8 [Cotesia glomerata] >KAH0564528.1 60S ribosomal protein L8 [Cotesia glomerata] >CAG5095185.1 Similar to RpL8: 60S ribosomal protein L8 (Spodoptera frugiperda) [Cotesia congregata]	2.000610128	1.000440047	6.91E-07	up	yes	0.9837	0.4917	0.485	0.494	0.496	1	0.979	0.972	BP:cellular biosynthetic process;BP:cellular macromolecule biosynthetic process;BP:organic substance biosynthetic process;BP:cellular nitrogen compound biosynthetic process;BP:cellular macromolecule metabolic process;BP:cellular amide metabolic process;BP:peptide biosynthetic process;BP:organic substance metabolic process;BP:organonitrogen compound biosynthetic process;BP:amide biosynthetic process;BP:cellular process;BP:biosynthetic process;BP:macromolecule biosynthetic process;BP:biological_process;BP:metabolic process;BP:organonitrogen compound metabolic process;BP:primary metabolic process;BP:protein metabolic process;BP:cellular nitrogen compound metabolic process;BP:cellular metabolic process;BP:macromolecule metabolic process;BP:nitrogen compound metabolic process;BP:peptide metabolic process;BP:translation;CC:large ribosomal subunit;CC:organelle;CC:intracellular organelle;CC:non-membrane-bounded organelle;CC:cellular anatomical entity;CC:ribosomal subunit;CC:macromolecular complex;CC:ribosome;CC:intracellular non-membrane-bounded organelle;CC:ribonucleoprotein complex;CC:cellular_component;MF:nucleic acid binding;MF:molecular_function;MF:binding;MF:heterocyclic compound binding;MF:structural molecule activity;MF:structural constituent of ribosome;MF:organic cyclic	GO:0044249;GO:0034645;GO:1901576;GO:0044271;GO:0044260;GO:0043603;GO:0043043;GO:0071704;GO:1901566;GO:0043604;GO:0009987;GO:0009058;GO:0009059;GO:0008150;GO:0008152;GO:1901564;GO:0044238;GO:0019538;GO:0034641;GO:0044237;GO:0043170;GO:0006807;GO:0006518;GO:0006412;GO:0015934;GO:0043226;GO:0043229;GO:0043228;GO:0110165;GO:0044391;GO:0032991;GO:0005840;GO:0043232;GO:1990904;GO:0005575;GO:0003676;GO:0003674;GO:0005488;GO:1901363;GO:0005198;GO:0003735;GO:0097159;GO:0003723	K02938	RP-L8e, RPL8	map03010;map	Ribosome;Coronavir	COG0090	J:Translation, ribosomal structure anc	PF03947.21;PF001	Ribosomal_L2_C;Ribosc	Ribosomal Proteins L2, C-terminal domain;Ribosomal Proteins L2, RNA binding domain	CYT	3	9	28.1	High
TRINITY_DN1326_c0_g1_i1_orf1	cuticle protein 7-like [Ostrinia furnacalis]	2.123912582	1.086724388	1.46E-05	up	yes	1.001	0.4713	0.458	0.447	0.509	1	1.017	0.987	MF:molecular_function;MF:structural constituent of cuticle;MF:structural molecule activity;	GO:0003674;GO:0042302;GO:0005198	------	------	------	------	ENOG410YSFI;ENOG	S:Function unknown;	PF00379.26	Chitin_bind_4	Insect cuticle protein	CYT	1	27	13.9	High
TRINITY_DN2082_c0_g1_i2_orf1	choline-phosphate cytidylyltransferase B-like isoform X1 [Ostrinia furnacalis]	2.055590256	1.039552719	6.23E-07	up	yes	0.9873	0.4803	0.49	0.482	0.469	1	0.985	0.977	MF:nucleotidyltransferase activity;MF:choline-phosphate cytidylyltransferase activity;MF:catalytic activity;MF:transferase activity, transferring phosphorus-containing groups;MF:cytidylyltransferase activity;MF:transferase activity;MF:molecular_function;	GO:0016779;GO:0004105;GO:0003824;GO:0016772;GO:0070567;GO:0016740;GO:0003674	K00968	PCYT1	map00564;map	Glycerophospholipic	COG0615;COG0615	I:Lipid transport and metabolism;M:C	PF01467.29	CTP_transf_like	Cytidylyltransferase-like	CYT	5	17	45.3	High
TRINITY_DN391_c5_g1_i1_orf1	hypothetical protein B5X24_HaOG210395 [Helicoverpa armigera]	2.178625623	1.123418304	4.11E-07	up	yes	1.005	0.4613	0.446	0.464	0.474	1	1.011	1.004	MF:oxidoreductase activity;MF:NAD binding;MF:nucleotide binding;MF:small molecule binding;MF:catalytic activity;MF:molecular_function;MF:binding;MF:organic cyclic compound binding;MF:quinone binding;MF:heterocyclic compound binding;MF:nucleoside phosphate binding;MF:oxidoreductase activity, acting on NA;	GO:0016491;GO:0051287;GO:0000166;GO:0036094;GO:0003824;GO:0003674;GO:0005488;GO:0097159;GO:0048038;GO:1901363;GO:1901265;GO:0016651	K03935	NDUFS2	map04714;map	Thermogenesis;Oxic	COG0649	C:Energy production and conversion	PF00346.22	Complex1_49kDa	Respiratory-chain NADH dehydrogenase, 49 Kd subunit	CYT	8	26	52.6	High
TRINITY_DN29448_c0_g1_i1_orf1	28S ribosomal protein S9, mitochondrial [Ostrinia furnacalis]	2.077754237	1.055025018	1.85E-06	up	yes	0.9807	0.472	0.478	0.467	0.471	1	0.982	0.96	BP:cellular biosynthetic process;BP:cellular nitrogen compound metabolic process;BP:nitrogen compound metabolic process;BP:cellular macromolecule biosynthetic process;BP:organic substance biosynthetic process;BP:cellular nitrogen compound biosynthetic process;BP:cellular macromolecule metabolic process;BP:peptide biosynthetic process;BP:organic substance metabolic process;BP:amide biosynthetic process;BP:cellular process;BP:biosynthetic process;BP:macromolecule biosynthetic process;BP:biological_process;BP:metabolic process;BP:organonitrogen compound metabolic process;BP:primary metabolic process;BP:cellular amide metabolic process;BP:organonitrogen compound biosynthetic process;BP:protein metabolic process;BP:cellular metabolic process;BP:macromolecule metabolic process;BP:peptide metabolic process;BP:translation;CC:intracellular non-membrane-bounded organelle;CC:organelle;CC:cellular anatomical entity;CC:ribosome;CC:macromolecular complex;CC:ribonucleoprotein complex;CC:intracellular organelle;CC:cellular_component;CC:non-membrane-bounded organelle;MF:molecular_function;MF:structural molecule activity;MF:structural constituent of ribosome;	GO:0044249;GO:0034641;GO:0006807;GO:0034645;GO:1901576;GO:0044271;GO:0044260;GO:0043043;GO:0071704;GO:0043604;GO:0009987;GO:0009058;GO:0009059;GO:0008150;GO:0008152;GO:1901564;GO:0044238;GO:0043603;GO:1901566;GO:0019538;GO:0044237;GO:0043170;GO:0006518;GO:0006412;GO:0043232;GO:0043226;GO:0110165;GO:0005840;GO:0032991;GO:1990904;GO:0043229;GO:0005575;GO:0043228;GO:0003674;GO:0005198;GO:0003735	K02996	RP-S9, MRPS!	map03010	Ribosome	COG0103	J:Translation, ribosomal structure anc	PF00380.22	Ribosomal_S9	Ribosomal protein S9/S16	CYT	2	5	45.9	High
TRINITY_DN7964_c0_g1_i1_orfp1	TRINITY_DN7964_c0_g1_i1_m.23483 TRINITY_DN7964_c0_g1::TRINITY_DN7964_c0_g1_i1::g.23483 ORF type:internal len:79 (+),score=44.25 TRINITY_DN7964_c0_g1_i1:3-236(+)	2.503028835	1.323674911	0.0001033	up	yes	1.033	0.4127	0.468	0.347	0.423	1	1.067	1.033	------	------	------	------	------	------	------	------	------	------	------	CYT	2	17	8.7	High
TRINITY_DN46173_c0_g3_i2_orf1	tropomyosin-1, isoforms 9A/A/B isoform X33 [Aedes aegypti] >EAT46020.1 AAEL002761-PB [Aedes aegypti]	2.299563519	1.201360049	6.78E-05	up	yes	1.001	0.4353	0.379	0.485	0.442	1	1.022	0.981	------	------	K10373	TPM1	map05206;map	MicroRNAs in cance	ENOG410XR5K	S:Function unknown	PF00261.23;PF127	Tropomyosin;Tropomy	Tropomyosin;Tropomyosin like	CYT	2	24	29	High
TRINITY_DN86956_c0_g5_i1_orf1	PREDICTED: protein sly1 homolog [Microplitis demolitor]	3.165697674	1.662523484	0.000283	up	yes	1.089	0.344	0.396	0.308	0.328	1	1.074	1.194	BP:transport;BP:biological_process;BP:vesicle-mediated transport;BP:establishment of localization;BP:localization;	GO:0006810;GO:0008150;GO:0016192;GO:0051234;GO:0051179	K19998	SCFD1, SLY1	------	------	COG5158	U:Intracellular trafficking, secretion, a	PF00995.26	Sec1	Sec1 family	CYT	1	1	71.3	High
TRINITY_DN16482_c0_g1_i6_orf1	transmembrane protein 120 homolog isoform X2 [Ostrinia furnacalis]	2.116851973	1.081920388	7.96E-05	up	yes	0.971	0.4587	0.47	0.439	0.467	1	0.912	1.001	CC:cellular_component;CC:integral component of membrane;CC:cellular anatomical entity;CC:intrinsic component of membrane;	GO:0005575;GO:0016021;GO:0110165;GO:0031224	------	------	------	------	ENOG410XT6G	S:Function unknown	PF07851.16	TMPIT	TMPIT-like protein	CYT	1	2	47.7	High
TRINITY_DN536_c0_g1_i7_orf1	polymerase delta-interacting protein 2 isoform X3 [Ostrinia furnacalis]	2.103275109	1.072637567	0.0005178	up	yes	0.9633	0.458	0.372	0.499	0.503	1	0.973	0.917	MF:organic cyclic compound binding;MF:molecular_function;MF:binding;MF:nucleic acid binding;MF:DNA binding;MF:heterocyclic compound binding;	GO:0097159;GO:0003674;GO:0005488;GO:0003676;GO:0003677;GO:1901363	K17809	POLDIP2	------	------	COG2967	P:Inorganic ion transport and metabo	PF04379.17;PF087	DUF525;YccV-like	ApaG domain;Hemimethylated DNA-binding protein YccV like	CYT	2	12	39.3	High
TRINITY_DN27994_c0_g1_i1_orf1	uncharacterized protein LOC114364076 [Ostrinia furnacalis]	2.051724138	1.036836768	0.0004393	up	yes	0.952	0.464	0.396	0.512	0.484	1	0.958	0.898	------	------	------	------	------	------	ENOG410XRSQ	S:Function unknown	PF00650.23	CRAL_TRIO	CRAL/TRIO domain	CYT	2	8	35	High
TRINITY_DN29934_c0_g1_i6_orf1	sodium/potassium-transporting ATPase subunit beta-2-like [Ostrinia furnacalis] >XP_028176258.1 sodium/potassium-transporting ATPase subunit beta-2-like [Ostrinia furnacalis]	3.71	1.891419187	0.000312	up	yes	1.113	0.3	0.296	0.255	0.349	1	1.223	1.117	BP:potassium ion transport;BP:cation transport;BP:ion transport;BP:transport;BP:sodium ion transport;BP:metal ion transport;BP:biological_process;BP:establishment of localization;BP:localization;CC:ATPase dependent transmembrane transport complex;CC:transmembrane transporter complex;CC:cellular_component;CC:transporter complex;CC:plasma membrane protein complex;CC:membrane protein complex;CC:sodiu;CC:macromolecular complex;CC:cation-transporting ATPase complex;	GO:0006813;GO:0006812;GO:0006811;GO:0006810;GO:0006814;GO:0030001;GO:0008150;GO:0051234;GO:0051179;GO:0098533;GO:1902495;GO:0005575;GO:1990351;GO:0098797;GO:0098796;GO:0005890;GO:0032991;GO:0090533	K01540	ATP1B, CD29(	map04918;map	Thyroid hormone sy	ENOG411150A	U:Intracellular trafficking, secretion, a	PF00287.21	Na_K-ATPase	Sodium / potassium ATPase beta chain	CYT	1	4	37.5	High
TRINITY_DN23343_c0_g1_i9_orf1	pre-mRNA-processing factor 6 isoform X1 [Ostrinia furnacalis] >XP_028175021.1 pre-mRNA-processing factor 6 isoform X2 [Ostrinia furnacalis]	2.295810906	1.199003819	0.0001524	up	yes	0.981	0.4273	0.471	0.427	0.384	1	1.022	0.921	BP:nucleic acid metabolic process;BP:cellular nitrogen compound metabolic process;BP:nitrogen compound metabolic process;BP:mRNA splicing, via spliceosome;BP:organic cyclic compound metabolic process;BP:nucleobase-containing compound metabolic process;BP:organic substance metabolic process;BP:RNA splicing;BP:mRNA metabolic process;BP:cellular process;BP:cellular aromatic compound metabolic process;BP:RNA splicing, via transesterification reactions;BP:RNA splicing, via transesterification reactions with bulged adenosine as nucleophile;BP:biological_process;BP:metabolic process;BP:heterocycle metabolic process;BP:RNA metabolic process;BP:primary metabolic process;BP:cellular metabolic process;BP:macromolecule metabolic process;BP:RNA processing;BP:mRNA processing;	GO:0090304;GO:0034641;GO:0006807;GO:0000398;GO:1901360;GO:0006139;GO:0071704;GO:0008380;GO:0016071;GO:0009987;GO:0006725;GO:0000375;GO:0000377;GO:0008150;GO:0008152;GO:0046483;GO:0016070;GO:0044238;GO:0044237;GO:0043170;GO:0006396;GO:0006397	K12855	PRPF6, PRP6	map03040	Spliceosome	ENOG410XRKD	A:RNA processing and modification	PF06424.15;PF134	PRP1_N;TPR_14;TPR_19	PRP1 splicing factor, N-terminal;Tetratricopeptide repeat;Tetratricopeptide repeat	CYT	1	1	104.7	High
TRINITY_DN30_c0_g1_i6_orf1	casein kinase I-like isoform X1 [Hyposmocoma kahamanoa]	2.09752166	1.068685709	0.0001369	up	yes	1.041	0.4963	0.446	0.508	0.535	1	1.031	1.093	BP:phosphorylation;BP:nitrogen compound metabolic process;BP:organic substance metabolic process;BP:protein phosphorylation;BP:cellular process;BP:macromolecule modification;BP:protein modification process;BP:biological_process;BP:metabolic process;BP:primary metabolic process;BP:organonitrogen compound metabolic process;BP:cellular metabolic process;BP:macromolecule metabolic process;BP:protein metabolic process;BP:phosphate-containing compound metabolic process;BP:phosphorus metabolic process;MF:purine ribonucleoside triphosphate binding;MF:molecular_function;MF:binding;MF:ion binding;MF:heterocyclic compound binding;MF:nucleoside phosphate binding;MF:anion binding;MF:carbohydrate derivative binding;MF:nucleotide binding;MF:protein serine/threonine kinase activity;MF:transferase activity;MF:protein kinase activity;MF:purine nucleotide binding;MF:ATP binding;MF:kinase activity;MF:catalytic activity, acting on a protein;MF:small molecule binding;MF:catalytic activity;MF:adenyl nucleotide binding;MF:phosphotransferase activity, alcohol group as acceptor;MF:transferase activity, transferring phosphorus-containing groups;MF:adenyl ribonucleotide binding;MF:purine ribonucleotide binding;MF:organic cyclic compound binding;MF:ribonucleotide	GO:0016310;GO:0006807;GO:0071704;GO:0006468;GO:0009987;GO:0043412;GO:0036211;GO:0008150;GO:0008152;GO:0044238;GO:1901564;GO:0044237;GO:0043170;GO:0019538;GO:0006796;GO:0006793;GO:0035639;GO:0003674;GO:0005488;GO:0043167;GO:1901363;GO:1901265;GO:0043168;GO:0097367;GO:0000166;GO:0004674;GO:0016740;GO:0004672;GO:0017076;GO:0005524;GO:0016301;GO:0140096;GO:0036094;GO:0003824;GO:0030554;GO:0016773;GO:0016772;GO:0032559;GO:0032555;GO:0097159;GO:0032553	K08957	CSNK1A	map05165;map	Human papillomavir	ENOG410XPGP	T:Signal transduction mechanisms	PF00069.28;PF077	Pkinase;PK_Tyr_Ser-Thi	Protein kinase domain;Protein tyrosine and serine/threonine kinase;Fungal protein kinase	CYT	1	11	43.4	High
TRINITY_DN9059_c0_g1_i1_orf1	sulfotransferase family cytosolic 1B member 1-like [Ostrinia furnacalis]	2.04367439	1.031165356	0.0002526	up	yes	0.9967	0.4877	0.418	0.56	0.485	1	0.985	1.005	MF:catalytic activity;MF:transferase activity;MF:transferase activity, transferring sulfur-containing groups;MF:sulfotransferase activity;MF:molecular_function;	GO:0003824;GO:0016740;GO:0016782;GO:0008146;GO:0003674	------	------	------	------	ENOG4111H56	S:Function unknown	PF00685.30	Sulfotransfer_1	Sulfotransferase domain	CYT	1	22	20.7	High
TRINITY_DN38301_c0_g1_i2_orf1	gamma-taxilin [Ostrinia furnacalis]	2.077919365	1.055139671	3.60E-06	up	yes	0.9947	0.4787	0.484	0.453	0.499	1	0.999	0.985	MF:molecular_function;MF:SNARE binding;MF:syntaxin binding;MF:binding;MF:protein binding;	GO:0003674;GO:0000149;GO:0019905;GO:0005488;GO:0005515	------	------	------	------	ENOG410XQH1	S:Function unknown	PF09728.12	Taxilin	Myosin-like coiled-coil protein	CYT	7	12	71.1	High
TRINITY_DN1313_c0_g1_i2_orf1	39S ribosomal protein L40, mitochondrial [Ostrinia furnacalis]	2.456264775	1.296466086	4.64E-05	up	yes	1.039	0.423	0.405	0.418	0.446	1	1.019	1.099	CC:large ribosomal subunit;CC:organellar large ribosomal subunit;CC:ribonucleoprotein complex;CC:ribosomal subunit;CC:cellular_component;CC:mitochondrial large ribosomal subunit;CC:macromolecular complex;CC:mitochondrial protein complex;	GO:0015934;GO:0000315;GO:1990904;GO:0044391;GO:0005575;GO:0005762;GO:0032991;GO:0098798	K17421	MRPL40	------	------	ENOG4111PQW	S:Function unknown	PF09812.12	MRP-L28	Mitochondrial ribosomal protein L28	CYT	1	11	23.8	High
TRINITY_DN32997_c0_g1_i8_orf1	RNA-binding protein squid isoform X1 [Ostrinia furnacalis]	2.365566582	1.242185768	1.71E-06	up	yes	1.025	0.4333	0.439	0.435	0.426	1	1.032	1.044	------	------	K03102	SQD	map04320	Dorso-ventral axis fc	COG0724	G:Carbohydrate transport and metab	PF00076.25;PF163	RRM_1;RRM_7	RNA recognition motif.;RNA recognition motif	CYT	8	21	30	High
TRINITY_DN4956_c0_g1_i6_orf1	nucleolar GTP-binding protein 1 [Ostrinia furnacalis]	2.309090909	1.207324973	6.91E-05	up	yes	1.016	0.44	0.377	0.475	0.468	1	1.039	1.008	BP:ribosome biogenesis;BP:cellular component organization or biogenesis;BP:ribonucleoprotein complex biogenesis;BP:cellular process;BP:biological_process;BP:cellular component biogenesis;CC:intracellular organelle;CC:non-membrane-bounded organelle;CC:organelle;CC:nucleolus;CC:cellular anatomical entity;CC:intracellular non-membrane-bounded organelle;CC:cellular_component;MF:purine ribonucleoside triphosphate binding;MF:heterocyclic compound binding;MF:molecular_function;MF:binding;MF:ion binding;MF:nucleoside phosphate binding;MF:anion binding;MF:nucleotide binding;MF:guanyl ribonucleotide binding;MF:purine nucleotide binding;MF:GTP binding;MF:small molecule binding;MF:carbohydrate derivative binding;MF:organic cyclic compound binding;MF:guanyl nucleotide binding;MF:purine ribonucleotide binding;MF:ribonucleotide binding;	GO:0042254;GO:0071840;GO:0022613;GO:0009987;GO:0008150;GO:0044085;GO:0043229;GO:0043228;GO:0043226;GO:0005730;GO:0110165;GO:0043232;GO:0005575;GO:0035639;GO:1901363;GO:0003674;GO:0005488;GO:0043167;GO:1901265;GO:0043168;GO:0000166;GO:0032561;GO:0017076;GO:0005525;GO:0036094;GO:0097367;GO:0097159;GO:0019001;GO:0032555;GO:0032553	K06943	NOG1	map03008	Ribosome biogenesi	COG1084	S:Function unknown	PF17835.4;PF0685	NOG1_N;NOG1;NOGC	NOG1 N-terminal helical domain;Nucleolar GTP-binding protein 1;NOGCT;50S ribosome-binding GTPase;Ferrous iron transport protein B	CYT	6	10	75.1	High
TRINITY_DN83005_c0_g1_i1_orf1	ATP synthase subunit O, mitochondrial [Danaus plexippus plexippus] >OWR53927.1 H+ transporting ATP synthase O subunit [Danaus plexippus plexippus]	2.194157698	1.133667219	1.77E-07	up	yes	0.999	0.4553	0.458	0.448	0.46	1	1.009	0.988	CC:cellular_component;CC:organelle membrane;CC:membrane;CC:cellular anatomical entity;CC:mitochondrial membrane;CC:organelle inner membrane;CC:mitochondrial inner membrane;MF:cation channel activity;MF:molecular_function;MF:proton-transporting ATP synthase activity, rotational mechanism;MF:inorganic solute uptake transmembrane transporter activity;MF:hydrogen ion channel activity;MF:passive transmembrane transporter activity;MF:transporter activity;MF:ion channel activity;MF:cation transmembrane transporter activity;MF:catalytic activity;MF:ligase activity;MF:inorganic cation transmembrane transporter activity;MF:ion transmembrane transporter activity;MF:transmembrane transporter activity;MF:channel activity;MF:hydrogen ion transmembrane transporter activity;	GO:0005575;GO:0031090;GO:0016020;GO:0110165;GO:0031966;GO:0019866;GO:0005743;GO:0005261;GO:0003674;GO:0046933;GO:0015318;GO:0015252;GO:0022803;GO:0005215;GO:0005216;GO:0008324;GO:0003824;GO:0016874;GO:0022890;GO:0015075;GO:0022857;GO:0015267;GO:0015078	K02137	ATPeF0O, ATI	map04714;map	Thermogenesis;Cher	COG0712;COG0442	C:Energy production and conversion;	PF00213.21	OSCP	ATP synthase delta	CYT	13	55	22.7	High
TRINITY_DN3733_c0_g1_i1_orf1	60S ribosomal protein L37, partial [Papilio machaon]	2.308219178	1.206780222	0.0001915	up	yes	1.011	0.438	0.486	0.411	0.417	1	1.079	0.955	BP:organonitrogen compound biosynthetic process;BP:cellular macromolecule biosynthetic process;BP:cellular biosynthetic process;BP:cellular nitrogen compound biosynthetic process;BP:cellular macromolecule metabolic process;BP:cellular amide metabolic process;BP:peptide biosynthetic process;BP:organic substance metabolic process;BP:macromolecule metabolic process;BP:organic substance biosynthetic process;BP:cellular process;BP:biosynthetic process;BP:macromolecule biosynthetic process;BP:biological_process;BP:metabolic process;BP:organonitrogen compound metabolic process;BP:amide biosynthetic process;BP:primary metabolic process;BP:protein metabolic process;BP:cellular nitrogen compound metabolic process;BP:cellular metabolic process;BP:nitrogen compound metabolic process;BP:peptide metabolic process;BP:translation;CC:organelle;CC:intracellular organelle;CC:non-membrane-bounded organelle;CC:cellular anatomical entity;CC:macromolecular complex;CC:ribosome;CC:intracellular non-membrane-bounded organelle;CC:ribonucleoprotein complex;CC:cellular_component;MF:molecular_function;MF:binding;MF:nucleic acid binding;MF:structural molecule activity;MF:heterocyclic compound binding;MF:cation binding;MF:ion binding;MF:structural constituent of ribosome;MF:metal ion binding;MF:organic cyclic compound binding;MF:rRNA binding;MF:RNA binding;	GO:1901566;GO:0034645;GO:0044249;GO:0044271;GO:0044260;GO:0043603;GO:0043043;GO:0071704;GO:0043170;GO:1901576;GO:0009987;GO:0009058;GO:0009059;GO:0008150;GO:0008152;GO:1901564;GO:0043604;GO:0044238;GO:0019538;GO:0034641;GO:0044237;GO:0006807;GO:0006518;GO:0006412;GO:0043226;GO:0043229;GO:0043228;GO:0110165;GO:0032991;GO:0005840;GO:0043232;GO:1990904;GO:0005575;GO:0003674;GO:0005488;GO:0003676;GO:0005198;GO:1901363;GO:0043169;GO:0043167;GO:0003735;GO:0046872;GO:0097159;GO:0019843;GO:0003723	K02922	RP-L37e, RPL:	map03010;map	Ribosome;Coronavir	COG2126	J:Translation, ribosomal structure anc	PF01907.22	Ribosomal_L37e	Ribosomal protein L37e	PLA	4	30	11.4	High
TRINITY_DN63914_c0_g1_i1_orf1	myophilin-like [Ostrinia furnacalis]	2.426488166	1.278869824	1.58E-06	up	yes	1.015	0.4183	0.433	0.423	0.399	1	1.031	1.013	------	------	K20526	TAGLN	------	------	COG5199	Z:Cytoskeleton	PF00307.34	CH	Calponin homology	CYT	9	51	20.7	High
TRINITY_DN753_c0_g1_i4_orf1	venom dipeptidyl peptidase 4-like isoform X2 [Ostrinia furnacalis]	2.404098165	1.265495806	4.35E-05	up	yes	1.009	0.4197	0.362	0.434	0.463	1	1.006	1.022	BP:primary metabolic process;BP:organonitrogen compound metabolic process;BP:protein metabolic process;BP:macromolecule metabolic process;BP:organic substance metabolic process;BP:nitrogen compound metabolic process;BP:biological_process;BP:metabolic process;BP:proteolysis;CC:integral component of membrane;CC:cellular_component;CC:cellular anatomical entity;CC:intrinsic component of membrane;MF:molecular_function;MF:serine hydrolase activity;MF:hydrolase activity;MF:catalytic activity, acting on a protein;MF:catalytic activity;MF:peptidase activity;MF:serine-type peptidase activity;	GO:0044238;GO:1901564;GO:0019538;GO:0043170;GO:0071704;GO:0006807;GO:0008150;GO:0008152;GO:0006508;GO:0016021;GO:0005575;GO:0110165;GO:0031224;GO:0003674;GO:0017171;GO:0016787;GO:0140096;GO:0003824;GO:0008233;GO:0008236	K01278	DPP4, CD26	map04974	Protein digestion an	COG1506;COG1506	J:Translation, ribosomal structure anc	PF00930.24;PF003	DPPIV_N;Peptidase_S9	Dipeptidyl peptidase IV;Prolyl oligopeptidase family	CYT	3	5	92.6	High
TRINITY_DN1079_c0_g1_i4_orf1	CD109 antigen-like [Ostrinia furnacalis]	2.46179966	1.299713361	1.95E-06	up	yes	1.015	0.4123	0.415	0.392	0.43	1	1.014	1.032	CC:cellular_component;CC:cellular anatomical entity;CC:extracellular space;MF:endopeptidase inhibitor activity;MF:endopeptidase regulator activity;MF:peptidase regulator activity;MF:molecular_function;MF:peptidase inhibitor activity;MF:enzyme inhibitor activity;MF:molecular function regulator;MF:enzyme regulator activity;	GO:0005575;GO:0110165;GO:0005615;GO:0004866;GO:0061135;GO:0061134;GO:0003674;GO:0030414;GO:0004857;GO:0098772;GO:0030234	K06530	CD109	------	------	ENOG410XRED;ENO(	S:Function unknown;	PF07678.17;PF018	TED_complement;MG2	A-macroglobulin TED domain;MG2 domain;Alpha-2-macroglobulin bait region domain;Alpha-2-macroglobulin family;Macroglobulin domain MG3	CYT	3	2	146.3	High
TRINITY_DN2780_c0_g1_i5_orf1	probable ATP-dependent RNA helicase DDX27 [Ostrinia furnacalis]	2.465218453	1.301715495	0.0002274	up	yes	1.01	0.4097	0.336	0.497	0.396	1	1.005	1.025	------	------	K13181	DDX27, DRS1	------	------	COG0513	O:Posttranslational modification, pro	PF00270.32;PF002	DEAD;Helicase_C;ResIII	DEAD/DEAH box helicase;Helicase conserved C-terminal domain;Type III restriction enzyme, res	CYT	4	7	90.9	High
TRINITY_DN18860_c0_g1_i1_orf1	DDB1- and CUL4-associated factor 13 [Ostrinia furnacalis]	2.286342593	1.193041598	9.91E-06	up	yes	0.9877	0.432	0.441	0.397	0.458	1	0.972	0.991	BP:nucleic acid metabolic process;BP:cellular nitrogen compound metabolic process;BP:nitrogen compound metabolic process;BP:ncRNA metabolic process;BP:organic cyclic compound metabolic process;BP:nucleobase-containing compound metabolic process;BP:protein modification by small protein conjugation;BP:protein modification by small protein conjugation or removal;BP:organic substance metabolic process;BP:rRNA processing;BP:ncRNA processing;BP:cellular process;BP:cellular aromatic compound metabolic process;BP:macromolecule modification;BP:protein modification process;BP:biological_process;BP:metabolic process;BP:heterocycle metabolic process;BP:RNA metabolic process;BP:primary metabolic process;BP:rRNA metabolic process;BP:organonitrogen compound metabolic process;BP:protein metabolic process;BP:cellular metabolic process;BP:macromolecule metabolic process;BP:protein ubiquitination;BP:RNA processing;CC:organelle;CC:cellular anatomical entity;CC:intracellular non-membrane-bounded organelle;CC:nucleolus;CC:intracellular organelle;CC:cellular_component;CC:non-membrane-bounded organelle;	GO:0090304;GO:0034641;GO:0006807;GO:0034660;GO:1901360;GO:0006139;GO:0032446;GO:0070647;GO:0071704;GO:0006364;GO:0034470;GO:0009987;GO:0006725;GO:0043412;GO:0036211;GO:0008150;GO:0008152;GO:0046483;GO:0016070;GO:0044238;GO:0016072;GO:1901564;GO:0019538;GO:0044237;GO:0043170;GO:0016567;GO:0006396;GO:0043226;GO:0110165;GO:0043232;GO:0005730;GO:0043229;GO:0005575;GO:0043228	K11806	DCAF13, WDS	------	------	ENOG410XNX6	S:Function unknown	PF04158.17;PF004	Sof1;WD40;ANAPC4_W	Sof1-like domain;WD domain, G-beta repeat;Anaphase-promoting complex subunit 4 WD40 domain	CYT	2	3	51.5	High
TRINITY_DN50571_c1_g1_i1_orf1	WD repeat-containing protein 46 [Ostrinia furnacalis]	2.206349206	1.141661149	0.0001464	up	yes	0.973	0.441	0.418	0.502	0.403	1	0.989	0.93	CC:intracellular non-membrane-bounded organelle;CC:nucleolus;CC:cellular_component;CC:intracellular organelle;CC:non-membrane-bounded organelle;CC:organelle;CC:cellular anatomical entity;	GO:0043232;GO:0005730;GO:0005575;GO:0043229;GO:0043228;GO:0043226;GO:0110165	K14768	UTP7, WDR46	------	------	ENOG410XP5D	S:Function unknown	PF08149.14	BING4CT	BING4CT	CYT	1	2	65.3	High
TRINITY_DN501_c1_g1_i1_orf1	sodium- and chloride-dependent GABA transporter ine isoform X1 [Ostrinia furnacalis]	2.443653327	1.289039629	1.37E-05	up	yes	0.9953	0.4073	0.434	0.395	0.393	1	1.025	0.961	BP:transmission of nerve impulse;BP:multicellular organismal signaling;BP:developmental cell growth;BP:developmental growth;BP:cell growth;BP:glial cell growth;BP:multicellular organismal process;BP:neurological system process;BP:biological_process;BP:growth;BP:system process;BP:signaling;BP:cellular process;CC:intrinsic component of membrane;CC:cellular_component;CC:integral component of membrane;CC:cellular anatomical entity;MF:neurotransmitte;MF:neurotransmitter transporter activity;MF:molecular transducer activity;MF:molecular_function;MF:signaling receptor activity;MF:sodium ion transmembrane transporter activity;MF:inorganic solute uptake transmembrane transporter activity;MF:transmembrane signaling receptor activity;MF:osmosensor activity;MF:solut;MF:transporter activity;MF:metal ion transmembrane transporter activity;MF:small molecule sensor activity;MF:active transmembrane transporter activity;MF:solut;MF:secondary active transmembrane transporter activity;MF:symporter activity;MF:cation transmembrane transporter activity;MF:inorganic cation transmembrane transporter activity;MF:ion transmembrane transporter activity;MF:transmembrane transporter activity;MF:active	GO:0019226;GO:0035637;GO:0048588;GO:0048589;GO:0016049;GO:0042065;GO:0032501;GO:0050877;GO:0008150;GO:0040007;GO:0003008;GO:0023052;GO:0009987;GO:0031224;GO:0005575;GO:0016021;GO:0110165;GO:0005328;GO:0005326;GO:0060089;GO:0003674;GO:0038023;GO:0015081;GO:0015318;GO:0004888;GO:0005034;GO:0015370;GO:0005215;GO:0046873;GO:0140299;GO:0022804;GO:0015294;GO:0015291;GO:0015293;GO:0008324;GO:0022890;GO:0015075;GO:0022857;GO:0022853	K05039	SLC6A6, TAUT	------	------	COG0733	S:Function unknown	PF00209.21	SNF	Sodiu	CYT	1	1	80.8	High
TRINITY_DN48641_c0_g1_i4_orf1	RNA-binding protein 45-like [Galleria mellonella]	2.625685013	1.392693856	0.0008442	up	yes	1.102	0.4197	0.501	0.362	0.396	1	1.218	1.089	MF:organic cyclic compound binding;MF:molecular_function;MF:binding;MF:nucleic acid binding;MF:heterocyclic compound binding;MF:RNA binding;	GO:0097159;GO:0003674;GO:0005488;GO:0003676;GO:1901363;GO:0003723	K25081	RBM45	------	------	ENOG410XP9Z	S:Function unknown	PF00076.25;PF087	RRM_1;RRM_3	RNA recognition motif.;RNA binding motif	CYT	1	2	53.8	High
TRINITY_DN783_c0_g1_i7_orf1	microtubule-associated protein Jupiter isoform X4 [Helicoverpa armigera]	2.060728745	1.043154614	8.62E-05	up	yes	1.018	0.494	0.467	0.49	0.525	1	0.981	1.072	------	------	------	------	------	------	ENOG410Z68G	S:Function unknown	PF17054.8	JUPITER	Microtubule-Associated protein Jupiter	EXC	2	12	24	High
TRINITY_DN26411_c0_g1_i2_orfp1	TRINITY_DN26411_c0_g1_i2_m.24123 TRINITY_DN26411_c0_g1::TRINITY_DN26411_c0_g1_i2::g.24123 ORF type:internal len:115 (-),score=72.83 TRINITY_DN26411_c0_g1_i2:3-344(-)	2.099856174	1.070290517	0.002086	up	yes	1.022	0.4867	0.377	0.472	0.611	1	1.086	0.979	------	------	------	------	------	------	------	------	------	------	------	CYT	1	46	10.9	High
TRINITY_DN19814_c0_g1_i4_orf1	general odorant-binding protein 28a-like [Ostrinia furnacalis]	2.030967223	1.022166957	9.99E-05	up	yes	1.01	0.4973	0.451	0.528	0.513	1	1.054	0.976	MF:odorant binding;MF:molecular_function;MF:binding;	GO:0005549;GO:0003674;GO:0005488	------	------	------	------	ENOG4110QNN;ENC	S:Function unknown;	PF01395.25	PBP_GOBP	PBP/GOBP family	CYT	1	5	16.7	High
TRINITY_DN1477_c0_g1_i5_orf1	mitochondrial import inner membrane translocase subunit Tim9 [Ostrinia furnacalis]	2.074739637	1.052930301	1.54E-05	up	yes	1.016	0.4897	0.507	0.5	0.462	1	1.048	1.001	------	------	K17777	TIM9	------	------	ENOG4111TUA	O:Posttranslational modification, pro	PF02953.18	zf-Tim10_DDP	Tim10/DDP family zinc finger	PLA	3	35	10.7	High
TRINITY_DN886_c0_g2_i4_orf1	collagenase-like [Ostrinia furnacalis]	2.065767635	1.046677984	1.03E-05	up	yes	0.9957	0.482	0.455	0.51	0.481	1	0.977	1.01	------	------	------	------	------	------	COG5640	S:Function unknown	PF00089.29	Trypsin	Trypsin	EXC	2	8	30.5	High
TRINITY_DN14705_c0_g2_i1_orf1	coiled-coil-helix-coiled-coil-helix domain-containing protein 10, mitochondrial [Ostrinia furnacalis]	2.129136924	1.090268732	0.0005003	up	yes	0.9843	0.4623	0.416	0.552	0.419	1	0.938	1.015	------	------	K22758	CHCHD2	------	------	ENOG41126E7;ENO(	S:Function unknown;	PF06747.16	CHCH	CHCH domain	CYT	1	7	15.2	High

TRINITY_DN19000_c0_g1_i4_orf1	NADH dehydrogenase [ubiquinone] 1 beta subcomplex subunit 9 [Ostrinia furnacalis]	2.036367458	1.025997916	2.12E-05	up	yes	0.9687	0.4757	0.456	0.467	0.504	1	0.956	0.95	BP:electron transport chain;BP:cellular process;BP:aerobic electron transport chain;BP:cellular metabolic process;BP:aerobic respiration;BP:cellular respiration;BP:metabolic process;BP:respiratory electron transport chain;BP:biological_process;BP:mitochondrial electron transport, NADH to ubiquinone;BP:generation of precursor metabolites and energy;BP:energy derivation by oxidation of organic compounds;CC:organelle membrane;CC:membrane;CC:organelle inner membrane;CC:mitochondrial membrane;CC:cellular_component;CC:mitochondrial inner membrane;CC:respiratory chain;CC:cellular anatomical entity;	GO:0022900;GO:0009987;GO:0019646;GO:0044237;GO:0009060;GO:0045333;GO:0008152;GO:0022904;GO:0008150;GO:0006120;GO:0006091;GO:0015980;GO:0031090;GO:0016020;GO:0019866;GO:0031966;GO:0005575;GO:0005743;GO:0070469;GO:0110165	K03965	NDUFB9	map04714;map	Thermogenesis;Oxic	ENOG4111PES	C:Energy production and conversion	------	------	------	CYT	6	43	17.6	High
TRINITY_DN2274_c0_g1_i6_orf1	membrane alanyl aminopeptidase-like [Ostrinia furnacalis]	2.257284843	1.174588481	5.63E-06	up	yes	0.9993	0.4427	0.425	0.471	0.432	1	0.982	1.016	BP:nitrogen compound metabolic process;BP:organic substance metabolic process;BP:proteolysis;BP:biological_process;BP:metabolic process;BP:primary metabolic process;BP:organonitrogen compound metabolic process;BP:protein metabolic process;BP:macromolecule metabolic process;MF:aminopeptidase activity;MF:exopeptidase activity;MF:molecular_function;MF:zinc ion binding;MF:peptidase activity;MF:metallopeptidase activity;MF:cation binding;MF:transition metal ion binding;MF:ion binding;MF:metal ion binding;MF:hydrolase activity;MF:catalytic activity, acting on a protein;MF:catalytic activity;MF:binding;	GO:0006807;GO:0071704;GO:0006508;GO:0008150;GO:0008152;GO:0044238;GO:1901564;GO:0019538;GO:0043170;GO:0004177;GO:0008238;GO:0003674;GO:0008270;GO:0008233;GO:0008237;GO:0043169;GO:0046914;GO:0043167;GO:0046872;GO:0016787;GO:0140096;GO:0003824;GO:0005488	------	------	------	------	COG0308	E:Amino acid transport and metaboli	PF01433.23;PF118	Peptidase_M1;ERAP1_C	Peptidase family M1 domain;ERAP1-like C-terminal domain;Peptidase M1 N-terminal domain	CYT	11	15	114.7	High
TRINITY_DN108122_c0_g1_i9_orf1	hypothetical protein SFRUCORN_003152 [Spodoptera frugiperda]	2.272727273	1.184424571	0.003661	up	yes	1	0.44	0.622	0.327	0.371	1	1.004	0.997	BP:cellular component organization;BP:cell death;BP:mitochondrion organization;BP:cellular component organization or biogenesis;BP:cellular process;BP:apoptotic process;BP:biological_process;BP:organelle organization;BP:programmed cell death;CC:intracellular organelle;CC:membrane-bounded organelle;CC:organelle;CC:mitochondrion;CC:cellular anatomical entity;CC:intracellular membrane-bounded organelle;CC:cellular_component;MF:molecular_function;MF:hydrolase activity;MF:GTPase activity;MF:nucleoside-triphosphatase activity;MF:catalytic activity;MF:hydrolase activity, acting on acid anhydrides, in phosphorus-containing anhydrides;MF:hydrolase activity, acting on acid anhydrides;MF:pyrophosphatase activity;	GO:0016043;GO:0008219;GO:0007005;GO:0071840;GO:0009987;GO:0006915;GO:0008150;GO:0006996;GO:0012501;GO:0043229;GO:0043227;GO:0043226;GO:0005739;GO:0110165;GO:0043231;GO:0005575;GO:0003674;GO:0016787;GO:0003924;GO:0017111;GO:0003824;GO:0016818;GO:0016817;GO:0016462	K17079	OPA1	map05017	Spinocerebellar atax	COG0699	S:Function unknown	PF19434.2	OPA1_C	Dynamin-like GTPase OPA1 C-terminal	CYT	1	20	10.8	High
TRINITY_DN7626_c0_g1_i1_orf1	NADH dehydrogenase [ubiquinone] flavoprotein 1, mitochondrial isoform X1 [Ostrinia furnacalis]	2.268863636	1.181969903	1.24E-06	up	yes	0.9983	0.44	0.44	0.438	0.442	1	1.018	0.977	CC:oxidoreductase complex;CC:mitochondrial inner membrane;CC:membrane;CC:respiratory chain complex;CC:cellular anatomical entity;CC:transmembrane transporter complex;CC:catalytic complex;CC:transporter complex;CC:mitochondrial membrane;CC:organelle membrane;CC:respiratory chain complex I;CC:macromolecular complex;CC:NADH dehydrogenase complex;CC:organelle inner membrane;CC:cellular_component;CC:membrane protein complex;MF:NAD binding;MF:metal ion binding;MF:molecular_function;MF:binding;MF:ion binding;MF:NA;MF:heterocyclic compound binding;MF:nucleoside phosphate binding;MF:oxidoreductase activity;MF:anion binding;MF:cation binding;MF:metal cluster binding;MF:transporter activity;MF:nucleotide binding;MF:primary active transmembrane transporter activity;MF:NADH dehydrogenas;MF:FMN binding;MF:electron carrier activity;MF:oxidoreductase activity, acting on NA;MF:oxidoreduction-driven active transmembrane transporter activity;MF:NADH dehydrogenas;MF:small molecule binding;MF:catalytic activity;MF:NADH dehydrogenase activity;MF:oxidoreductase activity, acting on NA;MF:4 iron, 4 sulfur cluster binding;MF:carbohydrate derivative binding;MF:organic cyclic compound binding;MF:transmembrane transporter activity;MF:active transmembrane	GO:1990204;GO:0005743;GO:0016020;GO:0098803;GO:0110165;GO:1902495;GO:1902494;GO:1990351;GO:0031966;GO:0031090;GO:0045271;GO:0032991;GO:0030964;GO:0019866;GO:0005575;GO:0098796;GO:0051287;GO:0046872;GO:0003674;GO:0005488;GO:0043167;GO:0003955;GO:1901363;GO:1901265;GO:0016491;GO:0043168;GO:0043169;GO:0051540;GO:0005215;GO:0000166;GO:0015399;GO:0050136;GO:0010181;GO:0009055;GO:0016651;GO:0015453;GO:0008137;GO:0036094;GO:0003824;GO:0003954;GO:0016655;GO:0051539;GO:0097367;GO:0097159;GO:0022857;GO:0022804;GO:0051536;GO:0032553	K03942	NDUFV1	map04714;map	Thermogenesis;Oxic	COG1894	C:Energy production and conversion	PF01512.20;PF105	Complex1_51K;NADH_	Respiratory-chain NADH dehydrogenase 51 Kd subunit;NADH-ubiquinone oxidoreductase-F iron-sulfur binding region	CYT	9	24	50.5	High
TRINITY_DN1173_c1_g1_i9_orf1	hypothetical protein evm_001011 [Chilo suppressalis]	2.042643923	1.030437733	6.41E-05	up	yes	0.958	0.469	0.503	0.462	0.442	1	0.946	0.928	BP:phosphorylation;BP:cell differentiation;BP:nitrogen compound metabolic process;BP:anatomical structure morphogenesis;BP:cellular developmental process;BP:organic substance metabolic process;BP:developmental process;BP:protein phosphorylation;BP:cellular process;BP:macromolecule modification;BP:protein modification process;BP:biological_process;BP:metabolic process;BP:primary metabolic process;BP:organonitrogen compound metabolic process;BP:protein metabolic process;BP:cellular metabolic process;BP:macromolecule metabolic process;BP:phosphate-containing compound metabolic process;BP:phosphorus metabolic process;MF:purine ribonucleoside triphosphate binding;MF:guanyl-nucleotide exchange factor activity;MF:molecular_function;MF:binding;MF:molecular function regulator;MF:heterocyclic compound binding;MF:nucleoside phosphate binding;MF:anion binding;MF:ion binding;MF:nucleotide binding;MF:transferase activity;MF:protein kinase activity;MF:purine nucleotide binding;MF:carbohydrate derivative binding;MF:ATP binding;MF:kinase activity;MF:enzyme regulator activity;MF:catalytic activity, acting on a protein;MF:small molecule binding;MF:GTPase regulator activity;MF:nucleoside-triphosphatase regulator activity;MF:catalytic activity;MF:transferase activity, transferring phosphorus-containing groups;MF:adenyl nucleotide binding;MF:phosphotransferase activity, alcohol group as acceptor;MF:adenyl ribonucleotide binding;MF:purine ribonucleotide binding;MF:organic cyclic compound binding;MF:ribonucleotide binding;	GO:0016310;GO:0030154;GO:0006807;GO:0009653;GO:0048869;GO:0071704;GO:0032502;GO:0006468;GO:0009987;GO:0043412;GO:0036211;GO:0008150;GO:0008152;GO:0044238;GO:1901564;GO:0019538;GO:0044237;GO:0043170;GO:0006796;GO:0006793;GO:0035639;GO:0005085;GO:0003674;GO:0005488;GO:0098772;GO:1901363;GO:1901265;GO:0043168;GO:0043167;GO:0000166;GO:0016740;GO:0004672;GO:0017076;GO:0097367;GO:0005524;GO:0016301;GO:0030234;GO:0140096;GO:0036094;GO:0030695;GO:0060589;GO:0003824;GO:0016772;GO:0030554;GO:0016773;GO:0032559;GO:0032555;GO:0097159;GO:0032553	------	------	------	------	ENOG410XQFD;ENO	S:Function unknown;T:Signal transdu	PF07679.19;PF139	I-set;Ig_3;ig;Ig_2;RhoGE	Immunoglobulin I-set domain;Immunoglobulin domain;Immunoglobulin domain;Immunoglobulin domain;RhoGEF domain;Immunoglobulin V-set domain;PH domain	CYT	1	18	160.4	High
TRINITY_DN2993_c0_g1_i4_orf1	heat shock 70 kDa protein cognate 5 [Ostrinia furnacalis]	2.378352718	1.249962688	5.42E-07	up	yes	1.002	0.4213	0.421	0.418	0.425	1	1.02	0.986	MF:purine ribonucleoside triphosphate binding;MF:molecular_function;MF:binding;MF:ion binding;MF:heterocyclic compound binding;MF:nucleoside phosphate binding;MF:anion binding;MF:unfolded protein binding;MF:nucleotide binding;MF:purine nucleotide binding;MF:ATP binding;MF:small molecule binding;MF:adenyl nucleotide binding;MF:carbohydrate derivative binding;MF:organic cyclic compound binding;MF:protein binding involved in protein folding;MF:adenyl ribonucleotide binding;MF:protein binding;MF:purine ribonucleotide binding;MF:ribonucleotide binding;	GO:0035639;GO:0003674;GO:0005488;GO:0043167;GO:1901363;GO:1901265;GO:0043168;GO:0051082;GO:0000166;GO:0017076;GO:0005524;GO:0036094;GO:0030554;GO:0097367;GO:0097159;GO:0044183;GO:0032559;GO:0005515;GO:0032555;GO:0032553	K04043	dnaK, HSPA9	map03018;map	RNA degradation;Tu	COG0443	O:Posttranslational modification, pro	PF00012.23;PF067	HSP70;MreB_Mbl	Hsp70 protein;MreB/Mbl protein	CYT	24	34	74.7	High
TRINITY_DN2117_c0_g1_i1_orf1	BUB3-interacting and GLEBS motif-containing protein ZNF207 [Chelonus insularis]	2.215677286	1.147747768	1.49E-05	up	yes	0.9667	0.4363	0.415	0.456	0.438	1	0.941	0.959	MF:cation binding;MF:organic cyclic compound binding;MF:ion binding;MF:molecular_function;MF:binding;MF:nucleic acid binding;MF:DNA binding;MF:metal ion binding;MF:heterocyclic compound binding;	GO:0043169;GO:0097159;GO:0043167;GO:0003674;GO:0005488;GO:0003676;GO:0003677;GO:0046872;GO:1901363	------	------	------	------	ENOG410ZJGQ	S:Function unknown	PF03153.16	TFIIA	Transcription factor IIA, alpha/beta subunit	CYT	2	4	52.5	High
TRINITY_DN106038_c0_g1_i1_orf1	ankyrin-3-like isoform X1 [Galleria mellonella]	2.004666537	1.003362274	5.43E-05	up	yes	1.031	0.5143	0.546	0.519	0.478	1	1.07	1.024	------	------	K10380	ANK	map05205;map	Proteoglycans in car	COG0666	F:Nucleotide transport and metabolis	PF12796.10;PF136	Ank_2;Ank_4;Ank;Ank_3	Ankyrin repeats;Ankyrin repeats;Ankyrin repeat;Ankyrin repeat;Ankyrin	CYT	3	8	47.2	High
TRINITY_DN4929_c1_g2_i5_orf1	guanylate kinase isoform X2 [Ostrinia furnacalis]	2.075315539	1.053330706	2.86E-06	up	yes	1.003	0.4833	0.49	0.502	0.458	1	1.011	0.999	BP:phosphorylation;BP:cellular process;BP:biological_process;BP:metabolic process;BP:cellular metabolic process;BP:phosphate-containing compound metabolic process;BP:phosphorus metabolic process;MF:nucleobase-containing compound kinase activity;MF:purine ribonucleoside triphosphate binding;MF:molecular_function;MF:binding;MF:ion binding;MF:heterocyclic compound binding;MF:nucleoside phosphate binding;MF:anion binding;MF:nucleotide binding;MF:transferase activity;MF:purine nucleotide binding;MF:ATP binding;MF:kinase activity;MF:guanylate kinase activity;MF:small molecule binding;MF:catalytic activity;MF:adenyl nucleotide binding;MF:carbohydrate derivative binding;MF:transferase activity, transferring phosphorus-containing groups;MF:phosphotransferase activity, phosphate group as acceptor;MF:nucleoside phosphate kinase activity;MF:adenyl ribonucleotide binding;MF:purine ribonucleotide binding;MF:organic cyclic compound binding;MF:ribonucleotide binding;	GO:0016310;GO:0009987;GO:0008150;GO:0008152;GO:0044237;GO:0006796;GO:0006793;GO:0019205;GO:0035639;GO:0003674;GO:0005488;GO:0043167;GO:1901363;GO:1901265;GO:0043168;GO:0000166;GO:0016740;GO:0017076;GO:0005524;GO:0016301;GO:0004385;GO:0036094;GO:0003824;GO:0030554;GO:0097367;GO:0016772;GO:0016776;GO:0050145;GO:0032559;GO:0032555;GO:0097159;GO:0032553	K00942	gmk, GUK1	map01232;map	Nucleotide metaboli	COG0194	U:Intracellular trafficking, secretion, a	PF00625.24;PF136	Guanylate_kin;AAA_33;	Guanylate kinase;AAA domain;AAA ATPase domain;AAA domain;AAA domain	CYT	2	12	22.7	High
TRINITY_DN825_c2_g1_i5_orf1	hypothetical protein SFRURICE_018365 [Spodoptera frugiperda] >KAG8107343.1 hypothetical protein SFRUCORN_012069 [Spodoptera frugiperda] >CAB3509414.1 unnamed protein product [Spodoptera littoralis] >CAH1638995.1 unnamed protein product [Spodoptera littoralis]	2.119484906	1.083713692	5.54E-06	up	yes	1.004	0.4737	0.505	0.466	0.45	1	1.005	1.007	CC:organelle lumen;CC:organelle envelope lumen;CC:membrane-enclosed lumen;CC:cellular_component;CC:intracellular organelle lumen;CC:mitochondrial intermembrane space;CC:cellular anatomical entity;MF:cation binding;MF:metal ion binding;MF:transition metal ion binding;MF:ion binding;MF:molecular_function;MF:binding;MF:copper chaperone activity;MF:metallochaperone activity;MF:copper ion binding;MF:molecular carrier activity;	GO:0043233;GO:0031970;GO:0031974;GO:0005575;GO:0070013;GO:0005758;GO:0110165;GO:0043169;GO:0046872;GO:0046914;GO:0043167;GO:0003674;GO:0005488;GO:0016531;GO:0016530;GO:0005507;GO:0140104	K02260	COX17	map00190;map	Oxidative phosphory	ENOG410XVYQ	O:Posttranslational modification, pro	PF05051.16	COX17	Cytochrome C oxidase copper chaperone	CYT	1	20	7.5	High
TRINITY_DN886_c0_g1_i1_orf1	collagenase-like isoform X1 [Ostrinia furnacalis]	2.087974684	1.06210422	5.44E-05	up	yes	0.9897	0.474	0.462	0.493	0.467	1	0.939	1.03	------	------	------	------	------	------	COG5640	O:Posttranslational modification, pro	PF00089.29	Trypsin	Trypsin	CYT	3	14	29.3	High
TRINITY_DN279_c0_g1_i10_orf1	RE1-silencing transcription factor-like isoform X1 [Ostrinia furnacalis]	2.563915267	1.358348584	3.81E-05	up	yes	1.053	0.4107	0.38	0.443	0.409	1	1.086	1.073	CC:intracellular membrane-bounded organelle;CC:nucleus;CC:cellular_component;CC:intracellular organelle;CC:membrane-bounded organelle;CC:organelle;CC:cellular anatomical entity;MF:cation binding;MF:transition metal ion binding;MF:ion binding;MF:molecular_function;MF:zinc ion binding;MF:metal ion binding;MF:binding;	GO:0043231;GO:0005634;GO:0005575;GO:0043229;GO:0043227;GO:0043226;GO:0110165;GO:0043169;GO:0046914;GO:0043167;GO:0003674;GO:0008270;GO:0046872;GO:0005488	------	------	------	------	COG5048	O:Posttranslational modification, pro	PF00096.29;PF138	zf-C2H2;zf-C2H2_4;zf-	Zinc finger, C2H2 type;C2H2-type zinc finger;Zinc-finger double domain;Zinc-finger associated domain	CYT	1	1	120.9	High
TRINITY_DN6143_c0_g2_i1_orf1	uncharacterized protein LOC114365036 [Ostrinia furnacalis]	2.190371991	1.131175904	1.93E-06	up	yes	1.001	0.457	0.462	0.475	0.434	1	1.009	0.993	------	------	------	------	------	------	ENOG41123CP	S:Function unknown	------	------	------	CYT	8	41	28.8	High
TRINITY_DN16900_c0_g2_i1_orf1	uncharacterized oxidoreductase dhs-27-like [Ostrinia furnacalis]	2.975047985	1.572912938	0.0002141	up	yes	1.085	0.3647	0.371	0.359	0.364	1	1.191	1.064	------	------	------	------	------	------	ENOG410XT8F;ENO(	S:Function unknown;	PF02958.23;PF079	EcKL;DUF1679;APH;Ch	Ecdysteroid kinase-like family;Uncharacterized oxidoreductase dhs-27;Phosphotransferase enzyme family;Choline/ethanolamine kinase	CYT	1	3	32.1	High
TRINITY_DN26649_c0_g1_i2_orf1	ATP synthase-coupling factor 6, mitochondrial [Ostrinia furnacalis]	2.174285714	1.120541532	1.04E-06	up	yes	0.9893	0.455	0.444	0.472	0.449	1	0.991	0.977	BP:nucleoside triphosphate metabolic process;BP:nucleoside triphosphate biosynthetic process;BP:purine nucleoside triphosphate metabolic process;BP:purine nucleoside triphosphate biosynthetic process;BP:cellular biosynthetic process;BP:cellular nitrogen compound metabolic process;BP:nucleotide biosynthetic process;BP:small molecule metabolic process;BP:purine-containing compound metabolic process;BP:purine-containing compound biosynthetic process;BP:organic cyclic compound biosynthetic process;BP:ribonucleotide metabolic process;BP:organic cyclic compound metabolic process;BP:nucleobase-containing compound metabolic process;BP:ribonucleoside triphosphate biosynthetic process;BP:organic substance metabolic process;BP:ribonucleoside triphosphate metabolic process;BP:heterocycle biosynthetic process;BP:purine ribonucleoside triphosphate biosynthetic process;BP:purine ribonucleoside triphosphate metabolic process;BP:ribose phosphate metabolic process;BP:organic substance biosynthetic process;BP:ATP metabolic process;BP:cellular process;BP:cellular aromatic compound metabolic process;BP:purine ribonucleotide biosynthetic process;BP:purine ribonucleotide metabolic process;BP:ribonucleotide biosynthetic process;BP:biosynthetic process;BP:nucleotide metabolic process;BP:metabolic process;BP:aromatic compound biosynthetic process;BP:nucleobase-containing compound biosynthetic process;BP:cellular nitrogen compound biosynthetic process;BP:organophosphate biosynthetic process;BP:nucleobase-containing small molecule metabolic process;BP:heterocycle metabolic process;BP:primary metabolic process;BP:ATP synthesis coupled proton transport;BP:organonitrogen compound metabolic process;BP:organonitrogen compound biosynthetic process;BP:carbohydrate derivative biosynthetic process;BP:carbohydrate derivative metabolic process;BP:purine nucleotide biosynthetic process;BP:ribose phosphate biosynthetic process;BP:cellular metabolic process;BP:purine nucleotide metabolic process;BP:phosphate-containing compound metabolic process;BP:nitrogen compound metabolic process;BP:nucleoside phosphate biosynthetic process;BP:phosphorus metabolic process;BP:organophosphate metabolic process;BP:biological_process;BP:nucleoside phosphate metabolic process;BP:ATP biosynthetic process;CC:proton-transporting ATP synthase complex, coupling factor;CC:cellular_component;CC:mitochondrial protein complex;CC:inner mitochondrial membrane protein complex;CC:macromolecular complex;CC:mitochondrial proton-transporting ATP synthase complex, coupling factor;CC:membrane protein complex;CC:proton-transporting two-sector ATPase complex, proton-transporting domain;MF:inorganic cation transmembrane transporter activity;MF:molecular_function;MF:inorganic solute uptake transmembrane transporter activity;MF:transmembrane transporter activity;MF:transporter activity;MF:cation transmembrane transporter activity;MF:ion transmembrane transporter activity;MF:hydrogen	GO:0009141;GO:0009142;GO:0009144;GO:0009145;GO:0044249;GO:0034641;GO:0009165;GO:0044281;GO:0072521;GO:0072522;GO:1901362;GO:0009259;GO:1901360;GO:0006139;GO:0009201;GO:0071704;GO:0009199;GO:0018130;GO:0009206;GO:0009205;GO:0019693;GO:1901576;GO:0046034;GO:0009987;GO:0006725;GO:0009152;GO:0009150;GO:0009260;GO:0009058;GO:0009117;GO:0008152;GO:0019438;GO:0034654;GO:0044271;GO:0090407;GO:0055086;GO:0046483;GO:0044238;GO:0015986;GO:1901564;GO:1901566;GO:1901137;GO:1901135;GO:0006164;GO:0046390;GO:0044237;GO:0006163;GO:0006796;GO:0006807;GO:1901293;GO:0006793;GO:0019637;GO:0008150;GO:0006753;GO:0006754;GO:0045263;GO:0005575;GO:0098798;GO:0098800;GO:0032991;GO:0000276;GO:0098796;GO:0033177;GO:0022890;GO:0003674;GO:0015318;GO:0022857;GO:0005215;GO:0008324;GO:0015075;GO:0015078	K02131	ATPeF0F6, AT	map04714;map	Thermogenesis;Cher	ENOG41122G1	C:Energy production and conversion	PF05511.14	ATP-synt_F6	Mitochondrial ATP synthase coupling factor 6	CYT	5	62	10.5	High
TRINITY_DN83327_c0_g1_i1_orf1	trypsin-like serine proteinase T22 [Ostrinia nubilalis]	3.458088463	1.789974776	0.0002515	up	yes	1.118	0.3233	0.278	0.332	0.36	1	1.16	1.195	BP:organic substance metabolic process;BP:organonitrogen compound metabolic process;BP:protein metabolic process;BP:macromolecule metabolic process;BP:biological_process;BP:nitrogen compound metabolic process;BP:metabolic process;BP:proteolysis;BP:primary metabolic process;MF:serine-type endopeptidase activity;MF:endopeptidase activity;MF:hydrolase activity;MF:serine hydrolase activity;MF:catalytic activity, acting on a protein;MF:catalytic activity;MF:molecular_function;MF:peptidase activity;MF:serine-type peptidase activity;	GO:0071704;GO:1901564;GO:0019538;GO:0043170;GO:0008150;GO:0006807;GO:0008152;GO:0006508;GO:0044238;GO:0004252;GO:0004175;GO:0016787;GO:0017171;GO:0140096;GO:0003824;GO:0003674;GO:0008233;GO:0008236	K09640;K01312	TMPRSS9;PRS	map04972;map	Pancreatic secretion	COG5640	O:Posttranslational modification, pro	PF00089.29	Trypsin	Trypsin	PLA	1	4	28.4	Medium
TRINITY_DN44219_c0_g1_i1_orf1	mitochondrial import inner membrane translocase subunit TIM50-C-like [Ostrinia furnacalis]	3.264942016	1.70705737	0.0007439	up	yes	1.098	0.3363	0.416	0.216	0.377	1	1.188	1.106	BP:protein localization;BP:cellular macromolecule localization;BP:nitrogen compound transport;BP:organic substance transport;BP:macromolecule localization;BP:transport;BP:cellular process;BP:establishment of protein localization;BP:biological_process;BP:establishment of localization;BP:localization;BP:cellular localization;BP:protein transport;CC:intrinsic component of membrane;CC:integral component of membrane;CC:inner mitochondrial membrane protein complex;CC:cellular anatomical entity;CC:macromolecular complex;CC:cellular_component;CC:membrane protein complex;CC:mitochondrial inner membrane presequence translocase complex;CC:mitochondrial protein complex;	GO:0008104;GO:0070727;GO:0071705;GO:0071702;GO:0033036;GO:0006810;GO:0009987;GO:0045184;GO:0008150;GO:0051234;GO:0051179;GO:0051641;GO:0015031;GO:0031224;GO:0016021;GO:0098800;GO:0110165;GO:0032991;GO:0005575;GO:0098796;GO:0005744;GO:0098798	K17496	TIM50	------	------	COG5190	K:Transcription	PF03031.21	NIF	NLI interacting factor-like phosphatase	CYT	1	3	44.9	High
TRINITY_DN29291_c0_g1_i1_orf1	carboxylesterase [Ostrinia furnacalis]	2.451240067	1.293511785	1.05E-05	up	yes	1.018	0.4153	0.39	0.435	0.421	1	1.001	1.053	MF:hydrolase activity;MF:molecular_function;MF:hydrolase activity, acting on ester bonds;MF:carboxylic ester hydrolase activity;MF:catalytic activity;	GO:0016787;GO:0003674;GO:0016788;GO:0052689;GO:0003824	K03927	CES2	map00983	Drug metabolism - (	COG2272	I:Lipid transport and metabolism	PF00135.31	COesterase	Carboxylesterase family	CYT	1	8	26.3	High
TRINITY_DN4938_c0_g1_i13_orf1	peroxisomal biogenesis factor 19 [Ostrinia furnacalis]	2.032606394	1.02333087	2.87E-05	up	yes	0.96	0.4723	0.481	0.463	0.473	1	0.957	0.923	CC:intracellular membrane-bounded organelle;CC:peroxisome;CC:cellular_component;CC:microbody;CC:intracellular organelle;CC:membrane-bounded organelle;CC:organelle;CC:cellular anatomical entity;	GO:0043231;GO:0005777;GO:0005575;GO:0042579;GO:0043229;GO:0043227;GO:0043226;GO:0110165	K13337	PEX19	map04146	Peroxisome	ENOG4111QGU	O:Posttranslational modification, pro	PF04614.15	Pex19	Pex19 protein family	CYT	4	11	31.8	High
TRINITY_DN5554_c0_g1_i2_orf1	double-stranded RNA-binding protein Staufen homolog 2 isoform X5 [Pectinophora gossypiella]	2.283681432	1.191361412	2.09E-06	up	yes	0.995	0.4357	0.432	0.442	0.433	1	0.97	1.015	------	------	K17597	STAU	------	------	ENOG410XSCK	K:Transcription	PF00035.29;PF164	dsrm;Staufen_C	Double-stranded RNA binding motif;Staufen C-terminal domain	CYT	1	2	56.8	High
TRINITY_DN556_c0_g2_i1_orf1	serine protease inhibitor dipetalogastin-like [Ostrinia furnacalis]	2.634807053	1.397697317	0.002607	up	yes	1.031	0.3913	0.558	0.344	0.272	1	0.978	1.114	------	------	K23615	SPIK5	------	------	ENOG410Z5MG;ENC	S:Function unknown;O:Posttranslatic	PF00050.24;PF076	Kazal_1;Kazal_2	Kazal-type serine protease inhibitor domain;Kazal-type serine protease inhibitor domain	EXC	1	19	30.5	High
TRINITY_DN6147_c0_g1_i2_orf1	uncharacterized protein LOC114352519 [Ostrinia furnacalis]	2.220711534	1.151022002	1.15E-06	up	yes	0.98	0.4413	0.432	0.442	0.45	1	0.971	0.969	------	------	------	------	------	------	ENOG410YKCN;ENO	S:Function unknown;	PF02958.23;PF079	EcKL;DUF1679;APH	Ecdysteroid kinase-like family;Uncharacterized oxidoreductase dhs-27;Phosphotransferase enzyme family	CYT	10	27	44.6	High
TRINITY_DN5956_c1_g1_i5_orf1	uncharacterized protein DDB_G0286299-like [Ostrinia furnacalis] >XP_028170990.1 uncharacterized protein DDB_G0286299-like [Ostrinia furnacalis] >XP_028170991.1 uncharacterized protein DDB_G0286299-like [Ostrinia furnacalis]	2.746460059	1.457573311	3.70E-05	up	yes	1.028	0.3743	0.363	0.427	0.333	1	1.06	1.025	MF:organic cyclic compound binding;MF:molecular_function;MF:binding;MF:nucleic acid binding;MF:heterocyclic compound binding;MF:RNA binding;	GO:0097159;GO:0003674;GO:0005488;GO:0003676;GO:1901363;GO:0003723	------	------	------	------	ENOG4111E9J;ENOG	S:Function unknown;	PF00076.25	RRM_1	RNA recognition motif.	CYT	3	7	66.4	High
TRINITY_DN45477_c0_g1_i1_orf1	putative E3 ubiquitin-protein ligase UBR7 [Ostrinia furnacalis]	2.482171613	1.311602864	0.0002939	up	yes	1.079	0.4347	0.412	0.463	0.429	1	1.058	1.178	------	------	K11979	UBR7	------	------	ENOG410XSV6	S:Function unknown	PF02207.23	zf-UBR	Putative zinc finger in N-recognin	CYT	2	4	80.9	High
TRINITY_DN100208_c0_g1_i1_orf1	neurofilament heavy polypeptide-like isoform X2 [Ostrinia furnacalis]	2.039540044	1.028243833	1.43E-05	up	yes	1.011	0.4957	0.461	0.517	0.509	1	1.002	1.032	------	------	------	------	------	------	------	------	------	------	------	CYT	1	88	8.4	High
TRINITY_DN2497_c0_g1_i2_orf1	protein stunted-like isoform X1 [Colias croceus]	2.268871003	1.181974587	1.98E-05	up	yes	1.043	0.4597	0.455	0.441	0.483	1	1.063	1.067	------	------	K02135	ATPeF1E, ATP	map04714;map	Thermogenesis;Cher	ENOG410Y5P5	C:Energy production and conversion	PF04627.16	ATP-synt_Eps	Mitochondrial ATP synthase epsilon chain	CYT	1	48	6.9	High
TRINITY_DN14601_c0_g1_i2_orf1	unnamed protein product [Chilo suppressalis]	2.019366552	1.01390281	5.24E-05	up	yes	1.001	0.4957	0.478	0.549	0.46	1	0.993	1.01	CC:cytoplasm;CC:integral component of membrane;CC:cellular_component;CC:cellular anatomical entity;CC:intrinsic component of membrane;MF:cation binding;MF:ion binding;MF:molecular_function;MF:metal ion binding;MF:binding;	GO:0005737;GO:0016021;GO:0005575;GO:0110165;GO:0031224;GO:0043169;GO:0043167;GO:0003674;GO:0046872;GO:0005488	K26089	ANKZF1, VMS	------	------	ENOG410XQAG	S:Function unknown	PF18826.4;PF1871	bVLRF1;VATC	Bacteroidetes VLRF1 release factor;Vms1-associating treble clef domain	CYT	2	2	76.4	High
TRINITY_DN34159_c0_g2_i1_orf1	guanine nucleotide exchange factor subunit Rich isoform X1 [Ostrinia furnacalis]	2.248533565	1.16898442	0.0002364	up	yes	1.035	0.4603	0.429	0.442	0.51	1	0.993	1.112	BP:protein localization;BP:cellular macromolecule localization;BP:intracellular protein transport;BP:nitrogen compound transport;BP:organic substance transport;BP:macromolecule localization;BP:transport;BP:cellular process;BP:establishment of protein localization;BP:biological_process;BP:establishment of localization in cell;BP:establishment of localization;BP:localization;BP:cellular localization;BP:intracellular transport;BP:protein transport;CC:guanyl-nucleotide exchange factor complex;CC:RIC1-RGP1 guanyl-nucleotide exchange factor complex;CC:membrane;CC:cellular anatomical entity;CC:macromolecular complex;CC:cellular_component;	GO:0008104;GO:0070727;GO:0006886;GO:0071705;GO:0071702;GO:0033036;GO:0006810;GO:0009987;GO:0045184;GO:0008150;GO:0051649;GO:0051234;GO:0051179;GO:0051641;GO:0046907;GO:0015031;GO:0032045;GO:0034066;GO:0016020;GO:0110165;GO:0032991;GO:0005575	K20476	RIC1	------	------	ENOG410XT5J	S:Function unknown	------	------	------	CYT	1	5	21.7	High
TRINITY_DN58413_c0_g1_i4_orf1	cysteine and histidine-rich protein 1 isoform X1 [Ostrinia furnacalis]	2.416475973	1.27290465	6.02E-05	up	yes	1.056	0.437	0.413	0.45	0.448	1	1.055	1.114	BP:cellular catabolic process;BP:nitrogen compound metabolic process;BP:modification-dependent macromolecule catabolic process;BP:organic substance catabolic process;BP:cellular macromolecule catabolic process;BP:cellular macromolecule metabolic process;BP:organic substance metabolic process;BP:protein modification by small protein conjugation or removal;BP:protein modification by small protein conjugation;BP:proteolysis;BP:proteolysis involved in cellular protein catabolic process;BP:cellular process;BP:modification-dependent protein catabolic process;BP:macromolecule modification;BP:protein modification process;BP:biological_process;BP:metabolic process;BP:catabolic process;BP:macromolecule catabolic process;BP:primary metabolic process;BP:organonitrogen compound metabolic process;BP:protein metabolic process;BP:cellular metabolic process;BP:macromolecule metabolic process;BP:protein ubiquitination;BP:ubiquitin-dependent protein catabolic process;CC:cytoplasm;CC:cellular anatomical entity;CC:cellular_component;MF:molecular_function;MF:binding;MF:cation binding;MF:ion binding;MF:metal ion binding;	GO:0044248;GO:0006807;GO:0043632;GO:1901575;GO:0044265;GO:0044260;GO:0071704;GO:0070647;GO:0032446;GO:0006508;GO:0051603;GO:0009987;GO:0019941;GO:0043412;GO:0036211;GO:0008150;GO:0008152;GO:0009056;GO:0009057;GO:0044238;GO:1901564;GO:0019538;GO:0044237;GO:0043170;GO:0016567;GO:0006511;GO:0005737;GO:0110165;GO:0005575;GO:0003674;GO:0005488;GO:0043169;GO:0043167;GO:0046872	K04506	SIAH1	map04310;map	Wnt signaling pathw	ENOG410XVP0	O:Posttranslational modification, pro	PF03145.19	Sina	Seven in absentia protein family	CYT	2	7	34.8	High
TRINITY_DN16258_c0_g1_i2_orf1	uncharacterized protein LOC114359911 [Ostrinia furnacalis]	2.171318986	1.118571686	6.98E-06	up	yes	1.019	0.4693	0.469	0.463	0.476	1	1.054	1.002	BP:organonitrogen compound metabolic process;BP:protein metabolic process;BP:nitrogen compound metabolic process;BP:macromolecule metabolic process;BP:biological_process;BP:metabolic process;BP:proteolysis;BP:primary metabolic process;BP:organic substance metabolic process;MF:ATP-dependent peptidase activity;MF:hydrolase activity;MF:endopeptidase activity;MF:serine-type endopeptidase activity;MF:serine hydrolase activity;MF:catalytic activity, acting on a protein;MF:catalytic activity;MF:molecular_function;MF:peptidase activity;MF:serine-type peptidase activity;	GO:1901564;GO:0019538;GO:0006807;GO:0043170;GO:0008150;GO:0008152;GO:0006508;GO:0044238;GO:0071704;GO:0004176;GO:0016787;GO:0004175;GO:0004252;GO:0017171;GO:0140096;GO:0003824;GO:0003674;GO:0008233;GO:0008236	K01358	clpP, CLPP	map04212	Longevity regulating	COG0740	O:Posttranslational modification, pro	PF00574.26	CLP_protease	Clp protease	CYT	3	15	27.3	High

TRINITY_DN1965_c0_g1_i7_orf1	CTP synthase isoform X1 [Ostrinia furnacalis]	2.058386631	1.041513992	1.16E-05	up	yes	0.9977	0.4847	0.452	0.506	0.496	1	0.98	1.013	BP:pyrimidine nucleoside triphosphate biosynthetic process;BP:pyrimidine ribonucleotide metabolic process;BP:nucleoside triphosphate metabolic process;BP:nucleoside triphosphate biosynthetic process;BP:nucleotide metabolic process;BP:carboxylic acid metabolic process;BP:pyrimidine nucleoside triphosphate metabolic process;BP:pyrimidine-containing compound biosynthetic process;BP:nucleobase-containing compound biosynthetic process;BP:cellular nitrogen compound metabolic process;BP:nitrogen compound metabolic process;BP:small molecule metabolic process;BP:glutamine family amino acid metabolic process;BP:organic cyclic compound biosynthetic process;BP:pyrimidine nucleotide biosynthetic process;BP:organic cyclic compound metabolic process;BP:pyrimidine-containing compound metabolic process;BP:pyrimidine nucleotide metabolic process;BP:nucleobase-containing compound metabolic process;BP:cellular amino acid metabolic process;BP:glutamine metabolic process;BP:ribose phosphate metabolic process;BP:ribonucleotide metabolic process;BP:organic substance metabolic process;BP:ribonucleoside triphosphate metabolic process;BP:CTP biosynthetic process;BP:alpha-amino acid metabolic process;BP:heterocycle biosynthetic process;BP:cellular process;BP:ribonucleoside triphosphate biosynthetic process;BP:organic substance biosynthetic process;BP:CTP metabolic process;BP:cellular aromatic compound metabolic process;BP:pyrimidine ribonucleotide biosynthetic process;BP:pyrimidine ribonucleoside triphosphate biosynthetic process;BP:pyrimidine ribonucleoside triphosphate metabolic process;BP:ribonucleotide biosynthetic process;BP:biosynthetic process;BP:biological_process;BP:metabolic process;BP:oxoacid metabolic process;BP:cellular nitrogen compound biosynthetic process;BP:organophosphate biosynthetic process;BP:nucleobase-containing small molecule metabolic process;BP:heterocycle metabolic process;BP:primary metabolic process;BP:'de novo' CTP biosynthetic process;BP:organonitrogen compound metabolic process;BP:organonitrogen compound biosynthetic process;BP:organic acid metabolic process;BP:cellular biosynthetic process;BP:carbohydrate derivative biosynthetic process;BP:carbohydrate derivative metabolic process;BP:ribose phosphate biosynthetic process;BP:cellular metabolic process;BP:phosphate-containing compound metabolic process;BP:nucleotide biosynthetic process;BP:nucleoside phosphate biosynthetic process;BP:phosphorus metabolic process;BP:organophosphate metabolic process;BP:aromatic compound biosynthetic process;BP:nucleoside phosphate metabolic process;MF:ATP binding;MF:molecular_function;MF:catalytic activity;MF:nucleotide binding;MF:ligase activity;MF:CTP synthase activity;MF:nucleoside phosphate binding;MF:purine nucleotide binding;MF:ligase activity, forming carbon-nitrogen bonds;MF:anion binding;MF:ribonucleotide binding;MF:purine ribonucleoside triphosphate	GO:0009148;GO:0009218;GO:0009141;GO:0009142;GO:0009117;GO:0019752;GO:0009147;GO:0072528;GO:0034654;GO:0034641;GO:0006807;GO:0044281;GO:0009064;GO:1901362;GO:0006221;GO:1901360;GO:0072527;GO:0006220;GO:0006139;GO:0006520;GO:0006541;GO:0019693;GO:0009259;GO:0071704;GO:0009199;GO:0006241;GO:1901605;GO:0018130;GO:0009987;GO:0009201;GO:1901576;GO:0046036;GO:0006725;GO:0009220;GO:0009209;GO:0009208;GO:0009260;GO:0009058;GO:0008150;GO:0008152;GO:0043436;GO:0044271;GO:0090407;GO:0055086;GO:0046483;GO:0044238;GO:0044210;GO:1901564;GO:1901566;GO:0006082;GO:0044249;GO:1901137;GO:1901135;GO:0046390;GO:0044237;GO:0006796;GO:0009165;GO:1901293;GO:0006793;GO:0019637;GO:0019438;GO:0006753;GO:0005524;GO:0003674;GO:0003824;GO:0000166;GO:0016874;GO:0003883;GO:1901265;GO:0017076;GO:0016879;GO:0043168;GO:0032553;GO:0035639;GO:0036094;GO:0032555;GO:0097159;GO:0030554;GO:0043167;GO:0097367;GO:0032559;GO:1901363;GO:0005488	K01937	pyrG, CTPS	map00240;map	Pyrimidine metaboli	COG0504	F:Nucleotide transport and metabolis	PF06418.17;PF001	CTP_synth_N;GATase	CTP synthase N-terminus;Glutamine amidotransferase class-I	CYT	6	10	68.1	High
TRINITY_DN1066_c0_g1_i4_orf1	ribosome biogenesis protein WDR12 homolog [Ostrinia furnacalis]	2.094643235	1.066704541	1.83E-05	up	yes	0.9893	0.4723	0.511	0.444	0.462	1	0.973	0.995	BP:maturation of 5.8S rRNA from tricistronic rRNA transcrip;BP:nucleic acid metabolic process;BP:maturation of LSU-rRNA;BP:ncRNA metabolic process;BP:organic cyclic compound metabolic process;BP:nucleobase-containing compound metabolic process;BP:organic substance metabolic process;BP:rRNA processing;BP:ncRNA processing;BP:cellular process;BP:cellular aromatic compound metabolic process;BP:maturation of 5.8S rRNA;BP:maturation of LSU-rRNA from tricistronic rRNA transcrip;BP:biological_process;BP:metabolic process;BP:heterocycle metabolic process;BP:RNA metabolic process;BP:primary metabolic process;BP:rRNA metabolic process;BP:cellular nitrogen compound metabolic process;BP:cellular metabolic process;BP:macromolecule metabolic process;BP:nitrogen compound metabolic process;BP:RNA processing;CC:preribosome, large subunit precursor;CC:preribosome;CC:intracellular organelle;CC:non-membrane-bounded organelle;CC:organelle;CC:nucleolus;CC:nucleoplasm;CC:cellular anatomical entity;CC:macromolecular complex;CC:intracellular non-membrane-bounded organelle;CC:ribonucleoprotein complex;CC:cellular_component;MF:macromolecular complex binding;MF:binding;MF:ribonucleoprotein complex binding;MF:molecular_function;	GO:0000466;GO:0090304;GO:0000470;GO:0034660;GO:1901360;GO:0006139;GO:0071704;GO:0006364;GO:0034470;GO:0009987;GO:0006725;GO:0000460;GO:0000463;GO:0008150;GO:0008152;GO:0046483;GO:0016070;GO:0044238;GO:0016072;GO:0034641;GO:0044237;GO:0043170;GO:0006807;GO:0006396;GO:0030687;GO:0030684;GO:0043229;GO:0043228;GO:0043226;GO:0005730;GO:0005654;GO:0110165;GO:0032991;GO:0043232;GO:1990904;GO:0005575;GO:0044877;GO:0005488;GO:0043021;GO:0003674	K14863	WDR12, YTM1	------	------	ENOG410XPW5	J:Translation, ribosomal structure anc	PF00400.35;PF081	WD40;NLE;NBCH_WD4	WD domain, G-beta repeat;NLE;Neurobeachin beta propeller domain	CYT	3	6	46.5	High
TRINITY_DN20582_c0_g1_i1_orf1	2-oxoglutarate dehydrogenase, mitochondrial isoform X3 [Ostrinia furnacalis] >XP_028167081.1 2-oxoglutarate dehydrogenase, mitochondrial isoform X3 [Ostrinia furnacalis]	2.480676329	1.310733509	3.42E-06	up	yes	1.027	0.414	0.411	0.423	0.408	1	1.056	1.024	------	------	K00164	OGDH, sucA	map00020	Citrate cycl	COG0567	Z:Cytoskeleton	PF16870.8;PF0277	OxoGdeHyase_C;Trans	2-oxoglutarate dehydrogenase C-terminal;Transketolase, pyrimidine binding domain	CYT	7	32	25.6	High
TRINITY_DN10637_c0_g1_i4_orf1	V-type proton ATPase subunit d [Bombyx mandarina]	2.698412698	1.432111013	1.09E-05	up	yes	1.037	0.3843	0.358	0.392	0.403	1	1.043	1.068	CC:cellular_component;CC:proton-transporting V-type ATPase, V0 domain;CC:macromolecular complex;CC:membrane protein complex;CC:proton-transporting two-sector ATPase complex, proton-transporting domain;MF:molecular_function;MF:ATPase activity, coupled to transmembrane movement of substances;MF:inorganic solute uptake transmembrane transporter activity;MF:hydrogen-translocating pyrophosphatase activity;MF:primary active transmembrane transporter activity;MF:active transmembrane transporter activity;MF:ATPase coupled ion transmembrane transporter activity;MF:transporter activity;MF:cation transmembrane transporter activity;MF:proton-transporting ATPase activity, rotational mechanism;MF:ATPase activity, coupled to transmembrane movement of ions, rotational mechanism;MF:inorganic cation transmembrane transporter activity;MF:cation-transporting ATPase activity;MF:ion transmembrane transporter activity;MF:transmembrane transporter activity;MF:active ion transmembrane transporter activity;MF:hydrogen ion transmembrane transporter activity;	GO:0005575;GO:0033179;GO:0032991;GO:0098796;GO:0033177;GO:0003674;GO:0042626;GO:0015318;GO:0009678;GO:0015399;GO:0022804;GO:0042625;GO:0005215;GO:0008324;GO:0046961;GO:0044769;GO:0022890;GO:0019829;GO:0015075;GO:0022857;GO:0022853;GO:0015078	K02146	ATPeV0D, ATI	map05152;map	Tuberculosis;Human	COG1527	S:Function unknown	PF01992.19	vATP-synt_AC39	ATP synthase	CYT	7	20	39.6	High
TRINITY_DN47123_c0_g1_i1_orf1	WD40 repeat-containing protein SMU1 [Ostrinia furnacalis]	2.452554745	1.29428534	7.98E-06	up	yes	1.008	0.411	0.399	0.395	0.439	1	0.987	1.036	BP:nucleic acid metabolic process;BP:cellular nitrogen compound metabolic process;BP:nitrogen compound metabolic process;BP:mRNA splicing, via spliceosome;BP:organic cyclic compound metabolic process;BP:nucleobase-containing compound metabolic process;BP:organic substance metabolic process;BP:RNA splicing;BP:mRNA metabolic process;BP:cellular process;BP:cellular aromatic compound metabolic process;BP:RNA splicing, via transesterification reactions;BP:RNA splicing, via transesterification reactions with bulged adenosine as nucleophile;BP:biological_process;BP:metabolic process;BP:heterocycle metabolic process;BP:RNA metabolic process;BP:primary metabolic process;BP:cellular metabolic process;BP:macromolecule metabolic process;BP:RNA processing;BP:mRNA processing;CC:nuclear speck;CC:nuclear body;CC:cellular anatomical entity;CC:cellular_component;	GO:0090304;GO:0034641;GO:0006807;GO:0000398;GO:1901360;GO:0006139;GO:0071704;GO:0008380;GO:0016071;GO:0009987;GO:0006725;GO:0000375;GO:0000377;GO:0008150;GO:0008152;GO:0046483;GO:0016070;GO:0044238;GO:0044237;GO:0043170;GO:0006396;GO:0006397;GO:0016607;GO:0016604;GO:0110165;GO:0005575	K13111	SMU1	------	------	ENOG410XP39	S:Function unknown	PF00400.35;PF128	WD40;ANAPC4_WD40;	WD domain, G-beta repeat;Anaphase-promoting complex subunit 4 WD40 domain;LisH-like dimerisation domain;Neurobeachin beta propeller domain;WD40 region of Ge1, enhancer of mRNA-decapping protein	CYT	2	4	56.4	High
TRINITY_DN130051_c0_g1_i1_orf1	5-methyltetrahydropteroyltriglutamate--homocysteine S-methyltransferase-like protein [Leptotrombidium deliense]	2.097000219	1.068327012	0.00205	up	yes	0.9577	0.4567	0.368	0.588	0.414	1	0.932	0.941	BP:one-carbon metabolic process;BP:carboxylic acid metabolic process;BP:cellular biosynthetic process;BP:nitrogen compound metabolic process;BP:aspartate family amino acid biosynthetic process;BP:small molecule metabolic process;BP:small molecule biosynthetic process;BP:tetrahydrofolate metabolic process;BP:macromolecule metabolic process;BP:organic substance biosynthetic process;BP:cellular amino acid metabolic process;BP:tetrahydrofolate interconversion;BP:pteridine-containing compound metabolic process;BP:organic substance metabolic process;BP:organic cyclic compound metabolic process;BP:proteolysis;BP:methylation;BP:alpha-amino acid metabolic process;BP:alpha-amino acid biosynthetic process;BP:sulfur amino acid metabolic process;BP:folic acid-containing compound metabolic process;BP:primary metabolic process;BP:sulfur amino acid biosynthetic process;BP:cellular process;BP:cellular aromatic compound metabolic process;BP:biosynthetic process;BP:biological_process;BP:metabolic process;BP:oxoacid metabolic process;BP:cellular modified amino acid metabolic process;BP:cellular amino acid biosynthetic process;BP:organonitrogen compound metabolic process;BP:organonitrogen compound biosynthetic process;BP:organic acid metabolic process;BP:sulfur compound biosynthetic process;BP:carboxylic acid biosynthetic process;BP:methionine metabolic process;BP:organic acid biosynthetic process;BP:cellular metabolic process;BP:heterocycle metabolic process;BP:protein metabolic process;BP:sulfur compound metabolic process;BP:methionine biosynthetic process;BP:aspartate family amino acid metabolic process;CC:intrinsic component of membrane;CC:integral component of membrane;CC:cellular_component;CC:cellular anatomical entity;MF:binding;MF:cation binding;MF:oxidoreductase activity, acting on the CH-NH group of donors, NAD or NADP as acceptor;MF:serine-type peptidase activity;MF:methyltransferase activity;MF:peptidase activity;MF:oxidoreductase activity;MF:oxidoreductase activity, acting on the CH-NH group of donors;MF:transition metal ion binding;MF:5-methyltetrahydropteroyltriglutamate-homocysteine S-methyltransferase activity;MF:zinc ion binding;MF:ion binding;MF:transferase activity;MF:transferase activity, transferring one-carbon groups;MF:hydrolase activity;MF:catalytic activity, acting on a protein;MF:methylenetetrahydrofolate reductas;MF:S-methyltransferase activity;MF:catalytic activity;MF:serine hydrolase activity;MF:metal ion binding;MF:5-methyltetrahydropteroyltri-L-glutamate-dependent	GO:0006730;GO:0019752;GO:0044249;GO:0006807;GO:0009067;GO:0044281;GO:0044283;GO:0046653;GO:0043170;GO:1901576;GO:0006520;GO:0035999;GO:0042558;GO:0071704;GO:1901360;GO:0006508;GO:0032259;GO:1901605;GO:1901607;GO:0000096;GO:0006760;GO:0044238;GO:0000097;GO:0009987;GO:0006725;GO:0009058;GO:0008150;GO:0008152;GO:0043436;GO:0006575;GO:0008652;GO:1901564;GO:1901566;GO:0006082;GO:0044272;GO:0046394;GO:0006555;GO:0016053;GO:0044237;GO:0046483;GO:0019538;GO:0006790;GO:0009086;GO:0009066;GO:0031224;GO:0016021;GO:0005575;GO:0110165;GO:0005488;GO:0043169;GO:0016646;GO:0008236;GO:0008168;GO:0008233;GO:0016491;GO:0016645;GO:0046914;GO:0003871;GO:0008270;GO:0043167;GO:0016740;GO:0016741;GO:0016787;GO:0140096;GO:0004489;GO:0008172;GO:0003824;GO:0017171;GO:0046872;GO:0042085;GO:0003674	K00549	metE	map00450;map	Selenocompound m	------	------	PF08267.15	Meth_synt_1	Cobalamin-independent synthase, N-terminal domain	CYT	1	10	13	High
TRINITY_DN1294_c0_g1_i3_orf1	46 kDa FK506-binding nuclear protein-like isoform X1 [Ostrinia furnacalis] >XP_028157904.1 46 kDa FK506-binding nuclear protein-like isoform X2 [Ostrinia furnacalis]	2.437317073	1.285293945	7.12E-07	up	yes	0.9993	0.41	0.399	0.424	0.407	1	1.013	0.985	CC:intracellular membrane-bounded organelle;CC:nucleus;CC:cellular_component;CC:intracellular organelle;CC:membrane-bounded organelle;CC:organelle;CC:cellular anatomical entity;MF:organic cyclic compound binding;MF:catalytic activity, acting on a protein;MF:heterocyclic compound binding;MF:cis-trans isomerase activity;MF:catalytic activity;MF:molecular_function;MF:binding;MF:peptidyl-prolyl cis-trans isomerase activity;MF:DNA binding;MF:nucleic acid binding;MF:isomerase activity;	GO:0043231;GO:0005634;GO:0005575;GO:0043229;GO:0043227;GO:0043226;GO:0110165;GO:0097159;GO:0140096;GO:1901363;GO:0016859;GO:0003824;GO:0003674;GO:0005488;GO:0003755;GO:0003677;GO:0003676;GO:0016853	K14826	FPR3_4	------	------	COG0545	C:Energy production and conversion	PF00254.31;PF178	FKBP_C;NPL	FKBP-type peptidyl-prolyl cis-trans isomerase;Nucleoplasmin-like domain	CYT	12	32	44.7	High
TRINITY_DN38540_c0_g1_i1_orf1	GSCOCG00000129001-RA-CDS [Cotesia congregata] >CAG5101050.1 Similar to LUC7L2: Putative RNA-binding protein Luc7-like 2 (Homo sapiens) [Cotesia congregata]	2.079840319	1.056472769	0.0003994	up	yes	1.042	0.501	0.457	0.558	0.488	1	1.121	1.005	BP:cellular component assembly;BP:macromolecular complex subunit organization;BP:cellular component organization or biogenesis;BP:ribonucleoprotein complex subunit organization;BP:cellular component organization;BP:macromolecular complex assembly;BP:ribonucleoprotein complex assembly;BP:cellular process;BP:biological_process;BP:mRNA splice site selection;CC:Sm-like protein family complex;CC:cellular_component;CC:spliceosomal snRNP complex;CC:macromolecular complex;CC:small nuclear ribonucleoprotein complex;CC:ribonucleoprotein complex;CC:U1 snRNP;MF:molecular_function;MF:binding;MF:nucleic acid binding;MF:heterocyclic compound binding;MF:organic cyclic compound binding;MF:RNA binding;MF:mRNA binding;	GO:0022607;GO:0043933;GO:0071840;GO:0071826;GO:0016043;GO:0065003;GO:0022618;GO:0009987;GO:0008150;GO:0006376;GO:0120114;GO:0005575;GO:0097525;GO:0032991;GO:0030532;GO:1990904;GO:0005685;GO:0003674;GO:0005488;GO:0003676;GO:1901363;GO:0097159;GO:0003723;GO:0003729	K13212	LUC7L2	------	------	COG5200	A:RNA processing and modification	PF03194.18	LUC7	LUC7 N_terminus	CYT	2	9	38.6	High
TRINITY_DN825_c8_g1_i5_orf1	ATP-binding cassette sub-family F member 2 [Ostrinia furnacalis] >XP_028169527.1 ATP-binding cassette sub-family F member 2 [Ostrinia furnacalis]	2.016590954	1.011918477	3.28E-06	up	yes	1.021	0.5063	0.518	0.501	0.5	1	1.019	1.044	MF:anion binding;MF:carbohydrate derivative binding;MF:molecular_function;MF:adenyl nucleotide binding;MF:purine ribonucleoside triphosphate binding;MF:organic cyclic compound binding;MF:nucleotide binding;MF:small molecule binding;MF:ribonucleotide binding;MF:adenyl ribonucleotide binding;MF:nucleoside phosphate binding;MF:purine ribonucleotide binding;MF:purine nucleotide binding;MF:ion binding;MF:heterocyclic compound binding;MF:ATP binding;MF:binding;	GO:0043168;GO:0097367;GO:0003674;GO:0030554;GO:0035639;GO:0097159;GO:0000166;GO:0036094;GO:0032553;GO:0032559;GO:1901265;GO:0032555;GO:0017076;GO:0043167;GO:1901363;GO:0005524;GO:0005488	K06185	ABCF2	map05130	Pathogenic Escheric	COG0488	S:Function unknown	PF00005.30;PF128	ABC_tran;ABC_tran_Xtr	ABC transporter;ABC transporter;AAA domain, putative AbiEii toxin, Type IV TA system;P-loop containing region of AAA domain;RsgA GTPase	CYT	15	26	70.8	High
TRINITY_DN5678_c0_g2_i3_orf1	coiled-coil domain-containing protein 115 [Ostrinia furnacalis]	2.038622337	1.027594535	0.0001467	up	yes	1.024	0.5023	0.533	0.485	0.489	1	1.09	0.981	BP:proton-transporting V-type ATPase complex assembly;BP:proton-transporting two-sector ATPase complex assembly;BP:vacuolar proton-transporting V-type ATPase complex assembly;BP:cellular component assembly;BP:macromolecular complex subunit organization;BP:cellular process;BP:cellular component organization;BP:macromolecular complex assembly;BP:biological_process;BP:cellular component organization or biogenesis;	GO:0070070;GO:0070071;GO:0070072;GO:0022607;GO:0043933;GO:0009987;GO:0016043;GO:0065003;GO:0008150;GO:0071840	K23543	CCDC115	------	------	ENOG41124X6	S:Function unknown	------	------	------	CYT	1	9	17.6	High
TRINITY_DN19286_c0_g1_i1_orf1	signal recognition particle 9 kDa protein [Ostrinia furnacalis]	2.077685616	1.05497737	0.009596	up	yes	1.027	0.4943	0.501	0.667	0.315	1	0.953	1.128	BP:establishment of protein localization to membrane;BP:negative regulation of translational elongation;BP:regulation of primary metabolic process;BP:regulation of metabolic process;BP:negative regulation of cellular biosynthetic process;BP:regulation of cellular macromolecule biosynthetic process;BP:cotranslational protein targeting to membrane;BP:negative regulation of cellular metabolic process;BP:regulation of cellular metabolic process;BP:SRP-dependent cotranslational protein targeting to membrane;BP:negative regulation of gene expression;BP:protein localization;BP:negative regulation of metabolic process;BP:regulation of biological process;BP:negative regulation of protein metabolic process;BP:establishment of localization;BP:negative regulation of macromolecule metabolic process;BP:negative regulation of biosynthetic process;BP:cellular macromolecule localization;BP:posttranscriptional regulation of gene expression;BP:protein targeting;BP:establishment of protein localization to endoplasmic reticulum;BP:regulation of protein metabolic process;BP:negative regulation of cellular macromolecule biosynthetic process;BP:nitrogen compound transport;BP:biological regulation;BP:transport;BP:regulation of cellular biosynthetic process;BP:negative regulation of biological process;BP:intracellular protein transport;BP:macromolecule localization;BP:regulation of gene expression;BP:protein targeting to membrane;BP:regulation of translational elongation;BP:regulation of macromolecule metabolic process;BP:establishment of protein localization to organelle;BP:regulation of biosynthetic process;BP:negative regulation of cellular amide metabolic process;BP:regulation of cellular amide metabolic process;BP:establishment of protein localization;BP:protein transport;BP:protein localization to endoplasmic reticulum;BP:biological_process;BP:regulation of nitrogen compound metabolic process;BP:negative regulation of nitrogen compound metabolic process;BP:establishment of localization in cell;BP:localization within membrane;BP:regulation of cellular process;BP:localization;BP:cellular localization;BP:organic substance transport;BP:intracellular transport;BP:protein targeting to ER;BP:protein localization to organelle;BP:regulation of macromolecule biosynthetic process;BP:negative regulation of translation;BP:regulation of translation;BP:cellular process;BP:negative regulation of macromolecule biosynthetic process;BP:negative regulation of cellular process;BP:protein localization to membrane;CC:signal recognition particle, endoplasmic reticulum targeting;CC:signal recognition particle;CC:macromolecular complex;CC:ribonucleoprotein complex;CC:cellular_component;MF:7S RNA binding;MF:binding;MF:nucleic acid binding;MF:heterocyclic compound binding;MF:organic cyclic compound binding;MF:RNA binding;MF:molecular_function;	GO:0090150;GO:0045900;GO:0080090;GO:0019222;GO:0031327;GO:2000112;GO:0006613;GO:0031324;GO:0031323;GO:0006614;GO:0010629;GO:0008104;GO:0009892;GO:0050789;GO:0051248;GO:0051234;GO:0010605;GO:0009890;GO:0070727;GO:0010608;GO:0006605;GO:0072599;GO:0051246;GO:2000113;GO:0071705;GO:0065007;GO:0006810;GO:0031326;GO:0048519;GO:0006886;GO:0033036;GO:0010468;GO:0006612;GO:0006448;GO:0060255;GO:0072594;GO:0009889;GO:0034249;GO:0034248;GO:0045184;GO:0015031;GO:0070972;GO:0008150;GO:0051171;GO:0051172;GO:0051649;GO:0051668;GO:0050794;GO:0051179;GO:0051641;GO:0071702;GO:0046907;GO:0045047;GO:0033365;GO:0010556;GO:0017148;GO:0006417;GO:0009987;GO:0010558;GO:0048523;GO:0072657;GO:0005786;GO:0048500;GO:0032991;GO:1990904;GO:0005575;GO:0008312;GO:0005488;GO:0003676;GO:1901363;GO:0097159;GO:0003723;GO:0003674	K03109	SRP9	map03060	Protein export	ENOG41123E8	U:Intracellular trafficking, secretion, a	PF05486.15	SRP9-21	Signal recognition particle 9 kDa protein	EXC	1	18	9.4	Medium
TRINITY_DN51252_c0_g2_i1_orf1	peroxidase-like [Ostrinia furnacalis]	2.091301666	1.064401183	4.14E-05	up	yes	1.017	0.4863	0.526	0.439	0.494	1	1.017	1.035	BP:response to stress;BP:response to oxidative stress;BP:response to stimulus;BP:biological_process;CC:chorion;CC:external encapsulating structure;CC:cellular_component;CC:cellular anatomical entity;MF:heme binding;MF:tetrapyrrole binding;MF:organic cyclic compound binding;MF:antioxidant activity;MF:heterocyclic compound binding;MF:catalytic activity;MF:molecular_function;MF:binding;MF:oxidoreductase activity, acting on peroxide as acceptor;MF:peroxidase activity;MF:oxidoreductase activity;	GO:0006950;GO:0006979;GO:0050896;GO:0008150;GO:0042600;GO:0030312;GO:0005575;GO:0110165;GO:0020037;GO:0046906;GO:0097159;GO:0016209;GO:1901363;GO:0003824;GO:0003674;GO:0005488;GO:0016684;GO:0004601;GO:0016491	K19511	PXDN, VPO1	------	------	ENOG410XPZ3	U:Intracellular trafficking, secretion, a	PF03098.18	An_peroxidase	Animal haem peroxidase	CYT	1	13	18.9	High
TRINITY_DN58636_c0_g1_i1_orf1	uncharacterized protein LOC114363665 [Ostrinia furnacalis]	2.625603865	1.392649268	0.0001245	up	yes	1.087	0.414	0.428	0.399	0.415	1	1.112	1.15	BP:nucleic acid metabolic process;BP:cellular nitrogen compound metabolic process;BP:nitrogen compound metabolic process;BP:ncRNA metabolic process;BP:organic cyclic compound metabolic process;BP:nucleobase-containing compound metabolic process;BP:organic substance metabolic process;BP:rRNA processing;BP:ncRNA processing;BP:cellular process;BP:cellular aromatic compound metabolic process;BP:biological_process;BP:metabolic process;BP:heterocycle metabolic process;BP:RNA metabolic process;BP:primary metabolic process;BP:rRNA metabolic process;BP:maturation of SSU-rRNA;BP:cellular metabolic process;BP:macromolecule metabolic process;BP:RNA processing;CC:organelle;CC:nucleolus;CC:cellular anatomical entity;CC:intracellular non-membrane-bounded organelle;CC:intracellular organelle;CC:cellular_component;CC:non-membrane-bounded organelle;	GO:0090304;GO:0034641;GO:0006807;GO:0034660;GO:1901360;GO:0006139;GO:0071704;GO:0006364;GO:0034470;GO:0009987;GO:0006725;GO:0008150;GO:0008152;GO:0046483;GO:0016070;GO:0044238;GO:0016072;GO:0030490;GO:0044237;GO:0043170;GO:0006396;GO:0043226;GO:0005730;GO:0110165;GO:0043232;GO:0043229;GO:0005575;GO:0043228	------	------	------	------	ENOG41118C1	S:Function unknown	------	------	------	CYT	1	1	66.5	Medium
TRINITY_DN42506_c0_g1_i1_orf1	28S ribosomal protein S7, mitochondrial [Ostrinia furnacalis]	2.309734513	1.207727034	4.67E-05	up	yes	1.044	0.452	0.425	0.441	0.49	1	1.085	1.046	BP:cellular biosynthetic process;BP:cellular macromolecule biosynthetic process;BP:organic substance biosynthetic process;BP:cellular nitrogen compound biosynthetic process;BP:cellular macromolecule metabolic process;BP:cellular amide metabolic process;BP:peptide biosynthetic process;BP:organic substance metabolic process;BP:organonitrogen compound biosynthetic process;BP:amide biosynthetic process;BP:cellular process;BP:biosynthetic process;BP:macromolecule biosynthetic process;BP:biological_process;BP:metabolic process;BP:organonitrogen compound metabolic process;BP:primary metabolic process;BP:protein metabolic process;BP:cellular nitrogen compound metabolic process;BP:cellular metabolic process;BP:macromolecule metabolic process;BP:nitrogen compound metabolic process;BP:peptide metabolic process;BP:translation;CC:organelle;CC:intracellular organelle;CC:non-membrane-bounded organelle;CC:cellular anatomical entity;CC:macromolecular complex;CC:ribosome;CC:intracellular non-membrane-bounded organelle;CC:ribonucleoprotein complex;CC:cellular_component;MF:nucleic acid binding;MF:molecular_function;MF:binding;MF:heterocyclic compound binding;MF:structural molecule activity;MF:structural constituent of ribosome;MF:organic cyclic compound binding;MF:RNA binding;	GO:0044249;GO:0034645;GO:1901576;GO:0044271;GO:0044260;GO:0043603;GO:0043043;GO:0071704;GO:1901566;GO:0043604;GO:0009987;GO:0009058;GO:0009059;GO:0008150;GO:0008152;GO:1901564;GO:0044238;GO:0019538;GO:0034641;GO:0044237;GO:0043170;GO:0006807;GO:0006518;GO:0006412;GO:0043226;GO:0043229;GO:0043228;GO:0110165;GO:0032991;GO:0005840;GO:0043232;GO:1990904;GO:0005575;GO:0003676;GO:0003674;GO:0005488;GO:1901363;GO:0005198;GO:0003735;GO:0097159;GO:0003723	K02992	RP-S7, MRPS	map03010	Ribosome	COG0049	S:Function unknown	PF00177.24	Ribosomal_S7	Ribosomal protein S7p/S5e	CYT	1	4	27.3	High
TRINITY_DN8676_c0_g1_i1_orf1	probable rRNA-processing protein EBP2 homolog [Ostrinia furnacalis]	2.002024292	1.001459479	1.75E-05	up	yes	0.989	0.494	0.491	0.485	0.506	1	1.016	0.951	BP:cellular process;BP:ribosome biogenesis;BP:cellular component biogenesis;BP:biological_process;BP:ribonucleoprotein complex biogenesis;BP:cellular component organization or biogenesis;CC:non-membrane-bounded organelle;CC:intracellular non-membrane-bounded organelle;CC:nucleolus;CC:cellular_component;CC:intracellular organelle;CC:cellular anatomical	GO:0009987;GO:0042254;GO:0044085;GO:0008150;GO:0022613;GO:0071840;GO:0043228;GO:0043232;GO:0005730;GO:0005575;GO:0043229;GO:0110165;GO:0043226	K14823	EBP2, EBNA1E	------	------	ENOG410YYHN	S:Function unknown	PF05890.15	Ebp2	Eukaryotic rRNA processing protein EBP2	CYT	3	10	36	High
TRINITY_DN10644_c0_g1_i2_orf1	carboxylesterase [Cnaphalocrocis medinalis]	2.275237464	1.186017126	6.37E-06	up	yes	1.03	0.4527	0.435	0.466	0.457	1	1.036	1.055	MF:hydrolase activity;MF:molecular_function;MF:hydrolase activity, acting on ester bonds;MF:carboxylic ester hydrolase activity;MF:catalytic activity;	GO:0016787;GO:0003674;GO:0016788;GO:0052689;GO:0003824	------	------	------	------	COG2272	I:Lipid transport and metabolism	PF00135.31;PF204	COesterase;BD-FAE;Ab	Carboxylesterase family;BD-FAE;alpha/beta hydrolase	CYT	6	17	55	High
TRINITY_DN2062_c0_g1_i9_orf1	uncharacterized protein LOC114350846 [Ostrinia furnacalis]	2.044187487	1.031527522	6.12E-07	up	yes	0.99	0.4843	0.473	0.497	0.483	1	0.99	0.98	------	------	------	------	------	------	COG1028	S:Function unknown	PF13561.9;PF0010	adh_short_C2;adh_shor	Enoyl;short chain dehydrogenase;KR domain	CYT	6	61	25.6	High
TRINITY_DN15965_c0_g1_i1_orf1	U3 small nucleolar RNA-associated protein 15 homolog [Ostrinia furnacalis]	2.073058405	1.051760762	8.76E-06	up	yes	0.9903	0.4777	0.51	0.47	0.453	1	0.991	0.98	BP:nucleic acid metabolic process;BP:cellular nitrogen compound metabolic process;BP:nitrogen compound metabolic process;BP:ncRNA metabolic process;BP:organic cyclic compound metabolic process;BP:nucleobase-containing compound metabolic process;BP:organic substance metabolic process;BP:rRNA processing;BP:ncRNA processing;BP:cellular process;BP:cellular aromatic compound metabolic process;BP:biological_process;BP:metabolic process;BP:heterocycle metabolic process;BP:RNA metabolic process;BP:primary metabolic process;BP:rRNA metabolic process;BP:cellular metabolic process;BP:macromolecule metabolic process;BP:RNA processing;CC:organelle;CC:nucleolus;CC:cellular anatomical entity;CC:intracellular non-membrane-bounded organelle;CC:intracellular organelle;CC:cellular_component;CC:non-membrane-bounded organelle;	GO:0090304;GO:0034641;GO:0006807;GO:0034660;GO:1901360;GO:0006139;GO:0071704;GO:0006364;GO:0034470;GO:0009987;GO:0006725;GO:0008150;GO:0008152;GO:0046483;GO:0016070;GO:0044238;GO:0016072;GO:0044237;GO:0043170;GO:0006396;GO:0043226;GO:0005730;GO:0110165;GO:0043232;GO:0043229;GO:0005575;GO:0043228	K14549	UTP15	map03008	Ribosome biogenesi	ENOG410XNZ0	I:Lipid transport and metabolism	PF09384.13;PF004	UTP15_C;WD40;ANAPC	UTP15 C terminal;WD domain, G-beta repeat;Anaphase-promoting complex subunit 4 WD40 domain	CYT	2	4	57.9	High

TRINITY_DN6563_c0_g1_i1_orf1	cytochrome c oxidase assembly protein COX15 homolog [Ostrinia furnacalis]	2.181186183	1.125112922	1.80E-05	up	yes	1.004	0.4603	0.436	0.442	0.503	1	0.994	1.019	BP:porphyrin-containing compound metabolic process;BP:porphyrin-containing compound biosynthetic process;BP:cellular biosynthetic process;BP:cellular nitrogen compound metabolic process;BP:nitrogen compound metabolic process;BP:organic cyclic compound biosynthetic process;BP:organic cyclic compound metabolic process;BP:organic substance biosynthetic process;BP:pigment metabolic process;BP:heme biosynthetic process;BP:heme a biosynthetic process;BP:organic substance metabolic process;BP:tetrapyrrole biosynthetic process;BP:tetrapyrrole metabolic process;BP:heterocycle biosynthetic process;BP:heme metabolic process;BP:cellular process;BP:cellular aromatic compound metabolic process;BP:heme a metabolic process;BP:biosynthetic process;BP:biological_process;BP:metabolic process;BP:cellular nitrogen compound biosynthetic process;BP:pigment biosynthetic process;BP:heterocycle metabolic process;BP:organonitrogen compound metabolic process;BP:organonitrogen compound biosynthetic process;BP:cellular metabolic process;BP:aromatic compound biosynthetic process;CC:integral component of membrane;CC:intrinsic component of membrane;CC:cellular anatomical	GO:0006778;GO:0006779;GO:0044249;GO:0034641;GO:0006807;GO:1901362;GO:1901360;GO:1901576;GO:0042440;GO:0006783;GO:0006784;GO:0071704;GO:0033014;GO:0033013;GO:0018130;GO:0042168;GO:0009987;GO:0006725;GO:0046160;GO:0009058;GO:0008150;GO:0008152;GO:0044271;GO:0046148;GO:0046483;GO:1901564;GO:1901566;GO:0044237;GO:0019438;GO:0016021;GO:0031224;GO:0110165;GO:0005575;GO:0003674;GO:0016491;GO:0003824;GO:0016651;GO:0016653	K02259	COX15, ctaA	map00190;map	Oxidative phosphory	COG5023;COG1612	T:Signal transduction mechanisms;O:	PF02628.18	COX15-CtaA	Cytochrome oxidase assembly protein	CYT	3	7	47.4	High
TRINITY_DN21539_c0_g1_i1_orf1	probable phenylalanine--tRNA ligase, mitochondrial [Ostrinia furnacalis]	2.10239559	1.072034155	0.003044	up	yes	0.9917	0.4717	0.52	0.389	0.506	1	0.867	1.108	BP:phenylalanyl-tRNA aminoacylation;BP:carboxylic acid metabolic process;BP:nucleic acid metabolic process;BP:cellular nitrogen compound metabolic process;BP:nitrogen compound metabolic process;BP:ncRNA metabolic process;BP:organic cyclic compound metabolic process;BP:nucleobase-containing compound metabolic process;BP:cellular amino acid metabolic process;BP:organic substance metabolic process;BP:cellular process;BP:cellular aromatic compound metabolic process;BP:biological_process;BP:metabolic process;BP:oxoacid metabolic process;BP:heterocycle metabolic process;BP:RNA metabolic process;BP:primary metabolic process;BP:organonitrogen compound metabolic process;BP:organic acid metabolic process;BP:cellular metabolic process;BP:macromolecule metabolic process;BP:tRNA metabolic process;BP:amino acid activation;BP:tRNA aminoacylation;BP:tRNA aminoacylation for protein translation;BP:small molecule metabolic process;CC:cytoplasm;CC:intracellular organelle lumen;CC:mitochondrial matrix;CC:cellular anatomical entity;CC:membrane-enclosed lumen;CC:organelle lumen;CC:cellular_component;MF:purine ribonucleoside triphosphate binding;MF:molecular_function;MF:binding;MF:nucleic acid binding;MF:nucleotide binding;MF:heterocyclic compound binding;MF:aminoacyl-tRNA ligase activity;MF:ATP binding;MF:nucleoside phosphate binding;MF:purine nucleotide binding;MF:anion binding;MF:catalytic activity, acting on RNA;MF:adenyl nucleotide binding;MF:small molecule binding;MF:catalytic activity;MF:tRNA binding;MF:ligase activity;MF:ligase activity, forming carbon-oxygen bonds;MF:phenylalanine-tRNA ligase activity;MF:carbohydrate derivative binding;MF:organic cyclic compound binding;MF:adenyl ribonucleotide binding;MF:RNA binding;MF:purine ribonucleotide binding;MF:catalytic activity, acting on a tRNA;MF:ion	GO:0006432;GO:0019752;GO:0090304;GO:0034641;GO:0006807;GO:0034660;GO:1901360;GO:0006139;GO:0006520;GO:0071704;GO:0009987;GO:0006725;GO:0008150;GO:0008152;GO:0043436;GO:0046483;GO:0016070;GO:0044238;GO:1901564;GO:0006082;GO:0044237;GO:0043170;GO:0006399;GO:0043038;GO:0043039;GO:0006418;GO:0044281;GO:0005737;GO:0070013;GO:0005759;GO:0110165;GO:0031974;GO:0043233;GO:0005575;GO:0035639;GO:0003674;GO:0005488;GO:0003676;GO:0000166;GO:1901363;GO:0004812;GO:0005524;GO:1901265;GO:0017076;GO:0043168;GO:0140098;GO:0030554;GO:0036094;GO:0003824;GO:0000049;GO:0016874;GO:0016875;GO:0004826;GO:0097367;GO:0097159;GO:0032559;GO:0003723;GO:0032555;GO:0140101;GO:0043167;GO:0032553	K01889	FARSA, pheS	map00970	Aminoacyl-tRNA bio	COG0072	S:Function unknown	PF01409.23;PF031	tRNA-synt_2d;FDX-AC	tRNA synthetases class II core domain;Ferredoxin-fold anticodon binding domain	CYT	1	3	51	High
TRINITY_DN46778_c0_g1_i2_orf1	Deoxycytidylate deaminase [Papilio xuthus]	2.513898961	1.329926666	2.57E-05	up	yes	1.04	0.4137	0.385	0.438	0.418	1	1.083	1.036	MF:hydrolase activity;MF:transition metal ion binding;MF:cation binding;MF:ion binding;MF:catalytic activity;MF:molecular_function;MF:zinc ion binding;MF:metal ion binding;MF:binding;	GO:0016787;GO:0046914;GO:0043169;GO:0043167;GO:0003824;GO:0003674;GO:0008270;GO:0046872;GO:0005488	K01493	comEB	map00240;map	Pyrimidine metaboli	COG2131	F:Nucleotide transport and metabolis	PF00383.26;PF144	dCMP_cyt_deam_1;Ma	Cytidine and deoxycytidylate deaminase zinc-binding region;MafB19-like deaminase	CYT	1	5	21.4	Medium
TRINITY_DN969_c0_g1_i3_orf1	protein UBASH3A homolog isoform X3 [Ostrinia furnacalis]	2.170571491	1.118074941	0.0004219	up	yes	0.9913	0.4567	0.437	0.425	0.508	1	0.914	1.06	------	------	K18993	UBASH3, STS	------	------	ENOG410YGUZ	O:Posttranslational modification, pro	PF00300.25;PF146	His_Phos_1;SH3_9;UBA	Histidine phosphatase superfamily;Variant SH3 domain;UBA/TS-N domain	CYT	2	3	77	High
TRINITY_DN14826_c0_g1_i1_orf1	uncharacterized protein LOC114350939 [Ostrinia furnacalis]	2.076923077	1.054447784	0.0001124	up	yes	0.9747	0.4693	0.433	0.531	0.444	1	0.962	0.962	CC:cellular_component;CC:integral component of membrane;CC:cellular anatomical entity;CC:intrinsic component of membrane;	GO:0005575;GO:0016021;GO:0110165;GO:0031224	------	------	------	------	ENOG410Z38Q	S:Function unknown	------	------	------	CYT	1	16	8.6	High
TRINITY_DN4040_c0_g1_i10_orf1	hypothetical protein evm_007488 [Chilo suppressalis]	2.111590762	1.078330259	2.00E-05	up	yes	0.9783	0.4633	0.428	0.494	0.468	1	0.969	0.966	CC:cellular anatomical entity;CC:intrinsic component of membrane;CC:cellular_component;CC:integral component of membrane;MF:antiporter activity;MF:secondary active transmembrane transporter activity;MF:inorganic anion transmembrane transporter activity;MF:inorganic solute uptake transmembrane transporter activity;MF:transporter activity;MF:anion transmembrane transporter activity;MF:solut;MF:ion transmembrane transporter activity;MF:transmembrane transporter activity;MF:molecular_function;MF:active ion transmembrane transporter activity;MF:inorganic anion exchanger activity;MF:active transmembrane transporter	GO:0110165;GO:0031224;GO:0005575;GO:0016021;GO:0015297;GO:0015291;GO:0015103;GO:0015318;GO:0005215;GO:0008509;GO:0140323;GO:0015075;GO:0022857;GO:0003674;GO:0022853;GO:0005452;GO:0022804	K13855	SLC4A2, AE2	map04972;map	Pancreatic secretion	ENOG410XPHD	U:Intracellular trafficking, secretion, a	PF00955.24;PF075	HCO3_cotransp;Band_3	HCO3- transporter family;Band 3 cytoplasmic domain	CYT	5	6	128.9	High
TRINITY_DN4070_c0_g1_i4_orf1	alpha-N-acetylgalactosaminidase isoform X3 [Ostrinia furnacalis]	2.099634173	1.070137983	8.58E-06	up	yes	0.9757	0.4647	0.449	0.488	0.457	1	0.972	0.955	BP:primary metabolic process;BP:organic substance metabolic process;BP:biological_process;BP:metabolic process;BP:carbohydrate metabolic process;BP:lipid metabolic process;CC:cellular_component;CC:lytic vacuole;CC:intracellular membrane-bounded organelle;CC:vacuole;CC:lysosome;CC:intracellular organelle;CC:membrane-bounded organelle;CC:organelle;CC:cellular anatomical entity;MF:molecular_function;MF:hydrolase activity;MF:hydrolase activity, acting on glycosyl bonds;MF:catalytic activity;MF:hydrolase activity, hydrolyzing O-glycosyl compounds;	GO:0044238;GO:0071704;GO:0008150;GO:0008152;GO:0005975;GO:0006629;GO:0005575;GO:0000323;GO:0043231;GO:0005773;GO:0005764;GO:0043229;GO:0043227;GO:0043226;GO:0110165;GO:0003674;GO:0016787;GO:0016798;GO:0003824;GO:0004553	K01204	NAGA	map00603;map	Glycosphingolipid bi	ENOG410XPF1	O:Posttranslational modification, pro	PF16499.8;PF1745	Melibiase_2;Melibiase_	Alpha galactosidase A;Alpha galactosidase A C-terminal beta sandwich domain;Alpha galactosidase C-terminal beta sandwich domain	CYT	5	15	49.7	High
TRINITY_DN2668_c0_g1_i7_orf1	unnamed protein product [Chrysodeixis includens]	2.572001002	1.362891205	1.53E-05	up	yes	1.027	0.3993	0.362	0.417	0.419	1	1.023	1.058	BP:organic substance metabolic process;BP:lipid metabolic process;BP:primary metabolic process;BP:metabolic process;BP:biological_process;CC:cellular_component;CC:extracellular region;CC:cellular anatomical entity;MF:hydrolase activity;MF:hydrolase activity, acting on ester bonds;MF:catalytic activity;MF:molecular_function;MF:lipase activity;	GO:0071704;GO:0006629;GO:0044238;GO:0008152;GO:0008150;GO:0005575;GO:0005576;GO:0110165;GO:0016787;GO:0016788;GO:0003824;GO:0003674;GO:0016298	------	------	------	------	ENOG410YGS3;ENO(	S:Function unknown;	PF00151.22	Lipase	Lipase	CYT	6	26	38.5	High
TRINITY_DN53136_c0_g1_i1_orf1	glutathione S transferase-E4 [Glyphodes pyloalis]	2.427231176	1.279311521	4.14E-06	up	yes	1.009	0.4157	0.414	0.445	0.388	1	1.015	1.013	MF:catalytic activity;MF:transferase activity;MF:glutathione transferase activity;MF:molecular_function;MF:transferase activity, transferring alkyl or ary;	GO:0003824;GO:0016740;GO:0004364;GO:0003674;GO:0016765	K00799	GST, gst	map05207;map	Chemical carcinoger	COG0625	U:Intracellular trafficking, secretion, a	PF13417.9;PF0004	GST_N_3;GST_C;GST_N	Glutathione S-transferase, N-terminal domain;Glutathione S-transferase, C-terminal domain;Glutathione S-transferase, N-terminal domain;Glutathione S-transferase, N-terminal domain;Glutathione S-transferase, C-terminal	CYT	3	15	25.1	High
TRINITY_DN10558_c0_g1_i4_orf1	unnamed protein product [Chrysodeixis includens]	2.041265769	1.029464031	0.003716	up	yes	0.9547	0.4677	0.487	0.328	0.588	1	0.956	0.908	CC:macromolecular complex;CC:ribonucleoprotein complex;CC:cellular_component;MF:organic cyclic compound binding;MF:molecular_function;MF:RNA binding;MF:nucleic acid binding;MF:heterocyclic compound binding;MF:binding;	GO:0032991;GO:1990904;GO:0005575;GO:0097159;GO:0003674;GO:0003723;GO:0003676;GO:1901363;GO:0005488	K14411	MSI	map03015	mRNA surveillance p	ENOG410YA8Z	O:Posttranslational modification, pro	------	------	------	CYT	1	44	18.3	High
TRINITY_DN3014_c0_g1_i4_orf1	putative inorganic phosphate cotransporter isoform X1 [Ostrinia furnacalis]	2.217687075	1.149055809	0.002491	up	yes	0.978	0.441	0.39	0.351	0.582	1	0.91	1.024	CC:integral component of membrane;CC:cellular_component;CC:cellular anatomical entity;CC:intrinsic component of membrane;MF:transporter activity;MF:transmembrane transporter activity;MF:molecular_function;	GO:0016021;GO:0005575;GO:0110165;GO:0031224;GO:0005215;GO:0022857;GO:0003674	K12301	SLC17A5	map04142	Lysosome	ENOG410XPWC	S:Function unknown	PF07690.19;PF000	MFS_1;Sugar_tr;MFS_4	Major Facilitator Superfamily;Sugar;Uncharacterised MFS-type transporter YbfB	CYT	1	1	56.6	Medium
TRINITY_DN4345_c0_g1_i9_orf1	uncharacterized protein LOC114357127 [Ostrinia furnacalis]	2.271527778	1.183662948	0.0002072	up	yes	0.9813	0.432	0.448	0.357	0.491	1	0.95	0.994	------	------	K13172;K22909	SRRM2, SRM3	------	------	ENOG4110R6X	S:Function unknown	------	------	------	CYT	1	2	92.7	High
TRINITY_DN4125_c1_g1_i5_orf1	angiotensin-converting enzyme-like isoform X2 [Ostrinia furnacalis]	2.387769784	1.255663746	3.36E-06	up	yes	0.9957	0.417	0.387	0.424	0.44	1	0.994	0.993	BP:nitrogen compound metabolic process;BP:organic substance metabolic process;BP:proteolysis;BP:biological_process;BP:metabolic process;BP:primary metabolic process;BP:organonitrogen compound metabolic process;BP:protein metabolic process;BP:macromolecule metabolic process;CC:membrane;CC:cellular anatomical entity;CC:cellular_component;MF:carboxypeptidase activity;MF:exopeptidase activity;MF:molecular_function;MF:binding;MF:peptidase activity;MF:metallopeptidase activity;MF:cation binding;MF:ion binding;MF:metal ion binding;MF:hydrolase activity;MF:peptidyl-dipeptidase activity;MF:catalytic activity, acting on a protein;MF:catalytic activity;	GO:0006807;GO:0071704;GO:0006508;GO:0008150;GO:0008152;GO:0044238;GO:1901564;GO:0019538;GO:0043170;GO:0016020;GO:0110165;GO:0005575;GO:0004180;GO:0008238;GO:0003674;GO:0005488;GO:0008233;GO:0008237;GO:0043169;GO:0043167;GO:0046872;GO:0016787;GO:0008241;GO:0140096;GO:0003824	K01283	ACE, CD143	map05142;map	Chagas disease;Reni	ENOG410XPJ3	S:Function unknown	PF01401.21	Peptidase_M2	Angiotensin-converting enzyme	CYT	10	21	74.4	High
TRINITY_DN4514_c0_g1_i1_orf1	enoyl-CoA delta isomerase 1, mitochondrial-like isoform X1 [Ostrinia furnacalis] >XP_028158560.1 enoyl-CoA delta isomerase 1, mitochondrial-like isoform X2 [Ostrinia furnacalis]	2.31056338	1.208244664	3.72E-05	up	yes	0.9843	0.426	0.441	0.435	0.402	1	0.935	1.018	------	------	K13238	ECI1, DCI	map00071	Fatty acid degradati	COG1024	I:Lipid transport and metabolism	PF00378.23;PF161	ECH_1;ECH_2	Enoyl-CoA hydratase/isomerase;Enoyl-CoA hydratase/isomerase	CYT	1	6	31	High
TRINITY_DN10476_c0_g1_i1_orf1	prohibitin-2 [Ostrinia furnacalis]	2.007062978	1.005085887	8.62E-06	up	yes	1.023	0.5097	0.508	0.49	0.531	1	1.023	1.046	CC:organelle membrane;CC:membrane;CC:organelle inner membrane;CC:mitochondrial membrane;CC:cellular_component;CC:mitochondrial inner membrane;CC:cellular anatomical entity;	GO:0031090;GO:0016020;GO:0019866;GO:0031966;GO:0005575;GO:0005743;GO:0110165	K17081	PHB2	------	------	COG0330	O:Posttranslational modification, pro	PF01145.28	Band_7	SPFH domain / Band 7 family	CYT	10	36	32.9	High
TRINITY_DN100821_c0_g1_i1_orf1	putative GMP synthase, partial [Operophtera brumata]	2.063293775	1.044949249	3.09E-06	up	yes	0.991	0.4803	0.455	0.493	0.493	1	0.982	0.991	BP:carboxylic acid metabolic process;BP:nitrogen compound metabolic process;BP:glutamine family amino acid metabolic process;BP:cellular amino acid metabolic process;BP:glutamine metabolic process;BP:organic substance metabolic process;BP:alpha-amino acid metabolic process;BP:cellular process;BP:biological_process;BP:metabolic process;BP:oxoacid metabolic process;BP:primary metabolic process;BP:organonitrogen compound metabolic process;BP:organic acid metabolic process;BP:cellular metabolic process;BP:small molecule metabolic process;MF:binding;MF:purine ribonucleoside triphosphate binding;MF:molecular_function;MF:carbon-nitrogen ligase activity, with glutamine as amido-N-donor;MF:ion binding;MF:heterocyclic compound binding;MF:nucleoside phosphate binding;MF:anion binding;MF:nucleotide binding;MF:purine nucleotide binding;MF:ATP binding;MF:hydrolase activity;MF:GMP synthas;MF:small molecule binding;MF:catalytic activity;MF:ligase activity;MF:hydrolase activity, acting on acid anhydrides, in phosphorus-containing anhydrides;MF:ligase activity, forming carbon-nitrogen bonds;MF:carbohydrate derivative binding;MF:organic cyclic compound binding;MF:adenyl nucleotide binding;MF:hydrolase activity, acting on acid anhydrides;MF:pyrophosphatase activity;MF:adenyl ribonucleotide binding;MF:purine ribonucleotide binding;MF:ribonucleotide binding;	GO:0019752;GO:0006807;GO:0009064;GO:0006520;GO:0006541;GO:0071704;GO:1901605;GO:0009987;GO:0008150;GO:0008152;GO:0043436;GO:0044238;GO:1901564;GO:0006082;GO:0044237;GO:0044281;GO:0005488;GO:0035639;GO:0003674;GO:0016884;GO:0043167;GO:1901363;GO:1901265;GO:0043168;GO:0000166;GO:0017076;GO:0005524;GO:0016787;GO:0003922;GO:0036094;GO:0003824;GO:0016874;GO:0016818;GO:0016879;GO:0097367;GO:0097159;GO:0030554;GO:0016817;GO:0016462;GO:0032559;GO:0032555;GO:0032553	K01951	guaA, GMPS	map01232;map	Nucleotide metaboli	COG0519	F:Nucleotide transport and metabolis	------	------	------	CYT	2	32	7.3	High
TRINITY_DN108051_c0_g1_i2_orf1	uncharacterized protein LOC114351921 [Ostrinia furnacalis]	2.160060645	1.111071818	3.36E-06	up	yes	0.9973	0.4617	0.488	0.444	0.453	1	1.006	0.986	BP:aerobic respiration;BP:generation of precursor metabolites and energy;BP:electron transport chain;BP:respiratory electron transport chain;BP:cellular process;BP:aerobic electron transport chain;BP:cellular respiration;BP:metabolic process;BP:energy derivation by oxidation of organic compounds;BP:cellular metabolic process;BP:mitochondrial electron transport, NADH to ubiquinone;BP:biological_process;CC:oxidoreductase complex;CC:respiratory chain complex;CC:inner mitochondrial membrane protein complex;CC:transmembrane transporter complex;CC:catalytic complex;CC:transporter complex;CC:respiratory chain complex I;CC:macromolecular complex;CC:NADH dehydrogenase complex;CC:cellular_component;CC:membrane protein complex;CC:mitochondrial respiratory chain complex I;CC:mitochondrial protein complex;	GO:0009060;GO:0006091;GO:0022900;GO:0022904;GO:0009987;GO:0019646;GO:0045333;GO:0008152;GO:0015980;GO:0044237;GO:0006120;GO:0008150;GO:1990204;GO:0098803;GO:0098800;GO:1902495;GO:1902494;GO:1990351;GO:0045271;GO:0032991;GO:0030964;GO:0005575;GO:0098796;GO:0005747;GO:0098798	K03962	NDUFB6	map04714;map	Thermogenesis;Oxid	ENOG4111RT5	U:Intracellular trafficking, secretion, a	PF09782.12	NDUF_B6	NAD	CYT	4	24	18.7	High
TRINITY_DN5417_c0_g1_i1_orf1	NADH dehydrogenase [ubiquinone] 1 alpha subcomplex subunit 10, mitochondrial isoform X1 [Ostrinia furnacalis] >XP_028175885.1 NADH dehydrogenase [ubiquinone] 1 alpha subcomplex subunit 10, mitochondrial isoform X2 [Ostrinia furnacalis]	2.227956024	1.155720757	1.84E-07	up	yes	0.993	0.4457	0.446	0.451	0.44	1	0.98	0.999	BP:aerobic respiration;BP:generation of precursor metabolites and energy;BP:electron transport chain;BP:respiratory electron transport chain;BP:cellular process;BP:aerobic electron transport chain;BP:cellular respiration;BP:metabolic process;BP:energy derivation by oxidation of organic compounds;BP:cellular metabolic process;BP:mitochondrial electron transport, NADH to ubiquinone;BP:biological_process;CC:membrane-enclosed lumen;CC:oxidoreductase complex;CC:intracellular organelle lumen;CC:respiratory chain complex;CC:inner mitochondrial membrane protein complex;CC:mitochondrial matrix;CC:cellular anatomical entity;CC:transmembrane transporter complex;CC:catalytic complex;CC:transporter complex;CC:respiratory chain complex I;CC:macromolecular complex;CC:organelle lumen;CC:NADH dehydrogenase complex;CC:cellular_component;CC:membrane protein complex;CC:mitochondrial respiratory chain complex	GO:0009060;GO:0006091;GO:0022900;GO:0022904;GO:0009987;GO:0019646;GO:0045333;GO:0008152;GO:0015980;GO:0044237;GO:0006120;GO:0008150;GO:0031974;GO:1990204;GO:0070013;GO:0098803;GO:0098800;GO:0005759;GO:0110165;GO:1902495;GO:1902494;GO:1990351;GO:0045271;GO:0032991;GO:0043233;GO:0030964;GO:0005575;GO:0098796;GO:0005747;GO:0098798	K03954	NDUFA10	map04714;map	Thermogenesis;Oxid	ENOG41101CN	F:Nucleotide transport and metabolis	PF01712.22	dNK	Deoxynucleoside kinase	CYT	9	22	45.9	High
TRINITY_DN2600_c0_g1_i7_orf1	mucin-5AC isoform X2 [Ostrinia furnacalis]	2.283105023	1.190997225	3.32E-07	up	yes	1	0.438	0.427	0.455	0.432	1	1.001	1	------	------	------	------	------	------	ENOG4112A7J	S:Function unknown	------	------	------	CYT	9	12	98	High
TRINITY_DN96170_c0_g1_i1_orf1	uncharacterized protein LOC114355569 [Ostrinia furnacalis]	2.395238095	1.260169072	0.000254	up	yes	1.006	0.42	0.459	0.367	0.434	1	0.941	1.076	BP:phosphorylation;BP:cellular process;BP:biological_process;BP:metabolic process;BP:cellular metabolic process;BP:phosphate-containing compound metabolic process;BP:phosphorus metabolic process;CC:intrinsic component of membrane;CC:integral component of membrane;CC:cellular_component;CC:cellular anatomical entity;MF:purine ribonucleoside triphosphate binding;MF:molecular_function;MF:binding;MF:ion binding;MF:heterocyclic compound binding;MF:anion binding;MF:nucleotide binding;MF:transferase activity;MF:nucleoside phosphate binding;MF:purine nucleotide binding;MF:ATP binding;MF:kinase activity;MF:small molecule binding;MF:catalytic activity;MF:adenyl nucleotide binding;MF:carbohydrate derivative binding;MF:transferase activity, transferring phosphorus-containing groups;MF:adenyl ribonucleotide binding;MF:purine ribonucleotide binding;MF:organic cyclic compound binding;MF:ribonucleotide binding;	GO:0016310;GO:0009987;GO:0008150;GO:0008152;GO:0044237;GO:0006796;GO:0006793;GO:0031224;GO:0016021;GO:0005575;GO:0110165;GO:0035639;GO:0003674;GO:0005488;GO:0043167;GO:1901363;GO:0043168;GO:0000166;GO:0016740;GO:1901265;GO:0017076;GO:0005524;GO:0016301;GO:0036094;GO:0003824;GO:0030554;GO:0097367;GO:0016772;GO:0032559;GO:0032555;GO:0097159;GO:0032553	------	------	------	------	COG0574	G:Carbohydrate transport and metab	PF01326.22	PPDK_N	Pyruvate phosphate dikinase, AMP/ATP-binding domain	CYT	3	4	87.6	High
TRINITY_DN5087_c0_g1_i6_orf1	nascent polypeptide-associated complex subunit alpha [Ostrinia furnacalis] >XP_028156807.1 nascent polypeptide-associated complex subunit alpha [Ostrinia furnacalis] >XP_028156808.1 nascent polypeptide-associated complex subunit alpha [Ostrinia furnacalis]	2.401393226	1.263871663	2.45E-06	up	yes	0.9997	0.4163	0.415	0.407	0.427	1	0.976	1.023	CC:cellular_component;CC:nascent polypeptide-associated complex;CC:macromolecular complex;	GO:0005575;GO:0005854;GO:0032991	K03626	EGD2, NACA	map04928	Parathyroid hormon	COG1308	K:Transcription	PF01849.21;PF190	NAC;HYPK_UBA	NAC domain;HYPK UBA domain	CYT	7	34	22.8	High
TRINITY_DN8019_c0_g1_i4_orf1	deoxyhypusine hydroxylase [Ostrinia furnacalis]	2.302085775	1.202941589	1.80E-05	up	yes	0.9823	0.4267	0.405	0.45	0.425	1	1.003	0.944	BP:nitrogen compound metabolic process;BP:peptidyl-amino acid modification;BP:organic substance metabolic process;BP:macromolecule modification;BP:protein modification process;BP:biological_process;BP:metabolic process;BP:peptidyl-lysine modification to peptidyl-hypusine;BP:primary metabolic process;BP:organonitrogen compound metabolic process;BP:protein maturation;BP:peptidyl-lysine modification;BP:biosynthetic process;BP:macromolecule metabolic process;BP:protein metabolic process;MF:monooxygenase activity;MF:deoxyhypusine monooxygenase activity;MF:molecular_function;MF:binding;MF:oxidoreductase activity;MF:cation binding;MF:oxidoreductase activity, acting on paired donors, with incorporation or reduction of molecular oxygen;MF:ion binding;MF:metal ion binding;MF:catalytic activity, acting on a protein;MF:catalytic activity;	GO:0006807;GO:0018193;GO:0071704;GO:0043412;GO:0036211;GO:0008150;GO:0008152;GO:0008612;GO:0044238;GO:1901564;GO:0051604;GO:0018205;GO:0009058;GO:0043170;GO:0019538;GO:0004497;GO:0019135;GO:0003674;GO:0005488;GO:0016491;GO:0043169;GO:0016705;GO:0043167;GO:0046872;GO:0140096;GO:0003824	K06072	DOHH	------	------	COG1413	C:Energy production and conversion	PF13646.9;PF0313	HEAT_2;HEAT_PBS;HEA	HEAT repeats;PBS lyase HEAT-like repeat;HEAT repeat;Family of unknown function	CYT	5	19	34.1	High
TRINITY_DN1109_c0_g1_i6_orf1	1-phosphatidylinositol phosphodiesterase-like [Cotesia glomerata]	2.438067206	1.285737895	4.25E-05	up	yes	0.994	0.4077	0.349	0.441	0.433	1	0.979	1.003	BP:primary metabolic process;BP:lipid metabolic process;BP:organic substance metabolic process;BP:biological_process;BP:metabolic process;MF:phosphoric diester hydrolase activity;MF:hydrolase activity;MF:hydrolase activity, acting on ester bonds;MF:phosphoric ester hydrolase activity;MF:molecular_function;MF:catalytic activity;	GO:0044238;GO:0006629;GO:0071704;GO:0008150;GO:0008152;GO:0008081;GO:0016787;GO:0016788;GO:0042578;GO:0003674;GO:0003824	K01771	plc	map00562	Inositol phosphate r	------	------	------	------	------	EXC	2	8	34.7	High
TRINITY_DN14336_c0_g3_i2_orf1	calbindin-32 isoform X1 [Ostrinia furnacalis]	2.07269757	1.051509626	0.0001873	up	yes	1.015	0.4897	0.547	0.464	0.458	1	0.977	1.068	------	------	K23908	CALB2	------	------	COG5126	O:Posttranslational modification, pro	PF00036.35;PF134	EF-hand_1;EF-hand_6;	EF hand;EF-hand domain;EF-hand domain pair;EF hand;EF-hand	CYT	1	5	36.7	High
TRINITY_DN23167_c0_g1_i4_orf1	uncharacterized protein LOC114363065 [Ostrinia furnacalis]	2.022177419	1.01590958	3.13E-05	up	yes	1.003	0.496	0.538	0.486	0.464	1	1.022	0.986	BP:organic substance metabolic process;BP:organonitrogen compound metabolic process;BP:protein metabolic process;BP:macromolecule metabolic process;BP:biological_process;BP:nitrogen compound metabolic process;BP:metabolic process;BP:proteolysis;BP:primary metabolic process;MF:serine-type endopeptidase activity;MF:endopeptidase activity;MF:hydrolase activity;MF:serine hydrolase activity;MF:catalytic activity, acting on a protein;MF:catalytic activity;MF:molecular_function;MF:peptidase activity;MF:serine-type peptidase activity;	GO:0071704;GO:1901564;GO:0019538;GO:0043170;GO:0008150;GO:0006807;GO:0008152;GO:0006508;GO:0044238;GO:0004252;GO:0004175;GO:0016787;GO:0017171;GO:0140096;GO:0003824;GO:0003674;GO:0008233;GO:0008236	------	------	------	------	COG5640	O:Posttranslational modification, pro	PF18322.4	CLIP_1	Serine protease Clip domain PPAF-2	CYT	7	14	69.4	High
TRINITY_DN69334_c0_g1_i1_orf1	PREDICTED: 15-hydroxyprostaglandin dehydrogenase [NAD(+)]-like [Papilio xuthus]	2.131009868	1.091537274	0.0001699	up	yes	1.015	0.4763	0.421	0.461	0.547	1	1.043	1.003	MF:catalytic activity;MF:molecular_function;MF:oxidoreductase activity;	GO:0003824;GO:0003674;GO:0016491	K00069	HPGD	map00590;map	Arachidonic acid me	COG1028	S:Function unknown	PF00106.28;PF135	adh_short;adh_short_C	short chain dehydrogenase;Enoyl;KR domain	CYT	3	11	29.1	High
TRINITY_DN4905_c0_g1_i6_orf1	uncharacterized protein LOC114351759 [Ostrinia furnacalis]	2.748302741	1.458540934	0.0001969	up	yes	1.093	0.3977	0.403	0.355	0.435	1	1.159	1.119	------	------	------	------	------	------	------	------	------	------	------	EXC	1	4	62.4	High
TRINITY_DN1422_c0_g1_i4_orf1	unnamed protein product [Chilo suppressalis]	2.134736842	1.094058234	6.05E-07	up	yes	1.014	0.475	0.484	0.466	0.475	1	1.028	1.014	BP:generation of precursor metabolites and energy;BP:electron transport chain;BP:cellular process;BP:respiratory electron transport chain;BP:biological_process;BP:metabolic process;BP:ATP synthesis coupled electron transport;BP:cellular metabolic process;CC:membrane;CC:cellular anatomical entity;CC:cellular_component;MF:molecular_function;MF:binding;MF:NA;MF:NADH dehydrogenase activity;MF:oxidoreductase activity;MF:metal cluster binding;MF:primary active transmembrane transporter activity;MF:NADH dehydrogenas;MF:transporter activity;MF:active transmembrane transporter activity;MF:oxidoreduction-driven active transmembrane transporter activity;MF:NADH dehydrogenas;MF:catalytic activity;MF:electron carrier activity;MF:4 iron, 4 sulfur cluster binding;MF:iron-sulfur cluster binding;MF:oxidoreductase activity, acting on NA;MF:transmembrane transporter activity;MF:oxidoreductase activity, acting on NA;	GO:0006091;GO:0022900;GO:0009987;GO:0022904;GO:0008150;GO:0008152;GO:0042773;GO:0044237;GO:0016020;GO:0110165;GO:0005575;GO:0003674;GO:0005488;GO:0003955;GO:0003954;GO:0016491;GO:0051540;GO:0015399;GO:0050136;GO:0005215;GO:0022804;GO:0015453;GO:0008137;GO:0003824;GO:0009055;GO:0051539;GO:0051536;GO:0016655;GO:0022857;GO:0016651	K03934	NDUFS1	map04714;map	Thermogenesis;Oxid	COG1034	C:Energy production and conversion	PF00384.25;PF135	Molybdopterin;Fer2_4;I	Molybdopterin oxidoreductase;2Fe-2S iron-sulfur cluster binding domain;NADH-ubiquinone oxidoreductase-G iron-sulfur binding region;NADH-ubiquinone oxidoreductase subunit G, C-terminal;2Fe-2S iron-sulfur cluster binding domain	CYT	23	40	79.7	High
TRINITY_DN53462_c0_g1_i1_orf1	uncharacterized protein LOC118072968 isoform X1 [Chelonus insularis] >XP_034949073.1 uncharacterized protein LOC118072968 isoform X1 [Chelonus insularis]	2.173447537	1.119985272	0.0005539	up	yes	1.015	0.467	0.36	0.533	0.508	1	1.017	1.028	------	------	------	------	------	------	ENOG41125KI	S:Function unknown	PF15376.9	DUF4603	Domain of unknown function	CYT	1	1	69.1	Medium

TRINITY_DN45924_c0_g1_i14_orf1	adenylosuccinate synthetase isoform X1 [Ostrinia furnacalis] >XP_028166048.1 adenylosuccinate synthetase isoform X2 [Ostrinia furnacalis]	2.167772309	1.116213232	6.53E-07	up	yes	1.013	0.4673	0.467	0.473	0.462	1	1.031	1.007	BP:nucleoside monophosphate biosynthetic process;BP:ribonucleotide biosynthetic process;BP:ribonucleoside monophosphate metabolic process;BP:purine nucleoside monophosphate biosynthetic process;BP:cellular biosynthetic process;BP:cellular nitrogen compound metabolic process;BP:nucleotide biosynthetic process;BP:purine-containing compound metabolic process;BP:purine nucleotide metabolic process;BP:purine-containing compound biosynthetic process;BP:organic cyclic compound biosynthetic process;BP:ribonucleotide metabolic process;BP:purine ribonucleoside monophosphate biosynthetic process;BP:'de novo' AMP biosynthetic process;BP:organophosphate biosynthetic process;BP:organic substance metabolic process;BP:organic cyclic compound metabolic process;BP:heterocycle biosynthetic process;BP:cellular process;BP:purine ribonucleoside monophosphate metabolic process;BP:ribose phosphate metabolic process;BP:nucleobase-containing compound metabolic process;BP:ribonucleoside monophosphate biosynthetic process;BP:phosphorus metabolic process;BP:cellular aromatic compound metabolic process;BP:purine ribonucleotide biosynthetic process;BP:AMP metabolic process;BP:purine ribonucleotide metabolic process;BP:biosynthetic process;BP:nucleotide metabolic process;BP:metabolic process;BP:aromatic compound biosynthetic process;BP:nucleobase-containing compound biosynthetic process;BP:cellular nitrogen compound biosynthetic process;BP:nucleobase-containing small molecule metabolic process;BP:heterocycle metabolic process;BP:primary metabolic process;BP:nucleoside monophosphate metabolic process;BP:organonitrogen compound metabolic process;BP:purine nucleoside monophosphate metabolic process;BP:organonitrogen compound biosynthetic process;BP:organic substance biosynthetic process;BP:carbohydrate derivative metabolic process;BP:ribose phosphate biosynthetic process;BP:cellular metabolic process;BP:carbohydrate derivative biosynthetic process;BP:phosphate-containing compound metabolic process;BP:nitrogen compound metabolic process;BP:AMP biosynthetic process;BP:nucleoside phosphate biosynthetic process;BP:purine nucleotide biosynthetic process;BP:organophosphate metabolic process;BP:biological_process;BP:nucleoside phosphate metabolic process;BP:small molecule metabolic process;CC:cytoplasm;CC:cellular anatomical entity;CC:cellular_component;MF:adenylosuccinate synthase activity;MF:nucleotide binding;MF:purine ribonucleoside triphosphate binding;MF:molecular_function;MF:binding;MF:guanyl nucleotide binding;MF:cation binding;MF:guanyl ribonucleotide binding;MF:magnesium ion binding;MF:GTP binding;MF:ion binding;MF:ribonucleotide binding;MF:purine nucleotide binding;MF:metal ion binding;MF:carbohydrate derivative binding;MF:anion binding;MF:nucleoside phosphate binding;MF:small molecule binding;MF:catalytic activity;MF:ligase activity;MF:purine ribonucleotide binding;MF:ligase activity, forming carbon-nitrogen	GO:0009124;GO:0009260;GO:0009161;GO:0009127;GO:0044249;GO:0034641;GO:0009165;GO:0072521;GO:0006163;GO:0072522;GO:1901362;GO:0009259;GO:0009168;GO:0044208;GO:0090407;GO:0071704;GO:1901360;GO:0018130;GO:0009987;GO:0009167;GO:0019693;GO:0006139;GO:0009156;GO:0006793;GO:0006725;GO:0009152;GO:0046033;GO:0009150;GO:0009058;GO:0009117;GO:0008152;GO:0019438;GO:0034654;GO:0044271;GO:0055086;GO:0046483;GO:0044238;GO:0009123;GO:1901564;GO:0009126;GO:1901566;GO:1901576;GO:1901135;GO:0046390;GO:0044237;GO:1901137;GO:0006796;GO:0006807;GO:0006167;GO:1901293;GO:0006164;GO:0019637;GO:0008150;GO:0006753;GO:0044281;GO:0005737;GO:0110165;GO:0005575;GO:0004019;GO:0000166;GO:0035639;GO:0003674;GO:0005488;GO:0019001;GO:0043169;GO:0032561;GO:0000287;GO:0005525;GO:0043167;GO:0032553;GO:0017076;GO:0046872;GO:0097367;GO:0043168;GO:1901265;GO:0036094;GO:0003824;GO:0016874;GO:0032555;GO:0016879;GO:0097159;GO:1901363	K01939	purA, ADSS	map00250;map	Alanine, aspartate a	COG0104	F:Nucleotide transport and metabolis	PF00709.24	Adenylsucc_synt	Adenylosuccinate synthetase	CYT	10	19	49.7	High
TRINITY_DN115082_c0_g1_i5_orf1	protein dj-1beta-like isoform X2 [Ostrinia furnacalis]	2.1037397	1.072956208	0.0001621	up	yes	0.9957	0.4733	0.413	0.525	0.482	1	1.027	0.96	------	------	K05687	PARK7	map05012;map	Parkinson disease;Pa	COG0693	S:Function unknown	PF01965.27	DJ-1_PfpI	DJ-1/PfpI family	CYT	1	45	11.4	High
TRINITY_DN24043_c0_g1_i1_orf1	uncharacterized protein LOC110377964 [Helicoverpa armigera] >XP_047026962.1 cytochrome c1-2, heme protein, mitochondrial [Helicoverpa zea] >PZC76159.1 hypothetical protein B5X24_HaOG204935 [Helicoverpa armigera]	2.182652392	1.126082386	1.76E-06	up	yes	0.999	0.4577	0.456	0.447	0.47	1	1.017	0.98	CC:organelle membrane;CC:integral component of membrane;CC:membrane;CC:organelle inner membrane;CC:mitochondrial membrane;CC:cellular_component;CC:mitochondrial inner membrane;CC:intrinsic component of membrane;CC:cellular anatomical entity;MF:oxidoreductase activity;MF:cation binding;MF:tetrapyrrole binding;MF:heme binding;MF:ion binding;MF:organic cyclic compound binding;MF:catalytic activity;MF:molecular_function;MF:electron carrier activity;MF:metal ion binding;MF:heterocyclic compound binding;MF:binding;	GO:0031090;GO:0016021;GO:0016020;GO:0019866;GO:0031966;GO:0005575;GO:0005743;GO:0031224;GO:0110165;GO:0016491;GO:0043169;GO:0046906;GO:0020037;GO:0043167;GO:0097159;GO:0003824;GO:0003674;GO:0009055;GO:0046872;GO:1901363;GO:0005488	K00413	CYC1, CYT1, p	map04714;map	Thermogenesis;Oxid	COG2857	C:Energy production and conversion	PF02167.18	Cytochrom_C1	Cytochrome C1 family	CYT	7	23	39.4	High
TRINITY_DN69049_c0_g2_i1_orf1	membrane alanyl aminopeptidase-like [Ostrinia furnacalis]	2.203389831	1.139724764	0.0005972	up	yes	1.001	0.4543	0.38	0.537	0.446	1	0.946	1.056	CC:integral component of membrane;CC:cellular_component;CC:cellular anatomical entity;CC:intrinsic component of membrane;MF:exopeptidase activity;MF:aminopeptidase activity;MF:transition metal ion binding;MF:hydrolase activity;MF:catalytic activity, acting on a protein;MF:cation binding;MF:ion binding;MF:catalytic activity;MF:metallopeptidase activity;MF:molecular_function;MF:binding;MF:peptidase activity;MF:metal ion binding;MF:zinc ion binding;	GO:0016021;GO:0005575;GO:0110165;GO:0031224;GO:0008238;GO:0004177;GO:0046914;GO:0016787;GO:0140096;GO:0043169;GO:0043167;GO:0003824;GO:0008237;GO:0003674;GO:0005488;GO:0008233;GO:0046872;GO:0008270	K11140	ANPEP, CD13	map04640;map	Hematopoietic cell l	COG0308	S:Function unknown	PF01433.23	Peptidase_M1	Peptidase family M1 domain	CYT	1	5	30.8	Medium
TRINITY_DN3665_c0_g1_i2_orf1	regucalcin-like isoform X2 [Ostrinia furnacalis] >XP_028175354.1 regucalcin-like isoform X2 [Ostrinia furnacalis]	3.056179775	1.61172941	0.0005496	up	yes	1.088	0.356	0.379	0.341	0.348	1	1.23	1.033	------	------	K01053	gnl, RGN	map00030;map	Pentose phosphate	COG3386	G:Carbohydrate transport and metab	PF08450.15	SGL	SMP-30/Gluconolactonase/LRE-like region	CYT	1	16	6.9	High
TRINITY_DN698_c0_g1_i5_orf1	PREDICTED: small nuclear ribonucleoprotein Sm D3 [Amyelois transitella]	2.226457399	1.154750008	6.82E-07	up	yes	0.993	0.446	0.46	0.442	0.436	1	0.979	1	BP:spliceosomal snRNP assembly;BP:cellular component assembly;BP:macromolecular complex subunit organization;BP:cellular process;BP:ribonucleoprotein complex subunit organization;BP:cellular component organization;BP:macromolecular complex assembly;BP:ribonucleoprotein complex assembly;BP:biological_process;BP:cellular component organization or biogenesis;CC:cellular_component;CC:macromolecular complex;CC:cytosol;CC:ribonucleoprotein complex;CC:cellular anatomical entity;CC:spliceosomal complex;	GO:0000387;GO:0022607;GO:0043933;GO:0009987;GO:0071826;GO:0016043;GO:0065003;GO:0022618;GO:0008150;GO:0071840;GO:0005575;GO:0032991;GO:0005829;GO:1990904;GO:0110165;GO:0005681	K11088	SNRPD3, SMD	map05322;map	Systemic lupus eryth	COG1958	S:Function unknown	PF01423.25	LSM	LSM domain	CYT	3	31	14.1	High
TRINITY_DN3053_c0_g1_i2_orf1	prostaglandin reductase 1-like [Ostrinia furnacalis]	2.220729796	1.151033866	2.27E-06	up	yes	0.992	0.4467	0.43	0.453	0.457	1	1.005	0.971	CC:cytoplasm;CC:cellular_component;CC:cellular anatomical entity;MF:13-prostaglandin reductase activity;MF:oxidoreductase activity, acting on CH-OH group of donors;MF:oxidoreductase activity, acting on the CH-CH group of donors, NAD or NADP as acceptor;MF:oxidoreductase activity, acting on the CH-OH group of donors, NAD or NADP as acceptor;MF:oxidoreductase activity, acting on the CH-CH group of donors;MF:catalytic activity;MF:molecular_function;MF:15-oxoprostaglandin 13-oxidase activity;MF:2-alkenal reductase [NA[illegible];MF:oxidoreductase activity;	GO:0005737;GO:0005575;GO:0110165;GO:0036132;GO:0016614;GO:0016628;GO:0016616;GO:0016627;GO:0003824;GO:0003674;GO:0047522;GO:0032440;GO:0016491	K13948	PTGR1, LTB4D	map00590	Arachidonic acid me	COG2130	S:Function unknown	PF16884.8;PF0010	ADH_N_2;ADH_zinc_N;	N-terminal domain of oxidoreductase;Zinc-binding dehydrogenase;Zinc-binding dehydrogenase	CYT	9	28	39.1	High
TRINITY_DN7112_c0_g1_i1_orf1	heterogeneous nuclear ribonucleoprotein K isoform X2 [Ostrinia furnacalis]	2.200445434	1.137795597	1.09E-05	up	yes	0.988	0.449	0.454	0.441	0.452	1	1.014	0.95	MF:organic cyclic compound binding;MF:molecular_function;MF:binding;MF:nucleic acid binding;MF:heterocyclic compound binding;MF:RNA binding;	GO:0097159;GO:0003674;GO:0005488;GO:0003676;GO:1901363;GO:0003723	K12886	HNRNPK	map05206;map	MicroRNAs in cance	ENOG4111GSB;ENO	K:Transcription;S:Function unknown	PF00013.32;PF076	KH_1;KH_2	KH domain;KH domain	CYT	7	48	22.2	High
TRINITY_DN32601_c0_g1_i2_orf1	uncharacterized protein LOC114363197 [Ostrinia furnacalis]	2.492647059	1.317678622	5.52E-05	up	yes	1.017	0.408	0.47	0.382	0.372	1	1.042	1.008	------	------	------	------	------	------	ENOG410XRFE	S:Function unknown	PF03022.19	MRJP	Major royal jelly protein	CYT	1	3	48.2	High
TRINITY_DN1465_c0_g2_i1_orf1	unnamed protein product, partial [Iphiclides podalirius]	2.506811989	1.325853798	1.08E-06	up	yes	1.012	0.4037	0.398	0.398	0.415	1	1.001	1.034	MF:catalytic activity;MF:monooxygenase activity;MF:molecular_function;MF:oxidoreductase activity;	GO:0003824;GO:0004497;GO:0003674;GO:0016491	K01904	4CL	map00130	Ubiquinone and oth	COG0318;COG0318	I:Lipid transport and metabolism;Q:S	PF00501.31;PF131	AMP-binding;AMP-bir	AMP-binding enzyme;AMP-binding enzyme C-terminal domain	CYT	8	23	57.9	High
TRINITY_DN7073_c0_g1_i1_orf1	unnamed protein product, partial [Brenthis ino]	2.657142857	1.409875794	9.08E-05	up	yes	1.023	0.385	0.446	0.326	0.383	1	1.063	1.006	CC:cellular_component;CC:macromolecular complex;CC:ribonucleoprotein complex;	GO:0005575;GO:0032991;GO:1990904	K11129	NHP2, NOLA2	map03008	Ribosome biogenesi	COG1358	L:Replication, recombination and rep	PF01248.29	Ribosomal_L7Ae	Ribosomal protein L7Ae/L30e/S12e/Gadd45 family	CYT	2	10	17.7	High
TRINITY_DN754_c1_g1_i6_orf1	28S ribosomal protein S2, mitochondrial [Ostrinia furnacalis]	2.513029685	1.329427713	0.000277	up	yes	1.109	0.4413	0.459	0.435	0.43	1	1.174	1.154	BP:cellular biosynthetic process;BP:cellular nitrogen compound metabolic process;BP:nitrogen compound metabolic process;BP:cellular macromolecule biosynthetic process;BP:organic substance biosynthetic process;BP:cellular macromolecule metabolic process;BP:peptide biosynthetic process;BP:organic substance metabolic process;BP:amide biosynthetic process;BP:cellular process;BP:biosynthetic process;BP:macromolecule biosynthetic process;BP:biological_process;BP:metabolic process;BP:cellular nitrogen compound biosynthetic process;BP:primary metabolic process;BP:organonitrogen compound metabolic process;BP:cellular amide metabolic process;BP:organonitrogen compound biosynthetic process;BP:protein metabolic process;BP:cellular metabolic process;BP:macromolecule metabolic process;BP:peptide metabolic process;BP:translation;CC:small ribosomal subunit;CC:macromolecular complex;CC:ribosomal subunit;CC:ribonucleoprotein complex;CC:cellular_component;MF:molecular_function;MF:structural molecule activity;MF:structural constituent of ribosome;	GO:0044249;GO:0034641;GO:0006807;GO:0034645;GO:1901576;GO:0044260;GO:0043043;GO:0071704;GO:0043604;GO:0009987;GO:0009058;GO:0009059;GO:0008150;GO:0008152;GO:0044271;GO:0044238;GO:1901564;GO:0043603;GO:1901566;GO:0019538;GO:0044237;GO:0043170;GO:0006518;GO:0006412;GO:0015935;GO:0032991;GO:0044391;GO:1990904;GO:0005575;GO:0003674;GO:0005198;GO:0003735	K02967	RP-S2, MRPS2	map03010	Ribosome	COG0052	S:Function unknown	PF00318.23	Ribosomal_S2	Ribosomal protein S2	CYT	1	5	28.4	High
TRINITY_DN3229_c0_g1_i1_orf1	uncharacterized protein LOC114358442 isoform X1 [Ostrinia furnacalis]	2.158362621	1.109937269	6.93E-05	up	yes	1.044	0.4837	0.469	0.507	0.475	1	1.029	1.103	MF:catalytic activity;MF:transferase activity;MF:molecular_function;	GO:0003824;GO:0016740;GO:0003674	------	------	------	------	ENOG41129KS	S:Function unknown	PF11901.11	DUF3421	Protein of unknown function	CYT	6	37	23.3	High
TRINITY_DN3457_c0_g1_i4_orf1	aryl hydrocarbon receptor nuclear translocator homolog [Ostrinia furnacalis]	2.102908277	1.072385925	0.006029	up	yes	1.034	0.4917	0.468	0.456	0.551	1	1.218	0.885	BP:positive regulation of nucleic acid-templated transcription;BP:regulation of RNA metabolic process;BP:regulation of primary metabolic process;BP:regulation of metabolic process;BP:positive regulation of cellular biosynthetic process;BP:regulation of cellular biosynthetic process;BP:positive regulation of cellular metabolic process;BP:positive regulation of nitrogen compound metabolic process;BP:regulation of cellular metabolic process;BP:positive regulation of metabolic process;BP:positive regulation of RNA biosynthetic process;BP:positive regulation of macromolecule metabolic process;BP:positive regulation of biosynthetic process;BP:regulation of biological process;BP:regulation of nucleobase-containing compound metabolic process;BP:regulation of macromolecule biosynthetic process;BP:biological regulation;BP:positive regulation of biological process;BP:regulation of gene expression;BP:developmental process;BP:positive regulation of nucleobase-containing compound metabolic process;BP:regulation of macromolecule metabolic process;BP:regulation of biosynthetic process;BP:regulation of nucleic acid-templated transcription;BP:regulation of cellular process;BP:biological_process;BP:positive regulation of transcription, DNA-templated;BP:regulation of RNA biosynthetic process;BP:anatomical structure development;BP:positive regulation of RNA metabolic process;BP:regulation of transcription, DNA-templated;BP:positive regulation of macromolecule biosynthetic process;BP:regulation of transcription from RNA polymerase II promoter;BP:positive regulation of transcription from RNA polymerase II promoter;BP:positive regulation of cellular process;BP:regulation of nitrogen compound metabolic process;CC:intracellular organelle;CC:membrane-bounded organelle;CC:organelle;CC:cytoplasm;CC:nucleus;CC:cellular anatomical entity;CC:macromolecular complex;CC:intracellular membrane-bounded organelle;CC:cellular_component;CC:transcription factor complex;MF:molecular_function;MF:binding;MF:nucleic acid binding;MF:DNA binding;MF:transcription regulator activity;MF:protein binding;MF:transcription factor activity, sequence-specific DNA binding;MF:organic cyclic compound binding;MF:protein dimerization activity;MF:heterocyclic compound binding;	GO:1903508;GO:0051252;GO:0080090;GO:0019222;GO:0031328;GO:0031326;GO:0031325;GO:0051173;GO:0031323;GO:0009893;GO:1902680;GO:0010604;GO:0009891;GO:0050789;GO:0019219;GO:0010556;GO:0065007;GO:0048518;GO:0010468;GO:0032502;GO:0045935;GO:0060255;GO:0009889;GO:1903506;GO:0050794;GO:0008150;GO:0045893;GO:2001141;GO:0048856;GO:0051254;GO:0006355;GO:0010557;GO:0006357;GO:0045944;GO:0048522;GO:0051171;GO:0043229;GO:0043227;GO:0043226;GO:0005737;GO:0005634;GO:0110165;GO:0032991;GO:0043231;GO:0005575;GO:0005667;GO:0003674;GO:0005488;GO:0003676;GO:0003677;GO:0140110;GO:0005515;GO:0003700;GO:0097159;GO:0046983;GO:1901363	K09097	ARNT	map05207;map	Chemical carcinoger	ENOG410XVHF;ENO	K:Transcription;	PF14598.9;PF0844	PAS_11;PAS_3;PAS;HLH	PAS domain;PAS fold;PAS fold;Helix-loop-helix DNA-binding domain;PAS domain;PAS fold	CYT	1	2	58.6	High
TRINITY_DN32479_c0_g1_i8_orf1	hypothetical protein evm_009815 [Chilo suppressalis] >CAB3525305.1 unnamed protein product [Chilo suppressalis] >CAH0402632.1 unnamed protein product [Chilo suppressalis]	2.141823444	1.09883956	7.31E-06	up	yes	1.036	0.4837	0.48	0.483	0.488	1	1.047	1.06	CC:integral component of membrane;CC:cellular_component;CC:cellular anatomical entity;CC:intrinsic component of membrane;MF:transporter activity;MF:transmembrane transporter activity;MF:molecular_function;	GO:0016021;GO:0005575;GO:0110165;GO:0031224;GO:0005215;GO:0022857;GO:0003674	------	------	------	------	COG0477	Q:Secondary metabolites biosynthesi	PF07690.19;PF000	MFS_1;Sugar_tr	Major Facilitator Superfamily;Sugar	CYT	1	2	56.4	High
TRINITY_DN1239_c0_g1_i3_orf1	uncharacterized protein LOC114355269 [Ostrinia furnacalis] >XP_028163822.1 uncharacterized protein LOC114355269 [Ostrinia furnacalis]	2.080536913	1.056955885	0.0007451	up	yes	1.023	0.4917	0.433	0.472	0.57	1	0.968	1.101	CC:integral component of membrane;CC:cellular_component;CC:cellular anatomical entity;CC:intrinsic component of membrane;MF:transporter activity;MF:transmembrane transporter activity;MF:molecular_function;	GO:0016021;GO:0005575;GO:0110165;GO:0031224;GO:0005215;GO:0022857;GO:0003674	K08190	SLC16A14	------	------	ENOG41116NW	P:Inorganic ion transport and metabo	PF07690.19	MFS_1	Major Facilitator Superfamily	CYT	1	2	63.4	High
TRINITY_DN59829_c0_g1_i1_orf1	putative mediator of RNA polymerase II transcription subunit 12 [Ostrinia furnacalis]	2.034412955	1.024612554	7.88E-05	up	yes	1.005	0.494	0.512	0.488	0.482	1	0.956	1.058	------	------	------	------	------	------	ENOG4110RMW	S:Function unknown	------	------	------	CYT	4	10	67.1	High
TRINITY_DN9002_c0_g1_i1_orf1	membrane magnesium transporter 1 [Ostrinia furnacalis]	2.217204301	1.148741711	0.001502	up	yes	1.031	0.465	0.401	0.459	0.535	1	0.943	1.151	BP:localization;BP:biological_process;BP:establishment of localization;CC:Golgi membrane;CC:macromolecular complex;CC:organelle membrane;CC:cytoplasmic vesicle membrane;CC:bounding membrane of organelle;CC:membrane;CC:ER membrane protein complex;CC:cellular_component;CC:membrane protein complex;CC:cellular anatomical entity;CC:vesicle membrane;CC:early endosome membrane;CC:endosome membrane;	GO:0051179;GO:0008150;GO:0051234;GO:0000139;GO:0032991;GO:0031090;GO:0030659;GO:0098588;GO:0016020;GO:0072546;GO:0005575;GO:0098796;GO:0110165;GO:0012506;GO:0031901;GO:0010008	K23566	MMGT1, EMC	------	------	ENOG4111TZW	U:Intracellular trafficking, secretion, a	PF10270.12	MMgT	Membrane magnesium transporter	CYT	1	8	13.1	High
TRINITY_DN338_c2_g1_i2_orf1	alpha-tocopherol transfer protein-like isoform X1 [Ostrinia furnacalis] >XP_028160444.1 alpha-tocopherol transfer protein-like isoform X1 [Ostrinia furnacalis] >XP_028160446.1 alpha-tocopherol transfer protein-like isoform X1 [Ostrinia furnacalis] >XP_028160447.1 alpha-tocopherol transfer protein-like isoform X1 [Ostrinia furnacalis] >XP_028160448.1 alpha-tocopherol transfer protein-like isoform X1 [Ostrinia furnacalis] >XP_028160449.1 alpha-tocopherol transfer protein-like isoform X1 [Ostrinia furnacalis]	2.021690655	1.015562263	3.93E-06	up	yes	0.9973	0.4933	0.492	0.47	0.518	1	1.002	0.99	------	------	------	------	------	------	ENOG410XRSQ	S:Function unknown	PF00650.23;PF137	CRAL_TRIO;CRAL_TRIO	CRAL/TRIO domain;Divergent CRAL/TRIO domain	CYT	2	14	35.6	High
TRINITY_DN14429_c0_g1_i2_orf1	NADH dehydrogenase [ubiquinone] 1 beta subcomplex subunit 11, mitochondrial [Ostrinia furnacalis]	2.432494807	1.282436725	0.0001209	up	yes	1.054	0.4333	0.454	0.382	0.464	1	1.114	1.049	CC:cellular_component;CC:integral component of membrane;CC:cellular anatomical entity;CC:intrinsic component of membrane;	GO:0005575;GO:0016021;GO:0110165;GO:0031224	K11351	NDUFB11	map04714;map	Thermogenesis;Oxid	ENOG41120A5	U:Intracellular trafficking, secretion, a	PF10183.12	ESSS	ESSS subunit of NAD	PLA	1	8	16.4	High
TRINITY_DN20369_c0_g1_i2_orf1	uncharacterized protein LOC114366225 [Ostrinia furnacalis]	2.262244674	1.177754973	8.91E-06	up	yes	1.03	0.4553	0.435	0.464	0.467	1	1.06	1.03	------	------	K17406	MRPS27	------	------	ENOG4110AQE	S:Function unknown	PF10037.12	MRP-S27	Mitochondrial 28S ribosomal protein S27	CYT	4	11	51.4	High
TRINITY_DN11153_c0_g1_i1_orf1	unnamed protein product [Chilo suppressalis]	2.064495798	1.045789482	8.32E-06	up	yes	0.9827	0.476	0.457	0.495	0.476	1	0.992	0.956	------	------	K20792	NAA15_16	------	------	ENOG410XR7D	S:Function unknown	PF12569.11;PF077	NatA_aux_su;TPR_2;TPI	N-terminal acetyltransferase A, auxiliary subunit;Tetratricopeptide repeat;Tetratricopeptide repeat;Tetratricopeptide repeat;Tetratricopeptide repeat;Anaphase-promoting complex, cyclosome, subunit 3;Tetratricopeptide repeat;Tetratricopeptide repeat	CYT	16	20	100.1	High
TRINITY_DN5867_c0_g1_i1_orf1	NADH dehydrogenase [ubiquinone] 1 alpha subcomplex subunit 7-like [Ostrinia furnacalis]	2.495654333	1.319418124	2.08E-07	up	yes	1.005	0.4027	0.396	0.409	0.403	1	1.019	0.995	BP:cellular process;BP:cellular metabolic process;BP:electron transport chain;BP:biological_process;BP:metabolic process;BP:respiratory electron transport chain;BP:generation of precursor metabolites and energy;BP:ATP synthesis coupled electron transport;CC:organelle membrane;CC:membrane;CC:organelle inner membrane;CC:mitochondrial membrane;CC:cellular_component;CC:mitochondrial inner membrane;CC:cellular	GO:0009987;GO:0044237;GO:0022900;GO:0008150;GO:0008152;GO:0022904;GO:0006091;GO:0042773;GO:0031090;GO:0016020;GO:0019866;GO:0031966;GO:0005575;GO:0005743;GO:0110165	K03951	NDUFA7	map04714;map	Thermogenesis;Oxid	ENOG410XW5P	C:Energy production and conversion	PF07347.15	CI-B14_5a	NAD	CYT	6	60	12.3	High
TRINITY_DN7289_c0_g1_i1_orf1	nuclear cap-binding protein subunit 2 [Ostrinia furnacalis]	2.08655946	1.061126033	0.0009338	up	yes	1.051	0.5037	0.429	0.484	0.598	1	1.029	1.125	BP:negative regulation of metabolic process;BP:regulation of metabolic process;BP:cellular metabolic process;BP:nucleic acid metabolic process;BP:regulation of biological process;BP:nitrogen compound metabolic process;BP:organic cyclic compound metabolic process;BP:nucleobase-containing compound metabolic process;BP:negative regulation of macromolecule metabolic process;BP:regulation of gene expression;BP:organic substance metabolic process;BP:biological regulation;BP:negative regulation of biological process;BP:RNA splicing;BP:mRNA metabolic process;BP:regulation of macromolecule metabolic process;BP:cellular process;BP:cellular aromatic compound metabolic process;BP:RNA splicing, via transesterification reactions;BP:RNA splicing, via transesterification reactions with bulged adenosine as nucleophile;BP:biological_process;BP:gene silencing by RNA;BP:metabolic process;BP:heterocycle metabolic process;BP:RNA metabolic process;BP:primary metabolic process;BP:mRNA cis splicing, via spliceosome;BP:cellular nitrogen compound metabolic process;BP:mRNA splicing, via spliceosome;BP:macromolecule metabolic process;BP:negative regulation of gene expression;BP:RNA processing;BP:mRNA processing;CC:intracellular organelle;CC:membrane-bounded organelle;CC:nucleus;CC:organelle;CC:RNA cap binding complex;CC:cellular anatomical entity;CC:nuclear cap binding complex;CC:macromolecular complex;CC:intracellular membrane-bounded organelle;CC:cellular_component;MF:molecular_function;MF:binding;MF:nucleic acid binding;MF:heterocyclic compound binding;MF:RNA cap binding;MF:organic cyclic compound binding;MF:RNA	GO:0009892;GO:0019222;GO:0044237;GO:0090304;GO:0050789;GO:0006807;GO:1901360;GO:0006139;GO:0010605;GO:0010468;GO:0071704;GO:0065007;GO:0048519;GO:0008380;GO:0016071;GO:0060255;GO:0009987;GO:0006725;GO:0000375;GO:0000377;GO:0008150;GO:0031047;GO:0008152;GO:0046483;GO:0016070;GO:0044238;GO:0045292;GO:0034641;GO:0000398;GO:0043170;GO:0010629;GO:0006396;GO:0006397;GO:0043229;GO:0043227;GO:0005634;GO:0043226;GO:0034518;GO:0110165;GO:0005846;GO:0032991;GO:0043231;GO:0005575;GO:0003674;GO:0005488;GO:0003676;GO:1901363;GO:0000339;GO:0097159;GO:0003723	K12883	NCBP2, CBP2(	map03013;map	Nucleocytoplasmic t	ENOG4111FJQ	A:RNA processing and modification	PF00076.25	RRM_1	RNA recognition motif.	CYT	1	9	18.8	High
TRINITY_DN8944_c0_g1_i1_orf1	actin, clone 403 [Trichonephila clavata]	2.583333333	1.36923381	0.001051	up	yes	1.116	0.432	0.488	0.353	0.455	1	1.24	1.107	------	------	K05692	ACTB_G1	map05164;map	Influenza A;Hippo si	COG5277	O:Posttranslational modification, pro	PF00022.22	Actin	Actin	CYT	1	64	42.8	High
TRINITY_DN86090_c0_g1_i1_orf1	ATP synthase subunit b, mitochondrial [Ostrinia furnacalis]	2.093877551	1.066177077	2.75E-05	up	yes	1.026	0.49	0.487	0.49	0.493	1	1.076	1.003	BP:nucleoside triphosphate metabolic process;BP:nucleoside triphosphate biosynthetic process;BP:purine nucleoside triphosphate metabolic process;BP:purine nucleoside triphosphate biosynthetic process;BP:cellular biosynthetic process;BP:cellular nitrogen compound metabolic process;BP:nucleotide biosynthetic process;BP:small molecule metabolic process;BP:purine-containing compound metabolic process;BP:purine-containing compound biosynthetic process;BP:organic cyclic compound biosynthetic process;BP:ribonucleotide metabolic process;BP:organic cyclic compound metabolic process;BP:nucleobase-containing compound metabolic process;BP:ribonucleoside triphosphate biosynthetic process;BP:organic substance metabolic process;BP:ribonucleoside triphosphate metabolic process;BP:heterocycle biosynthetic process;BP:purine ribonucleoside triphosphate biosynthetic process;BP:purine ribonucleoside triphosphate metabolic process;BP:ribose phosphate metabolic process;BP:organic substance biosynthetic process;BP:ATP metabolic process;BP:cellular process;BP:cellular aromatic compound metabolic process;BP:purine ribonucleotide biosynthetic process;BP:purine ribonucleotide metabolic process;BP:ribonucleotide biosynthetic process;BP:biosynthetic process;BP:nucleotide metabolic process;BP:metabolic process;BP:aromatic compound biosynthetic process;BP:nucleobase-containing compound biosynthetic process;BP:cellular nitrogen compound biosynthetic process;BP:organophosphate biosynthetic process;BP:nucleobase-containing small molecule metabolic process;BP:heterocycle metabolic process;BP:primary metabolic process;BP:ATP synthesis coupled proton transport;BP:organonitrogen compound metabolic process;BP:organonitrogen compound biosynthetic process;BP:carbohydrate derivative biosynthetic process;BP:carbohydrate derivative metabolic process;BP:purine nucleotide biosynthetic process;BP:ribose phosphate biosynthetic process;BP:cellular metabolic process;BP:purine nucleotide metabolic process;BP:phosphate-containing compound metabolic process;BP:nitrogen compound metabolic process;BP:nucleoside phosphate biosynthetic process;BP:phosphorus metabolic process;BP:organophosphate metabolic process;BP:biological_process;BP:nucleoside phosphate metabolic process;BP:ATP biosynthetic process;CC:proton-transporting ATP synthase complex, coupling factor;CC:cellular_component;CC:mitochondrial protein complex;CC:inner mitochondrial membrane protein complex;CC:macromolecular complex;CC:mitochondrial proton-transporting ATP synthase complex, coupling factor;CC:membrane protein complex;CC:proton-transporting two-sector ATPase complex, proton-transporting domain;MF:inorganic cation transmembrane transporter activity;MF:molecular_function;MF:inorganic solute uptake transmembrane transporter activity;MF:transmembrane transporter activity;MF:transporter activity;MF:cation transmembrane transporter activity;MF:ion transmembrane transporter activity;MF:hydrogen	GO:0009141;GO:0009142;GO:0009144;GO:0009145;GO:0044249;GO:0034641;GO:0009165;GO:0044281;GO:0072521;GO:0072522;GO:1901362;GO:0009259;GO:1901360;GO:0006139;GO:0009201;GO:0071704;GO:0009199;GO:0018130;GO:0009206;GO:0009205;GO:0019693;GO:1901576;GO:0046034;GO:0009987;GO:0006725;GO:0009152;GO:0009150;GO:0009260;GO:0009058;GO:0009117;GO:0008152;GO:0019438;GO:0034654;GO:0044271;GO:0090407;GO:0055086;GO:0046483;GO:0044238;GO:0015986;GO:1901564;GO:1901566;GO:1901137;GO:1901135;GO:0006164;GO:0046390;GO:0044237;GO:0006163;GO:0006796;GO:0006807;GO:1901293;GO:0006793;GO:0019637;GO:0008150;GO:0006753;GO:0006754;GO:0045263;GO:0005575;GO:0098798;GO:0098800;GO:0032991;GO:0000276;GO:0098796;GO:0033177;GO:0022890;GO:0003674;GO:0015318;GO:0022857;GO:0005215;GO:0008324;GO:0015075;GO:0015078	K02127	ATPeF0B, ATP	map04714;map	Thermogenesis;Cher	ENOG410ZUQH	I:Lipid transport and metabolism	PF05405.17	Mt_ATP-synt_B	Mitochondrial ATP synthase B chain precursor	CYT	12	46	27.4	High
TRINITY_DN11117_c0_g1_i1_orf1	venom carboxylesterase-6-like [Ostrinia furnacalis]	2.037299818	1.026658309	2.56E-06	up	yes	1.005	0.4933	0.494	0.496	0.49	1	1.03	0.986	MF:hydrolase activity;MF:molecular_function;MF:hydrolase activity, acting on ester bonds;MF:carboxylic ester hydrolase activity;MF:catalytic activity;	GO:0016787;GO:0003674;GO:0016788;GO:0052689;GO:0003824	------	------	------	------	COG2272;COG0580;I	S:Function unknown;G:Carbohydrate	PF00135.31	COesterase	Carboxylesterase family	CYT	4	11	63.8	High
TRINITY_DN3135_c0_g1_i6_orf1	acanthoscurrin-1-like [Ostrinia furnacalis]	2.009317399	1.006705476	0.0002718	up	yes	0.992	0.4937	0.501	0.49	0.49	1	1.059	0.917	------	------	------	------	------	------	------	------	------	------	------	EXC	1	7	25	High
TRINITY_DN34040_c0_g2_i1_orf1	uncharacterized protein LOC114352849 [Ostrinia furnacalis]	2.086819424	1.061305767	2.08E-05	up	yes	0.9927	0.4757	0.461	0.485	0.481	1	1.025	0.953	------	------	------	------	------	------	------	------	------	------	------	CYT	1	6	24	High
TRINITY_DN14507_c0_g1_i5_orf1	PTB domain-containing adapter protein ced-6 [Ostrinia furnacalis]	2.659494621	1.411152119	0.001271	up	yes	1.063	0.3997	0.544	0.305	0.35	1	1.127	1.061	------	------	K23285	GULP1, CED6	------	------	ENOG410ZHJT;COG(	S:Function unknown;	PF00640.26	PID	Phosphotyrosine interaction domain	CYT	1	3	57	High
TRINITY_DN5019_c0_g1_i2_orf1	RRP12-like protein isoform X4 [Ostrinia furnacalis] >XP_028175539.1 RRP12-like protein isoform X5 [Ostrinia furnacalis]	2.31884058	1.213403638	1.23E-07	up	yes	1.008	0.4347	0.438	0.44	0.426	1	1.018	1.006	CC:intracellular membrane-bounded organelle;CC:nucleus;CC:cellular_component;CC:intracellular organelle;CC:membrane-bounded organelle;CC:organelle;CC:cellular anatomical entity;	GO:0043231;GO:0005634;GO:0005575;GO:0043229;GO:0043227;GO:0043226;GO:0110165	K14794	RRP12	------	------	ENOG410XQ3D	S:Function unknown	PF08161.15	NUC173	NUC173 domain	CYT	8	6	153.7	High
TRINITY_DN3598_c0_g1_i1_orf1	esterase FE4-like [Ostrinia furnacalis]	2.357494722	1.237254541	5.44E-06	up	yes	1.005	0.4263	0.392	0.451	0.436	1	1.007	1.008	MF:hydrolase activity;MF:molecular_function;MF:hydrolase activity, acting on ester bonds;MF:carboxylic ester hydrolase activity;MF:catalytic activity;	GO:0016787;GO:0003674;GO:0016788;GO:0052689;GO:0003824	------	------	------	------	COG2272	T:Signal transduction mechanisms	PF00135.31;PF204	COesterase;BD-FAE;Ab	Carboxylesterase family;BD-FAE;alpha/beta hydrolase	CYT	4	10	61.6	High

TRINITY_DN747_c0_g1_i4_orf1	trypsin, alkaline C-like [Ostrinia furnacalis]	2.591939547	1.37403207	0.0001485	up	yes	1.029	0.397	0.422	0.396	0.373	1	0.974	1.113	BP:organic substance metabolic process;BP:organonitrogen compound metabolic process;BP:protein metabolic process;BP:macromolecule metabolic process;BP:biological_process;BP:nitrogen compound metabolic process;BP:metabolic process;BP:proteolysis;BP:primary metabolic process;MF:serine-type endopeptidase activity;MF:endopeptidase activity;MF:hydrolase activity;MF:serine hydrolase activity;MF:catalytic activity, acting on a protein;MF:catalytic activity;MF:molecular_function;MF:peptidase activity;MF:serine-type peptidase activity	GO:0071704;GO:1901564;GO:0019538;GO:0043170;GO:0008150;GO:0006807;GO:0008152;GO:0006508;GO:0044238;GO:0004252;GO:0004175;GO:0016787;GO:0017171;GO:0140096;GO:0003824;GO:0003674;GO:0008233;GO:0008236	K09628;K09640	PRSS27;TMPR	------	------	COG5640	O:Posttranslational modification, pro	PF00089.29	Trypsin	Trypsin	EXC	1	5	28	High
TRINITY_DN6231_c0_g1_i6_orf1	ran-binding protein 3 isoform X1 [Ostrinia furnacalis] >XP_028166372.1 ran-binding protein 3 isoform X2 [Ostrinia furnacalis]	2.240073445	1.163546035	1.24E-05	up	yes	0.976	0.4357	0.441	0.442	0.424	1	0.937	0.991	BP:cellular process;BP:intracellular transport;BP:transport;BP:biological_process;BP:establishment of localization in cell;BP:establishment of localization;BP:localization;BP:cellular localization;	GO:0009987;GO:0046907;GO:0006810;GO:0008150;GO:0051649;GO:0051234;GO:0051179;GO:0051641	K15304	RANBP3	map05166	Human T-cell leuker	ENOG4111NCK	U:Intracellular trafficking, secretion, a	PF00638.21	Ran_BP1	RanBP1 domain	CYT	4	11	48.6	High
TRINITY_DN17049_c0_g1_i6_orf1	unnamed protein product [Arctia plantaginis] >CAB3248215.1 unnamed protein product [Arctia plantaginis]	2.092220191	1.065034693	1.18E-05	up	yes	1.03	0.4923	0.481	0.491	0.505	1	1.065	1.025	BP:cellular component assembly;BP:macromolecular complex subunit organization;BP:ribonucleoprotein complex subunit organization;BP:cellular component organization;BP:macromolecular complex assembly;BP:ribonucleoprotein complex assembly;BP:cellular component organization or biogenesis;BP:cellular process;BP:biological_process;BP:formation of cytoplasmic translation initiation complex;CC:eukaryotic 48S preinitiation complex;CC:eukaryotic translation initiation factor 3 complex;CC:eukaryotic 43S preinitiation complex;CC:translation preinitiation complex;CC:macromolecular complex;CC:ribonucleoprotein complex;CC:cellular_component;MF:molecular_function;MF:binding;MF:nucleic acid binding;MF:peptidase activity;MF:heterocyclic compound binding;MF:metallopeptidase activity;MF:hydrolase activity;MF:translation factor activity, RNA binding;MF:catalytic activity, acting on a protein;MF:catalytic activity;MF:translation regulator activity;MF:translation regulator activity, nucleic acid binding;MF:organic cyclic compound binding;MF:translation initiation factor activity;	GO:0022607;GO:0043933;GO:0071826;GO:0016043;GO:0065003;GO:0022618;GO:0071840;GO:0009987;GO:0008150;GO:0001732;GO:0033290;GO:0005852;GO:0016282;GO:0070993;GO:0032991;GO:1990904;GO:0005575;GO:0003674;GO:0005488;GO:0003676;GO:0008233;GO:1901363;GO:0008237;GO:0016787;GO:0008135;GO:0140096;GO:0003824;GO:0045182;GO:0090079;GO:0097159;GO:0003743	K03247	EIF3H	map05162	Measles	ENOG410XSR9	J:Translation, ribosomal structure anc	PF19445.2;PF0139	eIF3h_C;JAB	C-terminal region of eIF3h;JAB1/Mov34/MPN/PAD-1 ubiquitin protease	CYT	5	16	38.7	High
TRINITY_DN76377_c0_g1_i1_orf1	uncharacterized protein LOC111357764, partial [Spodoptera litura]	2.039168181	1.027980767	2.00E-06	up	yes	1.01	0.4953	0.505	0.494	0.487	1	1.032	0.997	------	------	------	------	------	------	ENOG410Y7O4;ENO	S:Function unknown;	PF03564.18	DUF1759	Protein of unknown function	CYT	1	4	27.4	Medium
TRINITY_DN81715_c0_g1_i1_orf1	gamma-interferon-inducible lysosomal thiol reductase-like [Ostrinia furnacalis]	2.436104076	1.28457577	0.0001045	up	yes	1.058	0.4343	0.381	0.474	0.448	1	1.078	1.097	MF:catalytic activity;MF:oxidoreductase activity, acting on a sulfur group of donors, disulfide as acceptor;MF:molecular_function;MF:oxidoreductase activity, acting on a sulfur group of donors;MF:oxidoreductase activity;	GO:0003824;GO:0016671;GO:0003674;GO:0016667;GO:0016491	K08059	IFI30, GILT	map04612	Antigen processing	ENOG4111IDT	S:Function unknown	PF03227.19	GILT	Gamma interferon inducible lysosomal thiol reductase	CYT	1	3	26.1	High
TRINITY_DN1073_c0_g1_i1_orf1	carboxylesterase [Loxostege sticticalis]	2.388876225	1.256332105	2.55E-05	up	yes	1.048	0.4387	0.444	0.425	0.447	1	1.05	1.093	CC:integral component of membrane;CC:cellular_component;CC:intrinsic component of membrane;CC:cellular anatomical entity;MF:hydrolase activity;MF:hydrolase activity, acting on ester bonds;MF:carboxylic ester hydrolase activity;MF:catalytic activity;MF:molecular_function;	GO:0016021;GO:0005575;GO:0031224;GO:0110165;GO:0016787;GO:0016788;GO:0052689;GO:0003824;GO:0003674	------	------	------	------	COG2272;COG0580;I	I:Lipid transport and metabolism;G:C	PF00135.31;PF204	COesterase;BD-FAE;Ab	Carboxylesterase family;BD-FAE;alpha/beta hydrolase fold	CYT	1	8	30.1	High
TRINITY_DN50085_c0_g1_i1_orf1	hypothetical protein evm_013997 [Chilo suppressalis]	2.058091286	1.041306974	3.30E-06	up	yes	0.992	0.482	0.483	0.459	0.504	1	0.983	0.993	BP:cellular component assembly;BP:regulation of primary metabolic process;BP:regulation of metabolic process;BP:macromolecular complex subunit organization;BP:regulation of cellular metabolic process;BP:formation of cytoplasmic translation initiation complex;BP:regulation of biological process;BP:cellular component organization or biogenesis;BP:ribonucleoprotein complex subunit organization;BP:posttranscriptional regulation of gene expression;BP:regulation of protein metabolic process;BP:cellular component organization;BP:macromolecular complex assembly;BP:ribonucleoprotein complex assembly;BP:biological regulation;BP:regulation of cellular biosynthetic process;BP:regulation of cellular macromolecule biosynthetic process;BP:regulation of gene expression;BP:regulation of macromolecule metabolic process;BP:regulation of translational initiation;BP:cellular process;BP:regulation of biosynthetic process;BP:regulation of cellular amide metabolic process;BP:biological_process;BP:regulation of nitrogen compound metabolic process;BP:regulation of cellular process;BP:regulation of macromolecule biosynthetic process;BP:regulation of translation;CC:translation preinitiation complex;CC:eukaryotic translation initiation factor 3 complex;CC:eukaryotic 48S preinitiation complex;CC:macromolecular complex;CC:ribonucleoprotein complex;CC:cellular_component;CC:eukaryotic 43S preinitiation complex;MF:molecular_function;MF:binding;MF:nucleic acid binding;MF:translation initiation factor binding;MF:heterocyclic compound binding;MF:translation factor activity, RNA binding;MF:translation regulator activity;MF:translation regulator activity, nucleic acid binding;MF:organic cyclic compound binding;MF:RNA binding;MF:protein binding;MF:translation initiation factor activity;	GO:0022607;GO:0080090;GO:0019222;GO:0043933;GO:0031323;GO:0001732;GO:0050789;GO:0071840;GO:0071826;GO:0010608;GO:0051246;GO:0016043;GO:0065003;GO:0022618;GO:0065007;GO:0031326;GO:2000112;GO:0010468;GO:0060255;GO:0006446;GO:0009987;GO:0009889;GO:0034248;GO:0008150;GO:0051171;GO:0050794;GO:0010556;GO:0006417;GO:0070993;GO:0005852;GO:0033290;GO:0032991;GO:1990904;GO:0005575;GO:0016282;GO:0003674;GO:0005488;GO:0003676;GO:0031369;GO:1901363;GO:0008135;GO:0045182;GO:0090079;GO:0097159;GO:0003723;GO:0005515;GO:0003743	K03253	EIF3B	------	------	COG5354	S:Function unknown	PF08662.14;PF000	eIF2A;RRM_1	Eukaryotic translation initiation factor eIF2A;RNA recognition motif.	CYT	13	22	82.4	High
TRINITY_DN8754_c0_g1_i2_orf1	dnaJ homolog subfamily C member 11 [Ostrinia furnacalis]	2.305617978	1.20515349	3.34E-05	up	yes	1.026	0.445	0.425	0.424	0.486	1	1.064	1.013	------	------	K09531	DNAJC11	------	------	COG0484	J:Translation, ribosomal structure anc	PF11875.11;PF002	DnaJ-like_C11_C;DnaJ	DnaJ-like protein C11, C-terminal;DnaJ domain	CYT	2	3	65.1	High
TRINITY_DN620_c0_g1_i4_orf1	lysine--tRNA ligase isoform X1 [Ostrinia furnacalis]	2.04989605	1.035550753	1.56E-06	up	yes	0.986	0.481	0.481	0.493	0.469	1	0.989	0.969	BP:lysyl-tRNA aminoacylation;BP:RNA metabolic process;BP:carboxylic acid metabolic process;BP:nucleic acid metabolic process;BP:cellular nitrogen compound metabolic process;BP:nitrogen compound metabolic process;BP:ncRNA metabolic process;BP:nucleobase-containing compound metabolic process;BP:cellular amino acid metabolic process;BP:organic substance metabolic process;BP:organic cyclic compound metabolic process;BP:cellular process;BP:cellular aromatic compound metabolic process;BP:biological_process;BP:metabolic process;BP:oxoacid metabolic process;BP:heterocycle metabolic process;BP:primary metabolic process;BP:organonitrogen compound metabolic process;BP:organic acid metabolic process;BP:cellular metabolic process;BP:macromolecule metabolic process;BP:tRNA metabolic process;BP:amino acid activation;BP:tRNA aminoacylation;BP:tRNA aminoacylation for protein translation;BP:small molecule metabolic process;CC:cytoplasm;CC:cellular_component;CC:cellular anatomical entity;MF:purine ribonucleoside triphosphate binding;MF:molecular_function;MF:binding;MF:nucleic acid binding;MF:ion binding;MF:heterocyclic compound binding;MF:nucleoside phosphate binding;MF:aminoacyl-tRNA ligase activity;MF:nucleotide binding;MF:purine nucleotide binding;MF:ATP binding;MF:anion binding;MF:catalytic activity, acting on RNA;MF:small molecule binding;MF:catalytic activity;MF:ligase activity;MF:ligase activity, forming carbon-oxygen bonds;MF:lysine-tRNA ligase activity;MF:adenyl nucleotide binding;MF:carbohydrate derivative binding;MF:organic cyclic compound binding;MF:adenyl ribonucleotide binding;MF:purine ribonucleotide binding;MF:catalytic activity, acting on a tRNA;MF:ribonucleotide binding;	GO:0006430;GO:0016070;GO:0019752;GO:0090304;GO:0034641;GO:0006807;GO:0034660;GO:0006139;GO:0006520;GO:0071704;GO:1901360;GO:0009987;GO:0006725;GO:0008150;GO:0008152;GO:0043436;GO:0046483;GO:0044238;GO:1901564;GO:0006082;GO:0044237;GO:0043170;GO:0006399;GO:0043038;GO:0043039;GO:0006418;GO:0044281;GO:0005737;GO:0005575;GO:0110165;GO:0035639;GO:0003674;GO:0005488;GO:0003676;GO:0043167;GO:1901363;GO:1901265;GO:0004812;GO:0000166;GO:0017076;GO:0005524;GO:0043168;GO:0140098;GO:0036094;GO:0003824;GO:0016874;GO:0016875;GO:0004824;GO:0030554;GO:0097367;GO:0097159;GO:0032559;GO:0032555;GO:0140101;GO:0032553	K04567	KARS, lysS	map00970	Aminoacyl-tRNA bic	COG1190	J:Translation, ribosomal structure anc	PF00152.23;PF014	tRNA-synt_2;tRNA-syn	tRNA synthetases class II;tRNA synthetases class II core domain	CYT	13	42	50.6	High
TRINITY_DN14920_c0_g1_i1_orf1	anamorsin homolog [Ostrinia furnacalis]	2.362751911	1.240468154	5.10E-07	up	yes	1.02	0.4317	0.432	0.432	0.431	1	1.033	1.026	BP:cellular component assembly;BP:cellular component organization or biogenesis;BP:cellular component organization;BP:cellular process;BP:iron-sulfur cluster assembly;BP:metallo-sulfur cluster assembly;BP:biological_process;BP:metabolic process;BP:cellular metabolic process;BP:sulfur compound metabolic process;CC:organelle envelope lumen;CC:membrane-enclosed lumen;CC:mitochondrial intermembrane space;CC:cellular anatomical entity;CC:organelle lumen;CC:cellular_component;CC:intracellular organelle lumen;MF:molecular_function;MF:binding;MF:oxidoreductase activity;MF:cation binding;MF:metal cluster binding;MF:ion binding;MF:metal ion binding;MF:catalytic activity;MF:electron carrier activity;MF:4 iron, 4 sulfur cluster binding;MF:iron-sulfur cluster binding;MF:2 iron, 2 sulfur cluster binding;	GO:0022607;GO:0071840;GO:0016043;GO:0009987;GO:0016226;GO:0031163;GO:0008150;GO:0008152;GO:0044237;GO:0006790;GO:0031970;GO:0031974;GO:0005758;GO:0110165;GO:0043233;GO:0005575;GO:0070013;GO:0003674;GO:0005488;GO:0016491;GO:0043169;GO:0051540;GO:0043167;GO:0046872;GO:0003824;GO:0009055;GO:0051539;GO:0051536;GO:0051537	K22746	CIAPIN1, DRE:	------	------	COG5636	S:Function unknown	PF05093.16	CIAPIN1	Cytokine-induced anti-apoptosis inhibitor 1, Fe-S biogenesis	CYT	2	8	27.1	High
TRINITY_DN37699_c0_g1_i4_orfp1	TRINITY_DN37699_c0_g1_i4_m.58777 TRINITY_DN37699_c0_g1::TRINITY_DN37699_c0_g1_i4::g.58777 ORF type:internal len:122 (+),score=34.90 TRINITY_DN37699_c0_g1_i4:1-363(+)	2.426175289	1.278683788	0.002099	up	yes	1.027	0.4233	0.537	0.458	0.275	1	1.097	0.984	------	------	------	------	------	------	------	------	------	------	------	PLA	1	18	12.7	High
TRINITY_DN3401_c0_g1_i1_orf1	28S ribosomal protein S5, mitochondrial [Ostrinia furnacalis]	2.067456897	1.047857252	0.0004477	up	yes	0.9593	0.464	0.413	0.547	0.432	1	0.946	0.932	BP:cellular biosynthetic process;BP:cellular macromolecule biosynthetic process;BP:organic substance biosynthetic process;BP:cellular nitrogen compound biosynthetic process;BP:cellular macromolecule metabolic process;BP:cellular amide metabolic process;BP:peptide biosynthetic process;BP:organic substance metabolic process;BP:organonitrogen compound biosynthetic process;BP:amide biosynthetic process;BP:cellular process;BP:biosynthetic process;BP:macromolecule biosynthetic process;BP:biological_process;BP:metabolic process;BP:organonitrogen compound metabolic process;BP:primary metabolic process;BP:protein metabolic process;BP:cellular nitrogen compound metabolic process;BP:cellular metabolic process;BP:macromolecule metabolic process;BP:nitrogen compound metabolic process;BP:peptide metabolic process;BP:translation;CC:organelle;CC:intracellular organelle;CC:non-membrane-bounded organelle;CC:cellular anatomical entity;CC:macromolecular complex;CC:ribosome;CC:intracellular non-membrane-bounded organelle;CC:ribonucleoprotein complex;CC:cellular_component;MF:nucleic acid binding;MF:molecular_function;MF:binding;MF:heterocyclic compound binding;MF:structural molecule activity;MF:structural constituent of ribosome;MF:organic cyclic compound binding;MF:RNA binding;	GO:0044249;GO:0034645;GO:1901576;GO:0044271;GO:0044260;GO:0043603;GO:0043043;GO:0071704;GO:1901566;GO:0043604;GO:0009987;GO:0009058;GO:0009059;GO:0008150;GO:0008152;GO:1901564;GO:0044238;GO:0019538;GO:0034641;GO:0044237;GO:0043170;GO:0006807;GO:0006518;GO:0006412;GO:0043226;GO:0043229;GO:0043228;GO:0110165;GO:0032991;GO:0005840;GO:0043232;GO:1990904;GO:0005575;GO:0003676;GO:0003674;GO:0005488;GO:1901363;GO:0005198;GO:0003735;GO:0097159;GO:0003723	K02988	RP-S5, MRPS!	map03010	Ribosome	COG0098	J:Translation, ribosomal structure anc	PF03719.18;PF003	Ribosomal_S5_C;Ribosc	Ribosomal protein S5, C-terminal domain;Ribosomal protein S5, N-terminal domain	CYT	2	6	49	High
TRINITY_DN905_c0_g1_i4_orf1	(11Z)-hexadec-11-enoyl-CoA conjugase-like [Ostrinia furnacalis] >XP_028172978.1 (11Z)-hexadec-11-enoyl-CoA conjugase-like [Ostrinia furnacalis]	2.882513661	1.527327445	0.0002726	up	yes	1.055	0.366	0.386	0.323	0.389	1	1.161	1.004	BP:fatty acid biosynthetic process;BP:fatty acid metabolic process;BP:carboxylic acid metabolic process;BP:cellular biosynthetic process;BP:small molecule metabolic process;BP:small molecule biosynthetic process;BP:monocarboxylic acid biosynthetic process;BP:organic substance biosynthetic process;BP:organic substance metabolic process;BP:lipid metabolic process;BP:cellular process;BP:monocarboxylic acid metabolic process;BP:biosynthetic process;BP:biological_process;BP:metabolic process;BP:oxoacid metabolic process;BP:cellular lipid metabolic process;BP:lipid biosynthetic process;BP:primary metabolic process;BP:organic acid metabolic process;BP:carboxylic acid biosynthetic process;BP:organic acid biosynthetic process;BP:cellular metabolic process;CC:intrinsic component of membrane;CC:integral component of membrane;CC:cellular anatomical entity;CC:cellular_component;MF:molecular_function;MF:oxidoreductase activity;MF:oxidoreductase activity, acting on paired donors, with incorporation or reduction of molecular oxygen;MF:catalytic activity;MF:oxidoreductase activity, acting on paired donors, with oxidation of a pair of donors resulting in the reduction of molecular oxygen to two molecules of water;	GO:0006633;GO:0006631;GO:0019752;GO:0044249;GO:0044281;GO:0044283;GO:0072330;GO:1901576;GO:0071704;GO:0006629;GO:0009987;GO:0032787;GO:0009058;GO:0008150;GO:0008152;GO:0043436;GO:0044255;GO:0008610;GO:0044238;GO:0006082;GO:0046394;GO:0016053;GO:0044237;GO:0031224;GO:0016021;GO:0110165;GO:0005575;GO:0003674;GO:0016491;GO:0016705;GO:0003824;GO:0016717	K00507	SCD, desC	map04936;map	Alcoholic liver diseas	COG1398	J:Translation, ribosomal structure anc	PF00487.27	FA_desaturase	Fatty acid desaturase	CYT	2	8	41.9	High
TRINITY_DN31663_c0_g1_i2_orf1	PHD finger-like domain-containing protein 5A [Nasonia vitripennis] >XP_002427197.1 conserved hypothetical protein [Pediculus humanus corporis] >XP_003484388.1 PHD finger-like domain-containing protein 5A [Bombus impatiens] >XP_003701008.1 PREDICTED: PHD finger-like domain-containing protein 5A [Megachile rotundata] >XP_006623871.1 PHD finger-like domain-containing protein 5A [Apis dorsata] >XP_011068502.1 PREDICTED: PHD finger-like domain-containing protein 5A [Acromyrmex echinatior] >XP_011154391.1 PHD finger-like domain-containing protein 5A [Harpegnathos saltator] >XP_011164776.1 PHD finger-like domain-containing protein 5A [Solenopsis invicta] >XP_011262550.1 PHD finger-like domain-containing protein 5A [Camponotus floridanus] >XP_011297178.1 PREDICTED: PHD finger-like domain-containing protein 5A [Fopius arisanus] >XP_011334720.1 PHD finger-like domain-containing protein 5A [Ooceraea biroi] >XP_011506347.1 PREDICTED: PHD finger-like domain-containing protein 5A [Ceratosolen solmsi marchali] >XP_011506348.1 PREDICTED: PHD finger-like domain-containing protein 5A [Ceratosolen solmsi marchali] >XP_011638597.1 PHD finger-like domain-containing protein 5A isoform X2 [Pogonomyrmex barbatus] >XP_011686073.1 PREDICTED: PHD finger-like domain-containing protein 5A [Wasmannia auropunctata] >XP_011858255.1 PREDICTED: PHD finger-like domain-containing protein 5A [Vollenhovia emeryi] >XP_012058015.1 PREDICTED: PHD finger-like domain-containing protein 5A [Atta cephalotes] >XP_012135327.1 PREDICTED: PHD finger-like domain-containing protein 5A [Megachile rotundata] >XP_012135328.1 PREDICTED: PHD finger-like domain-containing protein 5A [Megachile rotundata] >XP_012222185.1 PREDICTED: PHD finger-like domain-containing protein 5A [Linepithema humile] >XP_012261946.1 PHD finger-like domain-containing protein 5A [Athalia rosae] >XP_012273120.1 PHD finger-like domain-containing protein 5A [Orussus abietinus] >XP_012526512.1 PHD finger-like domain-containing protein 5A [Monomorium pharaonis] >XP_014217558.1 PHD finger-like domain-containing protein 5A [Copidosoma floridanum] >XP_014484566.1 PREDICTED: PHD finger-like domain-containing protein 5A [Dinoponera quadriceps] >XP_014611099.1 PREDICTED: PHD finger-like domain-containing protein 5A [Polistes canadensis] >XP_015122018.1 PHD finger-like domain-containing protein 5A [Diachasma alloeum] >XP_015174163.1 PREDICTED: PHD finger-like domain-containing protein 5A [Polistes dominula] >XP_015433827.1 PREDICTED: PHD finger-like domain-containing protein 5A [Dufourea novaeangliae] >XP_015516165.1 PHD finger-like domain-containing protein 5A	2.050522992	1.03599192	3.48E-05	up	yes	1.039	0.5067	0.51	0.521	0.489	1	1.032	1.084	BP:nucleic acid metabolic process;BP:cellular nitrogen compound metabolic process;BP:nitrogen compound metabolic process;BP:cellular metabolic process;BP:organic cyclic compound metabolic process;BP:nucleobase-containing compound metabolic process;BP:mRNA metabolic process;BP:organic substance metabolic process;BP:RNA splicing;BP:primary metabolic process;BP:cellular process;BP:cellular aromatic compound metabolic process;BP:RNA splicing, via transesterification reactions;BP:RNA splicing, via transesterification reactions with bulged adenosine as nucleophile;BP:biological_process;BP:metabolic process;BP:heterocycle metabolic process;BP:RNA metabolic process;BP:mRNA splicing, via spliceosome;BP:macromolecule metabolic process;BP:RNA processing;BP:mRNA processing;CC:Sm-like protein family complex;CC:precatalytic spliceosome;CC:spliceosomal snRNP complex;CC:macromolecular complex;CC:small nuclear ribonucleoprotein complex;CC:ribonucleoprotein complex;CC:cellular_component;CC:spliceosomal complex;CC:U12-type spliceosomal complex;CC:U2 snRNP;	GO:0090304;GO:0034641;GO:0006807;GO:0044237;GO:1901360;GO:0006139;GO:0016071;GO:0071704;GO:0008380;GO:0044238;GO:0009987;GO:0006725;GO:0000375;GO:0000377;GO:0008150;GO:0008152;GO:0046483;GO:0016070;GO:0000398;GO:0043170;GO:0006396;GO:0006397;GO:0120114;GO:0071011;GO:0097525;GO:0032991;GO:0030532;GO:1990904;GO:0005575;GO:0005681;GO:0005689;GO:0005686	K12834	PHF5A	map03040	Spliceosome	ENOG4111JIM	A:RNA processing and modification	PF03660.17	PHF5	PHF5-like protein	CYT	2	18	12.5	High
TRINITY_DN2718_c0_g1_i6_orf1	cleavage stimulation factor subunit 2 isoform X1 [Ostrinia furnacalis]	2.008605515	1.006194251	2.66E-05	up	yes	1.027	0.5113	0.486	0.514	0.534	1	1.016	1.064	BP:nucleic acid metabolic process;BP:cellular nitrogen compound metabolic process;BP:nitrogen compound metabolic process;BP:RNA polyadenylation;BP:organic cyclic compound metabolic process;BP:nucleobase-containing compound metabolic process;BP:organic substance metabolic process;BP:mRNA metabolic process;BP:mRNA 3'-end processing;BP:cellular process;BP:cellular aromatic compound metabolic process;BP:RNA 3'-end processing;BP:biological_process;BP:metabolic process;BP:heterocycle metabolic process;BP:RNA metabolic process;BP:primary metabolic process;BP:cellular metabolic process;BP:macromolecule metabolic process;BP:mRNA polyadenylation;BP:RNA processing;BP:mRNA processing;CC:mRNA cleavage and polyadenylation specificity factor complex;CC:macromolecular complex;CC:mRNA cleavage factor complex;CC:cellular_component;MF:molecular_function;MF:binding;MF:nucleic acid binding;MF:heterocyclic compound binding;MF:organic cyclic compound binding;MF:RNA binding;	GO:0090304;GO:0034641;GO:0006807;GO:0043631;GO:1901360;GO:0006139;GO:0071704;GO:0016071;GO:0031124;GO:0009987;GO:0006725;GO:0031123;GO:0008150;GO:0008152;GO:0046483;GO:0016070;GO:0044238;GO:0044237;GO:0043170;GO:0006378;GO:0006396;GO:0006397;GO:0005847;GO:0032991;GO:0005849;GO:0005575;GO:0003674;GO:0005488;GO:0003676;GO:1901363;GO:0097159;GO:0003723	K14407	CSTF2, RNA15	map03015	mRNA surveillance p	ENOG410XQBV	S:Function unknown	PF14327.9;PF0007	CSTF2_hinge;RRM_1;CS	Hinge domain of cleavage stimulation factor subunit 2;RNA recognition motif.;Transcription termination and cleavage factor C-terminal	CYT	3	8	42.8	High
TRINITY_DN11584_c0_g1_i2_orf1	L-threonine 3-dehydrogenase, mitochondrial [Ostrinia furnacalis]	2.067433878	1.047841189	0.0008085	up	yes	1.024	0.4953	0.473	0.439	0.574	1	0.967	1.105	------	------	K15789	TDH	map00260	Glycine, serine and t	COG0451	Z:Cytoskeleton	PF01370.24;PF163	Epimerase;GDP_Man_D	NAD dependent epimerase/dehydratase family;GDP-mannose 4,6 dehydratase;RmlD substrate binding domain;3-beta hydroxysteroid dehydrogenase/isomerase family;Male sterility protein;Polysaccharide biosynthesis protein	CYT	1	4	39.3	High
TRINITY_DN10662_c0_g1_i4_orf1	HD domain-containing protein 2 [Ostrinia furnacalis]	2.0329119	1.023547695	1.57E-05	up	yes	1.013	0.4983	0.467	0.525	0.503	1	1.037	1.002	MF:hydrolase activity;MF:phosphatase activity;MF:hydrolase activity, acting on ester bonds;MF:phosphoric ester hydrolase activity;MF:catalytic activity;MF:molecular_function;MF:5'-deoxynucleotidase activity;	GO:0016787;GO:0016791;GO:0016788;GO:0042578;GO:0003824;GO:0003674;GO:0002953	K07023	YGK1, HDDC2	map00240;map	Pyrimidine metaboli	COG1896	S:Function unknown	PF13023.9;PF1291	HD_3;YfbR-like	HD domain;5'-deoxynucleotidase YfbR-like	CYT	2	10	24.1	High
TRINITY_DN2265_c0_g2_i1_orf1	LOW QUALITY PROTEIN: elongation factor G, mitochondrial-like [Leguminivora glycinivorella]	2.200300236	1.137700396	0.0004518	up	yes	1.026	0.4663	0.387	0.552	0.46	1	1.071	1.006	BP:cellular macromolecule biosynthetic process;BP:cellular biosynthetic process;BP:organic substance biosynthetic process;BP:cellular macromolecule metabolic process;BP:cellular process;BP:organic substance metabolic process;BP:mitochondrial translational elongation;BP:biosynthetic process;BP:macromolecule biosynthetic process;BP:biological_process;BP:metabolic process;BP:cellular metabolic process;BP:macromolecule metabolic process;BP:translational elongation;CC:intracellular organelle;CC:membrane-bounded organelle;CC:mitochondrion;CC:organelle;CC:cellular anatomical entity;CC:intracellular membrane-bounded organelle;CC:cellular_component;MF:purine ribonucleoside triphosphate binding;MF:molecular_function;MF:binding;MF:nucleic acid binding;MF:guanyl nucleotide binding;MF:heterocyclic compound binding;MF:nucleoside phosphate binding;MF:anion binding;MF:guanyl ribonucleotide binding;MF:purine nucleotide binding;MF:GTP binding;MF:hydrolase activity;MF:nucleotide binding;MF:translation factor activity, RNA binding;MF:GTPase activity;MF:nucleoside-triphosphatase activity;MF:small molecule binding;MF:catalytic activity;MF:hydrolase activity, acting on acid anhydrides, in phosphorus-containing anhydrides;MF:translation regulator activity, nucleic acid binding;MF:ion binding;MF:carbohydrate derivative binding;MF:organic cyclic compound binding;MF:hydrolase activity, acting on acid anhydrides;MF:pyrophosphatase activity;MF:purine ribonucleotide binding;MF:translation elongation factor activity;MF:translation regulator activity;MF:ribonucleotide binding;	GO:0034645;GO:0044249;GO:1901576;GO:0044260;GO:0009987;GO:0071704;GO:0070125;GO:0009058;GO:0009059;GO:0008150;GO:0008152;GO:0044237;GO:0043170;GO:0006414;GO:0043229;GO:0043227;GO:0005739;GO:0043226;GO:0110165;GO:0043231;GO:0005575;GO:0035639;GO:0003674;GO:0005488;GO:0003676;GO:0019001;GO:1901363;GO:1901265;GO:0043168;GO:0032561;GO:0017076;GO:0005525;GO:0016787;GO:0000166;GO:0008135;GO:0003924;GO:0017111;GO:0036094;GO:0003824;GO:0016818;GO:0090079;GO:0043167;GO:0097367;GO:0097159;GO:0016817;GO:0016462;GO:0032555;GO:0003746;GO:0045182;GO:0032553	K02355	fusA, GFM, EF	------	------	COG0480	U:Intracellular trafficking, secretion, a	PF00679.27;PF037	EFG_C;EFG_IV	Elongation factor G C-terminus;Elongation factor G, domain IV	CYT	1	7	18.1	High
TRINITY_DN15667_c0_g1_i2_orf1	coiled-coil domain-containing protein 25 [Ostrinia furnacalis]	2.312486089	1.209444687	0.0001373	up	yes	1.039	0.4493	0.438	0.497	0.413	1	1.104	1.013	CC:cellular_component;CC:endomembrane system;CC:cellular anatomical entity;	GO:0005575;GO:0012505;GO:0110165	------	------	------	------	ENOG410Z5JK	S:Function unknown	PF05670.16	NFACT-R_1	NFACT protein RNA binding domain	CYT	2	10	24.3	High
TRINITY_DN16343_c0_g1_i6_orf1	aminopeptidase N6 [Ostrinia nubilalis]	2.175965665	1.121655792	7.92E-07	up	yes	1.014	0.466	0.453	0.478	0.467	1	1.026	1.015	BP:nitrogen compound metabolic process;BP:organic substance metabolic process;BP:proteolysis;BP:biological_process;BP:metabolic process;BP:primary metabolic process;BP:organonitrogen compound metabolic process;BP:protein metabolic process;BP:macromolecule metabolic process;CC:integral component of membrane;CC:intrinsic component of membrane;CC:cellular anatomical entity;CC:cellular_component;MF:aminopeptidase activity;MF:exopeptidase activity;MF:molecular_function;MF:binding;MF:peptidase activity;MF:metallopeptidase activity;MF:cation binding;MF:transition metal ion binding;MF:ion binding;MF:metal ion binding;MF:hydrolase activity;MF:catalytic activity, acting on a protein;MF:catalytic activity;MF:zinc ion binding;	GO:0006807;GO:0071704;GO:0006508;GO:0008150;GO:0008152;GO:0044238;GO:1901564;GO:0019538;GO:0043170;GO:0016021;GO:0031224;GO:0110165;GO:0005575;GO:0004177;GO:0008238;GO:0003674;GO:0005488;GO:0008233;GO:0008237;GO:0043169;GO:0046914;GO:0043167;GO:0046872;GO:0016787;GO:0140096;GO:0003824;GO:0008270	K11140	ANPEP, CD13	map04640;map	Hematopoietic cell l	COG0308	E:Amino acid transport and metaboli	PF11838.11;PF014	ERAP1_C;Peptidase_M1	ERAP1-like C-terminal domain;Peptidase family M1 domain;Peptidase M1 N-terminal domain	CYT	5	7	100.2	High
TRINITY_DN45227_c0_g1_i3_orf1	uncharacterized protein LOC114359191 [Ostrinia furnacalis]	2.596107056	1.376349877	0.0001608	up	yes	1.067	0.411	0.377	0.466	0.39	1	1.134	1.067	CC:respiratory chain complex I;CC:transmembrane transporter complex;CC:catalytic complex;CC:transporter complex;CC:NADH dehydrogenase complex;CC:respiratory chain complex;CC:inner mitochondrial membrane protein complex;CC:cellular_component;CC:membrane protein complex;CC:mitochondrial respiratory chain complex I;CC:macromolecular complex;CC:oxidoreductase complex;CC:mitochondrial protein complex;	GO:0045271;GO:1902495;GO:1902494;GO:1990351;GO:0030964;GO:0098803;GO:0098800;GO:0005575;GO:0098796;GO:0005747;GO:0032991;GO:1990204;GO:0098798	------	------	------	------	ENOG410XXY6	S:Function unknown	PF15880.8	NDUFV3	NADH dehydrogenase [ubiquinone] flavoprotein 3, mitochondrial	CYT	1	8	11.2	High
TRINITY_DN58531_c0_g1_i1_orf1	uncharacterized protein LOC114357371 [Ostrinia furnacalis] >XP_028166768.1 uncharacterized protein LOC114357371 [Ostrinia furnacalis]	3.27696793	1.712361554	0.001257	up	yes	1.124	0.343	0.21	0.362	0.457	1	1.157	1.214	------	------	------	------	------	------	ENOG410Y4ER;ENO(	S:Function unknown;	------	------	------	CYT	1	3	29.7	Medium
TRINITY_DN43942_c0_g1_i1_orf1	LOW QUALITY PROTEIN: caprin homolog [Ostrinia furnacalis]	2.319037894	1.213526395	6.88E-06	up	yes	1.022	0.4407	0.471	0.426	0.425	1	1.039	1.027	------	------	K18743	CAPRIN1, GPI	------	------	ENOG4110M5Q	S:Function unknown	PF18293.4	Caprin-1_dimer	Caprin-1 dimerization domain	CYT	6	20	45.3	High
TRINITY_DN4213_c0_g1_i4_orf1	nardilysin-like isoform X1 [Ostrinia furnacalis] >XP_028157649.1 nardilysin-like isoform X2 [Ostrinia furnacalis] >XP_028157650.1 nardilysin-like isoform X3 [Ostrinia furnacalis] >XP_028157651.1 nardilysin-like isoform X4 [Ostrinia furnacalis]	2.435927581	1.284471243	5.57E-05	up	yes	1.036	0.4253	0.399	0.481	0.396	1	1.042	1.066	------	------	K01411	NRD1	------	------	COG1025	O:Posttranslational modification, pro	PF16187.8;PF0067	Peptidase_M16_M;Pep	Middle or third domain of peptidase_M16;Insulinase;Peptidase M16 inactive domain	CYT	3	3	131.4	High
TRINITY_DN27979_c0_g1_i2_orf1	zinc finger CCCH domain-containing protein 15 homolog [Ostrinia furnacalis]	2.262921348	1.178186442	8.48E-07	up	yes	1.007	0.445	0.443	0.462	0.43	1	1.018	1.002	MF:cation binding;MF:metal ion binding;MF:molecular_function;MF:binding;MF:ion binding;	GO:0043169;GO:0046872;GO:0003674;GO:0005488;GO:0043167	------	------	------	------	COG5252	S:Function unknown	PF16543.8;PF1613	DFRP_C;Torus	DRG Family Regulatory Proteins, Tma46;Torus domain	CYT	5	13	43.1	High

TRINITY_DN27641_c0_g1_i1_orf1	succinate dehydrogenase [ubiquinone] iron-sulfur subunit, mitochondrial [Ostrinia furnacalis]	2.309638831	1.207667269	2.77E-07	up	yes	1.004	0.4347	0.438	0.443	0.423	1	1.015	0.996	[illegible]	[illegible]	K00235	SDHB, SDH2	map04714;map	Thermogenesis;Oxic	COG0479	C:Energy production and conversion	PF13085.9;PF1353	Fer2_3;Fer4_17;Fer4_8;F	2Fe-2S iron-sulfur cluster binding domain;4Fe-4S dicluster domain;4Fe-4S dicluster domain;4Fe-4S dicluster domain	CYT	6	30	31.8	High
TRINITY_DN8833_c0_g1_i1_orf1	nucleolar protein 16 [Ostrinia furnacalis]	2.480582524	1.310678954	1.27E-06	up	yes	1.022	0.412	0.41	0.403	0.423	1	1.04	1.026	------	------	------	------	------	------	ENOG4111QB6	S:Function unknown	PF09420.13	Nop16	Ribosome biogenesis protein Nop16	CYT	2	8	24.1	High
TRINITY_DN14217_c0_g1_i1_orf1	putative serine protease K12H4.7 [Ostrinia furnacalis] >XP_028166339.1 putative serine protease K12H4.7 [Ostrinia furnacalis]	7.028248588	2.81316522	0.00135	up	yes	1.244	0.177	0.133	0.136	0.262	1	1.304	1.428	[illegible]	[illegible]	------	------	------	------	ENOG410XSGG	S:Function unknown	PF05577.15	Peptidase_S28	Serine carboxypeptidase S28	CYT	1	3	54.9	High
TRINITY_DN2070_c1_g1_i1_orf1	mitochondrial carrier homolog 2-like [Helicoverpa zea] >PZC82360.1 hypothetical protein B5X24_HaOG210663 [Helicoverpa armigera]	2.007936508	1.005713651	0.0002188	up	yes	1.012	0.504	0.499	0.502	0.511	1	0.95	1.086	[illegible]	GO:0005575;GO:0016021;GO:0110165;GO:0031224	K17885	MTCH	------	------	ENOG410XSXF	O:Posttranslational modification, pro	PF00153.30	Mito_carr	Mitochondrial carrier protein	CYT	3	12	33.1	High
TRINITY_DN51968_c0_g1_i1_orf1	splicing factor U2af 38 kDa subunit [Aphidius gifuensis] >KAF7990547.1 hypothetical protein HCN44_000352 [Aphidius gifuensis]	2.201271186	1.13833689	0.0001111	up	yes	1.039	0.472	0.487	0.451	0.478	1	1.111	1.007	[illegible]	[illegible]	K12836	U2AF1	map05131;map	Shigellosis;Spliceoso	ENOG410Z5PX	S:Function unknown	PF00642.27;PF000	zf-CCCH;RRM_1	Zinc finger C-x8-C-x5-C-x3-H type;RNA recognition motif.	CYT	1	6	28.6	High
TRINITY_DN23360_c0_g1_i3_orf1	protein PTCD3 homolog, mitochondrial [Ostrinia furnacalis]	2.027435949	1.019656337	2.17E-06	up	yes	1.005	0.4957	0.492	0.511	0.484	1	0.992	1.024	[illegible]	[illegible]	K17659	PTCD3	------	------	ENOG410XNQS	S:Function unknown	PF17177.7	PPR_long	Pentacotripeptide-repeat region of PRORP	CYT	5	8	78.8	High
TRINITY_DN124950_c0_g2_i1_orf1	TATA box-binding protein-like protein 1 [Ostrinia furnacalis] >XP_028155830.1 TATA box-binding protein-like protein 1 [Ostrinia furnacalis]	2.887084097	1.529613132	0.0002552	up	yes	1.102	0.3817	0.362	0.438	0.345	1	1.164	1.142	[illegible]	[illegible]	K03120	TBP, tbp	map05166;map	Human T-cell leuker	COG2101	K:Transcription	PF00352.24	TBP	Transcription factor TFIID	CYT	1	4	31.8	High
TRINITY_DN48413_c1_g1_i2_orf1	probable protein phosphatase 2C 11 isoform X1 [Manduca sexta] >KAG6442694.1 hypothetical protein O3G_MSEX002471 [Manduca sexta]	2.234302197	1.159824328	4.13E-06	up	yes	1.007	0.4507	0.454	0.45	0.448	1	1.037	0.983	------	------	K17499	PPM1G, PP2C	------	------	COG0631	T:Signal transduction mechanisms	PF00481.24	PP2C	Protein phosphatase 2C	CYT	6	13	70.5	High
TRINITY_DN3292_c2_g1_i4_orf1	ribosome biogenesis regulatory protein homolog [Ostrinia furnacalis]	2.096511877	1.067991003	5.01E-06	up	yes	0.9797	0.4673	0.47	0.474	0.458	1	0.951	0.988	[illegible]	[illegible]	K14852	RRS1	------	------	COG5225	J:Translation, ribosomal structure and	PF04939.15	RRS1	Ribosome biogenesis regulatory protein	CYT	4	12	40	High
TRINITY_DN430_c0_g1_i5_orf1	hypothetical protein NE865_02252 [Phthorimaea operculella]	2.302947224	1.20348135	7.21E-06	up	yes	1.008	0.4377	0.468	0.435	0.41	1	0.998	1.025	[illegible]	[illegible]	------	------	------	------	ENOG4110QHB	S:Function unknown	------	------	------	CYT	5	8	83.6	High
TRINITY_DN19361_c0_g1_i7_orf1	hydroxylysine kinase [Ostrinia furnacalis] >XP_028168144.1 hydroxylysine kinase [Ostrinia furnacalis]	2.638020833	1.399455958	4.93E-07	up	yes	1.013	0.384	0.37	0.388	0.394	1	1.027	1.011	------	------	K18201	AGPHD1	map00310	Lysine degradation	COG2334	S:Function unknown	PF01636.26	APH	Phosphotransferase enzyme family	CYT	9	31	42.1	High
TRINITY_DN76036_c0_g1_i1_orf1	cytochrome c oxidase subunit 6A1, mitochondrial-like [Ostrinia furnacalis]	2.869420571	1.520759439	5.56E-06	up	yes	1.035	0.3607	0.358	0.358	0.366	1	1.034	1.072	[illegible]	[illegible]	K02266	COX6A	map04714;map	Thermogenesis;Oxic	ENOG411253X	O:Posttranslational modification, pro	PF02046.18	COX6A	Cytochrome c oxidase subunit VIa	CYT	2	24	12	High
TRINITY_DN63662_c0_g4_i1_orf1	polyadenylate-binding protein 1 [Ostrinia furnacalis]	2.536139995	1.342634384	1.03E-07	up	yes	1	0.3943	0.386	0.4	0.397	1	1.01	0.991	[illegible]	[illegible]	K13126	PABPC	map03018;map	RNA degradation;m	ENOG410XR5X	S:Function unknown	PF00076.25;PF006	RRM_1;PABP;Nup35_R	RNA recognition motif.;Poly-adenylate binding protein, unique domain;Nup53/35/40-type RNA recognition motif	CYT	21	40	68.2	High
TRINITY_DN334_c0_g1_i3_orf1	chymotrypsin-like serine protease [Ostrinia nubilalis] >AAX62030.1 chymotrypsin-like serine protease [Ostrinia nubilalis]	2.451851852	1.29387181	1.32E-05	up	yes	0.993	0.405	0.446	0.394	0.375	1	1.002	0.977	[illegible]	[illegible]	------	------	------	------	COG5640	O:Posttranslational modification, pro	PF00089.29	Trypsin	Trypsin	PLA	1	35	29.6	High
TRINITY_DN327_c1_g1_i4_orf1	mitochondrial import receptor subunit TOM40 homolog 1-like [Ostrinia furnacalis]	2.47402436	1.306859706	2.21E-07	up	yes	0.9953	0.4023	0.393	0.407	0.407	1	0.982	1.004	[illegible]	[illegible]	K11518	TOM40	map05022;map	Pathways of neurod	ENOG410XS2U	O:Posttranslational modification, pro	PF01459.25	Porin_3	Eukaryotic porin	CYT	6	26	34	High
TRINITY_DN5578_c0_g1_i4_orf1	chromatin modification-related protein eaf-1-like [Ostrinia furnacalis]	2.226497696	1.154776119	0.0002709	up	yes	0.9663	0.434	0.453	0.43	0.419	1	1.018	0.881	[illegible]	[illegible]	K23390	BAG6	------	------	ENOG410XS9P;ENO(	O:Posttranslational modification, pro	PF12057.11;PF002	BAG6;ubiquitin	BCL2-associated athanogene 6;Ubiquitin family	CYT	5	6	150.2	High
TRINITY_DN12973_c0_g1_i1_orf1	mitochondrial-processing peptidase subunit alpha [Ostrinia furnacalis]	2.231552163	1.15804753	1.98E-05	up	yes	0.9647	0.4323	0.455	0.435	0.407	1	0.953	0.941	[illegible]	[illegible]	K01412	PMPCA, MAS	------	------	COG0612	O:Posttranslational modification, pro	PF05193.24;PF006	Peptidase_M16_C;Pept	Peptidase M16 inactive domain;Insulinase	CYT	5	10	59.2	High
TRINITY_DN53167_c0_g1_i2_orf1	uncharacterized protein LOC114359219 [Ostrinia furnacalis]	2.381875454	1.252097978	2.33E-05	up	yes	0.983	0.4127	0.394	0.459	0.385	1	0.984	0.965	[illegible]	[illegible]	------	------	------	------	COG1621	G:Carbohydrate transport and metab	PF00251.23;PF082	Glyco_hydro_32N;Glyco	Glycosyl hydrolases family 32 N-terminal domain;Glycosyl hydrolases family 32 C terminal	CYT	2	5	59.8	High
TRINITY_DN36538_c0_g1_i2_orf1	xaa-Pro dipeptidase isoform X1 [Ostrinia furnacalis] >XP_028156507.1 xaa-Pro dipeptidase isoform X2 [Ostrinia furnacalis]	2.667546174	1.415513244	4.65E-07	up	yes	1.011	0.379	0.38	0.389	0.368	1	1.028	1.005	[illegible]	[illegible]	K14213	PEPD	------	------	COG0006	E:Amino acid transport and metaboli	PF00557.27;PF051	Peptidase_M24;AMP_N	Metallopeptidase family M24;Aminopeptidase P, N-terminal domain	CYT	9	18	60.8	High
TRINITY_DN14967_c0_g2_i1_orf1	glyceraldehyde-3-phosphate dehydrogenase 2 [Holotrichia oblita]	2.815723446	1.493505643	1.22E-05	up	yes	1.01	0.3587	0.355	0.335	0.386	1	0.982	1.048	[illegible]	[illegible]	K05298	GAPA	------	------	COG0057	C:Energy production and conversion	PF02800.23;PF000	Gp_dh_C;Gp_dh_N	Glyceraldehyde 3-phosphate dehydrogenase, C-terminal domain;Glyceraldehyde 3-phosphate dehydrogenase, NAD binding domain	CYT	2	6	35.5	High
TRINITY_DN6685_c0_g1_i8_orf1	cleft lip and palate transmembrane protein 1 homolog [Ostrinia furnacalis]	2.842224104	1.507020313	7.30E-07	up	yes	1.007	0.3543	0.364	0.367	0.332	1	1.004	1.016	[illegible]	GO:0005575;GO:0016021;GO:0110165;GO:0031224	------	------	------	------	ENOG410XPEV	S:Function unknown	PF05602.15	CLPTM1	Cleft lip and palate transmembrane protein 1	CYT	3	7	72.4	High
TRINITY_DN13055_c0_g1_i5_orf1	116 kDa U5 small nuclear ribonucleoprotein component isoform X1 [Ostrinia furnacalis] >XP_028159219.1 116 kDa U5 small nuclear ribonucleoprotein component isoform X2 [Ostrinia furnacalis]	2.241831624	1.164677927	0.004605	up	yes	0.94	0.4193	0.445	0.405	0.408	1	0.763	1.057	[illegible]	[illegible]	K12852	EFTUD2	map03040	Spliceosome	COG0480	J:Translation, ribosomal structure and	PF03764.21;PF006	EFG_IV;EFG_C;GTP_EFT	Elongation factor G, domain IV;Elongation factor G C-terminus;Elongation factor Tu domain 2;Elongation Factor G, domain III	CYT	6	8	79.1	High
TRINITY_DN791_c0_g1_i2_orf1	peroxiredoxin 1 isoform X1 [Maniola jurtina]	2.477581348	1.308932427	5.09E-05	up	yes	0.967	0.3903	0.399	0.359	0.413	1	0.987	0.914	[illegible]	[illegible]	K13279	PRDX1	map04146;map	Peroxisome;Amoebi	COG0450	L:Replication, recombination and rep	PF00578.24;PF104	AhpC-TSA;1-cysPrx_C;	AhpC/TSA family;C-terminal domain of 1-Cys peroxiredoxin;Redoxin	CYT	7	32	26.5	High
TRINITY_DN48237_c0_g1_i5_orf1	myogenesis-regulating glycosidase-like [Ostrinia furnacalis]	3.622214362	1.856871927	8.87E-05	up	yes	1.024	0.2827	0.312	0.283	0.253	1	1.108	0.965	[illegible]	[illegible]	K24727	MYORG	------	------	COG1501	G:Carbohydrate transport and metab	PF01055.29	Glyco_hydro_31	Glycosyl hydrolases family 31	CYT	1	1	76.4	High
TRINITY_DN48020_c0_g1_i1_orf1	aminopeptidase N4 [Cnaphalocrocis medinalis]	2.559474979	1.355847903	0.0001268	up	yes	0.936	0.3657	0.335	0.388	0.374	1	0.877	0.931	[illegible]	[illegible]	K11140	ANPEP, CD13	map04640;map	Hematopoietic cell l	COG0308	E:Amino acid transport and metaboli	PF17900.4	Peptidase_M1_N	Peptidase M1 N-terminal domain	CYT	1	21	15.7	High
TRINITY_DN7556_c0_g1_i1_orf1	venom carboxylesterase-6-like [Ostrinia furnacalis]	3.714182082	1.893044543	1.10E-06	up	yes	1.024	0.2757	0.263	0.282	0.282	1	1.049	1.024	------	------	------	------	------	------	COG2272;COG0580;I	I:Lipid transport and metabolism;G:C	PF00135.31;PF078	COesterase;Abhydrolas	Carboxylesterase family;alpha/beta hydrolase fold;BD-FAE	CYT	1	1	54.8	Medium

TRINITY_DN79319_c0_g1_i8_orfp1	TRINITY_DN79319_c0_g1_i8_m.49956 TRINITY_DN79319_c0_g1::TRINITY_DN79319_c0_g1_i8::g.49956 ORF type:5prime_partial len:84 (+),score=1.39 TRINITY_DN79319_c0_g1_i8:1-252(+)	3.246795925	1.699016708	2.57E-06	up	yes	0.988	0.3043	0.284	0.295	0.334	1	0.993	0.971	------	------	------	------	------	------	------	------	------	------	------	CYT	2	20	9.6	High
TRINITY_DN7964_c0_g1_i6_orfp1	TRINITY_DN7964_c0_g1_i6_m.23478 TRINITY_DN7964_c0_g1::TRINITY_DN7964_c0_g1_i6::g.23478 ORF type:internal len:87 (+),score=67.94 TRINITY_DN7964_c0_g1_i6:2-259(+)	4.0969163	2.034538419	8.43E-06	up	yes	1.023	0.2497	0.258	0.226	0.265	1	1.071	0.999	------	------	------	------	------	------	------	------	------	------	------	CYT	2	30	9	High
TRINITY_DN1249_c0_g1_i10_orf1	venom carboxylesterase-6-like [Ostrinia furnacalis]	3.420537897	1.774223214	3.55E-06	up	yes	0.9793	0.2863	0.298	0.284	0.277	1	0.995	0.943	MF:hydrolase activity;MF:molecular_function;MF:hydrolase activity, acting on ester bonds;MF:carboxylic ester hydrolase activity;MF:catalytic activity;	GO:0016787;GO:0003674;GO:0016788;GO:0052689;GO:0003824	------	------	------	------	COG2272;ENOG410X	I:Lipid transport and metabolism;T:Si	PF00135.31;PF204	COesterase;BD-FAE;Ab	Carboxylesterase family;BD-FAE:alpha/beta hydrolase	PLA	1	6	55.8	High
TRINITY_DN43942_c0_g2_i1_orf1	LOW QUALITY PROTEIN: caprin homolog [Ostrinia furnacalis]	4.621585311	2.208387814	6.01E-06	up	yes	1.032	0.2233	0.242	0.224	0.204	1	1.077	1.019	------	------	K18743	CAPRIN1, GPI	------	------	ENOG4110M5Q	S:Function unknown	------	------	------	CYT	1	5	22.7	High
TRINITY_DN15380_c0_g1_i1_orf1	39S ribosomal protein L32, mitochondrial [Ostrinia furnacalis]	4.053278689	2.019089373	5.08E-05	up	yes	0.989	0.244	0.255	0.251	0.226	1	0.916	1.051	BP:cellular biosynthetic process;BP:cellular nitrogen compound metabolic process;BP:nitrogen compound metabolic process;BP:cellular macromolecule biosynthetic process;BP:organic substance biosynthetic process;BP:cellular nitrogen compound biosynthetic process;BP:cellular macromolecule metabolic process;BP:peptide biosynthetic process;BP:organic substance metabolic process;BP:amide biosynthetic process;BP:cellular process;BP:biosynthetic process;BP:macromolecule biosynthetic process;BP:biological_process;BP:metabolic process;BP:primary metabolic process;BP:organonitrogen compound metabolic process;BP:cellular amide metabolic process;BP:organonitrogen compound biosynthetic process;BP:protein metabolic process;BP:cellular metabolic process;BP:macromolecule metabolic process;BP:peptide metabolic process;BP:translation;CC:large ribosomal subunit;CC:intracellular non-membrane-bounded organelle;CC:organelle;CC:cellular anatomical entity;CC:ribosomal subunit;CC:macromolecular complex;CC:ribosome;CC:ribonucleoprotein complex;CC:intracellular organelle;CC:cellular_component;CC:non-membrane-bounded organelle;MF:molecular_function;MF:structural molecule activity;MF:structural constituent	GO:0044249;GO:0034641;GO:0006807;GO:0034645;GO:1901576;GO:0044271;GO:0044260;GO:0043043;GO:0071704;GO:0043604;GO:0009987;GO:0009058;GO:0009059;GO:0008150;GO:0008152;GO:0044238;GO:1901564;GO:0043603;GO:1901566;GO:0019538;GO:0044237;GO:0043170;GO:0006518;GO:0006412;GO:0015934;GO:0043232;GO:0043226;GO:0110165;GO:0044391;GO:0032991;GO:0005840;GO:1990904;GO:0043229;GO:0005575;GO:0043228;GO:0003674;GO:0005198;GO:0003735	K02911	RP-L32, MRPL	map03010	Ribosome	COG0333	J:Translation, ribosomal structure and	------	------	------	CYT	1	5	22.6	High
TRINITY_DN123396_c0_g1_i1_orf1	PREDICTED: delta-1-pyrroline-5-carboxylate dehydrogenase, mitochondrial isoform X1 [Megachile rotundata]	4.244782609	2.08569067	1.53E-05	up	yes	0.9763	0.23	0.215	0.227	0.248	1	1.009	0.92	BP:small molecule catabolic process;BP:carboxylic acid metabolic process;BP:cellular amino acid catabolic process;BP:nitrogen compound metabolic process;BP:small molecule metabolic process;BP:glutamine family amino acid catabolic process;BP:glutamine family amino acid metabolic process;BP:organic substance catabolic process;BP:proline catabolic process;BP:proline metabolic process;BP:cellular amino acid metabolic process;BP:organic substance metabolic process;BP:alpha-amino acid metabolic process;BP:alpha-amino acid catabolic process;BP:primary metabolic process;BP:cellular process;BP:proline catabolic process to glutamate;BP:metabolic process;BP:oxoacid metabolic process;BP:catabolic process;BP:cellular catabolic process;BP:dicarboxylic acid metabolic process;BP:organonitrogen compound metabolic process;BP:organonitrogen compound catabolic process;BP:organic acid metabolic process;BP:carboxylic acid catabolic process;BP:organic acid catabolic process;BP:cellular metabolic process;BP:glutamate metabolic process;BP:biological_process;MF:molecular_function;MF:oxidoreductase activity;MF:oxidoreductase activity, acting on the aldehyde or oxo group of donors, NAD or NADP as acceptor;MF:catalytic	GO:0044282;GO:0019752;GO:0009063;GO:0006807;GO:0044281;GO:0009065;GO:0009064;GO:1901575;GO:0006562;GO:0006560;GO:0006520;GO:0071704;GO:1901605;GO:1901606;GO:0044238;GO:0009987;GO:0010133;GO:0008152;GO:0043436;GO:0009056;GO:0044248;GO:0043648;GO:1901564;GO:1901565;GO:0006082;GO:0046395;GO:0016054;GO:0044237;GO:0006536;GO:0008150;GO:0003674;GO:0016491;GO:0016620;GO:0003824;GO:0016903;GO:0003842	K00294	E1.2.1.88	map00250;map	Alanine, aspartate a	COG1012	Z:Cytoskeleton	PF00171.25	Aldedh	Aldehyde dehydrogenase family	CYT	1	1	63.5	High
TRINITY_DN6074_c0_g1_i1_orf1	uncharacterized protein C1683.06c-like isoform X1 [Ostrinia furnacalis]	5.143753267	2.362821442	6.95E-07	up	yes	0.984	0.1913	0.172	0.202	0.2	1	0.989	0.963	MF:hydrolase activity;MF:molecular_function;MF:hydrolase activity, hydrolyzing N-glycosyl	GO:0016787;GO:0003674;GO:0016799;GO:0016798;GO:0003824	------	------	------	------	COG1957;COG1199	F:Nucleotide transport and metabolis	PF01156.22	IU_nuc_hydro	Inosine-uridine preferring nucleoside hydrolase	CYT	2	5	38.9	High
TRINITY_DN48410_c0_g1_i1_orf1	alpha-amylase 1-like [Ostrinia furnacalis]	5.339334424	2.416659914	6.95E-07	up	yes	0.9787	0.1833	0.169	0.196	0.185	1	0.979	0.957	BP:organic substance metabolic process;BP:primary metabolic process;BP:carbohydrate metabolic process;BP:metabolic process;BP:biological_process;MF:hydrolase activity;MF:cation binding;MF:amylase activity;MF:hydrolase activity, acting on glycosyl bonds;MF:ion binding;MF:catalytic activity;MF:alpha-amylase activity;MF:molecular_function;MF:binding;MF:hydrolase activity, hydrolyzing O-glycosyl compounds;MF:metal	GO:0071704;GO:0044238;GO:0005975;GO:0008152;GO:0008150;GO:0016787;GO:0043169;GO:0016160;GO:0016798;GO:0043167;GO:0003824;GO:0004556;GO:0003674;GO:0005488;GO:0004553;GO:0046872	K01176	AMY, amyA, r	map04972;map	Pancreatic secretion	COG0366	G:Carbohydrate transport and metab	PF00128.27;PF028	Alpha-amylase;Alpha-	Alpha amylase, catalytic domain;Alpha amylase, C-terminal all-beta domain	EXC	11	29	56	High
TRINITY_DN11587_c0_g1_i7_orf1	elongation of very long chain fatty acids protein AAEL008004-like isoform X1 [Danaus plexippus plexippus] >XP_032511006.1 elongation of very long chain fatty acids protein AAEL008004-like isoform X1 [Danaus plexippus plexippus] >XP_032511007.1 elongation of very long chain fatty acids protein AAEL008004-like isoform X1 [Danaus plexippus plexippus] >XP_032511008.1 elongation of very long chain fatty acids protein AAEL008004-like isoform X1 [Danaus plexippus plexippus] >XP_032511009.1 elongation of very long chain fatty acids protein AAEL008004-like isoform X1	6.644693474	2.732202648	6.31E-07	up	yes	1.008	0.1517	0.122	0.166	0.167	1	1.011	1.014	------	------	K10250	ELOVL7	map01040;map	Biosynthesis of unsa	ENOG410XRWT	C:Energy production and conversion	PF01151.21	ELO	GNS1/SUR4 family	CYT	1	4	36.4	High
TRINITY_DN52861_c0_g1_i1_orf1	protein MAK16 homolog A [Ostrinia furnacalis]	2.65535248	1.408903382	4.49E-05	up	yes	1.017	0.383	0.326	0.387	0.436	1	1.034	1.016	CC:intracellular non-membrane-bounded organelle;CC:nucleolus;CC:cellular_component;CC:intracellular organelle;CC:non-membrane-bounded organelle;CC:organelle;CC:cellular anatomical entity;	GO:0043232;GO:0005730;GO:0005575;GO:0043229;GO:0043228;GO:0043226;GO:0110165	K14831	MAK16	------	------	COG5129	S:Function unknown	PF01778.20;PF048	Ribosomal_L28e;Mak16	Ribosomal L28e protein family;Mak16 protein C-terminal region	CYT	1	2	35.4	Medium
TRINITY_DN8261_c0_g1_i1_orf1	UDP-N-acetylhexosamine pyrophosphorylase-like protein 1 [Ostrinia furnacalis]	2.250991369	1.170560525	1.29E-05	up	yes	0.965	0.4287	0.431	0.41	0.445	1	0.954	0.941	MF:nucleotidyltransferase activity;MF:transferase activity, transferring phosphorus-containing groups;MF:catalytic activity;MF:transferase activity;MF:UDP-N-acetylglucosamine diphosphorylase	GO:0016779;GO:0016772;GO:0003824;GO:0016740;GO:0003977;GO:0003674;GO:0070569	K00972	UAP1	map00520;map	Amino sugar and nu	COG4284	O:Posttranslational modification, pro	PF01704.21	UDPGP	UTP--glucose-1-phosphate uridylyltransferase	CYT	5	11	54.6	High
TRINITY_DN27848_c0_g1_i2_orf1	cystathionine beta-synthase-like [Ostrinia furnacalis] >XP_028159011.1 cystathionine beta-synthase-like [Ostrinia furnacalis]	2.526756277	1.337286513	4.17E-08	up	yes	0.9963	0.3943	0.391	0.4	0.392	1	1.002	0.987	BP:carboxylic acid metabolic process;BP:cellular biosynthetic process;BP:nitrogen compound metabolic process;BP:small molecule metabolic process;BP:small molecule biosynthetic process;BP:serine family amino acid metabolic process;BP:cysteine metabolic process;BP:L-serine metabolic process;BP:cellular amino acid metabolic process;BP:organic substance metabolic process;BP:cysteine biosynthetic process via cystathionine;BP:cellular amino acid biosynthetic process;BP:alpha-amino acid metabolic process;BP:sulfur amino acid metabolic process;BP:organic substance biosynthetic process;BP:sulfur amino acid biosynthetic process;BP:cellular process;BP:biosynthetic process;BP:biological_process;BP:metabolic process;BP:oxoacid metabolic process;BP:serine family amino acid biosynthetic process;BP:primary metabolic process;BP:organonitrogen compound metabolic process;BP:organonitrogen compound biosynthetic process;BP:organic acid metabolic process;BP:sulfur compound biosynthetic process;BP:carboxylic acid biosynthetic process;BP:organic acid biosynthetic process;BP:cellular metabolic process;BP:cysteine biosynthetic process from serine;BP:cysteine biosynthetic process;BP:sulfur compound metabolic process;BP:alpha-amino acid biosynthetic process;CC:cytoplasm;CC:cellular anatomical entity;CC:cellular_component;MF:molecular_function;MF:lyase activity;MF:cystathionine beta-synthase activity;MF:catalytic activity;MF:carbon-oxygen lyase activity;MF:hydro-	GO:0019752;GO:0044249;GO:0006807;GO:0044281;GO:0044283;GO:0009069;GO:0006534;GO:0006563;GO:0006520;GO:0071704;GO:0019343;GO:0008652;GO:1901605;GO:0000096;GO:1901576;GO:0000097;GO:0009987;GO:0009058;GO:0008150;GO:0008152;GO:0043436;GO:0009070;GO:0044238;GO:1901564;GO:1901566;GO:0006082;GO:0044272;GO:0046394;GO:0016053;GO:0044237;GO:0006535;GO:0019344;GO:0006790;GO:1901607;GO:0005737;GO:0110165;GO:0005575;GO:0003674;GO:0016829;GO:0004122;GO:0003824;GO:0016835;GO:0016836	K01697	CBS	map00270;map	Cysteine and methic	COG0031	E:Amino acid transport and metaboli	PF00291.28	PALP	Pyridoxal-phosphate dependent enzyme	CYT	12	31	54.7	High
TRINITY_DN16830_c0_g1_i5_orf1	adrenodoxin [Ostrinia furnacalis]	2.78630137	1.47835131	9.88E-05	up	yes	1.017	0.365	0.366	0.397	0.332	1	1.089	0.963	BP:cellular process;BP:cellular metabolic process;BP:electron transport chain;BP:metabolic process;BP:biological_process;BP:generation of precursor metabolites and energy;MF:metal cluster	GO:0009987;GO:0044237;GO:0022900;GO:0008152;GO:0008150;GO:0006091;GO:0051540;GO:0051536;GO:0051537;GO:0003674;GO:0005488	K22070	FDX1, ADX	------	------	COG0633	C:Energy production and conversion	PF00111.30	Fer2	2Fe-2S iron-sulfur cluster binding domain	CYT	1	6	17.6	High
TRINITY_DN43412_c0_g1_i2_orf1	U1 small nuclear ribonucleoprotein C [Ostrinia furnacalis]	2.278571429	1.188129597	0.0003495	up	yes	0.957	0.42	0.496	0.408	0.356	1	0.955	0.916	BP:macromolecular complex subunit organization;BP:cellular component assembly;BP:cellular component organization or biogenesis;BP:spliceosomal snRNP assembly;BP:cellular component organization;BP:macromolecular complex assembly;BP:ribonucleoprotein complex assembly;BP:cellular process;BP:ribonucleoprotein complex subunit organization;BP:biological_process;BP:mRNA 5'-splice site recognition;BP:mRNA splice site selection;CC:commitment complex;CC:U2-type prespliceosome;CC:Sm-like protein family complex;CC:cellular_component;CC:prespliceosome;CC:spliceosomal snRNP complex;CC:macromolecular complex;CC:small nuclear ribonucleoprotein complex;CC:ribonucleoprotein complex;CC:U1 snRNP;CC:U2-type spliceosomal complex;CC:spliceosomal complex;MF:molecular_function;MF:binding;MF:nucleic acid binding;MF:heterocyclic compound binding;MF:mRNA binding;MF:transition metal ion binding;MF:ion binding;MF:metal ion binding;MF:cation	GO:0043933;GO:0022607;GO:0071840;GO:0000387;GO:0016043;GO:0065003;GO:0022618;GO:0009987;GO:0071826;GO:0008150;GO:0000395;GO:0006376;GO:0000243;GO:0071004;GO:0120114;GO:0005575;GO:0071010;GO:0097525;GO:0032991;GO:0030532;GO:1990904;GO:0005685;GO:0005684;GO:0005681;GO:0003674;GO:0005488;GO:0003676;GO:1901363;GO:0003729;GO:0046914;GO:0043167;GO:0046872;GO:0043169;GO:0030619;GO:0097159;GO:0017069;GO:0003723;GO:0008270	K11095	SNRPC	map03040	Spliceosome	COG5136	A:RNA processing and modification	PF06220.15	zf-U1	U1 zinc finger	CYT	1	6	17.6	High
TRINITY_DN53167_c0_g1_i3_orf1	uncharacterized protein LOC114359219 [Ostrinia furnacalis]	2.869663557	1.520881603	1.15E-07	up	yes	1.015	0.3537	0.351	0.355	0.355	1	1.023	1.023	BP:primary metabolic process;BP:carbohydrate metabolic process;BP:organic substance metabolic process;BP:biological_process;BP:metabolic process;CC:cytoplasm;CC:cellular_component;CC:cellular anatomical entity;MF:hydrolase activity;MF:catalytic activity;MF:hydrolase activity, acting on glycosyl bonds;MF:beta-	GO:0044238;GO:0005975;GO:0071704;GO:0008150;GO:0008152;GO:0005737;GO:0005575;GO:0110165;GO:0016787;GO:0003824;GO:0016798;GO:0004564;GO:0004553;GO:0003674	------	------	------	------	COG1621	G:Carbohydrate transport and metab	PF00251.23;PF082	Glyco_hydro_32N;Glyco	Glycosyl hydrolases family 32 N-terminal domain;Glycosyl hydrolases family 32 C terminal	CYT	6	16	55.9	High
TRINITY_DN12242_c0_g1_i5_orf1	heterogeneous nuclear ribonucleoprotein 87F-like isoform X1 [Vanessa tameamea] >XP_046967652.1 heterogeneous nuclear ribonucleoprotein 87F-like isoform X1 [Vanessa cardui]	2.863636364	1.517848305	4.76E-08	up	yes	1.008	0.352	0.357	0.353	0.346	1	1.018	1.006	MF:organic cyclic compound binding;MF:molecular_function;MF:binding;MF:nucleic acid binding;MF:heterocyclic compound binding;MF:RNA binding;	GO:0097159;GO:0003674;GO:0005488;GO:0003676;GO:1901363;GO:0003723	K12741	HNRNPA1_3	map05014;map	Amyotrophic lateral	COG0724	S:Function unknown	PF00076.25;PF163	RRM_1;RRM_7	RNA recognition motif.;RNA recognition motif	CYT	8	41	34	High
TRINITY_DN96170_c0_g2_i1_orf1	uncharacterized protein LOC114355569 [Ostrinia furnacalis]	2.493690446	1.318282387	1.48E-05	up	yes	0.9683	0.3883	0.378	0.404	0.383	1	0.927	0.978	BP:phosphorylation;BP:cellular process;BP:biological_process;BP:metabolic process;BP:cellular metabolic process;BP:phosphate-containing compound metabolic process;BP:phosphorus metabolic process;CC:intrinsic component of membrane;CC:integral component of membrane;CC:cellular_component;CC:cellular anatomical entity;MF:purine ribonucleoside triphosphate binding;MF:molecular_function;MF:binding;MF:ion binding;MF:heterocyclic compound binding;MF:anion binding;MF:nucleotide binding;MF:transferase activity;MF:nucleoside phosphate binding;MF:purine nucleotide binding;MF:ATP binding;MF:kinase activity;MF:small molecule binding;MF:catalytic activity;MF:adenyl nucleotide binding;MF:carbohydrate derivative binding;MF:transferase activity, transferring phosphorus-containing groups;MF:adenyl ribonucleotide	GO:0016310;GO:0009987;GO:0008150;GO:0008152;GO:0044237;GO:0006796;GO:0006793;GO:0031224;GO:0016021;GO:0005575;GO:0110165;GO:0035639;GO:0003674;GO:0005488;GO:0043167;GO:1901363;GO:0043168;GO:0000166;GO:0016740;GO:1901265;GO:0017076;GO:0005524;GO:0016301;GO:0036094;GO:0003824;GO:0030554;GO:0097367;GO:0016772;GO:0032559;GO:0032555;GO:0097159;GO:0032553	------	------	------	------	COG0574	G:Carbohydrate transport and metab	PF00391.26	PEP-utilizers	PEP-utilising enzyme, mobile domain	CYT	1	4	31.7	High
TRINITY_DN2852_c0_g1_i9_orf1	golgin subfamily A member 4-like [Ostrinia furnacalis]	2.411276114	1.269796863	1.80E-05	up	yes	0.958	0.3973	0.417	0.392	0.383	1	0.94	0.934	------	------	K20283	GOLGA4	------	------	ENOG410XQQG	U:Intracellular trafficking, secretion, a	PF01465.23	GRIP	GRIP domain	CYT	4	3	155.2	High
TRINITY_DN3476_c0_g1_i5_orf1	maltase A1-like [Ostrinia furnacalis]	3.262658228	1.706047869	1.81E-06	up	yes	1.031	0.316	0.315	0.308	0.325	1	1.053	1.041	BP:primary metabolic process;BP:carbohydrate metabolic process;BP:organic substance metabolic process;BP:biological_process;BP:metabolic process;MF:hydrolase activity;MF:catalytic activity;MF:hydrolase	GO:0044238;GO:0005975;GO:0071704;GO:0008150;GO:0008152;GO:0016787;GO:0003824;GO:0016798;GO:0003674	K01187	malZ	map00500;map	Starch and sucrose r	COG0366	E:Amino acid transport and metaboli	PF00128.27	Alpha-amylase	Alpha amylase, catalytic domain	CYT	8	22	66.3	High
TRINITY_DN1505_c0_g1_i1_orf1	uncharacterized protein LOC114362816 isoform X1 [Ostrinia furnacalis] >XP_028174154.1	2.876550332	1.524339714	4.84E-05	up	yes	0.9973	0.3467	0.293	0.41	0.337	1	1.008	0.984	------	------	K16913	PELP1, MNAR	------	------	ENOG4111B29;ENOG	S:Function unknown;	------	------	------	CYT	2	4	56	High
TRINITY_DN17905_c0_g3_i1_orf1	zinc finger protein 706-like [Ostrinia furnacalis] >XP_028176219.1 zinc finger protein 706-like [Ostrinia furnacalis] >XP_028176220.1 zinc finger protein 706-like [Ostrinia furnacalis]	2.831001998	1.501312768	1.38E-05	up	yes	0.9917	0.3503	0.321	0.373	0.357	1	1.021	0.954	CC:cytoplasm;CC:intracellular membrane-bounded organelle;CC:nucleus;CC:cellular_component;CC:intracellular organelle;CC:membrane-bounded organelle;CC:organelle;CC:cellular anatomical entity;	GO:0005737;GO:0043231;GO:0005634;GO:0005575;GO:0043229;GO:0043227;GO:0043226;GO:0110165	------	------	------	------	ENOG411234V	S:Function unknown	PF04419.17	4F5	4F5 protein related disordered region	CYT	2	18	8.7	High
TRINITY_DN5244_c0_g1_i1_orf1	eukaryotic peptide chain release factor GTP-binding subunit-like [Ostrinia furnacalis]	2.852586207	1.512270486	1.41E-07	up	yes	0.9927	0.348	0.351	0.346	0.347	1	1.001	0.977	------	------	------	------	------	------	ENOG41116ZG;ENO	S:Function unknown;	PF03103.20	DUF243	Domain of unknown function	CYT	6	20	48.5	High
TRINITY_DN31047_c0_g1_i4_orf1	4-coumarate--CoA ligase 1-like isoform X1 [Ostrinia furnacalis] >XP_028160248.1 4-coumarate--CoA ligase 1-like isoform X1 [Ostrinia furnacalis] >XP_028160249.1 4-coumarate--CoA ligase 1-like isoform X1 [Ostrinia furnacalis] >XP_028160250.1 4-coumarate--CoA ligase 1-like isoform X1 [Ostrinia furnacalis] >XP_028160251.1 4-coumarate--CoA ligase 1-like isoform X1 [Ostrinia furnacalis]	2.61998362	1.389557792	2.61E-05	up	yes	0.9597	0.3663	0.368	0.352	0.379	1	0.968	0.911	------	------	K01904	4CL	map00130	Ubiquinone and oth	COG0318;COG0318	I:Lipid transport and metabolism;Q:S	PF00501.31;PF131	AMP-binding;AMP-bir	AMP-binding enzyme;AMP-binding enzyme C-terminal domain	CYT	4	8	59.4	High
TRINITY_DN12474_c0_g1_i6_orf1	aromatic-L-amino-acid decarboxylase [Ostrinia furnacalis]	3.327863472	1.734596247	9.19E-08	up	yes	1.014	0.3047	0.301	0.301	0.312	1	1.018	1.023	BP:carboxylic acid metabolic process;BP:nitrogen compound metabolic process;BP:cellular amino acid metabolic process;BP:organic substance metabolic process;BP:cellular process;BP:biological_process;BP:metabolic process;BP:oxoacid metabolic process;BP:primary metabolic process;BP:organonitrogen compound metabolic process;BP:organic acid metabolic process;BP:cellular metabolic process;BP:small molecule metabolic process;MF:binding;MF:aromatic-L-amino-acid decarboxylase activity;MF:vitamin B6 binding;MF:molecular_function;MF:pyridoxal phosphate binding;MF:heterocyclic compound binding;MF:anion binding;MF:lyase activity;MF:ion binding;MF:small molecule binding;MF:catalytic activity;MF:vitamin	GO:0019752;GO:0006807;GO:0006520;GO:0071704;GO:0009987;GO:0008150;GO:0008152;GO:0043436;GO:0044238;GO:1901564;GO:0006082;GO:0044237;GO:0044281;GO:0005488;GO:0004058;GO:0070279;GO:0003674;GO:0030170;GO:1901363;GO:0043168;GO:0016829;GO:0043167;GO:0036094;GO:0003824;GO:0019842;GO:0097159;GO:0016830;GO:0016831	K01593	DDC, TDC	map00350;map	Tyrosine metabolism	COG0076	S:Function unknown	PF00282.22	Pyridoxal_deC	Pyridoxal-dependent decarboxylase conserved domain	CYT	10	20	54.3	High
TRINITY_DN146544_c0_g1_i1_orf1	UPF0047 protein YjbQ [Aphidius gifuensis] >KAF7996225.1 hypothetical protein HCN44_001857	2.420297417	1.275184343	0.0001694	up	yes	0.9277	0.3833	0.412	0.353	0.385	1	0.888	0.895	------	------	------	------	------	------	COG0432	S:Function unknown	PF01894.20	UPF0047	Uncharacterised protein family UPF0047	CYT	1	9	16.6	High
TRINITY_DN43420_c0_g2_i1_orf1	collagenase-like [Ostrinia furnacalis]	2.972315181	1.571587106	1.05E-05	up	yes	0.977	0.3287	0.32	0.32	0.346	1	0.998	0.933	BP:organic substance metabolic process;BP:organonitrogen compound metabolic process;BP:protein metabolic process;BP:macromolecule metabolic process;BP:biological_process;BP:nitrogen compound metabolic process;BP:metabolic process;BP:proteolysis;BP:primary metabolic process;MF:serine-type endopeptidase activity;MF:endopeptidase activity;MF:hydrolase activity;MF:serine hydrolase activity;MF:catalytic activity, acting	GO:0071704;GO:1901564;GO:0019538;GO:0043170;GO:0008150;GO:0006807;GO:0008152;GO:0006508;GO:0044238;GO:0004252;GO:0004175;GO:0016787;GO:0017171;GO:0140096;GO:0003824;GO:0003674;GO:0008233;GO:0008236	------	------	------	------	COG5640	O:Posttranslational modification, pro	PF00089.29	Trypsin	Trypsin	CYT	4	24	29.8	High
TRINITY_DN46409_c0_g1_i1_orf1	unnamed protein product [Heterotrigona itama]	3.895102198	1.961661182	0.0001683	up	yes	1.01	0.2593	0.283	0.235	0.26	1	1.107	0.923	BP:regulation of T cell mediated immunity;BP:establishment of localization;BP:regulation of primary metabolic process;BP:regulation of metabolic process;BP:positive regulation of response to stimulus;BP:regulation of response to stimulus;BP:regulation of lymphocyte mediated immunity;BP:positive regulation of leukocyte mediated immunity;BP:nitrogen compound transport;BP:regulation of molecular function;BP:signal transduction;BP:T cell activation;BP:organic cyclic compound metabolic process;BP:positive regulation of lymphocyte mediated immunity;BP:leukocyte proliferation;BP:cellular response to stimulus;BP:response to unfolded protein;BP:positive regulation of cell adhesion;BP:cellular response to organic substance;BP:positive regulation of endopeptidase activity;BP:regulation of cytokine production;BP:interspecies interaction between organisms;BP:leukocyte activation involved in immune response;BP:isotype switching to IgG isotypes;BP:positive regulation of interferon-gamma production;BP:positive regulation of biological process;BP:positive regulation of interferon-alpha production;BP:macromolecule localization;BP:response to temperature stimulus;BP:mitochondrial unfolded protein response;BP:regulation of interleukin-12 production;BP:regulation of interleukin-10 production;BP:protein localization to mitochondrion;BP:regulation of macromolecule metabolic process;BP:negative regulation of cell death;BP:positive regulation of adaptive immune response based on somatic recombination of immune receptors built from immunoglobulin superfamily domains;BP:organic substance transport;BP:regulation of adaptive immune response based on somatic recombination of immune receptors built from immunoglobulin superfamily domains;BP:apoptotic mitochondrial changes;BP:positive regulation of adaptive immune response;BP:establishment of protein localization;BP:protein import into mitochondrial intermembrane space;BP:regulation of cell activation;BP:lymphocyte activation;BP:regulation of proteolysis;BP:regulation of adaptive immune response;BP:establishment of protein localization to mitochondrion;BP:mitochondrial transport;BP:interaction with symbiont;BP:response to abiotic stimulus;BP:response to organic substance;BP:heterocycle metabolic process;BP:intracellular transport;BP:positive regulation of response to biotic stimulus;BP:regulation of gene expression;BP:immune system process;BP:protein metabolic process;BP:somatic recombination of immunoglobulin genes involved in immune response;BP:cellular response to stress;BP:positive regulation of interleukin-12 production;BP:regulation of leukocyte mediated immunity;BP:regulation of macrophage activation;BP:somatic diversification of immunoglobulins;BP:somatic cell DNA recombination;BP:somatic recombination of immunoglobulin gene segments;BP:regulation of interferon-alpha production;BP:cellular component assembly;BP:protein folding;BP:positive regulation of metabolic	GO:0002709;GO:0051234;GO:0080090;GO:0019222;GO:0048584;GO:0048583;GO:0002706;GO:0002705;GO:0071705;GO:0065009;GO:0007165;GO:0042110;GO:1901360;GO:0002708;GO:0070661;GO:0051716;GO:0006986;GO:0045785;GO:0071310;GO:0010950;GO:0001817;GO:0044419;GO:0002366;GO:0048291;GO:0032729;GO:0048518;GO:0032727;GO:0033036;GO:0009266;GO:0034514;GO:0032655;GO:0032653;GO:0070585;GO:0060255;GO:0060548;GO:0002824;GO:0071702;GO:0002822;GO:0008637;GO:0002821;GO:0045184;GO:0045041;GO:0050865;GO:0046649;GO:0030162;GO:0002819;GO:0072655;GO:0006839;GO:0051702;GO:0009628;GO:0010033;GO:0046483;GO:0046907;GO:0002833;GO:0010468;GO:0002376;GO:0019538;GO:0002204;GO:0033554;GO:0032735;GO:0002703;GO:0043030;GO:0016445;GO:0016444;GO:0016447;GO:0032647;GO:0022607;GO:0006457;GO:0009893;GO:0051131;GO:0002834;GO:0002837;GO:0006458;GO:0032649;GO:0050867;GO:0008104;GO:0050821;GO:0045321;GO:0006807;GO:0046651;GO:0051179;GO:0042981;GO:0043280;GO:0044260;GO:0002285;GO:0002684;GO:0006886;GO:0052547;GO:0016043;GO:1990542;GO:0065003;GO:0065002;GO:0002208;GO:0035966;GO:0065007;GO:0071840;GO:0002221;GO:0032943;GO:0002200;GO:0065008;GO:0006810;GO:0006139;GO:0050790;GO:0050794;GO:0006950;GO:0051249;GO:0008150;GO:0008152;GO:0045862;GO:0032755;GO:0002694;GO:0010604;GO:0051336;GO:0002755;GO:0051604;GO:0050896;GO:0031647;GO:0001775;GO:0002697;GO:0002696;GO:0032733;GO:0032481;GO:0002840;GO:0002699;GO:0002263;GO:0051239;GO:0030155;GO:0034641;GO:0070887;GO:0002839;GO:0007005;GO:0043085;GO:0071806;GO:0051649;GO:0051240;GO:0051246;GO:0051247;GO:1903039;GO:0002224;GO:0051641;GO:1903037;GO:0006996;GO:0045190;GO:0044093;GO:0002764;GO:0044238;GO:0002831;GO:0009987;GO:0006725;GO:	K04077	groEL, HSPD1	map05152;map	Tuberculosis;Type I	COG0459	G:Carbohydrate transport and metab	PF00118.27	Cpn60_TCP1	TCP-1/cpn60 chaperonin family	CYT	2	7	54.5	High
TRINITY_DN25779_c0_g1_i6_orf1	aldo-keto reductase AKR2E4-like [Ostrinia furnacalis]	3.64503117	1.865931152	2.20E-06	up	yes	0.994	0.2727	0.273	0.277	0.268	1	1.021	0.961	MF:catalytic activity;MF:molecular_function;MF:oxidoreductase activity;	GO:0003824;GO:0003674;GO:0016491	K00011	AKR1B	map00051;map	Fructose and mann	COG0656	S:Function unknown	PF00248.24	Aldo_ket_red	Aldo/keto reductase family	CYT	5	23	35.9	High
TRINITY_DN17864_c0_g1_i1_orf1	PREDICTED: erlin-2-B [Microplitis demolitor]	2.879581152	1.52585898	0.0001852	up	yes	0.935	0.3247	0.3	0.344	0.33	1	0.954	0.851	CC:endoplasmic reticulum;CC:integral component of membrane;CC:intracellular membrane-bounded organelle;CC:cellular_component;CC:intracellular organelle;CC:intrinsic component of membrane;CC:membrane-bounded organelle;CC:organelle;CC:cellular anatomical entity;MF:ubiquitin protein ligase binding;MF:ubiquitin-	GO:0005783;GO:0016021;GO:0043231;GO:0005575;GO:0043229;GO:0031224;GO:0043227;GO:0043226;GO:0110165;GO:0031625;GO:0044389;GO:0019899;GO:0003674;GO:0005515;GO:0005488	K23341	ERLIN	------	------	ENOG410XQSH	O:Posttranslational modification, pro	PF01145.28	Band_7	SPFH domain / Band 7 family	CYT	1	2	37.3	Medium
TRINITY_DN141462_c0_g1_i1_orf1	mitochondrial-processing peptidase subunit beta [Diachasma alloeum] >THK33262.1 core protein 1, ubiquinol-cytochrome c reductase [Diachasma alloeum]	3.977047883	1.991697932	1.01E-06	up	yes	1.005	0.2527	0.265	0.225	0.268	1	1.018	0.998	BP:mitochondrial protein processing;BP:nitrogen compound metabolic process;BP:organic substance metabolic process;BP:proteolysis;BP:protein processing involved in protein targeting to mitochondrion;BP:biological_process;BP:metabolic process;BP:protein processing;BP:primary metabolic process;BP:organonitrogen compound metabolic process;BP:protein maturation;BP:protein metabolic process;BP:macromolecule metabolic process;CC:catalytic complex;CC:macromolecular complex;CC:cellular_component;CC:mitochondrial processing peptidase complex;CC:mitochondrial protein complex;MF:endopeptidase activity;MF:molecular_function;MF:binding;MF:peptidase activity;MF:metallopeptidase activity;MF:cation binding;MF:ion binding;MF:metal ion binding;MF:hydrolase	GO:0034982;GO:0006807;GO:0071704;GO:0006508;GO:0006627;GO:0008150;GO:0008152;GO:0016485;GO:0044238;GO:1901564;GO:0051604;GO:0019538;GO:0043170;GO:1902494;GO:0032991;GO:0005575;GO:0017087;GO:0098798;GO:0004175;GO:0003674;GO:0005488;GO:0008233;GO:0008237;GO:0043169;GO:0043167;GO:0046872;GO:0016787;GO:0004222;GO:0140096;GO:0003824	K17732	PMPCB, MAS	------	------	COG0612	S:Function unknown	PF00675.23;PF051	Peptidase_M16;Peptida	Insulinase;Peptidase M16 inactive domain	CYT	1	2	53.3	High
TRINITY_DN2083_c0_g1_i4_orf1	uncharacterized protein LOC114359113 [Ostrinia furnacalis]	4.29535865	2.102778597	5.36E-06	up	yes	1.018	0.237	0.248	0.23	0.233	1	1.064	0.989	------	------	------	------	------	------	ENOG410ZGJA	S:Function unknown	PF06585.14	JHBP	Haemolymph juvenile hormone binding protein	CYT	3	17	24.2	High
TRINITY_DN5107_c0_g1_i4_orf1	peptide methionine sulfoxide reductase [Ostrinia furnacalis]	4.132129668	2.046885527	1.73E-05	up	yes	1.007	0.2437	0.203	0.242	0.286	1	1.046	0.976	MF:peptide-methionin;MF:catalytic activity;MF:oxidoreductase activity, acting on a sulfur group of donors, disulfide as acceptor;MF:molecular_function;MF:oxidoreductase activity, acting on a sulfur group of	GO:0008113;GO:0003824;GO:0016671;GO:0003674;GO:0016667;GO:0016491	K07304	msrA	------	------	COG0225	O:Posttranslational modification, pro	PF01625.24	PMSR	Peptide methionine sulfoxide reductase	CYT	1	4	26.1	Medium
TRINITY_DN36061_c0_g4_i2_orf1	putative GPI-anchored protein pfl2 [Ostrinia furnacalis] >XP_028163002.1 putative GPI-anchored	4.012195122	2.004391769	1.89E-05	up	yes	0.987	0.246	0.243	0.26	0.235	1	0.929	1.032	CC:cellular_component;CC:extracellular region;CC:cellular anatomical entity;MF:carbohydrate derivative	GO:0005575;GO:0005576;GO:0110165;GO:0097367;GO:0003674;	------	------	------	------	ENOG4111P12	S:Function unknown	------	------	------	CYT	1	1	91.7	High
TRINITY_DN195_c4_g1_i1_orf1	beta-1,3-glucan-binding protein 1 [Ostrinia furnacalis]	2.874402295	1.523261992	0.0005637	up	yes	0.9017	0.3137	0.258	0.369	0.314	1	0.845	0.86	BP:immune system process;BP:response to external biotic stimulus;BP:interspecies interaction between organisms;BP:organic substance metabolic process;BP:innate immune response;BP:defense response;BP:response to stress;BP:biological_process;BP:metabolic process;BP:immune response;BP:carbohydrate metabolic process;BP:response to other organism;BP:primary metabolic process;BP:response to biotic stimulus;BP:response to external stimulus;BP:response to stimulus;BP:defense response to other organism;CC:cellular anatomical entity;CC:cellular_component;CC:extracellular region;MF:carbohydrate binding;MF:molecular_function;MF:binding;MF:hydrolase activity;MF:hydrolase activity, acting on glycosyl	GO:0002376;GO:0043207;GO:0044419;GO:0071704;GO:0045087;GO:0006952;GO:0006950;GO:0008150;GO:0008152;GO:0006955;GO:0005975;GO:0051707;GO:0044238;GO:0009607;GO:0009605;GO:0050896;GO:0098542;GO:0110165;GO:0005575;GO:0005576;GO:0030246;GO:0003674;GO:0005488;GO:0016787;GO:0016798;GO:0003824;GO:0004553	K20692	GNBP3	map04624	Toll and Imd signalir	ENOG410Z83Q;ENO	S:Function unknown;	PF15886.8	CBM39	Carbohydrate binding domain	CYT	1	19	14.1	High
TRINITY_DN57904_c0_g2_i1_orf1	cuticle protein 19 [Plutella xylostella] >CAG9138481.1 unnamed protein product [Plutella xylostella]	3.865983607	1.95083552	0.0001578	up	yes	0.9433	0.244	0.217	0.272	0.243	1	0.982	0.848	MF:molecular_function;MF:structural constituent of cuticle;MF:structural molecule activity;	GO:0003674;GO:0042302;GO:0005198	------	------	------	------	ENOG410YSFI	S:Function unknown	PF00379.26	Chitin_bind_4	Insect cuticle protein	CYT	1	16	14.1	High
TRINITY_DN334_c0_g1_i4_orf1	collagenase-like [Ostrinia furnacalis]	7.173462336	2.842669617	8.62E-06	up	yes	1.038	0.1447	0.101	0.182	0.151	1	1.068	1.047	BP:organic substance metabolic process;BP:organonitrogen compound metabolic process;BP:protein metabolic process;BP:macromolecule metabolic process;BP:biological_process;BP:nitrogen compound metabolic process;BP:metabolic process;BP:proteolysis;BP:primary metabolic process;MF:serine-type endopeptidase activity;MF:endopeptidase activity;MF:hydrolase activity;MF:serine hydrolase activity;MF:catalytic activity, acting	GO:0071704;GO:1901564;GO:0019538;GO:0043170;GO:0008150;GO:0006807;GO:0008152;GO:0006508;GO:0044238;GO:0004252;GO:0004175;GO:0016787;GO:0017171;GO:0140096;GO:0003824;GO:0003674;GO:0008233;GO:0008236	------	------	------	------	COG5640	O:Posttranslational modification, pro	PF00089.29	Trypsin	Trypsin	EXC	1	39	29.8	High

TRINITY_DN8087_c0_g1_i9_orf1	cysteine-rich with EGF-like domain protein 2 isoform X1 [Ostrinia furnacalis]	3.821576763	1.93416801	7.09E-05	up	yes	0.921	0.241	0.237	0.233	0.253	1	0.887	0.876	BP:developmental process;BP:cellular process;BP:cellular developmental process;BP:anatomical structure development;BP:cell development;BP:cellular component organization;BP:animal organ development;BP:biological_process;BP:cellular component organization or biogenesis;MF:cation binding;MF:ion binding;MF:calcium ion binding;MF:molecular_function;MF:metal ion binding;MF:binding;	GO:0032502;GO:0009987;GO:0048869;GO:0048856;GO:0048468;GO:0016043;GO:0048513;GO:0008150;GO:0071840;GO:0043169;GO:0043167;GO:0005509;GO:0003674;GO:0046872;GO:0005488	K24335	CRELD	------	------	ENOG41107X2	O:Posttranslational modification, pro	PF11938.11	DUF3456	TLR4 regulator and MIR-interacting MSAP	CYT	1	2	42.5	High
TRINITY_DN7688_c0_g1_i2_orf1	uncharacterized protein LOC114352518 [Ostrinia furnacalis]	7.537313433	2.914050387	1.61E-05	up	yes	1.01	0.134	0.176	0.098	0.128	1	1.062	0.969	BP:phosphorylation;BP:cellular process;BP:cellular metabolic process;BP:phosphate-containing compound metabolic process;BP:biological_process;BP:metabolic process;BP:phosphorus metabolic process;MF:kinase activity;MF:transferase activity;MF:catalytic activity;MF:molecular_function;MF:transferase activity, transferring phosphorus-containing groups;	GO:0016310;GO:0009987;GO:0044237;GO:0006796;GO:0008150;GO:0008152;GO:0006793;GO:0016301;GO:0016740;GO:0003824;GO:0003674;GO:0016772	------	------	------	------	ENOG410YKCN;ENO	S:Function unknown;	PF02958.23;PF079	EcKL;DUF1679	Ecdysteroid kinase-like family;Uncharacterized oxidoreductase dhs-27	CYT	1	10	47.9	High
TRINITY_DN41697_c0_g1_i1_orf1	5-formyltetrahydrofolate cyclo-ligase [Ostrinia furnacalis]	4.309178744	2.107412942	0.0003097	up	yes	0.892	0.207	0.221	0.175	0.225	1	0.809	0.867	MF:purine ribonucleoside triphosphate binding;MF:cyclo-ligase activity;MF:molecular_function;MF:binding;MF:ion binding;MF:heterocyclic compound binding;MF:nucleoside phosphate binding;MF:anion binding;MF:cation binding;MF:nucleotide binding;MF:ligase activity, forming carbon-nitrogen bonds;MF:purine nucleotide binding;MF:metal ion binding;MF:ATP binding;MF:small molecule binding;MF:catalytic activity;MF:ligase activity;MF:5-formyltetrahydrofolate cyclo-ligase activity;MF:adenyl nucleotide binding;MF:carbohydrate derivative binding;MF:organic cyclic compound binding;MF:adenyl ribonucleotide binding;MF:purine ribonucleotide binding;MF:ribonucleotide binding;	GO:0035639;GO:0016882;GO:0003674;GO:0005488;GO:0043167;GO:1901363;GO:1901265;GO:0043168;GO:0043169;GO:0000166;GO:0016879;GO:0017076;GO:0046872;GO:0005524;GO:0036094;GO:0003824;GO:0016874;GO:0030272;GO:0030554;GO:0097367;GO:0097159;GO:0032559;GO:0032555;GO:0032553	K01934	MTHFS	map00670	One carbon pool by	COG0212	H:Coenzyme transport and metabolis	------	------	------	CYT	1	38	4.5	High
TRINITY_DN2490_c0_g2_i1_orfp1	TRINITY_DN2490_c0_g2_i1_m.56872 TRINITY_DN2490_c0_g2::TRINITY_DN2490_c0_g2_i1::g.56872 ORF type:internal len:359 (-),score=123.59 TRINITY_DN2490_c0_g2_i1:2-1075(-)	10.84285714	3.438673058	1.26E-05	up	yes	0.9867	0.091	0.08	0.091	0.102	1	0.923	1.037	------	------	------	------	------	------	------	------	------	------	------	EXC	7	34	36.3	High
TRINITY_DN39200_c0_g1_i5_orf1	juvenile hormone epoxide hydrolase-like [Ostrinia furnacalis]	12.64487179	3.660480505	9.87E-07	up	yes	0.9863	0.078	0.053	0.081	0.1	1	0.962	0.997	CC:integral component of membrane;CC:cellular_component;CC:intrinsic component of membrane;CC:cellular anatomical entity;MF:hydrolase activity;MF:cis-stilbene-oxide hydrolase activity;MF:hydrolase activity, acting on ether bonds;MF:catalytic activity;MF:molecular_function;MF:ether hydrolase activity;	GO:0016021;GO:0005575;GO:0031224;GO:0110165;GO:0016787;GO:0033961;GO:0016801;GO:0003824;GO:0003674;GO:0016803	K10719	JHEH	map00981	Insect hormone bios	COG0596;COG0386	U:Intracellular trafficking, secretion, a	PF06441.15;PF005	EHN;Abhydrolase_1	Epoxide hydrolase N terminus;alpha/beta hydrolase fold	PLA	1	4	56.8	High
TRINITY_DN7920_c0_g1_i2_orf1	uncharacterized protein LOC114357268 [Ostrinia furnacalis] >XP_028166599.1 uncharacterized protein LOC114357268 [Ostrinia furnacalis]	2.354086538	1.235167356	2.21E-05	up	yes	0.9793	0.416	0.381	0.415	0.452	1	0.953	0.985	------	------	K03098	APOD	------	------	COG3040	K:Transcription	PF08212.15;PF000	Lipocalin_2;Lipocalin;Tr	Lipocalin-like domain;Lipocalin / cytosolic fatty-acid binding protein family;Triabin	CYT	3	14	22.3	High
TRINITY_DN4895_c0_g1_i2_orf1	coiled-coil domain-containing protein 86 [Ostrinia furnacalis]	2.633507853	1.396985762	4.38E-06	up	yes	1.006	0.382	0.348	0.405	0.393	1	1.017	1	CC:intracellular membrane-bounded organelle;CC:nucleus;CC:cellular_component;CC:intracellular organelle;CC:membrane-bounded organelle;CC:organelle;CC:cellular anatomical entity;	GO:0043231;GO:0005634;GO:0005575;GO:0043229;GO:0043227;GO:0043226;GO:0110165	K14822	CGR1	------	------	ENOG41129IK	S:Function unknown	PF03879.17	Cgr1	Cgr1 family	CYT	1	8	19.7	High
TRINITY_DN20294_c0_g2_i1_orf1	cytochrome b-c1 complex subunit 2, mitochondrial isoform X1 [Ostrinia furnacalis] >XP_028170208.1 cytochrome b-c1 complex subunit 2, mitochondrial isoform X2 [Ostrinia furnacalis]	2.689477955	1.427326164	2.02E-07	up	yes	0.9943	0.3697	0.368	0.363	0.378	1	1.003	0.98	MF:cation binding;MF:metal ion binding;MF:molecular_function;MF:binding;MF:ion binding;	GO:0043169;GO:0046872;GO:0003674;GO:0005488;GO:0043167	K00415	QCR2, UQCR	map04714;map	Thermogenesis;Oxid	COG0612	T:Signal transduction mechanisms	PF00675.23;PF051	Peptidase_M16;Peptida	Insulinase;Peptidase M16 inactive domain	CYT	17	43	45.7	High
TRINITY_DN17351_c0_g1_i3_orf1	V-type proton ATPase subunit F [Ostrinia furnacalis]	2.81512605	1.493199522	1.07E-05	up	yes	1.005	0.357	0.34	0.401	0.33	1	1.021	0.993	CC:proton-transporting V-type ATPase, V1 domain;CC:proton-transporting two-sector ATPase complex, catalytic domain;CC:macromolecular complex;CC:cellular_component;CC:membrane protein complex;MF:molecular_function;MF:ion transmembrane transporter activity;MF:ATPase activity, coupled to transmembrane movement of substances;MF:inorganic solute uptake transmembrane transporter activity;MF:hydrogen-translocating pyrophosphatase activity;MF:primary active transmembrane transporter activity;MF:active transmembrane transporter activity;MF:ATPase coupled ion transmembrane transporter activity;MF:transporter activity;MF:cation transmembrane transporter activity;MF:proton-transporting ATPase activity, rotational mechanism;MF:ATPase activity, coupled to transmembrane movement of ions, rotational mechanism;MF:inorganic cation transmembrane transporter activity;MF:cation-transporting ATPase activity;MF:transmembrane transporter activity;MF:active ion transmembrane transporter activity;MF:hydrogen ion transmembrane transporter activity;	GO:0033180;GO:0033178;GO:0032991;GO:0005575;GO:0098796;GO:0003674;GO:0015075;GO:0042626;GO:0015318;GO:0009678;GO:0015399;GO:0022804;GO:0042625;GO:0005215;GO:0008324;GO:0046961;GO:0044769;GO:0022890;GO:0019829;GO:0022857;GO:0022853;GO:0015078	K02151	ATPeV1F, ATF	map05323;map	Rheumatoid arthritis	COG1436	C:Energy production and conversion	PF01990.20	ATP-synt_F	ATP synthase	CYT	6	40	13.8	High
TRINITY_DN86844_c0_g2_i1_orf1	spermine oxidase-like isoform X1 [Ostrinia furnacalis]	2.543489583	1.346809186	0.0004237	up	yes	0.9767	0.384	0.294	0.48	0.378	1	0.97	0.96	MF:catalytic activity;MF:molecular_function;MF:oxidoreductase activity;	GO:0003824;GO:0003674;GO:0016491	K12259	SMOX, PAO5	map00330;map	Arginine and proline	ENOG410YT60;ENO(	F:Nucleotide transport and metabolis	PF01593.27	Amino_oxidase	Flavin containing amine oxidoreductase	CYT	1	5	46.8	High
TRINITY_DN2243_c0_g1_i4_orf1	WD repeat-containing protein 75 [Ostrinia furnacalis]	3.237410072	1.694840118	2.42E-05	up	yes	1.035	0.3197	0.287	0.367	0.305	1	1.073	1.031	------	------	K14552	NAN1, UTP17	map03008	Ribosome biogenesi	ENOG410YY9G	S:Function unknown	------	------	------	CYT	1	1	99.1	Medium
TRINITY_DN3959_c1_g2_i1_orf1	PREDICTED: probable isocitrate dehydrogenase [NAD] subunit alpha, mitochondrial isoform X3 [Papilio xuthus] >XP_014363280.1 probable isocitrate dehydrogenase [NAD] subunit alpha, mitochondrial isoform X3 [Papilio machaon]	3.141246477	1.651337148	1.62E-07	up	yes	1.003	0.3193	0.331	0.309	0.318	1	1.015	0.995	BP:tricarboxylic acid cycle;BP:biological_process;BP:metabolic process;BP:primary metabolic process;CC:oxidoreductase complex;CC:isocitrate dehydrogenase comple;CC:mitochondrial isocitrate dehydrogenase comple;CC:catalytic complex;CC:mitochondrial tricarboxylic acid cycle enzyme complex;CC:tricarboxylic acid cycle enzyme complex;CC:macromolecular complex;CC:cellular_component;CC:mitochondrial protein complex;MF:NAD binding;MF:molecular_function;MF:binding;MF:nucleotide binding;MF:heterocyclic compound binding;MF:nucleoside phosphate binding;MF:oxidoreductase activity;MF:cation binding;MF:magnesium ion binding;MF:ion binding;MF:isocitrate dehydrogenase activity;MF:metal ion binding;MF:small molecule binding;MF:oxidoreductase activity, acting on CH-OH group of donors;MF:isocitrate dehydrogenas;MF:oxidoreductase activity, acting on the CH-OH group of donors, NAD or NADP as acceptor;MF:catalytic activity;MF:organic cyclic compound binding;	GO:0006099;GO:0008150;GO:0008152;GO:0044238;GO:1990204;GO:0045242;GO:0005962;GO:1902494;GO:0030062;GO:0045239;GO:0032991;GO:0005575;GO:0098798;GO:0051287;GO:0003674;GO:0005488;GO:0000166;GO:1901363;GO:1901265;GO:0016491;GO:0043169;GO:0000287;GO:0043167;GO:0004448;GO:0046872;GO:0036094;GO:0016614;GO:0004449;GO:0016616;GO:0003824;GO:0097159	K00030	IDH3	map00020	Citrate cycl	COG0473	S:Function unknown	PF00180.23	Iso_dh	Isocitrate/isopropylmalate dehydrogenase	CYT	16	55	38.7	High
TRINITY_DN2594_c0_g2_i4_orf1	isocitrate dehydrogenase [NAD] subunit beta, mitochondrial isoform X2 [Ostrinia furnacalis]	3.481228669	1.799596582	4.53E-07	up	yes	1.02	0.293	0.287	0.299	0.293	1	1.02	1.04	BP:tricarboxylic acid cycle;BP:biological_process;BP:metabolic process;BP:primary metabolic process;CC:oxidoreductase complex;CC:isocitrate dehydrogenase comple;CC:mitochondrial isocitrate dehydrogenase comple;CC:catalytic complex;CC:mitochondrial tricarboxylic acid cycle enzyme complex;CC:tricarboxylic acid cycle enzyme complex;CC:macromolecular complex;CC:cellular_component;CC:mitochondrial protein complex;MF:NAD binding;MF:molecular_function;MF:binding;MF:nucleotide binding;MF:heterocyclic compound binding;MF:nucleoside phosphate binding;MF:oxidoreductase activity;MF:cation binding;MF:magnesium ion binding;MF:ion binding;MF:isocitrate dehydrogenase activity;MF:metal ion binding;MF:small molecule binding;MF:oxidoreductase activity, acting on CH-OH group of donors;MF:isocitrate dehydrogenas;MF:oxidoreductase activity, acting on the CH-OH group of donors, NAD or NADP as acceptor;MF:catalytic activity;MF:organic cyclic compound binding;	GO:0006099;GO:0008150;GO:0008152;GO:0044238;GO:1990204;GO:0045242;GO:0005962;GO:1902494;GO:0030062;GO:0045239;GO:0032991;GO:0005575;GO:0098798;GO:0051287;GO:0003674;GO:0005488;GO:0000166;GO:1901363;GO:1901265;GO:0016491;GO:0043169;GO:0000287;GO:0043167;GO:0004448;GO:0046872;GO:0036094;GO:0016614;GO:0004449;GO:0016616;GO:0003824;GO:0097159	K00030	IDH3	map00020	Citrate cycl	COG0473	E:Amino acid transport and metaboli	PF00180.23	Iso_dh	Isocitrate/isopropylmalate dehydrogenase	CYT	3	13	41.2	High
TRINITY_DN99_c0_g1_i3_orf1	uncharacterized protein LOC126375979 [Pectinophora gossypiella] >XP_049879066.1 uncharacterized protein LOC126375979 [Pectinophora gossypiella]	2.129186603	1.090302394	0.002168	up	yes	0.89	0.418	0.494	0.392	0.368	1	0.838	0.832	------	------	------	------	------	------	ENOG410YKH0;ENO(	S:Function unknown;	------	------	------	CYT	1	1	140.2	Medium
TRINITY_DN105901_c0_g1_i2_orfp1	contactin-like [Pectinophora gossypiella]	2.684507042	1.42465719	0.0001411	up	yes	0.953	0.355	0.335	0.384	0.346	1	0.875	0.984	------	------	------	------	------	------	------	------	------	------	------	CYT	2	16	16.7	High
TRINITY_DN4762_c0_g1_i2_orf1	ATPase family AAA domain-containing protein 1 isoform X2 [Ostrinia furnacalis]	3.781791375	1.919069778	0.0004062	up	yes	1.026	0.2713	0.34	0.286	0.188	1	1.128	0.949	MF:purine ribonucleoside triphosphate binding;MF:molecular_function;MF:binding;MF:ATPase activity;MF:ion binding;MF:heterocyclic compound binding;MF:nucleoside phosphate binding;MF:anion binding;MF:nucleotide binding;MF:purine nucleotide binding;MF:ATP binding;MF:hydrolase activity;MF:nucleoside-triphosphatase activity;MF:small molecule binding;MF:catalytic activity;MF:hydrolase activity, acting on acid anhydrides, in phosphorus-containing anhydrides;MF:adenyl nucleotide binding;MF:carbohydrate derivative binding;MF:organic cyclic compound binding;MF:hydrolase activity, acting on acid anhydrides;MF:pyrophosphatase activity;MF:adenyl ribonucleotide binding;MF:purine ribonucleotide binding;MF:ribonucleotide binding;	GO:0035639;GO:0003674;GO:0005488;GO:0016887;GO:0043167;GO:1901363;GO:1901265;GO:0043168;GO:0000166;GO:0017076;GO:0005524;GO:0016787;GO:0017111;GO:0036094;GO:0003824;GO:0016818;GO:0030554;GO:0097367;GO:0097159;GO:0016817;GO:0016462;GO:0032559;GO:0032555;GO:0032553	K22530	ATAD1	------	------	COG0464	S:Function unknown	PF00004.32;PF178	AAA;AAA_lid_3	ATPase family associated with various cellular activities;AAA+ lid domain	CYT	1	2	43.3	High
TRINITY_DN122170_c0_g1_i2_orfp1	TRINITY_DN122170_c0_g1_i2_m.81408 TRINITY_DN122170_c0_g1::TRINITY_DN122170_c0_g1_i2::g.81408 ORF type:internal len:87 (+),score=9.58,Baculo_E25,PF05274.12,1.7e-07 TRINITY_DN122170_c0_g1_i2:2-259(+)	5.92929834	2.567861389	9.23E-05	up	yes	1.107	0.1867	0.164	0.212	0.184	1	1.13	1.191	------	------	------	------	------	------	------	------	PF05274.14	Baculo_E25	Occlusion-derived virus envelope protein E25	CYT	1	12	9	Medium
TRINITY_DN1044_c0_g1_i2_orf1	V-type proton ATPase subunit H isoform X3 [Ostrinia furnacalis] >QRR19186.1 V-type proton ATPase subunit H [Ostrinia nubilalis]	4.007751938	2.002793215	3.15E-06	up	yes	1.034	0.258	0.259	0.245	0.27	1	1.068	1.034	CC:vacuolar proton-transporting V-type ATPase, V1 domain;CC:proton-transporting V-type ATPase, V1 domain;CC:proton-transporting two-sector ATPase complex, catalytic domain;CC:macromolecular complex;CC:cellular_component;CC:membrane protein complex;MF:molecular_function;MF:ion transmembrane transporter activity;MF:ATPase activity, coupled to transmembrane movement of substances;MF:inorganic solute uptake transmembrane transporter activity;MF:hydrogen-translocating pyrophosphatase activity;MF:primary active transmembrane transporter activity;MF:active transmembrane transporter activity;MF:ATPase coupled ion transmembrane transporter activity;MF:transporter activity;MF:cation transmembrane transporter activity;MF:proton-transporting ATPase activity, rotational mechanism;MF:ATPase activity, coupled to transmembrane movement of ions, rotational mechanism;MF:inorganic cation transmembrane transporter activity;MF:cation-transporting ATPase activity;MF:transmembrane transporter activity;MF:active ion transmembrane transporter activity;MF:hydrogen ion transmembrane transporter activity;	GO:0000221;GO:0033180;GO:0033178;GO:0032991;GO:0005575;GO:0098796;GO:0003674;GO:0015075;GO:0042626;GO:0015318;GO:0009678;GO:0015399;GO:0022804;GO:0042625;GO:0005215;GO:0008324;GO:0046961;GO:0044769;GO:0022890;GO:0019829;GO:0022857;GO:0022853;GO:0015078	K02144	ATPeV1H	map05152;map	Tuberculosis;Human	COG5231	C:Energy production and conversion	PF03224.17;PF116	V-ATPase_H_N;V-ATPa	V-ATPase subunit H;V-ATPase subunit H;Armadillo-like	CYT	15	38	55.2	High
TRINITY_DN30704_c0_g1_i1_orf1	cytochrome P450 monooxygenase CYP6AE134v2 [Ostrinia furnacalis]	3.926356589	1.973191203	1.68E-05	up	yes	1.013	0.258	0.23	0.271	0.273	1	0.973	1.066	CC:integral component of membrane;CC:cellular_component;CC:cellular anatomical entity;CC:intrinsic component of membrane;MF:cation binding;MF:monooxygenase activity;MF:heme binding;MF:transition metal ion binding;MF:tetrapyrrole binding;MF:organic cyclic compound binding;MF:oxidoreductase activity, acting on paired donors, with incorporation or reduction of molecular oxygen;MF:ion binding;MF:catalytic activity;MF:molecular_function;MF:binding;MF:iron ion binding;MF:metal ion binding;MF:heterocyclic compound binding;MF:oxidoreductase activity;	GO:0016021;GO:0005575;GO:0110165;GO:0031224;GO:0043169;GO:0004497;GO:0020037;GO:0046914;GO:0046906;GO:0097159;GO:0016705;GO:0043167;GO:0003824;GO:0003674;GO:0005488;GO:0005506;GO:0046872;GO:1901363;GO:0016491	K14999	CYP6	------	------	COG2124	Q:Secondary metabolites biosynthesi	PF00067.25	p450	Cytochrome P450	CYT	1	2	61.1	High
TRINITY_DN5740_c0_g1_i4_orf1	unconventional myosin IC isoform X1 [Ostrinia furnacalis]	4.193680755	2.06821704	2.74E-07	up	yes	1.022	0.2437	0.243	0.249	0.239	1	1.033	1.033	CC:cytoplasm;CC:myosin complex;CC:macromolecular complex;CC:cellular_component;CC:cellular anatomical entity;MF:purine ribonucleoside triphosphate binding;MF:molecular_function;MF:binding;MF:ion binding;MF:heterocyclic compound binding;MF:nucleoside phosphate binding;MF:nucleotide binding;MF:actin binding;MF:purine nucleotide binding;MF:motor activity;MF:ATP binding;MF:anion binding;MF:small molecule binding;MF:cytoskeletal protein binding;MF:carbohydrate derivative binding;MF:organic cyclic compound binding;MF:adenyl nucleotide binding;MF:adenyl ribonucleotide binding;MF:protein binding;MF:purine ribonucleotide binding;MF:ribonucleotide binding;	GO:0005737;GO:0016459;GO:0032991;GO:0005575;GO:0110165;GO:0035639;GO:0003674;GO:0005488;GO:0043167;GO:1901363;GO:1901265;GO:0000166;GO:0003779;GO:0017076;GO:0003774;GO:0005524;GO:0043168;GO:0036094;GO:0008092;GO:0097367;GO:0097159;GO:0030554;GO:0032559;GO:0005515;GO:0032555;GO:0032553	K10356	MYO1	map05130	Pathogenic Escheric	COG5022	S:Function unknown	PF00063.24;PF060	Myosin_head;Myosin_T	Myosin head;Unconventional myosin tail, actin- and lipid-binding;IQ calmodulin-binding motif	CYT	15	15	119.7	High
TRINITY_DN6221_c0_g1_i5_orf1	unnamed protein product [Diatraea saccharalis]	3.776012107	1.916863391	3.68E-05	up	yes	0.998	0.2643	0.238	0.251	0.304	1	0.944	1.05	CC:intrinsic component of membrane;CC:cellular_component;CC:proton-transporting V-type ATPase, V0 domain;CC:integral component of membrane;CC:cellular anatomical entity;CC:macromolecular complex;CC:membrane protein complex;CC:proton-transporting two-sector ATPase complex, proton-transporting domain;MF:molecular_function;MF:ATPase activity, coupled to transmembrane movement of substances;MF:inorganic solute uptake transmembrane transporter activity;MF:hydrogen-translocating pyrophosphatase activity;MF:primary active transmembrane transporter activity;MF:ATPase coupled ion transmembrane transporter activity;MF:transporter activity;MF:cation transmembrane transporter activity;MF:proton-transporting ATPase activity, rotational mechanism;MF:ATPase activity, coupled to transmembrane movement of ions, rotational mechanism;MF:inorganic cation transmembrane transporter activity;MF:cation-transporting ATPase activity;MF:ion transmembrane transporter activity;MF:transmembrane transporter activity;MF:active transmembrane transporter activity;MF:active ion transmembrane transporter activity;MF:hydrogen ion transmembrane transporter activity;	GO:0031224;GO:0005575;GO:0033179;GO:0016021;GO:0110165;GO:0032991;GO:0098796;GO:0033177;GO:0003674;GO:0042626;GO:0015318;GO:0009678;GO:0015399;GO:0042625;GO:0005215;GO:0008324;GO:0046961;GO:0044769;GO:0022890;GO:0019829;GO:0015075;GO:0022857;GO:0022804;GO:0022853;GO:0015078	K02153	ATPeV0E, ATF	map05165;map	Human papillomavir	ENOG41121M0	C:Energy production and conversion	PF05493.16	ATP_synt_H	ATP synthase subunit H	CYT	1	9	9.6	High
TRINITY_DN43611_c0_g1_i1_orf1	39S ribosomal protein L41, mitochondrial [Ostrinia furnacalis]	3.474042248	1.796615299	2.09E-06	up	yes	0.9703	0.2793	0.282	0.273	0.283	1	0.943	0.968	CC:cellular_component;CC:large ribosomal subunit;CC:organellar large ribosomal subunit;CC:ribonucleoprotein complex;CC:mitochondrial large ribosomal subunit;CC:macromolecular complex;CC:ribosomal subunit;CC:mitochondrial protein complex;MF:structural molecule activity;MF:structural constituent of ribosome;MF:molecular_function;	GO:0005575;GO:0015934;GO:0000315;GO:1990904;GO:0005762;GO:0032991;GO:0044391;GO:0098798;GO:0005198;GO:0003735;GO:0003674	K17422	MRPL41	------	------	ENOG4111ND6	S:Function unknown	PF09809.12	MRP-L27	Mitochondrial ribosomal protein L27	CYT	2	25	18.2	High
TRINITY_DN2986_c1_g1_i1_orf1	Troponin C, isoform 1 [Papilio xuthus]	4.167012879	2.059013559	8.49E-06	up	yes	1.003	0.2407	0.258	0.233	0.231	1	1.048	0.962	MF:cation binding;MF:ion binding;MF:molecular_function;MF:binding;MF:metal ion binding;MF:calcium ion binding;	GO:0043169;GO:0043167;GO:0003674;GO:0005488;GO:0046872;GO:0005509	------	------	------	------	COG5126	F:Nucleotide transport and metabolis	PF13499.9;PF0003	EF-hand_7;EF-hand_1;	EF-hand domain pair;EF hand;EF-hand domain;EF-hand domain pair;EF-hand domain;EF hand	CYT	5	22	20.2	High
TRINITY_DN3194_c0_g1_i6_orf1	uncharacterized protein LOC114361386 [Ostrinia furnacalis]	4.786729858	2.259040389	4.03E-06	up	yes	1.01	0.211	0.208	0.222	0.203	1	0.977	1.052	BP:nitrogen compound metabolic process;BP:organic substance metabolic process;BP:proteolysis;BP:biological_process;BP:metabolic process;BP:primary metabolic process;BP:organonitrogen compound metabolic process;BP:protein metabolic process;BP:macromolecule metabolic process;CC:integral component of membrane;CC:intrinsic component of membrane;CC:cellular anatomical entity;CC:cellular_component;MF:aminopeptidase activity;MF:exopeptidase activity;MF:molecular_function;MF:binding;MF:peptidase activity;MF:metallopeptidase activity;MF:cation binding;MF:transition metal ion binding;MF:ion binding;MF:metal ion binding;MF:hydrolase activity;MF:catalytic activity, acting on a protein;MF:catalytic activity;MF:zinc ion binding;	GO:0006807;GO:0071704;GO:0006508;GO:0008150;GO:0008152;GO:0044238;GO:1901564;GO:0019538;GO:0043170;GO:0016021;GO:0031224;GO:0110165;GO:0005575;GO:0004177;GO:0008238;GO:0003674;GO:0005488;GO:0008233;GO:0008237;GO:0043169;GO:0046914;GO:0043167;GO:0046872;GO:0016787;GO:0140096;GO:0003824;GO:0008270	K11140	ANPEP, CD13	map04640;map	Hematopoietic cell l	COG0308	E:Amino acid transport and metaboli	PF01433.23;PF118	Peptidase_M1;ERAP1_(	Peptidase family M1 domain;ERAP1-like C-terminal domain;Peptidase M1 N-terminal domain	CYT	4	7	109.4	High
TRINITY_DN26010_c0_g1_i2_orf1	cytochrome b-c1 complex subunit 10-like [Ostrinia furnacalis]	4.68627451	2.228441466	1.26E-05	up	yes	0.956	0.204	0.202	0.198	0.212	1	0.965	0.903	BP:aerobic respiration;BP:generation of precursor metabolites and energy;BP:electron transport chain;BP:respiratory electron transport chain;BP:cellular process;BP:aerobic electron transport chain;BP:cellular respiration;BP:metabolic process;BP:energy derivation by oxidation of organic compounds;BP:mitochondrial electron transport, ubiquinol to cytochrome c;BP:cellular metabolic process;BP:biological_process;CC:oxidoreductase complex;CC:respiratory chain complex;CC:cytochrome complex;CC:inner mitochondrial membrane protein complex;CC:mitochondrial respiratory chain complex III;CC:transmembrane transporter complex;CC:catalytic complex;CC:transporter complex;CC:macromolecular complex;CC:respiratory chain complex III;CC:cellular_component;CC:membrane protein complex;CC:mitochondrial	GO:0009060;GO:0006091;GO:0022900;GO:0022904;GO:0009987;GO:0019646;GO:0045333;GO:0008152;GO:0015980;GO:0006122;GO:0044237;GO:0008150;GO:1990204;GO:0098803;GO:0070069;GO:0098800;GO:0005750;GO:1902495;GO:1902494;GO:1990351;GO:0032991;GO:0045275;GO:0005575;GO:0098796;GO:0098798	K00420	QCR10, UQCF	map04714;map	Thermogenesis;Oxid	ENOG410XVMI	C:Energy production and conversion	PF08997.13	UCR_6-4kD	Ubiquinol-cytochrome C reductase complex, 6.4kD protein	PLA	1	14	6.3	High
TRINITY_DN4869_c0_g1_i10_orf1	estrogen sulfotransferase-like isoform X1 [Ostrinia furnacalis]	6.522875817	2.705508163	1.08E-06	up	yes	0.998	0.153	0.136	0.158	0.165	1	1.023	0.971	------	------	------	------	------	------	ENOG4111H56	S:Function unknown	PF00685.30;PF134	Sulfotransfer_1;Sulfotra	Sulfotransferase domain;Sulfotransferase family	CYT	9	34	39.9	High
TRINITY_DN6933_c0_g1_i2_orf1	Chlorophyll a-b binding protein 40, chloroplastic [Trichinella nelsoni] >KRY99282.1 Chlorophyll a-b binding protein 40, chloroplastic [Trichinella zimbabwensis]	8.426301853	3.074899598	9.24E-06	up	yes	0.9547	0.1133	0.114	0.125	0.101	1	0.901	0.963	CC:cellular_component;CC:membrane;CC:cellular anatomical entity;	GO:0005575;GO:0016020;GO:0110165	K08912	LHCB1	------	------	ENOG410ZHBU;ENO	J:Translation, ribosomal structure anc	PF00504.24	Chloroa_b-bind	Chlorophyll A-B binding protein	CYT	2	7	28.3	High
TRINITY_DN4923_c0_g1_i4_orf1	O-acyltransferase like protein-like [Ostrinia furnacalis]	2.41918077	1.274518577	8.64E-05	up	yes	0.9863	0.4077	0.381	0.453	0.389	1	0.933	1.026	------	------	------	------	------	------	ENOG410YD1T;ENO(	O:Posttranslational modification, pro	------	------	------	PLA	1	1	70.2	Medium
TRINITY_DN7336_c0_g1_i13_orf1	PREDICTED: calcium-transporting ATPase sarcoplasmic/endoplasmic reticulum type isoform X2 [Amyelois transitella]	2.54000508	1.344831382	2.12E-07	up	yes	1	0.3937	0.388	0.383	0.41	1	1.001	0.999	CC:endoplasmic reticulum membrane;CC:intrinsic component of membrane;CC:organelle membrane;CC:integral component of membrane;CC:membrane;CC:sarcoplasmic reticulum membrane;CC:bounding membrane of organelle;CC:cellular_component;CC:cellular anatomical entity;MF:purine ribonucleoside triphosphate binding;MF:molecular_function;MF:binding;MF:ATPase activity;MF:nucleotide binding;MF:heterocyclic compound binding;MF:anion binding;MF:calcium ion transmembrane transporter activity;MF:ATPase activity, coupled to transmembrane movement of substances;MF:ion binding;MF:inorganic solute uptake transmembrane transporter activity;MF:ATP binding;MF:primary active transmembrane transporter activity;MF:nucleoside phosphate binding;MF:purine nucleotide binding;MF:metal ion transmembrane transporter activity;MF:active transmembrane transporter activity;MF:hydrolase activity;MF:transporter activity;MF:cation transmembrane transporter activity;MF:P-type transmembrane transporter activity;MF:nucleoside-triphosphatase activity;MF:small molecule binding;MF:catalytic activity;MF:ATPase activity, coupled to transmembrane movement of ions, phosphorylative mechanism;MF:inorganic cation transmembrane transporter activity;MF:hydrolase activity, acting on acid anhydrides, in phosphorus-containing anhydrides;MF:adenyl nucleotide binding;MF:carbohydrate derivative binding;MF:organic cyclic compound binding;MF:ion transmembrane transporter activity;MF:hydrolase activity, acting on acid anhydrides;MF:cation-transporting ATPase activity;MF:pyrophosphatase activity;MF:transmembrane transporter activity;MF:adenyl ribonucleotide binding;MF:active ion transmembrane transporter activity;MF:purine ribonucleotide binding;MF:calcium-	GO:0005789;GO:0031224;GO:0031090;GO:0016021;GO:0016020;GO:0033017;GO:0098588;GO:0005575;GO:0110165;GO:0035639;GO:0003674;GO:0005488;GO:0016887;GO:0000166;GO:1901363;GO:0043168;GO:0015085;GO:0042626;GO:0043167;GO:0015318;GO:0005524;GO:0015399;GO:1901265;GO:0017076;GO:0046873;GO:0022804;GO:0016787;GO:0005215;GO:0008324;GO:0140358;GO:0017111;GO:0036094;GO:0003824;GO:0015662;GO:0022890;GO:0016818;GO:0030554;GO:0097367;GO:0097159;GO:0015075;GO:0016817;GO:0019829;GO:0016462;GO:0022857;GO:0032559;GO:0022853;GO:0032555;GO:0005388;GO:0032553	K05853	ATP2A	map04972;map	Pancreatic secretion	COG0474	G:Carbohydrate transport and metab	PF00122.23;PF006	E1-E2_ATPase;Cation_A	E1-E2 ATPase;Cation transporting ATPase, C-terminus;Cation transporter/ATPase, N-terminus;Cation transport ATPase;haloacid dehalogenase-like hydrolase;haloacid dehalogenase-like hydrolase	CYT	37	37	109.8	High
TRINITY_DN28152_c0_g1_i1_orf1	mitochondrial import inner membrane translocase subunit Tim29 [Ostrinia furnacalis]	2.740540541	1.454460476	2.76E-05	up	yes	1.014	0.37	0.346	0.342	0.422	1	1.044	0.999	CC:mitochondrial inner membrane protein insertion complex;CC:macromolecular complex;CC:inner mitochondrial membrane protein complex;CC:cellular_component;CC:membrane protein complex;CC:mitochondrial protein complex;	GO:0042721;GO:0032991;GO:0098800;GO:0005575;GO:0098796;GO:0098798	------	------	------	------	ENOG4111K8C	S:Function unknown	PF10171.12	Tim29	Translocase of the Inner Mitochondrial membrane 29	CYT	1	4	21.8	High
TRINITY_DN33452_c0_g1_i3_orf1	lethal(2) giant larvae protein isoform X8 [Ostrinia furnacalis]	2.712865968	1.439817772	0.0001328	up	yes	1.01	0.3723	0.3	0.439	0.378	1	1.045	0.985	BP:secretion;BP:transport;BP:vesicle-mediated transport;BP:export from cell;BP:exocytosis;BP:secretion by cell;BP:biological_process;BP:cellular process;BP:establishment of localization;BP:localization;	GO:0046903;GO:0006810;GO:0016192;GO:0140352;GO:0006887;GO:0032940;GO:0008150;GO:0009987;GO:0051234;GO:0051179	K06094	LLGL	map04530;map	Tight junction;Huma	ENOG410XS6Z	S:Function unknown	------	------	------	CYT	1	18	10.7	High
TRINITY_DN389_c0_g1_i2_orf1	uncharacterized protein LOC118068293 isoform X2 [Chelonus insularis]	2.914772727	1.543383397	2.63E-06	up	yes	1.026	0.352	0.347	0.369	0.34	1	1.052	1.026	------	------	------	------	------	------	ENOG410XX7G	S:Function unknown	PF01607.27;PF000	CBM_14;Ldl_recept_a	Chitin binding Peritrophin-A domain;Low-density lipoprotein receptor domain class A	PLA	4	11	60.2	High
TRINITY_DN3082_c1_g1_i7_orf1	ribosomal RNA processing protein 1 homolog [Ostrinia furnacalis]	3.928700036	1.974052019	0.0001094	up	yes	1.091	0.2777	0.251	0.248	0.334	1	1.138	1.135	BP:nucleic acid metabolic process;BP:cellular nitrogen compound metabolic process;BP:nitrogen compound metabolic process;BP:ncRNA metabolic process;BP:organic cyclic compound metabolic process;BP:nucleobase-containing compound metabolic process;BP:organic substance metabolic process;BP:rRNA processing;BP:ncRNA processing;BP:cellular process;BP:cellular aromatic compound metabolic process;BP:biological_process;BP:metabolic process;BP:heterocycle metabolic process;BP:RNA metabolic process;BP:primary metabolic process;BP:rRNA metabolic process;BP:cellular metabolic process;BP:macromolecule metabolic process;BP:RNA processing;CC:preribosome, small subunit precursor;CC:preribosome;CC:macromolecular complex;CC:ribonucleoprotein complex;CC:cellular_component;	GO:0090304;GO:0034641;GO:0006807;GO:0034660;GO:1901360;GO:0006139;GO:0071704;GO:0006364;GO:0034470;GO:0009987;GO:0006725;GO:0008150;GO:0008152;GO:0046483;GO:0016070;GO:0044238;GO:0016072;GO:0044237;GO:0043170;GO:0006396;GO:0030688;GO:0030684;GO:0032991;GO:1990904;GO:0005575	K14849	RRP1	------	------	ENOG4111GJ6	J:Translation, ribosomal structure anc	PF05997.15	Nop52	Nucleolar protein,Nop52	CYT	1	1	84.5	Medium
TRINITY_DN4550_c1_g1_i5_orfp2	TRINITY_DN4550_c1_g1_i5_m.14710 TRINITY_DN4550_c1_g1::TRINITY_DN4550_c1_g1_i5::g.14710 ORF type:5prime_partial len:168 (+),score=78.12 TRINITY_DN4550_c1_g1_i5:3-506(+)	2.45994898	1.298628394	3.01E-05	up	yes	0.9643	0.392	0.418	0.354	0.404	1	0.935	0.958	------	------	------	------	------	------	------	------	------	------	------	CYT	1	7	20.9	High
TRINITY_DN107035_c0_g1_i1_orf1	splicing factor 3A subunit 3 [Ostrinia furnacalis]	2.82991453	1.500758481	1.82E-05	up	yes	0.9933	0.351	0.367	0.34	0.346	1	1.034	0.946	BP:cellular metabolic process;BP:nucleic acid metabolic process;BP:cellular nitrogen compound metabolic process;BP:nitrogen compound metabolic process;BP:organic cyclic compound metabolic process;BP:nucleobase-containing compound metabolic process;BP:organic substance metabolic process;BP:RNA splicing;BP:mRNA metabolic process;BP:cellular process;BP:cellular aromatic compound metabolic process;BP:RNA splicing, via transesterification reactions;BP:RNA splicing, via transesterification reactions with bulged adenosine as nucleophile;BP:biological_process;BP:metabolic process;BP:heterocycle metabolic process;BP:RNA metabolic process;BP:primary metabolic process;BP:mRNA splicing, via spliceosome;BP:macromolecule metabolic process;BP:RNA processing;BP:mRNA processing;CC:spliceosomal complex;CC:macromolecular complex;CC:ribonucleoprotein complex;CC:cellular_component;MF:molecular_function;MF:binding;MF:nucleic acid binding;MF:heterocyclic compound binding;MF:transition metal ion binding;MF:zinc ion binding;MF:ion binding;MF:cation binding;MF:metal ion binding;MF:organic cyclic compound binding;MF:RNA binding;	GO:0044237;GO:0090304;GO:0034641;GO:0006807;GO:1901360;GO:0006139;GO:0071704;GO:0008380;GO:0016071;GO:0009987;GO:0006725;GO:0000375;GO:0000377;GO:0008150;GO:0008152;GO:0046483;GO:0016070;GO:0044238;GO:0000398;GO:0043170;GO:0006396;GO:0006397;GO:0005681;GO:0032991;GO:1990904;GO:0005575;GO:0003674;GO:0005488;GO:0003676;GO:1901363;GO:0046914;GO:0008270;GO:0043167;GO:0043169;GO:0046872;GO:0097159;GO:0003723	K12827	SF3A3, SAP61	map03040	Spliceosome	COG5188	A:RNA processing and modification	PF11931.11;PF132	SF3a60_Prp9_C;SDE2_2	SF3a60/Prp9 C-terminal;Replication stress response SDE2 C-terminal;Pre-mRNA-splicing factor SF3A3, of SF3a complex, Prp9;Splicing factor SF3a60 binding domain	CYT	1	1	57.7	High

TRINITY_DN4030_c0_g2_i1_orf1	putative trypsin 6 [Ostrinia nubilalis]	3.048338369	1.608023052	1.84E-06	up	yes	1.009	0.331	0.315	0.332	0.346	1	0.992	1.035	BP:organic substance metabolic process;BP:organonitrogen compound metabolic process;BP:protein metabolic process;BP:macromolecule metabolic process;BP:biological_process;BP:nitrogen compound metabolic process;BP:metabolic process;BP:proteolysis;BP:primary metabolic process;MF:serine-type endopeptidase activity;MF:endopeptidase activity;MF:hydrolase activity;MF:serine hydrolase activity;MF:catalytic activity, acting on a protein;MF:catalytic activity;MF:molecular_function;MF:peptidase activity;MF:serine-type peptidase activity	GO:0071704;GO:1901564;GO:0019538;GO:0043170;GO:0008150;GO:0006807;GO:0008152;GO:0006508;GO:0044238;GO:0004252;GO:0004175;GO:0016787;GO:0017171;GO:0140096;GO:0003824;GO:0003674;GO:0008233;GO:0008236	K09628;K09640	PRSS27;TMPR	------	------	COG5640	O:Posttranslational modification, pro	PF00089.29	Trypsin	Trypsin	CYT	3	20	28.7	High
TRINITY_DN3332_c0_g1_i2_orf1	glutathione S-transferase sigma3 [Glyphodes pyloalis]	3.23876315	1.695442968	1.94E-07	up	yes	1.016	0.3137	0.313	0.317	0.311	1	1.032	1.015	MF:catalytic activity;MF:transferase activity;MF:glutathione transferase activity;MF:molecular_function;MF:transferase activity, transferring alkyl or ary;	GO:0003824;GO:0016740;GO:0004364;GO:0003674;GO:0016765	K04097	HPGDS	map00590;map	Arachidonic acid me	ENOG41103AU;ENO	O:Posttranslational modification, pro	PF02798.23;PF144	GST_N;GST_C_3;GST_C	Glutathione S-transferase, N-terminal domain;Glutathione S-transferase, C-terminal domain;Glutathione S-transferase, C-terminal	CYT	3	50	20.6	High
TRINITY_DN36262_c0_g1_i1_orf1	trypsin, alkaline C-like [Maniola jurtina]	3.524004086	1.817215597	2.80E-05	up	yes	1.035	0.2937	0.292	0.255	0.334	1	1.086	1.02	BP:organic substance metabolic process;BP:organonitrogen compound metabolic process;BP:protein metabolic process;BP:macromolecule metabolic process;BP:biological_process;BP:nitrogen compound metabolic process;BP:metabolic process;BP:proteolysis;BP:primary metabolic process;MF:serine-type endopeptidase activity;MF:endopeptidase activity;MF:hydrolase activity;MF:serine hydrolase activity;MF:catalytic activity, acting on a protein;MF:catalytic activity;MF:molecular_function;MF:peptidase activity;MF:serine-type peptidase activity	GO:0071704;GO:1901564;GO:0019538;GO:0043170;GO:0008150;GO:0006807;GO:0008152;GO:0006508;GO:0044238;GO:0004252;GO:0004175;GO:0016787;GO:0017171;GO:0140096;GO:0003824;GO:0003674;GO:0008233;GO:0008236	K01312	PRSS1_2_3	map04972;map	Pancreatic secretion	COG5640	O:Posttranslational modification, pro	PF00089.29	Trypsin	Trypsin	CYT	1	9	11.8	High
TRINITY_DN23734_c0_g1_i1_orf1	histone-lysine N-methyltransferase SMYD3 [Ostrinia furnacalis]	2.247588424	1.168377875	0.008204	up	yes	0.9087	0.4043	0.261	0.533	0.419	1	0.778	0.948	CC:cellular_component;CC:intracellular organelle;CC:organelle;CC:cellular anatomical entity;MF:cation binding;MF:ion binding;MF:molecular_function;MF:binding;MF:metal ion binding;	GO:0005575;GO:0043229;GO:0043226;GO:0110165;GO:0043169;GO:0043167;GO:0003674;GO:0005488;GO:0046872	K11426	SMYD	map00310	Lysine degradation	COG2940	S:Function unknown	PF01753.21	zf-MYND	MYND finger	CYT	1	1	56.4	Medium
TRINITY_DN18404_c0_g1_i5_orf1	periodic tryptophan protein 1 homolog isoform X1 [Ostrinia furnacalis] >XP_028157695.1 periodic tryptophan protein 1 homolog isoform X2 [Ostrinia furnacalis]	3.222687722	1.688264398	1.25E-06	up	yes	1	0.3103	0.283	0.318	0.33	1	1.008	0.993	BP:RNA metabolic process;BP:nucleobase-containing compound metabolic process;BP:rRNA metabolic process;BP:ncRNA processing;BP:primary metabolic process;BP:RNA processing;BP:cellular process;BP:cellular aromatic compound metabolic process;BP:cellular metabolic process;BP:macromolecule metabolic process;BP:nucleic acid metabolic process;BP:organic substance metabolic process;BP:cellular nitrogen compound metabolic process;BP:nitrogen compound metabolic process;BP:biological_process;BP:rRNA processing;BP:metabolic process;BP:ncRNA metabolic process;BP:organic cyclic compound metabolic process;BP:heterocycle metabolic process;	GO:0016070;GO:0006139;GO:0016072;GO:0034470;GO:0044238;GO:0006396;GO:0009987;GO:0006725;GO:0044237;GO:0043170;GO:0090304;GO:0071704;GO:0034641;GO:0006807;GO:0008150;GO:0006364;GO:0008152;GO:0034660;GO:1901360;GO:0046483	K14791	PWP1	------	------	ENOG410XT1V	S:Function unknown	PF00400.35	WD40	WD domain, G-beta repeat	CYT	1	2	51.1	High
TRINITY_DN542_c0_g1_i4_orf1	uncharacterized protein LOC114364889 [Ostrinia furnacalis]	2.956841139	1.564056734	8.32E-06	up	yes	0.966	0.3267	0.324	0.329	0.327	1	0.925	0.973	BP:amino sugar catabolic process;BP:nitrogen compound metabolic process;BP:aminoglycan metabolic process;BP:organic substance catabolic process;BP:glucosamine-containing compound catabolic process;BP:organic substance metabolic process;BP:chitin metabolic process;BP:polysaccharide catabolic process;BP:biological_process;BP:metabolic process;BP:organonitrogen compound metabolic process;BP:catabolic process;BP:macromolecule catabolic process;BP:primary metabolic process;BP:chitin catabolic process;BP:carbohydrate metabolic process;BP:organonitrogen compound catabolic process;BP:polysaccharide metabolic process;BP:amino sugar metabolic process;BP:carbohydrate derivative catabolic process;BP:carbohydrate catabolic process;BP:macromolecule metabolic process;BP:glucosamine-containing compound metabolic process;BP:aminoglycan catabolic process;BP:carbohydrate derivative metabolic process;CC:extracellular space;CC:cellular anatomical entity;CC:cellular_component;MF:hydrolase activity, acting on glycosyl bonds;MF:pullulanase activity;MF:molecular_function;MF:zinc ion binding;MF:cation binding;MF:transition metal ion binding;MF:ion binding;MF:metal ion binding;MF:hydrolase activity;MF:deacetylase activity;MF:catalytic activity;MF:hydrolase activity, acting on carbon-nitroge;MF:hydrolase activity, acting on carbon-nitroge[illegible]	GO:0046348;GO:0006807;GO:0006022;GO:1901575;GO:1901072;GO:0071704;GO:0006030;GO:0000272;GO:0008150;GO:0008152;GO:1901564;GO:0009056;GO:0009057;GO:0044238;GO:0006032;GO:0005975;GO:1901565;GO:0005976;GO:0006040;GO:1901136;GO:0016052;GO:0043170;GO:1901071;GO:0006026;GO:1901135;GO:0005615;GO:0110165;GO:0005575;GO:0016798;GO:0051060;GO:0003674;GO:0008270;GO:0043169;GO:0046914;GO:0043167;GO:0046872;GO:0016787;GO:0019213;GO:0003824;GO:0016810;GO:0016811;GO:0004553;GO:0004099;GO:0005488	------	------	------	------	ENOG410ZWU3	S:Function unknown	PF01522.24	Polysacc_deac_1	Polysaccharide deacetylase	CYT	5	14	44.8	High
TRINITY_DN2953_c1_g1_i2_orf1	methionine--tRNA ligase, cytoplasmic isoform X6 [Ostrinia furnacalis]	5.348492338	2.419132274	0.0001205	up	yes	1.082	0.2023	0.195	0.249	0.163	1	1.183	1.062	BP:methionyl-tRNA aminoacylation;BP:carboxylic acid metabolic process;BP:nucleic acid metabolic process;BP:cellular nitrogen compound metabolic process;BP:nitrogen compound metabolic process;BP:small molecule metabolic process;BP:ncRNA metabolic process;BP:organic cyclic compound metabolic process;BP:nucleobase-containing compound metabolic process;BP:cellular amino acid metabolic process;BP:organic substance metabolic process;BP:primary metabolic process;BP:cellular process;BP:cellular aromatic compound metabolic process;BP:biological_process;BP:metabolic process;BP:oxoacid metabolic process;BP:organonitrogen compound metabolic process;BP:heterocycle metabolic process;BP:RNA metabolic process;BP:organic acid metabolic process;BP:cellular metabolic process;BP:macromolecule metabolic process;BP:tRNA metabolic process;BP:amino acid activation;BP:tRNA aminoacylation;BP:tRNA aminoacylation for protein translation;CC:aminoacyl-tRNA synthetase multienzyme complex;CC:catalytic complex;CC:macromolecular complex;CC:cellular_component;MF:purine ribonucleoside triphosphate binding;MF:molecular_function;MF:ion binding;MF:heterocyclic compound binding;MF:anion binding;MF:aminoacyl-tRNA ligase activity;MF:nucleotide binding;MF:nucleoside phosphate binding;MF:purine nucleotide binding;MF:ATP binding;MF:catalytic activity, acting on RNA;MF:small molecule binding;MF:catalytic activity;MF:ligase activity;MF:ligase activity, forming carbon-oxygen bonds;MF:methionine-tRNA ligase activity;MF:adenyl nucleotide binding;MF:carbohydrate derivative binding;MF:organic cyclic compound binding;MF:adenyl ribonucleotide binding;MF:purine ribonucleotide binding;MF:catalytic activity, acting on a [illegible]	GO:0006431;GO:0019752;GO:0090304;GO:0034641;GO:0006807;GO:0044281;GO:0034660;GO:1901360;GO:0006139;GO:0006520;GO:0071704;GO:0044238;GO:0009987;GO:0006725;GO:0008150;GO:0008152;GO:0043436;GO:1901564;GO:0046483;GO:0016070;GO:0006082;GO:0044237;GO:0043170;GO:0006399;GO:0043038;GO:0043039;GO:0006418;GO:0017101;GO:1902494;GO:0032991;GO:0005575;GO:0035639;GO:0003674;GO:0043167;GO:1901363;GO:0043168;GO:0004812;GO:0000166;GO:1901265;GO:0017076;GO:0005524;GO:0140098;GO:0036094;GO:0003824;GO:0016874;GO:0016875;GO:0004825;GO:0030554;GO:0097367;GO:0097159;GO:0032559;GO:0032555;GO:0140101;GO:0032553;GO:0005488	K01874	MARS, metG	map00450;map	Selenocompound m	COG0143	J:Translation, ribosomal structure anc	PF00458.23	WHEP-TRS	WHEP-TRS domain	CYT	1	43	10.3	High
TRINITY_DN2825_c0_g1_i3_orf1	carbonic anhydrase 2-like [Ostrinia furnacalis]	4.464055101	2.158354835	3.65E-06	up	yes	1.037	0.2323	0.25	0.212	0.235	1	1.067	1.043	MF:cation binding;MF:metal ion binding;MF:transition metal ion binding;MF:carbon-oxygen lyase activity;MF:hydro-lyase activity;MF:ion binding;MF:catalytic activity;MF:molecular_function;MF:zinc ion binding;MF:carbonate dehydratase activity;MF:lyase activity;MF:binding;	GO:0043169;GO:0046872;GO:0046914;GO:0016835;GO:0016836;GO:0043167;GO:0003824;GO:0003674;GO:0008270;GO:0004089;GO:0016829;GO:0005488	K01672	CA	map00910	Nitrogen metabolisr	COG3338	I:Lipid transport and metabolism	PF00194.24	Carb_anhydrase	Eukaryotic-type carbonic anhydrase	CYT	2	8	30.6	High
TRINITY_DN4731_c0_g1_i1_orf1	gelsolin-like [Ostrinia furnacalis]	3.86599923	1.95084135	1.97E-07	up	yes	1.004	0.2597	0.248	0.263	0.268	1	1.019	0.992	------	------	K05768	GSN	map04666;map	Fc gamma R-media	ENOG410XR0A	O:Posttranslational modification, pro	PF00626.25	Gelsolin	Gelsolin repeat	CYT	7	15	72	High
TRINITY_DN36494_c0_g1_i1_orf1	MKI67 FHA domain-interacting nucleolar phosphoprotein-like [Ostrinia furnacalis]	3.888242142	1.959118066	8.50E-07	up	yes	1.002	0.2577	0.265	0.243	0.265	1	1.024	0.981	MF:organic cyclic compound binding;MF:molecular_function;MF:binding;MF:nucleic acid binding;MF:heterocyclic compound binding;MF:RNA binding;	GO:0097159;GO:0003674;GO:0005488;GO:0003676;GO:1901363;GO:0003723	K14838	NOP15	------	------	ENOG4111NF9	S:Function unknown	PF00076.25	RRM_1	RNA recognition motif.	CYT	2	6	30.6	High
TRINITY_DN1353_c0_g1_i1_orf1	UDP-glucose 4-epimerase-like [Ostrinia furnacalis]	3.262671233	1.70605362	6.11E-05	up	yes	0.9527	0.292	0.336	0.269	0.271	1	0.962	0.896	BP:primary metabolic process;BP:carbohydrate metabolic process;BP:organic substance metabolic process;BP:monosaccharide metabolic process;BP:hexose metabolic process;BP:galactose metabolic process;BP:biological_process;BP:small molecule metabolic process;BP:metabolic process;MF:racemase and epimerase activity;MF:isomerase activity;MF:catalytic activity;MF:racemase and epimerase activity, acting on carbohydrates and derivatives;MF:UDP-N-acetylglucosamine 4-epimerase activity;MF:molecular_function;MF:UDP-glucose 4-epimerase activity;	GO:0044238;GO:0005975;GO:0071704;GO:0005996;GO:0019318;GO:0006012;GO:0008150;GO:0044281;GO:0008152;GO:0016854;GO:0016853;GO:0003824;GO:0016857;GO:0003974;GO:0003674;GO:0003978	K01784	galE, GALE	map00520;map	Amino sugar and nu	COG1087	M:Cell wall/membrane/envelope bio	PF16363.8;PF0137	GDP_Man_Dehyd;Epim	GDP-mannose 4,6 dehydratase;NAD dependent epimerase/dehydratase family;Polysaccharide biosynthesis protein;3-beta hydroxysteroid dehydrogenase/isomerase family;RmlD substrate binding domain;Male sterility	CYT	2	8	40.3	High
TRINITY_DN69049_c0_g1_i2_orf1	membrane alanyl aminopeptidase-like [Ostrinia furnacalis]	4.193100547	2.068017425	3.35E-06	up	yes	0.9967	0.2377	0.223	0.251	0.239	1	1.028	0.962	CC:integral component of membrane;CC:cellular_component;CC:cellular anatomical entity;CC:intrinsic component of membrane;MF:exopeptidase activity;MF:aminopeptidase activity;MF:transition metal ion binding;MF:hydrolase activity;MF:catalytic activity, acting on a protein;MF:cation binding;MF:ion binding;MF:catalytic activity;MF:metallopeptidase activity;MF:molecular_function;MF:binding;MF:peptidase activity;MF:metal ion binding;MF:zinc ion binding;	GO:0016021;GO:0005575;GO:0110165;GO:0031224;GO:0008238;GO:0004177;GO:0046914;GO:0016787;GO:0140096;GO:0043169;GO:0043167;GO:0003824;GO:0008237;GO:0003674;GO:0005488;GO:0008233;GO:0046872;GO:0008270	K11140	ANPEP, CD13	map04640;map	Hematopoietic cell l	COG0308	E:Amino acid transport and metaboli	PF11838.11	ERAP1_C	ERAP1-like C-terminal domain	CYT	1	2	51.3	High
TRINITY_DN57202_c0_g1_i1_orf1	PREDICTED: U4/U6 small nuclear ribonucleoprotein Prp31 [Amyelois transitella]	3.141946309	1.651658527	3.82E-05	up	yes	0.9363	0.298	0.29	0.305	0.299	1	0.908	0.901	BP:cellular component assembly;BP:spliceosomal tri-snRNP complex assembly;BP:macromolecular complex subunit organization;BP:cellular component organization or biogenesis;BP:spliceosomal snRNP assembly;BP:cellular component organization;BP:macromolecular complex assembly;BP:ribonucleoprotein complex assembly;BP:cellular process;BP:ribonucleoprotein complex subunit organization;BP:biological_process;CC:Sm-like protein family complex;CC:U4/U6 x U5 tri-snRNP complex;CC:spliceosomal snRNP complex;CC:macromolecular complex;CC:small nuclear ribonucleoprotein complex;CC:ribonucleoprotein complex;CC:cellular_component;CC:spliceosomal tri-snRNP complex;CC:spliceosomal complex;MF:molecular_function;MF:binding;MF:nucleic acid binding;MF:heterocyclic compound binding;MF:organic cyclic compound binding;MF:RNA binding;	GO:0022607;GO:0000244;GO:0043933;GO:0071840;GO:0000387;GO:0016043;GO:0065003;GO:0022618;GO:0009987;GO:0071826;GO:0008150;GO:0120114;GO:0046540;GO:0097525;GO:0032991;GO:0030532;GO:1990904;GO:0005575;GO:0097526;GO:0005681;GO:0003674;GO:0005488;GO:0003676;GO:1901363;GO:0097159;GO:0003723	K12844	PRPF31	map03040	Spliceosome	COG1498	S:Function unknown	PF01798.21;PF097	Nop;Prp31_C	snoRNA binding domain, fibrillarin;Prp31 C terminal domain	CYT	1	3	55.5	High
TRINITY_DN19160_c0_g1_i1_orf1	alkyldihydroxyacetonephosphate synthase [Ostrinia furnacalis]	6.693699314	2.742803745	3.06E-05	up	yes	1.073	0.1603	0.147	0.186	0.148	1	1.074	1.144	------	------	K00803	AGPS, agpS	map00565;map	Ether lipid metabolis	COG0277	C:Energy production and conversion	PF02913.22;PF015	FAD-oxidase_C;FAD_bi	FAD linked oxidases, C-terminal domain;FAD binding domain	CYT	1	1	69.6	Medium
TRINITY_DN19244_c0_g1_i7_orf1	uncharacterized protein LOC114350218 [Ostrinia furnacalis]	3.945968072	1.980379283	3.72E-06	up	yes	0.964	0.2443	0.255	0.252	0.226	1	0.946	0.946	------	------	K16830	AURKAIP1	------	------	ENOG410Y2DU;COG	S:Function unknown;C:Energy produ	------	------	------	CYT	1	4	25.6	High
TRINITY_DN144807_c0_g1_i1_orf1	hypothetical protein G9C98_004245 [Cotesia typhae]	4.770539621	2.254152466	9.73E-05	up	yes	0.9813	0.2057	0.131	0.294	0.192	1	0.954	0.99	BP:L-methionine salvage;BP:carboxylic acid metabolic process;BP:L-methionine biosynthetic process;BP:cellular biosynthetic process;BP:nitrogen compound metabolic process;BP:aspartate family amino acid biosynthetic process;BP:small molecule metabolic process;BP:small molecule biosynthetic process;BP:L-methionine biosynthetic process from S-adenosylmethionine;BP:organic substance biosynthetic process;BP:L-methionine biosynthetic process from methylthioadenosine;BP:cellular amino acid metabolic process;BP:organic substance metabolic process;BP:amino acid salvage;BP:alpha-amino acid metabolic process;BP:sulfur amino acid metabolic process;BP:primary metabolic process;BP:sulfur amino acid biosynthetic process;BP:cellular process;BP:biosynthetic process;BP:biological_process;BP:metabolic process;BP:oxoacid metabolic process;BP:cellular metabolic compound salvage;BP:cellular amino acid biosynthetic process;BP:organonitrogen compound metabolic process;BP:organonitrogen compound biosynthetic process;BP:organic acid metabolic process;BP:sulfur compound biosynthetic process;BP:carboxylic acid biosynthetic process;BP:methionine metabolic process;BP:organic acid biosynthetic process;BP:cellular metabolic process;BP:sulfur compound metabolic process;BP:alpha-amino acid biosynthetic process;BP:methionine biosynthetic process;BP:aspartate family amino acid metabolic process;CC:intracellular organelle;CC:membrane-bounded organelle;CC:cytoplasm;CC:nucleus;CC:cellular anatomical entity;CC:intracellular membrane-bounded organelle;CC:cellular_component;CC:organelle;MF:binding;MF:magnesium ion binding;MF:phosphatase activity;MF:acireductone synthase activity;MF:phosphoric ester hydrolase activity;MF:intramolecular oxidoreductase activity, interconverting keto- and enol-groups;MF:intramolecular oxidoreductase activity;MF:2-hydroxy-3-keto-5-methylthiopentenyl-1-phosphate phosphatase activity;MF:2,3-diketo-5-methylthiopentyl-1-phosphate enolase activity;MF:ion binding;MF:cation binding;MF:metal ion binding;MF:hydrolase activity;MF:hydrolase activity, acting on ester bonds;MF:catalytic activity;MF:isomerase	GO:0071267;GO:0019752;GO:0071265;GO:0044249;GO:0006807;GO:0009067;GO:0044281;GO:0044283;GO:0019284;GO:1901576;GO:0019509;GO:0006520;GO:0071704;GO:0043102;GO:1901605;GO:0000096;GO:0044238;GO:0000097;GO:0009987;GO:0009058;GO:0008150;GO:0008152;GO:0043436;GO:0043094;GO:0008652;GO:1901564;GO:1901566;GO:0006082;GO:0044272;GO:0046394;GO:0006555;GO:0016053;GO:0044237;GO:0006790;GO:1901607;GO:0009086;GO:0009066;GO:0043229;GO:0043227;GO:0005737;GO:0005634;GO:0110165;GO:0043231;GO:0005575;GO:0043226;GO:0005488;GO:0000287;GO:0016791;GO:0043874;GO:0042578;GO:0016862;GO:0016860;GO:0043716;GO:0043715;GO:0043167;GO:0043169;GO:0046872;GO:0016787;GO:0016788;GO:0003824;GO:0016853;GO:0003674	K09880	mtnC, ENOPH	map00270	Cysteine and methic	COG4229	C:Energy production and conversion	------	------	------	CYT	1	1	75.4	Medium
TRINITY_DN75188_c0_g1_i1_orf1	fatty acid-binding protein 1-like [Ostrinia furnacalis]	5.597636466	2.484817795	1.43E-05	up	yes	0.9947	0.1777	0.189	0.189	0.155	1	0.94	1.044	MF:molecular_function;MF:lipid binding;MF:binding;	GO:0003674;GO:0008289;GO:0005488	K08753;K08752	FABP4, aP2;F/	map04923;map	Regulation of lipolys	ENOG4111US8	I:Lipid transport and metabolism	------	------	------	CYT	1	7	14.7	High
TRINITY_DN61112_c0_g1_i4_orfp1	TRINITY_DN61112_c0_g1_i4_m.53012 TRINITY_DN61112_c0_g1::TRINITY_DN61112_c0_g1_i4::g.53012 ORF type:3prime_partial len:87 (-),score=12.29,HMMR_N PF15905.6 0.0011 TRINITY_DN61112_c0_g1_i4:2-232(-)	5.186539481	2.374772277	8.38E-07	up	yes	0.9787	0.1887	0.175	0.181	0.21	1	0.971	0.965	------	------	------	------	------	------	------	------	------	------	------	CYT	3	39	9.2	High
TRINITY_DN237_c1_g1_i1_orf1	PREDICTED: cytoplasmic protein NCK1 isoform X1 [Microplitis demolitor]	2.717593825	1.442329845	7.06E-07	up	yes	1.021	0.3757	0.374	0.367	0.386	1	1.035	1.027	BP:developmental process;BP:morphogenesis of an epithelium;BP:cellular process;BP:cellular developmental process;BP:anatomical structure development;BP:cell development;BP:animal organ development;BP:biological_process;BP:tissue morphogenesis;BP:anatomical structure morphogenesis;BP:localization;	GO:0032502;GO:0002009;GO:0009987;GO:0048869;GO:0048856;GO:0048468;GO:0048513;GO:0008150;GO:0048729;GO:0009653;GO:0051179	K19862	NCK2, GRB4	map04360;map	Axon guidance;Axor	ENOG410XRPF	T:Signal transduction mechanisms	PF00018.31;PF146	SH3_1;SH3_9;SH3_2;SH	SH3 domain;Variant SH3 domain;Variant SH3 domain;SH2 domain;Bacterial SH3 domain;SH3 domain	PLA	2	3	44.1	High
TRINITY_DN37538_c0_g4_i1_orf1	esterase FE4-like [Ostrinia furnacalis]	2.509571788	1.327441216	1.08E-06	up	yes	0.9963	0.397	0.393	0.414	0.384	1	1.009	0.98	------	------	------	------	------	------	COG2272;COG0580	I:Lipid transport and metabolism;G:C	PF00135.31	COesterase	Carboxylesterase family	CYT	1	12	12.7	High
TRINITY_DN1330_c0_g1_i1_orf1	pancreatic triacylglycerol lipase-like [Ostrinia furnacalis]	2.71263751	1.439696273	9.31E-06	up	yes	1.011	0.3727	0.356	0.38	0.382	1	0.982	1.052	CC:cellular_component;CC:extracellular region;CC:cellular anatomical entity;MF:hydrolase activity;MF:hydrolase activity, acting on ester bonds;MF:catalytic activity;MF:molecular_function;MF:lipase activity;	GO:0005575;GO:0005576;GO:0110165;GO:0016787;GO:0016788;GO:0003824;GO:0003674;GO:0016298	------	------	------	------	ENOG41116Y2;ENO(	S:Function unknown;	PF00151.22	Lipase	Lipase	CYT	7	15	65	High
TRINITY_DN6933_c1_g1_i1_orf1	Chlorophyll a-b binding protein 40, chloroplastic [Trichinella nelsoni] >KRY99282.1 Chlorophyll a-b binding protein 40, chloroplastic [Trichinella zimbabwensis]	2.509438711	1.327364711	0.0001128	up	yes	0.997	0.3973	0.413	0.401	0.378	1	0.929	1.062	CC:cellular_component;CC:membrane;CC:cellular anatomical entity;	GO:0005575;GO:0016020;GO:0110165	K08913	LHCB2	------	------	ENOG410ZHBU	S:Function unknown	PF00504.24	Chloroa_b-bind	Chlorophyll A-B binding protein	CYT	2	8	24	High
TRINITY_DN1277_c4_g1_i5_orf1	GTP cyclohydrolase 1 isoform X1 [Ostrinia furnacalis] >XP_028166842.1 GTP cyclohydrolase 1 isoform X1 [Ostrinia furnacalis]	2.791208791	1.480890047	1.42E-06	up	yes	1.016	0.364	0.354	0.386	0.352	1	1.032	1.015	BP:cellular modified amino acid biosynthetic process;BP:dihydrobiopterin metabolic process;BP:polyol biosynthetic process;BP:polyol metabolic process;BP:diol metabolic process;BP:cellular biosynthetic process;BP:cellular nitrogen compound metabolic process;BP:diol biosynthetic process;BP:small molecule metabolic process;BP:small molecule biosynthetic process;BP:tetrahydrofolate metabolic process;BP:organic cyclic compound biosynthetic process;BP:tetrahydrofolate biosynthetic process;BP:organic cyclic compound metabolic process;BP:organic substance biosynthetic process;BP:folic acid-containing compound biosynthetic process;BP:7,8-dihydroneopterin 3'-triphosphate biosynthetic process;BP:pteridine-containing compound metabolic process;BP:pteridine-containing compound biosynthetic process;BP:organic substance metabolic process;BP:heterocycle biosynthetic process;BP:tetrahydrobiopterin biosynthetic process;BP:folic acid-containing compound metabolic process;BP:alcohol biosynthetic process;BP:cellular process;BP:cellular aromatic compound metabolic process;BP:biosynthetic process;BP:tetrahydrobiopterin metabolic process;BP:biological_process;BP:metabolic process;BP:organonitrogen compound metabolic process;BP:organophosphate biosynthetic process;BP:heterocycle metabolic process;BP:cellular nitrogen compound biosynthetic process;BP:cellular modified amino acid metabolic process;BP:organonitrogen compound biosynthetic process;BP:cellular metabolic process;BP:alcohol metabolic process;BP:phosphate-containing compound metabolic process;BP:nitrogen compound metabolic process;BP:phosphorus metabolic process;BP:organophosphate metabolic process;BP:aromatic compound biosynthetic process;BP:organic hydroxy compound biosynthetic process;BP:organic hydroxy compound metabolic process;MF:GTP cyclohydrolase activity;MF:molecular_function;MF:hydrolase activity, acting on carbon-nitrogen;MF:cyclohydrolase activity;MF:hydrolase activity;MF:GTP cyclohydrolase I activity;MF:catalytic activity;MF:hydrolase activity, acting on carbon-nitroge;	GO:0042398;GO:0051066;GO:0046173;GO:0019751;GO:0034311;GO:0044249;GO:0034641;GO:0034312;GO:0044281;GO:0044283;GO:0046653;GO:1901362;GO:0046654;GO:1901360;GO:1901576;GO:0009396;GO:0035998;GO:0042558;GO:0042559;GO:0071704;GO:0018130;GO:0006729;GO:0006760;GO:0046165;GO:0009987;GO:0006725;GO:0009058;GO:0046146;GO:0008150;GO:0008152;GO:1901564;GO:0090407;GO:0046483;GO:0044271;GO:0006575;GO:1901566;GO:0044237;GO:0006066;GO:0006796;GO:0006807;GO:0006793;GO:0019637;GO:0019438;GO:1901617;GO:1901615;GO:0003933;GO:0003674;GO:0016814;GO:0019238;GO:0016787;GO:0003934;GO:0003824;GO:0016810	K01495	GCH1, folE	map00790;map	Folate biosynthesis;E	COG0302	H:Coenzyme transport and metaboli	PF01227.25	GTP_cyclohydrol	GTP cyclohydrolase I	CYT	5	19	32.6	High
TRINITY_DN6535_c0_g2_i1_orf1	NADH dehydrogenase [ubiquinone] 1 beta subcomplex subunit 3 [Ostrinia furnacalis] >XP_028166399.1 NADH dehydrogenase [ubiquinone] 1 beta subcomplex subunit 3 [Ostrinia furnacalis]	2.409346709	1.268642015	2.01E-05	up	yes	0.9847	0.4087	0.429	0.421	0.376	1	1.006	0.948	BP:cellular process;BP:cellular metabolic process;BP:electron transport chain;BP:biological_process;BP:metabolic process;BP:generation of precursor metabolites and energy;CC:organelle membrane;CC:integral component of membrane;CC:membrane;CC:organelle inner membrane;CC:mitochondrial membrane;CC:cellular_component;CC:mitochondrial inner membrane;CC:intrinsic component of membrane;CC:respiratory chain;CC:cellular anatomical entity;	GO:0009987;GO:0044237;GO:0022900;GO:0008150;GO:0008152;GO:0006091;GO:0031090;GO:0016021;GO:0016020;GO:0019866;GO:0031966;GO:0005575;GO:0005743;GO:0031224;GO:0070469;GO:0110165	K03959	NDUFB3	map04714;map	Thermogenesis;Oxic	ENOG411294X	I:Lipid transport and metabolism	PF08122.15	NDUF_B12	NADH-ubiquinone oxidoreductase B12 subunit family	CYT	4	39	11	High
TRINITY_DN948_c0_g1_i1_orf1	mitochondrial-processing peptidase subunit beta [Ostrinia furnacalis]	2.610191752	1.384155795	5.68E-08	up	yes	0.9937	0.3807	0.386	0.374	0.382	1	0.997	0.984	BP:primary metabolic process;BP:organonitrogen compound metabolic process;BP:protein metabolic process;BP:macromolecule metabolic process;BP:organic substance metabolic process;BP:nitrogen compound metabolic process;BP:biological_process;BP:metabolic process;BP:proteolysis;MF:molecular_function;MF:endopeptidase activity;MF:cation binding;MF:hydrolase activity;MF:ion binding;MF:catalytic activity, acting on a protein;MF:catalytic activity;MF:metalloendopeptidase activity;MF:binding;MF:peptidase activity;MF:metal ion binding;MF:metallopeptidase activity;	GO:0044238;GO:1901564;GO:0019538;GO:0043170;GO:0071704;GO:0006807;GO:0008150;GO:0008152;GO:0006508;GO:0003674;GO:0004175;GO:0043169;GO:0016787;GO:0043167;GO:0140096;GO:0003824;GO:0004222;GO:0005488;GO:0008233;GO:0046872;GO:0008237	K17732	PMPCB, MAS	------	------	COG0612	O:Posttranslational modification, pro	PF05193.24;PF006	Peptidase_M16_C;Pept	Peptidase M16 inactive domain;Insulinase	CYT	13	44	37.5	High
TRINITY_DN86149_c0_g1_i1_orf1	NADH dehydrogenase [ubiquinone] 1 alpha subcomplex subunit 8 [Galleria mellonella]	2.560102302	1.356201462	1.44E-07	up	yes	1.001	0.391	0.389	0.387	0.397	1	1.014	0.99	BP:electron transport chain;BP:cellular process;BP:aerobic electron transport chain;BP:mitochondrial electron transport, NADH to ubiquinone;BP:aerobic respiration;BP:cellular respiration;BP:cellular metabolic process;BP:metabolic process;BP:respiratory electron transport chain;BP:biological_process;BP:generation of precursor metabolites and energy;BP:energy derivation by oxidation of organic compounds;CC:intracellular membrane-bounded organelle;CC:mitochondrion;CC:cellular_component;CC:intracellular organelle;CC:membrane-bounded organelle;CC:organelle;CC:cellular anatomical entity;	GO:0022900;GO:0009987;GO:0019646;GO:0006120;GO:0009060;GO:0045333;GO:0044237;GO:0008152;GO:0022904;GO:0008150;GO:0006091;GO:0015980;GO:0043231;GO:0005739;GO:0005575;GO:0043229;GO:0043227;GO:0043226;GO:0110165	K03952	NDUFA8	map04714;map	Thermogenesis;Oxic	ENOG4111IST	I:Lipid transport and metabolism	------	------	------	EXC	6	42	20.1	High
TRINITY_DN30233_c0_g1_i2_orf1	39S ribosomal protein L10, mitochondrial [Ostrinia furnacalis]	2.22627907	1.15463445	0.0001114	up	yes	0.9573	0.43	0.382	0.477	0.431	1	0.931	0.941	CC:ribosome;CC:intracellular non-membrane-bounded organelle;CC:cellular_component;CC:intracellular organelle;CC:non-membrane-bounded organelle;CC:organelle;CC:cellular anatomical entity;	GO:0005840;GO:0043232;GO:0005575;GO:0043229;GO:0043228;GO:0043226;GO:0110165	K02864	RP-L10, MRPL	map03010	Ribosome	ENOG4111GA4	J:Translation, ribosomal structure anc	------	------	------	CYT	1	2	27.9	High
TRINITY_DN40704_c0_g1_i2_orf1	COX assembly mitochondrial protein homolog [Ostrinia furnacalis]	2.420763664	1.275462238	2.62E-05	up	yes	0.97	0.4007	0.408	0.433	0.361	1	0.961	0.949	CC:intracellular membrane-bounded organelle;CC:mitochondrion;CC:cellular_component;CC:intracellular organelle;CC:membrane-bounded organelle;CC:organelle;CC:cellular anatomical entity;	GO:0043231;GO:0005739;GO:0005575;GO:0043229;GO:0043227;GO:0043226;GO:0110165	K18171	CMC1	------	------	ENOG4111YK0	S:Function unknown	PF08583.13	Cmc1	Cytochrome c oxidase biogenesis protein Cmc1 like	CYT	1	9	13.6	High
TRINITY_DN12133_c0_g2_i1_orf1	O-acyltransferase like protein-like [Ostrinia furnacalis]	2.467171717	1.302858132	0.0001335	up	yes	0.977	0.396	0.336	0.412	0.44	1	1.005	0.926	CC:integral component of membrane;CC:cellular_component;CC:intrinsic component of membrane;CC:cellular anatomical entity;MF:catalytic activity;MF:transferase activity;MF:transferase activity, transferring acyl groups;MF:molecular_function;MF:transferase activity, transferring acyl groups other than amino-acyl groups;	GO:0016021;GO:0005575;GO:0031224;GO:0110165;GO:0003824;GO:0016740;GO:0016746;GO:0003674;GO:0016747	------	------	------	------	ENOG410YD1T;ENO(	S:Function unknown;	PF01757.25;PF201	Acyl_transf_3;NRF	Acyltransferase family;Nose resistant-to-fluoxetine protein, N-terminal domain	CYT	1	1	84.1	High
TRINITY_DN22664_c0_g1_i1_orf1	larval cuticle protein LCP-14-like [Ostrinia furnacalis]	3.02049302	1.594784053	5.15E-07	up	yes	1.017	0.3367	0.334	0.345	0.331	1	1.013	1.037	MF:molecular_function;MF:structural constituent of cuticle;MF:structural molecule activity;	GO:0003674;GO:0042302;GO:0005198	------	------	------	------	ENOG4111PJ1;ENOG	S:Function unknown;	PF00379.26	Chitin_bind_4	Insect cuticle protein	CYT	6	52	19.3	High
TRINITY_DN21494_c0_g1_i2_orf1	pancreatic triacylglycerol lipase-like [Ostrinia furnacalis]	2.799555185	1.485197619	1.66E-05	up	yes	1.007	0.3597	0.352	0.364	0.363	1	1.055	0.965	BP:organic substance metabolic process;BP:lipid metabolic process;BP:primary metabolic process;BP:metabolic process;BP:biological_process;CC:cellular_component;CC:extracellular region;CC:cellular anatomical entity;MF:hydrolase activity;MF:hydrolase activity, acting on ester bonds;MF:catalytic activity;MF:molecular_function;MF:lipase activity;	GO:0071704;GO:0006629;GO:0044238;GO:0008152;GO:0008150;GO:0005575;GO:0005576;GO:0110165;GO:0016787;GO:0016788;GO:0003824;GO:0003674;GO:0016298	------	------	------	------	ENOG41116Y2;ENO(	S:Function unknown;	PF00151.22	Lipase	Lipase	CYT	1	4	35.8	High
TRINITY_DN12497_c0_g1_i1_orf1	probable N-acetyltransferase san [Ostrinia furnacalis]	2.841328413	1.506565594	1.36E-06	up	yes	1.001	0.3523	0.328	0.353	0.376	1	1.007	0.997	MF:transferase activity;MF:catalytic activity;MF:acetyltransferase activity;MF:transferase activity, transferring acyl groups;MF:transferase activity, transferring acyl groups other than amino-acyl groups;MF:molecular_function;	GO:0016740;GO:0003824;GO:0016407;GO:0016746;GO:0016747;GO:0003674	K20793	NAA50, NAT5	------	------	COG0456	S:Function unknown	PF00583.28;PF135	Acetyltransf_1;Acetyltra	Acetyltransferase;Acetyltransferase;Acetyltransferase;FR47-like protein;Acetyltransferase	CYT	5	36	20.2	High
TRINITY_DN107288_c0_g1_i2_orf1	methionine-tRNA synthetase, partial [Papilio xuthus]	2.261046095	1.176990404	5.78E-05	up	yes	0.9467	0.4187	0.42	0.396	0.44	1	0.918	0.922	BP:methionyl-tRNA aminoacylation;BP:prolyl-tRNA aminoacylation;BP:carboxylic acid metabolic process;BP:nucleic acid metabolic process;BP:cellular nitrogen compound metabolic process;BP:nitrogen compound metabolic process;BP:small molecule metabolic process;BP:ncRNA metabolic process;BP:organic cyclic compound metabolic process;BP:nucleobase-containing compound metabolic process;BP:cellular amino acid metabolic process;BP:organic substance metabolic process;BP:primary metabolic process;BP:cellular process;BP:cellular aromatic compound metabolic process;BP:biological_process;BP:metabolic process;BP:oxoacid metabolic process;BP:organonitrogen compound metabolic process;BP:heterocycle metabolic process;BP:RNA metabolic process;BP:organic acid metabolic process;BP:cellular metabolic process;BP:macromolecule metabolic process;BP:tRNA metabolic process;BP:amino acid activation;BP:tRNA aminoacylation;BP:tRNA aminoacylation for protein translation;CC:aminoacyl-tRNA synthetase multienzyme complex;CC:cytoplasm;CC:cellular anatomical entity;CC:catalytic complex;CC:macromolecular complex;CC:cellular_component;MF:purine ribonucleoside triphosphate binding;MF:molecular_function;MF:ion binding;MF:heterocyclic compound binding;MF:aminoacyl-tRNA ligase activity;MF:nucleotide binding;MF:nucleoside phosphate binding;MF:purine nucleotide binding;MF:anion binding;MF:catalytic activity, acting on RNA;MF:small molecule binding;MF:catalytic activity;MF:ligase activity;MF:ligase activity, forming carbon-oxygen bonds;MF:methionine-tRNA ligase activity;MF:proline-tRNA ligase activity;MF:adenyl nucleotide binding;MF:carbohydrate derivative binding;MF:organic cyclic compound binding;MF:adenyl ribonucleotide binding;MF:ATP binding;MF:purine ribonucleotide binding;MF:catalytic activity, acting on a tRNA;MF:ribonucleotide binding;MF:binding;	GO:0006431;GO:0006433;GO:0019752;GO:0090304;GO:0034641;GO:0006807;GO:0044281;GO:0034660;GO:1901360;GO:0006139;GO:0006520;GO:0071704;GO:0044238;GO:0009987;GO:0006725;GO:0008150;GO:0008152;GO:0043436;GO:1901564;GO:0046483;GO:0016070;GO:0006082;GO:0044237;GO:0043170;GO:0006399;GO:0043038;GO:0043039;GO:0006418;GO:0017101;GO:0005737;GO:0110165;GO:1902494;GO:0032991;GO:0005575;GO:0035639;GO:0003674;GO:0043167;GO:1901363;GO:0004812;GO:0000166;GO:1901265;GO:0017076;GO:0043168;GO:0140098;GO:0036094;GO:0003824;GO:0016874;GO:0016875;GO:0004825;GO:0004827;GO:0030554;GO:0097367;GO:0097159;GO:0032559;GO:0005524;GO:0032555;GO:0140101;GO:0032553;GO:0005488	K01874	MARS, metG	map00450;map	Selenocompound m	COG0143	J:Translation, ribosomal structure anc	PF00458.23	WHEP-TRS	WHEP-TRS domain	CYT	1	44	7.7	High

TRINITY_DN4842_c0_g1_i5_orf1	cytochrome c oxidase assembly factor 4 homolog, mitochondrial isoform X1 [Ostrinia furnacalis] >XP_028162331.1 cytochrome c oxidase assembly factor 4 homolog, mitochondrial isoform X2 [Ostrinia furnacalis]	3.126344912	1.644476952	5.33E-06	up	yes	1.017	0.3253	0.328	0.302	0.346	1	1	1.051	BP:mitochondrial respiratory chain complex assembly;BP:mitochondrial respiratory chain complex IV assembly;BP:cellular component assembly;BP:macromolecular complex subunit organization;BP:cellular process;BP:respiratory chain complex IV assembly;BP:biological_process;BP:cytochrome complex assembly;BP:cellular component organization or biogenesis;BP:macromolecular complex assembly;BP:cellular component organization;CC:organelle lumen;CC:organelle envelope lumen;CC:membrane-enclosed lumen;CC:cellular_component;CC:intracellular organelle lumen;CC:mitochondrial intermembrane space;CC:cellular anatomical entity;	GO:0033108;GO:0033617;GO:0022607;GO:0043933;GO:0009987;GO:0008535;GO:0008150;GO:0017004;GO:0071840;GO:0065003;GO:0016043;GO:0043233;GO:0031970;GO:0031974;GO:0005575;GO:0070013;GO:0005758;GO:0110165	K18177	COA4	map04714	Thermogenesis	ENOG410XXMU;CO(	O:Posttranslational modification, pro	------	------	------	CYT	1	13	8.2	High
TRINITY_DN2579_c0_g1_i7_orf1	aminopeptidase N5 [Ostrinia nubilalis]	2.60111023	1.379127539	4.98E-07	up	yes	0.984	0.3783	0.366	0.386	0.383	1	0.973	0.979	BP:nitrogen compound metabolic process;BP:organic substance metabolic process;BP:proteolysis;BP:biological_process;BP:metabolic process;BP:primary metabolic process;BP:organonitrogen compound metabolic process;BP:protein metabolic process;BP:macromolecule metabolic process;CC:integral component of membrane;CC:intrinsic component of membrane;CC:cellular anatomical entity;CC:cellular_component;MF:aminopeptidase activity;MF:exopeptidase activity;MF:molecular_function;MF:binding;MF:peptidase activity;MF:metallopeptidase activity;MF:cation binding;MF:transition metal ion binding;MF:ion binding;MF:metal ion binding;MF:hydrolase activity;MF:catalytic activity, acting on a protein;MF:catalytic activity;MF:zinc ion binding;	GO:0006807;GO:0071704;GO:0006508;GO:0008150;GO:0008152;GO:0044238;GO:1901564;GO:0019538;GO:0043170;GO:0016021;GO:0031224;GO:0110165;GO:0005575;GO:0004177;GO:0008238;GO:0003674;GO:0005488;GO:0008233;GO:0008237;GO:0043169;GO:0046914;GO:0043167;GO:0046872;GO:0016787;GO:0140096;GO:0003824;GO:0008270	K11140	ANPEP, CD13	map04640;map	Hematopoietic cell l	COG0308	L:Replication, recombination and rep	PF01433.23;PF118	Peptidase_M1;ERAP1_(	Peptidase family M1 domain;ERAP1-like C-terminal domain;Peptidase M1 N-terminal domain	CYT	3	3	108	High
TRINITY_DN1285_c0_g2_i1_orf1	bifunctional 3'-phosphoadenosine 5'-phosphosulfate synthase isoform X3 [Ostrinia furnacalis]	2.588915792	1.372348041	7.06E-05	up	yes	0.9623	0.3717	0.351	0.361	0.403	1	0.901	0.986	------	------	K13811	PAPSS	map00450;map	Selenocompound m	COG0529;COG2046	P:Inorganic ion transport and metab	PF01583.23;PF143	APS_kinase;PUA_2;AAA	Adenylylsulphate kinase;PUA-like domain;AAA	CYT	3	14	32.9	High
TRINITY_DN15900_c0_g1_i6_orf1	unnamed protein product [Diatraea saccharalis]	3.081342607	1.623559101	0.0001051	up	yes	1.019	0.3307	0.302	0.355	0.335	1	1.1	0.958	BP:RNA metabolic process;BP:mRNA metabolic process;BP:primary metabolic process;BP:nucleobase-containing compound metabolic process;BP:cellular process;BP:cellular aromatic compound metabolic process;BP:cellular metabolic process;BP:macromolecule metabolic process;BP:nucleic acid metabolic process;BP:organic substance metabolic process;BP:cellular nitrogen compound metabolic process;BP:nitrogen compound metabolic process;BP:biological_process;BP:organic cyclic compound metabolic process;BP:metabolic process;BP:heterocycle metabolic process;BP:RNA processing;BP:mRNA processing;	GO:0016070;GO:0016071;GO:0044238;GO:0006139;GO:0009987;GO:0006725;GO:0044237;GO:0043170;GO:0090304;GO:0071704;GO:0034641;GO:0006807;GO:0008150;GO:1901360;GO:0008152;GO:0046483;GO:0006396;GO:0006397	K13171	SRRM1, SRM1	map03013;map	Nucleocytoplasmic t	ENOG4111IMU	S:Function unknown	PF01480.20	PWI	PWI domain	CYT	2	2	111.5	High
TRINITY_DN1775_c0_g1_i3_orf1	ATP-dependent RNA helicase dbp2-like isoform X1 [Ostrinia furnacalis]	3.140186916	1.650850436	2.04E-07	up	yes	1.008	0.321	0.336	0.318	0.309	1	1.017	1.008	------	------	K12823	DDX5, DBP2	map05205;map	Proteoglycans in car	COG0513	L:Replication, recombination and rep	PF00270.32;PF002	DEAD;Helicase_C	DEAD/DEAH box helicase;Helicase conserved C-terminal domain	CYT	13	24	92.9	High
TRINITY_DN3062_c0_g1_i1_orf1	HEAT repeat-containing protein 1 [Ostrinia furnacalis]	3.00297619	1.586393036	1.08E-06	up	yes	1.009	0.336	0.321	0.361	0.326	1	1.007	1.02	BP:nucleic acid metabolic process;BP:cellular nitrogen compound metabolic process;BP:nitrogen compound metabolic process;BP:ncRNA metabolic process;BP:organic cyclic compound metabolic process;BP:nucleobase-containing compound metabolic process;BP:organic substance metabolic process;BP:rRNA processing;BP:ncRNA processing;BP:cellular process;BP:cellular aromatic compound metabolic process;BP:biological_process;BP:metabolic process;BP:heterocycle metabolic process;BP:RNA metabolic process;BP:primary metabolic process;BP:rRNA metabolic process;BP:cellular metabolic process;BP:macromolecule metabolic process;BP:RNA processing;CC:organelle;CC:nucleolus;CC:cellular anatomical entity;CC:macromolecular complex;CC:intracellular non-membrane-bounded organelle;CC:ribonucleoprotein complex;CC:intracellular organelle;CC:cellular_component;CC:non-membrane-bounded organelle;	GO:0090304;GO:0034641;GO:0006807;GO:0034660;GO:1901360;GO:0006139;GO:0071704;GO:0006364;GO:0034470;GO:0009987;GO:0006725;GO:0008150;GO:0008152;GO:0046483;GO:0016070;GO:0044238;GO:0016072;GO:0044237;GO:0043170;GO:0006396;GO:0043226;GO:0005730;GO:0110165;GO:0032991;GO:0043232;GO:1990904;GO:0043229;GO:0005575;GO:0043228	K14550	UTP10, HEATR	map03008	Ribosome biogenesi	ENOG410XNQQ;ENC	J:Translation, ribosomal structure anc	PF08146.15;PF123	BP28CT;U3snoRNP10	BP28CT;U3 small nucleolar RNA-associated protein 10	CYT	4	2	240.8	High
TRINITY_DN11069_c0_g2_i1_orf1	fat storage-inducing transmembrane protein [Ostrinia furnacalis]	3.32248617	1.732263194	5.93E-05	up	yes	1.021	0.3073	0.307	0.313	0.302	1	0.965	1.099	BP:lipid storage;BP:cellular component organization;BP:cellular component organization or biogenesis;BP:cellular process;BP:biological_process;BP:maintenance of location;BP:localization;BP:organelle organization;BP:lipid particle organization;CC:endoplasmic reticulum membrane;CC:intrinsic component of membrane;CC:organelle membrane;CC:integral component of membrane;CC:membrane;CC:cellular anatomical entity;CC:cellular_component;MF:molecular_function;MF:hydrolase activity;MF:catalytic activity;MF:hydrolase activity, acting on acid anhydrides, in phosphorus-containing anhydrides;MF:hydrolase activity, acting on acid anhydrides;MF:pyrophosphatase activity;	GO:0019915;GO:0016043;GO:0071840;GO:0009987;GO:0008150;GO:0051235;GO:0051179;GO:0006996;GO:0034389;GO:0005789;GO:0031224;GO:0031090;GO:0016021;GO:0016020;GO:0110165;GO:0005575;GO:0003674;GO:0016787;GO:0003824;GO:0016818;GO:0016817;GO:0016462	------	------	------	------	ENOG4111CNJ	S:Function unknown	PF10261.12	Scs3p	Inositol phospholipid synthesis and fat-storage-inducing TM	CYT	2	6	42.2	High
TRINITY_DN55160_c0_g2_i1_orf1	esterase FE4-like isoform X2 [Ostrinia furnacalis]	6.1352657	2.617125824	0.0002089	up	yes	1.143	0.1863	0.165	0.169	0.225	1	1.217	1.213	------	------	------	------	------	------	COG2272	I:Lipid transport and metabolism	PF00135.31	COesterase	Carboxylesterase family	CYT	1	4	34.3	High
TRINITY_DN657_c0_g1_i2_orf1	cytochrome c-type heme lyase [Ostrinia furnacalis]	3.230130865	1.691592615	1.99E-07	up	yes	1.012	0.3133	0.325	0.315	0.3	1	1.02	1.015	------	------	K01764	HCCS	map00860	Porphyrin metabolis	ENOG410YAUW	O:Posttranslational modification, pro	PF01265.20	Cyto_heme_lyase	Cytochrome c/c1 heme lyase	CYT	2	7	31.6	High
TRINITY_DN9119_c0_g1_i3_orf1	actin-binding Rho-activating protein [Helicoverpa armigera] >XP_047029227.1 actin-binding Rho-activating protein-like [Helicoverpa zea] >PZC85229.1 hypothetical protein B5X24_HaOG202414 [Helicoverpa armigera]	2.605801105	1.38172697	0.0001492	up	yes	0.9433	0.362	0.345	0.353	0.388	1	0.962	0.868	BP:regulation of Rho protein signal transduction;BP:regulation of response to stimulus;BP:positive regulation of Ras protein signal transduction;BP:positive regulation of signaling;BP:positive regulation of Rho protein signal transduction;BP:regulation of signaling;BP:positive regulation of cell communication;BP:regulation of cell communication;BP:regulation of biological process;BP:regulation of signal transduction;BP:positive regulation of signal transduction;BP:biological regulation;BP:positive regulation of biological process;BP:positive regulation of small GTPase mediated signal transduction;BP:regulation of small GTPase mediated signal transduction;BP:positive regulation of response to stimulus;BP:regulation of Ras protein signal transduction;BP:regulation of cellular process;BP:biological_process;BP:positive regulation of intracellular signal transduction;BP:regulation of intracellular signal transduction;BP:positive regulation of cellular process;MF:molecular_function;MF:binding;MF:actin binding;MF:cytoskeletal protein binding;MF:protein binding;	GO:0035023;GO:0048583;GO:0046579;GO:0023056;GO:0035025;GO:0023051;GO:0010647;GO:0010646;GO:0050789;GO:0009966;GO:0009967;GO:0065007;GO:0048518;GO:0051057;GO:0051056;GO:0048584;GO:0046578;GO:0050794;GO:0008150;GO:1902533;GO:1902531;GO:0048522;GO:0003674;GO:0005488;GO:0003779;GO:0008092;GO:0005515	------	------	------	------	ENOG410XR7V	S:Function unknown	PF14705.9	Costars	Costars	CYT	1	4	21.7	High
TRINITY_DN5238_c0_g1_i2_orf1	DNA-(apurinic or apyrimidinic site) lyase [Ostrinia furnacalis]	3.242806337	1.697242867	9.62E-06	up	yes	1.003	0.3093	0.352	0.28	0.296	1	1.025	0.985	BP:nucleic acid metabolic process;BP:cellular nitrogen compound metabolic process;BP:nitrogen compound metabolic process;BP:organic cyclic compound metabolic process;BP:nucleobase-containing compound metabolic process;BP:cellular response to stimulus;BP:cellular macromolecule metabolic process;BP:organic substance metabolic process;BP:DNA repair;BP:cellular process;BP:cellular aromatic compound metabolic process;BP:cellular response to DNA damage stimulus;BP:response to stress;BP:biological_process;BP:metabolic process;BP:heterocycle metabolic process;BP:primary metabolic process;BP:response to stimulus;BP:cellular metabolic process;BP:macromolecule metabolic process;BP:cellular response to stress;BP:DNA metabolic process;MF:cation binding;MF:molecular_function;MF:binding;MF:nucleic acid binding;MF:DNA binding;MF:heterocyclic compound binding;MF:ion binding;MF:metal ion binding;MF:hydrolase activity;MF:hydrolase activity, acting on ester bonds;MF:catalytic activity;MF:nuclease activity;MF:endonuclease activity;MF:organic cyclic compound binding;	GO:0090304;GO:0034641;GO:0006807;GO:1901360;GO:0006139;GO:0051716;GO:0044260;GO:0071704;GO:0006281;GO:0009987;GO:0006725;GO:0006974;GO:0006950;GO:0008150;GO:0008152;GO:0046483;GO:0044238;GO:0050896;GO:0044237;GO:0043170;GO:0033554;GO:0006259;GO:0043169;GO:0003674;GO:0005488;GO:0003676;GO:0003677;GO:1901363;GO:0043167;GO:0046872;GO:0016787;GO:0016788;GO:0003824;GO:0004518;GO:0004519;GO:0097159	K10771	APEX1	map03410	Base excision repair	COG0708	L:Replication, recombination and rep	PF03372.26	Exo_endo_phos	Endonuclease/Exonuclease/phosphatase family	CYT	5	11	73.1	High
TRINITY_DN94248_c0_g2_i3_orf1	uncharacterized protein LOC114357292 isoform X4 [Ostrinia furnacalis]	3.008307692	1.588952135	1.86E-06	up	yes	0.9777	0.325	0.305	0.341	0.329	1	0.968	0.965	------	------	------	------	------	------	COG1028;ENOG410)	C:Energy production and conversion;	PF13561.9;PF0010	adh_short_C2;adh_shor	Enoyl;short chain dehydrogenase;KR domain	CYT	7	29	28.7	High
TRINITY_DN49508_c0_g2_i8_orf1	putative fatty acyl-CoA reductase CG5065 [Ostrinia furnacalis]	2.966230605	1.568630762	2.09E-05	up	yes	0.975	0.3287	0.292	0.355	0.339	1	0.991	0.934	BP:primary metabolic process;BP:lipid metabolic process;BP:fatty acid derivative metabolic process;BP:organic substance metabolic process;BP:biological_process;BP:metabolic process;CC:cellular_component;CC:cellular anatomical entity;CC:integral component of membrane;CC:intrinsic component of membrane;MF:fatty-acyl-CoA reductas;MF:alcohol-forming fatty acyl-CoA reductase activity;MF:catalytic activity;MF:oxidoreductase activity, acting on the aldehyde or oxo group of donors, NAD or NADP as acceptor;MF:oxidoreductase activity, acting on the aldehyde or oxo group of donors;MF:molecular_function;MF:oxidoreductase activity;	GO:0044238;GO:0006629;GO:1901568;GO:0071704;GO:0008150;GO:0008152;GO:0005575;GO:0110165;GO:0016021;GO:0031224;GO:0080019;GO:0102965;GO:0003824;GO:0016620;GO:0016903;GO:0003674;GO:0016491	K13356	FAR	map04146;map	Peroxisome;Cutin, su	ENOG410XS7R	S:Function unknown	PF07993.15;PF030	NAD_binding_4;Sterile;	Male sterility protein;Male sterility protein;NAD dependent epimerase/dehydratase family;GDP-mannose 4,6 dehydratase	CYT	1	2	60.6	High
TRINITY_DN2894_c0_g3_i1_orf1	lactase-phlorizin hydrolase-like [Ostrinia furnacalis]	3.44023424	1.782506799	2.22E-07	up	yes	0.9987	0.2903	0.295	0.284	0.292	1	1.014	0.982	BP:primary metabolic process;BP:carbohydrate metabolic process;BP:organic substance metabolic process;BP:biological_process;BP:metabolic process;MF:hydrolase activity;MF:catalytic activity;MF:hydrolase activity, acting on glycosyl bonds;MF:hydrolase activity, hydrolyzing O-glycosyl	GO:0044238;GO:0005975;GO:0071704;GO:0008150;GO:0008152;GO:0016787;GO:0003824;GO:0016798;GO:0004553;GO:0003674	------	------	------	------	COG2723	G:Carbohydrate transport and metab	PF00232.21	Glyco_hydro_1	Glycosyl hydrolase family 1	CYT	10	28	57.5	High
TRINITY_DN6418_c0_g1_i28_orf1	peritrophic membrane chitin binding protein [Loxostege sticticalis]	3.663437388	1.873197959	9.10E-06	up	yes	1.021	0.2787	0.267	0.283	0.286	1	1.072	0.992	CC:cellular_component;CC:extracellular region;CC:cellular anatomical entity;MF:carbohydrate derivative binding;MF:molecular_function;MF:chitin binding;MF:binding;	GO:0005575;GO:0005576;GO:0110165;GO:0097367;GO:0003674;GO:0008061;GO:0005488	------	------	------	------	ENOG41121EJ;ENOG	S:Function unknown;	PF01607.27	CBM_14	Chitin binding Peritrophin-A domain	CYT	1	3	48	High
TRINITY_DN3504_c0_g1_i4_orfp1	TRINITY_DN3504_c0_g1_i4_m.43930 TRINITY_DN3504_c0_g1::TRINITY_DN3504_c0_g1_i4::g.43930 ORF type:internal len:196 (-) score=84.82 TRINITY_DN3504_c0_g1_i4:3-587(-)	2.801775148	1.486341179	0.0003075	up	yes	0.947	0.338	0.312	0.284	0.418	1	0.888	0.953	------	------	------	------	------	------	------	------	------	------	------	EXC	1	8	21.5	High
TRINITY_DN49265_c0_g3_i2_orf1	cytochrome c [Ostrinia furnacalis] >XP_028160278.1 cytochrome c [Ostrinia furnacalis]	3.099778971	1.632165348	3.57E-07	up	yes	0.9817	0.3167	0.318	0.324	0.308	1	0.975	0.97	CC:organelle lumen;CC:organelle envelope lumen;CC:membrane-enclosed lumen;CC:cellular_component;CC:intracellular organelle lumen;CC:mitochondrial intermembrane space;CC:respiratory chain;CC:cellular anatomical entity;MF:oxidoreductase activity;MF:cation binding;MF:heme binding;MF:organic cyclic compound binding;MF:tetrapyrrole binding;MF:ion binding;MF:molecular_function;MF:binding;MF:catalytic activity;MF:metal ion binding;MF:heterocyclic compound binding;MF:electron carrier activity;	GO:0043233;GO:0031970;GO:0031974;GO:0005575;GO:0070013;GO:0005758;GO:0070469;GO:0110165;GO:0016491;GO:0043169;GO:0020037;GO:0097159;GO:0046906;GO:0043167;GO:0003674;GO:0005488;GO:0003824;GO:0046872;GO:1901363;GO:0009055	K08738	CYC	map05167;map	Kaposi sarcoma-ass	COG3474	C:Energy production and conversion	PF00034.24;PF134	Cytochrom_C;Cytochrc	Cytochrome c;Cytochrome C oxidase, cbb3-type, subunit III	PLA	7	50	11.8	High
TRINITY_DN1318_c0_g1_i5_orf1	uncharacterized protein LOC114360956 [Ostrinia furnacalis]	3.285332442	1.716039364	9.18E-06	up	yes	0.9833	0.2993	0.285	0.31	0.303	1	1.012	0.938	CC:cellular_component;CC:integral component of membrane;CC:cellular anatomical entity;CC:intrinsic component of membrane;	GO:0005575;GO:0016021;GO:0110165;GO:0031224	------	------	------	------	ENOG4110SBJ;COG4	S:Function unknown;	PF13855.9;PF1279	LRR_8;LRR_4	Leucine rich repeat;Leucine Rich repeats	CYT	5	21	47.9	High
TRINITY_DN6747_c0_g1_i7_orf1	retinol dehydrogenase 12-like [Ostrinia furnacalis]	3.277647845	1.712660858	6.42E-07	up	yes	0.981	0.2993	0.312	0.29	0.296	1	0.964	0.979	------	------	K11153	RDH12	map00830;map	Retinol metabolism;	COG1028	S:Function unknown	PF00106.28;PF135	adh_short;adh_short_C	short chain dehydrogenase;Enoyl;KR domain;NAD dependent epimerase/dehydratase	CYT	3	8	34.2	High
TRINITY_DN24476_c0_g1_i1_orf1	ensconsin-like isoform X1 [Ostrinia furnacalis]	4.257595208	2.090038792	4.12E-06	up	yes	0.995	0.2337	0.241	0.244	0.216	1	1.027	0.958	------	------	K24203;K16806	MAP7D2;MAF	------	------	ENOG4111S2A;ENO(	S:Function unknown;	------	------	------	CYT	4	6	92	High
TRINITY_DN1310_c0_g1_i4_orf1	trypsin-like isoform X1 [Ostrinia furnacalis] >XP_028159118.1 trypsin-like isoform X2 [Ostrinia furnacalis]	5.067941621	2.341399906	3.92E-07	up	yes	1.007	0.1987	0.194	0.203	0.199	1	1.032	0.99	BP:organic substance metabolic process;BP:organonitrogen compound metabolic process;BP:protein metabolic process;BP:macromolecule metabolic process;BP:biological_process;BP:nitrogen compound metabolic process;BP:metabolic process;BP:proteolysis;BP:primary metabolic process;MF:serine-type endopeptidase activity;MF:endopeptidase activity;MF:hydrolase activity;MF:serine hydrolase activity;MF:catalytic activity, acting on a protein;MF:catalytic activity;MF:molecular_function;MF:peptidase activity;MF:serine-type peptidase activity;	GO:0071704;GO:1901564;GO:0019538;GO:0043170;GO:0008150;GO:0006807;GO:0008152;GO:0006508;GO:0044238;GO:0004252;GO:0004175;GO:0016787;GO:0017171;GO:0140096;GO:0003824;GO:0003674;GO:0008233;GO:0008236	------	------	------	------	ENOG411152E;COG5	O:Posttranslational modification, pro	PF00089.29	Trypsin	Trypsin	CYT	3	26	26.7	High
TRINITY_DN23570_c0_g1_i2_orf1	putative trypsin 6 [Ostrinia nubilalis]	4.163793103	2.057898384	4.64E-05	up	yes	0.966	0.232	0.212	0.24	0.244	1	0.891	1.007	BP:organic substance metabolic process;BP:organonitrogen compound metabolic process;BP:protein metabolic process;BP:macromolecule metabolic process;BP:nitrogen compound metabolic process;BP:biological_process;BP:metabolic process;BP:proteolysis;BP:primary metabolic process;CC:extracellular space;CC:cellular_component;CC:cellular anatomical entity;MF:serine-type endopeptidase activity;MF:endopeptidase activity;MF:hydrolase activity;MF:serine hydrolase activity;MF:catalytic activity, acting on a protein;MF:catalytic activity;MF:molecular_function;MF:peptidase activity;MF:serine-type peptidase activity;	GO:0071704;GO:1901564;GO:0019538;GO:0043170;GO:0006807;GO:0008150;GO:0008152;GO:0006508;GO:0044238;GO:0005615;GO:0005575;GO:0110165;GO:0004252;GO:0004175;GO:0016787;GO:0017171;GO:0140096;GO:0003824;GO:0003674;GO:0008233;GO:0008236	K09628;K09640	PRSS27;TMPR	map04972;map	Pancreatic secretion	COG5640	O:Posttranslational modification, pro	PF00089.29	Trypsin	Trypsin	CYT	1	4	26.1	High
TRINITY_DN98091_c0_g1_i3_orf1	UDP-glycosyltransferase UGT40AP2, partial [Ostrinia furnacalis]	3.624521073	1.857790377	1.62E-05	up	yes	0.946	0.261	0.249	0.263	0.271	1	0.923	0.915	CC:integral component of membrane;CC:cellular_component;CC:cellular anatomical entity;CC:intrinsic component of membrane;MF:catalytic activity;MF:transferase activity;MF:molecular_function;MF:transferase activity, transferring glycosyl groups;MF:transferase activity, transferring hexosyl groups;MF:glucuronosyltransferase activity;MF:UDP-glycosyltransferase activity;	GO:0016021;GO:0005575;GO:0110165;GO:0031224;GO:0003824;GO:0016740;GO:0003674;GO:0016757;GO:0016758;GO:0015020;GO:0008194	K00699	UGT	map05207;map	Chemical carcinoger	COG1819	S:Function unknown	PF00201.21;PF041	UDPGT;Glyco_tran_28_(	UDP-glucoronosyl and UDP-glucosyl transferase;Glycosyltransferase family 28 C-terminal	CYT	4	11	57.4	High
TRINITY_DN47731_c0_g1_i2_orf1	nucleolar GTP-binding protein 2 [Ostrinia furnacalis]	6.47463995	2.694799968	0.000531	up	yes	1.034	0.1597	0.201	0.129	0.149	1	1.193	0.91	CC:organelle;CC:intracellular organelle;CC:non-membrane-bounded organelle;CC:nucleolus;CC:cellular anatomical entity;CC:intracellular non-membrane-bounded organelle;CC:cellular_component;MF:purine ribonucleoside triphosphate binding;MF:molecular_function;MF:binding;MF:ion binding;MF:heterocyclic compound binding;MF:nucleoside phosphate binding;MF:anion binding;MF:nucleotide binding;MF:guanyl ribonucleotide binding;MF:purine nucleotide binding;MF:GTP binding;MF:small molecule binding;MF:carbohydrate derivative binding;MF:organic cyclic compound binding;MF:guanyl nucleotide binding;MF:purine ribonucleotide binding;MF:ribonucleotide binding;	GO:0043226;GO:0043229;GO:0043228;GO:0005730;GO:0110165;GO:0043232;GO:0005575;GO:0035639;GO:0003674;GO:0005488;GO:0043167;GO:1901363;GO:1901265;GO:0043168;GO:0000166;GO:0032561;GO:0017076;GO:0005525;GO:0036094;GO:0097367;GO:0097159;GO:0019001;GO:0032555;GO:0032553	K14537	NUG2, GNL2	map03008	Ribosome biogenesi	COG1161;ENOG4111	S:Function unknown;	PF07572.15	BCNT	Bucentaur or craniofacial development	CYT	1	4	30.3	Medium
TRINITY_DN2178_c0_g1_i1_orf1	carboxypeptidase B-like [Ostrinia furnacalis]	5.032712632	2.331336222	7.55E-06	up	yes	1	0.1987	0.162	0.243	0.191	1	1.022	0.978	BP:nitrogen compound metabolic process;BP:organic substance metabolic process;BP:proteolysis;BP:biological_process;BP:metabolic process;BP:primary metabolic process;BP:organonitrogen compound metabolic process;BP:protein metabolic process;BP:macromolecule metabolic process;MF:exopeptidase activity;MF:molecular_function;MF:zinc ion binding;MF:peptidase activity;MF:metalloexopeptidase activity;MF:metallopeptidase activity;MF:cation binding;MF:transition metal ion binding;MF:metallocarboxypeptidase activity;MF:carboxypeptidase activity;MF:ion binding;MF:metal ion binding;MF:hydrolase activity;MF:catalytic activity, acting on a protein;MF:catalytic activity;MF:binding;	GO:0006807;GO:0071704;GO:0006508;GO:0008150;GO:0008152;GO:0044238;GO:1901564;GO:0019538;GO:0043170;GO:0008238;GO:0003674;GO:0008270;GO:0008233;GO:0008235;GO:0008237;GO:0043169;GO:0046914;GO:0004181;GO:0004180;GO:0043167;GO:0046872;GO:0016787;GO:0140096;GO:0003824;GO:0005488	K01291;K01298	CPB1;CPA2	map04972;map	Pancreatic secretion	COG2866	E:Amino acid transport and metaboli	PF00246.27;PF022	Peptidase_M14;Propep	Zinc carboxypeptidase;Carboxypeptidase activation peptide	CYT	1	2	48.1	High
TRINITY_DN4612_c0_g1_i1_orf1	uncharacterized protein LOC114362092 [Ostrinia furnacalis]	5.053926702	2.337404741	3.35E-05	up	yes	0.9653	0.191	0.195	0.191	0.187	1	1.006	0.89	CC:cellular_component;CC:integral component of membrane;CC:cellular anatomical entity;CC:intrinsic component of membrane;	GO:0005575;GO:0016021;GO:0110165;GO:0031224	------	------	------	------	ENOG4110SII	S:Function unknown	------	------	------	CYT	1	1	70.8	Medium
TRINITY_DN82320_c0_g1_i2_orf1	glutathione S-transferase sigma3 [Glyphodes pyloalis]	4.665548634	2.222046741	0.0001931	up	yes	0.9737	0.2087	0.194	0.243	0.189	1	0.867	1.054	MF:catalytic activity;MF:transferase activity;MF:glutathione transferase activity;MF:molecular_function;MF:transferase activity, transferring alkyl or aryl	GO:0003824;GO:0016740;GO:0004364;GO:0003674;GO:0016765	K04097	HPGDS	map00590;map	Arachidonic acid me	ENOG4110YU0;ENO(	O:Posttranslational modification, pro	PF14497.9	GST_C_3	Glutathione S-transferase, C-terminal domain	CYT	1	13	7.2	High
TRINITY_DN27592_c0_g1_i1_orf1	D-arabinitol dehydrogenase 1-like [Ostrinia furnacalis]	6.111320755	2.611484203	4.37E-06	up	yes	0.9717	0.159	0.125	0.172	0.18	1	0.972	0.943	MF:oxidoreductase activity;MF:cation binding;MF:transition metal ion binding;MF:ion binding;MF:catalytic activity;MF:molecular_function;MF:binding;MF:metal ion binding;MF:zinc ion binding;	GO:0016491;GO:0043169;GO:0046914;GO:0043167;GO:0003824;GO:0003674;GO:0005488;GO:0046872;GO:0008270	K17818	ARD1	map00040	Pentose and glucurc	COG1063	E:Amino acid transport and metaboli	PF00107.29	ADH_zinc_N	Zinc-binding dehydrogenase	CYT	1	59	15.1	High
TRINITY_DN144190_c0_g1_i1_orf1	PREDICTED: uncharacterized protein LOC103572804 isoform X2 [Microplitis demolitor]	11.77777778	3.557995453	1.17E-05	up	yes	0.954	0.081	0.078	0.086	0.079	1	0.971	0.891	------	------	------	------	------	------	ENOG4112B12	S:Function unknown	------	------	------	CYT	1	3	26.8	Medium
TRINITY_DN79804_c0_g1_i1_orf1	zinc finger protein on ecdysone puffs-like [Ostrinia furnacalis]	2.638020833	1.399455958	0.001656	up	yes	1.013	0.384	0.433	0.326	0.393	1	1.153	0.886	------	------	------	------	------	------	ENOG4111SKG	S:Function unknown	------	------	------	CYT	1	21	8.9	High
TRINITY_DN64510_c0_g1_i1_orf1	39S ribosomal protein L15, mitochondrial [Ostrinia furnacalis]	2.437888972	1.285632423	1.29E-05	up	yes	0.9793	0.4017	0.417	0.422	0.366	1	0.983	0.955	BP:cellular biosynthetic process;BP:cellular nitrogen compound metabolic process;BP:nitrogen compound metabolic process;BP:cellular macromolecule biosynthetic process;BP:organic substance biosynthetic process;BP:cellular nitrogen compound biosynthetic process;BP:cellular macromolecule metabolic process;BP:peptide biosynthetic process;BP:organic substance metabolic process;BP:amide biosynthetic process;BP:cellular process;BP:biosynthetic process;BP:macromolecule biosynthetic process;BP:biological_process;BP:metabolic process;BP:primary metabolic process;BP:organonitrogen compound metabolic process;BP:cellular amide metabolic process;BP:organonitrogen compound biosynthetic process;BP:protein metabolic process;BP:cellular metabolic process;BP:macromolecule metabolic process;BP:peptide metabolic process;BP:translation;CC:large ribosomal subunit;CC:ribosomal subunit;CC:macromolecular complex;CC:ribonucleoprotein complex;CC:cellular_component;MF:molecular_function;MF:structural molecule activity;MF:structural constituent of ribosome;	GO:0044249;GO:0034641;GO:0006807;GO:0034645;GO:1901576;GO:0044271;GO:0044260;GO:0043043;GO:0071704;GO:0043604;GO:0009987;GO:0009058;GO:0009059;GO:0008150;GO:0008152;GO:0044238;GO:1901564;GO:0043603;GO:1901566;GO:0019538;GO:0044237;GO:0043170;GO:0006518;GO:0006412;GO:0015934;GO:0044391;GO:0032991;GO:1990904;GO:0005575;GO:0003674;GO:0005198;GO:0003735	K02876	RP-L15, MRPL	map03010	Ribosome	COG0200	U:Intracellular trafficking, secretion, a	PF00828.22	Ribosomal_L27A	Ribosomal proteins 50S-L15, 50S-L18e, 60S-L27A	CYT	2	7	33.9	High
TRINITY_DN84357_c0_g1_i1_orf1	4-coumarate--CoA ligase 1-like [Ostrinia furnacalis]	2.634826939	1.397708205	0.0001287	up	yes	0.982	0.3727	0.294	0.428	0.396	1	0.963	0.983	MF:catalytic activity;MF:monooxygenase activity;MF:molecular_function;MF:oxidoreductase activity;	GO:0003824;GO:0004497;GO:0003674;GO:0016491	K01904	4CL	map00130	Ubiquinone and oth	COG0318;COG0318	I:Lipid transport and metabolism;Q:S(	PF00501.31;PF131	AMP-binding;AMP-bir	AMP-binding enzyme;AMP-binding enzyme C-terminal domain	CYT	1	2	58.8	High
TRINITY_DN6710_c0_g1_i6_orf1	multiple C2 and transmembrane domain-containing protein-like [Ostrinia furnacalis]	3.348519362	1.74352331	0.0001246	up	yes	1.029	0.3073	0.254	0.382	0.286	1	0.997	1.09	CC:cellular_component;CC:integral component of membrane;CC:cellular anatomical entity;CC:intrinsic component of membrane;	GO:0005575;GO:0016021;GO:0110165;GO:0031224	------	------	------	------	COG5038;ENOG410)	H:Coenzyme transport and metabolis	PF00168.33	C2	C2 domain	PLA	1	1	69.9	Medium
TRINITY_DN2266_c0_g1_i6_orf1	bilin-binding protein-like [Ostrinia furnacalis]	2.954054054	1.562696225	6.43E-06	up	yes	0.9837	0.333	0.346	0.327	0.326	1	1.007	0.944	MF:pigment binding;MF:molecular_function;MF:binding;	GO:0031409;GO:0003674;GO:0005488	K03098	APOD	------	------	COG3040	M:Cell wall/membrane/envelope bio(	PF00061.26;PF082	Lipocalin;Lipocalin_2;Tr	Lipocalin / cytosolic fatty-acid binding protein family;Lipocalin-like domain;Triabin	CYT	6	31	22.1	High
TRINITY_DN3134_c0_g1_i1_orf1	cytochrome c oxidase subunit 6C-1 isoform X1 [Hyposmocoma kahamanoa]	3.55017301	1.827889333	1.97E-06	up	yes	1.026	0.289	0.294	0.271	0.302	1	1.025	1.052	CC:organelle membrane;CC:integral component of membrane;CC:membrane;CC:organelle inner membrane;CC:mitochondrial membrane;CC:cellular_component;CC:mitochondrial inner membrane;CC:intrinsic component of membrane;CC:cellular anatomical entity;	GO:0031090;GO:0016021;GO:0016020;GO:0019866;GO:0031966;GO:0005575;GO:0005743;GO:0031224;GO:0110165	K02268	COX6C	map04714;map	Thermogenesis;Oxic	ENOG41124VH	O:Posttranslational modification, pro	PF02937.18	COX6C	Cytochrome c oxidase subunit VIc	CYT	3	21	11.6	High
TRINITY_DN43391_c0_g1_i5_orf1	myrosinase 1-like isoform X2 [Ostrinia furnacalis]	3.141955836	1.651662902	1.31E-05	up	yes	0.996	0.317	0.277	0.337	0.337	1	0.965	1.023	BP:primary metabolic process;BP:carbohydrate metabolic process;BP:organic substance metabolic process;BP:biological_process;BP:metabolic process;MF:hydrolase activity;MF:catalytic activity;MF:hydrolase activity, acting on glycosyl bonds;MF:hydrolase activity, hydrolyzing O-glycosyl	GO:0044238;GO:0005975;GO:0071704;GO:0008150;GO:0008152;GO:0016787;GO:0003824;GO:0016798;GO:0004553;GO:0003674	------	------	------	------	COG2723	G:Carbohydrate transport and metab	PF00232.21	Glyco_hydro_1	Glycosyl hydrolase family 1	CYT	3	6	59.3	High
TRINITY_DN35725_c0_g1_i1_orf1	mitochondrial import inner membrane translocase subunit Tim13-like [Bicyclus anynana] >CAG9745432.1 unnamed protein product [Diatraea saccharalis] >CAG9784117.1 unnamed protein product [Diatraea saccharalis]	3.128737803	1.645580762	6.08E-06	up	yes	0.994	0.3177	0.33	0.324	0.299	1	1.024	0.958	------	------	K17781	TIM13	------	------	ENOG41124YI	O:Posttranslational modification, pro	PF02953.18	zf-Tim10_DDP	Tim10/DDP family zinc finger	CYT	3	39	10.9	High
TRINITY_DN679_c0_g1_i2_orf1	cytochrome b-c1 complex subunit 7-like [Ostrinia furnacalis]	3.136263041	1.649046565	2.04E-06	up	yes	0.992	0.3163	0.318	0.311	0.32	1	1.015	0.961	BP:aerobic respiration;BP:generation of precursor metabolites and energy;BP:electron transport chain;BP:respiratory electron transport chain;BP:cellular process;BP:aerobic electron transport chain;BP:cellular respiration;BP:metabolic process;BP:energy derivation by oxidation of organic compounds;BP:mitochondrial electron transport, ubiquinol to cytochrome c;BP:cellular metabolic process;BP:biological_process;CC:oxidoreductase complex;CC:respiratory chain complex;CC:cytochrome complex;CC:inner mitochondrial membrane protein complex;CC:mitochondrial respiratory chain complex III;CC:transmembrane transporter complex;CC:catalytic complex;CC:transporter complex;CC:macromolecular complex;CC:respiratory chain complex III;CC:cellular_component;CC:membrane protein complex;CC:mitochondrial	GO:0009060;GO:0006091;GO:0022900;GO:0022904;GO:0009987;GO:0019646;GO:0045333;GO:0008152;GO:0015980;GO:0006122;GO:0044237;GO:0008150;GO:1990204;GO:0098803;GO:0070069;GO:0098800;GO:0005750;GO:1902495;GO:1902494;GO:1990351;GO:0032991;GO:0045275;GO:0005575;GO:0098796;GO:0098798	K00417	QCR7, UQCRE	map04714;map	Thermogenesis;Oxic	ENOG4111V1G	C:Energy production and conversion	PF02271.19	UCR_14kD	Ubiquinol-cytochrome C reductase complex 14kD subunit	CYT	6	44	13.2	High
TRINITY_DN7213_c0_g1_i2_orf1	probable ATP-dependent RNA helicase CG8611 [Ostrinia furnacalis]	2.88978979	1.530964551	1.01E-05	up	yes	0.9623	0.333	0.34	0.344	0.315	1	0.928	0.959	MF:purine ribonucleoside triphosphate binding;MF:molecular_function;MF:binding;MF:nucleic acid binding;MF:RNA-dependent ATPase activity;MF:ion binding;MF:heterocyclic compound binding;MF:nucleoside phosphate binding;MF:anion binding;MF:nucleotide binding;MF:purine nucleotide binding;MF:ATP binding;MF:catalytic activity, acting on RNA;MF:helicase activity;MF:small molecule binding;MF:catalytic activity;MF:adenyl nucleotide binding;MF:carbohydrate derivative binding;MF:organic cyclic compound binding;MF:RNA helicase activity;MF:adenyl ribonucleotide binding;MF:RNA binding;MF:purine ribonucleotide binding;MF:ribonucleotide binding;	GO:0035639;GO:0003674;GO:0005488;GO:0003676;GO:0008186;GO:0043167;GO:1901363;GO:1901265;GO:0043168;GO:0000166;GO:0017076;GO:0005524;GO:0140098;GO:0004386;GO:0036094;GO:0003824;GO:0030554;GO:0097367;GO:0097159;GO:0003724;GO:0032559;GO:0003723;GO:0032555;GO:0032553	K14806	DDX31, DBP7	------	------	ENOG410XNT7	L:Replication, recombination and rep	PF00270.32;PF002	DEAD;Helicase_C;DUF4	DEAD/DEAH box helicase;Helicase conserved C-terminal domain;Domain of unknown function;Type III restriction enzyme, res subunit	CYT	1	1	106.1	Medium
TRINITY_DN628_c0_g1_i7_orf1	prostamide/prostaglandin F synthase-like [Ostrinia furnacalis]	3.020427705	1.594752856	2.20E-05	up	yes	0.9463	0.3133	0.309	0.312	0.319	1	0.908	0.931	------	------	K15717	PRXL2B, FAM:	map00590	Arachidonic acid me	ENOG410YFY9	O:Posttranslational modification, pro	PF13911.9	AhpC-TSA_2	AhpC/TSA antioxidant enzyme	CYT	2	8	26.6	High
TRINITY_DN139212_c0_g1_i4_orf1	uncharacterized protein LOC114350112 [Ostrinia furnacalis]	4.403433476	2.138628871	1.77E-05	up	yes	1.026	0.233	0.273	0.235	0.191	1	1.072	1.007	MF:catalytic activity;MF:molecular_function;MF:oxidoreductase activity;	GO:0003824;GO:0003674;GO:0016491	------	------	------	------	COG1028	Z:Cytoskeleton	PF13561.9;PF0010	adh_short_C2;adh_shor	Enoyl;short chain dehydrogenase;KR domain	CYT	2	8	27.3	High
TRINITY_DN713_c0_g1_i4_orf1	periodic tryptophan protein 2 homolog isoform X1 [Ostrinia furnacalis] >XP_028176443.1 periodic tryptophan protein 2 homolog isoform X2 [Ostrinia furnacalis] >XP_028176445.1 periodic tryptophan protein 2 homolog isoform X3 [Ostrinia furnacalis]	4.449845338	2.153755194	1.03E-05	up	yes	1.007	0.2263	0.229	0.177	0.273	1	1.003	1.018	------	------	K14558	PWP2, UTP1	map03008	Ribosome biogenesi	ENOG410XP2P	S:Function unknown	PF00400.35;PF128	WD40;ANAPC4_WD40;	WD domain, G-beta repeat;Anaphase-promoting complex subunit 4 WD40 domain;Dip2/Utp12 Family;Eukaryotic translation initiation factor eIF2A	CYT	1	1	102.3	High
TRINITY_DN2114_c0_g1_i5_orf1	vegetative cell wall protein gp1-like isoform X1 [Ostrinia furnacalis]	4.54744186	2.185055193	1.07E-06	up	yes	0.9777	0.215	0.23	0.204	0.211	1	0.98	0.953	BP:biological_process;BP:metabolic process;BP:methylation;MF:catalytic activity;MF:transferase activity;MF:transferase activity, transferring one-carbon groups;MF:molecular_function;MF:methyltransferase	GO:0008150;GO:0008152;GO:0032259;GO:0003824;GO:0016740;GO:0016741;GO:0003674;GO:0008168	------	------	------	------	ENOG410Y1IK;ENOG	S:Function unknown;	PF11901.11	DUF3421	Protein of unknown function	CYT	4	16	25.5	High
TRINITY_DN348_c0_g2_i3_orf1	pancreatic triacylglycerol lipase-like [Ostrinia furnacalis]	11.14269663	3.478026515	3.64E-08	up	yes	0.9917	0.089	0.083	0.091	0.093	1	0.998	0.977	------	------	------	------	------	------	ENOG4110T03;ENO(	S:Function unknown;	PF00151.22	Lipase	Lipase	CYT	4	45	36.1	High
TRINITY_DN117_c0_g1_i4_orf1	lipase member I-like [Ostrinia furnacalis]	12.78311688	3.676167744	1.19E-07	up	yes	0.9843	0.077	0.071	0.082	0.078	1	0.988	0.965	BP:organic substance metabolic process;BP:lipid metabolic process;BP:primary metabolic process;BP:metabolic process;BP:biological_process;CC:cellular_component;CC:extracellular region;CC:cellular anatomical entity;MF:hydrolase activity;MF:hydrolase activity, acting on ester bonds;MF:catalytic activity;MF:molecular_function;MF:lipase activity;	GO:0071704;GO:0006629;GO:0044238;GO:0008152;GO:0008150;GO:0005575;GO:0005576;GO:0110165;GO:0016787;GO:0016788;GO:0003824;GO:0003674;GO:0016298	------	------	------	------	ENOG41116Y2;ENO(	S:Function unknown;	PF00151.22	Lipase	Lipase	CYT	5	82	16.1	High

TRINITY_DN8621_c0_g1_i5_orf1	aminopeptidase N-like isoform X2 [Ostrinia furnacalis]	2.296938049	1.199711946	3.14E-05	up	yes	0.9677	0.4213	0.424	0.445	0.395	1	0.926	0.977	MF:exopeptidase activity;MF:aminopeptidase activity;MF:transition metal ion binding;MF:hydrolase activity;MF:catalytic activity, acting on a protein;MF:cation binding;MF:ion binding;MF:catalytic activity;MF:molecular_function;MF:zinc ion binding;MF:peptidase activity;MF:metal ion binding;MF:metallopeptidase activity;MF:binding;	GO:0008238;GO:0004177;GO:0046914;GO:0016787;GO:0140096;GO:0043169;GO:0043167;GO:0003824;GO:0003674;GO:0008270;GO:0008233;GO:0046872;GO:0008237;GO:0005488	K11140;K11141	ANPEP, CD13	map04640;map	Hematopoietic cell l	COG0308	S:Function unknown	PF11838.11;PF014	ERAP1_C;Peptidase_M1	ERAP1-like C-terminal domain;Peptidase family M1 domain	CYT	1	2	69.5	High
TRINITY_DN17559_c0_g1_i4_orf1	GDP-mannose 4,6 dehydratase isoform X2 [Ostrinia furnacalis] >XP_028166204.1 GDP-mannose 4,6 dehydratase isoform X2 [Ostrinia furnacalis]	2.617571059	1.388228703	1.42E-05	up	yes	1.013	0.387	0.359	0.413	0.389	1	1.05	0.988	BP:GDP-mannose metabolic process;BP:GDP-L-fucose metabolic process;BP:cellular biosynthetic process;BP:cellular nitrogen compound metabolic process;BP:nitrogen compound metabolic process;BP:small molecule metabolic process;BP:organic cyclic compound biosynthetic process;BP:organic cyclic compound metabolic process;BP:nucleobase-containing compound metabolic process;BP:GDP-L-fucose biosynthetic process;BP:'de novo' GDP-L-fucose biosynthetic process;BP:organic substance metabolic process;BP:heterocycle biosynthetic process;BP:nucleotide-sugar biosynthetic process;BP:organic substance biosynthetic process;BP:nucleotide-sugar metabolic process;BP:cellular process;BP:cellular aromatic compound metabolic process;BP:biosynthetic process;BP:biological_process;BP:metabolic process;BP:nucleobase-containing compound biosynthetic process;BP:nucleobase-containing small molecule metabolic process;BP:heterocycle metabolic process;BP:primary metabolic process;BP:cellular nitrogen compound biosynthetic process;BP:carbohydrate derivative biosynthetic process;BP:cellular metabolic process;BP:phosphorus metabolic process;BP:aromatic compound biosynthetic process;BP:carbohydrate derivative metabolic process;MF:molecular_function;MF:GDP-mannose 4,6-dehydratase activity;MF:lyase activity;MF:catalytic activity;MF:carbon-oxygen lyase activity;MF:hydro-lyase activity;	GO:0019673;GO:0046368;GO:0044249;GO:0034641;GO:0006807;GO:0044281;GO:1901362;GO:1901360;GO:0006139;GO:0042350;GO:0042351;GO:0071704;GO:0018130;GO:0009226;GO:1901576;GO:0009225;GO:0009987;GO:0006725;GO:0009058;GO:0008150;GO:0008152;GO:0034654;GO:0055086;GO:0046483;GO:0044238;GO:0044271;GO:1901137;GO:0044237;GO:0006793;GO:0019438;GO:1901135;GO:0003674;GO:0008446;GO:0016829;GO:0003824;GO:0016835;GO:0016836	K01711	gmd, GMDS	map00520;map	Amino sugar and nu	COG1089	M:Cell wall/membrane/envelope bio	PF16363.8;PF0137	GDP_Man_Dehyd;Epim	GDP-mannose 4,6 dehydratase;NAD dependent epimerase/dehydratase family;RmlD substrate binding domain	CYT	4	16	40.5	High
TRINITY_DN85319_c0_g1_i1_orf1	cholinesterase 2-like [Ostrinia furnacalis]	2.581893216	1.368429334	1.73E-06	up	yes	1.001	0.3877	0.393	0.388	0.382	1	1.025	0.977	MF:hydrolase activity;MF:molecular_function;MF:hydrolase activity, acting on ester bonds;MF:carboxylic ester hydrolase activity;MF:catalytic activity;	GO:0016787;GO:0003674;GO:0016788;GO:0052689;GO:0003824	K12298	CEL	map04972;map	Pancreatic secretion	COG2272	I:Lipid transport and metabolism	PF00135.31;PF204	COesterase;BD-FAE;Ab	Carboxylesterase family;BD-FAE;alpha/beta hydrolase	CYT	9	18	61.4	High
TRINITY_DN19829_c0_g2_i1_orf1	28S ribosomal protein S35, mitochondrial [Ostrinia furnacalis]	2.331580206	1.221308059	5.45E-06	up	yes	0.9753	0.4183	0.419	0.435	0.401	1	0.975	0.951	BP:cellular biosynthetic process;BP:cellular nitrogen compound metabolic process;BP:nitrogen compound metabolic process;BP:cellular macromolecule biosynthetic process;BP:organic substance biosynthetic process;BP:cellular macromolecule metabolic process;BP:peptide biosynthetic process;BP:organic substance metabolic process;BP:mitochondrial translation;BP:amide biosynthetic process;BP:cellular process;BP:biosynthetic process;BP:macromolecule biosynthetic process;BP:biological_process;BP:metabolic process;BP:cellular nitrogen compound biosynthetic process;BP:primary metabolic process;BP:organonitrogen compound metabolic process;BP:cellular amide metabolic process;BP:organonitrogen compound biosynthetic process;BP:protein metabolic process;BP:cellular metabolic process;BP:macromolecule metabolic process;BP:peptide metabolic process;BP:translation;CC:membrane-bounded organelle;CC:organelle;CC:mitochondrion;CC:cellular anatomical entity;CC:intracellular membrane-bounded organelle;CC:intracellular organelle;CC:cellular_component;MF:molecular_function;MF:structural molecule activity;MF:structural constituent of ribosome;	GO:0044249;GO:0034641;GO:0006807;GO:0034645;GO:1901576;GO:0044260;GO:0043043;GO:0071704;GO:0032543;GO:0043604;GO:0009987;GO:0009058;GO:0009059;GO:0008150;GO:0008152;GO:0044271;GO:0044238;GO:1901564;GO:0043603;GO:1901566;GO:0019538;GO:0044237;GO:0043170;GO:0006518;GO:0006412;GO:0043227;GO:0043226;GO:0005739;GO:0110165;GO:0043231;GO:0043229;GO:0005575;GO:0003674;GO:0005198;GO:0003735	K17413	MRPS35	------	------	ENOG410ZX0Y	S:Function unknown	PF10213.12	MRP-S28	Mitochondrial ribosomal subunit protein	CYT	1	6	18.6	High
TRINITY_DN6248_c0_g1_i1_orf1	DNA topoisomerase I, mitochondrial [Ostrinia furnacalis]	2.662269129	1.412656421	3.10E-07	up	yes	1.009	0.379	0.373	0.383	0.381	1	1.027	1	BP:DNA conformation change;BP:nucleic acid metabolic process;BP:cellular nitrogen compound metabolic process;BP:nitrogen compound metabolic process;BP:cellular component organization or biogenesis;BP:nucleobase-containing compound metabolic process;BP:cellular macromolecule metabolic process;BP:DNA topological change;BP:cellular component organization;BP:organic substance metabolic process;BP:organic cyclic compound metabolic process;BP:cellular process;BP:cellular aromatic compound metabolic process;BP:biological_process;BP:metabolic process;BP:heterocycle metabolic process;BP:organelle organization;BP:primary metabolic process;BP:chromosome organization;BP:cellular metabolic process;BP:macromolecule metabolic process;BP:DNA metabolic process;CC:membrane-bounded organelle;CC:organelle;CC:nucleus;CC:chromosome;CC:cellular anatomical entity;CC:intracellular organelle;CC:intracellular membrane-bounded organelle;CC:non-membrane-bounded organelle;CC:intracellular non-membrane-bounded organelle;CC:cellular_component;MF:molecular_function;MF:binding;MF:nucleic acid binding;MF:DNA binding;MF:heterocyclic compound binding;MF:catalytic activity, acting on DNA;MF:catalytic activity;MF:isomerase activity;MF:organic cyclic compound binding;MF:DNA topoisomerase type I activity;MF:DNA topoisomerase activity;	GO:0071103;GO:0090304;GO:0034641;GO:0006807;GO:0071840;GO:0006139;GO:0044260;GO:0006265;GO:0016043;GO:0071704;GO:1901360;GO:0009987;GO:0006725;GO:0008150;GO:0008152;GO:0046483;GO:0006996;GO:0044238;GO:0051276;GO:0044237;GO:0043170;GO:0006259;GO:0043227;GO:0043226;GO:0005634;GO:0005694;GO:0110165;GO:0043229;GO:0043231;GO:0043228;GO:0043232;GO:0005575;GO:0003674;GO:0005488;GO:0003676;GO:0003677;GO:1901363;GO:0140097;GO:0003824;GO:0016853;GO:0097159;GO:0003917;GO:0003916	K03163	TOP1	------	------	COG3569	L:Replication, recombination and rep	PF01028.23;PF029	Topoisom_I;Topoisom_	Eukaryotic DNA topoisomerase I, catalytic core;Eukaryotic DNA topoisomerase I, DNA binding fragment;C-terminal topoisomerase domain	CYT	8	9	110.1	High
TRINITY_DN25856_c0_g1_i1_orf1	myrosinase 1-like [Ostrinia furnacalis]	2.67321476	1.418575745	1.49E-06	up	yes	1.007	0.3767	0.375	0.383	0.372	1	1.033	0.987	------	------	------	------	------	------	COG2723;ENOG4111	G:Carbohydrate transport and metab	PF00232.21	Glyco_hydro_1	Glycosyl hydrolase family 1	CYT	3	9	57.7	High
TRINITY_DN39673_c0_g1_i1_orf1	uncharacterized protein LOC114359357 isoform X1 [Ostrinia furnacalis]	2.241338675	1.164360662	9.62E-05	up	yes	0.951	0.4243	0.44	0.447	0.386	1	0.948	0.905	------	------	------	------	------	------	------	------	PF07294.14	Fibroin_P25	Fibroin P25	PLA	1	4	27.9	High
TRINITY_DN1427_c0_g1_i9_orf1	SAFB-like transcription modulator isoform X3 [Ostrinia furnacalis]	2.758241758	1.463748914	2.06E-05	up	yes	1.004	0.364	0.321	0.414	0.357	1	0.997	1.015	------	------	------	------	------	------	ENOG4111F1G;ENO(	K:Transcription;S:Function unknown;I	PF00076.25;PF020	RRM_1;SAP	RNA recognition motif.;SAP domain	CYT	1	2	71.9	High
TRINITY_DN50787_c0_g2_i2_orf1	40S ribosomal protein S29 [Hyposmocoma kahamanoa] >XP_028176503.1 40S ribosomal protein S29 [Ostrinia furnacalis] >XP_049877832.1 40S ribosomal protein S29 [Pectinophora gossypiella] >ADT80654.1 ribosomal protein S29 [Euphydryas aurinia] >CAB3523209.1 unnamed protein product [Chilo suppressalis] >CAH0400531.1 unnamed protein product [Chilo suppressalis]	2.605163043	1.381373666	2.09E-05	up	yes	0.9587	0.368	0.369	0.365	0.37	1	0.964	0.912	BP:organonitrogen compound biosynthetic process;BP:cellular biosynthetic process;BP:cellular macromolecule biosynthetic process;BP:cellular nitrogen compound biosynthetic process;BP:peptide biosynthetic process;BP:organic substance metabolic process;BP:amide biosynthetic process;BP:cytoplasmic translation;BP:organic substance biosynthetic process;BP:cellular macromolecule metabolic process;BP:cellular process;BP:biosynthetic process;BP:macromolecule biosynthetic process;BP:metabolic process;BP:organonitrogen compound metabolic process;BP:primary metabolic process;BP:protein metabolic process;BP:cellular nitrogen compound metabolic process;BP:cellular metabolic process;BP:macromolecule metabolic process;BP:nitrogen compound metabolic process;BP:cellular amide metabolic process;BP:biological_process;BP:peptide metabolic process;BP:translation;CC:endoplasmic reticulum;CC:intracellular organelle;CC:non-membrane-bounded organelle;CC:membrane-bounded organelle;CC:organelle;CC:cytosolic small ribosomal subunit;CC:cellular anatomical entity;CC:rough endoplasmic reticulum;CC:ribosomal subunit;CC:small ribosomal subunit;CC:intracellular membrane-bounded organelle;CC:polysomal ribosome;CC:macromolecular complex;CC:ribosome;CC:intracellular non-membrane-bounded organelle;CC:cytosol;CC:ribonucleoprotein complex;CC:cellular_component;MF:heterocyclic compound binding;MF:molecular_function;MF:zinc ion binding;MF:nucleic acid binding;MF:ion binding;MF:cation binding;MF:transition metal ion binding;MF:structural molecule activity;MF:structural constituent of ribosome;MF:metal ion binding;MF:RNA binding;MF:organic cyclic compound binding;MF:binding;	GO:1901566;GO:0044249;GO:0034645;GO:0044271;GO:0043043;GO:0071704;GO:0043604;GO:0002181;GO:1901576;GO:0044260;GO:0009987;GO:0009058;GO:0009059;GO:0008152;GO:1901564;GO:0044238;GO:0019538;GO:0034641;GO:0044237;GO:0043170;GO:0006807;GO:0043603;GO:0008150;GO:0006518;GO:0006412;GO:0005783;GO:0043229;GO:0043228;GO:0043227;GO:0043226;GO:0022627;GO:0110165;GO:0005791;GO:0044391;GO:0015935;GO:0043231;GO:0042788;GO:0032991;GO:0005840;GO:0043232;GO:0005829;GO:1990904;GO:0005575;GO:1901363;GO:0003674;GO:0008270;GO:0003676;GO:0043167;GO:0043169;GO:0046914;GO:0005198;GO:0003735;GO:0046872;GO:0003723;GO:0097159;GO:0005488	K02980	RP-S29e, RPS	map03010;map	Ribosome;Coronavir	COG0199	J:Translation, ribosomal structure an(	PF00253.24	Ribosomal_S14	Ribosomal protein S14p/S29e	CYT	2	38	6.6	High
TRINITY_DN13783_c0_g4_i2_orf1	hypothetical protein evm_010131 [Chilo suppressalis]	3.096875	1.630813152	4.33E-06	up	yes	0.991	0.32	0.323	0.309	0.328	1	1.018	0.955	BP:protein localization;BP:maintenance of protein localization in endoplasmic reticulum;BP:maintenance of location in cell;BP:cellular macromolecule localization;BP:nitrogen compound transport;BP:organic substance transport;BP:macromolecule localization;BP:transport;BP:maintenance of protein location in cell;BP:maintenance of protein localization in organelle;BP:cellular process;BP:protein retention in ER lumen;BP:establishment of protein localization;BP:maintenance of protein location;BP:biological_process;BP:maintenance of location;BP:establishment of localization;BP:localization;BP:cellular localization;BP:protein transport;CC:endoplasmic reticulum membrane;CC:intrinsic component of membrane;CC:organelle membrane;CC:integral component of membrane;CC:membrane;CC:cellular anatomical entity;CC:cellular_component;MF:molecular_function;MF:binding;MF:peptide binding;MF:amide binding;MF:ER retention sequence binding;MF:signal sequence binding;	GO:0008104;GO:0035437;GO:0051651;GO:0070727;GO:0071705;GO:0071702;GO:0033036;GO:0006810;GO:0032507;GO:0072595;GO:0009987;GO:0006621;GO:0045184;GO:0045185;GO:0008150;GO:0051235;GO:0051234;GO:0051179;GO:0051641;GO:0015031;GO:0005789;GO:0031224;GO:0031090;GO:0016021;GO:0016020;GO:0110165;GO:0005575;GO:0003674;GO:0005488;GO:0042277;GO:0033218;GO:0046923;GO:0005048	------	------	------	------	ENOG410Z4PE	S:Function unknown	PF14880.9	COX14	Cytochrome oxidase c assembly	CYT	1	10	11.2	High
TRINITY_DN117_c0_g1_i5_orf1	lipase member I-like [Ostrinia furnacalis]	3.253289474	1.701899197	1.52E-07	up	yes	0.989	0.304	0.298	0.317	0.297	1	0.986	0.981	BP:organic substance metabolic process;BP:lipid metabolic process;BP:primary metabolic process;BP:metabolic process;BP:biological_process;CC:cellular_component;CC:extracellular region;CC:cellular anatomical entity;MF:hydrolase activity;MF:hydrolase activity, acting on ester bonds;MF:catalytic activity;MF:molecular_function;MF:lipase activity;	GO:0071704;GO:0006629;GO:0044238;GO:0008152;GO:0008150;GO:0005575;GO:0005576;GO:0110165;GO:0016787;GO:0016788;GO:0003824;GO:0003674;GO:0016298	------	------	------	------	ENOG41116Y2;ENO(	S:Function unknown;	PF00151.22	Lipase	Lipase	CYT	6	21	46.9	High
TRINITY_DN3332_c0_g1_i11_orf1	glutathione S-transferase sigma3 [Glyphodes pyloalis]	3.567266187	1.834818873	1.54E-06	up	yes	0.9917	0.278	0.273	0.279	0.282	1	0.961	1.014	MF:catalytic activity;MF:transferase activity;MF:glutathione transferase activity;MF:molecular_function;MF:transferase activity, transferring alkyl or ary;	GO:0003824;GO:0016740;GO:0004364;GO:0003674;GO:0016765	K04097	HPGDS	map00590;map	Arachidonic acid me	ENOG41103AU;ENO(	O:Posttranslational modification, pro	PF14497.9;PF0279	GST_C_3;GST_N;GST_C	Glutathione S-transferase, C-terminal domain;Glutathione S-transferase, N-terminal domain;Glutathione S-transferase, C-terminal domain	CYT	2	45	23.1	High
TRINITY_DN3159_c0_g1_i4_orf1	uncharacterized protein LOC114362782 [Ostrinia furnacalis]	4.102776627	2.03660061	8.17E-08	up	yes	0.99	0.2413	0.235	0.251	0.238	1	0.992	0.978	BP:response to host;BP:response to defenses of other organism involved in symbiotic interaction;BP:response to biotic stimulus;BP:response to external stimulus;BP:response to external biotic stimulus;BP:evasion or tolerance of host defense response;BP:response to stimulus;BP:interspecies interaction between organisms;BP:biological_process;BP:symbiosis, encompassing mutualism through parasitism;BP:interaction with host;BP:response to host defenses;BP:response to other organism;MF:pigment binding;MF:molecular_function;MF:binding;	GO:0075136;GO:0052173;GO:0009607;GO:0009605;GO:0043207;GO:0030682;GO:0050896;GO:0044419;GO:0008150;GO:0044403;GO:0051701;GO:0052200;GO:0051707;GO:0031409;GO:0003674;GO:0005488	K18271	RBP4	------	------	COG3040	M:Cell wall/membrane/envelope bio	PF00061.26;PF082	Lipocalin;Lipocalin_2;Tr	Lipocalin / cytosolic fatty-acid binding protein family;Lipocalin-like domain;Triabin	CYT	22	10	323.1	High
TRINITY_DN108573_c0_g1_i1_orf1	uncharacterized protein LOC114366171 [Ostrinia furnacalis]	3.881018703	1.956435386	1.50E-06	up	yes	0.9753	0.2513	0.256	0.232	0.266	1	0.958	0.968	------	------	------	------	------	------	ENOG410XT8F;ENO(	G:Carbohydrate transport and metab	PF02958.23;PF079	EcKL;DUF1679	Ecdysteroid kinase-like family;Uncharacterized oxidoreductase dhs-27	CYT	3	7	47.8	High
TRINITY_DN20682_c0_g2_i1_orf1	glutathione S-transferase delta3 [Glyphodes pyloalis]	4.38223597	2.131667171	1.10E-05	up	yes	0.9917	0.2263	0.187	0.272	0.22	1	0.965	1.01	MF:catalytic activity;MF:transferase activity;MF:glutathione transferase activity;MF:molecular_function;MF:transferase activity, transferring alkyl or ary;	GO:0003824;GO:0016740;GO:0004364;GO:0003674;GO:0016765	K00799	GST, gst	map05207;map	Chemical carcinoger	COG0625	O:Posttranslational modification, pro	PF13417.9;PF0279	GST_N_3;GST_N;GST_N	Glutathione S-transferase, N-terminal domain;Glutathione S-transferase, N-terminal domain;Glutathione S-transferase, N-terminal	PLA	1	8	14.1	Medium
TRINITY_DN27721_c1_g1_i2_orf1	mitochondrial import receptor subunit TOM20 homolog [Ostrinia furnacalis]	2.46598349	1.302163141	0.001664	up	yes	0.8663	0.3513	0.373	0.342	0.339	1	0.783	0.816	BP:protein localization;BP:cellular macromolecule localization;BP:nitrogen compound transport;BP:organic substance transport;BP:macromolecule localization;BP:transport;BP:cellular process;BP:protein targeting;BP:establishment of protein localization;BP:biological_process;BP:establishment of localization in cell;BP:establishment of localization;BP:localization;BP:cellular localization;BP:intracellular transport;BP:protein transport;BP:intracellular protein transport;CC:intrinsic component of membrane;CC:mitochondrial outer membrane translocase complex;CC:integral component of membrane;CC:cellular anatomical entity;CC:macromolecular complex;CC:cellular_component;CC:membrane protein complex;CC:outer mitochondrial membrane protein complex;CC:mitochondrial protein complex;	GO:0008104;GO:0070727;GO:0071705;GO:0071702;GO:0033036;GO:0006810;GO:0009987;GO:0006605;GO:0045184;GO:0008150;GO:0051649;GO:0051234;GO:0051179;GO:0051641;GO:0046907;GO:0015031;GO:0006886;GO:0031224;GO:0005742;GO:0016021;GO:0110165;GO:0032991;GO:0005575;GO:0098796;GO:0098799;GO:0098798	K17770	TOM20	------	------	ENOG4111NAH	U:Intracellular trafficking, secretion, a	PF02064.18	MAS20	MAS20 protein import receptor	CYT	2	15	17.2	High
TRINITY_DN6693_c0_g1_i1_orf1	uncharacterized protein LOC114356358 [Ostrinia furnacalis]	4.933830846	2.302708254	4.40E-07	up	yes	0.9917	0.201	0.207	0.195	0.201	1	1.008	0.967	MF:catalytic activity;MF:transferase activity;MF:transferase activity, transferring sulfur-containing groups;MF:sulfotransferase activity;MF:molecular_function;	GO:0003824;GO:0016740;GO:0016782;GO:0008146;GO:0003674	------	------	------	------	ENOG4111H56	S:Function unknown	PF00685.30;PF134	Sulfotransfer_1;Sulfotra	Sulfotransferase domain;Sulfotransferase family	CYT	6	24	40.4	High
TRINITY_DN542_c0_g2_i1_orf1	uncharacterized protein LOC114364889 [Ostrinia furnacalis]	4.778804084	2.256649623	4.81E-06	up	yes	0.983	0.2057	0.203	0.207	0.207	1	1.012	0.937	BP:amino sugar catabolic process;BP:nitrogen compound metabolic process;BP:aminoglycan metabolic process;BP:organic substance catabolic process;BP:glucosamine-containing compound catabolic process;BP:organic substance metabolic process;BP:chitin metabolic process;BP:polysaccharide catabolic process;BP:biological_process;BP:metabolic process;BP:organonitrogen compound metabolic process;BP:catabolic process;BP:macromolecule catabolic process;BP:primary metabolic process;BP:chitin catabolic process;BP:carbohydrate metabolic process;BP:organonitrogen compound catabolic process;BP:polysaccharide metabolic process;BP:amino sugar metabolic process;BP:carbohydrate derivative catabolic process;BP:carbohydrate catabolic process;BP:macromolecule metabolic process;BP:glucosamine-containing compound metabolic process;BP:aminoglycan catabolic process;BP:carbohydrate derivative metabolic process;CC:extracellular space;CC:cellular anatomical entity;CC:cellular_component;MF:molecular_function;MF:zinc ion binding;MF:cation binding;MF:transition metal ion binding;MF:ion binding;MF:metal ion binding;MF:hydrolase activity;MF:deacetylase activity;MF:catalytic activity;MF:hydrolase activity, acting on carbon-nitroge;MF:hydrolase activity, acting on carbon-nitroge;MF:chitin	GO:0046348;GO:0006807;GO:0006022;GO:1901575;GO:1901072;GO:0071704;GO:0006030;GO:0000272;GO:0008150;GO:0008152;GO:1901564;GO:0009056;GO:0009057;GO:0044238;GO:0006032;GO:0005975;GO:1901565;GO:0005976;GO:0006040;GO:1901136;GO:0016052;GO:0043170;GO:1901071;GO:0006026;GO:1901135;GO:0005615;GO:0110165;GO:0005575;GO:0003674;GO:0008270;GO:0043169;GO:0046914;GO:0043167;GO:0046872;GO:0016787;GO:0019213;GO:0003824;GO:0016810;GO:0016811;GO:0004099;GO:0005488	------	------	------	------	ENOG410ZWU3	S:Function unknown	PF01522.24	Polysacc_deac_1	Polysaccharide deacetylase	PLA	4	16	44.5	High
TRINITY_DN64446_c0_g1_i1_orf1	uncharacterized protein LOC114364307 [Ostrinia furnacalis]	5.218518519	2.383640299	1.36E-07	up	yes	0.9863	0.189	0.182	0.193	0.192	1	0.99	0.969	------	------	------	------	------	------	ENOG410Y27Q	S:Function unknown	PF06757.16	Ins_allergen_rp	Insect allergen related repeat, nitrile-specifier	CYT	3	15	23.7	High
TRINITY_DN14679_c0_g1_i1_orf1	hypothetical protein evm_003043 [Chilo suppressalis]	10.29900332	3.364432823	3.23E-07	up	yes	1.023	0.09933	0.106	0.084	0.108	1	1.032	1.038	CC:cellular_component;CC:integral component of membrane;CC:cellular anatomical entity;CC:intrinsic component of membrane;	GO:0005575;GO:0016021;GO:0110165;GO:0031224	------	------	------	------	ENOG410ZMUH;ENC	S:Function unknown;	PF13927.9;PF0767	Ig_3;I-set;Ig_2;ig;C2-set	Immunoglobulin domain;Immunoglobulin I-set domain;Immunoglobulin domain;Immunoglobulin domain;CD80-like C2-set immunoglobulin domain;Immunoglobulin V-set domain	CYT	1	1	90	High
TRINITY_DN336_c0_g1_i6_orfp1	TRINITY_DN336_c0_g1_i6_m.64791 TRINITY_DN336_c0_g1::TRINITY_DN336_c0_g1_i6::g.64791 ORF type:complete len:61 (-),score=19.53 TRINITY_DN336_c0_g1_i6:236-418(-)	9.797492768	3.292412603	7.67E-08	up	yes	1.016	0.1037	0.105	0.098	0.108	1	1.017	1.032	------	------	------	------	------	------	------	------	------	------	------	CYT	1	12	6.3	High
TRINITY_DN94755_c0_g1_i5_orfp1	TRINITY_DN94755_c0_g1_i5_m.62794 TRINITY_DN94755_c0_g1::TRINITY_DN94755_c0_g1_i5::g.62794 ORF type:internal len:70 (+),score=5.17 TRINITY_DN94755_c0_g1_i5:2-208(+)	10.50537634	3.393055941	1.41E-05	up	yes	0.977	0.093	0.092	0.098	0.089	1	1.022	0.909	------	------	------	------	------	------	------	------	------	------	------	CYT	1	16	8	High
TRINITY_DN145647_c0_g1_i1_orf1	PREDICTED: U6 snRNA-associated Sm-like protein LSm5 isoform X1 [Fopius arisanus]	2.649594983	1.405771846	6.97E-05	up	yes	1.014	0.3827	0.31	0.415	0.423	1	1.022	1.019	BP:nucleic acid metabolic process;BP:cellular nitrogen compound metabolic process;BP:nitrogen compound metabolic process;BP:organic cyclic compound metabolic process;BP:nucleobase-containing compound metabolic process;BP:organic substance metabolic process;BP:RNA splicing;BP:mRNA metabolic process;BP:cellular process;BP:cellular aromatic compound metabolic process;BP:biological_process;BP:metabolic process;BP:heterocycle metabolic process;BP:RNA metabolic process;BP:primary metabolic process;BP:cellular metabolic process;BP:macromolecule metabolic process;BP:RNA processing;BP:mRNA processing;CC:spliceosomal complex;CC:macromolecular complex;CC:cellular_component;CC:ribonucleoprotein complex;MF:molecular_function;MF:binding;MF:nucleic acid binding;MF:heterocyclic compound binding;MF:organic cyclic compound binding;MF:RNA binding;	GO:0090304;GO:0034641;GO:0006807;GO:1901360;GO:0006139;GO:0071704;GO:0008380;GO:0016071;GO:0009987;GO:0006725;GO:0008150;GO:0008152;GO:0046483;GO:0016070;GO:0044238;GO:0044237;GO:0043170;GO:0006396;GO:0006397;GO:0005681;GO:0032991;GO:0005575;GO:1990904;GO:0003674;GO:0005488;GO:0003676;GO:1901363;GO:0097159;GO:0003723	K12624	LSM5	map03018;map	RNA degradation;Sp	COG1958	O:Posttranslational modification, pro	PF01423.25	LSM	LSM domain	CYT	1	6	10.5	High
TRINITY_DN12495_c0_g1_i2_orf1	probable ATP-dependent RNA helicase pitchoune [Manduca sexta] >KAG6441249.1 hypothetical protein O3G_MSEX001749 [Manduca sexta]	2.456048084	1.296338806	5.77E-07	up	yes	0.9807	0.3993	0.393	0.402	0.403	1	0.973	0.969	MF:purine ribonucleoside triphosphate binding;MF:molecular_function;MF:binding;MF:nucleic acid binding;MF:RNA-dependent ATPase activity;MF:ion binding;MF:heterocyclic compound binding;MF:nucleoside phosphate binding;MF:anion binding;MF:nucleotide binding;MF:purine nucleotide binding;MF:ATP binding;MF:hydrolase activity;MF:catalytic activity, acting on RNA;MF:helicase activity;MF:small molecule binding;MF:catalytic activity;MF:adenyl nucleotide binding;MF:carbohydrate derivative binding;MF:organic cyclic compound binding;MF:RNA helicase activity;MF:adenyl ribonucleotide binding;MF:RNA binding;MF:purine ribonucleotide binding;MF:ribonucleotide binding;	GO:0035639;GO:0003674;GO:0005488;GO:0003676;GO:0008186;GO:0043167;GO:1901363;GO:1901265;GO:0043168;GO:0000166;GO:0017076;GO:0005524;GO:0016787;GO:0140098;GO:0004386;GO:0036094;GO:0003824;GO:0030554;GO:0097367;GO:0097159;GO:0003724;GO:0032559;GO:0003723;GO:0032555;GO:0032553	K13179	DDX18, HAS1	------	------	COG0513	S:Function unknown	PF00270.32;PF002	DEAD;Helicase_C;DUF4	DEAD/DEAH box helicase;Helicase conserved C-terminal domain;Domain of unknown function	CYT	2	3	69	High
TRINITY_DN66302_c0_g1_i1_orf1	carboxypeptidase B-like [Ostrinia furnacalis]	3.365384615	1.750771394	1.74E-05	up	yes	1.05	0.312	0.293	0.343	0.3	1	1.089	1.06	BP:nitrogen compound metabolic process;BP:organic substance metabolic process;BP:proteolysis;BP:biological_process;BP:metabolic process;BP:primary metabolic process;BP:organonitrogen compound metabolic process;BP:protein metabolic process;BP:macromolecule metabolic process;CC:intrinsic component of membrane;CC:cellular anatomical entity;CC:cellular_component;CC:integral component of membrane;MF:exopeptidase activity;MF:molecular_function;MF:zinc ion binding;MF:peptidase activity;MF:metalloexopeptidase activity;MF:cation binding;MF:transition metal ion binding;MF:metallocarboxypeptidase activity;MF:carboxypeptidase activity;MF:ion binding;MF:metallopeptidase activity;MF:metal ion binding;MF:hydrolase activity;MF:catalytic activity, acting on a protein;MF:catalytic activity;MF:binding;	GO:0006807;GO:0071704;GO:0006508;GO:0008150;GO:0008152;GO:0044238;GO:1901564;GO:0019538;GO:0043170;GO:0031224;GO:0110165;GO:0005575;GO:0016021;GO:0008238;GO:0003674;GO:0008270;GO:0008233;GO:0008235;GO:0043169;GO:0046914;GO:0004181;GO:0004180;GO:0043167;GO:0008237;GO:0046872;GO:0016787;GO:0140096;GO:0003824;GO:0005488	K01298	CPA2	map04972;map	Pancreatic secretion	COG2866	E:Amino acid transport and metaboli	PF00246.27	Peptidase_M14	Zinc carboxypeptidase	CYT	1	3	47.1	High
TRINITY_DN11657_c0_g1_i2_orf1	trehalase-1 [Omphisa fuscidentalis]	2.793005402	1.481818364	2.94E-06	up	yes	0.9823	0.3517	0.332	0.37	0.353	1	0.958	0.989	BP:primary metabolic process;BP:cellular carbohydrate metabolic process;BP:oligosaccharide metabolic process;BP:cellular process;BP:disaccharide metabolic process;BP:trehalose metabolic process;BP:biological_process;BP:cellular metabolic process;BP:metabolic process;BP:carbohydrate metabolic process;BP:organic substance metabolic process;MF:trehalase activity;MF:hydrolase activity;MF:catalytic activity;MF:hydrolase activity, acting on glycosyl bonds;MF:alpha,alpha-trehalase activity;MF:molecular_function;MF:hydrolase activity, hydrolyzing O-glycosyl compounds;	GO:0044238;GO:0044262;GO:0009311;GO:0009987;GO:0005984;GO:0005991;GO:0008150;GO:0044237;GO:0008152;GO:0005975;GO:0071704;GO:0015927;GO:0016787;GO:0003824;GO:0016798;GO:0004555;GO:0003674;GO:0004553	K01194	TREH, treA, tr	map00500	Starch and sucrose r	COG1626	S:Function unknown	PF01204.21	Trehalase	Trehalase	CYT	2	5	67.2	High
TRINITY_DN35635_c0_g1_i1_orf1	probable NADH dehydrogenase [ubiquinone] 1 alpha subcomplex subunit 12 [Ostrinia furnacalis]	2.957185629	1.564224807	2.81E-07	up	yes	0.9877	0.334	0.345	0.335	0.322	1	0.987	0.976	BP:mitochondrial respiratory chain complex assembly;BP:cellular component assembly;BP:mitochondrial respiratory chain complex I assembly;BP:macromolecular complex subunit organization;BP:NADH dehydrogenase complex assembly;BP:cellular component organization;BP:cellular process;BP:biological_process;BP:cellular component organization or biogenesis;BP:macromolecular complex assembly;CC:organelle membrane;CC:membrane;CC:organelle inner membrane;CC:mitochondrial membrane;CC:cellular_component;CC:mitochondrial inner membrane;CC:respiratory chain;CC:cellular anatomical entity;	GO:0033108;GO:0022607;GO:0032981;GO:0043933;GO:0010257;GO:0016043;GO:0009987;GO:0008150;GO:0071840;GO:0065003;GO:0031090;GO:0016020;GO:0019866;GO:0031966;GO:0005575;GO:0005743;GO:0070469;GO:0110165	K11352	NDUFA12	map04714;map	Thermogenesis;Oxic	ENOG4111XVR	I:Lipid transport and metabolism	PF05071.19	NDUFA12	NADH ubiquinone oxidoreductase subunit NDUFA12	CYT	5	44	16.9	High
TRINITY_DN73923_c0_g1_i1_orf1	protein obstructor-E-like [Ostrinia furnacalis]	3.393393393	1.76272869	5.07E-07	up	yes	1.017	0.2997	0.296	0.311	0.292	1	1.037	1.014	CC:cellular_component;CC:extracellular region;CC:cellular anatomical entity;MF:carbohydrate derivative binding;MF:molecular_function;MF:chitin binding;MF:binding;	GO:0005575;GO:0005576;GO:0110165;GO:0097367;GO:0003674;GO:0008061;GO:0005488	------	------	------	------	ENOG410ZSCN	S:Function unknown	PF01607.27	CBM_14	Chitin binding Peritrophin-A domain	CYT	15	65	26.5	High
TRINITY_DN1901_c0_g1_i6_orf1	mitochondrial intermembrane space import and assembly protein 40 [Ostrinia furnacalis] >XP_028171079.1 mitochondrial intermembrane space import and assembly protein 40 [Ostrinia furnacalis] >XP_028171080.1 mitochondrial intermembrane space import and assembly protein 40 [Ostrinia furnacalis]	3.412671807	1.770901679	4.42E-06	up	yes	1.018	0.2983	0.266	0.297	0.332	1	1.029	1.025	BP:protein localization;BP:establishment of localization in cell;BP:protein transmembrane transport;BP:cellular macromolecule localization;BP:intracellular protein transport;BP:localization;BP:nitrogen compound transport;BP:intracellular protein transmembrane transport;BP:mitochondrial transmembrane transport;BP:organic substance transport;BP:macromolecule localization;BP:transport;BP:establishment of protein localization to organelle;BP:cellular process;BP:establishment of protein localization;BP:protein localization to organelle;BP:biological_process;BP:establishment of protein localization to mitochondrion;BP:mitochondrial transport;BP:establishment of localization;BP:transmembrane transport;BP:intracellular protein transmembrane import;BP:cellular localization;BP:protein localization to mitochondrion;BP:intracellular transport;BP:protein import into mitochondrial intermembrane space;BP:protein transport;MF:molecular_function;MF:oxidoreductase activity;MF:oxidoreductase activity, acting on a sulfur group of donors;MF:catalytic activity, acting on a protein;MF:catalytic activity;MF:protein disulfide oxidoreductase activity;MF:disulfide oxidoreductase activity;	GO:0008104;GO:0051649;GO:0071806;GO:0070727;GO:0006886;GO:0051179;GO:0071705;GO:0065002;GO:1990542;GO:0071702;GO:0033036;GO:0006810;GO:0072594;GO:0009987;GO:0045184;GO:0033365;GO:0008150;GO:0072655;GO:0006839;GO:0051234;GO:0055085;GO:0044743;GO:0051641;GO:0070585;GO:0046907;GO:0045041;GO:0015031;GO:0003674;GO:0016491;GO:0016667;GO:0140096;GO:0003824;GO:0015035;GO:0015036	K17782	MIA40, CHCH	------	------	ENOG4111V0I	O:Posttranslational modification, pro	PF06747.16	CHCH	CHCH domain	CYT	2	13	15	High

TRINITY_DN1707_c0_g1_i1_orf1	inositol oxygenase-like [Ostrinia furnacalis]	3.577178859	1.838822255	1.24E-05	up	yes	1.022	0.2857	0.252	0.287	0.318	1	1.063	1.003	BP:polyol catabolic process;BP:polyol metabolic process;BP:cellular catabolic process;BP:small molecule metabolic process;BP:small molecule catabolic process;BP:alcohol metabolic process;BP:organic substance catabolic process;BP:cellular carbohydrate metabolic process;BP:organic substance metabolic process;BP:alcohol catabolic process;BP:inositol catabolic process;BP:cellular process;BP:biological_process;BP:metabolic process;BP:catabolic process;BP:cellular carbohydrate catabolic process;BP:primary metabolic process;BP:carbohydrate metabolic process;BP:carbohydrate catabolic process;BP:cellular metabolic process;BP:inositol metabolic process;BP:organic hydroxy compound catabolic process;BP:organic hydroxy compound metabolic process;CC:cytoplasm;CC:cellular anatomical entity;CC:cellular_component;MF:binding;MF:monooxygenase activity;MF:molecular_function;MF:ion binding;MF:oxidoreductase activity;MF:transition metal ion binding;MF:oxidoreductase activity, acting on single donors with incorporation of molecular oxygen;MF:inositol oxygenase activity;MF:metal ion binding;MF:cation binding;MF:catalytic activity;MF:iron ion binding;	GO:0046174;GO:0019751;GO:0044248;GO:0044281;GO:0044282;GO:0006066;GO:1901575;GO:0044262;GO:0071704;GO:0046164;GO:0019310;GO:0009987;GO:0008150;GO:0008152;GO:0009056;GO:0044275;GO:0044238;GO:0005975;GO:0016052;GO:0044237;GO:0006020;GO:1901616;GO:1901615;GO:0005737;GO:0110165;GO:0005575;GO:0005488;GO:0004497;GO:0003674;GO:0043167;GO:0016491;GO:0046914;GO:0016701;GO:0050113;GO:0046872;GO:0043169;GO:0003824;GO:0005506	K00469	MIOX	map01250;map	Biosynthesis of nucl	ENOG410XQ4J	S:Function unknown	PF05153.18	MIOX	Myo-inositol oxygenase	CYT	4	21	34.5	High
TRINITY_DN130075_c1_g2_i1_orf1	60S ribosomal protein L23 [Microtus ochrogaster]	2.415509562	1.272327564	0.0003857	up	yes	0.922	0.3817	0.386	0.418	0.341	1	0.849	0.917	BP:regulation of primary metabolic process;BP:regulation of metabolic process;BP:positive regulation of response to stimulus;BP:regulation of response to stimulus;BP:cellular component organization or biogenesis;BP:cellular response to stimulus;BP:negative regulation of macromolecule metabolic process;BP:positive regulation of macromolecule metabolic process;BP:regulation of signal transduction;BP:positive regulation of signal transduction;BP:peptide biosynthetic process;BP:positive regulation of signal transduction by p53 class mediator;BP:positive regulation of biological process;BP:negative regulation of biological process;BP:regulation of cell proliferation;BP:regulation of macromolecule metabolic process;BP:cellular response to actinomycin D;BP:response to actinomycin D;BP:regulation of proteolysis;BP:regulation of RNA biosynthetic process;BP:response to organic substance;BP:organonitrogen compound metabolic process;BP:organonitrogen compound biosynthetic process;BP:protein metabolic process;BP:response to organonitrogen compound;BP:regulation of biological process;BP:regulation of catabolic process;BP:negative regulation of catabolic process;BP:negative regulation of metabolic process;BP:positive regulation of metabolic process;BP:negative regulation of biosynthetic process;BP:protein stabilization;BP:organic substance biosynthetic process;BP:cellular macromolecule metabolic process;BP:negative regulation of transferase activity;BP:cellular component organization;BP:cellular response to chemical stimulus;BP:biological regulation;BP:regulation of molecular function;BP:regulation of biological quality;BP:response to endogenous stimulus;BP:regulation of catalytic activity;BP:regulation of biosynthetic process;BP:regulation of cellular process;BP:biological_process;BP:metabolic process;BP:positive regulation of intracellular signal transduction;BP:regulation of intracellular signal transduction;BP:negative regulation of ubiquitin-protein transferase activity;BP:cellular nitrogen compound biosynthetic process;BP:negative regulation of protein modification process;BP:regulation of protein stability;BP:regulation of transferase activity;BP:regulation of transcription, DNA-templated;BP:regulation of transcription from RNA polymerase II promoter;BP:peptide metabolic process;BP:negative regulation of macromolecule biosynthetic process;BP:regulation of cell cycle process;BP:negative regulation of molecular function;BP:positive regulation of signaling;BP:cellular biosynthetic process;BP:cellular nitrogen compound metabolic process;BP:response to antibiotic;BP:cellular macromolecule biosynthetic process;BP:regulation of signaling;BP:regulation of G1 to G0 transition;BP:positive regulation of cell communication;BP:regulation of cell communication;BP:negative regulation of catalytic activity;BP:negative regulation of protein metabolic process;BP:negative regulation of transcription from RNA polymerase II promoter;BP:response to nitrogen compound;BP:regulation of protein metabolic process;BP:regulation of protein	GO:0080090;GO:0019222;GO:0048584;GO:0048583;GO:0071840;GO:0051716;GO:0010605;GO:0010604;GO:0009966;GO:0009967;GO:0043043;GO:1901798;GO:0048518;GO:0048519;GO:0042127;GO:0060255;GO:0072717;GO:0072716;GO:0030162;GO:2001141;GO:0010033;GO:1901564;GO:1901566;GO:0019538;GO:0010243;GO:0050789;GO:0009894;GO:0009895;GO:0009892;GO:0009893;GO:0009890;GO:0050821;GO:1901576;GO:0044260;GO:0051348;GO:0016043;GO:0070887;GO:0065007;GO:0065009;GO:0065008;GO:0009719;GO:0050790;GO:0009889;GO:0050794;GO:0008150;GO:0008152;GO:1902533;GO:1902531;GO:0051444;GO:0044271;GO:0031400;GO:0031647;GO:0051338;GO:0006355;GO:0006357;GO:0006518;GO:0010558;GO:0010564;GO:0044092;GO:0023056;GO:0044249;GO:0034641;GO:0046677;GO:0034645;GO:0023051;GO:1903450;GO:0010647;GO:0010646;GO:0043086;GO:0051248;GO:0000122;GO:1901698;GO:0051246;GO:1903320;GO:0043933;GO:0031399;GO:0002181;GO:0031397;GO:0008284;GO:1901701;GO:0031330;GO:1903050;GO:1903051;GO:1903506;GO:0031396;GO:0045892;GO:1901796;GO:0043604;GO:0043603;GO:0051253;GO:0051252;GO:0043170;GO:0010628;GO:0045861;GO:0050896;GO:0071495;GO:1901699;GO:0031329;GO:0031327;GO:0031326;GO:0031324;GO:0031323;GO:0070314;GO:0006807;GO:0022402;GO:0051438;GO:0071824;GO:0032984;GO:0071417;GO:2000059;GO:2000058;GO:0071704;GO:0071310;GO:0010556;GO:1904666;GO:1904667;GO:0010468;GO:0032986;GO:0009987;GO:0045934;GO:0019219;GO:1902679;GO:0009058;GO:0009059;GO:0022411;GO:0051171;GO:0051172;GO:0042221;GO:1903507;GO:0071236;GO:1901700;GO:0044238;GO:0051726;GO:1903321;GO:0044237;GO:1901652;GO:1901653;GO:0006412;GO:0048523;GO:0048522;GO:0005730;GO:0022626;GO:0022625;GO:0043232;GO:0005829;GO:0043229;GO:0043228;GO:0043226;GO:0005654;GO:0110165;GO:0005840;GO:0015934;GO:0005737;GO:0044391;GO:1990	K02894	RP-L23e, RPL:	map03010;map	Ribosome;Coronavir	COG0093	J:Translation, ribosomal structure and	PF00238.22	Ribosomal_L14	Ribosomal protein L14p/L23e	CYT	1	43	14.9	High
TRINITY_DN35662_c0_g1_i5_orf1	hypothetical protein evm_006436 [Chilo suppressalis] >CAB3522373.1 unnamed protein product [Chilo suppressalis] >CAH0399695.1 unnamed protein product [Chilo suppressalis]	4.107933951	2.038412986	1.42E-06	up	yes	1.02	0.2483	0.245	0.269	0.231	1	1.044	1.015	MF:hydrolase activity;MF:molecular_function;MF:catalytic activity;	GO:0016787;GO:0003674;GO:0003824	K21013	FPPP	map00981	Insect hormone bios	COG0647	G:Carbohydrate transport and metab	PF13344.9;PF1324	Hydrolase_6;Hydrolase_	Haloacid dehalogenase-like hydrolase;HAD-hyrolase-like	CYT	2	6	32.9	High
TRINITY_DN44288_c0_g1_i2_orf1	ATP-dependent RNA helicase p62 [Ostrinia furnacalis]	3.548073461	1.82703588	3.97E-07	up	yes	0.9853	0.2777	0.266	0.282	0.285	1	0.967	0.989	CC:intracellular organelle;CC:membrane-bounded organelle;CC:nucleus;CC:organelle;CC:intracellular membrane-bounded organelle;CC:cellular_component;CC:cellular anatomical entity;MF:purine ribonucleoside triphosphate binding;MF:molecular_function;MF:binding;MF:nucleic acid binding;MF:DNA binding;MF:RNA-dependent ATPase activity;MF:nucleotide binding;MF:heterocyclic compound binding;MF:nucleoside phosphate binding;MF:anion binding;MF:ion binding;MF:purine nucleotide binding;MF:ATP binding;MF:hydrolase activity;MF:catalytic activity, acting on RNA;MF:helicase activity;MF:small molecule binding;MF:catalytic activity;MF:adenyl nucleotide binding;MF:carbohydrate derivative binding;MF:organic cyclic compound binding;MF:RNA helicase activity;MF:adenyl ribonucleotide binding;MF:purine ribonucleotide binding;MF:ribonucleotide binding;	GO:0043229;GO:0043227;GO:0005634;GO:0043226;GO:0043231;GO:0005575;GO:0110165;GO:0035639;GO:0003674;GO:0005488;GO:0003676;GO:0003677;GO:0008186;GO:0000166;GO:1901363;GO:1901265;GO:0043168;GO:0043167;GO:0017076;GO:0005524;GO:0016787;GO:0140098;GO:0004386;GO:0036094;GO:0003824;GO:0030554;GO:0097367;GO:0097159;GO:0003724;GO:0032559;GO:0032555;GO:0032553	K12823	DDX5, DBP2	map05205;map	Proteoglycans in car	COG0513	I:Lipid transport and metabolism	PF00270.32;PF002	DEAD;Helicase_C;ResIII	DEAD/DEAH box helicase;Helicase conserved C-terminal domain;Type III restriction enzyme, res subunit	CYT	8	25	60.3	High
TRINITY_DN82801_c0_g1_i1_orf1	uncharacterized protein LOC114364712 [Ostrinia furnacalis]	4.490345757	2.166826537	1.60E-06	up	yes	1	0.2227	0.216	0.237	0.215	1	1.028	0.972	BP:organic substance metabolic process;BP:carbohydrate metabolic process;BP:primary metabolic process;BP:metabolic process;BP:biological_process;CC:cellular_component;CC:extracellular region;CC:cellular anatomical entity;MF:hydrolase activity;MF:deacetylase activity;MF:carbohydrate derivative binding;MF:hydrolase activity, acting on carbon-nitroge;MF:hydrolase activity, acting on carbon-nitroge;MF:catalytic activity;MF:molecular_function;MF:chitin binding;MF:chitin deacetylase activity;MF:binding;	GO:0071704;GO:0005975;GO:0044238;GO:0008152;GO:0008150;GO:0005575;GO:0005576;GO:0110165;GO:0016787;GO:0019213;GO:0097367;GO:0016810;GO:0016811;GO:0003824;GO:0003674;GO:0008061;GO:0004099;GO:0005488	------	------	------	------	ENOG410XX7G	Z:Cytoskeleton	PF01607.27;PF015	CBM_14;Polysacc_deac	Chitin binding Peritrophin-A domain;Polysaccharide deacetylase;Low-density lipoprotein receptor domain class A	CYT	10	27	61.3	High
TRINITY_DN8116_c0_g1_i2_orf1	uncharacterized protein LOC114350845 [Ostrinia furnacalis]	4.452232143	2.154528818	2.48E-08	up	yes	0.9973	0.224	0.229	0.228	0.215	1	1.003	0.989	------	------	------	------	------	------	COG1028	S:Function unknown	PF13561.9;PF0010	adh_short_C2;adh_shor	Enoyl;short chain dehydrogenase;KR domain	CYT	6	37	26.1	High
TRINITY_DN2894_c0_g1_i2_orf1	myrosinase 1-like isoform X1 [Ostrinia furnacalis]	4.97273178	2.314038617	5.32E-06	up	yes	1.003	0.2017	0.196	0.225	0.184	1	1.042	0.968	BP:primary metabolic process;BP:carbohydrate metabolic process;BP:organic substance metabolic process;BP:biological_process;BP:metabolic process;MF:hydrolase activity;MF:catalytic activity;MF:hydrolase activity, acting on glycosyl bonds;MF:hydrolase activity, hydrolyzing O-glycosyl	GO:0044238;GO:0005975;GO:0071704;GO:0008150;GO:0008152;GO:0016787;GO:0003824;GO:0016798;GO:0004553;GO:0003674	------	------	------	------	COG2723	G:Carbohydrate transport and metab	PF00232.21	Glyco_hydro_1	Glycosyl hydrolase family 1	CYT	6	17	59.3	High
TRINITY_DN7688_c0_g1_i10_orf1	uncharacterized protein LOC114352518 [Ostrinia furnacalis]	5.28113505	2.400848035	2.25E-05	up	yes	1.005	0.1903	0.162	0.228	0.181	1	1.059	0.955	BP:phosphorylation;BP:cellular process;BP:cellular metabolic process;BP:phosphate-containing compound metabolic process;BP:biological_process;BP:metabolic process;BP:phosphorus metabolic process;MF:kinase activity;MF:transferase activity;MF:catalytic activity;MF:molecular_function;MF:transferase activity, transferring phosphorus-containing groups;	GO:0016310;GO:0009987;GO:0044237;GO:0006796;GO:0008150;GO:0008152;GO:0006793;GO:0016301;GO:0016740;GO:0003824;GO:0003674;GO:0016772	------	------	------	------	ENOG410YKCN;ENO	S:Function unknown;	PF02958.23;PF079	EcKL;DUF1679	Ecdysteroid kinase-like family;Uncharacterized oxidoreductase dhs-27	CYT	1	10	47.9	High
TRINITY_DN117_c0_g1_i6_orf1	lipase member I-like [Ostrinia furnacalis]	8.166259169	3.029675354	5.58E-07	up	yes	1.002	0.1227	0.105	0.121	0.142	1	1.022	0.984	BP:organic substance metabolic process;BP:lipid metabolic process;BP:primary metabolic process;BP:metabolic process;BP:biological_process;CC:cellular_component;CC:extracellular region;CC:cellular anatomical entity;MF:hydrolase activity;MF:hydrolase activity, acting on ester bonds;MF:catalytic activity;MF:molecular_function;MF:lipase activity;	GO:0071704;GO:0006629;GO:0044238;GO:0008152;GO:0008150;GO:0005575;GO:0005576;GO:0110165;GO:0016787;GO:0016788;GO:0003824;GO:0003674;GO:0016298	------	------	------	------	ENOG41116Y2;ENO(	S:Function unknown;	PF00151.22	Lipase	Lipase	CYT	1	11	42.3	High
TRINITY_DN22815_c0_g1_i2_orf1	acyl carrier protein, mitochondrial isoform X1 [Ostrinia furnacalis]	2.150681818	1.104794102	4.99E-05	up	yes	0.9463	0.44	0.442	0.437	0.441	1	0.928	0.911	------	------	K03955	NDUFAB1	map04714;map	Thermogenesis;Oxid	COG0236	I:Lipid transport and metabolism	PF00550.28	PP-binding	Phosphopantetheine attachment site	CYT	2	15	17.3	High
TRINITY_DN220_c0_g1_i3_orf1	serine-arginine protein 55 isoform X6 [Pieris brassicae]	2.16368576	1.113490986	4.93E-05	up	yes	0.9557	0.4417	0.434	0.472	0.419	1	0.925	0.942	------	------	K12893	SRSF4_5_6, SF	map05168;map	Herpes simplex virus	COG0724	S:Function unknown	PF00076.25;PF087	RRM_1;RRM_3	RNA recognition motif.;RNA binding motif	CYT	6	28	26.5	High
TRINITY_DN6308_c0_g1_i6_orf1	myc box-dependent-interacting protein 1 isoform X2 [Ostrinia furnacalis]	2.605173765	1.381379604	2.64E-06	up	yes	0.997	0.3827	0.365	0.408	0.375	1	1.011	0.98	CC:cytoplasm;CC:cellular_component;CC:cellular anatomical entity;	GO:0005737;GO:0005575;GO:0110165	K12562	AMPH	map04666;map	Fc gamma R-mediat	ENOG410ZBW1	S:Function unknown	PF03114.21;PF146	BAR;SH3_9;SH3_1;SH3_	BAR domain;Variant SH3 domain;SH3 domain;Variant SH3 domain	CYT	3	33	52.4	High
TRINITY_DN54543_c0_g5_i2_orf1	reactive oxygen species modulator 1 [Papilio machaon] >XP_022123076.1 reactive oxygen species modulator 1 [Pieris rapae] >XP_022820703.1 reactive oxygen species modulator 1 [Spodoptera litura] >XP_028176790.1 reactive oxygen species modulator 1 [Ostrinia furnacalis] >XP_030021418.1 reactive oxygen species modulator 1 [Manduca sexta] >XP_034836896.1 reactive oxygen species modulator 1 [Maniola hyperantus] >XP_035446679.1 reactive oxygen species modulator 1-like [Spodoptera frugiperda] >XP_045458971.1 reactive oxygen species modulator 1 [Melitaea cinxia] >XP_045521377.1 reactive oxygen species modulator 1 [Pieris brassicae] >XP_045765649.1 reactive oxygen species modulator 1 [Maniola jurtina] >XP_047042173.1 reactive oxygen species modulator 1 [Helicoverpa zea] >XP_049691668.1 reactive oxygen species modulator 1 [Helicoverpa armigera] >KAF9414699.1 hypothetical protein HW555_007477 [Spodoptera exigua] >KPI93862.1 Reactive oxygen species modulator 1 [Papilio xuthus] >CAB3515149.1 unnamed protein product [Spodoptera littoralis] >CAG5021695.1 unnamed protein product [Parnassius apollo] >KAF9823017.1 hypothetical protein SFRURICE_018191 [Spodoptera frugiperda]	2.831932773	1.501787018	9.22E-05	up	yes	1.011	0.357	0.367	0.328	0.376	1	0.95	1.082	CC:cellular_component;CC:integral component of membrane;CC:cellular anatomical entity;CC:intrinsic component of membrane;	GO:0005575;GO:0016021;GO:0110165;GO:0031224	------	------	------	------	ENOG411279J	S:Function unknown	PF10247.12	Romo1	Reactive mitochondrial oxygen species modulator 1	CYT	1	23	8.2	High
TRINITY_DN144258_c0_g1_i1_orf1	PREDICTED: enhancer of rudimentary homolog [Microplitis demolitor] >XP_044577051.1 enhancer of rudimentary homolog [Cotesia glomerata] >KAG8041963.1 hypothetical protein G9C98_007267 [Cotesia typhae] >KAH0539785.1 hypothetical protein KQX54_008036 [Cotesia glomerata] >CAD6227368.1 GSCOCG00006137001-RA-CDS [Cotesia congregata]	2.038963964	1.027836278	0.00443	up	yes	0.9053	0.444	0.389	0.455	0.488	1	0.759	0.957	BP:cellular process;BP:biological_process;BP:cell cycle;	GO:0009987;GO:0008150;GO:0007049	------	------	------	------	ENOG4111Z48	S:Function unknown	PF01133.20	ER	Enhancer of rudimentary	CYT	1	10	12.1	Medium
TRINITY_DN56110_c0_g1_i1_orf1	pescadillo homolog [Ostrinia furnacalis]	2.697612732	1.431683251	7.60E-06	up	yes	1.017	0.377	0.342	0.39	0.399	1	1.041	1.011	BP:maturation of 5.8S rRNA from tricistronic rRNA transcrip;BP:nucleic acid metabolic process;BP:maturation of LSU-rRNA;BP:ncRNA metabolic process;BP:organic cyclic compound metabolic process;BP:nucleobase-containing compound metabolic process;BP:organic substance metabolic process;BP:rRNA processing;BP:ncRNA processing;BP:cellular process;BP:cellular aromatic compound metabolic process;BP:maturation of 5.8S rRNA;BP:maturation of LSU-rRNA from tricistronic rRNA transcrip;BP:biological_process;BP:metabolic process;BP:heterocycle metabolic process;BP:RNA metabolic process;BP:primary metabolic process;BP:rRNA metabolic process;BP:cellular nitrogen compound metabolic process;BP:cellular metabolic process;BP:macromolecule metabolic process;BP:nitrogen compound metabolic process;BP:RNA processing;CC:preribosome, large subunit precursor;CC:preribosome;CC:intracellular organelle;CC:non-membrane-bounded organelle;CC:organelle;CC:nucleolus;CC:nucleoplasm;CC:cellular anatomical entity;CC:macromolecular complex;CC:intracellular non-membrane-bounded organelle;CC:ribonucleoprotein complex;CC:cellular_component;MF:macromolecular complex binding;MF:binding;MF:ribonucleoprotein complex binding;MF:molecular_function;	GO:0000466;GO:0090304;GO:0000470;GO:0034660;GO:1901360;GO:0006139;GO:0071704;GO:0006364;GO:0034470;GO:0009987;GO:0006725;GO:0000460;GO:0000463;GO:0008150;GO:0008152;GO:0046483;GO:0016070;GO:0044238;GO:0016072;GO:0034641;GO:0044237;GO:0043170;GO:0006807;GO:0006396;GO:0030687;GO:0030684;GO:0043229;GO:0043228;GO:0043226;GO:0005730;GO:0005654;GO:0110165;GO:0032991;GO:0043232;GO:1990904;GO:0005575;GO:0044877;GO:0005488;GO:0043021;GO:0003674	K14843	PES1, NOP7	------	------	COG5163	J:Translation, ribosomal structure and	PF06732.14;PF165	Pescadillo_N;BRCT_2	Pescadillo N-terminus;BRCT domain, a BRCA1 C-terminus domain	CYT	2	3	71.4	High
TRINITY_DN11347_c0_g1_i1_orf1	N(4)-(Beta-N-acetylglucosaminyl)-L-asparaginase-like [Ostrinia furnacalis]	3.618374558	1.855341757	1.52E-05	up	yes	1.024	0.283	0.272	0.267	0.31	1	1.077	0.996	MF:hydrolase activity;MF:molecular_function;MF:catalytic activity;	GO:0016787;GO:0003674;GO:0003824	K01444	AGA, aspG	map00511;map	Other glycan degrad	COG1446	E:Amino acid transport and metaboli	PF01112.21	Asparaginase_2	Asparaginase	CYT	4	13	37	High
TRINITY_DN51045_c0_g1_i1_orf1	cell growth-regulating nucleolar protein [Ostrinia furnacalis]	2.816360143	1.493831831	4.90E-06	up	yes	1.026	0.3643	0.339	0.386	0.368	1	1.028	1.05	CC:intracellular membrane-bounded organelle;CC:nucleus;CC:cellular_component;CC:intracellular organelle;CC:membrane-bounded organelle;CC:organelle;CC:cellular anatomical entity;MF:cation binding;MF:organic cyclic compound binding;MF:heterocyclic compound binding;MF:ion binding;MF:molecular_function;MF:binding;MF:nucleic acid binding;MF:DNA binding;MF:metal ion binding;	GO:0043231;GO:0005634;GO:0005575;GO:0043229;GO:0043227;GO:0043226;GO:0110165;GO:0043169;GO:0097159;GO:1901363;GO:0043167;GO:0003674;GO:0005488;GO:0003676;GO:0003677;GO:0046872	K15263	LYER	------	------	ENOG411081U	S:Function unknown	PF08790.14	zf-LYAR	LYAR-type C2HC zinc finger	CYT	4	13	48.3	High
TRINITY_DN15607_c0_g1_i6_orf1	protein artichoke-like [Ostrinia furnacalis]	2.500630835	1.32229209	2.20E-06	up	yes	0.991	0.3963	0.407	0.397	0.385	1	1.008	0.965	CC:cellular_component;CC:integral component of membrane;CC:cellular anatomical entity;CC:intrinsic component of membrane;	GO:0005575;GO:0016021;GO:0110165;GO:0031224	------	------	------	------	COG4886	I:Lipid transport and metabolism	PF13855.9;PF1279	LRR_8;LRR_4;LRR_5;LRR	Leucine rich repeat;Leucine Rich repeats;BspA type Leucine rich repeat region;Leucine-rich repeat	CYT	5	6	109	High
TRINITY_DN28577_c0_g1_i6_orf1	delta-1-pyrroline-5-carboxylate dehydrogenase, mitochondrial [Nymphalis io]	2.993883792	1.582018224	7.45E-07	up	yes	0.979	0.327	0.324	0.321	0.336	1	0.961	0.976	BP:small molecule catabolic process;BP:carboxylic acid metabolic process;BP:cellular amino acid catabolic process;BP:nitrogen compound metabolic process;BP:small molecule metabolic process;BP:glutamine family amino acid catabolic process;BP:glutamine family amino acid metabolic process;BP:organic substance catabolic process;BP:proline catabolic process;BP:proline metabolic process;BP:cellular amino acid metabolic process;BP:organic substance metabolic process;BP:alpha-amino acid metabolic process;BP:alpha-amino acid catabolic process;BP:primary metabolic process;BP:cellular process;BP:proline catabolic process to glutamate;BP:metabolic process;BP:oxoacid metabolic process;BP:catabolic process;BP:cellular catabolic process;BP:dicarboxylic acid metabolic process;BP:organonitrogen compound metabolic process;BP:organonitrogen compound catabolic process;BP:organic acid metabolic process;BP:carboxylic acid catabolic process;BP:organic acid catabolic process;BP:cellular metabolic process;BP:glutamate metabolic process;BP:biological_process;MF:molecular_function;MF:oxidoreductase activity;MF:oxidoreductase activity, acting on the aldehyde or oxo group of donors, NAD or NADP as acceptor;MF:catalytic activity;MF:oxidoreductase activity, acting on the aldehyde or oxo group of donors;MF:1-pyrroline-5-carboxylate	GO:0044282;GO:0019752;GO:0009063;GO:0006807;GO:0044281;GO:0009065;GO:0009064;GO:1901575;GO:0006562;GO:0006560;GO:0006520;GO:0071704;GO:1901605;GO:1901606;GO:0044238;GO:0009987;GO:0010133;GO:0008152;GO:0043436;GO:0009056;GO:0044248;GO:0043648;GO:1901564;GO:1901565;GO:0006082;GO:0046395;GO:0016054;GO:0044237;GO:0006536;GO:0008150;GO:0003674;GO:0016491;GO:0016620;GO:0003824;GO:0016903;GO:0003842	K00294	E1.2.1.88	map00250;map	Alanine, aspartate a	COG1012	S:Function unknown	PF00171.25	Aldedh	Aldehyde dehydrogenase family	CYT	3	34	11.3	High
TRINITY_DN2574_c0_g1_i5_orf1	prion-like-(Q/N-rich) domain-bearing protein 25 [Ostrinia furnacalis] >XP_028158239.1 prion-like-(Q/N-rich) domain-bearing protein 25 [Ostrinia furnacalis] >XP_028158240.1 prion-like-(Q/N-rich) domain-bearing protein 25 [Ostrinia furnacalis] >XP_028158241.1 prion-like-(Q/N-rich) domain-bearing protein 25 [Ostrinia furnacalis]	2.574864236	1.364496366	3.22E-05	up	yes	0.9957	0.3867	0.363	0.436	0.361	1	1.021	0.966	------	------	------	------	------	------	ENOG410XTK6;ENO(	S:Function unknown;	PF01683.21	EB	EB module	CYT	4	20	34.9	High
TRINITY_DN8369_c0_g1_i1_orf1	39S ribosomal protein L37, mitochondrial [Ostrinia furnacalis]	3.066812363	1.616739901	7.35E-07	up	yes	0.9823	0.3203	0.316	0.309	0.336	1	0.968	0.979	------	------	K17418	MRPL37	------	------	ENOG410ZT8U	J:Translation, ribosomal structure and	PF07147.15	PDCD9	Mitochondrial 28S ribosomal protein S30	CYT	3	6	47.1	High
TRINITY_DN140669_c0_g1_i1_orf1	S-methyl-5'-thioadenosine phosphorylase-like isoform X1 [Hyposmocoma kahamanoa]	4.188	2.066261442	3.13E-05	up	yes	1.047	0.25	0.21	0.254	0.286	1	1.034	1.106	BP:nucleobase-containing small molecule biosynthetic process;BP:cellular biosynthetic process;BP:cellular nitrogen compound metabolic process;BP:nitrogen compound metabolic process;BP:nucleoside biosynthetic process;BP:small molecule biosynthetic process;BP:purine-containing compound metabolic process;BP:purine-containing compound biosynthetic process;BP:organic cyclic compound biosynthetic process;BP:purine nucleoside biosynthetic process;BP:organic cyclic compound metabolic process;BP:nucleobase-containing compound metabolic process;BP:purine nucleoside metabolic process;BP:organic substance metabolic process;BP:ribonucleoside biosynthetic process;BP:purine-containing compound salvage;BP:small molecule metabolic process;BP:heterocycle biosynthetic process;BP:ribonucleoside metabolic process;BP:organic substance biosynthetic process;BP:cellular process;BP:cellular aromatic compound metabolic process;BP:biosynthetic process;BP:biological_process;BP:nucleoside metabolic process;BP:metabolic process;BP:nucleobase-containing compound biosynthetic process;BP:organonitrogen compound metabolic process;BP:purine ribonucleoside biosynthetic process;BP:purine ribonucleoside metabolic process;BP:nucleobase-containing small molecule metabolic process;BP:heterocycle metabolic process;BP:cellular metabolic compound salvage;BP:primary metabolic process;BP:cellular nitrogen compound biosynthetic process;BP:organonitrogen compound biosynthetic process;BP:carbohydrate derivative biosynthetic process;BP:carbohydrate derivative metabolic process;BP:nucleoside salvage;BP:cellular metabolic process;BP:glycosyl compound metabolic process;BP:purine ribonucleoside salvage;BP:aromatic compound biosynthetic process;BP:glycosyl compound biosynthetic process;MF:molecular_function;MF:purine-nucleoside phosphorylase activity;MF:transferase activity;MF:transferase activity, transferring pentosyl groups;MF:catalytic activity;MF:S-methyl-5-thioadenosine phosphorylase activity;MF:transferase activity, transferring glycosyl groups;	GO:0034404;GO:0044249;GO:0034641;GO:0006807;GO:0009163;GO:0044283;GO:0072521;GO:0072522;GO:1901362;GO:0042451;GO:1901360;GO:0006139;GO:0042278;GO:0071704;GO:0042455;GO:0043101;GO:0044281;GO:0018130;GO:0009119;GO:1901576;GO:0009987;GO:0006725;GO:0009058;GO:0008150;GO:0009116;GO:0008152;GO:0034654;GO:1901564;GO:0046129;GO:0046128;GO:0055086;GO:0046483;GO:0043094;GO:0044238;GO:0044271;GO:1901566;GO:1901137;GO:1901135;GO:0043174;GO:0044237;GO:1901657;GO:0006166;GO:0019438;GO:1901659;GO:0003674;GO:0004731;GO:0016740;GO:0016763;GO:0003824;GO:0017061;GO:0016757	K00772	mtaP, MTAP	map00270	Cysteine and methic	COG0005	F:Nucleotide transport and metabolis	PF01048.23	PNP_UDP_1	Phosphorylase superfamily	CYT	1	10	8.1	Medium
TRINITY_DN14436_c0_g1_i7_orf1	V-type proton ATPase subunit C [Vanessa cardui]	2.703502579	1.434829733	1.93E-07	up	yes	0.9957	0.3683	0.357	0.365	0.383	1	0.998	0.989	------	------	K02148	ATPeV1C, ATF	map05323;map	Rheumatoid arthritis	COG5127	C:Energy production and conversion	PF03223.18	V-ATPase_C	V-ATPase subunit C	CYT	20	51	44.1	High
TRINITY_DN7966_c0_g1_i4_orf1	leucine-rich repeat neuronal protein 1-like [Ostrinia furnacalis]	3.14057508	1.651028759	7.14E-06	up	yes	0.983	0.313	0.311	0.322	0.306	1	0.94	1.009	BP:locomotion;BP:biological_process;CC:integral component of membrane;CC:cellular_component;CC:cell periphery;CC:intrinsic component of membrane;CC:cellular anatomical entity;	GO:0040011;GO:0008150;GO:0016021;GO:0005575;GO:0071944;GO:0031224;GO:0110165	------	------	------	------	COG4886	P:Inorganic ion transport and metab	PF13855.9;PF1279	LRR_8;LRR_4	Leucine rich repeat;Leucine Rich repeats	PLA	2	4	57.2	High
TRINITY_DN37538_c0_g2_i1_orf1	esterase FE4-like [Ostrinia furnacalis]	3.582797155	1.841086366	3.16E-05	up	yes	1.058	0.2953	0.285	0.327	0.274	1	1.059	1.114	------	------	K07378	NLGN	map04514	Cell adhesion molec	COG2272	I:Lipid transport and metabolism	PF00135.31	COesterase	Carboxylesterase family	CYT	3	31	15.9	High
TRINITY_DN96566_c0_g1_i1_orf1	NADH-ubiquinone oxidoreductase subunit 8-like [Ostrinia furnacalis]	2.581873784	1.368418475	0.0001376	up	yes	0.9287	0.3597	0.381	0.329	0.369	1	0.906	0.88	CC:membrane;CC:cellular_component;CC:cellular anatomical entity;MF:4 iron, 4 sulfur cluster binding;MF:oxidoreductase activity, acting on NA;MF:iron-sulfur cluster binding;MF:catalytic activity;MF:molecular_function;MF:binding;MF:oxidoreductase activity;MF:metal cluster binding;	GO:0016020;GO:0005575;GO:0110165;GO:0051539;GO:0016651;GO:0051536;GO:0003824;GO:0003674;GO:0005488;GO:0016491;GO:0051540	K03941	NDUFS8	map04714;map	Thermogenesis;Oxid	COG1143	C:Energy production and conversion	PF12838.10;PF000	Fer4_7;Fer4;Fer4_10;Fer	4Fe-4S dicluster domain;4Fe-4S binding domain;4Fe-4S dicluster domain;4Fe-4S double cluster binding domain;4Fe-4S dicluster domain;4Fe-4S dicluster domain;4Fe-4S dicluster domain	CYT	1	4	28.6	High
TRINITY_DN25975_c0_g3_i2_orf1	V-type proton ATPase subunit D isoform X2 [Ostrinia furnacalis]	2.958492788	1.564862378	9.97E-07	up	yes	1.005	0.3397	0.325	0.341	0.353	1	0.99	1.026	MF:inorganic cation transmembrane transporter activity;MF:ATPase activity, coupled to transmembrane movement of substances;MF:active ion transmembrane transporter activity;MF:ATPase coupled ion transmembrane transporter activity;MF:inorganic solute uptake transmembrane transporter activity;MF:transporter activity;MF:cation transmembrane transporter activity;MF:hydrogen-translocating pyrophosphatase activity;MF:cation-transporting ATPase activity;MF:ion transmembrane transporter activity;MF:primary active transmembrane transporter activity;MF:transmembrane transporter activity;MF:molecular_function;MF:active transmembrane transporter activity;MF:proton-transporting ATPase activity, rotational mechanism;MF:hydrogen ion transmembrane transporter activity;MF:ATPase activity, coupled	GO:0022890;GO:0042626;GO:0022853;GO:0042625;GO:0015318;GO:0005215;GO:0008324;GO:0009678;GO:0019829;GO:0015075;GO:0015399;GO:0022857;GO:0003674;GO:0022804;GO:0046961;GO:0015078;GO:0044769	K02149	ATPeV1D, ATF	map05323;map	Rheumatoid arthritis	COG1394	C:Energy production and conversion	PF01813.20	ATP-synt_D	ATP synthase subunit D	CYT	7	20	27.4	High
TRINITY_DN29440_c1_g1_i4_orf1	neutral lipase [Helicoverpa armigera]	2.916794596	1.544383794	0.0007983	up	yes	0.95	0.3257	0.275	0.452	0.25	1	0.93	0.92	BP:organic substance metabolic process;BP:lipid metabolic process;BP:primary metabolic process;BP:metabolic process;BP:biological_process;CC:cellular_component;CC:extracellular region;CC:cellular anatomical entity;MF:hydrolase activity;MF:hydrolase activity, acting on ester bonds;MF:catalytic activity;MF:molecular_function;MF:lipase activity;	GO:0071704;GO:0006629;GO:0044238;GO:0008152;GO:0008150;GO:0005575;GO:0005576;GO:0110165;GO:0016787;GO:0016788;GO:0003824;GO:0003674;GO:0016298	------	------	------	------	ENOG410YGS3;ENO(	S:Function unknown;	PF00151.22	Lipase	Lipase	PLA	1	10	11.7	High
TRINITY_DN1073_c0_g1_i4_orf1	carboxylesterase [Loxostege sticticalis]	2.748433048	1.458609336	1.52E-05	up	yes	0.9647	0.351	0.335	0.373	0.345	1	0.969	0.925	CC:integral component of membrane;CC:cellular_component;CC:intrinsic component of membrane;CC:cellular anatomical entity;MF:hydrolase activity;MF:hydrolase activity, acting on ester bonds;MF:carboxylic ester hydrolase activity;MF:catalytic activity;MF:molecular_function;	GO:0016021;GO:0005575;GO:0031224;GO:0110165;GO:0016787;GO:0016788;GO:0052689;GO:0003824;GO:0003674	------	------	------	------	COG2272;COG0580;I	I:Lipid transport and metabolism;G:C	PF00135.31;PF204	COesterase;BD-FAE;Ab	Carboxylesterase family;BD-FAE;alpha/beta hydrolase fold	EXC	5	14	65.7	High
TRINITY_DN2918_c0_g1_i1_orf1	28S ribosomal protein S10, mitochondrial [Ostrinia furnacalis] >XP_028175147.1 28S ribosomal protein S10, mitochondrial [Ostrinia furnacalis]	3.066390041	1.616541218	1.90E-05	up	yes	0.9607	0.3133	0.286	0.326	0.328	1	0.964	0.918	------	------	K02946	RP-S10, MRP!	map03010	Ribosome	COG0051	J:Translation, ribosomal structure and	PF00338.25	Ribosomal_S10	Ribosomal protein S10p/S20e	CYT	1	5	19.9	High
TRINITY_DN8543_c0_g1_i1_orf1	39S ribosomal protein L38, mitochondrial [Ostrinia furnacalis]	3.157174674	1.658634081	1.57E-06	up	yes	0.9923	0.3143	0.334	0.31	0.299	1	1.007	0.97	------	------	K17419	MRPL38	------	------	COG1881	S:Function unknown	PF01161.23	PBP	Phosphatidylethanolamine-binding protein	CYT	1	4	46.4	High
TRINITY_DN344_c1_g1_i1_orf1	chymotrypsin-like serine protease 16 [Ostrinia nubilalis]	3.853246254	1.946074389	1.75E-07	up	yes	1.003	0.2603	0.253	0.269	0.259	1	0.99	1.019	BP:organic substance metabolic process;BP:organonitrogen compound metabolic process;BP:protein metabolic process;BP:macromolecule metabolic process;BP:biological_process;BP:nitrogen compound metabolic process;BP:metabolic process;BP:proteolysis;BP:primary metabolic process;MF:serine-type endopeptidase activity;MF:endopeptidase activity;MF:hydrolase activity;MF:serine hydrolase activity;MF:catalytic activity, acting on a protein;MF:catalytic activity;MF:molecular_function;MF:peptidase activity;MF:serine-type peptidase activity;	GO:0071704;GO:1901564;GO:0019538;GO:0043170;GO:0008150;GO:0006807;GO:0008152;GO:0006508;GO:0044238;GO:0004252;GO:0004175;GO:0016787;GO:0017171;GO:0140096;GO:0003824;GO:0003674;GO:0008233;GO:0008236	K01312	PRSS1_2_3	map04972;map	Pancreatic secretion	COG5640	I:Lipid transport and metabolism	PF00089.29	Trypsin	Trypsin	CYT	5	29	30.7	High

TRINITY_DN42759_c0_g3_i1_orf1	fatty acid synthase-like [Ostrinia furnacalis]	3.226194345	1.689833349	2.13E-07	up	yes	0.9927	0.3077	0.315	0.312	0.296	1	0.978	1	[illegible]	[illegible]	K00665	FASN	map04910;map	Insulin signaling pat	COG3321	Q:Secondary metabolites biosynthesi	PF00698.24;PF001	Acyl_transf_1;ketoacyl-	Acyl transferase domain;Beta-ketoacyl synthase, N-terminal domain;Ketoacyl-synthetase C-terminal extension;Beta-ketoacyl synthase, C-terminal domain	CYT	2	3	84.3	High
TRINITY_DN29034_c0_g1_i2_orf1	trypsin-like serine protease [Ostrinia nubilalis]	3.868725869	1.951858506	3.27E-07	up	yes	1.002	0.259	0.278	0.257	0.242	1	1.011	0.996	[illegible]	[illegible]	K01312	PRSS1_2_3	map04972;map	Pancreatic secretion	COG5640	O:Posttranslational modification, pro	PF00089.29	Trypsin	Trypsin	CYT	1	8	15.3	High
TRINITY_DN76283_c0_g6_i1_orf1	fatty acid synthase-like [Ostrinia furnacalis]	3.557658605	1.830928075	5.10E-05	up	yes	1.015	0.2853	0.249	0.335	0.272	1	0.972	1.073	[illegible]	[illegible]	K00665	FASN	map04910;map	Insulin signaling pat	COG3321;ENOG4111	Q:Secondary metabolites biosynthesi	PF13602.9;PF0010	ADH_zinc_N_2;ADH_zir	Zinc-binding dehydrogenase;Zinc-binding dehydrogenase	CYT	1	2	95	High
TRINITY_DN13651_c0_g1_i2_orf1	40S ribosomal protein S12, mitochondrial [Ostrinia furnacalis]	4.13218632	2.046905307	1.00E-06	up	yes	0.9847	0.2383	0.22	0.253	0.242	1	0.992	0.962	[illegible]	[illegible]	K02950	RP-S12, MRPS	map03010	Ribosome	COG0048	S:Function unknown	PF00164.28	Ribosom_S12_S23	Ribosomal protein S12/S23	CYT	1	6	17.4	High
TRINITY_DN26375_c0_g1_i1_orf1	hypothetical protein O3G_MSEX007366 [Manduca sexta]	2.78552593	1.477949746	2.76E-05	up	yes	0.9507	0.3413	0.346	0.345	0.333	1	0.949	0.903	------	------	------	------	------	------	ENOG410Y78S;ENO(	S:Function unknown;	------	------	------	CYT	1	2	129.6	High
TRINITY_DN8691_c0_g1_i3_orf1	nucleolin-like [Melitaea cinxia]	4.926829268	2.300659478	8.95E-07	up	yes	1.01	0.205	0.222	0.195	0.198	1	1.036	0.993	------	------	------	------	------	------	ENOG4110RR1	S:Function unknown	------	------	------	CYT	2	15	14.4	High
TRINITY_DN17825_c1_g1_i1_orf1	39S ribosomal protein L1, mitochondrial [Ostrinia furnacalis]	3.009947156	1.589738158	1.18E-05	up	yes	0.9683	0.3217	0.336	0.329	0.3	1	0.978	0.927	CC:ribosome;CC:intracellular non-membrane-bounded organelle;[illegible]	[illegible]	K02863	RP-L1, MRPL1	map03010	Ribosome	COG0081	J:Translation, ribosomal structure anc	PF00687.24	Ribosomal_L1	Ribosomal protein L1p/L10e family	CYT	1	3	39.6	High
TRINITY_DN311_c0_g1_i4_orfp1	TRINITY_DN311_c0_g1_i4_m.65135 TRINITY_DN311_c0_g1::TRINITY_DN311_c0_g1_i4::g.65135 ORF type:5prime_partial len:126 (+),score=71.21,TRINITY_DN311_c0_g1_i4:1-378(+)	4.349579087	2.120875797	3.02E-07	up	yes	0.9817	0.2257	0.235	0.226	0.216	1	0.966	0.979	------	------	------	------	------	------	------	------	------	------	------	EXC	1	6	13.8	High
TRINITY_DN334_c0_g1_i2_orf1	chymotrypsin-like serine protease, partial [Ostrinia nubilalis]	4.71040724	2.235851794	7.34E-06	up	yes	1.041	0.221	0.253	0.204	0.206	1	1.076	1.047	[illegible]	[illegible]	------	------	------	------	COG5640	O:Posttranslational modification, pro	PF00089.29	Trypsin	Trypsin	CYT	1	18	13.2	High
TRINITY_DN97680_c0_g1_i1_orf1	39S ribosomal protein L52, mitochondrial [Ostrinia furnacalis]	3.499433321	1.807121319	0.0004326	up	yes	0.9263	0.2647	0.339	0.199	0.256	1	0.939	0.84	[illegible]	[illegible]	K17433	MRPL52	------	------	ENOG4111XV4;COG:	J:Translation, ribosomal structure anc	PF18699.4	MRPL52	Mitoribosomal protein mL52	CYT	1	20	14.8	Medium
TRINITY_DN11259_c0_g1_i1_orf1	uncharacterized protein LOC114357075 [Ostrinia furnacalis]	3.51352326	1.812918449	1.91E-06	up	yes	0.9743	0.2773	0.258	0.294	0.28	1	0.962	0.961	------	------	------	------	------	------	COG1028	S:Function unknown	PF13561.9;PF0010	adh_short_C2;adh_shor	Enoyl;short chain dehydrogenase;KR domain	CYT	6	46	26.7	High
TRINITY_DN15338_c0_g1_i7_orf1	methyltransferase-like protein 17, mitochondrial [Ostrinia furnacalis]	2.174390571	1.120611105	0.01399	up	yes	0.8117	0.3733	0.298	0.364	0.458	1	0.722	0.713	[illegible]	[illegible]	------	------	------	------	COG5459	J:Translation, ribosomal structure anc	PF09243.13	Rsm22	Mitochondrial small ribosomal subunit Rsm22	CYT	1	2	50.6	Medium
TRINITY_DN16939_c0_g1_i4_orf1	39S ribosomal protein L17, mitochondrial [Ostrinia furnacalis]	3.372183099	1.753682872	6.84E-06	up	yes	0.9577	0.284	0.279	0.284	0.289	1	0.946	0.927	[illegible]	[illegible]	K02879	RP-L17, MRPL	map03010	Ribosome	COG0203	J:Translation, ribosomal structure anc	PF01196.22	Ribosomal_L17	Ribosomal protein L17	CYT	2	7	24	High
TRINITY_DN717_c0_g1_i2_orfp1	TRINITY_DN717_c0_g1_i2_m.67915 TRINITY_DN717_c0_g1::TRINITY_DN717_c0_g1_i2::g.67915 ORF type:internal len:868 (+),score=265.71,Collagen PF01391.19 0.11,Collagen|PF01391.19|0.039,Collagen|PF01391.19|0.00054,Collagen|PF01391.19|0.0019,Collagen|PF01391.19|0.0005,Collagen|PF01391.19|9.9e-05,Collagen|PF01391.19|1.7e-07 TRINITY_DN717_c0_g1_i2:3-2603(+)	6.201598033	2.632640018	3.91E-06	up	yes	1.009	0.1627	0.174	0.147	0.167	1	1.052	0.975	------	------	------	------	------	------	------	------	PF01391.21	Collagen	Collagen triple helix repeat	PLA	8	20	90.1	High
TRINITY_DN26688_c0_g1_i2_orf1	myogenesis-regulating glycosidase-like [Ostrinia furnacalis]	4.271143221	2.094622276	3.24E-08	up	yes	1.005	0.2353	0.236	0.232	0.238	1	1.018	0.998	[illegible]	[illegible]	K24727	MYORG	------	------	COG1501	G:Carbohydrate transport and metat	PF01055.29	Glyco_hydro_31	Glycosyl hydrolases family 31	CYT	11	21	71.1	High
TRINITY_DN1914_c0_g1_i6_orf1	loricrin-like [Ostrinia furnacalis]	8.537387017	3.093794581	2.13E-05	up	yes	1.039	0.1217	0.121	0.127	0.117	1	1.119	0.999	------	------	------	------	------	------	ENOG4110OTU;ENO	S:Function unknown;	------	------	------	CYT	1	15	58.9	High
TRINITY_DN51766_c0_g1_i2_orf1	facilitated trehalose transporter Tret1-like [Ostrinia furnacalis]	5.764241399	2.527130755	2.76E-07	up	yes	1.022	0.1773	0.173	0.184	0.175	1	1.041	1.024	[illegible]	[illegible]	------	------	------	------	ENOG410XNQK	E:Amino acid transport and metaboli	PF00083.27;PF076	Sugar_tr;MFS_1	Sugar;Major Facilitator Superfamily	CYT	1	3	49.7	High
TRINITY_DN35051_c0_g1_i1_orf1	uncharacterized protein LOC114364307 [Ostrinia furnacalis]	6.477052359	2.695337407	2.67E-07	up	yes	1.002	0.1547	0.15	0.153	0.161	1	1.023	0.982	------	------	------	------	------	------	ENOG410Y27Q	S:Function unknown	PF06757.16	Ins_allergen_rp	Insect allergen related repeat, nitrile-specifier	CYT	5	30	14.8	High
TRINITY_DN30663_c0_g1_i1_orf1	surfeit locus protein 6 homolog [Ostrinia furnacalis]	4.70281892	2.233525783	1.96E-06	up	yes	0.9843	0.2093	0.225	0.205	0.198	1	1.002	0.951	[illegible]	[illegible]	------	------	------	------	ENOG4111G0Y	S:Function unknown	PF04935.15	SURF6	Surfeit locus protein 6	CYT	2	6	33.5	High
TRINITY_DN81803_c0_g2_i1_orf1	cathepsin K-like [Ostrinia furnacalis]	8.070904645	3.01273039	4.55E-08	up	yes	0.9903	0.1227	0.122	0.119	0.127	1	0.975	0.996	[illegible]	[illegible]	------	------	------	------	COG4870	O:Posttranslational modification, pro	------	------	------	CYT	1	8	13.3	High
TRINITY_DN10792_c0_g2_i5_orf1	uncharacterized protein LOC114366171 [Ostrinia furnacalis]	4.965	2.311793718	4.67E-07	up	yes	0.993	0.2	0.194	0.202	0.204	1	1.011	0.968	------	------	------	------	------	------	ENOG410XT8F;ENO(	S:Function unknown;	PF02958.23;PF079	EcKL;DUF1679;APH	Ecdysteroid kinase-like family;Uncharacterized oxidoreductase dhs-27;Phosphotransferase enzyme family	CYT	9	20	48	High
TRINITY_DN41086_c0_g1_i4_orf1	collagenase-like [Pectinophora gossypiella]	7.768745067	2.95768157	5.80E-07	up	yes	0.9843	0.1267	0.111	0.131	0.138	1	0.994	0.959	[illegible]	[illegible]	------	------	------	------	COG5640	O:Posttranslational modification, pro	PF00089.29;PF133	Trypsin;Trypsin_2	Trypsin;Trypsin-like peptidase domain	CYT	3	10	36.1	High
TRINITY_DN61674_c0_g1_i2_orf1	fatty acid-binding protein 1-like [Ostrinia furnacalis]	7.329504666	2.873715703	7.45E-06	up	yes	1.021	0.1393	0.137	0.148	0.133	1	0.985	1.079	[illegible]	[illegible]	K08753;K08752	FABP4, aP2;F/	map04923;map	Regulation of lipolys	ENOG4111US8	I:Lipid transport and metabolism	------	------	------	CYT	2	12	14.2	High
TRINITY_DN1249_c0_g1_i6_orf1	venom carboxylesterase-6-like [Ostrinia furnacalis]	6.390463027	2.675920467	6.55E-05	up	yes	0.9247	0.1447	0.172	0.102	0.16	1	0.908	0.866	------	------	------	------	------	------	COG2272;ENOG410)	I:Lipid transport and metabolism;T:Si(	PF00135.31;PF204	COesterase;BD-FAE;Ab	Carboxylesterase family;BD-FAE;alpha/beta hydrolase	PLA	1	6	64.7	High
TRINITY_DN36199_c0_g1_i1_orf1	fatty acid-binding protein 1-like [Ostrinia furnacalis]	19.17307692	4.261009976	2.31E-07	up	yes	0.997	0.052	0.039	0.062	0.055	1	1.015	0.976	[illegible]	[illegible]	K08753;K08752	FABP4, aP2;F/	map04923;map	Regulation of lipolys	ENOG4111US8	I:Lipid transport and metabolism	------	------	------	CYT	5	48	15.2	High
TRINITY_DN19917_c0_g1_i1_orf1	synaptic vesicle glycoprotein 2B-like isoform X4 [Ostrinia furnacalis]	13.88976604	3.795950394	7.16E-07	up	yes	0.9677	0.06967	0.066	0.078	0.065	1	0.951	0.952	[illegible]	[illegible]	K06258	SV2	map04512	ECM-receptor intera	ENOG410ZYFU;ENO(	G:Carbohydrate transport and metab	PF07690.19;PF000	MFS_1;Sugar_tr	Major Facilitator Superfamily;Sugar	CYT	1	2	57.3	High
TRINITY_DN3428_c0_g1_i1_orf1	10 kDa heat shock protein, mitochondrial [Ostrinia furnacalis]	2.375090514	1.247982495	5.32E-05	up	yes	0.984	0.4143	0.42	0.422	0.401	1	1.027	0.925	[illegible]	[illegible]	K04078	groES, HSPE1	------	------	COG0234	O:Posttranslational modification, pro	PF00166.24	Cpn10	Chaperonin 10 Kd subunit	CYT	8	64	11.1	High
TRINITY_DN4944_c0_g1_i2_orf1	bifunctional glutamate/proline--tRNA ligase [Ostrinia furnacalis]	2.342105263	1.227805918	9.09E-07	up	yes	0.979	0.418	0.423	0.418	0.413	1	0.965	0.972	[illegible]	[illegible]	K14163	EPRS	map00860;map	Porphyrin metabolis	COG0442	J:Translation, ribosomal structure anc	PF09180.14;PF005	ProRS-C_1;tRNA-synt_	Prolyl-tRNA synthetase, C-terminal;tRNA synthetase class II core domain;Anticodon binding domain	CYT	6	28	51	High
TRINITY_DN3759_c0_g1_i1_orf1	uncharacterized protein LOC114350416 [Ostrinia furnacalis] >XP_028157016.1 uncharacterized protein LOC114350416 [Ostrinia furnacalis] >XP_028157017.1 uncharacterized protein LOC114350416 [Ostrinia furnacalis] >XP_028157018.1 uncharacterized protein LOC114350416 [Ostrinia furnacalis] >XP_028157019.1 uncharacterized protein LOC114350416 [Ostrinia furnacalis]	2.459506604	1.298368929	4.05E-06	up	yes	0.987	0.4013	0.405	0.41	0.389	1	1.005	0.956	[illegible]	[illegible]	K22909	ZC3H13	------	------	ENOG4111GFX	S:Function unknown	PF01607.27	CBM_14	Chitin binding Peritrophin-A domain	CYT	2	2	132.4	High
TRINITY_DN17738_c0_g1_i2_orf1	unnamed protein product [Diatraea saccharalis]	2.508234102	1.326672006	3.02E-06	up	yes	0.99	0.3947	0.396	0.392	0.396	1	1.011	0.959	[illegible]	[illegible]	K11090	LA, SSB	map05322	Systemic lupus eryth	COG5193	G:Carbohydrate transport and metat	PF05383.20;PF087	La;RRM_3;RRM_1	La domain;RNA binding motif;RNA recognition motif.	CYT	10	28	43.9	High
TRINITY_DN2238_c0_g2_i1_orf1	mitochondrial import inner membrane translocase subunit Tim8 [Ostrinia furnacalis]	2.349648655	1.232445046	9.94E-05	up	yes	0.9697	0.4127	0.41	0.422	0.406	1	1.01	0.899	[illegible]	[illegible]	K17780	TIM8	------	------	ENOG41126D5	O:Posttranslational modification, pro	PF02953.18	zf-Tim10_DDP	Tim10/DDP family zinc finger	CYT	4	44	10.2	High
TRINITY_DN47_c0_g1_i2_orf1	uncharacterized protein LOC114356437 isoform X1 [Ostrinia furnacalis]	2.434253045	1.283479147	0.0001183	up	yes	0.9793	0.4023	0.475	0.38	0.352	1	0.964	0.974	------	------	------	------	------	------	------	------	PF00041.24	fn3	Fibronectin type III domain	CYT	1	8	17.9	High
TRINITY_DN18909_c0_g1_i8_orf1	unnamed protein product [Euphydryas editha]	2.464710347	1.301418111	0.0002402	up	yes	0.9743	0.3953	0.432	0.398	0.356	1	1.029	0.894	[illegible]	[illegible]	------	------	------	------	COG2272;COG0580;I	I:Lipid transport and metabolism;G:C:	PF00135.31;PF204	COesterase;BD-FAE;Ab	Carboxylesterase family;BD-FAE;alpha/beta hydrolase	CYT	1	4	60.6	High
TRINITY_DN34536_c0_g1_i6_orf1	clustered mitochondria protein homolog isoform X2 [Ostrinia furnacalis]	2.985422741	1.577935234	1.45E-06	up	yes	1.024	0.343	0.349	0.355	0.325	1	1.035	1.037	[illegible]	[illegible]	K03255	TIF31, CLU1	------	------	ENOG410XQUQ	S:Function unknown	PF13236.9;PF1280	CLU;eIF3_p135;CLU_N	Clustered mitochondria;Translation initiation factor eIF3 subunit 135;Mitochondrial function, CLU-N-term	CYT	9	8	124.7	High

TRINITY_DN14313_c0_g1_i1_orf1	25S rRNA (cytosine-C(5))-methyltransferase nop2 [Ostrinia furnacalis]	2.489863998	1.316066941	1.21E-05	up	yes	0.9703	0.3897	0.387	0.406	0.376	1	0.979	0.932	BP:RNA modification;BP:nucleic acid metabolic process;BP:cellular component organization or biogenesis;BP:nucleobase-containing compound metabolic process;BP:cellular macromolecule metabolic process;BP:ribosome biogenesis;BP:organic substance metabolic process;BP:organic cyclic compound metabolic process;BP:ribonucleoprotein complex biogenesis;BP:methylation;BP:cellular process;BP:cellular aromatic compound metabolic process;BP:macromolecule modification;BP:biological_process;BP:metabolic process;BP:RNA methylation;BP:heterocycle metabolic process;BP:RNA metabolic process;BP:primary metabolic process;BP:nitrogen compound metabolic process;BP:cellular nitrogen compound metabolic process;BP:cellular metabolic process;BP:macromolecule metabolic process;BP:cellular component biogenesis;BP:macromolecule methylation;BP:RNA processing;CC:intracellular organelle;CC:non-membrane-bounded organelle;CC:organelle;CC:nucleolus;CC:cellular anatomical entity;CC:intracellular non-membrane-bounded organelle;CC:cellular_component;MF:molecular_function;MF:binding;MF:nucleic acid binding;MF:methyltransferase activity;MF:heterocyclic compound binding;MF:transferase activity;MF:transferase activity, transferring one-carbon groups;MF:S-adenosylmethionine-dependent methyltransferase activity;MF:catalytic activity;MF:organic cyclic compound binding;MF:RNA binding;	GO:0009451;GO:0090304;GO:0071840;GO:0006139;GO:0044260;GO:0042254;GO:0071704;GO:1901360;GO:0022613;GO:0032259;GO:0009987;GO:0006725;GO:0043412;GO:0008150;GO:0008152;GO:0001510;GO:0046483;GO:0016070;GO:0044238;GO:0006807;GO:0034641;GO:0044237;GO:0043170;GO:0044085;GO:0043414;GO:0006396;GO:0043229;GO:0043228;GO:0043226;GO:0005730;GO:0110165;GO:0043232;GO:0005575;GO:0003674;GO:0005488;GO:0003676;GO:0008168;GO:1901363;GO:0016740;GO:0016741;GO:0008757;GO:0003824;GO:0097159;GO:0003723	K14835	NOP2	------	------	COG0144	O:Posttranslational modification, pro	PF01189.20;PF171	Methyltr_RsmB-F;Meth	16S rRNA methyltransferase RsmB/F;N-terminal domain of 16S rRNA methyltransferase RsmF	CYT	9	12	95.6	High
TRINITY_DN18164_c0_g1_i7_orf1	uncharacterized protein LOC114366518 isoform X5 [Ostrinia furnacalis]	2.685636856	1.425264241	0.00102	up	yes	0.991	0.369	0.503	0.296	0.308	1	0.939	1.034	CC:integral component of membrane;CC:cellular_component;CC:intrinsic component of membrane;CC:cellular anatomical entity;MF:binding;MF:cation binding;MF:transition metal ion binding;MF:ion binding;MF:molecular_function;MF:metal ion binding;MF:zinc ion binding;	GO:0016021;GO:0005575;GO:0031224;GO:0110165;GO:0005488;GO:0043169;GO:0046914;GO:0043167;GO:0003674;GO:0046872;GO:0008270	------	------	------	------	COG5183	O:Posttranslational modification, pro	PF12906.10;PF064	RINGv;THH1_TOM1-3_	RING-variant domain;THH1/TOM1/TOM3 domain	CYT	1	2	64.7	High
TRINITY_DN40_c0_g2_i1_orf1	trypsin CFT-1-like [Ostrinia furnacalis]	3.13803681	1.649862276	1.02E-05	up	yes	1.023	0.326	0.312	0.36	0.306	1	1.01	1.06	BP:organic substance metabolic process;BP:organonitrogen compound metabolic process;BP:protein metabolic process;BP:macromolecule metabolic process;BP:biological_process;BP:nitrogen compound metabolic process;BP:metabolic process;BP:proteolysis;BP:primary metabolic process;MF:serine-type endopeptidase activity;MF:endopeptidase activity;MF:hydrolase activity;MF:serine hydrolase activity;MF:catalytic activity, acting on a protein;MF:catalytic activity;MF:molecular_function;MF:peptidase activity;MF:serine-type peptidase activity;	GO:0071704;GO:1901564;GO:0019538;GO:0043170;GO:0008150;GO:0006807;GO:0008152;GO:0006508;GO:0044238;GO:0004252;GO:0004175;GO:0016787;GO:0017171;GO:0140096;GO:0003824;GO:0003674;GO:0008233;GO:0008236	K01312	PRSS1_2_3	map04972;map	Pancreatic secretion	COG5640	O:Posttranslational modification, pro	PF00089.29	Trypsin	Trypsin	CYT	1	3	28.5	High
TRINITY_DN3847_c1_g1_i1_orf1	ribosome production factor 2 homolog [Ostrinia furnacalis]	2.734903047	1.45148969	3.03E-05	up	yes	0.9873	0.361	0.338	0.387	0.358	1	0.937	1.025	BP:cellular component assembly;BP:macromolecular complex subunit organization;BP:nucleic acid metabolic process;BP:maturation of LSU-rRNA;BP:ncRNA metabolic process;BP:organic cyclic compound metabolic process;BP:ribosomal large subunit assembly;BP:nucleobase-containing compound metabolic process;BP:ribonucleoprotein complex subunit organization;BP:cellular component organization or biogenesis;BP:cellular component organization;BP:macromolecular complex assembly;BP:organic substance metabolic process;BP:ribonucleoprotein complex assembly;BP:rRNA processing;BP:ncRNA processing;BP:cellular process;BP:cellular aromatic compound metabolic process;BP:biological_process;BP:metabolic process;BP:heterocycle metabolic process;BP:RNA metabolic process;BP:primary metabolic process;BP:rRNA metabolic process;BP:cellular nitrogen compound metabolic process;BP:cellular metabolic process;BP:macromolecule metabolic process;BP:nitrogen compound metabolic process;BP:RNA processing;CC:intracellular organelle;CC:non-membrane-bounded organelle;CC:organelle;CC:nucleolus;CC:cellular anatomical entity;CC:intracellular non-membrane-bounded organelle;CC:cellular_component;MF:molecular_function;MF:binding;MF:nucleic acid binding;MF:rRNA binding;MF:heterocyclic compound binding;MF:organic cyclic compound binding;MF:RNA binding;	GO:0022607;GO:0043933;GO:0090304;GO:0000470;GO:0034660;GO:1901360;GO:0000027;GO:0006139;GO:0071826;GO:0071840;GO:0016043;GO:0065003;GO:0071704;GO:0022618;GO:0006364;GO:0034470;GO:0009987;GO:0006725;GO:0008150;GO:0008152;GO:0046483;GO:0016070;GO:0044238;GO:0016072;GO:0034641;GO:0044237;GO:0043170;GO:0006807;GO:0006396;GO:0043229;GO:0043228;GO:0043226;GO:0005730;GO:0110165;GO:0043232;GO:0005575;GO:0003674;GO:0005488;GO:0003676;GO:0019843;GO:1901363;GO:0097159;GO:0003723	K14847	RPF2	------	------	COG5106	S:Function unknown	PF04427.21	Brix	Brix domain	CYT	2	7	35.9	High
TRINITY_DN15624_c0_g1_i1_orf1	LOW QUALITY PROTEIN: V-type proton ATPase subunit S1-like [Ostrinia furnacalis]	4.227290602	2.079733293	3.03E-05	up	yes	1.075	0.2543	0.261	0.234	0.268	1	1.118	1.107	------	------	K03662	ATPeVS1, ATF	map05152;map	Tuberculosis;Human	ENOG410XTHY	U:Intracellular trafficking, secretion, a	PF20520.1;PF0582	Ac45-VOA1_TM;VAS1_	V0 complex accessory subunit Ac45/VOA1 transmembrane domain;V-type proton ATPase subunit S1, luminal domain	CYT	3	9	45.2	High
TRINITY_DN40_c0_g1_i3_orf1	trypsin CFT-1-like [Ostrinia furnacalis]	3.325581395	1.733606582	1.10E-06	up	yes	1.001	0.301	0.289	0.321	0.293	1	0.983	1.019	BP:organic substance metabolic process;BP:organonitrogen compound metabolic process;BP:protein metabolic process;BP:macromolecule metabolic process;BP:biological_process;BP:nitrogen compound metabolic process;BP:metabolic process;BP:proteolysis;BP:primary metabolic process;MF:serine-type endopeptidase activity;MF:endopeptidase activity;MF:hydrolase activity;MF:serine hydrolase activity;MF:catalytic activity, acting on a protein;MF:catalytic activity;MF:molecular_function;MF:peptidase activity;MF:serine-type peptidase activity;	GO:0071704;GO:1901564;GO:0019538;GO:0043170;GO:0008150;GO:0006807;GO:0008152;GO:0006508;GO:0044238;GO:0004252;GO:0004175;GO:0016787;GO:0017171;GO:0140096;GO:0003824;GO:0003674;GO:0008233;GO:0008236	K01312	PRSS1_2_3	map04972;map	Pancreatic secretion	COG5640	O:Posttranslational modification, pro	PF00089.29	Trypsin	Trypsin	CYT	4	18	28.1	High
TRINITY_DN468_c0_g1_i3_orf1	transmembrane protein 41 homolog isoform X2 [Ostrinia furnacalis]	3.190584416	1.673820705	6.60E-07	up	yes	0.9827	0.308	0.298	0.301	0.325	1	0.977	0.971	------	------	------	------	------	------	COG0398	S:Function unknown	PF09335.14	SNARE_assoc	SNARE associated Golgi protein	CYT	1	7	30.6	High
TRINITY_DN9715_c0_g1_i1_orf1	V-type proton ATPase subunit E [Manduca sexta] >P31402.1 RecName: Full=V-type proton ATPase subunit E; Short=V-ATPase subunit E; AltName: Full=V-ATPase 26 kDa subunit; AltName: Full=Vacuolar proton pump subunit E [Manduca sexta] >KAG6457535.1 hypothetical protein O3G_MSEX010354 [Manduca sexta] >CAA47610.1 H(+)-transporting ATPase [Manduca sexta]	3.31628852	1.729569528	1.04E-06	up	yes	0.9793	0.2953	0.296	0.283	0.307	1	0.98	0.958	CC:proton-transporting two-sector ATPase complex, catalytic domain;CC:macromolecular complex;CC:cellular_component;CC:membrane protein complex;MF:molecular_function;MF:ion transmembrane transporter activity;MF:ATPase activity, coupled to transmembrane movement of substances;MF:inorganic solute uptake transmembrane transporter activity;MF:hydrogen-translocating pyrophosphatase activity;MF:primary active transmembrane transporter activity;MF:active transmembrane transporter activity;MF:ATPase coupled ion transmembrane transporter activity;MF:transporter activity;MF:cation transmembrane transporter activity;MF:proton-transporting ATPase activity, rotational mechanism;MF:ATPase activity, coupled to transmembrane movement of ions, rotational mechanism;MF:inorganic cation transmembrane transporter activity;MF:cation-transporting ATPase activity;MF:transmembrane transporter activity;MF:active ion transmembrane transporter activity;MF:hydrogen ion transmembrane transporter activity;	GO:0033178;GO:0032991;GO:0005575;GO:0098796;GO:0003674;GO:0015075;GO:0042626;GO:0015318;GO:0009678;GO:0015399;GO:0022804;GO:0042625;GO:0005215;GO:0008324;GO:0046961;GO:0044769;GO:0022890;GO:0019829;GO:0022857;GO:0022853;GO:0015078	K02150	ATPeV1E, ATF	map05323;map	Rheumatoid arthritis	COG1390	C:Energy production and conversion	PF01991.21	vATP-synt_E	ATP synthase	CYT	12	44	26	High
TRINITY_DN3235_c0_g1_i1_orf1	SPARC [Trichoplusia ni]	3.744855967	1.904910231	6.16E-06	up	yes	1.001	0.2673	0.25	0.279	0.273	1	1.039	0.964	CC:extracellular space;CC:cellular_component;CC:cellular anatomical entity;MF:cation binding;MF:ion binding;MF:molecular_function;MF:binding;MF:metal ion binding;MF:calcium ion binding;	GO:0005615;GO:0005575;GO:0110165;GO:0043169;GO:0043167;GO:0003674;GO:0005488;GO:0046872;GO:0005509	K24262	SPARC	------	------	ENOG41101WW	S:Function unknown	PF10591.12	SPARC_Ca_bdg	Secreted protein acidic and rich in cysteine Ca binding region	CYT	6	24	36.9	High
TRINITY_DN72707_c0_g1_i1_orf1	uncharacterized protein LOC114357549 [Ostrinia furnacalis]	3.759742717	1.91063394	4.15E-08	up	yes	0.9937	0.2643	0.268	0.253	0.272	1	0.989	0.992	MF:hydrolase activity;MF:molecular_function;MF:hydrolase activity, acting on ester bonds;MF:carboxylic ester hydrolase activity;MF:catalytic activity;	GO:0016787;GO:0003674;GO:0016788;GO:0052689;GO:0003824	K03927;K01049	CES2;ACHE	map00564;map	Glycerophospholipic	COG2272	I:Lipid transport and metabolism	PF00135.31;PF204	COesterase;BD-FAE;Ab	Carboxylesterase family;BD-FAE;alpha/beta hydrolase	CYT	3	7	60.3	High
TRINITY_DN5459_c0_g1_i1_orf1	protein takeout-like isoform X2 [Ostrinia furnacalis]	4.471689701	2.16082008	2.22E-05	up	yes	1.003	0.2243	0.269	0.169	0.235	1	1.035	0.974	------	------	------	------	------	------	ENOG4111E83;ENOC	S:Function unknown;	PF06585.14	JHBP	Haemolymph juvenile hormone binding protein	CYT	1	4	21.2	Medium
TRINITY_DN7047_c0_g1_i1_orf1	hypothetical protein G9C98_004728 [Cotesia typhae]	4.173525668	2.061266644	4.05E-06	up	yes	0.9837	0.2357	0.226	0.262	0.219	1	1.001	0.95	CC:macromolecular complex;CC:ribosome;CC:intracellular non-membrane-bounded organelle;CC:ribonucleoprotein complex;CC:mitochondrion;CC:cellular_component;CC:intracellular organelle;CC:non-membrane-bounded organelle;CC:membrane-bounded organelle;CC:organelle;CC:cellular anatomical entity;CC:intracellular membrane-bounded organelle;	GO:0032991;GO:0005840;GO:0043232;GO:1990904;GO:0005739;GO:0005575;GO:0043229;GO:0043228;GO:0043227;GO:0043226;GO:0110165;GO:0043231	K17431	MRPL50	------	------	ENOG4111QY3	S:Function unknown	------	------	------	CYT	1	4	24	High
TRINITY_DN96_c0_g1_i1_orf1	collagenase-like [Ostrinia furnacalis]	4.950738916	2.307643869	0.000119	up	yes	1.005	0.203	0.168	0.188	0.253	1	0.925	1.089	BP:organic substance metabolic process;BP:organonitrogen compound metabolic process;BP:protein metabolic process;BP:macromolecule metabolic process;BP:biological_process;BP:nitrogen compound metabolic process;BP:metabolic process;BP:proteolysis;BP:primary metabolic process;MF:serine-type endopeptidase activity;MF:endopeptidase activity;MF:hydrolase activity;MF:serine hydrolase activity;MF:catalytic activity, acting on a protein;MF:catalytic activity;MF:molecular_function;MF:peptidase activity;MF:serine-type peptidase activity;	GO:0071704;GO:1901564;GO:0019538;GO:0043170;GO:0008150;GO:0006807;GO:0008152;GO:0006508;GO:0044238;GO:0004252;GO:0004175;GO:0016787;GO:0017171;GO:0140096;GO:0003824;GO:0003674;GO:0008233;GO:0008236	------	------	------	------	COG5640	O:Posttranslational modification, pro	PF00089.29	Trypsin	Trypsin	CYT	1	3	44.4	High
TRINITY_DN11376_c0_g2_i1_orf1	cathepsin K-like [Ostrinia furnacalis]	3.946502058	1.980574501	1.18E-05	up	yes	0.959	0.243	0.247	0.236	0.246	1	0.968	0.909	BP:primary metabolic process;BP:organonitrogen compound metabolic process;BP:protein metabolic process;BP:macromolecule metabolic process;BP:organic substance metabolic process;BP:nitrogen compound metabolic process;BP:biological_process;BP:metabolic process;BP:proteolysis;MF:molecular_function;MF:hydrolase activity;MF:catalytic activity;MF:catalytic activity, acting on a protein;MF:peptidase activity;MF:cysteine-type peptidase activity;	GO:0044238;GO:1901564;GO:0019538;GO:0043170;GO:0071704;GO:0006807;GO:0008150;GO:0008152;GO:0006508;GO:0003674;GO:0016787;GO:0003824;GO:0140096;GO:0008233;GO:0008234	------	------	------	------	COG4870	O:Posttranslational modification, pro	PF00112.26;PF082	Peptidase_C1;Inhibitor_	Papain family cysteine protease;Cathepsin propeptide inhibitor domain;Peptidase C1-like family	CYT	2	6	53.7	High
TRINITY_DN29120_c0_g1_i6_orf1	putative inorganic phosphate cotransporter [Ostrinia furnacalis]	5.781881131	2.531538949	5.05E-06	up	yes	1.002	0.1733	0.204	0.166	0.15	1	1.036	0.969	CC:integral component of membrane;CC:cellular_component;CC:cellular anatomical entity;CC:intrinsic component of membrane;MF:transporter activity;MF:transmembrane transporter activity;MF:molecular_function;	GO:0016021;GO:0005575;GO:0110165;GO:0031224;GO:0005215;GO:0022857;GO:0003674	K08193	SLC17A	------	------	ENOG410XPWC;ENC	G:Carbohydrate transport and metab	PF07690.19	MFS_1	Major Facilitator Superfamily	CYT	1	4	27.3	High
TRINITY_DN6140_c0_g3_i3_orf1	CD63 antigen-like [Ostrinia furnacalis]	4.372039015	2.128306275	3.45E-05	up	yes	0.9413	0.2153	0.22	0.192	0.234	1	0.884	0.94	CC:cellular_component;CC:integral component of membrane;CC:cellular anatomical entity;CC:intrinsic component of membrane;	GO:0005575;GO:0016021;GO:0110165;GO:0031224	K06497	CD63, MLA1,	map05205;map	Proteoglycans in car	ENOG4111IRY	O:Posttranslational modification, pro	PF00335.23	Tetraspanin	Tetraspanin family	CYT	1	4	32.2	High
TRINITY_DN37538_c0_g1_i1_orf1	esterase FE4-like [Ostrinia furnacalis]	8.222222222	3.039528364	3.97E-06	up	yes	0.962	0.117	0.12	0.094	0.137	1	0.957	0.929	------	------	K07378	NLGN	map04514	Cell adhesion molec	COG2272;ENOG410	I:Lipid transport and metabolism;T:Si	PF00135.31;PF204	COesterase;BD-FAE;Ab	Carboxylesterase family;BD-FAE;alpha/beta hydrolase	CYT	1	4	22.5	High
TRINITY_DN21909_c0_g1_i1_orf1	complement component 1 Q subcomponent-binding protein, mitochondrial [Ostrinia furnacalis]	2.692613185	1.42900699	6.93E-07	up	yes	1.017	0.3777	0.381	0.378	0.374	1	1.039	1.011	CC:organelle lumen;CC:membrane-enclosed lumen;CC:cellular_component;CC:intracellular organelle lumen;CC:mitochondrial matrix;CC:cellular anatomical entity;	GO:0043233;GO:0031974;GO:0005575;GO:0070013;GO:0005759;GO:0110165	K15414	C1QBP	------	------	ENOG4111G4Z	S:Function unknown	PF02330.19	MAM33	Mitochondrial glycoprotein	CYT	6	24	31.9	High
TRINITY_DN42759_c0_g2_i1_orf1	fatty acid synthase-like [Ostrinia furnacalis]	2.399461181	1.262710473	0.0001234	up	yes	0.9797	0.4083	0.375	0.461	0.389	1	1.015	0.924	BP:fatty acid biosynthetic process;BP:fatty acid metabolic process;BP:carboxylic acid metabolic process;BP:cellular biosynthetic process;BP:small molecule metabolic process;BP:small molecule biosynthetic process;BP:monocarboxylic acid biosynthetic process;BP:organic substance biosynthetic process;BP:organic substance metabolic process;BP:lipid metabolic process;BP:cellular process;BP:monocarboxylic acid metabolic process;BP:biosynthetic process;BP:biological_process;BP:metabolic process;BP:oxoacid metabolic process;BP:cellular lipid metabolic process;BP:lipid biosynthetic process;BP:primary metabolic process;BP:organic acid metabolic process;BP:carboxylic acid biosynthetic process;BP:organic acid biosynthetic process;BP:cellular metabolic process;MF:3-hydroxyoctanoyl-[acyl-carrier-protein] dehydratase activity;MF:thiolester hydrolase activity;MF:S-acyltransferase activity;MF:enoyl-[acyl-carrier-protein] reductas;MF:oxidoreductase activity, acting on the CH-CH group of donors;MF:molecular_function;MF:oxidoreductase activity;MF:oxidoreductase activity, acting on the CH-CH group of donors, NAD or NADP as acceptor;MF:[acyl-carrier-protein] S-acetyltransferase activity;MF:lyase activity;MF:oleoyl-[acyl-carrier-protein] hydrolase activity;MF:acetyltransferase activity;MF:transferase activity;MF:transferase activity, transferring acyl groups;MF:transferase activity, transferring acyl groups other than amino-acyl groups;MF:S-acetyltransferase activity;MF:hydrolase activity;MF:3-hydroxydecanoyl-[acyl-carrier-protein] dehydratase activity;MF:hydrolase activity, acting on ester bonds;MF:catalytic activity;MF:oxidoreductase activity, acting on CH-OH group of donors;MF:oxidoreductase activity, acting on the CH-OH group of donors, NAD or NADP as acceptor;MF:carbon-oxygen lyase activity;MF:hydro-lyase activity;MF:fatty acid synthase activity;MF:myristoyl-[acyl-carrier-protein] hydrolase activity;MF:palmitoyl-[acyl-carrier-protein] hydrolase activity;MF:acyl-[acyl-carrier-protein] hydrolase activity;MF:3-oxoacyl-[acyl-carrier-protein] reductas;MF:3-hydroxypalmitoyl-[acyl-carrier-protein] dehydratase activity;MF:3-oxoacyl-[acyl-carrier-protein] synthase activity;MF:3-hydroxyacyl-[acyl-carrier-protein]	GO:0006633;GO:0006631;GO:0019752;GO:0044249;GO:0044281;GO:0044283;GO:0072330;GO:1901576;GO:0071704;GO:0006629;GO:0009987;GO:0032787;GO:0009058;GO:0008150;GO:0008152;GO:0043436;GO:0044255;GO:0008610;GO:0044238;GO:0006082;GO:0046394;GO:0016053;GO:0044237;GO:0047451;GO:0016790;GO:0016417;GO:0047117;GO:0016627;GO:0003674;GO:0016491;GO:0016628;GO:0004313;GO:0016829;GO:0004320;GO:0016407;GO:0016740;GO:0016746;GO:0016747;GO:0016418;GO:0016787;GO:0008693;GO:0016788;GO:0003824;GO:0016614;GO:0016616;GO:0016835;GO:0016836;GO:0004312;GO:0016295;GO:0016296;GO:0016297;GO:0004316;GO:0004317;GO:0004315;GO:0019171;GO:0008659	K00665	FASN	map04910;map	Insulin signaling pat	COG3321	Q:Secondary metabolites biosynthesi	PF00975.23;PF147	Thioesterase;PS-DH	Thioesterase domain;Polyketide synthase dehydratase	CYT	5	3	166.4	High
TRINITY_DN5925_c0_g1_i5_orf1	isocitrate dehydrogenase [NAD] subunit gamma, mitochondrial-like isoform X1 [Ostrinia furnacalis]	2.681371246	1.42297098	1.21E-05	up	yes	1.009	0.3763	0.37	0.351	0.408	1	1.042	0.985	BP:primary metabolic process;BP:tricarboxylic acid cycle;BP:biological_process;BP:metabolic process;CC:intracellular membrane-bounded organelle;CC:intracellular organelle;CC:mitochondrion;CC:cellular_component;CC:membrane-bounded organelle;CC:organelle;CC:cellular anatomical entity;	GO:0044238;GO:0006099;GO:0008150;GO:0008152;GO:0043231;GO:0043229;GO:0005739;GO:0005575;GO:0043227;GO:0043226;GO:0110165	K00030	IDH3	map00020	Citrate cycl	COG0473	E:Amino acid transport and metaboli	PF00180.23	Iso_dh	Isocitrate/isopropylmalate dehydrogenase	CYT	2	6	42.4	High
TRINITY_DN2704_c0_g1_i5_orf1	hypothetical protein evm_009002 [Chilo suppressalis]	2.680440032	1.422469859	7.47E-08	up	yes	0.999	0.3727	0.38	0.369	0.369	1	1.008	0.989	------	------	K06569	MFI2, CD228	------	------	ENOG410ZEJE	S:Function unknown	PF00405.20	Transferrin	Transferrin	CYT	9	16	84.2	High
TRINITY_DN1706_c0_g1_i7_orf1	LOW QUALITY PROTEIN: RNA polymerase-associated protein CTR9 homolog [Ostrinia furnacalis]	2.176634862	1.12209941	0.0001232	up	yes	0.9353	0.4297	0.446	0.413	0.43	1	0.914	0.892	BP:regulation of primary metabolic process;BP:regulation of metabolic process;BP:regulation of cellular biosynthetic process;BP:regulation of cellular metabolic process;BP:nitrogen compound metabolic process;BP:regulation of biological process;BP:organic substance metabolic process;BP:regulation of macromolecule metabolic process;BP:biological regulation;BP:histone modification;BP:regulation of gene expression;BP:regulation of nucleobase-containing compound metabolic process;BP:regulation of biosynthetic process;BP:regulation of nucleic acid-templated transcription;BP:regulation of cellular process;BP:macromolecule modification;BP:protein modification process;BP:biological_process;BP:regulation of nitrogen compound metabolic process;BP:metabolic process;BP:regulation of RNA biosynthetic process;BP:primary metabolic process;BP:organonitrogen compound metabolic process;BP:protein metabolic process;BP:regulation of RNA metabolic process;BP:macromolecule metabolic process;BP:regulation of transcription, DNA-templated;BP:regulation of macromolecule biosynthetic process;CC:intracellular organelle;CC:membrane-bounded organelle;CC:organelle;CC:cellular anatomical entity;CC:intracellular membrane-bounded organelle;CC:cellular_component;	GO:0080090;GO:0019222;GO:0031326;GO:0031323;GO:0006807;GO:0050789;GO:0071704;GO:0060255;GO:0065007;GO:0016570;GO:0010468;GO:0019219;GO:0009889;GO:1903506;GO:0050794;GO:0043412;GO:0036211;GO:0008150;GO:0051171;GO:0008152;GO:2001141;GO:0044238;GO:1901564;GO:0019538;GO:0051252;GO:0043170;GO:0006355;GO:0010556;GO:0043229;GO:0043227;GO:0043226;GO:0110165;GO:0043231;GO:0005575	K15176	CTR9	------	------	COG0457	S:Function unknown	PF00515.31;PF077	TPR_1;TPR_2;TPR_8;TPR	Tetratricopeptide repeat;Tetratricopeptide repeat;Tetratricopeptide repeat;Tetratricopeptide repeat;Tetratricopeptide repeat;TPR repeat;Tetratricopeptide repeat;Tetratricopeptide repeat;Coatomer epsilon subunit	CYT	1	1	136.5	High
TRINITY_DN110888_c0_g1_i2_orf1	uncharacterized protein LOC114364502 [Ostrinia furnacalis]	2.598153034	1.37748641	1.36E-05	up	yes	0.9847	0.379	0.376	0.37	0.391	1	0.94	1.014	------	------	------	------	------	------	ENOG410XU1K	S:Function unknown	------	------	------	CYT	1	4	33.8	High
TRINITY_DN107261_c0_g1_i1_orf1	ATP synthase subunit g, mitochondrial [Ostrinia furnacalis]	2.273055622	1.184632988	7.14E-05	up	yes	0.944	0.4153	0.432	0.42	0.394	1	0.902	0.93	BP:nucleoside triphosphate metabolic process;BP:nucleoside triphosphate biosynthetic process;BP:purine nucleoside triphosphate metabolic process;BP:purine nucleoside triphosphate biosynthetic process;BP:cellular biosynthetic process;BP:cellular nitrogen compound metabolic process;BP:nucleotide biosynthetic process;BP:small molecule metabolic process;BP:purine-containing compound metabolic process;BP:purine-containing compound biosynthetic process;BP:organic cyclic compound biosynthetic process;BP:ribonucleotide metabolic process;BP:organic cyclic compound metabolic process;BP:nucleobase-containing compound metabolic process;BP:ribonucleoside triphosphate biosynthetic process;BP:organic substance metabolic process;BP:ribonucleoside triphosphate metabolic process;BP:heterocycle biosynthetic process;BP:purine ribonucleoside triphosphate biosynthetic process;BP:purine ribonucleoside triphosphate metabolic process;BP:ribose phosphate metabolic process;BP:organic substance biosynthetic process;BP:ATP metabolic process;BP:cellular process;BP:cellular aromatic compound metabolic process;BP:purine ribonucleotide biosynthetic process;BP:purine ribonucleotide metabolic process;BP:ribonucleotide biosynthetic process;BP:biosynthetic process;BP:nucleotide metabolic process;BP:metabolic process;BP:aromatic compound biosynthetic process;BP:nucleobase-containing compound biosynthetic process;BP:cellular nitrogen compound biosynthetic process;BP:organophosphate biosynthetic process;BP:nucleobase-containing small molecule metabolic process;BP:heterocycle metabolic process;BP:primary metabolic process;BP:ATP synthesis coupled proton transport;BP:organonitrogen compound metabolic process;BP:organonitrogen compound biosynthetic process;BP:carbohydrate derivative biosynthetic process;BP:carbohydrate derivative metabolic process;BP:purine nucleotide biosynthetic process;BP:ribose phosphate biosynthetic process;BP:cellular metabolic process;BP:purine nucleotide metabolic process;BP:phosphate-containing compound metabolic process;BP:nitrogen compound metabolic process;BP:nucleoside phosphate biosynthetic process;BP:phosphorus metabolic process;BP:organophosphate metabolic process;BP:biological_process;BP:nucleoside phosphate metabolic process;BP:ATP biosynthetic process;CC:proton-transporting ATP synthase complex, coupling factor;CC:cellular_component;CC:mitochondrial protein complex;CC:inner mitochondrial membrane protein complex;CC:macromolecular complex;CC:mitochondrial proton-transporting ATP synthase complex, coupling factor;CC:membrane protein complex;CC:proton-transporting two-sector ATPase complex, proton-transporting domain;MF:inorganic cation transmembrane transporter activity;MF:molecular_function;MF:inorganic solute uptake transmembrane transporter activity;MF:transmembrane transporter activity;MF:transporter activity;MF:cation transmembrane transporter activity;MF:ion transmembrane transporter activity;MF:hydrogen	GO:0009141;GO:0009142;GO:0009144;GO:0009145;GO:0044249;GO:0034641;GO:0009165;GO:0044281;GO:0072521;GO:0072522;GO:1901362;GO:0009259;GO:1901360;GO:0006139;GO:0009201;GO:0071704;GO:0009199;GO:0018130;GO:0009206;GO:0009205;GO:0019693;GO:1901576;GO:0046034;GO:0009987;GO:0006725;GO:0009152;GO:0009150;GO:0009260;GO:0009058;GO:0009117;GO:0008152;GO:0019438;GO:0034654;GO:0044271;GO:0090407;GO:0055086;GO:0046483;GO:0044238;GO:0015986;GO:1901564;GO:1901566;GO:1901137;GO:1901135;GO:0006164;GO:0046390;GO:0044237;GO:0006163;GO:0006796;GO:0006807;GO:1901293;GO:0006793;GO:0019637;GO:0008150;GO:0006753;GO:0006754;GO:0045263;GO:0005575;GO:0098798;GO:0098800;GO:0032991;GO:0000276;GO:0098796;GO:0033177;GO:0022890;GO:0003674;GO:0015318;GO:0022857;GO:0005215;GO:0008324;GO:0015075;GO:0015078	K02140	ATPeFG, ATP5	map00190;map	Oxidative phosphor	ENOG4111TZ1	I:Lipid transport and metabolism	PF04718.18	ATP-synt_G	Mitochondrial ATP synthase g subunit	CYT	3	30	10.7	High
TRINITY_DN478_c0_g1_i16_orf1	lipid storage droplets surface-binding protein 2 isoform X1 [Ostrinia furnacalis]	2.665227947	1.414258927	2.22E-06	up	yes	0.988	0.3707	0.353	0.374	0.385	1	1	0.964	------	------	K17284	PLIN2, ADRP	map03320	PPAR signaling path	ENOG410XYYG;ENO	S:Function unknown;U:Intracellular tr	PF03036.19	Perilipin	Perilipin family	CYT	3	9	33.9	High
TRINITY_DN10530_c0_g1_i1_orf1	cytochrome c oxidase subunit NDUFA4 [Ostrinia furnacalis]	2.764343689	1.466936996	8.59E-08	up	yes	0.9877	0.3573	0.359	0.359	0.354	1	0.986	0.977	CC:cellular_component;CC:integral component of membrane;CC:cellular anatomical entity;CC:intrinsic component of membrane;	GO:0005575;GO:0016021;GO:0110165;GO:0031224	K03948	NDUFA4	map04714;map	Thermogenesis;Oxid	ENOG41124SW	U:Intracellular trafficking, secretion, a	PF06522.14	B12D	NADH-ubiquinone reductase complex 1 MLRQ subunit	CYT	5	48	9.2	High
TRINITY_DN760_c1_g2_i6_orf1	ADP,ATP carrier protein [Pieris napi]	2.625790487	1.392751807	2.34E-05	up	yes	0.955	0.3637	0.352	0.363	0.376	1	0.912	0.953	BP:nucleobase-containing compound transport;BP:nucleotide transport;BP:anion transport;BP:carbohydrate derivative transport;BP:adenine nucleotide transport;BP:transmembrane transport;BP:nitrogen compound transport;BP:mitochondrial transmembrane transport;BP:mitochondrial ATP transmembrane transport;BP:organic substance transport;BP:purine nucleotide transport;BP:purine ribonucleotide transport;BP:ADP transport;BP:ATP transport;BP:ion transport;BP:transport;BP:organic anion transport;BP:ion transmembrane transport;BP:biological_process;BP:establishment of localization;BP:localization;BP:purine-containing compound transmembrane transport;BP:nucleotide transmembrane transport;BP:mitochondrial ADP transmembrane transport;BP:organophosphate ester transport;BP:anion transmembrane transport;BP:cellular process;CC:intrinsic component of membrane;CC:organelle membrane;CC:membrane;CC:cellular anatomical entity;CC:mitochondrial membrane;CC:organelle inner membrane;CC:cellular_component;CC:mitochondrial inner membrane;CC:integral component of membrane;MF:purine ribonucleotide transmembrane transporter activity;MF:nucleobase-containing compound transmembrane transporter activity;MF:purine nucleotide transmembrane transporter activity;MF:ATP transmembrane transporter activity;MF:solut;MF:organophosphate ester transmembrane transporter activity;MF:anion transmembrane transporter activity;MF:molecular_function;MF:ion transmembrane transporter activity;MF:nucleotide transmembrane transporter activity;MF:transporter activity;MF:carbohydrate derivative transporter activity;MF:antiporter activity;MF:secondary active transmembrane transporter activity;MF:adenine nucleotide transmembrane transporter activity;MF:organic anion transmembrane transporter activity;MF:AT;MF:transmembrane transporter activity;MF:active transmembrane transporter activity;MF:active ion transmembrane transporter activity;MF:ADP transmembrane transporter activity;	GO:0015931;GO:0006862;GO:0006820;GO:1901264;GO:0051503;GO:0055085;GO:0071705;GO:1990542;GO:1990544;GO:0071702;GO:0015865;GO:0015868;GO:0015866;GO:0015867;GO:0006811;GO:0006810;GO:0015711;GO:0034220;GO:0008150;GO:0051234;GO:0051179;GO:0072530;GO:1901679;GO:0140021;GO:0015748;GO:0098656;GO:0009987;GO:0031224;GO:0031090;GO:0016020;GO:0110165;GO:0031966;GO:0019866;GO:0005575;GO:0005743;GO:0016021;GO:0005346;GO:0015932;GO:0015216;GO:0005347;GO:0140323;GO:0015605;GO:0008509;GO:0003674;GO:0015075;GO:0015215;GO:0005215;GO:1901505;GO:0015297;GO:0015291;GO:0000295;GO:0008514;GO:0005471;GO:0022857;GO:0022804;GO:0022853;GO:0015217	K05863	SLC25A4S, AN	map05166;map	Human T-cell leuker	ENOG410XNW0	U:Intracellular trafficking, secretion, a	PF00153.30	Mito_carr	Mitochondrial carrier protein	CYT	21	57	33	High
TRINITY_DN15046_c0_g1_i8_orf1	epidermal retinol dehydrogenase 2-like isoform X1 [Ostrinia furnacalis] >XP_028169999.1 epidermal retinol dehydrogenase 2-like isoform X2 [Ostrinia furnacalis]	3.388110694	1.76048101	6.38E-07	up	yes	0.9917	0.2927	0.277	0.299	0.302	1	1.003	0.972	MF:catalytic activity;MF:molecular_function;MF:oxidoreductase activity;	GO:0003824;GO:0003674;GO:0016491	K15734	SDR16C5	map00830;map	Retinol metabolism;	COG1028;COG4221	S:Function unknown;	PF00106.28;PF135	adh_short;adh_short_C	short chain dehydrogenase;Enoyl-KR domain	CYT	3	13	34.7	High
TRINITY_DN16931_c0_g1_i1_orf1	pancreatic triacylglycerol lipase-like [Ostrinia furnacalis]	3.608995285	1.851597258	4.29E-08	up	yes	0.995	0.2757	0.266	0.28	0.281	1	0.986	0.999	------	------	------	------	------	------	ENOG4110RTX;ENOC	S:Function unknown;	PF00151.22	Lipase	Lipase	PLA	5	26	32.3	High

TRINITY_DN79210_c0_g1_i1_orf1	V-type proton ATPase 16 kDa proteolipid subunit [Frieseomelitta varia]	3.25952381	1.704661214	7.50E-06	up	yes	0.9583	0.294	0.283	0.29	0.309	1	0.939	0.936	[illegible]	[illegible]	K02155	ATPeV0C, ATI	map05152;map	Tuberculosis;Rheum	COG0636	J:Translation, ribosomal structure and	PF00137.24	ATP-synt_C	ATP synthase subunit C	CYT	1	11	16	High
TRINITY_DN23175_c0_g1_i6_orf1	myb-binding protein 1A-like protein [Ostrinia furnacalis]	3.878631118	1.955547573	2.05E-05	up	yes	0.9747	0.2513	0.236	0.271	0.247	1	0.916	1.008	------	------	K02331	POL5, MYBBP	------	------	ENOG410YFVF;ENO	S:Function unknown;L:Replication, re	PF04931.16	DNA_pol_phi	DNA polymerase phi	CYT	1	1	130.2	High
TRINITY_DN104586_c0_g1_i1_orf1	Chlorophyll a-b binding protein 37, chloroplastic, partial [Trichinella patagoniensis]	3.438867925	1.781933708	0.0001439	up	yes	0.9113	0.265	0.248	0.284	0.263	1	0.863	0.871	[illegible]	[illegible]	K08913	LHCB2	------	------	ENOG410ZHBU	L:Replication, recombination and rep	------	------	------	CYT	1	15	10.4	High
TRINITY_DN3862_c0_g1_i7_orf1	venom acid phosphatase Acph-1-like [Ostrinia furnacalis]	6.426751592	2.68408971	4.47E-08	up	yes	1.009	0.157	0.164	0.154	0.153	1	1.023	1.004	[illegible]	[illegible]	K19283	ACPP	------	------	ENOG410ZVBQ	C:Energy production and conversion	PF00328.25	His_Phos_2	Histidine phosphatase superfamily	CYT	1	2	43.7	Medium
TRINITY_DN22983_c0_g1_i2_orfp1	TRINITY_DN22983_c0_g1_i2_m.10495 TRINITY_DN22983_c0_g1::TRINITY_DN22983_c0_g1_i2::g.10495 ORF type:internal len:79 (-),score=15.95,Polyhedrin PF00738.19 7.6e-40 TRINITY_DN22983_c0_g1_i2:2-235(-)	5.402747253	2.433693192	1.02E-07	up	yes	0.9833	0.182	0.175	0.184	0.187	1	0.973	0.977	------	------	------	------	------	------	------	------	PF00738.21	Polyhedrin	Polyhedrin	CYT	3	21	9.2	High
TRINITY_DN5578_c0_g1_i10_orf1	unnamed protein product [Chilo suppressalis]	5.095638466	2.349262922	1.31E-06	up	yes	0.9697	0.1903	0.184	0.184	0.203	1	0.961	0.948	------	------	K23390	BAG6	------	------	ENOG410XS9P	O:Posttranslational modification, pro	PF00240.26;PF120	ubiquitin;BAG6;Rad60-	Ubiquitin family;BCL2-associated athanogene 6;Ubiquitin-2 like Rad60 SUMO-like	CYT	1	4	62.9	High
TRINITY_DN94355_c0_g1_i2_orf1	uncharacterized protein LOC126369488 [Pectinophora gossypiella]	14.65914154	3.873728715	2.89E-06	up	yes	0.987	0.06733	0.053	0.073	0.076	1	1.019	0.942	------	------	------	------	------	------	ENOG410Z1QR;ENO	S:Function unknown;L:Replication, re	PF05380.16;PF187	Peptidase_A17;DUF564	Pao retrotransposon peptidase;Family of unknown function;Protein of unknown function;Putative peptidase;Integrase core domain	CYT	1	0	179.7	High
TRINITY_DN17312_c0_g1_i1_orf1	mRNA cap guanine-N7 methyltransferase [Ostrinia furnacalis]	2.356780276	1.236817261	1.35E-05	up	yes	0.975	0.4137	0.413	0.43	0.398	1	0.989	0.936	[illegible]	[illegible]	K00565	RNMT	map03015	mRNA surveillance p	ENOG410Y7HG	A:RNA processing and modification	PF03291.19;PF136	Pox_MCEL;Methyltrans	mRNA capping enzyme;Methyltransferase domain;Methyltransferase domain;Methyltransferase domain;Methyltransferase domain;Methyltransferase domain	CYT	2	5	47.5	High
TRINITY_DN3749_c0_g1_i1_orf1	cytochrome c oxidase subunit 6B1 [Ostrinia furnacalis]	2.822222222	1.49683159	5.93E-06	up	yes	1.016	0.36	0.362	0.357	0.361	1	0.991	1.057	[illegible]	[illegible]	K02267	COX6B	map04714;map	Thermogenesis;Oxid	ENOG41121WM	S:Function unknown	PF02297.20	COX6B	Cytochrome oxidase c subunit VIb	CYT	5	43	9.9	High
TRINITY_DN1285_c0_g1_i6_orf1	bifunctional 3'-phosphoadenosine 5'-phosphosulfate synthase isoform X3 [Ostrinia furnacalis]	2.348791799	1.231918836	1.88E-05	up	yes	0.9623	0.4097	0.397	0.433	0.399	1	0.931	0.956	[illegible]	[illegible]	K13811	PAPSS	map00450;map	Selenocompound m	COG2046	P:Inorganic ion transport and metab	PF01747.20;PF143	ATP-sulfurylase;PUA_2	ATP-sulfurylase;PUA-like domain	CYT	5	16	43.5	High
TRINITY_DN18036_c0_g1_i7_orf1	pentatricopeptide repeat-containing protein 2, mitochondrial-like [Ostrinia furnacalis]	2.843137255	1.507483748	2.51E-07	up	yes	1.015	0.357	0.347	0.358	0.366	1	1.025	1.02	[illegible]	[illegible]	K17711	PTCD2	------	------	ENOG4111GFQ	S:Function unknown	------	------	------	CYT	6	16	44.3	High
TRINITY_DN2270_c0_g2_i1_orf1	integrin beta-nu [Ostrinia furnacalis]	3.146233848	1.653625905	2.05E-05	up	yes	0.9983	0.3173	0.372	0.274	0.306	1	0.988	1.007	[illegible]	[illegible]	------	------	------	------	ENOG410XP60	J:Translation, ribosomal structure and	PF00362.21;PF172	Integrin_beta;PSI_integ	Integrin beta chain VWA domain;Integrin plexin domain;EGF-like domain;Integrin beta epidermal growth factor like domain 1	CYT	2	3	89	High
TRINITY_DN1366_c0_g1_i5_orf1	unnamed protein product, partial [Iphiclides podalirius]	3.095416277	1.630133439	2.81E-08	up	yes	0.9927	0.3207	0.32	0.325	0.317	1	0.995	0.983	[illegible]	[illegible]	K02147	ATPeV1B, ATI	map05323;map	Rheumatoid arthritis	COG1156	C:Energy production and conversion	PF00006.28;PF028	ATP-synt_ab;ATP-synt_	ATP synthase alpha/beta family, nucleotide-binding domain;ATP synthase alpha/beta family, beta-barrel domain	CYT	16	45	54.8	High
TRINITY_DN10332_c0_g1_i2_orfp1	TRINITY_DN10332_c0_g1_i2_m.42894 TRINITY_DN10332_c0_g1::TRINITY_DN10332_c0_g1_i2::g.42894 ORF type:3prime_partial len:77 (+),score=1.70 TRINITY_DN10332_c0_g1_i2:1005-1232(+)	2.749421965	1.45912834	5.70E-05	up	yes	0.9513	0.346	0.381	0.302	0.355	1	0.932	0.922	------	------	------	------	------	------	------	------	------	------	------	CYT	1	8	9	Medium
TRINITY_DN47114_c0_g1_i5_orf1	nucleolar protein dao-5 isoform X2 [Ostrinia furnacalis]	3.55962012	1.831723286	1.45E-06	up	yes	1.012	0.2843	0.281	0.283	0.289	1	1.044	0.993	[illegible]	[illegible]	K25818	NOLC1	------	------	ENOG4111U5P;ENO	S:Function unknown;	PF05022.15	SRP40_C	SRP40, C-terminal domain	CYT	7	10	72.7	High
TRINITY_DN1013_c0_g1_i3_orf1	TELO2-interacting protein 1 homolog isoform X2 [Ostrinia furnacalis]	4.260162602	2.090908496	0.0001835	up	yes	1.048	0.246	0.24	0.183	0.315	1	1.141	1.003	------	[illegible]	K20403	TTI1	map04150	mTOR signaling pat	ENOG410XSN6;ENO	S:Function unknown;	------	------	------	CYT	1	1	124.9	Medium
TRINITY_DN1154_c0_g1_i1_orf1	calexcitin-1-like [Ostrinia furnacalis] >ADK94879.2 juvenile hormone diol kinase [Ostrinia furnacalis]	2.903712647	1.537898691	1.56E-05	up	yes	0.962	0.3313	0.301	0.334	0.359	1	0.945	0.941	[illegible]	[illegible]	------	------	------	------	ENOG4111I7F	S:Function unknown	PF00036.35;PF132	EF-hand_1;EF-hand_5;	EF hand;EF hand;EF-hand domain;EF-hand domain pair;EF-hand domain pair	CYT	5	35	20.6	High
TRINITY_DN9003_c0_g1_i20_orf1	RNA-binding protein Nova-2 isoform X4 [Ostrinia furnacalis]	3.093610224	1.629291437	2.10E-05	up	yes	0.9683	0.313	0.31	0.328	0.301	1	0.914	0.991	------	------	K14944	NOVA	------	------	ENOG410XRZD	S:Function unknown	PF00013.32;PF076	KH_1;KH_2;KH_4	KH domain;KH domain;KH domain	CYT	4	11	42.2	High
TRINITY_DN18172_c0_g1_i6_orf1	digestive cysteine proteinase 2-like [Ostrinia furnacalis]	3.645266594	1.866024329	1.54E-08	up	yes	1.005	0.2757	0.275	0.276	0.276	1	0.999	1.015	[illegible]	[illegible]	------	------	------	------	COG4870	O:Posttranslational modification, pro	PF00112.26;PF082	Peptidase_C1;Inhibitor_	Papain family cysteine protease;Cathepsin propeptide inhibitor domain	EXC	3	7	57.6	High
TRINITY_DN7024_c0_g1_i1_orf1	uncharacterized protein LOC114363116 [Ostrinia furnacalis]	20.23333333	4.338662111	0.0004755	up	yes	1.214	0.06	0.054	0.066	0.06	1	1.369	1.272	MF:molecular_function;MF:lipid binding;MF:binding;	GO:0003674;GO:0008289;GO:0005488	K08753;K08752	FABP4, aP2;F	map04923;map	Regulation of lipolys	ENOG4111US8	I:Lipid transport and metabolism	------	------	------	CYT	7	68	15.1	High
TRINITY_DN2267_c0_g1_i1_orf1	hypothetical protein evm_006312 [Chilo suppressalis]	3.565217391	1.833990049	1.02E-06	up	yes	0.984	0.276	0.275	0.257	0.296	1	0.983	0.969	[illegible]	[illegible]	K15102	SLC25A3, PHC	------	------	ENOG410XPST	S:Function unknown	PF00153.30	Mito_carr	Mitochondrial carrier protein	CYT	9	19	41.3	High
TRINITY_DN69713_c0_g1_i1_orf1	membrane-bound alkaline phosphatase-like [Ostrinia furnacalis]	3.700339239	1.88765754	2.75E-06	up	yes	0.9817	0.2653	0.245	0.259	0.292	1	0.987	0.958	[illegible]	[illegible]	K01077	E3.1.3.1, phoA	map00730;map	Thiamine metabolism	COG1785	P:Inorganic ion transport and metab	PF00245.23	Alk_phosphatase	Alkaline phosphatase	CYT	3	7	57.6	High
TRINITY_DN21722_c0_g1_i3_orf1	V-type proton ATPase 116 kDa subunit a isoform X1 [Ostrinia furnacalis] >XP_028177509.1 V-type proton ATPase 116 kDa subunit a isoform X1 [Ostrinia furnacalis]	3.768965517	1.914168596	7.09E-07	up	yes	0.9837	0.261	0.27	0.259	0.254	1	0.992	0.959	[illegible]	[illegible]	K02154	ATPeV0A, ATI	map05152;map	Tuberculosis;Rheum	COG1269	C:Energy production and conversion	PF01496.22	V_ATPase_I	V-type ATPase 116kDa subunit family	CYT	11	20	95.5	High
TRINITY_DN41259_c0_g1_i6_orf1	endocuticle structural glycoprotein SgAbd-8 [Ostrinia furnacalis]	6.03347535	2.59298925	6.94E-08	up	yes	0.9913	0.1643	0.154	0.174	0.165	1	0.995	0.979	[illegible]	[illegible]	------	------	------	------	ENOG4111PJ1	S:Function unknown	PF00379.26	Chitin_bind_4	Insect cuticle protein	CYT	7	44	24.7	High
TRINITY_DN109540_c0_g1_i3_orf1	4-coumarate--CoA ligase 1-like isoform X4 [Ostrinia furnacalis]	2.59125964	1.373653579	5.80E-06	up	yes	1.008	0.389	0.352	0.4	0.415	1	1.013	1.012	[illegible]	[illegible]	K01904	4CL	map00130	Ubiquinone and oth	COG0318;COG0318	I:Lipid transport and metabolism;Q:S	PF00501.31;PF131	AMP-binding;AMP-bin	AMP-binding enzyme;AMP-binding enzyme C-terminal domain	CYT	6	14	58.4	High
TRINITY_DN109733_c0_g1_i1_orf1	uncharacterized protein LOC112452128 [Temnothorax curvispinosus]	3.19504644	1.675836901	0.0003053	up	yes	1.032	0.323	0.309	0.327	0.333	1	1.148	0.947	[illegible]	[illegible]	------	------	------	------	COG0507;ENOG410Y	S:Function unknown;	------	------	------	CYT	1	2	37.9	High
TRINITY_DN21531_c0_g1_i1_orf1	viral IAP-associated factor homolog [Ostrinia furnacalis]	2.836717186	1.504222325	1.88E-07	up	yes	0.992	0.3497	0.35	0.351	0.348	1	1.001	0.975	------	------	------	------	------	------	ENOG4111H24	S:Function unknown	PF02114.19	Phosducin	Phosducin	CYT	4	21	26.4	High
TRINITY_DN4476_c0_g1_i5_orf1	trypsin, alkaline C-like isoform X1 [Ostrinia furnacalis]	2.383118524	1.252850706	5.50E-05	up	yes	0.943	0.3957	0.41	0.376	0.401	1	0.913	0.916	[illegible]	[illegible]	K01312	PRSS1_2_3	map04972;map	Pancreatic secretion	COG5640	O:Posttranslational modification, pro	PF00089.29	Trypsin	Trypsin	PLA	1	22	26.3	High
TRINITY_DN23266_c0_g2_i1_orf1	medium-chain acyl-CoA ligase ACSF2, mitochondrial [Chelonus insularis]	3.511683034	1.812162633	2.52E-06	up	yes	1.037	0.2953	0.294	0.3	0.292	1	1.059	1.053	------	------	K23617	ACSF2	------	------	COG0318	S:Function unknown	PF00501.31;PF131	AMP-binding;AMP-bin	AMP-binding enzyme;AMP-binding enzyme C-terminal domain	CYT	1	1	65	Medium
TRINITY_DN28592_c0_g1_i2_orf1	UDP-glucuronosyltransferase 2B14-like isoform X1 [Ostrinia furnacalis] >XP_028167291.1 UDP-glucuronosyltransferase 2B14-like isoform X2 [Ostrinia furnacalis]	2.98737296	1.578877363	8.09E-05	up	yes	0.97	0.3247	0.333	0.312	0.329	1	1.017	0.893	[illegible]	[illegible]	K00699	UGT	map05207;map	Chemical carcinoger	COG1819	S:Function unknown	PF00201.21	UDPGT	UDP-glucoronosyl and UDP-glucosyl transferase	CYT	1	4	58.6	High
TRINITY_DN37538_c0_g3_i1_orf1	esterase FE4-like [Ostrinia furnacalis]	2.517881518	1.332210397	0.0007334	up	yes	0.9223	0.3663	0.334	0.438	0.327	1	0.836	0.931	------	------	K07378	NLGN	map04514	Cell adhesion molec	COG2272;COG0580;E	I:Lipid transport and metabolism;G:C	PF00135.31	COesterase	Carboxylesterase family	CYT	1	13	13.4	High
TRINITY_DN6059_c0_g1_i1_orf1	brachyurin-like [Ostrinia furnacalis]	4.204918033	2.072077678	0.0001005	up	yes	1.026	0.244	0.208	0.245	0.279	1	0.962	1.115	[illegible]	[illegible]	K09634	TMPRSS3	------	------	COG5640	S:Function unknown	PF00089.29	Trypsin	Trypsin	CYT	1	4	30.1	High
TRINITY_DN2343_c1_g1_i8_orf1	receptor expression-enhancing protein 5-like isoform X3 [Ostrinia furnacalis]	4.419202743	2.14378612	1.86E-05	up	yes	1.031	0.2333	0.217	0.231	0.252	1	1.095	0.998	[illegible]	[illegible]	K17279	REEP5_6	------	------	COG5052	U:Intracellular trafficking, secretion, a	PF03134.22	TB2_DP1_HVA22	TB2/DP1, HVA22 family	CYT	1	21	22.4	High
TRINITY_DN2394_c0_g1_i4_orf1	uncharacterized protein LOC114363116 [Ostrinia furnacalis]	9.835212489	3.297956222	0.0001387	up	yes	1.134	0.1153	0.116	0.127	0.103	1	1.241	1.161	MF:molecular_function;MF:lipid binding;MF:binding;	GO:0003674;GO:0008289;GO:0005488	K08752	FABP3	map03320	PPAR signaling path	ENOG4111US8	I:Lipid transport and metabolism	PF14651.9	Lipocalin_7	Lipocalin / cytosolic fatty-acid binding protein family	CYT	6	33	18.8	High
TRINITY_DN700_c0_g1_i3_orf1	V-type proton ATPase subunit H isoform X1 [Chelonus insularis]	4.423160644	2.14507764	9.02E-07	up	yes	1.016	0.2297	0.24	0.204	0.245	1	1.029	1.02	[illegible]	[illegible]	K02144	ATPeV1H	map05152;map	Tuberculosis;Human	COG5231	C:Energy production and conversion	PF03224.17;PF116	V-ATPase_H_N;V-ATPa	V-ATPase subunit H;V-ATPase subunit H;Armadillo-like	CYT	1	3	59.4	High
TRINITY_DN2695_c0_g1_i8_orfp1	TRINITY_DN2695_c0_g1_i8_m.44478 TRINITY_DN2695_c0_g1::TRINITY_DN2695_c0_g1_i8::g.44478 ORF type:3prime_partial len:532 (+),score=80.62 TRINITY_DN2695_c0_g1_i8:101-1594(+)	2.818553459	1.494954931	0.007397	up	yes	0.8963	0.318	0.372	0.353	0.229	1	0.684	1.005	------	------	------	------	------	------	------	------	------	------	------	CYT	1	2	56.2	High

TRINITY_DN8116_c0_g1_i1_orf1	uncharacterized protein LOC114350845 [Ostrinia furnacalis]	4.0513252	2.018393895	8.12E-06	up	yes	0.963	0.2377	0.24	0.226	0.247	1	0.971	0.918	------	------	------	------	------	------	COG1028	S:Function unknown	PF13561.9;PF0010	adh_short_C2;adh_shor	Enoyl;short chain dehydrogenase;KR domain	CYT	4	26	25.5	High
TRINITY_DN30932_c0_g1_i2_orf1	delta(24)-sterol reductase-like isoform X2 [Ostrinia furnacalis]	4.149779736	2.053034762	4.38E-05	up	yes	0.942	0.227	0.267	0.194	0.22	1	0.93	0.896	CC:microbody;CC:intracellular organelle;CC:membrane-bounded organelle;CC:organelle;CC:integral component of membrane;CC:peroxisome;CC:intrinsic component of membrane;CC:cellular anatomical entity;CC:intracellular membrane-bounded organelle;CC:cellular_component;MF:heterocyclic compound binding;MF:molecular_function;MF:binding;MF:FAD binding;MF:ion binding;MF:nucleoside phosphate binding;MF:oxidoreductase activity;MF:anion binding;MF:flavin adenine dinucleotide binding;MF:oxidoreductase activity, acting on the CH-CH group of donors, NAD or NADP as acceptor;MF:nucleotide binding;MF:small molecule binding;MF:catalytic activity;MF:delta24-sterol reductase activity;MF:organic cyclic compound binding;MF:oxidoreductase activity, acting on the CH-CH group of donors;	GO:0042579;GO:0043229;GO:0043227;GO:0043226;GO:0016021;GO:0005777;GO:0031224;GO:0110165;GO:0043231;GO:0005575;GO:1901363;GO:0003674;GO:0005488;GO:0071949;GO:0043167;GO:1901265;GO:0016491;GO:0043168;GO:0050660;GO:0016628;GO:0000166;GO:0036094;GO:0003824;GO:0050614;GO:0097159;GO:0016627	K09828	DHCR24, DWI	map00100	Steroid biosynthesis	COG0277	O:Posttranslational modification, pro	PF01565.26	FAD_binding_4	FAD binding domain	CYT	1	2	59.2	High
TRINITY_DN29018_c0_g1_i4_orf1	prostaglandin reductase 1-like isoform X1 [Ostrinia furnacalis] >XP_028178925.1 prostaglandin reductase 1-like isoform X2 [Ostrinia furnacalis]	5.573142857	2.478491135	7.70E-07	up	yes	0.9753	0.175	0.171	0.172	0.182	1	0.977	0.949	CC:cytoplasm;CC:cellular_component;CC:cellular anatomical entity;MF:13-prostaglandin reductase activity;MF:oxidoreductase activity, acting on CH-OH group of donors;MF:oxidoreductase activity, acting on the CH-CH group of donors, NAD or NADP as acceptor;MF:oxidoreductase activity, acting on the CH-OH group of donors, NAD or NADP as acceptor;MF:oxidoreductase activity, acting on the CH-CH group of donors;MF:catalytic activity;MF:molecular_function;MF:15-oxoprostaglandin 13-oxidase activity;MF:2-alkenal reductase [NAD(P)]:MF:oxidoreductase activity;	GO:0005737;GO:0005575;GO:0110165;GO:0036132;GO:0016614;GO:0016628;GO:0016616;GO:0016627;GO:0003824;GO:0003674;GO:0047522;GO:0032440;GO:0016491	K13948	PTGR1, LTB4D	map00590	Arachidonic acid me	COG2130	S:Function unknown	PF16884.8;PF0010	ADH_N_2;ADH_zinc_N;	N-terminal domain of oxidoreductase;Zinc-binding dehydrogenase;Zinc-binding dehydrogenase	CYT	7	24	36.5	High
TRINITY_DN22430_c0_g3_i1_orf1	V-type proton ATPase 16 kDa proteolipid subunit c [Homo sapiens] >NP_001685.1 V-type proton ATPase 16 kDa proteolipid subunit c [Homo sapiens] >P27449.1 RecName: Full=V-type proton ATPase 16 kDa proteolipid subunit c; Short=V-ATPase 16 kDa proteolipid subunit c; AltName: Full=Vacuolar proton pump 16 kDa proteolipid subunit c [Homo sapiens] >6WLW_1 The Vo region of human V-ATPase in state 1 (focused refinement) [Homo sapiens] >6WLW_2 The Vo region of human V-ATPase in state 1 (focused refinement) [Homo sapiens] >6WLW_3 The Vo region of human V-ATPase in state 1 (focused refinement) [Homo sapiens] >6WLW_4 The Vo region of human V-ATPase in state 1 (focused refinement) [Homo sapiens] >6WLW_5 The Vo region of human V-ATPase in state 1 (focused refinement) [Homo sapiens] >6WLW_6 The Vo region of human V-ATPase in state 1 (focused refinement) [Homo sapiens] >6WLW_7 The Vo region of human V-ATPase in state 1 (focused refinement) [Homo sapiens] >6WLW_8 The Vo region of human V-ATPase in state 1 (focused refinement) [Homo sapiens] >6WLW_9 The Vo region of human V-ATPase in state 1 (focused refinement) [Homo sapiens] >6WM2_1 Human V-ATPase in state 1 with SidK and ADP [Homo sapiens] >6WM2_2 Human V-ATPase in state 1 with SidK and ADP [Homo sapiens] >6WM2_3 Human V-ATPase in state 1 with SidK and ADP [Homo sapiens] >6WM2_4 Human V-ATPase in state 1 with SidK and ADP [Homo sapiens] >6WM2_5 Human V-ATPase in state 1 with SidK and ADP [Homo sapiens] >6WM2_6 Human V-ATPase in state 1 with SidK and ADP [Homo sapiens] >6WM2_7 Human V-ATPase in state 1 with SidK and ADP [Homo sapiens] >6WM2_8 Human V-ATPase in state 1 with SidK and ADP [Homo sapiens] >6WM2_9 Human V-ATPase in state 1 with SidK and ADP [Homo sapiens] >6WM3_1 Human V-ATPase in state 2 with SidK and ADP [Homo sapiens] >6WM3_2 Human V-ATPase in state 2 with SidK and ADP [Homo sapiens] >6WM3_3 Human V-ATPase in state 2 with SidK and ADP [Homo sapiens] >6WM3_4 Human V-ATPase in state 2 with SidK and ADP [Homo sapiens] >6WM3_5 Human V-ATPase in state 2 with SidK and ADP [Homo sapiens] >6WM3_6 Human V-ATPase in state 2 with SidK and ADP [Homo sapiens] >6WM3_7 Human V-ATPase in state 2 with SidK and ADP [Homo sapiens] >6WM3_8 Human V-ATPase in state 2 with SidK and ADP [Homo sapiens] >6WM3_9 Human V-ATPase in state 2 with SidK and ADP [Homo sapiens] >6WM4_1 Human V-ATPase in state 3 with SidK and ADP [Homo sapiens] >6WM4_2 Human V-ATPase in state 3 with SidK and ADP [Homo sapiens] >6WM4_3 Human V-ATPase in state 3 with SidK and ADP [Homo sapiens]	8.417884781	3.073457763	3.18E-06	up	yes	0.979	0.1163	0.121	0.097	0.131	1	1	0.937	BP:regulation of metabolic process;BP:positive regulation of response to stimulus;BP:regulation of response to stimulus;BP:regulation of Wnt signaling pathway;BP:inorganic ion homeostasis;BP:regulation of signal transduction;BP:positive regulation of signal transduction;BP:positive regulation of biological process;BP:cellular homeostasis;BP:pH reduction;BP:chemical homeostasis;BP:regulation of catabolic process;BP:positive regulation of Wnt signaling pathway;BP:Golgi lumen acidification;BP:regulation of biological process;BP:regulation of pH;BP:biological regulation;BP:inorganic cation transmembrane transport;BP:inorganic ion transmembrane transport;BP:regulation of biological quality;BP:transport;BP:cation transport;BP:ion transport;BP:regulation of cellular process;BP:biological_process;BP:establishment of localization;BP:hydrogen ion transmembrane transport;BP:ion homeostasis;BP:positive regulation of signaling;BP:endosomal lumen acidification;BP:lysosomal lumen acidification;BP:regulation of signaling;BP:regulation of intracellular pH;BP:intracellular pH reduction;BP:positive regulation of cell communication;BP:regulation of cell communication;BP:cellular process;BP:cellular ion homeostasis;BP:cellular monovalent inorganic cation homeostasis;BP:cellular cation homeostasis;BP:cation homeostasis;BP:cellular chemical homeostasis;BP:transmembrane transport;BP:vacuolar acidification;BP:regulation of lysosomal lumen pH;BP:regulation of cellular catabolic process;BP:regulation of cellular metabolic process;BP:homeostatic process;BP:regulation of cellular pH;BP:regulation of autophagy;BP:regulation of macroautophagy;BP:ion transmembrane transport;BP:monovalent inorganic cation homeostasis;BP:localization;BP:cation transmembrane transport;BP:positive regulation of cellular process;CC:azurophil granule membrane;CC:cell junction;CC:cell-substrate junction;CC:vesicle;CC:integral component of membrane;CC:membrane;CC:vacuolar membrane;CC:exocytic vesicle membrane;CC:bounding membrane of organelle;CC:extracellular organelle;CC:extracellular membrane-bounded organelle;CC:lytic vacuole membrane;CC:focal adhesion;CC:membrane-bounded organelle;CC:cellular anatomical entity;CC:anchoring junction;CC:vesicle membrane;CC:synaptic vesicle membrane;CC:phagocytic vesicle membrane;CC:Golgi membrane;CC:proton-transporting V-type ATPase, V0 domain;CC:proton-transporting two-sector ATPase complex;CC:proton-transporting V-type ATPase complex;CC:proton-transporting two-sector ATPase complex, proton-transporting domain;CC:ficolin-1-rich granule membrane;CC:intrinsic component of membrane;CC:endosome membrane;CC:clathrin-coated vesicle membrane;CC:secretory granule membrane;CC:endocytic vesicle membrane;CC:organelle membrane;CC:coated vesicle membrane;CC:transport vesicle membrane;CC:vacuolar proton-transporting V-type ATPase, V0 domain;CC:tertiary granule membrane;CC:extracellular exosome;CC:membrane protein complex;CC:organelle;CC:plasma	GO:0019222;GO:0048584;GO:0048583;GO:0030111;GO:0098771;GO:0009966;GO:0009967;GO:0048518;GO:0019725;GO:0045851;GO:0048878;GO:0009894;GO:0030177;GO:0061795;GO:0050789;GO:0006885;GO:0065007;GO:0098662;GO:0098660;GO:0065008;GO:0006810;GO:0006812;GO:0006811;GO:0050794;GO:0008150;GO:0051234;GO:1902600;GO:0050801;GO:0023056;GO:0048388;GO:0007042;GO:0023051;GO:0051453;GO:0051452;GO:0010647;GO:0010646;GO:0009987;GO:0006873;GO:0030004;GO:0030003;GO:0055080;GO:0055082;GO:0055085;GO:0007035;GO:0035751;GO:0031329;GO:0031323;GO:0042592;GO:0030641;GO:0010506;GO:0016241;GO:0034220;GO:0055067;GO:0051179;GO:0098655;GO:0048522;GO:0035577;GO:0030054;GO:0030055;GO:0031982;GO:0016021;GO:0016020;GO:0005774;GO:0099501;GO:0098588;GO:0043230;GO:0065010;GO:0098852;GO:0005925;GO:0043227;GO:0110165;GO:0070161;GO:0012506;GO:0030672;GO:0030670;GO:0000139;GO:0033179;GO:0016469;GO:0033176;GO:0033177;GO:0101003;GO:0031224;GO:0010008;GO:0030665;GO:0030667;GO:0030666;GO:0031090;GO:0030662;GO:0030658;GO:0000220;GO:0070821;GO:0070062;GO:0098796;GO:0043226;GO:0005886;GO:1903561;GO:0032991;GO:0030659;GO:0005575;GO:0005765;GO:0005261;GO:0046961;GO:0022804;GO:0003674;GO:0005488;GO:0042625;GO:0042626;GO:0015318;GO:0009678;GO:0015399;GO:0015252;GO:0022803;GO:0016787;GO:0003824;GO:0022890;GO:0046933;GO:0015075;GO:0015267;GO:0015078;GO:0019899;GO:0019829;GO:0005215;GO:0005216;GO:0031625;GO:0005515;GO:0044389;GO:0008324;GO:0016874;GO:0044769;GO:0022857;GO:0022853	K02155	ATPeV0C, ATI	map05152;map	Tuberculosis;Rheum	COG0636	S:Function unknown	PF00137.24	ATP-synt_C	ATP synthase subunit C	CYT	1	12	15.7	Medium
TRINITY_DN1445_c0_g1_i1_orf1	leucine-rich PPR motif-containing protein, mitochondrial [Ostrinia furnacalis]	2.688829787	1.42697843	3.18E-07	up	yes	1.011	0.376	0.36	0.384	0.384	1	1.017	1.015	------	------	K17964	LRPPRC	------	------	ENOG410XSG9	S:Function unknown	PF01535.23;PF130	PPR;PPR_2;PPR_3;PPR_	PPR repeat;PPR repeat family;Pentatricopeptide repeat domain;Pentacotripeptide-repeat region of PRORP;PPR repeat	CYT	9	14	87.6	High
TRINITY_DN13216_c0_g1_i5_orf1	uncharacterized protein LOC114358344 isoform X1 [Ostrinia furnacalis]	3.303125	1.723831567	0.0001673	up	yes	1.057	0.32	0.319	0.32	0.321	1	1.165	1.006	BP:cellular process;BP:regulation of cellular process;BP:biological regulation;BP:cell surface receptor signaling pathway;BP:biological_process;BP:regulation of biological process;BP:signal transduction;CC:integral component of membrane;CC:cellular_component;CC:cellular anatomical entity;CC:intrinsic component of membrane;MF:G-protein coupled receptor activity;MF:signaling receptor activity;MF:molecular transducer activity;MF:transmembrane signaling receptor activity;MF:molecular_function;	GO:0009987;GO:0050794;GO:0065007;GO:0007166;GO:0008150;GO:0050789;GO:0007165;GO:0016021;GO:0005575;GO:0110165;GO:0031224;GO:0004930;GO:0038023;GO:0060089;GO:0004888;GO:0003674	K08451;K08455	ADGRG2, GPR	------	------	ENOG410XSD2	S:Function unknown	PF00002.27;PF018	7tm_2;GPS	7 transmembrane receptor;GPCR proteolysis site, GPS, motif	CYT	1	1	148.9	Medium
TRINITY_DN9464_c0_g1_i1_orf1	angio-associated migratory cell protein [Ostrinia furnacalis] >XP_028162594.1 angio-associated migratory cell protein [Ostrinia furnacalis]	2.869688385	1.520894086	8.04E-07	up	yes	1.013	0.353	0.349	0.345	0.365	1	1.035	1.004	------	------	K24725	AAMP	------	------	ENOG410XR6A	S:Function unknown	PF00400.35;PF128	WD40;ANAPC4_WD40;	WD domain, G-beta repeat;Anaphase-promoting complex subunit 4 WD40 domain;WD40-like domain;Neurobeachin beta propeller domain	CYT	6	20	44.7	High
TRINITY_DN7583_c0_g1_i1_orf1	39S ribosomal protein L21, mitochondrial [Ostrinia furnacalis]	2.796089385	1.483410482	8.19E-05	up	yes	1.001	0.358	0.428	0.292	0.354	1	1	1.003	BP:cellular biosynthetic process;BP:cellular macromolecule biosynthetic process;BP:organic substance biosynthetic process;BP:cellular nitrogen compound biosynthetic process;BP:cellular macromolecule metabolic process;BP:cellular amide metabolic process;BP:peptide biosynthetic process;BP:organic substance metabolic process;BP:organonitrogen compound biosynthetic process;BP:amide biosynthetic process;BP:cellular process;BP:biosynthetic process;BP:macromolecule biosynthetic process;BP:biological_process;BP:metabolic process;BP:organonitrogen compound metabolic process;BP:primary metabolic process;BP:protein metabolic process;BP:cellular nitrogen compound metabolic process;BP:cellular metabolic process;BP:macromolecule metabolic process;BP:nitrogen compound metabolic process;BP:peptide metabolic process;BP:translation;CC:organelle;CC:intracellular organelle;CC:non-membrane-bounded organelle;CC:cytoplasm;CC:cellular anatomical entity;CC:macromolecular complex;CC:ribosome;CC:intracellular non-membrane-bounded organelle;CC:ribonucleoprotein complex;CC:cellular_component;MF:nucleic acid binding;MF:molecular_function;MF:binding;MF:heterocyclic compound binding;MF:structural molecule activity;MF:structural constituent of ribosome;MF:organic cyclic compound binding;MF:RNA binding;	GO:0044249;GO:0034645;GO:1901576;GO:0044271;GO:0044260;GO:0043603;GO:0043043;GO:0071704;GO:1901566;GO:0043604;GO:0009987;GO:0009058;GO:0009059;GO:0008150;GO:0008152;GO:1901564;GO:0044238;GO:0019538;GO:0034641;GO:0044237;GO:0043170;GO:0006807;GO:0006518;GO:0006412;GO:0043226;GO:0043229;GO:0043228;GO:0005737;GO:0110165;GO:0032991;GO:0005840;GO:0043232;GO:1990904;GO:0005575;GO:0003676;GO:0003674;GO:0005488;GO:1901363;GO:0005198;GO:0003735;GO:0097159;GO:0003723	K02888	RP-L21, MRPL	map03010	Ribosome	ENOG4111TX4	J:Translation, ribosomal structure anc	PF00829.24	Ribosomal_L21p	Ribosomal prokaryotic L21 protein	CYT	1	5	19.8	High
TRINITY_DN26130_c0_g1_i1_orf1	membrane alanyl aminopeptidase-like [Ostrinia furnacalis]	3.107033639	1.635537861	4.72E-07	up	yes	1.016	0.327	0.329	0.329	0.323	1	1.038	1.01	BP:nitrogen compound metabolic process;BP:organic substance metabolic process;BP:proteolysis;BP:biological_process;BP:metabolic process;BP:primary metabolic process;BP:organonitrogen compound metabolic process;BP:protein metabolic process;BP:macromolecule metabolic process;CC:integral component of membrane;CC:intrinsic component of membrane;CC:cellular anatomical entity;CC:cellular_component;MF:aminopeptidase activity;MF:exopeptidase activity;MF:molecular_function;MF:binding;MF:peptidase activity;MF:metallopeptidase activity;MF:cation binding;MF:transition metal ion binding;MF:ion binding;MF:metal ion binding;MF:hydrolase activity;MF:catalytic activity, acting on a protein;MF:catalytic activity;MF:zinc ion binding;	GO:0006807;GO:0071704;GO:0006508;GO:0008150;GO:0008152;GO:0044238;GO:1901564;GO:0019538;GO:0043170;GO:0016021;GO:0031224;GO:0110165;GO:0005575;GO:0004177;GO:0008238;GO:0003674;GO:0005488;GO:0008233;GO:0008237;GO:0043169;GO:0046914;GO:0043167;GO:0046872;GO:0016787;GO:0140096;GO:0003824;GO:0008270	K11140	ANPEP, CD13	map04640;map	Hematopoietic cell li	COG0308	E:Amino acid transport and metaboli	PF01433.23;PF118	Peptidase_M1;ERAP1_C	Peptidase family M1 domain;ERAP1-like C-terminal domain;Peptidase M1 N-terminal domain	CYT	9	13	104.2	High
TRINITY_DN4143_c0_g1_i1_orf1	zinc finger protein 530-like isoform X8 [Ostrinia furnacalis]	3.213949105	1.684347083	0.000254	up	yes	1.023	0.3183	0.414	0.304	0.237	1	0.996	1.074	------	------	K09228	KRAB	map05168	Herpes simplex virus	COG5048;ENOG411(	S:Function unknown;	PF07776.18	zf-AD	Zinc-finger associated domain	CYT	1	5	20.3	High
TRINITY_DN24399_c0_g1_i1_orf1	retinol-binding protein pinta-like [Ostrinia furnacalis]	3.482956463	1.800312439	7.65E-06	up	yes	1.032	0.2963	0.272	0.299	0.318	1	1.071	1.024	------	------	------	------	------	------	ENOG410XRSO	S:Function unknown	PF00650.23	CRAL_TRIO	CRAL/TRIO domain	CYT	4	15	35.4	High
TRINITY_DN52768_c0_g1_i1_orf1	carboxypeptidase Q-like isoform X2 [Ostrinia furnacalis]	3.424749164	1.775998326	8.87E-06	up	yes	1.024	0.299	0.285	0.341	0.271	1	1.026	1.047	CC:lytic vacuole;CC:endoplasmic reticulum;CC:intracellular organelle;CC:organelle;CC:lysosome;CC:vacuole;CC:membrane-bounded organelle;CC:cellular anatomical entity;CC:Golgi apparatus;CC:intracellular membrane-bounded organelle;CC:cellular_component;CC:extracellular region;MF:hydrolase activity;MF:exopeptidase activity;MF:molecular_function;MF:peptidase activity;MF:metalloexopeptidase activity;MF:metallopeptidase activity;MF:dipeptidase activity;MF:metallodipeptidase activity;MF:catalytic activity, acting on a protein;MF:catalytic activity;	GO:0000323;GO:0005783;GO:0043229;GO:0043226;GO:0005764;GO:0005773;GO:0043227;GO:0110165;GO:0005794;GO:0043231;GO:0005575;GO:0005576;GO:0016787;GO:0008238;GO:0003674;GO:0008233;GO:0008235;GO:0008237;GO:0016805;GO:0070573;GO:0140096;GO:0003824	K01302	CPQ	------	------	COG2234	S:Function unknown	PF04389.20;PF022	Peptidase_M28;PA;Pep	Peptidase family M28;PA domain;Peptidase family M20/M25/M40	CYT	1	2	52	High
TRINITY_DN3614_c0_g2_i1_orf1	PC4 and SFRS1-interacting protein isoform X4 [Galleria mellonella]	2.942037646	1.556815707	3.26E-05	up	yes	0.9847	0.3347	0.321	0.313	0.37	1	0.934	1.02	CC:intracellular membrane-bounded organelle;CC:nucleus;CC:cellular_component;CC:intracellular organelle;CC:membrane-bounded organelle;CC:organelle;CC:cellular anatomical entity;	GO:0043231;GO:0005634;GO:0005575;GO:0043229;GO:0043227;GO:0043226;GO:0110165	K25057	PSIP1, LEDGF	map03250	Viral life cycle - HIV-	ENOG4111K9I	S:Function unknown	------	------	------	CYT	1	13	11.9	High
TRINITY_DN6087_c0_g1_i7_orf1	uncharacterized protein LOC114355564 [Ostrinia furnacalis]	2.721729173	1.444523518	3.74E-05	up	yes	0.9507	0.3493	0.319	0.356	0.373	1	0.914	0.938	MF:hydrolase activity;MF:methyl indole-3-acetate esterase activity;MF:hydrolase activity, acting on ester bonds;MF:catalytic activity;MF:molecular_function;MF:carboxylic ester hydrolase activity;	GO:0016787;GO:0080030;GO:0016788;GO:0003824;GO:0003674;GO:0052689	K03927	CES2	map00983	Drug metabolism - (	COG2272;ENOG410X	I:Lipid transport and metabolism;T:Sig	PF00135.31;PF204	COesterase;BD-FAE;Ab	Carboxylesterase family;BD-FAE;alpha/beta hydrolase	CYT	5	9	61.5	High
TRINITY_DN52296_c0_g1_i6_orf1	protein takeout-like [Ostrinia furnacalis]	2.655492958	1.408979703	0.0002278	up	yes	0.9427	0.355	0.311	0.415	0.339	1	0.948	0.88	------	------	------	------	------	------	ENOG4110RSP;ENO(	S:Function unknown;	PF06585.14	JHBP	Haemolymph juvenile hormone binding protein	CYT	1	3	19.5	High
TRINITY_DN18773_c0_g1_i3_orf1	keratin, type II cytoskeletal 68 kDa, component IB-like [Ostrinia furnacalis]	2.422163588	1.276296305	0.00049	up	yes	0.918	0.379	0.361	0.393	0.383	1	0.824	0.93	------	------	------	------	------	------	------	------	------	------	------	CYT	3	47	13.9	High
TRINITY_DN2894_c0_g2_i3_orf1	myrosinase 1-like isoform X1 [Ostrinia furnacalis]	2.880372708	1.526255502	2.83E-05	up	yes	0.9583	0.3327	0.331	0.299	0.368	1	0.946	0.929	BP:primary metabolic process;BP:carbohydrate metabolic process;BP:organic substance metabolic process;BP:biological_process;BP:metabolic process;MF:hydrolase activity;MF:catalytic activity;MF:hydrolase activity, acting on glycosyl bonds;MF:hydrolase activity, hydrolyzing O-glycosyl	GO:0044238;GO:0005975;GO:0071704;GO:0008150;GO:0008152;GO:0016787;GO:0003824;GO:0016798;GO:0004553;GO:0003674	------	------	------	------	COG2723	G:Carbohydrate transport and metab	PF00232.21	Glyco_hydro_1	Glycosyl hydrolase family 1	CYT	1	6	50.1	High
TRINITY_DN64403_c0_g2_i1_orf1	carboxylesterase [Ostrinia furnacalis]	3.324251598	1.733029577	1.01E-05	up	yes	0.9883	0.2973	0.268	0.344	0.28	1	0.973	0.992	MF:hydrolase activity;MF:molecular_function;MF:hydrolase activity, acting on ester bonds;MF:carboxylic ester hydrolase activity;MF:catalytic activity;	GO:0016787;GO:0003674;GO:0016788;GO:0052689;GO:0003824	K03927	CES2	map00983	Drug metabolism - (	COG2272	K:Transcription	PF00135.31	COesterase	Carboxylesterase family	CYT	1	14	10.6	High
TRINITY_DN15870_c0_g1_i3_orf1	PREDICTED: mitochondrial import inner membrane translocase subunit Tim23 isoform X1 [Fopius arisanus]	3.630740741	1.860263916	2.41E-05	up	yes	0.9803	0.27	0.237	0.252	0.321	1	0.998	0.943	CC:integral component of membrane;CC:cellular_component;CC:cellular anatomical entity;CC:intrinsic component of membrane;MF:transporter activity;MF:transmembrane transporter activity;MF:molecular_function;	GO:0016021;GO:0005575;GO:0110165;GO:0031224;GO:0005215;GO:0022857;GO:0003674	K17794	TIM23	map04212	Longevity regulating	COG5596	O:Posttranslational modification, pro	PF02466.22	Tim17	Tim17/Tim22/Tim23/Pmp24 family	CYT	1	5	22.1	High
TRINITY_DN10940_c0_g1_i10_orfp1	TRINITY_DN10940_c0_g1_i10_m.52163 TRINITY_DN10940_c0_g1::TRINITY_DN10940_c0_g1_i10::g.52163 ORF type:5prime_partial len:248 (-),score=128.24 TRINITY_DN10940_c0_g1_i10:121-864(-)	5.208955224	2.380994036	8.95E-06	up	yes	1.047	0.201	0.195	0.181	0.227	1	1.091	1.05	------	------	------	------	------	------	------	------	------	------	------	CYT	1	14	22.7	High
TRINITY_DN5891_c0_g2_i4_orf1	amino acid transporter AVT1A-like [Ostrinia furnacalis] >XP_028156666.1 amino acid transporter AVT1A-like [Ostrinia furnacalis]	4.588394062	2.197989299	7.81E-06	up	yes	1.02	0.2223	0.253	0.202	0.212	1	0.996	1.064	CC:cellular_component;CC:integral component of membrane;CC:cellular anatomical entity;CC:intrinsic component of membrane;	GO:0005575;GO:0016021;GO:0110165;GO:0031224	K15015	SLC32A, VGA1	map04727;map	GABAergic synapse;I	COG0814	E:Amino acid transport and metaboli	PF01490.21;PF032	Aa_trans;Trp_Tyr_perm	Transmembrane amino acid transporter protein;Tryptophan/tyrosine permease family	CYT	1	2	48.1	High
TRINITY_DN1199_c0_g1_i1_orf1	pupal cuticle protein 36a-like [Ostrinia furnacalis]	4.029185245	2.010488136	5.16E-06	up	yes	0.994	0.2467	0.231	0.288	0.221	1	1.006	0.976	------	------	------	------	------	------	ENOG4110OGB	S:Function unknown	------	------	------	CYT	1	2	23.9	High
TRINITY_DN48410_c0_g2_i1_orf1	alpha-amylase 2-like isoform X3 [Ostrinia furnacalis]	4.119396732	2.042433076	3.04E-06	up	yes	0.9833	0.2387	0.24	0.242	0.234	1	1.006	0.944	BP:organic substance metabolic process;BP:primary metabolic process;BP:carbohydrate metabolic process;BP:metabolic process;BP:biological_process;MF:hydrolase activity;MF:cation binding;MF:amylase activity;MF:hydrolase activity, acting on glycosyl bonds;MF:ion binding;MF:catalytic activity;MF:alpha-amylase activity;MF:molecular_function;MF:binding;MF:hydrolase activity, hydrolyzing O-glycosyl compounds;MF:metal ion binding;	GO:0071704;GO:0044238;GO:0005975;GO:0008152;GO:0008150;GO:0016787;GO:0043169;GO:0016160;GO:0016798;GO:0043167;GO:0003824;GO:0004556;GO:0003674;GO:0005488;GO:0004553;GO:0046872	K01176	AMY, amyA, r	map04972;map	Pancreatic secretion	COG0366	G:Carbohydrate transport and metab	PF00128.27;PF028	Alpha-amylase;Alpha-	Alpha amylase, catalytic domain;Alpha amylase, C-terminal all-beta domain	EXC	8	15	56.5	High
TRINITY_DN67623_c0_g1_i1_orf1	maltase A1-like [Ostrinia furnacalis]	4.891146589	2.290172703	3.01E-07	up	yes	1.011	0.2067	0.189	0.223	0.208	1	1.024	1.01	BP:primary metabolic process;BP:carbohydrate metabolic process;BP:organic substance metabolic process;BP:biological_process;BP:metabolic process;CC:integral component of membrane;CC:cellular_component;CC:intrinsic component of membrane;CC:cellular anatomical entity;MF:hydrolase activity;MF:hydrolase activity, acting on glycosyl bonds;MF:catalytic activity;MF:molecular_function;	GO:0044238;GO:0005975;GO:0071704;GO:0008150;GO:0008152;GO:0016021;GO:0005575;GO:0031224;GO:0110165;GO:0016787;GO:0016798;GO:0003824;GO:0003674	------	------	------	------	COG0366	G:Carbohydrate transport and metab	PF00128.27	Alpha-amylase	Alpha amylase, catalytic domain	CYT	4	9	68.6	High
TRINITY_DN334_c0_g1_i1_orf1	putative chymotrypsin 12 [Ostrinia nubilalis]	4.612575127	2.205572409	9.04E-07	up	yes	0.9977	0.2163	0.213	0.225	0.211	1	0.971	1.022	BP:organic substance metabolic process;BP:organonitrogen compound metabolic process;BP:protein metabolic process;BP:macromolecule metabolic process;BP:biological_process;BP:nitrogen compound metabolic process;BP:metabolic process;BP:proteolysis;BP:primary metabolic process;MF:serine-type endopeptidase activity;MF:endopeptidase activity;MF:hydrolase activity;MF:serine hydrolase activity;MF:catalytic activity, acting on a protein;MF:catalytic activity;MF:molecular_function;MF:peptidase activity;MF:serine-type peptidase activity;	GO:0071704;GO:1901564;GO:0019538;GO:0043170;GO:0008150;GO:0006807;GO:0008152;GO:0006508;GO:0044238;GO:0004252;GO:0004175;GO:0016787;GO:0017171;GO:0140096;GO:0003824;GO:0003674;GO:0008233;GO:0008236	------	------	------	------	COG5640	O:Posttranslational modification, pro	PF00089.29	Trypsin	Trypsin	CYT	5	53	21.6	High
TRINITY_DN48694_c0_g1_i1_orfp1	TRINITY_DN48694_c0_g1_i1_m.75338 TRINITY_DN48694_c0_g1::TRINITY_DN48694_c0_g1_i1::g.75338 ORF type:internal len:84 (+),score=16.02 TRINITY_DN48694_c0_g1_i1:2-250(+)	2.95049505	1.560957038	0.006801	up	yes	0.894	0.303	0.258	0.155	0.496	1	0.815	0.867	------	------	------	------	------	------	------	------	------	------	------	CYT	1	13	9.1	Medium
TRINITY_DN26408_c0_g1_i7_orf1	venom carboxylesterase-6-like [Ostrinia furnacalis]	4.969230769	2.313022542	6.32E-05	up	yes	0.969	0.195	0.13	0.203	0.252	1	0.916	0.991	CC:integral component of membrane;CC:cellular_component;CC:intrinsic component of membrane;CC:cellular anatomical entity;MF:hydrolase activity;MF:hydrolase activity, acting on ester bonds;MF:carboxylic ester hydrolase activity;MF:catalytic activity;MF:molecular_function;	GO:0016021;GO:0005575;GO:0031224;GO:0110165;GO:0016787;GO:0016788;GO:0052689;GO:0003824;GO:0003674	------	------	------	------	COG2272;ENOG410X	I:Lipid transport and metabolism;T:Sig	PF00135.31;PF204	COesterase;BD-FAE;Ab	Carboxylesterase family;BD-FAE;alpha/beta hydrolase fold	PLA	1	2	64.2	High
TRINITY_DN2815_c0_g1_i3_orf1	uncharacterized protein LOC114364075 [Ostrinia furnacalis]	4.39116126	2.134602517	0.0001544	up	yes	0.934	0.2127	0.188	0.244	0.206	1	0.964	0.838	------	------	------	------	------	------	ENOG410XRSQ	S:Function unknown	PF00650.23	CRAL_TRIO	CRAL/TRIO domain	CYT	1	3	35.8	High
TRINITY_DN3784_c0_g1_i1_orf1	pancreatic triacylglycerol lipase-like [Ostrinia furnacalis]	6.696005416	2.743300695	3.90E-08	up	yes	0.989	0.1477	0.14	0.154	0.149	1	0.989	0.978	BP:organic substance metabolic process;BP:lipid metabolic process;BP:primary metabolic process;BP:metabolic process;BP:biological_process;CC:cellular_component;CC:extracellular region;CC:cellular anatomical entity;MF:hydrolase activity;MF:hydrolase activity, acting on ester bonds;MF:catalytic activity;MF:molecular_function;MF:lipase activity;	GO:0071704;GO:0006629;GO:0044238;GO:0008152;GO:0008150;GO:0005575;GO:0005576;GO:0110165;GO:0016787;GO:0016788;GO:0003824;GO:0003674;GO:0016298	------	------	------	------	ENOG41116Y2;ENO(	S:Function unknown;	PF00151.22	Lipase	Lipase	PLA	13	30	61.9	High
TRINITY_DN6044_c0_g1_i4_orf1	acyl-CoA-binding protein-like [Ostrinia furnacalis]	8.542372881	3.094636874	2.38E-06	up	yes	1.008	0.118	0.115	0.121	0.118	1	1.05	0.974	MF:sulfur compound binding;MF:molecular_function;MF:fatty acid derivative binding;MF:carbohydrate derivative binding;MF:amide binding;MF:adenyl nucleotide binding;MF:ribonucleotide binding;MF:lipid binding;MF:fatty-acyl-CoA binding;MF:nucleotide binding;MF:small molecule binding;MF:purine ribonucleotide binding;MF:adenyl ribonucleotide binding;MF:binding;MF:organic cyclic compound binding;MF:acyl-CoA binding;MF:purine nucleotide binding;MF:heterocyclic compound binding;MF:nucleoside phosphate binding;	GO:1901681;GO:0003674;GO:1901567;GO:0097367;GO:0033218;GO:0030554;GO:0032553;GO:0008289;GO:0000062;GO:0000166;GO:0036094;GO:0032555;GO:0032559;GO:0005488;GO:0097159;GO:0120227;GO:0017076;GO:1901363;GO:1901265	K08762	DBI, ACBP	map03320	PPAR signaling path	COG4281	I:Lipid transport and metabolism	PF00887.22	ACBP	Acyl CoA binding protein	CYT	5	54	9.5	High
TRINITY_DN4959_c0_g1_i1_orf1	pancreatic triacylglycerol lipase-like [Ostrinia furnacalis]	8.065420561	3.011749763	7.03E-06	up	yes	0.9493	0.1177	0.104	0.129	0.12	1	0.937	0.911	------	------	------	------	------	------	ENOG410YGS3;ENO(	S:Function unknown;	PF00151.22	Lipase	Lipase	CYT	1	7	37.1	High
