# Supplementary material for: Comparative Proteomics and Metabonomics Analysis of Different Diapause Stages Revealed a New Regulation Mechanism of Diapause in Loxostege sticticalis (Lepidoptera: Pyralidae)
Source: Molecules. 2024 Jul 25;29(15):3472. doi: 10.3390/molecules29153472 (PMC11314584; doi:10.3390/molecules29153472)
Supplement: Supplementary file 1 [file molecules-29-03472-s001.zip › analysis process/proteomic/diffreential protein statistic table/NDvsPreD.pdf]

Accession	Description	FC(ND/PreD)	Log2FC(ND/PreD)	P_value(ND/PreD)	Regulate	Significant	ND	PreD	PreD1	PreD2	PreD3	ND1	ND2	ND3	GO Term	GO ID	KO ID	KO Name	Pathway ID	Pathway_defition	EggNOG ID	EggNOG_Functional Categories	Pfam ID	Domain	Domain Description	Subcellular Loc	Unique Peptides	Coverage %	MW [kDa]	FDR Confidence
TRINITY_DN38506_c0_g1_i4_orf1	C-1-tetrahydrofolate synthase, cytoplasmic isoform X1 [Ostrinia furnacalis] >XP_028166137.1 C-1-tetrahydrofolate synthase, cytoplasmic isoform X2 [Ostrinia furnacalis] >XP_028166140.1 C-1-tetrahydrofolate synthase, cytoplasmic isoform X4 [Ostrinia furnacalis]	0.203655352	-2.295798363	1.46E-05	down	yes	0.936	4.596	4.646	4.808	4.333	1	0.864	0.944	BP:one-carbon metabolic process;BP:nitrogen compound metabolic process;BP:cellular metabolic process;BP:tetrahydrofolate metabolic process;BP:organic cyclic compound metabolic process;BP:tetrahydrofolate interconversion;BP:pteridine-containing compound metabolic process;BP:organic substance metabolic process;BP:folic acid-containing compound metabolic process;BP:cellular process;BP:cellular aromatic compound metabolic process;BP:biological_process;BP:metabolic process;BP:heterocycle metabolic process;BP:organonitrogen compound metabolic process;BP:cellular modified amino acid metabolic process;BP:small molecule metabolic process;MF:binding;MF:purine ribonucleoside triphosphate binding;MF:oxidoreductase activity, acting on the CH-NH group of donors, NAD or NADP as acceptor;MF:molecular_function;MF:ion binding;MF:heterocyclic compound binding;MF:oxidoreductase activity;MF:anion binding;MF:formate-tetrahydrofolate ligase activity;MF:oxidoreductase activity, acting on the CH-NH group of	GO:00067	K00288	MTHFD	map00670;m	One carbon pool b	COG0190	H:Coenzyme transport and meta	PF00763.26	THF_DHG_CYH	Tetrahydrofolate dehydrogenase/cyclohydrolase, catalytic domain	CYT	3	61	16.2	High
TRINITY_DN2396_c0_g1_i9_orfp1	TRINITY_DN2396_c0_g1_i9_m.39038 TRINITY_DN2396_c0_g1::TRINITY_DN2396_c0_g1_i9::g.39038 ORF type:5prime_partial len:181 (+),score=90.33 TRINITY_DN2396_c0_g1_i9:3-545(+)	0.03346577	-4.901169973	3.09E-05	down	yes	1.095	32.72	30.425	35.527	32.211	1	0.759	1.526	------	------	------	------	------	------	------	------	------	------	------	CYT	1	6	19.3	High
TRINITY_DN20356_c0_g1_i5_orf1	uncharacterized protein LOC114362428 [Ostrinia furnacalis]	0.085314092	-3.551072133	3.66E-06	down	yes	1.005	11.78	11.209	12.224	11.917	1	1.05	0.964	CC:cellular_component;CC:integral component of membrane;CC:cellular anatomical entity;CC:intrinsic component of membrane;	GO:00055	------	------	------	------	ENOG4110QHP	S:Function unknown	------	------	------	CYT	1	2	60	High
TRINITY_DN56459_c0_g1_i2_orf1	aldo-keto reductase AKR2E4-like [Ostrinia furnacalis]	0.167242559	-2.579986071	8.45E-06	down	yes	0.9777	5.846	5.649	6.179	5.71	1	0.951	0.982	MF:catalytic activity;MF:molecular_function;MF:oxidoreductase activity;	GO:0003	------	------	------	------	COG0656	S:Function unknown	------	------	------	CYT	4	78	8.7	High
TRINITY_DN295_c3_g1_i1_orfp1	TRINITY_DN295_c3_g1_i1_m.18839 TRINITY_DN295_c3_g1::TRINITY_DN295_c3_g1_i1::g.18839 ORF type:5prime_partial len:118 (+),score=46.25 TRINITY_DN295_c3_g1_i1:1-354(+)	0.086654644	-3.528579124	1.25E-06	down	yes	0.961	11.09	10.965	10.796	11.508	1	0.975	0.908	------	------	------	------	------	------	------	------	------	------	------	PLA	3	49	12.6	High
TRINITY_DN10877_c0_g1_i1_orf1	spodomicin-like [Ostrinia furnacalis]	0.053492063	-4.224531332	0.001666	down	yes	1.011	18.9	19.726	22.526	14.44	1	1.278	0.754	CC:cellular_component;CC:extracellular region;CC:cellular anatomical entity;MF:molecular_function;MF:toxin activity;	GO:00055	------	------	------	------	------	------	PF08036.14	Antimicrobial_6	Diapausin family of antimicrobial peptide	CYT	1	19	6.9	Medium
TRINITY_DN1108_c3_g1_i1_orfp1	TRINITY_DN1108_c3_g1_i1_m.5561 TRINITY_DN1108_c3_g1::TRINITY_DN1108_c3_g1_i1::g.5561 ORF type:internal len:113 (+),score=89.07 TRINITY_DN1108_c3_g1_i1:1-336(+)	0.104130262	-3.263538692	0.003035	down	yes	1.311	12.59	14.8	13.607	9.364	1	2.486	0.447	------	------	------	------	------	------	------	------	------	------	------	PLA	1	11	12.3	High
TRINITY_DN2407_c0_g1_i2_orf1	uncharacterized protein LOC114366345 isoform X2 [Ostrinia furnacalis]	0.130177515	-2.941447818	1.75E-06	down	yes	0.968	7.436	7.327	7.724	7.256	1	0.892	1.012	BP:response to biotic stimulus;BP:response to external stimulus;BP:response to external biotic stimulus;BP:response to stimulus;BP:defense response;BP:response to stress;BP:biological_process;BP:interspecies interaction between organisms;BP:response to other organism;CC:cellular_component;CC:extr	GO:0009	------	------	------	------	ENOG4110S6N	S:Function unknown	PF00451.22	Toxin_2	Scorpion short toxin, BmKK2	CYT	1	36	21.5	High
TRINITY_DN5080_c0_g1_i5_orf1	storage protein [Ostrinia furnacalis]	0.14962406	-2.74058591	5.71E-05	down	yes	0.995	6.65	7.24	6.162	6.547	1	0.967	1.018	------	------	------	------	------	------	ENOG410XR2D	J:Translation, ribosomal structure	PF00372.22;PF03	Hemocyanin_M;Hemocyanin_N	Hemocyanin, copper containing domain;Hemocyanin, all-alpha domain	CYT	3	69	49.2	High
TRINITY_DN34423_c0_g1_i3_orf1	THAP domain-containing protein 4-like [Ostrinia furnacalis]	0.111040508	-3.170842017	3.91E-06	down	yes	0.699	6.295	6.247	6.327	6.312	1	0.628	0.469	------	------	K23205	THAP4	------	------	ENOG4111TBC	S:Function unknown	PF08768.14	THAP4_heme-bd	THAP4-like, heme-binding beta-barrel domain	CYT	1	23	12.2	High
TRINITY_DN1506_c0_g1_i6_orfp1	TRINITY_DN1506_c0_g1_i6_m.57691 TRINITY_DN1506_c0_g1::TRINITY_DN1506_c0_g1_i6::g.57691 ORF type:5prime_partial len:173 (+),score=30.49 TRINITY_DN1506_c0_g1_i6:1-519(+)	0.071375187	-3.808433573	1.48E-06	down	yes	0.955	13.38	13.483	13.775	12.871	1	0.807	1.058	------	------	------	------	------	------	------	------	------	------	------	CYT	1	7	19	High
TRINITY_DN84478_c0_g1_i8_orf1	uncharacterized protein LOC114359035 isoform X1 [Ostrinia furnacalis]	0.070625439	-3.823668255	5.20E-08	down	yes	1.005	14.23	14.06	14.464	14.167	1	0.936	1.078	BP:nitrogen compound metabolic process;BP:membrane lipid metabolic process;BP:organic substance metabolic process;BP:sphingolipid metabolic process;BP:lipid metabolic process;BP:cellular process;BP:biological_process;BP:metabolic process;BP:cellular lipid metabolic process;BP:primary metabolic process;BP:organonitrogen compound metabolic process;BP:cellular metabolic process;CC:lytic vacuole;CC:intracellular organelle;CC:membrane-bounded organelle;CC:organelle;CC:lysosome;CC:vacuole;CC:cellular anatomical entity;CC:intracellular membrane-bounded organelle;CC:cellular_component;CC:extr	GO:0006	K12382	PSAP, SGP1	map00600;m	Sphingolipid metab	ENOG410XSI5	O:Posttranslational modification,	PF02199.18	SapA	Saposin A-type domain	CYT	1	52	5.9	High
TRINITY_DN27300_c0_g1_i1_orfp1	TRINITY_DN27300_c0_g1_i1_m.71142 TRINITY_DN27300_c0_g1::TRINITY_DN27300_c0_g1_i1::g.71142 ORF type:internal len:82 (-),score=9.46 TRINITY_DN27300_c0_g1_i1:3-245(-)	0.09245614	-3.435087052	1.16E-06	down	yes	1.054	11.4	11.271	11.811	11.118	1	0.998	1.164	------	------	------	------	------	------	------	------	------	------	------	CYT	1	21	9.2	High
TRINITY_DN971_c0_g1_i10_orfp1	TRINITY_DN971_c0_g1_i10_m.54268 TRINITY_DN971_c0_g1::TRINITY_DN971_c0_g1_i10::g.54268 ORF type:internal len:187 (+),score=141.68 TRINITY_DN971_c0_g1_i10:1-	0.023815347	-5.391964621	3.15E-05	down	yes	0.779	32.71	31.231	35.761	31.133	1	0.834	0.503	------	------	------	------	------	------	------	------	------	------	------	CYT	2	20	20.5	High
TRINITY_DN14242_c0_g1_i2_orfp1	TRINITY_DN14242_c0_g1_i2_m.18449 TRINITY_DN14242_c0_g1::TRINITY_DN14242_c0_g1_i2::g.18449 ORF type:internal len:148 (-),score=102.30 TRINITY_DN14242_c0_g1_i2:2-	0.057051282	-4.131596883	4.21E-07	down	yes	1.335	23.4	23.089	24.022	23.09	1	1.386	1.618	------	------	------	------	------	------	------	------	------	------	------	CYT	1	8	16	High
TRINITY_DN57111_c0_g1_i1_orf1	trypsin-like serine proteinase T26 protein, partial [Chilo infuscatellus]	0.254120149	-1.976417326	1.12E-06	down	yes	0.956	3.762	3.679	3.857	3.751	1	0.961	0.907	BP:organic substance metabolic process;BP:organonitrogen compound metabolic process;BP:protein metabolic process;BP:macromolecule metabolic process;BP:biological_process;BP:nitrogen compound metabolic process;BP:metabolic process;BP:proteolysis;BP:primary metabolic process;MF:serine-type endopeptidase activity;MF:endopeptidase activity;MF:hydrolase activity;MF:serine hydrolase activity;MF:catalytic activity, acting on a protein;MF:catalytic activity;MF:molecular_function;MF:pepti	GO:0071	------	------	------	------	------	------	------	------	------	CYT	1	26	7.9	High
TRINITY_DN71698_c0_g1_i1_orfp1	TRINITY_DN71698_c0_g1_i1_m.1194 TRINITY_DN71698_c0_g1::TRINITY_DN71698_c0_g1_i1::g.1194 ORF type:internal len:134 (+),score=19.66,Toxin_2 PF00451.20 4.3e-05,Toxin_2|PF00451.20|0.037,Toxin_2|PF00451.20|7.5e-05,Gamma-thionin|PF00304.21|0.017,Gamma-thionin|PF00304.21|0.05,Gamma-thionin|PF00304.21|0.021,Toxin_38|PF14866.7|0.13,Toxin_38|PF14866.7|0.15,Toxin_38|PF14866.7|0.15,Defensin_2|PF01097.19|0.053,Defensin_2|PF01097.19|0.34,Defensin_2|PF01097.19|0.092 TRINITY_DN71698_c0_g1_i1:3-	0.2726603	-1.874823439	2.12E-05	down	yes	0.9993	3.665	3.61	3.886	3.499	1	1.024	0.974	------	------	------	------	------	------	------	------	PF00451.22;PF00	Toxin_2;Gamma-thionin	Scorpion short toxin, BmKK2;Gamma-thionin family	CYT	6	47	15.5	High
TRINITY_DN114890_c0_g1_i4_orf1	chemosensory protein 10 [Ostrinia furnacalis]	0.303287982	-1.721239764	9.65E-06	down	yes	1.07	3.528	3.397	3.519	3.669	1	1.077	1.134	------	------	------	------	------	------	ENOG4111ATT;E	S:Function unknown;	PF03392.16	OS-D	Insect pheromone-binding family, A10/OS-D	CYT	1	76	6.9	High
TRINITY_DN135780_c0_g1_i1_orf1	flotillin-1 isoform X1 [Pectinophora gossypiella]	0.282097649	-1.825733451	2.16E-07	down	yes	0.936	3.318	3.304	3.328	3.321	1	0.906	0.902	CC:cellular_component;CC:membrane;CC:cellular anatomical entity;	GO:00055	K07192	FLOT	map04910	Insulin signaling pa	COG2268	S:Function unknown	PF01145.28	Band_7	SPFH domain / Band 7 family	CYT	2	27	12.8	High
TRINITY_DN84938_c0_g1_i4_orf1	vinculin-like isoform X2 [Ostrinia furnacalis]	0.422416813	-1.243260838	0.0005804	down	yes	1.206	2.855	2.88	2.629	3.057	1	1.239	1.379	------	------	K05700;K	VCL;TLN	map05166;m	Human T-cell leuk	------	------	------	------	------	CYT	1	23	15.7	High
TRINITY_DN1175_c1_g1_i1_orf1	methanethiol oxidase [Ostrinia furnacalis]	0.336719541	-1.570380647	9.96E-06	down	yes	0.9977	2.963	3.037	2.823	3.03	1	0.982	1.011	MF:selenium binding;MF:molecular_function;MF:binding;	GO:0008	K17285	SELENBP1	map00920	Sulfur metabolism	ENOG410XP4F	S:Function unknown	PF05694.14	SBP56	56kDa selenium binding	CYT	11	46	53.7	High
TRINITY_DN5655_c0_g1_i2_orf1	uncharacterized protein LOC114359603 [Ostrinia furnacalis]	0.443946188	-1.17154328	1.25E-05	down	yes	1.089	2.453	2.48	2.476	2.402	1	1.147	1.121	------	------	------	------	------	------	ENOG4111C52	S:Function unknown	------	------	------	CYT	1	8	10.8	High
TRINITY_DN9400_c0_g1_i8_orf1	PREDICTED: monoacylglycerol lipase ABHD12-like [Amyelois transitella]	0.472955975	-1.080222198	0.0004642	down	yes	1.128	2.385	2.374	2.226	2.556	1	1.134	1.25	------	------	K13704	ABHD12	------	------	ENOG4111FHA;(	S:Function unknown;	PF12146.11;PF00	Hydrolase_4;Abhydrolase_1;Abhydrolase_6	Serine aminopeptidase, S33;alpha/beta hydrolase fold;Alpha/bet	PLA	1	5	24.6	High

TRINITY_DN21420_c0_g1_i2_orf1	glutathione peroxidase, partial [Ostrinia furnacalis]	0.402791262	-1.311895708	3.06E-06	down	yes	0.9957	2.472	2.487	2.503	2.425	1	0.939	1.048	BP:response to stimulus;BP:response to stress;BP:biological_process;BP:response to oxidative stress;MF:antioxidant activity;MF:peroxidase activity;MF:catalytic activity;MF:molecular_function;MF:oxidoreductase activity, acting on peroxide as acceptor;MF:glutathione peroxidase	GO:0050€	K05361;K	GPX4;gpx, bt	map00480;m	Glutathione metab	COG0386	U:Intracellular trafficking, secretic	------	------	------	CYT	5	76	8.3	High
TRINITY_DN9090_c0_g1_i9_orf1	CD63 antigen [Ostrinia furnacalis]	0.401480872	-1.31659684	6.37E-07	down	yes	0.976	2.431	2.402	2.417	2.474	1	0.95	0.978	CC:cellular_component;CC:integral component of membrane;CC:cellular anatomical entity;CC:intrinsic component of membrane;	GO:0005	K06497	CD63, MLA1,	map05205;m	Proteoglycans in ca	ENOG4111IRY	S:Function unknown	PF00335.23	Tetraspanin	Tetraspanin family	EXC	3	13	25.6	High
TRINITY_DN798_c1_g1_i3_orf1	protein goliath isoform X1 [Ostrinia furnacalis] >XP_028170876.1 protein goliath isoform X1 [Ostrinia furnacalis]	0.492989597	-1.02037089	0.001491	down	yes	1.09	2.211	1.942	2.367	2.323	1	1.18	1.089	CC:integral component of membrane;CC:cellular_component;CC:intrinsic component of membrane;CC:cellular anatomical entity;MF:cation binding;MF:ion binding;MF:molecular_function;MF:metal ion binding;MF:binding;	GO:0016(	------	------	------	------	ENOG41121N2	O:Posttranslational modification,	PF13639.9;PF171	zf-RING_2;zf-RING_11;zf-C3HC4;zf-C3HC4_2;zf-rbx1;PA	Ring finger domain;RING-like zinc finger;Zinc finger, C3HC4 type;Zinc finger, C3HC4 type;RING-H2 zinc finger domain;PA domain	CYT	1	3	47	High
TRINITY_DN121650_c0_g1_i1_orf1	carboxylesterase [Ostrinia furnacalis]	0.428440367	-1.22283368	0.0001394	down	yes	0.9807	2.289	2.47	2.206	2.192	1	0.952	0.99	CC:integral component of membrane;CC:cellular_component;CC:intrinsic component of membrane;CC:cellular anatomical entity;MF:hydrolase activity;MF:hydrolase activity, acting on ester bonds;MF:carboxylic ester hydrolase activity;MF:catalytic	GO:0016(	------	------	------	------	COG2272	I:Lipid transport and metabolism	------	------	------	CYT	1	19	7.6	High
TRINITY_DN140_c0_g1_i1_orf1	calcyphosin-like protein [Ostrinia furnacalis]	0.462969838	-1.11100989	1.81E-08	down	yes	0.9977	2.155	2.143	2.17	2.153	1	0.991	1.002	------	------	K23909	CAPS	------	------	ENOG410XRMJ	T:Signal transduction mechanism	PF13405.9;PF134	EF-hand_6;EF-hand_7;EF-hand_1;EF-hand_5;EF-hand_8	EF-hand domain;EF-hand domain pair;EF hand;EF hand;EF-hand domain pair	CYT	7	55	27.1	High
TRINITY_DN15812_c0_g1_i2_orf1	transferrin [Ostrinia furnacalis]	0.472369668	-1.082011765	2.10E-05	down	yes	0.9967	2.11	2.066	2.206	2.057	1	0.992	0.998	BP:ion homeostasis;BP:homeostatic process;BP:iron ion homeostasis;BP:transition metal ion homeostasis;BP:inorganic ion homeostasis;BP:biological regulation;BP:ion transport;BP:transport;BP:biological_process;BP:metal ion homeostasis;BP:cation homeostasis;BP:establishment of localization;BP:localization;BP:regulation of biological quality;BP:chemical homeostasis;CC:extracellular space;CC:cellular anatomical entity;CC:cellular_component;MF:molecular_function;MF:binding;MF:cation binding;MF:ion binding;MF:metal ion binding;	GO:0050€	K06569	MFI2, CD228	------	------	ENOG410XQ36	O:Posttranslational modification,	PF00405.20;PF12	Transferrin;Phosphonate-bd	Transferrin;ABC transporter, phosphonate, periplasmic substrate-binding protein	CYT	30	52	75.6	High
TRINITY_DN10138_c0_g1_i1_orf1	storage protein 1 [Omphisa fuscidentalis]	0.468892045	-1.09267229	1.30E-05	down	yes	0.9903	2.112	2.068	2.197	2.07	1	0.979	0.992	------	------	------	------	------	------	ENOG410XR2D	S:Function unknown	PF03723.17	Hemocyanin_C	Hemocyanin, ig-like domain	PLA	6	91	10.1	High
TRINITY_DN4255_c0_g1_i10_orf1	LOW QUALITY PROTEIN: lebocin-4-like [Ostrinia furnacalis]	0.389782016	-1.359260565	0.002697	down	yes	0.8583	2.202	2.185	2.532	1.89	1	0.715	0.86	------	------	------	------	------	------	ENOG4111ASS	S:Function unknown	------	------	------	CYT	2	21	22.7	High
TRINITY_DN811_c0_g1_i15_orf1	uncharacterized protein LOC114364160 [Ostrinia furnacalis]	0.4527394	-1.143247232	0.002793	down	yes	0.9503	2.099	2.431	1.977	1.888	1	0.853	0.998	------	------	------	------	------	------	ENOG4112C1R	S:Function unknown	------	------	------	PLA	1	4	37.7	High
TRINITY_DN6325_c0_g1_i9_orf1	fructose-bisphosphate aldolase isoform X2 [Pieris brassicae]	0.468514947	-1.09383302	3.54E-05	down	yes	0.9717	2.074	2.072	2.165	1.984	1	0.969	0.946	BP:ribonucleoside diphosphate metabolic process;BP:phosphorylation;BP:purine nucleoside triphosphate metabolic process;BP:carboxylic acid metabolic process;BP:cellular nitrogen compound metabolic process;BP:nitrogen compound metabolic process;BP:small molecule metabolic process;BP:cellular aromatic compound metabolic process;BP:purine-containing compound metabolic process;BP:generation of precursor metabolites and energy;BP:pyruvate metabolic process;BP:glycolytic process;BP:organic cyclic compound metabolic process;BP:nucleobase-containing compound metabolic process;BP:organic substance catabolic process;BP:purine nucleoside diphosphate metabolic process;BP:nucleotide phosphorylation;BP:ribonucleotide metabolic process;BP:organic substance metabolic process;BP:ribonucleoside triphosphate metabolic process;BP:nucleoside triphosphate metabolic process;BP:purine ribonucleotide metabolic	GO:0009	K01623	ALDO	map00010;m	Glycolysis / Glucon	COG3588	G:Carbohydrate transport and m	PF00274.22	Glycolytic	Fructose-bisphosphate aldolase class-I	CYT	5	82	42.3	High
TRINITY_DN45037_c0_g1_i1_orf1	trafficking protein particle complex subunit 1 [Ostrinia furnacalis]	0.43729064	-1.193335625	0.002546	down	yes	0.8877	2.03	2.196	1.734	2.159	1	0.726	0.937	BP:ER to Golgi vesicle-mediated transport;BP:localization;BP:cellular localization;BP:transport;BP:Golgi vesicle transport;BP:cellular process;BP:biological_process;BP:establishment of localization in cell;BP:establishment of localization;BP:vesicle-mediated transport;BP:intracellular transport;CC:endoplasmic reticulum;CC:intracellular organelle;CC:tethering complex;CC:membrane-bounded organelle;CC:organelle;CC:cellular anatomical entity;CC:Golgi apparatus;CC:TRAPP complex;CC:intracellular membrane-bounded organelle;CC:macromolecular complex;CC:cellular_component;	GO:0006	K20300	TRAPPC1, BE	------	------	ENOG4111X41	U:Intracellular trafficking, secretic	PF04099.15;PF04	Sybindin;Sedlin_N	Sybindin-like family;Sedlin, N-terminal conserved region	CYT	1	9	16.8	High
TRINITY_DN24723_c2_g1_i1_orf1	hypothetical protein evm_001103 [Chilo suppressalis]	0.263301163	-1.925214201	0.000263	down	yes	1.064	4.041	4.264	4.251	3.609	1	0.905	1.288	BP:small molecule biosynthetic process;BP:carboxylic acid metabolic process;BP:cellular biosynthetic process;BP:cellular nitrogen compound metabolic process;BP:nitrogen compound metabolic process;BP:small molecule metabolic process;BP:nitrogen cycle metabolic process;BP:glutamine family amino acid metabolic process;BP:organic substance biosynthetic process;BP:cellular amino acid metabolic process;BP:arginine biosynthetic process;BP:arginine metabolic process;BP:organic substance metabolic process;BP:organic acid metabolic process;BP:cellular amino acid biosynthetic process;BP:alpha-amino acid metabolic process;BP:alpha-amino acid biosynthetic process;BP:urea metabolic process;BP:primary metabolic process;BP:cellular process;BP:biosynthetic process;BP:biological_process;BP:metabolic process;BP:oxoacid metabolic process;BP:cellular nitrogen compound biosynthetic process;BP:amide biosynthetic process;BP:organonitrogen compound metabolic process;BP:organonitrogen compound	GO:0044	K01940	argG, ASS1	map00220;m	Arginine biosynthe	COG0137	E:Amino acid transport and meta	PF00764.22	Arginosuc_synth	Arginosuccinate synthase	CYT	1	9	11.6	High
TRINITY_DN66040_c0_g1_i2_orf1	serine protease inhibitor dipetalogastin-like isoform X2 [Ostrinia furnacalis]	0.270072993	-1.888578717	9.08E-05	down	yes	1.073	3.973	4.317	3.809	3.793	1	1.191	1.028	------	------	------	------	------	------	------	------	PF07648.18;PF00	Kazal_2;Kazal_1	Kazal-type serine protease inhibitor domain;Kazal-type serine protease inhibitor domain	CYT	1	21	6	High

TRINITY_DN64719_c0_g1_i2_orfp1	TRINITY_DN64719_c0_g1_i2_m.37745 TRINITY_DN64719_c0_g1::TRINITY_DN64719_c0_g1_i2::g.37745 ORF type:internal len:91 (+),score=41.89 TRINITY_DN64719_c0_g1_i2:1-270(+)	0.272703684	-1.874593902	0.0001635	down	yes	1.051	3.854	4.16	3.927	3.475	1	1.031	1.121	------	------	------	------	------	------	------	------	------	------	------	CYT	1	14	8.4 High
TRINITY_DN7226_c0_g1_i5_orf1	chemosensory protein [Dioryctria abietella]	0.230673126	-2.116078159	1.08E-05	down	yes	0.9047	3.922	3.845	4.12	3.8	1	0.854	0.86	------	------	------	------	------	------	ENOG4111U5H;E	S:Function unknown;	PF03392.16	OS-D	Insect pheromone-binding family, A10/OS-D	CYT	4	28	8.8 High
TRINITY_DN66287_c0_g1_i1_orfp1	tenascin-like isoform X4 [Trichoplusia ni]	0.310621242	-1.6867716	0.0006042	down	yes	1.085	3.493	3.548	3.879	3.053	1	1.177	1.077	BP:response to biotic stimulus;BP:response to external stimulus;BP:response to external biotic stimulus;BP:response to stimulus;BP:defense response;BP:response to stress;BP:biological_process;BP:interspecies interaction between organisms;BP:response to other organism;CC:cellular_component;CC:extr	GO:00096	------	------	------	------	------	------	------	------	------	CYT	2	25	11.9 High
TRINITY_DN106156_c1_g1_i1_orf1	arylphorin subunit alpha-like [Ostrinia furnacalis]	0.31930677	-1.646984952	0.0001357	down	yes	0.9857	3.087	3.358	2.86	3.042	1	0.96	0.997	------	------	------	------	------	------	ENOG410XR2D	S:Function unknown	PF03723.17	Hemocyanin_C	Hemocyanin, ig-like domain	CYT	12	59	13.5 High
TRINITY_DN295_c5_g1_i2_orf1	unnamed protein product [Chilo suppressalis]	0.343739621	-1.54061194	2.11E-06	down	yes	1.035	3.011	2.987	3.074	2.972	1	0.997	1.107	MF:hydrolase activity;MF:molecular_function;MF:catalytic activity;	GO:00167	K21013	FPPP	map00981	Insect hormone bio	COG0647	G:Carbohydrate transport and m	PF13344.9	Hydrolase_6	Haloacid dehalogenase-like hydrolase	CYT	5	24	19.9 High
TRINITY_DN5444_c0_g1_i1_orfp1	TRINITY_DN5444_c0_g1_i1_m.14077 TRINITY_DN5444_c0_g1::TRINITY_DN5444_c0_g1_i1::g.14077 ORF type:3prime_partial len:90 (-),score=25.11 TRINITY_DN5444_c0_g1_i1:2-268(-)	0.315560027	-1.66401363	0.0001268	down	yes	0.941	2.982	3.254	2.849	2.843	1	0.896	0.927	------	------	------	------	------	------	------	------	------	------	------	CYT	1	8	9.9 Medium
TRINITY_DN14185_c0_g1_i1_orf1	uncharacterized protein LOC114358675 [Ostrinia furnacalis] >XP_028168498.1 uncharacterized protein LOC114358675 [Ostrinia furnacalis]	0.399025852	-1.325445875	5.70E-05	down	yes	1.065	2.669	2.572	2.833	2.602	1	1.076	1.119	------	------	------	------	------	------	------	------	------	------	------	CYT	2	19	18.4 High
TRINITY_DN37307_c0_g1_i4_orf1	superoxide dismutase [Mn], mitochondrial [Ostrinia furnacalis]	0.382848392	-1.385154897	2.69E-05	down	yes	1	2.612	2.658	2.711	2.466	1	1.002	0.998	MF:oxidoreductase activity, acting on superoxide radicals as acceptor;MF:cation binding;MF:antioxidant activity;MF:ion binding;MF:catalytic activity;MF:molecular_function;MF:binding;MF:superoxide dismutase activity;MF:metal ion binding;MF:oxidoreductase activity;	GO:00167	K04564	SOD2	map04146;m	Peroxisome;MAPK s	COG0605	P:Inorganic ion transport and me	PF02777.21;PF00	Sod_Fe_C;Sod_Fe_N	Iron/manganese superoxide dismutases, C-terminal domain;Iron/manganese superoxide dismutases, alpha-hairpin domain	CYT	9	50	24.2 High
TRINITY_DN3821_c1_g1_i7_orf1	mitochondrial carrier protein Rim2 isoform X1 [Ostrinia furnacalis]	0.401276426	-1.317331691	8.40E-06	down	yes	1.006	2.507	2.564	2.466	2.491	1	0.936	1.083	BP:cellular process;BP:intracellular transport;BP:transport;BP:transmembrane transport;BP:biological_process;BP:establishment of localization in cell;BP:mitochondrial transport;BP:establishment of localization;BP:localization;BP:cellular localization;CC:membrane;CC:organelle membrane;CC:integral component of membrane;CC:cellular_component;CC:cellular anatomical entity;CC:mitochondrial	GO:00099	K15116	SLC25A33_36	------	------	ENOG410XS20	S:Function unknown	PF00153.30	Mito_carr	Mitochondrial carrier protein	CYT	1	4	40.2 High
TRINITY_DN230_c2_g1_i5_orf1	6-pyruvoyl tetrahydrobiopterin synthase [Ostrinia furnacalis]	0.424559933	-1.235959869	1.65E-06	down	yes	1.013	2.386	2.443	2.374	2.34	1	1.029	1.011	BP:polyol biosynthetic process;BP:polyol metabolic process;BP:diol metabolic process;BP:cellular biosynthetic process;BP:cellular nitrogen compound metabolic process;BP:diol biosynthetic process;BP:small molecule metabolic process;BP:small molecule biosynthetic process;BP:organic cyclic compound biosynthetic process;BP:organic cyclic compound metabolic process;BP:organic substance biosynthetic process;BP:pteridine-containing compound metabolic process;BP:pteridine-containing compound biosynthetic process;BP:organic substance metabolic process;BP:heterocycle biosynthetic process;BP:tetrahydrobiopterin biosynthetic process;BP:alcohol biosynthetic process;BP:cellular process;BP:cellular aromatic compound metabolic process;BP:biosynthetic process;BP:tetrahydrobiopterin metabolic process;BP:biological_process;BP:metabolic process;BP:organonitrogen compound metabolic process;BP:heterocycle metabolic process;BP:cellular nitrogen compound	GO:00461	K01737	queD, ptpS, F	map00790;m	Folate biosynthesis	COG0720	H:Coenzyme transport and meta	PF01242.22	PTPS	6-pyruvoyl tetrahydropterin synthase	CYT	8	62	18.7 High
TRINITY_DN30169_c0_g1_i1_orfp1	TRINITY_DN30169_c0_g1_i1_m.11367 TRINITY_DN30169_c0_g1::TRINITY_DN30169_c0_g1_i1::g.11367 ORF type:3prime_partial len:52 (+),score=0.98 TRINITY_DN30169_c0_g1_i1:72-224(+)	0.465563506	-1.10295012	0.005004	down	yes	1.041	2.236	2.378	1.819	2.51	1	1.034	1.09	------	------	------	------	------	------	------	------	------	------	------	CYT	1	12	6.1 High
TRINITY_DN10222_c0_g1_i2_orf1	glutathione S-transferase sigma 3 [Ostrinia furnacalis]	0.427871025	-1.224752111	3.42E-05	down	yes	0.9687	2.264	2.385	2.195	2.212	1	0.971	0.935	MF:catalytic activity;MF:transferase activity;MF:glutathione transferase activity;MF:molecular_function;MF:transferase activity, transferring alkyl or ary;	GO:0003	K04097	HPGDS	map00590;m	Arachidonic acid m	ENOG41103AU;E	O:Posttranslational modification,	PF14497.9;PF027	GST_C_3;GST_N;GST_C	Glutathione S-transferase, C-terminal domain;Glutathione S-transferase, N-terminal domain;Glutathione S-transferase, C-terminal	CYT	14	58	22.9 High
TRINITY_DN5099_c0_g1_i3_orf1	trans-1,2-dihydrobenzene-1,2-diol dehydrogenase-like [Ostrinia furnacalis]	0.46256316	-1.112277724	2.19E-05	down	yes	1.007	2.177	2.273	2.161	2.098	1	1.01	1.011	MF:organic cyclic compound binding;MF:nucleotide binding;MF:small molecule binding;MF:molecular_function;MF:binding;MF:heterocyclic compound binding;MF:nucleoside phosphate binding;	GO:00971	K00078	DHDH	map00040;m	Pentose and glucur	COG0673	S:Function unknown	PF01408.25;PF02	GFO_IDH_MocA;GFO_IDH_MocA_C	Oxidoreductase family, NAD-binding Rossmann fold;Oxidoreductase family, C-terminal alpha/beta domain	CYT	17	55	38.1 High
TRINITY_DN1304_c0_g1_i6_orf1	uncharacterized protein LOC114359545 [Ostrinia furnacalis]	0.489104116	-1.031786489	9.47E-06	down	yes	1.01	2.065	2.085	2.107	2.004	1	0.981	1.05	CC:cellular_component;CC:integral component of membrane;CC:cellular anatomical entity;CC:intrinsic component of membrane;	GO:00055	------	------	------	------	------	------	------	------	------	CYT	1	14	8.8 High
TRINITY_DN1491_c0_g1_i4_orf1	GILT-like protein 2 isoform X1 [Ostrinia furnacalis] >XP_028156245.1 GILT-like protein 2 isoform X2 [Ostrinia furnacalis] >XP_028156247.1 GILT-like protein 2 isoform X3 [Ostrinia furnacalis]	0.407458564	-1.295274743	0.0001917	down	yes	0.885	2.172	2.169	2.038	2.308	1	0.806	0.849	MF:catalytic activity;MF:oxidoreductase activity, acting on a sulfur group of donors, disulfide as acceptor;MF:molecular_function;MF:oxidoreductase activity, acting on a sulfur group of donors;MF:oxidoreductase	GO:0003	K08059	IFI30, GILT	map04612	Antigen processing	ENOG4111IDT	S:Function unknown	PF03227.19	GILT	Gamma interferon inducible lysosomal thiol reductase	CYT	1	16	26.5 High
TRINITY_DN33488_c0_g1_i2_orf1	semaphorin-1A isoform X3 [Trichoplusia ni]	0.471185617	-1.085632593	9.15E-05	down	yes	0.9697	2.058	1.984	2.191	1.999	1	0.961	0.948	------	------	K06842	SEMA6	map04360	Axon guidance	ENOG410XQZC	S:Function unknown	PF01403.22;PF01	Sema;PSI	Sema domain;Plexin repeat	CYT	5	7	83.1 High
TRINITY_DN3301_c0_g1_i2_orf1	hemicentin-2-like isoform X1 [Ostrinia furnacalis]	0.484266263	-1.046127595	0.0005301	down	yes	0.9603	1.983	1.949	2.169	1.832	1	0.943	0.938	MF:hydrolase activity;MF:cation binding;MF:organic cyclic compound binding;MF:ion binding;MF:catalytic activity;MF:molecular_function;MF:binding;MF:nucleic acid binding;MF:metal ion binding;MF:heterocyclic compound binding;	GO:00167	------	------	------	------	ENOG410ZN3C;E	S:Function unknown;	PF07679.19;PF13	I-set;Ig_3;Ig_2;ig;VWA;VWA_2	Immunoglobulin I-set domain;Immunoglobulin domain;Immunoglobulin domain;Immunoglobulin domain;von Willebrand factor type A domain;von Willebrand factor type A domain	CYT	3	3	129.9 High
TRINITY_DN21170_c0_g1_i5_orf1	twitchin-like [Ostrinia furnacalis]	0.493606138	-1.01856776	0.0002372	down	yes	0.965	1.955	1.801	2.032	2.033	1	0.937	0.958	------	------	------	------	------	------	ENOG410XP8U;E	S:Function unknown;	PF00041.24	fn3	Fibronectin type III domain	CYT	1	52	17 High
TRINITY_DN971_c0_g1_i5_orfp1	TRINITY_DN971_c0_g1_i5_m.54249 TRINITY_DN971_c0_g1::TRINITY_DN971_c0_g1_i5::g.54249 ORF type:internal len:108 (+),score=66.98 TRINITY_DN971_c0_g1_i5:1-321(+)	0.080267857	-3.639033806	2.02E-05	down	yes	0.899	11.2	12.061	10.866	10.683	1	0.708	0.989	------	------	------	------	------	------	------	------	------	------	------	CYT	1	9	11.5 High
TRINITY_DN22515_c0_g1_i10_orf1	hypothetical protein KGM_202621 [Danaus plexippus plexippus]	0.191240325	-2.386541335	1.90E-07	down	yes	1.013	5.297	5.191	5.317	5.382	1	1.004	1.035	------	------	------	------	------	------	ENOG41119TW	S:Function unknown	------	------	------	CYT	2	29	7.7 High

TRINITY_DN1880_c0_g1_i4_orf1	serine protease inhibitor dipetalogastin-like [Helicoverpa zea]	0.263831479	-1.922311388	8.85E-08	down	yes	0.9957	3.774	3.832	3.761	3.729	1	0.998	0.989	------	------	K23615	SPIK5	------	------	COG5640;ENOG	O:Posttranslational modification,	PF00050.24;PF07	Kazal_1;Kazal_2	Kazal-type serine protease inhibitor domain;Kazal-type serine protease inhibitor domain	CYT	12	52	42.6	High
TRINITY_DN14328_c0_g1_i12_orf1	larval cuticle protein LCP-30-like [Ostrinia furnacalis]	0.288064696	-1.795535234	9.57E-06	down	yes	1.033	3.586	3.454	3.75	3.555	1	1.012	1.086	MF:molecular_function;MF:structural constituent of cuticle;MF:structural molecule activity;	GO:00036	------	------	------	------	ENOG41112Q7;E	S:Function unknown;	PF00379.26	Chitin_bind_4	Insect cuticle protein	CYT	10	50	24.6	High
TRINITY_DN5439_c0_g1_i2_orf1	uncharacterized protein LOC114353087 [Ostrinia furnacalis]	0.294564582	-1.763344121	1.35E-06	down	yes	1.008	3.422	3.525	3.384	3.356	1	1.004	1.019	CC:membrane;CC:endoplasmic reticulum membrane;CC:organelle membrane;CC:cellular_component;CC:cellular anatomical entity;MF:cation binding;MF:monooxygenase activity;MF:heme binding;MF:transition metal ion binding;MF:tetrapyrrole binding;MF:organic cyclic compound binding;MF:oxidoreductase activity, acting on paired donors, with incorporation or reduction of molecular oxygen;MF:ion binding;MF:catalytic activity;MF:molecular_function;MF:binding;MF:iron ion binding;MF:metal ion binding;MF:heterocyclic compound binding;MF:oxidoreductase activity;	GO:0016(	K15003	CYP9	------	------	COG2124	S:Function unknown	PF00067.25	p450	Cytochrome P450	CYT	16	34	61.8	High
TRINITY_DN1450_c0_g2_i1_orf1	death-associated protein 1 [Ostrinia furnacalis]	0.348020434	-1.522756078	1.61E-05	down	yes	1.09	3.132	3.045	3.118	3.234	1	1.209	1.062	------	------	------	------	------	------	ENOG41122SE	S:Function unknown	PF15228.9	DAP	Death-associated protein	CYT	2	37	10.7	High
TRINITY_DN7040_c0_g2_i1_orf1	uncharacterized protein LOC114353763 [Ostrinia furnacalis]	0.309409938	-1.69240856	6.47E-06	down	yes	0.9963	3.22	3.305	3.078	3.278	1	1.003	0.986	------	------	------	------	------	------	ENOG4110RZK	O:Posttranslational modification,	------	------	------	CYT	8	32	22	High
TRINITY_DN5748_c0_g1_i6_orf1	glycine N-methyltransferase isoform X1 [Ostrinia furnacalis] >XP_028165118.1 glycine N-methyltransferase isoform X2 [Ostrinia furnacalis] >XP_028165119.1 glycine N-methyltransferase isoform X1 [Ostrinia furnacalis] >XP_028165120.1 glycine N-methyltransferase isoform X2 [Ostrinia furnacalis]	0.338693797	-1.561946529	2.01E-05	down	yes	1.032	3.047	3.151	3.112	2.879	1	1.045	1.051	BP:organic substance metabolic process;BP:biological_process;BP:metabolic process;BP:methylation;MF:glycine N-methyltransferase activity;MF:catalytic activity;MF:S-adenosylmethionine-dependent methyltransferase activity;MF:molecular_function;MF:transferase activity;MF:N-methyltransferase activity;MF:transferase activity, transferring one-carbon groups;MF:methyltransferase activity;	GO:0071;	K00552	GNMT	map00260	Glycine, serine and	ENOG410ZWXN	S:Function unknown	PF13649.9;PF082	Methyltransf_25;Methyltransf_12;Methyltransf_31;Methyltransf_11;Methyltransf_23	Methyltransferase domain;Methyltransferase domain;Methyltransferase domain;Methyltransferase domain;Methyltransferase domain	CYT	3	48	27.4	High
TRINITY_DN364_c1_g1_i2_orf1	talin-2-like, partial [Ostrinia furnacalis]	0.35459993	-1.495735845	3.20E-06	down	yes	1.006	2.837	2.758	2.918	2.836	1	1.04	0.979	CC:cytoskeleton;CC:cellular_component;CC:ruffle;CC:focal adhesion;CC:intracellular non-membrane-bounded organelle;CC:cell junction;CC:anchoring junction;CC:intracellular organelle;CC:non-membrane-bounded organelle;CC:plasma membrane bounded cell projection;CC:cell projection;CC:organelle;CC:cellular anatomical entity;CC:cell-substrate junction;MF:structural constituent of cytoskeleton;MF:cytoskeletal protein binding;MF:structural molecule activity;MF:actin	GO:0005(	K06271	TLN	map05166;m	Human T-cell leuk	ENOG410XQ0V;E	S:Function unknown;	PF09141.13	Talin_middle	Talin, middle domain	CYT	7	9	81.6	High
TRINITY_DN24121_c1_g1_i6_orf1	serine protease persephone-like [Ostrinia furnacalis]	0.343294615	-1.54248087	0.0001144	down	yes	0.9753	2.841	2.741	3.086	2.695	1	0.952	0.974	BP:organic substance metabolic process;BP:organonitrogen compound metabolic process;BP:protein metabolic process;BP:macromolecule metabolic process;BP:biological_process;BP:nitrogen compound metabolic process;BP:metabolic process;BP:proteolysis;BP:primary metabolic process;MF:serine-type endopeptidase activity;MF:endopeptidase activity;MF:hydrolase activity;MF:serine hydrolase activity;MF:catalytic activity, acting on a protein;MF:catalytic activity;MF:molecular_function;MF:pepti	GO:0071;	K20677;K	HAYAN;PRSS	map04624	Toll and Imd signal	COG5640	O:Posttranslational modification,	PF00089.29	Trypsin	Trypsin	CYT	7	29	28.9	High
TRINITY_DN25976_c0_g1_i4_orf1	hypothetical protein B566_EDAN014657 [Ephemera danica]	0.476859504	-1.068363824	0.0005406	down	yes	1.154	2.42	2.461	2.321	2.477	1	1.082	1.379	BP:developmental process;BP:cellular process;BP:cellular developmental process;BP:anatomical structure development;BP:cell development;BP:cellular component organization;BP:animal organ development;BP:biological_process;BP:cellular component organization or biogenesis;CC:integral component of membrane;CC:cellular_component;CC:intrinsic component of membrane;CC:cellular anatomical entity;	GO:0032!	K24068	MEGF10_11	------	------	ENOG410XQWV	S:Function unknown	PF00053.27;PF07	Laminin_EGF;EGF_2;hEGF;EGF	Laminin EGF domain;EGF-like domain;Human growth factor-like EGF;EGF-like domain	CYT	1	30	8.4	High
TRINITY_DN12014_c0_g1_i2_orf1	unnamed protein product [Chilo suppressalis]	0.49955397	-1.001287546	0.0007107	down	yes	1.12	2.242	2.436	2.204	2.085	1	1.186	1.173	------	------	K13023;K	CPN2;TL	map04624	Toll and Imd signal	COG4886	T:Signal transduction mechanism	PF13855.9;PF136	LRR_8;TIR_2;TIR;LRR_4	Leucine rich repeat;TIR domain;TIR domain;Leucine Rich repeats	CYT	1	1	99.8	High
TRINITY_DN585_c0_g1_i12_orf1	very low-density lipoprotein receptor isoform X3 [Galleria mellonella]	0.443375601	-1.173398713	0.0006251	down	yes	1.014	2.287	2.298	2.506	2.057	1	1.048	0.994	CC:integral component of membrane;CC:cellular_component;CC:intrinsic component of membrane;CC:cellular anatomical entity;MF:binding;MF:cation binding;MF:cargo receptor activity;MF:very-low-density lipoprotein particle receptor activity;MF:lipoprotein particle receptor activity;MF:ion binding;MF:molecular_function;MF:metal ion binding;MF:calcium ion binding;	GO:0016(	K20053	VLDLR	map05417;m	Lipid and atheroscl	ENOG410YQ6J	U:Intracellular trafficking, secretic	PF00057.21;PF00	Ldl_recept_a;Ldl_recept_b;FXa_inhibition;cEGF;EGF_CA;DUF5050	Low-density lipoprotein receptor domain class A;Low-density lipoprotein receptor repeat class B;Coagulation Factor Xa inhibitory site;Complement Clr-like EGF-like;Calcium-binding EGF domain;Domain of unknown	PLA	3	31	100.3	High
TRINITY_DN2803_c4_g1_i1_orf1	ornithine aminotransferase, mitochondrial isoform X2 [Ostrinia furnacalis]	0.448632634	-1.156393526	4.82E-06	down	yes	0.9843	2.194	2.223	2.234	2.124	1	0.967	0.986	BP:small molecule biosynthetic process;BP:carboxylic acid metabolic process;BP:nitrogen compound metabolic process;BP:small molecule metabolic process;BP:glutamine family amino acid metabolic process;BP:L-proline biosynthetic process;BP:organic cyclic compound biosynthetic process;BP:organic cyclic compound metabolic process;BP:organic substance biosynthetic process;BP:proline biosynthetic process;BP:proline metabolic process;BP:cellular amino acid metabolic process;BP:organic substance metabolic process;BP:alpha-amino acid metabolic process;BP:alpha-amino acid biosynthetic process;BP:heterocycle biosynthetic process;BP:cellular amino acid biosynthetic process;BP:cellular process;BP:biosynthetic process;BP:biological_process;BP:metabolic process;BP:oxoacid metabolic process;BP:heterocycle metabolic process;BP:primary metabolic process;BP:organonitrogen compound metabolic process;BP:organonitrogen compound biosynthetic process;BP:organic acid metabolic process;BP:cellular biosynthetic	GO:0044;	K00819	rocD, OAT	map00330	Arginine and prolin	COG4992	E:Amino acid transport and meta	PF00202.24	Aminotran_3	Aminotransferase class-III	CYT	25	68	44.7	High

TRINITY_DN805_c0_g1_i5_orf1	serine protease snake-like [Ostrinia furnacalis]	0.499763145	-1.00068358	6.63E-05	down	yes	1.055	2.111	2.198	2.101	2.034	1	1.035	1.129	BP:organic substance metabolic process;BP:organonitrogen compound metabolic process;BP:protein metabolic process;BP:macromolecule metabolic process;BP:biological_process;BP:nitrogen compound metabolic process;BP:metabolic process;BP:proteolysis;BP:primary metabolic process;MF:serine-type endopeptidase activity;MF:endopeptidase activity;MF:hydrolase activity;MF:serine hydrolase activity;MF:catalytic activity, acting on a protein;MF:catalytic activity;MF:molecular_function;MF:pepti [illegible]	GO:00717	------	------	------	------	COG5640	O:Posttranslational modification,	PF00089.29	Trypsin	Trypsin	EXC	6	23	43.9	High
TRINITY_DN7960_c0_g1_i2_orf1	uncharacterized protein LOC114364878 [Ostrinia furnacalis]	0.497572816	-1.007020428	6.43E-05	down	yes	1.025	2.06	2.146	1.949	2.085	1	1.038	1.037	------	------	------	------	------	------	COG0451	I:Lipid transport and metabolism	PF01370.24;PF16	Epimerase;GDP_Man_Dehyd;3Beta_HSD;NAD_binding_4;RmlD_sub_bind	NAD dependent epimerase/dehydratase family;GDP-mannose 4,6 dehydratase;3-beta hydroxysteroid dehydrogenase/isomerase family;Male sterility protein;RmlD substrate [illegible]	CYT	13	50	34.5	High
TRINITY_DN9044_c0_g1_i2_orf1	unnamed protein product [Euphydryas editha]	0.19729425	-2.341579182	4.42E-07	down	yes	1.05	5.322	5.23	5.411	5.325	1	1.005	1.144	BP:immune system process;BP:response to external biotic stimulus;BP:interspecies interaction between organisms;BP:organic substance metabolic process;BP:innate immune response;BP:defense response;BP:response to stress;BP:biological_process;BP:metabolic process;BP:immune response;BP:carbohydrate metabolic process;BP:response to other organism;BP:primary metabolic process;BP:response to biotic stimulus;BP:response to external stimulus;BP:response to stimulus;BP:defense response to other organism;CC:cellular anatomical entity;CC:cellular_component;CC:extracellular region;MF:carbohydrate binding;MF:molecular_function;MF:binding;MF:hydrolase activity;MF:hydrolase activity, acting on glycosyl bonds;MF:catalytic activity;MF:hydrolase [illegible]	GO:00023	K20692	GNBP3	map04624	Toll and Imd signal	ENOG410Z83Q;E	S:Function unknown;	PF15886.8	CBM39	Carbohydrate binding domain	CYT	1	10	11.4	High
TRINITY_DN7226_c0_g1_i2_orf1	chemosensory protein [Conogethes punctiferalis]	0.250367182	-1.99788263	1.82E-06	down	yes	0.9887	3.949	3.905	4.083	3.859	1	0.963	1.003	------	------	------	------	------	------	ENOG4111U5H;E	S:Function unknown;	PF03392.16	OS-D	Insect pheromone-binding family, A10/OS-D	CYT	5	54	9	High
TRINITY_DN48694_c0_g1_i1_orfp1	TRINITY_DN48694_c0_g1_i1_m.75338 TRINITY_DN48694_c0_g1::TRINITY_DN48694_c0_g1_i1::g.75338 ORF type:internal len:84 (+),score=16.02 TRINITY_DN48694_c0_g1_i1:2-250(+)	0.222831505	-2.165974869	1.55E-05	down	yes	0.894	4.012	3.795	4.173	4.067	1	0.815	0.867	------	------	------	------	------	------	------	------	------	------	------	CYT	1	13	9.1	Medium
TRINITY_DN5198_c0_g1_i5_orfp1	TRINITY_DN5198_c0_g1_i5_m.8637 TRINITY_DN5198_c0_g1::TRINITY_DN5198_c0_g1_i5::g.8637 ORF type:complete len:223 (-),score=54.99 TRINITY_DN5198_c0_g1_i5:319-	0.195183996	-2.35709333	3.75E-05	down	yes	0.7903	4.049	3.805	4.19	4.153	1	0.638	0.733	------	------	------	------	------	------	------	------	------	------	------	CYT	1	5	24.7	High
TRINITY_DN98692_c0_g3_i1_orf1	fatty acyl-CoA hydrolase precursor, medium chain [Ostrinia furnacalis]	0.265136641	-1.915192035	6.47E-06	down	yes	0.9993	3.769	3.794	3.909	3.604	1	0.983	1.015	------	------	------	------	------	------	------	------	------	------	------	CYT	1	13	7.2	High
TRINITY_DN142657_c0_g1_i1_orf1	sorting and assembly machinery component 50 homolog [Diachasma alloeum]	0.294083187	-1.765703789	3.88E-06	down	yes	1.004	3.414	3.523	3.288	3.432	1	1.007	1.006	CC:cellular_component;CC:membrane;CC:outer membrane;CC:cellular anatomical entity;	GO:00055	K07277	SAM50, TOB	------	------	COG4775	M:Cell wall/membrane/envelope	PF01103.26	Omp85	Omp85 superfamily domain	CYT	1	2	53.8	High
TRINITY_DN8008_c0_g1_i6_orf1	uncharacterized protein LOC114357965 isoform X1 [Ostrinia furnacalis] >XP_028167599.1 uncharacterized protein LOC114357965 isoform X1 [Ostrinia furnacalis] >XP_028167600.1 uncharacterized protein LOC114357965 isoform X2 [Ostrinia furnacalis] >XP_028167601.1 uncharacterized protein LOC114357965 isoform X3 [Ostrinia furnacalis]	0.351439791	-1.50865055	1.64E-05	down	yes	1.074	3.056	3.195	2.991	2.981	1	1.141	1.082	MF:organic cyclic compound binding;MF:molecular_function;MF:binding;MF:nucleic acid binding;MF:DNA binding;MF:heterocyclic compound binding;	GO:00971	------	------	------	------	ENOG410ZC30	S:Function unknown	------	------	------	CYT	1	0	506.7	Medium
TRINITY_DN13236_c0_g1_i4_orf1	zonadhesin-like isoform X4 [Ostrinia furnacalis]	0.325611368	-1.618777025	5.69E-05	down	yes	0.9853	3.026	2.806	3.167	3.105	1	1.016	0.94	------	------	------	------	------	------	COG4826;ENOG	O:Posttranslational modification,	PF01826.20	TIL	Trypsin Inhibitor like cysteine rich	CYT	2	48	10.2	High
TRINITY_DN325_c0_g1_i15_orf1	protein draper-like [Ostrinia furnacalis]	0.328042877	-1.6080437	7.89E-05	down	yes	0.9487	2.892	2.765	3.108	2.804	1	0.857	0.989	CC:cellular_component;CC:integral component of membrane;CC:cellular anatomical entity;CC:intrinsic component of membrane;	GO:00055	------	------	------	------	------	------	PF02363.22	C_tripleX	Cysteine rich repeat	PLA	2	16	51.6	High
TRINITY_DN5310_c2_g1_i2_orf1	serine protease persephone-like [Ostrinia furnacalis]	0.357394113	-1.484412223	8.10E-07	down	yes	0.9957	2.786	2.787	2.728	2.842	1	0.977	1.01	BP:organic substance metabolic process;BP:organonitrogen compound metabolic process;BP:protein metabolic process;BP:macromolecule metabolic process;BP:biological_process;BP:nitrogen compound metabolic process;BP:metabolic process;BP:proteolysis;BP:primary metabolic process;MF:serine-type endopeptidase activity;MF:endopeptidase activity;MF:hydrolase activity;MF:serine hydrolase activity;MF:catalytic activity, acting on a protein;MF:catalytic activity;MF:molecular_function;MF:pepti [illegible]	GO:00717	K20677;K	HAYAN;PSH	map04624	Toll and Imd signal	COG5640	O:Posttranslational modification,	PF00089.29	Trypsin	Trypsin	CYT	9	34	34.8	High
TRINITY_DN1421_c0_g1_i1_orf1	uncharacterized protein LOC114352615 [Ostrinia furnacalis]	0.387383901	-1.368164096	1.83E-06	down	yes	1.001	2.584	2.635	2.604	2.514	1	0.987	1.016	BP:nitrogen compound metabolic process;BP:organic substance metabolic process;BP:proteolysis;BP:biological_process;BP:metabolic process;BP:primary metabolic process;BP:organonitrogen compound metabolic process;BP:protein metabolic process;BP:macromolecule metabolic process;CC:cytoplasm;CC:cellular anatomical entity;CC:cellular_component;CC:extracellular region;MF:serine-type endopeptidase activity;MF:endopeptidase activity;MF:molecular_function;MF:binding;MF:peptidase activity;MF:serine-type peptidase activity;MF:cation binding;MF:ion binding;MF:metal ion binding;MF:hydrolase activity;MF:serine hydrolase activity;MF:catalytic activity, [illegible]	GO:00068	------	------	------	------	COG5640	U:Intracellular trafficking, secretic	PF00089.29;PF12	Trypsin;CLIP	Trypsin;Regulatory CLIP domain of proteinases	CYT	7	22	44.4	High
TRINITY_DN4443_c0_g1_i4_orf1	lysosome-associated membrane glycoprotein 1-like isoform X4 [Ostrinia furnacalis]	0.400160385	-1.321349744	9.52E-07	down	yes	0.998	2.494	2.481	2.545	2.455	1	0.974	1.02	CC:organelle membrane;CC:integral component of membrane;CC:membrane;CC:cellular_component;CC:cytoplasmic vesicle membrane;CC:intrinsic component of membrane;CC:vesicle membrane;CC:bounding membrane of organelle;CC:cellular anatomical entity;CC:endosome membrane;	GO:0031(	K06528	LAMP1_2, CD	map05152;m	Tuberculosis;Phago	ENOG410XQ96	U:Intracellular trafficking, secretic	PF01299.20	Lamp	Lysosome-associated membrane glycoprotein	CYT	1	3	24.7	High
TRINITY_DN4748_c0_g1_i5_orf1	unnamed protein product, partial [Brenthis ino]	0.414604462	-1.27019245	8.32E-06	down	yes	1.022	2.465	2.457	2.544	2.393	1	0.998	1.069	BP:response to biotic stimulus;BP:response to external stimulus;BP:response to external biotic stimulus;BP:response to other organism;BP:response to stimulus;BP:response to stress;BP:defense response;BP:biological_process;BP:interspecies interaction between organisms;CC:cellular_component;CC:extracellular region;CC:cellular anatomical entity;MF:ion channel regulator activity;MF:sodium channel inhibitor activity;MF:sodium channel regulator activity;MF:molecular_function;MF:channel inhibitor activity;MF:channel regulator [illegible]	GO:0009(	------	------	------	------	------	------	------	------	------	CYT	3	54	7.8	High

TRINITY_DN14262_c0_g1_i5_orf1	cytochrome P450 monooxygenase CYP9G18 [Cnaphalocrocis medinalis]	0.471035941	-1.086090951	0.0001957	down	yes	1.114	2.365	2.215	2.418	2.463	1	1.18	1.163	CC:endoplasmic reticulum membrane;CC:intrinsic component of membrane;CC:organelle membrane;CC:integral component of membrane;CC:membrane;CC:cellular anatomical entity;CC:cellular_component;MF:monooxygenase activity;MF:oxidoreductase activity, acting on paired donors, with incorporation or reduction of molecular oxygen;MF:molecular_function;MF:binding;MF:heterocyclic compound binding;MF:oxidoreductase activity;MF:cation binding;MF:transition metal ion binding;MF:ion binding;MF:iron ion binding;MF:metal ion binding;MF:heme binding;MF:catalytic activity;MF:tetrapyrrole	GO:00057	K15003	CYP9	------	------	COG2124	Q:Secondary metabolites biosynt	PF00067.25	p450	Cytochrome P450	CYT	1	39	12.9	High
TRINITY_DN15858_c0_g1_i2_orf1	15-hydroxyprostaglandin dehydrogenase [NAD(+)]-like [Ostrinia furnacalis]	0.425477707	-1.232844551	4.87E-06	down	yes	1.002	2.355	2.367	2.285	2.413	1	0.975	1.03	MF:catalytic activity;MF:molecular_function;MF:oxidoreductase activity;	GO:00038	K00069	HPGD	map00590;m	Arachidonic acid m	COG1028;COG0	S:Function unknown;	PF00106.28;PF13	adh_short;adh_short_C2;KR	short chain dehydrogenase;Enoyl;KR domain	CYT	3	30	30.1	High
TRINITY_DN122321_c0_g1_i1_orf1	uncharacterized protein LOC114356271 [Ostrinia furnacalis]	0.472998138	-1.080093591	0.0134	down	yes	1.016	2.148	2.603	1.706	2.135	1	1.141	0.906	CC:cellular_component;CC:cellular anatomical entity;CC:extracellular space;MF:endopeptidase inhibitor activity;MF:endopeptidase regulator activity;MF:peptidase regulator activity;MF:molecular_function;MF:peptidase inhibitor activity;MF:serine-type endopeptidase inhibitor activity;MF:enzyme inhibitor activity;MF:molecular function regulator;MF:enzyme regulator activity;	GO:00055	------	------	------	------	COG4826	J:Translation, ribosomal structure	PF00079.23	Serpin	Serpin	CYT	1	32	7.6	High
TRINITY_DN18388_c0_g1_i6_orf1	serine protease [Ostrinia furnacalis]	0.497441146	-1.007402248	2.77E-07	down	yes	0.972	1.954	1.949	1.959	1.955	1	0.955	0.961	BP:organic substance metabolic process;BP:organonitrogen compound metabolic process;BP:protein metabolic process;BP:nitrogen compound metabolic process;BP:macromolecule metabolic process;BP:biological_process;BP:metabolic process;BP:proteolysis;BP:primary metabolic process;CC:cellular_component;CC:extracellular region;CC:cellular anatomical entity;MF:serine-type endopeptidase activity;MF:endopeptidase activity;MF:hydrolase activity;MF:serine hydrolase activity;MF:catalytic activity, acting on a protein;MF:catalytic activity;MF:molecular_function;MF:pepti	GO:00717	------	------	------	------	COG5640	O:Posttranslational modification,	PF00089.29;PF12	Trypsin;CLIP;Trypsin_2	Trypsin;Regulatory CLIP domain of proteinases;Trypsin-like peptidase domain	CYT	4	13	47	High
TRINITY_DN636_c1_g1_i9_orf1	secretory phospholipase A2 receptor-like [Ostrinia furnacalis]	0.246872546	-2.018161685	0.0002985	down	yes	0.9433	3.821	4.093	3.337	4.034	1	0.931	0.899	MF:molecular_function;MF:carbohydrate binding;MF:binding;	GO:00036	------	------	------	------	ENOG410ZRYS;E	S:Function unknown;U:Intracellul	PF00059.24	Lectin_C	Lectin C-type domain	PLA	2	18	31	High
TRINITY_DN1363_c0_g1_i11_orf1	cytochrome P450 CYP12A2-like isoform X1 [Ostrinia furnacalis] >QPF77619.1 cytochrome P450 monooxygenase CYP333A20 [Ostrinia furnacalis]	0.298432416	-1.744523842	9.30E-06	down	yes	1.009	3.381	3.255	3.539	3.349	1	1.004	1.024	MF:cation binding;MF:monooxygenase activity;MF:heme binding;MF:transition metal ion binding;MF:tetrapyrrole binding;MF:organic cyclic compound binding;MF:oxidoreductase activity, acting on paired donors, with incorporation or reduction of molecular oxygen;MF:ion binding;MF:catalytic activity;MF:molecular_function;MF:binding;MF:iron ion binding;MF:metal ion binding;MF:heterocyclic compound binding;MF:oxidoreductase activity;	GO:00431	K15004	CYP12	------	------	COG2124	Q:Secondary metabolites biosynt	PF00067.25	p450	Cytochrome P450	CYT	8	17	57.7	High
TRINITY_DN4255_c0_g1_i11_orf1	LOW QUALITY PROTEIN: lebocin-4-like [Ostrinia furnacalis]	0.311618133	-1.68214891	4.45E-07	down	yes	1.038	3.331	3.355	3.274	3.365	1	1.03	1.084	------	------	------	------	------	------	ENOG4111ASS	S:Function unknown	------	------	------	CYT	1	13	22.2	High
TRINITY_DN335_c1_g1_i5_orf1	PREDICTED: perilipin-4 isoform X14 [Papilio polytes]	0.319646241	-1.645451965	2.72E-06	down	yes	1.012	3.166	3.089	3.272	3.137	1	1.035	1.002	------	------	K20254	PLIN4	map03320	PPAR signaling pat	ENOG4112C4W	S:Function unknown	------	------	------	CYT	13	74	15.1	High
TRINITY_DN7534_c0_g1_i15_orf1	protein-glucosylgalactosylhydroxylysine glucosidase isoform X2 [Ostrinia furnacalis]	0.384148518	-1.380263906	6.38E-06	down	yes	1.076	2.801	2.881	2.767	2.754	1	1.11	1.118	BP:primary metabolic process;BP:carbohydrate metabolic process;BP:organic substance metabolic process;BP:metabolic process;BP:biological_process;MF:catalytic activity;MF:molecular_function;	GO:00442	K22078	PGGHG, ATH	------	------	COG1554	G:Carbohydrate transport and m	PF03632.18	Glyco_hydro_65m	Glycosyl hydrolase family 65 central catalytic domain	CYT	2	5	48.7	High
TRINITY_DN6423_c0_g1_i6_orf1	phenoloxidase-activating factor 2-like isoform X1 [Ostrinia furnacalis]	0.376163207	-1.410569351	0.0001398	down	yes	1.051	2.794	2.802	2.997	2.584	1	1.07	1.084	BP:organic substance metabolic process;BP:organonitrogen compound metabolic process;BP:protein metabolic process;BP:macromolecule metabolic process;BP:biological_process;BP:nitrogen compound metabolic process;BP:metabolic process;BP:proteolysis;BP:primary metabolic process;MF:serine-type endopeptidase activity;MF:endopeptidase activity;MF:hydrolase activity;MF:serine hydrolase activity;MF:catalytic activity, acting on a protein;MF:catalytic activity;MF:molecular_function;MF:pepti	GO:00717	------	------	------	------	COG5640	O:Posttranslational modification,	PF00089.29;PF18	Trypsin;CLIP_1	Trypsin;Serine protease Clip domain PPAF-2	CYT	3	13	55.7	High
TRINITY_DN5177_c0_g1_i2_orf1	hemolin-like isoform X1 [Ostrinia furnacalis]	0.364613181	-1.45556138	7.57E-08	down	yes	1.018	2.792	2.802	2.811	2.764	1	1.013	1.041	------	------	------	------	------	------	ENOG410XSVG	S:Function unknown	PF07679.19;PF13	I-set;Ig_3;ig;Ig_2;V-set;Receptor_2B4	Immunoglobulin I-set domain;Immunoglobulin domain;Immunoglobulin domain;Immunoglobulin domain;Immunoglobulin V-set domain;Natural killer cell	CYT	22	58	47.3	High
TRINITY_DN43350_c0_g3_i1_orf1	uncharacterized protein LOC114355190 [Ostrinia furnacalis]	0.365494978	-1.45207651	1.13E-05	down	yes	1.019	2.788	2.898	2.79	2.676	1	1.011	1.047	------	------	------	------	------	------	ENOG4111DOA	S:Function unknown	------	------	------	CYT	3	25	25.8	High
TRINITY_DN60946_c0_g2_i3_orf1	protein yellow-like [Ostrinia furnacalis]	0.356086176	-1.489701667	1.89E-06	down	yes	0.9917	2.785	2.828	2.827	2.701	1	0.981	0.994	------	------	------	------	------	------	COG3386	S:Function unknown	PF03022.19	MRJP	Major royal jelly protein	CYT	13	36	49.2	High
TRINITY_DN18323_c0_g1_i5_orf1	GILT-like protein 1 isoform X1 [Ostrinia furnacalis]	0.35609493	-1.4896662	2.16E-06	down	yes	0.9903	2.781	2.765	2.863	2.716	1	0.996	0.975	------	------	K08059	IFI30, GILT	map04612	Antigen processing	ENOG4111IDT	U:Intracellular trafficking, secretic	PF03227.19	GILT	Gamma interferon inducible lysosomal thiol reductase	CYT	15	62	25	High
TRINITY_DN28503_c0_g1_i6_orf1	uncharacterized protein LOC114363584 [Ostrinia furnacalis] >AXY94663.1 seroin transcript 3 [Ostrinia nubilalis]	0.3572684	-1.484919781	0.00018	down	yes	0.9757	2.731	2.717	2.955	2.52	1	0.903	1.024	------	------	------	------	------	------	------	------	------	------	------	PLA	5	42	12.8	High
TRINITY_DN28221_c0_g2_i1_orf1	unnamed protein product [Chilo suppressalis]	0.361422657	-1.468241145	1.62E-07	down	yes	0.9603	2.657	2.657	2.671	2.642	1	0.935	0.946	BP:small molecule biosynthetic process;BP:carboxylic acid metabolic process;BP:cellular biosynthetic process;BP:cellular nitrogen compound metabolic process;BP:nitrogen compound metabolic process;BP:small molecule metabolic process;BP:nitrogen cycle metabolic process;BP:glutamine family amino acid metabolic process;BP:organic substance biosynthetic process;BP:cellular amino acid metabolic process;BP:arginine biosynthetic process;BP:arginine metabolic process;BP:organic substance metabolic process;BP:organic acid metabolic process;BP:cellular amino acid biosynthetic process;BP:alpha-amino acid metabolic process;BP:alpha-amino acid biosynthetic process;BP:urea metabolic process;BP:primary metabolic process;BP:cellular process;BP:biosynthetic process;BP:biological_process;BP:metabolic process;BP:oxoacid metabolic process;BP:cellular nitrogen compound biosynthetic process;BP:amide biosynthetic process;BP:organonitrogen compound metabolic process;BP:organonitrogen compound	GO:00442	K01940	argG, ASS1	map00220;m	Arginine biosynthe	COG0137	E:Amino acid transport and meta	PF00764.22	Arginosuc_synth	Arginosuccinate synthase	CYT	1	9	10.1	High
TRINITY_DN4245_c0_g2_i1_orf1	long-chain fatty acid transport protein 4-like [Ostrinia furnacalis]	0.384200311	-1.38006941	1.34E-05	down	yes	0.9897	2.576	2.691	2.487	2.55	1	0.966	1.003	------	------	K08745	SLC27A1_4, F	map04931;m	Insulin resistance;P	ENOG410XQ8T	I:Lipid transport and metabolism	PF00501.31	AMP-binding	AMP-binding enzyme	CYT	2	21	19.7	High

TRINITY_DN33346_c0_g1_i1_orf1	PREDICTED: U6 snRNA-associated Sm-like protein LSm3 [Papilio xuthus] >XP_028165558.1 U6 snRNA-associated Sm-like protein LSm3 [Ostrinia furnacalis] >KOB73597.1 LSM Sm-like protein family member [Operophtera brumata] >RVE45517.1 hypothetical protein evm_009856 [Chilo suppressalis] >CAB3523639.1 unnamed protein product [Chilo suppressalis] >CAH0400961.1 unnamed protein product [Chilo suppressalis]	0.359700346	-1.475132549	1.77E-05	down	yes	0.9363	2.603	2.711	2.568	2.53	1	0.952	0.857	BP:primary metabolic process;BP:cellular metabolic process;BP:nucleic acid metabolic process;BP:cellular nitrogen compound metabolic process;BP:nitrogen compound metabolic process;BP:organic cyclic compound metabolic process;BP:mRNA metabolic process;BP:organic substance metabolic process;BP:RNA splicing;BP:nucleobase-containing compound metabolic process;BP:cellular process;BP:cellular aromatic compound metabolic process;BP:RNA splicing, via transesterification reactions;BP:RNA splicing, via transesterification reactions with bulged adenosine as nucleophile;BP:biological_process;BP:metabolic process;BP:heterocycle metabolic process;BP:RNA metabolic process;BP:mRNA splicing, via spliceosome;BP:macromolecule metabolic process;BP:RNA processing;BP:mRNA processing;CC:Sm-like protein family complex;CC:spliceosomal complex;CC:U4/U6 x U5 tri-snRNP complex;CC:spliceosomal snRNP complex;CC:macromolecular complex;CC:small nuclear ribonucleoprotein	GO:00442	K12622	LSM3	map03018;m	RNA degradation;S	ENOG4111PB8	K:Transcription	PF01423.25	LSM	LSM domain	CYT	1	14	11.7	Medium
TRINITY_DN2024_c0_g1_i12_orfp1	unnamed protein product, partial [Brenthis ino]	0.423214286	-1.240539768	1.56E-06	down	yes	0.9717	2.296	2.263	2.348	2.277	1	0.954	0.961	------	------	------	------	------	------	------	------	PF13930.9	Endonuclea_NS_2	DNA/RNA non-specific endonuclease	CYT	2	6	46	High
TRINITY_DN15682_c0_g1_i4_orf1	seroin transcript 1B [Ostrinia nubilalis]	0.459252589	-1.122640241	3.91E-05	down	yes	1.02	2.221	2.21	2.297	2.156	1	1.106	0.954	------	------	------	------	------	------	------	------	------	------	------	CYT	2	36	5.9	High
TRINITY_DN11826_c0_g1_i4_orf1	aldehyde dehydrogenase X, mitochondrial-like [Ostrinia furnacalis]	0.442328278	-1.176810617	0.002069	down	yes	0.9917	2.242	2.553	1.947	2.225	1	1.014	0.961	MF:catalytic activity;MF:oxidoreductase activity, acting on the aldehyde or oxo group of donors;MF:molecular_function;MF:oxidoreductase activity, acting on the aldehyde or oxo group of donors, NAD or NADP as acceptor;MF:oxidoreductase	GO:0003	K00128	ALDH	map00310;m	Lysine degradation	COG1012	C:Energy production and convers	PF00171.25	Aldedh	Aldehyde dehydrogenase family	CYT	3	48	11.9	High
TRINITY_DN4565_c0_g2_i1_orf1	acid phosphatase type 7 isoform X2 [Ostrinia furnacalis]	0.433483958	-1.205949489	5.29E-05	down	yes	0.9593	2.213	2.309	2.149	2.182	1	1.015	0.863	MF:cation binding;MF:phosphatase activity;MF:hydrolase activity;MF:hydrolase activity, acting on ester bonds;MF:ion binding;MF:phosphoric ester hydrolase activity;MF:catalytic activity;MF:molecular_function;MF:binding;MF:metal ion binding;MF:acid	GO:00431	K22390	ACP7	------	------	COG1409	S:Function unknown	PF16656.8	Pur_ac_phosph_N	Purple acid Phosphatase, N-terminal domain	CYT	2	15	21.7	High
TRINITY_DN2472_c0_g1_i6_orf1	programmed cell death protein 6 isoform X1 [Colias croceus] >XP_045492459.1 programmed cell death protein 6 isoform X1 [Colias croceus]	0.448328691	-1.157371267	8.20E-07	down	yes	0.9657	2.154	2.138	2.143	2.18	1	0.936	0.961	MF:cation binding;MF:ion binding;MF:molecular_function;MF:binding;MF:metal ion binding;MF:calcium ion binding;	GO:00431	K23902	PDCD6	------	------	ENOG410YKQK	S:Function unknown	PF00036.35;PF13	EF-hand_1;EF-hand_6;EF-hand_7;EF-hand_8;EF-hand_5	EF hand;EF-hand domain;EF-hand domain pair;EF-hand domain pair;EF hand	CYT	3	22	21	High
TRINITY_DN9132_c0_g1_i5_orf1	ubiquitin-like-conjugating enzyme ATG3 [Spodoptera frugiperda]	0.459052533	-1.123268833	1.73E-07	down	yes	0.9787	2.132	2.121	2.15	2.124	1	0.959	0.977	MF:ubiquitin-like protein transferase activity;MF:catalytic activity;MF:transferase activity;MF:catalytic activity, acting on a	GO:0019	K08343	ATG3	map04136;m	Autophagy - other	ENOG410Y3BC	U:Intracellular trafficking, secretic	PF03987.18	Autophagy_act_C	Autophagocytosis associated protein, active-site domain	CYT	5	18	36.1	High
TRINITY_DN125441_c0_g1_i5_orf1	KH domain-containing, RNA-binding, signal transduction-associated protein 2-like isoform X12 [Ostrinia furnacalis]	0.498764212	-1.003570145	0.0002079	down	yes	1.009	2.023	2.016	2.162	1.891	1	1.022	1.004	------	------	K17843;K	KHDRBS2, SL	------	------	COG5176;ENOG	J:Translation, ribosomal structure	------	------	------	CYT	2	24	9.8	High
TRINITY_DN14754_c0_g1_i6_orf1	cathepsin L [Papilio xuthus]	0.14552145	-2.780696268	0.0001957	down	yes	0.8107	5.571	6.27	5.26	5.183	1	0.685	0.747	BP:primary metabolic process;BP:organonitrogen compound metabolic process;BP:protein metabolic process;BP:macromolecule metabolic process;BP:organic substance metabolic process;BP:nitrogen compound metabolic process;BP:biological_process;BP:metabolic process;BP:proteolysis;MF:molecular_function;MF:hydrolase activity;MF:catalytic activity;MF:catalytic activity, acting on a protein;MF:peptidase	GO:0044	------	------	------	------	COG4870	O:Posttranslational modification,	PF00112.26	Peptidase_C1	Papain family cysteine protease	CYT	1	41	12.8	High
TRINITY_DN100327_c0_g1_i1_orf1	arylphorin subunit alpha-like [Ostrinia furnacalis]	0.262469799	-1.929776668	0.0006026	down	yes	0.9777	3.725	4.166	3.207	3.802	1	0.967	0.966	------	------	------	------	------	------	ENOG410XR2D	S:Function unknown	PF00372.22;PF03	Hemocyanin_M;Hemocyanin_C;Hemocyanin_N	Hemocyanin, copper containing domain;Hemocyanin, ig-like domain;Hemocyanin, all-alpha domain	PLA	35	56	83.5	High
TRINITY_DN2406_c0_g1_i6_orf1	uncharacterized protein LOC114361672 [Ostrinia furnacalis]	0.314669653	-1.668090045	0.0002151	down	yes	1.124	3.572	3.42	3.919	3.378	1	1.098	1.274	------	------	------	------	------	------	ENOG4110RST	S:Function unknown	------	------	------	CYT	2	4	61.1	High
TRINITY_DN4816_c0_g2_i3_orf1	15-hydroxyprostaglandin dehydrogenase [NAD(+)]-like [Ostrinia furnacalis]	0.289965986	-1.786044416	2.80E-05	down	yes	1.023	3.528	3.615	3.652	3.316	1	0.954	1.116	MF:catalytic activity;MF:molecular_function;MF:oxidoreductase activity;	GO:0003	K00069	HPGD	map00590;m	Arachidonic acid m	COG1028;COG0	S:Function unknown;	PF00106.28;PF13	adh_short;adh_short_C2;KR;Epimerase;3Beta_HSD	short chain dehydrogenase;Enoyl;KR domain;NAD dependent epimerase/dehydratase family;3-beta hydroxysteroid dehydrogenase/isomerase	CYT	7	38	29.1	High
TRINITY_DN61042_c0_g2_i2_orfp1	TRINITY_DN61042_c0_g2_i2_m.5292 TRINITY_DN61042_c0_g2::TRINITY_DN61042_c0_g2_i2::g.5292 ORF type:5prime_partial len:63 (-),score=21.86 TRINITY_DN61042_c0_g2_i2:77-265(-)	0.42124183	-1.247279389	0.0003579	down	yes	1.289	3.06	3.154	2.945	3.082	1	1.457	1.411	------	------	------	------	------	------	------	------	------	------	------	CYT	1	10	7.1	Medium
TRINITY_DN1093_c0_g1_i4_orf1	uncharacterized protein LOC114361723 isoform X4 [Ostrinia furnacalis]	0.31803122	-1.652759698	0.000246	down	yes	0.9983	3.139	3.332	3.29	2.796	1	1.031	0.964	------	------	------	------	------	------	ENOG410XRJB	S:Function unknown	------	------	------	CYT	1	32	26.1	High
TRINITY_DN69_c0_g1_i1_orf1	glycerol-3-phosphate dehydrogenase [NAD(+)], cytoplasmic isoform X1 [Ostrinia furnacalis]	0.323539122	-1.627987924	4.98E-06	down	yes	0.98	3.029	3.139	2.928	3.02	1	0.966	0.974	------	------	K00006	GPD1	map00564	Glycerophospholipi	COG0240	C:Energy production and convers	PF01210.26;PF07	NAD_Gly3P_dh_N;NAD_Gly3P_dh_C	NAD-dependent glycerol-3-phosphate dehydrogenase N-terminus;NAD-dependent glycerol-3-phosphate dehydrogenase C-terminus	CYT	21	63	39.8	High
TRINITY_DN6098_c1_g1_i5_orf1	unnamed protein product, partial [Iphiclides podalirius]	0.361311239	-1.468685962	1.10E-05	down	yes	1.003	2.776	2.741	2.901	2.686	1	1.023	0.985	BP:immune system process;BP:response to external biotic stimulus;BP:response to other organism;BP:interspecies interaction between organisms;BP:innate immune response;BP:cellular process;BP:defense response;BP:response to stress;BP:defense response to other organism;BP:immune response;BP:cell killing;BP:defense response to fungus;BP:response to biotic stimulus;BP:response to external stimulus;BP:response to fungus;BP:response to stimulus;BP:killing of cells of other organism;BP:biological_process;CC:extracellular space;CC:cellular anatomical entity;CC:cellular_component;CC:extracellular region;MF:ion channel regulator activity;MF:molecular_function;MF:molecular function regulator;MF:sodium channel inhibitor activity;MF:channel inhibitor activity;MF:channel regulator activity;MF:ion channel inhibitor	GO:0002	------	------	------	------	------	------	------	------	------	CYT	2	28	7.7	High
TRINITY_DN9538_c1_g3_i1_orf1	cilia- and flagella-associated protein 410 isoform X2 [Aphidius gifuensis]	0.419178082	-1.254364811	8.76E-05	down	yes	1.071	2.555	2.693	2.562	2.41	1	1.065	1.148	------	------	K23456	CFAP410, C2	------	------	ENOG410Y9AE	S:Function unknown	PF14580.9;PF127	LRR_9;LRR_4	Leucine-rich repeat;Leucine Rich repeats	CYT	1	2	47.7	Medium

TRINITY_DN19980_c0_g1_i4_orf1	hypothetical protein evm_012507 [Chilo suppressalis]	0.412673267	-1.27692811	0.003535	down	yes	1.042	2.525	2.501	2.949	2.126	1	1.007	1.119	BP:developmental process;BP:cellular process;BP:cellular developmental process;BP:anatomical structure development;BP:cell development;BP:cellular component organization;BP:animal organ development;BP:biological_process;BP:cellular component organization or biogenesis;	GO:0032!	------	------	------	------	ENOG410Y7WV	S:Function unknown	PF07974.16;PF00	EGF_2;EGF;Laminin_EGF;DSL	EGF-like domain;EGF-like domain;Laminin EGF domain;Delta serrate ligand	PLA	1	4	37.3	High
TRINITY_DN33995_c0_g1_i5_orf1	unnamed protein product [Spodoptera exigua]	0.394491697	-1.341933159	0.0001	down	yes	0.974	2.469	2.401	2.468	2.539	1	1.111	0.811	CC:cellular_component;CC:integral component of membrane;CC:cellular anatomical entity;CC:intrinsic component of membrane;	GO:0005!	------	------	------	------	ENOG410XPW0	U:Intracellular trafficking, secretic	PF03798.19	TRAM_LAG1_CLN8	TLC domain	CYT	1	4	37.8	High
TRINITY_DN1750_c1_g1_i5_orf1	lipid droplet localized protein-like [Ostrinia furnacalis] >XP_028161280.1 lipid droplet localized protein-like [Ostrinia furnacalis]	0.423253869	-1.24040484	4.40E-05	down	yes	1.012	2.391	2.385	2.517	2.27	1	1.004	1.032	BP:nucleic acid metabolic process;BP:cellular biosynthetic process;BP:cellular nitrogen compound metabolic process;BP:nitrogen compound metabolic process;BP:organic cyclic compound biosynthetic process;BP:nucleobase-containing compound metabolic process;BP:cellular macromolecule metabolic process;BP:organic substance metabolic process;BP:organic cyclic compound metabolic process;BP:heterocycle biosynthetic process;BP:organic substance biosynthetic process;BP:cellular process;BP:cellular aromatic compound metabolic process;BP:biosynthetic process;BP:macromolecule biosynthetic process;BP:biological_process;BP:metabolic process;BP:nucleobase-containing compound biosynthetic process;BP:heterocycle metabolic process;BP:primary metabolic process;BP:cellular nitrogen compound biosynthetic process;BP:cellular metabolic process;BP:macromolecule metabolic process;BP:DNA metabolic process;BP:aromatic compound biosynthetic process;BP:DNA	GO:0090:	------	------	------	------	COG3268	S:Function unknown	PF03435.21	Sacchrp_dh_NADP	Saccharopine dehydrogenase NADP binding domain	CYT	15	35	47.8	High
TRINITY_DN5337_c0_g1_i6_orf1	epoxide hydrolase 1-like [Ostrinia furnacalis]	0.424883623	-1.23486036	0.000605	down	yes	1.004	2.363	2.465	2.089	2.535	1	0.999	1.012	CC:integral component of membrane;CC:cellular_component;CC:intrinsic component of membrane;CC:cellular anatomical entity;MF:hydrolase activity;MF:catalytic activity;MF:molecular_function;	GO:0016(	K22368;K:	EPHX3;EPHX	map05207;m	Chemical carcinoge	ENOG410XRPK;(	S:Function unknown;	PF00561.23;PF12	Abhydrolase_1;Abhydrolase_6;Hydrolase_4	alpha/beta hydrolase fold;Alpha/beta hydrolase family;Serine aminopeptidase. S33	CYT	11	39	39.7	High
TRINITY_DN5421_c0_g1_i1_orf1	arylphorin subunit alpha-like [Ostrinia furnacalis]	0.44411006	-1.171010843	6.39E-06	down	yes	1.033	2.326	2.264	2.384	2.33	1	1.021	1.077	------	------	------	------	------	------	------	------	------	------	------	EXC	1	21	17.2	High
TRINITY_DN64772_c0_g1_i1_orf1	aldehyde dehydrogenase, partial [Mythimna separata]	0.469669118	-1.09028336	1.54E-05	down	yes	1.022	2.176	2.242	2.091	2.194	1	1.023	1.044	MF:catalytic activity;MF:oxidoreductase activity, acting on the aldehyde or oxo group of donors;MF:molecular_function;MF:oxidoreductase activity, acting on the aldehyde or oxo group of donors, NAD or NADP as acceptor;MF:oxidoreductase	GO:0003!	K00128	ALDH	map00310;m	Lysine degradation	COG1012	C:Energy production and convers	PF00171.25	Aldedh	Aldehyde dehydrogenase family	CYT	2	42	5.9	High
TRINITY_DN64297_c0_g1_i1_orf1	vanin-like protein 2 isoform X2 [Ostrinia furnacalis]	0.218668065	-2.193185555	0.001626	down	yes	1.251	5.721	5.983	6.562	4.619	1	1.417	1.336	BP:nitrogen compound metabolic process;BP:biological_process;BP:metabolic process;	GO:0006!	------	------	------	------	COG0388	O:Posttranslational modification,	------	------	------	CYT	1	57	7.8	High
TRINITY_DN20558_c0_g1_i2_orf1	Transient receptor potential channel pyrexia [Operophtera brumata]	0.496089932	-1.011326418	9.28E-05	down	yes	1.015	2.046	2.149	2.027	1.963	1	0.963	1.083	BP:response to stimulus;BP:biological_process;CC:transporter complex;CC:transmembrane transporter complex;CC:ion channel complex;CC:cellular_component;CC:membrane protein complex;CC:cellular anatomical entity;CC:macromolecular complex;CC:integral component of membrane;CC:intrinsic component of membrane;CC:cation channel complex;MF:ion transmembrane transporter activity;MF:inorganic solute uptake transmembrane transporter activity;MF:transporter activity;MF:ion channel activity;MF:transmembrane transporter activity;MF:channel activity;MF:passive transmembrane transporter	GO:0050!	K04984	TRPA1, ANK1	map04750	Inflammatory medi	COG0666	Q:Secondary metabolites biosynt	------	------	------	CYT	1	14	8.5	High
TRINITY_DN10220_c1_g1_i7_orf1	uncharacterized protein LOC124645895 isoform X2 [Helicoverpa zea]	0.47897535	-1.061976683	0.001083	down	yes	0.991	2.069	2.235	1.818	2.155	1	0.99	0.983	CC:cellular_component;CC:integral component of membrane;CC:cellular anatomical entity;CC:intrinsic component of membrane;	GO:0005!	K14788	NOL10, ENP:	------	------	ENOG410ZV5F	S:Function unknown	------	------	------	CYT	1	2	52.5	Medium
TRINITY_DN4767_c0_g1_i4_orf1	cysteine protease XCP2-like [Ostrinia furnacalis]	0.203599242	-2.296195903	2.67E-06	down	yes	0.9673	4.751	4.639	4.937	4.676	1	0.912	0.99	BP:primary metabolic process;BP:organonitrogen compound metabolic process;BP:protein metabolic process;BP:macromolecule metabolic process;BP:organic substance metabolic process;BP:nitrogen compound metabolic process;BP:biological_process;BP:metabolic process;BP:proteolysis;MF:molecular_function;MF:hydrolase activity;MF:catalytic activity;MF:catalytic activity, acting on a	GO:0044:	------	------	------	------	ENOG4110R2S;E	S:Function unknown;	PF08246.15	Inhibitor_I29	Cathepsin propeptide inhibitor domain	CYT	6	38	11.7	High
TRINITY_DN710_c0_g1_i11_orfp1	TRINITY_DN710_c0_g1_i11_m.67699 TRINITY_DN710_c0_g1::TRINITY_DN710_c0_g1_i11::g.67699 ORF type:complete len:194 (-),score=34.99,Collagen PF01391.19 0.00029 TRINITY_DN710_c0_g1_i11:1283-1864(-)	0.233943036	-2.09577081	8.18E-05	down	yes	0.8953	3.827	3.498	4.069	3.914	1	0.874	0.812	------	------	------	------	------	------	------	------	------	------	------	CYT	1	7	21.3	High
TRINITY_DN85290_c0_g2_i1_orf1	unnamed protein product, partial [Brenthis ino]	0.264555943	-1.918355267	1.47E-07	down	yes	0.986	3.727	3.707	3.706	3.768	1	1.025	0.933	------	------	K23615;K:	SPIK5;SPINK!	------	------	ENOG4111TR7;C	O:Posttranslational modification,	PF00050.24;PF07	Kazal_1;Kazal_2	Kazal-type serine protease inhibitor domain;Kazal-type serine protease inhibitor domain	CYT	1	56	7.6	High
TRINITY_DN31348_c0_g1_i1_orf1	protein lethal(2)essential for life [Bombyx mori]	0.299220212	-1.740720463	1.25E-06	down	yes	0.9593	3.206	3.13	3.268	3.22	1	0.911	0.967	MF:cation binding;MF:metal ion binding;MF:molecular_function;MF:binding;MF:ion binding;	GO:0043:	K09542	CRYAB	map04213;m	Longevity regulatin	ENOG410YERS	O:Posttranslational modification,	------	------	------	CYT	1	22	7.2	High
TRINITY_DN4767_c0_g1_i6_orf1	cysteine protease XCP2-like [Ostrinia furnacalis]	0.319839228	-1.644581199	3.24E-05	down	yes	0.9947	3.11	2.985	3.311	3.033	1	1.013	0.971	BP:primary metabolic process;BP:organonitrogen compound metabolic process;BP:protein metabolic process;BP:macromolecule metabolic process;BP:organic substance metabolic process;BP:nitrogen compound metabolic process;BP:biological_process;BP:metabolic process;BP:proteolysis;MF:molecular_function;MF:hydrolase activity;MF:catalytic activity;MF:catalytic activity, acting on a	GO:0044:	------	------	------	------	ENOG4110R2S	S:Function unknown	PF08246.15	Inhibitor_I29	Cathepsin propeptide inhibitor domain	CYT	2	44	5.3	High
TRINITY_DN8783_c0_g1_i5_orf1	luciferin 4-monooxygenase-like [Ostrinia furnacalis] >XP_028165580.1 luciferin 4-monooxygenase-like [Ostrinia furnacalis]	0.441237624	-1.180372282	0.0001516	down	yes	0.8913	2.02	2.132	1.943	1.984	1	0.809	0.865	------	------	K01904	4CL	map00130	Ubiquinone and ot	COG0318;COG0:	I:Lipid transport and metabolism	PF00501.31;PF13	AMP-binding;AMP-binding_C	AMP-binding enzyme;AMP-binding enzyme C-terminal	CYT	1	8	58.1	High
TRINITY_DN135449_c0_g1_i5_orf1	larval cuticle protein LCP-17-like [Galleria mellonella]	0.35336856	-1.500754409	6.35E-05	down	yes	1.07	3.028	3.208	2.841	3.034	1	1.086	1.125	MF:molecular_function;MF:structural constituent of cuticle;MF:structural molecule activity;	GO:0003(	------	------	------	------	ENOG4110RUZ;E	S:Function unknown;	------	------	------	CYT	1	68	8.6	High
TRINITY_DN801_c0_g1_i2_orf1	cathepsin L [Ostrinia furnacalis] >XP_028165920.1 cathepsin L [Ostrinia furnacalis] >UKI61015.1 cathepsin L [Ostrinia furnacalis]	0.327275726	-1.611421495	7.46E-06	down	yes	0.9923	3.032	2.981	3.165	2.951	1	0.966	1.011	BP:primary metabolic process;BP:organonitrogen compound metabolic process;BP:protein metabolic process;BP:macromolecule metabolic process;BP:organic substance metabolic process;BP:nitrogen compound metabolic process;BP:biological_process;BP:metabolic process;BP:proteolysis;MF:molecular_function;MF:hydrolase activity;MF:catalytic activity;MF:catalytic activity, acting on a protein;MF:peptidase	GO:0044:	K01365	CTSL	map05205;m	Proteoglycans in ca	COG4870	S:Function unknown	PF00112.26;PF08:	Peptidase_C1;Inhibitor_I29	Papain family cysteine protease;Cathepsin propeptide inhibitor domain	CYT	16	47	38.1	High

TRINITY_DN1149_c0_g1_i4_orf1	circadian clock-controlled protein-like [Ostrinia furnacalis]	0.217041119	-2.203959703	1.09E-06	down	yes	1.024	4.718	4.797	4.767	4.589	1	0.97	1.102	------	------	------	------	------	------	ENOG410YQCZ;I	S:Function unknown;	PF06585.14	JHBP	Haemolymph juvenile hormone binding	CYT	7	35	27.4	High
TRINITY_DN12586_c0_g1_i4_orf1	zonadhesin-like isoform X4 [Ostrinia furnacalis]	0.241564006	-2.049522589	2.35E-05	down	yes	0.902	3.734	3.785	3.513	3.905	1	0.873	0.833	------	------	------	------	------	------	ENOG4110RF5;C	S:Function unknown;O:Posttransl	PF01826.20	TIL	Trypsin Inhibitor like cysteine rich	PLA	1	26	14.3	High
TRINITY_DN12920_c0_g3_i1_orf1	zonadhesin-like isoform X4 [Ostrinia furnacalis]	0.294801421	-1.762184618	0.000398	down	yes	0.913	3.097	2.962	3.478	2.851	1	0.916	0.823	------	------	------	------	------	------	------	------	PF01826.20	TIL	Trypsin Inhibitor like cysteine rich	CYT	1	13	8.8	High
TRINITY_DN45948_c1_g1_i1_orf1	unnamed protein product [Leptidea sinapis]	0.289018952	-1.790763996	1.89E-05	down	yes	1.037	3.588	3.749	3.39	3.625	1	1.032	1.079	BP:metabolic process;BP:nitrogen compound metabolic process;BP:organic substance metabolic process;BP:proteolysis;BP:biological_process;BP:primary metabolic process;BP:organonitrogen compound metabolic process;BP:protein metabolic process;BP:macromolecule metabolic process;MF:endopeptidase activity;MF:peptidase inhibitor activity;MF:molecular_function;MF:enzyme inhibitor activity;MF:molecular function regulator;MF:peptidase activity;MF:cysteine-type peptidase activity;MF:enzyme regulator activity;MF:hydrolase activity;MF:endopeptidase regulator activity;MF:peptidase regulator activity;MF:catalytic activity, acting on a protein;MF:catalytic activity;MF:endopeptidase inhibitor activity;MF:cysteine-type endopeptidase inhibitor activity;MF:cysteine-type endopeptidase activity;	GO:00081	K01373	CTSF	map04210;m	Apoptosis;Lysosom	COG4870	O:Posttranslational modification,	PF00112.26	Peptidase_C1	Papain family cysteine protease	CYT	2	68	3.7	High
TRINITY_DN2464_c0_g1_i12_orf1	uncharacterized protein LOC114362996 isoform X1 [Ostrinia furnacalis]	0.330431871	-1.597575248	1.13E-05	down	yes	0.987	2.987	2.932	3.133	2.895	1	0.981	0.98	MF:molecular_function;MF:structural constituent of cuticle;MF:structural molecule activity;	GO:0003	------	------	------	------	ENOG410YNFC	S:Function unknown	PF00379.26	Chitin_bind_4	Insect cuticle protein	CYT	8	45	37.2	High
TRINITY_DN42275_c0_g1_i1_orfp1	TRINITY_DN42275_c0_g1_i1_m.44265 TRINITY_DN42275_c0_g1::TRINITY_DN42275_c0_g1_i1::g.44265 ORF type:internal len:76 (+),score=8.67 TRINITY_DN42275_c0_g1_i1:1-225(+)	0.499470339	-1.001529089	0.001927	down	yes	0.943	1.888	1.774	1.983	1.908	1	1.108	0.721	------	------	------	------	------	------	------	------	------	------	------	CYT	1	16	7.9	Medium
TRINITY_DN12582_c0_g1_i5_orf1	uncharacterized protein LOC114355527 isoform X1 [Ostrinia furnacalis]	0.340288925	-1.555167898	0.002204	down	yes	1.06	3.115	3.046	3.649	2.651	1	1.025	1.154	MF:hydrolase activity;MF:hydrolase activity, acting on acid anhydrides, in phosphorus-containing anhydrides;MF:catalytic activity;MF:hydrolase activity, acting on acid anhydrides;MF:pyrophosphatase activity;MF:molecular_function;MF:nucle	GO:00167	------	------	------	------	COG0424	D:Cell cycle control, cell division,	PF02545.17	Maf	Maf-like protein	CYT	1	5	22.9	High
TRINITY_DN3707_c0_g1_i1_orf1	protein FAM160B1-like isoform X1 [Ostrinia furnacalis]	0.353604373	-1.499791978	4.36E-05	down	yes	1.035	2.927	3.047	2.999	2.736	1	1.069	1.037	------	------	------	------	------	------	ENOG410XTDG;I	S:Function unknown;	PF10257.12;PF19	RAI16-like;DUF5917;KELAA	Retinoic acid induced 16-like protein;Family of unknown function:KELAA	CYT	1	1	93.1	High
TRINITY_DN24_c0_g1_i1_orf1	hypothetical protein evm_007803 [Chilo suppressalis]	0.32262653	-1.632063017	1.49E-05	down	yes	0.9753	3.023	3.101	2.868	3.099	1	1.002	0.924	BP:organophosphate catabolic process;BP:nucleotide catabolic process;BP:cellular nitrogen compound metabolic process;BP:nitrogen compound metabolic process;BP:small molecule metabolic process;BP:organic cyclic compound metabolic process;BP:organic cyclic compound catabolic process;BP:nucleobase-containing compound metabolic process;BP:organic substance catabolic process;BP:organic substance metabolic process;BP:cellular catabolic process;BP:cellular process;BP:cellular aromatic compound metabolic process;BP:heterocycle catabolic process;BP:aromatic compound catabolic process;BP:biological_process;BP:metabolic process;BP:nucleobase-containing compound catabolic process;BP:catabolic process;BP:nucleobase-containing small molecule metabolic process;BP:heterocycle metabolic process;BP:primary metabolic process;BP:cellular nitrogen compound catabolic process;BP:cellular metabolic process;BP:phosphate-containing	GO:00464	------	------	------	------	COG0737	S:Function unknown	PF02872.21;PF00	5_nucleotid_C;Metallophos	5'-nucleotidase, C-terminal domain;Calcineurin-like phosphoesterase	CYT	9	19	60.2	High
TRINITY_DN13660_c0_g1_i1_orf1	Aliphatic nitrilase [Operophtera brumata]	0.377259036	-1.406372638	7.86E-07	down	yes	1.002	2.656	2.665	2.704	2.6	1	0.989	1.018	BP:nitrogen compound metabolic process;BP:metabolic process;BP:biological_process;MF:hydrolase activity;MF:catalytic activity;MF:hydrolase activity, acting on carbon-nitrogen;MF:hydrolase activity, acting on carbon-nitrogen;MF:beta-ureidopropionase	GO:00068	K01431	UPB1, pydC	map00240;m	Pyrimidine metabo	COG0388	S:Function unknown	PF00795.25	CN_hydrolase	Carbon-nitrogen hydrolase	CYT	17	50	44.5	High
TRINITY_DN29026_c0_g1_i4_orf1	TIL [Ostrinia furnacalis]	0.401488306	-1.316570127	0.001915	down	yes	1.133	2.822	2.456	3.106	2.903	1	1.003	1.396	------	------	------	------	------	------	ENOG4110ODA;	S:Function unknown;	------	------	------	CYT	1	14	9.9	High
TRINITY_DN2040_c0_g1_i6_orf1	trypsin-like serine proteinase T26 protein, partial [Chilo infuscatellus]	0.40168647	-1.315858227	5.47E-06	down	yes	1.048	2.609	2.635	2.557	2.636	1	1.016	1.129	BP:organic substance metabolic process;BP:organonitrogen compound metabolic process;BP:protein metabolic process;BP:macromolecule metabolic process;BP:biological_process;BP:nitrogen compound metabolic process;BP:metabolic process;BP:proteolysis;BP:primary metabolic process;MF:serine-type endopeptidase activity;MF:endopeptidase activity;MF:hydrolase activity;MF:serine hydrolase activity;MF:catalytic activity, acting on a protein;MF:catalytic activity;MF:molecular_function;MF:pepti	GO:00717	------	------	------	------	------	------	------	------	------	PLA	2	17	37.1	High
TRINITY_DN1772_c1_g2_i1_orf1	aldose reductase-like isoform X2 [Ostrinia furnacalis]	0.361616528	-1.467467477	2.24E-08	down	yes	0.9977	2.759	2.774	2.761	2.742	1	0.979	1.014	------	------	K00011	AKR1B	map00051;m	Fructose and mann	COG0656	Z:Cytoskeleton	PF00248.24	Aldo_ket_red	Aldo/keto reductase	CYT	13	77	36.1	High
TRINITY_DN955_c0_g1_i2_orf1	gloverin-like [Ostrinia furnacalis] >XP_028168251.1 gloverin-like [Ostrinia furnacalis] >AYM26645.1 gloverin [Ostrinia furnacalis]	0.38184261	-1.388949991	5.42E-05	down	yes	0.9947	2.605	2.521	2.766	2.529	1	1.057	0.927	------	------	------	------	------	------	ENOG4110QR6	S:Function unknown	PF10793.12	Gloverin	Gloverin-like protein	PLA	2	20	21.1	High
TRINITY_DN6415_c0_g2_i1_orf1	D-arabinitol dehydrogenase 1-like [Ostrinia furnacalis]	0.372850348	-1.423331408	2.99E-05	down	yes	1.019	2.733	2.875	2.605	2.719	1	0.995	1.063	MF:oxidoreductase activity;MF:cation binding;MF:transition metal ion binding;MF:ion binding;MF:catalytic activity;MF:molecular_function;MF:binding;MF:metal ion binding;MF:zinc ion binding;	GO:00164	K17818	ARD1	map00040	Pentose and glucur	COG1063	E:Amino acid transport and meta	PF08240.15;PF00	ADH_N;ADH_zinc_N;Glu_dehyd_C	Alcohol dehydrogenase GroES-like domain;Zinc-binding dehydrogenase;Glucose dehydrogenase C-terminus	CYT	13	61	37.5	High
TRINITY_DN57137_c0_g1_i1_orfp1	TRINITY_DN57137_c0_g1_i1_m.46420 TRINITY_DN57137_c0_g1::TRINITY_DN57137_c0_g1_i1::g.46420 ORF type:5prime_partial len:56 (-),score=4.65 TRINITY_DN57137_c0_g1_i1:82-249(-)	0.107804158	-3.213515275	8.34E-05	down	yes	0.949	8.803	8.355	9.766	8.288	1	0.936	0.911	------	------	------	------	------	------	------	------	------	------	------	CYT	3	33	5.8	High
TRINITY_DN50725_c0_g1_i6_orf1	BTB/POZ domain-containing protein 2-like [Ostrinia furnacalis]	0.410931174	-1.283031314	0.004101	down	yes	1.015	2.47	2.846	2.556	2.007	1	1.029	1.015	BP:developmental process;BP:cellular process;BP:cellular developmental process;BP:cell differentiation;BP:biological_process;BP:neurogenesis;CC:cytoplasm;CC:ribonucleoprotein granule;CC:intracellular non-membrane-bounded organelle;CC:cytosol;CC:cytoplasmic mRNA processing body;CC:cellular_component;CC:supramolecular complex;CC:intracellular organelle;CC:non-membrane-bounded organelle;CC:cytoplasmic ribonucleoprotein granule;CC:organelle;CC:cellular anatomical entity;	GO:00325	K10478	BTBD3_6	------	------	ENOG410XQ3X	S:Function unknown	PF00651.34;PF00	BTB;MATH;BACK	BTB/POZ domain;MATH domain;BTB And C-terminal Kelch	CYT	3	9	45.4	High

TRINITY_DN14670_c0_g1_i1_orf1	heat shock protein beta-1 isoform X1 [Helicoverpa armigera] >XP_022829066.1 heat shock protein beta-1 isoform X1 [Spodoptera litura] >XP_026747148.1 heat shock protein beta-1 isoform X3 [Trichoplusia ni] >XP_026748187.1 heat shock protein beta-1 isoform X2 [Galleria mellonella] >XP_028167756.1 heat shock protein beta-1 isoform X2 [Ostrinia furnacalis] >XP_035431734.1 heat shock protein beta-1-like isoform X3 [Spodoptera frugiperda] >XP_047023072.1 heat shock protein beta-1 isoform X1 [Helicoverpa zea] >XP_049865086.1 heat shock protein beta-1 [Pectinophora gossypiella] >KAH9640995.1 hypothetical protein HF086_015091 [Spodoptera exigua] >QGZ00460.1 heat shock protein 21.4 [Glyphodes pyloalis] >QKR72095.1 heat-shock protein 21.4 [Mythimna separata] >CAB3228281.1 unnamed protein product [Arctia plantaginis] >CAH0628881.1 unnamed protein product	0.377365492	-1.405965594	7.92E-05	down	yes	1.017	2.695	2.66	2.886	2.54	1	1.03	1.02	MF:cation binding;MF:metal ion binding;MF:molecular_function;MF:binding;MF:ion binding;	GO:00431	------	------	------	------	ENOG410YERS	K:Transcription	PF00011.24	HSP20	Hsp20/alpha crystallin family	CYT	13	72	21.4	High
TRINITY_DN28501_c0_g1_i2_orfp1	TRINITY_DN28501_c0_g1_i2_m.58934 TRINITY_DN28501_c0_g1::TRINITY_DN28501_c0_g1_i2::g.58934 ORF type:internal len:98 (+),score=13.70 TRINITY_DN28501_c0_g1_i2:3-293(+)	0.118614771	-3.075644417	4.87E-06	down	yes	0.9813	8.273	8.157	8.692	7.971	1	0.911	1.033	------	------	------	------	------	------	------	------	------	------	------	CYT	1	30	10.9	High
TRINITY_DN27021_c0_g1_i1_orf1	rab3 GTPase-activating protein catalytic subunit isoform X1 [Ostrinia furnacalis]	0.429454967	-1.219421237	0.001381	down	yes	1.111	2.587	2.659	2.838	2.265	1	1.069	1.263	BP:regulation of hydrolase activity;BP:positive regulation of catalytic activity;BP:positive regulation of GTPase activity;BP:positive regulation of hydrolase activity;BP:biological_process;BP:regulation of GTPase activity;BP:regulation of catalytic activity;BP:positive regulation of molecular function;BP:biological regulation;BP:regulation of molecular function;CC:cytoplasm;CC:cellular_component;CC:cellular anatomical entity;MF:enzyme regulator activity;MF:GTPase activator activity;MF:GTPase regulator activity;MF:molecular_function;MF:enzyme activator activity;MF:molecular function regulator;MF:nucleoside-	GO:00513	K18270	RAB3GAP1	------	------	ENOG410Y2R4;E	S:Function unknown;	PF13890.9	Rab3-GTPase_cat	Rab3 GTPase-activating protein catalytic subunit	CYT	1	1	89.6	High
TRINITY_DN2457_c0_g1_i8_orf1	uncharacterized protein LOC114355596 [Ostrinia furnacalis]	0.152975821	-2.708624449	4.07E-05	down	yes	0.987	6.452	6.429	6.946	5.98	1	0.94	1.021	BP:biological_process;BP:metabolic process;BP:methylation;MF:catalytic activity;MF:transferase activity;MF:transferase activity, transferring one-carbon groups;MF:molecular_function;MF:methyltransferase activity;	GO:00081	------	------	------	------	ENOG410Y1IK	M:Cell wall/membrane/envelope	PF12248.11	Methyltransf_FA	Farnesoic acid 0-methyl transferase	CYT	7	27	33.4	High
TRINITY_DN858_c0_g1_i3_orf1	uncharacterized protein LOC114351944 [Ostrinia furnacalis]	0.414244781	-1.271444574	6.95E-05	down	yes	1.012	2.443	2.289	2.577	2.464	1	1.02	1.015	CC:cellular_component;CC:extracellular space;CC:cellular anatomical entity;	GO:00055	------	------	------	------	------	------	PF16077.8	Spaetzle	Spaetzle	CYT	2	9	20.2	High
TRINITY_DN26209_c0_g1_i6_orf1	uncharacterized protein LOC114352357 [Ostrinia furnacalis]	0.375348137	-1.413698773	2.01E-06	down	yes	0.9973	2.657	2.679	2.711	2.58	1	0.985	1.007	------	------	------	------	------	------	COG1028	S:Function unknown	PF13561.9;PF001	adh_short_C2;adh_short;KR	Enoyl;short chain dehydrogenase:KR domain	CYT	11	54	27.3	High
TRINITY_DN5406_c0_g2_i1_orf1	uncharacterized protein LOC114350326 [Ostrinia furnacalis]	0.220834959	-2.17895952	0.0001214	down	yes	1.132	5.126	5.612	5.023	4.742	1	1.108	1.289	BP:positive regulation of ion transmembrane transporter activity;BP:positive regulation of transporter activity;BP:regulation of ion transmembrane transporter activity;BP:regulation of transport;BP:modulation of synaptic transmission;BP:regulation of potassium ion transport;BP:regulation of signaling;BP:positive regulation of transmembrane transport;BP:regulation of ion transmembrane transport;BP:positive regulation of ion transmembrane transport;BP:regulation of biological process;BP:regulation of transmembrane transport;BP:positive regulation of potassium ion transport;BP:regulation of cell communication;BP:rhythmic process;BP:regulation of transporter activity;BP:biological regulation;BP:regulation of ion transport;BP:positive regulation of molecular function;BP:regulation of trans-synaptic signaling;BP:positive regulation of biological process;BP:regulation of molecular function;BP:positive regulation of potassium ion transmembrane transport;BP:multicellular organismal	GO:00324	------	------	------	------	ENOG410ZEPJ;E	S:Function unknown;	PF17064.8	QVR	Sleepless protein	CYT	1	9	15.6	High
TRINITY_DN5682_c0_g1_i6_orf1	spodomicin-like [Ostrinia furnacalis] >QKV49445.1 diapausin [Ostrinia furnacalis]	0.44092827	-1.181384117	0.0001338	down	yes	1.045	2.37	2.277	2.548	2.285	1	1.065	1.069	------	------	------	------	------	------	------	------	PF08036.14	Antimicrobial_6	Diapausin family of antimicrobial	CYT	2	40	7	High
TRINITY_DN29414_c1_g2_i1_orf1	serine protease 44-like isoform X2 [Ostrinia furnacalis]	0.361297539	-1.468740666	0.004826	down	yes	0.969	2.682	3.277	2.3	2.468	1	0.911	0.996	BP:organic substance metabolic process;BP:organonitrogen compound metabolic process;BP:protein metabolic process;BP:macromolecule metabolic process;BP:biological_process;BP:nitrogen compound metabolic process;BP:metabolic process;BP:proteolysis;BP:primary metabolic process;MF:serine-type endopeptidase activity;MF:endopeptidase activity;MF:hydrolase activity;MF:serine hydrolase activity;MF:catalytic activity, acting on a protein;MF:catalytic activity;MF:molecular_function;MF:peptidase activity	GO:00717	------	------	------	------	COG5640	S:Function unknown	------	------	------	CYT	1	15	8.8	High
TRINITY_DN15400_c0_g1_i1_orf1	uncharacterized protein LOC114366781 [Ostrinia furnacalis]	0.232166593	-2.106767702	1.92E-05	down	yes	1.048	4.514	4.242	4.568	4.731	1	1.106	1.038	MF:odorant binding;MF:molecular_function;MF:binding;	GO:00055	------	------	------	------	ENOG4111CBI	S:Function unknown	------	------	------	CYT	1	36	10.3	High
TRINITY_DN1161_c0_g1_i2_orf1	fructose-1,6-bisphosphatase 1 [Ostrinia furnacalis]	0.429245283	-1.220125814	3.05E-06	down	yes	1.001	2.332	2.343	2.385	2.268	1	0.984	1.018	BP:monosaccharide biosynthetic process;BP:small molecule biosynthetic process;BP:gluconeogenesis;BP:organic substance metabolic process;BP:organic substance biosynthetic process;BP:hexose metabolic process;BP:hexose biosynthetic process;BP:biosynthetic process;BP:biological_process;BP:metabolic process;BP:primary metabolic process;BP:carbohydrate metabolic process;BP:monosaccharide metabolic process;BP:carbohydrate biosynthetic process;BP:glucose metabolic process;BP:small molecule metabolic process;MF:carbohydrate phosphatase activity;MF:fructose 1,6-bisphosphate 1-phosphatase activity;MF:phosphatase activity;MF:phosphoric ester hydrolase activity;MF:molecular_function;MF:binding;MF:cation binding;MF:ion binding;MF:metal ion binding;MF:hydrolase activity;MF:hydrolase activity, acting on	GO:00463	K03841	FBP, fbp	map00051;m	Fructose and mann	COG0158	G:Carbohydrate transport and m	PF00316.23;PF18	FBPase;FBPase_C;Inositol_P	Fructose-1-6-bisphosphatase, N-terminal domain;Fructose-1-6-bisphosphatase, C-terminal domain;Inositol monophosphatase family	CYT	12	41	36.3	High
TRINITY_DN4068_c1_g2_i1_orf1	larval cuticle protein LCP-17 [Helicoverpa armigera] >PZC82071.1 hypothetical protein B5X24_HaOG211161 [Helicoverpa armigera] >PZC87412.1 hypothetical protein B5X24_HaOG216859 [Helicoverpa armigera]	0.38792772	-1.366140225	1.84E-06	down	yes	1.009	2.601	2.592	2.613	2.597	1	1.077	0.95	MF:molecular_function;MF:structural constituent of cuticle;MF:structural molecule activity;	GO:0003	------	------	------	------	ENOG4110RUZ;E	S:Function unknown;	PF00379.26	Chitin_bind_4	Insect cuticle protein	CYT	4	64	17	High
TRINITY_DN3439_c0_g2_i2_orf1	histone H2A.Z-specific chaperone CHZ1-like [Ostrinia furnacalis]	0.210114155	-2.250754736	8.85E-07	down	yes	0.9203	4.38	4.486	4.35	4.303	1	0.884	0.877	------	------	------	------	------	------	------	------	------	------	------	PLA	4	14	31.9	High
TRINITY_DN27300_c0_g1_i6_orfp1	TRINITY_DN27300_c0_g1_i6_m.71140 TRINITY_DN27300_c0_g1::TRINITY_DN27300_c0_g1_i6::g.71140 ORF type:internal len:129 (-),score=20.75 TRINITY_DN27300_c0_g1_i6:1-384(-)	0.481955231	-1.053028956	9.89E-05	down	yes	1.055	2.189	2.295	2.149	2.122	1	1.013	1.153	------	------	------	------	------	------	------	------	------	------	------	CYT	1	9	14.4	High
TRINITY_DN703_c0_g1_i2_orf1	acidic juvenile hormone-suppressible protein 1-like [Ostrinia furnacalis]	0.229352886	-2.124359031	4.91E-09	down	yes	0.9853	4.296	4.327	4.274	4.287	1	0.982	0.974	------	------	------	------	------	------	ENOG410XR2D	S:Function unknown	PF03723.17	Hemocyanin_C	Hemocyanin, ig-like domain	CYT	6	97	10.1	High
TRINITY_DN40126_c0_g1_i1_orf1	aldehyde dehydrogenase X, mitochondrial-like [Ostrinia furnacalis]	0.408422725	-1.291864952	1.62E-06	down	yes	1.028	2.517	2.548	2.457	2.545	1	1.027	1.056	MF:catalytic activity;MF:oxidoreductase activity, acting on the aldehyde or oxo group of donors;MF:molecular_function;MF:oxidoreductase activity, acting on the aldehyde or oxo group of donors, NAD or NADP as acceptor;MF:oxidoreductase	GO:0003	K00128	ALDH	map00310;m	Lysine degradation	COG1012	C:Energy production and convers	PF00171.25	Aldedh	Aldehyde dehydrogenase family	CYT	19	65	45.5	High
TRINITY_DN56308_c0_g1_i2_orf1	storage protein 1 [Omphisa fuscidentalis]	0.395292722	-1.339006704	3.51E-06	down	yes	0.9993	2.528	2.565	2.569	2.449	1	0.972	1.026	CC:extracellular space;CC:cellular_component;CC:extracellular region;CC:cellular anatomical entity;MF:molecular_function;MF:nutrient reservoir activity;	GO:0005	------	------	------	------	ENOG410XR2D	S:Function unknown	PF03722.17	Hemocyanin_N	Hemocyanin, all-alpha domain	CYT	9	60	11.3	High
TRINITY_DN20560_c0_g1_i6_orf1	pupal cuticle protein C1B-like [Ostrinia furnacalis]	0.301949861	-1.727619087	6.87E-06	down	yes	1.084	3.59	3.608	3.692	3.471	1	1.077	1.176	------	------	------	------	------	------	ENOG410Y08D	S:Function unknown	------	------	------	CYT	3	30	22.6	High

TRINITY_DN16091_c0_g1_i1_orfp1	TRINITY_DN16091_c0_g1_i1_m.64010 TRINITY_DN16091_c0_g1::TRINITY_DN16091_c0_g1_i1::g.64010 ORF type:5prime_partial len:124 (-),score=7.29,Toxin_2 PF00451.20 0.00035,Toxin_2|PF00451.20|0.00013,Toxin_2|PF00451.20|0.00037,Gamma-thionin|PF00304.21|0.37,Gamma-thionin|PF00304.21|0.052,Gamma-thionin|PF00304.21|0.022,Defensin_2|PF01097.19|0.58,Defensin_2|PF01097.19|0.12,Defensin_2|PF01097.19|0.011,Toxin_38|PF14866.7|0.18,Toxin_38|PF14866.7|0.18,Toxin_38|PF14866.7|0.4 TRINITY_DN16091_c0_g1_i1:19-	0.468566639	-1.093673855	0.0001605	down	yes	1.118	2.386	2.342	2.524	2.292	1	1.171	1.183	------	------	------	------	------	------	------	------	PF00451.22;PF00	Toxin_2;Gamma-thionin;Defensin_2	Scorpion short toxin, BmKK2;Gamma-thionin family;Arthropod defensin	CYT	2	43	14.3	High
TRINITY_DN6497_c0_g1_i1_orf1	ommochrome-binding protein-like [Ostrinia furnacalis]	0.494859813	-1.014908207	0.0002036	down	yes	1.059	2.14	2.066	2.06	2.294	1	1.109	1.068	------	------	------	------	------	------	ENOG4110SZS;E	S:Function unknown;G:Carbohyd	------	------	------	CYT	2	15	17	High
TRINITY_DN29555_c0_g1_i8_orf1	fasciclin-1 [Ostrinia furnacalis]	0.38572	-1.374374142	1.26E-05	down	yes	0.9643	2.5	2.553	2.555	2.393	1	0.92	0.973	------	------	K19897	FAS1	------	------	COG2335	M:Cell wall/membrane/envelope	PF02469.25	Fasciclin	Fasciclin	CYT	9	15	76	High
TRINITY_DN15685_c0_g1_i5_orf1	uncharacterized protein LOC114352354 [Ostrinia furnacalis]	0.283985102	-1.816112846	2.37E-05	down	yes	0.915	3.222	3.22	3.382	3.063	1	0.905	0.84	------	------	------	------	------	------	ENOG4110T90;E	S:Function unknown;	PF06585.14	JHBP	Haemolymph juvenile hormone binding	CYT	3	14	29.1	High
TRINITY_DN26985_c0_g1_i5_orf1	secretory phospholipase A2 receptor-like [Helicoverpa zea]	0.416368515	-1.264067115	9.68E-06	down	yes	0.931	2.236	2.233	2.269	2.207	1	0.852	0.941	MF:molecular_function;MF:carbohydrate binding;MF:binding;	GO:0003(	------	------	------	------	ENOG410XPJ1;E	S:Function unknown;U:Intracellul	------	------	------	CYT	2	26	9.8	High
TRINITY_DN55147_c0_g1_i1_orfp1	TRINITY_DN55147_c0_g1_i1_m.59251 TRINITY_DN55147_c0_g1::TRINITY_DN55147_c0_g1_i1::g.59251 ORF type:5prime_partial len:331 (-),score=113.97,Cuticle_3 PF11018.9 0.29,Cuticle_3|PF11018.9|2.9e-05,Cuticle_3|PF11018.9|0.00037 TRINITY_DN55147_c0_g1_i1:21-1013(-)	0.369526522	-1.436250181	2.46E-05	down	yes	1.038	2.809	2.817	2.935	2.674	1	1.026	1.089	------	------	------	------	------	------	------	------	PF11018.11	Cuticle_3	Pupal cuticle protein C1	CYT	7	50	33.7	High
TRINITY_DN12526_c0_g1_i5_orf1	uncharacterized protein LOC114359035 isoform X3 [Ostrinia furnacalis]	0.454616805	-1.13727708	5.65E-05	down	yes	0.9847	2.166	2.156	2.093	2.248	1	0.895	1.059	BP:nitrogen compound metabolic process;BP:membrane lipid metabolic process;BP:organic substance metabolic process;BP:sphingolipid metabolic process;BP:lipid metabolic process;BP:cellular process;BP:biological_process;BP:metabolic process;BP:cellular lipid metabolic process;BP:primary metabolic process;BP:organonitrogen compound metabolic process;BP:cellular metabolic process;CC:lytic vacuole;CC:intracellular organelle;CC:membrane-bounded organelle;CC:organelle;CC:lysosome;CC:vacuole;CC:cellular anatomical entity;CC:intracellular membrane-bounded organelle;CC:cellular_component;CC:extr	GO:0006(	K12382	PSAP, SGP1	map00600;m	Sphingolipid metal	ENOG410XSI5	O:Posttranslational modification,	PF05184.18;PF03	SapB_1;SapB_2	Saposin-like type B, region 1;Saposin-like type B, region 2	CYT	2	37	15.2	High
TRINITY_DN2343_c1_g1_i2_orf1	receptor expression-enhancing protein 5-like isoform X1 [Ostrinia furnacalis] >XP_028170586.1 receptor expression-enhancing protein 5-like isoform X1 [Ostrinia furnacalis]	0.406036636	-1.300318189	1.22E-06	down	yes	0.9753	2.402	2.397	2.431	2.379	1	0.923	1.003	CC:cellular_component;CC:integral component of membrane;CC:cellular anatomical entity;CC:intrinsic component of membrane;	GO:0005(	K17279	REEP5_6	------	------	COG5052	U:Intracellular trafficking, secretic	PF03134.22	TB2_DP1_HVA22	TB2/DP1, HVA22 family	CYT	2	26	22.8	High
TRINITY_DN104663_c1_g1_i2_orf1	PREDICTED: gelsolin-like [Amyelois transitella]	0.447414549	-1.160315925	0.0002493	down	yes	1.021	2.282	2.302	2.097	2.448	1	1.027	1.036	MF:cytoskeletal protein binding;MF:actin filament binding;MF:actin binding;MF:macromolecular complex binding;MF:binding;MF:protein binding;MF:molecular_function;	GO:0008(	K05768	GSN	map04666;m	Fc gamma R-media	ENOG410XR0A	S:Function unknown	------	------	------	CYT	1	53	12.4	High
TRINITY_DN4802_c0_g1_i4_orf1	uncharacterized protein LOC114366345 isoform X2 [Ostrinia furnacalis]	0.326535542	-1.614688064	9.00E-06	down	yes	0.9463	2.898	2.81	2.864	3.02	1	0.921	0.918	BP:response to biotic stimulus;BP:response to external stimulus;BP:response to external biotic stimulus;BP:response to stimulus;BP:defense response;BP:response to stress;BP:biological_process;BP:interspecies interaction between organisms;BP:response to other organism;CC:cellular_component;CC:extr	GO:0009(	------	------	------	------	------	------	------	------	------	CYT	1	12	9.9	High
TRINITY_DN66453_c0_g1_i4_orfp1	TRINITY_DN66453_c0_g1_i4_m.7345 TRINITY_DN66453_c0_g1::TRINITY_DN66453_c0_g1_i4::g.7345 ORF type:internal len:93 (+),score=24.53 TRINITY_DN66453_c0_g1_i4:3-278(+)	0.443763676	-1.172136513	1.24E-06	down	yes	1.014	2.285	2.306	2.301	2.249	1	0.989	1.054	------	------	------	------	------	------	------	------	------	------	------	CYT	1	21	9.7	High
TRINITY_DN12231_c0_g1_i1_orf1	carbonyl reductase [NADPH] 3-like [Ostrinia furnacalis]	0.450892857	-1.149143439	3.76E-05	down	yes	1.01	2.24	2.36	2.165	2.196	1	0.995	1.035	------	------	K00079	CBR1	map00590;m	Arachidonic acid m	COG1028	K:Transcription	PF00106.28;PF13	adh_short;adh_short_C2;KR	short chain dehydrogenase;Enoyl;KR domain	CYT	10	39	32.2	High
TRINITY_DN8953_c0_g1_i4_orf1	gonadotropin-releasing hormone receptor [Ostrinia furnacalis] >AXF67446.1 adipokinetic hormone receptor 1 [Ostrinia furnacalis]	0.330399434	-1.597716878	0.00046	down	yes	0.9347	2.829	3.167	2.748	2.571	1	0.889	0.915	CC:integral component of membrane;CC:cellular_component;CC:cellular anatomical entity;CC:intrinsic component of membrane;MF:G-protein coupled receptor activity;MF:signaling receptor activity;MF:molecular transducer activity;MF:transmembrane signaling receptor	GO:0016(	K04280	GNRHR	map04080;m	Neuroactive ligand	ENOG410XRW9	U:Intracellular trafficking, secretic	PF00001.24	7tm_1	7 transmembrane receptor	CYT	2	6	45	High
TRINITY_DN812_c2_g1_i1_orf1	1,4-alpha-glucan-branching enzyme [Ostrinia furnacalis]	0.457466063	-1.128263372	2.79E-05	down	yes	1.011	2.21	2.309	2.119	2.202	1	1.002	1.031	BP:cellular biosynthetic process;BP:cellular macromolecule biosynthetic process;BP:organic substance biosynthetic process;BP:generation of precursor metabolites and energy;BP:cellular polysaccharide biosynthetic process;BP:cellular polysaccharide metabolic process;BP:cellular carbohydrate metabolic process;BP:cellular macromolecule metabolic process;BP:carbohydrate biosynthetic process;BP:organic substance metabolic process;BP:energy reserve metabolic process;BP:cellular glucan metabolic process;BP:glucan biosynthetic process;BP:cellular process;BP:polysaccharide biosynthetic process;BP:cellular carbohydrate biosynthetic process;BP:biosynthetic process;BP:macromolecule biosynthetic process;BP:biological_process;BP:metabolic process;BP:glucan metabolic process;BP:primary metabolic process;BP:carbohydrate metabolic process;BP:energy derivation by oxidation of organic compounds;BP:glycogen metabolic process;BP:polysaccharide metabolic	GO:0044(	K00700	GBE1, glgB	map00500	Starch and sucrose	COG0296	G:Carbohydrate transport and m	PF02806.21;PF00	Alpha-amylase_C;Alpha-amylase;CBM_48	Alpha amylase, C-terminal all-beta domain;Alpha amylase, catalytic domain;Carbohydrate-binding module 48	CYT	35	57	79.1	High
TRINITY_DN9560_c0_g1_i5_orf1	uncharacterized protein LOC114357350 [Ostrinia furnacalis]	0.47900323	-1.06189271	0.0001995	down	yes	1.038	2.167	2.071	2.312	2.118	1	0.985	1.128	------	------	------	------	------	------	ENOG410XV86;E	S:Function unknown;	------	------	------	CYT	1	11	27.1	High
TRINITY_DN13760_c1_g1_i1_orf1	pre-mRNA-processing factor 40 homolog A isoform X1 [Ostrinia furnacalis] >XP_028162665.1 pre-mRNA-processing factor 40 homolog A isoform X2 [Ostrinia furnacalis] >XP_028162667.1 pre-mRNA-processing factor 40 homolog A isoform X3 [Ostrinia furnacalis]	0.354764513	-1.495066392	0.0002373	down	yes	0.9717	2.739	2.939	2.626	2.652	1	1.129	0.786	BP:nucleic acid metabolic process;BP:cellular nitrogen compound metabolic process;BP:nitrogen compound metabolic process;BP:mRNA splicing, via spliceosome;BP:organic cyclic compound metabolic process;BP:nucleobase-containing compound metabolic process;BP:organic substance metabolic process;BP:RNA splicing;BP:mRNA metabolic process;BP:cellular process;BP:cellular aromatic compound metabolic process;BP:RNA splicing, via transesterification reactions;BP:RNA splicing, via transesterification reactions with bulged adenosine as nucleophile;BP:biological_process;BP:metabolic process;BP:heterocycle metabolic process;BP:RNA metabolic process;BP:primary metabolic process;BP:mRNA cis splicing, via spliceosome;BP:cellular metabolic process;BP:macromolecule metabolic process;BP:RNA processing;BP:mRNA processing;CC:membrane-bounded organelle;CC:organelle;CC:nucleus;CC:cellular anatomical entity;CC:intracellular membrane-bounded organelle;CC:intracellular	GO:0090(	K12821	PRPF40, PRP	map03040	Spliceosome	COG5104	A:RNA processing and modificati	PF01846.22;PF00	FF;WW;RhoGAP-FF1	FF domain;WW domain;p190-A and -B Rho GAPs FF domain	CYT	1	1	92.5	High
TRINITY_DN1048_c0_g1_i6_orf1	uncharacterized protein LOC114360661 [Ostrinia furnacalis]	0.403108581	-1.310759599	0.0002393	down	yes	0.9207	2.284	2.149	2.483	2.22	1	0.898	0.864	------	------	------	------	------	------	ENOG4111BN3;E	S:Function unknown;	PF15430.9	SVWC	Single domain von Willebrand factor type C	CYT	4	39	12.1	High
TRINITY_DN125521_c0_g2_i1_orf1	serpin transcript 1A2 [Ostrinia nubilalis]	0.447971781	-1.158520238	0.0003909	down	yes	1.016	2.268	2.444	2.245	2.116	1	0.916	1.131	------	------	------	------	------	------	------	------	------	------	------	CYT	1	35	8.1	High

TRINITY_DN1209_c0_g1_i9_orf1	NADP-dependent malic enzyme-like isoform X1 [Ostrinia furnacalis] >XP_028161889.1 NADP-dependent malic enzyme-like isoform X1 [Ostrinia furnacalis] >XP_028161891.1 NADP-dependent malic enzyme-like isoform X3 [Ostrinia furnacalis]	0.465581395	-1.102894686	8.62E-07	down	yes	1.001	2.15	2.175	2.155	2.12	1	0.974	1.028	MF:cation binding;MF:oxidoreductase activity, acting on CH-OH group of donors;MF:malate dehydrogenase activity;MF:oxidoreductase activity, acting on the CH-OH group of donors, NAD or NADP as acceptor;MF:ion binding;MF:NAD binding;MF:malate dehydrogenas;MF:malic enzyme activity;MF:nucleotide binding;MF:small molecule binding;MF:catalytic activity;MF:molecular_function;MF:binding;MF:organic cyclic compound binding;MF:metal ion binding;MF:heterocyclic compound binding;MF:nucleoside phosphate binding;MF:oxidoreductase activity;	GO:00431	K00029	E1.1.1.40, ma	map00620;m	Pyruvate metabolis	COG0281	S:Function unknown	PF03949.18;PF00	Malic_M;malic	Malic enzyme, NAD binding domain;Malic enzyme, N-terminal domain	CYT	25	41	70.7	High
TRINITY_DN13511_c0_g1_i4_orf1	alpha-2-macroglobulin receptor-associated protein isoform X1 [Ostrinia furnacalis]	0.47711826	-1.067581193	0.001353	down	yes	1.053	2.207	2.45	2.212	1.96	1	1.111	1.049	CC:cellular_component;CC:endoplasmic reticulum;CC:intracellular organelle;CC:membrane-bounded organelle;CC:organelle;CC:cellular anatomical entity;CC:intracellular membrane-bounded organelle;MF:sulfur compound binding;MF:carbohydrate derivative binding;MF:receptor regulator activity;MF:receptor inhibitor activity;MF:glycosaminoglycan binding;MF:heparin binding;MF:receptor antagonist activity;MF:molecular_function;MF:binding;MF:protein binding;MF:lipoprotein particle receptor binding;MF:low-density lipoprotein particle receptor binding;MF:receptor	GO:00055	K22290	LRPAP1	map04979	Cholesterol metab	ENOG410YJFP	S:Function unknown	PF06401.14;PF06	Alpha-2-MRAP_C;Alpha-2-MRAP_N	Alpha-2-macroglobulin RAP, C-terminal domain;Alpha-2-macroglobulin RAP, N-terminal domain	CYT	2	7	41.9	High
TRINITY_DN24789_c0_g1_i9_orfp1	TRINITY_DN24789_c0_g1_i9_m.25888 TRINITY_DN24789_c0_g1::TRINITY_DN24789_c0_g1_i9::g.25888 ORF type:internal len:114 (-),score=12.65 TRINITY_DN24789_c0_g1_i9:2-340(-)	0.174366099	-2.51980852	1.03E-05	down	yes	1.004	5.758	5.517	6.069	5.687	1	1.102	0.909	------	------	------	------	------	------	------	------	------	------	------	CYT	5	46	13.2	High
TRINITY_DN15327_c2_g1_i2_orf1	protein lethal(2)essential for life-like [Ostrinia furnacalis]	0.454545455	-1.137503524	5.09E-06	down	yes	1.005	2.211	2.215	2.268	2.151	1	1.032	0.984	MF:cation binding;MF:structural molecule activity;MF:structural constituent of eye lens;MF:ion binding;MF:molecular_function;MF:binding;MF:metal ion binding;	GO:00431	K09542	CRYAB	map04213;m	Longevity regulatin	ENOG410YERS	E:Amino acid transport and meta	PF00011.24;PF00	HSP20;Crystallin	Hsp20/alpha crystallin family;Alpha crystallin A chain, N terminal	CYT	7	44	22	High
TRINITY_DN29698_c0_g1_i3_orf1	15-hydroxyprostaglandin dehydrogenase [NAD(+)]-like [Ostrinia furnacalis]	0.467538023	-1.096844397	1.14E-05	down	yes	0.9837	2.104	2.18	2.043	2.088	1	0.964	0.987	------	------	K00069	HPGD	map00590;m	Arachidonic acid m	COG1028;COG0	S:Function unknown;	PF00106.28;PF13	adh_short;adh_short_C2;KR;Polysacc_synt_2	short chain dehydrogenase;Enoyl;KR domain;Polysaccharide biosynthesis protein	CYT	10	48	30.3	High
TRINITY_DN12009_c0_g1_i1_orf1	uncharacterized protein LOC114365631 [Ostrinia furnacalis]	0.297634609	-1.7483858	0.0002036	down	yes	0.9563	3.213	3.376	2.868	3.394	1	0.946	0.923	------	------	------	------	------	------	------	------	------	------	------	CYT	2	17	8.2	High
TRINITY_DN467_c3_g1_i5_orf1	lysozyme precursor [Loxostege sticticalis]	0.31527554	-1.66531485	2.42E-06	down	yes	0.9783	3.103	3.077	3.2	3.032	1	0.994	0.941	BP:metabolic process;BP:biological_process;MF:hydrolase activity;MF:catalytic activity;MF:hydrolase activity, acting on glycosyl bonds;MF:lysozyme activity;MF:molecular_function;MF:hydrolase activity, hydrolyzing O-glycosyl compounds;MF:peptidoglycan muralytic activity;	GO:00081	K13915	LYZ	map04970	Salivary secretion	ENOG4111QHM	G:Carbohydrate transport and m	PF00062.23	Lys	C-type lysozyme/alpha-lactalbumin family	CYT	7	52	16	High
TRINITY_DN4360_c0_g1_i4_orf1	glucose-6-phosphate isomerase-like, partial [Bicyclus anynana]	0.4835406	-1.048291066	0.002562	down	yes	1.322	2.734	2.772	2.941	2.49	1	1.537	1.43	BP:ribonucleoside diphosphate metabolic process;BP:phosphorylation;BP:purine nucleoside triphosphate metabolic process;BP:carboxylic acid metabolic process;BP:cellular nitrogen compound metabolic process;BP:nitrogen compound metabolic process;BP:small molecule biosynthetic process;BP:cellular aromatic compound metabolic process;BP:purine-containing compound metabolic process;BP:generation of precursor metabolites and energy;BP:pyruvate metabolic process;BP:glycolytic process;BP:nucleobase-containing compound metabolic process;BP:organic substance catabolic process;BP:purine nucleoside diphosphate metabolic process;BP:nucleotide phosphorylation;BP:ribonucleotide metabolic process;BP:organic substance metabolic process;BP:ribonucleoside triphosphate metabolic process;BP:organic cyclic compound metabolic process;BP:nucleoside triphosphate metabolic process;BP:gluconeogenesis;BP:small molecule metabolic process;BP:purine	GO:00091	K01810	GPI, pgi	map00520;m	Amino sugar and n	COG0166	G:Carbohydrate transport and m	PF00342.22	PGI	Phosphoglucose isomerase	CYT	1	60	4.9	High
TRINITY_DN38230_c0_g1_i4_orf1	hypothetical protein evm_007803 [Chilo suppressalis]	0.348790323	-1.519568083	0.004653	down	yes	1.038	2.976	3.151	2.326	3.452	1	1.127	0.986	BP:organophosphate catabolic process;BP:nucleotide catabolic process;BP:cellular nitrogen compound metabolic process;BP:nitrogen compound metabolic process;BP:small molecule metabolic process;BP:organic cyclic compound metabolic process;BP:organic cyclic compound catabolic process;BP:nucleobase-containing compound metabolic process;BP:organic substance catabolic process;BP:organic substance metabolic process;BP:cellular catabolic process;BP:cellular process;BP:cellular aromatic compound metabolic process;BP:heterocycle catabolic process;BP:aromatic compound catabolic process;BP:biological_process;BP:metabolic process;BP:nucleobase-containing compound catabolic process;BP:catabolic process;BP:nucleobase-containing small molecule metabolic process;BP:heterocycle metabolic process;BP:primary metabolic process;BP:cellular nitrogen compound catabolic process;BP:cellular metabolic process;BP:phosphate-containing	GO:0046	------	------	------	------	COG0737	F:Nucleotide transport and meta	PF02872.21	5_nucleotid_C	5'-nucleotidase, C-terminal domain	CYT	1	17	12.1	High
TRINITY_DN4069_c0_g1_i5_orf1	putative sulfiredoxin [Ostrinia furnacalis]	0.468284637	-1.094542386	4.65E-07	down	yes	0.9937	2.122	2.146	2.129	2.092	1	0.974	1.007	------	------	K12260	SRX1	------	------	COG5119	S:Function unknown	PF02195.21	ParBc	ParB/Sulfiredoxin domain	CYT	4	47	14.6	High
TRINITY_DN295_c2_g1_i2_orf1	phosphoglycolate phosphatase 1A, chloroplastic [Manduca sexta]	0.344200412	-1.538679271	1.96E-05	down	yes	1.003	2.914	3.068	2.883	2.791	1	1.002	1.008	MF:hydrolase activity;MF:molecular_function;MF:catalytic activity;	GO:00167	K21013	FPPP	map00981	Insect hormone bic	COG0647	G:Carbohydrate transport and m	PF13242.9	Hydrolase_like	HAD-hyrolase-like	PLA	4	28	13.9	High
TRINITY_DN12969_c0_g1_i3_orf1	queuosine salvage protein [Ostrinia furnacalis] >XP_028167327.1 queuosine salvage protein [Ostrinia furnacalis]	0.344483609	-1.537492754	7.05E-06	down	yes	0.9773	2.837	2.949	2.816	2.747	1	0.947	0.985	------	------	------	------	------	------	ENOG410XRIA	S:Function unknown	PF10343.12	Q_salvage	Potential Queuosine, Q, salvage protein family	CYT	5	17	38.6	High
TRINITY_DN2187_c0_g1_i1_orf1	flotillin-1 [Chelonus insularis] >XP_034947202.1 flotillin-1 [Chelonus insularis]	0.366398243	-1.448515509	1.20E-05	down	yes	1.001	2.732	2.719	2.851	2.626	1	1.003	1.001	------	------	K07192	FLOT	map04910	Insulin signaling pa	COG2268	S:Function unknown	PF01145.28;PF15	Band_7;Flot	SPFH domain / Band 7 family;Flotillin	CYT	3	10	46.9	High
TRINITY_DN38225_c0_g2_i1_orf1	uncharacterized protein LOC114354273 [Ostrinia furnacalis]	0.454387611	-1.138004594	4.89E-05	down	yes	0.9683	2.131	2.244	2.041	2.109	1	0.969	0.936	CC:cellular_component;CC:integral component of membrane;CC:cellular anatomical entity;CC:intrinsic component of membrane;	GO:00055	------	------	------	------	ENOG410ZPQS	S:Function unknown	------	------	------	CYT	2	4	46.6	High

TRINITY_DN95558_c0_g3_i1_orf1	cytochrome P450 monooxygenase CYP9G19 [Cnaphalocrocis medinalis]	0.413602214	-1.273684185	0.0002697	down	yes	1.046	2.529	2.653	2.637	2.298	1	1.01	1.128	CC:membrane;CC:endoplasmic reticulum membrane;CC:organelle membrane;CC:cellular_component;CC:cellular anatomical entity;MF:cation binding;MF:monooxygenase activity;MF:heme binding;MF:transition metal ion binding;MF:tetrapyrrole binding;MF:organic cyclic compound binding;MF:oxidoreductase activity, acting on paired donors, with incorporation or reduction of molecular oxygen;MF:ion binding;MF:catalytic activity;MF:molecular_function;MF:binding;MF:iron ion binding;MF:metal ion binding;MF:heterocyclic compound binding;MF:oxidoreductase activity;	GO:0016(	K15003	CYP9	------	------	COG2124	J:Translation, ribosomal structure	PF00067.25	p450	Cytochrome P450	CYT	1	16	8	High
TRINITY_DN1424_c0_g1_i5_orf1	insect group I lytic polysaccharide monooxygenase [Ostrinia furnacalis]	0.461684011	-1.115022324	6.38E-06	down	yes	0.976	2.114	2.169	2.066	2.107	1	0.934	0.994	------	------	------	------	------	------	ENOG410Y8ZQ	S:Function unknown	PF03067.18	LPMO_10	Lytic polysaccharide mono-oxygenase, cellulose-degrading	CYT	6	21	39	High
TRINITY_DN618_c0_g1_i3_orf1	triokinase/FMN cyclase-like isoform X1 [Ostrinia furnacalis]	0.492248062	-1.022542569	2.79E-05	down	yes	1.016	2.064	2.131	2.089	1.972	1	1.01	1.037	BP:phosphorylation;BP:polyol metabolic process;BP:small molecule metabolic process;BP:cellular carbohydrate metabolic process;BP:organic substance metabolic process;BP:alditol metabolic process;BP:glycerol metabolic process;BP:phosphorus metabolic process;BP:cellular process;BP:primary metabolic process;BP:biological_process;BP:metabolic process;BP:carbohydrate metabolic process;BP:cellular metabolic process;BP:alcohol metabolic process;BP:phosphate-containing compound metabolic process;BP:organic hydroxy compound metabolic process;MF:binding;MF:purine ribonucleoside triphosphate binding;MF:molecular_function;MF:ion binding;MF:heterocyclic compound binding;MF:nucleoside phosphate binding;MF:anion binding;MF:carbohydrate derivative binding;MF:lyase activity;MF:phosphorus-oxygen lyase activity;MF:nucleotide binding;MF:transferase activity;MF:ATP binding;MF:kinase activity;MF:small molecule binding;MF:catalytic	GO:0016:	K00863	DAK, TKFC	map04622;m	RIG-I-like receptor	COG2376	G:Carbohydrate transport and m	PF02733.20;PF02	Dak1;Dak2	Dak1 domain;DAK2 domain	CYT	12	25	62.6	High
TRINITY_DN15755_c0_g1_i1_orf1	cytochrome P450 monooxygenase CYP6AB141 [Ostrinia furnacalis]	0.428686219	-1.222006054	1.08E-05	down	yes	1.067	2.489	2.559	2.437	2.47	1	1.129	1.072	CC:integral component of membrane;CC:cellular_component;CC:cellular anatomical entity;CC:intrinsic component of membrane;MF:cation binding;MF:monooxygenase activity;MF:heme binding;MF:transition metal ion binding;MF:tetrapyrrole binding;MF:organic cyclic compound binding;MF:oxidoreductase activity, acting on paired donors, with incorporation or reduction of molecular oxygen;MF:ion binding;MF:catalytic activity;MF:molecular_function;MF:binding;MF:iron ion binding;MF:metal ion binding;MF:heterocyclic compound binding;MF:oxidoreductase activity;	GO:0016(	K14999	CYP6	------	------	COG2124	Q:Secondary metabolites biosynt	PF00067.25	p450	Cytochrome P450	CYT	3	28	27.4	High
TRINITY_DN11108_c0_g1_i4_orf1	peroxisomal leader peptide-processing protease [Ostrinia furnacalis] >XP_028165527.1 peroxisomal leader peptide-processing protease [Ostrinia furnacalis]	0.45855045	-1.124847626	0.0001777	down	yes	0.968	2.111	2.266	2.083	1.985	1	0.977	0.927	------	------	K23012	TYSND1	------	------	COG0265;ENOG	O:Posttranslational modification,	PF13365.9;PF000	Trypsin_2;Trypsin	Trypsin-like peptidase domain;Trypsin	CYT	6	17	58.1	High
TRINITY_DN41_c0_g1_i3_orf1	uncharacterized protein LOC114359035 isoform X3 [Ostrinia furnacalis]	0.390681998	-1.355933313	7.51E-06	down	yes	0.9853	2.522	2.522	2.608	2.436	1	0.958	0.998	BP:nitrogen compound metabolic process;BP:membrane lipid metabolic process;BP:organic substance metabolic process;BP:sphingolipid metabolic process;BP:lipid metabolic process;BP:cellular process;BP:biological_process;BP:metabolic process;BP:cellular lipid metabolic process;BP:primary metabolic process;BP:organonitrogen compound metabolic process;BP:cellular metabolic process;CC:lytic vacuole;CC:intracellular organelle;CC:membrane-bounded organelle;CC:organelle;CC:lysosome;CC:vacuole;CC:cellular anatomical entity;CC:intracellular membrane-bounded organelle;CC:cellular_component;CC:extr	GO:0006{	K12382	PSAP, SGP1	map00600;m	Sphingolipid metab	ENOG410XSI5	J:Translation, ribosomal structure	PF03489.20;PF05	SapB_2;SapB_1;SapA	Saposin-like type B, region 2;Saposin-like type B, region 1;Saposin A-type domain	CYT	40	46	125.9	High
TRINITY_DN7735_c0_g1_i4_orf1	calphotin-like [Ostrinia furnacalis]	0.485714286	-1.041820176	0.01473	down	yes	1.003	2.065	2.044	2.505	1.646	1	0.88	1.129	------	------	------	------	------	------	ENOG4110RAS;E	S:Function unknown;	------	------	------	CYT	1	3	22.5	High
TRINITY_DN136358_c0_g1_i1_orf1	acidic juvenile hormone-suppressible protein 1-like [Ostrinia furnacalis]	0.415147833	-1.268302927	8.79E-05	down	yes	1.025	2.469	2.347	2.642	2.419	1	1.026	1.049	------	------	------	------	------	------	ENOG410XR2D	S:Function unknown	PF03723.17	Hemocyanin_C	Hemocyanin, ig-like domain	CYT	2	55	9.7	High
TRINITY_DN12013_c0_g1_i6_orf1	tudor domain-containing protein 7 isoform X3 [Ostrinia furnacalis]	0.462579281	-1.112227445	0.000182	down	yes	1.094	2.365	2.337	2.253	2.504	1	1.206	1.076	CC:cytoplasm;CC:cellular_component;CC:cellular anatomical entity;	GO:0005;	K18405	TDRD1_4_6_7	------	------	ENOG41104YI;C(	S:Function unknown;	PF00567.27	TUDOR	Tudor domain	CYT	1	7	33.4	High
TRINITY_DN4235_c0_g1_i2_orf1	uncharacterized protein LOC114361536 [Ostrinia furnacalis]	0.430877483	-1.214650386	6.37E-05	down	yes	1.041	2.416	2.565	2.317	2.365	1	1.053	1.071	CC:cellular_component;CC:cellular anatomical entity;CC:extracellular space;MF:endopeptidase inhibitor activity;MF:endopeptidase regulator activity;MF:peptidase regulator activity;MF:molecular_function;MF:peptidase inhibitor activity;MF:serine-type endopeptidase inhibitor activity;MF:enzyme inhibitor activity;MF:molecular function regulator;MF:enzyme regulator activity;	GO:0005!	------	------	------	------	COG4826	P:Inorganic ion transport and me	PF00079.23	Serpin	Serpin	CYT	5	16	43.7	High
TRINITY_DN3712_c0_g1_i1_orf1	ribonuclease Oy [Ostrinia furnacalis]	0.453737864	-1.140069038	0.0002078	down	yes	0.9347	2.06	2.193	1.961	2.027	1	0.975	0.829	MF:ribonuclease T2 activity;MF:hydrolase activity;MF:catalytic activity, acting on RNA;MF:nuclease activity;MF:endoribonuclease activity;MF:lyase activity;MF:ribonuclease activity;MF:phosphorus-oxygen lyase activity;MF:hydrolase activity, acting on ester bonds;MF:catalytic activity;MF:molecular_function;MF:RNA binding;MF:nucleic acid binding;MF:endonuclease activity;MF:organic cyclic compound	GO:0033{	K01166	RNASET2	------	------	ENOG4111M7G	S:Function unknown	PF00445.21	Ribonuclease_T2	Ribonuclease T2 family	PLA	3	15	31.5	High
TRINITY_DN8095_c0_g1_i3_orf1	circadian clock-controlled protein-like [Ostrinia furnacalis]	0.470350404	-1.08819215	4.80E-06	down	yes	1.047	2.226	2.235	2.198	2.245	1	1.109	1.033	------	------	------	------	------	------	ENOG41114TE;E	S:Function unknown;	PF06585.14	JHBP	Haemolymph juvenile hormone binding	CYT	4	18	27.1	High
TRINITY_DN285_c0_g1_i4_orf1	catalase-like [Ostrinia furnacalis]	0.445118613	-1.167738265	9.45E-06	down	yes	0.9757	2.192	2.223	2.165	2.187	1	0.898	1.029	BP:cellular catabolic process;BP:hydrogen peroxide catabolic process;BP:cellular process;BP:reactive oxygen species metabolic process;BP:response to stress;BP:biological_process;BP:metabolic process;BP:catabolic process;BP:response to oxidative stress;BP:response to stimulus;BP:cellular metabolic process;BP:hydrogen peroxide metabolic process;MF:molecular_function;MF:binding;MF:heterocyclic compound binding;MF:oxidoreductase activity;MF:cation binding;MF:ion binding;MF:metal ion binding;MF:heme binding;MF:antioxidant activity;MF:catalytic activity;MF:oxidoreductase activity, acting on peroxide as acceptor;MF:tetrapyrrole	GO:0044;	K03781	katE, CAT, ca	map00630;m	Glyoxylate and dica	COG0753	P:Inorganic ion transport and me	PF00199.22;PF06	Catalase;Catalase-rel	Catalase;Catalase-related immune-responsive	CYT	9	23	58.6	High

TRINITY_DN8037_c0_g2_i1_orf1	2-oxo-4-hydroxy-4-carboxy-5-ureidoimidazoline decarboxylase-like [Ostrinia furnacalis]	0.45877193	-1.124150973	1.22E-05	down	yes	0.9937	2.166	2.212	2.2	2.087	1	0.957	1.024	BP:cellular catabolic process;BP:cellular nitrogen compound metabolic process;BP:nitrogen compound metabolic process;BP:small molecule metabolic process;BP:small molecule catabolic process;BP:purine-containing compound metabolic process;BP:purine-containing compound catabolic process;BP:organic cyclic compound metabolic process;BP:organic cyclic compound catabolic process;BP:nucleobase-containing compound metabolic process;BP:organic substance catabolic process;BP:urate catabolic process;BP:organic substance metabolic process;BP:allantoin metabolic process;BP:cellular process;BP:cellular aromatic compound metabolic process;BP:heterocycle catabolic process;BP:biological_process;BP:metabolic process;BP:catabolic process;BP:nucleobase-containing small molecule metabolic process;BP:heterocycle metabolic process;BP:primary metabolic process;BP:organonitrogen compound metabolic process;BP:cellular nitrogen compound catabolic process;BP:cellular	GO:00442	K13485	PRHOXNB, U	map00230	Purine metabolism	COG3195	S:Function unknown	PF09349.13	OHCU_decarbox	OHCU decarboxylase	CYT	4	24	19.9	High
TRINITY_DN3616_c0_g1_i4_orf1	conotoxin ArMKLT2-032-like [Ostrinia furnacalis]	0.422793449	-1.241975073	0.0002301	down	yes	0.9293	2.198	2.349	2.202	2.043	1	0.952	0.836	CC:cellular_component;CC:integral component of membrane;CC:cellular anatomical entity;CC:intrinsic component of membrane;	GO:00055	------	------	------	------	------	------	------	------	------	CYT	1	68	6.1	High
TRINITY_DN1466_c0_g1_i4_orf1	insecticyanin-A-like [Ostrinia furnacalis]	0.498556304	-1.004171651	9.10E-06	down	yes	1.036	2.078	2.106	2.018	2.111	1	1.035	1.072	MF:pigment binding;MF:molecular_function;MF:binding;	GO:00314	K03098	APOD	------	------	ENOG4110R73;E	S:Function unknown;M:Cell wall/	PF00061.26	Lipocalin	Lipocalin / cytosolic fatty-acid binding protein family	CYT	5	23	25.4	High
TRINITY_DN1175_c1_g1_i2_orf1	methanethiol oxidase [Ostrinia furnacalis]	0.483454282	-1.048548629	2.94E-07	down	yes	0.9993	2.067	2.053	2.098	2.049	1	1.003	0.995	MF:selenium binding;MF:molecular_function;MF:binding;	GO:00084	K17285	SELENBP1	map00920	Sulfur metabolism	ENOG410XP4F	S:Function unknown	PF05694.14	SBP56	56kDa selenium binding	CYT	10	45	53.7	High
TRINITY_DN30510_c0_g1_i6_orf1	spodomicin-like [Ostrinia furnacalis]	0.110557318	-3.177133577	6.58E-06	down	yes	0.976	8.828	8.65	9.33	8.505	1	0.961	0.967	------	------	------	------	------	------	------	------	PF08036.14	Antimicrobial_6	Diapausin family of antimicrobial	CYT	3	60	6.7	High
TRINITY_DN35809_c0_g1_i1_orf1	spodomicin-like [Ostrinia furnacalis] >QKV49445.1 diapausin [Ostrinia furnacalis]	0.117768344	-3.085976293	1.15E-05	down	yes	0.971	8.245	8.035	8.781	7.918	1	0.978	0.935	------	------	------	------	------	------	------	------	PF08036.14	Antimicrobial_6	Diapausin family of antimicrobial	CYT	3	60	6.8	High
TRINITY_DN34406_c0_g2_i9_orfp1	TRINITY_DN34406_c0_g2_i9_m.33755 TRINITY_DN34406_c0_g2::TRINITY_DN34406_c0_g2_i9::g.33755 ORF type:internal len:82 (-),score=12.88 TRINITY_DN34406_c0_g2_i9:3-245(-)	0.100466503	-3.315213536	0.0002441	down	yes	0.7753	7.717	7.68	8.685	6.786	1	0.702	0.624	------	------	------	------	------	------	------	------	------	------	------	CYT	1	11	9.3	High
TRINITY_DN47784_c0_g2_i1_orfp1	arylphorin subunit alpha-like [Ostrinia furnacalis]	0.151855047	-2.719233232	1.97E-06	down	yes	1.056	6.954	6.794	7.224	6.844	1	1.037	1.131	------	------	------	------	------	------	------	------	------	------	------	CYT	2	27	10.1	High
TRINITY_DN86772_c0_g1_i3_orfp1	x-tox [Spodoptera exigua]	0.137652391	-2.860898421	2.82E-07	down	yes	0.8807	6.398	6.342	6.479	6.373	1	0.884	0.758	BP:response to biotic stimulus;BP:response to external stimulus;BP:response to external biotic stimulus;BP:response to stimulus;BP:defense response;BP:response to stress;BP:biological_process;BP:interspecies interaction between organisms;BP:response to other organism;CC:cellular_component;CC:extr	GO:00096	------	------	------	------	------	------	------	------	------	CYT	1	9	8.5	High
TRINITY_DN12534_c0_g1_i4_orf1	antibacterial protein [Heliothis virescens]	0.197596533	-2.339370463	5.68E-06	down	yes	1.003	5.076	5.086	5.289	4.852	1	1.032	0.978	BP:response to biotic stimulus;BP:response to external stimulus;BP:response to external biotic stimulus;BP:response to stimulus;BP:defense response;BP:response to stress;BP:biological_process;BP:interspecies interaction between organisms;BP:response to other organism;CC:cellular_component;CC:extr	GO:00096	------	------	------	------	ENOG4110S6N	S:Function unknown	PF00451.22	Toxin_2	Scorpion short toxin, BmKK2	CYT	5	57	12	High
TRINITY_DN129863_c0_g1_i1_orf1	protein PFC0760c-like isoform X2 [Ostrinia furnacalis]	0.228027452	-2.132720576	0.0004332	down	yes	1.03	4.517	4.378	5.136	4.038	1	0.995	1.094	------	------	------	------	------	------	------	------	------	------	------	CYT	1	21	19.3	High
TRINITY_DN6330_c0_g1_i1_orfp1	TRINITY_DN6330_c0_g1_i1_m.42332 TRINITY_DN6330_c0_g1::TRINITY_DN6330_c0_g1_i1::g.42332 ORF type:3prime_partial len:51 (-),score=33.30 TRINITY_DN6330_c0_g1_i1:3-152(-)	0.165105054	-2.598543812	2.26E-05	down	yes	1.163	7.044	6.649	7.491	6.992	1	1.304	1.186	------	------	------	------	------	------	------	------	------	------	------	CYT	1	26	5.3	High
TRINITY_DN6025_c0_g2_i1_orfp1	TRINITY_DN6025_c0_g2_i1_m.7749 TRINITY_DN6025_c0_g2::TRINITY_DN6025_c0_g2_i1::g.7749 ORF type:5prime_partial len:106 (+),score=47.10 TRINITY_DN6025_c0_g2_i1:3-320(+)	0.185815909	-2.428054072	2.73E-05	down	yes	0.862	4.639	4.397	4.786	4.733	1	0.611	0.975	------	------	------	------	------	------	------	------	------	------	------	PLA	1	10	11.6	Medium
TRINITY_DN75086_c0_g1_i5_orf1	lysosome membrane protein 2-like [Ostrinia furnacalis]	0.240841215	-2.053845797	0.0001875	down	yes	0.9677	4.018	4.113	3.581	4.359	1	0.959	0.944	BP:multicellular organismal process;BP:sensory perception of smell;BP:neurological system process;BP:response to stimulus;BP:sensory perception of chemical stimulus;BP:sensory perception;BP:biological_process;BP:system process;CC:membrane;CC:cellular_component;CC:cellular anatomical entity;CC:plasma membrane;CC:integral component of membrane;CC:intrinsic	GO:00325	K13885	SCARB1	map04979;m	Cholesterol metab	ENOG410XS17	U:Intracellular trafficking, secretic	------	------	------	CYT	1	11	7.7	High
TRINITY_DN2044_c0_g1_i5_orfp1	TRINITY_DN2044_c0_g1_i5_m.4210 TRINITY_DN2044_c0_g1::TRINITY_DN2044_c0_g1_i5::g.4210 ORF type:complete len:151 (-),score=85.31 TRINITY_DN2044_c0_g1_i5:857-1309(-)	0.167196729	-2.580381474	0.0002079	down	yes	1.104	6.603	5.83	6.958	7.022	1	1.451	0.861	------	------	------	------	------	------	------	------	------	------	------	CYT	1	5	16.2	High
TRINITY_DN15545_c0_g1_i1_orf1	larval cuticle protein LCP-14-like [Ostrinia furnacalis]	0.286307922	-1.804360504	2.27E-05	down	yes	0.9577	3.345	3.184	3.538	3.313	1	0.934	0.939	MF:molecular_function;MF:structural constituent of cuticle;MF:structural molecule activity;	GO:00036	------	------	------	------	ENOG4110RUZ;E	S:Function unknown;	PF00379.26	Chitin_bind_4	Insect cuticle protein	EXC	3	48	14.6	High
TRINITY_DN67193_c0_g1_i1_orf1	A-kinase anchor protein 14-like [Ostrinia furnacalis]	0.352199224	-1.505536365	0.01804	down	yes	1.089	3.092	3.902	2.157	3.216	1	0.973	1.294	------	------	------	------	------	------	------	------	------	------	------	CYT	1	6	15.2	High
TRINITY_DN609_c0_g1_i1_orf1	zonadhesin-like isoform X1 [Ostrinia furnacalis]	0.191432634	-2.385091303	4.85E-06	down	yes	1.077	5.626	5.377	5.681	5.819	1	1.135	1.096	------	------	------	------	------	------	COG4826;ENOG	O:Posttranslational modification,	PF01826.20	TIL	Trypsin Inhibitor like cysteine rich	PLA	9	18	109	High
TRINITY_DN48548_c0_g1_i1_orf1	glutathione S-transferase sigma 2 [Heortia vitessoides]	0.309528362	-1.691856486	3.07E-06	down	yes	0.9713	3.138	3.205	3.182	3.028	1	0.942	0.972	MF:catalytic activity;MF:transferase activity;MF:glutathione transferase activity;MF:molecular_function;MF:transferase activity, transferring alkyl or ary;	GO:00038	K04097	HPGDS	map00590;m	Arachidonic acid m	ENOG41103AU;E	O:Posttranslational modification,	PF14497.9;PF027	GST_C_3;GST_N;GST_C	Glutathione S-transferase, C-terminal domain;Glutathione S-transferase, N-terminal domain;Glutathione S-transferase, C-terminal	CYT	18	60	23.2	High
TRINITY_DN42719_c0_g2_i1_orf1	inter-alpha-trypsin inhibitor heavy chain H4-like isoform X11 [Ostrinia furnacalis]	0.303941026	-1.71813667	0.0002372	down	yes	1.072	3.527	3.828	3.576	3.176	1	1.043	1.172	------	------	------	------	------	------	COG2304	S:Function unknown	------	------	------	CYT	1	12	12.1	High

TRINITY_DN16905_c0_g1_i1_orf1	unnamed protein product [Leptidea sinapis]	0.407735983	-1.294292814	0.003487	down	yes	1.149	2.818	3.187	2.875	2.392	1	1.428	1.018	BP:phosphorylation;BP:nitrogen compound metabolic process;BP:organic substance metabolic process;BP:phosphate-containing compound metabolic process;BP:protein phosphorylation;BP:cellular process;BP:macromolecule modification;BP:protein modification process;BP:biological_process;BP:metabolic process;BP:primary metabolic process;BP:organonitrogen compound metabolic process;BP:cellular metabolic process;BP:macromolecule metabolic process;BP:protein metabolic process;BP:phosphorus metabolic process;CC:intrinsic component of membrane;CC:integral component of membrane;CC:cellular anatomical entity;CC:cellular_component;MF:signaling receptor activity;MF:purine ribonucleoside triphosphate binding;MF:molecular transducer activity;MF:molecular_function;MF:binding;MF:nucleotide binding;MF:heterocyclic compound binding;MF:anion binding;MF:cation binding;MF:organic cyclic compound binding;MF:ion binding;MF:transmembrane receptor	GO:0016?	K13578	BMPR1B, ALK	map04390;m	Hippo signaling pa	ENOG410XQT0	T:Signal transduction mechanism	PF00069.28;PF07	Pkinase;PK_Tyr_Ser-Thr;TGF_beta_GS	Protein kinase domain;Protein tyrosine and serine/threonine kinase;Transforming growth factor beta type I GS-motif	CYT	1	2	40.3	High
TRINITY_DN5080_c0_g1_i1_orf1	basic juvenile hormone-suppressible protein 2-like [Ostrinia furnacalis]	0.286934244	-1.801207938	2.00E-05	down	yes	1.008	3.513	3.727	3.392	3.421	1	1.007	1.018	------	------	------	------	------	------	ENOG410XR2D	S:Function unknown	PF00372.22;PF03	Hemocyanin_M;Hemocyanin_C;Hemocyanin_N	Hemocyanin, copper containing domain;Hemocyanin, ig-like domain;Hemocyanin, all-alpha domain	CYT	46	75	89.5	High
TRINITY_DN184_c0_g1_i1_orf1	macrophage mannose receptor 1-like isoform X1 [Maniola jurtina]	0.336698177	-1.570472185	5.31E-06	down	yes	0.9973	2.962	3.042	2.844	3.001	1	0.995	0.997	------	------	------	------	------	------	ENOG410XRS3;E	U:Intracellular trafficking, secretic	PF00059.24	Lectin_C	Lectin C-type domain	CYT	1	14	35.7	High
TRINITY_DN214_c0_g1_i4_orf1	uncharacterized protein LOC114352813 [Ostrinia furnacalis]	0.293881335	-1.766694362	4.95E-07	down	yes	0.951	3.236	3.272	3.236	3.2	1	0.889	0.964	------	------	------	------	------	------	ENOG4110S0N;E	S:Function unknown;	PF06585.14	JHBP	Haemolymph juvenile hormone binding	CYT	4	31	29.6	High
TRINITY_DN628_c0_g1_i1_orf1	prostamide/prostaglandin F synthase-like [Ostrinia furnacalis]	0.337377492	-1.567564365	7.33E-06	down	yes	0.9983	2.959	3.058	2.984	2.836	1	0.993	1.002	------	------	K15717	PRXL2B, FAM	map00590	Arachidonic acid m	ENOG410YFY9	O:Posttranslational modification,	PF13911.9	AhpC-TSA_2	AhpC/TSA antioxidant enzyme	CYT	9	46	26.8	High
TRINITY_DN18338_c0_g1_i6_orf1	aquaporin AQPAn.G isoform X1 [Ostrinia furnacalis]	0.371201158	-1.429726885	0.01084	down	yes	1.026	2.764	2.841	2.064	3.387	1	1.113	0.965	CC:integral component of membrane;CC:cellular_component;CC:intrinsic component of membrane;CC:cellular anatomical entity;MF:transporter activity;MF:transmembrane transporter activity;MF:channel activity;MF:molecular_function;MF:passiv	GO:0016(	K09884	AQPN	------	------	COG0580	O:Posttranslational modification,	PF00230.23	MIP	Major intrinsic protein	CYT	1	8	29	High
TRINITY_DN1540_c0_g1_i7_orf1	alaserpin-like isoform X13 [Ostrinia furnacalis]	0.373280232	-1.421668987	4.90E-05	down	yes	1.031	2.762	2.638	2.933	2.715	1	1.089	1.005	CC:cellular_component;CC:cellular anatomical entity;CC:extracellular space;MF:endopeptidase inhibitor activity;MF:endopeptidase regulator activity;MF:peptidase regulator activity;MF:molecular_function;MF:peptidase inhibitor activity;MF:serine-type endopeptidase inhibitor activity;MF:enzyme inhibitor activity;MF:molecular function regulator;MF:enzyme regulator activity;	GO:0005!	------	------	------	------	COG4826	O:Posttranslational modification,	PF00079.23	Serpin	Serpin	CYT	1	35	42.9	High
TRINITY_DN40126_c0_g2_i1_orf1	aldehyde dehydrogenase X, mitochondrial-like [Ostrinia furnacalis]	0.447154472	-1.161154792	0.0003189	down	yes	1.155	2.583	2.472	2.763	2.515	1	1.288	1.176	MF:catalytic activity;MF:oxidoreductase activity, acting on the aldehyde or oxo group of donors;MF:molecular_function;MF:oxidoreductase activity, acting on the aldehyde or oxo group of donors, NAD or NADP as acceptor;MF:oxidoreductase	GO:0003{	K00128	ALDH	map00310;m	Lysine degradation	COG1012	C:Energy production and conver	PF00171.25	Aldedh	Aldehyde dehydrogenase family	CYT	3	62	15.4	High
TRINITY_DN1201_c0_g1_i4_orf1	triosephosphate isomerase [Ostrinia furnacalis] >XP_028170843.1 triosephosphate isomerase [Ostrinia furnacalis]	0.368727273	-1.439373966	1.96E-07	down	yes	1.014	2.75	2.784	2.757	2.708	1	1.019	1.023	BP:ribonucleoside diphosphate metabolic process;BP:phosphorylation;BP:purine nucleoside triphosphate metabolic process;BP:carboxylic acid metabolic process;BP:cellular nitrogen compound metabolic process;BP:nitrogen compound metabolic process;BP:small molecule metabolic process;BP:small molecule biosynthetic process;BP:cellular aromatic compound metabolic process;BP:purine-containing compound metabolic process;BP:generation of precursor metabolites and energy;BP:pyruvate metabolic process;BP:glycolytic process;BP:organic cyclic compound metabolic process;BP:nucleobase-containing compound metabolic process;BP:organic substance catabolic process;BP:purine nucleoside diphosphate metabolic process;BP:nucleotide phosphorylation;BP:ribonucleotide metabolic process;BP:organic substance metabolic process;BP:ribonucleoside triphosphate metabolic process;BP:nucleoside triphosphate metabolic	GO:0009	K01803	TPI, tpiA	map00010;m	Glycolysis / Glucon	COG0149	G:Carbohydrate transport and m	PF00121.21	TIM	Triosephosphate isomerase	CYT	16	71	26.4	High
TRINITY_DN31286_c0_g1_i6_orfp1	TRINITY_DN31286_c0_g1_i6_m.28438 TRINITY_DN31286_c0_g1::TRINITY_DN31286_c0_g1_i6::g.28438 ORF type:internal len:92 (-),score=3.10,Perilipin PF03036.17 2e-05 TRINITY_DN31286_c0_g1_i6:1-273(-)	0.38475976	-1.377970173	3.33E-06	down	yes	1.025	2.664	2.589	2.738	2.666	1	1.037	1.038	------	------	------	------	------	------	------	------	PF03036.19	Perilipin	Perilipin family	CYT	5	71	9.4	High
TRINITY_DN19662_c4_g1_i1_orf1	basic juvenile hormone-suppressible protein 1-like [Ostrinia furnacalis]	0.367302053	-1.444961136	1.42E-05	down	yes	1.002	2.728	2.829	2.756	2.599	1	0.999	1.007	CC:cellular_component;CC:membrane;CC:extracellular region;CC:cellular anatomical entity;MF:signaling receptor activity;MF:catalytic activity;MF:phosphotransferase activity, alcohol group as acceptor;MF:transferase activity, transferring phosphorus-containing groups;MF:kinase activity;MF:catalytic activity, acting on a protein;MF:transmembrane signaling receptor activity;MF:transmembrane receptor protein serine/threonine kinase activity;MF:protein serine/threonine kinase activity;MF:transferase activity;MF:molecular_function;MF:protein kinase activity;MF:transmembrane receptor protein kinase	GO:0005!	------	------	------	------	ENOG410XR2D	S:Function unknown	PF03723.17	Hemocyanin_C	Hemocyanin, ig-like domain	CYT	31	82	30.7	High
TRINITY_DN1108_c1_g2_i1_orfp1	TRINITY_DN1108_c1_g2_i1_m.5565 TRINITY_DN1108_c1_g2::TRINITY_DN1108_c1_g2_i1::g.5565 ORF type:internal len:205 (-),score=147.90 TRINITY_DN1108_c1_g2_i1:2-613(-)	0.427901524	-1.224649278	8.52E-05	down	yes	1.095	2.559	2.445	2.655	2.577	1	1.223	1.061	------	------	------	------	------	------	------	------	------	------	------	PLA	2	11	22.5	High
TRINITY_DN5408_c0_g1_i5_orf1	uncharacterized protein LOC114359912 [Ostrinia furnacalis]	0.414836303	-1.269385941	5.41E-05	down	yes	1.001	2.413	2.448	2.305	2.486	1	0.907	1.097	CC:cellular_component;CC:integral component of membrane;CC:cellular anatomical entity;CC:intrinsic component of membrane;	GO:0005!	------	------	------	------	ENOG4110RME	S:Function unknown	------	------	------	PLA	1	5	30.4	High
TRINITY_DN52316_c0_g1_i1_orf1	arylphorin subunit alpha-like [Ostrinia furnacalis]	0.377309008	-1.406181551	0.001337	down	yes	0.9927	2.631	3.005	2.299	2.589	1	0.967	1.011	------	------	------	------	------	------	ENOG410XR2D	J:Translation, ribosomal structure	PF03722.17	Hemocyanin_N	Hemocyanin, all-alpha domain	PLA	4	55	13.9	High
TRINITY_DN11060_c0_g1_i6_orf1	extracellular matrix protein A-like isoform X3 [Ostrinia furnacalis]	0.375958048	-1.411356411	0.0001027	down	yes	0.932	2.479	2.498	2.628	2.312	1	0.86	0.936	------	------	K19720	COL3A	map05146;m	Amoebiasis;Protein	ENOG4110SA3	S:Function unknown	------	------	------	PLA	3	4	105.7	High
TRINITY_DN5274_c0_g2_i2_orf1	lopap-like [Ostrinia furnacalis]	0.406779661	-1.297680549	2.03E-05	down	yes	1.008	2.478	2.518	2.556	2.359	1	0.98	1.044	------	------	K03098	APOD	------	------	ENOG4110TA7;E	S:Function unknown;	PF00061.26	Lipocalin	Lipocalin / cytosolic fatty-acid binding protein family	CYT	2	14	24.4	High
TRINITY_DN97883_c0_g1_i2_orf1	talin-2-like, partial [Ostrinia furnacalis]	0.437367304	-1.193082722	0.0003008	down	yes	1.03	2.355	2.248	2.536	2.282	1	0.932	1.158	------	------	K06271	TLN	map05166;m	Human T-cell leuke	ENOG410XQ0V	S:Function unknown	------	------	------	CYT	1	10	10.1	High
TRINITY_DN51995_c0_g3_i1_orf1	circadian clock-controlled protein-like [Ostrinia furnacalis]	0.440275981	-1.183519954	0.0002344	down	yes	1.021	2.319	2.467	2.121	2.37	1	1.02	1.042	------	------	------	------	------	------	ENOG4111G78	S:Function unknown	PF06585.14	JHBP	Haemolymph juvenile hormone binding	CYT	3	10	29.2	High

TRINITY_DN52761_c0_g1_i2_orf1	atlastin isoform X4 [Ostrinia furnacalis]	0.436648983	-1.195454114	3.67E-05	down	yes	0.9877	2.262	2.325	2.135	2.325	1	0.982	0.981	CC:integral component of membrane;CC:intrinsic component of membrane;CC:cellular anatomical entity;CC:cellular_component;MF:purine ribonucleoside triphosphate binding;MF:molecular_function;MF:binding;MF:ion binding;MF:heterocyclic compound binding;MF:nucleoside phosphate binding;MF:anion binding;MF:guanyl nucleotide binding;MF:nucleotide binding;MF:guanyl ribonucleotide binding;MF:purine nucleotide binding;MF:GTP binding;MF:hydrolase activity;MF:GTPase activity;MF:nucleoside-triphosphatase activity;MF:small molecule binding;MF:catalytic activity;MF:hydrolase activity, acting on acid anhydrides, in phosphorus-containing anhydrides;MF:carbohydrate derivative binding;MF:organic cyclic compound binding;MF:hydrolase activity, acting on acid anhydrides;MF:pyrophosphatase	GO:0016(	K17339	ATL	------	------	ENOG410XR6Z	S:Function unknown	PF02263.22	GBP	Guanylate-binding protein, N-terminal domain	CYT	2	31	12.4	High
TRINITY_DN14460_c0_g1_i6_orf1	scavenger receptor class B member 1-like [Ostrinia furnacalis]	0.499061914	-1.002709287	3.77E-05	down	yes	1.064	2.132	2.208	2.089	2.099	1	1.061	1.131	BP:multicellular organismal process;BP:sensory perception of smell;BP:neurological system process;BP:response to stimulus;BP:sensory perception of chemical stimulus;BP:sensory perception;BP:biological_process;BP:syst em process;CC:membrane;CC:cellular_component;CC:cellular anatomical entity;CC:plasma membrane;CC:integral component of membrane;CC:intrinsic	GO:0032!	K13885	SCARB1	map04979;m	Cholesterol metabo	ENOG410XS17	C:Energy production and convers	PF01130.24	CD36	CD36 family	CYT	3	6	62.9	High
TRINITY_DN4062_c0_g2_i1_orf1	venom peptide BmKAPI-like isoform X2 [Ostrinia furnacalis]	0.423932384	-1.238093916	1.07E-05	down	yes	0.953	2.248	2.174	2.299	2.272	1	0.957	0.902	------	------	------	------	------	------	COG4826;ENOG	O:Posttranslational modification,	PF01826.20	TIL	Trypsin Inhibitor like cysteine rich	CYT	1	9	10.4	High
TRINITY_DN31225_c0_g1_i1_orf1	ribosome biogenesis protein BMS1 homolog [Ostrinia furnacalis]	0.458048104	-1.126428979	5.41E-06	down	yes	0.9903	2.162	2.208	2.167	2.111	1	1.024	0.947	BP:ribosome biogenesis;BP:cellular component organization or biogenesis;BP:ribonucleoprotein complex biogenesis;BP:cellular process;BP:biological_process;BP:cellular component biogenesis;CC:intracellular organelle;CC:non-membrane-bounded organelle;CC:organelle;CC:nucleolus;CC:cellular anatomical entity;CC:intracellular non-membrane-bounded organelle;CC:cellular_component;MF:purine ribonucleoside triphosphate binding;MF:heterocyclic compound binding;MF:molecular_function;MF:binding;MF:ion binding;MF:nucleoside phosphate binding;MF:anion binding;MF:nucleotide binding;MF:guanyl ribonucleotide binding;MF:purine nucleotide binding;MF:GTP binding;MF:small molecule binding;MF:carbohydrate derivative binding;MF:organic cyclic compound binding;MF:guanyl	GO:0042	K14569	BMS1	map03008	Ribosome biogenes	COG5192	J:Translation, ribosomal structure	PF04950.15;PF08	RIBIOP_C;AARP2CN	40S ribosome biogenesis protein Tsr1 and BMS1 C-terminal;AARP2CN	CYT	2	2	124.6	High
TRINITY_DN1592_c0_g1_i1_orf1	serine protease 7-like isoform X2 [Ostrinia furnacalis]	0.482709616	-1.050772527	2.15E-05	down	yes	1.019	2.111	2.076	2.203	2.054	1	1.021	1.037	BP:organic substance metabolic process;BP:organonitrogen compound metabolic process;BP:protein metabolic process;BP:nitrogen compound metabolic process;BP:macromolecule metabolic process;BP:biological_process;BP:metabolic process;BP:proteolysis;BP:primary metabolic process;CC:cellular_component;CC:extracellular region;CC:cellular anatomical entity;MF:serine-type endopeptidase activity;MF:endopeptidase activity;MF:hydrolase activity;MF:serine hydrolase activity;MF:catalytic activity, acting on a protein;MF:catalytic activity;MF:molecular_function;MF:pepti	GO:0071	K24132	Sp7	------	------	COG5640	S:Function unknown	PF00089.29;PF12	Trypsin;CLIP;Trypsin_2	Trypsin;Regulatory CLIP domain of proteinases;Trypsin-like peptidase domain	CYT	9	29	41.6	High
TRINITY_DN80328_c0_g1_i5_orf1	arylphorin subunit alpha-like [Ostrinia furnacalis]	0.454545455	-1.137503524	1.75E-05	down	yes	0.98	2.156	2.23	2.069	2.168	1	0.955	0.985	------	------	------	------	------	------	ENOG410XR2D	S:Function unknown	PF00372.22	Hemocyanin_M	Hemocyanin, copper containing domain	CYT	7	74	12.5	High
TRINITY_DN32532_c0_g1_i1_orf1	fatty acyl-CoA hydrolase precursor, medium chain [Ostrinia furnacalis]	0.496183206	-1.011055189	3.62E-05	down	yes	1.04	2.096	2.123	2.028	2.137	1	1.001	1.12	------	------	------	------	------	------	ENOG410Y6DN;(	S:Function unknown;I:Lipid transp	------	------	------	CYT	2	33	8.6	High
TRINITY_DN1759_c0_g1_i4_orf1	protein PFC0760c-like isoform X1 [Ostrinia furnacalis]	0.483405483	-1.048694257	5.45E-06	down	yes	1.005	2.079	2.039	2.144	2.055	1	0.998	1.017	------	------	------	------	------	------	COG3752;ENOG	S:Function unknown;	------	------	------	CYT	9	12	100.6	High
TRINITY_DN3647_c1_g1_i5_orf1	unnamed protein product, partial [Iphiclides podalirius]	0.494463168	-1.016065035	6.49E-05	down	yes	1.027	2.077	1.963	2.154	2.114	1	1.022	1.058	------	------	------	------	------	------	ENOG4111RPK	S:Function unknown	PF04923.15	Niniurin	Niniurin	CYT	4	24	33.9	High
TRINITY_DN12256_c0_g1_i1_orf1	lysosome membrane protein 2-like [Ostrinia furnacalis]	0.491167812	-1.025712077	1.21E-06	down	yes	1.001	2.038	2.081	2.012	2.022	1	0.993	1.009	BP:multicellular organismal process;BP:sensory perception of smell;BP:neurological system process;BP:response to stimulus;BP:sensory perception of chemical stimulus;BP:sensory perception;BP:biological_process;BP:syst em process;CC:membrane;CC:cellular_component;CC:cellular anatomical entity;CC:plasma membrane;CC:integral component of membrane;CC:intrinsic	GO:0032!	K13885	SCARB1	map04979;m	Cholesterol metabo	ENOG410XS17	U:Intracellular trafficking, secretic	PF01130.24	CD36	CD36 family	CYT	3	22	17.6	High
TRINITY_DN2286_c2_g1_i1_orf1	coatomer subunit zeta-1 isoform X1 [Ostrinia furnacalis]	0.495835375	-1.012066892	0.0002756	down	yes	1.012	2.041	2.166	1.894	2.063	1	0.962	1.073	BP:protein localization;BP:cellular macromolecule localization;BP:nitrogen compound transport;BP:organic substance transport;BP:macromolecule localization;BP:transport;BP:Golgi vesicle transport;BP:cellular process;BP:establishment of protein localization;BP:biological_process;BP:establishment of localization;BP:localization;BP:cellular localization;BP:retrograde vesicle-mediated transport, Golgi to ER;BP:vesicle-mediated transport;BP:protein transport;CC:membrane coat;CC:organelle membrane;CC:membrane;CC:cellular anatomical entity;CC:bounding membrane of organelle;CC:vesicle coat;CC:COPI vesicle coat;CC:macromolecular	GO:0008	K20472	COPZ, RET3	------	------	COG5541	U:Intracellular trafficking, secretic	PF01217.23	Clat_adaptor_s	Clathrin adaptor complex small chain	CYT	2	21	20.6	High
TRINITY_DN26961_c0_g1_i1_orf1	uncharacterized protein LOC120424957 [Culex pipiens pallens]	0.480727092	-1.056709985	0.009397	down	yes	0.9653	2.008	1.616	2.383	2.025	1	0.932	0.964	CC:sarcomere;CC:myosin complex;CC:macromolecular complex;CC:cellular_component;CC:cellular anatomical entity;MF:purine ribonucleoside triphosphate binding;MF:macromolecular complex binding;MF:binding;MF:ion binding;MF:heterocyclic compound binding;MF:nucleoside phosphate binding;MF:anion binding;MF:nucleotide binding;MF:actin binding;MF:purine nucleotide binding;MF:motor activity;MF:ATP binding;MF:actin filament binding;MF:small molecule binding;MF:cytoskeletal protein binding;MF:carbohydrate derivative binding;MF:organic cyclic compound binding;MF:adenyl nucleotide binding;MF:adenyl ribonucleotide binding;MF:protein binding;MF:purine ribonucleotide binding;MF:molecular_function;MF:ribonucleotide binding;	GO:0030(	K17751	MYH6_7	map04260;m	Cardiac muscle cor	COG5022	E:Amino acid transport and meta	PF01576.22	Myosin_tail_1	Myosin tail	CYT	5	64	12.3	High

TRINITY_DN2813_c0_g1_i10_orf1	arylphorin subunit alpha-like [Ostrinia furnacalis]	0.132518898	-2.915729978	6.05E-07	down	yes	0.9817	7.408	7.49	7.548	7.186	1	0.936	1.009	------	------	------	------	------	------	ENOG4110QFU;[illegible]	S:Function unknown;	PF03723.17;PF00[illegible]	Hemocyanin_C;Hemocyanin_M;Hemocyanin_N	Hemocyanin, ig-like domain;Hemocyanin, copper containing domain;Hemocyanin, all-alpha domain	CYT	1	33	139.5	High
TRINITY_DN64181_c0_g1_i1_orf1	uncharacterized protein LOC114356431 isoform X2 [Ostrinia furnacalis]	0.240153504	-2.057971234	0.002638	down	yes	1.189	4.951	5.928	4.924	4	1	1.297	1.269	------	------	------	------	------	------	------	------	------	------	------	PLA	1	18	7.9	High
TRINITY_DN59429_c0_g1_i6_orf1	uncharacterized protein LOC114366345 isoform X2 [Ostrinia furnacalis]	0.187522869	-2.41486155	9.51E-06	down	yes	1.025	5.466	5.774	5.269	5.355	1	1.074	1.002	BP:response to biotic stimulus;BP:response to external stimulus;BP:response to external biotic stimulus;BP:response to stimulus;BP:defense response;BP:response to stress;BP:biological_process;BP:interspecies interaction between organisms;BP:response to other organism;CC:cellular_component;CC:extr	GO:0009[illegible]	------	------	------	------	ENOG4110S6N	S:Function unknown	PF00451.22	Toxin_2	Scorpion short toxin, BmKK2	CYT	2	22	12.3	High
TRINITY_DN3275_c0_g2_i3_orf1	hypothetical protein B5X24_HaOG216046 [Helicoverpa armigera]	0.105466182	-3.245147621	5.41E-05	down	yes	0.6097	5.781	5.49	5.69	6.163	1	0.513	0.316	MF:cation binding;MF:ion binding;MF:molecular_function;MF:binding;MF:metal ion binding;MF:calcium ion binding;	GO:0043[illegible]	K02183	CALM	map05214;m[illegible]	Glioma;Pathways o[illegible]	COG5126;COG5[illegible]	D:Cell cycle control, cell division,	------	------	------	CYT	1	36	2.8	Medium
TRINITY_DN3166_c1_g1_i6_orf1	hypothetical protein evm_013813 [Chilo suppressalis]	0.202970703	-2.300656594	5.99E-07	down	yes	0.9907	4.881	4.917	4.751	4.975	1	0.958	1.014	BP:response to biotic stimulus;BP:response to external stimulus;BP:response to external biotic stimulus;BP:response to stimulus;BP:defense response;BP:response to stress;BP:biological_process;BP:interspecies interaction between organisms;BP:response to other organism;CC:cellular_component;CC:extr	GO:0009[illegible]	------	------	------	------	------	------	PF00451.22	Toxin_2	Scorpion short toxin, BmKK2	CYT	1	13	10.5	High
TRINITY_DN143895_c0_g1_i1_orf1	cathepsin L-like [Aphidius gifuensis] >KAF7988186.1 hypothetical protein HCN44_007680 [Aphidius gifuensis]	0.201272534	-2.312777779	0.0009712	down	yes	0.949	4.715	5.558	4.445	4.143	1	0.834	1.013	BP:primary metabolic process;BP:organonitrogen compound metabolic process;BP:protein metabolic process;BP:macromolecule metabolic process;BP:organic substance metabolic process;BP:nitrogen compound metabolic process;BP:biological_process;BP:metabolic process;BP:proteolysis;MF:molecular_function;MF:hydrolase activity;MF:catalytic activity;MF:catalytic activity, acting on a protein;MF:peptidase	GO:0044[illegible]	K01365	CTSL	map05205;m[illegible]	Proteoglycans in ca[illegible]	COG4870	O:Posttranslational modification,	PF00112.26;PF08[illegible]	Peptidase_C1;Inhibitor_I29;Peptidase_C1_2	Papain family cysteine protease;Cathepsin propeptide inhibitor domain;Peptidase C1-like family	CYT	1	2	38.3	High
TRINITY_DN17247_c0_g1_i14_orf1	uncharacterized protein LOC114356308 [Ostrinia furnacalis]	0.31068525	-1.686474346	0.001677	down	yes	1.07	3.444	2.854	3.582	3.895	1	1.206	1.005	------	------	------	------	------	------	------	------	------	------	------	CYT	1	5	24.8	Medium
TRINITY_DN15578_c0_g2_i1_orfp1	uncharacterized protein LOC125235519 [Leguminivora glycinivorella]	0.244728578	-2.030745515	1.09E-05	down	yes	1.091	4.458	4.536	4.278	4.56	1	1.012	1.26	------	------	------	------	------	------	------	------	------	------	------	CYT	1	9	10.9	High
TRINITY_DN1593_c0_g1_i1_orf1	chemosensory protein csp11 [Helopeltis theivora]	0.302104942	-1.72687831	7.94E-06	down	yes	0.9903	3.278	3.177	3.429	3.228	1	1.003	0.968	------	------	------	------	------	------	ENOG4110RV8;E[illegible]	S:Function unknown;	PF03392.16	OS-D	Insect pheromone-binding family, A10/OS-D	CYT	8	39	15.4	High
TRINITY_DN1370_c0_g1_i2_orf1	hypothetical protein evm_000756 [Chilo suppressalis]	0.266037279	-1.910299674	9.47E-06	down	yes	1.099	4.131	4.269	3.988	4.136	1	1.06	1.236	------	------	------	------	------	------	------	------	------	------	------	CYT	2	35	23	High
TRINITY_DN36476_c1_g1_i1_orfp1	TRINITY_DN36476_c1_g1_i1_m.70910 TRINITY_DN36476_c1_g1::TRINITY_DN36476_c1_g1_i1::g.70910 ORF type:5prime_partial len:88 (-),score=0.50 TRINITY_DN36476_c1_g1_i1:49-312(-)	0.112216242	-3.155646597	3.77E-06	down	yes	0.959	8.546	8.606	8.881	8.151	1	0.942	0.935	------	------	------	------	------	------	------	------	------	------	------	CYT	2	21	9.5	High
TRINITY_DN8685_c0_g1_i5_orf1	macrophage mannose receptor 1-like [Zerene cesonia]	0.312421973	-1.678432174	4.04E-06	down	yes	1.001	3.204	3.088	3.273	3.251	1	1.043	0.959	BP:detection of biotic stimulus;BP:detection of chemical stimulus;BP:positive regulation of response to external stimulus;BP:regulation of response to external stimulus;BP:positive regulation of response to stimulus;BP:regulation of response to biotic stimulus;BP:positive regulation of response to biotic stimulus;BP:positive regulation of defense response;BP:defense response to bacterium;BP:detection of molecule of bacterial origin;BP:regulation of biological process;BP:regulation of defense response;BP:response to bacterial lipoprotein;BP:response to external biotic stimulus;BP:response to bacterium;BP:interspecies interaction between organisms;BP:positive regulation of immune system process;BP:biological regulation;BP:positive regulation of biological process;BP:regulation of immune system process;BP:detection of external biotic stimulus;BP:positive regulation of innate immune response;BP:regulation of innate immune response;BP:regulation of response to stimulus;BP:innate immune	GO:0009[illegible]	------	------	------	------	ENOG410XRS3;E[illegible]	U:Intracellular trafficking, secretic[illegible]	PF00059.24	Lectin_C	Lectin C-type domain	PLA	2	6	40.6	High
TRINITY_DN14904_c0_g1_i1_orf1	attacin [Ostrinia furnacalis]	0.363109444	-1.461523641	2.28E-06	down	yes	1.065	2.933	2.871	2.981	2.948	1	1.089	1.107	BP:interspecies interaction between organisms;BP:response to biotic stimulus;BP:response to external stimulus;BP:response to external biotic stimulus;BP:response to stimulus;BP:response to bacterium;BP:defense response;BP:response to stress;BP:defense response to other organism;BP:defense response to bacterium;BP:biological_process;BP:response to other organism;CC:cellular_component;CC:extracellular region;CC:cellular anatomical	GO:0044[illegible]	------	------	------	------	ENOG410ZBQ8;[illegible]	S:Function unknown;	PF03769.18	Attacin_C	Attacin, C-terminal region	CYT	3	26	17.3	High
TRINITY_DN380_c0_g2_i2_orf1	chemosensory protein 10 [Ostrinia furnacalis]	0.251948705	-1.988798054	5.28E-09	down	yes	1.002	3.977	3.952	3.971	4.007	1	1.005	1.001	------	------	------	------	------	------	ENOG4111ATT;E[illegible]	S:Function unknown;	PF03392.16	OS-D	Insect pheromone-binding family, A10/OS-D	CYT	3	54	13.6	High
TRINITY_DN136031_c0_g1_i7_orf1	ferritin, lower subunit isoform X3 [Spodoptera litura]	0.149802746	-2.738864027	5.19E-05	down	yes	0.9493	6.337	6.291	5.855	6.865	1	0.913	0.935	BP:ion homeostasis;BP:homeostatic process;BP:iron ion homeostasis;BP:transition metal ion homeostasis;BP:inorganic ion homeostasis;BP:cellular transition metal ion homeostasis;BP:transition metal ion transport;BP:biological regulation;BP:cellular homeostasis;BP:metal ion transport;BP:transport;BP:cellular iron ion homeostasis;BP:cellular metal ion homeostasis;BP:cation transport;BP:ion transport;BP:cellular process;BP:cellular ion homeostasis;BP:biological_process;BP:cellular cation homeostasis;BP:metal ion homeostasis;BP:cation homeostasis;BP:cellular chemical homeostasis;BP:establishment of localization;BP:localization;BP:regulation of biological quality;BP:chemical homeostasis;BP:iron ion transport;MF:molecular_function;MF:binding;MF:oxidoreductase activity;MF:cation binding;MF:oxidoreductase activity, oxidizing metal ions;MF:transition metal ion binding;MF:ferroxidase activity;MF:ion binding;MF:iron ion	GO:0050[illegible]	------	------	------	------	ENOG4111Q26;[illegible]	P:Inorganic ion transport and me[illegible]	------	------	------	EXC	1	28	11.1	High
TRINITY_DN27300_c0_g1_i7_orfp1	TRINITY_DN27300_c0_g1_i7_m.71141 TRINITY_DN27300_c0_g1::TRINITY_DN27300_c0_g1_i7::g.71141 ORF type:internal len:82 (-),score=6.59 TRINITY_DN27300_c0_g1_i7:3-245(-)	0.272182907	-1.877351626	5.26E-05	down	yes	1	3.674	3.72	3.899	3.403	1	0.968	1.032	------	------	------	------	------	------	------	------	------	------	------	CYT	2	35	9.2	High
TRINITY_DN418_c1_g1_i3_orf1	hypothetical protein evm_003996 [Chilo suppressalis]	0.364433532	-1.456272385	0.0001616	down	yes	1.039	2.851	2.696	3.098	2.759	1	0.995	1.123	MF:molecular_function;MF:carbohydrate binding;MF:binding;	GO:0003[illegible]	------	------	------	------	------	------	------	------	------	PLA	1	46	8.8	High

TRINITY_DN51813_c0_g1_i1_orf1	uncharacterized protein LOC114350216 [Ostrinia furnacalis]	0.258206381	-1.95340344	0.001542	down	yes	0.8983	3.479	3.248	4.133	3.055	1	0.85	0.845	BP: de novo NAD biosynthetic process from tryptophan;BP:aromatic amino acid family catabolic process;BP:nucleotide biosynthetic process;BP:small molecule metabolic process;BP:small molecule catabolic process;BP:small molecule biosynthetic process;BP:organic cyclic compound biosynthetic process;BP:organic cyclic compound metabolic process;BP:organic cyclic compound catabolic process;BP:dicarboxylic acid biosynthetic process;BP:benzene-containing compound metabolic process;BP:nicotinamide nucleotide biosynthetic process;BP:nicotinamide nucleotide metabolic process;BP:monocarboxylic acid metabolic process;BP:oxoacid metabolic process;BP:aromatic amino acid family metabolic process;BP:heterocycle metabolic process;BP:dicarboxylic acid metabolic process;BP:organonitrogen compound metabolic process;BP:organonitrogen compound catabolic process;BP:organonitrogen compound biosynthetic process;BP:organic acid catabolic process;BP:organic acid biosynthetic	GO:0034	K01556	KYNU, kynU	map00380;m	Tryptophan metab	COG3844	E:Amino acid transport and meta	PF00266.22	Aminotran_5	Aminotransferase class-V	CYT	1	1	48.1	High
TRINITY_DN17615_c0_g1_i3_orf1	hypothetical protein SFRUCORN_008858 [Spodoptera frugiperda]	0.228206203	-2.131590089	3.90E-05	down	yes	1.089	4.772	4.47	4.759	5.087	1	1.076	1.192	------	------	K20674	MODSP	map04624	Toll and Imd signal	COG5640	O:Posttranslational modification,	------	------	------	CYT	1	20	8.4	High
TRINITY_DN6122_c0_g1_i6_orf1	deubiquitinase DESI2 isoform X1 [Helicoverpa armigera] >XP_049707835.1 deubiquitinase DESI2 isoform X1 [Helicoverpa armigera]	0.412110092	-1.278898302	0.002225	down	yes	1.123	2.725	2.744	2.333	3.099	1	1.182	1.188	MF:hydrolase activity;MF:peptidase activity;MF:catalytic activity, acting on a protein;MF:molecular_function;MF:catalytic activity;	GO:0016	K22763	DESI2, PPPDI	------	------	ENOG4111H3Z	S:Function unknown	PF05903.17	Peptidase_C97	PPPDE putative peptidase domain	CYT	1	8	23.8	High
TRINITY_DN9079_c0_g1_i5_orf1	UDP-glucuronosyltransferase-like [Ostrinia furnacalis]	0.274338942	-1.865968668	1.91E-05	down	yes	0.913	3.328	3.507	3.266	3.212	1	0.902	0.837	CC:integral component of membrane;CC:cellular_component;CC:cellular anatomical entity;CC:intrinsic component of membrane;MF:catalytic activity;MF:transferase activity;MF:molecular_function;MF:transferase activity, transferring glycosyl groups;MF:transferase activity, transferring hexosyl groups;MF:glucuronosyltransferase activity;MF:UDP-glycosyltransferase	GO:0016	K00699	UGT	map05207;m	Chemical carcinoge	COG1819	S:Function unknown	PF00201.21	UDPGT	UDP-glucoronosyl and UDP-glucosyl transferase	CYT	5	14	48.5	High
TRINITY_DN25234_c0_g1_i1_orf1	uncharacterized protein LOC114353853 [Ostrinia furnacalis]	0.195492263	-2.354816585	5.72E-05	down	yes	0.8717	4.459	4.515	4.122	4.739	1	0.705	0.91	------	------	------	------	------	------	------	------	------	------	------	CYT	1	15	10	High
TRINITY_DN20344_c0_g1_i5_orf1	uncharacterized protein LOC114351483 [Ostrinia furnacalis]	0.348501264	-1.520764206	4.69E-06	down	yes	0.965	2.769	2.836	2.703	2.767	1	0.89	1.005	MF:molecular_function;MF:structural constituent of cuticle;MF:structural molecule activity;	GO:0003	------	------	------	------	------	------	PF00379.26	Chitin_bind_4	Insect cuticle protein	CYT	4	57	13.5	High
TRINITY_DN15202_c0_g1_i6_orf1	uncharacterized protein LOC114364499 isoform X2 [Ostrinia furnacalis]	0.245792842	-2.024485193	2.40E-05	down	yes	1.037	4.219	3.951	4.372	4.335	1	1.132	0.98	------	------	------	------	------	------	ENOG410XU1K	S:Function unknown	------	------	------	CYT	2	41	14	High
TRINITY_DN19662_c0_g2_i1_orf1	storage protein [Ostrinia furnacalis]	0.331006979	-1.595066459	5.48E-06	down	yes	0.996	3.009	3.132	2.935	2.959	1	0.985	1.003	------	------	------	------	------	------	ENOG410XR2D	S:Function unknown	PF00372.22;PF03	Hemocyanin_M;Hemocyanin_N;Hemocyanin_C	Hemocyanin, copper containing domain;Hemocyanin, all-alpha domain;Hemocyanin, ig-like	CYT	43	69	59	High
TRINITY_DN20658_c0_g1_i1_orf1	prostaglandin reductase 1-like [Ostrinia furnacalis]	0.403884265	-1.307986155	8.43E-07	down	yes	1.019	2.523	2.546	2.553	2.471	1	1.014	1.043	------	------	K13948	PTGR1, LTB4	map00590	Arachidonic acid m	COG2130	S:Function unknown	PF16884.8	ADH_N_2	N-terminal domain of oxidoreductase	CYT	8	49	17.6	High
TRINITY_DN38307_c0_g1_i1_orfp1	TRINITY_DN38307_c0_g1_i1_m.10661 TRINITY_DN38307_c0_g1::TRINITY_DN38307_c0_g1_i1::g.10661 ORF type:5prime_partial len:66 (+),score=5.90 TRINITY_DN38307_c0_g1_i1:2-199(+)	0.27245509	-1.875909652	0.005468	down	yes	1.001	3.674	4.581	3.035	3.407	1	0.741	1.262	------	------	------	------	------	------	------	------	------	------	------	CYT	1	17	7.9	High
TRINITY_DN1597_c0_g1_i5_orfp1	TRINITY_DN1597_c0_g1_i5_m.57494 TRINITY_DN1597_c0_g1::TRINITY_DN1597_c0_g1_i5::g.57494 ORF type:complete len:86 (+),score=7.19 TRINITY_DN1597_c0_g1_i5:134-	0.346102528	-1.530728615	2.77E-06	down	yes	0.9857	2.848	2.84	2.934	2.77	1	0.991	0.966	------	------	------	------	------	------	------	------	------	------	------	CYT	2	27	9.2	High
TRINITY_DN1575_c0_g1_i10_orf1	uncharacterized protein LOC114359245 [Ostrinia furnacalis]	0.239686337	-2.060780422	0.0004337	down	yes	0.9017	3.762	3.377	4.233	3.675	1	0.716	0.989	------	------	------	------	------	------	ENOG4111CB2;E	S:Function unknown;	PF15868.8	MBF2	Transcription activator MBF2	CYT	1	2	45.9	High
TRINITY_DN26439_c0_g1_i2_orf1	uncharacterized protein LOC114351853 [Ostrinia furnacalis]	0.435775862	-1.198341808	0.0001867	down	yes	1.011	2.32	2.138	2.413	2.41	1	0.951	1.081	------	------	------	------	------	------	ENOG4110RUT;E	S:Function unknown;	------	------	------	CYT	3	50	10.9	High
TRINITY_DN960_c1_g1_i6_orf1	hypothetical protein evm_007130 [Chilo suppressalis]	0.367727772	-1.443289958	5.51E-07	down	yes	1.005	2.733	2.746	2.695	2.757	1	1.048	0.968	BP:transport;BP:intracellular transport;BP:sterol transport;BP:lipid transport;BP:intracellular sterol transport;BP:intracellular cholesterol transport;BP:intracellular lipid transport;BP:organic hydroxy compound transport;BP:cholesterol transport;BP:biological_process;BP:organic substance transport;BP:cellular process;BP:establishment of localization;BP:localization;BP:establishment of localization in cell;BP:cellular localization;CC:cellular_component;CC:extracellular region;CC:cellular anatomical entity;	GO:0006	K13443	NPC2	map04979;m	Cholesterol metab	ENOG4111Q8S	S:Function unknown	PF02221.18	E1_DerP2_DerF2	ML domain	PLA	5	38	15.5	High
TRINITY_DN11981_c0_g1_i7_orf1	luciferin 4-monooxygenase-like isoform X2 [Ostrinia furnacalis]	0.26240022	-1.930159164	4.65E-05	down	yes	0.9533	3.633	3.753	3.79	3.356	1	0.898	0.962	------	------	K01904	4CL	map00130	Ubiquinone and ot	COG0365;COG0	I:Lipid transport and metabolism	PF00501.31;PF13	AMP-binding;AMP-binding_C	AMP-binding enzyme;AMP-binding enzyme C-terminal	CYT	3	7	62.4	High
TRINITY_DN11817_c0_g1_i4_orf1	glycogen phosphorylase [Heortia vitessoides]	0.432327586	-1.209803199	1.85E-06	down	yes	1.003	2.32	2.371	2.324	2.264	1	1.002	1.007	BP:cellular metabolic process;BP:generation of precursor metabolites and energy;BP:cellular polysaccharide metabolic process;BP:cellular carbohydrate metabolic process;BP:cellular macromolecule metabolic process;BP:organic substance metabolic process;BP:energy reserve metabolic process;BP:cellular glucan metabolic process;BP:cellular process;BP:biological_process;BP:metabolic process;BP:glucan metabolic process;BP:primary metabolic process;BP:carbohydrate metabolic process;BP:energy derivation by oxidation of organic compounds;BP:glycogen metabolic process;BP:polysaccharide metabolic process;BP:macromolecule metabolic process;MF:vitamin B6 binding;MF:molecular_function;MF:binding;MF:glycogen phosphorylase activity;MF:heterocyclic compound binding;MF:anion binding;MF:ion binding;MF:transferase activity;MF:small molecule binding;MF:catalytic activity;MF:SHG alpha-glucan phosphorylase activity;MF:organic cyclic	GO:0044	K00688	PYG, glgP	map04910;m	Insulin signaling pa	COG0058	G:Carbohydrate transport and m	PF00343.23	Phosphorylase	Carbohydrate phosphorylase	CYT	48	62	96.7	High
TRINITY_DN350_c0_g1_i5_orf1	tau-like protein isoform X6 [Bombyx mori]	0.383002621	-1.384573831	0.0002036	down	yes	1.023	2.671	2.472	2.882	2.66	1	0.957	1.111	CC:cytoplasm;CC:supramolecular complex;CC:supramolecular fiber;CC:polymeric cytoskeletal fiber;CC:cellular_component;CC:supramolecular polymer;CC:microtubule;CC:cellular anatomical entity;MF:tubulin binding;MF:cytoskeletal protein binding;MF:molecular_function;MF:bindi	GO:0005	K04380	MAPT, TAU	map05012;m	Parkinson disease;A	ENOG4111J07	O:Posttranslational modification,	PF00418.22	Tubulin-binding	Tau and MAP protein, tubulin-binding repeat	CYT	1	16	26.7	High
TRINITY_DN33272_c0_g1_i5_orf1	Low-density lipoprotein receptor-related protein 1 [Papilio xuthus]	0.267197541	-1.904021363	0.000487	down	yes	0.9563	3.579	3.081	3.877	3.779	1	0.892	0.977	BP:endocytosis;BP:vesicle-mediated transport;BP:transport;BP:biological_process;BP:establishment of localization;BP:localization;CC:integral component of membrane;CC:cellular_component;CC:intrinsic component of membrane;CC:cellular anatomical entity;MF:binding;MF:cation binding;MF:metal ion binding;MF:ion binding;MF:molecular_function;MF:calciu	GO:0006	K04550;K	LRP1, CD91;S	map05010;m	Alzheimer disease;(	ENOG410XP34	U:Intracellular trafficking, secretic	PF00057.21	Ldl_recept_a	Low-density lipoprotein receptor domain class A	CYT	1	13	9.2	High
TRINITY_DN1226_c0_g1_i11_orfp1	TRINITY_DN1226_c0_g1_i11_m.52385 TRINITY_DN1226_c0_g1::TRINITY_DN1226_c0_g1_i11::g.52385 ORF type:internal len:92 (-),score=5.77 TRINITY_DN1226_c0_g1_i11:2-274(-)	0.449934412	-1.152213384	2.23E-05	down	yes	1.029	2.287	2.261	2.383	2.216	1	1.077	1.011	------	------	------	------	------	------	------	------	------	------	------	PLA	2	53	9.1	High

TRINITY_DN8595_c0_g1_i3_orf1	aldose reductase-like isoform X4 [Trichoplusia ni]	0.283946981	-1.816306523	4.39E-08	down	yes	0.964	3.395	3.42	3.375	3.391	1	0.951	0.941	MF:catalytic activity;MF:molecular_function;MF:oxidor eductase activity;	GO:0003[illegible]	K00011	AKR1B	map00051;m[illegible]	Fructose and mann[illegible]	COG0656	S:Function unknown	PF00248.24	Aldo_ket_red	Aldo/keto reductase family	CYT	1	67	13.9	High
TRINITY_DN5770_c0_g1_i4_orf1	rotatin-like [Ostrinia furnacalis]	0.427279874	-1.22674673	0.0002804	down	yes	1.087	2.544	2.607	2.681	2.344	1	1.043	1.217	------	------	------	------	------	------	ENOG4110T3E;E[illegible]	S:Function unknown;O:Secondar[illegible]	------	------	------	CYT	1	1	111.2	Medium
TRINITY_DN3609_c0_g1_i6_orf1	leukocyte elastase inhibitor-like [Ostrinia furnacalis]	0.45179667	-1.146254459	6.32E-05	down	yes	1.031	2.282	2.372	2.144	2.329	1	1.048	1.045	CC:cellular_component;CC:cellular anatomical entity;CC:extracellular space;MF:endopeptidase inhibitor activity;MF:endopeptidase regulator activity;MF:peptidase regulator activity;MF:molecular_function;MF:peptidase inhibitor activity;MF:serine-type endopeptidase inhibitor activity;MF:enzyme inhibitor activity;MF:molecular function regulator;MF:enzyme regulator activity;	GO:0005[illegible]	------	------	------	------	COG4826	S:Function unknown	PF00079.23	Serpin	Serpin	CYT	8	17	56.7	High
TRINITY_DN394_c0_g1_i2_orf1	uncharacterized protein LOC114351483 [Ostrinia furnacalis]	0.290804954	-1.781876251	1.01E-05	down	yes	0.9393	3.23	3.247	3.352	3.092	1	0.883	0.935	MF:molecular_function;MF:structural constituent of cuticle;MF:structural molecule activity;	GO:0003[illegible]	------	------	------	------	ENOG41113X7;E[illegible]	S:Function unknown;	PF00379.26	Chitin_bind_4	Insect cuticle protein	CYT	1	28	12	High
TRINITY_DN22962_c0_g1_i1_orf1	lysosomal acid glucosylceramidase-like isoform X2 [Ostrinia furnacalis]	0.335809313	-1.574285855	4.43E-06	down	yes	0.9087	2.706	2.748	2.66	2.71	1	0.875	0.851	------	------	K01201	GBA, srfJ	map00600;m[illegible]	Sphingolipid metab[illegible]	COG5520	G:Carbohydrate transport and m[illegible]	PF02055.19;PF17[illegible]	Glyco_hydro_30;Glyco_hydro_30C;Glyco_hydro_59	Glycosyl hydrolase family 30 TIM-barrel domain;Glycosyl hydrolase family 30 beta sandwich domain;Glycosyl hydrolase [illegible]	CYT	1	4	53.5	High
TRINITY_DN59388_c0_g1_i1_orf1	uncharacterized protein LOC114353759 [Ostrinia furnacalis]	0.432361896	-1.209688709	8.24E-05	down	yes	0.994	2.299	2.289	2.441	2.168	1	0.97	1.012	------	------	------	------	------	------	------	------	------	------	------	CYT	5	36	20.8	High
TRINITY_DN140_c0_g1_i5_orf1	calcyphosin-like protein isoform X4 [Helicoverpa armigera]	0.327794767	-1.609135271	1.25E-05	down	yes	0.9647	2.943	2.885	3.088	2.856	1	0.936	0.958	MF:cation binding;MF:ion binding;MF:molecular_function;MF:binding;MF:metal ion binding;MF:calcium ion binding;	GO:0043[illegible]	K23909	CAPS	------	------	ENOG410XRMJ	T:Signal transduction mechanism[illegible]	PF13405.9;PF00[illegible]	EF-hand_6;EF-hand_1;EF-hand_7;EF-hand_5;EF-hand_8	EF-hand domain;EF hand;EF-hand domain pair;EF hand;EF-hand domain pair	CYT	2	73	12	High
TRINITY_DN33885_c0_g1_i1_orf1	inhibitor of nuclear factor kappa-B kinase subunit alpha [Ostrinia furnacalis]	0.36450737	-1.45598011	0.002725	down	yes	0.9397	2.578	2.18	3.026	2.528	1	0.876	0.943	------	------	K07209	IKBKB, IKKB	map05166;m[illegible]	Human T-cell leuk[illegible]	ENOG410XRMU	T:Signal transduction mechanism[illegible]	PF00069.28;PF07[illegible]	Pkinase;PK_Tyr_Ser-Thr;Pkinase_fungal	Protein kinase domain;Protein tyrosine and serine/threonine kinase:Fungal	CYT	1	2	89.6	High
TRINITY_DN1503_c0_g1_i6_orf1	ecdysteroid-regulated 16 kDa protein [Ostrinia furnacalis]	0.393341404	-1.346146038	6.39E-05	down	yes	0.9747	2.478	2.437	2.641	2.355	1	0.976	0.948	------	------	K13443	NPC2	map04979;m[illegible]	Cholesterol metab[illegible]	ENOG4111Q8S	O:Posttranslational modification,[illegible]	PF02221.18	E1_DerP2_DerF	ML domain	CYT	6	38	17.1	High
TRINITY_DN20717_c0_g1_i1_orf1	putative uncharacterized protein DDB_G0282133 isoform X1 [Ostrinia furnacalis]	0.399022189	-1.325459121	0.0003057	down	yes	1.061	2.659	2.395	2.763	2.82	1	1.1	1.082	------	------	------	------	------	------	ENOG410ZIVR	S:Function unknown	------	------	------	PLA	1	17	5.2	High
TRINITY_DN1024_c0_g4_i1_orf1	superoxide dismutase [Cu-Zn]-like [Ostrinia furnacalis]	0.415739596	-1.266247934	0.0001187	down	yes	1.009	2.427	2.318	2.616	2.346	1	1.023	1.003	MF:oxidoreductase activity, acting on superoxide radicals as acceptor;MF:cation binding;MF:antioxidant activity;MF:ion binding;MF:catalytic activity;MF:molecular_function;MF:binding;MF:superoxide dismutase [illegible]	GO:0016[illegible]	K04565	SOD1	map05208;m[illegible]	Chemical carcinoge[illegible]	COG2032	S:Function unknown	PF00080.23	Sod_Cu	Copper/zinc superoxide dismutase	PLA	8	58	18.2	High
TRINITY_DN6243_c0_g1_i5_orf1	sorting nexin-20 [Ostrinia furnacalis]	0.475074331	-1.073774837	0.0004931	down	yes	0.9587	2.018	2.165	2.053	1.836	1	0.985	0.891	BP:protein localization;BP:cellular macromolecule localization;BP:nitrogen compound transport;BP:organic substance transport;BP:macromolecule localization;BP:cellular process;BP:transport;BP:establishment of protein localization;BP:biological_process;BP:establishment of localization;BP:localization;BP:cellular localization;BP:protein transport;CC:endosome membrane;CC:membrane;CC:organelle membrane;CC:early endosome membrane;CC:bounding membrane of organelle;CC:cytoplasmic vesicle membrane;CC:vesicle membrane;CC:cellular_component;CC:cellular anatomical entity;MF:molecular_function;MF:binding;MF:phospholipid binding;MF:lipid binding;MF:phosphatidylinositol [illegible]	GO:0008[illegible]	K17931	SNX20	------	------	ENOG4111IVA;E[illegible]	S:Function unknown;U:Intracellul[illegible]	PF00787.27	PX	PX domain	CYT	1	5	28.8	High
TRINITY_DN19731_c0_g1_i1_orf1	allergen Tha p 1-like [Ostrinia furnacalis] >XP_028174916.1 allergen Tha p 1-like [Ostrinia furnacalis] >BAV56808.1 chemosensory protein 4 [Ostrinia furnacalis]	0.387072243	-1.369325238	3.29E-05	down	yes	1.018	2.63	2.495	2.632	2.764	1	1.02	1.035	------	------	------	------	------	------	ENOG4111U5H;[illegible]	S:Function unknown;	PF03392.16	OS-D	Insect pheromone-binding family, A10/OS-D	CYT	3	23	14.9	High
TRINITY_DN7040_c0_g1_i4_orf1	uncharacterized protein LOC114353763 [Ostrinia furnacalis]	0.400127119	-1.321469684	3.45E-05	down	yes	0.9443	2.36	2.457	2.251	2.373	1	0.878	0.955	------	------	------	------	------	------	------	------	------	------	------	CYT	2	39	7.4	High
TRINITY_DN2880_c0_g1_i2_orf1	sialomucin core protein 24 [Pectinophora gossypiella]	0.442333186	-1.17679461	3.15E-05	down	yes	1.001	2.263	2.145	2.3	2.345	1	0.998	1.006	CC:cellular_component;CC:integral component of membrane;CC:cellular anatomical entity;CC:intrinsic component of membrane;	GO:0005[illegible]	K06546	CD164, MGC[illegible]	map04142	Lysosome	ENOG410XVJX	U:Intracellular trafficking, secretio[illegible]	PF05283.14	MGC-24	Multi-glycosylated core protein 24	CYT	3	11	22.5	High
TRINITY_DN4998_c0_g1_i21_orf1	phenoloxidase subunit 2-like [Ostrinia furnacalis]	0.382922201	-1.384876787	7.51E-06	down	yes	1.009	2.635	2.625	2.734	2.547	1	1.007	1.019	CC:cellular_component;CC:extracellular region;CC:cellular anatomical entity;MF:cation binding;MF:monooxygenase activity;MF:oxidoreductase activity, acting on paired donors, with incorporation or reduction of molecular oxygen;MF:monophenol monooxygenase activity;MF:oxidoreductase activity, acting on paired donors, with incorporation or reduction of molecular oxygen, another compound as one donor, and incorporation of one atom of oxygen;MF:ion binding;MF:catalytic activity;MF:molecular_function;MF:binding;MF:metal ion [illegible]	GO:0005[illegible]	------	------	------	------	ENOG4111KTW	S:Function unknown	PF03723.17;PF00[illegible]	Hemocyanin_C;Hemocyanin_M;Hemocyanin_N;Tyrosinase	Hemocyanin, ig-like domain;Hemocyanin, copper containing domain;Hemocyanin, all-alpha domain;Common central domain of tyrosinase	PLA	16	38	80	High
TRINITY_DN4464_c0_g2_i1_orf1	glypican-6 [Pectinophora gossypiella]	0.498567775	-1.004138458	1.70E-05	down	yes	0.9747	1.955	1.898	2.014	1.952	1	0.93	0.994	BP:regulation of signal transduction;BP:regulation of response to stimulus;BP:regulation of cellular process;BP:biological regulation;BP:regulation of signaling;BP:biological_process;BP:regulation of cell communication;BP:regulation of biological process;CC:cellular_component;CC:anchored component of plasma membrane;CC:external encapsulating structure;CC:collagen-containing extracellular matrix;CC:cellular anatomical entity;CC:extracellular matrix;CC:intrinsic component of plasma membrane;CC:intrinsic component of membrane;CC:anchored component of membrane;	GO:0009[illegible]	K08110	GPC4	map04310	Wnt signaling path[illegible]	ENOG410XST2	B:Chromatin structure and dynar[illegible]	PF01153.22	Glypican	Glypican	CYT	11	20	84.3	High
TRINITY_DN184_c0_g1_i10_orf1	C-type mannose receptor 2-like isoform X1 [Leguminivora glycinivorella]	0.3245329	-1.623563354	0.0002018	down	yes	0.799	2.462	2.313	2.579	2.494	1	0.716	0.681	MF:molecular_function;MF:carbohydrate binding;MF:binding;	GO:0003[illegible]	------	------	------	------	ENOG410XPJ1;E[illegible]	S:Function unknown;U:Intracellul[illegible]	PF00059.24	Lectin_C	Lectin C-type domain	CYT	1	27	30.8	High

TRINITY_DN768_c0_g1_i7_orf1	neutral ceramidase [Leguminivora glycinivorella]	0.370069876	-1.434130392	1.68E-05	down	yes	0.9533	2.576	2.54	2.697	2.49	1	0.932	0.928	BP:sphingolipid catabolic process;BP:cellular catabolic process;BP:cellular nitrogen compound metabolic process;BP:nitrogen compound metabolic process;BP:cellular lipid catabolic process;BP:lipid metabolic process;BP:organic substance catabolic process;BP:lipid catabolic process;BP:organic substance metabolic process;BP:membrane lipid catabolic process;BP:sphingolipid metabolic process;BP:membrane lipid metabolic process;BP:cellular process;BP:biological_process;BP:metabolic process;BP:cellular lipid metabolic process;BP:catabolic process;BP:primary metabolic process;BP:organonitrogen compound metabolic process;BP:organonitrogen compound catabolic process;BP:cellular amide metabolic process;BP:cellular metabolic process;BP:ceramide catabolic process;BP:ceramide metabolic process;MF:ceramidase activity;MF:molecular_function;MF:ceramidase activity;MF:hydrolase activity;MF:catalytic activity;MF:hydrolase activity, acting on carbon-nitroge;MF:hydrolase activity, [illegible]	GO:00301	K12349	ASAH2	map00600;m	Sphingolipid metab	ENOG410XQWE	S:Function unknown	PF04734.16;PF17	Ceramidase_alk;Ceramidse_alk_C	Neutral/alkaline non-lysosomal ceramidase, N-terminal;Neutral/alkaline non-lysosomal ceramidase, C-terminal	CYT	10	20	77.2	High
TRINITY_DN26879_c0_g1_i1_orf1	hypothetical protein O3G_MSEX014401 [Manduca sexta]	0.431539479	-1.212435544	3.78E-05	down	yes	0.9783	2.267	2.279	2.34	2.183	1	1.045	0.89	BP:nucleic acid metabolic process;BP:cellular biosynthetic process;BP:cellular nitrogen compound metabolic process;BP:nitrogen compound metabolic process;BP:organic cyclic compound biosynthetic process;BP:organic cyclic compound metabolic process;BP:nucleobase-containing compound metabolic process;BP:cellular macromolecule metabolic process;BP:organic substance metabolic process;BP:heterocycle biosynthetic process;BP:organic substance biosynthetic process;BP:cellular process;BP:cellular aromatic compound metabolic process;BP:biosynthetic process;BP:macromolecule biosynthetic process;BP:biological_process;BP:metabolic process;BP:nucleobase-containing compound biosynthetic process;BP:heterocycle metabolic process;BP:primary metabolic process;BP:cellular nitrogen compound biosynthetic process;BP:cellular metabolic process;BP:macromolecule metabolic process;BP:DNA metabolic process;BP:aromatic compound biosynthetic process;BP:DNA [illegible]	GO:00903	------	------	------	------	ENOG410Y65V;E	O:Posttranslational modification,	PF00078.30	RVT_1	Reverse transcriptase	EXC	1	1	97.8	Medium
TRINITY_DN73900_c0_g1_i1_orf1	carbonic anhydrase 7 [Ostrinia furnacalis]	0.384127609	-1.380342433	3.39E-05	down	yes	0.9753	2.539	2.427	2.668	2.523	1	0.914	1.012	MF:cation binding;MF:metal ion binding;MF:transition metal ion binding;MF:carbon-oxygen lyase activity;MF:hydro-lyase activity;MF:ion binding;MF:catalytic activity;MF:molecular_function;MF:zinc ion binding;MF:carbonate dehydratase activity;MF:lyase activity;MF:binding;	GO:00431	K01672	CA	map00910	Nitrogen metabolis	COG3338	U:Intracellular trafficking, secretic	PF00194.24	Carb_anhydrase	Eukaryotic-type carbonic anhydrase	CYT	1	3	36.5	High
TRINITY_DN1206_c0_g1_i6_orf1	sorbitol dehydrogenase-like [Spodoptera frugiperda] >KAG8104768.1 hypothetical protein SFRUCORN_013827 [Spodoptera frugiperda]	0.452684258	-1.143422956	0.0001633	down	yes	0.995	2.198	2.367	2.154	2.074	1	0.982	1.003	MF:oxidoreductase activity;MF:cation binding;MF:oxidoreductase activity, acting on CH-OH group of donors;MF:oxidoreductase activity, acting on the CH-OH group of donors, NAD or NADP as acceptor;MF:transition metal ion binding;MF:ion binding;MF:catalytic activity;MF:molecular_function;MF:bindi [illegible]	GO:0016	K00008	SORD, gutB	map00040;m	Pentose and glucur	COG1063	E:Amino acid transport and meta	PF08240.15;PF00	ADH_N;ADH_zinc_N;Glu_dehyd_C	Alcohol dehydrogenase GroES-like domain;Zinc-binding dehydrogenase;Glucose dehydrogenase C-terminus	CYT	12	46	38.7	High
TRINITY_DN20676_c0_g1_i6_orf1	aldo-keto reductase AKR2E4-like isoform X1 [Ostrinia furnacalis]	0.405438312	-1.302445673	2.78E-06	down	yes	0.999	2.464	2.503	2.501	2.388	1	0.991	1.006	MF:catalytic activity;MF:molecular_function;MF:oxidoreductase activity;	GO:0003	------	------	------	------	COG0656	S:Function unknown	PF00248.24	Aldo_ket_red	Aldo/keto reductase family	CYT	13	47	40.6	High
TRINITY_DN80424_c0_g1_i1_orf1	PREDICTED: cytoplasmic FMR1-interacting protein [Dufourea novaeangliae] >KZC10094.1 Cytoplasmic FMR1-interacting protein [Dufourea novaeangliae]	0.474270684	-1.076217401	1.59E-06	down	yes	0.9917	2.091	2.076	2.114	2.083	1	1.025	0.95	BP:regulation of organelle organization;BP:regulation of cellular component organization;BP:regulation of actin polymerization or depolymerization;BP:regulation of biological process;BP:cellular component organization or biogenesis;BP:regulation of actin filament length;BP:regulation of actin filament polymerization;BP:regulation of cytoskeleton organization;BP:cellular component organization;BP:regulation of anatomical structure size;BP:biological regulation;BP:regulation of protein polymerization;BP:regulation of biological quality;BP:regulation of actin filament-based process;BP:cellular process;BP:regulation of cellular process;BP:biological_process;BP:regulation of actin cytoskeleton organization;BP:regulation of protein complex assembly;BP:regulation of fibril organization;BP:regulation of cellular component biogenesis;BP:regulation of actin filament organization;BP:regulation of cellular component size;CC:cytoplasm;CC:cellular [illegible]	GO:0033	K05749	CYFIP	map05130;m	Pathogenic Escheri	ENOG410XPKW	S:Function unknown	PF05994.14	FragX_IP	Cytoplasmic Fragile-X interacting family	CYT	1	2	69.2	High
TRINITY_DN7740_c0_g1_i2_orf1	D-arabinitol dehydrogenase 1 [Eumeta japonica]	0.412633306	-1.277067821	2.05E-05	down	yes	1.006	2.438	2.343	2.544	2.428	1	0.974	1.044	MF:oxidoreductase activity;MF:cation binding;MF:transition metal ion binding;MF:ion binding;MF:catalytic activity;MF:molecular_function;MF:binding;MF:metal ion binding;MF:zinc ion	GO:0016	K17818	ARD1	map00040	Pentose and glucur	COG1063	E:Amino acid transport and meta	------	------	------	CYT	1	69	9.3	High
TRINITY_DN7183_c0_g1_i2_orf1	seminal fluid protein CSSFP028 [Chilo suppressalis]	0.484305151	-1.046011749	2.13E-05	down	yes	0.9967	2.058	2.128	2.073	1.973	1	0.98	1.01	BP:primary metabolic process;BP:carbohydrate metabolic process;BP:organic substance metabolic process;BP:biological_process;BP:metabolic process;MF:hydrolase activity;MF:catalytic activity;MF:hydrolase activity, acting on glycosyl bonds;MF:hydrolase activity, hydrolyzing O-glycosyl [illegible]	GO:0044	------	------	------	------	COG2723	U:Intracellular trafficking, secretic	PF00232.21	Glyco_hydro_1	Glycosyl hydrolase family 1	CYT	12	25	58.9	High
TRINITY_DN44517_c0_g1_i4_orf1	regucalcin-like [Ostrinia furnacalis]	0.416631843	-1.263154987	0.0002824	down	yes	0.997	2.393	2.452	2.533	2.194	1	0.897	1.094	CC:cytoplasm;CC:cellular_component;CC:cellular anatomical entity;MF:hydrolase activity;MF:metal ion binding;MF:cation binding;MF:enzyme regulator activity;MF:gluconolactonase activity;MF:carboxylic ester hydrolase activity;MF:ion binding;MF:catalytic activity;MF:molecular_function;MF:hydrolase activity, acting on ester bonds;MF:binding;MF:molecular function regulator;MF:calcium ion [illegible]	GO:0005	K01053	gnl, RGN	map00030;m	Pentose phosphate	COG3386;COG3	I:Lipid transport and metabolism	PF08450.15;PF07	SGL;Reg_prop	SMP-30/Gluconolactonase/LRE-like region;Two component regulator propeller	CYT	1	4	36.9	High

TRINITY_DN3732_c1_g1_i5_orf1	cytochrome P450 6B2-like [Ostrinia furnacalis]	0.466811384	-1.099088349	3.46E-06	down	yes	0.9677	2.073	2.086	2.039	2.094	1	0.918	0.985	MF:cation binding;MF:monooxygenase activity;MF:heme binding;MF:transition metal ion binding;MF:tetrapyrrole binding;MF:organic cyclic compound binding;MF:oxidoreductase activity, acting on paired donors, with incorporation or reduction of molecular oxygen;MF:ion binding;MF:catalytic activity;MF:molecular_function;MF:binding;MF:iron ion binding;MF:metal ion binding;MF:heterocyclic compound binding;MF:oxidoreductase activity;	GO:00431	K14999	CYP6	------	------	COG2124	Q:Secondary metabolites biosynt	PF00067.25	p450	Cytochrome P450	CYT	5	23	55.1	High
TRINITY_DN2798_c0_g1_i5_orf1	arylsulfatase B-like isoform X1 [Ostrinia furnacalis]	0.458232738	-1.125847561	3.83E-05	down	yes	0.949	2.071	2.155	2.077	1.981	1	0.915	0.932	MF:hydrolase activity;MF:molecular_function;MF:hydrolase activity, acting on ester bonds;MF:sulfuric ester hydrolase activity;MF:catalytic activity;	GO:00167	K01135	ARSB	map04142;m	Lysosome;Glycosan	COG3119	S:Function unknown	PF00884.26;PF01	Sulfatase;Phosphodiest	Sulfatase;Type I phosphodiesterase / nucleotide	CYT	3	7	66.8	High
TRINITY_DN20658_c0_g2_i3_orf1	prostaglandin reductase 1-like [Ostrinia furnacalis]	0.415394295	-1.267446691	0.0001718	down	yes	0.9903	2.384	2.464	2.181	2.507	1	0.968	1.003	CC:cytoplasm;CC:cellular_component;CC:cellular anatomical entity;MF:13-prostaglandin reductase activity;MF:oxidoreductase activity, acting on CH-OH group of donors;MF:oxidoreductase activity, acting on the CH-CH group of donors, NAD or NADP as acceptor;MF:oxidoreductase activity, acting on the CH-OH group of donors, NAD or NADP as acceptor;MF:oxidoreductase activity, acting on the CH-CH group of donors;MF:catalytic activity;MF:molecular_function;MF:15-oxoprostaglandin 13-oxidase activity;MF:2-alkenal reductase	GO:00057	K13948	PTGR1, LTB4	map00590	Arachidonic acid m	COG2130	S:Function unknown	PF00107.29;PF13	ADH_zinc_N;ADH_zinc_N_2	Zinc-binding dehydrogenase;Zinc-binding dehydrogenase	CYT	7	27	25.7	High
TRINITY_DN9608_c0_g1_i3_orf1	cytochrome P450 monooxygenase CYP9G18 [Cnaphalocrocis medinalis]	0.409903076	-1.286645278	9.23E-06	down	yes	0.9727	2.373	2.453	2.292	2.375	1	0.942	0.976	CC:endoplasmic reticulum membrane;CC:intrinsic component of membrane;CC:organelle membrane;CC:integral component of membrane;CC:membrane;CC:cellular anatomical entity;CC:cellular_component;MF:monooxygenase activity;MF:oxidoreductase activity, acting on paired donors, with incorporation or reduction of molecular oxygen;MF:molecular_function;MF:binding;MF:heterocyclic compound binding;MF:oxidoreductase activity;MF:cation binding;MF:transition metal ion binding;MF:ion binding;MF:iron ion binding;MF:metal ion binding;MF:heme binding;MF:catalytic activity;MF:tetrapyrrole	GO:00057	K15003	CYP9	------	------	COG2124	K:Transcription	PF00067.25	p450	Cytochrome P450	CYT	7	17	58.5	High
TRINITY_DN4343_c0_g1_i2_orf1	uncharacterized protein LOC114365231 isoform X3 [Ostrinia furnacalis]	0.439446367	-1.186240996	1.57E-05	down	yes	1.016	2.312	2.282	2.383	2.27	1	0.959	1.088	------	------	------	------	------	------	ENOG410YXRP	S:Function unknown	PF16091.8	DUF4820	Domain of unknown function	CYT	9	28	43.4	High
TRINITY_DN42856_c0_g1_i1_orf1	GSCOCG00007769001-RA-CDS [Cotesia congregata] >CAG5103393.1 Similar to GLDC: Glycine dehydrogenase (decarboxylating) [Cotesia congregata]	0.47309217	-1.07980681	0.0003817	down	yes	0.9547	2.018	1.923	2.192	1.939	1	0.873	0.991	BP:glycine catabolic process;BP:carboxylic acid metabolic process;BP:cellular amino acid catabolic process;BP:nitrogen compound metabolic process;BP:small molecule metabolic process;BP:small molecule catabolic process;BP:serine family amino acid metabolic process;BP:organic substance catabolic process;BP:glycine metabolic process;BP:cellular amino acid metabolic process;BP:organic substance metabolic process;BP:alpha-amino acid metabolic process;BP:alpha-amino acid catabolic process;BP:cellular process;BP:biological_process;BP:metabolic process;BP:oxoacid metabolic process;BP:catabolic process;BP:serine family amino acid catabolic process;BP:cellular catabolic process;BP:primary metabolic process;BP:organonitrogen compound metabolic process;BP:organonitrogen compound catabolic process;BP:organic acid metabolic process;BP:carboxylic acid catabolic process;BP:organic acid catabolic process;BP:cellular metabolic process;CC:intracellular organelle;CC:membrane-bounded organelle;CC:organelle;CC:mitochondrio	GO:00065	K00281	GLDC, gcvP	map00630;m	Glyoxylate and dica	COG0403	E:Amino acid transport and meta	PF02347.19	GDC-P	Glycine cleavage system P-protein	CYT	1	4	110.7	High
TRINITY_DN3822_c0_g1_i7_orf1	hypothetical protein HF086_002539 [Spodoptera exigua] >CAH0699327.1 unnamed protein product [Spodoptera exigua]	0.435942282	-1.197790956	7.09E-07	down	yes	0.997	2.287	2.329	2.286	2.247	1	0.99	1.001	BP:nucleoside monophosphate biosynthetic process;BP:ribose phosphate biosynthetic process;BP:ribonucleoside monophosphate metabolic process;BP:cellular biosynthetic process;BP:cellular nitrogen compound metabolic process;BP:nucleotide biosynthetic process;BP:biological_process;BP:nucleoside monophosphate metabolic process;BP:purine-containing compound metabolic process;BP:purine-containing compound biosynthetic process;BP:organic cyclic compound biosynthetic process;BP:ribonucleotide metabolic process;BP:purine ribonucleoside monophosphate biosynthetic process;BP:nucleobase-containing compound metabolic process;BP:organic substance metabolic process;BP:organophosphate biosynthetic process;BP:purine nucleoside monophosphate biosynthetic process;BP:organic cyclic compound metabolic process;BP:IMP metabolic process;BP:heterocycle biosynthetic process;BP:cellular	GO:00091	K01587	PAICS	map00230	Purine metabolism	COG0152	F:Nucleotide transport and meta	PF01259.21;PF00	SAICAR_synt;AIRC	SAICAR synthetase;AIR carboxylase	CYT	28	60	47.1	High
TRINITY_DN103475_c0_g1_i4_orf1	lipid storage droplets surface-binding protein 1 isoform X3 [Ostrinia furnacalis]	0.497879859	-1.006130441	0.0001722	down	yes	0.9863	1.981	1.99	2.097	1.855	1	0.943	1.016	------	------	K17284	PLIN2, ADRP	map03320	PPAR signaling pat	ENOG410ZHRU	S:Function unknown	PF03036.19	Perilipin	Perilipin family	CYT	3	23	18	High
TRINITY_DN59422_c0_g1_i2_orf1	larval cuticle protein LCP-22-like isoform X2 [Pectinophora gossypiella]	0.467713004	-1.096304552	2.82E-05	down	yes	1.043	2.23	2.21	2.314	2.167	1	1.019	1.111	MF:molecular_function;MF:structural constituent of cuticle;MF:structural molecule activity;	GO:0003(	------	------	------	------	ENOG4111CQ7;E	S:Function unknown;	PF00379.26	Chitin_bind_4	Insect cuticle protein	CYT	4	38	19.5	High
TRINITY_DN28741_c0_g1_i3_orf1	uncharacterized protein LOC114351652 [Ostrinia furnacalis]	0.483598875	-1.048117205	5.57E-05	down	yes	1.032	2.134	2.078	2.092	2.231	1	1.105	0.99	BP:organic substance metabolic process;BP:primary metabolic process;BP:carbohydrate metabolic process;BP:metabolic process;BP:biological_process;MF:glucosidase activity;MF:hydrolase activity;MF:hydrolase activity, acting on glycosyl bonds;MF:carbohydrate binding;MF:alpha-glucosidase activity;MF:catalytic activity;MF:molecular_function;MF:binding;MF:hydrolase activity, hydrolyzing O-glycosyl compounds;	GO:00717	K05546;K	GANAB;nag3	map00510;m	N-Glycan biosynth	COG1501	J:Translation, ribosomal structure	PF01055.29;PF17	Glyco_hydro_31;DUF5110;F5_F8_type_C;Gal_mutarotas_2	Glycosyl hydrolases family 31;Domain of unknown function;F5/8 type C domain;Galactose mutarotase-like	CYT	3	4	123.5	High

TRINITY_DN1352_c0_g1_i5_orf1	uncharacterized protein LOC113491815 [Trichoplusia ni]	0.439611291	-1.185699654	4.18E-05	down	yes	0.95	2.161	2.085	2.271	2.128	1	0.906	0.944	BP:positive regulation of ion transmembrane transporter activity;BP:positive regulation of transporter activity;BP:regulation of ion transmembrane transporter activity;BP:regulation of transport;BP:modulation of synaptic transmission;BP:regulation of potassium ion transport;BP:regulation of signaling;BP:positive regulation of transmembrane transport;BP:regulation of ion transmembrane transport;BP:positive regulation of ion transmembrane transport;BP:regulation of biological process;BP:regulation of transmembrane transport;BP:positive regulation of potassium ion transport;BP:regulation of cell communication;BP:rhythmic process;BP:regulation of transporter activity;BP:biological regulation;BP:regulation of ion transport;BP:positive regulation of molecular function;BP:regulation of trans-synaptic signaling;BP:positive regulation of biological process;BP:regulation of molecular function;BP:positive regulation of potassium ion transmembrane [illegible]	GO:0032[illegible]	------	------	------	------	ENOG410Z7FK	S:Function unknown	PF17064.8	QVR	Sleepless protein	CYT	3	28	16.5	High
TRINITY_DN1230_c1_g1_i5_orf1	uncharacterized protein LOC114353440 [Ostrinia furnacalis]	0.425452856	-1.232928816	7.43E-05	down	yes	0.916	2.153	2.169	2.224	2.066	1	0.942	0.806	------	------	------	------	------	------	ENOG4110OI3;E[illegible]	S:Function unknown;	------	------	------	CYT	1	3	35.6	High
TRINITY_DN22513_c0_g1_i4_orf1	DNA-directed RNA polymerase II subunit RPB1 [Ostrinia furnacalis] >XP_028179194.1 DNA-directed RNA polymerase II subunit RPB1 [Ostrinia furnacalis] >XP_028179195.1 DNA-directed RNA polymerase II subunit RPB1 [Ostrinia furnacalis]	0.46559655	-1.102847727	0.001373	down	yes	0.9717	2.087	1.829	2.232	2.199	1	1.051	0.864	MF:molecular_function;MF:structural constituent of cuticle;MF:structural molecule activity;	GO:0003[illegible]	------	------	------	------	ENOG4111M49	S:Function unknown	PF00379.26	Chitin_bind_4	Insect cuticle protein	CYT	1	5	40.9	Medium
TRINITY_DN36434_c0_g2_i3_orf1	clotting factor B isoform X1 [Ostrinia furnacalis]	0.493037648	-1.02023028	0.0002904	down	yes	0.956	1.939	1.82	1.907	2.091	1	0.926	0.942	[illegible] process;BP:organonitrogen compound metabolic process;BP:protein metabolic process;BP:macromolecule metabolic process;BP:biological_process;BP:nitrogen compound metabolic process;BP:metabolic process;BP:proteolysis;BP:primary metabolic process;MF:serine-type endopeptidase activity;MF:endopeptidase activity;MF:hydrolase activity;MF:serine hydrolase activity;MF:catalytic activity, acting on a protein;MF:catalytic activity;MF:molecular_function;MF:pepti	GO:0071[illegible]	------	------	------	------	COG5640	U:Intracellular trafficking, secretic	PF00089.29	Trypsin	Trypsin	CYT	3	9	48.8	High
TRINITY_DN45097_c0_g1_i5_orf1	ras-related protein Rab-24-like [Ostrinia furnacalis]	0.490557276	-1.027506505	1.41E-05	down	yes	0.9507	1.938	1.96	1.957	1.896	1	0.889	0.963	[illegible] binding;MF:molecular_function;MF:binding;MF:ion binding;MF:heterocyclic compound binding;MF:nucleoside phosphate binding;MF:anion binding;MF:guanyl nucleotide binding;MF:nucleotide binding;MF:guanyl ribonucleotide binding;MF:purine nucleotide binding;MF:GTP binding;MF:hydrolase activity;MF:GTPase activity;MF:nucleoside-triphosphatase activity;MF:small molecule binding;MF:catalytic activity;MF:hydrolase activity, acting on acid anhydrides, in phosphorus-containing anhydrides;MF:carbohydrate derivative binding;MF:organic cyclic compound binding;MF:hydrolase activity, acting on acid anhydrides;MF:pyrophosphatase [illegible]	GO:0035[illegible]	K07912	RAB24	------	------	ENOG410YCCP	S:Function unknown	PF00071.25;PF08[illegible]	Ras;Roc;Arf	Ras family;Ras of Complex, Roc, domain of DAPkinase;ADP-ribosylation factor family	CYT	2	9	23.2	High
TRINITY_DN2813_c0_g1_i7_orf1	arylphorin subunit alpha-like [Ostrinia furnacalis]	0.166841553	-2.583449449	1.38E-06	down	yes	0.954	5.718	5.597	5.919	5.637	1	0.948	0.914	------	------	------	------	------	------	ENOG4110QFU;[illegible]	S:Function unknown;	PF03723.17;PF00[illegible]	Hemocyanin_C;Hemocyanin_M;Hemocyanin_N	Hemocyanin, ig-like domain;Hemocyanin, copper containing domain;Hemocyanin, all-alpha domain	CYT	6	36	139.8	High
TRINITY_DN7064_c0_g1_i19_orf1	unnamed protein product [Chilo suppressalis]	0.271902131	-1.878840636	0.0003009	down	yes	1.378	5.068	5	5.356	4.848	1	1.915	1.219	------	------	------	------	------	------	------	------	PF00050.24;PF07[illegible]	Kazal_1;Kazal_2	Kazal-type serine protease inhibitor domain;Kazal-type serine protease inhibitor domain	EXC	1	2	73	High
TRINITY_DN30306_c0_g2_i1_orf1	perilipin-4-like isoform X3 [Ostrinia furnacalis]	0.197851563	-2.337509636	1.21E-05	down	yes	1.013	5.12	4.939	5.428	4.992	1	1.032	1.006	------	------	K20254	PLIN4	map03320	PPAR signaling pat	ENOG4112C4W	S:Function unknown	------	------	------	CYT	6	64	7.2	High
TRINITY_DN103107_c0_g1_i2_orf1	superoxide dismutase [Cu-Zn] [Ostrinia furnacalis] >XP_028177872.1 superoxide dismutase [Cu-Zn] [Ostrinia furnacalis]	0.2185193	-2.19416739	5.65E-05	down	yes	1.036	4.741	4.549	5.113	4.56	1	1.205	0.904	[illegible] superoxide radicals as acceptor;MF:cation binding;MF:antioxidant activity;MF:ion binding;MF:catalytic activity;MF:molecular_function;MF:binding;MF:superoxide dismutase [illegible]	GO:0016[illegible]	K04565	SOD1	map05208;m[illegible]	Chemical carcinoge	COG2032	P:Inorganic ion transport and me	PF00080.23	Sod_Cu	Copper/zinc superoxide dismutase	PLA	1	47	8.5	High
TRINITY_DN1391_c0_g1_i29_orfp1	TRINITY_DN1391_c0_g1_i29_m.70767 TRINITY_DN1391_c0_g1::TRINITY_DN1391_c0_g1_i29::g.70767 ORF type:complete len:495 (+),score=158.49 TRINITY_DN1391_c0_g1_i29:728-2212(+)	0.271781534	-1.879480656	5.12E-06	down	yes	1.045	3.845	3.833	3.989	3.713	1	1.035	1.101	------	------	------	------	------	------	------	------	------	------	------	CYT	5	14	52.3	High
TRINITY_DN59885_c0_g1_i3_orf1	TGF-beta-activated kinase 1 and MAP3K7-binding protein 1-like [Ostrinia furnacalis]	0.265053905	-1.9156423	4.38E-05	down	yes	1.008	3.803	3.617	4.083	3.708	1	1.065	0.959	[illegible] dephosphorylation;BP:nitrogen compound metabolic process;BP:organic substance metabolic process;BP:cellular process;BP:macromolecule modification;BP:protein modification process;BP:biological_process;BP:metabolic process;BP:primary metabolic process;BP:organonitrogen compound metabolic process;BP:protein metabolic process;BP:cellular metabolic process;BP:macromolecule metabolic process;BP:phosphate-containing compound metabolic process;BP:phosphorus metabolic process;MF:phosphatase activity;MF:phosphoric ester hydrolase activity;MF:molecular_function;MF:phosphoprotein phosphatase activity;MF:hydrolase activity;MF:catalytic activity, acting on a protein;MF:hydrolase activity, acting on ester bonds;MF:catalytic [illegible]	GO:0016[illegible]	K04403	MAP3K7IP1,	map05140;m[illegible]	Leishmaniasis;Hum	COG0631	S:Function unknown	PF00481.24	PP2C	Protein phosphatase 2C	CYT	1	3	51	High
TRINITY_DN5153_c1_g1_i1_orf1	nose resistant to fluoxetine protein 6-like isoform X1 [Ostrinia furnacalis]	0.277531821	-1.8492749	6.61E-05	down	yes	1.003	3.614	3.781	3.662	3.4	1	0.83	1.18	[illegible] membrane;CC:cellular_component;CC:intrinsic component of membrane;CC:cellular anatomical entity;MF:catalytic activity;MF:transferase activity;MF:transferase activity, transferring acyl groups;MF:molecular_function;MF:transferase activity, transferring acyl groups [illegible]	GO:0016[illegible]	------	------	------	------	ENOG410YD1T	S:Function unknown	------	------	------	CYT	1	1	71.6	High
TRINITY_DN7900_c0_g1_i4_orf1	uncharacterized protein LOC114366119 [Ostrinia furnacalis]	0.243063664	-2.040593855	0.0002413	down	yes	0.8743	3.597	3.987	3.323	3.48	1	0.92	0.703	------	------	K20701	PIRK	map04624	Toll and Imd signal	ENOG410Y5Y5	S:Function unknown	------	------	------	CYT	1	9	13.5	Medium

TRINITY_DN15247_c0_g1_i2_orf1	probable G-protein coupled receptor Mth-like 3 isoform X1 [Ostrinia furnacalis]	0.320748731	-1.640484538	0.0001007	down	yes	1.011	3.152	3.426	2.998	3.032	1	1.037	0.996	BP:cellular process;BP:regulation of cellular process;BP:biological regulation;BP:cell surface receptor signaling pathway;BP:biological_process;BP:regulation of biological process;BP:signal transduction;CC:integral component of membrane;CC:cellular_component;CC:cellular anatomical entity;CC:intrinsic component of membrane;MF:G-protein coupled receptor activity;MF:signaling receptor activity;MF:molecular transducer activity;MF:transmembrane	GO:00099	K04599	MTH	------	------	ENOG410XSJF;EI	S:Function unknown;	PF00002.27	7tm_2	7 transmembrane receptor	CYT	1	2	46.6	High
TRINITY_DN8158_c0_g1_i2_orf1	hypothetical protein G9C98_000136 [Cotesia typhae]	0.333007494	-1.58637345	0.03583	down	yes	1.022	3.069	4.062	1.825	3.32	1	1.036	1.03	------	------	------	------	------	------	ENOG410XR2D	S:Function unknown	PF00372.22;PF03	Hemocyanin_M;Hemocyanin_C;Hemocyanin_N	Hemocyanin, copper containing domain;Hemocyanin, ig-like domain;Hemocyanin, all-alpha domain	CYT	2	1	88	High
TRINITY_DN121802_c0_g1_i6_orfp	TRINITY_DN121802_c0_g1_i6_m.78506 TRINITY_DN121802_c0_g1::TRINITY_DN121802_c0_g1_i6::g.78506 ORF type:3prime_partial len:123 (+),score=6.04 TRINITY_DN121802_c0_g1_i6:35-367(+)	0.367560042	-1.443948158	0.0002446	down	yes	1.056	2.873	2.659	3.142	2.817	1	1.043	1.124	------	------	------	------	------	------	------	------	------	------	------	PLA	1	32	12.2	High
TRINITY_DN703_c13_g1_i1_orf1	acidic juvenile hormone-suppressible protein 1-like [Ostrinia furnacalis]	0.339538885	-1.558351289	0.0004306	down	yes	0.9867	2.906	3.241	2.632	2.846	1	0.952	1.008	------	------	------	------	------	------	ENOG410XR2D	S:Function unknown	PF00372.22;PF03	Hemocyanin_M;Hemocyanin_N;Hemocyanin_C	Hemocyanin, copper containing domain;Hemocyanin, all-alpha domain;Hemocyanin, ig-like	CYT	15	47	57.7	High
TRINITY_DN214_c0_g1_i3_orf1	uncharacterized protein LOC114352813 [Ostrinia furnacalis]	0.368943151	-1.438529559	0.0008784	down	yes	0.967	2.621	2.923	2.285	2.655	1	0.946	0.955	------	------	------	------	------	------	ENOG4110S0N;E	S:Function unknown;	PF06585.14	JHBP	Haemolymph juvenile hormone binding	CYT	3	38	19.4	High
TRINITY_DN130575_c0_g1_i1_orfp	TRINITY_DN130575_c0_g1_i1_m.77798 TRINITY_DN130575_c0_g1::TRINITY_DN130575_c0_g1_i1::g.77798 ORF type:internal len:70 (+),score=15.12 TRINITY_DN130575_c0_g1_i1:3-	0.39558121	-1.337954194	2.40E-05	down	yes	0.9937	2.512	2.471	2.645	2.421	1	0.982	0.999	------	------	------	------	------	------	------	------	------	------	------	CYT	2	74	6.7	High
TRINITY_DN11948_c0_g1_i8_orf1	cystathionine gamma-lyase [Ostrinia furnacalis]	0.449820789	-1.152577758	6.49E-06	down	yes	1.004	2.232	2.303	2.225	2.167	1	0.999	1.013	BP:carboxylic acid metabolic process;BP:cellular biosynthetic process;BP:nitrogen compound metabolic process;BP:small molecule metabolic process;BP:small molecule biosynthetic process;BP:serine family amino acid metabolic process;BP:organic substance biosynthetic process;BP:homocysteine metabolic process;BP:cellular amino acid metabolic process;BP:organic substance metabolic process;BP:homoserine metabolic process;BP:sulfur amino acid biosynthetic process;BP:alpha-amino acid biosynthetic process;BP:sulfur amino acid metabolic process;BP:cellular amino acid biosynthetic process;BP:cellular process;BP:biosynthetic process;BP:biological_process;BP:metabolic process;BP:oxoacid metabolic process;BP:serine family amino acid biosynthetic process;BP:primary metabolic process;BP:organonitrogen compound metabolic process;BP:organonitrogen compound biosynthetic process;BP:organic acid metabolic process;BP:sulfur compound biosynthetic process;BP:carboxylic acid	GO:00197	K01758	CTH	map00450;m	Selenocompound r	COG0626	E:Amino acid transport and meta	PF01053.23;PF00	Cys_Met_Meta_PP;Aminotran_1_2;Met_gamma_lyase	Cys/Met metabolism PLP-dependent enzyme;Aminotransferase class I and II;Methionine gamma-lyase	CYT	16	56	43	High
TRINITY_DN80328_c0_g1_i9_orf1	arylphorin subunit alpha-like [Ostrinia furnacalis]	0.437867975	-1.191432158	8.86E-05	down	yes	0.9817	2.242	2.394	2.135	2.197	1	0.969	0.976	------	------	------	------	------	------	ENOG410XR2D	S:Function unknown	PF00372.22	Hemocyanin_M	Hemocyanin, copper containing domain	CYT	4	84	9.1	High
TRINITY_DN4068_c0_g2_i4_orf1	larval cuticle protein LCP-17-like precursor [Papilio polytes] >BAM18876.1 cuticular protein PpolCPR2 [Papilio polytes]	0.489082969	-1.031848866	0.0001604	down	yes	1.008	2.061	1.912	2.147	2.125	1	0.988	1.037	MF:molecular_function;MF:structural constituent of cuticle;MF:structural molecule activity;	GO:0003€	------	------	------	------	ENOG4110RUZ;E	S:Function unknown;	PF00379.26	Chitin_bind_4	Insect cuticle protein	CYT	2	56	13.4	High
TRINITY_DN9492_c0_g1_i7_orf1	aldo-keto reductase AKR2E4-like [Ostrinia furnacalis]	0.464672092	-1.105715095	4.68E-05	down	yes	0.9707	2.089	2.13	2.156	1.98	1	0.926	0.986	MF:catalytic activity;MF:molecular_function;MF:oxidoreductase activity;	GO:0003ε	------	------	------	------	COG0656	S:Function unknown	PF00248.24	Aldo_ket_red	Aldo/keto reductase family	CYT	7	30	38.3	High
TRINITY_DN14611_c0_g1_i5_orf1	hsc70-interacting protein-like [Galleria mellonella]	0.45642754	-1.131542252	0.0004312	down	yes	0.948	2.077	1.94	2.227	2.064	1	0.819	1.025	CC:integral component of membrane;CC:cellular anatomical entity;CC:cellular_component;CC:intrinsic component of membrane;MF:molecular_function;MF:binding;MF:protein binding;MF:protein dimerization activity;	GO:0016(	K09560	ST13	------	------	ENOG410XR19	S:Function unknown	PF17830.4	STI1	STI1 domain	CYT	1	28	11.5	High
TRINITY_DN20133_c0_g1_i1_orf1	fructose-bisphosphate aldolase A isoform X2 [Microcebus murinus] >XP_012619765.1 fructose-bisphosphate aldolase A isoform X2 [Microcebus murinus] >XP_012619766.1 fructose-bisphosphate aldolase A isoform X2 [Microcebus murinus] >XP_012619767.1 fructose-bisphosphate aldolase A isoform X2 [Microcebus murinus] >XP_012619768.1 fructose-bisphosphate aldolase A isoform X2 [Microcebus murinus]	0.485678767	-1.04192568	0.0001799	down	yes	0.9767	2.011	1.89	1.992	2.152	1	0.957	0.973	BP:organonitrogen compound biosynthetic process;BP:protein homotetramerization;BP:negative regulation of organelle organization;BP:canonical glycolysis;BP:glycolytic process through glucose-6-phosphate;BP:small molecule metabolic process;BP:small molecule catabolic process;BP:small molecule biosynthetic process;BP:organic cyclic compound biosynthetic process;BP:organic cyclic compound metabolic process;BP:regulation of cytoskeleton organization;BP:ribonucleoside triphosphate metabolic process;BP:positive regulation of biological process;BP:negative regulation of biological process;BP:system process;BP:cellular homeostasis;BP:purine ribonucleoside triphosphate biosynthetic process;BP:purine ribonucleoside triphosphate metabolic process;BP:muscle contraction;BP:ATP metabolic process;BP:ADP metabolic process;BP:monocarboxylic acid metabolic process;BP:regulation of cell shape;BP:negative regulation of cellular	GO:1901!	K01623	ALDO	map00010;m	Glycolysis / Glucon	COG3588	U:Intracellular trafficking, secretic	PF00274.22	Glycolytic	Fructose-bisphosphate aldolase class-I	CYT	1	4	39.3	High
TRINITY_DN10373_c0_g1_i1_orf1	homocysteine S-methyltransferase 1-like [Ostrinia furnacalis] >XP_028162778.1 homocysteine S-methyltransferase 1-like [Ostrinia furnacalis]	0.486908359	-1.038277828	1.47E-05	down	yes	0.967	1.986	2.059	1.956	1.942	1	0.948	0.953	------	------	K00547	mmuM, BHM	map00270	Cysteine and methi	COG2040	E:Amino acid transport and meta	PF02574.19	S-methyl_trans	Homocysteine S-methyltransferase	CYT	4	16	38.2	High

TRINITY_DN2943_c2_g2_i1_orf1	protein phosphatase inhibitor 2-like [Ostrinia furnacalis]	0.476115486	-1.070616542	0.0001379	down	yes	0.907	1.905	1.927	1.888	1.899	1	0.773	0.948	BP:regulation of phosphate metabolic process;BP:regulation of primary metabolic process;BP:regulation of metabolic process;BP:regulation of phosphatase activity;BP:regulation of response to stimulus;BP:regulation of signaling;BP:regulation of biological process;BP:regulation of cell communication;BP:regulation of signal transduction;BP:regulation of protein metabolic process;BP:regulation of macromolecule metabolic process;BP:biological regulation;BP:regulation of protein modification process;BP:regulation of molecular function;BP:regulation of cellular metabolic process;BP:regulation of catalytic activity;BP:regulation of cellular process;BP:regulation of phosphorus metabolic process;BP:biological_process;BP:regulation of nitrogen compound metabolic process;BP:regulation of dephosphorylation;BP:regulation of protein dephosphorylation;BP:regulation of hydrolase activity;BP:regulation of phosphoprotein phosphatase activity;MF:phosphatase regulator activity;MF:molecular_function;MF:enzy	GO:00192	K16833	PPP1R2, IPP2	------	------	ENOG4111SYV	T:Signal transduction mechanism	PF04979.17	IPP-2	Protein phosphatase inhibitor 2	CYT	1	9	18.8	High
TRINITY_DN2049_c1_g1_i2_orf1	luciferin 4-monooxygenase-like [Ostrinia furnacalis]	0.495883362	-1.011927275	0.009279	down	yes	0.8673	1.749	1.464	1.955	1.827	1	0.966	0.636	------	------	K01904	4CL	map00130	Ubiquinone and ot	COG0318;COG0	I:Lipid transport and metabolism	PF00501.31;PF13	AMP-binding;AMP-binding_C	AMP-binding enzyme;AMP-binding enzyme C-terminal	CYT	1	32	59.6	High
TRINITY_DN106479_c1_g1_i1_orf1	secretory phospholipase A2 receptor-like [Ostrinia furnacalis]	0.48298573	-1.04994753	0.0001221	down	yes	0.88	1.822	1.79	1.859	1.816	1	0.807	0.833	MF:molecular_function;MF:carbohydrate binding;MF:binding;	GO:00036	------	------	------	------	------	------	------	------	------	CYT	1	20	7.8	High
TRINITY_DN1091_c0_g1_i1_orf1	macrophage mannose receptor 1-like [Pararge aegeria]	0.475385463	-1.072830309	0.001719	down	yes	0.8633	1.816	1.811	1.993	1.644	1	0.861	0.729	BP:response to extracellular stimulus;BP:nucleic acid metabolic process;BP:cellular nitrogen compound metabolic process;BP:nitrogen compound metabolic process;BP:DNA protection;BP:organic cyclic compound metabolic process;BP:nucleobase-containing compound metabolic process;BP:cellular response to stimulus;BP:cellular macromolecule metabolic process;BP:organic substance metabolic process;BP:cellular response to external stimulus;BP:cellular process;BP:cellular aromatic compound metabolic process;BP:cellular response to extracellular stimulus;BP:response to stress;BP:biological_process;BP:metabolic process;BP:cell communication;BP:heterocycle metabolic process;BP:primary metabolic process;BP:response to external stimulus;BP:cellular metabolic process;BP:macromolecule metabolic process;BP:cellular response to stress;BP:DNA metabolic process;BP:response to stimulus;MF:molecular_function;MF:hydrolase activity;MF:catalytic activity;MF:nucleoside-triphosphate	GO:00099	------	------	------	------	ENOG410XRS3;E	U:Intracellular trafficking, secretic	PF00059.24	Lectin_C	Lectin C-type domain	CYT	1	6	33.2	High
TRINITY_DN29969_c0_g1_i5_orf1	twitchin-like [Ostrinia furnacalis]	0.48594132	-1.041145983	0.02845	down	yes	0.795	1.636	1.302	2.069	1.538	1	0.746	0.639	------	------	------	------	------	------	ENOG410XQFD;I	S:Function unknown;	PF00041.24	fn3	Fibronectin type III domain	CYT	1	38	16.3	High
TRINITY_DN5696_c0_g1_i4_orf1	serine protease snake-like isoform X1 [Ostrinia furnacalis]	0.415028018	-1.26871936	0.003858	down	yes	0.8147	1.963	2.191	1.644	2.055	1	0.67	0.774	BP:organic substance metabolic process;BP:organonitrogen compound metabolic process;BP:protein metabolic process;BP:macromolecule metabolic process;BP:biological_process;BP:nitrogen compound metabolic process;BP:metabolic process;BP:proteolysis;BP:primary metabolic process;MF:serine-type endopeptidase activity;MF:endopeptidase activity;MF:hydrolase activity;MF:serine hydrolase activity;MF:catalytic activity, acting on a protein;MF:catalytic activity;MF:molecular_function;MF:pepti	GO:00717	K20677;K	HAYAN;TMP	map05164	Influenza A	COG5640	O:Posttranslational modification,	PF00089.29	Trypsin	Trypsin	CYT	1	3	43.2	High
TRINITY_DN3109_c0_g1_i5_orf1	protein takeout isoform X2 [Ostrinia furnacalis]	0.492497262	-1.021812392	0.0002177	down	yes	0.8993	1.826	1.767	1.78	1.93	1	0.853	0.845	------	------	------	------	------	------	ENOG4111H46	S:Function unknown	PF06585.14	JHBP	Haemolymph juvenile hormone binding	CYT	2	11	27.6	High
TRINITY_DN1267_c0_g2_i10_orf1	secretory phospholipase A2 receptor-like [Ostrinia furnacalis]	0.499557767	-1.00127658	0.0002553	down	yes	0.9037	1.809	1.893	1.803	1.73	1	0.803	0.908	MF:molecular_function;MF:carbohydrate binding;MF:binding;	GO:00036	------	------	------	------	ENOG410ZPTE;E	S:Function unknown;	PF00059.24	Lectin_C	Lectin C-type domain	CYT	2	5	34.1	High
TRINITY_DN6698_c0_g2_i2_orf1	protein mesh isoform X1 [Ostrinia furnacalis]	0.455731643	-1.13374355	0.0002251	down	yes	0.8627	1.893	1.89	1.916	1.874	1	0.867	0.721	------	------	K23820	SUSD2	------	------	ENOG410YZ7Z	S:Function unknown	PF03782.20;PF00	AMOP;VWD;NIDO	AMOP domain;von Willebrand factor type D domain;Nidogen-like	PLA	1	18	155	High
TRINITY_DN4123_c0_g1_i1_orf1	uncharacterized protein LOC114355030 [Ostrinia furnacalis]	0.482320442	-1.051936138	0.008494	down	yes	0.873	1.81	1.457	2.048	1.926	1	0.748	0.871	------	------	------	------	------	------	------	------	------	------	------	CYT	1	4	15.8	Medium
TRINITY_DN142652_c0_g1_i1_orf1	pre-mRNA-splicing factor RBM22 [Chelonus insularis]	2.033274956	1.023805322	0.004559	up	yes	1.161	0.571	0.509	0.668	0.536	1	1.17	1.312	CC:macromolecular complex;CC:cellular_component;CC:ribonucleoprotein complex;CC:spliceosomal complex;MF:cation binding;MF:organic cyclic compound binding;MF:ion binding;MF:molecular_function;MF:RNA binding;MF:nucleic acid binding;MF:metal ion binding;MF:heterocyclic compound	GO:00329	K12872	RBM22, SLT1	map03040	Spliceosome	ENOG410XSFK	S:Function unknown	PF00076.25;PF18	RRM_1;zf_CCCH_4	RNA recognition motif.;Zinc finger domain	CYT	1	2	45.3	High
TRINITY_DN31232_c1_g1_i9_orf1	PREDICTED: elongation factor 1-alpha 1, partial [Haliaeetus albicilla]	2.086317012	1.060958389	0.0008654	up	yes	1.078	0.5167	0.504	0.469	0.577	1	1.182	1.052	CC:cytoplasm;CC:cellular anatomical entity;CC:cellular_component;MF:purine ribonucleoside triphosphate binding;MF:molecular_function;MF:binding;MF:nucleic acid binding;MF:ion binding;MF:heterocyclic compound binding;MF:nucleoside phosphate binding;MF:guanyl nucleotide binding;MF:nucleotide binding;MF:guanyl ribonucleotide binding;MF:purine nucleotide binding;MF:GTP binding;MF:hydrolase activity;MF:translation factor activity, RNA binding;MF:GTPase activity;MF:nucleoside-triphosphatase activity;MF:small molecule binding;MF:catalytic activity;MF:translation regulator activity;MF:hydrolase activity, acting on acid anhydrides, in phosphorus-containing anhydrides;MF:translation regulator activity, nucleic acid binding;MF:carbohydrate derivative binding;MF:organic cyclic compound binding;MF:hydrolase activity, acting on acid anhydrides;MF:anion binding;MF:pyrophosphatase activity;MF:purine ribonucleotide	GO:00057	K03231	EEF1A	map05140;m	Leishmaniasis;Legic	COG5256	U:Intracellular trafficking, secretic	PF00009.30;PF03	GTP_EFTU;GTP_EFTU_D3;GTP_EFTU_D2	Elongation factor Tu GTP binding domain;Elongation factor Tu C-terminal domain;Elongation factor Tu domain 2	CYT	1	14	45.4	High
TRINITY_DN14730_c0_g1_i7_orf1	titin homolog [Ostrinia furnacalis]	2.10261194	1.07218261	0.0008348	up	yes	1.127	0.536	0.509	0.56	0.539	1	1.18	1.201	------	------	------	------	------	------	ENOG410ZVTG;E	S:Function unknown;	PF07679.19;PF13	I-set;Ig_3;Ig_2	Immunoglobulin I-set domain;Immunoglobulin domain;Immunoglobulin domain	CYT	1	0	324.7	High

TRINITY_DN72934_c0_g1_i1_orf1	carboxylesterase 8 [Streltzoviella insularis]	2.633613828	1.397043816	0.001208	up	yes	1.158	0.4397	0.419	0.483	0.417	1	1.291	1.183	MF:hydrolase activity;MF:molecular_function;MF:hydrolase activity, acting on ester bonds;MF:carboxylic ester hydrolase activity;MF:catalytic activity;	GO:0016	------	------	------	------	COG2272	I:Lipid transport and metabolism	PF00135.31;PF20	COesterase;BD-FAE;Abhydrolase_3	Carboxylesterase family;BD-FAE;alpha/beta hydrolase fold	CYT	1	5	61.5	High
TRINITY_DN57798_c0_g1_i1_orf1	ubiquitin carboxyl-terminal hydrolase 36 [Ostrinia furnacalis]	3.699439032	1.887306523	0.002947	up	yes	1.253	0.3387	0.324	0.242	0.45	1	1.346	1.412	BP:cellular catabolic process;BP:nitrogen compound metabolic process;BP:modification-dependent macromolecule catabolic process;BP:organic substance catabolic process;BP:cellular macromolecule catabolic process;BP:cellular macromolecule metabolic process;BP:protein modification by small protein conjugation or removal;BP:protein modification by small protein removal;BP:organic substance metabolic process;BP:proteolysis;BP:protein deubiquitination;BP:primary metabolic process;BP:cellular process;BP:modification-dependent protein catabolic process;BP:macromolecule modification;BP:protein modification process;BP:biological_process;BP:metabolic process;BP:catabolic process;BP:macromolecule catabolic process;BP:proteolysis involved in cellular protein catabolic process;BP:organonitrogen compound metabolic process;BP:protein metabolic process;BP:cellular metabolic process;BP:macromolecule metabolic	GO:0044	K11855	USP36_42	------	------	ENOG410XQ92	O:Posttranslational modification,	PF00443.32;PF13	UCH;UCH_1	Ubiquitin carboxyl-terminal hydrolase;Ubiquitin carboxyl-terminal hydrolase	CYT	1	2	79	High
TRINITY_DN3638_c0_g1_i1_orf1	DNA replication licensing factor Mcm3 [Ostrinia furnacalis]	2.330039526	1.220354428	0.004285	up	yes	1.179	0.506	0.514	0.518	0.486	1	1.144	1.393	------	------	K02541	MCM3	map04110;m	Cell cycle;DNA repl	COG1241	L:Replication, recombination and	PF00493.26;PF17	MCM;MCM_OB;MCM_lid;MCM_N;Mg_chelatase	MCM P-loop domain;MCM OB domain;MCM AAA-lid domain;MCM N-terminal domain;Magnesium chelatase, subunit ChlI	CYT	1	1	90.7	High
TRINITY_DN4497_c0_g1_i4_orf1	cytochrome P450 9e2-like [Ostrinia furnacalis] >QPF77612.1 cytochrome P450 monooxygenase CYP9A185 [Ostrinia furnacalis]	2.413276231	1.27099306	0.0005283	up	yes	1.127	0.467	0.473	0.486	0.442	1	1.2	1.18	CC:intrinsic component of membrane;CC:integral component of membrane;CC:cellular anatomical entity;CC:cellular_component;MF:monooxygenase activity;MF:NADPH-hemoprotein reductase activity;MF:molecular_function;MF:binding;MF:heterocyclic compound binding;MF:oxidoreductase activity;MF:cation binding;MF:transition metal ion binding;MF:oxidoreductase activity, acting on paired donors, with incorporation or reduction of molecular oxygen;MF:ion binding;MF:iron ion binding;MF:metal ion binding;MF:heme binding;MF:catalytic activity;MF:tetrapyrrole binding;MF:organic cyclic compound binding;MF:oxidoreductase activity, acting on NA;MF:oxidoreductase	GO:0031	K15003	CYP9	------	------	COG2124	Q:Secondary metabolites biosyn	PF00067.25	p450	Cytochrome P450	CYT	1	15	31.2	High
TRINITY_DN3814_c1_g1_i1_orf1	39S ribosomal protein L11, mitochondrial [Ostrinia furnacalis]	2.366666667	1.242856524	0.00558	up	yes	1.136	0.48	0.514	0.441	0.485	1	1.373	1.035	BP:cellular biosynthetic process;BP:cellular nitrogen compound metabolic process;BP:nitrogen compound metabolic process;BP:cellular macromolecule biosynthetic process;BP:organic substance biosynthetic process;BP:cellular nitrogen compound biosynthetic process;BP:cellular macromolecule metabolic process;BP:peptide biosynthetic process;BP:organic substance metabolic process;BP:amide biosynthetic process;BP:cellular process;BP:biosynthetic process;BP:macromolecule biosynthetic process;BP:biological_process;BP:metabolic process;BP:organonitrogen compound metabolic process;BP:primary metabolic process;BP:cellular amide metabolic process;BP:organonitrogen compound biosynthetic process;BP:protein metabolic process;BP:cellular metabolic process;BP:macromolecule metabolic process;BP:peptide metabolic process;BP:translation;CC:intracellular non-membrane-bounded organelle;CC:organelle;CC:cellular anatomical	GO:0044	K02867	RP-L11, MRF	map03010	Ribosome	COG0080	J:Translation, ribosomal structure	PF00298.22;PF03	Ribosomal_L11;Ribosomal_L11_N	Ribosomal protein L11, RNA binding domain;Ribosomal protein L11, N-terminal domain	CYT	1	5	22	High
TRINITY_DN58636_c0_g1_i1_orf1	uncharacterized protein LOC114363665 [Ostrinia furnacalis]	2.228829198	1.156286063	0.0002593	up	yes	1.087	0.4877	0.471	0.527	0.465	1	1.112	1.15	BP:nucleic acid metabolic process;BP:cellular nitrogen compound metabolic process;BP:nitrogen compound metabolic process;BP:ncRNA metabolic process;BP:organic cyclic compound metabolic process;BP:nucleobase-containing compound metabolic process;BP:organic substance metabolic process;BP:rRNA processing;BP:ncRNA processing;BP:cellular process;BP:cellular aromatic compound metabolic process;BP:biological_process;BP:metabolic process;BP:heterocycle metabolic process;BP:RNA metabolic process;BP:primary metabolic process;BP:rRNA metabolic process;BP:maturation of SSU-rRNA;BP:cellular metabolic process;BP:macromolecule metabolic process;BP:RNA processing;CC:organelle;CC:nucleolus;CC:cellular anatomical entity;CC:intracellular non-membrane-bounded organelle;CC:intracellular organelle;CC:cellular_component;CC:no	GO:0090	------	------	------	------	ENOG41118C1	S:Function unknown	------	------	------	CYT	1	1	66.5	Medium

TRINITY_DN1757_c0_g1_i4_orf1	F-box/LRR-repeat protein 2 isoform X1 [Ostrinia furnacalis]	2.042201835	1.030125458	0.002438	up	yes	1.113	0.545	0.586	0.431	0.618	1	1.206	1.134	BP:cellular catabolic process;BP:nitrogen compound metabolic process;BP:modification-dependent macromolecule catabolic process;BP:organic substance catabolic process;BP:cellular macromolecule catabolic process;BP:cellular macromolecule metabolic process;BP:proteasome-mediated ubiquitin-dependent protein catabolic process;BP:organic substance metabolic process;BP:proteolysis;BP:primary metabolic process;BP:cellular process;BP:modification-dependent protein catabolic process;BP:SCF-dependent proteasomal ubiquitin-dependent protein catabolic process;BP:biological_process;BP:protein catabolic process;BP:metabolic process;BP:catabolic process;BP:macromolecule catabolic process;BP:proteolysis involved in cellular protein catabolic process;BP:organonitrogen compound metabolic process;BP:organonitrogen compound catabolic process;BP:protein metabolic process;BP:proteasomal protein catabolic process;BP:cellular metabolic process;BP:macromolecule metabolic process;BP:ubiquitin-	GO:0044[illegible]	------	------	------	------	ENOG410YRGC;[illegible]	S:Function unknown;	PF12937.10;PF00[illegible]	F-box-like;F-box	F-box-like;F-box domain	CYT	1	2	59.1	High
TRINITY_DN37699_c0_g1_i3_orfp1	TRINITY_DN37699_c0_g1_i3_m.58788 TRINITY_DN37699_c0_g1::TRINITY_DN37699_c0_g1_i3::g.58788 ORF type:internal len:122 (+),score=39.86 TRINITY_DN37699_c0_g1_i3:1-	2.753083547	1.461048391	0.001557	up	yes	1.183	0.4297	0.406	0.446	0.437	1	1.333	1.215	------	------	------	------	------	------	------	------	------	------	------	CYT	1	12	12.6	High
TRINITY_DN24917_c0_g2_i1_orf1	hypothetical protein L3Q82_022586 [Scortum barcoo]	2.347086875	1.230871243	0.00105	up	yes	1.132	0.4823	0.44	0.539	0.468	1	1.241	1.155	CC:DNA packaging complex;CC:intracellular organelle;CC:nucleosome;CC:membrane-bounded organelle;CC:nucleus;CC:nucleoplasm;CC:cellular anatomical entity;CC:macromolecular complex;CC:intracellular membrane-bounded organelle;CC:protein-DNA complex;CC:cellular_component;CC:organelle;MF:DNA binding;MF:molecular_function;MF:binding;MF:nucleic acid binding;MF:heterocyclic compound binding;MF:structural constituent of chromatin;MF:structural molecule activity;MF:protein binding;MF:organic cyclic compound binding;MF:protein dimerization activity;MF:protein heterodimerization activity;	GO:0044[illegible]	K11254	H4	map05034;m[illegible]	Alcoholism;Neutrop[illegible]	COG2036	B:Chromatin structure and dynar[illegible]	PF15511.9	CENP-T_C	Centromere kinetochore component CENP-T histone fold	CYT	1	44	13.1	High
TRINITY_DN57765_c0_g1_i1_orf1	cytochrome P450 6B6-like [Ostrinia furnacalis]	2.082619456	1.058399249	0.0004486	up	yes	1.094	0.5253	0.486	0.56	0.53	1	1.116	1.166	CC:membrane;CC:endoplasmic reticulum membrane;CC:organelle membrane;CC:cellular_component;CC:cellular anatomical entity;MF:cation binding;MF:monooxygenase activity;MF:heme binding;MF:transition metal ion binding;MF:tetrapyrrole binding;MF:organic cyclic compound binding;MF:oxidoreductase activity, acting on paired donors, with incorporation or reduction of molecular oxygen;MF:ion binding;MF:catalytic activity;MF:molecular_function;MF:binding;MF:iron ion binding;MF:metal ion binding;MF:heterocyclic compound binding;MF:oxidoreductase activity;	GO:0016[illegible]	K14999	CYP6	------	------	COG2124	Q:Secondary metabolites biosyn[illegible]	PF00067.25	p450	Cytochrome P450	CYT	1	5	58.8	High
TRINITY_DN8944_c0_g1_i1_orf1	actin, clone 403 [Trichonephila clavata]	2.260482074	1.176630477	0.001934	up	yes	1.116	0.4937	0.534	0.393	0.554	1	1.24	1.107	------	------	K05692	ACTB_G1	map05164;m[illegible]	Influenza A;Hippo s[illegible]	COG5277	O:Posttranslational modification,	PF00022.22	Actin	Actin	CYT	1	64	42.8	High
TRINITY_DN21181_c0_g1_i6_orf1	unnamed protein product, partial [Brenthis ino]	2.198396794	1.136451805	0.001755	up	yes	1.097	0.499	0.613	0.481	0.403	1	1.111	1.179	BP:phosphorylation;BP:nitrogen compound metabolic process;BP:organic substance metabolic process;BP:protein phosphorylation;BP:cellular process;BP:macromolecule modification;BP:protein modification process;BP:biological_process;BP:metabolic process;BP:primary metabolic process;BP:organonitrogen compound metabolic process;BP:protein metabolic process;BP:cellular metabolic process;BP:macromolecule metabolic process;BP:phosphate-containing compound metabolic process;BP:phosphorus metabolic process;MF:purine ribonucleoside triphosphate binding;MF:molecular_function;MF:binding;MF:ion binding;MF:heterocyclic compound binding;MF:nucleoside phosphate binding;MF:anion binding;MF:carbohydrate derivative binding;MF:nucleotide binding;MF:protein serine/threonine kinase activity;MF:transferase activity;MF:protein kinase activity;MF:purine nucleotide binding;MF:AMP-activated protein kinase activity;MF:ATP binding;MF:kinase activity;MF:catalytic	GO:0016[illegible]	K07198	PRKAA, AMP	map04152;m[illegible]	AMPK signaling pa[illegible]	ENOG410XNQ0	O:Posttranslational modification,	PF00069.28;PF07[illegible]	Pkinase;PK_Tyr_Ser-Thr;AdenylateSensor;Kinase-like	Protein kinase domain;Protein tyrosine and serine/threonine kinase;Adenylate sensor of SNF1-like protein kinase;Kinase-like	CYT	1	3	58.3	High
TRINITY_DN46140_c0_g1_i1_orf1	protein PRRC2A-like isoform X4 [Ostrinia furnacalis]	2.028093131	1.020123903	0.0003136	up	yes	1.054	0.5197	0.522	0.515	0.522	1	1.145	1.016	------	------	------	------	------	------	ENOG410XQRI;E[illegible]	S:Function unknown;	PF07001.14	BAT2_N	BAT2 N-terminus	CYT	1	10	24.1	High
TRINITY_DN3027_c0_g1_i4_orf1	ribosome biogenesis protein BOP1 homolog [Ostrinia furnacalis]	2.006707658	1.004830457	0.0002956	up	yes	1.077	0.5367	0.532	0.534	0.544	1	1.158	1.073	------	------	K14824	ERB1, BOP1	------	------	ENOG410XRBX	J:Translation, ribosomal structure	PF08145.15;PF00[illegible]	BOP1NT;WD40;ANAPC4_WD40	BOP1NT;WD domain, G-beta repeat;Anaphase-promoting complex	CYT	4	6	95.8	High
TRINITY_DN14168_c0_g1_i1_orf1	transmembrane 7 superfamily member 3-like [Ostrinia furnacalis]	2.146534653	1.102009464	0.001695	up	yes	1.084	0.505	0.557	0.536	0.422	1	1.041	1.212	CC:cellular_component;CC:integral component of membrane;CC:cellular anatomical entity;CC:intrinsic component of membrane;	GO:0005[illegible]	------	------	------	------	ENOG41117R0	S:Function unknown	PF13886.9	DUF4203	Domain of unknown function	CYT	1	3	62.2	High
TRINITY_DN659_c0_g1_i3_orf1	probable malonyl-CoA-acyl carrier protein transacylase, mitochondrial [Ostrinia furnacalis]	2.146596859	1.102051271	0.0006059	up	yes	1.107	0.5157	0.46	0.539	0.548	1	1.156	1.164	BP:fatty acid biosynthetic process;BP:fatty acid metabolic process;BP:carboxylic acid metabolic process;BP:cellular biosynthetic process;BP:small molecule metabolic process;BP:small molecule biosynthetic process;BP:monocarboxylic acid biosynthetic process;BP:organic substance biosynthetic process;BP:organic substance metabolic process;BP:lipid metabolic process;BP:cellular process;BP:monocarboxylic acid metabolic process;BP:biosynthetic process;BP:biological_process;BP:metabolic process;BP:oxoacid metabolic process;BP:cellular lipid metabolic process;BP:lipid biosynthetic process;BP:primary metabolic process;BP:organic acid metabolic process;BP:carboxylic acid biosynthetic process;BP:organic acid biosynthetic process;BP:cellular metabolic	GO:0006[illegible]	K00645	fabD, MCAT,	map00061	Fatty acid biosynth[illegible]	COG0331	I:Lipid transport and metabolism	PF00698.24	Acyl_transf_1	Acyl transferase domain	CYT	1	3	33	High
TRINITY_DN3889_c0_g1_i7_orfp1	TRINITY_DN3889_c0_g1_i7_m.1657 TRINITY_DN3889_c0_g1::TRINITY_DN3889_c0_g1_i7::g.1657 ORF type:5prime_partial len:235 (+),score=70.90 TRINITY_DN3889_c0_g1_i7:1-705(+)	8.025626092	3.004613944	0.005129	up	yes	1.378	0.1717	0.191	0.208	0.116	1	1.745	1.388	------	------	------	------	------	------	------	------	------	------	------	EXC	1	38	24.3	High

TRINITY_DN29034_c0_g1_i1_orf1	trypsin-like serine protease [Ostrinia nubilalis]	2.114181965	1.080099553	3.13E-05	up	yes	1.048	0.4957	0.477	0.515	0.495	1	1.076	1.068	BP:organic substance metabolic process;BP:organonitrogen compound metabolic process;BP:protein metabolic process;BP:macromolecule metabolic process;BP:biological_process;BP:nitrogen compound metabolic process;BP:metabolic process;BP:proteolysis;BP:primary metabolic process;MF:serine-type endopeptidase activity;MF:endopeptidase activity;MF:hydrolase activity;MF:serine hydrolase activity;MF:catalytic activity, acting on a protein;MF:catalytic activity;MF:molecular_function;MF:pepti	GO:0071	K01312	PRSS1_2_3	map04972;m	Pancreatic secretio	COG5640	O:Posttranslational modification,	PF00089.29	Trypsin	Trypsin	CYT	2	16	27.5	High
TRINITY_DN21619_c0_g1_i1_orf1	28S ribosomal protein S15, mitochondrial [Ostrinia furnacalis]	2.044	1.031395196	7.90E-06	up	yes	1.022	0.5	0.476	0.524	0.5	1	1.029	1.036	BP:cellular biosynthetic process;BP:cellular nitrogen compound metabolic process;BP:nitrogen compound metabolic process;BP:cellular macromolecule biosynthetic process;BP:organic substance biosynthetic process;BP:cellular nitrogen compound biosynthetic process;BP:cellular macromolecule metabolic process;BP:peptide biosynthetic process;BP:organic substance metabolic process;BP:amide biosynthetic process;BP:cellular process;BP:biosynthetic process;BP:macromolecule biosynthetic process;BP:biological_process;BP:metabolic process;BP:organonitrogen compound metabolic process;BP:primary metabolic process;BP:cellular amide metabolic process;BP:organonitrogen compound biosynthetic process;BP:protein metabolic process;BP:cellular metabolic process;BP:macromolecule metabolic process;BP:peptide metabolic process;BP:translation;CC:intracellular non-membrane-bounded organelle;CC:organelle;CC:cellular anatomical	GO:0044	K02956	RP-S15, MRF	map03010	Ribosome	COG0184	S:Function unknown	PF00312.25	Ribosomal_S15	Ribosomal protein S15	CYT	3	14	32.8	High
TRINITY_DN94337_c0_g1_i1_orf1	hypothetical protein evm_006136 [Chilo suppressalis]	2.093877551	1.066177077	0.0004973	up	yes	1.026	0.49	0.527	0.413	0.53	1	1.095	0.983	CC:cellular_component;CC:integral component of membrane;CC:cellular anatomical entity;CC:intrinsic component of membrane;	GO:0005	------	------	------	------	ENOG410ZPQS	S:Function unknown	------	------	------	CYT	2	7	47.2	High
TRINITY_DN1375_c0_g1_i5_orf1	glutaminase [Chilo suppressalis] >CAB3528726.1 unnamed protein product [Chilo suppressalis] >CAH0405319.1 unnamed protein product [Chilo suppressalis]	2.03006135	1.021523327	6.08E-05	up	yes	0.9927	0.489	0.504	0.487	0.476	1	0.942	1.036	BP:carboxylic acid metabolic process;BP:nitrogen compound metabolic process;BP:small molecule metabolic process;BP:glutamine family amino acid metabolic process;BP:cellular amino acid metabolic process;BP:glutamine metabolic process;BP:organic substance metabolic process;BP:alpha-amino acid metabolic process;BP:cellular process;BP:biological_process;BP:metabolic process;BP:oxoacid metabolic process;BP:primary metabolic process;BP:organonitrogen compound metabolic process;BP:organic acid metabolic process;BP:cellular metabolic process;MF:molecular_function;MF:hydrolase activity;MF:catalytic activity;MF:glutaminase activity;MF:hydrolase activity, acting on carbon-nitroge;MF:hydrolase activity,	GO:0019	K01425	glsA, GLS	map05206;m	MicroRNAs in canc	COG2066	E:Amino acid transport and meta	PF04960.18;PF17	Glutaminase;EF-hand_14;Ank_2	Glutaminase;EF-hand domain;Ankyrin repeats	CYT	2	3	71.8	High
TRINITY_DN5417_c0_g1_i1_orf1	NADH dehydrogenase [ubiquinone] 1 alpha subcomplex subunit 10, mitochondrial isoform X1 [Ostrinia furnacalis] >XP_028175885.1 NADH dehydrogenase [ubiquinone] 1 alpha subcomplex subunit 10, mitochondrial isoform X2 [Ostrinia furnacalis]	2.073068894	1.051768062	1.81E-06	up	yes	0.993	0.479	0.459	0.486	0.492	1	0.98	0.999	BP:aerobic respiration;BP:generation of precursor metabolites and energy;BP:electron transport chain;BP:respiratory electron transport chain;BP:cellular process;BP:aerobic electron transport chain;BP:cellular respiration;BP:metabolic process;BP:energy derivation by oxidation of organic compounds;BP:cellular metabolic process;BP:mitochondrial electron transport, NADH to ubiquinone;BP:biological_process;CC:membrane-enclosed lumen;CC:oxidoreductase complex;CC:intracellular organelle lumen;CC:respiratory chain complex;CC:inner mitochondrial membrane protein complex;CC:mitochondrial matrix;CC:cellular anatomical entity;CC:transmembrane transporter complex;CC:catalytic complex;CC:transporter complex;CC:respiratory chain complex I;CC:macromolecular complex;CC:organelle lumen;CC:NADH dehydrogenase complex;CC:cellular_component;CC:me	GO:0009	K03954	NDUFA10	map04714;m	Thermogenesis;Oxi	ENOG41101CN	F:Nucleotide transport and meta	PF01712.22	dNK	Deoxynucleoside kinase	CYT	9	22	45.9	High
TRINITY_DN45449_c0_g1_i1_orf1	ATP-dependent helicase brm [Ostrinia furnacalis]	2.100845666	1.070970181	1.08E-07	up	yes	0.9937	0.473	0.47	0.466	0.483	1	0.99	0.991	BP:regulation of primary metabolic process;BP:regulation of metabolic process;BP:regulation of cellular biosynthetic process;BP:regulation of cellular metabolic process;BP:regulation of biological process;BP:regulation of nucleobase-containing compound metabolic process;BP:biological regulation;BP:regulation of gene expression;BP:regulation of macromolecule metabolic process;BP:regulation of biosynthetic process;BP:regulation of nucleic acid-templated transcription;BP:biological_process;BP:regulation of nitrogen compound metabolic process;BP:regulation of RNA biosynthetic process;BP:regulation of cellular process;BP:regulation of transcription, DNA-templated;BP:regulation of macromolecule biosynthetic process;BP:regulation of RNA metabolic process;CC:cellular_component;CC:SWI/SNF superfamily-type complex;CC:SWI/SNF complex;CC:catalytic complex;CC:ATPase complex;CC:macromolecular	GO:0080	K11647	SMARCA2_4	map05225;m	Hepatocellular carc	COG5076;ENOG	I:Lipid transport and metabolism	PF08880.14	QLQ	QLQ	CYT	3	20	15.3	High
TRINITY_DN112120_c0_g1_i1_orf1	juvenile hormone esterase-like [Ostrinia furnacalis]	2.008213552	1.005912693	0.0001309	up	yes	0.978	0.487	0.474	0.442	0.545	1	0.95	0.984	MF:hydrolase activity;MF:molecular_function;MF:hydrolase activity, acting on ester bonds;MF:carboxylic ester hydrolase activity;MF:catalytic activity;	GO:0016	------	------	------	------	COG2272	I:Lipid transport and metabolism	PF00135.31;PF20	COesterase;BD-FAE;Abhydrolase_3	Carboxylesterase family;BD-FAE;alpha/beta hydrolase fold	CYT	5	17	60.2	High
TRINITY_DN10644_c0_g1_i2_orf1	carboxylesterase [Cnaphalocrocis medinalis]	2.43498818	1.283914769	9.11E-06	up	yes	1.03	0.423	0.416	0.403	0.45	1	1.036	1.055	MF:hydrolase activity;MF:molecular_function;MF:hydrolase activity, acting on ester bonds;MF:carboxylic ester hydrolase activity;MF:catalytic activity;	GO:0016	------	------	------	------	COG2272	I:Lipid transport and metabolism	PF00135.31;PF20	COesterase;BD-FAE;Abhydrolase_3	Carboxylesterase family;BD-FAE;alpha/beta hydrolase fold	CYT	6	17	55	High

TRINITY_DN24873_c0_g1_i4_orf1	uncharacterized protein LOC114365742 [Ostrinia furnacalis]	2.485436893	1.313499473	2.14E-05	up	yes	1.024	0.412	0.432	0.365	0.439	1	1.029	1.042	CC:endoplasmic reticulum membrane;CC:intrinsic component of membrane;CC:organelle membrane;CC:integral component of membrane;CC:membrane;CC:cellular anatomical entity;CC:cellular_component;MF:monooxygenase activity;MF:oxidoreductase activity, acting on paired donors, with incorporation or reduction of molecular oxygen;MF:molecular_function;MF:binding;MF:heterocyclic compound binding;MF:oxidoreductase activity;MF:cation binding;MF:transition metal ion binding;MF:ion binding;MF:iron ion binding;MF:metal ion binding;MF:heme binding;MF:catalytic activity;MF:tetrapyrrole	GO:00057	K15001	CYP4	------	------	COG2124	S:Function unknown	PF00067.25	p450	Cytochrome P450	CYT		2	3	59.3	High
TRINITY_DN17559_c0_g1_i4_orf1	GDP-mannose 4,6 dehydratase isoform X2 [Ostrinia furnacalis] >XP_028166204.1 GDP-mannose 4,6 dehydratase isoform X2 [Ostrinia furnacalis]	2.511777833	1.328708863	1.95E-05	up	yes	1.013	0.4033	0.368	0.423	0.419	1	1.05	0.988	BP:GDP-mannose metabolic process;BP:GDP-L-fucose metabolic process;BP:cellular biosynthetic process;BP:cellular nitrogen compound metabolic process;BP:nitrogen compound metabolic process;BP:small molecule metabolic process;BP:organic cyclic compound biosynthetic process;BP:organic cyclic compound metabolic process;BP:nucleobase-containing compound metabolic process;BP:GDP-L-fucose biosynthetic process;BP:'de novo' GDP-L-fucose biosynthetic process;BP:organic substance metabolic process;BP:heterocycle biosynthetic process;BP:nucleotide-sugar biosynthetic process;BP:organic substance biosynthetic process;BP:nucleotide-sugar metabolic process;BP:cellular process;BP:cellular aromatic compound metabolic process;BP:biosynthetic process;BP:biological_process;BP:metabolic process;BP:nucleobase-containing compound biosynthetic process;BP:nucleobase-containing small molecule metabolic process;BP:heterocycle metabolic	GO:00196	K01711	gmd, GMDS	map00520;m	Amino sugar and n	COG1089	M:Cell wall/membrane/envelope	PF16363.8;PF013	GDP_Man_Dehyd;Epimerase;RmlD_sub_bind	GDP-mannose 4,6 dehydratase;NAD dependent epimerase/dehydratase family;RmlD substrate binding domain	CYT		4	16	40.5	High
TRINITY_DN3229_c0_g1_i1_orf1	uncharacterized protein LOC114358442 isoform X1 [Ostrinia furnacalis]	2.208588957	1.143124942	9.94E-05	up	yes	1.044	0.4727	0.452	0.453	0.513	1	1.029	1.103	MF:catalytic activity;MF:transferase activity;MF:molecular_function;	GO:00038	------	------	------	------	ENOG41129KS	S:Function unknown	PF11901.11	DUF3421	Protein of unknown function	CYT		6	37	23.3	High
TRINITY_DN24399_c0_g1_i1_orf1	retinol-binding protein pinta-like [Ostrinia furnacalis]	2.23038686	1.157293967	2.74E-05	up	yes	1.032	0.4627	0.454	0.44	0.494	1	1.071	1.024	------	------	------	------	------	------	ENOG410XRSQ	S:Function unknown	PF00650.23	CRAL_TRIO	CRAL/TRIO domain	CYT		4	15	35.4	High
TRINITY_DN6563_c0_g1_i1_orf1	cytochrome c oxidase assembly protein COX15 homolog [Ostrinia furnacalis]	2.242573152	1.165155046	7.99E-05	up	yes	1.004	0.4477	0.432	0.4	0.511	1	0.994	1.019	BP:porphyrin-containing compound metabolic process;BP:porphyrin-containing compound biosynthetic process;BP:cellular biosynthetic process;BP:cellular nitrogen compound metabolic process;BP:nitrogen compound metabolic process;BP:organic cyclic compound biosynthetic process;BP:organic cyclic compound metabolic process;BP:organic substance biosynthetic process;BP:pigment metabolic process;BP:heme biosynthetic process;BP:heme a biosynthetic process;BP:organic substance metabolic process;BP:tetrapyrrole biosynthetic process;BP:tetrapyrrole metabolic process;BP:heterocycle biosynthetic process;BP:heme metabolic process;BP:cellular process;BP:cellular aromatic compound metabolic process;BP:heme a metabolic process;BP:biosynthetic process;BP:biological_process;BP:metabolic process;BP:cellular nitrogen compound biosynthetic process;BP:pigment biosynthetic process;BP:heterocycle metabolic process;BP:organonitrogen compound metabolic process;BP:organonitrogen	GO:00067	K02259	COX15, ctaA	map00190;m	Oxidative phospho	COG5023;COG1	T:Signal transduction mechanism	PF02628.18	COX15-CtaA	Cytochrome oxidase assembly protein	CYT		3	7	47.4	High
TRINITY_DN4905_c0_g1_i6_orf1	uncharacterized protein LOC114351759 [Ostrinia furnacalis]	3.168115942	1.663625134	0.0003795	up	yes	1.093	0.345	0.25	0.402	0.383	1	1.159	1.119	------	------	------	------	------	------	------	------	------	------	------	EXC		1	4	62.4	High
TRINITY_DN14107_c0_g1_i4_orf1	bifunctional methylenetetrahydrofolate dehydrogenase/cyclohydrolase, mitochondrial isoform X1 [Ostrinia furnacalis]	2.061324786	1.043571837	0.000164	up	yes	0.9647	0.468	0.448	0.468	0.488	1	0.896	0.998	BP:one-carbon metabolic process;BP:cellular process;BP:cellular metabolic process;BP:biological_process;BP:small molecule metabolic process;BP:metabolic process;MF:methylenetetrahydrofolate dehydrogenase [NA;MF:hydrolase activity;MF:oxidoreductase activity, acting on the CH-NH group of donors;MF:methylenetetrahydrofolate dehydrogenas;MF:methenyltetrahydrofolate cyclohydrolase activity;MF:hydrolase activity, acting on carbon-nitroge;MF:oxidoreductase activity, acting on the CH-NH group of donors, NAD or NADP as acceptor;MF:catalytic activity;MF:cyclohydrolase activity;MF:molecular_function;MF:hydro	GO:00067	K13403	MTHFD2	map00670;m	One carbon pool by	COG0190	S:Function unknown	PF02882.22;PF00	THF_DHG_CYH_C;THF_DHG_CYH	Tetrahydrofolate dehydrogenase/cyclohydrolase, NA;Tetrahydrofolate dehydrogenase/cyclohydrolase, catalytic domain	CYT		2	5	36.5	High
TRINITY_DN3929_c0_g1_i1_orf1	glutathione S-transferase 1-1-like [Ostrinia furnacalis]	2.264840183	1.179409251	5.65E-05	up	yes	0.992	0.438	0.468	0.379	0.467	1	0.974	1.002	MF:catalytic activity;MF:transferase activity;MF:molecular_function;	GO:00038	K00799	GST, gst	map05207;m	Chemical carcinoge	COG0625	O:Posttranslational modification,	PF00043.28;PF14	GST_C;GST_C_3;GST_C_2	Glutathione S-transferase, C-terminal domain;Glutathione S-transferase, C-terminal domain;Glutathione S-transferase, C-terminal	CYT		3	23	18.9	High
TRINITY_DN19361_c0_g1_i7_orf1	hydroxylysine kinase [Ostrinia furnacalis] >XP_028168144.1 hydroxylysine kinase [Ostrinia furnacalis]	2.440963855	1.287450933	2.52E-06	up	yes	1.013	0.415	0.396	0.409	0.44	1	1.027	1.011	------	------	K18201	AGPHD1	map00310	Lysine degradation	COG2334	S:Function unknown	PF01636.26	APH	Phosphotransferase enzyme family	CYT		9	31	42.1	High
TRINITY_DN15965_c0_g1_i1_orf1	U3 small nucleolar RNA-associated protein 15 homolog [Ostrinia furnacalis]	2.312164371	1.209243962	0.0001152	up	yes	0.9903	0.4283	0.385	0.398	0.502	1	0.991	0.98	BP:nucleic acid metabolic process;BP:cellular nitrogen compound metabolic process;BP:nitrogen compound metabolic process;BP:ncRNA metabolic process;BP:organic cyclic compound metabolic process;BP:nucleobase-containing compound metabolic process;BP:organic substance metabolic process;BP:rRNA processing;BP:ncRNA processing;BP:cellular process;BP:cellular aromatic compound metabolic process;BP:biological_process;BP:metabolic process;BP:heterocycle metabolic process;BP:RNA metabolic process;BP:primary metabolic process;BP:rRNA metabolic process;BP:cellular metabolic process;BP:macromolecule metabolic process;BP:RNA processing;CC:organelle;CC:nucleolus;CC:cellular anatomical entity;CC:intracellular non-membrane-bounded organelle;CC:intracellular organelle;CC:cellular_component;CC:no	GO:00903	K14549	UTP15	map03008	Ribosome biogenes	ENOG410XNZ0	I:Lipid transport and metabolism	PF09384.13;PF00	UTP15_C;WD40;ANAPC4_WD40	UTP15 C terminal;WD domain, G-beta repeat;Anaphase-promoting complex subunit 4 WD40 domain	CYT		2	4	57.9	High

TRINITY_DN14019_c0_g1_i5_orf1	hypothetical protein evm_009768 [Chilo suppressalis]	2.067792939	1.048091727	5.73E-05	up	yes	0.9547	0.4617	0.491	0.438	0.456	1	0.929	0.935	BP:interspecies interaction between organisms;BP:response to biotic stimulus;BP:response to external stimulus;BP:response to external biotic stimulus;BP:response to stimulus;BP:response to bacterium;BP:defense response;BP:response to stress;BP:defense response to other organism;BP:defense response to bacterium;BP:biological_process;BP:response to other organism;CC:cellular_component;CC:extracellular region;CC:cellular anatomical	GO:0044	------	------	------	------	------	------	------	------	------	CYT	1	14	10.8	High
TRINITY_DN32997_c0_g1_i8_orf1	RNA-binding protein squid isoform X1 [Ostrinia furnacalis]	2.067782933	1.048084746	6.99E-06	up	yes	1.025	0.4957	0.473	0.51	0.504	1	1.032	1.044	------	------	K03102	SQD	map04320	Dorso-ventral axis	COG0724	G:Carbohydrate transport and m	PF00076.25;PF16	RRM_1;RRM_7	RNA recognition motif.;RNA recognition	CYT	8	21	30	High
TRINITY_DN10662_c0_g1_i4_orf1	HD domain-containing protein 2 [Ostrinia furnacalis]	2.04770568	1.03400837	3.91E-05	up	yes	1.013	0.4947	0.462	0.482	0.54	1	1.037	1.002	MF:hydrolase activity;MF:phosphatase activity;MF:hydrolase activity, acting on ester bonds;MF:phosphoric ester hydrolase activity;MF:catalytic activity;MF:molecular_function;MF:5'-deoxynucleotidase activity;	GO:0016	K07023	YGK1, HDDC	map00240;m	Pyrimidine metabo	COG1896	S:Function unknown	PF13023.9;PF129	HD_3;YfbR-like	HD domain;5'-deoxynucleotidase YfbR-like	CYT	2	10	24.1	High
TRINITY_DN57462_c0_g1_i1_orf1	glutathione S transferase-S5 [Glyphodes pyloalis]	2.356347439	1.236552277	0.002871	up	yes	1.058	0.449	0.443	0.399	0.505	1	1.231	0.942	MF:catalytic activity;MF:transferase activity;MF:glutathione transferase activity;MF:molecular_function;MF:transferase activity, transferring alkyl or ary;	GO:0003	K04097	HPGDS	map00590;m	Arachidonic acid m	ENOG41103AU;E	O:Posttranslational modification,	------	------	------	CYT	1	28	9.2	High
TRINITY_DN94625_c0_g1_i1_orf1	uncharacterized protein LOC114354112 [Ostrinia furnacalis]	2.098969072	1.069680909	8.49E-06	up	yes	1.018	0.485	0.48	0.46	0.515	1	1.027	1.027	CC:cytoplasm;CC:cellular anatomical entity;CC:cellular_component;MF:purine ribonucleoside triphosphate binding;MF:molecular_function;MF:binding;MF:nucleic acid binding;MF:ion binding;MF:heterocyclic compound binding;MF:nucleoside phosphate binding;MF:guanyl nucleotide binding;MF:nucleotide binding;MF:guanyl ribonucleotide binding;MF:purine nucleotide binding;MF:GTP binding;MF:hydrolase activity;MF:translation factor activity, RNA binding;MF:GTPase activity;MF:nucleoside-triphosphatase activity;MF:small molecule binding;MF:catalytic activity;MF:translation regulator activity;MF:hydrolase activity, acting on acid anhydrides, in phosphorus-containing anhydrides;MF:translation regulator activity, nucleic acid binding;MF:carbohydrate derivative binding;MF:organic cyclic compound binding;MF:hydrolase activity, acting on acid anhydrides;MF:anion binding;MF:pyrophosphatase activity;MF:purine ribonucleotide	GO:0005	K02358	tuf, TUFM	------	------	COG0050	J:Translation, ribosomal structure	PF00009.30;PF03	GTP_EFTU;GTP_EFTU_D3;GTP_EFTU_D2	Elongation factor Tu GTP binding domain;Elongation factor Tu C-terminal domain;Elongation factor Tu domain 2	CYT	12	36	50.9	High
TRINITY_DN754_c1_g1_i6_orf1	28S ribosomal protein S2, mitochondrial [Ostrinia furnacalis]	2.916118854	1.544049521	0.0002613	up	yes	1.109	0.3803	0.344	0.372	0.425	1	1.174	1.154	BP:cellular biosynthetic process;BP:cellular nitrogen compound metabolic process;BP:nitrogen compound metabolic process;BP:cellular macromolecule biosynthetic process;BP:organic substance biosynthetic process;BP:cellular macromolecule metabolic process;BP:peptide biosynthetic process;BP:organic substance metabolic process;BP:amide biosynthetic process;BP:cellular process;BP:biosynthetic process;BP:macromolecule biosynthetic process;BP:biological_process;BP:metabolic process;BP:cellular nitrogen compound biosynthetic process;BP:primary metabolic process;BP:organonitrogen compound metabolic process;BP:cellular amide metabolic process;BP:organonitrogen compound biosynthetic process;BP:protein metabolic process;BP:cellular metabolic process;BP:macromolecule metabolic process;BP:peptide metabolic process;BP:translation;CC:small ribosomal subunit;CC:macromolecular complex;CC:ribosomal	GO:0044	K02967	RP-S2, MRPS	map03010	Ribosome	COG0052	S:Function unknown	PF00318.23	Ribosomal_S2	Ribosomal protein S2	CYT	1	5	28.4	High
TRINITY_DN14073_c0_g1_i1_orf1	cytochrome c oxidase subunit 4 isoform 1, mitochondrial-like [Ostrinia furnacalis] >XP_028164918.1 cytochrome c oxidase subunit 4 isoform 1, mitochondrial-like [Ostrinia furnacalis]	2.12394958	1.086749519	9.69E-06	up	yes	1.011	0.476	0.476	0.451	0.501	1	1.036	0.997	BP:aerobic respiration;BP:generation of precursor metabolites and energy;BP:electron transport chain;BP:respiratory electron transport chain;BP:cellular process;BP:aerobic electron transport chain;BP:cellular respiration;BP:metabolic process;BP:energy derivation by oxidation of organic compounds;BP:mitochondrial electron transport, cytochrome c to oxygen;BP:cellular metabolic process;BP:biological_process;CC:intrinsic component of membrane;CC:integral component of membrane;CC:respiratory chain complex;CC:cytochrome complex;CC:inner mitochondrial membrane protein complex;CC:mitochondrial respiratory chain complex IV;CC:cellular anatomical entity;CC:catalytic complex;CC:respiratory chain complex IV;CC:macromolecular complex;CC:cellular_component;CC:me	GO:0009	K02263	COX4	map04714;m	Thermogenesis;Oxi	ENOG4111T97	O:Posttranslational modification,	PF02936.17	COX4	Cytochrome c oxidase subunit IV	CYT	6	47	20.2	High
TRINITY_DN69236_c0_g1_i1_orf1	peroxiredoxin [Ostrinia furnacalis]	2.103303177	1.07265682	3.83E-07	up	yes	0.9997	0.4753	0.489	0.476	0.461	1	0.996	1.003	MF:antioxidant activity;MF:peroxiredoxin activity;MF:catalytic activity;MF:molecular_function;MF:oxidoreductase activity, acting on peroxide as acceptor;MF:peroxidase activity;MF:oxidoreductase activity;	GO:0016	K03386	PRDX2_4, ah	map04214	Apoptosis - fly	COG0450;COG0	I:Lipid transport and metabolism	PF00578.24;PF10	AhpC-TSA;1-cysPrx_C;Redoxin	AhpC/TSA family;C-terminal domain of 1-Cys peroxiredoxin;R	CYT	2	14	27.8	High
TRINITY_DN57496_c0_g1_i1_orf1	NADPH:adrenodoxin oxidoreductase, mitochondrial [Ostrinia furnacalis]	2.164483573	1.114022852	4.06E-06	up	yes	1.008	0.4657	0.441	0.47	0.486	1	1	1.025	------	------	K18914	FDXR	------	------	COG0493	E:Amino acid transport and meta	PF07992.17;PF13	Pyr_redox_2;NAD_binding_8	Pyridine nucleotide-disulphide oxidoreductase:NA	CYT	2	3	52.8	High
TRINITY_DN5925_c0_g1_i5_orf1	isocitrate dehydrogenase [NAD] subunit gamma, mitochondrial-like isoform X1 [Ostrinia furnacalis]	2.174568966	1.120729464	2.72E-05	up	yes	1.009	0.464	0.442	0.449	0.501	1	1.042	0.985	BP:primary metabolic process;BP:tricarboxylic acid cycle;BP:biological_process;BP:metabolic process;CC:intracellular membrane-bounded organelle;CC:intracellular organelle;CC:mitochondrion;CC:cellular_component;CC:membrane-bounded organelle;CC:organelle;CC:cellular anatomical entity;	GO:0044	K00030	IDH3	map00020	Citrate cycl	COG0473	E:Amino acid transport and meta	PF00180.23	Iso_dh	Isocitrate/isopropylmalate dehydrogenase	CYT	2	6	42.4	High
TRINITY_DN54150_c0_g1_i1_orf1	uncharacterized protein LOC114351648 [Ostrinia furnacalis]	2.001222743	1.000881753	0.0003099	up	yes	0.982	0.4907	0.43	0.494	0.548	1	0.933	1.013	------	------	------	------	------	------	ENOG41121D5;E	S:Function unknown;	------	------	------	CYT	1	6	25.9	High

TRINITY_DN86149_c0_g1_i1_orf1	NADH dehydrogenase [ubiquinone] 1 alpha subcomplex subunit 8 [Galleria mellonella]	2.17750707	1.122677403	5.53E-06	up	yes	1.001	0.4597	0.433	0.46	0.486	1	1.014	0.99	BP:electron transport chain;BP:cellular process;BP:aerobic electron transport chain;BP:mitochondrial electron transport, NADH to ubiquinone;BP:aerobic respiration;BP:cellular respiration;BP:cellular metabolic process;BP:metabolic process;BP:respiratory electron transport chain;BP:biological_process;BP:generation of precursor metabolites and energy;BP:energy derivation by oxidation of organic compounds;CC:intracellular membrane-bounded organelle;CC:mitochondrion;CC:cellular_component;CC:intracellular	GO:0022!	K03952	NDUFA8	map04714;m	Thermogenesis;Oxi	ENOG4111IST	I:Lipid transport and metabolism	------	------	------	EXC	6	42	20.1	High
TRINITY_DN8676_c0_g1_i1_orf1	probable rRNA-processing protein EBP2 homolog [Ostrinia furnacalis]	2.104255319	1.073309764	3.23E-05	up	yes	0.989	0.47	0.45	0.459	0.501	1	1.016	0.951	BP:cellular process;BP:ribosome biogenesis;BP:cellular component biogenesis;BP:biological_process;BP:ribonucleoprotein complex biogenesis;BP:cellular component organization or biogenesis;CC:non-membrane-bounded organelle;CC:intracellular non-membrane-bounded organelle;CC:nucleolus;CC:cellular_component;CC:intracellular	GO:0009!	K14823	EBP2, EBNA1	------	------	ENOG410YYHN	S:Function unknown	PF05890.15	Ebp2	Eukaryotic rRNA processing protein EBP2	CYT	3	10	36	High
TRINITY_DN83005_c0_g1_i1_orf1	ATP synthase subunit O, mitochondrial [Danaus plexippus plexippus] >OWR53927.1 H+ transporting ATP synthase O subunit [Danaus plexippus plexippus]	2.210176991	1.144161905	7.60E-06	up	yes	0.999	0.452	0.45	0.423	0.483	1	1.009	0.988	CC:cellular_component;CC:organelle membrane;CC:membrane;CC:cellular anatomical entity;CC:mitochondrial membrane;CC:organelle inner membrane;CC:mitochondrial inner membrane;MF:cation channel activity;MF:molecular_function;MF:proton-transporting ATP synthase activity, rotational mechanism;MF:inorganic solute uptake transmembrane transporter activity;MF:hydrogen ion channel activity;MF:passive transmembrane transporter activity;MF:transporter activity;MF:ion channel activity;MF:cation transmembrane transporter activity;MF:catalytic activity;MF:ligase activity;MF:inorganic cation transmembrane transporter activity;MF:ion transmembrane transporter activity;MF:transmembrane transporter activity;MF:channel	GO:0005!	K02137	ATPeF0O, AT	map04714;m	Thermogenesis;Che	COG0712;COG0	C:Energy production and conver	PF00213.21	OSCP	ATP synthase delta	CYT	13	55	22.7	High
TRINITY_DN42506_c0_g1_i1_orf1	28S ribosomal protein S7, mitochondrial [Ostrinia furnacalis]	2.66530508	1.414300679	5.32E-05	up	yes	1.044	0.3917	0.393	0.346	0.436	1	1.085	1.046	BP:cellular biosynthetic process;BP:cellular macromolecule biosynthetic process;BP:organic substance biosynthetic process;BP:cellular nitrogen compound biosynthetic process;BP:cellular macromolecule metabolic process;BP:cellular amide metabolic process;BP:peptide biosynthetic process;BP:organic substance metabolic process;BP:organonitrogen compound biosynthetic process;BP:amide biosynthetic process;BP:cellular process;BP:biosynthetic process;BP:macromolecule biosynthetic process;BP:biological_process;BP:metabolic process;BP:organonitrogen compound metabolic process;BP:primary metabolic process;BP:protein metabolic process;BP:cellular nitrogen compound metabolic process;BP:cellular metabolic process;BP:macromolecule metabolic process;BP:nitrogen compound metabolic process;BP:peptide metabolic process;BP:translation;CC:organelle;CC:intracellular organelle;CC:non-membrane-bounded organelle;CC:cellular anatomical	GO:0044:	K02992	RP-S7, MRPS	map03010	Ribosome	COG0049	S:Function unknown	PF00177.24	Ribosomal_S7	Ribosomal protein S7p/S5e	CYT	1	4	27.3	High
TRINITY_DN14565_c0_g1_i11_orf1	4-aminobutyrate aminotransferase, mitochondrial [Galleria mellonella]	2.267926246	1.181373724	1.08E-06	up	yes	0.9963	0.4393	0.441	0.44	0.437	1	0.975	1.014	BP:neurotransmitter metabolic process;BP:neurotransmitter catabolic process;BP:carboxylic acid metabolic process;BP:cellular catabolic process;BP:nitrogen compound metabolic process;BP:regulation of neurotransmitter levels;BP:cellular amino acid metabolic process;BP:organic substance metabolic process;BP:biological regulation;BP:regulation of biological quality;BP:small molecule metabolic process;BP:gamma-aminobutyric acid metabolic process;BP:cellular process;BP:monocarboxylic acid metabolic process;BP:biological_process;BP:metabolic process;BP:oxoacid metabolic process;BP:catabolic process;BP:primary metabolic process;BP:organonitrogen compound metabolic process;BP:organic acid metabolic process;BP:cellular metabolic process;MF:binding;MF:vitamin B6 binding;MF:transaminase activity;MF:molecular_function;MF:heterocyclic compound binding;MF:anion binding;MF:ion binding;MF:transferase activity;MF:;MF:transferase activity,	GO:0042:	K13524	ABAT	map00640;m	Propanoate metab	COG0160	S:Function unknown	PF00202.24	Aminotran_3	Aminotransferase class-III	CYT	6	18	52.2	High
TRINITY_DN19186_c0_g1_i1_orf1	39S ribosomal protein L9, mitochondrial [Ostrinia furnacalis]	2.10021645	1.070538021	2.69E-05	up	yes	0.9703	0.462	0.439	0.464	0.483	1	0.933	0.978	CC:macromolecular complex;CC:ribosome;CC:intracellular non-membrane-bounded organelle;CC:ribonucleoprotein complex;CC:cellular_component;CC:intracellular organelle;CC:non-membrane-bounded organelle;CC:organelle;CC:cellular anatomical entity;	GO:0032!	K02939	RP-L9, MRPL	map03010	Ribosome	ENOG4111HFB	J:Translation, ribosomal structure	PF01281.22	Ribosomal_L9_N	Ribosomal protein L9, N-terminal domain	CYT	2	10	29.2	High
TRINITY_DN22815_c0_g1_i2_orf1	acyl carrier protein, mitochondrial isoform X1 [Ostrinia furnacalis]	2.010410028	1.007489772	0.000113	up	yes	0.9463	0.4707	0.443	0.498	0.471	1	0.928	0.911	------	------	K03955	NDUFAB1	map04714;m	Thermogenesis;Oxi	COG0236	I:Lipid transport and metabolism	PF00550.28	PP-binding	Phosphopantetheine attachment	CYT	2	15	17.3	High

TRINITY_DN10637_c0_g1_i4_orf1	V-type proton ATPase subunit d [Bombyx mandarina]	2.767547371	1.468608011	8.88E-06	up	yes	1.037	0.3747	0.377	0.353	0.394	1	1.043	1.068	CC:cellular_component;CC:proton-transporting V-type ATPase, V0 domain;CC:macromolecular complex;CC:membrane protein complex;CC:proton-transporting two-sector ATPase complex, proton-transporting domain;MF:molecular_function;MF:ATPase activity, coupled to transmembrane movement of substances;MF:inorganic solute uptake transmembrane transporter activity;MF:hydrogen-translocating pyrophosphatase activity;MF:primary active transmembrane transporter activity;MF:active transmembrane transporter activity;MF:ATPase coupled ion transmembrane transporter activity;MF:transporter activity;MF:cation transmembrane transporter activity;MF:proton-transporting ATPase activity, rotational mechanism;MF:ATPase activity, coupled to transmembrane movement of ions, rotational mechanism;MF:inorganic cation transmembrane transporter activity;MF:cation-transporting ATPase activity;MF:ion transmembrane transporter activity;MF:transmembrane	GO:0005	K02146	ATPeV0D, AT	map05152;m	Tuberculosis;Huma	COG1527	S:Function unknown	PF01992.19	vATP-synt_AC39	ATP synthase	CYT	7	20	39.6	High
TRINITY_DN34159_c0_g2_i1_orf1	guanine nucleotide exchange factor subunit Rich isoform X1 [Ostrinia furnacalis]	2.090909091	1.064130337	0.0007399	up	yes	1.035	0.495	0.581	0.461	0.443	1	0.993	1.112	BP:protein localization;BP:cellular macromolecule localization;BP:intracellular protein transport;BP:nitrogen compound transport;BP:organic substance transport;BP:macromolecule localization;BP:transport;BP:cellular process;BP:establishment of protein localization;BP:biological_process;BP:establishment of localization in cell;BP:establishment of localization;BP:localization;BP:cellular localization;BP:intracellular transport;BP:protein transport;CC:guanyl-nucleotide exchange factor complex;CC:RIC1-RGP1 guanyl-nucleotide exchange factor complex;CC:membrane;CC:cellular anatomical entity;CC:macromolecular	GO:0008	K20476	RIC1	------	------	ENOG410XT5J	S:Function unknown	------	------	------	CYT	1	5	21.7	High
TRINITY_DN32479_c0_g1_i8_orf1	hypothetical protein evm_009815 [Chilo suppressalis] >CAB3525305.1 unnamed protein product [Chilo suppressalis] >CAH0402632.1 unnamed protein product [Chilo suppressalis]	2.236132096	1.161005416	1.49E-05	up	yes	1.036	0.4633	0.477	0.436	0.477	1	1.047	1.06	CC:integral component of membrane;CC:cellular_component;CC:cellular anatomical entity;CC:intrinsic component of membrane;MF:transporter activity;MF:transmembrane transporter	GO:0016	------	------	------	------	COG0477	Q:Secondary metabolites biosynt	PF07690.19;PF00	MFS_1;Sugar_tr	Major Facilitator Superfamily;Sugar	CYT	1	2	56.4	High
TRINITY_DN20582_c0_g1_i1_orf1	2-oxoglutarate dehydrogenase, mitochondrial isoform X3 [Ostrinia furnacalis] >XP_028167081.1 2-oxoglutarate dehydrogenase, mitochondrial isoform X3 [Ostrinia furnacalis]	2.174465382	1.120660741	5.45E-06	up	yes	1.027	0.4723	0.474	0.462	0.481	1	1.056	1.024	------	------	K00164	OGDH, sucA	map00020	Citrate cycl	COG0567	Z:Cytoskeleton	PF16870.8;PF027	OxoGdeHyase_C;Transket_pyr	2-oxoglutarate dehydrogenase C-terminal;Transketolase, pyrimidine binding	CYT	7	32	25.6	High
TRINITY_DN15959_c0_g1_i1_orf1	dnaJ homolog subfamily A member 2-like [Ostrinia furnacalis]	2.022213165	1.015935082	2.15E-06	up	yes	0.9923	0.4907	0.47	0.505	0.497	1	0.997	0.98	BP:protein folding;BP:cellular process;BP:response to stress;BP:biological_process;BP:response to temperature stimulus;BP:response to heat;BP:response to abiotic stimulus;BP:response to stimulus;CC:membrane;CC:cellular_component;CC:cellular anatomical entity;MF:purine ribonucleoside triphosphate binding;MF:heat shock protein binding;MF:molecular_function;MF:binding;MF:nucleotide binding;MF:heterocyclic compound binding;MF:nucleoside phosphate binding;MF:anion binding;MF:cation binding;MF:Hsp70 protein binding;MF:unfolded protein binding;MF:ion binding;MF:purine nucleotide binding;MF:metal ion binding;MF:ATP binding;MF:small molecule binding;MF:adenyl nucleotide binding;MF:carbohydrate derivative binding;MF:organic cyclic compound binding;MF:adenyl ribonucleotide binding;MF:protein binding;MF:purine ribonucleotide	GO:0006	K09503	DNAJA2	map04141	Protein processing	COG0484	O:Posttranslational modification,	PF01556.21;PF00	DnaJ_C;DnaJ;DnaJ_CXXCXGXG	DnaJ C terminal domain;DnaJ domain;DnaJ central domain	CYT	10	33	44.9	High
TRINITY_DN1073_c0_g1_i1_orf1	carboxylesterase [Loxostege sticticalis]	2.471698113	1.305502547	3.86E-05	up	yes	1.048	0.424	0.442	0.391	0.439	1	1.05	1.093	CC:integral component of membrane;CC:cellular_component;CC:intrinsic component of membrane;CC:cellular anatomical entity;MF:hydrolase activity;MF:hydrolase activity, acting on ester bonds;MF:carboxylic ester hydrolase activity;MF:catalytic	GO:0016	------	------	------	------	COG2272;COG0	I:Lipid transport and metabolism	PF00135.31;PF20	COesterase;BD-FAE;Abhydrolase_3	Carboxylesterase family;BD-FAE;alpha/beta hydrolase fold	CYT	1	8	30.1	High
TRINITY_DN12683_c0_g1_i3_orf1	sulfated surface glycoprotein 185-like [Ostrinia furnacalis]	2.098315789	1.069231815	1.30E-07	up	yes	0.9967	0.475	0.478	0.481	0.466	1	1.002	0.988	------	------	------	------	------	------	ENOG4110QPT;E	S:Function unknown;	PF11901.11	DUF3421	Protein of unknown function	CYT	2	9	27.1	High
TRINITY_DN1656_c2_g1_i5_orf1	15-hydroxyprostaglandin dehydrogenase [NAD(+)]-like [Ostrinia furnacalis]	2.017018659	1.01222443	4.55E-05	up	yes	0.9837	0.4877	0.482	0.448	0.533	1	0.971	0.98	MF:catalytic activity;MF:molecular_function;MF:oxidoreductase activity;	GO:0003	K00069	HPGD	map00590;m	Arachidonic acid m	COG1028;COG0	S:Function unknown;	PF00106.28;PF13	adh_short;adh_short_C2;KR	short chain dehydrogenase;Enoyl;KR domain	CYT	3	17	29.5	High
TRINITY_DN2062_c0_g1_i9_orf1	uncharacterized protein LOC114350846 [Ostrinia furnacalis]	2.066805846	1.047402869	2.59E-07	up	yes	0.99	0.479	0.47	0.485	0.482	1	0.99	0.98	------	------	------	------	------	------	COG1028	S:Function unknown	PF13561.9;PF001	adh_short_C2;adh_short;KR	Enoyl;short chain dehydrogenase;KR domain	CYT	6	61	25.6	High
TRINITY_DN36538_c0_g1_i2_orf1	xaa-Pro dipeptidase isoform X1 [Ostrinia furnacalis] >XP_028156507.1 xaa-Pro dipeptidase isoform X2 [Ostrinia furnacalis]	2.309801234	1.207768708	2.74E-06	up	yes	1.011	0.4377	0.42	0.432	0.461	1	1.028	1.005	MF:cation binding;MF:transition metal ion binding;MF:aminopeptidase activity;MF:hydrolase activity;MF:catalytic activity, acting on a protein;MF:manganese ion binding;MF:catalytic activity;MF:ion binding;MF:exopeptidase activity;MF:molecular_function;MF:binding;MF:metalloaminopeptidase activity;MF:peptidase activity;MF:metal ion binding;MF:metalloexopeptidase	GO:0043	K14213	PEPD	------	------	COG0006	E:Amino acid transport and meta	PF00557.27;PF05	Peptidase_M24;AMP_N	Metallopeptidase family M24;Aminopeptidase P, N-terminal domain	CYT	9	18	60.8	High

TRINITY_DN2265_c0_g2_i1_orf1	LOW QUALITY PROTEIN: elongation factor G, mitochondrial-like [Leguminivora glycinivorella]	2.466346154	1.302375297	3.78E-05	up	yes	1.026	0.416	0.392	0.399	0.457	1	1.071	1.006	BP:cellular macromolecule biosynthetic process;BP:cellular biosynthetic process;BP:organic substance biosynthetic process;BP:cellular macromolecule metabolic process;BP:cellular process;BP:organic substance metabolic process;BP:mitochondrial translational elongation;BP:biosynthetic process;BP:macromolecule biosynthetic process;BP:biological_process;BP:metabolic process;BP:cellular metabolic process;BP:macromolecule metabolic process;BP:translational elongation;CC:intracellular organelle;CC:membrane-bounded organelle;CC:mitochondrion;CC:organelle;CC:cellular anatomical entity;CC:intracellular membrane-bounded organelle;CC:cellular_component;MF:purine ribonucleoside triphosphate binding;MF:molecular_function;MF:binding;MF:nucleic acid binding;MF:guanyl nucleotide binding;MF:heterocyclic compound binding;MF:nucleoside phosphate binding;MF:anion binding;MF:guanyl ribonucleotide binding;MF:purine nucleotide [illegible]	GO:0034€	K02355	fusA, GFM, E	------	------	COG0480	U:Intracellular trafficking, secretic	PF00679.27;PF03	EFG_C;EFG_IV	Elongation factor G C-terminus;Elongation factor G, domain IV	CYT	1	7	18.1	High
TRINITY_DN1868_c0_g1_i1_orf1	protein obstructor-E isoform X1 [Ostrinia furnacalis]	2.465521413	1.301892783	3.17E-06	up	yes	1.019	0.4133	0.422	0.396	0.422	1	1.046	1.011	------	------	------	------	------	------	ENOG410XQ49	S:Function unknown	PF01607.27	CBM_14	Chitin binding Peritrophin-A domain	CYT	16	68	30.6	High
TRINITY_DN32161_c0_g1_i1_orf1	uncharacterized protein LOC114352518 [Ostrinia furnacalis]	2.080551368	1.056965908	2.16E-05	up	yes	0.966	0.4643	0.442	0.468	0.483	1	0.937	0.961	------	------	------	------	------	------	ENOG410YKCN;I	S:Function unknown;	------	------	------	CYT	1	6	15.2	High
TRINITY_DN23343_c0_g1_i9_orf1	pre-mRNA-processing factor 6 isoform X1 [Ostrinia furnacalis] >XP_028175021.1 pre-mRNA-processing factor 6 isoform X2 [Ostrinia furnacalis]	2.226004084	1.15445624	0.0002412	up	yes	0.981	0.4407	0.379	0.472	0.471	1	1.022	0.921	BP:nucleic acid metabolic process;BP:cellular nitrogen compound metabolic process;BP:nitrogen compound metabolic process;BP:mRNA splicing, via spliceosome;BP:organic cyclic compound metabolic process;BP:nucleobase-containing compound metabolic process;BP:organic substance metabolic process;BP:RNA splicing;BP:mRNA metabolic process;BP:cellular process;BP:cellular aromatic compound metabolic process;BP:RNA splicing, via transesterification reactions;BP:RNA splicing, via transesterification reactions with bulged adenosine as nucleophile;BP:biological_process;BP:metabolic process;BP:heterocycle metabolic process;BP:RNA metabolic process;BP:primary metabolic process;BP:cellular metabolic process;BP:macromolecule metabolic process;BP:RNA processing;BP:mRNA [illegible]	GO:0090€	K12855	PRPF6, PRP6	map03040	Spliceosome	ENOG410XRKD	A:RNA processing and modificati	PF06424.15;PF13	PRP1_N;TPR_14;TPR_19	PRP1 splicing factor, N-terminal;Tetratricopeptide repeat;Tetratricopeptide repeat	CYT	1	1	104.7	High
TRINITY_DN47389_c0_g1_i2_orf1	non-specific lipid-transfer protein-like [Ostrinia furnacalis]	2.430605841	1.281315958	8.11E-08	up	yes	1.007	0.4143	0.409	0.412	0.422	1	1.017	1.005	BP:lipid transport;BP:organic substance transport;BP:transport;BP:biological_process;BP:establishment of localization;BP:localization;CC:microbody;CC:intracellular organelle;CC:membrane-bounded organelle;CC:organelle;CC:peroxisome;CC:mitochondrion;CC:cellular anatomical entity;CC:intracellular membrane-bounded organelle;CC:cellular_component;MF:molecular_function;MF:binding;MF:propanoyl-CoA C-acyltransferase activity;MF:acetyl-CoA C-myristoyltransferase activity;MF:transferase activity;MF:transferase activity, transferring acyl groups;MF:transferase activity, transferring acyl groups other than amino-acyl groups;MF:acetyl-CoA C-acyltransferase activity;MF:lipid binding;MF:myristoyltransferase activity;MF:catalytic [illegible]	GO:0006€	K08764	SCP2, SCPX	map04146;m	Peroxisome;Primary	ENOG410XPRW	I:Lipid transport and metabolism	PF00108.26;PF02	Thiolase_N;Thiolase_C	Thiolase, N-terminal domain;Thiolase, C-terminal domain	CYT	10	38	43.9	High
TRINITY_DN334_c0_g1_i3_orf1	chymotrypsin-like serine protease [Ostrinia nubilalis] >AAX62030.1 chymotrypsin-like serine protease [Ostrinia nubilalis]	2.351408951	1.233525471	0.0001779	up	yes	0.993	0.4223	0.371	0.391	0.505	1	1.002	0.977	BP:organic substance metabolic process;BP:organonitrogen compound metabolic process;BP:protein metabolic process;BP:macromolecule metabolic process;BP:biological_process;BP:nitrogen compound metabolic process;BP:metabolic process;BP:proteolysis;BP:primary metabolic process;MF:serine-type endopeptidase activity;MF:endopeptidase activity;MF:hydrolase activity;MF:serine hydrolase activity;MF:catalytic activity, acting on a protein;MF:catalytic activity;MF:molecular_function;MF:pepti [illegible]	GO:0071€	------	------	------	------	COG5640	O:Posttranslational modification,	PF00089.29	Trypsin	Trypsin	PLA	1	35	29.6	High
TRINITY_DN18860_c0_g1_i1_orf1	DDB1- and CUL4-associated factor 13 [Ostrinia furnacalis]	2.320178529	1.21423582	6.82E-06	up	yes	0.9877	0.4257	0.393	0.445	0.439	1	0.972	0.991	BP:nucleic acid metabolic process;BP:cellular nitrogen compound metabolic process;BP:nitrogen compound metabolic process;BP:ncRNA metabolic process;BP:organic cyclic compound metabolic process;BP:nucleobase-containing compound metabolic process;BP:protein modification by small protein conjugation;BP:protein modification by small protein conjugation or removal;BP:organic substance metabolic process;BP:rRNA processing;BP:ncRNA processing;BP:cellular process;BP:cellular aromatic compound metabolic process;BP:macromolecule modification;BP:protein modification process;BP:biological_process;BP:metabolic process;BP:heterocycle metabolic process;BP:RNA metabolic process;BP:primary metabolic process;BP:rRNA metabolic process;BP:organonitrogen compound metabolic process;BP:protein metabolic process;BP:cellular metabolic process;BP:macromolecule metabolic process;BP:protein ubiquitination;BP:RNA processing;CC:organelle;CC:cellular [illegible]	GO:0090€	K11806	DCAF13, WD	------	------	ENOG410XNX6	S:Function unknown	PF04158.17;PF00	Sof1;WD40;ANAPC4_WD40	Sof1-like domain;WD domain, G-beta repeat;Anaphase-promoting complex subunit 4 WD40 domain	CYT	2	3	51.5	High
TRINITY_DN4842_c0_g1_i5_orf1	cytochrome c oxidase assembly factor 4 homolog, mitochondrial isoform X1 [Ostrinia furnacalis] >XP_028162331.1 cytochrome c oxidase assembly factor 4 homolog, mitochondrial isoform X2 [Ostrinia furnacalis]	2.572729572	1.363299819	4.71E-05	up	yes	1.017	0.3953	0.355	0.45	0.381	1	1	1.051	BP:mitochondrial respiratory chain complex assembly;BP:mitochondrial respiratory chain complex IV assembly;BP:cellular component assembly;BP:macromolecular complex subunit organization;BP:cellular process;BP:respiratory chain complex IV assembly;BP:biological_process;BP:cytochrome complex assembly;BP:cellular component organization or biogenesis;BP:macromolecular complex assembly;BP:cellular component organization;CC:organelle lumen;CC:organelle envelope lumen;CC:membrane-enclosed lumen;CC:cellular_component;CC:intracellular organelle lumen;CC:mitochondrial intermembrane space;CC:cellular anatomical entity;	GO:0033€	K18177	COA4	map04714	Thermogenesis	ENOG410XXMU;	O:Posttranslational modification,	------	------	------	CYT	1	13	8.2	High

TRINITY_DN7583_c0_g1_i1_orf1	39S ribosomal protein L21, mitochondrial [Ostrinia furnacalis]	2.463696776	1.300824704	1.37E-05	up	yes	1.001	0.4063	0.44	0.362	0.417	1	1	1.003	BP:cellular biosynthetic process;BP:cellular macromolecule biosynthetic process;BP:organic substance biosynthetic process;BP:cellular nitrogen compound biosynthetic process;BP:cellular macromolecule metabolic process;BP:cellular amide metabolic process;BP:peptide biosynthetic process;BP:organic substance metabolic process;BP:organonitrogen compound biosynthetic process;BP:amide biosynthetic process;BP:cellular process;BP:biosynthetic process;BP:macromolecule biosynthetic process;BP:biological_process;BP:metabolic process;BP:organonitrogen compound metabolic process;BP:primary metabolic process;BP:protein metabolic process;BP:cellular nitrogen compound metabolic process;BP:cellular metabolic process;BP:macromolecule metabolic process;BP:nitrogen compound metabolic process;BP:peptide metabolic process;BP:translation;CC:organelle;CC:intracellular organelle;CC:non-membrane-bounded organelle;CC:cytoplasm;CC:cellular	GO:00442	K02888	RP-L21, MRF	map03010	Ribosome	ENOG4111TX4	J:Translation, ribosomal structure	PF00829.24	Ribosomal_L21p	Ribosomal prokaryotic L21 protein	CYT	1	5	19.8	High
TRINITY_DN2852_c0_g1_i9_orf1	golgin subfamily A member 4-like [Ostrinia furnacalis]	2.135056831	1.094274472	9.26E-05	up	yes	0.958	0.4487	0.411	0.441	0.494	1	0.94	0.934	------	------	K20283	GOLGA4	------	------	ENOG410XQQG	U:Intracellular trafficking, secretic	PF01465.23	GRIP	GRIP domain	CYT	4	3	155.2	High
TRINITY_DN1285_c0_g1_i6_orf1	bifunctional 3'-phosphoadenosine 5'-phosphosulfate synthase isoform X3 [Ostrinia furnacalis]	2.177149321	1.122440359	1.74E-05	up	yes	0.9623	0.442	0.436	0.433	0.457	1	0.931	0.956	BP:sulfate assimilation;BP:sulfur compound metabolic process;BP:cellular process;BP:biological_process;BP:metabolic process;BP:cellular metabolic process;MF:purine ribonucleoside triphosphate binding;MF:molecular_function;MF:binding;MF:ion binding;MF:heterocyclic compound binding;MF:nucleoside phosphate binding;MF:anion binding;MF:nucleotide binding;MF:transferase activity;MF:sulfate adenylyltransferase activity;MF:ATP binding;MF:kinase activity;MF:adenylylsulfate kinase activity;MF:small molecule binding;MF:catalytic activity;MF:purine nucleotide binding;MF:sulfate adenylyltransferas;MF:nucleotidyltransferase activity;MF:adenyl nucleotide binding;MF:phosphotransferase activity, alcohol group as acceptor;MF:transferase activity, transferring phosphorus-containing groups;MF:adenylyltransferase activity;MF:carbohydrate derivative binding;MF:adenyl ribonucleotide binding;MF:organic cyclic compound binding;MF:purine ribonucleotide	GO:00001	K13811	PAPSS	map00450;m	Selenocompound r	COG2046	P:Inorganic ion transport and me	PF01747.20;PF14	ATP-sulfurylase;PUA_2	ATP-sulfurylase;PUA-like domain	CYT	5	16	43.5	High
TRINITY_DN1266_c2_g1_i1_orf1	serine/threonine-protein kinase RIO3 [Ostrinia furnacalis]	2.577024122	1.365706042	0.003334	up	yes	1.079	0.4187	0.5	0.445	0.311	1	1.257	0.979	BP:phosphorylation;BP:nitrogen compound metabolic process;BP:organic substance metabolic process;BP:protein phosphorylation;BP:cellular process;BP:macromolecule modification;BP:protein modification process;BP:biological_process;BP:metabolic process;BP:primary metabolic process;BP:organonitrogen compound metabolic process;BP:cellular metabolic process;BP:macromolecule metabolic process;BP:protein metabolic process;BP:phosphate-containing compound metabolic process;BP:phosphorus metabolic process;MF:purine ribonucleoside triphosphate binding;MF:molecular_function;MF:binding;MF:nucleotide binding;MF:heterocyclic compound binding;MF:nucleoside phosphate binding;MF:anion binding;MF:cation binding;MF:carbohydrate derivative binding;MF:ion binding;MF:protein serine/threonine kinase activity;MF:transferase activity;MF:protein kinase activity;MF:purine nucleotide binding;MF:metal ion binding;MF:ATP	GO:00163	K08872	RIOK3, SUDD	------	------	COG1718	S:Function unknown	PF01163.25	RIO1	RIO1 family	CYT	1	2	57.3	Medium
TRINITY_DN755_c0_g1_i3_orf1	uncharacterized protein LOC114358844 [Ostrinia furnacalis]	2.149473684	1.103983448	7.99E-06	up	yes	1.021	0.475	0.465	0.498	0.462	1	1.049	1.014	CC:cellular_component;CC:integral component of membrane;CC:cellular anatomical entity;CC:intrinsic component of membrane;	GO:0005	------	------	------	------	ENOG411170P	S:Function unknown	------	------	------	CYT	2	37	7	High
TRINITY_DN30070_c0_g1_i6_orf1	uncharacterized protein LOC114361440 isoform X1 [Ostrinia furnacalis] >XP_028172261.1 uncharacterized protein LOC114361440 isoform X2 [Ostrinia furnacalis]	2.320675105	1.21454456	8.09E-05	up	yes	1.045	0.4503	0.464	0.405	0.482	1	1.04	1.096	MF:catalytic activity;MF:molecular_function;MF:oxidoreductase activity;	GO:0003	------	------	------	------	COG0656	S:Function unknown	PF00248.24	Aldo_ket_red	Aldo/keto reductase family	CYT	2	7	55	High
TRINITY_DN11013_c0_g1_i3_orf1	glutamine:fructose-6-phosphate aminotransferase 1 [Heortia vitessoides]	2.018027142	1.012945578	1.11E-05	up	yes	0.9963	0.4937	0.48	0.487	0.514	1	0.968	1.021	BP:amino sugar biosynthetic process;BP:carbohydrate derivative biosynthetic process;BP:cellular nitrogen compound metabolic process;BP:nitrogen compound metabolic process;BP:glutamine family amino acid metabolic process;BP:organic cyclic compound biosynthetic process;BP:organic cyclic compound metabolic process;BP:nucleobase-containing compound metabolic process;BP:cellular amino acid metabolic process;BP:glutamine metabolic process;BP:organic substance metabolic process;BP:alpha-amino acid metabolic process;BP:heterocycle biosynthetic process;BP:nucleotide-sugar biosynthetic process;BP:organic substance biosynthetic process;BP:nucleotide-sugar metabolic process;BP:cellular process;BP:cellular aromatic compound metabolic process;BP:biosynthetic process;BP:biological_process;BP:carboxylic acid metabolic process;BP:metabolic process;BP:nucleobase-containing compound biosynthetic process;BP:organonitrogen compound	GO:00463	K00820	glmS, GFPT	map00250;m	Alanine, aspartate a	COG0449	M:Cell wall/membrane/envelope	PF01380.25;PF13	SIS;GATase_6;GATase_7;GATase_4	SIS domain;Glutamine amidotransferase domain;Glutamine amidotransferase domain;Glutamine amidotransferases class-II	CYT	15	29	75.4	High
TRINITY_DN35277_c0_g1_i1_orf1	luciferin 4-monooxygenase-like, partial [Ostrinia furnacalis]	2.124210526	1.086926756	2.32E-05	up	yes	1.009	0.475	0.489	0.44	0.496	1	0.987	1.04	CC:cellular_component;CC:integral component of membrane;CC:cellular anatomical entity;CC:intrinsic component of membrane;	GO:0005	K01904	4CL	map00130	Ubiquinone and ot	COG0365;COG0	I:Lipid transport and metabolism	PF00501.31;PF13	AMP-binding;AMP-binding_C	AMP-binding enzyme;AMP-binding enzyme C-terminal	CYT	5	10	61.2	High
TRINITY_DN8367_c0_g1_i1_orf1	uncharacterized protein LOC114357075 [Ostrinia furnacalis]	2.2275397	1.155451144	1.52E-05	up	yes	1.024	0.4597	0.443	0.454	0.482	1	1.062	1.009	------	------	------	------	------	------	COG1028	S:Function unknown	PF00106.28;PF13	adh_short;adh_short_C2;KR	short chain dehydrogenase;Enoyl;KR domain	CYT	3	36	23.4	High

TRINITY_DN108051_c0_g1_i2_orf1	uncharacterized protein LOC114351921 [Ostrinia furnacalis]	2.083350742	1.058905744	1.15E-05	up	yes	0.9973	0.4787	0.494	0.442	0.5	1	1.006	0.986	BP:aerobic respiration;BP:generation of precursor metabolites and energy;BP:electron transport chain;BP:respiratory electron transport chain;BP:cellular process;BP:aerobic electron transport chain;BP:cellular respiration;BP:metabolic process;BP:energy derivation by oxidation of organic compounds;BP:cellular metabolic process;BP:mitochondrial electron transport, NADH to ubiquinone;BP:biological_process;CC:oxidoreductase complex;CC:respiratory chain complex;CC:inner mitochondrial membrane protein complex;CC:transmembrane transporter complex;CC:catalytic complex;CC:transporter complex;CC:respiratory chain complex I;CC:macromolecular complex;CC:NADH dehydrogenase complex;CC:cellular_component;CC:membrane protein complex;CC:mitochondrial respiratory	GO:0009(	K03962	NDUFB6	map04714;m	Thermogenesis;Oxi	ENOG4111RT5	U:Intracellular trafficking, secretic	PF09782.12	NDUF_B6	NAD	CYT	4	24	18.7	High
TRINITY_DN886_c0_g2_i4_orf1	collagenase-like [Ostrinia furnacalis]	2.118510638	1.083050373	3.28E-05	up	yes	0.9957	0.47	0.438	0.456	0.516	1	0.977	1.01	------	------	------	------	------	------	COG5640	S:Function unknown	PF00089.29	Trypsin	Trypsin	EXC	2	8	30.5	High
TRINITY_DN3428_c0_g1_i1_orf1	10 kDa heat shock protein, mitochondrial [Ostrinia furnacalis]	2.06159648	1.043761979	0.000112	up	yes	0.984	0.4773	0.457	0.471	0.504	1	1.027	0.925	MF:binding;MF:carbohydrate derivative binding;MF:anion binding;MF:adenyl nucleotide binding;MF:purine ribonucleoside triphosphate binding;MF:organic cyclic compound binding;MF:protein binding involved in protein folding;MF:nucleotide binding;MF:small molecule binding;MF:ribonucleotide binding;MF:molecular_function;MF:nucleoside phosphate binding;MF:purine nucleotide binding;MF:purine ribonucleotide binding;MF:adenyl ribonucleotide binding;MF:ion binding;MF:heterocyclic compound binding;MF:ATP binding;	GO:0005	K04078	groES, HSPE1	------	------	COG0234	O:Posttranslational modification,	PF00166.24	Cpn10	Chaperonin 10 Kd subunit	CYT	8	64	11.1	High
TRINITY_DN14601_c0_g1_i2_orf1	unnamed protein product [Chilo suppressalis]	2.201451506	1.138455065	1.07E-05	up	yes	1.001	0.4547	0.416	0.472	0.476	1	0.993	1.01	CC:cytoplasm;CC:integral component of membrane;CC:cellular_component;CC:cellular anatomical entity;CC:intrinsic component of membrane;MF:cation binding;MF:ion binding;MF:molecular_function;MF:metal ion binding;MF:binding;	GO:0005	K26089	ANKZF1, VM	------	------	ENOG410XQAG	S:Function unknown	PF18826.4;PF187	bVLRF1;VATC	Bacteroidetes VLRF1 release factor;Vms1-associating treble clef domain	CYT	2	2	76.4	High
TRINITY_DN2600_c0_g1_i7_orf1	mucin-5AC isoform X2 [Ostrinia furnacalis]	2.351281448	1.23344724	2.47E-05	up	yes	1	0.4253	0.377	0.466	0.433	1	1.001	1	------	------	------	------	------	------	ENOG4112A7J	S:Function unknown	------	------	------	CYT	9	12	98	High
TRINITY_DN6933_c1_g1_i1_orf1	Chlorophyll a-b binding protein 40, chloroplastic [Trichinella nelsoni] >KRY99282.1 Chlorophyll a-b binding protein 40, chloroplastic [Trichinella zimbabwensis]	2.334894614	1.223357435	0.0002016	up	yes	0.997	0.427	0.403	0.409	0.469	1	0.929	1.062	CC:cellular_component;CC:membrane;CC:cellular anatomical entity;	GO:0005	K08913	LHCB2	------	------	ENOG410ZHBU	S:Function unknown	PF00504.24	Chloroa_b-bind	Chlorophyll A-B binding protein	CYT	2	8	24	High
TRINITY_DN45477_c0_g1_i1_orf1	putative E3 ubiquitin-protein ligase UBR7 [Ostrinia furnacalis]	3.26277593	1.706099914	0.000154	up	yes	1.079	0.3307	0.354	0.32	0.318	1	1.058	1.178	------	------	K11979	UBR7	------	------	ENOG410XSV6	S:Function unknown	PF02207.23	zf-UBR	Putative zinc finger in N-recognin	CYT	2	4	80.9	High
TRINITY_DN20369_c0_g1_i2_orf1	uncharacterized protein LOC114366225 [Ostrinia furnacalis]	2.154811715	1.107561814	6.29E-06	up	yes	1.03	0.478	0.473	0.485	0.476	1	1.06	1.03	------	------	K17406	MRPS27	------	------	ENOG4110AQE	S:Function unknown	PF10037.12	MRP-S27	Mitochondrial 28S ribosomal protein S27	CYT	4	11	51.4	High
TRINITY_DN53136_c0_g1_i1_orf1	glutathione S transferase-E4 [Glyphodes pyloalis]	2.060445171	1.042956073	7.90E-05	up	yes	1.009	0.4897	0.528	0.428	0.513	1	1.015	1.013	MF:catalytic activity;MF:transferase activity;MF:glutathione transferase activity;MF:molecular_function;MF:transferase activity, transferring alkyl or ary;	GO:0003	K00799	GST, gst	map05207;m	Chemical carcinoge	COG0625	U:Intracellular trafficking, secretic	PF13417.9;PF000	GST_N_3;GST_C;GST_N_2;GST_N;GST_C_2	Glutathione S-transferase, N-terminal domain;Glutathione S-transferase, C-terminal domain;Glutathione S-transferase, N-terminal domain;Glutathione S-transferase, N-terminal domain;Glutathione S-transferase, C-terminal	CYT	3	15	25.1	High
TRINITY_DN4929_c0_g1_i1_orf1	unnamed protein product [Danaus chrysippus]	2.19303865	1.132931239	0.0005365	up	yes	1.027	0.4683	0.458	0.425	0.522	1	1.119	0.962	CC:intracellular membrane-bounded organelle;CC:nucleus;CC:cellular_component;CC:intracellular organelle;CC:membrane-bounded organelle;CC:organelle;CC:cellular anatomical entity;MF:organic cyclic compound binding;MF:heterocyclic compound binding;MF:molecular_function;MF:binding;MF:nucleic acid binding;MF:DNA	GO:0043	K14970	MEN1, MNN	map04934;m	Cushing syndrome;	ENOG410ZNZF	K:Transcription	PF05053.16	Menin	Menin	CYT	1	2	65	High
TRINITY_DN15607_c0_g1_i6_orf1	protein artichoke-like [Ostrinia furnacalis]	2.002020202	1.001456532	1.16E-05	up	yes	0.991	0.495	0.473	0.494	0.518	1	1.008	0.965	CC:cellular_component;CC:integral component of membrane;CC:cellular anatomical entity;CC:intrinsic component of membrane;	GO:0005	------	------	------	------	COG4886	I:Lipid transport and metabolism	PF13855.9;PF127	LRR_8;LRR_4;LRR_5;LRR_9	Leucine rich repeat;Leucine Rich repeats;BspA type Leucine rich repeat region;Leucine-rich repeat	CYT	5	6	109	High
TRINITY_DN4451_c0_g1_i1_orf1	uncharacterized protein LOC114361986 isoform X1 [Ostrinia furnacalis] >XP_028173022.1 uncharacterized protein LOC114361986 isoform X2 [Ostrinia furnacalis]	2.329660445	1.220119693	1.47E-05	up	yes	1.036	0.4447	0.445	0.457	0.432	1	1.032	1.077	BP:carboxylic acid metabolic process;BP:organonitrogen compound metabolic process;BP:cellular amino acid catabolic process;BP:nitrogen compound metabolic process;BP:small molecule catabolic process;BP:cellular amino acid metabolic process;BP:organic substance metabolic process;BP:alpha-amino acid metabolic process;BP:alpha-amino acid catabolic process;BP:cellular process;BP:organic substance catabolic process;BP:biological_process;BP:metabolic process;BP:oxoacid metabolic process;BP:catabolic process;BP:cellular catabolic process;BP:organonitrogen compound catabolic process;BP:primary metabolic process;BP:valine metabolic process;BP:valine catabolic process;BP:organic acid metabolic process;BP:organic acid catabolic process;BP:cellular metabolic process;BP:carboxylic acid catabolic process;BP:branched-chain amino acid metabolic process;BP:branched-chain amino acid catabolic process;BP:small molecule metabolic process;MF:NAD binding;MF:molecular_function;MF:binding;MF:heterocyclic compound binding;MF:nucleoside phosphate	GO:0019	K23146	HPD1	map00280;m	Valine, leucine and	COG2084	I:Lipid transport and metabolism	PF14833.9;PF034	NAD_binding_11;NAD_binding_2	NAD-binding of NADP-dependent 3-hydroxyisobutyrate dehydrogenase;NAD binding domain of 6-phosphogluconate dehydrogenase	CYT	3	39	20.6	High

TRINITY_DN76036_c0_g1_i1_orf1	cytochrome c oxidase subunit 6A1, mitochondrial-like [Ostrinia furnacalis]	2.327411738	1.218726458	9.27E-06	up	yes	1.035	0.4447	0.442	0.444	0.448	1	1.034	1.072	BP:aerobic respiration;BP:generation of precursor metabolites and energy;BP:oxidative phosphorylation;BP:cellular process;BP:cellular respiration;BP:metabolic process;BP:cellular metabolic process;BP:energy derivation by oxidation of organic compounds;BP:biological_process;CC:intrinsic component of membrane;CC:cellular_component;CC:integral component of membrane;CC:respiratory chain complex;CC:cytochrome complex;CC:inner mitochondrial membrane protein complex;CC:mitochondrial respiratory chain complex IV;CC:catalytic complex;CC:macromolecular complex;CC:respiratory chain complex IV;CC:membrane protein complex;CC:cellular anatomical entity;CC:mitochondrial protein complex;MF:oxidoreduction-driven active transmembrane transporter activity;MF:molecular_function;MF:oxidoreductase activity;MF:inorganic solute uptake transmembrane transporter	GO:0009(	K02266	COX6A	map04714;m	Thermogenesis;Oxi	ENOG411253X	O:Posttranslational modification,	PF02046.18	COX6A	Cytochrome c oxidase subunit VIa	CYT	2	24	12	High
TRINITY_DN5678_c0_g2_i3_orf1	coiled-coil domain-containing protein 115 [Ostrinia furnacalis]	2.459764593	1.298520252	8.88E-05	up	yes	1.024	0.4163	0.389	0.411	0.449	1	1.09	0.981	BP:proton-transporting V-type ATPase complex assembly;BP:proton-transporting two-sector ATPase complex assembly;BP:vacuolar proton-transporting V-type ATPase complex assembly;BP:cellular component assembly;BP:macromolecular complex subunit organization;BP:cellular process;BP:cellular component organization;BP:macromolecular complex assembly;BP:biological_process;BP:cellul	GO:0070(	K23543	CCDC115	------	------	ENOG41124X6	S:Function unknown	------	------	------	CYT	1	9	17.6	High
TRINITY_DN103457_c0_g1_i1_orf1	28S ribosomal protein S22, mitochondrial [Ostrinia furnacalis]	2.240252423	1.163661299	4.57E-06	up	yes	0.994	0.4437	0.459	0.46	0.412	1	0.987	0.995	------	------	K17401	MRPS22	------	------	ENOG4111I44	S:Function unknown	PF10245.12	MRP-S22	Mitochondrial 28S ribosomal protein S22	CYT	2	7	43.6	High
TRINITY_DN7073_c0_g1_i1_orf1	unnamed protein product, partial [Brenthis ino]	2.50919794	1.327226283	1.11E-05	up	yes	1.023	0.4077	0.428	0.392	0.403	1	1.063	1.006	CC:cellular_component;CC:macromolecular complex;CC:ribonucleoprotein complex;	GO:0005!	K11129	NHP2, NOLA	map03008	Ribosome biogenes	COG1358	L:Replication, recombination and	PF01248.29	Ribosomal_L7Ae	Ribosomal protein L7Ae/L30e/S12e/Gadd45	CYT	2	10	17.7	High
TRINITY_DN5867_c0_g1_i1_orf1	NADH dehydrogenase [ubiquinone] 1 alpha subcomplex subunit 7-like [Ostrinia furnacalis]	2.463235294	1.300554444	8.30E-06	up	yes	1.005	0.408	0.373	0.412	0.439	1	1.019	0.995	BP:cellular process;BP:cellular metabolic process;BP:electron transport chain;BP:biological_process;BP:metabolic process;BP:respiratory electron transport chain;BP:generation of precursor metabolites and energy;BP:ATP synthesis coupled electron transport;CC:organelle membrane;CC:membrane;CC:organelle inner membrane;CC:mitochondrial membrane;CC:cellular_component;CC:mitochondrial inner membrane;CC:cellular	GO:0009!	K03951	NDUFA7	map04714;m	Thermogenesis;Oxi	ENOG410XW5P	C:Energy production and convers	PF07347.15	CI-B14_5a	NAD	CYT	6	60	12.3	High
TRINITY_DN14920_c0_g1_i1_orf1	anamorsin homolog [Ostrinia furnacalis]	2.01183432	1.0085115	8.45E-06	up	yes	1.02	0.507	0.495	0.49	0.536	1	1.033	1.026	BP:cellular component assembly;BP:cellular component organization or biogenesis;BP:cellular component organization;BP:cellular process;BP:iron-sulfur cluster assembly;BP:metallo-sulfur cluster assembly;BP:biological_process;BP:metabolic process;BP:cellular metabolic process;BP:sulfur compound metabolic process;CC:organelle envelope lumen;CC:membrane-enclosed lumen;CC:mitochondrial intermembrane space;CC:cellular anatomical entity;CC:organelle lumen;CC:cellular_component;CC:intracellular organelle lumen;MF:molecular_function;MF:binding;MF:oxidoreductase activity;MF:cation binding;MF:metal cluster binding;MF:ion binding;MF:metal ion binding;MF:catalytic activity;MF:electron carrier activity;MF:4 iron, 4 sulfur cluster binding;MF:iron-sulfur cluster binding;MF:2 iron, 2 sulfur cluster	GO:0022(	K22746	CIAPIN1, DRI	------	------	COG5636	S:Function unknown	PF05093.16	CIAPIN1	Cytokine-induced anti-apoptosis inhibitor 1, Fe-S biogenesis	CYT	2	8	27.1	High
TRINITY_DN11584_c0_g1_i2_orf1	L-threonine 3-dehydrogenase, mitochondrial [Ostrinia furnacalis]	2.080032501	1.056606071	0.0002814	up	yes	1.024	0.4923	0.487	0.468	0.522	1	0.967	1.105	------	------	K15789	TDH	map00260	Glycine, serine and	COG0451	Z:Cytoskeleton	PF01370.24;PF16	Epimerase;GDP_Man_Dehyd;RmlD_sub_bind;3Beta_HSD;NAD_binding_4;Polysacc_synt_2	NAD dependent epimerase/dehydratase family;GDP-mannose 4,6 dehydratase;RmlD substrate binding domain;3-beta hydroxysteroid dehydrogenase/isomerase family;Male sterility protein;Polysaccharide biosynthesis	CYT	1	4	39.3	High
TRINITY_DN1239_c0_g1_i3_orf1	uncharacterized protein LOC114355269 [Ostrinia furnacalis] >XP_028163822.1 uncharacterized protein LOC114355269 [Ostrinia furnacalis]	2.140167364	1.097723622	0.0003039	up	yes	1.023	0.478	0.431	0.506	0.497	1	0.968	1.101	CC:integral component of membrane;CC:cellular_component;CC:cellular anatomical entity;CC:intrinsic component of membrane;MF:transporter activity;MF:transmembrane transporter	GO:0016(	K08190	SLC16A14	------	------	ENOG41116NW	P:Inorganic ion transport and me	PF07690.19	MFS_1	Major Facilitator Superfamily	CYT	1	2	63.4	High
TRINITY_DN1313_c0_g1_i2_orf1	39S ribosomal protein L40, mitochondrial [Ostrinia furnacalis]	2.287034999	1.193478444	9.22E-05	up	yes	1.039	0.4543	0.481	0.469	0.413	1	1.019	1.099	CC:large ribosomal subunit;CC:organellar large ribosomal subunit;CC:ribonucleoprotein complex;CC:ribosomal subunit;CC:cellular_component;CC:mitochondrial large ribosomal subunit;CC:macromolecular complex;CC:mitochondrial protein complex;	GO:0015!	K17421	MRPL40	------	------	ENOG4111PQW	S:Function unknown	PF09812.12	MRP-L28	Mitochondrial ribosomal protein L28	CYT	1	11	23.8	High
TRINITY_DN391_c5_g1_i1_orf1	hypothetical protein B5X24_HaOG210395 [Helicoverpa armigera]	2.067901235	1.048167282	1.69E-05	up	yes	1.005	0.486	0.485	0.45	0.523	1	1.011	1.004	MF:oxidoreductase activity;MF:NAD binding;MF:nucleotide binding;MF:small molecule binding;MF:catalytic activity;MF:molecular_function;MF:binding;MF:organic cyclic compound binding;MF:quinone binding;MF:heterocyclic compound binding;MF:nucleoside phosphate binding;MF:oxidoreductase activity, acting on NA;	GO:0016	K03935	NDUFS2	map04714;m	Thermogenesis;Oxi	COG0649	C:Energy production and convers	PF00346.22	Complex1_49kDa	Respiratory-chain NADH dehydrogenase, 49 Kd subunit	CYT	8	26	52.6	High
TRINITY_DN1557_c0_g1_i9_orf1	carboxylesterase CXE18 [Ostrinia furnacalis]	2.100770352	1.070918461	1.84E-05	up	yes	1.009	0.4803	0.485	0.442	0.514	1	1.024	1.004	MF:hydrolase activity;MF:molecular_function;MF:hydrolase activity, acting on ester bonds;MF:carboxylic ester hydrolase activity;MF:catalytic activity;	GO:0016	------	------	------	------	COG2272	S:Function unknown	PF00135.31;PF20	COesterase;BD-FAE;Abhydrolase_3	Carboxylesterase family;BD-FAE;alpha/beta hydrolase fold	CYT	4	7	63.7	High

TRINITY_DN4631_c0_g1_i7_orf1	uncharacterized protein LOC114366284 [Ostrinia furnacalis]	2.040451745	1.028888593	2.97E-06	up	yes	0.9937	0.487	0.467	0.488	0.506	1	1.002	0.979	BP:primary metabolic process;BP:organonitrogen compound metabolic process;BP:protein metabolic process;BP:macromolecule metabolic process;BP:organic substance metabolic process;BP:nitrogen compound metabolic process;BP:biological_process;BP:metabolic process;BP:proteolysis;MF:molecular_function;MF:serine hydrolase activity;MF:hydrolase activity;MF:catalytic activity, acting on a protein;MF:catalytic	GO:00442	------	------	------	------	COG1506	E:Amino acid transport and meta	PF00930.24;PF00	DPPIV_N;Peptidase_S9;Esterase	Dipeptidyl peptidase IV;Prolyl oligopeptidase family;Putative esterase	PLA	5	10	84.8	High
TRINITY_DN3053_c0_g1_i2_orf1	prostaglandin reductase 1-like [Ostrinia furnacalis]	2.049586777	1.035333073	1.21E-05	up	yes	0.992	0.484	0.468	0.468	0.516	1	1.005	0.971	CC:cytoplasm;CC:cellular_component;CC:cellular anatomical entity;MF:13-prostaglandin reductase activity;MF:oxidoreductase activity, acting on CH-OH group of donors;MF:oxidoreductase activity, acting on the CH-CH group of donors, NAD or NADP as acceptor;MF:oxidoreductase activity, acting on the CH-OH group of donors, NAD or NADP as acceptor;MF:oxidoreductase activity, acting on the CH-CH group of donors;MF:catalytic activity;MF:molecular_function;MF:15-oxoprostaglandin 13-oxidase activity;MF:2-alkenal reductase	GO:00057	K13948	PTGR1, LTB4	map00590	Arachidonic acid m	COG2130	S:Function unknown	PF16884.8;PF001	ADH_N_2;ADH_zinc_N;ADH_zinc_N_2	N-terminal domain of oxidoreductase;Zinc-binding dehydrogenase;Zinc-binding dehydrogenase	CYT	9	28	39.1	High
TRINITY_DN2840_c0_g1_i5_orf1	hypothetical protein evm_002181 [Chilo suppressalis]	2.019958848	1.014325902	0.0003092	up	yes	0.9817	0.486	0.474	0.462	0.522	1	1.037	0.908	------	------	------	------	------	------	ENOG41129M9	S:Function unknown	------	------	------	PLA	3	23	16.5	High
TRINITY_DN11799_c0_g1_i4_orf1	V-type proton ATPase 116 kDa subunit a1 isoform X1 [Manduca sexta]	2.271406215	1.183585739	2.20E-06	up	yes	1.016	0.4473	0.431	0.442	0.469	1	1.027	1.022	------	------	K02154	ATPeV0A, AT	map05152;m	Tuberculosis;Rheun	COG1269	F:Nucleotide transport and metal	PF01496.22	V_ATPase_I	V-type ATPase 116kDa subunit family	CYT	8	12	97.5	High
TRINITY_DN14705_c0_g2_i1_orf1	coiled-coil-helix-coiled-coil-helix domain-containing protein 10, mitochondrial [Ostrinia furnacalis]	2.073520118	1.052082045	0.0001768	up	yes	0.9843	0.4747	0.444	0.534	0.446	1	0.938	1.015	------	------	K22758	CHCHD2	------	------	ENOG41126E7;E	S:Function unknown;	PF06747.16	CHCH	CHCH domain	CYT	1	7	15.2	High
TRINITY_DN28981_c0_g1_i1_orf1	uncharacterized protein C6orf203 homolog [Ostrinia furnacalis]	2.500603427	1.322276278	0.0001175	up	yes	1.036	0.4143	0.44	0.384	0.419	1	0.996	1.113	BP:regulation of transcription from mitochondrial promoter;BP:regulation of primary metabolic process;BP:regulation of metabolic process;BP:regulation of cellular biosynthetic process;BP:regulation of cellular metabolic process;BP:regulation of biological process;BP:regulation of nucleobase-containing compound metabolic process;BP:biological regulation;BP:regulation of gene expression;BP:regulation of macromolecule metabolic process;BP:regulation of biosynthetic process;BP:regulation of nucleic acid-templated transcription;BP:regulation of cellular process;BP:biological_process;BP:regulation of nitrogen compound metabolic process;BP:regulation of RNA biosynthetic process;BP:regulation of mitochondrial gene expression;BP:regulation of RNA metabolic process;BP:regulation of transcription, DNA-templated;BP:regulation of macromolecule biosynthetic process;CC:organelle;CC:intracellular organelle;CC:membrane-bounded	GO:19031	------	------	------	------	ENOG4111QEK	S:Function unknown	------	------	------	CYT	1	5	22.6	High
TRINITY_DN86844_c0_g2_i1_orf1	spermine oxidase-like isoform X1 [Ostrinia furnacalis]	2.092779087	1.065420029	3.52E-05	up	yes	0.9767	0.4667	0.423	0.495	0.482	1	0.97	0.96	MF:catalytic activity;MF:molecular_function;MF:oxidoreductase activity;	GO:00038	K12259	SMOX, PAO5	map00330;m	Arginine and prolin	ENOG410YT60;E	F:Nucleotide transport and metal	PF01593.27	Amino_oxidase	Flavin containing amine oxidoreductase	CYT	1	5	46.8	High
TRINITY_DN11117_c0_g1_i1_orf1	venom carboxylesterase-6-like [Ostrinia furnacalis]	2.357494722	1.237254541	4.46E-06	up	yes	1.005	0.4263	0.419	0.412	0.448	1	1.03	0.986	MF:hydrolase activity;MF:molecular_function;MF:hydrolase activity, acting on ester bonds;MF:carboxylic ester hydrolase activity;MF:catalytic activity;	GO:00167	------	------	------	------	COG2272;COG0	S:Function unknown;G:Carbohyd	PF00135.31	COesterase	Carboxylesterase family	CYT	4	11	63.8	High
TRINITY_DN2065_c1_g2_i1_orf1	2-amino-3-ketobutyrate coenzyme A ligase, mitochondrial [Ostrinia furnacalis]	2.537128713	1.343196712	1.82E-05	up	yes	1.025	0.404	0.362	0.42	0.43	1	1.052	1.023	BP:carboxylic acid metabolic process;BP:cellular amino acid catabolic process;BP:nitrogen compound metabolic process;BP:aspartate family amino acid metabolic process;BP:small molecule catabolic process;BP:aspartate family amino acid catabolic process;BP:threonine catabolic process;BP:threonine metabolic process;BP:organic substance catabolic process;BP:cellular amino acid metabolic process;BP:organic substance metabolic process;BP:alpha-amino acid metabolic process;BP:alpha-amino acid catabolic process;BP:cellular process;BP:biosynthetic process;BP:biological_process;BP:metabolic process;BP:oxoacid metabolic process;BP:catabolic process;BP:cellular catabolic process;BP:primary metabolic process;BP:organonitrogen compound metabolic process;BP:organonitrogen compound catabolic process;BP:organic acid metabolic process;BP:carboxylic acid catabolic process;BP:organic acid catabolic process;BP:cellular metabolic process;BP:small molecule metabolic process;MF:pyridoxal phosphate binding;MF:vitamin B6	GO:00197	K00639	kbl, GCAT	map00260	Glycine, serine and	COG0156;COG0	J:Translation, ribosomal structure	PF00155.24	Aminotran_1_2	Aminotransferase class I and II	CYT	4	13	46.2	High
TRINITY_DN6308_c0_g1_i6_orf1	myc box-dependent-interacting protein 1 isoform X2 [Ostrinia furnacalis]	2.377772478	1.249610674	1.34E-05	up	yes	0.997	0.4193	0.395	0.46	0.403	1	1.011	0.98	CC:cytoplasm;CC:cellular_component;CC:cellular anatomical entity;	GO:00057	K12562	AMPH	map04666;m	Fc gamma R-media	ENOG410ZBW1	S:Function unknown	PF03114.21;PF14	BAR;SH3_9;SH3_1;SH3_2	BAR domain;Variant SH3 domain;SH3 domain;Variant SH3 domain	CYT	3	33	52.4	High
TRINITY_DN4550_c1_g1_i5_orfp2	TRINITY_DN4550_c1_g1_i5_m.14710 TRINITY_DN4550_c1_g1::TRINITY_DN4550_c1_g1_i5::g.14710 ORF type:5prime_partial len:168 (+),score=78.12 TRINITY_DN4550_c1_g1_i5:3-506(+)	2.168428154	1.116649643	1.80E-05	up	yes	0.9643	0.4447	0.426	0.462	0.446	1	0.935	0.958	------	------	------	------	------	------	------	------	------	------	------	CYT	1	7	20.9	High
TRINITY_DN2668_c0_g1_i7_orf1	unnamed protein product [Chrysodeixis includens]	2.721971906	1.444652176	3.40E-06	up	yes	1.027	0.3773	0.371	0.389	0.372	1	1.023	1.058	BP:organic substance metabolic process;BP:lipid metabolic process;BP:primary metabolic process;BP:metabolic process;BP:biological_process;CC:cellular_component;CC:extracellular region;CC:cellular anatomical entity;MF:hydrolase activity;MF:hydrolase activity, acting on ester bonds;MF:catalytic activity;MF:molecular_function;MF:lipase	GO:00717	------	------	------	------	ENOG410YGS3;E	S:Function unknown;	PF00151.22	Lipase	Lipase	CYT	6	26	38.5	High
TRINITY_DN15667_c0_g1_i2_orf1	coiled-coil domain-containing protein 25 [Ostrinia furnacalis]	2.13654123	1.095277158	0.0001012	up	yes	1.039	0.4863	0.458	0.502	0.499	1	1.104	1.013	CC:cellular_component;CC:endomembrane system;CC:cellular anatomical entity;	GO:0005	------	------	------	------	ENOG410Z5JK	S:Function unknown	PF05670.16	NFACT-R_1	NFACT protein RNA binding domain	CYT	2	10	24.3	High
TRINITY_DN83327_c0_g1_i1_orf1	trypsin-like serine proteinase T22 [Ostrinia nubilalis]	3.068899259	1.617721288	0.0006555	up	yes	1.118	0.3643	0.264	0.425	0.404	1	1.16	1.195	BP:organic substance metabolic process;BP:organonitrogen compound metabolic process;BP:protein metabolic process;BP:macromolecule metabolic process;BP:biological_process;BP:nitrogen compound metabolic process;BP:metabolic process;BP:proteolysis;BP:primary metabolic process;MF:serine-type endopeptidase activity;MF:endopeptidase activity;MF:hydrolase activity;MF:serine hydrolase activity;MF:catalytic activity, acting on a protein;MF:catalytic activity;MF:molecular_function;MF:pepti	GO:00717	K09640;K	TMPRSS9;PR	map04972;m	Pancreatic secretio	COG5640	O:Posttranslational modification,	PF00089.29	Trypsin	Trypsin	PLA	1	4	28.4	Medium

TRINITY_DN3665_c0_g1_i2_orf1	regucalcin-like isoform X2 [Ostrinia furnacalis] >XP_028175354.1 regucalcin-like isoform X2 [Ostrinia furnacalis]	2.770562771	1.470179053	0.000727	up	yes	1.088	0.3927	0.359	0.394	0.425	1	1.23	1.033	------	------	K01053	gnl, RGN	map00030;m	Pentose phosphate	COG3386	G:Carbohydrate transport and m	PF08450.15	SGL	SMP-30/Gluconolactonase/LRE-like region	CYT	1	16	6.9	High
TRINITY_DN58413_c0_g1_i4_orf1	cysteine and histidine-rich protein 1 isoform X1 [Ostrinia furnacalis]	2.488805091	1.315453251	5.54E-05	up	yes	1.056	0.4243	0.448	0.41	0.415	1	1.055	1.114	BP:cellular catabolic process;BP:nitrogen compound metabolic process;BP:modification-dependent macromolecule catabolic process;BP:organic substance catabolic process;BP:cellular macromolecule catabolic process;BP:cellular macromolecule metabolic process;BP:organic substance metabolic process;BP:protein modification by small protein conjugation or removal;BP:protein modification by small protein conjugation;BP:proteolysis;BP:proteolysis involved in cellular protein catabolic process;BP:cellular process;BP:modification-dependent protein catabolic process;BP:macromolecule modification;BP:protein modification process;BP:biological_process;BP:metabolic process;BP:catabolic process;BP:macromolecule catabolic process;BP:primary metabolic process;BP:organonitrogen compound metabolic process;BP:protein metabolic process;BP:cellular metabolic process;BP:macromolecule metabolic process;BP:protein ubiquitination;BP:ubiquitin-dependent	GO:0044	K04506	SIAH1	map04310;m	Wnt signaling path	ENOG410XVP0	O:Posttranslational modification,	PF03145.19	Sina	Seven in absentia protein family	CYT	2	7	34.8	High
TRINITY_DN26649_c0_g1_i2_orf1	ATP synthase-coupling factor 6, mitochondrial [Ostrinia furnacalis]	2.041056324	1.029315994	4.20E-06	up	yes	0.9893	0.4847	0.462	0.485	0.507	1	0.991	0.977	BP:nucleoside triphosphate metabolic process;BP:nucleoside triphosphate biosynthetic process;BP:purine nucleoside triphosphate metabolic process;BP:purine nucleoside triphosphate biosynthetic process;BP:cellular biosynthetic process;BP:cellular nitrogen compound metabolic process;BP:nucleotide biosynthetic process;BP:small molecule metabolic process;BP:purine-containing compound metabolic process;BP:purine-containing compound biosynthetic process;BP:organic cyclic compound biosynthetic process;BP:ribonucleotide metabolic process;BP:organic cyclic compound metabolic process;BP:nucleobase-containing compound metabolic process;BP:ribonucleoside triphosphate biosynthetic process;BP:organic substance metabolic process;BP:ribonucleoside triphosphate metabolic process;BP:heterocycle biosynthetic process;BP:purine ribonucleoside triphosphate biosynthetic process;BP:purine ribonucleoside triphosphate metabolic process;BP:ribose phosphate metabolic	GO:0009	K02131	ATPeF0F6, A	map04714;m	Thermogenesis;Che	ENOG41122G1	C:Energy production and convers	PF05511.14	ATP-synt_F6	Mitochondrial ATP synthase coupling factor 6	CYT	5	62	10.5	High
TRINITY_DN2593_c0_g1_i1_orf1	midgut carboxypeptidase [Loxostege sticticalis]	2.088204484	1.062262992	8.34E-06	up	yes	0.9967	0.4773	0.468	0.454	0.51	1	0.985	1.005	BP:nitrogen compound metabolic process;BP:organic substance metabolic process;BP:proteolysis;BP:biological_process;BP:metabolic process;BP:primary metabolic process;BP:organonitrogen compound metabolic process;BP:protein metabolic process;BP:macromolecule metabolic process;MF:exopeptidase activity;MF:molecular_function;MF:zinc ion binding;MF:peptidase activity;MF:metalloexopeptidase activity;MF:metallopeptidase activity;MF:cation binding;MF:transition metal ion binding;MF:metallocarboxypeptidase activity;MF:carboxypeptidase activity;MF:ion binding;MF:metal ion binding;MF:hydrolase activity;MF:catalytic activity, acting on a protein;MF:catalytic activity;MF:binding;	GO:0006	K01298	CPA2	map04972;m	Pancreatic secretio	COG2866	E:Amino acid transport and meta	PF00246.27;PF02	Peptidase_M14;Propep_M14	Zinc carboxypeptidase;Carboxypeptidase activation peptide	CYT	4	14	49.1	High
TRINITY_DN27704_c0_g1_i1_orf1	PREDICTED: tRNA (guanine-N(7)-)-methyltransferase [Amyelois transitella]	2.062761506	1.044577028	5.15E-05	up	yes	0.986	0.478	0.469	0.449	0.516	1	1.01	0.948	------	------	K03439	trmB, METTL	------	------	COG0220	S:Function unknown	PF02390.20	Methyltransf_4	Putative methyltransferase	CYT	2	7	28	High
TRINITY_DN38301_c0_g1_i2_orf1	gamma-taxilin [Ostrinia furnacalis]	2.176586433	1.122067311	2.01E-05	up	yes	0.9947	0.457	0.437	0.432	0.502	1	0.999	0.985	MF:molecular_function;MF:SNARE binding;MF:syntaxin binding;MF:binding;MF:protein binding;	GO:0003	------	------	------	------	ENOG410XQH1	S:Function unknown	PF09728.12	Taxilin	Myosin-like coiled-coil protein	CYT	7	12	71.1	High
TRINITY_DN27979_c0_g1_i2_orf1	zinc finger CCCH domain-containing protein 15 homolog [Ostrinia furnacalis]	2.264447942	1.179159374	9.71E-07	up	yes	1.007	0.4447	0.433	0.437	0.464	1	1.018	1.002	MF:cation binding;MF:metal ion binding;MF:molecular_function;MF:binding;MF:ion binding;	GO:0043	------	------	------	------	COG5252	S:Function unknown	PF16543.8;PF161	DFRP_C;Torus	DRG Family Regulatory Proteins, Tma46;Torus domain	CYT	5	13	43.1	High
TRINITY_DN21539_c0_g1_i1_orf1	probable phenylalanine--tRNA ligase, mitochondrial [Ostrinia furnacalis]	2.165283843	1.114556157	0.002529	up	yes	0.9917	0.458	0.517	0.469	0.388	1	0.867	1.108	BP:phenylalanyl-tRNA aminoacylation;BP:carboxylic acid metabolic process;BP:nucleic acid metabolic process;BP:cellular nitrogen compound metabolic process;BP:nitrogen compound metabolic process;BP:ncRNA metabolic process;BP:organic cyclic compound metabolic process;BP:nucleobase-containing compound metabolic process;BP:cellular amino acid metabolic process;BP:organic substance metabolic process;BP:cellular process;BP:cellular aromatic compound metabolic process;BP:biological_process;BP:metabolic process;BP:oxoacid metabolic process;BP:heterocycle metabolic process;BP:RNA metabolic process;BP:primary metabolic process;BP:organonitrogen compound metabolic process;BP:organic acid metabolic process;BP:cellular metabolic process;BP:macromolecule metabolic process;BP:tRNA metabolic process;BP:amino acid activation;BP:tRNA aminoacylation;BP:tRNA aminoacylation for protein translation;BP:small molecule metabolic process;CC:cytoplasm;CC:intracellular	GO:0006	K01889	FARSA, pheS	map00970	Aminoacyl-tRNA bi	COG0072	S:Function unknown	PF01409.23;PF03	tRNA-synt_2d;FDX-ACB	tRNA synthetases class II core domain;Ferredoxin-fold anticodon binding domain	CYT	1	3	51	High

TRINITY_DN7336_c0_g1_i13_orf1	PREDICTED: calcium-transporting ATPase sarcoplasmic/endoplasmic reticulum type isoform X2 [Amyelois transitella]	2.235636038	1.160685337	1.09E-06	up	yes	1	0.4473	0.451	0.426	0.465	1	1.001	0.999	CC:endoplasmic reticulum membrane;CC:intrinsic component of membrane;CC:organelle membrane;CC:integral component of membrane;CC:membrane;CC:sarcoplasmic reticulum membrane;CC:bounding membrane of organelle;CC:cellular_component;CC:cellular anatomical entity;MF:purine ribonucleoside triphosphate binding;MF:molecular_function;MF:binding;MF:ATPase activity;MF:nucleotide binding;MF:heterocyclic compound binding;MF:anion binding;MF:calcium ion transmembrane transporter activity;MF:ATPase activity, coupled to transmembrane movement of substances;MF:ion binding;MF:inorganic solute uptake transmembrane transporter activity;MF:ATP binding;MF:primary active transmembrane transporter activity;MF:nucleoside phosphate binding;MF:purine nucleotide binding;MF:metal ion transmembrane transporter activity;MF:active transmembrane transporter activity;MF:hydrolase activity;MF:transporter activity;MF:cation	GO:00057	K05853	ATP2A	map04972;m	Pancreatic secretio	COG0474	G:Carbohydrate transport and m	PF00122.23;PF00	E1-E2_ATPase;Cation_ATPase_C;Cation_ATPase_N;Cation_ATPase;Hydrolase;Hydrolase_3	E1-E2 ATPase;Cation transporting ATPase, C-terminus;Cation transporter/ATPase, N-terminus;Cation transport ATPase;haloacid dehalogenase-like hydrolase;halo acid dehalogenase-like hydrolase	CYT	37	37	109.8	High
TRINITY_DN3135_c0_g1_i6_orf1	acanthoscurrin-1-like [Ostrinia furnacalis]	2.245868236	1.167273288	0.0002123	up	yes	0.992	0.4417	0.461	0.42	0.444	1	1.059	0.917	------	------	------	------	------	------	------	------	------	------	------	EXC	1	7	25	High
TRINITY_DN44219_c0_g1_i1_orf1	mitochondrial import inner membrane translocase subunit TIM50-C-like [Ostrinia furnacalis]	2.6438719	1.402652277	0.0002858	up	yes	1.098	0.4153	0.435	0.379	0.432	1	1.188	1.106	BP:protein localization;BP:cellular macromolecule localization;BP:nitrogen compound transport;BP:organic substance transport;BP:macromolecule localization;BP:transport;BP:cellular process;BP:establishment of protein localization;BP:biological_process;BP:establishment of localization;BP:localization;BP:cellular localization;BP:protein transport;CC:intrinsic component of membrane;CC:integral component of membrane;CC:inner mitochondrial membrane protein complex;CC:cellular anatomical entity;CC:macromolecular complex;CC:cellular_component;CC:membrane protein complex;CC:mitochondrial inner membrane presequence translocase complex;CC:mitochondrial protein	GO:00081	K17496	TIM50	------	------	COG5190	K:Transcription	PF03031.21	NIF	NLI interacting factor-like phosphatase	CYT	1	3	44.9	High
TRINITY_DN6535_c0_g2_i1_orf1	NADH dehydrogenase [ubiquinone] 1 beta subcomplex subunit 3 [Ostrinia furnacalis] >XP_028166399.1 NADH dehydrogenase [ubiquinone] 1 beta subcomplex subunit 3 [Ostrinia furnacalis]	2.199463927	1.13715194	6.49E-05	up	yes	0.9847	0.4477	0.401	0.456	0.486	1	1.006	0.948	BP:cellular process;BP:cellular metabolic process;BP:electron transport chain;BP:biological_process;BP:metabolic process;BP:generation of precursor metabolites and energy;CC:organelle membrane;CC:integral component of membrane;CC:membrane;CC:organelle inner membrane;CC:mitochondrial membrane;CC:cellular_component;CC:mitochondrial inner membrane;CC:intrinsic component of membrane;CC:respiratory	GO:00099	K03959	NDUFB3	map04714;m	Thermogenesis;Oxi	ENOG411294X	I:Lipid transport and metabolism	PF08122.15	NDUF_B12	NADH-ubiquinone oxidoreductase B12 subunit family	CYT	4	39	11	High
TRINITY_DN29291_c0_g1_i1_orf1	carboxylesterase [Ostrinia furnacalis]	2.545	1.347665656	0.0006074	up	yes	1.018	0.4	0.288	0.496	0.416	1	1.001	1.053	MF:hydrolase activity;MF:molecular_function;MF:hydrolase activity, acting on ester bonds;MF:carboxylic ester hydrolase activity;MF:catalytic activity;	GO:00167	K03927	CES2	map00983	Drug metabolism -	COG2272	I:Lipid transport and metabolism	PF00135.31	COesterase	Carboxylesterase family	CYT	1	8	26.3	High
TRINITY_DN43328_c0_g1_i1_orf1	tubulin--tyrosine ligase-like protein 12 [Ostrinia furnacalis]	2.171247357	1.118524093	0.001841	up	yes	1.027	0.473	0.382	0.446	0.591	1	1.112	0.969	------	------	K16609	TTLL12	------	------	ENOG410XQ0A	S:Function unknown	PF03133.18	TTL	Tubulin-tyrosine ligase	CYT	1	2	71.9	High
TRINITY_DN30_c0_g1_i6_orf1	casein kinase I-like isoform X1 [Hyposmocoma kahamanoa]	2.32885906	1.219623332	2.74E-05	up	yes	1.041	0.447	0.443	0.444	0.454	1	1.031	1.093	BP:phosphorylation;BP:nitrogen compound metabolic process;BP:organic substance metabolic process;BP:protein phosphorylation;BP:cellular process;BP:macromolecule modification;BP:protein modification process;BP:biological_process;BP:metabolic process;BP:primary metabolic process;BP:organonitrogen compound metabolic process;BP:cellular metabolic process;BP:macromolecule metabolic process;BP:protein metabolic process;BP:phosphate-containing compound metabolic process;BP:phosphorus metabolic process;MF:purine ribonucleoside triphosphate binding;MF:molecular_function;MF:binding;MF:ion binding;MF:heterocyclic compound binding;MF:nucleoside phosphate binding;MF:anion binding;MF:carbohydrate derivative binding;MF:nucleotide binding;MF:protein serine/threonine kinase activity;MF:transferase activity;MF:protein kinase activity;MF:purine nucleotide binding;MF:ATP binding;MF:kinase activity;MF:catalytic activity, acting on a	GO:00163	K08957	CSNK1A	map05165;m	Human papillomav	ENOG410XPGP	T:Signal transduction mechanism	PF00069.28;PF07	Pkinase;PK_Tyr_Ser-Thr;Pkinase_fungal	Protein kinase domain;Protein tyrosine and serine/threonine kinase;Fungal protein kinase	CYT	1	11	43.4	High
TRINITY_DN27641_c0_g1_i1_orf1	succinate dehydrogenase [ubiquinone] iron-sulfur subunit, mitochondrial [Ostrinia furnacalis]	2.177874187	1.122920614	1.89E-05	up	yes	1.004	0.461	0.434	0.444	0.505	1	1.015	0.996	BP:tricarboxylic acid cycle;BP:biological_process;BP:metabolic process;BP:primary metabolic process;CC:organelle membrane;CC:membrane;CC:cellular anatomical entity;CC:mitochondrial membrane;CC:organelle inner membrane;CC:cellular_component;CC:mitochondrial inner membrane;MF:oxidoreductase activity, acting on the CH-CH group of donors, quinone or related compound as acceptor;MF:molecular_function;MF:binding;MF:succinate dehydrogenase activity;MF:oxidoreductase activity;MF:cation binding;MF:oxidoreductase activity, acting on the CH-CH group of donors;MF:ion binding;MF:metal ion binding;MF:catalytic activity;MF:succinate dehydrogenas;MF:metal cluster binding;MF:electron carrier activity;MF:3 iron, 4 sulfur cluster binding;MF:4 iron, 4 sulfur cluster binding;MF:iron-sulfur	GO:0006(	K00235	SDHB, SDH2	map04714;m	Thermogenesis;Oxi	COG0479	C:Energy production and conver	PF13085.9;PF135	Fer2_3;Fer4_17;Fer4_8;Fer4_10	2Fe-2S iron-sulfur cluster binding domain;4Fe-4S dicluster domain;4Fe-4S dicluster domain;4Fe-4S dicluster domain	CYT	6	30	31.8	High
TRINITY_DN754_c1_g1_i8_orf1	lysophospholipid acyltransferase 5 [Ostrinia furnacalis] >XP_028169982.1 lysophospholipid acyltransferase 5 [Ostrinia furnacalis]	2.075949367	1.053771256	4.61E-05	up	yes	0.984	0.474	0.488	0.476	0.458	1	0.934	1.018	CC:integral component of membrane;CC:cellular_component;CC:cellular anatomical entity;CC:intrinsic component of membrane;MF:catalytic activity;MF:transferase activity;MF:molecular_function;	GO:0016(	K13515	LPCAT3, MB(	map00564;m	Glycerophospholipi	COG5202	I:Lipid transport and metabolism	PF03062.22	MBOAT	MBOAT, membrane-bound O-acyltransferase family	CYT	2	3	55.3	High
TRINITY_DN5087_c0_g1_i6_orf1	nascent polypeptide-associated complex subunit alpha [Ostrinia furnacalis] >XP_028156807.1 nascent polypeptide-associated complex subunit alpha [Ostrinia furnacalis] >XP_028156808.1 nascent polypeptide-associated complex subunit alpha [Ostrinia furnacalis]	2.19376783	1.133410851	3.95E-05	up	yes	0.9997	0.4557	0.45	0.417	0.5	1	0.976	1.023	CC:cellular_component;CC:nascent polypeptide-associated complex;CC:macromolecular complex;	GO:0005!	K03626	EGD2, NACA	map04928	Parathyroid hormo	COG1308	K:Transcription	PF01849.21;PF19	NAC;HYPK_UBA	NAC domain;HYPK UBA domain	CYT	7	34	22.8	High
TRINITY_DN8754_c0_g1_i2_orf1	dnaJ homolog subfamily C member 11 [Ostrinia furnacalis]	2.427253371	1.279324713	2.84E-05	up	yes	1.026	0.4227	0.385	0.428	0.455	1	1.064	1.013	------	------	K09531	DNAJC11	------	------	COG0484	J:Translation, ribosomal structure	PF11875.11;PF00	DnaJ-like_C11_C;DnaJ	DnaJ-like protein C11, C-terminal;DnaJ	CYT	2	3	65.1	High

TRINITY_DN7512_c0_g1_i1_orf1	hypothetical protein evm_010529 [Chilo suppressalis] >CAB3530682.1 unnamed protein product [Chilo suppressalis] >CAH0407273.1 unnamed protein product [Chilo suppressalis]	2.188396206	1.129873959	6.89E-06	up	yes	0.992	0.4533	0.458	0.425	0.477	1	0.974	1.002	BP:one-carbon metabolic process;BP:cellular biosynthetic process;BP:small molecule metabolic process;BP:organic substance biosynthetic process;BP:organic substance metabolic process;BP:sulfur compound metabolic process;BP:S-adenosylmethionine metabolic process;BP:cellular process;BP:biosynthetic process;BP:biological_process;BP:metabolic process;BP:sulfur compound biosynthetic process;BP:S-adenosylmethionine biosynthetic process;BP:cellular metabolic process;MF:purine ribonucleoside triphosphate binding;MF:molecular_function;MF:nucleotide binding;MF:heterocyclic compound binding;MF:nucleoside phosphate binding;MF:anion binding;MF:transferase activity, transferring alkyl or ary;MF:ion binding;MF:transferase activity;MF:metal ion binding;MF:ATP binding;MF:cation binding;MF:small molecule binding;MF:catalytic activity;MF:purine nucleotide binding;MF:methionine adenosyltransferase activity;MF:organic	GO:00067	K00789	metK, MAT	map00270;m	Cysteine and methi	COG0192	H:Coenzyme transport and meta	PF02773.19;PF02	S-AdoMet_synt_C;S-AdoMet_synt_M	S-adenosylmethionine synthetase, C-terminal domain;S-adenosylmethionine synthetase, central domain	CYT	5	50	22.1	High
TRINITY_DN8543_c0_g1_i1_orf1	39S ribosomal protein L38, mitochondrial [Ostrinia furnacalis]	2.193897855	1.133496358	1.68E-05	up	yes	0.9923	0.4523	0.429	0.438	0.49	1	1.007	0.97	------	------	K17419	MRPL38	------	------	COG1881	S:Function unknown	PF01161.23	PBP	Phosphatidylethanolamine-binding	CYT	1	4	46.4	High
TRINITY_DN108122_c0_g1_i9_orf1	hypothetical protein SFRUCORN_003152 [Spodoptera frugiperda]	2.309468822	1.20756107	0.00373	up	yes	1	0.433	0.341	0.338	0.62	1	1.004	0.997	BP:cellular component organization;BP:cell death;BP:mitochondrion organization;BP:cellular component organization or biogenesis;BP:cellular process;BP:apoptotic process;BP:biological_process;BP:organelle organization;BP:programmed cell death;CC:intracellular organelle;CC:membrane-bounded organelle;CC:organelle;CC:mitochondrion;CC:cellular anatomical entity;CC:intracellular membrane-bounded organelle;CC:cellular_component;MF:molecular_function;MF:hydrolase activity;MF:GTPase activity;MF:nucleoside-triphosphatase activity;MF:catalytic activity;MF:hydrolase activity, acting on acid anhydrides, in phosphorus-	GO:0016(	K17079	OPA1	map05017	Spinocerebellar ata	COG0699	S:Function unknown	PF19434.2	OPA1_C	Dynamin-like GTPase OPA1 C-terminal	CYT	1	20	10.8	High
TRINITY_DN12242_c0_g1_i5_orf1	heterogeneous nuclear ribonucleoprotein 87F-like isoform X1 [Vanessa tameamea] >XP_046967652.1 heterogeneous nuclear ribonucleoprotein 87F-like isoform X1 [Vanessa cardui] >XP_047532045.1 heterogeneous nuclear ribonucleoprotein 87F-like [Vanessa atalanta]	2.398286938	1.262004277	1.88E-06	up	yes	1.008	0.4203	0.406	0.409	0.446	1	1.018	1.006	MF:organic cyclic compound binding;MF:molecular_function;MF:binding;MF:nucleic acid binding;MF:heterocyclic compound	GO:00971	K12741	HNRNPA1_3	map05014;m	Amyotrophic latera	COG0724	S:Function unknown	PF00076.25;PF16	RRM_1;RRM_7	RNA recognition motif.;RNA recognition	CYT	8	41	34	High
TRINITY_DN17738_c0_g1_i2_orf1	unnamed protein product [Diatraea saccharalis]	2.309307208	1.207460108	1.20E-05	up	yes	0.99	0.4287	0.424	0.407	0.455	1	1.011	0.959	BP:nucleic acid metabolic process;BP:cellular nitrogen compound metabolic process;BP:nitrogen compound metabolic process;BP:organic cyclic compound metabolic process;BP:nucleobase-containing compound metabolic process;BP:organic substance metabolic process;BP:cellular process;BP:cellular aromatic compound metabolic process;BP:biological_process;BP:metabolic process;BP:heterocycle metabolic process;BP:RNA metabolic process;BP:primary metabolic process;BP:cellular metabolic process;BP:macromolecule metabolic process;BP:RNA processing;CC:membrane-bounded organelle;CC:nucleus;CC:organelle;CC:cellular anatomical entity;CC:macromolecular complex;CC:intracellular membrane-bounded organelle;CC:ribonucleoprotein complex;CC:intracellular organelle;CC:cellular_component;MF:molecular_function;MF:binding;MF:nucleic acid binding;MF:heterocyclic compound	GO:0090	K11090	LA, SSB	map05322	Systemic lupus eryt	COG5193	G:Carbohydrate transport and m	PF05383.20;PF08	La;RRM_3;RRM_1	La domain;RNA binding motif;RNA recognition motif.	CYT	10	28	43.9	High
TRINITY_DN28638_c0_g1_i1_orf1	uncharacterized protein LOC114364075 [Ostrinia furnacalis]	2.326352941	1.218069991	1.67E-05	up	yes	0.9887	0.425	0.396	0.413	0.466	1	0.996	0.97	------	------	------	------	------	------	ENOG410XRSQ	G:Carbohydrate transport and m	PF00650.23	CRAL_TRIO	CRAL/TRIO domain	CYT	7	25	34.8	High
TRINITY_DN12495_c0_g1_i2_orf1	probable ATP-dependent RNA helicase pitchoune [Manduca sexta] >KAG6441249.1 hypothetical protein O3G_MSEX001749 [Manduca sexta]	2.270138889	1.182780565	1.32E-05	up	yes	0.9807	0.432	0.429	0.401	0.466	1	0.973	0.969	MF:purine ribonucleoside triphosphate binding;MF:molecular_function;MF:binding;MF:nucleic acid binding;MF:RNA-dependent ATPase activity;MF:ion binding;MF:heterocyclic compound binding;MF:nucleoside phosphate binding;MF:anion binding;MF:nucleotide binding;MF:purine nucleotide binding;MF:ATP binding;MF:hydrolase activity;MF:catalytic activity, acting on RNA;MF:helicase activity;MF:small molecule binding;MF:catalytic activity;MF:adenyl nucleotide binding;MF:carbohydrate derivative binding;MF:organic cyclic compound binding;MF:RNA helicase activity;MF:adenyl ribonucleotide binding;MF:RNA binding;MF:purine ribonucleotide	GO:0035(	K13179	DDX18, HAS	------	------	COG0513	S:Function unknown	PF00270.32;PF00	DEAD;Helicase_C;DUF4217	DEAD/DEAH box helicase;Helicase conserved C-terminal domain;Domain of unknown function	CYT	2	3	69	High
TRINITY_DN81926_c0_g1_i1_orf1	membrane-bound alkaline phosphatase-like isoform X3 [Ostrinia furnacalis]	2.077998818	1.055194834	5.70E-05	up	yes	1.055	0.5077	0.508	0.494	0.521	1	1.065	1.101	MF:hydrolase activity;MF:phosphatase activity;MF:alkaline phosphatase activity;MF:hydrolase activity, acting on ester bonds;MF:phosphoric ester hydrolase activity;MF:catalytic activity;MF:molecular_function;	GO:00167	K01077	E3.1.3.1, pho	map00730;m	Thiamine metaboli	COG1785	P:Inorganic ion transport and me	PF00245.23	Alk_phosphatase	Alkaline phosphatase	CYT	1	4	59.1	High
TRINITY_DN38540_c0_g1_i1_orf1	GSCOCG00000129001-RA-CDS [Cotesia congregata] >CAG5101050.1 Similar to LUC7L2: Putative RNA-binding protein Luc7-like 2 (Homo sapiens) [Cotesia congregata]	2.066230418	1.047001147	0.000242	up	yes	1.042	0.5043	0.469	0.524	0.52	1	1.121	1.005	BP:cellular component assembly;BP:macromolecular complex subunit organization;BP:cellular component organization or biogenesis;BP:ribonucleoprotein complex subunit organization;BP:cellular component organization;BP:macromolecular complex assembly;BP:ribonucleoprotein complex assembly;BP:cellular process;BP:biological_process;BP:mRNA splice site selection;CC:Sm-like protein family complex;CC:cellular_component;CC:spliceosomal snRNP complex;CC:macromolecular complex;CC:small nuclear ribonucleoprotein complex;CC:ribonucleoprotein complex;CC:U1 snRNP;MF:molecular_function;MF:binding;MF:nucleic acid binding;MF:heterocyclic compound binding;MF:organic cyclic compound binding;MF:RNA binding;MF:mRNA	GO:0022(	K13212	LUC7L2	------	------	COG5200	A:RNA processing and modificati	PF03194.18	LUC7	LUC7 N_terminus	CYT	2	9	38.6	High

TRINITY_DN23360_c0_g1_i3_orf1	protein PTCD3 homolog, mitochondrial [Ostrinia furnacalis]	2.026209677	1.018783476	5.31E-06	up	yes	1.005	0.496	0.52	0.479	0.489	1	0.992	1.024	BP:regulation of primary metabolic process;BP:regulation of metabolic process;BP:regulation of cellular macromolecule biosynthetic process;BP:regulation of cellular metabolic process;BP:nitrogen compound metabolic process;BP:cellular biosynthetic process;BP:peptide biosynthetic process;BP:organic substance biosynthetic process;BP:cellular nitrogen compound biosynthetic process;BP:cellular macromolecule metabolic process;BP:posttranscriptional regulation of gene expression;BP:cellular amide metabolic process;BP:regulation of protein metabolic process;BP:regulation of biological process;BP:organic substance metabolic process;BP:amide biosynthetic process;BP:biological regulation;BP:regulation of cellular biosynthetic process;BP:organonitrogen compound biosynthetic process;BP:macromolecule metabolic process;BP:primary metabolic process;BP:regulation of macromolecule metabolic process;BP:cellular process;BP:regulation of biosynthetic process;BP:regulation of cellular amide [illegible]	GO:0080(	K17659	PTCD3	------	------	ENOG410XNQS	S:Function unknown	PF17177.7	PPR_long	Pentacotripeptide-repeat region of PRORP	CYT	5	8	78.8	High
TRINITY_DN783_c0_g1_i7_orf1	microtubule-associated protein Jupiter isoform X4 [Helicoverpa armigera]	2.036	1.025737561	5.51E-05	up	yes	1.018	0.5	0.513	0.499	0.488	1	0.981	1.072	------	------	------	------	------	------	ENOG410Z68G	S:Function unknown	PF17054.8	JUPITER	Microtubule-Associated protein Jupiter	EXC	2	12	24	High
TRINITY_DN60821_c0_g1_i1_orf1	nucleolar GTP-binding protein 2 [Ostrinia furnacalis]	2.227923883	1.155699944	0.000617	up	yes	1.042	0.4677	0.474	0.37	0.559	1	1.052	1.073	CC:organelle;CC:intracellular organelle;CC:non-membrane-bounded organelle;CC:nucleolus;CC:cellular anatomical entity;CC:intracellular non-membrane-bounded organelle;CC:cellular_component;MF:purine ribonucleoside triphosphate binding;MF:molecular_function;MF:binding;MF:ion binding;MF:heterocyclic compound binding;MF:nucleoside phosphate binding;MF:anion binding;MF:nucleotide binding;MF:guanyl ribonucleotide binding;MF:purine nucleotide binding;MF:GTP binding;MF:small molecule binding;MF:carbohydrate derivative binding;MF:organic cyclic compound binding;MF:guanyl nucleotide binding;MF:purine ribonucleotide [illegible]	GO:0043(	K14537	NUG2, GNL2	map03008	Ribosome biogenes	COG1161	S:Function unknown	PF08153.15;PF01(	NGP1NT;MMR_HSR1	NGP1NT;50S ribosome-binding GTPase	CYT	2	3	79.9	High
TRINITY_DN143603_c0_g1_i1_orf1	hypothetical protein KR044_005587 [Drosophila immigrans]	2.9079884	1.540021514	0.0003658	up	yes	1.103	0.3793	0.39	0.326	0.422	1	1.108	1.202	BP:response to extracellular stimulus;BP:carbon utilization;BP:organic substance metabolic process;BP:biological_process;BP:metabolic process;BP:primary metabolic process;BP:carbohydrate metabolic process;BP:response to external stimulus;BP:response to stimulus;BP:response to nutrient levels;CC:membrane;CC:cellular anatomical entity;CC:cellular_component;MF:hydrolase activity;MF:carbohydrate binding;MF:lyase activity;MF:molecular_function;MF:binding;MF:ion binding;MF:heterocyclic compound binding;MF:nucleoside phosphate binding;MF:oxidoreductase activity;MF:anion binding;MF:cation binding;MF:flavin adenine dinucleotide binding;MF:transition metal ion binding;MF:oxidoreductase activity, acting on the CH-CH group of donors;MF:nucleotide binding;MF:alpha-glucosidase activity;MF:carbon-oxygen lyase activity;MF:tRNA dihydrouridine synthase activity;MF:metal ion binding;MF:glucosidase [illegible]	GO:0009(	K01673	cynT, can	map00910	Nitrogen metabolis	COG0288	P:Inorganic ion transport and me	PF00484.22	Pro_CA	Carbonic anhydrase	CYT	1	5	26.9	High
TRINITY_DN698_c0_g1_i5_orf1	PREDICTED: small nuclear ribonucleoprotein Sm D3 [Amyelois transitella]	2.036087759	1.025799745	6.31E-06	up	yes	0.993	0.4877	0.507	0.459	0.497	1	0.979	1	BP:spliceosomal snRNP assembly;BP:cellular component assembly;BP:macromolecular complex subunit organization;BP:cellular process;BP:ribonucleoprotein complex subunit organization;BP:cellular component organization;BP:macromolecular complex assembly;BP:ribonucleoprotein complex assembly;BP:biological_process;BP:cellular component organization or biogenesis;CC:cellular_component;CC:macromolecular complex;CC:cytosol;CC:ribonucleoprotein complex;CC:cellular anatomical [illegible]	GO:0000(	K11088	SNRPD3, SM	map05322;m	Systemic lupus eryt	COG1958	S:Function unknown	PF01423.25	LSM	LSM domain	CYT	3	31	14.1	High
TRINITY_DN36928_c0_g1_i5_orf1	actin-interacting protein 1 isoform X2 [Ostrinia furnacalis]	2.126066154	1.088186488	0.0004995	up	yes	1.022	0.4807	0.426	0.583	0.433	1	1.04	1.027	------	------	K24736	WDR1, AIP1	------	------	ENOG410XQN1	S:Function unknown	PF00400.35	WD40	WD domain, G-beta repeat	CYT	1	38	10.3	High
TRINITY_DN96080_c0_g2_i1_orf1	ATP synthase subunit delta, mitochondrial [Ostrinia furnacalis]	2.134831461	1.094122177	1.39E-05	up	yes	1.007	0.4717	0.453	0.46	0.502	1	1.034	0.986	CC:proton-transporting ATP synthase complex, catalytic core;CC:cellular_component;CC:macromolecular complex;CC:proton-transporting two-sector ATPase complex, catalytic domain;CC:membrane protein complex;MF:cation transmembrane transporter activity;MF:proton-transporting ATP synthase activity, rotational mechanism;MF:inorganic solute uptake transmembrane transporter activity;MF:transporter activity;MF:ion channel activity;MF:passive transmembrane transporter activity;MF:ion transmembrane transporter activity;MF:catalytic activity;MF:transmembrane transporter activity;MF:hydrogen ion channel activity;MF:molecular_function;MF:channel activity;MF:cation channel activity;MF:inorganic cation [illegible]	GO:0045(	K02134	ATPeF1D, AT	map04714;m	Thermogenesis;Che	COG0355	C:Energy production and conver	PF02823.19	ATP-synt_DE_N	ATP synthase, Delta/Epsilon chain, beta-sandwich domain	CYT	6	53	17.2	High
TRINITY_DN6710_c0_g1_i6_orf1	multiple C2 and transmembrane domain-containing protein-like [Ostrinia furnacalis]	2.174096767	1.120416155	0.004138	up	yes	1.029	0.4733	0.364	0.65	0.406	1	0.997	1.09	CC:cellular_component;CC:integral component of membrane;CC:cellular anatomical entity;CC:intrinsic component of membrane;	GO:0005(	------	------	------	------	COG5038;ENOG	H:Coenzyme transport and meta	PF00168.33	C2	C2 domain	PLA	1	1	69.9	Medium

TRINITY_DN15370_c0_g1_i4_orf1	DNA replication licensing factor Mcm5 [Spodoptera litura]	2.136643327	1.095346097	6.32E-05	up	yes	1.007	0.4713	0.431	0.475	0.508	1	1.047	0.975	BP:nucleic acid metabolic process;BP:nitrogen compound metabolic process;BP:organic cyclic compound metabolic process;BP:nucleobase-containing compound metabolic process;BP:cellular macromolecule metabolic process;BP:organic substance metabolic process;BP:cell cycle;BP:cellular process;BP:cellular aromatic compound metabolic process;BP:biological_process;BP:metabolic process;BP:heterocycle metabolic process;BP:primary metabolic process;BP:DNA replication initiation;BP:cellular nitrogen compound metabolic process;BP:cellular metabolic process;BP:macromolecule metabolic process;BP:DNA metabolic process;CC:intracellular organelle;CC:membrane-bounded organelle;CC:organelle;CC:MCM complex;CC:nucleus;CC:cellular anatomical entity;CC:macromolecular complex;CC:intracellular membrane-bounded organelle;CC:cellular_component;MF:ATPase activity;MF:purine ribonucleoside triphosphate binding;MF:hydrolase	GO:0090[illegible]	K02209	MCM5, CDC[illegible]	map04110;m[illegible]	Cell cycle;DNA repl[illegible]	COG1241	O:Posttranslational modification,	PF00493.26;PF17[illegible]	MCM;MCM_OB;MCM_lid;MCM_N;Mg_chelatase	MCM P-loop domain;MCM OB domain;MCM AAA-lid domain;MCM N-terminal domain;Magnesium chelatase, subunit ChlI	CYT	2	3	81.3	High
TRINITY_DN825_c2_g1_i5_orf1	hypothetical protein SFRURICE_018365 [Spodoptera frugiperda] >KAG8107343.1 hypothetical protein SFRUCORN_012069 [Spodoptera frugiperda] >CAB3509414.1 unnamed protein product [Spodoptera littoralis] >CAH1638995.1 unnamed protein product [Spodoptera littoralis]	2.027054311	1.019384743	0.0001176	up	yes	1.004	0.4953	0.447	0.561	0.478	1	1.005	1.007	CC:organelle lumen;CC:organelle envelope lumen;CC:membrane-enclosed lumen;CC:cellular_component;CC:intracellular organelle lumen;CC:mitochondrial intermembrane space;CC:cellular anatomical entity;MF:cation binding;MF:metal ion binding;MF:transition metal ion binding;MF:ion binding;MF:molecular_function;MF:binding;MF:copper chaperone activity;MF:metallochaperone	GO:0043[illegible]	K02260	COX17	map00190;m[illegible]	Oxidative phospho[illegible]	ENOG410XVYQ	O:Posttranslational modification,	PF05051.16	COX17	Cytochrome C oxidase copper chaperone	CYT	1	20	7.5	High
TRINITY_DN86956_c0_g5_i1_orf1	PREDICTED: protein sly1 homolog [Microplitis demolitor]	2.904	1.538041453	0.0002478	up	yes	1.089	0.375	0.381	0.351	0.393	1	1.074	1.194	BP:transport;BP:biological_process;BP:vesicle-mediated transport;BP:establishment of	GO:0006[illegible]	K19998	SCFD1, SLY1	------	------	COG5158	U:Intracellular trafficking, secretic	PF00995.26	Sec1	Sec1 family	CYT	1	1	71.3	High
TRINITY_DN9059_c0_g1_i1_orf1	sulfotransferase family cytosolic 1B member 1-like [Ostrinia furnacalis]	2.020474356	1.014694041	0.000938	up	yes	0.9967	0.4933	0.393	0.496	0.591	1	0.985	1.005	MF:catalytic activity;MF:transferase activity;MF:transferase activity, transferring sulfur-containing groups;MF:sulfotransferase activity;MF:molecular_function;	GO:0003[illegible]	------	------	------	------	ENOG4111H56	S:Function unknown	PF00685.30	Sulfotransfer_1	Sulfotransferase domain	CYT	1	22	20.7	High
TRINITY_DN11153_c0_g1_i1_orf1	unnamed protein product [Chilo suppressalis]	2.114697654	1.080451411	9.62E-06	up	yes	0.9827	0.4647	0.447	0.458	0.489	1	0.992	0.956	------	------	K20792	NAA15_16	------	------	ENOG410XR7D	S:Function unknown	PF12569.11;PF07[illegible]	NatA_aux_su;TPR_2;TPR_8;TPR_1;TPR_14;ANAPC3;TPR_19;TPR_16	N-terminal acetyltransferase A, auxiliary subunit;Tetratricopeptide repeat;Tetratricopeptide repeat;Tetratricopeptide repeat;Tetratricopeptide repeat;Anaphase-promoting complex, cyclosome, subunit 3;Tetratricopeptide repeat;Tetratricopeptide	CYT	16	20	100.1	High
TRINITY_DN8019_c0_g1_i4_orf1	deoxyhypusine hydroxylase [Ostrinia furnacalis]	2.165087062	1.114425039	3.13E-05	up	yes	0.9823	0.4537	0.423	0.458	0.48	1	1.003	0.944	BP:nitrogen compound metabolic process;BP:peptidyl-amino acid modification;BP:organic substance metabolic process;BP:macromolecule modification;BP:protein modification process;BP:biological_process;BP:metabolic process;BP:peptidyl-lysine modification to peptidyl-hypusine;BP:primary metabolic process;BP:organonitrogen compound metabolic process;BP:protein maturation;BP:peptidyl-lysine modification;BP:biosynthetic process;BP:macromolecule metabolic process;BP:protein metabolic process;MF:monooxygenase activity;MF:deoxyhypusine monooxygenase activity;MF:molecular_function;MF:binding;MF:oxidoreductase activity;MF:cation binding;MF:oxidoreductase activity, acting on paired donors, with incorporation or reduction of molecular oxygen;MF:ion binding;MF:metal ion binding;MF:catalytic activity, acting on a	GO:0006[illegible]	K06072	DOHH	------	------	COG1413	C:Energy production and convers	PF13646.9;PF031[illegible]	HEAT_2;HEAT_PBS;HEAT;DUF5691	HEAT repeats;PBS lyase HEAT-like repeat;HEAT repeat;Family of unknown function	CYT	5	19	34.1	High
TRINITY_DN7626_c0_g1_i1_orf1	NADH dehydrogenase [ubiquinone] flavoprotein 1, mitochondrial isoform X1 [Ostrinia furnacalis]	2.074174112	1.052537003	1.28E-05	up	yes	0.9983	0.4813	0.454	0.481	0.509	1	1.018	0.977	CC:oxidoreductase complex;CC:mitochondrial inner membrane;CC:membrane;CC:respiratory chain complex;CC:cellular anatomical entity;CC:transmembrane transporter complex;CC:catalytic complex;CC:transporter complex;CC:mitochondrial membrane;CC:organelle membrane;CC:respiratory chain complex I;CC:macromolecular complex;CC:NADH dehydrogenase complex;CC:organelle inner membrane;CC:cellular_component;CC:membrane protein complex;MF:NAD binding;MF:metal ion binding;MF:molecular_function;MF:binding;MF:ion binding;MF:NA;MF:heterocyclic compound binding;MF:nucleoside phosphate binding;MF:oxidoreductase activity;MF:anion binding;MF:cation binding;MF:metal cluster binding;MF:transporter activity;MF:nucleotide binding;MF:primary active transmembrane transporter activity;MF:NADH dehydrogenas;MF:FMN	GO:1990[illegible]	K03942	NDUFV1	map04714;m[illegible]	Thermogenesis;Oxi[illegible]	COG1894	C:Energy production and convers	PF01512.20;PF10[illegible]	Complex1_51K;NADH_4Fe-4S	Respiratory-chain NADH dehydrogenase 51 Kd subunit;NADH-ubiquinone oxidoreductase-F iron-sulfur binding region	CYT	9	24	50.5	High
TRINITY_DN52861_c0_g1_i1_orf1	protein MAK16 homolog A [Ostrinia furnacalis]	2.427207637	1.27929753	4.64E-05	up	yes	1.017	0.419	0.404	0.376	0.477	1	1.034	1.016	CC:intracellular non-membrane-bounded organelle;CC:nucleolus;CC:cellular_component;CC:intracellular organelle;CC:non-membrane-bounded organelle;CC:organelle;CC:cellular	GO:0043[illegible]	K14831	MAK16	------	------	COG5129	S:Function unknown	PF01778.20;PF04[illegible]	Ribosomal_L28e;Mak16	Ribosomal L28e protein family;Mak16 protein C-terminal region	CYT	1	2	35.4	Medium

TRINITY_DN12476_c0_g1_i4_orf1	guanine nucleotide-binding protein-like 3 homolog [Ostrinia furnacalis]	2.25607064	1.173812241	2.48E-05	up	yes	1.022	0.453	0.428	0.433	0.498	1	1.043	1.023	MF:anion binding;MF:carbohydrate derivative binding;MF:guanyl nucleotide binding;MF:purine ribonucleoside triphosphate binding;MF:organic cyclic compound binding;MF:ribonucleotide binding;MF:nucleotide binding;MF:small molecule binding;MF:guanyl ribonucleotide binding;MF:molecular_function;MF:binding;MF:purine ribonucleotide binding;MF:purine nucleotide binding;MF:ion binding;MF:heterocyclic compound binding;MF:nucleoside phosphate binding;MF:GTP binding;	GO:00431	K14538	NUG1, GNL3	map03008	Ribosome biogenes	COG1161	S:Function unknown	PF08701.14;PF01	GN3L_Grn1;MMR_HSR1;FeoB_N	GNL3L/Grn1 putative GTPase;50S ribosome-binding GTPase;Ferrous iron transport protein B	CYT	4	7	64.8	High
TRINITY_DN76377_c0_g1_i1_orf1	uncharacterized protein LOC111357764, partial [Spodoptera litura]	2.568014238	1.360653202	2.12E-05	up	yes	1.01	0.3933	0.367	0.442	0.371	1	1.032	0.997	------	------	------	------	------	------	ENOG410Y7Q4;E	S:Function unknown;	PF03564.18	DUF1759	Protein of unknown function	CYT	1	4	27.4	Medium
TRINITY_DN23586_c0_g1_i3_orf1	myrosinase 1-like isoform X1 [Ostrinia furnacalis]	2.318047	1.212909818	8.45E-07	up	yes	1.016	0.4383	0.442	0.444	0.429	1	1.014	1.035	BP:primary metabolic process;BP:carbohydrate metabolic process;BP:organic substance metabolic process;BP:biological_process;BP:metabolic process;MF:hydrolase activity;MF:catalytic activity;MF:hydrolase activity, acting on glycosyl bonds;MF:hydrolase activity, hydrolyzing O-glycosyl	GO:00442	------	------	------	------	COG2723	G:Carbohydrate transport and m	PF00232.21	Glyco_hydro_1	Glycosyl hydrolase family 1	CYT	5	29	39.2	High
TRINITY_DN14063_c0_g1_i7_orf1	probable phosphorylase b kinase regulatory subunit beta isoform X1 [Ostrinia furnacalis] >XP_028175664.1 probable phosphorylase b kinase regulatory subunit beta isoform X2 [Ostrinia furnacalis] >XP_028175665.1 probable phosphorylase b kinase regulatory subunit beta isoform X3 [Ostrinia furnacalis]	2.31884058	1.213403638	0.0001017	up	yes	1.008	0.4347	0.43	0.481	0.393	1	0.966	1.058	------	------	K07190	PHKA_B	map04910;m	Insulin signaling pa	ENOG410XPJZ	S:Function unknown	PF00723.24;PF19	Glyco_hydro_15;KPBB_C	Glycosyl hydrolases family 15;Phosphorylase b kinase C-terminal	CYT	1	1	134.3	High
TRINITY_DN3749_c0_g1_i1_orf1	cytochrome c oxidase subunit 6B1 [Ostrinia furnacalis]	2.464225079	1.301134036	1.15E-05	up	yes	1.016	0.4123	0.396	0.414	0.427	1	0.991	1.057	CC:macromolecular complex;CC:respiratory chain complex IV;CC:respiratory chain complex;CC:cytochrome complex;CC:intracellular membrane-bounded organelle;CC:cellular_component;CC:membrane protein complex;CC:intracellular organelle;CC:mitochondrion;CC:membrane-bounded organelle;CC:organelle;CC:cellular anatomical entity;CC:catalytic complex;	GO:0032	K02267	COX6B	map04714;m	Thermogenesis;Oxi	ENOG41121WM	S:Function unknown	PF02297.20	COX6B	Cytochrome oxidase c subunit VIb	CYT	5	43	9.9	High
TRINITY_DN1173_c1_g1_i9_orf1	hypothetical protein evm_001011 [Chilo suppressalis]	2.057118317	1.040624774	8.04E-05	up	yes	0.958	0.4657	0.496	0.426	0.475	1	0.946	0.928	BP:phosphorylation;BP:cell differentiation;BP:nitrogen compound metabolic process;BP:anatomical structure morphogenesis;BP:cellular developmental process;BP:organic substance metabolic process;BP:developmental process;BP:protein phosphorylation;BP:cellular process;BP:macromolecule modification;BP:protein modification process;BP:biological_process;BP:metabolic process;BP:primary metabolic process;BP:organonitrogen compound metabolic process;BP:protein metabolic process;BP:cellular metabolic process;BP:macromolecule metabolic process;BP:phosphate-containing compound metabolic process;BP:phosphorus metabolic process;MF:purine ribonucleoside triphosphate binding;MF:guanyl-nucleotide exchange factor activity;MF:molecular_function;MF:binding;MF:molecular function regulator;MF:heterocyclic compound binding;MF:nucleoside phosphate binding;MF:anion binding;MF:ion binding;MF:nucleotide	GO:0016	------	------	------	------	ENOG410XQFD;	S:Function unknown;T:Signal trar	PF07679.19;PF13	I-set;Ig_3;ig;Ig_2;RhoGEF;V-set;PH	Immunoglobulin I-set domain;Immunoglobulin domain;Immunoglobulin domain;Immunoglobulin domain;RhoGEF domain;Immunoglobulin V-set domain;PH domain	CYT	1	18	160.4	High
TRINITY_DN79804_c0_g1_i1_orf1	zinc finger protein on ecdysone puffs-like [Ostrinia furnacalis]	2.487110238	1.314470455	0.001997	up	yes	1.013	0.4073	0.407	0.349	0.466	1	1.153	0.886	------	------	------	------	------	------	ENOG4111SKG	S:Function unknown	------	------	------	CYT	1	21	8.9	High
TRINITY_DN25856_c0_g1_i1_orf1	myrosinase 1-like [Ostrinia furnacalis]	2.480295567	1.310512051	2.68E-06	up	yes	1.007	0.406	0.409	0.392	0.417	1	1.033	0.987	------	------	------	------	------	------	COG2723;ENOG	G:Carbohydrate transport and m	PF00232.21	Glyco_hydro_1	Glycosyl hydrolase family 1	CYT	3	9	57.7	High
TRINITY_DN10520_c0_g1_i2_orf1	probable 39S ribosomal protein L45, mitochondrial [Ostrinia furnacalis]	2.233610933	1.159377908	0.0003749	up	yes	1.046	0.4683	0.488	0.421	0.496	1	1	1.139	CC:ribosome;CC:intracellular non-membrane-bounded organelle;CC:cellular_component;CC:intracellular organelle;CC:non-membrane-bounded organelle;CC:organelle;CC:cellular anatomical entity;	GO:0005	K17426	MRPL45	------	------	ENOG4110198	S:Function unknown	PF04280.18	Tim44	Tim44-like domain	CYT	4	16	30	High
TRINITY_DN47123_c0_g1_i1_orf1	WD40 repeat-containing protein SMU1 [Ostrinia furnacalis]	2.041725744	1.029789089	0.0001313	up	yes	1.008	0.4937	0.505	0.543	0.433	1	0.987	1.036	BP:nucleic acid metabolic process;BP:cellular nitrogen compound metabolic process;BP:nitrogen compound metabolic process;BP:mRNA splicing, via spliceosome;BP:organic cyclic compound metabolic process;BP:nucleobase-containing compound metabolic process;BP:organic substance metabolic process;BP:RNA splicing;BP:mRNA metabolic process;BP:cellular process;BP:cellular aromatic compound metabolic process;BP:RNA splicing, via transesterification reactions;BP:RNA splicing, via transesterification reactions with bulged adenosine as nucleophile;BP:biological_process;BP:metabolic process;BP:heterocycle metabolic process;BP:RNA metabolic process;BP:primary metabolic process;BP:cellular metabolic process;BP:macromolecule metabolic process;BP:RNA processing;BP:mRNA processing;CC:nuclear speck;CC:nuclear body;CC:cellular anatomical	GO:0090	K13111	SMU1	------	------	ENOG410XP39	S:Function unknown	PF00400.35;PF12	WD40;ANAPC4_WD40;LisH_TPL;NBCH_WD40;Ge1_WD40	WD domain, G-beta repeat;Anaphase-promoting complex subunit 4 WD40 domain;LisH-like dimerisation domain;Neurobeachin beta propeller domain;WD40 region of Ge1, enhancer of mRNA-decapping protein	CYT	2	4	56.4	High
TRINITY_DN2265_c0_g1_i5_orf1	elongation factor G, mitochondrial [Ostrinia furnacalis]	2.071091021	1.050390959	0.0002148	up	yes	1.008	0.4867	0.49	0.427	0.543	1	1.052	0.973	BP:cellular macromolecule biosynthetic process;BP:cellular biosynthetic process;BP:organic substance biosynthetic process;BP:cellular macromolecule metabolic process;BP:cellular process;BP:organic substance metabolic process;BP:mitochondrial translational elongation;BP:biosynthetic process;BP:macromolecule biosynthetic process;BP:biological_process;BP:metabolic process;BP:cellular metabolic process;BP:macromolecule metabolic process;BP:translational elongation;CC:intracellular organelle;CC:membrane-bounded organelle;CC:mitochondrion;CC:organelle;CC:cellular anatomical entity;CC:intracellular membrane-bounded organelle;CC:cellular_component;MF:purine ribonucleoside triphosphate binding;MF:molecular_function;MF:binding;MF:nucleic acid binding;MF:guanyl nucleotide binding;MF:heterocyclic compound binding;MF:nucleoside phosphate binding;MF:anion binding;MF:guanyl ribonucleotide binding;MF:purine nucleotide	GO:0034	K02355	fusA, GFM, E	------	------	COG0480	J:Translation, ribosomal structure	PF00009.30;PF03	GTP_EFTU;EFG_IV;EFG_III;GTP_EFTU_D2;RF3_C	Elongation factor Tu GTP binding domain;Elongation factor G, domain IV;Elongation Factor G, domain III;Elongation factor Tu domain 2;Class II release factor RF3, C-terminal domain	CYT	3	5	71.3	High
TRINITY_DN14301_c0_g2_i1_orf1	unnamed protein product [Chrysodeixis includens]	2.22631693	1.154658984	4.85E-05	up	yes	1.027	0.4613	0.47	0.466	0.448	1	0.995	1.086	------	------	K04727	AIFM1, PDCD	map04210;m	Apoptosis;Necropto	COG0446	S:Function unknown	------	------	------	CYT	2	11	19.5	High

TRINITY_DN3949_c1_g1_i1_orf1	probable cytochrome P450 304a1 isoform X2 [Ostrinia furnacalis]	2.397260274	1.261386553	0.0001187	up	yes	1.05	0.438	0.416	0.48	0.418	1	1.031	1.118	CC:integral component of membrane;CC:cellular_component;CC:cellular anatomical entity;CC:intrinsic component of membrane;MF:cation binding;MF:monooxygenase activity;MF:heme binding;MF:transition metal ion binding;MF:tetrapyrrole binding;MF:organic cyclic compound binding;MF:oxidoreductase activity, acting on paired donors, with incorporation or reduction of molecular oxygen;MF:ion binding;MF:catalytic activity;MF:molecular_function;MF:binding;MF:iron ion binding;MF:metal ion binding;MF:heterocyclic compound binding;MF:oxidoreductase activity;	GO:0016(	------	------	------	------	COG2124	Q:Secondary metabolites biosyn	PF00067.25	p450	Cytochrome P450	CYT	2	17	16.8	High
TRINITY_DN16343_c0_g1_i6_orf1	aminopeptidase N6 [Ostrinia nubilalis]	2.232005283	1.158340442	6.56E-06	up	yes	1.014	0.4543	0.456	0.425	0.482	1	1.026	1.015	BP:nitrogen compound metabolic process;BP:organic substance metabolic process;BP:proteolysis;BP:biological_process;BP:metabolic process;BP:primary metabolic process;BP:organonitrogen compound metabolic process;BP:protein metabolic process;BP:macromolecule metabolic process;CC:integral component of membrane;CC:intrinsic component of membrane;CC:cellular anatomical entity;CC:cellular_component;MF:aminopeptidase activity;MF:exopeptidase activity;MF:molecular_function;MF:binding;MF:peptidase activity;MF:metallopeptidase activity;MF:cation binding;MF:transition metal ion binding;MF:ion binding;MF:metal ion binding;MF:hydrolase	GO:0006{	K11140	ANPEP, CD1:	map04640;m:	Hematopoietic cell	COG0308	E:Amino acid transport and meta	PF11838.11;PF01	ERAP1_C;Peptidase_M1;Peptidase_M1_N	ERAP1-like C-terminal domain;Peptidase family M1 domain;Peptidase M1 N-terminal domain	CYT	5	7	100.2	High
TRINITY_DN1578_c0_g3_i1_orf1	S-adenosylmethionine synthase isoform X1 [Ostrinia furnacalis]	2.395833333	1.26052755	5.82E-05	up	yes	1.035	0.432	0.381	0.465	0.45	1	1.075	1.03	BP:one-carbon metabolic process;BP:cellular biosynthetic process;BP:small molecule metabolic process;BP:organic substance biosynthetic process;BP:organic substance metabolic process;BP:sulfur compound metabolic process;BP:S-adenosylmethionine metabolic process;BP:cellular process;BP:biosynthetic process;BP:biological_process;BP:metabolic process;BP:sulfur compound biosynthetic process;BP:S-adenosylmethionine biosynthetic process;BP:cellular metabolic process;MF:purine ribonucleoside triphosphate binding;MF:molecular_function;MF:nucleotide binding;MF:heterocyclic compound binding;MF:nucleoside phosphate binding;MF:anion binding;MF:transferase activity, transferring alkyl or ary;MF:ion binding;MF:transferase activity;MF:metal ion binding;MF:ATP binding;MF:cation binding;MF:small molecule binding;MF:catalytic activity;MF:purine nucleotide binding;MF:methionine adenosyltransferase activity;MF:organic	GO:0006/	K00789	metK, MAT	map00270;m:	Cysteine and methi	COG0192	H:Coenzyme transport and meta	PF02772.19;PF00	S-AdoMet_synt_M;S-AdoMet_synt_N	S-adenosylmethionine synthetase, central domain;S-adenosylmethionine synthetase, N-terminal domain	CYT	7	42	28.2	High
TRINITY_DN33038_c0_g1_i1_orf1	39S ribosomal protein L46, mitochondrial [Ostrinia furnacalis]	2.427867699	1.279689807	0.0002077	up	yes	1.035	0.4263	0.431	0.358	0.49	1	1.016	1.09	CC:cellular_component;CC:macromolecular complex;CC:ribosome;CC:intracellular non-membrane-bounded organelle;CC:ribonucleoprotein complex;CC:mitochondrion;CC:intracellular organelle;CC:non-membrane-bounded organelle;CC:membrane-bounded organelle;CC:organelle;CC:cellular anatomical entity;CC:intracellular membrane-bounded organelle;MF:structural molecule activity;MF:structural constituent of	GO:0005!	K17427	MRPL46	------	------	ENOG4111HSF	S:Function unknown	PF11788.11	MRP-L46	39S mitochondrial ribosomal protein L46	CYT	1	5	29.9	High
TRINITY_DN21984_c0_g1_i6_orf1	venom serine protease 34-like [Ostrinia furnacalis]	2.15952381	1.110713223	6.90E-05	up	yes	0.9977	0.462	0.489	0.404	0.493	1	1.017	0.976	BP:organic substance metabolic process;BP:organonitrogen compound metabolic process;BP:protein metabolic process;BP:macromolecule metabolic process;BP:biological_process;BP:nitrogen compound metabolic process;BP:metabolic process;BP:proteolysis;BP:primary metabolic process;MF:serine-type endopeptidase activity;MF:endopeptidase activity;MF:hydrolase activity;MF:serine hydrolase activity;MF:catalytic activity, acting on a protein;MF:catalytic activity;MF:molecular_function;MF:pepti	GO:0071/	K01312	PRSS1_2_3	map04972;m:	Pancreatic secretio	COG5640	V:Defense mechanisms	PF00089.29;PF00	Trypsin;CUB	Trypsin;CUB domain	CYT	3	18	44.3	High
TRINITY_DN6231_c0_g1_i6_orf1	ran-binding protein 3 isoform X1 [Ostrinia furnacalis] >XP_028166372.1 ran-binding protein 3 isoform X2 [Ostrinia furnacalis]	2.021959809	1.015754321	2.14E-05	up	yes	0.976	0.4827	0.468	0.482	0.498	1	0.937	0.991	BP:cellular process;BP:intracellular transport;BP:transport;BP:biological_process;BP:establishment of localization in cell;BP:establishment of localization;BP:localization;BP:cellular localization;	GO:0009!	K15304	RANBP3	map05166	Human T-cell leuk	ENOG4111NCK	U:Intracellular trafficking, secretic	PF00638.21	Ran_BP1	RanBP1 domain	CYT	4	11	48.6	High
TRINITY_DN6685_c0_g1_i8_orf1	cleft lip and palate transmembrane protein 1 homolog [Ostrinia furnacalis]	2.261396811	1.177214166	0.0001256	up	yes	1.007	0.4453	0.465	0.372	0.499	1	1.004	1.016	CC:cellular_component;CC:integral component of membrane;CC:cellular anatomical entity;CC:intrinsic component of membrane;	GO:0005!	------	------	------	------	ENOG410XPEV	S:Function unknown	PF05602.15	CLPTM1	Cleft lip and palate transmembrane protein 1	CYT	3	7	72.4	High
TRINITY_DN3355_c0_g2_i4_orf1	UDP-glycosyltransferase UGT33AL1 [Ostrinia furnacalis]	2.351851852	1.233797185	7.11E-06	up	yes	1.016	0.432	0.412	0.441	0.443	1	1.049	0.999	CC:integral component of membrane;CC:cellular_component;CC:cellular anatomical entity;CC:intrinsic component of membrane;MF:catalytic activity;MF:transferase activity;MF:molecular_function;MF:transferase activity, transferring glycosyl groups;MF:transferase activity, transferring hexosyl groups;MF:glucuronosyltransferase activity;MF:UDP-glycosyltransferase	GO:0016(	K00699	UGT	map05207;m:	Chemical carcinoge	COG1819	S:Function unknown	PF00201.21	UDPGT	UDP-glucoronosyl and UDP-glucosyl transferase	CYT	4	13	60.5	High
TRINITY_DN5019_c0_g1_i2_orf1	RRP12-like protein isoform X4 [Ostrinia furnacalis] >XP_028175539.1 RRP12-like protein isoform X5 [Ostrinia furnacalis]	2.31884058	1.213403638	1.05E-07	up	yes	1.008	0.4347	0.437	0.427	0.44	1	1.018	1.006	CC:intracellular membrane-bounded organelle;CC:nucleus;CC:cellular_component;CC:intracellular organelle;CC:membrane-bounded organelle;CC:organelle;CC:cellular anatomical entity;	GO:0043:	K14794	RRP12	------	------	ENOG410XQ3D	S:Function unknown	PF08161.15	NUC173	NUC173 domain	CYT	8	6	153.7	High

TRINITY_DN2749_c4_g1_i2_orf1	RNA exonuclease 4-like [Ostrinia furnacalis] >QEE79882.1 REX4 [Ostrinia furnacalis]	2.286634461	1.193225756	0.00141	up	yes	0.994	0.4347	0.339	0.572	0.393	1	1.007	0.975	BP:nucleic acid metabolic process;BP:cellular nitrogen compound metabolic process;BP:nitrogen compound metabolic process;BP:ncRNA metabolic process;BP:organic cyclic compound metabolic process;BP:nucleobase-containing compound metabolic process;BP:organic substance metabolic process;BP:rRNA processing;BP:ncRNA processing;BP:cellular process;BP:cellular aromatic compound metabolic process;BP:biological_process;BP:metabolic process;BP:heterocycle metabolic process;BP:RNA metabolic process;BP:primary metabolic process;BP:rRNA metabolic process;BP:cellular metabolic process;BP:macromolecule metabolic process;BP:RNA processing;MF:molecular_function;MF:binding;MF:nucleic acid binding;MF:heterocyclic compound binding;MF:3'-5' exonuclease activity;MF:exonuclease activity;MF:hydrolase activity;MF:hydrolase activity, acting on	GO:00903	K18327	REXO4, REX4	------	------	COG0847	S:Function unknown	PF00929.27	RNase_T	Exonuclease	CYT	3	53	9.2	High
TRINITY_DN3647_c2_g1_i3_orf1	unnamed protein product, partial [Iphiclides podalirius]	2.183705357	1.12677821	9.56E-05	up	yes	0.9783	0.448	0.447	0.394	0.503	1	0.958	0.977	CC:cellular_component;CC:integral component of membrane;CC:cellular anatomical entity;CC:intrinsic component of membrane;	GO:00055	------	------	------	------	ENOG410ZUNN	S:Function unknown	PF10961.11	SelK_SelG	Selenoprotein SelK_SelG	EXC	1	13	9.5	High
TRINITY_DN4125_c1_g1_i5_orf1	angiotensin-converting enzyme-like isoform X2 [Ostrinia furnacalis]	2.337323944	1.2248577	1.57E-06	up	yes	0.9957	0.426	0.426	0.404	0.448	1	0.994	0.993	BP:nitrogen compound metabolic process;BP:organic substance metabolic process;BP:proteolysis;BP:biological_process;BP:metabolic process;BP:primary metabolic process;BP:organonitrogen compound metabolic process;BP:protein metabolic process;BP:macromolecule metabolic process;CC:membrane;CC:cellular anatomical entity;CC:cellular_component;MF:carboxypeptidase activity;MF:exopeptidase activity;MF:molecular_function;MF:binding;MF:peptidase activity;MF:metallopeptidase activity;MF:cation binding;MF:ion binding;MF:metal ion binding;MF:hydrolase activity;MF:peptidyl-dipeptidase	GO:00068	K01283	ACE, CD143	map05142;m	Chagas disease;Rer	ENOG410XPJ3	S:Function unknown	PF01401.21	Peptidase_M2	Angiotensin-converting enzyme	CYT	10	21	74.4	High
TRINITY_DN501_c1_g1_i1_orf1	sodium- and chloride-dependent GABA transporter ine isoform X1 [Ostrinia furnacalis]	2.343536614	1.228687335	5.35E-05	up	yes	0.9953	0.4247	0.409	0.391	0.474	1	1.025	0.961	BP:transmission of nerve impulse;BP:multicellular organismal signaling;BP:developmental cell growth;BP:developmental growth;BP:cell growth;BP:glial cell growth;BP:multicellular organismal process;BP:neurological system process;BP:biological_process;BP:growth;BP:system process;BP:signaling;BP:cellular process;CC:intrinsic component of membrane;CC:cellular_component;CC:integral component of membrane;CC:cellular anatomical entity;MF:neurotransmitte;MF:neurotransmitter transporter activity;MF:molecular transducer activity;MF:molecular_function;MF:signaling receptor activity;MF:sodium ion transmembrane transporter activity;MF:inorganic solute uptake transmembrane transporter activity;MF:transmembrane signaling receptor activity;MF:osmosensor activity;MF:solut;MF:transporter activity;MF:metal ion transmembrane transporter activity;MF:small molecule sensor activity;MF:active transmembrane transporter	GO:00192	K05039	SLC6A6, TAU	------	------	COG0733	S:Function unknown	PF00209.21	SNF	Sodiu	CYT	1	1	80.8	High
TRINITY_DN753_c0_g1_i4_orf1	venom dipeptidyl peptidase 4-like isoform X2 [Ostrinia furnacalis]	2.530724856	1.339550664	1.70E-06	up	yes	1.009	0.3987	0.376	0.401	0.419	1	1.006	1.022	BP:primary metabolic process;BP:organonitrogen compound metabolic process;BP:protein metabolic process;BP:macromolecule metabolic process;BP:organic substance metabolic process;BP:nitrogen compound metabolic process;BP:biological_process;BP:metabolic process;BP:proteolysis;CC:integral component of membrane;CC:cellular_component;CC:cellular anatomical entity;CC:intrinsic component of membrane;MF:molecular_function;MF:serine hydrolase activity;MF:hydrolase activity;MF:catalytic activity, acting on a protein;MF:catalytic	GO:00442	K01278	DPP4, CD26	map04974	Protein digestion a	COG1506;COG1!	J:Translation, ribosomal structure	PF00930.24;PF00	DPPIV_N;Peptidase_S9	Dipeptidyl peptidase IV;Prolyl oligopeptidase family	CYT	3	5	92.6	High
TRINITY_DN3929_c0_g3_i3_orf1	Glutathione S-transferase 1, isoform D [Papilio machaon]	2.635257572	1.397943979	2.12E-06	up	yes	1.018	0.3863	0.369	0.381	0.409	1	1.034	1.02	MF:catalytic activity;MF:transferase activity;MF:glutathione transferase activity;MF:molecular_function;MF:transferase activity, transferring alkyl or ary;	GO:00038	K00799	GST, gst	map05207;m	Chemical carcinoge	COG0625	O:Posttranslational modification,	PF13417.9;PF027	GST_N_3;GST_N;GST_N_2	transferase, N-terminal domain;Glutathione S-transferase, N-terminal domain;Glutathione S-transferase, N-terminal	CYT	1	23	12.3	High
TRINITY_DN45227_c0_g1_i3_orf1	uncharacterized protein LOC114359191 [Ostrinia furnacalis]	2.265392781	1.179761211	0.0001918	up	yes	1.067	0.471	0.425	0.486	0.502	1	1.134	1.067	CC:respiratory chain complex I;CC:transmembrane transporter complex;CC:catalytic complex;CC:transporter complex;CC:NADH dehydrogenase complex;CC:respiratory chain complex;CC:inner mitochondrial membrane protein complex;CC:cellular_component;CC:membrane protein complex;CC:mitochondrial respiratory chain complex I;CC:macromolecular complex;CC:oxidoreductase complex;CC:mitochondrial protein complex;	GO:00452	------	------	------	------	ENOG410XXY6	S:Function unknown	PF15880.8	NDUFV3	NADH dehydrogenase [ubiquinone] flavoprotein 3, mitochondrial	CYT	1	8	11.2	High
TRINITY_DN43942_c0_g1_i1_orf1	LOW QUALITY PROTEIN: caprin homolog [Ostrinia furnacalis]	2.010623647	1.00764306	1.01E-05	up	yes	1.022	0.5083	0.482	0.511	0.532	1	1.039	1.027	------	------	K18743	CAPRIN1, GP	------	------	ENOG4110M5Q	S:Function unknown	PF18293.4	Caprin-1_dimer	Caprin-1 dimerization domain	CYT	6	20	45.3	High

TRINITY_DN81248_c0_g1_i1_orf1	39S ribosomal protein L12, mitochondrial [Ostrinia furnacalis]	2.103965482	1.073111036	0.0002176	up	yes	1.024	0.4867	0.411	0.512	0.537	1	1.057	1.015	BP:cellular biosynthetic process;BP:cellular nitrogen compound metabolic process;BP:nitrogen compound metabolic process;BP:cellular macromolecule biosynthetic process;BP:organic substance biosynthetic process;BP:cellular nitrogen compound biosynthetic process;BP:cellular macromolecule metabolic process;BP:peptide biosynthetic process;BP:organic substance metabolic process;BP:amide biosynthetic process;BP:cellular process;BP:biosynthetic process;BP:macromolecule biosynthetic process;BP:biological_process;BP:metabolic process;BP:organonitrogen compound metabolic process;BP:primary metabolic process;BP:cellular amide metabolic process;BP:organonitrogen compound biosynthetic process;BP:protein metabolic process;BP:cellular metabolic process;BP:macromolecule metabolic process;BP:peptide metabolic process;BP:translation;CC:intracellular non-membrane-bounded organelle;CC:organelle;CC:cellular anatomical	GO:00442	K02935	RP-L7, MRPL	map03010	Ribosome	COG0222	J:Translation, ribosomal structure	PF00542.22;PF16	Ribosomal_L12;Ribosomal_L12_N	Ribosomal protein L7/L12 C-terminal domain;Ribosomal protein L7/L12 dimerisation domain	CYT	1	7	19.8	High
TRINITY_DN3355_c0_g1_i1_orf1	UDP-glucuronosyltransferase 2B2-like [Ostrinia furnacalis]	2.184204863	1.127108177	0.001933	up	yes	1.015	0.4647	0.515	0.552	0.327	1	1.073	0.971	CC:integral component of membrane;CC:cellular_component;CC:cellular anatomical entity;CC:intrinsic component of membrane;MF:catalytic activity;MF:transferase activity;MF:molecular_function;MF:transferase activity, transferring glycosyl groups;MF:transferase activity, transferring hexosyl groups;MF:glucuronosyltransferase activity;MF:UDP-glycosyltransferase	GO:0016(	K00699	UGT	map05207;m	Chemical carcinoge	COG1819	S:Function unknown	PF00201.21	UDPGT	UDP-glucoronosyl and UDP-glucosyl transferase	CYT	1	4	60.2	High
TRINITY_DN430_c0_g1_i5_orf1	hypothetical protein NE865_02252 [Phthorimaea operculella]	2.140127389	1.097696674	3.23E-06	up	yes	1.008	0.471	0.448	0.486	0.479	1	0.998	1.025	MF:cation binding;MF:transition metal ion binding;MF:organic cyclic compound binding;MF:ion binding;MF:molecular_function;MF:binding;MF:nucleic acid binding;MF:metal ion binding;MF:heterocyclic compound binding;MF:zinc ion binding;	GO:00431	------	------	------	------	ENOG4110QHB	S:Function unknown	------	------	------	CYT	5	8	83.6	High
TRINITY_DN46173_c0_g3_i2_orf1	tropomyosin-1, isoforms 9A/A/B isoform X33 [Aedes aegypti] >EAT46020.1 AAEL002761-PB [Aedes aegypti]	2.140260851	1.09778664	4.61E-06	up	yes	1.001	0.4677	0.465	0.451	0.487	1	1.022	0.981	------	------	K10373	TPM1	map05206;m	MicroRNAs in canc	ENOG410XR5K	S:Function unknown	PF00261.23;PF12	Tropomyosin;Tropomyosin_1	Tropomyosin;Tropomyosin	CYT	2	24	29	High
TRINITY_DN113626_c0_g1_i3_orfp	TRINITY_DN113626_c0_g1_i3_m.80721 TRINITY_DN113626_c0_g1::TRINITY_DN113626_c0_g1_i3::g.80721 ORF type:internal len:118 (-),score=87.34 TRINITY_DN113626_c0_g1_i3:2-	2.312785388	1.209631399	0.002841	up	yes	1.013	0.438	0.282	0.542	0.49	1	0.955	1.085	------	------	------	------	------	------	------	------	------	------	------	PLA	1	9	11.6	High
TRINITY_DN1386_c0_g1_i6_orf1	ras-related protein Rab-36 [Ostrinia furnacalis]	2.141921397	1.098905538	7.42E-06	up	yes	0.981	0.458	0.447	0.44	0.487	1	0.973	0.97	------	------	K07918	RAB32	------	------	ENOG410YITC	S:Function unknown	PF00071.25;PF08	Ras;Roc;Arf	Ras family;Ras of Complex, Roc, domain of DAPkinase;ADP-ribosylation factor family	CYT	7	13	54.1	High
TRINITY_DN2082_c0_g1_i2_orf1	choline-phosphate cytidylyltransferase B-like isoform X1 [Ostrinia furnacalis]	2.210208193	1.144182273	1.16E-05	up	yes	0.9873	0.4467	0.411	0.453	0.476	1	0.985	0.977	MF:nucleotidyltransferase activity;MF:choline-phosphate cytidylyltransferase activity;MF:catalytic activity;MF:transferase activity, transferring phosphorus-containing groups;MF:cytidylyltransferase activity;MF:transferase activity;MF:molecular_function;	GO:00167	K00968	PCYT1	map00564;m	Glycerophospholipi	COG0615;COG0	I:Lipid transport and metabolism	PF01467.29	CTP_transf_like	Cytidylyltransferase-like	CYT	5	17	45.3	High
TRINITY_DN3028_c0_g1_i1_orf1	pre-rRNA processing protein FTSJ3 [Ostrinia furnacalis]	2.122336921	1.085653702	6.71E-05	up	yes	0.9663	0.4553	0.459	0.492	0.415	1	0.932	0.967	BP:metabolic process;BP:RNA modification;BP:nucleic acid metabolic process;BP:cellular nitrogen compound metabolic process;BP:nitrogen compound metabolic process;BP:ncRNA metabolic process;BP:rRNA 2'-O-methylation;BP:enzyme-directed rRNA 2'-O-methylation;BP:nucleobase-containing compound metabolic process;BP:cellular macromolecule metabolic process;BP:organic cyclic compound metabolic process;BP:organic substance metabolic process;BP:rRNA processing;BP:methylation;BP:ncRNA processing;BP:cellular process;BP:cellular aromatic compound metabolic process;BP:macromolecule modification;BP:biological_process;BP:rRNA methylation;BP:RNA methylation;BP:heterocycle metabolic process;BP:RNA metabolic process;BP:primary metabolic process;BP:rRNA metabolic process;BP:rRNA modification;BP:cellular metabolic process;BP:macromolecule metabolic process;BP:macromolecule methylation;BP:RNA processing;CC:organelle;CC:nucleolus;CC:cellular anatomical	GO:00081	K14857	SPB1, FTSJ3	------	------	COG0293	J:Translation, ribosomal structure	PF07780.15;PF01	Spb1_C;FtsJ;DUF3381	Spb1 C-terminal domain;FtsJ-like methyltransferase;Domain of unknown function	CYT	2	3	99	High
TRINITY_DN12442_c0_g1_i4_orf1	midasin-like [Ostrinia furnacalis]	2.365105776	1.241904708	2.05E-05	up	yes	0.995	0.4207	0.377	0.446	0.439	1	0.973	1.012	BP:cellular component assembly;BP:ribosomal large subunit assembly;BP:macromolecular complex assembly;BP:ribonucleoprotein complex subunit organization;BP:cellular component organization;BP:macromolecular complex subunit organization;BP:ribonucleoprotein complex assembly;BP:cellular component organization or biogenesis;BP:cellular process;BP:biological_process;CC:intracellular organelle;CC:non-membrane-bounded organelle;CC:organelle;CC:nucleolus;CC:nucleoplasm;CC:intracellular non-membrane-bounded organelle;CC:cellular_component;CC:cellular anatomical entity;MF:purine ribonucleoside triphosphate binding;MF:heterocyclic compound binding;MF:molecular_function;MF:binding;MF:ATPase activity;MF:ion binding;MF:nucleoside phosphate binding;MF:anion binding;MF:nucleotide binding;MF:purine nucleotide binding;MF:ATP binding;MF:hydrolase activity;MF:nucleoside-triphosphatase	GO:0022(	K14572	MDN1, REA1	map03008	Ribosome biogenes	COG5271	S:Function unknown	PF13519.9;PF000	VWA_2;VWA	von Willebrand factor type A domain;von Willebrand factor type A domain	CYT	2	1	270.7	High
TRINITY_DN63662_c0_g4_i1_orf1	polyadenylate-binding protein 1 [Ostrinia furnacalis]	2.431315342	1.281737023	7.84E-07	up	yes	1	0.4113	0.397	0.407	0.43	1	1.01	0.991	CC:cytoplasm;CC:cellular_component;CC:cellular anatomical entity;MF:organic cyclic compound binding;MF:molecular_function;MF:RNA binding;MF:nucleic acid binding;MF:heterocyclic compound binding;MF:binding;	GO:00057	K13126	PABPC	map03018;m	RNA degradation;m	ENOG410XR5X	S:Function unknown	PF00076.25;PF00	RRM_1;PABP;Nup35_RRM_2	recognition motif.;Poly-adenylate binding protein, unique domain;Nup53/35/40-type RNA	CYT	21	40	68.2	High
TRINITY_DN2780_c0_g1_i5_orf1	probable ATP-dependent RNA helicase DDX27 [Ostrinia furnacalis]	2.516820334	1.331602232	1.40E-06	up	yes	1.01	0.4013	0.421	0.4	0.383	1	1.005	1.025	------	------	K13181	DDX27, DRS1	------	------	COG0513	O:Posttranslational modification,	PF00270.32;PF00	DEAD;Helicase_C;ResIII	DEAD/DEAH box helicase;Helicase conserved C-terminal domain;Type III restriction enzyme, res subunit	CYT	4	7	90.9	High

TRINITY_DN948_c0_g1_i1_orf1	mitochondrial-processing peptidase subunit beta [Ostrinia furnacalis]	2.406053269	1.266668583	2.55E-06	up	yes	0.9937	0.413	0.417	0.387	0.435	1	0.997	0.984	BP:primary metabolic process;BP:organonitrogen compound metabolic process;BP:protein metabolic process;BP:macromolecule metabolic process;BP:organic substance metabolic process;BP:nitrogen compound metabolic process;BP:biological_process;BP:metabolic process;BP:proteolysis;MF:molecular_function;MF:endopeptidase activity;MF:cation binding;MF:hydrolase activity;MF:ion binding;MF:catalytic activity, acting on a protein;MF:catalytic activity;MF:metalloendopeptidase activity;MF:binding;MF:peptidase activity;MF:metal ion [illegible]	GO:00442	K17732	PMPCB, MAS	------	------	COG0612	O:Posttranslational modification,	PF05193.24;PF00	Peptidase_M16_C;Peptidase_M16	Peptidase M16 inactive domain;Insulinase	CYT	13	44	37.5	High
TRINITY_DN71465_c0_g1_i1_orf1	adenylate kinase isoenzyme 6 [Ostrinia furnacalis]	2.217866722	1.149172672	0.0006626	up	yes	1.075	0.4847	0.466	0.517	0.471	1	1.192	1.032	BP:phosphorylation;BP:cellular process;BP:phosphorus metabolic process;BP:biological_process;BP:metabolic process;BP:cellular metabolic process;BP:phosphate-containing compound metabolic process;CC:intracellular organelle;CC:membrane-bounded organelle;CC:organelle;CC:nucleus;CC:intracellular membrane-bounded organelle;CC:cellular_component;CC:cellular anatomical entity;MF:nucleobase-containing compound kinase activity;MF:purine ribonucleoside triphosphate binding;MF:heterocyclic compound binding;MF:adenylate kinase activity;MF:phosphotransferase activity, phosphate group as acceptor;MF:molecular_function;MF:binding;MF:ATPase activity;MF:ion binding;MF:nucleoside phosphate binding;MF:anion binding;MF:nucleotide binding;MF:transferase activity;MF:purine nucleotide binding;MF:ATP binding;MF:hydrolase activity;MF:kinase activity;MF:nucleoside-triphosphatase activity;MF:small molecule binding;MF:catalytic [illegible]	GO:0016	K18532	AK6, FAP7	map03008;m	Ribosome biogenes	COG1936	F:Nucleotide transport and meta	PF13238.9	AAA_18	AAA domain	CYT	1	9	20.6	High
TRINITY_DN2062_c0_g1_i11_orf1	uncharacterized protein LOC114350846 [Ostrinia furnacalis]	2.026630116	1.019082804	0.0002415	up	yes	1.035	0.5107	0.55	0.44	0.542	1	1.078	1.026	------	------	------	------	------	------	COG1028	V:Defense mechanisms	PF13561.9;PF00	adh_short_C2;adh_short	Enoyl;short chain dehydrogenas	CYT	1	61	15.4	High
TRINITY_DN12323_c0_g2_i2_orf1	polyribonucleotide nucleotidyltransferase 1, mitochondrial [Ostrinia furnacalis]	2.104081633	1.073190678	0.0003204	up	yes	1.031	0.49	0.477	0.456	0.537	1	1.11	0.982	BP:negative regulation of metabolic process;BP:regulation of metabolic process;BP:nucleic acid metabolic process;BP:cellular nitrogen compound metabolic process;BP:nitrogen compound metabolic process;BP:regulation of biological process;BP:organic cyclic compound metabolic process;BP:organic cyclic compound catabolic process;BP:nucleobase-containing compound metabolic process;BP:organic substance catabolic process;BP:cellular macromolecule catabolic process;BP:negative regulation of macromolecule metabolic process;BP:cellular macromolecule metabolic process;BP:organic substance metabolic process;BP:biological regulation;BP:RNA catabolic process;BP:mRNA catabolic process;BP:regulation of gene expression;BP:primary metabolic process;BP:regulation of macromolecule metabolic process;BP:cellular process;BP:cellular aromatic compound metabolic process;BP:heterocycle catabolic process;BP:biological_process;BP:metabo [illegible]	GO:0009	K00962	pnp, PNPT1	map03018	RNA degradation	COG1185	J:Translation, ribosomal structure	PF01138.24;PF03	RNase_PH;RNase_PH_C;PNPase	3' exoribonuclease family, domain 1;3' exoribonuclease family, domain 2;Polyribonucleotide nucleotidyltransferase, RNA binding domain	CYT	1	1	82.8	Medium
TRINITY_DN22664_c0_g1_i1_orf1	larval cuticle protein LCP-14-like [Ostrinia furnacalis]	2.154661017	1.107460915	2.67E-06	up	yes	1.017	0.472	0.454	0.481	0.481	1	1.013	1.037	MF:molecular_function;MF:structural constituent of cuticle;MF:structural molecule activity;	GO:0003	------	------	------	------	ENOG4111PJ1;E	S:Function unknown;	PF00379.26	Chitin_bind_4	Insect cuticle protein	CYT	6	52	19.3	High
TRINITY_DN1884_c0_g2_i2_orf1	phosphotriesterase-related protein [Ostrinia furnacalis]	2.112350095	1.078848963	0.0001442	up	yes	1.004	0.4753	0.437	0.447	0.542	1	1.035	0.978	BP:biological_process;BP:metabolic process;BP:catabolic process;MF:cation binding;MF:transition metal ion binding;MF:hydrolase activity;MF:hydrolase activity, acting on ester bonds;MF:ion binding;MF:catalytic activity;MF:zinc ion binding;MF:molecular_function;MF:metal [illegible]	GO:0008	K07048	PTER, php	------	------	COG1735	S:Function unknown	PF02126.21	PTE	Phosphotriesterase family	CYT	2	20	20.9	High
TRINITY_DN4956_c0_g1_i6_orf1	nucleolar GTP-binding protein 1 [Ostrinia furnacalis]	2.295007906	1.198499124	9.05E-06	up	yes	1.016	0.4427	0.429	0.475	0.424	1	1.039	1.008	BP:ribosome biogenesis;BP:cellular component organization or biogenesis;BP:ribonucleoprotein complex biogenesis;BP:cellular process;BP:biological_process;BP:cellular component biogenesis;CC:intracellular organelle;CC:non-membrane-bounded organelle;CC:organelle;CC:nucleolus;CC:cellular anatomical entity;CC:intracellular non-membrane-bounded organelle;CC:cellular_component;MF:purine ribonucleoside triphosphate binding;MF:heterocyclic compound binding;MF:molecular_function;MF:binding;MF:ion binding;MF:nucleoside phosphate binding;MF:anion binding;MF:nucleotide binding;MF:guanyl ribonucleotide binding;MF:purine nucleotide binding;MF:GTP binding;MF:small molecule binding;MF:carbohydrate derivative binding;MF:organic cyclic compound binding;MF:guanyl [illegible]	GO:0042	K06943	NOG1	map03008	Ribosome biogenes	COG1084	S:Function unknown	PF17835.4;PF068	NOG1_N;NOG1;NOGCT;MMR_HSR1;FeoB_N	NOG1 N-terminal helical domain;Nucleolar GTP-binding protein 1;NOGCT;50S ribosome-binding GTPase;Ferrous iron transport protein B	CYT	6	10	75.1	High
TRINITY_DN139212_c0_g1_i4_orf1	uncharacterized protein LOC114350112 [Ostrinia furnacalis]	2.480058013	1.310373868	2.99E-05	up	yes	1.026	0.4137	0.388	0.405	0.448	1	1.072	1.007	MF:catalytic activity;MF:molecular_function;MF:oxidoreductase activity;	GO:0003	------	------	------	------	COG1028	Z:Cytoskeleton	PF13561.9;PF00	adh_short_C2;adh_short;KR	Enoyl;short chain dehydrogenase:KR domain	CYT	2	8	27.3	High
TRINITY_DN227_c0_g1_i1_orf1	double-stranded ribonuclease 2 [Ostrinia nubilalis]	2.09687064	1.068237861	0.001004	up	yes	1.052	0.5017	0.502	0.474	0.529	1	0.98	1.175	MF:hydrolase activity;MF:cation binding;MF:nuclease activity;MF:organic cyclic compound binding;MF:hydrolase activity, acting on ester bonds;MF:ion binding;MF:catalytic activity;MF:molecular_function;MF:binding;MF:nucleic acid binding;MF:endonuclease activity;MF:metal ion binding;MF:heterocyclic compound [illegible]	GO:0016	------	------	------	------	ENOG4111S9Z	O:Posttranslational modification,	PF01223.26	Endonuclease_NS	DNA/RNA non-specific endonuclease	CYT	1	3	50.9	High
TRINITY_DN23824_c0_g1_i1_orf1	28S ribosomal protein S30, mitochondrial [Ostrinia furnacalis]	2.123965274	1.086760179	0.000298	up	yes	1.052	0.4953	0.488	0.455	0.543	1	1.13	1.026	------	------	K17409	MRPS30	------	------	ENOG410ZG5G	J:Translation, ribosomal structure	PF07147.15	PDCD9	Mitochondrial 28S ribosomal protein S30	CYT	2	3	65.3	High
TRINITY_DN15513_c0_g1_i6_orf1	uncharacterized protein LOC114350859 [Ostrinia furnacalis]	2.012873025	1.009256168	2.31E-05	up	yes	1.032	0.5127	0.506	0.521	0.511	1	1.021	1.076	------	------	------	------	------	------	COG1028	S:Function unknown	PF13561.9;PF00	adh_short_C2;adh_short;KR	Enoyl;short chain dehydrogenase:KR domain	CYT	3	22	25.8	High
TRINITY_DN2425_c0_g1_i1_orf1	thyroid receptor-interacting protein 11 [Ostrinia furnacalis]	2.182531489	1.126002469	0.0005732	up	yes	1.057	0.4843	0.418	0.582	0.453	1	1.092	1.079	------	------	------	------	------	------	ENOG410XUHJ	S:Function unknown	PF11365.11	SOGA	Protein SOGA	CYT	1	1	262.5	Medium
TRINITY_DN17417_c0_g1_i11_orf1	sodium/hydrogen exchanger 9B2-like isoform X4 [Ostrinia furnacalis]	2.135724475	1.09472554	0.001289	up	yes	1.048	0.4907	0.362	0.569	0.541	1	1.069	1.076	------	------	K23994	SLC9B1_2	------	------	COG0025;COG0	E:Amino acid transport and meta	PF00999.24	Na_H_Exchanger	Sodium/hydrogen exchanger family	CYT	1	3	75.4	High
TRINITY_DN58531_c0_g1_i1_orf1	uncharacterized protein LOC114357371 [Ostrinia furnacalis] >XP_028166768.1 uncharacterized protein LOC114357371 [Ostrinia furnacalis]	3.081985193	1.623859931	0.000713	up	yes	1.124	0.3647	0.334	0.299	0.461	1	1.157	1.214	------	------	------	------	------	------	ENOG410Y4ER;E	S:Function unknown;	------	------	------	CYT	1	3	29.7	Medium

TRINITY_DN10458_c0_g1_i1_orf1	V-type proton ATPase 21 kDa proteolipid subunit [Ostrinia furnacalis]	2.452830189	1.294447358	0.0002943	up	yes	1.053	0.4293	0.464	0.359	0.465	1	1.029	1.13	CC:intrinsic component of membrane;CC:cellular_component;CC:proton-transporting V-type ATPase, V0 domain;CC:integral component of membrane;CC:cellular anatomical entity;CC:macromolecular complex;CC:membrane protein complex;CC:proton-transporting two-sector ATPase complex, proton-transporting domain;MF:molecular_function;MF:ATPase activity, coupled to transmembrane movement of substances;MF:inorganic solute uptake transmembrane transporter activity;MF:hydrogen-translocating pyrophosphatase activity;MF:primary active transmembrane transporter activity;MF:ATPase coupled ion transmembrane transporter activity;MF:transporter activity;MF:cation transmembrane transporter activity;MF:proton-transporting ATPase activity, rotational mechanism;MF:ATPase activity, coupled to transmembrane movement of ions, rotational mechanism;MF:inorganic cation transmembrane transporter activity;MF:cation-transporting ATPase	GO:00312	K03661	ATPeV0B, AT	map05152;m	Tuberculosis;Huma	COG0636	C:Energy production and convers	PF00137.24	ATP-synt_C	ATP synthase subunit C	CYT	1	5	21.6	High
TRINITY_DN4213_c0_g1_i4_orf1	nardilysin-like isoform X1 [Ostrinia furnacalis] >XP_028157649.1 nardilysin-like isoform X2 [Ostrinia furnacalis] >XP_028157650.1 nardilysin-like isoform X3 [Ostrinia furnacalis] >XP_028157651.1 nardilysin-like isoform X4 [Ostrinia furnacalis]	2.414918415	1.27197445	6.21E-06	up	yes	1.036	0.429	0.425	0.431	0.431	1	1.042	1.066	------	------	K01411	NRD1	------	------	COG1025	O:Posttranslational modification,	PF16187.8;PF006	Peptidase_M16_M;Peptidase_M16;Peptidase_M16_C	Middle or third domain of peptidase_M16;Insulinase;Peptidase M16 inactive	CYT	3	3	131.4	High
TRINITY_DN10796_c0_g2_i1_orf1	F-BAR domain only protein 2 [Ostrinia furnacalis]	2.082437276	1.058273042	0.0001348	up	yes	0.9877	0.4743	0.464	0.42	0.539	1	0.973	0.99	CC:cellular_component;CC:plasma membrane region;CC:membrane;CC:clathrin-coated pit;CC:cellular anatomical entity;	GO:00055	K20042	FCHO	------	------	ENOG410Y7RY	S:Function unknown	PF10291.12;PF00	muHD;FCH	Muniscin C-terminal mu homology domain;Fes/CIP4, and EFC/F-BAR homology domain	CYT	3	3	99.2	High
TRINITY_DN29448_c0_g1_i1_orf1	28S ribosomal protein S9, mitochondrial [Ostrinia furnacalis]	2.041848844	1.029876069	5.69E-06	up	yes	0.9807	0.4803	0.477	0.464	0.5	1	0.982	0.96	BP:cellular biosynthetic process;BP:cellular nitrogen compound metabolic process;BP:nitrogen compound metabolic process;BP:cellular macromolecule biosynthetic process;BP:organic substance biosynthetic process;BP:cellular nitrogen compound biosynthetic process;BP:cellular macromolecule metabolic process;BP:peptide biosynthetic process;BP:organic substance metabolic process;BP:amide biosynthetic process;BP:cellular process;BP:biosynthetic process;BP:macromolecule biosynthetic process;BP:biological_process;BP:metabolic process;BP:organonitrogen compound metabolic process;BP:primary metabolic process;BP:cellular amide metabolic process;BP:organonitrogen compound biosynthetic process;BP:protein metabolic process;BP:cellular metabolic process;BP:macromolecule metabolic process;BP:peptide metabolic process;BP:translation;CC:intracellular non-membrane-bounded organelle;CC:organelle;CC:cellular anatomical	GO:00442	K02996	RP-S9, MRPS	map03010	Ribosome	COG0103	J:Translation, ribosomal structure	PF00380.22	Ribosomal_S9	Ribosomal protein S9/S16	CYT	2	5	45.9	High
TRINITY_DN4944_c0_g1_i2_orf1	bifunctional glutamate/proline--tRNA ligase [Ostrinia furnacalis]	2.056722689	1.040347286	1.43E-06	up	yes	0.979	0.476	0.482	0.474	0.472	1	0.965	0.972	BP:prolyl-tRNA aminoacylation;BP:carboxylic acid metabolic process;BP:nucleic acid metabolic process;BP:cellular nitrogen compound metabolic process;BP:nitrogen compound metabolic process;BP:small molecule metabolic process;BP:ncRNA metabolic process;BP:organic cyclic compound metabolic process;BP:nucleobase-containing compound metabolic process;BP:cellular amino acid metabolic process;BP:organic substance metabolic process;BP:cellular process;BP:cellular aromatic compound metabolic process;BP:biological_process;BP:metabolic process;BP:oxoacid metabolic process;BP:heterocycle metabolic process;BP:RNA metabolic process;BP:primary metabolic process;BP:organonitrogen compound metabolic process;BP:organic acid metabolic process;BP:cellular metabolic process;BP:macromolecule metabolic process;BP:tRNA metabolic process;BP:amino acid activation;BP:tRNA aminoacylation;BP:tRNA aminoacylation for protein	GO:00064	K14163	EPRS	map00860;m	Porphyrin metaboli	COG0442	J:Translation, ribosomal structure	PF09180.14;PF00	ProRS-C_1;tRNA-synt_2b;HGTP_anticodon	Prolyl-tRNA synthetase, C-terminal;tRNA synthetase class II core domain;Anticodon binding domain	CYT	6	28	51	High
TRINITY_DN27087_c0_g1_i1_orf1	2',5'-phosphodiesterase 12 [Ostrinia furnacalis]	2.010618364	1.007639269	4.46E-05	up	yes	0.9657	0.4803	0.482	0.457	0.502	1	0.972	0.925	MF:catalytic activity;MF:molecular_function;	GO:00038	K19612	PDE12	------	------	COG5239	O:Posttranslational modification,	PF03372.26	Exo_endo_phos	Endonuclease/Exonuclease/phosphatase	CYT	2	3	67.3	High
TRINITY_DN80560_c0_g1_i1_orf1	ATP synthase subunit alpha, mitochondrial [Ostrinia furnacalis]	2.249155976	1.169383713	3.52E-06	up	yes	0.9993	0.4443	0.441	0.42	0.472	1	1.004	0.994	CC:proton-transporting ATP synthase complex, catalytic core;CC:proton-transporting two-sector ATPase complex, catalytic domain;CC:macromolecular complex;CC:cellular_component;CC:membrane protein complex;MF:cation channel activity;MF:purine ribonucleoside triphosphate binding;MF:molecular_function;MF:binding;MF:ion binding;MF:heterocyclic compound binding;MF:nucleoside phosphate binding;MF:proton-transporting ATP synthase activity, rotational mechanism;MF:inorganic solute uptake transmembrane transporter activity;MF:nucleotide binding;MF:hydrogen ion channel activity;MF:purine nucleotide binding;MF:ATP binding;MF:anion binding;MF:transporter activity;MF:ion channel activity;MF:cation transmembrane transporter activity;MF:small molecule binding;MF:catalytic activity;MF:passive transmembrane transporter activity;MF:ligase activity;MF:inorganic cation transmembrane transporter activity;MF:adenyl nucleotide	GO:00452	K02132	ATPeF1A, AT	map04714;m	Thermogenesis;Che	COG0056	C:Energy production and convers	PF00006.28;PF00	ATP-synt_ab;ATP-synt_ab_C;ATP-synt_ab_N	ATP synthase alpha/beta family, nucleotide-binding domain;ATP synthase alpha/beta chain, C terminal domain;ATP synthase alpha/beta family, beta-barrel domain	CYT	23	45	59.4	High
TRINITY_DN1803_c0_g1_i3_orf1	translocator protein-like isoform X1 [Ostrinia furnacalis] >XP_028178947.1 translocator protein-like isoform X1 [Ostrinia furnacalis]	2.033212689	1.02376114	0.0002753	up	yes	0.955	0.4697	0.434	0.446	0.529	1	0.959	0.906	CC:cellular_component;CC:integral component of membrane;CC:cellular anatomical entity;CC:intrinsic component of membrane;	GO:00055	K05770	TSPO, BZRP	map05166;m	Human T-cell leuk	COG3476;ENOG	T:Signal transduction mechanism	PF03073.18	TspO_MBR	TspO/MBR family	CYT	3	12	21.3	High

TRINITY_DN1294_c0_g1_i3_orf1	46 kDa FK506-binding nuclear protein-like isoform X1 [Ostrinia furnacalis] >XP_028157904.1 46 kDa FK506-binding nuclear protein-like isoform X2 [Ostrinia furnacalis]	2.362411348	1.240260191	2.68E-06	up	yes	0.9993	0.423	0.412	0.409	0.448	1	1.013	0.985	CC:intracellular membrane-bounded organelle;CC:nucleus;CC:cellular_component;CC:intracellular organelle;CC:membrane-bounded organelle;CC:organelle;CC:cellular anatomical entity;MF:organic cyclic compound binding;MF:catalytic activity, acting on a protein;MF:heterocyclic compound binding;MF:cis-trans isomerase activity;MF:catalytic activity;MF:molecular_function;MF:binding;MF:peptidyl-prolyl cis-trans isomerase activity;MF:DNA binding;MF:nucleic acid	GO:00432	K14826	FPR3_4	------	------	COG0545	C:Energy production and convers	PF00254.31;PF17	FKBP_C;NPL	FKBP-type peptidyl-prolyl cis-trans isomerase;Nucleoplasmin-like domain	CYT	12	32	44.7	High
TRINITY_DN21909_c0_g1_i1_orf1	complement component 1 Q subcomponent-binding protein, mitochondrial [Ostrinia furnacalis]	2.550790068	1.350944169	1.96E-06	up	yes	1.017	0.3987	0.39	0.389	0.417	1	1.039	1.011	CC:organelle lumen;CC:membrane-enclosed lumen;CC:cellular_component;CC:intracellular organelle lumen;CC:mitochondrial matrix;CC:cellular anatomical entity;	GO:00432	K15414	C1QBP	------	------	ENOG4111G4Z	S:Function unknown	PF02330.19	MAM33	Mitochondrial glycoprotein	CYT	6	24	31.9	High
TRINITY_DN12973_c0_g1_i1_orf1	mitochondrial-processing peptidase subunit alpha [Ostrinia furnacalis]	2.143777778	1.100155365	1.48E-05	up	yes	0.9647	0.45	0.431	0.463	0.456	1	0.953	0.941	BP:mitochondrial protein processing;BP:nitrogen compound metabolic process;BP:organic substance metabolic process;BP:proteolysis;BP:protein processing involved in protein targeting to mitochondrion;BP:biological_process;BP:metabolic process;BP:protein processing;BP:primary metabolic process;BP:organonitrogen compound metabolic process;BP:protein maturation;BP:protein metabolic process;BP:macromolecule metabolic process;CC:membrane-enclosed lumen;CC:mitochondrial matrix;CC:cellular anatomical entity;CC:organelle lumen;CC:cellular_component;CC:intracellular organelle lumen;MF:molecular_function;MF:bindin	GO:00349	K01412	PMPCA, MAS	------	------	COG0612	O:Posttranslational modification,	PF05193.24;PF00	Peptidase_M16_C;Peptidase_M16	Peptidase M16 inactive domain;Insulinase	CYT	5	10	59.2	High
TRINITY_DN33883_c0_g1_i1_orf1	probable 28S ribosomal protein S6, mitochondrial [Ostrinia furnacalis]	2.211740042	1.145181828	0.00011	up	yes	1.055	0.477	0.486	0.461	0.484	1	1.039	1.126	BP:cellular biosynthetic process;BP:cellular macromolecule biosynthetic process;BP:organic substance biosynthetic process;BP:cellular nitrogen compound biosynthetic process;BP:cellular macromolecule metabolic process;BP:peptide biosynthetic process;BP:organic substance metabolic process;BP:organonitrogen compound biosynthetic process;BP:amide biosynthetic process;BP:cellular process;BP:biosynthetic process;BP:macromolecule biosynthetic process;BP:biological_process;BP:metabolic process;BP:organonitrogen compound metabolic process;BP:primary metabolic process;BP:protein metabolic process;BP:cellular nitrogen compound metabolic process;BP:cellular metabolic process;BP:macromolecule metabolic process;BP:nitrogen compound metabolic process;BP:cellular amide metabolic process;BP:peptide metabolic process;BP:translation;CC:intracellular organelle;CC:non-membrane-bounded organelle;CC:organelle;CC:cellular anatomical entity;CC:macromolecular	GO:00442	K02990	RP-S6, MRPS	map03010	Ribosome	COG0360	J:Translation, ribosomal structure	PF01250.20	Ribosomal_S6	Ribosomal protein S6	CYT	2	17	17.7	High
TRINITY_DN279_c0_g1_i10_orf1	RE1-silencing transcription factor-like isoform X1 [Ostrinia furnacalis]	2.210791518	1.144562983	7.08E-05	up	yes	1.053	0.4763	0.443	0.515	0.471	1	1.086	1.073	CC:intracellular membrane-bounded organelle;CC:nucleus;CC:cellular_component;CC:intracellular organelle;CC:membrane-bounded organelle;CC:organelle;CC:cellular anatomical entity;MF:cation binding;MF:transition metal ion binding;MF:ion binding;MF:molecular_function;MF:zinc ion binding;MF:metal ion	GO:00432	------	------	------	------	COG5048	O:Posttranslational modification,	PF00096.29;PF13	zf-C2H2;zf-C2H2_4;zf-H2C2_2;zf-AD	Zinc finger, C2H2 type;C2H2-type zinc finger;Zinc-finger double domain;Zinc-finger associated domain	CYT	1	1	120.9	High
TRINITY_DN81715_c0_g1_i1_orf1	gamma-interferon-inducible lysosomal thiol reductase-like [Ostrinia furnacalis]	2.331937404	1.221529063	6.26E-05	up	yes	1.058	0.4537	0.434	0.438	0.489	1	1.078	1.097	MF:catalytic activity;MF:oxidoreductase activity, acting on a sulfur group of donors, disulfide as acceptor;MF:molecular_function;MF:oxidoreductase activity, acting on a sulfur group of donors;MF:oxidoreductase	GO:00038	K08059	IFI30, GILT	map04612	Antigen processing	ENOG4111IDT	S:Function unknown	PF03227.19	GILT	Gamma interferon inducible lysosomal thiol reductase	CYT	1	3	26.1	High
TRINITY_DN969_c0_g1_i3_orf1	protein UBASH3A homolog isoform X3 [Ostrinia furnacalis]	2.02017526	1.014480459	0.0003288	up	yes	0.9913	0.4907	0.511	0.484	0.477	1	0.914	1.06	------	------	K18993	UBASH3, STS	------	------	ENOG410YGUZ	O:Posttranslational modification,	PF00300.25;PF14	His_Phos_1;SH3_9;UBA	Histidine phosphatase superfamily;Variant SH3 domain;UBA/TS-N domain	CYT	2	3	77	High
TRINITY_DN108200_c0_g1_i1_orf1	uncharacterized protein LOC114350842 [Ostrinia furnacalis]	2.008757637	1.006303509	0.0001166	up	yes	0.9863	0.491	0.485	0.47	0.518	1	0.929	1.03	MF:catalytic activity;MF:molecular_function;MF:oxidoreductase activity;	GO:00038	------	------	------	------	COG1028	S:Function unknown	PF13561.9;PF001	adh_short_C2;adh_short;KR	Enoyl;short chain dehydrogenase:KR domain	CYT	3	16	26	High
TRINITY_DN905_c0_g1_i4_orf1	(11Z)-hexadec-11-enoyl-CoA conjugase-like [Ostrinia furnacalis] >XP_028172978.1 (11Z)-hexadec-11-enoyl-CoA conjugase-like [Ostrinia furnacalis]	2.519703845	1.333254176	0.0002814	up	yes	1.055	0.4187	0.427	0.42	0.409	1	1.161	1.004	BP:fatty acid biosynthetic process;BP:fatty acid metabolic process;BP:carboxylic acid metabolic process;BP:cellular biosynthetic process;BP:small molecule metabolic process;BP:small molecule biosynthetic process;BP:monocarboxylic acid biosynthetic process;BP:organic substance biosynthetic process;BP:organic substance metabolic process;BP:lipid metabolic process;BP:cellular process;BP:monocarboxylic acid metabolic process;BP:biosynthetic process;BP:biological_process;BP:metabolic process;BP:oxoacid metabolic process;BP:cellular lipid metabolic process;BP:lipid biosynthetic process;BP:primary metabolic process;BP:organic acid metabolic process;BP:carboxylic acid biosynthetic process;BP:organic acid biosynthetic process;BP:cellular metabolic process;CC:intrinsic component of membrane;CC:integral component of membrane;CC:cellular anatomical entity;CC:cellular_component;MF:molecular_function;MF:oxidoreductase activity;MF:oxidoreductase activity,	GO:00066	K00507	SCD, desC	map04936;m	Alcoholic liver dise	COG1398	J:Translation, ribosomal structure	PF00487.27	FA_desaturase	Fatty acid desaturase	CYT	2	8	41.9	High
TRINITY_DN4725_c0_g1_i4_orf1	uncharacterized protein LOC114354375 [Ostrinia furnacalis]	2.11212766	1.078697036	2.02E-07	up	yes	0.9927	0.47	0.474	0.461	0.475	1	0.982	0.996	------	------	------	------	------	------	ENOG410Y8DA;	S:Function unknown;	PF02958.23;PF07	EcKL;DUF1679;APH	Ecdysteroid kinase-like family;Uncharacterized oxidoreductase dhs-27;Phosphotransferase enzyme family	CYT	5	15	49.1	High
TRINITY_DN21792_c0_g1_i1_orf1	probable 28S ribosomal protein S25, mitochondrial [Ostrinia furnacalis]	2.130040975	1.090881183	6.21E-06	up	yes	0.9877	0.4637	0.493	0.44	0.458	1	0.981	0.982	------	------	K17404	MRPS25	------	------	ENOG410YI66	S:Function unknown	PF05047.19	L51_S25_CI-B8	Mitochondrial ribosomal protein L51 / S25 / CI-B8 domain	CYT	4	23	18.7	High

TRINITY_DN237_c1_g1_i1_orf1	PREDICTED: cytoplasmic protein NCK1 isoform X1 [Microplitis demolitor]	2.376076332	1.248581184	0.0003254	up	yes	1.021	0.4297	0.332	0.458	0.499	1	1.035	1.027	BP:developmental process;BP:morphogenesis of an epithelium;BP:cellular process;BP:cellular developmental process;BP:anatomical structure development;BP:cell development;BP:animal organ development;BP:biological_process;BP:tissue morphogenesis;BP:anatomical structure morphogenesis;BP:localization;	GO:0032	K19862	NCK2, GRB4	map04360;m	Axon guidance;Axo	ENOG410XRPF	T:Signal transduction mechanism	PF00018.31;PF14	SH3_1;SH3_9;SH3_2;SH2;SH3_3;SH3_10	SH3 domain;Variant SH3 domain;Variant SH3 domain;SH2 domain;Bacterial SH3 domain;SH3 domain	PLA	2	3	44.1	High
TRINITY_DN2993_c0_g1_i4_orf1	heat shock 70 kDa protein cognate 5 [Ostrinia furnacalis]	2.260320325	1.176527241	2.12E-06	up	yes	1.002	0.4433	0.436	0.432	0.462	1	1.02	0.986	MF:purine ribonucleoside triphosphate binding;MF:molecular_function;MF:binding;MF:ion binding;MF:heterocyclic compound binding;MF:nucleoside phosphate binding;MF:anion binding;MF:unfolded protein binding;MF:nucleotide binding;MF:purine nucleotide binding;MF:ATP binding;MF:small molecule binding;MF:adenyl nucleotide binding;MF:carbohydrate derivative binding;MF:organic cyclic compound binding;MF:protein binding involved in protein folding;MF:adenyl ribonucleotide binding;MF:protein binding;MF:purine ribonucleotide binding;MF:ribonucleotide binding;	GO:0035	K04043	dnaK, HSPA	map03018;m	RNA degradation;T	COG0443	O:Posttranslational modification,	PF00012.23;PF06	HSP70;MreB_Mbl	Hsp70 protein;MreB/Mbl protein	CYT	24	34	74.7	High
TRINITY_DN2593_c0_g2_i1_orf1	midgut carboxypeptidase [Loxostege sticticalis]	2.254163815	1.172592363	0.002348	up	yes	0.988	0.4383	0.447	0.314	0.554	1	1.05	0.914	BP:nitrogen compound metabolic process;BP:organic substance metabolic process;BP:proteolysis;BP:biological_process;BP:metabolic process;BP:primary metabolic process;BP:organonitrogen compound metabolic process;BP:protein metabolic process;BP:macromolecule metabolic process;MF:exopeptidase activity;MF:molecular_function;MF:zinc ion binding;MF:peptidase activity;MF:metalloexopeptidase activity;MF:metallopeptidase activity;MF:cation binding;MF:transition metal ion binding;MF:metallocarboxypeptidase activity;MF:carboxypeptidase activity;MF:ion binding;MF:metal ion binding;MF:hydrolase activity;MF:catalytic activity, acting on a protein;MF:catalytic activity;MF:binding;	GO:0006	K01298	CPA2	map04972;m	Pancreatic secretio	COG2866	E:Amino acid transport and meta	PF00246.27;PF02	Peptidase_M14;Propep_M14	Zinc carboxypeptidase;Carboxypeptidase activation peptide	CYT	1	6	48.9	High
TRINITY_DN35635_c0_g1_i1_orf1	probable NADH dehydrogenase [ubiquinone] 1 alpha subcomplex subunit 12 [Ostrinia furnacalis]	2.258632518	1.175449562	1.83E-06	up	yes	0.9877	0.4373	0.429	0.424	0.459	1	0.987	0.976	BP:mitochondrial respiratory chain complex assembly;BP:cellular component assembly;BP:mitochondrial respiratory chain complex I assembly;BP:macromolecular complex subunit organization;BP:NADH dehydrogenase complex assembly;BP:cellular component organization;BP:cellular process;BP:biological_process;BP:cellular component organization or biogenesis;BP:macromolecular complex assembly;CC:organelle membrane;CC:membrane;CC:organelle inner membrane;CC:mitochondrial membrane;CC:cellular_component;CC:mitochondrial inner membrane;CC:respiratory [illegible]	GO:0033	K11352	NDUFA12	map04714;m	Thermogenesis;Oxi	ENOG4111XVR	I:Lipid transport and metabolism	PF05071.19	NDUFA12	NADH ubiquinone oxidoreductase subunit NDUFA12	CYT	5	44	16.9	High
TRINITY_DN20294_c0_g2_i1_orf1	cytochrome b-c1 complex subunit 2, mitochondrial isoform X1 [Ostrinia furnacalis] >XP_028170208.1 cytochrome b-c1 complex subunit 2, mitochondrial isoform X2 [Ostrinia furnacalis]	2.405758529	1.266491843	6.04E-07	up	yes	0.9943	0.4133	0.404	0.408	0.428	1	1.003	0.98	MF:cation binding;MF:metal ion binding;MF:molecular_function;MF:binding;MF:ion binding;	GO:0043	K00415	QCR2, UQCR	map04714;m	Thermogenesis;Oxi	COG0612	T:Signal transduction mechanism	PF00675.23;PF05	Peptidase_M16;Peptidase_M16_C	Insulinase;Peptidase M16 inactive	CYT	17	43	45.7	High
TRINITY_DN327_c1_g1_i4_orf1	mitochondrial import receptor subunit TOM40 homolog 1-like [Ostrinia furnacalis]	2.437668381	1.285501876	1.22E-06	up	yes	0.9953	0.4083	0.401	0.395	0.429	1	0.982	1.004	BP:protein localization;BP:establishment of localization in cell;BP:protein import into mitochondrial matrix;BP:protein transmembrane transport;BP:cellular macromolecule localization;BP:localization;BP:nitrogen compound transport;BP:intracellular protein transmembrane transport;BP:mitochondrial transmembrane transport;BP:organic substance transport;BP:macromolecule localization;BP:transport;BP:establishment of protein localization to organelle;BP:cellular process;BP:establishment of protein localization;BP:protein localization to organelle;BP:biological_process;BP:establishment of protein localization to mitochondrion;BP:mitochondrial transport;BP:establishment of localization;BP:transmembrane transport;BP:intracellular protein transmembrane import;BP:cellular localization;BP:protein localization to mitochondrion;BP:intracellular transport;BP:protein transport;BP:intracellular protein transport;CC:intrinsic component of membrane;CC:organelle [illegible]	GO:0008	K11518	TOM40	map05022;m	Pathways of neuro	ENOG410XS2U	O:Posttranslational modification,	PF01459.25	Porin_3	Eukaryotic porin	CYT	6	26	34	High
TRINITY_DN46778_c0_g1_i2_orf1	Deoxycytidylate deaminase [Papilio xuthus]	2.867383513	1.519734878	1.62E-05	up	yes	1.04	0.3627	0.345	0.354	0.389	1	1.083	1.036	MF:hydrolase activity;MF:transition metal ion binding;MF:cation binding;MF:ion binding;MF:catalytic activity;MF:molecular_function;MF:zinc ion binding;MF:metal ion binding;MF:binding;	GO:0016	K01493	comEB	map00240;m	Pyrimidine metabo	COG2131	F:Nucleotide transport and metal	PF00383.26;PF14	dCMP_cyt_deam_1;MafB19-deam	Cytidine and deoxycytidylate deaminase zinc-binding region;MafB19-like	CYT	1	5	21.4	Medium
TRINITY_DN5238_c0_g1_i2_orf1	DNA-(apurinic or apyrimidinic site) lyase [Ostrinia furnacalis]	2.618798956	1.388905309	3.05E-06	up	yes	1.003	0.383	0.39	0.36	0.399	1	1.025	0.985	BP:nucleic acid metabolic process;BP:cellular nitrogen compound metabolic process;BP:nitrogen compound metabolic process;BP:organic cyclic compound metabolic process;BP:nucleobase-containing compound metabolic process;BP:cellular response to stimulus;BP:cellular macromolecule metabolic process;BP:organic substance metabolic process;BP:DNA repair;BP:cellular process;BP:cellular aromatic compound metabolic process;BP:cellular response to DNA damage stimulus;BP:response to stress;BP:biological_process;BP:metabolic process;BP:heterocycle metabolic process;BP:primary metabolic process;BP:response to stimulus;BP:cellular metabolic process;BP:macromolecule metabolic process;BP:cellular response to stress;BP:DNA metabolic process;MF:cation binding;MF:molecular_function;MF:binding;MF:nucleic acid binding;MF:DNA binding;MF:heterocyclic compound binding;MF:ion binding;MF:metal ion binding;MF:hydrolase activity;MF:hydrolase activity, acting on [illegible]	GO:0090	K10771	APEX1	map03410	Base excision repai	COG0708	L:Replication, recombination and	PF03372.26	Exo_endo_phos	Endonuclease/Exonuclease/phosphatase family	CYT	5	11	73.1	High
TRINITY_DN8261_c0_g1_i1_orf1	UDP-N-acetylhexosamine pyrophosphorylase-like protein 1 [Ostrinia furnacalis]	2.235348622	1.16049985	4.26E-05	up	yes	0.965	0.4317	0.43	0.396	0.469	1	0.954	0.941	MF:nucleotidyltransferase activity;MF:transferase activity, transferring phosphorus-containing groups;MF:catalytic activity;MF:transferase activity;MF:UDP-N-acetylglucosamine diphosphorylase activity;MF:molecular_function;MF:uridylyltransferase activity;	GO:0016	K00972	UAP1	map00520;m	Amino sugar and n	COG4284	O:Posttranslational modification,	PF01704.21	UDPGP	UTP--glucose-1-phosphate uridylyltransferase	CYT	5	11	54.6	High

TRINITY_DN9464_c0_g1_i1_orf1	angio-associated migratory cell protein [Ostrinia furnacalis] >XP_028162594.1 angio-associated migratory cell protein [Ostrinia furnacalis]	2.829608939	1.500602682	3.55E-06	up	yes	1.013	0.358	0.333	0.358	0.383	1	1.035	1.004	------	------	K24725	AAMP	------	------	ENOG410XR6A	S:Function unknown	PF00400.35;PF12	WD40;ANAPC4_WD40;WD40_like;NBCH_WD40	WD domain, G-beta repeat;Anaphase-promoting complex subunit 4 WD40 domain;WD40-like domain;Neurobeachin beta propeller	CYT	6	20	44.7	High
TRINITY_DN1666_c0_g1_i2_orf1	putative defense protein Hdd11 [Ostrinia furnacalis] >XP_028179344.1 putative defense protein Hdd11 [Ostrinia furnacalis] >AGV28583.1 immune-induced protein [Ostrinia furnacalis]	2.603550296	1.380480277	1.10E-06	up	yes	1.012	0.3887	0.378	0.382	0.406	1	1.031	1.006	BP:defense response;BP:response to biotic stimulus;BP:response to external stimulus;BP:response to external biotic stimulus;BP:innate immune response;BP:response to bacterium;BP:immune system process;BP:response to stress;BP:biological_process;BP:defense response to bacterium;BP:immune response;BP:defense response to other organism;BP:response to other organism;BP:response to stimulus;BP:interspecies interaction between organisms;CC:cellular_component;CC:extracellular region;CC:cellular anatomical entity;	GO:00069	------	------	------	------	ENOG41123CK;E	S:Function unknown;	PF02014.19	Reeler	Reeler domain	CYT	6	52	17.7	High
TRINITY_DN8173_c0_g1_i3_orf1	dihydroceramide fatty acyl 2-hydroxylase FAH1 [Ostrinia furnacalis]	3.36677116	1.751365664	0.0009465	up	yes	1.074	0.319	0.336	0.265	0.356	1	0.985	1.238	BP:fatty acid biosynthetic process;BP:fatty acid metabolic process;BP:carboxylic acid metabolic process;BP:cellular biosynthetic process;BP:small molecule metabolic process;BP:small molecule biosynthetic process;BP:monocarboxylic acid biosynthetic process;BP:organic substance biosynthetic process;BP:organic substance metabolic process;BP:lipid metabolic process;BP:cellular process;BP:monocarboxylic acid metabolic process;BP:biosynthetic process;BP:biological_process;BP:metabolic process;BP:oxoacid metabolic process;BP:cellular lipid metabolic process;BP:lipid biosynthetic process;BP:primary metabolic process;BP:organic acid metabolic process;BP:carboxylic acid biosynthetic process;BP:organic acid biosynthetic process;BP:cellular metabolic process;CC:endoplasmic reticulum membrane;CC:intrinsic component of membrane;CC:organelle membrane;CC:integral component of membrane;CC:membrane;CC:cellular anatomical	GO:00066	K19703	FA2H, SCS7	------	------	COG5274;COG5	C:Energy production and convers	PF04116.16;PF00	FA_hydroxylase;Cyt-b5	Fatty acid hydroxylase;Cytochrome b5-like Heme/Steroid binding domain	CYT	1	4	39.3	High
TRINITY_DN787_c0_g1_i7_orf1	YLP motif-containing protein 1-like isoform X1 [Ostrinia furnacalis]	2.042146426	1.030086314	0.0007726	up	yes	0.94	0.4603	0.436	0.418	0.527	1	0.955	0.865	CC:intracellular membrane-bounded organelle;CC:nucleus;CC:cellular_component;CC:intracellular organelle;CC:membrane-bounded organelle;CC:organelle;CC:cellular anatomical entity;	GO:0043	K17602	YLPM1	------	------	ENOG410YIR5	S:Function unknown	------	------	------	CYT	1	1	137.9	Medium
TRINITY_DN24317_c0_g1_i7_orf1	peptidyl-tRNA hydrolase ICT1, mitochondrial [Ostrinia furnacalis]	2.206206897	1.141568092	5.50E-05	up	yes	0.9597	0.435	0.414	0.419	0.472	1	0.956	0.923	MF:translation termination factor activity;MF:translation regulator activity, nucleic acid binding;MF:nucleic acid binding;MF:organic cyclic compound binding;MF:translation factor activity, RNA binding;MF:molecular_function;MF:binding;MF:translation regulator activity;MF:translation release factor activity;MF:heterocyclic compound	GO:0008	K15033	ICT1, MRPL5	------	------	COG1186	J:Translation, ribosomal structure	PF00472.23	RF-1	RF-1 domain	CYT	3	16	22.9	High
TRINITY_DN32601_c0_g1_i2_orf1	uncharacterized protein LOC114363197 [Ostrinia furnacalis]	2.639501687	1.400265588	6.14E-05	up	yes	1.017	0.3853	0.358	0.346	0.452	1	1.042	1.008	------	------	------	------	------	------	ENOG410XRFE	S:Function unknown	PF03022.19	MRJP	Major royal jelly protein	CYT	1	3	48.2	High
TRINITY_DN8621_c0_g1_i5_orf1	aminopeptidase N-like isoform X2 [Ostrinia furnacalis]	2.226132965	1.154539766	3.26E-05	up	yes	0.9677	0.4347	0.439	0.409	0.456	1	0.926	0.977	MF:exopeptidase activity;MF:aminopeptidase activity;MF:transition metal ion binding;MF:hydrolase activity;MF:catalytic activity, acting on a protein;MF:cation binding;MF:ion binding;MF:catalytic activity;MF:molecular_function;MF:zinc ion binding;MF:peptidase activity;MF:metal ion	GO:0008	K11140;K	ANPEP, CD1	map04640;m	Hematopoietic cell	COG0308	S:Function unknown	PF11838.11;PF01	ERAP1_C;Peptidase_M1	ERAP1-like C-terminal domain;Peptidase family M1 domain	CYT	1	2	69.5	High
TRINITY_DN50787_c0_g2_i2_orf1	40S ribosomal protein S29 [Hyposmocoma kahamanoa] >XP_028176503.1 40S ribosomal protein S29 [Ostrinia furnacalis] >XP_049877832.1 40S ribosomal protein S29 [Pectinophora gossypiella] >ADT80654.1 ribosomal protein S29 [Euphydryas aurinia] >CAB3523209.1 unnamed protein product [Chilo suppressalis] >CAH0400531.1 unnamed protein product [Chilo suppressalis]	2.236295778	1.161111015	0.0001694	up	yes	0.9587	0.4287	0.378	0.428	0.48	1	0.964	0.912	BP:organonitrogen compound biosynthetic process;BP:cellular biosynthetic process;BP:cellular macromolecule biosynthetic process;BP:cellular nitrogen compound biosynthetic process;BP:peptide biosynthetic process;BP:organic substance metabolic process;BP:amide biosynthetic process;BP:cytoplasmic translation;BP:organic substance biosynthetic process;BP:cellular macromolecule metabolic process;BP:cellular process;BP:biosynthetic process;BP:macromolecule biosynthetic process;BP:metabolic process;BP:organonitrogen compound metabolic process;BP:primary metabolic process;BP:protein metabolic process;BP:cellular nitrogen compound metabolic process;BP:cellular metabolic process;BP:macromolecule metabolic process;BP:nitrogen compound metabolic process;BP:cellular amide metabolic process;BP:biological_process;BP:peptide metabolic process;BP:translation;CC:endoplasmic reticulum;CC:intracellular	GO:1901	K02980	RP-S29e, RP	map03010;m	Ribosome;Coronavi	COG0199	J:Translation, ribosomal structure	PF00253.24	Ribosomal_S14	Ribosomal protein S14p/S29e	CYT	2	38	6.6	High

TRINITY_DN4434_c0_g1_i7_orf1	V-type proton ATPase catalytic subunit A [Ostrinia furnacalis] >XP_028155921.1 V-type proton ATPase catalytic subunit A [Ostrinia furnacalis] >XP_028155922.1 V-type proton ATPase catalytic subunit A [Ostrinia furnacalis] >ADP23923.1 V-ATPase subunit A [Ostrinia furnacalis] >ADT80587.1 V-type proton ATPase catalytic subunit A [Ostrinia furnacalis] >CBY05457.1 V-type proton ATPase catalytic subunit A [Ostrinia furnacalis]	2.453290609	1.294718141	1.92E-06	up	yes	0.9953	0.4057	0.399	0.388	0.43	1	1.003	0.983	CC:proton-transporting V-type ATPase, V1 domain;CC:proton-transporting two-sector ATPase complex, catalytic domain;CC:macromolecular complex;CC:cellular_component;CC:membrane protein complex;MF:cation channel activity;MF:purine ribonucleoside triphosphate binding;MF:molecular_function;MF:binding;MF:ion binding;MF:heterocyclic compound binding;MF:nucleoside phosphate binding;MF:anion binding;MF:ion transmembrane transporter activity;MF:ATPase activity, coupled to transmembrane movement of substances;MF:inorganic solute uptake transmembrane transporter activity;MF:hydrogen-translocating pyrophosphatase activity;MF:nucleotide binding;MF:primary active transmembrane transporter activity;MF:purine nucleotide binding;MF:active transmembrane transporter activity;MF:hydrogen ion channel activity;MF:ATPase coupled ion transmembrane transporter activity;MF:transporter activity;MF:ion channel activity;MF:cation transmembrane transporter activity;MF:small molecule	GO:00331	K02145	ATPeV1A, AT	map05323;m	Rheumatoid arthrit	COG1155	C:Energy production and convers	PF00006.28;PF16	ATP-synt_ab;ATP-synt_ab_Xtn;ATP-synt_ab_N	ATP synthase alpha/beta family, nucleotide-binding domain;ATPsynthase alpha/beta subunit N-term extension;ATP synthase alpha/beta family, beta-barrel domain	CYT	29	62	68.1	High
TRINITY_DN31047_c0_g1_i4_orf1	4-coumarate--CoA ligase 1-like isoform X1 [Ostrinia furnacalis] >XP_028160248.1 4-coumarate--CoA ligase 1-like isoform X1 [Ostrinia furnacalis] >XP_028160249.1 4-coumarate--CoA ligase 1-like isoform X1 [Ostrinia furnacalis] >XP_028160250.1 4-coumarate--CoA ligase 1-like isoform X1 [Ostrinia furnacalis] >XP_028160251.1 4-coumarate--CoA ligase 1-like isoform X1 [Ostrinia furnacalis] >XP_028160253.1 4-coumarate--CoA ligase 1-like isoform X2 [Ostrinia furnacalis]	2.187599726	1.129348787	7.67E-05	up	yes	0.9597	0.4387	0.431	0.413	0.472	1	0.968	0.911	------	------	K01904	4CL	map00130	Ubiquinone and ot	COG0318;COG0	I:Lipid transport and metabolism	PF00501.31;PF13	AMP-binding;AMP-binding_C	AMP-binding enzyme;AMP-binding enzyme C-terminal domain	CYT	4	8	59.4	High
TRINITY_DN886_c0_g1_i1_orf1	collagenase-like isoform X1 [Ostrinia furnacalis]	2.526678581	1.33724215	5.55E-05	up	yes	0.9897	0.3917	0.353	0.416	0.406	1	0.939	1.03	------	------	------	------	------	------	COG5640	O:Posttranslational modification,	PF00089.29	Trypsin	Trypsin	CYT	3	14	29.3	High
TRINITY_DN40704_c0_g1_i2_orf1	COX assembly mitochondrial protein homolog [Ostrinia furnacalis]	2.411135968	1.269713009	2.47E-05	up	yes	0.97	0.4023	0.362	0.416	0.429	1	0.961	0.949	CC:intracellular membrane-bounded organelle;CC:mitochondrion;CC:cellular_component;CC:intracellular organelle;CC:membrane-bounded organelle;CC:organelle;CC:cellular anatomical entity;	GO:0043	K18171	CMC1	------	------	ENOG4111YK0	S:Function unknown	PF08583.13	Cmc1	Cytochrome c oxidase biogenesis protein Cmc1 like	CYT	1	9	13.6	High
TRINITY_DN106730_c0_g1_i1_orf1	Photosystem I reaction center subunit II, chloroplastic, partial [Trichinella zimbabwensis]	2.853528254	1.512746848	4.92E-05	up	yes	1.015	0.3557	0.317	0.328	0.422	1	1.039	1.006	------	------	K02692	psaD	------	------	ENOG4111HAV	C:Energy production and convers	PF02531.19	PsaD	PsaD	CYT	2	11	22.6	High
TRINITY_DN34040_c0_g2_i1_orf1	uncharacterized protein LOC114352849 [Ostrinia furnacalis]	2.67574124	1.419938606	4.07E-05	up	yes	0.9927	0.371	0.359	0.337	0.417	1	1.025	0.953	------	------	------	------	------	------	------	------	------	------	------	CYT	1	6	24	High
TRINITY_DN5956_c1_g1_i5_orf1	uncharacterized protein DDB_G0286299-like [Ostrinia furnacalis] >XP_028170990.1 uncharacterized protein DDB_G0286299-like [Ostrinia furnacalis] >XP_028170991.1 uncharacterized protein DDB_G0286299-like [Ostrinia furnacalis]	3.146617692	1.653801905	3.42E-06	up	yes	1.028	0.3267	0.327	0.312	0.341	1	1.06	1.025	MF:organic cyclic compound binding;MF:molecular_function;MF:binding;MF:nucleic acid binding;MF:heterocyclic compound	GO:00971	------	------	------	------	ENOG4111E9J;E	S:Function unknown;	PF00076.25	RRM_1	RNA recognition motif.	CYT	3	7	66.4	High
TRINITY_DN12474_c0_g1_i6_orf1	aromatic-L-amino-acid decarboxylase [Ostrinia furnacalis]	3.292207792	1.719055396	3.28E-06	up	yes	1.014	0.308	0.319	0.273	0.332	1	1.018	1.023	BP:carboxylic acid metabolic process;BP:nitrogen compound metabolic process;BP:cellular amino acid metabolic process;BP:organic substance metabolic process;BP:cellular process;BP:biological_process;BP:metabolic process;BP:oxoacid metabolic process;BP:primary metabolic process;BP:organonitrogen compound metabolic process;BP:organic acid metabolic process;BP:cellular metabolic process;BP:small molecule metabolic process;MF:binding;MF:aromatic-L-amino-acid decarboxylase activity;MF:vitamin B6 binding;MF:molecular_function;MF:pyridoxal phosphate binding;MF:heterocyclic compound binding;MF:anion binding;MF:lyase activity;MF:ion binding;MF:small molecule binding;MF:catalytic activity;MF:vitamin binding;MF:organic cyclic compound binding;MF:carbon-carbon lyase activity;MF:carboxy-lyase activity;	GO:00197	K01593	DDC, TDC	map00350;m	Tyrosine metabolis	COG0076	S:Function unknown	PF00282.22	Pyridoxal_deC	Pyridoxal-dependent decarboxylase conserved domain	CYT	10	20	54.3	High
TRINITY_DN3959_c1_g2_i1_orf1	PREDICTED: probable isocitrate dehydrogenase [NAD] subunit alpha, mitochondrial isoform X3 [Papilio xuthus] >XP_014363280.1 probable isocitrate dehydrogenase [NAD] subunit alpha, mitochondrial isoform X3 [Papilio machaon]	3.235483871	1.693981485	3.04E-06	up	yes	1.003	0.31	0.291	0.294	0.345	1	1.015	0.995	BP:tricarboxylic acid cycle;BP:biological_process;BP:metabolic process;BP:primary metabolic process;CC:oxidoreductase complex;CC:isocitrate dehydrogenase comple;CC:mitochondrial isocitrate dehydrogenase comple;CC:catalytic complex;CC:mitochondrial tricarboxylic acid cycle enzyme complex;CC:tricarboxylic acid cycle enzyme complex;CC:macromolecular complex;CC:cellular_component;CC:mitochondrial protein complex;MF:NAD binding;MF:molecular_function;MF:binding;MF:nucleotide binding;MF:heterocyclic compound binding;MF:nucleoside phosphate binding;MF:oxidoreductase activity;MF:cation binding;MF:magnesium ion binding;MF:ion binding;MF:isocitrate dehydrogenase activity;MF:metal ion binding;MF:small molecule binding;MF:oxidoreductase activity, acting on CH-OH group of donors;MF:isocitrate dehydrogenas;MF:oxidoreductase activity, acting on the CH-OH group of	GO:0006	K00030	IDH3	map00020	Citrate cycl	COG0473	S:Function unknown	PF00180.23	Iso_dh	Isocitrate/isopropylmalate dehydrogenase	CYT	16	55	38.7	High
TRINITY_DN6418_c0_g1_i28_orf1	peritrophic membrane chitin binding protein [Loxostege sticticalis]	3.616719802	1.854681832	5.24E-05	up	yes	1.021	0.2823	0.252	0.345	0.25	1	1.072	0.992	CC:cellular_component;CC:extracellular region;CC:cellular anatomical entity;MF:carbohydrate derivative binding;MF:molecular_function;MF:chitin binding;MF:binding;	GO:00055	------	------	------	------	ENOG41121EJ;E	S:Function unknown;	PF01607.27	CBM_14	Chitin binding Peritrophin-A domain	CYT	1	3	48	High
TRINITY_DN19244_c0_g1_i7_orf1	uncharacterized protein LOC114350218 [Ostrinia furnacalis]	3.102671387	1.633510905	6.37E-05	up	yes	0.964	0.3107	0.245	0.34	0.347	1	0.946	0.946	------	------	K16830	AURKAIP1	------	------	ENOG410Y2DU;	S:Function unknown;C:Energy pr	------	------	------	CYT	1	4	25.6	High
TRINITY_DN140669_c0_g1_i1_orf1	S-methyl-5'-thioadenosine phosphorylase-like isoform X1 [Hyposmocoma kahamanoa]	5.882022472	2.556312296	0.0001126	up	yes	1.047	0.178	0.195	0.087	0.252	1	1.034	1.106	BP:nucleobase-containing small molecule biosynthetic process;BP:cellular biosynthetic process;BP:cellular nitrogen compound metabolic process;BP:nitrogen compound metabolic process;BP:nucleoside biosynthetic process;BP:small molecule biosynthetic process;BP:purine-containing compound metabolic process;BP:purine-containing compound biosynthetic process;BP:organic cyclic compound biosynthetic process;BP:purine nucleoside biosynthetic process;BP:organic cyclic compound metabolic process;BP:nucleobase-containing compound metabolic process;BP:purine nucleoside metabolic process;BP:organic substance metabolic process;BP:ribonucleoside biosynthetic process;BP:purine-containing compound salvage;BP:small molecule metabolic process;BP:heterocycle biosynthetic process;BP:ribonucleoside metabolic process;BP:organic substance biosynthetic process;BP:cellular process;BP:cellular aromatic compound metabolic process;BP:biosynthetic	GO:0034	K00772	mtaP, MTAP	map00270	Cysteine and meth	COG0005	F:Nucleotide transport and metal	PF01048.23	PNP_UDP_1	Phosphorylase superfamily	CYT	1	10	8.1	Medium

TRINITY_DN1353_c0_g1_i1_orf1	UDP-glucose 4-epimerase-like [Ostrinia furnacalis]	3.707003891	1.890253629	2.94E-05	up	yes	0.9527	0.257	0.26	0.234	0.277	1	0.962	0.896	BP:primary metabolic process;BP:carbohydrate metabolic process;BP:organic substance metabolic process;BP:monosaccharide metabolic process;BP:hexose metabolic process;BP:galactose metabolic process;BP:biological_process;BP:small molecule metabolic process;BP:metabolic process;MF:racemase and epimerase activity;MF:isomerase activity;MF:catalytic activity;MF:racemase and epimerase activity, acting on carbohydrates and derivatives;MF:UDP-N-acetylglucosamine 4-epimerase activity;MF:molecular_function;MF:UDP-glucose 4-epimerase activity;	GO:0044[illegible]	K01784	galE, GALE	map00520;m[illegible]	Amino sugar and n[illegible]	COG1087	M:Cell wall/membrane/envelope	PF16363.8;PF013[illegible]	GDP_Man_Dehyd;Epimerase;Polysacc_synt_2;3Beta_HSD;RmlD_sub_bind;NAD_binding_4	GDP-mannose 4,6 dehydratase;NAD dependent epimerase/dehydratase family;Polysaccharide biosynthesis protein;3-beta hydroxysteroid dehydrogenase/isomerase family;RmlD substrate binding domain;Male sterility protein	CYT	2	8	40.3	High
TRINITY_DN57904_c0_g2_i1_orf1	cuticle protein 19 [Plutella xylostella] >CAG9138481.1 unnamed protein product [Plutella xylostella]	3.870742716	1.952610417	0.0001296	up	yes	0.9433	0.2437	0.246	0.237	0.248	1	0.982	0.848	MF:molecular_function;MF:structural constituent of cuticle;MF:structural molecule activity;	GO:0003[illegible]	------	------	------	------	ENOG410YSFI	S:Function unknown	PF00379.26	Chitin_bind_4	Insect cuticle protein	CYT	1	16	14.1	High
TRINITY_DN26010_c0_g1_i2_orf1	cytochrome b-c1 complex subunit 10-like [Ostrinia furnacalis]	4.198506807	2.069876327	2.39E-05	up	yes	0.956	0.2277	0.227	0.2	0.256	1	0.965	0.903	BP:aerobic respiration;BP:generation of precursor metabolites and energy;BP:electron transport chain;BP:respiratory electron transport chain;BP:cellular process;BP:aerobic electron transport chain;BP:cellular respiration;BP:metabolic process;BP:energy derivation by oxidation of organic compounds;BP:mitochondrial electron transport, ubiquinol to cytochrome c;BP:cellular metabolic process;BP:biological_process;CC:oxidoreductase complex;CC:respiratory chain complex;CC:cytochrome complex;CC:inner mitochondrial membrane protein complex;CC:mitochondrial respiratory chain complex III;CC:transmembrane transporter complex;CC:catalytic complex;CC:transporter complex;CC:macromolecular complex;CC:respiratory chain complex III;CC:cellular_component;CC:membrane	GO:0009[illegible]	K00420	QCR10, UQC[illegible]	map04714;m[illegible]	Thermogenesis;Oxi[illegible]	ENOG410XVMI	C:Energy production and conver[illegible]	PF08997.13	UCR_6-4kD	Ubiquinol-cytochrome C reductase complex, 6.4kD protein	PLA	1	14	6.3	High
TRINITY_DN1310_c0_g1_i4_orf1	trypsin-like isoform X1 [Ostrinia furnacalis] >XP_028159118.1 trypsin-like isoform X2 [Ostrinia furnacalis]	6.227581942	2.638672099	6.64E-07	up	yes	1.007	0.1617	0.145	0.165	0.175	1	1.032	0.99	BP:organic substance metabolic process;BP:organonitrogen compound metabolic process;BP:protein metabolic process;BP:macromolecule metabolic process;BP:biological_process;BP:nitrogen compound metabolic process;BP:metabolic process;BP:proteolysis;BP:primary metabolic process;MF:serine-type endopeptidase activity;MF:endopeptidase activity;MF:hydrolase activity;MF:serine hydrolase activity;MF:catalytic activity, acting on a protein;MF:catalytic activity;MF:molecular_function;MF:pepti	GO:0071[illegible]	------	------	------	------	ENOG411152E;C	O:Posttranslational modification,	PF00089.29	Trypsin	Trypsin	CYT	3	26	26.7	High
TRINITY_DN64446_c0_g1_i1_orf1	uncharacterized protein LOC114364307 [Ostrinia furnacalis]	6.175954915	2.626662221	2.13E-06	up	yes	0.9863	0.1597	0.152	0.133	0.194	1	0.99	0.969	------	------	------	------	------	------	ENOG410Y27Q	S:Function unknown	PF06757.16	Ins_allergen_rp	Insect allergen related repeat, nitrile-specifier detoxification	CYT	3	15	23.7	High
TRINITY_DN3733_c0_g1_i1_orf1	60S ribosomal protein L37, partial [Papilio machaon]	2.607686355	1.382770357	7.47E-05	up	yes	1.011	0.3877	0.379	0.379	0.405	1	1.079	0.955	BP:organonitrogen compound biosynthetic process;BP:cellular macromolecule biosynthetic process;BP:cellular biosynthetic process;BP:cellular nitrogen compound biosynthetic process;BP:cellular macromolecule metabolic process;BP:cellular amide metabolic process;BP:peptide biosynthetic process;BP:organic substance metabolic process;BP:macromolecule metabolic process;BP:organic substance biosynthetic process;BP:cellular process;BP:biosynthetic process;BP:macromolecule biosynthetic process;BP:biological_process;BP:metabolic process;BP:organonitrogen compound metabolic process;BP:amide biosynthetic process;BP:primary metabolic process;BP:protein metabolic process;BP:cellular nitrogen compound metabolic process;BP:cellular metabolic process;BP:nitrogen compound metabolic process;BP:peptide metabolic process;BP:translation;CC:organelle;CC:intracellular organelle;CC:non-membrane-bounded organelle;CC:cellular anatomical entity;CC:macromolecular	GO:1901[illegible]	K02922	RP-L37e, RPL[illegible]	map03010;m[illegible]	Ribosome;Coronavi[illegible]	COG2126	J:Translation, ribosomal structure	PF01907.22	Ribosomal_L37e	Ribosomal protein L37e	PLA	4	30	11.4	High
TRINITY_DN53167_c0_g1_i3_orf1	uncharacterized protein LOC114359219 [Ostrinia furnacalis]	2.666141319	1.414753252	1.03E-06	up	yes	1.015	0.3807	0.362	0.398	0.382	1	1.023	1.023	BP:primary metabolic process;BP:carbohydrate metabolic process;BP:organic substance metabolic process;BP:biological_process;BP:metabolic process;CC:cytoplasm;CC:cellular_component;CC:cellular anatomical entity;MF:hydrolase activity;MF:catalytic activity;MF:hydrolase activity, acting on glycosyl bonds;MF:beta-fructofuranosidase activity;MF:hydrolase activity, hydrolyzing O-glycosyl compounds;MF:molecular_function;	GO:0044[illegible]	------	------	------	------	COG1621	G:Carbohydrate transport and m	PF00251.23;PF08[illegible]	Glyco_hydro_32N;Glyco_hydro_32C	Glycosyl hydrolases family 32 N-terminal domain;Glycosyl hydrolases family 32 C terminal	CYT	6	16	55.9	High
TRINITY_DN145647_c0_g1_i1_orf1	PREDICTED: U6 snRNA-associated Sm-like protein LSm5 isoform X1 [Fopius arisanus]	2.933179057	1.552465144	1.35E-07	up	yes	1.014	0.3457	0.344	0.339	0.354	1	1.022	1.019	BP:nucleic acid metabolic process;BP:cellular nitrogen compound metabolic process;BP:nitrogen compound metabolic process;BP:organic cyclic compound metabolic process;BP:nucleobase-containing compound metabolic process;BP:organic substance metabolic process;BP:RNA splicing;BP:mRNA metabolic process;BP:cellular process;BP:cellular aromatic compound metabolic process;BP:biological_process;BP:metabolic process;BP:heterocycle metabolic process;BP:RNA metabolic process;BP:primary metabolic process;BP:cellular metabolic process;BP:macromolecule metabolic process;BP:RNA processing;BP:mRNA processing;CC:spliceosomal complex;CC:macromolecular complex;CC:cellular_component;CC:ribonucleoprotein complex;MF:molecular_function;MF:binding;MF:nucleic acid binding;MF:heterocyclic compound binding;MF:organic cyclic compound	GO:0090[illegible]	K12624	LSM5	map03018;m[illegible]	RNA degradation;S[illegible]	COG1958	O:Posttranslational modification,	PF01423.25	LSM	LSM domain	CYT	1	6	10.5	High
TRINITY_DN38498_c0_g3_i1_orf1	unnamed protein product [Parnassius apollo]	2.110344828	1.077478753	0.02379	up	yes	0.918	0.435	0.554	0.453	0.298	1	0.693	1.061	------	------	------	------	------	------	ENOG410ZSXY;E	S:Function unknown;	------	------	------	CYT	1	3	36.4	Medium
TRINITY_DN17825_c1_g1_i1_orf1	39S ribosomal protein L1, mitochondrial [Ostrinia furnacalis]	2.931577354	1.551677125	3.77E-05	up	yes	0.9683	0.3303	0.29	0.329	0.372	1	0.978	0.927	CC:ribosome;CC:intracellular non-membrane-bounded organelle;CC:cellular_component;CC:intracellular organelle;CC:non-membrane-bounded organelle;CC:organelle;CC:cellular anatomical entity;	GO:0005[illegible]	K02863	RP-L1, MRPL	map03010	Ribosome	COG0081	J:Translation, ribosomal structure	PF00687.24	Ribosomal_L1	Ribosomal protein L1p/L10e family	CYT	1	3	39.6	High

TRINITY_DN7966_c0_g1_i4_orf1	leucine-rich repeat neuronal protein 1-like [Ostrinia furnacalis]	3.130573248	1.646426857	2.44E-05	up	yes	0.983	0.314	0.305	0.283	0.354	1	0.94	1.009	BP:locomotion;BP:biological_process;CC:integral component of membrane;CC:cellular_component;CC:cell periphery;CC:intrinsic component of membrane;CC:cellular anatomical entity;	GO:0040(	------	------	------	------	COG4886	P:Inorganic ion transport and me	PF13855.9;PF127!	LRR_8;LRR_4	Leucine rich repeat;Leucine Rich repeats	PLA	2	4	57.2	High
TRINITY_DN52768_c0_g1_i1_orf1	carboxypeptidase Q-like isoform X2 [Ostrinia furnacalis]	3.989092326	1.996060514	6.31E-06	up	yes	1.024	0.2567	0.22	0.259	0.291	1	1.026	1.047	CC:lytic vacuole;CC:endoplasmic reticulum;CC:intracellular organelle;CC:organelle;CC:lysosome;CC:vacuole;CC:membrane-bounded organelle;CC:cellular anatomical entity;CC:Golgi apparatus;CC:intracellular membrane-bounded organelle;CC:cellular_component;CC:extracellular region;MF:hydrolase activity;MF:exopeptidase activity;MF:molecular_function;MF:peptidase activity;MF:metalloexopeptidase activity;MF:metallopeptidase activity;MF:dipeptidase activity;MF:metallodipeptidase activity;MF:catalytic activity, acting on a	GO:0000:	K01302	CPQ	------	------	COG2234	S:Function unknown	PF04389.20;PF02:	Peptidase_M28;PA;Peptidase_M20	Peptidase family M28;PA domain;Peptidase family M20/M25/M40	CYT	1	2	52	High
TRINITY_DN66302_c0_g1_i1_orf1	carboxypeptidase B-like [Ostrinia furnacalis]	4.861111111	2.28128611	7.11E-06	up	yes	1.05	0.216	0.219	0.2	0.229	1	1.089	1.06	BP:nitrogen compound metabolic process;BP:organic substance metabolic process;BP:proteolysis;BP:biological_process;BP:metabolic process;BP:primary metabolic process;BP:organonitrogen compound metabolic process;BP:protein metabolic process;BP:macromolecule metabolic process;CC:intrinsic component of membrane;CC:cellular anatomical entity;CC:cellular_component;CC:integral component of membrane;MF:exopeptidase activity;MF:molecular_function;MF:zinc ion binding;MF:peptidase activity;MF:metalloexopeptidase activity;MF:cation binding;MF:transition metal ion binding;MF:metallocarboxypeptidase activity;MF:carboxypeptidase activity;MF:ion binding;MF:metallopeptidase activity;MF:metal ion binding;MF:hydrolase	GO:0006{	K01298	CPA2	map04972;m:	Pancreatic secretio	COG2866	E:Amino acid transport and meta	PF00246.27	Peptidase_M14	Zinc carboxypeptidase	CYT	1	3	47.1	High
TRINITY_DN30663_c0_g1_i1_orf1	surfeit locus protein 6 homolog [Ostrinia furnacalis]	3.575372321	1.838093485	1.44E-05	up	yes	0.9843	0.2753	0.231	0.304	0.291	1	1.002	0.951	CC:intracellular membrane-bounded organelle;CC:nucleus;CC:cellular_component;CC:intracellular organelle;CC:membrane-bounded organelle;CC:organelle;CC:cellular anatomical entity;	GO:0043:	------	------	------	------	ENOG4111G0Y	S:Function unknown	PF04935.15	SURF6	Surfeit locus protein 6	CYT	2	6	33.5	High
TRINITY_DN7024_c0_g1_i1_orf1	uncharacterized protein LOC114363116 [Ostrinia furnacalis]	30.86702263	4.947994425	0.000467	up	yes	1.214	0.03933	0.02	0.022	0.076	1	1.369	1.272	MF:molecular_function;MF:lipid binding;MF:binding;	GO:0003(	K08753;K(	FABP4, aP2;F	map04923;m:	Regulation of lipoly	ENOG4111US8	I:Lipid transport and metabolism	------	------	------	CYT	7	68	15.1	High
TRINITY_DN18909_c0_g1_i8_orf1	unnamed protein product [Euphydryas editha]	3.564946945	1.833880606	8.05E-05	up	yes	0.9743	0.2733	0.291	0.251	0.278	1	1.029	0.894	MF:hydrolase activity;MF:molecular_function;MF:hydrolase activity, acting on ester bonds;MF:carboxylic ester hydrolase activity;MF:catalytic activity;	GO:0016:	------	------	------	------	COG2272;COG0!	I:Lipid transport and metabolism	PF00135.31;PF20:	COesterase;BD-FAE;Abhydrolase_3	Carboxylesterase family;BD-FAE;alpha/beta hydrolase fold	CYT	1	4	60.6	High
TRINITY_DN2953_c1_g1_i2_orf1	methionine--tRNA ligase, cytoplasmic isoform X6 [Ostrinia furnacalis]	7.071895425	2.822096941	0.0001941	up	yes	1.082	0.153	0.174	0.065	0.22	1	1.183	1.062	BP:methionyl-tRNA aminoacylation;BP:carboxylic acid metabolic process;BP:nucleic acid metabolic process;BP:cellular nitrogen compound metabolic process;BP:nitrogen compound metabolic process;BP:small molecule metabolic process;BP:ncRNA metabolic process;BP:organic cyclic compound metabolic process;BP:nucleobase-containing compound metabolic process;BP:cellular amino acid metabolic process;BP:organic substance metabolic process;BP:primary metabolic process;BP:cellular process;BP:cellular aromatic compound metabolic process;BP:biological_process;BP:metabolic process;BP:oxoacid metabolic process;BP:organonitrogen compound metabolic process;BP:heterocycle metabolic process;BP:RNA metabolic process;BP:organic acid metabolic process;BP:cellular metabolic process;BP:macromolecule metabolic process;BP:tRNA metabolic process;BP:amino acid activation;BP:tRNA aminoacylation;BP:tRNA aminoacylation for protein translation;CC:aminoacyl-	GO:0006:	K01874	MARS, metG	map00450;m:	Selenocompound r	COG0143	J:Translation, ribosomal structure	PF00458.23	WHEP-TRS	WHEP-TRS domain	CYT	1	43	10.3	High
TRINITY_DN51766_c0_g1_i2_orf1	facilitated trehalose transporter Tret1-like [Ostrinia furnacalis]	5.796937039	2.535290817	1.38E-06	up	yes	1.022	0.1763	0.153	0.174	0.202	1	1.041	1.024	BP:transport;BP:carbohydrate transport;BP:organic substance transport;BP:biological_process;BP:establishment of localization;BP:localization;CC:integral component of membrane;CC:cellular_component;CC:cellular anatomical entity;CC:intrinsic component of membrane;MF:transporter activity;MF:transmembrane transporter	GO:0006{	------	------	------	------	ENOG410XNQK	E:Amino acid transport and meta	PF00083.27;PF07(	Sugar_tr;MFS_1	Sugar;Major Facilitator Superfamily	CYT	1	3	49.7	High
TRINITY_DN11259_c0_g1_i1_orf1	uncharacterized protein LOC114357075 [Ostrinia furnacalis]	4.684134615	2.227782537	3.19E-06	up	yes	0.9743	0.208	0.197	0.187	0.24	1	0.962	0.961	------	------	------	------	------	------	COG1028	S:Function unknown	PF13561.9;PF001(	adh_short_C2;adh_short;KR	Enoyl;short chain dehydrogenase;KR domain	CYT	6	46	26.7	High
TRINITY_DN33452_c0_g1_i1_orf1	lethal(2) giant larvae protein isoform X8 [Ostrinia furnacalis]	8.874656908	3.149691346	9.00E-06	up	yes	0.97	0.1093	0.076	0.091	0.161	1	0.955	0.955	BP:secretion;BP:transport;BP:vesicle-mediated transport;BP:export from cell;BP:exocytosis;BP:secretion by cell;BP:biological_process;BP:cellular process;BP:establishment of localization;BP:localization;	GO:0046!	K06094	LLGL	map04530;m:	Tight junction;Hum	ENOG410XS6Z	S:Function unknown	------	------	------	CYT	1	14	13.7	High
TRINITY_DN117_c0_g1_i4_orf1	lipase member I-like [Ostrinia furnacalis]	20.64820642	4.367944564	1.74E-07	up	yes	0.9843	0.04767	0.036	0.048	0.059	1	0.988	0.965	BP:organic substance metabolic process;BP:lipid metabolic process;BP:primary metabolic process;BP:metabolic process;BP:biological_process;CC:cellular_component;CC:extracellular region;CC:cellular anatomical entity;MF:hydrolase activity;MF:hydrolase activity, acting on ester bonds;MF:catalytic activity;MF:molecular_function;MF:lipase	GO:0071:	------	------	------	------	ENOG41116Y2;E	S:Function unknown;	PF00151.22	Lipase	Lipase	CYT	5	82	16.1	High

TRINITY_DN49936_c0_g2_i1_orf1	39S ribosomal protein L20, mitochondrial [Ostrinia furnacalis]	2.271028037	1.183345517	8.58E-05	up	yes	0.9477	0.4173	0.403	0.393	0.456	1	0.916	0.927	BP:cellular biosynthetic process;BP:cellular macromolecule biosynthetic process;BP:organic substance biosynthetic process;BP:cellular nitrogen compound biosynthetic process;BP:cellular macromolecule metabolic process;BP:peptide biosynthetic process;BP:organic substance metabolic process;BP:organonitrogen compound biosynthetic process;BP:amide biosynthetic process;BP:cellular process;BP:biosynthetic process;BP:macromolecule biosynthetic process;BP:biological_process;BP:metabolic process;BP:organonitrogen compound metabolic process;BP:primary metabolic process;BP:protein metabolic process;BP:cellular nitrogen compound metabolic process;BP:cellular metabolic process;BP:macromolecule metabolic process;BP:nitrogen compound metabolic process;BP:cellular amide metabolic process;BP:peptide metabolic process;BP:translation;CC:intracellular organelle;CC:non-membrane-bounded organelle;CC:organelle;CC:cellular anatomical entity;CC:macromolecular [illegible]	GO:00442	K02887	RP-L20, MRF	map03010	Ribosome	COG0292	E:Amino acid transport and meta	PF00453.21	Ribosomal_L20	Ribosomal protein L20	CYT	2	12	18.6	High
TRINITY_DN14217_c0_g1_i1_orf1	putative serine protease K12H4.7 [Ostrinia furnacalis] >XP_028166339.1 putative serine protease K12H4.7 [Ostrinia furnacalis]	10.45378151	3.385953007	0.001006	up	yes	1.244	0.119	0.14	0.058	0.159	1	1.304	1.428	[illegible] BP:primary metabolic process;BP:organonitrogen compound metabolic process;BP:protein metabolic process;BP:macromolecule metabolic process;BP:organic substance metabolic process;BP:nitrogen compound metabolic process;BP:biological_process;BP:metabolic process;BP:proteolysis;MF:molecular_function;MF:serine hydrolase activity;MF:hydrolase activity;MF:catalytic activity, acting on a protein;MF:catalytic [illegible]	GO:00442	------	------	------	------	ENOG410XSGG	S:Function unknown	PF05577.15	Peptidase_S28	Serine carboxypeptidase S28	CYT	1	3	54.9	High
TRINITY_DN20346_c0_g1_i1_orf1	NADH dehydrogenase [ubiquinone] 1 alpha subcomplex subunit 6 [Ostrinia furnacalis]	3.072524827	1.619424668	5.49E-06	up	yes	1.021	0.3323	0.33	0.314	0.353	1	1.057	1.006	CC:respiratory chain complex I;CC:transmembrane transporter complex;CC:catalytic complex;CC:transporter complex;CC:NADH dehydrogenase complex;CC:respiratory chain complex;CC:inner mitochondrial membrane protein complex;CC:cellular_component;CC:membrane protein complex;CC:mitochondrial respiratory chain complex I;CC:macromolecular complex;CC:oxidoreductase complex;CC:mitochondrial protein complex;	GO:00452	K03950	NDUFA6	map04714;m	Thermogenesis;Oxi	ENOG4111S2F	I:Lipid transport and metabolism	PF05347.18	Complex1_LYR	Complex 1 protein	CYT	2	12	14.8	High
TRINITY_DN7213_c0_g1_i2_orf1	probable ATP-dependent RNA helicase CG8611 [Ostrinia furnacalis]	2.480154639	1.310430076	2.79E-05	up	yes	0.9623	0.388	0.36	0.418	0.386	1	0.928	0.959	MF:purine ribonucleoside triphosphate binding;MF:molecular_function;MF:binding;MF:nucleic acid binding;MF:RNA-dependent ATPase activity;MF:ion binding;MF:heterocyclic compound binding;MF:nucleoside phosphate binding;MF:anion binding;MF:nucleotide binding;MF:purine nucleotide binding;MF:ATP binding;MF:catalytic activity, acting on RNA;MF:helicase activity;MF:small molecule binding;MF:catalytic activity;MF:adenyl nucleotide binding;MF:carbohydrate derivative binding;MF:organic cyclic compound binding;MF:RNA helicase activity;MF:adenyl ribonucleotide binding;MF:RNA binding;MF:purine ribonucleotide binding;MF:ribonucleotide binding;	GO:0035€	K14806	DDX31, DBP7	------	------	ENOG410XNT7	L:Replication, recombination and	PF00270.32;PF00	DEAD;Helicase_C;DUF4217;ResIII	DEAD/DEAH box helicase;Helicase conserved C-terminal domain;Domain of unknown function;Type III restriction enzyme, res subunit	CYT	1	1	106.1	Medium
TRINITY_DN2749_c0_g1_i4_orf1	RNA exonuclease 4-like [Ostrinia furnacalis] >QEE79882.1 REX4 [Ostrinia furnacalis]	2.233333333	1.159198595	0.0005533	up	yes	0.9313	0.417	0.42	0.48	0.351	1	0.884	0.91	BP:nucleic acid metabolic process;BP:cellular nitrogen compound metabolic process;BP:nitrogen compound metabolic process;BP:ncRNA metabolic process;BP:organic cyclic compound metabolic process;BP:nucleobase-containing compound metabolic process;BP:organic substance metabolic process;BP:rRNA processing;BP:ncRNA processing;BP:cellular process;BP:cellular aromatic compound metabolic process;BP:biological_process;BP:metabolic process;BP:heterocycle metabolic process;BP:RNA metabolic process;BP:primary metabolic process;BP:rRNA metabolic process;BP:cellular metabolic process;BP:macromolecule metabolic process;BP:RNA processing;MF:molecular_function;MF:binding;MF:nucleic acid binding;MF:heterocyclic compound binding;MF:3'-5' exonuclease activity;MF:exonuclease activity;MF:hydrolase activity;MF:hydrolase activity, acting on [illegible]	GO:0090:	K18327	REXO4, REX4	------	------	COG0847	L:Replication, recombination and	PF00929.27	RNase_T	Exonuclease	CYT	2	27	12.4	High
TRINITY_DN2704_c0_g1_i5_orf1	hypothetical protein evm_009002 [Chilo suppressalis]	3.024523161	1.596707708	1.35E-06	up	yes	0.999	0.3303	0.335	0.305	0.351	1	1.008	0.989	------	------	K06569	MFI2, CD228	------	------	ENOG410ZEJE	S:Function unknown	PF00405.20	Transferrin	Transferrin	CYT	9	16	84.2	High
TRINITY_DN542_c0_g1_i4_orf1	uncharacterized protein LOC114364889 [Ostrinia furnacalis]	2.907011736	1.539536896	3.20E-05	up	yes	0.966	0.3323	0.323	0.301	0.373	1	0.925	0.973	BP:amino sugar catabolic process;BP:nitrogen compound metabolic process;BP:aminoglycan metabolic process;BP:organic substance catabolic process;BP:glucosamine-containing compound catabolic process;BP:organic substance metabolic process;BP:chitin metabolic process;BP:polysaccharide catabolic process;BP:biological_process;BP:metabolic process;BP:organonitrogen compound metabolic process;BP:catabolic process;BP:macromolecule catabolic process;BP:primary metabolic process;BP:chitin catabolic process;BP:carbohydrate metabolic process;BP:organonitrogen compound catabolic process;BP:polysaccharide metabolic process;BP:amino sugar metabolic process;BP:carbohydrate derivative catabolic process;BP:carbohydrate catabolic process;BP:macromolecule metabolic process;BP:glucosamine-containing compound metabolic process;BP:aminoglycan catabolic process;BP:carbohydrate derivative metabolic process;CC:extracellular [illegible]	GO:0046:	------	------	------	------	ENOG410ZWU3	S:Function unknown	PF01522.24	Polysacc_deac_1	Polysaccharide deacetylase	CYT	5	14	44.8	High

TRINITY_DN42759_c0_g2_i1_orf1	fatty acid synthase-like [Ostrinia furnacalis]	3.449647887	1.786449111	1.82E-05	up	yes	0.9797	0.284	0.281	0.273	0.298	1	1.015	0.924	BP:fatty acid biosynthetic process;BP:fatty acid metabolic process;BP:carboxylic acid metabolic process;BP:cellular biosynthetic process;BP:small molecule metabolic process;BP:small molecule biosynthetic process;BP:monocarboxylic acid biosynthetic process;BP:organic substance biosynthetic process;BP:organic substance metabolic process;BP:lipid metabolic process;BP:cellular process;BP:monocarboxylic acid metabolic process;BP:biosynthetic process;BP:biological_process;BP:metabolic process;BP:oxoacid metabolic process;BP:cellular lipid metabolic process;BP:lipid biosynthetic process;BP:primary metabolic process;BP:organic acid metabolic process;BP:carboxylic acid biosynthetic process;BP:organic acid biosynthetic process;BP:cellular metabolic process;MF:3-hydroxyoctanoyl-[acyl-carrier-protein] dehydratase activity;MF:thiolester hydrolase activity;MF:S-acyltransferase activity;MF:enoyl-[acyl-carrier-protein] reductas;MF:oxidoreductase activity, acting on the CH-CH group of	GO:0006	K00665	FASN	map04910;m	Insulin signaling pa	COG3321	Q:Secondary metabolites biosyn	PF00975.23;PF14	Thioesterase;PS-DH	Thioesterase domain;Polyketide synthase dehydratase	CYT	5	3	166.4	High
TRINITY_DN8369_c0_g1_i1_orf1	39S ribosomal protein L37, mitochondrial [Ostrinia furnacalis]	3.581115567	1.840409077	1.90E-06	up	yes	0.9823	0.2743	0.252	0.271	0.3	1	0.968	0.979	------	------	K17418	MRPL37	------	------	ENOG410ZT8U	J:Translation, ribosomal structure	PF07147.15	PDCD9	Mitochondrial 28S ribosomal protein S30	CYT	3	6	47.1	High
TRINITY_DN868_c0_g1_i4_orf1	uncharacterized protein LOC114359357 isoform X1 [Ostrinia furnacalis]	3.94488189	1.979982106	3.74E-05	up	yes	1.002	0.254	0.215	0.221	0.326	1	1.021	0.985	CC:cellular_component;CC:extracellular region;CC:cellular anatomical entity;MF:molecular_function;MF:structural molecule activity;	GO:0005	------	------	------	------	------	------	PF07294.14	Fibroin_P25	Fibroin P25	CYT	1	5	22.6	High
TRINITY_DN26375_c0_g1_i1_orf1	hypothetical protein O3G_MSEX007366 [Manduca sexta]	3.407526882	1.768725038	2.64E-05	up	yes	0.9507	0.279	0.256	0.301	0.28	1	0.949	0.903	------	------	------	------	------	------	ENOG410Y78S;E	S:Function unknown;	------	------	------	CYT	1	2	129.6	High
TRINITY_DN23570_c0_g1_i2_orf1	putative trypsin 6 [Ostrinia nubilalis]	3.744186047	1.904652123	7.49E-05	up	yes	0.966	0.258	0.285	0.22	0.269	1	0.891	1.007	BP:organic substance metabolic process;BP:organonitrogen compound metabolic process;BP:protein metabolic process;BP:macromolecule metabolic process;BP:nitrogen compound metabolic process;BP:biological_process;BP:metabolic process;BP:proteolysis;BP:primary metabolic process;CC:extracellular space;CC:cellular_component;CC:cellular anatomical entity;MF:serine-type endopeptidase activity;MF:endopeptidase activity;MF:hydrolase activity;MF:serine hydrolase activity;MF:catalytic activity, acting on a protein;MF:catalytic	GO:0071	K09628;K	PRSS27;TMP	map04972;m	Pancreatic secretio	COG5640	O:Posttranslational modification,	PF00089.29	Trypsin	Trypsin	CYT	1	4	26.1	High
TRINITY_DN1707_c0_g1_i1_orf1	inositol oxygenase-like [Ostrinia furnacalis]	5.066931086	2.341112207	1.45E-05	up	yes	1.022	0.2017	0.169	0.185	0.251	1	1.063	1.003	BP:polyol catabolic process;BP:polyol metabolic process;BP:cellular catabolic process;BP:small molecule metabolic process;BP:small molecule catabolic process;BP:alcohol metabolic process;BP:organic substance catabolic process;BP:cellular carbohydrate metabolic process;BP:organic substance metabolic process;BP:alcohol catabolic process;BP:inositol catabolic process;BP:cellular process;BP:biological_process;BP:metabolic process;BP:catabolic process;BP:cellular carbohydrate catabolic process;BP:primary metabolic process;BP:carbohydrate metabolic process;BP:carbohydrate catabolic process;BP:cellular metabolic process;BP:inositol metabolic process;BP:organic hydroxy compound catabolic process;BP:organic hydroxy compound metabolic process;CC:cytoplasm;CC:cellular anatomical entity;CC:cellular_component;MF:binding;MF:monooxygenase activity;MF:molecular_function;MF:ion binding;MF:oxidoreductase activity;MF:transition metal ion	GO:0046	K00469	MIOX	map01250;m	Biosynthesis of nuc	ENOG410XQ4J	S:Function unknown	PF05153.18	MIOX	Myo-inositol oxygenase	CYT	4	21	34.5	High
TRINITY_DN2894_c0_g1_i2_orf1	myrosinase 1-like isoform X1 [Ostrinia furnacalis]	4.715561824	2.237429668	1.15E-05	up	yes	1.003	0.2127	0.189	0.196	0.253	1	1.042	0.968	BP:primary metabolic process;BP:carbohydrate metabolic process;BP:organic substance metabolic process;BP:biological_process;BP:metabolic process;MF:hydrolase activity;MF:catalytic activity;MF:hydrolase activity, acting on glycosyl bonds;MF:hydrolase activity, hydrolyzing O-glycosyl	GO:0044	------	------	------	------	COG2723	G:Carbohydrate transport and m	PF00232.21	Glyco_hydro_1	Glycosyl hydrolase family 1	CYT	6	17	59.3	High
TRINITY_DN6693_c0_g1_i1_orf1	uncharacterized protein LOC114356358 [Ostrinia furnacalis]	5.157046282	2.366544993	1.92E-06	up	yes	0.9917	0.1923	0.188	0.17	0.219	1	1.008	0.967	MF:catalytic activity;MF:transferase activity;MF:transferase activity, transferring sulfur-containing groups;MF:sulfotransferase activity;MF:molecular_function;	GO:0003	------	------	------	------	ENOG4111H56	S:Function unknown	PF00685.30;PF13	Sulfotransfer_1;Sulfotransfer_3	Sulfotransferase domain;Sulfotransferase family	CYT	6	24	40.4	High
TRINITY_DN48410_c0_g1_i1_orf1	alpha-amylase 1-like [Ostrinia furnacalis]	6.671438309	2.737997829	7.53E-07	up	yes	0.9787	0.1467	0.131	0.145	0.164	1	0.979	0.957	BP:organic substance metabolic process;BP:primary metabolic process;BP:carbohydrate metabolic process;BP:metabolic process;BP:biological_process;MF:hydrolase activity;MF:cation binding;MF:amylase activity;MF:hydrolase activity, acting on glycosyl bonds;MF:ion binding;MF:catalytic activity;MF:alpha-amylase activity;MF:molecular_function;MF:bindi	GO:0071	K01176	AMY, amyA,	map04972;m	Pancreatic secretio	COG0366	G:Carbohydrate transport and m	PF00128.27;PF02	Alpha-amylase;Alpha-amylase_C	Alpha amylase, catalytic domain;Alpha amylase, C-terminal all-beta domain	EXC	11	29	56	High
TRINITY_DN2695_c0_g1_i8_orfp1	TRINITY_DN2695_c0_g1_i8_m.44478 TRINITY_DN2695_c0_g1::TRINITY_DN2695_c0_g1_i8::g.44478 ORF type:3prime_partial len:532 (+),score=80.62 TRINITY_DN2695_c0_g1_i8:101-1594(+)	3.978251221	1.992134383	0.003303	up	yes	0.8963	0.2253	0.209	0.215	0.252	1	0.684	1.005	------	------	------	------	------	------	------	------	------	------	------	CYT	1	2	56.2	High
TRINITY_DN117362_c0_g1_i5_orf1	uncharacterized protein LOC114354191 [Ostrinia furnacalis]	2.050873362	1.036238411	0.0003925	up	yes	0.9393	0.458	0.447	0.517	0.41	1	0.901	0.917	BP:primary metabolic process;BP:organonitrogen compound metabolic process;BP:protein metabolic process;BP:macromolecule metabolic process;BP:organic substance metabolic process;BP:nitrogen compound metabolic process;BP:biological_process;BP:metabolic process;BP:proteolysis;MF:molecular_function;MF:exopeptidase activity;MF:hydrolase activity;MF:carboxypeptidase activity;MF:catalytic activity, acting on a protein;MF:serine hydrolase activity;MF:serine-type carboxypeptidase activity;MF:serine-	GO:0044	K09646	SCPEP1	------	------	COG2939	C:Energy production and conver	PF00450.25	Peptidase_S10	Serine carboxypeptidase	CYT	1	5	48.5	High
TRINITY_DN51045_c0_g1_i1_orf1	cell growth-regulating nucleolar protein [Ostrinia furnacalis]	2.904047552	1.538065077	8.00E-06	up	yes	1.026	0.3533	0.318	0.374	0.368	1	1.028	1.05	CC:intracellular membrane-bounded organelle;CC:nucleus;CC:cellular_component;CC:intracellular organelle;CC:membrane-bounded organelle;CC:organelle;CC:cellular anatomical entity;MF:cation binding;MF:organic cyclic compound binding;MF:heterocyclic compound binding;MF:ion binding;MF:molecular_function;MF:binding;MF:nucleic acid binding;MF:DNA binding;MF:metal ion binding;	GO:0043	K15263	LYER	------	------	ENOG411081U	S:Function unknown	PF08790.14	zf-LYAR	LYAR-type C2HC zinc finger	CYT	4	13	48.3	High

TRINITY_DN114198_c0_g1_i1_orf1	catalase isozyme 1, partial [Sturnira hondurensis]	2.198746227	1.136681102	0.0003642	up	yes	0.947	0.4307	0.401	0.4	0.491	1	0.96	0.881	BP:regulation of biological process;BP:response to reactive oxygen species;BP:cellular catabolic process;BP:determination of adult lifespan;BP:cellular component organization or biogenesis;BP:cellular component organization;BP:biological regulation;BP:positive regulation of biological process;BP:response to oxygen-containing compound;BP:multicellular organismal process;BP:cellular process;BP:reactive oxygen species metabolic process;BP:regulation of cellular process;BP:response to stress;BP:biological_process;BP:metabolic process;BP:response to chemical;BP:response to inorganic substance;BP:catabolic process;BP:response to oxidative stress;BP:organelle organization;BP:peroxisome organization;BP:response to stimulus;BP:response to hydrogen peroxide;BP:cellular metabolic process;BP:positive regulation of cell division;BP:hydrogen peroxide catabolic process;BP:regulation of cell division;BP:hydrogen peroxide	GO:00507	K03781	katE, CAT, ca	map00630;m	Glyoxylate and dica	COG0753	J:Translation, ribosomal structure	PF00199.22;PF06	Catalase;Catalase-rel	Catalase;Catalase-related immune-responsive	CYT	1	3	56.9	High
TRINITY_DN1465_c0_g2_i1_orf1	unnamed protein product, partial [Iphiclides podalirius]	2.78021978	1.475198935	2.96E-06	up	yes	1.012	0.364	0.345	0.358	0.389	1	1.001	1.034	MF:catalytic activity;MF:monooxygenase activity;MF:molecular_function;MF:oxidoreductase activity;	GO:0003	K01904	4CL	map00130	Ubiquinone and ot	COG0318;COG0	I:Lipid transport and metabolism	PF00501.31;PF13	AMP-binding;AMP-binding_C	AMP-binding enzyme;AMP-binding enzyme C-terminal	CYT	8	23	57.9	High
TRINITY_DN3292_c2_g1_i4_orf1	ribosome biogenesis regulatory protein homolog [Ostrinia furnacalis]	2.582915898	1.369000669	2.19E-06	up	yes	0.9797	0.3793	0.381	0.379	0.378	1	0.951	0.988	BP:cellular process;BP:ribosome biogenesis;BP:cellular component biogenesis;BP:biological_process;BP:cellular component organization or biogenesis;BP:ribonucleoprotein complex biogenesis;CC:intracellular membrane-bounded organelle;CC:nucleus;CC:cellular_component;CC:intracellular organelle;CC:membrane-bounded organelle;CC:organelle;CC:cellular anatomical entity;	GO:0009	K14852	RRS1	------	------	COG5225	J:Translation, ribosomal structure	PF04939.15	RRS1	Ribosome biogenesis regulatory protein	CYT	4	12	40	High
TRINITY_DN17905_c0_g3_i1_orf1	zinc finger protein 706-like [Ostrinia furnacalis] >XP_028176219.1 zinc finger protein 706-like [Ostrinia furnacalis] >XP_028176220.1 zinc finger protein 706-like [Ostrinia furnacalis] >XP_028176221.1 zinc finger protein 706-like [Ostrinia furnacalis]	2.795883846	1.483304425	1.50E-05	up	yes	0.9917	0.3547	0.323	0.368	0.373	1	1.021	0.954	CC:cytoplasm;CC:intracellular membrane-bounded organelle;CC:nucleus;CC:cellular_component;CC:intracellular organelle;CC:membrane-bounded organelle;CC:organelle;CC:cellular anatomical entity;	GO:0005	------	------	------	------	ENOG411234V	S:Function unknown	PF04419.17	4F5	4F5 protein related disordered region	CYT	2	18	8.7	High
TRINITY_DN11069_c0_g2_i1_orf1	fat storage-inducing transmembrane protein [Ostrinia furnacalis]	3.138641254	1.650140139	6.76E-05	up	yes	1.021	0.3253	0.319	0.337	0.32	1	0.965	1.099	organization;BP:cellular component organization or biogenesis;BP:cellular process;BP:biological_process;BP:maintenance of location;BP:localization;BP:organelle organization;BP:lipid particle organization;CC:endoplasmic reticulum membrane;CC:intrinsic component of membrane;CC:organelle membrane;CC:integral component of membrane;CC:membrane;CC:cellular anatomical entity;CC:cellular_component;MF:molecular_function;MF:hydrolase activity;MF:catalytic activity;MF:hydrolase activity, acting on acid anhydrides, in phosphorus-containing anhydrides;MF:hydrolase	GO:0019	------	------	------	------	ENOG4111CNJ	S:Function unknown	PF10261.12	Scs3p	Inositol phospholipid synthesis and fat-storage-inducing TM	CYT	2	6	42.2	High
TRINITY_DN4476_c0_g1_i5_orf1	trypsin, alkaline C-like isoform X1 [Ostrinia furnacalis]	2.532903572	1.340792155	0.0001454	up	yes	0.943	0.3723	0.323	0.422	0.372	1	0.913	0.916	process;BP:organonitrogen compound metabolic process;BP:protein metabolic process;BP:macromolecule metabolic process;BP:biological_process;BP:nitrogen compound metabolic process;BP:metabolic process;BP:proteolysis;BP:primary metabolic process;MF:serine-type endopeptidase activity;MF:endopeptidase activity;MF:hydrolase activity;MF:serine hydrolase activity;MF:catalytic activity, acting on a protein;MF:catalytic activity;MF:molecular_function;MF:pepti	GO:0071	K01312	PRSS1_2_3	map04972;m	Pancreatic secretio	COG5640	O:Posttranslational modification,	PF00089.29	Trypsin	Trypsin	PLA	1	22	26.3	High
TRINITY_DN8691_c0_g1_i3_orf1	nucleolin-like [Melitaea cinxia]	3.438883214	1.781940122	5.21E-06	up	yes	1.01	0.2937	0.259	0.309	0.313	1	1.036	0.993	------	------	------	------	------	------	ENOG4110RR1	S:Function unknown	------	------	------	CYT	2	15	14.4	High
TRINITY_DN43369_c0_g2_i1_orf1	cytochrome P450 monooxygenase 304 [Glyphodes pyloalis]	2.858858859	1.515439396	3.21E-05	up	yes	0.952	0.333	0.338	0.302	0.359	1	0.917	0.939	CC:integral component of membrane;CC:cellular_component;CC:cellular anatomical entity;CC:intrinsic component of membrane;MF:cation binding;MF:monooxygenase activity;MF:heme binding;MF:transition metal ion binding;MF:tetrapyrrole binding;MF:organic cyclic compound binding;MF:oxidoreductase activity, acting on paired donors, with incorporation or reduction of molecular oxygen;MF:ion binding;MF:catalytic activity;MF:molecular_function;MF:binding;MF:iron ion binding;MF:metal ion binding;MF:heterocyclic compound binding;MF:oxidoreductase activity;	GO:0016	------	------	------	------	COG2124	S:Function unknown	------	------	------	CYT	1	8	13	High
TRINITY_DN40_c0_g1_i3_orf1	trypsin CFT-1-like [Ostrinia furnacalis]	3.578834466	1.839489815	1.66E-05	up	yes	1.001	0.2797	0.229	0.286	0.324	1	0.983	1.019	process;BP:organonitrogen compound metabolic process;BP:protein metabolic process;BP:macromolecule metabolic process;BP:biological_process;BP:nitrogen compound metabolic process;BP:metabolic process;BP:proteolysis;BP:primary metabolic process;MF:serine-type endopeptidase activity;MF:endopeptidase activity;MF:hydrolase activity;MF:serine hydrolase activity;MF:catalytic activity, acting on a protein;MF:catalytic activity;MF:molecular_function;MF:pepti	GO:0071	K01312	PRSS1_2_3	map04972;m	Pancreatic secretio	COG5640	O:Posttranslational modification,	PF00089.29	Trypsin	Trypsin	CYT	4	18	28.1	High
TRINITY_DN4762_c0_g1_i2_orf1	ATPase family AAA domain-containing protein 1 isoform X2 [Ostrinia furnacalis]	4.082769598	2.029548155	0.0002566	up	yes	1.026	0.2513	0.196	0.244	0.314	1	1.128	0.949	binding;MF:molecular_function;MF:binding;MF:ATPase activity;MF:ion binding;MF:heterocyclic compound binding;MF:nucleoside phosphate binding;MF:anion binding;MF:nucleotide binding;MF:purine nucleotide binding;MF:ATP binding;MF:hydrolase activity;MF:nucleoside-triphosphatase activity;MF:small molecule binding;MF:catalytic activity;MF:hydrolase activity, acting on acid anhydrides, in phosphorus-containing anhydrides;MF:adenyl nucleotide binding;MF:carbohydrate derivative binding;MF:organic cyclic compound binding;MF:hydrolase activity, acting on acid anhydrides;MF:pyrophosphatase activity;MF:adenyl ribonucleotide binding;MF:purine ribonucleotide	GO:0035	K22530	ATAD1	------	------	COG0464	S:Function unknown	PF00004.32;PF17	AAA;AAA_lid_3	ATPase family associated with various cellular activities;AAA+ lid domain	CYT	1	2	43.3	High

TRINITY_DN29440_c1_g1_i4_orf1	neutral lipase [Helicoverpa armigera]	2.941176471	1.556393349	0.0007783	up	yes	0.95	0.323	0.199	0.408	0.362	1	0.93	0.92	BP:organic substance metabolic process;BP:lipid metabolic process;BP:primary metabolic process;BP:metabolic process;BP:biological_process;CC:cellular_component;CC:extracellular region;CC:cellular anatomical entity;MF:hydrolase activity;MF:hydrolase activity, acting on ester bonds;MF:catalytic activity;MF:molecular_function;MF:lipase	GO:00717	------	------	------	------	ENOG410YGS3;E	S:Function unknown;	PF00151.22	Lipase	Lipase	PLA	1	10	11.7	High
TRINITY_DN4030_c0_g2_i1_orf1	putative trypsin 6 [Ostrinia nubilalis]	3.840883137	1.941438069	1.51E-06	up	yes	1.009	0.2627	0.282	0.247	0.259	1	0.992	1.035	BP:organic substance metabolic process;BP:organonitrogen compound metabolic process;BP:protein metabolic process;BP:macromolecule metabolic process;BP:biological_process;BP:nitrogen compound metabolic process;BP:metabolic process;BP:proteolysis;BP:primary metabolic process;MF:serine-type endopeptidase activity;MF:endopeptidase activity;MF:hydrolase activity;MF:serine hydrolase activity;MF:catalytic activity, acting on a protein;MF:catalytic activity;MF:molecular_function;MF:pepti	GO:00717	K09628;K	PRSS27;TMPI	------	------	COG5640	O:Posttranslational modification,	PF00089.29	Trypsin	Trypsin	CYT	3	20	28.7	High
TRINITY_DN344_c1_g1_i1_orf1	chymotrypsin-like serine protease 16 [Ostrinia nubilalis]	3.996015936	1.998562337	9.77E-06	up	yes	1.003	0.251	0.223	0.228	0.302	1	0.99	1.019	BP:organic substance metabolic process;BP:organonitrogen compound metabolic process;BP:protein metabolic process;BP:macromolecule metabolic process;BP:biological_process;BP:nitrogen compound metabolic process;BP:metabolic process;BP:proteolysis;BP:primary metabolic process;MF:serine-type endopeptidase activity;MF:endopeptidase activity;MF:hydrolase activity;MF:serine hydrolase activity;MF:catalytic activity, acting on a protein;MF:catalytic activity;MF:molecular_function;MF:pepti	GO:00717	K01312	PRSS1_2_3	map04972;m	Pancreatic secretion	COG5640	I:Lipid transport and metabolism	PF00089.29	Trypsin	Trypsin	CYT	5	29	30.7	High
TRINITY_DN69049_c0_g1_i2_orf1	membrane alanyl aminopeptidase-like [Ostrinia furnacalis]	3.95515873	1.983735595	1.26E-05	up	yes	0.9967	0.252	0.246	0.219	0.291	1	1.028	0.962	CC:integral component of membrane;CC:cellular_component;CC:cellular anatomical entity;CC:intrinsic component of membrane;MF:exopeptidase activity;MF:aminopeptidase activity;MF:transition metal ion binding;MF:hydrolase activity;MF:catalytic activity, acting on a protein;MF:cation binding;MF:ion binding;MF:catalytic activity;MF:metallopeptidase activity;MF:molecular_function;MF:binding;MF:peptidase activity;MF:metal ion	GO:0016(	K11140	ANPEP, CD1:	map04640;m	Hematopoietic cell	COG0308	E:Amino acid transport and meta	PF11838.11	ERAP1_C	ERAP1-like C-terminal domain	CYT	1	2	51.3	High
TRINITY_DN104586_c0_g1_i1_orf1	Chlorophyll a-b binding protein 37, chloroplastic, partial [Trichinella patagoniensis]	4.019850022	2.007141676	0.0001774	up	yes	0.9113	0.2267	0.195	0.209	0.276	1	0.863	0.871	CC:cellular_component;CC:membrane;CC:cellular anatomical entity;	GO:0005!	K08913	LHCB2	------	------	ENOG410ZHBU	L:Replication, recombination and	------	------	------	CYT	1	15	10.4	High
TRINITY_DN94755_c0_g1_i5_orfp1	TRINITY_DN94755_c0_g1_i5_m.62794 TRINITY_DN94755_c0_g1::TRINITY_DN94755_c0_g1_i5::g.62794 ORF type:internal len:70 (+),score=5.17 TRINITY_DN94755_c0_g1_i5:2-208(+)	10.73626374	3.424420112	2.57E-05	up	yes	0.977	0.091	0.06	0.082	0.131	1	1.022	0.909	------	------	------	------	------	------	------	------	------	------	------	CYT	1	16	8	High
TRINITY_DN348_c0_g2_i3_orf1	pancreatic triacylglycerol lipase-like [Ostrinia furnacalis]	15.9104765	3.991905138	2.69E-07	up	yes	0.9917	0.06233	0.05	0.052	0.085	1	0.998	0.977	------	------	------	------	------	------	ENOG4110T03;E	S:Function unknown;	PF00151.22	Lipase	Lipase	CYT	4	45	36.1	High
TRINITY_DN6140_c0_g3_i3_orf1	CD63 antigen-like [Ostrinia furnacalis]	11.76625	3.55658269	1.91E-05	up	yes	0.9413	0.08	0.051	0.095	0.094	1	0.884	0.94	CC:cellular_component;CC:integral component of membrane;CC:cellular anatomical entity;CC:intrinsic component of membrane;	GO:0005!	K06497	CD63, MLA1,	map05205;m	Proteoglycans in ca	ENOG4111IRY	O:Posttranslational modification,	PF00335.23	Tetraspanin	Tetraspanin family	CYT	1	4	32.2	High
TRINITY_DN10530_c0_g1_i1_orf1	cytochrome c oxidase subunit NDUFA4 [Ostrinia furnacalis]	2.528026619	1.338011654	7.30E-06	up	yes	0.9877	0.3907	0.374	0.37	0.428	1	0.986	0.977	CC:cellular_component;CC:integral component of membrane;CC:cellular anatomical entity;CC:intrinsic component of membrane;	GO:0005!	K03948	NDUFA4	map04714;m	Thermogenesis;Oxi	ENOG41124SW	U:Intracellular trafficking, secretic	PF06522.14	B12D	NADH-ubiquinone reductase complex 1 MLRO subunit	CYT	5	48	9.2	High
TRINITY_DN3062_c0_g1_i1_orf1	HEAT repeat-containing protein 1 [Ostrinia furnacalis]	2.784984819	1.477669464	9.78E-06	up	yes	1.009	0.3623	0.337	0.343	0.407	1	1.007	1.02	BP:nucleic acid metabolic process;BP:cellular nitrogen compound metabolic process;BP:nitrogen compound metabolic process;BP:ncRNA metabolic process;BP:organic cyclic compound metabolic process;BP:nucleobase-containing compound metabolic process;BP:organic substance metabolic process;BP:rRNA processing;BP:ncRNA processing;BP:cellular process;BP:cellular aromatic compound metabolic process;BP:biological_process;BP:metabolic process;BP:heterocycle metabolic process;BP:RNA metabolic process;BP:primary metabolic process;BP:rRNA metabolic process;BP:cellular metabolic process;BP:macromolecule metabolic process;BP:RNA processing;CC:organelle;CC:nucleolus;CC:cellular anatomical entity;CC:macromolecular complex;CC:intracellular non-membrane-bounded organelle;CC:ribonucleoprotein complex;CC:intracellular	GO:0090:	K14550	UTP10, HEAT	map03008	Ribosome biogenes	ENOG410XNQQ	J:Translation, ribosomal structure	PF08146.15;PF12	BP28CT;U3snoRNP10	BP28CT;U3 small nucleolar RNA-associated protein 10	CYT	4	2	240.8	High
TRINITY_DN2243_c0_g1_i4_orf1	WD repeat-containing protein 75 [Ostrinia furnacalis]	3.214285714	1.684498174	6.11E-06	up	yes	1.035	0.322	0.333	0.306	0.327	1	1.073	1.031	------	------	K14552	NAN1, UTP1	map03008	Ribosome biogenes	ENOG410YY9G	S:Function unknown	------	------	------	CYT	1	1	99.1	Medium
TRINITY_DN15624_c0_g1_i1_orf1	LOW QUALITY PROTEIN: V-type proton ATPase subunit S1-like [Ostrinia furnacalis]	3.839285714	1.940837928	5.33E-05	up	yes	1.075	0.28	0.237	0.31	0.293	1	1.118	1.107	------	------	K03662	ATPeVS1, AT	map05152;m	Tuberculosis;Huma	ENOG410XTHY	U:Intracellular trafficking, secretic	PF20520.1;PF058	Ac45-VOA1_TM;VAS1_LD	V0 complex accessory subunit Ac45/VOA1 transmembrane domain;V-type proton ATPase subunit S1,	CYT	3	9	45.2	High
TRINITY_DN30233_c0_g1_i2_orf1	39S ribosomal protein L10, mitochondrial [Ostrinia furnacalis]	2.450217558	1.292909854	2.42E-05	up	yes	0.9573	0.3907	0.364	0.409	0.399	1	0.931	0.941	CC:ribosome;CC:intracellular non-membrane-bounded organelle;CC:cellular_component;CC:intracellular organelle;CC:non-membrane-bounded organelle;CC:organelle;CC:cellular anatomical entity;	GO:0005{	K02864	RP-L10, MRF	map03010	Ribosome	ENOG4111GA4	J:Translation, ribosomal structure	------	------	------	CYT	1	2	27.9	High
TRINITY_DN54543_c0_g5_i2_orf1	reactive oxygen species modulator 1 [Papilio machaon] >XP_022123076.1 reactive oxygen species modulator 1 [Pieris rapae] >XP_022820703.1 reactive oxygen species modulator 1 [Spodoptera litura] >XP_028176790.1 reactive oxygen species modulator 1 [Ostrinia furnacalis] >XP_030021418.1 reactive oxygen species modulator 1 [Manduca sexta] >XP_034836896.1 reactive oxygen species modulator 1 [Maniola hyperantus] >XP_035446679.1 reactive oxygen species modulator 1-like [Spodoptera frugiperda] >XP_045458971.1 reactive oxygen species modulator 1 [Melitaea cinxia] >XP_045521377.1 reactive oxygen species modulator 1 [Pieris brassicae] >XP_045765649.1 reactive oxygen species modulator 1 [Maniola jurtina] >XP_047042173.1 reactive oxygen species modulator 1 [Helicoverpa zea] >XP_049691668.1 reactive oxygen species modulator 1 [Helicoverpa armigera] >KAF9414699.1 hypothetical protein HW555_007477 [Spodoptera exigua] >KPI93862.1 Reactive oxygen species modulator 1 [Papilio xuthus] >CAB3515149.1 unnamed protein product [Spodoptera littoralis] >CAG5021695.1 unnamed protein product [Parnassius apollo] >KAF9823017.1	3.159375	1.659639187	0.0001815	up	yes	1.011	0.32	0.352	0.251	0.357	1	0.95	1.082	CC:cellular_component;CC:integral component of membrane;CC:cellular anatomical entity;CC:intrinsic component of membrane;	GO:0005!	------	------	------	------	ENOG411279J	S:Function unknown	PF10247.12	Romo1	Reactive mitochondrial oxygen species modulator 1	CYT	1	23	8.2	High

TRINITY_DN3082_c1_g1_i7_orf1	ribosomal RNA processing protein 1 homolog [Ostrinia furnacalis]	4.636634084	2.213077876	5.19E-05	up	yes	1.091	0.2353	0.219	0.254	0.233	1	1.138	1.135	BP:nucleic acid metabolic process;BP:cellular nitrogen compound metabolic process;BP:nitrogen compound metabolic process;BP:ncRNA metabolic process;BP:organic cyclic compound metabolic process;BP:nucleobase-containing compound metabolic process;BP:organic substance metabolic process;BP:rRNA processing;BP:ncRNA processing;BP:cellular process;BP:cellular aromatic compound metabolic process;BP:biological_process;BP:metabolic process;BP:heterocycle metabolic process;BP:RNA metabolic process;BP:primary metabolic process;BP:rRNA metabolic process;BP:cellular metabolic process;BP:macromolecule metabolic process;BP:RNA processing;CC:preribosome, small subunit precursor;CC:preribosome;CC:macromol	GO:00903	K14849	RRP1	------	------	ENOG4111GJ6	J:Translation, ribosomal structure	PF05997.15	Nop52	Nucleolar protein,Nop52	CYT	1	1	84.5	Medium
TRINITY_DN110888_c0_g1_i2_orf1	uncharacterized protein LOC114364502 [Ostrinia furnacalis]	2.890226005	1.531182311	1.37E-05	up	yes	0.9847	0.3407	0.343	0.358	0.321	1	0.94	1.014	------	------	------	------	------	------	ENOG410XU1K	S:Function unknown	------	------	------	CYT	1	4	33.8	High
TRINITY_DN1318_c0_g1_i5_orf1	uncharacterized protein LOC114360956 [Ostrinia furnacalis]	2.995126409	1.582616893	2.05E-05	up	yes	0.9833	0.3283	0.316	0.308	0.361	1	1.012	0.938	CC:cellular_component;CC:integral component of membrane;CC:cellular anatomical entity;CC:intrinsic component of membrane;	GO:00055	------	------	------	------	ENOG4110SBJ;C	S:Function unknown;	PF13855.9;PF127	LRR_8;LRR_4	Leucine rich repeat;Leucine Rich repeats	CYT	5	21	47.9	High
TRINITY_DN2918_c0_g1_i1_orf1	28S ribosomal protein S10, mitochondrial [Ostrinia furnacalis] >XP_028175147.1 28S ribosomal protein S10, mitochondrial [Ostrinia furnacalis]	3.023921939	1.596420898	4.86E-05	up	yes	0.9607	0.3177	0.286	0.3	0.367	1	0.964	0.918	------	------	K02946	RP-S10, MRF	map03010	Ribosome	COG0051	J:Translation, ribosomal structure	PF00338.25	Ribosomal_S10	Ribosomal protein S10p/S20e	CYT	1	5	19.9	High
TRINITY_DN11347_c0_g1_i1_orf1	N(4)-(Beta-N-acetylglucosaminyl)-L-asparaginase-like [Ostrinia furnacalis]	4.363016617	2.125325968	3.79E-05	up	yes	1.024	0.2347	0.209	0.201	0.294	1	1.077	0.996	MF:hydrolase activity;MF:molecular_function;MF:catalytic activity;	GO:00167	K01444	AGA, aspG	map00511;m	Other glycan degra	COG1446	E:Amino acid transport and meta	PF01112.21	Asparaginase_2	Asparaginase	CYT	4	13	37	High
TRINITY_DN39673_c0_g1_i1_orf1	uncharacterized protein LOC114359357 isoform X1 [Ostrinia furnacalis]	3.135509397	1.648699843	2.10E-05	up	yes	0.951	0.3033	0.299	0.296	0.315	1	0.948	0.905	------	------	------	------	------	------	------	------	PF07294.14	Fibroin_P25	Fibroin P25	PLA	1	4	27.9	High
TRINITY_DN18172_c0_g1_i6_orf1	digestive cysteine proteinase 2-like [Ostrinia furnacalis]	4.394403148	2.135667231	9.69E-08	up	yes	1.005	0.2287	0.226	0.218	0.242	1	0.999	1.015	BP:primary metabolic process;BP:organonitrogen compound metabolic process;BP:protein metabolic process;BP:macromolecule metabolic process;BP:organic substance metabolic process;BP:nitrogen compound metabolic process;BP:biological_process;BP:metabolic process;BP:proteolysis;MF:molecular_function;MF:hydrolase activity;MF:catalytic activity;MF:catalytic activity, acting on a protein;MF:peptidase	GO:00442	------	------	------	------	COG4870	O:Posttranslational modification,	PF00112.26;PF08	Peptidase_C1;Inhibitor_I29	Papain family cysteine protease;Cathepsin propeptide inhibitor domain	EXC	3	7	57.6	High
TRINITY_DN79319_c0_g1_i8_orfp1	TRINITY_DN79319_c0_g1_i8_m.49956 TRINITY_DN79319_c0_g1::TRINITY_DN79319_c0_g1_i8::g.49956 ORF type:5prime_partial len:84 (+),score=1.39 TRINITY_DN79319_c0_g1_i8:1-252(+)	4.198895028	2.070009721	2.42E-06	up	yes	0.988	0.2353	0.22	0.217	0.269	1	0.993	0.971	------	------	------	------	------	------	------	------	------	------	------	CYT	2	20	9.6	High
TRINITY_DN21722_c0_g1_i3_orf1	V-type proton ATPase 116 kDa subunit a isoform X1 [Ostrinia furnacalis] >XP_028177509.1 V-type proton ATPase 116 kDa subunit a isoform X1 [Ostrinia furnacalis]	4.145385588	2.051506302	1.10E-06	up	yes	0.9837	0.2373	0.223	0.235	0.254	1	0.992	0.959	CC:vacuolar proton-transporting V-type ATPase, V0 domain;CC:intrinsic component of membrane;CC:cellular_component;CC:proton-transporting V-type ATPase, V0 domain;CC:integral component of membrane;CC:cellular anatomical entity;CC:macromolecular complex;CC:membrane protein complex;CC:proton-transporting two-sector ATPase complex, proton-transporting domain;MF:molecular_function;MF:ATPase activity, coupled to transmembrane movement of substances;MF:inorganic solute uptake transmembrane transporter activity;MF:hydrogen-translocating pyrophosphatase activity;MF:primary active transmembrane transporter activity;MF:ATPase coupled ion transmembrane transporter activity;MF:transporter activity;MF:cation transmembrane transporter activity;MF:proton-transporting ATPase activity, rotational mechanism;MF:ATPase activity, coupled to transmembrane movement of ions, rotational mechanism;MF:inorganic	GO:00002	K02154	ATPeV0A, AT	map05152;m	Tuberculosis;Rheun	COG1269	C:Energy production and convers	PF01496.22	V_ATPase_I	V-type ATPase 116kDa subunit family	CYT	11	20	95.5	High
TRINITY_DN96566_c0_g1_i1_orf1	NADH-ubiquinone oxidoreductase subunit 8-like [Ostrinia furnacalis]	3.188122211	1.672706933	0.0005877	up	yes	0.9287	0.2913	0.286	0.202	0.386	1	0.906	0.88	CC:membrane;CC:cellular_component;CC:cellular anatomical entity;MF:4 iron, 4 sulfur cluster binding;MF:oxidoreductase activity, acting on NA;MF:iron-sulfur cluster binding;MF:catalytic activity;MF:molecular_function;MF:binding;MF:oxidoreductase activity;MF:metal cluster binding;	GO:0016(	K03941	NDUFS8	map04714;m	Thermogenesis;Oxi	COG1143	C:Energy production and convers	PF12838.10;PF00	Fer4_7;Fer4;Fer4_10;Fer4_16;Fer4_9;Fer4_21;Fer4_8	4Fe-4S dicluster domain;4Fe-4S binding domain;4Fe-4S dicluster domain;4Fe-4S double cluster binding domain;4Fe-4S dicluster domain;4Fe-4S dicluster domain;4Fe-4S dicluster	CYT	1	4	28.6	High
TRINITY_DN2394_c0_g1_i4_orf1	uncharacterized protein LOC114363116 [Ostrinia furnacalis]	13.5	3.754887502	0.0001273	up	yes	1.134	0.084	0.075	0.07	0.107	1	1.241	1.161	MF:molecular_function;MF:lipid binding;MF:binding;	GO:0003(	K08752	FABP3	map03320	PPAR signaling pat	ENOG4111US8	I:Lipid transport and metabolism	PF14651.9	Lipocalin_7	Lipocalin / cytosolic fatty-acid binding protein family	CYT	6	33	18.8	High
TRINITY_DN3159_c0_g1_i4_orf1	uncharacterized protein LOC114362782 [Ostrinia furnacalis]	4.465493911	2.158819755	3.19E-07	up	yes	0.99	0.2217	0.21	0.214	0.241	1	0.992	0.978	BP:response to host;BP:response to defenses of other organism involved in symbiotic interaction;BP:response to biotic stimulus;BP:response to external stimulus;BP:response to external biotic stimulus;BP:evasion or tolerance of host defense response;BP:response to stimulus;BP:interspecies interaction between organisms;BP:biological_process;BP:symbiosis, encompassing mutualism through parasitism;BP:interaction with host;BP:response to host defenses;BP:response to other organism;MF:pigment binding;MF:molecular_function;MF:binding;	GO:00751	K18271	RBP4	------	------	COG3040	M:Cell wall/membrane/envelope	PF00061.26;PF08	Lipocalin;Lipocalin_2;Triabin	Lipocalin / cytosolic fatty-acid binding protein family;Lipocalin-like domain;Triabin	CYT	22	10	323.1	High
TRINITY_DN6074_c0_g1_i1_orf1	uncharacterized protein C1683.06c-like isoform X1 [Ostrinia furnacalis]	5.098445596	2.350057468	8.28E-07	up	yes	0.984	0.193	0.18	0.185	0.214	1	0.989	0.963	MF:hydrolase activity;MF:molecular_function;MF:hydrolase activity, hydrolyzing N-glycosyl compounds;MF:hydrolase activity, acting on glycosyl bonds;MF:catalytic activity;	GO:00167	------	------	------	------	COG1957;COG1	F:Nucleotide transport and metal	PF01156.22	IU_nuc_hydro	Inosine-uridine preferring nucleoside hydrolase	CYT	2	5	38.9	High
TRINITY_DN20682_c0_g2_i1_orf1	glutathione S-transferase delta3 [Glyphodes pyloalis]	5.479005525	2.453914058	1.11E-05	up	yes	0.9917	0.181	0.149	0.16	0.234	1	0.965	1.01	MF:catalytic activity;MF:transferase activity;MF:glutathione transferase activity;MF:molecular_function;MF:transferase activity, transferring alkyl or ary;	GO:00038	K00799	GST, gst	map05207;m	Chemical carcinoge	COG0625	O:Posttranslational modification,	PF13417.9;PF027	GST_N_3;GST_N;GST_N_2	Glutathione S-transferase, N-terminal domain;Glutathione S-transferase, N-terminal domain;Glutathione S-transferase, N-terminal	PLA	1	8	14.1	Medium
TRINITY_DN8116_c0_g1_i2_orf1	uncharacterized protein LOC114350845 [Ostrinia furnacalis]	5.774753909	2.529759467	9.64E-08	up	yes	0.9973	0.1727	0.176	0.157	0.185	1	1.003	0.989	------	------	------	------	------	------	COG1028	S:Function unknown	PF13561.9;PF001	adh_short_C2;adh_short;KR	Enoyl;short chain dehydrogenase:KR domain	CYT	6	37	26.1	High

TRINITY_DN7688_c0_g1_i10_orf1	uncharacterized protein LOC114352518 [Ostrinia furnacalis]	7.730769231	2.950611973	3.74E-05	up	yes	1.005	0.13	0.119	0.081	0.19	1	1.059	0.955	BP:phosphorylation;BP:cellular process;BP:cellular metabolic process;BP:phosphate-containing compound metabolic process;BP:biological_process;BP:metabolic process;BP:phosphorus metabolic process;MF:kinase activity;MF:transferase activity;MF:catalytic activity;MF:molecular_function;MF:transferase activity, transferring phosphorus-	GO:0016:	------	------	------	------	ENOG410YKCN;I	S:Function unknown;	PF02958.23;PF07	EcKL;DUF1679	Ecdysteroid kinase-like family;Uncharacterized oxidoreductase dhs-27	CYT	1	10	47.9	High
TRINITY_DN4194_c0_g1_i1_orf1	hornerin-like [Ostrinia furnacalis]	2.47330519	1.30644027	0.002157	up	yes	0.996	0.4027	0.387	0.324	0.497	1	1.111	0.877	------	------	K19720;K	COL3A;COL5	map05146;m	Amoebiasis;Protein	ENOG4111IEE;EN	S:Function unknown;	PF16058.8	Mucin-like	Mucin-like	CYT	2	1	184.6	High
TRINITY_DN4895_c0_g1_i2_orf1	coiled-coil domain-containing protein 86 [Ostrinia furnacalis]	2.617746552	1.388325424	8.29E-06	up	yes	1.006	0.3843	0.374	0.424	0.355	1	1.017	1	CC:intracellular membrane-bounded organelle;CC:nucleus;CC:cellular_component;CC:intracellular organelle;CC:membrane-bounded organelle;CC:organelle;CC:cellular anatomical entity;	GO:0043:	K14822	CGR1	------	------	ENOG41129IK	S:Function unknown	PF03879.17	Cgr1	Cgr1 family	CYT	1	8	19.7	High
TRINITY_DN3598_c0_g1_i1_orf1	esterase FE4-like [Ostrinia furnacalis]	2.65171504	1.406925748	5.24E-06	up	yes	1.005	0.379	0.36	0.36	0.417	1	1.007	1.008	MF:hydrolase activity;MF:molecular_function;MF:hydrolase activity, acting on ester bonds;MF:carboxylic ester hydrolase activity;MF:catalytic activity;	GO:0016:	------	------	------	------	COG2272	T:Signal transduction mechanism	PF00135.31;PF20	COesterase;BD-FAE;Abhydrolase_3	Carboxylesterase family;BD-FAE;alpha/beta hydrolase fold	CYT	4	10	61.6	High
TRINITY_DN76283_c0_g2_i1_orf1	fatty acid synthase-like [Ostrinia furnacalis]	2.67321476	1.418575745	1.76E-06	up	yes	1.007	0.3767	0.395	0.362	0.373	1	1.029	0.993	BP:fatty acid biosynthetic process;BP:fatty acid metabolic process;BP:carboxylic acid metabolic process;BP:cellular biosynthetic process;BP:small molecule metabolic process;BP:small molecule biosynthetic process;BP:monocarboxylic acid biosynthetic process;BP:organic substance biosynthetic process;BP:organic substance metabolic process;BP:lipid metabolic process;BP:cellular process;BP:monocarboxylic acid metabolic process;BP:biosynthetic process;BP:biological_process;BP:metabolic process;BP:oxoacid metabolic process;BP:cellular lipid metabolic process;BP:lipid biosynthetic process;BP:primary metabolic process;BP:organic acid metabolic process;BP:carboxylic acid biosynthetic process;BP:organic acid biosynthetic process;BP:cellular metabolic process;MF:molecular_function;MF:oxidoreductase activity;MF:transferase	GO:0006	K00665	FASN	map04910;m	Insulin signaling pa	COG3321	Q:Secondary metabolites biosynt	PF00698.24	Acyl_transf_1	Acyl transferase domain	CYT	3	8	32	High
TRINITY_DN27848_c0_g1_i2_orf1	cystathionine beta-synthase-like [Ostrinia furnacalis] >XP_028159011.1 cystathionine beta-synthase-like [Ostrinia furnacalis]	2.7675	1.468583317	1.16E-06	up	yes	0.9963	0.36	0.347	0.348	0.385	1	1.002	0.987	BP:carboxylic acid metabolic process;BP:cellular biosynthetic process;BP:nitrogen compound metabolic process;BP:small molecule metabolic process;BP:small molecule biosynthetic process;BP:serine family amino acid metabolic process;BP:cysteine metabolic process;BP:L-serine metabolic process;BP:cellular amino acid metabolic process;BP:organic substance metabolic process;BP:cysteine biosynthetic process via cystathionine;BP:cellular amino acid biosynthetic process;BP:alpha-amino acid metabolic process;BP:sulfur amino acid metabolic process;BP:organic substance biosynthetic process;BP:sulfur amino acid biosynthetic process;BP:cellular process;BP:biosynthetic process;BP:biological_process;BP:metabolic process;BP:oxoacid metabolic process;BP:serine family amino acid biosynthetic process;BP:primary metabolic process;BP:organonitrogen compound metabolic process;BP:organonitrogen compound biosynthetic process;BP:organic acid metabolic process;BP:sulfur compound	GO:0019	K01697	CBS	map00270;m	Cysteine and meth	COG0031	E:Amino acid transport and meta	PF00291.28	PALP	Pyridoxal-phosphate dependent enzyme	CYT	12	31	54.7	High
TRINITY_DN15900_c0_g1_i6_orf1	unnamed protein product [Diatraea saccharalis]	3.266025641	1.707536117	0.0002457	up	yes	1.019	0.312	0.246	0.31	0.38	1	1.1	0.958	BP:RNA metabolic process;BP:mRNA metabolic process;BP:primary metabolic process;BP:nucleobase-containing compound metabolic process;BP:cellular process;BP:cellular aromatic compound metabolic process;BP:cellular metabolic process;BP:macromolecule metabolic process;BP:nucleic acid metabolic process;BP:organic substance metabolic process;BP:cellular nitrogen compound metabolic process;BP:nitrogen compound metabolic process;BP:biological_process;BP:organic cyclic compound metabolic process;BP:metabolic process;BP:heterocycle metabolic process;BP:RNA processing;BP:mRNA processing;	GO:0016	K13171	SRRM1, SRM	map03013;m	Nucleocytoplasmic	ENOG4111IMU	S:Function unknown	PF01480.20	PWI	PWI domain	CYT	2	2	111.5	High
TRINITY_DN23734_c0_g1_i1_orf1	histone-lysine N-methyltransferase SMYD3 [Ostrinia furnacalis]	2.300506329	1.201951426	0.003571	up	yes	0.9087	0.395	0.493	0.364	0.328	1	0.778	0.948	CC:cellular_component;CC:intracellular organelle;CC:organelle;CC:cellular anatomical entity;MF:cation binding;MF:ion binding;MF:molecular_function;MF:bindi	GO:0005	K11426	SMYD	map00310	Lysine degradation	COG2940	S:Function unknown	PF01753.21	zf-MYND	MYND finger	CYT	1	1	56.4	Medium
TRINITY_DN747_c0_g2_i1_orf1	trypsin, alkaline C-like [Ostrinia furnacalis]	2.213702696	1.146461479	0.001988	up	yes	0.895	0.4043	0.317	0.449	0.447	1	0.843	0.842	BP:organic substance metabolic process;BP:organonitrogen compound metabolic process;BP:protein metabolic process;BP:macromolecule metabolic process;BP:biological_process;BP:nitrogen compound metabolic process;BP:metabolic process;BP:proteolysis;BP:primary metabolic process;MF:serine-type endopeptidase activity;MF:endopeptidase activity;MF:hydrolase activity;MF:serine hydrolase activity;MF:catalytic activity, acting on a protein;MF:catalytic activity;MF:molecular_function;MF:pepti	GO:0071	K09628;K	PRSS27;TMP	------	------	COG5640	O:Posttranslational modification,	PF00089.29	Trypsin	Trypsin	CYT	1	5	30.5	High
TRINITY_DN96170_c0_g2_i1_orf1	uncharacterized protein LOC114355569 [Ostrinia furnacalis]	3.01933271	1.594229741	1.16E-05	up	yes	0.9683	0.3207	0.337	0.325	0.3	1	0.927	0.978	BP:phosphorylation;BP:cellular process;BP:biological_process;BP:metabolic process;BP:cellular metabolic process;BP:phosphate-containing compound metabolic process;BP:phosphorus metabolic process;CC:intrinsic component of membrane;CC:integral component of membrane;CC:cellular_component;CC:cellular anatomical entity;MF:purine ribonucleoside triphosphate binding;MF:molecular_function;MF:binding;MF:ion binding;MF:heterocyclic compound binding;MF:anion binding;MF:nucleotide binding;MF:transferase activity;MF:nucleoside phosphate binding;MF:purine nucleotide binding;MF:ATP binding;MF:kinase activity;MF:small molecule binding;MF:catalytic activity;MF:adenyl nucleotide binding;MF:carbohydrate derivative binding;MF:transferase activity, transferring phosphorus-containing groups;MF:adenyl ribonucleotide binding;MF:purine ribonucleotide binding;MF:organic cyclic compound binding;MF:ribonucleotide	GO:0016	------	------	------	------	COG0574	G:Carbohydrate transport and m	PF00391.26	PEP-utilizers	PEP-utilising enzyme, mobile domain	CYT	1	4	31.7	High

TRINITY_DN1044_c0_g1_i2_orf1	V-type proton ATPase subunit H isoform X3 [Ostrinia furnacalis] >QRR19186.1 V-type proton ATPase subunit H [Ostrinia nubilalis]	4.357353561	2.12345218	1.42E-05	up	yes	1.034	0.2373	0.232	0.198	0.282	1	1.068	1.034	CC:vacuolar proton-transporting V-type ATPase, V1 domain;CC:proton-transporting V-type ATPase, V1 domain;CC:proton-transporting two-sector ATPase complex, catalytic domain;CC:macromolecular complex;CC:cellular_component;CC:membrane protein complex;MF:molecular_function;MF:ion transmembrane transporter activity;MF:ATPase activity, coupled to transmembrane movement of substances;MF:inorganic solute uptake transmembrane transporter activity;MF:hydrogen-translocating pyrophosphatase activity;MF:primary active transmembrane transporter activity;MF:active transmembrane transporter activity;MF:ATPase coupled ion transmembrane transporter activity;MF:transporter activity;MF:cation transmembrane transporter activity;MF:proton-transporting ATPase activity, rotational mechanism;MF:ATPase activity, coupled to transmembrane movement of ions, rotational mechanism;MF:inorganic cation transmembrane transporter activity;MF:cation-transporting ATPase	GO:00002	K02144	ATPeV1H	map05152;m	Tuberculosis;Huma	COG5231	C:Energy production and conver	PF03224.17;PF11	V-ATPase_H_N;V-ATPase_H_C;Arm_2	V-ATPase subunit H;V-ATPase subunit H;Armadillo-like	CYT	15	38	55.2	High
TRINITY_DN17864_c0_g1_i1_orf1	PREDICTED: erlin-2-B [Microplitis demolitor]	2.931034483	1.551409941	0.0002825	up	yes	0.935	0.319	0.281	0.305	0.371	1	0.954	0.851	CC:endoplasmic reticulum;CC:integral component of membrane;CC:intracellular membrane-bounded organelle;CC:cellular_component;CC:intracellular organelle;CC:intrinsic component of membrane;CC:membrane-bounded organelle;CC:organelle;CC:cellular anatomical entity;MF:ubiquitin protein ligase binding;MF:ubiquitin-like protein ligase binding;MF:enzyme	GO:00057	K23341	ERLIN	------	------	ENOG410XQSH	O:Posttranslational modification,	PF01145.28	Band_7	SPFH domain / Band 7 family	CYT	1	2	37.3	Medium
TRINITY_DN49508_c0_g2_i8_orf1	putative fatty acyl-CoA reductase CG5065 [Ostrinia furnacalis]	3.82803298	1.936603259	1.51E-05	up	yes	0.975	0.2547	0.259	0.218	0.287	1	0.991	0.934	BP:primary metabolic process;BP:lipid metabolic process;BP:fatty acid derivative metabolic process;BP:organic substance metabolic process;BP:biological_process;BP:metabolic process;CC:cellular_component;CC:cellular anatomical entity;CC:integral component of membrane;CC:intrinsic component of membrane;MF:fatty-acyl-CoA reductas;MF:alcohol-forming fatty acyl-CoA reductase activity;MF:catalytic activity;MF:oxidoreductase activity, acting on the aldehyde or oxo group of donors, NAD or NADP as acceptor;MF:oxidoreductase activity, acting on the aldehyde or oxo group of donors;MF:molecular_function;MF:oxidoreductase activity;	GO:00442	K13356	FAR	map04146;m	Peroxisome;Cutin, s	ENOG410XS7R	S:Function unknown	PF07993.15;PF03	NAD_binding_4;Sterile;Epimerase;GDP_Man_Dehyd	Male sterility protein;Male sterility protein;NAD dependent epimerase/dehydratase family;GDP-mannose 4,6 dehydratase	CYT	1	2	60.6	High
TRINITY_DN4731_c0_g1_i1_orf1	gelsolin-like [Ostrinia furnacalis]	4.945812808	2.306207637	1.72E-07	up	yes	1.004	0.203	0.199	0.194	0.216	1	1.019	0.992	------	------	K05768	GSN	map04666;m	Fc gamma R-medi	ENOG410XR0A	O:Posttranslational modification,	PF00626.25	Gelsolin	Gelsolin repeat	CYT	7	15	72	High
TRINITY_DN67623_c0_g1_i1_orf1	maltase A1-like [Ostrinia furnacalis]	5.515548282	2.463504306	5.82E-08	up	yes	1.011	0.1833	0.18	0.178	0.192	1	1.024	1.01	BP:primary metabolic process;BP:carbohydrate metabolic process;BP:organic substance metabolic process;BP:biological_process;BP:metabolic process;CC:integral component of membrane;CC:cellular_component;CC:intrinsic component of membrane;CC:cellular anatomical entity;MF:hydrolase activity;MF:hydrolase activity, acting on glycosyl bonds;MF:catalytic	GO:00442	------	------	------	------	COG0366	G:Carbohydrate transport and m	PF00128.27	Alpha-amylase	Alpha amylase, catalytic domain	CYT	4	9	68.6	High
TRINITY_DN2343_c1_g1_i8_orf1	receptor expression-enhancing protein 5-like isoform X3 [Ostrinia furnacalis]	6.403726708	2.678911739	2.90E-05	up	yes	1.031	0.161	0.134	0.137	0.212	1	1.095	0.998	CC:cellular_component;CC:integral component of membrane;CC:cellular anatomical entity;CC:intrinsic component of membrane;	GO:00055	K17279	REEP5_6	------	------	COG5052	U:Intracellular trafficking, secretic	PF03134.22	TB2_DP1_HVA22	TB2/DP1, HVA22 family	CYT	1	21	22.4	High
TRINITY_DN22983_c0_g1_i2_orfp1	TRINITY_DN22983_c0_g1_i2_m.10495 TRINITY_DN22983_c0_g1::TRINITY_DN22983_c0_g1_i2::g.10495 ORF type:internal len:79 (-),score=15.95,Polyhedrin PF00738.19 7.6e-40 TRINITY_DN22983_c0_g1_i2:2-235(-)	4.884749131	2.288284471	1.10E-06	up	yes	0.9833	0.2013	0.187	0.188	0.229	1	0.973	0.977	------	------	------	------	------	------	------	------	PF00738.21	Polyhedrin	Polyhedrin	CYT	3	21	9.2	High
TRINITY_DN64403_c0_g2_i1_orf1	carboxylesterase [Ostrinia furnacalis]	5.481419856	2.454549644	1.29E-06	up	yes	0.9883	0.1803	0.173	0.158	0.21	1	0.973	0.992	MF:hydrolase activity;MF:molecular_function;MF:hydrolase activity, acting on ester bonds;MF:carboxylic ester hydrolase activity;MF:catalytic activity;	GO:00167	K03927	CES2	map00983	Drug metabolism -	COG2272	K:Transcription	PF00135.31	COesterase	Carboxylesterase family	CYT	1	14	10.6	High
TRINITY_DN1249_c0_g1_i10_orf1	venom carboxylesterase-6-like [Ostrinia furnacalis]	5.322282609	2.412045118	1.97E-05	up	yes	0.9793	0.184	0.15	0.161	0.241	1	0.995	0.943	MF:hydrolase activity;MF:molecular_function;MF:hydrolase activity, acting on ester bonds;MF:carboxylic ester hydrolase activity;MF:catalytic activity;	GO:00167	------	------	------	------	COG2272;ENOG	I:Lipid transport and metabolism	PF00135.31;PF20	COesterase;BD-FAE;Abhydrolase_3	Carboxylesterase family;BD-FAE;alpha/beta hydrolase fold	PLA	1	6	55.8	High
TRINITY_DN542_c0_g2_i1_orf1	uncharacterized protein LOC114364889 [Ostrinia furnacalis]	5.806261075	2.537609443	4.95E-06	up	yes	0.983	0.1693	0.167	0.157	0.184	1	1.012	0.937	BP:amino sugar catabolic process;BP:nitrogen compound metabolic process;BP:aminoglycan metabolic process;BP:organic substance catabolic process;BP:glucosamine-containing compound catabolic process;BP:organic substance metabolic process;BP:chitin metabolic process;BP:polysaccharide catabolic process;BP:biological_process;BP:metabolic process;BP:organonitrogen compound metabolic process;BP:catabolic process;BP:macromolecule catabolic process;BP:primary metabolic process;BP:chitin catabolic process;BP:carbohydrate metabolic process;BP:organonitrogen compound catabolic process;BP:polysaccharide metabolic process;BP:amino sugar metabolic process;BP:carbohydrate derivative catabolic process;BP:carbohydrate catabolic process;BP:macromolecule metabolic process;BP:glucosamine-containing compound metabolic process;BP:aminoglycan catabolic process;BP:carbohydrate derivative metabolic process;CC:extracellular space;CC:cellular anatomical	GO:00463	------	------	------	------	ENOG410ZWU3	S:Function unknown	PF01522.24	Polysacc_deac_1	Polysaccharide deacetylase	PLA	4	16	44.5	High
TRINITY_DN30932_c0_g1_i2_orf1	delta(24)-sterol reductase-like isoform X2 [Ostrinia furnacalis]	5.37364518	2.425901064	4.96E-05	up	yes	0.942	0.1753	0.174	0.128	0.224	1	0.93	0.896	CC:microbody;CC:intracellular organelle;CC:membrane-bounded organelle;CC:organelle;CC:integral component of membrane;CC:peroxisome;CC:intrinsic component of membrane;CC:cellular anatomical entity;CC:intracellular membrane-bounded organelle;CC:cellular_component;MF:heterocyclic compound binding;MF:molecular_function;MF:binding;MF:FAD binding;MF:ion binding;MF:nucleoside phosphate binding;MF:oxidoreductase activity;MF:anion binding;MF:flavin adenine dinucleotide binding;MF:oxidoreductase activity, acting on the CH-CH group of donors, NAD or NADP as acceptor;MF:nucleotide binding;MF:small molecule binding;MF:catalytic activity;MF:delta24-sterol reductase activity;MF:organic cyclic compound	GO:00425	K09828	DHCR24, DW	map00100	Steroid biosynthesi	COG0277	O:Posttranslational modification,	PF01565.26	FAD_binding_4	FAD binding domain	CYT	1	2	59.2	High

TRINITY_DN311_c0_g1_i4_orfp1	TRINITY_DN311_c0_g1_i4_m.65135 TRINITY_DN311_c0_g1::TRINITY_DN311_c0_g1_i4::g.65135 ORF type:5prime_partial len:126 (+),score=71.21 TRINITY_DN311_c0_g1_i4:1-378(+)	8.765178571	3.131783483	1.08E-05	up	yes	0.9817	0.112	0.09	0.074	0.172	1	0.966	0.979	------	------	------	------	------	------	------	------	------	------	------	EXC	1	6	13.8	High
TRINITY_DN4959_c0_g1_i1_orf1	pancreatic triacylglycerol lipase-like [Ostrinia furnacalis]	26.12992018	4.707630812	9.54E-06	up	yes	0.9493	0.03633	0.026	0.01	0.073	1	0.937	0.911	------	------	------	------	------	------	ENOG410YGS3;E	S:Function unknown;	PF00151.22	Lipase	Lipase	CYT	1	7	37.1	High
TRINITY_DN1445_c0_g1_i1_orf1	leucine-rich PPR motif-containing protein, mitochondrial [Ostrinia furnacalis]	2.603656966	1.380539385	5.50E-06	up	yes	1.011	0.3883	0.355	0.391	0.419	1	1.017	1.015	------	------	K17964	LRPPRC	------	------	ENOG410XSG9	S:Function unknown	PF01535.23;PF13	PPR;PPR_2;PPR_3;PPR_long;PPR_1	PPR repeat;PPR repeat family;Pentatricopeptide repeat domain;Pentacotripeptide-repeat region of PRORP;PPR	CYT	9	14	87.6	High
TRINITY_DN107261_c0_g1_i1_orf1	ATP synthase subunit g, mitochondrial [Ostrinia furnacalis]	2.085266181	1.060231553	8.03E-05	up	yes	0.944	0.4527	0.447	0.445	0.466	1	0.902	0.93	BP:nucleoside triphosphate metabolic process;BP:nucleoside triphosphate biosynthetic process;BP:purine nucleoside triphosphate metabolic process;BP:purine nucleoside triphosphate biosynthetic process;BP:cellular biosynthetic process;BP:cellular nitrogen compound metabolic process;BP:nucleotide biosynthetic process;BP:small molecule metabolic process;BP:purine-containing compound metabolic process;BP:purine-containing compound biosynthetic process;BP:organic cyclic compound biosynthetic process;BP:ribonucleotide metabolic process;BP:organic cyclic compound metabolic process;BP:nucleobase-containing compound metabolic process;BP:ribonucleoside triphosphate biosynthetic process;BP:organic substance metabolic process;BP:ribonucleoside triphosphate metabolic process;BP:heterocycle biosynthetic process;BP:purine ribonucleoside triphosphate biosynthetic process;BP:purine ribonucleoside triphosphate metabolic	GO:00091	K02140	ATPeFG, ATP	map00190;m	Oxidative phospho	ENOG4111TZ1	I:Lipid transport and metabolism	PF04718.18	ATP-synt_G	Mitochondrial ATP synthase g subunit	CYT	3	30	10.7	High
TRINITY_DN85319_c0_g1_i1_orf1	cholinesterase 2-like [Ostrinia furnacalis]	2.593264249	1.374769222	1.36E-05	up	yes	1.001	0.386	0.378	0.357	0.423	1	1.025	0.977	MF:hydrolase activity;MF:molecular_function;MF:hydrolase activity, acting on ester bonds;MF:carboxylic ester hydrolase activity;MF:catalytic activity;	GO:00167	K12298	CEL	map04972;m	Pancreatic secretio	COG2272	I:Lipid transport and metabolism	PF00135.31;PF20	COesterase;BD-FAE;Abhydrolase_3	Carboxylesterase family;BD-FAE;alpha/beta hydrolase fold	CYT	9	18	61.4	High
TRINITY_DN49265_c0_g3_i2_orf1	cytochrome c [Ostrinia furnacalis] >XP_028160278.1 cytochrome c [Ostrinia furnacalis]	2.491624365	1.317086586	7.04E-06	up	yes	0.9817	0.394	0.376	0.378	0.428	1	0.975	0.97	CC:organelle lumen;CC:organelle envelope lumen;CC:membrane-enclosed lumen;CC:cellular_component;CC:intracellular organelle lumen;CC:mitochondrial intermembrane space;CC:respiratory chain;CC:cellular anatomical entity;MF:oxidoreductase activity;MF:cation binding;MF:heme binding;MF:organic cyclic compound binding;MF:tetrapyrrole binding;MF:ion binding;MF:molecular_function;MF:binding;MF:catalytic activity;MF:metal ion binding;MF:heterocyclic compound	GO:00432	K08738	CYC	map05167;m	Kaposi sarcoma-as	COG3474	C:Energy production and convers	PF00034.24;PF13	Cytochrom_C;Cytochrome_CBB3	Cytochrome c;Cytochrome C oxidase, cbb3-type, subunit III	PLA	7	50	11.8	High
TRINITY_DN6747_c0_g1_i7_orf1	retinol dehydrogenase 12-like [Ostrinia furnacalis]	2.55668491	1.354274372	7.65E-06	up	yes	0.981	0.3837	0.368	0.365	0.418	1	0.964	0.979	------	------	K11153	RDH12	map00830;m	Retinol metabolism	COG1028	S:Function unknown	PF00106.28;PF13	adh_short;adh_short_C2;KR;Epimerase	short chain dehydrogenase;Enoyl;KR domain;NAD dependent epimerase/dehydratase family	CYT	3	8	34.2	High
TRINITY_DN56110_c0_g1_i1_orf1	pescadillo homolog [Ostrinia furnacalis]	3	1.584962501	4.84E-06	up	yes	1.017	0.339	0.318	0.328	0.371	1	1.041	1.011	BP:maturation of 5.8S rRNA from tricistronic rRNA transcrip;BP:nucleic acid metabolic process;BP:maturation of LSU-rRNA;BP:ncRNA metabolic process;BP:organic cyclic compound metabolic process;BP:nucleobase-containing compound metabolic process;BP:organic substance metabolic process;BP:rRNA processing;BP:ncRNA processing;BP:cellular process;BP:cellular aromatic compound metabolic process;BP:maturation of 5.8S rRNA;BP:maturation of LSU-rRNA from tricistronic rRNA transcrip;BP:biological_process;BP:metabolic process;BP:heterocycle metabolic process;BP:RNA metabolic process;BP:primary metabolic process;BP:rRNA metabolic process;BP:cellular nitrogen compound metabolic process;BP:cellular metabolic process;BP:macromolecule metabolic process;BP:nitrogen compound metabolic process;BP:RNA processing;CC:preribosome, large subunit precursor;CC:preribosome;CC:intracellular organelle;CC:non-membrane-bounded	GO:0000	K14843	PES1, NOP7	------	------	COG5163	J:Translation, ribosomal structure	PF06732.14;PF16	Pescadillo_N;BRCT_2	Pescadillo N-terminus;BRCT domain, a BRCA1 C-terminus domain	CYT	2	3	71.4	High
TRINITY_DN29229_c0_g1_i5_orfp1	TRINITY_DN29229_c0_g1_i5_m.11187 TRINITY_DN29229_c0_g1::TRINITY_DN29229_c0_g1_i5::g.11187 ORF type:internal len:133 (+),score=48.64 TRINITY_DN29229_c0_g1_i5:3-	2.15234102	1.105906679	0.0007424	up	yes	0.924	0.4293	0.373	0.495	0.42	1	0.867	0.905	------	------	------	------	------	------	------	------	------	------	------	CYT	1	10	14.2	High
TRINITY_DN50571_c1_g1_i1_orf1	WD repeat-containing protein 46 [Ostrinia furnacalis]	2.743163237	1.455840475	1.66E-05	up	yes	0.973	0.3547	0.335	0.35	0.379	1	0.989	0.93	CC:intracellular non-membrane-bounded organelle;CC:nucleolus;CC:cellular_component;CC:intracellular organelle;CC:non-membrane-bounded organelle;CC:organelle;CC:cellular	GO:00432	K14768	UTP7, WDR4	------	------	ENOG410XP5D	S:Function unknown	PF08149.14	BING4CT	BING4CT	CYT	1	2	65.3	High
TRINITY_DN37538_c0_g2_i1_orf1	esterase FE4-like [Ostrinia furnacalis]	3.958099514	1.984807885	1.98E-05	up	yes	1.058	0.2673	0.252	0.274	0.276	1	1.059	1.114	------	------	K07378	NLGN	map04514	Cell adhesion mole	COG2272	I:Lipid transport and metabolism	PF00135.31	COesterase	Carboxylesterase family	CYT	3	31	15.9	High
TRINITY_DN1277_c4_g1_i5_orf1	GTP cyclohydrolase 1 isoform X1 [Ostrinia furnacalis] >XP_028166842.1 GTP cyclohydrolase 1 isoform X1 [Ostrinia furnacalis]	3.345406651	1.742181588	2.55E-06	up	yes	1.016	0.3037	0.275	0.307	0.329	1	1.032	1.015	BP:cellular modified amino acid biosynthetic process;BP:dihydrobiopterin metabolic process;BP:polyol biosynthetic process;BP:polyol metabolic process;BP:diol metabolic process;BP:cellular biosynthetic process;BP:cellular nitrogen compound metabolic process;BP:diol biosynthetic process;BP:small molecule metabolic process;BP:small molecule biosynthetic process;BP:tetrahydrofolate metabolic process;BP:organic cyclic compound biosynthetic process;BP:tetrahydrofolate biosynthetic process;BP:organic cyclic compound metabolic process;BP:organic substance biosynthetic process;BP:folic acid-containing compound biosynthetic process;BP:7,8-dihydroneopterin 3'-triphosphate biosynthetic process;BP:pteridine-containing compound metabolic process;BP:pteridine-containing compound biosynthetic process;BP:organic substance metabolic process;BP:heterocycle biosynthetic process;BP:tetrahydrobiopterin biosynthetic process;BP:folic acid-containing compound metabolic process;BP:alcohol biosynthetic	GO:00423	K01495	GCH1, folE	map00790;m	Folate biosynthesis	COG0302	H:Coenzyme transport and meta	PF01227.25	GTP_cyclohydrol	GTP cyclohydrolase I	CYT	5	19	32.6	High

TRINITY_DN9119_c0_g1_i3_orf1	actin-binding Rho-activating protein [Helicoverpa armigera] >XP_047029227.1 actin-binding Rho-activating protein-like [Helicoverpa zea] >PZC85229.1 hypothetical protein B5X24_HaOG202414 [Helicoverpa armigera]	2.563315217	1.358010901	0.000311	up	yes	0.9433	0.368	0.421	0.317	0.366	1	0.962	0.868	BP:regulation of Rho protein signal transduction;BP:regulation of response to stimulus;BP:positive regulation of Ras protein signal transduction;BP:positive regulation of signaling;BP:positive regulation of Rho protein signal transduction;BP:regulation of signaling;BP:positive regulation of cell communication;BP:regulation of cell communication;BP:regulation of biological process;BP:regulation of signal transduction;BP:positive regulation of signal transduction;BP:biological regulation;BP:positive regulation of biological process;BP:positive regulation of small GTPase mediated signal transduction;BP:regulation of small GTPase mediated signal transduction;BP:positive regulation of response to stimulus;BP:regulation of Ras protein signal transduction;BP:regulation of cellular process;BP:biological_process;BP:positive regulation of intracellular signal transduction;BP:regulation of intracellular signal transduction;BP:positive regulation of cellular	GO:0035	------	------	------	------	ENOG410XR7V	S:Function unknown	PF14705.9	Costars	Costars	CYT	1	4	21.7	High
TRINITY_DN13221_c0_g1_i3_orf1	fasciclin-3-like [Ostrinia furnacalis]	3.170028818	1.664495956	1.27E-05	up	yes	0.99	0.3123	0.31	0.315	0.312	1	0.941	1.029	------	------	------	------	------	------	ENOG4111K6M	S:Function unknown	PF08205.15;PF13	C2-set_2;Ig_3	CD80-like C2-set immunoglobulin domain;Immunoglobulin domain	CYT	1	46	19	High
TRINITY_DN28592_c0_g1_i2_orf1	UDP-glucuronosyltransferase 2B14-like isoform X1 [Ostrinia furnacalis] >XP_028167291.1 UDP-glucuronosyltransferase 2B14-like isoform X2 [Ostrinia furnacalis]	3.057043807	1.612137226	7.49E-05	up	yes	0.97	0.3173	0.316	0.311	0.325	1	1.017	0.893	CC:integral component of membrane;CC:cellular_component;CC:cellular anatomical entity;CC:intrinsic component of membrane;MF:catalytic activity;MF:transferase activity;MF:molecular_function;MF:transferase activity, transferring glycosyl groups;MF:transferase activity, transferring hexosyl groups;MF:glucuronosyltransferase activity;MF:UDP-glycosyltransferase	GO:0016	K00699	UGT	map05207;m	Chemical carcinoge	COG1819	S:Function unknown	PF00201.21	UDPGT	UDP-glucoronosyl and UDP-glucosyl transferase	CYT	1	4	58.6	High
TRINITY_DN16939_c0_g1_i4_orf1	39S ribosomal protein L17, mitochondrial [Ostrinia furnacalis]	3.076453582	1.621268225	9.88E-06	up	yes	0.9577	0.3113	0.321	0.296	0.317	1	0.946	0.927	BP:cellular biosynthetic process;BP:cellular nitrogen compound metabolic process;BP:nitrogen compound metabolic process;BP:cellular macromolecule biosynthetic process;BP:organic substance biosynthetic process;BP:cellular nitrogen compound biosynthetic process;BP:cellular macromolecule metabolic process;BP:peptide biosynthetic process;BP:organic substance metabolic process;BP:amide biosynthetic process;BP:cellular process;BP:biosynthetic process;BP:macromolecule biosynthetic process;BP:biological_process;BP:metabolic process;BP:organonitrogen compound metabolic process;BP:primary metabolic process;BP:cellular amide metabolic process;BP:organonitrogen compound biosynthetic process;BP:protein metabolic process;BP:cellular metabolic process;BP:macromolecule metabolic process;BP:peptide metabolic process;BP:translation;CC:intracellular non-membrane-bounded organelle;CC:organelle;CC:cellular anatomical	GO:0044	K02879	RP-L17, MRF	map03010	Ribosome	COG0203	J:Translation, ribosomal structure	PF01196.22	Ribosomal_L17	Ribosomal protein L17	CYT	2	7	24	High
TRINITY_DN47_c0_g1_i2_orf1	uncharacterized protein LOC114356437 isoform X1 [Ostrinia furnacalis]	3.432527164	1.779271138	6.44E-06	up	yes	0.9793	0.2853	0.251	0.286	0.319	1	0.964	0.974	------	------	------	------	------	------	------	------	PF00041.24	fn3	Fibronectin type III domain	CYT	1	8	17.9	High
TRINITY_DN109733_c0_g1_i1_orf1	uncharacterized protein LOC112452128 [Temnothorax curvispinosus]	4.51246174	2.1739147	0.0008853	up	yes	1.032	0.2287	0.102	0.332	0.252	1	1.148	0.947	BP:nucleic acid metabolic process;BP:cellular nitrogen compound metabolic process;BP:nitrogen compound metabolic process;BP:organic cyclic compound metabolic process;BP:telomere maintenance;BP:nucleobase-containing compound metabolic process;BP:cellular response to stimulus;BP:cellular macromolecule metabolic process;BP:DNA repair;BP:cellular component organization;BP:organic substance metabolic process;BP:cellular component organization or biogenesis;BP:primary metabolic process;BP:cellular process;BP:cellular aromatic compound metabolic process;BP:cellular response to DNA damage stimulus;BP:response to stress;BP:biological_process;BP:metabolic process;BP:heterocycle metabolic process;BP:organelle organization;BP:chromosome organization;BP:response to stimulus;BP:telomere organization;BP:cellular metabolic process;BP:macromolecule metabolic process;BP:cellular response to stress;BP:DNA metabolic	GO:0090	------	------	------	------	COG0507;ENOG	S:Function unknown;	------	------	------	CYT	1	2	37.9	High
TRINITY_DN9715_c0_g1_i1_orf1	V-type proton ATPase subunit E [Manduca sexta] >P31402.1 RecName: Full=V-type proton ATPase subunit E; Short=V-ATPase subunit E; AltName: Full=V-ATPase 26 kDa subunit; AltName: Full=Vacuolar proton pump subunit E [Manduca sexta] >KAG6457535.1 hypothetical protein O3G_MSEX010354 [Manduca sexta] >CAA47610.1 H(+)-transporting ATPase [Manduca sexta]	3.548188406	1.827082618	2.81E-06	up	yes	0.9793	0.276	0.268	0.257	0.303	1	0.98	0.958	CC:proton-transporting two-sector ATPase complex, catalytic domain;CC:macromolecular complex;CC:cellular_component;CC:membrane protein complex;MF:molecular_function;MF:ion transmembrane transporter activity;MF:ATPase activity, coupled to transmembrane movement of substances;MF:inorganic solute uptake transmembrane transporter activity;MF:hydrogen-translocating pyrophosphatase activity;MF:primary active transmembrane transporter activity;MF:active transmembrane transporter activity;MF:ATPase coupled ion transmembrane transporter activity;MF:transporter activity;MF:cation transmembrane transporter activity;MF:proton-transporting ATPase activity, rotational mechanism;MF:ATPase activity, coupled to transmembrane movement of ions, rotational mechanism;MF:inorganic cation transmembrane transporter activity;MF:cation-transporting ATPase activity;MF:transmembrane transporter activity;MF:active ion transmembrane transporter activity;MF:hydrogen ion	GO:0033	K02150	ATPeV1E, AT	map05323;m	Rheumatoid arthriti	COG1390	C:Energy production and convers	PF01991.21	vATP-synt_E	ATP synthase	CYT	12	44	26	High
TRINITY_DN43942_c0_g2_i1_orf1	LOW QUALITY PROTEIN: caprin homolog [Ostrinia furnacalis]	4.733944954	2.243042931	1.61E-05	up	yes	1.032	0.218	0.198	0.191	0.265	1	1.077	1.019	------	------	K18743	CAPRIN1, GP	------	------	ENOG4110M5O	S:Function unknown	------	------	------	CYT	1	5	22.7	High

TRINITY_DN700_c0_g1_i3_orf1	V-type proton ATPase subunit H isoform X1 [Chelonus insularis]	5.012333498	2.325482408	2.05E-05	up	yes	1.016	0.2027	0.211	0.14	0.257	1	1.029	1.02	CC:vacuolar proton-transporting V-type ATPase, V1 domain;CC:proton-transporting V-type ATPase, V1 domain;CC:proton-transporting two-sector ATPase complex, catalytic domain;CC:macromolecular complex;CC:cellular_component;CC:membrane protein complex;MF:molecular_function;MF:ion transmembrane transporter activity;MF:ATPase activity, coupled to transmembrane movement of substances;MF:inorganic solute uptake transmembrane transporter activity;MF:hydrogen-translocating pyrophosphatase activity;MF:primary active transmembrane transporter activity;MF:active transmembrane transporter activity;MF:ATPase coupled ion transmembrane transporter activity;MF:transporter activity;MF:cation transmembrane transporter activity;MF:proton-transporting ATPase activity, rotational mechanism;MF:ATPase activity, coupled to transmembrane movement of ions, rotational mechanism;MF:inorganic cation transmembrane transporter activity;MF:cation-transporting ATPase	GO:00002	K02144	ATPeV1H	map05152;m	Tuberculosis;Huma	COG5231	C:Energy production and convers	PF03224.17;PF11	V-ATPase_H_N;V-ATPase_H_C;Arm_2	V-ATPase subunit H;V-ATPase subunit H;Armadillo-like	CYT	1	3	59.4	High
TRINITY_DN27721_c1_g1_i2_orf1	mitochondrial import receptor subunit TOM20 homolog [Ostrinia furnacalis]	2.468091168	1.303395687	0.001703	up	yes	0.8663	0.351	0.338	0.337	0.378	1	0.783	0.816	BP:protein localization;BP:cellular macromolecule localization;BP:nitrogen compound transport;BP:organic substance transport;BP:macromolecule localization;BP:transport;BP:cellular process;BP:protein targeting;BP:establishment of protein localization;BP:biological_process;BP:establishment of localization in cell;BP:establishment of localization;BP:localization;BP:cellular localization;BP:intracellular transport;BP:protein transport;BP:intracellular protein transport;CC:intrinsic component of membrane;CC:mitochondrial outer membrane translocase complex;CC:integral component of membrane;CC:cellular anatomical entity;CC:macromolecular complex;CC:cellular_component;CC:membrane protein complex;CC:outer mitochondrial membrane protein complex;CC:mitochondrial protein complex;	GO:00081	K17770	TOM20	------	------	ENOG4111NAH	U:Intracellular trafficking, secretic	PF02064.18	MAS20	MAS20 protein import receptor	CYT	2	15	17.2	High
TRINITY_DN48020_c0_g1_i1_orf1	aminopeptidase N4 [Cnaphalocrocis medinalis]	3.528081417	1.818883854	0.0001233	up	yes	0.936	0.2653	0.262	0.218	0.316	1	0.877	0.931	BP:nitrogen compound metabolic process;BP:organic substance metabolic process;BP:proteolysis;BP:biological_process;BP:metabolic process;BP:primary metabolic process;BP:organonitrogen compound metabolic process;BP:protein metabolic process;BP:macromolecule metabolic process;CC:integral component of membrane;CC:intrinsic component of membrane;CC:cellular anatomical entity;CC:cellular_component;MF:aminopeptidase activity;MF:exopeptidase activity;MF:molecular_function;MF:binding;MF:peptidase activity;MF:metallopeptidase activity;MF:cation binding;MF:transition metal ion binding;MF:ion binding;MF:metal ion binding;MF:hydrolase	GO:00068	K11140	ANPEP, CD1:	map04640;m	Hematopoietic cell	COG0308	E:Amino acid transport and meta	PF17900.4	Peptidase_M1_N	Peptidase M1 N-terminal domain	CYT	1	21	15.7	High
TRINITY_DN48410_c0_g2_i1_orf1	alpha-amylase 2-like isoform X3 [Ostrinia furnacalis]	4.773300971	2.254987305	5.35E-06	up	yes	0.9833	0.206	0.193	0.192	0.233	1	1.006	0.944	BP:organic substance metabolic process;BP:primary metabolic process;BP:carbohydrate metabolic process;BP:metabolic process;BP:biological_process;MF:hydrolase activity;MF:cation binding;MF:amylase activity;MF:hydrolase activity, acting on glycosyl bonds;MF:ion binding;MF:catalytic activity;MF:alpha-amylase activity;MF:molecular_function;MF:bindi	GO:00717	K01176	AMY, amyA,	map04972;m	Pancreatic secretio	COG0366	G:Carbohydrate transport and m	PF00128.27;PF02	Alpha-amylase;Alpha-amylase_C	Alpha amylase, catalytic domain;Alpha amylase, C-terminal all-beta domain	EXC	8	15	56.5	High
TRINITY_DN8116_c0_g1_i1_orf1	uncharacterized protein LOC114350845 [Ostrinia furnacalis]	4.261061947	2.091213025	1.10E-05	up	yes	0.963	0.226	0.218	0.209	0.251	1	0.971	0.918	------	------	------	------	------	------	COG1028	S:Function unknown	PF13561.9;PF00	adh_short_C2;adh_short;KR	Enoyl;short chain dehydrogenase:KR domain	CYT	4	26	25.5	High
TRINITY_DN7047_c0_g1_i1_orf1	hypothetical protein G9C98_004728 [Cotesia typhae]	4.814977974	2.267529198	1.67E-05	up	yes	0.9837	0.2043	0.184	0.171	0.258	1	1.001	0.95	CC:macromolecular complex;CC:ribosome;CC:intracellular non-membrane-bounded organelle;CC:ribonucleoprotein complex;CC:mitochondrion;CC:cellular_component;CC:intracellular organelle;CC:non-membrane-bounded organelle;CC:membrane-bounded organelle;CC:organelle;CC:cellular anatomical entity;CC:intracellular membrane-bounded organelle;	GO:0032	K17431	MRPL50	------	------	ENOG4111QY3	S:Function unknown	------	------	------	CYT	1	4	24	High
TRINITY_DN8087_c0_g1_i9_orf1	cysteine-rich with EGF-like domain protein 2 isoform X1 [Ostrinia furnacalis]	3.779236767	1.918094905	0.0001766	up	yes	0.921	0.2437	0.196	0.233	0.302	1	0.887	0.876	BP:developmental process;BP:cellular process;BP:cellular developmental process;BP:anatomical structure development;BP:cell development;BP:cellular component organization;BP:animal organ development;BP:biological_process;BP:cellular component organization or biogenesis;MF:cation binding;MF:ion binding;MF:calcium ion binding;MF:molecular_function;MF:metal ion binding;MF:binding;	GO:0032	K24335	CRELD	------	------	ENOG41107X2	O:Posttranslational modification,	PF11938.11	DUF3456	TLR4 regulator and MIR-interacting MSAP	CYT	1	2	42.5	High
TRINITY_DN61112_c0_g1_i4_orfp1	TRINITY_DN61112_c0_g1_i4_m.53012 TRINITY_DN61112_c0_g1::TRINITY_DN61112_c0_g1_i4::g.53012 ORF type:3prime_partial len:87 (-),score=12.29,HMMR_N PF15905.6 0.0011 TRINITY_DN61112_c0_g1_i4:2-232(-)	5.767236299	2.527880135	9.03E-07	up	yes	0.9787	0.1697	0.16	0.156	0.193	1	0.971	0.965	------	------	------	------	------	------	------	------	------	------	------	CYT	3	39	9.2	High
TRINITY_DN334_c0_g1_i4_orf1	collagenase-like [Ostrinia furnacalis]	10.8125	3.434628228	3.04E-06	up	yes	1.038	0.096	0.092	0.072	0.124	1	1.068	1.047	BP:organic substance metabolic process;BP:organonitrogen compound metabolic process;BP:protein metabolic process;BP:macromolecule metabolic process;BP:biological_process;BP:nitrogen compound metabolic process;BP:metabolic process;BP:proteolysis;BP:primary metabolic process;MF:serine-type endopeptidase activity;MF:endopeptidase activity;MF:hydrolase activity;MF:serine hydrolase activity;MF:catalytic activity, acting on a protein;MF:catalytic activity;MF:molecular_function;MF:pepti	GO:00717	------	------	------	------	COG5640	O:Posttranslational modification,	PF00089.29	Trypsin	Trypsin	EXC	1	39	29.8	High
TRINITY_DN26411_c0_g1_i2_orfp1	TRINITY_DN26411_c0_g1_i2_m.24123 TRINITY_DN26411_c0_g1::TRINITY_DN26411_c0_g1_i2::g.24123 ORF type:internal len:115 (-),score=72.83 TRINITY_DN26411_c0_g1_i2:3-344(-)	10.4638067	3.387335889	4.25E-05	up	yes	1.022	0.09767	0.098	0.037	0.158	1	1.086	0.979	------	------	------	------	------	------	------	------	------	------	------	CYT	1	46	10.9	High
TRINITY_DN1249_c0_g1_i6_orf1	venom carboxylesterase-6-like [Ostrinia furnacalis]	4.77387713	2.255161435	0.0001013	up	yes	0.9247	0.1937	0.194	0.238	0.149	1	0.908	0.866	------	------	------	------	------	------	COG2272;ENOG	I:Lipid transport and metabolism	PF00135.31;PF20	COesterase;BD-FAE;Abhydrolase_3	Carboxylesterase family;BD-FAE;alpha/beta hydrolase fold	PLA	1	6	64.7	High
TRINITY_DN2815_c0_g1_i3_orf1	uncharacterized protein LOC114364075 [Ostrinia furnacalis]	5.140341222	2.36186413	0.0001957	up	yes	0.934	0.1817	0.155	0.149	0.241	1	0.964	0.838	------	------	------	------	------	------	ENOG410XRSQ	S:Function unknown	PF00650.23	CRAL_TRIO	CRAL/TRIO domain	CYT	1	3	35.8	High

TRINITY_DN7688_c0_g1_i2_orf1	uncharacterized protein LOC114352518 [Ostrinia furnacalis]	12.12048482	3.599375503	1.25E-05	up	yes	1.01	0.08333	0.06	0.062	0.128	1	1.062	0.969	BP:phosphorylation;BP:cellular process;BP:cellular metabolic process;BP:phosphate-containing compound metabolic process;BP:biological_process;BP:metabolic process;BP:phosphorus metabolic process;MF:kinase activity;MF:transferase activity;MF:catalytic activity;MF:molecular_function;MF:transferase activity, transferring phosphorus-	GO:0016:	------	------	------	------	ENOG410YKCN;I	S:Function unknown;	PF02958.23;PF07!	EcKL;DUF1679	Ecdysteroid kinase-like family;Uncharacterized oxidoreductase dhs-27	CYT	1	10	47.9	High
TRINITY_DN6933_c0_g1_i2_orf1	Chlorophyll a-b binding protein 40, chloroplastic [Trichinella nelsoni] >KRY99282.1 Chlorophyll a-b binding protein 40, chloroplastic [Trichinella zimbabwensis]	9.741836735	3.284193805	1.03E-05	up	yes	0.9547	0.098	0.088	0.085	0.121	1	0.901	0.963	CC:cellular_component;CC:membrane;CC:cellular anatomical entity;	GO:0005!	K08912	LHCB1	------	------	ENOG410ZHBU;I	J:Translation, ribosomal structure	PF00504.24	Chloroa_b-bind	Chlorophyll A-B binding protein	CYT	2	7	28.3	High
TRINITY_DN21494_c0_g1_i2_orf1	pancreatic triacylglycerol lipase-like [Ostrinia furnacalis]	2.570845034	1.36224265	0.000613	up	yes	1.007	0.3917	0.465	0.279	0.431	1	1.055	0.965	BP:organic substance metabolic process;BP:lipid metabolic process;BP:primary metabolic process;BP:metabolic process;BP:biological_process;CC:cellular_component;CC:extracellular region;CC:cellular anatomical entity;MF:hydrolase activity;MF:hydrolase activity, acting on ester bonds;MF:catalytic activity;MF:molecular_function;MF:lipase	GO:0071:	------	------	------	------	ENOG41116Y2;E	S:Function unknown;	PF00151.22	Lipase	Lipase	CYT	1	4	35.8	High
TRINITY_DN16122_c0_g1_i4_orf1	cytochrome P450 6k1-like [Ostrinia furnacalis]	2.274004684	1.185235226	1.42E-05	up	yes	0.971	0.427	0.414	0.417	0.45	1	0.938	0.975	CC:integral component of membrane;CC:cellular_component;CC:cellular anatomical entity;CC:intrinsic component of membrane;MF:cation binding;MF:monooxygenase activity;MF:heme binding;MF:transition metal ion binding;MF:tetrapyrrole binding;MF:organic cyclic compound binding;MF:oxidoreductase activity, acting on paired donors, with incorporation or reduction of molecular oxygen;MF:ion binding;MF:catalytic activity;MF:molecular_function;MF:binding;MF:iron ion binding;MF:metal ion binding;MF:heterocyclic compound binding;MF:oxidoreductase activity;	GO:0016(	K14999	CYP6	------	------	COG2124	S:Function unknown	PF00067.25	p450	Cytochrome P450	CYT	5	12	56.9	High
TRINITY_DN11657_c0_g1_i2_orf1	trehalase-1 [Omphisa fuscidentalis]	2.378450363	1.250021917	3.40E-06	up	yes	0.9823	0.413	0.4	0.408	0.431	1	0.958	0.989	BP:primary metabolic process;BP:cellular carbohydrate metabolic process;BP:oligosaccharide metabolic process;BP:cellular process;BP:disaccharide metabolic process;BP:trehalose metabolic process;BP:biological_process;BP:cellular metabolic process;BP:metabolic process;BP:carbohydrate metabolic process;BP:organic substance metabolic process;MF:trehalase activity;MF:hydrolase activity;MF:catalytic activity;MF:hydrolase activity, acting on glycosyl bonds;MF:alpha,alpha-trehalase	GO:0044:	K01194	TREH, treA, t	map00500	Starch and sucrose	COG1626	S:Function unknown	PF01204.21	Trehalase	Trehalase	CYT	2	5	67.2	High
TRINITY_DN4040_c0_g1_i10_orf1	hypothetical protein evm_007488 [Chilo suppressalis]	2.361332368	1.239601121	1.92E-06	up	yes	0.9783	0.4143	0.405	0.408	0.43	1	0.969	0.966	CC:cellular anatomical entity;CC:intrinsic component of membrane;CC:cellular_component;CC:integral component of membrane;MF:antiporter activity;MF:secondary active transmembrane transporter activity;MF:inorganic anion transmembrane transporter activity;MF:inorganic solute uptake transmembrane transporter activity;MF:transporter activity;MF:anion transmembrane transporter activity;MF:solut;MF:ion transmembrane transporter activity;MF:transmembrane transporter activity;MF:molecular_function;MF:active ion transmembrane transporter activity;MF:inorganic anion exchanger activity;MF:active transmembrane	GO:0110:	K13855	SLC4A2, AE2	map04972;m	Pancreatic secretio	ENOG410XPHD	U:Intracellular trafficking, secretic	PF00955.24;PF07	HCO3_cotransp;Band_3_cyto	HCO3-transporter family;Band 3 cytoplasmic domain	CYT	5	6	128.9	High
TRINITY_DN18036_c0_g1_i7_orf1	pentatricopeptide repeat-containing protein 2, mitochondrial-like [Ostrinia furnacalis]	2.730696798	1.449269134	1.42E-05	up	yes	1.015	0.3717	0.36	0.337	0.418	1	1.025	1.02	BP:regulation of primary metabolic process;BP:regulation of metabolic process;BP:regulation of nucleobase-containing compound metabolic process;BP:regulation of mRNA processing;BP:regulation of RNA metabolic process;BP:regulation of cellular metabolic process;BP:regulation of cellular process;BP:regulation of macromolecule metabolic process;BP:biological regulation;BP:regulation of nitrogen compound metabolic process;BP:biological_process;BP:regulation of mRNA metabolic process;BP:regulation of biological process;CC:cellular_component;CC:intracellular organelle;CC:mitochondrion;CC:organelle;CC:membrane-bounded organelle;CC:cellular anatomical entity;CC:intracellular membrane-	GO:0080(	K17711	PTCD2	------	------	ENOG4111GFQ	S:Function unknown	------	------	------	CYT	6	16	44.3	High
TRINITY_DN13216_c0_g1_i5_orf1	uncharacterized protein LOC114358344 isoform X1 [Ostrinia furnacalis]	3.341764148	1.740609916	0.0002352	up	yes	1.057	0.3163	0.315	0.275	0.359	1	1.165	1.006	BP:cellular process;BP:regulation of cellular process;BP:biological regulation;BP:cell surface receptor signaling pathway;BP:biological_process;BP:regulation of biological process;BP:signal transduction;CC:integral component of membrane;CC:cellular_component;CC:cellular anatomical entity;CC:intrinsic component of membrane;MF:G-protein coupled receptor activity;MF:signaling receptor activity;MF:molecular transducer activity;MF:transmembrane	GO:0009!	K08451;K	ADGRG2, GP	------	------	ENOG410XSD2	S:Function unknown	PF00002.27;PF01	7tm_2;GPS	7 transmembrane receptor;GPCR proteolysis site, GPS, motif	CYT	1	1	148.9	Medium
TRINITY_DN2749_c0_g2_i3_orf1	RNA exonuclease 4-like [Ostrinia furnacalis] >QEE79882.1 REX4 [Ostrinia furnacalis]	2.750758202	1.459829329	1.19E-06	up	yes	0.9977	0.3627	0.348	0.351	0.389	1	1	0.993	BP:nucleic acid metabolic process;BP:cellular nitrogen compound metabolic process;BP:nitrogen compound metabolic process;BP:ncRNA metabolic process;BP:organic cyclic compound metabolic process;BP:nucleobase-containing compound metabolic process;BP:organic substance metabolic process;BP:rRNA processing;BP:ncRNA processing;BP:cellular process;BP:cellular aromatic compound metabolic process;BP:biological_process;BP:metabolic process;BP:heterocycle metabolic process;BP:RNA metabolic process;BP:primary metabolic process;BP:rRNA metabolic process;BP:cellular metabolic process;BP:macromolecule metabolic process;BP:RNA processing;MF:molecular_function;MF:binding;MF:nucleic acid binding;MF:heterocyclic compound binding;MF:3'-5' exonuclease activity;MF:exonuclease activity;MF:hydrolase activity;MF:hydrolase activity, acting on	GO:0090:	K18327	REXO4, REX4	------	------	COG0847	L:Replication, recombination and	PF00929.27	RNase_T	Exonuclease	CYT	5	45	17.8	High

TRINITY_DN2594_c0_g2_i4_orf1	isocitrate dehydrogenase [NAD] subunit beta, mitochondrial isoform X2 [Ostrinia furnacalis]	3.060306031	1.61367593	1.45E-06	up	yes	1.02	0.3333	0.339	0.314	0.347	1	1.02	1.04	BP:tricarboxylic acid cycle;BP:biological_process;BP:metabolic process;BP:primary metabolic process;CC:oxidoreductase complex;CC:isocitrate dehydrogenase comple;CC:mitochondrial isocitrate dehydrogenase comple;CC:catalytic complex;CC:mitochondrial tricarboxylic acid cycle enzyme complex;CC:tricarboxylic acid cycle enzyme complex;CC:macromolecular complex;CC:cellular_component;CC:mitochondrial protein complex;MF:NAD binding;MF:molecular_function;MF:binding;MF:nucleotide binding;MF:heterocyclic compound binding;MF:nucleoside phosphate binding;MF:oxidoreductase activity;MF:cation binding;MF:magnesium ion binding;MF:ion binding;MF:isocitrate dehydrogenase activity;MF:metal ion binding;MF:small molecule binding;MF:oxidoreductase activity, acting on CH-OH group of donors;MF:isocitrate dehydrogenas;MF:oxidoreductase activity, acting on the CH-OH group of	GO:0006(	K00030	IDH3	map00020	Citrate cycl	COG0473	E:Amino acid transport and meta	PF00180.23	Iso_dh	Isocitrate/isopropylmalate dehydrogenase	CYT	3	13	41.2	High
TRINITY_DN12497_c0_g1_i1_orf1	probable N-acetyltransferase san [Ostrinia furnacalis]	2.846175718	1.509024734	4.89E-06	up	yes	1.001	0.3517	0.363	0.314	0.378	1	1.007	0.997	MF:transferase activity;MF:catalytic activity;MF:acetyltransferase activity;MF:transferase activity, transferring acyl groups;MF:transferase activity, transferring acyl groups other than amino-acyl	GO:0016	K20793	NAA50, NAT!	------	------	COG0456	S:Function unknown	PF00583.28;PF13	Acetyltransf_1; Acetyltransf_7; Acetyltransf_10; FR47;Acetyltransf_4	Acetyltransferase;Acetyltransferase;Acetyltransferase;FR47-like protein;Acetyltr	CYT	5	36	20.2	High
TRINITY_DN2304_c0_g1_i4_orf1	clustered mitochondria protein homolog isoform X2 [Ostrinia furnacalis]	2.704414587	1.435316334	1.53E-05	up	yes	0.9863	0.3647	0.328	0.36	0.406	1	0.993	0.966	BP:organelle organization;BP:cellular component organization or biogenesis;BP:cellular process;BP:cellular component organization;BP:biological_process;BP:mitochondrion organization;CC:cytoplasm;CC:cellular_component;CC:cellular anatomical entity;MF:organic cyclic compound binding;MF:molecular_function;MF:RNA binding;MF:nucleic acid binding;MF:heterocyclic compound binding;MF:binding;	GO:0006	K03255	TIF31, CLU1	------	------	ENOG410XQUQ	S:Function unknown	PF13424.9;PF133	TPR_12;TPR_10; eIF3_p135	Tetratricopeptide repeat;Tetratricopeptide repeat;Translation initiation factor eIF3 subunit 135	CYT	4	11	48	High
TRINITY_DN2574_c0_g1_i5_orf1	prion-like-(Q/N-rich) domain-bearing protein 25 [Ostrinia furnacalis] >XP_028158239.1 prion-like-(Q/N-rich) domain-bearing protein 25 [Ostrinia furnacalis] >XP_028158240.1 prion-like-(Q/N-rich) domain-bearing protein 25 [Ostrinia furnacalis] >XP_028158241.1 prion-like-(Q/N-rich) domain-bearing protein 25 [Ostrinia furnacalis]	3.079802041	1.622837623	4.95E-06	up	yes	0.9957	0.3233	0.314	0.308	0.348	1	1.021	0.966	------	------	------	------	------	------	ENOG410XTK6;E	S:Function unknown;	PF01683.21	EB	EB module	CYT	4	20	34.9	High
TRINITY_DN3476_c0_g1_i5_orf1	maltase A1-like [Ostrinia furnacalis]	3.583594022	1.841407207	8.38E-06	up	yes	1.031	0.2877	0.274	0.262	0.327	1	1.053	1.041	BP:primary metabolic process;BP:carbohydrate metabolic process;BP:organic substance metabolic process;BP:biological_process;BP:metabolic process;MF:hydrolase activity;MF:catalytic activity;MF:hydrolase activity, acting on	GO:0044	K01187	malZ	map00500;m	Starch and sucrose	COG0366	E:Amino acid transport and meta	PF00128.27	Alpha-amylase	Alpha amylase, catalytic domain	CYT	8	22	66.3	High
TRINITY_DN1775_c0_g1_i3_orf1	ATP-dependent RNA helicase dbp2-like isoform X1 [Ostrinia furnacalis]	3.323442137	1.732678236	2.76E-06	up	yes	1.008	0.3033	0.277	0.296	0.337	1	1.017	1.008	------	------	K12823	DDX5, DBP2	map05205;m	Proteoglycans in ca	COG0513	L:Replication, recombination and	PF00270.32;PF00	DEAD;Helicase_C	DEAD/DEAH box helicase;Helicase conserved C-terminal	CYT	13	24	92.9	High
TRINITY_DN8366_c0_g1_i4_orf1	luciferin 4-monooxygenase-like [Ostrinia furnacalis]	2.484637991	1.313035668	0.0001466	up	yes	0.93	0.3743	0.4	0.36	0.363	1	0.873	0.917	------	------	------	------	------	------	COG0318;COG0	I:Lipid transport and metabolism	PF00501.31;PF13	AMP-binding;AMP-binding_C	AMP-binding enzyme;AMP-binding enzyme C-terminal	CYT	3	5	61.5	High
TRINITY_DN36262_c0_g1_i1_orf1	trypsin, alkaline C-like [Maniola jurtina]	3.895370719	1.961760635	3.37E-05	up	yes	1.035	0.2657	0.236	0.241	0.32	1	1.086	1.02	BP:organic substance metabolic process;BP:organonitrogen compound metabolic process;BP:protein metabolic process;BP:macromolecule metabolic process;BP:biological_process;BP:nitrogen compound metabolic process;BP:metabolic process;BP:proteolysis;BP:primary metabolic process;MF:serine-type endopeptidase activity;MF:endopeptidase activity;MF:hydrolase activity;MF:serine hydrolase activity;MF:catalytic activity, acting on a protein;MF:catalytic activity;MF:molecular_function;MF:pepti	GO:0071	K01312	PRSS1_2_3	map04972;m	Pancreatic secretio	COG5640	O:Posttranslational modification,	PF00089.29	Trypsin	Trypsin	CYT	1	9	11.8	High
TRINITY_DN34821_c0_g1_i4_orf1	acetylcholine receptor subunit alpha-L1-like [Ostrinia furnacalis]	3.150159744	1.655424989	3.39E-05	up	yes	0.986	0.313	0.321	0.303	0.315	1	0.924	1.034	CC:cellular_component;CC:integral component of membrane;CC:cellular anatomical entity;CC:intrinsic component of membrane;MF:signaling receptor activity;MF:molecular transducer activity;MF:inorganic solute uptake transmembrane transporter activity;MF:transporter activity;MF:extracellular ligand-gated ion channel activity;MF:ligand-gated ion channel activity;MF:ion transmembrane transporter activity;MF:transmembrane signaling receptor activity;MF:transmembrane transporter activity;MF:channel activity;MF:molecular_function;MF:gated channel activity;MF:ligand-gated channel activity;MF:passive transmembrane transporter	GO:0005	K04803;K	CHRNA1;CH	map04080	Neuroactive ligand	ENOG410XQGR	S:Function unknown	PF02931.26	Neur_chan_LBD	Neurotransmitter-gated ion-channel ligand binding domain	PLA	1	3	47.7	High
TRINITY_DN628_c0_g1_i7_orf1	prostamide/prostaglandin F synthase-like [Ostrinia furnacalis]	2.696011396	1.430826595	2.95E-05	up	yes	0.9463	0.351	0.343	0.349	0.361	1	0.908	0.931	------	------	K15717	PRXL2B, FAM	map00590	Arachidonic acid m	ENOG410YFY9	O:Posttranslational modification,	PF13911.9	AhpC-TSA_2	AhpC/TSA antioxidant enzyme	CYT	2	8	26.6	High
TRINITY_DN2267_c0_g1_i1_orf1	hypothetical protein evm_006312 [Chilo suppressalis]	3.177268324	1.667786933	1.46E-05	up	yes	0.984	0.3097	0.323	0.261	0.345	1	0.983	0.969	BP:phosphate ion transmembrane transport;BP:anion transport;BP:transmembrane transport;BP:mitochondrial transmembrane transport;BP:inorganic anion transmembrane transport;BP:mitochondrial phosphate ion transmembrane transport;BP:inorganic anion transport;BP:cellular process;BP:ion transport;BP:transport;BP:phosphate ion transport;BP:ion transmembrane transport;BP:biological_process;BP:establishment of localization;BP:localization;BP:inorganic ion transmembrane transport;BP:anion transmembrane transport;CC:intrinsic component of membrane;CC:organelle membrane;CC:integral component of membrane;CC:membrane;CC:cellular anatomical entity;CC:mitochondrial membrane;CC:organelle inner membrane;CC:cellular_component;CC:mitochondrial inner membrane;MF:molecular_function;MF:inorganic solute uptake transmembrane transporter activity;MF:transporter activity;MF:secondary active transmembrane transporter	GO:0035	K15102	SLC25A3, PH	------	------	ENOG410XPST	S:Function unknown	PF00153.30	Mito_carr	Mitochondrial carrier protein	CYT	9	19	41.3	High

TRINITY_DN6221_c0_g1_i5_orf1	unnamed protein product [Diatraea saccharalis]	3.398025196	1.76469655	0.0002441	up	yes	0.998	0.2937	0.2	0.323	0.358	1	0.944	1.05	CC:intrinsic component of membrane;CC:cellular_component;CC:proton-transporting V-type ATPase, V0 domain;CC:integral component of membrane;CC:cellular anatomical entity;CC:macromolecular complex;CC:membrane protein complex;CC:proton-transporting two-sector ATPase complex, proton-transporting domain;MF:molecular_function;MF:ATPase activity, coupled to transmembrane movement of substances;MF:inorganic solute uptake transmembrane transporter activity;MF:hydrogen-translocating pyrophosphatase activity;MF:primary active transmembrane transporter activity;MF:ATPase coupled ion transmembrane transporter activity;MF:transporter activity;MF:cation transmembrane transporter activity;MF:proton-transporting ATPase activity, rotational mechanism;MF:ATPase activity, coupled to transmembrane movement of ions, rotational mechanism;MF:inorganic cation transmembrane transporter activity;MF:cation-transporting ATPase	GO:00312	K02153	ATPeV0E, AT	map05165;m	Human papillomav	ENOG41121M0	C:Energy production and conver	PF05493.16	ATP_synt_H	ATP synthase subunit H	CYT	1	9	9.6	High
TRINITY_DN43611_c0_g1_i1_orf1	39S ribosomal protein L41, mitochondrial [Ostrinia furnacalis]	3.119935691	1.641516292	2.46E-05	up	yes	0.9703	0.311	0.265	0.318	0.35	1	0.943	0.968	CC:cellular_component;CC:large ribosomal subunit;CC:organellar large ribosomal subunit;CC:ribonucleoprotein complex;CC:mitochondrial large ribosomal subunit;CC:macromolecular complex;CC:ribosomal subunit;CC:mitochondrial protein complex;MF:structural molecule activity;MF:structural constituent of ribosome;MF:molecular_function;	GO:00055	K17422	MRPL41	------	------	ENOG4111ND6	S:Function unknown	PF09809.12	MRP-L27	Mitochondrial ribosomal protein L27	CYT	2	25	18.2	High
TRINITY_DN15380_c0_g1_i1_orf1	39S ribosomal protein L32, mitochondrial [Ostrinia furnacalis]	3.736305251	1.901612326	5.17E-05	up	yes	0.989	0.2647	0.26	0.265	0.269	1	0.916	1.051	BP:cellular biosynthetic process;BP:cellular nitrogen compound metabolic process;BP:nitrogen compound metabolic process;BP:cellular macromolecule biosynthetic process;BP:organic substance biosynthetic process;BP:cellular nitrogen compound biosynthetic process;BP:cellular macromolecule metabolic process;BP:peptide biosynthetic process;BP:organic substance metabolic process;BP:amide biosynthetic process;BP:cellular process;BP:biosynthetic process;BP:macromolecule biosynthetic process;BP:biological_process;BP:metabolic process;BP:primary metabolic process;BP:organonitrogen compound metabolic process;BP:cellular amide metabolic process;BP:organonitrogen compound biosynthetic process;BP:protein metabolic process;BP:cellular metabolic process;BP:macromolecule metabolic process;BP:peptide metabolic process;BP:translation;CC:large ribosomal subunit;CC:intracellular non-membrane-bounded organelle;CC:organelle;CC:cellular	GO:00442	K02911	RP-L32, MRF	map03010	Ribosome	COG0333	J:Translation, ribosomal structure	------	------	------	CYT	1	5	22.6	High
TRINITY_DN94248_c0_g2_i3_orf1	uncharacterized protein LOC114357292 isoform X4 [Ostrinia furnacalis]	3.546245919	1.826292585	2.13E-06	up	yes	0.9777	0.2757	0.258	0.268	0.301	1	0.968	0.965	------	------	------	------	------	------	COG1028;ENOG	C:Energy production and conver	PF13561.9;PF001	adh_short_C2;adh_short;KR	Enoyl;short chain dehydrogenase:KR domain	CYT	7	29	28.7	High
TRINITY_DN48237_c0_g1_i5_orf1	myogenesis-regulating glycosidase-like [Ostrinia furnacalis]	4.485326325	2.165212951	7.34E-05	up	yes	1.024	0.2283	0.192	0.233	0.26	1	1.108	0.965	BP:primary metabolic process;BP:carbohydrate metabolic process;BP:organic substance metabolic process;BP:biological_process;BP:metabolic process;CC:integral component of membrane;CC:cellular_component;CC:intrinsic component of membrane;CC:cellular anatomical entity;MF:hydrolase activity;MF:hydrolase activity, acting on glycosyl bonds;MF:catalytic activity;MF:hydrolase activity, hydrolyzing O-glycosyl	GO:00442	K24727	MYORG	------	------	COG1501	G:Carbohydrate transport and m	PF01055.29	Glyco_hydro_31	Glycosyl hydrolases family 31	CYT	1	1	76.4	High
TRINITY_DN117_c0_g1_i5_orf1	lipase member I-like [Ostrinia furnacalis]	3.837795887	1.940277984	1.63E-06	up	yes	0.989	0.2577	0.244	0.24	0.289	1	0.986	0.981	BP:organic substance metabolic process;BP:lipid metabolic process;BP:primary metabolic process;BP:metabolic process;BP:biological_process;CC:cellular_component;CC:extracellular region;CC:cellular anatomical entity;MF:hydrolase activity;MF:hydrolase activity, acting on ester bonds;MF:catalytic activity;MF:molecular_function;MF:lipase	GO:00717	------	------	------	------	ENOG41116Y2;E	S:Function unknown;	PF00151.22	Lipase	Lipase	CYT	6	21	46.9	High
TRINITY_DN3504_c0_g1_i4_orfp1	TRINITY_DN3504_c0_g1_i4_m.43930 TRINITY_DN3504_c0_g1::TRINITY_DN3504_c0_g1_i4::g.43930 ORF type:internal len:196 (-),score=84.82 TRINITY_DN3504_c0_g1_i4:3-587(-)	3.206908229	1.681183069	6.69E-05	up	yes	0.947	0.2953	0.272	0.334	0.28	1	0.888	0.953	------	------	------	------	------	------	------	------	------	------	------	EXC	1	8	21.5	High
TRINITY_DN96_c0_g1_i1_orf1	collagenase-like [Ostrinia furnacalis]	6.177012907	2.626909345	7.56E-05	up	yes	1.005	0.1627	0.132	0.164	0.192	1	0.925	1.089	BP:organic substance metabolic process;BP:organonitrogen compound metabolic process;BP:protein metabolic process;BP:macromolecule metabolic process;BP:biological_process;BP:nitrogen compound metabolic process;BP:metabolic process;BP:proteolysis;BP:primary metabolic process;MF:serine-type endopeptidase activity;MF:endopeptidase activity;MF:hydrolase activity;MF:serine hydrolase activity;MF:catalytic activity, acting on a protein;MF:catalytic activity;MF:molecular_function;MF:pepti	GO:00717	------	------	------	------	COG5640	O:Posttranslational modification,	PF00089.29	Trypsin	Trypsin	CYT	1	3	44.4	High
TRINITY_DN10792_c0_g2_i5_orf1	uncharacterized protein LOC114366171 [Ostrinia furnacalis]	6.296766011	2.654611058	4.96E-06	up	yes	0.993	0.1577	0.137	0.135	0.201	1	1.011	0.968	------	------	------	------	------	------	ENOG410XT8F;E	S:Function unknown;	PF02958.23;PF07	EcKL;DUF1679;APH	Ecdysteroid kinase-like family;Uncharacterized oxidoreductase dhs-27;Phosphotransferase enzyme family	CYT	9	20	48	High
TRINITY_DN5554_c0_g1_i2_orf1	double-stranded RNA-binding protein Staufen homolog 2 isoform X5 [Pectinophora gossypiella]	2.557840617	1.35492637	4.51E-06	up	yes	0.995	0.389	0.4	0.402	0.365	1	0.97	1.015	------	------	K17597	STAU	------	------	ENOG410XSCK	K:Transcription	PF00035.29;PF16	dsrm;Staufen_C	Double-stranded RNA binding motif;Staufen C-terminal	CYT	1	2	56.8	High
TRINITY_DN14429_c0_g1_i2_orf1	NADH dehydrogenase [ubiquinone] 1 beta subcomplex subunit 11, mitochondrial [Ostrinia furnacalis]	3.220287198	1.687189359	0.0001422	up	yes	1.054	0.3273	0.268	0.313	0.401	1	1.114	1.049	CC:cellular_component;CC:integral component of membrane;CC:cellular anatomical entity;CC:intrinsic component of membrane;	GO:00055	K11351	NDUFB11	map04714;m	Thermogenesis;Oxi	ENOG41120A5	U:Intracellular trafficking, secretic	PF10183.12	ESSS	ESSS subunit of NAD	PLA	1	8	16.4	High
TRINITY_DN3014_c0_g1_i4_orf1	putative inorganic phosphate cotransporter isoform X1 [Ostrinia furnacalis]	2.490450726	1.316406867	0.0001118	up	yes	0.978	0.3927	0.378	0.373	0.427	1	0.91	1.024	CC:integral component of membrane;CC:cellular_component;CC:cellular anatomical entity;CC:intrinsic component of membrane;MF:transporter activity;MF:transmembrane transporter	GO:0016(	K12301	SLC17A5	map04142	Lysosome	ENOG410XPWC	S:Function unknown	PF07690.19;PF00	MFS_1;Sugar_tr;MFS_4	Major Facilitator Superfamily;Sugar;Uncharacterised MFS-type transporter	CYT	1	1	56.6	Medium

TRINITY_DN2270_c0_g2_i1_orf1	integrin beta-nu [Ostrinia furnacalis]	2.732822338	1.450391673	4.30E-05	up	yes	0.9983	0.3653	0.303	0.381	0.412	1	0.988	1.007	BP:cell-cell adhesion;BP:integrin-mediated signaling pathway;BP:cellular process;BP:regulation of cellular process;BP:biological_process;BP:signal transduction;BP:cell surface receptor signaling pathway;BP:cell adhesion;BP:biological regulation;BP:heterophilic cell-cell adhesion via plasma membrane cell adhesion molecules;BP:regulation of biological process;BP:cell-cell adhesion via plasma-membrane adhesion molecules;CC:cellular anatomical entity;CC:integral component of membrane;CC:cellular_component;CC:intrinsic component of membrane;	GO:0098	------	------	------	------	ENOG410XP60	J:Translation, ribosomal structure	PF00362.21;PF17	Integrin_beta;PSI_integrin;EGF_2;I-EGF_1	Integrin beta chain VWA domain;Integrin plexin domain;EGF-like domain;Integrin beta epidermal growth factor like domain 1	CYT	2	3	89 High
TRINITY_DN34536_c0_g1_i6_orf1	clustered mitochondria protein homolog isoform X2 [Ostrinia furnacalis]	3.063116961	1.615000455	5.10E-06	up	yes	1.024	0.3343	0.3	0.353	0.35	1	1.035	1.037	BP:organelle organization;BP:cellular component organization or biogenesis;BP:cellular process;BP:cellular component organization;BP:biological_process;BP:mitochondrion organization;CC:cytoplasm;CC:cellular_component;CC:cellular anatomical entity;MF:organic cyclic compound binding;MF:molecular_function;MF:RNA binding;MF:nucleic acid binding;MF:heterocyclic compound binding;MF:binding;	GO:0006	K03255	TIF31, CLU1	------	------	ENOG410XQUQ	S:Function unknown	PF13236.9;PF128	CLU;eIF3_p135;CLU_N	Clustered mitochondria;Translation initiation factor eIF3 subunit 135;Mitochondrial function, CLU-N-term	CYT	9	8	124.7 High
TRINITY_DN4143_c0_g1_i1_orf1	zinc finger protein 530-like isoform X8 [Ostrinia furnacalis]	3.125572869	1.644120638	0.0001523	up	yes	1.023	0.3273	0.257	0.32	0.405	1	0.996	1.074	------	------	K09228	KRAB	map05168	Herpes simplex viru	COG5048;ENOG	S:Function unknown;	PF07776.18	zf-AD	Zinc-finger associated domain	CYT	1	5	20.3 High
TRINITY_DN747_c0_g1_i4_orf1	trypsin, alkaline C-like [Ostrinia furnacalis]	3.212613175	1.683747279	9.75E-05	up	yes	1.029	0.3203	0.29	0.338	0.333	1	0.974	1.113	BP:organic substance metabolic process;BP:organonitrogen compound metabolic process;BP:protein metabolic process;BP:macromolecule metabolic process;BP:biological_process;BP:nitrogen compound metabolic process;BP:metabolic process;BP:proteolysis;BP:primary metabolic process;MF:serine-type endopeptidase activity;MF:endopeptidase activity;MF:hydrolase activity;MF:serine hydrolase activity;MF:catalytic activity, acting on a protein;MF:catalytic activity;MF:molecular_function;MF:pepti	GO:0071	K09628;K	PRSS27;TMP	------	------	COG5640	O:Posttranslational modification,	PF00089.29	Trypsin	Trypsin	EXC	1	5	28 High
TRINITY_DN16900_c0_g2_i1_orf1	uncharacterized oxidoreductase dhs-27-like [Ostrinia furnacalis]	4.23828125	2.083479327	0.0002112	up	yes	1.085	0.256	0.318	0.237	0.213	1	1.191	1.064	------	------	------	------	------	------	ENOG410XT8F;E	S:Function unknown;	PF02958.23;PF07	EcKL;DUF1679;APH;Choline_kinase	Ecdysteroid kinase-like family;Uncharacterized oxidoreductase dhs-27;Phosphotransferase enzyme family;Choline/ethanolamine	CYT	1	3	32.1 High
TRINITY_DN17351_c0_g1_i3_orf1	V-type proton ATPase subunit F [Ostrinia furnacalis]	3.114347691	1.638930018	2.10E-06	up	yes	1.005	0.3227	0.305	0.312	0.351	1	1.021	0.993	CC:proton-transporting V-type ATPase, V1 domain;CC:proton-transporting two-sector ATPase complex, catalytic domain;CC:macromolecular complex;CC:cellular_component;CC:membrane protein complex;MF:molecular_function;MF:ion transmembrane transporter activity;MF:ATPase activity, coupled to transmembrane movement of substances;MF:inorganic solute uptake transmembrane transporter activity;MF:hydrogen-translocating pyrophosphatase activity;MF:primary active transmembrane transporter activity;MF:active transmembrane transporter activity;MF:ATPase coupled ion transmembrane transporter activity;MF:transporter activity;MF:cation transmembrane transporter activity;MF:proton-transporting ATPase activity, rotational mechanism;MF:ATPase activity, coupled to transmembrane movement of ions, rotational mechanism;MF:inorganic cation transmembrane transporter activity;MF:cation-transporting ATPase activity;MF:transmembrane transporter activity;MF:active ion transmembrane	GO:0033	K02151	ATPeV1F, AT	map05323;m	Rheumatoid arthrit	COG1436	C:Energy production and conver	PF01990.20	ATP-synt_F	ATP synthase	CYT	6	40	13.8 High
TRINITY_DN51252_c0_g2_i1_orf1	peroxidase-like [Ostrinia furnacalis]	2.712	1.439357178	0.0002549	up	yes	1.017	0.375	0.446	0.275	0.404	1	1.017	1.035	BP:response to stress;BP:response to oxidative stress;BP:response to stimulus;BP:biological_process;CC:chorion;CC:external encapsulating structure;CC:cellular_component;CC:cellular anatomical entity;MF:heme binding;MF:tetrapyrrole binding;MF:organic cyclic compound binding;MF:antioxidant activity;MF:heterocyclic compound binding;MF:catalytic activity;MF:molecular_function;MF:binding;MF:oxidoreductase activity, acting on peroxide as acceptor;MF:peroxidase activity;MF:oxidoreductase activity;	GO:0006	K19511	PXDN, VPO1	------	------	ENOG410XPZ3	U:Intracellular trafficking, secretic	PF03098.18	An_peroxidase	Animal haem peroxidase	CYT	1	13	18.9 High
TRINITY_DN42759_c0_g3_i1_orf1	fatty acid synthase-like [Ostrinia furnacalis]	3.247301276	1.69924124	3.00E-06	up	yes	0.9927	0.3057	0.305	0.277	0.335	1	0.978	1	BP:fatty acid biosynthetic process;BP:fatty acid metabolic process;BP:carboxylic acid metabolic process;BP:cellular biosynthetic process;BP:small molecule metabolic process;BP:small molecule biosynthetic process;BP:monocarboxylic acid biosynthetic process;BP:organic substance biosynthetic process;BP:organic substance metabolic process;BP:lipid metabolic process;BP:cellular process;BP:monocarboxylic acid metabolic process;BP:biosynthetic process;BP:biological_process;BP:metabolic process;BP:oxoacid metabolic process;BP:cellular lipid metabolic process;BP:lipid biosynthetic process;BP:primary metabolic process;BP:organic acid metabolic process;BP:carboxylic acid biosynthetic process;BP:organic acid biosynthetic process;BP:cellular metabolic process;MF:3-hydroxyoctanoyl-[acyl-carrier-protein] dehydratase activity;MF:thiolester hydrolase activity;MF:S-acyltransferase activity;MF:enoyl-[acyl-carrier-protein] reductas;MF:oxidoreductase activity,	GO:0006	K00665	FASN	map04910;m	Insulin signaling pa	COG3321	Q:Secondary metabolites biosyn	PF00698.24;PF00	Acyl_transf_1;ketoacyl-synt;KAsynt_C_assoc;Ketoacyl-synt_C	Acyl transferase domain;Beta-ketoacyl synthase, N-terminal domain;Ketoacyl-synthetase C-terminal extension;Beta-ketoacyl synthase, C-terminal domain	CYT	2	3	84.3 High
TRINITY_DN389_c0_g1_i2_orf1	uncharacterized protein LOC118068293 isoform X2 [Chelonus insularis]	2.871536524	1.521822912	7.95E-06	up	yes	1.026	0.3573	0.325	0.383	0.364	1	1.052	1.026	------	------	------	------	------	------	ENOG410XX7G	S:Function unknown	PF01607.27;PF00	CBM_14;Ldl_recept_a	Chitin binding Peritrophin-A domain;Low-density lipoprotein receptor domain class A	PLA	4	11	60.2 High
TRINITY_DN35725_c0_g1_i1_orf1	mitochondrial import inner membrane translocase subunit Tim13-like [Bicyclus anynana] >CAG9745432.1 unnamed protein product [Diatraea saccharalis] >CAG9784117.1 unnamed protein product [Diatraea	3.346801347	1.742782921	3.54E-05	up	yes	0.994	0.297	0.279	0.259	0.353	1	1.024	0.958	------	------	K17781	TIM13	------	------	ENOG41124YI	O:Posttranslational modification,	PF02953.18	zf-Tim10_DDP	Tim10/DDP family zinc finger	CYT	3	39	10.9 High

TRINITY_DN6147_c0_g1_i2_orf1	uncharacterized protein LOC114352519 [Ostrinia furnacalis]	2.43357338	1.283076277	1.09E-06	up	yes	0.98	0.4027	0.393	0.4	0.415	1	0.971	0.969	------	------	------	------	------	------	ENOG410YKCN;l	S:Function unknown;	PF02958.23;PF07	EcKL;DUF1679;APH	Ecdysteroid kinase-like family;Uncharacterized oxidoreductase dhs-27;Phosphotransferase enzyme family	CYT	10	27	44.6	High
TRINITY_DN29034_c0_g1_i2_orf1	trypsin-like serine protease [Ostrinia nubilalis]	3.600431189	1.848169695	2.81E-06	up	yes	1.002	0.2783	0.263	0.257	0.315	1	1.011	0.996	BP:organic substance metabolic process;BP:organonitrogen compound metabolic process;BP:protein metabolic process;BP:macromolecule metabolic process;BP:biological_process;BP:nitrogen compound metabolic process;BP:metabolic process;BP:proteolysis;BP:primary metabolic process;MF:serine-type endopeptidase activity;MF:endopeptidase activity;MF:hydrolase activity;MF:serine hydrolase activity;MF:catalytic activity, acting on a protein;MF:catalytic activity;MF:molecular_function;MF:pepti	GO:0071	K01312	PRSS1_2_3	map04972;m	Pancreatic secretio	COG5640	O:Posttranslational modification,	PF00089.29	Trypsin	Trypsin	CYT	1	8	15.3	High
TRINITY_DN3759_c0_g1_i1_orf1	uncharacterized protein LOC114350416 [Ostrinia furnacalis] >XP_028157016.1 uncharacterized protein LOC114350416 [Ostrinia furnacalis] >XP_028157017.1 uncharacterized protein LOC114350416 [Ostrinia furnacalis] >XP_028157018.1 uncharacterized protein LOC114350416 [Ostrinia furnacalis] >XP_028157019.1 uncharacterized protein LOC114350416 [Ostrinia furnacalis]	2.556994819	1.354449237	7.06E-06	up	yes	0.987	0.386	0.367	0.409	0.382	1	1.005	0.956	CC:cellular_component;CC:extracellular region;CC:cellular anatomical entity;MF:carbohydrate derivative binding;MF:molecular_function;MF:chitin binding;MF:binding;	GO:0005	K22909	ZC3H13	------	------	ENOG4111GFX	S:Function unknown	PF01607.27	CBM_14	Chitin binding Peritrophin-A domain	CYT	2	2	132.4	High
TRINITY_DN13783_c0_g4_i2_orf1	hypothetical protein evm_010131 [Chilo suppressalis]	3.465034965	1.79286991	3.37E-06	up	yes	0.991	0.286	0.293	0.277	0.288	1	1.018	0.955	BP:protein localization;BP:maintenance of protein localization in endoplasmic reticulum;BP:maintenance of location in cell;BP:cellular macromolecule localization;BP:nitrogen compound transport;BP:organic substance transport;BP:macromolecule localization;BP:transport;BP:maintenance of protein location in cell;BP:maintenance of protein localization in organelle;BP:cellular process;BP:protein retention in ER lumen;BP:establishment of protein localization;BP:maintenance of protein location;BP:biological_process;BP:maintenance of location;BP:establishment of localization;BP:localization;BP:cellular localization;BP:protein transport;CC:endoplasmic reticulum membrane;CC:intrinsic component of membrane;CC:organelle membrane;CC:integral component of membrane;CC:membrane;CC:cellular anatomical entity;CC:cellular_component;MF:molecular_function;MF:binding;MF:peptide binding;MF:amide binding;MF:ER retention sequence binding;MF:signal	GO:0008	------	------	------	------	ENOG410Z4PE	S:Function unknown	PF14880.9	COX14	Cytochrome oxidase c assembly	CYT	1	10	11.2	High
TRINITY_DN109540_c0_g1_i3_orf1	4-coumarate--CoA ligase 1-like isoform X4 [Ostrinia furnacalis]	2.763915547	1.466713534	4.96E-07	up	yes	1.008	0.3647	0.36	0.35	0.384	1	1.013	1.012	CC:integral component of membrane;CC:cellular_component;CC:intrinsic component of membrane;CC:cellular anatomical entity;MF:monooxygenase activity;MF:catalytic activity;MF:molecular_function;MF:oxidor	GO:0016	K01904	4CL	map00130	Ubiquinone and ot	COG0318;COG0	I:Lipid transport and metabolism	PF00501.31;PF13	AMP-binding;AMP-binding_C	AMP-binding enzyme;AMP-binding enzyme C-terminal domain	CYT	6	14	58.4	High
TRINITY_DN657_c0_g1_i2_orf1	cytochrome c-type heme lyase [Ostrinia furnacalis]	3.867023309	1.951223461	5.23E-08	up	yes	1.012	0.2617	0.269	0.255	0.261	1	1.02	1.015	------	------	K01764	HCCS	map00860	Porphyrin metaboli	ENOG410YAUW	O:Posttranslational modification,	PF01265.20	Cyto_heme_lyase	Cytochrome c/c1 heme	CYT	2	7	31.6	High
TRINITY_DN23266_c0_g2_i1_orf1	medium-chain acyl-CoA ligase ACSF2, mitochondrial [Chelonus insularis]	3.10758166	1.635792303	2.75E-05	up	yes	1.037	0.3337	0.283	0.344	0.374	1	1.059	1.053	------	------	K23617	ACSF2	------	------	COG0318	S:Function unknown	PF00501.31;PF13	AMP-binding;AMP-binding_C	AMP-binding enzyme;AMP-binding enzyme C-terminal	CYT	1	1	65	Medium
TRINITY_DN43391_c0_g1_i5_orf1	myrosinase 1-like isoform X2 [Ostrinia furnacalis]	3.648351648	1.867244791	1.40E-05	up	yes	0.996	0.273	0.241	0.261	0.317	1	0.965	1.023	BP:primary metabolic process;BP:carbohydrate metabolic process;BP:organic substance metabolic process;BP:biological_process;BP:metabolic process;MF:hydrolase activity;MF:catalytic activity;MF:hydrolase activity, acting on glycosyl bonds;MF:hydrolase activity, hydrolyzing O-glycosyl	GO:0044	------	------	------	------	COG2723	G:Carbohydrate transport and m	PF00232.21	Glyco_hydro_1	Glycosyl hydrolase family 1	CYT	3	6	59.3	High
TRINITY_DN3134_c0_g1_i1_orf1	cytochrome c oxidase subunit 6C-1 isoform X1 [Hyposmocoma kahamanoa]	3.059946317	1.613506343	8.50E-06	up	yes	1.026	0.3353	0.347	0.299	0.36	1	1.025	1.052	CC:organelle membrane;CC:integral component of membrane;CC:membrane;CC:organelle inner membrane;CC:mitochondrial membrane;CC:cellular_component;CC:mitochondrial inner membrane;CC:intrinsic component of membrane;CC:cellular anatomical entity;	GO:0031	K02268	COX6C	map04714;m	Thermogenesis;Oxi	ENOG41124VH	O:Posttranslational modification,	PF02937.18	COX6C	Cytochrome c oxidase subunit VIc	CYT	3	21	11.6	High
TRINITY_DN5107_c0_g1_i4_orf1	peptide methionine sulfoxide reductase [Ostrinia furnacalis]	3.847917463	1.944077854	0.0001063	up	yes	1.007	0.2617	0.184	0.264	0.337	1	1.046	0.976	MF:peptide-methionin;MF:catalytic activity;MF:oxidoreductase activity, acting on a sulfur group of donors, disulfide as acceptor;MF:molecular_function;MF:oxidoreductase activity, acting on a sulfur	GO:0008	K07304	msrA	------	------	COG0225	O:Posttranslational modification,	PF01625.24	PMSR	Peptide methionine sulfoxide reductase	CYT	1	4	26.1	Medium
TRINITY_DN7964_c0_g1_i1_orfp1	TRINITY_DN7964_c0_g1_i1_m.23483 TRINITY_DN7964_c0_g1::TRINITY_DN7964_c0_g1_i1::g.23483 ORF type:internal len:79 (+),score=44.25 TRINITY_DN7964_c0_g1_i1:3-236(+)	3.181398214	1.669660964	1.38E-05	up	yes	1.033	0.3247	0.337	0.286	0.351	1	1.067	1.033	------	------	------	------	------	------	------	------	------	------	------	CYT	2	17	8.7	High
TRINITY_DN46409_c0_g1_i1_orf1	unnamed protein product [Heterotrigona itama]	3.929961089	1.974515028	0.0001887	up	yes	1.01	0.257	0.226	0.251	0.294	1	1.107	0.923	BP:regulation of T cell mediated immunity;BP:establishment of localization;BP:regulation of primary metabolic process;BP:regulation of metabolic process;BP:positive regulation of response to stimulus;BP:regulation of response to stimulus;BP:regulation of lymphocyte mediated immunity;BP:positive regulation of leukocyte mediated immunity;BP:nitrogen compound transport;BP:regulation of molecular function;BP:signal transduction;BP:T cell activation;BP:organic cyclic compound metabolic process;BP:positive regulation of lymphocyte mediated immunity;BP:leukocyte proliferation;BP:cellular response to stimulus;BP:response to unfolded protein;BP:positive regulation of cell adhesion;BP:cellular response to organic substance;BP:positive regulation of endopeptidase activity;BP:regulation of cytokine production;BP:interspecies interaction between organisms;BP:leukocyte activation involved in immune response;BP:isotype switching to IgG isotypes;BP:positive regulation of interferon-gamma	GO:0002	K04077	groEL, HSPD	map05152;m	Tuberculosis;Type I	COG0459	G:Carbohydrate transport and m	PF00118.27	Cpn60_TCP1	TCP-1/cpn60 chaperonin family	CYT	2	7	54.5	High

TRINITY_DN1617_c0_g1_i5_orf1	hypothetical protein evm_009822 [Chilo suppressalis] >CAB3525311.1 unnamed protein product [Chilo suppressalis] >CAH0402638.1 unnamed protein product [Chilo suppressalis]	2.686125441	1.42552668	2.52E-06	up	yes	0.9893	0.3683	0.343	0.394	0.368	1	0.98	0.988	CC:integral component of membrane;CC:intrinsic component of membrane;CC:cellular_component;CC:cellular anatomical entity;MF:purine ribonucleoside triphosphate binding;MF:molecular_function;MF:binding;MF:ion binding;MF:heterocyclic compound binding;MF:nucleoside phosphate binding;MF:anion binding;MF:nucleotide binding;MF:purine nucleotide binding;MF:ATP binding;MF:hydrolase activity;MF:small molecule binding;MF:catalytic activity;MF:adenyl nucleotide binding;MF:carbohydrate derivative binding;MF:organic cyclic compound binding;MF:adenyl ribonucleotide binding;MF:purine ribonucleotide	GO:0016(	K01511	ENTPD5_6	map00240;m	Pyrimidine metabo	COG5371;COG5:	G:Carbohydrate transport and m	PF01150.20	GDA1_CD39	GDA1/CD39	CYT	3	7	51.5	High
TRINITY_DN3235_c0_g1_i1_orf1	SPARC [Trichoplusia ni]	4.024929634	2.008963562	6.79E-06	up	yes	1.001	0.2487	0.246	0.23	0.27	1	1.039	0.964	CC:extracellular space;CC:cellular_component;CC:cellular anatomical entity;MF:cation binding;MF:ion binding;MF:molecular_function;MF:binding;MF:metal ion binding;MF:calcium ion	GO:0005(	K24262	SPARC	------	------	ENOG41101WW	S:Function unknown	PF10591.12	SPARC_Ca_bdg	Secreted protein acidic and rich in cysteine Ca binding region	CYT	6	24	36.9	High
TRINITY_DN2266_c0_g1_i6_orf1	bilin-binding protein-like [Ostrinia furnacalis]	2.80816443	1.489627414	1.28E-05	up	yes	0.9837	0.3503	0.337	0.336	0.378	1	1.007	0.944	MF:pigment binding;MF:molecular_function;MF:binding;	GO:0031	K03098	APOD	------	------	COG3040	M:Cell wall/membrane/envelope	PF00061.26;PF08	Lipocalin;Lipocalin_2;Triabin	Lipocalin / cytosolic fatty-acid binding protein family;Lipocalin-like domain:Triabin	CYT	6	31	22.1	High
TRINITY_DN5459_c0_g1_i1_orf1	protein takeout-like isoform X2 [Ostrinia furnacalis]	4.341991342	2.118356849	1.06E-05	up	yes	1.003	0.231	0.233	0.192	0.268	1	1.035	0.974	------	------	------	------	------	------	ENOG4111E83;E	S:Function unknown;	PF06585.14	JHBP	Haemolymph juvenile hormone binding	CYT	1	4	21.2	Medium
TRINITY_DN97680_c0_g1_i1_orf1	39S ribosomal protein L52, mitochondrial [Ostrinia furnacalis]	2.474752872	1.307284465	0.001079	up	yes	0.9263	0.3743	0.437	0.285	0.401	1	0.939	0.84	BP:cellular biosynthetic process;BP:cellular nitrogen compound metabolic process;BP:nitrogen compound metabolic process;BP:cellular macromolecule biosynthetic process;BP:organic substance biosynthetic process;BP:cellular macromolecule metabolic process;BP:peptide biosynthetic process;BP:organic substance metabolic process;BP:mitochondrial translation;BP:amide biosynthetic process;BP:cellular process;BP:biosynthetic process;BP:macromolecule biosynthetic process;BP:biological_process;BP:metabolic process;BP:cellular nitrogen compound biosynthetic process;BP:primary metabolic process;BP:organonitrogen compound metabolic process;BP:cellular amide metabolic process;BP:organonitrogen compound biosynthetic process;BP:protein metabolic process;BP:cellular metabolic process;BP:macromolecule metabolic process;BP:peptide metabolic process;BP:translation;CC:large ribosomal subunit;CC:organellar large	GO:0044	K17433	MRPL52	------	------	ENOG4111XV4;C	J:Translation, ribosomal structure	PF18699.4	MRPL52	Mitoribosomal protein mL52	CYT	1	20	14.8	Medium
TRINITY_DN3194_c0_g1_i6_orf1	uncharacterized protein LOC114361386 [Ostrinia furnacalis]	6.234567901	2.640289575	5.05E-06	up	yes	1.01	0.162	0.161	0.14	0.185	1	0.977	1.052	BP:nitrogen compound metabolic process;BP:organic substance metabolic process;BP:proteolysis;BP:biological_process;BP:metabolic process;BP:primary metabolic process;BP:organonitrogen compound metabolic process;BP:protein metabolic process;BP:macromolecule metabolic process;CC:integral component of membrane;CC:intrinsic component of membrane;CC:cellular anatomical entity;CC:cellular_component;MF:aminopeptidase activity;MF:exopeptidase activity;MF:molecular_function;MF:binding;MF:peptidase activity;MF:metallopeptidase activity;MF:cation binding;MF:transition metal ion binding;MF:ion binding;MF:metal ion binding;MF:hydrolase	GO:0006	K11140	ANPEP, CD1:	map04640;m	Hematopoietic cell	COG0308	E:Amino acid transport and meta	PF01433.23;PF11	Peptidase_M1;ERAP1_C;Peptidase_M1_N	Peptidase family M1 domain;ERAP1-like C-terminal domain;Peptidase M1 N-terminal domain	CYT	4	7	109.4	High
TRINITY_DN3332_c0_g1_i2_orf1	glutathione S-transferase sigma3 [Glyphodes pyloalis]	3.650736615	1.868187588	1.52E-05	up	yes	1.016	0.2783	0.245	0.256	0.334	1	1.032	1.015	MF:catalytic activity;MF:transferase activity;MF:glutathione transferase activity;MF:molecular_function;MF:transferase activity, transferring alkyl or ary;	GO:0003	K04097	HPGDS	map00590;m	Arachidonic acid m	ENOG41103AU;E	O:Posttranslational modification,	PF02798.23;PF14	GST_N;GST_C_3;GST_C	Glutathione S-transferase, N-terminal domain;Glutathione S-transferase, C-terminal domain;Glutathione S-transferase, C-terminal	CYT	3	50	20.6	High
TRINITY_DN94355_c0_g1_i2_orf1	uncharacterized protein LOC126369488 [Pectinophora gossypiella]	5.672413793	2.503962779	7.99E-06	up	yes	0.987	0.174	0.15	0.17	0.202	1	1.019	0.942	------	------	------	------	------	------	ENOG410Z1QR;E	S:Function unknown;L:Replicatio	PF05380.16;PF18	Peptidase_A17;DUF5641;DUF1759;DUF1758;rve	Pao retrotransposon peptidase;Family of unknown function;Protein of unknown function;Putative	CYT	1	0	179.7	High
TRINITY_DN141462_c0_g1_i1_orf1	mitochondrial-processing peptidase subunit beta [Diachasma alloeum] >THK33262.1 core protein 1, ubiquinol-cytochrome c reductase [Diachasma alloeum]	3.547476174	1.826792995	4.03E-06	up	yes	1.005	0.2833	0.28	0.251	0.319	1	1.018	0.998	BP:mitochondrial protein processing;BP:nitrogen compound metabolic process;BP:organic substance metabolic process;BP:proteolysis;BP:protein processing involved in protein targeting to mitochondrion;BP:biological_process;BP:metabolic process;BP:protein processing;BP:primary metabolic process;BP:organonitrogen compound metabolic process;BP:protein maturation;BP:protein metabolic process;BP:macromolecule metabolic process;CC:catalytic complex;CC:macromolecular complex;CC:cellular_component;CC:mitochondrial processing peptidase complex;CC:mitochondrial protein complex;MF:endopeptidase activity;MF:molecular_function;MF:binding;MF:peptidase activity;MF:metallopeptidase activity;MF:cation binding;MF:ion binding;MF:metal ion binding;MF:hydrolase activity;MF:metalloendopeptidase	GO:0034	K17732	PMPCB, MA	------	------	COG0612	S:Function unknown	PF00675.23;PF05	Peptidase_M16;Peptidase_M16_C	Insulinase;Peptidase M16 inactive domain	CYT	1	2	53.3	High

TRINITY_DN29018_c0_g1_i4_orf1	prostaglandin reductase 1-like isoform X1 [Ostrinia furnacalis] >XP_028178925.1 prostaglandin reductase 1-like isoform X2 [Ostrinia furnacalis]	5.271891892	2.398320786	3.74E-06	up	yes	0.9753	0.185	0.154	0.19	0.211	1	0.977	0.949	CC:cytoplasm;CC:cellular_component;CC :cellular anatomical entity;MF:13-prostaglandin reductase activity;MF:oxidoreductase activity, acting on CH-OH group of donors;MF:oxidoreductase activity, acting on the CH-CH group of donors, NAD or NADP as acceptor;MF:oxidoreductase activity, acting on the CH-OH group of donors, NAD or NADP as acceptor;MF:oxidoreductase activity, acting on the CH-CH group of donors;MF:catalytic activity;MF:molecular_function;MF:15-oxoprostaglandin 13-oxidase activity;MF:2-alkenal reductase	GO:00057	K13948	PTGR1, LTB4	map00590	Arachidonic acid m	COG2130	S:Function unknown	PF16884.8;PF001	ADH_N_2;ADH_zinc_N;ADH_zinc_N_2	N-terminal domain of oxidoreductase;Zinc-binding dehydrogenase;Zinc-binding dehydrogenase	CYT	7	24	36.5	High
TRINITY_DN28577_c0_g1_i6_orf1	delta-1-pyrroline-5-carboxylate dehydrogenase, mitochondrial [Nymphalis io]	3.178571429	1.668378509	4.43E-06	up	yes	0.979	0.308	0.295	0.289	0.34	1	0.961	0.976	BP:small molecule catabolic process;BP:carboxylic acid metabolic process;BP:cellular amino acid catabolic process;BP:nitrogen compound metabolic process;BP:small molecule metabolic process;BP:glutamine family amino acid catabolic process;BP:glutamine family amino acid metabolic process;BP:organic substance catabolic process;BP:proline catabolic process;BP:proline metabolic process;BP:cellular amino acid metabolic process;BP:organic substance metabolic process;BP:alpha-amino acid metabolic process;BP:alpha-amino acid catabolic process;BP:primary metabolic process;BP:cellular process;BP:proline catabolic process to glutamate;BP:metabolic process;BP:oxoacid metabolic process;BP:catabolic process;BP:cellular catabolic process;BP:dicarboxylic acid metabolic process;BP:organonitrogen compound metabolic process;BP:organonitrogen compound catabolic process;BP:organic acid metabolic process;BP:carboxylic acid catabolic process;BP:organic acid catabolic process;BP:cellular metabolic	GO:00442	K00294	E1.2.1.88	map00250;m	Alanine, aspartate a	COG1012	S:Function unknown	PF00171.25	Aldedh	Aldehyde dehydrogenase family	CYT	3	34	11.3	High
TRINITY_DN41259_c0_g1_i6_orf1	endocuticle structural glycoprotein SgAbd-8 [Ostrinia furnacalis]	6.274050633	2.649397172	3.35E-07	up	yes	0.9913	0.158	0.15	0.144	0.18	1	0.995	0.979	MF:molecular_function;MF:structural constituent of cuticle;MF:structural molecule activity;	GO:00036	------	------	------	------	ENOG4111PJ1	S:Function unknown	PF00379.26	Chitin_bind_4	Insect cuticle protein	CYT	7	44	24.7	High
TRINITY_DN76283_c0_g6_i1_orf1	fatty acid synthase-like [Ostrinia furnacalis]	3.934108527	1.976036757	2.18E-05	up	yes	1.015	0.258	0.231	0.266	0.277	1	0.972	1.073	BP:fatty acid biosynthetic process;BP:fatty acid metabolic process;BP:carboxylic acid metabolic process;BP:cellular biosynthetic process;BP:small molecule metabolic process;BP:small molecule biosynthetic process;BP:monocarboxylic acid biosynthetic process;BP:organic substance biosynthetic process;BP:organic substance metabolic process;BP:lipid metabolic process;BP:cellular process;BP:monocarboxylic acid metabolic process;BP:biosynthetic process;BP:biological_process;BP:metabolic process;BP:oxoacid metabolic process;BP:cellular lipid metabolic process;BP:lipid biosynthetic process;BP:primary metabolic process;BP:organic acid metabolic process;BP:carboxylic acid biosynthetic process;BP:organic acid biosynthetic process;BP:cellular metabolic process;MF:thiolester hydrolase activity;MF:molecular_function;MF:oxidoreductase activity;MF:oleoyl-[acyl-carrier-protein] hydrolase activity;MF:transferase activity;MF:transferase activity,	GO:00066	K00665	FASN	map04910;m	Insulin signaling pa	COG3321;ENOG	Q:Secondary metabolites biosynt	PF13602.9;PF001	ADH_zinc_N_2;ADH_zinc_N	Zinc-binding dehydrogenase;Zinc-binding dehydrogenase	CYT	1	2	95	High
TRINITY_DN61674_c0_g1_i2_orf1	fatty acid-binding protein 1-like [Ostrinia furnacalis]	9.03539823	3.175588188	7.21E-06	up	yes	1.021	0.113	0.099	0.115	0.125	1	0.985	1.079	MF:molecular_function;MF:lipid binding;MF:binding;	GO:00036	K08753;K	FABP4, aP2;F	map04923;m	Regulation of lipoly	ENOG4111US8	I:Lipid transport and metabolism	------	------	------	CYT	2	12	14.2	High
TRINITY_DN1199_c0_g1_i1_orf1	pupal cuticle protein 36a-like [Ostrinia furnacalis]	3.566559024	1.834532849	4.36E-07	up	yes	0.994	0.2787	0.264	0.285	0.287	1	1.006	0.976	------	------	------	------	------	------	ENOG4110QGB	S:Function unknown	------	------	------	CYT	1	2	23.9	High
TRINITY_DN82320_c0_g1_i2_orf1	glutathione S-transferase sigma3 [Glyphodes pyloalis]	7.415841584	2.890610426	0.0001459	up	yes	0.9737	0.1313	0.129	0.095	0.17	1	0.867	1.054	MF:catalytic activity;MF:transferase activity;MF:glutathione transferase activity;MF:molecular_function;MF:transferase activity, transferring alkyl or aryl	GO:00038	K04097	HPGDS	map00590;m	Arachidonic acid m	ENOG4110YU0;E	O:Posttranslational modification,	PF14497.9	GST_C_3	Glutathione S-transferase, C-terminal domain	CYT	1	13	7.2	High
TRINITY_DN37538_c0_g3_i1_orf1	esterase FE4-like [Ostrinia furnacalis]	2.667920162	1.415715494	0.0004272	up	yes	0.9223	0.3457	0.365	0.296	0.376	1	0.836	0.931	------	------	K07378	NLGN	map04514	Cell adhesion mole	COG2272;COG0	I:Lipid transport and metabolism	PF00135.31	COesterase	Carboxylesterase family	CYT	1	13	13.4	High
TRINITY_DN117_c0_g1_i6_orf1	lipase member I-like [Ostrinia furnacalis]	12.95745506	3.695710485	6.67E-07	up	yes	1.002	0.07733	0.059	0.071	0.102	1	1.022	0.984	BP:organic substance metabolic process;BP:lipid metabolic process;BP:primary metabolic process;BP:metabolic process;BP:biological_process;CC:cellular_component;CC:extracellular region;CC:cellular anatomical entity;MF:hydrolase activity;MF:hydrolase activity, acting on ester bonds;MF:catalytic activity;MF:molecular_function;MF:lipase	GO:00717	------	------	------	------	ENOG41116Y2;E	S:Function unknown;	PF00151.22	Lipase	Lipase	CYT	1	11	42.3	High
TRINITY_DN19160_c0_g1_i1_orf1	alkyldihydroxyacetonephosphate synthase [Ostrinia furnacalis]	5.6266387	2.492273327	7.41E-05	up	yes	1.073	0.1907	0.132	0.197	0.243	1	1.074	1.144	------	------	K00803	AGPS, agpS	map00565;m	Ether lipid metabol	COG0277	C:Energy production and convers	PF02913.22;PF01	FAD-oxidase_C;FAD_binding_4	FAD linked oxidases, C-terminal domain;FAD binding	CYT	1	1	69.6	Medium
TRINITY_DN81803_c0_g2_i1_orf1	cathepsin K-like [Ostrinia furnacalis]	12.12562753	3.599987506	1.02E-06	up	yes	0.9903	0.08167	0.081	0.053	0.111	1	0.975	0.996	BP:primary metabolic process;BP:organonitrogen compound metabolic process;BP:protein metabolic process;BP:macromolecule metabolic process;BP:organic substance metabolic process;BP:nitrogen compound metabolic process;BP:biological_process;BP:metabolic process;BP:proteolysis;MF:molecular_function;MF:hydrolase activity;MF:catalytic activity;MF:catalytic activity, acting on a protein;MF:peptidase	GO:00442	------	------	------	------	COG4870	O:Posttranslational modification,	------	------	------	CYT	1	8	13.3	High
TRINITY_DN99_c0_g1_i3_orf1	uncharacterized protein LOC126375979 [Pectinophora gossypiella] >XP_049879066.1 uncharacterized protein LOC126375979 [Pectinophora gossypiella]	2.433688816	1.283144709	0.001549	up	yes	0.89	0.3657	0.34	0.312	0.445	1	0.838	0.832	------	------	------	------	------	------	ENOG410YKH0;E	S:Function unknown;	------	------	------	CYT	1	1	140.2	Medium
TRINITY_DN41086_c0_g1_i4_orf1	collagenase-like [Pectinophora gossypiella]	13.60846122	3.766432038	3.30E-06	up	yes	0.9843	0.07233	0.051	0.051	0.115	1	0.994	0.959	BP:organic substance metabolic process;BP:organonitrogen compound metabolic process;BP:protein metabolic process;BP:macromolecule metabolic process;BP:biological_process;BP:nitrogen compound metabolic process;BP:metabolic process;BP:proteolysis;BP:primary metabolic process;MF:serine-type endopeptidase activity;MF:endopeptidase activity;MF:hydrolase activity;MF:serine hydrolase activity;MF:catalytic activity, acting on a protein;MF:catalytic activity;MF:molecular_function;MF:pepti	GO:00717	------	------	------	------	COG5640	O:Posttranslational modification,	PF00089.29;PF13	Trypsin;Trypsin_2	Trypsin;Trypsin-like peptidase domain	CYT	3	10	36.1	High

TRINITY_DN5740_c0_g1_i4_orf1	unconventional myosin IC isoform X1 [Ostrinia furnacalis]	4.813942534	2.26721892	2.35E-06	up	yes	1.022	0.2123	0.2	0.191	0.246	1	1.033	1.033	CC:cytoplasm;CC:myosin complex;CC:macromolecular complex;CC:cellular_component;CC:cellular anatomical entity;MF:purine ribonucleoside triphosphate binding;MF:molecular_function;MF:binding;MF:ion binding;MF:heterocyclic compound binding;MF:nucleoside phosphate binding;MF:nucleotide binding;MF:actin binding;MF:purine nucleotide binding;MF:motor activity;MF:ATP binding;MF:anion binding;MF:small molecule binding;MF:cytoskeletal protein binding;MF:carbohydrate derivative binding;MF:organic cyclic compound binding;MF:adenyl nucleotide binding;MF:adenyl ribonucleotide binding;MF:protein binding;MF:purine ribonucleotide binding;MF:ribonucleotide binding;	GO:00057	K10356	MYO1	map05130	Pathogenic Escheri	COG5022	S:Function unknown	PF00063.24;PF06	Myosin_head; Myosin_TH1;IQ	Myosin head;Unconventional myosin tail, actin- and lipid-binding;IQ calmodulin-binding motif	CYT	15	15	119.7	High
TRINITY_DN69713_c0_g1_i1_orf1	membrane-bound alkaline phosphatase-like [Ostrinia furnacalis]	4.765533981	2.252637878	5.67E-07	up	yes	0.9817	0.206	0.202	0.199	0.217	1	0.987	0.958	MF:hydrolase activity;MF:phosphatase activity;MF:alkaline phosphatase activity;MF:hydrolase activity, acting on ester bonds;MF:phosphoric ester hydrolase activity;MF:catalytic activity;MF:molecular_function;	GO:00167	K01077	E3.1.3.1, pho	map00730;m	Thiamine metaboli	COG1785	P:Inorganic ion transport and me	PF00245.23	Alk_phosphatase	Alkaline phosphatase	CYT	3	7	57.6	High
TRINITY_DN11587_c0_g1_i7_orf1	elongation of very long chain fatty acids protein AAEL008004-like isoform X1 [Danaus plexippus plexippus] >XP_032511006.1 elongation of very long chain fatty acids protein AAEL008004-like isoform X1 [Danaus plexippus plexippus] >XP_032511007.1 elongation of very long chain fatty acids protein AAEL008004-like isoform X1 [Danaus plexippus plexippus] >XP_032511008.1 elongation of very long chain fatty acids protein AAEL008004-like isoform X1 [Danaus plexippus plexippus] >XP_032511009.1 elongation of very long chain fatty acids protein AAEL008004-like isoform X1 [Danaus plexippus plexippus]	6.857142857	2.777607579	1.21E-06	up	yes	1.008	0.147	0.155	0.113	0.173	1	1.011	1.014	------	------	K10250	ELOVL7	map01040;m	Biosynthesis of uns	ENOG410XRWT	C:Energy production and convers	PF01151.21	ELO	GNS1/SUR4 family	CYT	1	4	36.4	High
TRINITY_DN16931_c0_g1_i1_orf1	pancreatic triacylglycerol lipase-like [Ostrinia furnacalis]	6.876295784	2.781631604	1.41E-06	up	yes	0.995	0.1447	0.126	0.127	0.181	1	0.986	0.999	------	------	------	------	------	------	ENOG4110RTX;E	S:Function unknown;	PF00151.22	Lipase	Lipase	PLA	5	26	32.3	High
TRINITY_DN39200_c0_g1_i5_orf1	juvenile hormone epoxide hydrolase-like [Ostrinia furnacalis]	11.78797657	3.559244194	4.74E-06	up	yes	0.9863	0.08367	0.08	0.044	0.127	1	0.962	0.997	CC:integral component of membrane;CC:cellular_component;CC:intrinsic component of membrane;CC:cellular anatomical entity;MF:hydrolase activity;MF:cis-stilbene-oxide hydrolase activity;MF:hydrolase activity, acting on ether bonds;MF:catalytic activity;MF:molecular_function;MF:ether	GO:0016(	K10719	JHEH	map00981	Insect hormone bic	COG0596;COG0	U:Intracellular trafficking, secretic	PF06441.15;PF00	EHN;Abhydrolase_1	Epoxide hydrolase N terminus;alpha/beta hydrolase fold	PLA	1	4	56.8	High
TRINITY_DN37538_c0_g1_i1_orf1	esterase FE4-like [Ostrinia furnacalis]	10.64983948	3.41275978	3.33E-06	up	yes	0.962	0.09033	0.08	0.077	0.114	1	0.957	0.929	------	------	K07378	NLGN	map04514	Cell adhesion mole	COG2272;ENOG	I:Lipid transport and metabolism	PF00135.31;PF20	COesterase;BD-FAE;Abhydrolase_3	Carboxylesterase family;BD-FAE;alpha/beta hydrolase fold	CYT	1	4	22.5	High
TRINITY_DN36199_c0_g1_i1_orf1	fatty acid-binding protein 1-like [Ostrinia furnacalis]	19.17307692	4.261009976	3.41E-07	up	yes	0.997	0.052	0.04	0.046	0.07	1	1.015	0.976	MF:molecular_function;MF:lipid binding;MF:binding;	GO:0003(	K08753;K	FABP4, aP2;F	map04923;m	Regulation of lipoly	ENOG4111US8	I:Lipid transport and metabolism	------	------	------	CYT	5	48	15.2	High
TRINITY_DN10332_c0_g1_i2_orfp1	TRINITY_DN10332_c0_g1_i2_m.42894 TRINITY_DN10332_c0_g1::TRINITY_DN10332_c0_g1_i2::g.42894 ORF type:3prime_partial len:77 (+),score=1.70 TRINITY_DN10332_c0_g1_i2:1005-1232(+)	2.129617193	1.090594124	6.36E-05	up	yes	0.9513	0.4467	0.465	0.416	0.459	1	0.932	0.922	------	------	------	------	------	------	------	------	------	------	------	CYT	1	8	9	Medium
TRINITY_DN6248_c0_g1_i1_orf1	DNA topoisomerase I, mitochondrial [Ostrinia furnacalis]	2.605215595	1.381402768	1.21E-05	up	yes	1.009	0.3873	0.377	0.356	0.429	1	1.027	1	BP:DNA conformation change;BP:nucleic acid metabolic process;BP:cellular nitrogen compound metabolic process;BP:nitrogen compound metabolic process;BP:cellular component organization or biogenesis;BP:nucleobase-containing compound metabolic process;BP:cellular macromolecule metabolic process;BP:DNA topological change;BP:cellular component organization;BP:organic substance metabolic process;BP:organic cyclic compound metabolic process;BP:cellular process;BP:cellular aromatic compound metabolic process;BP:biological_process;BP:metabolic process;BP:heterocycle metabolic process;BP:organelle organization;BP:primary metabolic process;BP:chromosome organization;BP:cellular metabolic process;BP:macromolecule metabolic process;BP:DNA metabolic process;CC:membrane-bounded organelle;CC:organelle;CC:nucleus;CC:chromosome;CC:cellular anatomical entity;CC:intracellular organelle;CC:intracellular membrane-bounded organelle;CC:non- membrane	GO:00711	K03163	TOP1	------	------	COG3569	L:Replication, recombination and	PF01028.23;PF02	Topoisom_I;Topoisom_I_N;Topo_C_assoc	Eukaryotic DNA topoisomerase I, catalytic core;Eukaryotic DNA topoisomerase I, DNA binding fragment;C-terminal topoisomerase domain	CYT	8	9	110.1	High
TRINITY_DN2274_c0_g1_i6_orf1	membrane alanyl aminopeptidase-like [Ostrinia furnacalis]	2.519032014	1.332869457	1.14E-06	up	yes	0.9993	0.3967	0.399	0.382	0.409	1	0.982	1.016	BP:nitrogen compound metabolic process;BP:organic substance metabolic process;BP:proteolysis;BP:biological_process;BP:metabolic process;BP:primary metabolic process;BP:organonitrogen compound metabolic process;BP:protein metabolic process;BP:macromolecule metabolic process;MF:aminopeptidase activity;MF:exopeptidase activity;MF:molecular_function;MF:zinc ion binding;MF:peptidase activity;MF:metallopeptidase activity;MF:cation binding;MF:transition metal ion binding;MF:ion binding;MF:metal ion binding;MF:hydrolase activity;MF:catalytic activity, acting on a protein;MF:catalytic activity;MF:binding;	GO:00068	------	------	------	------	COG0308	E:Amino acid transport and meta	PF01433.23;PF11	Peptidase_M1;ERAP1_C;Peptidase_M1_N	Peptidase family M1 domain;ERAP1-like C-terminal domain;Peptidase M1 N-terminal domain	CYT	11	15	114.7	High
TRINITY_DN53167_c0_g1_i2_orf1	uncharacterized protein LOC114359219 [Ostrinia furnacalis]	2.47794303	1.309143019	1.83E-05	up	yes	0.983	0.3967	0.41	0.353	0.427	1	0.984	0.965	BP:primary metabolic process;BP:carbohydrate metabolic process;BP:organic substance metabolic process;BP:biological_process;BP:metabolic process;CC:cytoplasm;CC:cellular_component;CC:cellular anatomical entity;MF:hydrolase activity;MF:catalytic activity;MF:hydrolase activity, acting on glycosyl bonds;MF:beta-fructofuranosidase activity;MF:hydrolase activity, hydrolyzing O-glycosyl compounds;MF:molecular_function;	GO:00442	------	------	------	------	COG1621	G:Carbohydrate transport and m	PF00251.23;PF08	Glyco_hydro_32N;Glyco_hydro_32C	Glycosyl hydrolases family 32 N-terminal domain;Glycosyl hydrolases family 32 C terminal	CYT	2	5	59.8	High
TRINITY_DN2579_c0_g1_i7_orf1	aminopeptidase N5 [Ostrinia nubilalis]	2.542635659	1.346324749	6.31E-05	up	yes	0.984	0.387	0.348	0.36	0.453	1	0.973	0.979	BP:nitrogen compound metabolic process;BP:organic substance metabolic process;BP:proteolysis;BP:biological_process;BP:metabolic process;BP:primary metabolic process;BP:organonitrogen compound metabolic process;BP:protein metabolic process;BP:macromolecule metabolic process;CC:integral component of membrane;CC:intrinsic component of membrane;CC:cellular anatomical entity;CC:cellular_component;MF:aminopeptidase activity;MF:exopeptidase activity;MF:molecular_function;MF:binding;MF:peptidase activity;MF:metallopeptidase activity;MF:cation binding;MF:transition metal ion binding;MF:ion binding;MF:metal ion binding;MF:hydrolase	GO:00068	K11140	ANPEP, CD1	map04640;m	Hematopoietic cell	COG0308	L:Replication, recombination and	PF01433.23;PF11	Peptidase_M1;ERAP1_C;Peptidase_M1_N	Peptidase family M1 domain;ERAP1-like C-terminal domain;Peptidase M1 N-terminal domain	CYT	3	3	108	High
TRINITY_DN37538_c0_g4_i1_orf1	esterase FE4-like [Ostrinia furnacalis]	2.727347386	1.44749847	8.44E-06	up	yes	0.9963	0.3653	0.404	0.355	0.337	1	1.009	0.98	------	------	------	------	------	------	COG2272;COG0	I:Lipid transport and metabolism	PF00135.31	COesterase	Carboxylesterase family	CYT	1	12	12.7	High

TRINITY_DN92232_c0_g1_i1_orf1	protein SDA1 homolog [Ostrinia furnacalis]	3.318196562	1.730399351	7.98E-05	up	yes	1.023	0.3083	0.226	0.362	0.337	1	1.03	1.039	BP:nuclear transport;BP:protein localization;BP:ribosome localization;BP:actin cytoskeleton organization;BP:nuclear export;BP:establishment of organelle localization;BP:cellular component organization or biogenesis;BP:ribosomal large subunit biogenesis;BP:cellular macromolecule localization;BP:cellular component organization;BP:nitrogen compound transport;BP:organic substance transport;BP:ribonucleoprotein complex biogenesis;BP:macromolecule localization;BP:organelle organization;BP:transport;BP:actin filament-based process;BP:cellular process;BP:nucleocytoplasmic transport;BP:establishment of protein localization;BP:biological_process;BP:establishment of localization in cell;BP:establishment of localization;BP:localization;BP:organelle localization;BP:cellular localization;BP:protein complex localization;BP:cytoskeleton organization;BP:intracellular transport;BP:ribosomal large subunit export from nucleus;BP:ribosomal	GO:00511	K14856	SDA1, SDAD	------	------	ENOG410XPHI	S:Function unknown	PF05285.15;PF08	SDA1;NUC130_3NT	SDA1;NUC130/3NT domain	CYT	1	1	84.7	Medium
TRINITY_DN36494_c0_g1_i1_orf1	MKI67 FHA domain-interacting nucleolar phosphoprotein-like [Ostrinia furnacalis]	3.23957323	1.69580377	1.27E-05	up	yes	1.002	0.3093	0.275	0.299	0.354	1	1.024	0.981	MF:organic cyclic compound binding;MF:molecular_function;MF:binding;MF:nucleic acid binding;MF:heterocyclic compound	GO:00971	K14838	NOP15	------	------	ENOG4111NF9	S:Function unknown	PF00076.25	RRM_1	RNA recognition motif.	CYT	2	6	30.6	High
TRINITY_DN2894_c0_g3_i1_orf1	lactase-phlorizin hydrolase-like [Ostrinia furnacalis]	3.221612903	1.687783156	7.34E-07	up	yes	0.9987	0.31	0.295	0.309	0.326	1	1.014	0.982	BP:primary metabolic process;BP:carbohydrate metabolic process;BP:organic substance metabolic process;BP:biological_process;BP:metabolic process;MF:hydrolase activity;MF:catalytic activity;MF:hydrolase activity, acting on glycosyl bonds;MF:hydrolase activity, hydrolyzing O-glycosyl	GO:00442	------	------	------	------	COG2723	G:Carbohydrate transport and m	PF00232.21	Glyco_hydro_1	Glycosyl hydrolase family 1	CYT	10	28	57.5	High
TRINITY_DN73923_c0_g1_i1_orf1	protein obstructor-E-like [Ostrinia furnacalis]	3.780669145	1.918641601	9.88E-07	up	yes	1.017	0.269	0.257	0.26	0.29	1	1.037	1.014	CC:cellular_component;CC:extracellular region;CC:cellular anatomical entity;MF:carbohydrate derivative binding;MF:molecular_function;MF:chitin binding;MF:binding;	GO:0005!	------	------	------	------	ENOG410ZSCN	S:Function unknown	PF01607.27	CBM_14	Chitin binding Peritrophin-A domain	CYT	15	65	26.5	High
TRINITY_DN2825_c0_g1_i3_orf1	carbonic anhydrase 2-like [Ostrinia furnacalis]	4.186515947	2.06575012	5.44E-06	up	yes	1.037	0.2477	0.228	0.239	0.276	1	1.067	1.043	MF:cation binding;MF:metal ion binding;MF:transition metal ion binding;MF:carbon-oxygen lyase activity;MF:hydro-lyase activity;MF:ion binding;MF:catalytic activity;MF:molecular_function;MF:zinc ion binding;MF:carbonate dehydratase activity;MF:lyase activity;MF:binding;	GO:00431	K01672	CA	map00910	Nitrogen metabolis	COG3338	I:Lipid transport and metabolism	PF00194.24	Carb_anhydrase	Eukaryotic-type carbonic anhydrase	CYT	2	8	30.6	High
TRINITY_DN35662_c0_g1_i5_orf1	hypothetical protein evm_006436 [Chilo suppressalis] >CAB3522373.1 unnamed protein product [Chilo suppressalis] >CAH0399695.1 unnamed protein product [Chilo suppressalis]	4.124545087	2.044235007	1.57E-05	up	yes	1.02	0.2473	0.217	0.221	0.304	1	1.044	1.015	MF:hydrolase activity;MF:molecular_function;MF:catalytic activity;	GO:00167	K21013	FPPP	map00981	Insect hormone bic	COG0647	G:Carbohydrate transport and m	PF13344.9;PF132	Hydrolase_6;Hydrolase_like	Haloacid dehalogenase-like hydrolase;HAD-hyrolase-like	CYT	2	6	32.9	High
TRINITY_DN6059_c0_g1_i1_orf1	brachyurin-like [Ostrinia furnacalis]	4.441558442	2.151065974	7.83E-05	up	yes	1.026	0.231	0.254	0.206	0.233	1	0.962	1.115	BP:organic substance metabolic process;BP:organonitrogen compound metabolic process;BP:protein metabolic process;BP:macromolecule metabolic process;BP:biological_process;BP:nitrogen compound metabolic process;BP:metabolic process;BP:proteolysis;BP:primary metabolic process;MF:serine-type endopeptidase activity;MF:endopeptidase activity;MF:hydrolase activity;MF:serine hydrolase activity;MF:catalytic activity, acting on a protein;MF:catalytic activity;MF:molecular_function;MF:pepti	GO:00717	K09634	TMPRSS3	------	------	COG5640	S:Function unknown	PF00089.29	Trypsin	Trypsin	CYT	1	4	30.1	High
TRINITY_DN15870_c0_g1_i3_orf1	PREDICTED: mitochondrial import inner membrane translocase subunit Tim23 isoform X1 [Fopius arisanus]	3.577737226	1.83904743	3.59E-05	up	yes	0.9803	0.274	0.314	0.216	0.292	1	0.998	0.943	CC:integral component of membrane;CC:cellular_component;CC:cellular anatomical entity;CC:intrinsic component of membrane;MF:transporter activity;MF:transmembrane transporter	GO:0016(	K17794	TIM23	map04212	Longevity regulatin	COG5596	O:Posttranslational modification,	PF02466.22	Tim17	Tim17/Tim22/Tim23/Pmp24 family	CYT	1	5	22.1	High
TRINITY_DN713_c0_g1_i4_orf1	periodic tryptophan protein 2 homolog isoform X1 [Ostrinia furnacalis] >XP_028176443.1 periodic tryptophan protein 2 homolog isoform X2 [Ostrinia furnacalis] >XP_028176445.1 periodic tryptophan protein 2 homolog isoform X3 [Ostrinia furnacalis]	4.475555556	2.162066777	0.0001013	up	yes	1.007	0.225	0.168	0.182	0.325	1	1.003	1.018	------	------	K14558	PWP2, UTP1	map03008	Ribosome biogenes	ENOG410XP2P	S:Function unknown	PF00400.35;PF12	WD40;ANAPC4_WD40;Utp12;eIF2A	WD domain, G-beta repeat;Anaphase-promoting complex subunit 4 WD40 domain;Dip2/Utp12 Family;Eukaryotic translation initiation factor	CYT	1	1	102.3	High
TRINITY_DN8603_c0_g1_i1_orf1	adenosine kinase 2 isoform X2 [Cotesia glomerata] >XP_044591805.1 adenosine kinase 2 isoform X4 [Cotesia glomerata]	2.254383599	1.172733021	0.008714	up	yes	0.8357	0.3707	0.422	0.27	0.42	1	0.733	0.774	BP:purine ribonucleoside monophosphate metabolic process;BP:nucleotide biosynthetic process;BP:small molecule metabolic process;BP:ribonucleoside monophosphate metabolic process;BP:organic cyclic compound biosynthetic process;BP:purine nucleoside biosynthetic process;BP:purine ribonucleoside monophosphate biosynthetic process;BP:AMP salvage;BP:AMP metabolic process;BP:purine ribonucleoside biosynthetic process;BP:purine ribonucleoside metabolic process;BP:ribonucleoside biosynthetic process;BP:heterocycle metabolic process;BP:organonitrogen compound metabolic process;BP:organonitrogen compound biosynthetic process;BP:purine nucleotide metabolic process;BP:AMP biosynthetic process;BP:purine ribonucleoside salvage;BP:purine nucleotide biosynthetic process;BP:organophosphate metabolic process;BP:aromatic compound biosynthetic process;BP:purine nucleotide salvage;BP:nucleoside	GO:00091	K00856	ADK, adoK	map01232;m	Nucleotide metabo	COG0524	G:Carbohydrate transport and m	PF00294.27	PfkB	pfkB family carbohydrate kinase	CYT	1	7	38.5	Medium
TRINITY_DN11376_c0_g2_i1_orf1	cathepsin K-like [Ostrinia furnacalis]	4.581939799	2.195958504	2.13E-05	up	yes	0.959	0.2093	0.191	0.19	0.247	1	0.968	0.909	BP:primary metabolic process;BP:organonitrogen compound metabolic process;BP:protein metabolic process;BP:macromolecule metabolic process;BP:organic substance metabolic process;BP:nitrogen compound metabolic process;BP:biological_process;BP:metabolic process;BP:proteolysis;MF:molecular_function;MF:hydrolase activity;MF:catalytic activity;MF:catalytic activity, acting on a protein;MF:peptidase	GO:00442	------	------	------	------	COG4870	O:Posttranslational modification,	PF00112.26;PF08	Peptidase_C1;Inhibitor_I29;Peptidase_C1_2	Papain family cysteine protease;Cathepsin propeptide inhibitor domain;Peptidase C1-like family	CYT	2	6	53.7	High
TRINITY_DN1914_c0_g1_i6_orf1	loricrin-like [Ostrinia furnacalis]	12.72192972	3.669245616	1.99E-05	up	yes	1.039	0.08167	0.07	0.074	0.101	1	1.119	0.999	------	------	------	------	------	------	ENOG4110OTU;I	S:Function unknown;	------	------	------	CYT	1	15	58.9	High
TRINITY_DN144190_c0_g1_i1_orf1	PREDICTED: uncharacterized protein LOC103572804 isoform X2 [Microplitis demolitor]	24.67028704	4.624702597	1.42E-05	up	yes	0.954	0.03867	0.012	0.039	0.065	1	0.971	0.891	------	------	------	------	------	------	ENOG4112B12	S:Function unknown	------	------	------	CYT	1	3	26.8	Medium

TRINITY_DN791_c0_g1_i2_orf1	peroxiredoxin 1 isoform X1 [Maniola jurtina]	2.311737987	1.208977892	9.28E-05	up	yes	0.967	0.4183	0.421	0.379	0.455	1	0.987	0.914	MF:antioxidant activity;MF:peroxiredoxin activity;MF:catalytic activity;MF:molecular_function;MF:oxidoreductase activity, acting on peroxide as acceptor;MF:peroxidase activity;MF:oxidoreductase activity;	GO:00162	K13279	PRDX1	map04146;m	Peroxisome;Amoeb	COG0450	L:Replication, recombination and	PF00578.24;PF10	AhpC-TSA;1-cysPrx_C;Redoxin	AhpC/TSA family;C-terminal domain of 1-Cys peroxiredoxin;R	CYT	7	32	26.5	High
TRINITY_DN3401_c0_g1_i1_orf1	28S ribosomal protein S5, mitochondrial [Ostrinia furnacalis]	2.361063254	1.239436692	0.000288	up	yes	0.9593	0.4063	0.451	0.323	0.445	1	0.946	0.932	BP:cellular biosynthetic process;BP:cellular macromolecule biosynthetic process;BP:organic substance biosynthetic process;BP:cellular nitrogen compound biosynthetic process;BP:cellular macromolecule metabolic process;BP:cellular amide metabolic process;BP:peptide biosynthetic process;BP:organic substance metabolic process;BP:organonitrogen compound biosynthetic process;BP:amide biosynthetic process;BP:cellular process;BP:biosynthetic process;BP:macromolecule biosynthetic process;BP:biological_process;BP:metabolic process;BP:organonitrogen compound metabolic process;BP:primary metabolic process;BP:protein metabolic process;BP:cellular nitrogen compound metabolic process;BP:cellular metabolic process;BP:macromolecule metabolic process;BP:nitrogen compound metabolic process;BP:peptide metabolic process;BP:translation;CC:organelle;CC:intracellular organelle;CC:non-membrane-bounded organelle;CC:cellular anatomical	GO:00442	K02988	RP-S5, MRPS	map03010	Ribosome	COG0098	J:Translation, ribosomal structure	PF03719.18;PF00	Ribosomal_S5_C;Ribosomal_S5	Ribosomal protein S5, C-terminal domain;Ribosomal protein S5, N-terminal domain	CYT	2	6	49	High
TRINITY_DN16830_c0_g1_i5_orf1	adrenodoxin [Ostrinia furnacalis]	2.916547175	1.54426141	8.17E-05	up	yes	1.017	0.3487	0.324	0.342	0.38	1	1.089	0.963	BP:cellular process;BP:cellular metabolic process;BP:electron transport chain;BP:metabolic process;BP:biological_process;BP:generation of precursor metabolites and energy;MF:metal cluster binding;MF:iron-sulfur cluster binding;MF:2 iron, 2 sulfur cluster binding;MF:molecular_function;MF:binding;	GO:00099	K22070	FDX1, ADX	------	------	COG0633	C:Energy production and convers	PF00111.30	Fer2	2Fe-2S iron-sulfur cluster binding domain	CYT	1	6	17.6	High
TRINITY_DN3503_c0_g1_i1_orfp1	uncharacterized protein LOC114356429 [Ostrinia furnacalis]	2.278593788	1.188143754	0.0001567	up	yes	0.9463	0.4153	0.396	0.45	0.4	1	0.883	0.956	------	------	------	------	------	------	------	------	------	------	------	CYT	1	9	14.4	High
TRINITY_DN12133_c0_g2_i1_orf1	O-acyltransferase like protein-like [Ostrinia furnacalis]	2.544270833	1.347252251	0.0002113	up	yes	0.977	0.384	0.361	0.332	0.459	1	1.005	0.926	CC:integral component of membrane;CC:cellular_component;CC:intrinsic component of membrane;CC:cellular anatomical entity;MF:catalytic activity;MF:transferase activity;MF:transferase activity, transferring acyl groups;MF:molecular_function;MF:transferase activity, transferring acyl groups	GO:0016(	------	------	------	------	ENOG410YD1T;E	S:Function unknown;	PF01757.25;PF20	Acyl_transf_3;NRF	Acyltransferase family;Nose resistant-to-fluoxetine protein, N-terminal domain	CYT	1	1	84.1	High
TRINITY_DN679_c0_g1_i2_orf1	cytochrome b-c1 complex subunit 7-like [Ostrinia furnacalis]	2.717808219	1.442443657	6.78E-06	up	yes	0.992	0.365	0.356	0.349	0.39	1	1.015	0.961	BP:aerobic respiration;BP:generation of precursor metabolites and energy;BP:electron transport chain;BP:respiratory electron transport chain;BP:cellular process;BP:aerobic electron transport chain;BP:cellular respiration;BP:metabolic process;BP:energy derivation by oxidation of organic compounds;BP:mitochondrial electron transport, ubiquinol to cytochrome c;BP:cellular metabolic process;BP:biological_process;CC:oxidoreductase complex;CC:respiratory chain complex;CC:cytochrome complex;CC:inner mitochondrial membrane protein complex;CC:mitochondrial respiratory chain complex III;CC:transmembrane transporter complex;CC:catalytic complex;CC:transporter complex;CC:macromolecular complex;CC:respiratory chain complex III;CC:cellular_component;CC:membrane	GO:0009(	K00417	QCR7, UQCR	map04714;m	Thermogenesis;Oxi	ENOG4111V1G	C:Energy production and convers	PF02271.19	UCR_14kD	Ubiquinol-cytochrome C reductase complex 14kD subunit	CYT	6	44	13.2	High
TRINITY_DN146544_c0_g1_i1_orf1	UPF0047 protein YjbQ [Aphidius gifuensis] >KAF7996225.1 hypothetical protein HCN44_001857 [Aphidius gifuensis]	2.303700025	1.203952869	0.0002011	up	yes	0.9277	0.4027	0.407	0.431	0.37	1	0.888	0.895	------	------	------	------	------	------	COG0432	S:Function unknown	PF01894.20	UPF0047	Uncharacterised protein family	CYT	1	9	16.6	High
TRINITY_DN16487_c0_g1_i1_orf1	p21-activated protein kinase-interacting protein 1-like [Ostrinia furnacalis]	2.287135922	1.193542106	0.0002286	up	yes	0.9423	0.412	0.363	0.465	0.408	1	0.928	0.899	BP:phosphorylation;BP:cellular process;BP:cellular metabolic process;BP:phosphate-containing compound metabolic process;BP:biological_process;BP:metabolic process;BP:phosphorus metabolic process;MF:kinase activity;MF:transferase activity;MF:catalytic activity;MF:molecular_function;MF:transferase activity, transferring phosphorus-	GO:0016	K14830	MAK11, PAK	------	------	ENOG410XRDQ	S:Function unknown	PF00400.35;PF17	WD40;WD40_like	WD domain, G-beta repeat;WD40-like domain	CYT	1	2	42.5	High
TRINITY_DN760_c1_g2_i6_orf1	ADP,ATP carrier protein [Pieris napi]	2.434361458	1.283543398	2.74E-05	up	yes	0.955	0.3923	0.388	0.385	0.404	1	0.912	0.953	BP:nucleobase-containing compound transport;BP:nucleotide transport;BP:anion transport;BP:carbohydrate derivative transport;BP:adenine nucleotide transport;BP:transmembrane transport;BP:nitrogen compound transport;BP:mitochondrial transmembrane transport;BP:mitochondrial ATP transmembrane transport;BP:organic substance transport;BP:purine nucleotide transport;BP:purine ribonucleotide transport;BP:ADP transport;BP:ATP transport;BP:ion transport;BP:transport;BP:organic anion transport;BP:ion transmembrane transport;BP:biological_process;BP:establishment of localization;BP:localization;BP:purine-containing compound transmembrane transport;BP:nucleotide transmembrane transport;BP:mitochondrial ADP transmembrane transport;BP:organophosphate ester transport;BP:anion transmembrane transport;BP:cellular process;CC:intrinsic component of membrane;CC:organelle membrane;CC:membrane;CC:cellular	GO:0015	K05863	SLC25A4S, A	map05166;m	Human T-cell leuk	ENOG410XNW0	U:Intracellular trafficking, secretio	PF00153.30	Mito_carr	Mitochondrial carrier protein	CYT	21	57	33	High

TRINITY_DN1901_c0_g1_i6_orf1	mitochondrial intermembrane space import and assembly protein 40 [Ostrinia furnacalis] >XP_028171079.1 mitochondrial intermembrane space import and assembly protein 40 [Ostrinia furnacalis] >XP_028171080.1 mitochondrial intermembrane space import and assembly protein 40 [Ostrinia furnacalis]	3.297700032	1.721460173	1.49E-05	up	yes	1.018	0.3087	0.327	0.256	0.343	1	1.029	1.025	BP:protein localization;BP:establishment of localization in cell;BP:protein transmembrane transport;BP:cellular macromolecule localization;BP:intracellular protein transport;BP:localization;BP:nitrogen compound transport;BP:intracellular protein transmembrane transport;BP:mitochondrial transmembrane transport;BP:organic substance transport;BP:macromolecule localization;BP:transport;BP:establishment of protein localization to organelle;BP:cellular process;BP:establishment of protein localization;BP:protein localization to organelle;BP:biological_process;BP:establishment of protein localization to mitochondrion;BP:mitochondrial transport;BP:establishment of localization;BP:transmembrane transport;BP:intracellular protein transmembrane import;BP:cellular localization;BP:protein localization to mitochondrion;BP:intracellular transport;BP:protein import into mitochondrial intermembrane space;BP:protein transport;MF:molecular_function;MF:oxid	GO:00081	K17782	MIA40, CHCH	------	------	ENOG4111V0I	O:Posttranslational modification,	PF06747.16	CHCH	CHCH domain	CYT	2	13	15	High
TRINITY_DN7964_c0_g1_i6_orfp1	TRINITY_DN7964_c0_g1_i6_m.23478 TRINITY_DN7964_c0_g1::TRINITY_DN7964_c0_g1_i6::g.23478 ORF type:internal len:87 (+),score=67.94 TRINITY_DN7964_c0_g1_i6:2-259(+)	3.924050633	1.972343657	7.40E-06	up	yes	1.023	0.2607	0.276	0.261	0.245	1	1.071	0.999	------	------	------	------	------	------	------	------	------	------	------	CYT	2	30	9	High
TRINITY_DN2184_c0_g1_i4_orf1	uncharacterized protein LOC114359356 [Ostrinia furnacalis]	2.852595354	1.512275113	1.08E-06	up	yes	0.9947	0.3487	0.353	0.331	0.362	1	0.976	1.008	CC:cellular_component;CC:extracellular region;CC:cellular anatomical entity;MF:molecular_function;MF:structural molecule activity;	GO:00055	------	------	------	------	------	------	PF07294.14	Fibroin_P25	Fibroin P25	CYT	2	8	28	High
TRINITY_DN55160_c0_g2_i1_orf1	esterase FE4-like isoform X2 [Ostrinia furnacalis]	9.195494771	3.200927202	0.0002352	up	yes	1.143	0.1243	0.129	0.055	0.189	1	1.217	1.213	------	------	------	------	------	------	COG2272	I:Lipid transport and metabolism	PF00135.31	COesterase	Carboxylesterase family	CYT	1	4	34.3	High
TRINITY_DN1109_c0_g1_i6_orf1	1-phosphatidylinositol phosphodiesterase-like [Cotesia glomerata]	3.005745389	1.587722806	4.43E-05	up	yes	0.994	0.3307	0.32	0.278	0.394	1	0.979	1.003	BP:primary metabolic process;BP:lipid metabolic process;BP:organic substance metabolic process;BP:biological_process;BP:metabolic process;MF:phosphoric diester hydrolase activity;MF:hydrolase activity;MF:hydrolase activity, acting on ester bonds;MF:phosphoric ester hydrolase activity;MF:molecular_function;MF:cataly	GO:00442	K01771	plc	map00562	Inositol phosphate	------	------	------	------	------	EXC	2	8	34.7	High
TRINITY_DN1154_c0_g1_i1_orf1	calexcitin-1-like [Ostrinia furnacalis] >ADK94879.2 juvenile hormone diol kinase [Ostrinia furnacalis]	3.768115942	1.913843356	1.44E-05	up	yes	0.962	0.2553	0.238	0.232	0.296	1	0.945	0.941	BP:phosphorylation;BP:cellular process;BP:cellular metabolic process;BP:phosphate-containing compound metabolic process;BP:biological_process;BP:metabolic process;BP:phosphorus metabolic process;MF:cation binding;MF:kinase activity;MF:metal ion binding;MF:transferase activity;MF:ion binding;MF:catalytic activity;MF:calcium ion binding;MF:molecular_function;MF:transferase activity, transferring phosphorus-	GO:00163	------	------	------	------	ENOG4111I7F	S:Function unknown	PF00036.35;PF13	EF-hand_1;EF-hand_5;EF-hand_6;EF-hand_7;EF-hand_8	EF hand;EF hand;EF-hand domain;EF-hand domain pair;EF-hand domain pair	CYT	5	35	20.6	High
TRINITY_DN3847_c1_g1_i1_orf1	ribosome production factor 2 homolog [Ostrinia furnacalis]	3.154313099	1.65732587	2.95E-05	up	yes	0.9873	0.313	0.287	0.348	0.304	1	0.937	1.025	BP:cellular component assembly;BP:macromolecular complex subunit organization;BP:nucleic acid metabolic process;BP:maturation of LSU-rRNA;BP:ncRNA metabolic process;BP:organic cyclic compound metabolic process;BP:ribosomal large subunit assembly;BP:nucleobase-containing compound metabolic process;BP:ribonucleoprotein complex subunit organization;BP:cellular component organization or biogenesis;BP:cellular component organization;BP:macromolecular complex assembly;BP:organic substance metabolic process;BP:ribonucleoprotein complex assembly;BP:rRNA processing;BP:ncRNA processing;BP:cellular process;BP:cellular aromatic compound metabolic process;BP:biological_process;BP:metabolic process;BP:heterocycle metabolic process;BP:RNA metabolic process;BP:primary metabolic process;BP:rRNA metabolic process;BP:cellular nitrogen compound metabolic process;BP:cellular metabolic process;BP:macromolecule metabolic process;BP:nitrogen compound	GO:00226	K14847	RPF2	------	------	COG5106	S:Function unknown	PF04427.21	Brix	Brix domain	CYT	2	7	35.9	High
TRINITY_DN57202_c0_g1_i1_orf1	PREDICTED: U4/U6 small nuclear ribonucleoprotein Prp31 [Amyelois transitella]	3.577760795	1.839056934	3.18E-05	up	yes	0.9363	0.2617	0.256	0.274	0.255	1	0.908	0.901	BP:cellular component assembly;BP:spliceosomal tri-snRNP complex assembly;BP:macromolecular complex subunit organization;BP:cellular component organization or biogenesis;BP:spliceosomal snRNP assembly;BP:cellular component organization;BP:macromolecular complex assembly;BP:ribonucleoprotein complex assembly;BP:cellular process;BP:ribonucleoprotein complex subunit organization;BP:biological_process;CC:Sm-like protein family complex;CC:U4/U6 x U5 tri-snRNP complex;CC:spliceosomal snRNP complex;CC:macromolecular complex;CC:small nuclear ribonucleoprotein complex;CC:ribonucleoprotein complex;CC:cellular_component;CC:spliceosomal tri-snRNP complex;CC:spliceosomal complex;MF:molecular_function;MF:binding;MF:nucleic acid binding;MF:heterocyclic compound	GO:00226	K12844	PRPF31	map03040	Spliceosome	COG1498	S:Function unknown	PF01798.21;PF09	Nop;Prp31_C	snoRNA binding domain, fibrillarin;Prp31 C terminal domain	CYT	1	3	55.5	High
TRINITY_DN25975_c0_g3_i2_orf1	V-type proton ATPase subunit D isoform X2 [Ostrinia furnacalis]	3.43354971	1.779700851	4.38E-06	up	yes	1.005	0.2927	0.299	0.259	0.32	1	0.99	1.026	MF:inorganic cation transmembrane transporter activity;MF:ATPase activity, coupled to transmembrane movement of substances;MF:active ion transmembrane transporter activity;MF:ATPase coupled ion transmembrane transporter activity;MF:inorganic solute uptake transmembrane transporter activity;MF:transporter activity;MF:cation transmembrane transporter activity;MF:hydrogen-translocating pyrophosphatase activity;MF:cation-transporting ATPase activity;MF:ion transmembrane transporter activity;MF:primary active transmembrane transporter activity;MF:transmembrane transporter activity;MF:molecular_function;MF:active transmembrane transporter activity;MF:proton-transporting ATPase activity, rotational mechanism;MF:hydrogen ion transmembrane transporter activity;MF:ATPase activity, coupled to transmembrane movement of ions,	GO:00228	K02149	ATPeV1D, AT	map05323;m	Rheumatoid arthriti	COG1394	C:Energy production and convers	PF01813.20	ATP-synt_D	ATP synthase subunit D	CYT	7	20	27.4	High

TRINITY_DN468_c0_g1_i3_orf1	transmembrane protein 41 homolog isoform X2 [Ostrinia furnacalis]	4.620122238	2.207931023	7.58E-06	up	yes	0.9827	0.2127	0.193	0.184	0.261	1	0.977	0.971	------	------	------	------	------	------	COG0398	S:Function unknown	PF09335.14	SNARE_assoc	SNARE associated Golgi protein	CYT	1	7	30.6	High
TRINITY_DN8838_c0_g1_i1_orf1	mannose-P-dolichol utilization defect 1 protein homolog [Ostrinia furnacalis]	2.310069534	1.207936278	0.003473	up	yes	0.897	0.3883	0.419	0.266	0.48	1	0.832	0.859	CC:cellular_component;CC:integral component of membrane;CC:cellular anatomical entity;CC:intrinsic component of membrane;	GO:0005!	K09660	MPDU1	------	------	ENOG4111FE3	S:Function unknown	PF04193.17	PQ-loop	PQ loop repeat	CYT	1	4	26.8	High
TRINITY_DN3862_c0_g1_i7_orf1	venom acid phosphatase Acph-1-like [Ostrinia furnacalis]	6.682119205	2.74030572	2.11E-06	up	yes	1.009	0.151	0.127	0.136	0.19	1	1.023	1.004	BP:nucleic acid metabolic process;BP:cellular nitrogen compound metabolic process;BP:nitrogen compound metabolic process;BP:organic cyclic compound metabolic process;BP:nucleobase-containing compound metabolic process;BP:cellular macromolecule metabolic process;BP:organic substance metabolic process;BP:cellular process;BP:cellular aromatic compound metabolic process;BP:biological_process;BP:metabolic process;BP:heterocycle metabolic process;BP:primary metabolic process;BP:cellular metabolic process;BP:macromolecule metabolic process;BP:DNA integration;BP:DNA metabolic process;MF:phosphatase activity;MF:phosphoric ester hydrolase activity;MF:molecular_function;MF:binding;MF:nucleic acid binding;MF:heterocyclic compound binding;MF:hydrolase activity;MF:hydrolase activity, acting on ester bonds;MF:catalytic activity;MF:organic cyclic compound	GO:0090:	K19283	ACPP	------	------	ENOG410ZVBQ	C:Energy production and convers	PF00328.25	His_Phos_2	Histidine phosphatase superfamily	CYT	1	2	43.7	Medium
TRINITY_DN6087_c0_g1_i7_orf1	uncharacterized protein LOC114355564 [Ostrinia furnacalis]	3.027707006	1.598225601	2.92E-05	up	yes	0.9507	0.314	0.286	0.316	0.34	1	0.914	0.938	MF:hydrolase activity;MF:methyl indole-3-acetate esterase activity;MF:hydrolase activity, acting on ester bonds;MF:catalytic activity;MF:molecular_function;MF:carbo	GO:0016	K03927	CES2	map00983	Drug metabolism -	COG2272;ENOG	I:Lipid transport and metabolism	PF00135.31;PF20	COesterase;BD-FAE;Abhydrolase_3	Carboxylesterase family;BD-FAE;alpha/beta hydrolase fold	CYT	5	9	61.5	High
TRINITY_DN19917_c0_g1_i1_orf1	synaptic vesicle glycoprotein 2B-like isoform X4 [Ostrinia furnacalis]	15.44119994	3.948712964	7.01E-07	up	yes	0.9677	0.06267	0.055	0.07	0.063	1	0.951	0.952	CC:integral component of membrane;CC:cellular_component;CC:cellular anatomical entity;CC:intrinsic component of membrane;MF:transporter activity;MF:transmembrane transporter	GO:0016(	K06258	SV2	map04512	ECM-receptor inte	ENOG410ZYFU;E	G:Carbohydrate transport and m	PF07690.19;PF00	MFS_1;Sugar_tr	Major Facilitator Superfamily;Sugar	CYT	1	2	57.3	High
TRINITY_DN72707_c0_g1_i1_orf1	uncharacterized protein LOC114357549 [Ostrinia furnacalis]	3.745571052	1.905185689	5.52E-08	up	yes	0.9937	0.2653	0.259	0.259	0.278	1	0.989	0.992	MF:hydrolase activity;MF:molecular_function;MF:hydrolase activity, acting on ester bonds;MF:carboxylic ester hydrolase activity;MF:catalytic activity;	GO:0016	K03927;K	CES2;ACHE	map00564;m	Glycerophospholipi	COG2272	I:Lipid transport and metabolism	PF00135.31;PF20	COesterase;BD-FAE;Abhydrolase_3	Carboxylesterase family;BD-FAE;alpha/beta hydrolase fold	CYT	3	7	60.3	High
TRINITY_DN13651_c0_g1_i2_orf1	40S ribosomal protein S12, mitochondrial [Ostrinia furnacalis]	3.797531816	1.925062052	8.75E-06	up	yes	0.9847	0.2593	0.233	0.241	0.304	1	0.992	0.962	BP:cellular biosynthetic process;BP:cellular nitrogen compound metabolic process;BP:nitrogen compound metabolic process;BP:cellular macromolecule biosynthetic process;BP:organic substance biosynthetic process;BP:cellular macromolecule metabolic process;BP:peptide biosynthetic process;BP:organic substance metabolic process;BP:amide biosynthetic process;BP:cellular process;BP:biosynthetic process;BP:macromolecule biosynthetic process;BP:biological_process;BP:metabolic process;BP:cellular nitrogen compound biosynthetic process;BP:primary metabolic process;BP:organonitrogen compound metabolic process;BP:cellular amide metabolic process;BP:organonitrogen compound biosynthetic process;BP:protein metabolic process;BP:cellular metabolic process;BP:macromolecule metabolic process;BP:peptide metabolic process;BP:translation;CC:small ribosomal subunit;CC:macromolecular complex;CC:ribosomal	GO:0044:	K02950	RP-S12, MRF	map03010	Ribosome	COG0048	S:Function unknown	PF00164.28	Ribosom_S12_S23	Ribosomal protein S12/S23	CYT	1	6	17.4	High
TRINITY_DN3332_c0_g1_i11_orf1	glutathione S-transferase sigma3 [Glyphodes pyloalis]	3.946279347	1.980493085	4.68E-06	up	yes	0.9917	0.2513	0.263	0.221	0.27	1	0.961	1.014	MF:catalytic activity;MF:transferase activity;MF:glutathione transferase activity;MF:molecular_function;MF:transferase activity, transferring alkyl or ary;	GO:0003	K04097	HPGDS	map00590;m	Arachidonic acid m	ENOG41103AU;E	O:Posttranslational modification,	PF14497.9;PF02	GST_C_3;GST_N;GST_C	Glutathione S-transferase, C-terminal domain;Glutathione S-transferase, N-terminal domain;Glutathione S-transferase, C-terminal	CYT	2	45	23.1	High
TRINITY_DN2083_c0_g1_i4_orf1	uncharacterized protein LOC114359113 [Ostrinia furnacalis]	5.24742268	2.391609004	1.00E-05	up	yes	1.018	0.194	0.197	0.161	0.224	1	1.064	0.989	------	------	------	------	------	------	ENOG410ZGJA	S:Function unknown	PF06585.14	JHBP	Haemolymph juvenile hormone binding	CYT	3	17	24.2	High
TRINITY_DN4356_c0_g1_i6_orf1	mulatexin-like [Ostrinia furnacalis]	4.417761667	2.143315589	5.20E-05	up	yes	0.975	0.2207	0.181	0.182	0.299	1	0.961	0.964	------	------	------	------	------	------	------	------	------	------	------	CYT	1	5	20.1	High
TRINITY_DN336_c0_g1_i6_orfp1	TRINITY_DN336_c0_g1_i6_m.64791 TRINITY_DN336_c0_g1::TRINITY_DN336_c0_g1_i6::g.64791 ORF type:complete len:61 (-),score=19.53 TRINITY_DN336_c0_g1_i6:236-418(-	9.433611885	3.237810247	1.05E-05	up	yes	1.016	0.1077	0.095	0.06	0.168	1	1.017	1.032	------	------	------	------	------	------	------	------	------	------	------	CYT	1	12	6.3	High
TRINITY_DN3784_c0_g1_i1_orf1	pancreatic triacylglycerol lipase-like [Ostrinia furnacalis]	8.622493461	3.10810513	4.71E-07	up	yes	0.989	0.1147	0.103	0.1	0.141	1	0.989	0.978	BP:organic substance metabolic process;BP:lipid metabolic process;BP:primary metabolic process;BP:metabolic process;BP:biological_process;CC:cellular_component;CC:extracellular region;CC:cellular anatomical entity;MF:hydrolase activity;MF:hydrolase activity, acting on ester bonds;MF:catalytic activity;MF:molecular_function;MF:lipase	GO:0071	------	------	------	------	ENOG41116Y2;E	S:Function unknown;	PF00151.22	Lipase	Lipase	PLA	13	30	61.9	High
TRINITY_DN22430_c0_g3_i1_orf1	V-type proton ATPase 16 kDa proteolipid subunit c [Homo sapiens] >NP_001685.1 V-type proton ATPase 16 kDa proteolipid subunit c [Homo sapiens] >P27449.1 RecName: Full=V-type proton ATPase 16 kDa proteolipid subunit c; Short=V-ATPase 16 kDa proteolipid subunit c; AltName: Full=Vacuolar proton pump 16 kDa proteolipid subunit c [Homo sapiens] >6WLW_1 The Vo region of human V-ATPase in state 1 (focused refinement) [Homo sapiens] >6WLW_2 The Vo region of human V-ATPase in state 1 (focused refinement) [Homo sapiens] >6WLW_3 The Vo region of human V-ATPase in state 1 (focused refinement) [Homo sapiens] >6WLW_4 The Vo region of human V-ATPase in state 1 (focused refinement) [Homo sapiens] >6WLW_5 The Vo region of human V-ATPase in state 1 (focused refinement) [Homo sapiens] >6WLW_6 The Vo region of human V-ATPase in state 1 (focused refinement) [Homo sapiens] >6WLW_7 The Vo region of human V-ATPase in state 1 (focused refinement) [Homo sapiens] >6WLW_8 The Vo region of human V-ATPase in state 1 (focused refinement) [Homo sapiens] >6WLW_9 The Vo region of human V-ATPase in state 1 (focused refinement) [Homo sapiens] >6WM2_1 Human V-ATPase in state 1 with SidK and ADP [Homo sapiens] >6WM2_2 Human V-ATPase in state 1 with SidK and ADP [Homo sapiens] >6WM2_3 Human V-ATPase in state 1 with SidK and ADP [Homo sapiens] >6WM2_4 Human V-ATPase in state 1 with SidK and ADP [Homo sapiens] >6WM2_5 Human V-ATPase in state 1 with SidK and ADP [Homo sapiens] >6WM2_6 Human V-ATPase in state 1 with SidK and ADP [Homo sapiens] >6WM2_7 Human V-ATPase in state 1 with SidK and ADP [Homo sapiens] >6WM2_8 Human V-ATPase in state 1 with SidK and ADP [Homo sapiens] >6WM2_9 Human V-ATPase in state 1 with SidK and ADP [Homo sapiens] >6WM3_1 Human V-ATPase in state 2 with SidK and ADP [Homo sapiens] >6WM3_2 Human V-ATPase in state 2 with SidK and ADP [Homo sapiens]	8.764547896	3.131679674	2.73E-05	up	yes	0.979	0.1117	0.089	0.067	0.179	1	1	0.937	BP:regulation of metabolic process;BP:positive regulation of response to stimulus;BP:regulation of response to stimulus;BP:regulation of Wnt signaling pathway;BP:inorganic ion homeostasis;BP:regulation of signal transduction;BP:positive regulation of signal transduction;BP:positive regulation of biological process;BP:cellular homeostasis;BP:pH reduction;BP:chemical homeostasis;BP:regulation of catabolic process;BP:positive regulation of Wnt signaling pathway;BP:Golgi lumen acidification;BP:regulation of biological process;BP:regulation of pH;BP:biological regulation;BP:inorganic cation transmembrane transport;BP:inorganic ion transmembrane transport;BP:regulation of biological quality;BP:transport;BP:cation transport;BP:ion transport;BP:regulation of cellular process;BP:biological_process;BP:establishment of localization;BP:hydrogen ion transmembrane transport;BP:ion homeostasis;BP:positive regulation of signaling;BP:endosomal lumen	GO:0019:	K02155	ATPeV0C, AT	map05152;m	Tuberculosis;Rheun	COG0636	S:Function unknown	PF00137.24	ATP-synt_C	ATP synthase subunit C	CYT	1	12	15.7	Medium
TRINITY_DN4612_c0_g1_i1_orf1	uncharacterized protein LOC114362092 [Ostrinia furnacalis]	7.7224	2.949049284	5.34E-05	up	yes	0.9653	0.125	0.102	0.095	0.178	1	1.006	0.89	CC:cellular_component;CC:integral component of membrane;CC:cellular anatomical entity;CC:intrinsic component of membrane;	GO:0005!	------	------	------	------	ENOG4110SII	S:Function unknown	------	------	------	CYT	1	1	70.8	Medium

TRINITY_DN14679_c0_g1_i1_orf1	hypothetical protein evm_003043 [Chilo suppressalis]	18.48906561	4.208600411	6.17E-07	up	yes	1.023	0.05533	0.066	0.07	0.03	1	1.032	1.038	CC:cellular_component;CC:integral component of membrane;CC:cellular anatomical entity;CC:intrinsic component of membrane;	GO:0005	------	------	------	------	ENOG410ZMUH	S:Function unknown;	PF13927.9;PF076	Ig_3;I-set;Ig_2;ig;C2-set_2;V-set	Immunoglobulin domain;Immunoglobulin I-set domain;Immunoglobulin domain;Immunoglobulin domain;CD80-like C2-set immunoglobulin domain;Immunoglobulin V-set domain	CYT	1	1	90	High
TRINITY_DN21531_c0_g1_i1_orf1	viral IAP-associated factor homolog [Ostrinia furnacalis]	2.513301241	1.329583601	3.43E-06	up	yes	0.992	0.3947	0.367	0.413	0.404	1	1.001	0.975	------	------	------	------	------	------	ENOG4111H24	S:Function unknown	PF02114.19	Phosducin	Phosducin	CYT	4	21	26.4	High
TRINITY_DN64510_c0_g1_i1_orf1	39S ribosomal protein L15, mitochondrial [Ostrinia furnacalis]	2.498214286	1.32089723	1.29E-05	up	yes	0.9793	0.392	0.363	0.387	0.426	1	0.983	0.955	BP:cellular biosynthetic process;BP:cellular nitrogen compound metabolic process;BP:nitrogen compound metabolic process;BP:cellular macromolecule biosynthetic process;BP:organic substance biosynthetic process;BP:cellular nitrogen compound biosynthetic process;BP:cellular macromolecule metabolic process;BP:peptide biosynthetic process;BP:organic substance metabolic process;BP:amide biosynthetic process;BP:cellular process;BP:biosynthetic process;BP:macromolecule biosynthetic process;BP:biological_process;BP:metabolic process;BP:primary metabolic process;BP:organonitrogen compound metabolic process;BP:cellular amide metabolic process;BP:organonitrogen compound biosynthetic process;BP:protein metabolic process;BP:cellular metabolic process;BP:macromolecule metabolic process;BP:peptide metabolic process;BP:translation;CC:large ribosomal subunit;CC:ribosomal subunit;CC:macromolecular complex;CC:ribonucleoprotein	GO:0044	K02876	RP-L15, MRF	map03010	Ribosome	COG0200	U:Intracellular trafficking, secretic	PF00828.22	Ribosomal_L27A	Ribosomal proteins 50S-L15, 50S-L18e, 60S-L27A	CYT	2	7	33.9	High
TRINITY_DN28152_c0_g1_i1_orf1	mitochondrial import inner membrane translocase subunit Tim29 [Ostrinia furnacalis]	3.045045045	1.60646357	7.78E-06	up	yes	1.014	0.333	0.367	0.308	0.324	1	1.044	0.999	CC:mitochondrial inner membrane protein insertion complex;CC:macromolecular complex;CC:inner mitochondrial membrane protein complex;CC:cellular_component;CC:membrane protein	GO:0042	------	------	------	------	ENOG4111K8C	S:Function unknown	PF10171.12	Tim29	Translocase of the Inner Mitochondrial membrane 29	CYT	1	4	21.8	High
TRINITY_DN107035_c0_g1_i1_orf1	splicing factor 3A subunit 3 [Ostrinia furnacalis]	2.879130435	1.52563315	4.90E-05	up	yes	0.9933	0.345	0.331	0.313	0.391	1	1.034	0.946	BP:cellular metabolic process;BP:nucleic acid metabolic process;BP:cellular nitrogen compound metabolic process;BP:nitrogen compound metabolic process;BP:organic cyclic compound metabolic process;BP:nucleobase-containing compound metabolic process;BP:organic substance metabolic process;BP:RNA splicing;BP:mRNA metabolic process;BP:cellular process;BP:cellular aromatic compound metabolic process;BP:RNA splicing, via transesterification reactions;BP:RNA splicing, via transesterification reactions with bulged adenosine as nucleophile;BP:biological_process;BP:metabolic process;BP:heterocycle metabolic process;BP:RNA metabolic process;BP:primary metabolic process;BP:mRNA splicing, via spliceosome;BP:macromolecule metabolic process;BP:RNA processing;BP:mRNA processing;CC:spliceosomal complex;CC:macromolecular complex;CC:ribonucleoprotein complex;CC:cellular_component;MF:molecular_function;MF:binding;MF:nucleic	GO:0044	K12827	SF3A3, SAP6	map03040	Spliceosome	COG5188	A:RNA processing and modificati	PF11931.11;PF13	SF3a60_Prp9_C;SDE2_2C;SF3A3;SF3a60_bindingd	SF3a60/Prp9 C-terminal;Replication stress response SDE2 C-terminal;Pre-mRNA-splicing factor SF3A3, of SF3a complex, Prp9;Splicing factor SF3a60 binding domain	CYT	1	1	57.7	High
TRINITY_DN1330_c0_g1_i1_orf1	pancreatic triacylglycerol lipase-like [Ostrinia furnacalis]	3.12326228	1.643053727	5.37E-06	up	yes	1.011	0.3237	0.318	0.327	0.326	1	0.982	1.052	CC:cellular_component;CC:extracellular region;CC:cellular anatomical entity;MF:hydrolase activity;MF:hydrolase activity, acting on ester bonds;MF:catalytic activity;MF:molecular_function;MF:lipase	GO:0005	------	------	------	------	ENOG41116Y2;E	S:Function unknown;	PF00151.22	Lipase	Lipase	CYT	7	15	65	High
TRINITY_DN2695_c0_g1_i14_orfp1	TRINITY_DN2695_c0_g1_i14_m.44485 TRINITY_DN2695_c0_g1::TRINITY_DN2695_c0_g1_i14::g.44485 ORF type:3prime_partial len:698 (+),score=187.51 TRINITY_DN2695_c0_g1_i14:101-2092(+)	3.155597131	1.657913031	9.00E-05	up	yes	1.012	0.3207	0.316	0.3	0.346	1	1.089	0.948	------	------	------	------	------	------	------	------	------	------	------	CYT	2	5	73.5	High
TRINITY_DN5244_c0_g1_i1_orf1	eukaryotic peptide chain release factor GTP-binding subunit-like [Ostrinia furnacalis]	3.023758757	1.596343042	1.44E-06	up	yes	0.9927	0.3283	0.313	0.319	0.353	1	1.001	0.977	------	------	------	------	------	------	ENOG41116ZG;E	S:Function unknown;	PF03103.20	DUF243	Domain of unknown function	CYT	6	20	48.5	High
TRINITY_DN4923_c0_g1_i4_orf1	O-acyltransferase like protein-like [Ostrinia furnacalis]	2.973470003	1.572147522	0.0002536	up	yes	0.9863	0.3317	0.271	0.421	0.303	1	0.933	1.026	------	------	------	------	------	------	ENOG410YD1T;E	O:Posttranslational modification,	------	------	------	PLA	1	1	70.2	Medium
TRINITY_DN105901_c0_g1_i2_orfp	contactin-like [Pectinophora gossypiella]	2.740868565	1.454633147	0.0001486	up	yes	0.953	0.3477	0.33	0.383	0.33	1	0.875	0.984	------	------	------	------	------	------	------	------	------	------	------	CYT	2	16	16.7	High
TRINITY_DN18404_c0_g1_i5_orf1	periodic tryptophan protein 1 homolog isoform X1 [Ostrinia furnacalis] >XP_028157695.1 periodic tryptophan protein 1 homolog isoform X2 [Ostrinia furnacalis]	3.571428571	1.836501268	2.57E-06	up	yes	1	0.28	0.25	0.278	0.312	1	1.008	0.993	BP:RNA metabolic process;BP:nucleobase-containing compound metabolic process;BP:rRNA metabolic process;BP:ncRNA processing;BP:primary metabolic process;BP:RNA processing;BP:cellular process;BP:cellular aromatic compound metabolic process;BP:cellular metabolic process;BP:macromolecule metabolic process;BP:nucleic acid metabolic process;BP:organic substance metabolic process;BP:cellular nitrogen compound metabolic process;BP:nitrogen compound metabolic process;BP:biological_process;BP:rRNA processing;BP:metabolic process;BP:ncRNA metabolic process;BP:organic cyclic compound metabolic process;BP:heterocycle	GO:0016	K14791	PWP1	------	------	ENOG410XT1V	S:Function unknown	PF00400.35	WD40	WD domain, G-beta repeat	CYT	1	2	51.1	High
TRINITY_DN7920_c0_g1_i2_orf1	uncharacterized protein LOC114357268 [Ostrinia furnacalis] >XP_028166599.1 uncharacterized protein LOC114357268 [Ostrinia furnacalis]	3.501251341	1.807870631	4.05E-06	up	yes	0.9793	0.2797	0.253	0.283	0.303	1	0.953	0.985	------	------	K03098	APOD	------	------	COG3040	K:Transcription	PF08212.15;PF00	Lipocalin_2;Lipocalin;Triabin	Lipocalin-like domain;Lipocalin / cytosolic fatty-acid binding protein	CYT	3	14	22.3	High

TRINITY_DN123396_c0_g1_i1_orf1	PREDICTED: delta-1-pyrroline-5-carboxylate dehydrogenase, mitochondrial isoform X1 [Megachile rotundata]	3.490525563	1.803444277	3.15E-05	up	yes	0.9763	0.2797	0.249	0.279	0.311	1	1.009	0.92	BP:small molecule catabolic process;BP:carboxylic acid metabolic process;BP:cellular amino acid catabolic process;BP:nitrogen compound metabolic process;BP:small molecule metabolic process;BP:glutamine family amino acid catabolic process;BP:glutamine family amino acid metabolic process;BP:organic substance catabolic process;BP:proline catabolic process;BP:proline metabolic process;BP:cellular amino acid metabolic process;BP:organic substance metabolic process;BP:alpha-amino acid metabolic process;BP:alpha-amino acid catabolic process;BP:primary metabolic process;BP:cellular process;BP:proline catabolic process to glutamate;BP:metabolic process;BP:oxoacid metabolic process;BP:catabolic process;BP:cellular catabolic process;BP:dicarboxylic acid metabolic process;BP:organonitrogen compound metabolic process;BP:organonitrogen compound catabolic process;BP:organic acid metabolic process;BP:carboxylic acid catabolic process;BP:organic acid catabolic process;BP:cellular metabolic process;BP:glutamate metabolic	GO:00442	K00294	E1.2.1.88	map00250;m	Alanine, aspartate a	COG1012	Z:Cytoskeleton	PF00171.25	Aldedh	Aldehyde dehydrogenase family	CYT	1	1	63.5	High
TRINITY_DN108573_c0_g1_i1_orf1	uncharacterized protein LOC114366171 [Ostrinia furnacalis]	4.180454351	2.063659749	1.42E-06	up	yes	0.9753	0.2333	0.221	0.225	0.254	1	0.958	0.968	------	------	------	------	------	------	ENOG410XT8F;E	G:Carbohydrate transport and m	PF02958.23;PF07	EcKL;DUF1679	Ecdysteroid kinase-like family;Uncharacterized oxidoreductase dhs-27	CYT	3	7	47.8	High
TRINITY_DN98091_c0_g1_i3_orf1	UDP-glycosyltransferase UGT40AP2, partial [Ostrinia furnacalis]	4.065320155	2.023368973	1.51E-05	up	yes	0.946	0.2327	0.221	0.227	0.25	1	0.923	0.915	CC:integral component of membrane;CC:cellular_component;CC:cellular anatomical entity;CC:intrinsic component of membrane;MF:catalytic activity;MF:transferase activity;MF:molecular_function;MF:transferase activity, transferring glycosyl groups;MF:transferase activity, transferring hexosyl groups;MF:glucuronosyltransferase activity;MF:UDP-glycosyltransferase	GO:0016(	K00699	UGT	map05207;m	Chemical carcinoge	COG1819	S:Function unknown	PF00201.21;PF04	UDPGT;Glyco_tran_28_C	UDP-glucoronosyl and UDP-glucosyl transferase;Glycosyltransferase family 28 C-terminal domain	CYT	4	11	57.4	High
TRINITY_DN82801_c0_g1_i1_orf1	uncharacterized protein LOC114364712 [Ostrinia furnacalis]	5.659309564	2.500626055	1.73E-06	up	yes	1	0.1767	0.167	0.166	0.197	1	1.028	0.972	BP:organic substance metabolic process;BP:carbohydrate metabolic process;BP:primary metabolic process;BP:metabolic process;BP:biological_process;CC:cellular_component;CC:extracellular region;CC:cellular anatomical entity;MF:hydrolase activity;MF:deacetylase activity;MF:carbohydrate derivative binding;MF:hydrolase activity, acting on carbon-nitroge;MF:hydrolase activity, acting on carbon-nitroge;MF:catalytic activity;MF:molecular_function;MF:chitin binding;MF:chitin deacetylase	GO:00717	------	------	------	------	ENOG410XX7G	Z:Cytoskeleton	PF01607.27;PF01	CBM_14;Polysacc_deac_1;Ldl_recept_a	Chitin binding Peritrophin-A domain;Polysaccharide deacetylase;Low-density lipoprotein receptor domain class A	CYT	10	27	61.3	High
TRINITY_DN29120_c0_g1_i6_orf1	putative inorganic phosphate cotransporter [Ostrinia furnacalis]	7.438752784	2.895060753	2.52E-06	up	yes	1.002	0.1347	0.119	0.13	0.155	1	1.036	0.969	CC:integral component of membrane;CC:cellular_component;CC:cellular anatomical entity;CC:intrinsic component of membrane;MF:transporter activity;MF:transmembrane transporter	GO:0016(	K08193	SLC17A	------	------	ENOG410XPWC;	G:Carbohydrate transport and m	PF07690.19	MFS_1	Major Facilitator Superfamily	CYT	1	4	27.3	High
TRINITY_DN2490_c0_g2_i1_orfp1	TRINITY_DN2490_c0_g2_i1_m.56872 TRINITY_DN2490_c0_g2::TRINITY_DN2490_c0_g2_i1::g.56872 ORF type:internal len:359 (-),score=123.59 TRINITY_DN2490_c0_g2_i1:2-	17.31052632	4.113577685	1.01E-05	up	yes	0.9867	0.057	0.058	0.057	0.056	1	0.923	1.037	------	------	------	------	------	------	------	------	------	------	------	EXC	7	34	36.3	High
TRINITY_DN75746_c0_g1_i1_orfp1	TRINITY_DN75746_c0_g1_i1_m.54871 TRINITY_DN75746_c0_g1::TRINITY_DN75746_c0_g1_i1::g.54871 ORF type:internal len:83 (+),score=-1.09,Polyhedrin PF00738.19 1.7e-25 TRINITY_DN75746_c0_g1_i1:2-247(+)	4.842899036	2.275870926	0.0004711	up	yes	1.156	0.2387	0.225	0.213	0.278	1	1.294	1.174	------	------	------	------	------	------	------	------	PF00738.21	Polyhedrin	Polyhedrin	CYT	2	29	9.6	High
TRINITY_DN2986_c1_g1_i1_orf1	Troponin C, isoform 1 [Papilio xuthus]	2.563250703	1.357974591	5.75E-05	up	yes	1.003	0.3913	0.363	0.373	0.438	1	1.048	0.962	MF:cation binding;MF:ion binding;MF:molecular_function;MF:binding;MF:metal ion binding;MF:calcium ion binding;	GO:00431	------	------	------	------	COG5126	F:Nucleotide transport and metal	PF13499.9;PF000	EF-hand_7;EF-hand_1;EF-hand_9;EF-hand_8;EF-hand_6;EF-hand_5	EF-hand domain pair;EF hand;EF-hand domain;EF-hand domain pair;EF-hand domain;EF	CYT	5	22	20.2	High
TRINITY_DN96170_c0_g1_i1_orf1	uncharacterized protein LOC114355569 [Ostrinia furnacalis]	2.635577679	1.398119213	0.0001772	up	yes	1.006	0.3817	0.369	0.346	0.43	1	0.941	1.076	BP:phosphorylation;BP:cellular process;BP:biological_process;BP:metabolic process;BP:cellular metabolic process;BP:phosphate-containing compound metabolic process;BP:phosphorus metabolic process;CC:intrinsic component of membrane;CC:integral component of membrane;CC:cellular_component;CC:cellular anatomical entity;MF:purine ribonucleoside triphosphate binding;MF:molecular_function;MF:binding;MF:ion binding;MF:heterocyclic compound binding;MF:anion binding;MF:nucleotide binding;MF:transferase activity;MF:nucleoside phosphate binding;MF:purine nucleotide binding;MF:ATP binding;MF:kinase activity;MF:small molecule binding;MF:catalytic activity;MF:adenyl nucleotide binding;MF:carbohydrate derivative binding;MF:transferase activity, transferring phosphorus-containing groups;MF:adenyl ribonucleotide binding;MF:purine ribonucleotide binding;MF:organic cyclic compound binding;MF:ribonucleotide	GO:00163	------	------	------	------	COG0574	G:Carbohydrate transport and m	PF01326.22	PPDK_N	Pyruvate phosphate dikinase, AMP/ATP-binding domain	CYT	3	4	87.6	High
TRINITY_DN9003_c0_g1_i20_orf1	RNA-binding protein Nova-2 isoform X4 [Ostrinia furnacalis]	2.314845804	1.210916096	4.81E-05	up	yes	0.9683	0.4183	0.405	0.44	0.41	1	0.914	0.991	------	------	K14944	NOVA	------	------	ENOG410XRZD	S:Function unknown	PF00013.32;PF07	KH_1;KH_2;KH_4	KH domain;KH domain;KH domain	CYT	4	11	42.2	High
TRINITY_DN1079_c0_g1_i4_orf1	CD109 antigen-like [Ostrinia furnacalis]	2.837573386	1.504657704	9.16E-07	up	yes	1.015	0.3577	0.344	0.354	0.375	1	1.014	1.032	CC:cellular_component;CC:cellular anatomical entity;CC:extracellular space;MF:endopeptidase inhibitor activity;MF:endopeptidase regulator activity;MF:peptidase regulator activity;MF:molecular_function;MF:peptidase inhibitor activity;MF:enzyme inhibitor activity;MF:molecular function regulator;MF:enzyme regulator activity;	GO:00055	K06530	CD109	------	------	ENOG410XRED;E	S:Function unknown;	PF07678.17;PF01	TED_complement;MG2;A2M_BRD;A2M;MG3	A-macroglobulin TED domain;MG2 domain;Alpha-2-macroglobulin bait region domain;Alpha-2-macroglobulin family;Macroglobulin domain MG3	CYT	3	2	146.3	High

TRINITY_DN14313_c0_g1_i1_orf1	25S rRNA (cytosine-C(5))-methyltransferase nop2 [Ostrinia furnacalis]	2.444080605	1.289291865	1.10E-05	up	yes	0.9703	0.397	0.384	0.402	0.405	1	0.979	0.932	BP:RNA modification;BP:nucleic acid metabolic process;BP:cellular component organization or biogenesis;BP:nucleobase-containing compound metabolic process;BP:cellular macromolecule metabolic process;BP:ribosome biogenesis;BP:organic substance metabolic process;BP:organic cyclic compound metabolic process;BP:ribonucleoprotein complex biogenesis;BP:methylation;BP:cellular process;BP:cellular aromatic compound metabolic process;BP:macromolecule modification;BP:biological_process;BP:metabolic process;BP:RNA methylation;BP:heterocycle metabolic process;BP:RNA metabolic process;BP:primary metabolic process;BP:nitrogen compound metabolic process;BP:cellular nitrogen compound metabolic process;BP:cellular metabolic process;BP:macromolecule metabolic process;BP:cellular component biogenesis;BP:macromolecule methylation;BP:RNA processing;CC:intracellular organelle;CC:non-membrane-bounded organelle;CC:organelle;CC:nucleolus;CC:	GO:00094	K14835	NOP2	------	------	COG0144	O:Posttranslational modification,	PF01189.20;PF17	Methyltr_RsmB-F;Methyltr_RsmF_N	16S rRNA methyltransferase RsmB/F;N-terminal domain of 16S rRNA methyltransferase RsmF	CYT	9	12	95.6	High
TRINITY_DN14436_c0_g1_i7_orf1	V-type proton ATPase subunit C [Vanessa cardui]	2.818284744	1.494817381	7.18E-07	up	yes	0.9957	0.3533	0.339	0.345	0.376	1	0.998	0.989	------	------	K02148	ATPeV1C, AT	map05323;m	Rheumatoid arthriti	COG5127	C:Energy production and convers	PF03223.18	V-ATPase_C	V-ATPase subunit C	CYT	20	51	44.1	High
TRINITY_DN14937_c0_g1_i7_orf1	multidrug resistance protein homolog 49-like [Ostrinia furnacalis] >XP_028159925.1 multidrug resistance protein homolog 49-like [Ostrinia furnacalis]	2.589033804	1.372413802	2.03E-05	up	yes	0.9727	0.3757	0.379	0.34	0.408	1	0.943	0.975	CC:integral component of membrane;CC:intrinsic component of membrane;CC:cellular_component;CC:cellular anatomical entity;MF:purine ribonucleoside triphosphate binding;MF:molecular_function;MF:binding;MF:ion binding;MF:heterocyclic compound binding;MF:nucleoside phosphate binding;MF:anion binding;MF:ATPase activity, coupled to transmembrane movement of substances;MF:nucleotide binding;MF:primary active transmembrane transporter activity;MF:purine nucleotide binding;MF:ATP binding;MF:active transmembrane transporter activity;MF:transporter activity;MF:ABC-type transmembrane transporter activity;MF:small molecule binding;MF:adenyl nucleotide binding;MF:carbohydrate derivative binding;MF:organic cyclic compound binding;MF:transmembrane transporter activity;MF:adenyl ribonucleotide binding;MF:purine ribonucleotide	GO:0016(	K05658	ABCB1, CD2	map05206;m	MicroRNAs in canc	COG1132	S:Function unknown	PF00664.26;PF00	ABC_membrane;ABC_tran;SMC_N;ABC_ATPase;AAA_22;AAA_16;RsgA_GTPase;Zeta_toxin	ABC transporter transmembrane region;ABC transporter;RecF/RecN/SMC N terminal domain;ATPase of the ABC class;AAA domain;AAA ATPase domain;RsgA GTPase;Zeta toxin	CYT	3	2	143.2	High
TRINITY_DN1505_c0_g1_i1_orf1	uncharacterized protein LOC114362816 isoform X1 [Ostrinia furnacalis] >XP_028174154.1 uncharacterized protein LOC114362816 isoform X2 [Ostrinia furnacalis]	2.86005162	1.516041186	5.39E-05	up	yes	0.9973	0.3487	0.29	0.345	0.411	1	1.008	0.984	------	------	K16913	PELP1, MNA	------	------	ENOG4111B29;E	S:Function unknown;	------	------	------	CYT	2	4	56	High
TRINITY_DN2238_c0_g2_i1_orf1	mitochondrial import inner membrane translocase subunit Tim8 [Ostrinia furnacalis]	2.578989362	1.366805822	0.0001079	up	yes	0.9697	0.376	0.363	0.357	0.408	1	1.01	0.899	BP:protein localization;BP:cellular macromolecule localization;BP:nitrogen compound transport;BP:organic substance transport;BP:macromolecule localization;BP:transport;BP:cellular process;BP:establishment of protein localization;BP:biological_process;BP:establishment of localization;BP:localization;BP:cellular localization;BP:protein transport;CC:organelle envelope lumen;CC:membrane-enclosed lumen;CC:organelle membrane;CC:membrane;CC:mitochondrial intermembrane space;CC:cellular anatomical entity;CC:mitochondrial membrane;CC:intracellular organelle lumen;CC:organelle lumen;CC:organelle inner membrane;CC:cellular_component;CC:mitochondrial inner membrane:	GO:00081	K17780	TIM8	------	------	ENOG41126D5	O:Posttranslational modification,	PF02953.18	zf-Tim10_DDP	Tim10/DDP family zinc finger	CYT	4	44	10.2	High
TRINITY_DN47114_c0_g1_i5_orf1	nucleolar protein dao-5 isoform X2 [Ostrinia furnacalis]	3.165467626	1.662418641	3.99E-06	up	yes	1.012	0.3197	0.306	0.31	0.343	1	1.044	0.993	CC:intracellular non-membrane-bounded organelle;CC:nucleolus;CC:cellular_component;CC:intracellular organelle;CC:non-membrane-bounded organelle;CC:organelle;CC:cellular	GO:0043	K25818	NOLC1	------	------	ENOG4111U5P;E	S:Function unknown;	PF05022.15	SRP40_C	SRP40, C-terminal domain	CYT	7	10	72.7	High
TRINITY_DN43420_c0_g2_i1_orf1	collagenase-like [Ostrinia furnacalis]	2.817998269	1.494670726	2.90E-05	up	yes	0.977	0.3467	0.322	0.332	0.386	1	0.998	0.933	BP:organic substance metabolic process;BP:organonitrogen compound metabolic process;BP:protein metabolic process;BP:macromolecule metabolic process;BP:biological_process;BP:nitrogen compound metabolic process;BP:metabolic process;BP:proteolysis;BP:primary metabolic process;MF:serine-type endopeptidase activity;MF:endopeptidase activity;MF:hydrolase activity;MF:serine hydrolase activity;MF:catalytic activity, acting on a protein;MF:catalytic activity;MF:molecular_function;MF:pepti	GO:0071	------	------	------	------	COG5640	O:Posttranslational modification,	PF00089.29	Trypsin	Trypsin	CYT	4	24	29.8	High
TRINITY_DN18773_c0_g1_i3_orf1	keratin, type II cytoskeletal 68 kDa, component IB-like [Ostrinia	2.341836735	1.227640499	0.0005475	up	yes	0.918	0.392	0.4	0.371	0.405	1	0.824	0.93	------	------	------	------	------	------	------	------	------	------	------	CYT	3	47	13.9	High
TRINITY_DN26130_c0_g1_i1_orf1	membrane alanyl aminopeptidase-like [Ostrinia furnacalis]	3.519224108	1.815257389	1.33E-06	up	yes	1.016	0.2887	0.288	0.27	0.308	1	1.038	1.01	BP:nitrogen compound metabolic process;BP:organic substance metabolic process;BP:proteolysis;BP:biological_process;BP:metabolic process;BP:primary metabolic process;BP:organonitrogen compound metabolic process;BP:protein metabolic process;BP:macromolecule metabolic process;CC:integral component of membrane;CC:intrinsic component of membrane;CC:cellular anatomical entity;CC:cellular_component;MF:aminopeptidase activity;MF:exopeptidase activity;MF:molecular_function;MF:binding;MF:peptidase activity;MF:metallopeptidase activity;MF:cation binding;MF:transition metal ion binding;MF:ion binding;MF:metal ion binding;MF:hydrolase	GO:0006	K11140	ANPEP, CD1	map04640;m	Hematopoietic cell	COG0308	E:Amino acid transport and meta	PF01433.23;PF11	Peptidase_M1;ERAP1_C;Peptidase_M1_N	Peptidase family M1 domain;ERAP1-like C-terminal domain;Peptidase M1 N-terminal domain	CYT	9	13	104.2	High

TRINITY_DN1366_c0_g1_i5_orf1	unnamed protein product, partial [Iphiclides podalirius]	3.209505335	1.682350959	1.34E-06	up	yes	0.9927	0.3093	0.292	0.299	0.337	1	0.995	0.983	BP:nucleoside triphosphate metabolic process;BP:purine nucleoside triphosphate metabolic process;BP:cellular nitrogen compound metabolic process;BP:nitrogen compound metabolic process;BP:small molecule metabolic process;BP:purine-containing compound metabolic process;BP:ribonucleotide metabolic process;BP:organic cyclic compound metabolic process;BP:nucleobase-containing compound metabolic process;BP:organic substance metabolic process;BP:ribonucleoside triphosphate metabolic process;BP:purine ribonucleoside triphosphate metabolic process;BP:ribose phosphate metabolic process;BP:ATP metabolic process;BP:cellular process;BP:cellular aromatic compound metabolic process;BP:purine ribonucleotide metabolic process;BP:nucleotide metabolic process;BP:metabolic process;BP:nucleobase-containing small molecule metabolic process;BP:heterocycle metabolic process;BP:primary metabolic process;BP:organonitrogen compound metabolic process;BP:carbohydrate	GO:00091	K02147	ATPeV1B, AT	map05323;m	Rheumatoid arthriti	COG1156	C:Energy production and convers	PF00006.28;PF02	ATP-synt_ab;ATP-synt_ab_N	ATP synthase alpha/beta family, nucleotide-binding domain;ATP synthase alpha/beta family, beta-barrel domain	CYT	16	45	54.8	High
TRINITY_DN26186_c0_g1_i7_orf1	sodium- and chloride-dependent glycine transporter 1-like [Ostrinia furnacalis]	3.931194434	1.97496772	2.92E-05	up	yes	1.017	0.2587	0.239	0.235	0.302	1	0.978	1.072	CC:integral component of membrane;CC:cellular_component;CC:intrinsic component of membrane;CC:cellular anatomical entity;MF:secondary active transmembrane transporter activity;MF:symporter activity;MF:transporter activity;MF:transmembrane transporter activity;MF:molecular_function;MF:active	GO:0016	K05038	SLC6A5_9, G	map04721	Synaptic vesicle cy	COG0733	U:Intracellular trafficking, secreti	PF00209.21	SNF	Sodiu	CYT	1	2	71.2	High
TRINITY_DN26688_c0_g1_i2_orf1	myogenesis-regulating glycosidase-like [Ostrinia furnacalis]	4.080389769	2.028706968	2.01E-06	up	yes	1.005	0.2463	0.239	0.221	0.279	1	1.018	0.998	BP:primary metabolic process;BP:carbohydrate metabolic process;BP:organic substance metabolic process;BP:biological_process;BP:metabolic process;MF:hydrolase activity;MF:catalytic activity;MF:hydrolase activity, acting on glycosyl bonds;MF:hydrolase activity, hydrolyzing O-glycosyl	GO:0044	K24727	MYORG	------	------	COG1501	G:Carbohydrate transport and m	PF01055.29	Glyco_hydro_31	Glycosyl hydrolases family 31	CYT	11	21	71.1	High
TRINITY_DN79210_c0_g1_i1_orf1	V-type proton ATPase 16 kDa proteolipid subunit [Frieseomelitta varia]	3.323968089	1.732906532	7.45E-06	up	yes	0.9583	0.2883	0.298	0.272	0.295	1	0.939	0.936	CC:intrinsic component of membrane;CC:cellular_component;CC:organelle membrane;CC:proton-transporting V-type ATPase, V0 domain;CC:integral component of membrane;CC:membrane;CC:vacuolar membrane;CC:cellular anatomical entity;CC:bounding membrane of organelle;CC:macromolecular complex;CC:membrane protein complex;CC:proton-transporting two-sector ATPase complex, proton-transporting domain;MF:molecular_function;MF:ATPase activity, coupled to transmembrane movement of substances;MF:inorganic solute uptake transmembrane transporter activity;MF:hydrogen-translocating pyrophosphatase activity;MF:primary active transmembrane transporter activity;MF:ATPase coupled ion transmembrane transporter activity;MF:transporter activity;MF:cation transmembrane transporter activity;MF:proton-transporting ATPase activity, rotational mechanism;MF:ATPase activity, coupled to transmembrane movement of ions,	GO:0031	K02155	ATPeV0C, AT	map05152;m	Tuberculosis;Rheun	COG0636	J:Translation, ribosomal structure	PF00137.24	ATP-synt_C	ATP synthase subunit C	CYT	1	11	16	High
TRINITY_DN36061_c0_g4_i2_orf1	putative GPI-anchored protein pfl2 [Ostrinia furnacalis] >XP_028163002.1 putative GPI-anchored protein pfl2 [Ostrinia furnacalis]	4.254310345	2.088925279	4.55E-05	up	yes	0.987	0.232	0.202	0.283	0.211	1	0.929	1.032	CC:cellular_component;CC:extracellular region;CC:cellular anatomical entity;MF:carbohydrate derivative binding;MF:molecular_function;MF:chitin binding;MF:binding;	GO:0005	------	------	------	------	ENOG4111P12	S:Function unknown	------	------	------	CYT	1	1	91.7	High
TRINITY_DN44288_c0_g1_i2_orf1	ATP-dependent RNA helicase p62 [Ostrinia furnacalis]	4.398660714	2.137064325	2.22E-06	up	yes	0.9853	0.224	0.211	0.205	0.256	1	0.967	0.989	CC:intracellular organelle;CC:membrane-bounded organelle;CC:nucleus;CC:organelle;CC:intracellular membrane-bounded organelle;CC:cellular_component;CC:cellular anatomical entity;MF:purine ribonucleoside triphosphate binding;MF:molecular_function;MF:binding;MF:nucleic acid binding;MF:DNA binding;MF:RNA-dependent ATPase activity;MF:nucleotide binding;MF:heterocyclic compound binding;MF:nucleoside phosphate binding;MF:anion binding;MF:ion binding;MF:purine nucleotide binding;MF:ATP binding;MF:hydrolase activity;MF:catalytic activity, acting on RNA;MF:helicase activity;MF:small molecule binding;MF:catalytic activity;MF:adenyl nucleotide binding;MF:carbohydrate derivative binding;MF:organic cyclic compound binding;MF:RNA helicase activity;MF:adenyl ribonucleotide binding;MF:purine ribonucleotide	GO:0043	K12823	DDX5, DBP2	map05205;m	Proteoglycans in ca	COG0513	I:Lipid transport and metabolism	PF00270.32;PF00	DEAD;Helicase_C;ResIII	DEAD/DEAH box helicase;Helicase conserved C-terminal domain;Type III restriction enzyme, res subunit	CYT	8	25	60.3	High
TRINITY_DN26408_c0_g1_i7_orf1	venom carboxylesterase-6-like [Ostrinia furnacalis]	4.404545455	2.138993142	1.31E-05	up	yes	0.969	0.22	0.224	0.199	0.237	1	0.916	0.991	CC:integral component of membrane;CC:cellular_component;CC:intrinsic component of membrane;CC:cellular anatomical entity;MF:hydrolase activity;MF:hydrolase activity, acting on ester bonds;MF:carboxylic ester hydrolase activity;MF:catalytic	GO:0016	------	------	------	------	COG2272;ENOG	I:Lipid transport and metabolism	PF00135.31;PF20	COesterase;BD-FAE;Abhydrolase_3	Carboxylesterase family;BD-FAE;alpha/beta hydrolase fold	PLA	1	2	64.2	High
TRINITY_DN334_c0_g1_i1_orf1	putative chymotrypsin 12 [Ostrinia nubilalis]	5.460864806	2.449129441	1.50E-06	up	yes	0.9977	0.1827	0.173	0.171	0.204	1	0.971	1.022	BP:organic substance metabolic process;BP:organonitrogen compound metabolic process;BP:protein metabolic process;BP:macromolecule metabolic process;BP:biological_process;BP:nitrogen compound metabolic process;BP:metabolic process;BP:proteolysis;BP:primary metabolic process;MF:serine-type endopeptidase activity;MF:endopeptidase activity;MF:hydrolase activity;MF:serine hydrolase activity;MF:catalytic activity, acting on a protein;MF:catalytic activity;MF:molecular_function;MF:pepti	GO:0071	------	------	------	------	COG5640	O:Posttranslational modification,	PF00089.29	Trypsin	Trypsin	CYT	5	53	21.6	High

TRINITY_DN47731_c0_g1_i2_orf1	nucleolar GTP-binding protein 2 [Ostrinia furnacalis]	7.116311081	2.831129578	0.0005345	up	yes	1.034	0.1453	0.092	0.18	0.164	1	1.193	0.91	organelle;CC:non-membrane-bounded organelle;CC:nucleolus;CC:cellular anatomical entity;CC:intracellular non-membrane-bounded organelle;CC:cellular_component;MF:pur ine ribonucleoside triphosphate binding;MF:molecular_function;MF:bindi ng;MF:ion binding;MF:heterocyclic compound binding;MF:nucleoside phosphate binding;MF:anion binding;MF:nucleotide binding;MF:guanyl ribonucleotide binding;MF:purine nucleotide binding;MF:GTP binding;MF:small molecule binding;MF:carbohydrate derivative binding;MF:organic cyclic compound binding;MF:guanyl nucleotide binding;MF:purine ribonucleotide	GO:0043[illegible]	K14537	NUG2, GNL2	map03008	Ribosome biogenes	COG1161;ENOG	S:Function unknown;	PF07572.15	BCNT	Bucentaur or craniofacial development	CYT	1	4	30.3	Medium
TRINITY_DN30704_c0_g1_i1_orf1	cytochrome P450 monooxygenase CYP6AE134v2 [Ostrinia furnacalis]	6.264687693	2.64724259	0.0001636	up	yes	1.013	0.1617	0.086	0.129	0.27	1	0.973	1.066	CC:integral component of membrane;CC:cellular_component;CC:ce llular anatomical entity;CC:intrinsic component of membrane;MF:cation binding;MF:monooxygenase activity;MF:heme binding;MF:transition metal ion binding;MF:tetrapyrrole binding;MF:organic cyclic compound binding;MF:oxidoreductase activity, acting on paired donors, with incorporation or reduction of molecular oxygen;MF:ion binding;MF:catalytic activity;MF:molecular_function;MF:bindi ng;MF:iron ion binding;MF:metal ion binding;MF:heterocyclic compound binding;MF:oxidoreductase activity;	GO:0016[illegible]	K14999	CYP6	------	------	COG2124	Q:Secondary metabolites biosynt	PF00067.25	p450	Cytochrome P450	CYT	1	2	61.1	High
TRINITY_DN2178_c0_g1_i1_orf1	carboxypeptidase B-like [Ostrinia furnacalis]	6.882312457	2.782893392	9.77E-07	up	yes	1	0.1453	0.166	0.126	0.144	1	1.022	0.978	BP:nitrogen compound metabolic process;BP:organic substance metabolic process;BP:proteolysis;BP:biological_proc ess;BP:metabolic process;BP:primary metabolic process;BP:organonitrogen compound metabolic process;BP:protein metabolic process;BP:macromolecule metabolic process;MF:exopeptidase activity;MF:molecular_function;MF:zinc ion binding;MF:peptidase activity;MF:metalloexopeptidase activity;MF:metallopeptidase activity;MF:cation binding;MF:transition metal ion binding;MF:metallocarboxypeptidase activity;MF:carboxypeptidase activity;MF:ion binding;MF:metal ion binding;MF:hydrolase activity;MF:catalytic activity, acting on a protein;MF:catalytic activity;MF:binding;	GO:0006[illegible]	K01291;K[illegible]	CPB1;CPA2	map04972;m[illegible]	Pancreatic secretio	COG2866	E:Amino acid transport and meta	PF00246.27;PF02	Peptidase_M14 ;Propep_M14	Zinc carboxypeptida se;Carboxypept idase activation peptide	CYT	1	2	48.1	High
TRINITY_DN5578_c0_g1_i10_orf1	unnamed protein product [Chilo suppressalis]	6.379605263	2.67346716	5.14E-06	up	yes	0.9697	0.152	0.117	0.155	0.184	1	0.961	0.948	------	------	K23390	BAG6	------	------	ENOG410XS9P	O:Posttranslational modification,	PF00240.26;PF12	ubiquitin;BAG6; Rad60-SLD	Ubiquitin family;BCL2-associated athanogene 6;Ubiquitin-2 like Rad60 SUMO-like	CYT	1	4	62.9	High
TRINITY_DN35051_c0_g1_i1_orf1	uncharacterized protein LOC114364307 [Ostrinia furnacalis]	9.134001823	3.191247078	2.59E-07	up	yes	1.002	0.1097	0.102	0.108	0.119	1	1.023	0.982	------	------	------	------	------	------	ENOG410Y27Q	S:Function unknown	PF06757.16	Ins_allergen_rp	Insect allergen related repeat, nitrile-specifier detoxification	CYT	5	30	14.8	High
TRINITY_DN63914_c0_g1_i1_orf1	myophilin-like [Ostrinia furnacalis]	2.733638567	1.450822507	1.03E-06	up	yes	1.015	0.3713	0.355	0.371	0.388	1	1.031	1.013	------	------	K20526	TAGLN	------	------	COG5199	Z:Cytoskeleton	PF00307.34	CH	Calponin homology	CYT	9	51	20.7	High
TRINITY_DN8833_c0_g1_i1_orf1	nucleolar protein 16 [Ostrinia furnacalis]	2.884561106	1.528351826	4.20E-06	up	yes	1.022	0.3543	0.325	0.377	0.361	1	1.04	1.026	------	------	------	------	------	------	ENOG4111QB6	S:Function unknown	PF09420.13	Nop16	Ribosome biogenesis protein Nop16	CYT	2	8	24.1	High
TRINITY_DN14967_c0_g2_i1_orf1	glyceraldehyde-3-phosphate dehydrogenase 2 [Holotrichia oblita]	3.114400247	1.638954364	0.0001245	up	yes	1.01	0.3243	0.272	0.293	0.408	1	0.982	1.048	BP:small molecule metabolic process;BP:organic cyclic compound metabolic process;BP:purine ribonucleoside triphosphate metabolic process;BP:ATP metabolic process;BP:ADP metabolic process;BP:monocarboxylic acid metabolic process;BP:oxoacid metabolic process;BP:purine ribonucleoside diphosphate metabolic process;BP:heterocycle metabolic process;BP:organonitrogen compound metabolic process;BP:carbohydrate catabolic process;BP:purine nucleotide metabolic process;BP:nucleoside diphosphate phosphorylation;BP:organophosphate metabolic process;BP:nucleoside triphosphate metabolic process;BP:purine nucleoside triphosphate metabolic process;BP:organic substance catabolic process;BP:ribonucleoside triphosphate metabolic process;BP:hexose metabolic process;BP:purine ribonucleotide metabolic process;BP:programmed cell death;BP:biological_process;BP:metaboli c process;BP:nucleoside phosphate metabolic process;BP:ATP generation	GO:0044[illegible]	K05298	GAPA	------	------	COG0057	C:Energy production and convers	PF02800.23;PF00	Gp_dh_C;Gp_d h_N	Glyceraldehyd e 3-phosphate dehydrogenas e, C-terminal domain;Glycer aldehyde 3-phosphate dehydrogenas e, NAD binding domain	CYT	2	6	35.5	High
TRINITY_DN1285_c0_g2_i1_orf1	bifunctional 3'-phosphoadenosine 5'-phosphosulfate synthase isoform X3 [Ostrinia furnacalis]	2.591704821	1.373901414	0.0001009	up	yes	0.9623	0.3713	0.369	0.334	0.411	1	0.901	0.986	------	------	K13811	PAPSS	map00450;m[illegible]	Selenocompound r	COG0529;COG2[illegible]	P:Inorganic ion transport and me	PF01583.23;PF14	APS_kinase;PU A_2;AAA_33	Adenylylsulpha te kinase;PUA-like domain;AAA	CYT	3	14	32.9	High
TRINITY_DN40_c0_g2_i1_orf1	trypsin CFT-1-like [Ostrinia furnacalis]	3.318196562	1.730399351	2.38E-05	up	yes	1.023	0.3083	0.313	0.261	0.351	1	1.01	1.06	process;BP:organonitrogen compound metabolic process;BP:protein metabolic process;BP:macromolecule metabolic process;BP:biological_process;BP:nitroge n compound metabolic process;BP:metabolic process;BP:proteolysis;BP:primary metabolic process;MF:serine-type endopeptidase activity;MF:endopeptidase activity;MF:hydrolase activity;MF:serine hydrolase activity;MF:catalytic activity, acting on a protein;MF:catalytic activity;MF:molecular_function;MF:pepti	GO:0071[illegible]	K01312	PRSS1_2_3	map04972;m[illegible]	Pancreatic secretio	COG5640	O:Posttranslational modification,	PF00089.29	Trypsin	Trypsin	CYT	1	3	28.5	High
TRINITY_DN25779_c0_g1_i6_orf1	aldo-keto reductase AKR2E4-like [Ostrinia furnacalis]	3.024034074	1.596474395	5.29E-06	up	yes	0.994	0.3287	0.311	0.328	0.347	1	1.021	0.961	MF:catalytic activity;MF:molecular_function;MF:oxidor eductase activity;	GO:0003[illegible]	K00011	AKR1B	map00051;m[illegible]	Fructose and mann	COG0656	S:Function unknown	PF00248.24	Aldo_ket_red	Aldo/keto reductase family	CYT	5	23	35.9	High

TRINITY_DN130075_c1_g2_i1_orf1	60S ribosomal protein L23 [Microtus ochrogaster]	2.314838062	1.210911271	0.0003914	up	yes	0.922	0.3983	0.361	0.425	0.409	1	0.849	0.917	BP:regulation of primary metabolic process;BP:regulation of metabolic process;BP:positive regulation of response to stimulus;BP:regulation of response to stimulus;BP:cellular component organization or biogenesis;BP:cellular response to stimulus;BP:negative regulation of macromolecule metabolic process;BP:positive regulation of macromolecule metabolic process;BP:regulation of signal transduction;BP:positive regulation of signal transduction;BP:peptide biosynthetic process;BP:positive regulation of signal transduction by p53 class mediator;BP:positive regulation of biological process;BP:negative regulation of biological process;BP:regulation of cell proliferation;BP:regulation of macromolecule metabolic process;BP:cellular response to actinomycin D;BP:response to actinomycin D;BP:regulation of proteolysis;BP:regulation of RNA biosynthetic process;BP:response to organic substance;BP:organonitrogen compound metabolic	GO:0080(	K02894	RP-L23e, RPl	map03010;m	Ribosome;Coronavi	COG0093	J:Translation, ribosomal structure	PF00238.22	Ribosomal_L14	Ribosomal protein L14p/L23e	CYT	1	43	14.9	High
TRINITY_DN24476_c0_g1_i1_orf1	ensconsin-like isoform X1 [Ostrinia furnacalis]	3.148734177	1.654771967	3.61E-05	up	yes	0.995	0.316	0.318	0.268	0.362	1	1.027	0.958	------	------	K24203;K	MAP7D2;MA	------	------	ENOG4111S2A;E	S:Function unknown;	------	------	------	CYT	4	6	92	High
TRINITY_DN122170_c0_g1_i2_orfp	TRINITY_DN122170_c0_g1_i2_m.81408 TRINITY_DN122170_c0_g1::TRINITY_DN122170_c0_g1_i2::g.81408 ORF type:internal len:87 (+),score=9.58,Baculo_E25 PF05274.12 1.7e-07 TRINITY_DN122170_c0_g1_i2:2-259(+)	5.715023232	2.514759363	0.0002942	up	yes	1.107	0.1937	0.098	0.281	0.202	1	1.13	1.191	------	------	------	------	------	------	------	------	PF05274.14	Baculo_E25	Occlusion-derived virus envelope protein E25	CYT	1	12	9	Medium
TRINITY_DN7556_c0_g1_i1_orf1	venom carboxylesterase-6-like [Ostrinia furnacalis]	3.953667954	1.983191712	1.13E-05	up	yes	1.024	0.259	0.23	0.239	0.308	1	1.049	1.024	------	------	------	------	------	------	COG2272;COG0!	I:Lipid transport and metabolism	PF00135.31;PF07	COesterase;Abhydrolase_3;BD-FAE	Carboxylesterase family;alpha/beta hydrolase fold;BD-FAE	CYT	1	1	54.8	Medium
TRINITY_DN130051_c0_g1_i1_orf1	5-methyltetrahydropteroyltriglutamate--homocysteine S-methyltransferase-like protein [Leptotrombidium deliense]	2.949491839	1.560466417	0.0002846	up	yes	0.9577	0.3247	0.278	0.422	0.274	1	0.932	0.941	BP:one-carbon metabolic process;BP:carboxylic acid metabolic process;BP:cellular biosynthetic process;BP:nitrogen compound metabolic process;BP:aspartate family amino acid biosynthetic process;BP:small molecule metabolic process;BP:small molecule biosynthetic process;BP:tetrahydrofolate metabolic process;BP:macromolecule metabolic process;BP:organic substance biosynthetic process;BP:cellular amino acid metabolic process;BP:tetrahydrofolate interconversion;BP:pteridine-containing compound metabolic process;BP:organic substance metabolic process;BP:organic cyclic compound metabolic process;BP:proteolysis;BP:methylation;BP:alpha-amino acid metabolic process;BP:alpha-amino acid biosynthetic process;BP:sulfur amino acid metabolic process;BP:folic acid-containing compound metabolic process;BP:primary metabolic process;BP:sulfur amino acid biosynthetic process;BP:cellular process;BP:cellular aromatic compound metabolic process;BP:biosynthetic	GO:00067	K00549	metE	map00450;m	Selenocompound r	------	------	PF08267.15	Meth_synt_1	Cobalamin-independent synthase, N-terminal domain	CYT	1	10	13	High
TRINITY_DN1073_c0_g1_i4_orf1	carboxylesterase [Loxostege sticticalis]	3.208180911	1.681755499	2.46E-05	up	yes	0.9647	0.3007	0.273	0.288	0.341	1	0.969	0.925	CC:integral component of membrane;CC:cellular_component;CC:intrinsic component of membrane;CC:cellular anatomical entity;MF:hydrolase activity;MF:hydrolase activity, acting on ester bonds;MF:carboxylic ester hydrolase activity;MF:catalytic	GO:0016(	------	------	------	------	COG2272;COG0!	I:Lipid transport and metabolism	PF00135.31;PF20	COesterase;BD-FAE;Abhydrolase_3	Carboxylesterase family;BD-FAE;alpha/beta hydrolase fold	EXC	5	14	65.7	High
TRINITY_DN10940_c0_g1_i10_orfp	TRINITY_DN10940_c0_g1_i10_m.52163 TRINITY_DN10940_c0_g1::TRINITY_DN10940_c0_g1_i10::g.52163 ORF type:5prime_partial len:248 (-),score=128.24 TRINITY_DN10940_c0_g1_i10:121-864(-)	5.360983103	2.422497588	5.78E-06	up	yes	1.047	0.1953	0.2	0.186	0.2	1	1.091	1.05	------	------	------	------	------	------	------	------	------	------	------	CYT	1	14	22.7	High
TRINITY_DN334_c0_g1_i2_orf1	chymotrypsin-like serine protease, partial [Ostrinia nubilalis]	5.575790037	2.479176236	3.50E-06	up	yes	1.041	0.1867	0.181	0.176	0.203	1	1.076	1.047	BP:organic substance metabolic process;BP:organonitrogen compound metabolic process;BP:protein metabolic process;BP:macromolecule metabolic process;BP:biological_process;BP:nitrogen compound metabolic process;BP:metabolic process;BP:proteolysis;BP:primary metabolic process;MF:serine-type endopeptidase activity;MF:endopeptidase activity;MF:hydrolase activity;MF:serine hydrolase activity;MF:catalytic activity, acting on a protein;MF:catalytic activity;MF:molecular_function;MF:pepti	GO:00717	------	------	------	------	COG5640	O:Posttranslational modification,	PF00089.29	Trypsin	Trypsin	CYT	1	18	13.2	High
TRINITY_DN23175_c0_g1_i6_orf1	myb-binding protein 1A-like protein [Ostrinia furnacalis]	3.999589659	1.999851993	1.97E-05	up	yes	0.9747	0.2437	0.225	0.245	0.261	1	0.916	1.008	------	------	K02331	POL5, MYBBl	------	------	ENOG410YFVF;E	S:Function unknown;L:Replicatio	PF04931.16	DNA_pol_phi	DNA polymerase phi	CYT	1	1	130.2	High
TRINITY_DN15046_c0_g1_i8_orf1	epidermal retinol dehydrogenase 2-like isoform X1 [Ostrinia furnacalis] >XP_028169999.1 epidermal retinol dehydrogenase 2-like isoform X2 [Ostrinia furnacalis]	4.534522177	2.180950535	5.09E-07	up	yes	0.9917	0.2187	0.205	0.216	0.235	1	1.003	0.972	MF:catalytic activity;MF:molecular_function;MF:oxidoreductase activity;	GO:0003{	K15734	SDR16C5	map00830;m	Retinol metabolism	COG1028;COG4	S:Function unknown;	PF00106.28;PF13	adh_short;adh_short_C2;KR	short chain dehydrogenase;Enoyl;KR domain	CYT	3	13	34.7	High
TRINITY_DN5891_c0_g2_i4_orf1	amino acid transporter AVT1A-like [Ostrinia furnacalis] >XP_028156666.1 amino acid transporter AVT1A-like [Ostrinia furnacalis]	5.657237937	2.50009785	1.65E-05	up	yes	1.02	0.1803	0.18	0.135	0.226	1	0.996	1.064	CC:cellular_component;CC:integral component of membrane;CC:cellular anatomical entity;CC:intrinsic component of membrane;	GO:0005!	K15015	SLC32A, VGA	map04727;m	GABAergic synapse	COG0814	E:Amino acid transport and meta	PF01490.21;PF03	Aa_trans;Trp_Tyr_perm	Transmembrane amino acid transporter protein;Tryptophan/tyrosine permease	CYT	1	2	48.1	High
TRINITY_DN144807_c0_g1_i1_orf1	hypothetical protein G9C98_004245 [Cotesia typhae]	4.501376147	2.170366125	6.10E-06	up	yes	0.9813	0.218	0.189	0.209	0.256	1	0.954	0.99	BP:L-methionine salvage;BP:carboxylic acid metabolic process;BP:L-methionine biosynthetic process;BP:cellular biosynthetic process;BP:nitrogen compound metabolic process;BP:aspartate family amino acid biosynthetic process;BP:small molecule metabolic process;BP:small molecule biosynthetic process;BP:L-methionine biosynthetic process from S-adenosylmethionine;BP:organic substance biosynthetic process;BP:L-methionine biosynthetic process from methylthioadenosine;BP:cellular amino acid metabolic process;BP:organic substance metabolic process;BP:amino acid salvage;BP:alpha-amino acid metabolic process;BP:sulfur amino acid metabolic process;BP:primary metabolic process;BP:sulfur amino acid biosynthetic process;BP:cellular process;BP:biosynthetic process;BP:biological_process;BP:metabolic process;BP:oxoacid metabolic process;BP:cellular metabolic compound salvage;BP:cellular amino acid biosynthetic process;BP:organonitrogen compound metabolic process;BP:organonitrogen compound	GO:0071:	K09880	mtnC, ENOPl	map00270	Cysteine and methi	COG4229	C:Energy production and convers	------	------	------	CYT	1	1	75.4	Medium

TRINITY_DN41697_c0_g1_i1_orf1	5-formyltetrahydrofolate cyclo-ligase [Ostrinia furnacalis]	2.953642384	1.562495161	0.0006055	up	yes	0.892	0.302	0.274	0.342	0.29	1	0.809	0.867	MF:purine ribonucleoside triphosphate binding;MF:cyclo-ligase activity;MF:molecular_function;MF:binding;MF:ion binding;MF:heterocyclic compound binding;MF:nucleoside phosphate binding;MF:anion binding;MF:cation binding;MF:nucleotide binding;MF:ligase activity, forming carbon-nitrogen bonds;MF:purine nucleotide binding;MF:metal ion binding;MF:ATP binding;MF:small molecule binding;MF:catalytic activity;MF:ligase activity;MF:5-formyltetrahydrofolate cyclo-ligase activity;MF:adenyl nucleotide binding;MF:carbohydrate derivative binding;MF:organic cyclic compound binding;MF:adenyl ribonucleotide binding;MF:purine [illegible]	GO:0035€	K01934	MTHFS	map00670	One carbon pool b	COG0212	H:Coenzyme transport and meta	------	------	------	CYT	1	38	4.5	High
TRINITY_DN75188_c0_g1_i1_orf1	fatty acid-binding protein 1-like [Ostrinia furnacalis]	5.108885465	2.353008593	2.23E-05	up	yes	0.9947	0.1947	0.231	0.18	0.173	1	0.94	1.044	MF:molecular_function;MF:lipid binding;MF:binding;	GO:0003€	K08753;K	FABP4, aP2;F	map04923;m	Regulation of lipoly	ENOG4111US8	I:Lipid transport and metabolism	------	------	------	CYT	1	7	14.7	High
TRINITY_DN15338_c0_g1_i7_orf1	methyltransferase-like protein 17, mitochondrial [Ostrinia furnacalis]	2.364404311	1.241476756	0.009486	up	yes	0.8117	0.3433	0.302	0.411	0.317	1	0.722	0.713	BP:cellular biosynthetic process;BP:cellular nitrogen compound metabolic process;BP:nitrogen compound metabolic process;BP:cellular macromolecule biosynthetic process;BP:organic substance biosynthetic process;BP:cellular macromolecule metabolic process;BP:peptide biosynthetic process;BP:organic substance metabolic process;BP:methylation;BP:amide biosynthetic process;BP:cellular process;BP:biosynthetic process;BP:macromolecule biosynthetic process;BP:biological_process;BP:metabolic process;BP:cellular nitrogen compound biosynthetic process;BP:primary metabolic process;BP:organonitrogen compound metabolic process;BP:cellular amide metabolic process;BP:organonitrogen compound biosynthetic process;BP:protein metabolic process;BP:cellular metabolic process;BP:macromolecule metabolic process;BP:peptide metabolic process;BP:translation;CC:membrane-bounded organelle;CC:organelle;CC:mitochondrio [illegible]	GO:0044	------	------	------	------	COG5459	J:Translation, ribosomal structure	PF09243.13	Rsm22	Mitochondrial small ribosomal subunit Rsm22	CYT	1	2	50.6	Medium
TRINITY_DN2114_c0_g1_i5_orf1	vegetative cell wall protein gp1-like isoform X1 [Ostrinia furnacalis]	5.545660805	2.471359378	5.28E-06	up	yes	0.9777	0.1763	0.152	0.16	0.217	1	0.98	0.953	BP:biological_process;BP:metabolic process;BP:methylation;MF:catalytic activity;MF:transferase activity;MF:transferase activity, transferring one-carbon groups;MF:molecular_function;MF:methyltransferase activity;	GO:0008	------	------	------	------	ENOG410Y1IK;EI	S:Function unknown;	PF11901.11	DUF3421	Protein of unknown function	CYT	4	16	25.5	High
TRINITY_DN717_c0_g1_i2_orfp1	TRINITY_DN717_c0_g1_i2_m.67915 TRINITY_DN717_c0_g1::TRINITY_DN717_c0_g1_i2::g.67915 ORF type:internal len:868 (+),score=265.71,Collagen PF01391.19 0.11,Collagen|PF01391.19|0.039,Collagen|PF01391.19|0.00054,Collagen|PF01391.19|0.0019,Collagen|PF01391.19|0.0005,Collagen|PF01391.19|9.9e-05,Collagen|PF01391.19|1.7e-07 TRINITY_DN717_c0_g1_i2:3-2603(+)	7.684691546	2.941987353	5.52E-06	up	yes	1.009	0.1313	0.121	0.112	0.161	1	1.052	0.975	------	------	------	------	------	------	------	------	PF01391.21	Collagen	Collagen triple helix repeat	PLA	8	20	90.1	High
TRINITY_DN6044_c0_g1_i4_orf1	acyl-CoA-binding protein-like [Ostrinia furnacalis]	8.637532134	3.110619173	4.42E-06	up	yes	1.008	0.1167	0.103	0.103	0.144	1	1.05	0.974	MF:sulfur compound binding;MF:molecular_function;MF:fatty acid derivative binding;MF:carbohydrate derivative binding;MF:amide binding;MF:adenyl nucleotide binding;MF:ribonucleotide binding;MF:lipid binding;MF:fatty-acyl-CoA binding;MF:nucleotide binding;MF:small molecule binding;MF:purine ribonucleotide binding;MF:adenyl ribonucleotide binding;MF:binding;MF:organic cyclic compound binding;MF:acyl-CoA binding;MF:purine nucleotide binding;MF:heterocyclic compound binding;MF:nucleoside phosphate [illegible]	GO:1901	K08762	DBI, ACBP	map03320	PPAR signaling pat	COG4281	I:Lipid transport and metabolism	PF00887.22	ACBP	Acyl CoA binding protein	CYT	5	54	9.5	High
TRINITY_DN4869_c0_g1_i10_orf1	estrogen sulfotransferase-like isoform X1 [Ostrinia furnacalis]	8.028962188	3.005213519	7.66E-07	up	yes	0.998	0.1243	0.117	0.118	0.138	1	1.023	0.971	------	------	------	------	------	------	ENOG4111H56	S:Function unknown	PF00685.30;PF13	Sulfotransfer_1; Sulfotransfer_3	Sulfotransferase domain:Sulfotr	CYT	9	34	39.9	High
