# Supplementary material for: Comparative Proteomics and Metabonomics Analysis of Different Diapause Stages Revealed a New Regulation Mechanism of Diapause in Loxostege sticticalis (Lepidoptera: Pyralidae)
Source: Molecules. 2024 Jul 25;29(15):3472. doi: 10.3390/molecules29153472 (PMC11314584; doi:10.3390/molecules29153472)
Supplement: Supplementary file 1 [file molecules-29-03472-s001.zip › analysis process/proteomic/diffreential protein statistic table/NDvsRD.pdf]

Accession	Description	FC(ND/RD)	Log2FC(ND/RD)	P_value(ND/RD)	Regulate	Significant	ND	RD	RD1	RD2	RD3	ND1	ND2	ND3
TRINITY_DN98334_c0_g1_i1_orf1	unnamed protein product [Spodoptera littoralis] >CAH1638738.1 unnamed protein product [Spodoptera littoralis]	0.197404399	-2.340773958	7.05E-07	down	yes	0.9963	5.047	5.053	5.172	4.917	1	0.97	1.019
TRINITY_DN381_c0_g1_i1_orf1	cuticle protein 8-like [Ostrinia furnacalis]	0.109601634	-3.189658784	5.08E-05	down	yes	0.9657	8.811	9.122	9.341	7.971	1	0.945	0.952
TRINITY_DN32514_c0_g2_i1_orf1	mucin-5AC-like [Ostrinia furnacalis]	0.051495017	-4.279423366	8.41E-05	down	yes	1.085	21.07	21.41	23.006	18.79	1	1.062	1.194
TRINITY_DN66040_c0_g1_i2_orf1	serine protease inhibitor dipetalogastin-like isoform X2 [Ostrinia furnacalis]	0.055595855	-4.168878866	5.65E-07	down	yes	1.073	19.3	19.572	19.656	18.675	1	1.191	1.028
TRINITY_DN64297_c0_g1_i1_orf1	vanin-like protein 2 isoform X2 [Ostrinia furnacalis]	0.088347458	-3.500667571	4.10E-06	down	yes	1.251	14.16	13.563	14.15	14.772	1	1.417	1.336
TRINITY_DN14242_c0_g1_i2_orfp1	TRINITY_DN14242_c0_g1_i2_m.18449 TRINITY_DN14242_c0_g1::TRINITY_DN14242_c0_g1_i2::g.18449 ORF type:internal len:148 (-	0.136698751	-2.870928036	0.0003337	down	yes	1.335	9.766	9.949	8.446	10.902	1	1.386	1.618
TRINITY_DN129835_c0_g1_i2_orf1	alpha-tocopherol transfer protein-like [Chelonus insularis]	0.108047819	-3.210258149	1.66E-07	down	yes	0.8767	8.114	8.168	8.09	8.084	1	0.927	0.703
TRINITY_DN1124_c0_g1_i7_orf1	PREDICTED: cuticle protein 18.6, isoform B [Amyelois transitella]	0.081658692	-3.614249728	5.26E-06	down	yes	1.024	12.54	12.45	13.19	11.979	1	1.062	1.011
TRINITY_DN56155_c0_g1_i1_orf1	hypothetical protein evm_004679 [Chilo suppressalis] >CAH2989683.1 unnamed protein product [Chilo suppressalis]	0.068956044	-3.858179181	3.52E-07	down	yes	1.004	14.56	14.637	14.883	14.164	1	1.001	1.011
TRINITY_DN40669_c0_g2_i1_orf1	uncharacterized protein LOC114355531 [Ostrinia furnacalis]	0.050716757	-4.301393684	4.83E-06	down	yes	1.026	20.23	20.114	21.28	19.295	1	1.048	1.029
TRINITY_DN84478_c0_g1_i8_orf1	uncharacterized protein LOC114359035 isoform X1 [Ostrinia furnacalis]	0.072931785	-3.777308482	3.16E-05	down	yes	1.005	13.78	13.599	14.927	12.827	1	0.936	1.078
TRINITY_DN5080_c0_g1_i5_orf1	storage protein [Ostrinia furnacalis]	0.0982231	-3.347793838	8.69E-07	down	yes	0.995	10.13	10.019	10.48	9.896	1	0.967	1.018
TRINITY_DN18624_c0_g1_i5_orf1	uncharacterized protein LOC114353135 [Ostrinia furnacalis]	0.095108184	-3.394286695	1.19E-06	down	yes	1.011	10.63	10.956	10.681	10.262	1	1.06	0.974
TRINITY_DN12671_c0_g1_i6_orf1	hemicentin-1-like isoform X1 [Ostrinia furnacalis]	0.189873418	-2.396890153	7.39E-06	down	yes	0.975	5.135	4.951	5.396	5.058	1	0.904	1.021
TRINITY_DN52553_c0_g2_i1_orf1	hemocyte protein-glutamine gamma-glutamyltransferase-like [Ostrinia furnacalis]	0.123942709	-3.012254689	6.30E-05	down	yes	1.099	8.867	9.519	9.05	8.031	1	1.064	1.232
TRINITY_DN4255_c0_g1_i11_orf1	LOW QUALITY PROTEIN: lebocin-4-like [Ostrinia furnacalis]	0.209612278	-2.254204871	1.23E-05	down	yes	1.038	4.952	5.024	5.162	4.67	1	1.03	1.084
TRINITY_DN77425_c0_g1_i2_orf1	anosmin-1 [Ostrinia furnacalis] >XP_028178811.1 anosmin-1 [Ostrinia furnacalis]	0.203819095	-2.294638873	8.04E-07	down	yes	1.014	4.975	5.038	5.061	4.827	1	1	1.043
TRINITY_DN4384_c0_g1_i5_orf1	chemosensory protein 5 [Conogethes punctiferalis]	0.211461197	-2.241535142	6.06E-07	down	yes	1	4.729	4.778	4.807	4.602	1	0.969	1.031
TRINITY_DN27264_c0_g1_i1_orf1	uncharacterized protein LOC114353424 [Ostrinia furnacalis]	0.210082719	-2.250970605	7.35E-05	down	yes	0.9397	4.473	4.421	4.857	4.142	1	0.916	0.903
TRINITY_DN19990_c0_g1_i1_orf1	carboxypeptidase B-like [Ostrinia furnacalis]	0.267689906	-1.901365354	1.89E-07	down	yes	1.029	3.844	3.847	3.886	3.798	1	1.003	1.084
TRINITY_DN135449_c0_g1_i5_orf1	larval cuticle protein LCP-17-like [Galleria mellonella]	0.312135356	-1.679756313	0.0004675	down	yes	1.07	3.428	3.799	3.452	3.032	1	1.086	1.125
TRINITY_DN2044_c0_g1_i5_orfp1	TRINITY_DN2044_c0_g1_i5_m.4210 TRINITY_DN2044_c0_g1::TRINITY_DN2044_c0_g1_i5::g.4210 ORF type:complete len:151 (-),score=85.31 TRINITY_DN2044_c0_g1_i5:857-1309(-)	0.33263031	-1.588008457	0.0012	down	yes	1.104	3.319	3.451	3.585	2.921	1	1.451	0.861
TRINITY_DN59965_c0_g4_i1_orf1	TKT protein, partial [Homo sapiens]	0.419854881	-1.252037334	0.01443	down	yes	1.273	3.032	3.285	3.542	2.269	1	1.593	1.226
TRINITY_DN28221_c0_g2_i1_orf1	unnamed protein product [Chilo suppressalis]	0.295658867	-1.75799455	8.03E-06	down	yes	0.9603	3.248	3.099	3.343	3.301	1	0.935	0.946
TRINITY_DN19980_c0_g1_i4_orf1	hypothetical protein evm_012507 [Chilo suppressalis]	0.341863517	-1.548507625	8.60E-06	down	yes	1.042	3.048	3.164	2.995	2.986	1	1.007	1.119
TRINITY_DN9733_c0_g1_i2_orf1	acidic juvenile hormone-suppressible protein 1-like [Ostrinia furnacalis]	0.31480763	-1.667457586	6.19E-05	down	yes	0.9737	3.093	3.122	3.282	2.876	1	0.923	0.998
TRINITY_DN18502_c0_g1_i1_orf1	uncharacterized protein LOC114359515 [Ostrinia furnacalis]	0.319553806	-1.645869225	4.01E-05	down	yes	0.974	3.048	3.119	3.18	2.846	1	1	0.922
TRINITY_DN1597_c0_g1_i5_orfp1	TRINITY_DN1597_c0_g1_i5_m.57494 TRINITY_DN1597_c0_g1::TRINITY_DN1597_c0_g1_i5::g.57494 ORF type:complete len:86 (+),score=7.19 TRINITY_DN1597_c0_g1_i5:134-391(+)	0.325313531	-1.620097263	1.24E-05	down	yes	0.9857	3.03	3.041	3.157	2.891	1	0.991	0.966
TRINITY_DN80328_c0_g1_i9_orf1	arylphorin subunit alpha-like [Ostrinia furnacalis]	0.324958623	-1.621672064	1.24E-07	down	yes	0.9817	3.021	3.018	3.062	2.984	1	0.969	0.976
TRINITY_DN31417_c0_g1_i3_orf1	titin-like [Ostrinia furnacalis]	0.288710733	-1.792303353	0.0004339	down	yes	0.8823	3.056	3.336	3.149	2.682	1	0.811	0.836
TRINITY_DN18323_c0_g1_i5_orf1	GILT-like protein 1 isoform X1 [Ostrinia furnacalis]	0.350797024	-1.511291584	1.03E-06	down	yes	0.9903	2.823	2.776	2.895	2.798	1	0.996	0.975
TRINITY_DN376_c1_g1_i1_orf1	matrix metalloproteinase-25-like [Ostrinia furnacalis]	0.423033067	-1.241157656	9.39E-05	down	yes	1.113	2.631	2.483	2.726	2.684	1	1.134	1.205
TRINITY_DN1407_c0_g1_i12_orf1	hypothetical protein evm_012298 [Chilo suppressalis]	0.34204793	-1.547729595	1.02E-05	down	yes	0.942	2.754	2.674	2.861	2.728	1	0.946	0.88

Accession	GO Term	GO ID	KO ID	KO Name	Pathway ID	Pathway_defition	EggNOG ID	EggNOG_Functional Categories	Pfam ID	Domain	Domain Descriptio	Subcellular Loc	Unique Peptides	Coverage %	MW [kDa]	FDR Confidence
TRINITY_DN98334_c0_g1_i1_orf1	------	------	------	------	------	------	ENOG4110QDI	S:Function unknown	------	------	------	CYT	8	52	34	High
TRINITY_DN381_c0_g1_i1_orf1	MF:molecular_function;MF:structural constituent of cuticle;MF:structural molecule activity;	GO:0003674;GO:0042302;GO:0005198	------	------	------	------	ENOG410ZM6M;ENOG410YSFI;ENOG4110SG6;ENOG4111NR3	S:Function unknown;	PF00379.26	Chitin_bind_4	Insect cuticle prote	CYT	4	22	25	High
TRINITY_DN32514_c0_g2_i1_orf1	------	------	------	------	------	------	------	------	------	------	------	CYT	1	9	14.6	High
TRINITY_DN66040_c0_g1_i2_orf1	------	------	------	------	------	------	------	------	PF07648.18;PF0(	Kazal_2;Kazal	Kazal-type serine	CYT	1	21	6	High
TRINITY_DN64297_c0_g1_i1_orf1	BP:nitrogen compound metabolic process;BP:biological_process;BP:metabolic process;	GO:0006807;GO:0008150;GO:0008152	------	------	------	------	COG0388	O:Posttranslational modificatio	------	------	------	CYT	1	57	7.8	High
TRINITY_DN14242_c0_g1_i2_orfp1	------	------	------	------	------	------	------	------	------	------	------	CYT	1	8	16	High
TRINITY_DN129835_c0_g1_i2_orf1	------	------	------	------	------	------	ENOG410XRSQ	S:Function unknown	PF00650.23;PF1:	CRAL_TRIO;Cl	CRAL/TRIO domair	CYT	1	2	35.4	Medium
TRINITY_DN1124_c0_g1_i7_orf1	MF:molecular_function;MF:structural constituent of cuticle;MF:structural molecule activity;	GO:0003674;GO:0042302;GO:0005198	------	------	------	------	ENOG4111Y6E	S:Function unknown	PF00379.26	Chitin_bind_4	Insect cuticle prote	CYT	4	18	27.2	High
TRINITY_DN56155_c0_g1_i1_orf1	MF:pigment binding;MF:molecular_function;MF:binding;	GO:0031409;GO:0003674;GO:0005488	K03098	APOD	------	------	COG3040	Z:Cytoskeleton	PF00061.26;PF0(	Lipocalin;Lipc	Lipocalin / cytosoli	CYT	19	58	34.1	High
TRINITY_DN40669_c0_g2_i1_orf1	------	------	------	------	------	------	ENOG4110S60	S:Function unknown	------	------	------	CYT	6	59	9.5	High
TRINITY_DN84478_c0_g1_i8_orf1	BP:nitrogen compound metabolic process;BP:membrane lipid metabolic process;BP:organic substance metabolic process;BP:sphingolipid metabolic process;BP:lipid metabolic process;BP:cellular process;BP:biological_process;BP:metabolic process;BP:cellular lipid metabolic process;BP:primary metabolic process;BP:organonitrogen compound metabolic process;BP:cellular metabolic process;CC:lytic vacuole;CC:intracellular organelle;CC:membrane-bounded organelle;CC:organelle;CC:lysosome;CC:vacuole;CC:cellular anatomical entity;CC:intracellular membrane-bounded organelle;CC:cellular_component;CC:extracellular region;	GO:0006807;GO:0006643;GO:0071704;GO:0006665;GO:0006629;GO:0009987;GO:0008150;GO:0008152;GO:0044255;GO:0044238;GO:1901564;GO:0044237;GO:0000323;GO:0043229;GO:0043227;GO:0043226;GO:0005764;GO:0005773;GO:0110165;GO:0043231;GO:0005575;GO:0005576	K12382	PSAP, SGP1	map00600;ma	Sphingolipid met	ENOG410XSI5	O:Posttranslational modificatio	PF02199.18	SapA	Saposin A-type do	CYT	1	52	5.9	High
TRINITY_DN5080_c0_g1_i5_orf1	------	------	------	------	------	------	ENOG410XR2D	J:Translation, ribosomal structu	PF00372.22;PF0:	Hemocyanin_	Hemocyanin, copp	CYT	3	69	49.2	High
TRINITY_DN18624_c0_g1_i5_orf1	------	------	------	------	------	------	ENOG4110T9R	S:Function unknown	------	------	------	CYT	1	5	34.8	High
TRINITY_DN12671_c0_g1_i6_orf1	BP:biological_process;BP:localization;	GO:0008150;GO:0051179	------	------	------	------	ENOG410XPQS;ENOG410YQ1Q;ENOG410ZN3C;ENOG410XSTY	S:Function unknown;U:Intracell	PF07679.19;PF1:	I-set;Ig_3;ig;Iç	Immunoglobulin I-	CYT	1	13	10.3	High
TRINITY_DN52553_c0_g2_i1_orf1	BP:primary metabolic process;BP:peptide cross-linking;BP:organonitrogen compound metabolic process;BP:organic substance metabolic process;BP:nitrogen compound metabolic process;BP:macromolecule modification;BP:macromolecule metabolic process;BP:biological_process;BP:protein modification process;BP:metabolic process;BP:protein metabolic process;MF:molecular_function;MF:protein-glutamine gamma-glutamyltransferase activity;MF:catalytic activity, acting on a protein;MF:catalytic activity;MF:transferase activity;MF:transferase activity, transferring acyl	GO:0044238;GO:0018149;GO:1901564;GO:0071704;GO:0006807;GO:0043412;GO:0043170;GO:0008150;GO:0036211;GO:0008152;GO:0019538;GO:0003674;GO:0003810;GO:0140096;GO:0003824;GO:0016740;GO:0016746;GO:0016755	K05619	TGM1	------	------	ENOG410XQEZ	S:Function unknown	PF00868.23	Transglut_N	Transglutaminase	CYT	2	11	25.2	High
TRINITY_DN4255_c0_g1_i11_orf1	------	------	------	------	------	------	ENOG4111ASS	S:Function unknown	------	------	------	CYT	1	13	22.2	High
TRINITY_DN77425_c0_g1_i2_orf1	CC:cellular_component;CC:extracellular region;CC:cellular anatomical entity;MF:enzyme regulator activity;MF:peptidase regulator activity;MF:molecular_function;MF:peptidase inhibitor activity;MF:enzyme inhibitor activity;MF:molecular function regulator;	GO:0005575;GO:0005576;GO:0110165;GO:0030234;GO:0061134;GO:0003674;GO:0030414;GO:0004857;GO:0098772	K23413	ANOS1, KAL1	------	------	ENOG4111T0F	S:Function unknown	PF00041.24;PF0(	fn3;WAP	Fibronectin type III	EXC	5	16	47.5	High
TRINITY_DN4384_c0_g1_i5_orf1	------	------	------	------	------	------	ENOG410YU7V;ENOG4110RV8	U:Intracellular trafficking, secre	PF03392.16	OS-D	Insect pheromone-	CYT	2	14	16.9	High
TRINITY_DN27264_c0_g1_i1_orf1	MF:molecular_function;MF:structural constituent of cuticle;MF:structural molecule activity;	GO:0003674;GO:0042302;GO:0005198	------	------	------	------	ENOG4111DJS;ENOG4111VJY	S:Function unknown;	PF00379.26	Chitin_bind_4	Insect cuticle prote	CYT	2	6	29.3	High
TRINITY_DN19990_c0_g1_i1_orf1	BP:nitrogen compound metabolic process;BP:organic substance metabolic process;BP:proteolysis;BP:biological_process;BP:metabolic process;BP:primary metabolic process;BP:organonitrogen compound metabolic process;BP:protein metabolic process;BP:macromolecule metabolic process;MF:exopeptidase activity;MF:molecular_function;MF:zinc ion binding;MF:peptidase activity;MF:metalloexopeptidase activity;MF:metallopeptidase activity;MF:cation binding;MF:transition metal ion binding;MF:metallocarboxypeptidase activity;MF:carboxypeptidase activity;MF:ion binding;MF:metal	GO:0006807;GO:0071704;GO:0006508;GO:0008150;GO:0008152;GO:0044238;GO:1901564;GO:0019538;GO:0043170;GO:0008238;GO:0003674;GO:0008270;GO:0008233;GO:0008235;GO:0008237;GO:0043169;GO:0046914;GO:0004181;GO:0004180;GO:0043167;GO:0046872;GO:0016787;GO:0140096;GO:0003824;GO:0005488	------	------	------	------	COG2866	U:Intracellular trafficking, secre	PF00246.27;PF0:	Peptidase_M1	Zinc carboxypeptic	CYT	7	16	57.6	High
TRINITY_DN135449_c0_g1_i5_orf1	MF:molecular_function;MF:structural constituent of cuticle;MF:structural molecule activity;	GO:0003674;GO:0042302;GO:0005198	------	------	------	------	ENOG4110RUZ;ENOG4111PJ1;ENOG410YZKX;ENOG4110T0M	S:Function unknown;	------	------	------	CYT	1	68	8.6	High
TRINITY_DN2044_c0_g1_i5_orfp1	------	------	------	------	------	------	------	------	------	------	------	CYT	1	5	16.2	High
TRINITY_DN59965_c0_g4_i1_orf1	BP:small molecule metabolic process;BP:organic cyclic compound metabolic process;BP:heterocycle metabolic process;BP:organonitrogen compound metabolic process;BP:organophosphate metabolic process;BP:nitrogen compound metabolic process;BP:regulation of biological process;BP:organic substance biosynthetic process;BP:biological regulation;BP:biological_process;BP:metabolic process;BP:organophosphate biosynthetic process;BP:ribose phosphate biosynthetic process;BP:ribose phosphate metabolic process;BP:nucleoside phosphate metabolic process;BP:NADP metabolic process;BP:cellular biosynthetic process;BP:cellular nitrogen compound metabolic process;BP:nucleobase-containing compound metabolic process;BP:glyceraldehyde-3-phosphate biosynthetic process;BP:cellular process;BP:cellular aromatic compound metabolic process;BP:glucose 6-phosphate metabolic process;BP:nucleobase-containing small molecule metabolic process;BP:cellular aldehyde metabolic process;BP:carbohydrate derivative biosynthetic process;BP:carbohydrate derivative metabolic process;BP:NADPH regeneration;BP:nicotinamide nucleotide metabolic process;BP:cellular metabolic process;BP:pentose-phosphate shunt;BP:generation of precursor metabolites and energy;BP:pyridine-containing compound metabolic process;BP:regulation of growth;BP:glyceraldehyde-3-phosphate metabolic process;BP:organic substance metabolic process;BP:aldehyde biosynthetic process;BP:biosynthetic process;BP:nucleotide metabolic process;BP:pentose-phosphate shunt, non-oxidative branch;BP:primary metabolic process;BP:phosphate-containing compound metabolic	GO:0044281;GO:1901360;GO:0046483;GO:1901564;GO:0019637;GO:0006807;GO:0050789;GO:1901576;GO:0065007;GO:0008150;GO:0008152;GO:0090407;GO:0046390;GO:0019693;GO:0006753;GO:0006739;GO:0044249;GO:0034641;GO:0006139;GO:0046166;GO:0009987;GO:0006725;GO:0051156;GO:0055086;GO:0006081;GO:1901137;GO:1901135;GO:0006740;GO:0046496;GO:0044237;GO:0006098;GO:0006091;GO:0072524;GO:0040008;GO:0019682;GO:0071704;GO:0046184;GO:0009058;GO:0009117;GO:0009052;GO:0044238;GO:0006796;GO:0006793;GO:0019362;GO:0005789;GO:0031982;GO:0016020;GO:0005777;GO:0043230;GO:0043231;GO:0042579;GO:0005829;GO:0065010;GO:0043229;GO:0043227;GO:0016604;GO:0005654;GO:0110165;GO:0031090;GO:0070062;GO:0043226;GO:1903561;GO:0005575;GO:1901363;GO:0016740;GO:0016744;GO:0046872;GO:0003674;GO:0005488;GO:0030976;GO:1901681;GO:0043169;GO:0003824;GO:0097159;GO:0046983;GO:0043168;GO:0000287;GO:0043167;GO:0005509;GO:0042802;GO:0042803;GO:0005515;GO:0019842;GO:0036094;GO:0004802:GO:0050997	K00615	E2.2.1.1, tktA,	map00030	Pentose phospha	COG0021	S:Function unknown	PF02779.27;PF0:	Transket_pyr;	Transketolase, pyri	CYT	1	4	45.1	High
TRINITY_DN28221_c0_g2_i1_orf1	BP:small molecule biosynthetic process;BP:carboxylic acid metabolic process;BP:cellular biosynthetic process;BP:cellular nitrogen compound metabolic process;BP:nitrogen compound metabolic process;BP:small molecule metabolic process;BP:nitrogen cycle metabolic process;BP:glutamine family amino acid metabolic process;BP:organic substance biosynthetic process;BP:cellular amino acid metabolic process;BP:arginine biosynthetic process;BP:arginine metabolic process;BP:organic substance metabolic process;BP:organic acid metabolic process;BP:cellular amino acid biosynthetic process;BP:alpha-amino acid metabolic process;BP:alpha-amino acid biosynthetic process;BP:urea metabolic process;BP:primary metabolic process;BP:cellular process;BP:biosynthetic process;BP:biological_process;BP:metabolic process;BP:oxoacid metabolic process;BP:cellular nitrogen compound biosynthetic process;BP:amide biosynthetic process;BP:organonitrogen compound metabolic process;BP:organonitrogen compound biosynthetic process;BP:cellular amide metabolic process;BP:carboxylic acid biosynthetic process;BP:urea cycle;BP:organic acid biosynthetic process;BP:cellular metabolic process;BP:glutamine family amino acid biosynthetic process;MF:argininosuccinate synthase activity;MF:purine ribonucleoside triphosphate binding;MF:molecular_function;MF:ion binding;MF:heterocyclic compound binding;MF:nucleoside phosphate binding;MF:anion binding;MF:nucleotide binding;MF:purine nucleotide binding;MF:small molecule binding;MF:catalytic activity;MF:ligase activity;MF:ligase activity, forming carbon-	GO:0044283;GO:0019752;GO:0044249;GO:0034641;GO:0006807;GO:0044281;GO:0071941;GO:0009064;GO:1901576;GO:0006520;GO:0006526;GO:0006525;GO:0071704;GO:0006082;GO:0008652;GO:1901605;GO:1901607;GO:0019627;GO:0044238;GO:0009987;GO:0009058;GO:0008150;GO:0008152;GO:0043436;GO:0044271;GO:0043604;GO:1901564;GO:1901566;GO:0043603;GO:0046394;GO:0000050;GO:0016053;GO:0044237;GO:0009084;GO:0004055;GO:0035639;GO:0003674;GO:0043167;GO:1901363;GO:1901265;GO:0043168;GO:0000166;GO:0017076;GO:0036094;GO:0003824;GO:0016874;GO:0016879;GO:0030554;GO:0097367;GO:0032559;GO:0005524;GO:0097159;GO:0032555;GO:0032553;GO:0005488	K01940	argG, ASS1	map00220;ma	Arginine biosynt	COG0137	E:Amino acid transport and me	PF00764.22	Arginosuc_syı	Arginosuccinate sy	CYT	1	9	10.1	High
TRINITY_DN19980_c0_g1_i4_orf1	BP:developmental process;BP:cellular process;BP:cellular developmental process;BP:anatomical structure development;BP:cell development;BP:cellular component organization;BP:animal organ development;BP:biological_process;BP:cellular component	GO:0032502;GO:0009987;GO:0048869;GO:0048856;GO:0048468;GO:0016043;GO:0048513;GO:0008150;GO:0071840	------	------	------	------	ENOG410Y7WV	S:Function unknown	PF07974.16;PF0(	EGF_2;EGF;La	EGF-like domain;E	PLA	1	4	37.3	High
TRINITY_DN9733_c0_g1_i2_orf1	------	------	------	------	------	------	ENOG410XR2D	S:Function unknown	PF00372.22	Hemocyanin	Hemocyanin, copp	CYT	3	54	12.1	High
TRINITY_DN18502_c0_g1_i1_orf1	------	------	------	------	------	------	ENOG41117F9	S:Function unknown	------	------	------	CYT	6	15	59.7	High
TRINITY_DN1597_c0_g1_i5_orfp1	------	------	------	------	------	------	------	------	------	------	------	CYT	2	27	9.2	High
TRINITY_DN80328_c0_g1_i9_orf1	------	------	------	------	------	------	ENOG410XR2D	S:Function unknown	PF00372.22	Hemocyanin_	Hemocyanin, copp	CYT	4	84	9.1	High
TRINITY_DN31417_c0_g1_i3_orf1	------	------	------	------	------	------	ENOG410ZKJ3;ENOG4111M3T	S:Function unknown;	PF00093.21	VWC	von Willebrand fac	CYT	2	17	25.3	High
TRINITY_DN18323_c0_g1_i5_orf1	------	------	K08059	IFI30, GILT	map04612	Antigen processi	ENOG4111IDT	U:Intracellular trafficking, secre	PF03227.19	GILT	Gamma interferon	CYT	15	62	25	High
TRINITY_DN376_c1_g1_i1_orf1	BP:nitrogen compound metabolic process;BP:organic substance metabolic process;BP:proteolysis;BP:biological_process;BP:metabolic process;BP:primary metabolic process;BP:organonitrogen compound metabolic process;BP:protein metabolic process;BP:macromolecule metabolic process;CC:extracellular matrix;CC:cellular anatomical entity;CC:external encapsulating structure;CC:cellular_component;MF:endopeptidase activity;MF:molecular_function;MF:zinc ion binding;MF:peptidase activity;MF:metallopeptidase activity;MF:cation binding;MF:transition metal ion binding;MF:ion binding;MF:metal ion binding;MF:hydrolase activity;MF:metalloendopeptidase activity;MF:catalytic activity	GO:0006807;GO:0071704;GO:0006508;GO:0008150;GO:0008152;GO:0044238;GO:1901564;GO:0019538;GO:0043170;GO:0031012;GO:0110165;GO:0030312;GO:0005575;GO:0004175;GO:0003674;GO:0008270;GO:0008233;GO:0008237;GO:0043169;GO:0046914;GO:0043167;GO:0046872;GO:0016787;GO:0004222;GO:0140096;GO:0003824;GO:0005488	K08003	MMP25	map04928	Parathyroid horr	ENOG410XQ5D	O:Posttranslational modificatio	PF00413.27;PF0(	Peptidase_M1	Matrixin;Hemopexi	EXC	1	2	59.2	High
TRINITY_DN1407_c0_g1_i12_orf1	BP:lipid transport;BP:transport;BP:organic substance transport;BP:biological_process;BP:establishment of localization;BP:localization;CC:cellular_component;CC:extracellular region;CC:cellular anatomical entity;MF:lipid binding;MF:molecular_function;MF:binding;	GO:0006869;GO:0006810;GO:0071702;GO:0008150;GO:0051234;GO:0051179;GO:0005575;GO:0005576;GO:0110165;GO:0008289;GO:0003674;GO:0005488	------	------	------	------	------	------	PF07464.14	ApoLp-III	Apolipophorin-III p	CYT	3	29	8	High

TRINITY_DN16905_c0_g1_i1_orf1	unnamed protein product [Leptidea sinapis]	0.451296151	-1.147853621	0.03288	down	yes	1.149	2.546	2.908	1.721	3.01	1	1.428	1.018	BP:phosphorylation;BP:nitrogen compound metabolic process;BP:organic substance metabolic process;BP:phosphate-containing compound metabolic process;BP:protein phosphorylation;BP:cellular process;BP:macromolecule modification;BP:protein modification process;BP:biological_process;BP:metabolic process;BP:primary metabolic process;BP:organonitrogen compound metabolic process;BP:cellular metabolic process;BP:macromolecule metabolic process;BP:protein metabolic process;BP:phosphorus metabolic process;CC:intrinsic component of membrane;CC:integral component of membrane;CC:cellular anatomical entity;CC:cellular_component;MF:signaling receptor activity;MF:purine ribonucleoside triphosphate binding;MF:molecular transducer activity;MF:molecular_function;MF:binding;MF:nucleotide binding;MF:heterocyclic compound binding;MF:anion binding;MF:cation binding;MF:organic cyclic compound binding;MF:ion binding;MF:transmembrane receptor protein serine/threonine kinase activity;MF:protein serine/threonine kinase activity;MF:transferase activity;MF:nucleoside phosphate binding;MF:protein kinase activity;MF:purine nucleotide binding;MF:metal ion binding;MF:carbohydrate derivative binding;MF:kinase activity;MF:catalytic activity, acting on a protein;MF:small molecule binding;MF:catalytic activity;MF:adenyl nucleotide binding;MF:phosphotransferase activity, alcohol group as acceptor;MF:transferase activity, transferring phosphorus-containing groups;MF:transmembrane signaling receptor activity;MF:adenyl ribonucleotide binding;MF:ATP binding;MF:purine ribonucleotide [illegible]	GO:0016310;GO:0006807;GO:0071704;GO:0006796;GO:0006468;GO:0009987;GO:0043412;GO:0036211;GO:0008150;GO:0008152;GO:0044238;GO:1901564;GO:0044237;GO:0043170;GO:0019538;GO:0006793;GO:0031224;GO:0016021;GO:0110165;GO:0005575;GO:0038023;GO:0035639;GO:0060089;GO:0003674;GO:0005488;GO:0000166;GO:1901363;GO:0043168;GO:0043169;GO:0097159;GO:0043167;GO:0004675;GO:0004674;GO:0016740;GO:1901265;GO:0004672;GO:0017076;GO:0046872;GO:0097367;GO:0016301;GO:0140096;GO:0036094;GO:0003824;GO:0030554;GO:0016773;GO:0016772;GO:0004888;GO:0032559;GO:0005524;GO:0032555;GO:0019199;GO:0032553	K13578	BMPR1B, ALK	map04390;maı	Hippo signaling ı	ENOG410XQT0	T:Signal transduction mechanis	PF00069.28;PF0	Pkinase;PK_Ty	Protein kinase don	CYT	1	2	40.3	High
TRINITY_DN7534_c0_g1_i15_orf1	protein-glucosylgalactosylhydroxylysine glucosidase isoform X2 [Ostrinia furnacalis]	0.41194487	-1.279476819	0.0001313	down	yes	1.076	2.612	2.774	2.433	2.628	1	1.11	1.118	BP:primary metabolic process;BP:carbohydrate metabolic process;BP:organic substance metabolic process;BP:metabolic process;BP:biological_process;MF:catalytic activity;MF:molecular_function;	GO:0044238;GO:0005975;GO:0071704;GO:0008152;GO:0008150;GO:0003824;GO:0003674	K22078	PGGHG, ATHI	------	------	COG1554	G:Carbohydrate transport and ı	PF03632.18	Glyco_hydro_	Glycosyl hydrolase	CYT	2	5	48.7	High
TRINITY_DN364_c1_g1_i2_orf1	talin-2-like, partial [Ostrinia furnacalis]	0.381060606	-1.391907624	9.99E-07	down	yes	1.006	2.64	2.637	2.689	2.593	1	1.04	0.979	CC:cytoskeleton;CC:cellular_component;CC:ruffle;CC:focal adhesion;CC:intracellular non-membrane-bounded organelle;CC:cell junction;CC:anchoring junction;CC:intracellular organelle;CC:non-membrane-bounded organelle;CC:plasma membrane bounded cell projection;CC:cell projection;CC:organelle;CC:cellular anatomical entity;CC:cell-substrate junction;MF:structural constituent of cytoskeleton;MF:cytoskeletal protein binding;MF:structural molecule activity;MF:actin binding;MF:molecular_function;MF:binding;MF:protein binding;	GO:0005856;GO:0005575;GO:0001726;GO:0005925;GO:0043232;GO:0030054;GO:0070161;GO:0043229;GO:0043228;GO:0120025;GO:0042995;GO:0043226;GO:0110165;GO:0030055;GO:0005200;GO:0008092;GO:0005198;GO:0003779;GO:0003674;GO:0005488;GO:0005515	K06271	TLN	map05166;maı	Human T-cell leı	ENOG410XQ0V;ENOG410Z6MR	S:Function unknown;	PF09141.13	Talin_middle	Talin, middle doma	CYT	7	9	81.6	High
TRINITY_DN12231_c0_g1_i1_orf1	carbonyl reductase [NADPH] 3-like [Ostrinia furnacalis]	0.401111994	-1.31792299	2.29E-06	down	yes	1.01	2.518	2.525	2.576	2.454	1	0.995	1.035	------	------	K00079	CBR1	map00590;maı	Arachidonic acid	COG1028	K:Transcription	PF00106.28;PF1:	adh_short;adh	short chain dehydr	CYT	10	39	32.2	High
TRINITY_DN36899_c0_g1_i1_orf1	glucose dehydrogenase [FAD, quinone]-like [Ostrinia furnacalis]	0.454116638	-1.138865199	0.0003346	down	yes	1.059	2.332	2.492	2.367	2.136	1	1.044	1.133	MF:anion binding;MF:flavin adenine dinucleotide binding;MF:oxidoreductase activity, acting on CH-OH group of donors;MF:organic cyclic compound binding;MF:nucleotide binding;MF:small molecule binding;MF:catalytic activity;MF:molecular_function;MF:binding;MF:ion binding;MF:heterocyclic compound binding;MF:nucleoside phosphate binding;MF:oxidoreductase activity;	GO:0043168;GO:0050660;GO:0016614;GO:0097159;GO:0000166;GO:0036094;GO:0003824;GO:0003674;GO:0005488;GO:0043167;GO:1901363;GO:1901265;GO:0016491	------	------	------	------	COG2303	E:Amino acid transport and me	PF00732.22;PF0!	GMC_oxred_N	GMC oxidoreducta	CYT	1	2	70	High
TRINITY_DN23167_c0_g2_i1_orf1	hypothetical protein evm_003712 [Chilo suppressalis]	0.45518146	-1.135486297	3.95E-05	down	yes	1.041	2.287	2.208	2.401	2.253	1	1.038	1.086	BP:organic substance metabolic process;BP:organonitrogen compound metabolic process;BP:protein metabolic process;BP:macromolecule metabolic process;BP:biological_process;BP:nitrogen compound metabolic process;BP:metabolic process;BP:proteolysis;BP:primary metabolic process;MF:serine-type endopeptidase activity;MF:endopeptidase activity;MF:hydrolase activity;MF:serine hydrolase activity;MF:catalytic activity, acting on a protein;MF:catalytic activity;MF:molecular_function;MF:peptidase activity;MF:serine-	GO:0071704;GO:1901564;GO:0019538;GO:0043170;GO:0008150;GO:0006807;GO:0008152;GO:0006508;GO:0044238;GO:0004252;GO:0004175;GO:0016787;GO:0017171;GO:0140096;GO:0003824;GO:0003674;GO:0008233;GO:0008236	------	------	------	------	COG5640	O:Posttranslational modificatio	PF18399.4	CLIP_SPH_Sca	Clip-domain serine	CYT	4	14	25.2	High
TRINITY_DN48590_c0_g1_i1_orf1	acyl-CoA Delta(11) desaturase isoform X1 [Ostrinia furnacalis] >XP_028172999.1 acyl-CoA Delta(11) desaturase isoform X2 [Ostrinia furnacalis] >XP_028173000.1 acyl-CoA Delta(11) desaturase isoform X1 [Ostrinia furnacalis]	0.407946735	-1.2935473	7.98E-05	down	yes	0.9497	2.328	2.331	2.426	2.226	1	0.829	1.02	BP:fatty acid biosynthetic process;BP:fatty acid metabolic process;BP:carboxylic acid metabolic process;BP:cellular biosynthetic process;BP:small molecule metabolic process;BP:small molecule biosynthetic process;BP:monocarboxylic acid biosynthetic process;BP:organic substance biosynthetic process;BP:organic substance metabolic process;BP:lipid metabolic process;BP:cellular process;BP:monocarboxylic acid metabolic process;BP:biosynthetic process;BP:biological_process;BP:metabolic process;BP:oxoacid metabolic process;BP:cellular lipid metabolic process;BP:lipid biosynthetic process;BP:primary metabolic process;BP:organic acid metabolic process;BP:carboxylic acid biosynthetic process;BP:organic acid biosynthetic process;BP:cellular metabolic process;CC:intrinsic component of membrane;CC:integral component of membrane;CC:cellular anatomical entity;CC:cellular_component;MF:molecular_function;MF:oxidoreductase activity;MF:oxidoreductase activity, acting on paired donors, with incorporation or reduction of molecular	GO:0006633;GO:0006631;GO:0019752;GO:0044249;GO:0044281;GO:0044283;GO:0072330;GO:1901576;GO:0071704;GO:0006629;GO:0009987;GO:0032787;GO:0009058;GO:0008150;GO:0008152;GO:0043436;GO:0044255;GO:0008610;GO:0044238;GO:0006082;GO:0046394;GO:0016053;GO:0044237;GO:0031224;GO:0016021;GO:0110165;GO:0005575;GO:0003674;GO:0016491;GO:0016705;GO:0003824;GO:0016717	K00507	SCD, desC	map04936;maı	Alcoholic liver dis	COG1398	J:Translation, ribosomal structu	------	------	------	CYT	4	14	36.9	High
TRINITY_DN1407_c0_g1_i5_orf1	unnamed protein product [Chrysodeixis includens]	0.437141597	-1.193827429	7.75E-06	down	yes	0.991	2.267	2.233	2.351	2.217	1	0.974	0.999	BP:lipid transport;BP:transport;BP:organic substance transport;BP:biological_process;BP:establishment of localization;BP:localization;CC:cellular_component;CC:extracellular region;CC:cellular anatomical entity;MF:lipid binding;MF:molecular_function;MF:binding;	GO:0006869;GO:0006810;GO:0071702;GO:0008150;GO:0051234;GO:0051179;GO:0005575;GO:0005576;GO:0110165;GO:0008289;GO:0003674;GO:0005488	------	------	------	------	ENOG410Z7P2;ENOG4110QP2	S:Function unknown;	PF07464.14	ApoLp-III	Apolipophorin-III ı	CYT	4	26	17	High
TRINITY_DN5421_c0_g1_i1_orf1	arylphorin subunit alpha-like [Ostrinia furnacalis]	0.468268359	-1.094592537	9.53E-05	down	yes	1.033	2.206	2.065	2.263	2.29	1	1.021	1.077	------	------	------	------	------	------	------	------	------	------	------	EXC	1	21	17.2	High
TRINITY_DN97097_c0_g1_i4_orf1	plectin-like, partial [Ostrinia furnacalis]	0.483233808	-1.049206703	0.0002233	down	yes	1.052	2.177	2.018	2.289	2.224	1	1.039	1.118	BP:organelle organization;BP:cytoskeleton organization;BP:intermediate filament cytoskeleton organization;BP:cellular process;BP:cellular component organization;BP:intermediate filament-based process;BP:biological_process;BP:cellular component organization or biogenesis;CC:cytoskeleton;CC:cellular_component;CC:intracellular non-membrane-bounded organelle;CC:cellular anatomical entity;CC:cytoplasm;CC:intracellular organelle;CC:non-membrane-bounded organelle;CC:organelle;MF:cytoskeletal protein binding;MF:actin binding;MF:molecular_function;MF:binding;MF:protein binding;	GO:0006996;GO:0007010;GO:0045104;GO:0009987;GO:0016043;GO:0045103;GO:0008150;GO:0071840;GO:0005856;GO:0005575;GO:0043232;GO:0110165;GO:0005737;GO:0043229;GO:0043228;GO:0043226;GO:0008092;GO:0003779;GO:0003674;GO:0005488;GO:0005515	K10382	DST	------	------	COG5069;ENOG4111KM4	S:Function unknown;Z:Cytoskel	------	------	------	CYT	3	24	13.9	High
TRINITY_DN6243_c0_g1_i5_orf1	sorting nexin-20 [Ostrinia furnacalis]	0.432236249	-1.210108028	5.80E-05	down	yes	0.9587	2.218	2.217	2.325	2.111	1	0.985	0.891	BP:protein localization;BP:cellular macromolecule localization;BP:nitrogen compound transport;BP:organic substance transport;BP:macromolecule localization;BP:cellular process;BP:transport;BP:establishment of protein localization;BP:biological_process;BP:establishment of localization;BP:localization;BP:cellular localization;BP:protein transport;CC:endosome membrane;CC:membrane;CC:organelle membrane;CC:early endosome membrane;CC:bounding membrane of organelle;CC:cytoplasmic vesicle membrane;CC:vesicle membrane;CC:cellular_component;CC:cellular anatomical entity;MF:molecular_function;MF:binding;MF:phospholipid	GO:0008104;GO:0070727;GO:0071705;GO:0071702;GO:0033036;GO:0009987;GO:0006810;GO:0045184;GO:0008150;GO:0051234;GO:0051179;GO:0051641;GO:0015031;GO:0010008;GO:0016020;GO:0031090;GO:0031901;GO:0098588;GO:0030659;GO:0012506;GO:0005575;GO:0110165;GO:0003674;GO:0005488;GO:0005543;GO:0008289;GO:0035091	K17931	SNX20	------	------	ENOG4111IVA;ENOG41114JN	S:Function unknown;U:Intracell	PF00787.27	PX	PX domain	CYT	1	5	28.8	High
TRINITY_DN22053_c0_g1_i13_orf1	uncharacterized protein LOC114355104 [Ostrinia furnacalis]	0.448559671	-1.156628179	3.59E-05	down	yes	0.981	2.187	2.256	2.07	2.236	1	0.963	0.98	CC:cellular_component;CC:integral component of membrane;CC:cellular anatomical entity;CC:intrinsic component of membrane;	GO:0005575;GO:0016021;GO:0110165;GO:0031224	------	------	------	------	ENOG4112A18	S:Function unknown	PF08357.14	SEFIR	SEFIR domain	EXC	2	3	61.5	High
TRINITY_DN14856_c0_g1_i1_orf1	upstream activation factor subunit spp27 [Ostrinia furnacalis]	0.477485929	-1.066469877	3.22E-06	down	yes	1.018	2.132	2.093	2.186	2.116	1	1.039	1.015	------	------	K15223	UAF30, SPP27	------	------	COG5531	B:Chromatin structure and dyn	PF02201.21;PF0	SWIB;DEK_C	SWIB/MDM2 doma	CYT	3	12	25.1	High
TRINITY_DN644_c0_g1_i1_orf1	cuticle protein 19-like [Ostrinia furnacalis]	0.124654555	-3.003992496	4.09E-05	down	yes	0.9743	7.816	7.811	8.425	7.211	1	0.989	0.934	MF:molecular_function;MF:structural constituent of cuticle;MF:structural molecule activity;	GO:0003674;GO:0042302;GO:0005198	------	------	------	------	ENOG410YSFI	S:Function unknown	PF00379.26	Chitin_bind_4	Insect cuticle prote	CYT	4	34	14.8	High
TRINITY_DN5581_c0_g1_i1_orf1	mucin-5AC-like [Ostrinia furnacalis]	0.15258216	-2.712341807	7.59E-06	down	yes	0.975	6.39	6.404	6.695	6.071	1	0.926	0.999	------	------	------	------	------	------	ENOG410YZWK	S:Function unknown	------	------	------	CYT	2	2	128.1	High
TRINITY_DN6423_c0_g1_i6_orf1	phenoloxidase-activating factor 2-like isoform X1 [Ostrinia furnacalis]	0.181238145	-2.464041469	3.71E-05	down	yes	1.051	5.799	5.771	6.222	5.405	1	1.07	1.084	BP:organic substance metabolic process;BP:organonitrogen compound metabolic process;BP:protein metabolic process;BP:macromolecule metabolic process;BP:biological_process;BP:nitrogen compound metabolic process;BP:metabolic process;BP:proteolysis;BP:primary metabolic process;MF:serine-type endopeptidase activity;MF:endopeptidase activity;MF:hydrolase activity;MF:serine hydrolase activity;MF:catalytic activity, acting on a protein;MF:catalytic activity;MF:molecular_function;MF:peptidase activity;MF:serine-	GO:0071704;GO:1901564;GO:0019538;GO:0043170;GO:0008150;GO:0006807;GO:0008152;GO:0006508;GO:0044238;GO:0004252;GO:0004175;GO:0016787;GO:0017171;GO:0140096;GO:0003824;GO:0003674;GO:0008233;GO:0008236	------	------	------	------	COG5640	O:Posttranslational modificatio	PF00089.29;PF1	Trypsin;CLIP_	Trypsin;Serine prot	CYT	3	13	55.7	High
TRINITY_DN2908_c0_g1_i1_orf1	uncharacterized protein LOC114361337 [Ostrinia furnacalis]	0.149663359	-2.740207032	4.79E-05	down	yes	0.8447	5.644	6.058	5.658	5.216	1	0.806	0.728	------	------	------	------	------	------	ENOG4111MU3	S:Function unknown	PF00354.20;PF1:	Pentaxin;Lam	Pentaxin family;Co	CYT	1	2	61.6	High
TRINITY_DN3616_c0_g1_i4_orf1	conotoxin ArMKLT2-032-like [Ostrinia furnacalis]	0.168138231	-2.5722803	0.0006937	down	yes	0.9293	5.527	6.181	5.815	4.584	1	0.952	0.836	CC:cellular_component;CC:integral component of membrane;CC:cellular anatomical entity;CC:intrinsic component of membrane;	GO:0005575;GO:0016021;GO:0110165;GO:0031224	------	------	------	------	------	------	------	------	------	CYT	1	68	6.1	High
TRINITY_DN276_c0_g1_i2_orf1	protein lethal(2)essential for life-like [Ostrinia furnacalis] >UTU55753.1 small heat shock protein Hsp20.7 [Ostrinia furnacalis]	0.08093617	-3.627071605	3.10E-05	down	yes	0.951	11.75	11.772	12.626	10.844	1	0.991	0.862	MF:cation binding;MF:metal ion binding;MF:molecular_function;MF:binding;MF:ion binding;	GO:0043169;GO:0046872;GO:0003674;GO:0005488;GO:0043167	K09542	CRYAB	map04213;maı	Longevity regulat	ENOG410YERS	O:Posttranslational modificatio	PF00011.24	HSP20	Hsp20/alpha crysta	CYT	2	43	20.8	High
TRINITY_DN2813_c0_g1_i10_orf1	arylphorin subunit alpha-like [Ostrinia furnacalis]	0.196026358	-2.350880442	4.71E-06	down	yes	0.9817	5.008	5.211	5.009	4.803	1	0.936	1.009	------	------	------	------	------	------	ENOG4110QFU;ENOG410XR2D	S:Function unknown;	PF03723.17;PF0	Hemocyanin_	Hemocyanin, ig-lik	CYT	1	33	139.5	High
TRINITY_DN138481_c0_g1_i5_orf1	hypothetical protein evm_003901 [Chilo suppressalis]	0.148548278	-2.750996212	4.03E-05	down	yes	1.1	7.405	7.965	7.389	6.862	1	1.153	1.146	MF:molecular_function;MF:structural constituent of cuticle;MF:structural molecule activity;	GO:0003674;GO:0042302;GO:0005198	------	------	------	------	ENOG410YRDB;ENOG410YM5B	S:Function unknown;	PF00379.26	Chitin_bind_4	Insect cuticle prote	CYT	2	32	22.6	High
TRINITY_DN138481_c0_g1_i2_orf1	cuticle protein 8-like isoform X2 [Vanessa tameamea]	0.204539749	-2.28954686	2.88E-07	down	yes	0.9777	4.78	4.769	4.874	4.696	1	0.933	1	MF:molecular_function;MF:structural constituent of cuticle;MF:structural molecule activity;	GO:0003674;GO:0042302;GO:0005198	------	------	------	------	ENOG410YRDB;ENOG410YM5B	S:Function unknown;	PF00379.26	Chitin_bind_4	Insect cuticle prote	CYT	2	27	24.2	High
TRINITY_DN3310_c0_g1_i1_orf1	hypothetical protein evm_010516 [Chilo suppressalis]	0.179345677	-2.479185126	2.01E-05	down	yes	0.9977	5.563	5.56	5.902	5.226	1	0.967	1.026	CC:integral component of membrane;CC:cellular_component;CC:intrinsic component of membrane;CC:cellular anatomical entity;MF:structural molecule activity;MF:molecular_function;MF:structural constituent of	GO:0016021;GO:0005575;GO:0031224;GO:0110165;GO:0005198;GO:0003674;GO:0042302	------	------	------	------	ENOG4111PCZ	S:Function unknown	------	------	------	CYT	5	17	40.4	High
TRINITY_DN971_c0_g1_i5_orfp1	TRINITY_DN971_c0_g1_i5_m.54249 TRINITY_DN971_c0_g1::TRINITY_DN971_c0_g1_i5::g.54249 ORF type:internal len:108 (+),score=66.98 TRINITY_DN971_c0_g1_i5:1-321(+)	0.217149758	-2.203237747	0.001105	down	yes	0.899	4.14	4.683	3.422	4.314	1	0.708	0.989	------	------	------	------	------	------	------	------	------	------	------	CYT	1	9	11.5	High
TRINITY_DN42719_c0_g2_i1_orf1	inter-alpha-trypsin inhibitor heavy chain H4-like isoform X11 [Ostrinia furnacalis]	0.225731733	-2.14731885	6.30E-07	down	yes	1.072	4.749	4.674	4.756	4.817	1	1.043	1.172	------	------	------	------	------	------	COG2304	S:Function unknown	------	------	------	CYT	1	12	12.1	High

TRINITY_DN1749_c0_g2_i2_orf1	putative GPI-anchored protein pfl2 isoform X1 [Ostrinia furnacalis]	0.277806653	-1.847846946	1.70E-06	down	yes	1.069	3.848	3.828	3.95	3.767	1	1.114	1.093	BP:developmental process;BP:dephosphorylation;BP:protein dephosphorylation;BP:cell development;BP:nitrogen compound metabolic process;BP:anatomical structure morphogenesis;BP:cellular component organization or biogenesis;BP:cellular developmental process;BP:cellular component organization;BP:organic substance metabolic process;BP:neuron development;BP:cellular process;BP:macromolecule modification;BP:protein modification process;BP:biological_process;BP:metabolic process;BP:response to chemical;BP:locomotion;BP:primary metabolic process;BP:organonitrogen compound metabolic process;BP:protein metabolic process;BP:anatomical structure development;BP:cellular metabolic process;BP:macromolecule metabolic process;BP:phosphate-containing compound metabolic process;BP:phosphorus metabolic process;BP:response to stimulus;CC:integral component of membrane;CC:intrinsic component of membrane;CC:cellular anatomical entity;CC:cellular_component;MF:phosphatase activity;MF:phosphoric ester hydrolase activity;MF:molecular_function;MF:phosphoprotein phosphatase activity;MF:hydrolase activity;MF:catalytic activity, acting on a protein;MF:hydrolase activity, acting on ester bonds;MF:catalytic activity;MF:protein tyrosine phosphatase	GO:0032502;GO:0016311;GO:0006470;GO:0048468;GO:0006807;GO:0009653;GO:0071840;GO:0048869;GO:0016043;GO:0071704;GO:0048666;GO:0009987;GO:0043412;GO:0036211;GO:0008150;GO:0008152;GO:0042221;GO:0040011;GO:0044238;GO:1901564;GO:0019538;GO:0048856;GO:0044237;GO:0043170;GO:0006796;GO:0006793;GO:0050896;GO:0016021;GO:0031224;GO:0110165;GO:0005575;GO:0016791;GO:0042578;GO:0003674;GO:0004721;GO:0016787;GO:0140096;GO:0016788;GO:0003824;GO:0004725	------	------	------	------	COG5599	T:Signal transduction mechanis	------	------	------	CYT	4	5	116.8	High
TRINITY_DN49147_c0_g2_i1_orf1	glutenin, high molecular weight subunit PW212-like [Ostrinia furnacalis]	0.230504834	-2.117131092	1.04E-06	down	yes	1.073	4.655	4.613	4.572	4.779	1	1.106	1.114	------	------	------	------	------	------	ENOG4110R9Z	S:Function unknown	------	------	------	PLA	1	5	31.6	High
TRINITY_DN7785_c0_g1_i1_orf1	uncharacterized protein LOC114364098 [Ostrinia furnacalis]	0.262914439	-1.927334722	5.71E-06	down	yes	0.9833	3.74	3.602	3.897	3.72	1	0.968	0.982	------	------	------	------	------	------	ENOG4110X3U;ENOG410XPJ1	S:Function unknown;	PF00059.24	Lectin_C	Lectin C-type dom	CYT	2	16	29.9	High
TRINITY_DN467_c3_g1_i5_orf1	lysozyme precursor [Loxostege sticticalis]	0.207310871	-2.270132325	1.31E-06	down	yes	0.9783	4.719	4.795	4.801	4.562	1	0.994	0.941	BP:metabolic process;BP:biological_process;MF:hydrolase activity;MF:catalytic activity;MF:hydrolase activity, acting on glycosyl bonds;MF:lysozyme activity;MF:molecular_function;MF:hydrolase activity, hydrolyzing O-glycosyl compounds;MF:peptidoglycan muralytic	GO:0008152;GO:0008150;GO:0016787;GO:0003824;GO:0016798;GO:0003796;GO:0003674;GO:0004553;GO:0061783	K13915	LYZ	map04970	Salivary secretior	ENOG4111QHM	G:Carbohydrate transport and r	PF00062.23	Lys	C-type lysozyme/a	CYT	7	52	16	High
TRINITY_DN29414_c1_g2_i1_orf1	serine protease 44-like isoform X2 [Ostrinia furnacalis]	0.259576748	-1.945766938	0.0005316	down	yes	0.969	3.733	3.995	3.191	4.012	1	0.911	0.996	BP:organic substance metabolic process;BP:organonitrogen compound metabolic process;BP:protein metabolic process;BP:macromolecule metabolic process;BP:biological_process;BP:nitrogen compound metabolic process;BP:metabolic process;BP:proteolysis;BP:primary metabolic process;MF:serine-type endopeptidase activity;MF:endopeptidase activity;MF:hydrolase activity;MF:serine hydrolase activity;MF:catalytic activity, acting on a protein;MF:catalytic activity;MF:molecular_function;MF:peptidase activity;MF:serine-	GO:0071704;GO:1901564;GO:0019538;GO:0043170;GO:0008150;GO:0006807;GO:0008152;GO:0006508;GO:0044238;GO:0004252;GO:0004175;GO:0016787;GO:0017171;GO:0140096;GO:0003824;GO:0003674;GO:0008233;GO:0008236	------	------	------	------	COG5640	S:Function unknown	------	------	------	CYT	1	15	8.8	High
TRINITY_DN19662_c0_g2_i1_orf1	storage protein [Ostrinia furnacalis]	0.232655922	-2.103730188	3.27E-06	down	yes	0.996	4.281	4.232	4.157	4.454	1	0.985	1.003	------	------	------	------	------	------	ENOG410XR2D	S:Function unknown	PF00372.22;PF0:	Hemocyanin_	Hemocyanin, copp	CYT	43	69	59	High
TRINITY_DN53866_c0_g1_i1_orf1	larval/pupal cuticle protein H1C-like [Ostrinia furnacalis]	0.3156708	-1.663507277	8.23E-06	down	yes	1.12	3.548	3.571	3.625	3.447	1	1.138	1.221	------	------	------	------	------	------	ENOG4110SXG	S:Function unknown	------	------	------	CYT	2	22	21.8	High
TRINITY_DN1110_c1_g1_i9_orf1	MD-2-related lipid-recognition protein-like [Ostrinia furnacalis]	0.259309791	-1.947251413	5.62E-07	down	yes	0.9693	3.738	3.694	3.702	3.818	1	0.915	0.993	------	------	K13443	NPC2	map04979;map	Cholesterol meta	ENOG4111D1U;ENOG41122NY	S:Function unknown;	PF02221.18	E1_DerP2_Der	ML domain	CYT	2	17	17.2	High
TRINITY_DN11826_c0_g1_i4_orf1	aldehyde dehydrogenase X, mitochondrial-like [Ostrinia furnacalis]	0.283748212	-1.817316795	1.24E-07	down	yes	0.9917	3.495	3.504	3.447	3.534	1	1.014	0.961	MF:catalytic activity;MF:oxidoreductase activity, acting on the aldehyde or oxo group of donors;MF:molecular_function;MF:oxidoreductase activity, acting on the aldehyde or oxo group of donors, NAD or NADP as acceptor;MF:oxidoreductase activity;	GO:0003824;GO:0016903;GO:0003674;GO:0016620;GO:0016491	K00128	ALDH	map00310;map	Lysine degradatic	COG1012	C:Energy production and conve	PF00171.25	Aldedh	Aldehyde dehydro	CYT	3	48	11.9	High
TRINITY_DN57137_c0_g1_i1_orfp1	TRINITY_DN57137_c0_g1_i1_m.46420 TRINITY_DN57137_c0_g1::TRINITY_DN57137_c0_g1_i1::g.46420 ORF type:5prime_partial len:56 (-	0.256417185	-1.963435143	5.43E-05	down	yes	0.949	3.701	3.461	3.667	3.975	1	0.936	0.911	------	------	------	------	------	------	------	------	------	------	------	CYT	3	33	5.8	High
TRINITY_DN696_c1_g1_i10_orf1	titin-like [Ostrinia furnacalis]	0.30967142	-1.691189855	1.40E-07	down	yes	0.999	3.226	3.198	3.28	3.199	1	0.991	1.006	------	------	------	------	------	------	ENOG410ZKJ3;ENOG4111M3T	S:Function unknown;	------	------	------	CYT	9	5	288.8	High
TRINITY_DN6470_c0_g3_i2_orf1	trypsin CFT-1-like [Ostrinia furnacalis]	0.247456258	-2.014754565	3.04E-05	down	yes	0.9193	3.715	3.514	3.948	3.683	1	0.859	0.899	BP:organic substance metabolic process;BP:organonitrogen compound metabolic process;BP:protein metabolic process;BP:macromolecule metabolic process;BP:biological_process;BP:nitrogen compound metabolic process;BP:metabolic process;BP:proteolysis;BP:primary metabolic process;MF:serine-type endopeptidase activity;MF:endopeptidase activity;MF:hydrolase activity;MF:serine hydrolase activity;MF:catalytic activity, acting on a protein;MF:catalytic activity;MF:molecular_function;MF:peptidase activity;MF:serine-	GO:0071704;GO:1901564;GO:0019538;GO:0043170;GO:0008150;GO:0006807;GO:0008152;GO:0006508;GO:0044238;GO:0004252;GO:0004175;GO:0016787;GO:0017171;GO:0140096;GO:0003824;GO:0003674;GO:0008233;GO:0008236	K01312	PRSS1_2_3	map04972;map	Pancreatic secret	COG5640	O:Posttranslational modificatio	PF00089.29;PF1:	Trypsin;Trypsi	Trypsin;Trypsin-like	CYT	1	5	27.4	High
TRINITY_DN13799_c0_g1_i1_orf1	uncharacterized protein LOC116345248 [Contarinia nasturtii]	0.320712909	-1.640645668	7.72E-07	down	yes	0.9987	3.114	3.059	3.191	3.092	1	1.008	0.988	------	------	------	------	------	------	------	------	PF06355.16	Aegerolysin	Aegerolysin	EXC	6	44	15.8	High
TRINITY_DN33728_c0_g2_i1_orf1	uncharacterized protein LOC114350200 [Ostrinia furnacalis]	0.410153397	-1.285764519	0.002004	down	yes	1.123	2.738	2.886	2.993	2.334	1	1.058	1.31	------	------	------	------	------	------	ENOG4111CDX	S:Function unknown	------	------	------	CYT	1	4	23	High
TRINITY_DN9239_c0_g2_i2_orf1	apolipophorins-like [Ostrinia furnacalis]	0.339779927	-1.557327471	6.62E-05	down	yes	1.019	2.999	3.18	2.8	3.017	1	0.974	1.082	BP:lipid transport;BP:transport;BP:organic substance transport;BP:biological_process;BP:establishment of localization;BP:localization;CC:cellular_component;CC:extracellular region;CC:cellular anatomical entity;MF:lipid transporter activity;MF:transporter activity;MF:lipid binding;MF:molecular_function;MF:binding;	GO:0006869;GO:0006810;GO:0071702;GO:0008150;GO:0051234;GO:0051179;GO:0005575;GO:0005576;GO:0110165;GO:0005319;GO:0005215;GO:0008289;GO:0003674;GO:0005488	------	------	------	------	ENOG41112S0;ENOG410Z6MR	S:Function unknown;	------	------	------	CYT	5	90	7.6	High
TRINITY_DN38307_c0_g1_i1_orfp1	TRINITY_DN38307_c0_g1_i1_m.10661 TRINITY_DN38307_c0_g1::TRINITY_DN38307_c0_g1_i1::g.10661 ORF type:5prime_partial len:66	0.06922545	-3.852553673	4.69E-05	down	yes	1.001	14.46	13.389	14.227	15.776	1	0.741	1.262	------	------	------	------	------	------	------	------	------	------	------	CYT	1	17	7.9	High
TRINITY_DN7040_c0_g1_i4_orf1	uncharacterized protein LOC114353763 [Ostrinia furnacalis]	0.310625	-1.686754148	0.0007562	down	yes	0.9443	3.04	3	3.445	2.674	1	0.878	0.955	------	------	------	------	------	------	------	------	------	------	------	CYT	2	39	7.4	High
TRINITY_DN11670_c0_g1_i1_orf1	teneurin-m isoform X1 [Ostrinia furnacalis]	0.364462209	-1.456158864	3.78E-08	down	yes	1.003	2.752	2.76	2.728	2.769	1	0.989	1.021	CC:integral component of membrane;CC:membrane;CC:cellular_component;CC:intrinsic component of membrane;CC:plasma membrane;CC:cellular anatomical entity;	GO:0016021;GO:0016020;GO:0005575;GO:0031224;GO:0005886;GO:0110165	K24473	TENM, ODZ	------	------	ENOG410XQQD	S:Function unknown	PF15636.9;PF07:	Tox-GHH;EGF	GHH signature cor	CYT	4	2	344.5	High
TRINITY_DN661_c0_g1_i1_orf1	hypothetical protein evm_002822 [Chilo suppressalis]	0.171193416	-2.54630088	0.0003199	down	yes	1.04	6.075	6.613	6.396	5.217	1	1.121	0.999	MF:molecular_function;MF:structural constituent of cuticle;MF:structural molecule activity;	GO:0003674;GO:0042302;GO:0005198	------	------	------	------	ENOG4111NR3;ENOG410YSFI;ENOG410ZM6M;ENOG4110SG6	S:Function unknown;	PF00379.26	Chitin_bind_4	Insect cuticle prote	CYT	1	22	17.6	High
TRINITY_DN2953_c1_g1_i10_orf1	methionine--tRNA ligase, cytoplasmic isoform X2 [Ostrinia furnacalis] >XP_028156683.1 methionine--tRNA ligase, cytoplasmic isoform X4 [Ostrinia furnacalis] >XP_028156684.1 methionine--tRNA ligase, cytoplasmic isoform X5 [Ostrinia furnacalis]	0.341032609	-1.552018402	0.0002395	down	yes	1.004	2.944	2.887	3.215	2.729	1	0.9	1.113	BP:methionyl-tRNA aminoacylation;BP:carboxylic acid metabolic process;BP:nucleic acid metabolic process;BP:cellular nitrogen compound metabolic process;BP:nitrogen compound metabolic process;BP:small molecule metabolic process;BP:ncRNA metabolic process;BP:organic cyclic compound metabolic process;BP:nucleobase-containing compound metabolic process;BP:cellular amino acid metabolic process;BP:organic substance metabolic process;BP:primary metabolic process;BP:cellular process;BP:cellular aromatic compound metabolic process;BP:biological_process;BP:metabolic process;BP:oxoacid metabolic process;BP:organonitrogen compound metabolic process;BP:heterocycle metabolic process;BP:RNA metabolic process;BP:organic acid metabolic process;BP:cellular metabolic process;BP:macromolecule metabolic process;BP:tRNA metabolic process;BP:amino acid activation;BP:tRNA aminoacylation;BP:tRNA aminoacylation for protein translation;CC:aminoacyl-tRNA synthetase multienzyme complex;CC:catalytic complex;CC:macromolecular complex;CC:cellular_component;MF:purine ribonucleoside triphosphate binding;MF:molecular_function;MF:ion binding;MF:heterocyclic compound binding;MF:anion binding;MF:aminoacyl-tRNA ligase activity;MF:nucleotide binding;MF:nucleoside phosphate binding;MF:purine nucleotide binding;MF:ATP binding;MF:catalytic activity, acting on RNA;MF:small molecule binding;MF:catalytic activity;MF:ligase activity;MF:ligase activity, forming carbon-oxygen bonds;MF:methionine-tRNA ligase activity;MF:adenyl nucleotide binding;MF:carbohydrate derivative [illegible]	GO:0006431;GO:0019752;GO:0090304;GO:0034641;GO:0006807;GO:0044281;GO:0034660;GO:1901360;GO:0006139;GO:0006520;GO:0071704;GO:0044238;GO:0009987;GO:0006725;GO:0008150;GO:0008152;GO:0043436;GO:1901564;GO:0046483;GO:0016070;GO:0006082;GO:0044237;GO:0043170;GO:0006399;GO:0043038;GO:0043039;GO:0006418;GO:0017101;GO:1902494;GO:0032991;GO:0005575;GO:0035639;GO:0003674;GO:0043167;GO:1901363;GO:0043168;GO:0004812;GO:0000166;GO:1901265;GO:0017076;GO:0005524;GO:0140098;GO:0036094;GO:0003824;GO:0016874;GO:0016875;GO:0004825;GO:0030554;GO:0097367;GO:0097159;GO:0032559;GO:0032555;GO:0140101;GO:0032553;GO:0005488	K01874	MARS, metG	map00450;map	Selenocompounc	COG0143	J:Translation, ribosomal structu	PF00458.23	WHEP-TRS	WHEP-TRS domair	CYT	2	57	17.6	High
TRINITY_DN81031_c0_g1_i1_orf1	aldehyde dehydrogenase, partial [Ectropis obliqua]	0.349746377	-1.515618983	3.00E-06	down	yes	0.9653	2.76	2.673	2.819	2.789	1	0.95	0.946	MF:catalytic activity;MF:oxidoreductase activity, acting on the aldehyde or oxo group of donors;MF:molecular_function;MF:oxidoreductase activity, acting on the aldehyde or oxo group of donors, NAD or NADP as acceptor;MF:oxidoreductase activity;	GO:0003824;GO:0016903;GO:0003674;GO:0016620;GO:0016491	K00128	ALDH	map00310;map	Lysine degradatic	COG1012	C:Energy production and conve	PF00171.25	Aldedh	Aldehyde dehydro	CYT	5	57	8.6	High
TRINITY_DN51813_c0_g1_i1_orf1	uncharacterized protein LOC114350216 [Ostrinia furnacalis]	0.147262295	-2.763540004	0.0005104	down	yes	0.8983	6.1	6.033	5.261	7.006	1	0.85	0.845	BP:'de novo' NAD biosynthetic process from tryptophan;BP:aromatic amino acid family catabolic process;BP:nucleotide biosynthetic process;BP:small molecule metabolic process;BP:small molecule catabolic process;BP:small molecule biosynthetic process;BP:organic cyclic compound biosynthetic process;BP:organic cyclic compound metabolic process;BP:organic cyclic compound catabolic process;BP:dicarboxylic acid biosynthetic process;BP:benzene-containing compound metabolic process;BP:nicotinamide nucleotide biosynthetic process;BP:nicotinamide nucleotide metabolic process;BP:monocarboxylic acid metabolic process;BP:oxoacid metabolic process;BP:aromatic amino acid family metabolic process;BP:heterocycle metabolic process;BP:dicarboxylic acid metabolic process;BP:organonitrogen compound metabolic process;BP:organonitrogen compound catabolic process;BP:organonitrogen compound biosynthetic process;BP:organic acid catabolic process;BP:organic acid biosynthetic process;BP:aromatic compound catabolic process;BP:aromatic compound biosynthetic process;BP:cellular amino acid catabolic process;BP:nitrogen compound metabolic process;BP:anthranilate metabolic process;BP:organic substance biosynthetic process;BP:organic substance catabolic process;BP:kynurenine metabolic process;BP:tryptophan catabolic process;BP:tryptophan metabolic process;BP:organophosphate metabolic process;BP:heterocycle biosynthetic process;BP:amine metabolic process;BP:nucleobase-containing compound metabolic process;BP:indolalkylamine metabolic [illegible]	GO:0034354;GO:0009074;GO:0009165;GO:0044281;GO:0044282;GO:0044283;GO:1901362;GO:1901360;GO:1901361;GO:0043650;GO:0042537;GO:0019359;GO:0046496;GO:0032787;GO:0043436;GO:0009072;GO:0046483;GO:0043648;GO:1901564;GO:1901565;GO:1901566;GO:0016054;GO:0016053;GO:0019439;GO:0019438;GO:0009063;GO:0006807;GO:0043420;GO:1901576;GO:1901575;GO:0070189;GO:0006569;GO:0006568;GO:0019637;GO:0018130;GO:0009308;GO:0006139;GO:0006586;GO:0008150;GO:0008152;GO:0034654;GO:0042436;GO:0090407;GO:0042430;GO:0044271;GO:0044270;GO:0046394;GO:0046395;GO:1901293;GO:0006753;GO:0006576;GO:0044248;GO:0044249;GO:0034641;GO:1901605;GO:1901606;GO:0009987;GO:0006725;GO:0044106;GO:0055086;GO:0006082;GO:0046700;GO:0046874;GO:0019752;GO:0009435;GO:0034627;GO:0044237;GO:0072524;GO:0072525;GO:0019805;GO:0006520;GO:0071704;GO:0009058;GO:0009117;GO:0009056;GO:0044238;GO:0042180;GO:0042182;GO:0097053;GO:0097052;GO:0006796;GO:0006793;GO:0019363;GO:0019362;GO:0110165;GO:0005737;GO:0005575; [illegible]	K01556	KYNU, kynU	map00380;map	Tryptophan meta	COG3844	E:Amino acid transport and me	PF00266.22	Aminotran_5	Aminotransferase c	CYT	1	1	48.1	High
TRINITY_DN2407_c0_g1_i6_orf1	uncharacterized protein LOC114366345 isoform X2 [Ostrinia furnacalis]	0.372968981	-1.422872446	0.0001158	down	yes	1.01	2.708	2.694	2.902	2.528	1	0.957	1.073	BP:response to biotic stimulus;BP:response to external stimulus;BP:response to external biotic stimulus;BP:response to stimulus;BP:defense response;BP:response to stress;BP:biological_process;BP:interspecies interaction between organisms;BP:response to other organism;CC:cellular_component;CC:extracellular region;CC:cellular anatomical entity;	GO:0009607;GO:0009605;GO:0043207;GO:0050896;GO:0006952;GO:0006950;GO:0008150;GO:0044419;GO:0051707;GO:0005575;GO:0005576;GO:0110165	------	------	------	------	ENOG4110S6N	S:Function unknown	PF00451.22	Toxin_2	Scorpion short toxi	CYT	5	24	37.6	High
TRINITY_DN2049_c1_g1_i3_orf1	luciferin 4-monooxygenase-like [Ostrinia furnacalis]	0.357539958	-1.48382361	0.00149	down	yes	1.029	2.878	3.026	2.515	3.092	1	0.778	1.308	------	------	K01904	4CL	map00130	Ubiquinone and	COG0318;COG0318	I:Lipid transport and metabolisi	PF00501.31;PF1:	AMP-binding	AMP-binding enzy	CYT	1	33	59.6	High
TRINITY_DN28922_c0_g1_i2_orf1	uncharacterized protein LOC119829283 isoform X2 [Zerene cesonia]	0.19009195	-2.395230659	4.54E-05	down	yes	1.013	5.329	5.421	5.665	4.902	1	0.963	1.076	CC:cellular_component;CC:cellular anatomical entity;	GO:0005575;GO:0110165	------	------	------	------	------	------	------	------	------	CYT	1	10	12.5	High
TRINITY_DN48878_c0_g2_i1_orf1	codanin-1 [Ostrinia furnacalis]	0.43423953	-1.203437031	0.0001735	down	yes	1.182	2.722	2.689	2.852	2.625	1	1.291	1.256	------	------	K19531	CDAN1	------	------	ENOG410XXWY	S:Function unknown	PF15296.9	Codanin-1_C	Codanin-1 C-term	CYT	1	1	83.1	Medium
TRINITY_DN578_c0_g1_i5_orf1	charged multivesicular body protein 7 [Ostrinia furnacalis]	0.461723447	-1.114899099	0.0001374	down	yes	1.152	2.495	2.445	2.455	2.585	1	1.173	1.282	BP:cellular process;BP:vacuolar transport;BP:intracellular transport;BP:transport;BP:biological_process;BP:establishment of localization in cell;BP:establishment of localization;BP:localization;BP:cellular localization;	GO:0009987;GO:0007034;GO:0046907;GO:0006810;GO:0008150;GO:0051649;GO:0051234;GO:0051179;GO:0051641	K15053	CHMP7	map04144;map	Endocytosis;Necr	ENOG4110IXB	U:Intracellular trafficking, secre	PF03357.24	Snf7	Snf7	CYT	1	10	43.1	High

TRINITY_DN4125_c0_g1_i14_orf1	angiotensin-converting enzyme-like isoform X1 [Ostrinia furnacalis]	0.345950643	-1.531361872	8.99E-05	down	yes	0.9953	2.877	3.012	2.976	2.642	1	0.993	0.993	BP:nitrogen compound metabolic process;BP:organic substance metabolic process;BP:proteolysis;BP:biological_process;BP:metabolic process;BP:primary metabolic process;BP:organonitrogen compound metabolic process;BP:protein metabolic process;BP:macromolecule metabolic process;CC:membrane;CC:intrinsic component of membrane;CC:integral component of membrane;CC:cellular anatomical entity;CC:cellular_component;MF:exopeptidase activity;MF:molecular_function;MF:binding;MF:peptidase activity;MF:cation binding;MF:carboxypeptidase activity;MF:ion binding;MF:metallopeptidase activity;MF:metal ion binding;MF:hydrolase activity;MF:peptidyl-dipeptidase	GO:0006807;GO:0071704;GO:0006508;GO:0008150;GO:0008152;GO:0044238;GO:1901564;GO:0019538;GO:0043170;GO:0016020;GO:0031224;GO:0016021;GO:0110165;GO:0005575;GO:0008238;GO:0003674;GO:0005488;GO:0008233;GO:0043169;GO:0004180;GO:0043167;GO:0008237;GO:0046872;GO:0016787;GO:0008241;GO:0140096;GO:0003824	K01283	ACE, CD143	map05142;ma	Chagas disease;F	ENOG410XPJ3	S:Function unknown	PF01401.21	Peptidase_M2	Angiotensin-conve	CYT	1	12	74.3	High
TRINITY_DN17247_c0_g1_i14_orf1	uncharacterized protein LOC114356308 [Ostrinia furnacalis]	0.417642467	-1.259659679	0.002228	down	yes	1.07	2.562	2.953	2.271	2.463	1	1.206	1.005	------	------	------	------	------	------	------	------	------	------	------	CYT	1	5	24.8	Medium
TRINITY_DN45037_c0_g1_i1_orf1	trafficking protein particle complex subunit 1 [Ostrinia furnacalis]	0.30060955	-1.734037254	0.0008202	down	yes	0.8877	2.953	2.609	2.909	3.34	1	0.726	0.937	BP:ER to Golgi vesicle-mediated transport;BP:localization;BP:cellular localization;BP:transport;BP:Golgi vesicle transport;BP:cellular process;BP:biological_process;BP:establishment of localization in cell;BP:establishment of localization;BP:vesicle-mediated transport;BP:intracellular transport;CC:endoplasmic reticulum;CC:intracellular organelle;CC:tethering complex;CC:membrane-bounded organelle;CC:organelle;CC:cellular anatomical entity;CC:Golgi apparatus;CC:TRAPP complex;CC:intracellular membrane-	GO:0006888;GO:0051179;GO:0051641;GO:0006810;GO:0048193;GO:0009987;GO:0008150;GO:0051649;GO:0051234;GO:0016192;GO:0046907;GO:0005783;GO:0043229;GO:0099023;GO:0043227;GO:0043226;GO:0110165;GO:0005794;GO:0030008;GO:0043231;GO:0032991;GO:0005575	K20300	TRAPPC1, BE	------	------	ENOG4111X41	U:Intracellular trafficking, secre	PF04099.15;PF0	Sybindin;Sedl	Sybindin-like famil	CYT	1	9	16.8	High
TRINITY_DN15222_c0_g1_i4_orf1	lysosomal alpha-mannosidase-like [Ostrinia furnacalis]	0.182769172	-2.451905346	2.43E-06	down	yes	0.9557	5.229	5.079	5.425	5.184	1	0.889	0.978	BP:nucleotide biosynthetic process;BP:small molecule metabolic process;BP:organic cyclic compound biosynthetic process;BP:organic cyclic compound metabolic process;BP:ribonucleoside triphosphate metabolic process;BP:purine ribonucleoside triphosphate biosynthetic process;BP:purine ribonucleoside triphosphate metabolic process;BP:ribonucleoside triphosphate biosynthetic process;BP:ATP metabolic process;BP:heterocycle metabolic process;BP:organonitrogen compound metabolic process;BP:organonitrogen compound biosynthetic process;BP:purine nucleotide metabolic process;BP:organophosphate metabolic process;BP:aromatic compound biosynthetic process;BP:nucleoside triphosphate metabolic process;BP:nucleoside triphosphate biosynthetic process;BP:purine nucleoside triphosphate metabolic process;BP:purine nucleoside triphosphate biosynthetic process;BP:nitrogen compound metabolic process;BP:organic substance biosynthetic process;BP:heterocycle biosynthetic process;BP:purine nucleotide biosynthetic process;BP:hexose metabolic process;BP:purine ribonucleotide biosynthetic process;BP:purine ribonucleotide metabolic process;BP:biological_process;BP:metabolic process;BP:nucleobase-containing compound biosynthetic process;BP:organophosphate biosynthetic process;BP:ATP synthesis coupled proton transport;BP:cellular nitrogen compound biosynthetic process;BP:ribonucleotide biosynthetic process;BP:ribose phosphate biosynthetic process;BP:nucleoside phosphate biosynthetic process;BP:nucleoside phosphate metabolic process;BP:ATP	GO:0009165;GO:0044281;GO:1901362;GO:1901360;GO:0009199;GO:0009206;GO:0009205;GO:0009201;GO:0046034;GO:0046483;GO:1901564;GO:1901566;GO:0006163;GO:0019637;GO:0019438;GO:0009141;GO:0009142;GO:0009144;GO:0009145;GO:0006807;GO:1901576;GO:0018130;GO:0006164;GO:0019318;GO:0009152;GO:0009150;GO:0008150;GO:0008152;GO:0034654;GO:0090407;GO:0015986;GO:0044271;GO:0009260;GO:0046390;GO:1901293;GO:0006753;GO:0006754;GO:0044249;GO:0034641;GO:0009259;GO:0006139;GO:0009987;GO:0006725;GO:0005975;GO:0055086;GO:1901137;GO:0005996;GO:1901135;GO:0044237;GO:0072521;GO:0072522;GO:0006796;GO:0006013;GO:0071704;GO:0009058;GO:0009117;GO:0044238;GO:0019693;GO:0006793;GO:0045263;GO:0033177;GO:0098800;GO:0000276;GO:0032991;GO:0005575;GO:0098796;GO:0098798;GO:0046872;GO:0004553;GO:0004559;GO:0003674;GO:0005488;GO:0015318;GO:0016787;GO:0043169;GO:0003824;GO:0022890;GO:0015075;GO:0015078;GO:0016798;GO:0043167;GO:0015923;GO:0005215;GO:0030246;GO:0008324;GO:0022857	K12311	MAN2B1, LAN	map00511;ma	Other glycan deg	ENOG410XQMZ	U:Intracellular trafficking, secre	------	------	------	CYT	1	17	11.9	High
TRINITY_DN18128_c0_g1_i4_orf1	arylsulfatase B [Ostrinia furnacalis]	0.39678179	-1.33358228	3.28E-06	down	yes	1.011	2.548	2.485	2.622	2.538	1	0.996	1.036	MF:hydrolase activity;MF:molecular_function;MF:hydrolase activity, acting on ester bonds;MF:sulfuric ester hydrolase activity;MF:catalytic activity;	GO:0016787;GO:0003674;GO:0016788;GO:0008484;GO:0003824	K01135	ARSB	map04142;ma	Lysosome;Glycos	COG3119	P:Inorganic ion transport and n	------	------	------	CYT	1	14	9.5	High
TRINITY_DN27035_c0_g1_i1_orf1	glucose-6-phosphate isomerase-like [Ostrinia furnacalis]	0.392512973	-1.349187759	8.69E-06	down	yes	1.059	2.698	2.674	2.788	2.632	1	1.065	1.113	BP:ribonucleoside diphosphate metabolic process;BP:phosphorylation;BP:purine nucleoside triphosphate metabolic process;BP:carboxylic acid metabolic process;BP:cellular nitrogen compound metabolic process;BP:nitrogen compound metabolic process;BP:small molecule biosynthetic process;BP:cellular aromatic compound metabolic process;BP:purine-containing compound metabolic process;BP:generation of precursor metabolites and energy;BP:pyruvate metabolic process;BP:glycolytic process;BP:nucleobase-containing compound metabolic process;BP:organic substance catabolic process;BP:purine nucleoside diphosphate metabolic process;BP:nucleotide phosphorylation;BP:ribonucleotide metabolic process;BP:organic substance metabolic process;BP:ribonucleoside triphosphate metabolic process;BP:organic cyclic compound metabolic process;BP:nucleoside triphosphate metabolic process;BP:gluconeogenesis;BP:small molecule metabolic process;BP:purine ribonucleotide metabolic process;BP:monosaccharide biosynthetic process;BP:purine ribonucleoside triphosphate metabolic process;BP:hexose metabolic process;BP:ribose phosphate metabolic process;BP:organic substance biosynthetic process;BP:ATP metabolic process;BP:cellular process;BP:ADP metabolic process;BP:hexose biosynthetic process;BP:monocarboxylic acid metabolic process;BP:biosynthetic process;BP:biological_process;BP:metabolic process;BP:oxoacid metabolic process;BP:carbohydrate metabolic process;BP:nucleoside diphosphate metabolic	GO:0009185;GO:0016310;GO:0009144;GO:0019752;GO:0034641;GO:0006807;GO:0044283;GO:0006725;GO:0072521;GO:0006091;GO:0006090;GO:0006096;GO:0006139;GO:1901575;GO:0009135;GO:0046939;GO:0009259;GO:0071704;GO:0009199;GO:1901360;GO:0009141;GO:0006094;GO:0044281;GO:0009150;GO:0046364;GO:0009205;GO:0019318;GO:0019693;GO:1901576;GO:0046034;GO:0009987;GO:0046031;GO:0019319;GO:0032787;GO:0009058;GO:0008150;GO:0008152;GO:0043436;GO:0005975;GO:0009132;GO:0009179;GO:0055086;GO:0046483;GO:0044238;GO:1901564;GO:0006082;GO:0005996;GO:1901135;GO:0016052;GO:0044237;GO:0016051;GO:0006163;GO:0006796;GO:0006165;GO:0006793;GO:0019637;GO:0009117;GO:0006006;GO:0006753;GO:0009056;GO:0006757;GO:0003674;GO:0005488;GO:0016861;GO:0016860;GO:0004347;GO:0003824;GO:0016853;GO:0097367	K01810	GPI, pgi	map00520;ma	Amino sugar and	COG0166	S:Function unknown	PF00342.22	PGI	Phosphoglucose is	CYT	3	39	13.3	High
TRINITY_DN4767_c0_g1_i6_orf1	cysteine protease XCP2-like [Ostrinia furnacalis]	0.193634417	-2.368592692	3.64E-06	down	yes	0.9947	5.137	5.013	5.367	5.031	1	1.013	0.971	BP:primary metabolic process;BP:organonitrogen compound metabolic process;BP:protein metabolic process;BP:macromolecule metabolic process;BP:organic substance metabolic process;BP:nitrogen compound metabolic process;BP:biological_process;BP:metabolic process;BP:proteolysis;MF:molecular_function;MF:hydrolase activity;MF:catalytic activity;MF:catalytic activity, acting on a protein;MF:peptidase activity;	GO:0044238;GO:1901564;GO:0019538;GO:0043170;GO:0071704;GO:0006807;GO:0008150;GO:0008152;GO:0006508;GO:0003674;GO:0016787;GO:0003824;GO:0140096;GO:0008233	------	------	------	------	ENOG4110R2S	S:Function unknown	PF08246.15	Inhibitor_I29	Cathepsin propept	CYT	2	44	5.3	High
TRINITY_DN87803_c0_g1_i2_orf1	hypothetical protein NF865_05903 [Phthorimaea operculella]	0.13480715	-2.891031082	6.49E-06	down	yes	1.433	10.63	10.94	10.257	10.691	1	1.718	1.581	MF:catalytic activity;MF:transferase	GO:0003824;GO:0016740;GO:0003674	K10532	HGSNAT	map04142;ma	Lysosome;Glycos	COG4299	S:Function unknown	------	------	------	CYT	1	23	9.7	High
TRINITY_DN33272_c0_g1_i1_orf1	unnamed protein product, partial [Iphiclides podalirius]	0.349432442	-1.516914536	0.0007446	down	yes	0.9543	2.731	2.752	2.392	3.048	1	0.927	0.936	------	------	K04550	LRP1, CD91	map05010;ma	Alzheimer diseas	ENOG410YM97;ENOG410XP34	S:Function unknown;	PF00057.21	Ldl_recept_a	Low-density lipopr	CYT	1	4	47.8	High
TRINITY_DN218_c0_g1_i1_orf1	altered inheritance of mitochondria protein 3-like isoform X2 [Ostrinia furnacalis]	0.393654524	-1.344998036	0.0001805	down	yes	1.005	2.553	2.577	2.732	2.349	1	1.062	0.952	MF:molecular_function;MF:structural constituent of cuticle;MF:structural molecule activity;	GO:0003674;GO:0042302;GO:0005198	------	------	------	------	ENOG4111QXK	S:Function unknown	PF00379.26	Chitin_bind_4	Insect cuticle prote	CYT	2	10	18	High
TRINITY_DN24971_c0_g1_i3_orf1	uncharacterized protein LOC114352370 [Ostrinia furnacalis]	0.244003916	-2.035023794	1.43E-06	down	yes	0.997	4.086	4.175	4.13	3.953	1	0.981	1.01	------	------	------	------	------	------	ENOG4111G1R	S:Function unknown	------	------	------	CYT	17	34	62.1	High
TRINITY_DN4592_c0_g1_i1_orf1	hypothetical protein KGM_205563 [Danaus plexippus plexippus]	0.136847222	-2.869361944	3.57E-06	down	yes	0.9853	7.2	7.378	7.365	6.858	1	0.939	1.017	------	------	------	------	------	------	ENOG4111M7I	S:Function unknown	------	------	------	CYT	17	56	42.8	High
TRINITY_DN23364_c0_g1_i1_orf1	PREDICTED: uncharacterized protein LOC106134920 [Amyelois transitella]	0.392857143	-1.347923303	0.001903	down	yes	1.023	2.604	2.682	2.934	2.197	1	1.07	0.999	------	------	------	------	------	------	ENOG4110SXM;ENOG4110SF2	S:Function unknown;	------	------	------	CYT	3	34	15.7	High
TRINITY_DN1986_c0_g1_i1_orf1	serine protease inhibitor 77Ba-like [Ostrinia furnacalis] >XP_028164032.1 serine protease inhibitor 77Ba-like [Ostrinia furnacalis]	0.392857143	-1.347923303	1.06E-05	down	yes	1.001	2.548	2.507	2.66	2.478	1	0.995	1.007	CC:cellular_component;CC:cellular anatomical entity;CC:extracellular space;MF:endopeptidase inhibitor activity;MF:endopeptidase regulator activity;MF:peptidase regulator activity;MF:molecular_function;MF:peptidase inhibitor activity;MF:serine-type endopeptidase inhibitor activity;MF:enzyme inhibitor activity;MF:molecular function regulator;MF:enzyme regulator activity;	GO:0005575;GO:0110165;GO:0005615;GO:0004866;GO:0061135;GO:0061134;GO:0003674;GO:0030414;GO:0004867;GO:0004857;GO:0098772;GO:0030234	------	------	------	------	COG4826	O:Posttranslational modificatio	PF00079.23	Serpin	Serpin	PLA	3	8	44.7	High
TRINITY_DN15400_c0_g1_i1_orf1	uncharacterized protein LOC114366781 [Ostrinia furnacalis]	0.156231366	-2.678243972	0.000763	down	yes	1.048	6.708	6.464	7.869	5.792	1	1.106	1.038	MF:odorant binding;MF:molecular_function;MF:binding;	GO:0005549;GO:0003674;GO:0005488	------	------	------	------	ENOG4111CBI	S:Function unknown	------	------	------	CYT	1	36	10.3	High
TRINITY_DN993_c0_g1_i7_orf1	apolipophorins-like [Ostrinia furnacalis]	0.245549219	-2.025915861	4.30E-09	down	yes	0.9903	4.033	4.022	4.015	4.062	1	0.98	0.991	MF:lipid transporter activity;MF:transporter activity;MF:molecular_function;	GO:0005319;GO:0005215;GO:0003674	------	------	------	------	ENOG41112S0;ENOG410Z6MR	S:Function unknown;	PF00094.28	VWD	von Willebrand fac	CYT	27	59	66.5	High
TRINITY_DN380_c0_g2_i2_orf1	chemosensory protein 10 [Ostrinia furnacalis]	0.415595189	-1.266749142	6.25E-05	down	yes	1.002	2.411	2.252	2.512	2.469	1	1.005	1.001	------	------	------	------	------	------	ENOG4111ATT;ENOG41116N7;ENOG410YU7V;ENOG4110RV8;ENOG4110R3	S:Function unknown;	PF03392.16	OS-D	Insect pheromone-	CYT	3	54	13.6	High
TRINITY_DN18539_c0_g1_i1_orf1	pupal cuticle protein PCP52-like [Ostrinia furnacalis]	0.14958478	-2.740964701	1.24E-05	down	yes	0.9907	6.623	6.602	7.003	6.263	1	0.983	0.989	MF:molecular_function;MF:structural constituent of cuticle;MF:structural molecule activity;	GO:0003674;GO:0042302;GO:0005198	------	------	------	------	ENOG4110SXG	S:Function unknown	------	------	------	CYT	13	54	28.3	High
TRINITY_DN26985_c0_g1_i5_orf1	secretory phospholipase A2 receptor-like [Helicoverpa zea]	0.347258486	-1.525918146	2.29E-06	down	yes	0.931	2.681	2.692	2.669	2.683	1	0.852	0.941	MF:molecular_function;MF:carbohydrate binding;MF:binding;	GO:0003674;GO:0030246;GO:0005488	------	------	------	------	ENOG410XPJ1;ENOG410XRS3	S:Function unknown;U:Intracell	------	------	------	CYT	2	26	9.8	High
TRINITY_DN13511_c0_g1_i4_orf1	alpha-2-macroglobulin receptor-associated protein isoform X1 [Ostrinia furnacalis]	0.450963597	-1.148917114	0.003174	down	yes	1.053	2.335	2.614	2.442	1.948	1	1.111	1.049	CC:cellular_component;CC:endoplasmic reticulum;CC:intracellular organelle;CC:membrane-bounded organelle;CC:organelle;CC:cellular anatomical entity;CC:intracellular membrane-bounded organelle;MF:sulfur compound binding;MF:carbohydrate derivative binding;MF:receptor regulator activity;MF:receptor inhibitor activity;MF:glycosaminoglycan binding;MF:heparin binding;MF:receptor antagonist activity;MF:molecular_function;MF:binding;MF:protein binding;MF:lipoprotein particle receptor binding;MF:low-density lipoprotein particle receptor binding;MF:receptor	GO:0005575;GO:0005783;GO:0043229;GO:0043227;GO:0043226;GO:0110165;GO:0043231;GO:1901681;GO:0097367;GO:0030545;GO:0030547;GO:0005539;GO:0008201;GO:0048019;GO:0003674;GO:0005488;GO:0005515;GO:0070325;GO:0050750;GO:0005102;GO:0098772	K22290	LRPAP1	map04979	Cholesterol meta	ENOG410YJFP	S:Function unknown	PF06401.14;PF0	Alpha-2-MRA	Alpha-2-macroglo	CYT	2	7	41.9	High
TRINITY_DN661_c0_g2_i2_orf1	cuticle protein 7-like [Ostrinia furnacalis]	0.268068331	-1.899327302	2.82E-06	down	yes	1.02	3.805	3.929	3.8	3.687	1	0.998	1.062	MF:molecular_function;MF:structural constituent of cuticle;MF:structural molecule activity;	GO:0003674;GO:0042302;GO:0005198	------	------	------	------	ENOG4110SG6;ENOG410YSFI;ENOG4110T8H;ENOG410Z	S:Function unknown;	PF00379.26	Chitin_bind_4	Insect cuticle prote	CYT	2	19	19.4	High
TRINITY_DN537_c0_g1_i1_orf1	pupal cuticle protein C1B-like precursor [Papilio xuthus] >BAM18715.1 cuticular protein PxutCPFL6Ba [Papilio xuthus]	0.425116674	-1.23406925	0.001107	down	yes	1.002	2.357	2.635	2.359	2.076	1	1.016	0.991	------	------	------	------	------	------	ENOG4112AQ8	S:Function unknown	------	------	------	CYT	4	32	27.7	High
TRINITY_DN13576_c0_g1_i1_orf1	uncharacterized protein LOC114350099 [Ostrinia furnacalis]	0.156786875	-2.673123301	1.46E-06	down	yes	1.013	6.461	6.633	6.522	6.228	1	1.02	1.02	------	------	------	------	------	------	ENOG4111QIW	S:Function unknown	PF00379.26	Chitin_bind_4	Insect cuticle prote	CYT	9	48	29.1	High
TRINITY_DN9593_c0_g1_i2_orf1	uncharacterized protein LOC113518937 [Galleria mellonella]	0.471052632	-1.086039831	2.22E-05	down	yes	1.074	2.28	2.307	2.329	2.205	1	1.113	1.108	------	------	------	------	------	------	------	------	------	------	------	CYT	3	32	15	High
TRINITY_DN41_c0_g1_i3_orf1	uncharacterized protein LOC114359035 isoform X3 [Ostrinia furnacalis]	0.266946627	-1.905376775	9.66E-07	down	yes	0.9853	3.691	3.719	3.764	3.589	1	0.958	0.998	BP:nitrogen compound metabolic process;BP:membrane lipid metabolic process;BP:organic substance metabolic process;BP:sphingolipid metabolic process;BP:lipid metabolic process;BP:cellular process;BP:biological_process;BP:metabolic process;BP:cellular lipid metabolic process;BP:primary metabolic process;BP:organonitrogen compound metabolic process;BP:cellular metabolic process;CC:lytic vacuole;CC:intracellular organelle;CC:membrane-bounded organelle;CC:organelle;CC:lysosome;CC:vacuole;CC:cellular anatomical entity;CC:intracellular membrane-bounded organelle;CC:cellular_component;CC:extracellular region;	GO:0006807;GO:0006643;GO:0071704;GO:0006665;GO:0006629;GO:0009987;GO:0008150;GO:0008152;GO:0044255;GO:0044238;GO:1901564;GO:0044237;GO:0000323;GO:0043229;GO:0043227;GO:0043226;GO:0005764;GO:0005773;GO:0110165;GO:0043231;GO:0005575;GO:0005576	K12382	PSAP, SGP1	map00600;ma	Sphingolipid met	ENOG410XSI5	J:Translation, ribosomal structu	PF03489.20;PF0	SapB_2;SapB_	Saposin-like type I	CYT	40	46	125.9	High
TRINITY_DN6004_c0_g1_i1_orf1	endocuticle structural glycoprotein ABD-4-like [Ostrinia furnacalis]	0.272295442	-1.876755261	2.45E-06	down	yes	0.9917	3.642	3.515	3.705	3.706	1	0.951	1.024	MF:molecular_function;MF:structural constituent of cuticle;MF:structural molecule activity;	GO:0003674;GO:0042302;GO:0005198	------	------	------	------	ENOG4111PJ1;ENOG410YR66	O:Posttranslational modificatio	PF00379.26	Chitin_bind_4	Insect cuticle prote	CYT	2	23	16.7	High
TRINITY_DN2946_c0_g1_i1_orf1	histidine-rich glycoprotein [Ostrinia furnacalis]	0.165848068	-2.592065888	2.23E-05	down	yes	1.013	6.108	6.303	6.359	5.662	1	1.051	0.987	------	------	------	------	------	------	ENOG410YTNT	S:Function unknown	------	------	------	PLA	9	28	23.4	High
TRINITY_DN361_c0_g1_i5_orf1	hexosaminidase [Ostrinia furnacalis]	0.434471134	-1.202667768	2.23E-06	down	yes	1.031	2.373	2.406	2.347	2.365	1	1.087	1.005	BP:organic substance metabolic process;BP:carbohydrate metabolic process;BP:primary metabolic process;BP:metabolic process;BP:biological_process;MF:hydrolase activity;MF:N-acetyl-beta-D-galactosaminidase activity;MF:hydrolase activity, acting on glycosyl bonds;MF:hexosaminidase activity;MF:catalytic activity;MF:beta-N-acetylhexosaminidase activity;MF:molecular_function;MF:hydrolase activity, hydrolyzing O-glycosyl compounds;	GO:0071704;GO:0005975;GO:0044238;GO:0008152;GO:0008150;GO:0016787;GO:0102148;GO:0016798;GO:0015929;GO:0003824;GO:0004563;GO:0003674;GO:0004553	K12373	HEXA_B	map00600;ma	Sphingolipid met	COG3525	S:Function unknown	PF00728.25;PF1	Glyco_hydro_	Glycosyl hydrolase	CYT	5	11	70.1	High

TRINITY_DN2652_c0_g2_i1_orf1	peroxidase-like isoform X1 [Ostrinia furnacalis]	0.294100802	-1.765617377	1.15E-06	down	yes	1.027	3.492	3.432	3.501	3.544	1	1.106	0.976	BP:response to stimulus;BP:cell development;BP:female gamete generation;BP:gamete generation;BP:cellular developmental process;BP:oogenesis;BP:developmental process;BP:multicellular organismal reproductive process;BP:cellular process;BP:germ cell development;BP:reproductive process;BP:response to stress;BP:biological_process;BP:cellular process involved in reproduction in multicellular organism;BP:response to oxidative stress;BP:developmental process involved in reproduction;BP:anatomical structure development;CC:integral component of membrane;CC:intrinsic component of membrane;CC:cellular anatomical entity;CC:cellular_component;MF:molecular_function;MF:binding;MF:heterocyclic compound binding;MF:oxidoreductase activity;MF:oxidoreductase activity, acting on peroxide as acceptor;MF:heme binding;MF:antioxidant activity;MF:organic cyclic compound binding;MF:catalytic activity;MF:peroxidase	GO:0050896;GO:0048468;GO:0007292;GO:0007276;GO:0048869;GO:0048477;GO:0032502;GO:0048609;GO:0009987;GO:0007281;GO:0022414;GO:0006950;GO:0008150;GO:0022412;GO:0006979;GO:0003006;GO:0048856;GO:0016021;GO:0031224;GO:0110165;GO:0005575;GO:0003674;GO:0005488;GO:1901363;GO:0016491;GO:0016684;GO:0020037;GO:0016209;GO:0097159;GO:0003824;GO:0004601;GO:0046906	K19511	PXDN, VPO1	------	------	ENOG410XPZ3	S:Function unknown	PF03098.18	An_peroxidas	Animal haem pero	CYT	11	19	88.2	High
TRINITY_DN53358_c0_g1_i3_orf1	arylphorin subunit alpha-like [Ostrinia furnacalis]	0.42028181	-1.250571076	0.001277	down	yes	0.9843	2.342	2.297	2.078	2.65	1	0.941	1.012	CC:cellular_component;CC:extracellular space;CC:cellular anatomical entity;MF:nutrient reservoir	GO:0005575;GO:0005615;GO:0110165;GO:0045735;GO:0003674	------	------	------	------	ENOG410XR2D	S:Function unknown	PF00372.22	Hemocyanin_	Hemocyanin, copp	CYT	4	39	13.3	High
TRINITY_DN35351_c0_g1_i3_orf1	adult-specific cuticular protein ACP-20-like [Ostrinia furnacalis]	0.186434959	-2.423255688	3.99E-05	down	yes	1.105	5.927	5.556	6.377	5.849	1	1.166	1.149	MF:molecular_function;MF:structural constituent of cuticle;MF:structural molecule activity;	GO:0003674;GO:0042302;GO:0005198	------	------	------	------	ENOG410YWJF;ENOG410YTXG;ENOG4110QVC;ENOG410YSFI;ENOG410ZUHH;ENOG41117GA	Q:Secondary metabolites biosy	PF00379.26	Chitin_bind_4	Insect cuticle prote	CYT	2	16	19.2	High
TRINITY_DN5132_c0_g1_i4_orf1	small heat shock protein Hsp24.2 [Ostrinia furnacalis]	0.437766411	-1.191766832	8.55E-07	down	yes	1.027	2.346	2.325	2.384	2.329	1	1.059	1.023	------	------	K09541	CRYAA	map04141	Protein processin	ENOG410YERS	J:Translation, ribosomal structu	PF00011.24	HSP20	Hsp20/alpha cryst	CYT	5	21	24.7	High
TRINITY_DN143497_c0_g1_i1_orf1	fibroin heavy chain-like [Ostrinia furnacalis]	0.281927024	-1.826606322	5.17E-07	down	yes	0.989	3.508	3.52	3.574	3.43	1	0.969	0.998	------	------	------	------	------	------	ENOG4111C6D	S:Function unknown	------	------	------	CYT	7	39	19.4	High
TRINITY_DN1575_c0_g1_i10_orf1	uncharacterized protein LOC114359245 [Ostrinia furnacalis]	0.366246954	-1.449111335	0.001324	down	yes	0.9017	2.462	2.461	2.759	2.165	1	0.716	0.989	------	------	------	------	------	------	ENOG4111CB2;ENOG41118JD;ENOG4110T1G	S:Function unknown;	PF15868.8	MBF2	Transcription activ	CYT	1	2	45.9	High
TRINITY_DN25534_c0_g1_i1_orf1	venom serine carboxypeptidase-like [Ostrinia furnacalis]	0.223170472	-2.163781943	2.22E-07	down	yes	1.046	4.687	4.655	4.691	4.714	1	0.997	1.141	BP:primary metabolic process;BP:organonitrogen compound metabolic process;BP:protein metabolic process;BP:macromolecule metabolic process;BP:organic substance metabolic process;BP:nitrogen compound metabolic process;BP:biological_process;BP:metabolic process;BP:proteolysis;MF:molecular_function;MF:exopeptidase activity;MF:hydrolase activity;MF:carboxypeptidase activity;MF:catalytic activity, acting on a protein;MF:serine hydrolase activity;MF:serine-type carboxypeptidase activity;MF:serine-type exopeptidase activity;MF:peptidase activity;MF:catalytic activity;MF:serine-type peptidase activity;	GO:0044238;GO:1901564;GO:0019538;GO:0043170;GO:0071704;GO:0006807;GO:0008150;GO:0008152;GO:0006508;GO:0003674;GO:0008238;GO:0016787;GO:0004180;GO:0140096;GO:0017171;GO:0004185;GO:0070008;GO:0008233;GO:0003824;GO:0008236	K09645	CPVL	------	------	ENOG410XPIW;COG2939	S:Function unknown;E:Amino a	PF00450.25	Peptidase_S1	Serine carboxypep	CYT	4	12	55.9	High
TRINITY_DN4235_c0_g1_i2_orf1	uncharacterized protein LOC114361536 [Ostrinia furnacalis]	0.454188482	-1.138636975	3.80E-05	down	yes	1.041	2.292	2.359	2.174	2.344	1	1.053	1.071	CC:cellular_component;CC:cellular anatomical entity;CC:extracellular space;MF:endopeptidase inhibitor activity;MF:endopeptidase regulator activity;MF:peptidase regulator activity;MF:molecular_function;MF:peptidase inhibitor activity;MF:serine-type endopeptidase inhibitor activity;MF:enzyme inhibitor activity;MF:molecular function regulator;MF:enzyme regulator activity;	GO:0005575;GO:0110165;GO:0005615;GO:0004866;GO:0061135;GO:0061134;GO:0003674;GO:0030414;GO:0004867;GO:0004857;GO:0098772;GO:0030234	------	------	------	------	COG4826	P:Inorganic ion transport and n	PF00079.23	Serpin	Serpin	CYT	5	16	43.7	High
TRINITY_DN113353_c0_g1_i1_orf1	unnamed protein product [Parnassius apollo]	0.323615257	-1.627648469	0.0003448	down	yes	0.9757	3.015	3.362	2.766	2.918	1	0.99	0.937	BP:cellular process;BP:transport;BP:lipid transport;BP:autophagy;BP:cellular metabolic process;BP:localization;BP:process utilizing autophagic mechanism;BP:metabolic process;BP:organic substance transport;BP:biological_process;BP:establishment of localization;BP:catabolic process;BP:cellular catabolic process;CC:cellular_component;CC:membrane;CC:pre-autophagosomal structure membrane;CC:intrinsic component of membrane;CC:cellular anatomical entity;CC:integral component of membrane;	GO:0009987;GO:0006810;GO:0006869;GO:0006914;GO:0044237;GO:0051179;GO:0061919;GO:0008152;GO:0071702;GO:0008150;GO:0051234;GO:0009056;GO:0044248;GO:0005575;GO:0016020;GO:0034045;GO:0031224;GO:0110165;GO:0016021	K17907	ATG9	map04136;ma	Autophagy - oth	ENOG410XQBE	U:Intracellular trafficking, secre	------	------	------	CYT	1	19	7.5	High
TRINITY_DN83374_c0_g1_i1_orf1	uncharacterized protein LOC114350302, partial [Ostrinia furnacalis]	0.445851528	-1.165364732	5.71E-05	down	yes	1.021	2.29	2.415	2.282	2.173	1	1.037	1.027	------	------	------	------	------	------	ENOG410Y20T	S:Function unknown	------	------	------	CYT	2	7	63	High
TRINITY_DN44073_c0_g1_i3_orf1	inter-alpha-trypsin inhibitor heavy chain H4-like isoform X11 [Ostrinia furnacalis]	0.386896047	-1.369982107	6.19E-05	down	yes	1.057	2.732	2.816	2.822	2.558	1	1.039	1.132	------	------	------	------	------	------	COG2304;ENOG410XRSO	S:Function unknown;	------	------	------	CYT	2	18	8.9	High
TRINITY_DN3833_c0_g1_i4_orf1	division abnormally delayed protein [Ostrinia furnacalis]	0.423719328	-1.238819154	1.60E-05	down	yes	0.9843	2.323	2.397	2.283	2.29	1	0.909	1.044	BP:regulation of smoothened signaling pathway;BP:positive regulation of canonical Wnt signaling pathway;BP:positive regulation of response to stimulus;BP:regulation of response to stimulus;BP:positive regulation of signaling;BP:regulation of Wnt signaling pathway;BP:positive regulation of smoothened signaling pathway;BP:regulation of signaling;BP:positive regulation of cell communication;BP:regulation of cell communication;BP:regulation of biological process;BP:regulation of cellular response to growth factor stimulus;BP:positive regulation of transmembrane receptor protein serine/threonine kinase signaling pathway;BP:regulation of signal transduction;BP:positive regulation of signal transduction;BP:biological regulation;BP:positive regulation of biological process;BP:regulation of transmembrane receptor protein serine/threonine kinase signaling pathway;BP:regulation of cellular process;BP:biological_process;BP:positive regulation of BMP signaling pathway;BP:regulation of BMP signaling pathway;BP:positive regulation of Wnt signaling pathway;BP:regulation of canonical Wnt signaling pathway;BP:positive regulation of cellular process;CC:extracellular matrix;CC:anchored component of plasma membrane;CC:intrinsic component of plasma membrane;CC:intrinsic component of membrane;CC:anchored component of membrane;CC:cellular anatomical	GO:0008589;GO:0090263;GO:0048584;GO:0048583;GO:0023056;GO:0030111;GO:0045880;GO:0023051;GO:0010647;GO:0010646;GO:0050789;GO:0090287;GO:0090100;GO:0009966;GO:0009967;GO:0065007;GO:0048518;GO:0090092;GO:0050794;GO:0008150;GO:0030513;GO:0030510;GO:0030177;GO:0060828;GO:0048522;GO:0031012;GO:0046658;GO:0031226;GO:0031224;GO:0031225;GO:0110165;GO:0062023;GO:0030312;GO:0005575	K02306	DALLY	map04310;ma	Wnt signaling pa	ENOG410XST2	O:Posttranslational modificatio	PF01153.22	Glypican	Glypican	CYT	2	3	60	High
TRINITY_DN126127_c0_g1_i1_orf1	prolow-density lipoprotein receptor-related protein 1, partial [Ostrinia furnacalis]	0.422362136	-1.243447588	2.31E-06	down	yes	0.9727	2.303	2.34	2.323	2.245	1	0.972	0.946	------	------	K04550	LRP1, CD91	map05010;ma	Alzheimer diseas	ENOG410XP34	S:Function unknown	PF00058.20;PF1	Ldl_recept_b;	Low-density lipop	CYT	9	25	56.4	High
TRINITY_DN47784_c0_g2_i1_orfp1	arylphorin subunit alpha-like [Ostrinia furnacalis]	0.396396396	-1.334984248	3.02E-05	down	yes	1.056	2.664	2.635	2.79	2.568	1	1.037	1.131	------	------	------	------	------	------	------	------	------	------	------	CYT	2	27	10.1	High
TRINITY_DN4748_c0_g1_i5_orf1	unnamed protein product, partial [Brenthis ino]	0.220877458	-2.178681903	6.48E-05	down	yes	1.022	4.627	4.379	5.037	4.465	1	0.998	1.069	BP:response to biotic stimulus;BP:response to external stimulus;BP:response to external biotic stimulus;BP:response to other organism;BP:response to stimulus;BP:response to stress;BP:defense response;BP:biological_process;BP:interspecies interaction between organisms;CC:cellular_component;CC:extracellular region;CC:cellular anatomical entity;MF:ion channel regulator activity;MF:sodium channel inhibitor activity;MF:sodium channel regulator activity;MF:molecular_function;MF:channel inhibitor activity;MF:channel regulator activity;MF:molecular function regulator;MF:ion channel inhibitor activity;	GO:0009607;GO:0009605;GO:0043207;GO:0051707;GO:0050896;GO:0006950;GO:0006952;GO:0008150;GO:0044419;GO:0005575;GO:0005576;GO:0110165;GO:0099106;GO:0019871;GO:0017080;GO:0003674;GO:0016248;GO:0016247;GO:0098772;GO:0008200	------	------	------	------	------	------	------	------	------	CYT	3	54	7.8	High
TRINITY_DN37585_c0_g2_i1_orf1	cuticle protein 19.8-like [Ostrinia furnacalis]	0.491788321	-1.02389062	0.004672	down	yes	1.078	2.192	2.184	2.528	1.865	1	1.105	1.128	MF:molecular_function;MF:structural constituent of cuticle;MF:structural molecule activity;	GO:0003674;GO:0042302;GO:0005198	------	------	------	------	ENOG410YTXG;ENOG410YSFI	S:Function unknown;	PF00379.26	Chitin_bind_4	Insect cuticle prote	CYT	1	6	14.7	High
TRINITY_DN2170_c1_g1_i3_orf1	beta-1,3-glucan-binding protein-like [Ostrinia furnacalis]	0.4	-1.321928095	1.45E-05	down	yes	1.002	2.505	2.459	2.601	2.455	1	1.063	0.942	BP:positive regulation of response to external stimulus;BP:regulation of response to external stimulus;BP:positive regulation of response to stimulus;BP:regulation of response to stimulus;BP:positive regulation of response to biotic stimulus;BP:positive regulation of defense response;BP:signal transduction;BP:cell surface receptor signaling pathway;BP:regulation of biological process;BP:regulation of defense response;BP:immune response-regulating signaling pathway;BP:response to external biotic stimulus;BP:immune response-regulating cell surface receptor signaling pathway;BP:interspecies interaction between organisms;BP:organic substance metabolic process;BP:positive regulation of immune system process;BP:regulation of immune system process;BP:positive regulation of biological process;BP:biological regulation;BP:innate immune response activating cell surface receptor signaling pathway;BP:pattern recognition receptor signaling pathway;BP:positive regulation of innate immune response;BP:regulation of innate immune response;BP:immune response-activating signal transduction;BP:regulation of response to biotic stimulus;BP:innate immune response;BP:cellular process;BP:regulation of cellular process;BP:defense response;BP:response to stress;BP:immune response-activating cell surface receptor signaling pathway;BP:biological_process;BP:metabolic process;BP:immune response;BP:response to biotic stimulus;BP:response to other organism;BP:primary metabolic process;BP:regulation of immune response;BP:carbohydrate metabolic process;BP:response to external stimulus;BP:cell	GO:0032103;GO:0032101;GO:0048584;GO:0048583;GO:0002833;GO:0031349;GO:0007165;GO:0007166;GO:0050789;GO:0031347;GO:0002764;GO:0043207;GO:0002768;GO:0044419;GO:0071704;GO:0002684;GO:0002682;GO:0048518;GO:0065007;GO:0002220;GO:0002221;GO:0045089;GO:0045088;GO:0002757;GO:0002831;GO:0045087;GO:0009987;GO:0050794;GO:0006952;GO:0006950;GO:0002429;GO:0008150;GO:0008152;GO:0006955;GO:0009607;GO:0051707;GO:0044238;GO:0050776;GO:0005975;GO:0009605;GO:0002752;GO:0080134;GO:0002758;GO:0050778;GO:0002376;GO:0002218;GO:0002253;GO:0098542;GO:0050896;GO:0005575;GO:0110165;GO:0005576;GO:0038023;GO:0030246;GO:0060089;GO:0003674;GO:0038187;GO:0016787;GO:0003824;GO:0004553;GO:0016798;GO:0005488	K20692	GNBP3	map04624	Toll and Imd sign	ENOG410Z83Q;ENOG4110NFS	S:Function unknown;	PF15886.8	CBM39	Carbohydrate bind	CYT	4	30	21.5	High
TRINITY_DN3005_c0_g1_i7_orf1	lachesin-like isoform X3 [Ostrinia furnacalis]	0.456482321	-1.131369108	2.45E-07	down	yes	1.007	2.206	2.224	2.215	2.178	1	0.995	1.026	CC:cellular_component;CC:integral component of membrane;CC:cellular anatomical entity;CC:intrinsic component of membrane;	GO:0005575;GO:0016021;GO:0110165;GO:0031224	K06774	HNT	------	------	ENOG410XT1Y	S:Function unknown	PF13927.9;PF07	Ig_3;I-set;ig;I	Immunoglobulin d	CYT	3	7	48.5	High
TRINITY_DN9239_c0_g1_i1_orf1	apolipophorins-like [Ostrinia furnacalis]	0.209124564	-2.257565559	4.87E-05	down	yes	0.9603	4.592	4.499	4.313	4.963	1	0.917	0.964	BP:lipid transport;BP:transport;BP:organic substance transport;BP:biological_process;BP:establishment of localization;BP:localization;CC:cellular_component;CC:extracellular region;CC:cellular anatomical entity;MF:lipid transporter activity;MF:transporter activity;MF:lipid binding;MF:molecular_function;MF:binding;	GO:0006869;GO:0006810;GO:0071702;GO:0008150;GO:0051234;GO:0051179;GO:0005575;GO:0005576;GO:0110165;GO:0005319;GO:0005215;GO:0008289;GO:0003674;GO:0005488	------	------	------	------	ENOG410Z6MR;ENOG41112S0	S:Function unknown;	------	------	------	CYT	10	78	18	High
TRINITY_DN12256_c0_g1_i1_orf1	lysosome membrane protein 2-like [Ostrinia furnacalis]	0.45582878	-1.13343608	5.25E-08	down	yes	1.001	2.196	2.175	2.21	2.202	1	0.993	1.009	BP:multicellular organismal process;BP:sensory perception of smell;BP:neurological system process;BP:response to stimulus;BP:sensory perception of chemical stimulus;BP:sensory perception;BP:biological_process;BP:system process;CC:membrane;CC:cellular_component;CC:cellular anatomical entity;CC:plasma membrane;CC:integral component of membrane;CC:intrinsic component of membrane;	GO:0032501;GO:0007608;GO:0050877;GO:0050896;GO:0007606;GO:0007600;GO:0008150;GO:0003008;GO:0016020;GO:0005575;GO:0110165;GO:0005886;GO:0016021;GO:0031224	K13885	SCARB1	map04979;ma	Cholesterol meta	ENOG410XS17	U:Intracellular trafficking, secre	PF01130.24	CD36	CD36 family	CYT	3	22	17.6	High
TRINITY_DN768_c0_g1_i7_orf1	neutral ceramidase [Leguminivora glycinivorella]	0.377395091	-1.405852438	5.77E-07	down	yes	0.9533	2.526	2.514	2.509	2.556	1	0.932	0.928	BP:sphingolipid catabolic process;BP:cellular catabolic process;BP:cellular nitrogen compound metabolic process;BP:nitrogen compound metabolic process;BP:cellular lipid catabolic process;BP:lipid metabolic process;BP:organic substance catabolic process;BP:lipid catabolic process;BP:organic substance metabolic process;BP:membrane lipid catabolic process;BP:sphingolipid metabolic process;BP:membrane lipid metabolic process;BP:cellular process;BP:biological_process;BP:metabolic process;BP:cellular lipid metabolic process;BP:catabolic process;BP:primary metabolic process;BP:organonitrogen compound metabolic process;BP:organonitrogen compound catabolic process;BP:cellular amide metabolic process;BP:cellular metabolic process;BP:ceramide catabolic process;BP:ceramide metabolic process;MF:ceramidase activity;MF:molecular_function;MF:ceramidase activity;MF:hydrolase activity;MF:catalytic activity;MF:hydrolase	GO:0030149;GO:0044248;GO:0034641;GO:0006807;GO:0044242;GO:0006629;GO:1901575;GO:0016042;GO:0071704;GO:0046466;GO:0006665;GO:0006643;GO:0009987;GO:0008150;GO:0008152;GO:0044255;GO:0009056;GO:0044238;GO:1901564;GO:1901565;GO:0043603;GO:0044237;GO:0046514;GO:0006672;GO:0017040;GO:0003674;GO:0102121;GO:0016787;GO:0003824;GO:0016810;GO:0016811	K12349	ASAH2	map00600;ma	Sphingolipid met	ENOG410XQWE	S:Function unknown	PF04734.16;PF1	Ceramidase_a	Neutral/alkaline nc	CYT	10	20	77.2	High
TRINITY_DN7539_c0_g1_i2_orf1	serine protease inhibitor 3/4 [Ostrinia furnacalis]	0.216258897	-2.209168609	1.28E-07	down	yes	0.9723	4.496	4.448	4.576	4.464	1	0.962	0.955	CC:cellular_component;CC:cellular anatomical entity;CC:extracellular space;MF:endopeptidase inhibitor activity;MF:endopeptidase regulator activity;MF:peptidase regulator activity;MF:molecular_function;MF:peptidase inhibitor activity;MF:serine-type endopeptidase inhibitor activity;MF:enzyme inhibitor activity;MF:molecular function regulator;MF:enzyme regulator activity;	GO:0005575;GO:0110165;GO:0005615;GO:0004866;GO:0061135;GO:0061134;GO:0003674;GO:0030414;GO:0004867;GO:0004857;GO:0098772;GO:0030234	------	------	------	------	COG4826	O:Posttranslational modificatio	PF00079.23	Serpin	Serpin	CYT	9	18	58.6	High
TRINITY_DN14009_c0_g1_i1_orf1	proline-rich extensin-like protein EPR1 [Manduca sexta]	0.484619025	-1.045077052	3.34E-06	down	yes	1.024	2.113	2.069	2.113	2.157	1	1.053	1.018	------	------	------	------	------	------	ENOG4111PWF	S:Function unknown	PF03103.20	DUF243	Domain of unknow	CYT	3	12	26.8	High

TRINITY_DN5444_c0_g1_i1_orfp1	TRINITY_DN5444_c0_g1_i1_m.14077 TRINITY_DN5444_c0_g1::TRINITY_DN5444_c0_g1_i1::g.14077 ORF type:3prime_partial len:90 (-),score=25.11 TRINITY_DN5444_c0_g1_i1:2-268(-)	0.126751078	-2.979930082	8.04E-05	down	yes	0.941	7.424	6.953	8.205	7.113	1	0.896	0.927	------	------	------	------	------	------	------	------	------	------	------	CYT	1	8	9.9	Medium
TRINITY_DN399_c3_g2_i6_orf1	proline-rich extensin-like protein EPR1 [Ostrinia furnacalis]	0.454871795	-1.136468114	7.51E-06	down	yes	0.9757	2.145	2.203	2.148	2.083	1	0.94	0.987	CC:cellular_component;CC:extracellular region;CC:cellular anatomical entity;MF:endopeptidase inhibitor activity;MF:endopeptidase regulator activity;MF:peptidase regulator activity;MF:molecular_function;MF:peptidase inhibitor activity;MF:serine-type endopeptidase inhibitor activity;MF:enzyme inhibitor activity;MF:molecular function regulator;MF:enzyme regulator activity;	GO:0005575;GO:0005576;GO:0110165;GO:0004866;GO:0061135;GO:0061134;GO:0003674;GO:0030414;GO:0004867;GO:0004857;GO:0098772;GO:0030234	------	------	------	------	ENOG410ZDQ9;ENOG4110QW6	S:Function unknown;	PF00050.24	Kazal_1	Kazal-type serine	PLA	3	33	18.8	High
TRINITY_DN10304_c0_g2_i1_orf1	glycine-rich cell wall structural protein [Ostrinia furnacalis]	0.369726949	-1.435467891	4.75E-05	down	yes	0.9343	2.527	2.562	2.642	2.378	1	0.909	0.894	------	------	------	------	------	------	ENOG4111PWE;ENOG4111WYB	S:Function unknown;	PF03103.20	DUF243	Domain of unknow	CYT	2	16	27.6	High
TRINITY_DN57998_c1_g1_i1_orf1	uncharacterized protein LOC113509309, partial [Galleria mellonella]	0.194960686	-2.358744861	0.0005368	down	yes	1.091	5.596	5.526	6.395	4.866	1	1.146	1.128	------	------	------	------	------	------	ENOG4111SVQ	S:Function unknown	------	------	------	CYT	1	14	9.1	High
TRINITY_DN661_c1_g2_i1_orf1	larval/pupal cuticle protein H1C-like [Ostrinia furnacalis]	0.419661297	-1.252702677	2.88E-05	down	yes	1.016	2.421	2.408	2.54	2.314	1	1.024	1.023	MF:molecular_function;MF:structural constituent of cuticle;MF:structural molecule activity;	GO:0003674;GO:0042302;GO:0005198	------	------	------	------	ENOG4111EA8;ENOG410YSFI;ENOG4110T8H	S:Function unknown;	PF00379.26	Chitin_bind_4	Insect cuticle prote	CYT	4	18	25.4	High
TRINITY_DN49785_c1_g1_i3_orf1	uncharacterized protein LOC114365444, partial [Ostrinia furnacalis]	0.242716165	-2.042657897	9.15E-07	down	yes	1.033	4.256	4.168	4.359	4.242	1	1.003	1.095	------	------	------	------	------	------	ENOG4110SB1	S:Function unknown	------	------	------	CYT	1	6	26.6	High
TRINITY_DN778_c0_g1_i1_orf1	uncharacterized protein LOC114363281 [Ostrinia furnacalis]	0.194359756	-2.363198568	1.56E-05	down	yes	1.02	5.248	5.326	4.936	5.483	1	0.944	1.115	------	------	------	------	------	------	COG2335	O:Posttranslational modificatio	PF02469.25	Fasciclin	Fasciclin domain	CYT	1	1	115.4	High
TRINITY_DN628_c0_g1_i1_orf1	prostamide/prostaglandin F synthase-like [Ostrinia furnacalis]	0.414232365	-1.271487815	5.27E-05	down	yes	0.9983	2.41	2.348	2.318	2.563	1	0.993	1.002	------	------	K15717	PRXL2B, FAM	map00590	Arachidonic acid	ENOG410YFY9	O:Posttranslational modificatio	PF13911.9	AhpC-TSA_2	AhpC/TSA antioxid	CYT	9	46	26.8	High
TRINITY_DN33272_c0_g1_i5_orf1	Low-density lipoprotein receptor-related protein 1 [Papilio xuthus]	0.191797032	-2.382347702	4.37E-06	down	yes	0.9563	4.986	4.788	5.18	4.989	1	0.892	0.977	BP:endocytosis;BP:vesicle-mediated transport;BP:transport;BP:biological_process;BP:establishment of localization;BP:localization;CC:integral component of membrane;CC:cellular_component;CC:intrinsic component of membrane;CC:cellular anatomical entity;MF:binding;MF:cation binding;MF:metal ion binding;MF:ion binding;MF:molecular_function;MF:calcium ion binding;	GO:0006897;GO:0016192;GO:0006810;GO:0008150;GO:0051234;GO:0051179;GO:0016021;GO:0005575;GO:0031224;GO:0110165;GO:0005488;GO:0043169;GO:0046872;GO:0043167;GO:0003674;GO:0005509	K04550;K244	LRP1, CD91;S	map05010;ma	Alzheimer diseas	ENOG410XP34	U:Intracellular trafficking, secre	PF00057.21	Ldl_recept_a	Low-density lipopr	CYT	1	13	9.2	High
TRINITY_DN15247_c0_g1_i2_orf1	probable G-protein coupled receptor Mth-like 3 isoform X1 [Ostrinia furnacalis]	0.245209799	-2.027911464	6.63E-06	down	yes	1.011	4.123	3.933	4.273	4.164	1	1.037	0.996	BP:cellular process;BP:regulation of cellular process;BP:biological regulation;BP:cell surface receptor signaling pathway;BP:biological_process;BP:regulation of biological process;BP:signal transduction;CC:integral component of membrane;CC:cellular_component;CC:cellular anatomical entity;CC:intrinsic component of membrane;MF:G-protein coupled receptor activity;MF:signaling receptor activity;MF:molecular transducer activity;MF:transmembrane signaling receptor activity;MF:molecular_function;	GO:0009987;GO:0050794;GO:0065007;GO:0007166;GO:0008150;GO:0050789;GO:0007165;GO:0016021;GO:0005575;GO:0110165;GO:0031224;GO:0004930;GO:0038023;GO:0060089;GO:0004888;GO:0003674	K04599	MTH	------	------	ENOG410XSJF;ENOG4111SC6;ENOG4111I4H;ENOG410YFKG	S:Function unknown;	PF00002.27	7tm_2	7 transmembrane	CYT	1	2	46.6	High
TRINITY_DN4464_c0_g2_i1_orf1	glypican-6 [Pectinophora gossypiella]	0.422679965	-1.242362363	8.21E-05	down	yes	0.9747	2.306	2.454	2.278	2.187	1	0.93	0.994	BP:regulation of signal transduction;BP:regulation of response to stimulus;BP:regulation of cellular process;BP:biological regulation;BP:regulation of signaling;BP:biological_process;BP:regulation of cell communication;BP:regulation of biological process;CC:cellular_component;CC:anchored component of plasma membrane;CC:external encapsulating structure;CC:collagen-containing extracellular matrix;CC:cellular anatomical entity;CC:extracellular matrix;CC:intrinsic component of plasma membrane;CC:intrinsic component of membrane;CC:anchored component of membrane;	GO:0009966;GO:0048583;GO:0050794;GO:0065007;GO:0023051;GO:0008150;GO:0010646;GO:0050789;GO:0005575;GO:0046658;GO:0030312;GO:0062023;GO:0110165;GO:0031012;GO:0031226;GO:0031224;GO:0031225	K08110	GPC4	map04310	Wnt signaling pa	ENOG410XST2	B:Chromatin structure and dyn	PF01153.22	Glypican	Glypican	CYT	11	20	84.3	High
TRINITY_DN20717_c0_g1_i1_orf1	putative uncharacterized protein DDB_G0282133 isoform X1 [Ostrinia furnacalis]	0.23385497	-2.096314003	2.15E-05	down	yes	1.061	4.537	4.271	4.554	4.785	1	1.1	1.082	------	------	------	------	------	------	ENOG410ZIVR	S:Function unknown	------	------	------	PLA	1	17	5.2	High
TRINITY_DN68397_c0_g1_i2_orf1	clavesin-1-like [Ostrinia furnacalis]	0.245296781	-2.027399795	2.54E-06	down	yes	0.9753	3.976	3.897	3.908	4.124	1	0.935	0.991	------	------	K19625	RLBP1	------	------	ENOG410XRSQ	S:Function unknown	PF00650.23;PF1	CRAL_TRIO;C	CRAL/TRIO domain	CYT	2	8	34.6	High
TRINITY_DN6122_c0_g1_i6_orf1	deubiquitinase DESI2 isoform X1 [Helicoverpa armigera] >XP_049707835.1 deubiquitinase DESI2 isoform X1 [Helicoverpa armigera]	0.27060241	-1.885753409	0.0001395	down	yes	1.123	4.15	3.819	4.518	4.112	1	1.182	1.188	MF:hydrolase activity;MF:peptidase activity;MF:catalytic activity, acting on a protein;MF:molecular_function;MF:catalytic activity;	GO:0016787;GO:0008233;GO:0140096;GO:0003674;GO:0003824	K22763	DESI2, PPPDE	------	------	ENOG4111H3Z	S:Function unknown	PF05903.17	Peptidase_C9	PPPDE putative pe	CYT	1	8	23.8	High
TRINITY_DN1672_c0_g1_i6_orf1	cystinosin homolog isoform X1 [Ostrinia furnacalis] >XP_028162341.1 cystinosin homolog isoform X1 [Ostrinia furnacalis] >XP_028162342.1 cystinosin homolog isoform X1 [Ostrinia furnacalis] >XP_028162343.1 cystinosin homolog isoform X1 [Ostrinia furnacalis] >XP_028162344.1 cystinosin homolog isoform X1 [Ostrinia furnacalis]	0.472387038	-1.081958717	7.45E-05	down	yes	1.035	2.191	2.234	2.088	2.25	1	0.979	1.126	CC:cellular_component;CC:integral component of membrane;CC:cellular anatomical entity;CC:intrinsic component of membrane;	GO:0005575;GO:0016021;GO:0110165;GO:0031224	K12386	CTNS	map04142	Lysosome	ENOG410XQSD	U:Intracellular trafficking, secre	PF04193.17	PQ-loop	PQ loop repeat	PLA	1	2	48.7	High
TRINITY_DN391_c0_g1_i4_orf1	phenoloxidase-activating enzyme-like [Ostrinia furnacalis]	0.268095114	-1.89918317	8.21E-06	down	yes	1.026	3.827	3.834	3.988	3.659	1	1.031	1.046	BP:organic substance metabolic process;BP:organonitrogen compound metabolic process;BP:protein metabolic process;BP:nitrogen compound metabolic process;BP:macromolecule metabolic process;BP:biological_process;BP:metabolic process;BP:proteolysis;BP:primary metabolic process;CC:cellular_component;CC:extracellular region;CC:cellular anatomical entity;MF:serine-type endopeptidase activity;MF:endopeptidase activity;MF:hydrolase activity;MF:serine hydrolase activity;MF:catalytic activity, acting on a protein;MF:catalytic activity;MF:molecular_function;MF:peptidase activity;MF:serine-	GO:0071704;GO:1901564;GO:0019538;GO:0006807;GO:0043170;GO:0008150;GO:0008152;GO:0006508;GO:0044238;GO:0005575;GO:0005576;GO:0110165;GO:0004252;GO:0004175;GO:0016787;GO:0017171;GO:0140096;GO:0003824;GO:0003674;GO:0008233;GO:0008236	------	------	------	------	COG5640	O:Posttranslational modificatio	PF00089.29;PF1	Trypsin;CLIP	Trypsin;Regulatory	CYT	11	39	45.8	High
TRINITY_DN875_c0_g1_i3_orf1	secernin-3 [Ostrinia furnacalis]	0.453345471	-1.141317222	7.00E-05	down	yes	0.996	2.197	2.213	2.309	2.07	1	1.016	0.972	BP:primary metabolic process;BP:organonitrogen compound metabolic process;BP:protein metabolic process;BP:macromolecule metabolic process;BP:organic substance metabolic process;BP:nitrogen compound metabolic process;BP:biological_process;BP:metabolic process;BP:proteolysis;MF:molecular_function;MF:exopeptidase activity;MF:catalytic activity, acting on a protein;MF:hydrolase activity;MF:dipeptidase activity;MF:catalytic activity;MF:cysteine-type exopeptidase activity;MF:peptidase activity;MF:cysteine-type peptidase activity;	GO:0044238;GO:1901564;GO:0019538;GO:0043170;GO:0071704;GO:0006807;GO:0008150;GO:0008152;GO:0006508;GO:0003674;GO:0008238;GO:0140096;GO:0016787;GO:0016805;GO:0003824;GO:0070004;GO:0008233;GO:0008234	K14358	SCRN	------	------	COG4690	E:Amino acid transport and me	PF03577.18	Peptidase_C6	Peptidase family C	CYT	5	15	44.7	High
TRINITY_DN21555_c0_g1_i4_orf1	uncharacterized protein LOC114351844 [Ostrinia furnacalis] >XP_028158981.1 uncharacterized protein LOC114351844 [Ostrinia furnacalis] >XP_028158982.1 uncharacterized protein LOC114351844 [Ostrinia furnacalis] >XP_028158983.1 uncharacterized protein LOC114351844 [Ostrinia furnacalis] >XP_028158984.1 uncharacterized protein LOC114351844 [Ostrinia furnacalis] >XP_028158985.1 uncharacterized protein LOC114351844 [Ostrinia furnacalis] >5GPR_A Crystal structure of chitinase-h from Ostrinia furnacalis [Ostrinia furnacalis] >5GQB_A Crystal structure of chitinase-h from O. furnacalis in complex with chitohepatose [Ostrinia furnacalis] >6JMN_A Crystal structure of Ostrinia furnacalis Chitinase h complexed with compound 2-8-s2 [Ostrinia furnacalis] >BAE16587.1 chitinase [Ostrinia furnacalis]	0.259798729	-1.944533723	9.02E-08	down	yes	0.981	3.776	3.826	3.778	3.724	1	0.971	0.972	BP:amino sugar catabolic process;BP:nitrogen compound metabolic process;BP:aminoglycan metabolic process;BP:organic substance catabolic process;BP:organic substance metabolic process;BP:chitin metabolic process;BP:chitin catabolic process;BP:biological_process;BP:metabolic process;BP:carbohydrate metabolic process;BP:catabolic process;BP:macromolecule catabolic process;BP:primary metabolic process;BP:organonitrogen compound metabolic process;BP:organonitrogen compound catabolic process;BP:amino sugar metabolic process;BP:carbohydrate derivative catabolic process;BP:carbohydrate derivative metabolic process;BP:macromolecule metabolic process;BP:glucosamine-containing compound catabolic process;BP:glucosamine-containing compound metabolic process;BP:aminoglycan catabolic process;MF:hydrolase activity, acting on glycosyl bonds;MF:molecular_function;MF:chitin binding;MF:hydrolase activity;MF:catalytic activity;MF:chitinase	GO:0046348;GO:0006807;GO:0006022;GO:1901575;GO:0071704;GO:0006030;GO:0006032;GO:0008150;GO:0008152;GO:0005975;GO:0009056;GO:0009057;GO:0044238;GO:1901564;GO:1901565;GO:0006040;GO:1901136;GO:1901135;GO:0043170;GO:1901072;GO:1901071;GO:0006026;GO:0016798;GO:0003674;GO:0008061;GO:0016787;GO:0003824;GO:0004568;GO:0097367;GO:0004553;GO:0005488	K01183	E3.2.1.14	map00520	Amino sugar and	COG3325	S:Function unknown	PF00704.31;PF0	Glyco_hydro_	Glycosyl hydrolase	CYT	12	28	60.8	High
TRINITY_DN2489_c0_g1_i1_orf1	uncharacterized protein LOC114354692 [Ostrinia furnacalis]	0.297064927	-1.75114981	5.77E-07	down	yes	1.002	3.373	3.422	3.296	3.402	1	0.977	1.028	------	------	------	------	------	------	ENOG41127D5	S:Function unknown	PF05335.16	DUF745	Protein of unknow	CYT	5	24	31.8	High
TRINITY_DN125441_c0_g1_i5_orf1	KH domain-containing, RNA-binding, signal transduction-associated protein 2-like isoform X12 [Ostrinia furnacalis]	0.465405904	-1.103438582	4.33E-06	down	yes	1.009	2.168	2.153	2.119	2.231	1	1.022	1.004	------	------	K17843;K14	KHDRBS2, SL	------	------	COG5176;ENOG4111HTF	J:Translation, ribosomal structu	------	------	------	CYT	2	24	9.8	High
TRINITY_DN26301_c0_g1_i1_orf1	uncharacterized protein LOC114359193 [Ostrinia furnacalis]	0.278498482	-1.844258631	1.38E-06	down	yes	1.009	3.623	3.708	3.642	3.518	1	1.035	0.991	CC:cellular_component;CC:extracellular region;CC:cellular anatomical entity;MF:carbohydrate derivative binding;MF:molecular_function;MF:chitin binding;MF:binding;	GO:0005575;GO:0005576;GO:0110165;GO:0097367;GO:0003674;GO:0008061;GO:0005488	------	------	------	------	ENOG410ZPH9;ENOG410XQ49;ENOG4111HJZ	S:Function unknown;	PF01607.27	CBM_14	Chitin binding Peri	CYT	3	16	28.5	High
TRINITY_DN10877_c0_g1_i1_orf1	spodomicin-like [Ostrinia furnacalis]	0.322796935	-1.631301215	0.0003166	down	yes	1.011	3.132	3.104	2.969	3.322	1	1.278	0.754	CC:cellular_component;CC:extracellular region;CC:cellular anatomical entity;MF:molecular_function;MF:toxin activity;	GO:0005575;GO:0005576;GO:0110165;GO:0003674;GO:0090729	------	------	------	------	------	------	PF08036.14	Antimicrobial_	Diapausin family o	CYT	1	19	6.9	Medium
TRINITY_DN52553_c0_g1_i1_orf1	hemocyte protein-glutamine gamma-glutamyltransferase-like [Ostrinia furnacalis]	0.267727527	-1.901162617	3.08E-06	down	yes	0.959	3.582	3.703	3.489	3.555	1	0.899	0.978	BP:primary metabolic process;BP:peptide cross-linking;BP:organonitrogen compound metabolic process;BP:organic substance metabolic process;BP:nitrogen compound metabolic process;BP:macromolecule modification;BP:macromolecule metabolic process;BP:biological_process;BP:protein modification process;BP:metabolic process;BP:protein metabolic process;MF:molecular_function;MF:protein-glutamine gamma-glutamyltransferase activity;MF:catalytic activity, acting on a protein;MF:catalytic activity;MF:transferase activity;MF:transferase activity, transferring acyl	GO:0044238;GO:0018149;GO:1901564;GO:0071704;GO:0006807;GO:0043412;GO:0043170;GO:0008150;GO:0036211;GO:0008152;GO:0019538;GO:0003674;GO:0003810;GO:0140096;GO:0003824;GO:0016740;GO:0016746;GO:0016755	K05619	TGM1	------	------	ENOG410XQEZ	O:Posttranslational modificatio	PF00927.25;PF0	Transglut_C;T	Transglutaminase	CYT	4	8	80	High
TRINITY_DN20680_c0_g1_i5_orf1	tsukushin isoform X2 [Ostrinia furnacalis]	0.32717849	-1.61185019	1.00E-06	down	yes	1.01	3.087	3.135	3.005	3.122	1	1.006	1.025	CC:cellular_component;CC:integral component of membrane;CC:cellular anatomical entity;CC:intrinsic component of membrane;	GO:0005575;GO:0016021;GO:0110165;GO:0031224	------	------	------	------	COG4886	S:Function unknown	PF13855.9;PF12	LRR_8;LRR_4	Leucine rich repea	CYT	5	15	40.6	High
TRINITY_DN27968_c0_g2_i2_orf1	nucleolin-like [Ostrinia furnacalis]	0.257329917	-1.958308899	8.48E-06	down	yes	0.904	3.513	3.39	3.652	3.496	1	0.847	0.865	------	------	------	------	------	------	ENOG4111NPU	S:Function unknown	------	------	------	CYT	2	8	33.3	High
TRINITY_DN17615_c0_g1_i3_orf1	hypothetical protein SFRUCORN_008858 [Spodoptera frugiperda]	0.327420325	-1.610784215	0.001374	down	yes	1.089	3.326	2.773	3.617	3.589	1	1.076	1.192	------	------	K20674	MODSP	map04624	Toll and Imd sign	COG5640	O:Posttranslational modificatio	------	------	------	CYT	1	20	8.4	High
TRINITY_DN1423_c0_g1_i4_orf1	hypothetical protein evm_003306 [Chilo suppressalis] >CAB3526495.1 unnamed protein product [Chilo suppressalis] >CAH0403823.1 unnamed protein product [Chilo suppressalis]	0.326445466	-1.615086089	1.98E-07	down	yes	0.9937	3.044	3.095	3.037	3.001	1	0.987	0.994	BP:ion homeostasis;BP:homeostatic process;BP:iron ion homeostasis;BP:transition metal ion homeostasis;BP:inorganic ion homeostasis;BP:cellular transition metal ion homeostasis;BP:transition metal ion transport;BP:biological regulation;BP:cellular homeostasis;BP:metal ion transport;BP:transport;BP:cellular iron ion homeostasis;BP:cellular metal ion homeostasis;BP:cation transport;BP:ion transport;BP:cellular process;BP:cellular ion homeostasis;BP:biological_process;BP:cellular cation homeostasis;BP:metal ion homeostasis;BP:cation homeostasis;BP:cellular chemical homeostasis;BP:establishment of localization;BP:localization;BP:regulation of biological quality;BP:chemical homeostasis;BP:iron ion transport;MF:molecular_function;MF:binding;MF:oxidoreductase activity;MF:cation binding;MF:oxidoreductase activity, oxidizing metal ions;MF:transition metal ion binding;MF:ferroxidase activity;MF:ion binding;MF:iron ion binding;MF:metal ion binding;MF:catalytic activity;MF:oxidoreductase activity, oxidizing metal ions, oxygen as acceptor;MF:ferric iron binding;	GO:0050801;GO:0042592;GO:0055072;GO:0055076;GO:0098771;GO:0046916;GO:0000041;GO:0065007;GO:0019725;GO:0030001;GO:0006810;GO:0006879;GO:0006875;GO:0006812;GO:0006811;GO:0009987;GO:0006873;GO:0008150;GO:0030003;GO:0055065;GO:0055080;GO:0055082;GO:0051234;GO:0051179;GO:0065008;GO:0048878;GO:0006826;GO:0003674;GO:0005488;GO:0016491;GO:0043169;GO:0016722;GO:0046914;GO:0004322;GO:0043167;GO:0005506;GO:0046872;GO:0003824;GO:0016724;GO:0008199	------	------	------	------	ENOG4111Q26	P:Inorganic ion transport and n	PF00210.27	Ferritin	Ferritin-like domai	CYT	9	44	26.4	High
TRINITY_DN129863_c0_g1_i1_orf1	protein PFC0760c-like isoform X2 [Ostrinia furnacalis]	0.330022429	-1.59936402	0.0001033	down	yes	1.03	3.121	3.143	2.883	3.338	1	0.995	1.094	------	------	------	------	------	------	------	------	------	------	------	CYT	1	21	19.3	High
TRINITY_DN113272_c0_g1_i1_orf1	altered inheritance of mitochondria protein 3-like [Ostrinia furnacalis]	0.324991875	-1.621524444	9.93E-05	down	yes	1	3.077	3.34	2.966	2.924	1	1.03	0.97	------	------	------	------	------	------	ENOG41113B8	S:Function unknown	------	------	------	PLA	1	5	33	High
TRINITY_DN11970_c0_g1_i4_orf1	myb-like protein AA [Ostrinia furnacalis]	0.323539345	-1.627986929	1.06E-05	down	yes	0.958	2.961	2.887	3.101	2.895	1	0.944	0.93	------	------	------	------	------	------	ENOG41117A2	S:Function unknown	------	------	------	CYT	3	5	126.8	High
TRINITY_DN5310_c2_g1_i2_orf1	serine protease persephone-like [Ostrinia furnacalis]	0.336158001	-1.572788605	3.68E-07	down	yes	0.9957	2.962	2.976	2.906	3.005	1	0.977	1.01	BP:organic substance metabolic process;BP:organonitrogen compound metabolic process;BP:protein metabolic process;BP:macromolecule metabolic process;BP:biological_process;BP:nitrogen compound metabolic process;BP:metabolic process;BP:proteolysis;BP:primary metabolic process;MF:serine-type endopeptidase activity;MF:endopeptidase activity;MF:hydrolase activity;MF:serine hydrolase activity;MF:catalytic activity, acting on a protein;MF:catalytic activity;MF:molecular_function;MF:peptidase activity;MF:serine-	GO:0071704;GO:1901564;GO:0019538;GO:0043170;GO:0008150;GO:0006807;GO:0008152;GO:0006508;GO:0044238;GO:0004252;GO:0004175;GO:0016787;GO:0017171;GO:0140096;GO:0003824;GO:0003674;GO:0008233;GO:0008236	K20677;K20	HAYAN;PSH	map04624	Toll and Imd sign	COG5640	O:Posttranslational modificatio	PF00089.29	Trypsin	Trypsin	CYT	9	34	34.8	High
TRINITY_DN13973_c0_g1_i6_orf1	27 kDa glycoprotein-like [Ostrinia furnacalis]	0.367973373	-1.442326721	9.27E-06	down	yes	0.995	2.704	2.787	2.592	2.734	1	0.965	1.02	------	------	------	------	------	------	ENOG4111POK	S:Function unknown	PF07165.14	DUF1397	Protein of unknow	CYT	6	35	29.1	High
TRINITY_DN1308_c0_g1_i4_orf1	serine proteinase stubble-like [Ostrinia furnacalis]	0.336860068	-1.569778675	0.0002322	down	yes	0.987	2.93	3.128	2.748	2.914	1	1.169	0.792	BP:organic substance metabolic process;BP:organonitrogen compound metabolic process;BP:protein metabolic process;BP:macromolecule metabolic process;BP:biological_process;BP:nitrogen compound metabolic process;BP:metabolic process;BP:proteolysis;BP:primary metabolic process;MF:serine-type endopeptidase activity;MF:endopeptidase activity;MF:hydrolase activity;MF:serine hydrolase activity;MF:catalytic activity, acting on a protein;MF:catalytic activity;MF:molecular_function;MF:peptidase activity;MF:serine-	GO:0071704;GO:1901564;GO:0019538;GO:0043170;GO:0008150;GO:0006807;GO:0008152;GO:0006508;GO:0044238;GO:0004252;GO:0004175;GO:0016787;GO:0017171;GO:0140096;GO:0003824;GO:0003674;GO:0008233;GO:0008236	------	------	------	------	COG5640	C:Energy production and conve	PF00089.29	Trypsin	Trypsin	CYT	1	3	28.8	Medium

TRINITY_DN98242_c0_g1_i1_orf1	adenosine deaminase 2-A-like [Galleria mellonella]	0.41003528	-1.286180047	0.0001892	down	yes	1.046	2.551	2.335	2.707	2.61	1	1.07	1.069	BP:cellular metabolic process;BP:cellular catabolic process;BP:cellular nitrogen compound metabolic process;BP:nitrogen compound metabolic process;BP:small molecule metabolic process;BP:small molecule catabolic process;BP:purine-containing compound metabolic process;BP:purine-containing compound catabolic process;BP:purine ribonucleoside catabolic process;BP:adenosine metabolic process;BP:organic cyclic compound metabolic process;BP:organic cyclic compound catabolic process;BP:nucleobase-containing compound metabolic process;BP:organic substance catabolic process;BP:purine nucleoside metabolic process;BP:organic substance metabolic process;BP:purine nucleoside catabolic process;BP:glycosyl compound catabolic process;BP:ribonucleoside catabolic process;BP:adenosine catabolic process;BP:cellular nitrogen compound catabolic process;BP:cellular process;BP:cellular aromatic compound metabolic process;BP:heterocycle catabolic process;BP:biological_process;BP:nucleoside metabolic process;BP:metabolic process;BP:nucleobase-containing compound catabolic process;BP:ribonucleoside metabolic process;BP:purine ribonucleoside metabolic process;BP:catabolic process;BP:nucleobase-containing small molecule metabolic process;BP:heterocycle metabolic process;BP:primary metabolic process;BP:organonitrogen compound metabolic process;BP:organonitrogen compound catabolic process;BP:carbohydrate derivative catabolic process;BP:nucleoside catabolic process;BP:glycosyl compound metabolic process;BP:aromatic compound catabolic [illegible]	GO:0044237;GO:0044248;GO:0034641;GO:0006807;GO:0044281;GO:0044282;GO:0072521;GO:0072523;GO:0046130;GO:0046085;GO:1901360;GO:1901361;GO:0006139;GO:1901575;GO:0042278;GO:0071704;GO:0006152;GO:1901658;GO:0042454;GO:0006154;GO:0044270;GO:0009987;GO:0006725;GO:0046700;GO:0008150;GO:0009116;GO:0008152;GO:0034655;GO:0009119;GO:0046128;GO:0009056;GO:0055086;GO:0046483;GO:0044238;GO:1901564;GO:1901565;GO:1901136;GO:0009164;GO:1901657;GO:0019439;GO:1901135;GO:0034656;GO:0005615;GO:0005575;GO:0110165;GO:0003674;GO:0019239;GO:0016787;GO:0004000;GO:0003824;GO:0016810;GO:0016814	K19572	CECR1, ADA2	map01232;ma	Nucleotide metal	COG1816	F:Nucleotide transport and me	PF00962.25;PF0	A_deaminase	Adenosine deamin	CYT	1	1	57.4	High
TRINITY_DN6330_c0_g1_i1_orfp1	TRINITY_DN6330_c0_g1_i1_m.42332 TRINITY_DN6330_c0_g1::TRINITY_DN6330_c0_g1_i1::g.42332 ORF type:3prime_partial len:51 (-) score=33.30 TRINITY_DN6330_c0_g1_i1:3-152(-)	0.426475981	-1.229463603	0.002167	down	yes	1.163	2.727	2.914	2.949	2.319	1	1.304	1.186	------	------	------	------	------	------	------	------	------	------	------	CYT	1	26	5.3	High
TRINITY_DN619_c0_g1_i1_orf1	putative uncharacterized protein DDB_G0271606 [Ostrinia furnacalis]	0.152862363	-2.709694858	1.12E-05	down	yes	1.004	6.568	6.655	6.873	6.177	1	0.985	1.028	CC:cellular_component;CC:extracellular region;CC:cellular anatomical entity;MF:carbohydrate derivative binding;MF:molecular_function;MF:chitin binding;MF:binding;	GO:0005575;GO:0005576;GO:0110165;GO:0097367;GO:0003674;GO:0008061;GO:0005488	------	------	------	------	ENOG4111K2A	S:Function unknown	PF01607.27	CBM_14	Chitin binding Peri	CYT	3	16	39.1	High
TRINITY_DN29026_c0_g1_i4_orf1	TIL [Ostrinia furnacalis]	0.480288258	-1.058027557	0.002215	down	yes	1.133	2.359	2.129	2.506	2.441	1	1.003	1.396	------	------	------	------	------	------	ENOG4110QDA;ENOG4110RUT	S:Function unknown;	------	------	------	CYT	1	14	9.9	High
TRINITY_DN3822_c0_g1_i7_orf1	hypothetical protein HF086_002539 [Spodoptera exigua] >CAH0699327.1 unnamed protein product [Spodoptera exigua]	0.36816839	-1.441562329	1.35E-07	down	yes	0.997	2.708	2.728	2.73	2.667	1	0.99	1.001	BP:nucleoside monophosphate biosynthetic process;BP:ribose phosphate biosynthetic process;BP:ribonucleoside monophosphate metabolic process;BP:cellular biosynthetic process;BP:cellular nitrogen compound metabolic process;BP:nucleotide biosynthetic process;BP:biological_process;BP:nucleoside monophosphate metabolic process;BP:purine-containing compound metabolic process;BP:purine-containing compound biosynthetic process;BP:organic cyclic compound biosynthetic process;BP:ribonucleotide metabolic process;BP:purine ribonucleoside monophosphate biosynthetic process;BP:nucleobase-containing compound metabolic process;BP:organic substance metabolic process;BP:organophosphate biosynthetic process;BP:purine nucleoside monophosphate biosynthetic process;BP:organic cyclic compound metabolic process;BP:IMP metabolic process;BP:heterocycle biosynthetic process;BP:cellular process;BP:purine ribonucleoside monophosphate metabolic process;BP:ribose phosphate metabolic process;BP:organic substance biosynthetic process;BP:ribonucleoside monophosphate biosynthetic process;BP:phosphorus metabolic process;BP:cellular aromatic compound metabolic process;BP:purine ribonucleotide biosynthetic process;BP:purine ribonucleotide metabolic process;BP:ribonucleotide biosynthetic process;BP:biosynthetic process;BP:'de novo' IMP biosynthetic process;BP:IMP biosynthetic process;BP:metabolic process;BP:aromatic compound biosynthetic process;BP:nucleobase-containing compound biosynthetic process;BP:cellular nitrogen compound biosynthetic [illegible]	GO:0009124;GO:0046390;GO:0009161;GO:0044249;GO:0034641;GO:0009165;GO:0008150;GO:0009123;GO:0072521;GO:0072522;GO:1901362;GO:0009259;GO:0009168;GO:0006139;GO:0071704;GO:0090407;GO:0009127;GO:1901360;GO:0046040;GO:0018130;GO:0009987;GO:0009167;GO:0019693;GO:1901576;GO:0009156;GO:0006793;GO:0006725;GO:0009152;GO:0009150;GO:0009260;GO:0009058;GO:0006189;GO:0006188;GO:0008152;GO:0019438;GO:0034654;GO:0044271;GO:0055086;GO:0046483;GO:0044238;GO:1901564;GO:0009126;GO:1901566;GO:1901137;GO:0044237;GO:0006163;GO:0006796;GO:0006807;GO:1901293;GO:0006164;GO:0019637;GO:0009117;GO:0006753;GO:1901135;GO:0044281;GO:0005488;GO:0035639;GO:1901363;GO:0043727;GO:0016881;GO:0003674;GO:0000166;GO:0043168;GO:0016829;GO:0004639;GO:0004638;GO:0017076;GO:0005524;GO:1901265;GO:0036094;GO:0016874;GO:0032555;GO:0016879;GO:0097159;GO:0003824;GO:0030554;GO:0016830;GO:0016831;GO:0043167;GO:0097367;GO:0032559;GO:0032553	K01587	PAICS	map00230	Purine metabolis	COG0152	F:Nucleotide transport and me	PF01259.21;PF0	SAICAR_synt;	SAICAR synthetase	CYT	28	60	47.1	High
TRINITY_DN67193_c0_g1_i1_orf1	A-kinase anchor protein 14-like [Ostrinia furnacalis]	0.189688208	-2.398298102	0.000157	down	yes	1.089	5.741	5.311	6.365	5.546	1	0.973	1.294	------	------	------	------	------	------	------	------	------	------	------	CYT	1	6	15.2	High
TRINITY_DN206_c0_g1_i11_orf1	A-kinase anchor protein 200-like [Ostrinia furnacalis] >XP_028173114.1 A-kinase anchor protein 200-like [Ostrinia furnacalis] >XP_028173115.1 A-kinase anchor protein 200-like [Ostrinia	0.375476009	-1.413207365	3.35E-06	down	yes	0.986	2.626	2.547	2.697	2.633	1	0.964	0.994	------	------	------	------	------	------	ENOG4110RAQ	S:Function unknown	------	------	------	CYT	3	12	67.6	High
TRINITY_DN97138_c0_g1_i2_orf1	tubulin beta chain-like isoform X2 [Ostrinia furnacalis]	0.396621078	-1.334166745	1.33E-07	down	yes	0.986	2.486	2.491	2.513	2.455	1	0.979	0.979	BP:cellular process;BP:biological_process;BP:microtubule-based process;CC:supramolecular fiber;CC:polymeric cytoskeletal fiber;CC:microtubule;CC:supramolecular complex;CC:supramolecular polymer;CC:cellular anatomical entity;CC:cellular_component;MF:structural constituent of cytoskeleton;MF:purine ribonucleoside triphosphate binding;MF:molecular_function;MF:binding;MF:guanyl nucleotide binding;MF:heterocyclic compound binding;MF:nucleoside phosphate binding;MF:anion binding;MF:structural molecule activity;MF:guanyl ribonucleotide binding;MF:purine nucleotide binding;MF:GTP binding;MF:hydrolase activity;MF:nucleotide binding;MF:GTPase activity;MF:nucleoside-triphosphatase activity;MF:small molecule binding;MF:catalytic activity;MF:hydrolase activity, acting on acid anhydrides, in phosphorus-containing anhydrides;MF:ion binding;MF:carbohydrate derivative binding;MF:organic cyclic compound binding;MF:hydrolase activity, acting on acid anhydrides;MF:pyrophosphatase	GO:0009987;GO:0008150;GO:0007017;GO:0099512;GO:0099513;GO:0005874;GO:0099080;GO:0099081;GO:0110165;GO:0005575;GO:0005200;GO:0035639;GO:0003674;GO:0005488;GO:0019001;GO:1901363;GO:1901265;GO:0043168;GO:0005198;GO:0032561;GO:0017076;GO:0005525;GO:0016787;GO:0000166;GO:0003924;GO:0017111;GO:0036094;GO:0003824;GO:0016818;GO:0043167;GO:0097367;GO:0097159;GO:0016817;GO:0016462;GO:0032555;GO:0032553	K07375	TUBB	map04540;ma	Gap junction;Par	COG5023	Z:Cytoskeleton	PF00091.28	Tubulin	Tubulin/FtsZ family	CYT	4	52	31.2	High
TRINITY_DN26882_c0_g1_i1_orf1	uncharacterized protein LOC114349648 [Ostrinia furnacalis]	0.16874351	-2.56709608	6.74E-06	down	yes	0.975	5.778	5.81	6.028	5.496	1	0.922	1.003	------	------	------	------	------	------	ENOG41120B2;ENOG4111JSP	S:Function unknown;	PF15998.8	DUF4773	Domain of unknow	PLA	2	8	28.6	High
TRINITY_DN114834_c0_g1_i1_orf1	uncharacterized protein LOC115444227 [Manduca sexta] >XP_030025790.1 uncharacterized protein LOC115444227 [Manduca sexta] >XP_037296791.1 uncharacterized protein LOC115444227 [Manduca sexta] >XP_037296792.1 uncharacterized protein LOC115444227 [Manduca sexta] >KAG6441350.1 hypothetical protein O3G_MSEX001778 [Manduca sexta]	0.398104265	-1.328781766	5.71E-06	down	yes	1.008	2.532	2.519	2.62	2.458	1	1.003	1.021	CC:cellular_component;CC:integral component of membrane;CC:cellular anatomical entity;CC:intrinsic component of membrane;	GO:0005575;GO:0016021;GO:0110165;GO:0031224	------	------	------	------	ENOG410XTAH	S:Function unknown	PF00024.29	PAN_1	PAN domain	CYT	10	19	81.4	High
TRINITY_DN338_c0_g1_i1_orf1	chorion protein S36-like [Ostrinia furnacalis]	0.208849198	-2.259466489	2.19E-06	down	yes	1.029	4.927	4.819	5.114	4.849	1	1.019	1.068	MF:molecular_function;MF:structural constituent of cuticle;MF:structural molecule activity;	GO:0003674;GO:0042302;GO:0005198	------	------	------	------	ENOG4111NR3	S:Function unknown	PF00379.26	Chitin_bind_4	Insect cuticle prote	CYT	1	3	50.9	High
TRINITY_DN798_c1_g1_i3_orf1	protein goliath isoform X1 [Ostrinia furnacalis] >XP_028170876.1 protein goliath isoform X1 [Ostrinia furnacalis]	0.462648557	-1.112011404	0.0001912	down	yes	1.09	2.356	2.318	2.239	2.511	1	1.18	1.089	CC:integral component of membrane;CC:cellular_component;CC:intrinsic component of membrane;CC:cellular anatomical entity;MF:cation binding;MF:ion binding;MF:molecular_function;MF:metal ion	GO:0016021;GO:0005575;GO:0031224;GO:0110165;GO:0043169;GO:0043167;GO:0003674;GO:0046872;GO:0005488	------	------	------	------	ENOG41121N2	O:Posttranslational modificatio	PF13639.9;PF17	zf-RING_2;zf-	Ring finger domair	CYT	1	3	47	High
TRINITY_DN906_c0_g1_i4_orf1	uncharacterized protein LOC114360441, partial [Ostrinia furnacalis]	0.201432607	-2.311630856	2.06E-09	down	yes	0.9983	4.956	4.988	4.946	4.933	1	0.991	1.004	------	------	K17339	ATL	------	------	ENOG410XR6Z	S:Function unknown	PF02263.22;PF0	GBP;GBP_C	Guanylate-bindinc	CYT	22	38	87.8	High
TRINITY_DN1391_c0_g1_i29_orfp1	TRINITY_DN1391_c0_g1_i29_m.70767 TRINITY_DN1391_c0_g1::TRINITY_DN1391_c0_g1_i29::g.70767 ORF type:complete len:495 (+),score=158.49 TRINITY_DN1391_c0_g1_i29:728-2212(+)	0.423076923	-1.2410081	9.78E-05	down	yes	1.045	2.47	2.562	2.551	2.298	1	1.035	1.101	------	------	------	------	------	------	------	------	------	------	------	CYT	5	14	52.3	High
TRINITY_DN42964_c0_g1_i1_orf1	protein lethal(2)essential for life-like [Galleria mellonella]	0.425910596	-1.231377472	4.38E-05	down	yes	1.029	2.416	2.447	2.519	2.281	1	1.028	1.058	------	------	K09542	CRYAB	map04213;ma	Longevity regulat	ENOG410YERS	O:Posttranslational modificatio	PF00011.24	HSP20	Hsp20/alpha cryst	CYT	1	5	23.2	High
TRINITY_DN15578_c0_g2_i1_orfp1	uncharacterized protein LOC125235519 [Leguminivora glycinivorella]	0.260942358	-1.938196942	0.0002757	down	yes	1.091	4.181	3.739	4.226	4.577	1	1.012	1.26	------	------	------	------	------	------	------	------	------	------	------	CYT	1	9	10.9	High
TRINITY_DN105574_c0_g1_i1_orf1	prolow-density lipoprotein receptor-related protein 1, partial [Ostrinia furnacalis]	0.431687243	-1.211941636	0.0001328	down	yes	1.049	2.43	2.334	2.349	2.607	1	1.118	1.028	BP:endocytosis;BP:vesicle-mediated transport;BP:transport;BP:biological_process;BP:establishment of localization;BP:localization;CC:membrane;CC:cellular_compone	GO:0006897;GO:0016192;GO:0006810;GO:0008150;GO:0051234;GO:0051179;GO:0016020;GO:0005575;GO:0110165	K04550	LRP1, CD91	map05010;ma	Alzheimer diseas	ENOG410YM97;ENOG410Y3W5;ENOG410XP34	S:Function unknown;U:Intracell	PF00057.21	Ldl_recept_a	Low-density lipop	PLA	1	6	20.1	High
TRINITY_DN2835_c0_g1_i6_orf1	probable isoaspartyl peptidase/L-asparaginase GA20639 [Ostrinia furnacalis]	0.42519685	-1.233797185	2.37E-07	down	yes	1.026	2.413	2.425	2.419	2.396	1	1.018	1.059	MF:hydrolase activity;MF:hydrolase activity, acting on carbon-nitroge;MF:hydrolase activity, acting on carbon-nitroge;MF:catalytic	GO:0016787;GO:0016810;GO:0016811;GO:0003824;GO:0003674;GO:0004067	K13051	ASRGL1, iaaA	map00250	Alanine, aspartat	COG1446	S:Function unknown	PF01112.21	Asparaginase	Asparaginase	CYT	9	37	32.4	High
TRINITY_DN261_c0_g1_i5_orfp1	TRINITY_DN261_c0_g1_i5_m.18559 TRINITY_DN261_c0_g1::TRINITY_DN261_c0_g1_i5::g.18559 ORF type:internal len:190 (+),score=69.81 TRINITY_DN261_c0_g1_i5:3-569(+)	0.466149202	-1.101136297	0.004324	down	yes	1.081	2.319	2.4	2.555	2.002	1	0.899	1.343	------	------	------	------	------	------	------	------	------	------	------	CYT	2	11	18.7	High
TRINITY_DN20676_c0_g1_i6_orf1	aldo-keto reductase AKR2E4-like isoform X1 [Ostrinia furnacalis]	0.235446618	-2.086528096	1.12E-07	down	yes	0.999	4.243	4.258	4.3	4.172	1	0.991	1.006	MF:catalytic activity;MF:molecular_function;MF:oxidoreductase activity;	GO:0003824;GO:0003674;GO:0016491	------	------	------	------	COG0656	S:Function unknown	PF00248.24	Aldo_ket_red	Aldo/keto reducta	CYT	13	47	40.6	High
TRINITY_DN86772_c0_g1_i3_orfp1	x-tox [Spodoptera exigua]	0.346187107	-1.5303761	4.84E-05	down	yes	0.8807	2.544	2.561	2.63	2.441	1	0.884	0.758	BP:response to biotic stimulus;BP:response to external stimulus;BP:response to external biotic stimulus;BP:response to stimulus;BP:defense response;BP:response to stress;BP:biological_process;BP:interspecies interaction between organisms;BP:response to other organism;CC:cellular_component;CC:extracellular region;CC:cellular anatomical entity;	GO:0009607;GO:0009605;GO:0043207;GO:0050896;GO:0006952;GO:0006950;GO:0008150;GO:0044419;GO:0051707;GO:0005575;GO:0005576;GO:0110165	------	------	------	------	------	------	------	------	------	CYT	1	9	8.5	High
TRINITY_DN81488_c0_g1_i1_orf1	apolipophorins-like [Ostrinia furnacalis]	0.27104777	-1.883380958	5.07E-05	down	yes	1.027	3.789	3.834	4.022	3.512	1	1.031	1.05	BP:lipid transport;BP:transport;BP:organic substance transport;BP:biological_process;BP:establishment of localization;BP:localization;CC:cellular_component;CC:extracellular region;CC:cellular anatomical entity;MF:lipid transporter activity;MF:transporter activity;MF:lipid binding;MF:molecular_function;MF:binding;	GO:0006869;GO:0006810;GO:0071702;GO:0008150;GO:0051234;GO:0051179;GO:0005575;GO:0005576;GO:0110165;GO:0005319;GO:0005215;GO:0008289;GO:0003674;GO:0005488	------	------	------	------	ENOG410Z6MR;ENOG41112S0	S:Function unknown;	------	------	------	CYT	6	75	8.8	High
TRINITY_DN31619_c0_g1_i2_orf1	endocuticle structural glycoprotein ABD-4-like [Ostrinia furnacalis]	0.405051335	-1.303823333	0.0009716	down	yes	0.9863	2.435	2.45	2.714	2.14	1	1.01	0.949	MF:molecular_function;MF:structural constituent of cuticle;MF:structural molecule activity;	GO:0003674;GO:0042302;GO:0005198	------	------	------	------	ENOG4111ZVI	S:Function unknown	PF00379.26	Chitin_bind_4	Insect cuticle prote	CYT	1	7	14.7	High
TRINITY_DN10824_c0_g1_i3_orf1	endochitinase isoform X2 [Ostrinia furnacalis]	0.132387399	-2.917162285	2.32E-07	down	yes	1.017	7.682	7.558	7.789	7.698	1	0.914	1.137	BP:amino sugar catabolic process;BP:nitrogen compound metabolic process;BP:aminoglycan metabolic process;BP:organic substance catabolic process;BP:organic substance metabolic process;BP:chitin metabolic process;BP:chitin catabolic process;BP:biological_process;BP:metabolic process;BP:carbohydrate metabolic process;BP:catabolic process;BP:macromolecule catabolic process;BP:primary metabolic process;BP:organonitrogen compound metabolic process;BP:organonitrogen compound catabolic process;BP:amino sugar metabolic process;BP:carbohydrate derivative catabolic process;BP:carbohydrate derivative metabolic process;BP:macromolecule metabolic process;BP:glucosamine-containing compound catabolic process;BP:glucosamine-containing compound metabolic process;BP:aminoglycan catabolic process;CC:cellular anatomical entity;CC:cellular_component;CC:extracellular region;MF:hydrolase activity, acting on glycosyl bonds;MF:molecular_function;MF:chitin binding;MF:hydrolase activity;MF:catalytic activity;MF:chitinase	GO:0046348;GO:0006807;GO:0006022;GO:1901575;GO:0071704;GO:0006030;GO:0006032;GO:0008150;GO:0008152;GO:0005975;GO:0009056;GO:0009057;GO:0044238;GO:1901564;GO:1901565;GO:0006040;GO:1901136;GO:1901135;GO:0043170;GO:1901072;GO:1901071;GO:0006026;GO:0110165;GO:0005575;GO:0005576;GO:0016798;GO:0003674;GO:0008061;GO:0016787;GO:0003824;GO:0004568;GO:0097367;GO:0004553;GO:0005488	K01183	E3.2.1.14	map00520	Amino sugar and	COG3325	S:Function unknown	PF00704.31;PF0	Glyco_hydro_	Glycosyl hydrolase	EXC	4	9	62.8	High
TRINITY_DN49143_c0_g1_i1_orf1	uncharacterized protein LOC107270465 [Cephus cinctus]	0.269837029	-1.889839755	6.83E-07	down	yes	1.01	3.743	3.687	3.705	3.836	1	1.045	0.986	CC:cellular_component;CC:integral component of membrane;CC:cellular anatomical entity;CC:intrinsic component of membrane;	GO:0005575;GO:0016021;GO:0110165;GO:0031224	------	------	------	------	ENOG410YYS2	S:Function unknown	PF00100.26	Zona_pelluci	Zona pellucida-lik	CYT	5	7	69.1	High

TRINITY_DN20009_c0_g1_i1_orf1	vimentin [Homo sapiens] >XP_003831224.1 vimentin [Pan paniscus] >XP_018890043.1 vimentin [Gorilla gorilla gorilla] >XP_024109584.1 vimentin [Pongo abelii] >XP_030675100.1 vimentin [Nomascus leucogenys] >XP_032020652.1 vimentin [Hylobates moloch] >P08670.4 RecName: Full=Vimentin [Homo sapiens] >AIC49963.1 VIM, partial [synthetic construct] >MXR00191.1 hypothetical protein [Bos mutus] >PNI30483.1 VIM isoform 1 [Pan troglodytes] >SJX39704.1 unnamed protein product, partial [Human ORFeome Gateway entry vector] >AAH00163.2 Vimentin [Homo sapiens]	0.482119205	-1.052538194	0.009543	down	yes	1.092	2.265	1.921	2.644	2.229	1	0.912	1.365	BP:regulation of primary metabolic process;BP:regulation of metabolic process;BP:signal transduction;BP:negative regulation of cell projection organization;BP:cellular component organization or biogenesis;BP:cellular response to stimulus;BP:response to external biotic stimulus;BP:posttranscriptional regulation of gene expression;BP:positive regulation of biological process;BP:negative regulation of biological process;BP:epithelial cell development;BP:regulation of macromolecule metabolic process;BP:cell development;BP:regulation of mRNA catabolic process;BP:negative regulation of neuron projection development;BP:regulation of neuron projection development;BP:response to organic substance;BP:positive regulation of translation;BP:response to biotic stimulus;BP:response to external stimulus;BP:intermediate filament cytoskeleton organization;BP:SMAD protein signal transduction;BP:response to organonitrogen compound;BP:intermediate filament-based process;BP:positive regulation of collagen biosynthetic process;BP:regulation of catabolic process;BP:cellular response to chemical stimulus;BP:glial cell development;BP:positive regulation of biosynthetic process;BP:neuron projection development;BP:plasma membrane bounded cell projection organization;BP:response to muramyl dipeptide;BP:response to lipopolysaccharide;BP:cellular component organization;BP:regulation of cell projection organization;BP:biological regulation;BP:cellular response to molecule of bacterial origin;BP:cellular response to biotic	GO:0080090;GO:0019222;GO:0007165;GO:0031345;GO:0071840;GO:0051716;GO:0043207;GO:0010608;GO:0048518;GO:0048519;GO:0002064;GO:0060255;GO:0048468;GO:0061013;GO:0010977;GO:0010975;GO:0010033;GO:0045727;GO:0009607;GO:0009605;GO:0045104;GO:0060395;GO:0010243;GO:0045103;GO:0032967;GO:0009894;GO:0070887;GO:0021782;GO:0009891;GO:0031175;GO:0120036;GO:0032495;GO:0032496;GO:0016043;GO:0031344;GO:0065007;GO:0071219;GO:0071216;GO:0065008;GO:0034097;GO:0009889;GO:0048708;GO:0050794;GO:0008150;GO:0014002;GO:0010604;GO:0071345;GO:1901698;GO:0010714;GO:0010712;GO:0010557;GO:0010556;GO:0048869;GO:1903311;GO:0034250;GO:0030154;GO:0051129;GO:0051128;GO:0032965;GO:0071225;GO:0010001;GO:0009893;GO:0009719;GO:0043488;GO:0043487;GO:0097435;GO:0051246;GO:0051247;GO:0045109;GO:0071495;GO:0006996;GO:0071346;GO:1901699;GO:0009987;GO:0071396;GO:0070307;GO:0034248;GO:0007010;GO:0051252;GO:0002237;GO:0050896;GO:0032502;GO:0071222;GO:0031329;GO:0031328;GO:0030030;GO:0031326;	K07606	VIM	map05206;map	MicroRNAs in car	ENOG410XRBS	Z:Cytoskeleton	PF00038.24;PF04	Filament;Filan	Intermediate filam	CYT	1	5	52.1	High
TRINITY_DN143895_c0_g1_i1_orf1	cathepsin L-like [Aphidius gifuensis] >KAF7988186.1 hypothetical protein HCN44_007680 [Aphidius gifuensis]	0.126786907	-2.979522324	1.90E-06	down	yes	0.949	7.485	7.773	7.336	7.346	1	0.834	1.013	BP:primary metabolic process;BP:organonitrogen compound metabolic process;BP:protein metabolic process;BP:macromolecule metabolic process;BP:organic substance metabolic process;BP:nitrogen compound metabolic process;BP:biological_process;BP:metabolic process;BP:proteolysis;MF:molecular_function;MF:hydrolase activity;MF:catalytic activity;MF:catalytic activity, acting on a protein;MF:peptidase activity;MF:cysteine-type peptidase	GO:0044238;GO:1901564;GO:0019538;GO:0043170;GO:0071704;GO:0006807;GO:0008150;GO:0008152;GO:0006508;GO:0003674;GO:0016787;GO:0003824;GO:0140096;GO:0008233;GO:0008234	K01365	CTSL	map05205;map	Proteoglycans in	COG4870	O:Posttranslational modificatio	PF00112.26;PF08	Peptidase_C1	Papain family cyste	CYT	1	2	38.3	High
TRINITY_DN104_c0_g1_i4_orf1	PREDICTED: heparan-alpha-glucosaminide N-acetyltransferase [Amyelois transitella]	0.410245731	-1.285439774	7.89E-07	down	yes	0.985	2.401	2.437	2.366	2.401	1	0.95	1.005	------	------	K10532	HGSNAT	map04142;map	Lysosome;Glycos	COG4299	S:Function unknown	PF16401.8;PF07	DUF5009;HGS	Domain of unknow	CYT	4	11	64.2	High
TRINITY_DN9090_c0_g1_i9_orf1	CD63 antigen [Ostrinia furnacalis]	0.417094017	-1.261555477	3.46E-06	down	yes	0.976	2.34	2.277	2.397	2.345	1	0.95	0.978	CC:cellular_component;CC:integral component of membrane;CC:cellular anatomical entity;CC:intrinsic component of membrane;	GO:0005575;GO:0016021;GO:0110165;GO:0031224	K06497	CD63, MLA1,	map05205;map	Proteoglycans in	ENOG4111IRY	S:Function unknown	PF00335.23	Tetraspanin	Tetraspanin family	EXC	3	13	25.6	High
TRINITY_DN1326_c0_g1_i2_orf1	cuticle protein 7 [Plutella xylostella] >CAG9138501.1 unnamed protein product [Plutella xylostella]	0.267791411	-1.900818405	0.0004493	down	yes	0.9603	3.586	3.647	3.979	3.131	1	0.919	0.962	MF:molecular_function;MF:structural constituent of cuticle;MF:structural molecule activity;	GO:0003674;GO:0042302;GO:0005198	------	------	------	------	ENOG410ZM6M;ENOG4111NR3;ENOG410YSFI;ENOG4110SG6	S:Function unknown;	PF00379.26	Chitin_bind_4	Insect cuticle prote	CYT	1	24	18.5	High
TRINITY_DN12009_c0_g1_i1_orf1	uncharacterized protein LOC114365631 [Ostrinia furnacalis]	0.158144534	-2.660684398	0.000419	down	yes	0.9563	6.047	5.886	6.932	5.323	1	0.946	0.923	------	------	------	------	------	------	------	------	------	------	------	CYT	2	17	8.2	High
TRINITY_DN23229_c0_g1_i2_orf1	uncharacterized protein LOC114362553 [Ostrinia furnacalis]	0.453028973	-1.142324776	2.63E-05	down	yes	1.032	2.278	2.191	2.347	2.297	1	1.1	0.996	CC:cellular_component;CC:integral component of membrane;CC:cellular anatomical entity;CC:intrinsic component of membrane;	GO:0005575;GO:0016021;GO:0110165;GO:0031224	------	------	------	------	ENOG4111M74	S:Function unknown	------	------	------	CYT	1	2	56.8	High
TRINITY_DN1012_c0_g1_i2_orf1	teneurin-a isoform X1 [Ostrinia furnacalis]	0.315556865	-1.664028085	9.28E-05	down	yes	1.071	3.394	3.117	3.55	3.514	1	1.161	1.053	CC:integral component of membrane;CC:membrane;CC:cellular_component;CC:intrinsic component of membrane;CC:plasma membrane;CC:cellular anatomical entity;	GO:0016021;GO:0016020;GO:0005575;GO:0031224;GO:0005886;GO:0110165	K24473	TENM, ODZ	------	------	ENOG410ZZMY;ENOG410XQQD	S:Function unknown;	PF07974.16;PF01	EGF_2;NHL	EGF-like domain;N	CYT	4	2	222.3	High
TRINITY_DN5406_c0_g2_i1_orf1	uncharacterized protein LOC114350326 [Ostrinia furnacalis]	0.197867506	-2.337393386	2.01E-05	down	yes	1.132	5.721	5.994	5.387	5.781	1	1.108	1.289	BP:positive regulation of ion transmembrane transporter activity;BP:positive regulation of transporter activity;BP:regulation of ion transmembrane transporter activity;BP:regulation of transport;BP:modulation of synaptic transmission;BP:regulation of potassium ion transport;BP:regulation of signaling;BP:positive regulation of transmembrane transport;BP:regulation of ion transmembrane transport;BP:positive regulation of ion transmembrane transport;BP:regulation of biological process;BP:regulation of transmembrane transport;BP:positive regulation of potassium ion transport;BP:regulation of cell communication;BP:rhythmic process;BP:regulation of transporter activity;BP:biological regulation;BP:regulation of ion transport;BP:positive regulation of molecular function;BP:regulation of trans-synaptic signaling;BP:positive regulation of biological process;BP:regulation of molecular function;BP:positive regulation of potassium ion transmembrane transport;BP:multicellular organismal process;BP:positive regulation of transport;BP:regulation of cellular process;BP:positive regulation of ion transport;BP:biological_process;BP:sleep;BP:positive regulation of voltage-gated potassium channel activity;BP:regulation of cation channel activity;BP:regulation of localization;BP:positive regulation of potassium ion transmembrane transporter activity;BP:regulation of metal ion transport;BP:regulation of synaptic transmission, cholinergic;BP:regulation of transmembrane transporter activity;BP:positive regulation of cation transmembrane transport;BP:regulation of cation transmembrane transport;BP:regulation of potassium ion	GO:0032414;GO:0032411;GO:0032412;GO:0051049;GO:0050804;GO:0043266;GO:0023051;GO:0034764;GO:0034765;GO:0034767;GO:0050789;GO:0034762;GO:0043268;GO:0010646;GO:0048511;GO:0032409;GO:0065007;GO:0043269;GO:0044093;GO:0099177;GO:0048518;GO:0065009;GO:1901381;GO:0032501;GO:0051050;GO:0050794;GO:0043270;GO:0008150;GO:0030431;GO:1903818;GO:2001257;GO:0032879;GO:1901018;GO:0010959;GO:0032222;GO:0022898;GO:1904064;GO:1904062;GO:1901016;GO:2001259;GO:1901379;GO:0048522;GO:0005886;GO:0031224;GO:0016020;GO:0031225;GO:0110165;GO:0005575	------	------	------	------	ENOG410ZEPJ;ENOG4111Y2E	S:Function unknown;	PF17064.8	QVR	Sleepless protein	CYT	1	9	15.6	High
TRINITY_DN2400_c0_g1_i1_orf1	uncharacterized protein LOC114351021 [Ostrinia furnacalis]	0.178171802	-2.488659062	1.08E-06	down	yes	1.035	5.809	5.975	5.801	5.651	1	1.095	1.009	------	------	------	------	------	------	ENOG41113AM	S:Function unknown	------	------	------	PLA	1	11	12.9	High
TRINITY_DN18804_c0_g1_i5_orf1	zinc finger protein Xfin-like [Ostrinia furnacalis]	0.36934409	-1.436962603	1.70E-05	down	yes	1.053	2.851	2.892	2.91	2.75	1	0.998	1.161	CC:intracellular membrane-bounded organelle;CC:nucleus;CC:cellular_component;CC:intracellular organelle;CC:membrane-bounded organelle;CC:organelle;CC:cellular anatomical entity;MF:cation binding;MF:transition metal ion binding;MF:organic cyclic compound binding;MF:heterocyclic compound binding;MF:ion binding;MF:molecular_function;MF:zinc ion binding;MF:nucleic acid binding;MF:metal ion binding;MF:binding;	GO:0043231;GO:0005634;GO:0005575;GO:0043229;GO:0043227;GO:0043226;GO:0110165;GO:0043169;GO:0046914;GO:0097159;GO:1901363;GO:0043167;GO:0003674;GO:0008270;GO:0003676;GO:0046872;GO:0005488	K09228	KRAB	map05168	Herpes simplex v	COG5048	I:Lipid transport and metabolisı	PF13894.9;PF00(	zf-C2H2_4;zf-	C2H2-type zinc fin	CYT	3	2	179.5	High
TRINITY_DN1091_c0_g1_i1_orf1	macrophage mannose receptor 1-like [Pararge aegeria]	0.363494737	-1.45999362	0.0002591	down	yes	0.8633	2.375	2.308	2.564	2.252	1	0.861	0.729	BP:response to extracellular stimulus;BP:nucleic acid metabolic process;BP:cellular nitrogen compound metabolic process;BP:nitrogen compound metabolic process;BP:DNA protection;BP:organic cyclic compound metabolic process;BP:nucleobase-containing compound metabolic process;BP:cellular response to stimulus;BP:cellular macromolecule metabolic process;BP:organic substance metabolic process;BP:cellular response to external stimulus;BP:cellular process;BP:cellular aromatic compound metabolic process;BP:cellular response to extracellular stimulus;BP:response to stress;BP:biological_process;BP:metabolic process;BP:cell communication;BP:heterocycle metabolic process;BP:primary metabolic process;BP:response to external stimulus;BP:cellular metabolic process;BP:macromolecule metabolic process;BP:cellular response to stress;BP:DNA metabolic process;BP:response to stimulus;MF:molecular_function;MF:hydrolase activity;MF:catalytic activity;MF:nucleoside-triphosphate diphosphatase activity;MF:hydrolase activity, acting on acid anhydrides, in phosphorus-containing anhydrides;MF:8-oxo-7,8-dihydroguanosine triphosphate pyrophosphatase	GO:0009991;GO:0090304;GO:0034641;GO:0006807;GO:0042262;GO:1901360;GO:0006139;GO:0051716;GO:0044260;GO:0071704;GO:0071496;GO:0009987;GO:0006725;GO:0031668;GO:0006950;GO:0008150;GO:0008152;GO:0007154;GO:0046483;GO:0044238;GO:0009605;GO:0044237;GO:0043170;GO:0033554;GO:0006259;GO:0050896;GO:0003674;GO:0016787;GO:0003824;GO:0047429;GO:0016818;GO:0008413;GO:0016817;GO:0016462	------	------	------	------	ENOG410XRS3;ENOG410XPJ1;ENOG410ZPTE;ENOG410ZRYS;ENOG410XNWT;ENOG410ZQRX	U:Intracellular trafficking, secre	PF00059.24	Lectin_C	Lectin C-type dom	CYT	1	6	33.2	High
TRINITY_DN22515_c0_g1_i10_orf1	hypothetical protein KGM_202621 [Danaus plexippus plexippus]	0.179387285	-2.478850457	8.30E-05	down	yes	1.013	5.647	5.775	6.062	5.103	1	1.004	1.035	------	------	------	------	------	------	ENOG41119TW	S:Function unknown	------	------	------	CYT	2	29	7.7	High
TRINITY_DN8480_c0_g1_i1_orf1	lysosomal Pro-X carboxypeptidase [Ostrinia furnacalis]	0.451208594	-1.148133547	0.0002179	down	yes	1.008	2.234	2.093	2.249	2.359	1	1.111	0.913	BP:primary metabolic process;BP:organonitrogen compound metabolic process;BP:protein metabolic process;BP:macromolecule metabolic process;BP:organic substance metabolic process;BP:nitrogen compound metabolic process;BP:biological_process;BP:metabolic process;BP:proteolysis;MF:molecular_function;MF:serine hydrolase activity;MF:hydrolase activity;MF:catalytic activity, acting on a protein;MF:catalytic activity;MF:peptidase activity;MF:serine-type peptidase activity;	GO:0044238;GO:1901564;GO:0019538;GO:0043170;GO:0071704;GO:0006807;GO:0008150;GO:0008152;GO:0006508;GO:0003674;GO:0017171;GO:0016787;GO:0140096;GO:0003824;GO:0008233;GO:0008236	K01285	PRCP	map04614;map	Renin-angiotens	ENOG410Y38A	S:Function unknown	PF05577.15;PF12	Peptidase_S28	Serine carboxypep	CYT	1	2	53.1	Medium
TRINITY_DN5080_c0_g1_i1_orf1	basic juvenile hormone-suppressible protein 2-like [Ostrinia furnacalis]	0.181458146	-2.462291273	9.71E-07	down	yes	1.008	5.555	5.495	5.436	5.734	1	1.007	1.018	------	------	------	------	------	------	ENOG410XR2D	S:Function unknown	PF00372.22;PF03	Hemocyanin_	Hemocyanin, copp	CYT	46	75	89.5	High
TRINITY_DN48548_c0_g1_i1_orf1	glutathione S-transferase siama 2 [Heortia vitessoides]	0.335162181	-1.577068728	0.0003459	down	yes	0.9713	2.898	2.824	2.649	3.221	1	0.942	0.972	MF:catalytic activity;MF:transferase activity;MF:glutathione transferase activity;MF:molecular_function;MF:transferase activity, transferring alkyl or ary;	GO:0003824;GO:0016740;GO:0004364;GO:0003674;GO:0016765	K04097	HPGDS	map00590;map	Arachidonic acid	ENOG41103AU;ENOG4110YU0;ENOG4111I69;ENOG4111VAU	O:Posttranslational modificatio	PF14497.9;PF02	GST_C_3;GST_	Glutathione S-tran	CYT	18	60	23.2	High
TRINITY_DN25345_c0_g1_i1_orf1	chromodomain-helicase-DNA-binding protein 1 isoform X3 [Ostrinia furnacalis]	0.418002628	-1.258416082	0.0004699	down	yes	0.9543	2.283	2.244	2.516	2.089	1	0.93	0.933	CC:intracellular organelle;CC:membrane-bounded organelle;CC:organelle;CC:nucleus;CC:intracellular membrane-bounded organelle;CC:cellular_component;CC:cellular anatomical entity;MF:purine ribonucleoside triphosphate binding;MF:heterocyclic compound binding;MF:molecular_function;MF:binding;MF:DNA helicase activity;MF:ion binding;MF:nucleoside phosphate binding;MF:anion binding;MF:nucleotide binding;MF:purine nucleotide binding;MF:ATP binding;MF:hydrolase activity;MF:catalytic activity, acting on DNA;MF:helicase activity;MF:small molecule binding;MF:catalytic activity;MF:DNA-dependent ATPase activity;MF:adenyl nucleotide binding;MF:carbohydrate derivative binding;MF:organic cyclic compound binding;MF:adenyl ribonucleotide binding;MF:purine ribonucleotide	GO:0043229;GO:0043227;GO:0043226;GO:0005634;GO:0043231;GO:0005575;GO:0110165;GO:0035639;GO:1901363;GO:0003674;GO:0005488;GO:0003678;GO:0043167;GO:1901265;GO:0043168;GO:0000166;GO:0017076;GO:0005524;GO:0016787;GO:0140097;GO:0004386;GO:0036094;GO:0003824;GO:0008094;GO:0030554;GO:0097367;GO:0097159;GO:0032559;GO:0032555;GO:0003682;GO:0032553	K11367	CHD1	------	------	COG0553;ENOG410XNUT	U:Intracellular trafficking, secre	PF00176.26;PF18	SNF2-rel_don	SNF2-related dom	CYT	2	1	208.3	High
TRINITY_DN20560_c0_g1_i6_orf1	pupal cuticle protein C1B-like [Ostrinia furnacalis]	0.201636905	-2.310168381	1.55E-07	down	yes	1.084	5.376	5.338	5.399	5.392	1	1.077	1.176	------	------	------	------	------	------	ENOG410Y08D	S:Function unknown	------	------	------	CYT	3	30	22.6	High
TRINITY_DN10138_c0_g1_i1_orf1	storage protein 1 [Omphisa fuscidentalis]	0.346379853	-1.529573076	3.47E-08	down	yes	0.9903	2.859	2.856	2.887	2.835	1	0.979	0.992	------	------	------	------	------	------	ENOG410XR2D	S:Function unknown	PF03723.17	Hemocyanin_	Hemocyanin, ig-lik	PLA	6	91	10.1	High
TRINITY_DN14721_c0_g1_i2_orf1	protein masquerade-like isoform X2 [Ostrinia furnacalis]	0.498565966	-1.004143694	0.000474	down	yes	1.043	2.092	2.27	1.973	2.033	1	0.999	1.129	------	------	------	------	------	------	ENOG411279V	S:Function unknown	------	------	------	CYT	1	3	38	High
TRINITY_DN25273_c0_g1_i1_orf1	skin secretory protein xP2-like [Ostrinia furnacalis]	0.223469604	-2.161849484	4.66E-06	down	yes	1.055	4.721	4.83	4.817	4.516	1	1.04	1.125	------	------	------	------	------	------	ENOG4111DG8	S:Function unknown	------	------	------	CYT	3	16	29	High
TRINITY_DN7549_c0_g1_i1_orf1	uncharacterized protein LOC114355006 [Ostrinia furnacalis]	0.217480778	-2.201040201	1.01E-05	down	yes	0.9617	4.422	4.355	4.649	4.262	1	0.872	1.013	------	------	------	------	------	------	ENOG4111H56;ENOG4110SWC	C:Energy production and conve	------	------	------	CYT	2	14	20	High
TRINITY_DN14904_c0_g1_i1_orf1	attacin [Ostrinia furnacalis]	0.494199536	-1.016834439	3.13E-05	down	yes	1.065	2.155	2.209	2.181	2.076	1	1.089	1.107	BP:interspecies interaction between organisms;BP:response to biotic stimulus;BP:response to external stimulus;BP:response to external biotic stimulus;BP:response to stimulus;BP:response to bacterium;BP:defense response;BP:response to stress;BP:defense response to other organism;BP:defense response to bacterium;BP:biological_process;BP:response to other organism;CC:cellular_component;CC:extracellular region;CC:cellular anatomical entity;	GO:0044419;GO:0009607;GO:0009605;GO:0043207;GO:0050896;GO:0009617;GO:0006952;GO:0006950;GO:0098542;GO:0042742;GO:0008150;GO:0051707;GO:0005575;GO:0005576;GO:0110165	------	------	------	------	ENOG410ZBQ8;ENOG4110N1X	S:Function unknown;	PF03769.18	Attacin_C	Attacin, C-termina	CYT	3	26	17.3	High
TRINITY_DN54269_c0_g1_i3_orf1	lopap-like [Ostrinia furnacalis]	0.421923077	-1.244948098	8.49E-05	down	yes	1.097	2.6	2.725	2.619	2.457	1	1.122	1.169	------	------	K03098	APOD	------	------	ENOG4110TA7	S:Function unknown	------	------	------	CYT	1	26	9.2	High
TRINITY_DN56459_c0_g1_i2_orf1	aldo-keto reductase AKR2E4-like [Ostrinia furnacalis]	0.44400545	-1.171350711	3.64E-05	down	yes	0.9777	2.202	2.237	2.086	2.282	1	0.951	0.982	MF:catalytic activity;MF:molecular_function;MF:oxidoreductase activity;	GO:0003824;GO:0003674;GO:0016491	------	------	------	------	COG0656	S:Function unknown	------	------	------	CYT	4	78	8.7	High
TRINITY_DN3616_c0_g2_i1_orf1	conotoxin ArMKLT2-032-like [Ostrinia furnacalis]	0.237512008	-2.073927643	9.71E-06	down	yes	0.989	4.164	4.093	4.37	4.028	1	0.909	1.058	CC:cellular_component;CC:integral component of membrane;CC:cellular anatomical entity;CC:intrinsic component of membrane;	GO:0005575;GO:0016021;GO:0110165;GO:0031224	------	------	------	------	------	------	------	------	------	CYT	2	64	8.7	High
TRINITY_DN91533_c0_g1_i1_orf1	uncharacterized protein LOC114361550 [Ostrinia furnacalis]	0.246695043	-2.019199371	5.52E-07	down	yes	0.9853	3.994	4.006	4.076	3.901	1	0.96	0.996	CC:cellular_component;CC:integral component of membrane;CC:cellular anatomical entity;CC:intrinsic component of membrane;	GO:0005575;GO:0016021;GO:0110165;GO:0031224	------	------	------	------	ENOG410ZKIK	S:Function unknown	PF00024.29;PF14	PAN_1;PAN_4	PAN domain;PAN c	CYT	10	18	78.9	High

TRINITY_DN1030_c0_g1_i6_orf1	gamma-glutamyl hydrolase A-like isoform X1 [Ostrinia furnacalis]	0.492951128	-1.020483473	0.0001191	down	yes	1.049	2.128	2.017	2.141	2.227	1	1.127	1.02	CC:extracellular space;CC:cellular_component;CC:cellular anatomical entity;MF:hydrolase activity;MF:gamma-glutamyl-peptidase activity;MF:catalytic activity, acting on a protein;MF:cysteine-type peptidase activity;MF:catalytic activity;MF:molecular_function;MF:peptidase activity;MF:omega peptidase activity;	GO:0005615;GO:0005575;GO:0110165;GO:0016787;GO:0034722;GO:0140096;GO:0008234;GO:0003824;GO:0003674;GO:0008233;GO:0008242	K01307	GGH	map01523;ma	Antifolate resista	ENOG410XQKI	S:Function unknown	PF07722.16;PF0	Peptidase_C2	Peptidase C26;Glut	CYT	2	6	48.4	High
TRINITY_DN1902_c0_g1_i4_orf1	chemosensory protein 10 [Conogethes pinicolalis]	0.280190418	-1.835520477	6.24E-06	down	yes	0.9123	3.256	3.218	3.358	3.193	1	0.812	0.925	------	------	------	------	------	------	ENOG4112163;ENOG410Y8R4	S:Function unknown;	PF03392.16	OS-D	Insect pheromone-	CYT	2	20	11.8	High
TRINITY_DN2069_c1_g1_i8_orf1	lysosomal aspartic protease [Trichoplusia ni]	0.372155237	-1.426023556	3.83E-05	down	yes	0.9877	2.654	2.672	2.501	2.789	1	0.97	0.993	BP:primary metabolic process;BP:organonitrogen compound metabolic process;BP:protein metabolic process;BP:macromolecule metabolic process;BP:organic substance metabolic process;BP:nitrogen compound metabolic process;BP:biological_process;BP:metabolic process;BP:proteolysis;MF:molecular_function;MF:endopeptidase activity;MF:hydrolase activity;MF:aspartic-type endopeptidase activity;MF:catalytic activity;MF:catalytic activity, acting on a protein;MF:aspartic-type peptidase activity;MF:peptidase	GO:0044238;GO:1901564;GO:0019538;GO:0043170;GO:0071704;GO:0006807;GO:0008150;GO:0008152;GO:0006508;GO:0003674;GO:0004175;GO:0016787;GO:0004190;GO:0003824;GO:0140096;GO:0070001;GO:0008233	K01379	CTSD	map05152;ma	Tuberculosis;Apo	ENOG410XNV7	O:Posttranslational modificatio	PF00026.26;PF1	Asp;TAXi_N;T	Eukaryotic aspartyl	CYT	13	48	41.7	High
TRINITY_DN7123_c0_g1_i1_orf1	activating signal cointegrator 1 complex subunit 2 homolog isoform X1 [Ostrinia furnacalis]	0.320810193	-1.640208115	1.55E-06	down	yes	0.982	3.061	3.068	3.135	2.981	1	0.953	0.993	MF:molecular_function;MF:structural constituent of cuticle;MF:structural molecule activity;	GO:0003674;GO:0042302;GO:0005198	------	------	------	------	ENOG4111QXK	S:Function unknown	------	------	------	CYT	5	46	17.6	High
TRINITY_DN125427_c0_g1_i1_orf1	heat shock protein 19.8 [Chilo suppressalis] >AGM90553.1 HSP19.8 [Chilo suppressalis] >BAE94664.1 small heat shock protein 19.7 [Chilo suppressalis]	0.394034537	-1.343606008	6.66E-06	down	yes	1.004	2.548	2.54	2.621	2.484	1	1.059	0.953	MF:cation binding;MF:structural molecule activity;MF:structural constituent of eye lens;MF:ion binding;MF:molecular_function;MF:binding;MF:metal ion	GO:0043169;GO:0005198;GO:0005212;GO:0043167;GO:0003674;GO:0005488;GO:0046872	K09542	CRYAB	map04213;ma	Longevity regula	ENOG410YERS	O:Posttranslational modificatio	PF00011.24	HSP20	Hsp20/alpha cryst	CYT	3	39	21.2	High
TRINITY_DN4367_c0_g1_i1_orf1	heat shock protein 21.7c [Chilo suppressalis] >AWT57938.1 heat shock protein 21.7c [Chilo suppressalis]	0.299613277	-1.738826536	3.97E-05	down	yes	0.9297	3.103	3.254	3.022	3.034	1	1.021	0.768	MF:cation binding;MF:metal ion binding;MF:molecular_function;MF:binding;MF:ion binding;	GO:0043169;GO:0046872;GO:0003674;GO:0005488;GO:0043167	K09542	CRYAB	map04213;ma	Longevity regula	ENOG410YERS	S:Function unknown	PF00011.24	HSP20	Hsp20/alpha cryst	CYT	1	19	21.6	High
TRINITY_DN5022_c0_g1_i4_orf1	syntenin-1-like [Ostrinia furnacalis]	0.448529412	-1.156725504	0.000493	down	yes	1.098	2.448	2.492	2.632	2.22	1	1.15	1.144	------	------	K17254	SDCBP	------	------	ENOG410YNPQ	U:Intracellular trafficking, secre	PF00595.27;PF1	PDZ;PDZ_6;P	PDZ domain;PDZ c	CYT	4	20	30.8	High
TRINITY_DN3949_c0_g1_i1_orf1	probable cytochrome P450 304a1 [Ostrinia furnacalis]	0.330687831	-1.59645814	1.41E-09	down	yes	1	3.024	3.011	3.024	3.038	1	0.998	1.003	CC:integral component of membrane;CC:cellular_component;CC:cellular anatomical entity;CC:intrinsic component of membrane;MF:cation binding;MF:monooxygenase activity;MF:heme binding;MF:transition metal ion binding;MF:tetrapyrrole binding;MF:organic cyclic compound binding;MF:oxidoreductase activity, acting on paired donors, with incorporation or reduction of molecular oxygen;MF:ion binding;MF:catalytic activity;MF:molecular_function;MF:binding;MF:iron ion binding;MF:metal ion binding;MF:heterocyclic compound	GO:0016021;GO:0005575;GO:0110165;GO:0031224;GO:0043169;GO:0004497;GO:0020037;GO:0046914;GO:0046906;GO:0097159;GO:0016705;GO:0043167;GO:0003824;GO:0003674;GO:0005488;GO:0005506;GO:0046872;GO:1901363;GO:0016491	------	------	------	------	COG2124	Q:Secondary metabolites biosy	PF00067.25	p450	Cytochrome P450	CYT	3	5	59	High
TRINITY_DN147458_c0_g1_i1_orf1	60S ribosomal protein L5, partial [Cotesia chilonis]	0.457179378	-1.129167766	4.39E-05	down	yes	1.073	2.347	2.323	2.447	2.271	1	1.075	1.143	BP:cellular macromolecule biosynthetic process;BP:cellular amide metabolic process;BP:cellular biosynthetic process;BP:organic substance biosynthetic process;BP:cellular nitrogen compound biosynthetic process;BP:cellular macromolecule metabolic process;BP:peptide biosynthetic process;BP:organic substance metabolic process;BP:organonitrogen compound biosynthetic process;BP:macromolecule metabolic process;BP:amide biosynthetic process;BP:cellular process;BP:biosynthetic process;BP:macromolecule biosynthetic process;BP:biological_process;BP:metabolic process;BP:organonitrogen compound metabolic process;BP:primary metabolic process;BP:protein metabolic process;BP:cellular nitrogen compound metabolic process;BP:cellular metabolic process;BP:nitrogen compound metabolic process;BP:peptide metabolic process;BP:translation;CC:intracellular organelle;CC:non-membrane-bounded organelle;CC:membrane-bounded organelle;CC:organelle;CC:cytoplasm;CC:nucleus;CC:cellular anatomical entity;CC:intracellular membrane-bounded organelle;CC:macromolecular complex;CC:ribosome;CC:intracellular non-membrane-bounded organelle;CC:ribonucleoprotein complex;CC:cellular_component;MF:molecular_function;MF:binding;MF:nucleic acid binding;MF:structural molecule activity;MF:heterocyclic compound binding;MF:structural	GO:0034645;GO:0043603;GO:0044249;GO:1901576;GO:0044271;GO:0044260;GO:0043043;GO:0071704;GO:1901566;GO:0043170;GO:0043604;GO:0009987;GO:0009058;GO:0009059;GO:0008150;GO:0008152;GO:1901564;GO:0044238;GO:0019538;GO:0034641;GO:0044237;GO:0006807;GO:0006518;GO:0006412;GO:0043229;GO:0043228;GO:0043227;GO:0043226;GO:0005737;GO:0005634;GO:0110165;GO:0043231;GO:0032991;GO:0005840;GO:0043232;GO:1990904;GO:0005575;GO:0003674;GO:0005488;GO:0003676;GO:0005198;GO:1901363;GO:0003735;GO:0008097;GO:0097159;GO:0019843;GO:0003723	K02932	RP-L5e, RPL5	map03010;ma	Ribosome;Coron	COG0256	J:Translation, ribosomal structu	PF17144.7;PF14	Ribosomal_L5	Ribosomal large su	CYT	1	13	37	High
TRINITY_DN2803_c4_g1_i1_orf1	ornithine aminotransferase, mitochondrial isoform X2 [Ostrinia furnacalis]	0.324102733	-1.625476909	4.20E-07	down	yes	0.9843	3.037	3.068	2.973	3.07	1	0.967	0.986	BP:small molecule biosynthetic process;BP:carboxylic acid metabolic process;BP:nitrogen compound metabolic process;BP:small molecule metabolic process;BP:glutamine family amino acid metabolic process;BP:L-proline biosynthetic process;BP:organic cyclic compound biosynthetic process;BP:organic cyclic compound metabolic process;BP:organic substance biosynthetic process;BP:proline biosynthetic process;BP:proline metabolic process;BP:cellular amino acid metabolic process;BP:organic substance metabolic process;BP:alpha-amino acid metabolic process;BP:alpha-amino acid biosynthetic process;BP:heterocycle biosynthetic process;BP:cellular amino acid biosynthetic process;BP:cellular process;BP:biosynthetic process;BP:biological_process;BP:metabolic process;BP:oxoacid metabolic process;BP:heterocycle metabolic process;BP:primary metabolic process;BP:organonitrogen compound metabolic process;BP:organonitrogen compound biosynthetic process;BP:organic acid metabolic process;BP:cellular biosynthetic process;BP:carboxylic acid biosynthetic process;BP:organic acid biosynthetic process;BP:cellular metabolic process;BP:glutamine family amino acid biosynthetic process;MF:binding;MF:vitamin B6 binding;MF:transaminase activity;MF:molecular_function;MF:heterocyclic compound binding;MF:anion binding;MF:ion binding;MF:ornithin;MF:transferase activity;MF:ornithine-oxo-acid transaminase activity;MF:transferase activity, transferring nitrogenous groups;MF:small molecule binding;MF:catalytic	GO:0044283;GO:0019752;GO:0006807;GO:0044281;GO:0009064;GO:0055129;GO:1901362;GO:1901360;GO:1901576;GO:0006561;GO:0006560;GO:0006520;GO:0071704;GO:1901605;GO:1901607;GO:0018130;GO:0008652;GO:0009987;GO:0009058;GO:0008150;GO:0008152;GO:0043436;GO:0046483;GO:0044238;GO:1901564;GO:1901566;GO:0006082;GO:0044249;GO:0046394;GO:0016053;GO:0044237;GO:0009084;GO:0005488;GO:0070279;GO:0008483;GO:0003674;GO:1901363;GO:0043168;GO:0043167;GO:0050155;GO:0016740;GO:0004587;GO:0016769;GO:0036094;GO:0003824;GO:0019842;GO:0097159;GO:0030170	K00819	rocD, OAT	map00330	Arginine and pro	COG4992	E:Amino acid transport and me	PF00202.24	Aminotran_3	Aminotransferase	CYT	25	68	44.7	High
TRINITY_DN15202_c0_g1_i6_orf1	uncharacterized protein LOC114364499 isoform X2 [Ostrinia furnacalis]	0.452048823	-1.145449497	1.49E-05	down	yes	1.037	2.294	2.318	2.266	2.297	1	1.132	0.98	------	------	------	------	------	------	ENOG410XU1K	S:Function unknown	------	------	------	CYT	2	41	14	High
TRINITY_DN1491_c0_g1_i4_orf1	GILT-like protein 2 isoform X1 [Ostrinia furnacalis] >XP_028156245.1 GILT-like protein 2 isoform X2 [Ostrinia furnacalis] >XP_028156247.1 GILT-like protein 2 isoform X3 [Ostrinia furnacalis]	0.28761781	-1.797775083	3.04E-05	down	yes	0.885	3.077	2.968	3.015	3.247	1	0.806	0.849	MF:catalytic activity;MF:oxidoreductase activity, acting on a sulfur group of donors, disulfide as acceptor;MF:molecular_function;MF:oxidoreductase activity, acting on a sulfur group of donors;MF:oxidoreductase activity;	GO:0003824;GO:0016671;GO:0003674;GO:0016667;GO:0016491	K08059	IFI30, GILT	map04612	Antigen processi	ENOG4111IDT	S:Function unknown	PF03227.19	GILT	Gamma interferon	CYT	1	16	26.5	High
TRINITY_DN198_c2_g1_i2_orf1	solute carrier organic anion transporter family member 5A1-like isoform X1 [Ostrinia furnacalis]	0.476593848	-1.069167767	8.87E-06	down	yes	1.069	2.243	2.265	2.207	2.258	1	1.125	1.083	BP:ion transport;BP:transport;BP:transmembrane transport;BP:biological_process;BP:cellular process;BP:establishment of localization;BP:localization;CC:membrane;CC:integral component of membrane;CC:cellular_component;CC:cellular	GO:0006811;GO:0006810;GO:0055085;GO:0008150;GO:0009987;GO:0051234;GO:0051179;GO:0016020;GO:0016021;GO:0005575;GO:0110165;GO:0005886;GO:0031224	K14356	SLCO5A	------	------	ENOG410XRSF	P:Inorganic ion transport and n	PF03137.23;PF0	OATP;MFS_1;	Organic Anion Tra	CYT	1	1	81.6	High
TRINITY_DN7183_c0_g1_i2_orf1	seminal fluid protein CSSFP028 [Chilo suppressalis]	0.352315306	-1.505060943	5.80E-06	down	yes	0.9967	2.829	2.854	2.72	2.912	1	0.98	1.01	BP:primary metabolic process;BP:carbohydrate metabolic process;BP:organic substance metabolic process;BP:biological_process;BP:metabolic process;MF:hydrolase activity;MF:catalytic activity;MF:hydrolase activity, acting on glycosyl bonds;MF:hydrolase activity, hydrolyzing O-glycosyl compounds;MF:molecular_function;	GO:0044238;GO:0005975;GO:0071704;GO:0008150;GO:0008152;GO:0016787;GO:0003824;GO:0016798;GO:0004553;GO:0003674	------	------	------	------	COG2723	U:Intracellular trafficking, secre	PF00232.21	Glyco_hydro_	Glycosyl hydrolase	CYT	12	25	58.9	High
TRINITY_DN7565_c0_g1_i3_orf1	acylphosphatase-2-like [Ostrinia furnacalis]	0.471040724	-1.086076301	0.001026	down	yes	1.041	2.21	2.064	2.478	2.087	1	1.035	1.087	MF:hydrolase activity;MF:hydrolase activity, acting on acid anhydrides, in phosphorus-containing anhydrides;MF:catalytic activity;MF:hydrolase activity, acting on acid anhydrides;MF:pyrophosphatase activity;MF:molecular_function;MF:acylphosphatase activity;	GO:0016787;GO:0016818;GO:0003824;GO:0016817;GO:0016462;GO:0003674;GO:0003998	K01512	acyP	map00620	Pyruvate metabo	ENOG41126ER	O:Posttranslational modificatio	PF00708.21	Acylphosphat	Acylphosphatase	CYT	1	9	13.7	High
TRINITY_DN2684_c0_g2_i3_orf1	glutamate decarboxylase 1-like isoform X1 [Ostrinia furnacalis]	0.385466617	-1.375322171	3.15E-05	down	yes	1.045	2.711	2.662	2.852	2.618	1	1.021	1.115	BP:cellular metabolic process;BP:carboxylic acid metabolic process;BP:organic substance metabolic process;BP:small molecule metabolic process;BP:cellular process;BP:biological_process;BP:metabolic process;BP:oxoacid metabolic process;BP:organic acid metabolic process;MF:vitamin B6 binding;MF:molecular_function;MF:pyridoxal phosphate binding;MF:heterocyclic compound binding;MF:anion binding;MF:lyase activity;MF:ion binding;MF:small molecule binding;MF:catalytic activity;MF:organic cyclic compound binding;MF:carbon-carbon lyase activity;MF:carboxy-lyase	GO:0044237;GO:0019752;GO:0071704;GO:0044281;GO:0009987;GO:0008150;GO:0008152;GO:0043436;GO:0006082;GO:0070279;GO:0003674;GO:0030170;GO:1901363;GO:0043168;GO:0016829;GO:0043167;GO:0036094;GO:0003824;GO:0097159;GO:0016830;GO:0016831;GO:0019842;GO:0005488	K01580;K18	E4.1.1.15, gad	map00770;ma	Pantothenate an	COG0076	J:Translation, ribosomal structu	PF00282.22	Pyridoxal_de	Pyridoxal-depende	CYT	6	18	54.5	High
TRINITY_DN72999_c0_g1_i1_orf1	protein obstructor-E-like isoform X1 [Ostrinia furnacalis] >XP_028169319.1 protein obstructor-E-like isoform X2 [Ostrinia furnacalis]	0.450313339	-1.150998881	1.71E-05	down	yes	1.006	2.234	2.17	2.333	2.2	1	1.02	0.997	CC:cellular_component;CC:extracellular region;CC:cellular anatomical entity;MF:carbohydrate derivative binding;MF:molecular_function;MF:chitin binding;MF:binding;	GO:0005575;GO:0005576;GO:0110165;GO:0097367;GO:0003674;GO:0008061;GO:0005488	------	------	------	------	ENOG4111EXD	S:Function unknown	PF01607.27	CBM_14	Chitin binding Peri	CYT	6	24	32.9	High
TRINITY_DN2290_c0_g1_i2_orfp1	TRINITY_DN2290_c0_g1_i2_m.69732 TRINITY_DN2290_c0_g1::TRINITY_DN2290_c0_g1_i2::g.69732 ORF type:complete len:234 (+),score=14.43 TRINITY_DN2290_c0_g1_i2:60-761(+)	0.340143113	-1.555786218	0.001116	down	yes	0.9507	2.795	3.127	2.877	2.382	1	0.911	0.941	------	------	------	------	------	------	------	------	------	------	------	CYT	2	16	26.1	High
TRINITY_DN15961_c0_g1_i1_orf1	uncharacterized protein LOC113522423 [Galleria mellonella]	0.483233808	-1.049206703	1.76E-05	down	yes	1.052	2.177	2.16	2.139	2.232	1	1.031	1.124	------	------	------	------	------	------	ENOG4111CHX	S:Function unknown	------	------	------	CYT	1	8	12.2	High
TRINITY_DN4189_c0_g2_i1_orf1	unnamed protein product [Chilo suppressalis]	0.39168242	-1.352243719	9.52E-06	down	yes	1.036	2.645	2.743	2.573	2.618	1	1.021	1.087	BP:nitrogen compound metabolic process;BP:organic substance metabolic process;BP:proteolysis;BP:biological_process;BP:metabolic process;BP:primary metabolic process;BP:organonitrogen compound metabolic process;BP:protein metabolic process;BP:macromolecule metabolic process;MF:endopeptidase activity;MF:molecular_function;MF:binding;MF:peptidase activity;MF:metallopeptidase activity;MF:cation binding;MF:transition metal ion binding;MF:ion binding;MF:calcium ion binding;MF:metal ion	GO:0006807;GO:0071704;GO:0006508;GO:0008150;GO:0008152;GO:0044238;GO:1901564;GO:0019538;GO:0043170;GO:0004175;GO:0003674;GO:0005488;GO:0008233;GO:0008237;GO:0043169;GO:0046914;GO:0043167;GO:0005509;GO:0046872;GO:0016787;GO:0004222;GO:0140096;GO:0003824;GO:0008270	K13046	tok	------	------	ENOG410ZPX7	S:Function unknown	PF00431.23;PF0	CUB;Astacin;	CUB domain;Astac	EXC	6	6	126.1	High
TRINITY_DN42275_c0_g1_i1_orfp1	TRINITY_DN42275_c0_g1_i1_m.44265 TRINITY_DN42275_c0_g1::TRINITY_DN42275_c0_g1_i1::g.44265 ORF type:internal len:76	0.361164305	-1.469272782	0.007216	down	yes	0.943	2.611	2.803	3.025	2.006	1	1.108	0.721	------	------	------	------	------	------	------	------	------	------	------	CYT	1	16	7.9	Medium
TRINITY_DN5074_c0_g1_i7_orf1	zonadhesin-like [Ostrinia furnacalis]	0.44119229	-1.180520514	3.14E-07	down	yes	0.9843	2.231	2.21	2.265	2.218	1	0.976	0.977	------	------	------	------	------	------	COG4826;ENOG410ZAEW;ENOG410XP6K;ENOG410Y2QH;ENOG4110S7R	O:Posttranslational modificatio	PF01826.20	TIL	Trypsin Inhibitor lik	CYT	4	21	25.8	High
TRINITY_DN1897_c0_g2_i4_orf1	phenoloxidase-activating factor 2-like [Hyposmocoma kahamanoa]	0.379233477	-1.398841772	0.001315	down	yes	0.9697	2.557	2.559	2.893	2.219	1	1.013	0.896	------	------	------	------	------	------	------	------	PF18322.4	CLIP_1	Serine protease Cli	CYT	1	7	13.9	Medium
TRINITY_DN1868_c0_g1_i1_orf1	protein obstructor-E isoform X1 [Ostrinia furnacalis]	0.468290441	-1.094524505	1.91E-05	down	yes	1.019	2.176	2.199	2.244	2.086	1	1.046	1.011	------	------	------	------	------	------	ENOG410XQ49	S:Function unknown	PF01607.27	CBM_14	Chitin binding Peri	CYT	16	68	30.6	High
TRINITY_DN25234_c0_g1_i1_orf1	uncharacterized protein LOC114353853 [Ostrinia furnacalis]	0.328819313	-1.604633056	3.70E-05	down	yes	0.8717	2.651	2.686	2.631	2.636	1	0.705	0.91	------	------	------	------	------	------	------	------	------	------	------	CYT	1	15	10	High
TRINITY_DN13648_c0_g1_i6_orf1	neurexin-4 [Ostrinia furnacalis]	0.472067039	-1.082936341	2.75E-05	down	yes	1.014	2.148	2.153	2.056	2.235	1	1.033	1.009	------	------	K07380	CNTNAP2	map04514	Cell adhesion mo	COG2091;ENOG410XPHG	H:Coenzyme transport and me	PF02210.27;PF0	Laminin_G_2;	Laminin G domain	CYT	5	4	142.6	High
TRINITY_DN4816_c0_g2_i3_orf1	15-hydroxyprostaglandin dehydrogenase [NAD(+)]-like [Ostrinia furnacalis]	0.432741117	-1.20842389	4.25E-05	down	yes	1.023	2.364	2.401	2.265	2.426	1	0.954	1.116	MF:catalytic activity;MF:molecular_function;MF:oxidoreductase activity;	GO:0003824;GO:0003674;GO:0016491	K00069	HPGD	map00590;ma	Arachidonic acid	COG1028;COG0300	S:Function unknown;	PF00106.28;PF1	adh_short;ad	short chain dehyd	CYT	7	38	29.1	High
TRINITY_DN19731_c0_g1_i1_orf1	allergen Tha p 1-like [Ostrinia furnacalis] >XP_028174916.1 allergen Tha p 1-like [Ostrinia furnacalis] >BAV56808.1 chemosensory protein 4 [Ostrinia furnacalis]	0.477485929	-1.066469877	0.0004156	down	yes	1.018	2.132	2.091	2.327	1.979	1	1.02	1.035	------	------	------	------	------	------	ENOG4111U5H;ENOG410YU7V	S:Function unknown;	PF03392.16	OS-D	Insect pheromone-	CYT	3	23	14.9	High
TRINITY_DN7102_c0_g1_i5_orf1	protein wings apart-like [Ostrinia furnacalis]	0.33332008	-1.585019865	0.0002251	down	yes	0.8383	2.515	2.361	2.714	2.47	1	0.736	0.779	BP:regulation of chromosome organization;BP:regulation of cell cycle;BP:regulation of organelle organization;BP:regulation of cell cycle process;BP:regulation of cellular process;BP:biological regulation;BP:biological_process;BP:regulation of cellular component organization;BP:regulation of biological process;BP:regulation of sister chromatid cohesion;	GO:0033044;GO:0051726;GO:0033043;GO:0010564;GO:0050794;GO:0065007;GO:0008150;GO:0051128;GO:0050789;GO:0007063	K25163	WAPL	------	------	ENOG410XSDS	S:Function unknown	PF07814.16	WAPL	Wings apart-like p	CYT	1	1	119.6	Medium
TRINITY_DN5439_c0_g1_i2_orf1	uncharacterized protein LOC114353087 [Ostrinia furnacalis]	0.432432432	-1.209453366	7.69E-06	down	yes	1.008	2.331	2.336	2.252	2.405	1	1.004	1.019	CC:membrane;CC:endoplasmic reticulum membrane;CC:organelle membrane;CC:cellular_component;CC:cellular anatomical entity;MF:cation binding;MF:monooxygenase activity;MF:heme binding;MF:transition metal ion binding;MF:tetrapyrrole binding;MF:organic cyclic compound binding;MF:oxidoreductase activity, acting on paired donors, with incorporation or reduction of molecular oxygen;MF:ion binding;MF:catalytic activity;MF:molecular_function;MF:binding;MF:iron ion	GO:0016020;GO:0005789;GO:0031090;GO:0005575;GO:0110165;GO:0043169;GO:0004497;GO:0020037;GO:0046914;GO:0046906;GO:0097159;GO:0016705;GO:0043167;GO:0003824;GO:0003674;GO:0005488;GO:0005506;GO:0046872;GO:1901363;GO:0016491	K15003	CYP9	------	------	COG2124	S:Function unknown	PF00067.25	p450	Cytochrome P450	CYT	16	34	61.8	High

TRINITY_DN26337_c0_g1_i3_orf1	lysosome membrane protein 2-like [Ostrinia furnacalis]	0.463134658	-1.110496372	1.61E-06	down	yes	1.049	2.265	2.289	2.254	2.251	1	1.069	1.079	BP:multicellular organismal process;BP:sensory perception of smell;BP:neurological system process;BP:response to stimulus;BP:sensory perception of chemical stimulus;BP:sensory perception;BP:biological_process;BP:system process;CC:membrane;CC:cellular_component;CC:cellular anatomical entity;CC:plasma membrane;CC:integral component of membrane;CC:intrinsic component of membrane;	GO:0032501;GO:0007608;GO:0050877;GO:0050896;GO:0007606;GO:0007600;GO:0008150;GO:0003008;GO:0016020;GO:0005575;GO:0110165;GO:0005886;GO:0016021;GO:0031224	K13885	SCARB1	map04979;map	Cholesterol meta	ENOG410XS17	Q:Secondary metabolites biosy	PF01130.24	CD36	CD36 family	PLA	3	4	84.8	High
TRINITY_DN1048_c0_g1_i6_orf1	uncharacterized protein LOC114360661 [Ostrinia furnacalis]	0.396	-1.336427665	1.11E-05	down	yes	0.9207	2.325	2.29	2.389	2.296	1	0.898	0.864	------	------	------	------	------	------	ENOG4111BN3;ENOG4110R95;ENOG4110MRV	S:Function unknown;	PF15430.9	SVWC	Single domain von	CYT	4	39	12.1	High
TRINITY_DN6423_c0_g1_i5_orf1	phenoloxidase-activating factor 2-like isoform X2 [Ostrinia furnacalis]	0.438059371	-1.190801681	2.15E-05	down	yes	0.9887	2.257	2.358	2.178	2.235	1	0.958	1.008	BP:organic substance metabolic process;BP:organonitrogen compound metabolic process;BP:protein metabolic process;BP:macromolecule metabolic process;BP:biological_process;BP:nitrogen compound metabolic process;BP:metabolic process;BP:proteolysis;BP:primary metabolic process;MF:serine-type endopeptidase activity;MF:endopeptidase activity;MF:hydrolase activity;MF:serine hydrolase activity;MF:catalytic activity, acting on a protein;MF:catalytic activity;MF:molecular_function;MF:peptidase activity;MF:serine-	GO:0071704;GO:1901564;GO:0019538;GO:0043170;GO:0008150;GO:0006807;GO:0008152;GO:0006508;GO:0044238;GO:0004252;GO:0004175;GO:0016787;GO:0017171;GO:0140096;GO:0003824;GO:0003674;GO:0008233;GO:0008236	------	------	------	------	------	------	PF18322.4	CLIP_1	Serine protease Cli	CYT	1	14	25.1	High
TRINITY_DN10364_c0_g1_i5_orf1	uncharacterized protein LOC114352615 [Ostrinia furnacalis]	0.451967063	-1.145710454	1.17E-06	down	yes	0.988	2.186	2.142	2.221	2.196	1	0.969	0.995	BP:organic substance metabolic process;BP:organonitrogen compound metabolic process;BP:protein metabolic process;BP:nitrogen compound metabolic process;BP:macromolecule metabolic process;BP:biological_process;BP:metabolic process;BP:proteolysis;BP:primary metabolic process;CC:cellular_component;CC:extracellular region;CC:cellular anatomical entity;MF:serine-type endopeptidase activity;MF:endopeptidase activity;MF:hydrolase activity;MF:serine hydrolase activity;MF:catalytic activity, acting on a protein;MF:catalytic activity;MF:molecular_function;MF:peptidase activity;MF:serine-	GO:0071704;GO:1901564;GO:0019538;GO:0006807;GO:0043170;GO:0008150;GO:0008152;GO:0006508;GO:0044238;GO:0005575;GO:0005576;GO:0110165;GO:0004252;GO:0004175;GO:0016787;GO:0017171;GO:0140096;GO:0003824;GO:0003674;GO:0008233;GO:0008236	------	------	------	------	COG5640	I:Lipid transport and metabolisı	PF00089.29;PF1	Trypsin;CLIP;T	Trypsin;Regulatory	CYT	3	6	46.8	High
TRINITY_DN1304_c0_g1_i6_orf1	uncharacterized protein LOC114359545 [Ostrinia furnacalis]	0.468677494	-1.093332576	5.05E-06	down	yes	1.01	2.155	2.209	2.139	2.116	1	0.981	1.05	CC:cellular_component;CC:integral component of membrane;CC:cellular anatomical entity;CC:intrinsic component of membrane;	GO:0005575;GO:0016021;GO:0110165;GO:0031224	------	------	------	------	------	------	------	------	------	CYT	1	14	8.8	High
TRINITY_DN23746_c0_g1_i2_orf1	protein 4.1 homolog isoform X1 [Ostrinia furnacalis]	0.496190476	-1.01103405	0.0008855	down	yes	1.042	2.1	2.183	1.872	2.244	1	1.025	1.1	BP:cell differentiation;BP:anatomical structure morphogenesis;BP:cellular developmental process;BP:developmental process;BP:animal organ morphogenesis;BP:neuron differentiation;BP:cellular process;BP:biological_process;CC:intracellular organelle;CC:cell periphery;CC:organelle;CC:cell junction;CC:cytoskeleton;CC:cellular anatomical entity;CC:anchoring junction;CC:adherens junction;CC:cell-cell junction;CC:intracellular non-membrane-bounded organelle;CC:cellular_component;CC:non-membrane-bounded organelle;MF:molecular_function;MF:binding;MF:cytoskeletal protein binding;MF:protein binding;	GO:0030154;GO:0009653;GO:0048869;GO:0032502;GO:0009887;GO:0030182;GO:0009987;GO:0008150;GO:0043229;GO:0071944;GO:0043226;GO:0030054;GO:0005856;GO:0110165;GO:0070161;GO:0005912;GO:0005911;GO:0043232;GO:0005575;GO:0043228;GO:0003674;GO:0005488;GO:0008092;GO:0005515	------	------	------	------	ENOG410Y7NQ	G:Carbohydrate transport and r	PF09380.13;PF0	FERM_C;FERM	FERM C-terminal F	CYT	1	8	39.5	High
TRINITY_DN1326_c0_g1_i1_orf1	cuticle protein 7-like [Ostrinia furnacalis]	0.114269406	-3.129488896	0.0009267	down	yes	1.001	8.76	9.675	9.612	6.994	1	1.017	0.987	MF:molecular_function;MF:structural constituent of cuticle;MF:structural molecule activity;	GO:0003674;GO:0042302;GO:0005198	------	------	------	------	ENOG410YSFI;ENOG4111NR3	S:Function unknown;	PF00379.26	Chitin_bind_4	Insect cuticle prote	CYT	1	27	13.9	High
TRINITY_DN15175_c0_g1_i1_orf1	zinc carboxypeptidase-like [Ostrinia furnacalis]	0.123568984	-3.016611427	2.66E-08	down	yes	1.047	8.473	8.516	8.539	8.363	1	1.056	1.085	------	------	K01298	CPA2	map04972;map	Pancreatic secret	COG2866	O:Posttranslational modificatio	PF00246.27	Peptidase_M1	Zinc carboxypeptic	CYT	3	7	45.8	High
TRINITY_DN4021_c0_g1_i1_orf1	leech-derived tryptase inhibitor C-like [Ostrinia furnacalis]	0.129427793	-2.949780645	4.44E-05	down	yes	1.045	8.074	7.663	8.807	7.751	1	1.08	1.055	------	------	K23417	SPINK1	------	------	ENOG410Z5MG;ENOG410XW7S;ENOG4112ADZ;COG1404;ENOG4111TR7	S:Function unknown;	PF07648.18;PF0	Kazal_2;Kazal	Kazal-type serine p	PLA	1	11	7.2	High
TRINITY_DN21719_c0_g1_i2_orf1	chymotrypsin-2-like [Ostrinia furnacalis]	0.155029407	-2.68938619	4.31E-06	down	yes	1.028	6.631	6.673	6.89	6.331	1	1.048	1.036	BP:nitrogen compound metabolic process;BP:organic substance metabolic process;BP:proteolysis;BP:biological_process;BP:metabolic process;BP:primary metabolic process;BP:organonitrogen compound metabolic process;BP:protein metabolic process;BP:macromolecule metabolic process;CC:intrinsic component of membrane;CC:integral component of membrane;CC:cellular anatomical entity;CC:cellular_component;MF:serine-type endopeptidase activity;MF:endopeptidase activity;MF:molecular_function;MF:peptidase activity;MF:serine-type peptidase activity;MF:hydrolase activity;MF:serine	GO:0006807;GO:0071704;GO:0006508;GO:0008150;GO:0008152;GO:0044238;GO:1901564;GO:0019538;GO:0043170;GO:0031224;GO:0016021;GO:0110165;GO:0005575;GO:0004252;GO:0004175;GO:0003674;GO:0008233;GO:0008236;GO:0016787;GO:0017171;GO:0140096;GO:0003824	K01312	PRSS1_2_3	map04972;map	Pancreatic secret	COG5640	O:Posttranslational modificatio	PF00089.29	Trypsin	Trypsin	CYT	2	14	15.6	High
TRINITY_DN13760_c1_g1_i1_orf1	pre-mRNA-processing factor 40 homolog A isoform X1 [Ostrinia furnacalis] >XP_028162665.1 pre-mRNA-processing factor 40 homolog A isoform X2 [Ostrinia furnacalis] >XP_028162667.1 pre-mRNA-processing factor 40 homolog A isoform X3 [Ostrinia furnacalis]	0.200102965	-2.321185548	6.15E-06	down	yes	0.9717	4.856	4.744	4.831	4.993	1	1.129	0.786	BP:nucleic acid metabolic process;BP:cellular nitrogen compound metabolic process;BP:nitrogen compound metabolic process;BP:mRNA splicing, via spliceosome;BP:organic cyclic compound metabolic process;BP:nucleobase-containing compound metabolic process;BP:organic substance metabolic process;BP:RNA splicing;BP:mRNA metabolic process;BP:cellular process;BP:cellular aromatic compound metabolic process;BP:RNA splicing, via transesterification reactions;BP:RNA splicing, via transesterification reactions with bulged adenosine as nucleophile;BP:biological_process;BP:metabolic process;BP:heterocycle metabolic process;BP:RNA metabolic process;BP:primary metabolic process;BP:mRNA cis splicing, via spliceosome;BP:cellular metabolic process;BP:macromolecule metabolic process;BP:RNA processing;BP:mRNA processing;CC:membrane-bounded organelle;CC:organelle;CC:nucleus;CC:cellular anatomical entity;CC:intracellular membrane-bounded	GO:0090304;GO:0034641;GO:0006807;GO:0000398;GO:1901360;GO:0006139;GO:0071704;GO:0008380;GO:0016071;GO:0009987;GO:0006725;GO:0000375;GO:0000377;GO:0008150;GO:0008152;GO:0046483;GO:0016070;GO:0044238;GO:0045292;GO:0044237;GO:0043170;GO:0006396;GO:0006397;GO:0043227;GO:0043226;GO:0005634;GO:0110165;GO:0043231;GO:0043229;GO:0005575	K12821	PRPF40, PRP4	map03040	Spliceosome	COG5104	A:RNA processing and modifica	PF01846.22;PF0	FF;WW;RhoGA	FF domain;WW do	CYT	1	1	92.5	High
TRINITY_DN34406_c0_g2_i9_orfp1	TRINITY_DN34406_c0_g2_i9_m.33755 TRINITY_DN34406_c0_g2::TRINITY_DN34406_c0_g2_i9::g.33755 ORF type:internal len:82 (-	0.156342004	-2.677222655	2.94E-05	down	yes	0.7753	4.959	5.226	4.98	4.67	1	0.702	0.624	------	------	------	------	------	------	------	------	------	------	------	CYT	1	11	9.3	High
TRINITY_DN9538_c1_g3_i1_orf1	cilia- and flagella-associated protein 410 isoform X2 [Aphidius gifuensis]	0.243188011	-2.039855989	3.21E-06	down	yes	1.071	4.404	4.508	4.248	4.456	1	1.065	1.148	------	------	K23456	CFAP410, C21	------	------	ENOG410Y9AF	S:Function unknown	PF14580.9;PF12	LRR_9;LRR_4	Leucine-rich repea	CYT	1	2	47.7	Medium
TRINITY_DN42719_c0_g1_i1_orf1	inter-alpha-trypsin inhibitor heavy chain H4-like isoform X11 [Ostrinia furnacalis]	0.26267172	-1.928667213	0.0003201	down	yes	1.109	4.222	4.229	4.658	3.78	1	1.29	1.036	------	------	------	------	------	------	------	------	------	------	------	CYT	1	23	7.4	High
TRINITY_DN98692_c0_g3_i1_orf1	fatty acyl-CoA hydrolase precursor, medium chain [Ostrinia furnacalis]	0.242725285	-2.042603689	1.70E-05	down	yes	0.9993	4.117	4.115	4.34	3.897	1	0.983	1.015	------	------	------	------	------	------	------	------	------	------	------	CYT	1	13	7.2	High
TRINITY_DN30154_c0_g1_i1_orf1	serine/threonine-protein kinase SIK2 [Ostrinia furnacalis] >XP_028174514.1 serine/threonine-protein kinase SIK2 [Ostrinia furnacalis]	0.073172086	-3.772562809	9.73E-08	down	yes	0.9227	12.61	12.645	12.793	12.382	1	0.818	0.95	BP:phosphorylation;BP:nitrogen compound metabolic process;BP:organic substance metabolic process;BP:protein phosphorylation;BP:cellular process;BP:macromolecule modification;BP:protein modification process;BP:biological_process;BP:metabolic process;BP:primary metabolic process;BP:organonitrogen compound metabolic process;BP:cellular metabolic process;BP:macromolecule metabolic process;BP:protein metabolic process;BP:phosphate-containing compound metabolic process;BP:phosphorus metabolic process;MF:purine ribonucleoside triphosphate binding;MF:molecular_function;MF:binding;MF:ion binding;MF:heterocyclic compound binding;MF:nucleoside phosphate binding;MF:anion binding;MF:carbohydrate derivative binding;MF:nucleotide binding;MF:protein serine/threonine kinase activity;MF:transferase activity;MF:protein kinase activity;MF:purine nucleotide binding;MF:ATP binding;MF:kinase activity;MF:catalytic activity, acting on a protein;MF:small molecule binding;MF:catalytic activity;MF:adenyl nucleotide binding;MF:phosphotransferase activity, alcohol group as acceptor;MF:transferase activity, transferring phosphorus-containing groups;MF:adenyl ribonucleotide binding;MF:purine ribonucleotide binding;MF:organic cyclic compound binding;MF:ribonucleotide	GO:0016310;GO:0006807;GO:0071704;GO:0006468;GO:0009987;GO:0043412;GO:0036211;GO:0008150;GO:0008152;GO:0044238;GO:1901564;GO:0044237;GO:0043170;GO:0019538;GO:0006796;GO:0006793;GO:0035639;GO:0003674;GO:0005488;GO:0043167;GO:1901363;GO:1901265;GO:0043168;GO:0097367;GO:0000166;GO:0004674;GO:0016740;GO:0004672;GO:0017076;GO:0005524;GO:0016301;GO:0140096;GO:0036094;GO:0003824;GO:0030554;GO:0016773;GO:0016772;GO:0032559;GO:0032555;GO:0097159;GO:0032553	K16311	SIK2	map04922	Glucagon signali	ENOG410XNQ0	T:Signal transduction mechanis	PF00069.28;PF0	Pkinase;PK_Ty	Protein kinase don	CYT	1	1	136.1	Medium
TRINITY_DN1093_c0_g1_i4_orf1	uncharacterized protein LOC114361723 isoform X4 [Ostrinia furnacalis]	0.245705144	-2.025000033	1.36E-05	down	yes	0.9983	4.063	4.207	3.83	4.153	1	1.031	0.964	------	------	------	------	------	------	ENOG410XRJB	S:Function unknown	------	------	------	CYT	1	32	26.1	High
TRINITY_DN2140_c0_g1_i1_orf1	uncharacterized protein LOC126367764 [Pectinophora gossypiella]	0.266375546	-1.908466451	2.58E-05	down	yes	1.037	3.893	3.658	4.102	3.92	1	1.037	1.073	CC:intracellular membrane-bounded organelle;CC:mitochondrion;CC:organelle;CC:intracellular organelle;CC:cellular_component;CC:membrane-bounded organelle;CC:cellular anatomical entity;MF:cation binding;MF:hydroquinon;MF:transition metal ion binding;MF:metal ion binding;MF:ion binding;MF:catalytic activity;MF:molecular_function;MF:binding;MF:copper ion binding;MF:oxidoreductase activity, acting on diphenols and related substances as donors;MF:oxidoreductase activity, acting on diphenols and related substances as donors, oxygen as acceptor;MF:oxidoreductase activity;	GO:0043231;GO:0005739;GO:0043226;GO:0043229;GO:0005575;GO:0043227;GO:0110165;GO:0043169;GO:0052716;GO:0046914;GO:0046872;GO:0043167;GO:0003824;GO:0003674;GO:0005488;GO:0005507;GO:0016679;GO:0016682;GO:0016491	------	------	------	------	COG2132	Q:Secondary metabolites biosy	PF07732.18;PF0	Cu-oxidase_3	Multicopper oxidas	CYT	4	7	84	High
TRINITY_DN699_c0_g2_i1_orf1	TPA_exp: putative parasitoid killing factor [Trichoplusia ni]	0.265306122	-1.914270126	6.06E-05	down	yes	1.014	3.822	3.807	4.104	3.554	1	1.014	1.028	------	------	------	------	------	------	------	------	------	------	------	CYT	1	3	43.9	High
TRINITY_DN2396_c0_g1_i9_orfp1	TRINITY_DN2396_c0_g1_i9_m.39038 TRINITY_DN2396_c0_g1::TRINITY_DN2396_c0_g1_i9::g.39038 ORF type:5prime_partial len:181 (+),score=90.33 TRINITY_DN2396_c0_g1_i9:3-545(+)	0.092016807	-3.441958799	0.0001502	down	yes	1.095	11.9	10.922	13.343	11.434	1	0.759	1.526	------	------	------	------	------	------	------	------	------	------	------	CYT	1	6	19.3	High
TRINITY_DN1175_c1_g1_i1_orf1	methanethiol oxidase [Ostrinia furnacalis]	0.260700287	-1.939535921	7.28E-06	down	yes	0.9977	3.827	3.816	3.994	3.67	1	0.982	1.011	MF:selenium binding;MF:molecular_function;MF:binding;	GO:0008430;GO:0003674;GO:0005488	K17285	SELENBP1	map00920	Sulfur metabolisı	ENOG410XP4F	S:Function unknown	PF05694.14	SBP56	56kDa selenium bi	CYT	11	46	53.7	High
TRINITY_DN64181_c0_g1_i1_orf1	uncharacterized protein LOC114356431 isoform X2 [Ostrinia furnacalis]	0.128637888	-2.958612468	0.0002742	down	yes	1.189	9.243	10.081	9.715	7.934	1	1.297	1.269	------	------	------	------	------	------	------	------	------	------	------	PLA	1	18	7.9	High
TRINITY_DN703_c13_g1_i1_orf1	acidic juvenile hormone-suppressible protein 1-like [Ostrinia furnacalis]	0.261654733	-1.934263736	3.39E-05	down	yes	0.9867	3.771	3.745	3.551	4.017	1	0.952	1.008	------	------	------	------	------	------	ENOG410XR2D	S:Function unknown	PF00372.22;PF0	Hemocyanin_	Hemocyanin, copp	CYT	15	47	57.7	High
TRINITY_DN74538_c0_g1_i1_orf1	tetra-peptide repeat homeobox protein 1-like [Ostrinia furnacalis]	0.139442775	-2.842254907	4.39E-06	down	yes	1.021	7.322	7.562	6.96	7.444	1	1.026	1.038	------	------	------	------	------	------	------	------	------	------	------	CYT	3	32	23.1	High
TRINITY_DN3056_c0_g1_i1_orf1	spidroin-2-like [Ostrinia furnacalis]	0.283238559	-1.819910411	1.75E-06	down	yes	1.046	3.693	3.678	3.743	3.658	1	1.157	0.98	MF:molecular_function;MF:structural constituent of cuticle;MF:structural molecule activity;	GO:0003674;GO:0042302;GO:0005198	------	------	------	------	ENOG4110SY1;ENOG4111PJ1;ENOG410XTK4;ENOG4110T	S:Function unknown;	PF00379.26	Chitin_bind_4	Insect cuticle prote	CYT	1	4	76.6	High
TRINITY_DN57998_c1_g3_i1_orf1	uncharacterized protein LOC114363305 isoform X2 [Ostrinia furnacalis]	0.20213819	-2.306586176	2.37E-06	down	yes	1.021	5.051	5.008	5.241	4.905	1	1.004	1.059	------	------	------	------	------	------	ENOG4111SVQ	S:Function unknown	------	------	------	CYT	2	17	23.2	High
TRINITY_DN34423_c0_g1_i3_orf1	THAP domain-containing protein 4-like [Ostrinia furnacalis]	0.175231888	-2.512662763	3.97E-05	down	yes	0.699	3.989	3.877	4.02	4.069	1	0.628	0.469	------	------	K23205	THAP4	------	------	ENOG4111TBC	S:Function unknown	PF08768.14	THAP4_heme	THAP4-like, heme-	CYT	1	23	12.2	High
TRINITY_DN12387_c0_g1_i1_orf1	repetitive proline-rich cell wall protein 2-like [Ostrinia furnacalis]	0.206411863	-2.276402207	2.32E-06	down	yes	0.9883	4.788	4.822	4.931	4.61	1	0.968	0.997	------	------	------	------	------	------	ENOG410YH2C;ENOG4110N1S	S:Function unknown;	------	------	------	CYT	18	64	40.7	High
TRINITY_DN76216_c0_g2_i3_orf1	lysosomal alpha-mannosidase isoform X1 [Pieris rapae]	0.265428021	-1.913607413	7.42E-05	down	yes	0.9643	3.633	3.404	3.862	3.633	1	0.796	1.097	------	------	K12311	MAN2B1, LAN	map00511;map	Other glycan deg	ENOG410XQMZ;COG0564	G:Carbohydrate transport and r	PF01074.25	Glyco_hydro_	Glycosyl hydrolase	CYT	1	25	12	High
TRINITY_DN1935_c0_g1_i1_orf1	adult-specific cuticular protein ACP-22-like [Ostrinia furnacalis]	0.224734343	-2.153707485	2.28E-06	down	yes	0.994	4.423	4.49	4.522	4.258	1	0.959	1.023	MF:molecular_function;MF:structural constituent of cuticle;MF:structural molecule activity;	GO:0003674;GO:0042302;GO:0005198	------	------	------	------	ENOG410YSFI	I:Lipid transport and metabolisı	PF00379.26	Chitin_bind_4	Insect cuticle prote	CYT	6	38	19.3	High
TRINITY_DN3255_c0_g1_i1_orf1	uncharacterized protein LOC114351042 [Ostrinia furnacalis]	0.302080099	-1.726996951	2.39E-05	down	yes	0.973	3.221	3.035	3.37	3.258	1	0.931	0.988	------	------	------	------	------	------	ENOG41114RQ	S:Function unknown	------	------	------	CYT	1	6	13.9	High
TRINITY_DN1749_c0_g1_i1_orf1	putative GPI-anchored protein pfl2 isoform X2 [Ostrinia furnacalis]	0.272870575	-1.873711264	5.19E-05	down	yes	0.9867	3.616	3.429	3.875	3.545	1	1.069	0.891	------	------	------	------	------	------	COG5599	S:Function unknown	PF00102.30	Y_phosphatas	Protein-tyrosine pl	CYT	2	6	59	High
TRINITY_DN3715_c0_g1_i2_orf1	uncharacterized protein LOC114356437 isoform X1 [Ostrinia furnacalis]	0.351490236	-1.50844348	8.07E-05	down	yes	1.026	2.919	2.845	3.143	2.77	1	1.018	1.061	------	------	------	------	------	------	------	------	PF00041.24	fn3	Fibronectin type III	CYT	3	19	19.1	High
TRINITY_DN3609_c0_g1_i6_orf1	leukocyte elastase inhibitor-like [Ostrinia furnacalis]	0.315773354	-1.663038659	1.86E-08	down	yes	1.031	3.265	3.275	3.254	3.266	1	1.048	1.045	CC:cellular_component;CC:cellular anatomical entity;CC:extracellular space;MF:endopeptidase inhibitor activity;MF:endopeptidase regulator activity;MF:peptidase regulator activity;MF:molecular_function;MF:peptidase inhibitor activity;MF:serine-type endopeptidase inhibitor activity;MF:enzyme inhibitor activity;MF:molecular function regulator;MF:enzyme regulator activity;	GO:0005575;GO:0110165;GO:0005615;GO:0004866;GO:0061135;GO:0061134;GO:0003674;GO:0030414;GO:0004867;GO:0004857;GO:0098772;GO:0030234	------	------	------	------	COG4826	S:Function unknown	PF00079.23	Serpin	Serpin	CYT	8	17	56.7	High
TRINITY_DN139537_c0_g1_i1_orf1	uncharacterized protein LOC114351440 [Ostrinia furnacalis]	0.261221067	-1.936656844	0.002259	down	yes	0.7787	2.981	2.458	3.257	3.229	1	0.424	0.912	BP:nucleic acid metabolic process;BP:cellular nitrogen compound metabolic process;BP:nitrogen compound metabolic process;BP:nucleobase-containing compound metabolic process;BP:cellular macromolecule metabolic process;BP:transposition;BP:organic substance metabolic process;BP:organic cyclic compound metabolic process;BP:cellular process;BP:cellular aromatic compound metabolic process;BP:biological_process;BP:metabolic process;BP:heterocycle metabolic process;BP:DNA recombination;BP:primary metabolic process;BP:transposition, DNA-mediated;BP:cellular metabolic process;BP:macromolecule metabolic process;BP:DNA integration;BP:DNA metabolic process;CC:intrinsic component of membrane;CC:integral component of membrane;CC:cellular anatomical entity;CC:cellular_component;MF:molecular_function;MF:binding;MF:nucleic acid binding;MF:DNA binding;MF:heterocyclic compound binding;MF:organic cyclic compound binding;	GO:0090304;GO:0034641;GO:0006807;GO:0006139;GO:0044260;GO:0032196;GO:0071704;GO:1901360;GO:0009987;GO:0006725;GO:0008150;GO:0008152;GO:0046483;GO:0006310;GO:0044238;GO:0006313;GO:0044237;GO:0043170;GO:0015074;GO:0006259;GO:0031224;GO:0016021;GO:0110165;GO:0005575;GO:0003674;GO:0005488;GO:0003676;GO:0003677;GO:1901363;GO:0097159	------	------	------	------	ENOG410XQD5	S:Function unknown	------	------	------	PLA	1	5	23.3	High

TRINITY_DN48694_c0_g1_i1_orfp1	TRINITY_DN48694_c0_g1_i1_m.75338 TRINITY_DN48694_c0_g1::TRINITY_DN48694_c0_g1_i1::g.75338 ORF type:internal len:84	0.263250883	-1.925489722	1.55E-05	down	yes	0.894	3.396	3.523	3.429	3.237	1	0.815	0.867	------	------	------	------	------	------	------	------	------	------	------	CYT	1	13	9.1	Medium
TRINITY_DN960_c1_g1_i6_orf1	hypothetical protein evm_007130 [Chilo suppressalis]	0.415632754	-1.266618743	1.51E-05	down	yes	1.005	2.418	2.466	2.472	2.315	1	1.048	0.968	BP:transport;BP:intracellular transport;BP:sterol transport;BP:lipid transport;BP:intracellular sterol transport;BP:intracellular cholesterol transport;BP:intracellular lipid transport;BP:organic hydroxy compound transport;BP:cholesterol transport;BP:biological_process;BP:organic substance transport;BP:cellular process;BP:establishment of localization;BP:localization;BP:establishment of localization in cell;BP:cellular	GO:0006810;GO:0046907;GO:0015918;GO:0006869;GO:0032366;GO:0032367;GO:0032365;GO:0015850;GO:0030301;GO:0008150;GO:0071702;GO:0009987;GO:0051234;GO:0051179;GO:0051649;GO:0051641;GO:0005575;GO:0005576;GO:0110165	K13443	NPC2	map04979;maן	Cholesterol meta	ENOG4111Q8S	S:Function unknown	PF02221.18	E1_DerP2_Der	ML domain	PLA	5	38	15.5	High
TRINITY_DN5467_c0_g1_i5_orf1	synaptic vesicle glycoprotein 2B-like isoform X2 [Ostrinia furnacalis] >XP_028161209.1 synaptic vesicle glycoprotein 2B-like isoform X2 [Ostrinia furnacalis] >XP_028161210.1 synaptic vesicle glycoprotein 2B-like isoform X2 [Ostrinia furnacalis]	0.354341267	-1.496788601	2.62E-06	down	yes	1.057	2.983	3.026	3.013	2.909	1	1.057	1.114	CC:integral component of membrane;CC:cellular_component;CC:cellular anatomical entity;CC:intrinsic component of membrane;MF:transporter activity;MF:transmembrane transporter activity;MF:molecular_function;	GO:0016021;GO:0005575;GO:0110165;GO:0031224;GO:0005215;GO:0022857;GO:0003674	------	------	------	------	COG0477;ENOG410YD9D;ENOG410ZVDQ	S:Function unknown;G:Carbohy	PF07690.19;PF0(	MFS_1;Sugar_	Major Facilitator Su	CYT	1	2	57.3	High
TRINITY_DN1287_c0_g1_i5_orf1	probable chitinase 10 isoform X6 [Ostrinia furnacalis]	0.433548387	-1.205735077	1.03E-06	down	yes	1.008	2.325	2.373	2.317	2.285	1	1	1.023	BP:amino sugar catabolic process;BP:nitrogen compound metabolic process;BP:aminoglycan metabolic process;BP:organic substance catabolic process;BP:organic substance metabolic process;BP:chitin metabolic process;BP:chitin catabolic process;BP:biological_process;BP:metabolic process;BP:carbohydrate metabolic process;BP:catabolic process;BP:macromolecule catabolic process;BP:primary metabolic process;BP:organonitrogen compound metabolic process;BP:organonitrogen compound catabolic process;BP:amino sugar metabolic process;BP:carbohydrate derivative catabolic process;BP:carbohydrate derivative metabolic process;BP:macromolecule metabolic process;BP:glucosamine-containing compound catabolic process;BP:glucosamine-containing compound metabolic process;BP:aminoglycan catabolic process;CC:cellular anatomical entity;CC:cellular_component;CC:extracellular region;MF:hydrolase activity, acting on glycosyl bonds;MF:molecular_function;MF:chitin binding;MF:hydrolase activity;MF:catalytic activity;MF:chitinase	GO:0046348;GO:0006807;GO:0006022;GO:1901575;GO:0071704;GO:0006030;GO:0006032;GO:0008150;GO:0008152;GO:0005975;GO:0009056;GO:0009057;GO:0044238;GO:1901564;GO:1901565;GO:0006040;GO:1901136;GO:1901135;GO:0043170;GO:1901072;GO:1901071;GO:0006026;GO:0110165;GO:0005575;GO:0005576;GO:0016798;GO:0003674;GO:0008061;GO:0016787;GO:0003824;GO:0004568;GO:0097367;GO:0004553;GO:0005488	K01183	E3.2.1.14	map00520	Amino sugar and	COG3325	S:Function unknown	PF00704.31;PF0:	Glyco_hydro_	Glycosyl hydrolase	CYT	51	24	327.2	High
TRINITY_DN80328_c0_g1_i5_orf1	arylphorin subunit alpha-like [Ostrinia furnacalis]	0.32183908	-1.635588574	8.85E-08	down	yes	0.98	3.045	3.017	3.038	3.08	1	0.955	0.985	------	------	------	------	------	------	ENOG410XR2D	S:Function unknown	PF00372.22	Hemocyanin_	Hemocyanin, copp	CYT	7	74	12.5	High
TRINITY_DN6497_c0_g1_i1_orf1	ommochrome-binding protein-like [Ostrinia furnacalis]	0.466931217	-1.098718051	3.26E-05	down	yes	1.059	2.268	2.187	2.357	2.26	1	1.109	1.068	------	------	------	------	------	------	ENOG4110SZS;ENOG4110QPA;ENOG4110QI3;ENOG41112QX;ENOG4110SGZ;ENOG410XYVZ	S:Function unknown;G:Carbohy	------	------	------	CYT	2	15	17	High
TRINITY_DN33365_c0_g1_i1_orf1	mucin-5AC isoform X1 [Ostrinia furnacalis]	0.336508475	-1.571285257	3.58E-07	down	yes	0.9927	2.95	2.946	2.986	2.918	1	0.949	1.029	------	------	------	------	------	------	ENOG41120DH	S:Function unknown	------	------	------	CYT	3	2	204.2	High
TRINITY_DN4689_c0_g1_i5_orf1	pericentriolar material 1 protein-like isoform X3 [Ostrinia furnacalis]	0.380681346	-1.393344217	0.002372	down	yes	0.9163	2.407	2.373	2.059	2.789	1	0.824	0.925	BP:cellular component assembly;BP:cell projection organization;BP:cell projection assembly;BP:protein localization to cytoskeleton;BP:protein localization;BP:microtubule cytoskeleton organization;BP:plasma membrane bounded cell projection organization;BP:cellular component organization or biogenesis;BP:plasma membrane bounded cell projection assembly;BP:microtubule anchoring at centrosome;BP:protein localization to centrosome;BP:microtubule anchoring at microtubule organizing center;BP:cellular component organization;BP:protein localization to microtubule organizing center;BP:macromolecule localization;BP:microtubule anchoring;BP:cilium assembly;BP:cellular process;BP:biological_process;BP:organelle assembly;BP:localization;BP:cellular localization;BP:organelle organization;BP:microtubule-based process;BP:cytoskeleton organization;BP:cellular macromolecule localization;BP:protein localization to microtubule cytoskeleton;BP:protein localization to organelle;BP:cilium organization;CC:centrosome;CC:microtubule organizing	GO:0022607;GO:0030030;GO:0030031;GO:0044380;GO:0008104;GO:0000226;GO:0120036;GO:0071840;GO:0120031;GO:0034454;GO:0071539;GO:0072393;GO:0016043;GO:1905508;GO:0033036;GO:0034453;GO:0060271;GO:0009987;GO:0008150;GO:0070925;GO:0051179;GO:0051641;GO:0006996;GO:0007017;GO:0007010;GO:0070727;GO:0072698;GO:0033365;GO:0044782;GO:0005813;GO:0005815;GO:0110165;GO:0005575	K16537	PCM1	------	------	ENOG410YNFX	S:Function unknown	PF15717.8	PCM1_C	Pericentriolar mate	CYT	2	1	193.5	High
TRINITY_DN3464_c0_g1_i1_orf1	putative mitochondrial aconitate hydratase isoform X1-likeprotein, partial [Cotesia chilonis]	0.37562724	-1.412626405	8.53E-05	down	yes	1.048	2.79	2.905	2.59	2.875	1	1.125	1.019	BP:tricarboxylic acid cycle;BP:biological_process;BP:metabolic process;BP:primary metabolic process;CC:membrane-bounded organelle;CC:organelle;CC:mitochondrion;CC:cellular anatomical entity;CC:intracellular membrane-bounded organelle;CC:intracellular organelle;CC:cellular_component;MF:molecular_function;MF:binding;MF:cation binding;MF:metal cluster binding;MF:lyase activity;MF:ion binding;MF:metal ion binding;MF:catalytic activity;MF:citrate dehydratase activity;MF:4 iron, 4 sulfur cluster binding;MF:carbon-oxygen lyase activity;MF:hydro-lyase activity;MF:aconitate hydratase activity;MF:iron-sulfur cluster	GO:0006099;GO:0008150;GO:0008152;GO:0044238;GO:0043227;GO:0043226;GO:0005739;GO:0110165;GO:0043231;GO:0043229;GO:0005575;GO:0003674;GO:0005488;GO:0043169;GO:0051540;GO:0016829;GO:0043167;GO:0046872;GO:0003824;GO:0047780;GO:0051539;GO:0016835;GO:0016836;GO:0003994;GO:0051536	K01681	ACO, acnA	map00020;maן	Citrate cycl;Glyox	COG1048	C:Energy production and conve	PF00330.23;PF0(	Aconitase;Acc	Aconitase family;A	CYT	1	6	89.4	High
TRINITY_DN40439_c0_g1_i5_orf1	ommochrome binding protein 1, partial [Ostrinia nubilalis]	0.475111111	-1.073663148	0.00068	down	yes	1.069	2.25	2.186	2.086	2.478	1	1.07	1.137	------	------	------	------	------	------	ENOG4110SZS;ENOG4110QPA;ENOG4110T7I;ENOG4110QI3;ENOG4110SGZ	S:Function unknown;	------	------	------	CYT	1	8	15.4	High
TRINITY_DN32532_c0_g1_i1_orf1	fatty acyl-CoA hydrolase precursor, medium chain [Ostrinia furnacalis]	0.38137147	-1.390731172	4.29E-05	down	yes	1.04	2.727	2.659	2.64	2.883	1	1.001	1.12	------	------	------	------	------	------	ENOG410Y6DN;COG2272	S:Function unknown;I:Lipid trar	------	------	------	CYT	2	33	8.6	High
TRINITY_DN20244_c0_g1_i1_orfp1	uncharacterized protein LOC125235519 [Leguminivora glycinivorella]	0.484480432	-1.045489698	0.004862	down	yes	1.077	2.223	1.93	2.581	2.157	1	1.216	1.016	------	------	------	------	------	------	------	------	------	------	------	CYT	1	7	12.3	High
TRINITY_DN3301_c0_g1_i2_orf1	hemicentin-2-like isoform X1 [Ostrinia furnacalis]	0.356062291	-1.489798439	5.72E-07	down	yes	0.9603	2.697	2.652	2.71	2.729	1	0.943	0.938	MF:hydrolase activity;MF:cation binding;MF:organic cyclic compound binding;MF:ion binding;MF:catalytic activity;MF:molecular_function;MF:binding;MF:nucleic acid binding;MF:metal ion binding;MF:heterocyclic compound	GO:0016787;GO:0043169;GO:0097159;GO:0043167;GO:0003824;GO:0003674;GO:0005488;GO:0003676;GO:0046872;GO:1901363	------	------	------	------	ENOG410ZN3C;ENOG410ZVGI;ENOG4110087	S:Function unknown;	PF07679.19;PF1:	I-set;Ig_3;Ig_2	Immunoglobulin I-	CYT	3	3	129.9	High
TRINITY_DN2566_c0_g1_i5_orf1	uncharacterized protein LOC114349936 [Ostrinia furnacalis]	0.453421634	-1.141074869	2.55E-05	down	yes	1.027	2.265	2.292	2.344	2.16	1	1.045	1.035	------	------	------	------	------	------	ENOG410XZ3K	S:Function unknown	------	------	------	PLA	2	21	12.4	High
TRINITY_DN806_c0_g2_i1_orf1	uncharacterized protein LOC114355167 [Ostrinia furnacalis]	0.391871166	-1.351548673	3.17E-06	down	yes	1.022	2.608	2.529	2.645	2.649	1	1.055	1.011	MF:molecular_function;MF:structural constituent of cuticle;MF:structural molecule activity;	GO:0003674;GO:0042302;GO:0005198	------	------	------	------	ENOG410YR66;ENOG4111PJ1	S:Function unknown;	PF00379.26	Chitin_bind_4	Insect cuticle prote	CYT	1	6	17.8	High
TRINITY_DN15597_c0_g1_i1_orf1	microsomal glutathione S-transferase 1-like [Ostrinia furnacalis]	0.422005208	-1.24466729	2.22E-06	down	yes	0.9723	2.304	2.269	2.316	2.327	1	0.917	1	CC:integral component of membrane;CC:cellular_component;CC:intrinsic component of membrane;CC:cellular anatomical entity;MF:glutathione transferase activity;MF:catalytic activity;MF:transferase activity, transferring alkyl or aryl;MF:transferase	GO:0016021;GO:0005575;GO:0031224;GO:0110165;GO:0004364;GO:0003824;GO:0016765;GO:0016740;GO:0003674	K00799	GST, gst	map05207;maן	Chemical carcinc	ENOG4111VJG	S:Function unknown	PF01124.21	MAPEG	MAPEG family	CYT	1	7	16.4	High
TRINITY_DN23354_c0_g1_i7_orf1	TBC1 domain family member 20 [Ostrinia furnacalis]	0.447072975	-1.161417755	0.01406	down	yes	1.115	2.494	2.726	1.856	2.9	1	1.095	1.249	BP:regulation of hydrolase activity;BP:establishment of localization;BP:transport;BP:positive regulation of GTPase activity;BP:positive regulation of hydrolase activity;BP:regulation of GTPase activity;BP:regulation of catalytic activity;BP:biological_process;BP:positive regulation of molecular function;BP:biological regulation;BP:regulation of molecular function;BP:localization;BP:positive regulation of catalytic activity;BP:vesicle-mediated transport;MF:enzyme regulator activity;MF:GTPase activator activity;MF:GTPase regulator activity;MF:molecular_function;MF:enzyme activator activity;MF:molecular function regulator;MF:nucleoside-triphosphatase regulator activity;	GO:0051336;GO:0051234;GO:0006810;GO:0043547;GO:0051345;GO:0043087;GO:0050790;GO:0008150;GO:0044093;GO:0065007;GO:0065009;GO:0051179;GO:0043085;GO:0016192;GO:0030234;GO:0005096;GO:0030695;GO:0003674;GO:0008047;GO:0098772;GO:0060589	K20372	TBC1D20, GYI	------	------	ENOG410XQXS	S:Function unknown	PF00566.21	RabGAP-TBC	Rab-GTPase-TBC (	CYT	1	1	49.4	Medium
TRINITY_DN2798_c0_g1_i5_orf1	arylsulfatase B-like isoform X1 [Ostrinia furnacalis]	0.408523461	-1.291509162	2.18E-05	down	yes	0.949	2.323	2.431	2.259	2.279	1	0.915	0.932	MF:hydrolase activity;MF:molecular_function;MF:hydrolase activity, acting on ester bonds;MF:sulfuric ester hydrolase activity;MF:catalytic activity;	GO:0016787;GO:0003674;GO:0016788;GO:0008484;GO:0003824	K01135	ARSB	map04142;maן	Lysosome;Glycos	COG3119	S:Function unknown	PF00884.26;PF0:	Sulfatase;Pho	Sulfatase;Type I ph	CYT	3	7	66.8	High
TRINITY_DN4688_c0_g1_i2_orf1	uncharacterized protein LOC114359411 [Ostrinia furnacalis]	0.375114679	-1.414596376	7.87E-06	down	yes	0.9813	2.616	2.531	2.678	2.64	1	0.916	1.028	------	------	------	------	------	------	ENOG4110SAV	S:Function unknown	------	------	------	CYT	6	10	71.4	High
TRINITY_DN2314_c0_g1_i7_orf1	protein dj-1beta-like isoform X1 [Ostrinia furnacalis]	0.483167378	-1.049405044	9.63E-07	down	yes	1.019	2.109	2.116	2.106	2.105	1	1.062	0.995	------	------	K05687	PARK7	map05012;maן	Parkinson diseas	COG0693	S:Function unknown	PF01965.27	DJ-1_PfpI	DJ-1/PfpI family	CYT	4	45	24.5	High
TRINITY_DN18388_c0_g1_i6_orf1	serine protease [Ostrinia furnacalis]	0.378652123	-1.401055077	3.89E-05	down	yes	0.972	2.567	2.676	2.613	2.412	1	0.955	0.961	BP:organic substance metabolic process;BP:organonitrogen compound metabolic process;BP:protein metabolic process;BP:nitrogen compound metabolic process;BP:macromolecule metabolic process;BP:biological_process;BP:metabolic process;BP:proteolysis;BP:primary metabolic process;CC:cellular_component;CC:extracellular region;CC:cellular anatomical entity;MF:serine-type endopeptidase activity;MF:endopeptidase activity;MF:hydrolase activity;MF:serine hydrolase activity;MF:catalytic activity, acting on a protein;MF:catalytic activity;MF:molecular_function;MF:peptidase activity;MF:serine-	GO:0071704;GO:1901564;GO:0019538;GO:0006807;GO:0043170;GO:0008150;GO:0008152;GO:0006508;GO:0044238;GO:0005575;GO:0005576;GO:0110165;GO:0004252;GO:0004175;GO:0016787;GO:0017171;GO:0140096;GO:0003824;GO:0003674;GO:0008233;GO:0008236	------	------	------	------	COG5640	O:Posttranslational modificatio	PF00089.29;PF1:	Trypsin;CLIP;T	Trypsin;Regulatory	CYT	4	13	47	High
TRINITY_DN95558_c0_g3_i1_orf1	cytochrome P450 monooxygenase CYP9G19 [Cnaphalocrocis medinalis]	0.43729097	-1.193334538	3.03E-05	down	yes	1.046	2.392	2.475	2.305	2.397	1	1.01	1.128	CC:membrane;CC:endoplasmic reticulum membrane;CC:organelle membrane;CC:cellular_component;CC:cellular anatomical entity;MF:cation binding;MF:monooxygenase activity;MF:heme binding;MF:transition metal ion binding;MF:tetrapyrrole binding;MF:organic cyclic compound binding;MF:oxidoreductase activity, acting on paired donors, with incorporation or reduction of molecular oxygen;MF:ion binding;MF:catalytic activity;MF:molecular_function;MF:binding;MF:iron ion	GO:0016020;GO:0005789;GO:0031090;GO:0005575;GO:0110165;GO:0043169;GO:0004497;GO:0020037;GO:0046914;GO:0046906;GO:0097159;GO:0016705;GO:0043167;GO:0003824;GO:0003674;GO:0005488;GO:0005506;GO:0046872;GO:1901363;GO:0016491	K15003	CYP9	------	------	COG2124	J:Translation, ribosomal structu	PF00067.25	p450	Cytochrome P450	CYT	1	16	8	High
TRINITY_DN39266_c0_g1_i1_orf1	PREDICTED: NECAP-like protein CG9132 [Microplitis demolitor]	0.433104631	-1.207212496	0.0009353	down	yes	1.01	2.332	2.17	2.192	2.633	1	1.013	1.016	BP:endocytosis;BP:vesicle-mediated transport;BP:transport;BP:biological_process;BP:establishment of localization;BP:localization;CC:membrane;CC:cellular_compone	GO:0006897;GO:0016192;GO:0006810;GO:0008150;GO:0051234;GO:0051179;GO:0016020;GO:0005575;GO:0110165	K20069	NECAP1_2	------	------	ENOG41113PS	S:Function unknown	PF07933.17	DUF1681	Protein of unknow	CYT	1	6	28.7	High
TRINITY_DN1703_c0_g1_i6_orf1	leucine-rich repeat-containing protein 15-like [Ostrinia furnacalis] >XP_028171914.1 leucine-rich repeat-containing protein 15-like [Ostrinia furnacalis] >XP_028171921.1 leucine-rich repeat-containing protein 15-like [Ostrinia furnacalis]	0.436936132	-1.194505681	0.000229	down	yes	0.9783	2.239	2.408	2.186	2.123	1	0.882	1.053	MF:hydrolase activity;MF:catalytic activity;MF:carboxypeptidase activity;MF:catalytic activity, acting on a protein;MF:exopeptidase	GO:0016787;GO:0003824;GO:0004180;GO:0140096;GO:0008238;GO:0003674;GO:0008233	K06839;K062	SLIT2;CHAD	map05165;maן	Human papillom	COG4886	O:Posttranslational modificatio	PF13855.9;PF12	LRR_8;LRR_4;L	Leucine rich repea	CYT	2	4	47.7	High
TRINITY_DN3647_c1_g1_i5_orf1	unnamed protein product, partial [Iphiclides podalirius]	0.469807868	-1.089857219	3.42E-05	down	yes	1.027	2.186	2.216	2.262	2.081	1	1.022	1.058	------	------	------	------	------	------	ENOG4111RPK	S:Function unknown	PF04923.15	Ninjurin	Ninjurin	CYT	4	24	33.9	High
TRINITY_DN37585_c0_g1_i1_orf1	cuticle protein 19.8-like [Ostrinia furnacalis]	0.442497754	-1.176257963	0.0002641	down	yes	0.985	2.226	2.305	2.349	2.024	1	0.968	0.987	------	------	------	------	------	------	------	------	------	------	------	EXC	1	10	17.7	High
TRINITY_DN25987_c0_g1_i5_orf1	GILT-like protein 2 isoform X1 [Ostrinia furnacalis] >XP_028156245.1 GILT-like protein 2 isoform X2 [Ostrinia furnacalis] >XP_028156247.1 GILT-like protein 2 isoform X3 [Ostrinia furnacalis]	0.475348837	-1.072941464	1.92E-05	down	yes	1.022	2.15	2.153	2.229	2.068	1	1.035	1.032	MF:catalytic activity;MF:oxidoreductase activity, acting on a sulfur group of donors, disulfide as acceptor;MF:molecular_function;MF:oxidoreductase activity, acting on a sulfur group of donors;MF:oxidoreductase activity;	GO:0003824;GO:0016671;GO:0003674;GO:0016667;GO:0016491	K08059	IFI30, GILT	map04612	Antigen processi	ENOG4111IDT	S:Function unknown	------	------	------	CYT	1	12	10.3	High
TRINITY_DN2058_c0_g1_i2_orf1	proteasomal ubiquitin receptor ADRM1 [Ostrinia furnacalis]	0.437052154	-1.194122646	0.00204	down	yes	0.9637	2.205	2.531	2.14	1.945	1	0.974	0.917	CC:cytoplasm;CC:macromolecular complex;CC:catalytic complex;CC:membrane-bounded organelle;CC:nucleus;CC:intracellular organelle;CC:cellular_component;CC:peptidase complex;CC:endopeptidase complex;CC:proteasome complex;CC:organelle;CC:cellular anatomical entity;CC:intracellular membrane-bounded organelle;	GO:0005737;GO:0032991;GO:1902494;GO:0043227;GO:0005634;GO:0043229;GO:0005575;GO:1905368;GO:1905369;GO:0000502;GO:0043226;GO:0110165;GO:0043231	K06691	RPN13	map03050;maן	Proteasome;Epst	ENOG410XSJJ	O:Posttranslational modificatio	PF16550.8	RPN13_C	UCH-binding dom	CYT	1	10	13.8	High

TRINITY_DN38274_c0_g1_i1_orf1	uncharacterized protein LOC114360402 [Ostrinia furnacalis]	0.160868881	-2.636042823	3.92E-06	down	yes	1.022	6.353	6.44	6.555	6.064	1	0.98	1.086	BP:nucleic acid metabolic process;BP:nitrogen compound metabolic process;BP:organic cyclic compound metabolic process;BP:nucleobase-containing compound metabolic process;BP:cellular response to stimulus;BP:cellular macromolecule metabolic process;BP:organic substance metabolic process;BP:DNA repair;BP:cellular process;BP:cellular aromatic compound metabolic process;BP:cellular response to DNA damage stimulus;BP:response to stress;BP:biological_process;BP:metabolic process;BP:heterocycle metabolic process;BP:primary metabolic process;BP:response to stimulus;BP:cellular nitrogen compound metabolic process;BP:cellular metabolic process;BP:macromolecule metabolic process;BP:cellular response to stress;BP:DNA metabolic process;CC:intracellular organelle;CC:membrane-bounded organelle;CC:organelle;CC:nucleus;CC:cellular anatomical entity;CC:intracellular membrane-bounded organelle;CC:cellular_component;MF:phosphoric ester hydrolase activity;MF:molecular_function;MF:phosphoric diester hydrolase activity;MF:structural molecule activity;MF:hydrolase	GO:0090304;GO:0006807;GO:1901360;GO:0006139;GO:0051716;GO:0044260;GO:0071704;GO:0006281;GO:0009987;GO:0006725;GO:0006974;GO:0006950;GO:0008150;GO:0008152;GO:0046483;GO:0044238;GO:0050896;GO:0034641;GO:0044237;GO:0043170;GO:0033554;GO:0006259;GO:0043229;GO:0043227;GO:0043226;GO:0005634;GO:0110165;GO:0043231;GO:0005575;GO:0042578;GO:0003674;GO:0008081;GO:0005198;GO:0016787;GO:0016788;GO:0003824;GO:0042302;GO:0004518	------	------	------	------	ENOG4112648;ENOG4111QIW	S:Function unknown;	PF00379.26	Chitin_bind_4	Insect cuticle prote	CYT	5	34	29.3	High
TRINITY_DN295_c3_g1_i1_orfp1	TRINITY_DN295_c3_g1_i1_m.18839 TRINITY_DN295_c3_g1::TRINITY_DN295_c3_g1_i1::g.18839 ORF type:5prime_partial len:118 (+),score=46.25 TRINITY_DN295_c3_g1_i1:1-354(+)	0.173873711	-2.523888276	8.60E-07	down	yes	0.961	5.527	5.686	5.496	5.398	1	0.975	0.908	------	------	------	------	------	------	------	------	------	------	------	PLA	3	49	12.6	High
TRINITY_DN19639_c0_g2_i1_orf1	basic juvenile hormone-suppressible protein 1-like [Hyposmocoma kahamanoa]	0.19669934	-2.345935979	0.0001427	down	yes	0.9833	4.999	4.955	4.538	5.504	1	1.045	0.905	------	------	------	------	------	------	ENOG410XR2D	S:Function unknown	PF03722.17	Hemocyanin_	Hemocyanin, all-a	CYT	2	72	8.6	High
TRINITY_DN54524_c0_g1_i6_orf1	serine protease inhibitor dipetalogastin [Ostrinia furnacalis]	0.237531895	-2.073806847	5.27E-06	down	yes	1.024	4.311	4.171	4.274	4.487	1	0.973	1.1	------	------	------	------	------	------	ENOG410ZXUD;ENOG410YKSA	S:Function unknown;	PF07648.18;PF0	Kazal_2;Kazal	Kazal-type serine	PLA	3	7	55.3	High
TRINITY_DN5655_c0_g1_i2_orf1	uncharacterized protein LOC114359603 [Ostrinia furnacalis]	0.263170614	-1.925929691	0.0001378	down	yes	1.089	4.138	4.099	4.516	3.799	1	1.147	1.121	------	------	------	------	------	------	ENOG4111C52	S:Function unknown	------	------	------	CYT	1	8	10.8	High
TRINITY_DN142657_c0_g1_i1_orf1	sorting and assembly machinery component 50 homolog [Diachasma alloeum]	0.245117188	-2.028456446	0.0005566	down	yes	1.004	4.096	4.316	4.485	3.487	1	1.007	1.006	CC:cellular_component;CC:membrane;CC:outer membrane;CC:cellular anatomical entity;	GO:0005575;GO:0016020;GO:0019867;GO:0110165	K07277	SAM50, TOB5	------	------	COG4775	M:Cell wall/membrane/envelop	PF01103.26	Omp85	Omp85 superfamil	CYT	1	2	53.8	High
TRINITY_DN7854_c0_g1_i4_orf1	failed axon connections [Ostrinia furnacalis]	0.270824115	-1.884571888	1.37E-08	down	yes	0.9793	3.616	3.597	3.623	3.628	1	0.949	0.989	------	------	------	------	------	------	ENOG4111HS1	S:Function unknown	PF17172.7;PF17	GST_N_4;GST	Glutathione S-tran	CYT	22	54	42.6	High
TRINITY_DN418_c1_g1_i3_orf1	hypothetical protein evm_003996 [Chilo suppressalis]	0.294084348	-1.765698095	0.0006844	down	yes	1.039	3.533	3.283	3.265	4.051	1	0.995	1.123	MF:molecular_function;MF:carbohydrate binding;MF:binding;	GO:0003674;GO:0030246;GO:0005488	------	------	------	------	------	------	------	------	------	PLA	1	46	8.8	High
TRINITY_DN5444_c0_g2_i1_orf1	phenoloxidase-activating factor 2-like [Ostrinia furnacalis]	0.294864397	-1.761876458	1.44E-06	down	yes	1.022	3.466	3.511	3.521	3.365	1	1.004	1.061	BP:organic substance metabolic process;BP:organonitrogen compound metabolic process;BP:protein metabolic process;BP:macromolecule metabolic process;BP:biological_process;BP:nitrogen compound metabolic process;BP:metabolic process;BP:proteolysis;BP:primary metabolic process;MF:serine-type endopeptidase activity;MF:endopeptidase activity;MF:hydrolase activity;MF:serine hydrolase activity;MF:catalytic activity, acting on a protein;MF:catalytic activity;MF:molecular_function;MF:peptidase activity;MF:serine-	GO:0071704;GO:1901564;GO:0019538;GO:0043170;GO:0008150;GO:0006807;GO:0008152;GO:0006508;GO:0044238;GO:0004252;GO:0004175;GO:0016787;GO:0017171;GO:0140096;GO:0003824;GO:0003674;GO:0008233;GO:0008236	------	------	------	------	COG5640	E:Amino acid transport and me	PF00089.29	Trypsin	Trypsin	CYT	3	14	27.9	High
TRINITY_DN19662_c4_g1_i1_orf1	basic juvenile hormone-suppressible protein 1-like [Ostrinia furnacalis]	0.295488057	-1.758828276	3.64E-07	down	yes	1.002	3.391	3.379	3.333	3.461	1	0.999	1.007	CC:cellular_component;CC:membrane;CC:extracellular region;CC:cellular anatomical entity;MF:signaling receptor activity;MF:catalytic activity;MF:phosphotransferase activity, alcohol group as acceptor;MF:transferase activity, transferring phosphorus-containing groups;MF:kinase activity;MF:catalytic activity, acting on a protein;MF:transmembrane signaling receptor activity;MF:transmembrane receptor protein serine/threonine kinase activity;MF:protein serine/threonine kinase activity;MF:transferase activity;MF:molecular_function;MF:protein kinase activity;MF:transmembrane receptor protein kinase	GO:0005575;GO:0016020;GO:0005576;GO:0110165;GO:0038023;GO:0003824;GO:0016773;GO:0016772;GO:0016301;GO:0140096;GO:0004888;GO:0004675;GO:0004674;GO:0016740;GO:0003674;GO:0004672;GO:0019199;GO:0060089	------	------	------	------	ENOG410XR2D	S:Function unknown	PF03723.17	Hemocyanin_	Hemocyanin, ig-lik	CYT	31	82	30.7	High
TRINITY_DN15812_c0_g1_i2_orf1	transferrin [Ostrinia furnacalis]	0.302030303	-1.727234791	5.23E-07	down	yes	0.9967	3.3	3.328	3.35	3.222	1	0.992	0.998	BP:ion homeostasis;BP:homeostatic process;BP:iron ion homeostasis;BP:transition metal ion homeostasis;BP:inorganic ion homeostasis;BP:biological regulation;BP:ion transport;BP:transport;BP:biological_process;BP:metal ion homeostasis;BP:cation homeostasis;BP:establishment of localization;BP:localization;BP:regulation of biological quality;BP:chemical homeostasis;CC:extracellular space;CC:cellular anatomical entity;CC:cellular_component;MF:molecular_function;MF:binding;MF:cation binding;MF:ion binding;MF:metal ion binding;	GO:0050801;GO:0042592;GO:0055072;GO:0055076;GO:0098771;GO:0065007;GO:0006811;GO:0006810;GO:0008150;GO:0055065;GO:0055080;GO:0051234;GO:0051179;GO:0065008;GO:0048878;GO:0005615;GO:0110165;GO:0005575;GO:0003674;GO:0005488;GO:0043169;GO:0043167;GO:0046872	K06569	MFI2, CD228	------	------	ENOG410XQ36	O:Posttranslational modificatio	PF00405.20;PF1	Transferrin;Ph	Transferrin;ABC tra	CYT	30	52	75.6	High
TRINITY_DN52944_c0_g1_i1_orf1	apolipophorins-like [Ostrinia furnacalis]	0.314461538	-1.669044522	3.24E-06	down	yes	1.022	3.25	3.317	3.283	3.15	1	0.98	1.086	MF:lipid transporter activity;MF:transporter activity;MF:molecular_function;	GO:0005319;GO:0005215;GO:0003674	------	------	------	------	ENOG410ZRUY;ENOG410Z6MR	S:Function unknown;	PF01347.25	Vitellogenin_l	Lipoprotein amino	CYT	5	59	11	High
TRINITY_DN26209_c0_g1_i6_orf1	uncharacterized protein LOC114352357 [Ostrinia furnacalis]	0.33761002	-1.566570371	3.49E-06	down	yes	0.9973	2.954	3.018	2.996	2.847	1	0.985	1.007	------	------	------	------	------	------	COG1028	S:Function unknown	PF13561.9;PF00	adh_short_C2	Enoyl;short chain	CYT	11	54	27.3	High
TRINITY_DN19110_c0_g1_i2_orf1	peroxidase [Ostrinia furnacalis]	0.374953687	-1.415215687	0.0002319	down	yes	1.012	2.699	2.502	2.955	2.639	1	1.008	1.028	------	------	K19511	PXDN, VPO1	------	------	ENOG410XPZ3	M:Cell wall/membrane/envelop	PF03098.18	An_peroxidas	Animal haem pero	CYT	1	2	84.9	High
TRINITY_DN805_c0_g1_i5_orf1	serine protease snake-like [Ostrinia furnacalis]	0.414375491	-1.27098942	0.0007836	down	yes	1.055	2.546	2.508	2.836	2.293	1	1.035	1.129	BP:organic substance metabolic process;BP:organonitrogen compound metabolic process;BP:protein metabolic process;BP:macromolecule metabolic process;BP:biological_process;BP:nitrogen compound metabolic process;BP:metabolic process;BP:proteolysis;BP:primary metabolic process;MF:serine-type endopeptidase activity;MF:endopeptidase activity;MF:hydrolase activity;MF:serine hydrolase activity;MF:catalytic activity, acting on a protein;MF:catalytic activity;MF:molecular_function;MF:peptidase activity;MF:serine-	GO:0071704;GO:1901564;GO:0019538;GO:0043170;GO:0008150;GO:0006807;GO:0008152;GO:0006508;GO:0044238;GO:0004252;GO:0004175;GO:0016787;GO:0017171;GO:0140096;GO:0003824;GO:0003674;GO:0008233;GO:0008236	------	------	------	------	COG5640	O:Posttranslational modificatio	PF00089.29	Trypsin	Trypsin	EXC	6	23	43.9	High
TRINITY_DN11060_c0_g1_i6_orf1	extracellular matrix protein A-like isoform X3 [Ostrinia furnacalis]	0.378707842	-1.400842798	1.69E-05	down	yes	0.932	2.461	2.477	2.535	2.371	1	0.86	0.936	------	------	K19720	COL3A	map05146;ma	Amoebiasis;Prote	ENOG4110SA3	S:Function unknown	------	------	------	PLA	3	4	105.7	High
TRINITY_DN8771_c0_g1_i5_orf1	regucalcin-like [Ostrinia furnacalis]	0.407682724	-1.294481272	1.12E-05	down	yes	0.9817	2.408	2.431	2.485	2.309	1	0.97	0.975	CC:cytoplasm;CC:cellular_component;CC:cellular anatomical entity;MF:cation binding;MF:metal ion binding;MF:enzyme regulator activity;MF:ion binding;MF:molecular_function;MF:calcium ion	GO:0005737;GO:0005575;GO:0110165;GO:0043169;GO:0046872;GO:0030234;GO:0043167;GO:0003674;GO:0005509;GO:0098772;GO:0005488	K01053	gnl, RGN	map00030;ma	Pentose phospha	COG3386	G:Carbohydrate transport and	PF08450.15	SGL	SMP-30/Gluconola	CYT	7	32	33.2	High
TRINITY_DN36281_c0_g1_i2_orf1	putative uncharacterized protein DDB_G0282499 isoform X1 [Ostrinia furnacalis]	0.474433081	-1.075723485	0.01236	down	yes	1.067	2.249	2.481	2.531	1.734	1	1.245	0.957	------	------	------	------	------	------	ENOG41113JC	S:Function unknown	------	------	------	EXC	1	2	81.1	High
TRINITY_DN7633_c0_g1_i1_orf1	prolow-density lipoprotein receptor-related protein 1, partial [Ostrinia furnacalis]	0.470561798	-1.087543894	0.000113	down	yes	1.047	2.225	2.28	2.081	2.315	1	1.098	1.043	BP:endocytosis;BP:vesicle-mediated transport;BP:transport;BP:biological_process;BP:establishment of localization;BP:localization;CC:integral component of membrane;CC:cellular_component;CC:intrinsic component of membrane;CC:cellular anatomical entity;MF:binding;MF:cation binding;MF:metal ion binding;MF:ion binding;MF:molecular_function;MF:calcium ion binding;	GO:0006897;GO:0016192;GO:0006810;GO:0008150;GO:0051234;GO:0051179;GO:0016021;GO:0005575;GO:0031224;GO:0110165;GO:0005488;GO:0043169;GO:0046872;GO:0043167;GO:0003674;GO:0005509	K04550	LRP1, CD91	map05010;ma	Alzheimer diseas	ENOG410YM97;ENOG410XP34	S:Function unknown;	PF00058.20;PF1	Ldl_recept_b;l	Low-density lipop	CYT	6	13	62.4	High
TRINITY_DN9383_c0_g1_i3_orf1	uncharacterized protein LOC114361502 [Ostrinia furnacalis]	0.435736264	-1.19847291	1.06E-05	down	yes	0.9913	2.275	2.206	2.333	2.287	1	1.036	0.938	BP:cellular process;BP:biological_process;BP:cell adhesion;CC:plasma membrane bounded cell projection;CC:intracellular organelle;CC:non-membrane-bounded organelle;CC:focal adhesion;CC:organelle;CC:cell junction;CC:cell-substrate junction;CC:cytoskeleton;CC:ruffle;CC:cellular anatomical entity;CC:cytoplasm;CC:anchoring junction;CC:cell projection;CC:intracellular non-membrane-bounded organelle;CC:cellular_component;MF:structural constituent of cytoskeleton;MF:molecular_function;MF:binding;MF:structural molecule activity;MF:actin binding;MF:actin filament binding;MF:cytoskeletal protein binding;MF:protein	GO:0009987;GO:0008150;GO:0007155;GO:0120025;GO:0043229;GO:0043228;GO:0005925;GO:0043226;GO:0030054;GO:0030055;GO:0005856;GO:0001726;GO:0110165;GO:0005737;GO:0070161;GO:0042995;GO:0043232;GO:0005575;GO:0005200;GO:0003674;GO:0005488;GO:0005198;GO:0003779;GO:0051015;GO:0008092;GO:0005515;GO:0044877	K06271	TLN	map05166;ma	Human T-cell leu	------	------	------	------	------	CYT	1	5	19.3	High
TRINITY_DN86833_c0_g3_i1_orf1	PREDICTED: glycerol-3-phosphate acyltransferase 1, mitochondrial isoform X1 [Microplitis demolitor]	0.45769577	-1.127539138	3.01E-07	down	yes	1.017	2.222	2.195	2.22	2.25	1	1.026	1.025	BP:primary metabolic process;BP:lipid metabolic process;BP:organic substance metabolic process;BP:cellular process;BP:cellular metabolic process;BP:biological_process;BP:metabolic process;BP:cellular lipid metabolic process;CC:cellular_component;CC:cellular anatomical entity;MF:catalytic activity;MF:transferase activity;MF:transferase activity, transferring acyl groups;MF:transferase activity, transferring acyl groups other than amino-acyl groups;MF:O-acyltransferase	GO:0044238;GO:0006629;GO:0071704;GO:0009987;GO:0044237;GO:0008150;GO:0008152;GO:0044255;GO:0005575;GO:0110165;GO:0003824;GO:0016740;GO:0016746;GO:0016747;GO:0008374;GO:0003674	K00629	GPAT1_2	map00564;ma	Glycerophosphol	COG2937	I:Lipid transport and metabolis	PF19277.2;PF01	GPAT_C;Acylt	Glycerol-3-phosph	CYT	1	1	98.9	Medium
TRINITY_DN280_c0_g1_i8_orf1	Tubulin beta-1 chain [Papilio xuthus]	0.448729583	-1.156081798	1.45E-05	down	yes	0.989	2.204	2.209	2.277	2.127	1	1.018	0.949	BP:cellular process;BP:biological_process;BP:microtubule-based process;CC:supramolecular fiber;CC:polymeric cytoskeletal fiber;CC:microtubule;CC:cytoplasm;CC:cellular_component;CC:cellular anatomical entity;CC:supramolecular complex;CC:supramolecular polymer;MF:structural constituent of cytoskeleton;MF:purine ribonucleoside triphosphate binding;MF:molecular_function;MF:binding;MF:guanyl nucleotide binding;MF:heterocyclic compound binding;MF:nucleoside phosphate binding;MF:pyrophosphatase activity;MF:structural molecule activity;MF:guanyl ribonucleotide binding;MF:purine nucleotide binding;MF:GTP binding;MF:hydrolase activity;MF:nucleotide binding;MF:GTPase activity;MF:nucleoside-triphosphatase activity;MF:small molecule binding;MF:catalytic activity;MF:hydrolase activity, acting on acid anhydrides, in phosphorus-containing anhydrides;MF:ion binding;MF:carbohydrate derivative binding;MF:organic cyclic compound binding;MF:hydrolase activity, acting on acid anhydrides;MF:anion binding;MF:purine ribonucleotide binding;MF:ribonucleotide binding;	GO:0009987;GO:0008150;GO:0007017;GO:0099512;GO:0099513;GO:0005874;GO:0005737;GO:0005575;GO:0110165;GO:0099080;GO:0099081;GO:0005200;GO:0035639;GO:0003674;GO:0005488;GO:0019001;GO:1901363;GO:1901265;GO:0016462;GO:0005198;GO:0032561;GO:0017076;GO:0005525;GO:0016787;GO:0000166;GO:0003924;GO:0017111;GO:0036094;GO:0003824;GO:0016818;GO:0043167;GO:0097367;GO:0097159;GO:0016817;GO:0043168;GO:0032555;GO:0032553	K07375	TUBB	map04540;ma	Gap junction;Parl	COG5023	Z:Cytoskeleton	PF00091.28;PF0	Tubulin;Tubul	Tubulin/FtsZ family	CYT	4	53	50.6	High
TRINITY_DN1630_c0_g1_i6_orf1	major facilitator superfamily domain-containing protein 1-like [Ostrinia furnacalis]	0.49084077	-1.026673007	0.0002878	down	yes	1.045	2.129	2.249	2.097	2.042	1	0.958	1.177	CC:integral component of membrane;CC:cellular_component;CC:cellular anatomical entity;CC:intrinsic component of membrane;MF:transporter activity;MF:transmembrane transporter activity;MF:molecular_function;	GO:0016021;GO:0005575;GO:0110165;GO:0031224;GO:0005215;GO:0022857;GO:0003674	------	------	------	------	COG0477	T:Signal transduction mechanis	PF07690.19	MFS_1	Major Facilitator Su	CYT	1	3	57.8	High
TRINITY_DN4602_c0_g1_i4_orf1	2-iminobutanoate/2-iminopropanoate deaminase [Ostrinia furnacalis]	0.489361702	-1.031026896	0.0004847	down	yes	1.035	2.115	2.218	1.911	2.216	1	1.034	1.07	------	------	K09022	ridA, tdcF, RID	------	------	COG0251	J:Translation, ribosomal structu	PF01042.24	Ribonuc_L-PS	Endoribonuclease	CYT	2	22	18.2	High
TRINITY_DN1196_c0_g1_i5_orf1	glucosamine-6-phosphate isomerase isoform X1 [Ostrinia furnacalis]	0.481411765	-1.054656696	3.73E-05	down	yes	1.023	2.125	2.226	2.108	2.041	1	1.037	1.031	BP:primary metabolic process;BP:carbohydrate metabolic process;BP:amino sugar metabolic process;BP:carbohydrate derivative metabolic process;BP:N-acetylglucosamine metabolic process;BP:organic substance metabolic process;BP:biological_process;BP:metabolic process;BP:glucosamine-containing compound metabolic process;CC:cytoplasm;CC:cellular_component;CC:cellular anatomical entity;MF:isomerase activity;MF:catalytic activity;MF:hydrolase activity;MF:glucosamine-6-phosphate deaminase activity;MF:deaminase activity;MF:intramolecular oxidoreductase activity, interconverting aldoses and ketoses;MF:molecular_function;MF:intramolecular oxidoreductase activity;	GO:0044238;GO:0005975;GO:0006040;GO:1901135;GO:0006044;GO:0071704;GO:0008150;GO:0008152;GO:1901071;GO:0005737;GO:0005575;GO:0110165;GO:0016853;GO:0003824;GO:0016787;GO:0004342;GO:0019239;GO:0016861;GO:0003674;GO:0016860	K02564	nagB, GNPDA	map00520	Amino sugar and	COG0363	G:Carbohydrate transport and	PF01182.23	Glucosamine_	Glucosamine-6-ph	CYT	1	45	31.2	High
TRINITY_DN8083_c0_g1_i1_orf1	solute carrier family 35 member F6 [Ostrinia furnacalis]	0.469158879	-1.091851527	1.67E-06	down	yes	1.004	2.14	2.102	2.131	2.187	1	1.019	0.993	CC:integral component of membrane;CC:cellular_component;CC:cellular anatomical entity;CC:intrinsic component of membrane;MF:transporter activity;MF:transmembrane transporter activity;MF:molecular_function;	GO:0016021;GO:0005575;GO:0110165;GO:0031224;GO:0005215;GO:0022857;GO:0003674	------	------	------	------	COG0697;COG0697	E:Amino acid transport and me	PF00892.23	EamA	EamA-like transpo	CYT	1	2	39.9	High
TRINITY_DN971_c0_g1_i10_orfp1	TRINITY_DN971_c0_g1_i10_m.54268 TRINITY_DN971_c0_g1::TRINITY_DN971_c0_g1_i10::g.54268 ORF type:internal len:187 (+),score=141.68 TRINITY_DN971_c0_g1_i10:1-558(+)	0.091431925	-3.451158197	2.51E-06	down	yes	0.779	8.52	8.668	8.635	8.256	1	0.834	0.503	------	------	------	------	------	------	------	------	------	------	------	CYT	2	20	20.5	High
TRINITY_DN18218_c0_g1_i7_orf1	inhibin beta B chain [Ostrinia furnacalis]	0.127854928	-2.967420328	0.004466	down	yes	0.9483	7.417	8.375	5.186	8.689	1	0.846	0.999	BP:response to stimulus;BP:biological_process;BP:response to chemical;BP:response to organic substance;CC:cellular_component;CC:extracellular region;CC:cellular anatomical entity;MF:growth factor activity;MF:receptor regulator activity;MF:receptor activator activity;MF:receptor agonist activity;MF:molecular_function;MF:binding;MF:protein	GO:0050896;GO:0008150;GO:0042221;GO:0010033;GO:0005575;GO:0005576;GO:0110165;GO:0008083;GO:0030545;GO:0030546;GO:0048018;GO:0003674;GO:0005488;GO:0005515;GO:0005102;GO:0098772	K04669	TGFBRX	------	------	ENOG410XT8Z	O:Posttranslational modificatio	PF00019.23;PF0	TGF_beta;TGF	Transforming grow	CYT	1	4	41.4	High
TRINITY_DN10747_c0_g1_i5_orf1	unnamed protein product [Plutella xylostella]	0.210215281	-2.250060551	6.01E-06	down	yes	0.996	4.738	4.781	4.915	4.517	1	0.96	1.028	------	------	------	------	------	------	ENOG410XRSQ	S:Function unknown	PF00650.23	CRAL_TRIO	CRAL/TRIO domair	CYT	10	36	41.8	High
TRINITY_DN75086_c0_g1_i5_orf1	lysosome membrane protein 2-like [Ostrinia furnacalis]	0.203084995	-2.299844447	2.73E-05	down	yes	0.9677	4.765	4.81	5.043	4.441	1	0.959	0.944	BP:multicellular organismal process;BP:sensory perception of smell;BP:neurological system process;BP:response to stimulus;BP:sensory perception of chemical stimulus;BP:sensory perception;BP:biological_process;BP:system process;CC:membrane;CC:cellular_component;CC:cellular anatomical entity;CC:plasma membrane;CC:integral component of membrane;CC:intrinsic component of membrane;	GO:0032501;GO:0007608;GO:0050877;GO:0050896;GO:0007606;GO:0007600;GO:0008150;GO:0003008;GO:0016020;GO:0005575;GO:0110165;GO:0005886;GO:0016021;GO:0031224	K13885	SCARB1	map04979;ma	Cholesterol meta	ENOG410XS17	U:Intracellular trafficking, secre	------	------	------	CYT	1	11	7.7	High

TRINITY_DN52316_c0_g1_i1_orf1	arylphorin subunit alpha-like [Ostrinia furnacalis]	0.229155125	-2.125603545	2.49E-05	down	yes	0.9927	4.332	4.327	4.074	4.596	1	0.967	1.011	------	------	------	------	------	------	ENOG410XR2D	J:Translation, ribosomal structu	PF03722.17	Hemocyanin_	Hemocyanin, all-a	PLA	4	55	13.9	High
TRINITY_DN12387_c1_g2_i1_orf1	skin secretory protein xP2-like [Ostrinia furnacalis]	0.224619991	-2.154441764	2.59E-05	down	yes	0.9753	4.342	4.353	4.602	4.071	1	0.954	0.972	------	------	------	------	------	------	ENOG410ZKDQ;ENOG4111M7J;ENOG410YJ7D	S:Function unknown;	------	------	------	CYT	10	50	39.7	High
TRINITY_DN2002_c0_g1_i5_orfp1	TRINITY_DN2002_c0_g1_i5_m.4230 TRINITY_DN2002_c0_g1::TRINITY_DN2002_c0_g1_i5::g.4230 ORF type:3prime_partial len:259 (+),score=29.98,Peptidase_C39 PF03412.16 1.7,Peptidase_C39|PF03412.16|0.049 TRINITY_DN2002_c0_g1_i5:65-838(+)	0.214612188	-2.220196082	8.81E-05	down	yes	0.9297	4.332	3.941	4.432	4.623	1	0.98	0.809	------	------	------	------	------	------	------	------	------	------	------	CYT	1	5	29.4	Medium
TRINITY_DN5595_c0_g1_i1_orf1	keratin, type I cytoskeletal 10-like [Ostrinia furnacalis]	0.237209302	-2.075767508	1.88E-05	down	yes	0.969	4.085	3.938	4.345	3.971	1	0.946	0.961	------	------	------	------	------	------	ENOG4112BJP	S:Function unknown	PF16058.8	Mucin-like	Mucin-like	EXC	1	14	16.6	High
TRINITY_DN1423_c0_g1_i8_orf1	ferritin subunit-like [Ostrinia furnacalis] >XP_028168186.1 ferritin subunit-like [Ostrinia furnacalis]	0.248310388	-2.009783477	1.51E-05	down	yes	0.992	3.995	3.931	4.227	3.827	1	0.99	0.986	BP:ion homeostasis;BP:homeostatic process;BP:iron ion homeostasis;BP:transition metal ion homeostasis;BP:inorganic ion homeostasis;BP:cellular transition metal ion homeostasis;BP:transition metal ion transport;BP:biological regulation;BP:regulation of biological quality;BP:cellular homeostasis;BP:metal ion transport;BP:transport;BP:cellular iron ion homeostasis;BP:cellular metal ion homeostasis;BP:cation transport;BP:ion transport;BP:cellular process;BP:cellular ion homeostasis;BP:biological_process;BP:cellular cation homeostasis;BP:metal ion homeostasis;BP:cation homeostasis;BP:cellular chemical homeostasis;BP:establishment of localization;BP:localization;BP:chemical homeostasis;BP:iron ion transport;MF:molecular_function;MF:binding;MF:oxidoreductase activity;MF:cation binding;MF:oxidoreductase activity, oxidizing metal ions;MF:transition metal ion binding;MF:ferroxidase activity;MF:ion binding;MF:iron ion binding;MF:ferric iron binding;MF:oxidoreductase activity, oxidizing metal ions	GO:0050801;GO:0042592;GO:0055072;GO:0055076;GO:0098771;GO:0046916;GO:0000041;GO:0065007;GO:0065008;GO:0019725;GO:0030001;GO:0006810;GO:0006879;GO:0006875;GO:0006812;GO:0006811;GO:0009987;GO:0006873;GO:0008150;GO:0030003;GO:0055065;GO:0055080;GO:0055082;GO:0051234;GO:0051179;GO:0048878;GO:0006826;GO:0003674;GO:0005488;GO:0016491;GO:0043169;GO:0016722;GO:0046914;GO:0004322;GO:0043167;GO:0005506;GO:0008199;GO:0016724;GO:0046872;GO:0003824	K00522	FTH1	map04978;ma	Mineral absorptic	ENOG4111MSG	P:Inorganic ion transport and n	PF00210.27	Ferritin	Ferritin-like domai	CYT	6	34	23.7	High
TRINITY_DN30169_c0_g1_i1_orfp1	TRINITY_DN30169_c0_g1_i1_m.11367 TRINITY_DN30169_c0_g1::TRINITY_DN30169_c0_g1_i1::g.11367 ORF type:3prime_partial len:52	0.271447197	-1.881256509	3.63E-05	down	yes	1.041	3.835	3.582	3.871	4.051	1	1.034	1.09	------	------	------	------	------	------	------	------	------	------	------	CYT	1	12	6.1	High
TRINITY_DN31314_c0_g1_i4_orf1	uncharacterized protein LOC114355976 [Ostrinia furnacalis]	0.268903088	-1.89484177	4.98E-07	down	yes	1.01	3.756	3.774	3.82	3.675	1	0.984	1.046	BP:mitotic cell cycle;BP:cellular process;BP:biological_process;BP:cell division;BP:cell cycle;CC:cytoplasm;CC:supramolecular complex;CC:chromosomal region;CC:intracellular non-membrane-bounded organelle;CC:nucleus;CC:chromosome, centromeric region;CC:cellular_component;CC:intracellular organelle;CC:non-membrane-bounded organelle;CC:cellular anatomical entity;CC:kinetochore;CC:membrane-bounded organelle;CC:organelle;CC:intracellular membrane-bounded	GO:0000278;GO:0009987;GO:0008150;GO:0051301;GO:0007049;GO:0005737;GO:0099080;GO:0098687;GO:0043232;GO:0005634;GO:0000775;GO:0005575;GO:0043229;GO:0043228;GO:0110165;GO:0000776;GO:0043227;GO:0043226;GO:0043231	K11578	ZW10, DSL1	------	------	ENOG410XQ7J;ENOG4110SRI	S:Function unknown;	------	------	------	CYT	7	9	86.8	High
TRINITY_DN60946_c0_g2_i3_orf1	protein yellow-like [Ostrinia furnacalis]	0.267232552	-1.90383234	2.78E-08	down	yes	0.9917	3.711	3.715	3.747	3.672	1	0.981	0.994	------	------	------	------	------	------	COG3386	S:Function unknown	PF03022.19	MRJP	Major royal jelly pr	CYT	13	36	49.2	High
TRINITY_DN59388_c0_g1_i1_orf1	uncharacterized protein LOC114353759 [Ostrinia furnacalis]	0.272254177	-1.876973912	2.62E-08	down	yes	0.994	3.651	3.681	3.653	3.62	1	0.97	1.012	------	------	------	------	------	------	------	------	------	------	------	CYT	5	36	20.8	High
TRINITY_DN2024_c0_g1_i12_orfp1	unnamed protein product, partial [Brenthis ino]	0.290580144	-1.782991973	9.08E-06	down	yes	0.9717	3.344	3.275	3.508	3.25	1	0.954	0.961	------	------	------	------	------	------	------	------	PF13930.9	Endonuclea_N	DNA/RNA non-spe	CYT	2	6	46	High
TRINITY_DN64772_c0_g1_i1_orf1	aldehyde dehydrogenase, partial [Mythimna separata]	0.310355299	-1.688007316	2.97E-06	down	yes	1.022	3.293	3.406	3.264	3.208	1	1.023	1.044	MF:catalytic activity;MF:oxidoreductase activity, acting on the aldehyde or oxo group of donors;MF:molecular_function;MF:oxidoreductase activity, acting on the aldehyde or oxo group of donors, NAD or NADP as acceptor;MF:oxidoreductase activity;	GO:0003824;GO:0016903;GO:0003674;GO:0016620;GO:0016491	K00128	ALDH	map00310;ma	Lysine degradatic	COG1012	C:Energy production and conve	PF00171.25	Aldedh	Aldehyde dehydro	CYT	2	42	5.9	High
TRINITY_DN28711_c1_g1_i1_orf1	apolipophorins-like [Ostrinia furnacalis]	0.300952674	-1.732391461	8.32E-07	down	yes	0.9793	3.254	3.333	3.2	3.229	1	0.945	0.993	MF:lipid transporter activity;MF:transporter activity;MF:molecular_function;	GO:0005319;GO:0005215;GO:0003674	------	------	------	------	ENOG410ZRUY;ENOG410Z6MR	S:Function unknown;	PF01347.25	Vitellogenin_N	Lipoprotein amino	CYT	7	86	9.8	High
TRINITY_DN71699_c0_g1_i1_orf1	apolipophorins-like [Ostrinia furnacalis]	0.3171875	-1.656592178	5.04E-08	down	yes	1.015	3.2	3.235	3.172	3.192	1	1.033	1.013	MF:lipid transporter activity;MF:transporter activity;MF:molecular_function;	GO:0005319;GO:0005215;GO:0003674	------	------	------	------	ENOG410Z6MR;ENOG41112S0	S:Function unknown;	------	------	------	CYT	6	63	8.2	High
TRINITY_DN267_c0_g1_i1_orf1	keratin, type I cytoskeletal 9-like [Ostrinia furnacalis]	0.292027406	-1.775824327	2.65E-05	down	yes	0.9377	3.211	3.027	3.37	3.237	1	0.904	0.909	------	------	------	------	------	------	ENOG4110QQM	S:Function unknown	------	------	------	CYT	3	9	52.5	High
TRINITY_DN13221_c0_g1_i3_orf1	fasciclin-3-like [Ostrinia furnacalis]	0.322685789	-1.631798053	2.52E-07	down	yes	0.99	3.068	3.076	3.04	3.089	1	0.941	1.029	------	------	------	------	------	------	ENOG4111K6M	S:Function unknown	PF08205.15;PF1	C2-set_2;Ig_3	CD80-like C2-set i	CYT	1	46	19	High
TRINITY_DN206_c0_g1_i8_orf1	A-kinase anchor protein 200-like [Ostrinia furnacalis] >XP_028173114.1 A-kinase anchor protein 200-like [Ostrinia furnacalis] >XP_028173115.1 A-kinase anchor protein 200-like [Ostrinia	0.363044224	-1.461782797	0.0005666	down	yes	1.059	2.917	2.823	3.265	2.663	1	1.153	1.024	------	------	------	------	------	------	ENOG4110RAQ	S:Function unknown	------	------	------	CYT	1	15	34.3	High
TRINITY_DN688_c0_g1_i8_orf1	lysosomal alpha-mannosidase-like [Ostrinia furnacalis]	0.344991511	-1.535367232	3.72E-07	down	yes	1.016	2.945	2.969	2.979	2.888	1	1.015	1.034	------	------	K12311	MAN2B1, LAN	map00511;ma	Other glycan deg	ENOG410XQMZ	G:Carbohydrate transport and r	PF01074.25;PF0	Glyco_hydro_	Glycosyl hydrolase	EXC	15	20	126.4	High
TRINITY_DN2912_c0_g1_i1_orf1	uncharacterized protein LOC114352866 [Ostrinia furnacalis] >XP_028160411.1 uncharacterized protein LOC114352866 [Ostrinia furnacalis]	0.416122841	-1.264918616	0.04589	down	yes	1.084	2.605	2.533	3.551	1.731	1	1.231	1.02	------	------	------	------	------	------	ENOG41115A0	S:Function unknown	------	------	------	CYT	1	3	48.8	High
TRINITY_DN2425_c0_g1_i3_orf1	sialic acid synthase [Ostrinia furnacalis]	0.381275953	-1.391092551	9.18E-05	down	yes	1.01	2.649	2.634	2.833	2.481	1	1.042	0.989	BP:organic substance biosynthetic process;BP:carbohydrate metabolic process;BP:primary metabolic process;BP:carbohydrate biosynthetic process;BP:organic substance metabolic process;BP:biosynthetic process;BP:biological_process;BP:metabolic process;	GO:1901576;GO:0005975;GO:0044238;GO:0016051;GO:0071704;GO:0009058;GO:0008150;GO:0008152	K05304	NANS, SAS	map00520;ma	Amino sugar and	COG2089	M:Cell wall/membrane/envelop	PF03102.17	NeuB	NeuB family	CYT	12	38	38.8	High
TRINITY_DN843_c0_g1_i2_orf1	unnamed protein product [Diatraea saccharalis]	0.418803419	-1.255654875	0.0008376	down	yes	1.078	2.574	2.877	2.506	2.338	1	1.072	1.161	------	------	------	------	------	------	ENOG4111K6M	S:Function unknown	PF08205.15;PF1	C2-set_2;Ig_3	CD80-like C2-set i	CYT	2	32	48.6	High
TRINITY_DN23398_c0_g1_i1_orf1	cytochrome P450 6B7-like [Ostrinia furnacalis]	0.434090004	-1.203933894	2.58E-05	down	yes	1.09	2.511	2.584	2.524	2.425	1	1.146	1.124	CC:integral component of membrane;CC:cellular_component;CC:cellular anatomical entity;CC:intrinsic component of membrane;MF:cation binding;MF:monooxygenase activity;MF:heme binding;MF:transition metal ion binding;MF:tetrapyrrole binding;MF:organic cyclic compound binding;MF:oxidoreductase activity, acting on paired donors, with incorporation or reduction of molecular oxygen;MF:ion binding;MF:catalytic activity;MF:molecular_function;MF:binding;MF:iron ion binding;MF:metal ion binding;MF:heterocyclic compound	GO:0016021;GO:0005575;GO:0110165;GO:0031224;GO:0043169;GO:0004497;GO:0020037;GO:0046914;GO:0046906;GO:0097159;GO:0016705;GO:0043167;GO:0003824;GO:0003674;GO:0005488;GO:0005506;GO:0046872;GO:1901363;GO:0016491	K14999	CYP6	------	------	COG2124	Q:Secondary metabolites biosy	PF00067.25	p450	Cytochrome P450	CYT	6	12	57.2	High
TRINITY_DN668_c0_g1_i4_orf1	fatty acid synthase-like isoform X1 [Ostrinia furnacalis]	0.41097561	-1.282875318	7.16E-07	down	yes	1.011	2.46	2.495	2.409	2.477	1	1.012	1.022	------	------	K00665	FASN	map04910;ma	Insulin signaling	COG3321	Q:Secondary metabolites biosy	PF00698.24;PF0	Acyl_transf_1;	Acyl transferase dc	CYT	55	29	268.3	High
TRINITY_DN9920_c0_g1_i1_orf1	uncharacterized protein LOC114351526 [Ostrinia furnacalis]	0.403029028	-1.311044342	3.17E-05	down	yes	0.958	2.377	2.37	2.268	2.492	1	0.946	0.928	MF:catalytic activity;MF:transferase	GO:0003824;GO:0016740;GO:0003674	K00681	ggt	map00480;ma	Glutathione meta	COG0405	F:Nucleotide transport and met	PF01019.24	G_glu_transpe	Gamma-glutamylt	CYT	5	9	57.9	High
TRINITY_DN2880_c0_g1_i2_orf1	sialomucin core protein 24 [Pectinophora gossypiella]	0.439227731	-1.18695895	3.32E-06	down	yes	1.001	2.279	2.222	2.342	2.272	1	0.998	1.006	CC:cellular_component;CC:integral component of membrane;CC:cellular anatomical entity;CC:intrinsic component of membrane;	GO:0005575;GO:0016021;GO:0110165;GO:0031224	K06546	CD164, MGC2	map04142	Lysosome	ENOG410XVJX	U:Intracellular trafficking, secre	PF05283.14	MGC-24	Multi-glycosylated	CYT	3	11	22.5	High
TRINITY_DN14250_c0_g1_i1_orf1	apolipophorins-like [Ostrinia furnacalis]	0.410869565	-1.283247627	6.91E-06	down	yes	0.945	2.3	2.32	2.343	2.237	1	0.941	0.894	MF:lipid transporter activity;MF:transporter activity;MF:molecular_function;	GO:0005319;GO:0005215;GO:0003674	------	------	------	------	ENOG41112S0;ENOG410Z6MR	S:Function unknown;	------	------	------	CYT	2	22	9.7	High
TRINITY_DN10090_c0_g1_i1_orf1	clotting factor B-like isoform X1 [Ostrinia furnacalis] >XP_028163447.1 clotting factor B-like isoform X2 [Ostrinia furnacalis] >XP_028163448.1 clotting factor B-like isoform X3 [Ostrinia furnacalis]	0.456036446	-1.132778966	3.42E-06	down	yes	1.001	2.195	2.203	2.248	2.135	1	0.999	1.003	BP:organic substance metabolic process;BP:organonitrogen compound metabolic process;BP:protein metabolic process;BP:macromolecule metabolic process;BP:biological_process;BP:nitrogen compound metabolic process;BP:metabolic process;BP:proteolysis;BP:primary metabolic process;MF:serine-type endopeptidase activity;MF:endopeptidase activity;MF:hydrolase activity;MF:serine hydrolase activity;MF:catalytic activity, acting on a protein;MF:catalytic activity;MF:molecular_function;MF:peptidase activity;MF:serine-	GO:0071704;GO:1901564;GO:0019538;GO:0043170;GO:0008150;GO:0006807;GO:0008152;GO:0006508;GO:0044238;GO:0004252;GO:0004175;GO:0016787;GO:0017171;GO:0140096;GO:0003824;GO:0003674;GO:0008233;GO:0008236	K01324	KLKB1	map04610	Complement and	COG5640	F:Nucleotide transport and met	PF00089.29	Trypsin	Trypsin	PLA	9	28	45.8	High
TRINITY_DN585_c0_g1_i5_orf1	very low-density lipoprotein receptor isoform X2 [Galleria mellonella]	0.432714092	-1.208513991	6.24E-05	down	yes	0.955	2.207	2.207	2.304	2.109	1	0.998	0.867	CC:integral component of membrane;CC:cellular_component;CC:intrinsic component of membrane;CC:cellular anatomical entity;MF:binding;MF:cation binding;MF:cargo receptor activity;MF:very-low-density lipoprotein particle receptor activity;MF:lipoprotein particle receptor activity;MF:ion binding;MF:molecular_function;MF:metal ion	GO:0016021;GO:0005575;GO:0031224;GO:0110165;GO:0005488;GO:0043169;GO:0038024;GO:0030229;GO:0030228;GO:0043167;GO:0003674;GO:0046872;GO:0005509	K20053	VLDLR	map05417;ma	Lipid and atheros	ENOG410YQ6J;ENOG410YEXK	U:Intracellular trafficking, secre	PF00057.21;PF0	Ldl_recept_a;l	Low-density lipopr	PLA	2	33	94.4	High
TRINITY_DN30498_c0_g1_i3_orf1	lipase 3-like [Ostrinia furnacalis]	0.483703354	-1.047805554	0.0003854	down	yes	1.024	2.117	2.162	2.024	2.164	1	0.885	1.186	------	------	K01052	LIPA	map04979;ma	Cholesterol meta	COG0596	S:Function unknown	PF00561.23;PF0	Abhydrolase_	alpha/beta hydrola	CYT	1	3	56.9	High
TRINITY_DN33346_c0_g1_i1_orf1	PREDICTED: U6 snRNA-associated Sm-like protein LSm3 [Papilio xuthus] >XP_028165558.1 U6 snRNA-associated Sm-like protein LSm3 [Ostrinia furnacalis] >KOB73597.1 LSM Sm-like protein family member [Operophtera brumata] >RVE45517.1 hypothetical protein evm_009856 [Chilo suppressalis] >CAB3523639.1 unnamed protein product [Chilo suppressalis] >CAH0400961.1 unnamed protein product [Chilo suppressalis]	0.42520436	-1.233771706	4.10E-05	down	yes	0.9363	2.202	2.158	2.301	2.147	1	0.952	0.857	BP:primary metabolic process;BP:cellular metabolic process;BP:nucleic acid metabolic process;BP:cellular nitrogen compound metabolic process;BP:nitrogen compound metabolic process;BP:organic cyclic compound metabolic process;BP:mRNA metabolic process;BP:organic substance metabolic process;BP:RNA splicing;BP:nucleobase-containing compound metabolic process;BP:cellular process;BP:cellular aromatic compound metabolic process;BP:RNA splicing, via transesterification reactions;BP:RNA splicing, via transesterification reactions with bulged adenosine as nucleophile;BP:biological_process;BP:metabolic process;BP:heterocycle metabolic process;BP:RNA metabolic process;BP:mRNA splicing, via spliceosome;BP:macromolecule metabolic process;BP:RNA processing;BP:mRNA processing;CC:Sm-like protein family complex;CC:spliceosomal complex;CC:U4/U6 x U5 tri-snRNP complex;CC:spliceosomal snRNP complex;CC:macromolecular complex;CC:small nuclear ribonucleoprotein complex;CC:ribonucleoprotein complex;CC:cellular_component;CC:spliceosomal tri-snRNP complex;CC:U6 snRNP;MF:molecular_function;MF:binding;MF:nucleic acid binding;MF:heterocyclic compound binding;MF:organic cyclic compound binding;MF:RNA binding;	GO:0044238;GO:0044237;GO:0090304;GO:0034641;GO:0006807;GO:1901360;GO:0016071;GO:0071704;GO:0008380;GO:0006139;GO:0009987;GO:0006725;GO:0000375;GO:0000377;GO:0008150;GO:0008152;GO:0046483;GO:0016070;GO:0000398;GO:0043170;GO:0006396;GO:0006397;GO:0120114;GO:0005681;GO:0046540;GO:0097525;GO:0032991;GO:0030532;GO:1990904;GO:0005575;GO:0097526;GO:0005688;GO:0003674;GO:0005488;GO:0003676;GO:1901363;GO:0097159;GO:0003723	K12622	LSM3	map03018;ma	RNA degradation	ENOG4111PB8	K:Transcription	PF01423.25	LSM	LSM domain	CYT	1	14	11.7	Medium
TRINITY_DN9282_c0_g1_i2_orf1	uncharacterized protein LOC114363102 isoform X2 [Ostrinia furnacalis]	0.177405615	-2.494876425	7.00E-07	down	yes	0.9163	5.165	5.158	5.07	5.268	1	0.816	0.933	------	------	------	------	------	------	ENOG41120R5	S:Function unknown	------	------	------	CYT	1	10	15.1	High
TRINITY_DN121650_c0_g1_i1_orf1	carboxylesterase [Ostrinia furnacalis]	0.201210505	-2.313222468	7.40E-08	down	yes	0.9807	4.874	4.906	4.919	4.798	1	0.952	0.99	CC:integral component of membrane;CC:cellular_component;CC:intrinsic component of membrane;CC:cellular anatomical entity;MF:hydrolase activity;MF:hydrolase activity, acting on ester bonds;MF:carboxylic ester hydrolase activity;MF:catalytic	GO:0016021;GO:0005575;GO:0031224;GO:0110165;GO:0016787;GO:0016788;GO:0052689;GO:0003824;GO:0003674	------	------	------	------	COG2272	I:Lipid transport and metabolis	------	------	------	CYT	1	19	7.6	High
TRINITY_DN31_c0_g1_i3_orfp1	TRINITY_DN31_c0_g1_i3_m.1394 TRINITY_DN31_c0_g1::TRINITY_DN31_c0_g1_i3::g.1394 ORF type:complete len:118 (+),score=43.35 TRINITY_DN31_c0_g1_i3:56-409(+)	0.201425278	-2.31168335	1.33E-06	down	yes	0.961	4.771	4.916	4.655	4.741	1	0.9	0.983	------	------	------	------	------	------	------	------	------	------	------	CYT	2	15	12.6	High
TRINITY_DN106156_c1_g1_i1_orf1	arylphorin subunit alpha-like [Ostrinia furnacalis]	0.236890171	-2.077709757	3.61E-06	down	yes	0.9857	4.161	4.17	4.005	4.308	1	0.96	0.997	------	------	------	------	------	------	ENOG410XR2D	S:Function unknown	PF03723.17	Hemocyanin_	Hemocyanin, ig-lik	CYT	12	59	13.5	High
TRINITY_DN56308_c0_g1_i2_orf1	storage protein 1 [Omphisa fuscidentalis]	0.271475143	-1.88110799	2.12E-05	down	yes	0.9993	3.681	3.661	3.492	3.891	1	0.972	1.026	CC:extracellular space;CC:cellular_component;CC:extracellular region;CC:cellular anatomical entity;MF:molecular_function;MF:nutrient reservoir activity;	GO:0005615;GO:0005575;GO:0005576;GO:0110165;GO:0003674;GO:0045735	------	------	------	------	ENOG410XR2D	S:Function unknown	PF03722.17	Hemocyanin_	Hemocyanin, all-a	CYT	9	60	11.3	High
TRINITY_DN2896_c0_g1_i2_orf1	general odorant-binding protein 56d-like isoform X2 [Ostrinia furnacalis]	0.293914047	-1.766533785	3.90E-05	down	yes	1.019	3.467	3.481	3.674	3.246	1	1.022	1.034	CC:integral component of membrane;CC:cellular anatomical entity;CC:cellular_component;CC:intrinsic component of membrane;MF:odorant	GO:0016021;GO:0110165;GO:0005575;GO:0031224;GO:0005549;GO:0003674;GO:0005488	------	------	------	------	ENOG410ZCHW;ENOG4110S21	S:Function unknown;	PF01395.25	PBP_GOBP	PBP/GOBP family	CYT	7	46	15	High
TRINITY_DN5664_c0_g1_i1_orf1	CDGSH iron-sulfur domain-containing protein 3, mitochondrial-like [Ostrinia furnacalis]	0.251912723	-1.989004106	3.97E-06	down	yes	0.889	3.529	3.621	3.511	3.454	1	0.858	0.809	CC:cytoplasm;CC:cellular_component;CC:intracellular organelle;CC:membrane-bounded organelle;CC:organelle;CC:cellular anatomical entity;CC:intracellular membrane-bounded organelle;MF:cation binding;MF:metal cluster binding;MF:iron-sulfur cluster binding;MF:2 iron, 2 sulfur cluster binding;MF:ion binding;MF:molecular_function;MF:binding;MF:metal ion	GO:0005737;GO:0005575;GO:0043229;GO:0043227;GO:0043226;GO:0110165;GO:0043231;GO:0043169;GO:0051540;GO:0051536;GO:0051537;GO:0043167;GO:0003674;GO:0005488;GO:0046872	K23886	CISD3	------	------	ENOG41122TT	S:Function unknown	PF09360.13	zf-CDGSH	Iron-binding zinc f	CYT	1	12	15.7	High
TRINITY_DN54366_c0_g1_i1_orf1	protein obstructor-E-like [Ostrinia furnacalis]	0.329864396	-1.600055026	9.77E-06	down	yes	1.046	3.171	3.04	3.29	3.184	1	1.071	1.068	CC:cellular_component;CC:extracellular region;CC:cellular anatomical entity;MF:carbohydrate derivative binding;MF:molecular_function;MF:chitin binding;MF:binding;	GO:0005575;GO:0005576;GO:0110165;GO:0097367;GO:0003674;GO:0008061;GO:0005488	------	------	------	------	ENOG4111EK0	S:Function unknown	PF01607.27	CBM_14	Chitin binding Peri	PLA	6	28	26.9	High
TRINITY_DN124654_c0_g1_i1_orf1	protein lethal(2)essential for life [Manduca sexta] >KAG6441919.1 hypothetical protein O3G_MSEX002019 [Manduca sexta]	0.317180617	-1.656623486	4.40E-07	down	yes	1.008	3.178	3.122	3.232	3.181	1	0.984	1.039	------	------	K09542	CRYAB	map04213;ma	Longevity regulat	ENOG410YERS	P:Inorganic ion transport and n	PF00011.24	HSP20	Hsp20/alpha cryst	CYT	6	44	22.4	High
TRINITY_DN17574_c0_g1_i2_orf1	heat shock protein Hsp-12.2-like [Ostrinia furnacalis]	0.309294266	-1.692948008	1.39E-07	down	yes	0.9817	3.174	3.218	3.174	3.13	1	0.975	0.97	------	------	K09542	CRYAB	map04213;ma	Longevity regulat	ENOG410YERS	Q:Secondary metabolites biosy	PF00011.24	HSP20	Hsp20/alpha cryst	CYT	7	51	19.4	High
TRINITY_DN7618_c0_g1_i4_orf1	uncharacterized protein LOC114366712 isoform X1 [Ostrinia furnacalis]	0.348425197	-1.521079137	0.0002275	down	yes	1.062	3.048	2.838	3.313	2.993	1	1.206	0.979	------	------	------	------	------	------	ENOG4111M89;COG2866	S:Function unknown;	------	------	------	CYT	1	4	36.6	High
TRINITY_DN858_c0_g1_i3_orf1	uncharacterized protein LOC114351944 [Ostrinia furnacalis]	0.341776427	-1.5488752	6.40E-05	down	yes	1.012	2.961	3.076	3.069	2.737	1	1.02	1.015	CC:cellular_component;CC:extracellular space;CC:cellular anatomical entity;	GO:0005575;GO:0005615;GO:0110165	------	------	------	------	------	------	PF16077.8	Spaetzle	Spaetzle	CYT	2	9	20.2	High
TRINITY_DN3166_c1_g1_i6_orf1	hypothetical protein evm_013813 [Chilo suppressalis]	0.337546848	-1.566840346	5.33E-05	down	yes	0.9907	2.935	2.99	2.731	3.083	1	0.958	1.014	BP:response to biotic stimulus;BP:response to external stimulus;BP:response to external biotic stimulus;BP:response to stimulus;BP:defense response;BP:response to stress;BP:biological_process;BP:interspecies interaction between organisms;BP:response to other organism;CC:cellular_component;CC:extracellular region;CC:cellular anatomical entity;	GO:0009607;GO:0009605;GO:0043207;GO:0050896;GO:0006952;GO:0006950;GO:0008150;GO:0044419;GO:0051707;GO:0005575;GO:0005576;GO:0110165	------	------	------	------	------	------	PF00451.22	Toxin_2	Scorpion short toxi	CYT	1	13	10.5	High
TRINITY_DN104663_c1_g1_i2_orf1	PREDICTED: gelsolin-like [Amyelois transitella]	0.359127682	-1.477431233	0.0002573	down	yes	1.021	2.843	2.753	2.642	3.133	1	1.027	1.036	MF:cytoskeletal protein binding;MF:actin filament binding;MF:actin binding;MF:macromolecular complex binding;MF:binding;MF:protein binding;MF:molecular_function;	GO:0008092;GO:0051015;GO:0003779;GO:0044877;GO:0005488;GO:0005515;GO:0003674	K05768	GSN	map04666;ma	Fc gamma R-me	ENOG410XR0A	S:Function unknown	------	------	------	CYT	1	53	12.4	High
TRINITY_DN5420_c0_g1_i2_orf1	DNA-directed RNA polymerase II subunit RPB1-like [Ostrinia furnacalis]	0.42791411	-1.224606842	0.0008226	down	yes	1.116	2.608	2.613	2.37	2.842	1	1.05	1.299	------	------	------	------	------	------	------	------	------	------	------	EXC	1	2	58	High
TRINITY_DN136358_c0_g1_i1_orf1	acidic juvenile hormone-suppressible protein 1-like [Ostrinia furnacalis]	0.389882084	-1.358890232	7.03E-08	down	yes	1.025	2.629	2.611	2.637	2.638	1	1.026	1.049	------	------	------	------	------	------	ENOG410XR2D	S:Function unknown	PF03723.17	Hemocyanin_	Hemocyanin, ig-lik	CYT	2	55	9.7	High

TRINITY_DN2097_c1_g2_i2_orf1	serine protease inhibitor 3 [Ostrinia furnacalis]	0.391421947	-1.353203443	3.17E-07	down	yes	1.013	2.588	2.579	2.554	2.631	1	1.013	1.026	CC:cellular_component;CC:cellular anatomical entity;CC:extracellular space;MF:endopeptidase inhibitor activity;MF:endopeptidase regulator activity;MF:peptidase regulator activity;MF:molecular_function;MF:peptidase inhibitor activity;MF:serine-type endopeptidase inhibitor activity;MF:enzyme inhibitor activity;MF:molecular function regulator;MF:enzyme regulator activity;	GO:0005575;GO:0110165;GO:0005615;GO:0004866;GO:0061135;GO:0061134;GO:0003674;GO:0030414;GO:0004867;GO:0004857;GO:0098772;GO:0030234	------	------	------	------	COG4826	O:Posttranslational modificatio	PF00079.23	Serpin	Serpin	CYT	12	30	50.5	High
TRINITY_DN38431_c0_g1_i1_orf1	neprilysin-2 isoform X1 [Ostrinia furnacalis]	0.397648466	-1.330434491	1.62E-06	down	yes	0.9977	2.509	2.574	2.472	2.48	1	0.978	1.015	BP:nitrogen compound metabolic process;BP:organic substance metabolic process;BP:proteolysis;BP:biological_process;BP:metabolic process;BP:primary metabolic process;BP:organonitrogen compound metabolic process;BP:protein metabolic process;BP:macromolecule metabolic process;CC:integral component of membrane;CC:membrane;CC:intrinsic component of membrane;CC:cellular anatomical entity;CC:plasma membrane;CC:cellular_component;MF:endopeptidase activity;MF:molecular_function;MF:binding;MF:peptidase activity;MF:metallopeptidase activity;MF:cation binding;MF:ion binding;MF:metal ion binding;MF:hydrolase	GO:0006807;GO:0071704;GO:0006508;GO:0008150;GO:0008152;GO:0044238;GO:1901564;GO:0019538;GO:0043170;GO:0016021;GO:0016020;GO:0031224;GO:0110165;GO:0005886;GO:0005575;GO:0004175;GO:0003674;GO:0005488;GO:0008233;GO:0008237;GO:0043169;GO:0043167;GO:0046872;GO:0016787;GO:0004222;GO:0140096;GO:0003824	K08635	MMEL1, NEP	------	------	COG3590	O:Posttranslational modificatio	PF05649.16;PF0[illegible]	Peptidase_M1	Peptidase family M	CYT	10	16	87.5	High
TRINITY_DN2406_c0_g1_i6_orf1	uncharacterized protein LOC114361672 [Ostrinia furnacalis]	0.474061577	-1.076853627	0.0008502	down	yes	1.124	2.371	2.597	2.278	2.239	1	1.098	1.274	------	------	------	------	------	------	ENOG4110RST	S:Function unknown	------	------	------	CYT	2	4	61.1	High
TRINITY_DN5757_c0_g1_i1_orf1	ATP-dependent DNA helicase 2 subunit 1 [Ostrinia furnacalis]	0.410194175	-1.285621091	0.004381	down	yes	1.014	2.472	2.133	2.329	2.953	1	0.947	1.096	BP:nucleic acid metabolic process;BP:cellular nitrogen compound metabolic process;BP:nitrogen compound metabolic process;BP:organic cyclic compound metabolic process;BP:nucleobase-containing compound metabolic process;BP:cellular response to stimulus;BP:double-strand break repair via nonhomologous end joining;BP:double-strand break repair;BP:cellular macromolecule metabolic process;BP:cellular component organization;BP:organic substance metabolic process;BP:cellular component organization or biogenesis;BP:telomere maintenance;BP:DNA repair;BP:cellular process;BP:cellular aromatic compound metabolic process;BP:cellular response to DNA damage stimulus;BP:response to stress;BP:biological_process;BP:metabolic process;BP:DNA recombination;BP:heterocycle metabolic process;BP:organelle organization;BP:primary metabolic process;BP:chromosome organization;BP:response to stimulus;BP:telomere organization;BP:cellular metabolic process;BP:macromolecule metabolic process;BP:cellular response to stress;BP:DNA metabolic process;CC:Ku7;CC:macromolecular complex;CC:cellular_component;MF:hydrolase activity;MF:purine ribonucleoside triphosphate binding;MF:small molecule binding;MF:molecular_function;MF:binding;MF:nucleic acid binding;MF:DNA binding;MF:DNA helicase activity;MF:ion binding;MF:heterocyclic compound binding;MF:sequence-specific DNA binding;MF:nucleotide binding;MF:purine ribonucleotide binding;MF:purine nucleotide binding;MF:DNA-dependent ATPase activity;MF:ATP binding;MF:telomeric DNA binding;MF:catalytic activity, acting on DNA;MF:nucleoside [illegible]	GO:0090304;GO:0034641;GO:0006807;GO:1901360;GO:0006139;GO:0051716;GO:0006303;GO:0006302;GO:0044260;GO:0016043;GO:0071704;GO:0071840;GO:0000723;GO:0006281;GO:0009987;GO:0006725;GO:0006974;GO:0006950;GO:0008150;GO:0008152;GO:0006310;GO:0046483;GO:0006996;GO:0044238;GO:0051276;GO:0050896;GO:0032200;GO:0044237;GO:0043170;GO:0033554;GO:0006259;GO:0043564;GO:0032991;GO:0005575;GO:0016787;GO:0035639;GO:0036094;GO:0003674;GO:0005488;GO:0003676;GO:0003677;GO:0003678;GO:0043167;GO:1901363;GO:0043565;GO:0000166;GO:0032555;GO:0017076;GO:0008094;GO:0005524;GO:0042162;GO:0140097;GO:1901265;GO:0004386;GO:0003824;GO:0030554;GO:0097367;GO:0097159;GO:0043168;GO:0032559;GO:0003684;GO:0032553	K10884	XRCC6, KU70	map03450	Non-homologou	ENOG410XNXU	L:Replication, recombination ar	PF02735.19;PF0[illegible]	Ku;Ku_N;Ku_C	Ku70/Ku80 beta-b	CYT	1	2	66.8	High
TRINITY_DN1196_c0_g1_i4_orf1	glucosamine-6-phosphate isomerase isoform X2 [Ostrinia furnacalis]	0.482173913	-1.052374496	0.0004397	down	yes	1.109	2.3	2.495	2.195	2.21	1	1.167	1.16	------	------	K02564	nagB, GNPDA	map00520	Amino sugar and	COG0363	S:Function unknown	PF01182.23	Glucosamine	Glucosamine-6-ph	CYT	1	45	30.4	High
TRINITY_DN8985_c0_g1_i4_orf1	cytochrome P450 6B6-like [Ostrinia furnacalis]	0.44893617	-1.155417758	1.20E-05	down	yes	1.055	2.35	2.298	2.349	2.404	1	1.037	1.128	MF:cation binding;MF:monooxygenase activity;MF:heme binding;MF:transition metal ion binding;MF:tetrapyrrole binding;MF:organic cyclic compound binding;MF:oxidoreductase activity, acting on paired donors, with incorporation or reduction of molecular oxygen;MF:ion binding;MF:catalytic activity;MF:molecular_function;MF:binding;MF:iron ion binding;MF:metal ion binding;MF:heterocyclic compound	GO:0043169;GO:0004497;GO:0020037;GO:0046914;GO:0046906;GO:0097159;GO:0016705;GO:0043167;GO:0003824;GO:0003674;GO:0005488;GO:0005506;GO:0046872;GO:1901363;GO:0016491	K14999	CYP6	------	------	COG2124	Q:Secondary metabolites biosy	PF00067.25	p450	Cytochrome P450	CYT	3	8	59.5	High
TRINITY_DN114982_c0_g1_i1_orf1	uncharacterized protein LOC114357708 [Ostrinia furnacalis]	0.402289759	-1.313693084	0.001935	down	yes	0.9663	2.402	2.678	2.019	2.508	1	0.938	0.961	CC:cellular_component;CC:ribosome;CC:intracellular non-membrane-bounded organelle;CC:mitochondrion;CC:intracellular organelle;CC:non-membrane-bounded organelle;CC:membrane-bounded organelle;CC:organelle;CC:cellular anatomical entity;CC:intracellular membrane-bounded organelle;MF:structural molecule activity;MF:structural	GO:0005575;GO:0005840;GO:0043232;GO:0005739;GO:0043229;GO:0043228;GO:0043227;GO:0043226;GO:0110165;GO:0043231;GO:0005198;GO:0003735;GO:0003674	K17412	MRPS34	------	------	ENOG4111Y87	S:Function unknown	PF16053.8	MRP-S34	Mitochondrial 28S	CYT	2	13	22.6	High
TRINITY_DN1421_c0_g1_i1_orf1	uncharacterized protein LOC114352615 [Ostrinia furnacalis]	0.423793395	-1.238566991	1.69E-07	down	yes	1.001	2.362	2.347	2.393	2.346	1	0.987	1.016	BP:nitrogen compound metabolic process;BP:organic substance metabolic process;BP:proteolysis;BP:biological_process;BP:metabolic process;BP:primary metabolic process;BP:organonitrogen compound metabolic process;BP:protein metabolic process;BP:macromolecule metabolic process;CC:cytoplasm;CC:cellular anatomical entity;CC:cellular_component;CC:extracellular region;MF:serine-type endopeptidase activity;MF:endopeptidase activity;MF:molecular_function;MF:binding;MF:peptidase activity;MF:serine-type peptidase activity;MF:cation binding;MF:ion binding;MF:metal ion binding;MF:hydrolase activity;MF:serine hydrolase activity;MF:catalytic activity, acting	GO:0006807;GO:0071704;GO:0006508;GO:0008150;GO:0008152;GO:0044238;GO:1901564;GO:0019538;GO:0043170;GO:0005737;GO:0110165;GO:0005575;GO:0005576;GO:0004252;GO:0004175;GO:0003674;GO:0005488;GO:0008233;GO:0008236;GO:0043169;GO:0043167;GO:0046872;GO:0016787;GO:0017171;GO:0140096;GO:0003824	------	------	------	------	COG5640	U:Intracellular trafficking, secre	PF00089.29;PF1[illegible]	Trypsin;CLIP	Trypsin;Regulatory	CYT	7	22	44.4	High
TRINITY_DN1507_c0_g1_i5_orf1	27 kDa hemolymph protein-like, partial [Ostrinia furnacalis]	0.471334276	-1.085177494	0.0003807	down	yes	0.9997	2.121	2.126	2.294	1.944	1	1.015	0.984	------	------	------	------	------	------	ENOG4111POK	S:Function unknown	PF07165.14	DUF1397	Protein of unknow	CYT	6	23	31.9	High
TRINITY_DN3190_c0_g1_i1_orf1	repetitive proline-rich cell wall protein 1 precursor [Papilio polytes] >XP_013147838.1 PREDICTED: repetitive proline-rich cell wall protein 1 [Papilio polytes] >BAM19190.1 cuticular protein PpolCPG24 [Papilio polytes]	0.098635478	-3.341749536	2.30E-06	down	yes	1.012	10.26	10.242	10.661	9.866	1	0.988	1.047	------	------	------	------	------	------	ENOG4111M7J	S:Function unknown	------	------	------	CYT	17	44	44	High
TRINITY_DN5408_c0_g1_i5_orf1	uncharacterized protein LOC114359912 [Ostrinia furnacalis]	0.150413223	-2.732996692	0.0001745	down	yes	1.001	6.655	6.785	7.3	5.879	1	0.907	1.097	CC:cellular_component;CC:integral component of membrane;CC:cellular anatomical entity;CC:intrinsic component of membrane;	GO:0005575;GO:0016021;GO:0110165;GO:0031224	------	------	------	------	ENOG4110RME	S:Function unknown	------	------	------	PLA	1	5	30.4	High
TRINITY_DN5829_c0_g1_i1_orf1	uncharacterized protein LOC114365758 isoform X3 [Ostrinia furnacalis]	0.165294592	-2.596888567	2.31E-07	down	yes	1.024	6.195	6.162	6.33	6.094	1	1.012	1.06	------	------	------	------	------	------	ENOG410XT4C	S:Function unknown	PF00059.24	Lectin_C	Lectin C-type dom	PLA	5	39	23.4	High
TRINITY_DN18338_c0_g1_i6_orf1	aquaporin AQPAn.G isoform X1 [Ostrinia furnacalis]	0.173486642	-2.527103513	4.24E-05	down	yes	1.026	5.914	6.357	5.495	5.89	1	1.113	0.965	CC:integral component of membrane;CC:cellular_component;CC:intrinsic component of membrane;CC:cellular anatomical entity;MF:transporter activity;MF:transmembrane transporter activity;MF:channel activity;MF:molecular_function;MF:passive transmembrane transporter activity;	GO:0016021;GO:0005575;GO:0031224;GO:0110165;GO:0005215;GO:0022857;GO:0015267;GO:0003674;GO:0022803	K09884	AQPN	------	------	COG0580	O:Posttranslational modificatio	PF00230.23	MIP	Major intrinsic pro	CYT	1	8	29	High
TRINITY_DN19043_c0_g2_i1_orf1	hypothetical protein EVAR_60653_1 [Eumeta japonica]	0.191585274	-2.383941419	2.23E-05	down	yes	1.02	5.324	5.314	5.657	5.002	1	1.014	1.047	------	------	------	------	------	------	ENOG410YRTV	S:Function unknown	------	------	------	PLA	6	32	21	High
TRINITY_DN59885_c0_g1_i3_orf1	TGF-beta-activated kinase 1 and MAP3K7-binding protein 1-like [Ostrinia furnacalis]	0.192771084	-2.375039431	1.75E-06	down	yes	1.008	5.229	5.116	5.157	5.414	1	1.065	0.959	BP:dephosphorylation;BP:protein dephosphorylation;BP:nitrogen compound metabolic process;BP:organic substance metabolic process;BP:cellular process;BP:macromolecule modification;BP:protein modification process;BP:biological_process;BP:metabolic process;BP:primary metabolic process;BP:organonitrogen compound metabolic process;BP:protein metabolic process;BP:cellular metabolic process;BP:macromolecule metabolic process;BP:phosphate-containing compound metabolic process;BP:phosphorus metabolic process;MF:phosphatase activity;MF:phosphoric ester hydrolase activity;MF:molecular_function;MF:phosphoprotein phosphatase activity;MF:hydrolase activity;MF:catalytic activity, acting on a protein;MF:hydrolase activity, acting on ester bonds;MF:catalytic activity;MF:protein serine/threonine	GO:0016311;GO:0006470;GO:0006807;GO:0071704;GO:0009987;GO:0043412;GO:0036211;GO:0008150;GO:0008152;GO:0044238;GO:1901564;GO:0019538;GO:0044237;GO:0043170;GO:0006796;GO:0006793;GO:0016791;GO:0042578;GO:0003674;GO:0004721;GO:0016787;GO:0140096;GO:0016788;GO:0003824;GO:0004722	K04403	MAP3K7IP1, T	map05140;ma	Leishmaniasis;Hu	COG0631	S:Function unknown	PF00481.24	PP2C	Protein phosphata	CYT	1	3	51	High
TRINITY_DN45948_c1_g1_i1_orf1	unnamed protein product [Leptidea sinapis]	0.207027351	-2.272106717	0.00011	down	yes	1.037	5.009	4.77	5.53	4.727	1	1.032	1.079	BP:metabolic process;BP:nitrogen compound metabolic process;BP:organic substance metabolic process;BP:proteolysis;BP:biological_process;BP:primary metabolic process;BP:organonitrogen compound metabolic process;BP:protein metabolic process;BP:macromolecule metabolic process;MF:endopeptidase activity;MF:peptidase inhibitor activity;MF:molecular_function;MF:enzyme inhibitor activity;MF:molecular function regulator;MF:peptidase activity;MF:cysteine-type peptidase activity;MF:enzyme regulator activity;MF:hydrolase activity;MF:endopeptidase regulator activity;MF:peptidase regulator activity;MF:catalytic activity, acting on a protein;MF:catalytic activity;MF:endopeptidase inhibitor activity;MF:cysteine-type endopeptidase inhibitor activity;MF:cysteine-type	GO:0008152;GO:0006807;GO:0071704;GO:0006508;GO:0008150;GO:0044238;GO:1901564;GO:0019538;GO:0043170;GO:0004175;GO:0030414;GO:0003674;GO:0004857;GO:0098772;GO:0008233;GO:0008234;GO:0030234;GO:0016787;GO:0061135;GO:0061134;GO:0140096;GO:0003824;GO:0004866;GO:0004869;GO:0004197	K01373	CTSF	map04210;ma	Apoptosis;Lysosc	COG4870	O:Posttranslational modificatio	PF00112.26	Peptidase_C1	Papain family cyste	CYT	2	68	3.7	High
TRINITY_DN13686_c0_g2_i1_orf1	transmembrane protease serine 9-like [Ostrinia furnacalis]	0.219525127	-2.187542015	1.13E-06	down	yes	0.9523	4.338	4.372	4.394	4.249	1	0.844	1.013	BP:organic substance metabolic process;BP:organonitrogen compound metabolic process;BP:protein metabolic process;BP:macromolecule metabolic process;BP:biological_process;BP:nitrogen compound metabolic process;BP:metabolic process;BP:proteolysis;BP:primary metabolic process;MF:serine-type endopeptidase activity;MF:endopeptidase activity;MF:hydrolase activity;MF:serine hydrolase activity;MF:catalytic activity, acting on a protein;MF:catalytic activity;MF:molecular_function;MF:peptidase activity;MF:serine-	GO:0071704;GO:1901564;GO:0019538;GO:0043170;GO:0008150;GO:0006807;GO:0008152;GO:0006508;GO:0044238;GO:0004252;GO:0004175;GO:0016787;GO:0017171;GO:0140096;GO:0003824;GO:0003674;GO:0008233;GO:0008236	K20677;K09[illegible]	HAYAN;TMPR	map05164	Influenza A	COG5640	O:Posttranslational modificatio	PF00089.29	Trypsin	Trypsin	CYT	2	6	43.9	High
TRINITY_DN85412_c0_g1_i1_orf1	unnamed protein product [Diatraea saccharalis]	0.246544916	-2.020077591	2.26E-07	down	yes	0.999	4.052	4.089	4.099	3.967	1	0.993	1.004	MF:lipid transporter activity;MF:transporter activity;MF:molecular_function;	GO:0005319;GO:0005215;GO:0003674	------	------	------	------	ENOG410Z6MR;ENOG41112S0	S:Function unknown;	------	------	------	CYT	11	87	12	High
TRINITY_DN28711_c0_g1_i1_orf1	hypothetical protein evm_000299 [Chilo suppressalis]	0.24599451	-2.023301979	1.30E-06	down	yes	0.9857	4.007	3.926	3.962	4.132	1	0.956	1.001	MF:lipid transporter activity;MF:transporter activity;MF:molecular_function;	GO:0005319;GO:0005215;GO:0003674	------	------	------	------	ENOG410Z6MR;ENOG410ZRUY	S:Function unknown;	------	------	------	CYT	7	83	7.5	High
TRINITY_DN5907_c0_g1_i4_orf1	hypothetical protein evm_010265 [Chilo suppressalis] >CAB3524755.1 unnamed protein product [Chilo suppressalis] >CAH0397522.1 unnamed protein product [Chilo suppressalis]	0.261813538	-1.933388398	1.68E-07	down	yes	1.025	3.915	3.893	3.869	3.983	1	1.028	1.048	------	------	------	------	------	------	ENOG410YTKH;ENOG410ZN7P;ENOG41119GI;ENOG410ZNC7;ENOG4110RJ7;ENOG4111E83;ENOG41122YZ	S:Function unknown;	PF06585.14	JHBP	Haemolymph juve	CYT	5	23	28.1	High
TRINITY_DN12582_c0_g1_i5_orf1	uncharacterized protein LOC114355527 isoform X1 [Ostrinia furnacalis]	0.288200109	-1.794857215	0.007949	down	yes	1.06	3.678	4.101	4.309	2.624	1	1.025	1.154	MF:hydrolase activity;MF:hydrolase activity, acting on acid anhydrides, in phosphorus-containing anhydrides;MF:catalytic activity;MF:hydrolase activity, acting on acid anhydrides;MF:pyrophosphatase activity;MF:molecular_function;MF:nucleoside-triphosphate diphosphatase activity;	GO:0016787;GO:0016818;GO:0003824;GO:0016817;GO:0016462;GO:0003674;GO:0047429	------	------	------	------	COG0424	D:Cell cycle control, cell divisio	PF02545.17	Maf	Maf-like protein	CYT	1	5	22.9	High
TRINITY_DN8158_c0_g1_i2_orf1	hypothetical protein G9C98_000136 [Cotesia typhae]	0.275249125	-1.861190119	0.0001616	down	yes	1.022	3.713	4.103	3.541	3.496	1	1.036	1.03	------	------	------	------	------	------	ENOG410XR2D	S:Function unknown	PF00372.22;PF0[illegible]	Hemocyanin	Hemocyanin, copp	CYT	2	1	88	High
TRINITY_DN20793_c0_g2_i1_orf1	mucin-2-like [Ostrinia furnacalis]	0.290512175	-1.78332947	3.41E-06	down	yes	1.038	3.573	3.644	3.632	3.444	1	1.087	1.027	------	------	------	------	------	------	ENOG41115RA	S:Function unknown	------	------	------	EXC	4	9	52.5	High
TRINITY_DN1540_c0_g1_i7_orf1	alaserpin-like isoform X13 [Ostrinia furnacalis]	0.298667439	-1.743388132	1.93E-05	down	yes	1.031	3.452	3.26	3.507	3.588	1	1.089	1.005	CC:cellular_component;CC:cellular anatomical entity;CC:extracellular space;MF:endopeptidase inhibitor activity;MF:endopeptidase regulator activity;MF:peptidase regulator activity;MF:molecular_function;MF:peptidase inhibitor activity;MF:serine-type endopeptidase inhibitor activity;MF:enzyme inhibitor activity;MF:molecular function regulator;MF:enzyme regulator activity;	GO:0005575;GO:0110165;GO:0005615;GO:0004866;GO:0061135;GO:0061134;GO:0003674;GO:0030414;GO:0004867;GO:0004857;GO:0098772;GO:0030234	------	------	------	------	COG4826	O:Posttranslational modificatio	PF00079.23	Serpin	Serpin	CYT	1	35	42.9	High
TRINITY_DN26149_c0_g1_i5_orf1	thymosin beta isoform X3 [Ostrinia furnacalis]	0.292632815	-1.772836538	1.02E-07	down	yes	0.997	3.407	3.434	3.414	3.372	1	1.031	0.96	------	------	------	------	------	------	ENOG410Y3I4	O:Posttranslational modificatio	PF01290.23	Thymosin	Thymosin beta-4 f	CYT	1	35	10.4	High

TRINITY_DN2515_c0_g1_i6_orf1	chitooligosaccharidolytic beta-N-acetylglucosaminidase isoform X1 [Ostrinia furnacalis]	0.324786325	-1.622437206	6.55E-06	down	yes	1.064	3.276	3.334	3.346	3.148	1	1.101	1.09	BP:amino sugar catabolic process;BP:nitrogen compound metabolic process;BP:aminoglycan metabolic process;BP:organic substance catabolic process;BP:organic substance metabolic process;BP:chitin metabolic process;BP:chitin catabolic process;BP:polysaccharide catabolic process;BP:biological_process;BP:metabolic process;BP:carbohydrate metabolic process;BP:catabolic process;BP:macromolecule catabolic process;BP:primary metabolic process;BP:organonitrogen compound metabolic process;BP:organonitrogen compound catabolic process;BP:polysaccharide metabolic process;BP:amino sugar metabolic process;BP:carbohydrate derivative catabolic process;BP:carbohydrate derivative metabolic process;BP:carbohydrate catabolic process;BP:macromolecule metabolic process;BP:glucosamine-containing compound catabolic process;BP:glucosamine-containing compound metabolic process;BP:aminoglycan catabolic process;MF:hydrolase activity, acting on glycosyl bonds;MF:molecular_function;MF:beta-N-acetylhexosaminidase activity;MF:hydrolase activity;MF:N-acetyl-beta-D-galactosaminidase activity;MF:hexosaminidase	GO:0046348;GO:0006807;GO:0006022;GO:1901575;GO:0071704;GO:0006030;GO:0006032;GO:0000272;GO:0008150;GO:0008152;GO:0005975;GO:0009056;GO:0009057;GO:0044238;GO:1901564;GO:1901565;GO:0005976;GO:0006040;GO:1901136;GO:1901135;GO:0016052;GO:0043170;GO:1901072;GO:1901071;GO:0006026;GO:0016798;GO:0003674;GO:0004563;GO:0016787;GO:0102148;GO:0015929;GO:0003824;GO:0004553	K12373	HEXA_B	map00600;maj	Sphingolipid met	COG3525	G:Carbohydrate transport and r	PF00728.25;PF1	Glyco_hydro_	Glycosyl hydrolase	CYT	16	35	70.4	High
TRINITY_DN25976_c0_g1_i4_orf1	hypothetical protein B566_EDAN014657 [Ephemera danica]	0.371299871	-1.42934328	0.0001913	down	yes	1.154	3.108	3.118	3.264	2.941	1	1.082	1.379	BP:developmental process;BP:cellular process;BP:cellular developmental process;BP:anatomical structure development;BP:cell development;BP:cellular component organization;BP:animal organ development;BP:biological_process;BP:cellular component organization or biogenesis;CC:integral component of membrane;CC:cellular_component;CC:intrinsic component of	GO:0032502;GO:0009987;GO:0048869;GO:0048856;GO:0048468;GO:0016043;GO:0048513;GO:0008150;GO:0071840;GO:0016021;GO:0005575;GO:0031224;GO:0110165	K24068	MEGF10_11	------	------	ENOG410XQWV	S:Function unknown	PF00053.27;PF0	Laminin_EGF;	Laminin EGF doma	CYT	1	30	8.4	High
TRINITY_DN40191_c2_g1_i1_orf1	stress-activated map kinase-interacting protein 1 [Ostrinia furnacalis]	0.345827901	-1.531873827	0.001012	down	yes	1.061	3.068	3.439	3.072	2.693	1	0.942	1.24	CC:cellular_component;CC:TORC2 complex;CC:macromolecular complex;CC:TOR complex;	GO:0005575;GO:0031932;GO:0032991;GO:0038201	K20410	MAPKAP1, SII	map04150	mTOR signaling	ENOG410XR57	S:Function unknown	PF05422.15;PF1	SIN1;SIN1_PH	Stress-activated m	CYT	1	1	60.4	Medium
TRINITY_DN5177_c0_g1_i2_orf1	hemolin-like isoform X1 [Ostrinia furnacalis]	0.331164606	-1.594379604	4.09E-07	down	yes	1.018	3.074	3.065	3.132	3.026	1	1.013	1.041	------	------	------	------	------	------	ENOG410XSVG	S:Function unknown	PF07679.19;PF1	I-set;Ig_3;ig;Ic	Immunoglobulin I-	CYT	22	58	47.3	High
TRINITY_DN110519_c0_g1_i1_orf1	uncharacterized protein LOC114366601 [Ostrinia furnacalis]	0.324701195	-1.6228154	0.0001491	down	yes	0.978	3.012	2.724	3.151	3.161	1	0.949	0.985	------	------	------	------	------	------	------	------	------	------	------	CYT	1	5	20	High
TRINITY_DN42337_c0_g1_i5_orf1	hypothetical protein evm_002829 [Chilo suppressalis]	0.333104396	-1.585953703	0.0001881	down	yes	0.97	2.912	2.975	3.124	2.637	1	0.915	0.995	MF:molecular_function;MF:structural constituent of cuticle;MF:structural molecule activity;	GO:0003674;GO:0042302;GO:0005198	------	------	------	------	ENOG410YSFI;ENOG4111EA8;ENOG4110T8H	S:Function unknown;	PF00379.26	Chitin_bind_4	Insect cuticle prote	CYT	1	31	17.6	High
TRINITY_DN3952_c0_g1_i3_orf1	protein Skeletor, isoforms D/E-like isoform X1 [Ostrinia furnacalis] >XP_028176405.1 protein Skeletor, isoforms D/E-like isoform X2 [Ostrinia furnacalis] >XP_028176406.1 protein Skeletor, isoforms D/E-like isoform X3 [Ostrinia furnacalis] >XP_028176407.1 protein Skeletor, isoforms D/E-like isoform X4 [Ostrinia furnacalis]	0.387995292	-1.365888947	5.78E-06	down	yes	0.989	2.549	2.638	2.474	2.534	1	0.969	0.998	------	------	------	------	------	------	ENOG410XSWZ	U:Intracellular trafficking, secre	PF10517.12;PF0	DM13;DOMO	Electron transfer D	CYT	4	4	162.2	High
TRINITY_DN10220_c1_g1_i7_orf1	uncharacterized protein LOC124645895 isoform X2 [Helicoverpa zea]	0.404324765	-1.306413522	8.72E-07	down	yes	0.991	2.451	2.419	2.427	2.507	1	0.99	0.983	CC:cellular_component;CC:integral component of membrane;CC:cellular anatomical entity;CC:intrinsic component of membrane;	GO:0005575;GO:0016021;GO:0110165;GO:0031224	K14788	NOL10, ENP2	------	------	ENOG410ZV5F	S:Function unknown	------	------	------	CYT	1	2	52.5	Medium
TRINITY_DN467_c0_g3_i1_orf1	histone-lysine N-methyltransferase 2B-like, partial [Ostrinia furnacalis]	0.410976121	-1.282873525	1.58E-06	down	yes	0.981	2.387	2.413	2.42	2.329	1	0.957	0.986	MF:molecular_function;MF:structural constituent of cuticle;MF:structural molecule activity;	GO:0003674;GO:0042302;GO:0005198	------	------	------	------	ENOG4111I81	S:Function unknown	PF00379.26	Chitin_bind_4	Insect cuticle prote	CYT	7	19	58.3	High
TRINITY_DN35809_c0_g1_i1_orf1	spodomicin-like [Ostrinia furnacalis] >OKV49445.1 diapausin [Ostrinia furnacalis]	0.421075455	-1.247849312	1.18E-05	down	yes	0.971	2.306	2.392	2.294	2.233	1	0.978	0.935	------	------	------	------	------	------	------	------	PF08036.14	Antimicrobial	Diapausin family o	CYT	3	60	6.8	High
TRINITY_DN1201_c0_g1_i4_orf1	triosephosphate isomerase [Ostrinia furnacalis] >XP_028170843.1 triosephosphate isomerase [Ostrinia furnacalis]	0.465778594	-1.102283755	2.35E-07	down	yes	1.014	2.177	2.148	2.197	2.185	1	1.019	1.023	BP:ribonucleoside diphosphate metabolic process;BP:phosphorylation;BP:purine nucleoside triphosphate metabolic process;BP:carboxylic acid metabolic process;BP:cellular nitrogen compound metabolic process;BP:nitrogen compound metabolic process;BP:small molecule metabolic process;BP:small molecule biosynthetic process;BP:cellular aromatic compound metabolic process;BP:purine-containing compound metabolic process;BP:generation of precursor metabolites and energy;BP:pyruvate metabolic process;BP:glycolytic process;BP:organic cyclic compound metabolic process;BP:nucleobase-containing compound metabolic process;BP:organic substance catabolic process;BP:purine nucleoside diphosphate metabolic process;BP:nucleotide phosphorylation;BP:ribonucleotide metabolic process;BP:organic substance metabolic process;BP:ribonucleoside triphosphate metabolic process;BP:nucleoside triphosphate metabolic process;BP:gluconeogenesis;BP:purine ribonucleotide metabolic process;BP:monosaccharide biosynthetic process;BP:purine ribonucleoside triphosphate metabolic process;BP:hexose metabolic process;BP:ribose phosphate metabolic process;BP:organic substance biosynthetic process;BP:ATP metabolic process;BP:cellular process;BP:ADP metabolic process;BP:hexose biosynthetic process;BP:monocarboxylic acid metabolic process;BP:biosynthetic process;BP:biological_process;BP:metabolic process;BP:oxoacid metabolic process;BP:carbohydrate metabolic process;BP:nucleoside diphosphate metabolic	GO:0009185;GO:0016310;GO:0009144;GO:0019752;GO:0034641;GO:0006807;GO:0044281;GO:0044283;GO:0006725;GO:0072521;GO:0006091;GO:0006090;GO:0006096;GO:1901360;GO:0006139;GO:1901575;GO:0009135;GO:0046939;GO:0009259;GO:0071704;GO:0009199;GO:0009141;GO:0006094;GO:0009150;GO:0046364;GO:0009205;GO:0019318;GO:0019693;GO:1901576;GO:0046034;GO:0009987;GO:0046031;GO:0019319;GO:0032787;GO:0009058;GO:0008150;GO:0008152;GO:0043436;GO:0005975;GO:0009132;GO:0009179;GO:0055086;GO:0046483;GO:0044238;GO:1901564;GO:0006082;GO:0005996;GO:1901135;GO:0016052;GO:0044237;GO:0016051;GO:0006163;GO:0006796;GO:0006165;GO:0006793;GO:0019637;GO:0009117;GO:0006006;GO:0006753;GO:0009056;GO:0006757;GO:0003674;GO:0016860;GO:0016861;GO:0003824;GO:0004807;GO:0016853	K01803	TPI, tpiA	map00010;maj	Glycolysis / Gluco	COG0149	G:Carbohydrate transport and r	PF00121.21	TIM	Triosephosphate is	CYT	16	71	26.4	High
TRINITY_DN4443_c0_g1_i4_orf1	lysosome-associated membrane glycoprotein 1-like isoform X4 [Ostrinia furnacalis]	0.455915943	-1.133160234	1.08E-06	down	yes	0.998	2.189	2.194	2.151	2.222	1	0.974	1.02	CC:organelle membrane;CC:integral component of membrane;CC:membrane;CC:cellular_component;CC:cytoplasmic vesicle membrane;CC:intrinsic component of membrane;CC:vesicle membrane;CC:bounding membrane of organelle;CC:cellular anatomical entity;CC:endosome	GO:0031090;GO:0016021;GO:0016020;GO:0005575;GO:0030659;GO:0031224;GO:0012506;GO:0098588;GO:0110165;GO:0010008	K06528	LAMP1_2, CD	map05152;maj	Tuberculosis;Pha	ENOG410XQ96	U:Intracellular trafficking, secre	PF01299.20	Lamp	Lysosome-associa	CYT	1	3	24.7	High
TRINITY_DN2101_c0_g1_i6_orf1	protein obstructor-E-like [Ostrinia furnacalis]	0.124264706	-3.0085115	2.07E-05	down	yes	1.014	8.16	8.411	8.523	7.546	1	1.033	1.008	------	------	------	------	------	------	ENOG410ZKXC	S:Function unknown	PF01607.27	CBM_14	Chitin binding Peri	EXC	5	20	28.1	High
TRINITY_DN9311_c0_g1_i1_orf1	cuticle protein 8-like [Ostrinia furnacalis]	0.152575462	-2.712405138	0.0001001	down	yes	1.016	6.659	6.733	7.248	5.997	1	1.032	1.017	MF:molecular_function;MF:structural constituent of cuticle;MF:structural molecule activity;	GO:0003674;GO:0042302;GO:0005198	------	------	------	------	ENOG410YSFI	S:Function unknown	PF00379.26	Chitin_bind_4	Insect cuticle prote	CYT	4	52	15	High
TRINITY_DN4767_c0_g1_i4_orf1	cysteine protease XCP2-like [Ostrinia furnacalis]	0.145524297	-2.78066805	1.70E-05	down	yes	0.9673	6.647	6.643	7.051	6.247	1	0.912	0.99	BP:primary metabolic process;BP:organonitrogen compound metabolic process;BP:protein metabolic process;BP:macromolecule metabolic process;BP:organic substance metabolic process;BP:nitrogen compound metabolic process;BP:biological_process;BP:metabolic process;BP:proteolysis;MF:molecular_function;MF:hydrolase activity;MF:catalytic activity;MF:catalytic activity, acting on a protein;MF:peptidase activity;	GO:0044238;GO:1901564;GO:0019538;GO:0043170;GO:0071704;GO:0006807;GO:0008150;GO:0008152;GO:0006508;GO:0003674;GO:0016787;GO:0003824;GO:0140096;GO:0008233	------	------	------	------	ENOG4110R2S;ENOG41119YM	S:Function unknown;	PF08246.15	Inhibitor_I29	Cathepsin propept	CYT	6	38	11.7	High
TRINITY_DN12526_c0_g1_i5_orf1	uncharacterized protein LOC114359035 isoform X3 [Ostrinia furnacalis]	0.15405194	-2.698511245	2.49E-05	down	yes	0.9847	6.392	6.359	5.993	6.823	1	0.895	1.059	BP:nitrogen compound metabolic process;BP:membrane lipid metabolic process;BP:organic substance metabolic process;BP:sphingolipid metabolic process;BP:lipid metabolic process;BP:cellular process;BP:biological_process;BP:metabolic process;BP:cellular lipid metabolic process;BP:primary metabolic process;BP:organonitrogen compound metabolic process;BP:cellular metabolic process;CC:lytic vacuole;CC:intracellular organelle;CC:membrane-bounded organelle;CC:organelle;CC:lysosome;CC:vacuole;CC:cellular anatomical entity;CC:intracellular membrane-bounded organelle;CC:cellular_component;CC:extracellular region;	GO:0006807;GO:0006643;GO:0071704;GO:0006665;GO:0006629;GO:0009987;GO:0008150;GO:0008152;GO:0044255;GO:0044238;GO:1901564;GO:0044237;GO:0000323;GO:0043229;GO:0043227;GO:0043226;GO:0005764;GO:0005773;GO:0110165;GO:0043231;GO:0005575;GO:0005576	K12382	PSAP, SGP1	map00600;maj	Sphingolipid met	ENOG410XSI5	O:Posttranslational modificatio	PF05184.18;PF0	SapB_1;SapB_	Saposin-like type I	CYT	2	37	15.2	High
TRINITY_DN98147_c0_g2_i1_orf1	hypothetical protein evm_002297 [Chilo suppressalis]	0.192139738	-2.379772169	1.38E-05	down	yes	1.188	6.183	6.353	6.353	5.842	1	1.303	1.262	MF:molecular_function;MF:structural constituent of cuticle;MF:structural molecule activity;	GO:0003674;GO:0042302;GO:0005198	------	------	------	------	ENOG4110RRZ;ENOG410XTK4;ENOG4111PJ1;ENOG4110T	S:Function unknown;	PF00379.26	Chitin_bind_4	Insect cuticle prote	EXC	3	18	31.2	High
TRINITY_DN20442_c0_g2_i1_orf1	hypothetical protein evm_008218 [Chilo suppressalis]	0.159963254	-2.644187563	0.0002762	down	yes	0.9577	5.987	5.788	6.782	5.391	1	0.827	1.046	BP:nucleosome organization;BP:cellular component assembly;BP:regulation of metabolic process;BP:macromolecular complex subunit organization;BP:regulation of cellular biosynthetic process;BP:regulation of cellular metabolic process;BP:cellular component organization or biogenesis;BP:regulation of primary metabolic process;BP:protein-DNA complex subunit organization;BP:chromatin organization;BP:cellular component organization;BP:regulation of biological process;BP:macromolecular complex assembly;BP:regulation of nucleobase-containing compound metabolic process;BP:protein-DNA complex assembly;BP:biological regulation;BP:regulation of gene expression;BP:regulation of macromolecule metabolic process;BP:regulation of biosynthetic process;BP:cellular process;BP:regulation of nucleic acid-templated transcription;BP:regulation of cellular process;BP:biological_process;BP:regulation of nitrogen compound metabolic process;BP:regulation of RNA biosynthetic process;BP:chromatin remodeling;BP:nucleosome assembly;BP:regulation of RNA metabolic process;BP:regulation of transcription, DNA-templated;BP:regulation of macromolecule biosynthetic process;CC:nucleosome;CC:DNA packaging complex;CC:macromolecular complex;CC:protein-DNA complex;CC:cellular_component;MF:nucleic acid binding;MF:peptide-lysine-N-acetyltransferase activity;MF:molecular_function;MF:binding;MF:peptide N-acetyltransferase activity;MF:DNA binding;MF:ion binding;MF:heterocyclic compound binding;MF:cation binding;MF:N-acetyltransferase activity;MF:histone	GO:0034728;GO:0022607;GO:0019222;GO:0043933;GO:0031326;GO:0031323;GO:0071840;GO:0080090;GO:0071824;GO:0006325;GO:0016043;GO:0050789;GO:0065003;GO:0019219;GO:0065004;GO:0065007;GO:0010468;GO:0060255;GO:0009889;GO:0009987;GO:1903506;GO:0050794;GO:0008150;GO:0051171;GO:2001141;GO:0006338;GO:0006334;GO:0051252;GO:0006355;GO:0010556;GO:0000786;GO:0044815;GO:0032991;GO:0032993;GO:0005575;GO:0003676;GO:0061733;GO:0003674;GO:0005488;GO:0034212;GO:0003677;GO:0043167;GO:1901363;GO:0043169;GO:0008080;GO:0004402;GO:0016740;GO:0016746;GO:0016747;GO:0016410;GO:0046872;GO:0140096;GO:0003824;GO:0097159;GO:0016407	K11306	MYST4, KAT6	------	------	COG5027	B:Chromatin structure and dyn	------	------	------	CYT	1	7	13.7	Medium
TRINITY_DN703_c0_g1_i2_orf1	acidic juvenile hormone-suppressible protein 1-like [Ostrinia furnacalis]	0.174050521	-2.522421961	1.78E-07	down	yes	0.9853	5.661	5.555	5.663	5.766	1	0.982	0.974	------	------	------	------	------	------	ENOG410XR2D	S:Function unknown	PF03723.17	Hemocyanin	Hemocyanin, ig-lik	CYT	6	97	10.1	High
TRINITY_DN636_c1_g1_i9_orf1	secretory phospholipase A2 receptor-like [Ostrinia furnacalis]	0.17566108	-2.509133515	2.00E-06	down	yes	0.9433	5.37	5.192	5.546	5.372	1	0.931	0.899	MF:molecular_function;MF:carbohydrate binding;MF:binding;	GO:0003674;GO:0030246;GO:0005488	------	------	------	------	ENOG410ZRYS;ENOG410XNWT;ENOG410XPJ1;ENOG410ZPTE;ENOG410XRS3	S:Function unknown;U:Intracell	PF00059.24	Lectin_C	Lectin C-type dom	PLA	2	18	31	High
TRINITY_DN2813_c0_g1_i7_orf1	arylphorin subunit alpha-like [Ostrinia furnacalis]	0.192688346	-2.375658778	1.99E-07	down	yes	0.954	4.951	4.864	4.959	5.029	1	0.948	0.914	------	------	------	------	------	------	ENOG4110QFU;ENOG410XR2D	S:Function unknown;	PF03723.17;PF0	Hemocyanin_	Hemocyanin, ig-lik	CYT	6	36	139.8	High
TRINITY_DN7900_c0_g1_i4_orf1	uncharacterized protein LOC114366119 [Ostrinia furnacalis]	0.175456552	-2.51081427	0.0003657	down	yes	0.8743	4.983	4.937	4.389	5.623	1	0.92	0.703	------	------	K20701	PIRK	map04624	Toll and Imd sigr	ENOG410Y5Y5	S:Function unknown	------	------	------	CYT	1	9	13.5	Medium
TRINITY_DN801_c0_g1_i2_orf1	cathepsin L [Ostrinia furnacalis] >XP_028165920.1 cathepsin L [Ostrinia furnacalis] >UKI61015.1 cathepsin L [Ostrinia furnacalis]	0.210948129	-2.245039802	6.04E-09	down	yes	0.9923	4.704	4.706	4.675	4.73	1	0.966	1.011	BP:primary metabolic process;BP:organonitrogen compound metabolic process;BP:protein metabolic process;BP:macromolecule metabolic process;BP:organic substance metabolic process;BP:nitrogen compound metabolic process;BP:biological_process;BP:metabolic process;BP:proteolysis;MF:molecular_function;MF:hydrolase activity;MF:catalytic activity;MF:catalytic activity, acting on a protein;MF:peptidase activity;MF:cysteine-type peptidase	GO:0044238;GO:1901564;GO:0019538;GO:0043170;GO:0071704;GO:0006807;GO:0008150;GO:0008152;GO:0006508;GO:0003674;GO:0016787;GO:0003824;GO:0140096;GO:0008233;GO:0008234	K01365	CTSL	map05205;maj	Proteoglycans in	COG4870	S:Function unknown	PF00112.26;PF0	Peptidase_C1	Papain family cyste	CYT	16	47	38.1	High
TRINITY_DN2311_c0_g3_i1_orf1	uncharacterized protein LOC114364231 isoform X1 [Ostrinia furnacalis] >XP_028176108.1 uncharacterized protein LOC114364231 isoform X2 [Ostrinia furnacalis]	0.211902674	-2.2385263	4.94E-05	down	yes	0.9667	4.562	4.518	4.914	4.253	1	0.979	0.921	------	------	------	------	------	------	ENOG410Y2YK	S:Function unknown	------	------	------	CYT	1	2	41.4	High
TRINITY_DN4676_c0_g1_i16_orf1	meiosis-specific nuclear structural protein 1-like isoform X2 [Ostrinia furnacalis]	0.274176121	-1.866825168	2.32E-06	down	yes	1.015	3.702	3.712	3.808	3.585	1	0.994	1.051	BP:regulation of microtubule polymerization or depolymerization;BP:regulation of cellular component organization;BP:regulation of organelle organization;BP:regulation of cytoskeleton organization;BP:regulation of cellular process;BP:biological_process;BP:biological regulation;BP:regulation of microtubule-based	GO:0031110;GO:0051128;GO:0033043;GO:0051493;GO:0050794;GO:0008150;GO:0065007;GO:0032886;GO:0070507;GO:0050789	K04381	STMN1	map05206;maj	MicroRNAs in car	ENOG4111K15	S:Function unknown	PF00836.22	Stathmin	Stathmin family	CYT	9	34	33.5	High
TRINITY_DN4324_c0_g1_i1_orf1	uncharacterized protein LOC114354985 isoform X1 [Ostrinia furnacalis]	0.2847178	-1.812395403	3.33E-07	down	yes	0.9837	3.455	3.384	3.473	3.508	1	0.981	0.97	CC:cellular_component;CC:integral component of membrane;CC:cellular anatomical entity;CC:intrinsic component of membrane;	GO:0005575;GO:0016021;GO:0110165;GO:0031224	------	------	------	------	ENOG41113AM;ENOG4110NNY	S:Function unknown;	------	------	------	CYT	4	20	16	High

TRINITY_DN58125_c0_g1_i1_orf1	protein yellow-like [Ostrinia furnacalis]	0.301719028	-1.728722412	1.56E-05	down	yes	1.018	3.374	3.532	3.371	3.219	1	0.98	1.074	BP:male mating behavior;BP:mating behavior;BP:behavior;BP:cellular biosynthetic process;BP:organic cyclic compound biosynthetic process;BP:organic cyclic compound metabolic process;BP:organic substance biosynthetic process;BP:pigment metabolic process;BP:secondary metabolite biosynthetic process;BP:organic substance metabolic process;BP:multicellular organismal process;BP:reproductive behavior;BP:phenol-containing compound biosynthetic process;BP:melanin metabolic process;BP:cellular aromatic compound metabolic process;BP:reproductive process;BP:biosynthetic process;BP:biological_process;BP:melanin biosynthetic process;BP:metabolic process;BP:phenol-containing compound metabolic process;BP:pigment biosynthetic process;BP:secondary metabolic process;BP:cellular metabolic	GO:0060179;GO:0007617;GO:0007610;GO:0044249;GO:1901362;GO:1901360;GO:1901576;GO:0042440;GO:0044550;GO:0071704;GO:0032501;GO:0019098;GO:0046189;GO:0006582;GO:0006725;GO:0022414;GO:0009058;GO:0008150;GO:0042438;GO:0008152;GO:0018958;GO:0046148;GO:0019748;GO:0044237;GO:0019438;GO:0009987;GO:1901617;GO:1901615	------	------	------	------	COG3386	S:Function unknown	PF03022.19	MRJP	Major royal jelly pr	CYT	1	9	19.2	High
TRINITY_DN2442_c0_g1_i2_orf1	digestive cysteine proteinase 2 [Ostrinia furnacalis]	0.29610814	-1.755803943	2.48E-07	down	yes	0.9967	3.366	3.36	3.427	3.312	1	0.986	1.004	BP:primary metabolic process;BP:organonitrogen compound metabolic process;BP:protein metabolic process;BP:macromolecule metabolic process;BP:organic substance metabolic process;BP:nitrogen compound metabolic process;BP:biological_process;BP:metabolic process;BP:proteolysis;MF:molecular_function;MF:hydrolase activity;MF:catalytic activity;MF:catalytic activity, acting on a protein;MF:peptidase activity;MF:cysteine-type peptidase	GO:0044238;GO:1901564;GO:0019538;GO:0043170;GO:0071704;GO:0006807;GO:0008150;GO:0008152;GO:0006508;GO:0003674;GO:0016787;GO:0003824;GO:0140096;GO:0008233;GO:0008234	------	------	------	------	COG4870	O:Posttranslational modificatio	PF00112.26;PF0(	Peptidase_C1	Papain family cyste	CYT	16	37	62.9	High
TRINITY_DN110231_c0_g1_i1_orf1	protein singed [Ostrinia furnacalis] >XP_028161434.1 protein singed [Ostrinia furnacalis]	0.319202784	-1.647454861	8.41E-08	down	yes	1.009	3.161	3.206	3.135	3.142	1	1.021	1.007	BP:cellular component organization or biogenesis;BP:fibril organization;BP:cellular component organization;BP:cell migration;BP:cellular process;BP:biological_process;BP:actin filament organization;BP:cell motility;CC:intracellular organelle;CC:non-membrane-bounded organelle;CC:organelle;CC:cytoplasm;CC:cellular anatomical entity;CC:cytoskeleton;CC:intracellular non-membrane-bounded organelle;CC:cellular_component;MF:macromolecular complex binding;MF:binding;MF:actin binding;MF:actin filament binding;MF:binding, bridging;MF:protein binding, bridging;MF:cytoskeletal protein binding;MF:protein binding;MF:molecular_function;	GO:0071840;GO:0097435;GO:0016043;GO:0016477;GO:0009987;GO:0008150;GO:0007015;GO:0048870;GO:0043229;GO:0043228;GO:0043226;GO:0005737;GO:0110165;GO:0005856;GO:0043232;GO:0005575;GO:0044877;GO:0005488;GO:0003779;GO:0051015;GO:0060090;GO:0030674;GO:0008092;GO:0005515;GO:0003674	K23551	FSCN1	map05206	MicroRNAs in car	ENOG410XPHV	O:Posttranslational modificatio	PF06268.16;PF0(	Fascin;FRG1;F	Fascin domain;FRG	CYT	11	29	57	High
TRINITY_DN12865_c0_g1_i1_orf1	synaptic vesicle membrane protein VAT-1 homolog-like [Ostrinia furnacalis]	0.313758283	-1.672274547	8.01E-09	down	yes	0.9943	3.169	3.193	3.166	3.149	1	0.99	0.993	------	------	------	------	------	------	COG0604	O:Posttranslational modificatio	PF13602.9;PF08;	ADH_zinc_N_;	Zinc-binding dehy	CYT	17	41	50.1	High
TRINITY_DN71308_c0_g1_i4_orf1	uncharacterized protein LOC114361536 [Ostrinia furnacalis]	0.412526242	-1.277442198	6.15E-05	down	yes	1.179	2.858	2.838	2.858	2.879	1	1.323	1.213	CC:cellular_component;CC:cellular anatomical entity;CC:extracellular space;MF:endopeptidase inhibitor activity;MF:endopeptidase regulator activity;MF:peptidase regulator activity;MF:molecular_function;MF:peptidase inhibitor activity;MF:serine-type endopeptidase inhibitor activity;MF:enzyme inhibitor activity;MF:molecular function regulator;MF:enzyme regulator activity;	GO:0005575;GO:0110165;GO:0005615;GO:0004866;GO:0061135;GO:0061134;GO:0003674;GO:0030414;GO:0004867;GO:0004857;GO:0098772;GO:0030234	------	------	------	------	COG4826	O:Posttranslational modificatio	PF00079.23	Serpin	Serpin	CYT	1	25	9.4	High
TRINITY_DN295_c2_g1_i2_orf1	phosphoglycolate phosphatase 1A, chloroplastic [Manduca sexta]	0.339884785	-1.556882315	5.44E-05	down	yes	1.003	2.951	2.948	3.139	2.767	1	1.002	1.008	MF:hydrolase activity;MF:molecular_function;MF:catalytic	GO:0016787;GO:0003674;GO:0003824	K21013	FPPP	map00981	Insect hormone b	COG0647	G:Carbohydrate transport and r	PF13242.9	Hydrolase_like	HAD-hyrolase-like	PLA	4	28	13.9	High
TRINITY_DN82426_c0_g1_i6_orfp1	lysosome-associated membrane glycoprotein 1-like isoform X4 [Ostrinia furnacalis]	0.352663787	-1.503634654	3.07E-05	down	yes	0.9797	2.778	2.734	2.66	2.941	1	0.994	0.945	------	------	K06528	LAMP1_2, CD	map05152;maj	Tuberculosis;Pha	------	------	------	------	------	CYT	1	12	12.6	High
TRINITY_DN4228_c0_g1_i5_orf1	phenoloxidase-activating enzyme-like [Ostrinia furnacalis]	0.37754724	-1.405270925	1.88E-06	down	yes	1.019	2.699	2.727	2.625	2.744	1	1.011	1.047	BP:organic substance metabolic process;BP:organonitrogen compound metabolic process;BP:protein metabolic process;BP:nitrogen compound metabolic process;BP:macromolecule metabolic process;BP:biological_process;BP:metabolic process;BP:proteolysis;BP:primary metabolic process;CC:cellular_component;CC:extracellular region;CC:cellular anatomical entity;MF:serine-type endopeptidase activity;MF:endopeptidase activity;MF:hydrolase activity;MF:serine hydrolase activity;MF:catalytic activity, acting on a protein;MF:catalytic activity;MF:molecular_function;MF:peptidase activity;MF:serine-	GO:0071704;GO:1901564;GO:0019538;GO:0006807;GO:0043170;GO:0008150;GO:0008152;GO:0006508;GO:0044238;GO:0005575;GO:0005576;GO:0110165;GO:0004252;GO:0004175;GO:0016787;GO:0017171;GO:0140096;GO:0003824;GO:0003674;GO:0008233;GO:0008236	------	------	------	------	COG5640	E:Amino acid transport and me	PF00089.29	Trypsin	Trypsin	CYT	3	16	47.5	High
TRINITY_DN5553_c0_g1_i4_orf1	uncharacterized protein LOC114353828 [Ostrinia furnacalis]	0.375786646	-1.412014294	2.73E-05	down	yes	0.9793	2.606	2.708	2.464	2.645	1	0.943	0.995	BP:positive regulation of ion transmembrane transporter activity;BP:positive regulation of transporter activity;BP:regulation of ion transmembrane transporter activity;BP:regulation of transport;BP:modulation of synaptic transmission;BP:regulation of potassium ion transport;BP:regulation of signaling;BP:positive regulation of transmembrane transport;BP:regulation of ion transmembrane transport;BP:regulation of cell communication;BP:regulation of biological process;BP:positive regulation of potassium ion transport;BP:positive regulation of ion transmembrane transport;BP:rhythmic process;BP:regulation of transporter activity;BP:biological regulation;BP:regulation of transmembrane transport;BP:positive regulation of molecular function;BP:regulation of trans-synaptic signaling;BP:positive regulation of biological process;BP:regulation of molecular function;BP:positive regulation of potassium ion transmembrane transport;BP:multicellular organismal process;BP:positive regulation of transport;BP:regulation of cellular process;BP:positive regulation of ion transport;BP:biological_process;BP:sleep;BP:positive regulation of voltage-gated potassium channel activity;BP:regulation of cation channel activity;BP:regulation of localization;BP:positive regulation of potassium ion transmembrane transporter activity;BP:regulation of metal ion transport;BP:regulation of synaptic transmission, cholinergic;BP:regulation of transmembrane transporter activity;BP:positive regulation of cation transmembrane transport;BP:regulation of cation transmembrane transport;BP:regulation of potassium ion transmembrane transporter activity;BP:regulation of ion	GO:0032414;GO:0032411;GO:0032412;GO:0051049;GO:0050804;GO:0043266;GO:0023051;GO:0034764;GO:0034765;GO:0010646;GO:0050789;GO:0043268;GO:0034767;GO:0048511;GO:0032409;GO:0065007;GO:0034762;GO:0044093;GO:0099177;GO:0048518;GO:0065009;GO:1901381;GO:0032501;GO:0051050;GO:0050794;GO:0043270;GO:0008150;GO:0030431;GO:1903818;GO:2001257;GO:0032879;GO:1901018;GO:0010959;GO:0032222;GO:0022898;GO:1904064;GO:1904062;GO:1901016;GO:0043269;GO:2001259;GO:1901379;GO:0048522;GO:0005886;GO:0031224;GO:0016021;GO:0016020;GO:0031225;GO:0110165;GO:0005575	------	------	------	------	ENOG4111SVR	S:Function unknown	PF17064.8	QVR	Sleepless protein	CYT	1	6	16.5	High
TRINITY_DN14532_c0_g1_i1_orf1	pupal cuticle protein-like [Trichoplusia ni]	0.378004622	-1.403524218	9.54E-07	down	yes	0.9813	2.596	2.547	2.647	2.595	1	0.953	0.991	MF:molecular_function;MF:structural constituent of cuticle;MF:structural molecule activity;	GO:0003674;GO:0042302;GO:0005198	------	------	------	------	ENOG4110QXC;ENOG410Z4W3	S:Function unknown;	------	------	------	CYT	4	26	25.9	High
TRINITY_DN1563_c0_g1_i4_orf1	pupal cuticle protein 36-like [Ostrinia furnacalis]	0.380565371	-1.393783803	1.51E-05	down	yes	0.9693	2.547	2.624	2.588	2.429	1	0.933	0.975	MF:molecular_function;MF:structural constituent of cuticle;MF:structural molecule activity;	GO:0003674;GO:0042302;GO:0005198	------	------	------	------	ENOG4111PJ1;ENOG410XTK4;ENOG4110T67;ENOG4110S	S:Function unknown;	PF00379.26	Chitin_bind_4	Insect cuticle prote	CYT	5	41	30.1	High
TRINITY_DN14460_c0_g1_i6_orf1	scavenger receptor class B member 1-like [Ostrinia furnacalis]	0.437140509	-1.193831017	0.0001728	down	yes	1.064	2.434	2.468	2.577	2.257	1	1.061	1.131	BP:multicellular organismal process;BP:sensory perception of smell;BP:neurological system process;BP:response to stimulus;BP:sensory perception of chemical stimulus;BP:sensory perception;BP:biological_process;BP:system process;CC:membrane;CC:cellular_component;CC:cellular anatomical entity;CC:plasma membrane;CC:integral component of membrane;CC:intrinsic component of membrane;	GO:0032501;GO:0007608;GO:0050877;GO:0050896;GO:0007606;GO:0007600;GO:0008150;GO:0003008;GO:0016020;GO:0005575;GO:0110165;GO:0005886;GO:0016021;GO:0031224	K13885	SCARB1	map04979;maj	Cholesterol meta	ENOG410XS17	C:Energy production and conve	PF01130.24	CD36	CD36 family	CYT	3	6	62.9	High
TRINITY_DN2227_c0_g1_i5_orf1	protein 60A [Ostrinia furnacalis]	0.402942395	-1.311354489	9.71E-07	down	yes	0.9723	2.413	2.422	2.452	2.366	1	0.953	0.964	BP:response to stimulus;BP:biological_process;BP:response to chemical;BP:response to organic substance;CC:cellular_component;CC:extracellular region;CC:cellular anatomical entity;MF:growth factor activity;MF:receptor regulator activity;MF:receptor activator activity;MF:receptor agonist activity;MF:molecular_function;MF:binding;MF:protein	GO:0050896;GO:0008150;GO:0042221;GO:0010033;GO:0005575;GO:0005576;GO:0110165;GO:0008083;GO:0030545;GO:0030546;GO:0048018;GO:0003674;GO:0005488;GO:0005515;GO:0005102;GO:0098772	K16621	BMP7	map04060;maj	Cytokine-cytokin	ENOG410XT8Z	S:Function unknown	PF00688.21;PF0(	TGFb_propep	TGF-beta propepti	CYT	4	11	46.3	High
TRINITY_DN12534_c0_g1_i4_orf1	antibacterial protein [Heliothis virescens]	0.432514015	-1.209181214	2.33E-06	down	yes	1.003	2.319	2.269	2.318	2.369	1	1.032	0.978	BP:response to biotic stimulus;BP:response to external stimulus;BP:response to external biotic stimulus;BP:response to stimulus;BP:defense response;BP:response to stress;BP:biological_process;BP:interspecies interaction between organisms;BP:response to other organism;CC:cellular_component;CC:extracellular region;CC:cellular anatomical entity;	GO:0009607;GO:0009605;GO:0043207;GO:0050896;GO:0006952;GO:0006950;GO:0008150;GO:0044419;GO:0051707;GO:0005575;GO:0005576;GO:0110165	------	------	------	------	ENOG4110S6N	S:Function unknown	PF00451.22	Toxin_2	Scorpion short toxi	CYT	5	57	12	High
TRINITY_DN64616_c0_g1_i1_orf1	uncharacterized protein LOC114366101 [Ostrinia furnacalis]	0.488745981	-1.032843257	1.83E-05	down	yes	1.064	2.177	2.241	2.163	2.128	1	1.085	1.107	CC:cellular_component;CC:integral component of membrane;CC:cellular anatomical entity;CC:intrinsic component of membrane;	GO:0005575;GO:0016021;GO:0110165;GO:0031224	K10089	M6PR	map04145;maj	Phagosome;Lyso	ENOG4112253;ENOG4111VXF	S:Function unknown;	PF02157.18;PF0!	Man-6-P_rec	Mannose-6-phosp	CYT	1	9	18.6	Medium
TRINITY_DN24723_c2_g1_i1_orf1	hypothetical protein evm_001103 [Chilo suppressalis]	0.499530516	-1.00135528	0.001561	down	yes	1.064	2.13	2.208	2.209	1.974	1	0.905	1.288	BP:small molecule biosynthetic process;BP:carboxylic acid metabolic process;BP:cellular biosynthetic process;BP:cellular nitrogen compound metabolic process;BP:nitrogen compound metabolic process;BP:small molecule metabolic process;BP:nitrogen cycle metabolic process;BP:glutamine family amino acid metabolic process;BP:organic substance biosynthetic process;BP:cellular amino acid metabolic process;BP:arginine biosynthetic process;BP:arginine metabolic process;BP:organic substance metabolic process;BP:organic acid metabolic process;BP:cellular amino acid biosynthetic process;BP:alpha-amino acid metabolic process;BP:alpha-amino acid biosynthetic process;BP:urea metabolic process;BP:primary metabolic process;BP:cellular process;BP:biosynthetic process;BP:biological_process;BP:metabolic process;BP:oxoacid metabolic process;BP:cellular nitrogen compound biosynthetic process;BP:amide biosynthetic process;BP:organonitrogen compound metabolic process;BP:organonitrogen compound biosynthetic process;BP:cellular amide metabolic process;BP:carboxylic acid biosynthetic process;BP:urea cycle;BP:organic acid biosynthetic process;BP:cellular metabolic process;BP:glutamine family amino acid biosynthetic process;MF:argininosuccinate synthase activity;MF:purine ribonucleoside triphosphate binding;MF:molecular_function;MF:ion binding;MF:heterocyclic compound binding;MF:nucleoside phosphate binding;MF:anion binding;MF:nucleotide binding;MF:purine nucleotide binding;MF:small molecule binding;MF:catalytic activity;MF:ligase activity;MF:ligase activity, forming carbon-	GO:0044283;GO:0019752;GO:0044249;GO:0034641;GO:0006807;GO:0044281;GO:0071941;GO:0009064;GO:1901576;GO:0006520;GO:0006526;GO:0006525;GO:0071704;GO:0006082;GO:0008652;GO:1901605;GO:1901607;GO:0019627;GO:0044238;GO:0009987;GO:0009058;GO:0008150;GO:0008152;GO:0043436;GO:0044271;GO:0043604;GO:1901564;GO:1901566;GO:0043603;GO:0046394;GO:0000050;GO:0016053;GO:0044237;GO:0009084;GO:0004055;GO:0035639;GO:0003674;GO:0043167;GO:1901363;GO:1901265;GO:0043168;GO:0000166;GO:0017076;GO:0036094;GO:0003824;GO:0016874;GO:0016879;GO:0030554;GO:0097367;GO:0032559;GO:0005524;GO:0097159;GO:0032555;GO:0032553;GO:0005488	K01940	argG, ASS1	map00220;maj	Arginine biosynth	COG0137	E:Amino acid transport and me	PF00764.22	Arginosuc_syr	Arginosuccinate sy	CYT	1	9	11.6	High
TRINITY_DN2109_c0_g1_i4_orf1	mucin-2-like isoform X2 [Ostrinia furnacalis]	0.465213315	-1.104035707	4.28E-06	down	yes	0.9923	2.133	2.072	2.172	2.156	1	1.008	0.969	CC:cellular_component;CC:integral component of membrane;CC:cellular anatomical entity;CC:intrinsic component of membrane;	GO:0005575;GO:0016021;GO:0110165;GO:0031224	------	------	------	------	ENOG410Y7ID	S:Function unknown	------	------	------	EXC	3	2	236.3	High
TRINITY_DN1370_c0_g1_i2_orf1	hypothetical protein evm_000756 [Chilo suppressalis]	0.13987527	-2.837787174	3.11E-06	down	yes	1.099	7.857	8.119	7.905	7.546	1	1.06	1.236	------	------	------	------	------	------	------	------	------	------	------	CYT	2	35	23	High
TRINITY_DN1108_c3_g1_i1_orfp1	TRINITY_DN1108_c3_g1_i1_m.5561 TRINITY_DN1108_c3_g1::TRINITY_DN1108_c3_g1_i1::g.5561 ORF type:internal len:113 (+),score=89.07 TRINITY_DN1108_c3_g1_i1:1-336(+)	0.175173704	-2.513141873	0.006735	down	yes	1.311	7.484	8.895	8.082	5.474	1	2.486	0.447	------	------	------	------	------	------	------	------	------	------	------	PLA	1	11	12.3	High
TRINITY_DN29604_c0_g2_i2_orf1	neurofilament heavy polypeptide-like isoform X2 [Ostrinia furnacalis]	0.178776325	-2.483772396	2.18E-06	down	yes	0.9847	5.508	5.473	5.714	5.338	1	0.952	1.002	------	------	------	------	------	------	------	------	------	------	------	CYT	22	77	40.5	High

TRINITY_DN46625_c0_g1_i1_orf1	ferritin subunit isoform X1 [Belonocnema kinseyi]	0.19887165	-2.330090465	2.20E-05	down	yes	0.987	4.963	5.03	4.635	5.224	1	0.945	1.016	BP:ion homeostasis;BP:homeostatic process;BP:iron ion homeostasis;BP:transition metal ion homeostasis;BP:inorganic ion homeostasis;BP:cellular transition metal ion homeostasis;BP:transition metal ion transport;BP:biological regulation;BP:regulation of biological quality;BP:cellular homeostasis;BP:metal ion transport;BP:transport;BP:cellular iron ion homeostasis;BP:cellular metal ion homeostasis;BP:cation transport;BP:ion transport;BP:cellular process;BP:cellular ion homeostasis;BP:biological_process;BP:cellular cation homeostasis;BP:metal ion homeostasis;BP:cation homeostasis;BP:cellular chemical homeostasis;BP:establishment of localization;BP:localization;BP:chemical homeostasis;BP:iron ion transport;MF:molecular_function;MF:binding;MF:oxidoreductase activity;MF:cation binding;MF:oxidoreductase activity, oxidizing metal ions;MF:transition metal ion binding;MF:ferroxidase activity;MF:ion binding;MF:iron ion binding;MF:ferric iron binding;MF:oxidoreductase activity, oxidizing metal ions,	GO:0050801;GO:0042592;GO:0055072;GO:0055076;GO:0098771;GO:0046916;GO:0000041;GO:0065007;GO:0065008;GO:0019725;GO:0030001;GO:0006810;GO:0006879;GO:0006875;GO:0006812;GO:0006811;GO:0009987;GO:0006873;GO:0008150;GO:0030003;GO:0055065;GO:0055080;GO:0055082;GO:0051234;GO:0051179;GO:0048878;GO:0006826;GO:0003674;GO:0005488;GO:0016491;GO:0043169;GO:0016722;GO:0046914;GO:0004322;GO:0043167;GO:0005506;GO:0008199;GO:0016724;GO:0046872;GO:0003824	K00522	FTH1	map04978;ma	Mineral absorptic	ENOG4111MSG	P:Inorganic ion transport and n	PF00210.27	Ferritin	Ferritin-like domai	CYT	1	4	24.1	High
TRINITY_DN65681_c0_g1_i1_orf1	ferritin subunit-like [Ostrinia furnacalis] >XP_028168186.1 ferritin subunit-like [Ostrinia furnacalis]	0.194084163	-2.365245691	2.10E-06	down	yes	0.9547	4.919	5.099	4.851	4.808	1	0.89	0.974	BP:ion homeostasis;BP:homeostatic process;BP:iron ion homeostasis;BP:transition metal ion homeostasis;BP:inorganic ion homeostasis;BP:cellular transition metal ion homeostasis;BP:transition metal ion transport;BP:biological regulation;BP:regulation of biological quality;BP:cellular homeostasis;BP:metal ion transport;BP:transport;BP:cellular iron ion homeostasis;BP:cellular metal ion homeostasis;BP:cation transport;BP:ion transport;BP:cellular process;BP:cellular ion homeostasis;BP:biological_process;BP:cellular cation homeostasis;BP:metal ion homeostasis;BP:cation homeostasis;BP:cellular chemical homeostasis;BP:establishment of localization;BP:localization;BP:chemical homeostasis;BP:iron ion transport;MF:molecular_function;MF:binding;MF:oxidoreductase activity;MF:cation binding;MF:oxidoreductase activity, oxidizing metal ions;MF:transition metal ion binding;MF:ferroxidase activity;MF:ion binding;MF:iron ion binding;MF:ferric iron binding;MF:oxidoreductase activity, oxidizing metal ions	GO:0050801;GO:0042592;GO:0055072;GO:0055076;GO:0098771;GO:0046916;GO:0000041;GO:0065007;GO:0065008;GO:0019725;GO:0030001;GO:0006810;GO:0006879;GO:0006875;GO:0006812;GO:0006811;GO:0009987;GO:0006873;GO:0008150;GO:0030003;GO:0055065;GO:0055080;GO:0055082;GO:0051234;GO:0051179;GO:0048878;GO:0006826;GO:0003674;GO:0005488;GO:0016491;GO:0043169;GO:0016722;GO:0046914;GO:0004322;GO:0043167;GO:0005506;GO:0008199;GO:0016724;GO:0046872;GO:0003824	K00522	FTH1	map04978;ma	Mineral absorptic	ENOG4111MSG;ENOG41124IC	P:Inorganic ion transport and n	PF00210.27	Ferritin	Ferritin-like domai	CYT	1	31	8.1	High
TRINITY_DN45220_c0_g1_i1_orf1	delta(3,5)-Delta(2,4)-dienoyl-CoA isomerase, mitochondrial isoform X1 [Ostrinia furnacalis]	0.268976522	-1.894447847	3.41E-05	down	yes	1.18	4.387	4.459	4.564	4.138	1	1.245	1.295	BP:lipid oxidation;BP:fatty acid metabolic process;BP:fatty acid beta-oxidation;BP:carboxylic acid metabolic process;BP:cellular catabolic process;BP:fatty acid catabolic process;BP:small molecule metabolic process;BP:small molecule catabolic process;BP:cellular lipid catabolic process;BP:organic substance catabolic process;BP:lipid modification;BP:lipid catabolic process;BP:organic substance metabolic process;BP:lipid metabolic process;BP:cellular process;BP:monocarboxylic acid metabolic process;BP:biological_process;BP:metabolic process;BP:monocarboxylic acid catabolic process;BP:oxoacid metabolic process;BP:fatty acid oxidation;BP:cellular lipid metabolic process;BP:catabolic process;BP:primary metabolic process;BP:organic acid metabolic process;BP:carboxylic acid catabolic process;BP:organic acid catabolic process;BP:cellular metabolic process;MF:molecular_function;MF:catalytic activity;MF:isomerase activity;	GO:0034440;GO:0006631;GO:0006635;GO:0019752;GO:0044248;GO:0009062;GO:0044281;GO:0044282;GO:0044242;GO:1901575;GO:0030258;GO:0016042;GO:0071704;GO:0006629;GO:0009987;GO:0032787;GO:0008150;GO:0008152;GO:0072329;GO:0043436;GO:0019395;GO:0044255;GO:0009056;GO:0044238;GO:0006082;GO:0046395;GO:0016054;GO:0044237;GO:0003674;GO:0003824;GO:0016853	K12663	ECH1	map04146	Peroxisome	ENOG410XTHX	S:Function unknown	PF00378.23	ECH_1	Enoyl-CoA hydrata	CYT	1	59	7.4	High
TRINITY_DN7040_c0_g2_i1_orf1	uncharacterized protein LOC114353763 [Ostrinia furnacalis]	0.230251907	-2.118714992	2.12E-06	down	yes	0.9963	4.327	4.225	4.268	4.487	1	1.003	0.986	------	------	------	------	------	------	ENOG4110RZK	O:Posttranslational modificatio	------	------	------	CYT	8	32	22	High
TRINITY_DN3707_c0_g1_i1_orf1	protein FAM160B1-like isoform X1 [Ostrinia furnacalis]	0.242502343	-2.043929408	2.26E-05	down	yes	1.035	4.268	4.549	4.098	4.157	1	1.069	1.037	------	------	------	------	------	------	ENOG410XTDG;ENOG410XY4X	S:Function unknown;	PF10257.12;PF1	RAI16-like;DU	Retinoic acid induc	CYT	1	1	93.1	High
TRINITY_DN12671_c0_g1_i4_orf1	hemicentin-1-like isoform X1 [Ostrinia furnacalis]	0.253426752	-1.980359272	6.36E-06	down	yes	0.9947	3.925	4.081	3.938	3.756	1	1	0.984	BP:biological_process;BP:localization;	GO:0008150;GO:0051179	------	------	------	------	ENOG410XPQS;ENOG4111FBT;ENOG410ZN3C	S:Function unknown;	PF07679.19;PF1	I-set;Ig_3;Ig_2	Immunoglobulin I-	CYT	5	9	89.1	High
TRINITY_DN97042_c0_g1_i6_orf1	apolipophorins-like [Ostrinia furnacalis]	0.287445887	-1.798637705	3.35E-07	down	yes	0.996	3.465	3.539	3.427	3.428	1	0.981	1.007	MF:lipid transporter activity;MF:transporter activity;MF:molecular_function;	GO:0005319;GO:0005215;GO:0003674	------	------	------	------	ENOG410Z6MR;ENOG41112S0	S:Function unknown;	PF09172.14	Vit_open_b-s	Vitellinogen, open	CYT	2	29	12.7	High
TRINITY_DN74086_c0_g1_i1_orf1	UPF0489 protein C5orf22 homolog [Ostrinia furnacalis]	0.330456227	-1.597468914	2.25E-05	down	yes	1.072	3.244	3.193	3.122	3.417	1	1.101	1.114	------	------	------	------	------	------	ENOG41109E9	S:Function unknown	PF12640.10	UPF0489	UPF0489 domain	CYT	1	4	42.6	High
TRINITY_DN100327_c0_g1_i1_orf1	arylphorin subunit alpha-like [Ostrinia furnacalis]	0.294310656	-1.764588315	8.40E-09	down	yes	0.9777	3.322	3.338	3.32	3.307	1	0.967	0.966	------	------	------	------	------	------	ENOG410XR2D	S:Function unknown	PF00372.22;PF0	Hemocyanin_	Hemocyanin, copp	PLA	35	56	83.5	High
TRINITY_DN1465_c2_g1_i2_orf1	transcription initiation factor TFIID subunit 1-like [Ostrinia furnacalis]	0.299263578	-1.74051139	1.12E-05	down	yes	0.9753	3.259	3.243	3.409	3.126	1	0.935	0.991	------	------	------	------	------	------	ENOG410YRBN;ENOG410YX73	S:Function unknown;	PF16009.8	DUF4779	Domain of unknow	PLA	7	26	36.9	High
TRINITY_DN47842_c0_g1_i1_orf1	protein lethal(2)essential for life-like [Helicoverpa armigera] >PZC74790.1 hypothetical protein B5X24_HaOG207163 [Helicoverpa armigera]	0.3627484	-1.462958843	9.53E-06	down	yes	1.077	2.969	3.065	2.966	2.875	1	1.105	1.126	MF:cation binding;MF:metal ion binding;MF:molecular_function;MF:binding;MF:ion binding;	GO:0043169;GO:0046872;GO:0003674;GO:0005488;GO:0043167	K09542	CRYAB	map04213;ma	Longevity regulat	ENOG410YERS	O:Posttranslational modificatio	PF00011.24	HSP20	Hsp20/alpha cryst	CYT	6	43	20.7	High
TRINITY_DN9044_c0_g1_i2_orf1	unnamed protein product [Euphydryas editha]	0.351523267	-1.50830791	2.75E-06	down	yes	1.05	2.987	3.02	2.981	2.959	1	1.005	1.144	BP:immune system process;BP:response to external biotic stimulus;BP:interspecies interaction between organisms;BP:organic substance metabolic process;BP:innate immune response;BP:defense response;BP:response to stress;BP:biological_process;BP:metabolic process;BP:immune response;BP:carbohydrate metabolic process;BP:response to other organism;BP:primary metabolic process;BP:response to biotic stimulus;BP:response to external stimulus;BP:response to stimulus;BP:defense response to other organism;CC:cellular anatomical entity;CC:cellular_component;CC:extracellular region;MF:carbohydrate binding;MF:molecular_function;MF:binding;MF:hydrolase activity;MF:hydrolase activity, acting on glycosyl bonds;MF:catalytic activity;MF:hydrolase activity, hydrolyzing	GO:0002376;GO:0043207;GO:0044419;GO:0071704;GO:0045087;GO:0006952;GO:0006950;GO:0008150;GO:0008152;GO:0006955;GO:0005975;GO:0051707;GO:0044238;GO:0009607;GO:0009605;GO:0050896;GO:0098542;GO:0110165;GO:0005575;GO:0005576;GO:0030246;GO:0003674;GO:0005488;GO:0016787;GO:0016798;GO:0003824;GO:0004553	K20692	GNBP3	map04624	Toll and Imd sigr	ENOG410Z83Q;ENOG4110NFS	S:Function unknown;	PF15886.8	CBM39	Carbohydrate bind	CYT	1	10	11.4	High
TRINITY_DN585_c0_g1_i12_orf1	very low-density lipoprotein receptor isoform X3 [Galleria mellonella]	0.34548552	-1.533302851	5.18E-07	down	yes	1.014	2.935	2.918	2.897	2.99	1	1.048	0.994	CC:integral component of membrane;CC:cellular_component;CC:intrinsic component of membrane;CC:cellular anatomical entity;MF:binding;MF:cation binding;MF:cargo receptor activity;MF:very-low-density lipoprotein particle receptor activity;MF:lipoprotein particle receptor activity;MF:ion binding;MF:molecular_function;MF:metal ion	GO:0016021;GO:0005575;GO:0031224;GO:0110165;GO:0005488;GO:0043169;GO:0038024;GO:0030229;GO:0030228;GO:0043167;GO:0003674;GO:0046872;GO:0005509	K20053	VLDLR	map05417;ma	Lipid and athero	ENOG410YQ6J	U:Intracellular trafficking, secre	PF00057.21;PF0	Ldl_recept_a;l	Low-density lipopr	PLA	3	31	100.3	High
TRINITY_DN2794_c1_g1_i8_orf1	carboxypeptidase D [Ostrinia furnacalis]	0.363830545	-1.458661428	1.27E-05	down	yes	1.022	2.809	2.761	2.941	2.726	1	1.013	1.052	BP:cellular nitrogen compound metabolic process;BP:nitrogen compound metabolic process;BP:organic substance metabolic process;BP:proteolysis;BP:cellular process;BP:biological_process;BP:metabolic process;BP:primary metabolic process;BP:organonitrogen compound metabolic process;BP:cellular amide metabolic process;BP:protein metabolic process;BP:cellular metabolic process;BP:macromolecule metabolic process;BP:peptide metabolic process;CC:intrinsic component of membrane;CC:integral component of membrane;CC:cellular anatomical entity;CC:cellular_component;MF:exopeptidase activity;MF:molecular_function;MF:zinc ion binding;MF:peptidase activity;MF:metalloexopeptidase activity;MF:cation binding;MF:transition metal ion binding;MF:carboxypeptidase activity;MF:ion binding;MF:metallopeptidase activity;MF:metal ion binding;MF:hydrolase activity;MF:catalytic activity, acting on a	GO:0034641;GO:0006807;GO:0071704;GO:0006508;GO:0009987;GO:0008150;GO:0008152;GO:0044238;GO:1901564;GO:0043603;GO:0019538;GO:0044237;GO:0043170;GO:0006518;GO:0031224;GO:0016021;GO:0110165;GO:0005575;GO:0008238;GO:0003674;GO:0008270;GO:0008233;GO:0008235;GO:0043169;GO:0046914;GO:0004180;GO:0043167;GO:0008237;GO:0046872;GO:0016787;GO:0140096;GO:0003824;GO:0004181;GO:0005488	K07752	CPD	------	------	ENOG410XX0H	E:Amino acid transport and me	PF00246.27;PF1	Peptidase_M1	Zinc carboxypeptic	CYT	12	8	155.8	High
TRINITY_DN64141_c0_g1_i4_orf1	probable salivary secreted peptide [Ostrinia furnacalis]	0.367870036	-1.442731925	0.001018	down	yes	1.019	2.77	2.723	2.443	3.143	1	1.056	1.002	------	------	------	------	------	------	ENOG410ZS6V;ENOG410Y5FW;ENOG4110QNX	S:Function unknown;	PF15868.8	MBF2	Transcription activ	CYT	3	33	13.3	High
TRINITY_DN268_c1_g1_i7_orf1	unnamed protein product [Spodoptera littoralis] >CAH1645252.1 unnamed protein product [Spodoptera littoralis]	0.383121733	-1.38412523	7.52E-08	down	yes	1.026	2.678	2.675	2.66	2.698	1	1.031	1.046	CC:integral component of membrane;CC:cellular_component;CC:intrinsic component of membrane;CC:cellular anatomical entity;MF:sulfate transmembrane transporter activity;MF:sulfur compound transmembrane transporter activity;MF:secondary active transmembrane transporter activity;MF:inorganic anion transmembrane transporter activity;MF:inorganic solute uptake transmembrane transporter activity;MF:transporter activity;MF:ion transmembrane transporter activity;MF:transmembrane transporter activity;MF:secondary active sulfate transmembrane transporter activity;MF:active ion transmembrane transporter activity;MF:molecular_function;MF:anion transmembrane	GO:0016021;GO:0005575;GO:0031224;GO:0110165;GO:0015116;GO:1901682;GO:0015291;GO:0015103;GO:0015318;GO:0005215;GO:0015075;GO:0022857;GO:0008271;GO:0022853;GO:0003674;GO:0008509;GO:0022804	K14708	SLC26A11	------	------	COG0659	S:Function unknown	PF00916.23	Sulfate_trans	Sulfate permease f	CYT	2	4	62.2	High
TRINITY_DN955_c0_g1_i2_orf1	gloverin-like [Ostrinia furnacalis] >XP_028168251.1 gloverin-like [Ostrinia furnacalis] >AYM26645.1 gloverin [Ostrinia furnacalis]	0.39069128	-1.355899037	5.84E-06	down	yes	0.9947	2.546	2.532	2.605	2.5	1	1.057	0.927	------	------	------	------	------	------	ENOG4110QR6	S:Function unknown	PF10793.12	Gloverin	Gloverin-like prote	PLA	2	20	21.1	High
TRINITY_DN479_c6_g1_i2_orf1	beta-1,3-glucan-binding protein-like [Ostrinia furnacalis]	0.414308812	-1.271221591	4.81E-07	down	yes	1.025	2.474	2.434	2.485	2.504	1	1.04	1.035	BP:regulation of response to external stimulus;BP:regulation of response to stimulus;BP:regulation of biological process;BP:regulation of defense response;BP:response to external biotic stimulus;BP:response to bacterium;BP:interspecies interaction between organisms;BP:organic substance metabolic process;BP:regulation of immune system process;BP:biological regulation;BP:regulation of innate immune response;BP:regulation of response to biotic stimulus;BP:innate immune response;BP:defense response;BP:response to stress;BP:defense response to other organism;BP:metabolic process;BP:immune response;BP:response to biotic stimulus;BP:response to other organism;BP:primary metabolic process;BP:regulation of immune response;BP:carbohydrate metabolic process;BP:response to external stimulus;BP:regulation of response to stress;BP:immune system process;BP:biological_process;BP:response to stimulus;BP:defense response to bacterium;CC:cellular_component;CC:cellular anatomical entity;CC:extracellular region;MF:carbohydrate binding;MF:molecular_function;MF:binding;MF:hydrolase	GO:0032101;GO:0048583;GO:0050789;GO:0031347;GO:0043207;GO:0009617;GO:0044419;GO:0071704;GO:0002682;GO:0065007;GO:0045088;GO:0002831;GO:0045087;GO:0006952;GO:0006950;GO:0098542;GO:0008152;GO:0006955;GO:0009607;GO:0051707;GO:0044238;GO:0050776;GO:0005975;GO:0009605;GO:0080134;GO:0002376;GO:0008150;GO:0050896;GO:0042742;GO:0005575;GO:0110165;GO:0005576;GO:0030246;GO:0003674;GO:0005488;GO:0016787;GO:0016798;GO:0003824;GO:0004553	K20697;K20	GNBP1;GNBP	map04624	Toll and Imd sigr	COG2273;ENOG410Z83Q;ENOG410YK4D	S:Function unknown;	PF15886.8;PF00	CBM39;Glyco_	Carbohydrate bind	CYT	5	11	53.4	High
TRINITY_DN10766_c0_g1_i1_orf1	hypothetical protein evm_008559 [Chilo suppressalis]	0.422964509	-1.241391482	1.30E-05	down	yes	1.013	2.395	2.41	2.477	2.297	1	1.027	1.012	BP:primary metabolic process;BP:organonitrogen compound metabolic process;BP:protein metabolic process;BP:macromolecule metabolic process;BP:organic substance metabolic process;BP:nitrogen compound metabolic process;BP:biological_process;BP:metabolic process;BP:proteolysis;MF:molecular_function;MF:serine hydrolase activity;MF:hydrolase activity;MF:catalytic activity, acting on a protein;MF:catalytic activity;MF:peptidase activity;MF:serine-type peptidase activity;	GO:0044238;GO:1901564;GO:0019538;GO:0043170;GO:0071704;GO:0006807;GO:0008150;GO:0008152;GO:0006508;GO:0003674;GO:0017171;GO:0016787;GO:0140096;GO:0003824;GO:0008233;GO:0008236	------	------	------	------	ENOG410XSGG	S:Function unknown	PF05577.15	Peptidase_S2	Serine carboxypep	CYT	3	6	56.2	High
TRINITY_DN5337_c0_g1_i6_orf1	epoxide hydrolase 1-like [Ostrinia furnacalis]	0.426508071	-1.229355051	1.20E-07	down	yes	1.004	2.354	2.383	2.349	2.33	1	0.999	1.012	CC:integral component of membrane;CC:cellular_component;CC:intrinsic component of membrane;CC:cellular anatomical entity;MF:hydrolase activity;MF:catalytic activity;MF:molecular_function;	GO:0016021;GO:0005575;GO:0031224;GO:0110165;GO:0016787;GO:0003824;GO:0003674	K22368;K223	EPHX3;EPHX4	map05207;ma	Chemical carcinc	ENOG410XRPK;COG0596	S:Function unknown;	PF00561.23;PF1	Abhydrolase_	alpha/beta hydrola	CYT	11	39	39.7	High
TRINITY_DN71863_c0_g1_i2_orf1	unnamed protein product [Diatraea saccharalis]	0.447623239	-1.159643156	1.42E-05	down	yes	1.017	2.272	2.326	2.175	2.314	1	1.02	1.032	BP:organic substance metabolic process;BP:organonitrogen compound metabolic process;BP:protein metabolic process;BP:macromolecule metabolic process;BP:biological_process;BP:nitrogen compound metabolic process;BP:metabolic process;BP:proteolysis;BP:primary metabolic process;MF:serine-type endopeptidase activity;MF:endopeptidase activity;MF:hydrolase activity;MF:serine hydrolase activity;MF:catalytic activity, acting on a protein;MF:catalytic activity;MF:molecular_function;MF:peptidase activity;MF:serine-	GO:0071704;GO:1901564;GO:0019538;GO:0043170;GO:0008150;GO:0006807;GO:0008152;GO:0006508;GO:0044238;GO:0004252;GO:0004175;GO:0016787;GO:0017171;GO:0140096;GO:0003824;GO:0003674;GO:0008233;GO:0008236	------	------	------	------	COG5640	O:Posttranslational modificatio	PF16030.8	GD_N	Serine protease gc	CYT	3	25	17.8	High
TRINITY_DN10231_c0_g2_i1_orf1	uncharacterized protein LOC114361472 [Ostrinia furnacalis]	0.387462301	-1.367872149	0.0001792	down	yes	0.8993	2.321	2.176	2.292	2.495	1	0.858	0.84	CC:cellular_component;CC:integral component of membrane;CC:cellular anatomical entity;CC:intrinsic component of membrane;	GO:0005575;GO:0016021;GO:0110165;GO:0031224	------	------	------	------	ENOG410XYE8	S:Function unknown	------	------	------	CYT	1	14	7.7	High

TRINITY_DN2205_c0_g1_i3_orf1	probable chitinase 2 [Ostrinia furnacalis]	0.477799135	-1.065523853	5.22E-07	down	yes	0.9943	2.081	2.088	2.108	2.046	1	0.996	0.987	BP:primary metabolic process;BP:carbohydrate metabolic process;BP:organic substance metabolic process;BP:biological_process;BP:metabolic process;MF:hydrolase activity;MF:carbohydrate derivative binding;MF:catalytic activity;MF:hydrolase activity, acting on glycosyl bonds;MF:chitin binding;MF:hydrolase activity, hydrolyzing O-glycosyl	GO:0044238;GO:0005975;GO:0071704;GO:0008150;GO:0008152;GO:0016787;GO:0097367;GO:0003824;GO:0016798;GO:0008061;GO:0004553;GO:0003674;GO:0005488	K01183	E3.2.1.14	map00520	Amino sugar and	COG3325	J:Translation, ribosomal structu	PF00704.31	Glyco_hydro_	Glycosyl hydrolase	CYT	14	29	57.1	High
TRINITY_DN9079_c0_g1_i5_orf1	UDP-glucuronosyltransferase-like [Ostrinia furnacalis]	0.41575592	-1.266191289	0.0002871	down	yes	0.913	2.196	2.067	2.135	2.386	1	0.902	0.837	CC:integral component of membrane;CC:cellular_component;CC:cellular anatomical entity;CC:intrinsic component of membrane;MF:catalytic activity;MF:transferase activity;MF:molecular_function;MF:transferase activity, transferring glycosyl groups;MF:transferase activity, transferring hexosyl groups;MF:glucuronosyltransferase activity;MF:UDP-	GO:0016021;GO:0005575;GO:0110165;GO:0031224;GO:0003824;GO:0016740;GO:0003674;GO:0016757;GO:0016758;GO:0015020;GO:0008194	K00699	UGT	map05207;ma	Chemical carcino	COG1819	S:Function unknown	PF00201.21	UDPGT	UDP-glucoronosyl	CYT	5	14	48.5	High
TRINITY_DN6988_c0_g1_i3_orf1	cuticle protein 1-like [Ostrinia furnacalis]	0.444702602	-1.169087248	1.23E-05	down	yes	0.957	2.152	2.073	2.201	2.182	1	0.937	0.934	MF:molecular_function;MF:structural constituent of cuticle;MF:structural molecule activity;	GO:0003674;GO:0042302;GO:0005198	------	------	------	------	ENOG4110SHP	S:Function unknown	PF17223.6	CPCFC	Cuticle protein CP	CYT	3	59	10.1	High
TRINITY_DN15420_c0_g3_i2_orf1	elongation factor 1-alpha 2-like [Galleria mellonella] >XP_031769625.1 elongation factor 1-alpha 2-like [Galleria mellonella]	0.462122642	-1.113652319	2.69E-05	down	yes	0.9797	2.12	2.062	2.214	2.084	1	1.005	0.934	CC:cytoplasm;CC:cellular anatomical entity;CC:cellular_component;MF:purine ribonucleoside triphosphate binding;MF:molecular_function;MF:binding;MF:nucleic acid binding;MF:ion binding;MF:heterocyclic compound binding;MF:nucleoside phosphate binding;MF:guanyl nucleotide binding;MF:nucleotide binding;MF:guanyl ribonucleotide binding;MF:purine nucleotide binding;MF:GTP binding;MF:hydrolase activity;MF:translation factor activity, RNA binding;MF:GTPase activity;MF:nucleoside-triphosphatase activity;MF:small molecule binding;MF:catalytic activity;MF:translation regulator activity;MF:hydrolase activity, acting on acid anhydrides, in phosphorus-containing anhydrides;MF:translation regulator activity, nucleic acid binding;MF:carbohydrate derivative binding;MF:organic cyclic compound binding;MF:hydrolase activity, acting on acid anhydrides;MF:anion binding;MF:pyrophosphatase	GO:0005737;GO:0110165;GO:0005575;GO:0035639;GO:0003674;GO:0005488;GO:0003676;GO:0043167;GO:1901363;GO:1901265;GO:0019001;GO:0000166;GO:0032561;GO:0017076;GO:0005525;GO:0016787;GO:0008135;GO:0003924;GO:0017111;GO:0036094;GO:0003824;GO:0045182;GO:0016818;GO:0090079;GO:0097367;GO:0097159;GO:0016817;GO:0043168;GO:0016462;GO:0032555;GO:0003746;GO:0032553	K03231	EEF1A	map05140;ma	Leishmaniasis;Le	COG5256	S:Function unknown	PF00009.30;PF0	GTP_EFTU;GT	Elongation factor T	CYT	3	30	50.6	High
TRINITY_DN13088_c0_g1_i5_orf1	beta-hexosaminidase subunit alpha-like isoform X2 [Ostrinia furnacalis]	0.482228626	-1.052210799	1.80E-07	down	yes	1.004	2.082	2.088	2.056	2.103	1	1.002	1.01	BP:organic substance metabolic process;BP:carbohydrate metabolic process;BP:primary metabolic process;BP:metabolic process;BP:biological_process;MF:hydrolase activity;MF:N-acetyl-beta-D-galactosaminidase activity;MF:hydrolase activity, acting on glycosyl bonds;MF:hexosaminidase activity;MF:catalytic activity;MF:beta-N-acetylhexosaminidase activity;MF:molecular_function;MF:hydrolase activity, hydrolyzing O-glycosyl compounds;	GO:0071704;GO:0005975;GO:0044238;GO:0008152;GO:0008150;GO:0016787;GO:0102148;GO:0016798;GO:0015929;GO:0003824;GO:0004563;GO:0003674;GO:0004553	K12373	HEXA_B	map00600;ma	Sphingolipid met	COG3525	F:Nucleotide transport and me	PF00728.25;PF1	Glyco_hydro_	Glycosyl hydrolase	CYT	9	17	62.8	High
TRINITY_DN15291_c0_g1_i11_orf1	uncharacterized protein LOC114353772 [Ostrinia furnacalis]	0.488220676	-1.034394701	1.84E-05	down	yes	0.9823	2.012	1.954	1.993	2.09	1	0.952	0.995	------	------	------	------	------	------	------	------	------	------	------	CYT	2	10	21.5	High
TRINITY_DN712_c0_g2_i1_orf1	serine protease inhibitor 77Ba-like [Ostrinia furnacalis]	0.48461165	-1.045099006	2.04E-06	down	yes	0.9983	2.06	2.061	2.102	2.018	1	1.012	0.983	CC:cellular_component;CC:cellular anatomical entity;CC:extracellular space;MF:endopeptidase inhibitor activity;MF:endopeptidase regulator activity;MF:peptidase regulator activity;MF:molecular_function;MF:peptidase inhibitor activity;MF:serine-type endopeptidase inhibitor activity;MF:enzyme inhibitor activity;MF:molecular function regulator;MF:enzyme regulator activity;	GO:0005575;GO:0110165;GO:0005615;GO:0004866;GO:0061135;GO:0061134;GO:0003674;GO:0030414;GO:0004867;GO:0004857;GO:0098772;GO:0030234	------	------	------	------	COG4826;ENOG4111MUA	S:Function unknown;	PF00079.23	Serpin	Serpin	CYT	7	17	46.4	High
TRINITY_DN98016_c0_g1_i1_orf1	methanethiol oxidase [Ostrinia furnacalis]	0.464240964	-1.107054266	4.58E-05	down	yes	0.9633	2.075	2.017	2.021	2.186	1	0.95	0.94	MF:selenium binding;MF:molecular_function;MF:binding;	GO:0008430;GO:0003674;GO:0005488	K17285	SELENBP1	map00920	Sulfur metabolisr	ENOG410XP4F	S:Function unknown	PF05694.14	SBP56	56kDa selenium bi	CYT	1	34	12.1	High
TRINITY_DN1194_c0_g1_i5_orf1	sequestosome-1-like isoform X4 [Ostrinia furnacalis]	0.495906141	-1.011861005	0.0005187	down	yes	0.9933	2.003	1.867	1.95	2.193	1	0.965	1.015	MF:cation binding;MF:transition metal ion binding;MF:ion binding;MF:molecular_function;MF:binding;MF:metal ion binding;MF:zinc ion binding;	GO:0043169;GO:0046914;GO:0043167;GO:0003674;GO:0005488;GO:0046872;GO:0008270	K14381	SQSTM1	map04137;ma	Mitophagy - anir	ENOG410XYAV;ENOG410Y3GC	S:Function unknown;	PF00569.20;PF1	ZZ;UBA_5;PB1	Zinc finger, ZZ typ	CYT	6	12	64.8	High
TRINITY_DN5667_c0_g1_i4_orf1	spodomicin-like [Ostrinia furnacalis] >QKV49445.1 diapausin [Ostrinia furnacalis]	0.479308617	-1.060973217	0.009518	down	yes	0.9567	1.996	2.16	1.588	2.24	1	0.791	1.079	BP:antifungal humoral response;BP:antimicrobial humoral response;BP:immune system process;BP:response to external biotic stimulus;BP:interspecies interaction between organisms;BP:cell killing;BP:cellular process;BP:defense response to fungus;BP:response to stress;BP:biological_process;BP:immune response;BP:humoral immune response;BP:response to other organism;BP:defense response;BP:response to biotic stimulus;BP:response to external stimulus;BP:response to fungus;BP:response to stimulus;BP:killing of cells of other organism;BP:defense response to other organism;CC:cellular anatomical entity;CC:cellular_component;CC:extracellular region;MF:ion channel regulator activity;MF:toxin activity;MF:molecular_function;MF:molecular function regulator;MF:ion channel inhibitor activity;MF:calcium channel regulator activity;MF:channel inhibitor activity;MF:channel	GO:0019732;GO:0019730;GO:0002376;GO:0043207;GO:0044419;GO:0001906;GO:0009987;GO:0050832;GO:0006950;GO:0008150;GO:0006955;GO:0006959;GO:0051707;GO:0006952;GO:0009607;GO:0009605;GO:0009620;GO:0050896;GO:0031640;GO:0098542;GO:0110165;GO:0005575;GO:0005576;GO:0099106;GO:0090729;GO:0003674;GO:0098772;GO:0008200;GO:0005246;GO:0016248;GO:0016247;GO:0019855	------	------	------	------	------	------	PF08036.14	Antimicrobial_	Diapausin family o	CYT	1	16	7.1	High
TRINITY_DN143637_c0_g1_i1_orf1	PX domain-containing protein kinase-like protein isoform X1 [Chelonus insularis]	0.458402923	-1.125311852	0.0001786	down	yes	0.8783	1.916	1.834	1.915	1.999	1	0.811	0.824	BP:phosphorylation;BP:nitrogen compound metabolic process;BP:organic substance metabolic process;BP:protein phosphorylation;BP:cellular process;BP:macromolecule modification;BP:protein modification process;BP:biological_process;BP:metabolic process;BP:primary metabolic process;BP:organonitrogen compound metabolic process;BP:cellular metabolic process;BP:macromolecule metabolic process;BP:protein metabolic process;BP:phosphate-containing compound metabolic process;BP:phosphorus metabolic process;MF:purine ribonucleoside triphosphate binding;MF:molecular_function;MF:binding;MF:ion binding;MF:heterocyclic compound binding;MF:nucleoside phosphate binding;MF:anion binding;MF:carbohydrate derivative binding;MF:phospholipid binding;MF:nucleotide binding;MF:transferase activity;MF:protein kinase activity;MF:purine nucleotide binding;MF:ATP binding;MF:kinase activity;MF:catalytic activity, acting on a protein;MF:lipid binding;MF:small molecule binding;MF:catalytic activity;MF:transferase activity, transferring phosphorus-containing groups;MF:adenyl nucleotide binding;MF:phosphatidylinositol binding;MF:organic cyclic compound binding;MF:adenyl ribonucleotide binding;MF:purine ribonucleotide binding;MF:phosphotransferase activity, alcohol	GO:0016310;GO:0006807;GO:0071704;GO:0006468;GO:0009987;GO:0043412;GO:0036211;GO:0008150;GO:0008152;GO:0044238;GO:1901564;GO:0044237;GO:0043170;GO:0019538;GO:0006796;GO:0006793;GO:0035639;GO:0003674;GO:0005488;GO:0043167;GO:1901363;GO:1901265;GO:0043168;GO:0097367;GO:0005543;GO:0000166;GO:0016740;GO:0004672;GO:0017076;GO:0005524;GO:0016301;GO:0140096;GO:0008289;GO:0036094;GO:0003824;GO:0016772;GO:0030554;GO:0035091;GO:0097159;GO:0032559;GO:0032555;GO:0016773;GO:0032553	K17543	PXK	------	------	ENOG410Z20X	T:Signal transduction mechanis	PF00787.27;PF0	PX;Pkinase;Pk	PX domain;Protein	CYT	1	2	65.7	Medium
TRINITY_DN82104_c0_g1_i5_orf1	uncharacterized protein LOC114349939 [Ostrinia furnacalis] >XP_028156338.1 uncharacterized protein LOC114349939 [Ostrinia furnacalis]	0.422912317	-1.241569515	0.000331	down	yes	0.8103	1.916	1.936	1.906	1.906	1	0.69	0.741	CC:cellular_component;CC:integral component of membrane;CC:cellular anatomical entity;CC:intrinsic component of membrane;	GO:0005575;GO:0016021;GO:0110165;GO:0031224	------	------	------	------	ENOG4112CWD;ENOG410Y3W5	S:Function unknown;	------	------	------	CYT	1	1	108	High
TRINITY_DN135780_c0_g1_i1_orf1	flotillin-1 isoform X1 [Pectinophora gossypiella]	0.431336406	-1.213114608	3.45E-06	down	yes	0.936	2.17	2.184	2.147	2.178	1	0.906	0.902	CC:cellular_component;CC:membrane;CC:cellular anatomical	GO:0005575;GO:0016020;GO:0110165	K07192	FLOT	map04910	Insulin signaling	COG2268	S:Function unknown	PF01145.28	Band_7	SPFH domain / Ba	CYT	2	27	12.8	High
TRINITY_DN6025_c0_g2_i1_orfp1	TRINITY_DN6025_c0_g2_i1_m.7749 TRINITY_DN6025_c0_g2::TRINITY_DN6025_c0_g2_i1::g.7749 ORF type:5prime_partial len:106 (+),score=47.10 TRINITY_DN6025_c0_g2_i1:3-320(+)	0.387067804	-1.369341784	0.01401	down	yes	0.862	2.227	2.373	2.661	1.646	1	0.611	0.975	------	------	------	------	------	------	------	------	------	------	------	PLA	1	10	11.6	Medium
TRINITY_DN29555_c0_g1_i8_orf1	fasciclin-1 [Ostrinia furnacalis]	0.479751244	-1.059641548	2.96E-06	down	yes	0.9643	2.01	2.009	1.985	2.036	1	0.92	0.973	------	------	K19897	FAS1	------	------	COG2335	M:Cell wall/membrane/envelop	PF02469.25	Fasciclin	Fasciclin domain	CYT	9	15	76	High
TRINITY_DN8953_c0_g1_i4_orf1	gonadotropin-releasing hormone receptor [Ostrinia furnacalis] >AXF67446.1 adipokinetic hormone receptor 1 [Ostrinia furnacalis]	0.488604286	-1.033261575	0.0003814	down	yes	0.9347	1.913	1.782	1.894	2.064	1	0.889	0.915	CC:integral component of membrane;CC:cellular_component;CC:cellular anatomical entity;CC:intrinsic component of membrane;MF:G-protein coupled receptor activity;MF:signaling receptor activity;MF:molecular transducer activity;MF:transmembrane signaling receptor activity;MF:molecular_function;	GO:0016021;GO:0005575;GO:0110165;GO:0031224;GO:0004930;GO:0038023;GO:0060089;GO:0004888;GO:0003674	K04280	GNRHR	map04080;ma	Neuroactive ligar	ENOG410XRW9	U:Intracellular trafficking, secre	PF00001.24	7tm_1	7 transmembrane	CYT	2	6	45	High
TRINITY_DN7590_c0_g1_i4_orf1	innexin inx1-like [Pectinophora gossypiella]	0.44505102	-1.16795736	0.0007792	down	yes	0.8723	1.96	1.947	1.799	2.135	1	0.847	0.77	BP:ion transport;BP:transport;BP:biological_process;BP:establishment of localization;BP:localization;CC:anchoring junction;CC:membrane;CC:integral component of membrane;CC:cellular_component;CC:intrinsic component of membrane;CC:cell-cell junction;CC:plasma membrane;CC:cell	GO:0006811;GO:0006810;GO:0008150;GO:0051234;GO:0051179;GO:0070161;GO:0016020;GO:0016021;GO:0005575;GO:0031224;GO:0005911;GO:0005886;GO:0030054;GO:0110165;GO:0005921	K22037	inx, zpg, ogre	------	------	ENOG4111F8E	S:Function unknown	PF00876.21	Innexin	Innexin	CYT	1	3	42.1	High
TRINITY_DN2943_c2_g2_i1_orf1	protein phosphatase inhibitor 2-like [Ostrinia furnacalis]	0.478627968	-1.063023393	0.0001617	down	yes	0.907	1.895	1.919	1.853	1.912	1	0.773	0.948	BP:regulation of phosphate metabolic process;BP:regulation of primary metabolic process;BP:regulation of metabolic process;BP:regulation of phosphatase activity;BP:regulation of response to stimulus;BP:regulation of signaling;BP:regulation of biological process;BP:regulation of cell communication;BP:regulation of signal transduction;BP:regulation of protein metabolic process;BP:regulation of macromolecule metabolic process;BP:biological regulation;BP:regulation of protein modification process;BP:regulation of molecular function;BP:regulation of cellular metabolic process;BP:regulation of catalytic activity;BP:regulation of cellular process;BP:regulation of phosphorus metabolic process;BP:biological_process;BP:regulation of nitrogen compound metabolic process;BP:regulation of dephosphorylation;BP:regulation of protein dephosphorylation;BP:regulation of hydrolase activity;BP:regulation of phosphoprotein phosphatase activity;MF:phosphatase regulator activity;MF:molecular_function;MF:enzyme inhibitor	GO:0019220;GO:0080090;GO:0019222;GO:0010921;GO:0048583;GO:0023051;GO:0050789;GO:0010646;GO:0009966;GO:0051246;GO:0060255;GO:0065007;GO:0031399;GO:0065009;GO:0031323;GO:0050790;GO:0050794;GO:0051174;GO:0008150;GO:0051171;GO:0035303;GO:0035304;GO:0051336;GO:0043666;GO:0019208;GO:0003674;GO:0004857;GO:0098772;GO:0019212;GO:0019888;GO:0030234;GO:0004864	K16833	PPP1R2, IPP2	------	------	ENOG4111SYV	T:Signal transduction mechanis	PF04979.17	IPP-2	Protein phosphata	CYT	1	9	18.8	High
TRINITY_DN4076_c1_g2_i2_orf1	vacuole membrane protein 1 [Ostrinia furnacalis]	0.433426573	-1.206140487	0.0007659	down	yes	0.9297	2.145	2.033	2.004	2.398	1	0.904	0.885	CC:cellular_component;CC:integral component of membrane;CC:cellular anatomical entity;CC:intrinsic component of membrane;	GO:0005575;GO:0016021;GO:0110165;GO:0031224	K21248	VMP1	map04140	Autophagy - ani	ENOG410XQHA	S:Function unknown	PF09335.14	SNARE_assoc	SNARE associated	CYT	1	4	44.2	High
TRINITY_DN1093_c0_g1_i6_orf1	uncharacterized protein LOC114361723 isoform X4 [Ostrinia furnacalis]	0.49003403	-1.029046155	5.67E-06	down	yes	1.008	2.057	2.096	2.079	1.995	1	1.028	0.997	------	------	------	------	------	------	ENOG410XRJB	S:Function unknown	PF16061.8	DUF4803	Domain of unknow	CYT	4	20	47.1	High
TRINITY_DN2171_c0_g1_i1_orf1	probable pterin-4-alpha-carbinolamine dehydratase isoform X1 [Ostrinia furnacalis]	0.496086106	-1.011337544	6.43E-07	down	yes	1.014	2.044	2.057	2.06	2.014	1	1.036	1.005	------	------	K01724	PCBD, phhB	map00790	Folate biosynthes	COG2154	H:Coenzyme transport and me	PF01329.22	Pterin_4a	Pterin 4 alpha carb	CYT	6	41	16.5	High
TRINITY_DN12286_c1_g1_i2_orf1	sideroflexin-1-3 [Galleria mellonella] >XP_026754161.1 sideroflexin-1-3 [Galleria mellonella]	0.491707317	-1.024128271	0.001523	down	yes	1.008	2.05	2.32	1.928	1.903	1	1.003	1.021	BP:amino acid transport;BP:organic substance transport;BP:nitrogen compound transport;BP:transport;BP:organic acid transport;BP:biological_process;BP:establishment of localization;BP:localization;CC:cellular_component;CC:integral component of membrane;CC:membrane;CC:mitochondrial membrane;CC:intrinsic component of membrane;CC:cellular anatomical entity;CC:organelle membrane;MF:transporter activity;MF:ion transmembrane transporter activity;MF:transmembrane transporter	GO:0006865;GO:0071702;GO:0071705;GO:0006810;GO:0015849;GO:0008150;GO:0051234;GO:0051179;GO:0005575;GO:0016021;GO:0016020;GO:0031966;GO:0031224;GO:0110165;GO:0031090;GO:0005215;GO:0015075;GO:0022857;GO:0003674	K23500	SFXN1_3	------	------	ENOG410YU8V	U:Intracellular trafficking, secre	PF03820.20	SFXNs	Sideroflexins	CYT	1	4	35.3	High
TRINITY_DN7735_c1_g1_i1_orf1	cuticular protein CPH [Spodoptera litura]	0.480107527	-1.058570541	0.002558	down	yes	0.9823	2.046	2.284	2.103	1.75	1	0.939	1.008	CC:cellular_component;CC:integral component of membrane;CC:cellular anatomical entity;CC:intrinsic component of membrane;	GO:0005575;GO:0016021;GO:0110165;GO:0031224	------	------	------	------	ENOG4110SF2;ENOG4110SXM	S:Function unknown;	------	------	------	CYT	1	5	15.2	High
TRINITY_DN2472_c0_g1_i6_orf1	programmed cell death protein 6 isoform X1 [Colias croceus] >XP_045492459.1 programmed cell death protein 6 isoform X1 [Colias croceus]	0.477596439	-1.066136015	6.73E-06	down	yes	0.9657	2.022	1.968	2.032	2.067	1	0.936	0.961	MF:cation binding;MF:ion binding;MF:molecular_function;MF:binding;MF:metal ion binding;MF:calcium ion binding;	GO:0043169;GO:0043167;GO:0003674;GO:0005488;GO:0046872;GO:0005509	K23902	PDCD6	------	------	ENOG410YKQK	S:Function unknown	PF00036.35;PF1	EF-hand_1;EF	EF hand;EF-hand c	CYT	3	22	21	High
TRINITY_DN3109_c0_g1_i5_orf1	protein takeout isoform X2 [Ostrinia furnacalis]	0.452364185	-1.144443383	5.07E-05	down	yes	0.8993	1.988	2.007	2.029	1.928	1	0.853	0.845	------	------	------	------	------	------	ENOG4111H46	S:Function unknown	PF06585.14	JHBP	Haemolymph juve	CYT	2	11	27.6	High
TRINITY_DN4256_c0_g1_i1_orf1	chitinase-3-like protein 1 [Ostrinia furnacalis]	0.446651163	-1.162779577	0.000645	down	yes	0.9603	2.15	2.392	2.051	2.007	1	0.938	0.943	------	------	K01183	E3.2.1.14	map00520	Amino sugar and	COG3325	G:Carbohydrate transport and	PF00704.31	Glyco_hydro_	Glycosyl hydrolase	CYT	1	2	53.8	High
TRINITY_DN276_c0_g1_i1_orf1	protein lethal(2)essential for life-like [Helicoverpa zea] >XP_049705426.1 protein lethal(2)essential for life [Helicoverpa armigera] >ATB54993.1 heat shock protein 20.8 [Helicoverpa armigera] >PZC74337.1 hypothetical protein B5X24_HaOG207971 [Helicoverpa armigera]	0.471635311	-1.084256359	4.69E-05	down	yes	0.9777	2.073	2.134	2.125	1.96	1	0.953	0.98	MF:cation binding;MF:metal ion binding;MF:molecular_function;MF:binding;MF:ion binding;	GO:0043169;GO:0046872;GO:0003674;GO:0005488;GO:0043167	K09542	CRYAB	map04213;ma	Longevity regulat	ENOG410YERS	O:Posttranslational modificatio	PF00011.24	HSP20	Hsp20/alpha cryst	CYT	5	65	21.3	High
TRINITY_DN7711_c1_g1_i3_orf1	long-chain fatty acid transport protein 1-like [Ostrinia furnacalis]	0.492064315	-1.023081199	4.28E-06	down	yes	0.9487	1.928	1.948	1.906	1.931	1	0.919	0.927	------	------	K08745	SLC27A1_4, F	map04931;ma	Insulin resistance	ENOG410XQ8T	S:Function unknown	PF00501.31;PF1	AMP-binding	AMP-binding enzy	CYT	5	8	73.6	High
TRINITY_DN58872_c0_g1_i1_orfp1	cuticle protein 64-like [Pectinophora gossypiella]	0.467197802	-1.097894607	0.001871	down	yes	0.8503	1.82	1.671	2.021	1.769	1	0.835	0.716	------	------	------	------	------	------	------	------	------	------	------	CYT	1	9	8.7	High
TRINITY_DN1424_c0_g1_i5_orf1	insect group I lytic polysaccharide monooxygenase [Ostrinia furnacalis]	0.4765625	-1.069262662	7.62E-06	down	yes	0.976	2.048	2.029	2.009	2.105	1	0.934	0.994	------	------	------	------	------	------	ENOG410Y8ZQ	S:Function unknown	PF03067.18	LPMO_10	Lytic polysaccharic	CYT	6	21	39	High

TRINITY_DN28661_c0_g1_i1_orf1	cathepsin B [Ostrinia furnacalis]	0.496837349	-1.009154464	5.62E-07	down	yes	0.9897	1.992	1.992	2.021	1.963	1	0.983	0.986	BP:biological regulation;BP:primary metabolic process;BP:organonitrogen compound metabolic process;BP:regulation of catalytic activity;BP:protein metabolic process;BP:macromolecule metabolic process;BP:regulation of molecular function;BP:organic substance metabolic process;BP:nitrogen compound metabolic process;BP:biological_process;BP:metabolic process;BP:proteolysis;MF:molecular_function;MF:endopeptidase activity;MF:hydrolase activity;MF:catalytic activity;MF:cysteine-type endopeptidase activity;MF:catalytic activity, acting on a protein;MF:peptidase activity;MF:cysteine-type peptidase	GO:0065007;GO:0044238;GO:1901564;GO:0050790;GO:0019538;GO:0043170;GO:0065009;GO:0071704;GO:0006807;GO:0008150;GO:0008152;GO:0006508;GO:0003674;GO:0004175;GO:0016787;GO:0003824;GO:0004197;GO:0140096;GO:0008233;GO:0008234	K01363	CTSB	map04142;ma	Lysosome;NOD-l	COG4870	S:Function unknown	PF00112.26;PF0	Peptidase_C1	Papain family cyst	CYT	10	38	37.1	High
TRINITY_DN4273_c1_g1_i5_orf1	tetraspanin-13 isoform X1 [Ostrinia furnacalis]	0.46038864	-1.11907586	0.0002597	down	yes	0.924	2.007	1.875	2.152	1.994	1	0.881	0.891	CC:cellular_component;CC:integral component of membrane;CC:cellular anatomical entity;CC:intrinsic component of membrane;	GO:0005575;GO:0016021;GO:0110165;GO:0031224	K17356	TSPAN13_31	------	------	ENOG4111IRY	I:Lipid transport and metabolis	PF00335.23	Tetraspanin	Tetraspanin family	CYT	1	4	25.3	High
TRINITY_DN325_c0_g1_i15_orf1	protein draper-like [Ostrinia furnacalis]	0.492831169	-1.020834594	8.55E-05	down	yes	0.9487	1.925	2.003	1.892	1.88	1	0.857	0.989	CC:cellular_component;CC:integral component of membrane;CC:cellular anatomical entity;CC:intrinsic component of membrane;	GO:0005575;GO:0016021;GO:0110165;GO:0031224	------	------	------	------	------	------	PF02363.22	C_tripleX	Cysteine rich repe	PLA	2	16	51.6	High
TRINITY_DN184_c0_g1_i10_orf1	C-type mannose receptor 2-like isoform X1 [Leguminivora glycinivorella]	0.408904811	-1.290163059	0.001079	down	yes	0.799	1.954	1.935	1.804	2.122	1	0.716	0.681	MF:molecular_function;MF:carbohydrate binding;MF:binding;	GO:0003674;GO:0030246;GO:0005488	------	------	------	------	ENOG410XPJ1;ENOG410XRS3;ENOG410ZPTE;ENOG410ZRYS;ENOG410XNWT	S:Function unknown;U:Intracell	PF00059.24	Lectin_C	Lectin C-type dom	CYT	1	27	30.8	High
TRINITY_DN13887_c0_g1_i5_orf1	transmembrane protein 184B isoform X3 [Ostrinia furnacalis]	0.494180407	-1.016890281	1.26E-05	down	yes	1.019	2.062	2.131	2.021	2.033	1	0.999	1.057	------	------	------	------	------	------	ENOG410XQ3V	S:Function unknown	PF03619.19	Solute_trans_	Organic solute tra	CYT	2	7	46	High
TRINITY_DN19923_c0_g1_i1_orf1	uncharacterized protein LOC114350958 [Ostrinia furnacalis]	0.49851925	-1.004278881	0.0001606	down	yes	1.01	2.026	2.055	2.136	1.887	1	1.004	1.025	------	------	------	------	------	------	ENOG4111MP7	S:Function unknown	PF15430.9	SVWC	Single domain von	CYT	7	71	16.2	High
TRINITY_DN19821_c0_g2_i4_orf1	uncharacterized protein LOC114359393 isoform X1 [Ostrinia furnacalis]	0.456581439	-1.131055881	1.51E-05	down	yes	0.9643	2.112	2.14	2.036	2.161	1	0.976	0.917	BP:autophagy;BP:cellular process;BP:cellular metabolic process;BP:cellular catabolic process;BP:process utilizing autophagic mechanism;BP:metabolic process;BP:biological_process;BP:catabolic process;	GO:0006914;GO:0009987;GO:0044237;GO:0044248;GO:0061919;GO:0008152;GO:0008150;GO:0009056	K10435	MAP1LC	map04137;ma	Mitophagy - anir	ENOG4111JAT	U:Intracellular trafficking, secre	PF02991.19;PF0	ATG8;APG12	Autophagy protein	CYT	4	17	29.1	High
TRINITY_DN664_c0_g1_i18_orf1	chitinase-like protein EN03 isoform X2 [Ostrinia furnacalis]	0.465472779	-1.103231293	4.37E-06	down	yes	0.9747	2.094	2.083	2.15	2.048	1	0.969	0.955	BP:primary metabolic process;BP:carbohydrate metabolic process;BP:organic substance metabolic process;BP:biological_process;BP:metabolic process;CC:cellular_component;CC:extracellular region;CC:cellular anatomical entity;MF:carbohydrate derivative binding;MF:chitin binding;MF:molecular_function;MF:binding;	GO:0044238;GO:0005975;GO:0071704;GO:0008150;GO:0008152;GO:0005575;GO:0005576;GO:0110165;GO:0097367;GO:0008061;GO:0003674;GO:0005488	------	------	------	------	COG3325	S:Function unknown	PF00704.31	Glyco_hydro_	Glycosyl hydrolase	CYT	19	48	48	High
TRINITY_DN501_c0_g1_i5_orf1	hypothetical protein evm_002550 [Chilo suppressalis]	0.496321726	-1.010652485	1.46E-06	down	yes	1.012	2.039	2.024	2.082	2.01	1	1.016	1.019	CC:integral component of membrane;CC:cellular_component;CC:extracellular region;CC:intrinsic component of membrane;CC:cellular anatomical entity;MF:binding;MF:cation binding;MF:molecular function regulator;MF:peptidase regulator activity;MF:ion binding;MF:molecular_function;MF:peptidase inhibitor activity;MF:enzyme inhibitor activity;MF:metal ion binding;MF:enzyme regulator activity;MF:calcium ion binding;	GO:0016021;GO:0005575;GO:0005576;GO:0031224;GO:0110165;GO:0005488;GO:0043169;GO:0098772;GO:0061134;GO:0043167;GO:0003674;GO:0030414;GO:0004857;GO:0046872;GO:0030234;GO:0005509	------	------	------	------	ENOG410XSTY;ENOG410XP6K	S:Function unknown;	PF12947.10;PF0	EGF_3;EGF_C	EGF domain;Calciu	PLA	90	8	1895.1	High
TRINITY_DN19951_c0_g1_i5_orf1	protein croquemort-like [Ostrinia furnacalis]	0.478453307	-1.063549957	0.001363	down	yes	0.9837	2.056	2.263	2.085	1.821	1	0.904	1.047	BP:multicellular organismal process;BP:sensory perception of smell;BP:neurological system process;BP:response to stimulus;BP:sensory perception of chemical stimulus;BP:sensory perception;BP:biological_process;BP:system process;CC:membrane;CC:cellular_component;CC:cellular anatomical entity;CC:plasma membrane;CC:integral component of membrane;CC:intrinsic component of membrane;	GO:0032501;GO:0007608;GO:0050877;GO:0050896;GO:0007606;GO:0007600;GO:0008150;GO:0003008;GO:0016020;GO:0005575;GO:0110165;GO:0005886;GO:0016021;GO:0031224	K13885	SCARB1	map04979;ma	Cholesterol meta	ENOG410XS17	O:Posttranslational modificatio	PF01130.24	CD36	CD36 family	CYT	1	3	56.6	High
TRINITY_DN5064_c0_g1_i4_orf1	sortilin-related receptor-like [Ostrinia furnacalis]	0.48671294	-1.038856964	5.76E-06	down	yes	0.9817	2.017	2.066	1.962	2.022	1	0.959	0.986	BP:metabolic process;BP:cellular biosynthetic process;BP:cellular macromolecule biosynthetic process;BP:macromolecule metabolic process;BP:organic substance biosynthetic process;BP:cellular macromolecule metabolic process;BP:peptide biosynthetic process;BP:organic substance metabolic process;BP:organonitrogen compound biosynthetic process;BP:organonitrogen compound metabolic process;BP:cellular process;BP:amide biosynthetic process;BP:cellular nitrogen compound biosynthetic process;BP:transport;BP:biosynthetic process;BP:macromolecule biosynthetic process;BP:biological_process;BP:establishment of localization;BP:localization;BP:primary metabolic process;BP:endocytosis;BP:vesicle-mediated transport;BP:protein metabolic process;BP:nitrogen compound metabolic process;BP:cellular nitrogen compound metabolic process;BP:cellular metabolic process;BP:cellular amide metabolic process;BP:peptide metabolic process;BP:translation;CC:multivesicular body membrane;CC:recycling endosome membrane;CC:endoplasmic reticulum membrane;CC:intracellular organelle;CC:non-membrane-bounded organelle;CC:organelle;CC:intrinsic component of membrane;CC:endosome membrane;CC:organelle membrane;CC:integral component of membrane;CC:membrane;CC:late endosome membrane;CC:cellular anatomical entity;CC:bounding membrane of organelle;CC:transport vesicle membrane;CC:cytoplasmic vesicle membrane;CC:vesicle membrane;CC:plasma membrane;CC:early endosome membrane;CC:macromolecular	GO:0008152;GO:0044249;GO:0034645;GO:0043170;GO:1901576;GO:0044260;GO:0043043;GO:0071704;GO:1901566;GO:1901564;GO:0009987;GO:0043604;GO:0044271;GO:0006810;GO:0009058;GO:0009059;GO:0008150;GO:0051234;GO:0051179;GO:0044238;GO:0006897;GO:0016192;GO:0019538;GO:0006807;GO:0034641;GO:0044237;GO:0043603;GO:0006518;GO:0006412;GO:0032585;GO:0055038;GO:0005789;GO:0043229;GO:0043228;GO:0043226;GO:0031224;GO:0010008;GO:0031090;GO:0016021;GO:0016020;GO:0031902;GO:0110165;GO:0098588;GO:0030658;GO:0030659;GO:0012506;GO:0005886;GO:0031901;GO:0032991;GO:0005840;GO:0043232;GO:1990904;GO:0000139;GO:0005575;GO:0005576;GO:0003674;GO:0005198;GO:0003735	K24498	SORL1	------	------	ENOG410Y3W5	S:Function unknown	PF15902.8;PF00	Sortilin-Vps1	Sortilin, neurotens	CYT	19	10	243.8	High
TRINITY_DN4013_c0_g1_i4_orf1	uncharacterized protein LOC114353190 isoform X1 [Ostrinia furnacalis] >XP_028160984.1 uncharacterized protein LOC114353190 isoform X2 [Ostrinia furnacalis] >XP_028161062.1 uncharacterized protein LOC114353190 isoform X1 [Ostrinia furnacalis] >XP_028161142.1 uncharacterized protein LOC114353190 isoform X1 [Ostrinia furnacalis]	0.467074963	-1.098273981	0.0003579	down	yes	0.9533	2.041	2.156	2.111	1.856	1	0.948	0.912	CC:cellular_component;CC:membrane protein complex;CC:macromolecular complex;CC:ESCRT complex;CC:ESCRT I complex;	GO:0005575;GO:0098796;GO:0032991;GO:0036452;GO:0000813	K12186	FAM125	map04144	Endocytosis	ENOG4110KHY	U:Intracellular trafficking, secre	PF10240.12	DUF2464	Multivesicular bod	CYT	1	10	10.9	High
TRINITY_DN110402_c0_g2_i1_orf1	apolipophorins-like [Ostrinia furnacalis]	0.494246436	-1.016697533	5.27E-06	down	yes	0.9707	1.964	1.943	1.936	2.013	1	0.938	0.974	BP:lipid transport;BP:transport;BP:organic substance transport;BP:biological_process;BP:establishment of localization;BP:localization;CC:cellular_component;CC:extracellular region;CC:cellular anatomical entity;MF:lipid transporter activity;MF:transporter activity;MF:lipid binding;MF:molecular_function;MF:binding;	GO:0006869;GO:0006810;GO:0071702;GO:0008150;GO:0051234;GO:0051179;GO:0005575;GO:0005576;GO:0110165;GO:0005319;GO:0005215;GO:0008289;GO:0003674;GO:0005488	------	------	------	------	ENOG41112S0	S:Function unknown	------	------	------	CYT	1	8	12.3	High
TRINITY_DN140_c1_g1_i2_orf1	modular serine protease-like isoform X1 [Ostrinia furnacalis]	0.487897757	-1.035349244	0.0001137	down	yes	0.9353	1.917	1.91	1.857	1.983	1	0.828	0.978	BP:nitrogen compound metabolic process;BP:organic substance metabolic process;BP:proteolysis;BP:biological_process;BP:metabolic process;BP:primary metabolic process;BP:organonitrogen compound metabolic process;BP:protein metabolic process;BP:macromolecule metabolic process;CC:intrinsic component of membrane;CC:integral component of membrane;CC:cellular anatomical entity;CC:cellular_component;MF:serine-type endopeptidase activity;MF:endopeptidase activity;MF:molecular_function;MF:peptidase activity;MF:serine-type peptidase activity;MF:hydrolase activity;MF:serine	GO:0006807;GO:0071704;GO:0006508;GO:0008150;GO:0008152;GO:0044238;GO:1901564;GO:0019538;GO:0043170;GO:0031224;GO:0016021;GO:0110165;GO:0005575;GO:0004252;GO:0004175;GO:0003674;GO:0008233;GO:0008236;GO:0016787;GO:0017171;GO:0140096;GO:0003824	K20674	MODSP	map04624	Toll and Imd sigr	COG5640	O:Posttranslational modificatio	PF00057.21;PF0	Ldl_recept_a;	Low-density lipop	CYT	3	5	83	High
TRINITY_DN7226_c0_g1_i5_orf1	chemosensory protein [Dioryctria abietella]	0.460407125	-1.119017936	0.0001349	down	yes	0.9047	1.965	2.025	1.853	2.017	1	0.854	0.86	------	------	------	------	------	------	ENOG4111U5H;ENOG410YU7V	S:Function unknown;	PF03392.16	OS-D	Insect pheromone	CYT	4	28	8.8	High
TRINITY_DN41761_c0_g1_i4_orf1	transmembrane protease serine 9 [Ostrinia furnacalis]	0.467843691	-1.095901496	0.000248	down	yes	0.9937	2.124	2.232	2.175	1.966	1	0.916	1.065	BP:organic substance metabolic process;BP:organonitrogen compound metabolic process;BP:protein metabolic process;BP:macromolecule metabolic process;BP:biological_process;BP:nitrogen compound metabolic process;BP:metabolic process;BP:proteolysis;BP:primary metabolic process;MF:serine-type endopeptidase activity;MF:endopeptidase activity;MF:hydrolase activity;MF:serine hydrolase activity;MF:catalytic activity, acting on a protein;MF:catalytic activity;MF:molecular_function;MF:peptidase activity;MF:serine-	GO:0071704;GO:1901564;GO:0019538;GO:0043170;GO:0008150;GO:0006807;GO:0008152;GO:0006508;GO:0044238;GO:0004252;GO:0004175;GO:0016787;GO:0017171;GO:0140096;GO:0003824;GO:0003674;GO:0008233;GO:0008236	------	------	------	------	COG5640	O:Posttranslational modificatio	PF00089.29;PF0	Trypsin;DUF1	Trypsin;Domain of	CYT	3	10	58.5	High
TRINITY_DN125150_c0_g1_i1_orf1	aldehyde dehydrogenase, dimeric NADP-preferring isoform X5 [Ostrinia furnacalis]	0.413824466	-1.272909154	0.0002559	down	yes	0.91	2.199	2.143	2.095	2.358	1	0.952	0.778	BP:cellular aldehyde metabolic process;BP:cellular process;BP:cellular metabolic process;BP:organic substance metabolic process;BP:biological_process;BP:metabolic process;MF:catalytic	GO:0006081;GO:0009987;GO:0044237;GO:0071704;GO:0008150;GO:0008152;GO:0003824;GO:0003674;GO:0016491	K00129	ALDH3	map00982;ma	Drug metabolism	------	------	------	------	------	CYT	2	22	8.3	High
TRINITY_DN34786_c0_g1_i1_orf1	small heat shock protein Hsp29.7 [Ostrinia furnacalis]	0.434699714	-1.201908948	5.29E-05	down	yes	0.912	2.098	2.081	2.052	2.162	1	0.808	0.928	------	------	K09542	CRYAB	map04213;ma	Longevity regula	ENOG410YFRS	O:Posttranslational modificatio	PF00011.24	HSP20	Hsp20/alpha cryst	CYT	2	8	28.2	High
TRINITY_DN2650_c0_g1_i1_orf1	hypothetical protein HW555_002849 [Spodoptera exigua] >CAH0691914.1 unnamed protein product [Spodoptera exigua]	0.471378968	-1.085040704	0.0001095	down	yes	0.9503	2.016	1.974	1.937	2.138	1	0.887	0.964	------	------	------	------	------	------	ENOG4111T57	S:Function unknown	PF12974.10	Phosphonate	ABC transporter, p	CYT	5	16	31.9	High
TRINITY_DN3275_c0_g2_i3_orf1	hypothetical protein B5X24_HaOG216046 [Helicoverpa armigera]	0.314602683	-1.66839712	0.01138	down	yes	0.6097	1.938	1.661	1.78	2.372	1	0.513	0.316	MF:cation binding;MF:ion binding;MF:molecular_function;MF:binding;MF:metal ion binding;MF:calcium ion binding;	GO:0043169;GO:0043167;GO:0003674;GO:0005488;GO:0046872;GO:0005509	K02183	CALM	map05214;ma	Glioma;Pathways	COG5126;COG5126;COG5126;ENOG410XTI5	D:Cell cycle control, cell divisio	------	------	------	CYT	1	36	2.8	Medium
TRINITY_DN40126_c0_g1_i1_orf1	aldehyde dehydrogenase X, mitochondrial-like [Ostrinia furnacalis]	0.496138996	-1.011183738	8.72E-06	down	yes	1.028	2.072	2.102	2.007	2.107	1	1.027	1.056	MF:catalytic activity;MF:oxidoreductase activity, acting on the aldehyde or oxo group of donors;MF:molecular_function;MF:oxidoreductase activity, acting on the aldehyde or oxo group of donors, NAD or NADP as acceptor;MF:oxidoreductase activity;	GO:0003824;GO:0016903;GO:0003674;GO:0016620;GO:0016491	K00128	ALDH	map00310;ma	Lysine degradati	COG1012	C:Energy production and conve	PF00171.25	Aldedh	Aldehyde dehydro	CYT	19	65	45.5	High
TRINITY_DN3383_c0_g1_i5_orf1	uncharacterized protein LOC114357426 [Ostrinia furnacalis]	0.483638114	-1.048000153	1.99E-06	down	yes	1.005	2.078	2.105	2.036	2.094	1	0.983	1.032	------	------	------	------	------	------	ENOG4110QG2;ENOG4111FAF	S:Function unknown;	------	------	------	CYT	4	14	35.5	High
TRINITY_DN9492_c0_g1_i7_orf1	aldo-keto reductase AKR2E4-like [Ostrinia furnacalis]	0.487788945	-1.035671034	3.44E-05	down	yes	0.9707	1.99	2.007	1.906	2.058	1	0.926	0.986	MF:catalytic activity;MF:molecular_function;MF:oxidoreductase activity;	GO:0003824;GO:0003674;GO:0016491	------	------	------	------	COG0656	S:Function unknown	PF00248.24	Aldo_ket_red	Aldo/keto reducta	CYT	7	30	38.3	High
TRINITY_DN20614_c0_g1_i1_orf1	hypothetical protein evm_003552 [Chilo suppressalis]	0.391153082	-1.354194764	0.0116	down	yes	0.787	2.012	1.936	1.611	2.49	1	0.681	0.68	CC:cytoplasm;CC:cellular_component;CC:cellular anatomical entity;MF:enzyme regulator activity;MF:guanyl-nucleotide exchange factor activity;MF:GTPase regulator activity;MF:molecular_function;MF:molecular function regulator;MF:nucleoside-triphosphatase regulator activity;	GO:0005737;GO:0005575;GO:0110165;GO:0030234;GO:0005085;GO:0030695;GO:0003674;GO:0098772;GO:0060589	K21066	ARHGEF18	map04530	Tight junction	ENOG410XT68;COG5422	T:Signal transduction mechanis	PF00621.23;PF1	RhoGEF;PH_1	RhoGEF domain;P	CYT	2	1	143	High
TRINITY_DN7226_c0_g1_i2_orf1	chemosensory protein [Conogethes punctiferalis]	0.495588972	-1.012784009	8.00E-05	down	yes	0.9887	1.995	1.916	1.956	2.112	1	0.963	1.003	------	------	------	------	------	------	ENOG4111U5H;ENOG410YU7V	S:Function unknown;	PF03392.16	OS-D	Insect pheromone	CYT	5	54	9	High
TRINITY_DN45097_c0_g1_i5_orf1	ras-related protein Rab-24-like [Ostrinia furnacalis]	0.49387013	-1.01779638	0.0001166	down	yes	0.9507	1.925	1.814	1.998	1.963	1	0.889	0.963	MF:purine ribonucleoside triphosphate binding;MF:molecular_function;MF:binding;MF:ion binding;MF:heterocyclic compound binding;MF:nucleoside phosphate binding;MF:anion binding;MF:guanyl nucleotide binding;MF:nucleotide binding;MF:guanyl ribonucleotide binding;MF:purine nucleotide binding;MF:GTP binding;MF:hydrolase activity;MF:GTPase activity;MF:nucleoside-triphosphatase activity;MF:small molecule binding;MF:catalytic activity;MF:hydrolase activity, acting on acid anhydrides, in phosphorus-containing anhydrides;MF:carbohydrate derivative binding;MF:organic cyclic compound binding;MF:hydrolase activity, acting on acid anhydrides;MF:pyrophosphatase activity;MF:purine	GO:0035639;GO:0003674;GO:0005488;GO:0043167;GO:1901363;GO:1901265;GO:0043168;GO:0019001;GO:0000166;GO:0032561;GO:0017076;GO:0005525;GO:0016787;GO:0003924;GO:0017111;GO:0036094;GO:0003824;GO:0016818;GO:0097367;GO:0097159;GO:0016817;GO:0016462;GO:0032555;GO:0032553	K07912	RAB24	------	------	ENOG410YCCP	S:Function unknown	PF00071.25;PF0	Ras;Roc;Arf	Ras family;Ras of C	CYT	2	9	23.2	High
TRINITY_DN36476_c1_g1_i1_orfp1	TRINITY_DN36476_c1_g1_i1_m.70910 TRINITY_DN36476_c1_g1::TRINITY_DN36476_c1_g1_i1::g.70910 ORF type:5prime_partial len:88 (-	0.452999528	-1.142418549	1.52E-05	down	yes	0.959	2.117	2.061	2.092	2.198	1	0.942	0.935	------	------	------	------	------	------	------	------	------	------	------	CYT	2	21	9.5	High
TRINITY_DN2416_c0_g1_i5_orf1	somatomedin-B and thrombospondin type-1 domain-containing protein [Ostrinia furnacalis] >XP_028177886.1 somatomedin-B and thrombospondin type-1 domain-containing protein [Ostrinia furnacalis]	0.484775254	-1.044612039	3.97E-05	down	yes	1.003	2.069	2.069	2.161	1.977	1	1.024	0.986	------	------	------	------	------	------	ENOG4111K8M;ENOG411278B;ENOG41100PZ	S:Function unknown;	PF19028.3;PF00	TSP1_spondir	Spondin-like TSP1	EXC	7	33	30.5	High
TRINITY_DN21420_c0_g1_i2_orf1	glutathione peroxidase, partial [Ostrinia furnacalis]	0.499348044	-1.001882374	2.15E-05	down	yes	0.9957	1.994	1.937	2.007	2.039	1	0.939	1.048	BP:response to stimulus;BP:response to stress;BP:biological_process;BP:response to oxidative stress;MF:antioxidant activity;MF:peroxidase activity;MF:catalytic activity;MF:molecular_function;MF:oxidoreductase activity, acting on peroxide as acceptor;MF:glutathione peroxidase activity;MF:oxidoreductase activity;	GO:0050896;GO:0006950;GO:0008150;GO:0006979;GO:0016209;GO:0004601;GO:0003824;GO:0003674;GO:0016684;GO:0004602;GO:0016491	K05361;K00	GPX4;gpx, bt	map00480;ma	Glutathione met	COG0386	U:Intracellular trafficking, secre	------	------	------	CYT	5	76	8.3	High
TRINITY_DN69_c0_g1_i1_orf1	glycerol-3-phosphate dehydrogenase [NAD(+)], cytoplasmic isoform X1 [Ostrinia furnacalis]	0.474576271	-1.075288127	1.04E-06	down	yes	0.98	2.065	2.03	2.066	2.098	1	0.966	0.974	------	------	K00006	GPD1	map00564	Glycerophosphol	COG0240	C:Energy production and conve	PF01210.26;PF0	NAD_Gly3P_d	NAD-dependent g	CYT	21	63	39.8	High
TRINITY_DN7868_c0_g1_i2_orf1	uncharacterized protein LOC114353432 isoform X4 [Ostrinia furnacalis]	0.468981247	-1.09239786	0.006127	down	yes	0.9253	1.973	1.862	1.712	2.344	1	0.954	0.822	------	------	------	------	------	------	ENOG410ZHS3	S:Function unknown	PF02995.20;PF0	DUF229;Sulfa	Protein of unknow	EXC	1	5	36.1	High

TRINITY_DN4998_c0_g1_i21_orf1	phenoloxidase subunit 2-like [Ostrinia furnacalis]	0.498271605	-1.004995734	1.62E-07	down	yes	1.009	2.025	2.01	2.016	2.048	1	1.007	1.019	CC:cellular_component;CC:extracellular region;CC:cellular anatomical entity;MF:cation binding;MF:monooxygenase activity;MF:oxidoreductase activity, acting on paired donors, with incorporation or reduction of molecular oxygen;MF:monophenol monooxygenase activity;MF:oxidoreductase activity, acting on paired donors, with incorporation or reduction of molecular oxygen, another compound as one donor, and incorporation of one atom of oxygen;MF:ion binding;MF:catalytic activity;MF:molecular_function;MF:binding;MF:metal ion	GO:0005575;GO:0005576;GO:0110165;GO:0043169;GO:0004497;GO:0016705;GO:0004503;GO:0016716;GO:0043167;GO:0003824;GO:0003674;GO:0005488;GO:0046872;GO:0016491	------	------	------	------	ENOG4111KTW	S:Function unknown	PF03723.17;PF0(	Hemocyanin_	Hemocyanin, ig-lik	PLA	16	38	80	High
TRINITY_DN3017_c0_g1_i6_orf1	uncharacterized protein LOC114364875 isoform X1 [Ostrinia furnacalis] >XP_028177022.1 uncharacterized protein LOC114364875 isoform X1 [Ostrinia furnacalis]	0.483870968	-1.047305715	0.01784	down	yes	0.975	2.015	1.989	2.489	1.566	1	1.001	0.924	CC:integral component of membrane;CC:cellular_component;CC:cellular anatomical entity;CC:intrinsic component of membrane;MF:transporter activity;MF:transmembrane transporter activity;MF:molecular_function;	GO:0016021;GO:0005575;GO:0110165;GO:0031224;GO:0005215;GO:0022857;GO:0003674	------	------	------	------	ENOG410YMHR	G:Carbohydrate transport and r	PF12832.10;PF07	MFS_1_like;M	MFS_1 like family;M	CYT	2	5	73	High
TRINITY_DN64892_c0_g1_i1_orf1	aldehyde dehydrogenase X, mitochondrial [Manduca sexta] >KAG6450704.1 hypothetical protein O3G_MSEX006722 [Manduca sexta]	0.482996973	-1.049913948	0.000804	down	yes	0.9573	1.982	1.995	1.786	2.166	1	0.917	0.955	MF:catalytic activity;MF:oxidoreductase activity, acting on the aldehyde or oxo group of donors;MF:molecular_function;MF:oxidoreductase activity, acting on the aldehyde or oxo group of donors, NAD or NADP as acceptor;MF:oxidoreductase activity;	GO:0003824;GO:0016903;GO:0003674;GO:0016620;GO:0016491	K00128	ALDH	map00310;maj	Lysine degradatic	COG1012	U:Intracellular trafficking, secre	PF00171.25	Aldedh	Aldehyde dehydro	CYT	3	59	5.7	High
TRINITY_DN89083_c0_g1_i1_orf1	lysine-specific demethylase 4A isoform X2 [Diachasma alloeum]	0.431740443	-1.211763853	0.0002032	down	yes	0.8583	1.988	1.958	1.92	2.087	1	0.792	0.783	BP:cellular component organization or biogenesis;BP:cellular component organization;BP:cellular process;BP:biological_process;BP:chromatin organization;CC:intracellular organelle;CC:membrane-bounded organelle;CC:organelle;CC:nucleus;CC:cellular anatomical entity;CC:intracellular membrane-bounded organelle;CC:cellular_component;MF:molecular_function;MF:binding;MF:oxidoreductase activity;MF:cation binding;MF:demethylase activity;MF:oxidoreductase activity, acting on paired donors, with incorporation or reduction of molecular oxygen, 2-oxoglutarate as one donor, and incorporation of one atom each of oxygen into both donors;MF:oxidoreductase activity, acting on paired donors, with incorporation or reduction of molecular oxygen;MF:ion binding;MF:metal ion binding;MF:catalytic activity, acting on a protein;MF:catalytic activity;MF:dioxygenase activity;MF:histone demethylase activity;MF:histone demethylase activit;MF:protein	GO:0071840;GO:0016043;GO:0009987;GO:0008150;GO:0006325;GO:0043229;GO:0043227;GO:0043226;GO:0005634;GO:0110165;GO:0043231;GO:0005575;GO:0003674;GO:0005488;GO:0016491;GO:0043169;GO:0032451;GO:0016706;GO:0016705;GO:0043167;GO:0046872;GO:0140096;GO:0003824;GO:0051213;GO:0032452;GO:0032454;GO:0140457	K06709	KDM4, JMJD2	------	------	COG5141;ENOG410XP0T	S:Function unknown;	PF18104.4	Tudor_2	Jumonji domain-c	PLA	1	6	12.5	Medium
TRINITY_DN6462_c0_g1_i5_orf1	probable histone-lysine N-methyltransferase CG1716 [Ostrinia furnacalis]	0.479183884	-1.061348706	0.0005333	down	yes	0.9277	1.936	2.065	1.768	1.976	1	0.942	0.841	BP:protein methylation;BP:histone H3-K36 trimethylation;BP:regulation of primary metabolic process;BP:regulation of metabolic process;BP:peptidyl-lysine methylation;BP:regulation of cellular biosynthetic process;BP:regulation of cellular metabolic process;BP:protein alkylation;BP:nitrogen compound metabolic process;BP:regulation of biological process;BP:peptidyl-lysine modification;BP:peptidyl-lysine trimethylation;BP:cellular macromolecule metabolic process;BP:organic substance metabolic process;BP:peptidyl-amino acid modification;BP:regulation of macromolecule metabolic process;BP:histone lysine methylation;BP:biological regulation;BP:histone modification;BP:histone methylation;BP:methylation;BP:regulation of gene expression;BP:regulation of nucleobase-containing compound metabolic process;BP:cellular process;BP:regulation of biosynthetic process;BP:regulation of nucleic acid-templated transcription;BP:regulation of cellular process;BP:macromolecule modification;BP:protein modification process;BP:biological_process;BP:regulation of nitrogen compound metabolic process;BP:metabolic process;BP:regulation of RNA biosynthetic process;BP:primary metabolic process;BP:organonitrogen compound metabolic process;BP:protein metabolic process;BP:histone H3-K36 methylation;BP:regulation of RNA metabolic process;BP:cellular metabolic process;BP:macromolecule metabolic process;BP:regulation of transcription, DNA-templated;BP:regulation of macromolecule biosynthetic process;BP:macromolecule methylation;CC:intracellular	GO:0006479;GO:0097198;GO:0080090;GO:0019222;GO:0018022;GO:0031326;GO:0031323;GO:0008213;GO:0006807;GO:0050789;GO:0018205;GO:0018023;GO:0044260;GO:0071704;GO:0018193;GO:0060255;GO:0034968;GO:0065007;GO:0016570;GO:0016571;GO:0032259;GO:0010468;GO:0019219;GO:0009987;GO:0009889;GO:1903506;GO:0050794;GO:0043412;GO:0036211;GO:0008150;GO:0051171;GO:0008152;GO:2001141;GO:0044238;GO:1901564;GO:0019538;GO:0010452;GO:0051252;GO:0044237;GO:0043170;GO:0006355;GO:0010556;GO:0043414;GO:0043229;GO:0043228;GO:0043227;GO:0005634;GO:0043226;GO:0005694;GO:0110165;GO:0043231;GO:0043232;GO:0005575;GO:0018024;GO:0003674;GO:0042054;GO:0016278;GO:0016279;GO:0046975;GO:0008168;GO:0016740;GO:0016741;GO:0008757;GO:0140096;GO:0003824;GO:0008170;GO:0008276	K11423	SETD2	map00310	Lysine degradatic	COG2940	S:Function unknown	PF08236.14;PF0(	SRI;SET;AWS;\	SRI;SET domain;AV	CYT	1	1	177	Medium
TRINITY_DN42159_c0_g1_i6_orf1	regucalcin-like [Nymphalis io] >XP_050348255.1 regucalcin-like [Nymphalis io]	0.468410976	-1.094153212	0.01044	down	yes	0.734	1.567	1.438	1.495	1.769	1	0.473	0.729	CC:cytoplasm;CC:cellular_component;CC:cellular anatomical entity;MF:hydrolase activity;MF:metal ion binding;MF:cation binding;MF:enzyme regulator activity;MF:gluconolactonase activity;MF:carboxylic ester hydrolase activity;MF:ion binding;MF:catalytic activity;MF:molecular_function;MF:hydrolase activity, acting on ester bonds;MF:binding;MF:molecular function	GO:0005737;GO:0005575;GO:0110165;GO:0016787;GO:0046872;GO:0043169;GO:0030234;GO:0004341;GO:0052689;GO:0043167;GO:0003824;GO:0003674;GO:0016788;GO:0005488;GO:0098772;GO:0005509	------	------	------	------	COG3386;COG1071	S:Function unknown;C:Energy p	PF08450.15	SGL	SMP-30/Gluconola	CYT	1	22	12.3	High
TRINITY_DN146006_c0_g1_i1_orf1	unnamed protein product [Chrysodeixis includens]	2.241902026	1.164723232	0.02157	up	yes	1.405	0.6267	0.515	0.676	0.689	1	1.664	1.552	MF:molecular_function;MF:structural constituent of cuticle;MF:structural molecule activity;	GO:0003674;GO:0042302;GO:0005198	------	------	------	------	ENOG4111PJ1	S:Function unknown	PF00379.26	Chitin_bind_4	Insect cuticle prote	CYT	1	17	10.9	High
TRINITY_DN64_c0_g1_i4_orf1	unnamed protein product [Chilo suppressalis]	2.362235829	1.240153	0.01141	up	yes	1.196	0.5063	0.567	0.613	0.339	1	1.144	1.443	BP:nucleobase-containing compound transport;BP:nuclear transport;BP:nuclear export;BP:nucleic acid metabolic process;BP:cellular nitrogen compound metabolic process;BP:nitrogen compound metabolic process;BP:mRNA transport;BP:organic cyclic compound metabolic process;BP:nitrogen compound transport;BP:organic substance metabolic process;BP:organic substance transport;BP:heterocycle metabolic process;BP:RNA export from nucleus;BP:mRNA export from nucleus;BP:cellular process;BP:nucleobase-containing compound metabolic process;BP:transport;BP:cellular aromatic compound metabolic process;BP:nucleocytoplasmic transport;BP:biological_process;BP:metabolic process;BP:establishment of localization in cell;BP:establishment of RNA localization;BP:establishment of localization;BP:primary metabolic process;BP:localization;BP:cellular localization;BP:RNA metabolic process;BP:mRNA metabolic process;BP:RNA transport;BP:intracellular transport;BP:nucleic acid transport;BP:cellular metabolic process;BP:macromolecule	GO:0015931;GO:0051169;GO:0051168;GO:0090304;GO:0034641;GO:0006807;GO:0051028;GO:1901360;GO:0071705;GO:0071704;GO:0071702;GO:0046483;GO:0006405;GO:0006406;GO:0009987;GO:0006139;GO:0006810;GO:0006725;GO:0006913;GO:0008150;GO:0008152;GO:0051649;GO:0051236;GO:0051234;GO:0044238;GO:0051179;GO:0051641;GO:0016070;GO:0016071;GO:0050658;GO:0046907;GO:0050657;GO:0044237;GO:0043170;GO:0006396;GO:0006397;GO:0000347;GO:0032991;GO:0005575	K12879	THOC2	map03013;maj	Nucleocytoplasm	ENOG410XQ2A	S:Function unknown	PF11262.11;PF1(	Tho2;THOC2_	Transcription facto	CYT	1	0	161.7	High
TRINITY_DN3889_c0_g1_i7_orfp1	TRINITY_DN3889_c0_g1_i7_m.1657 TRINITY_DN3889_c0_g1::TRINITY_DN3889_c0_g1_i7::g.1657 ORF type:5prime_partial len:235 (+),score=70.90 TRINITY_DN3889_c0_g1_i7:1-705(+)	4.522481129	2.177114483	0.007583	up	yes	1.378	0.3047	0.298	0.292	0.324	1	1.745	1.388	------	------	------	------	------	------	------	------	------	------	------	EXC	1	38	24.3	High
TRINITY_DN49742_c0_g1_i4_orf1	neuroglobin-like [Ostrinia furnacalis]	2.087227414	1.061587798	0.00287	up	yes	1.139	0.5457	0.554	0.606	0.477	1	1.287	1.129	MF:oxygen binding;MF:heme binding;MF:tetrapyrrole binding;MF:organic cyclic compound binding;MF:small molecule binding;MF:molecular_function;MF:binding;MF:heterocyclic	GO:0019825;GO:0020037;GO:0046906;GO:0097159;GO:0036094;GO:0003674;GO:0005488;GO:1901363	K21893	NGB	------	------	ENOG4111QDK	S:Function unknown	PF00042.25	Globin	Globin	CYT	1	11	21.5	High
TRINITY_DN4497_c0_g1_i4_orf1	cytochrome P450 9e2-like [Ostrinia furnacalis] >QPF77612.1 cytochrome P450 monooxygenase CYP9A185 [Ostrinia furnacalis]	2.270803949	1.183203156	0.0006993	up	yes	1.127	0.4963	0.502	0.459	0.528	1	1.2	1.18	CC:intrinsic component of membrane;CC:integral component of membrane;CC:cellular anatomical entity;CC:cellular_component;MF:monooxygenase activity;MF:NADPH-hemoprotein reductase activity;MF:molecular_function;MF:binding;MF:heterocyclic compound binding;MF:oxidoreductase activity;MF:cation binding;MF:transition metal ion binding;MF:oxidoreductase activity, acting on paired donors, with incorporation or reduction of molecular oxygen;MF:ion binding;MF:iron ion binding;MF:metal ion binding;MF:heme binding;MF:catalytic activity;MF:tetrapyrrole binding;MF:organic cyclic compound binding;MF:oxidoreductase activity, acting on	GO:0031224;GO:0016021;GO:0110165;GO:0005575;GO:0004497;GO:0003958;GO:0003674;GO:0005488;GO:1901363;GO:0016491;GO:0043169;GO:0046914;GO:0016705;GO:0043167;GO:0005506;GO:0046872;GO:0020037;GO:0003824;GO:0046906;GO:0097159;GO:0016651;GO:0016653	K15003	CYP9	------	------	COG2124	Q:Secondary metabolites biosy	PF00067.25	p450	Cytochrome P450	CYT	1	15	31.2	High
TRINITY_DN44219_c0_g1_i1_orf1	mitochondrial import inner membrane translocase subunit TIM50-C-like [Ostrinia furnacalis]	2.174257426	1.120522761	0.0005318	up	yes	1.098	0.505	0.538	0.512	0.465	1	1.188	1.106	BP:protein localization;BP:cellular macromolecule localization;BP:nitrogen compound transport;BP:organic substance transport;BP:macromolecule localization;BP:transport;BP:cellular process;BP:establishment of protein localization;BP:biological_process;BP:establishment of localization;BP:localization;BP:cellular localization;BP:protein transport;CC:intrinsic component of membrane;CC:integral component of membrane;CC:inner mitochondrial membrane protein complex;CC:cellular anatomical entity;CC:macromolecular complex;CC:cellular_component;CC:membrane protein complex;CC:mitochondrial inner membrane presequence	GO:0008104;GO:0070727;GO:0071705;GO:0071702;GO:0033036;GO:0006810;GO:0009987;GO:0045184;GO:0008150;GO:0051234;GO:0051179;GO:0051641;GO:0015031;GO:0031224;GO:0016021;GO:0098800;GO:0110165;GO:0032991;GO:0005575;GO:0098796;GO:0005744;GO:0098798	K17496	TIM50	------	------	COG5190	K:Transcription	PF03031.21	NIF	NLI interacting fact	CYT	1	3	44.9	High
TRINITY_DN71465_c0_g1_i1_orf1	adenylate kinase isoenzyme 6 [Ostrinia furnacalis]	2.121570949	1.085132926	0.0007409	up	yes	1.075	0.5067	0.479	0.524	0.517	1	1.192	1.032	BP:phosphorylation;BP:cellular process;BP:phosphorus metabolic process;BP:biological_process;BP:metabolic process;BP:cellular metabolic process;BP:phosphate-containing compound metabolic process;CC:intracellular organelle;CC:membrane-bounded organelle;CC:organelle;CC:nucleus;CC:intracellular membrane-bounded organelle;CC:cellular_component;CC:cellular anatomical entity;MF:nucleobase-containing compound kinase activity;MF:purine ribonucleoside triphosphate binding;MF:heterocyclic compound binding;MF:adenylate kinase activity;MF:phosphotransferase activity, phosphate group as acceptor;MF:molecular_function;MF:binding;MF:ATPase activity;MF:ion binding;MF:nucleoside phosphate binding;MF:anion binding;MF:nucleotide binding;MF:transferase activity;MF:purine nucleotide binding;MF:ATP binding;MF:hydrolase activity;MF:kinase activity;MF:nucleoside-triphosphatase activity;MF:small molecule binding;MF:catalytic activity;MF:hydrolase activity, acting on acid anhydrides, in phosphorus-containing anhydrides;MF:adenyl nucleotide binding;MF:carbohydrate derivative binding;MF:organic cyclic compound binding;MF:hydrolase activity, acting on acid anhydrides;MF:pyrophosphatase activity;MF:nucleoside	GO:0016310;GO:0009987;GO:0006793;GO:0008150;GO:0008152;GO:0044237;GO:0006796;GO:0043229;GO:0043227;GO:0043226;GO:0005634;GO:0043231;GO:0005575;GO:0110165;GO:0019205;GO:0035639;GO:1901363;GO:0004017;GO:0016776;GO:0003674;GO:0005488;GO:0016887;GO:0043167;GO:1901265;GO:0043168;GO:0000166;GO:0016740;GO:0017076;GO:0005524;GO:0016787;GO:0016301;GO:0017111;GO:0036094;GO:0003824;GO:0016818;GO:0030554;GO:0097367;GO:0097159;GO:0016817;GO:0016462;GO:0050145;GO:0032559;GO:0016772;GO:0032555;GO:0032553	K18532	AK6, FAP7	map03008;maj	Ribosome biogen	COG1936	F:Nucleotide transport and met	PF13238.9	AAA_18	AAA domain	CYT	1	9	20.6	High
TRINITY_DN747_c0_g1_i1_orf1	trypsin, alkaline C-like [Ostrinia furnacalis]	2.096436059	1.067938829	0.00156	up	yes	1.1	0.5247	0.549	0.482	0.543	1	1.061	1.24	BP:organic substance metabolic process;BP:organonitrogen compound metabolic process;BP:protein metabolic process;BP:macromolecule metabolic process;BP:biological_process;BP:nitrogen compound metabolic process;BP:metabolic process;BP:proteolysis;BP:primary metabolic process;MF:serine-type endopeptidase activity;MF:endopeptidase activity;MF:hydrolase activity;MF:serine hydrolase activity;MF:catalytic activity, acting on a protein;MF:catalytic activity;MF:molecular_function;MF:peptidase activity;MF:serine-	GO:0071704;GO:1901564;GO:0019538;GO:0043170;GO:0008150;GO:0006807;GO:0008152;GO:0006508;GO:0044238;GO:0004252;GO:0004175;GO:0016787;GO:0017171;GO:0140096;GO:0003824;GO:0003674;GO:0008233;GO:0008236	K09628;K096	PRSS27;TMPR	------	------	COG5640	O:Posttranslational modificatio	PF00089.29	Trypsin	Trypsin	CYT	1	6	30.5	High
TRINITY_DN14730_c0_g1_i7_orf1	titin homolog [Ostrinia furnacalis]	2.303290415	1.203696328	0.001176	up	yes	1.127	0.4893	0.489	0.565	0.414	1	1.18	1.201	------	------	------	------	------	------	ENOG410ZVTG;ENOG410XQFD;ENOG410XPX0;ENOG410YNSF	S:Function unknown;	PF07679.19;PF1:	I-set;Ig_3;Ig_2	Immunoglobulin I-	CYT	1	0	324.7	High
TRINITY_DN72934_c0_g1_i1_orf1	carboxylesterase 8 [Streltzoviella insularis]	2.358452138	1.237840324	0.001554	up	yes	1.158	0.491	0.455	0.518	0.5	1	1.291	1.183	MF:hydrolase activity;MF:molecular_function;MF:hydrolase activity, acting on ester bonds;MF:carboxylic ester hydrolase activity;MF:catalytic activity;	GO:0016787;GO:0003674;GO:0016788;GO:0052689;GO:0003824	------	------	------	------	COG2272	I:Lipid transport and metabolis	PF00135.31;PF2(	COesterase;B	Carboxylesterase fa	CYT	1	5	61.5	High

TRINITY_DN3814_c1_g1_i1_orf1	39S ribosomal protein L11, mitochondrial [Ostrinia furnacalis]	2.473328979	1.306454146	0.004811	up	yes	1.136	0.4593	0.438	0.458	0.482	1	1.373	1.035	BP:cellular biosynthetic process;BP:cellular nitrogen compound metabolic process;BP:nitrogen compound metabolic process;BP:cellular macromolecule biosynthetic process;BP:organic substance biosynthetic process;BP:cellular nitrogen compound biosynthetic process;BP:cellular macromolecule metabolic process;BP:peptide biosynthetic process;BP:organic substance metabolic process;BP:amide biosynthetic process;BP:cellular process;BP:biosynthetic process;BP:macromolecule biosynthetic process;BP:biological_process;BP:metabolic process;BP:organonitrogen compound metabolic process;BP:primary metabolic process;BP:cellular amide metabolic process;BP:organonitrogen compound biosynthetic process;BP:protein metabolic process;BP:cellular metabolic process;BP:macromolecule metabolic process;BP:peptide metabolic process;BP:translation;CC:intracellular non-membrane-bounded organelle;CC:organelle;CC:cellular anatomical entity;CC:ribosome;CC:macromolecular complex;CC:ribonucleoprotein complex;CC:intracellular organelle;CC:cellular_component;CC:non-membrane-bounded organelle;MF:molecular_function;MF:structural molecule	GO:0044249;GO:0034641;GO:0006807;GO:0034645;GO:1901576;GO:0044271;GO:0044260;GO:0043043;GO:0071704;GO:0043604;GO:0009987;GO:0009058;GO:0009059;GO:0008150;GO:0008152;GO:1901564;GO:0044238;GO:0043603;GO:1901566;GO:0019538;GO:0044237;GO:0043170;GO:0006518;GO:0006412;GO:0043232;GO:0043226;GO:0110165;GO:0005840;GO:0032991;GO:1990904;GO:0043229;GO:0005575;GO:0043228;GO:0003674;GO:0005198;GO:0003735	K02867	RP-L11, MRP	map03010	Ribosome	COG0080	J:Translation, ribosomal structu	PF00298.22;PF0	Ribosomal_L1	Ribosomal protein	CYT	1	5	22	High
TRINITY_DN21181_c0_g1_i6_orf1	unnamed protein product, partial [Brenthis ino]	2.258595841	1.175426134	0.0003343	up	yes	1.097	0.4857	0.474	0.474	0.509	1	1.111	1.179	BP:phosphorylation;BP:nitrogen compound metabolic process;BP:organic substance metabolic process;BP:protein phosphorylation;BP:cellular process;BP:macromolecule modification;BP:protein modification process;BP:biological_process;BP:metabolic process;BP:primary metabolic process;BP:organonitrogen compound metabolic process;BP:protein metabolic process;BP:cellular metabolic process;BP:macromolecule metabolic process;BP:phosphate-containing compound metabolic process;BP:phosphorus metabolic process;MF:purine ribonucleoside triphosphate binding;MF:molecular_function;MF:binding;MF:ion binding;MF:heterocyclic compound binding;MF:nucleoside phosphate binding;MF:anion binding;MF:carbohydrate derivative binding;MF:nucleotide binding;MF:protein serine/threonine kinase activity;MF:transferase activity;MF:protein kinase activity;MF:purine nucleotide binding;MF:AMP-activated protein kinase activity;MF:ATP binding;MF:kinase activity;MF:catalytic activity, acting on a protein;MF:small molecule binding;MF:catalytic activity;MF:adenyl nucleotide binding;MF:transferase activity, transferring phosphorus-containing groups;MF:phosphotransferase activity, alcohol group as acceptor;MF:adenyl ribonucleotide binding;MF:purine	GO:0016310;GO:0006807;GO:0071704;GO:0006468;GO:0009987;GO:0043412;GO:0036211;GO:0008150;GO:0008152;GO:0044238;GO:1901564;GO:0019538;GO:0044237;GO:0043170;GO:0006796;GO:0006793;GO:0035639;GO:0003674;GO:0005488;GO:0043167;GO:1901363;GO:1901265;GO:0043168;GO:0097367;GO:0000166;GO:0004674;GO:0016740;GO:0004672;GO:0017076;GO:0004679;GO:0005524;GO:0016301;GO:0140096;GO:0036094;GO:0003824;GO:0030554;GO:0016772;GO:0016773;GO:0032559;GO:0032555;GO:0097159;GO:0032553	K07198	PRKAA, AMPK	map04152;ma	AMPK signaling	ENOG410XNQ0	O:Posttranslational modificatio	PF00069.28;PF0	Pkinase;PK_Ty	Protein kinase don	CYT	1	3	58.3	High
TRINITY_DN3299_c0_g1_i2_orf1	metaxin-1 isoform X3 [Ostrinia furnacalis] >XP_028170907.1 metaxin-1 isoform X4 [Ostrinia furnacalis]	2.334064182	1.222844233	0.003187	up	yes	1.171	0.5017	0.54	0.517	0.448	1	1.353	1.16	BP:nitrogen compound transport;BP:macromolecule localization;BP:protein localization;BP:cellular macromolecule localization;BP:cellular process;BP:establishment of protein localization;BP:biological_process;BP:transport;BP:organic substance transport;BP:protein transport;BP:establishment of localization;BP:localization;BP:cellular localization;CC:macromolecular complex;CC:membrane protein complex;CC:mitochondrial sorting and assembly machinery complex;CC:cellular_component;CC:mitochondrial outer membrane translocase complex;CC:mitochondrial protein complex;CC:outer mitochondrial membrane protein complex;	GO:0071705;GO:0033036;GO:0008104;GO:0070727;GO:0009987;GO:0045184;GO:0008150;GO:0006810;GO:0071702;GO:0015031;GO:0051234;GO:0051179;GO:0051641;GO:0032991;GO:0098796;GO:0001401;GO:0005575;GO:0005742;GO:0098798;GO:0098799	K17776	MTX	------	------	ENOG41100SS	U:Intracellular trafficking, secre	PF10568.12;PF1	Tom37;GST_C	Outer mitochondri	CYT	1	4	36.5	High
TRINITY_DN1814_c0_g1_i11_orf1	titin-like, partial [Ostrinia furnacalis]	2.025655753	1.018389019	7.33E-05	up	yes	1.058	0.5223	0.515	0.506	0.546	1	1.08	1.095	------	------	K12567	TTN	map05414;ma	Dilated cardiomy	ENOG410XQFD;ENOG410XTJ8;ENOG410Y6GZ	S:Function unknown;	------	------	------	CYT	4	15	66.6	High
TRINITY_DN18696_c0_g1_i1_orf1	rap guanine nucleotide exchange factor 2 [Ostrinia furnacalis] >XP_028159576.1 rap guanine nucleotide exchange factor 2 [Ostrinia furnacalis]	2.322488234	1.215671288	0.01212	up	yes	1.135	0.4887	0.456	0.562	0.448	1	0.983	1.423	BP:small GTPase mediated signal transduction;BP:cellular process;BP:regulation of cellular process;BP:biological regulation;BP:signal transduction;BP:biological_process;BP:intracellular signal transduction;BP:regulation of biological process;MF:GTPase activator activity;MF:enzyme regulator activity;MF:guanyl-nucleotide exchange factor activity;MF:GTPase regulator activity;MF:molecular_function;MF:molecular function regulator;MF:nucleoside-triphosphatase regulator activity;MF:enzyme activator activity;	GO:0007264;GO:0009987;GO:0050794;GO:0065007;GO:0007165;GO:0008150;GO:0035556;GO:0050789;GO:0005096;GO:0030234;GO:0005085;GO:0030695;GO:0003674;GO:0098772;GO:0060589;GO:0008047	K08018	RAPGEF2, PD	map04015;ma	Rap1 signaling p	ENOG410XS6B	T:Signal transduction mechanis	PF00617.22;PF0	RasGEF;PDZ;R	RasGEF domain;PD	CYT	1	1	132.4	High
TRINITY_DN110400_c0_g1_i1_orf1	DNA replication factor Cdt1 [Chelonus insularis]	2.020522388	1.014728337	0.003067	up	yes	1.083	0.536	0.406	0.552	0.65	1	1.084	1.166	BP:cellular component assembly;BP:regulation of primary metabolic process;BP:DNA replication preinitiation complex assembly;BP:macromolecular complex subunit organization;BP:regulation of cellular metabolic process;BP:regulation of DNA-dependent DNA replication initiation;BP:cell cycle process;BP:regulation of DNA-dependent DNA replication;BP:cellular component organization or biogenesis;BP:protein-DNA complex subunit organization;BP:regulation of metabolic process;BP:cellular component organization;BP:regulation of biological process;BP:macromolecular complex assembly;BP:regulation of macromolecule metabolic process;BP:biological regulation;BP:protein-DNA complex assembly;BP:regulation of DNA metabolic process;BP:regulation of nucleobase-containing compound metabolic process;BP:cellular process;BP:regulation of cellular process;BP:biological_process;BP:regulation of nitrogen compound metabolic process;BP:regulation of DNA replication;CC:intracellular organelle;CC:membrane-bounded organelle;CC:organelle;CC:nucleus;CC:cellular anatomical entity;CC:intracellular membrane-bounded organelle;CC:cellular_component;MF:binding;MF:nucleic acid binding;MF:DNA binding;MF:organic cyclic compound binding;MF:molecular_function;MF:heterocyclic compound	GO:0022607;GO:0080090;GO:0071163;GO:0043933;GO:0031323;GO:0030174;GO:0022402;GO:0090329;GO:0071840;GO:0071824;GO:0019222;GO:0016043;GO:0050789;GO:0065003;GO:0060255;GO:0065007;GO:0065004;GO:0051052;GO:0019219;GO:0009987;GO:0050794;GO:0008150;GO:0051171;GO:0006275;GO:0043229;GO:0043227;GO:0043226;GO:0005634;GO:0110165;GO:0043231;GO:0005575;GO:0005488;GO:0003676;GO:0003677;GO:0097159;GO:0003674;GO:1901363	K10727	CDT1	------	------	ENOG410XT37	L:Replication, recombination ar	PF08839.14;PF1	CDT1;CDT1_C	DNA replication fa	CYT	1	1	82.5	High
TRINITY_DN29229_c0_g1_i4_orf1	uncharacterized protein LOC114351433 isoform X1 [Ostrinia furnacalis]	2.181451613	1.125288473	0.002111	up	yes	1.082	0.496	0.491	0.46	0.537	1	1.242	1.005	MF:molecular_function;MF:lipid binding;MF:binding;	GO:0003674;GO:0008289;GO:0005488	K24141	STARD7	------	------	ENOG4111HC0;ENOG41102B9	U:Intracellular trafficking, secre	PF01852.22	START	START domain	CYT	1	2	69.3	High
TRINITY_DN1791_c0_g1_i3_orf1	succinate dehydrogenase assembly factor 2-B, mitochondrial-like [Ostrinia furnacalis]	2.075879693	1.053722835	6.11E-05	up	yes	1.056	0.5087	0.484	0.523	0.519	1	1.094	1.073	BP:nitrogen compound metabolic process;BP:cellular metabolic process;BP:generation of precursor metabolites and energy;BP:organic substance metabolic process;BP:electron transport chain;BP:respiratory electron transport chain;BP:protein-FAD linkage;BP:cellular process;BP:aerobic electron transport chain;BP:macromolecule modification;BP:protein modification process;BP:biological_process;BP:metabolic process;BP:primary metabolic process;BP:organonitrogen compound metabolic process;BP:energy derivation by oxidation of organic compounds;BP:protein metabolic process;BP:mitochondrial electron transport, succinate to ubiquinone;BP:macromolecule metabolic process;BP:aerobic respiration;BP:cellular respiration;CC:membrane-enclosed lumen;CC:mitochondrial matrix;CC:cellular anatomical entity;CC:organelle	GO:0006807;GO:0044237;GO:0006091;GO:0071704;GO:0022900;GO:0022904;GO:0018293;GO:0009987;GO:0019646;GO:0043412;GO:0036211;GO:0008150;GO:0008152;GO:0044238;GO:1901564;GO:0015980;GO:0019538;GO:0006121;GO:0043170;GO:0009060;GO:0045333;GO:0031974;GO:0005759;GO:0110165;GO:0043233;GO:0005575;GO:0070013	K18168	SDHAF2, SDH	------	------	COG2938	S:Function unknown	PF03937.19	Sdh5	Flavinator of succi	CYT	1	4	16.8	Medium
TRINITY_DN45227_c0_g1_i3_orf1	uncharacterized protein LOC114359191 [Ostrinia furnacalis]	2.273598977	1.184977811	0.0003118	up	yes	1.067	0.4693	0.529	0.467	0.412	1	1.134	1.067	CC:respiratory chain complex I;CC:transmembrane transporter complex;CC:catalytic complex;CC:transporter complex;CC:NADH dehydrogenase complex;CC:respiratory chain complex;CC:inner mitochondrial membrane protein complex;CC:cellular_component;CC:membrane protein complex;CC:mitochondrial respiratory chain complex I;CC:macromolecular complex;CC:oxidoreductase complex;CC:mitochondrial protein complex;	GO:0045271;GO:1902495;GO:1902494;GO:1990351;GO:0030964;GO:0098803;GO:0098800;GO:0005575;GO:0098796;GO:0005747;GO:0032991;GO:1990204;GO:0098798	------	------	------	------	ENOG410XXY6	S:Function unknown	PF15880.8	NDUFV3	NADH dehydroger	CYT	1	8	11.2	High
TRINITY_DN4762_c0_g1_i2_orf1	ATPase family AAA domain-containing protein 1 isoform X2 [Ostrinia furnacalis]	2.069800282	1.049491567	0.0006588	up	yes	1.026	0.4957	0.524	0.475	0.488	1	1.128	0.949	MF:purine ribonucleoside triphosphate binding;MF:molecular_function;MF:binding;MF:ATPase activity;MF:ion binding;MF:heterocyclic compound binding;MF:nucleoside phosphate binding;MF:anion binding;MF:nucleotide binding;MF:purine nucleotide binding;MF:ATP binding;MF:hydrolase activity;MF:nucleoside-triphosphatase activity;MF:small molecule binding;MF:catalytic activity;MF:hydrolase activity, acting on acid anhydrides, in phosphorus-containing anhydrides;MF:adenyl nucleotide binding;MF:carbohydrate derivative binding;MF:organic cyclic compound binding;MF:hydrolase activity, acting on acid anhydrides;MF:pyrophosphatase activity;MF:adenyl ribonucleotide binding;MF:purine ribonucleotide binding;MF:ribonucleotide binding;	GO:0035639;GO:0003674;GO:0005488;GO:0016887;GO:0043167;GO:1901363;GO:1901265;GO:0043168;GO:0000166;GO:0017076;GO:0005524;GO:0016787;GO:0017111;GO:0036094;GO:0003824;GO:0016818;GO:0030554;GO:0097367;GO:0097159;GO:0016817;GO:0016462;GO:0032559;GO:0032555;GO:0032553	K22530	ATAD1	------	------	COG0464	S:Function unknown	PF00004.32;PF1	AAA;AAA_lid_	ATPase family assc	CYT	1	2	43.3	High
TRINITY_DN825_c8_g1_i5_orf1	ATP-binding cassette sub-family F member 2 [Ostrinia furnacalis] >XP_028169527.1 ATP-binding cassette sub-family F member 2 [Ostrinia furnacalis]	2.072254922	1.051201489	2.45E-06	up	yes	1.021	0.4927	0.492	0.5	0.486	1	1.019	1.044	MF:anion binding;MF:carbohydrate derivative binding;MF:molecular_function;MF:adenyl nucleotide binding;MF:purine ribonucleoside triphosphate binding;MF:organic cyclic compound binding;MF:nucleotide binding;MF:small molecule binding;MF:ribonucleotide binding;MF:adenyl ribonucleotide binding;MF:nucleoside phosphate binding;MF:purine ribonucleotide binding;MF:purine nucleotide binding;MF:ion binding;MF:heterocyclic compound binding;MF:ATP binding;MF:binding;	GO:0043168;GO:0097367;GO:0003674;GO:0030554;GO:0035639;GO:0097159;GO:0000166;GO:0036094;GO:0032553;GO:0032559;GO:1901265;GO:0032555;GO:0017076;GO:0043167;GO:1901363;GO:0005524;GO:0005488	K06185	ABCF2	map05130	Pathogenic Esch	COG0488	S:Function unknown	PF00005.30;PF1	ABC_tran;ABC	ABC transporter;AE	CYT	15	26	70.8	High
TRINITY_DN98538_c0_g1_i1_orf1	ATP synthase subunit d, mitochondrial [Ostrinia furnacalis]	2.031910569	1.022836906	1.40E-08	up	yes	0.9997	0.492	0.497	0.492	0.487	1	1.003	0.996	BP:nucleoside triphosphate metabolic process;BP:nucleoside triphosphate biosynthetic process;BP:purine nucleoside triphosphate metabolic process;BP:purine nucleoside triphosphate biosynthetic process;BP:cellular biosynthetic process;BP:cellular nitrogen compound metabolic process;BP:nucleotide biosynthetic process;BP:small molecule metabolic process;BP:purine-containing compound metabolic process;BP:purine-containing compound biosynthetic process;BP:organic cyclic compound biosynthetic process;BP:ribonucleotide metabolic process;BP:organic cyclic compound metabolic process;BP:nucleobase-containing compound metabolic process;BP:ribonucleoside triphosphate biosynthetic process;BP:organic substance metabolic process;BP:ribonucleoside triphosphate metabolic process;BP:heterocycle biosynthetic process;BP:purine ribonucleoside triphosphate biosynthetic process;BP:purine ribonucleoside triphosphate metabolic process;BP:ribose phosphate metabolic process;BP:organic substance biosynthetic process;BP:ATP metabolic process;BP:cellular process;BP:cellular aromatic compound metabolic process;BP:purine ribonucleotide biosynthetic process;BP:purine ribonucleotide metabolic process;BP:ribonucleotide biosynthetic process;BP:biosynthetic process;BP:nucleotide metabolic process;BP:metabolic process;BP:aromatic compound biosynthetic process;BP:nucleobase-containing compound biosynthetic process;BP:cellular nitrogen compound biosynthetic process;BP:organophosphate biosynthetic process;BP:nucleobase-containing small molecule metabolic process;BP:heterocycle metabolic process;BP:primary metabolic process;BP:ATP synthesis coupled proton	GO:0009141;GO:0009142;GO:0009144;GO:0009145;GO:0044249;GO:0034641;GO:0009165;GO:0044281;GO:0072521;GO:0072522;GO:1901362;GO:0009259;GO:1901360;GO:0006139;GO:0009201;GO:0071704;GO:0009199;GO:0018130;GO:0009206;GO:0009205;GO:0019693;GO:1901576;GO:0046034;GO:0009987;GO:0006725;GO:0009152;GO:0009150;GO:0009260;GO:0009058;GO:0009117;GO:0008152;GO:0019438;GO:0034654;GO:0044271;GO:0090407;GO:0055086;GO:0046483;GO:0044238;GO:0015986;GO:1901564;GO:1901566;GO:1901137;GO:1901135;GO:0006164;GO:0046390;GO:0044237;GO:0006163;GO:0006796;GO:0006807;GO:1901293;GO:0006793;GO:0019637;GO:0008150;GO:0006753;GO:0006754;GO:0045263;GO:0005575;GO:0098798;GO:0098800;GO:0032991;GO:0000276;GO:0098796;GO:0033177;GO:0022890;GO:0003674;GO:0015318;GO:0022857;GO:0005215;GO:0008324;GO:0015075;GO:0015078	K02138	ATPeF0D, ATF	map04714;ma	Thermogenesis;C	ENOG4111IHJ	S:Function unknown	PF05873.15	Mt_ATP-synt_	ATP synthase D ch	CYT	12	60	19.6	High

TRINITY_DN9871_c0_g1_i11_orf1	PEST proteolytic signal-containing nuclear protein-like [Ostrinia furnacalis]	2.4098323	1.268932753	0.0003853	up	yes	1.049	0.4353	0.417	0.471	0.418	1	1.154	0.992	BP:nitrogen compound metabolic process;BP:cell cycle;BP:protein modification by small protein conjugation;BP:protein modification by small protein conjugation or removal;BP:organic substance metabolic process;BP:cellular process;BP:macromolecule modification;BP:protein modification process;BP:biological_process;BP:metabolic process;BP:primary metabolic process;BP:organonitrogen compound metabolic process;BP:protein metabolic process;BP:macromolecule metabolic process;BP:protein ubiquitination;CC:intracellular organelle;CC:membrane-bounded organelle;CC:organelle;CC:nucleus;CC:cellular anatomical entity;CC:intracellular membrane-bounded	GO:0006807;GO:0007049;GO:0032446;GO:0070647;GO:0071704;GO:0009987;GO:0043412;GO:0036211;GO:0008150;GO:0008152;GO:0044238;GO:1901564;GO:0019538;GO:0043170;GO:0016567;GO:0043229;GO:0043227;GO:0043226;GO:0005634;GO:0110165;GO:0043231;GO:0005575	------	------	------	------	ENOG4111S9J	S:Function unknown	PF15473.9	PCNP	PEST, proteolytic s	CYT	1	6	24.7	High
TRINITY_DN1294_c0_g1_i3_orf1	46 kDa FK506-binding nuclear protein-like isoform X1 [Ostrinia furnacalis] >XP_028157904.1 46 kDa FK506-binding nuclear protein-like isoform X2 [Ostrinia furnacalis]	2.153663793	1.106793049	4.37E-07	up	yes	0.9993	0.464	0.464	0.47	0.458	1	1.013	0.985	CC:intracellular membrane-bounded organelle;CC:nucleus;CC:cellular_component;CC:intracellular organelle;CC:membrane-bounded organelle;CC:organelle;CC:cellular anatomical entity;MF:organic cyclic compound binding;MF:catalytic activity, acting on a protein;MF:heterocyclic compound binding;MF:cis-trans isomerase activity;MF:catalytic activity;MF:molecular_function;MF:binding;MF:peptidyl-prolyl cis-trans isomerase activity;MF:DNA binding;MF:nucleic acid binding;MF:isomerase activity;	GO:0043231;GO:0005634;GO:0005575;GO:0043229;GO:0043227;GO:0043226;GO:0110165;GO:0097159;GO:0140096;GO:1901363;GO:0016859;GO:0003824;GO:0003674;GO:0005488;GO:0003755;GO:0003677;GO:0003676;GO:0016853	K14826	FPR3_4	------	------	COG0545	C:Energy production and conve	PF00254.31;PF1	FKBP_C;NPL	FKBP-type peptidy	CYT	12	32	44.7	High
TRINITY_DN19829_c0_g2_i1_orf1	28S ribosomal protein S35, mitochondrial [Ostrinia furnacalis]	2.018001241	1.012927062	1.07E-05	up	yes	0.9753	0.4833	0.495	0.494	0.461	1	0.975	0.951	BP:cellular biosynthetic process;BP:cellular nitrogen compound metabolic process;BP:nitrogen compound metabolic process;BP:cellular macromolecule biosynthetic process;BP:organic substance biosynthetic process;BP:cellular macromolecule metabolic process;BP:peptide biosynthetic process;BP:organic substance metabolic process;BP:mitochondrial translation;BP:amide biosynthetic process;BP:cellular process;BP:biosynthetic process;BP:macromolecule biosynthetic process;BP:biological_process;BP:metabolic process;BP:cellular nitrogen compound biosynthetic process;BP:primary metabolic process;BP:organonitrogen compound metabolic process;BP:cellular amide metabolic process;BP:organonitrogen compound biosynthetic process;BP:protein metabolic process;BP:cellular metabolic process;BP:macromolecule metabolic process;BP:peptide metabolic process;BP:translation;CC:membrane-bounded organelle;CC:organelle;CC:mitochondrion;CC:cellular anatomical entity;CC:intracellular membrane-bounded organelle;CC:intracellular	GO:0044249;GO:0034641;GO:0006807;GO:0034645;GO:1901576;GO:0044260;GO:0043043;GO:0071704;GO:0032543;GO:0043604;GO:0009987;GO:0009058;GO:0009059;GO:0008150;GO:0008152;GO:0044271;GO:0044238;GO:1901564;GO:0043603;GO:1901566;GO:0019538;GO:0044237;GO:0043170;GO:0006518;GO:0006412;GO:0043227;GO:0043226;GO:0005739;GO:0110165;GO:0043231;GO:0043229;GO:0005575;GO:0003674;GO:0005198;GO:0003735	K17413	MRPS35	------	------	ENOG410ZX0Y	S:Function unknown	PF10213.12	MRP-S28	Mitochondrial ribo	CYT	1	6	18.6	High
TRINITY_DN26649_c0_g1_i2_orf1	ATP synthase-coupling factor 6, mitochondrial [Ostrinia furnacalis]	2.221648327	1.151630465	3.00E-07	up	yes	0.9893	0.4453	0.437	0.453	0.446	1	0.991	0.977	BP:nucleoside triphosphate metabolic process;BP:nucleoside triphosphate biosynthetic process;BP:purine nucleoside triphosphate metabolic process;BP:purine nucleoside triphosphate biosynthetic process;BP:cellular biosynthetic process;BP:cellular nitrogen compound metabolic process;BP:nucleotide biosynthetic process;BP:small molecule metabolic process;BP:purine-containing compound metabolic process;BP:purine-containing compound biosynthetic process;BP:organic cyclic compound biosynthetic process;BP:ribonucleotide metabolic process;BP:organic cyclic compound metabolic process;BP:nucleobase-containing compound metabolic process;BP:ribonucleoside triphosphate biosynthetic process;BP:organic substance metabolic process;BP:ribonucleoside triphosphate metabolic process;BP:heterocycle biosynthetic process;BP:purine ribonucleoside triphosphate biosynthetic process;BP:purine ribonucleoside triphosphate metabolic process;BP:ribose phosphate metabolic process;BP:organic substance biosynthetic process;BP:ATP metabolic process;BP:cellular process;BP:cellular aromatic compound metabolic process;BP:purine ribonucleotide biosynthetic process;BP:purine ribonucleotide metabolic process;BP:ribonucleotide biosynthetic process;BP:biosynthetic process;BP:nucleotide metabolic process;BP:metabolic process;BP:aromatic compound biosynthetic process;BP:nucleobase-containing compound biosynthetic process;BP:cellular nitrogen compound biosynthetic process;BP:organophosphate biosynthetic process;BP:nucleobase-containing small molecule metabolic process;BP:heterocycle metabolic process;BP:primary metabolic [illegible]	GO:0009141;GO:0009142;GO:0009144;GO:0009145;GO:0044249;GO:0034641;GO:0009165;GO:0044281;GO:0072521;GO:0072522;GO:1901362;GO:0009259;GO:1901360;GO:0006139;GO:0009201;GO:0071704;GO:0009199;GO:0018130;GO:0009206;GO:0009205;GO:0019693;GO:1901576;GO:0046034;GO:0009987;GO:0006725;GO:0009152;GO:0009150;GO:0009260;GO:0009058;GO:0009117;GO:0008152;GO:0019438;GO:0034654;GO:0044271;GO:0090407;GO:0055086;GO:0046483;GO:0044238;GO:0015986;GO:1901564;GO:1901566;GO:1901137;GO:1901135;GO:0006164;GO:0046390;GO:0044237;GO:0006163;GO:0006796;GO:0006807;GO:1901293;GO:0006793;GO:0019637;GO:0008150;GO:0006753;GO:0006754;GO:0045263;GO:0005575;GO:0098798;GO:0098800;GO:0032991;GO:0000276;GO:0098796;GO:0033177;GO:0022890;GO:0003674;GO:0015318;GO:0022857;GO:0005215;GO:0008324;GO:0015075;GO:0015078	K02131	ATPeF0F6, AT	map04714;map	Thermogenesis;C	ENOG41122G1	C:Energy production and conve	PF05511.14	ATP-synt_F6	Mitochondrial ATP	CYT	5	62	10.5	High
TRINITY_DN17559_c0_g1_i4_orf1	GDP-mannose 4,6 dehydratase isoform X2 [Ostrinia furnacalis] >XP_028166204.1 GDP-mannose 4,6 dehydratase isoform X2 [Ostrinia furnacalis]	2.407891609	1.267770451	6.73E-06	up	yes	1.013	0.4207	0.427	0.42	0.415	1	1.05	0.988	BP:GDP-mannose metabolic process;BP:GDP-L-fucose metabolic process;BP:cellular biosynthetic process;BP:cellular nitrogen compound metabolic process;BP:nitrogen compound metabolic process;BP:small molecule metabolic process;BP:organic cyclic compound biosynthetic process;BP:organic cyclic compound metabolic process;BP:nucleobase-containing compound metabolic process;BP:GDP-L-fucose biosynthetic process;BP:'de novo' GDP-L-fucose biosynthetic process;BP:organic substance metabolic process;BP:heterocycle biosynthetic process;BP:nucleotide-sugar biosynthetic process;BP:organic substance biosynthetic process;BP:nucleotide-sugar metabolic process;BP:cellular process;BP:cellular aromatic compound metabolic process;BP:biosynthetic process;BP:biological_process;BP:metabolic process;BP:nucleobase-containing compound biosynthetic process;BP:nucleobase-containing small molecule metabolic process;BP:heterocycle metabolic process;BP:primary metabolic process;BP:cellular nitrogen compound biosynthetic process;BP:carbohydrate derivative biosynthetic process;BP:cellular metabolic process;BP:phosphorus metabolic process;BP:aromatic compound biosynthetic process;BP:carbohydrate derivative metabolic	GO:0019673;GO:0046368;GO:0044249;GO:0034641;GO:0006807;GO:0044281;GO:1901362;GO:1901360;GO:0006139;GO:0042350;GO:0042351;GO:0071704;GO:0018130;GO:0009226;GO:1901576;GO:0009225;GO:0009987;GO:0006725;GO:0009058;GO:0008150;GO:0008152;GO:0034654;GO:0055086;GO:0046483;GO:0044238;GO:0044271;GO:1901137;GO:0044237;GO:0006793;GO:0019438;GO:1901135;GO:0003674;GO:0008446;GO:0016829;GO:0003824;GO:0016835;GO:0016836	K01711	gmd, GMDS	map00520;map	Amino sugar and	COG1089	M:Cell wall/membrane/envelop	PF16363.8;PF01	GDP_Man_De	GDP-mannose 4,6	CYT	4	16	40.5	High
TRINITY_DN4622_c0_g1_i1_orf1	keratin-associated protein 19-2-like [Ostrinia furnacalis]	2.352941176	1.234465254	3.98E-05	up	yes	1.004	0.4267	0.407	0.398	0.475	1	1.034	0.977	------	------	------	------	------	------	------	------	------	------	------	EXC	1	6	22.1	High
TRINITY_DN55160_c0_g1_i1_orf1	esterase FE4-like isoform X2 [Ostrinia furnacalis]	2.03328355	1.023811419	0.0002235	up	yes	0.953	0.4687	0.484	0.418	0.504	1	0.956	0.903	------	------	------	------	------	------	COG2272	D:Cell cycle control, cell divisio	PF00135.31	COesterase	Carboxylesterase f	CYT	2	8	34	High
TRINITY_DN3082_c1_g1_i7_orf1	ribosomal RNA processing protein 1 homolog [Ostrinia furnacalis]	3.43081761	1.778552431	7.07E-05	up	yes	1.091	0.318	0.322	0.315	0.317	1	1.138	1.135	BP:nucleic acid metabolic process;BP:cellular nitrogen compound metabolic process;BP:nitrogen compound metabolic process;BP:ncRNA metabolic process;BP:organic cyclic compound metabolic process;BP:nucleobase-containing compound metabolic process;BP:organic substance metabolic process;BP:rRNA processing;BP:ncRNA processing;BP:cellular process;BP:cellular aromatic compound metabolic process;BP:biological_process;BP:metabolic process;BP:heterocycle metabolic process;BP:RNA metabolic process;BP:primary metabolic process;BP:rRNA metabolic process;BP:cellular metabolic process;BP:macromolecule metabolic process;BP:RNA processing;CC:preribosome, small subunit precursor;CC:preribosome;CC:macromolecular complex;CC:ribonucleoprotein complex;CC:cellular_component;	GO:0090304;GO:0034641;GO:0006807;GO:0034660;GO:1901360;GO:0006139;GO:0071704;GO:0006364;GO:0034470;GO:0009987;GO:0006725;GO:0008150;GO:0008152;GO:0046483;GO:0016070;GO:0044238;GO:0016072;GO:0044237;GO:0043170;GO:0006396;GO:0030688;GO:0030684;GO:0032991;GO:1990904;GO:0005575	K14849	RRP1	------	------	ENOG4111GJ6	J:Translation, ribosomal structu	PF05997.15	Nop52	Nucleolar protein,N	CYT	1	1	84.5	Medium
TRINITY_DN3292_c2_g1_i4_orf1	ribosome biogenesis regulatory protein homolog [Ostrinia furnacalis]	2.342098972	1.227802042	1.84E-05	up	yes	0.9797	0.4183	0.455	0.402	0.398	1	0.951	0.988	BP:cellular process;BP:ribosome biogenesis;BP:cellular component biogenesis;BP:biological_process;BP:cellular component organization or biogenesis;BP:ribonucleoprotein complex biogenesis;CC:intracellular membrane-bounded organelle;CC:nucleus;CC:cellular_component;CC:intracellular organelle;CC:membrane-bounded organelle;CC:organelle;CC:cellular anatomical entity;	GO:0009987;GO:0042254;GO:0044085;GO:0008150;GO:0071840;GO:0022613;GO:0043231;GO:0005634;GO:0005575;GO:0043229;GO:0043227;GO:0043226;GO:0110165	K14852	RRS1	------	------	COG5225	J:Translation, ribosomal structu	PF04939.15	RRS1	Ribosome biogene	CYT	4	12	40	High
TRINITY_DN14094_c0_g1_i1_orfp1	TRINITY_DN14094_c0_g1_i1_m.76391 TRINITY_DN14094_c0_g1::TRINITY_DN14094_c0_g1_i1::g.76391 ORF type:5prime_partial len:120	2.306600426	1.205768106	8.52E-05	up	yes	0.975	0.4227	0.4	0.4	0.468	1	0.924	1.001	------	------	------	------	------	------	------	------	------	------	------	CYT	5	48	13.5	High
TRINITY_DN3027_c0_g1_i4_orf1	ribosome biogenesis protein BOP1 homolog [Ostrinia furnacalis]	2.231199503	1.157819518	0.0002069	up	yes	1.077	0.4827	0.485	0.472	0.491	1	1.158	1.073	------	------	K14824	ERB1, BOP1	------	------	ENOG410XRBX	J:Translation, ribosomal structu	PF08145.15;PF0	BOP1NT;WD4	BOP1NT;WD doma	CYT	4	6	95.8	High
TRINITY_DN10476_c0_g1_i1_orf1	prohibitin-2 [Ostrinia furnacalis]	2.089034103	1.062836044	2.89E-06	up	yes	1.023	0.4897	0.489	0.482	0.498	1	1.023	1.046	CC:organelle membrane;CC:membrane;CC:organelle inner membrane;CC:mitochondrial membrane;CC:cellular_component;CC:mitochondrial inner membrane;CC:cellular anatomical entity;	GO:0031090;GO:0016020;GO:0019866;GO:0031966;GO:0005575;GO:0005743;GO:0110165	K17081	PHB2	------	------	COG0330	O:Posttranslational modificatio	PF01145.28	Band_7	SPFH domain / Ba	CYT	10	36	32.9	High
TRINITY_DN79804_c0_g1_i1_orf1	zinc finger protein on ecdysone puffs-like [Ostrinia furnacalis]	2.028840377	1.020655362	0.004491	up	yes	1.013	0.4993	0.526	0.559	0.413	1	1.153	0.886	------	------	------	------	------	------	ENOG4111SKG	S:Function unknown	------	------	------	CYT	1	21	8.9	High
TRINITY_DN6685_c0_g1_i8_orf1	cleft lip and palate transmembrane protein 1 homolog [Ostrinia furnacalis]	2.015209125	1.01092956	1.26E-07	up	yes	1.007	0.4997	0.5	0.493	0.506	1	1.004	1.016	CC:cellular_component;CC:integral component of membrane;CC:cellular anatomical entity;CC:intrinsic component of membrane;	GO:0005575;GO:0016021;GO:0110165;GO:0031224	------	------	------	------	ENOG410XPEV	S:Function unknown	PF05602.15	CLPTM1	Cleft lip and palate	CYT	3	7	72.4	High
TRINITY_DN11448_c0_g1_i15_orf1	unnamed protein product [Chilo suppressalis]	2.047802309	1.034076447	2.37E-05	up	yes	1.011	0.4937	0.527	0.486	0.468	1	1.041	0.992	------	------	K23353	PDLIM1_2_3_	------	------	ENOG4111KMK;ENOG410YX4U;ENOG4110362	S:Function unknown;	PF00595.27	PDZ	PDZ domain	CYT	2	52	27	High
TRINITY_DN4532_c0_g1_i1_orf1	3-hydroxyacyl-CoA dehydrogenase type-2 [Ostrinia furnacalis]	2.012753036	1.009170165	1.45E-06	up	yes	0.9943	0.494	0.493	0.493	0.496	1	0.973	1.01	MF:catalytic activity;MF:molecular_function;MF:oxidoreductase activity;	GO:0003824;GO:0003674;GO:0016491	K08683	HSD17B10	map05010;map	Alzheimer diseas	ENOG410XNNW	S:Function unknown	PF00106.28;PF1	adh_short;adh	short chain dehydr	CYT	9	44	27	High
TRINITY_DN549_c0_g1_i7_orf1	titin-like, partial [Ostrinia furnacalis]	2.454991817	1.295718216	3.34E-05	up	yes	1.05	0.4277	0.395	0.436	0.452	1	1.076	1.075	BP:developmental process;BP:cell differentiation;BP:cellular developmental process;BP:cellular process;BP:biological_process;BP:anatomical structure morphogenesis;	GO:0032502;GO:0030154;GO:0048869;GO:0009987;GO:0008150;GO:0009653	K12567	TTN	map05414;map	Dilated cardiomy	ENOG410XQFD;ENOG410XTJ8;ENOG410Y6GZ	S:Function unknown;	------	------	------	CYT	2	4	48.1	High
TRINITY_DN14597_c0_g1_i5_orf1	UDP-glucuronosyltransferase 2B1-like isoform X3 [Ostrinia furnacalis]	2.042946058	1.030651112	1.13E-06	up	yes	0.9847	0.482	0.482	0.47	0.494	1	0.98	0.974	CC:integral component of membrane;CC:cellular_component;CC:cellular anatomical entity;CC:intrinsic component of membrane;MF:catalytic activity;MF:transferase activity;MF:molecular_function;MF:transferase activity, transferring glycosyl groups;MF:transferase activity, transferring hexosyl groups;MF:glucuronosyltransferase activity;MF:UDP-	GO:0016021;GO:0005575;GO:0110165;GO:0031224;GO:0003824;GO:0016740;GO:0003674;GO:0016757;GO:0016758;GO:0015020;GO:0008194	K00699	UGT	map05207;map	Chemical carcino	COG1819	S:Function unknown	PF00201.21	UDPGT	UDP-glucoronosyl	CYT	5	14	58.6	High
TRINITY_DN4956_c0_g1_i6_orf1	nucleolar GTP-binding protein 1 [Ostrinia furnacalis]	2.261295348	1.177149435	3.07E-06	up	yes	1.016	0.4493	0.468	0.438	0.442	1	1.039	1.008	BP:ribosome biogenesis;BP:cellular component organization or biogenesis;BP:ribonucleoprotein complex biogenesis;BP:cellular process;BP:biological_process;BP:cellular component biogenesis;CC:intracellular organelle;CC:non-membrane-bounded organelle;CC:organelle;CC:nucleolus;CC:cellular anatomical entity;CC:intracellular non-membrane-bounded organelle;CC:cellular_component;MF:purine ribonucleoside triphosphate binding;MF:heterocyclic compound binding;MF:molecular_function;MF:binding;MF:ion binding;MF:nucleoside phosphate binding;MF:anion binding;MF:nucleotide binding;MF:guanyl ribonucleotide binding;MF:purine nucleotide binding;MF:GTP binding;MF:small molecule binding;MF:carbohydrate derivative binding;MF:organic cyclic compound binding;MF:guanyl nucleotide binding;MF:purine ribonucleotide	GO:0042254;GO:0071840;GO:0022613;GO:0009987;GO:0008150;GO:0044085;GO:0043229;GO:0043228;GO:0043226;GO:0005730;GO:0110165;GO:0043232;GO:0005575;GO:0035639;GO:1901363;GO:0003674;GO:0005488;GO:0043167;GO:1901265;GO:0043168;GO:0000166;GO:0032561;GO:0017076;GO:0005525;GO:0036094;GO:0097367;GO:0097159;GO:0019001;GO:0032555;GO:0032553	K06943	NOG1	map03008	Ribosome bioger	COG1084	S:Function unknown	PF17835.4;PF06	NOG1_N;NOC	NOG1 N-terminal	CYT	6	10	75.1	High
TRINITY_DN35725_c0_g1_i1_orf1	mitochondrial import inner membrane translocase subunit Tim13-like [Bicyclus anynana] >CAG9745432.1 unnamed protein product [Diatraea saccharalis] >CAG9784117.1 unnamed protein product [Diatraea saccharalis]	2.128479657	1.089823302	1.25E-05	up	yes	0.994	0.467	0.468	0.476	0.457	1	1.024	0.958	------	------	K17781	TIM13	------	------	ENOG41124YI	O:Posttranslational modificatio	PF02953.18	zf-Tim10_DD	Tim10/DDP family	CYT	3	39	10.9	High
TRINITY_DN4036_c0_g2_i1_orf1	microvitellogenin-like [Ostrinia furnacalis]	2.161933666	1.112322258	6.26E-06	up	yes	0.9973	0.4613	0.484	0.463	0.437	1	1.014	0.978	CC:cellular_component;CC:extracellular region;CC:cellular anatomical entity;	GO:0005575;GO:0005576;GO:0110165	------	------	------	------	ENOG410Z5ZD	S:Function unknown	PF03260.16	Lipoprotein_1	Lepidopteran low r	CYT	5	20	33.5	High

TRINITY_DN12476_c0_g1_i4_orf1	guanine nucleotide-binding protein-like 3 homolog [Ostrinia furnacalis]	2.356467604	1.236625848	3.74E-05	up	yes	1.022	0.4337	0.474	0.383	0.444	1	1.043	1.023	MF:anion binding;MF:carbohydrate derivative binding;MF:guanyl nucleotide binding;MF:purine ribonucleoside triphosphate binding;MF:organic cyclic compound binding;MF:ribonucleotide binding;MF:nucleotide binding;MF:small molecule binding;MF:guanyl ribonucleotide binding;MF:molecular_function;MF:binding;MF:purine ribonucleotide binding;MF:purine nucleotide binding;MF:ion binding;MF:heterocyclic compound binding;MF:nucleoside phosphate binding;MF:GTP binding;	GO:0043168;GO:0097367;GO:0019001;GO:0035639;GO:0097159;GO:0032553;GO:0000166;GO:0036094;GO:0032561;GO:0003674;GO:0005488;GO:0032555;GO:0017076;GO:0043167;GO:1901363;GO:1901265;GO:0005525	K14538	NUG1, GNL3	map03008	Ribosome bioger	COG1161	S:Function unknown	PF08701.14;PF0	GN3L_Grn1;M	GNL3L/Grn1 putati	CYT	4	7	64.8	High
TRINITY_DN12442_c0_g1_i4_orf1	midasin-like [Ostrinia furnacalis]	2.169173752	1.117145618	0.0001217	up	yes	0.995	0.4587	0.468	0.513	0.395	1	0.973	1.012	BP:cellular component assembly;BP:ribosomal large subunit assembly;BP:macromolecular complex assembly;BP:ribonucleoprotein complex subunit organization;BP:cellular component organization;BP:macromolecular complex subunit organization;BP:ribonucleoprotein complex assembly;BP:cellular component organization or biogenesis;BP:cellular process;BP:biological_process;CC:intracellular organelle;CC:non-membrane-bounded organelle;CC:organelle;CC:nucleolus;CC:nucleoplasm;CC:intracellular non-membrane-bounded organelle;CC:cellular_component;CC:cellular anatomical entity;MF:purine ribonucleoside triphosphate binding;MF:heterocyclic compound binding;MF:molecular_function;MF:binding;MF:ATPase activity;MF:ion binding;MF:nucleoside phosphate binding;MF:anion binding;MF:nucleotide binding;MF:purine nucleotide binding;MF:ATP binding;MF:hydrolase activity;MF:nucleoside-triphosphatase activity;MF:small molecule binding;MF:catalytic activity;MF:hydrolase activity, acting on acid anhydrides, in phosphorus-containing anhydrides;MF:adenyl nucleotide binding;MF:carbohydrate derivative binding;MF:organic cyclic compound binding;MF:hydrolase activity, acting on acid anhydrides;MF:pyrophosphatase activity;MF:adenyl ribonucleotide binding;MF:purine ribonucleotide	GO:0022607;GO:0000027;GO:0065003;GO:0071826;GO:0016043;GO:0043933;GO:0022618;GO:0071840;GO:0009987;GO:0008150;GO:0043229;GO:0043228;GO:0043226;GO:0005730;GO:0005654;GO:0043232;GO:0005575;GO:0110165;GO:0035639;GO:1901363;GO:0003674;GO:0005488;GO:0016887;GO:0043167;GO:1901265;GO:0043168;GO:0000166;GO:0017076;GO:0005524;GO:0016787;GO:0017111;GO:0036094;GO:0003824;GO:0016818;GO:0030554;GO:0097367;GO:0097159;GO:0016817;GO:0016462;GO:0032559;GO:0032555;GO:0032553	K14572	MDN1, REA1	map03008	Ribosome bioger	COG5271	S:Function unknown	PF13519.9;PF00	VWA_2;VWA	von Willebrand fac	CYT	2	1	270.7	High
TRINITY_DN1578_c0_g3_i1_orf1	S-adenosylmethionine synthase isoform X1 [Ostrinia furnacalis]	2.520087655	1.333473915	9.56E-06	up	yes	1.035	0.4107	0.416	0.402	0.414	1	1.075	1.03	BP:one-carbon metabolic process;BP:cellular biosynthetic process;BP:small molecule metabolic process;BP:organic substance biosynthetic process;BP:organic substance metabolic process;BP:sulfur compound metabolic process;BP:S-adenosylmethionine metabolic process;BP:cellular process;BP:biosynthetic process;BP:biological_process;BP:metabolic process;BP:sulfur compound biosynthetic process;BP:S-adenosylmethionine biosynthetic process;BP:cellular metabolic process;MF:purine ribonucleoside triphosphate binding;MF:molecular_function;MF:nucleotide binding;MF:heterocyclic compound binding;MF:nucleoside phosphate binding;MF:anion binding;MF:transferase activity, transferring alkyl or ary;MF:ion binding;MF:transferase activity;MF:metal ion binding;MF:ATP binding;MF:cation binding;MF:small molecule binding;MF:catalytic activity;MF:purine nucleotide binding;MF:methionine adenosyltransferase activity;MF:organic cyclic compound binding;MF:adenyl nucleotide binding;MF:carbohydrate derivative binding;MF:adenyl ribonucleotide binding;MF:purine	GO:0006730;GO:0044249;GO:0044281;GO:1901576;GO:0071704;GO:0006790;GO:0046500;GO:0009987;GO:0009058;GO:0008150;GO:0008152;GO:0044272;GO:0006556;GO:0044237;GO:0035639;GO:0003674;GO:0000166;GO:1901363;GO:1901265;GO:0043168;GO:0016765;GO:0043167;GO:0016740;GO:0046872;GO:0005524;GO:0043169;GO:0036094;GO:0003824;GO:0017076;GO:0004478;GO:0097159;GO:0030554;GO:0097367;GO:0032559;GO:0032555;GO:0032553;GO:0005488	K00789	metK, MAT	map00270;ma	Cysteine and me	COG0192	H:Coenzyme transport and me	PF02772.19;PF0	S-AdoMet_sy	S-adenosylmethio	CYT	7	42	28.2	High
TRINITY_DN14436_c0_g1_i7_orf1	V-type proton ATPase subunit C [Vanessa cardui]	2.261412673	1.177224286	7.97E-08	up	yes	0.9957	0.4403	0.434	0.45	0.437	1	0.998	0.989	------	------	K02148	ATPeV1C, ATI	map05323;ma	Rheumatoid arth	COG5127	C:Energy production and conve	PF03223.18	V-ATPase_C	V-ATPase subunit	CYT	20	51	44.1	High
TRINITY_DN16900_c0_g2_i1_orf1	uncharacterized oxidoreductase dhs-27-like [Ostrinia furnacalis]	3.248502994	1.699775035	0.0003827	up	yes	1.085	0.334	0.395	0.263	0.344	1	1.191	1.064	------	------	------	------	------	------	ENOG410XT8F;ENOG4110RTE;ENOG410ZI5B;ENOG410YKCN;ENOG410ZQUX	S:Function unknown;	PF02958.23;PF0	EcKL;DUF167	Ecdysteroid kinase	CYT	1	3	32.1	High
TRINITY_DN57111_c0_g1_i1_orf1	trypsin-like serine proteinase T26 protein, partial [Chilo infuscatellus]	2.07826087	1.055376757	0.0002417	up	yes	0.956	0.46	0.41	0.458	0.512	1	0.961	0.907	BP:organic substance metabolic process;BP:organonitrogen compound metabolic process;BP:protein metabolic process;BP:macromolecule metabolic process;BP:biological_process;BP:nitrogen compound metabolic process;BP:metabolic process;BP:proteolysis;BP:primary metabolic process;MF:serine-type endopeptidase activity;MF:endopeptidase activity;MF:hydrolase activity;MF:serine hydrolase activity;MF:catalytic activity, acting on a protein;MF:catalytic activity;MF:molecular_function;MF:peptidase activity;MF:serine-	GO:0071704;GO:1901564;GO:0019538;GO:0043170;GO:0008150;GO:0006807;GO:0008152;GO:0006508;GO:0044238;GO:0004252;GO:0004175;GO:0016787;GO:0017171;GO:0140096;GO:0003824;GO:0003674;GO:0008233;GO:0008236	------	------	------	------	------	------	------	------	------	CYT	1	26	7.9	High
TRINITY_DN107035_c0_g1_i1_orf1	splicing factor 3A subunit 3 [Ostrinia furnacalis]	2.355465971	1.23601249	3.48E-05	up	yes	0.9933	0.4217	0.411	0.445	0.409	1	1.034	0.946	BP:cellular metabolic process;BP:nucleic acid metabolic process;BP:cellular nitrogen compound metabolic process;BP:nitrogen compound metabolic process;BP:organic cyclic compound metabolic process;BP:nucleobase-containing compound metabolic process;BP:organic substance metabolic process;BP:RNA splicing;BP:mRNA metabolic process;BP:cellular process;BP:cellular aromatic compound metabolic process;BP:RNA splicing, via transesterification reactions;BP:RNA splicing, via transesterification reactions with bulged adenosine as nucleophile;BP:biological_process;BP:metabolic process;BP:heterocycle metabolic process;BP:RNA metabolic process;BP:primary metabolic process;BP:mRNA splicing, via spliceosome;BP:macromolecule metabolic process;BP:RNA processing;BP:mRNA processing;CC:spliceosomal complex;CC:macromolecular complex;CC:ribonucleoprotein complex;CC:cellular_component;MF:molecular_function;MF:binding;MF:nucleic acid binding;MF:heterocyclic compound binding;MF:transition metal ion binding;MF:zinc ion binding;MF:ion binding;MF:cation binding;MF:metal ion	GO:0044237;GO:0090304;GO:0034641;GO:0006807;GO:1901360;GO:0006139;GO:0071704;GO:0008380;GO:0016071;GO:0009987;GO:0006725;GO:0000375;GO:0000377;GO:0008150;GO:0008152;GO:0046483;GO:0016070;GO:0044238;GO:0000398;GO:0043170;GO:0006396;GO:0006397;GO:0005681;GO:0032991;GO:1990904;GO:0005575;GO:0003674;GO:0005488;GO:0003676;GO:1901363;GO:0046914;GO:0008270;GO:0043167;GO:0043169;GO:0046872;GO:0097159;GO:0003723	K12827	SF3A3, SAP61	map03040	Spliceosome	COG5188	A:RNA processing and modifica	PF11931.11;PF1	SF3a60_Prp9_	SF3a60/Prp9 C-ter	CYT	1	1	57.7	High
TRINITY_DN3929_c0_g1_i1_orf1	glutathione S-transferase 1-1-like [Ostrinia furnacalis]	2.388634722	1.256186249	1.63E-06	up	yes	0.992	0.4153	0.413	0.433	0.4	1	0.974	1.002	MF:catalytic activity;MF:transferase	GO:0003824;GO:0016740;GO:0003674	K00799	GST, gst	map05207;ma	Chemical carcinc	COG0625	O:Posttranslational modificatio	PF00043.28;PF1	GST_C;GST_C	Glutathione S-tran	CYT	3	23	18.9	High
TRINITY_DN948_c0_g1_i1_orf1	mitochondrial-processing peptidase subunit beta [Ostrinia furnacalis]	2.445729756	1.290265	3.29E-08	up	yes	0.9937	0.4063	0.408	0.407	0.404	1	0.997	0.984	BP:primary metabolic process;BP:organonitrogen compound metabolic process;BP:protein metabolic process;BP:macromolecule metabolic process;BP:organic substance metabolic process;BP:nitrogen compound metabolic process;BP:biological_process;BP:metabolic process;BP:proteolysis;MF:molecular_function;MF:endopeptidase activity;MF:cation binding;MF:hydrolase activity;MF:ion binding;MF:catalytic activity, acting on a protein;MF:catalytic activity;MF:metalloendopeptidase activity;MF:binding;MF:peptidase activity;MF:metal ion	GO:0044238;GO:1901564;GO:0019538;GO:0043170;GO:0071704;GO:0006807;GO:0008150;GO:0008152;GO:0006508;GO:0003674;GO:0004175;GO:0043169;GO:0016787;GO:0043167;GO:0140096;GO:0003824;GO:0004222;GO:0005488;GO:0008233;GO:0046872;GO:0008237	K17732	PMPCB, MAS	------	------	COG0612	O:Posttranslational modificatio	PF05193.24;PF0	Peptidase_M1	Peptidase M16 ina	CYT	13	44	37.5	High
TRINITY_DN5925_c0_g1_i5_orf1	isocitrate dehydrogenase [NAD] subunit gamma, mitochondrial-like isoform X1 [Ostrinia furnacalis]	2.600515464	1.378797617	5.53E-06	up	yes	1.009	0.388	0.387	0.404	0.373	1	1.042	0.985	BP:primary metabolic process;BP:tricarboxylic acid cycle;BP:biological_process;BP:metabolic process;CC:intracellular membrane-bounded organelle;CC:intracellular organelle;CC:mitochondrion;CC:cellular_component;CC:membra	GO:0044238;GO:0006099;GO:0008150;GO:0008152;GO:0043231;GO:0043229;GO:0005739;GO:0005575;GO:0043227;GO:0043226;GO:0110165	K00030	IDH3	map00020	Citrate cycl	COG0473	E:Amino acid transport and me	PF00180.23	Iso_dh	Isocitrate/isopropy	CYT	2	6	42.4	High
TRINITY_DN10831_c1_g1_i1_orf1	40S ribosomal protein S16 [Ostrinia furnacalis]	2.035087719	1.025090981	3.81E-05	up	yes	1.044	0.513	0.517	0.493	0.529	1	1.047	1.085	BP:cellular biosynthetic process;BP:cellular nitrogen compound metabolic process;BP:nitrogen compound metabolic process;BP:cellular macromolecule biosynthetic process;BP:organic substance biosynthetic process;BP:cellular nitrogen compound biosynthetic process;BP:cellular macromolecule metabolic process;BP:peptide biosynthetic process;BP:organic substance metabolic process;BP:amide biosynthetic process;BP:cellular process;BP:biosynthetic process;BP:macromolecule biosynthetic process;BP:biological_process;BP:metabolic process;BP:organonitrogen compound metabolic process;BP:primary metabolic process;BP:cellular amide metabolic process;BP:organonitrogen compound biosynthetic process;BP:protein metabolic process;BP:cellular metabolic process;BP:macromolecule metabolic process;BP:peptide metabolic process;BP:translation;CC:intracellular non-membrane-bounded organelle;CC:organelle;CC:cellular anatomical entity;CC:ribosome;CC:macromolecular complex;CC:ribonucleoprotein complex;CC:intracellular organelle;CC:cellular_component;CC:non-membrane-bounded organelle;MF:molecular_function;MF:structural molecule	GO:0044249;GO:0034641;GO:0006807;GO:0034645;GO:1901576;GO:0044271;GO:0044260;GO:0043043;GO:0071704;GO:0043604;GO:0009987;GO:0009058;GO:0009059;GO:0008150;GO:0008152;GO:1901564;GO:0044238;GO:0043603;GO:1901566;GO:0019538;GO:0044237;GO:0043170;GO:0006518;GO:0006412;GO:0043232;GO:0043226;GO:0110165;GO:0005840;GO:0032991;GO:1990904;GO:0043229;GO:0005575;GO:0043228;GO:0003674;GO:0005198;GO:0003735	K02960	RP-S16e, RPS	map03010;ma	Ribosome;Corona	COG0103	S:Function unknown	PF00380.22	Ribosomal_S	Ribosomal protein	CYT	5	40	17	High
TRINITY_DN5857_c0_g1_i13_orf1	uncharacterized protein LOC114353981 isoform X1 [Ostrinia furnacalis]	2	1	4.05E-06	up	yes	1.026	0.513	0.512	0.507	0.52	1	1.049	1.029	CC:cellular_component;CC:macromolecular complex;CC:aminoacyl-tRNA synthetase multienzyme	GO:0005575;GO:0032991;GO:0017101;GO:1902494	K15438	AIMP2	------	------	ENOG4110B04	J:Translation, ribosomal structu	PF18569.4	Thioredoxin_1	Thioredoxin-like d	CYT	3	9	32.8	High
TRINITY_DN15624_c0_g1_i1_orf1	LOW QUALITY PROTEIN: V-type proton ATPase subunit S1-like [Ostrinia furnacalis]	2.34204793	1.227770601	0.0003714	up	yes	1.075	0.459	0.467	0.525	0.385	1	1.118	1.107	------	------	K03662	ATPeVS1, ATI	map05152;ma	Tuberculosis;Hun	ENOG410XTHY	U:Intracellular trafficking, secre	PF20520.1;PF05	Ac45-VOA1_1	V0 complex access	CYT	3	9	45.2	High
TRINITY_DN23740_c1_g1_i1_orf1	NADH dehydrogenase [ubiquinone] iron-sulfur protein 6, mitochondrial isoform X1 [Ostrinia	2.01470296	1.010567149	0.0001122	up	yes	1.014	0.5033	0.482	0.542	0.486	1	1.067	0.974	------	------	K03939	NDUFS6	map04714;ma	Thermogenesis;C	ENOG4111W6X	I:Lipid transport and metabolis	PF10276.12	zf-CHCC	Zinc-finger domai	CYT	1	12	13.7	High
TRINITY_DN2953_c1_g1_i2_orf1	methionine--tRNA ligase, cytoplasmic isoform X6 [Ostrinia furnacalis]	2.705	1.435628594	0.0002776	up	yes	1.082	0.4	0.378	0.437	0.385	1	1.183	1.062	BP:methionyl-tRNA aminoacylation;BP:carboxylic acid metabolic process;BP:nucleic acid metabolic process;BP:cellular nitrogen compound metabolic process;BP:nitrogen compound metabolic process;BP:small molecule metabolic process;BP:ncRNA metabolic process;BP:organic cyclic compound metabolic process;BP:nucleobase-containing compound metabolic process;BP:cellular amino acid metabolic process;BP:organic substance metabolic process;BP:primary metabolic process;BP:cellular process;BP:cellular aromatic compound metabolic process;BP:biological_process;BP:metabolic process;BP:oxoacid metabolic process;BP:organonitrogen compound metabolic process;BP:heterocycle metabolic process;BP:RNA metabolic process;BP:organic acid metabolic process;BP:cellular metabolic process;BP:macromolecule metabolic process;BP:tRNA metabolic process;BP:amino acid activation;BP:tRNA aminoacylation;BP:tRNA aminoacylation for protein translation;CC:aminoacyl-tRNA synthetase multienzyme complex;CC:catalytic complex;CC:macromolecular complex;CC:cellular_component;MF:purine ribonucleoside triphosphate binding;MF:molecular_function;MF:ion binding;MF:heterocyclic compound binding;MF:anion binding;MF:aminoacyl-tRNA ligase activity;MF:nucleotide binding;MF:nucleoside phosphate binding;MF:purine nucleotide binding;MF:ATP binding;MF:catalytic activity, acting on RNA;MF:small molecule binding;MF:catalytic activity;MF:ligase activity;MF:ligase activity, forming carbon-oxygen bonds;MF:methionine-tRNA ligase activity;MF:adenyl nucleotide binding;MF:carbohydrate derivative	GO:0006431;GO:0019752;GO:0090304;GO:0034641;GO:0006807;GO:0044281;GO:0034660;GO:1901360;GO:0006139;GO:0006520;GO:0071704;GO:0044238;GO:0009987;GO:0006725;GO:0008150;GO:0008152;GO:0043436;GO:1901564;GO:0046483;GO:0016070;GO:0006082;GO:0044237;GO:0043170;GO:0006399;GO:0043038;GO:0043039;GO:0006418;GO:0017101;GO:1902494;GO:0032991;GO:0005575;GO:0035639;GO:0003674;GO:0043167;GO:1901363;GO:0043168;GO:0004812;GO:0000166;GO:1901265;GO:0017076;GO:0005524;GO:0140098;GO:0036094;GO:0003824;GO:0016874;GO:0016875;GO:0004825;GO:0030554;GO:0097367;GO:0097159;GO:0032559;GO:0032555;GO:0140101;GO:0032553;GO:0005488	K01874	MARS, metG	map00450;ma	Selenocompounc	COG0143	J:Translation, ribosomal structu	PF00458.23	WHEP-TRS	WHEP-TRS domai	CYT	1	43	10.3	High
TRINITY_DN2497_c0_g1_i2_orf1	protein stunted-like isoform X1 [Colias croceus]	2.431235431	1.281689605	4.56E-05	up	yes	1.043	0.429	0.381	0.456	0.45	1	1.063	1.067	------	------	K02135	ATPeF1E, ATF	map04714;ma	Thermogenesis;C	ENOG410Y5P5	C:Energy production and conve	PF04627.16	ATP-synt_Eps	Mitochondrial ATP	CYT	1	48	6.9	High
TRINITY_DN94337_c0_g1_i1_orf1	hypothetical protein evm_006136 [Chilo suppressalis]	2.316027088	1.211652127	0.0001344	up	yes	1.026	0.443	0.444	0.478	0.407	1	1.095	0.983	CC:cellular_component;CC:integral component of membrane;CC:cellular anatomical entity;CC:intrinsic component of membrane;	GO:0005575;GO:0016021;GO:0110165;GO:0031224	------	------	------	------	ENOG410ZPQS	S:Function unknown	------	------	------	CYT	2	7	47.2	High
TRINITY_DN30208_c0_g1_i3_orf1	unnamed protein product [Timema cristinae]	2.186956522	1.128924539	5.45E-08	up	yes	1.006	0.46	0.456	0.461	0.463	1	1.016	1.003	------	------	K10374	TPM2	map05414;ma	Dilated cardiomy	ENOG410XR5K	Z:Cytoskeleton	PF00261.23;PF1	Tropomyosin;	Tropomyosin;Trop	CYT	4	57	7.7	High
TRINITY_DN11153_c0_g1_i1_orf1	unnamed protein product [Chilo suppressalis]	2.19499665	1.134218738	3.54E-06	up	yes	0.9827	0.4477	0.438	0.446	0.459	1	0.992	0.956	------	------	K20792	NAA15_16	------	------	ENOG410XR7D	S:Function unknown	PF12569.11;PF0	NatA_aux_su	N-terminal acetylt	CYT	16	20	100.1	High

TRINITY_DN29_c0_g1_i4_orf1	sodium-dependent nutrient amino acid transporter 1-like [Ostrinia furnacalis] >XP_028167707.1 sodium-dependent nutrient amino acid transporter 1-like [Ostrinia furnacalis]	2.342241186	1.227889641	0.0001308	up	yes	1.043	0.4453	0.386	0.503	0.447	1	1.081	1.048	CC:integral component of membrane;CC:cellular_component;CC:intrinsic component of membrane;CC:cellular anatomical entity;MF:secondary active transmembrane transporter activity;MF:symporter activity;MF:transporter activity;MF:transmembrane transporter activity;MF:molecular_function;MF:active transmembrane transporter activity;	GO:0016021;GO:0005575;GO:0031224;GO:0110165;GO:0015291;GO:0015293;GO:0005215;GO:0022857;GO:0003674;GO:0022804	K05038	SLC6A5_9, GL	map04721	Synaptic vesicle c	COG0733	P:Inorganic ion transport and n	PF00209.21	SNF	Sodiu	CYT	1	1	63	Medium
TRINITY_DN6147_c0_g1_i2_orf1	uncharacterized protein LOC114352519 [Ostrinia furnacalis]	2.190923318	1.131538991	6.33E-06	up	yes	0.98	0.4473	0.475	0.432	0.435	1	0.971	0.969	------	------	------	------	------	------	ENOG410YKCN;ENOG4111746	S:Function unknown;	PF02958.23;PF0	EcKL;DUF167	Ecdysteroid kinase	CYT	10	27	44.6	High
TRINITY_DN51934_c0_g2_i1_orf1	SCAN domain-containing protein 3-like [Pieris napi] >XP_047520696.1 SCAN domain-containing protein 3-like [Pieris napi]	2.490779444	1.316597278	8.29E-06	up	yes	1.013	0.4067	0.42	0.421	0.379	1	0.995	1.044	BP:nucleic acid metabolic process;BP:cellular nitrogen compound metabolic process;BP:nitrogen compound metabolic process;BP:organic cyclic compound metabolic process;BP:nucleobase-containing compound metabolic process;BP:cellular macromolecule metabolic process;BP:organic substance metabolic process;BP:cellular process;BP:cellular aromatic compound metabolic process;BP:biological_process;BP:metabolic process;BP:heterocycle metabolic process;BP:primary metabolic process;BP:cellular metabolic process;BP:macromolecule metabolic process;BP:DNA integration;BP:DNA metabolic process;MF:molecular_function;MF:binding;MF:nucleic acid binding;MF:heterocyclic compound binding;MF:organic cyclic	GO:0090304;GO:0034641;GO:0006807;GO:1901360;GO:0006139;GO:0044260;GO:0071704;GO:0009987;GO:0006725;GO:0008150;GO:0008152;GO:0046483;GO:0044238;GO:0044237;GO:0043170;GO:0015074;GO:0006259;GO:0003674;GO:0005488;GO:0003676;GO:1901363;GO:0097159	------	------	------	------	COG2801;ENOG410YZQA;ENOG410Y65V	E:Amino acid transport and me	------	------	------	CYT	1	4	17.4	Medium
TRINITY_DN29100_c0_g1_i2_orf1	endocuticle structural glycoprotein ABD-5-like [Galleria mellonella]	2.29144447	1.196257325	0.0001011	up	yes	0.9883	0.4313	0.422	0.378	0.494	1	1.001	0.964	MF:molecular_function;MF:structural constituent of cuticle;MF:structural molecule activity;	GO:0003674;GO:0042302;GO:0005198	------	------	------	------	ENOG41113X7;ENOG41125BY;ENOG410YVGC	S:Function unknown;	PF00379.26	Chitin_bind_4	Insect cuticle prote	CYT	3	61	11.2	High
TRINITY_DN48610_c0_g1_i2_orf1	hypothetical protein evm_002298 [Chilo suppressalis] >CAH0682062.1 unnamed protein product [Chilo suppressalis]	2.398753894	1.262285148	5.56E-07	up	yes	1.001	0.4173	0.435	0.413	0.404	1	0.994	1.009	MF:molecular_function;MF:structural constituent of cuticle;MF:structural molecule activity;	GO:0003674;GO:0042302;GO:0005198	------	------	------	------	ENOG410XTK4;ENOG4111PJ1;ENOG4110RRZ;ENOG4110	S:Function unknown;	PF00379.26	Chitin_bind_4	Insect cuticle prote	CYT	5	41	18.6	High
TRINITY_DN24142_c0_g1_i1_orf1	arylalkylamine N-acetyltransferase [Chilo suppressalis]	2.214175993	1.146769899	0.0007318	up	yes	1.009	0.4557	0.524	0.505	0.338	1	1.012	1.016	MF:N-acetyltransferase activity;MF:aralkylamine N-acetyltransferase activity;MF:transferase activity;MF:catalytic activity;MF:acetyltransferase activity;MF:molecular_function;MF:transferase activity, transferring acyl groups other than amino-acyl groups;MF:N-acyltransferase activity;MF:transferase activity, transferring acyl	GO:0008080;GO:0004059;GO:0016740;GO:0003824;GO:0016407;GO:0003674;GO:0016747;GO:0016410;GO:0016746	------	------	------	------	ENOG4111REY	S:Function unknown	------	------	------	CYT	1	3	29.3	High
TRINITY_DN2274_c0_g1_i6_orf1	membrane alanyl aminopeptidase-like [Ostrinia furnacalis]	2.392386881	1.258450711	9.56E-07	up	yes	0.9993	0.4177	0.43	0.41	0.413	1	0.982	1.016	BP:nitrogen compound metabolic process;BP:organic substance metabolic process;BP:proteolysis;BP:biological_process;BP:metabolic process;BP:primary metabolic process;BP:organonitrogen compound metabolic process;BP:protein metabolic process;BP:macromolecule metabolic process;MF:aminopeptidase activity;MF:exopeptidase activity;MF:molecular_function;MF:zinc ion binding;MF:peptidase activity;MF:metallopeptidase activity;MF:cation binding;MF:transition metal ion	GO:0006807;GO:0071704;GO:0006508;GO:0008150;GO:0008152;GO:0044238;GO:1901564;GO:0019538;GO:0043170;GO:0004177;GO:0008238;GO:0003674;GO:0008270;GO:0008233;GO:0008237;GO:0043169;GO:0046914;GO:0043167;GO:0046872;GO:0016787;GO:0140096;GO:0003824;GO:0005488	------	------	------	------	COG0308	E:Amino acid transport and me	PF01433.23;PF1	Peptidase_M1	Peptidase family M	CYT	11	15	114.7	High
TRINITY_DN48097_c0_g1_i1_orf1	unnamed protein product [Homo sapiens]	2.18655538	1.128659889	0.0007981	up	yes	0.9693	0.4433	0.419	0.443	0.468	1	0.861	1.047	BP:positive regulation of cellular amide metabolic process;BP:positive regulation of RNA binding;BP:regulation of primary metabolic process;BP:regulation of metabolic process;BP:positive regulation of cellular biosynthetic process;BP:macromolecular complex subunit organization;BP:regulation of cellular macromolecule biosynthetic process;BP:positive regulation of cellular metabolic process;BP:regulation of cellular metabolic process;BP:positive regulation of gene expression;BP:formation of cytoplasmic translation initiation complex;BP:regulation of biological process;BP:cellular component organization or biogenesis;BP:positive regulation of metabolic process;BP:ribonucleoprotein complex subunit organization;BP:positive regulation of macromolecule metabolic process;BP:posttranscriptional regulation of gene expression;BP:positive regulation of biosynthetic process;BP:regulation of protein metabolic process;BP:positive regulation of protein metabolic process;BP:cellular component organization;BP:macromolecular complex assembly;BP:ribonucleoprotein complex assembly;BP:biological regulation;BP:positive regulation of molecular function;BP:regulation of cellular biosynthetic process;BP:positive regulation of biological process;BP:regulation of molecular function;BP:positive regulation of translation;BP:regulation of gene expression;BP:cellular component assembly;BP:regulation of macromolecule metabolic process;BP:cellular process;BP:regulation of biosynthetic process;BP:regulation of cellular amide metabolic	GO:0034250;GO:1905216;GO:0080090;GO:0019222;GO:0031328;GO:0043933;GO:2000112;GO:0031325;GO:0031323;GO:0010628;GO:0001732;GO:0050789;GO:0071840;GO:0009893;GO:0071826;GO:0010604;GO:0010608;GO:0009891;GO:0051246;GO:0051247;GO:0016043;GO:0065003;GO:0022618;GO:0065007;GO:0044093;GO:0031326;GO:0048518;GO:0065009;GO:0045727;GO:0010468;GO:0022607;GO:0060255;GO:0009987;GO:0009889;GO:0034248;GO:0008150;GO:0051171;GO:0008152;GO:0051173;GO:0050794;GO:1902415;GO:1902416;GO:0051099;GO:0051098;GO:0044237;GO:0010557;GO:0010556;GO:0006417;GO:1905214;GO:0006413;GO:0048522;GO:0070993;GO:0005852;GO:0110165;GO:0033290;GO:0032991;GO:1990904;GO:0005575;GO:0016282;GO:0005829;GO:0003674;GO:0005488;GO:0003676;GO:0031369;GO:1901363;GO:0008135;GO:0043021;GO:0090079;GO:0043022;GO:0045182;GO:0097159;GO:0003723;GO:0005515;GO:0044877;GO:0003743	K03252	EIF3C	------	------	ENOG410XRU3	S:Function unknown	PF05470.15;PF0	eIF-3c_N;PCI	Eukaryotic translat	CYT	1	1	70.4	Medium
TRINITY_DN12133_c0_g2_i1_orf1	O-acyltransferase like protein-like [Ostrinia furnacalis]	2.25478883	1.172992326	2.89E-05	up	yes	0.977	0.4333	0.435	0.433	0.432	1	1.005	0.926	CC:integral component of membrane;CC:cellular_component;CC:intrinsic component of membrane;CC:cellular anatomical entity;MF:catalytic activity;MF:transferase activity;MF:transferase activity, transferring acyl groups;MF:molecular_function;MF:transferase activity, transferring acyl groups other than amino-acyl groups;	GO:0016021;GO:0005575;GO:0031224;GO:0110165;GO:0003824;GO:0016740;GO:0016746;GO:0003674;GO:0016747	------	------	------	------	ENOG410YD1T;ENOG410YT8E	S:Function unknown;	PF01757.25;PF2	Acyl_transf_3;	Acyltransferase fan	CYT	1	1	84.1	High
TRINITY_DN327_c1_g1_i4_orf1	mitochondrial import receptor subunit TOM40 homolog 1-like [Ostrinia furnacalis]	2.371455802	1.24577298	2.32E-07	up	yes	0.9953	0.4197	0.413	0.418	0.428	1	0.982	1.004	BP:protein localization;BP:establishment of localization in cell;BP:protein import into mitochondrial matrix;BP:protein transmembrane transport;BP:cellular macromolecule localization;BP:localization;BP:nitrogen compound transport;BP:intracellular protein transmembrane transport;BP:mitochondrial transmembrane transport;BP:organic substance transport;BP:macromolecule localization;BP:transport;BP:establishment of protein localization to organelle;BP:cellular process;BP:establishment of protein localization;BP:protein localization to organelle;BP:biological_process;BP:establishment of protein localization to mitochondrion;BP:mitochondrial transport;BP:establishment of localization;BP:transmembrane transport;BP:intracellular protein transmembrane import;BP:cellular localization;BP:protein localization to mitochondrion;BP:intracellular transport;BP:protein transport;BP:intracellular protein transport;CC:intrinsic component of membrane;CC:organelle membrane;CC:integral component of membrane;CC:membrane;CC:cellular anatomical entity;CC:organelle outer membrane;CC:bounding membrane of organelle;CC:mitochondrial membrane;CC:outer membrane;CC:cellular_component;CC:mitochondrial outer membrane;MF:protein transporter activity;MF:molecular_function;MF:macromolecule transmembrane transporter activity;MF:transporter activity;MF:protein transmembrane transporter activity;MF:transmembrane transporter activity;	GO:0008104;GO:0051649;GO:0030150;GO:0071806;GO:0070727;GO:0051179;GO:0071705;GO:0065002;GO:1990542;GO:0071702;GO:0033036;GO:0006810;GO:0072594;GO:0009987;GO:0045184;GO:0033365;GO:0008150;GO:0072655;GO:0006839;GO:0051234;GO:0055085;GO:0044743;GO:0051641;GO:0070585;GO:0046907;GO:0015031;GO:0006886;GO:0031224;GO:0031090;GO:0016021;GO:0016020;GO:0110165;GO:0031968;GO:0098588;GO:0031966;GO:0019867;GO:0005575;GO:0005741;GO:0140318;GO:0003674;GO:0022884;GO:0005215;GO:0008320;GO:0022857	K11518	TOM40	map05022;ma	Pathways of neu	ENOG410XS2U	O:Posttranslational modificatio	PF01459.25	Porin_3	Eukaryotic porin	CYT	6	26	34	High
TRINITY_DN30131_c0_g1_i1_orf1	PREDICTED: 60S ribosomal protein L44 [Amyelois transitella] >XP_021198018.1 60S ribosomal protein L44 [Helicoverpa armigera] >XP_022814294.1 60S ribosomal protein L44 [Spodoptera litura] >XP_026732397.1 60S ribosomal protein L44 [Trichoplusia ni] >XP_026752106.1 60S ribosomal protein L44 [Galleria mellonella] >XP_028158932.1 60S ribosomal protein L44 [Ostrinia furnacalis] >XP_035434364.1 60S ribosomal protein L44 [Spodoptera frugiperda] >XP_035434370.1 60S ribosomal protein L44 [Spodoptera frugiperda] >XP_047019234.1 60S ribosomal protein L44 [Helicoverpa zea] >XP_049868501.1 60S ribosomal protein L44 [Pectinophora gossypiella] >AAM53948.1 ribosomal protein L44 [Choristoneura parallela] >KAF9418375.1 hypothetical protein HW555_004805 [Spodoptera exigua] >RVE50750.1 hypothetical protein evm_004660 [Chilo suppressalis] >CAB3235328.1 unnamed protein product [Arctia plantaginis] >CAB3516516.1 unnamed protein product [Spodoptera littoralis] >CAG9747186.1 unnamed protein product [Diatraea saccharalis] >CAH0581656.1 unnamed protein product [Chrysodeixis includens]	2.373112868	1.246780719	1.41E-06	up	yes	0.9903	0.4173	0.408	0.428	0.416	1	1.003	0.968	BP:cellular biosynthetic process;BP:cellular nitrogen compound metabolic process;BP:nitrogen compound metabolic process;BP:cellular macromolecule biosynthetic process;BP:organic substance biosynthetic process;BP:cellular nitrogen compound biosynthetic process;BP:cellular macromolecule metabolic process;BP:peptide biosynthetic process;BP:organic substance metabolic process;BP:amide biosynthetic process;BP:cellular process;BP:biosynthetic process;BP:macromolecule biosynthetic process;BP:biological_process;BP:metabolic process;BP:organonitrogen compound metabolic process;BP:primary metabolic process;BP:cellular amide metabolic process;BP:organonitrogen compound biosynthetic process;BP:protein metabolic process;BP:cellular metabolic process;BP:macromolecule metabolic process;BP:peptide metabolic process;BP:translation;CC:intracellular non-membrane-bounded organelle;CC:organelle;CC:cellular anatomical entity;CC:ribosome;CC:macromolecular complex;CC:ribonucleoprotein complex;CC:intracellular organelle;CC:cellular_component;CC:non-membrane-bounded organelle;MF:molecular_function;MF:structural molecule	GO:0044249;GO:0034641;GO:0006807;GO:0034645;GO:1901576;GO:0044271;GO:0044260;GO:0043043;GO:0071704;GO:0043604;GO:0009987;GO:0009058;GO:0009059;GO:0008150;GO:0008152;GO:1901564;GO:0044238;GO:0043603;GO:1901566;GO:0019538;GO:0044237;GO:0043170;GO:0006518;GO:0006412;GO:0043232;GO:0043226;GO:0110165;GO:0005840;GO:0032991;GO:1990904;GO:0043229;GO:0005575;GO:0043228;GO:0003674;GO:0005198;GO:0003735	K02929	RP-L44e, RPL	map03010;ma	Ribosome;Coron	COG1631	J:Translation, ribosomal structu	PF00935.22	Ribosomal_L4	Ribosomal protein	CYT	3	28	12.3	High
TRINITY_DN14937_c0_g1_i7_orf1	multidrug resistance protein homolog 49-like [Ostrinia furnacalis] >XP_028159925.1 multidrug resistance protein homolog 49-like [Ostrinia furnacalis]	2.202173421	1.138928085	8.17E-06	up	yes	0.9727	0.4417	0.43	0.456	0.439	1	0.943	0.975	CC:integral component of membrane;CC:intrinsic component of membrane;CC:cellular_component;CC:cellular anatomical entity;MF:purine ribonucleoside triphosphate binding;MF:molecular_function;MF:binding;MF:ion binding;MF:heterocyclic compound binding;MF:nucleoside phosphate binding;MF:anion binding;MF:ATPase activity, coupled to transmembrane movement of substances;MF:nucleotide binding;MF:primary active transmembrane transporter activity;MF:purine nucleotide binding;MF:ATP binding;MF:active transmembrane transporter activity;MF:transporter activity;MF:ABC-type transmembrane transporter activity;MF:small molecule binding;MF:adenyl nucleotide binding;MF:carbohydrate derivative binding;MF:organic cyclic compound binding;MF:transmembrane transporter activity;MF:adenyl	GO:0016021;GO:0031224;GO:0005575;GO:0110165;GO:0035639;GO:0003674;GO:0005488;GO:0043167;GO:1901363;GO:1901265;GO:0043168;GO:0042626;GO:0000166;GO:0015399;GO:0017076;GO:0005524;GO:0022804;GO:0005215;GO:0140359;GO:0036094;GO:0030554;GO:0097367;GO:0097159;GO:0022857;GO:0032559;GO:0032555;GO:0032553	K05658	ABCB1, CD24	map05206;ma	MicroRNAs in ca	COG1132	S:Function unknown	PF00664.26;PF0	ABC_membra	ABC transporter tr	CYT	3	2	143.2	High
TRINITY_DN29440_c1_g1_i4_orf1	neutral lipase [Helicoverpa armigera]	2.089289642	1.063012509	4.46E-05	up	yes	0.95	0.4547	0.459	0.442	0.463	1	0.93	0.92	BP:organic substance metabolic process;BP:lipid metabolic process;BP:primary metabolic process;BP:metabolic process;BP:biological_process;CC:cellular_component;CC:extracellular region;CC:cellular anatomical entity;MF:hydrolase activity;MF:hydrolase activity, acting on ester bonds;MF:catalytic activity;MF:molecular_function;MF:lipase activity;	GO:0071704;GO:0006629;GO:0044238;GO:0008152;GO:0008150;GO:0005575;GO:0005576;GO:0110165;GO:0016787;GO:0016788;GO:0003824;GO:0003674;GO:0016298	------	------	------	------	ENOG410YGS3;ENOG4110T03	S:Function unknown;	PF00151.22	Lipase	Lipase	PLA	1	10	11.7	High
TRINITY_DN45949_c0_g1_i1_orf1	uncharacterized protein LOC114355167 [Ostrinia furnacalis]	2.284037559	1.191586375	1.01E-05	up	yes	0.973	0.426	0.423	0.409	0.446	1	0.976	0.943	MF:molecular_function;MF:structural constituent of cuticle;MF:structural molecule activity;	GO:0003674;GO:0042302;GO:0005198	------	------	------	------	ENOG410ZET2;ENOG410YR66;ENOG410YXXU;ENOG4111P	O:Posttranslational modificatio	PF00379.26	Chitin_bind_4	Insect cuticle prote	CYT	3	18	22.7	High
TRINITY_DN10637_c0_g1_i4_orf1	V-type proton ATPase subunit d [Bombyx mandarina]	2.777926601	1.47400848	5.75E-06	up	yes	1.037	0.3733	0.371	0.364	0.385	1	1.043	1.068	CC:cellular_component;CC:proton-transporting V-type ATPase, V0 domain;CC:macromolecular complex;CC:membrane protein complex;CC:proton-transporting two-sector ATPase complex, proton-transporting domain;MF:molecular_function;MF:ATPase activity, coupled to transmembrane movement of substances;MF:inorganic solute uptake transmembrane transporter activity;MF:hydrogen-translocating pyrophosphatase activity;MF:primary active transmembrane transporter activity;MF:active transmembrane transporter activity;MF:ATPase coupled ion transmembrane transporter activity;MF:transporter activity;MF:cation transmembrane transporter activity;MF:proton-transporting ATPase activity, rotational mechanism;MF:ATPase activity, coupled to transmembrane movement of ions, rotational mechanism;MF:inorganic cation transmembrane transporter activity;MF:cation-transporting ATPase activity;MF:ion transmembrane transporter activity;MF:transmembrane transporter activity;MF:active ion transmembrane transporter	GO:0005575;GO:0033179;GO:0032991;GO:0098796;GO:0033177;GO:0003674;GO:0042626;GO:0015318;GO:0009678;GO:0015399;GO:0022804;GO:0042625;GO:0005215;GO:0008324;GO:0046961;GO:0044769;GO:0022890;GO:0019829;GO:0015075;GO:0022857;GO:0022853;GO:0015078	K02146	ATPeV0D, AT	map05152;ma	Tuberculosis;Hur	COG1527	S:Function unknown	PF01992.19	vATP-synt_A	ATP synthase	CYT	7	20	39.6	High

TRINITY_DN141381_c0_g1_i1_orf1	very long-chain specific acyl-CoA dehydrogenase, mitochondrial [Chelonus insularis]	2.436609337	1.284874961	5.13E-05	up	yes	0.9917	0.407	0.434	0.364	0.423	1	0.948	1.027	MF:anion binding;MF:flavin adenine dinucleotide binding;MF:organic cyclic compound binding;MF:oxidoreductase activity, acting on the CH-CH group of donors, with a flavin as acceptor;MF:heterocyclic compound binding;MF:nucleotide binding;MF:small molecule binding;MF:catalytic activity;MF:molecular_function;MF:binding;MF:acyl-CoA dehydrogenase activity;MF:ion binding;MF:oxidoreductase activity, acting on the CH-CH group of donors;MF:nucleoside	GO:0043168;GO:0050660;GO:0097159;GO:0052890;GO:1901363;GO:0000166;GO:0036094;GO:0003824;GO:0003674;GO:0005488;GO:0003995;GO:0043167;GO:0016627;GO:1901265;GO:0016491	K09479	ACADVL	map04936;ma	Alcoholic liver di	COG1960	S:Function unknown	PF00441.27;PF0	Acyl-CoA_dh_	Acyl-CoA dehydro	CYT	2	4	69	High
TRINITY_DN501_c1_g1_i1_orf1	sodium- and chloride-dependent GABA transporter ine isoform X1 [Ostrinia furnacalis]	2.451477833	1.293651716	2.16E-05	up	yes	0.9953	0.406	0.372	0.432	0.414	1	1.025	0.961	BP:transmission of nerve impulse;BP:multicellular organismal signaling;BP:developmental cell growth;BP:developmental growth;BP:cell growth;BP:glial cell growth;BP:multicellular organismal process;BP:neurological system process;BP:biological_process;BP:growth;BP:system process;BP:signaling;BP:cellular process;CC:intrinsic component of membrane;CC:cellular_component;CC:integral component of membrane;CC:cellular anatomical entity;MF:neurotransmitte;MF:neurotransmitter transporter activity;MF:molecular transducer activity;MF:molecular_function;MF:signaling receptor activity;MF:sodium ion transmembrane transporter activity;MF:inorganic solute uptake transmembrane transporter activity;MF:transmembrane signaling receptor activity;MF:osmosensor activity;MF:solut;MF:transporter activity;MF:metal ion transmembrane transporter activity;MF:small molecule sensor activity;MF:active transmembrane transporter activity;MF:solut;MF:secondary active transmembrane transporter activity;MF:symporter activity;MF:cation transmembrane transporter activity;MF:inorganic cation transmembrane transporter activity;MF:ion transmembrane transporter activity;MF:transmembrane transporter activity;MF:active ion	GO:0019226;GO:0035637;GO:0048588;GO:0048589;GO:0016049;GO:0042065;GO:0032501;GO:0050877;GO:0008150;GO:0040007;GO:0003008;GO:0023052;GO:0009987;GO:0031224;GO:0005575;GO:0016021;GO:0110165;GO:0005328;GO:0005326;GO:0060089;GO:0003674;GO:0038023;GO:0015081;GO:0015318;GO:0004888;GO:0005034;GO:0015370;GO:0005215;GO:0046873;GO:0140299;GO:0022804;GO:0015294;GO:0015291;GO:0015293;GO:0008324;GO:0022890;GO:0015075;GO:0022857;GO:0022853	K05039	SLC6A6, TAU	------	------	COG0733	S:Function unknown	PF00209.21	SNF	Sodiu	CYT	1	1	80.8	High
TRINITY_DN13530_c0_g1_i1_orf1	unnamed protein product [Euphydryas editha]	2.081769702	1.057810478	8.09E-05	up	yes	1.054	0.5063	0.529	0.523	0.467	1	1.077	1.084	------	------	K16815	PNPLA8	------	------	ENOG410XQXI	S:Function unknown	PF01734.25	Patatin	Patatin-like phosp	CYT	1	2	63.4	High
TRINITY_DN10520_c0_g1_i2_orf1	probable 39S ribosomal protein L45, mitochondrial [Ostrinia furnacalis]	2.055009823	1.03914529	0.0003562	up	yes	1.046	0.509	0.517	0.524	0.486	1	1	1.139	CC:ribosome;CC:intracellular non-membrane-bounded organelle;CC:cellular_component;CC:intracellular organelle;CC:non-membrane-bounded organelle;CC:organelle;CC:cellular anatomical entity;	GO:0005840;GO:0043232;GO:0005575;GO:0043229;GO:0043228;GO:0043226;GO:0110165	K17426	MRPL45	------	------	ENOG411019B	S:Function unknown	PF04280.18	Tim44	Tim44-like domain	CYT	4	16	30	High
TRINITY_DN83948_c0_g1_i3_orf1	carbonyl reductase [NADPH] 1-like [Ostrinia furnacalis]	2.15738648	1.109284648	5.88E-05	up	yes	1.05	0.4867	0.518	0.47	0.472	1	1.095	1.055	MF:catalytic activity;MF:molecular_function;MF:oxidoreductase activity, acting on CH-OH group of donors;MF:oxidoreductase activity, acting on the CH-OH group of donors, NAD or NADP as acceptor;MF:oxidoreductase activity;	GO:0003824;GO:0003674;GO:0016614;GO:0016616;GO:0016491	K00079	CBR1	map00590;ma	Arachidonic acid	COG1028	S:Function unknown	PF00106.28;PF1	adh_short;adh	short chain dehyd	CYT	4	19	36.4	High
TRINITY_DN42738_c0_g1_i1_orf1	arf-GAP domain and FG repeat-containing protein 1 [Ostrinia furnacalis]	2.155876807	1.108274741	8.94E-06	up	yes	1.029	0.4773	0.457	0.474	0.501	1	1.046	1.04	BP:carboxylic acid metabolic process;BP:cellular biosynthetic process;BP:nitrogen compound metabolic process;BP:aspartate family amino acid biosynthetic process;BP:aspartate family amino acid metabolic process;BP:small molecule biosynthetic process;BP:organic substance biosynthetic process;BP:asparagine biosynthetic process;BP:asparagine metabolic process;BP:cellular amino acid metabolic process;BP:organic substance metabolic process;BP:alpha-amino acid metabolic process;BP:alpha-amino acid biosynthetic process;BP:cellular amino acid biosynthetic process;BP:cellular process;BP:biosynthetic process;BP:biological_process;BP:metabolic process;BP:oxoacid metabolic process;BP:primary metabolic process;BP:organonitrogen compound metabolic process;BP:organic acid metabolic process;BP:organonitrogen compound biosynthetic process;BP:carboxylic acid biosynthetic process;BP:organic acid biosynthetic process;BP:cellular metabolic process;BP:small molecule metabolic process;MF:enzyme activator activity;MF:molecular function regulator;MF:ligase activity;MF:enzyme regulator activity;MF:GTPase activator activity;MF:GTPase regulator activity;MF:nucleoside-triphosphatase regulator activity;MF:carbon-nitrogen ligase activity, with glutamine as amido-N-donor;MF:ligase activity, forming carbon-nitrogen	GO:0019752;GO:0044249;GO:0006807;GO:0009067;GO:0009066;GO:0044283;GO:1901576;GO:0006529;GO:0006528;GO:0006520;GO:0071704;GO:1901605;GO:1901607;GO:0008652;GO:0009987;GO:0009058;GO:0008150;GO:0008152;GO:0043436;GO:0044238;GO:1901564;GO:0006082;GO:1901566;GO:0046394;GO:0016053;GO:0044237;GO:0044281;GO:0008047;GO:0098772;GO:0016874;GO:0030234;GO:0005096;GO:0030695;GO:0060589;GO:0016884;GO:0016879;GO:0003824;GO:0004066;GO:0003674	K15044	AGFG1	------	------	ENOG4111QGH;ENOG41122WA;COG0367;COG5347	U:Intracellular trafficking, secre	PF01412.21	ArfGap	Putative GTPase a	EXC	1	5	38.3	High
TRINITY_DN11194_c0_g1_i4_orf1	ATPase family AAA domain-containing protein 3A homolog [Ostrinia furnacalis]	2.10460251	1.073547782	9.91E-09	up	yes	1.006	0.478	0.476	0.477	0.481	1	1.009	1.009	BP:mitochondrion organization;BP:cellular component organization;BP:cellular component organization or biogenesis;BP:cellular process;BP:biological_process;BP:organelle organization;CC:intracellular organelle;CC:mitochondrion;CC:membrane-bounded organelle;CC:organelle;CC:cellular anatomical entity;CC:intracellular membrane-bounded organelle;CC:cellular_component;MF:purine ribonucleoside triphosphate binding;MF:heterocyclic compound binding;MF:molecular_function;MF:ATPase activity;MF:ion binding;MF:anion binding;MF:nucleotide binding;MF:purine nucleotide binding;MF:hydrolase activity;MF:nucleoside-triphosphatase activity;MF:nucleoside phosphate binding;MF:small molecule binding;MF:catalytic activity;MF:hydrolase activity, acting on acid anhydrides, in phosphorus-containing anhydrides;MF:adenyl nucleotide binding;MF:carbohydrate derivative binding;MF:organic cyclic compound binding;MF:hydrolase activity, acting on acid anhydrides;MF:pyrophosphatase activity;MF:adenyl	GO:0007005;GO:0016043;GO:0071840;GO:0009987;GO:0008150;GO:0006996;GO:0043229;GO:0005739;GO:0043227;GO:0043226;GO:0110165;GO:0043231;GO:0005575;GO:0035639;GO:1901363;GO:0003674;GO:0016887;GO:0043167;GO:0043168;GO:0000166;GO:0017076;GO:0016787;GO:0017111;GO:1901265;GO:0036094;GO:0003824;GO:0016818;GO:0030554;GO:0097367;GO:0097159;GO:0016817;GO:0016462;GO:0032559;GO:0005524;GO:0032555;GO:0032553;GO:0005488	K17681	ATAD3A_B	------	------	COG1223;COG0464	S:Function unknown;O:Posttrar	PF12037.11;PF0	ATAD3_N;AA	ATPase family AA	CYT	13	21	70.3	High
TRINITY_DN6535_c0_g2_i1_orf1	NADH dehydrogenase [ubiquinone] 1 beta subcomplex subunit 3 [Ostrinia furnacalis] >XP_028166399.1 NADH dehydrogenase [ubiquinone] 1 beta subcomplex subunit 3 [Ostrinia furnacalis]	2.017827869	1.01280311	1.25E-05	up	yes	0.9847	0.488	0.489	0.495	0.48	1	1.006	0.948	BP:cellular process;BP:cellular metabolic process;BP:electron transport chain;BP:biological_process;BP:metabolic process;BP:generation of precursor metabolites and energy;CC:organelle membrane;CC:integral component of membrane;CC:membrane;CC:organelle inner membrane;CC:mitochondrial membrane;CC:cellular_component;CC:mitochondrial inner membrane;CC:intrinsic component of membrane;CC:respiratory chain;CC:cellular anatomical entity;	GO:0009987;GO:0044237;GO:0022900;GO:0008150;GO:0008152;GO:0006091;GO:0031090;GO:0016021;GO:0016020;GO:0019866;GO:0031966;GO:0005575;GO:0005743;GO:0031224;GO:0070469;GO:0110165	K03959	NDUFB3	map04714;ma	Thermogenesis;C	ENOG411294X	I:Lipid transport and metabolis	PF08122.15	NDUF_B12	NADH-ubiquinone	CYT	4	39	11	High
TRINITY_DN2993_c0_g1_i4_orf1	heat shock 70 kDa protein cognate 5 [Ostrinia furnacalis]	2.136460554	1.095222681	1.62E-06	up	yes	1.002	0.469	0.464	0.483	0.46	1	1.02	0.986	MF:purine ribonucleoside triphosphate binding;MF:molecular_function;MF:binding;MF:ion binding;MF:heterocyclic compound binding;MF:nucleoside phosphate binding;MF:anion binding;MF:unfolded protein binding;MF:nucleotide binding;MF:purine nucleotide binding;MF:ATP binding;MF:small molecule binding;MF:adenyl nucleotide binding;MF:carbohydrate derivative binding;MF:organic cyclic compound binding;MF:protein binding involved in protein folding;MF:adenyl ribonucleotide binding;MF:protein binding;MF:purine ribonucleotide binding;MF:ribonucleotide binding;	GO:0035639;GO:0003674;GO:0005488;GO:0043167;GO:1901363;GO:1901265;GO:0043168;GO:0051082;GO:0000166;GO:0017076;GO:0005524;GO:0036094;GO:0030554;GO:0097367;GO:0097159;GO:0044183;GO:0032559;GO:0005515;GO:0032555;GO:0032553	K04043	dnaK, HSPA9	map03018;ma	RNA degradation	COG0443	O:Posttranslational modificatio	PF00012.23;PF0	HSP70;MreB_	Hsp70 protein;Mre	CYT	24	34	74.7	High
TRINITY_DN4842_c0_g1_i5_orf1	cytochrome c oxidase assembly factor 4 homolog, mitochondrial isoform X1 [Ostrinia furnacalis] >XP_028162331.1 cytochrome c oxidase assembly factor 4 homolog, mitochondrial isoform X2 [Ostrinia furnacalis]	2.25	1.169925001	0.0001511	up	yes	1.017	0.452	0.503	0.381	0.472	1	1	1.051	BP:mitochondrial respiratory chain complex assembly;BP:mitochondrial respiratory chain complex IV assembly;BP:cellular component assembly;BP:macromolecular complex subunit organization;BP:cellular process;BP:respiratory chain complex IV assembly;BP:biological_process;BP:cytochrome complex assembly;BP:cellular component organization or biogenesis;BP:macromolecular complex assembly;BP:cellular component organization;CC:organelle lumen;CC:organelle envelope lumen;CC:membrane-enclosed lumen;CC:cellular_component;CC:intracellular organelle lumen;CC:mitochondrial intermembrane space;CC:cellular	GO:0033108;GO:0033617;GO:0022607;GO:0043933;GO:0009987;GO:0008535;GO:0008150;GO:0017004;GO:0071840;GO:0065003;GO:0016043;GO:0043233;GO:0031970;GO:0031974;GO:0005575;GO:0070013;GO:0005758;GO:0110165	K18177	COA4	map04714	Thermogenesis	ENOG410XXMU;COG1990	O:Posttranslational modificatio	------	------	------	CYT	1	13	8.2	High
TRINITY_DN2749_c0_g2_i3_orf1	RNA exonuclease 4-like [Ostrinia furnacalis] >QEE79882.1 REX4 [Ostrinia furnacalis]	2.218590171	1.149643191	1.57E-07	up	yes	0.9977	0.4497	0.459	0.453	0.437	1	1	0.993	BP:nucleic acid metabolic process;BP:cellular nitrogen compound metabolic process;BP:nitrogen compound metabolic process;BP:ncRNA metabolic process;BP:organic cyclic compound metabolic process;BP:nucleobase-containing compound metabolic process;BP:organic substance metabolic process;BP:rRNA processing;BP:ncRNA processing;BP:cellular process;BP:cellular aromatic compound metabolic process;BP:biological_process;BP:metabolic process;BP:heterocycle metabolic process;BP:RNA metabolic process;BP:primary metabolic process;BP:rRNA metabolic process;BP:cellular metabolic process;BP:macromolecule metabolic process;BP:RNA processing;MF:molecular_function;MF:binding;MF:nucleic acid binding;MF:heterocyclic compound binding;MF:3'-5' exonuclease activity;MF:exonuclease activity;MF:hydrolase activity;MF:hydrolase activity, acting on ester bonds;MF:catalytic	GO:0090304;GO:0034641;GO:0006807;GO:0034660;GO:1901360;GO:0006139;GO:0071704;GO:0006364;GO:0034470;GO:0009987;GO:0006725;GO:0008150;GO:0008152;GO:0046483;GO:0016070;GO:0044238;GO:0016072;GO:0044237;GO:0043170;GO:0006396;GO:0003674;GO:0005488;GO:0003676;GO:1901363;GO:0008408;GO:0004527;GO:0016787;GO:0016788;GO:0003824;GO:0004518;GO:0097159	K18327	REXO4, REX4	------	------	COG0847	L:Replication, recombination ar	PF00929.27	RNase_T	Exonuclease	CYT	5	45	17.8	High
TRINITY_DN27641_c0_g1_i1_orf1	succinate dehydrogenase [ubiquinone] iron-sulfur subunit, mitochondrial [Ostrinia furnacalis]	2.287015945	1.193466424	9.31E-07	up	yes	1.004	0.439	0.458	0.427	0.432	1	1.015	0.996	BP:tricarboxylic acid cycle;BP:biological_process;BP:metabolic process;BP:primary metabolic process;CC:organelle membrane;CC:membrane;CC:cellular anatomical entity;CC:mitochondrial membrane;CC:organelle inner membrane;CC:cellular_component;CC:mitochondrial inner membrane;MF:oxidoreductase activity, acting on the CH-CH group of donors, quinone or related compound as acceptor;MF:molecular_function;MF:binding;MF:succinate dehydrogenase activity;MF:oxidoreductase activity;MF:cation binding;MF:oxidoreductase activity, acting on the CH-CH group of donors;MF:ion binding;MF:metal ion binding;MF:catalytic activity;MF:succinate dehydrogenas;MF:metal cluster binding;MF:electron carrier activity;MF:3 iron, 4 sulfur cluster binding;MF:4 iron, 4 sulfur cluster binding;MF:iron-sulfur cluster binding;MF:2 iron, 2 sulfur cluster binding;	GO:0006099;GO:0008150;GO:0008152;GO:0044238;GO:0031090;GO:0016020;GO:0110165;GO:0031966;GO:0019866;GO:0005575;GO:0005743;GO:0016635;GO:0003674;GO:0005488;GO:0000104;GO:0016491;GO:0043169;GO:0016627;GO:0043167;GO:0046872;GO:0003824;GO:0008177;GO:0051540;GO:0009055;GO:0051538;GO:0051539;GO:0051536;GO:0051537	K00235	SDHB, SDH2	map04714;ma	Thermogenesis;C	COG0479	C:Energy production and conve	PF13085.9;PF13	Fer2_3;Fer4_1	2Fe-2S iron-sulfur	CYT	6	30	31.8	High
TRINITY_DN1330_c0_g1_i1_orf1	pancreatic triacylglycerol lipase-like [Ostrinia furnacalis]	2.363806406	1.241111885	1.19E-05	up	yes	1.011	0.4277	0.429	0.416	0.438	1	0.982	1.052	CC:cellular_component;CC:extracellular region;CC:cellular anatomical entity;MF:hydrolase activity;MF:hydrolase activity, acting on ester bonds;MF:catalytic activity;MF:molecular_function;MF:lipase activity;	GO:0005575;GO:0005576;GO:0110165;GO:0016787;GO:0016788;GO:0003824;GO:0003674;GO:0016298	------	------	------	------	ENOG41116Y2;ENOG4110RTX;ENOG410YGS3;ENOG4111JY5	S:Function unknown;	PF00151.22	Lipase	Lipase	CYT	7	15	65	High
TRINITY_DN35301_c0_g1_i3_orf1	ATP synthase subunit gamma, mitochondrial-like [Ostrinia furnacalis] >XP_028164649.1 ATP synthase subunit gamma, mitochondrial-like [Ostrinia furnacalis]	2.352803738	1.234380982	6.09E-07	up	yes	1.007	0.428	0.433	0.429	0.422	1	0.994	1.026	------	------	K02136	ATPeF1G, ATI	map04714;ma	Thermogenesis;C	COG0224	C:Energy production and conve	PF00231.22	ATP-synt	ATP synthase	CYT	10	32	32.5	High

TRINITY_DN8173_c0_g1_i3_orf1	dihydroceramide fatty acyl 2-hydroxylase FAH1 [Ostrinia furnacalis]	3.039909425	1.604028339	0.001133	up	yes	1.074	0.3533	0.405	0.345	0.31	1	0.985	1.238	BP:fatty acid biosynthetic process;BP:fatty acid metabolic process;BP:carboxylic acid metabolic process;BP:cellular biosynthetic process;BP:small molecule metabolic process;BP:small molecule biosynthetic process;BP:monocarboxylic acid biosynthetic process;BP:organic substance biosynthetic process;BP:organic substance metabolic process;BP:lipid metabolic process;BP:cellular process;BP:monocarboxylic acid metabolic process;BP:biosynthetic process;BP:biological_process;BP:metabolic process;BP:oxoacid metabolic process;BP:cellular lipid metabolic process;BP:lipid biosynthetic process;BP:primary metabolic process;BP:organic acid metabolic process;BP:carboxylic acid biosynthetic process;BP:organic acid biosynthetic process;BP:cellular metabolic process;CC:endoplasmic reticulum membrane;CC:intrinsic component of membrane;CC:organelle membrane;CC:integral component of membrane;CC:membrane;CC:cellular anatomical entity;CC:cellular_component;MF:molecular_function;MF:ion binding;MF:oxidoreductase activity;MF:transition metal ion binding;MF:heterocyclic compound binding;MF:iron ion binding;MF:metal ion binding;MF:heme binding;MF:cation	GO:0006633;GO:0006631;GO:0019752;GO:0044249;GO:0044281;GO:0044283;GO:0072330;GO:1901576;GO:0071704;GO:0006629;GO:0009987;GO:0032787;GO:0009058;GO:0008150;GO:0008152;GO:0043436;GO:0044255;GO:0008610;GO:0044238;GO:0006082;GO:0046394;GO:0016053;GO:0044237;GO:0005789;GO:0031224;GO:0031090;GO:0016021;GO:0016020;GO:0110165;GO:0005575;GO:0003674;GO:0043167;GO:0016491;GO:0046914;GO:1901363;GO:0005506;GO:0046872;GO:0020037;GO:0043169;GO:0003824;GO:0046906;GO:0097159;GO:0080132;GO:0005488	K19703	FA2H, SCS7	------	------	COG5274;COG5274;COG3000	C:Energy production and conve	PF04116.16;PF0(	FA_hydroxyla:	Fatty acid hydroxyl	CYT	1	4	39.3	High
TRINITY_DN3062_c0_g1_i1_orf1	HEAT repeat-containing protein 1 [Ostrinia furnacalis]	2.441325913	1.287664906	5.03E-06	up	yes	1.009	0.4133	0.444	0.411	0.385	1	1.007	1.02	BP:nucleic acid metabolic process;BP:cellular nitrogen compound metabolic process;BP:nitrogen compound metabolic process;BP:ncRNA metabolic process;BP:organic cyclic compound metabolic process;BP:nucleobase-containing compound metabolic process;BP:organic substance metabolic process;BP:rRNA processing;BP:ncRNA processing;BP:cellular process;BP:cellular aromatic compound metabolic process;BP:biological_process;BP:metabolic process;BP:heterocycle metabolic process;BP:RNA metabolic process;BP:primary metabolic process;BP:rRNA metabolic process;BP:cellular metabolic process;BP:macromolecule metabolic process;BP:RNA processing;CC:organelle;CC:nucleolus;CC:cellular anatomical entity;CC:macromolecular complex;CC:intracellular non-membrane-bounded organelle;CC:ribonucleoprotein complex;CC:intracellular	GO:0090304;GO:0034641;GO:0006807;GO:0034660;GO:1901360;GO:0006139;GO:0071704;GO:0006364;GO:0034470;GO:0009987;GO:0006725;GO:0008150;GO:0008152;GO:0046483;GO:0016070;GO:0044238;GO:0016072;GO:0044237;GO:0043170;GO:0006396;GO:0043226;GO:0005730;GO:0110165;GO:0032991;GO:0043232;GO:1990904;GO:0043229;GO:0005575;GO:0043228	K14550	UTP10, HEAT	map03008	Ribosome bioger	ENOG410XNQQ;ENOG410ZVW6	J:Translation, ribosomal structu	PF08146.15;PF1:	BP28CT;U3sn	BP28CT;U3 small n	CYT	4	2	240.8	High
TRINITY_DN1666_c0_g1_i2_orf1	putative defense protein Hdd11 [Ostrinia furnacalis] >XP_028179344.1 putative defense protein Hdd11 [Ostrinia furnacalis] >AGV28583.1 immune-induced protein [Ostrinia furnacalis]	2.536340852	1.342748638	3.15E-06	up	yes	1.012	0.399	0.387	0.384	0.426	1	1.031	1.006	BP:defense response;BP:response to biotic stimulus;BP:response to external stimulus;BP:response to external biotic stimulus;BP:innate immune response;BP:response to bacterium;BP:immune system process;BP:response to stress;BP:biological_process;BP:defense response to bacterium;BP:immune response;BP:defense response to other organism;BP:response to other organism;BP:response to stimulus;BP:interspecies interaction between organisms;CC:cellular_component;CC:extracellular	GO:0006952;GO:0009607;GO:0009605;GO:0043207;GO:0045087;GO:0009617;GO:0002376;GO:0006950;GO:0008150;GO:0042742;GO:0006955;GO:0098542;GO:0051707;GO:0050896;GO:0044419;GO:0005575;GO:0005576;GO:0110165	------	------	------	------	ENOG41123CK;ENOG410XR70	S:Function unknown;	PF02014.19	Reeler	Reeler domain	CYT	6	52	17.7	High
TRINITY_DN2594_c0_g2_i4_orf1	isocitrate dehydrogenase [NAD] subunit beta, mitochondrial isoform X2 [Ostrinia furnacalis]	2.613374327	1.385913784	1.08E-06	up	yes	1.02	0.3903	0.399	0.393	0.379	1	1.02	1.04	BP:tricarboxylic acid cycle;BP:biological_process;BP:metabolic process;BP:primary metabolic process;CC:oxidoreductase complex;CC:isocitrate dehydrogenase comple;CC:mitochondrial isocitrate dehydrogenase comple;CC:catalytic complex;CC:mitochondrial tricarboxylic acid cycle enzyme complex;CC:tricarboxylic acid cycle enzyme complex;CC:macromolecular complex;CC:cellular_component;CC:mitochondrial protein complex;MF:NAD binding;MF:molecular_function;MF:binding;MF:nucleotide binding;MF:heterocyclic compound binding;MF:nucleoside phosphate binding;MF:oxidoreductase activity;MF:cation binding;MF:magnesium ion binding;MF:ion binding;MF:isocitrate dehydrogenase activity;MF:metal ion binding;MF:small molecule binding;MF:oxidoreductase activity, acting on CH-OH group of donors;MF:isocitrate dehydrogenas;MF:oxidoreductase activity, acting on the CH-OH group of donors, NAD or NADP as acceptor;MF:catalytic	GO:0006099;GO:0008150;GO:0008152;GO:0044238;GO:1990204;GO:0045242;GO:0005962;GO:1902494;GO:0030062;GO:0045239;GO:0032991;GO:0005575;GO:0098798;GO:0051287;GO:0003674;GO:0005488;GO:0000166;GO:1901363;GO:1901265;GO:0016491;GO:0043169;GO:0000287;GO:0043167;GO:0004448;GO:0046872;GO:0036094;GO:0016614;GO:0004449;GO:0016616;GO:0003824;GO:0097159	K00030	IDH3	map00020	Citrate cycl	COG0473	E:Amino acid transport and me	PF00180.23	Iso_dh	Isocitrate/isopropy	CYT	3	13	41.2	High
TRINITY_DN45924_c0_g1_i14_orf1	adenylosuccinate synthetase isoform X1 [Ostrinia furnacalis] >XP_028166048.1 adenylosuccinate synthetase isoform X2 [Ostrinia furnacalis]	2.02074606	1.014888035	7.66E-07	up	yes	1.013	0.5013	0.506	0.499	0.499	1	1.031	1.007	BP:nucleoside monophosphate biosynthetic process;BP:ribonucleotide biosynthetic process;BP:ribonucleoside monophosphate metabolic process;BP:purine nucleoside monophosphate biosynthetic process;BP:cellular biosynthetic process;BP:cellular nitrogen compound metabolic process;BP:nucleotide biosynthetic process;BP:purine-containing compound metabolic process;BP:purine nucleotide metabolic process;BP:purine-containing compound biosynthetic process;BP:organic cyclic compound biosynthetic process;BP:ribonucleotide metabolic process;BP:purine ribonucleoside monophosphate biosynthetic process;BP:'de novo' AMP biosynthetic process;BP:organophosphate biosynthetic process;BP:organic substance metabolic process;BP:organic cyclic compound metabolic process;BP:heterocycle biosynthetic process;BP:cellular process;BP:purine ribonucleoside monophosphate metabolic process;BP:ribose phosphate metabolic process;BP:nucleobase-containing compound metabolic process;BP:ribonucleoside monophosphate biosynthetic process;BP:phosphorus metabolic process;BP:cellular aromatic compound metabolic process;BP:purine ribonucleotide biosynthetic process;BP:AMP metabolic process;BP:purine ribonucleotide metabolic process;BP:biosynthetic process;BP:nucleotide metabolic process;BP:metabolic process;BP:aromatic compound biosynthetic process;BP:nucleobase-containing compound biosynthetic process;BP:cellular nitrogen compound biosynthetic process;BP:nucleobase-containing small molecule metabolic process;BP:heterocycle metabolic process;BP:primary	GO:0009124;GO:0009260;GO:0009161;GO:0009127;GO:0044249;GO:0034641;GO:0009165;GO:0072521;GO:0006163;GO:0072522;GO:1901362;GO:0009259;GO:0009168;GO:0044208;GO:0090407;GO:0071704;GO:1901360;GO:0018130;GO:0009987;GO:0009167;GO:0019693;GO:0006139;GO:0009156;GO:0006793;GO:0006725;GO:0009152;GO:0046033;GO:0009150;GO:0009058;GO:0009117;GO:0008152;GO:0019438;GO:0034654;GO:0044271;GO:0055086;GO:0046483;GO:0044238;GO:0009123;GO:1901564;GO:0009126;GO:1901566;GO:1901576;GO:1901135;GO:0046390;GO:0044237;GO:1901137;GO:0006796;GO:0006807;GO:0006167;GO:1901293;GO:0006164;GO:0019637;GO:0008150;GO:0006753;GO:0044281;GO:0005737;GO:0110165;GO:0005575;GO:0004019;GO:0000166;GO:0035639;GO:0003674;GO:0005488;GO:0019001;GO:0043169;GO:0032561;GO:0000287;GO:0005525;GO:0043167;GO:0032553;GO:0017076;GO:0046872;GO:0097367;GO:0043168;GO:1901265;GO:0036094;GO:0003824;GO:0016874;GO:0032555;GO:0016879;GO:0097159;GO:1901363	K01939	purA, ADSS	map00250;ma	Alanine, aspartat	COG0104	F:Nucleotide transport and met	PF00709.24	Adenylsucc_s	Adenylosuccinate :	CYT	10	19	49.7	High
TRINITY_DN4451_c0_g1_i1_orf1	uncharacterized protein LOC114361986 isoform X1 [Ostrinia furnacalis] >XP_028173022.1 uncharacterized protein LOC114361986 isoform X2 [Ostrinia furnacalis]	2.177843178	1.122900073	1.91E-05	up	yes	1.036	0.4757	0.477	0.489	0.461	1	1.032	1.077	BP:carboxylic acid metabolic process;BP:organonitrogen compound metabolic process;BP:cellular amino acid catabolic process;BP:nitrogen compound metabolic process;BP:small molecule catabolic process;BP:cellular amino acid metabolic process;BP:organic substance metabolic process;BP:alpha-amino acid metabolic process;BP:alpha-amino acid catabolic process;BP:cellular process;BP:organic substance catabolic process;BP:biological_process;BP:metabolic process;BP:oxoacid metabolic process;BP:catabolic process;BP:cellular catabolic process;BP:organonitrogen compound catabolic process;BP:primary metabolic process;BP:valine metabolic process;BP:valine catabolic process;BP:organic acid metabolic process;BP:organic acid catabolic process;BP:cellular metabolic process;BP:carboxylic acid catabolic process;BP:branched-chain amino acid metabolic process;BP:branched-chain amino acid catabolic process;BP:small molecule metabolic process;MF:NAD binding;MF:molecular_function;MF:binding;MF:heterocyclic compound binding;MF:nucleoside phosphate binding;MF:oxidoreductase activity;MF:NADP binding;MF:3-hydroxyisobutyrate dehydrogenase activity;MF:nucleotide binding;MF:small molecule binding;MF:catalytic activity;MF:oxidoreductase activity, acting on CH-OH group of donors;MF:oxidoreductase activity, acting on the CH-OH group of donors, NAD or NADP as acceptor;MF:organic cyclic compound binding;	GO:0019752;GO:1901564;GO:0009063;GO:0006807;GO:0044282;GO:0006520;GO:0071704;GO:1901605;GO:1901606;GO:0009987;GO:1901575;GO:0008150;GO:0008152;GO:0043436;GO:0009056;GO:0044248;GO:1901565;GO:0044238;GO:0006573;GO:0006574;GO:0006082;GO:0016054;GO:0044237;GO:0046395;GO:0009081;GO:0009083;GO:0044281;GO:0051287;GO:0003674;GO:0005488;GO:1901363;GO:1901265;GO:0016491;GO:0050661;GO:0008442;GO:0000166;GO:0036094;GO:0003824;GO:0016614;GO:0016616;GO:0097159	K23146	HPD1	map00280;ma	Valine, leucine ar	COG2084	I:Lipid transport and metabolis	PF14833.9;PF03	NAD_binding_	NAD-binding of N.	CYT	3	39	20.6	High
TRINITY_DN9002_c0_g1_i1_orf1	membrane magnesium transporter 1 [Ostrinia furnacalis]	2.234988077	1.160267135	0.0008011	up	yes	1.031	0.4613	0.466	0.471	0.447	1	0.943	1.151	BP:localization;BP:biological_process;BP:establishment of localization;CC:Golgi membrane;CC:macromolecular complex;CC:organelle membrane;CC:cytoplasmic vesicle membrane;CC:bounding membrane of organelle;CC:membrane;CC:ER membrane protein complex;CC:cellular_component;CC:membrane protein complex;CC:cellular anatomical entity;CC:vesicle membrane;CC:early endosome membrane;CC:endosome	GO:0051179;GO:0008150;GO:0051234;GO:0000139;GO:0032991;GO:0031090;GO:0030659;GO:0098588;GO:0016020;GO:0072546;GO:0005575;GO:0098796;GO:0110165;GO:0012506;GO:0031901;GO:0010008	K23566	MMGT1, EMC	------	------	ENOG4111TZW	U:Intracellular trafficking, secre	PF10270.12	MMgT	Membrane magne	CYT	1	8	13.1	High
TRINITY_DN17299_c0_g1_i4_orf1	RNA cytidine acetyltransferase isoform X1 [Ostrinia furnacalis] >XP_028171321.1 RNA cytidine acetyltransferase isoform X2 [Ostrinia furnacalis] >XP_028171329.1 RNA cytidine acetyltransferase isoform X3 [Ostrinia furnacalis]	2.056592167	1.040255728	6.25E-07	up	yes	1.003	0.4877	0.49	0.499	0.474	1	0.995	1.014	BP:RNA modification;BP:nucleic acid metabolic process;BP:cellular nitrogen compound metabolic process;BP:nitrogen compound metabolic process;BP:ncRNA metabolic process;BP:organic cyclic compound metabolic process;BP:ncRNA processing;BP:nucleobase-containing compound metabolic process;BP:ribonucleoprotein complex biogenesis;BP:cellular component organization or biogenesis;BP:organic substance metabolic process;BP:cellular component biogenesis;BP:tRNA modification;BP:rRNA processing;BP:tRNA acetylation;BP:tRNA processing;BP:cellular process;BP:cellular aromatic compound metabolic process;BP:macromolecule modification;BP:biological_process;BP:metabolic process;BP:heterocycle metabolic process;BP:RNA metabolic process;BP:primary metabolic process;BP:rRNA metabolic process;BP:rRNA modification;BP:ribosomal small subunit biogenesis;BP:cellular metabolic process;BP:macromolecule metabolic process;BP:tRNA metabolic process;BP:RNA acetylation;BP:RNA processing;CC:organelle;CC:nucleolus;CC:cellular anatomical entity;CC:intracellular non-membrane-bounded organelle;CC:intracellular organelle;CC:cellular_component;CC:non-membrane-bounded organelle;MF:anion binding;MF:purine ribonucleoside triphosphate binding;MF:heterocyclic compound binding;MF:ribonucleotide binding;MF:molecular_function;MF:binding;MF:ion binding;MF:N-acetyltransferase activity;MF:carbohydrate derivative binding;MF:nucleotide binding;MF:transferase	GO:0009451;GO:0090304;GO:0034641;GO:0006807;GO:0034660;GO:1901360;GO:0034470;GO:0006139;GO:0022613;GO:0071840;GO:0071704;GO:0044085;GO:0006400;GO:0006364;GO:0051391;GO:0008033;GO:0009987;GO:0006725;GO:0043412;GO:0008150;GO:0008152;GO:0046483;GO:0016070;GO:0044238;GO:0016072;GO:0000154;GO:0042274;GO:0044237;GO:0043170;GO:0006399;GO:1990884;GO:0006396;GO:0043226;GO:0005730;GO:0110165;GO:0043232;GO:0043229;GO:0005575;GO:0043228;GO:0043168;GO:0035639;GO:1901363;GO:0032553;GO:0003674;GO:0005488;GO:0043167;GO:0008080;GO:0097367;GO:0000166;GO:0016740;GO:0016746;GO:0016747;GO:0016410;GO:0017076;GO:1901265;GO:0036094;GO:0003824;GO:0030554;GO:0097159;GO:0016407;GO:0032559;GO:0005524;GO:0032555	K14521	NAT10, KRE3:	map03008	Ribosome bioger	COG1444	S:Function unknown	PF13718.9;PF13	GNAT_acetylt	GNAT acetyltransfe	CYT	10	9	108.8	High

TRINITY_DN15882_c0_g1_i1_orf1	succinate--CoA ligase [ADP-forming] subunit beta, mitochondrial [Ostrinia furnacalis]	2.075358107	1.053360298	9.03E-07	up	yes	0.9997	0.4817	0.494	0.482	0.469	1	1.012	0.987	BP:tricarboxylic acid cycle;BP:biological_process;BP:metabolic process;BP:primary metabolic process;CC:membrane-bounded organelle;CC:organelle;CC:mitochondrion;CC:cellular anatomical entity;CC:intracellular membrane-bounded organelle;CC:intracellular organelle;CC:cellular_component;MF:purine ribonucleoside triphosphate binding;MF:heterocyclic compound binding;MF:small molecule binding;MF:molecular_function;MF:binding;MF:nucleotide binding;MF:nucleoside phosphate binding;MF:anion binding;MF:cation binding;MF:magnesium ion binding;MF:ion binding;MF:succinate-CoA ligase activity;MF:succinate-CoA ligas;MF:metal ion binding;MF:acid-thiol ligase activity;MF:catalytic activity;MF:purine nucleotide binding;MF:ligase activity;MF:ligase activity, forming carbon-sulfur bonds;MF:adenyl nucleotide binding;MF:carbohydrate derivative binding;MF:organic cyclic compound binding;MF:adenyl ribonucleotide binding;MF:ATP	GO:0006099;GO:0008150;GO:0008152;GO:0044238;GO:0043227;GO:0043226;GO:0005739;GO:0110165;GO:0043231;GO:0043229;GO:0005575;GO:0035639;GO:1901363;GO:0036094;GO:0003674;GO:0005488;GO:0000166;GO:1901265;GO:0043168;GO:0043169;GO:0000287;GO:0043167;GO:0004774;GO:0004775;GO:0046872;GO:0016878;GO:0003824;GO:0017076;GO:0016874;GO:0016877;GO:0030554;GO:0097367;GO:0097159;GO:0032559;GO:0005524;GO:0032555;GO:0032553	K01900	LSC2	map00020;map	Citrate cycl;Propa	COG0045	L:Replication, recombination ar	PF08442.13;PF0	ATP-grasp_2;	ATP-grasp domair	CYT		9	22	51.8	High
TRINITY_DN6933_c1_g1_i1_orf1	Chlorophyll a-b binding protein 40, chloroplastic [Trichinella nelsoni] >KRY99282.1 Chlorophyll a-b binding protein 40, chloroplastic [Trichinella zimbabwensis]	2.075785967	1.053657696	0.000223	up	yes	0.997	0.4803	0.456	0.482	0.503	1	0.929	1.062	CC:cellular_component;CC:membrane;CC:cellular anatomical entity;	GO:0005575;GO:0016020;GO:0110165	K08913	LHCB2	------	------	ENOG410ZHBU	S:Function unknown	PF00504.24	Chloroa_b-bi	Chlorophyll A-B bi	CYT		2	8	24	High
TRINITY_DN8019_c0_g1_i4_orf1	deoxyhypusine hydroxylase [Ostrinia furnacalis]	2.017043121	1.012241927	2.56E-05	up	yes	0.9823	0.487	0.464	0.493	0.504	1	1.003	0.944	BP:nitrogen compound metabolic process;BP:peptidyl-amino acid modification;BP:organic substance metabolic process;BP:macromolecule modification;BP:protein modification process;BP:biological_process;BP:metabolic process;BP:peptidyl-lysine modification to peptidyl-hypusine;BP:primary metabolic process;BP:organonitrogen compound metabolic process;BP:protein maturation;BP:peptidyl-lysine modification;BP:biosynthetic process;BP:macromolecule metabolic process;BP:protein metabolic process;MF:monooxygenase activity;MF:deoxyhypusine monooxygenase activity;MF:molecular_function;MF:binding;MF:oxidoreductase activity;MF:cation binding;MF:oxidoreductase activity, acting on paired donors, with incorporation or reduction of molecular oxygen;MF:ion binding;MF:metal ion binding;MF:catalytic	GO:0006807;GO:0018193;GO:0071704;GO:0043412;GO:0036211;GO:0008150;GO:0008152;GO:0008612;GO:0044238;GO:1901564;GO:0051604;GO:0018205;GO:0009058;GO:0043170;GO:0019538;GO:0004497;GO:0019135;GO:0003674;GO:0005488;GO:0016491;GO:0043169;GO:0016705;GO:0043167;GO:0046872;GO:0140096;GO:0003824	K06072	DOHH	------	------	COG1413	C:Energy production and conve	PF13646.9;PF03	HEAT_2;HEAT	HEAT repeats;PBS	CYT		5	19	34.1	High
TRINITY_DN5962_c0_g1_i1_orf1	tRNA (cytosine(34)-C(5))-methyltransferase [Ostrinia furnacalis]	2.076890844	1.054425394	7.51E-07	up	yes	0.9913	0.4773	0.482	0.483	0.467	1	0.999	0.975	CC:intracellular organelle;CC:membrane-bounded organelle;CC:nucleus;CC:organelle;CC:cellular anatomical entity;CC:intracellular membrane-bounded organelle;CC:cellular_component;MF:molecular_function;MF:binding;MF:nucleic acid binding;MF:methyltransferase activity;MF:heterocyclic compound binding;MF:tRNA binding;MF:transferase activity;MF:transferase activity, transferring one-carbon groups;MF:catalytic activity, acting on RNA;MF:S-adenosylmethionine-dependent methyltransferase activity;MF:RNA methyltransferase activity;MF:tRNA methyltransferase activity;MF:tRN;MF:tRN;MF:organic cyclic compound binding;MF:catalytic activity;MF:RNA binding;MF:catalytic activity, acting on a tRNA;	GO:0043229;GO:0043227;GO:0005634;GO:0043226;GO:0110165;GO:0043231;GO:0005575;GO:0003674;GO:0005488;GO:0003676;GO:0008168;GO:1901363;GO:0000049;GO:0016740;GO:0016741;GO:0140098;GO:0008757;GO:0008173;GO:0008175;GO:0016428;GO:0016427;GO:0097159;GO:0003824;GO:0003723;GO:0140101	K15335	NSUN2, TRM4	------	------	COG0144	J:Translation, ribosomal structu	PF01189.20	Methyltr_Rsm	16S rRNA methyltr	CYT		6	10	83.8	High
TRINITY_DN9820_c0_g1_i1_orf1	endocuticle structural glycoprotein SgAbd-2-like [Ostrinia furnacalis]	2.275262101	1.186032748	2.09E-05	up	yes	1.02	0.4483	0.469	0.404	0.472	1	1.038	1.021	MF:molecular_function;MF:structural constituent of cuticle;MF:structural molecule activity;	GO:0003674;GO:0042302;GO:0005198	------	------	------	------	ENOG4111PJ1;ENOG410YXXU;ENOG410YR66	S:Function unknown;	PF00379.26	Chitin_bind_4	Insect cuticle prote	CYT		4	56	16	High
TRINITY_DN2505_c0_g1_i1_orf1	uncharacterized protein LOC114349853 [Ostrinia furnacalis]	2.097101756	1.068396866	8.95E-06	up	yes	0.9913	0.4727	0.456	0.488	0.474	1	1.013	0.961	------	------	------	------	------	------	ENOG4111VFJ	S:Function unknown	PF06585.14	JHBP	Haemolymph juvei	CYT		1	3	29.1	High
TRINITY_DN45446_c0_g1_i2_orf1	peptide transporter family 1-like isoform X1 [Ostrinia furnacalis]	2.153159041	1.106454887	0.0002118	up	yes	0.9883	0.459	0.433	0.48	0.464	1	1.049	0.916	BP:oligopeptide transport;BP:amide transport;BP:transport;BP:nitrogen compound transport;BP:peptide transport;BP:biological_process;BP:organic substance transport;BP:establishment of localization;BP:localization;CC:cellular_component;CC:integral component of membrane;CC:cellular anatomical entity;CC:intrinsic component of membrane;MF:transporter activity;MF:transmembrane transporter activity;MF:molecular_function;	GO:0006857;GO:0042886;GO:0006810;GO:0071705;GO:0015833;GO:0008150;GO:0071702;GO:0051234;GO:0051179;GO:0005575;GO:0016021;GO:0110165;GO:0031224;GO:0005215;GO:0022857;GO:0003674	K14206	SLC15A1, PEF	map04974	Protein digestion	COG3104	E:Amino acid transport and me	PF00854.24;PF0	PTR2;MFS_1	POT family;Major F	CYT		2	3	78.5	High
TRINITY_DN4040_c0_g1_i10_orf1	hypothetical protein evm_007488 [Chilo suppressalis]	2.087262641	1.061612147	1.10E-05	up	yes	0.9783	0.4687	0.441	0.494	0.471	1	0.969	0.966	CC:cellular anatomical entity;CC:intrinsic component of membrane;CC:cellular_component;CC:integral component of membrane;MF:antiporter activity;MF:secondary active transmembrane transporter activity;MF:inorganic anion transmembrane transporter activity;MF:inorganic solute uptake transmembrane transporter activity;MF:transporter activity;MF:anion transmembrane transporter activity;MF:solut;MF:ion transmembrane transporter activity;MF:transmembrane transporter activity;MF:molecular_function;MF:active ion transmembrane transporter activity;MF:inorganic anion exchanger	GO:0110165;GO:0031224;GO:0005575;GO:0016021;GO:0015297;GO:0015291;GO:0015103;GO:0015318;GO:0005215;GO:0008509;GO:0140323;GO:0015075;GO:0022857;GO:0003674;GO:0022853;GO:0005452;GO:0022804	K13855	SLC4A2, AE2	map04972;map	Pancreatic secret	ENOG410XPHD	U:Intracellular trafficking, secre	PF00955.24;PF0	HCO3_cotran	HCO3- transporter	CYT		5	6	128.9	High
TRINITY_DN23429_c0_g2_i1_orf1	muscle-specific protein 20 [Zerene cesonia]	2.228117305	1.155825189	2.99E-07	up	yes	0.9953	0.4467	0.457	0.434	0.449	1	1	0.986	------	------	K20526	TAGLN	------	------	COG5199	Z:Cytoskeleton	PF00307.34;PF0	CH;Calponin	Calponin homolog	CYT		12	75	20.3	High
TRINITY_DN21494_c0_g1_i2_orf1	pancreatic triacylglycerol lipase-like [Ostrinia furnacalis]	2.413132039	1.270906858	7.88E-05	up	yes	1.007	0.4173	0.459	0.418	0.375	1	1.055	0.965	BP:organic substance metabolic process;BP:lipid metabolic process;BP:primary metabolic process;BP:metabolic process;BP:biological_process;CC:cellular_component;CC:extracellular region;CC:cellular anatomical entity;MF:hydrolase activity;MF:hydrolase activity, acting on ester bonds;MF:catalytic activity;MF:molecular_function;MF:lipase activity;	GO:0071704;GO:0006629;GO:0044238;GO:0008152;GO:0008150;GO:0005575;GO:0005576;GO:0110165;GO:0016787;GO:0016788;GO:0003824;GO:0003674;GO:0016298	------	------	------	------	ENOG41116Y2;ENOG4110RTX;ENOG4110T03;ENOG410YGS3;ENOG4111JY5	S:Function unknown;	PF00151.22	Lipase	Lipase	CYT		1	4	35.8	High
TRINITY_DN4356_c0_g1_i6_orf1	mulatexin-like [Ostrinia furnacalis]	2.205882353	1.141355849	3.75E-05	up	yes	0.975	0.442	0.461	0.395	0.47	1	0.961	0.964	------	------	------	------	------	------	------	------	------	------	------	CYT		1	5	20.1	High
TRINITY_DN11117_c0_g1_i1_orf1	venom carboxylesterase-6-like [Ostrinia furnacalis]	2.457212714	1.297022753	4.56E-06	up	yes	1.005	0.409	0.412	0.428	0.387	1	1.03	0.986	MF:hydrolase activity;MF:molecular_function;MF:hydrolase activity, acting on ester bonds;MF:carboxylic ester hydrolase activity;MF:catalytic activity;	GO:0016787;GO:0003674;GO:0016788;GO:0052689;GO:0003824	------	------	------	------	COG2272;COG0580;ENOG410XSWS	S:Function unknown;G:Carbohy	PF00135.31	COesterase	Carboxylesterase fa	CYT		4	11	63.8	High
TRINITY_DN4434_c0_g1_i7_orf1	V-type proton ATPase catalytic subunit A [Ostrinia furnacalis] >XP_028155921.1 V-type proton ATPase catalytic subunit A [Ostrinia furnacalis] >XP_028155922.1 V-type proton ATPase catalytic subunit A [Ostrinia furnacalis] >ADP23923.1 V-ATPase subunit A [Ostrinia furnacalis] >ADT80587.1 V-type proton ATPase catalytic subunit A [Ostrinia furnacalis] >CBY05457.1 V-type proton ATPase catalytic subunit A [Ostrinia furnacalis]	2.411679186	1.270038005	1.46E-07	up	yes	0.9953	0.4127	0.419	0.413	0.406	1	1.003	0.983	CC:proton-transporting V-type ATPase, V1 domain;CC:proton-transporting two-sector ATPase complex, catalytic domain;CC:macromolecular complex;CC:cellular_component;CC:membrane protein complex;MF:cation channel activity;MF:purine ribonucleoside triphosphate binding;MF:molecular_function;MF:binding;MF:ion binding;MF:heterocyclic compound binding;MF:nucleoside phosphate binding;MF:anion binding;MF:ion transmembrane transporter activity;MF:ATPase activity, coupled to transmembrane movement of substances;MF:inorganic solute uptake transmembrane transporter activity;MF:hydrogen-translocating pyrophosphatase activity;MF:nucleotide binding;MF:primary active transmembrane transporter activity;MF:purine nucleotide binding;MF:active transmembrane transporter activity;MF:hydrogen ion channel activity;MF:ATPase coupled ion transmembrane transporter activity;MF:transporter activity;MF:ion channel activity;MF:cation transmembrane transporter activity;MF:small molecule binding;MF:catalytic activity;MF:proton-transporting ATPase activity, rotational mechanism;MF:passive transmembrane transporter activity;MF:ligase activity;MF:ATPase activity, coupled to transmembrane movement of ions, rotational mechanism;MF:inorganic cation transmembrane transporter activity;MF:adenyl nucleotide binding;MF:carbohydrate derivative binding;MF:organic cyclic compound binding;MF:proton-transporting ATP synthase activity, rotational mechanism;MF:cation-transporting ATPase activity;MF:transmembrane transporter activity;MF:channel activity;MF:adenyl ribonucleotide binding;MF:ATP	GO:0033180;GO:0033178;GO:0032991;GO:0005575;GO:0098796;GO:0005261;GO:0035639;GO:0003674;GO:0005488;GO:0043167;GO:1901363;GO:1901265;GO:0043168;GO:0015075;GO:0042626;GO:0015318;GO:0009678;GO:0000166;GO:0015399;GO:0017076;GO:0022804;GO:0015252;GO:0042625;GO:0005215;GO:0005216;GO:0008324;GO:0036094;GO:0003824;GO:0046961;GO:0022803;GO:0016874;GO:0044769;GO:0022890;GO:0030554;GO:0097367;GO:0097159;GO:0046933;GO:0019829;GO:0022857;GO:0015267;GO:0032559;GO:0005524;GO:0022853;GO:0032555;GO:0015078;GO:0032553	K02145	ATPeV1A, ATI	map05323;map	Rheumatoid arth	COG1155	C:Energy production and conve	PF00006.28;PF1	ATP-synt_ab;	ATP synthase alph	CYT		29	62	68.1	High
TRINITY_DN14063_c0_g1_i7_orf1	probable phosphorylase b kinase regulatory subunit beta isoform X1 [Ostrinia furnacalis] >XP_028175664.1 probable phosphorylase b kinase regulatory subunit beta isoform X2 [Ostrinia furnacalis] >XP_028175665.1 probable phosphorylase b kinase regulatory subunit beta isoform X3 [Ostrinia furnacalis]	2.534573799	1.341743172	3.81E-05	up	yes	1.008	0.3977	0.423	0.371	0.399	1	0.966	1.058	------	------	K07190	PHKA_B	map04910;map	Insulin signaling	ENOG410XPJZ	S:Function unknown	PF00723.24;PF1	Glyco_hydro_	Glycosyl hydrolase	CYT		1	1	134.3	High
TRINITY_DN35277_c0_g1_i1_orf1	luciferin 4-monooxygenase-like, partial [Ostrinia furnacalis]	2.066352652	1.047086491	2.82E-05	up	yes	1.009	0.4883	0.525	0.472	0.468	1	0.987	1.04	CC:cellular_component;CC:integral component of membrane;CC:cellular anatomical entity;CC:intrinsic component of membrane;	GO:0005575;GO:0016021;GO:0110165;GO:0031224	K01904	4CL	map00130	Ubiquinone and	COG0365;COG0318;COG0318	I:Lipid transport and metabolis	PF00501.31;PF1	AMP-binding	AMP-binding enzy	CYT		5	10	61.2	High
TRINITY_DN5704_c0_g1_i6_orf1	2-oxoglutarate dehydrogenase, mitochondrial isoform X2 [Ostrinia furnacalis]	2.070208333	1.049775959	3.04E-07	up	yes	0.9937	0.48	0.472	0.474	0.494	1	0.991	0.99	------	------	K00164	OGDH, sucA	map00020	Citrate cycl	COG0567	C:Energy production and conve	PF00676.23;PF0	E1_dh;Transk	Dehydrogenase E1	CYT		18	29	98.3	High
TRINITY_DN74889_c0_g1_i1_orf1	probable 28S ribosomal protein S23, mitochondrial [Ostrinia furnacalis]	2.206235012	1.141586478	1.09E-05	up	yes	1.012	0.4587	0.462	0.489	0.425	1	1.007	1.028	BP:cellular biosynthetic process;BP:cellular nitrogen compound metabolic process;BP:nitrogen compound metabolic process;BP:cellular macromolecule biosynthetic process;BP:organic substance biosynthetic process;BP:cellular nitrogen compound biosynthetic process;BP:cellular macromolecule metabolic process;BP:peptide biosynthetic process;BP:organic substance metabolic process;BP:amide biosynthetic process;BP:cellular process;BP:biosynthetic process;BP:macromolecule biosynthetic process;BP:biological_process;BP:metabolic process;BP:organonitrogen compound metabolic process;BP:primary metabolic process;BP:cellular amide metabolic process;BP:organonitrogen compound biosynthetic process;BP:protein metabolic process;BP:cellular metabolic process;BP:macromolecule metabolic process;BP:peptide metabolic process;BP:translation;CC:intracellular non-membrane-bounded organelle;CC:membrane-bounded organelle;CC:organelle;CC:mitochondrion;CC:cellular anatomical entity;CC:intracellular membrane-bounded organelle;CC:macromolecular complex;CC:ribosome;CC:ribonucleoprotein complex;CC:intracellular organelle;CC:cellular_component;CC:non-membrane-bounded	GO:0044249;GO:0034641;GO:0006807;GO:0034645;GO:1901576;GO:0044271;GO:0044260;GO:0043043;GO:0071704;GO:0043604;GO:0009987;GO:0009058;GO:0009059;GO:0008150;GO:0008152;GO:1901564;GO:0044238;GO:0043603;GO:1901566;GO:0019538;GO:0044237;GO:0043170;GO:0006518;GO:0006412;GO:0043232;GO:0043227;GO:0043226;GO:0005739;GO:0110165;GO:0043231;GO:0032991;GO:0005840;GO:1990904;GO:0043229;GO:0005575;GO:0043228;GO:0003674;GO:0005198;GO:0003735	K17402	MRPS23	------	------	ENOG4111IJ9	J:Translation, ribosomal structu	PF10484.12	MRP-S23	Mitochondrial ribo	CYT		1	6	17.8	Medium

TRINITY_DN97589_c0_g1_i3_orf1	ribosomal protein L37A [Bombyx mori] >XP_013189707.1 PREDICTED: 60S ribosomal protein L37a [Amyelois transitella] >XP_021198447.1 60S ribosomal protein L37a [Helicoverpa armigera] >XP_022122377.1 60S ribosomal protein L37a [Pieris rapae] >XP_022822835.1 60S ribosomal protein L37a [Spodoptera litura] >XP_023937141.1 60S ribosomal protein L37a [Bicyclus anynana] >XP_026321523.1 60S ribosomal protein L37a [Hyposmocoma kahamanoa] >XP_026495655.1 60S ribosomal protein L37a [Vanessa tameamea] >XP_026746489.1 60S ribosomal protein L37a [Trichoplusia ni] >XP_026756267.1 60S ribosomal protein L37a [Galleria mellonella] >XP_028041705.1 60S ribosomal protein L37a [Bombyx mandarina] >XP_028161757.1 60S ribosomal protein L37a [Ostrinia furnacalis] >XP_030020263.1 LOW QUALITY PROTEIN: 60S ribosomal protein L37a [Manduca sexta] >XP_032518929.1 60S ribosomal protein L37a [Danaus plexippus plexippus] >XP_034834514.1 60S ribosomal protein L37a [Maniola hyperantus] >XP_035444256.1 60S ribosomal protein L37a [Spodoptera frugiperda] >XP_038222439.1 60S ribosomal protein L37a [Zerene cesonia] >XP_039756348.1 60S ribosomal protein L37a [Pararge aegeria] >XP_041981914.1 60S ribosomal protein L37a [Aricia agestis] >XP_045451710.1 60S ribosomal protein L37a [Melitaea cinxia] >XP_045500579.1 60S ribosomal protein L37a [Colias croceus] >XP_045517305.1 60S ribosomal protein L37a [Pieris brassicae] >XP_045775103.1 60S ribosomal protein L37a [Maniola jurtina] >XP_046969745.1 60S ribosomal protein L37a [Vanessa cardui] >XP_047032252.1 60S ribosomal protein L37a [Helicoverpa zea] >XP_047525321.1 60S ribosomal protein L37a [Pieris napi] >XP_047535357.1 60S ribosomal protein L37a [Vanessa atalanta] >XP_049875744.1 60S ribosomal protein L37a [Pectinophora gossypiella] >XP_050348149.1 60S ribosomal protein L37a [Nymphalis io] >ADO95156.1 ribosomal protein L37A [Antheraea yamamai] >ADT80705.1 ribosomal protein L37A [Euphydryas aurinia] >AEL28885.1 ribosomal protein L37A [Heliconius melpomene cythera] >KAF9418899.1 hypothetical protein HW555_004419 [Spodoptera exigua] >KOB75009.1 Ribosomal protein L37A [Operophtera brumata] >RVE49828.1 hypothetical protein evm_005558 [Chilo suppressalis] >CAB3234150.1 unnamed protein product [Arctia plantaginis] >CAB3509616.1 unnamed protein product [Spodoptera littoralis] >CAF4811073.1 unnamed protein product [Pieris macdunnoughi] >CAG4956733.1 unnamed protein product [Parnassius apollo] >CAG9564640.1 unnamed protein product [Danaus chrysippus] >CAG9750098.1 unnamed protein product [Diatraea saccharalis] [illegible]	2.152360515	1.105919746	6.83E-06	up	yes	1.003	0.466	0.464	0.471	0.463	1	0.975	1.035	BP:cellular biosynthetic process;BP:cellular nitrogen compound metabolic process;BP:nitrogen compound metabolic process;BP:cellular macromolecule biosynthetic process;BP:organic substance biosynthetic process;BP:cellular macromolecule metabolic process;BP:peptide biosynthetic process;BP:organic substance metabolic process;BP:amide biosynthetic process;BP:cellular process;BP:biosynthetic process;BP:macromolecule biosynthetic process;BP:biological_process;BP:metabolic process;BP:cellular nitrogen compound biosynthetic process;BP:primary metabolic process;BP:organonitrogen compound metabolic process;BP:cellular amide metabolic process;BP:organonitrogen compound biosynthetic process;BP:protein metabolic process;BP:cellular metabolic process;BP:macromolecule metabolic process;BP:peptide metabolic process;BP:translation;CC:intracellular non-membrane-bounded organelle;CC:organelle;CC:cellular anatomical entity;CC:ribosome;CC:intracellular organelle;CC:cellular_component;CC:non-membrane-bounded organelle;MF:molecular_function;MF:structural molecule activity;MF:structural constituent of ribosome;	GO:0044249;GO:0034641;GO:0006807;GO:0034645;GO:1901576;GO:0044260;GO:0043043;GO:0071704;GO:0043604;GO:0009987;GO:0009058;GO:0009059;GO:0008150;GO:0008152;GO:0044271;GO:0044238;GO:1901564;GO:0043603;GO:1901566;GO:0019538;GO:0044237;GO:0043170;GO:0006518;GO:0006412;GO:0043232;GO:0043226;GO:0110165;GO:0005840;GO:0043229;GO:0005575;GO:0043228;GO:0003674;GO:0005198;GO:0003735	K02921	RP-L37Ae, RF	map03010;ma	Ribosome;Coron	COG1997	J:Translation, ribosomal structu	PF01780.22	Ribosomal_L3	Ribosomal L37ae p	CYT	5	31	12	High
TRINITY_DN8226_c0_g1_i1_orf1	myosin heavy chain, muscle isoform X16 [Helicoverpa armigera]	2.220759271	1.151053014	1.26E-07	up	yes	1.012	0.4557	0.456	0.452	0.459	1	1.013	1.022	------	------	K17751	MYH6_7	map04260;ma	Cardiac muscle c	COG5022	G:Carbohydrate transport and	------	------	------	CYT	1	56	6.3	High
TRINITY_DN8754_c0_g1_i2_orf1	dnaJ homolog subfamily C member 11 [Ostrinia furnacalis]	2.389937107	1.256972653	4.81E-05	up	yes	1.026	0.4293	0.382	0.468	0.438	1	1.064	1.013	------	------	K09531	DNAJC11	------	------	COG0484	J:Translation, ribosomal structu	PF11875.11;PF0	DnaJ-like_C1	DnaJ-like protein (	CYT	2	3	65.1	High
TRINITY_DN2780_c0_g1_i5_orf1	probable ATP-dependent RNA helicase DDX27 [Ostrinia furnacalis]	2.279909707	1.188976689	6.56E-06	up	yes	1.01	0.443	0.422	0.431	0.476	1	1.005	1.025	------	------	K13181	DDX27, DRS1	------	------	COG0513	O:Posttranslational modificatio	PF00270.32;PF0	DEAD;Helicas	DEAD/DEAH box h	CYT	4	7	90.9	High
TRINITY_DN3008_c0_g1_i12_orf1	reticulon-4-interacting protein 1 homolog, mitochondrial [Ostrinia furnacalis]	2.176470588	1.121990524	1.46E-05	up	yes	0.9953	0.4573	0.438	0.438	0.496	1	0.978	1.008	------	------	K23164	RTN4IP1	------	------	COG0604	S:Function unknown	PF13602.9;PF00	ADH_zinc_N_	Zinc-binding dehy	CYT	2	9	39	High
TRINITY_DN14313_c0_g1_i1_orf1	25S rRNA (cytosine-C(5))-methyltransferase nop2 [Ostrinia furnacalis]	2.024410599	1.017501933	6.37E-05	up	yes	0.9703	0.4793	0.518	0.467	0.453	1	0.979	0.932	BP:RNA modification;BP:nucleic acid metabolic process;BP:cellular component organization or biogenesis;BP:nucleobase-containing compound metabolic process;BP:cellular macromolecule metabolic process;BP:ribosome biogenesis;BP:organic substance metabolic process;BP:organic cyclic compound metabolic process;BP:ribonucleoprotein complex biogenesis;BP:methylation;BP:cellular process;BP:cellular aromatic compound metabolic process;BP:macromolecule modification;BP:biological_process;BP:metabolic process;BP:RNA methylation;BP:heterocycle metabolic process;BP:RNA metabolic process;BP:primary metabolic process;BP:nitrogen compound metabolic process;BP:cellular nitrogen compound metabolic process;BP:cellular metabolic process;BP:macromolecule metabolic process;BP:cellular component biogenesis;BP:macromolecule methylation;BP:RNA processing;CC:intracellular organelle;CC:non-membrane-bounded organelle;CC:organelle;CC:nucleolus;CC:cellular anatomical entity;CC:intracellular non-membrane-bounded organelle;CC:cellular_component;MF:molecular_function;MF:binding;MF:nucleic acid binding;MF:methyltransferase activity;MF:heterocyclic compound binding;MF:transferase activity;MF:transferase activity, transferring one-carbon groups;MF:S-adenosylmethionine-dependent methyltransferase activity;MF:catalytic activity;MF:organic cyclic [illegible]	GO:0009451;GO:0090304;GO:0071840;GO:0006139;GO:0044260;GO:0042254;GO:0071704;GO:1901360;GO:0022613;GO:0032259;GO:0009987;GO:0006725;GO:0043412;GO:0008150;GO:0008152;GO:0001510;GO:0046483;GO:0016070;GO:0044238;GO:0006807;GO:0034641;GO:0044237;GO:0043170;GO:0044085;GO:0043414;GO:0006396;GO:0043229;GO:0043228;GO:0043226;GO:0005730;GO:0110165;GO:0043232;GO:0005575;GO:0003674;GO:0005488;GO:0003676;GO:0008168;GO:1901363;GO:0016740;GO:0016741;GO:0008757;GO:0003824;GO:0097159;GO:0003723	K14835	NOP2	------	------	COG0144	O:Posttranslational modificatio	PF01189.20;PF1	Methyltr_Rsm	16S rRNA methyltr	CYT	9	12	95.6	High
TRINITY_DN59422_c0_g1_i2_orf1	larval cuticle protein LCP-22-like isoform X2 [Pectinophora gossypiella]	2.586164146	1.370813847	6.57E-05	up	yes	1.043	0.4033	0.393	0.386	0.431	1	1.019	1.111	MF:molecular_function;MF:structural constituent of cuticle;MF:structural molecule activity;	GO:0003674;GO:0042302;GO:0005198	------	------	------	------	ENOG4111CQ7;ENOG41119AX;ENOG4111DQA;ENOG410YZKX;ENOG4111PJ1;ENOG410YR66	S:Function unknown;	PF00379.26	Chitin_bind_4	Insect cuticle prote	CYT	4	38	19.5	High
TRINITY_DN18036_c0_g1_i7_orf1	pentatricopeptide repeat-containing protein 2, mitochondrial-like [Ostrinia furnacalis]	2.382629108	1.252554392	1.83E-07	up	yes	1.015	0.426	0.423	0.427	0.428	1	1.025	1.02	BP:regulation of primary metabolic process;BP:regulation of metabolic process;BP:regulation of nucleobase-containing compound metabolic process;BP:regulation of mRNA processing;BP:regulation of RNA metabolic process;BP:regulation of cellular metabolic process;BP:regulation of cellular process;BP:regulation of macromolecule metabolic process;BP:biological regulation;BP:regulation of nitrogen compound metabolic process;BP:biological_process;BP:regulation of mRNA metabolic process;BP:regulation of biological process;CC:cellular_component;CC:intracellular organelle;CC:mitochondrion;CC:organelle;CC:membrane-bounded organelle;CC:cellular anatomical entity;CC:intracellular [illegible]	GO:0080090;GO:0019222;GO:0019219;GO:0050684;GO:0051252;GO:0031323;GO:0050794;GO:0060255;GO:0065007;GO:0051171;GO:0008150;GO:1903311;GO:0050789;GO:0005575;GO:0043229;GO:0005739;GO:0043226;GO:0043227;GO:0110165;GO:0043231	K17711	PTCD2	------	------	ENOG4111GFQ	S:Function unknown	------	------	------	CYT	6	16	44.3	High
TRINITY_DN16343_c0_g1_i6_orf1	aminopeptidase N6 [Ostrinia nubilalis]	2.378606615	1.250116692	1.14E-06	up	yes	1.014	0.4263	0.436	0.436	0.407	1	1.026	1.015	BP:nitrogen compound metabolic process;BP:organic substance metabolic process;BP:proteolysis;BP:biological_process;BP:metabolic process;BP:primary metabolic process;BP:organonitrogen compound metabolic process;BP:protein metabolic process;BP:macromolecule metabolic process;CC:integral component of membrane;CC:intrinsic component of membrane;CC:cellular anatomical entity;CC:cellular_component;MF:aminopeptidase activity;MF:exopeptidase activity;MF:molecular_function;MF:binding;MF:peptidase activity;MF:metallopeptidase activity;MF:cation binding;MF:transition metal ion binding;MF:ion binding;MF:metal ion binding;MF:hydrolase activity;MF:catalytic [illegible]	GO:0006807;GO:0071704;GO:0006508;GO:0008150;GO:0008152;GO:0044238;GO:1901564;GO:0019538;GO:0043170;GO:0016021;GO:0031224;GO:0110165;GO:0005575;GO:0004177;GO:0008238;GO:0003674;GO:0005488;GO:0008233;GO:0008237;GO:0043169;GO:0046914;GO:0043167;GO:0046872;GO:0016787;GO:0140096;GO:0003824;GO:0008270	K11140	ANPEP, CD13	map04640;ma	Hematopoietic c	COG0308	E:Amino acid transport and me	PF11838.11;PF0	ERAP1_C;Pep	ERAP1-like C-term	CYT	5	7	100.2	High
TRINITY_DN32681_c0_g1_i3_orf1	long-chain-fatty-acid--CoA ligase ACSBG2 isoform X2 [Ostrinia furnacalis]	2.287671233	1.193879734	2.01E-07	up	yes	1.002	0.438	0.434	0.438	0.442	1	1.015	0.99	------	------	K15013	ACSBG	map04920;ma	Adipocytokine si	COG1022	G:Carbohydrate transport and	PF00501.31	AMP-binding	AMP-binding enzy	CYT	16	32	74.7	High
TRINITY_DN48619_c0_g1_i1_orf1	PREDICTED: lysine--tRNA ligase isoform X2 [Fopius arisanus]	2.336361519	1.224263528	3.17E-09	up	yes	1.003	0.4293	0.431	0.431	0.426	1	1.001	1.007	BP:lysyl-tRNA aminoacylation;BP:carboxylic acid metabolic process;BP:nucleic acid metabolic process;BP:cellular nitrogen compound metabolic process;BP:nitrogen compound metabolic process;BP:ncRNA metabolic process;BP:nucleobase-containing compound metabolic process;BP:cellular amino acid metabolic process;BP:organic substance metabolic process;BP:organic cyclic compound metabolic process;BP:RNA metabolic process;BP:cellular process;BP:cellular aromatic compound metabolic process;BP:biological_process;BP:metabolic process;BP:oxoacid metabolic process;BP:heterocycle metabolic process;BP:primary metabolic process;BP:organonitrogen compound metabolic process;BP:organic acid metabolic process;BP:cellular metabolic process;BP:macromolecule metabolic process;BP:tRNA metabolic process;BP:amino acid activation;BP:tRNA aminoacylation;BP:tRNA aminoacylation for protein translation;BP:small molecule metabolic process;MF:purine ribonucleoside triphosphate binding;MF:molecular_function;MF:binding;MF:nucleic acid binding;MF:ion binding;MF:heterocyclic compound binding;MF:nucleoside phosphate binding;MF:anion binding;MF:aminoacyl-tRNA ligase activity;MF:nucleotide binding;MF:purine nucleotide binding;MF:ATP binding;MF:catalytic activity, acting on RNA;MF:small molecule binding;MF:catalytic activity;MF:ligase activity;MF:ligase activity, forming carbon-oxygen bonds;MF:lysine-tRNA ligase activity;MF:adenyl nucleotide binding;MF:carbohydrate derivative binding;MF:organic cyclic compound binding;MF:adenyl ribonucleotide binding;MF:purine [illegible]	GO:0006430;GO:0019752;GO:0090304;GO:0034641;GO:0006807;GO:0034660;GO:0006139;GO:0006520;GO:0071704;GO:1901360;GO:0016070;GO:0009987;GO:0006725;GO:0008150;GO:0008152;GO:0043436;GO:0046483;GO:0044238;GO:1901564;GO:0006082;GO:0044237;GO:0043170;GO:0006399;GO:0043038;GO:0043039;GO:0006418;GO:0044281;GO:0035639;GO:0003674;GO:0005488;GO:0003676;GO:0043167;GO:1901363;GO:1901265;GO:0043168;GO:0004812;GO:0000166;GO:0017076;GO:0005524;GO:0140098;GO:0036094;GO:0003824;GO:0016874;GO:0016875;GO:0004824;GO:0030554;GO:0097367;GO:0097159;GO:0032559;GO:0032555;GO:0140101;GO:0032553	K04567	KARS, lysS	map00970	Aminoacyl-tRNA	COG1190	J:Translation, ribosomal structu	PF00152.23;PF0	tRNA-synt_2;	tRNA synthetases	CYT	1	5	66.7	High
TRINITY_DN145647_c0_g1_i1_orf1	PREDICTED: U6 snRNA-associated Sm-like protein LSm5 isoform X1 [Fopius arisanus]	2.45699055	1.296892309	4.22E-07	up	yes	1.014	0.4127	0.4	0.414	0.424	1	1.022	1.019	BP:nucleic acid metabolic process;BP:cellular nitrogen compound metabolic process;BP:nitrogen compound metabolic process;BP:organic cyclic compound metabolic process;BP:nucleobase-containing compound metabolic process;BP:organic substance metabolic process;BP:RNA splicing;BP:mRNA metabolic process;BP:cellular process;BP:cellular aromatic compound metabolic process;BP:biological_process;BP:metabolic process;BP:heterocycle metabolic process;BP:RNA metabolic process;BP:primary metabolic process;BP:cellular metabolic process;BP:macromolecule metabolic process;BP:RNA processing;BP:mRNA processing;CC:spliceosomal complex;CC:macromolecular complex;CC:cellular_component;CC:ribonucleoprotein complex;MF:molecular_function;MF:binding;MF:nucleic acid binding;MF:heterocyclic compound binding;MF:organic cyclic [illegible]	GO:0090304;GO:0034641;GO:0006807;GO:1901360;GO:0006139;GO:0071704;GO:0008380;GO:0016071;GO:0009987;GO:0006725;GO:0008150;GO:0008152;GO:0046483;GO:0016070;GO:0044238;GO:0044237;GO:0043170;GO:0006396;GO:0006397;GO:0005681;GO:0032991;GO:0005575;GO:1990904;GO:0003674;GO:0005488;GO:0003676;GO:1901363;GO:0097159;GO:0003723	K12624	LSM5	map03018;ma	RNA degradation	COG1958	O:Posttranslational modificatio	PF01423.25	LSM	LSM domain	CYT	1	6	10.5	High
TRINITY_DN1239_c0_g1_i3_orf1	uncharacterized protein LOC114355269 [Ostrinia furnacalis] >XP_028163822.1 uncharacterized protein LOC114355269 [Ostrinia furnacalis]	2.542878449	1.346462502	0.0001054	up	yes	1.023	0.4023	0.411	0.392	0.404	1	0.968	1.101	CC:integral component of membrane;CC:cellular_component;CC:cellular anatomical entity;CC:intrinsic component of membrane;MF:transporter activity;MF:transmembrane transporter activity;MF:molecular_function;	GO:0016021;GO:0005575;GO:0110165;GO:0031224;GO:0005215;GO:0022857;GO:0003674	K08190	SLC16A14	------	------	ENOG41116NW	P:Inorganic ion transport and n	PF07690.19	MFS_1	Major Facilitator S	CYT	1	2	63.4	High
TRINITY_DN20346_c0_g1_i1_orf1	NADH dehydrogenase [ubiquinone] 1 alpha subcomplex subunit 6 [Ostrinia furnacalis]	2.529105772	1.338627374	1.81E-05	up	yes	1.021	0.4037	0.398	0.438	0.375	1	1.057	1.006	CC:respiratory chain complex I;CC:transmembrane transporter complex;CC:catalytic complex;CC:transporter complex;CC:NADH dehydrogenase complex;CC:respiratory chain complex;CC:inner mitochondrial membrane protein complex;CC:cellular_component;CC:membrane protein complex;CC:mitochondrial respiratory chain complex I;CC:macromolecular complex;CC:oxidoreductase complex;CC:mitochondrial protein complex;	GO:0045271;GO:1902495;GO:1902494;GO:1990351;GO:0030964;GO:0098803;GO:0098800;GO:0005575;GO:0098796;GO:0005747;GO:0032991;GO:1990204;GO:0098798	K03950	NDUFA6	map04714;ma	Thermogenesis;C	ENOG4111S2F	I:Lipid transport and metabolis	PF05347.18	Complex1_LY	Complex 1 protein	CYT	2	12	14.8	High
TRINITY_DN2852_c0_g1_i9_orf1	golgin subfamily A member 4-like [Ostrinia furnacalis]	2.078091106	1.055258905	2.88E-05	up	yes	0.958	0.461	0.473	0.469	0.441	1	0.94	0.934	------	------	K20283	GOLGA4	------	------	ENOG410XQQG	U:Intracellular trafficking, secre	PF01465.23	GRIP	GRIP domain	CYT	4	3	155.2	High
TRINITY_DN15965_c0_g1_i1_orf1	U3 small nucleolar RNA-associated protein 15 homolog [Ostrinia furnacalis]	2.328474018	1.219384784	1.37E-05	up	yes	0.9903	0.4253	0.386	0.431	0.459	1	0.991	0.98	BP:nucleic acid metabolic process;BP:cellular nitrogen compound metabolic process;BP:nitrogen compound metabolic process;BP:ncRNA metabolic process;BP:organic cyclic compound metabolic process;BP:nucleobase-containing compound metabolic process;BP:organic substance metabolic process;BP:rRNA processing;BP:ncRNA processing;BP:cellular process;BP:cellular aromatic compound metabolic process;BP:biological_process;BP:metabolic process;BP:heterocycle metabolic process;BP:RNA metabolic process;BP:primary metabolic process;BP:rRNA metabolic process;BP:cellular metabolic process;BP:macromolecule metabolic process;BP:RNA processing;CC:organelle;CC:nucleolus;CC:cellular anatomical entity;CC:intracellular non-membrane-bounded organelle;CC:intracellular [illegible]	GO:0090304;GO:0034641;GO:0006807;GO:0034660;GO:1901360;GO:0006139;GO:0071704;GO:0006364;GO:0034470;GO:0009987;GO:0006725;GO:0008150;GO:0008152;GO:0046483;GO:0016070;GO:0044238;GO:0016072;GO:0044237;GO:0043170;GO:0006396;GO:0043226;GO:0005730;GO:0110165;GO:0043232;GO:0043229;GO:0005575;GO:0043228	K14549	UTP15	map03008	Ribosome bioger	ENOG410XNZ0	I:Lipid transport and metabolis	PF09384.13;PF0	UTP15_C;WD	UTP15 C terminal;	CYT	2	4	57.9	High
TRINITY_DN120439_c1_g1_i1_orf1	myosin heavy chain variant, partial [Bombyx mori]	2.582544152	1.368793014	1.10E-06	up	yes	1.009	0.3907	0.393	0.369	0.41	1	1.016	1.011	CC:cellular_component;CC:myosin complex;CC:macromolecular complex;	GO:0005575;GO:0016459;GO:0032991	K17751	MYH6_7	map04260;ma	Cardiac muscle c	COG5022	Z:Cytoskeleton	PF01576.22	Myosin_tail_1	Myosin tail	CYT	7	65	12.3	High

TRINITY_DN17738_c0_g1_i2_orf1	unnamed protein product [Diatraea saccharalis]	2.420537897	1.275327682	4.78E-06	up	yes	0.99	0.409	0.423	0.405	0.399	1	1.011	0.959	BP:nucleic acid metabolic process;BP:cellular nitrogen compound metabolic process;BP:nitrogen compound metabolic process;BP:organic cyclic compound metabolic process;BP:nucleobase-containing compound metabolic process;BP:organic substance metabolic process;BP:cellular process;BP:cellular aromatic compound metabolic process;BP:biological_process;BP:metabolic process;BP:heterocycle metabolic process;BP:RNA metabolic process;BP:primary metabolic process;BP:cellular metabolic process;BP:macromolecule metabolic process;BP:RNA processing;CC:membrane-bounded organelle;CC:nucleus;CC:organelle;CC:cellular anatomical entity;CC:macromolecular complex;CC:intracellular membrane-bounded organelle;CC:ribonucleoprotein complex;CC:intracellular organelle;CC:cellular_component;MF:molecular_function;MF:bin	GO:0090304;GO:0034641;GO:0006807;GO:1901360;GO:0006139;GO:0071704;GO:0009987;GO:0006725;GO:0008150;GO:0008152;GO:0046483;GO:0016070;GO:0044238;GO:0044237;GO:0043170;GO:0006396;GO:0043227;GO:0005634;GO:0043226;GO:0110165;GO:0032991;GO:0043231;GO:1990904;GO:0043229;GO:0005575;GO:0003674;GO:0005488;GO:0003676;GO:1901363;GO:0097159;GO:0003723	K11090	LA, SSB	map05322	Systemic lupus e	COG5193	G:Carbohydrate transport and	PF05383.20;PF0	La;RRM_3;RRM	La domain;RNA bir	CYT	10	28	43.9	High
TRINITY_DN4589_c0_g1_i1_orf1	protein penguin [Ostrinia furnacalis]	2.044418005	1.031690202	1.73E-05	up	yes	1.031	0.5043	0.513	0.478	0.522	1	1.059	1.035	MF:cation binding;MF:organic cyclic compound binding;MF:ion binding;MF:molecular_function;MF:RNA binding;MF:nucleic acid binding;MF:DNA binding;MF:metal ion binding;MF:heterocyclic compound binding;MF:binding;	GO:0043169;GO:0097159;GO:0043167;GO:0003674;GO:0003723;GO:0003676;GO:0003677;GO:0046872;GO:1901363;GO:0005488	K14844	PUF6	------	------	ENOG410XSHY	S:Function unknown	PF08144.14;PF0	CPL;PUF	CPL;Pumilio-family	CYT	2	3	70.9	High
TRINITY_DN754_c1_g1_i6_orf1	28S ribosomal protein S2, mitochondrial [Ostrinia furnacalis]	2.72280874	1.445095646	0.0003997	up	yes	1.109	0.4073	0.474	0.372	0.376	1	1.174	1.154	BP:cellular biosynthetic process;BP:cellular nitrogen compound metabolic process;BP:nitrogen compound metabolic process;BP:cellular macromolecule biosynthetic process;BP:organic substance biosynthetic process;BP:cellular macromolecule metabolic process;BP:peptide biosynthetic process;BP:organic substance metabolic process;BP:amide biosynthetic process;BP:cellular process;BP:biosynthetic process;BP:macromolecule biosynthetic process;BP:biological_process;BP:metabolic process;BP:cellular nitrogen compound biosynthetic process;BP:primary metabolic process;BP:organonitrogen compound metabolic process;BP:cellular amide metabolic process;BP:organonitrogen compound biosynthetic process;BP:protein metabolic process;BP:cellular metabolic process;BP:macromolecule metabolic process;BP:peptide metabolic process;BP:translation;CC:small ribosomal subunit;CC:macromolecular complex;CC:ribosomal subunit;CC:ribonucleoprotein	GO:0044249;GO:0034641;GO:0006807;GO:0034645;GO:1901576;GO:0044260;GO:0043043;GO:0071704;GO:0043604;GO:0009987;GO:0009058;GO:0009059;GO:0008150;GO:0008152;GO:0044271;GO:0044238;GO:1901564;GO:0043603;GO:1901566;GO:0019538;GO:0044237;GO:0043170;GO:0006518;GO:0006412;GO:0015935;GO:0032991;GO:0044391;GO:1990904;GO:0005575;GO:0003674;GO:0005198;GO:0003735	K02967	RP-S2, MRPS	map03010	Ribosome	COG0052	S:Function unknown	PF00318.23	Ribosomal_S2	Ribosomal protein	CYT	1	5	28.4	High
TRINITY_DN2803_c2_g1_i8_orf1	trans-1,2-dihydrobenzene-1,2-diol dehydrogenase-like isoform X1 [Ostrinia furnacalis]	2.068057525	1.048276316	1.26E-05	up	yes	1.021	0.4937	0.478	0.507	0.496	1	1.057	1.005	MF:organic cyclic compound binding;MF:nucleotide binding;MF:small molecule binding;MF:molecular_function;MF:binding;MF:heterocyclic compound binding;MF:nucleoside phosphate binding;	GO:0097159;GO:0000166;GO:0036094;GO:0003674;GO:0005488;GO:1901363;GO:1901265	K00078	DHDH	map00040;ma	Pentose and gluc	COG0673	S:Function unknown	PF01408.25;PF0	GFO_IDH_Mo	Oxidoreductase fa	CYT	1	3	38.6	High
TRINITY_DN45598_c0_g1_i2_orf1	heat shock protein 60A-like [Ostrinia furnacalis]	2.040733198	1.029087579	8.43E-09	up	yes	1.002	0.491	0.491	0.486	0.496	1	1.001	1.004	BP:protein folding;BP:cellular process;BP:biological_process;BP:protein refolding;MF:purine ribonucleoside triphosphate binding;MF:molecular_function;MF:binding;MF:ion binding;MF:heterocyclic compound binding;MF:nucleoside phosphate binding;MF:anion binding;MF:nucleotide binding;MF:purine nucleotide binding;MF:ATP binding;MF:small molecule binding;MF:adenyl nucleotide binding;MF:carbohydrate derivative binding;MF:organic cyclic compound binding;MF:protein binding involved in protein folding;MF:adenyl ribonucleotide binding;MF:purine ribonucleotide binding;MF:ribonucleotide binding;	GO:0006457;GO:0009987;GO:0008150;GO:0042026;GO:0035639;GO:0003674;GO:0005488;GO:0043167;GO:1901363;GO:1901265;GO:0043168;GO:0000166;GO:0017076;GO:0005524;GO:0036094;GO:0030554;GO:0097367;GO:0097159;GO:0044183;GO:0032559;GO:0032555;GO:0032553	K04077	groEL, HSPD1	map05152;ma	Tuberculosis;Typ	COG0459	O:Posttranslational modificatio	PF00118.27	Cpn60_TCP1	TCP-1/cpn60 chap	CYT	24	53	60.8	High
TRINITY_DN108051_c0_g1_i2_orf1	uncharacterized protein LOC114351921 [Ostrinia furnacalis]	2.02826927	1.020249195	2.60E-06	up	yes	0.9973	0.4917	0.472	0.512	0.491	1	1.006	0.986	BP:aerobic respiration;BP:generation of precursor metabolites and energy;BP:electron transport chain;BP:respiratory electron transport chain;BP:cellular process;BP:aerobic electron transport chain;BP:cellular respiration;BP:metabolic process;BP:energy derivation by oxidation of organic compounds;BP:cellular metabolic process;BP:mitochondrial electron transport, NADH to ubiquinone;BP:biological_process;CC:oxidoreductase complex;CC:respiratory chain complex;CC:inner mitochondrial membrane protein complex;CC:transmembrane transporter complex;CC:catalytic complex;CC:transporter complex;CC:respiratory chain complex I;CC:macromolecular complex;CC:NADH dehydrogenase complex;CC:cellular_component;CC:membrane protein complex;CC:mitochondrial respiratory chain complex I;CC:mitochondrial protein complex;	GO:0009060;GO:0006091;GO:0022900;GO:0022904;GO:0009987;GO:0019646;GO:0045333;GO:0008152;GO:0015980;GO:0044237;GO:0006120;GO:0008150;GO:1990204;GO:0098803;GO:0098800;GO:1902495;GO:1902494;GO:1990351;GO:0045271;GO:0032991;GO:0030964;GO:0005575;GO:0098796;GO:0005747;GO:0098798	K03962	NDUFB6	map04714;ma	Thermogenesis;C	ENOG4111RT5	U:Intracellular trafficking, secre	PF09782.12	NDUF_B6	NAD	CYT	4	24	18.7	High
TRINITY_DN5126_c0_g2_i1_orf1	cytochrome P450 monooxygenase CYP4L47 [Ostrinia furnacalis]	2.004894962	1.003526655	0.000646	up	yes	0.983	0.4903	0.588	0.455	0.428	1	0.958	0.991	MF:cation binding;MF:monooxygenase activity;MF:heme binding;MF:transition metal ion binding;MF:tetrapyrrole binding;MF:organic cyclic compound binding;MF:oxidoreductase activity, acting on paired donors, with incorporation or reduction of molecular oxygen;MF:ion binding;MF:catalytic activity;MF:molecular_function;MF:binding;MF:iron ion binding;MF:metal ion binding;MF:heterocyclic compound	GO:0043169;GO:0004497;GO:0020037;GO:0046914;GO:0046906;GO:0097159;GO:0016705;GO:0043167;GO:0003824;GO:0003674;GO:0005488;GO:0005506;GO:0046872;GO:1901363;GO:0016491	K15001	CYP4	------	------	COG2124	I:Lipid transport and metabolis	PF00067.25	p450	Cytochrome P450	CYT	1	7	13	High
TRINITY_DN86090_c0_g1_i1_orf1	ATP synthase subunit b, mitochondrial [Ostrinia furnacalis]	2.353211009	1.234630691	1.95E-05	up	yes	1.026	0.436	0.442	0.437	0.429	1	1.076	1.003	BP:nucleoside triphosphate metabolic process;BP:nucleoside triphosphate biosynthetic process;BP:purine nucleoside triphosphate metabolic process;BP:purine nucleoside triphosphate biosynthetic process;BP:cellular biosynthetic process;BP:cellular nitrogen compound metabolic process;BP:nucleotide biosynthetic process;BP:small molecule metabolic process;BP:purine-containing compound metabolic process;BP:purine-containing compound biosynthetic process;BP:organic cyclic compound biosynthetic process;BP:ribonucleotide metabolic process;BP:organic cyclic compound metabolic process;BP:nucleobase-containing compound metabolic process;BP:ribonucleoside triphosphate biosynthetic process;BP:organic substance metabolic process;BP:ribonucleoside triphosphate metabolic process;BP:heterocycle biosynthetic process;BP:purine ribonucleoside triphosphate biosynthetic process;BP:purine ribonucleoside triphosphate metabolic process;BP:ribose phosphate metabolic process;BP:organic substance biosynthetic process;BP:ATP metabolic process;BP:cellular process;BP:cellular aromatic compound metabolic process;BP:purine ribonucleotide biosynthetic process;BP:purine ribonucleotide metabolic process;BP:ribonucleotide biosynthetic process;BP:biosynthetic process;BP:nucleotide metabolic process;BP:metabolic process;BP:aromatic compound biosynthetic process;BP:nucleobase-containing compound biosynthetic process;BP:cellular nitrogen compound biosynthetic process;BP:organophosphate biosynthetic process;BP:nucleobase-containing small molecule metabolic process;BP:heterocycle metabolic process;BP:primary metabolic process;BP:ATP synthesis coupled proton	GO:0009141;GO:0009142;GO:0009144;GO:0009145;GO:0044249;GO:0034641;GO:0009165;GO:0044281;GO:0072521;GO:0072522;GO:1901362;GO:0009259;GO:1901360;GO:0006139;GO:0009201;GO:0071704;GO:0009199;GO:0018130;GO:0009206;GO:0009205;GO:0019693;GO:1901576;GO:0046034;GO:0009987;GO:0006725;GO:0009152;GO:0009150;GO:0009260;GO:0009058;GO:0009117;GO:0008152;GO:0019438;GO:0034654;GO:0044271;GO:0090407;GO:0055086;GO:0046483;GO:0044238;GO:0015986;GO:1901564;GO:1901566;GO:1901137;GO:1901135;GO:0006164;GO:0046390;GO:0044237;GO:0006163;GO:0006796;GO:0006807;GO:1901293;GO:0006793;GO:0019637;GO:0008150;GO:0006753;GO:0006754;GO:0045263;GO:0005575;GO:0098798;GO:0098800;GO:0032991;GO:0000276;GO:0098796;GO:0033177;GO:0022890;GO:0003674;GO:0015318;GO:0022857;GO:0005215;GO:0008324;GO:0015075;GO:0015078	K02127	ATPeF0B, ATF	map04714;ma	Thermogenesis;C	ENOG410ZUQH	I:Lipid transport and metabolis	PF05405.17	Mt_ATP-synt_	Mitochondrial ATP	CYT	12	46	27.4	High
TRINITY_DN3665_c0_g1_i2_orf1	regucalcin-like isoform X2 [Ostrinia furnacalis] >XP_028175354.1 regucalcin-like isoform X2 [Ostrinia furnacalis]	2.914545942	1.543271143	0.0005919	up	yes	1.088	0.3733	0.372	0.358	0.39	1	1.23	1.033	------	------	K01053	gnl, RGN	map00030;ma	Pentose phospha	COG3386	G:Carbohydrate transport and	PF08450.15	SGL	SMP-30/Gluconola	CYT	1	16	6.9	High
TRINITY_DN7626_c0_g1_i1_orf1	NADH dehydrogenase [ubiquinone] flavoprotein 1, mitochondrial isoform X1 [Ostrinia furnacalis]	2.216966467	1.148586949	1.42E-06	up	yes	0.9983	0.4503	0.451	0.454	0.446	1	1.018	0.977	CC:oxidoreductase complex;CC:mitochondrial inner membrane;CC:membrane;CC:respiratory chain complex;CC:cellular anatomical entity;CC:transmembrane transporter complex;CC:catalytic complex;CC:transporter complex;CC:mitochondrial membrane;CC:organelle membrane;CC:respiratory chain complex I;CC:macromolecular complex;CC:NADH dehydrogenase complex;CC:organelle inner membrane;CC:cellular_component;CC:membrane protein complex;MF:NAD binding;MF:metal ion binding;MF:molecular_function;MF:binding;MF:ion binding;MF:NA;MF:heterocyclic compound binding;MF:nucleoside phosphate binding;MF:oxidoreductase activity;MF:anion binding;MF:cation binding;MF:metal cluster binding;MF:transporter activity;MF:nucleotide binding;MF:primary active transmembrane transporter activity;MF:NADH dehydrogenas;MF:FMN binding;MF:electron carrier activity;MF:oxidoreductase activity, acting on NA;MF:oxidoreduction-driven active transmembrane transporter activity;MF:NADH dehydrogenas;MF:small molecule binding;MF:catalytic activity;MF:NADH dehydrogenase activity;MF:oxidoreductase activity, acting on NA;MF:4 iron, 4 sulfur cluster binding;MF:carbohydrate derivative binding;MF:organic cyclic compound binding;MF:transmembrane transporter activity;MF:active	GO:1990204;GO:0005743;GO:0016020;GO:0098803;GO:0110165;GO:1902495;GO:1902494;GO:1990351;GO:0031966;GO:0031090;GO:0045271;GO:0032991;GO:0030964;GO:0019866;GO:0005575;GO:0098796;GO:0051287;GO:0046872;GO:0003674;GO:0005488;GO:0043167;GO:0003955;GO:1901363;GO:1901265;GO:0016491;GO:0043168;GO:0043169;GO:0051540;GO:0005215;GO:0000166;GO:0015399;GO:0050136;GO:0010181;GO:0009055;GO:0016651;GO:0015453;GO:0008137;GO:0036094;GO:0003824;GO:0003954;GO:0016655;GO:0051539;GO:0097367;GO:0097159;GO:0022857;GO:0022804;GO:0051536;GO:0032553	K03942	NDUFV1	map04714;ma	Thermogenesis;C	COG1894	C:Energy production and conve	PF01512.20;PF1	Complex1_51	Respiratory-chain	CYT	9	24	50.5	High
TRINITY_DN791_c0_g1_i2_orf1	peroxiredoxin 1 isoform X1 [Maniola jurtina]	2.115973742	1.081321724	4.55E-05	up	yes	0.967	0.457	0.454	0.455	0.462	1	0.987	0.914	MF:antioxidant activity;MF:peroxiredoxin activity;MF:catalytic activity;MF:molecular_function;MF:oxidoreductase activity, acting on peroxide as acceptor;MF:peroxidase activity;MF:oxidoreductase activity;	GO:0016209;GO:0051920;GO:0003824;GO:0003674;GO:0016684;GO:0004601;GO:0016491	K13279	PRDX1	map04146;ma	Peroxisome;Amo	COG0450	L:Replication, recombination ar	PF00578.24;PF1	AhpC-TSA;1-	AhpC/TSA family;C	CYT	7	32	26.5	High
TRINITY_DN108200_c0_g1_i1_orf1	uncharacterized protein LOC114350842 [Ostrinia furnacalis]	2.27415264	1.185329091	9.82E-05	up	yes	0.9863	0.4337	0.396	0.454	0.451	1	0.929	1.03	MF:catalytic activity;MF:molecular_function;MF:oxidoreductase activity;	GO:0003824;GO:0003674;GO:0016491	------	------	------	------	COG1028	S:Function unknown	PF13561.9;PF00	adh_short_C2	Enoyl;short chain	CYT	3	16	26	High
TRINITY_DN8691_c0_g1_i3_orf1	nucleolin-like [Melitaea cinxia]	2.022831965	1.016376481	4.11E-05	up	yes	1.01	0.4993	0.54	0.496	0.462	1	1.036	0.993	------	------	------	------	------	------	ENOG4110RR1	S:Function unknown	------	------	------	CYT	2	15	14.4	High
TRINITY_DN905_c0_g1_i4_orf1	(11Z)-hexadec-11-enoyl-CoA conjugase-like [Ostrinia furnacalis] >XP_028172978.1 (11Z)-hexadec-11-enoyl-CoA conjugase-like [Ostrinia furnacalis]	2.327377013	1.218704933	0.00041	up	yes	1.055	0.4533	0.486	0.437	0.437	1	1.161	1.004	BP:fatty acid biosynthetic process;BP:fatty acid metabolic process;BP:carboxylic acid metabolic process;BP:cellular biosynthetic process;BP:small molecule metabolic process;BP:small molecule biosynthetic process;BP:monocarboxylic acid biosynthetic process;BP:organic substance biosynthetic process;BP:organic substance metabolic process;BP:lipid metabolic process;BP:cellular process;BP:monocarboxylic acid metabolic process;BP:biosynthetic process;BP:biological_process;BP:metabolic process;BP:oxoacid metabolic process;BP:cellular lipid metabolic process;BP:lipid biosynthetic process;BP:primary metabolic process;BP:organic acid metabolic process;BP:carboxylic acid biosynthetic process;BP:organic acid biosynthetic process;BP:cellular metabolic process;CC:intrinsic component of membrane;CC:integral component of membrane;CC:cellular anatomical entity;CC:cellular_component;MF:molecular_function;MF:oxidoreductase activity;MF:oxidoreductase activity, acting on paired donors, with incorporation or reduction of molecular	GO:0006633;GO:0006631;GO:0019752;GO:0044249;GO:0044281;GO:0044283;GO:0072330;GO:1901576;GO:0071704;GO:0006629;GO:0009987;GO:0032787;GO:0009058;GO:0008150;GO:0008152;GO:0043436;GO:0044255;GO:0008610;GO:0044238;GO:0006082;GO:0046394;GO:0016053;GO:0044237;GO:0031224;GO:0016021;GO:0110165;GO:0005575;GO:0003674;GO:0016491;GO:0016705;GO:0003824;GO:0016717	K00507	SCD, desC	map04936;ma	Alcoholic liver di	COG1398	J:Translation, ribosomal structu	PF00487.27	FA_desaturas	Fatty acid desatura	CYT	2	8	41.9	High
TRINITY_DN11894_c1_g1_i5_orf1	39S ribosomal protein L33, mitochondrial [Ostrinia furnacalis]	2.089582905	1.063214999	0.0009286	up	yes	1.017	0.4867	0.561	0.431	0.468	1	1.104	0.946	------	------	K02913	RP-L33, MRP	map03010	Ribosome	ENOG4112B1M	J:Translation, ribosomal structu	------	------	------	CYT	1	18	7.9	High
TRINITY_DN4010_c0_g2_i1_orf1	myophilin [Ostrinia furnacalis]	2.128413592	1.089778522	1.16E-06	up	yes	1.021	0.4797	0.481	0.478	0.48	1	1.039	1.023	BP:organelle organization;BP:cytoskeleton organization;BP:actomyosin structure organization;BP:actin filament-based process;BP:cellular process;BP:actin cytoskeleton organization;BP:cellular component organization;BP:biological_process;BP:cellular component organization or biogenesis;MF:cytoskeletal protein binding;MF:actin binding;MF:molecular_function;MF:calmodulin binding;MF:protein binding;MF:binding;	GO:0006996;GO:0007010;GO:0031032;GO:0030029;GO:0009987;GO:0030036;GO:0016043;GO:0008150;GO:0071840;GO:0008092;GO:0003779;GO:0003674;GO:0005516;GO:0005515;GO:0005488	K20526	TAGLN	------	------	COG5199	S:Function unknown	PF00307.34;PF0	CH;Calponin	Calponin homolog	CYT	7	47	19.2	High
TRINITY_DN416_c0_g1_i1_orf1	unnamed protein product [Diatraea saccharalis]	2.110341934	1.077476775	1.93E-07	up	yes	1.006	0.4767	0.471	0.47	0.489	1	1.012	1.005	------	------	K05699	ACTN1_4	map04510;ma	Focal adhesion;R	COG5069	S:Function unknown	PF00435.24;PF0	Spectrin;CH;E	Spectrin repeat;Ca	CYT	42	55	103.8	High

TRINITY_DN1445_c0_g1_i1_orf1	leucine-rich PPR motif-containing protein, mitochondrial [Ostrinia furnacalis]	2.147864882	1.102903239	2.04E-06	up	yes	1.011	0.4707	0.473	0.449	0.49	1	1.017	1.015	------	------	K17964	LRPPRC	------	------	ENOG410XSG9	S:Function unknown	PF01535.23;PF1:	PPR;PPR_2;PP	PPR repeat;PPR re	CYT	9	14	87.6	High
TRINITY_DN18860_c0_g1_i1_orf1	DDB1- and CUL4-associated factor 13 [Ostrinia furnacalis]	2.00222988	1.001607623	3.57E-06	up	yes	0.9877	0.4933	0.475	0.513	0.492	1	0.972	0.991	BP:nucleic acid metabolic process;BP:cellular nitrogen compound metabolic process;BP:nitrogen compound metabolic process;BP:ncRNA metabolic process;BP:organic cyclic compound metabolic process;BP:nucleobase-containing compound metabolic process;BP:protein modification by small protein conjugation;BP:protein modification by small protein conjugation or removal;BP:organic substance metabolic process;BP:rRNA processing;BP:ncRNA processing;BP:cellular process;BP:cellular aromatic compound metabolic process;BP:macromolecule modification;BP:protein modification process;BP:biological_process;BP:metabolic process;BP:heterocycle metabolic process;BP:RNA metabolic process;BP:primary metabolic process;BP:rRNA metabolic process;BP:organonitrogen compound metabolic process;BP:protein metabolic process;BP:cellular metabolic process;BP:macromolecule metabolic process;BP:protein ubiquitination;BP:RNA processing;CC:organelle;CC:cellular anatomical entity;CC:intracellular non-membrane-bounded organelle;CC:nucleolus;CC:intracellular organelle;CC:cellular_component;CC:non-membrane-bounded	GO:0090304;GO:0034641;GO:0006807;GO:0034660;GO:1901360;GO:0006139;GO:0032446;GO:0070647;GO:0071704;GO:0006364;GO:0034470;GO:0009987;GO:0006725;GO:0043412;GO:0036211;GO:0008150;GO:0008152;GO:0046483;GO:0016070;GO:0044238;GO:0016072;GO:1901564;GO:0019538;GO:0044237;GO:0043170;GO:0016567;GO:0006396;GO:0043226;GO:0110165;GO:0043232;GO:0005730;GO:0043229;GO:0005575;GO:0043228	K11806	DCAF13, WDS	------	------	ENOG410XNX6	S:Function unknown	PF04158.17;PF0(	Sof1;WD40;AI	Sof1-like domain;V	CYT	2	3	51.5	High
TRINITY_DN886_c0_g1_i1_orf1	collagenase-like isoform X1 [Ostrinia furnacalis]	2.021033286	1.015093083	5.23E-05	up	yes	0.9897	0.4897	0.487	0.5	0.482	1	0.939	1.03	------	------	------	------	------	------	COG5640	O:Posttranslational modificatio	PF00089.29	Trypsin	Trypsin	CYT	3	14	29.3	High
TRINITY_DN38435_c0_g1_i1_orf1	UDP-glucuronosyltransferase 2B20-like [Ostrinia furnacalis]	2.307865675	1.206559257	4.15E-06	up	yes	1.024	0.4437	0.439	0.433	0.459	1	1.021	1.051	CC:integral component of membrane;CC:cellular_component;CC:cellular anatomical entity;CC:intrinsic component of membrane;MF:catalytic activity;MF:transferase activity;MF:molecular_function;MF:transferase activity, transferring glycosyl groups;MF:transferase activity, transferring hexosyl groups;MF:glucuronosyltransferase activity;MF:UDP-	GO:0016021;GO:0005575;GO:0110165;GO:0031224;GO:0003824;GO:0016740;GO:0003674;GO:0016757;GO:0016758;GO:0015020;GO:0008194	K00699	UGT	map05207;ma	Chemical carcinc	COG1819	S:Function unknown	PF00201.21;PF0	UDPGT;Glyco_	UDP-glucoronosyl	CYT	5	11	60.4	High
TRINITY_DN53167_c0_g1_i2_orf1	uncharacterized protein LOC114359219 [Ostrinia furnacalis]	2.169977925	1.117680366	1.81E-06	up	yes	0.983	0.453	0.454	0.465	0.44	1	0.984	0.965	BP:primary metabolic process;BP:carbohydrate metabolic process;BP:organic substance metabolic process;BP:biological_process;BP:metabolic process;CC:cytoplasm;CC:cellular_component;CC:cellular anatomical entity;MF:hydrolase activity;MF:catalytic activity;MF:hydrolase activity, acting on glycosyl bonds;MF:beta-fructofuranosidase activity;MF:hydrolase activity, hydrolyzing O-glycosyl	GO:0044238;GO:0005975;GO:0071704;GO:0008150;GO:0008152;GO:0005737;GO:0005575;GO:0110165;GO:0016787;GO:0003824;GO:0016798;GO:0004564;GO:0004553;GO:0003674	------	------	------	------	COG1621	G:Carbohydrate transport and	PF00251.23;PF0	Glyco_hydro_	Glycosyl hydrolase	CYT	2	5	59.8	High
TRINITY_DN620_c0_g1_i4_orf1	lysine--tRNA ligase isoform X1 [Ostrinia furnacalis]	2.275559658	1.18622141	4.95E-07	up	yes	0.986	0.4333	0.437	0.434	0.429	1	0.989	0.969	BP:lysyl-tRNA aminoacylation;BP:RNA metabolic process;BP:carboxylic acid metabolic process;BP:nucleic acid metabolic process;BP:cellular nitrogen compound metabolic process;BP:nitrogen compound metabolic process;BP:ncRNA metabolic process;BP:nucleobase-containing compound metabolic process;BP:cellular amino acid metabolic process;BP:organic substance metabolic process;BP:organic cyclic compound metabolic process;BP:cellular process;BP:cellular aromatic compound metabolic process;BP:biological_process;BP:metabolic process;BP:oxoacid metabolic process;BP:heterocycle metabolic process;BP:primary metabolic process;BP:organonitrogen compound metabolic process;BP:organic acid metabolic process;BP:cellular metabolic process;BP:macromolecule metabolic process;BP:tRNA metabolic process;BP:amino acid activation;BP:tRNA aminoacylation;BP:tRNA aminoacylation for protein translation;BP:small molecule metabolic process;CC:cytoplasm;CC:cellular_component;CC:cellular anatomical entity;MF:purine ribonucleoside triphosphate binding;MF:molecular_function;MF:binding;MF:nucleic acid binding;MF:ion binding;MF:heterocyclic compound binding;MF:nucleoside phosphate binding;MF:aminoacyl-tRNA ligase activity;MF:nucleotide binding;MF:purine nucleotide binding;MF:ATP binding;MF:anion binding;MF:catalytic activity, acting on RNA;MF:small molecule binding;MF:catalytic activity;MF:ligase activity;MF:ligase activity, forming carbon-oxygen bonds;MF:lysine-tRNA ligase activity;MF:adenyl nucleotide binding;MF:carbohydrate derivative binding;MF:organic cyclic compound binding;MF:adenyl	GO:0006430;GO:0016070;GO:0019752;GO:0090304;GO:0034641;GO:0006807;GO:0034660;GO:0006139;GO:0006520;GO:0071704;GO:1901360;GO:0009987;GO:0006725;GO:0008150;GO:0008152;GO:0043436;GO:0046483;GO:0044238;GO:1901564;GO:0006082;GO:0044237;GO:0043170;GO:0006399;GO:0043038;GO:0043039;GO:0006418;GO:0044281;GO:0005737;GO:0005575;GO:0110165;GO:0035639;GO:0003674;GO:0005488;GO:0003676;GO:0043167;GO:1901363;GO:1901265;GO:0004812;GO:0000166;GO:0017076;GO:0005524;GO:0043168;GO:0140098;GO:0036094;GO:0003824;GO:0016874;GO:0016875;GO:0004824;GO:0030554;GO:0097367;GO:0097159;GO:0032559;GO:0032555;GO:0140101;GO:0032553	K04567	KARS, lysS	map00970	Aminoacyl-tRNA	COG1190	J:Translation, ribosomal structu	PF00152.23;PF0	tRNA-synt_2;	tRNA synthetases	CYT	13	42	50.6	High
TRINITY_DN3733_c0_g1_i1_orf1	60S ribosomal protein L37, partial [Papilio machaon]	2.477941176	1.30914194	8.78E-05	up	yes	1.011	0.408	0.426	0.406	0.392	1	1.079	0.955	BP:organonitrogen compound biosynthetic process;BP:cellular macromolecule biosynthetic process;BP:cellular biosynthetic process;BP:cellular nitrogen compound biosynthetic process;BP:cellular macromolecule metabolic process;BP:cellular amide metabolic process;BP:peptide biosynthetic process;BP:organic substance metabolic process;BP:macromolecule metabolic process;BP:organic substance biosynthetic process;BP:cellular process;BP:biosynthetic process;BP:macromolecule biosynthetic process;BP:biological_process;BP:metabolic process;BP:organonitrogen compound metabolic process;BP:amide biosynthetic process;BP:primary metabolic process;BP:protein metabolic process;BP:cellular nitrogen compound metabolic process;BP:cellular metabolic process;BP:nitrogen compound metabolic process;BP:peptide metabolic process;BP:translation;CC:organelle;CC:intracellular organelle;CC:non-membrane-bounded organelle;CC:cellular anatomical entity;CC:macromolecular complex;CC:ribosome;CC:intracellular non-membrane-bounded organelle;CC:ribonucleoprotein complex;CC:cellular_component;MF:molecular_function;MF:binding;MF:nucleic acid binding;MF:structural molecule activity;MF:heterocyclic compound binding;MF:cation binding;MF:ion binding;MF:structural constituent of ribosome;MF:metal ion binding;MF:organic cyclic compound binding;MF:rRNA binding;MF:RNA binding;	GO:1901566;GO:0034645;GO:0044249;GO:0044271;GO:0044260;GO:0043603;GO:0043043;GO:0071704;GO:0043170;GO:1901576;GO:0009987;GO:0009058;GO:0009059;GO:0008150;GO:0008152;GO:1901564;GO:0043604;GO:0044238;GO:0019538;GO:0034641;GO:0044237;GO:0006807;GO:0006518;GO:0006412;GO:0043226;GO:0043229;GO:0043228;GO:0110165;GO:0032991;GO:0005840;GO:0043232;GO:1990904;GO:0005575;GO:0003674;GO:0005488;GO:0003676;GO:0005198;GO:1901363;GO:0043169;GO:0043167;GO:0003735;GO:0046872;GO:0097159;GO:0019843;GO:0003723	K02922	RP-L37e, RPL	map03010;ma	Ribosome;Coron	COG2126	J:Translation, ribosomal structu	PF01907.22	Ribosomal_L3	Ribosomal protein	PLA	4	30	11.4	High
TRINITY_DN434_c0_g1_i4_orf1	uncharacterized protein LOC126367148 [Pectinophora gossypiella]	2.108262108	1.07605424	0.0006157	up	yes	0.962	0.4563	0.411	0.435	0.523	1	0.884	1.002	------	------	------	------	------	------	COG5022;ENOG4111V1C;COG2801;ENOG410YRP6	Z:Cytoskeleton;K:Transcription;	------	------	------	CYT	1	2	79.9	Medium
TRINITY_DN21909_c0_g1_i1_orf1	complement component 1 Q subcomponent-binding protein, mitochondrial [Ostrinia furnacalis]	2.538058398	1.343725264	2.80E-06	up	yes	1.017	0.4007	0.39	0.423	0.389	1	1.039	1.011	CC:organelle lumen;CC:membrane-enclosed lumen;CC:cellular_component;CC:intracellular organelle lumen;CC:mitochondrial matrix;CC:cellular anatomical entity;	GO:0043233;GO:0031974;GO:0005575;GO:0070013;GO:0005759;GO:0110165	K15414	C1QBP	------	------	ENOG4111G4Z	S:Function unknown	PF02330.19	MAM33	Mitochondrial glyc	CYT	6	24	31.9	High
TRINITY_DN3749_c0_g1_i1_orf1	cytochrome c oxidase subunit 6B1 [Ostrinia furnacalis]	2.54	1.344828497	7.84E-06	up	yes	1.016	0.4	0.405	0.395	0.4	1	0.991	1.057	CC:macromolecular complex;CC:respiratory chain complex IV;CC:respiratory chain complex;CC:cytochrome complex;CC:intracellular membrane-bounded organelle;CC:cellular_component;CC:membrane protein complex;CC:intracellular organelle;CC:mitochondrion;CC:membrane-bounded organelle;CC:organelle;CC:cellular anatomical entity;CC:catalytic complex;	GO:0032991;GO:0045277;GO:0098803;GO:0070069;GO:0043231;GO:0005575;GO:0098796;GO:0043229;GO:0005739;GO:0043227;GO:0043226;GO:0110165;GO:1902494	K02267	COX6B	map04714;ma	Thermogenesis;C	ENOG41121WM	S:Function unknown	PF02297.20	COX6B	Cytochrome oxida	CYT	5	43	9.9	High
TRINITY_DN1353_c0_g1_i1_orf1	UDP-glucose 4-epimerase-like [Ostrinia furnacalis]	2.080131004	1.056674391	8.35E-05	up	yes	0.9527	0.458	0.46	0.458	0.456	1	0.962	0.896	BP:primary metabolic process;BP:carbohydrate metabolic process;BP:organic substance metabolic process;BP:monosaccharide metabolic process;BP:hexose metabolic process;BP:galactose metabolic process;BP:biological_process;BP:small molecule metabolic process;BP:metabolic process;MF:racemase and epimerase activity;MF:isomerase activity;MF:catalytic activity;MF:racemase and epimerase activity, acting on carbohydrates and derivatives;MF:UDP-N-acetylglucosamine 4-epimerase activity;MF:molecular_function;MF:UDP-glucose 4-epimerase activity;	GO:0044238;GO:0005975;GO:0071704;GO:0005996;GO:0019318;GO:0006012;GO:0008150;GO:0044281;GO:0008152;GO:0016854;GO:0016853;GO:0003824;GO:0016857;GO:0003974;GO:0003674;GO:0003978	K01784	galE, GALE	map00520;ma	Amino sugar and	COG1087	M:Cell wall/membrane/envelop	PF16363.8;PF01:	GDP_Man_De	GDP-mannose 4,6	CYT	2	8	40.3	High
TRINITY_DN3028_c0_g1_i1_orf1	pre-rRNA processing protein FTSJ3 [Ostrinia furnacalis]	2.24199536	1.164783292	6.62E-05	up	yes	0.9663	0.431	0.385	0.466	0.442	1	0.932	0.967	BP:metabolic process;BP:RNA modification;BP:nucleic acid metabolic process;BP:cellular nitrogen compound metabolic process;BP:nitrogen compound metabolic process;BP:ncRNA metabolic process;BP:rRNA 2'-O-methylation;BP:enzyme-directed rRNA 2'-O-methylation;BP:nucleobase-containing compound metabolic process;BP:cellular macromolecule metabolic process;BP:organic cyclic compound metabolic process;BP:organic substance metabolic process;BP:rRNA processing;BP:methylation;BP:ncRNA processing;BP:cellular process;BP:cellular aromatic compound metabolic process;BP:macromolecule modification;BP:biological_process;BP:rRNA methylation;BP:RNA methylation;BP:heterocycle metabolic process;BP:RNA metabolic process;BP:primary metabolic process;BP:rRNA metabolic process;BP:rRNA modification;BP:cellular metabolic process;BP:macromolecule metabolic process;BP:macromolecule methylation;BP:RNA processing;CC:organelle;CC:nucleolus;CC:cellular anatomical entity;CC:intracellular non-membrane-bounded organelle;CC:intracellular organelle;CC:cellular_component;CC:non-membrane-bounded organelle;MF:molecular_function;MF:methyltransferase activity;MF:rRNA methyltransferase activity;MF:transferase activity;MF:transferase activity, transferring one-carbon groups;MF:catalytic activity, acting on RNA;MF:S-adenosylmethionine-dependent methyltransferase activity;MF:catalytic activity;MF:RNA methyltransferase activity;MF:catalytic activity, acting on a rRNA;	GO:0008152;GO:0009451;GO:0090304;GO:0034641;GO:0006807;GO:0034660;GO:0000451;GO:0000453;GO:0006139;GO:0044260;GO:1901360;GO:0071704;GO:0006364;GO:0032259;GO:0034470;GO:0009987;GO:0006725;GO:0043412;GO:0008150;GO:0031167;GO:0001510;GO:0046483;GO:0016070;GO:0044238;GO:0016072;GO:0000154;GO:0044237;GO:0043170;GO:0043414;GO:0006396;GO:0043226;GO:0005730;GO:0110165;GO:0043232;GO:0043229;GO:0005575;GO:0043228;GO:0003674;GO:0008168;GO:0008649;GO:0016740;GO:0016741;GO:0140098;GO:0008757;GO:0003824;GO:0008173;GO:0140102	K14857	SPB1, FTSJ3	------	------	COG0293	J:Translation, ribosomal structu	PF07780.15;PF0	Spb1_C;FtsJ;D	Spb1 C-terminal d	CYT	2	3	99	High
TRINITY_DN52395_c0_g2_i2_orf1	twitchin isoform X20 [Zerene cesonia]	2.124974965	1.087445844	0.0009078	up	yes	1.061	0.4993	0.414	0.579	0.505	1	1.042	1.142	BP:developmental process;BP:cell differentiation;BP:cellular developmental process;BP:cellular process;BP:biological_process;BP:anatomical structure morphogenesis;	GO:0032502;GO:0030154;GO:0048869;GO:0009987;GO:0008150;GO:0009653	------	------	------	------	ENOG410XP8U;ENOG410XQFD;ENOG410XRMJ	S:Function unknown;	PF07679.19	I-set	Immunoglobulin I-	CYT	1	73	10.1	High
TRINITY_DN1073_c0_g1_i3_orf1	carboxylesterase [Loxostege sticticalis]	2.420608483	1.275369752	0.0006657	up	yes	1.09	0.4503	0.44	0.393	0.518	1	1.193	1.078	CC:integral component of membrane;CC:cellular_component;CC:intrinsic component of membrane;CC:cellular anatomical entity;MF:hydrolase activity;MF:hydrolase activity, acting on ester bonds;MF:carboxylic ester hydrolase activity;MF:catalytic	GO:0016021;GO:0005575;GO:0031224;GO:0110165;GO:0016787;GO:0016788;GO:0052689;GO:0003824;GO:0003674	------	------	------	------	COG2272;COG0580;ENOG410XSWS	I:Lipid transport and metabolis	PF00135.31;PF2	COesterase;B	Carboxylesterase f	CYT	3	7	68.1	High
TRINITY_DN86956_c0_g5_i1_orf1	PREDICTED: protein sly1 homolog [Microplitis demolitor]	2.340425532	1.226770862	0.001091	up	yes	1.089	0.4653	0.561	0.424	0.411	1	1.074	1.194	BP:transport;BP:biological_process;BP:vesicle-mediated transport;BP:establishment of localization;BP:localization;	GO:0006810;GO:0008150;GO:0016192;GO:0051234;GO:0051179	K19998	SCFD1, SLY1	------	------	COG5158	U:Intracellular trafficking, secre	PF00995.26	Sec1	Sec1 family	CYT	1	1	71.3	High
TRINITY_DN27979_c0_g1_i2_orf1	zinc finger CCCH domain-containing protein 15 homolog [Ostrinia furnacalis]	2.024934647	1.017875347	3.48E-07	up	yes	1.007	0.4973	0.497	0.488	0.507	1	1.018	1.002	MF:cation binding;MF:metal ion binding;MF:molecular_function;MF:binding;MF:ion binding;	GO:0043169;GO:0046872;GO:0003674;GO:0005488;GO:0043167	------	------	------	------	COG5252	S:Function unknown	PF16543.8;PF16	DFRP_C;Torus	DRG Family Regula	CYT	5	13	43.1	High
TRINITY_DN14168_c0_g1_i1_orf1	transmembrane 7 superfamily member 3-like [Ostrinia furnacalis]	2.371991247	1.246098686	0.0007825	up	yes	1.084	0.457	0.498	0.447	0.426	1	1.041	1.212	CC:cellular_component;CC:integral component of membrane;CC:cellular anatomical entity;CC:intrinsic component of membrane;	GO:0005575;GO:0016021;GO:0110165;GO:0031224	------	------	------	------	ENOG41117R0	S:Function unknown	PF13886.9	DUF4203	Domain of unknow	CYT	1	3	62.2	High
TRINITY_DN2070_c1_g1_i1_orf1	mitochondrial carrier homolog 2-like [Helicoverpa zea] >PZC82360.1 hypothetical protein B5X24_HaOG210663 [Helicoverpa armigera]	2.076749436	1.054327162	0.0002364	up	yes	1.012	0.4873	0.491	0.462	0.509	1	0.95	1.086	CC:cellular_component;CC:integral component of membrane;CC:cellular anatomical entity;CC:intrinsic component of membrane;	GO:0005575;GO:0016021;GO:0110165;GO:0031224	K17885	MTCH	------	------	ENOG410XSXF	O:Posttranslational modificatio	PF00153.30	Mito_carr	Mitochondrial carr	CYT	3	12	33.1	High
TRINITY_DN9117_c0_g1_i1_orf1	spherulin-2A-like [Ostrinia furnacalis]	2.114583333	1.080373416	0.0004975	up	yes	1.015	0.48	0.476	0.462	0.502	1	0.936	1.109	------	------	------	------	------	------	ENOG4111T7I	S:Function unknown	------	------	------	CYT	1	4	30	High
TRINITY_DN81926_c0_g1_i1_orf1	membrane-bound alkaline phosphatase-like isoform X3 [Ostrinia furnacalis]	2.298474946	1.20067694	0.0002855	up	yes	1.055	0.459	0.463	0.527	0.387	1	1.065	1.101	MF:hydrolase activity;MF:phosphatase activity;MF:alkaline phosphatase activity;MF:hydrolase activity, acting on ester bonds;MF:phosphoric ester hydrolase activity;MF:catalytic activity;MF:molecular_function;	GO:0016787;GO:0016791;GO:0004035;GO:0016788;GO:0042578;GO:0003824;GO:0003674	K01077	E3.1.3.1, pho	map00730;ma	Thiamine metab	COG1785	P:Inorganic ion transport and n	PF00245.23	Alk_phosphat	Alkaline phosphata	CYT	1	4	59.1	High

TRINITY_DN21506_c0_g1_i4_orf1	glutamate dehydrogenase, mitochondrial isoform X2 [Ostrinia furnacalis]	2.10712022	1.075272628	4.93E-08	up	yes	0.9973	0.4733	0.467	0.471	0.482	1	0.999	0.993	BP:primary metabolic process;BP:cellular process;BP:organonitrogen compound metabolic process;BP:organic acid metabolic process;BP:cellular amino acid metabolic process;BP:carboxylic acid metabolic process;BP:organic substance metabolic process;BP:nitrogen compound metabolic process;BP:biological_process;BP:cellular metabolic process;BP:metabolic process;BP:oxoacid metabolic process;BP:small molecule metabolic process;MF:oxidoreductase activity, acting on the CH-NH2 group of donors;MF:oxidoreductase activity, acting on the CH-NH2 group of donors, NAD or NADP as acceptor;MF:catalytic activity;MF:glutamate dehydrogenase	GO:0044238;GO:0009987;GO:1901564;GO:0006082;GO:0006520;GO:0019752;GO:0071704;GO:0006807;GO:0008150;GO:0044237;GO:0008152;GO:0043436;GO:0044281;GO:0016638;GO:0016639;GO:0003824;GO:0004353;GO:0003674;GO:0016491	K00261	GLUD1_2, gdl	map04964;ma	Proximal tubule l	COG0334	E:Amino acid transport and me	PF02812.21;PF0	ELFV_dehydr	Glu/Leu/Phe/Val d	CYT	22	49	61.3	High
TRINITY_DN94625_c0_g1_i1_orf1	uncharacterized protein LOC114354112 [Ostrinia furnacalis]	2.273844092	1.185133338	1.43E-06	up	yes	1.018	0.4477	0.465	0.442	0.436	1	1.027	1.027	CC:cytoplasm;CC:cellular anatomical entity;CC:cellular_component;MF:purine ribonucleoside triphosphate binding;MF:molecular_function;MF:binding;MF:nucleic acid binding;MF:ion binding;MF:heterocyclic compound binding;MF:nucleoside phosphate binding;MF:guanyl nucleotide binding;MF:nucleotide binding;MF:guanyl ribonucleotide binding;MF:purine nucleotide binding;MF:GTP binding;MF:hydrolase activity;MF:translation factor activity, RNA binding;MF:GTPase activity;MF:nucleoside-triphosphatase activity;MF:small molecule binding;MF:catalytic activity;MF:translation regulator activity;MF:hydrolase activity, acting on acid anhydrides, in phosphorus-containing anhydrides;MF:translation regulator activity, nucleic acid binding;MF:carbohydrate derivative binding;MF:organic cyclic compound binding;MF:hydrolase activity, acting on acid anhydrides;MF:anion binding;MF:pyrophosphatase	GO:0005737;GO:0110165;GO:0005575;GO:0035639;GO:0003674;GO:0005488;GO:0003676;GO:0043167;GO:1901363;GO:1901265;GO:0019001;GO:0000166;GO:0032561;GO:0017076;GO:0005525;GO:0016787;GO:0008135;GO:0003924;GO:0017111;GO:0036094;GO:0003824;GO:0045182;GO:0016818;GO:0090079;GO:0097367;GO:0097159;GO:0016817;GO:0043168;GO:0016462;GO:0032555;GO:0003746;GO:0032553	K02358	tuf, TUFM	------	------	COG0050	J:Translation, ribosomal structu	PF00009.30;PF0	GTP_EFTU;GT	Elongation factor T	CYT	12	36	50.9	High
TRINITY_DN15900_c0_g1_i6_orf1	unnamed protein product [Diatraea saccharalis]	2.326484018	1.218151277	0.0002416	up	yes	1.019	0.438	0.477	0.429	0.408	1	1.1	0.958	BP:RNA metabolic process;BP:mRNA metabolic process;BP:primary metabolic process;BP:nucleobase-containing compound metabolic process;BP:cellular process;BP:cellular aromatic compound metabolic process;BP:cellular metabolic process;BP:macromolecule metabolic process;BP:nucleic acid metabolic process;BP:organic substance metabolic process;BP:cellular nitrogen compound metabolic process;BP:nitrogen compound metabolic process;BP:biological_process;BP:organic cyclic compound metabolic process;BP:metabolic process;BP:heterocycle	GO:0016070;GO:0016071;GO:0044238;GO:0006139;GO:0009987;GO:0006725;GO:0044237;GO:0043170;GO:0090304;GO:0071704;GO:0034641;GO:0006807;GO:0008150;GO:1901360;GO:0008152;GO:0046483;GO:0006396;GO:0006397	K13171	SRRM1, SRM1	map03013;ma	Nucleocytoplasm	ENOG4111IMU	S:Function unknown	PF01480.20	PWI	PWI domain	CYT	2	2	111.5	High
TRINITY_DN23343_c0_g1_i9_orf1	pre-mRNA-processing factor 6 isoform X1 [Ostrinia furnacalis] >XP_028175021.1 pre-mRNA-processing factor 6 isoform X2 [Ostrinia furnacalis]	2.069620253	1.049366077	0.0001833	up	yes	0.981	0.474	0.519	0.45	0.453	1	1.022	0.921	BP:nucleic acid metabolic process;BP:cellular nitrogen compound metabolic process;BP:nitrogen compound metabolic process;BP:mRNA splicing, via spliceosome;BP:organic cyclic compound metabolic process;BP:nucleobase-containing compound metabolic process;BP:organic substance metabolic process;BP:RNA splicing;BP:mRNA metabolic process;BP:cellular process;BP:cellular aromatic compound metabolic process;BP:RNA splicing, via transesterification reactions;BP:RNA splicing, via transesterification reactions with bulged adenosine as nucleophile;BP:biological_process;BP:metabolic process;BP:heterocycle metabolic process;BP:RNA metabolic process;BP:primary metabolic process;BP:cellular metabolic process;BP:macromolecule metabolic process;BP:RNA	GO:0090304;GO:0034641;GO:0006807;GO:0000398;GO:1901360;GO:0006139;GO:0071704;GO:0008380;GO:0016071;GO:0009987;GO:0006725;GO:0000375;GO:0000377;GO:0008150;GO:0008152;GO:0046483;GO:0016070;GO:0044238;GO:0044237;GO:0043170;GO:0006396;GO:0006397	K12855	PRPF6, PRP6	map03040	Spliceosome	ENOG410XRKD	A:RNA processing and modifica	PF06424.15;PF1	PRP1_N;TPR_	PRP1 splicing facto	CYT	1	1	104.7	High
TRINITY_DN1814_c0_g2_i4_orfp1	TRINITY_DN1814_c0_g2_i4_m.63284 TRINITY_DN1814_c0_g2::TRINITY_DN1814_c0_g2_i4::g.63284 ORF type:internal len:258 (-),score=126.31 TRINITY_DN1814_c0_g2_i4:3-773(-)	2.636067218	1.398387159	3.47E-05	up	yes	1.051	0.3987	0.415	0.382	0.399	1	1.106	1.047	------	------	------	------	------	------	------	------	------	------	------	CYT	3	34	30.5	High
TRINITY_DN14826_c0_g1_i1_orf1	uncharacterized protein LOC114350939 [Ostrinia furnacalis]	2.068109484	1.048312563	2.52E-06	up	yes	0.9747	0.4713	0.468	0.475	0.471	1	0.962	0.962	CC:cellular_component;CC:integral component of membrane;CC:cellular anatomical entity;CC:intrinsic component of membrane;	GO:0005575;GO:0016021;GO:0110165;GO:0031224	------	------	------	------	ENOG410Z38Q	S:Function unknown	------	------	------	CYT	1	16	8.6	High
TRINITY_DN63662_c0_g4_i1_orf1	polyadenylate-binding protein 1 [Ostrinia furnacalis]	2.029632636	1.021218623	9.37E-08	up	yes	1	0.4927	0.49	0.495	0.493	1	1.01	0.991	CC:cytoplasm;CC:cellular_component;CC:cellular anatomical entity;MF:organic cyclic compound binding;MF:molecular_function;MF:RNA binding;MF:nucleic acid binding;MF:heterocyclic compound binding;MF:binding;	GO:0005737;GO:0005575;GO:0110165;GO:0097159;GO:0003674;GO:0003723;GO:0003676;GO:1901363;GO:0005488	K13126	PABPC	map03018;ma	RNA degradation	ENOG410XR5X	S:Function unknown	PF00076.25;PF0	RRM_1;PABP;	RNA recognition m	CYT	21	40	68.2	High
TRINITY_DN19829_c0_g1_i1_orf1	28S ribosomal protein S35, mitochondrial [Ostrinia furnacalis]	2.159973667	1.111013724	7.75E-06	up	yes	0.9843	0.4557	0.443	0.472	0.452	1	1	0.953	BP:cellular biosynthetic process;BP:cellular nitrogen compound metabolic process;BP:nitrogen compound metabolic process;BP:cellular macromolecule biosynthetic process;BP:organic substance biosynthetic process;BP:cellular macromolecule metabolic process;BP:peptide biosynthetic process;BP:organic substance metabolic process;BP:mitochondrial translation;BP:amide biosynthetic process;BP:cellular process;BP:biosynthetic process;BP:macromolecule biosynthetic process;BP:biological_process;BP:metabolic process;BP:cellular nitrogen compound biosynthetic process;BP:primary metabolic process;BP:organonitrogen compound metabolic process;BP:cellular amide metabolic process;BP:organonitrogen compound biosynthetic process;BP:protein metabolic process;BP:cellular metabolic process;BP:macromolecule metabolic process;BP:peptide metabolic process;BP:translation;CC:membrane-bounded organelle;CC:organelle;CC:mitochondrion;CC:cellular anatomical entity;CC:intracellular membrane-bounded organelle;CC:intracellular	GO:0044249;GO:0034641;GO:0006807;GO:0034645;GO:1901576;GO:0044260;GO:0043043;GO:0071704;GO:0032543;GO:0043604;GO:0009987;GO:0009058;GO:0009059;GO:0008150;GO:0008152;GO:0044271;GO:0044238;GO:1901564;GO:0043603;GO:1901566;GO:0019538;GO:0044237;GO:0043170;GO:0006518;GO:0006412;GO:0043227;GO:0043226;GO:0005739;GO:0110165;GO:0043231;GO:0043229;GO:0005575;GO:0003674;GO:0005198;GO:0003735	K17413	MRPS35	------	------	ENOG410ZX0Y	S:Function unknown	PF10213.12	MRP-S28	Mitochondrial ribo	CYT	2	8	27.2	High
TRINITY_DN33926_c0_g1_i1_orf1	ribosomal protein S9 [Ailuropoda melanoleuca] >AEA39538.1 ribosomal protein S9 [Ailuropoda melanoleuca]	2.068869936	1.04884295	0.0004221	up	yes	0.9703	0.469	0.52	0.417	0.47	1	1.012	0.899	BP:positive regulation of cellular amide metabolic process;BP:regulation of translational fidelity;BP:regulation of metabolic process;BP:positive regulation of translational fidelity;BP:regulation of cellular macromolecule biosynthetic process;BP:positive regulation of cellular metabolic process;BP:regulation of cellular metabolic process;BP:peptide biosynthetic process;BP:cellular biosynthetic process;BP:cellular nitrogen compound metabolic process;BP:positive regulation of gene expression;BP:regulation of biological process;BP:regulation of primary metabolic process;BP:positive regulation of metabolic process;BP:organic substance biosynthetic process;BP:positive regulation of macromolecule metabolic process;BP:cellular macromolecule metabolic process;BP:posttranscriptional regulation of gene expression;BP:positive regulation of cellular biosynthetic process;BP:regulation of protein metabolic process;BP:positive regulation of protein metabolic process;BP:organic substance metabolic process;BP:biological regulation;BP:regulation of cellular biosynthetic process;BP:positive regulation of biological process;BP:regulation of biological quality;BP:regulation of gene expression;BP:macromolecule metabolic process;BP:amide biosynthetic process;BP:regulation of macromolecule metabolic process;BP:cellular nitrogen compound biosynthetic process;BP:cellular process;BP:regulation of biosynthetic process;BP:regulation of cellular amide metabolic process;BP:nitrogen compound metabolic process;BP:cellular macromolecule biosynthetic process;BP:macromolecule biosynthetic process;BP:biological_process;BP:regulation of nitrogen compound metabolic process;BP:metabolic	GO:0034250;GO:0006450;GO:0019222;GO:0045903;GO:2000112;GO:0031325;GO:0031323;GO:0043043;GO:0044249;GO:0034641;GO:0010628;GO:0050789;GO:0080090;GO:0009893;GO:1901576;GO:0010604;GO:0044260;GO:0010608;GO:0031328;GO:0051246;GO:0051247;GO:0071704;GO:0065007;GO:0031326;GO:0048518;GO:0065008;GO:0010468;GO:0043170;GO:0043604;GO:0060255;GO:0044271;GO:0009987;GO:0009889;GO:0034248;GO:0006807;GO:0034645;GO:0009059;GO:0008150;GO:0051171;GO:0008152;GO:0051173;GO:1901564;GO:0009891;GO:0050794;GO:0044238;GO:0045727;GO:0043603;GO:1901566;GO:0019538;GO:0009058;GO:0044237;GO:0010557;GO:0010556;GO:0006417;GO:0006518;GO:0006412;GO:0048522;GO:0015935;GO:0022627;GO:0044391;GO:0032991;GO:1990904;GO:0005575;GO:0003674;GO:0005488;GO:0003676;GO:0005198;GO:1901363;GO:0003735;GO:0097159;GO:0019843;GO:0003723	K02997	RP-S9e, RPS9	map03010;ma	Ribosome;Coron	COG0522	J:Translation, ribosomal structu	PF00163.22;PF0	Ribosomal_S4	Ribosomal protein	CYT	1	21	22.6	High
TRINITY_DN106476_c0_g1_i3_orf1	mitochondrial import inner membrane translocase subunit TIM44 [Ostrinia furnacalis]	2.171010123	1.118366453	1.40E-06	up	yes	1.008	0.4643	0.448	0.469	0.476	1	0.999	1.025	BP:establishment of localization in cell;BP:protein import into mitochondrial matrix;BP:protein localization;BP:protein transmembrane transport;BP:cellular macromolecule localization;BP:intracellular protein transport;BP:transmembrane transport;BP:nitrogen compound transport;BP:intracellular protein transmembrane transport;BP:mitochondrial transmembrane transport;BP:organic substance transport;BP:macromolecule localization;BP:cellular process;BP:establishment of protein localization to organelle;BP:transport;BP:establishment of protein localization;BP:protein localization to organelle;BP:biological_process;BP:establishment of protein localization to mitochondrion;BP:mitochondrial transport;BP:establishment of localization;BP:localization;BP:intracellular protein transmembrane import;BP:cellular localization;BP:protein localization to mitochondrion;BP:intracellular transport;BP:protein transport;CC:membrane;CC:organelle membrane;CC:cellular anatomical entity;CC:mitochondrial membrane;CC:organelle inner membrane;CC:cellular_component;CC:mitochondrial inner	GO:0051649;GO:0030150;GO:0008104;GO:0071806;GO:0070727;GO:0006886;GO:0055085;GO:0071705;GO:0065002;GO:1990542;GO:0071702;GO:0033036;GO:0009987;GO:0072594;GO:0006810;GO:0045184;GO:0033365;GO:0008150;GO:0072655;GO:0006839;GO:0051234;GO:0051179;GO:0044743;GO:0051641;GO:0070585;GO:0046907;GO:0015031;GO:0016020;GO:0031090;GO:0110165;GO:0031966;GO:0019866;GO:0005575;GO:0005743;GO:0003674;GO:0005488;GO:0051087;GO:0005515	K17804	TIM44	------	------	ENOG410Y5IK	O:Posttranslational modificatio	PF04280.18	Tim44	Tim44-like domain	CYT	7	18	49.7	High
TRINITY_DN23586_c0_g1_i3_orf1	myrosinase 1-like isoform X1 [Ostrinia furnacalis]	2.413301663	1.271008264	3.99E-06	up	yes	1.016	0.421	0.424	0.396	0.443	1	1.014	1.035	BP:primary metabolic process;BP:carbohydrate metabolic process;BP:organic substance metabolic process;BP:biological_process;BP:metabolic process;MF:hydrolase activity;MF:catalytic activity;MF:hydrolase activity, acting on glycosyl bonds;MF:hydrolase activity, hydrolyzing O-glycosyl compounds;MF:molecular_function;	GO:0044238;GO:0005975;GO:0071704;GO:0008150;GO:0008152;GO:0016787;GO:0003824;GO:0016798;GO:0004553;GO:0003674	------	------	------	------	COG2723	G:Carbohydrate transport and r	PF00232.21	Glyco_hydro_	Glycosyl hydrolase	CYT	5	29	39.2	High
TRINITY_DN53136_c0_g1_i1_orf1	glutathione S transferase-E4 [Glyphodes pyloalis]	2.400666191	1.263434813	2.10E-06	up	yes	1.009	0.4203	0.4	0.446	0.415	1	1.015	1.013	MF:catalytic activity;MF:transferase activity;MF:glutathione transferase activity;MF:molecular_function;MF:transferase activity, transferring alkyl or aryl	GO:0003824;GO:0016740;GO:0004364;GO:0003674;GO:0016765	K00799	GST, gst	map05207;ma	Chemical carcino	COG0625	U:Intracellular trafficking, secre	PF13417.9;PF00	GST_N_3;GST_	Glutathione S-tran	CYT	3	15	25.1	High
TRINITY_DN391_c5_g1_i1_orf1	hypothetical protein B5X24_HaOG210395 [Helicoverpa armigera]	2.181462991	1.125295998	1.72E-06	up	yes	1.005	0.4607	0.483	0.458	0.441	1	1.011	1.004	MF:oxidoreductase activity;MF:NAD binding;MF:nucleotide binding;MF:small molecule binding;MF:catalytic activity;MF:molecular_function;MF:binding;MF:organic cyclic compound binding;MF:quinone binding;MF:heterocyclic compound binding;MF:nucleoside phosphate binding;MF:oxidoreductase activity, acting on NA	GO:0016491;GO:0051287;GO:0000166;GO:0036094;GO:0003824;GO:0003674;GO:0005488;GO:0097159;GO:0048038;GO:1901363;GO:1901265;GO:0016651	K03935	NDUFS2	map04714;ma	Thermogenesis;C	COG0649	C:Energy production and conv	PF00346.22	Complex1_49	Respiratory-chain	CYT	8	26	52.6	High
TRINITY_DN11655_c0_g1_i1_orf1	ribosome biogenesis protein BRX1 homolog [Ostrinia furnacalis]	2.019661734	1.014113681	7.40E-05	up	yes	0.9553	0.473	0.458	0.475	0.486	1	0.905	0.961	BP:nucleic acid metabolic process;BP:cellular nitrogen compound metabolic process;BP:nitrogen compound metabolic process;BP:ncRNA metabolic process;BP:organic cyclic compound metabolic process;BP:nucleobase-containing compound metabolic process;BP:organic substance metabolic process;BP:rRNA processing;BP:ncRNA processing;BP:cellular process;BP:cellular aromatic compound metabolic process;BP:biological_process;BP:metabolic process;BP:heterocycle metabolic process;BP:RNA metabolic process;BP:primary metabolic process;BP:rRNA metabolic process;BP:cellular metabolic process;BP:macromolecule metabolic process;BP:RNA processing;MF:molecular_function;MF:binding;MF:nucleic acid binding;MF:rRNA binding;MF:heterocyclic compound	GO:0090304;GO:0034641;GO:0006807;GO:0034660;GO:1901360;GO:0006139;GO:0071704;GO:0006364;GO:0034470;GO:0009987;GO:0006725;GO:0008150;GO:0008152;GO:0046483;GO:0016070;GO:0044238;GO:0016072;GO:0044237;GO:0043170;GO:0006396;GO:0003674;GO:0005488;GO:0003676;GO:0019843;GO:1901363;GO:0097159;GO:0003723	K14820	BRX1, BRIX1	------	------	COG5154	J:Translation, ribosomal structu	PF04427.21	Brix	Brix domain	CYT	5	16	42.7	High

TRINITY_DN2593_c0_g2_i1_orf1	midgut carboxypeptidase [Loxostege sticticalis]	2.293940098	1.197827718	0.0002133	up	yes	0.988	0.4307	0.413	0.413	0.466	1	1.05	0.914	BP:nitrogen compound metabolic process;BP:organic substance metabolic process;BP:proteolysis;BP:biological_process;BP:metabolic process;BP:primary metabolic process;BP:organonitrogen compound metabolic process;BP:protein metabolic process;BP:macromolecule metabolic process;MF:exopeptidase activity;MF:molecular_function;MF:zinc ion binding;MF:peptidase activity;MF:metalloexopeptidase activity;MF:metallopeptidase activity;MF:cation binding;MF:transition metal ion binding;MF:metallocarboxypeptidase activity;MF:carboxypeptidase activity;MF:ion binding;MF:metal	GO:0006807;GO:0071704;GO:0006508;GO:0008150;GO:0008152;GO:0044238;GO:1901564;GO:0019538;GO:0043170;GO:0008238;GO:0003674;GO:0008270;GO:0008233;GO:0008235;GO:0008237;GO:0043169;GO:0046914;GO:0004181;GO:0004180;GO:0043167;GO:0046872;GO:0016787;GO:0140096;GO:0003824;GO:0005488	K01298	CPA2	map04972;ma	Pancreatic secret	COG2866	E:Amino acid transport and me	PF00246.27;PF0	Peptidase_M1	Zinc carboxypeptic	CYT	1	6	48.9	High
TRINITY_DN2084_c0_g1_i1_orf1	40S ribosomal protein S24 [Helicoverpa armigera] >XP_022830907.1 40S ribosomal protein S24 [Spodoptera litura] >XP_026729116.1 40S ribosomal protein S24 [Trichoplusia ni] >XP_035432908.1 40S ribosomal protein S24 [Spodoptera frugiperda] >XP_047024473.1 40S ribosomal protein S24 [Helicoverpa zea] >Q962Q6.1 RecName: Full=40S ribosomal protein S24 [Spodoptera frugiperda] >KAF9418537.1 hypothetical protein HW555_004686 [Spodoptera exigua] >CAB3515448.1 unnamed protein product [Spodoptera littoralis] >CAH0579501.1 unnamed protein product [Chrysodeixis includens] >AAK92192.1 ribosomal protein S24 [Spodoptera frugiperda] >KAF9808794.1 hypothetical protein SFRURICE_013056 [Spodoptera frugiperda]	2.170995671	1.118356849	2.26E-06	up	yes	1.003	0.462	0.468	0.451	0.467	1	1.026	0.984	BP:cellular biosynthetic process;BP:cellular nitrogen compound metabolic process;BP:nitrogen compound metabolic process;BP:cellular macromolecule biosynthetic process;BP:organic substance biosynthetic process;BP:cellular nitrogen compound biosynthetic process;BP:cellular macromolecule metabolic process;BP:peptide biosynthetic process;BP:organic substance metabolic process;BP:amide biosynthetic process;BP:cellular process;BP:biosynthetic process;BP:macromolecule biosynthetic process;BP:biological_process;BP:metabolic process;BP:organonitrogen compound metabolic process;BP:primary metabolic process;BP:cellular amide metabolic process;BP:organonitrogen compound biosynthetic process;BP:protein metabolic process;BP:cellular metabolic process;BP:macromolecule metabolic process;BP:peptide metabolic process;BP:translation;CC:intracellular non-membrane-bounded organelle;CC:organelle;CC:cellular anatomical entity;CC:ribosome;CC:macromolecular complex;CC:ribonucleoprotein complex;CC:intracellular organelle;CC:cellular_component;CC:non-membrane-bounded organelle;MF:molecular_function;MF:structural molecule	GO:0044249;GO:0034641;GO:0006807;GO:0034645;GO:1901576;GO:0044271;GO:0044260;GO:0043043;GO:0071704;GO:0043604;GO:0009987;GO:0009058;GO:0009059;GO:0008150;GO:0008152;GO:1901564;GO:0044238;GO:0043603;GO:1901566;GO:0019538;GO:0044237;GO:0043170;GO:0006518;GO:0006412;GO:0043232;GO:0043226;GO:0110165;GO:0005840;GO:0032991;GO:1990904;GO:0043229;GO:0005575;GO:0043228;GO:0003674;GO:0005198;GO:0003735	K02974	RP-S24e, RPS	map03010;ma	Ribosome;Coron	COG2004	J:Translation, ribosomal structu	PF01282.22	Ribosomal_S2	Ribosomal protein	CYT	2	13	15.2	High
TRINITY_DN2238_c0_g2_i1_orf1	mitochondrial import inner membrane translocase subunit Tim8 [Ostrinia furnacalis]	2.177633056	1.122760872	0.0001242	up	yes	0.9697	0.4453	0.438	0.449	0.449	1	1.01	0.899	BP:protein localization;BP:cellular macromolecule localization;BP:nitrogen compound transport;BP:organic substance transport;BP:macromolecule localization;BP:transport;BP:cellular process;BP:establishment of protein localization;BP:biological_process;BP:establishment of localization;BP:localization;BP:cellular localization;BP:protein transport;CC:organelle envelope lumen;CC:membrane-enclosed lumen;CC:organelle membrane;CC:membrane;CC:mitochondrial intermembrane space;CC:cellular anatomical entity;CC:mitochondrial membrane;CC:intracellular organelle lumen;CC:organelle lumen;CC:organelle inner membrane;CC:cellular_component;CC:mitochondrial inner membrane;	GO:0008104;GO:0070727;GO:0071705;GO:0071702;GO:0033036;GO:0006810;GO:0009987;GO:0045184;GO:0008150;GO:0051234;GO:0051179;GO:0051641;GO:0015031;GO:0031970;GO:0031974;GO:0031090;GO:0016020;GO:0005758;GO:0110165;GO:0031966;GO:0070013;GO:0043233;GO:0019866;GO:0005575;GO:0005743	K17780	TIM8	------	------	ENOG41126D5	O:Posttranslational modificatio	PF02953.18	zf-Tim10_DDI	Tim10/DDP family	CYT	4	44	10.2	High
TRINITY_DN7512_c0_g1_i1_orf1	hypothetical protein evm_010529 [Chilo suppressalis] >CAB3530682.1 unnamed protein product [Chilo suppressalis] >CAH0407273.1 unnamed protein product [Chilo suppressalis]	2.352383211	1.234123099	2.41E-06	up	yes	0.992	0.4217	0.412	0.409	0.444	1	0.974	1.002	BP:one-carbon metabolic process;BP:cellular biosynthetic process;BP:small molecule metabolic process;BP:organic substance biosynthetic process;BP:organic substance metabolic process;BP:sulfur compound metabolic process;BP:S-adenosylmethionine metabolic process;BP:cellular process;BP:biosynthetic process;BP:biological_process;BP:metabolic process;BP:sulfur compound biosynthetic process;BP:S-adenosylmethionine biosynthetic process;BP:cellular metabolic process;MF:purine ribonucleoside triphosphate binding;MF:molecular_function;MF:nucleotide binding;MF:heterocyclic compound binding;MF:nucleoside phosphate binding;MF:anion binding;MF:transferase activity, transferring alkyl or ary;MF:ion binding;MF:transferase activity;MF:metal ion binding;MF:ATP binding;MF:cation binding;MF:small molecule binding;MF:catalytic activity;MF:purine nucleotide binding;MF:methionine adenosyltransferase activity;MF:organic cyclic compound binding;MF:adenyl nucleotide binding;MF:carbohydrate derivative binding;MF:adenyl ribonucleotide binding;MF:purine	GO:0006730;GO:0044249;GO:0044281;GO:1901576;GO:0071704;GO:0006790;GO:0046500;GO:0009987;GO:0009058;GO:0008150;GO:0008152;GO:0044272;GO:0006556;GO:0044237;GO:0035639;GO:0003674;GO:0000166;GO:1901363;GO:1901265;GO:0043168;GO:0016765;GO:0043167;GO:0016740;GO:0046872;GO:0005524;GO:0043169;GO:0036094;GO:0003824;GO:0017076;GO:0004478;GO:0097159;GO:0030554;GO:0097367;GO:0032559;GO:0032555;GO:0032553;GO:0005488	K00789	metK, MAT	map00270;ma	Cysteine and me	COG0192	H:Coenzyme transport and me	PF02773.19;PF0	S-AdoMet_sy	S-adenosylmethio	CYT	5	50	22.1	High
TRINITY_DN2283_c0_g2_i1_orf1	H/ACA ribonucleoprotein complex subunit 4 [Ostrinia furnacalis]	2.156566746	1.108736368	7.94E-07	up	yes	1	0.4637	0.468	0.471	0.452	1	0.986	1.015	BP:RNA modification;BP:nucleic acid metabolic process;BP:cellular nitrogen compound metabolic process;BP:nitrogen compound metabolic process;BP:pseudouridine synthesis;BP:organic cyclic compound metabolic process;BP:nucleobase-containing compound metabolic process;BP:organic substance metabolic process;BP:cellular process;BP:cellular aromatic compound metabolic process;BP:macromolecule modification;BP:biological_process;BP:metabolic process;BP:heterocycle metabolic process;BP:RNA metabolic process;BP:primary metabolic process;BP:cellular metabolic process;BP:macromolecule metabolic process;BP:RNA processing;CC:macromolecular complex;CC:ribonucleoprotein complex;CC:cellular_component;MF:molecular_function;MF:binding;MF:nucleic acid binding;MF:intramolecular transferase activity;MF:heterocyclic compound binding;MF:pseudouridine synthase activity;MF:catalytic activity;MF:isomerase	GO:0009451;GO:0090304;GO:0034641;GO:0006807;GO:0001522;GO:1901360;GO:0006139;GO:0071704;GO:0009987;GO:0006725;GO:0043412;GO:0008150;GO:0008152;GO:0046483;GO:0016070;GO:0044238;GO:0044237;GO:0043170;GO:0006396;GO:0032991;GO:1990904;GO:0005575;GO:0003674;GO:0005488;GO:0003676;GO:0016866;GO:1901363;GO:0009982;GO:0003824;GO:0016853;GO:0097159;GO:0003723	K11131	DKC1, NOLA4	map03008	Ribosome bioger	COG0130	J:Translation, ribosomal structu	PF08068.15;PF0	DKCLD;TruB_I	DKCLD;TruB family	CYT	9	16	57.3	High
TRINITY_DN44557_c0_g1_i4_orf1	serine hydrolase-like protein [Ostrinia furnacalis]	2.033284488	1.023812085	8.88E-05	up	yes	0.9713	0.4777	0.457	0.531	0.445	1	0.965	0.949	MF:catalytic activity;MF:transferase activity;MF:molecular_function;	GO:0003824;GO:0016740;GO:0003674	------	------	------	------	COG0596;ENOG4111NO7	S:Function unknown;	------	------	------	PLA	2	16	10.4	High
TRINITY_DN1073_c0_g1_i1_orf1	carboxylesterase [Loxostege sticticalis]	2.653164557	1.407714158	6.34E-05	up	yes	1.048	0.395	0.447	0.371	0.367	1	1.05	1.093	CC:integral component of membrane;CC:cellular_component;CC:intrinsic component of membrane;CC:cellular anatomical entity;MF:hydrolase activity;MF:hydrolase activity, acting on ester bonds;MF:carboxylic ester hydrolase activity;MF:catalytic	GO:0016021;GO:0005575;GO:0031224;GO:0110165;GO:0016787;GO:0016788;GO:0052689;GO:0003824;GO:0003674	------	------	------	------	COG2272;COG0580;ENOG410XSWS	I:Lipid transport and metabolis	PF00135.31;PF2	COesterase;B	Carboxylesterase f	CYT	1	8	30.1	High
TRINITY_DN3461_c0_g1_i1_orf1	protein SCO1 homolog, mitochondrial [Ostrinia furnacalis]	2.267926246	1.181373724	3.61E-06	up	yes	0.9963	0.4393	0.462	0.414	0.442	1	0.983	1.006	BP:cellular component assembly;BP:macromolecular complex subunit organization;BP:ion homeostasis;BP:cytochrome complex assembly;BP:homeostatic process;BP:copper ion homeostasis;BP:transition metal ion homeostasis;BP:inorganic ion homeostasis;BP:respiratory chain complex IV assembly;BP:cellular transition metal ion homeostasis;BP:cellular component organization;BP:macromolecular complex assembly;BP:biological regulation;BP:cellular component organization or biogenesis;BP:regulation of biological quality;BP:cellular homeostasis;BP:cellular copper ion homeostasis;BP:cellular metal ion homeostasis;BP:cellular process;BP:cellular ion homeostasis;BP:biological_process;BP:cellular cation homeostasis;BP:metal ion homeostasis;BP:cation homeostasis;BP:cellular chemical homeostasis;BP:chemical homeostasis;CC:intrinsic component of membrane;CC:organelle membrane;CC:integral component of membrane;CC:membrane;CC:cellular anatomical entity;CC:mitochondrial membrane;CC:organelle inner membrane;CC:cellular_component;CC:mitochondrial inner membrane;MF:molecular_function;MF:binding;MF:cation binding;MF:transition metal ion binding;MF:ion	GO:0022607;GO:0043933;GO:0050801;GO:0017004;GO:0042592;GO:0055070;GO:0055076;GO:0098771;GO:0008535;GO:0046916;GO:0016043;GO:0065003;GO:0065007;GO:0071840;GO:0065008;GO:0019725;GO:0006878;GO:0006875;GO:0009987;GO:0006873;GO:0008150;GO:0030003;GO:0055065;GO:0055080;GO:0055082;GO:0048878;GO:0031224;GO:0031090;GO:0016021;GO:0016020;GO:0110165;GO:0031966;GO:0019866;GO:0005575;GO:0005743;GO:0003674;GO:0005488;GO:0043169;GO:0046914;GO:0043167;GO:0016531;GO:0016530;GO:0005507;GO:0046872;GO:0140104	K07152	SCO1	------	------	COG1999	S:Function unknown	PF02630.17;PF0	SCO1-SenC;A	SCO1/SenC;AhpC/	CYT	4	25	28.2	High
TRINITY_DN4121_c0_g1_i1_orf1	uncharacterized protein LOC114358001, partial [Ostrinia furnacalis]	2.230405405	1.157305963	1.20E-06	up	yes	0.9903	0.444	0.431	0.439	0.462	1	0.994	0.977	BP:cellular catabolic process;BP:nitrogen compound metabolic process;BP:modification-dependent macromolecule catabolic process;BP:organic substance catabolic process;BP:cellular macromolecule catabolic process;BP:cellular macromolecule metabolic process;BP:protein modification by small protein conjugation or removal;BP:protein modification by small protein removal;BP:organic substance metabolic process;BP:proteolysis;BP:protein deubiquitination;BP:primary metabolic process;BP:cellular process;BP:modification-dependent protein catabolic process;BP:macromolecule modification;BP:protein modification process;BP:biological_process;BP:metabolic process;BP:catabolic process;BP:macromolecule catabolic process;BP:proteolysis involved in cellular protein catabolic process;BP:organonitrogen compound metabolic process;BP:protein metabolic process;BP:cellular metabolic process;BP:macromolecule metabolic process;BP:ubiquitin-dependent protein catabolic process;MF:ubiquitinyl hydrolase activity;MF:molecular_function;MF:cysteine-type peptidase activity;MF:ubiquitin-like protein-specific protease activity;MF:thiol-dependent ubiquitin-specific protease activity;MF:hydrolase activity;MF:catalytic activity, acting on a	GO:0044248;GO:0006807;GO:0043632;GO:1901575;GO:0044265;GO:0044260;GO:0070647;GO:0070646;GO:0071704;GO:0006508;GO:0016579;GO:0044238;GO:0009987;GO:0019941;GO:0043412;GO:0036211;GO:0008150;GO:0008152;GO:0009056;GO:0009057;GO:0051603;GO:1901564;GO:0019538;GO:0044237;GO:0043170;GO:0006511;GO:0101005;GO:0003674;GO:0008234;GO:0019783;GO:0004843;GO:0016787;GO:0140096;GO:0003824;GO:0008233	K11833	USP2	------	------	ENOG410XP8T	S:Function unknown	PF00443.32;PF1	UCH;UCH_1	Ubiquitin carboxyl-	CYT	3	4	104.2	High
TRINITY_DN753_c0_g1_i4_orf1	venom dipeptidyl peptidase 4-like isoform X2 [Ostrinia furnacalis]	2.413875598	1.271351327	1.72E-06	up	yes	1.009	0.418	0.394	0.431	0.429	1	1.006	1.022	BP:primary metabolic process;BP:organonitrogen compound metabolic process;BP:protein metabolic process;BP:macromolecule metabolic process;BP:organic substance metabolic process;BP:nitrogen compound metabolic process;BP:biological_process;BP:metabolic process;BP:proteolysis;CC:integral component of membrane;CC:cellular_component;CC:cellular anatomical entity;CC:intrinsic component of membrane;MF:molecular_function;MF:serine hydrolase activity;MF:hydrolase activity;MF:catalytic activity, acting on a protein;MF:catalytic activity;MF:peptidase activity;MF:serine-type peptidase activity;	GO:0044238;GO:1901564;GO:0019538;GO:0043170;GO:0071704;GO:0006807;GO:0008150;GO:0008152;GO:0006508;GO:0016021;GO:0005575;GO:0110165;GO:0031224;GO:0003674;GO:0017171;GO:0016787;GO:0140096;GO:0003824;GO:0008233;GO:0008236	K01278	DPP4, CD26	map04974	Protein digestion	COG1506;COG1506	J:Translation, ribosomal structu	PF00930.24;PF0	DPPIV_N;Pept	Dipeptidyl peptida	CYT	3	5	92.6	High
TRINITY_DN20294_c0_g2_i1_orf1	cytochrome b-c1 complex subunit 2, mitochondrial isoform X1 [Ostrinia furnacalis] >XP_028170208.1 cytochrome b-c1 complex subunit 2, mitochondrial isoform X2 [Ostrinia	2.429269484	1.28052254	2.90E-07	up	yes	0.9943	0.4093	0.412	0.416	0.4	1	1.003	0.98	MF:cation binding;MF:metal ion binding;MF:molecular_function;MF:binding;MF:ion binding;	GO:0043169;GO:0046872;GO:0003674;GO:0005488;GO:0043167	K00415	QCR2, UQCR	map04714;ma	Thermogenesis;C	COG0612	T:Signal transduction mechanis	PF00675.23;PF0	Peptidase_M1	Insulinase;Peptidas	CYT	17	43	45.7	High
TRINITY_DN7964_c0_g1_i1_orfp1	TRINITY_DN7964_c0_g1_i1_m.23483 TRINITY_DN7964_c0_g1::TRINITY_DN7964_c0_g1_i1::g.23483 ORF type:internal len:79 (+),score=44.25 TRINITY_DN7964_c0_g1_i1:3-236(+)	2.822404372	1.496924701	5.89E-05	up	yes	1.033	0.366	0.404	0.392	0.302	1	1.067	1.033	------	------	------	------	------	------	------	------	------	------	------	CYT	2	17	8.7	High
TRINITY_DN20_c0_g1_i11_orf1	plasma membrane calcium-transporting ATPase 2 [Ostrinia furnacalis]	2.182234242	1.125805969	4.63E-05	up	yes	1.049	0.4807	0.511	0.472	0.459	1	1.087	1.06	------	------	K05850	ATP2B	map04978;ma	Mineral absorptic	ENOG410XNNC	P:Inorganic ion transport and n	PF00689.24;PF0	Cation_ATPas	Cation transportin	CYT	9	22	125	High
TRINITY_DN1445_c0_g2_i4_orf1	leucine-rich PPR motif-containing protein, mitochondrial [Ostrinia furnacalis]	2.116147901	1.081440463	0.0002106	up	yes	1.013	0.4787	0.402	0.521	0.513	1	1.045	0.995	------	------	K17964	LRPPRC	------	------	ENOG410XSG9	S:Function unknown	------	------	------	CYT	2	28	60.7	High
TRINITY_DN36538_c0_g1_i2_orf1	xaa-Pro dipeptidase isoform X1 [Ostrinia furnacalis] >XP_028156507.1 xaa-Pro dipeptidase isoform X2 [Ostrinia furnacalis]	2.103182858	1.072574289	6.99E-07	up	yes	1.011	0.4807	0.482	0.472	0.488	1	1.028	1.005	MF:cation binding;MF:transition metal ion binding;MF:aminopeptidase activity;MF:hydrolase activity;MF:catalytic activity, acting on a protein;MF:manganese ion binding;MF:catalytic activity;MF:ion binding;MF:exopeptidase activity;MF:molecular_function;MF:binding;MF:metalloaminopeptidase activity;MF:peptidase activity;MF:metal ion binding;MF:metalloexopeptidase activity;MF:metallopeptidase	GO:0043169;GO:0046914;GO:0004177;GO:0016787;GO:0140096;GO:0030145;GO:0003824;GO:0043167;GO:0008238;GO:0003674;GO:0005488;GO:0070006;GO:0008233;GO:0046872;GO:0008235;GO:0008237	K14213	PEPD	------	------	COG0006	E:Amino acid transport and me	PF00557.27;PF0	Peptidase_M2	Metallopeptidase f	CYT	9	18	60.8	High

TRINITY_DN35635_c0_g1_i1_orf1	probable NADH dehydrogenase [ubiquinone] 1 alpha subcomplex subunit 12 [Ostrinia furnacalis]	2.036494845	1.026088163	1.44E-06	up	yes	0.9877	0.485	0.501	0.483	0.471	1	0.987	0.976	BP:mitochondrial respiratory chain complex assembly;BP:cellular component assembly;BP:mitochondrial respiratory chain complex I assembly;BP:macromolecular complex subunit organization;BP:NADH dehydrogenase complex assembly;BP:cellular component organization;BP:cellular process;BP:biological_process;BP:cellular component organization or biogenesis;BP:macromolecular complex assembly;CC:organelle membrane;CC:membrane;CC:organelle inner membrane;CC:mitochondrial membrane;CC:cellular_component;CC:mitochondrial inner	GO:0033108;GO:0022607;GO:0032981;GO:0043933;GO:0010257;GO:0016043;GO:0009987;GO:0008150;GO:0071840;GO:0065003;GO:0031090;GO:0016020;GO:0019866;GO:0031966;GO:0005575;GO:0005743;GO:0070469;GO:0110165	K11352	NDUFA12	map04714;ma[illegible]	Thermogenesis;[illegible]	ENOG4111XVR	I:Lipid transport and metabolis[illegible]	PF05071.19	NDUFA12	NADH ubiquinone	CYT	5	44	16.9	High
TRINITY_DN100821_c0_g1_i1_orf1	putative GMP synthase, partial [Operophtera brumata]	2.083245743	1.058833032	5.97E-06	up	yes	0.991	0.4757	0.483	0.498	0.446	1	0.982	0.991	BP:carboxylic acid metabolic process;BP:nitrogen compound metabolic process;BP:glutamine family amino acid metabolic process;BP:cellular amino acid metabolic process;BP:glutamine metabolic process;BP:organic substance metabolic process;BP:alpha-amino acid metabolic process;BP:cellular process;BP:biological_process;BP:metabolic process;BP:oxoacid metabolic process;BP:primary metabolic process;BP:organonitrogen compound metabolic process;BP:organic acid metabolic process;BP:cellular metabolic process;BP:small molecule metabolic process;MF:binding;MF:purine ribonucleoside triphosphate binding;MF:molecular_function;MF:carbon-nitrogen ligase activity, with glutamine as amido-N-donor;MF:ion binding;MF:heterocyclic compound binding;MF:nucleoside phosphate binding;MF:anion binding;MF:nucleotide binding;MF:purine nucleotide binding;MF:ATP binding;MF:hydrolase activity;MF:GMP synthas;MF:small molecule binding;MF:catalytic activity;MF:ligase activity;MF:hydrolase activity, acting on acid anhydrides, in phosphorus-containing anhydrides;MF:ligase activity, forming carbon-nitrogen bonds;MF:carbohydrate derivative binding;MF:organic cyclic compound binding;MF:adenyl nucleotide binding;MF:hydrolase activity, acting on acid anhydrides;MF:pyrophosphatase activity;MF:adenyl	GO:0019752;GO:0006807;GO:0009064;GO:0006520;GO:0006541;GO:0071704;GO:1901605;GO:0009987;GO:0008150;GO:0008152;GO:0043436;GO:0044238;GO:1901564;GO:0006082;GO:0044237;GO:0044281;GO:0005488;GO:0035639;GO:0003674;GO:0016884;GO:0043167;GO:1901363;GO:1901265;GO:0043168;GO:0000166;GO:0017076;GO:0005524;GO:0016787;GO:0003922;GO:0036094;GO:0003824;GO:0016874;GO:0016818;GO:0016879;GO:0097367;GO:0097159;GO:0030554;GO:0016817;GO:0016462;GO:0032559;GO:0032555;GO:0032553	K01951	guaA, GMPS	map01232;ma[illegible]	Nucleotide metal	COG0519	F:Nucleotide transport and me[illegible]	------	------	------	CYT	2	32	7.3	High
TRINITY_DN235_c0_g1_i2_orf1	unnamed protein product [Parnassius apollo]	2.097457627	1.068641666	2.90E-07	up	yes	0.99	0.472	0.473	0.476	0.467	1	0.976	0.994	CC:organelle;CC:intracellular organelle;CC:non-membrane-bounded organelle;CC:cytoplasm;CC:cytoskeleton;CC:intracellular non-membrane-bounded organelle;CC:cellular_component;CC:cellular anatomical entity;MF:purine ribonucleoside triphosphate binding;MF:molecular_function;MF:binding;MF:ion binding;MF:heterocyclic compound binding;MF:nucleoside phosphate binding;MF:nucleotide binding;MF:purine nucleotide binding;MF:ATP binding;MF:small molecule binding;MF:adenyl nucleotide binding;MF:carbohydrate derivative binding;MF:organic cyclic compound binding;MF:anion binding;MF:adenyl ribonucleotide binding;MF:purine	GO:0043226;GO:0043229;GO:0043228;GO:0005737;GO:0005856;GO:0043232;GO:0005575;GO:0110165;GO:0035639;GO:0003674;GO:0005488;GO:0043167;GO:1901363;GO:1901265;GO:0000166;GO:0017076;GO:0005524;GO:0036094;GO:0030554;GO:0097367;GO:0097159;GO:0043168;GO:0032559;GO:0032555;GO:0032553	------	------	------	------	COG5277	S:Function unknown	PF00022.22	Actin	Actin	CYT	5	70	44.3	High
TRINITY_DN2704_c0_g1_i5_orf1	hypothetical protein evm_009002 [Chilo suppressalis]	2.25	1.169925001	8.23E-05	up	yes	0.999	0.444	0.413	0.511	0.408	1	1.008	0.989	------	------	K06569	MFI2, CD228	------	------	ENOG410ZEJF	S:Function unknown	PF00405.20	Transferrin	Transferrin	CYT	9	16	84.2	High
TRINITY_DN4905_c0_g1_i6_orf1	uncharacterized protein LOC114351759 [Ostrinia furnacalis]	3.186588921	1.67201292	0.0001392	up	yes	1.093	0.343	0.34	0.307	0.382	1	1.159	1.119	------	------	------	------	------	------	------	------	------	------	------	EXC	1	4	62.4	High
TRINITY_DN75746_c0_g1_i1_orfp1	TRINITY_DN75746_c0_g1_i1_m.54871 TRINITY_DN75746_c0_g1::TRINITY_DN75746_c0_g1_i1::g.54871 ORF type:internal len:83 (+),score=-1.09,Polyhedrin,PF00738.19,1.7e-25,TRINITY_DN75746_c0_g1_i1:2-247(+)	4.138918725	2.049253919	0.0005098	up	yes	1.156	0.2793	0.279	0.288	0.271	1	1.294	1.174	------	------	------	------	------	------	------	------	PF00738.21	Polyhedrin	Polyhedrin	CYT	2	29	9.6	High
TRINITY_DN15513_c0_g1_i6_orf1	uncharacterized protein LOC114350859 [Ostrinia furnacalis]	2.623951182	1.391740879	1.62E-05	up	yes	1.032	0.3933	0.415	0.371	0.394	1	1.021	1.076	------	------	------	------	------	------	COG1028	S:Function unknown	PF13561.9;PF00[illegible]	adh_short_C2	Enoyl;short chain [illegible]	CYT	3	22	25.8	High
TRINITY_DN43942_c0_g2_i1_orf1	LOW QUALITY PROTEIN: caprin homolog [Ostrinia furnacalis]	2.648190916	1.405007134	3.43E-05	up	yes	1.032	0.3897	0.402	0.419	0.348	1	1.077	1.019	------	------	K18743	CAPRIN1, GPI	------	------	ENOG4110M5O	S:Function unknown	------	------	------	CYT	1	5	22.7	High
TRINITY_DN76529_c0_g1_i1_orfp1	TRINITY_DN76529_c0_g1_i1_m.64079 TRINITY_DN76529_c0_g1::TRINITY_DN76529_c0_g1_i1::g.64079 ORF type:internal len:70	2.17184265	1.118919584	5.54E-05	up	yes	1.049	0.483	0.499	0.469	0.481	1	1.044	1.104	------	------	------	------	------	------	------	------	------	------	------	CYT	1	23	7.7	High
TRINITY_DN3836_c0_g1_i4_orf1	2-oxoisovalerate dehydrogenase subunit alpha, mitochondrial [Ostrinia furnacalis]	2.384375749	1.253611605	3.02E-06	up	yes	0.995	0.4173	0.426	0.429	0.397	1	1.012	0.973	BP:carboxylic acid metabolic process;BP:cellular amino acid catabolic process;BP:nitrogen compound metabolic process;BP:small molecule metabolic process;BP:small molecule catabolic process;BP:organic substance catabolic process;BP:cellular amino acid metabolic process;BP:organic substance metabolic process;BP:cellular process;BP:biological_process;BP:metabolic process;BP:oxoacid metabolic process;BP:catabolic process;BP:cellular catabolic process;BP:primary metabolic process;BP:organonitrogen compound metabolic process;BP:organonitrogen compound catabolic process;BP:organic acid metabolic process;BP:carboxylic acid catabolic process;BP:organic acid catabolic process;BP:cellular metabolic process;BP:branched-chain amino acid metabolic process;BP:branched-chain amino acid catabolic process;MF:molecular_function;MF:oxidoreductase activity;MF:oxidoreductase activity, acting on the aldehyde or oxo group of donors, disulfide as acceptor;MF:catalytic activity;MF:oxidoreductase activity, acting on the aldehyde or	GO:0019752;GO:0009063;GO:0006807;GO:0044281;GO:0044282;GO:1901575;GO:0006520;GO:0071704;GO:0009987;GO:0008150;GO:0008152;GO:0043436;GO:0009056;GO:0044248;GO:0044238;GO:1901564;GO:1901565;GO:0006082;GO:0046395;GO:0016054;GO:0044237;GO:0009081;GO:0009083;GO:0003674;GO:0016491;GO:0016624;GO:0003824;GO:0016903;GO:0003863	K00166	BCKDHA, bkd	map00280;ma[illegible]	Valine, leucine a[illegible]	COG1071	S:Function unknown	PF00676.23	E1_dh	Dehydrogenase E1	CYT	8	21	48.9	High
TRINITY_DN38835_c0_g3_i1_orf1	protein transport protein Sec61 subunit alpha [Spodoptera litura] >XP_035429226.1 protein transport protein Sec61 subunit alpha [Spodoptera frugiperda] >XP_047985890.1 protein transport protein Sec61 subunit alpha [Leguminivora glycinivorella] >KAF9413961.1 hypothetical protein HW555_007991 [Spodoptera exigua] >CAB3514725.1 unnamed protein product [Spodoptera littoralis] >KAF9810869.1 hypothetical protein SFRURICE_005295 [Spodoptera frugiperda] >KAG8115796.1 hypothetical protein SFRUCORN_012373 [Spodoptera frugiperda] >CAH0700181.1 unnamed protein product [Spodoptera exigua]	2.150515464	1.104682505	9.53E-05	up	yes	1.043	0.485	0.508	0.496	0.451	1	1.103	1.026	BP:nitrogen compound transport;BP:macromolecule localization;BP:protein localization;BP:cellular macromolecule localization;BP:cellular process;BP:establishment of protein localization;BP:biological_process;BP:transport;BP:organic substance transport;BP:protein transport;BP:establishment of localization;BP:localization;BP:cellular localization;CC:cellular_component;CC:integral component of membrane;CC:cellular anatomical entity;CC:membrane;CC:intrinsic component of membrane;	GO:0071705;GO:0033036;GO:0008104;GO:0070727;GO:0009987;GO:0045184;GO:0008150;GO:0006810;GO:0071702;GO:0015031;GO:0051234;GO:0051179;GO:0051641;GO:0005575;GO:0016021;GO:0110165;GO:0016020;GO:0031224	K10956	SEC61A	map04145;ma[illegible]	Phagosome;Prot[illegible]	COG0201	U:Intracellular trafficking, secre[illegible]	PF00344.23;PF1[illegible]	SecY;Plug_tra[illegible]	SecY;Plug domain	CYT	3	11	46.1	High
TRINITY_DN63914_c0_g1_i1_orf1	myophilin-like [Ostrinia furnacalis]	2.636363636	1.398549376	2.89E-07	up	yes	1.015	0.385	0.38	0.387	0.388	1	1.031	1.013	------	------	K20526	TAGLN	------	------	COG5199	Z:Cytoskeleton	PF00307.34	CH	Calponin homolog	CYT	9	51	20.7	High
TRINITY_DN1617_c0_g1_i5_orf1	hypothetical protein evm_009822 [Chilo suppressalis] >CAB3525311.1 unnamed protein product [Chilo suppressalis] >CAH0402638.1 unnamed protein product [Chilo suppressalis]	2.418826406	1.274307234	2.22E-07	up	yes	0.9893	0.409	0.416	0.398	0.413	1	0.98	0.988	CC:integral component of membrane;CC:intrinsic component of membrane;CC:cellular_component;CC:cellular anatomical entity;MF:purine ribonucleoside triphosphate binding;MF:molecular_function;MF:binding;MF:ion binding;MF:heterocyclic compound binding;MF:nucleoside phosphate binding;MF:anion binding;MF:nucleotide binding;MF:purine nucleotide binding;MF:ATP binding;MF:hydrolase activity;MF:small molecule binding;MF:catalytic activity;MF:adenyl nucleotide binding;MF:carbohydrate derivative binding;MF:organic cyclic compound binding;MF:adenyl ribonucleotide binding;MF:purine ribonucleotide binding;MF:ribonucleotide binding;	GO:0016021;GO:0031224;GO:0005575;GO:0110165;GO:0035639;GO:0003674;GO:0005488;GO:0043167;GO:1901363;GO:1901265;GO:0043168;GO:0000166;GO:0017076;GO:0005524;GO:0016787;GO:0036094;GO:0003824;GO:0030554;GO:0097367;GO:0097159;GO:0032559;GO:0032555;GO:0032553	K01511	ENTPD5_6	map00240;ma[illegible]	Pyrimidine meta[illegible]	COG5371;COG5371	G:Carbohydrate transport and [illegible]	PF01150.20	GDA1_CD39	GDA1/CD39	CYT	3	7	51.5	High
TRINITY_DN33038_c0_g1_i1_orf1	39S ribosomal protein L46, mitochondrial [Ostrinia furnacalis]	2.174369748	1.120597289	0.000105	up	yes	1.035	0.476	0.429	0.504	0.495	1	1.016	1.09	CC:cellular_component;CC:macromolecular complex;CC:ribosome;CC:intracellular non-membrane-bounded organelle;CC:ribonucleoprotein complex;CC:mitochondrion;CC:intracellular organelle;CC:non-membrane-bounded organelle;CC:membrane-bounded organelle;CC:organelle;CC:cellular anatomical entity;CC:intracellular membrane-bounded organelle;MF:structural molecule activity;MF:structural constituent of ribosome;MF:molecular_function;	GO:0005575;GO:0032991;GO:0005840;GO:0043232;GO:1990904;GO:0005739;GO:0043229;GO:0043228;GO:0043227;GO:0043226;GO:0110165;GO:0043231;GO:0005198;GO:0003735;GO:0003674	K17427	MRPL46	------	------	ENOG4111HSF	S:Function unknown	PF11788.11	MRP-L46	39S mitochondrial	CYT	1	5	29.9	High
TRINITY_DN42646_c0_g2_i1_orf1	40S ribosomal protein S3 [Helicoverpa armigera] >XP_026740562.1 40S ribosomal protein S3 [Trichoplusia ni] >XP_026751545.1 40S ribosomal protein S3 [Galleria mellonella] >XP_047027704.1 40S ribosomal protein S3 [Helicoverpa zea] >CAH0591481.1 unnamed protein product [Chrysodeixis includens] >AIR07416.1 ribosomal protein S3 [Helicoverpa armigera] >AND95944.1 ribosomal protein S3 [Helicoverpa armigera] >AXY94820.1 ribosomal ribosomal protein S3 [Galleria mellonella] >PZC80336.1 hypothetical protein B5X24_HaOG214853 [Helicoverpa armigera]	2.058284084	1.041442116	1.12E-05	up	yes	1.01	0.4907	0.491	0.487	0.494	1	0.983	1.047	BP:cellular biosynthetic process;BP:cellular nitrogen compound metabolic process;BP:nitrogen compound metabolic process;BP:cellular macromolecule biosynthetic process;BP:organic substance biosynthetic process;BP:cellular nitrogen compound biosynthetic process;BP:cellular macromolecule metabolic process;BP:peptide biosynthetic process;BP:organic substance metabolic process;BP:amide biosynthetic process;BP:cellular process;BP:biosynthetic process;BP:macromolecule biosynthetic process;BP:biological_process;BP:metabolic process;BP:organonitrogen compound metabolic process;BP:primary metabolic process;BP:cellular amide metabolic process;BP:organonitrogen compound biosynthetic process;BP:protein metabolic process;BP:cellular metabolic process;BP:macromolecule metabolic process;BP:peptide metabolic process;BP:translation;CC:small ribosomal subunit;CC:cytoplasm;CC:cellular anatomical entity;CC:ribosomal subunit;CC:macromolecular complex;CC:ribonucleoprotein complex;CC:cellular_component;MF:molecular_function;MF:binding;MF:heterocyclic compound binding;MF:structural molecule	GO:0044249;GO:0034641;GO:0006807;GO:0034645;GO:1901576;GO:0044271;GO:0044260;GO:0043043;GO:0071704;GO:0043604;GO:0009987;GO:0009058;GO:0009059;GO:0008150;GO:0008152;GO:1901564;GO:0044238;GO:0043603;GO:1901566;GO:0019538;GO:0044237;GO:0043170;GO:0006518;GO:0006412;GO:0015935;GO:0005737;GO:0110165;GO:0044391;GO:0032991;GO:1990904;GO:0005575;GO:0003674;GO:0005488;GO:1901363;GO:0005198;GO:0003735;GO:0003676;GO:0097159;GO:0003723	K02985	RP-S3e, RPS3	map03010;ma[illegible]	Ribosome;Coron[illegible]	COG0092	J:Translation, ribosomal structu[illegible]	PF00189.23;PF0[illegible]	Ribosomal_S[illegible]	Ribosomal protein	CYT	13	56	26.8	High
TRINITY_DN5867_c0_g1_i1_orf1	NADH dehydrogenase [ubiquinone] 1 alpha subcomplex subunit 7-like [Ostrinia furnacalis]	2.033178232	1.02373669	3.77E-06	up	yes	1.005	0.4943	0.48	0.519	0.484	1	1.019	0.995	BP:cellular process;BP:cellular metabolic process;BP:electron transport chain;BP:biological_process;BP:metabolic process;BP:respiratory electron transport chain;BP:generation of precursor metabolites and energy;BP:ATP synthesis coupled electron transport;CC:organelle membrane;CC:membrane;CC:organelle inner membrane;CC:mitochondrial membrane;CC:cellular_component;CC:mitochondrial inner	GO:0009987;GO:0044237;GO:0022900;GO:0008150;GO:0008152;GO:0022904;GO:0006091;GO:0042773;GO:0031090;GO:0016020;GO:0019866;GO:0031966;GO:0005575;GO:0005743;GO:0110165	K03951	NDUFA7	map04714;ma[illegible]	Thermogenesis;[illegible]	ENOG410XW5P	C:Energy production and conv[illegible]	PF07347.15	CI-B14_5a	NAD	CYT	6	60	12.3	High
TRINITY_DN14336_c0_g3_i2_orf1	calbindin-32 isoform X1 [Ostrinia furnacalis]	2.104499274	1.073477012	4.78E-05	up	yes	1.015	0.4823	0.47	0.497	0.48	1	0.977	1.068	------	------	K23908	CALB2	------	------	COG5126	O:Posttranslational modificatio[illegible]	PF00036.35;PF1[illegible]	EF-hand_1;EF[illegible]	EF hand;EF-hand [illegible]	CYT	1	5	36.7	High
TRINITY_DN2064_c1_g1_i1_orf1	hypothetical protein evm_007509 [Chilo suppressalis] >CAB3521498.1 unnamed protein product [Chilo suppressalis]	2.149057802	1.103704287	6.73E-05	up	yes	1.015	0.4723	0.48	0.419	0.518	1	1.005	1.041	BP:metabolic process;BP:protein acetylation;BP:N-terminal protein amino acid acetylation;BP:nitrogen compound metabolic process;BP:N-terminal protein amino acid modification;BP:protein acylation;BP:organic substance metabolic process;BP:macromolecule modification;BP:protein modification process;BP:biological_process;BP:organonitrogen compound metabolic process;BP:primary metabolic process;BP:protein maturation;BP:protein metabolic process;BP:macromolecule metabolic process;CC:protein acetyltransferase complex;CC:NatA complex;CC:N-terminal protein acetyltransferase complex;CC:acetyltransferase complex;CC:catalytic complex;CC:transferase complex;CC:macromolecular complex;CC:cellular_component;MF:molecular_function;MF:peptide N-acetyltransferase activity;MF:N-acetyltransferase activity;MF:transferase activity;MF:transferase activity, transferring acyl groups;MF:transferase activity, transferring acyl groups other than amino-acyl groups;MF:N-acyltransferase activity;MF:catalytic activity;MF:acetyltransferase	GO:0008152;GO:0006473;GO:0006474;GO:0006807;GO:0031365;GO:0043543;GO:0071704;GO:0043412;GO:0036211;GO:0008150;GO:1901564;GO:0044238;GO:0051604;GO:0019538;GO:0043170;GO:0031248;GO:0031415;GO:0031414;GO:1902493;GO:1902494;GO:1990234;GO:0032991;GO:0005575;GO:0003674;GO:0034212;GO:0008080;GO:0016740;GO:0016746;GO:0016747;GO:0016410;GO:0003824;GO:0016407;GO:0004596	K20791	NAA10_11, A[illegible]	------	------	COG0456	G:Carbohydrate transport and [illegible]	PF00583.28;PF1[illegible]	Acetyltransf_1	Acetyltransferase;[illegible]	CYT	3	21	20.8	High
TRINITY_DN20582_c0_g1_i1_orf1	2-oxoglutarate dehydrogenase, mitochondrial isoform X3 [Ostrinia furnacalis] >XP_028167081.1 2-oxoglutarate dehydrogenase, mitochondrial isoform X3 [Ostrinia furnacalis]	2.243827835	1.165961985	6.40E-06	up	yes	1.027	0.4577	0.441	0.469	0.463	1	1.056	1.024	------	------	K00164	OGDH, sucA	map00020	Citrate cycl	COG0567	Z:Cytoskeleton	PF16870.8;PF02[illegible]	OxoGdeHyas[illegible]	2-oxoglutarate de[illegible]	CYT	7	32	25.6	High
TRINITY_DN24043_c0_g1_i1_orf1	uncharacterized protein LOC110377964 [Helicoverpa armigera] >XP_047026962.1 cytochrome c1-2, heme protein, mitochondrial [Helicoverpa zea] >PZC76159.1 hypothetical protein B5X24_HaOG204935 [Helicoverpa armigera]	2.153017241	1.106359873	1.34E-06	up	yes	0.999	0.464	0.461	0.473	0.458	1	1.017	0.98	CC:organelle membrane;CC:integral component of membrane;CC:membrane;CC:organelle inner membrane;CC:mitochondrial membrane;CC:cellular_component;CC:mitochondrial inner membrane;CC:intrinsic component of membrane;CC:cellular anatomical entity;MF:oxidoreductase activity;MF:cation binding;MF:tetrapyrrole binding;MF:heme binding;MF:ion binding;MF:organic cyclic compound binding;MF:catalytic activity;MF:molecular_function;MF:electron carrier activity;MF:metal ion binding;MF:heterocyclic compound	GO:0031090;GO:0016021;GO:0016020;GO:0019866;GO:0031966;GO:0005575;GO:0005743;GO:0031224;GO:0110165;GO:0016491;GO:0043169;GO:0046906;GO:0020037;GO:0043167;GO:0097159;GO:0003824;GO:0003674;GO:0009055;GO:0046872;GO:1901363;GO:0005488	K00413	CYC1, CYT1, [illegible]	map04714;ma[illegible]	Thermogenesis;[illegible]	COG2857	C:Energy production and conv[illegible]	PF02167.18	Cytochrom_C	Cytochrome C1 fa[illegible]	CYT	7	23	39.4	High

TRINITY_DN2918_c0_g1_i1_orf1	28S ribosomal protein S10, mitochondrial [Ostrinia furnacalis] >XP_028175147.1 28S ribosomal protein S10, mitochondrial [Ostrinia furnacalis]	2.000208203	1.000150179	3.87E-05	up	yes	0.9607	0.4803	0.47	0.487	0.484	1	0.964	0.918	------	------	K02946	RP-S10, MRP	map03010	Ribosome	COG0051	J:Translation, ribosomal structu	PF00338.25	Ribosomal_S1	Ribosomal protein	CYT	1	5	19.9	High
TRINITY_DN52861_c0_g1_i1_orf1	protein MAK16 homolog A [Ostrinia furnacalis]	2.4231594	1.276889311	0.0002607	up	yes	1.017	0.4197	0.481	0.453	0.325	1	1.034	1.016	CC:intracellular non-membrane-bounded organelle;CC:nucleolus;CC:cellular_component;CC:intracellular organelle;CC:non-membrane-bounded organelle;CC:organelle;CC:cellular anatomical entity;	GO:0043232;GO:0005730;GO:0005575; GO:0043229;GO:0043228;GO:0043226; GO:0110165	K14831	MAK16	------	------	COG5129	S:Function unknown	PF01778.20;PF0	Ribosomal_L2	Ribosomal L28e pr	CYT	1	2	35.4	Medium
TRINITY_DN12973_c0_g1_i1_orf1	mitochondrial-processing peptidase subunit alpha [Ostrinia furnacalis]	2.045155819	1.032210766	1.57E-05	up	yes	0.9647	0.4717	0.47	0.458	0.487	1	0.953	0.941	BP:mitochondrial protein processing;BP:nitrogen compound metabolic process;BP:organic substance metabolic process;BP:proteolysis;BP:protein processing involved in protein targeting to mitochondrion;BP:biological_process;BP:metabolic process;BP:protein processing;BP:primary metabolic process;BP:organonitrogen compound metabolic process;BP:protein maturation;BP:protein metabolic process;BP:macromolecule metabolic process;CC:membrane-enclosed lumen;CC:mitochondrial matrix;CC:cellular anatomical entity;CC:organelle lumen;CC:cellular_component;CC:intracellular organelle lumen;MF:molecular_function;MF:binding;MF:cation	GO:0034982;GO:0006807;GO:0071704; GO:0006508;GO:0006627;GO:0008150; GO:0008152;GO:0016485;GO:0044238; GO:1901564;GO:0051604;GO:0019538; GO:0043170;GO:0031974;GO:0005759; GO:0110165;GO:0043233;GO:0005575; GO:0070013;GO:0003674;GO:0005488; GO:0043169;GO:0043167;GO:0046872	K01412	PMPCA, MAS	------	------	COG0612	O:Posttranslational modificatio	PF05193.24;PF0	Peptidase_M1	Peptidase M16 ina	CYT	5	10	59.2	High
TRINITY_DN58413_c0_g1_i4_orf1	cysteine and histidine-rich protein 1 isoform X1 [Ostrinia furnacalis]	2.851741831	1.51184338	4.81E-05	up	yes	1.056	0.3703	0.398	0.342	0.371	1	1.055	1.114	BP:cellular catabolic process;BP:nitrogen compound metabolic process;BP:modification-dependent macromolecule catabolic process;BP:organic substance catabolic process;BP:cellular macromolecule catabolic process;BP:cellular macromolecule metabolic process;BP:organic substance metabolic process;BP:protein modification by small protein conjugation or removal;BP:protein modification by small protein conjugation;BP:proteolysis;BP:proteolysis involved in cellular protein catabolic process;BP:cellular process;BP:modification-dependent protein catabolic process;BP:macromolecule modification;BP:protein modification process;BP:biological_process;BP:metabolic process;BP:catabolic process;BP:macromolecule catabolic process;BP:primary metabolic process;BP:organonitrogen compound metabolic process;BP:protein metabolic process;BP:cellular metabolic process;BP:macromolecule metabolic process;BP:protein ubiquitination;BP:ubiquitin-dependent protein catabolic process;CC:cytoplasm;CC:cellular anatomical	GO:0044248;GO:0006807;GO:0043632; GO:1901575;GO:0044265;GO:0044260; GO:0071704;GO:0070647;GO:0032446; GO:0006508;GO:0051603;GO:0009987; GO:0019941;GO:0043412;GO:0036211; GO:0008150;GO:0008152;GO:0009056; GO:0009057;GO:0044238;GO:1901564; GO:0019538;GO:0044237;GO:0043170; GO:0016567;GO:0006511;GO:0005737; GO:0110165;GO:0005575;GO:0003674; GO:0005488;GO:0043169;GO:0043167; GO:0046872	K04506	SIAH1	map04310;ma	Wnt signaling pa	ENOG410XVP0	O:Posttranslational modificatio	PF03145.19	Sina	Seven in absentia	CYT	2	7	34.8	High
TRINITY_DN4408_c6_g1_i1_orf1	polyprotein, partial [Bemisia tabaci]	2.609137056	1.38357273	1.38E-05	up	yes	1.028	0.394	0.429	0.368	0.385	1	1.058	1.025	BP:viral RNA genome replication;BP:RNA biosynthetic process;BP:nucleic acid-protein covalent cross-linking;BP:RNA-protein covalent cross-linking;BP:nucleic acid metabolic process;BP:cellular biosynthetic process;BP:cellular nitrogen compound metabolic process;BP:nitrogen compound metabolic process;BP:organic cyclic compound biosynthetic process;BP:organic cyclic compound metabolic process;BP:nucleobase-containing compound metabolic process;BP:cellular macromolecule metabolic process;BP:organic substance metabolic process;BP:nucleic acid-templated transcription;BP:proteolysis;BP:heterocycle biosynthetic process;BP:organic substance biosynthetic process;BP:viral genome replication;BP:cellular aromatic compound metabolic process;BP:cellular process;BP:macromolecule modification;BP:protein modification process;BP:biological_process;BP:metabolic process;BP:aromatic compound biosynthetic process;BP:nucleobase-containing compound biosynthetic process;BP:cellular nitrogen compound biosynthetic process;BP:heterocycle metabolic process;BP:RNA metabolic process;BP:primary metabolic process;BP:transcription, DNA-templated;BP:organonitrogen compound metabolic process;BP:protein metabolic process;BP:biosynthetic process;BP:macromolecule biosynthetic process;BP:macromolecule metabolic process;BP:viral process;BP:DNA metabolic process;BP:cellular metabolic process;BP:DNA biosynthetic process;MF:RNA polymerase activity;MF:molecular_function;MF:binding;MF:nucleic acid binding;MF:RNA-dependent ATPase activity;MF:peptidase activity;MF:cysteine-type peptidase activity;MF:heterocyclic	GO:0039694;GO:0032774;GO:0018143; GO:0018144;GO:0090304;GO:0044249; GO:0034641;GO:0006807;GO:1901362; GO:1901360;GO:0006139;GO:0044260; GO:0071704;GO:0097659;GO:0006508; GO:0018130;GO:1901576;GO:0019079; GO:0006725;GO:0009987;GO:0043412; GO:0036211;GO:0008150;GO:0008152; GO:0019438;GO:0034654;GO:0044271; GO:0046483;GO:0016070;GO:0044238; GO:0006351;GO:1901564;GO:0019538; GO:0009058;GO:0009059;GO:0043170; GO:0016032;GO:0006259;GO:0044237; GO:0071897;GO:0034062;GO:0003674; GO:0005488;GO:0003676;GO:0008186; GO:0008233;GO:0008234;GO:1901363; GO:0016740;GO:0016787;GO:0140098; GO:0140096;GO:0004386;GO:0003824; GO:0016779;GO:0097747;GO:0016772; GO:0003724;GO:0004197;GO:0003723; GO:0097159;GO:0003968;GO:0004175	------	------	------	------	------	------	PF00680.23	RdRP_1	Viral RNA-depend	CYT	6	10	76.2	High
TRINITY_DN1079_c0_g1_i4_orf1	CD109 antigen-like [Ostrinia furnacalis]	2.545773765	1.348104217	5.69E-07	up	yes	1.015	0.3987	0.388	0.407	0.401	1	1.014	1.032	CC:cellular_component;CC:cellular anatomical entity;CC:extracellular space;MF:endopeptidase inhibitor activity;MF:endopeptidase regulator activity;MF:peptidase regulator activity;MF:molecular_function;MF:peptidase inhibitor activity;MF:enzyme inhibitor activity;MF:molecular function regulator;MF:enzyme regulator activity;	GO:0005575;GO:0110165;GO:0005615; GO:0004866;GO:0061135;GO:0061134; GO:0003674;GO:0030414;GO:0004857; GO:0098772;GO:0030234	K06530	CD109	------	------	ENOG410XRED;ENOG410ZKZQ;COG2373	S:Function unknown;	PF07678.17;PF0	TED_complen	A-macroglobulin T	CYT	3	2	146.3	High
TRINITY_DN15607_c0_g1_i6_orf1	protein artichoke-like [Ostrinia furnacalis]	2.378209743	1.249875957	3.16E-06	up	yes	0.991	0.4167	0.402	0.418	0.43	1	1.008	0.965	CC:cellular_component;CC:integral component of membrane;CC:cellular anatomical entity;CC:intrinsic component of membrane;	GO:0005575;GO:0016021;GO:0110165; GO:0031224	------	------	------	------	COG4886	I:Lipid transport and metabolis	PF13855.9;PF12	LRR_8;LRR_4;L	Leucine rich repea	CYT	5	6	109	High
TRINITY_DN12497_c0_g1_i1_orf1	probable N-acetyltransferase san [Ostrinia furnacalis]	2.469775475	1.304379894	1.76E-07	up	yes	1.001	0.4053	0.418	0.405	0.393	1	1.007	0.997	MF:transferase activity;MF:catalytic activity;MF:acetyltransferase activity;MF:transferase activity, transferring acyl groups;MF:transferase activity, transferring acyl groups other than amino-acyl groups;MF:molecular_function;	GO:0016740;GO:0003824;GO:0016407; GO:0016746;GO:0016747;GO:0003674	K20793	NAA50, NAT5	------	------	COG0456	S:Function unknown	PF00583.28;PF1	Acetyltransf_1	Acetyltransferase;A	CYT	5	36	20.2	High
TRINITY_DN25975_c0_g3_i2_orf1	V-type proton ATPase subunit D isoform X2 [Ostrinia furnacalis]	2.510617037	1.328041981	3.25E-06	up	yes	1.005	0.4003	0.376	0.417	0.408	1	0.99	1.026	MF:inorganic cation transmembrane transporter activity;MF:ATPase activity, coupled to transmembrane movement of substances;MF:active ion transmembrane transporter activity;MF:ATPase coupled ion transmembrane transporter activity;MF:inorganic solute uptake transmembrane transporter activity;MF:transporter activity;MF:cation transmembrane transporter activity;MF:hydrogen-translocating pyrophosphatase activity;MF:cation-transporting ATPase activity;MF:ion transmembrane transporter activity;MF:primary active transmembrane transporter activity;MF:transmembrane transporter activity;MF:molecular_function;MF:active transmembrane transporter activity;MF:proton-transporting ATPase activity, rotational mechanism;MF:hydrogen ion transmembrane transporter activity;MF:ATPase activity, coupled to transmembrane movement of ions, rotational mechanism;	GO:0022890;GO:0042626;GO:0022853; GO:0042625;GO:0015318;GO:0005215; GO:0008324;GO:0009678;GO:0019829; GO:0015075;GO:0015399;GO:0022857; GO:0003674;GO:0022804;GO:0046961; GO:0015078;GO:0044769	K02149	ATPeV1D, AT	map05323;ma	Rheumatoid arth	COG1394	C:Energy production and conv	PF01813.20	ATP-synt_D	ATP synthase subu	CYT	7	20	27.4	High
TRINITY_DN12323_c0_g2_i2_orf1	polyribonucleotide nucleotidyltransferase 1, mitochondrial [Ostrinia furnacalis]	2.063237943	1.04491021	0.0005281	up	yes	1.031	0.4997	0.476	0.566	0.457	1	1.11	0.982	BP:negative regulation of metabolic process;BP:regulation of metabolic process;BP:nucleic acid metabolic process;BP:cellular nitrogen compound metabolic process;BP:nitrogen compound metabolic process;BP:regulation of biological process;BP:organic cyclic compound metabolic process;BP:organic cyclic compound catabolic process;BP:nucleobase-containing compound metabolic process;BP:organic substance catabolic process;BP:cellular macromolecule catabolic process;BP:negative regulation of macromolecule metabolic process;BP:cellular macromolecule metabolic process;BP:organic substance metabolic process;BP:biological regulation;BP:RNA catabolic process;BP:mRNA catabolic process;BP:regulation of gene expression;BP:primary metabolic process;BP:regulation of macromolecule metabolic process;BP:cellular process;BP:cellular aromatic compound metabolic process;BP:heterocycle catabolic process;BP:biological_process;BP:metabolic process;BP:negative regulation of biological process;BP:nucleobase-containing compound catabolic process;BP:catabolic process;BP:macromolecule catabolic process;BP:cellular catabolic process;BP:heterocycle metabolic process;BP:RNA metabolic process;BP:mRNA metabolic process;BP:cellular nitrogen compound catabolic process;BP:negative regulation of gene expression;BP:cellular metabolic process;BP:macromolecule metabolic process;BP:aromatic compound catabolic process;BP:RNA processing;MF:molecular_function;MF:binding;MF:nucleic acid binding;MF:polyribonucleotide nucleotidyltransferase	GO:0009892;GO:0019222;GO:0090304; GO:0034641;GO:0006807;GO:0050789; GO:1901360;GO:1901361;GO:0006139; GO:1901575;GO:0044265;GO:0010605; GO:0044260;GO:0071704;GO:0065007; GO:0006401;GO:0006402;GO:0010468; GO:0044238;GO:0060255;GO:0009987; GO:0006725;GO:0046700;GO:0008150; GO:0008152;GO:0048519;GO:0034655; GO:0009056;GO:0009057;GO:0044248; GO:0046483;GO:0016070;GO:0016071; GO:0044270;GO:0010629;GO:0044237; GO:0043170;GO:0019439;GO:0006396; GO:0003674;GO:0005488;GO:0003676; GO:0004654;GO:1901363;GO:0016740; GO:0003824;GO:0016779;GO:0016772; GO:0003723;GO:0097159	K00962	pnp, PNPT1	map03018	RNA degradation	COG1185	J:Translation, ribosomal structu	PF01138.24;PF0	RNase_PH;RN	3' exoribonuclease	CYT	1	1	82.8	Medium
TRINITY_DN76036_c0_g1_i1_orf1	cytochrome c oxidase subunit 6A1, mitochondrial-like [Ostrinia furnacalis]	2.205412316	1.141048402	3.28E-05	up	yes	1.035	0.4693	0.448	0.505	0.455	1	1.034	1.072	BP:aerobic respiration;BP:generation of precursor metabolites and energy;BP:oxidative phosphorylation;BP:cellular process;BP:cellular respiration;BP:metabolic process;BP:cellular metabolic process;BP:energy derivation by oxidation of organic compounds;BP:biological_process;CC:intrinsic component of membrane;CC:cellular_component;CC:integral component of membrane;CC:respiratory chain complex;CC:cytochrome complex;CC:inner mitochondrial membrane protein complex;CC:mitochondrial respiratory chain complex IV;CC:catalytic complex;CC:macromolecular complex;CC:respiratory chain complex IV;CC:membrane protein complex;CC:cellular anatomical entity;CC:mitochondrial protein complex;MF:oxidoreduction-driven active transmembrane transporter activity;MF:molecular_function;MF:oxidoreductase activity;MF:inorganic solute uptake transmembrane transporter activity;MF:primary active transmembrane transporter activity;MF:active transmembrane transporter activity;MF:cytochrome-c oxidase activity;MF:transporter activity;MF:cation transmembrane transporter activity;MF:catalytic activity;MF:electron carrier activity;MF:inorganic cation transmembrane transporter activity;MF:ion transmembrane transporter activity;MF:transmembrane transporter activity;MF:active ion transmembrane transporter activity;MF:oxidoreductase activity, acting on a heme group of donors;MF:hydrogen ion	GO:0009060;GO:0006091;GO:0006119; GO:0009987;GO:0045333;GO:0008152; GO:0044237;GO:0015980;GO:0008150; GO:0031224;GO:0005575;GO:0016021; GO:0098803;GO:0070069;GO:0098800; GO:0005751;GO:1902494;GO:0032991; GO:0045277;GO:0098796;GO:0110165; GO:0098798;GO:0015453;GO:0003674; GO:0016491;GO:0015318;GO:0015399; GO:0022804;GO:0004129;GO:0005215; GO:0008324;GO:0003824;GO:0009055; GO:0022890;GO:0015075;GO:0022857; GO:0022853;GO:0016675;GO:0015078	K02266	COX6A	map04714;ma	Thermogenesis;C	ENOG411253X	O:Posttranslational modificatio	PF02046.18	COX6A	Cytochrome c oxid	CYT	2	24	12	High
TRINITY_DN4724_c0_g1_i4_orf1	paramyosin, long form isoform X1 [Manduca sexta] >KAG6443143.1 hypothetical protein O3G_MSEX002737 [Manduca sexta]	2.108970375	1.07653883	3.12E-06	up	yes	1.018	0.4827	0.492	0.465	0.491	1	1.015	1.039	CC:cellular_component;CC:myosin complex;CC:macromolecular complex;	GO:0005575;GO:0016459;GO:0032991	------	------	------	------	COG5022	S:Function unknown	PF01576.22	Myosin_tail_1	Myosin tail	CYT	59	59	102.5	High
TRINITY_DN1375_c0_g1_i5_orf1	glutaminase [Chilo suppressalis] >CAB3528726.1 unnamed protein product [Chilo suppressalis] >CAH0405319.1 unnamed protein product [Chilo suppressalis]	2.00141129	1.001017672	0.001303	up	yes	0.9927	0.496	0.591	0.498	0.399	1	0.942	1.036	BP:carboxylic acid metabolic process;BP:nitrogen compound metabolic process;BP:small molecule metabolic process;BP:glutamine family amino acid metabolic process;BP:cellular amino acid metabolic process;BP:glutamine metabolic process;BP:organic substance metabolic process;BP:alpha-amino acid metabolic process;BP:cellular process;BP:biological_process;BP:metabolic process;BP:oxoacid metabolic process;BP:primary metabolic process;BP:organonitrogen compound metabolic process;BP:organic acid metabolic process;BP:cellular metabolic process;MF:molecular_function;MF:hydrolase activity;MF:catalytic activity;MF:glutaminase activity;MF:hydrolase activity, acting on carbon-	GO:0019752;GO:0006807;GO:0044281; GO:0009064;GO:0006520;GO:0006541; GO:0071704;GO:1901605;GO:0009987; GO:0008150;GO:0008152;GO:0043436; GO:0044238;GO:1901564;GO:0006082; GO:0044237;GO:0003674;GO:0016787; GO:0003824;GO:0004359;GO:0016810; GO:0016811	K01425	glsA, GLS	map05206;ma	MicroRNAs in ca	COG2066	E:Amino acid transport and me	PF04960.18;PF1	Glutaminase;I	Glutaminase;EF-ha	CYT	2	3	71.8	High
TRINITY_DN2691_c0_g1_i1_orf1	WD repeat-containing protein 18 [Ostrinia furnacalis]	2.106471816	1.074828613	5.09E-05	up	yes	1.009	0.479	0.525	0.449	0.463	1	0.985	1.041	MF:hydrolase activity;MF:molecular_function;MF:catalytic activity;	GO:0016787;GO:0003674;GO:0003824	K14829	IPI3	------	------	COG2319;ENOG410XRMS	S:Function unknown;	PF00400.35;PF2	WD40;NBCH_	WD domain, G-be	CYT	1	4	49.4	High

TRINITY_DN4944_c0_g1_i2_orf1	bifunctional glutamate/proline--tRNA ligase [Ostrinia furnacalis]	2.022727273	1.016301812	3.77E-06	up	yes	0.979	0.484	0.483	0.5	0.469	1	0.965	0.972	BP:prolyl-tRNA aminoacylation;BP:carboxylic acid metabolic process;BP:nucleic acid metabolic process;BP:cellular nitrogen compound metabolic process;BP:nitrogen compound metabolic process;BP:small molecule metabolic process;BP:ncRNA metabolic process;BP:organic cyclic compound metabolic process;BP:nucleobase-containing compound metabolic process;BP:cellular amino acid metabolic process;BP:organic substance metabolic process;BP:cellular process;BP:cellular aromatic compound metabolic process;BP:biological_process;BP:metabolic process;BP:oxoacid metabolic process;BP:heterocycle metabolic process;BP:RNA metabolic process;BP:primary metabolic process;BP:organonitrogen compound metabolic process;BP:organic acid metabolic process;BP:cellular metabolic process;BP:macromolecule metabolic process;BP:tRNA metabolic process;BP:amino acid activation;BP:tRNA aminoacylation;BP:tRNA aminoacylation for protein translation;CC:cytoplasm;CC:cellular anatomical entity;CC:cellular_component;MF:purine ribonucleoside triphosphate binding;MF:molecular_function;MF:ion binding;MF:heterocyclic compound binding;MF:aminoacyl-tRNA ligase activity;MF:nucleotide binding;MF:nucleoside phosphate binding;MF:purine nucleotide binding;MF:anion binding;MF:catalytic activity, acting on RNA;MF:small molecule binding;MF:catalytic activity;MF:ligase activity;MF:ligase activity, forming carbon-oxygen bonds;MF:proline-tRNA ligase activity;MF:adenyl nucleotide binding;MF:carbohydrate derivative binding;MF:organic cyclic compound	GO:0006433;GO:0019752;GO:0090304;GO:0034641;GO:0006807;GO:0044281;GO:0034660;GO:1901360;GO:0006139;GO:0006520;GO:0071704;GO:0009987;GO:0006725;GO:0008150;GO:0008152;GO:0043436;GO:0046483;GO:0016070;GO:0044238;GO:1901564;GO:0006082;GO:0044237;GO:0043170;GO:0006399;GO:0043038;GO:0043039;GO:0006418;GO:0005737;GO:0110165;GO:0005575;GO:0035639;GO:0003674;GO:0043167;GO:1901363;GO:0004812;GO:0000166;GO:1901265;GO:0017076;GO:0043168;GO:0140098;GO:0036094;GO:0003824;GO:0016874;GO:0016875;GO:0004827;GO:0030554;GO:0097367;GO:0097159;GO:0032559;GO:0005524;GO:0032555;GO:0140101;GO:0032553;GO:0005488	K14163	EPRS	map00860;ma[illegible]	Porphyrin metab[illegible]	COG0442	J:Translation, ribosomal structu[illegible]	PF09180.14;PF0[illegible]	ProRS-C_1;tR[illegible]	Prolyl-tRNA synth[illegible]	CYT	6	28	51	High
TRINITY_DN10458_c0_g1_i1_orf1	V-type proton ATPase 21 kDa proteolipid subunit [Ostrinia furnacalis]	2.604501608	1.381007328	0.0001848	up	yes	1.053	0.4043	0.347	0.437	0.429	1	1.029	1.13	CC:intrinsic component of membrane;CC:cellular_component;CC:proton-transporting V-type ATPase, V0 domain;CC:integral component of membrane;CC:cellular anatomical entity;CC:macromolecular complex;CC:membrane protein complex;CC:proton-transporting two-sector ATPase complex, proton-transporting domain;MF:molecular_function;MF:ATPase activity, coupled to transmembrane movement of substances;MF:inorganic solute uptake transmembrane transporter activity;MF:hydrogen-translocating pyrophosphatase activity;MF:primary active transmembrane transporter activity;MF:ATPase coupled ion transmembrane transporter activity;MF:transporter activity;MF:cation transmembrane transporter activity;MF:proton-transporting ATPase activity, rotational mechanism;MF:ATPase activity, coupled to transmembrane movement of ions, rotational mechanism;MF:inorganic cation transmembrane transporter activity;MF:cation-transporting ATPase activity;MF:ion transmembrane transporter activity;MF:transmembrane transporter activity;MF:active transmembrane transporter activity;MF:active ion transmembrane transporter activity;MF:hydrogen ion	GO:0031224;GO:0005575;GO:0033179;GO:0016021;GO:0110165;GO:0032991;GO:0098796;GO:0033177;GO:0003674;GO:0042626;GO:0015318;GO:0009678;GO:0015399;GO:0042625;GO:0005215;GO:0008324;GO:0046961;GO:0044769;GO:0022890;GO:0019829;GO:0015075;GO:0022857;GO:0022804;GO:0022853;GO:0015078	K03661	ATPeV0B, AT[illegible]	map05152;ma[illegible]	Tuberculosis;Hu[illegible]	COG0636	C:Energy production and conv[illegible]	PF00137.24	ATP-synt_C	ATP synthase sub[illegible]	CYT	1	5	21.6	High
TRINITY_DN143603_c0_g1_i1_orf1	hypothetical protein KR044_005587 [Drosophila immigrans]	3.121992641	1.642467137	0.0002836	up	yes	1.103	0.3533	0.389	0.362	0.309	1	1.108	1.202	BP:response to extracellular stimulus;BP:carbon utilization;BP:organic substance metabolic process;BP:biological_process;BP:metabolic process;BP:primary metabolic process;BP:carbohydrate metabolic process;BP:response to external stimulus;BP:response to stimulus;BP:response to nutrient levels;CC:membrane;CC:cellular anatomical entity;CC:cellular_component;MF:hydrolase activity;MF:carbohydrate binding;MF:lyase activity;MF:molecular_function;MF:binding;MF:ion binding;MF:heterocyclic compound binding;MF:nucleoside phosphate binding;MF:oxidoreductase activity;MF:anion binding;MF:cation binding;MF:flavin adenine dinucleotide binding;MF:transition metal ion binding;MF:oxidoreductase activity, acting on the CH-CH group of donors;MF:nucleotide binding;MF:alpha-glucosidase activity;MF:carbon-oxygen lyase activity;MF:tRNA dihydrouridine synthase activity;MF:metal ion binding;MF:glucosidase activity;MF:catalytic activity, acting on RNA;MF:hydrolase activity, acting on glycosyl bonds;MF:small molecule binding;MF:catalytic activity;MF:carbonate dehydratase activity;MF:organic cyclic compound	GO:0009991;GO:0015976;GO:0071704;GO:0008150;GO:0008152;GO:0044238;GO:0005975;GO:0009605;GO:0050896;GO:0031667;GO:0016020;GO:0110165;GO:0005575;GO:0016787;GO:0030246;GO:0016829;GO:0003674;GO:0005488;GO:0043167;GO:1901363;GO:1901265;GO:0016491;GO:0043168;GO:0043169;GO:0050660;GO:0046914;GO:0016627;GO:0000166;GO:0090599;GO:0016835;GO:0017150;GO:0046872;GO:0015926;GO:0140098;GO:0016798;GO:0036094;GO:0003824;GO:0004089;GO:0097159;GO:0004553;GO:0140101;GO:0016836;GO:0008270	K01673	cynT, can	map00910	Nitrogen metab[illegible]	COG0288	P:Inorganic ion transport and [illegible]	PF00484.22	Pro_CA	Carbonic anhydras[illegible]	CYT	1	5	26.9	High
TRINITY_DN16122_c0_g1_i4_orf1	cytochrome P450 6k1-like [Ostrinia furnacalis]	2.124726477	1.08727713	1.47E-05	up	yes	0.971	0.457	0.458	0.44	0.473	1	0.938	0.975	CC:integral component of membrane;CC:cellular_component;CC:cellular anatomical entity;CC:intrinsic component of membrane;MF:cation binding;MF:monooxygenase activity;MF:heme binding;MF:transition metal ion binding;MF:tetrapyrrole binding;MF:organic cyclic compound binding;MF:oxidoreductase activity, acting on paired donors, with incorporation or reduction of molecular oxygen;MF:ion binding;MF:catalytic activity;MF:molecular_function;MF:binding;MF:iron ion binding;MF:metal ion binding;MF:heterocyclic compound	GO:0016021;GO:0005575;GO:0110165;GO:0031224;GO:0043169;GO:0004497;GO:0020037;GO:0046914;GO:0046906;GO:0097159;GO:0016705;GO:0043167;GO:0003824;GO:0003674;GO:0005488;GO:0005506;GO:0046872;GO:1901363;GO:0016491	K14999	CYP6	------	------	COG2124	S:Function unknown	PF00067.25	p450	Cytochrome P450	CYT	5	12	56.9	High
TRINITY_DN7580_c0_g1_i1_orf1	cytochrome P450 monooxygenase CYP6AB141 [Ostrinia furnacalis]	2.281474612	1.189966599	1.97E-05	up	yes	0.984	0.4313	0.407	0.432	0.455	1	1.006	0.946	MF:cation binding;MF:monooxygenase activity;MF:heme binding;MF:transition metal ion binding;MF:tetrapyrrole binding;MF:organic cyclic compound binding;MF:oxidoreductase activity, acting on paired donors, with incorporation or reduction of molecular oxygen;MF:ion binding;MF:catalytic activity;MF:molecular_function;MF:binding;MF:iron ion binding;MF:metal ion binding;MF:heterocyclic compound	GO:0043169;GO:0004497;GO:0020037;GO:0046914;GO:0046906;GO:0097159;GO:0016705;GO:0043167;GO:0003824;GO:0003674;GO:0005488;GO:0005506;GO:0046872;GO:1901363;GO:0016491	K14999	CYP6	------	------	COG2124	S:Function unknown	PF00067.25	p450	Cytochrome P450	CYT	4	14	52.1	High
TRINITY_DN83005_c0_g1_i1_orf1	ATP synthase subunit O, mitochondrial [Danaus plexippus plexippus] >OWR53927.1 H+ transporting ATP synthase O subunit [Danaus plexippus plexippus]	2.434803802	1.283805524	1.34E-07	up	yes	0.999	0.4103	0.416	0.412	0.403	1	1.009	0.988	CC:cellular_component;CC:organelle membrane;CC:membrane;CC:cellular anatomical entity;CC:mitochondrial membrane;CC:organelle inner membrane;CC:mitochondrial inner membrane;MF:cation channel activity;MF:molecular_function;MF:proton-transporting ATP synthase activity, rotational mechanism;MF:inorganic solute uptake transmembrane transporter activity;MF:hydrogen ion channel activity;MF:passive transmembrane transporter activity;MF:transporter activity;MF:ion channel activity;MF:cation transmembrane transporter activity;MF:catalytic activity;MF:ligase activity;MF:inorganic cation transmembrane transporter activity;MF:ion transmembrane transporter activity;MF:transmembrane transporter activity;MF:channel activity;MF:hydrogen ion transmembrane transporter activity;	GO:0005575;GO:0031090;GO:0016020;GO:0110165;GO:0031966;GO:0019866;GO:0005743;GO:0005261;GO:0003674;GO:0046933;GO:0015318;GO:0015252;GO:0022803;GO:0005215;GO:0005216;GO:0008324;GO:0003824;GO:0016874;GO:0022890;GO:0015075;GO:0022857;GO:0015267;GO:0015078	K02137	ATPeF0O, AT[illegible]	map04714;ma[illegible]	Thermogenesis;[illegible]	COG0712;COG0442	C:Energy production and conv[illegible]	PF00213.21	OSCP	ATP synthase delt[illegible]	CYT	13	55	22.7	High
TRINITY_DN16830_c0_g1_i5_orf1	adrenodoxin [Ostrinia furnacalis]	2.664396123	1.413808588	9.93E-05	up	yes	1.017	0.3817	0.396	0.4	0.349	1	1.089	0.963	BP:cellular process;BP:cellular metabolic process;BP:electron transport chain;BP:metabolic process;BP:biological_process;BP:generation of precursor metabolites and energy;MF:metal cluster binding;MF:iron-sulfur cluster binding;MF:2 iron, 2 sulfur cluster binding;MF:molecular_function;MF:binding;	GO:0009987;GO:0044237;GO:0022900;GO:0008152;GO:0008150;GO:0006091;GO:0051540;GO:0051536;GO:0051537;GO:0003674;GO:0005488	K22070	FDX1, ADX	------	------	COG0633	C:Energy production and conv[illegible]	PF00111.30	Fer2	2Fe-2S iron-sulfu[illegible]	CYT	1	6	17.6	High
TRINITY_DN31232_c1_g1_i9_orf1	PREDICTED: elongation factor 1-alpha 1, partial [Haliaeetus albicilla]	2.314794932	1.210884391	0.00136	up	yes	1.078	0.4657	0.432	0.392	0.573	1	1.182	1.052	CC:cytoplasm;CC:cellular anatomical entity;CC:cellular_component;MF:purine ribonucleoside triphosphate binding;MF:molecular_function;MF:binding;MF:nucleic acid binding;MF:ion binding;MF:heterocyclic compound binding;MF:nucleoside phosphate binding;MF:guanyl nucleotide binding;MF:nucleotide binding;MF:guanyl ribonucleotide binding;MF:purine nucleotide binding;MF:GTP binding;MF:hydrolase activity;MF:translation factor activity, RNA binding;MF:GTPase activity;MF:nucleoside-triphosphatase activity;MF:small molecule binding;MF:catalytic activity;MF:translation regulator activity;MF:hydrolase activity, acting on acid anhydrides, in phosphorus-containing anhydrides;MF:translation regulator activity, nucleic acid binding;MF:carbohydrate derivative binding;MF:organic cyclic compound binding;MF:hydrolase activity, acting on acid anhydrides;MF:anion binding;MF:pyrophosphatase	GO:0005737;GO:0110165;GO:0005575;GO:0035639;GO:0003674;GO:0005488;GO:0003676;GO:0043167;GO:1901363;GO:1901265;GO:0019001;GO:0000166;GO:0032561;GO:0017076;GO:0005525;GO:0016787;GO:0008135;GO:0003924;GO:0017111;GO:0036094;GO:0003824;GO:0045182;GO:0016818;GO:0090079;GO:0097367;GO:0097159;GO:0016817;GO:0043168;GO:0016462;GO:0032555;GO:0003746;GO:0032553	K03231	EEF1A	map05140;ma[illegible]	Leishmaniasis;Le[illegible]	COG5256	U:Intracellular trafficking, secre[illegible]	PF00009.30;PF0[illegible]	GTP_EFTU;GT[illegible]	Elongation factor [illegible]	CYT	1	14	45.4	High
TRINITY_DN1212_c0_g1_i8_orf1	extensin isoform X5 [Ostrinia furnacalis] >XP_028175473.1 extensin isoform X5 [Ostrinia furnacalis] >XP_028175474.1 extensin isoform X5 [Ostrinia furnacalis]	2.156619018	1.108771337	2.26E-05	up	yes	1.041	0.4827	0.477	0.462	0.509	1	1.058	1.064	------	------	------	------	------	------	ENOG4111WKY;ENOG4110SQB	S:Function unknown;	PF15936.8	DUF4749	Domain of unknow[illegible]	CYT	7	26	44.1	High
TRINITY_DN100_c0_g1_i9_orf1	uncharacterized protein LOC114353052 [Ostrinia furnacalis]	2.100980597	1.071062838	2.94E-07	up	yes	1.007	0.4793	0.474	0.475	0.489	1	1.001	1.019	------	------	------	------	------	------	ENOG4111QMX	S:Function unknown	------	------	------	CYT	1	9	17.7	High
TRINITY_DN29291_c0_g1_i1_orf1	carboxylesterase [Ostrinia furnacalis]	2.187835805	1.129504469	3.88E-05	up	yes	1.018	0.4653	0.471	0.425	0.5	1	1.001	1.053	MF:hydrolase activity;MF:molecular_function;MF:hydrolase activity, acting on ester bonds;MF:carboxylic ester hydrolase activity;MF:catalytic activity;	GO:0016787;GO:0003674;GO:0016788;GO:0052689;GO:0003824	K03927	CES2	map00983	Drug metabolism[illegible]	COG2272	I:Lipid transport and metabolis[illegible]	PF00135.31	COesterase	Carboxylesterase f[illegible]	CYT	1	8	26.3	High
TRINITY_DN1422_c0_g1_i4_orf1	unnamed protein product [Chilo suppressalis]	2.190064795	1.130973554	1.24E-06	up	yes	1.014	0.463	0.47	0.473	0.446	1	1.028	1.014	BP:generation of precursor metabolites and energy;BP:electron transport chain;BP:cellular process;BP:respiratory electron transport chain;BP:biological_process;BP:metabolic process;BP:ATP synthesis coupled electron transport;BP:cellular metabolic process;CC:membrane;CC:cellular anatomical entity;CC:cellular_component;MF:molecular_function;MF:binding;MF:NA;MF:NADH dehydrogenase activity;MF:oxidoreductase activity;MF:metal cluster binding;MF:primary active transmembrane transporter activity;MF:NADH dehydrogenas;MF:transporter activity;MF:active transmembrane transporter activity;MF:oxidoreduction-driven active transmembrane transporter activity;MF:NADH dehydrogenas;MF:catalytic activity;MF:electron carrier activity;MF:4 iron, 4 sulfur cluster binding;MF:iron-sulfur cluster binding;MF:oxidoreductase activity, acting on NA;MF:transmembrane transporter activity;MF:oxidoreductase	GO:0006091;GO:0022900;GO:0009987;GO:0022904;GO:0008150;GO:0008152;GO:0042773;GO:0044237;GO:0016020;GO:0110165;GO:0005575;GO:0003674;GO:0005488;GO:0003955;GO:0003954;GO:0016491;GO:0051540;GO:0015399;GO:0050136;GO:0005215;GO:0022804;GO:0015453;GO:0008137;GO:0003824;GO:0009055;GO:0051539;GO:0051536;GO:0016655;GO:0022857;GO:0016651	K03934	NDUFS1	map04714;ma[illegible]	Thermogenesis;[illegible]	COG1034	C:Energy production and conv[illegible]	PF00384.25;PF1[illegible]	Molybdopteri[illegible]	Molybdopterin oxi[illegible]	CYT	23	40	79.7	High
TRINITY_DN26130_c0_g1_i1_orf1	membrane alanyl aminopeptidase-like [Ostrinia furnacalis]	2.226605304	1.154845843	6.73E-06	up	yes	1.016	0.4563	0.46	0.43	0.479	1	1.038	1.01	BP:nitrogen compound metabolic process;BP:organic substance metabolic process;BP:proteolysis;BP:biological_process;BP:metabolic process;BP:primary metabolic process;BP:organonitrogen compound metabolic process;BP:protein metabolic process;BP:macromolecule metabolic process;CC:integral component of membrane;CC:intrinsic component of membrane;CC:cellular anatomical entity;CC:cellular_component;MF:aminopeptidase activity;MF:exopeptidase activity;MF:molecular_function;MF:binding;MF:peptidase activity;MF:metallopeptidase activity;MF:cation binding;MF:transition metal ion binding;MF:ion binding;MF:metal ion binding;MF:hydrolase activity;MF:catalytic	GO:0006807;GO:0071704;GO:0006508;GO:0008150;GO:0008152;GO:0044238;GO:1901564;GO:0019538;GO:0043170;GO:0016021;GO:0031224;GO:0110165;GO:0005575;GO:0004177;GO:0008238;GO:0003674;GO:0005488;GO:0008233;GO:0008237;GO:0043169;GO:0046914;GO:0043167;GO:0046872;GO:0016787;GO:0140096;GO:0003824;GO:0008270	K11140	ANPEP, CD13	map04640;ma[illegible]	Hematopoietic c[illegible]	COG0308	E:Amino acid transport and me[illegible]	PF01433.23;PF1[illegible]	Peptidase_M1	Peptidase family [illegible]	CYT	9	13	104.2	High

TRINITY_DN4451_c0_g2_i4_orf1	uncharacterized protein LOC114361986 isoform X1 [Ostrinia furnacalis] >XP_028173022.1 uncharacterized protein LOC114361986 isoform X2 [Ostrinia furnacalis]	2.141327623	1.098505545	1.87E-06	up	yes	1	0.467	0.463	0.461	0.477	1	1.02	0.98	BP:carboxylic acid metabolic process;BP:organonitrogen compound metabolic process;BP:cellular amino acid catabolic process;BP:nitrogen compound metabolic process;BP:small molecule catabolic process;BP:cellular amino acid metabolic process;BP:organic substance metabolic process;BP:alpha-amino acid metabolic process;BP:alpha-amino acid catabolic process;BP:cellular process;BP:organic substance catabolic process;BP:biological_process;BP:metabolic process;BP:oxoacid metabolic process;BP:catabolic process;BP:cellular catabolic process;BP:organonitrogen compound catabolic process;BP:primary metabolic process;BP:valine metabolic process;BP:valine catabolic process;BP:organic acid metabolic process;BP:organic acid catabolic process;BP:cellular metabolic process;BP:carboxylic acid catabolic process;BP:branched-chain amino acid metabolic process;BP:branched-chain amino acid catabolic process;BP:small molecule metabolic process;MF:NAD binding;MF:molecular_function;MF:binding;MF:heterocyclic compound binding;MF:nucleoside phosphate binding;MF:oxidoreductase activity;MF:NADP binding;MF:3-hydroxyisobutyrate dehydrogenase activity;MF:nucleotide binding;MF:small molecule binding;MF:catalytic activity;MF:oxidoreductase activity, acting on CH-OH group of donors;MF:oxidoreductase activity, acting on the CH-OH group of donors, NAD or NADP as acceptor;MF:organic cyclic compound binding;	GO:0019752;GO:1901564;GO:0009063;GO:0006807;GO:0044282;GO:0006520;GO:0071704;GO:1901605;GO:1901606;GO:0009987;GO:1901575;GO:0008150;GO:0008152;GO:0043436;GO:0009056;GO:0044248;GO:1901565;GO:0044238;GO:0006573;GO:0006574;GO:0006082;GO:0016054;GO:0044237;GO:0046395;GO:0009081;GO:0009083;GO:0044281;GO:0051287;GO:0003674;GO:0005488;GO:1901363;GO:1901265;GO:0016491;GO:0050661;GO:0008442;GO:0000166;GO:0036094;GO:0003824;GO:0016614;GO:0016616;GO:0097159	K23146	HPD1	map00280;ma	Valine, leucine ar	COG2084	I:Lipid transport and metabolis	PF03446.18;PF0	NAD_binding_	NAD binding doma	CYT	6	60	18.6	High
TRINITY_DN695_c0_g1_i12_orf1	seroin transcript 2A, partial [Ostrinia nubilalis]	2.508433735	1.326786827	6.21E-05	up	yes	1.041	0.415	0.361	0.424	0.46	1	1.068	1.056	------	------	------	------	------	------	ENOG4110R3H;ENOG4110N4H	S:Function unknown;	------	------	------	CYT	1	30	15.1	High
TRINITY_DN5417_c0_g1_i1_orf1	NADH dehydrogenase [ubiquinone] 1 alpha subcomplex subunit 10, mitochondrial isoform X1 [Ostrinia furnacalis] >XP_028175885.1 NADH dehydrogenase [ubiquinone] 1 alpha subcomplex subunit 10, mitochondrial isoform X2 [Ostrinia furnacalis]	2.152612183	1.106088425	1.63E-06	up	yes	0.993	0.4613	0.477	0.442	0.465	1	0.98	0.999	BP:aerobic respiration;BP:generation of precursor metabolites and energy;BP:electron transport chain;BP:respiratory electron transport chain;BP:cellular process;BP:aerobic electron transport chain;BP:cellular respiration;BP:metabolic process;BP:energy derivation by oxidation of organic compounds;BP:cellular metabolic process;BP:mitochondrial electron transport, NADH to ubiquinone;BP:biological_process;CC:membrane-enclosed lumen;CC:oxidoreductase complex;CC:intracellular organelle lumen;CC:respiratory chain complex;CC:inner mitochondrial membrane protein complex;CC:mitochondrial matrix;CC:cellular anatomical entity;CC:transmembrane transporter complex;CC:catalytic complex;CC:transporter complex;CC:respiratory chain complex I;CC:macromolecular complex;CC:organelle lumen;CC:NADH dehydrogenase complex;CC:cellular_component;CC:membrane protein complex;CC:mitochondrial respiratory chain complex I;CC:mitochondrial protein complex;	GO:0009060;GO:0006091;GO:0022900;GO:0022904;GO:0009987;GO:0019646;GO:0045333;GO:0008152;GO:0015980;GO:0044237;GO:0006120;GO:0008150;GO:0031974;GO:1990204;GO:0070013;GO:0098803;GO:0098800;GO:0005759;GO:0110165;GO:1902495;GO:1902494;GO:1990351;GO:0045271;GO:0032991;GO:0043233;GO:0030964;GO:0005575;GO:0098796;GO:0005747;GO:0098798	K03954	NDUFA10	map04714;ma	Thermogenesis;C	ENOG41101CN	F:Nucleotide transport and met	PF01712.22	dNK	Deoxynucleoside k	CYT	9	22	45.9	High
TRINITY_DN19000_c0_g1_i4_orf1	NADH dehydrogenase [ubiquinone] 1 beta subcomplex subunit 9 [Ostrinia furnacalis]	2.064138078	1.045539481	6.59E-06	up	yes	0.9687	0.4693	0.474	0.462	0.472	1	0.956	0.95	BP:electron transport chain;BP:cellular process;BP:aerobic electron transport chain;BP:cellular metabolic process;BP:aerobic respiration;BP:cellular respiration;BP:metabolic process;BP:respiratory electron transport chain;BP:biological_process;BP:mitochondrial electron transport, NADH to ubiquinone;BP:generation of precursor metabolites and energy;BP:energy derivation by oxidation of organic compounds;CC:organelle membrane;CC:membrane;CC:organelle inner membrane;CC:mitochondrial membrane;CC:cellular_component;CC:mitochondrial inner	GO:0022900;GO:0009987;GO:0019646;GO:0044237;GO:0009060;GO:0045333;GO:0008152;GO:0022904;GO:0008150;GO:0006120;GO:0006091;GO:0015980;GO:0031090;GO:0016020;GO:0019866;GO:0031966;GO:0005575;GO:0005743;GO:0070469;GO:0110165	K03965	NDUFB9	map04714;ma	Thermogenesis;C	ENOG4111PES	C:Energy production and conv	------	------	------	CYT	6	43	17.6	High
TRINITY_DN1982_c0_g1_i17_orf1	unnamed protein product, partial [Iphiclides podalirius]	2.380047506	1.25099037	1.95E-06	up	yes	1.002	0.421	0.441	0.395	0.427	1	1.007	0.998	CC:cellular_component;CC:integral component of membrane;CC:cellular anatomical entity;CC:intrinsic component of membrane;	GO:0005575;GO:0016021;GO:0110165;GO:0031224	------	------	------	------	COG0526	O:Posttranslational modificatio	PF00085.23;PF1	Thioredoxin;T	Thioredoxin;Thiore	CYT	6	17	124.3	High
TRINITY_DN430_c0_g1_i5_orf1	hypothetical protein NE865_02252 [Phthorimaea operculella]	2.582628747	1.368840271	8.76E-06	up	yes	1.008	0.3903	0.41	0.41	0.351	1	0.998	1.025	MF:cation binding;MF:transition metal ion binding;MF:organic cyclic compound binding;MF:ion binding;MF:molecular_function;MF:binding;MF:nucleic acid binding;MF:metal ion binding;MF:heterocyclic compound binding;MF:zinc ion binding;	GO:0043169;GO:0046914;GO:0097159;GO:0043167;GO:0003674;GO:0005488;GO:0003676;GO:0046872;GO:1901363;GO:0008270	------	------	------	------	ENOG4110QHB	S:Function unknown	------	------	------	CYT	5	8	83.6	High
TRINITY_DN3976_c0_g1_i6_orf1	grpE protein homolog, mitochondrial [Ostrinia furnacalis]	2.06833435	1.048469419	2.28E-06	up	yes	1.017	0.4917	0.48	0.49	0.505	1	1.037	1.013	------	------	K03687	GRPE	------	------	COG0576	O:Posttranslational modificatio	PF01025.22;PF1	GrpE;HAUS5	GrpE;HAUS augmi	CYT	3	23	24.4	High
TRINITY_DN1757_c0_g1_i4_orf1	F-box/LRR-repeat protein 2 isoform X1 [Ostrinia furnacalis]	2.888658188	1.530399502	0.0006952	up	yes	1.113	0.3853	0.435	0.431	0.29	1	1.206	1.134	BP:cellular catabolic process;BP:nitrogen compound metabolic process;BP:modification-dependent macromolecule catabolic process;BP:organic substance catabolic process;BP:cellular macromolecule catabolic process;BP:cellular macromolecule metabolic process;BP:proteasome-mediated ubiquitin-dependent protein catabolic process;BP:organic substance metabolic process;BP:proteolysis;BP:primary metabolic process;BP:cellular process;BP:modification-dependent protein catabolic process;BP:SCF-dependent proteasomal ubiquitin-dependent protein catabolic process;BP:biological_process;BP:protein catabolic process;BP:metabolic process;BP:catabolic process;BP:macromolecule catabolic process;BP:proteolysis involved in cellular protein catabolic process;BP:organonitrogen compound metabolic process;BP:organonitrogen compound catabolic process;BP:protein metabolic process;BP:proteasomal protein catabolic process;BP:cellular metabolic process;BP:macromolecule metabolic process;BP:ubiquitin-dependent protein catabolic process;CC:transferase complex;CC:macromolecular complex;CC:catalytic complex;CC:cullin-RING ubiquitin ligase complex;CC:ubiquitin	GO:0044248;GO:0006807;GO:0043632;GO:1901575;GO:0044265;GO:0044260;GO:0043161;GO:0071704;GO:0006508;GO:0044238;GO:0009987;GO:0019941;GO:0031146;GO:0008150;GO:0030163;GO:0008152;GO:0009056;GO:0009057;GO:0051603;GO:1901564;GO:1901565;GO:0019538;GO:0010498;GO:0044237;GO:0043170;GO:0006511;GO:1990234;GO:0032991;GO:1902494;GO:0031461;GO:0000151;GO:0005575;GO:0019005	------	------	------	------	ENOG410YRGC;ENOG410XQ54	S:Function unknown;	PF12937.10;PF0	F-box-like;F-	F-box-like;F-box d	CYT	1	2	59.1	High
TRINITY_DN5678_c0_g2_i3_orf1	coiled-coil domain-containing protein 115 [Ostrinia furnacalis]	2.178723404	1.123483053	0.0001295	up	yes	1.024	0.47	0.503	0.465	0.442	1	1.09	0.981	BP:proton-transporting V-type ATPase complex assembly;BP:proton-transporting two-sector ATPase complex assembly;BP:vacuolar proton-transporting V-type ATPase complex assembly;BP:cellular component assembly;BP:macromolecular complex subunit organization;BP:cellular process;BP:cellular component organization;BP:macromolecular complex assembly;BP:biological_process;BP:cellular component	GO:0070070;GO:0070071;GO:0070072;GO:0022607;GO:0043933;GO:0009987;GO:0016043;GO:0065003;GO:0008150;GO:0071840	K23543	CCDC115	------	------	ENOG41124X6	S:Function unknown	------	------	------	CYT	1	9	17.6	High
TRINITY_DN17417_c0_g1_i11_orf1	sodium/hydrogen exchanger 9B2-like isoform X4 [Ostrinia furnacalis]	2.433248201	1.282883488	1.50E-05	up	yes	1.048	0.4307	0.427	0.426	0.439	1	1.069	1.076	------	------	K23994	SLC9B1_2	------	------	COG0025;COG0025	E:Amino acid transport and me	PF00999.24	Na_H_Exchan	Sodium/hydrogen	CYT	1	3	75.4	High
TRINITY_DN6308_c0_g1_i6_orf1	myc box-dependent-interacting protein 1 isoform X2 [Ostrinia furnacalis]	2.071473094	1.050657081	1.44E-05	up	yes	0.997	0.4813	0.483	0.512	0.449	1	1.011	0.98	CC:cytoplasm;CC:cellular_component;CC:cellular anatomical	GO:0005737;GO:0005575;GO:0110165	K12562	AMPH	map04666;ma	Fc gamma R-me	ENOG410ZBW1	S:Function unknown	PF03114.21;PF1	BAR;SH3_9;SH	BAR domain;Variar	CYT	3	33	52.4	High
TRINITY_DN86149_c0_g1_i1_orf1	NADH dehydrogenase [ubiquinone] 1 alpha subcomplex subunit 8 [Galleria mellonella]	2.1104786	1.077570201	3.87E-07	up	yes	1.001	0.4743	0.465	0.478	0.48	1	1.014	0.99	BP:electron transport chain;BP:cellular process;BP:aerobic electron transport chain;BP:mitochondrial electron transport, NADH to ubiquinone;BP:aerobic respiration;BP:cellular respiration;BP:cellular metabolic process;BP:metabolic process;BP:respiratory electron transport chain;BP:biological_process;BP:generation of precursor metabolites and energy;BP:energy derivation by oxidation of organic compounds;CC:intracellular membrane-bounded organelle;CC:mitochondrion;CC:cellular_component;CC:intracellular organelle;CC:membrane-bounded	GO:0022900;GO:0009987;GO:0019646;GO:0006120;GO:0009060;GO:0045333;GO:0044237;GO:0008152;GO:0022904;GO:0008150;GO:0006091;GO:0015980;GO:0043231;GO:0005739;GO:0005575;GO:0043229;GO:0043227;GO:0043226;GO:0110165	K03952	NDUFA8	map04714;ma	Thermogenesis;C	ENOG4111IST	I:Lipid transport and metabolis	------	------	------	EXC	6	42	20.1	High
TRINITY_DN51045_c0_g1_i1_orf1	cell growth-regulating nucleolar protein [Ostrinia furnacalis]	2.288645996	1.194494326	3.83E-06	up	yes	1.026	0.4483	0.451	0.46	0.434	1	1.028	1.05	CC:intracellular membrane-bounded organelle;CC:nucleus;CC:cellular_component;CC:intracellular organelle;CC:membrane-bounded organelle;CC:organelle;CC:cellular anatomical entity;MF:cation binding;MF:organic cyclic compound binding;MF:heterocyclic compound binding;MF:ion binding;MF:molecular_function;MF:binding;MF:nucleic acid binding;MF:DNA binding;MF:metal ion binding;	GO:0043231;GO:0005634;GO:0005575;GO:0043229;GO:0043227;GO:0043226;GO:0110165;GO:0043169;GO:0097159;GO:1901363;GO:0043167;GO:0003674;GO:0005488;GO:0003676;GO:0003677;GO:0046872	K15263	LYER	------	------	ENOG411081U	S:Function unknown	PF08790.14	zf-LYAR	LYAR-type C2HC z	CYT	4	13	48.3	High
TRINITY_DN26186_c0_g1_i7_orf1	sodium- and chloride-dependent glycine transporter 1-like [Ostrinia furnacalis]	2.292089249	1.196663221	3.98E-05	up	yes	1.017	0.4437	0.43	0.449	0.452	1	0.978	1.072	CC:integral component of membrane;CC:cellular_component;CC:intrinsic component of membrane;CC:cellular anatomical entity;MF:secondary active transmembrane transporter activity;MF:symporter activity;MF:transporter activity;MF:transmembrane transporter activity;MF:molecular_function;MF:active transmembrane transporter activity;	GO:0016021;GO:0005575;GO:0031224;GO:0110165;GO:0015291;GO:0015293;GO:0005215;GO:0022857;GO:0003674;GO:0022804	K05038	SLC6A5_9, GL	map04721	Synaptic vesicle c	COG0733	U:Intracellular trafficking, secre	PF00209.21	SNF	Sodiu	CYT	1	2	71.2	High
TRINITY_DN2748_c0_g1_i6_orf1	uncharacterized protein LOC114352811 [Ostrinia furnacalis]	2.399254601	1.26258626	1.05E-05	up	yes	1.03	0.4293	0.43	0.422	0.436	1	1.072	1.019	------	------	------	------	------	------	ENOG41117PX	S:Function unknown	------	------	------	CYT	3	8	48	High
TRINITY_DN14235_c0_g1_i1_orf1	maltase A1 [Helicoverpa armigera]	2.542001461	1.345964859	9.96E-06	up	yes	1.044	0.4107	0.402	0.422	0.408	1	1.07	1.061	BP:primary metabolic process;BP:carbohydrate metabolic process;BP:organic substance metabolic process;BP:biological_process;BP:metabolic process;MF:hydrolase activity;MF:catalytic activity;MF:hydrolase activity, acting on glycosyl bonds;MF:molecular_function;	GO:0044238;GO:0005975;GO:0071704;GO:0008150;GO:0008152;GO:0016787;GO:0003824;GO:0016798;GO:0003674	K01187	malZ	map00500;ma	Starch and sucro	COG0366	G:Carbohydrate transport and	PF00128.27	Alpha-amylas	Alpha amylase, cat	CYT	10	29	66.7	High
TRINITY_DN3949_c1_g1_i1_orf1	probable cytochrome P450 304a1 isoform X2 [Ostrinia furnacalis]	2.600941293	1.379033836	6.71E-05	up	yes	1.05	0.4037	0.41	0.422	0.379	1	1.031	1.118	CC:integral component of membrane;CC:cellular_component;CC:cellular anatomical entity;CC:intrinsic component of membrane;MF:cation binding;MF:monooxygenase activity;MF:heme binding;MF:transition metal ion binding;MF:tetrapyrrole binding;MF:organic cyclic compound binding;MF:oxidoreductase activity, acting on paired donors, with incorporation or reduction of molecular oxygen;MF:ion binding;MF:catalytic activity;MF:molecular_function;MF:binding;MF:iron ion binding;MF:metal ion binding;MF:heterocyclic compound	GO:0016021;GO:0005575;GO:0110165;GO:0031224;GO:0043169;GO:0004497;GO:0020037;GO:0046914;GO:0046906;GO:0097159;GO:0016705;GO:0043167;GO:0003824;GO:0003674;GO:0005488;GO:0005506;GO:0046872;GO:1901363;GO:0016491	------	------	------	------	COG2124	Q:Secondary metabolites biosy	PF00067.25	p450	Cytochrome P450	CYT	2	17	16.8	High
TRINITY_DN47731_c0_g1_i2_orf1	nucleolar GTP-binding protein 2 [Ostrinia furnacalis]	2.473684211	1.306661338	0.001799	up	yes	1.034	0.418	0.419	0.412	0.423	1	1.193	0.91	CC:organelle;CC:intracellular organelle;CC:non-membrane-bounded organelle;CC:nucleolus;CC:cellular anatomical entity;CC:intracellular non-membrane-bounded organelle;CC:cellular_component;MF:purine ribonucleoside triphosphate binding;MF:molecular_function;MF:binding;MF:ion binding;MF:heterocyclic compound binding;MF:nucleoside phosphate binding;MF:anion binding;MF:nucleotide binding;MF:guanyl ribonucleotide binding;MF:purine nucleotide binding;MF:GTP binding;MF:small molecule binding;MF:carbohydrate derivative binding;MF:organic cyclic compound binding;MF:guanyl nucleotide binding;MF:purine ribonucleotide binding;MF:ribonucleotide binding;	GO:0043226;GO:0043229;GO:0043228;GO:0005730;GO:0110165;GO:0043232;GO:0005575;GO:0035639;GO:0003674;GO:0005488;GO:0043167;GO:1901363;GO:1901265;GO:0043168;GO:0000166;GO:0032561;GO:0017076;GO:0005525;GO:0036094;GO:0097367;GO:0097159;GO:0019001;GO:0032555;GO:0032553	K14537	NUG2, GNL2	map03008	Ribosome bioger	COG1161;ENOG4111H9K	S:Function unknown;	PF07572.15	BCNT	Bucentaur or crani	CYT	1	4	30.3	Medium
TRINITY_DN5019_c0_g1_i2_orf1	RRP12-like protein isoform X4 [Ostrinia furnacalis] >XP_028175539.1 RRP12-like protein isoform X5 [Ostrinia furnacalis]	2.275395034	1.186117035	9.62E-07	up	yes	1.008	0.443	0.423	0.453	0.453	1	1.018	1.006	CC:intracellular membrane-bounded organelle;CC:nucleus;CC:cellular_component;CC:intracellular organelle;CC:membrane-bounded organelle;CC:organelle;CC:cellular anatomical entity;	GO:0043231;GO:0005634;GO:0005575;GO:0043229;GO:0043227;GO:0043226;GO:0110165	K14794	RRP12	------	------	ENOG410XQ3D	S:Function unknown	PF08161.15	NUC173	NUC173 domain	CYT	8	6	153.7	High
TRINITY_DN83327_c0_g1_i1_orf1	trypsin-like serine proteinase T22 [Ostrinia nubilalis]	3.519043122	1.815183193	0.0002288	up	yes	1.118	0.3177	0.359	0.303	0.291	1	1.16	1.195	BP:organic substance metabolic process;BP:organonitrogen compound metabolic process;BP:protein metabolic process;BP:macromolecule metabolic process;BP:biological_process;BP:nitrogen compound metabolic process;BP:metabolic process;BP:proteolysis;BP:primary metabolic process;MF:serine-type endopeptidase activity;MF:endopeptidase activity;MF:hydrolase activity;MF:serine hydrolase activity;MF:catalytic activity, acting on a protein;MF:catalytic activity;MF:molecular_function;MF:peptidase activity;MF:serine-	GO:0071704;GO:1901564;GO:0019538;GO:0043170;GO:0008150;GO:0006807;GO:0008152;GO:0006508;GO:0044238;GO:0004252;GO:0004175;GO:0016787;GO:0017171;GO:0140096;GO:0003824;GO:0003674;GO:0008233;GO:0008236	K09640;K01	TMPRSS9;PRS	map04972;ma	Pancreatic secret	COG5640	O:Posttranslational modificatio	PF00089.29	Trypsin	Trypsin	PLA	1	4	28.4	Medium
TRINITY_DN109931_c0_g1_i1_orf1	hydroxymethylglutaryl-CoA lyase, mitochondrial isoform X1 [Ostrinia furnacalis]	2.572151899	1.362975844	1.48E-05	up	yes	1.016	0.395	0.424	0.379	0.382	1	1.056	0.993	MF:hydroxymethylglutaryl-CoA lyase activity;MF:lyase activity;MF:carbon-carbon lyase activity;MF:oxo-acid-lyase activity;MF:catalytic activity;MF:molecular_function;	GO:0004419;GO:0016829;GO:0016830;GO:0016833;GO:0003824;GO:0003674	K01640	HMGCL, hmg	map04146;ma	Peroxisome;Valin	COG0119	E:Amino acid transport and me	------	------	------	CYT	1	15	5.9	High

TRINITY_DN58207_c0_g1_i1_orf1	60S ribosomal protein L6 [Ostrinia furnacalis] >XP_028170357.1 60S ribosomal protein L6 [Ostrinia furnacalis]	2.374969974	1.247909274	1.09E-05	up	yes	0.9887	0.4163	0.417	0.428	0.404	1	1.016	0.95	BP:cellular biosynthetic process;BP:cellular nitrogen compound metabolic process;BP:nitrogen compound metabolic process;BP:cellular macromolecule biosynthetic process;BP:organic substance biosynthetic process;BP:cellular nitrogen compound biosynthetic process;BP:cellular macromolecule metabolic process;BP:peptide biosynthetic process;BP:organic substance metabolic process;BP:amide biosynthetic process;BP:cellular process;BP:biosynthetic process;BP:macromolecule biosynthetic process;BP:biological_process;BP:metabolic process;BP:organonitrogen compound metabolic process;BP:primary metabolic process;BP:cellular amide metabolic process;BP:organonitrogen compound biosynthetic process;BP:protein metabolic process;BP:cellular metabolic process;BP:macromolecule metabolic process;BP:peptide metabolic process;BP:translation;CC:intracellular non-membrane-bounded organelle;CC:organelle;CC:cellular anatomical entity;CC:ribosome;CC:macromolecular complex;CC:ribonucleoprotein complex;CC:intracellular organelle;CC:cellular_component;CC:non-membrane-bounded organelle;MF:molecular_function;MF:structural molecule	GO:0044249;GO:0034641;GO:0006807; GO:0034645;GO:1901576;GO:0044271; GO:0044260;GO:0043043;GO:0071704; GO:0043604;GO:0009987;GO:0009058; GO:0009059;GO:0008150;GO:0008152; GO:1901564;GO:0044238;GO:0043603; GO:1901566;GO:0019538;GO:0044237; GO:0043170;GO:0006518;GO:0006412; GO:0043232;GO:0043226;GO:0110165; GO:0005840;GO:0032991;GO:1990904; GO:0043229;GO:0005575;GO:0043228; GO:0003674;GO:0005198;GO:0003735	K02934	RP-L6e, RPL6	map03010;ma	Ribosome;Coron	COG2163	J:Translation, ribosomal structu	PF01159.22;PF0	Ribosomal_L6	Ribosomal protein	CYT	9	32	28.2	High
TRINITY_DN37538_c0_g2_i1_orf1	esterase FE4-like [Ostrinia furnacalis]	3.05515449	1.611245335	2.74E-05	up	yes	1.058	0.3463	0.343	0.351	0.345	1	1.059	1.114	------	------	K07378	NLGN	map04514	Cell adhesion mo	COG2272	I:Lipid transport and metabolis	PF00135.31	COesterase	Carboxylesterase f	CYT	3	31	15.9	High
TRINITY_DN10332_c0_g1_i2_orfp1	TRINITY_DN10332_c0_g1_i2_m.42894 TRINITY_DN10332_c0_g1::TRINITY_DN10332_c0_g1_i2::g.42894 ORF type:3prime_partial len:77	2.128187919	1.089625547	4.78E-05	up	yes	0.9513	0.447	0.464	0.451	0.426	1	0.932	0.922	------	------	------	------	------	------	------	------	------	------	------	CYT	1	8	9	Medium
TRINITY_DN1505_c0_g1_i1_orf1	uncharacterized protein LOC114362816 isoform X1 [Ostrinia furnacalis] >XP_028174154.1 uncharacterized protein LOC114362816 isoform X2 [Ostrinia furnacalis]	2.507669097	1.326346988	5.80E-06	up	yes	0.9973	0.3977	0.386	0.375	0.432	1	1.008	0.984	------	------	K16913	PELP1, MNAR	------	------	ENOG4111B29;ENOG4110K7C	S:Function unknown;	------	------	------	CYT	2	4	56	High
TRINITY_DN2184_c0_g1_i4_orf1	uncharacterized protein LOC114359356 [Ostrinia furnacalis]	2.516316721	1.331313521	8.99E-07	up	yes	0.9947	0.3953	0.389	0.388	0.409	1	0.976	1.008	CC:cellular_component;CC:extracellular region;CC:cellular anatomical entity;MF:molecular_function;MF:structural molecule activity;	GO:0005575;GO:0005576;GO:0110165; GO:0003674;GO:0005198	------	------	------	------	------	------	PF07294.14	Fibroin_P25	Fibroin P25	CYT	2	8	28	High
TRINITY_DN1285_c0_g1_i6_orf1	bifunctional 3'-phosphoadenosine 5'-phosphosulfate synthase isoform X3 [Ostrinia furnacalis]	2.232714617	1.158798859	1.67E-05	up	yes	0.9623	0.431	0.429	0.446	0.418	1	0.931	0.956	BP:sulfate assimilation;BP:sulfur compound metabolic process;BP:cellular process;BP:biological_process;BP:metabolic process;BP:cellular metabolic process;MF:purine ribonucleoside triphosphate binding;MF:molecular_function;MF:binding;MF:ion binding;MF:heterocyclic compound binding;MF:nucleoside phosphate binding;MF:anion binding;MF:nucleotide binding;MF:transferase activity;MF:sulfate adenylyltransferase activity;MF:ATP binding;MF:kinase activity;MF:adenylylsulfate kinase activity;MF:small molecule binding;MF:catalytic activity;MF:purine nucleotide binding;MF:sulfate adenylyltransferas;MF:nucleotidyltransferase activity;MF:adenyl nucleotide binding;MF:phosphotransferase activity, alcohol group as acceptor;MF:transferase activity, transferring phosphorus-containing groups;MF:adenylyltransferase activity;MF:carbohydrate derivative binding;MF:adenyl ribonucleotide binding;MF:organic cyclic compound binding;MF:purine ribonucleotide binding;MF:ribonucleotide	GO:0000103;GO:0006790;GO:0009987; GO:0008150;GO:0008152;GO:0044237; GO:0035639;GO:0003674;GO:0005488; GO:0043167;GO:1901363;GO:1901265; GO:0043168;GO:0000166;GO:0016740; GO:0004779;GO:0005524;GO:0016301; GO:0004020;GO:0036094;GO:0003824; GO:0017076;GO:0004781;GO:0016779; GO:0030554;GO:0016773;GO:0016772; GO:0070566;GO:0097367;GO:0032559; GO:0097159;GO:0032555;GO:0032553	K13811	PAPSS	map00450;ma	Selenocompounc	COG2046	P:Inorganic ion transport and n	PF01747.20;PF1	ATP-sulfuryla	ATP-sulfurylase;PL	CYT	5	16	43.5	High
TRINITY_DN3355_c0_g2_i4_orf1	UDP-glycosyltransferase UGT33AL1 [Ostrinia furnacalis]	2.673684211	1.418829078	7.88E-06	up	yes	1.016	0.38	0.357	0.378	0.405	1	1.049	0.999	CC:integral component of membrane;CC:cellular_component;CC:cellular anatomical entity;CC:intrinsic component of membrane;MF:catalytic activity;MF:transferase activity;MF:molecular_function;MF:transferase activity, transferring glycosyl groups;MF:transferase activity, transferring hexosyl groups;MF:glucuronosyltransferase activity;MF:UDP-	GO:0016021;GO:0005575;GO:0110165; GO:0031224;GO:0003824;GO:0016740; GO:0003674;GO:0016757;GO:0016758; GO:0015020;GO:0008194	K00699	UGT	map05207;ma	Chemical carcinc	COG1819	S:Function unknown	PF00201.21	UDPGT	UDP-glucoronosyl	CYT	4	13	60.5	High
TRINITY_DN30233_c0_g1_i2_orf1	39S ribosomal protein L10, mitochondrial [Ostrinia furnacalis]	2.219568746	1.150279393	1.98E-05	up	yes	0.9573	0.4313	0.441	0.419	0.434	1	0.931	0.941	CC:ribosome;CC:intracellular non-membrane-bounded organelle;CC:cellular_component;CC:intracellular organelle;CC:non-membrane-bounded organelle;CC:organelle;CC:cellular anatomical entity;	GO:0005840;GO:0043232;GO:0005575; GO:0043229;GO:0043228;GO:0043226; GO:0110165	K02864	RP-L10, MRP	map03010	Ribosome	ENOG4111GA4	J:Translation, ribosomal structu	------	------	------	CYT	1	2	27.9	High
TRINITY_DN2172_c0_g2_i5_orf1	4-hydroxyphenylpyruvate dioxygenase [Ostrinia furnacalis]	2.770718232	1.470260004	2.02E-07	up	yes	1.003	0.362	0.359	0.357	0.37	1	0.992	1.018	------	------	K00457	HPD, hppD	map00130;ma	Ubiquinone and	COG3185	E:Amino acid transport and me	PF00903.28;PF1	Glyoxalase;Gl	Glyoxalase/Bleomy	CYT	9	26	43.6	High
TRINITY_DN85319_c0_g1_i1_orf1	cholinesterase 2-like [Ostrinia furnacalis]	2.568642546	1.361006138	1.66E-06	up	yes	1.001	0.3897	0.388	0.394	0.387	1	1.025	0.977	MF:hydrolase activity;MF:molecular_function;MF:hydrolase activity, acting on ester bonds;MF:carboxylic ester hydrolase activity;MF:catalytic activity;	GO:0016787;GO:0003674;GO:0016788; GO:0052689;GO:0003824	K12298	CEL	map04972;ma	Pancreatic secret	COG2272	I:Lipid transport and metabolis	PF00135.31;PF2	COesterase;B	Carboxylesterase f	CYT	9	18	61.4	High
TRINITY_DN47389_c0_g1_i2_orf1	non-specific lipid-transfer protein-like [Ostrinia furnacalis]	2.723830133	1.445636735	4.06E-07	up	yes	1.007	0.3697	0.357	0.365	0.387	1	1.017	1.005	BP:lipid transport;BP:organic substance transport;BP:transport;BP:biological_process;BP:establishment of localization;BP:localization;CC:microbody;CC:intracellular organelle;CC:membrane-bounded organelle;CC:organelle;CC:peroxisome;CC:mitochondrion;CC:cellular anatomical entity;CC:intracellular membrane-bounded organelle;CC:cellular_component;MF:molecular_function;MF:binding;MF:propanoyl-CoA C-acyltransferase activity;MF:acetyl-CoA C-myristoyltransferase activity;MF:transferase activity;MF:transferase activity, transferring acyl groups;MF:transferase activity, transferring acyl groups other than amino-acyl groups;MF:acetyl-CoA C-acyltransferase activity;MF:lipid binding;MF:myristoyltransferase activity;MF:catalytic activity;MF:propionyl-CoA C2-	GO:0006869;GO:0071702;GO:0006810; GO:0008150;GO:0051234;GO:0051179; GO:0042579;GO:0043229;GO:0043227; GO:0043226;GO:0005777;GO:0005739; GO:0110165;GO:0043231;GO:0005575; GO:0003674;GO:0005488;GO:0033814; GO:0050633;GO:0016740;GO:0016746; GO:0016747;GO:0003988;GO:0008289; GO:0019107;GO:0003824;GO:0050632; GO:0016408	K08764	SCP2, SCPX	map04146;ma	Peroxisome;Prim	ENOG410XPRW	I:Lipid transport and metabolis	PF00108.26;PF0	Thiolase_N;Th	Thiolase, N-termin	CYT	10	38	43.9	High
TRINITY_DN34536_c0_g1_i6_orf1	clustered mitochondria protein homolog isoform X2 [Ostrinia furnacalis]	2.784878977	1.477614634	1.26E-06	up	yes	1.024	0.3677	0.37	0.354	0.379	1	1.035	1.037	BP:organelle organization;BP:cellular component organization or biogenesis;BP:cellular process;BP:cellular component organization;BP:biological_process;BP:mitochondrion organization;CC:cytoplasm;CC:cellular_component;CC:cellular anatomical entity;MF:organic cyclic compound binding;MF:molecular_function;MF:RNA binding;MF:nucleic acid binding;MF:heterocyclic compound binding;MF:binding;	GO:0006996;GO:0071840;GO:0009987; GO:0016043;GO:0008150;GO:0007005; GO:0005737;GO:0005575;GO:0110165; GO:0097159;GO:0003674;GO:0003723; GO:0003676;GO:1901363;GO:0005488	K03255	TIF31, CLU1	------	------	ENOG410XQUQ	S:Function unknown	PF13236.9;PF12	CLU;eIF3_p13	Clustered mitocho	CYT	9	8	124.7	High
TRINITY_DN107617_c3_g1_i1_orf1	NADH dehydrogenase [ubiquinone] 1 alpha subcomplex subunit 9, mitochondrial [Ostrinia	2.384298522	1.253564877	3.58E-06	up	yes	0.984	0.4127	0.405	0.394	0.439	1	0.972	0.98	------	------	K03953	NDUFA9	map04714;ma	Thermogenesis;C	COG0702;COG0702	G:Carbohydrate transport and	PF01073.22;PF0	3Beta_HSD;Ep	3-beta hydroxyste	CYT	8	16	45.2	High
TRINITY_DN27087_c0_g1_i1_orf1	2',5'-phosphodiesterase 12 [Ostrinia furnacalis]	2.249475891	1.169588905	2.87E-05	up	yes	0.9657	0.4293	0.45	0.431	0.407	1	0.972	0.925	MF:catalytic activity;MF:molecular_function;	GO:0003824;GO:0003674	K19612	PDE12	------	------	COG5239	O:Posttranslational modificatio	PF03372.26	Exo_endo_ph	Endonuclease/Exo	CYT	2	3	67.3	High
TRINITY_DN36262_c0_g1_i1_orf1	trypsin, alkaline C-like [Maniola jurtina]	3.062130178	1.614535616	1.33E-05	up	yes	1.035	0.338	0.339	0.35	0.325	1	1.086	1.02	BP:organic substance metabolic process;BP:organonitrogen compound metabolic process;BP:protein metabolic process;BP:macromolecule metabolic process;BP:biological_process;BP:nitrogen compound metabolic process;BP:metabolic process;BP:proteolysis;BP:primary metabolic process;MF:serine-type endopeptidase activity;MF:endopeptidase activity;MF:hydrolase activity;MF:serine hydrolase activity;MF:catalytic activity, acting on a protein;MF:catalytic activity;MF:molecular_function;MF:peptidase activity;MF:serine-	GO:0071704;GO:1901564;GO:0019538; GO:0043170;GO:0008150;GO:0006807; GO:0008152;GO:0006508;GO:0044238; GO:0004252;GO:0004175;GO:0016787; GO:0017171;GO:0140096;GO:0003824; GO:0003674;GO:0008233;GO:0008236	K01312	PRSS1_2_3	map04972;ma	Pancreatic secret	COG5640	O:Posttranslational modificatio	PF00089.29	Trypsin	Trypsin	CYT	1	9	11.8	High
TRINITY_DN1465_c0_g2_i1_orf1	unnamed protein product, partial [Iphiclides podalirius]	2.832353764	1.502001471	5.68E-07	up	yes	1.012	0.3573	0.36	0.352	0.36	1	1.001	1.034	MF:catalytic activity;MF:monooxygenase activity;MF:molecular_function;MF:oxidoreductase activity;	GO:0003824;GO:0004497;GO:0003674; GO:0016491	K01904	4CL	map00130	Ubiquinone and	COG0318;COG0318	I:Lipid transport and metabolis	PF00501.31;PF1	AMP-binding	AMP-binding enzy	CYT	8	23	57.9	High
TRINITY_DN18404_c0_g1_i5_orf1	periodic tryptophan protein 1 homolog isoform X1 [Ostrinia furnacalis] >XP_028157695.1 periodic tryptophan protein 1 homolog isoform X2 [Ostrinia furnacalis]	2.754820937	1.461958547	2.29E-08	up	yes	1	0.363	0.36	0.361	0.368	1	1.008	0.993	BP:RNA metabolic process;BP:nucleobase-containing compound metabolic process;BP:rRNA metabolic process;BP:ncRNA processing;BP:primary metabolic process;BP:RNA processing;BP:cellular process;BP:cellular aromatic compound metabolic process;BP:cellular metabolic process;BP:macromolecule metabolic process;BP:nucleic acid metabolic process;BP:organic substance metabolic process;BP:cellular nitrogen compound metabolic process;BP:nitrogen compound metabolic process;BP:biological_process;BP:rRNA processing;BP:metabolic process;BP:ncRNA metabolic process;BP:organic cyclic	GO:0016070;GO:0006139;GO:0016072; GO:0034470;GO:0044238;GO:0006396; GO:0009987;GO:0006725;GO:0044237; GO:0043170;GO:0090304;GO:0071704; GO:0034641;GO:0006807;GO:0008150; GO:0006364;GO:0008152;GO:0034660; GO:1901360;GO:0046483	K14791	PWP1	------	------	ENOG410XT1V	S:Function unknown	PF00400.35	WD40	WD domain, G-be	CYT	1	2	51.1	High
TRINITY_DN22815_c0_g1_i2_orf1	acyl carrier protein, mitochondrial isoform X1 [Ostrinia furnacalis]	2.276401251	1.186754877	4.28E-05	up	yes	0.9463	0.4157	0.414	0.423	0.41	1	0.928	0.911	------	------	K03955	NDUFAB1	map04714;ma	Thermogenesis;C	COG0236	I:Lipid transport and metabolis	PF00550.28	PP-binding	Phosphopantethei	CYT	2	15	17.3	High
TRINITY_DN20957_c0_g1_i1_orf1	adenylate kinase isoenzyme 1 isoform X2 [Ostrinia furnacalis]	2.930088496	1.550944238	2.39E-07	up	yes	0.9933	0.339	0.342	0.333	0.342	1	1.004	0.976	------	------	K00939	adk, AK	map00730;ma	Thiamine metab	COG0563	S:Function unknown	PF00406.25;PF1	ADK;AAA_17	Adenylate kinase;A	CYT	6	30	26.2	High
TRINITY_DN37699_c0_g1_i4_orfp1	TRINITY_DN37699_c0_g1_i4_m.58777 TRINITY_DN37699_c0_g1::TRINITY_DN37699_c0_g1_i4::g.58777 ORF type:internal len:122	3.378289474	1.756292953	4.85E-05	up	yes	1.027	0.304	0.278	0.301	0.333	1	1.097	0.984	------	------	------	------	------	------	------	------	------	------	------	PLA	1	18	12.7	High
TRINITY_DN42759_c0_g2_i1_orf1	fatty acid synthase-like [Ostrinia furnacalis]	2.89595034	1.534036863	2.50E-05	up	yes	0.9797	0.3383	0.347	0.324	0.344	1	1.015	0.924	BP:fatty acid biosynthetic process;BP:fatty acid metabolic process;BP:carboxylic acid metabolic process;BP:cellular biosynthetic process;BP:small molecule metabolic process;BP:small molecule biosynthetic process;BP:monocarboxylic acid biosynthetic process;BP:organic substance biosynthetic process;BP:organic substance metabolic process;BP:lipid metabolic process;BP:cellular process;BP:monocarboxylic acid metabolic process;BP:biosynthetic process;BP:biological_process;BP:metabolic process;BP:oxoacid metabolic process;BP:cellular lipid metabolic process;BP:lipid biosynthetic process;BP:primary metabolic process;BP:organic acid metabolic process;BP:carboxylic acid biosynthetic process;BP:organic acid biosynthetic process;BP:cellular metabolic process;MF:3-hydroxyoctanoyl-[acyl-carrier-protein] dehydratase activity;MF:thiolester hydrolase activity;MF:S-acyltransferase activity;MF:enoyl-[acyl-carrier-protein] reductas;MF:oxidoreductase activity, acting on the CH-CH group of donors;MF:molecular_function;MF:oxidoreductase activity;MF:oxidoreductase activity, acting on the CH-CH group of donors, NAD or NADP as acceptor;MF:[acyl-carrier-protein] S-acetyltransferase activity;MF:lyase activity;MF:oleoyl-[acyl-carrier-protein] hydrolase activity;MF:acetyltransferase activity;MF:transferase activity;MF:transferase activity, transferring acyl groups;MF:transferase activity, transferring acyl groups other than amino-acyl groups;MF:S-acetyltransferase activity;MF:hydrolase activity;MF:3-hydroxydecanoyl-[acyl-carrier-protein] dehydratase activity;MF:hydrolase activity, acting on ester bonds;MF:catalytic activity;MF:oxidoreductase [illegible]	GO:0006633;GO:0006631;GO:0019752; GO:0044249;GO:0044281;GO:0044283; GO:0072330;GO:1901576;GO:0071704; GO:0006629;GO:0009987;GO:0032787; GO:0009058;GO:0008150;GO:0008152; GO:0043436;GO:0044255;GO:0008610; GO:0044238;GO:0006082;GO:0046394; GO:0016053;GO:0044237;GO:0047451; GO:0016790;GO:0016417;GO:0047117; GO:0016627;GO:0003674;GO:0016491; GO:0016628;GO:0004313;GO:0016829; GO:0004320;GO:0016407;GO:0016740; GO:0016746;GO:0016747;GO:0016418; GO:0016787;GO:0008693;GO:0016788; GO:0003824;GO:0016614;GO:0016616; GO:0016835;GO:0016836;GO:0004312; GO:0016295;GO:0016296;GO:0016297; GO:0004316;GO:0004317;GO:0004315; GO:0019171;GO:0008659	K00665	FASN	map04910;ma	Insulin signaling	COG3321	Q:Secondary metabolites biosy	PF00975.23;PF1	Thioesterase;	Thioesterase doma	CYT	5	3	166.4	High
TRINITY_DN115082_c0_g1_i5_orf1	protein dj-1beta-like isoform X2 [Ostrinia furnacalis]	3.1115625	1.637639225	4.14E-06	up	yes	0.9957	0.32	0.32	0.322	0.318	1	1.027	0.96	------	------	K05687	PARK7	map05012;ma	Parkinson diseas	COG0693	S:Function unknown	PF01965.27	DJ-1_PfpI	DJ-1/PfpI family	CYT	1	45	11.4	High
TRINITY_DN66302_c0_g1_i1_orf1	carboxypeptidase B-like [Ostrinia furnacalis]	4.085603113	2.030549063	4.67E-05	up	yes	1.05	0.257	0.194	0.272	0.305	1	1.089	1.06	BP:nitrogen compound metabolic process;BP:organic substance metabolic process;BP:proteolysis;BP:biological_process;BP:metabolic process;BP:primary metabolic process;BP:organonitrogen compound metabolic process;BP:protein metabolic process;BP:macromolecule metabolic process;CC:intrinsic component of membrane;CC:cellular anatomical entity;CC:cellular_component;CC:integral component of membrane;MF:exopeptidase activity;MF:molecular_function;MF:zinc ion binding;MF:peptidase activity;MF:metalloexopeptidase activity;MF:cation binding;MF:transition metal ion binding;MF:metallocarboxypeptidase activity;MF:carboxypeptidase activity;MF:ion	GO:0006807;GO:0071704;GO:0006508; GO:0008150;GO:0008152;GO:0044238; GO:1901564;GO:0019538;GO:0043170; GO:0031224;GO:0110165;GO:0005575; GO:0016021;GO:0008238;GO:0003674; GO:0008270;GO:0008233;GO:0008235; GO:0043169;GO:0046914;GO:0004181; GO:0004180;GO:0043167;GO:0008237; GO:0046872;GO:0016787;GO:0140096; GO:0003824;GO:0005488	K01298	CPA2	map04972;ma	Pancreatic secret	COG2866	E:Amino acid transport and me	PF00246.27	Peptidase_M1	Zinc carboxypeptic	CYT	1	3	47.1	High

TRINITY_DN109733_c0_g1_i1_orf1	uncharacterized protein LOC112452128 [Temnothorax curvispinosus]	3.860830528	1.948911228	0.0007053	up	yes	1.032	0.2673	0.161	0.34	0.301	1	1.148	0.947	BP:nucleic acid metabolic process;BP:cellular nitrogen compound metabolic process;BP:nitrogen compound metabolic process;BP:organic cyclic compound metabolic process;BP:telomere maintenance;BP:nucleobase-containing compound metabolic process;BP:cellular response to stimulus;BP:cellular macromolecule metabolic process;BP:DNA repair;BP:cellular component organization;BP:organic substance metabolic process;BP:cellular component organization or biogenesis;BP:primary metabolic process;BP:cellular process;BP:cellular aromatic compound metabolic process;BP:cellular response to DNA damage stimulus;BP:response to stress;BP:biological_process;BP:metabolic process;BP:heterocycle metabolic process;BP:organelle organization;BP:chromosome organization;BP:response to stimulus;BP:telomere organization;BP:cellular metabolic process;BP:macromolecule metabolic process;BP:cellular response to stress;BP:DNA metabolic	GO:0090304;GO:0034641;GO:0006807;GO:1901360;GO:0000723;GO:0006139;GO:0051716;GO:0044260;GO:0006281;GO:0016043;GO:0071704;GO:0071840;GO:0044238;GO:0009987;GO:0006725;GO:0006974;GO:0006950;GO:0008150;GO:0008152;GO:0046483;GO:0006996;GO:0051276;GO:0050896;GO:0032200;GO:0044237;GO:0043170;GO:0033554;GO:0006259;GO:0003674;GO:0003678;GO:0140097;GO:0004386;GO:0003824;GO:0008094	------	------	------	------	COG0507;ENOG410Y4KS	S:Function unknown;	------	------	------	CYT	1	2	37.9	High
TRINITY_DN1044_c0_g1_i2_orf1	V-type proton ATPase subunit H isoform X3 [Ostrinia furnacalis] >QRR19186.1 V-type proton ATPase subunit H [Ostrinia nubilalis]	4.108065157	2.038459064	2.67E-06	up	yes	1.034	0.2517	0.257	0.242	0.256	1	1.068	1.034	CC:vacuolar proton-transporting V-type ATPase, V1 domain;CC:proton-transporting V-type ATPase, V1 domain;CC:proton-transporting two-sector ATPase complex, catalytic domain;CC:macromolecular complex;CC:cellular_component;CC:membrane protein complex;MF:molecular_function;MF:ion transmembrane transporter activity;MF:ATPase activity, coupled to transmembrane movement of substances;MF:inorganic solute uptake transmembrane transporter activity;MF:hydrogen-translocating pyrophosphatase activity;MF:primary active transmembrane transporter activity;MF:active transmembrane transporter activity;MF:ATPase coupled ion transmembrane transporter activity;MF:transporter activity;MF:cation transmembrane transporter activity;MF:proton-transporting ATPase activity, rotational mechanism;MF:ATPase activity, coupled to transmembrane movement of ions, rotational mechanism;MF:inorganic cation transmembrane transporter activity;MF:cation-transporting ATPase activity;MF:transmembrane transporter activity;MF:active ion	GO:0000221;GO:0033180;GO:0033178;GO:0032991;GO:0005575;GO:0098796;GO:0003674;GO:0015075;GO:0042626;GO:0015318;GO:0009678;GO:0015399;GO:0022804;GO:0042625;GO:0005215;GO:0008324;GO:0046961;GO:0044769;GO:0022890;GO:0019829;GO:0022857;GO:0022853;GO:0015078	K02144	ATPeV1H	map05152;ma[illegible]	Tuberculosis;Hun	COG5231	C:Energy production and conve	PF03224.17;PF1[illegible]	V-ATPase_H_	V-ATPase subunit	CYT	15	38	55.2	High
TRINITY_DN6074_c0_g1_i1_orf1	uncharacterized protein C1683.06c-like isoform X1 [Ostrinia furnacalis]	3.947051745	1.980775433	2.72E-06	up	yes	0.984	0.2493	0.279	0.243	0.226	1	0.989	0.963	MF:hydrolase activity;MF:molecular_function;MF:hydrolase activity, hydrolyzing N-glycosyl compounds;MF:hydrolase activity, acting on glycosyl bonds;MF:catalytic activity;	GO:0016787;GO:0003674;GO:0016799;GO:0016798;GO:0003824	------	------	------	------	COG1957;COG1199	F:Nucleotide transport and met	PF01156.22	IU_nuc_hydro	Inosine-uridine pr	CYT	2	5	38.9	High
TRINITY_DN2695_c0_g1_i14_orfp1	TRINITY_DN2695_c0_g1_i14_m.44485 TRINITY_DN2695_c0_g1::TRINITY_DN2695_c0_g1_i14::g.44485 ORF type:3prime_partial len:698 (+),score=187.51 TRINITY_DN2695_c0_g1_i14:101-2092(+)	4.85837734	2.280474546	5.34E-05	up	yes	1.012	0.2083	0.177	0.229	0.219	1	1.089	0.948	------	------	------	------	------	------	------	------	------	------	------	CYT	2	5	73.5	High
TRINITY_DN48410_c0_g2_i1_orf1	alpha-amylase 2-like isoform X3 [Ostrinia furnacalis]	4.558646268	2.188605466	2.78E-06	up	yes	0.9833	0.2157	0.215	0.21	0.222	1	1.006	0.944	BP:organic substance metabolic process;BP:primary metabolic process;BP:carbohydrate metabolic process;BP:metabolic process;BP:biological_process;MF:hydrolase activity;MF:cation binding;MF:amylase activity;MF:hydrolase activity, acting on glycosyl bonds;MF:ion binding;MF:catalytic activity;MF:alpha-amylase activity;MF:molecular_function;MF:binding;MF:hydrolase	GO:0071704;GO:0044238;GO:0005975;GO:0008152;GO:0008150;GO:0016787;GO:0043169;GO:0016160;GO:0016798;GO:0043167;GO:0003824;GO:0004556;GO:0003674;GO:0005488;GO:0004553;GO:0046872	K01176	AMY, amyA, r	map04972;ma[illegible]	Pancreatic secret	COG0366	G:Carbohydrate transport and r	PF00128.27;PF0[illegible]	Alpha-amylas	Alpha amylase, cat	EXC	8	15	56.5	High
TRINITY_DN29034_c0_g1_i2_orf1	trypsin-like serine protease [Ostrinia nubilalis]	5.191709845	2.376209756	2.44E-07	up	yes	1.002	0.193	0.197	0.209	0.173	1	1.011	0.996	BP:organic substance metabolic process;BP:organonitrogen compound metabolic process;BP:protein metabolic process;BP:macromolecule metabolic process;BP:biological_process;BP:nitrogen compound metabolic process;BP:metabolic process;BP:proteolysis;BP:primary metabolic process;MF:serine-type endopeptidase activity;MF:endopeptidase activity;MF:hydrolase activity;MF:serine hydrolase activity;MF:catalytic activity, acting on a protein;MF:catalytic activity;MF:molecular_function;MF:peptidase activity;MF:serine-	GO:0071704;GO:1901564;GO:0019538;GO:0043170;GO:0008150;GO:0006807;GO:0008152;GO:0006508;GO:0044238;GO:0004252;GO:0004175;GO:0016787;GO:0017171;GO:0140096;GO:0003824;GO:0003674;GO:0008233;GO:0008236	K01312	PRSS1_2_3	map04972;ma[illegible]	Pancreatic secret	COG5640	O:Posttranslational modificatio	PF00089.29	Trypsin	Trypsin	CYT	1	8	15.3	High
TRINITY_DN2178_c0_g1_i1_orf1	carboxypeptidase B-like [Ostrinia furnacalis]	5.356186395	2.421206167	1.89E-06	up	yes	1	0.1867	0.158	0.205	0.197	1	1.022	0.978	BP:nitrogen compound metabolic process;BP:organic substance metabolic process;BP:proteolysis;BP:biological_process;BP:metabolic process;BP:primary metabolic process;BP:organonitrogen compound metabolic process;BP:protein metabolic process;BP:macromolecule metabolic process;MF:exopeptidase activity;MF:molecular_function;MF:zinc ion binding;MF:peptidase activity;MF:metalloexopeptidase activity;MF:metallopeptidase activity;MF:cation binding;MF:transition metal ion binding;MF:metallocarboxypeptidase activity;MF:carboxypeptidase activity;MF:ion binding;MF:metal	GO:0006807;GO:0071704;GO:0006508;GO:0008150;GO:0008152;GO:0044238;GO:1901564;GO:0019538;GO:0043170;GO:0008238;GO:0003674;GO:0008270;GO:0008233;GO:0008235;GO:0008237;GO:0043169;GO:0046914;GO:0004181;GO:0004180;GO:0043167;GO:0046872;GO:0016787;GO:0140096;GO:0003824;GO:0005488	K01291;K012[illegible]	CPB1;CPA2	map04972;ma[illegible]	Pancreatic secret	COG2866	E:Amino acid transport and me	PF00246.27;PF0[illegible]	Peptidase_M1	Zinc carboxypeptic	CYT	1	2	48.1	High
TRINITY_DN10940_c0_g1_i10_orfp1	TRINITY_DN10940_c0_g1_i10_m.52163 TRINITY_DN10940_c0_g1::TRINITY_DN10940_c0_g1_i10::g.52163 ORF type:5prime_partial len:248 (-),score=128.24 TRINITY_DN10940_c0_g1_i10:121-864(-)	13.19803353	3.722251083	3.57E-06	up	yes	1.047	0.07933	0.085	0.085	0.068	1	1.091	1.05	------	------	------	------	------	------	------	------	------	------	------	CYT	1	14	22.7	High
TRINITY_DN348_c0_g2_i3_orf1	pancreatic triacylglycerol lipase-like [Ostrinia furnacalis]	10.36584091	3.373765252	3.53E-08	up	yes	0.9917	0.09567	0.101	0.094	0.092	1	0.998	0.977	------	------	------	------	------	------	ENOG4110T03;ENOG410YGS3;ENOG4110QHK	S:Function unknown;	PF00151.22	Lipase	Lipase	CYT	4	45	36.1	High
TRINITY_DN76815_c0_g1_i3_orf1	5-formyltetrahydrofolate cyclo-ligase [Ostrinia furnacalis]	9.406163612	3.233606427	5.78E-05	up	yes	0.9187	0.09767	0.133	0.079	0.081	1	0.857	0.899	MF:purine ribonucleoside triphosphate binding;MF:cyclo-ligase activity;MF:molecular_function;MF:binding;MF:ion binding;MF:heterocyclic compound binding;MF:nucleoside phosphate binding;MF:anion binding;MF:cation binding;MF:nucleotide binding;MF:ligase activity, forming carbon-nitrogen bonds;MF:purine nucleotide binding;MF:metal ion binding;MF:ATP binding;MF:small molecule binding;MF:catalytic activity;MF:ligase activity;MF:5-formyltetrahydrofolate cyclo-ligase activity;MF:adenyl nucleotide binding;MF:carbohydrate derivative binding;MF:organic cyclic compound binding;MF:adenyl ribonucleotide binding;MF:purine ribonucleotide	GO:0035639;GO:0016882;GO:0003674;GO:0005488;GO:0043167;GO:1901363;GO:1901265;GO:0043168;GO:0043169;GO:0000166;GO:0016879;GO:0017076;GO:0046872;GO:0005524;GO:0036094;GO:0003824;GO:0016874;GO:0030272;GO:0030554;GO:0097367;GO:0097159;GO:0032559;GO:0032555;GO:0032553	K01934	MTHFS	map00670	One carbon pool	COG0212	H:Coenzyme transport and me	PF01812.23	5-FTHF_cyc-l	5-formyltetrahydrc	CYT	1	61	14.2	High
TRINITY_DN19244_c0_g1_i7_orf1	uncharacterized protein LOC114350218 [Ostrinia furnacalis]	2.250758814	1.170411469	0.0002173	up	yes	0.964	0.4283	0.491	0.434	0.36	1	0.946	0.946	------	------	K16830	AURKAIP1	------	------	ENOG410Y2DU;COG0022	S:Function unknown;C:Energy p	------	------	------	CYT	1	4	25.6	High
TRINITY_DN24476_c0_g1_i1_orf1	ensconsin-like isoform X1 [Ostrinia furnacalis]	2.573054047	1.363481761	1.32E-05	up	yes	0.995	0.3867	0.375	0.411	0.374	1	1.027	0.958	------	------	K24203;K16[illegible]	MAP7D2;MA[illegible]	------	------	ENOG4111S2A;ENOG4111CQ3	S:Function unknown;	------	------	------	CYT	4	6	92	High
TRINITY_DN24873_c0_g1_i4_orf1	uncharacterized protein LOC114365742 [Ostrinia furnacalis]	3.090854211	1.628005607	1.14E-06	up	yes	1.024	0.3313	0.335	0.342	0.317	1	1.029	1.042	CC:endoplasmic reticulum membrane;CC:intrinsic component of membrane;CC:organelle membrane;CC:integral component of membrane;CC:membrane;CC:cellular anatomical entity;CC:cellular_component;MF:monooxygenase activity;MF:oxidoreductase activity, acting on paired donors, with incorporation or reduction of molecular oxygen;MF:molecular_function;MF:binding;MF:heterocyclic compound binding;MF:oxidoreductase activity;MF:cation binding;MF:transition metal ion binding;MF:ion binding;MF:iron ion binding;MF:metal ion binding;MF:heme binding;MF:catalytic activity;MF:tetrapyrrole binding;MF:organic cyclic compound binding;	GO:0005789;GO:0031224;GO:0031090;GO:0016021;GO:0016020;GO:0110165;GO:0005575;GO:0004497;GO:0016705;GO:0003674;GO:0005488;GO:1901363;GO:0016491;GO:0043169;GO:0046914;GO:0043167;GO:0005506;GO:0046872;GO:0020037;GO:0003824;GO:0046906;GO:0097159	K15001	CYP4	------	------	COG2124	S:Function unknown	PF00067.25	p450	Cytochrome P450	CYT	2	3	59.3	High
TRINITY_DN1123_c2_g1_i3_orf1	troponin I isoform X8 [Ostrinia furnacalis]	3.082798656	1.624240667	4.21E-06	up	yes	1.009	0.3273	0.299	0.36	0.323	1	1.026	1	CC:cellular_component;CC:macromolecular complex;CC:troponin complex;	GO:0005575;GO:0032991;GO:0005861	K12044	TNNI3	map04260;ma[illegible]	Cardiac muscle c	ENOG410Y9IX	Z:Cytoskeleton	PF00992.23	Troponin	Troponin	CYT	3	55	10.3	High
TRINITY_DN146544_c0_g1_i1_orf1	UPF0047 protein YjbQ [Aphidius gifuensis] >KAF7996225.1 hypothetical protein HCN44_001857 [Aphidius gifuensis]	2.29458323	1.198232138	0.0005636	up	yes	0.9277	0.4043	0.469	0.338	0.406	1	0.888	0.895	------	------	------	------	------	------	COG0432	S:Function unknown	PF01894.20	UPF0047	Uncharacterised pr	CYT	1	9	16.6	High
TRINITY_DN40_c0_g1_i3_orf1	trypsin CFT-1-like [Ostrinia furnacalis]	3.051829268	1.609674254	3.70E-07	up	yes	1.001	0.328	0.329	0.324	0.331	1	0.983	1.019	BP:organic substance metabolic process;BP:organonitrogen compound metabolic process;BP:protein metabolic process;BP:macromolecule metabolic process;BP:biological_process;BP:nitrogen compound metabolic process;BP:metabolic process;BP:proteolysis;BP:primary metabolic process;MF:serine-type endopeptidase activity;MF:endopeptidase activity;MF:hydrolase activity;MF:serine hydrolase activity;MF:catalytic activity, acting on a protein;MF:catalytic activity;MF:molecular_function;MF:peptidase activity;MF:serine-	GO:0071704;GO:1901564;GO:0019538;GO:0043170;GO:0008150;GO:0006807;GO:0008152;GO:0006508;GO:0044238;GO:0004252;GO:0004175;GO:0016787;GO:0017171;GO:0140096;GO:0003824;GO:0003674;GO:0008233;GO:0008236	K01312	PRSS1_2_3	map04972;ma[illegible]	Pancreatic secret	COG5640	O:Posttranslational modificatio	PF00089.29	Trypsin	Trypsin	CYT	4	18	28.1	High
TRINITY_DN53462_c0_g1_i1_orf1	uncharacterized protein LOC118072968 isoform X1 [Chelonus insularis] >XP_034949073.1 uncharacterized protein LOC118072968 isoform X1 [Chelonus insularis]	3.42558218	1.776349196	2.77E-05	up	yes	1.015	0.2963	0.361	0.268	0.26	1	1.017	1.028	------	------	------	------	------	------	ENOG41125KI	S:Function unknown	PF15376.9	DUF4603	Domain of unknow	CYT	1	1	69.1	Medium
TRINITY_DN4145_c0_g1_i1_orf1	uncharacterized protein LOC114353175 isoform X1 [Ostrinia furnacalis]	2.552552553	1.351940664	0.0005433	up	yes	0.935	0.3663	0.39	0.293	0.416	1	0.949	0.856	BP:cellular catabolic process;BP:cellular nitrogen compound metabolic process;BP:nitrogen compound metabolic process;BP:organic cyclic compound metabolic process;BP:organic cyclic compound catabolic process;BP:organic substance catabolic process;BP:organic substance metabolic process;BP:heterocycle metabolic process;BP:allantoin catabolic process;BP:allantoin metabolic process;BP:cellular process;BP:heterocycle catabolic process;BP:biological_process;BP:metabolic process;BP:catabolic process;BP:organonitrogen compound catabolic process;BP:cellular amide catabolic process;BP:organonitrogen compound metabolic process;BP:cellular nitrogen compound catabolic process;BP:cellular amide metabolic process;BP:cellular metabolic process;MF:hydrolase activity;MF:allantoinase activity;MF:molecular_function;MF:zinc ion binding;MF:cation binding;MF:transition metal ion binding;MF:ion binding;MF:catalytic activity;MF:metal ion binding;MF:hydrolase	GO:0044248;GO:0034641;GO:0006807;GO:1901360;GO:1901361;GO:1901575;GO:0071704;GO:0046483;GO:0000256;GO:0000255;GO:0009987;GO:0046700;GO:0008150;GO:0008152;GO:0009056;GO:1901565;GO:0043605;GO:1901564;GO:0044270;GO:0043603;GO:0044237;GO:0016787;GO:0004038;GO:0003674;GO:0008270;GO:0043169;GO:0046914;GO:0043167;GO:0003824;GO:0046872;GO:0016812;GO:0016810;GO:0050897;GO:0005488	K01466	allB	map00230	Purine metabolis	COG0044	F:Nucleotide transport and met	PF01979.23;PF0[illegible]	Amidohydro_	Amidohydrolase fa	CYT	1	2	73.6	High
TRINITY_DN84357_c0_g1_i1_orf1	4-coumarate--CoA ligase 1-like [Ostrinia furnacalis]	3.110547989	1.637168764	5.93E-06	up	yes	0.982	0.3157	0.291	0.351	0.305	1	0.963	0.983	MF:catalytic activity;MF:monooxygenase activity;MF:molecular_function;MF:oxidoreductase activity;	GO:0003824;GO:0004497;GO:0003674;GO:0016491	K01904	4CL	map00130	Ubiquinone and	COG0318;COG0318	I:Lipid transport and metabolis	PF00501.31;PF1[illegible]	AMP-binding	AMP-binding enzy	CYT	1	2	58.8	High
TRINITY_DN1073_c0_g1_i4_orf1	carboxylesterase [Loxostege sticticalis]	3.072292994	1.619315807	7.83E-06	up	yes	0.9647	0.314	0.308	0.32	0.314	1	0.969	0.925	CC:integral component of membrane;CC:cellular_component;CC:intrinsic component of membrane;CC:cellular anatomical entity;MF:hydrolase activity;MF:hydrolase activity, acting on ester bonds;MF:carboxylic ester hydrolase activity;MF:catalytic	GO:0016021;GO:0005575;GO:0031224;GO:0110165;GO:0016787;GO:0016788;GO:0052689;GO:0003824;GO:0003674	------	------	------	------	COG2272;COG0580;ENOG410XSWS	I:Lipid transport and metabolis	PF00135.31;PF2[illegible]	COesterase;B	Carboxylesterase fa	EXC	5	14	65.7	High

TRINITY_DN96557_c0_g1_i1_orf1	charged multivesicular body protein 4B [Phyllostomus discolor]	4.381685922	2.131486076	1.40E-06	up	yes	1.024	0.2337	0.212	0.253	0.236	1	1.043	1.029	BP:protein localization;BP:regulation of metabolic process;BP:anatomical structure homeostasis;BP:membrane organization;BP:plasma membrane organization;BP:establishment of organelle localization;BP:cellular component organization or biogenesis;BP:regulation of spindle assembly;BP:cellular macromolecule localization;BP:regulation of cytoskeleton organization;BP:interspecies interaction between organisms;BP:mitotic metaphase plate congression;BP:late endosome to lysosome transport;BP:negative regulation of biological process;BP:macromolecule localization;BP:protein polymerization;BP:negative regulation of cell death;BP:protein targeting;BP:establishment of protein localization;BP:protein localization to membrane;BP:localization within membrane;BP:mitotic cytokinetic process;BP:interaction with host;BP:organonitrogen compound metabolic process;BP:vesicle-mediated transport;BP:protein metabolic process;BP:regulation of autophagy;BP:negative regulation of organelle organization;BP:negative regulation of autophagy;BP:ubiquitin-independent protein catabolic process via the multivesicular body sorting pathway;BP:regulation of microtubule cytoskeleton organization;BP:regulation of catabolic process;BP:regulation of mitotic spindle assembly;BP:negative regulation of metabolic process;BP:negative regulation of neuron death;BP:protein targeting to membrane;BP:nitrogen compound metabolic process;BP:regulation of biological process;BP:organic substance catabolic process;BP:cellular macromolecule catabolic process;BP:cellular macromolecule metabolic	GO:0008104;GO:0019222;GO:0060249; GO:0061024;GO:0007009;GO:0051656; GO:0071840;GO:0090169;GO:0070727; GO:0051493;GO:0044419;GO:0007080; GO:1902774;GO:0048519;GO:0033036; GO:0051258;GO:0060548;GO:0006605; GO:0045184;GO:0072657;GO:0051668; GO:1902410;GO:0051701;GO:1901564; GO:0016192;GO:0019538;GO:0010506; GO:0010639;GO:0010507;GO:0090611; GO:0070507;GO:0009894;GO:1901673; GO:0009892;GO:1901215;GO:0006612; GO:0006807;GO:0050789;GO:1901575; GO:0044265;GO:0044260;GO:0006886; GO:0016043;GO:0065003;GO:0065007; GO:0071985;GO:0065008;GO:0006810; GO:0006620;GO:0060236;GO:0070972; GO:0008150;GO:0008152;GO:1901214; GO:0050794;GO:0001778;GO:0051603; GO:0009895;GO:0046907;GO:0039702; GO:0045047;GO:0006511;GO:0033043; GO:0051129;GO:0051128;GO:0044248; GO:0022607;GO:0007041;GO:0032886; GO:0051234;GO:0043162;GO:0010564; GO:0009057;GO:0071763;GO:0044000; GO:0031468;GO:0006508;GO:0072599; GO:0036438;GO:0019076;GO:0031330; GO:0072594;GO:0009987;GO:0019941; GO:0046605;GO:0051310;GO:0001894;	K12194	CHMP4A_B, S	map03250;ma	Viral life cycle - H	ENOG410YE9I	I:Lipid transport and metabolis	PF03357.24	Snf7	Snf7	CYT	1	14	8	High
TRINITY_DN20682_c0_g2_i1_orf1	glutathione S-transferase delta3 [Glyphodes pyloalis]	3.738032416	1.90227908	2.17E-06	up	yes	0.9917	0.2653	0.272	0.281	0.243	1	0.965	1.01	MF:catalytic activity;MF:transferase activity;MF:glutathione transferase activity;MF:molecular_function;MF:transferase activity, transferring alkyl or aryl	GO:0003824;GO:0016740;GO:0004364; GO:0003674;GO:0016765	K00799	GST, gst	map05207;ma	Chemical carcinc	COG0625	O:Posttranslational modificatio	PF13417.9;PF02	GST_N_3;GST_	Glutathione S-tran	PLA	1	8	14.1	Medium
TRINITY_DN75188_c0_g1_i1_orf1	fatty acid-binding protein 1-like [Ostrinia furnacalis]	4.356986421	2.123330617	2.08E-05	up	yes	0.9947	0.2283	0.252	0.204	0.229	1	0.94	1.044	MF:molecular_function;MF:lipid binding;MF:binding;	GO:0003674;GO:0008289;GO:0005488	K08753;K087	FABP4, aP2;F	map04923;ma	Regulation of lip	ENOG4111US8	I:Lipid transport and metabolis	------	------	------	CYT	1	7	14.7	High
TRINITY_DN1232_c0_g1_i1_orf1	acanthoscurrin-2-like isoform X1 [Ostrinia furnacalis]	2.25728948	1.174591445	1.69E-05	up	yes	0.9677	0.4287	0.411	0.453	0.422	1	0.937	0.966	CC:cellular_component;CC:integral component of membrane;CC:cellular anatomical entity;CC:intrinsic component of membrane;	GO:0005575;GO:0016021;GO:0110165; GO:0031224	------	------	------	------	------	------	------	------	------	CYT	2	15	16.6	High
TRINITY_DN1310_c0_g1_i4_orf1	trypsin-like isoform X1 [Ostrinia furnacalis] >XP_028159118.1 trypsin-like isoform X2 [Ostrinia furnacalis]	4.888349515	2.289347441	1.22E-06	up	yes	1.007	0.206	0.222	0.184	0.212	1	1.032	0.99	BP:organic substance metabolic process;BP:organonitrogen compound metabolic process;BP:protein metabolic process;BP:macromolecule metabolic process;BP:biological_process;BP:nitrogen compound metabolic process;BP:metabolic process;BP:proteolysis;BP:primary metabolic process;MF:serine-type endopeptidase activity;MF:endopeptidase activity;MF:hydrolase activity;MF:serine hydrolase activity;MF:catalytic activity, acting on a protein;MF:catalytic	GO:0071704;GO:1901564;GO:0019538; GO:0043170;GO:0008150;GO:0006807; GO:0008152;GO:0006508;GO:0044238; GO:0004252;GO:0004175;GO:0016787; GO:0017171;GO:0140096;GO:0003824; GO:0003674;GO:0008233;GO:0008236	------	------	------	------	ENOG411152E;COG5640	O:Posttranslational modificatio	PF00089.29	Trypsin	Trypsin	CYT	3	26	26.7	High
TRINITY_DN67623_c0_g1_i1_orf1	maltase A1-like [Ostrinia furnacalis]	5.357710652	2.421616669	3.48E-08	up	yes	1.011	0.1887	0.186	0.188	0.192	1	1.024	1.01	BP:primary metabolic process;BP:carbohydrate metabolic process;BP:organic substance metabolic process;BP:biological_process;BP:metabolic process;CC:integral component of membrane;CC:cellular_component;CC:intrinsic component of membrane;CC:cellular anatomical entity;MF:hydrolase activity;MF:hydrolase activity, acting on glycosyl bonds;MF:catalytic activity;MF:molecular_function;	GO:0044238;GO:0005975;GO:0071704; GO:0008150;GO:0008152;GO:0016021; GO:0005575;GO:0031224;GO:0110165; GO:0016787;GO:0016798;GO:0003824; GO:0003674	------	------	------	------	COG0366	G:Carbohydrate transport and	PF00128.27	Alpha-amylas	Alpha amylase, cat	CYT	4	9	68.6	High
TRINITY_DN146841_c0_g1_i1_orf1	muscle-specific protein 20 [Temnothorax curvispinosus]	4.698029557	2.23205579	3.24E-05	up	yes	0.9537	0.203	0.211	0.236	0.162	1	0.9	0.961	------	------	K20526	TAGLN	------	------	COG5199	S:Function unknown	PF00307.34;PF0	CH;Calponin	Calponin homolog	CYT	1	10	20.6	High
TRINITY_DN31118_c1_g1_i1_orf1	endocuticle structural glycoprotein ABD-4-like [Ostrinia furnacalis]	5.772114821	2.529099999	2.31E-06	up	yes	0.9853	0.1707	0.193	0.137	0.182	1	0.992	0.964	MF:molecular_function;MF:structural constituent of cuticle;MF:structural molecule activity;	GO:0003674;GO:0042302;GO:0005198	------	------	------	------	ENOG4111PJ1;ENOG410YR66	S:Function unknown;	PF00379.26	Chitin_bind_4	Insect cuticle prote	CYT	1	23	12	High
TRINITY_DN2695_c0_g1_i8_orfp1	TRINITY_DN2695_c0_g1_i8_m.44478 TRINITY_DN2695_c0_g1::TRINITY_DN2695_c0_g1_i8::g.44478 ORF type:3prime_partial len:532 (+),score=80.62 TRINITY_DN2695_c0_g1_i8:101-1594(+)	4.863266413	2.281925626	0.002895	up	yes	0.8963	0.1843	0.185	0.231	0.137	1	0.684	1.005	------	------	------	------	------	------	------	------	------	------	------	CYT	1	2	56.2	High
TRINITY_DN94355_c0_g1_i2_orf1	uncharacterized protein LOC126369488 [Pectinophora gossypiella]	10.92660246	3.449772971	3.19E-06	up	yes	0.987	0.09033	0.097	0.076	0.098	1	1.019	0.942	------	------	------	------	------	------	ENOG410Z1QR;ENOG410Y7Q4;COG2801;ENOG410YP2D	S:Function unknown;L:Replicati	PF05380.16;PF1	Peptidase_A1	Pao retrotransposo	CYT	1	0	179.7	High
TRINITY_DN3504_c0_g1_i4_orfp1	TRINITY_DN3504_c0_g1_i4_m.43930 TRINITY_DN3504_c0_g1::TRINITY_DN3504_c0_g1_i4::g.43930 ORF type:internal len:196 (-),score=84.82 TRINITY_DN3504_c0_g1_i4:3-587(-)	9.729785267	3.282407966	2.71E-05	up	yes	0.947	0.09733	0.121	0.053	0.118	1	0.888	0.953	------	------	------	------	------	------	------	------	------	------	------	EXC	1	8	21.5	High
TRINITY_DN8369_c0_g1_i1_orf1	39S ribosomal protein L37, mitochondrial [Ostrinia furnacalis]	2.391770149	1.258078752	4.57E-07	up	yes	0.9823	0.4107	0.413	0.411	0.408	1	0.968	0.979	------	------	K17418	MRPL37	------	------	ENOG410ZT8U	J:Translation, ribosomal structu	PF07147.15	PDCD9	Mitochondrial 28S	CYT	3	6	47.1	High
TRINITY_DN1952_c0_g1_i2_orf1	uncharacterized protein LOC114354403 [Ostrinia furnacalis] >AYE20402.1 RNAi efficiency-related nuclease REase [Ostrinia furnacalis]	2.625032869	1.392335487	1.84E-07	up	yes	0.9983	0.3803	0.386	0.384	0.371	1	0.986	1.009	CC:intracellular membrane-bounded organelle;CC:nucleus;CC:cellular_component;CC:intracellular organelle;CC:membrane-bounded organelle;CC:organelle;CC:cellular anatomical entity;MF:hydrolase activity;MF:cation binding;MF:nuclease activity;MF:hydrolase activity, acting on ester bonds;MF:ion binding;MF:catalytic	GO:0043231;GO:0005634;GO:0005575; GO:0043229;GO:0043227;GO:0043226; GO:0110165;GO:0016787;GO:0043169; GO:0004518;GO:0016788;GO:0043167; GO:0003824;GO:0003674;GO:0005488; GO:0046872	------	------	------	------	ENOG4111F5P	S:Function unknown	------	------	------	CYT	11	17	70.8	High
TRINITY_DN54543_c0_g5_i2_orf1	reactive oxygen species modulator 1 [Papilio machaon] >XP_022123076.1 reactive oxygen species modulator 1 [Pieris rapae] >XP_022820703.1 reactive oxygen species modulator 1 [Spodoptera litura] >XP_028176790.1 reactive oxygen species modulator 1 [Ostrinia furnacalis] >XP_030021418.1 reactive oxygen species modulator 1 [Manduca sexta] >XP_034836896.1 reactive oxygen species modulator 1 [Maniola hyperantus] >XP_035446679.1 reactive oxygen species modulator 1-like [Spodoptera frugiperda] >XP_045458971.1 reactive oxygen species modulator 1 [Melitaea cinxia] >XP_045521377.1 reactive oxygen species modulator 1 [Pieris brassicae] >XP_045765649.1 reactive oxygen species modulator 1 [Maniola jurtina] >XP_047042173.1 reactive oxygen species modulator 1 [Helicoverpa zea] >XP_049691668.1 reactive oxygen species modulator 1 [Helicoverpa armigera] >KAF9414699.1 hypothetical protein HW555_007477 [Spodoptera exigua] >KPI93862.1 Reactive oxygen species modulator 1 [Papilio xuthus] >CAB3515149.1 unnamed protein product [Spodoptera littoralis] >CAG5021695.1 unnamed protein product [Parnassius apollo] >KAF9823017.1 hypothetical protein	2.628021835	1.393977262	9.81E-05	up	yes	1.011	0.3847	0.368	0.406	0.38	1	0.95	1.082	CC:cellular_component;CC:integral component of membrane;CC:cellular anatomical entity;CC:intrinsic component of membrane;	GO:0005575;GO:0016021;GO:0110165; GO:0031224	------	------	------	------	ENOG411279J	S:Function unknown	PF10247.12	Romo1	Reactive mitochon	CYT	1	23	8.2	High
TRINITY_DN106534_c0_g1_i1_orf1	nucleolar complex protein 2 homolog [Ostrinia furnacalis]	2.752491247	1.460737976	0.0003382	up	yes	1.022	0.3713	0.41	0.382	0.322	1	1.119	0.947	CC:intracellular membrane-bounded organelle;CC:nucleus;CC:cellular_component;CC:intracellular organelle;CC:membrane-bounded organelle;CC:organelle;CC:cellular anatomical entity;	GO:0043231;GO:0005634;GO:0005575; GO:0043229;GO:0043227;GO:0043226; GO:0110165	K14833	NOC2	------	------	COG5604	S:Function unknown	PF03715.16	Noc2	Noc2p family	CYT	1	2	78.2	High
TRINITY_DN8833_c0_g1_i1_orf1	nucleolar protein 16 [Ostrinia furnacalis]	2.759924386	1.464628742	1.16E-05	up	yes	1.022	0.3703	0.334	0.408	0.369	1	1.04	1.026	------	------	------	------	------	------	ENOG4111QB6	S:Function unknown	PF09420.13	Nop16	Ribosome biogene	CYT	2	8	24.1	High
TRINITY_DN536_c0_g1_i7_orf1	polymerase delta-interacting protein 2 isoform X3 [Ostrinia furnacalis]	2.247025892	1.168016749	3.07E-05	up	yes	0.9633	0.4287	0.437	0.414	0.435	1	0.973	0.917	MF:organic cyclic compound binding;MF:molecular_function;MF:binding;MF:nucleic acid binding;MF:DNA binding;MF:heterocyclic compound binding;	GO:0097159;GO:0003674;GO:0005488; GO:0003676;GO:0003677;GO:1901363	K17809	POLDIP2	------	------	COG2967	P:Inorganic ion transport and n	PF04379.17;PF0	DUF525;YccV	ApaG domain;Hem	CYT	2	12	39.3	High
TRINITY_DN2579_c0_g1_i7_orf1	aminopeptidase N5 [Ostrinia nubilalis]	2.527613666	1.337775971	1.31E-05	up	yes	0.984	0.3893	0.377	0.431	0.36	1	0.973	0.979	BP:nitrogen compound metabolic process;BP:organic substance metabolic process;BP:proteolysis;BP:biological_process;BP:metabolic process;BP:primary metabolic process;BP:organonitrogen compound metabolic process;BP:protein metabolic process;BP:macromolecule metabolic process;CC:integral component of membrane;CC:intrinsic component of membrane;CC:cellular anatomical entity;CC:cellular_component;MF:aminopeptidase activity;MF:exopeptidase activity;MF:molecular_function;MF:binding;MF:peptidase activity;MF:metallopeptidase activity;MF:cation binding;MF:transition metal ion binding;MF:ion binding;MF:metal ion binding;MF:hydrolase activity;MF:catalytic	GO:0006807;GO:0071704;GO:0006508; GO:0008150;GO:0008152;GO:0044238; GO:1901564;GO:0019538;GO:0043170; GO:0016021;GO:0031224;GO:0110165; GO:0005575;GO:0004177;GO:0008238; GO:0003674;GO:0005488;GO:0008233; GO:0008237;GO:0043169;GO:0046914; GO:0043167;GO:0046872;GO:0016787; GO:0140096;GO:0003824;GO:0008270	K11140	ANPEP, CD13	map04640;ma	Hematopoietic c	COG0308	L:Replication, recombination ar	PF01433.23;PF1	Peptidase_M1	Peptidase family M	CYT	3	3	108	High
TRINITY_DN6351_c0_g1_i4_orf1	cytochrome P450 CYP12A2-like [Ostrinia furnacalis]	2.62133615	1.390302371	1.92E-06	up	yes	0.9927	0.3787	0.371	0.395	0.37	1	1.009	0.969	MF:cation binding;MF:monooxygenase activity;MF:heme binding;MF:transition metal ion binding;MF:tetrapyrrole binding;MF:organic cyclic compound binding;MF:oxidoreductase activity, acting on paired donors, with incorporation or reduction of molecular oxygen;MF:ion binding;MF:catalytic activity;MF:molecular_function;MF:binding;MF:iron ion binding;MF:metal ion binding;MF:heterocyclic compound	GO:0043169;GO:0004497;GO:0020037; GO:0046914;GO:0046906;GO:0097159; GO:0016705;GO:0043167;GO:0003824; GO:0003674;GO:0005488;GO:0005506; GO:0046872;GO:1901363;GO:0016491	K15004	CYP12	------	------	COG2124;ENOG410XP8T	G:Carbohydrate transport and	PF00067.25	p450	Cytochrome P450	CYT	2	4	57.5	High
TRINITY_DN56110_c0_g1_i1_orf1	pescadillo homolog [Ostrinia furnacalis]	3.103448276	1.633872101	1.42E-06	up	yes	1.017	0.3277	0.342	0.311	0.33	1	1.041	1.011	BP:maturation of 5.8S rRNA from tricistronic rRNA transcrip;BP:nucleic acid metabolic process;BP:maturation of LSU-rRNA;BP:ncRNA metabolic process;BP:organic cyclic compound metabolic process;BP:nucleobase-containing compound metabolic process;BP:organic substance metabolic process;BP:rRNA processing;BP:ncRNA processing;BP:cellular process;BP:cellular aromatic compound metabolic process;BP:maturation of 5.8S rRNA;BP:maturation of LSU-rRNA from tricistronic rRNA transcrip;BP:biological_process;BP:metabolic process;BP:heterocycle metabolic process;BP:RNA metabolic process;BP:primary metabolic process;BP:rRNA metabolic process;BP:cellular nitrogen compound metabolic process;BP:cellular metabolic process;BP:macromolecule metabolic process;BP:nitrogen compound metabolic process;BP:RNA processing;CC:preribosome, large subunit precursor;CC:preribosome;CC:intracellular organelle;CC:non-membrane-bounded organelle;CC:organelle;CC:nucleolus;CC:nucleoplasm;CC:cellular anatomical entity;CC:macromolecular complex;CC:intracellular non-membrane-bounded organelle;CC:ribonucleoprotein complex;CC:cellular_component;MF:macromolecular complex	GO:0000466;GO:0090304;GO:0000470; GO:0034660;GO:1901360;GO:0006139; GO:0071704;GO:0006364;GO:0034470; GO:0009987;GO:0006725;GO:0000460; GO:0000463;GO:0008150;GO:0008152; GO:0046483;GO:0016070;GO:0044238; GO:0016072;GO:0034641;GO:0044237; GO:0043170;GO:0006807;GO:0006396; GO:0030687;GO:0030684;GO:0043229; GO:0043228;GO:0043226;GO:0005730; GO:0005654;GO:0110165;GO:0032991; GO:0043232;GO:1990904;GO:0005575; GO:0044877;GO:0005488;GO:0043021; GO:0003674	K14843	PES1, NOP7	------	------	COG5163	J:Translation, ribosomal structu	PF06732.14;PF1	Pescadillo_N;	Pescadillo N-termi	CYT	2	3	71.4	High
TRINITY_DN31001_c0_g1_i1_orf1	endocuticle structural glycoprotein ABD-5-like [Ostrinia furnacalis]	2.903320599	1.537703891	2.75E-05	up	yes	0.988	0.3403	0.332	0.371	0.318	1	1.025	0.939	MF:molecular_function;MF:structural constituent of cuticle;MF:structural molecule activity;	GO:0003674;GO:0042302;GO:0005198	------	------	------	------	ENOG4111EHD;ENOG410YVGC;ENOG41113X7	S:Function unknown;	PF00379.26	Chitin_bind_4	Insect cuticle prote	CYT	3	41	12.2	High
TRINITY_DN17351_c0_g1_i3_orf1	V-type proton ATPase subunit F [Ostrinia furnacalis]	3.391832602	1.76206497	1.43E-07	up	yes	1.005	0.2963	0.296	0.292	0.301	1	1.021	0.993	CC:proton-transporting V-type ATPase, V1 domain;CC:proton-transporting two-sector ATPase complex, catalytic domain;CC:macromolecular complex;CC:cellular_component;CC:membrane protein complex;MF:molecular_function;MF:ion transmembrane transporter activity;MF:ATPase activity, coupled to transmembrane movement of substances;MF:inorganic solute uptake transmembrane transporter activity;MF:hydrogen-translocating pyrophosphatase activity;MF:primary active transmembrane transporter activity;MF:active transmembrane transporter activity;MF:ATPase coupled ion transmembrane transporter activity;MF:transporter activity;MF:cation transmembrane transporter activity;MF:proton-transporting ATPase activity, rotational mechanism;MF:ATPase activity, coupled to transmembrane movement of ions, rotational mechanism;MF:inorganic cation transmembrane transporter activity;MF:cation-transporting ATPase activity;MF:transmembrane transporter activity;MF:active ion	GO:0033180;GO:0033178;GO:0032991; GO:0005575;GO:0098796;GO:0003674; GO:0015075;GO:0042626;GO:0015318; GO:0009678;GO:0015399;GO:0022804; GO:0042625;GO:0005215;GO:0008324; GO:0046961;GO:0044769;GO:0022890; GO:0019829;GO:0022857;GO:0022853; GO:0015078	K02151	ATPeV1F, ATI	map05323;ma	Rheumatoid arth	COG1436	C:Energy production and conve	PF01990.20	ATP-synt_F	ATP synthase	CYT	6	40	13.8	High

TRINITY_DN1366_c0_g1_i5_orf1	unnamed protein product, partial [Iphiclides podalirius]	3.287086093	1.716809243	1.42E-07	up	yes	0.9927	0.302	0.302	0.29	0.314	1	0.995	0.983	BP:nucleoside triphosphate metabolic process;BP:purine nucleoside triphosphate metabolic process;BP:cellular nitrogen compound metabolic process;BP:nitrogen compound metabolic process;BP:small molecule metabolic process;BP:purine-containing compound metabolic process;BP:ribonucleotide metabolic process;BP:organic cyclic compound metabolic process;BP:nucleobase-containing compound metabolic process;BP:organic substance metabolic process;BP:ribonucleoside triphosphate metabolic process;BP:purine ribonucleoside triphosphate metabolic process;BP:ribose phosphate metabolic process;BP:ATP metabolic process;BP:cellular process;BP:cellular aromatic compound metabolic process;BP:purine ribonucleotide metabolic process;BP:nucleotide metabolic process;BP:metabolic process;BP:nucleobase-containing small molecule metabolic process;BP:heterocycle metabolic process;BP:primary metabolic process;BP:organonitrogen compound metabolic process;BP:carbohydrate derivative metabolic process;BP:cellular metabolic process;BP:purine nucleotide metabolic process;BP:phosphate-containing compound metabolic process;BP:phosphorus metabolic process;BP:organophosphate metabolic process;BP:biological_process;BP:nucleoside phosphate metabolic process;CC:proton-transporting two-sector ATPase complex, catalytic domain;CC:macromolecular complex;CC:cellular_component;CC:proton-transporting V-type ATPase, V1 domain;CC:membrane protein complex;MF:proton-transporting ATPase activity, rotational mechanism;MF:purine ribonucleoside triphosphate binding;MF:ribonucleotide	GO:0009141;GO:0009144;GO:0034641;GO:0006807;GO:0044281;GO:0072521;GO:0009259;GO:1901360;GO:0006139;GO:0071704;GO:0009199;GO:0009205;GO:0019693;GO:0046034;GO:0009987;GO:0006725;GO:0009150;GO:0009117;GO:0008152;GO:0055086;GO:0046483;GO:0044238;GO:1901564;GO:1901135;GO:0044237;GO:0006163;GO:0006796;GO:0006793;GO:0019637;GO:0008150;GO:0006753;GO:0033178;GO:0032991;GO:0005575;GO:0033180;GO:0098796;GO:0046961;GO:0035639;GO:0032553;GO:0003674;GO:0043167;GO:0043168;GO:0042626;GO:0015399;GO:0015318;GO:0022857;GO:0009678;GO:0000166;GO:0044769;GO:0005215;GO:0017076;GO:0022804;GO:0008324;GO:0022890;GO:1901265;GO:0036094;GO:0032555;GO:0042625;GO:0030554;GO:0097367;GO:0097159;GO:0015075;GO:0019829;GO:0032559;GO:0005524;GO:0022853;GO:0015078;GO:1901363;GO:0005488	K02147	ATPeV1B, ATI	map05323;ma	Rheumatoid arth	COG1156	C:Energy production and conv	PF00006.28;PF0	ATP-synt_ab;	ATP synthase alph	CYT		16	45	54.8	High
TRINITY_DN31118_c0_g1_i1_orf1	CPR9 [Ostrinia furnacalis]	3.250082156	1.700476187	3.00E-06	up	yes	0.989	0.3043	0.309	0.285	0.319	1	1.008	0.959	CC:integral component of membrane;CC:cellular_component;CC:intrinsic component of membrane;CC:cellular anatomical entity;MF:structural molecule activity;MF:molecular_function;MF:structural constituent of	GO:0016021;GO:0005575;GO:0031224;GO:0110165;GO:0005198;GO:0003674;GO:0042302	------	------	------	------	ENOG41125BY;ENOG410YVGC	S:Function unknown;	PF00379.26	Chitin_bind_4	Insect cuticle prote	EXC		8	78	11.4	High
TRINITY_DN122170_c0_g1_i2_orfp1	TRINITY_DN122170_c0_g1_i2_m.81408 TRINITY_DN122170_c0_g1::TRINITY_DN122170_c0_g1_i2::g.81408 ORF type:internal len:87 (+),score=9.58,Baculo_E25,PF05274.12,1.7e-07 TRINITY_DN122170_c0_g1_i2:2-259(+)	6.485061511	2.697120259	7.90E-05	up	yes	1.107	0.1707	0.184	0.167	0.161	1	1.13	1.191	------	------	------	------	------	------	------	------	PF05274.14	Baculo_E25	Occlusion-derived	CYT		1	12	9	Medium
TRINITY_DN7047_c0_g1_i1_orf1	hypothetical protein G9C98_004728 [Cotesia typhae]	3.500711744	1.807648272	5.31E-06	up	yes	0.9837	0.281	0.254	0.294	0.295	1	1.001	0.95	CC:macromolecular complex;CC:ribosome;CC:intracellular non-membrane-bounded organelle;CC:ribonucleoprotein complex;CC:mitochondrion;CC:cellular_component;CC:intracellular organelle;CC:non-membrane-bounded organelle;CC:membrane-bounded organelle;CC:organelle;CC:cellular anatomical	GO:0032991;GO:0005840;GO:0043232;GO:1990904;GO:0005739;GO:0005575;GO:0043229;GO:0043228;GO:0043227;GO:0043226;GO:0110165;GO:0043231	K17431	MRPL50	------	------	ENOG4111QY3	S:Function unknown	------	------	------	CYT		1	4	24	High
TRINITY_DN468_c0_g1_i3_orf1	transmembrane protein 41 homolog isoform X2 [Ostrinia furnacalis]	3.58649635	1.842575163	6.11E-07	up	yes	0.9827	0.274	0.285	0.281	0.256	1	0.977	0.971	------	------	------	------	------	------	COG0398	S:Function unknown	PF09335.14	SNARE_assoc	SNARE associated	CYT		1	7	30.6	High
TRINITY_DN94248_c0_g2_i3_orf1	uncharacterized protein LOC114357292 isoform X4 [Ostrinia furnacalis]	3.607749077	1.851099001	1.03E-06	up	yes	0.9777	0.271	0.281	0.253	0.279	1	0.968	0.965	------	------	------	------	------	------	COG1028;ENOG410XOCY	C:Energy production and conv	PF13561.9;PF00	adh_short_C2	Enoyl;short chain	CYT		7	29	28.7	High
TRINITY_DN29229_c0_g1_i5_orfp1	TRINITY_DN29229_c0_g1_i5_m.11187 TRINITY_DN29229_c0_g1::TRINITY_DN29229_c0_g1_i5::g.11187 ORF type:internal len:133	2.942675159	1.557128293	0.001133	up	yes	0.924	0.314	0.328	0.413	0.201	1	0.867	0.905	------	------	------	------	------	------	------	------	------	------	------	CYT		1	10	14.2	High
TRINITY_DN2825_c0_g1_i3_orf1	carbonic anhydrase 2-like [Ostrinia furnacalis]	5.486772487	2.455957755	3.38E-06	up	yes	1.037	0.189	0.166	0.192	0.209	1	1.067	1.043	MF:cation binding;MF:metal ion binding;MF:transition metal ion binding;MF:carbon-oxygen lyase activity;MF:hydro-lyase activity;MF:ion binding;MF:catalytic activity;MF:molecular_function;MF:zinc ion binding;MF:carbonate dehydratase activity;MF:lyase	GO:0043169;GO:0046872;GO:0046914;GO:0016835;GO:0016836;GO:0043167;GO:0003824;GO:0003674;GO:0008270;GO:0004089;GO:0016829;GO:0005488	K01672	CA	map00910	Nitrogen metabo	COG3338	I:Lipid transport and metabolis	PF00194.24	Carb_anhydra	Eukaryotic-type ca	CYT		2	8	30.6	High
TRINITY_DN868_c0_g1_i4_orf1	uncharacterized protein LOC114359357 isoform X1 [Ostrinia furnacalis]	4.985074627	2.317615102	1.26E-06	up	yes	1.002	0.201	0.207	0.175	0.221	1	1.021	0.985	CC:cellular_component;CC:extracellular region;CC:cellular anatomical entity;MF:molecular_function;MF:structural molecule activity;	GO:0005575;GO:0005576;GO:0110165;GO:0003674;GO:0005198	------	------	------	------	------	------	PF07294.14	Fibroin_P25	Fibroin P25	CYT		1	5	22.6	High
TRINITY_DN11376_c0_g2_i1_orf1	cathepsin K-like [Ostrinia furnacalis]	3.979253112	1.992497669	1.17E-05	up	yes	0.959	0.241	0.239	0.236	0.248	1	0.968	0.909	BP:primary metabolic process;BP:organonitrogen compound metabolic process;BP:protein metabolic process;BP:macromolecule metabolic process;BP:organic substance metabolic process;BP:nitrogen compound metabolic process;BP:biological_process;BP:metabolic process;BP:proteolysis;MF:molecular_function;MF:hydrolase activity;MF:catalytic activity;MF:catalytic activity, acting on a protein;MF:peptidase activity;MF:cysteine-type peptidase	GO:0044238;GO:1901564;GO:0019538;GO:0043170;GO:0071704;GO:0006807;GO:0008150;GO:0008152;GO:0006508;GO:0003674;GO:0016787;GO:0003824;GO:0140096;GO:0008233;GO:0008234	------	------	------	------	COG4870	O:Posttranslational modificatio	PF00112.26;PF0	Peptidase_C1	Papain family cyste	CYT		2	6	53.7	High
TRINITY_DN6881_c0_g1_i1_orf1	putative protein TPRXL [Ostrinia furnacalis]	5.511858798	2.46253893	1.49E-06	up	yes	0.9993	0.1813	0.186	0.15	0.208	1	1.011	0.987	MF:molecular_function;MF:structural constituent of cuticle;MF:structural molecule activity;	GO:0003674;GO:0042302;GO:0005198	------	------	------	------	ENOG410ZF0W;ENOG410YXXU;ENOG4111PJ1	S:Function unknown;	PF00379.26	Chitin_bind_4	Insect cuticle prote	CYT		5	23	22.4	High
TRINITY_DN3862_c0_g1_i7_orf1	venom acid phosphatase Acph-1-like [Ostrinia furnacalis]	6.771812081	2.759541939	2.65E-07	up	yes	1.009	0.149	0.143	0.169	0.135	1	1.023	1.004	BP:nucleic acid metabolic process;BP:cellular nitrogen compound metabolic process;BP:nitrogen compound metabolic process;BP:organic cyclic compound metabolic process;BP:nucleobase-containing compound metabolic process;BP:cellular macromolecule metabolic process;BP:organic substance metabolic process;BP:cellular process;BP:cellular aromatic compound metabolic process;BP:biological_process;BP:metabolic process;BP:heterocycle metabolic process;BP:primary metabolic process;BP:cellular metabolic process;BP:macromolecule metabolic process;BP:DNA integration;BP:DNA metabolic process;MF:phosphatase activity;MF:phosphoric ester hydrolase activity;MF:molecular_function;MF:binding;MF:nucleic acid binding;MF:heterocyclic compound binding;MF:hydrolase activity;MF:hydrolase activity, acting on ester bonds;MF:catalytic activity;MF:organic cyclic compound binding;MF:acid	GO:0090304;GO:0034641;GO:0006807;GO:1901360;GO:0006139;GO:0044260;GO:0071704;GO:0009987;GO:0006725;GO:0008150;GO:0008152;GO:0046483;GO:0044238;GO:0044237;GO:0043170;GO:0015074;GO:0006259;GO:0016791;GO:0042578;GO:0003674;GO:0005488;GO:0003676;GO:1901363;GO:0016787;GO:0016788;GO:0003824;GO:0097159;GO:0003993	K19283	ACPP	------	------	ENOG410ZVBQ	C:Energy production and conv	PF00328.25	His_Phos_2	Histidine phosphat	CYT		1	2	43.7	Medium
TRINITY_DN36199_c0_g1_i1_orf1	fatty acid-binding protein 1-like [Ostrinia furnacalis]	16.43316301	4.038538288	1.41E-07	up	yes	0.997	0.06067	0.057	0.06	0.065	1	1.015	0.976	MF:molecular_function;MF:lipid binding;MF:binding;	GO:0003674;GO:0008289;GO:0005488	K08753;K087	FABP4, aP2;F	map04923;ma	Regulation of lip	ENOG4111US8	I:Lipid transport and metabolis	------	------	------	CYT		5	48	15.2	High
TRINITY_DN248_c0_g1_i12_orf1	twitchin-like [Ostrinia furnacalis]	2.297650131	1.200159132	1.46E-05	up	yes	0.968	0.4213	0.41	0.406	0.448	1	0.942	0.962	------	------	------	------	------	------	ENOG410YKFP;ENOG410XQFD;ENOG410XRM1	S:Function unknown;T:Signal tr	PF07679.19;PF1	I-set;Ig_3;Ig_2	Immunoglobulin I-	CYT		3	21	226.6	High
TRINITY_DN28152_c0_g1_i1_orf1	mitochondrial import inner membrane translocase subunit Tim29 [Ostrinia furnacalis]	2.740540541	1.454460476	5.16E-05	up	yes	1.014	0.37	0.308	0.413	0.389	1	1.044	0.999	CC:mitochondrial inner membrane protein insertion complex;CC:macromolecular complex;CC:inner mitochondrial membrane protein complex;CC:cellular_component;CC:membrane protein	GO:0042721;GO:0032991;GO:0098800;GO:0005575;GO:0098796;GO:0098798	------	------	------	------	ENOG4111K8C	S:Function unknown	PF10171.12	Tim29	Translocase of the	CYT		1	4	21.8	High
TRINITY_DN7556_c0_g1_i1_orf1	venom carboxylesterase-6-like [Ostrinia furnacalis]	3.047619048	1.607682577	0.0001291	up	yes	1.024	0.336	0.304	0.279	0.425	1	1.049	1.024	------	------	------	------	------	------	COG2272;COG0580;ENOG410XSWS	I:Lipid transport and metabolis	PF00135.31;PF0	COesterase;A	Carboxylesterase f	CYT		1	1	54.8	Medium
TRINITY_DN55160_c0_g2_i1_orf1	esterase FE4-like isoform X2 [Ostrinia furnacalis]	5.299026426	2.405727322	0.0002479	up	yes	1.143	0.2157	0.185	0.26	0.202	1	1.217	1.213	------	------	------	------	------	------	COG2272	I:Lipid transport and metabolis	PF00135.31	COesterase	Carboxylesterase f	CYT		1	4	34.3	High
TRINITY_DN4929_c1_g2_i5_orf1	guanylate kinase isoform X2 [Ostrinia furnacalis]	2.941348974	1.556477962	3.97E-06	up	yes	1.003	0.341	0.369	0.348	0.306	1	1.011	0.999	BP:phosphorylation;BP:cellular process;BP:biological_process;BP:metabolic process;BP:cellular metabolic process;BP:phosphate-containing compound metabolic process;BP:phosphorus metabolic process;MF:nucleobase-containing compound kinase activity;MF:purine ribonucleoside triphosphate binding;MF:molecular_function;MF:binding;MF:ion binding;MF:heterocyclic compound binding;MF:nucleoside phosphate binding;MF:anion binding;MF:nucleotide binding;MF:transferase activity;MF:purine nucleotide binding;MF:ATP binding;MF:kinase activity;MF:guanylate kinase activity;MF:small molecule binding;MF:catalytic activity;MF:adenyl nucleotide binding;MF:carbohydrate derivative binding;MF:transferase activity, transferring phosphorus-containing groups;MF:phosphotransferase activity, phosphate group as acceptor;MF:nucleoside phosphate kinase activity;MF:adenyl ribonucleotide binding;MF:purine	GO:0016310;GO:0009987;GO:0008150;GO:0008152;GO:0044237;GO:0006796;GO:0006793;GO:0019205;GO:0035639;GO:0003674;GO:0005488;GO:0043167;GO:1901363;GO:1901265;GO:0043168;GO:0000166;GO:0016740;GO:0017076;GO:0005524;GO:0016301;GO:0004385;GO:0036094;GO:0003824;GO:0030554;GO:0097367;GO:0016772;GO:0016776;GO:0050145;GO:0032559;GO:0032555;GO:0097159;GO:0032553	K00942	gmk, GUK1	map01232;ma	Nucleotide metal	COG0194	U:Intracellular trafficking, secre	PF00625.24;PF1	Guanylate_kir	Guanylate kinase;A	CYT		2	12	22.7	High
TRINITY_DN107261_c0_g1_i1_orf1	ATP synthase subunit g, mitochondrial [Ostrinia furnacalis]	2.451948052	1.293928414	0.0001064	up	yes	0.944	0.385	0.365	0.361	0.429	1	0.902	0.93	BP:nucleoside triphosphate metabolic process;BP:nucleoside triphosphate biosynthetic process;BP:purine nucleoside triphosphate metabolic process;BP:purine nucleoside triphosphate biosynthetic process;BP:cellular biosynthetic process;BP:cellular nitrogen compound metabolic process;BP:nucleotide biosynthetic process;BP:small molecule metabolic process;BP:purine-containing compound metabolic process;BP:purine-containing compound biosynthetic process;BP:organic cyclic compound biosynthetic process;BP:ribonucleotide metabolic process;BP:organic cyclic compound metabolic process;BP:nucleobase-containing compound metabolic process;BP:ribonucleoside triphosphate biosynthetic process;BP:organic substance metabolic process;BP:ribonucleoside triphosphate metabolic process;BP:heterocycle biosynthetic process;BP:purine ribonucleoside triphosphate biosynthetic process;BP:purine ribonucleoside triphosphate metabolic process;BP:ribose phosphate metabolic process;BP:organic substance biosynthetic process;BP:ATP metabolic process;BP:cellular process;BP:cellular aromatic compound metabolic process;BP:purine ribonucleotide biosynthetic process;BP:purine ribonucleotide metabolic process;BP:ribonucleotide biosynthetic process;BP:biosynthetic process;BP:nucleotide metabolic process;BP:metabolic process;BP:aromatic compound biosynthetic process;BP:nucleobase-containing compound biosynthetic process;BP:cellular nitrogen compound biosynthetic process;BP:organophosphate biosynthetic process;BP:nucleobase-containing small molecule metabolic process;BP:heterocycle metabolic process;BP:primary metabolic	GO:0009141;GO:0009142;GO:0009144;GO:0009145;GO:0044249;GO:0034641;GO:0009165;GO:0044281;GO:0072521;GO:0072522;GO:1901362;GO:0009259;GO:1901360;GO:0006139;GO:0009201;GO:0071704;GO:0009199;GO:0018130;GO:0009206;GO:0009205;GO:0019693;GO:1901576;GO:0046034;GO:0009987;GO:0006725;GO:0009152;GO:0009150;GO:0009260;GO:0009058;GO:0009117;GO:0008152;GO:0019438;GO:0034654;GO:0044271;GO:0090407;GO:0055086;GO:0046483;GO:0044238;GO:0015986;GO:1901564;GO:1901566;GO:1901137;GO:1901135;GO:0006164;GO:0046390;GO:0044237;GO:0006163;GO:0006796;GO:0006807;GO:1901293;GO:0006793;GO:0019637;GO:0008150;GO:0006753;GO:0006754;GO:0045263;GO:0005575;GO:0098798;GO:0098800;GO:0032991;GO:0000276;GO:0098796;GO:0033177;GO:0022890;GO:0003674;GO:0015318;GO:0022857;GO:0005215;GO:0008324;GO:0015075;GO:0015078	K02140	ATPeFG, ATP	map00190;ma	Oxidative phosph	ENOG4111TZ1	I:Lipid transport and metabolis	PF04718.18	ATP-synt_G	Mitochondrial ATP	CYT		3	30	10.7	High
TRINITY_DN97680_c0_g1_i1_orf1	39S ribosomal protein L52, mitochondrial [Ostrinia furnacalis]	2.376956633	1.249115582	0.0004311	up	yes	0.9263	0.3897	0.361	0.424	0.384	1	0.939	0.84	BP:cellular biosynthetic process;BP:cellular nitrogen compound metabolic process;BP:nitrogen compound metabolic process;BP:cellular macromolecule biosynthetic process;BP:organic substance biosynthetic process;BP:cellular macromolecule metabolic process;BP:peptide biosynthetic process;BP:organic substance metabolic process;BP:mitochondrial translation;BP:amide biosynthetic process;BP:cellular process;BP:biosynthetic process;BP:macromolecule biosynthetic process;BP:biological_process;BP:metabolic process;BP:cellular nitrogen compound biosynthetic process;BP:primary metabolic process;BP:organonitrogen compound metabolic process;BP:cellular amide metabolic process;BP:organonitrogen compound biosynthetic process;BP:protein metabolic process;BP:cellular metabolic process;BP:macromolecule metabolic process;BP:peptide metabolic process;BP:translation;CC:large ribosomal subunit;CC:organellar large ribosomal subunit;CC:macromolecular complex;CC:ribosomal subunit;CC:ribonucleoprotein complex;CC:cellular_component;CC:mitochondrial large ribosomal subunit;CC:mitochondrial protein	GO:0044249;GO:0034641;GO:0006807;GO:0034645;GO:1901576;GO:0044260;GO:0043043;GO:0071704;GO:0032543;GO:0043604;GO:0009987;GO:0009058;GO:0009059;GO:0008150;GO:0008152;GO:0044271;GO:0044238;GO:1901564;GO:0043603;GO:1901566;GO:0019538;GO:0044237;GO:0043170;GO:0006518;GO:0006412;GO:0015934;GO:0000315;GO:0032991;GO:0044391;GO:1990904;GO:0005575;GO:0005762;GO:0098798;GO:0003674;GO:0005198;GO:0003735	K17433	MRPL52	------	------	ENOG4111XV4;COG1428	J:Translation, ribosomal structu	PF18699.4	MRPL52	Mitoribosomal pro	CYT		1	20	14.8	Medium
TRINITY_DN5578_c0_g1_i10_orf1	unnamed protein product [Chilo suppressalis]	2.944731248	1.558135972	9.68E-06	up	yes	0.9697	0.3293	0.362	0.307	0.319	1	0.961	0.948	------	------	K23390	BAG6	------	------	ENOG410XS9P	O:Posttranslational modificatio	PF00240.26;PF1	ubiquitin;BAG	Ubiquitin family;B	CYT		1	4	62.9	High
TRINITY_DN15870_c0_g1_i3_orf1	PREDICTED: mitochondrial import inner membrane translocase subunit Tim23 isoform X1 [Fopius arisanus]	3.092429022	1.628740483	1.16E-05	up	yes	0.9803	0.317	0.321	0.287	0.343	1	0.998	0.943	CC:integral component of membrane;CC:cellular_component;CC:cellular anatomical entity;CC:intrinsic component of membrane;MF:transporter activity;MF:transmembrane transporter activity;MF:molecular_function;	GO:0016021;GO:0005575;GO:0110165;GO:0031224;GO:0005215;GO:0022857;GO:0003674	K17794	TIM23	map04212	Longevity regula	COG5596	O:Posttranslational modificatio	PF02466.22	Tim17	Tim17/Tim22/Tim2	CYT		1	5	22.1	High
TRINITY_DN7735_c0_g1_i4_orf1	calphotin-like [Ostrinia furnacalis]	3.6875	1.882643049	0.001388	up	yes	1.003	0.272	0.156	0.339	0.321	1	0.88	1.129	------	------	------	------	------	------	ENOG4110RAS;ENOG4110R6G	S:Function unknown;	------	------	------	CYT		1	3	22.5	High

TRINITY_DN18909_c0_g1_i8_orf1	unnamed protein product [Euphydryas editha]	3.269463087	1.709053735	8.35E-05	up	yes	0.9743	0.298	0.286	0.309	0.299	1	1.029	0.894	MF:hydrolase activity;MF:molecular_function;MF:hydrolase activity, acting on ester bonds;MF:carboxylic ester hydrolase activity;MF:catalytic activity;	GO:0016787;GO:0003674;GO:0016788; GO:0052689;GO:0003824	------	------	------	------	COG2272;COG0580 ;ENOG410XSWS	I:Lipid transport and metabolis	PF00135.31;PF2(	COesterase;B	Carboxylesterase fa	CYT	1	4	60.6	High
TRINITY_DN27721_c1_g1_i2_orf1	mitochondrial import receptor subunit TOM20 homolog [Ostrinia furnacalis]	2.158734114	1.110185561	0.00256	up	yes	0.8663	0.4013	0.373	0.405	0.426	1	0.783	0.816	BP:protein localization;BP:cellular macromolecule localization;BP:nitrogen compound transport;BP:organic substance transport;BP:macromolecule localization;BP:transport;BP:cellular process;BP:protein targeting;BP:establishment of protein localization;BP:biological_process;BP:establishment of localization in cell;BP:establishment of localization;BP:localization;BP:cellular localization;BP:intracellular transport;BP:protein transport;BP:intracellular protein transport;CC:intrinsic component of membrane;CC:mitochondrial outer membrane translocase complex;CC:integral component of membrane;CC:cellular anatomical entity;CC:macromolecular complex;CC:cellular_component;CC:membrane protein	GO:0008104;GO:0070727;GO:0071705; GO:0071702;GO:0033036;GO:0006810; GO:0009987;GO:0006605;GO:0045184; GO:0008150;GO:0051649;GO:0051234; GO:0051179;GO:0051641;GO:0046907; GO:0015031;GO:0006886;GO:0031224; GO:0005742;GO:0016021;GO:0110165; GO:0032991;GO:0005575;GO:0098796; GO:0098799;GO:0098798	K17770	TOM20	------	------	ENOG4111NAH	U:Intracellular trafficking, secre	PF02064.18	MAS20	MAS20 protein imp	CYT	2	15	17.2	High
TRINITY_DN2267_c0_g1_i1_orf1	hypothetical protein evm_006312 [Chilo suppressalis]	3.498044792	1.806548763	1.67E-07	up	yes	0.984	0.2813	0.281	0.279	0.284	1	0.983	0.969	BP:phosphate ion transmembrane transport;BP:anion transport;BP:transmembrane transport;BP:mitochondrial transmembrane transport;BP:inorganic anion transmembrane transport;BP:mitochondrial phosphate ion transmembrane transport;BP:inorganic anion transport;BP:cellular process;BP:ion transport;BP:transport;BP:phosphate ion transport;BP:ion transmembrane transport;BP:biological_process;BP:establishment of localization;BP:localization;BP:inorganic ion transmembrane transport;BP:anion transmembrane transport;CC:intrinsic component of membrane;CC:organelle membrane;CC:integral component of membrane;CC:membrane;CC:cellular anatomical entity;CC:mitochondrial membrane;CC:organelle inner membrane;CC:cellular_component;CC:mitochondrial inner membrane;MF:molecular_function;MF:inorganic solute uptake transmembrane transporter activity;MF:transporter activity;MF:secondary active transmembrane transporter activity;MF:inorganic phosphate transmembrane transporter activity;MF:transmembrane transporter activity;MF:active	GO:0035435;GO:0006820;GO:0055085; GO:1990542;GO:0098661;GO:1990547; GO:0015698;GO:0009987;GO:0006811; GO:0006810;GO:0006817;GO:0034220; GO:0008150;GO:0051234;GO:0051179; GO:0098660;GO:0098656;GO:0031224; GO:0031090;GO:0016021;GO:0016020; GO:0110165;GO:0031966;GO:0019866; GO:0005575;GO:0005743;GO:0003674; GO:0015318;GO:0005215;GO:0015291; GO:0005315;GO:0022857;GO:0022804	K15102	SLC25A3, PH(	------	------	ENOG410XPST	S:Function unknown	PF00153.30	Mito_carr	Mitochondrial carr	CYT	9	19	41.3	High
TRINITY_DN47_c0_g1_i2_orf1	uncharacterized protein LOC114356437 isoform X1 [Ostrinia furnacalis]	3.427721386	1.777249848	4.59E-07	up	yes	0.9793	0.2857	0.294	0.28	0.283	1	0.964	0.974	------	------	------	------	------	------	------	------	PF00041.24	fn3	Fibronectin type III	CYT	1	8	17.9	High
TRINITY_DN116951_c0_g3_i2_orf1	spermine oxidase-like isoform X2 [Ostrinia furnacalis]	3.411538462	1.770422481	2.29E-05	up	yes	0.9757	0.286	0.281	0.262	0.315	1	1.004	0.923	MF:catalytic activity;MF:molecular_function;MF:oxidoreductase activity;	GO:0003824;GO:0003674;GO:0016491	K12259	SMOX, PAO5	map00330;ma	Arginine and pro	ENOG4111HZN;COG1231;ENOG410XSNC	S:Function unknown;E:Amino a	PF13450.9;PF01	NAD_binding_	NA;Flavin containi	CYT	2	38	8.6	High
TRINITY_DN1180_c0_g1_i4_orf1	larval cuticle protein LCP-30-like [Ostrinia furnacalis]	4.208106979	2.073171381	8.57E-08	up	yes	1.007	0.2393	0.247	0.225	0.246	1	1.006	1.015	MF:molecular_function;MF:structural constituent of cuticle;MF:structural molecule activity;	GO:0003674;GO:0042302;GO:0005198	------	------	------	------	ENOG410YZ4Z;ENOG4110S48	S:Function unknown;	PF00379.26	Chitin_bind_4	Insect cuticle prote	CYT	13	61	31	High
TRINITY_DN7964_c0_g1_i6_orfp1	TRINITY_DN7964_c0_g1_i6_m.23478 TRINITY_DN7964_c0_g1::TRINITY_DN7964_c0_g1_i6::g.23478 ORF type:internal len:87 (+),score=67.94 TRINITY_DN7964_c0_g1_i6:2-259(+)	5.158850227	2.367049562	1.27E-05	up	yes	1.023	0.1983	0.172	0.184	0.239	1	1.071	0.999	------	------	------	------	------	------	------	------	------	------	------	CYT	2	30	9	High
TRINITY_DN2894_c0_g1_i2_orf1	myrosinase 1-like isoform X1 [Ostrinia furnacalis]	4.737836561	2.244228431	3.48E-06	up	yes	1.003	0.2117	0.205	0.22	0.21	1	1.042	0.968	BP:primary metabolic process;BP:carbohydrate metabolic process;BP:organic substance metabolic process;BP:biological_process;BP:metabolic process;MF:hydrolase activity;MF:catalytic activity;MF:hydrolase activity, acting on glycosyl bonds;MF:hydrolase activity, hydrolyzing O-glycosyl compounds;MF:molecular_function;	GO:0044238;GO:0005975;GO:0071704; GO:0008150;GO:0008152;GO:0016787; GO:0003824;GO:0016798;GO:0004553; GO:0003674	------	------	------	------	COG2723	G:Carbohydrate transport and i	PF00232.21	Glyco_hydro_	Glycosyl hydrolase	CYT	6	17	59.3	High
TRINITY_DN49508_c0_g2_i8_orf1	putative fatty acyl-CoA reductase CG5065 [Ostrinia furnacalis]	4.425783023	2.145932724	3.52E-06	up	yes	0.975	0.2203	0.22	0.226	0.215	1	0.991	0.934	BP:primary metabolic process;BP:lipid metabolic process;BP:fatty acid derivative metabolic process;BP:organic substance metabolic process;BP:biological_process;BP:metabolic process;CC:cellular_component;CC:cellular anatomical entity;CC:integral component of membrane;CC:intrinsic component of membrane;MF:fatty-acyl-CoA reductas;MF:alcohol-forming fatty acyl-CoA reductase activity;MF:catalytic activity;MF:oxidoreductase activity, acting on the aldehyde or oxo group of donors, NAD or NADP as acceptor;MF:oxidoreductase activity, acting on the aldehyde or	GO:0044238;GO:0006629;GO:1901568; GO:0071704;GO:0008150;GO:0008152; GO:0005575;GO:0110165;GO:0016021; GO:0031224;GO:0080019;GO:0102965; GO:0003824;GO:0016620;GO:0016903; GO:0003674;GO:0016491	K13356	FAR	map04146;ma	Peroxisome;Cutir	ENOG410XS7R	S:Function unknown	PF07993.15;PF0	NAD_binding_	Male sterility prote	CYT	1	2	60.6	High
TRINITY_DN6140_c0_g3_i3_orf1	CD63 antigen-like [Ostrinia furnacalis]	5.857498444	2.550284666	0.0001955	up	yes	0.9413	0.1607	0.116	0.259	0.107	1	0.884	0.94	CC:cellular_component;CC:integral component of membrane;CC:cellular anatomical entity;CC:intrinsic component of membrane;	GO:0005575;GO:0016021;GO:0110165; GO:0031224	K06497	CD63, MLA1,	map05205;ma	Proteoglycans in	ENOG4111IRY	O:Posttranslational modificatio	PF00335.23	Tetraspanin	Tetraspanin family	CYT	1	4	32.2	High
TRINITY_DN4612_c0_g1_i1_orf1	uncharacterized protein LOC114362092 [Ostrinia furnacalis]	7.679395386	2.940992729	2.68E-05	up	yes	0.9653	0.1257	0.141	0.11	0.126	1	1.006	0.89	CC:cellular_component;CC:integral component of membrane;CC:cellular anatomical entity;CC:intrinsic component of membrane;	GO:0005575;GO:0016021;GO:0110165; GO:0031224	------	------	------	------	ENOG4110SII	S:Function unknown	------	------	------	CYT	1	1	70.8	Medium
TRINITY_DN129869_c0_g4_i1_orf1	putative myosin heavy chain, muscle, partial [Cotesia chilonis]	2.901926949	1.537011202	3.96E-07	up	yes	1.009	0.3477	0.35	0.343	0.35	1	1.03	0.998	CC:sarcomere;CC:myosin complex;CC:cellular anatomical entity;CC:macromolecular complex;CC:cellular_component;MF:purine ribonucleoside triphosphate binding;MF:molecular_function;MF:binding;MF:ion binding;MF:heterocyclic compound binding;MF:nucleoside phosphate binding;MF:anion binding;MF:nucleotide binding;MF:actin binding;MF:purine nucleotide binding;MF:motor activity;MF:actin filament binding;MF:small molecule binding;MF:adenyl nucleotide binding;MF:carbohydrate derivative binding;MF:organic cyclic compound binding;MF:cytoskeletal protein binding;MF:adenyl ribonucleotide binding;MF:ATP binding;MF:protein binding;MF:purine ribonucleotide binding;MF:macromolecular complex binding;MF:ribonucleotide binding;	GO:0030017;GO:0016459;GO:0110165; GO:0032991;GO:0005575;GO:0035639; GO:0003674;GO:0005488;GO:0043167; GO:1901363;GO:1901265;GO:0043168; GO:0000166;GO:0003779;GO:0017076; GO:0003774;GO:0051015;GO:0036094; GO:0030554;GO:0097367;GO:0097159; GO:0008092;GO:0032559;GO:0005524; GO:0005515;GO:0032555;GO:0044877; GO:0032553	K17751	MYH6_7	map04260;ma	Cardiac muscle c	COG5022	Z:Cytoskeleton	PF00063.24;PF0	Myosin_head	Myosin head;Myos	CYT	42	22	225.6	High
TRINITY_DN2266_c0_g1_i6_orf1	bilin-binding protein-like [Ostrinia furnacalis]	2.658648649	1.410693132	9.40E-06	up	yes	0.9837	0.37	0.356	0.386	0.368	1	1.007	0.944	MF:pigment binding;MF:molecular_function;MF:binding;	GO:0031409;GO:0003674;GO:0005488	K03098	APOD	------	------	COG3040	M:Cell wall/membrane/envelop	PF00061.26;PF0	Lipocalin;Lipo	Lipocalin / cytosoli	CYT	6	31	22.1	High
TRINITY_DN113626_c0_g1_i3_orfp1	TRINITY_DN113626_c0_g1_i3_m.80721 TRINITY_DN113626_c0_g1::TRINITY_DN113626_c0_g1_i3::g.80721 ORF type:internal len:118 (-),score=87.34 TRINITY_DN113626_c0_g1_i3:2-352(-)	3.09124199	1.628186596	0.0003757	up	yes	1.013	0.3277	0.391	0.36	0.232	1	0.955	1.085	------	------	------	------	------	------	------	------	------	------	------	PLA	1	9	11.6	High
TRINITY_DN6087_c0_g1_i7_orf1	uncharacterized protein LOC114355564 [Ostrinia furnacalis]	2.45468629	1.295538659	8.60E-05	up	yes	0.9507	0.3873	0.41	0.34	0.412	1	0.914	0.938	MF:hydrolase activity;MF:methyl indole-3-acetate esterase activity;MF:hydrolase activity, acting on ester bonds;MF:catalytic activity;MF:molecular_function;MF:carboxylic ester hydrolase	GO:0016787;GO:0080030;GO:0016788; GO:0003824;GO:0003674;GO:0052689	K03927	CES2	map00983	Drug metabolism	COG2272;ENOG410XSWS;COG0580	I:Lipid transport and metabolis	PF00135.31;PF2(	COesterase;B	Carboxylesterase fa	CYT	5	9	61.5	High
TRINITY_DN26375_c0_g1_i1_orf1	hypothetical protein O3G_MSEX007366 [Manduca sexta]	2.458494957	1.297775396	0.000149	up	yes	0.9507	0.3867	0.444	0.362	0.354	1	0.949	0.903	------	------	------	------	------	------	ENOG410Y78S;ENOG410XPFI	S:Function unknown;	------	------	------	CYT	1	2	129.6	High
TRINITY_DN49265_c0_g3_i2_orf1	cytochrome c [Ostrinia furnacalis] >XP_028160278.1 cytochrome c [Ostrinia furnacalis]	2.826662828	1.499099805	3.12E-07	up	yes	0.9817	0.3473	0.352	0.346	0.344	1	0.975	0.97	CC:organelle lumen;CC:organelle envelope lumen;CC:membrane-enclosed lumen;CC:cellular_component;CC:intracellular organelle lumen;CC:mitochondrial intermembrane space;CC:respiratory chain;CC:cellular anatomical entity;MF:oxidoreductase activity;MF:cation binding;MF:heme binding;MF:organic cyclic compound binding;MF:tetrapyrrole binding;MF:ion binding;MF:molecular_function;MF:binding;MF:catalytic activity;MF:metal ion binding;MF:heterocyclic compound	GO:0043233;GO:0031970;GO:0031974; GO:0005575;GO:0070013;GO:0005758; GO:0070469;GO:0110165;GO:0016491; GO:0043169;GO:0020037;GO:0097159; GO:0046906;GO:0043167;GO:0003674; GO:0005488;GO:0003824;GO:0046872; GO:1901363;GO:0009055	K08738	CYC	map05167;ma	Kaposi sarcoma-	COG3474	C:Energy production and conve	PF00034.24;PF1	Cytochrom_C	Cytochrome c;Cytc	PLA	7	50	11.8	High
TRINITY_DN7966_c0_g1_i4_orf1	leucine-rich repeat neuronal protein 1-like [Ostrinia furnacalis]	2.84350593	1.507670813	1.09E-05	up	yes	0.983	0.3457	0.346	0.361	0.33	1	0.94	1.009	BP:locomotion;BP:biological_process;CC:integral component of membrane;CC:cellular_component;CC:cell periphery;CC:intrinsic component of membrane;CC:cellular anatomical entity;	GO:0040011;GO:0008150;GO:0016021; GO:0005575;GO:0071944;GO:0031224; GO:0110165	------	------	------	------	COG4886	P:Inorganic ion transport and n	PF13855.9;PF12	LRR_8;LRR_4	Leucine rich repea	PLA	2	4	57.2	High
TRINITY_DN8366_c0_g1_i4_orf1	luciferin 4-monooxygenase-like [Ostrinia furnacalis]	2.348484848	1.231730286	0.0001446	up	yes	0.93	0.396	0.407	0.395	0.386	1	0.873	0.917	------	------	------	------	------	------	COG0318;COG0318	I:Lipid transport and metabolis	PF00501.31;PF1	AMP-binding	AMP-binding enzy	CYT	3	5	61.5	High
TRINITY_DN110888_c0_g1_i2_orf1	uncharacterized protein LOC114364502 [Ostrinia furnacalis]	3.023334357	1.596140538	1.31E-05	up	yes	0.9847	0.3257	0.322	0.308	0.347	1	0.94	1.014	------	------	------	------	------	------	ENOG410XU1K	S:Function unknown	------	------	------	CYT	1	4	33.8	High
TRINITY_DN1123_c2_g1_i5_orf1	troponin I isoform X4 [Leguminivora glycinivorella]	2.5513968	1.351287288	7.53E-05	up	yes	0.9407	0.3687	0.358	0.347	0.401	1	0.903	0.919	------	------	K12044	TNNI3	map04260;ma	Cardiac muscle c	ENOG410Y9IX	Z:Cytoskeleton	PF00992.23	Troponin	Troponin	CYT	2	40	16.3	High
TRINITY_DN43391_c0_g1_i5_orf1	myrosinase 1-like isoform X2 [Ostrinia furnacalis]	3.368278661	1.752011499	4.67E-06	up	yes	0.996	0.2957	0.273	0.315	0.299	1	0.965	1.023	BP:primary metabolic process;BP:carbohydrate metabolic process;BP:organic substance metabolic process;BP:biological_process;BP:metabolic process;MF:hydrolase activity;MF:catalytic activity;MF:hydrolase activity, acting on glycosyl bonds;MF:hydrolase activity, hydrolyzing O-glycosyl compounds;MF:molecular_function;	GO:0044238;GO:0005975;GO:0071704; GO:0008150;GO:0008152;GO:0016787; GO:0003824;GO:0016798;GO:0004553; GO:0003674	------	------	------	------	COG2723	G:Carbohydrate transport and i	PF00232.21	Glyco_hydro_	Glycosyl hydrolase	CYT	3	6	59.3	High
TRINITY_DN19814_c0_g1_i4_orf1	general odorant-binding protein 28a-like [Ostrinia furnacalis]	3.731067603	1.8995885	9.70E-06	up	yes	1.01	0.2707	0.296	0.262	0.254	1	1.054	0.976	MF:odorant binding;MF:molecular_function;MF:binding;	GO:0005549;GO:0003674;GO:0005488	------	------	------	------	ENOG4110QNN;ENOG41117B2;ENOG41113VF;ENOG4110SS7;ENOG410ZC7X	S:Function unknown;	PF01395.25	PBP_GOBP	PBP/GOBP family	CYT	1	5	16.7	High
TRINITY_DN3159_c0_g1_i4_orf1	uncharacterized protein LOC114362782 [Ostrinia furnacalis]	3.465173259	1.792927489	1.04E-07	up	yes	0.99	0.2857	0.294	0.286	0.277	1	0.992	0.978	BP:response to host;BP:response to defenses of other organism involved in symbiotic interaction;BP:response to biotic stimulus;BP:response to external stimulus;BP:response to external biotic stimulus;BP:evasion or tolerance of host defense response;BP:response to stimulus;BP:interspecies interaction between organisms;BP:biological_process;BP:symbiosis, encompassing mutualism through parasitism;BP:interaction with host;BP:response to host defenses;BP:response to other organism;MF:pigment	GO:0075136;GO:0052173;GO:0009607; GO:0009605;GO:0043207;GO:0030682; GO:0050896;GO:0044419;GO:0008150; GO:0044403;GO:0051701;GO:0052200; GO:0051707;GO:0031409;GO:0003674; GO:0005488	K18271	RBP4	------	------	COG3040	M:Cell wall/membrane/envelop	PF00061.26;PF0	Lipocalin;Lipo	Lipocalin / cytosoli	CYT	22	10	323.1	High
TRINITY_DN8116_c0_g1_i1_orf1	uncharacterized protein LOC114350845 [Ostrinia furnacalis]	3.340270552	1.739964961	1.07E-05	up	yes	0.963	0.2883	0.297	0.291	0.277	1	0.971	0.918	------	------	------	------	------	------	COG1028	S:Function unknown	PF13561.9;PF00	adh_short_C2	Enoyl;short chain c	CYT	4	26	25.5	High
TRINITY_DN72707_c0_g1_i1_orf1	uncharacterized protein LOC114357549 [Ostrinia furnacalis]	3.923016186	1.971963288	1.59E-08	up	yes	0.9937	0.2533	0.258	0.245	0.257	1	0.989	0.992	MF:hydrolase activity;MF:molecular_function;MF:hydrolase activity, acting on ester bonds;MF:carboxylic ester hydrolase activity;MF:catalytic activity;	GO:0016787;GO:0003674;GO:0016788; GO:0052689;GO:0003824	K03927;K01(	CES2;ACHE	map00564;ma	Glycerophosphol	COG2272	I:Lipid transport and metabolis	PF00135.31;PF2(	COesterase;B	Carboxylesterase fa	CYT	3	7	60.3	High
TRINITY_DN141462_c0_g1_i1_orf1	mitochondrial-processing peptidase subunit beta [Diachasma alloeum] >THK33262.1 core protein 1, ubiquinol-cytochrome c reductase [Diachasma alloeum]	4.192740926	2.067893687	6.12E-08	up	yes	1.005	0.2397	0.244	0.244	0.231	1	1.018	0.998	BP:mitochondrial protein processing;BP:nitrogen compound metabolic process;BP:organic substance metabolic process;BP:proteolysis;BP:protein processing involved in protein targeting to mitochondrion;BP:biological_process;BP:metabolic process;BP:protein processing;BP:primary metabolic process;BP:organonitrogen compound metabolic process;BP:protein maturation;BP:protein metabolic process;BP:macromolecule metabolic process;CC:catalytic complex;CC:macromolecular complex;CC:cellular_component;CC:mitochondrial processing peptidase complex;CC:mitochondrial protein complex;MF:endopeptidase activity;MF:molecular_function;MF:binding;MF:peptidase activity;MF:metallopeptidase activity;MF:cation binding;MF:ion binding;MF:metal ion binding;MF:hydrolase activity;MF:metalloendopeptidase activity;MF:catalytic activity, acting on a protein;MF:catalytic activity;	GO:0034982;GO:0006807;GO:0071704; GO:0006508;GO:0006627;GO:0008150; GO:0008152;GO:0016485;GO:0044238; GO:1901564;GO:0051604;GO:0019538; GO:0043170;GO:1902494;GO:0032991; GO:0005575;GO:0017087;GO:0098798; GO:0004175;GO:0003674;GO:0005488; GO:0008233;GO:0008237;GO:0043169; GO:0043167;GO:0046872;GO:0016787; GO:0004222;GO:0140096;GO:0003824	K17732	PMPCB, MAS	------	------	COG0612	S:Function unknown	PF00675.23;PF0	Peptidase_M1	Insulinase;Peptidas	CYT	1	2	53.3	High
TRINITY_DN98091_c0_g1_i3_orf1	UDP-glycosyltransferase UGT40AP2, partial [Ostrinia furnacalis]	3.265447014	1.707280499	1.91E-05	up	yes	0.946	0.2897	0.302	0.285	0.282	1	0.923	0.915	CC:integral component of membrane;CC:cellular_component;CC:cellular anatomical entity;CC:intrinsic component of membrane;MF:catalytic activity;MF:transferase activity;MF:molecular_function;MF:transferase activity, transferring glycosyl groups;MF:transferase activity, transferring hexosyl groups;MF:glucuronosyltransferase activity;MF:UDP-	GO:0016021;GO:0005575;GO:0110165; GO:0031224;GO:0003824;GO:0016740; GO:0003674;GO:0016757;GO:0016758; GO:0015020;GO:0008194	K00699	UGT	map05207;ma	Chemical carcinc	COG1819	S:Function unknown	PF00201.21;PF0	UDPGT;Glyco_	UDP-glucoronosyl	CYT	4	11	57.4	High
TRINITY_DN3194_c0_g1_i6_orf1	uncharacterized protein LOC114361386 [Ostrinia furnacalis]	4.704238472	2.233961197	4.50E-06	up	yes	1.01	0.2147	0.201	0.227	0.216	1	0.977	1.052	BP:nitrogen compound metabolic process;BP:organic substance metabolic process;BP:proteolysis;BP:biological_process;BP:metabolic process;BP:primary metabolic process;BP:organonitrogen compound metabolic process;BP:protein metabolic process;BP:macromolecule metabolic process;CC:integral component of membrane;CC:intrinsic component of membrane;CC:cellular anatomical entity;CC:cellular_component;MF:aminopeptidase activity;MF:exopeptidase activity;MF:molecular_function;MF:binding;MF:peptidase activity;MF:metallopeptidase activity;MF:cation binding;MF:transition metal ion binding;MF:ion binding;MF:metal ion binding;MF:hydrolase activity;MF:catalytic	GO:0006807;GO:0071704;GO:0006508; GO:0008150;GO:0008152;GO:0044238; GO:1901564;GO:0019538;GO:0043170; GO:0016021;GO:0031224;GO:0110165; GO:0005575;GO:0004177;GO:0008238; GO:0003674;GO:0005488;GO:0008233; GO:0008237;GO:0043169;GO:0046914; GO:0043167;GO:0046872;GO:0016787; GO:0140096;GO:0003824;GO:0008270	K11140	ANPEP, CD13	map04640;ma	Hematopoietic c	COG0308	E:Amino acid transport and me	PF01433.23;PF1	Peptidase_M1	Peptidase family M	CYT	4	7	109.4	High
TRINITY_DN22983_c0_g1_i2_orfp1	TRINITY_DN22983_c0_g1_i2_m.10495 TRINITY_DN22983_c0_g1::TRINITY_DN22983_c0_g1_i2::g.10495 ORF type:internal len:79 (-),score=15.95,Polyhedrin,PF00738.19,7.6e-40 TRINITY_DN22983_c0_g1_i2:2-235(-)	4.780262518	2.257089849	8.24E-08	up	yes	0.9833	0.2057	0.205	0.206	0.206	1	0.973	0.977	------	------	------	------	------	------	------	------	PF00738.21	Polyhedrin	Polyhedrin	CYT	3	21	9.2	High

TRINITY_DN22664_c0_g1_i1_orf1	larval cuticle protein LCP-14-like [Ostrinia furnacalis]	6.531791908	2.70747883	2.02E-07	up	yes	1.017	0.1557	0.156	0.148	0.163	1	1.013	1.037	MF:molecular_function;MF:structural constituent of cuticle;MF:structural molecule activity;	GO:0003674;GO:0042302;GO:0005198	------	------	------	------	ENOG4111PJ1;ENOG4110T0M;ENOG4110RUZ	S:Function unknown;	PF00379.26	Chitin_bind_4	Insect cuticle prote	CYT	6	52	19.3	High
TRINITY_DN130051_c0_g1_i1_orf1	5-methyltetrahydropteroyltriglutamate--homocysteine S-methyltransferase-like protein [Leptotrombidium deliense]	4.567000477	2.19124694	0.0004311	up	yes	0.9577	0.2097	0.314	0.086	0.229	1	0.932	0.941	BP:one-carbon metabolic process;BP:carboxylic acid metabolic process;BP:cellular biosynthetic process;BP:nitrogen compound metabolic process;BP:aspartate family amino acid biosynthetic process;BP:small molecule metabolic process;BP:small molecule biosynthetic process;BP:tetrahydrofolate metabolic process;BP:macromolecule metabolic process;BP:organic substance biosynthetic process;BP:cellular amino acid metabolic process;BP:tetrahydrofolate interconversion;BP:pteridine-containing compound metabolic process;BP:organic substance metabolic process;BP:organic cyclic compound metabolic process;BP:proteolysis;BP:methylation;BP:alpha-amino acid metabolic process;BP:alpha-amino acid biosynthetic process;BP:sulfur amino acid metabolic process;BP:folic acid-containing compound metabolic process;BP:primary metabolic process;BP:sulfur amino acid biosynthetic process;BP:cellular process;BP:cellular aromatic compound metabolic process;BP:biosynthetic process;BP:biological_process;BP:metabolic process;BP:oxoacid metabolic process;BP:cellular modified amino acid metabolic process;BP:cellular amino acid biosynthetic process;BP:organonitrogen compound metabolic process;BP:organonitrogen compound biosynthetic process;BP:organic acid metabolic process;BP:sulfur compound biosynthetic process;BP:carboxylic acid biosynthetic process;BP:methionine metabolic process;BP:organic acid biosynthetic process;BP:cellular metabolic process;BP:heterocycle metabolic process;BP:protein metabolic process;BP:sulfur compound metabolic process;BP:methionine	GO:0006730;GO:0019752;GO:0044249;GO:0006807;GO:0009067;GO:0044281;GO:0044283;GO:0046653;GO:0043170;GO:1901576;GO:0006520;GO:0035999;GO:0042558;GO:0071704;GO:1901360;GO:0006508;GO:0032259;GO:1901605;GO:1901607;GO:0000096;GO:0006760;GO:0044238;GO:0000097;GO:0009987;GO:0006725;GO:0009058;GO:0008150;GO:0008152;GO:0043436;GO:0006575;GO:0008652;GO:1901564;GO:1901566;GO:0006082;GO:0044272;GO:0046394;GO:0006555;GO:0016053;GO:0044237;GO:0046483;GO:0019538;GO:0006790;GO:0009086;GO:0009066;GO:0031224;GO:0016021;GO:0005575;GO:0110165;GO:0005488;GO:0043169;GO:0016646;GO:0008236;GO:0008168;GO:0008233;GO:0016491;GO:0016645;GO:0046914;GO:0003871;GO:0008270;GO:0043167;GO:0016740;GO:0016741;GO:0016787;GO:0140096;GO:0004489;GO:0008172;GO:0003824;GO:0017171;GO:0046872;GO:0042085;GO:0003674	K00549	metE	map00450;ma	Selenocompoun	------	------	PF08267.15	Meth_synt_1	Cobalamin-indepe	CYT	1	10	13	High
TRINITY_DN61674_c0_g1_i2_orf1	fatty acid-binding protein 1-like [Ostrinia furnacalis]	6.978810663	2.802981192	7.52E-06	up	yes	1.021	0.1463	0.14	0.149	0.15	1	0.985	1.079	MF:molecular_function;MF:lipid binding;MF:binding;	GO:0003674;GO:0008289;GO:0005488	K08753;K087	FABP4, aP2;F	map04923;ma	Regulation of lip	ENOG4111US8	I:Lipid transport and metabolis	------	------	------	CYT	2	12	14.2	High
TRINITY_DN2490_c0_g2_i1_orfp1	TRINITY_DN2490_c0_g2_i1_m.56872 TRINITY_DN2490_c0_g2::TRINITY_DN2490_c0_g2_i1::g.56872 ORF type:internal len:359 (-),score=123.59 TRINITY_DN2490_c0_g2_i1:2-1075(-)	9.514946962	3.250195615	1.40E-05	up	yes	0.9867	0.1037	0.088	0.107	0.116	1	0.923	1.037	------	------	------	------	------	------	------	------	------	------	------	EXC	7	34	36.3	High
TRINITY_DN96080_c0_g2_i1_orf1	ATP synthase subunit delta, mitochondrial [Ostrinia furnacalis]	2.753623188	1.461331152	2.06E-06	up	yes	1.007	0.3657	0.359	0.36	0.378	1	1.034	0.986	CC:proton-transporting ATP synthase complex, catalytic core;CC:cellular_component;CC:macromolecular complex;CC:proton-transporting two-sector ATPase complex, catalytic domain;CC:membrane protein complex;MF:cation transmembrane transporter activity;MF:proton-transporting ATP synthase activity, rotational mechanism;MF:inorganic solute uptake transmembrane transporter activity;MF:transporter activity;MF:ion channel activity;MF:passive transmembrane transporter activity;MF:ion transmembrane transporter activity;MF:catalytic activity;MF:transmembrane transporter activity;MF:hydrogen ion channel activity;MF:molecular_function;MF:channel activity;MF:cation channel activity;MF:inorganic cation transmembrane transporter activity;MF:ligase	GO:0045261;GO:0005575;GO:0032991;GO:0033178;GO:0098796;GO:0008324;GO:0046933;GO:0015318;GO:0005215;GO:0005216;GO:0022803;GO:0015075;GO:0003824;GO:0022857;GO:0015252;GO:0003674;GO:0015267;GO:0005261;GO:0022890;GO:0016874;GO:0015078	K02134	ATPeF1D, ATI	map04714;ma	Thermogenesis;C	COG0355	C:Energy production and conve	PF02823.19	ATP-synt_DE_	ATP synthase, Delt	CYT	6	53	17.2	High
TRINITY_DN12683_c0_g1_i3_orf1	sulfated surface glycoprotein 185-like [Ostrinia furnacalis]	2.669970533	1.41682382	3.25E-07	up	yes	0.9967	0.3733	0.36	0.389	0.371	1	1.002	0.988	------	------	------	------	------	------	ENOG4110QPT;ENOG410Y1IK;ENOG41129KS	S:Function unknown;	PF11901.11	DUF3421	Protein of unknow	CYT	2	9	27.1	High
TRINITY_DN7920_c0_g1_i2_orf1	uncharacterized protein LOC114357268 [Ostrinia furnacalis] >XP_028166599.1 uncharacterized protein LOC114357268 [Ostrinia furnacalis]	2.550260417	1.350644574	2.79E-05	up	yes	0.9793	0.384	0.43	0.373	0.349	1	0.953	0.985	------	------	K03098	APOD	------	------	COG3040	K:Transcription	PF08212.15;PF0	Lipocalin_2;Li	Lipocalin-like dom	CYT	3	14	22.3	High
TRINITY_DN10636_c0_g1_i1_orf1	unnamed protein product [Arctia plantaginis] >CAB3253774.1 unnamed protein product [Arctia plantaginis]	2.065941536	1.046799428	0.000538	up	yes	0.9117	0.4413	0.435	0.447	0.442	1	0.843	0.892	BP:regulation of primary metabolic process;BP:regulation of metabolic process;BP:regulation of cellular biosynthetic process;BP:regulation of cellular metabolic process;BP:regulation of biological process;BP:cellular component organization or biogenesis;BP:chromatin organization;BP:cellular component organization;BP:regulation of macromolecule metabolic process;BP:biological regulation;BP:regulation of gene expression;BP:regulation of nucleobase-containing compound metabolic process;BP:cellular process;BP:regulation of biosynthetic process;BP:regulation of nucleic acid-templated transcription;BP:regulation of cellular process;BP:biological_process;BP:regulation of nitrogen compound metabolic process;BP:regulation of RNA biosynthetic process;BP:regulation of RNA metabolic process;BP:regulation of transcription, DNA-templated;BP:regulation of macromolecule biosynthetic process;CC:protein acetyltransferase complex;CC:H4 histone acetyltransferase complex;CC:Sin3-type complex;CC:histone acetyltransferase complex;CC:H4/H2A histone acetyltransferase complex;CC:acetyltransferase complex;CC:catalytic complex;CC:histone deacetylase complex;CC:transferase complex;CC:macromolecular complex;CC:Sin3	GO:0080090;GO:0019222;GO:0031326;GO:0031323;GO:0050789;GO:0071840;GO:0006325;GO:0016043;GO:0060255;GO:0065007;GO:0010468;GO:0019219;GO:0009987;GO:0009889;GO:1903506;GO:0050794;GO:0008150;GO:0051171;GO:2001141;GO:0051252;GO:0006355;GO:0010556;GO:0031248;GO:1902562;GO:0070822;GO:0000123;GO:0043189;GO:1902493;GO:1902494;GO:0000118;GO:1990234;GO:0032991;GO:0016580;GO:0005575;GO:0035267;GO:0003674;GO:0005488;GO:0003682	K11339	MORF4L1, MI	------	------	ENOG410XR9F	K:Transcription	PF05712.16;PF1	MRG;Tudor-k	MRG;RNA binding	CYT	3	9	38.3	High
TRINITY_DN7213_c0_g1_i2_orf1	probable ATP-dependent RNA helicase CG8611 [Ostrinia furnacalis]	2.519109948	1.332914091	3.01E-05	up	yes	0.9623	0.382	0.402	0.398	0.346	1	0.928	0.959	MF:purine ribonucleoside triphosphate binding;MF:molecular_function;MF:binding;MF:nucleic acid binding;MF:RNA-dependent ATPase activity;MF:ion binding;MF:heterocyclic compound binding;MF:nucleoside phosphate binding;MF:anion binding;MF:nucleotide binding;MF:purine nucleotide binding;MF:ATP binding;MF:catalytic activity, acting on RNA;MF:helicase activity;MF:small molecule binding;MF:catalytic activity;MF:adenyl nucleotide binding;MF:carbohydrate derivative binding;MF:organic cyclic compound binding;MF:RNA helicase activity;MF:adenyl ribonucleotide binding;MF:RNA binding;MF:purine ribonucleotide binding;MF:ribonucleotide	GO:0035639;GO:0003674;GO:0005488;GO:0003676;GO:0008186;GO:0043167;GO:1901363;GO:1901265;GO:0043168;GO:0000166;GO:0017076;GO:0005524;GO:0140098;GO:0004386;GO:0036094;GO:0003824;GO:0030554;GO:0097367;GO:0097159;GO:0003724;GO:0032559;GO:0003723;GO:0032555;GO:0032553	K14806	DDX31, DBP7	------	------	ENOG410XNT7	L:Replication, recombination ar	PF00270.32;PF0	DEAD;Helicas	DEAD/DEAH box h	CYT	1	1	106.1	Medium
TRINITY_DN3134_c0_g1_i1_orf1	cytochrome c oxidase subunit 6C-1 isoform X1 [Hyposmocoma kahamanoa]	3.229461756	1.691293736	2.82E-06	up	yes	1.026	0.3177	0.303	0.311	0.339	1	1.025	1.052	CC:organelle membrane;CC:integral component of membrane;CC:membrane;CC:organelle inner membrane;CC:mitochondrial membrane;CC:cellular_component;CC:mitochondrial inner membrane;CC:intrinsic component of membrane;CC:cellular anatomical entity;	GO:0031090;GO:0016021;GO:0016020;GO:0019866;GO:0031966;GO:0005575;GO:0005743;GO:0031224;GO:0110165	K02268	COX6C	map04714;ma	Thermogenesis;C	ENOG41124VH	O:Posttranslational modificatio	PF02937.18	COX6C	Cytochrome c oxid	CYT	3	21	11.6	High
TRINITY_DN6747_c0_g1_i7_orf1	retinol dehydrogenase 12-like [Ostrinia furnacalis]	2.816537468	1.493922664	5.30E-07	up	yes	0.981	0.3483	0.343	0.354	0.348	1	0.964	0.979	------	------	K11153	RDH12	map00830;ma	Retinol metabolis	COG1028	S:Function unknown	PF00106.28;PF1	adh_short;adl	short chain dehydr	CYT	3	8	34.2	High
TRINITY_DN53167_c0_g1_i3_orf1	uncharacterized protein LOC114359219 [Ostrinia furnacalis]	3.232484076	1.692643263	1.07E-07	up	yes	1.015	0.314	0.31	0.313	0.319	1	1.023	1.023	BP:primary metabolic process;BP:carbohydrate metabolic process;BP:organic substance metabolic process;BP:biological_process;BP:metabolic process;CC:cytoplasm;CC:cellular_component;CC:cellular anatomical entity;MF:hydrolase activity;MF:catalytic activity;MF:hydrolase activity, acting on glycosyl bonds;MF:beta-fructofuranosidase activity;MF:hydrolase activity, hydrolyzing O-glycosyl	GO:0044238;GO:0005975;GO:0071704;GO:0008150;GO:0008152;GO:0005737;GO:0005575;GO:0110165;GO:0016787;GO:0003824;GO:0016798;GO:0004564;GO:0004553;GO:0003674	------	------	------	------	COG1621	G:Carbohydrate transport and i	PF00251.23;PF0	Glyco_hydro_	Glycosyl hydrolase	CYT	6	16	55.9	High
TRINITY_DN4923_c0_g1_i4_orf1	O-acyltransferase like protein-like [Ostrinia furnacalis]	2.915459651	1.543723357	3.54E-05	up	yes	0.9863	0.3383	0.31	0.366	0.339	1	0.933	1.026	------	------	------	------	------	------	ENOG410YD1T;ENOG410YWG8	O:Posttranslational modificatio	------	------	------	PLA	1	1	70.2	Medium
TRINITY_DN9464_c0_g1_i1_orf1	angio-associated migratory cell protein [Ostrinia furnacalis] >XP_028162594.1 angio-associated migratory cell protein [Ostrinia furnacalis]	3.343234323	1.741244475	4.14E-06	up	yes	1.013	0.303	0.337	0.281	0.291	1	1.035	1.004	------	------	K24725	AAMP	------	------	ENOG410XR6A	S:Function unknown	PF00400.35;PF1	WD40;ANAPC	WD domain, G-be	CYT	6	20	44.7	High
TRINITY_DN12474_c0_g1_i6_orf1	aromatic-L-amino-acid decarboxylase [Ostrinia furnacalis]	3.38	1.757023247	3.62E-07	up	yes	1.014	0.3	0.288	0.295	0.317	1	1.018	1.023	BP:carboxylic acid metabolic process;BP:nitrogen compound metabolic process;BP:cellular amino acid metabolic process;BP:organic substance metabolic process;BP:cellular process;BP:biological_process;BP:metabolic process;BP:oxoacid metabolic process;BP:primary metabolic process;BP:organonitrogen compound metabolic process;BP:organic acid metabolic process;BP:cellular metabolic process;BP:small molecule metabolic process;MF:binding;MF:aromatic-L-amino-acid decarboxylase activity;MF:vitamin B6 binding;MF:molecular_function;MF:pyridoxal phosphate binding;MF:heterocyclic compound binding;MF:anion binding;MF:lyase activity;MF:ion binding;MF:small molecule binding;MF:catalytic activity;MF:vitamin binding;MF:organic	GO:0019752;GO:0006807;GO:0006520;GO:0071704;GO:0009987;GO:0008150;GO:0008152;GO:0043436;GO:0044238;GO:1901564;GO:0006082;GO:0044237;GO:0044281;GO:0005488;GO:0004058;GO:0070279;GO:0003674;GO:0030170;GO:1901363;GO:0043168;GO:0016829;GO:0043167;GO:0036094;GO:0003824;GO:0019842;GO:0097159;GO:0016830;GO:0016831	K01593	DDC, TDC	map00350;ma	Tyrosine metabo	COG0076	S:Function unknown	PF00282.22	Pyridoxal_de(	Pyridoxal-depende	CYT	10	20	54.3	High
TRINITY_DN9003_c0_g1_i20_orf1	RNA-binding protein Nova-2 isoform X4 [Ostrinia furnacalis]	2.936912345	1.554300207	3.37E-05	up	yes	0.9683	0.3297	0.3	0.339	0.35	1	0.914	0.991	------	------	K14944	NOVA	------	------	ENOG410XR7D	S:Function unknown	PF00013.32;PF0	KH_1;KH_2;KI	KH domain;KH dor	CYT	4	11	42.2	High
TRINITY_DN17825_c1_g1_i1_orf1	39S ribosomal protein L1, mitochondrial [Ostrinia furnacalis]	3.057467635	1.612337227	7.43E-06	up	yes	0.9683	0.3167	0.312	0.317	0.321	1	0.978	0.927	CC:ribosome;CC:intracellular non-membrane-bounded organelle;CC:cellular_component;CC:intracellular organelle;CC:non-membrane-bounded organelle;CC:organelle;CC:cellular anatomical entity;	GO:0005840;GO:0043232;GO:0005575;GO:0043229;GO:0043228;GO:0043226;GO:0110165	K02863	RP-L1, MRPL	map03010	Ribosome	COG0081	J:Translation, ribosomal structu	PF00687.24	Ribosomal_L1	Ribosomal protein	CYT	1	3	39.6	High
TRINITY_DN15380_c0_g1_i1_orf1	39S ribosomal protein L32, mitochondrial [Ostrinia furnacalis]	3.466526463	1.793490774	8.33E-05	up	yes	0.989	0.2853	0.266	0.321	0.269	1	0.916	1.051	BP:cellular biosynthetic process;BP:cellular nitrogen compound metabolic process;BP:nitrogen compound metabolic process;BP:cellular macromolecule biosynthetic process;BP:organic substance biosynthetic process;BP:cellular nitrogen compound biosynthetic process;BP:cellular macromolecule metabolic process;BP:peptide biosynthetic process;BP:organic substance metabolic process;BP:amide biosynthetic process;BP:cellular process;BP:biosynthetic process;BP:macromolecule biosynthetic process;BP:biological_process;BP:metabolic process;BP:primary metabolic process;BP:organonitrogen compound metabolic process;BP:cellular amide metabolic process;BP:organonitrogen compound biosynthetic process;BP:protein metabolic process;BP:cellular metabolic process;BP:macromolecule metabolic process;BP:peptide metabolic process;BP:translation;CC:large ribosomal subunit;CC:intracellular non-membrane-bounded organelle;CC:organelle;CC:cellular anatomical entity;CC:ribosomal subunit;CC:macromolecular complex;CC:ribosome;CC:ribonucleoprotein complex;CC:intracellular	GO:0044249;GO:0034641;GO:0006807;GO:0034645;GO:1901576;GO:0044271;GO:0044260;GO:0043043;GO:0071704;GO:0043604;GO:0009987;GO:0009058;GO:0009059;GO:0008150;GO:0008152;GO:0044238;GO:1901564;GO:0043603;GO:1901566;GO:0019538;GO:0044237;GO:0043170;GO:0006518;GO:0006412;GO:0015934;GO:0043232;GO:0043226;GO:0110165;GO:0044391;GO:0032991;GO:0005840;GO:1990904;GO:0043229;GO:0005575;GO:0043228;GO:0003674;GO:0005198;GO:0003735	K02911	RP-L32, MRPI	map03010	Ribosome	COG0333	J:Translation, ribosomal structu	------	------	------	CYT	1	5	22.6	High

TRINITY_DN123396_c0_g1_i1_orf1	PREDICTED: delta-1-pyrroline-5-carboxylate dehydrogenase, mitochondrial isoform X1 [Megachile rotundata]	3.580124679	1.840009831	1.72E-05	up	yes	0.9763	0.2727	0.282	0.26	0.276	1	1.009	0.92	BP:small molecule catabolic process;BP:carboxylic acid metabolic process;BP:cellular amino acid catabolic process;BP:nitrogen compound metabolic process;BP:small molecule metabolic process;BP:glutamine family amino acid catabolic process;BP:glutamine family amino acid metabolic process;BP:organic substance catabolic process;BP:proline catabolic process;BP:proline metabolic process;BP:cellular amino acid metabolic process;BP:organic substance metabolic process;BP:alpha-amino acid metabolic process;BP:alpha-amino acid catabolic process;BP:primary metabolic process;BP:cellular process;BP:proline catabolic process to glutamate;BP:metabolic process;BP:oxoacid metabolic process;BP:catabolic process;BP:cellular catabolic process;BP:dicarboxylic acid metabolic process;BP:organonitrogen compound metabolic process;BP:organonitrogen compound catabolic process;BP:organic acid metabolic process;BP:carboxylic acid catabolic process;BP:organic acid catabolic process;BP:cellular metabolic process;BP:glutamate metabolic process;BP:biological_process;MF:molecular_function;MF:oxidoreductase activity;MF:oxidoreductase activity, acting on the aldehyde or oxo group of donors, NAD or NADP as acceptor;MF:catalytic activity;MF:oxidoreductase activity, acting on the aldehyde or oxo group of donors;MF:1-pyrroline-5-	GO:0044282;GO:0019752;GO:0009063; GO:0006807;GO:0044281;GO:0009065; GO:0009064;GO:1901575;GO:0006562; GO:0006560;GO:0006520;GO:0071704; GO:1901605;GO:1901606;GO:0044238; GO:0009987;GO:0010133;GO:0008152; GO:0043436;GO:0009056;GO:0044248; GO:0043648;GO:1901564;GO:1901565; GO:0006082;GO:0046395;GO:0016054; GO:0044237;GO:0006536;GO:0008150; GO:0003674;GO:0016491;GO:0016620; GO:0003824;GO:0016903;GO:0003842	K00294	E1.2.1.88	map00250;ma[	Alanine, aspartat	COG1012	Z:Cytoskeleton	PF00171.25	Aldedh	Aldehyde dehydro	CYT	1	1	63.5	High
TRINITY_DN99_c0_g1_i3_orf1	uncharacterized protein LOC126375979 [Pectinophora gossypiella] >XP_049879066.1 uncharacterized protein LOC126375979 [Pectinophora gossypiella]	2.602339181	1.379809011	0.0005736	up	yes	0.89	0.342	0.347	0.338	0.341	1	0.838	0.832	------	------	------	------	------	------	ENOG410YKH0;ENOG41115H7	S:Function unknown;	------	------	------	CYT	1	1	140.2	Medium
TRINITY_DN15046_c0_g1_i8_orf1	epidermal retinol dehydrogenase 2-like isoform X1 [Ostrinia furnacalis] >XP_028169999.1 epidermal retinol dehydrogenase 2-like isoform X2 [Ostrinia furnacalis]	4.184388186	2.065016697	2.49E-06	up	yes	0.9917	0.237	0.243	0.206	0.262	1	1.003	0.972	MF:catalytic activity;MF:molecular_function;MF:oxidoreductase activity;	GO:0003824;GO:0003674;GO:0016491	K15734	SDR16C5	map00830;ma[	Retinol metabolis	COG1028;COG4221	S:Function unknown;	PF00106.28;PF1	adh_short;adl	short chain dehydr	CYT	3	13	34.7	High
TRINITY_DN4731_c0_g1_i1_orf1	gelsolin-like [Ostrinia furnacalis]	4.706985466	2.234803399	1.18E-07	up	yes	1.004	0.2133	0.208	0.209	0.223	1	1.019	0.992	------	------	K05768	GSN	map04666;ma[	Fc gamma R-me	ENOG410XR0A	O:Posttranslational modificatio	PF00626.25	Gelsolin	Gelsolin repeat	CYT	7	15	72	High
TRINITY_DN36494_c0_g1_i1_orf1	MKI67 FHA domain-interacting nucleolar phosphoprotein-like [Ostrinia furnacalis]	4.94326591	2.305464515	9.05E-07	up	yes	1.002	0.2027	0.193	0.222	0.193	1	1.024	0.981	MF:organic cyclic compound binding;MF:molecular_function;MF:binding;MF:nucleic acid binding;MF:heterocyclic compound binding;MF:RNA binding;	GO:0097159;GO:0003674;GO:0005488; GO:0003676;GO:1901363;GO:0003723	K14838	NOP15	------	------	ENOG4111NF9	S:Function unknown	PF00076.25	RRM_1	RNA recognition m	CYT	2	6	30.6	High
TRINITY_DN344_c1_g1_i1_orf1	chymotrypsin-like serine protease 16 [Ostrinia nubilalis]	5.306878307	2.407863466	2.94E-07	up	yes	1.003	0.189	0.2	0.172	0.195	1	0.99	1.019	BP:organic substance metabolic process;BP:organonitrogen compound metabolic process;BP:protein metabolic process;BP:macromolecule metabolic process;BP:biological_process;BP:nitrogen compound metabolic process;BP:metabolic process;BP:proteolysis;BP:primary metabolic process;MF:serine-type endopeptidase activity;MF:endopeptidase activity;MF:hydrolase activity;MF:serine hydrolase activity;MF:catalytic activity, acting on a protein;MF:catalytic activity;MF:molecular_function;MF:peptidase activity;MF:serine-	GO:0071704;GO:1901564;GO:0019538; GO:0043170;GO:0008150;GO:0006807; GO:0008152;GO:0006508;GO:0044238; GO:0004252;GO:0004175;GO:0016787; GO:0017171;GO:0140096;GO:0003824; GO:0003674;GO:0008233;GO:0008236	K01312	PRSS1_2_3	map04972;ma[	Pancreatic secret	COG5640	I:Lipid transport and metabolisi	PF00089.29	Trypsin	Trypsin	CYT	5	29	30.7	High
TRINITY_DN46216_c0_g3_i1_orf1	unnamed protein product, partial [Brenthis ino]	4.578665368	2.194927129	3.10E-05	up	yes	0.94	0.2053	0.22	0.169	0.227	1	0.908	0.912	------	------	------	------	------	------	ENOG410Y7Q4;ENOG410XW69;ENOG4110RGQ	J:Translation, ribosomal structu	PF03564.18	DUF1759	Protein of unknow	CYT	1	11	9.6	Medium
TRINITY_DN2304_c0_g1_i4_orf1	clustered mitochondria protein homolog isoform X2 [Ostrinia furnacalis]	2.61826387	1.3886105	7.50E-07	up	yes	0.9863	0.3767	0.385	0.377	0.368	1	0.993	0.966	BP:organelle organization;BP:cellular component organization or biogenesis;BP:cellular process;BP:cellular component organization;BP:biological_process;BP:mitochondrion organization;CC:cytoplasm;CC:cellular_component;CC:cellular anatomical entity;MF:organic cyclic compound binding;MF:molecular_function;MF:RNA binding;MF:nucleic acid binding;MF:heterocyclic compound binding;MF:binding;	GO:0006996;GO:0071840;GO:0009987; GO:0016043;GO:0008150;GO:0007005; GO:0005737;GO:0005575;GO:0110165; GO:0097159;GO:0003674;GO:0003723; GO:0003676;GO:1901363;GO:0005488	K03255	TIF31, CLU1	------	------	ENOG410XQUQ	S:Function unknown	PF13424.9;PF13	TPR_12;TPR_1	Tetratricopeptide r	CYT	4	11	48	High
TRINITY_DN1656_c2_g1_i5_orf1	15-hydroxyprostaglandin dehydrogenase [NAD(+)]-like [Ostrinia furnacalis]	2.689909762	1.427557776	5.58E-07	up	yes	0.9837	0.3657	0.353	0.375	0.369	1	0.971	0.98	MF:catalytic activity;MF:molecular_function;MF:oxidoreductase activity;	GO:0003824;GO:0003674;GO:0016491	K00069	HPGD	map00590;ma[	Arachidonic acid	COG1028;COG0300	S:Function unknown;	PF00106.28;PF1	adh_short;adl	short chain dehydr	CYT	3	17	29.5	High
TRINITY_DN3332_c0_g1_i2_orf1	glutathione S-transferase sigma3 [Glyphodes pyloalis]	3.060240964	1.613645255	1.61E-06	up	yes	1.016	0.332	0.321	0.318	0.357	1	1.032	1.015	MF:catalytic activity;MF:transferase activity;MF:glutathione transferase activity;MF:molecular_function;MF:transferase activity, transferring alkyl or ary;	GO:0003824;GO:0016740;GO:0004364; GO:0003674;GO:0016765	K04097	HPGDS	map00590;ma[	Arachidonic acid	ENOG41103AU;ENOG4110YU0;ENOG4111KFX;ENOG4111VAU;ENOG4111I69;ENOG4111Y4Q	O:Posttranslational modificatio	PF02798.23;PF1	GST_N;GST_C	Glutathione S-tran	CYT	3	50	20.6	High
TRINITY_DN1557_c0_g1_i9_orf1	carboxylesterase CXE18 [Ostrinia furnacalis]	2.97640118	1.573568996	3.81E-06	up	yes	1.009	0.339	0.311	0.335	0.371	1	1.024	1.004	MF:hydrolase activity;MF:molecular_function;MF:hydrolase activity, acting on ester bonds;MF:carboxylic ester hydrolase activity;MF:catalytic activity;	GO:0016787;GO:0003674;GO:0016788; GO:0052689;GO:0003824	------	------	------	------	COG2272	S:Function unknown	PF00135.31;PF2	COesterase;B	Carboxylesterase f	CYT	4	7	63.7	High
TRINITY_DN8543_c0_g1_i1_orf1	39S ribosomal protein L38, mitochondrial [Ostrinia furnacalis]	2.824651295	1.498072777	2.11E-06	up	yes	0.9923	0.3513	0.364	0.33	0.36	1	1.007	0.97	------	------	K17419	MRPL38	------	------	COG1881	S:Function unknown	PF01161.23	PBP	Phosphatidylethan	CYT	1	4	46.4	High
TRINITY_DN25856_c0_g1_i1_orf1	myrosinase 1-like [Ostrinia furnacalis]	3.024024024	1.596469601	2.01E-06	up	yes	1.007	0.333	0.347	0.317	0.335	1	1.033	0.987	------	------	------	------	------	------	COG2723;ENOG4111IRY	G:Carbohydrate transport and	PF00232.21	Glyco_hydro_	Glycosyl hydrolase	CYT	3	9	57.7	High
TRINITY_DN35662_c0_g1_i5_orf1	hypothetical protein evm_006436 [Chilo suppressalis] >CAB3522373.1 unnamed protein product [Chilo suppressalis] >CAH0399695.1 unnamed protein product [Chilo suppressalis]	3.224786595	1.689203691	1.16E-06	up	yes	1.02	0.3163	0.313	0.33	0.306	1	1.044	1.015	MF:hydrolase activity;MF:molecular_function;MF:catalytic activity;	GO:0016787;GO:0003674;GO:0003824	K21013	FPPP	map00981	Insect hormone	COG0647	G:Carbohydrate transport and	PF13344.9;PF13	Hydrolase_6;H	Haloacid dehaloge	CYT	2	6	32.9	High
TRINITY_DN18172_c0_g1_i6_orf1	digestive cysteine proteinase 2-like [Ostrinia furnacalis]	3.121118012	1.642062908	4.37E-08	up	yes	1.005	0.322	0.315	0.327	0.324	1	0.999	1.015	BP:primary metabolic process;BP:organonitrogen compound metabolic process;BP:protein metabolic process;BP:macromolecule metabolic process;BP:organic substance metabolic process;BP:nitrogen compound metabolic process;BP:biological_process;BP:metabolic process;BP:proteolysis;MF:molecular_function;MF:hydrolase activity;MF:catalytic activity;MF:catalytic activity, acting on a protein;MF:peptidase activity;MF:cysteine-type peptidase	GO:0044238;GO:1901564;GO:0019538; GO:0043170;GO:0071704;GO:0006807; GO:0008150;GO:0008152;GO:0006508; GO:0003674;GO:0016787;GO:0003824; GO:0140096;GO:0008233;GO:0008234	------	------	------	------	COG4870	O:Posttranslational modificatio	PF00112.26;PF0	Peptidase_C1	Papain family cyst	EXC	3	7	57.6	High
TRINITY_DN76283_c0_g6_i1_orf1	fatty acid synthase-like [Ostrinia furnacalis]	3.929539295	1.974360179	1.49E-05	up	yes	1.015	0.2583	0.256	0.259	0.26	1	0.972	1.073	BP:fatty acid biosynthetic process;BP:fatty acid metabolic process;BP:carboxylic acid metabolic process;BP:cellular biosynthetic process;BP:small molecule metabolic process;BP:small molecule biosynthetic process;BP:monocarboxylic acid biosynthetic process;BP:organic substance biosynthetic process;BP:organic substance metabolic process;BP:lipid metabolic process;BP:cellular process;BP:monocarboxylic acid metabolic process;BP:biosynthetic process;BP:biological_process;BP:metabolic process;BP:oxoacid metabolic process;BP:cellular lipid metabolic process;BP:lipid biosynthetic process;BP:primary metabolic process;BP:organic acid metabolic process;BP:carboxylic acid biosynthetic process;BP:organic acid biosynthetic process;BP:cellular metabolic process;MF:thiolester hydrolase activity;MF:molecular_function;MF:oxidoreductase activity;MF:oleoyl-[acyl-carrier-protein] hydrolase activity;MF:transferase activity;MF:transferase activity, transferring acyl groups;MF:transferase activity, transferring acyl groups other than amino-acyl groups;MF:hydrolase activity;MF:hydrolase activity, acting on ester bonds;MF:catalytic activity;MF:fatty acid synthase activity;MF:myristoyl-[acyl-	GO:0006633;GO:0006631;GO:0019752; GO:0044249;GO:0044281;GO:0044283; GO:0072330;GO:1901576;GO:0071704; GO:0006629;GO:0009987;GO:0032787; GO:0009058;GO:0008150;GO:0008152; GO:0043436;GO:0044255;GO:0008610; GO:0044238;GO:0006082;GO:0046394; GO:0016053;GO:0044237;GO:0016790; GO:0003674;GO:0016491;GO:0004320; GO:0016740;GO:0016746;GO:0016747; GO:0016787;GO:0016788;GO:0003824; GO:0004312;GO:0016295;GO:0016296; GO:0016297	K00665	FASN	map04910;ma[	Insulin signaling	COG3321;ENOG4111ANH	Q:Secondary metabolites biosy	PF13602.9;PF00	ADH_zinc_N_	Zinc-binding dehy	CYT	1	2	95	High
TRINITY_DN40_c0_g2_i1_orf1	trypsin CFT-1-like [Ostrinia furnacalis]	4.103489771	2.036851357	9.16E-06	up	yes	1.023	0.2493	0.242	0.219	0.287	1	1.01	1.06	BP:organic substance metabolic process;BP:organonitrogen compound metabolic process;BP:protein metabolic process;BP:macromolecule metabolic process;BP:biological_process;BP:nitrogen compound metabolic process;BP:metabolic process;BP:proteolysis;BP:primary metabolic process;MF:serine-type endopeptidase activity;MF:endopeptidase activity;MF:hydrolase activity;MF:serine hydrolase activity;MF:catalytic activity, acting on a protein;MF:catalytic activity;MF:molecular_function;MF:peptidase activity;MF:serine-	GO:0071704;GO:1901564;GO:0019538; GO:0043170;GO:0008150;GO:0006807; GO:0008152;GO:0006508;GO:0044238; GO:0004252;GO:0004175;GO:0016787; GO:0017171;GO:0140096;GO:0003824; GO:0003674;GO:0008233;GO:0008236	K01312	PRSS1_2_3	map04972;ma[	Pancreatic secret	COG5640	O:Posttranslational modificatio	PF00089.29	Trypsin	Trypsin	CYT	1	3	28.5	High
TRINITY_DN5740_c0_g1_i4_orf1	unconventional myosin IC isoform X1 [Ostrinia furnacalis]	4.13263243	2.047061051	2.60E-07	up	yes	1.022	0.2473	0.251	0.244	0.247	1	1.033	1.033	CC:cytoplasm;CC:myosin complex;CC:macromolecular complex;CC:cellular_component;CC:cellular anatomical entity;MF:purine ribonucleoside triphosphate binding;MF:molecular_function;MF:binding;MF:ion binding;MF:heterocyclic compound binding;MF:nucleoside phosphate binding;MF:nucleotide binding;MF:actin binding;MF:purine nucleotide binding;MF:motor activity;MF:ATP binding;MF:anion binding;MF:small molecule binding;MF:cytoskeletal protein binding;MF:carbohydrate derivative binding;MF:organic cyclic compound binding;MF:adenyl nucleotide binding;MF:adenyl ribonucleotide binding;MF:protein binding;MF:purine ribonucleotide binding;MF:ribonucleotide binding;	GO:0005737;GO:0016459;GO:0032991; GO:0005575;GO:0110165;GO:0035639; GO:0003674;GO:0005488;GO:0043167; GO:1901363;GO:1901265;GO:0000166; GO:0003779;GO:0017076;GO:0003774; GO:0005524;GO:0043168;GO:0036094; GO:0008092;GO:0097367;GO:0097159; GO:0030554;GO:0032559;GO:0005515; GO:0032555;GO:0032553	K10356	MYO1	map05130	Pathogenic Esch	COG5022	S:Function unknown	PF00063.24;PF0	Myosin_head	Myosin head;Unco	CYT	15	15	119.7	High
TRINITY_DN6221_c0_g1_i5_orf1	unnamed protein product [Diatraea saccharalis]	4.095199015	2.033933565	2.00E-05	up	yes	0.998	0.2437	0.255	0.223	0.253	1	0.944	1.05	CC:intrinsic component of membrane;CC:cellular_component;CC:proton-transporting V-type ATPase, V0 domain;CC:integral component of membrane;CC:cellular anatomical entity;CC:macromolecular complex;CC:membrane protein complex;CC:proton-transporting two-sector ATPase complex, proton-transporting domain;MF:molecular_function;MF:ATPase activity, coupled to transmembrane movement of substances;MF:inorganic solute uptake transmembrane transporter activity;MF:hydrogen-translocating pyrophosphatase activity;MF:primary active transmembrane transporter activity;MF:ATPase coupled ion transmembrane transporter activity;MF:transporter activity;MF:cation transmembrane transporter activity;MF:proton-transporting ATPase activity, rotational mechanism;MF:ATPase activity, coupled to transmembrane movement of ions, rotational mechanism;MF:inorganic cation transmembrane transporter activity;MF:cation-transporting ATPase activity;MF:ion transmembrane transporter activity;MF:transmembrane transporter activity;MF:active transmembrane transporter activity;MF:active ion transmembrane transporter activity;MF:hydrogen ion	GO:0031224;GO:0005575;GO:0033179; GO:0016021;GO:0110165;GO:0032991; GO:0098796;GO:0033177;GO:0003674; GO:0042626;GO:0015318;GO:0009678; GO:0015399;GO:0042625;GO:0005215; GO:0008324;GO:0046961;GO:0044769; GO:0022890;GO:0019829;GO:0015075; GO:0022857;GO:0022804;GO:0022853; GO:0015078	K02153	ATPeV0E, ATI	map05165;ma[	Human papillom	ENOG41121M0	C:Energy production and conve	PF05493.16	ATP_synt_H	ATP synthase subu	CYT	1	9	9.6	High
TRINITY_DN79319_c0_g1_i8_orfp1	TRINITY_DN79319_c0_g1_i8_m.49956 TRINITY_DN79319_c0_g1::TRINITY_DN79319_c0_g1_i8::g.49956 ORF type:5prime_partial len:84	3.963096671	1.986628159	2.61E-07	up	yes	0.988	0.2493	0.259	0.251	0.238	1	0.993	0.971	------	------	------	------	------	------	------	------	------	------	------	CYT	2	20	9.6	High
TRINITY_DN1914_c0_g1_i6_orf1	loricrin-like [Ostrinia furnacalis]	5.586021505	2.481821128	3.39E-05	up	yes	1.039	0.186	0.175	0.211	0.172	1	1.119	0.999	------	------	------	------	------	------	ENOG4110QTU;ENOG4110QBC	S:Function unknown;	------	------	------	CYT	1	15	58.9	High
TRINITY_DN31118_c0_g2_i1_orf1	unnamed protein product [Spodoptera exigua]	4.182011031	2.064196867	2.34E-06	up	yes	0.9857	0.2357	0.252	0.212	0.243	1	1	0.957	MF:molecular_function;MF:structural constituent of cuticle;MF:structural molecule activity;	GO:0003674;GO:0042302;GO:0005198	------	------	------	------	ENOG410YVGC;ENOG41125BY;ENOG410YVKS	S:Function unknown;	PF00379.26	Chitin_bind_4	Insect cuticle prot	CYT	2	53	12.9	High
TRINITY_DN96_c0_g1_i1_orf1	collagenase-like [Ostrinia furnacalis]	6.320754717	2.660096831	6.14E-05	up	yes	1.005	0.159	0.172	0.144	0.161	1	0.925	1.089	BP:organic substance metabolic process;BP:organonitrogen compound metabolic process;BP:protein metabolic process;BP:macromolecule metabolic process;BP:biological_process;BP:nitrogen compound metabolic process;BP:metabolic process;BP:proteolysis;BP:primary metabolic process;MF:serine-type endopeptidase activity;MF:endopeptidase activity;MF:hydrolase activity;MF:serine hydrolase activity;MF:catalytic activity, acting on a protein;MF:catalytic activity;MF:molecular_function;MF:peptidase activity;MF:serine-	GO:0071704;GO:1901564;GO:0019538; GO:0043170;GO:0008150;GO:0006807; GO:0008152;GO:0006508;GO:0044238; GO:0004252;GO:0004175;GO:0016787; GO:0017171;GO:0140096;GO:0003824; GO:0003674;GO:0008233;GO:0008236	------	------	------	------	COG5640	O:Posttranslational modificatio	PF00089.29	Trypsin	Trypsin	CYT	1	3	44.4	High
TRINITY_DN48410_c0_g1_i1_orf1	alpha-amylase 1-like [Ostrinia furnacalis]	5.680208938	2.505943998	3.55E-07	up	yes	0.9787	0.1723	0.172	0.169	0.176	1	0.979	0.957	BP:organic substance metabolic process;BP:primary metabolic process;BP:carbohydrate metabolic process;BP:metabolic process;BP:biological_process;MF:hydrolase activity;MF:cation binding;MF:amylase activity;MF:hydrolase activity, acting on glycosyl bonds;MF:ion binding;MF:catalytic activity;MF:alpha-amylase activity;MF:molecular_function;MF:binding;MF:hydrolase	GO:0071704;GO:0044238;GO:0005975; GO:0008152;GO:0008150;GO:0016787; GO:0043169;GO:0016160;GO:0016798; GO:0043167;GO:0003824;GO:0004556; GO:0003674;GO:0005488;GO:0004553; GO:0046872	K01176	AMY, amyA, r	map04972;ma[	Pancreatic secret	COG0366	G:Carbohydrate transport and	PF00128.27;PF0	Alpha-amylas	Alpha amylase, cat	EXC	11	29	56	High
TRINITY_DN33452_c0_g1_i1_orf1	lethal(2) giant larvae protein isoform X8 [Ostrinia furnacalis]	5.501985252	2.459952273	1.02E-05	up	yes	0.97	0.1763	0.211	0.129	0.189	1	0.955	0.955	BP:secretion;BP:transport;BP:vesicle-mediated transport;BP:export from cell;BP:exocytosis;BP:secretion by cell;BP:biological_process;BP:cellular process;BP:establishment of localization;BP:localization;	GO:0046903;GO:0006810;GO:0016192; GO:0140352;GO:0006887;GO:0032940; GO:0008150;GO:0009987;GO:0051234; GO:0051179	K06094	LLGL	map04530;ma[	Tight junction;Hu	ENOG410XS6Z	S:Function unknown	------	------	------	CYT	1	14	13.7	High

TRINITY_DN6044_c0_g1_i4_orf1	acyl-CoA-binding protein-like [Ostrinia furnacalis]	7.653758542	2.936168388	2.54E-06	up	yes	1.008	0.1317	0.129	0.135	0.131	1	1.05	0.974	MF:sulfur compound binding;MF:molecular_function;MF:fatty acid derivative binding;MF:carbohydrate derivative binding;MF:amide binding;MF:adenyl nucleotide binding;MF:ribonucleotide binding;MF:lipid binding;MF:fatty-acyl-CoA binding;MF:nucleotide binding;MF:small molecule binding;MF:purine ribonucleotide binding;MF:adenyl ribonucleotide binding;MF:binding;MF:organic cyclic compound binding;MF:acyl-CoA binding;MF:purine nucleotide binding;MF:heterocyclic compound binding;MF:nucleoside	GO:1901681;GO:0003674;GO:1901567;GO:0097367;GO:0033218;GO:0030554;GO:0032553;GO:0008289;GO:0000062;GO:0000166;GO:0036094;GO:0032555;GO:0032559;GO:0005488;GO:0097159;GO:0120227;GO:0017076;GO:1901363;GO:1901265	K08762	DBI, ACBP	map03320	PPAR signaling p	COG4281	I:Lipid transport and metabolis	PF00887.22	ACBP	Acyl CoA binding p	CYT	5	54	9.5	High
TRINITY_DN26411_c0_g1_i2_orfp1	TRINITY_DN26411_c0_g1_i2_m.24123 TRINITY_DN26411_c0_g1::TRINITY_DN26411_c0_g1_i2::g.24123 ORF type:internal len:115 (-	10.1894317	3.349001685	1.18E-05	up	yes	1.022	0.1003	0.103	0.118	0.08	1	1.086	0.979	------	------	------	------	------	------	------	------	------	------	------	CYT	1	46	10.9	High
TRINITY_DN2114_c0_g1_i5_orf1	vegetative cell wall protein gp1-like isoform X1 [Ostrinia furnacalis]	6.789583333	2.763323041	4.33E-07	up	yes	0.9777	0.144	0.144	0.142	0.146	1	0.98	0.953	BP:biological_process;BP:metabolic process;BP:methylation;MF:catalytic activity;MF:transferase activity;MF:transferase activity, transferring one-carbon groups;MF:molecular_function;MF:methyltransferase activity;	GO:0008150;GO:0008152;GO:0032259;GO:0003824;GO:0016740;GO:0016741;GO:0003674;GO:0008168	------	------	------	------	ENOG410Y1IK;ENOG4110QPT;ENOG41129KS	S:Function unknown;	PF11901.11	DUF3421	Protein of unknow	CYT	4	16	25.5	High
TRINITY_DN39200_c0_g1_i5_orf1	juvenile hormone epoxide hydrolase-like [Ostrinia furnacalis]	10.38210526	3.376027115	2.97E-07	up	yes	0.9863	0.095	0.105	0.087	0.093	1	0.962	0.997	CC:integral component of membrane;CC:cellular_component;CC:intrinsic component of membrane;CC:cellular anatomical entity;MF:hydrolase activity;MF:cis-stilbene-oxide hydrolase activity;MF:hydrolase activity, acting on ether bonds;MF:catalytic activity;MF:molecular_function;MF:ether hydrolase activity;	GO:0016021;GO:0005575;GO:0031224;GO:0110165;GO:0016787;GO:0033961;GO:0016801;GO:0003824;GO:0003674;GO:0016803	K10719	JHEH	map00981	Insect hormone b	COG0596;COG0386	U:Intracellular trafficking, secre	PF06441.15;PF0	EHN;Abhydro	Epoxide hydrolase	PLA	1	4	56.8	High
TRINITY_DN23266_c0_g2_i1_orf1	medium-chain acyl-CoA ligase ACSF2, mitochondrial [Chelonus insularis]	3.217499224	1.685939801	3.50E-06	up	yes	1.037	0.3223	0.31	0.331	0.326	1	1.059	1.053	------	------	K23617	ACSF2	------	------	COG0318	S:Function unknown	PF00501.31;PF1	AMP-binding	AMP-binding enzy	CYT	1	1	65	Medium
TRINITY_DN109540_c0_g1_i3_orf1	4-coumarate--CoA ligase 1-like isoform X4 [Ostrinia furnacalis]	2.894056848	1.533093261	1.26E-07	up	yes	1.008	0.3483	0.353	0.335	0.357	1	1.013	1.012	CC:integral component of membrane;CC:cellular_component;CC:intrinsic component of membrane;CC:cellular anatomical entity;MF:monooxygenase activity;MF:catalytic activity;MF:molecular_function;MF:oxidoreductase activity;	GO:0016021;GO:0005575;GO:0031224;GO:0110165;GO:0004497;GO:0003824;GO:0003674;GO:0016491	K01904	4CL	map00130	Ubiquinone and	COG0318;COG0318	I:Lipid transport and metabolis	PF00501.31;PF1	AMP-binding	AMP-binding enzy	CYT	6	14	58.4	High
TRINITY_DN107288_c0_g1_i2_orf1	methionine-tRNA synthetase, partial [Papilio xuthus]	2.32433096	1.216815508	0.0002378	up	yes	0.9467	0.4073	0.39	0.473	0.359	1	0.918	0.922	BP:methionyl-tRNA aminoacylation;BP:prolyl-tRNA aminoacylation;BP:carboxylic acid metabolic process;BP:nucleic acid metabolic process;BP:cellular nitrogen compound metabolic process;BP:nitrogen compound metabolic process;BP:small molecule metabolic process;BP:ncRNA metabolic process;BP:organic cyclic compound metabolic process;BP:nucleobase-containing compound metabolic process;BP:cellular amino acid metabolic process;BP:organic substance metabolic process;BP:primary metabolic process;BP:cellular process;BP:cellular aromatic compound metabolic process;BP:biological_process;BP:metabolic process;BP:oxoacid metabolic process;BP:organonitrogen compound metabolic process;BP:heterocycle metabolic process;BP:RNA metabolic process;BP:organic acid metabolic process;BP:cellular metabolic process;BP:macromolecule metabolic process;BP:tRNA metabolic process;BP:amino acid activation;BP:tRNA aminoacylation;BP:tRNA aminoacylation for protein translation;CC:aminoacyl-tRNA synthetase multienzyme complex;CC:cytoplasm;CC:cellular anatomical entity;CC:catalytic complex;CC:macromolecular complex;CC:cellular_component;MF:purine ribonucleoside triphosphate binding;MF:molecular_function;MF:ion binding;MF:heterocyclic compound binding;MF:aminoacyl-tRNA ligase activity;MF:nucleotide binding;MF:nucleoside phosphate binding;MF:purine nucleotide binding;MF:anion binding;MF:catalytic activity, acting on RNA;MF:small molecule binding;MF:catalytic activity;MF:ligase activity;MF:ligase activity, forming carbon-oxygen bonds;MF:methionine-tRNA ligase activity;MF:proline-tRNA ligase activity;MF:adenyl nucleotide	GO:0006431;GO:0006433;GO:0019752;GO:0090304;GO:0034641;GO:0006807;GO:0044281;GO:0034660;GO:1901360;GO:0006139;GO:0006520;GO:0071704;GO:0044238;GO:0009987;GO:0006725;GO:0008150;GO:0008152;GO:0043436;GO:1901564;GO:0046483;GO:0016070;GO:0006082;GO:0044237;GO:0043170;GO:0006399;GO:0043038;GO:0043039;GO:0006418;GO:0017101;GO:0005737;GO:0110165;GO:1902494;GO:0032991;GO:0005575;GO:0035639;GO:0003674;GO:0043167;GO:1901363;GO:0004812;GO:0000166;GO:1901265;GO:0017076;GO:0043168;GO:0140098;GO:0036094;GO:0003824;GO:0016874;GO:0016875;GO:0004825;GO:0004827;GO:0030554;GO:0097367;GO:0097159;GO:0032559;GO:0005524;GO:0032555;GO:0140101;GO:0032553;GO:0005488	K01874	MARS, metG	map00450;ma	Selenocompoun	COG0143	J:Translation, ribosomal structu	PF00458.23	WHEP-TRS	WHEP-TRS domair	CYT	1	44	7.7	High
TRINITY_DN1277_c4_g1_i5_orf1	GTP cyclohydrolase 1 isoform X1 [Ostrinia furnacalis] >XP_028166842.1 GTP cyclohydrolase 1 isoform X1 [Ostrinia furnacalis]	3.044650884	1.60627681	3.70E-07	up	yes	1.016	0.3337	0.334	0.324	0.343	1	1.032	1.015	BP:cellular modified amino acid biosynthetic process;BP:dihydrobiopterin metabolic process;BP:polyol biosynthetic process;BP:polyol metabolic process;BP:diol metabolic process;BP:cellular biosynthetic process;BP:cellular nitrogen compound metabolic process;BP:diol biosynthetic process;BP:small molecule metabolic process;BP:small molecule biosynthetic process;BP:tetrahydrofolate metabolic process;BP:organic cyclic compound biosynthetic process;BP:tetrahydrofolate biosynthetic process;BP:organic cyclic compound metabolic process;BP:organic substance biosynthetic process;BP:folic acid-containing compound biosynthetic process;BP:7,8-dihydroneopterin 3'-triphosphate biosynthetic process;BP:pteridine-containing compound metabolic process;BP:pteridine-containing compound biosynthetic process;BP:organic substance metabolic process;BP:heterocycle biosynthetic process;BP:tetrahydrobiopterin biosynthetic process;BP:folic acid-containing compound metabolic process;BP:alcohol biosynthetic process;BP:cellular process;BP:cellular aromatic compound metabolic process;BP:biosynthetic process;BP:tetrahydrobiopterin metabolic process;BP:biological_process;BP:metabolic process;BP:organonitrogen compound metabolic process;BP:organophosphate biosynthetic process;BP:heterocycle metabolic process;BP:cellular nitrogen compound biosynthetic process;BP:cellular modified amino acid metabolic process;BP:organonitrogen compound biosynthetic process;BP:cellular metabolic process;BP:alcohol metabolic process;BP:phosphate-containing compound metabolic	GO:0042398;GO:0051066;GO:0046173;GO:0019751;GO:0034311;GO:0044249;GO:0034641;GO:0034312;GO:0044281;GO:0044283;GO:0046653;GO:1901362;GO:0046654;GO:1901360;GO:1901576;GO:0009396;GO:0035998;GO:0042558;GO:0042559;GO:0071704;GO:0018130;GO:0006729;GO:0006760;GO:0046165;GO:0009987;GO:0006725;GO:0009058;GO:0046146;GO:0008150;GO:0008152;GO:1901564;GO:0090407;GO:0046483;GO:0044271;GO:0006575;GO:1901566;GO:0044237;GO:0006066;GO:0006796;GO:0006807;GO:0006793;GO:0019637;GO:0019438;GO:1901617;GO:1901615;GO:0003933;GO:0003674;GO:0016814;GO:0019238;GO:0016787;GO:0003934;GO:0003824;GO:0016810	K01495	GCH1, folE	map00790;ma	Folate biosynthe	COG0302	H:Coenzyme transport and me	PF01227.25	GTP_cyclohy	GTP cyclohydrolas	CYT	5	19	32.6	High
TRINITY_DN96170_c0_g2_i1_orf1	uncharacterized protein LOC114355569 [Ostrinia furnacalis]	2.546147778	1.348316155	2.10E-05	up	yes	0.9683	0.3803	0.38	0.404	0.357	1	0.927	0.978	BP:phosphorylation;BP:cellular process;BP:biological_process;BP:metabolic process;BP:cellular metabolic process;BP:phosphate-containing compound metabolic process;BP:phosphorus metabolic process;CC:intrinsic component of membrane;CC:integral component of membrane;CC:cellular_component;CC:cellular anatomical entity;MF:purine ribonucleoside triphosphate binding;MF:molecular_function;MF:binding;MF:ion binding;MF:heterocyclic compound binding;MF:anion binding;MF:nucleotide binding;MF:transferase activity;MF:nucleoside phosphate binding;MF:purine nucleotide binding;MF:ATP binding;MF:kinase activity;MF:small molecule binding;MF:catalytic activity;MF:adenyl nucleotide binding;MF:carbohydrate derivative binding;MF:transferase activity, transferring phosphorus-containing groups;MF:adenyl ribonucleotide binding;MF:purine ribonucleotide binding;MF:organic cyclic compound binding;MF:ribonucleotide	GO:0016310;GO:0009987;GO:0008150;GO:0008152;GO:0044237;GO:0006796;GO:0006793;GO:0031224;GO:0016021;GO:0005575;GO:0110165;GO:0035639;GO:0003674;GO:0005488;GO:0043167;GO:1901363;GO:0043168;GO:0000166;GO:0016740;GO:1901265;GO:0017076;GO:0005524;GO:0016301;GO:0036094;GO:0003824;GO:0030554;GO:0097367;GO:0016772;GO:0032559;GO:0032555;GO:0097159;GO:0032553	------	------	------	------	COG0574	G:Carbohydrate transport and	PF00391.26	PEP-utilizers	PEP-utilising enzy	CYT	1	4	31.7	High
TRINITY_DN44288_c0_g1_i2_orf1	ATP-dependent RNA helicase p62 [Ostrinia furnacalis]	2.788848005	1.479669308	3.67E-07	up	yes	0.9853	0.3533	0.349	0.355	0.356	1	0.967	0.989	CC:intracellular organelle;CC:membrane-bounded organelle;CC:nucleus;CC:organelle;CC:intracellular membrane-bounded organelle;CC:cellular_component;CC:cellular anatomical entity;MF:purine ribonucleoside triphosphate binding;MF:molecular_function;MF:binding;MF:nucleic acid binding;MF:DNA binding;MF:RNA-dependent ATPase activity;MF:nucleotide binding;MF:heterocyclic compound binding;MF:nucleoside phosphate binding;MF:anion binding;MF:ion binding;MF:purine nucleotide binding;MF:ATP binding;MF:hydrolase activity;MF:catalytic activity, acting on RNA;MF:helicase activity;MF:small molecule binding;MF:catalytic activity;MF:adenyl nucleotide binding;MF:carbohydrate derivative binding;MF:organic cyclic compound binding;MF:RNA helicase activity;MF:adenyl ribonucleotide binding;MF:purine ribonucleotide binding;MF:ribonucleotide binding;	GO:0043229;GO:0043227;GO:0005634;GO:0043226;GO:0043231;GO:0005575;GO:0110165;GO:0035639;GO:0003674;GO:0005488;GO:0003676;GO:0003677;GO:0008186;GO:0000166;GO:1901363;GO:1901265;GO:0043168;GO:0043167;GO:0017076;GO:0005524;GO:0016787;GO:0140098;GO:0004386;GO:0036094;GO:0003824;GO:0030554;GO:0097367;GO:0097159;GO:0003724;GO:0032559;GO:0032555;GO:0032553	K12823	DDX5, DBP2	map05205;ma	Proteoglycans in	COG0513	I:Lipid transport and metabolis	PF00270.32;PF0	DEAD;Helicas	DEAD/DEAH box h	CYT	8	25	60.3	High
TRINITY_DN24399_c0_g1_i1_orf1	retinol-binding protein pinta-like [Ostrinia furnacalis]	3.394736842	1.763299742	9.69E-06	up	yes	1.032	0.304	0.287	0.335	0.29	1	1.071	1.024	------	------	------	------	------	------	ENOG410XRSQ	S:Function unknown	PF00650.23	CRAL_TRIO	CRAL/TRIO domair	CYT	4	15	35.4	High
TRINITY_DN140669_c0_g1_i1_orf1	S-methyl-5'-thioadenosine phosphorylase-like isoform X1 [Hyposmocoma kahamanoa]	3.708820404	1.890960408	2.62E-05	up	yes	1.047	0.2823	0.31	0.255	0.282	1	1.034	1.106	BP:nucleobase-containing small molecule biosynthetic process;BP:cellular biosynthetic process;BP:cellular nitrogen compound metabolic process;BP:nitrogen compound metabolic process;BP:nucleoside biosynthetic process;BP:small molecule biosynthetic process;BP:purine-containing compound metabolic process;BP:purine-containing compound biosynthetic process;BP:organic cyclic compound biosynthetic process;BP:purine nucleoside biosynthetic process;BP:organic cyclic compound metabolic process;BP:nucleobase-containing compound metabolic process;BP:purine nucleoside metabolic process;BP:organic substance metabolic process;BP:ribonucleoside biosynthetic process;BP:purine-containing compound salvage;BP:small molecule metabolic process;BP:heterocycle biosynthetic process;BP:ribonucleoside metabolic process;BP:organic substance biosynthetic process;BP:cellular process;BP:cellular aromatic compound metabolic process;BP:biosynthetic process;BP:biological_process;BP:nucleoside metabolic process;BP:metabolic process;BP:nucleobase-containing compound biosynthetic process;BP:organonitrogen compound metabolic process;BP:purine ribonucleoside biosynthetic process;BP:purine ribonucleoside metabolic process;BP:nucleobase-containing small molecule metabolic process;BP:heterocycle metabolic process;BP:cellular metabolic compound salvage;BP:primary metabolic process;BP:cellular nitrogen compound biosynthetic process;BP:organonitrogen compound biosynthetic process;BP:carbohydrate derivative biosynthetic process;BP:carbohydrate derivative metabolic process;BP:nucleoside salvage;BP:cellular metabolic	GO:0034404;GO:0044249;GO:0034641;GO:0006807;GO:0009163;GO:0044283;GO:0072521;GO:0072522;GO:1901362;GO:0042451;GO:1901360;GO:0006139;GO:0042278;GO:0071704;GO:0042455;GO:0043101;GO:0044281;GO:0018130;GO:0009119;GO:1901576;GO:0009987;GO:0006725;GO:0009058;GO:0008150;GO:0009116;GO:0008152;GO:0034654;GO:1901564;GO:0046129;GO:0046128;GO:0055086;GO:0046483;GO:0043094;GO:0044238;GO:0044271;GO:1901566;GO:1901137;GO:1901135;GO:0043174;GO:0044237;GO:1901657;GO:0006166;GO:0019438;GO:1901659;GO:0003674;GO:0004731;GO:0016740;GO:0016763;GO:0003824;GO:0017061;GO:0016757	K00772	mtaP, MTAP	map00270	Cysteine and me	COG0005	F:Nucleotide transport and me	PF01048.23	PNP_UDP_1	Phosphorylase sup	CYT	1	10	8.1	Medium
TRINITY_DN37699_c0_g1_i3_orfp1	TRINITY_DN37699_c0_g1_i3_m.58788 TRINITY_DN37699_c0_g1::TRINITY_DN37699_c0_g1_i3::g.58788 ORF type:internal len:122	8.102739726	3.018409799	0.0004672	up	yes	1.183	0.146	0.114	0.156	0.168	1	1.333	1.215	------	------	------	------	------	------	------	------	------	------	------	CYT	1	12	12.6	High
TRINITY_DN2243_c0_g1_i4_orf1	WD repeat-containing protein 75 [Ostrinia furnacalis]	3.901243875	1.963934187	9.04E-06	up	yes	1.035	0.2653	0.235	0.268	0.293	1	1.073	1.031	------	------	K14552	NAN1, UTP17	map03008	Ribosome bioger	ENOG410YY9G	S:Function unknown	------	------	------	CYT	1	1	99.1	Medium
TRINITY_DN3504_c0_g1_i3_orfp2	TRINITY_DN3504_c0_g1_i3_m.43947 TRINITY_DN3504_c0_g1::TRINITY_DN3504_c0_g1_i3::g.43947 ORF type:5prime_partial len:208 (-),score=77.75 TRINITY_DN3504_c0_g1_i3:185-808(-)	4.795689268	2.261738184	0.0001066	up	yes	1.068	0.2227	0.225	0.263	0.18	1	1.165	1.039	------	------	------	------	------	------	------	------	------	------	------	EXC	1	3	22.4	High
TRINITY_DN39673_c0_g1_i1_orf1	uncharacterized protein LOC114359357 isoform X1 [Ostrinia furnacalis]	2.919864906	1.545901621	2.43E-05	up	yes	0.951	0.3257	0.338	0.32	0.319	1	0.948	0.905	------	------	------	------	------	------	------	------	PF07294.14	Fibroin_P25	Fibroin P25	PLA	1	4	27.9	High
TRINITY_DN13119_c0_g1_i4_orf1	endocuticle structural glycoprotein ABD-5-like [Bicyclus anynana]	3.399305556	1.765240048	1.85E-06	up	yes	0.979	0.288	0.266	0.289	0.309	1	0.97	0.967	------	------	------	------	------	------	ENOG410YVGC;ENOG4110QDR;ENOG410YVKS;ENOG41125BY	S:Function unknown;	PF00379.26	Chitin_bind_4	Insect cuticle prote	CYT	3	49	12.3	High

TRINITY_DN1707_c0_g1_i1_orf1	inositol oxygenase-like [Ostrinia furnacalis]	4.193680755	2.06821704	6.29E-06	up	yes	1.022	0.2437	0.252	0.216	0.263	1	1.063	1.003	BP:polyol catabolic process;BP:polyol metabolic process;BP:cellular catabolic process;BP:small molecule metabolic process;BP:small molecule catabolic process;BP:alcohol metabolic process;BP:organic substance catabolic process;BP:cellular carbohydrate metabolic process;BP:organic substance metabolic process;BP:alcohol catabolic process;BP:inositol catabolic process;BP:cellular process;BP:biological_process;BP:metabolic process;BP:catabolic process;BP:cellular carbohydrate catabolic process;BP:primary metabolic process;BP:carbohydrate metabolic process;BP:carbohydrate catabolic process;BP:cellular metabolic process;BP:inositol metabolic process;BP:organic hydroxy compound catabolic process;BP:organic hydroxy compound metabolic process;CC:cytoplasm;CC:cellular anatomical entity;CC:cellular_component;MF:binding;MF:monooxygenase activity;MF:molecular_function;MF:ion binding;MF:oxidoreductase activity;MF:transition metal ion binding;MF:oxidoreductase activity, acting on single donors	GO:0046174;GO:0019751;GO:0044248;GO:0044281;GO:0044282;GO:0006066;GO:1901575;GO:0044262;GO:0071704;GO:0046164;GO:0019310;GO:0009987;GO:0008150;GO:0008152;GO:0009056;GO:0044275;GO:0044238;GO:0005975;GO:0016052;GO:0044237;GO:0006020;GO:1901616;GO:1901615;GO:0005737;GO:0110165;GO:0005575;GO:0005488;GO:0004497;GO:0003674;GO:0043167;GO:0016491;GO:0046914;GO:0016701;GO:0050113;GO:0046872;GO:0043169;GO:0003824;GO:0005506	K00469	MIOX	map01250;ma	Biosynthesis of n	ENOG410XQ4J	S:Function unknown	PF05153.18	MIOX	Myo-inositol oxyg	CYT	4	21	34.5	High
TRINITY_DN42759_c0_g3_i1_orf1	fatty acid synthase-like [Ostrinia furnacalis]	3.822487486	1.934511778	6.62E-06	up	yes	0.9927	0.2597	0.301	0.223	0.255	1	0.978	1	BP:fatty acid biosynthetic process;BP:fatty acid metabolic process;BP:carboxylic acid metabolic process;BP:cellular biosynthetic process;BP:small molecule metabolic process;BP:small molecule biosynthetic process;BP:monocarboxylic acid biosynthetic process;BP:organic substance biosynthetic process;BP:organic substance metabolic process;BP:lipid metabolic process;BP:cellular process;BP:monocarboxylic acid metabolic process;BP:biosynthetic process;BP:biological_process;BP:metabolic process;BP:oxoacid metabolic process;BP:cellular lipid metabolic process;BP:lipid biosynthetic process;BP:primary metabolic process;BP:organic acid metabolic process;BP:carboxylic acid biosynthetic process;BP:organic acid biosynthetic process;BP:cellular metabolic process;MF:3-hydroxyoctanoyl-[acyl-carrier-protein] dehydratase activity;MF:thiolester hydrolase activity;MF:S-acyltransferase activity;MF:enoyl-[acyl-carrier-protein] reductas;MF:oxidoreductase activity, acting on the CH-CH group of donors;MF:molecular_function;MF:oxidoreductase activity;MF:oxidoreductase activity, acting on the CH-CH group of donors, NAD or NADP as acceptor;MF:[acyl-carrier-protein] S-acetyltransferase activity;MF:lyase activity;MF:oleoyl-[acyl-carrier-protein] hydrolase activity;MF:acetyltransferase activity;MF:transferase activity;MF:transferase activity, transferring acyl groups;MF:transferase activity, transferring acyl groups other than amino-acyl groups;MF:S-acetyltransferase activity;MF:hydrolase activity;MF:3-hydroxydecanoyl-[acyl-carrier-protein] dehydratase activity;MF:hydrolase activity, acting on ester bonds;MF:catalytic activity;MF:oxidoreductase	GO:0006633;GO:0006631;GO:0019752;GO:0044249;GO:0044281;GO:0044283;GO:0072330;GO:1901576;GO:0071704;GO:0006629;GO:0009987;GO:0032787;GO:0009058;GO:0008150;GO:0008152;GO:0043436;GO:0044255;GO:0008610;GO:0044238;GO:0006082;GO:0046394;GO:0016053;GO:0044237;GO:0047451;GO:0016790;GO:0016417;GO:0047117;GO:0016627;GO:0003674;GO:0016491;GO:0016628;GO:0004313;GO:0016829;GO:0004320;GO:0016407;GO:0016740;GO:0016746;GO:0016747;GO:0016418;GO:0016787;GO:0008693;GO:0016788;GO:0003824;GO:0016614;GO:0016616;GO:0016835;GO:0016836;GO:0004312;GO:0016295;GO:0016296;GO:0016297;GO:0004316;GO:0004317;GO:0004315;GO:0019171;GO:0008659	K00665	FASN	map04910;ma	Insulin signaling	COG3321	Q:Secondary metabolites biosy	PF00698.24;PF0	Acyl_transf_1;	Acyl transferase dc	CYT	2	3	84.3	High
TRINITY_DN6693_c0_g1_i1_orf1	uncharacterized protein LOC114356358 [Ostrinia furnacalis]	3.982730924	1.993758013	7.39E-07	up	yes	0.9917	0.249	0.237	0.254	0.256	1	1.008	0.967	MF:catalytic activity;MF:transferase activity;MF:transferase activity, transferring sulfur-containing groups;MF:sulfotransferase activity;MF:molecular_function;	GO:0003824;GO:0016740;GO:0016782;GO:0008146;GO:0003674	------	------	------	------	ENOG4111H56	S:Function unknown	PF00685.30;PF1	Sulfotransfer_	Sulfotransferase dc	CYT	6	24	40.4	High
TRINITY_DN23570_c0_g1_i2_orf1	putative trypsin 6 [Ostrinia nubilalis]	3.573806881	1.837461677	5.61E-05	up	yes	0.966	0.2703	0.278	0.252	0.281	1	0.891	1.007	BP:organic substance metabolic process;BP:organonitrogen compound metabolic process;BP:protein metabolic process;BP:macromolecule metabolic process;BP:nitrogen compound metabolic process;BP:biological_process;BP:metabolic process;BP:proteolysis;BP:primary metabolic process;CC:extracellular space;CC:cellular_component;CC:cellular anatomical entity;MF:serine-type endopeptidase activity;MF:endopeptidase activity;MF:hydrolase activity;MF:serine hydrolase activity;MF:catalytic activity, acting on a protein;MF:catalytic	GO:0071704;GO:1901564;GO:0019538;GO:0043170;GO:0006807;GO:0008150;GO:0008152;GO:0006508;GO:0044238;GO:0005615;GO:0005575;GO:0110165;GO:0004252;GO:0004175;GO:0016787;GO:0017171;GO:0140096;GO:0003824;GO:0003674;GO:0008233;GO:0008236	K09628;K096	PRSS27;TMPR	map04972;ma	Pancreatic secret	COG5640	O:Posttranslational modificatio	PF00089.29	Trypsin	Trypsin	CYT	1	4	26.1	High
TRINITY_DN717_c0_g1_i2_orfp1	TRINITY_DN717_c0_g1_i2_m.67915 TRINITY_DN717_c0_g1::TRINITY_DN717_c0_g1_i2::g.67915 ORF type:internal len:868 (+),score=265.71,Collagen PF01391.19 0.11,Collagen|PF01391.19|0.039,Collagen|PF01391.19|0.00054,Collagen|PF01391.19|0.0019,Collagen|PF01391.19|0.0005,Collagen|PF01391.19|9.9e-05,Collagen|PF01391.19|1.7e-07 TRINITY_DN717_c0_g1_i2:3-2603(+)	4.450816056	2.154069878	5.13E-06	up	yes	1.009	0.2267	0.213	0.229	0.238	1	1.052	0.975	------	------	------	------	------	------	------	------	PF01391.21	Collagen	Collagen triple heli	PLA	8	20	90.1	High
TRINITY_DN1154_c0_g1_i1_orf1	calexcitin-1-like [Ostrinia furnacalis] >ADK94879.2 juvenile hormone diol kinase [Ostrinia furnacalis]	4.081459482	2.029085135	3.19E-06	up	yes	0.962	0.2357	0.229	0.245	0.233	1	0.945	0.941	BP:phosphorylation;BP:cellular process;BP:cellular metabolic process;BP:phosphate-containing compound metabolic process;BP:biological_process;BP:metabolic process;BP:phosphorus metabolic process;MF:cation binding;MF:kinase activity;MF:metal ion binding;MF:transferase activity;MF:ion binding;MF:catalytic activity;MF:calcium ion binding;MF:molecular_function;MF:transferase activity, transferring phosphorus-containing groups;MF:binding;	GO:0016310;GO:0009987;GO:0044237;GO:0006796;GO:0008150;GO:0008152;GO:0006793;GO:0043169;GO:0016301;GO:0046872;GO:0016740;GO:0043167;GO:0003824;GO:0005509;GO:0003674;GO:0016772;GO:0005488	------	------	------	------	ENOG4111I7F	S:Function unknown	PF00036.35;PF1	EF-hand_1;EF	EF hand;EF hand;E	CYT	5	35	20.6	High
TRINITY_DN117_c0_g1_i4_orf1	lipase member I-like [Ostrinia furnacalis]	5.990870359	2.582765614	7.26E-07	up	yes	0.9843	0.1643	0.172	0.142	0.179	1	0.988	0.965	BP:organic substance metabolic process;BP:lipid metabolic process;BP:primary metabolic process;BP:metabolic process;BP:biological_process;CC:cellular_component;CC:extracellular region;CC:cellular anatomical entity;MF:hydrolase activity;MF:hydrolase activity, acting on ester bonds;MF:catalytic activity;MF:molecular_function;MF:lipase activity;	GO:0071704;GO:0006629;GO:0044238;GO:0008152;GO:0008150;GO:0005575;GO:0005576;GO:0110165;GO:0016787;GO:0016788;GO:0003824;GO:0003674;GO:0016298	------	------	------	------	ENOG41116Y2;ENOG4110RTX;ENOG4111JY5;ENOG410YGS3;ENOG410ZKVQ;ENOG4110RNC	S:Function unknown;	PF00151.22	Lipase	Lipase	CYT	5	82	16.1	High
TRINITY_DN30704_c0_g1_i1_orf1	cytochrome P450 monooxygenase CYP6AE134v2 [Ostrinia furnacalis]	11.77906977	3.558153704	8.98E-06	up	yes	1.013	0.086	0.117	0.083	0.058	1	0.973	1.066	CC:integral component of membrane;CC:cellular_component;CC:cellular anatomical entity;CC:intrinsic component of membrane;MF:cation binding;MF:monooxygenase activity;MF:heme binding;MF:transition metal ion binding;MF:tetrapyrrole binding;MF:organic cyclic compound binding;MF:oxidoreductase activity, acting on paired donors, with incorporation or reduction of molecular oxygen;MF:ion binding;MF:catalytic activity;MF:molecular_function;MF:binding;MF:iron ion binding;MF:metal ion binding;MF:heterocyclic compound	GO:0016021;GO:0005575;GO:0110165;GO:0031224;GO:0043169;GO:0004497;GO:0020037;GO:0046914;GO:0046906;GO:0097159;GO:0016705;GO:0043167;GO:0003824;GO:0003674;GO:0005488;GO:0005506;GO:0046872;GO:1901363;GO:0016491	K14999	CYP6	------	------	COG2124	Q:Secondary metabolites biosy	PF00067.25	p450	Cytochrome P450	CYT	1	2	61.1	High
TRINITY_DN3229_c0_g1_i1_orf1	uncharacterized protein LOC114358442 isoform X1 [Ostrinia furnacalis]	3.151222457	1.655911603	2.10E-05	up	yes	1.044	0.3313	0.337	0.324	0.333	1	1.029	1.103	MF:catalytic activity;MF:transferase	GO:0003824;GO:0016740;GO:0003674	------	------	------	------	ENOG41129KS	S:Function unknown	PF11901.11	DUF3421	Protein of unknow	CYT	6	37	23.3	High
TRINITY_DN96170_c0_g1_i1_orf1	uncharacterized protein LOC114355569 [Ostrinia furnacalis]	2.794444444	1.482561493	9.04E-05	up	yes	1.006	0.36	0.381	0.347	0.352	1	0.941	1.076	BP:phosphorylation;BP:cellular process;BP:biological_process;BP:metabolic process;BP:cellular metabolic process;BP:phosphate-containing compound metabolic process;BP:phosphorus metabolic process;CC:intrinsic component of membrane;CC:integral component of membrane;CC:cellular_component;CC:cellular anatomical entity;MF:purine ribonucleoside triphosphate binding;MF:molecular_function;MF:binding;MF:ion binding;MF:heterocyclic compound binding;MF:anion binding;MF:nucleotide binding;MF:transferase activity;MF:nucleoside phosphate binding;MF:purine nucleotide binding;MF:ATP binding;MF:kinase activity;MF:small molecule binding;MF:catalytic activity;MF:adenyl nucleotide binding;MF:carbohydrate derivative binding;MF:transferase activity, transferring phosphorus-containing groups;MF:adenyl ribonucleotide binding;MF:purine ribonucleotide binding;MF:organic cyclic compound binding;MF:ribonucleotide	GO:0016310;GO:0009987;GO:0008150;GO:0008152;GO:0044237;GO:0006796;GO:0006793;GO:0031224;GO:0016021;GO:0005575;GO:0110165;GO:0035639;GO:0003674;GO:0005488;GO:0043167;GO:1901363;GO:0043168;GO:0000166;GO:0016740;GO:1901265;GO:0017076;GO:0005524;GO:0016301;GO:0036094;GO:0003824;GO:0030554;GO:0097367;GO:0016772;GO:0032559;GO:0032555;GO:0097159;GO:0032553	------	------	------	------	COG0574	G:Carbohydrate transport and	PF01326.22	PPDK_N	Pyruvate phosphat	CYT	3	4	87.6	High
TRINITY_DN4476_c0_g1_i5_orf1	trypsin, alkaline C-like isoform X1 [Ostrinia furnacalis]	2.255980861	1.173754829	0.0005316	up	yes	0.943	0.418	0.368	0.382	0.504	1	0.913	0.916	BP:organic substance metabolic process;BP:organonitrogen compound metabolic process;BP:protein metabolic process;BP:macromolecule metabolic process;BP:biological_process;BP:nitrogen compound metabolic process;BP:metabolic process;BP:proteolysis;BP:primary metabolic process;MF:serine-type endopeptidase activity;MF:endopeptidase activity;MF:hydrolase activity;MF:serine hydrolase activity;MF:catalytic activity, acting on a protein;MF:catalytic activity;MF:molecular_function;MF:peptidase activity;MF:serine-	GO:0071704;GO:1901564;GO:0019538;GO:0043170;GO:0008150;GO:0006807;GO:0008152;GO:0006508;GO:0044238;GO:0004252;GO:0004175;GO:0016787;GO:0017171;GO:0140096;GO:0003824;GO:0003674;GO:0008233;GO:0008236	K01312	PRSS1_2_3	map04972;ma	Pancreatic secret	COG5640	O:Posttranslational modificatio	PF00089.29	Trypsin	Trypsin	PLA	1	22	26.3	High
TRINITY_DN38366_c0_g1_i4_orfp1	TRINITY_DN38366_c0_g1_i4_m.10666 TRINITY_DN38366_c0_g1::TRINITY_DN38366_c0_g1_i4::g.10666 ORF type:internal len:143	3.328030544	1.734668674	0.0001745	up	yes	1.046	0.3143	0.28	0.335	0.328	1	1.149	0.99	------	------	------	------	------	------	------	------	------	------	------	CYT	1	8	13.5	High
TRINITY_DN141353_c0_g1_i1_orf1	uncharacterized protein LOC123263755 [Cotesia glomerata] >KAH0554923.1 hypothetical protein KQX54_013880 [Cotesia glomerata]	2.231657445	1.158115594	0.004011	up	yes	0.9277	0.4157	0.394	0.461	0.392	1	1.021	0.762	BP:nucleoside triphosphate metabolic process;BP:nucleoside triphosphate biosynthetic process;BP:purine nucleoside triphosphate metabolic process;BP:purine nucleoside triphosphate biosynthetic process;BP:cellular biosynthetic process;BP:cellular nitrogen compound metabolic process;BP:nucleotide biosynthetic process;BP:small molecule metabolic process;BP:purine-containing compound metabolic process;BP:purine-containing compound biosynthetic process;BP:organic cyclic compound biosynthetic process;BP:ribonucleotide metabolic process;BP:organic cyclic compound metabolic process;BP:nucleobase-containing compound metabolic process;BP:ribonucleoside triphosphate biosynthetic process;BP:organic substance metabolic process;BP:ribonucleoside triphosphate metabolic process;BP:heterocycle biosynthetic process;BP:purine ribonucleoside triphosphate biosynthetic process;BP:purine ribonucleoside triphosphate metabolic process;BP:ribose phosphate metabolic process;BP:organic substance biosynthetic process;BP:ATP metabolic process;BP:cellular process;BP:cellular aromatic compound metabolic process;BP:purine ribonucleotide biosynthetic process;BP:purine ribonucleotide metabolic process;BP:ribonucleotide biosynthetic process;BP:biosynthetic process;BP:nucleotide metabolic process;BP:metabolic process;BP:aromatic compound biosynthetic process;BP:nucleobase-containing compound biosynthetic process;BP:cellular nitrogen compound biosynthetic process;BP:organophosphate biosynthetic process;BP:nucleobase-containing small molecule metabolic process;BP:heterocycle metabolic process;BP:primary metabolic	GO:0009141;GO:0009142;GO:0009144;GO:0009145;GO:0044249;GO:0034641;GO:0009165;GO:0044281;GO:0072521;GO:0072522;GO:1901362;GO:0009259;GO:1901360;GO:0006139;GO:0009201;GO:0071704;GO:0009199;GO:0018130;GO:0009206;GO:0009205;GO:0019693;GO:1901576;GO:0046034;GO:0009987;GO:0006725;GO:0009152;GO:0009150;GO:0009260;GO:0009058;GO:0009117;GO:0008152;GO:0019438;GO:0034654;GO:0044271;GO:0090407;GO:0055086;GO:0046483;GO:0044238;GO:0015986;GO:1901564;GO:1901566;GO:1901137;GO:1901135;GO:0006164;GO:0046390;GO:0044237;GO:0006163;GO:0006796;GO:0006807;GO:1901293;GO:0006793;GO:0019637;GO:0008150;GO:0006753;GO:0006754;GO:0045263;GO:0005575;GO:0098798;GO:0098800;GO:0032991;GO:0000276;GO:0098796;GO:0033177;GO:0022890;GO:0003674;GO:0015318;GO:0022857;GO:0005215;GO:0008324;GO:0015075;GO:0015078	K02129	ATPeF0E, ATF	map00190;ma	Oxidative phosp	ENOG410XYSS	Z:Cytoskeleton	PF05680.15	ATP-synt_E	ATP synthase E ch	CYT	1	14	9.1	High
TRINITY_DN36817_c0_g1_i1_orf1	uncharacterized protein LOC114357350 [Ostrinia furnacalis]	3.108359133	1.636153199	0.000141	up	yes	1.004	0.323	0.232	0.385	0.352	1	1.025	0.987	MF:translation regulator activity, nucleic acid binding;MF:nucleic acid binding;MF:organic cyclic compound binding;MF:translation factor activity, RNA binding;MF:molecular_function;MF:binding;MF:translation regulator activity;MF:heterocyclic compound binding;MF:translation initiation factor activity;	GO:0090079;GO:0003676;GO:0097159;GO:0008135;GO:0003674;GO:0005488;GO:0045182;GO:1901363;GO:0003743	------	------	------	------	ENOG410XV86;ENOG4111ASB	S:Function unknown;	------	------	------	CYT	1	29	7.6	High
TRINITY_DN23167_c0_g1_i4_orf1	uncharacterized protein LOC114363065 [Ostrinia furnacalis]	3.102381689	1.633376193	6.57E-07	up	yes	1.003	0.3233	0.314	0.336	0.32	1	1.022	0.986	BP:organic substance metabolic process;BP:organonitrogen compound metabolic process;BP:protein metabolic process;BP:macromolecule metabolic process;BP:biological_process;BP:nitrogen compound metabolic process;BP:metabolic process;BP:proteolysis;BP:primary metabolic process;MF:serine-type endopeptidase activity;MF:endopeptidase activity;MF:hydrolase activity;MF:serine hydrolase activity;MF:catalytic activity, acting on a protein;MF:catalytic activity;MF:molecular_function;MF:peptidase activity;MF:serine-	GO:0071704;GO:1901564;GO:0019538;GO:0043170;GO:0008150;GO:0006807;GO:0008152;GO:0006508;GO:0044238;GO:0004252;GO:0004175;GO:0016787;GO:0017171;GO:0140096;GO:0003824;GO:0003674;GO:0008233;GO:0008236	------	------	------	------	COG5640	O:Posttranslational modificatio	PF18322.4	CLIP_1	Serine protease Cli	CYT	7	14	69.4	High

TRINITY_DN43420_c0_g2_i1_orf1	collagenase-like [Ostrinia furnacalis]	2.81556196	1.493422899	1.92E-05	up	yes	0.977	0.347	0.34	0.376	0.325	1	0.998	0.933	BP:organic substance metabolic process;BP:organonitrogen compound metabolic process;BP:protein metabolic process;BP:macromolecule metabolic process;BP:biological_process;BP:nitrogen compound metabolic process;BP:metabolic process;BP:proteolysis;BP:primary metabolic process;MF:serine-type endopeptidase activity;MF:endopeptidase activity;MF:hydrolase activity;MF:serine hydrolase activity;MF:catalytic activity, acting on a protein;MF:catalytic activity;MF:molecular_function;MF:peptidase activity;MF:serine-	GO:0071704;GO:1901564;GO:0019538;GO:0043170;GO:0008150;GO:0006807;GO:0008152;GO:0006508;GO:0044238;GO:0004252;GO:0004175;GO:0016787;GO:0017171;GO:0140096;GO:0003824;GO:0003674;GO:0008233;GO:0008236	------	------	------	------	COG5640	O:Posttranslational modificatio	PF00089.29	Trypsin	Trypsin	CYT	4	24	29.8	High
TRINITY_DN4550_c1_g1_i5_orfp2	TRINITY_DN4550_c1_g1_i5_m.14710 TRINITY_DN4550_c1_g1::TRINITY_DN4550_c1_g1_i5::g.14710 ORF type:5prime_partial len:168 (+),score=78.12 TRINITY_DN4550_c1_g1_i5:3-506(+)	2.830349281	1.500980101	1.40E-05	up	yes	0.9643	0.3407	0.314	0.367	0.341	1	0.935	0.958	------	------	------	------	------	------	------	------	------	------	------	CYT	1	7	20.9	High
TRINITY_DN1123_c2_g1_i4_orf1	troponin I isoform X16 [Ostrinia furnacalis]	3.087702574	1.62653379	0.0001169	up	yes	0.9717	0.3147	0.307	0.297	0.34	1	1.026	0.889	CC:cellular_component;CC:macromolecular complex;CC:troponin complex;	GO:0005575;GO:0032991;GO:0005861	K12044	TNNI3	map04260;ma	Cardiac muscle c	ENOG410Y9IX	Z:Cytoskeleton	PF00992.23	Troponin	Troponin	CYT	8	46	24.4	High
TRINITY_DN13783_c0_g4_i2_orf1	hypothetical protein evm_010131 [Chilo suppressalis]	3.501766784	1.808083004	3.96E-06	up	yes	0.991	0.283	0.271	0.297	0.281	1	1.018	0.955	BP:protein localization;BP:maintenance of protein localization in endoplasmic reticulum;BP:maintenance of location in cell;BP:cellular macromolecule localization;BP:nitrogen compound transport;BP:organic substance transport;BP:macromolecule localization;BP:transport;BP:maintenance of protein location in cell;BP:maintenance of protein localization in organelle;BP:cellular process;BP:protein retention in ER lumen;BP:establishment of protein localization;BP:maintenance of protein location;BP:biological_process;BP:maintenance of location;BP:establishment of localization;BP:localization;BP:cellular localization;BP:protein transport;CC:endoplasmic reticulum membrane;CC:intrinsic component of membrane;CC:organelle membrane;CC:integral component of membrane;CC:membrane;CC:cellular anatomical entity;CC:cellular_component;MF:molecular_function;MF:binding;MF:peptide binding;MF:amide binding;MF:ER retention	GO:0008104;GO:0035437;GO:0051651;GO:0070727;GO:0071705;GO:0071702;GO:0033036;GO:0006810;GO:0032507;GO:0072595;GO:0009987;GO:0006621;GO:0045184;GO:0045185;GO:0008150;GO:0051235;GO:0051234;GO:0051179;GO:0051641;GO:0015031;GO:0005789;GO:0031224;GO:0031090;GO:0016021;GO:0016020;GO:0110165;GO:0005575;GO:0003674;GO:0005488;GO:0042277;GO:0033218;GO:0046923;GO:0005048	------	------	------	------	ENOG410Z4PE	S:Function unknown	PF14880.9	COX14	Cytochrome oxida	CYT	1	10	11.2	High
TRINITY_DN34040_c0_g2_i1_orf1	uncharacterized protein LOC114352849 [Ostrinia furnacalis]	3.813676527	1.931182481	5.34E-06	up	yes	0.9927	0.2603	0.258	0.275	0.248	1	1.025	0.953	------	------	------	------	------	------	------	------	------	------	------	CYT	1	6	24	High
TRINITY_DN2186_c0_g1_i17_orf1	paxillin isoform X6 [Leguminivora glycinivorella]	4.781382228	2.257427741	4.47E-06	up	yes	1.017	0.2127	0.196	0.215	0.227	1	1.06	0.99	BP:cell-matrix adhesion;BP:cell-substrate adhesion;BP:cellular process;BP:biological_process;BP:cell adhesion;CC:intracellular organelle;CC:non-membrane-bounded organelle;CC:focal adhesion;CC:organelle;CC:cell junction;CC:cell-substrate junction;CC:cytoskeleton;CC:cellular anatomical entity;CC:cytoplasm;CC:anchoring junction;CC:cell cortex;CC:intracellular non-membrane-bounded organelle;CC:cellular_component;MF:molecular_function;MF:binding;MF:cation binding;MF:ion binding;MF:metal ion binding;	GO:0007160;GO:0031589;GO:0009987;GO:0008150;GO:0007155;GO:0043229;GO:0043228;GO:0005925;GO:0043226;GO:0030054;GO:0030055;GO:0005856;GO:0110165;GO:0005737;GO:0070161;GO:0005938;GO:0043232;GO:0005575;GO:0003674;GO:0005488;GO:0043169;GO:0043167;GO:0046872	K05760	PXN	map05205;ma	Proteoglycans in	ENOG410XRD4	S:Function unknown	PF00412.25	LIM	LIM domain	CYT	2	65	26.4	High
TRINITY_DN334_c0_g1_i1_orf1	putative chymotrypsin 12 [Ostrinia nubilalis]	5.15074858	2.364782121	7.33E-07	up	yes	0.9977	0.1937	0.197	0.188	0.196	1	0.971	1.022	BP:organic substance metabolic process;BP:organonitrogen compound metabolic process;BP:protein metabolic process;BP:macromolecule metabolic process;BP:biological_process;BP:nitrogen compound metabolic process;BP:metabolic process;BP:proteolysis;BP:primary metabolic process;MF:serine-type endopeptidase activity;MF:endopeptidase activity;MF:hydrolase activity;MF:serine hydrolase activity;MF:catalytic activity, acting on a protein;MF:catalytic activity;MF:molecular_function;MF:peptidase activity;MF:serine-	GO:0071704;GO:1901564;GO:0019538;GO:0043170;GO:0008150;GO:0006807;GO:0008152;GO:0006508;GO:0044238;GO:0004252;GO:0004175;GO:0016787;GO:0017171;GO:0140096;GO:0003824;GO:0003674;GO:0008233;GO:0008236	------	------	------	------	COG5640	O:Posttranslational modificatio	PF00089.29	Trypsin	Trypsin	CYT	5	53	21.6	High
TRINITY_DN10792_c0_g2_i5_orf1	uncharacterized protein LOC114366171 [Ostrinia furnacalis]	5.007564297	2.32410904	5.31E-07	up	yes	0.993	0.1983	0.207	0.191	0.197	1	1.011	0.968	------	------	------	------	------	------	ENOG410XT8F;ENOG410YKCN;ENOG410ZQUX;ENOG410ZI5B	S:Function unknown;	PF02958.23;PF0	EcKL;DUF167	Ecdysteroid kinase	CYT	9	20	48	High
TRINITY_DN311_c0_g1_i4_orfp1	TRINITY_DN311_c0_g1_i4_m.65135 TRINITY_DN311_c0_g1::TRINITY_DN311_c0_g1_i4::g.65135 ORF type:5prime_partial len:126 (+),score=71.21 TRINITY_DN311_c0_g1_i4:1-378(+)	5.269457864	2.397654541	5.30E-07	up	yes	0.9817	0.1863	0.205	0.174	0.18	1	0.966	0.979	------	------	------	------	------	------	------	------	------	------	------	EXC	1	6	13.8	High
TRINITY_DN144807_c0_g1_i1_orf1	hypothetical protein G9C98_004245 [Cotesia typhae]	5.544067797	2.4709449	6.64E-07	up	yes	0.9813	0.177	0.182	0.168	0.181	1	0.954	0.99	BP:L-methionine salvage;BP:carboxylic acid metabolic process;BP:L-methionine biosynthetic process;BP:cellular biosynthetic process;BP:nitrogen compound metabolic process;BP:aspartate family amino acid biosynthetic process;BP:small molecule metabolic process;BP:small molecule biosynthetic process;BP:L-methionine biosynthetic process from S-adenosylmethionine;BP:organic substance biosynthetic process;BP:L-methionine biosynthetic process from methylthioadenosine;BP:cellular amino acid metabolic process;BP:organic substance metabolic process;BP:amino acid salvage;BP:alpha-amino acid metabolic process;BP:sulfur amino acid metabolic process;BP:primary metabolic process;BP:sulfur amino acid biosynthetic process;BP:cellular process;BP:biosynthetic process;BP:biological_process;BP:metabolic process;BP:oxoacid metabolic process;BP:cellular metabolic compound salvage;BP:cellular amino acid biosynthetic process;BP:organonitrogen compound metabolic process;BP:organonitrogen compound biosynthetic process;BP:organic acid metabolic process;BP:sulfur compound biosynthetic process;BP:carboxylic acid biosynthetic process;BP:methionine metabolic process;BP:organic acid biosynthetic process;BP:cellular metabolic process;BP:sulfur compound metabolic process;BP:alpha-amino acid biosynthetic process;BP:methionine biosynthetic process;BP:aspartate family amino acid metabolic process;CC:intracellular organelle;CC:membrane-bounded organelle;CC:cytoplasm;CC:nucleus;CC:cellular anatomical entity;CC:intracellular membrane-bounded [illegible]	GO:0071267;GO:0019752;GO:0071265;GO:0044249;GO:0006807;GO:0009067;GO:0044281;GO:0044283;GO:0019284;GO:1901576;GO:0019509;GO:0006520;GO:0071704;GO:0043102;GO:1901605;GO:0000096;GO:0044238;GO:0000097;GO:0009987;GO:0009058;GO:0008150;GO:0008152;GO:0043436;GO:0043094;GO:0008652;GO:1901564;GO:1901566;GO:0006082;GO:0044272;GO:0046394;GO:0006555;GO:0016053;GO:0044237;GO:0006790;GO:1901607;GO:0009086;GO:0009066;GO:0043229;GO:0043227;GO:0005737;GO:0005634;GO:0110165;GO:0043231;GO:0005575;GO:0043226;GO:0005488;GO:0000287;GO:0016791;GO:0043874;GO:0042578;GO:0016862;GO:0016860;GO:0043716;GO:0043715;GO:0043167;GO:0043169;GO:0046872;GO:0016787;GO:0016788;GO:0003824;GO:0016853;GO:0003674	K09880	mtnC, ENOPH	map00270	Cysteine and me	COG4229	C:Energy production and conve	------	------	------	CYT	1	1	75.4	Medium
TRINITY_DN5244_c0_g1_i1_orf1	eukaryotic peptide chain release factor GTP-binding subunit-like [Ostrinia furnacalis]	6.404516129	2.679089577	6.63E-08	up	yes	0.9927	0.155	0.159	0.148	0.158	1	1.001	0.977	------	------	------	------	------	------	ENOG41116ZG;ENOG4111PWE;ENOG4111WYB	S:Function unknown;	PF03103.20	DUF243	Domain of unknow	CYT	6	20	48.5	High
TRINITY_DN37538_c0_g1_i1_orf1	esterase FE4-like [Ostrinia furnacalis]	8.745454545	3.12853337	2.76E-06	up	yes	0.962	0.11	0.121	0.094	0.115	1	0.957	0.929	------	------	K07378	NLGN	map04514	Cell adhesion mo	COG2272;ENOG410XSWS;COG0580	I:Lipid transport and metabolis	PF00135.31;PF2	COesterase;B	Carboxylesterase f	CYT	1	4	22.5	High
TRINITY_DN3401_c0_g1_i1_orf1	28S ribosomal protein S5, mitochondrial [Ostrinia furnacalis]	2.284047619	1.191592729	1.84E-05	up	yes	0.9593	0.42	0.402	0.427	0.431	1	0.946	0.932	BP:cellular biosynthetic process;BP:cellular macromolecule biosynthetic process;BP:organic substance biosynthetic process;BP:cellular nitrogen compound biosynthetic process;BP:cellular macromolecule metabolic process;BP:cellular amide metabolic process;BP:peptide biosynthetic process;BP:organic substance metabolic process;BP:organonitrogen compound biosynthetic process;BP:amide biosynthetic process;BP:cellular process;BP:biosynthetic process;BP:macromolecule biosynthetic process;BP:biological_process;BP:metabolic process;BP:organonitrogen compound metabolic process;BP:primary metabolic process;BP:protein metabolic process;BP:cellular nitrogen compound metabolic process;BP:cellular metabolic process;BP:macromolecule metabolic process;BP:nitrogen compound metabolic process;BP:peptide metabolic process;BP:translation;CC:organelle;CC:intracellular organelle;CC:non-membrane-bounded organelle;CC:cellular anatomical entity;CC:macromolecular complex;CC:ribosome;CC:intracellular non-membrane-bounded organelle;CC:ribonucleoprotein complex;CC:cellular_component;MF:nucleic acid binding;MF:molecular_function;MF:binding;MF:heterocyclic compound binding;MF:structural molecule activity;MF:structural constituent of ribosome;MF:organic cyclic compound	GO:0044249;GO:0034645;GO:1901576;GO:0044271;GO:0044260;GO:0043603;GO:0043043;GO:0071704;GO:1901566;GO:0043604;GO:0009987;GO:0009058;GO:0009059;GO:0008150;GO:0008152;GO:1901564;GO:0044238;GO:0019538;GO:0034641;GO:0044237;GO:0043170;GO:0006807;GO:0006518;GO:0006412;GO:0043226;GO:0043229;GO:0043228;GO:0110165;GO:0032991;GO:0005840;GO:0043232;GO:1990904;GO:0005575;GO:0003676;GO:0003674;GO:0005488;GO:1901363;GO:0005198;GO:0003735;GO:0097159;GO:0003723	K02988	RP-S5, MRPS	map03010	Ribosome	COG0098	J:Translation, ribosomal structu	PF03719.18;PF0	Ribosomal_S	Ribosomal protein	CYT	2	6	49	High
TRINITY_DN4501_c0_g1_i3_orf1	methylcrotonoyl-CoA carboxylase subunit alpha, mitochondrial [Ostrinia furnacalis]	2.480344915	1.310540755	5.47E-05	up	yes	0.978	0.3943	0.423	0.409	0.351	1	1.003	0.931	MF:anion binding;MF:cation binding;MF:ribonucleotide binding;MF:adenyl nucleotide binding;MF:purine ribonucleoside triphosphate binding;MF:nucleotide binding;MF:organic cyclic compound binding;MF:nucleoside phosphate binding;MF:ion binding;MF:small molecule binding;MF:catalytic activity;MF:carbohydrate derivative binding;MF:molecular_function;MF:binding;MF:purine nucleotide binding;MF:purine ribonucleotide binding;MF:adenyl ribonucleotide binding;MF:metal ion binding;MF:ligase activity;MF:heterocyclic compound binding;MF:ATP binding;	GO:0043168;GO:0043169;GO:0032553;GO:0030554;GO:0035639;GO:0000166;GO:0097159;GO:1901265;GO:0043167;GO:0036094;GO:0003824;GO:0097367;GO:0003674;GO:0005488;GO:0017076;GO:0032555;GO:0032559;GO:0046872;GO:0016874;GO:1901363;GO:0005524	K01968	E6.4.1.4A	map00280	Valine, leucine a	COG4770	I:Lipid transport and metabolis	PF00364.25	Biotin_lipoyl	Biotin-requiring e	CYT	1	18	15.5	High
TRINITY_DN4938_c0_g1_i13_orf1	peroxisomal biogenesis factor 19 [Ostrinia furnacalis]	2.253521127	1.172180975	3.44E-05	up	yes	0.96	0.426	0.426	0.45	0.402	1	0.957	0.923	CC:intracellular membrane-bounded organelle;CC:peroxisome;CC:cellular_component;CC:microbody;CC:intracellular organelle;CC:membrane-bounded organelle;CC:organelle;CC:cellular anatomical entity;	GO:0043231;GO:0005777;GO:0005575;GO:0042579;GO:0043229;GO:0043227;GO:0043226;GO:0110165	K13337	PEX19	map04146	Peroxisome	ENOG4111QGU	O:Posttranslational modificatio	PF04614.15	Pex19	Pex19 protein fami	CYT	4	11	31.8	High
TRINITY_DN1901_c0_g1_i6_orf1	mitochondrial intermembrane space import and assembly protein 40 [Ostrinia furnacalis] >XP_028171079.1 mitochondrial intermembrane space import and assembly protein 40 [Ostrinia furnacalis] >XP_028171080.1 mitochondrial intermembrane space import and assembly protein 40 [Ostrinia furnacalis]	2.894512369	1.533320321	6.90E-06	up	yes	1.018	0.3517	0.374	0.312	0.369	1	1.029	1.025	BP:protein localization;BP:establishment of localization in cell;BP:protein transmembrane transport;BP:cellular macromolecule localization;BP:intracellular protein transport;BP:localization;BP:nitrogen compound transport;BP:intracellular protein transmembrane transport;BP:mitochondrial transmembrane transport;BP:organic substance transport;BP:macromolecule localization;BP:transport;BP:establishment of protein localization to organelle;BP:cellular process;BP:establishment of protein localization;BP:protein localization to organelle;BP:biological_process;BP:establishment of protein localization to mitochondrion;BP:mitochondrial transport;BP:establishment of localization;BP:transmembrane transport;BP:intracellular protein transmembrane import;BP:cellular localization;BP:protein localization to mitochondrion;BP:intracellular transport;BP:protein import into mitochondrial intermembrane space;BP:protein transport;MF:molecular_function;MF:oxidoreductase activity;MF:oxidoreductase activity, acting on a sulfur group of donors;MF:catalytic activity, acting on a protein;MF:catalytic activity;MF:protein disulfide oxidoreductase activity;MF:disulfide oxidoreductase activity;	GO:0008104;GO:0051649;GO:0071806;GO:0070727;GO:0006886;GO:0051179;GO:0071705;GO:0065002;GO:1990542;GO:0071702;GO:0033036;GO:0006810;GO:0072594;GO:0009987;GO:0045184;GO:0033365;GO:0008150;GO:0072655;GO:0006839;GO:0051234;GO:0055085;GO:0044743;GO:0051641;GO:0070585;GO:0046907;GO:0045041;GO:0015031;GO:0003674;GO:0016491;GO:0016667;GO:0140096;GO:0003824;GO:0015035;GO:0015036	K17782	MIA40, CHCH	------	------	ENOG4111V0I	O:Posttranslational modificatio	PF06747.16	CHCH	CHCH domain	CYT	2	13	15	High
TRINITY_DN2425_c0_g1_i1_orf1	thyroid receptor-interacting protein 11 [Ostrinia furnacalis]	3.488448845	1.802585678	2.34E-05	up	yes	1.057	0.303	0.307	0.271	0.331	1	1.092	1.079	------	------	------	------	------	------	ENOG410XUH1	S:Function unknown	PF11365.11	SOGA	Protein SOGA	CYT	1	1	262.5	Medium
TRINITY_DN117362_c0_g1_i5_orf1	uncharacterized protein LOC114354191 [Ostrinia furnacalis]	2.194626168	1.133975213	0.0004083	up	yes	0.9393	0.428	0.413	0.375	0.496	1	0.901	0.917	BP:primary metabolic process;BP:organonitrogen compound metabolic process;BP:protein metabolic process;BP:macromolecule metabolic process;BP:organic substance metabolic process;BP:nitrogen compound metabolic process;BP:biological_process;BP:metabolic process;BP:proteolysis;MF:molecular_function;MF:exopeptidase activity;MF:hydrolase activity;MF:carboxypeptidase activity;MF:catalytic activity, acting on a protein;MF:serine hydrolase activity;MF:serine-type carboxypeptidase activity;MF:serine-type exopeptidase activity;MF:peptidase activity;MF:catalytic activity;MF:serine-type peptidase activity;	GO:0044238;GO:1901564;GO:0019538;GO:0043170;GO:0071704;GO:0006807;GO:0008150;GO:0008152;GO:0006508;GO:0003674;GO:0008238;GO:0016787;GO:0004180;GO:0140096;GO:0017171;GO:0004185;GO:0070008;GO:0008233;GO:0003824;GO:0008236	K09646	SCPEP1	------	------	COG2939	C:Energy production and conve	PF00450.25	Peptidase_S1	Serine carboxypep	CYT	1	5	48.5	High

TRINITY_DN11069_c0_g2_i1_orf1	fat storage-inducing transmembrane protein [Ostrinia furnacalis]	3.078082605	1.622031949	0.0001029	up	yes	1.021	0.3317	0.371	0.313	0.311	1	0.965	1.099	BP:lipid storage;BP:cellular component organization;BP:cellular component organization or biogenesis;BP:cellular process;BP:biological_process;BP:maintenance of location;BP:localization;BP:organelle organization;BP:lipid particle organization;CC:endoplasmic reticulum membrane;CC:intrinsic component of membrane;CC:organelle membrane;CC:integral component of membrane;CC:membrane;CC:cellular anatomical entity;CC:cellular_component;MF:molecular_function;MF:hydrolase activity;MF:catalytic activity;MF:hydrolase activity, acting on acid anhydrides, in phosphorus-containing	GO:0019915;GO:0016043;GO:0071840;GO:0009987;GO:0008150;GO:0051235;GO:0051179;GO:0006996;GO:0034389;GO:0005789;GO:0031224;GO:0031090;GO:0016021;GO:0016020;GO:0110165;GO:0005575;GO:0003674;GO:0016787;GO:0003824;GO:0016818;GO:0016817;GO:0016462	------	------	------	------	ENOG4111CNJ	S:Function unknown	PF10261.12	Scs3p	Inositol phospholip	CYT	2	6	42.2	High
TRINITY_DN12495_c0_g1_i2_orf1	probable ATP-dependent RNA helicase pitchoune [Manduca sexta] >KAG6441249.1 hypothetical protein O3G_MSEX001749 [Manduca sexta]	2.565262883	1.359106678	1.62E-06	up	yes	0.9807	0.3823	0.365	0.384	0.398	1	0.973	0.969	MF:purine ribonucleoside triphosphate binding;MF:molecular_function;MF:binding;MF:nucleic acid binding;MF:RNA-dependent ATPase activity;MF:ion binding;MF:heterocyclic compound binding;MF:nucleoside phosphate binding;MF:anion binding;MF:nucleotide binding;MF:purine nucleotide binding;MF:ATP binding;MF:hydrolase activity;MF:catalytic activity, acting on RNA;MF:helicase activity;MF:small molecule binding;MF:catalytic activity;MF:adenyl nucleotide binding;MF:carbohydrate derivative binding;MF:organic cyclic compound binding;MF:RNA helicase activity;MF:adenyl ribonucleotide binding;MF:RNA binding;MF:purine ribonucleotide binding;MF:ribonucleotide	GO:0035639;GO:0003674;GO:0005488;GO:0003676;GO:0008186;GO:0043167;GO:1901363;GO:1901265;GO:0043168;GO:0000166;GO:0017076;GO:0005524;GO:0016787;GO:0140098;GO:0004386;GO:0036094;GO:0003824;GO:0030554;GO:0097367;GO:0097159;GO:0003724;GO:0032559;GO:0003723;GO:0032555;GO:0032553	K13179	DDX18, HAS1	------	------	COG0513	S:Function unknown	PF00270.32;PF0(	DEAD;Helicas	DEAD/DEAH box h	CYT	2	3	69	High
TRINITY_DN4920_c0_g1_i5_orf1	titin homolog [Ostrinia furnacalis]	2.619585687	1.389338654	2.39E-06	up	yes	0.9737	0.3717	0.363	0.385	0.367	1	0.966	0.955	------	------	------	------	------	------	ENOG4112AA8	S:Function unknown	------	------	------	CYT	19	26	125.1	High
TRINITY_DN76283_c0_g2_i1_orf1	fatty acid synthase-like [Ostrinia furnacalis]	2.999702115	1.584819241	2.06E-06	up	yes	1.007	0.3357	0.317	0.332	0.358	1	1.029	0.993	BP:fatty acid biosynthetic process;BP:fatty acid metabolic process;BP:carboxylic acid metabolic process;BP:cellular biosynthetic process;BP:small molecule metabolic process;BP:small molecule biosynthetic process;BP:monocarboxylic acid biosynthetic process;BP:organic substance biosynthetic process;BP:organic substance metabolic process;BP:lipid metabolic process;BP:cellular process;BP:monocarboxylic acid metabolic process;BP:biosynthetic process;BP:biological_process;BP:metabolic process;BP:oxoacid metabolic process;BP:cellular lipid metabolic process;BP:lipid biosynthetic process;BP:primary metabolic process;BP:organic acid metabolic process;BP:carboxylic acid biosynthetic process;BP:organic acid biosynthetic process;BP:cellular metabolic process;MF:molecular_function;MF:oxidoreductase	GO:0006633;GO:0006631;GO:0019752;GO:0044249;GO:0044281;GO:0044283;GO:0072330;GO:1901576;GO:0071704;GO:0006629;GO:0009987;GO:0032787;GO:0009058;GO:0008150;GO:0008152;GO:0043436;GO:0044255;GO:0008610;GO:0044238;GO:0006082;GO:0046394;GO:0016053;GO:0044237;GO:0003674;GO:0016491;GO:0016740;GO:0016746;GO:0003824	K00665	FASN	map04910;map	Insulin signaling	COG3321	Q:Secondary metabolites biosy	PF00698.24	Acyl_transf_1	Acyl transferase do	CYT	3	8	32	High
TRINITY_DN27848_c0_g1_i2_orf1	cystathionine beta-synthase-like [Ostrinia furnacalis] >XP_028159011.1 cystathionine beta-synthase-like [Ostrinia furnacalis]	2.8441336	1.507989235	5.76E-08	up	yes	0.9963	0.3503	0.358	0.35	0.343	1	1.002	0.987	BP:carboxylic acid metabolic process;BP:cellular biosynthetic process;BP:nitrogen compound metabolic process;BP:small molecule metabolic process;BP:small molecule biosynthetic process;BP:serine family amino acid metabolic process;BP:cysteine metabolic process;BP:L-serine metabolic process;BP:cellular amino acid metabolic process;BP:organic substance metabolic process;BP:cysteine biosynthetic process via cystathionine;BP:cellular amino acid biosynthetic process;BP:alpha-amino acid metabolic process;BP:sulfur amino acid metabolic process;BP:organic substance biosynthetic process;BP:sulfur amino acid biosynthetic process;BP:cellular process;BP:biosynthetic process;BP:biological_process;BP:metabolic process;BP:oxoacid metabolic process;BP:serine family amino acid biosynthetic process;BP:primary metabolic process;BP:organonitrogen compound metabolic process;BP:organonitrogen compound biosynthetic process;BP:organic acid metabolic process;BP:sulfur compound biosynthetic process;BP:carboxylic acid biosynthetic process;BP:organic acid biosynthetic process;BP:cellular metabolic process;BP:cysteine biosynthetic process from serine;BP:cysteine biosynthetic process;BP:sulfur compound metabolic process;BP:alpha-amino acid biosynthetic process;CC:cytoplasm;CC:cellular anatomical entity;CC:cellular_component;MF:molecular_function;MF:lyase activity;MF:cystathionine beta-synthase activity;MF:catalytic	GO:0019752;GO:0044249;GO:0006807;GO:0044281;GO:0044283;GO:0009069;GO:0006534;GO:0006563;GO:0006520;GO:0071704;GO:0019343;GO:0008652;GO:1901605;GO:0000096;GO:1901576;GO:0000097;GO:0009987;GO:0009058;GO:0008150;GO:0008152;GO:0043436;GO:0009070;GO:0044238;GO:1901564;GO:1901566;GO:0006082;GO:0044272;GO:0046394;GO:0016053;GO:0044237;GO:0006535;GO:0019344;GO:0006790;GO:1901607;GO:0005737;GO:0110165;GO:0005575;GO:0003674;GO:0016829;GO:0004122;GO:0003824;GO:0016835;GO:0016836	K01697	CBS	map00270;map	Cysteine and me	COG0031	E:Amino acid transport and me	PF00291.28	PALP	Pyridoxal-phospha	CYT	12	31	54.7	High
TRINITY_DN51766_c0_g1_i2_orf1	facilitated trehalose transporter Tret1-like [Ostrinia furnacalis]	3.36516299	1.750676383	9.43E-06	up	yes	1.022	0.3037	0.305	0.342	0.264	1	1.041	1.024	BP:transport;BP:carbohydrate transport;BP:organic substance transport;BP:biological_process;BP:establishment of localization;BP:localization;CC:integral component of membrane;CC:cellular_component;CC:cellular anatomical entity;CC:intrinsic component of membrane;MF:transporter activity;MF:transmembrane transporter activity;MF:molecular_function;	GO:0006810;GO:0008643;GO:0071702;GO:0008150;GO:0051234;GO:0051179;GO:0016021;GO:0005575;GO:0110165;GO:0031224;GO:0005215;GO:0022857;GO:0003674	------	------	------	------	ENOG410XNQK	E:Amino acid transport and me	PF00083.27;PF07	Sugar_tr;MFS_	Sugar;Major Facilit	CYT	1	3	49.7	High
TRINITY_DN23734_c0_g1_i1_orf1	histone-lysine N-methyltransferase SMYD3 [Ostrinia furnacalis]	2.111782477	1.078461239	0.003274	up	yes	0.9087	0.4303	0.359	0.475	0.457	1	0.778	0.948	CC:cellular_component;CC:intracellular organelle;CC:organelle;CC:cellular anatomical entity;MF:cation binding;MF:ion binding;MF:molecular_function;MF:binding;MF:metal ion	GO:0005575;GO:0043229;GO:0043226;GO:0110165;GO:0043169;GO:0043167;GO:0003674;GO:0005488;GO:0046872	K11426	SMYD	map00310	Lysine degradatio	COG2940	S:Function unknown	PF01753.21	zf-MYND	MYND finger	CYT	1	1	56.4	Medium
TRINITY_DN1285_c0_g2_i1_orf1	bifunctional 3'-phosphoadenosine 5'-phosphosulfate synthase isoform X3 [Ostrinia furnacalis]	2.687988827	1.426527141	5.44E-05	up	yes	0.9623	0.358	0.379	0.336	0.359	1	0.901	0.986	------	------	K13811	PAPSS	map00450;map	Selenocompound	COG0529;COG2046	P:Inorganic ion transport and m	PF01583.23;PF14	APS_kinase;PU	Adenylylsulphate k	CYT	3	14	32.9	High
TRINITY_DN3053_c0_g1_i2_orf1	prostaglandin reductase 1-like [Ostrinia furnacalis]	2.519685039	1.333243408	6.85E-07	up	yes	0.992	0.3937	0.398	0.388	0.395	1	1.005	0.971	CC:cytoplasm;CC:cellular_component;CC:cellular anatomical entity;MF:13-prostaglandin reductase activity;MF:oxidoreductase activity, acting on CH-OH group of donors;MF:oxidoreductase activity, acting on the CH-CH group of donors, NAD or NADP as acceptor;MF:oxidoreductase activity, acting on the CH-OH group of donors, NAD or NADP as acceptor;MF:oxidoreductase activity, acting on the CH-CH group of donors;MF:catalytic activity;MF:molecular_function;MF:15-oxoprostaglandin 13-	GO:0005737;GO:0005575;GO:0110165;GO:0036132;GO:0016614;GO:0016628;GO:0016616;GO:0016627;GO:0003824;GO:0003674;GO:0047522;GO:0032440;GO:0016491	K13948	PTGR1, LTB4D	map00590	Arachidonic acid	COG2130	S:Function unknown	PF16884.8;PF00	ADH_N_2;ADH	N-terminal domain	CYT	9	28	39.1	High
TRINITY_DN2924_c0_g1_i2_orf1	cuticular protein RR-2 [Spodoptera litura]	3.124367089	1.643563969	1.89E-06	up	yes	0.9873	0.316	0.295	0.31	0.343	1	0.975	0.987	MF:molecular_function;MF:structural constituent of cuticle;MF:structural molecule activity;	GO:0003674;GO:0042302;GO:0005198	------	------	------	------	ENOG410YSFI;ENOG4111EA8;ENOG4110T8H	S:Function unknown;	------	------	------	CYT	2	14	11.6	High
TRINITY_DN5956_c1_g1_i5_orf1	uncharacterized protein DDB_G0286299-like [Ostrinia furnacalis] >XP_028170990.1 uncharacterized protein DDB_G0286299-like [Ostrinia furnacalis] >XP_028170991.1 uncharacterized protein DDB_G0286299-like [Ostrinia furnacalis]	3.351809586	1.744940193	4.12E-06	up	yes	1.028	0.3067	0.316	0.32	0.284	1	1.06	1.025	MF:organic cyclic compound binding;MF:molecular_function;MF:binding;MF:nucleic acid binding;MF:heterocyclic compound binding;MF:RNA binding;	GO:0097159;GO:0003674;GO:0005488;GO:0003676;GO:1901363;GO:0003723	------	------	------	------	ENOG4111E9J;ENOG4110R6H	S:Function unknown;	PF00076.25	RRM_1	RNA recognition m	CYT	3	7	66.4	High
TRINITY_DN108122_c0_g1_i9_orf1	hypothetical protein SFRUCORN_003152 [Spodoptera frugiperda]	2.643404705	1.402397319	4.20E-06	up	yes	1	0.3783	0.409	0.379	0.347	1	1.004	0.997	BP:cellular component organization;BP:cell death;BP:mitochondrion organization;BP:cellular component organization or biogenesis;BP:cellular process;BP:apoptotic process;BP:biological_process;BP:organelle organization;BP:programmed cell death;CC:intracellular organelle;CC:membrane-bounded organelle;CC:organelle;CC:mitochondrion;CC:cellular anatomical entity;CC:intracellular membrane-bounded organelle;CC:cellular_component;MF:molecular_function;MF:hydrolase activity;MF:GTPase activity;MF:nucleoside-triphosphatase activity;MF:catalytic activity;MF:hydrolase activity, acting on acid anhydrides, in phosphorus-containing anhydrides;MF:hydrolase activity, acting on acid	GO:0016043;GO:0008219;GO:0007005;GO:0071840;GO:0009987;GO:0006915;GO:0008150;GO:0006996;GO:0012501;GO:0043229;GO:0043227;GO:0043226;GO:0005739;GO:0110165;GO:0043231;GO:0005575;GO:0003674;GO:0016787;GO:0003924;GO:0017111;GO:0003824;GO:0016818;GO:0016817;GO:0016462	K17079	OPA1	map05017	Spinocerebellar a	COG0699	S:Function unknown	PF19434.2	OPA1_C	Dynamin-like GTPa	CYT	1	20	10.8	High
TRINITY_DN313_c0_g1_i5_orf1	collagen alpha-1(X) chain-like [Ostrinia furnacalis]	3.275167785	1.711568817	2.58E-05	up	yes	0.976	0.298	0.309	0.244	0.341	1	0.966	0.962	CC:macromolecular complex;CC:collagen trimer;CC:cellular_component;MF:structural molecule activity;MF:molecular_function;MF:structural constituent of	GO:0032991;GO:0005581;GO:0005575;GO:0005198;GO:0003674;GO:0042302	K06237	COL4A	map05165;map	Human papilloma	ENOG410XNMM;ENOG410YS80	S:Function unknown;	PF01391.21	Collagen	Collagen triple heli	PLA	1	2	36.1	High
TRINITY_DN64472_c0_g2_i1_orf1	repressed by EFG1 protein 1-like isoform X3 [Ostrinia furnacalis]	2.277381245	1.187375826	0.01197	up	yes	0.9253	0.4063	0.588	0.372	0.259	1	0.787	0.989	CC:cellular_component;CC:extracellular region;CC:cellular anatomical entity;	GO:0005575;GO:0005576;GO:0110165	K24835	GLIPR2	------	------	COG2340	G:Carbohydrate transport and m	PF00188.29	CAP	Cysteine-rich secre	PLA	1	3	21.3	High
TRINITY_DN1455_c0_g1_i8_orf1	troponin T, skeletal muscle isoform X1 [Galleria mellonella]	2.739118457	1.453711659	1.73E-06	up	yes	0.9943	0.363	0.348	0.355	0.386	1	0.977	1.006	BP:regulation of system process;BP:regulation of muscle contraction;BP:biological regulation;BP:regulation of muscle system process;BP:regulation of multicellular organismal process;BP:biological_process;BP:regulation of biological process;CC:macromolecular complex;CC:cellular_component;CC:troponin complex;	GO:0044057;GO:0006937;GO:0065007;GO:0090257;GO:0051239;GO:0008150;GO:0050789;GO:0032991;GO:0005575;GO:0005861	K12046	TNNT3	------	------	ENOG410XS6A	Z:Cytoskeleton	------	------	------	CYT	5	41	43.1	High
TRINITY_DN3959_c1_g2_i1_orf1	PREDICTED: probable isocitrate dehydrogenase [NAD] subunit alpha, mitochondrial isoform X3 [Papilio xuthus] >XP_014363280.1 probable isocitrate dehydrogenase [NAD] subunit alpha, mitochondrial isoform X3 [Papilio machaon]	3.095679012	1.630255888	8.55E-08	up	yes	1.003	0.324	0.316	0.325	0.331	1	1.015	0.995	BP:tricarboxylic acid cycle;BP:biological_process;BP:metabolic process;BP:primary metabolic process;CC:oxidoreductase complex;CC:isocitrate dehydrogenase comple;CC:mitochondrial isocitrate dehydrogenase comple;CC:catalytic complex;CC:mitochondrial tricarboxylic acid cycle enzyme complex;CC:tricarboxylic acid cycle enzyme complex;CC:macromolecular complex;CC:cellular_component;CC:mitochondrial protein complex;MF:NAD binding;MF:molecular_function;MF:binding;MF:nucleotide binding;MF:heterocyclic compound binding;MF:nucleoside phosphate binding;MF:oxidoreductase activity;MF:cation binding;MF:magnesium ion binding;MF:ion binding;MF:isocitrate dehydrogenase activity;MF:metal ion binding;MF:small molecule binding;MF:oxidoreductase activity, acting on CH-OH group of donors;MF:isocitrate dehydrogenas;MF:oxidoreductase activity, acting on the CH-OH group of donors, NAD or NADP as acceptor;MF:catalytic	GO:0006099;GO:0008150;GO:0008152;GO:0044238;GO:1990204;GO:0045242;GO:0005962;GO:1902494;GO:0030062;GO:0045239;GO:0032991;GO:0005575;GO:0098798;GO:0051287;GO:0003674;GO:0005488;GO:0000166;GO:1901363;GO:1901265;GO:0016491;GO:0043169;GO:0000287;GO:0043167;GO:0004448;GO:0046872;GO:0036094;GO:0016614;GO:0004449;GO:0016616;GO:0003824;GO:0097159	K00030	IDH3	map00020	Citrate cycl	COG0473	S:Function unknown	PF00180.23	Iso_dh	Isocitrate/isopropy	CYT	16	55	38.7	High
TRINITY_DN3332_c0_g1_i11_orf1	glutathione S-transferase sigma3 [Glyphodes pyloalis]	2.94272997	1.557155164	4.59E-06	up	yes	0.9917	0.337	0.354	0.316	0.341	1	0.961	1.014	MF:catalytic activity;MF:transferase activity;MF:glutathione transferase activity;MF:molecular_function;MF:transferase activity, transferring alkyl or ary;	GO:0003824;GO:0016740;GO:0004364;GO:0003674;GO:0016765	K04097	HPGDS	map00590;map	Arachidonic acid	ENOG41103AU;ENOG4110YU0;ENOG4111VAU;ENOG4111I69;ENOG4111KFX	O:Posttranslational modificatio	PF14497.9;PF02	GST_C_3;GST_	Glutathione S-tran	CYT	2	45	23.1	High
TRINITY_DN27500_c0_g1_i4_orf1	hemicentin-1-like [Ostrinia furnacalis]	3.565730142	1.834197523	2.80E-05	up	yes	1.028	0.2883	0.266	0.297	0.302	1	0.991	1.093	BP:developmental process;BP:anatomical structure development;BP:biological_process;CC:integral component of membrane;CC:cellular_component;CC:intrinsic component of membrane;CC:cellular anatomical entity;	GO:0032502;GO:0048856;GO:0008150;GO:0016021;GO:0005575;GO:0031224;GO:0110165	------	------	------	------	ENOG4110SA1;ENOG4111UKY	S:Function unknown;	PF13927.9;PF07	Ig_3;I-set;Ig_2	Immunoglobulin d	CYT	1	2	57.4	High
TRINITY_DN657_c0_g1_i2_orf1	cytochrome c-type heme lyase [Ostrinia furnacalis]	4.306382979	2.106476628	3.87E-08	up	yes	1.012	0.235	0.228	0.239	0.238	1	1.02	1.015	------	------	K01764	HCCS	map00860	Porphyrin metab	ENOG410YAUW	O:Posttranslational modificatio	PF01265.20	Cyto_heme_ly	Cytochrome c/c1 h	CYT	2	7	31.6	High
TRINITY_DN747_c0_g1_i4_orf1	trypsin, alkaline C-like [Ostrinia furnacalis]	3.468149646	1.79416615	7.27E-05	up	yes	1.029	0.2967	0.291	0.313	0.286	1	0.974	1.113	BP:organic substance metabolic process;BP:organonitrogen compound metabolic process;BP:protein metabolic process;BP:macromolecule metabolic process;BP:biological_process;BP:nitrogen compound metabolic process;BP:metabolic process;BP:proteolysis;BP:primary metabolic process;MF:serine-type endopeptidase activity;MF:endopeptidase activity;MF:hydrolase activity;MF:serine hydrolase activity;MF:catalytic activity, acting on a protein;MF:catalytic activity;MF:molecular_function;MF:peptidase activity;MF:serine-	GO:0071704;GO:1901564;GO:0019538;GO:0043170;GO:0008150;GO:0006807;GO:0008152;GO:0006508;GO:0044238;GO:0004252;GO:0004175;GO:0016787;GO:0017171;GO:0140096;GO:0003824;GO:0003674;GO:0008233;GO:0008236	K09628;K096	PRSS27;TMPR	------	------	COG5640	O:Posttranslational modificatio	PF00089.29	Trypsin	Trypsin	EXC	1	5	28	High

TRINITY_DN16939_c0_g1_i4_orf1	39S ribosomal protein L17, mitochondrial [Ostrinia furnacalis]	3.457400722	1.789687826	1.10E-05	up	yes	0.9577	0.277	0.27	0.301	0.26	1	0.946	0.927	BP:cellular biosynthetic process;BP:cellular nitrogen compound metabolic process;BP:nitrogen compound metabolic process;BP:cellular macromolecule biosynthetic process;BP:organic substance biosynthetic process;BP:cellular nitrogen compound biosynthetic process;BP:cellular macromolecule metabolic process;BP:peptide biosynthetic process;BP:organic substance metabolic process;BP:amide biosynthetic process;BP:cellular process;BP:biosynthetic process;BP:macromolecule biosynthetic process;BP:biological_process;BP:metabolic process;BP:organonitrogen compound metabolic process;BP:primary metabolic process;BP:cellular amide metabolic process;BP:organonitrogen compound biosynthetic process;BP:protein metabolic process;BP:cellular metabolic process;BP:macromolecule metabolic process;BP:peptide metabolic process;BP:translation;CC:intracellular non-membrane-bounded organelle;CC:organelle;CC:cellular anatomical entity;CC:ribosome;CC:macromolecular complex;CC:ribonucleoprotein complex;CC:intracellular organelle;CC:cellular_component;CC:non-membrane-bounded organelle;MF:molecular_function;MF:structural molecule	GO:0044249;GO:0034641;GO:0006807; GO:0034645;GO:1901576;GO:0044271; GO:0044260;GO:0043043;GO:0071704; GO:0043604;GO:0009987;GO:0009058; GO:0009059;GO:0008150;GO:0008152; GO:1901564;GO:0044238;GO:0043603; GO:1901566;GO:0019538;GO:0044237; GO:0043170;GO:0006518;GO:0006412; GO:0043232;GO:0043226;GO:0110165; GO:0005840;GO:0032991;GO:1990904; GO:0043229;GO:0005575;GO:0043228; GO:0003674;GO:0005198;GO:0003735	K02879	RP-L17, MRP	map03010	Ribosome	COG0203	J:Translation, ribosomal structu	PF01196.22	Ribosomal_L1	Ribosomal protein	CYT	2	7	24	High
TRINITY_DN30932_c0_g1_i2_orf1	delta(24)-sterol reductase-like isoform X2 [Ostrinia furnacalis]	2.699140401	1.432500023	9.19E-05	up	yes	0.942	0.349	0.377	0.307	0.363	1	0.93	0.896	CC:microbody;CC:intracellular organelle;CC:membrane-bounded organelle;CC:organelle;CC:integral component of membrane;CC:peroxisome;CC:intrinsic component of membrane;CC:cellular anatomical entity;CC:intracellular membrane-bounded organelle;CC:cellular_component;MF:heterocyclic compound binding;MF:molecular_function;MF:binding;MF:FAD binding;MF:ion binding;MF:nucleoside phosphate binding;MF:oxidoreductase activity;MF:anion binding;MF:flavin adenine dinucleotide binding;MF:oxidoreductase activity, acting on the CH-CH group of donors, NAD or NADP as acceptor;MF:nucleotide binding;MF:small molecule binding;MF:catalytic activity;MF:delta24-sterol reductase activity;MF:organic cyclic compound binding;MF:oxidoreductase	GO:0042579;GO:0043229;GO:0043227; GO:0043226;GO:0016021;GO:0005777; GO:0031224;GO:0110165;GO:0043231; GO:0005575;GO:1901363;GO:0003674; GO:0005488;GO:0071949;GO:0043167; GO:1901265;GO:0016491;GO:0043168; GO:0050660;GO:0016628;GO:0000166; GO:0036094;GO:0003824;GO:0050614; GO:0097159;GO:0016627	K09828	DHCR24, DW	map00100	Steroid biosynthe	COG0277	O:Posttranslational modificatio	PF01565.26	FAD_binding_	FAD binding doma	CYT	1	2	59.2	High
TRINITY_DN16931_c0_g1_i1_orf1	pancreatic triacylglycerol lipase-like [Ostrinia furnacalis]	5.958083832	2.574848423	8.41E-09	up	yes	0.995	0.167	0.171	0.163	0.167	1	0.986	0.999	------	------	------	------	------	------	ENOG4110RTX;ENOG410YGS3;ENOG41116Y2;ENOG411	S:Function unknown;	PF00151.22	Lipase	Lipase	PLA	5	26	32.3	High
TRINITY_DN4016_c0_g1_i1_orf1	60S acidic ribosomal protein P0 [Homo sapiens] >NP_444505.1 60S acidic ribosomal protein P0 [Homo sapiens] >XP_002823894.1 60S acidic ribosomal protein P0 [Pongo abelii] >XP_003280010.1 60S acidic ribosomal protein P0 [Nomascus leucogenys] >XP_004054038.1 60S acidic ribosomal protein P0 [Gorilla gorilla gorilla] >XP_004054039.1 60S acidic ribosomal protein P0 [Gorilla gorilla gorilla] >XP_008956032.1 60S acidic ribosomal protein P0 [Pan paniscus] >XP_008956033.1 60S acidic ribosomal protein P0 [Pan paniscus] >XP_012611945.1 60S acidic ribosomal protein P0 [Microcebus murinus] >XP_016802006.1 60S acidic ribosomal protein P0 [Pan troglodytes] >XP_016802007.1 60S acidic ribosomal protein P0 [Pan troglodytes] >XP_025256707.1 60S acidic ribosomal protein P0 isoform X1 [Theropithecus gelada] >XP_025256708.1 60S acidic ribosomal protein P0 isoform X1 [Theropithecus gelada] >XP_032024425.1 60S acidic ribosomal protein P0 [Hylobates moloch] >XP_032657670.1 60S acidic ribosomal protein P0 [Chelonoidis abingdonii] >XP_045390642.1 60S acidic ribosomal protein P0 [Lemur catta] >P05388.1 RecName: Full=60S acidic ribosomal protein P0; AltName: Full=60S ribosomal protein L10E; AltName: Full=Large ribosomal subunit protein uL10 [Homo sapiens] >3J92_s Structure and assembly pathway of the ribosome quality control complex [Oryctolagus cuniculus] >4V5Z_Bg Chain Bg, 60S acidic ribosomal protein P0 [Canis lupus familiaris] >4V6X_Cq Chain Cq, 60S acidic ribosomal protein P0 [Homo sapiens] >5AJ0_AK Chain AK, 60S acidic ribosomal protein P0 [Homo sapiens] >6ZM7_Ls Chain Ls, 60S acidic ribosomal protein P0 [Homo sapiens] >6ZME_Ls Chain Ls, 60S acidic ribosomal protein P0 [Homo sapiens] >6ZMI_Ls Chain Ls, 60S acidic ribosomal protein P0 [Homo sapiens] >6ZMO_Ls Chain Ls, 60S acidic ribosomal protein P0 [Homo sapiens] >ABM82739.1 ribosomal protein, large, P0 [synthetic construct] >SJX33952.1 unnamed protein product, partial [Human ORFeome Gateway entry vector] >AAA36470.1 acidic ribosomal phosphoprotein (P0) [Homo sapiens] >AAC05176.1 60S ACIDIC RIBOSOMAL PROTEIN; match to P05388 (PID:g133041) [Homo sapiens] >AAH00087.1 Ribosomal protein, large, P0 [Homo sapiens]	2.669529086	1.416585268	0.0001169	up	yes	0.9637	0.361	0.34	0.367	0.376	1	0.886	1.005	BP:lactation;BP:cellular response to interleukin-4;BP:cellular response to stimulus;BP:response to inorganic substance;BP:peptide biosynthetic process;BP:response to phorbol 13-acetate 12-myristate;BP:cellular response to phorbol 13-acetate 12-myristate;BP:response to cAMP;BP:organonitrogen compound metabolic process;BP:response to organic substance;BP:protein metabolic process;BP:response to organonitrogen compound;BP:cellular response to cAMP;BP:response to glycoprotein;BP:cellular response to glycoprotein;BP:cellular component assembly;BP:cellular macromolecule biosynthetic process;BP:ribosomal large subunit assembly;BP:organic substance biosynthetic process;BP:cellular macromolecule metabolic process;BP:ribosome biogenesis;BP:cellular component organization;BP:macromolecular complex assembly;BP:biological regulation;BP:cellular component organization or biogenesis;BP:regulation of biological quality;BP:response to cytokine;BP:transport;BP:biological_process;BP:metabolic process;BP:establishment of localization;BP:secretion;BP:cellular nitrogen compound biosynthetic process;BP:cellular response to cytokine stimulus;BP:response to nitrogen compound;BP:cellular response to nitrogen compound;BP:response to selenium ion;BP:peptide metabolic process;BP:response to alcohol;BP:cellular response to alcohol;BP:cellular biosynthetic process;BP:cellular nitrogen compound metabolic process;BP:cellular response to chemical stimulus;BP:response to purine-containing compound;BP:organonitrogen compound biosynthetic	GO:0007595;GO:0071353;GO:0051716; GO:0010035;GO:0043043;GO:1904627; GO:1904628;GO:0051591;GO:1901564; GO:0010033;GO:0019538;GO:0010243; GO:0071320;GO:1904587;GO:1904588; GO:0022607;GO:0034645;GO:0000027; GO:1901576;GO:0044260;GO:0042254; GO:0016043;GO:0065003;GO:0065007; GO:0071840;GO:0065008;GO:0034097; GO:0006810;GO:0008150;GO:0008152; GO:0051234;GO:0046903;GO:0044271; GO:0071345;GO:1901698;GO:1901699; GO:0010269;GO:0006518;GO:0097305; GO:0097306;GO:0044249;GO:0034641; GO:0070887;GO:0014074;GO:1901566; GO:0014070;GO:0009719;GO:1904400; GO:1904401;GO:0022618;GO:0022613; GO:0071495;GO:0050878;GO:0044238; GO:0009987;GO:0071396;GO:0002181; GO:0043604;GO:0043603;GO:0071407; GO:0043170;GO:0070670;GO:0006807; GO:0050896;GO:0043933;GO:0071826; GO:0033993;GO:0071417;GO:0071704; GO:0071310;GO:1901655;GO:0046683; GO:0007589;GO:0009058;GO:0009059; GO:0042221;GO:0051179;GO:1901700; GO:1901701;GO:0044237;GO:1901654; GO:0044085;GO:0006412;GO:0022626; GO:0005783;GO:0022625;GO:0030425;	K02941	RP-LP0, RPLP	map03010;ma	Ribosome;Coron	COG0244	S:Function unknown	PF17777.4;PF00	RL10P_insert;	Insertion domain i	CYT	1	4	34.3	Medium
TRINITY_DN6143_c0_g2_i1_orf1	uncharacterized protein LOC114365036 [Ostrinia furnacalis]	3.662641786	1.872884609	9.94E-07	up	yes	1.001	0.2733	0.253	0.3	0.267	1	1.009	0.993	------	------	------	------	------	------	ENOG41123CP	S:Function unknown	------	------	------	CYT	8	41	28.8	High
TRINITY_DN6933_c0_g1_i2_orf1	Chlorophyll a-b binding protein 40, chloroplastic [Trichinella nelsoni] >KRY99282.1 Chlorophyll a-b binding protein 40, chloroplastic [Trichinella zimbabwensis]	8.138959932	3.024844446	9.41E-06	up	yes	0.9547	0.1173	0.104	0.121	0.127	1	0.901	0.963	CC:cellular_component;CC:membrane;CC:cellular anatomical entity;	GO:0005575;GO:0016020;GO:0110165	K08912	LHCB1	------	------	ENOG410ZHBU;ENOG410YAFK	J:Translation, ribosomal structu	PF00504.24	Chloroa_b-bi	Chlorophyll A-B bi	CYT	2	7	28.3	High
TRINITY_DN4895_c0_g1_i2_orf1	coiled-coil domain-containing protein 86 [Ostrinia furnacalis]	3.252505658	1.701551567	6.60E-06	up	yes	1.006	0.3093	0.321	0.267	0.34	1	1.017	1	CC:intracellular membrane-bounded organelle;CC:nucleus;CC:cellular_component;CC:intracellular organelle;CC:membrane-bounded organelle;CC:organelle;CC:cellular anatomical entity;	GO:0043231;GO:0005634;GO:0005575; GO:0043229;GO:0043227;GO:0043226; GO:0110165	K14822	CGR1	------	------	ENOG41129IK	S:Function unknown	PF03879.17	Cgr1	Cgr1 family	CYT	1	8	19.7	High
TRINITY_DN8621_c0_g1_i5_orf1	aminopeptidase N-like isoform X2 [Ostrinia furnacalis]	3.183223684	1.670488538	4.49E-05	up	yes	0.9677	0.304	0.282	0.358	0.272	1	0.926	0.977	MF:exopeptidase activity;MF:aminopeptidase activity;MF:transition metal ion binding;MF:hydrolase activity;MF:catalytic activity, acting on a protein;MF:cation binding;MF:ion binding;MF:catalytic activity;MF:molecular_function;MF:zinc ion binding;MF:peptidase activity;MF:metal ion	GO:0008238;GO:0004177;GO:0046914; GO:0016787;GO:0140096;GO:0043169; GO:0043167;GO:0003824;GO:0003674; GO:0008270;GO:0008233;GO:0046872; GO:0008237;GO:0005488	K11140;K111	ANPEP, CD13	map04640;ma	Hematopoietic c	COG0308	S:Function unknown	PF11838.11;PF0	ERAP1_C;Pep	ERAP1-like C-term	CYT	1	2	69.5	High
TRINITY_DN76333_c0_g1_i2_orf1	larval cuticle protein 65Ag1-like [Ostrinia furnacalis]	3.828244275	1.936682889	1.20E-05	up	yes	1.003	0.262	0.274	0.252	0.26	1	1.051	0.957	MF:molecular_function;MF:structural constituent of cuticle;MF:structural molecule activity;	GO:0003674;GO:0042302;GO:0005198	------	------	------	------	ENOG410YVGC;ENOG410YVKS;ENOG4111EHD	S:Function unknown;	PF00379.26	Chitin_bind_4	Insect cuticle prote	CYT	4	43	15.6	High
TRINITY_DN760_c1_g2_i6_orf1	ADP,ATP carrier protein [Pieris napi]	2.667597765	1.415541146	2.70E-05	up	yes	0.955	0.358	0.357	0.34	0.377	1	0.912	0.953	BP:nucleobase-containing compound transport;BP:nucleotide transport;BP:anion transport;BP:carbohydrate derivative transport;BP:adenine nucleotide transport;BP:transmembrane transport;BP:nitrogen compound transport;BP:mitochondrial transmembrane transport;BP:mitochondrial ATP transmembrane transport;BP:organic substance transport;BP:purine nucleotide transport;BP:purine ribonucleotide transport;BP:ADP transport;BP:ATP transport;BP:ion transport;BP:transport;BP:organic anion transport;BP:ion transmembrane transport;BP:biological_process;BP:establishment of localization;BP:localization;BP:purine-containing compound transmembrane transport;BP:nucleotide transmembrane transport;BP:mitochondrial ADP transmembrane transport;BP:organophosphate ester transport;BP:anion transmembrane transport;BP:cellular process;CC:intrinsic component of membrane;CC:organelle membrane;CC:membrane;CC:cellular anatomical entity;CC:mitochondrial membrane;CC:organelle inner membrane;CC:cellular_component;CC:mitochondrial inner membrane;CC:integral component of membrane;MF:purine ribonucleotide transmembrane transporter activity;MF:nucleobase-containing compound transmembrane transporter activity;MF:purine nucleotide transmembrane transporter activity;MF:ATP transmembrane transporter activity;MF:solut;MF:organophosphate ester transmembrane transporter activity;MF:anion transmembrane transporter activity;MF:molecular_function;MF:ion transmembrane transporter activity;MF:nucleotide transmembrane transporter	GO:0015931;GO:0006862;GO:0006820; GO:1901264;GO:0051503;GO:0055085; GO:0071705;GO:1990542;GO:1990544; GO:0071702;GO:0015865;GO:0015868; GO:0015866;GO:0015867;GO:0006811; GO:0006810;GO:0015711;GO:0034220; GO:0008150;GO:0051234;GO:0051179; GO:0072530;GO:1901679;GO:0140021; GO:0015748;GO:0098656;GO:0009987; GO:0031224;GO:0031090;GO:0016020; GO:0110165;GO:0031966;GO:0019866; GO:0005575;GO:0005743;GO:0016021; GO:0005346;GO:0015932;GO:0015216; GO:0005347;GO:0140323;GO:0015605; GO:0008509;GO:0003674;GO:0015075; GO:0015215;GO:0005215;GO:1901505; GO:0015297;GO:0015291;GO:0000295; GO:0008514;GO:0005471;GO:0022857; GO:0022804;GO:0022853;GO:0015217	K05863	SLC25A4S, A	map05166;ma	Human T-cell le	ENOG410XNW0	U:Intracellular trafficking, secre	PF00153.30	Mito_carr	Mitochondrial carr	CYT	21	57	33	High
TRINITY_DN713_c0_g1_i4_orf1	periodic tryptophan protein 2 homolog isoform X1 [Ostrinia furnacalis] >XP_028176443.1 periodic tryptophan protein 2 homolog isoform X2 [Ostrinia furnacalis] >XP_028176445.1 periodic tryptophan protein 2 homolog isoform X3 [Ostrinia furnacalis]	4.105177334	2.037444544	1.94E-06	up	yes	1.007	0.2453	0.254	0.27	0.212	1	1.003	1.018	------	------	K14558	PWP2, UTP1	map03008	Ribosome bioger	ENOG410XP2P	S:Function unknown	PF00400.35;PF1	WD40;ANAPC	WD domain, G-be	CYT	1	1	102.3	High
TRINITY_DN17864_c0_g1_i1_orf1	PREDICTED: erlin-2-B [Microplitis demolitor]	3.006430868	1.588051785	0.0002479	up	yes	0.935	0.311	0.269	0.309	0.355	1	0.954	0.851	CC:endoplasmic reticulum;CC:integral component of membrane;CC:intracellular membrane-bounded organelle;CC:cellular_component;CC:intracellular organelle;CC:intrinsic component of membrane;CC:membrane-bounded organelle;CC:organelle;CC:cellular anatomical entity;MF:ubiquitin protein ligase binding;MF:ubiquitin-like protein ligase binding;MF:enzyme binding;MF:molecular_function;MF:protein binding;MF:binding;	GO:0005783;GO:0016021;GO:0043231; GO:0005575;GO:0043229;GO:0031224; GO:0043227;GO:0043226;GO:0110165; GO:0031625;GO:0044389;GO:0019899; GO:0003674;GO:0005515;GO:0005488	K23341	ERLIN	------	------	ENOG410XQSH	O:Posttranslational modificatio	PF01145.28	Band_7	SPFH domain / Ba	CYT	1	2	37.3	Medium
TRINITY_DN29120_c0_g1_i6_orf1	putative inorganic phosphate cotransporter [Ostrinia furnacalis]	3.359034529	1.748046626	7.62E-06	up	yes	1.002	0.2983	0.3	0.321	0.274	1	1.036	0.969	CC:integral component of membrane;CC:cellular_component;CC:cellular anatomical entity;CC:intrinsic component of membrane;MF:transporter activity;MF:transmembrane transporter activity;MF:molecular_function;	GO:0016021;GO:0005575;GO:0110165; GO:0031224;GO:0005215;GO:0022857; GO:0003674	K08193	SLC17A	------	------	ENOG410XPWC;ENOG4110JNM;COG0477;ENOG410XRH7	G:Carbohydrate transport and	PF07690.19	MFS_1	Major Facilitator S	CYT	1	4	27.3	High
TRINITY_DN3014_c0_g1_i4_orf1	putative inorganic phosphate cotransporter isoform X1 [Ostrinia furnacalis]	3.790697674	1.9224634	3.86E-05	up	yes	0.978	0.258	0.28	0.249	0.245	1	0.91	1.024	CC:integral component of membrane;CC:cellular_component;CC:cellular anatomical entity;CC:intrinsic component of membrane;MF:transporter activity;MF:transmembrane transporter activity;MF:molecular_function;	GO:0016021;GO:0005575;GO:0110165; GO:0031224;GO:0005215;GO:0022857; GO:0003674	K12301	SLC17A5	map04142	Lysosome	ENOG410XPWC	S:Function unknown	PF07690.19;PF0	MFS_1;Sugar_	Major Facilitator S	CYT	1	1	56.6	Medium
TRINITY_DN2574_c0_g1_i5_orf1	prion-like-(Q/N-rich) domain-bearing protein 25 [Ostrinia furnacalis] >XP_028158239.1 prion-like-(Q/N-rich) domain-bearing protein 25 [Ostrinia furnacalis] >XP_028158240.1 prion-like-(Q/N-rich) domain-bearing protein 25 [Ostrinia furnacalis] >XP_028158241.1 prion-like-(Q/N-rich) domain-bearing protein 25 [Ostrinia furnacalis]	3.375254237	1.754996176	2.04E-06	up	yes	0.9957	0.295	0.294	0.305	0.286	1	1.021	0.966	------	------	------	------	------	------	ENOG410XTK6;ENOG4111NZ9	S:Function unknown;	PF01683.21	EB	EB module	CYT	4	20	34.9	High
TRINITY_DN542_c0_g1_i4_orf1	uncharacterized protein LOC114364889 [Ostrinia furnacalis]	3.608516997	1.85140605	5.84E-06	up	yes	0.966	0.2677	0.265	0.269	0.269	1	0.925	0.973	BP:amino sugar catabolic process;BP:nitrogen compound metabolic process;BP:aminoglycan metabolic process;BP:organic substance catabolic process;BP:glucosamine-containing compound catabolic process;BP:organic substance metabolic process;BP:chitin metabolic process;BP:polysaccharide catabolic process;BP:biological_process;BP:metabolic process;BP:organonitrogen compound metabolic process;BP:catabolic process;BP:macromolecule catabolic process;BP:primary metabolic process;BP:chitin catabolic process;BP:carbohydrate metabolic process;BP:organonitrogen compound catabolic process;BP:polysaccharide metabolic process;BP:amino sugar metabolic process;BP:carbohydrate derivative catabolic process;BP:carbohydrate catabolic process;BP:macromolecule metabolic process;BP:glucosamine-containing compound metabolic process;BP:aminoglycan catabolic process;BP:carbohydrate derivative metabolic process;CC:extracellular space;CC:cellular anatomical entity;CC:cellular_component;MF:hydrolase activity, acting on glycosyl bonds;MF:pullulanase activity;MF:molecular_function;MF:zinc ion binding;MF:cation binding;MF:transition metal ion binding;MF:ion binding;MF:metal ion binding;MF:hydrolase activity;MF:deacetylase activity;MF:catalytic activity;MF:hydrolase activity, acting on carbon-nitroge;MF:hydrolase activity, acting on carbon-	GO:0046348;GO:0006807;GO:0006022; GO:1901575;GO:1901072;GO:0071704; GO:0006030;GO:0000272;GO:0008150; GO:0008152;GO:1901564;GO:0009056; GO:0009057;GO:0044238;GO:0006032; GO:0005975;GO:1901565;GO:0005976; GO:0006040;GO:1901136;GO:0016052; GO:0043170;GO:1901071;GO:0006026; GO:1901135;GO:0005615;GO:0110165; GO:0005575;GO:0016798;GO:0051060; GO:0003674;GO:0008270;GO:0043169; GO:0046914;GO:0043167;GO:0046872; GO:0016787;GO:0019213;GO:0003824; GO:0016810;GO:0016811;GO:0004553; GO:0004099;GO:0005488	------	------	------	------	ENOG410ZWU3	S:Function unknown	PF01522.24	Polysacc_dea	Polysaccharide de	CYT	5	14	44.8	High
TRINITY_DN23175_c0_g1_i6_orf1	myb-binding protein 1A-like protein [Ostrinia furnacalis]	3.117045091	1.640179025	2.33E-05	up	yes	0.9747	0.3127	0.311	0.311	0.316	1	0.916	1.008	------	------	K02331	POL5, MYBBF	------	------	ENOG410YFVF;ENOG410ZVK4	S:Function unknown;L:Replicati	PF04931.16	DNA_pol_phi	DNA polymerase p	CYT	1	1	130.2	High

TRINITY_DN2215_c0_g2_i1_orf1	PREDICTED: larval cuticle protein LCP-22-like [Amyelois transitella]	4.154811715	2.0547831	1.88E-06	up	yes	0.993	0.239	0.264	0.206	0.247	1	0.984	0.995	------	------	------	------	------	------	ENOG41119AX;ENOG4111CQ7;ENOG4111DQA;ENOG410YZKX;ENOG4111PJ	S:Function unknown;	PF00379.26	Chitin_bind_4	Insect cuticle prote	CYT	3	33	16.5	High
TRINITY_DN700_c0_g1_i3_orf1	V-type proton ATPase subunit H isoform X1 [Chelonus insularis]	3.888251052	1.959121372	2.11E-07	up	yes	1.016	0.2613	0.251	0.271	0.262	1	1.029	1.02	CC:vacuolar proton-transporting V-type ATPase, V1 domain;CC:proton-transporting V-type ATPase, V1 domain;CC:proton-transporting two-sector ATPase complex, catalytic domain;CC:macromolecular complex;CC:cellular_component;CC:membrane protein complex;MF:molecular_function;MF:ion transmembrane transporter activity;MF:ATPase activity, coupled to transmembrane movement of substances;MF:inorganic solute uptake transmembrane transporter activity;MF:hydrogen-translocating pyrophosphatase activity;MF:primary active transmembrane transporter activity;MF:active transmembrane transporter activity;MF:ATPase coupled ion transmembrane transporter activity;MF:transporter activity;MF:cation transmembrane transporter activity;MF:proton-transporting ATPase activity, rotational mechanism;MF:ATPase activity, coupled to transmembrane movement of ions, rotational mechanism;MF:inorganic cation transmembrane transporter activity;MF:cation-transporting ATPase activity;MF:transmembrane transporter activity;MF:active ion	GO:0000221;GO:0033180;GO:0033178;GO:0032991;GO:0005575;GO:0098796;GO:0003674;GO:0015075;GO:0042626;GO:0015318;GO:0009678;GO:0015399;GO:0022804;GO:0042625;GO:0005215;GO:0008324;GO:0046961;GO:0044769;GO:0022890;GO:0019829;GO:0022857;GO:0022853;GO:0015078	K02144	ATPeV1H	map05152;ma	Tuberculosis;Hun	COG5231	C:Energy production and conve	PF03224.17;PF1	V-ATPase_H_	V-ATPase subunit	CYT	1	3	59.4	High
TRINITY_DN5891_c0_g2_i4_orf1	amino acid transporter AVT1A-like [Ostrinia furnacalis] >XP_028156666.1 amino acid transporter AVT1A-like [Ostrinia furnacalis]	5.143721634	2.36281257	5.26E-06	up	yes	1.02	0.1983	0.201	0.176	0.218	1	0.996	1.064	CC:cellular_component;CC:integral component of membrane;CC:cellular anatomical entity;CC:intrinsic component of membrane;	GO:0005575;GO:0016021;GO:0110165;GO:0031224	K15015	SLC32A, VGA	map04727;ma	GABAergic synap	COG0814	E:Amino acid transport and me	PF01490.21;PF0	Aa_trans;Trp_	Transmembrane ar	CYT	1	2	48.1	High
TRINITY_DN79210_c0_g1_i1_orf1	V-type proton ATPase 16 kDa proteolipid subunit [Frieseomelitta varia]	3.121498371	1.642238713	1.05E-05	up	yes	0.9583	0.307	0.31	0.286	0.325	1	0.939	0.936	CC:intrinsic component of membrane;CC:cellular_component;CC:organelle membrane;CC:proton-transporting V-type ATPase, V0 domain;CC:integral component of membrane;CC:membrane;CC:vacuolar membrane;CC:cellular anatomical entity;CC:bounding membrane of organelle;CC:macromolecular complex;CC:membrane protein complex;CC:proton-transporting two-sector ATPase complex, proton-transporting domain;MF:molecular_function;MF:ATPase activity, coupled to transmembrane movement of substances;MF:inorganic solute uptake transmembrane transporter activity;MF:hydrogen-translocating pyrophosphatase activity;MF:primary active transmembrane transporter activity;MF:ATPase coupled ion transmembrane transporter activity;MF:transporter activity;MF:cation transmembrane transporter activity;MF:proton-transporting ATPase activity, rotational mechanism;MF:ATPase activity, coupled to transmembrane movement of ions, rotational mechanism;MF:inorganic cation transmembrane transporter activity;MF:cation-transporting ATPase activity;MF:ion transmembrane transporter activity;MF:transmembrane transporter activity;MF:active transmembrane transporter activity;MF:active ion transmembrane transporter activity;MF:hydrogen ion transmembrane transporter activity;	GO:0031224;GO:0005575;GO:0031090;GO:0033179;GO:0016021;GO:0016020;GO:0005774;GO:0110165;GO:0098588;GO:0032991;GO:0098796;GO:0033177;GO:0003674;GO:0042626;GO:0015318;GO:0009678;GO:0015399;GO:0042625;GO:0005215;GO:0008324;GO:0046961;GO:0044769;GO:0022890;GO:0019829;GO:0015075;GO:0022857;GO:0022804;GO:0022853;GO:0015078	K02155	ATPeV0C, ATI	map05152;ma	Tuberculosis;Rhe	COG0636	J:Translation, ribosomal structu	PF00137.24	ATP-synt_C	ATP synthase subu	CYT	1	11	16	High
TRINITY_DN21035_c0_g1_i14_orf1	mitochondrial amidoxime reducing component 2-like [Ostrinia furnacalis]	3.774180813	1.916163541	1.06E-05	up	yes	0.956	0.2533	0.248	0.244	0.268	1	0.953	0.915	MF:anion binding;MF:cation binding;MF:molybdenum ion binding;MF:metal ion binding;MF:transition metal ion binding;MF:organic cyclic compound binding;MF:vitamin binding;MF:vitamin B6 binding;MF:small molecule binding;MF:catalytic activity;MF:transferase activity;MF:molecular_function;MF:pyridoxal phosphate binding;MF:ion binding;MF:heterocyclic compound binding;MF:binding;	GO:0043168;GO:0043169;GO:0030151;GO:0046872;GO:0046914;GO:0097159;GO:0019842;GO:0070279;GO:0036094;GO:0003824;GO:0016740;GO:0003674;GO:0030170;GO:0043167;GO:1901363;GO:0005488	------	------	------	------	COG3217	S:Function unknown	PF03476.19	MOSC_N	MOSC N-terminal	CYT	1	41	15.6	High
TRINITY_DN61112_c0_g1_i4_orfp1	TRINITY_DN61112_c0_g1_i4_m.53012 TRINITY_DN61112_c0_g1::TRINITY_DN61112_c0_g1_i4::g.53012 ORF type:3prime_partial len:87 (-),score=12.29,HMMR_N,PF15905.6,0.0011 TRINITY_DN61112_c0_g1_i4:2-232(-)	5.037056099	2.332580799	4.27E-07	up	yes	0.9787	0.1943	0.204	0.181	0.198	1	0.971	0.965	------	------	------	------	------	------	------	------	------	------	------	CYT	3	39	9.2	High
TRINITY_DN117_c0_g1_i5_orf1	lipase member I-like [Ostrinia furnacalis]	4.125990822	2.044740612	3.81E-08	up	yes	0.989	0.2397	0.233	0.241	0.245	1	0.986	0.981	BP:organic substance metabolic process;BP:lipid metabolic process;BP:primary metabolic process;BP:metabolic process;BP:biological_process;CC:cellular_component;CC:extracellular region;CC:cellular anatomical entity;MF:hydrolase activity;MF:hydrolase activity, acting on ester bonds;MF:catalytic activity;MF:molecular_function;MF:lipase activity;	GO:0071704;GO:0006629;GO:0044238;GO:0008152;GO:0008150;GO:0005575;GO:0005576;GO:0110165;GO:0016787;GO:0016788;GO:0003824;GO:0003674;GO:0016298	------	------	------	------	ENOG41116Y2;ENOG4110RTX;ENOG410YGS3;ENOG4111JY5	S:Function unknown;	PF00151.22	Lipase	Lipase	CYT	6	21	46.9	High
TRINITY_DN4869_c0_g1_i10_orf1	estrogen sulfotransferase-like isoform X1 [Ostrinia furnacalis]	6.775288527	2.760282385	6.65E-07	up	yes	0.998	0.1473	0.14	0.153	0.149	1	1.023	0.971	------	------	------	------	------	------	ENOG4111H56	S:Function unknown	PF00685.30;PF1	Sulfotransfer_	Sulfotransferase d	CYT	9	34	39.9	High
TRINITY_DN96566_c0_g1_i1_orf1	NADH-ubiquinone oxidoreductase subunit 8-like [Ostrinia furnacalis]	3.123780693	1.643293172	7.22E-05	up	yes	0.9287	0.2973	0.282	0.311	0.299	1	0.906	0.88	CC:membrane;CC:cellular_component;CC:cellular anatomical entity;MF:4 iron, 4 sulfur cluster binding;MF:oxidoreductase activity, acting on NA;MF:iron-sulfur cluster binding;MF:catalytic activity;MF:molecular_function;MF:binding;MF:oxidoreductase	GO:0016020;GO:0005575;GO:0110165;GO:0051539;GO:0016651;GO:0051536;GO:0003824;GO:0003674;GO:0005488;GO:0016491;GO:0051540	K03941	NDUFS8	map04714;ma	Thermogenesis;C	COG1143	C:Energy production and conve	PF12838.10;PF0	Fer4_7;Fer4;Fe	4Fe-4S dicluster d	CYT	1	4	28.6	High
TRINITY_DN22430_c0_g3_i1_orf1	V-type proton ATPase 16 kDa proteolipid subunit c [Homo sapiens] >NP_001685.1 V-type proton ATPase 16 kDa proteolipid subunit c [Homo sapiens] >P27449.1 RecName: Full=V-type proton ATPase 16 kDa proteolipid subunit c; Short=V-ATPase 16 kDa proteolipid subunit c; AltName: Full=Vacuolar proton pump 16 kDa proteolipid subunit c [Homo sapiens] >6WLW_1 The Vo region of human V-ATPase in state 1 (focused refinement) [Homo sapiens] >6WLW_2 The Vo region of human V-ATPase in state 1 (focused refinement) [Homo sapiens] >6WLW_3 The Vo region of human V-ATPase in state 1 (focused refinement) [Homo sapiens] >6WLW_4 The Vo region of human V-ATPase in state 1 (focused refinement) [Homo sapiens] >6WLW_5 The Vo region of human V-ATPase in state 1 (focused refinement) [Homo sapiens] >6WLW_6 The Vo region of human V-ATPase in state 1 (focused refinement) [Homo sapiens] >6WLW_7 The Vo region of human V-ATPase in state 1 (focused refinement) [Homo sapiens] >6WLW_8 The Vo region of human V-ATPase in state 1 (focused refinement) [Homo sapiens] >6WLW_9 The Vo region of human V-ATPase in state 1 (focused refinement) [Homo sapiens] >6WM2_1 Human V-ATPase in state 1 with SidK and ADP [Homo sapiens] >6WM2_2 Human V-ATPase in state 1 with SidK and ADP [Homo sapiens] >6WM2_3 Human V-ATPase in state 1 with SidK and ADP [Homo sapiens] >6WM2_4 Human V-ATPase in state 1 with SidK and ADP [Homo sapiens] >6WM2_5 Human V-ATPase in state 1 with SidK and ADP [Homo sapiens] >6WM2_6 Human V-ATPase in state 1 with SidK and ADP [Homo sapiens] >6WM2_7 Human V-ATPase in state 1 with SidK and ADP [Homo sapiens] >6WM2_8 Human V-ATPase in state 1 with SidK and ADP [Homo sapiens] >6WM2_9 Human V-ATPase in state 1 with SidK and ADP [Homo sapiens] >6WM3_1 Human V-ATPase in state 2 with SidK and ADP [Homo sapiens] >6WM3_2 Human V-ATPase in state 2 with SidK and ADP [Homo sapiens] >6WM3_3 Human V-ATPase in state 2 with SidK and ADP [Homo sapiens] >6WM3_4 Human V-ATPase in state 2 with SidK and ADP [Homo sapiens] >6WM3_5 Human V-ATPase in state 2 with SidK and ADP [Homo sapiens] >6WM3_6 Human V-ATPase in state 2 with SidK and ADP [Homo sapiens] >6WM3_7 Human V-ATPase in state 2 with SidK and ADP [Homo sapiens] >6WM3_8 Human V-ATPase in state 2 with SidK and ADP [Homo sapiens] >6WM3_9 Human V-ATPase in state 2 with SidK and ADP [Homo sapiens] >6WM4_1 Human V-ATPase in state 3 with SidK and ADP [Homo sapiens] >6WM4_2 Human V-ATPase in state 3 with SidK and ADP [Homo sapiens] >6WM4_3 Human V-ATPase in state 3 with SidK and ADP [Homo	8.158333333	3.028274454	2.16E-06	up	yes	0.979	0.12	0.12	0.123	0.117	1	1	0.937	BP:regulation of metabolic process;BP:positive regulation of response to stimulus;BP:regulation of response to stimulus;BP:regulation of Wnt signaling pathway;BP:inorganic ion homeostasis;BP:regulation of signal transduction;BP:positive regulation of signal transduction;BP:positive regulation of biological process;BP:cellular homeostasis;BP:pH reduction;BP:chemical homeostasis;BP:regulation of catabolic process;BP:positive regulation of Wnt signaling pathway;BP:Golgi lumen acidification;BP:regulation of biological process;BP:regulation of pH;BP:biological regulation;BP:inorganic cation transmembrane transport;BP:inorganic ion transmembrane transport;BP:regulation of biological quality;BP:transport;BP:cation transport;BP:ion transport;BP:regulation of cellular process;BP:biological_process;BP:establishment of localization;BP:hydrogen ion transmembrane transport;BP:ion homeostasis;BP:positive regulation of signaling;BP:endosomal lumen acidification;BP:lysosomal lumen acidification;BP:regulation of signaling;BP:regulation of intracellular pH;BP:intracellular pH reduction;BP:positive regulation of cell communication;BP:regulation of cell communication;BP:cellular process;BP:cellular ion homeostasis;BP:cellular monovalent inorganic cation homeostasis;BP:cellular cation homeostasis;BP:cation homeostasis;BP:cellular chemical homeostasis;BP:transmembrane transport;BP:vacuolar acidification;BP:regulation of lysosomal lumen pH;BP:regulation of cellular catabolic process;BP:regulation of cellular metabolic	GO:0019222;GO:0048584;GO:0048583;GO:0030111;GO:0098771;GO:0009966;GO:0009967;GO:0048518;GO:0019725;GO:0045851;GO:0048878;GO:0009894;GO:0030177;GO:0061795;GO:0050789;GO:0006885;GO:0065007;GO:0098662;GO:0098660;GO:0065008;GO:0006810;GO:0006812;GO:0006811;GO:0050794;GO:0008150;GO:0051234;GO:1902600;GO:0050801;GO:0023056;GO:0048388;GO:0007042;GO:0023051;GO:0051453;GO:0051452;GO:0010647;GO:0010646;GO:0009987;GO:0006873;GO:0030004;GO:0030003;GO:0055080;GO:0055082;GO:0055085;GO:0007035;GO:0035751;GO:0031329;GO:0031323;GO:0042592;GO:0030641;GO:0010506;GO:0016241;GO:0034220;GO:0055067;GO:0051179;GO:0098655;GO:0048522;GO:0035577;GO:0030054;GO:0030055;GO:0031982;GO:0016021;GO:0016020;GO:0005774;GO:0099501;GO:0098588;GO:0043230;GO:0065010;GO:0098852;GO:0005925;GO:0043227;GO:0110165;GO:0070161;GO:0012506;GO:0030672;GO:0030670;GO:0000139;GO:0033179;GO:0016469;GO:0033176;GO:0033177;GO:0101003;GO:0031224;GO:0010008;GO:0030665;GO:0030667;GO:0030666;GO:0031090;	K02155	ATPeV0C, ATI	map05152;ma	Tuberculosis;Rhe	COG0636	S:Function unknown	PF00137.24	ATP-synt_C	ATP synthase subu	CYT	1	12	15.7	Medium
TRINITY_DN8116_c0_g1_i2_orf1	uncharacterized protein LOC114350845 [Ostrinia furnacalis]	4.937128713	2.303672257	8.24E-09	up	yes	0.9973	0.202	0.206	0.198	0.202	1	1.003	0.989	------	------	------	------	------	------	COG1028	S:Function unknown	PF13561.9;PF00	adh_short_C2	Enoyl;short chain c	CYT	6	37	26.1	High
TRINITY_DN334_c0_g1_i4_orf1	collagenase-like [Ostrinia furnacalis]	20.08902651	4.32833575	1.50E-06	up	yes	1.038	0.05167	0.042	0.07	0.043	1	1.068	1.047	BP:organic substance metabolic process;BP:organonitrogen compound metabolic process;BP:protein metabolic process;BP:macromolecule metabolic process;BP:biological_process;BP:nitrogen compound metabolic process;BP:metabolic process;BP:proteolysis;BP:primary metabolic process;MF:serine-type endopeptidase activity;MF:endopeptidase activity;MF:hydrolase activity;MF:serine hydrolase activity;MF:catalytic activity, acting on a protein;MF:catalytic activity;MF:molecular_function;MF:peptidase activity;MF:serine-	GO:0071704;GO:1901564;GO:0019538;GO:0043170;GO:0008150;GO:0006807;GO:0008152;GO:0006508;GO:0044238;GO:0004252;GO:0004175;GO:0016787;GO:0017171;GO:0140096;GO:0003824;GO:0003674;GO:0008233;GO:0008236	------	------	------	------	COG5640	O:Posttranslational modificatio	PF00089.29	Trypsin	Trypsin	EXC	1	39	29.8	High
TRINITY_DN542_c0_g2_i1_orf1	uncharacterized protein LOC114364889 [Ostrinia furnacalis]	4.674274845	2.224742567	5.05E-06	up	yes	0.983	0.2103	0.211	0.205	0.215	1	1.012	0.937	BP:amino sugar catabolic process;BP:nitrogen compound metabolic process;BP:aminoglycan metabolic process;BP:organic substance catabolic process;BP:glucosamine-containing compound catabolic process;BP:organic substance metabolic process;BP:chitin metabolic process;BP:polysaccharide catabolic process;BP:biological_process;BP:metabolic process;BP:organonitrogen compound metabolic process;BP:catabolic process;BP:macromolecule catabolic process;BP:primary metabolic process;BP:chitin catabolic process;BP:carbohydrate metabolic process;BP:organonitrogen compound catabolic process;BP:polysaccharide metabolic process;BP:amino sugar metabolic process;BP:carbohydrate derivative catabolic process;BP:carbohydrate catabolic process;BP:macromolecule metabolic process;BP:glucosamine-containing compound metabolic process;BP:aminoglycan catabolic process;BP:carbohydrate derivative metabolic process;CC:extracellular space;CC:cellular anatomical entity;CC:cellular_component;MF:molecular_function;MF:zinc ion binding;MF:cation binding;MF:transition metal ion binding;MF:ion binding;MF:metal ion binding;MF:hydrolase activity;MF:deacetylase activity;MF:catalytic activity;MF:hydrolase activity, acting on carbon-	GO:0046348;GO:0006807;GO:0006022;GO:1901575;GO:1901072;GO:0071704;GO:0006030;GO:0000272;GO:0008150;GO:0008152;GO:1901564;GO:0009056;GO:0009057;GO:0044238;GO:0006032;GO:0005975;GO:1901565;GO:0005976;GO:0006040;GO:1901136;GO:0016052;GO:0043170;GO:1901071;GO:0006026;GO:1901135;GO:0005615;GO:0110165;GO:0005575;GO:0003674;GO:0008270;GO:0043169;GO:0046914;GO:0043167;GO:0046872;GO:0016787;GO:0019213;GO:0003824;GO:0016810;GO:0016811;GO:0004099;GO:0005488	------	------	------	------	ENOG410ZWU3	S:Function unknown	PF01522.24	Polysacc_dea	Polysaccharide de	PLA	4	16	44.5	High
TRINITY_DN82320_c0_g1_i2_orf1	glutathione S-transferase sigma3 [Glyphodes pyloalis]	4.756717147	2.24996624	0.0001793	up	yes	0.9737	0.2047	0.225	0.177	0.212	1	0.867	1.054	MF:catalytic activity;MF:transferase activity;MF:glutathione transferase activity;MF:molecular_function;MF:transferase activity, transferring alkyl or ary;	GO:0003824;GO:0016740;GO:0004364;GO:0003674;GO:0016765	K04097	HPGDS	map00590;ma	Arachidonic acid	ENOG4110YU0;ENOG41103AU	O:Posttranslational modificatio	PF14497.9	GST_C_3	Glutathione S-tran	CYT	1	13	7.2	High
TRINITY_DN144190_c0_g1_i1_orf1	PREDICTED: uncharacterized protein LOC103572804 isoform X2 [Microplitis demolitor]	12.83465626	3.681972753	1.13E-05	up	yes	0.954	0.07433	0.078	0.072	0.073	1	0.971	0.891	------	------	------	------	------	------	ENOG4112B12	S:Function unknown	------	------	------	CYT	1	3	26.8	Medium
TRINITY_DN92232_c0_g1_i1_orf1	protein SDA1 homolog [Ostrinia furnacalis]	2.762624899	1.46603969	3.89E-06	up	yes	1.023	0.3703	0.366	0.348	0.397	1	1.03	1.039	BP:nuclear transport;BP:protein localization;BP:ribosome localization;BP:actin cytoskeleton organization;BP:nuclear export;BP:establishment of organelle localization;BP:cellular component organization or biogenesis;BP:ribosomal large subunit biogenesis;BP:cellular macromolecule localization;BP:cellular component organization;BP:nitrogen compound transport;BP:organic substance transport;BP:ribonucleoprotein complex biogenesis;BP:macromolecule localization;BP:organelle organization;BP:transport;BP:actin filament-based process;BP:cellular process;BP:nucleocytoplasmic transport;BP:establishment of protein localization;BP:biological_process;BP:establishment of localization in cell;BP:establishment of localization;BP:localization;BP:organelle localization;BP:cellular localization;BP:protein complex localization;BP:cytoskeleton organization;BP:intracellular transport;BP:ribosomal large subunit export from nucleus;BP:ribosomal subunit export from nucleus;BP:cellular component biogenesis;BP:protein transport;CC:intracellular organelle;CC:non-membrane-bounded organelle;CC:organelle;CC:cellular anatomical entity;CC:intracellular non-membrane-bounded	GO:0051169;GO:0008104;GO:0033750;GO:0030036;GO:0051168;GO:0051656;GO:0071840;GO:0042273;GO:0070727;GO:0016043;GO:0071705;GO:0071702;GO:0022613;GO:0033036;GO:0006996;GO:0006810;GO:0030029;GO:0009987;GO:0006913;GO:0045184;GO:0008150;GO:0051649;GO:0051234;GO:0051179;GO:0051640;GO:0051641;GO:0031503;GO:0007010;GO:0046907;GO:0000055;GO:0000054;GO:0044085;GO:0015031;GO:0043229;GO:0043228;GO:0043226;GO:0110165;GO:0043232;GO:0005730;GO:0005575	K14856	SDA1, SDAD1	------	------	ENOG410XPHI	S:Function unknown	PF05285.15;PF0	SDA1;NUC13	SDA1;NUC130/3NT	CYT	1	1	84.7	Medium

TRINITY_DN80560_c0_g1_i1_orf1	ATP synthase subunit alpha, mitochondrial [Ostrinia furnacalis]	2.575515464	1.364861202	1.80E-08	up	yes	0.9993	0.388	0.382	0.388	0.394	1	1.004	0.994	CC:proton-transporting ATP synthase complex, catalytic core;CC:proton-transporting two-sector ATPase complex, catalytic domain;CC:macromolecular complex;CC:cellular_component;CC:membrane protein complex;MF:cation channel activity;MF:purine ribonucleoside triphosphate binding;MF:molecular_function;MF:binding;MF:ion binding;MF:heterocyclic compound binding;MF:nucleoside phosphate binding;MF:proton-transporting ATP synthase activity, rotational mechanism;MF:inorganic solute uptake transmembrane transporter activity;MF:nucleotide binding;MF:hydrogen ion channel activity;MF:purine nucleotide binding;MF:ATP binding;MF:anion binding;MF:transporter activity;MF:ion channel activity;MF:cation transmembrane transporter activity;MF:small molecule binding;MF:catalytic activity;MF:passive transmembrane transporter activity;MF:ligase activity;MF:inorganic cation transmembrane transporter activity;MF:adenyl nucleotide binding;MF:carbohydrate derivative binding;MF:organic cyclic compound binding;MF:ion transmembrane transporter activity;MF:transmembrane transporter activity;MF:channel activity;MF:adenyl ribonucleotide binding;MF:purine	GO:0045261;GO:0033178;GO:0032991;GO:0005575;GO:0098796;GO:0005261;GO:0035639;GO:0003674;GO:0005488;GO:0043167;GO:1901363;GO:1901265;GO:0046933;GO:0015318;GO:0000166;GO:0015252;GO:0017076;GO:0005524;GO:0043168;GO:0005215;GO:0005216;GO:0008324;GO:0036094;GO:0003824;GO:0022803;GO:0016874;GO:0022890;GO:0030554;GO:0097367;GO:0097159;GO:0015075;GO:0022857;GO:0015267;GO:0032559;GO:0032555;GO:0015078;GO:0032553	K02132	ATPeF1A, ATF	map04714;ma	Thermogenesis;C	COG0056	C:Energy production and conv	PF00006.28;PF0	ATP-synt_ab;	ATP synthase alph	CYT	23	45	59.4	High
TRINITY_DN106730_c0_g1_i1_orf1	Photosystem I reaction center subunit II, chloroplastic, partial [Trichinella zimbabwensis]	2.743243243	1.455882552	1.37E-05	up	yes	1.015	0.37	0.366	0.41	0.334	1	1.039	1.006	------	------	K02692	psaD	------	------	ENOG4111HAV	C:Energy production and conv	PF02531.19	PsaD	PsaD	CYT	2	11	22.6	High
TRINITY_DN64510_c0_g1_i1_orf1	39S ribosomal protein L15, mitochondrial [Ostrinia furnacalis]	2.434253045	1.283479147	3.80E-06	up	yes	0.9793	0.4023	0.412	0.383	0.412	1	0.983	0.955	BP:cellular biosynthetic process;BP:cellular nitrogen compound metabolic process;BP:nitrogen compound metabolic process;BP:cellular macromolecule biosynthetic process;BP:organic substance biosynthetic process;BP:cellular nitrogen compound biosynthetic process;BP:cellular macromolecule metabolic process;BP:peptide biosynthetic process;BP:organic substance metabolic process;BP:amide biosynthetic process;BP:cellular process;BP:biosynthetic process;BP:macromolecule biosynthetic process;BP:biological_process;BP:metabolic process;BP:primary metabolic process;BP:organonitrogen compound metabolic process;BP:cellular amide metabolic process;BP:organonitrogen compound biosynthetic process;BP:protein metabolic process;BP:cellular metabolic process;BP:macromolecule metabolic process;BP:peptide metabolic process;BP:translation;CC:large ribosomal subunit;CC:ribosomal subunit;CC:macromolecular complex;CC:ribonucleoprotein complex;CC:cellular_component;MF:molecular_function;MF:stru	GO:0044249;GO:0034641;GO:0006807;GO:0034645;GO:1901576;GO:0044271;GO:0044260;GO:0043043;GO:0071704;GO:0043604;GO:0009987;GO:0009058;GO:0009059;GO:0008150;GO:0008152;GO:0044238;GO:1901564;GO:0043603;GO:1901566;GO:0019538;GO:0044237;GO:0043170;GO:0006518;GO:0006412;GO:0015934;GO:0044391;GO:0032991;GO:1990904;GO:0005575;GO:0003674;GO:0005198;GO:0003735	K02876	RP-L15, MRPI	map03010	Ribosome	COG0200	U:Intracellular trafficking, secre	PF00828.22	Ribosomal_L2	Ribosomal protein:	CYT	2	7	33.9	High
TRINITY_DN1215_c0_g1_i2_orf1	PI-stichotoxin-She2a-like [Ostrinia furnacalis]	2.142007775	1.098963717	0.0002509	up	yes	0.9367	0.4373	0.427	0.44	0.445	1	0.948	0.862	BP:envenomation resulting in modulation of voltage-gated potassium channel activity in other organism;BP:interspecies interaction between organisms;BP:biological_process;BP:envenomation resulting in modulation of ion channel activity in other organism;BP:envenomation resulting in negative regulation of voltage-gated potassium channel activity in other organism;BP:envenomation resulting in modification of morphology or physiology of other organism;BP:modification of morphology or physiology of other organism;MF:enzyme regulator activity;MF:peptidase regulator activity;MF:peptidase inhibitor activity;MF:endopeptidase regulator activity;MF:serine-type endopeptidase inhibitor activity;MF:endopeptidase inhibitor activity;MF:molecular_function;MF:enzyme inhibitor activity;MF:molecular function regulator;	GO:0044559;GO:0044419;GO:0008150;GO:0044560;GO:0044562;GO:0035738;GO:0035821;GO:0030234;GO:0061134;GO:0030414;GO:0061135;GO:0004867;GO:0004866;GO:0003674;GO:0004857;GO:0098772	K03909	TFPI	map04610	Complement anc	ENOG410XQNP	O:Posttranslational modificatio	PF00014.26	Kunitz_BPTI	Kunitz/Bovine pan	CYT	2	31	8.6	High
TRINITY_DN9647_c0_g1_i1_orf1	cytochrome P450 6B2-like [Ostrinia furnacalis]	2.42997729	1.280942831	2.84E-05	up	yes	0.963	0.3963	0.407	0.371	0.411	1	0.92	0.969	MF:cation binding;MF:monooxygenase activity;MF:heme binding;MF:transition metal ion binding;MF:tetrapyrrole binding;MF:organic cyclic compound binding;MF:oxidoreductase activity, acting on paired donors, with incorporation or reduction of molecular oxygen;MF:ion binding;MF:catalytic activity;MF:molecular_function;MF:binding;MF:iron ion binding;MF:metal ion binding;MF:heterocyclic compound	GO:0043169;GO:0004497;GO:0020037;GO:0046914;GO:0046906;GO:0097159;GO:0016705;GO:0043167;GO:0003824;GO:0003674;GO:0005488;GO:0005506;GO:0046872;GO:1901363;GO:0016491	K14999	CYP6	------	------	COG2124	Q:Secondary metabolites biosy	PF00067.25	p450	Cytochrome P450	CYT	5	11	59.3	High
TRINITY_DN9119_c0_g1_i3_orf1	actin-binding Rho-activating protein [Helicoverpa armigera] >XP_047029227.1 actin-binding Rho-activating protein-like [Helicoverpa zea] >PZC85229.1 hypothetical protein B5X24_HaOG202414 [Helicoverpa armigera]	2.274656378	1.18564862	0.0002863	up	yes	0.9433	0.4147	0.404	0.455	0.385	1	0.962	0.868	BP:regulation of Rho protein signal transduction;BP:regulation of response to stimulus;BP:positive regulation of Ras protein signal transduction;BP:positive regulation of signaling;BP:positive regulation of Rho protein signal transduction;BP:regulation of signaling;BP:positive regulation of cell communication;BP:regulation of cell communication;BP:regulation of biological process;BP:regulation of signal transduction;BP:positive regulation of signal transduction;BP:biological regulation;BP:positive regulation of biological process;BP:positive regulation of small GTPase mediated signal transduction;BP:regulation of small GTPase mediated signal transduction;BP:positive regulation of response to stimulus;BP:regulation of Ras protein signal transduction;BP:regulation of cellular process;BP:biological_process;BP:positive regulation of intracellular signal transduction;BP:regulation of intracellular signal transduction;BP:positive regulation of cellular	GO:0035023;GO:0048583;GO:0046579;GO:0023056;GO:0035025;GO:0023051;GO:0010647;GO:0010646;GO:0050789;GO:0009966;GO:0009967;GO:0065007;GO:0048518;GO:0051057;GO:0051056;GO:0048584;GO:0046578;GO:0050794;GO:0008150;GO:1902533;GO:1902531;GO:0048522;GO:0003674;GO:0005488;GO:0003779;GO:0008092;GO:0005515	------	------	------	------	ENOG410XR7V	S:Function unknown	PF14705.9	Costars	Costars	CYT	1	4	21.7	High
TRINITY_DN11347_c0_g1_i1_orf1	N(4)-(Beta-N-acetylglucosaminyl)-L-asparaginase-like [Ostrinia furnacalis]	3.131498471	1.646853175	2.14E-05	up	yes	1.024	0.327	0.357	0.316	0.308	1	1.077	0.996	MF:hydrolase activity;MF:molecular_function;MF:catalytic	GO:0016787;GO:0003674;GO:0003824	K01444	AGA, aspG	map00511;ma	Other glycan deg	COG1446	E:Amino acid transport and me	PF01112.21	Asparaginase	Asparaginase	CYT	4	13	37	High
TRINITY_DN42461_c0_g1_i4_orf1	obscurin [Ostrinia furnacalis]	3.35163061	1.744863156	2.44E-05	up	yes	1.038	0.3097	0.276	0.336	0.317	1	1.023	1.092	BP:phosphorylation;BP:cell differentiation;BP:nitrogen compound metabolic process;BP:anatomical structure morphogenesis;BP:cellular developmental process;BP:organic substance metabolic process;BP:developmental process;BP:protein phosphorylation;BP:cellular process;BP:macromolecule modification;BP:protein modification process;BP:biological_process;BP:metabolic process;BP:primary metabolic process;BP:organonitrogen compound metabolic process;BP:protein metabolic process;BP:cellular metabolic process;BP:macromolecule metabolic process;BP:phosphate-containing compound metabolic process;BP:phosphorus metabolic process;MF:purine ribonucleoside triphosphate binding;MF:guanyl-nucleotide exchange factor activity;MF:molecular_function;MF:binding;MF:molecular function regulator;MF:heterocyclic compound binding;MF:nucleoside phosphate binding;MF:anion binding;MF:ion binding;MF:nucleotide binding;MF:transferase activity;MF:protein kinase activity;MF:purine nucleotide binding;MF:carbohydrate derivative binding;MF:ATP binding;MF:kinase activity;MF:enzyme regulator activity;MF:catalytic activity, acting on a protein;MF:small molecule binding;MF:GTPase regulator activity;MF:nucleoside-triphosphatase regulator activity;MF:catalytic activity;MF:transferase activity, transferring phosphorus-containing groups;MF:adenyl nucleotide binding;MF:phosphotransferase activity, alcohol group as acceptor;MF:adenyl ribonucleotide binding;MF:purine	GO:0016310;GO:0030154;GO:0006807;GO:0009653;GO:0048869;GO:0071704;GO:0032502;GO:0006468;GO:0009987;GO:0043412;GO:0036211;GO:0008150;GO:0008152;GO:0044238;GO:1901564;GO:0019538;GO:0044237;GO:0043170;GO:0006796;GO:0006793;GO:0035639;GO:0005085;GO:0003674;GO:0005488;GO:0098772;GO:1901363;GO:1901265;GO:0043168;GO:0043167;GO:0000166;GO:0016740;GO:0004672;GO:0017076;GO:0097367;GO:0005524;GO:0016301;GO:0030234;GO:0140096;GO:0036094;GO:0030695;GO:0060589;GO:0003824;GO:0016772;GO:0030554;GO:0016773;GO:0032559;GO:0032555;GO:0097159;GO:0032553	------	------	------	------	ENOG410XQFD;ENOG4111QEW;ENOG410XSWY	S:Function unknown;	PF07679.19;PF1	I-set;Ig_3	Immunoglobulin I-	CYT	1	37	11.1	High
TRINITY_DN52296_c0_g1_i6_orf1	protein takeout-like [Ostrinia furnacalis]	2.350872818	1.233196491	0.0001612	up	yes	0.9427	0.401	0.437	0.376	0.39	1	0.948	0.88	------	------	------	------	------	------	ENOG4110RSP;ENOG4111E83;ENOG410ZNC7;ENOG4110QW6;ENOG41119GI;ENOG4110RJ7;ENOG410ZN7P;ENOG4110SYW;ENOG411	S:Function unknown;	PF06585.14	JHBP	Haemolymph juve	CYT	1	3	19.5	High
TRINITY_DN4514_c0_g1_i1_orf1	enoyl-CoA delta isomerase 1, mitochondrial-like isoform X1 [Ostrinia furnacalis] >XP_028158560.1 enoyl-CoA delta isomerase 1, mitochondrial-like isoform X2 [Ostrinia	2.762559641	1.466005611	1.71E-05	up	yes	0.9843	0.3563	0.345	0.36	0.364	1	0.935	1.018	------	------	K13238	ECI1, DCI	map00071	Fatty acid degrac	COG1024	I:Lipid transport and metabolis	PF00378.23;PF1	ECH_1;ECH_2	Enoyl-CoA hydrata	CYT	1	6	31	High
TRINITY_DN28592_c0_g1_i2_orf1	UDP-glucuronosyltransferase 2B14-like isoform X1 [Ostrinia furnacalis] >XP_028167291.1 UDP-glucuronosyltransferase 2B14-like isoform X2 [Ostrinia furnacalis]	2.655351766	1.408902994	0.0001163	up	yes	0.97	0.3653	0.349	0.36	0.387	1	1.017	0.893	CC:integral component of membrane;CC:cellular_component;CC:cellular anatomical entity;CC:intrinsic component of membrane;MF:catalytic activity;MF:transferase activity;MF:molecular_function;MF:transferase activity, transferring glycosyl groups;MF:transferase activity, transferring hexosyl groups;MF:glucuronosyltransferase activity;MF:UDP-	GO:0016021;GO:0005575;GO:0110165;GO:0031224;GO:0003824;GO:0016740;GO:0003674;GO:0016757;GO:0016758;GO:0015020;GO:0008194	K00699	UGT	map05207;ma	Chemical carcinc	COG1819	S:Function unknown	PF00201.21	UDPGT	UDP-glucoronosyl	CYT	1	4	58.6	High
TRINITY_DN1173_c1_g1_i9_orf1	hypothetical protein evm_001011 [Chilo suppressalis]	2.575268817	1.364723035	0.0009886	up	yes	0.958	0.372	0.5	0.319	0.297	1	0.946	0.928	BP:phosphorylation;BP:cell differentiation;BP:nitrogen compound metabolic process;BP:anatomical structure morphogenesis;BP:cellular developmental process;BP:organic substance metabolic process;BP:developmental process;BP:protein phosphorylation;BP:cellular process;BP:macromolecule modification;BP:protein modification process;BP:biological_process;BP:metabolic process;BP:primary metabolic process;BP:organonitrogen compound metabolic process;BP:protein metabolic process;BP:cellular metabolic process;BP:macromolecule metabolic process;BP:phosphate-containing compound metabolic process;BP:phosphorus metabolic process;MF:purine ribonucleoside triphosphate binding;MF:guanyl-nucleotide exchange factor activity;MF:molecular_function;MF:binding;MF:molecular function regulator;MF:heterocyclic compound binding;MF:nucleoside phosphate binding;MF:anion binding;MF:ion binding;MF:nucleotide binding;MF:transferase activity;MF:protein kinase activity;MF:purine nucleotide binding;MF:carbohydrate derivative binding;MF:ATP binding;MF:kinase activity;MF:enzyme regulator activity;MF:catalytic activity, acting on a protein;MF:small molecule binding;MF:GTPase regulator activity;MF:nucleoside-triphosphatase regulator activity;MF:catalytic activity;MF:transferase activity, transferring phosphorus-containing groups;MF:adenyl nucleotide binding;MF:phosphotransferase activity, alcohol group as acceptor;MF:adenyl ribonucleotide binding;MF:purine	GO:0016310;GO:0030154;GO:0006807;GO:0009653;GO:0048869;GO:0071704;GO:0032502;GO:0006468;GO:0009987;GO:0043412;GO:0036211;GO:0008150;GO:0008152;GO:0044238;GO:1901564;GO:0019538;GO:0044237;GO:0043170;GO:0006796;GO:0006793;GO:0035639;GO:0005085;GO:0003674;GO:0005488;GO:0098772;GO:1901363;GO:1901265;GO:0043168;GO:0043167;GO:0000166;GO:0016740;GO:0004672;GO:0017076;GO:0097367;GO:0005524;GO:0016301;GO:0030234;GO:0140096;GO:0036094;GO:0030695;GO:0060589;GO:0003824;GO:0016772;GO:0030554;GO:0016773;GO:0032559;GO:0032555;GO:0097159;GO:0032553	------	------	------	------	ENOG410XQFD;ENOG410XSWY	S:Function unknown;T:Signal tr	PF07679.19;PF1	I-set;Ig_3;ig;Iç	Immunoglobulin I-	CYT	1	18	160.4	High
TRINITY_DN48237_c0_g1_i5_orf1	myogenesis-regulating glycosidase-like [Ostrinia furnacalis]	3.479442746	1.798856268	7.26E-05	up	yes	1.024	0.2943	0.304	0.289	0.29	1	1.108	0.965	BP:primary metabolic process;BP:carbohydrate metabolic process;BP:organic substance metabolic process;BP:biological_process;BP:metabolic process;CC:integral component of membrane;CC:cellular_component;CC:intrinsic component of membrane;CC:cellular anatomical entity;MF:hydrolase activity;MF:hydrolase activity, acting on glycosyl bonds;MF:catalytic activity;MF:hydrolase activity, hydrolyzing O-glycosyl compounds;MF:molecular_function;	GO:0044238;GO:0005975;GO:0071704;GO:0008150;GO:0008152;GO:0016021;GO:0005575;GO:0031224;GO:0110165;GO:0016787;GO:0016798;GO:0003824;GO:0004553;GO:0003674	K24727	MYORG	------	------	COG1501	G:Carbohydrate transport and	PF01055.29	Glyco_hydro_	Glycosyl hydrolase	CYT	1	1	76.4	High

TRINITY_DN13651_c0_g1_i2_orf1	40S ribosomal protein S12, mitochondrial [Ostrinia furnacalis]	3.129011757	1.64570708	9.59E-06	up	yes	0.9847	0.3147	0.273	0.338	0.333	1	0.992	0.962	BP:cellular biosynthetic process;BP:cellular nitrogen compound metabolic process;BP:nitrogen compound metabolic process;BP:cellular macromolecule biosynthetic process;BP:organic substance biosynthetic process;BP:cellular macromolecule metabolic process;BP:peptide biosynthetic process;BP:organic substance metabolic process;BP:amide biosynthetic process;BP:cellular process;BP:biosynthetic process;BP:macromolecule biosynthetic process;BP:biological_process;BP:metabolic process;BP:cellular nitrogen compound biosynthetic process;BP:primary metabolic process;BP:organonitrogen compound metabolic process;BP:cellular amide metabolic process;BP:organonitrogen compound biosynthetic process;BP:protein metabolic process;BP:cellular metabolic process;BP:macromolecule metabolic process;BP:peptide metabolic process;BP:translation;CC:small ribosomal subunit;CC:macromolecular complex;CC:ribosomal subunit;CC:ribonucleoprotein	GO:0044249;GO:0034641;GO:0006807; GO:0034645;GO:1901576;GO:0044260; GO:0043043;GO:0071704;GO:0043604; GO:0009987;GO:0009058;GO:0009059; GO:0008150;GO:0008152;GO:0044271; GO:0044238;GO:1901564;GO:0043603; GO:1901566;GO:0019538;GO:0044237; GO:0043170;GO:0006518;GO:0006412; GO:0015935;GO:0032991;GO:0044391; GO:1990904;GO:0005575;GO:0003674; GO:0005198;GO:0003735	K02950	RP-S12, MRP	map03010	Ribosome	COG0048	S:Function unknown	PF00164.28	Ribosom_S12_	Ribosomal protein	CYT	1	6	17.4	High
TRINITY_DN130075_c1_g2_i1_orf1	60S ribosomal protein L23 [Microtus ochrogaster]	2.487186404	1.314514636	0.000826	up	yes	0.922	0.3707	0.36	0.303	0.449	1	0.849	0.917	BP:regulation of primary metabolic process;BP:regulation of metabolic process;BP:positive regulation of response to stimulus;BP:regulation of response to stimulus;BP:cellular component organization or biogenesis;BP:cellular response to stimulus;BP:negative regulation of macromolecule metabolic process;BP:positive regulation of macromolecule metabolic process;BP:regulation of signal transduction;BP:positive regulation of signal transduction;BP:peptide biosynthetic process;BP:positive regulation of signal transduction by p53 class mediator;BP:positive regulation of biological process;BP:negative regulation of biological process;BP:regulation of cell proliferation;BP:regulation of macromolecule metabolic process;BP:cellular response to actinomycin D;BP:response to actinomycin D;BP:regulation of proteolysis;BP:regulation of RNA biosynthetic process;BP:response to organic substance;BP:organonitrogen compound metabolic process;BP:organonitrogen compound biosynthetic process;BP:protein metabolic process;BP:response to organonitrogen compound;BP:regulation of biological process;BP:regulation of catabolic process;BP:negative regulation of catabolic process;BP:negative regulation of metabolic process;BP:positive regulation of metabolic process;BP:negative regulation of biosynthetic process;BP:protein stabilization;BP:organic substance biosynthetic process;BP:cellular macromolecule metabolic process;BP:negative regulation of transferase activity;BP:cellular component organization;BP:cellular response to chemical stimulus;BP:biological regulation;BP:regulation of molecular function;BP:regulation of biological quality;BP:response to	GO:0080090;GO:0019222;GO:0048584; GO:0048583;GO:0071840;GO:0051716; GO:0010605;GO:0010604;GO:0009966; GO:0009967;GO:0043043;GO:1901798; GO:0048518;GO:0048519;GO:0042127; GO:0060255;GO:0072717;GO:0072716; GO:0030162;GO:2001141;GO:0010033; GO:1901564;GO:1901566;GO:0019538; GO:0010243;GO:0050789;GO:0009894; GO:0009895;GO:0009892;GO:0009893; GO:0009890;GO:0050821;GO:1901576; GO:0044260;GO:0051348;GO:0016043; GO:0070887;GO:0065007;GO:0065009; GO:0065008;GO:0009719;GO:0050790; GO:0009889;GO:0050794;GO:0008150; GO:0008152;GO:1902533;GO:1902531; GO:0051444;GO:0044271;GO:0031400; GO:0031647;GO:0051338;GO:0006355; GO:0006357;GO:0006518;GO:0010558; GO:0010564;GO:0044092;GO:0023056; GO:0044249;GO:0034641;GO:0046677; GO:0034645;GO:0023051;GO:1903450; GO:0010647;GO:0010646;GO:0043086; GO:0051248;GO:0000122;GO:1901698; GO:0051246;GO:1903320;GO:0043933; GO:0031399;GO:0002181;GO:0031397; GO:0008284;GO:1901701;GO:0031330; GO:1903050;GO:1903051;GO:1903506; GO:0031396;GO:0045892;GO:1901796;	K02894	RP-L23e, RPL	map03010;ma	Ribosome;Coron	COG0093	J:Translation, ribosomal structu	PF00238.22	Ribosomal_L1	Ribosomal protein	CYT	1	43	14.9	High
TRINITY_DN3929_c0_g3_i3_orf1	Glutathione S-transferase 1, isoform D [Papilio machaon]	3.705860939	1.889808746	1.80E-06	up	yes	1.018	0.2747	0.289	0.246	0.289	1	1.034	1.02	MF:catalytic activity;MF:transferase activity;MF:glutathione transferase activity;MF:molecular_function;MF:transferase activity, transferring alkyl or ary	GO:0003824;GO:0016740;GO:0004364; GO:0003674;GO:0016765	K00799	GST, gst	map05207;ma	Chemical carcin	COG0625	O:Posttranslational modificatio	PF13417.9;PF02	GST_N_3;GST_	Glutathione S-tran	CYT	1	23	12.3	High
TRINITY_DN3476_c0_g1_i5_orf1	maltase A1-like [Ostrinia furnacalis]	4.063854947	2.022848908	1.14E-06	up	yes	1.031	0.2537	0.254	0.249	0.258	1	1.053	1.041	BP:primary metabolic process;BP:carbohydrate metabolic process;BP:organic substance metabolic process;BP:biological_process;BP:metabolic process;MF:hydrolase activity;MF:catalytic activity;MF:hydrolase activity, acting on glycosyl bonds;MF:molecular_function;	GO:0044238;GO:0005975;GO:0071704; GO:0008150;GO:0008152;GO:0016787; GO:0003824;GO:0016798;GO:0003674	K01187	malZ	map00500;ma	Starch and sucro	COG0366	E:Amino acid transport and me	PF00128.27	Alpha-amylas	Alpha amylase, cat	CYT	8	22	66.3	High
TRINITY_DN2668_c0_g1_i7_orf1	unnamed protein product [Chrysodeixis includens]	4.162950953	2.057606561	1.63E-06	up	yes	1.027	0.2467	0.243	0.258	0.239	1	1.023	1.058	BP:organic substance metabolic process;BP:lipid metabolic process;BP:primary metabolic process;BP:metabolic process;BP:biological_process;CC:cellular_component;CC:extracellular region;CC:cellular anatomical entity;MF:hydrolase activity;MF:hydrolase activity, acting on ester bonds;MF:catalytic activity;MF:molecular_function;MF:lipase activity;	GO:0071704;GO:0006629;GO:0044238; GO:0008152;GO:0008150;GO:0005575; GO:0005576;GO:0110165;GO:0016787; GO:0016788;GO:0003824;GO:0003674; GO:0016298	------	------	------	------	ENOG410YGS3;ENOG4110T03	S:Function unknown;	PF00151.22	Lipase	Lipase	CYT	6	26	38.5	High
TRINITY_DN2394_c0_g1_i4_orf1	uncharacterized protein LOC114363116 [Ostrinia furnacalis]	10.52924791	3.396330485	0.0001328	up	yes	1.134	0.1077	0.113	0.101	0.109	1	1.241	1.161	MF:molecular_function;MF:lipid binding;MF:binding;	GO:0003674;GO:0008289;GO:0005488	K08752	FABP3	map03320	PPAR signaling p	ENOG4111US8	I:Lipid transport and metabolis	PF14651.9	Lipocalin_7	Lipocalin / cytosoli	CYT	6	33	18.8	High
TRINITY_DN69049_c0_g1_i2_orf1	membrane alanyl aminopeptidase-like [Ostrinia furnacalis]	4.635813953	2.212822669	2.86E-06	up	yes	0.9967	0.215	0.224	0.2	0.221	1	1.028	0.962	CC:integral component of membrane;CC:cellular_component;CC:cellular anatomical entity;CC:intrinsic component of membrane;MF:exopeptidase activity;MF:aminopeptidase activity;MF:transition metal ion binding;MF:hydrolase activity;MF:catalytic activity, acting on a protein;MF:cation binding;MF:ion binding;MF:catalytic activity;MF:metallopeptidase activity;MF:molecular_function;MF:binding;MF:peptidase activity;MF:metal ion binding;MF:zinc ion binding;	GO:0016021;GO:0005575;GO:0110165; GO:0031224;GO:0008238;GO:0004177; GO:0046914;GO:0016787;GO:0140096; GO:0043169;GO:0043167;GO:0003824; GO:0008237;GO:0003674;GO:0005488; GO:0008233;GO:0046872;GO:0008270	K11140	ANPEP, CD13	map04640;ma	Hematopoietic c	COG0308	E:Amino acid transport and me	PF11838.11	ERAP1_C	ERAP1-like C-term	CYT	1	2	51.3	High
TRINITY_DN2343_c1_g1_i8_orf1	receptor expression-enhancing protein 5-like isoform X3 [Ostrinia furnacalis]	6.064705882	2.600437681	1.32E-05	up	yes	1.031	0.17	0.169	0.155	0.186	1	1.095	0.998	CC:cellular_component;CC:integral component of membrane;CC:cellular anatomical entity;CC:intrinsic component of membrane;	GO:0005575;GO:0016021;GO:0110165; GO:0031224	K17279	REEP5_6	------	------	COG5052	U:Intracellular trafficking, secre	PF03134.22	TB2_DP1_HV	TB2/DP1, HVA22 fa	CYT	1	21	22.4	High
TRINITY_DN26408_c0_g1_i7_orf1	venom carboxylesterase-6-like [Ostrinia furnacalis]	5.028541775	2.330140094	2.29E-05	up	yes	0.969	0.1927	0.234	0.184	0.16	1	0.916	0.991	CC:integral component of membrane;CC:cellular_component;CC:intrinsic component of membrane;CC:cellular anatomical entity;MF:hydrolase activity;MF:hydrolase activity, acting on ester bonds;MF:carboxylic ester hydrolase activity;MF:catalytic	GO:0016021;GO:0005575;GO:0031224; GO:0110165;GO:0016787;GO:0016788; GO:0052689;GO:0003824;GO:0003674	------	------	------	------	COG2272;ENOG410XSWS;COG0580	I:Lipid transport and metabolis	PF00135.31;PF2	COesterase;B	Carboxylesterase fa	PLA	1	2	64.2	High
TRINITY_DN64403_c0_g2_i1_orf1	carboxylesterase [Ostrinia furnacalis]	6.149968886	2.620579112	4.36E-06	up	yes	0.9883	0.1607	0.184	0.115	0.183	1	0.973	0.992	MF:hydrolase activity;MF:molecular_function;MF:hydrolase activity, acting on ester bonds;MF:carboxylic ester hydrolase activity;MF:catalytic activity;	GO:0016787;GO:0003674;GO:0016788; GO:0052689;GO:0003824	K03927	CES2	map00983	Drug metabolism	COG2272	K:Transcription	PF00135.31	COesterase	Carboxylesterase fa	CYT	1	14	10.6	High
TRINITY_DN69713_c0_g1_i1_orf1	membrane-bound alkaline phosphatase-like [Ostrinia furnacalis]	5.996945632	2.584227894	8.75E-07	up	yes	0.9817	0.1637	0.183	0.149	0.159	1	0.987	0.958	MF:hydrolase activity;MF:phosphatase activity;MF:alkaline phosphatase activity;MF:hydrolase activity, acting on ester bonds;MF:phosphoric ester hydrolase activity;MF:catalytic activity;MF:molecular_function;	GO:0016787;GO:0016791;GO:0004035; GO:0016788;GO:0042578;GO:0003824; GO:0003674	K01077	E3.1.3.1, pho	map00730;ma	Thiamine metab	COG1785	P:Inorganic ion transport and m	PF00245.23	Alk_phosphat	Alkaline phosphata	CYT	3	7	57.6	High
TRINITY_DN29018_c0_g1_i4_orf1	prostaglandin reductase 1-like isoform X1 [Ostrinia furnacalis] >XP_028178925.1 prostaglandin reductase 1-like isoform X2 [Ostrinia furnacalis]	6.458940397	2.691297507	6.61E-07	up	yes	0.9753	0.151	0.146	0.151	0.156	1	0.977	0.949	CC:cytoplasm;CC:cellular_component;CC:cellular anatomical entity;MF:13-prostaglandin reductase activity;MF:oxidoreductase activity, acting on CH-OH group of donors;MF:oxidoreductase activity, acting on the CH-CH group of donors, NAD or NADP as acceptor;MF:oxidoreductase activity, acting on the CH-OH group of donors, NAD or NADP as acceptor;MF:oxidoreductase activity, acting on the CH-CH group of donors;MF:catalytic activity;MF:molecular_function;MF:15-oxoprostaglandin 13-	GO:0005737;GO:0005575;GO:0110165; GO:0036132;GO:0016614;GO:0016628; GO:0016616;GO:0016627;GO:0003824; GO:0003674;GO:0047522;GO:0032440; GO:0016491	K13948	PTGR1, LTB4D	map00590	Arachidonic acid	COG2130	S:Function unknown	PF16884.8;PF00	ADH_N_2;ADH	N-terminal domai	CYT	7	24	36.5	High
TRINITY_DN336_c0_g1_i6_orfp1	TRINITY_DN336_c0_g1_i6_m.64791 TRINITY_DN336_c0_g1::TRINITY_DN336_c0_g1_i6::g.64791 ORF type:complete len:61 (-),score=19.53 TRINITY_DN336_c0_g1_i6:236-418(-)	18.70053378	4.225007545	8.25E-08	up	yes	1.016	0.05433	0.045	0.061	0.057	1	1.017	1.032	------	------	------	------	------	------	------	------	------	------	------	CYT	1	12	6.3	High
TRINITY_DN19917_c0_g1_i1_orf1	synaptic vesicle glycoprotein 2B-like isoform X4 [Ostrinia furnacalis]	12.67784619	3.664237766	6.99E-07	up	yes	0.9677	0.07633	0.082	0.071	0.076	1	0.951	0.952	CC:integral component of membrane;CC:cellular_component;CC:cellular anatomical entity;CC:intrinsic component of membrane;MF:transporter activity;MF:transmembrane transporter activity;MF:molecular_function;	GO:0016021;GO:0005575;GO:0110165; GO:0031224;GO:0005215;GO:0022857; GO:0003674	K06258	SV2	map04512	ECM-receptor in	ENOG410ZYFU;ENOG410XNVV;COG0477	G:Carbohydrate transport and	PF07690.19;PF0	MFS_1;Sugar_	Major Facilitator S	CYT	1	2	57.3	High
TRINITY_DN3598_c0_g1_i1_orf1	esterase FE4-like [Ostrinia furnacalis]	2.65171504	1.406925748	6.14E-08	up	yes	1.005	0.379	0.379	0.369	0.389	1	1.007	1.008	MF:hydrolase activity;MF:molecular_function;MF:hydrolase activity, acting on ester bonds;MF:carboxylic ester hydrolase activity;MF:catalytic activity;	GO:0016787;GO:0003674;GO:0016788; GO:0052689;GO:0003824	------	------	------	------	COG2272	T:Signal transduction mechanis	PF00135.31;PF2	COesterase;B	Carboxylesterase fa	CYT	4	10	61.6	High
TRINITY_DN14967_c0_g2_i1_orf1	glyceraldehyde-3-phosphate dehydrogenase 2 [Holotrichia oblita]	2.927536232	1.549687026	5.62E-06	up	yes	1.01	0.345	0.355	0.347	0.333	1	0.982	1.048	BP:small molecule metabolic process;BP:organic cyclic compound metabolic process;BP:purine ribonucleoside triphosphate metabolic process;BP:ATP metabolic process;BP:ADP metabolic process;BP:monocarboxylic acid metabolic process;BP:oxoacid metabolic process;BP:purine ribonucleoside diphosphate metabolic process;BP:heterocycle metabolic process;BP:organonitrogen compound metabolic process;BP:carbohydrate catabolic process;BP:purine nucleotide metabolic process;BP:nucleoside diphosphate phosphorylation;BP:organophosphate metabolic process;BP:nucleoside triphosphate metabolic process;BP:purine nucleoside triphosphate metabolic process;BP:organic substance catabolic process;BP:ribonucleoside triphosphate metabolic process;BP:hexose metabolic process;BP:purine ribonucleotide metabolic process;BP:programmed cell death;BP:biological_process;BP:metabolic process;BP:nucleoside phosphate metabolic process;BP:ATP generation from ADP;BP:phosphorylation;BP:cellular nitrogen compound metabolic process;BP:ribonucleotide metabolic process;BP:nucleobase-containing compound metabolic process;BP:nucleotide phosphorylation;BP:cellular process;BP:cellular aromatic compound metabolic process;BP:nucleoside diphosphate metabolic process;BP:purine nucleoside diphosphate metabolic process;BP:nucleobase-containing small molecule metabolic process;BP:organic acid metabolic process;BP:monosaccharide metabolic process;BP:carbohydrate derivative metabolic process;BP:nitrogen compound metabolic process;BP:glucose	GO:0044281;GO:1901360;GO:0009205; GO:0046034;GO:0046031;GO:0032787; GO:0043436;GO:0009179;GO:0046483; GO:1901564;GO:0016052;GO:0006163; GO:0006165;GO:0019637;GO:0009141; GO:0009144;GO:1901575;GO:0009199; GO:0019318;GO:0009150;GO:0012501; GO:0008150;GO:0008152;GO:0006753; GO:0006757;GO:0016310;GO:0034641; GO:0009259;GO:0006139;GO:0046939; GO:0009987;GO:0006725;GO:0009132; GO:0009135;GO:0055086;GO:0006082; GO:0005996;GO:1901135;GO:0006807; GO:0006006;GO:0009185;GO:0019752; GO:0019693;GO:0072521;GO:0008219; GO:0006090;GO:0006096;GO:0071704; GO:0006915;GO:0009117;GO:0009056; GO:0006091;GO:0044238;GO:0005975; GO:0044237;GO:0006796;GO:0006793; GO:0043231;GO:0043232;GO:0005829; GO:0043229;GO:0043228;GO:0043227; GO:0043226;GO:0005856;GO:0110165; GO:0005634;GO:0005575;GO:0051287; GO:1901363;GO:0004365;GO:0043891; GO:0000166;GO:0016740;GO:0016744; GO:0046872;GO:0016209;GO:0004784; GO:0003674;GO:0005488;GO:1901265; GO:0043169;GO:0004743;GO:0003824; GO:0016773;GO:0097159;GO:0016620;	K05298	GAPA	------	------	COG0057	C:Energy production and conve	PF02800.23;PF0	Gp_dh_C;Gp_	Glyceraldehyde 3-	CYT	2	6	35.5	High
TRINITY_DN4030_c0_g2_i1_orf1	putative trypsin 6 [Ostrinia nubilalis]	3.014639976	1.591985718	1.59E-06	up	yes	1.009	0.3347	0.325	0.329	0.35	1	0.992	1.035	BP:organic substance metabolic process;BP:organonitrogen compound metabolic process;BP:protein metabolic process;BP:macromolecule metabolic process;BP:biological_process;BP:nitrogen compound metabolic process;BP:metabolic process;BP:proteolysis;BP:primary metabolic process;MF:serine-type endopeptidase activity;MF:endopeptidase activity;MF:hydrolase activity;MF:serine hydrolase activity;MF:catalytic activity, acting on a protein;MF:catalytic activity;MF:molecular_function;MF:peptidase activity;MF:serine-	GO:0071704;GO:1901564;GO:0019538; GO:0043170;GO:0008150;GO:0006807; GO:0008152;GO:0006508;GO:0044238; GO:0004252;GO:0004175;GO:0016787; GO:0017171;GO:0140096;GO:0003824; GO:0003674;GO:0008233;GO:0008236	K09628;K09	PRSS27;TMPR	------	------	COG5640	O:Posttranslational modificatio	PF00089.29	Trypsin	Trypsin	CYT	3	20	28.7	High
TRINITY_DN679_c0_g1_i2_orf1	cytochrome b-c1 complex subunit 7-like [Ostrinia furnacalis]	2.928845586	1.550332134	5.42E-06	up	yes	0.992	0.3387	0.326	0.363	0.327	1	1.015	0.961	BP:aerobic respiration;BP:generation of precursor metabolites and energy;BP:electron transport chain;BP:respiratory electron transport chain;BP:cellular process;BP:aerobic electron transport chain;BP:cellular respiration;BP:metabolic process;BP:energy derivation by oxidation of organic compounds;BP:mitochondrial electron transport, ubiquinol to cytochrome c;BP:cellular metabolic process;BP:biological_process;CC:oxidoreductase complex;CC:respiratory chain complex;CC:cytochrome complex;CC:inner mitochondrial membrane protein complex;CC:mitochondrial respiratory chain complex III;CC:transmembrane transporter complex;CC:catalytic complex;CC:transporter complex;CC:macromolecular complex;CC:respiratory chain complex III;CC:cellular_component;CC:membrane protein complex;CC:mitochondrial protein complex;	GO:0009060;GO:0006091;GO:0022900; GO:0022904;GO:0009987;GO:0019646; GO:0045333;GO:0008152;GO:0015980; GO:0006122;GO:0044237;GO:0008150; GO:1990204;GO:0098803;GO:0070069; GO:0098800;GO:0005750;GO:1902495; GO:1902494;GO:1990351;GO:0032991; GO:0045275;GO:0005575;GO:0098796; GO:0098798	K00417	QCR7, UQCR	map04714;ma	Thermogenesis;C	ENOG4111V1G	C:Energy production and conve	PF02271.19	UCR_14kD	Ubiquinol-cytochr	CYT	6	44	13.2	High
TRINITY_DN86844_c0_g2_i1_orf1	spermine oxidase-like isoform X1 [Ostrinia furnacalis]	2.806609195	1.488828191	8.40E-06	up	yes	0.9767	0.348	0.373	0.358	0.313	1	0.97	0.96	MF:catalytic activity;MF:molecular_function;MF:oxidoreductase activity;	GO:0003824;GO:0003674;GO:0016491	K12259	SMOX, PAO5	map00330;ma	Arginine and pro	ENOG410YT60;ENOG410XQW0;ENOG4110H7V;ENOG410XSNC	F:Nucleotide transport and me	PF01593.27	Amino_oxidas	Flavin containing a	CYT	1	5	46.8	High

TRINITY_DN46409_c0_g1_i1_orf1	unnamed protein product [Heterotrigona itama]	2.646057113	1.403844201	0.0003268	up	yes	1.01	0.3817	0.392	0.395	0.358	1	1.107	0.923	BP:regulation of T cell mediated immunity;BP:establishment of localization;BP:regulation of primary metabolic process;BP:regulation of metabolic process;BP:positive regulation of response to stimulus;BP:regulation of response to stimulus;BP:regulation of lymphocyte mediated immunity;BP:positive regulation of leukocyte mediated immunity;BP:nitrogen compound transport;BP:regulation of molecular function;BP:signal transduction;BP:T cell activation;BP:organic cyclic compound metabolic process;BP:positive regulation of lymphocyte mediated immunity;BP:leukocyte proliferation;BP:cellular response to stimulus;BP:response to unfolded protein;BP:positive regulation of cell adhesion;BP:cellular response to organic substance;BP:positive regulation of endopeptidase activity;BP:regulation of cytokine production;BP:interspecies interaction between organisms;BP:leukocyte activation involved in immune response;BP:isotype switching to IgG isotypes;BP:positive regulation of interferon-gamma production;BP:positive regulation of biological process;BP:positive regulation of interferon-alpha production;BP:macromolecule localization;BP:response to temperature stimulus;BP:mitochondrial unfolded protein response;BP:regulation of interleukin-12 production;BP:regulation of interleukin-10 production;BP:protein localization to mitochondrion;BP:regulation of macromolecule metabolic process;BP:negative regulation of cell death;BP:positive regulation of adaptive immune response based on somatic recombination of immune receptors built from immunoglobulin	GO:0002709;GO:0051234;GO:0080090;GO:0019222;GO:0048584;GO:0048583;GO:0002706;GO:0002705;GO:0071705;GO:0065009;GO:0007165;GO:0042110;GO:1901360;GO:0002708;GO:0070661;GO:0051716;GO:0006986;GO:0045785;GO:0071310;GO:0010950;GO:0001817;GO:0044419;GO:0002366;GO:0048291;GO:0032729;GO:0048518;GO:0032727;GO:0033036;GO:0009266;GO:0034514;GO:0032655;GO:0032653;GO:0070585;GO:0060255;GO:0060548;GO:0002824;GO:0071702;GO:0002822;GO:0008637;GO:0002821;GO:0045184;GO:0045041;GO:0050865;GO:0046649;GO:0030162;GO:0002819;GO:0072655;GO:0006839;GO:0051702;GO:0009628;GO:0010033;GO:0046483;GO:0046907;GO:0002833;GO:0010468;GO:0002376;GO:0019538;GO:0002204;GO:0033554;GO:0032735;GO:0002703;GO:0043030;GO:0016445;GO:0016444;GO:0016447;GO:0032647;GO:0022607;GO:0006457;GO:0009893;GO:0051131;GO:0002834;GO:0002837;GO:0006458;GO:0032649;GO:0050867;GO:0008104;GO:0050821;GO:0045321;GO:0006807;GO:0046651;GO:0051179;GO:0042981;GO:0043280;GO:0044260;GO:0002285;GO:0002684;GO:0006886;	K04077	groEL, HSPD1	map05152;ma	Tuberculosis;Typ	COG0459	G:Carbohydrate transport and	PF00118.27	Cpn60_TCP1	TCP-1/cpn60 chap	CYT	2	7	54.5	High
TRINITY_DN28577_c0_g1_i6_orf1	delta-1-pyrroline-5-carboxylate dehydrogenase, mitochondrial [Nymphalis io]	2.868444184	1.520268445	1.43E-06	up	yes	0.979	0.3413	0.332	0.334	0.358	1	0.961	0.976	BP:small molecule catabolic process;BP:carboxylic acid metabolic process;BP:cellular amino acid catabolic process;BP:nitrogen compound metabolic process;BP:small molecule metabolic process;BP:glutamine family amino acid catabolic process;BP:glutamine family amino acid metabolic process;BP:organic substance catabolic process;BP:proline catabolic process;BP:proline metabolic process;BP:cellular amino acid metabolic process;BP:organic substance metabolic process;BP:alpha-amino acid metabolic process;BP:alpha-amino acid catabolic process;BP:primary metabolic process;BP:cellular process;BP:proline catabolic process to glutamate;BP:metabolic process;BP:oxoacid metabolic process;BP:catabolic process;BP:cellular catabolic process;BP:dicarboxylic acid metabolic process;BP:organonitrogen compound metabolic process;BP:organonitrogen compound catabolic process;BP:organic acid metabolic process;BP:carboxylic acid catabolic process;BP:organic acid catabolic process;BP:cellular metabolic process;BP:glutamate metabolic process;BP:biological_process;MF:molecular_function;MF:oxidoreductase activity;MF:oxidoreductase activity, acting on the aldehyde or oxo group of donors, NAD or NADP as acceptor;MF:catalytic activity;MF:oxidoreductase activity, acting on the aldehyde or oxo group of donors;MF:1-pyrroline-5-	GO:0044282;GO:0019752;GO:0009063;GO:0006807;GO:0044281;GO:0009065;GO:0009064;GO:1901575;GO:0006562;GO:0006560;GO:0006520;GO:0071704;GO:1901605;GO:1901606;GO:0044238;GO:0009987;GO:0010133;GO:0008152;GO:0043436;GO:0009056;GO:0044248;GO:0043648;GO:1901564;GO:1901565;GO:0006082;GO:0046395;GO:0016054;GO:0044237;GO:0006536;GO:0008150;GO:0003674;GO:0016491;GO:0016620;GO:0003824;GO:0016903;GO:0003842	K00294	E1.2.1.88	map00250;ma	Alanine, aspartat	COG1012	S:Function unknown	PF00171.25	Aldedh	Aldehyde dehydro	CYT	3	34	11.3	High
TRINITY_DN52768_c0_g1_i1_orf1	carboxypeptidase Q-like isoform X2 [Ostrinia furnacalis]	3.741322616	1.903548376	2.26E-06	up	yes	1.024	0.2737	0.298	0.268	0.255	1	1.026	1.047	CC:lytic vacuole;CC:endoplasmic reticulum;CC:intracellular organelle;CC:organelle;CC:lysosome;CC:vacuole;CC:membrane-bounded organelle;CC:cellular anatomical entity;CC:Golgi apparatus;CC:intracellular membrane-bounded organelle;CC:cellular_component;CC:extracellular region;MF:hydrolase activity;MF:exopeptidase activity;MF:molecular_function;MF:peptidase activity;MF:metalloexopeptidase activity;MF:metallopeptidase activity;MF:dipeptidase activity;MF:metallodipeptidase activity;MF:catalytic activity, acting on a protein;MF:catalytic	GO:0000323;GO:0005783;GO:0043229;GO:0043226;GO:0005764;GO:0005773;GO:0043227;GO:0110165;GO:0005794;GO:0043231;GO:0005575;GO:0005576;GO:0016787;GO:0008238;GO:0003674;GO:0008233;GO:0008235;GO:0008237;GO:0016805;GO:0070573;GO:0140096;GO:0003824	K01302	CPQ	------	------	COG2234	S:Function unknown	PF04389.20;PF0	Peptidase_M2	Peptidase family M	CYT	1	2	52	High
TRINITY_DN47114_c0_g1_i5_orf1	nucleolar protein dao-5 isoform X2 [Ostrinia furnacalis]	3.670656511	1.876038118	1.41E-06	up	yes	1.012	0.2757	0.275	0.271	0.281	1	1.044	0.993	CC:intracellular non-membrane-bounded organelle;CC:nucleolus;CC:cellular_component;CC:intracellular organelle;CC:non-membrane-bounded organelle;CC:organelle;CC:cellular anatomical entity;	GO:0043232;GO:0005730;GO:0005575;GO:0043229;GO:0043228;GO:0043226;GO:0110165	K25818	NOLC1	------	------	ENOG4111U5P;ENOG4110S3V	S:Function unknown;	PF05022.15	SRP40_C	SRP40, C-terminal	CYT	7	10	72.7	High
TRINITY_DN7336_c0_g1_i13_orf1	PREDICTED: calcium-transporting ATPase sarcoplasmic/endoplasmic reticulum type isoform X2 [Amyelois transitella]	3.567606136	1.834956351	1.35E-11	up	yes	1	0.2803	0.281	0.279	0.281	1	1.001	0.999	CC:endoplasmic reticulum membrane;CC:intrinsic component of membrane;CC:organelle membrane;CC:integral component of membrane;CC:membrane;CC:sarcoplasmic reticulum membrane;CC:bounding membrane of organelle;CC:cellular_component;CC:cellular anatomical entity;MF:purine ribonucleoside triphosphate binding;MF:molecular_function;MF:binding;MF:ATPase activity;MF:nucleotide binding;MF:heterocyclic compound binding;MF:anion binding;MF:calcium ion transmembrane transporter activity;MF:ATPase activity, coupled to transmembrane movement of substances;MF:ion binding;MF:inorganic solute uptake transmembrane transporter activity;MF:ATP binding;MF:primary active transmembrane transporter activity;MF:nucleoside phosphate binding;MF:purine nucleotide binding;MF:metal ion transmembrane transporter activity;MF:active transmembrane transporter activity;MF:hydrolase activity;MF:transporter activity;MF:cation transmembrane transporter activity;MF:P-type transmembrane transporter activity;MF:nucleoside-triphosphatase activity;MF:small molecule binding;MF:catalytic activity;MF:ATPase activity, coupled to transmembrane movement of ions, phosphorylative mechanism;MF:inorganic cation transmembrane transporter activity;MF:hydrolase activity, acting on acid anhydrides, in phosphorus-containing anhydrides;MF:adenyl nucleotide binding;MF:carbohydrate derivative binding;MF:organic cyclic compound binding;MF:ion transmembrane transporter activity;MF:hydrolase activity, acting on acid anhydrides;MF:cation-transporting ATPase activity;MF:pyrophosphatase activity;MF:transmembrane	GO:0005789;GO:0031224;GO:0031090;GO:0016021;GO:0016020;GO:0033017;GO:0098588;GO:0005575;GO:0110165;GO:0035639;GO:0003674;GO:0005488;GO:0016887;GO:0000166;GO:1901363;GO:0043168;GO:0015085;GO:0042626;GO:0043167;GO:0015318;GO:0005524;GO:0015399;GO:1901265;GO:0017076;GO:0046873;GO:0022804;GO:0016787;GO:0005215;GO:0008324;GO:0140358;GO:0017111;GO:0036094;GO:0003824;GO:0015662;GO:0022890;GO:0016818;GO:0030554;GO:0097367;GO:0097159;GO:0015075;GO:0016817;GO:0019829;GO:0016462;GO:0022857;GO:0032559;GO:0022853;GO:0032555;GO:0005388;GO:0032553	K05853	ATP2A	map04972;ma	Pancreatic secret	COG0474	G:Carbohydrate transport and	PF00122.23;PF0	E1-E2_ATPas	E1-E2 ATPase;Cati	CYT	37	37	109.8	High
TRINITY_DN110460_c0_g2_i1_orf1	Similar to chaf1a-b: Chromatin assembly factor 1 subunit A-B (Xenopus laevis) [Cotesia congregata]	2.968063287	1.569521855	3.35E-06	up	yes	1.013	0.3413	0.323	0.335	0.366	1	1.039	0.999	CC:intracellular membrane-bounded organelle;CC:nucleus;CC:cellular_component;CC:intracellular organelle;CC:membrane-bounded organelle;CC:organelle;CC:cellular anatomical entity;	GO:0043231;GO:0005634;GO:0005575;GO:0043229;GO:0043227;GO:0043226;GO:0110165	K10750	CHAF1A	------	------	ENOG4111K9K	K:Transcription	PF15539.9	CAF1-p150_C	CAF1 complex sub	CYT	1	3	29.2	High
TRINITY_DN5012_c0_g1_i6_orf1	putative serine protease K12H4.7 [Ostrinia furnacalis]	2.362556113	1.240348595	0.000975	up	yes	0.8947	0.3787	0.36	0.411	0.365	1	0.881	0.803	BP:primary metabolic process;BP:organonitrogen compound metabolic process;BP:protein metabolic process;BP:macromolecule metabolic process;BP:organic substance metabolic process;BP:nitrogen compound metabolic process;BP:biological_process;BP:metabolic process;BP:proteolysis;CC:integral component of membrane;CC:cellular_component;CC:cellular anatomical entity;CC:intrinsic component of membrane;MF:molecular_function;MF:serine hydrolase activity;MF:hydrolase activity;MF:catalytic activity, acting on a protein;MF:catalytic activity;MF:peptidase activity;MF:serine-type peptidase activity;	GO:0044238;GO:1901564;GO:0019538;GO:0043170;GO:0071704;GO:0006807;GO:0008150;GO:0008152;GO:0006508;GO:0016021;GO:0005575;GO:0110165;GO:0031224;GO:0003674;GO:0017171;GO:0016787;GO:0140096;GO:0003824;GO:0008233;GO:0008236	K09649	PRSS16	------	------	ENOG410XSGG	O:Posttranslational modificatio	PF05577.15	Peptidase_S2	Serine carboxypep	CYT	1	3	55	High
TRINITY_DN2270_c0_g2_i1_orf1	integrin beta-nu [Ostrinia furnacalis]	2.913043478	1.542527234	1.81E-06	up	yes	0.9983	0.3427	0.314	0.357	0.357	1	0.988	1.007	BP:cell-cell adhesion;BP:integrin-mediated signaling pathway;BP:cellular process;BP:regulation of cellular process;BP:biological_process;BP:signal transduction;BP:cell surface receptor signaling pathway;BP:cell adhesion;BP:biological regulation;BP:heterophilic cell-cell adhesion via plasma membrane cell adhesion molecules;BP:regulation of biological process;BP:cell-cell adhesion via plasma-membrane adhesion molecules;CC:cellular anatomical entity;CC:integral component of membrane;CC:cellular_component;CC:intrinsic component of	GO:0098609;GO:0007229;GO:0009987;GO:0050794;GO:0008150;GO:0007165;GO:0007166;GO:0007155;GO:0065007;GO:0007157;GO:0050789;GO:0098742;GO:0110165;GO:0016021;GO:0005575;GO:0031224	------	------	------	------	ENOG410XP60	J:Translation, ribosomal structu	PF00362.21;PF1	Integrin_beta;	Integrin beta chair	CYT	2	3	89	High
TRINITY_DN1199_c0_g1_i1_orf1	pupal cuticle protein 36a-like [Ostrinia furnacalis]	4.717607973	2.238055538	2.22E-06	up	yes	0.994	0.2107	0.211	0.181	0.24	1	1.006	0.976	------	------	------	------	------	------	ENOG4110OGB	S:Function unknown	------	------	------	CYT	1	2	23.9	High
TRINITY_DN26254_c0_g1_i1_orf1	hypothetical protein evm_003664 [Chilo suppressalis] >CAB3521132.1 unnamed protein product [Chilo suppressalis] >CAH0398453.1 unnamed protein product [Chilo suppressalis]	3.14797361	1.654423446	3.80E-06	up	yes	1.002	0.3183	0.325	0.304	0.326	1	1.034	0.972	MF:molecular_function;MF:structural constituent of cuticle;MF:structural molecule activity;	GO:0003674;GO:0042302;GO:0005198	------	------	------	------	ENOG410ZNDD;ENOG4110RUZ;ENOG4111PJ1;ENOG4110T0M	S:Function unknown;	PF00379.26	Chitin_bind_4	Insect cuticle prote	CYT	3	36	14.7	High
TRINITY_DN195_c4_g1_i1_orf1	beta-1,3-glucan-binding protein 1 [Ostrinia furnacalis]	3.008675342	1.589128438	0.0003361	up	yes	0.9017	0.2997	0.335	0.292	0.272	1	0.845	0.86	BP:immune system process;BP:response to external biotic stimulus;BP:interspecies interaction between organisms;BP:organic substance metabolic process;BP:innate immune response;BP:defense response;BP:response to stress;BP:biological_process;BP:metabolic process;BP:immune response;BP:carbohydrate metabolic process;BP:response to other organism;BP:primary metabolic process;BP:response to biotic stimulus;BP:response to external stimulus;BP:response to stimulus;BP:defense response to other organism;CC:cellular anatomical entity;CC:cellular_component;CC:extracellular region;MF:carbohydrate binding;MF:molecular_function;MF:binding;MF:hydrolase activity;MF:hydrolase activity, acting on glycosyl bonds;MF:catalytic activity;MF:hydrolase activity, hydrolyzing	GO:0002376;GO:0043207;GO:0044419;GO:0071704;GO:0045087;GO:0006952;GO:0006950;GO:0008150;GO:0008152;GO:0006955;GO:0005975;GO:0051707;GO:0044238;GO:0009607;GO:0009605;GO:0050896;GO:0098542;GO:0110165;GO:0005575;GO:0005576;GO:0030246;GO:0003674;GO:0005488;GO:0016787;GO:0016798;GO:0003824;GO:0004553	K20692	GNBP3	map04624	Toll and Imd sigr	ENOG410Z83Q;ENOG4110NFS	S:Function unknown;	PF15886.8	CBM39	Carbohydrate bind	CYT	1	19	14.1	High
TRINITY_DN83295_c0_g1_i3_orf1	SSSX-APN4 [Ostrinia furnacalis]	2.678977273	1.421682342	0.0001063	up	yes	0.943	0.352	0.356	0.395	0.305	1	0.915	0.914	BP:nitrogen compound metabolic process;BP:organic substance metabolic process;BP:proteolysis;BP:biological_process;BP:metabolic process;BP:primary metabolic process;BP:organonitrogen compound metabolic process;BP:protein metabolic process;BP:macromolecule metabolic process;CC:integral component of membrane;CC:intrinsic component of membrane;CC:cellular anatomical entity;CC:cellular_component;MF:aminopeptidase activity;MF:exopeptidase activity;MF:molecular_function;MF:binding;MF:peptidase activity;MF:metallopeptidase activity;MF:cation binding;MF:transition metal ion binding;MF:ion binding;MF:metal ion binding;MF:hydrolase activity;MF:catalytic	GO:0006807;GO:0071704;GO:0006508;GO:0008150;GO:0008152;GO:0044238;GO:1901564;GO:0019538;GO:0043170;GO:0016021;GO:0031224;GO:0110165;GO:0005575;GO:0004177;GO:0008238;GO:0003674;GO:0005488;GO:0008233;GO:0008237;GO:0043169;GO:0046914;GO:0043167;GO:0046872;GO:0016787;GO:0140096;GO:0003824;GO:0008270	K11140	ANPEP, CD13	map04640;ma	Hematopoietic c	COG0308	E:Amino acid transport and me	PF01433.23	Peptidase_M1	Peptidase family M	CYT	1	3	35.8	High

TRINITY_DN8603_c0_g1_i1_orf1	adenosine kinase 2 isoform X2 [Cotesia glomerata] >XP_044591805.1 adenosine kinase 2 isoform X4 [Cotesia glomerata]	2.362736783	1.240458917	0.01106	up	yes	0.8357	0.3537	0.312	0.261	0.488	1	0.733	0.774	BP:purine ribonucleoside monophosphate metabolic process;BP:nucleotide biosynthetic process;BP:small molecule metabolic process;BP:ribonucleoside monophosphate metabolic process;BP:organic cyclic compound biosynthetic process;BP:purine nucleoside biosynthetic process;BP:purine ribonucleoside monophosphate biosynthetic process;BP:AMP salvage;BP:AMP metabolic process;BP:purine ribonucleoside biosynthetic process;BP:purine ribonucleoside metabolic process;BP:ribonucleoside biosynthetic process;BP:heterocycle metabolic process;BP:organonitrogen compound metabolic process;BP:organonitrogen compound biosynthetic process;BP:purine nucleotide metabolic process;BP:AMP biosynthetic process;BP:purine ribonucleoside salvage;BP:purine nucleotide biosynthetic process;BP:organophosphate metabolic process;BP:aromatic compound biosynthetic process;BP:purine nucleotide salvage;BP:nucleoside biosynthetic process;BP:small molecule biosynthetic process;BP:nitrogen compound metabolic process;BP:organic substance biosynthetic process;BP:purine nucleoside monophosphate biosynthetic process;BP:organic cyclic compound metabolic process;BP:heterocycle biosynthetic process;BP:ribonucleoside monophosphate biosynthetic process;BP:purine ribonucleotide biosynthetic process;BP:purine ribonucleotide metabolic process;BP:biological_process;BP:metabolic process;BP:nucleobase-containing compound biosynthetic process;BP:organophosphate biosynthetic process;BP:cellular metabolic compound salvage;BP:cellular nitrogen compound biosynthetic process;BP:ribonucleotide biosynthetic [illegible]	GO:0009167;GO:0009165;GO:0044281;GO:0009161;GO:1901362;GO:0042451;GO:0009168;GO:0044209;GO:0046033;GO:0046129;GO:0046128;GO:0042455;GO:0046483;GO:1901564;GO:1901566;GO:0006163;GO:0006167;GO:0006166;GO:0006164;GO:0019637;GO:0019438;GO:0032261;GO:0009163;GO:0044283;GO:0006807;GO:1901576;GO:0009127;GO:1901360;GO:0018130;GO:0009156;GO:0009152;GO:0009150;GO:0008150;GO:0008152;GO:0034654;GO:0090407;GO:0043094;GO:0044271;GO:0009260;GO:0046390;GO:1901293;GO:0006753;GO:0016310;GO:0044249;GO:0034641;GO:0009123;GO:0009126;GO:0009259;GO:0009124;GO:0006139;GO:0042278;GO:0009987;GO:0006725;GO:0055086;GO:1901137;GO:1901135;GO:0043174;GO:0043173;GO:0034404;GO:0044237;GO:0072521;GO:0072522;GO:0071704;GO:0043101;GO:0009058;GO:0009117;GO:0009116;GO:0009119;GO:0044238;GO:0019693;GO:1901657;GO:0006796;GO:0006793;GO:1901659;GO:0043231;GO:0043229;GO:0043227;GO:0043226;GO:0110165;GO:0005634;GO:0005575;GO:0000166;GO:0016740;GO:0097367;GO:0003674;GO:0005488;GO:1901265;[illegible]	K00856	ADK, adoK	map01232;ma[	Nucleotide metal	COG0524	G:Carbohydrate transport and [	PF00294.27	PfkB	pfkB family carboh	CYT	1	7	38.5	Medium
TRINITY_DN2894_c0_g3_i1_orf1	lactase-phlorizin hydrolase-like [Ostrinia furnacalis]	3.381984423	1.757870015	2.99E-07	up	yes	0.9987	0.2953	0.297	0.286	0.303	1	1.014	0.982	BP:primary metabolic process;BP:carbohydrate metabolic process;BP:organic substance metabolic process;BP:biological_process;BP:metabolic process;MF:hydrolase activity;MF:catalytic activity;MF:hydrolase activity, acting on glycosyl bonds;MF:hydrolase activity, hydrolyzing O-glycosyl compounds;MF:molecular_function;	GO:0044238;GO:0005975;GO:0071704;GO:0008150;GO:0008152;GO:0016787;GO:0003824;GO:0016798;GO:0004553;GO:0003674	------	------	------	------	COG2723	G:Carbohydrate transport and [	PF00232.21	Glyco_hydro_	Glycosyl hydrolase	CYT	10	28	57.5	High
TRINITY_DN18773_c0_g1_i3_orf1	keratin, type II cytoskeletal 68 kDa, component IB-like [Ostrinia furnacalis]	3.5859375	1.842350343	0.0002438	up	yes	0.918	0.256	0.235	0.247	0.286	1	0.824	0.93	------	------	------	------	------	------	------	------	------	------	------	CYT	3	47	13.9	High
TRINITY_DN32479_c0_g1_i8_orf1	hypothetical protein evm_009815 [Chilo suppressalis] >CAB3525305.1 unnamed protein product [Chilo suppressalis] >CAH0402632.1 unnamed protein product [Chilo suppressalis]	4.015503876	2.005581032	6.40E-05	up	yes	1.036	0.258	0.29	0.307	0.177	1	1.047	1.06	CC:integral component of membrane;CC:cellular_component;CC:cellular anatomical entity;CC:intrinsic component of membrane;MF:transporter activity;MF:transmembrane transporter activity;MF:molecular_function;	GO:0016021;GO:0005575;GO:0110165;GO:0031224;GO:0005215;GO:0022857;GO:0003674	------	------	------	------	COG0477	Q:Secondary metabolites biosy	PF07690.19;PF0(	MFS_1;Sugar_	Major Facilitator Su	CYT	1	2	56.4	High
TRINITY_DN1249_c0_g1_i10_orf1	venom carboxylesterase-6-like [Ostrinia furnacalis]	5.081992735	2.345394313	1.92E-06	up	yes	0.9793	0.1927	0.2	0.185	0.193	1	0.995	0.943	MF:hydrolase activity;MF:molecular_function;MF:hydrolase activity, acting on ester bonds;MF:carboxylic ester hydrolase activity;MF:catalytic activity;	GO:0016787;GO:0003674;GO:0016788;GO:0052689;GO:0003824	------	------	------	------	COG2272;ENOG410XSWS;COG0580	I:Lipid transport and metabolis	PF00135.31;PF2(	COesterase;B	Carboxylesterase fa	PLA	1	6	55.8	High
TRINITY_DN50787_c0_g2_i2_orf1	40S ribosomal protein S29 [Hyposmocoma kahamanoa] >XP_028176503.1 40S ribosomal protein S29 [Ostrinia furnacalis] >XP_049877832.1 40S ribosomal protein S29 [Pectinophora gossypiella] >ADT80654.1 ribosomal protein S29 [Euphydryas aurinia] >CAB3523209.1 unnamed protein product [Chilo suppressalis] >CAH0400531.1 unnamed protein product [Chilo suppressalis]	2.861791045	1.516918337	2.07E-05	up	yes	0.9587	0.335	0.318	0.346	0.341	1	0.964	0.912	BP:organonitrogen compound biosynthetic process;BP:cellular biosynthetic process;BP:cellular macromolecule biosynthetic process;BP:cellular nitrogen compound biosynthetic process;BP:peptide biosynthetic process;BP:organic substance metabolic process;BP:amide biosynthetic process;BP:cytoplasmic translation;BP:organic substance biosynthetic process;BP:cellular macromolecule metabolic process;BP:cellular process;BP:biosynthetic process;BP:macromolecule biosynthetic process;BP:metabolic process;BP:organonitrogen compound metabolic process;BP:primary metabolic process;BP:protein metabolic process;BP:cellular nitrogen compound metabolic process;BP:cellular metabolic process;BP:macromolecule metabolic process;BP:nitrogen compound metabolic process;BP:cellular amide metabolic process;BP:biological_process;BP:peptide metabolic process;BP:translation;CC:endoplasmic reticulum;CC:intracellular organelle;CC:non-membrane-bounded organelle;CC:membrane-bounded organelle;CC:organelle;CC:cytosolic small ribosomal subunit;CC:cellular anatomical entity;CC:rough endoplasmic reticulum;CC:ribosomal subunit;CC:small ribosomal subunit;CC:intracellular membrane-bounded organelle;CC:polysomal ribosome;CC:macromolecular complex;CC:ribosome;CC:intracellular non-membrane-bounded organelle;CC:cytosol;CC:ribonucleoprotein complex;CC:cellular_component;MF:heterocyclic compound binding;MF:molecular_function;MF:zinc ion binding;MF:nucleic acid binding;MF:ion binding;MF:cation binding;MF:transition [illegible]	GO:1901566;GO:0044249;GO:0034645;GO:0044271;GO:0043043;GO:0071704;GO:0043604;GO:0002181;GO:1901576;GO:0044260;GO:0009987;GO:0009058;GO:0009059;GO:0008152;GO:1901564;GO:0044238;GO:0019538;GO:0034641;GO:0044237;GO:0043170;GO:0006807;GO:0043603;GO:0008150;GO:0006518;GO:0006412;GO:0005783;GO:0043229;GO:0043228;GO:0043227;GO:0043226;GO:0022627;GO:0110165;GO:0005791;GO:0044391;GO:0015935;GO:0043231;GO:0042788;GO:0032991;GO:0005840;GO:0043232;GO:0005829;GO:1990904;GO:0005575;GO:1901363;GO:0003674;GO:0008270;GO:0003676;GO:0043167;GO:0043169;GO:0046914;GO:0005198;GO:0003735;GO:0046872;GO:0003723;GO:0097159;GO:0005488	K02980	RP-S29e, RPS	map03010;ma[	Ribosome;Coron	COG0199	J:Translation, ribosomal structu	PF00253.24	Ribosomal_S1	Ribosomal protein	CYT	2	38	6.6	High
TRINITY_DN104586_c0_g1_i1_orf1	Chlorophyll a-b binding protein 37, chloroplastic, partial [Trichinella patagoniensis]	3.845147679	1.943039008	0.0003232	up	yes	0.9113	0.237	0.312	0.188	0.211	1	0.863	0.871	CC:cellular_component;CC:membrane;CC:cellular anatomical [illegible]	GO:0005575;GO:0016020;GO:0110165 [illegible]	K08913	LHCB2	------	------	ENOG410ZHBU	L:Replication, recombination ar	------	------	------	CYT	1	15	10.4	High
TRINITY_DN43611_c0_g1_i1_orf1	39S ribosomal protein L41, mitochondrial [Ostrinia furnacalis]	3.0321875	1.600358968	5.31E-05	up	yes	0.9703	0.32	0.293	0.383	0.284	1	0.943	0.968	CC:cellular_component;CC:large ribosomal subunit;CC:organellar large ribosomal subunit;CC:ribonucleoprotein complex;CC:mitochondrial large ribosomal subunit;CC:macromolecular complex;CC:ribosomal subunit;CC:mitochondrial protein complex;MF:structural molecule activity;MF:structural constituent of ribosome;MF:molecular_function;	GO:0005575;GO:0015934;GO:0000315;GO:1990904;GO:0005762;GO:0032991;GO:0044391;GO:0098798;GO:0005198;GO:0003735;GO:0003674	K17422	MRPL41	------	------	ENOG4111ND6	S:Function unknown	PF09809.12	MRP-L27	Mitochondrial ribo	CYT	2	25	18.2	High
TRINITY_DN35051_c0_g1_i1_orf1	uncharacterized protein LOC114364307 [Ostrinia furnacalis]	7.749419954	2.954088328	2.19E-07	up	yes	1.002	0.1293	0.131	0.125	0.132	1	1.023	0.982	------	------	------	------	------	------	ENOG410Y27O	S:Function unknown	PF06757.16	Ins_allergen_r	Insect allergen rela	CYT	5	30	14.8	High
TRINITY_DN9715_c0_g1_i1_orf1	V-type proton ATPase subunit E [Manduca sexta] >P31402.1 RecName: Full=V-type proton ATPase subunit E; Short=V-ATPase subunit E; AltName: Full=V-ATPase 26 kDa subunit; AltName: Full=Vacuolar proton pump subunit E [Manduca sexta] >KAG6457535.1 hypothetical protein O3G_MSEX010354 [Manduca sexta] >CAA47610.1 H(+)-transporting ATPase [Manduca sexta]	3.385067404	1.759184561	6.17E-07	up	yes	0.9793	0.2893	0.294	0.288	0.286	1	0.98	0.958	CC:proton-transporting two-sector ATPase complex, catalytic domain;CC:macromolecular complex;CC:cellular_component;CC:membrane protein complex;MF:molecular_function;MF:ion transmembrane transporter activity;MF:ATPase activity, coupled to transmembrane movement of substances;MF:inorganic solute uptake transmembrane transporter activity;MF:hydrogen-translocating pyrophosphatase activity;MF:primary active transmembrane transporter activity;MF:active transmembrane transporter activity;MF:ATPase coupled ion transmembrane transporter activity;MF:transporter activity;MF:cation transmembrane transporter activity;MF:proton-transporting ATPase activity, rotational mechanism;MF:ATPase activity, coupled to transmembrane movement of ions, rotational mechanism;MF:inorganic cation transmembrane transporter activity;MF:cation-transporting ATPase activity;MF:transmembrane transporter activity;MF:active ion [illegible]	GO:0033178;GO:0032991;GO:0005575;GO:0098796;GO:0003674;GO:0015075;GO:0042626;GO:0015318;GO:0009678;GO:0015399;GO:0022804;GO:0042625;GO:0005215;GO:0008324;GO:0046961;GO:0044769;GO:0022890;GO:0019829;GO:0022857;GO:0022853;GO:0015078	K02150	ATPeV1E, AT[	map05323;ma[	Rheumatoid arth	COG1390	C:Energy production and conv	PF01991.21	vATP-synt_E	ATP synthase	CYT	12	44	26	High
TRINITY_DN2986_c1_g1_i1_orf1	Troponin C, isoform 1 [Papilio xuthus]	10.03	3.326249701	3.46E-06	up	yes	1.003	0.1	0.103	0.098	0.099	1	1.048	0.962	MF:cation binding;MF:ion binding;MF:molecular_function;MF:binding;MF:metal ion binding;MF:calcium ion binding;	GO:0043169;GO:0043167;GO:0003674;GO:0005488;GO:0046872;GO:0005509	------	------	------	------	COG5126	F:Nucleotide transport and me	PF13499.9;PF00(	EF-hand_7;EF	EF-hand domain p	CYT	5	22	20.2	High
TRINITY_DN105901_c0_g1_i2_orfp1	contactin-like [Pectinophora gossypiella]	3.022518237	1.595751044	0.0001093	up	yes	0.953	0.3153	0.333	0.286	0.327	1	0.875	0.984	------	------	------	------	------	------	------	------	------	------	------	CYT	2	16	16.7	High
TRINITY_DN1318_c0_g1_i5_orf1	uncharacterized protein LOC114360956 [Ostrinia furnacalis]	3.796525097	1.924679545	6.99E-06	up	yes	0.9833	0.259	0.253	0.252	0.272	1	1.012	0.938	CC:cellular_component;CC:integral component of membrane;CC:cellular anatomical entity;CC:intrinsic component of membrane;	GO:0005575;GO:0016021;GO:0110165;GO:0031224	------	------	------	------	ENOG4110SBJ;COG4886	S:Function unknown;	PF13855.9;PF12	LRR_8;LRR_4	Leucine rich repea	CYT	5	21	47.9	High
TRINITY_DN6059_c0_g1_i1_orf1	brachyurin-like [Ostrinia furnacalis]	5.677919203	2.50536232	7.93E-05	up	yes	1.026	0.1807	0.176	0.144	0.222	1	0.962	1.115	BP:organic substance metabolic process;BP:organonitrogen compound metabolic process;BP:protein metabolic process;BP:macromolecule metabolic process;BP:biological_process;BP:nitrogen compound metabolic process;BP:metabolic process;BP:proteolysis;BP:primary metabolic process;MF:serine-type endopeptidase activity;MF:endopeptidase activity;MF:hydrolase activity;MF:serine hydrolase activity;MF:catalytic activity, acting on a protein;MF:catalytic activity;MF:molecular_function;MF:peptidase activity;MF:serine- [illegible]	GO:0071704;GO:1901564;GO:0019538;GO:0043170;GO:0008150;GO:0006807;GO:0008152;GO:0006508;GO:0044238;GO:0004252;GO:0004175;GO:0016787;GO:0017171;GO:0140096;GO:0003824;GO:0003674;GO:0008233;GO:0008236	K09634	TMPRSS3	------	------	COG5640	S:Function unknown	PF00089.29	Trypsin	Trypsin	CYT	1	4	30.1	High
TRINITY_DN64446_c0_g1_i1_orf1	uncharacterized protein LOC114364307 [Ostrinia furnacalis]	4.811219512	2.266402624	2.31E-07	up	yes	0.9863	0.205	0.2	0.198	0.217	1	0.99	0.969	------	------	------	------	------	------	ENOG410Y27O	S:Function unknown	PF06757.16	Ins_allergen_r	Insect allergen rela	CYT	3	15	23.7	High
TRINITY_DN11259_c0_g1_i1_orf1	uncharacterized protein LOC114357075 [Ostrinia furnacalis]	4.661722488	2.220863124	6.24E-07	up	yes	0.9743	0.209	0.2	0.21	0.217	1	0.962	0.961	------	------	------	------	------	------	COG1028	S:Function unknown	PF13561.9;PF00	adh_short_C2	Enoyl;short chain c	CYT	6	46	26.7	High
TRINITY_DN3135_c0_g1_i6_orf1	acanthoscurrin-1-like [Ostrinia furnacalis]	5.71099597	2.513742367	4.78E-05	up	yes	0.992	0.1737	0.145	0.192	0.184	1	1.059	0.917	------	------	------	------	------	------	------	------	------	------	------	EXC	1	7	25	High
TRINITY_DN26010_c0_g1_i2_orf1	cytochrome b-c1 complex subunit 10-like [Ostrinia furnacalis]	4.625060474	2.209472229	1.25E-05	up	yes	0.956	0.2067	0.201	0.208	0.211	1	0.965	0.903	BP:aerobic respiration;BP:generation of precursor metabolites and energy;BP:electron transport chain;BP:respiratory electron transport chain;BP:cellular process;BP:aerobic electron transport chain;BP:cellular respiration;BP:metabolic process;BP:energy derivation by oxidation of organic compounds;BP:mitochondrial electron transport, ubiquinol to cytochrome c;BP:cellular metabolic process;BP:biological_process;CC:oxidoreductase complex;CC:respiratory chain complex;CC:cytochrome complex;CC:inner mitochondrial membrane protein complex;CC:mitochondrial respiratory chain complex III;CC:transmembrane transporter complex;CC:catalytic complex;CC:transporter complex;CC:macromolecular complex;CC:respiratory chain complex III;CC:cellular_component;CC:membrane protein complex;CC:mitochondrial protein complex;	GO:0009060;GO:0006091;GO:0022900;GO:0022904;GO:0009987;GO:0019646;GO:0045333;GO:0008152;GO:0015980;GO:0006122;GO:0044237;GO:0008150;GO:1990204;GO:0098803;GO:0070069;GO:0098800;GO:0005750;GO:1902495;GO:1902494;GO:1990351;GO:0032991;GO:0045275;GO:0005575;GO:0098796;GO:0098798	K00420	QCR10, UQC[	map04714;ma[	Thermogenesis;C	ENOG410XVMI	C:Energy production and conv	PF08997.13	UCR_6-4kD	Ubiquinol-cytochr	PLA	1	14	6.3	High
TRINITY_DN81803_c0_g2_i1_orf1	cathepsin K-like [Ostrinia furnacalis]	6.049480757	2.596811317	9.69E-08	up	yes	0.9903	0.1637	0.159	0.174	0.158	1	0.975	0.996	BP:primary metabolic process;BP:organonitrogen compound metabolic process;BP:protein metabolic process;BP:macromolecule metabolic process;BP:organic substance metabolic process;BP:nitrogen compound metabolic process;BP:biological_process;BP:metabolic process;BP:proteolysis;MF:molecular_function;MF:hydrolase activity;MF:catalytic activity;MF:catalytic activity, acting on a protein;MF:peptidase activity;MF:cysteine-type peptidase [illegible]	GO:0044238;GO:1901564;GO:0019538;GO:0043170;GO:0071704;GO:0006807;GO:0008150;GO:0008152;GO:0006508;GO:0003674;GO:0016787;GO:0003824;GO:0140096;GO:0008233;GO:0008234	------	------	------	------	COG4870	O:Posttranslational modificatio	------	------	------	CYT	1	8	13.3	High
TRINITY_DN7688_c0_g1_i2_orf1	uncharacterized protein LOC114352518 [Ostrinia furnacalis]	10.27153463	3.36057984	8.37E-06	up	yes	1.01	0.09833	0.091	0.076	0.128	1	1.062	0.969	BP:phosphorylation;BP:cellular process;BP:cellular metabolic process;BP:phosphate-containing compound metabolic process;BP:biological_process;BP:metabolic process;BP:phosphorus metabolic process;MF:kinase activity;MF:transferase activity;MF:catalytic activity;MF:molecular_function;MF:transferase activity, [illegible]	GO:0016310;GO:0009987;GO:0044237;GO:0006796;GO:0008150;GO:0008152;GO:0006793;GO:0016301;GO:0016740;GO:0003824;GO:0003674;GO:0016772	------	------	------	------	ENOG410YKCN;ENOG4111746	S:Function unknown;	PF02958.23;PF0	EcKL;DUF167	Ecdysteroid kinase	CYT	1	10	47.9	High
TRINITY_DN4959_c0_g1_i1_orf1	pancreatic triacylglycerol lipase-like [Ostrinia furnacalis]	7.358914729	2.879493018	6.63E-06	up	yes	0.9493	0.129	0.123	0.135	0.129	1	0.937	0.911	------	------	------	------	------	------	ENOG410YGS3;ENOG4110T03;ENOG4110QHK	S:Function unknown;	PF00151.22	Lipase	Lipase	CYT	1	7	37.1	High
TRINITY_DN38498_c0_g3_i1_orf1	unnamed protein product [Parnassius apollo]	5.821179455	2.541311494	0.002619	up	yes	0.918	0.1577	0.164	0.154	0.155	1	0.693	1.061	------	------	------	------	------	------	ENOG410ZSXY;ENOG410YWUP;ENOG410ZA5J;ENOG410ZHWV;ENOG410ZQ[illegible]	S:Function unknown;	------	------	------	CYT	1	3	36.4	Medium
TRINITY_DN67231_c0_g1_i1_orf1	endocuticle structural glycoprotein SgAbd-8-like [Ostrinia furnacalis]	38.5984998	5.270472871	4.15E-05	up	yes	0.9777	0.02533	0.024	0.02	0.032	1	1.049	0.884	MF:molecular_function;MF:structural constituent of cuticle;MF:structural molecule activity;	GO:0003674;GO:0042302;GO:0005198	------	------	------	------	ENOG4111PJ1;ENOG410YR66	S:Function unknown;	PF00379.26	Chitin_bind_4	Insect cuticle prote	CYT	1	20	13.9	High
TRINITY_DN10530_c0_g1_i1_orf1	cytochrome c oxidase subunit NDUFA4 [Ostrinia furnacalis]	2.572135417	1.362966599	4.80E-07	up	yes	0.9877	0.384	0.394	0.389	0.369	1	0.986	0.977	CC:cellular_component;CC:integral component of membrane;CC:cellular anatomical entity;CC:intrinsic component of membrane;	GO:0005575;GO:0016021;GO:0110165;GO:0031224	K03948	NDUFA4	map04714;ma[	Thermogenesis;C	ENOG41124SW	U:Intracellular trafficking, secre	PF06522.14	B12D	NADH-ubiquinone	CYT	5	48	9.2	High
TRINITY_DN20682_c0_g1_i2_orf1	hypothetical protein B5X24_HaOG200252 [Helicoverpa armigera]	2.540253689	1.344972583	1.54E-05	up	yes	0.9813	0.3863	0.427	0.378	0.354	1	0.964	0.98	MF:catalytic activity;MF:transferase [illegible]	GO:0003824;GO:0016740;GO:0003674	K00799	GST, gst	map05207;ma[	Chemical carcino	COG0625	O:Posttranslational modificatio	PF00043.28;PF1	GST_C;GST_C	Glutathione S-tran	CYT	2	40	11	High

TRINITY_DN3847_c1_g1_i1_orf1	ribosome production factor 2 homolog [Ostrinia furnacalis]	2.616750596	1.387776427	5.61E-05	up	yes	0.9873	0.3773	0.347	0.419	0.366	1	0.937	1.025	BP:cellular component assembly;BP:macromolecular complex subunit organization;BP:nucleic acid metabolic process;BP:maturation of LSU-rRNA;BP:ncRNA metabolic process;BP:organic cyclic compound metabolic process;BP:ribosomal large subunit assembly;BP:nucleobase-containing compound metabolic process;BP:ribonucleoprotein complex subunit organization;BP:cellular component organization or biogenesis;BP:cellular component organization;BP:macromolecular complex assembly;BP:organic substance metabolic process;BP:ribonucleoprotein complex assembly;BP:rRNA processing;BP:ncRNA processing;BP:cellular process;BP:cellular aromatic compound metabolic process;BP:biological_process;BP:metabolic process;BP:heterocycle metabolic process;BP:RNA metabolic process;BP:primary metabolic process;BP:rRNA metabolic process;BP:cellular nitrogen compound metabolic process;BP:cellular metabolic process;BP:macromolecule metabolic process;BP:nitrogen compound metabolic process;BP:RNA processing;CC:intracellular organelle;CC:non-membrane-bounded organelle;CC:organelle;CC:nucleolus;CC:cellular anatomical entity;CC:intracellular non-membrane-bounded organelle;CC:cellular_component;MF:molecular_function;MF:binding;MF:nucleic acid binding;MF:rRNA binding;MF:heterocyclic compound binding;MF:organic cyclic compound	GO:0022607;GO:0043933;GO:0090304;GO:0000470;GO:0034660;GO:1901360;GO:0000027;GO:0006139;GO:0071826;GO:0071840;GO:0016043;GO:0065003;GO:0071704;GO:0022618;GO:0006364;GO:0034470;GO:0009987;GO:0006725;GO:0008150;GO:0008152;GO:0046483;GO:0016070;GO:0044238;GO:0016072;GO:0034641;GO:0044237;GO:0043170;GO:0006807;GO:0006396;GO:0043229;GO:0043228;GO:0043226;GO:0005730;GO:0110165;GO:0043232;GO:0005575;GO:0003674;GO:0005488;GO:0003676;GO:0019843;GO:1901363;GO:0097159;GO:0003723	K14847	RPF2	------	------	COG5106	S:Function unknown	PF04427.21	Brix	Brix domain	CYT	2	7	35.9	High
TRINITY_DN7073_c0_g1_i1_orf1	unnamed protein product, partial [Brenthis ino]	3.065627809	1.616182553	9.06E-06	up	yes	1.023	0.3337	0.339	0.354	0.308	1	1.063	1.006	CC:cellular_component;CC:macromolecular complex;CC:ribonucleoprotein complex;	GO:0005575;GO:0032991;GO:1990904	K11129	NHP2, NOLA2	map03008	Ribosome biogen	COG1358	L:Replication, recombination ar	PF01248.29	Ribosomal_L7	Ribosomal protein	CYT	2	10	17.7	High
TRINITY_DN1173_c1_g1_i10_orf1	hypothetical protein evm_001011 [Chilo suppressalis]	2.920922089	1.546423877	5.86E-05	up	yes	1.001	0.3427	0.32	0.352	0.356	1	0.941	1.063	BP:phosphorylation;BP:cell differentiation;BP:nitrogen compound metabolic process;BP:anatomical structure morphogenesis;BP:cellular developmental process;BP:organic substance metabolic process;BP:developmental process;BP:protein phosphorylation;BP:cellular process;BP:macromolecule modification;BP:protein modification process;BP:biological_process;BP:metabolic process;BP:primary metabolic process;BP:organonitrogen compound metabolic process;BP:protein metabolic process;BP:cellular metabolic process;BP:macromolecule metabolic process;BP:phosphate-containing compound metabolic process;BP:phosphorus metabolic process;MF:purine ribonucleoside triphosphate binding;MF:guanyl-nucleotide exchange factor activity;MF:molecular_function;MF:binding;MF:molecular function regulator;MF:heterocyclic compound binding;MF:nucleoside phosphate binding;MF:anion binding;MF:ion binding;MF:nucleotide binding;MF:transferase activity;MF:protein kinase activity;MF:purine nucleotide binding;MF:carbohydrate derivative binding;MF:ATP binding;MF:kinase activity;MF:enzyme regulator activity;MF:catalytic activity, acting on a protein;MF:small molecule binding;MF:GTPase regulator activity;MF:nucleoside-triphosphatase regulator activity;MF:catalytic activity;MF:transferase activity, transferring phosphorus-containing groups;MF:adenyl nucleotide binding;MF:phosphotransferase activity, alcohol group as acceptor;MF:adenyl ribonucleotide binding;MF:purine	GO:0016310;GO:0030154;GO:0006807;GO:0009653;GO:0048869;GO:0071704;GO:0032502;GO:0006468;GO:0009987;GO:0043412;GO:0036211;GO:0008150;GO:0008152;GO:0044238;GO:1901564;GO:0019538;GO:0044237;GO:0043170;GO:0006796;GO:0006793;GO:0035639;GO:0005085;GO:0003674;GO:0005488;GO:0098772;GO:1901363;GO:1901265;GO:0043168;GO:0043167;GO:0000166;GO:0016740;GO:0004672;GO:0017076;GO:0097367;GO:0005524;GO:0016301;GO:0030234;GO:0140096;GO:0036094;GO:0030695;GO:0060589;GO:0003824;GO:0016772;GO:0030554;GO:0016773;GO:0032559;GO:0032555;GO:0097159;GO:0032553	------	------	------	------	ENOG410XQFD;ENOG410XSWY	S:Function unknown;T:Signal tr	PF07679.19;PF1:	I-set;Ig_3;ig;Ig	Immunoglobulin I-	CYT	1	17	169.8	High
TRINITY_DN28638_c0_g1_i1_orf1	uncharacterized protein LOC114364075 [Ostrinia furnacalis]	2.841091954	1.506445526	9.61E-07	up	yes	0.9887	0.348	0.362	0.35	0.332	1	0.996	0.97	------	------	------	------	------	------	ENOG410XRSO	G:Carbohydrate transport and r	PF00650.23	CRAL_TRIO	CRAL/TRIO domain	CYT	7	25	34.8	High
TRINITY_DN8087_c0_g1_i9_orf1	cysteine-rich with EGF-like domain protein 2 isoform X1 [Ostrinia furnacalis]	2.285359801	1.192421318	0.0003421	up	yes	0.921	0.403	0.401	0.443	0.365	1	0.887	0.876	BP:developmental process;BP:cellular process;BP:cellular developmental process;BP:anatomical structure development;BP:cell development;BP:cellular component organization;BP:animal organ development;BP:biological_process;BP:cellular component organization or biogenesis;MF:cation binding;MF:ion binding;MF:calcium ion	GO:0032502;GO:0009987;GO:0048869;GO:0048856;GO:0048468;GO:0016043;GO:0048513;GO:0008150;GO:0071840;GO:0043169;GO:0043167;GO:0005509;GO:0003674;GO:0046872;GO:0005488	K24335	CRELD	------	------	ENOG41107X2	O:Posttranslational modificatio	PF11938.11	DUF3456	TLR4 regulator and	CYT	1	2	42.5	High
TRINITY_DN26688_c0_g1_i2_orf1	myogenesis-regulating glycosidase-like [Ostrinia furnacalis]	3.273615635	1.710884941	1.18E-07	up	yes	1.005	0.307	0.315	0.309	0.297	1	1.018	0.998	BP:primary metabolic process;BP:carbohydrate metabolic process;BP:organic substance metabolic process;BP:biological_process;BP:metabolic process;MF:hydrolase activity;MF:catalytic activity;MF:hydrolase activity, acting on glycosyl bonds;MF:hydrolase activity, hydrolyzing O-glycosyl compounds;MF:molecular_function;	GO:0044238;GO:0005975;GO:0071704;GO:0008150;GO:0008152;GO:0016787;GO:0003824;GO:0016798;GO:0004553;GO:0003674	K24727	MYORG	------	------	COG1501	G:Carbohydrate transport and r	PF01055.29	Glyco_hydro_	Glycosyl hydrolase	CYT	11	21	71.1	High
TRINITY_DN2083_c0_g1_i4_orf1	uncharacterized protein LOC114359113 [Ostrinia furnacalis]	3.506717189	1.810121084	9.78E-06	up	yes	1.018	0.2903	0.268	0.306	0.297	1	1.064	0.989	------	------	------	------	------	------	ENOG410ZGJA	S:Function unknown	PF06585.14	JHBP	Haemolymph juve	CYT	3	17	24.2	High
TRINITY_DN18396_c0_g1_i1_orf1	uncharacterized protein LOC114359424 [Ostrinia furnacalis]	3.575438596	1.838120227	1.06E-06	up	yes	1.019	0.285	0.277	0.286	0.292	1	1.047	1.009	MF:molecular_function;MF:structural constituent of cuticle;MF:structural molecule activity;	GO:0003674;GO:0042302;GO:0005198	------	------	------	------	ENOG410YSFI;ENOG410YWJF;ENOG4110QVC;ENOG410ZUHH;ENOG410ZM6	K:Transcription;S:Function unkr	PF00379.26	Chitin_bind_4	Insect cuticle prote	CYT	1	4	39.7	High
TRINITY_DN1914_c0_g1_i4_orf1	loricrin-like [Ostrinia furnacalis]	3.168709677	1.663895484	2.55E-05	up	yes	0.9823	0.31	0.366	0.268	0.296	1	0.968	0.979	------	------	------	------	------	------	ENOG4110QTU;ENOG4110QBC	S:Function unknown;	------	------	------	CYT	1	15	58.5	High
TRINITY_DN31047_c0_g1_i4_orf1	4-coumarate--CoA ligase 1-like isoform X1 [Ostrinia furnacalis] >XP_028160248.1 4-coumarate--CoA ligase 1-like isoform X1 [Ostrinia furnacalis] >XP_028160249.1 4-coumarate--CoA ligase 1-like isoform X1 [Ostrinia furnacalis] >XP_028160250.1 4-coumarate--CoA ligase 1-like isoform X1 [Ostrinia furnacalis] >XP_028160251.1 4-coumarate--CoA ligase 1-like isoform X1 [Ostrinia furnacalis] >XP_028160253.1 4-coumarate--CoA ligase 1-like isoform X2 [Ostrinia furnacalis]	2.893277058	1.532704481	2.26E-05	up	yes	0.9597	0.3317	0.337	0.313	0.345	1	0.968	0.911	------	------	K01904	4CL	map00130	Ubiquinone and	COG0318;COG0318	I:Lipid transport and metabolisı	PF00501.31;PF1:	AMP-binding	AMP-binding enzy	CYT	4	8	59.4	High
TRINITY_DN7024_c0_g1_i1_orf1	uncharacterized protein LOC114363116 [Ostrinia furnacalis]	19.16942997	4.260735532	0.000481	up	yes	1.214	0.06333	0.07	0.057	0.063	1	1.369	1.272	MF:molecular_function;MF:lipid binding;MF:binding;	GO:0003674;GO:0008289;GO:0005488	K08753;K087	FABP4, aP2;F	map04923;ma	Regulation of lip	ENOG4111US8	I:Lipid transport and metabolisı	------	------	------	CYT	7	68	15.1	High
TRINITY_DN108573_c0_g1_i1_orf1	uncharacterized protein LOC114366171 [Ostrinia furnacalis]	3.437786394	1.781479906	9.23E-07	up	yes	0.9753	0.2837	0.277	0.28	0.294	1	0.958	0.968	------	------	------	------	------	------	ENOG410XT8F;ENOG410XT8F;ENOG410ZI5B;ENOG410YKCN;ENOG4111FI2	G:Carbohydrate transport and r	PF02958.23;PF0	EcKL;DUF167	Ecdysteroid kinase	CYT	3	7	47.8	High
TRINITY_DN2709_c0_g1_i4_orf1	ATP-dependent RNA helicase dbp2-like [Ostrinia furnacalis]	2.609061865	1.383531153	0.0008065	up	yes	0.8983	0.3443	0.363	0.332	0.338	1	0.903	0.792	CC:intracellular organelle;CC:organelle;CC:cellular_component;CC:cellular anatomical entity;MF:purine ribonucleoside triphosphate binding;MF:molecular_function;MF:binding;MF:nucleic acid binding;MF:RNA-dependent ATPase activity;MF:nucleotide binding;MF:heterocyclic compound binding;MF:nucleoside phosphate binding;MF:anion binding;MF:ion binding;MF:purine nucleotide binding;MF:ATP binding;MF:hydrolase activity;MF:catalytic activity, acting on RNA;MF:helicase activity;MF:small molecule binding;MF:catalytic activity;MF:adenyl nucleotide binding;MF:carbohydrate derivative binding;MF:organic cyclic compound binding;MF:RNA helicase activity;MF:adenyl ribonucleotide binding;MF:purine ribonucleotide binding;MF:ribonucleotide binding;	GO:0043229;GO:0043226;GO:0005575;GO:0110165;GO:0035639;GO:0003674;GO:0005488;GO:0003676;GO:0008186;GO:0000166;GO:1901363;GO:1901265;GO:0043168;GO:0043167;GO:0017076;GO:0005524;GO:0016787;GO:0140098;GO:0004386;GO:0036094;GO:0003824;GO:0030554;GO:0097367;GO:0097159;GO:0003724;GO:0032559;GO:0032555;GO:0032553	K12823	DDX5, DBP2	map05205;ma	Proteoglycans in	COG0513	S:Function unknown	PF00270.32;PF0	DEAD;Helicas	DEAD/DEAH box h	CYT	1	4	58.7	High
TRINITY_DN6418_c0_g1_i28_orf1	peritrophic membrane chitin binding protein [Loxostege sticticalis]	4.626189397	2.209824331	9.76E-06	up	yes	1.021	0.2207	0.247	0.209	0.206	1	1.072	0.992	CC:cellular_component;CC:extracellular region;CC:cellular anatomical entity;MF:carbohydrate derivative binding;MF:molecular_function;MF:chitin binding;MF:binding;	GO:0005575;GO:0005576;GO:0110165;GO:0097367;GO:0003674;GO:0008061;GO:0005488	------	------	------	------	ENOG41121EJ;ENOG4112A9I;ENOG41126RR;ENOG4110R	S:Function unknown;	PF01607.27	CBM_14	Chitin binding Peri	CYT	1	3	48	High
TRINITY_DN5107_c0_g1_i4_orf1	peptide methionine sulfoxide reductase [Ostrinia furnacalis]	4.321888412	2.111661823	1.18E-05	up	yes	1.007	0.233	0.27	0.199	0.23	1	1.046	0.976	MF:peptide-methionin;MF:catalytic activity;MF:oxidoreductase activity, acting on a sulfur group of donors, disulfide as acceptor;MF:molecular_function;MF:oxidoreductase activity, acting on a sulfur group of donors;MF:oxidoreductase activity;	GO:0008113;GO:0003824;GO:0016671;GO:0003674;GO:0016667;GO:0016491	K07304	msrA	------	------	COG0225	O:Posttranslational modificatio	PF01625.24	PMSR	Peptide methionin	CYT	1	4	26.1	Medium
TRINITY_DN37538_c0_g3_i1_orf1	esterase FE4-like [Ostrinia furnacalis]	2.981894601	1.576229264	0.0002324	up	yes	0.9223	0.3093	0.307	0.291	0.33	1	0.836	0.931	------	------	K07378	NLGN	map04514	Cell adhesion mo	COG2272;COG0580;ENOG410XSWS	I:Lipid transport and metabolisı	PF00135.31	COesterase	Carboxylesterase f	CYT	1	13	13.4	High
TRINITY_DN334_c0_g1_i2_orf1	chymotrypsin-like serine protease, partial [Ostrinia nubilalis]	5.636166757	2.494714297	6.13E-06	up	yes	1.041	0.1847	0.211	0.156	0.187	1	1.076	1.047	BP:organic substance metabolic process;BP:organonitrogen compound metabolic process;BP:protein metabolic process;BP:macromolecule metabolic process;BP:biological_process;BP:nitrogen compound metabolic process;BP:metabolic process;BP:proteolysis;BP:primary metabolic process;MF:serine-type endopeptidase activity;MF:endopeptidase activity;MF:hydrolase activity;MF:serine hydrolase activity;MF:catalytic activity, acting on a protein;MF:catalytic activity;MF:molecular_function;MF:peptidase activity;MF:serine-	GO:0071704;GO:1901564;GO:0019538;GO:0043170;GO:0008150;GO:0006807;GO:0008152;GO:0006508;GO:0044238;GO:0004252;GO:0004175;GO:0016787;GO:0017171;GO:0140096;GO:0003824;GO:0003674;GO:0008233;GO:0008236	------	------	------	------	COG5640	O:Posttranslational modificatio	PF00089.29	Trypsin	Trypsin	CYT	1	18	13.2	High
TRINITY_DN21722_c0_g1_i3_orf1	V-type proton ATPase 116 kDa subunit a isoform X1 [Ostrinia furnacalis] >XP_028177509.1 V-type proton ATPase 116 kDa subunit a isoform X1 [Ostrinia furnacalis]	4.168220339	2.059431543	1.02E-06	up	yes	0.9837	0.236	0.253	0.226	0.229	1	0.992	0.959	CC:vacuolar proton-transporting V-type ATPase, V0 domain;CC:intrinsic component of membrane;CC:cellular_component;CC:proton-transporting V-type ATPase, V0 domain;CC:integral component of membrane;CC:cellular anatomical entity;CC:macromolecular complex;CC:membrane protein complex;CC:proton-transporting two-sector ATPase complex, proton-transporting domain;MF:molecular_function;MF:ATPase activity, coupled to transmembrane movement of substances;MF:inorganic solute uptake transmembrane transporter activity;MF:hydrogen-translocating pyrophosphatase activity;MF:primary active transmembrane transporter activity;MF:ATPase coupled ion transmembrane transporter activity;MF:transporter activity;MF:cation transmembrane transporter activity;MF:proton-transporting ATPase activity, rotational mechanism;MF:ATPase activity, coupled to transmembrane movement of ions, rotational mechanism;MF:inorganic cation transmembrane transporter activity;MF:cation-transporting ATPase activity;MF:ion transmembrane transporter activity;MF:transmembrane transporter activity;MF:active transmembrane transporter activity;MF:active ion transmembrane transporter activity;MF:hydrogen ion	GO:0000220;GO:0031224;GO:0005575;GO:0033179;GO:0016021;GO:0110165;GO:0032991;GO:0098796;GO:0033177;GO:0003674;GO:0042626;GO:0015318;GO:0009678;GO:0015399;GO:0042625;GO:0005215;GO:0008324;GO:0046961;GO:0044769;GO:0022890;GO:0019829;GO:0015075;GO:0022857;GO:0022804;GO:0022853;GO:0015078	K02154	ATPeV0A, ATI	map05152;ma	Tuberculosis;Rhe	COG1269	C:Energy production and conve	PF01496.22	V_ATPase_I	V-type ATPase 11	CYT	11	20	95.5	High
TRINITY_DN48020_c0_g1_i1_orf1	aminopeptidase N4 [Cnaphalocrocis medinalis]	3.391304348	1.761840263	5.74E-05	up	yes	0.936	0.276	0.27	0.263	0.295	1	0.877	0.931	BP:nitrogen compound metabolic process;BP:organic substance metabolic process;BP:proteolysis;BP:biological_process;BP:metabolic process;BP:primary metabolic process;BP:organonitrogen compound metabolic process;BP:protein metabolic process;BP:macromolecule metabolic process;CC:integral component of membrane;CC:intrinsic component of membrane;CC:cellular anatomical entity;CC:cellular_component;MF:aminopeptidase activity;MF:exopeptidase activity;MF:molecular_function;MF:binding;MF:peptidase activity;MF:metallopeptidase activity;MF:cation binding;MF:transition metal ion binding;MF:ion binding;MF:metal ion binding;MF:hydrolase activity;MF:catalytic	GO:0006807;GO:0071704;GO:0006508;GO:0008150;GO:0008152;GO:0044238;GO:1901564;GO:0019538;GO:0043170;GO:0016021;GO:0031224;GO:0110165;GO:0005575;GO:0004177;GO:0008238;GO:0003674;GO:0005488;GO:0008233;GO:0008237;GO:0043169;GO:0046914;GO:0043167;GO:0046872;GO:0016787;GO:0140096;GO:0003824;GO:0008270	K11140	ANPEP, CD13	map04640;ma	Hematopoietic c	COG0308	E:Amino acid transport and me	PF17900.4	Peptidase_M1	Peptidase M1 N-te	CYT	1	21	15.7	High
TRINITY_DN11587_c0_g1_i7_orf1	elongation of very long chain fatty acids protein AAEL008004-like isoform X1 [Danaus plexippus plexippus] >XP_032511006.1 elongation of very long chain fatty acids protein AAEL008004-like isoform X1 [Danaus plexippus plexippus] >XP_032511007.1 elongation of very long chain fatty acids protein AAEL008004-like isoform X1 [Danaus plexippus plexippus] >XP_032511008.1 elongation of very long chain fatty acids protein AAEL008004-like isoform X1 [Danaus plexippus plexippus] >XP_032511009.1 elongation of very long chain fatty acids protein AAEL008004-like isoform X1 [Danaus plexippus plexippus]	5.090909091	2.347923303	6.26E-08	up	yes	1.008	0.198	0.211	0.187	0.196	1	1.011	1.014	------	------	K10250	ELOVL7	map01040;ma	Biosynthesis of u	ENOG410XRWT	C:Energy production and conve	PF01151.21	ELO	GNS1/SUR4 family	CYT	1	4	36.4	High

TRINITY_DN57202_c0_g1_i1_orf1	PREDICTED: U4/U6 small nuclear ribonucleoprotein Prp31 [Amyelois transitella]	3.484555266	1.800974537	0.0001086	up	yes	0.9363	0.2687	0.264	0.323	0.219	1	0.908	0.901	BP:cellular component assembly;BP:spliceosomal tri-snRNP complex assembly;BP:macromolecular complex subunit organization;BP:cellular component organization or biogenesis;BP:spliceosomal snRNP assembly;BP:cellular component organization;BP:macromolecular complex assembly;BP:ribonucleoprotein complex assembly;BP:cellular process;BP:ribonucleoprotein complex subunit organization;BP:biological_process;CC:Sm-like protein family complex;CC:U4/U6 x U5 tri-snRNP complex;CC:spliceosomal snRNP complex;CC:macromolecular complex;CC:small nuclear ribonucleoprotein complex;CC:ribonucleoprotein complex;CC:cellular_component;CC:spliceosomal tri-snRNP complex;CC:spliceosomal complex;MF:molecular_function;MF:binding;MF:nucleic acid binding;MF:heterocyclic compound binding;MF:organic cyclic compound binding;MF:RNA binding;	GO:0022607;GO:0000244;GO:0043933;GO:0071840;GO:0000387;GO:0016043;GO:0065003;GO:0022618;GO:0009987;GO:0071826;GO:0008150;GO:0120114;GO:0046540;GO:0097525;GO:0032991;GO:0030532;GO:1990904;GO:0005575;GO:0097526;GO:0005681;GO:0003674;GO:0005488;GO:0003676;GO:1901363;GO:0097159;GO:0003723	K12844	PRPF31	map03040	Spliceosome	COG1498	S:Function unknown	PF01798.21;PF0	Nop;Prp31_C	snoRNA binding d	CYT	1	3	55.5	High
TRINITY_DN30663_c0_g1_i1_orf1	surfeit locus protein 6 homolog [Ostrinia furnacalis]	4.571760334	2.192749775	7.90E-06	up	yes	0.9843	0.2153	0.176	0.242	0.228	1	1.002	0.951	CC:intracellular membrane-bounded organelle;CC:nucleus;CC:cellular_component;CC:intracellular organelle;CC:membrane-bounded organelle;CC:organelle;CC:cellular anatomical entity;	GO:0043231;GO:0005634;GO:0005575;GO:0043229;GO:0043227;GO:0043226;GO:0110165	------	------	------	------	ENOG4111G0Y	S:Function unknown	PF04935.15	SURF6	Surfeit locus protei	CYT	2	6	33.5	High
TRINITY_DN117_c0_g1_i6_orf1	lipase member I-like [Ostrinia furnacalis]	5.407447383	2.434947722	2.56E-07	up	yes	1.002	0.1853	0.192	0.178	0.186	1	1.022	0.984	BP:organic substance metabolic process;BP:lipid metabolic process;BP:primary metabolic process;BP:metabolic process;BP:biological_process;CC:cellular_component;CC:extracellular region;CC:cellular anatomical entity;MF:hydrolase activity;MF:hydrolase activity, acting on ester bonds;MF:catalytic activity;MF:molecular_function;MF:lipase activity;	GO:0071704;GO:0006629;GO:0044238;GO:0008152;GO:0008150;GO:0005575;GO:0005576;GO:0110165;GO:0016787;GO:0016788;GO:0003824;GO:0003674;GO:0016298	------	------	------	------	ENOG41116Y2;ENOG4110RTX;ENOG410YGS3;ENOG410Y92X;ENOG4111AHY	S:Function unknown;	PF00151.22	Lipase	Lipase	CYT	1	11	42.3	High
TRINITY_DN3784_c0_g1_i1_orf1	pancreatic triacylglycerol lipase-like [Ostrinia furnacalis]	5.443037975	2.444412101	3.88E-08	up	yes	0.989	0.1817	0.181	0.188	0.176	1	0.989	0.978	BP:organic substance metabolic process;BP:lipid metabolic process;BP:primary metabolic process;BP:metabolic process;BP:biological_process;CC:cellular_component;CC:extracellular region;CC:cellular anatomical entity;MF:hydrolase activity;MF:hydrolase activity, acting on ester bonds;MF:catalytic activity;MF:molecular_function;MF:lipase activity;	GO:0071704;GO:0006629;GO:0044238;GO:0008152;GO:0008150;GO:0005575;GO:0005576;GO:0110165;GO:0016787;GO:0016788;GO:0003824;GO:0003674;GO:0016298	------	------	------	------	ENOG41116Y2;ENOG4110RTX;ENOG410YGS3;ENOG4111JY5	S:Function unknown;	PF00151.22	Lipase	Lipase	PLA	13	30	61.9	High
TRINITY_DN7688_c0_g1_i10_orf1	uncharacterized protein LOC114352518 [Ostrinia furnacalis]	6.731413262	2.750909431	1.04E-05	up	yes	1.005	0.1493	0.136	0.149	0.163	1	1.059	0.955	BP:phosphorylation;BP:cellular process;BP:cellular metabolic process;BP:phosphate-containing compound metabolic process;BP:biological_process;BP:metabolic process;BP:phosphorus metabolic process;MF:kinase activity;MF:transferase activity;MF:catalytic activity;MF:molecular_function;MF:transferase activity,	GO:0016310;GO:0009987;GO:0044237;GO:0006796;GO:0008150;GO:0008152;GO:0006793;GO:0016301;GO:0016740;GO:0003824;GO:0003674;GO:0016772	------	------	------	------	ENOG410YKCN;ENOG4111746	S:Function unknown;	PF02958.23;PF0	EcKL;DUF167	Ecdysteroid kinase	CYT	1	10	47.9	High
TRINITY_DN2815_c0_g1_i3_orf1	uncharacterized protein LOC114364075 [Ostrinia furnacalis]	4.556097561	2.18779864	0.0001607	up	yes	0.934	0.205	0.203	0.24	0.172	1	0.964	0.838	------	------	------	------	------	------	ENOG410XRSQ	S:Function unknown	PF00650.23	CRAL_TRIO	CRAL/TRIO domair	CYT	1	3	35.8	High
TRINITY_DN27592_c0_g1_i1_orf1	D-arabinitol dehydrogenase 1-like [Ostrinia furnacalis]	5.818562874	2.540662866	1.35E-06	up	yes	0.9717	0.167	0.155	0.172	0.174	1	0.972	0.943	MF:oxidoreductase activity;MF:cation binding;MF:transition metal ion binding;MF:ion binding;MF:catalytic activity;MF:molecular_function;MF:binding;MF:metal ion binding;MF:zinc ion binding;	GO:0016491;GO:0043169;GO:0046914;GO:0043167;GO:0003824;GO:0003674;GO:0005488;GO:0046872;GO:0008270	K17818	ARD1	map00040	Pentose and gluc	COG1063	E:Amino acid transport and me	PF00107.29	ADH_zinc_N	Zinc-binding dehy	CYT	1	59	15.1	High
TRINITY_DN14679_c0_g1_i1_orf1	hypothetical protein evm_003043 [Chilo suppressalis]	13.34289813	3.738000155	1.75E-07	up	yes	1.023	0.07667	0.077	0.07	0.083	1	1.032	1.038	CC:cellular_component;CC:integral component of membrane;CC:cellular anatomical entity;CC:intrinsic component of membrane;	GO:0005575;GO:0016021;GO:0110165;GO:0031224	------	------	------	------	ENOG410ZMUH;ENOG410Z1X1;ENOG410XRIN	S:Function unknown;	PF13927.9;PF07	Ig_3;I-set;Ig_2	Immunoglobulin d	CYT	1	1	90	High
TRINITY_DN41086_c0_g1_i4_orf1	collagenase-like [Pectinophora gossypiella]	10.58387097	3.403795473	2.57E-07	up	yes	0.9843	0.093	0.095	0.092	0.092	1	0.994	0.959	BP:organic substance metabolic process;BP:organonitrogen compound metabolic process;BP:protein metabolic process;BP:macromolecule metabolic process;BP:biological_process;BP:nitrogen compound metabolic process;BP:metabolic process;BP:proteolysis;BP:primary metabolic process;MF:serine-type endopeptidase activity;MF:endopeptidase activity;MF:hydrolase activity;MF:serine hydrolase activity;MF:catalytic activity, acting on a protein;MF:catalytic activity;MF:molecular_function;MF:peptidase activity;MF:serine-	GO:0071704;GO:1901564;GO:0019538;GO:0043170;GO:0008150;GO:0006807;GO:0008152;GO:0006508;GO:0044238;GO:0004252;GO:0004175;GO:0016787;GO:0017171;GO:0140096;GO:0003824;GO:0003674;GO:0008233;GO:0008236	------	------	------	------	COG5640	O:Posttranslational modificatio	PF00089.29;PF1	Trypsin;Trypsi	Trypsin;Trypsin-lik	CYT	3	10	36.1	High
TRINITY_DN94755_c0_g1_i5_orfp1	TRINITY_DN94755_c0_g1_i5_m.62794<br>TRINITY_DN94755_c0_g1::TRINITY_DN94755_c0_g1_i5::g.62794 ORF type:internal len:70	11.27264336	3.494753952	1.48E-05	up	yes	0.977	0.08667	0.073	0.098	0.089	1	1.022	0.909	------	------	------	------	------	------	------	------	------	------	------	CYT	1	16	8	High
