# Supplementary material for: Comparative Proteomics and Metabonomics Analysis of Different Diapause Stages Revealed a New Regulation Mechanism of Diapause in Loxostege sticticalis (Lepidoptera: Pyralidae)
Source: Molecules. 2024 Jul 25;29(15):3472. doi: 10.3390/molecules29153472 (PMC11314584; doi:10.3390/molecules29153472)
Supplement: Supplementary file 1 [file molecules-29-03472-s001.zip › analysis process/proteomic/diffreential protein statistic table/PreDvsD.pdf]

Accession	Description	FC(PreD/D)	Log2FC(PreD/D)	P_value(PreD/D)	Regulate	Significant	PreD	D	D1	D2	D3	PreD1	PreD2	PreD3
TRINITY_DN34406_c0_g2_i9_orfp1	TRINITY_DN34406_c0_g2_i9_m.33755 TRINITY_DN34406_c0_g2::TRINITY_DN34406_c0_g2_i9::g.33755 ORF type:internal len:82 (-).score=12.88 TRINITY_DN34406_c0_g2_i9:3-245(-)	2.32930878	1.219901902	0.001446	up	yes	7.717	3.313	3.269	3.114	3.555	7.68	8.685	6.786
TRINITY_DN131603_c0_g1_i4_orfp1	TRINITY_DN131603_c0_g1_i4_m.86149 TRINITY_DN131603_c0_g1::TRINITY_DN131603_c0_g1_i4::g.86149 ORF type:internal len:112 (-).score=8.40 TRINITY_DN131603_c0_g1_i4:2-334(-)	0.10936749	-3.192744088	0.0001032	down	yes	1.366	12.49	13.197	13.229	11.052	1.289	1.394	1.416
TRINITY_DN27300_c0_g1_i1_orfp1	TRINITY_DN27300_c0_g1_i1_m.71142 TRINITY_DN27300_c0_g1::TRINITY_DN27300_c0_g1_i1::g.71142 ORF type:internal len:82 (-).score=9.46 TRINITY_DN27300_c0_g1_i1:3-245(-)	3.60417325	1.849668363	6.84E-06	up	yes	11.4	3.163	2.949	3.043	3.496	11.271	11.811	11.118
TRINITY_DN14242_c0_g1_i2_orfp1	TRINITY_DN14242_c0_g1_i2_m.18449 TRINITY_DN14242_c0_g1::TRINITY_DN14242_c0_g1_i2::g.18449 ORF type:internal len:148 (-).score=102.30 TRINITY_DN14242_c0_g1_i2:2-442(-)	2.32373386	1.216444846	0.0004537	up	yes	23.4	10.07	12.447	8.38	9.395	23.089	24.022	23.09
TRINITY_DN9044_c0_g1_i2_orf1	unnamed protein product [Euphydryas editha]	2.69604863	1.430846521	6.80E-06	up	yes	5.322	1.974	1.952	1.819	2.15	5.23	5.411	5.325
TRINITY_DN64297_c0_g1_i1_orf1	vanin-like protein 2 isoform X2 [Ostrinia furnacalis]	2.06608884	1.046902291	0.01049	up	yes	5.721	2.769	2.526	3.369	2.411	5.983	6.562	4.619
TRINITY_DN20442_c0_g2_i1_orf1	hypothetical protein evm_008218 [Chilo suppressalis]	0.27596384	-1.857448832	2.44E-05	down	yes	1.496	5.421	5.673	5.108	5.481	1.496	1.389	1.602
TRINITY_DN1091_c0_g3_i1_orf1	macrophage mannose receptor 1-like [Ostrinia furnacalis]	0.42778793	-1.225032303	0.0001413	down	yes	1.404	3.282	3.37	3.445	3.032	1.341	1.464	1.408
TRINITY_DN325_c0_g1_i15_orf1	protein draper-like [Ostrinia furnacalis]	2.44670051	1.290837517	0.000143	up	yes	2.892	1.182	1.085	1.265	1.197	2.765	3.108	2.804
TRINITY_DN19639_c0_g2_i1_orf1	basic juvenile hormone-suppressible protein 1-like [Hyposmocoma kahamanoa]	0.36375962	-1.458942697	0.003614	down	yes	0.9927	2.729	2.838	2.194	3.154	1.045	0.956	0.977
TRINITY_DN30177_c0_g2_i1_orf1	uncharacterized protein LOC114365032 [Ostrinia furnacalis]	0.28223256	-1.825043669	1.80E-05	down	yes	1.517	5.375	5.423	5.596	5.106	1.428	1.662	1.461
TRINITY_DN25234_c0_g1_i1_orf1	uncharacterized protein LOC114353853 [Ostrinia furnacalis]	2.21070897	1.144509116	0.0002963	up	yes	4.459	2.017	1.88	1.954	2.216	4.515	4.122	4.739
TRINITY_DN17574_c0_g1_i2_orf1	heat shock protein Hsp-12.2-like [Ostrinia furnacalis]	0.41512973	-1.268365846	1.75E-05	down	yes	1.328	3.199	3.271	3.279	3.046	1.319	1.354	1.31
TRINITY_DN307_c1_g1_i1_orf1	uncharacterized protein LOC114356704 [Ostrinia furnacalis]	0.41260841	-1.277154883	7.95E-05	down	yes	1.237	2.998	3.168	3.022	2.803	1.24	1.213	1.258
TRINITY_DN85290_c0_g2_i1_orf1	unnamed protein product, partial [Brenthis ino]	2.75461936	1.46185298	4.64E-06	up	yes	3.727	1.353	1.444	1.221	1.393	3.707	3.706	3.768
TRINITY_DN18568_c0_g1_i2_orfp1	TRINITY_DN18568_c0_g1_i2_m.13844 TRINITY_DN18568_c0_g1::TRINITY_DN18568_c0_g1_i2::g.13844 ORF type:5prime_partial len:77 (+).score=12.07 TRINITY_DN18568_c0_g1_i2:1-231(+)	0.21971866	-2.186270713	0.0001424	down	yes	0.8903	4.052	4.39	3.7	4.065	0.828	0.759	1.084
TRINITY_DN1575_c0_g1_i10_orf1	uncharacterized protein LOC114359245 [Ostrinia furnacalis]	3.33510638	1.737732781	0.0005411	up	yes	3.762	1.128	1.028	1.09	1.266	3.377	4.233	3.675
TRINITY_DN103107_c0_g1_i2_orf1	superoxide dismutase [Cu-Zn] [Ostrinia furnacalis] >XP_028177872.1 superoxide dismutase [Cu-Zn] [Ostrinia furnacalis]	6.45912807	2.691339425	4.52E-05	up	yes	4.741	0.734	0.554	0.753	0.895	4.549	5.113	4.56
TRINITY_DN5177_c0_g1_i2_orf1	hemolin-like isoform X1 [Ostrinia furnacalis]	0.42073538	-1.249014945	6.65E-06	down	yes	2.792	6.636	6.835	6.665	6.408	2.802	2.811	2.764
TRINITY_DN24789_c0_g1_i9_orfp1	TRINITY_DN24789_c0_g1_i9_m.25888 TRINITY_DN24789_c0_g1::TRINITY_DN24789_c0_g1_i9::g.25888 ORF type:internal len:114 (-).score=12.65 TRINITY_DN24789_c0_g1_i9:2-340(-)	2.72761724	1.44764121	2.56E-05	up	yes	5.758	2.111	2.05	2.128	2.156	5.517	6.069	5.687
TRINITY_DN4204_c0_g1_i1_orf1	uncharacterized protein LOC114359352 [Ostrinia furnacalis]	0.34665309	-1.528435464	5.98E-05	down	yes	1.362	3.929	4.197	3.714	3.875	1.345	1.419	1.322
TRINITY_DN2406_c0_g1_i6_orf1	uncharacterized protein LOC114361672 [Ostrinia furnacalis]	2.084014	1.059364971	0.0005078	up	yes	3.572	1.714	1.626	1.802	1.714	3.42	3.919	3.378
TRINITY_DN14904_c0_g1_i1_orf1	attacin [Ostrinia furnacalis]	0.33424501	-1.581022054	4.51E-05	down	yes	2.933	8.775	8.823	9.278	8.223	2.871	2.981	2.948
TRINITY_DN12526_c0_g1_i5_orf1	uncharacterized protein LOC114359035 isoform X3 [Ostrinia furnacalis]	0.44540407	-1.166813349	0.0001052	down	yes	2.166	4.863	4.838	5.169	4.582	2.156	2.093	2.248
TRINITY_DN276_c0_g1_i2_orf1	protein lethal(2)essential for life-like [Ostrinia furnacalis] >UTU55753.1 small heat shock protein Hsp20.7 [Ostrinia furnacalis]	0.46503713	-1.104582189	4.00E-06	down	yes	1.503	3.232	3.171	3.258	3.266	1.507	1.433	1.568
TRINITY_DN1897_c0_g2_i4_orf1	phenoloxidase-activating factor 2-like [Hyposmocoma kahamanoa]	0.4333644	-1.206347455	0.008721	down	yes	1.395	3.219	3.95	3.015	2.691	1.306	1.482	1.397
TRINITY_DN10877_c0_g1_i1_orf1	spodomicin-like [Ostrinia furnacalis]	4.6735905	2.224531332	0.00346	up	yes	18.9	4.044	3.708	3.645	4.779	19.726	22.526	14.44
TRINITY_DN2407_c0_g1_i2_orf1	uncharacterized protein LOC114366345 isoform X2 [Ostrinia furnacalis]	4.98725687	2.318246513	5.93E-06	up	yes	7.436	1.491	1.34	1.409	1.725	7.327	7.724	7.256
TRINITY_DN9608_c0_g1_i3_orf1	cytochrome P450 monooxygenase CYP9G18 [Cnaphalocrocis medinalis]	0.45364175	-1.140374669	4.99E-05	down	yes	2.373	5.231	5.455	4.954	5.284	2.453	2.292	2.375
TRINITY_DN48694_c0_g1_i1_orfp1	TRINITY_DN48694_c0_g1_i1_m.75338 TRINITY_DN48694_c0_g1::TRINITY_DN48694_c0_g1_i1::g.75338 ORF type:internal len:84 (+).score=16.02 TRINITY_DN48694_c0_g1_i1:2-250(+)	13.2409241	3.726931907	1.64E-05	up	yes	4.012	0.303	0.258	0.155	0.496	3.795	4.173	4.067
TRINITY_DN276_c0_g1_i1_orf1	protein lethal(2)essential for life-like [Helicoverpa zea] >XP_049705426.1 protein lethal(2)essential for life [Helicoverpa armigera] >ATB54993.1 heat shock protein 20.8 [Helicoverpa armigera] >PZC74337.1 hypothetical protein B5X24_HaOG207971 [Helicoverpa	0.47105173	-1.086042592	6.99E-06	down	yes	1.375	2.919	2.998	2.924	2.835	1.381	1.405	1.34
TRINITY_DN66287_c0_g1_i1_orfp1	tenascin-like isoform X4 [Trichoplusia ni]	3.871218	1.952787552	0.0004216	up	yes	3.493	0.9023	0.875	0.933	0.899	3.548	3.879	3.053
TRINITY_DN28501_c0_g1_i2_orfp1	TRINITY_DN28501_c0_g1_i2_m.58934 TRINITY_DN28501_c0_g1::TRINITY_DN28501_c0_g1_i2::g.58934 ORF type:internal len:98 (+).score=13.70 TRINITY_DN28501_c0_g1_i2:3-293(+)	2.673021	1.418471173	2.00E-05	up	yes	8.273	3.095	3.149	2.994	3.141	8.157	8.692	7.971
TRINITY_DN14754_c0_g1_i6_orf1	cathepsin L [Papilio xuthus]	2.54383562	1.347005446	0.0006846	up	yes	5.571	2.19	2.306	2.174	2.09	6.27	5.26	5.183
TRINITY_DN16234_c0_g2_i3_orf1	uncharacterized protein LOC114363370 [Ostrinia furnacalis]	0.41689856	-1.262231713	8.99E-05	down	yes	1.648	3.953	4.11	4.07	3.679	1.561	1.7	1.684
TRINITY_DN57111_c0_g1_i1_orf1	trypsin-like serine proteinase T26 protein, partial [Chilo infuscatellus]	2.08305648	1.058701956	4.97E-05	up	yes	3.762	1.806	1.788	1.973	1.656	3.679	3.857	3.751
TRINITY_DN609_c0_g1_i1_orf1	zonadhesin-like isoform X1 [Ostrinia furnacalis]	2.05703839	1.04056872	4.70E-05	up	yes	5.626	2.735	2.803	2.829	2.574	5.377	5.681	5.819
TRINITY_DN20356_c0_g1_i5_orf1	uncharacterized protein LOC114362428 [Ostrinia furnacalis]	17.9847328	4.168700822	8.85E-06	up	yes	11.78	0.655	0.267	0.586	1.112	11.209	12.224	11.917
TRINITY_DN12920_c0_g3_i1_orf1	zonadhesin-like isoform X4 [Ostrinia furnacalis]	2.52610114	1.336912404	0.0006625	up	yes	3.097	1.226	1.242	1.171	1.265	2.962	3.478	2.851
TRINITY_DN124654_c0_g1_i1_orf1	protein lethal(2)essential for life [Manduca sexta] >KAG6441919.1 hypothetical protein O3G_MSEX002019 [Manduca sexta]	0.4090705	-1.289578599	0.001114	down	yes	1.822	4.454	4.577	4.922	3.863	1.828	1.889	1.749
TRINITY_DN4360_c0_g1_i4_orf1	glucose-6-phosphate isomerase-like, partial [Bicyclus anynana]	2.0870229	1.061446431	0.02037	up	yes	2.734	1.31	1.931	0.688	1.312	2.772	2.941	2.49
TRINITY_DN184_c0_g1_i1_orf1	macrophage mannose receptor 1-like isoform X1 [Maniola jurtina]	2.99706567	1.583550695	0.0003041	up	yes	2.962	0.9883	0.823	0.839	1.303	3.042	2.844	3.001
TRINITY_DN15000_c0_g1_i4_orf1	15-hydroxyprostaglandin dehydrogenase [NAD(+)]-like [Ostrinia furnacalis]	0.46293671	-1.111113128	0.000291	down	yes	0.9143	1.975	1.973	1.835	2.116	0.91	0.851	0.982
TRINITY_DN311_c0_g1_i8_orfp1	TRINITY_DN311_c0_g1_i8_m.65152 TRINITY_DN311_c0_g1::TRINITY_DN311_c0_g1_i8::g.65152 ORF type:5prime_partial len:138 (+).score=74.30 TRINITY_DN311_c0_g1_i8:2-415(+)	2.35398972	1.235108023	0.001045	up	yes	2.245	0.9537	0.972	0.882	1.007	2.518	2.014	2.202
TRINITY_DN26985_c0_g1_i5_orf1	secretory phospholipase A2 receptor-like [Helicoverpa zea]	3.18972896	1.673433839	1.49E-06	up	yes	2.236	0.701	0.759	0.666	0.678	2.233	2.269	2.207
TRINITY_DN1293_c1_g1_i4_orf1	putative fatty acyl-CoA reductase CG5065 [Ostrinia furnacalis]	2.19905629	1.136884536	0.00264	up	yes	2.004	0.9113	0.758	0.762	1.214	2.126	1.975	1.912
TRINITY_DN5770_c0_g1_i4_orf1	rotatin-like [Ostrinia furnacalis]	3.42857143	1.777607579	0.000114	up	yes	2.544	0.742	0.677	0.867	0.682	2.607	2.681	2.344
TRINITY_DN80424_c0_g1_i1_orf1	PREDICTED: cytoplasmic FMR1-interacting protein [Dufourea novaeangliae] >KZC10094.1 Cytoplasmic FMR1-interacting protein [Dufourea novaeangliae]	2.21106059	1.14473856	0.0007785	up	yes	2.091	0.9457	1.171	0.743	0.923	2.076	2.114	2.083
TRINITY_DN29190_c0_g1_i4_orf1	gloverin-like [Ostrinia furnacalis]	0.26660833	-1.907206235	7.76E-06	down	yes	0.7617	2.857	2.872	2.966	2.732	0.754	0.799	0.732
TRINITY_DN27300_c0_g1_i6_orfp1	TRINITY_DN27300_c0_g1_i6_m.71140 TRINITY_DN27300_c0_g1::TRINITY_DN27300_c0_g1_i6::g.71140 ORF type:internal len:129 (-).score=20.75 TRINITY_DN27300_c0_g1_i6:1-384(-)	2.37676439	1.248998893	3.43E-05	up	yes	2.189	0.921	0.932	0.862	0.969	2.295	2.149	2.122
TRINITY_DN1305_c0_g1_i6_orf1	glutathione S-transferase sigma 3 [Ostrinia furnacalis]	0.49733914	-1.007698124	4.86E-05	down	yes	1.028	2.067	1.999	2.031	2.172	1.06	1.004	1.019
TRINITY_DN50725_c0_g1_i6_orf1	BTB/POZ domain-containing protein 2-like [Ostrinia furnacalis]	3.11083123	1.637300129	0.002541	up	yes	2.47	0.794	0.736	0.867	0.779	2.846	2.556	2.007
TRINITY_DN1491_c0_g1_i4_orf1	GILT-like protein 2 isoform X1 [Ostrinia furnacalis] >XP_028156245.1 GILT-like protein 2 isoform X2 [Ostrinia furnacalis] >XP_028156247.1 GILT-like protein 2 isoform X3 [Ostrinia	2.5704142	1.362000857	0.0007069	up	yes	2.172	0.845	1.027	0.626	0.882	2.169	2.038	2.308
TRINITY_DN21170_c0_g1_i5_orf1	twitchin-like [Ostrinia furnacalis]	2.29540918	1.198751353	0.0002061	up	yes	1.955	0.8517	0.892	0.779	0.884	1.801	2.032	2.033
TRINITY_DN42964_c0_g1_i1_orf1	protein lethal(2)essential for life-like [Galleria mellonella]	0.39808429	-1.328854153	0.0001188	down	yes	0.7273	1.827	1.9	1.884	1.698	0.729	0.665	0.788
TRINITY_DN48497_c0_g1_i1_orf1	unnamed protein product [Chrysodeixis includens]	0.43885613	-1.188180029	0.0004926	down	yes	0.7443	1.696	1.797	1.742	1.55	0.677	0.85	0.706
TRINITY_DN9400_c0_g1_i1_orf1	lysophosphatidylserine lipase ABHD12 isoform X2 [Maniola hyperantus]	0.46209767	-1.113730282	1.12E-05	down	yes	0.8327	1.802	1.753	1.829	1.823	0.884	0.817	0.797
TRINITY_DN65681_c0_g1_i1_orf1	ferritin subunit-like [Ostrinia furnacalis] >XP_028168186.1 ferritin subunit-like [Ostrinia	0.37638436	-1.409721396	0.0008888	down	yes	0.6933	1.842	2.081	1.737	1.708	0.646	0.643	0.791
TRINITY_DN5126_c0_g2_i1_orf1	cytochrome P450 monooxygenase CYP4L47 [Ostrinia furnacalis]	0.27358843	-1.869920888	9.06E-05	down	yes	0.5863	2.143	2.237	1.957	2.236	0.548	0.567	0.644
TRINITY_DN65974_c0_g1_i2_orf1	uncharacterized protein LOC114362364 [Ostrinia furnacalis]	0.47672043	-1.068784641	0.0006118	down	yes	0.8867	1.86	1.909	1.684	1.987	0.919	0.935	0.806
TRINITY_DN41280_c0_g1_i2_orf1	unnamed protein product [Plutella xylostella]	0.49849669	-1.004344162	0.002249	down	yes	0.829	1.663	1.877	1.561	1.552	0.769	0.938	0.78
TRINITY_DN195_c8_g1_i1_orf1	hypothetical protein evm_009768 [Chilo suppressalis]	3.90911096	1.966840538	0.001136	up	yes	1.772	0.4533	0.505	0.409	0.446	1.532	2.064	1.719
TRINITY_DN29969_c0_g1_i5_orf1	twitchin-like [Ostrinia furnacalis]	2.07878018	1.055737207	0.02861	up	yes	1.636	0.787	0.762	0.604	0.995	1.302	2.069	1.538
TRINITY_DN40911_c0_g1_i1_orf1	peroxisomal membrane protein PEX16 [Ostrinia furnacalis]	2.47874016	1.309607044	5.65E-05	up	yes	1.574	0.635	0.573	0.649	0.683	1.583	1.499	1.64
TRINITY_DN920_c0_g1_i6_orf1	glutathione S-transferase omega 2 [Ostrinia furnacalis]	0.40685608	-1.29740953	3.32E-05	down	yes	0.6587	1.619	1.69	1.635	1.532	0.656	0.649	0.671
TRINITY_DN81181_c0_g1_i6_orfp1	C-type lectin domain family 4 member E [Pieris rapae]	2.10630199	1.074712295	0.001195	up	yes	1.494	0.7093	0.545	0.808	0.775	1.454	1.439	1.589
TRINITY_DN124711_c0_g1_i1_orf1	muskelin isoform X1 [Ostrinia furnacalis] >XP_028163274.1 muskelin isoform X2 [Ostrinia furnacalis]	0.47494852	-1.074156934	0.00185	down	yes	0.692	1.457	1.618	1.317	1.435	0.715	0.777	0.584
TRINITY_DN51938_c0_g3_i1_orf1	unnamed protein product [Mus musculus]	0.48923636	-1.031396455	0.0006633	down	yes	0.6727	1.375	1.36	1.499	1.266	0.646	0.729	0.643
TRINITY_DN52244_c1_g1_i1_orf1	triokinase/FMN cyclase-like isoform X2 [Ostrinia furnacalis]	3.35162602	1.744861178	2.93E-05	up	yes	1.649	0.492	0.439	0.462	0.575	1.597	1.715	1.634
TRINITY_DN32687_c0_g1_i2_orf1	protein D2-like isoform X2 [Ostrinia furnacalis] >XP_028164613.1 protein D2-like isoform X2 [Ostrinia furnacalis]	2.12502095	1.087477063	3.45E-05	up	yes	1.268	0.5967	0.567	0.563	0.66	1.264	1.285	1.254
TRINITY_DN3307_c1_g1_i2_orf1	BTB/POZ domain-containing protein 2-like [Ostrinia furnacalis]	2.15453195	1.10737449	0.0004249	up	yes	1.305	0.6057	0.644	0.546	0.627	1.387	1.335	1.194
TRINITY_DN100208_c0_g1_i1_orf1	neurofilament heavy polypeptide-like isoform X2 [Ostrinia furnacalis]	2.85454912	1.513262889	5.43E-06	up	yes	1.415	0.4957	0.461	0.517	0.509	1.439	1.435	1.37
TRINITY_DN1914_c0_g1_i4_orf1	loricrin-like [Ostrinia furnacalis]	0.41143533	-1.281262407	5.39E-06	down	yes	0.5217	1.268	1.261	1.306	1.236	0.501	0.535	0.529
TRINITY_DN198_c0_g1_i2_orf1	retinol dehydrogenase 13-like [Ostrinia furnacalis]	2.36603774	1.242473083	0.0001148	up	yes	1.254	0.53	0.531	0.532	0.527	1.275	1.325	1.162
TRINITY_DN5962_c0_g1_i1_orf1	tRNA (cytosine(34)-C(5))-methyltransferase [Ostrinia furnacalis]	0.43934295	-1.186580555	9.74E-05	down	yes	0.5483	1.248	1.33	1.183	1.232	0.541	0.533	0.571
TRINITY_DN69334_c0_g1_i1_orf1	PREDICTED: 15-hydroxyprostaglandin dehydrogenase [NAD(+)]-like [Papilio xuthus]	2.67268528	1.418289965	0.0001437	up	yes	1.273	0.4763	0.421	0.461	0.547	1.276	1.199	1.345
TRINITY_DN115082_c0_g1_i5_orf1	protein dj-1beta-like isoform X2 [Ostrinia furnacalis]	2.33044581	1.220605964	0.0001512	up	yes	1.103	0.4733	0.413	0.525	0.482	1.164	1.082	1.063
TRINITY_DN129835_c0_g1_i2_orf1	alpha-tocopherol transfer protein-like [Chelonus insularis]	2.01711068	1.012290246	0.00229	up	yes	0.9313	0.4617	0.533	0.451	0.401	0.881	0.87	1.043
TRINITY_DN2749_c4_g1_i2_orf1	RNA exonuclease 4-like [Ostrinia furnacalis] >OEE79882.1 REX4 [Ostrinia furnacalis]	0.42743363	-1.226227678	0.001944	down	yes	0.4347	1.017	1.052	1.061	0.939	0.339	0.572	0.393
TRINITY_DN33452_c0_g1_i3_orf1	lethal(2) giant larvae protein isoform X8 [Ostrinia furnacalis]	2.95997851	1.565586703	0.0003872	up	yes	1.102	0.3723	0.3	0.439	0.378	1.029	1.072	1.204
TRINITY_DN2749_c0_g2_i3_orf1	RNA exonuclease 4-like [Ostrinia furnacalis] >OEE79882.1 REX4 [Ostrinia furnacalis]	0.37010204	-1.434005004	3.41E-05	down	yes	0.3627	0.98	0.947	1.034	0.959	0.348	0.351	0.389
TRINITY_DN195_c4_g1_i1_orf1	beta-1,3-glucan-binding protein 1 [Ostrinia furnacalis]	3.2738285	1.710978747	0.003182	up	yes	1.027	0.3137	0.258	0.369	0.314	1.212	1.031	0.838
TRINITY_DN556_c0_g2_i1_orf1	serine protease inhibitor dipetalogastin-like [Ostrinia furnacalis]	2.36391515	1.241178255	0.004652	up	yes	0.925	0.3913	0.558	0.344	0.272	0.995	0.87	0.91
TRINITY_DN1013_c0_g1_i3_orf1	TELO2-interacting protein 1 homolog isoform X2 [Ostrinia furnacalis]	3.75081301	1.907203341	0.001018	up	yes	0.9227	0.246	0.24	0.183	0.315	0.811	1.049	0.908
TRINITY_DN27592_c0_g1_i1_orf1	D-arabinitol dehydrogenase 1-like [Ostrinia furnacalis]	4.80314465	2.263979258	0.0005743	up	yes	0.7637	0.159	0.125	0.172	0.18	0.851	0.653	0.787
TRINITY_DN43369_c0_g2_i1_orf1	cytochrome P450 monooxygenase 304 [Glyphodes pyloalis]	0.47280988	-1.080667905	0.00741	down	yes	0.333	0.7043	0.692	0.586	0.835	0.338	0.302	0.359
TRINITY_DN13221_c0_g1_i3_orf1	fasciclin-3-like [Ostrinia furnacalis]	0.41092105	-1.283066849	1.26E-05	down	yes	0.3123	0.76	0.742	0.794	0.744	0.31	0.315	0.312
TRINITY_DN311_c0_g1_i4_orfp1	TRINITY_DN311_c0_g1_i4_m.65135 TRINITY_DN311_c0_g1::TRINITY_DN311_c0_g1_i4::g.65135 ORF type:5prime_partial len:126 (+).score=71.21 TRINITY_DN311_c0_g1_i4:1-378(+)	0.49623394	-1.010907686	0.02111	down	yes	0.112	0.2257	0.235	0.226	0.216	0.09	0.074	0.172
TRINITY_DN6140_c0_g3_i3_orf1	CD63 antigen-like [Ostrinia furnacalis]	0.37157455	-1.428276414	0.002073	down	yes	0.08	0.2153	0.22	0.192	0.234	0.051	0.095	0.094
TRINITY_DN4959_c0_g1_i1_orf1	pancreatic triacylglycerol lipase-like [Ostrinia furnacalis]	0.3086661	-1.69588105	0.01596	down	yes	0.03633	0.1177	0.104	0.129	0.12	0.026	0.01	0.073
TRINITY_DN4356_c0_g1_i6_orf1	mulatexin-like [Ostrinia furnacalis]	0.39837545	-1.327799347	0.003799	down	yes	0.2207	0.554	0.538	0.496	0.628	0.181	0.182	0.299
TRINITY_DN33452_c0_g1_i1_orf1	lethal(2) giant larvae protein isoform X8 [Ostrinia furnacalis]	0.20289586	-2.301188664	0.0009452	down	yes	0.1093	0.5387	0.538	0.611	0.467	0.076	0.091	0.161
TRINITY_DN26411_c0_g1_i2_orfp1	TRINITY_DN26411_c0_g1_i2_m.24123 TRINITY_DN26411_c0_g1::TRINITY_DN26411_c0_g1_i2::g.24123 ORF type:internal len:115 (-).score=72.83 TRINITY_DN26411_c0_g1_i2:3-344(-)	0.20067804	-2.317045373	0.007023	down	yes	0.09767	0.4867	0.377	0.472	0.611	0.098	0.037	0.158
TRINITY_DN94355_c0_g1_i2_orf1	uncharacterized protein LOC126369488 [Pectinophora gossypiella]	2.58428635	1.369765936	0.003136	up	yes	0.174	0.06733	0.053	0.073	0.076	0.15	0.17	0.202

Accession	GO Term	GO ID	KO ID	KO Name	Pathway ID	Pathway_defition	EggNOG ID	EggNOG_Functional Categories	Pfam ID	Domain	Domain Description	Subcellular Loc	Unique Peptides	Coverage %	MW [kDa]	FDR Confidence
TRINITY_DN34406_c0_g2_i9_orfp1	------	------	------	------	------	------	------	------	------	------	------	CYT	1	11	9.3	High
TRINITY_DN131603_c0_g1_i4_orfp1	------	------	------	------	------	------	------	------	------	------	------	PLA	1	22	12.9	High
TRINITY_DN27300_c0_g1_i1_orfp1	------	------	------	------	------	------	------	------	------	------	------	CYT	1	21	9.2	High
TRINITY_DN14242_c0_g1_i2_orfp1	------	------	------	------	------	------	------	------	------	------	------	CYT	1	8	16	High
TRINITY_DN9044_c0_g1_i2_orf1	BP:immune s	GO:00023	K20692	GNBP3	map04624	Toll and Imd signali	ENOG410Z83	S:Function unknown;	PF15886.8	CBM39	Carbohydrate binding	CYT	1	10	11.4	High
TRINITY_DN64297_c0_g1_i1_orf1	BP:nitrogen c	GO:00068	------	------	------	------	COG0388	O:Posttranslational modification, p	------	------	------	CYT	1	57	7.8	High
TRINITY_DN20442_c0_g2_i1_orf1	BP:nucleoson	GO:00347	K11306	MYST4, KAT	------	------	COG5027	B:Chromatin structure and dynami	------	------	------	CYT	1	7	13.7	Medium
TRINITY_DN1091_c0_g3_i1_orf1	BP:response t	GO:00099	------	------	------	------	ENOG410XR	U:Intracellular trafficking, secretion	PF00059.24	Lectin_C	Lectin C-type domain	CYT	9	34	38.3	High
TRINITY_DN325_c0_g1_i15_orf1	CC:cellular cc	GO:00055	------	------	------	------	------	------	PF02363.22	C_tripleX	Cysteine rich repeat	PLA	2	16	51.6	High
TRINITY_DN19639_c0_g2_i1_orf1	------	------	------	------	------	------	ENOG410XR	S:Function unknown	PF03722.17	Hemocyanin	Hemocyanin, all-alph	CYT	2	72	8.6	High
TRINITY_DN30177_c0_g2_i1_orf1	------	------	------	------	------	------	ENOG4111N	S:Function unknown;	------	------	------	CYT	1	2	63	Medium
TRINITY_DN25234_c0_g1_i1_orf1	------	------	------	------	------	------	------	------	------	------	------	CYT	1	15	10	High
TRINITY_DN17574_c0_g1_i2_orf1	------	------	K09542	CRYAB	map04213;m	Longevity regulatinc	ENOG410YEI	O:Secondary metabolites biosynth	PF00011.24	HSP20	Hsp20/alpha crystallin	CYT	7	51	19.4	High
TRINITY_DN307_c1_g1_i1_orf1	------	------	------	------	------	------	ENOG410Y4	S:Function unknown	PF07648.18	Kazal_2	Kazal-type serine pro	CYT	3	35	10.4	High
TRINITY_DN85290_c0_g2_i1_orf1	------	------	K23615;K2342	SPIK5;SPINK	------	------	ENOG4111TI	O:Posttranslational modification, p	PF00050.24;	Kazal_1;Kazal	Kazal-type serine pro	CYT	1	56	7.6	High
TRINITY_DN18568_c0_g1_i2_orfp1	------	------	------	------	------	------	------	------	------	------	------	CYT	1	13	8.5	High
TRINITY_DN1575_c0_g1_i10_orf1	------	------	------	------	------	------	ENOG4111CI	S:Function unknown;	PF15868.8	MBF2	Transcription activato	CYT	1	2	45.9	High
TRINITY_DN103107_c0_g1_i2_orf1	MF:oxidoredu	GO:00167	K04565	SOD1	map05208;m	Chemical carcinoge	COG2032	P:Inorganic ion transport and meta	PF00080.23	Sod_Cu	Copper/zinc superoxic	PLA	1	47	8.5	High
TRINITY_DN5177_c0_g1_i2_orf1	------	------	------	------	------	------	ENOG410XS	S:Function unknown	PF07679.19;	I-set;Ig_3;ig;Ic	Immunoglobulin I-set	CYT	22	58	47.3	High
TRINITY_DN24789_c0_g1_i9_orfp1	------	------	------	------	------	------	------	------	------	------	------	CYT	5	46	13.2	High
TRINITY_DN4204_c0_g1_i1_orf1	CC:cellular cc	GO:00055	------	------	------	------	------	------	------	------	------	EXC	1	4	32.1	High
TRINITY_DN2406_c0_g1_i6_orf1	------	------	------	------	------	------	ENOG4110R	S:Function unknown	------	------	------	CYT	2	4	61.1	High
TRINITY_DN14904_c0_g1_i1_orf1	BP:interspecie	GO:00444	------	------	------	------	ENOG410ZB	S:Function unknown;	PF03769.18	Attacin_C	Attacin, C-terminal re	CYT	3	26	17.3	High
TRINITY_DN12526_c0_g1_i5_orf1	BP:nitrogen c	GO:00068	K12382	PSAP, SGP1	map00600;m	Sphingolipid metab	ENOG410XSI	O:Posttranslational modification, p	PF05184.18;	SapB_1;SapB_	Saposin-like type B, r	CYT	2	37	15.2	High
TRINITY_DN276_c0_g1_i2_orf1	MF:cation bir	GO:00431	K09542	CRYAB	map04213;m	Longevity regulating	ENOG410YEI	O:Posttranslational modification, p	PF00011.24	HSP20	Hsp20/alpha crystallin	CYT	2	43	20.8	High
TRINITY_DN1897_c0_g2_i4_orf1	------	------	------	------	------	------	------	------	PF18322.4	CLIP_1	Serine protease Clip d	CYT	1	7	13.9	Medium
TRINITY_DN10877_c0_g1_i1_orf1	CC:cellular cc	GO:00055	------	------	------	------	------	------	PF08036.14	Antimicrobial	Diapausin family of ar	CYT	1	19	6.9	Medium
TRINITY_DN2407_c0_g1_i2_orf1	BP:response t	GO:00096	------	------	------	------	ENOG4110S	S:Function unknown	PF00451.22	Toxin_2	Scorpion short toxin, E	CYT	1	36	21.5	High
TRINITY_DN9608_c0_g1_i3_orf1	CC:endoplasr	GO:00057	K15003	CYP9	------	------	COG2124	K:Transcription	PF00067.25	p450	Cytochrome P450	CYT	7	17	58.5	High
TRINITY_DN48694_c0_g1_i1_orfp1	------	------	------	------	------	------	------	------	------	------	------	CYT	1	13	9.1	Medium
TRINITY_DN276_c0_g1_i1_orf1	MF:cation bir	GO:00431	K09542	CRYAB	map04213;m	Longevity regulating	ENOG410YEI	O:Posttranslational modification, p	PF00011.24	HSP20	Hsp20/alpha crystallin	CYT	5	65	21.3	High
TRINITY_DN66287_c0_g1_i1_orfp1	BP:response t	GO:00096	------	------	------	------	------	------	------	------	------	CYT	2	25	11.9	High
TRINITY_DN28501_c0_g1_i2_orfp1	------	------	------	------	------	------	------	------	------	------	------	CYT	1	30	10.9	High
TRINITY_DN14754_c0_g1_i6_orf1	BP:primary m	GO:00442	------	------	------	------	COG4870	O:Posttranslational modification, p	PF00112.26	Peptidase_C1	Papain family cysteine	CYT	1	41	12.8	High
TRINITY_DN16234_c0_g2_i3_orf1	CC:cellular cc	GO:00055	------	------	------	------	ENOG410Y67	S:Function unknown	------	------	------	CYT	3	51	7.4	High
TRINITY_DN57111_c0_g1_i1_orf1	BP:organic su	GO:00717	------	------	------	------	------	------	------	------	------	CYT	1	26	7.9	High
TRINITY_DN609_c0_g1_i1_orf1	------	------	------	------	------	------	COG4826;EN	O:Posttranslational modification, p	PF01826.20	TIL	Trypsin Inhibitor like c	PLA	9	18	109	High
TRINITY_DN20356_c0_g1_i5_orf1	CC:cellular cc	GO:00055	------	------	------	------	ENOG4110O	S:Function unknown	------	------	------	CYT	1	2	60	High
TRINITY_DN12920_c0_g3_i1_orf1	------	------	------	------	------	------	------	------	PF01826.20	TIL	Trypsin Inhibitor like c	CYT	1	13	8.8	High
TRINITY_DN124654_c0_g1_i1_orf1	------	------	K09542	CRYAB	map04213;m	Longevity regulating	ENOG410YEI	P:Inorganic ion transport and meta	PF00011.24	HSP20	Hsp20/alpha crystallin	CYT	6	44	22.4	High
TRINITY_DN4360_c0_g1_i4_orf1	BP:ribonuclec	GO:00091	K01810	GPI, pgi	map00520;m	Amino sugar and nu	COG0166	G:Carbohydrate transport and met	PF00342.22	PGI	Phosphoglucose isom	CYT	1	60	4.9	High
TRINITY_DN184_c0_g1_i1_orf1	------	------	------	------	------	------	ENOG410XR	U:Intracellular trafficking, secretion	PF00059.24	Lectin_C	Lectin C-type domain	CYT	1	14	35.7	High
TRINITY_DN15000_c0_g1_i4_orf1	------	------	K00069	HPGD	map00590;m	Arachidonic acid me	COG1028	O:Posttranslational modification, p	PF00106.28;	adh_short;adh	short chain dehydroge	CYT	5	18	27.4	High
TRINITY_DN311_c0_g1_i8_orfp1	------	------	------	------	------	------	------	------	------	------	------	PLA	1	18	15	High
TRINITY_DN26985_c0_g1_i5_orf1	MF:moleculai	GO:00036	------	------	------	------	ENOG410XP	S:Function unknown;U:Intracellulai	------	------	------	CYT	2	26	9.8	High
TRINITY_DN1293_c1_g1_i4_orf1	BP:primary m	GO:00442	K13356	FAR	map04146;m	Peroxisome;Cutin, su	ENOG410XS	S:Function unknown	PF07993.15;	NAD_binding	Male sterility protein;N	CYT	1	3	56.9	High
TRINITY_DN5770_c0_g1_i4_orf1	------	------	------	------	------	------	ENOG4110T	S:Function unknown;O:Secondary	------	------	------	CYT	1	1	111.2	Medium
TRINITY_DN80424_c0_g1_i1_orf1	BP:regulation	GO:0033	K05749	CYFIP	map05130;m	Pathogenic Escheric	ENOG410XPI	S:Function unknown	PF05994.14	FragX_IP	Cytoplasmic Fragile-X	CYT	1	2	69.2	High
TRINITY_DN29190_c0_g1_i4_orf1	BP:interspecie	GO:00444	------	------	------	------	ENOG4110O	S:Function unknown	PF10793.12	Gloverin	Gloverin-like protein	PLA	1	28	11.5	High
TRINITY_DN27300_c0_g1_i6_orfp1	------	------	------	------	------	------	------	------	------	------	------	CYT	1	9	14.4	High
TRINITY_DN1305_c0_g1_i6_orf1	MF:catalytic a	GO:00038	K04097	HPGDS	map00590;m	Arachidonic acid me	ENOG41103	O:Posttranslational modification, p	PF02798.23;	GST_N;GST_C	Glutathione S-transfe	CYT	2	26	19.4	High
TRINITY_DN50725_c0_g1_i6_orf1	BP:developm	GO:00325	K10478	BTBD3_6	------	------	ENOG410XQ	S:Function unknown	PF00651.34;	BTB;MATH;BA	BTB/POZ domain;MA	CYT	3	9	45.4	High
TRINITY_DN1491_c0_g1_i4_orf1	MF:catalytic a	GO:00038	K08059	IFI30, GILT	map04612	Antigen processing	ENOG4111ID	S:Function unknown	PF03227.19	GILT	Gamma interferon inc	CYT	1	16	26.5	High
TRINITY_DN21170_c0_g1_i5_orf1	------	------	------	------	------	------	ENOG410XP8	S:Function unknown;	PF00041.24	fn3	Fibronectin type III do	CYT	1	52	17	High
TRINITY_DN42964_c0_g1_i1_orf1	------	------	K09542	CRYAB	map04213;m	Longevity regulatinc	ENOG410YEI	O:Posttranslational modification, p	PF00011.24	HSP20	Hsp20/alpha crystallin	CYT	1	5	23.2	High
TRINITY_DN48497_c0_g1_i1_orf1	------	------	K11493	RCC1	------	------	COG5184	O:Posttranslational modification, p	PF00415.21;	RCC1;RCC1_2	Regulator of chromos	CYT	1	12	10	High
TRINITY_DN9400_c0_g1_i1_orf1	------	------	K13704	ABHD12	------	------	ENOG4111FI	S:Function unknown;	PF00561.23;	Abhydrolase	alpha/beta hydrolase	CYT	1	3	29.8	High
TRINITY_DN65681_c0_g1_i1_orf1	BP:ion home	GO:00508	K00522	FTH1	map04978;m	Mineral absorption;I	ENOG4111M	P:Inorganic ion transport and meta	PF00210.27	Ferritin	Ferritin-like domain	CYT	1	31	8.1	High
TRINITY_DN5126_c0_g2_i1_orf1	MF:cation bir	GO:00431	K15001	CYP4	------	------	COG2124	I:Lipid transport and metabolism	PF00067.25	p450	Cytochrome P450	CYT	1	7	13	High
TRINITY_DN65974_c0_g1_i2_orf1	------	------	------	------	------	------	ENOG410Y2I	S:Function unknown	------	------	------	CYT	2	26	13	High
TRINITY_DN41280_c0_g1_i2_orf1	MF:moleculai	GO:00036	------	------	------	------	ENOG4111ZI	S:Function unknown	------	------	------	CYT	1	35	8	High
TRINITY_DN195_c8_g1_i1_orf1	BP:interspecie	GO:00444	------	------	------	------	ENOG410ZB	S:Function unknown	PF03769.18	Attacin_C	Attacin, C-terminal re	CYT	1	4	21.6	Medium
TRINITY_DN29969_c0_g1_i5_orf1	------	------	------	------	------	------	ENOG410XQ	S:Function unknown;	PF00041.24	fn3	Fibronectin type III do	CYT	1	38	16.3	High
TRINITY_DN40911_c0_g1_i1_orf1	BP:organelle	GO:00069	K13335	PEX16	map04146	Peroxisome	ENOG4111GI	S:Function unknown	PF08610.13	Pex16	Peroxisomal membrar	CYT	1	4	38.2	High
TRINITY_DN920_c0_g1_i6_orf1	BP:metabolic	GO:00081	K00310	SE	map00480;m	Glutathione metabc	ENOG410XSI	O:Posttranslational modification, p	PF13417.9;F	GST_N_3;GST	Glutathione S-transfe	CYT	1	11	32.3	High
TRINITY_DN81181_c0_g1_i6_orfp1	MF:moleculai	GO:00036	------	------	------	------	------	------	------	------	------	CYT	1	14	9.7	High
TRINITY_DN124711_c0_g1_i1_orf1	------	------	K23337	MKLN1	------	------	ENOG410YJK	S:Function unknown	PF06588.14	Muskelin_N	Muskelin N-terminus	CYT	1	4	31.3	High
TRINITY_DN51938_c0_g3_i1_orf1	BP:positive re	GO:20012	K08056	PDIA3, GRP5	map05163;m	Human cytomegalo	COG0526	E:Amino acid transport and metab	PF00085.23;	Thioredoxin;T	Thioredoxin;Thioredo	CYT	1	4	40.6	High
TRINITY_DN52244_c1_g1_i1_orf1	BP:phosphory	GO:00163	------	------	------	------	COG2376	G:Carbohydrate transport and met	PF02733.20	Dak1	Dak1 domain	CYT	1	22	8.2	High
TRINITY_DN32687_c0_g1_i2_orf1	------	------	K06910	PEBP, TFS1	------	------	COG1881	S:Function unknown	------	------	------	CYT	1	66	11.6	High
TRINITY_DN3307_c1_g1_i2_orf1	------	------	------	------	------	------	ENOG410XO	S:Function unknown	PF00651.34;	BTB;MATH;BA	BTB/POZ domain;MA	CYT	2	7	47	High
TRINITY_DN100208_c0_g1_i1_orf1	------	------	------	------	------	------	------	------	------	------	------	CYT	1	88	8.4	High
TRINITY_DN1914_c0_g1_i4_orf1	------	------	------	------	------	------	ENOG4110O	S:Function unknown;	------	------	------	CYT	1	15	58.5	High
TRINITY_DN198_c0_g1_i2_orf1	------	------	K11153	RDH12	map00830;m	Retinol metabolism;	COG1028	V:Defense mechanisms	PF00106.28;	adh_short;adh	short chain dehydroge	CYT	1	4	35.3	High
TRINITY_DN5962_c0_g1_i1_orf1	CC:intracellul	GO:00432	K15335	NSUN2, TRM	------	------	COG0144	J:Translation, ribosomal structure a	PF01189.20	Methyltr_Rsm	16S rRNA methyltrans	CYT	6	10	83.8	High
TRINITY_DN69334_c0_g1_i1_orf1	MF:catalytic a	GO:00038	K00069	HPGD	map00590;m	Arachidonic acid me	COG1028	S:Function unknown	PF00106.28;	adh_short;adh	short chain dehydroge	CYT	3	11	29.1	High
TRINITY_DN115082_c0_g1_i5_orf1	------	------	K05687	PARK7	map05012;m	Parkinson disease;P	COG0693	S:Function unknown	PF01965.27	DJ-1_PfpI	DJ-1/PfpI family	CYT	1	45	11.4	High
TRINITY_DN129835_c0_g1_i2_orf1	------	------	------	------	------	------	ENOG410XR	S:Function unknown	PF00650.23;	CRAL_TRIO;C	CRAL/TRIO domain;Di	CYT	1	2	35.4	Medium
TRINITY_DN2749_c4_g1_i2_orf1	BP:nucleic ac	GO:00903	K18327	REXO4, REX4	------	------	COG0847	S:Function unknown	PF00929.27	RNase_T	Exonuclease	CYT	3	53	9.2	High
TRINITY_DN33452_c0_g1_i3_orf1	BP:secretion;I	GO:00469	K06094	LLGL	map04530;m	Tight junction;Huma	ENOG410XS	S:Function unknown	------	------	------	CYT	1	18	10.7	High
TRINITY_DN2749_c0_g2_i3_orf1	BP:nucleic ac	GO:00903	K18327	REXO4, REX4	------	------	COG0847	L:Replication, recombination and r	PF00929.27	RNase_T	Exonuclease	CYT	5	45	17.8	High
TRINITY_DN195_c4_g1_i1_orf1	BP:immune s	GO:00023	K20692	GNBP3	map04624	Toll and Imd signali	ENOG410Z83	S:Function unknown;	PF15886.8	CBM39	Carbohydrate binding	CYT	1	19	14.1	High
TRINITY_DN556_c0_g2_i1_orf1	------	------	K23615	SPIK5	------	------	ENOG410Z5I	S:Function unknown;O:Posttransla	PF00050.24;	Kazal_1;Kazal	Kazal-type serine pro	EXC	1	19	30.5	High
TRINITY_DN1013_c0_g1_i3_orf1	------	------	K20403	TTI1	map04150	mTOR signaling pat	ENOG410XSI	S:Function unknown;	------	------	------	CYT	1	1	124.9	Medium
TRINITY_DN27592_c0_g1_i1_orf1	MF:oxidoredu	GO:00164	K17818	ARD1	map00040	Pentose and glucurc	COG1063	E:Amino acid transport and metab	PF00107.29	ADH_zinc_N	Zinc-binding dehydro	CYT	1	59	15.1	High
TRINITY_DN43369_c0_g2_i1_orf1	CC:integral cc	GO:00160	------	------	------	------	COG2124	S:Function unknown	------	------	------	CYT	1	8	13	High
TRINITY_DN13221_c0_g1_i3_orf1	------	------	------	------	------	------	ENOG4111K	S:Function unknown	PF08205.15;	C2-set_2;Ig_3	CD80-like C2-set imn	CYT	1	46	19	High
TRINITY_DN311_c0_g1_i4_orfp1	------	------	------	------	------	------	------	------	------	------	------	EXC	1	6	13.8	High
TRINITY_DN6140_c0_g3_i3_orf1	CC:cellular_cc	GO:00055	K06497	CD63, MLA1	map05205;m	Proteoglycans in car	ENOG4111IR	O:Posttranslational modification, p	PF00335.23	Tetraspanin	Tetraspanin family	CYT	1	4	32.2	High
TRINITY_DN4959_c0_g1_i1_orf1	------	------	------	------	------	------	ENOG410YG	S:Function unknown;	PF00151.22	Lipase	Lipase	CYT	1	7	37.1	High
TRINITY_DN4356_c0_g1_i6_orf1	------	------	------	------	------	------	------	------	------	------	------	CYT	1	5	20.1	High
TRINITY_DN33452_c0_g1_i1_orf1	BP:secretion;I	GO:00469	K06094	LLGL	map04530;m	Tight junction;Huma	ENOG410XS	S:Function unknown	------	------	------	CYT	1	14	13.7	High
TRINITY_DN26411_c0_g1_i2_orfp1	------	------	------	------	------	------	------	------	------	------	------	CYT	1	46	10.9	High
TRINITY_DN94355_c0_g1_i2_orf1	------	------	------	------	------	------	ENOG410Z1	S:Function unknown;L:Replication,	PF05380.16;	Peptidase_A1	Pao retrotransposon p	CYT	1	0	179.7	High
