# Supplementary material for: Comparative Proteomics and Metabonomics Analysis of Different Diapause Stages Revealed a New Regulation Mechanism of Diapause in Loxostege sticticalis (Lepidoptera: Pyralidae)
Source: Molecules. 2024 Jul 25;29(15):3472. doi: 10.3390/molecules29153472 (PMC11314584; doi:10.3390/molecules29153472)
Supplement: Supplementary file 1 [file molecules-29-03472-s001.zip › analysis process/proteomic/diffreential protein statistic table/PreDvsRD.pdf]

Accession	Description	FC(PreD/RD)	Log2FC(PreD/RD)	P_value(PreD/RD)	Regulate	Significant	PreD	RD	RD1	RD2	RD3	PreD1	PreD2	PreD3	GO Term	GO ID	KO ID	KO Name	Pathway ID	Pathway defition	EggNOG ID	EggNOG Functional Categories	Pfam ID	Domain	Domain Description	Subcellular Loc	Unique Peptides	Coverage %	MW [kDa]	FDR Confidence
TRINITY_DN20356_c0_g1_i5_orf1	uncharacterized protein LOC114362428 [Ostrinia furnacalis]	7.853333333	2.973305133	6.56E-06	up	yes	11.78	1.5	1.765	1.464	1.271	11.209	12.224	11.917	CC:cellular_component;CC:integral component of membrane;CC:cellular anatomical entity;CC:intrinsic component of membrane;	GO:0005575;GO:0016021;GO:0110165;GO:0031224	------	------	------	------	ENOG4110QHP	S:Function unknown	------	------	------	CYT	1	2	60	High
TRINITY_DN56155_c0_g1_i1_orf1	hypothetical protein evm_004679 [Chilo suppressalis] >CAH2989683.1 unnamed protein product [Chilo suppressalis]	0.061627747	-4.020276134	3.55E-07	down	yes	0.8973	14.56	14.637	14.883	14.164	0.958	0.856	0.878	MF:pigment binding;MF:molecular_function;MF:binding;	GO:0031409;GO:0003674;GO:0005488	K03098	APOD	------	------	COG3040	Z:Cytoskeleton	PF00061.26;PF08212.15	Lipocalin;Lipocalin_2	Lipocalin / cytosolic fatty-acid binding protein family;Lipocalin-like domain	CYT	19	58	34.1	High
TRINITY_DN276_c0_g1_i2_orf1	protein lethal(2)essential for life-like [Ostrinia furnacalis] >UTU55753.1 small heat shock protein Hsp20.7 [Ostrinia furnacalis]	0.127914894	-2.966743842	3.80E-05	down	yes	1.503	11.75	11.772	12.626	10.844	1.507	1.433	1.568	MF:cation binding;MF:metal ion binding;MF:molecular_function;MF:binding;MF:ion binding;	GO:0043169;GO:0046872;GO:0003674;GO:0005488;GO:0043167	K09542	CRYAB	map04213;ma	Longevity regulating pathway - multiple species;Protein processing in	ENOG410YERS	O:Posttranslational modification, protein turnover, chaperones	PF00011.24	HSP20	Hsp20/alpha crystallin family	CYT	2	43	20.8	High
TRINITY_DN1124_c0_g1_i7_orf1	PREDICTED: cuticle protein 18.6, isoform B [Amyelois transitella]	0.070199362	-3.83239827	5.05E-06	down	yes	0.8803	12.54	12.45	13.19	11.979	0.933	0.833	0.875	MF:molecular_function;MF:structural constituent of cuticle;MF:structural molecule activity;	GO:0003674;GO:0042302;GO:0005198	------	------	------	------	ENOG4111Y6E	S:Function unknown	PF00379.26	Chitin_bind_4	Insect cuticle protein	CYT	4	18	27.2	High
TRINITY_DN30154_c0_g1_i1_orf1	serine/threonine-protein kinase SIK2 [Ostrinia furnacalis] >XP_028174514.1 serine/threonine-protein kinase SIK2 [Ostrinia furnacalis]	0.111736717	-3.161824759	3.85E-07	down	yes	1.409	12.61	12.645	12.793	12.382	1.435	1.169	1.623	BP:phosphorylation;BP:nitrogen compound metabolic process;BP:organic substance metabolic process;BP:protein phosphorylation;BP:cellular process;BP:macromolecule modification;BP:protein modification process;BP:biological_process;BP:metabolic process;BP:primary metabolic process;BP:organonitrogen compound metabolic process;BP:cellular metabolic process;BP:macromolecule metabolic process;BP:protein metabolic process;BP:phosphate-containing compound metabolic process;BP:phosphorus metabolic process;MF:purine ribonucleoside triphosphate binding;MF:molecular_function;MF:binding;MF:ion binding;MF:heterocyclic compound binding;MF:nucleoside phosphate binding;MF:anion binding;MF:carbohydrate derivative binding;MF:nucleotide binding;MF:protein serine/threonine kinase activity;MF:transferase activity;MF:protein kinase activity;MF:purine nucleotide binding;MF:ATP binding;MF:kinase activity;MF:catalytic activity, acting on a protein;MF:small molecule binding;MF:catalytic activity;MF:adenyl nucleotide binding;MF:phosphotransferase activity, alcohol group as acceptor;MF:transferase activity, transferring phosphorus-containing groups;MF:adenyl ribonucleotide binding;MF:purine ribonucleotide binding;MF:organic cyclic compound binding;MF:ribonucleotide binding;	GO:0016310;GO:0006807;GO:0071704;GO:0006468;GO:0009987;GO:0043412;GO:0036211;GO:0008150;GO:0008152;GO:0044238;GO:1901564;GO:0044237;GO:0043170;GO:0019538;GO:0006796;GO:0006793;GO:0035639;GO:0003674;GO:0005488;GO:0043167;GO:1901363;GO:1901265;GO:0043168;GO:0097367;GO:0000166;GO:0004674;GO:0016740;GO:0004672;GO:0017076;GO:0005524;GO:0016301;GO:0140096;GO:0036094;GO:0003824;GO:0030554;GO:0016773;GO:0016772;GO:0032559;GO:0032555;GO:0097159;GO:0032553	K16311	SIK2	map04922	Glucagon signaling pathway	ENOG410XNQ0	T:Signal transduction mechanisms	PF00069.28;PF07714.20;PF14531.9	Pkinase;PK_Tyr_Ser-Thr;Kinase-like	Protein kinase domain;Protein tyrosine and serine/threonine kinase;Kinase-like	CYT	1	1	136.1	Medium
TRINITY_DN40669_c0_g2_i1_orf1	uncharacterized protein LOC114355531 [Ostrinia furnacalis]	0.046431043	-4.4287665	4.77E-06	down	yes	0.9393	20.23	20.114	21.28	19.295	1	0.913	0.905	------	------	------	------	------	------	ENOG4110S60	S:Function unknown	------	------	------	CYT	6	59	9.5	High
TRINITY_DN2396_c0_g1_i9_orfp1	TRINITY_DN2396_c0_g1_i9_m.39038 TRINITY_DN2396_c0_g1::TRINITY_DN2396_c0_g1_i9::g.39038 ORF type:5prime_partial len:181 (+),score=90.33 TRINITY_DN2396_c0_g1_i9:3-545(+)	2.749579832	1.459211175	0.000236	up	yes	32.72	11.9	10.922	13.343	11.434	30.425	35.527	32.211	------	------	------	------	------	------	------	------	------	------	------	CYT	1	6	19.3	High
TRINITY_DN1506_c0_g1_i6_orfp1	TRINITY_DN1506_c0_g1_i6_m.57691 TRINITY_DN1506_c0_g1::TRINITY_DN1506_c0_g1_i6::g.57691 ORF type:5prime_partial len:173 (+),score=30.49 TRINITY_DN1506_c0_g1_i6:1-519(+)	18.95989797	4.244879296	1.38E-06	up	yes	13.38	0.7057	0.75	0.552	0.815	13.483	13.775	12.871	------	------	------	------	------	------	------	------	------	------	------	CYT	1	7	19	High
TRINITY_DN971_c0_g1_i10_orfp1	TRINITY_DN971_c0_g1_i10_m.54268 TRINITY_DN971_c0_g1::TRINITY_DN971_c0_g1_i10::g.54268 ORF type:internal len:187 (+),score=141.68 TRINITY_DN971_c0_g1_i10:1-558(+)	3.839201878	1.940806424	9.41E-05	up	yes	32.71	8.52	8.668	8.635	8.256	31.231	35.761	31.133	------	------	------	------	------	------	------	------	------	------	------	CYT	2	20	20.5	High
TRINITY_DN32514_c0_g2_i1_orf1	mucin-5AC-like [Ostrinia furnacalis]	0.060037969	-4.057981024	8.91E-05	down	yes	1.265	21.07	21.41	23.006	18.79	1.076	1.543	1.175	------	------	------	------	------	------	------	------	------	------	------	CYT	1	9	14.6	High
TRINITY_DN5444_c0_g1_i1_orfp1	TRINITY_DN5444_c0_g1_i1_m.14077 TRINITY_DN5444_c0_g1::TRINITY_DN5444_c0_g1_i1::g.14077 ORF type:3prime_partial len:90 (-),score=25.11 TRINITY_DN5444_c0_g1_i1:2-268(-)	0.401670259	-1.315916453	0.0004368	down	yes	2.982	7.424	6.953	8.205	7.113	3.254	2.849	2.843	------	------	------	------	------	------	------	------	------	------	------	CYT	1	8	9.9	Medium
TRINITY_DN14754_c0_g1_i6_orf1	cathepsin L [Papilio xuthus]	8.654652789	3.113475942	0.000162	up	yes	5.571	0.6437	0.569	0.567	0.795	6.27	5.26	5.183	BP:primary metabolic process;BP:organonitrogen compound metabolic process;BP:protein metabolic process;BP:macromolecule metabolic process;BP:organic substance metabolic process;BP:nitrogen compound metabolic process;BP:biological_process;BP:metabolic process;BP:proteolysis;MF:molecular_function;MF:hydrolase activity;MF:catalytic activity;MF:catalytic activity, acting on a protein;MF:peptidase activity;MF:cysteine-type peptidase activity;	GO:0044238;GO:1901564;GO:0019538;GO:0043170;GO:0071704;GO:0006807;GO:0008150;GO:0008152;GO:0006508;GO:0003674;GO:0016787;GO:0003824;GO:0140096;GO:0008233;GO:0008234	------	------	------	------	COG4870	O:Posttranslational modification, protein turnover, chaperones	PF00112.26	Peptidase_C1	Papain family cysteine protease	CYT	1	41	12.8	High
TRINITY_DN10747_c0_g1_i5_orf1	unnamed protein product [Plutella xylostella]	0.22794428	-2.133246886	7.04E-06	down	yes	1.08	4.738	4.781	4.915	4.517	1.056	1.046	1.139	------	------	------	------	------	------	ENOG410XRSO	S:Function unknown	PF00650.23	CRAL_TRIO	CRAL/TRIO domain	CYT	10	36	41.8	High
TRINITY_DN138481_c0_g1_i2_orf1	cuticle protein 8-like isoform X2 [Vanessa tameamea]	0.174121339	-2.521835076	3.16E-07	down	yes	0.8323	4.78	4.769	4.874	4.696	0.891	0.815	0.791	MF:molecular_function;MF:structural constituent of cuticle;MF:structural molecule activity;	GO:0003674;GO:0042302;GO:0005198	------	------	------	------	ENOG410YRDB;EN	S:Function unknown;	PF00379.26	Chitin_bind_4	Insect cuticle protein	CYT	2	27	24.2	High
TRINITY_DN74086_c0_g1_i1_orf1	UPF0489 protein C5orf22 homolog [Ostrinia furnacalis]	0.444512947	-1.169702655	0.0002125	down	yes	1.442	3.244	3.193	3.122	3.417	1.275	1.405	1.646	------	------	------	------	------	------	ENOG41109E9	S:Function unknown	PF12640.10	UPF0489	UPF0489 domain	CYT	1	4	42.6	High
TRINITY_DN14328_c0_g1_i12_orf1	larval cuticle protein LCP-30-like [Ostrinia furnacalis]	3.415238095	1.77198616	1.09E-05	up	yes	3.586	1.05	1.048	0.991	1.11	3.454	3.75	3.555	MF:molecular_function;MF:structural constituent of cuticle;MF:structural molecule activity;	GO:0003674;GO:0042302;GO:0005198	------	------	------	------	ENOG41112O7;EN	S:Function unknown;	PF00379.26	Chitin_bind_4	Insect cuticle protein	CYT	10	50	24.6	High
TRINITY_DN143497_c0_g1_i1_orf1	fibroin heavy chain-like [Ostrinia furnacalis]	0.223574686	-2.161171242	7.20E-07	down	yes	0.7843	3.508	3.52	3.574	3.43	0.73	0.826	0.797	------	------	------	------	------	------	ENOG4111C6D	S:Function unknown	------	------	------	CYT	7	39	19.4	High
TRINITY_DN57111_c0_g1_i1_orf1	trypsin-like serine proteinase T26 protein, partial [Chilo infuscatellus]	8.17826087	3.031794083	6.31E-07	up	yes	3.762	0.46	0.41	0.458	0.512	3.679	3.857	3.751	BP:organic substance metabolic process;BP:organonitrogen compound metabolic process;BP:protein metabolic process;BP:macromolecule metabolic process;BP:biological_process;BP:nitrogen compound metabolic process;BP:metabolic process;BP:proteolysis;BP:primary metabolic process;MF:serine-type endopeptidase activity;MF:endopeptidase activity;MF:hydrolase activity;MF:serine hydrolase activity;MF:catalytic activity, acting on a protein;MF:catalytic activity;MF:molecular_function;MF:peptidase activity;MF:serine-type peptidase activity;	GO:0071704;GO:1901564;GO:0019538;GO:0043170;GO:0008150;GO:0006807;GO:0008152;GO:0006508;GO:0044238;GO:0004252;GO:0004175;GO:0016787;GO:0017171;GO:0140096;GO:0003824;GO:0003674;GO:0008233;GO:0008236	------	------	------	------	------	------	------	------	------	CYT	1	26	7.9	High
TRINITY_DN121802_c0_g1_i6_orfp1	TRINITY_DN121802_c0_g1_i6_m.78506 TRINITY_DN121802_c0_g1::TRINITY_DN121802_c0_g1_i6::g.78506 ORF type:3prime_partial len:123 (+),score=6.04 TRINITY_DN121802_c0_g1_i6:35-367(+)	2.535745808	1.342410132	0.0004589	up	yes	2.873	1.133	1.113	0.998	1.287	2.659	3.142	2.817	------	------	------	------	------	------	------	------	------	------	------	PLA	1	32	12.2	High
TRINITY_DN19110_c0_g1_i2_orf1	peroxidase [Ostrinia furnacalis]	0.483141904	-1.049481107	0.0007768	down	yes	1.304	2.699	2.502	2.955	2.639	1.389	1.359	1.163	------	------	K19511	PXDN, VPO1	------	------	ENOG410XPZ3	M:Cell wall/membrane/envelope	PF03098.18	An_peroxidase	Animal haem peroxidase	CYT	1	2	84.9	High
TRINITY_DN52553_c0_g2_i1_orf1	hemocyte protein-glutamine gamma-glutamyltransferase-like [Ostrinia furnacalis]	0.108413218	-3.205387437	5.93E-05	down	yes	0.9613	8.867	9.519	9.05	8.031	1.109	0.864	0.911	BP:primary metabolic process;BP:peptide cross-linking;BP:organonitrogen compound metabolic process;BP:organic substance metabolic process;BP:nitrogen compound metabolic process;BP:macromolecule modification;BP:macromolecule metabolic process;BP:biological_process;BP:protein modification process;BP:metabolic process;BP:protein metabolic process;MF:molecular_function;MF:protein-glutamine gamma-glutamyltransferase activity;MF:catalytic activity, acting on a protein;MF:catalytic activity;MF:transferase activity;MF:transferase activity, transferring acyl groups;MF:transferase activity, transferring amino-acyl groups;	GO:0044238;GO:0018149;GO:1901564;GO:0071704;GO:0006807;GO:0043412;GO:0043170;GO:0008150;GO:0036211;GO:0008152;GO:0019538;GO:0003674;GO:0003810;GO:0140096;GO:0003824;GO:0016740;GO:0016746;GO:0016755	K05619	TGM1	------	------	ENOG410XQEZ	S:Function unknown	PF00868.23	Transglut_N	Transglutaminase family	CYT	2	11	25.2	High
TRINITY_DN129835_c0_g1_i2_orf1	alpha-tocopherol transfer protein-like [Chelonus insularis]	0.114776929	-3.123095419	3.36E-08	down	yes	0.9313	8.114	8.168	8.09	8.084	0.881	0.87	1.043	------	------	------	------	------	------	ENOG410XRSQ	S:Function unknown	PF00650.23;PF13716.9	CRAL_TRIO;CRAL_TRIO_2	CRAL/TRIO domain;Divergent CRAL/TRIO domain	CYT	1	2	35.4	Medium
TRINITY_DN138481_c0_g1_i5_orf1	hypothetical protein evm_003901 [Chilo suppressalis]	0.176772451	-2.500034638	4.59E-05	down	yes	1.309	7.405	7.965	7.389	6.862	1.384	1.22	1.324	MF:molecular_function;MF:structural constituent of cuticle;MF:structural molecule activity;	GO:0003674;GO:0042302;GO:0005198	------	------	------	------	ENOG410YRDB;EN	S:Function unknown;	PF00379.26	Chitin_bind_4	Insect cuticle protein	CYT	2	32	22.6	High
TRINITY_DN4592_c0_g1_i1_orf1	hypothetical protein KGM_205563 [Danaus plexippus plexippus]	0.146527778	-2.770753908	3.65E-06	down	yes	1.055	7.2	7.378	7.365	6.858	1.084	1.042	1.039	------	------	------	------	------	------	ENOG4111M7J	S:Function unknown	------	------	------	CYT	17	56	42.8	High
TRINITY_DN9311_c0_g1_i1_orf1	cuticle protein 8-like [Ostrinia furnacalis]	0.132106923	-2.920222023	9.13E-05	down	yes	0.8797	6.659	6.733	7.248	5.997	0.854	0.91	0.875	MF:molecular_function;MF:structural constituent of cuticle;MF:structural molecule activity;	GO:0003674;GO:0042302;GO:0005198	------	------	------	------	ENOG410YSFI	S:Function unknown	PF00379.26	Chitin_bind_4	Insect cuticle protein	CYT	4	52	15	High
TRINITY_DN121650_c0_g1_i1_orf1	carboxylesterase [Ostrinia furnacalis]	0.469634797	-1.090388788	1.24E-05	down	yes	2.289	4.874	4.906	4.919	4.798	2.47	2.206	2.192	CC:integral component of membrane;CC:cellular_component;CC:intrinsic component of membrane;CC:cellular anatomical entity;MF:hydrolase activity;MF:hydrolase activity, acting on ester bonds;MF:carboxylic ester hydrolase activity;MF:catalytic activity;MF:molecular_function;	GO:0016021;GO:0005575;GO:0031224;GO:0110165;GO:0016787;GO:0016788;GO:0052689;GO:0003824;GO:0003674	------	------	------	------	COG2272	I:Lipid transport and metabolism	------	------	------	CYT	1	19	7.6	High
TRINITY_DN5829_c0_g1_i1_orf1	uncharacterized protein LOC114365758 isoform X3 [Ostrinia furnacalis]	0.148135593	-2.75500977	2.18E-07	down	yes	0.9177	6.195	6.162	6.33	6.094	0.957	0.892	0.904	------	------	------	------	------	------	ENOG410XT4C	S:Function unknown	PF00059.24	Lectin_C	Lectin C-type domain	PLA	5	39	23.4	High
TRINITY_DN12671_c0_g1_i6_orf1	hemicentin-1-like isoform X1 [Ostrinia furnacalis]	0.283349562	-1.819345123	1.75E-05	down	yes	1.455	5.135	4.951	5.396	5.058	1.518	1.537	1.311	BP:biological_process;BP:localization;	GO:0008150;GO:0051179	------	------	------	------	ENOG410XPQS;EN	S:Function unknown;U:Intracellular trafficking, secretion, and vesicular transport;U:Intracellular trafficking, secretion, and vesicular transport;S:Function	PF07679.19;PF13927.9;PF00047.28;PF13895.9	I-set;Ig_3;ig;Ig_2	Immunoglobulin I-set domain;Immunoglobulin domain;Immunoglobulin domain;Immunoglobulin domain	CYT	1	13	10.3	High
TRINITY_DN7064_c0_g1_i19_orf1	unnamed protein product [Chilo suppressalis]	3.485557084	1.801389255	4.84E-05	up	yes	5.068	1.454	1.553	1.212	1.597	5	5.356	4.848	------	------	------	------	------	------	------	------	PF00050.24;PF07648.18	Kazal_1;Kazal_2	Kazal-type serine protease inhibitor domain;Kazal-type serine protease inhibitor domain	EXC	1	2	73	High
TRINITY_DN77425_c0_g1_i2_orf1	anosmin-1 [Ostrinia furnacalis] >XP_028178811.1 anosmin-1 [Ostrinia furnacalis]	0.221507538	-2.174572302	1.30E-06	down	yes	1.102	4.975	5.038	5.061	4.827	1.174	1.044	1.087	CC:cellular_component;CC:extracellular region;CC:cellular anatomical entity;MF:enzyme regulator activity;MF:peptidase regulator activity;MF:molecular_function;MF:peptidase inhibitor activity;MF:enzyme inhibitor activity;MF:molecular function regulator;	GO:0005575;GO:0005576;GO:0110165;GO:0030234;GO:0061134;GO:0003674;GO:0030414;GO:0004857;GO:0098772	K23413	ANOS1, KAL1	------	------	ENOG4111T0F	S:Function unknown	PF00041.24;PF00095.24	fn3;WAP	Fibronectin type III domain;WAP-type	EXC	5	16	47.5	High
TRINITY_DN9239_c0_g1_i1_orf1	apolipophorins-like [Ostrinia furnacalis]	0.31162892	-1.68209897	0.0001062	down	yes	1.431	4.592	4.499	4.313	4.963	1.576	1.36	1.356	BP:lipid transport;BP:transport;BP:organic substance transport;BP:biological_process;BP:establishment of localization;BP:localization;CC:cellular_component;CC:extracellular region;CC:cellular anatomical entity;MF:lipid transporter activity;MF:transporter activity;MF:lipid binding;MF:molecular_function;MF:binding;	GO:0006869;GO:0006810;GO:0071702;GO:0008150;GO:0051234;GO:0051179;GO:0005575;GO:0005576;GO:0110165;GO:0005319;GO:0005215;GO:0008289;GO:0003674;GO:0005488	------	------	------	------	ENOG410Z6MR;EI	S:Function unknown;	------	------	------	CYT	10	78	18	High
TRINITY_DN98334_c0_g1_i1_orf1	unnamed protein product [Spodoptera littoralis] >CAH1638738.1 unnamed protein product [Spodoptera littoralis]	0.180760848	-2.467845864	6.66E-07	down	yes	0.9123	5.047	5.053	5.172	4.917	0.944	0.905	0.888	------	------	------	------	------	------	ENOG4110QDI	S:Function unknown	------	------	------	CYT	8	52	34	High
TRINITY_DN26301_c0_g1_i1_orf1	uncharacterized protein LOC114359193 [Ostrinia furnacalis]	0.38807618	-1.365588211	3.56E-06	down	yes	1.406	3.623	3.708	3.642	3.518	1.436	1.429	1.354	CC:cellular_component;CC:extracellular region;CC:cellular anatomical entity;MF:carbohydrate derivative binding;MF:molecular_function;MF:chitin binding;MF:binding;	GO:0005575;GO:0005576;GO:0110165;GO:0097367;GO:0003674;GO:0008061;GO:0005488	------	------	------	------	ENOG410ZPH9;EN	S:Function unknown;	PF01607.27	CBM_14	Chitin binding Peritrophin-A domain	CYT	3	16	28.5	High
TRINITY_DN26149_c0_g1_i5_orf1	thymosin beta isoform X3 [Ostrinia furnacalis]	0.450836513	-1.149323731	7.36E-08	down	yes	1.536	3.407	3.434	3.414	3.372	1.521	1.544	1.542	------	------	------	------	------	------	ENOG410Y3I4	O:Posttranslational modification, protein turnover,	PF01290.23	Thymosin	Thymosin beta-4 family	CYT	1	35	10.4	High
TRINITY_DN58125_c0_g1_i1_orf1	protein yellow-like [Ostrinia furnacalis]	0.373740368	-1.419891698	0.0001866	down	yes	1.261	3.374	3.532	3.371	3.219	1.124	1.523	1.135	BP:male mating behavior;BP:mating behavior;BP:behavior;BP:cellular biosynthetic process;BP:organic cyclic compound biosynthetic process;BP:organic cyclic compound metabolic process;BP:organic substance biosynthetic process;BP:pigment metabolic process;BP:secondary metabolite biosynthetic process;BP:organic substance metabolic process;BP:multicellular organismal process;BP:reproductive behavior;BP:phenol-containing compound biosynthetic process;BP:melanin metabolic process;BP:cellular aromatic compound metabolic process;BP:reproductive process;BP:biosynthetic process;BP:biological_process;BP:melanin biosynthetic process;BP:metabolic process;BP:phenol-containing compound metabolic process;BP:pigment biosynthetic process;BP:secondary metabolic process;BP:cellular metabolic process;BP:aromatic compound biosynthetic process;BP:cellular process;BP:organic hydroxy compound biosynthetic process;BP:organic hydroxy compound metabolic process;	GO:0060179;GO:0007617;GO:0007610;GO:0044249;GO:1901362;GO:1901360;GO:1901576;GO:0042440;GO:0044550;GO:0071704;GO:0032501;GO:0019098;GO:0046189;GO:0006582;GO:0006725;GO:0022414;GO:0009058;GO:0008150;GO:0042438;GO:0008152;GO:0018958;GO:0046148;GO:0019748;GO:0044237;GO:0019438;GO:0009987;GO:1901617;GO:1901615	------	------	------	------	COG3386	S:Function unknown	PF03022.19	MRJP	Major royal jelly protein	CYT	1	9	19.2	High
TRINITY_DN9239_c0_g2_i2_orf1	apolipophorins-like [Ostrinia furnacalis]	0.490163388	-1.028665367	0.0003091	down	yes	1.47	2.999	3.18	2.8	3.017	1.446	1.604	1.361	BP:lipid transport;BP:transport;BP:organic substance transport;BP:biological_process;BP:establishment of localization;BP:localization;CC:cellular_component;CC:extracellular region;CC:cellular anatomical entity;MF:lipid transporter activity;MF:transporter activity;MF:lipid binding;MF:molecular_function;MF:binding;	GO:0006869;GO:0006810;GO:0071702;GO:0008150;GO:0051234;GO:0051179;GO:0005575;GO:0005576;GO:0110165;GO:0005319;GO:0005215;GO:0008289;GO:0003674;GO:0005488	------	------	------	------	ENOG41112S0;EN	S:Function unknown;	------	------	------	CYT	5	90	7.6	High
TRINITY_DN394_c0_g1_i2_orf1	uncharacterized protein LOC114351483 [Ostrinia furnacalis]	3.872901679	1.953414876	8.26E-06	up	yes	3.23	0.834	0.782	0.826	0.894	3.247	3.352	3.092	MF:molecular_function;MF:structural constituent of cuticle;MF:structural molecule activity;	GO:0003674;GO:0042302;GO:0005198	------	------	------	------	ENOG41113X7;EN	S:Function unknown;	PF00379.26	Chitin_bind_4	Insect cuticle protein	CYT	1	28	12	High
TRINITY_DN295_c3_g1_i1_orfp1	TRINITY_DN295_c3_g1_i1_m.18839 TRINITY_DN295_c3_g1::TRINITY_DN295_c3_g1_i1::g.18839 ORF type:5prime_partial len:118 (+),score=46.25 TRINITY_DN295_c3_g1_i1:1-354(+)	2.006513479	1.004690848	1.76E-05	up	yes	11.09	5.527	5.686	5.496	5.398	10.965	10.796	11.508	------	------	------	------	------	------	------	------	------	------	------	PLA	3	49	12.6	High
TRINITY_DN381_c0_g1_i1_orf1	cuticle protein 8-like [Ostrinia furnacalis]	0.105924413	-3.238892966	5.06E-05	down	yes	0.9333	8.811	9.122	9.341	7.971	0.936	0.996	0.868	MF:molecular_function;MF:structural constituent of cuticle;MF:structural molecule activity;	GO:0003674;GO:0042302;GO:0005198	------	------	------	------	ENOG410ZM6M;E	S:Function unknown;	PF00379.26	Chitin_bind_4	Insect cuticle protein	CYT	4	22	25	High
TRINITY_DN5408_c0_g1_i5_orf1	uncharacterized protein LOC114359912 [Ostrinia furnacalis]	0.362584523	-1.463610751	0.0005359	down	yes	2.413	6.655	6.785	7.3	5.879	2.448	2.305	2.486	CC:cellular_component;CC:integral component of membrane;CC:cellular anatomical entity;CC:intrinsic component of membrane;	GO:0005575;GO:0016021;GO:0110165;GO:0031224	------	------	------	------	ENOG4110RME	S:Function unknown	------	------	------	PLA	1	5	30.4	High
TRINITY_DN18338_c0_g1_i6_orf1	aquaporin AQPAn.G isoform X1 [Ostrinia furnacalis]	0.467365573	-1.097376628	0.002334	down	yes	2.764	5.914	6.357	5.495	5.89	2.841	2.064	3.387	CC:integral component of membrane;CC:cellular_component;CC:intrinsic component of membrane;CC:cellular anatomical entity;MF:transporter activity;MF:transmembrane transporter activity;MF:channel activity;MF:molecular_function;MF:passive transmembrane transporter activity;	GO:0016021;GO:0005575;GO:0031224;GO:0110165;GO:0005215;GO:0022857;GO:0015267;GO:0003674;GO:0022803	K09884	AQPN	------	------	COG0580	O:Posttranslational modification, protein turnover, chaperones	PF00230.23	MIP	Major intrinsic protein	CYT	1	8	29	High
TRINITY_DN38274_c0_g1_i1_orf1	uncharacterized protein LOC114360402 [Ostrinia furnacalis]	0.153990241	-2.699089172	5.27E-06	down	yes	0.9783	6.353	6.44	6.555	6.064	1.107	0.97	0.858	BP:nucleic acid metabolic process;BP:nitrogen compound metabolic process;BP:organic cyclic compound metabolic process;BP:nucleobase-containing compound metabolic process;BP:cellular response to stimulus;BP:cellular macromolecule metabolic process;BP:organic substance metabolic process;BP:DNA repair;BP:cellular process;BP:cellular aromatic compound metabolic process;BP:cellular response to DNA damage stimulus;BP:response to stress;BP:biological_process;BP:metabolic process;BP:heterocycle metabolic process;BP:primary metabolic process;BP:response to stimulus;BP:cellular nitrogen compound metabolic process;BP:cellular metabolic process;BP:macromolecule metabolic process;BP:cellular response to stress;BP:DNA metabolic process;CC:intracellular organelle;CC:membrane-bounded organelle;CC:organelle;CC:nucleus;CC:cellular anatomical entity;CC:intracellular membrane-bounded organelle;CC:cellular_component;MF:phosphoric ester hydrolase activity;MF:molecular_function;MF:phosphoric diester hydrolase activity;MF:structural molecule activity;MF:hydrolase activity;MF:hydrolase activity, acting on ester bonds;MF:catalytic activity;MF:structural constituent of cuticle;MF:nuclease	GO:0090304;GO:0006807;GO:1901360;GO:0006139;GO:0051716;GO:0044260;GO:0071704;GO:0006281;GO:0009987;GO:0006725;GO:0006974;GO:0006950;GO:0008150;GO:0008152;GO:0046483;GO:0044238;GO:0050896;GO:0034641;GO:0044237;GO:0043170;GO:0033554;GO:0006259;GO:0043229;GO:0043227;GO:0043226;GO:0005634;GO:0110165;GO:0043231;GO:0005575;GO:0042578;GO:0003674;GO:0008081;GO:0005198;GO:0016787;GO:0016788;GO:0003824;GO:0042302;GO:0004518	------	------	------	------	ENOG4112648;EN	S:Function unknown;	PF00379.26	Chitin_bind_4	Insect cuticle protein	CYT	5	34	29.3	High
TRINITY_DN28922_c0_g1_i2_orf1	uncharacterized protein LOC119829283 isoform X2 [Zerene cesonia]	0.2585851	-1.951288945	9.89E-05	down	yes	1.378	5.329	5.421	5.665	4.902	1.513	1.145	1.475	CC:cellular_component;CC:cellular anatomical entity;	GO:0005575;GO:0110165	------	------	------	------	------	------	------	------	------	CYT	1	10	12.5	High
TRINITY_DN2002_c0_g1_i5_orfp1	TRINITY_DN2002_c0_g1_i5_m.4230 TRINITY_DN2002_c0_g1::TRINITY_DN2002_c0_g1_i5::g.4230 ORF type:3prime_partial len:259 (+),score=29.98,Peptidase_C39 PF03412.16 1.7,Peptidase_C39|PF03412.16|0.049 TRINITY_DN2002_c0_g1_i5:65-838(+)	0.346722068	-1.52814843	0.0001933	down	yes	1.502	4.332	3.941	4.432	4.623	1.616	1.371	1.519	------	------	------	------	------	------	------	------	------	------	------	CYT	1	5	29.4	Medium
TRINITY_DN1423_c0_g1_i8_orf1	ferritin subunit-like [Ostrinia furnacalis] >XP_028168186.1 ferritin subunit-like [Ostrinia furnacalis]	0.440550688	-1.182620074	5.76E-05	down	yes	1.76	3.995	3.931	4.227	3.827	1.78	1.691	1.81	BP:ion homeostasis;BP:homeostatic process;BP:iron ion homeostasis;BP:transition metal ion homeostasis;BP:inorganic ion homeostasis;BP:cellular transition metal ion homeostasis;BP:transition metal ion transport;BP:biological regulation;BP:regulation of biological quality;BP:cellular homeostasis;BP:metal ion transport;BP:transport;BP:cellular iron ion homeostasis;BP:cellular metal ion homeostasis;BP:cation transport;BP:ion transport;BP:cellular process;BP:cellular ion homeostasis;BP:biological_process;BP:cellular cation homeostasis;BP:metal ion homeostasis;BP:cation homeostasis;BP:cellular chemical homeostasis;BP:establishment of localization;BP:localization;BP:chemical homeostasis;BP:iron ion transport;MF:molecular_function;MF:binding;MF:oxidoreductase activity;MF:cation binding;MF:oxidoreductase activity, oxidizing metal ions;MF:transition metal ion binding;MF:ferroxidase activity;MF:ion binding;MF:iron ion binding;MF:ferric iron binding;MF:oxidoreductase activity, oxidizing metal ions, oxygen as acceptor;MF:metal ion binding;MF:catalytic activity;	GO:0050801;GO:0042592;GO:0055072;GO:0055076;GO:0098771;GO:0046916;GO:0000041;GO:0065007;GO:0065008;GO:0019725;GO:0030001;GO:0006810;GO:0006879;GO:0006875;GO:0006812;GO:0006811;GO:0009987;GO:0006873;GO:0008150;GO:0030003;GO:0055065;GO:0055080;GO:0055082;GO:0051234;GO:0051179;GO:0048878;GO:0006826;GO:0003674;GO:0005488;GO:0016491;GO:0043169;GO:0016722;GO:0046914;GO:0004322;GO:0043167;GO:0005506;GO:0008199;GO:0016724;GO:0046872;GO:0003824	K00522	FTH1	map04978;ma	Mineral absorption;Necroptosis;Ferroptosis	ENOG4111MSG	P:Inorganic ion transport and metabolism	PF00210.27	Ferritin	Ferritin-like domain	CYT	6	34	23.7	High
TRINITY_DN993_c0_g1_i7_orf1	apolipophorins-like [Ostrinia furnacalis]	0.425737664	-1.231963366	8.06E-08	down	yes	1.717	4.033	4.022	4.015	4.062	1.736	1.739	1.677	MF:lipid transporter activity;MF:transporter activity;MF:molecular_function;	GO:0005319;GO:0005215;GO:0003674	------	------	------	------	ENOG41112S0;EN	S:Function unknown;	PF00094.28	VWD	von Willebrand factor type D domain	CYT	27	59	66.5	High
TRINITY_DN5153_c1_g1_i1_orf1	nose resistant to fluoxetine protein 6-like isoform X1 [Ostrinia furnacalis]	2.221266134	1.151382255	6.33E-05	up	yes	3.614	1.627	1.597	1.658	1.625	3.781	3.662	3.4	CC:integral component of membrane;CC:cellular_component;CC:intrinsic component of membrane;CC:cellular anatomical entity;MF:catalytic activity;MF:transferase activity;MF:transferase activity, transferring acyl groups;MF:molecular_function;MF:transferase activity, transferring acyl groups other than amino-acyl groups;	GO:0016021;GO:0005575;GO:0031224;GO:0110165;GO:0003824;GO:0016740;GO:0016746;GO:0003674;GO:0016747	------	------	------	------	ENOG410YD1T	S:Function unknown	------	------	------	CYT	1	1	71.6	High
TRINITY_DN5595_c0_g1_i1_orf1	keratin, type I cytoskeletal 10-like [Ostrinia furnacalis]	0.240391677	-2.056541149	5.29E-05	down	yes	0.982	4.085	3.938	4.345	3.971	1.171	0.981	0.794	------	------	------	------	------	------	ENOG4112BJP	S:Function unknown	PF16058.8	Mucin-like	Mucin-like	EXC	1	14	16.6	High
TRINITY_DN2515_c0_g1_i6_orf1	chitooligosaccharidolytic beta-N-acetylglucosaminidase isoform X1 [Ostrinia furnacalis]	0.486568987	-1.039283728	1.27E-05	down	yes	1.594	3.276	3.334	3.346	3.148	1.59	1.604	1.587	BP:amino sugar catabolic process;BP:nitrogen compound metabolic process;BP:aminoglycan metabolic process;BP:organic substance catabolic process;BP:organic substance metabolic process;BP:chitin metabolic process;BP:chitin catabolic process;BP:polysaccharide catabolic process;BP:biological_process;BP:metabolic process;BP:carbohydrate metabolic process;BP:catabolic process;BP:macromolecule catabolic process;BP:primary metabolic process;BP:organonitrogen compound metabolic process;BP:organonitrogen compound catabolic process;BP:polysaccharide metabolic process;BP:amino sugar metabolic process;BP:carbohydrate derivative catabolic process;BP:carbohydrate derivative metabolic process;BP:carbohydrate catabolic process;BP:macromolecule metabolic process;BP:glucosamine-containing compound catabolic process;BP:glucosamine-containing compound metabolic process;BP:aminoglycan catabolic process;MF:hydrolase activity, acting on glycosyl bonds;MF:molecular_function;MF:beta-N-acetylhexosaminidase activity;MF:hydrolase activity;MF:N-acetyl-beta-D-galactosaminidase activity;MF:hexosaminidase activity;MF:catalytic activity;MF:hydrolase activity, hydrolyzing O-glycosyl compounds;	GO:0046348;GO:0006807;GO:0006022;GO:1901575;GO:0071704;GO:0006030;GO:0006032;GO:0000272;GO:0008150;GO:0008152;GO:0005975;GO:0009056;GO:0009057;GO:0044238;GO:1901564;GO:1901565;GO:0005976;GO:0006040;GO:1901136;GO:1901135;GO:0016052;GO:0043170;GO:1901072;GO:1901071;GO:0006026;GO:0016798;GO:0003674;GO:0004563;GO:0016787;GO:0102148;GO:0015929;GO:0003824;GO:0004553	K12373	HEXA_B	map00600;ma	Sphingolipid metabolism;Amino sugar and nucleotide sugar metabolism;Glycosphingolipid biosynthesis - globo and isoglobo series;Glycosphingolipid biosynthesis - ganglio series;Other glycan degradation;Various types of N-glycan biosynthesis;Glycosaminoglycan degradation;Lysosome	COG3525	G:Carbohydrate transport and metabolism	PF00728.25;PF14845.9	Glyco_hydro_20;Glycohydro_20b2	Glycosyl hydrolase family 20, catalytic domain;beta-acetyl hexosaminidase like	CYT	16	35	70.4	High
TRINITY_DN8685_c0_g1_i5_orf1	macrophage mannose receptor 1-like [Zerene cesonia]	2.447669977	1.29140905	2.17E-05	up	yes	3.204	1.309	1.405	1.202	1.321	3.088	3.273	3.251	BP:detection of biotic stimulus;BP:detection of chemical stimulus;BP:positive regulation of response to external stimulus;BP:regulation of response to external stimulus;BP:positive regulation of response to stimulus;BP:regulation of response to biotic stimulus;BP:positive regulation of response to biotic stimulus;BP:positive regulation of defense response;BP:defense response to bacterium;BP:detection of molecule of bacterial origin;BP:regulation of biological process;BP:regulation of defense response;BP:response to bacterial lipoprotein;BP:response to external biotic stimulus;BP:response to bacterium;BP:interspecies interaction between organisms;BP:positive regulation of immune system process;BP:biological regulation;BP:positive regulation of biological process;BP:regulation of immune system process;BP:detection of external biotic stimulus;BP:positive regulation of innate immune response;BP:regulation of innate immune response;BP:regulation of response to stimulus;BP:innate immune response;BP:defense response;BP:response to stress;BP:biological_process;BP:immune response;BP:response to chemical;BP:response to other organism;BP:response to organic substance;BP:regulation of immune response;BP:response to biotic stimulus;BP:detection of stimulus;BP:response to external stimulus;BP:regulation of response to stress;BP:detection of bacterial lipoprotein;BP:positive regulation of immune response;BP:immune system process;BP:activation of innate immune response;BP:response to molecule of bacterial origin;BP:activation of immune response;BP:defense response to other organism;BP:response to stimulus;BP:antibacterial innate immune response;	GO:0009595;GO:0009593;GO:0032103;GO:0032101;GO:0048584;GO:0002831;GO:0002833;GO:0031349;GO:0042742;GO:0032490;GO:0050789;GO:0031347;GO:0032493;GO:0043207;GO:0009617;GO:0044419;GO:0002684;GO:0065007;GO:0048518;GO:0002682;GO:0098581;GO:0045089;GO:0045088;GO:0048583;GO:0045087;GO:0006952;GO:0006950;GO:0008150;GO:0006955;GO:0042221;GO:0051707;GO:0010033;GO:0050776;GO:0009607;GO:0051606;GO:0009605;GO:0080134;GO:0042494;GO:0050778;GO:0002376;GO:0002218;GO:0002237;GO:0002253;GO:0098542;GO:0050896;GO:0140367	------	------	------	------	ENOG410XRS3;EN	U:Intracellular trafficking, secretion, and vesicular transport;S:Function unknown;S:Function unknown;S:Function unknown	PF00059.24	Lectin_C	Lectin C-type domain	PLA	2	6	40.6	High
TRINITY_DN86772_c0_g1_i3_orfp1	x-tox [Spodoptera exigua]	2.514937107	1.330522322	6.17E-07	up	yes	6.398	2.544	2.561	2.63	2.441	6.342	6.479	6.373	BP:response to biotic stimulus;BP:response to external stimulus;BP:response to external biotic stimulus;BP:response to stimulus;BP:defense response;BP:response to stress;BP:biological_process;BP:interspecies interaction between organisms;BP:response to other organism;CC:cellular_component;CC:extracellular region;CC:cellular anatomical entity;	GO:0009607;GO:0009605;GO:0043207;GO:0050896;GO:0006952;GO:0006950;GO:0008150;GO:0044419;GO:0051707;GO:0005575;GO:0005576;GO:0110165	------	------	------	------	------	------	------	------	------	CYT	1	9	8.5	High
TRINITY_DN2101_c0_g1_i6_orf1	protein obstructor-E-like [Ostrinia furnacalis]	0.092120098	-3.440340243	1.79E-05	down	yes	0.7517	8.16	8.411	8.523	7.546	0.769	0.74	0.746	------	------	------	------	------	------	ENOG410ZKXC	S:Function unknown	PF01607.27	CBM_14	Chitin binding Peritrophin-A domain	EXC	5	20	28.1	High

TRINITY_DN20442_c0_g2_i1_orf1	hypothetical protein evm_008218 [Chilo suppressalis]	0.249874729	-2.000723095	0.0004262	down	yes	1.496	5.987	5.788	6.782	5.391	1.496	1.389	1.602	BP:nucleosome organization;BP:cellular component assembly;BP:regulation of metabolic process;BP:macromolecular complex subunit organization;BP:regulation of cellular biosynthetic process;BP:regulation of cellular metabolic process;BP:cellular component organization or biogenesis;BP:regulation of primary metabolic process;BP:protein-DNA complex subunit organization;BP:chromatin organization;BP:cellular component organization;BP:regulation of biological process;BP:macromolecular complex assembly;BP:regulation of nucleobase-containing compound metabolic process;BP:protein-DNA complex assembly;BP:biological regulation;BP:regulation of gene expression;BP:regulation of macromolecule metabolic process;BP:regulation of biosynthetic process;BP:cellular process;BP:regulation of nucleic acid-templated transcription;BP:regulation of cellular process;BP:biological_process;BP:regulation of nitrogen compound metabolic process;BP:regulation of RNA biosynthetic process;BP:chromatin remodeling;BP:nucleosome assembly;BP:regulation of RNA metabolic process;BP:regulation of transcription, DNA-templated;BP:regulation of macromolecule biosynthetic process;CC:nucleosome;CC:DNA packaging complex;CC:macromolecular complex;CC:protein-DNA complex;CC:cellular_component;MF:nucleic acid binding;MF:peptide-lysine-N-acetyltransferase activity;MF:molecular_function;MF:binding;MF:peptide N-acetyltransferase activity;MF:DNA binding;MF:ion binding;MF:heterocyclic compound binding;MF:cation binding;MF:N-acetyltransferase activity;MF:histone acetyltransferase activity;MF:transferase activity;MF:transferase activity, transferring acyl groups;MF:transferase activity, transferring acyl groups other than amino-acyl groups;MF:N-acyltransferase activity;MF:metal ion binding;MF:catalytic activity, acting on a protein;MF:catalytic activity;MF:organic cyclic compound binding;MF:acetyltransferase activity;	GO:0034728;GO:0022607;GO:0019222;GO:0043933;GO:0031326;GO:0031323;GO:0071840;GO:0080090;GO:0071824;GO:0006325;GO:0016043;GO:0050789;GO:0065003;GO:0019219;GO:0065004;GO:0065007;GO:0010468;GO:0060255;GO:0009889;GO:0009987;GO:1903506;GO:0050794;GO:0008150;GO:0051171;GO:2001141;GO:0006338;GO:0006334;GO:0051252;GO:0006355;GO:0010556;GO:0000786;GO:0044815;GO:0032991;GO:0032993;GO:0005575;GO:0003676;GO:0061733;GO:0003674;GO:0005488;GO:0034212;GO:0003677;GO:0043167;GO:1901363;GO:0043169;GO:0008080;GO:0004402;GO:0016740;GO:0016746;GO:0016747;GO:0016410;GO:0046872;GO:0140096;GO:0003824;GO:0097159;GO:0016407	K11306	MYST4, KAT6	------	------	COG5027	B:Chromatin structure and dynamics	------	------	------	CYT	1	7	13.7	Medium
TRINITY_DN778_c0_g1_i1_orf1	uncharacterized protein LOC114363281 [Ostrinia furnacalis]	0.13351753	-2.904898919	1.01E-05	down	yes	0.7007	5.248	5.326	4.936	5.483	0.656	0.731	0.715	------	------	------	------	------	------	COG2335	O:Posttranslational modification, protein turnover	PF02469.25	Fasciclin	Fasciclin domain	CYT	1	1	115.4	High
TRINITY_DN25534_c0_g1_i1_orf1	venom serine carboxypeptidase-like [Ostrinia furnacalis]	0.169127374	-2.563817913	2.48E-09	down	yes	0.7927	4.687	4.655	4.691	4.714	0.8	0.789	0.789	BP:primary metabolic process;BP:organonitrogen compound metabolic process;BP:protein metabolic process;BP:macromolecule metabolic process;BP:organic substance metabolic process;BP:nitrogen compound metabolic process;BP:biological_process;BP:metabolic process;BP:proteolysis;MF:molecular_function;MF:exopeptidase activity;MF:hydrolase activity;MF:carboxypeptidase activity;MF:catalytic activity, acting on a protein;MF:serine hydrolase activity;MF:serine-type carboxypeptidase activity;MF:serine-type exopeptidase activity;MF:peptidase activity;MF:catalytic activity;MF:serine-type peptidase activity;	GO:0044238;GO:1901564;GO:0019538;GO:0043170;GO:0071704;GO:0006807;GO:0008150;GO:0008152;GO:0006508;GO:0003674;GO:0008238;GO:0016787;GO:0004180;GO:0140096;GO:0017171;GO:0004185;GO:0070008;GO:0008233;GO:0003824;GO:0008236	K09645	CPVL	------	------	ENOG410XPIW;CO	S:Function unknown;E:Amino acid transport and metabolism	PF00450.25	Peptidase_S10	Serine carboxypeptidase	CYT	4	12	55.9	High
TRINITY_DN53866_c0_g1_i1_orf1	larval/pupal cuticle protein H1C-like [Ostrinia furnacalis]	0.43686584	-1.194737794	3.50E-05	down	yes	1.55	3.548	3.571	3.625	3.447	1.476	1.716	1.457	------	------	------	------	------	------	ENOG4110SXG	S:Function unknown	------	------	------	CYT	2	22	21.8	High
TRINITY_DN6004_c0_g1_i1_orf1	endocuticle structural glycoprotein ABD-4-like [Ostrinia furnacalis]	0.35118067	-1.509714658	3.55E-06	down	yes	1.279	3.642	3.515	3.705	3.706	1.309	1.275	1.252	MF:molecular_function;MF:structural constituent of cuticle;MF:structural molecule activity;	GO:0003674;GO:0042302;GO:0005198	------	------	------	------	ENOG4111PJ1;EN	O:Posttranslational modification, protein turnover, chaperones;S:Function unknown	PF00379.26	Chitin_bind_4	Insect cuticle protein	CYT	2	23	16.7	High
TRINITY_DN17574_c0_g1_i2_orf1	heat shock protein Hsp-12.2-like [Ostrinia furnacalis]	0.418399496	-1.257046981	3.51E-07	down	yes	1.328	3.174	3.218	3.174	3.13	1.319	1.354	1.31	------	------	K09542	CRYAB	map04213;ma	Longevity regulating pathway - multiple species;Protein processing in	ENOG410YERS	Q:Secondary metabolites biosynthesis, transport and catabolism	PF00011.24	HSP20	Hsp20/alpha crystallin family	CYT	7	51	19.4	High
TRINITY_DN6470_c0_g3_i2_orf1	trypsin CFT-1-like [Ostrinia furnacalis]	0.193001346	-2.373317187	1.88E-05	down	yes	0.717	3.715	3.514	3.948	3.683	0.73	0.706	0.715	BP:organic substance metabolic process;BP:organonitrogen compound metabolic process;BP:protein metabolic process;BP:macromolecule metabolic process;BP:biological_process;BP:nitrogen compound metabolic process;BP:metabolic process;BP:proteolysis;BP:primary metabolic process;MF:serine-type endopeptidase activity;MF:endopeptidase activity;MF:hydrolase activity;MF:serine hydrolase activity;MF:catalytic activity, acting on a protein;MF:catalytic activity;MF:molecular_function;MF:peptidase activity;MF:serine-type peptidase activity;	GO:0071704;GO:1901564;GO:0019538;GO:0043170;GO:0008150;GO:0006807;GO:0008152;GO:0006508;GO:0044238;GO:0004252;GO:0004175;GO:0016787;GO:0017171;GO:0140096;GO:0003824;GO:0003674;GO:0008233;GO:0008236	K01312	PRSS1_2_3	map04972;ma	Pancreatic secretion;Influenza A;Neuroactive ligand-receptor interaction;Protein digestion and absorption	COG5640	O:Posttranslational modification, protein turnover, chaperones	PF00089.29;PF13365.9	Trypsin;Trypsin_2	Trypsin;Trypsin-like peptidase domain	CYT	1	5	27.4	High
TRINITY_DN2953_c1_g1_i10_orf1	methionine--tRNA ligase, cytoplasmic isoform X2 [Ostrinia furnacalis] >XP_028156683.1 methionine--tRNA ligase, cytoplasmic isoform X4 [Ostrinia furnacalis] >XP_028156684.1 methionine--tRNA ligase, cytoplasmic isoform X5 [Ostrinia furnacalis]	0.400475543	-1.320213953	0.0003474	down	yes	1.179	2.944	2.887	3.215	2.729	1.173	1.289	1.075	BP:methionyl-tRNA aminoacylation;BP:carboxylic acid metabolic process;BP:nucleic acid metabolic process;BP:cellular nitrogen compound metabolic process;BP:nitrogen compound metabolic process;BP:small molecule metabolic process;BP:ncRNA metabolic process;BP:organic cyclic compound metabolic process;BP:nucleobase-containing compound metabolic process;BP:cellular amino acid metabolic process;BP:organic substance metabolic process;BP:primary metabolic process;BP:cellular process;BP:cellular aromatic compound metabolic process;BP:biological_process;BP:metabolic process;BP:oxoacid metabolic process;BP:organonitrogen compound metabolic process;BP:heterocycle metabolic process;BP:RNA metabolic process;BP:organic acid metabolic process;BP:cellular metabolic process;BP:macromolecule metabolic process;BP:tRNA metabolic process;BP:amino acid activation;BP:tRNA aminoacylation;BP:tRNA aminoacylation for protein translation;CC:aminoacyl-tRNA synthetase multienzyme complex;CC:catalytic complex;CC:macromolecular complex;CC:cellular_component;MF:purine ribonucleoside triphosphate binding;MF:molecular_function;MF:ion binding;MF:heterocyclic compound binding;MF:anion binding;MF:aminoacyl-tRNA ligase activity;MF:nucleotide binding;MF:nucleoside phosphate binding;MF:purine nucleotide binding;MF:ATP binding;MF:catalytic activity, acting on RNA;MF:small molecule binding;MF:catalytic activity;MF:ligase activity;MF:ligase activity, forming carbon-oxygen bonds;MF:methionine-tRNA ligase activity;MF:adenyl nucleotide binding;MF:carbohydrate derivative binding;MF:organic cyclic compound binding;MF:adenyl ribonucleotide binding;MF:purine ribonucleotide binding;MF:catalytic activity, acting on a tRNA;MF:ribonucleotide binding;MF:binding;	GO:0006431;GO:0019752;GO:0090304;GO:0034641;GO:0006807;GO:0044281;GO:0034660;GO:1901360;GO:0006139;GO:0006520;GO:0071704;GO:0044238;GO:0009987;GO:0006725;GO:0008150;GO:0008152;GO:0043436;GO:1901564;GO:0046483;GO:0016070;GO:0006082;GO:0044237;GO:0043170;GO:0006399;GO:0043038;GO:0043039;GO:0006418;GO:0017101;GO:1902494;GO:0032991;GO:0005575;GO:0035639;GO:0003674;GO:0043167;GO:1901363;GO:0043168;GO:0004812;GO:0000166;GO:1901265;GO:0017076;GO:0005524;GO:0140098;GO:0036094;GO:0003824;GO:0016874;GO:0016875;GO:0004825;GO:0030554;GO:0097367;GO:0097159;GO:0032559;GO:0032555;GO:0140101;GO:0032553;GO:0005488	K01874	MARS, metG	map00450;ma	Selenocompound metabolism;Aminoacyl-tRNA biosynthesis	COG0143	J:Translation, ribosomal structure and biogenesis	PF00458.23	WHEP-TRS	WHEP-TRS domain	CYT	2	57	17.6	High
TRINITY_DN18804_c0_g1_i5_orf1	zinc finger protein Xfin-like [Ostrinia furnacalis]	0.435285865	-1.199964924	1.07E-05	down	yes	1.241	2.851	2.892	2.91	2.75	1.181	1.265	1.277	CC:intracellular membrane-bounded organelle;CC:nucleus;CC:cellular_component;CC:intracellular organelle;CC:membrane-bounded organelle;CC:organelle;CC:cellular anatomical entity;MF:cation binding;MF:transition metal ion binding;MF:organic cyclic compound binding;MF:heterocyclic compound binding;MF:ion binding;MF:molecular_function;MF:zinc ion binding;MF:nucleic acid binding;MF:metal ion binding;MF:binding;	GO:0043231;GO:0005634;GO:0005575;GO:0043229;GO:0043227;GO:0043226;GO:0110165;GO:0043169;GO:0046914;GO:0097159;GO:1901363;GO:0043167;GO:0003674;GO:0008270;GO:0003676;GO:0046872;GO:0005488	K09228	KRAB	map05168	Herpes simplex virus 1 infection	COG5048	I:Lipid transport and metabolism	PF13894.9;PF00096.29;PF07776.18;PF18868.4;PF13912.9;PF12874.10;PF12171.11;PF13465.9	zf-C2H2_4;zf-C2H2;zf-AD;zf-C2H2_3rep;zf-C2H2_6;zf-met;zf-C2H2_jaz;zf-H2C2_2	C2H2-type zinc finger;Zinc finger, C2H2 type;Zinc-finger associated domain;Zinc finger C2H2-type, 3 repeats;C2H2-type zinc finger;Zinc-finger of C2H2 type;Zinc-finger double-stranded RNA-binding;Zinc-finger double domain	CYT	3	2	179.5	High
TRINITY_DN14242_c0_g1_i2_orfp1	TRINITY_DN14242_c0_g1_i2_m.18449 TRINITY_DN14242_c0_g1::TRINITY_DN14242_c0_g1_i2::g.18449 ORF type:internal len:148 (-) score=102.30 TRINITY_DN14242_c0_g1_i2:2-442(-)	2.396067991	1.260668847	6.27E-05	up	yes	23.4	9.766	9.949	8.446	10.902	23.089	24.022	23.09	------	------	------	------	------	------	------	------	------	------	------	CYT	1	8	16	High
TRINITY_DN2407_c0_g1_i2_orf1	uncharacterized protein LOC114366345 isoform X2 [Ostrinia furnacalis]	6.323129252	2.66063871	3.67E-06	up	yes	7.436	1.176	1.32	0.99	1.219	7.327	7.724	7.256	BP:response to biotic stimulus;BP:response to external stimulus;BP:response to external biotic stimulus;BP:response to stimulus;BP:defense response;BP:response to stress;BP:biological_process;BP:interspecies interaction between organisms;BP:response to other organism;CC:cellular_component;CC:extracellular region;CC:cellular anatomical entity;	GO:0009607;GO:0009605;GO:0043207;GO:0050896;GO:0006952;GO:0006950;GO:0008150;GO:0044419;GO:0051707;GO:0005575;GO:0005576;GO:0110165	------	------	------	------	ENOG4110S6N	S:Function unknown	PF00451.22	Toxin_2	Scorpion short toxin, BmKK2	CYT	1	36	21.5	High
TRINITY_DN6423_c0_g1_i6_orf1	phenoloxidase-activating factor 2-like isoform X1 [Ostrinia furnacalis]	0.481807208	-1.053472118	0.0003432	down	yes	2.794	5.799	5.771	6.222	5.405	2.802	2.997	2.584	BP:organic substance metabolic process;BP:organonitrogen compound metabolic process;BP:protein metabolic process;BP:macromolecule metabolic process;BP:biological_process;BP:nitrogen compound metabolic process;BP:metabolic process;BP:proteolysis;BP:primary metabolic process;MF:serine-type endopeptidase activity;MF:endopeptidase activity;MF:hydrolase activity;MF:serine hydrolase activity;MF:catalytic activity, acting on a protein;MF:catalytic activity;MF:molecular_function;MF:peptidase activity;MF:serine-type peptidase activity;	GO:0071704;GO:1901564;GO:0019538;GO:0043170;GO:0008150;GO:0006807;GO:0008152;GO:0006508;GO:0044238;GO:0004252;GO:0004175;GO:0016787;GO:0017171;GO:0140096;GO:0003824;GO:0003674;GO:0008233;GO:0008236	------	------	------	------	COG5640	O:Posttranslational modification, protein turnover, chaperones	PF00089.29;PF18322.4	Trypsin;CLIP_1	Trypsin;Serine protease Clip domain PPAF-2	CYT	3	13	55.7	High
TRINITY_DN661_c0_g1_i1_orf1	hypothetical protein evm_002822 [Chilo suppressalis]	0.27473251	-1.863900454	0.0005356	down	yes	1.669	6.075	6.613	6.396	5.217	1.729	1.673	1.606	MF:molecular_function;MF:structural constituent of cuticle;MF:structural molecule activity;	GO:0003674;GO:0042302;GO:0005198	------	------	------	------	ENOG4111NR3;EN	S:Function unknown;	PF00379.26	Chitin_bind_4	Insect cuticle protein	CYT	1	22	17.6	High
TRINITY_DN3275_c0_g2_i3_orf1	hypothetical protein B5X24_HaOG216046 [Helicoverpa armigera]	2.982972136	1.576750502	0.0002055	up	yes	5.781	1.938	1.661	1.78	2.372	5.49	5.69	6.163	MF:cation binding;MF:ion binding;MF:molecular_function;MF:binding;MF:metal ion binding;MF:calcium ion binding;	GO:0043169;GO:0043167;GO:0003674;GO:0005488;GO:0046872;GO:0005509	K02183	CALM	map05214;ma	Glioma;Pathways of neurodegeneration - multiple diseases;Oocyte meiosis;Human cytomegalovirus infection;Inflammatory mediator regulation of TRP channels;Kaposi sarcoma-associated herpesvirus infection;Rap1 signaling pathway;Ras signaling pathway;Vascular smooth muscle contraction;Cellular senescence;Apelin signaling pathway;cGMP-PKG signaling pathway;Phosphatidylinositol signaling system;Parkinson disease;Oxytocin signaling pathway;Alzheimer disease;Glucagon signaling pathway;Aldosterone synthesis and	COG5126;COG512	D:Cell cycle control, cell division, chromosome partitioning;T:Signal transduction mechanisms;Z:Cytoskeleton;S:Function unknown	------	------	------	CYT	1	36	2.8	Medium
TRINITY_DN619_c0_g1_i1_orf1	putative uncharacterized protein DDB_G0271606 [Ostrinia furnacalis]	0.103577954	-3.271211133	9.75E-06	down	yes	0.6803	6.568	6.655	6.873	6.177	0.667	0.766	0.608	CC:cellular_component;CC:extracellular region;CC:cellular anatomical entity;MF:carbohydrate derivative binding;MF:molecular_function;MF:chitin binding;MF:binding;	GO:0005575;GO:0005576;GO:0110165;GO:0097367;GO:0003674;GO:0008061;GO:0005488	------	------	------	------	ENOG4111K2A	S:Function unknown	PF01607.27	CBM_14	Chitin binding Peritrophin-A domain	CYT	3	16	39.1	High
TRINITY_DN609_c0_g1_i1_orf1	zonadhesin-like isoform X1 [Ostrinia furnacalis]	5.1900369	2.375744796	4.27E-06	up	yes	5.626	1.084	1.117	1.048	1.088	5.377	5.681	5.819	------	------	------	------	------	------	COG4826;ENOG4	O:Posttranslational modification, protein turnover, chaperones;S:Function unknown;S:Function unknown;S:Function unknown	PF01826.20	TIL	Trypsin Inhibitor like cysteine rich domain	PLA	9	18	109	High
TRINITY_DN45220_c0_g1_i1_orf1	delta(3,5)-Delta(2,4)-dienoyl-CoA isomerase, mitochondrial isoform X1 [Ostrinia furnacalis]	0.459311602	-1.122454868	0.0001538	down	yes	2.015	4.387	4.459	4.564	4.138	1.844	2.226	1.976	BP:lipid oxidation;BP:fatty acid metabolic process;BP:fatty acid beta-oxidation;BP:carboxylic acid metabolic process;BP:cellular catabolic process;BP:fatty acid catabolic process;BP:small molecule metabolic process;BP:small molecule catabolic process;BP:cellular lipid catabolic process;BP:organic substance catabolic process;BP:lipid modification;BP:lipid catabolic process;BP:organic substance metabolic process;BP:lipid metabolic process;BP:cellular process;BP:monocarboxylic acid metabolic process;BP:biological_process;BP:metabolic process;BP:monocarboxylic acid catabolic process;BP:oxoacid metabolic process;BP:fatty acid oxidation;BP:cellular lipid metabolic process;BP:catabolic process;BP:primary metabolic process;BP:organic acid metabolic process;BP:carboxylic acid catabolic process;BP:organic acid catabolic process;BP:cellular metabolic process;MF:molecular_function;MF:catalytic activity;MF:isomerase activity;	GO:0034440;GO:0006631;GO:0006635;GO:0019752;GO:0044248;GO:0009062;GO:0044281;GO:0044282;GO:0044242;GO:1901575;GO:0030258;GO:0016042;GO:0071704;GO:0006629;GO:0009987;GO:0032787;GO:0008150;GO:0008152;GO:0072329;GO:0043436;GO:0019395;GO:0044255;GO:0009056;GO:0044238;GO:0006082;GO:0046395;GO:0016054;GO:0044237;GO:0003674;GO:0003824;GO:0016853	K12663	ECH1	map04146	Peroxisome	ENOG410XTHX	S:Function unknown	PF00378.23	ECH_1	Enoyl-CoA hydratase/isomerase	CYT	1	59	7.4	High
TRINITY_DN85412_c0_g1_i1_orf1	unnamed protein product [Diatraea saccharalis]	0.381293189	-1.391027336	6.96E-07	down	yes	1.545	4.052	4.089	4.099	3.967	1.543	1.578	1.514	MF:lipid transporter activity;MF:transporter activity;MF:molecular_function;	GO:0005319;GO:0005215;GO:0003674	------	------	------	------	ENOG410Z6MR;E	S:Function unknown;	------	------	------	CYT	11	87	12	High
TRINITY_DN27264_c0_g1_i1_orf1	uncharacterized protein LOC114353424 [Ostrinia furnacalis]	0.242790074	-2.042218655	0.0001088	down	yes	1.086	4.473	4.421	4.857	4.142	1.067	0.96	1.232	MF:molecular_function;MF:structural constituent of cuticle;MF:structural molecule activity;	GO:0003674;GO:0042302;GO:0005198	------	------	------	------	ENOG4111DJS;EN	S:Function unknown;	PF00379.26	Chitin_bind_4	Insect cuticle protein	CYT	2	6	29.3	High
TRINITY_DN28711_c0_g1_i1_orf1	hypothetical protein evm_000299 [Chilo suppressalis]	0.381582231	-1.389934103	3.68E-06	down	yes	1.529	4.007	3.926	3.962	4.132	1.54	1.571	1.476	MF:lipid transporter activity;MF:transporter activity;MF:molecular_function;	GO:0005319;GO:0005215;GO:0003674	------	------	------	------	ENOG410Z6MR;E	S:Function unknown;	------	------	------	CYT	7	83	7.5	High
TRINITY_DN19990_c0_g1_i1_orf1	carboxypeptidase B-like [Ostrinia furnacalis]	0.279136316	-1.84095826	5.66E-08	down	yes	1.073	3.844	3.847	3.886	3.798	1.062	1.065	1.093	BP:nitrogen compound metabolic process;BP:organic substance metabolic process;BP:proteolysis;BP:biological_process;BP:metabolic process;BP:primary metabolic process;BP:organonitrogen compound metabolic process;BP:protein metabolic process;BP:macromolecule metabolic process;MF:exopeptidase activity;MF:molecular_function;MF:zinc ion binding;MF:peptidase activity;MF:metalloexopeptidase activity;MF:metallopeptidase activity;MF:cation binding;MF:transition metal ion binding;MF:metallocarboxypeptidase activity;MF:carboxypeptidase activity;MF:ion binding;MF:metal ion binding;MF:hydrolase activity;MF:catalytic activity, acting on a protein;MF:catalytic activity;MF:binding;	GO:0006807;GO:0071704;GO:0006508;GO:0008150;GO:0008152;GO:0044238;GO:1901564;GO:0019538;GO:0043170;GO:0008238;GO:0003674;GO:0008270;GO:0008233;GO:0008235;GO:0008237;GO:0043169;GO:0046914;GO:0004181;GO:0004180;GO:0043167;GO:0046872;GO:0016787;GO:0140096;GO:0003824;GO:0005488	------	------	------	------	COG2866	U:Intracellular trafficking, secretion, and vesicular transport	PF00246.27;PF02244.19	Peptidase_M14;Propep_M14	Zinc carboxypeptidase;Carboxypeptidase activation peptide	CYT	7	16	57.6	High
TRINITY_DN71699_c0_g1_i1_orf1	apolipophorins-like [Ostrinia furnacalis]	0.4415625	-1.179310439	5.75E-07	down	yes	1.413	3.2	3.235	3.172	3.192	1.444	1.433	1.363	MF:lipid transporter activity;MF:transporter activity;MF:molecular_function;	GO:0005319;GO:0005215;GO:0003674	------	------	------	------	ENOG410Z6MR;E	S:Function unknown;	------	------	------	CYT	6	63	8.2	High
TRINITY_DN18624_c0_g1_i5_orf1	uncharacterized protein LOC114353135 [Ostrinia furnacalis]	0.105738476	-3.241427656	1.39E-06	down	yes	1.124	10.63	10.956	10.681	10.262	1.23	1.066	1.077	------	------	------	------	------	------	ENOG4110T9R	S:Function unknown	------	------	------	CYT	1	5	34.8	High
TRINITY_DN3190_c0_g1_i1_orf1	repetitive proline-rich cell wall protein 1 precursor [Papilio polytes] >XP_013147838.1 PREDICTED: repetitive proline-rich cell wall protein 1 [Papilio polytes] >BAM19190.1 cuticular protein PpolCPG24 [Papilio polytes]	0.094902534	-3.397409579	2.24E-06	down	yes	0.9737	10.26	10.242	10.661	9.866	0.979	0.978	0.964	------	------	------	------	------	------	ENOG4111M7J	S:Function unknown	------	------	------	CYT	17	44	44	High
TRINITY_DN1326_c0_g1_i1_orf1	cuticle protein 7-like [Ostrinia furnacalis]	0.0675	-3.888968688	0.000765	down	yes	0.5913	8.76	9.675	9.612	6.994	0.688	0.571	0.515	MF:molecular_function;MF:structural constituent of cuticle;MF:structural molecule activity;	GO:0003674;GO:0042302;GO:0005198	------	------	------	------	ENOG410YSFI;EN	S:Function unknown;	PF00379.26	Chitin_bind_4	Insect cuticle protein	CYT	1	27	13.9	High
TRINITY_DN3616_c0_g1_i4_orf1	conotoxin ArMKLT2-032-like [Ostrinia furnacalis]	0.397684096	-1.330305226	0.002472	down	yes	2.198	5.527	6.181	5.815	4.584	2.349	2.202	2.043	CC:cellular_component;CC:integral component of membrane;CC:cellular anatomical entity;CC:intrinsic component of membrane;	GO:0005575;GO:0016021;GO:0110165;GO:0031224	------	------	------	------	------	------	------	------	------	CYT	1	68	6.1	High
TRINITY_DN2908_c0_g1_i1_orf1	uncharacterized protein LOC114361337 [Ostrinia furnacalis]	0.130634302	-2.936394326	3.87E-05	down	yes	0.7373	5.644	6.058	5.658	5.216	0.837	0.672	0.703	------	------	------	------	------	------	ENOG4111MU3	S:Function unknown	PF00354.20;PF13385.9	Pentaxin;Laminin_G_3	Pentaxin family;Concanavalin A-like lectin/glucanases superfamily	CYT	1	2	61.6	High
TRINITY_DN57998_c1_g3_i1_orf1	uncharacterized protein LOC114363305 isoform X2 [Ostrinia furnacalis]	0.250049495	-1.999714403	3.35E-06	down	yes	1.263	5.051	5.008	5.241	4.905	1.321	1.246	1.223	------	------	------	------	------	------	ENOG4111SVO	S:Function unknown	------	------	------	CYT	2	17	23.2	High
TRINITY_DN25273_c0_g1_i1_orf1	skin secretory protein xP2-like [Ostrinia furnacalis]	0.292099132	-1.775470026	7.55E-06	down	yes	1.379	4.721	4.83	4.817	4.516	1.292	1.445	1.399	------	------	------	------	------	------	ENOG4111DG8	S:Function unknown	------	------	------	CYT	3	16	29	High
TRINITY_DN3439_c0_g2_i2_orf1	histone H2A.Z-specific chaperone CHZ1-like [Ostrinia furnacalis]	3.208791209	1.682029919	9.03E-07	up	yes	4.38	1.365	1.32	1.384	1.392	4.486	4.35	4.303	------	------	------	------	------	------	------	------	------	------	------	PLA	4	14	31.9	High
TRINITY_DN24971_c0_g1_i3_orf1	uncharacterized protein LOC114352370 [Ostrinia furnacalis]	0.370044053	-1.434231065	3.42E-06	down	yes	1.512	4.086	4.175	4.13	3.953	1.548	1.511	1.477	------	------	------	------	------	------	ENOG4111G1R	S:Function unknown	------	------	------	CYT	17	34	62.1	High
TRINITY_DN85290_c0_g2_i1_orf1	unnamed protein product, partial [Brenthis ino]	2.020054201	1.014394003	1.91E-06	up	yes	3.727	1.845	1.771	1.858	1.907	3.707	3.706	3.768	------	------	K23615;K2342	SPIK5;SPINK5	------	------	ENOG4111TR7;CC	O:Posttranslational modification, protein turnover, chaperones;	PF00050.24;PF07648.18	Kazal_1;Kazal_2	Kazal-type serine protease inhibitor domain;Kazal-type serine protease inhibitor domain	CYT	1	56	7.6	High
TRINITY_DN42719_c0_g1_i1_orf1	inter-alpha-trypsin inhibitor heavy chain H4-like isoform X11 [Ostrinia furnacalis]	0.31241118	-1.678482014	0.0005232	down	yes	1.319	4.222	4.229	4.658	3.78	1.389	1.501	1.066	------	------	------	------	------	------	------	------	------	------	------	CYT	1	23	7.4	High
TRINITY_DN49785_c1_g1_i3_orf1	uncharacterized protein LOC114365444, partial [Ostrinia furnacalis]	0.292763158	-1.772194082	1.01E-06	down	yes	1.246	4.256	4.168	4.359	4.242	1.206	1.241	1.292	------	------	------	------	------	------	ENOG4110SB1	S:Function unknown	------	------	------	CYT	1	6	26.6	High
TRINITY_DN699_c0_g2_i1_orf1	TPA_exp: putative parasitoid killing factor [Trichoplusia ni]	0.397959184	-1.329307625	0.0001519	down	yes	1.521	3.822	3.807	4.104	3.554	1.457	1.602	1.504	------	------	------	------	------	------	------	------	------	------	------	CYT	1	3	43.9	High
TRINITY_DN1935_c0_g1_i1_orf1	adult-specific cuticular protein ACP-22-like [Ostrinia furnacalis]	0.185620619	-2.429571115	1.87E-06	down	yes	0.821	4.423	4.49	4.522	4.258	0.857	0.811	0.795	MF:molecular_function;MF:structural constituent of cuticle;MF:structural molecule activity;	GO:0003674;GO:0042302;GO:0005198	------	------	------	------	ENOG410YSFI	I:Lipid transport and metabolism	PF00379.26	Chitin_bind_4	Insect cuticle protein	CYT	6	38	19.3	High
TRINITY_DN5907_c0_g1_i4_orf1	hypothetical protein evm_010265 [Chilo suppressalis] >CAB3524755.1 unnamed protein product [Chilo suppressalis] >CAH0397522.1 unnamed protein product [Chilo suppressalis]	0.285823755	-1.806802271	2.92E-07	down	yes	1.119	3.915	3.893	3.869	3.983	1.081	1.16	1.117	------	------	------	------	------	------	ENOG410YTKH;EN	S:Function unknown;	PF06585.14	JHBP	Haemolymph juvenile hormone binding protein	CYT	5	23	28.1	High
TRINITY_DN1110_c1_g1_i9_orf1	MD-2-related lipid-recognition protein-like [Ostrinia furnacalis]	0.272338149	-1.876529008	8.96E-07	down	yes	1.018	3.738	3.694	3.702	3.818	1.03	0.951	1.072	------	------	K13443	NPC2	map04979;ma	Cholesterol metabolism;Lysosome	ENOG4111D1U;EN	S:Function unknown;	PF02221.18	E1_DerP2_DerF2	ML domain	CYT	2	17	17.2	High
TRINITY_DN661_c0_g2_i2_orf1	cuticle protein 7-like [Ostrinia furnacalis]	0.224888305	-2.152719458	2.07E-06	down	yes	0.8557	3.805	3.929	3.8	3.687	0.886	0.845	0.836	MF:molecular_function;MF:structural constituent of cuticle;MF:structural molecule activity;	GO:0003674;GO:0042302;GO:0005198	------	------	------	------	ENOG4110SG6;EN	S:Function unknown;	PF00379.26	Chitin_bind_4	Insect cuticle protein	CYT	2	19	19.4	High
TRINITY_DN2896_c0_g1_i2_orf1	general odorant-binding protein 56d-like isoform X2 [Ostrinia furnacalis]	0.31843092	-1.650947664	4.48E-05	down	yes	1.104	3.467	3.481	3.674	3.246	1.106	1.119	1.086	CC:integral component of membrane;CC:cellular anatomical entity;CC:cellular_component;CC:intrinsic component of membrane;MF:odorant binding;MF:molecular_function;MF:binding;	GO:0016021;GO:0110165;GO:0005575;GO:0031224;GO:0005549;GO:0003674;GO:0005488	------	------	------	------	ENOG410ZCHW;E	S:Function unknown;	PF01395.25	PBP_GOBP	PBP/GOBP family	CYT	7	46	15	High
TRINITY_DN7785_c0_g1_i1_orf1	uncharacterized protein LOC114364098 [Ostrinia furnacalis]	0.194572193	-2.361622554	4.36E-06	down	yes	0.7277	3.74	3.602	3.897	3.72	0.693	0.763	0.727	------	------	------	------	------	------	ENOG4110X3U;EN	S:Function unknown;	PF00059.24	Lectin_C	Lectin C-type domain	CYT	2	16	29.9	High
TRINITY_DN5748_c0_g1_i6_orf1	glycine N-methyltransferase isoform X1 [Ostrinia furnacalis] >XP_028165118.1 glycine N-methyltransferase isoform X2 [Ostrinia furnacalis] >XP_028165119.1 glycine N-methyltransferase isoform X1 [Ostrinia furnacalis] >XP_028165120.1 glycine N-methyltransferase isoform X2 [Ostrinia furnacalis]	2.340245776	1.226660052	5.20E-05	up	yes	3.047	1.302	1.254	1.388	1.265	3.151	3.112	2.879	BP:organic substance metabolic process;BP:biological_process;BP:metabolic process;BP:methylation;MF:glycine N-methyltransferase activity;MF:catalytic activity;MF:S-adenosylmethionine-dependent methyltransferase activity;MF:molecular_function;MF:transferase activity;MF:N-methyltransferase activity;MF:transferase activity, transferring one-carbon groups;MF:methyltransferase activity;	GO:0071704;GO:0008150;GO:0008152;GO:0032259;GO:0017174;GO:0003824;GO:0008757;GO:0003674;GO:0016740;GO:0008170;GO:0016741;GO:0008168	K00552	GNMT	map00260	Glycine, serine and threonine metabolism	ENOG410ZWXN	S:Function unknown	PF13649.9;PF08242.15;PF13847.9;PF08241.15;PF13489.9	Methyltransf_25;Methyltransf_12;Methyltransf_31;Methyltransf_11;Methyltransf_23	Methyltransferase domain;Methyltransferase domain;Methyltransferase domain;Methyltransferase domain;Methyltransferase domain	CYT	3	48	27.4	High
TRINITY_DN6415_c0_g2_i1_orf1	D-arabinitol dehydrogenase 1-like [Ostrinia furnacalis]	2.073596358	1.05213509	7.77E-05	up	yes	2.733	1.318	1.304	1.267	1.383	2.875	2.605	2.719	MF:oxidoreductase activity;MF:cation binding;MF:transition metal ion binding;MF:ion binding;MF:catalytic activity;MF:molecular_function;MF:binding;MF:metal ion binding;MF:zinc ion binding;	GO:0016491;GO:0043169;GO:0046914;GO:0043167;GO:0003824;GO:0003674;GO:0005488;GO:0046872;GO:0008270	K17818	ARD1	map00040	Pentose and glucuronate interconversions	COG1063	E:Amino acid transport and metabolism	PF08240.15;PF00107.29;PF16912.8	ADH_N;ADH_zinc_N;Glu_dehyd_C	Alcohol dehydrogenase GroES-like domain;Zinc-binding dehydrogenase;Glucose dehydrogenase C-terminus	CYT	13	61	37.5	High
TRINITY_DN33728_c0_g2_i1_orf1	uncharacterized protein LOC114350200 [Ostrinia furnacalis]	0.469685902	-1.090231804	0.004844	down	yes	1.286	2.738	2.886	2.993	2.334	1.208	1.063	1.587	------	------	------	------	------	------	ENOG4111CDX	S:Function unknown	------	------	------	CYT	1	4	23	High
TRINITY_DN4367_c0_g1_i1_orf1	heat shock protein 21.7c [Chilo suppressalis] >AWT57938.1 heat shock protein 21.7c [Chilo suppressalis]	0.294779246	-1.762293141	1.44E-05	down	yes	0.9147	3.103	3.254	3.022	3.034	0.972	0.939	0.833	MF:cation binding;MF:metal ion binding;MF:molecular_function;MF:binding;MF:ion binding;	GO:0043169;GO:0046872;GO:0003674;GO:0005488;GO:0043167	K09542	CRYAB	map04213;ma	Longevity regulating pathway - multiple species;Protein processing in	ENOG410YERS	S:Function unknown	PF00011.24	HSP20	Hsp20/alpha crystallin family	CYT	1	19	21.6	High
TRINITY_DN27300_c0_g1_i1_orfp1	TRINITY_DN27300_c0_g1_i1_m.71142 TRINITY_DN27300_c0_g1::TRINITY_DN27300_c0_g1_i1::g.71142 ORF type:internal len:82 (-) score=9.46 TRINITY_DN27300_c0_g1_i1:3-245(-)	8.539325843	3.094122177	1.73E-06	up	yes	11.4	1.335	1.448	1.132	1.425	11.271	11.811	11.118	------	------	------	------	------	------	------	------	------	------	------	CYT	1	21	9.2	High
TRINITY_DN36476_c1_g1_i1_orfp1	TRINITY_DN36476_c1_g1_i1_m.70910 TRINITY_DN36476_c1_g1::TRINITY_DN36476_c1_g1_i1::g.70910 ORF type:5prime_partial len:88 (-) score=0.50 TRINITY_DN36476_c1_g1_i1:49-312(-)	4.036844591	2.013228048	7.71E-06	up	yes	8.546	2.117	2.061	2.092	2.198	8.606	8.881	8.151	------	------	------	------	------	------	------	------	------	------	------	CYT	2	21	9.5	High
TRINITY_DN35809_c0_g1_i1_orf1	spodomicin-like [Ostrinia furnacalis] >QKV49445.1 diapausin [Ostrinia furnacalis]	3.575455334	1.838126981	2.69E-05	up	yes	8.245	2.306	2.392	2.294	2.233	8.035	8.781	7.918	------	------	------	------	------	------	------	------	PF08036.14	Antimicrobial_6	Diapausin family of antimicrobial peptide	CYT	3	60	6.8	High

TRINITY_DN24789_c0_g1_i9_orfp1	TRINITY_DN24789_c0_g1_i9_m.25888 TRINITY_DN24789_c0_g1::TRINITY_DN24789_c0_g1_i9::g.25888 ORF type:internal len:114 (-),score=12.65 TRINITY_DN24789_c0_g1_i9:2-340(-)	5.77127393	2.528889809	8.31E-06	up	yes	5.758	0.9977	0.976	1.014	1.003	5.517	6.069	5.687	------	------	------	------	------	------	------	------	------	------	------	CYT	5	46	13.2	High
TRINITY_DN46625_c0_g1_i1_orf1	ferritin subunit isoform X1 [Belonocnema kinseyi]	0.343945194	-1.539749396	0.000185	down	yes	1.707	4.963	5.03	4.635	5.224	1.946	1.371	1.805	BP:ion homeostasis;BP:homeostatic process;BP:iron ion homeostasis;BP:transition metal ion homeostasis;BP:inorganic ion homeostasis;BP:cellular transition metal ion homeostasis;BP:transition metal ion transport;BP:biological regulation;BP:regulation of biological quality;BP:cellular homeostasis;BP:metal ion transport;BP:transport;BP:cellular iron ion homeostasis;BP:cellular metal ion homeostasis;BP:cation transport;BP:ion transport;BP:cellular process;BP:cellular ion homeostasis;BP:biological_process;BP:cellular cation homeostasis;BP:metal ion homeostasis;BP:cation homeostasis;BP:cellular chemical homeostasis;BP:establishment of localization;BP:localization;BP:chemical homeostasis;BP:iron ion transport;MF:molecular_function;MF:binding;MF:oxidoreductase activity;MF:cation binding;MF:oxidoreductase activity, oxidizing metal ions;MF:transition metal ion binding;MF:ferroxidase activity;MF:ion binding;MF:iron ion binding;MF:ferric iron binding;MF:oxidoreductase activity, oxidizing metal ions, oxygen as acceptor;MF:metal ion binding;MF:catalytic activity;	GO:0050801;GO:0042592;GO:0055072;GO:0055076;GO:0098771;GO:0046916;GO:0000041;GO:0065007;GO:0065008;GO:0019725;GO:0030001;GO:0006810;GO:0006879;GO:0006875;GO:0006812;GO:0006811;GO:0009987;GO:0006873;GO:0008150;GO:0030003;GO:0055065;GO:0055080;GO:0055082;GO:0051234;GO:0051179;GO:0048878;GO:0006826;GO:0003674;GO:0005488;GO:0016491;GO:0043169;GO:0016722;GO:0046914;GO:0004322;GO:0043167;GO:0005506;GO:0008199;GO:0016724;GO:0046872;GO:0003824	K00522	FTH1	map04978;ma	Mineral absorption;Necroptosis;Ferroptosis	ENOG4111MSG	P:Inorganic ion transport and metabolism	PF00210.27	Ferritin	Ferritin-like domain	CYT	1	4	24.1	High
TRINITY_DN11981_c0_g1_i7_orf1	luciferin 4-monooxygenase-like isoform X2 [Ostrinia furnacalis]	2.273466834	1.184893957	0.0001652	up	yes	3.633	1.598	1.698	1.578	1.517	3.753	3.79	3.356	------	------	K01904	4CL	map00130	Ubiquinone and other terpenoid-quinone biosynthesis	COG0365;COG03	I:Lipid transport and metabolism;Q:Secondary metabolites biosynthesis, transport and catabolism	PF00501.31;PF13193.9	AMP-binding;AMP-binding_C	AMP-binding enzyme;AMP-binding enzyme C-terminal domain	CYT	3	7	62.4	High
TRINITY_DN76216_c0_g2_i3_orf1	lysosomal alpha-mannosidase isoform X1 [Pieris rapae]	0.431599229	-1.212235806	0.0003552	down	yes	1.568	3.633	3.404	3.862	3.633	1.591	1.337	1.776	------	------	K12311	MAN2B1, LAM	map00511;ma	Other glycan degradation;Lysosome	ENOG410XQMZ;C	G:Carbohydrate transport and metabolism;J:Translation, ribosomal structure and biogenesis	PF01074.25	Glyco_hydro_38N	Glycosyl hydrolases family 38 N-terminal domain	CYT	1	25	12	High
TRINITY_DN20793_c0_g2_i1_orf1	mucin-2-like [Ostrinia furnacalis]	0.210747271	-2.246414144	1.94E-06	down	yes	0.753	3.573	3.644	3.632	3.444	0.787	0.748	0.724	------	------	------	------	------	------	ENOG41115RA	S:Function unknown	------	------	------	EXC	4	9	52.5	High
TRINITY_DN84938_c0_g1_i4_orf1	vinculin-like isoform X2 [Ostrinia furnacalis]	2.011980268	1.008616156	0.005836	up	yes	2.855	1.419	1.082	1.299	1.877	2.88	2.629	3.057	------	------	K05700;K062	VCL;TLN	map05166;ma	Human T-cell leukemia virus 1 infection;Focal adhesion;Regulation of actin cytoskeleton;Amoebiasis;Rap1 signaling pathway;Shigellosis;Leukocyte transendothelial migration;Bacterial invasion of epithelial cells;Adherens junction;Platelet	------	------	------	------	------	CYT	1	23	15.7	High
TRINITY_DN87803_c0_g1_i2_orf1	hypothetical protein NE865_05903 [Phthorimaea operculella]	0.15042333	-2.732899753	3.19E-06	down	yes	1.599	10.63	10.94	10.257	10.691	1.837	1.609	1.35	MF:catalytic activity;MF:transferase activity;MF:molecular_function;	GO:0003824;GO:0016740;GO:0003674	K10532	HGSNAT	map04142;ma	Lysosome;Glycosaminoglycan degradation	COG4299	S:Function unknown	------	------	------	CYT	1	23	9.7	High
TRINITY_DN74538_c0_g1_i1_orf1	tetra-peptide repeat homeobox protein 1-like [Ostrinia furnacalis]	0.14190112	-2.817042119	5.23E-06	down	yes	1.039	7.322	7.562	6.96	7.444	1.141	1.026	0.951	------	------	------	------	------	------	------	------	------	------	------	CYT	3	32	23.1	High
TRINITY_DN5581_c0_g1_i1_orf1	mucin-5AC-like [Ostrinia furnacalis]	0.147621283	-2.760027358	8.97E-06	down	yes	0.9433	6.39	6.404	6.695	6.071	1.019	0.819	0.992	------	------	------	------	------	------	ENOG410YZWK	S:Function unknown	------	------	------	CYT	2	2	128.1	High
TRINITY_DN136031_c0_g1_i7_orf1	ferritin, lower subunit isoform X3 [Spodoptera litura]	7.787882512	2.96123112	5.33E-05	up	yes	6.337	0.8137	0.878	0.656	0.907	6.291	5.855	6.865	BP:ion homeostasis;BP:homeostatic process;BP:iron ion homeostasis;BP:transition metal ion homeostasis;BP:inorganic ion homeostasis;BP:cellular transition metal ion homeostasis;BP:transition metal ion transport;BP:biological regulation;BP:cellular homeostasis;BP:metal ion transport;BP:transport;BP:cellular iron ion homeostasis;BP:cellular metal ion homeostasis;BP:cation transport;BP:ion transport;BP:cellular process;BP:cellular ion homeostasis;BP:biological_process;BP:cellular cation homeostasis;BP:metal ion homeostasis;BP:cation homeostasis;BP:cellular chemical homeostasis;BP:establishment of localization;BP:localization;BP:regulation of biological quality;BP:chemical homeostasis;BP:iron ion transport;MF:molecular_function;MF:binding;MF:oxidoreductase activity;MF:cation binding;MF:oxidoreductase activity, oxidizing metal ions;MF:transition metal ion binding;MF:ferroxidase activity;MF:ion binding;MF:iron ion binding;MF:metal ion binding;MF:catalytic activity;MF:oxidoreductase activity, oxidizing metal ions, oxygen as acceptor;MF:ferric iron binding;	GO:0050801;GO:0042592;GO:0055072;GO:0055076;GO:0098771;GO:0046916;GO:0000041;GO:0065007;GO:0019725;GO:0030001;GO:0006810;GO:0006879;GO:0006875;GO:0006812;GO:0006811;GO:0009987;GO:0006873;GO:0008150;GO:0030003;GO:0055065;GO:0055080;GO:0055082;GO:0051234;GO:0051179;GO:0065008;GO:0048878;GO:0006826;GO:0003674;GO:0005488;GO:0016491;GO:0043169;GO:0016722;GO:0046914;GO:0004322;GO:0043167;GO:0005506;GO:0046872;GO:0003824;GO:0016724;GO:0008199	------	------	------	------	ENOG4111Q26;C	P:Inorganic ion transport and metabolism;	------	------	------	EXC	1	28	11.1	High
TRINITY_DN35351_c0_g1_i3_orf1	adult-specific cuticular protein ACP-20-like [Ostrinia furnacalis]	0.187447275	-2.415443241	4.00E-05	down	yes	1.111	5.927	5.556	6.377	5.849	1.205	1.026	1.102	MF:molecular_function;MF:structural constituent of cuticle;MF:structural molecule activity;	GO:0003674;GO:0042302;GO:0005198	------	------	------	------	ENOG410YWJF;EN	Q:Secondary metabolites biosynthesis, transport and catabolism;S:Function unknown;S:Function unknown;S:Function unknown;S:Function unknown	PF00379.26	Chitin_bind_4	Insect cuticle protein	CYT	2	16	19.2	High
TRINITY_DN29604_c0_g2_i2_orf1	neurofilament heavy polypeptide-like isoform X2 [Ostrinia furnacalis]	0.261801017	-1.933457395	3.33E-06	down	yes	1.442	5.508	5.473	5.714	5.338	1.421	1.473	1.431	------	------	------	------	------	------	------	------	------	------	------	CYT	22	77	40.5	High
TRINITY_DN26882_c0_g1_i1_orf1	uncharacterized protein LOC114349648 [Ostrinia furnacalis]	0.161941848	-2.626452245	6.24E-06	down	yes	0.9357	5.778	5.81	6.028	5.496	0.914	0.956	0.937	------	------	------	------	------	------	ENOG41120B2;EN	S:Function unknown;	PF15998.8	DUF4773	Domain of unknown function	PLA	2	8	28.6	High
TRINITY_DN57137_c0_g1_i1_orfp1	TRINITY_DN57137_c0_g1_i1_m.46420 TRINITY_DN57137_c0_g1::TRINITY_DN57137_c0_g1_i1::g.46420 ORF type:5prime_partial len:56 (-),score=4.65 TRINITY_DN57137_c0_g1_i1:82-249(-)	2.378546339	1.250080132	0.0005381	up	yes	8.803	3.701	3.461	3.667	3.975	8.355	9.766	8.288	------	------	------	------	------	------	------	------	------	------	------	CYT	3	33	5.8	High
TRINITY_DN906_c0_g1_i4_orf1	uncharacterized protein LOC114360441, partial [Ostrinia furnacalis]	0.288942696	-1.791144695	1.36E-08	down	yes	1.432	4.956	4.988	4.946	4.933	1.455	1.444	1.397	------	------	K17339	ATL	------	------	ENOG410XR6Z	S:Function unknown	PF02263.22;PF02841.17	GBP;GBP_C	Guanylate-binding protein, N-terminal domain;Guanylate-binding protein, C-terminal domain	CYT	22	38	87.8	High
TRINITY_DN49147_c0_g2_i1_orf1	glutenin, high molecular weight subunit PW212-like [Ostrinia furnacalis]	0.287003222	-1.80086116	1.64E-05	down	yes	1.336	4.655	4.613	4.572	4.779	1.421	1.1	1.487	------	------	------	------	------	------	ENOG4110R9Z	S:Function unknown	------	------	------	PLA	1	5	31.6	High
TRINITY_DN2311_c0_g3_i1_orf1	uncharacterized protein LOC114364231 isoform X1 [Ostrinia furnacalis] >XP_028176108.1 uncharacterized protein LOC114364231 isoform X2 [Ostrinia furnacalis]	0.309951776	-1.689884327	9.99E-05	down	yes	1.414	4.562	4.518	4.914	4.253	1.395	1.314	1.533	------	------	------	------	------	------	ENOG410Y2YK	S:Function unknown	------	------	------	CYT	1	2	41.4	High
TRINITY_DN6330_c0_g1_i1_orfp1	TRINITY_DN6330_c0_g1_i1_m.42332 TRINITY_DN6330_c0_g1::TRINITY_DN6330_c0_g1_i1::g.42332 ORF type:3prime_partial len:51 (-),score=33.30 TRINITY_DN6330_c0_g1_i1:3-152(-)	2.583058306	1.369080209	0.0001719	up	yes	7.044	2.727	2.914	2.949	2.319	6.649	7.491	6.992	------	------	------	------	------	------	------	------	------	------	------	CYT	1	26	5.3	High
TRINITY_DN12387_c0_g1_i1_orf1	repetitive proline-rich cell wall protein 2-like [Ostrinia furnacalis]	0.20628655	-2.277278337	2.29E-06	down	yes	0.9877	4.788	4.822	4.931	4.61	0.972	0.994	0.997	------	------	------	------	------	------	ENOG410YH2C;EN	S:Function unknown;	------	------	------	CYT	18	64	40.7	High
TRINITY_DN47784_c0_g2_i1_orfp1	arylphorin subunit alpha-like [Ostrinia furnacalis]	2.61036036	1.384248985	9.10E-06	up	yes	6.954	2.664	2.635	2.79	2.568	6.794	7.224	6.844	------	------	------	------	------	------	------	------	------	------	------	CYT	2	27	10.1	High
TRINITY_DN71698_c0_g1_i1_orfp1	TRINITY_DN71698_c0_g1_i1_m.1194 TRINITY_DN71698_c0_g1::TRINITY_DN71698_c0_g1_i1::g.1194 ORF type:internal len:134 (+),score=19.66,Toxin_2 PF00451.20 4.3e-05,Toxin_2|PF00451.20|0.037,Toxin_2|PF00451.20|7.5e-05,Gamma-thionin|PF00304.21|0.017,Gamma-thionin|PF00304.21|0.05,Gamma-thionin|PF00304.21|0.021,Toxin_38|PF14866.7|0.13,Toxin_38|PF14866.7|0.15,Toxin_38|PF14866.7|0.15,Defensin_2|PF01097.19|0.053,Defensin_2|PF01097.19|0.34,Defensin_2|PF01097.19|0.092 TRINITY_DN71698_c0_g1_i1:3-401(+)	2.091894977	1.064810423	8.01E-05	up	yes	3.665	1.752	1.785	1.727	1.744	3.61	3.886	3.499	------	------	------	------	------	------	------	------	PF00451.22;PF00304.23	Toxin_2;Gamma-thionin	Scorpion short toxin, BmKK2;Gamma-thionin family	CYT	6	47	15.5	High
TRINITY_DN4021_c0_g1_i1_orf1	leech-derived tryptase inhibitor C-like [Ostrinia furnacalis]	0.138593015	-2.851073551	4.94E-05	down	yes	1.119	8.074	7.663	8.807	7.751	1.014	1.091	1.253	------	------	K23417	SPINK1	------	------	ENOG410Z5MG;E	S:Function unknown;	PF07648.18;PF00050.24	Kazal_2;Kazal_1	Kazal-type serine protease inhibitor domain;Kazal-type serine protease inhibitor domain	PLA	1	11	7.2	High
TRINITY_DN8595_c0_g1_i3_orf1	aldose reductase-like isoform X4 [Trichoplusia ni]	2.019631172	1.01409185	6.79E-07	up	yes	3.395	1.681	1.681	1.631	1.73	3.42	3.375	3.391	MF:catalytic activity;MF:molecular_function;MF:oxidoreductase activity;	GO:0003824;GO:0003674;GO:0016491	K00011	AKR1B	map00051;ma	Fructose and mannose metabolism;Pentose and glucuronate interconversions;Glycerolipid metabolism;Folate biosynthesis;Galactose metabolism	COG0656	S:Function unknown	PF00248.24	Aldo_ket_red	Aldo/keto reductase family	CYT	1	67	13.9	High
TRINITY_DN710_c0_g1_i11_orfp1	TRINITY_DN710_c0_g1_i11_m.67699 TRINITY_DN710_c0_g1::TRINITY_DN710_c0_g1_i11::g.67699 ORF type:complete len:194 (-),score=34.99,Collagen PF01391.19 0.00029 TRINITY_DN710_c0_g1_i11:1283-1864(-)	3.441546763	1.783057113	9.61E-05	up	yes	3.827	1.112	1.096	1.071	1.168	3.498	4.069	3.914	------	------	------	------	------	------	------	------	------	------	------	CYT	1	7	21.3	High
TRINITY_DN3310_c0_g1_i1_orf1	hypothetical protein evm_010516 [Chilo suppressalis]	0.114200971	-3.130353181	1.47E-05	down	yes	0.6353	5.563	5.56	5.902	5.226	0.642	0.617	0.647	CC:integral component of membrane;CC:cellular_component;CC:intrinsic component of membrane;CC:cellular anatomical entity;MF:structural molecule activity;MF:molecular_function;MF:structural constituent of cuticle;	GO:0016021;GO:0005575;GO:0031224;GO:0110165;GO:0005198;GO:0003674;GO:0042302	------	------	------	------	ENOG4111PCZ	S:Function unknown	------	------	------	CYT	5	17	40.4	High
TRINITY_DN3056_c0_g1_i1_orf1	spidroin-2-like [Ostrinia furnacalis]	0.303818034	-1.718720587	8.88E-07	down	yes	1.122	3.693	3.678	3.743	3.658	1.067	1.092	1.208	MF:molecular_function;MF:structural constituent of cuticle;MF:structural molecule activity;	GO:0003674;GO:0042302;GO:0005198	------	------	------	------	ENOG4110SY1;EN	S:Function unknown;	PF00379.26	Chitin_bind_4	Insect cuticle protein	CYT	1	4	76.6	High
TRINITY_DN1149_c0_g1_i4_orf1	circadian clock-controlled protein-like [Ostrinia furnacalis]	3.233721727	1.693195535	1.17E-06	up	yes	4.718	1.459	1.479	1.415	1.483	4.797	4.767	4.589	------	------	------	------	------	------	ENOG410YQCZ;E	S:Function unknown;	PF06585.14	JHBP	Haemolymph juvenile hormone binding protein	CYT	7	35	27.4	High
TRINITY_DN19639_c0_g2_i1_orf1	basic juvenile hormone-suppressible protein 1-like [Hyposmocoma kahamanoa]	0.198579716	-2.33220983	0.0001406	down	yes	0.9927	4.999	4.955	4.538	5.504	1.045	0.956	0.977	------	------	------	------	------	------	ENOG410XR2D	S:Function unknown	PF03722.17	Hemocyanin_N	Hemocyanin, all-alpha domain	CYT	2	72	8.6	High
TRINITY_DN8008_c0_g1_i6_orf1	uncharacterized protein LOC114357965 isoform X1 [Ostrinia furnacalis] >XP_028167599.1 uncharacterized protein LOC114357965 isoform X1 [Ostrinia furnacalis] >XP_028167600.1 uncharacterized protein LOC114357965 isoform X2 [Ostrinia furnacalis] >XP_028167601.1 uncharacterized protein LOC114357965 isoform X3 [Ostrinia furnacalis]	2	1	0.0001594	up	yes	3.056	1.528	1.688	1.394	1.501	3.195	2.991	2.981	MF:organic cyclic compound binding;MF:molecular_function;MF:binding;MF:nucleic acid binding;MF:DNA binding;MF:heterocyclic compound binding;	GO:0097159;GO:0003674;GO:0005488;GO:0003676;GO:0003677;GO:1901363	------	------	------	------	ENOG410ZC30	S:Function unknown	------	------	------	CYT	1	0	506.7	Medium
TRINITY_DN13686_c0_g2_i1_orf1	transmembrane protease serine 9-like [Ostrinia furnacalis]	0.323420931	-1.628515044	1.41E-06	down	yes	1.403	4.338	4.372	4.394	4.249	1.357	1.357	1.496	BP:organic substance metabolic process;BP:organonitrogen compound metabolic process;BP:protein metabolic process;BP:macromolecule metabolic process;BP:biological_process;BP:nitrogen compound metabolic process;BP:metabolic process;BP:proteolysis;BP:primary metabolic process;MF:serine-type endopeptidase activity;MF:endopeptidase activity;MF:hydrolase activity;MF:serine hydrolase activity;MF:catalytic activity, acting on a protein;MF:catalytic activity;MF:molecular_function;MF:peptidase activity;MF:serine-type peptidase activity;	GO:0071704;GO:1901564;GO:0019538;GO:0043170;GO:0008150;GO:0006807;GO:0008152;GO:0006508;GO:0044238;GO:0004252;GO:0004175;GO:0016787;GO:0017171;GO:0140096;GO:0003824;GO:0003674;GO:0008233;GO:0008236	K20677;K096	HAYAN;TMPR	map05164	Influenza A	COG5640	O:Posttranslational modification, protein turnover, chaperones	PF00089.29	Trypsin	Trypsin	CYT	2	6	43.9	High
TRINITY_DN391_c0_g1_i4_orf1	phenoloxidase-activating enzyme-like [Ostrinia furnacalis]	0.446302587	-1.163905926	2.48E-05	down	yes	1.708	3.827	3.834	3.988	3.659	1.706	1.731	1.686	BP:organic substance metabolic process;BP:organonitrogen compound metabolic process;BP:protein metabolic process;BP:nitrogen compound metabolic process;BP:macromolecule metabolic process;BP:biological_process;BP:metabolic process;BP:proteolysis;BP:primary metabolic process;CC:cellular_component;CC:extracellular region;CC:cellular anatomical entity;MF:serine-type endopeptidase activity;MF:endopeptidase activity;MF:hydrolase activity;MF:serine hydrolase activity;MF:catalytic activity, acting on a protein;MF:catalytic activity;MF:molecular_function;MF:peptidase activity;MF:serine-type peptidase activity;	GO:0071704;GO:1901564;GO:0019538;GO:0006807;GO:0043170;GO:0008150;GO:0008152;GO:0006508;GO:0044238;GO:0005575;GO:0005576;GO:0110165;GO:0004252;GO:0004175;GO:0016787;GO:0017171;GO:0140096;GO:0003824;GO:0003674;GO:0008233;GO:0008236	------	------	------	------	COG5640	O:Posttranslational modification, protein turnover, chaperones	PF00089.29;PF12032.11	Trypsin;CLIP	Trypsin;Regulatory CLIP domain of proteinases	CYT	11	39	45.8	High
TRINITY_DN7539_c0_g1_i2_orf1	serine protease inhibitor 3/4 [Ostrinia furnacalis]	0.231094306	-2.113446381	1.20E-07	down	yes	1.039	4.496	4.448	4.576	4.464	1.048	1.047	1.022	CC:cellular_component;CC:cellular anatomical entity;CC:extracellular space;MF:endopeptidase inhibitor activity;MF:endopeptidase regulator activity;MF:peptidase regulator activity;MF:molecular_function;MF:peptidase inhibitor activity;MF:serine-type endopeptidase inhibitor activity;MF:enzyme inhibitor activity;MF:molecular function regulator;MF:enzyme regulator activity;	GO:0005575;GO:0110165;GO:0005615;GO:0004866;GO:0061135;GO:0061134;GO:0003674;GO:0030414;GO:0004867;GO:0004857;GO:0098772;GO:0030234	------	------	------	------	COG4826	O:Posttranslational modification, protein turnover, chaperones	PF00079.23	Serpin	Serpin	CYT	9	18	58.6	High
TRINITY_DN1880_c0_g1_i4_orf1	serine protease inhibitor dipetalogastin-like [Helicoverpa zea]	2.288659794	1.194503024	3.14E-07	up	yes	3.774	1.649	1.667	1.631	1.648	3.832	3.761	3.729	------	------	K23615	SPIK5	------	------	COG5640;ENOG4	O:Posttranslational modification, protein turnover, chaperones;S:Function unknown	PF00050.24;PF07648.18	Kazal_1;Kazal_2	Kazal-type serine protease inhibitor domain;Kazal-type serine protease inhibitor domain	CYT	12	52	42.6	High
TRINITY_DN696_c1_g1_i10_orf1	titin-like [Ostrinia furnacalis]	0.301301922	-1.73071822	1.44E-07	down	yes	0.972	3.226	3.198	3.28	3.199	0.977	0.981	0.958	------	------	------	------	------	------	ENOG410ZKJ3;EN	S:Function unknown;	------	------	------	CYT	9	5	288.8	High
TRINITY_DN15545_c0_g1_i1_orf1	larval cuticle protein LCP-14-like [Ostrinia furnacalis]	4.150124069	2.053154467	1.68E-05	up	yes	3.345	0.806	0.804	0.829	0.785	3.184	3.538	3.313	MF:molecular_function;MF:structural constituent of cuticle;MF:structural molecule activity;	GO:0003674;GO:0042302;GO:0005198	------	------	------	------	ENOG4110RUZ;EN	S:Function unknown;	PF00379.26	Chitin_bind_4	Insect cuticle protein	EXC	3	48	14.6	High
TRINITY_DN1749_c0_g2_i2_orf1	putative GPI-anchored protein pfl2 isoform X1 [Ostrinia furnacalis]	0.271049896	-1.883369641	8.98E-07	down	yes	1.043	3.848	3.828	3.95	3.767	1.058	1.018	1.052	BP:developmental process;BP:dephosphorylation;BP:protein dephosphorylation;BP:cell development;BP:nitrogen compound metabolic process;BP:anatomical structure morphogenesis;BP:cellular component organization or biogenesis;BP:cellular developmental process;BP:cellular component organization;BP:organic substance metabolic process;BP:neuron development;BP:cellular process;BP:macromolecule modification;BP:protein modification process;BP:biological_process;BP:metabolic process;BP:response to chemical;BP:locomotion;BP:primary metabolic process;BP:organonitrogen compound metabolic process;BP:protein metabolic process;BP:anatomical structure development;BP:cellular metabolic process;BP:macromolecule metabolic process;BP:phosphate-containing compound metabolic process;BP:phosphorus metabolic process;BP:response to stimulus;CC:integral component of membrane;CC:intrinsic component of membrane;CC:cellular anatomical entity;CC:cellular_component;MF:phosphatase activity;MF:phosphoric ester hydrolase activity;MF:molecular_function;MF:phosphoprotein phosphatase activity;MF:hydrolase activity;MF:catalytic activity, acting on a protein;MF:hydrolase activity, acting on ester bonds;MF:catalytic activity;MF:protein tyrosine phosphatase activity;	GO:0032502;GO:0016311;GO:0006470;GO:0048468;GO:0006807;GO:0009653;GO:0071840;GO:0048869;GO:0016043;GO:0071704;GO:0048666;GO:0009987;GO:0043412;GO:0036211;GO:0008150;GO:0008152;GO:0042221;GO:0040011;GO:0044238;GO:1901564;GO:0019538;GO:0048856;GO:0044237;GO:0043170;GO:0006796;GO:0006793;GO:0050896;GO:0016021;GO:0031224;GO:0110165;GO:0005575;GO:0016791;GO:0042578;GO:0003674;GO:0004721;GO:0016787;GO:0140096;GO:0016788;GO:0003824;GO:0004725	------	------	------	------	COG5599	T:Signal transduction mechanisms	------	------	------	CYT	4	5	116.8	High
TRINITY_DN2652_c0_g2_i1_orf1	peroxidase-like isoform X1 [Ostrinia furnacalis]	0.382302405	-1.387213817	6.22E-07	down	yes	1.335	3.492	3.432	3.501	3.544	1.353	1.358	1.293	BP:response to stimulus;BP:cell development;BP:female gamete generation;BP:gamete generation;BP:cellular developmental process;BP:oogenesis;BP:developmental process;BP:multicellular organismal reproductive process;BP:cellular process;BP:germ cell development;BP:reproductive process;BP:response to stress;BP:biological_process;BP:cellular process involved in reproduction in multicellular organism;BP:response to oxidative stress;BP:developmental process involved in reproduction;BP:anatomical structure development;CC:integral component of membrane;CC:intrinsic component of membrane;CC:cellular anatomical entity;CC:cellular_component;MF:molecular_function;MF:binding;MF:heterocyclic compound binding;MF:oxidoreductase activity;MF:oxidoreductase activity, acting on peroxide as acceptor;MF:heme binding;MF:antioxidant activity;MF:organic cyclic compound binding;MF:catalytic activity;MF:peroxidase activity;MF:tetrapyrrole binding;	GO:0050896;GO:0048468;GO:0007292;GO:0007276;GO:0048869;GO:0048477;GO:0032502;GO:0048609;GO:0009987;GO:0007281;GO:0022414;GO:0006950;GO:0008150;GO:0022412;GO:0006979;GO:0003006;GO:0048856;GO:0016021;GO:0031224;GO:0110165;GO:0005575;GO:0003674;GO:0005488;GO:1901363;GO:0016491;GO:0016684;GO:0020037;GO:0016209;GO:0097159;GO:0003824;GO:0004601;GO:0046906	K19511	PXDN, VPO1	------	------	ENOG410XPZ3	S:Function unknown	PF03098.18	An_peroxidase	Animal haem peroxidase	CYT	11	19	88.2	High
TRINITY_DN644_c0_g1_i1_orf1	cuticle protein 19-like [Ostrinia furnacalis]	0.073822927	-3.759787243	3.31E-05	down	yes	0.577	7.816	7.811	8.425	7.211	0.647	0.545	0.539	MF:molecular_function;MF:structural constituent of cuticle;MF:structural molecule activity;	GO:0003674;GO:0042302;GO:0005198	------	------	------	------	ENOG410YSFI	S:Function unknown	PF00379.26	Chitin_bind_4	Insect cuticle protein	CYT	4	34	14.8	High
TRINITY_DN28711_c1_g1_i1_orf1	apolipophorins-like [Ostrinia furnacalis]	0.434234788	-1.203452785	5.13E-06	down	yes	1.413	3.254	3.333	3.2	3.229	1.391	1.489	1.36	MF:lipid transporter activity;MF:transporter activity;MF:molecular_function;	GO:0005319;GO:0005215;GO:0003674	------	------	------	------	ENOG410ZRUY;EN	S:Function unknown;	PF01347.25	Vitellogenin_N	Lipoprotein amino terminal region	CYT	7	86	9.8	High
TRINITY_DN2457_c0_g1_i8_orf1	uncharacterized protein LOC114355596 [Ostrinia furnacalis]	4.779259259	2.256787031	5.41E-05	up	yes	6.452	1.35	1.372	1.286	1.393	6.429	6.946	5.98	BP:biological_process;BP:metabolic process;BP:methylation;MF:catalytic activity;MF:transferase activity;MF:transferase activity, transferring one-carbon groups;MF:molecular_function;MF:methyltransferase activity;	GO:0008150;GO:0008152;GO:0032259;GO:0003824;GO:0016740;GO:0016741;GO:0003674;GO:0008168	------	------	------	------	ENOG410Y1IK	M:Cell wall/membrane/envelope biogenesis	PF12248.11	Methyltransf_FA	Farnesoic acid O-methyl transferase	CYT	7	27	33.4	High
TRINITY_DN2489_c0_g1_i1_orf1	uncharacterized protein LOC114354692 [Ostrinia furnacalis]	0.366439371	-1.448353575	8.58E-07	down	yes	1.236	3.373	3.422	3.296	3.402	1.254	1.208	1.246	------	------	------	------	------	------	ENOG41127D5	S:Function unknown	PF05335.16	DUF745	Protein of unknown function	CYT	5	24	31.8	High
TRINITY_DN9282_c0_g1_i2_orf1	uncharacterized protein LOC114363102 isoform X2 [Ostrinia furnacalis]	0.115837367	-3.109827381	2.01E-07	down	yes	0.5983	5.165	5.158	5.07	5.268	0.619	0.624	0.552	------	------	------	------	------	------	ENOG41120R5	S:Function unknown	------	------	------	CYT	1	10	15.1	High
TRINITY_DN12920_c0_g3_i1_orf1	zonadhesin-like isoform X4 [Ostrinia furnacalis]	2.593802345	1.375068546	0.0006351	up	yes	3.097	1.194	1.199	1.131	1.253	2.962	3.478	2.851	------	------	------	------	------	------	------	------	PF01826.20	TIL	Trypsin Inhibitor like cysteine rich domain	CYT	1	13	8.8	High
TRINITY_DN65681_c0_g1_i1_orf1	ferritin subunit-like [Ostrinia furnacalis] >XP_028168186.1 ferritin subunit-like [Ostrinia furnacalis]	0.140943281	-2.826813389	2.11E-06	down	yes	0.6933	4.919	5.099	4.851	4.808	0.646	0.643	0.791	BP:ion homeostasis;BP:homeostatic process;BP:iron ion homeostasis;BP:transition metal ion homeostasis;BP:inorganic ion homeostasis;BP:cellular transition metal ion homeostasis;BP:transition metal ion transport;BP:biological regulation;BP:regulation of biological quality;BP:cellular homeostasis;BP:metal ion transport;BP:transport;BP:cellular iron ion homeostasis;BP:cellular metal ion homeostasis;BP:cation transport;BP:ion transport;BP:cellular process;BP:cellular ion homeostasis;BP:biological_process;BP:cellular cation homeostasis;BP:metal ion homeostasis;BP:cation homeostasis;BP:cellular chemical homeostasis;BP:establishment of localization;BP:localization;BP:chemical homeostasis;BP:iron ion transport;MF:molecular_function;MF:binding;MF:oxidoreductase activity;MF:cation binding;MF:oxidoreductase activity, oxidizing metal ions;MF:transition metal ion binding;MF:ferroxidase activity;MF:ion binding;MF:iron ion binding;MF:ferric iron binding;MF:oxidoreductase activity, oxidizing metal ions, oxygen as acceptor;MF:metal ion binding;MF:catalytic activity;	GO:0050801;GO:0042592;GO:0055072;GO:0055076;GO:0098771;GO:0046916;GO:0000041;GO:0065007;GO:0065008;GO:0019725;GO:0030001;GO:0006810;GO:0006879;GO:0006875;GO:0006812;GO:0006811;GO:0009987;GO:0006873;GO:0008150;GO:0030003;GO:0055065;GO:0055080;GO:0055082;GO:0051234;GO:0051179;GO:0048878;GO:0006826;GO:0003674;GO:0005488;GO:0016491;GO:0043169;GO:0016722;GO:0046914;GO:0004322;GO:0043167;GO:0005506;GO:0008199;GO:0016724;GO:0046872;GO:0003824	K00522	FTH1	map04978;ma	Mineral absorption;Necroptosis;Ferroptosis	ENOG4111MSG;E	P:Inorganic ion transport and metabolism;	PF00210.27	Ferritin	Ferritin-like domain	CYT	1	31	8.1	High
TRINITY_DN4676_c0_g1_i16_orf1	meiosis-specific nuclear structural protein 1-like isoform X2 [Ostrinia furnacalis]	0.428146948	-1.223822055	6.81E-06	down	yes	1.585	3.702	3.712	3.808	3.585	1.543	1.629	1.584	BP:regulation of microtubule polymerization or depolymerization;BP:regulation of cellular component organization;BP:regulation of organelle organization;BP:regulation of cytoskeleton organization;BP:regulation of cellular process;BP:biological_process;BP:biological regulation;BP:regulation of microtubule-based process;BP:regulation of microtubule cytoskeleton organization;BP:regulation of biological process;	GO:0031110;GO:0051128;GO:0033043;GO:0051493;GO:0050794;GO:0008150;GO:0065007;GO:0032886;GO:0070507;GO:0050789	K04381	STMN1	map05206;ma	MicroRNAs in cancer;MAPK signaling pathway	ENOG4111K15	S:Function unknown	PF00836.22	Stathmin	Stathmin family	CYT	9	34	33.5	High
TRINITY_DN12671_c0_g1_i4_orf1	hemicentin-1-like isoform X1 [Ostrinia furnacalis]	0.322802548	-1.63127613	1.17E-05	down	yes	1.267	3.925	4.081	3.938	3.756	1.213	1.264	1.325	BP:biological_process;BP:localization;	GO:0008150;GO:0051179	------	------	------	------	ENOG410XPQS;EN	S:Function unknown;	PF07679.19;PF13927.9;PF13895.9;PF00047.28	I-set;Ig_3;Ig_2;ig	Immunoglobulin I-set domain;Immunoglobulin domain;Immunoglobulin	CYT	5	9	89.1	High
TRINITY_DN21555_c0_g1_i4_orf1	uncharacterized protein LOC114351844 [Ostrinia furnacalis] >XP_028158981.1 uncharacterized protein LOC114351844 [Ostrinia furnacalis] >XP_028158982.1 uncharacterized protein LOC114351844 [Ostrinia furnacalis] >XP_028158983.1 uncharacterized protein LOC114351844 [Ostrinia furnacalis] >XP_028158984.1 uncharacterized protein LOC114351844 [Ostrinia furnacalis] >XP_028158985.1 uncharacterized protein LOC114351844 [Ostrinia furnacalis] >5GPR_A Crystal structure of chitinase-h from Ostrinia furnacalis [Ostrinia furnacalis] >5GQB_A Crystal structure of chitinase-h from O. furnacalis in complex with chitohepatose [Ostrinia furnacalis] >6JMN_A Crystal structure of Ostrinia furnacalis Chitinase h complexed with compound 2-8-s2 [Ostrinia furnacalis] >BAE16587.1 chitinase [Ostrinia furnacalis]	0.348252119	-1.521795965	2.45E-07	down	yes	1.315	3.776	3.826	3.778	3.724	1.343	1.323	1.279	BP:amino sugar catabolic process;BP:nitrogen compound metabolic process;BP:aminoglycan metabolic process;BP:organic substance catabolic process;BP:organic substance metabolic process;BP:chitin metabolic process;BP:chitin catabolic process;BP:biological_process;BP:metabolic process;BP:carbohydrate metabolic process;BP:catabolic process;BP:macromolecule catabolic process;BP:primary metabolic process;BP:organonitrogen compound metabolic process;BP:organonitrogen compound catabolic process;BP:amino sugar metabolic process;BP:carbohydrate derivative catabolic process;BP:carbohydrate derivative metabolic process;BP:macromolecule metabolic process;BP:glucosamine-containing compound catabolic process;BP:glucosamine-containing compound metabolic process;BP:aminoglycan catabolic process;MF:hydrolase activity, acting on glycosyl bonds;MF:molecular_function;MF:chitin binding;MF:hydrolase activity;MF:catalytic activity;MF:chitinase activity;MF:carbohydrate derivative binding;MF:hydrolase activity, hydrolyzing O-glycosyl compounds;MF:binding;	GO:0046348;GO:0006807;GO:0006022;GO:1901575;GO:0071704;GO:0006030;GO:0006032;GO:0008150;GO:0008152;GO:0005975;GO:0009056;GO:0009057;GO:0044238;GO:1901564;GO:1901565;GO:0006040;GO:1901136;GO:1901135;GO:0043170;GO:1901072;GO:1901071;GO:0006026;GO:0016798;GO:0003674;GO:0008061;GO:0016787;GO:0003824;GO:0004568;GO:0097367;GO:0004553;GO:0005488	K01183	E3.2.1.14	map00520	Amino sugar and nucleotide sugar metabolism	COG3325	S:Function unknown	PF00704.31;PF08329.13	Glyco_hydro_18;ChitinaseA_N	Glycosyl hydrolases family 18;Chitinase A, N-terminal domain	CYT	12	28	60.8	High

TRINITY_DN12586_c0_g1_i4_orf1	zonadhesin-like isoform X4 [Ostrinia furnacalis]	4.548112058	2.1852678	2.88E-05	up	yes	3.734	0.821	0.682	0.853	0.928	3.785	3.513	3.905	------	------	------	------	------	------	ENOG4110RF5;CC	S:Function unknown;O:Posttranslational modification, protein turnover, chaperones	PF01826.20	TIL	Trypsin Inhibitor like cysteine rich domain	PLA	1	26	14.3	High
TRINITY_DN43350_c0_g3_i1_orf1	uncharacterized protein LOC114355190 [Ostrinia furnacalis]	2.306038048	1.205416317	3.15E-05	up	yes	2.788	1.209	1.143	1.202	1.283	2.898	2.79	2.676	------	------	------	------	------	------	ENOG4111DOA	S:Function unknown	------	------	------	CYT	3	25	25.8	High
TRINITY_DN10877_c0_g1_i1_orf1	spodomicin-like [Ostrinia furnacalis]	6.034482759	2.593230117	0.002664	up	yes	18.9	3.132	3.104	2.969	3.322	19.726	22.526	14.44	CC:cellular_component;CC:extracellular region;CC:cellular anatomical entity;MF:molecular_function;MF:toxin activity;	GO:0005575;GO:0005576;GO:0110165;GO:0003674;GO:0090729	------	------	------	------	------	------	PF08036.14	Antimicrobial_6	Diapausin family of antimicrobial peptide	CYT	1	19	6.9	Medium
TRINITY_DN28501_c0_g1_i2_orfp1	TRINITY_DN28501_c0_g1_i2_m.58934 TRINITY_DN28501_c0_g1::TRINITY_DN28501_c0_g1_i2::g.58934 ORF type:internal len:98 (+) score=13.70 TRINITY_DN28501_c0_g1_i2:3-293(+)	11.51747181	3.525752162	4.09E-06	up	yes	8.273	0.7183	0.765	0.688	0.702	8.157	8.692	7.971	------	------	------	------	------	------	------	------	------	------	------	CYT	1	30	10.9	High
TRINITY_DN56459_c0_g1_i2_orf1	aldo-keto reductase AKR2E4-like [Ostrinia furnacalis]	2.654859219	1.40863536	3.33E-05	up	yes	5.846	2.202	2.237	2.086	2.282	5.649	6.179	5.71	MF:catalytic activity;MF:molecular_function;MF:oxidoreductase activity;	GO:0003824;GO:0003674;GO:0016491	------	------	------	------	COG0656	S:Function unknown	------	------	------	CYT	4	78	8.7	High
TRINITY_DN38506_c0_g1_i4_orf1	C-1-tetrahydrofolate synthase, cytoplasmic isoform X1 [Ostrinia furnacalis] >XP_028166137.1 C-1-tetrahydrofolate synthase, cytoplasmic isoform X2 [Ostrinia furnacalis] >XP_028166140.1 C-1-tetrahydrofolate synthase, cytoplasmic isoform X4 [Ostrinia furnacalis]	3.313626532	1.72841101	0.0001076	up	yes	4.596	1.387	1.12	1.376	1.665	4.646	4.808	4.333	BP:one-carbon metabolic process;BP:nitrogen compound metabolic process;BP:cellular metabolic process;BP:tetrahydrofolate metabolic process;BP:organic cyclic compound metabolic process;BP:tetrahydrofolate interconversion;BP:pteridine-containing compound metabolic process;BP:organic substance metabolic process;BP:folic acid-containing compound metabolic process;BP:cellular process;BP:cellular aromatic compound metabolic process;BP:biological_process;BP:metabolic process;BP:heterocycle metabolic process;BP:organonitrogen compound metabolic process;BP:cellular modified amino acid metabolic process;BP:small molecule metabolic process;MF:binding;MF:purine ribonucleoside triphosphate binding;MF:oxidoreductase activity, acting on the CH-NH group of donors, NAD or NADP as acceptor;MF:molecular_function;MF:ion binding;MF:heterocyclic compound binding;MF:oxidoreductase activity;MF:anion binding;MF:formate-tetrahydrofolate ligase activity;MF:oxidoreductase activity, acting on the CH-NH group of donors;MF:nucleotide binding;MF:cyclohydrolase activity;MF:nucleoside phosphate binding;MF:purine nucleotide binding;MF:ATP binding;MF:methylenetetrahydrofolate dehydrogenase [NA;MF:hydrolase activity;MF:methylenetetrahydrofolate dehydrogenas;MF:small molecule binding;MF:catalytic activity;MF:ligase activity;MF:ligase activity, forming carbon-nitrogen bonds;MF:carbohydrate derivative binding;MF:organic cyclic compound binding;MF:hydrolase activity, acting on carbon-nitroge;MF:adenyl nucleotide binding;MF:methenyltetrahydrofolate cyclohydrolase activity;MF:hydrolase activity, acting on carbon-nitroge;MF:adenyl ribonucleotide binding;MF:purine ribonucleotide binding;MF:ribonucleotide binding;	GO:0006730;GO:0006807;GO:0044237;GO:0046653;GO:1901360;GO:0035999;GO:0042558;GO:0071704;GO:0006760;GO:0009987;GO:0006725;GO:0008150;GO:0008152;GO:0046483;GO:1901564;GO:0006575;GO:0044281;GO:0005488;GO:0035639;GO:0016646;GO:0003674;GO:0043167;GO:1901363;GO:0016491;GO:0043168;GO:0004329;GO:0016645;GO:0000166;GO:0019238;GO:1901265;GO:0017076;GO:0005524;GO:0004486;GO:0016787;GO:0004488;GO:0036094;GO:0003824;GO:0016874;GO:0016879;GO:0097367;GO:0097159;GO:0016810;GO:0030554;GO:0004477;GO:0016814;GO:0032559;GO:0032555;GO:0032553	K00288	MTHFD	map00670;ma	One carbon pool by folate;Biosynthesis of cofactors	COG0190	H:Coenzyme transport and metabolism	PF00763.26	THF_DHG_CYH	Tetrahydrofolate dehydrogenase/cyclohydrolase, catalytic domain	CYT	3	61	16.2	High
TRINITY_DN81488_c0_g1_i1_orf1	apolipophorins-like [Ostrinia furnacalis]	0.428873054	-1.221377422	0.0002241	down	yes	1.625	3.789	3.834	4.022	3.512	1.758	1.47	1.648	BP:lipid transport;BP:transport;BP:organic substance transport;BP:biological_process;BP:establishment of localization;BP:localization;CC:cellular_component;CC:extracellular region;CC:cellular anatomical entity;MF:lipid transporter activity;MF:transporter activity;MF:lipid binding;MF:molecular_function;MF:binding;	GO:0006869;GO:0006810;GO:0071702;GO:0008150;GO:0051234;GO:0051179;GO:0005575;GO:0005576;GO:0110165;GO:0005319;GO:0005215;GO:0008289;GO:0003674;GO:0005488	------	------	------	------	ENOG410Z6MR;EI	S:Function unknown;	------	------	------	CYT	6	75	8.8	High
TRINITY_DN12387_c1_g2_i1_orf1	skin secretory protein xP2-like [Ostrinia furnacalis]	0.208959005	-2.258708162	2.43E-05	down	yes	0.9073	4.342	4.353	4.602	4.071	0.882	0.946	0.894	------	------	------	------	------	------	ENOG410ZKDO;EI	S:Function unknown;	------	------	------	CYT	10	50	39.7	High
TRINITY_DN114890_c0_g1_i4_orf1	chemosensory protein 10 [Ostrinia furnacalis]	2.149908592	1.104275322	2.48E-05	up	yes	3.528	1.641	1.654	1.691	1.578	3.397	3.519	3.669	------	------	------	------	------	------	ENOG4111ATT;EN	S:Function unknown;	PF03392.16	OS-D	Insect pheromone-binding family, A10/OS-D	CYT	1	76	6.9	High
TRINITY_DN1326_c0_g1_i2_orf1	cuticle protein 7 [Plutella xylostella] >CAG9138501.1 unnamed protein product [Plutella xylostella]	0.261767987	-1.933639424	0.0004811	down	yes	0.9387	3.586	3.647	3.979	3.131	0.825	1.037	0.954	MF:molecular_function;MF:structural constituent of cuticle;MF:structural molecule activity;	GO:0003674;GO:0042302;GO:0005198	------	------	------	------	ENOG410ZM6M;E	S:Function unknown;	PF00379.26	Chitin_bind_4	Insect cuticle protein	CYT	1	24	18.5	High
TRINITY_DN4324_c0_g1_i1_orf1	uncharacterized protein LOC114354985 isoform X1 [Ostrinia furnacalis]	0.281910275	-1.826692033	4.60E-07	down	yes	0.974	3.455	3.384	3.473	3.508	0.995	0.99	0.937	CC:cellular_component;CC:integral component of membrane;CC:cellular anatomical entity;CC:intrinsic component of membrane;	GO:0005575;GO:0016021;GO:0110165;GO:0031224	------	------	------	------	ENOG41113AM;EI	S:Function unknown;	------	------	------	CYT	4	20	16	High
TRINITY_DN31417_c0_g1_i3_orf1	titin-like [Ostrinia furnacalis]	0.392015707	-1.351016635	0.0006987	down	yes	1.198	3.056	3.336	3.149	2.682	1.157	1.252	1.186	------	------	------	------	------	------	ENOG410ZKJ3;EN	S:Function unknown;	PF00093.21	VWC	von Willebrand factor type C domain	CYT	2	17	25.3	High
TRINITY_DN267_c0_g1_i1_orf1	keratin, type I cytoskeletal 9-like [Ostrinia furnacalis]	0.28847711	-1.793471246	2.28E-05	down	yes	0.9263	3.211	3.027	3.37	3.237	0.927	0.955	0.897	------	------	------	------	------	------	ENOG4110QQM	S:Function unknown	------	------	------	CYT	3	9	52.5	High
TRINITY_DN18539_c0_g1_i1_orf1	pupal cuticle protein PCP52-like [Ostrinia furnacalis]	0.132674015	-2.914042259	1.15E-05	down	yes	0.8787	6.623	6.602	7.003	6.263	0.894	0.855	0.887	MF:molecular_function;MF:structural constituent of cuticle;MF:structural molecule activity;	GO:0003674;GO:0042302;GO:0005198	------	------	------	------	ENOG4110SXG	S:Function unknown	------	------	------	CYT	13	54	28.3	High
TRINITY_DN98147_c0_g2_i1_orf1	hypothetical protein evm_002297 [Chilo suppressalis]	0.190845868	-2.389520146	9.24E-06	down	yes	1.18	6.183	6.353	6.353	5.842	1.264	1.103	1.174	MF:molecular_function;MF:structural constituent of cuticle;MF:structural molecule activity;	GO:0003674;GO:0042302;GO:0005198	------	------	------	------	ENOG4110RRZ;EN	S:Function unknown;	PF00379.26	Chitin_bind_4	Insect cuticle protein	EXC	3	18	31.2	High
TRINITY_DN59429_c0_g1_i6_orf1	uncharacterized protein LOC114366345 isoform X2 [Ostrinia furnacalis]	3.316747573	1.729769217	2.75E-05	up	yes	5.466	1.648	1.763	1.484	1.698	5.774	5.269	5.355	BP:response to biotic stimulus;BP:response to external stimulus;BP:response to external biotic stimulus;BP:response to stimulus;BP:defense response;BP:response to stress;BP:biological_process;BP:interspecies interaction between organisms;BP:response to other organism;CC:cellular_component;CC:extracellular region;CC:cellular anatomical entity;	GO:0009607;GO:0009605;GO:0043207;GO:0050896;GO:0006952;GO:0006950;GO:0008150;GO:0044419;GO:0051707;GO:0005575;GO:0005576;GO:0110165	------	------	------	------	ENOG4110S6N	S:Function unknown	PF00451.22	Toxin_2	Scorpion short toxin, BmKK2	CYT	2	22	12.3	High
TRINITY_DN2946_c0_g1_i1_orf1	histidine-rich glycoprotein [Ostrinia furnacalis]	0.131024885	-2.932087248	1.88E-05	down	yes	0.8003	6.108	6.303	6.359	5.662	0.777	0.803	0.821	------	------	------	------	------	------	ENOG410YTNT	S:Function unknown	------	------	------	PLA	9	28	23.4	High
TRINITY_DN338_c0_g1_i1_orf1	chorion protein S36-like [Ostrinia furnacalis]	0.24193221	-2.047325236	1.19E-05	down	yes	1.192	4.927	4.819	5.114	4.849	1.085	1.401	1.089	MF:molecular_function;MF:structural constituent of cuticle;MF:structural molecule activity;	GO:0003674;GO:0042302;GO:0005198	------	------	------	------	ENOG4111NR3	S:Function unknown	PF00379.26	Chitin_bind_4	Insect cuticle protein	CYT	1	3	50.9	High
TRINITY_DN31_c0_g1_i3_orfp1	TRINITY_DN31_c0_g1_i3_m.1394 TRINITY_DN31_c0_g1::TRINITY_DN31_c0_g1_i3::g.1394 ORF type:complete len:118 (+) score=43.35 TRINITY_DN31_c0_g1_i3:56-409(+)	0.177740516	-2.492155516	4.38E-06	down	yes	0.848	4.771	4.916	4.655	4.741	1.016	0.738	0.79	------	------	------	------	------	------	------	------	------	------	------	CYT	2	15	12.6	High
TRINITY_DN54524_c0_g1_i6_orf1	serine protease inhibitor dipetalogastin [Ostrinia furnacalis]	0.208304338	-2.263235212	5.04E-06	down	yes	0.898	4.311	4.171	4.274	4.487	0.924	0.81	0.96	------	------	------	------	------	------	ENOG410ZXUD;EI	S:Function unknown;	PF07648.18;PF00050.24	Kazal_2;Kazal_1	Kazal-type serine protease inhibitor domain;Kazal-type serine protease inhibitor domain	PLA	3	7	55.3	High
TRINITY_DN5198_c0_g1_i5_orfp1	TRINITY_DN5198_c0_g1_i5_m.8637 TRINITY_DN5198_c0_g1::TRINITY_DN5198_c0_g1_i5::g.8637 ORF type:complete len:223 (-) score=54.99 TRINITY_DN5198_c0_g1_i5:319-987(-)	3.981317601	1.993245964	1.63E-05	up	yes	4.049	1.017	1.041	1.018	0.992	3.805	4.19	4.153	------	------	------	------	------	------	------	------	------	------	------	CYT	1	5	24.7	High
TRINITY_DN97042_c0_g1_i6_orf1	apolipophorins-like [Ostrinia furnacalis]	0.452525253	-1.143929793	2.73E-06	down	yes	1.568	3.465	3.539	3.427	3.428	1.628	1.517	1.558	MF:lipid transporter activity;MF:transporter activity;MF:molecular_function;	GO:0005319;GO:0005215;GO:0003674	------	------	------	------	ENOG410Z6MR;EI	S:Function unknown;	PF09172.14	Vit_open_b-sht	Vitellinogen, open beta-sheet	CYT	2	29	12.7	High
TRINITY_DN66287_c0_g1_i1_orfp1	tenascin-like isoform X4 [Trichoplusia ni]	3.975190623	1.991024044	0.0004554	up	yes	3.493	0.8787	0.8	0.837	0.999	3.548	3.879	3.053	BP:response to biotic stimulus;BP:response to external stimulus;BP:response to external biotic stimulus;BP:response to stimulus;BP:defense response;BP:response to stress;BP:biological_process;BP:interspecies interaction between organisms;BP:response to other organism;CC:cellular_component;CC:extracellular region;CC:cellular anatomical entity;	GO:0009607;GO:0009605;GO:0043207;GO:0050896;GO:0006952;GO:0006950;GO:0008150;GO:0044419;GO:0051707;GO:0005575;GO:0005576;GO:0110165	------	------	------	------	------	------	------	------	------	CYT	2	25	11.9	High
TRINITY_DN15685_c0_g1_i5_orf1	uncharacterized protein LOC114352354 [Ostrinia furnacalis]	3.875857091	1.954515377	1.55E-05	up	yes	3.222	0.8313	0.778	0.848	0.868	3.22	3.382	3.063	------	------	------	------	------	------	ENOG4110T90;EN	S:Function unknown;	PF06585.14	JHBP	Haemolymph juvenile hormone binding protein	CYT	3	14	29.1	High
TRINITY_DN1407_c0_g1_i12_orf1	hypothetical protein evm_012298 [Chilo suppressalis]	0.4513435	-1.147702263	2.54E-05	down	yes	1.243	2.754	2.674	2.861	2.728	1.207	1.324	1.198	BP:lipid transport;BP:transport;BP:organic substance transport;BP:biological_process;BP:establishment of localization;BP:localization;CC:cellular_component;CC:extracellular region;CC:cellular anatomical entity;MF:lipid binding;MF:molecular_function;MF:binding;	GO:0006869;GO:0006810;GO:0071702;GO:0008150;GO:0051234;GO:0051179;GO:0005575;GO:0005576;GO:0110165;GO:0008289;GO:0003674;GO:0005488	------	------	------	------	------	------	PF07464.14	ApoLp-III	Apolipophorin-III precursor	CYT	3	29	8	High
TRINITY_DN2464_c0_g1_i12_orf1	uncharacterized protein LOC114362996 isoform X1 [Ostrinia furnacalis]	2.981037924	1.575814729	1.36E-05	up	yes	2.987	1.002	0.966	1.042	0.999	2.932	3.133	2.895	MF:molecular_function;MF:structural constituent of cuticle;MF:structural molecule activity;	GO:0003674;GO:0042302;GO:0005198	------	------	------	------	ENOG410YNFC	S:Function unknown	PF00379.26	Chitin_bind_4	Insect cuticle protein	CYT	8	45	37.2	High
TRINITY_DN66040_c0_g1_i2_orf1	serine protease inhibitor dipetalogastin-like isoform X2 [Ostrinia furnacalis]	0.205854922	-2.280300149	1.78E-06	down	yes	3.973	19.3	19.572	19.656	18.675	4.317	3.809	3.793	------	------	------	------	------	------	------	------	PF07648.18;PF00050.24	Kazal_2;Kazal_1	Kazal-type serine protease inhibitor domain;Kazal-type serine protease inhibitor domain	CYT	1	21	6	High
TRINITY_DN971_c0_g1_i5_orfp1	TRINITY_DN971_c0_g1_i5_m.54249 TRINITY_DN971_c0_g1::TRINITY_DN971_c0_g1_i5::g.54249 ORF type:internal len:108 (+) score=66.98 TRINITY_DN971_c0_g1_i5:1-321(+)	2.70531401	1.435796059	0.0002465	up	yes	11.2	4.14	4.683	3.422	4.314	12.061	10.866	10.683	------	------	------	------	------	------	------	------	------	------	------	CYT	1	9	11.5	High
TRINITY_DN15175_c0_g1_i1_orf1	zinc carboxypeptidase-like [Ostrinia furnacalis]	0.130768323	-2.934914988	3.99E-08	down	yes	1.108	8.473	8.516	8.539	8.363	1.08	1.062	1.181	------	------	K01298	CPA2	map04972;ma	Pancreatic secretion;Protein digestion and absorption	COG2866	O:Posttranslational modification, protein turnover, chaperones	PF00246.27	Peptidase_M14	Zinc carboxypeptidase	CYT	3	7	45.8	High
TRINITY_DN12526_c0_g1_i5_orf1	uncharacterized protein LOC114359035 isoform X3 [Ostrinia furnacalis]	0.338861076	-1.561234165	6.56E-05	down	yes	2.166	6.392	6.359	5.993	6.823	2.156	2.093	2.248	BP:nitrogen compound metabolic process;BP:membrane lipid metabolic process;BP:organic substance metabolic process;BP:sphingolipid metabolic process;BP:lipid metabolic process;BP:cellular process;BP:biological_process;BP:metabolic process;BP:cellular lipid metabolic process;BP:primary metabolic process;BP:organonitrogen compound metabolic process;BP:cellular metabolic process;CC:lytic vacuole;CC:intracellular organelle;CC:membrane-bounded organelle;CC:organelle;CC:lysosome;CC:vacuole;CC:cellular anatomical entity;CC:intracellular membrane-bounded organelle;CC:cellular_component;CC:extracellular region;	GO:0006807;GO:0006643;GO:0071704;GO:0006665;GO:0006629;GO:0009987;GO:0008150;GO:0008152;GO:0044255;GO:0044238;GO:1901564;GO:0044237;GO:0000323;GO:0043229;GO:0043227;GO:0043226;GO:0005764;GO:0005773;GO:0110165;GO:0043231;GO:0005575;GO:0005576	K12382	PSAP, SGP1	map00600;ma	Sphingolipid metabolism;Lysosome	ENOG410XSI5	O:Posttranslational modification, protein turnover, chaperones	PF05184.18;PF03489.20	SapB_1;SapB_2	Saposin-like type B, region 1;Saposin-like type B, region 2	CYT	2	37	15.2	High
TRINITY_DN13576_c0_g1_i1_orf1	uncharacterized protein LOC114350099 [Ostrinia furnacalis]	0.152654388	-2.711659036	1.43E-06	down	yes	0.9863	6.461	6.633	6.522	6.228	0.998	0.989	0.972	------	------	------	------	------	------	ENOG4111OIW	S:Function unknown	PF00379.26	Chitin_bind_4	Insect cuticle protein	CYT	9	48	29.1	High
TRINITY_DN2400_c0_g1_i1_orf1	uncharacterized protein LOC114351021 [Ostrinia furnacalis]	0.226200723	-2.144324554	5.61E-06	down	yes	1.314	5.809	5.975	5.801	5.651	1.18	1.243	1.519	------	------	------	------	------	------	ENOG41113AM	S:Function unknown	------	------	------	PLA	1	11	12.9	High
TRINITY_DN30306_c0_g2_i1_orf1	perilipin-4-like isoform X3 [Ostrinia furnacalis]	2.730666667	1.449253215	3.63E-05	up	yes	5.12	1.875	1.792	1.881	1.951	4.939	5.428	4.992	------	------	K20254	PLIN4	map03320	PPAR signaling pathway	ENOG4112C4W	S:Function unknown	------	------	------	CYT	6	64	7.2	High
TRINITY_DN103107_c0_g1_i2_orf1	superoxide dismutase [Cu-Zn] [Ostrinia furnacalis] >XP_028177872.1 superoxide dismutase [Cu-Zn] [Ostrinia furnacalis]	4.973250813	2.314189192	3.95E-05	up	yes	4.741	0.9533	1.026	0.975	0.859	4.549	5.113	4.56	MF:oxidoreductase activity, acting on superoxide radicals as acceptor;MF:cation binding;MF:antioxidant activity;MF:ion binding;MF:catalytic activity;MF:molecular_function;MF:binding;MF:superoxide dismutase activity;MF:metal ion binding;MF:oxidoreductase activity;	GO:0016721;GO:0043169;GO:0016209;GO:0043167;GO:0003824;GO:0003674;GO:0005488;GO:0004784;GO:0046872;GO:0016491	K04565	SOD1	map05208;ma	Chemical carcinogenesis - reactive oxygen species;Parkinson disease;Huntington disease;Amyotrophic lateral sclerosis;Longevity regulating pathway - multiple species;Peroxisome;Pathways of neurodegeneration - multiple diseases;Prion	COG2032	P:Inorganic ion transport and metabolism	PF00080.23	Sod_Cu	Copper/zinc superoxide dismutase	PLA	1	47	8.5	High
TRINITY_DN7549_c0_g1_i1_orf1	uncharacterized protein LOC114355006 [Ostrinia furnacalis]	0.178426052	-2.48660182	7.21E-06	down	yes	0.789	4.422	4.355	4.649	4.262	0.729	0.811	0.827	------	------	------	------	------	------	ENOG4111H56;EN	C:Energy production and conversion;S:Function unknown	------	------	------	CYT	2	14	20	High
TRINITY_DN2140_c0_g1_i1_orf1	uncharacterized protein LOC126367764 [Pectinophora gossypiella]	0.330850244	-1.595749751	6.25E-05	down	yes	1.288	3.893	3.658	4.102	3.92	1.269	1.425	1.169	CC:intracellular membrane-bounded organelle;CC:mitochondrion;CC:organelle;CC:intracellular organelle;CC:cellular_component;CC:membrane-bounded organelle;CC:cellular anatomical entity;MF:cation binding;MF:hydroquinon;MF:transition metal ion binding;MF:metal ion binding;MF:ion binding;MF:catalytic activity;MF:molecular_function;MF:binding;MF:copper ion binding;MF:oxidoreductase activity, acting on diphenols and related substances as donors;MF:oxidoreductase activity, acting on diphenols and related substances as donors, oxygen as acceptor;MF:oxidoreductase activity;	GO:0043231;GO:0005739;GO:0043226;GO:0043229;GO:0005575;GO:0043227;GO:0110165;GO:0043169;GO:0052716;GO:0046914;GO:0046872;GO:0043167;GO:0003824;GO:0003674;GO:0005488;GO:0005507;GO:0016679;GO:0016682;GO:0016491	------	------	------	------	COG2132	Q:Secondary metabolites biosynthesis, transport and catabolism	PF07732.18;PF07731.17;PF00394.25	Cu-oxidase_3;Cu-oxidase_2;Cu-oxidase	Multicopper oxidase;Multicopper oxidase;Multicopper oxidase	CYT	4	7	84	High
TRINITY_DN5444_c0_g2_i1_orf1	phenoloxidase-activating factor 2-like [Ostrinia furnacalis]	0.388920946	-1.362451158	6.27E-06	down	yes	1.348	3.466	3.511	3.521	3.365	1.434	1.28	1.33	BP:organic substance metabolic process;BP:organonitrogen compound metabolic process;BP:protein metabolic process;BP:macromolecule metabolic process;BP:biological_process;BP:nitrogen compound metabolic process;BP:metabolic process;BP:proteolysis;BP:primary metabolic process;MF:serine-type endopeptidase activity;MF:endopeptidase activity;MF:hydrolase activity;MF:serine hydrolase activity;MF:catalytic activity, acting on a protein;MF:catalytic activity;MF:molecular_function;MF:peptidase activity;MF:serine-type peptidase activity;	GO:0071704;GO:1901564;GO:0019538;GO:0043170;GO:0008150;GO:0006807;GO:0008152;GO:0006508;GO:0044238;GO:0004252;GO:0004175;GO:0016787;GO:0017171;GO:0140096;GO:0003824;GO:0003674;GO:0008233;GO:0008236	------	------	------	------	COG5640	E:Amino acid transport and metabolism	PF00089.29	Trypsin	Trypsin	CYT	3	14	27.9	High
TRINITY_DN52553_c0_g1_i1_orf1	hemocyte protein-glutamine gamma-glutamyltransferase-like [Ostrinia furnacalis]	0.28866555	-1.792529152	2.34E-06	down	yes	1.034	3.582	3.703	3.489	3.555	1.019	1.04	1.043	BP:primary metabolic process;BP:peptide cross-linking;BP:organonitrogen compound metabolic process;BP:organic substance metabolic process;BP:nitrogen compound metabolic process;BP:macromolecule modification;BP:macromolecule metabolic process;BP:biological_process;BP:protein modification process;BP:metabolic process;BP:protein metabolic process;MF:molecular_function;MF:protein-glutamine gamma-glutamyltransferase activity;MF:catalytic activity, acting on a protein;MF:catalytic activity;MF:transferase activity;MF:transferase activity, transferring acyl groups;MF:transferase activity, transferring amino-acyl groups;	GO:0044238;GO:0018149;GO:1901564;GO:0071704;GO:0006807;GO:0043412;GO:0043170;GO:0008150;GO:0036211;GO:0008152;GO:0019538;GO:0003674;GO:0003810;GO:0140096;GO:0003824;GO:0016740;GO:0016746;GO:0016755	K05619	TGM1	------	------	ENOG410XQEZ	O:Posttranslational modification, protein turnover, chaperones	PF00927.25;PF01841.22;PF00868.23	Transglut_C;Transglut_core;Transglut_N	Transglutaminase family, C-terminal ig like domain;Transglutaminase-like superfamily;Transglutaminase family	CYT	4	8	80	High
TRINITY_DN1902_c0_g1_i4_orf1	chemosensory protein 10 [Conogethes pinicolalis]	0.328624079	-1.605489903	1.95E-06	down	yes	1.07	3.256	3.218	3.358	3.193	1.054	1.087	1.068	------	------	------	------	------	------	ENOG4112163;EN	S:Function unknown;	PF03392.16	OS-D	Insect pheromone-binding family, A10/OS-D	CYT	2	20	11.8	High
TRINITY_DN5664_c0_g1_i1_orf1	CDGSH iron-sulfur domain-containing protein 3, mitochondrial-like [Ostrinia furnacalis]	0.154066308	-2.698376697	1.56E-06	down	yes	0.5437	3.529	3.621	3.511	3.454	0.602	0.577	0.452	CC:cytoplasm;CC:cellular_component;CC:intracellular organelle;CC:membrane-bounded organelle;CC:organelle;CC:cellular anatomical entity;CC:intracellular membrane-bounded organelle;MF:cation binding;MF:metal cluster binding;MF:iron-sulfur cluster binding;MF:2 iron, 2 sulfur cluster binding;MF:ion binding;MF:molecular_function;MF:binding;MF:metal ion binding;	GO:0005737;GO:0005575;GO:0043229;GO:0043227;GO:0043226;GO:0110165;GO:0043231;GO:0043169;GO:0051540;GO:0051536;GO:0051537;GO:0043167;GO:0003674;GO:0005488;GO:0046872	K23886	CISD3	------	------	ENOG41122TT	S:Function unknown	PF09360.13	zf-CDGSH	Iron-binding zinc finger CDGSH type	CYT	1	12	15.7	High
TRINITY_DN18218_c0_g1_i7_orf1	inhibin beta B chain [Ostrinia furnacalis]	0.187946609	-2.411605208	0.005803	down	yes	1.394	7.417	8.375	5.186	8.689	1.44	1.487	1.256	BP:response to stimulus;BP:biological_process;BP:response to chemical;BP:response to organic substance;CC:cellular_component;CC:extracellular region;CC:cellular anatomical entity;MF:growth factor activity;MF:receptor regulator activity;MF:receptor activator activity;MF:receptor agonist activity;MF:molecular_function;MF:binding;MF:protein binding;MF:receptor binding;MF:molecular function regulator;	GO:0050896;GO:0008150;GO:0042221;GO:0010033;GO:0005575;GO:0005576;GO:0110165;GO:0008083;GO:0030545;GO:0030546;GO:0048018;GO:0003674;GO:0005488;GO:0005515;GO:0005102;GO:0098772	K04669	TGFBRX	------	------	ENOG410XT8Z	O:Posttranslational modification, protein turnover, chaperones	PF00019.23;PF00688.21	TGF_beta;TGFb_propeptide	Transforming growth factor beta like domain;TGF-beta propeptide	CYT	1	4	41.4	High
TRINITY_DN19043_c0_g2_i1_orf1	hypothetical protein FVAR_60653_1 [Eumeta japonica]	0.354244929	-1.497180895	5.59E-05	down	yes	1.886	5.324	5.314	5.657	5.002	1.887	1.931	1.84	------	------	------	------	------	------	ENOG410YRTV	S:Function unknown	------	------	------	PLA	6	32	21	High
TRINITY_DN91533_c0_g1_i1_orf1	uncharacterized protein LOC114361550 [Ostrinia furnacalis]	0.257386079	-1.957994068	6.36E-07	down	yes	1.028	3.994	4.006	4.076	3.901	1.058	1	1.026	CC:cellular_component;CC:integral component of membrane;CC:cellular anatomical entity;CC:intrinsic component of membrane;	GO:0005575;GO:0016021;GO:0110165;GO:0031224	------	------	------	------	ENOG410ZKIK	S:Function unknown	PF00024.29;PF14295.9	PAN_1;PAN_4	PAN domain;PAN domain	CYT	10	18	78.9	High
TRINITY_DN10824_c0_g1_i3_orf1	endochitinase isoform X2 [Ostrinia furnacalis]	0.202030721	-2.307353406	1.03E-07	down	yes	1.552	7.682	7.558	7.789	7.698	1.525	1.592	1.539	BP:amino sugar catabolic process;BP:nitrogen compound metabolic process;BP:aminoglycan metabolic process;BP:organic substance catabolic process;BP:organic substance metabolic process;BP:chitin metabolic process;BP:chitin catabolic process;BP:biological_process;BP:metabolic process;BP:carbohydrate metabolic process;BP:catabolic process;BP:macromolecule catabolic process;BP:primary metabolic process;BP:organonitrogen compound metabolic process;BP:organonitrogen compound catabolic process;BP:amino sugar metabolic process;BP:carbohydrate derivative catabolic process;BP:carbohydrate derivative metabolic process;BP:macromolecule metabolic process;BP:glucosamine-containing compound catabolic process;BP:glucosamine-containing compound metabolic process;BP:aminoglycan catabolic process;CC:cellular anatomical entity;CC:cellular_component;CC:extracellular region;MF:hydrolase activity, acting on glycosyl bonds;MF:molecular_function;MF:chitin binding;MF:hydrolase activity;MF:catalytic activity;MF:chitinase activity;MF:carbohydrate derivative binding;MF:hydrolase activity, hydrolyzing O-glycosyl compounds;MF:binding;	GO:0046348;GO:0006807;GO:0006022;GO:1901575;GO:0071704;GO:0006030;GO:0006032;GO:0008150;GO:0008152;GO:0005975;GO:0009056;GO:0009057;GO:0044238;GO:1901564;GO:1901565;GO:0006040;GO:1901136;GO:1901135;GO:0043170;GO:1901072;GO:1901071;GO:0006026;GO:0110165;GO:0005575;GO:0005576;GO:0016798;GO:0003674;GO:0008061;GO:0016787;GO:0003824;GO:0004568;GO:0097367;GO:0004553;GO:0005488	K01183	E3.2.1.14	map00520	Amino sugar and nucleotide sugar metabolism	COG3325	S:Function unknown	PF00704.31;PF01607.27	Glyco_hydro_18;CBM_14	Glycosyl hydrolases family 18;Chitin binding Peritrophin-A domain	EXC	4	9	62.8	High
TRINITY_DN52944_c0_g1_i1_orf1	apolipophorins-like [Ostrinia furnacalis]	0.455076923	-1.135817666	6.13E-06	down	yes	1.479	3.25	3.317	3.283	3.15	1.469	1.525	1.443	MF:lipid transporter activity;MF:transporter activity;MF:molecular_function;	GO:0005319;GO:0005215;GO:0003674	------	------	------	------	ENOG410ZRUY;EN	S:Function unknown;	PF01347.25	Vitellogenin_N	Lipoprotein amino terminal region	CYT	5	59	11	High
TRINITY_DN1593_c0_g1_i1_orf1	chemosensory protein csp11 [Helopeltis theivora]	3.063551402	1.615205058	9.21E-06	up	yes	3.278	1.07	1.048	1.072	1.09	3.177	3.429	3.228	------	------	------	------	------	------	ENOG4110RV8;EN	S:Function unknown;	PF03392.16	OS-D	Insect pheromone-binding family, A10/OS-D	CYT	8	39	15.4	High
TRINITY_DN21719_c0_g1_i2_orf1	chymotrypsin-2-like [Ostrinia furnacalis]	0.134866536	-2.890395674	3.89E-06	down	yes	0.8943	6.631	6.673	6.89	6.331	0.912	0.893	0.878	BP:nitrogen compound metabolic process;BP:organic substance metabolic process;BP:proteolysis;BP:biological_process;BP:metabolic process;BP:primary metabolic process;BP:organonitrogen compound metabolic process;BP:protein metabolic process;BP:macromolecule metabolic process;CC:intrinsic component of membrane;CC:integral component of membrane;CC:cellular anatomical entity;CC:cellular_component;MF:serine-type endopeptidase activity;MF:endopeptidase activity;MF:molecular_function;MF:peptidase activity;MF:serine-type peptidase activity;MF:hydrolase activity;MF:serine hydrolase activity;MF:catalytic activity, acting on a protein;MF:catalytic activity;	GO:0006807;GO:0071704;GO:0006508;GO:0008150;GO:0008152;GO:0044238;GO:1901564;GO:0019538;GO:0043170;GO:0031224;GO:0016021;GO:0110165;GO:0005575;GO:0004252;GO:0004175;GO:0003674;GO:0008233;GO:0008236;GO:0016787;GO:0017171;GO:0140096;GO:0003824	K01312	PRSS1_2_3	map04972;ma	Pancreatic secretion;Influenza A;Neuroactive ligand-receptor interaction;Protein digestion and absorption	COG5640	O:Posttranslational modification, protein turnover, chaperones	PF00089.29	Trypsin	Trypsin	CYT	2	14	15.6	High
TRINITY_DN12534_c0_g1_i4_orf1	antibacterial protein [Heliothis virescens]	2.188874515	1.130189249	2.88E-05	up	yes	5.076	2.319	2.269	2.318	2.369	5.086	5.289	4.852	BP:response to biotic stimulus;BP:response to external stimulus;BP:response to external biotic stimulus;BP:response to stimulus;BP:defense response;BP:response to stress;BP:biological_process;BP:interspecies interaction between organisms;BP:response to other organism;CC:cellular_component;CC:extracellular region;CC:cellular anatomical entity;	GO:0009607;GO:0009605;GO:0043207;GO:0050896;GO:0006952;GO:0006950;GO:0008150;GO:0044419;GO:0051707;GO:0005575;GO:0005576;GO:0110165	------	------	------	------	ENOG4110S6N	S:Function unknown	PF00451.22	Toxin_2	Scorpion short toxin, BmKK2	CYT	5	57	12	High
TRINITY_DN57998_c1_g1_i1_orf1	uncharacterized protein LOC113509309, partial [Galleria mellonella]	0.235346676	-2.087140617	0.0006479	down	yes	1.317	5.596	5.526	6.395	4.866	1.261	1.371	1.319	------	------	------	------	------	------	ENOG4111SVO	S:Function unknown	------	------	------	CYT	1	14	9.1	High
TRINITY_DN6025_c0_g2_i1_orfp1	TRINITY_DN6025_c0_g2_i1_m.7749 TRINITY_DN6025_c0_g2::TRINITY_DN6025_c0_g2_i1::g.7749 ORF type:5prime_partial len:106 (+) score=47.10 TRINITY_DN6025_c0_g2_i1:3-320(+)	2.083071397	1.058712288	0.001772	up	yes	4.639	2.227	2.373	2.661	1.646	4.397	4.786	4.733	------	------	------	------	------	------	------	------	------	------	------	PLA	1	10	11.6	Medium
TRINITY_DN3616_c0_g2_i1_orf1	conotoxin ArMKLT2-032-like [Ostrinia furnacalis]	0.394092219	-1.34339483	2.65E-05	down	yes	1.641	4.164	4.093	4.37	4.028	1.542	1.696	1.686	CC:cellular_component;CC:integral component of membrane;CC:cellular anatomical entity;CC:intrinsic component of membrane;	GO:0005575;GO:0016021;GO:0110165;GO:0031224	------	------	------	------	------	------	------	------	------	CYT	2	64	8.7	High
TRINITY_DN49143_c0_g1_i1_orf1	uncharacterized protein LOC107270465 [Cephus cinctus]	0.281592306	-1.828320181	5.65E-07	down	yes	1.054	3.743	3.687	3.705	3.836	1.05	1.062	1.051	CC:cellular_component;CC:integral component of membrane;CC:cellular anatomical entity;CC:intrinsic component of membrane;	GO:0005575;GO:0016021;GO:0110165;GO:0031224	------	------	------	------	ENOG410YYS2	S:Function unknown	PF00100.26	Zona_pellucida	Zona pellucida-like domain	CYT	5	7	69.1	High
TRINITY_DN31314_c0_g1_i4_orf1	uncharacterized protein LOC114355976 [Ostrinia furnacalis]	0.262326944	-1.930562101	1.12E-06	down	yes	0.9853	3.756	3.774	3.82	3.675	0.988	0.917	1.051	BP:mitotic cell cycle;BP:cellular process;BP:biological_process;BP:cell division;BP:cell cycle;CC:cytoplasm;CC:supramolecular complex;CC:chromosomal region;CC:intracellular non-membrane-bounded organelle;CC:nucleus;CC:chromosome, centromeric region;CC:cellular_component;CC:intracellular organelle;CC:non-membrane-bounded organelle;CC:cellular anatomical entity;CC:kinetochore;CC:membrane-bounded organelle;CC:organelle;CC:intracellular membrane-bounded organelle;	GO:0000278;GO:0009987;GO:0008150;GO:0051301;GO:0007049;GO:0005737;GO:0099080;GO:0098687;GO:0043232;GO:0005634;GO:0000775;GO:0005575;GO:0043229;GO:0043228;GO:0110165;GO:0000776;GO:0043227;GO:0043226;GO:0043231	K11578	ZW10, DSL1	------	------	ENOG410XQ7J;EN	S:Function unknown;	------	------	------	CYT	7	9	86.8	High
TRINITY_DN64719_c0_g1_i2_orfp1	TRINITY_DN64719_c0_g1_i2_m.37745 TRINITY_DN64719_c0_g1::TRINITY_DN64719_c0_g1_i2::g.37745 ORF type:internal len:91 (+) score=41.89 TRINITY_DN64719_c0_g1_i2:1-270(+)	4.7387188	2.244497053	0.0001578	up	yes	3.854	0.8133	0.866	0.641	0.933	4.16	3.927	3.475	------	------	------	------	------	------	------	------	------	------	------	CYT	1	14	8.4	High
TRINITY_DN1465_c2_g1_i2_orf1	transcription initiation factor TFIID subunit 1-like [Ostrinia furnacalis]	0.416692237	-1.262945872	2.51E-05	down	yes	1.358	3.259	3.243	3.409	3.126	1.33	1.411	1.334	------	------	------	------	------	------	ENOG410YRBN;EI	S:Function unknown;	PF16009.8	DUF4779	Domain of unknown function	PLA	7	26	36.9	High
TRINITY_DN335_c1_g1_i5_orf1	PREDICTED: perilipin-4 isoform X14 [Papilio polytes]	2.520700637	1.333824791	4.41E-06	up	yes	3.166	1.256	1.234	1.259	1.274	3.089	3.272	3.137	------	------	K20254	PLIN4	map03320	PPAR signaling pathway	ENOG4112C4W	S:Function unknown	------	------	------	CYT	13	74	15.1	High
TRINITY_DN184_c0_g1_i1_orf1	macrophage mannose receptor 1-like isoform X1 [Maniola jurtina]	2.048409405	1.034504088	0.002007	up	yes	2.962	1.446	1.05	1.72	1.568	3.042	2.844	3.001	------	------	------	------	------	------	ENOG410XRS3;EN	U:Intracellular trafficking, secretion, and vesicular transport;S:Function unknown;S:Function unknown;S:Function unknown;S:Function unknown	PF00059.24	Lectin_C	Lectin C-type domain	CYT	1	14	35.7	High
TRINITY_DN27968_c0_g2_i2_orf1	nucleolin-like [Ostrinia furnacalis]	0.231226872	-2.112619027	5.23E-06	down	yes	0.8123	3.513	3.39	3.652	3.496	0.875	0.768	0.794	------	------	------	------	------	------	ENOG4111NPU	S:Function unknown	------	------	------	CYT	2	8	33.3	High
TRINITY_DN214_c0_g1_i4_orf1	uncharacterized protein LOC114352813 [Ostrinia furnacalis]	3.826415987	1.935993724	2.43E-06	up	yes	3.236	0.8457	0.869	0.738	0.93	3.272	3.236	3.2	------	------	------	------	------	------	ENOG4110S0N;EN	S:Function unknown;	PF06585.14	JHBP	Haemolymph juvenile hormone binding protein	CYT	4	31	29.6	High
TRINITY_DN33365_c0_g1_i1_orf1	mucin-5AC isoform X1 [Ostrinia furnacalis]	0.370508475	-1.432421553	3.13E-06	down	yes	1.093	2.95	2.946	2.986	2.918	1.091	1.173	1.014	------	------	------	------	------	------	ENOG41120DH	S:Function unknown	------	------	------	CYT	3	2	204.2	High

TRINITY_DN14670_c0_g1_i1_orf1	heat shock protein beta-1 isoform X1 [Helicoverpa armigera] >XP_022829066.1 heat shock protein beta-1 isoform X1 [Spodoptera litura] >XP_026747148.1 heat shock protein beta-1 isoform X3 [Trichoplusia ni] >XP_026748187.1 heat shock protein beta-1 isoform X2 [Galleria mellonella] >XP_028167756.1 heat shock protein beta-1 isoform X2 [Ostrinia furnacalis] >XP_035431734.1 heat shock protein beta-1-like isoform X3 [Spodoptera frugiperda] >XP_047023072.1 heat shock protein beta-1 isoform X1 [Helicoverpa zea] >XP_049865086.1 heat shock protein beta-1 [Pectinophora gossypiella] >KAH9640995.1 hypothetical protein HF086_015091 [Spodoptera exigua] >QGZ00460.1 heat shock protein 21.4 [Glyphodes pyloalis] >QKR72095.1 heat-shock protein 21.4 [Mythimna separata] >CAB3228281.1 unnamed protein product [Arctia plantaginis] >CAH0628881.1 unnamed protein product [Chrysodeixis includens]	2.090768037	1.064033009	0.0001576	up	yes	2.695	1.289	1.281	1.294	1.293	2.66	2.886	2.54	MF:cation binding;MF:metal ion binding;MF:molecular_function;MF:binding;MF:ion binding;	GO:0043169;GO:0046872;GO:0003674;GO:0005488;GO:0043167	------	------	------	------	ENOG410YERS	K:Transcription	PF00011.24	HSP20	Hsp20/alpha crystallin family	CYT	13	72	21.4	High
TRINITY_DN64297_c0_g1_i1_orf1	vanin-like protein 2 isoform X2 [Ostrinia furnacalis]	0.404025424	-1.307482016	0.0002332	down	yes	5.721	14.16	13.563	14.15	14.772	5.983	6.562	4.619	BP:nitrogen compound metabolic process;BP:biological_process;BP:metabolic process;	GO:0006807;GO:0008150;GO:0008152	------	------	------	------	COG0388	O:Posttranslational modification, protein turnover	------	------	------	CYT	1	57	7.8	High
TRINITY_DN38307_c0_g1_i1_orfp1	TRINITY_DN38307_c0_g1_i1_m.10661 TRINITY_DN38307_c0_g1::TRINITY_DN38307_c0_g1_i1::g.10661 ORF type:5prime_partial len:66 (+),score=5.90 TRINITY_DN38307_c0_g1_i1:2-199(+)	0.254080221	-1.976644021	0.000212	down	yes	3.674	14.46	13.389	14.227	15.776	4.581	3.035	3.407	------	------	------	------	------	------	------	------	------	------	------	CYT	1	17	7.9	High
TRINITY_DN30510_c0_g1_i6_orf1	spodomicin-like [Ostrinia furnacalis]	7.314001657	2.870660954	7.73E-06	up	yes	8.828	1.207	1.233	1.131	1.257	8.65	9.33	8.505	------	------	------	------	------	------	------	------	PF08036.14	Antimicrobial_6	Diapausin family of antimicrobial peptide	CYT	3	60	6.7	High
TRINITY_DN15222_c0_g1_i4_orf1	lysosomal alpha-mannosidase-like [Ostrinia furnacalis]	0.172690763	-2.533737177	2.13E-06	down	yes	0.903	5.229	5.079	5.425	5.184	0.855	0.944	0.91	BP:nucleotide biosynthetic process;BP:small molecule metabolic process;BP:organic cyclic compound biosynthetic process;BP:organic cyclic compound metabolic process;BP:ribonucleoside triphosphate metabolic process;BP:purine ribonucleoside triphosphate biosynthetic process;BP:purine ribonucleoside triphosphate metabolic process;BP:ribonucleoside triphosphate biosynthetic process;BP:ATP metabolic process;BP:heterocycle metabolic process;BP:organonitrogen compound metabolic process;BP:organonitrogen compound biosynthetic process;BP:purine nucleotide metabolic process;BP:organophosphate metabolic process;BP:aromatic compound biosynthetic process;BP:nucleoside triphosphate metabolic process;BP:nucleoside triphosphate biosynthetic process;BP:purine nucleoside triphosphate metabolic process;BP:purine nucleoside triphosphate biosynthetic process;BP:nitrogen compound metabolic process;BP:organic substance biosynthetic process;BP:heterocycle biosynthetic process;BP:purine nucleotide biosynthetic process;BP:hexose metabolic process;BP:purine ribonucleotide biosynthetic process;BP:purine ribonucleotide metabolic process;BP:biological_process;BP:metabolic process;BP:nucleobase-containing compound biosynthetic process;BP:organophosphate biosynthetic process;BP:ATP synthesis coupled proton transport;BP:cellular nitrogen compound biosynthetic process;BP:ribonucleotide biosynthetic process;BP:ribose phosphate biosynthetic process;BP:nucleoside phosphate biosynthetic process;BP:nucleoside phosphate metabolic process;BP:ATP biosynthetic process;BP:cellular biosynthetic process;BP:cellular nitrogen compound metabolic process;BP:ribonucleotide metabolic process;BP:nucleobase-containing compound metabolic process;BP:cellular process;BP:cellular aromatic compound metabolic process;BP:carbohydrate metabolic process;BP:nucleobase-containing small molecule metabolic process;BP:carbohydrate derivative biosynthetic process;BP:monosaccharide metabolic process;BP:carbohydrate derivative metabolic process;BP:cellular metabolic process;BP:purine-containing compound metabolic process;BP:purine-containing compound biosynthetic process;BP:phosphate-containing compound metabolic process;BP:mannose metabolic process;BP:organic substance metabolic process;BP:biosynthetic process;BP:nucleotide metabolic process;BP:primary metabolic process;BP:ribose phosphate metabolic process;BP:phosphorus metabolic process;CC:proton-transporting ATP synthase complex, coupling factor;CC:proton-transporting two-sector ATPase complex, proton-transporting domain;CC:inner mitochondrial membrane protein complex;CC:mitochondrial proton-transporting ATP synthase complex, coupling factor;CC:macromolecular complex;CC:cellular_component;CC:membrane protein complex;CC:mitochondrial protein complex;MF:metal ion binding;MF:hydrolase activity, hydrolyzing O-glycosyl compounds;MF:alpha-mannosidase activity;MF:molecular_function;MF:binding;MF:inorganic solute uptake transmembrane transporter activity;MF:hydrolase activity;MF:cation binding;MF:catalytic activity;MF:inorganic cation transmembrane transporter activity;MF:ion transmembrane transporter activity;MF:hydrogen ion transmembrane transporter activity;MF:hydrolase activity, acting on	GO:0009165;GO:0044281;GO:1901362;GO:1901360;GO:0009199;GO:0009206;GO:0009205;GO:0009201;GO:0046034;GO:0046483;GO:1901564;GO:1901566;GO:0006163;GO:0019637;GO:0019438;GO:0009141;GO:0009142;GO:0009144;GO:0009145;GO:0006807;GO:1901576;GO:0018130;GO:0006164;GO:0019318;GO:0009152;GO:0009150;GO:0008152;GO:0034654;GO:0090407;GO:0015986;GO:0044271;GO:0009260;GO:0046390;GO:1901293;GO:0006753;GO:0006754;GO:0044249;GO:0034641;GO:0009259;GO:0006139;GO:0009987;GO:0006725;GO:0005975;GO:0055086;GO:1901137;GO:0005996;GO:1901135;GO:0044237;GO:0072521;GO:0072522;GO:0006796;GO:0006013;GO:0071704;GO:0009058;GO:0009117;GO:0044238;GO:0019693;GO:0006793;GO:0045263;GO:0033177;GO:0098800;GO:0000276;GO:0032991;GO:0005575;GO:0098796;GO:0098798;GO:0046872;GO:0004553;GO:0004559;GO:0003674;GO:0005488;GO:0015318;GO:0016787;GO:0043169;GO:0003824;GO:0022890;GO:0015075;GO:0015078;GO:0016798;GO:0043167;GO:0015923;GO:0005215;GO:0030246;GO:0008324;GO:0022857	K12311	MAN2B1, LAN	map00511;ma	Other glycan degradation;Lysosome	ENOG410XQMZ	U:Intracellular trafficking, secretion, and vesicular transport	------	------	------	CYT	1	17	11.9	High
TRINITY_DN4384_c0_g1_i5_orf1	chemosensory protein 5 [Conogethes punctiferalis]	0.281031931	-1.831194037	1.39E-06	down	yes	1.329	4.729	4.778	4.807	4.602	1.321	1.399	1.267	------	------	------	------	------	------	ENOG410YU7V;EN	U:Intracellular trafficking, secretion, and vesicular transport;S:Function unknown	PF03392.16	OS-D	Insect pheromone-binding family, A10/OS-D	CYT	2	14	16.9	High
TRINITY_DN27300_c0_g1_i7_orfp1	TRINITY_DN27300_c0_g1_i7_m.71141 TRINITY_DN27300_c0_g1::TRINITY_DN27300_c0_g1_i7::g.71141 ORF type:internal len:82 (-),score=6.59 TRINITY_DN27300_c0_g1_i7:3-245(-)	2.387264457	1.255358395	0.0001346	up	yes	3.674	1.539	1.508	1.509	1.599	3.72	3.899	3.403	------	------	------	------	------	------	------	------	------	------	------	CYT	2	35	9.2	High
TRINITY_DN68397_c0_g1_i2_orf1	clavesin-1-like [Ostrinia furnacalis]	0.22945171	-2.123737535	4.45E-06	down	yes	0.9123	3.976	3.897	3.908	4.124	1.003	0.909	0.825	------	------	K19625	RLBP1	------	------	ENOG410XRSQ	S:Function unknown	PF00650.23;PF13716.9	CRAL_TRIO;CRAL_TRIO_2	CRAL/TRIO domain;Divergent CRAL/TRIO domain	CYT	2	8	34.6	High
TRINITY_DN1749_c0_g1_i1_orf1	putative GPI-anchored protein pfl2 isoform X2 [Ostrinia furnacalis]	0.293418142	-1.768970022	4.65E-05	down	yes	1.061	3.616	3.429	3.875	3.545	1.104	1.029	1.051	------	------	------	------	------	------	COG5599	S:Function unknown	PF00102.30	Y_phosphatase	Protein-tyrosine phosphatase	CYT	2	6	59	High
TRINITY_DN139537_c0_g1_i1_orf1	uncharacterized protein LOC114351440 [Ostrinia furnacalis]	0.393492117	-1.345593362	0.003631	down	yes	1.173	2.981	2.458	3.257	3.229	1.29	1.331	0.898	BP:nucleic acid metabolic process;BP:cellular nitrogen compound metabolic process;BP:nitrogen compound metabolic process;BP:nucleobase-containing compound metabolic process;BP:cellular macromolecule metabolic process;BP:transposition;BP:organic substance metabolic process;BP:organic cyclic compound metabolic process;BP:cellular process;BP:cellular aromatic compound metabolic process;BP:biological_process;BP:metabolic process;BP:heterocycle metabolic process;BP:DNA recombination;BP:primary metabolic process;BP:transposition, DNA-mediated;BP:cellular metabolic process;BP:macromolecule metabolic process;BP:DNA integration;BP:DNA metabolic process;CC:intrinsic component of membrane;CC:integral component of membrane;CC:cellular anatomical entity;CC:cellular_component;MF:molecular_function;MF:binding;MF:nucleic acid binding;MF:DNA binding;MF:heterocyclic compound binding;MF:organic cyclic compound binding;	GO:0090304;GO:0034641;GO:0006807;GO:0006139;GO:0044260;GO:0032196;GO:0071704;GO:1901360;GO:0009987;GO:0006725;GO:0008150;GO:0008152;GO:0046483;GO:0006310;GO:0044238;GO:0006313;GO:0044237;GO:0043170;GO:0015074;GO:0006259;GO:0031224;GO:0016021;GO:0110165;GO:0005575;GO:0003674;GO:0005488;GO:0003676;GO:0003677;GO:1901363;GO:0097159	------	------	------	------	ENOG410XQD5	S:Function unknown	------	------	------	PLA	1	5	23.3	High
TRINITY_DN7123_c0_g1_i1_orf1	activating signal cointegrator 1 complex subunit 2 homolog isoform X1 [Ostrinia furnacalis]	0.285200915	-1.809949486	1.12E-06	down	yes	0.873	3.061	3.068	3.135	2.981	0.89	0.871	0.858	MF:molecular_function;MF:structural constituent of cuticle;MF:structural molecule activity;	GO:0003674;GO:0042302;GO:0005198	------	------	------	------	ENOG4111QXK	S:Function unknown	------	------	------	CYT	5	46	17.6	High
TRINITY_DN206_c0_g1_i8_orf1	A-kinase anchor protein 200-like [Ostrinia furnacalis] >XP_028173114.1 A-kinase anchor protein 200-like [Ostrinia furnacalis] >XP_028173115.1 A-kinase anchor protein 200-like [Ostrinia furnacalis]	0.316660953	-1.658989113	0.0004024	down	yes	0.9237	2.917	2.823	3.265	2.663	0.887	0.988	0.896	------	------	------	------	------	------	ENOG4110RAQ	S:Function unknown	------	------	------	CYT	1	15	34.3	High
TRINITY_DN42337_c0_g1_i5_orf1	hypothetical protein evm_002829 [Chilo suppressalis]	0.290968407	-1.781065581	0.0002115	down	yes	0.8473	2.912	2.975	3.124	2.637	0.801	0.987	0.754	MF:molecular_function;MF:structural constituent of cuticle;MF:structural molecule activity;	GO:0003674;GO:0042302;GO:0005198	------	------	------	------	ENOG410YSFI;EN	S:Function unknown;	PF00379.26	Chitin_bind_4	Insect cuticle protein	CYT	1	31	17.6	High
TRINITY_DN140_c0_g1_i5_orf1	calcyphosin-like protein isoform X4 [Helicoverpa armigera]	3.11527469	1.639359379	1.25E-05	up	yes	2.943	0.9447	0.902	0.967	0.965	2.885	3.088	2.856	MF:cation binding;MF:ion binding;MF:molecular_function;MF:binding;MF:metal ion binding;MF:calcium ion binding;	GO:0043169;GO:0043167;GO:0003674;GO:0005488;GO:0046872;GO:0005509	K23909	CAPS	------	------	ENOG410XRMJ	T:Signal transduction mechanisms	PF13405.9;PF00036.35;PF13499.9;PF13202.9;PF13833.9	EF-hand_6;EF-hand_1;EF-hand_7;EF-hand_5;EF-hand_8	EF-hand domain;EF hand;EF-hand domain pair;EF hand;EF-hand domain pair	CYT	2	73	12	High
TRINITY_DN268_c1_g1_i7_orf1	unnamed protein product [Spodoptera littoralis] >CAH1645252.1 unnamed protein product [Spodoptera littoralis]	0.424570575	-1.235923706	4.92E-07	down	yes	1.137	2.678	2.675	2.66	2.698	1.172	1.147	1.092	CC:integral component of membrane;CC:cellular_component;CC:intrinsic component of membrane;CC:cellular anatomical entity;MF:sulfate transmembrane transporter activity;MF:sulfur compound transmembrane transporter activity;MF:secondary active transmembrane transporter activity;MF:inorganic anion transmembrane transporter activity;MF:inorganic solute uptake transmembrane transporter activity;MF:transporter activity;MF:ion transmembrane transporter activity;MF:transmembrane transporter activity;MF:secondary active sulfate transmembrane transporter activity;MF:active ion transmembrane transporter activity;MF:molecular_function;MF:anion transmembrane transporter	GO:0016021;GO:0005575;GO:0031224;GO:0110165;GO:0015116;GO:1901682;GO:0015291;GO:0015103;GO:0015318;GO:0005215;GO:0015075;GO:0022857;GO:0008271;GO:0022853;GO:0003674;GO:0008509;GO:0022804	K14708	SLC26A11	------	------	COG0659	S:Function unknown	PF00916.23	Sulfate_transp	Sulfate permease family	CYT	2	4	62.2	High
TRINITY_DN82426_c0_g1_i6_orfp1	lysosome-associated membrane glycoprotein 1-like isoform X4 [Ostrinia furnacalis]	0.342692585	-1.545013121	3.14E-05	down	yes	0.952	2.778	2.734	2.66	2.941	0.996	0.95	0.91	------	------	K06528	LAMP1_2, CD	map05152;ma	Tuberculosis;Phagosome;Lysosome;Autophagy - animal	------	------	------	------	------	CYT	1	12	12.6	High
TRINITY_DN55147_c0_g1_i1_orfp1	TRINITY_DN55147_c0_g1_i1_m.59251 TRINITY_DN55147_c0_g1::TRINITY_DN55147_c0_g1_i1::g.59251 ORF type:5prime_partial len:331 (-),score=113.97,Cuticle_3 PF11018.9 0.29,Cuticle_3|PF11018.9|2.9e-05,Cuticle_3|PF11018.9|0.00037 TRINITY_DN55147_c0_g1_i1:21-1013(-)	3.254924681	1.70262416	1.39E-05	up	yes	2.809	0.863	0.86	0.847	0.882	2.817	2.935	2.674	------	------	------	------	------	------	------	------	PF11018.11	Cuticle_3	Pupal cuticle protein C1	CYT	7	50	33.7	High
TRINITY_DN54269_c0_g1_i3_orf1	lopap-like [Ostrinia furnacalis]	0.382423077	-1.386758511	5.55E-05	down	yes	0.9943	2.6	2.725	2.619	2.457	1.068	0.919	0.996	------	------	K03098	APOD	------	------	ENOG4110TA7	S:Function unknown	------	------	------	CYT	1	26	9.2	High
TRINITY_DN230_c2_g1_i5_orf1	6-pyruvoyl tetrahydrobiopterin synthase [Ostrinia furnacalis]	2.184981685	1.127621187	2.28E-06	up	yes	2.386	1.092	1.101	1.071	1.105	2.443	2.374	2.34	BP:polyol biosynthetic process;BP:polyol metabolic process;BP:diol metabolic process;BP:cellular biosynthetic process;BP:cellular nitrogen compound metabolic process;BP:diol biosynthetic process;BP:small molecule metabolic process;BP:small molecule biosynthetic process;BP:organic cyclic compound biosynthetic process;BP:organic cyclic compound metabolic process;BP:organic substance biosynthetic process;BP:pteridine-containing compound metabolic process;BP:pteridine-containing compound biosynthetic process;BP:organic substance metabolic process;BP:heterocycle biosynthetic process;BP:tetrahydrobiopterin biosynthetic process;BP:alcohol biosynthetic process;BP:cellular process;BP:cellular aromatic compound metabolic process;BP:biosynthetic process;BP:tetrahydrobiopterin metabolic process;BP:biological_process;BP:metabolic process;BP:organonitrogen compound metabolic process;BP:heterocycle metabolic process;BP:cellular nitrogen compound biosynthetic process;BP:organonitrogen compound biosynthetic process;BP:cellular metabolic process;BP:alcohol metabolic process;BP:nitrogen compound metabolic process;BP:aromatic compound biosynthetic process;BP:organic hydroxy compound biosynthetic process;BP:organic hydroxy compound metabolic process;MF:binding;MF:molecular_function;MF:ion binding;MF:carbon-oxygen lyase activity, acting on phosphates;MF:lyase activity;MF:6-pyruvoyltetrahydropterin synthase activity;MF:metal ion binding;MF:cation binding;MF:catalytic activity;MF:carbon-oxygen lyase activity;	GO:0046173;GO:0019751;GO:0034311;GO:0044249;GO:0034641;GO:0034312;GO:0044281;GO:0044283;GO:1901362;GO:1901360;GO:1901576;GO:0042558;GO:0042559;GO:0071704;GO:0018130;GO:0006729;GO:0046165;GO:0009987;GO:0006725;GO:0009058;GO:0046146;GO:0008150;GO:0008152;GO:1901564;GO:0046483;GO:0044271;GO:1901566;GO:0044237;GO:0006066;GO:0006807;GO:0019438;GO:1901617;GO:1901615;GO:0005488;GO:0003674;GO:0043167;GO:0016838;GO:0016829;GO:0003874;GO:0046872;GO:0043169;GO:0003824;GO:0016835	K01737	queD, ptpS, P	map00790;ma	Folate biosynthesis;Biosynthesis of cofactors	COG0720	H:Coenzyme transport and metabolism	PF01242.22	PTPS	6-pyruvoyl tetrahydropterin synthase	CYT	8	62	18.7	High
TRINITY_DN218_c0_g1_i1_orf1	altered inheritance of mitochondria protein 3-like isoform X2 [Ostrinia furnacalis]	0.336349393	-1.571967441	0.0001157	down	yes	0.8587	2.553	2.577	2.732	2.349	0.83	0.898	0.848	MF:molecular_function;MF:structural constituent of cuticle;MF:structural molecule activity;	GO:0003674;GO:0042302;GO:0005198	------	------	------	------	ENOG4111QXK	S:Function unknown	PF00379.26	Chitin_bind_4	Insect cuticle protein	CYT	2	10	18	High
TRINITY_DN64616_c0_g1_i1_orf1	uncharacterized protein LOC114366101 [Ostrinia furnacalis]	0.464859899	-1.105132117	1.21E-05	down	yes	1.012	2.177	2.241	2.163	2.128	1.069	0.989	0.978	CC:cellular_component;CC:integral component of membrane;CC:cellular anatomical entity;CC:intrinsic component of membrane;	GO:0005575;GO:0016021;GO:0110165;GO:0031224	K10089	M6PR	map04145;ma	Phagosome;Lysosome;Salmonella infection	ENOG4112253;EN	S:Function unknown;	PF02157.18;PF09451.13	Man-6-P_recep;ATG27	Mannose-6-phosphate receptor;Autophagy-related protein 27	CYT	1	9	18.6	Medium
TRINITY_DN280_c0_g1_i8_orf1	Tubulin beta-1 chain [Papilio xuthus]	0.400952813	-1.318495635	7.20E-06	down	yes	0.8837	2.204	2.209	2.277	2.127	0.884	0.894	0.873	BP:cellular process;BP:biological_process;BP:microtubule-based process;CC:supramolecular fiber;CC:polymeric cytoskeletal fiber;CC:microtubule;CC:cytoplasm;CC:cellular_component;CC:cellular anatomical entity;CC:supramolecular complex;CC:supramolecular polymer;MF:structural constituent of cytoskeleton;MF:purine ribonucleoside triphosphate binding;MF:molecular_function;MF:binding;MF:guanyl nucleotide binding;MF:heterocyclic compound binding;MF:nucleoside phosphate binding;MF:pyrophosphatase activity;MF:structural molecule activity;MF:guanyl ribonucleotide binding;MF:purine nucleotide binding;MF:GTP binding;MF:hydrolase activity;MF:nucleotide binding;MF:GTPase activity;MF:nucleoside-triphosphatase activity;MF:small molecule binding;MF:catalytic activity;MF:hydrolase activity, acting on acid anhydrides, in phosphorus-containing anhydrides;MF:ion binding;MF:carbohydrate derivative binding;MF:organic cyclic compound binding;MF:hydrolase activity, acting on acid anhydrides;MF:anion binding;MF:purine ribonucleotide binding;MF:ribonucleotide binding;	GO:0009987;GO:0008150;GO:0007017;GO:0099512;GO:0099513;GO:0005874;GO:0005737;GO:0005575;GO:0110165;GO:0099080;GO:0099081;GO:0005200;GO:0035639;GO:0003674;GO:0005488;GO:0019001;GO:1901363;GO:1901265;GO:0016462;GO:0005198;GO:0032561;GO:0017076;GO:0005525;GO:0016787;GO:0000166;GO:0003924;GO:0017111;GO:0036094;GO:0003824;GO:0016818;GO:0043167;GO:0097367;GO:0097159;GO:0016817;GO:0043168;GO:0032555;GO:0032553	K07375	TUBB	map04540;ma	Gap junction;Parkinson disease;Alzheimer disease;Huntington disease;Pathogenic Escherichia coli infection;Amyotrophic lateral sclerosis;Salmonella infection;Phagosome;Pathways of neurodegeneration - multiple diseases;Prion	COG5023	Z:Cytoskeleton	PF00091.28;PF03953.20	Tubulin;Tubulin_C	Tubulin/FtsZ family, GTPase domain;Tubulin C-terminal domain	CYT	4	53	50.6	High
TRINITY_DN501_c0_g1_i5_orf1	hypothetical protein evm_002550 [Chilo suppressalis]	0.479646886	-1.059955405	1.88E-06	down	yes	0.978	2.039	2.024	2.082	2.01	0.991	0.989	0.954	CC:integral component of membrane;CC:cellular_component;CC:extracellular region;CC:intrinsic component of membrane;CC:cellular anatomical entity;MF:binding;MF:cation binding;MF:molecular function regulator;MF:peptidase regulator activity;MF:ion binding;MF:molecular_function;MF:peptidase inhibitor activity;MF:enzyme inhibitor activity;MF:metal ion binding;MF:enzyme regulator activity;MF:calcium ion binding;	GO:0016021;GO:0005575;GO:0005576;GO:0031224;GO:0110165;GO:0005488;GO:0043169;GO:0098772;GO:0061134;GO:0043167;GO:0003674;GO:0030414;GO:0004857;GO:0046872;GO:0030234;GO:0005509	------	------	------	------	ENOG410XSTY;EN	S:Function unknown;	PF12947.10;PF07645.18	EGF_3;EGF_CA	EGF domain;Calcium-binding EGF domain	PLA	90	8	1895.1	High
TRINITY_DN110519_c0_g1_i1_orf1	uncharacterized protein LOC114366601 [Ostrinia furnacalis]	0.257071713	-1.959757223	0.0001062	down	yes	0.7743	3.012	2.724	3.151	3.161	0.824	0.751	0.748	------	------	------	------	------	------	------	------	------	------	------	CYT	1	5	20	High
TRINITY_DN10304_c0_g2_i1_orf1	glycine-rich cell wall structural protein [Ostrinia furnacalis]	0.363949347	-1.458190419	5.92E-05	down	yes	0.9197	2.527	2.562	2.642	2.378	0.932	0.992	0.835	------	------	------	------	------	------	ENOG4111PWE;EN	S:Function unknown;	PF03103.20	DUF243	Domain of unknown function	CYT	2	16	27.6	High
TRINITY_DN50725_c0_g1_i6_orf1	BTB/POZ domain-containing protein 2-like [Ostrinia furnacalis]	2.769989907	1.46988072	0.00312	up	yes	2.47	0.8917	0.932	0.833	0.91	2.846	2.556	2.007	BP:developmental process;BP:cellular process;BP:cellular developmental process;BP:cell differentiation;BP:biological_process;BP:neurogenesis;CC:cytoplasm;CC:ribonucleoprotein granule;CC:intracellular non-membrane-bounded organelle;CC:cytosol;CC:cytoplasmic mRNA processing body;CC:cellular_component;CC:supramolecular complex;CC:intracellular organelle;CC:non-membrane-bounded organelle;CC:cytoplasmic ribonucleoprotein granule;CC:organelle;CC:cellular anatomical entity;	GO:0032502;GO:0009987;GO:0048869;GO:0030154;GO:0008150;GO:0022008;GO:0005737;GO:0035770;GO:0043232;GO:0005829;GO:0000932;GO:0005575;GO:0099080;GO:0043229;GO:0043228;GO:0036464;GO:0043226;GO:0110165	K10478	BTBD3_6	------	------	ENOG410XQ3X	S:Function unknown	PF00651.34;PF00917.29;PF07707.18	BTB;MATH;BACK	BTB/POZ domain;MATH domain;BTB And C-terminal Kelch	CYT	3	9	45.4	High
TRINITY_DN98242_c0_g1_i1_orf1	adenosine deaminase 2-A-like [Galleria mellonella]	0.310858487	-1.685670128	9.64E-05	down	yes	0.793	2.551	2.335	2.707	2.61	0.785	0.778	0.816	BP:cellular metabolic process;BP:cellular catabolic process;BP:cellular nitrogen compound metabolic process;BP:nitrogen compound metabolic process;BP:small molecule metabolic process;BP:small molecule catabolic process;BP:purine-containing compound metabolic process;BP:purine-containing compound catabolic process;BP:purine ribonucleoside catabolic process;BP:adenosine metabolic process;BP:organic cyclic compound metabolic process;BP:organic cyclic compound catabolic process;BP:nucleobase-containing compound metabolic process;BP:organic substance catabolic process;BP:purine nucleoside metabolic process;BP:organic substance metabolic process;BP:purine nucleoside catabolic process;BP:glycosyl compound catabolic process;BP:ribonucleoside catabolic process;BP:adenosine catabolic process;BP:cellular nitrogen compound catabolic process;BP:cellular process;BP:cellular aromatic compound metabolic process;BP:heterocycle catabolic process;BP:biological_process;BP:nucleoside metabolic process;BP:metabolic process;BP:nucleobase-containing compound catabolic process;BP:ribonucleoside metabolic process;BP:purine ribonucleoside metabolic process;BP:catabolic process;BP:nucleobase-containing small molecule metabolic process;BP:heterocycle metabolic process;BP:primary metabolic process;BP:organonitrogen compound metabolic process;BP:organonitrogen compound catabolic process;BP:carbohydrate derivative catabolic process;BP:nucleoside catabolic process;BP:glycosyl compound metabolic process;BP:aromatic compound catabolic process;BP:carbohydrate derivative metabolic process;BP:nucleobase-containing small molecule catabolic process;CC:extracellular space;CC:cellular_component;CC:cellular anatomical entity;MF:molecular_function;MF:deaminase activity;MF:hydrolase activity;MF:adenosine deaminase activity;MF:catalytic activity;MF:hydrolase activity, acting on carbon-nitroge;MF:hydrolase activity, acting on carbon-nitroge;	GO:0044237;GO:0044248;GO:0034641;GO:0006807;GO:0044281;GO:0044282;GO:0072521;GO:0072523;GO:0046130;GO:0046085;GO:1901360;GO:1901361;GO:0006139;GO:1901575;GO:0042278;GO:0071704;GO:0006152;GO:1901658;GO:0042454;GO:0006154;GO:0044270;GO:0009987;GO:0006725;GO:0046700;GO:0008150;GO:0009116;GO:0008152;GO:0034655;GO:0009119;GO:0046128;GO:0009056;GO:0055086;GO:0046483;GO:0044238;GO:1901564;GO:1901565;GO:1901136;GO:0009164;GO:1901657;GO:0019439;GO:1901135;GO:0034656;GO:0005615;GO:0005575;GO:0110165;GO:0003674;GO:0019239;GO:0016787;GO:0004000;GO:0003824;GO:0016810;GO:0016814	K19572	CECR1, ADA2	map01232;ma	Nucleotide metabolism;Purine metabolism	COG1816	F:Nucleotide transport and metabolism	PF00962.25;PF08451.14	A_deaminase;A_deaminase_N	Adenosine deaminase;Adenosine/AMP deaminase N-terminal	CYT	1	1	57.4	High
TRINITY_DN26879_c0_g1_i1_orf1	hypothetical protein O3G_MSEX014401 [Manduca sexta]	2.116713352	1.081825911	0.002401	up	yes	2.267	1.071	1.408	0.879	0.926	2.279	2.34	2.183	BP:nucleic acid metabolic process;BP:cellular biosynthetic process;BP:cellular nitrogen compound metabolic process;BP:nitrogen compound metabolic process;BP:organic cyclic compound biosynthetic process;BP:organic cyclic compound metabolic process;BP:nucleobase-containing compound metabolic process;BP:cellular macromolecule metabolic process;BP:organic substance metabolic process;BP:heterocycle biosynthetic process;BP:organic substance biosynthetic process;BP:cellular process;BP:cellular aromatic compound metabolic process;BP:biosynthetic process;BP:macromolecule biosynthetic process;BP:biological_process;BP:metabolic process;BP:nucleobase-containing compound biosynthetic process;BP:heterocycle metabolic process;BP:primary metabolic process;BP:cellular nitrogen compound biosynthetic process;BP:cellular metabolic process;BP:macromolecule metabolic process;BP:DNA metabolic process;BP:aromatic compound biosynthetic process;BP:DNA biosynthetic process;MF:molecular_function;MF:catalytic	GO:0090304;GO:0044249;GO:0034641;GO:0006807;GO:1901362;GO:1901360;GO:0006139;GO:0044260;GO:0071704;GO:0018130;GO:1901576;GO:0009987;GO:0006725;GO:0009058;GO:0009059;GO:0008150;GO:0008152;GO:0034654;GO:0046483;GO:0044238;GO:0044271;GO:0044237;GO:0043170;GO:0006259;GO:0019438;GO:0071897;GO:0003674;GO:0003824	------	------	------	------	ENOG410Y65V;EN	O:Posttranslational modification, protein turnover, chaperones;S:Function unknown	PF00078.30	RVT_1	Reverse transcriptase	EXC	1	1	97.8	Medium
TRINITY_DN33885_c0_g1_i1_orf1	inhibitor of nuclear factor kappa-B kinase subunit alpha [Ostrinia furnacalis]	2.443601896	1.289009265	0.005927	up	yes	2.578	1.055	1.151	0.769	1.244	2.18	3.026	2.528	------	------	K07209	IKBKB, IKKB	map05166;ma	Human T-cell leukemia virus 1 infection;Prostate cancer;Chagas disease;Human papillomavirus infection;IL-17 signaling pathway;Toxoplasmosis;Hepatitis C;Kaposi sarcoma-associated herpesvirus infection;Ras signaling pathway;Th17 cell differentiation;Th1 and Th2 cell differentiation;MAPK signaling pathway;Herpes simplex virus 1 infection;Epstein-Barr virus infection;Measles;Apoptosis;Human cytomegalovirus infection;Yersinia infection;Pancreatic cancer;Adipocytokine signaling pathway;Hepatitis B;Alzheimer disease;RIG-I-like receptor signaling pathway;Cytosolic DNA-	ENOG410XRMU	T:Signal transduction mechanisms	PF00069.28;PF07714.20;PF17667.4	Pkinase;PK_Tyr_Ser-Thr;Pkinase_fungal	Protein kinase domain;Protein tyrosine and serine/threonine kinase;Fungal protein kinase	CYT	1	2	89.6	High
TRINITY_DN31225_c0_g1_i1_orf1	ribosome biogenesis protein BMS1 homolog [Ostrinia furnacalis]	2.576877235	1.365623807	5.89E-06	up	yes	2.162	0.839	0.792	0.897	0.828	2.208	2.167	2.111	BP:ribosome biogenesis;BP:cellular component organization or biogenesis;BP:ribonucleoprotein complex biogenesis;BP:cellular process;BP:biological_process;BP:cellular component biogenesis;CC:intracellular organelle;CC:non-membrane-bounded organelle;CC:organelle;CC:nucleolus;CC:cellular anatomical entity;CC:intracellular non-membrane-bounded organelle;CC:cellular_component;MF:purine ribonucleoside triphosphate binding;MF:heterocyclic compound binding;MF:molecular_function;MF:binding;MF:guanyl ribonucleotide binding;MF:purine nucleotide binding;MF:GTP binding;MF:small molecule binding;MF:carbohydrate derivative binding;MF:organic cyclic compound binding;MF:guanyl nucleotide binding;MF:purine ribonucleotide binding;MF:ribonucleotide binding;	GO:0042254;GO:0071840;GO:0022613;GO:0009987;GO:0008150;GO:0044085;GO:0043229;GO:0043228;GO:0043226;GO:0005730;GO:0110165;GO:0043232;GO:0005575;GO:0035639;GO:1901363;GO:0003674;GO:0005488;GO:0043167;GO:1901265;GO:0043168;GO:0000166;GO:0032561;GO:0017076;GO:0005525;GO:0036094;GO:0097367;GO:0097159;GO:0019001;GO:0032555;GO:0032553	K14569	BMS1	map03008	Ribosome biogenesis in eukaryotes	COG5192	J:Translation, ribosomal structure and biogenesis	PF04950.15;PF08142.15	RIBIOP_C;AARP2CN	40S ribosome biogenesis protein Tsr1 and BMS1 C-terminal;AARP2CN	CYT	2	2	124.6	High

TRINITY_DN2684_c0_g2_i3_orf1	glutamate decarboxylase 1-like isoform X1 [Ostrinia furnacalis]	0.43526374	-1.200038254	3.24E-05	down	yes	1.18	2.711	2.662	2.852	2.618	1.215	1.171	1.154	BP:cellular metabolic process;BP:carboxylic acid metabolic process;BP:organic substance metabolic process;BP:small molecule metabolic process;BP:cellular process;BP:biological_process;BP:metabolic process;BP:oxoacid metabolic process;BP:organic acid metabolic process;MF:vitamin B6 binding;MF:molecular_function;MF:pyridoxal phosphate binding;MF:heterocyclic compound binding;MF:anion binding;MF:lyase activity;MF:ion binding;MF:small molecule binding;MF:catalytic activity;MF:organic cyclic compound binding;MF:carbon-carbon lyase activity;MF:carboxy-lyase activity;MF:vitamin binding;MF:binding;	GO:0044237;GO:0019752;GO:0071704;GO:0044281;GO:0009987;GO:0008150;GO:0008152;GO:0043436;GO:0006082;GO:0070279;GO:0003674;GO:0030170;GO:1901363;GO:0043168;GO:0016829;GO:0043167;GO:0036094;GO:0003824;GO:0097159;GO:0016830;GO:0016831;GO:0019842;GO:0005488	K01580;K189	E4.1.1.15, gad	map00770;ma	Pantothenate and CoA biosynthesis;Taurine and hypotaurine metabolism;Butanoate metabolism;Alanine, aspartate and glutamate metabolism;Type I diabetes mellitus;GABAergic synapse;beta-Alanine metabolism	COG0076	J:Translation, ribosomal structure and biogenesis	PF00282.22	Pyridoxal_deC	Pyridoxal-dependent decarboxylase conserved domain	CYT	6	18	54.5	High
TRINITY_DN73900_c0_g1_i1_orf1	carbonic anhydrase 7 [Ostrinia furnacalis]	2.144425676	1.100591314	0.0002699	up	yes	2.539	1.184	1.349	1.152	1.05	2.427	2.668	2.523	MF:cation binding;MF:metal ion binding;MF:transition metal ion binding;MF:carbon-oxygen lyase activity;MF:hydro-lyase activity;MF:ion binding;MF:catalytic activity;MF:molecular_function;MF:zinc ion binding;MF:carbonate dehydratase activity;MF:lyase activity;MF:binding;	GO:0043169;GO:0046872;GO:0046914;GO:0016835;GO:0016836;GO:0043167;GO:0003824;GO:0003674;GO:0008270;GO:0004089;GO:0016829;GO:0005488	K01672	CA	map00910	Nitrogen metabolism	COG3338	U:Intracellular trafficking, secretion, and vesicular transport	PF00194.24	Carb_anhydrase	Eukaryotic-type carbonic anhydrase	CYT	1	3	36.5	High
TRINITY_DN20344_c0_g1_i5_orf1	uncharacterized protein LOC114351483 [Ostrinia furnacalis]	3.057303743	1.612259891	4.37E-06	up	yes	2.769	0.9057	0.935	0.829	0.953	2.836	2.703	2.767	MF:molecular_function;MF:structural constituent of cuticle;MF:structural molecule activity;	GO:0003674;GO:0042302;GO:0005198	------	------	------	------	------	------	PF00379.26	Chitin_bind_4	Insect cuticle protein	CYT	4	57	13.5	High
TRINITY_DN24121_c1_g1_i6_orf1	serine protease persephone-like [Ostrinia furnacalis]	3.465902159	1.793230929	8.57E-05	up	yes	2.841	0.8197	0.809	0.791	0.859	2.741	3.086	2.695	BP:organic substance metabolic process;BP:organonitrogen compound metabolic process;BP:protein metabolic process;BP:macromolecule metabolic process;BP:biological_process;BP:nitrogen compound metabolic process;BP:metabolic process;BP:proteolysis;BP:primary metabolic process;MF:serine-type endopeptidase activity;MF:endopeptidase activity;MF:hydrolase activity;MF:serine hydrolase activity;MF:catalytic activity, acting on a protein;MF:catalytic activity;MF:molecular_function;MF:peptidase activity;MF:serine-type peptidase activity;	GO:0071704;GO:1901564;GO:0019538;GO:0043170;GO:0008150;GO:0006807;GO:0008152;GO:0006508;GO:0044238;GO:0004252;GO:0004175;GO:0016787;GO:0017171;GO:0140096;GO:0003824;GO:0003674;GO:0008233;GO:0008236	K20677;K0962	HAYAN;PRSS	map04624	Toll and Imd signaling pathway	COG5640	O:Posttranslational modification, protein turnover, chaperones	PF00089.29	Trypsin	Trypsin	CYT	7	29	28.9	High
TRINITY_DN1563_c0_g1_i4_orf1	pupal cuticle protein 36-like [Ostrinia furnacalis]	0.39497448	-1.340168654	3.65E-05	down	yes	1.006	2.547	2.624	2.588	2.429	1.065	1.043	0.911	MF:molecular_function;MF:structural constituent of cuticle;MF:structural molecule activity;	GO:0003674;GO:0042302;GO:0005198	------	------	------	------	ENOG4111PJ1;EN	S:Function unknown;	PF00379.26	Chitin_bind_4	Insect cuticle protein	CYT	5	41	30.1	High
TRINITY_DN38431_c0_g1_i1_orf1	neprilysin-2 isoform X1 [Ostrinia furnacalis]	0.394061379	-1.343507733	1.50E-06	down	yes	0.9887	2.509	2.574	2.472	2.48	0.975	0.985	1.006	BP:nitrogen compound metabolic process;BP:organic substance metabolic process;BP:proteolysis;BP:biological_process;BP:metabolic process;BP:primary metabolic process;BP:organonitrogen compound metabolic process;BP:protein metabolic process;BP:macromolecule metabolic process;CC:integral component of membrane;CC:membrane;CC:intrinsic component of membrane;CC:cellular anatomical entity;CC:plasma membrane;CC:cellular_component;MF:endopeptidase activity;MF:molecular_function;MF:binding;MF:peptidase activity;MF:metallopeptidase activity;MF:cation binding;MF:ion binding;MF:metal ion binding;MF:hydrolase activity;MF:metalloendopeptidase activity;MF:catalytic activity, acting on a protein;MF:catalytic activity;	GO:0006807;GO:0071704;GO:0006508;GO:0008150;GO:0008152;GO:0044238;GO:1901564;GO:0019538;GO:0043170;GO:0016021;GO:0016020;GO:0031224;GO:0110165;GO:0005886;GO:0005575;GO:0004175;GO:0003674;GO:0005488;GO:0008233;GO:0008237;GO:0043169;GO:0043167;GO:0046872;GO:0016787;GO:0004222;GO:0140096;GO:0003824	K08635	MMEL1, NEP	------	------	COG3590	O:Posttranslational modification, protein turnover, chaperones	PF05649.16;PF01431.24	Peptidase_M13_N;Peptidase_M13	Peptidase family M13;Peptidase family M13	CYT	10	16	87.5	High
TRINITY_DN114982_c0_g1_i1_orf1	uncharacterized protein LOC114357708 [Ostrinia furnacalis]	0.450457952	-1.150535652	0.007559	down	yes	1.082	2.402	2.678	2.019	2.508	0.915	1.434	0.897	CC:cellular_component;CC:ribosome;CC:intracellular non-membrane-bounded organelle;CC:mitochondrion;CC:intracellular organelle;CC:non-membrane-bounded organelle;CC:membrane-bounded organelle;CC:organelle;CC:cellular anatomical entity;CC:intracellular membrane-bounded organelle;MF:structural molecule activity;MF:structural constituent of ribosome;MF:molecular_function;	GO:0005575;GO:0005840;GO:0043232;GO:0005739;GO:0043229;GO:0043228;GO:0043227;GO:0043226;GO:0110165;GO:0043231;GO:0005198;GO:0003735;GO:0003674	K17412	MRPS34	------	------	ENOG4111Y87	S:Function unknown	PF16053.8	MRP-S34	Mitochondrial 28S ribosomal protein S34	CYT	2	13	22.6	High
TRINITY_DN83374_c0_g1_i1_orf1	uncharacterized protein LOC114350302, partial [Ostrinia furnacalis]	0.366069869	-1.449809064	3.47E-05	down	yes	0.8383	2.29	2.415	2.282	2.173	0.841	0.812	0.862	------	------	------	------	------	------	ENOG410Y20T	S:Function unknown	------	------	------	CYT	2	7	63	High
TRINITY_DN1308_c0_g1_i4_orf1	serine proteinase stubble-like [Ostrinia furnacalis]	0.327542662	-1.610245266	7.76E-05	down	yes	0.9597	2.93	3.128	2.748	2.914	0.9	1.048	0.931	BP:organic substance metabolic process;BP:organonitrogen compound metabolic process;BP:protein metabolic process;BP:macromolecule metabolic process;BP:biological_process;BP:nitrogen compound metabolic process;BP:metabolic process;BP:proteolysis;BP:primary metabolic process;MF:serine-type endopeptidase activity;MF:endopeptidase activity;MF:hydrolase activity;MF:serine hydrolase activity;MF:catalytic activity, acting on a protein;MF:catalytic activity;MF:molecular_function;MF:peptidase activity;MF:serine-type peptidase activity;	GO:0071704;GO:1901564;GO:0019538;GO:0043170;GO:0008150;GO:0006807;GO:0008152;GO:0006508;GO:0044238;GO:0004252;GO:0004175;GO:0016787;GO:0017171;GO:0140096;GO:0003824;GO:0003674;GO:0008233;GO:0008236	------	------	------	------	COG5640	C:Energy production and conversion	PF00089.29	Trypsin	Trypsin	CYT	1	3	28.8	Medium
TRINITY_DN5757_c0_g1_i1_orf1	ATP-dependent DNA helicase 2 subunit 1 [Ostrinia furnacalis]	0.441343042	-1.180027642	0.00578	down	yes	1.091	2.472	2.133	2.329	2.953	1.226	0.996	1.051	BP:nucleic acid metabolic process;BP:cellular nitrogen compound metabolic process;BP:nitrogen compound metabolic process;BP:organic cyclic compound metabolic process;BP:nucleobase-containing compound metabolic process;BP:cellular response to stimulus;BP:double-strand break repair via nonhomologous end joining;BP:double-strand break repair;BP:cellular macromolecule metabolic process;BP:cellular component organization;BP:organic substance metabolic process;BP:cellular component organization or biogenesis;BP:telomere maintenance;BP:DNA repair;BP:cellular process;BP:cellular aromatic compound metabolic process;BP:cellular response to DNA damage stimulus;BP:response to stress;BP:biological_process;BP:metabolic process;BP:DNA recombination;BP:heterocycle metabolic process;BP:organelle organization;BP:primary metabolic process;BP:chromosome organization;BP:response to stimulus;BP:telomere organization;BP:cellular metabolic process;BP:macromolecule metabolic process;BP:cellular response to stress;BP:DNA metabolic process;CC:Ku7;CC:macromolecular complex;CC:cellular_component;MF:hydrolase activity;MF:purine ribonucleoside triphosphate binding;MF:small molecule binding;MF:molecular_function;MF:binding;MF:nucleic acid binding;MF:DNA binding;MF:DNA helicase activity;MF:ion binding;MF:heterocyclic compound binding;MF:sequence-specific DNA binding;MF:nucleotide binding;MF:purine ribonucleotide binding;MF:purine nucleotide binding;MF:DNA-dependent ATPase activity;MF:ATP binding;MF:telomeric DNA binding;MF:catalytic activity, acting on DNA;MF:nucleoside phosphate binding;MF:helicase activity;MF:catalytic activity;MF:adenyl nucleotide binding;MF:carbohydrate derivative binding;MF:organic cyclic compound binding;MF:anion binding;MF:adenyl ribonucleotide binding;MF:damaged DNA binding;MF:ribonucleotide binding;	GO:0090304;GO:0034641;GO:0006807;GO:1901360;GO:0006139;GO:0051716;GO:0006303;GO:0006302;GO:0044260;GO:0016043;GO:0071704;GO:0071840;GO:0000723;GO:0006281;GO:0009987;GO:0006725;GO:0006974;GO:0006950;GO:0008150;GO:0008152;GO:0006310;GO:0046483;GO:0006996;GO:0044238;GO:0051276;GO:0050896;GO:0032200;GO:0044237;GO:0043170;GO:0033554;GO:0006259;GO:0043564;GO:0032991;GO:0005575;GO:0016787;GO:0035639;GO:0036094;GO:0003674;GO:0005488;GO:0003676;GO:0003677;GO:0003678;GO:0043167;GO:1901363;GO:0043565;GO:0000166;GO:0032555;GO:0017076;GO:0008094;GO:0005524;GO:0042162;GO:0140097;GO:1901265;GO:0004386;GO:0003824;GO:0030554;GO:0097367;GO:0097159;GO:0043168;GO:0032559;GO:0003684;GO:0032553	K10884	XRCC6, KU70,	map03450	Non-homologous end-joining	ENOG410XNXU	L:Replication, recombination and repair	PF02735.19;PF03731.18;PF03730.17	Ku;Ku_N;Ku_C	Ku70/Ku80 beta-barrel domain;Ku70/Ku80 N-terminal alpha/beta domain;Ku70/Ku80 C-terminal arm	CYT	1	2	66.8	High
TRINITY_DN3833_c0_g1_i4_orf1	division abnormally delayed protein [Ostrinia furnacalis]	0.414679294	-1.269932083	4.16E-06	down	yes	0.9633	2.323	2.397	2.283	2.29	0.958	0.943	0.989	BP:regulation of smoothened signaling pathway;BP:positive regulation of canonical Wnt signaling pathway;BP:positive regulation of response to stimulus;BP:regulation of response to stimulus;BP:positive regulation of signaling;BP:regulation of Wnt signaling pathway;BP:positive regulation of smoothened signaling pathway;BP:regulation of signaling;BP:positive regulation of cell communication;BP:regulation of cell communication;BP:regulation of biological process;BP:regulation of cellular response to growth factor stimulus;BP:positive regulation of transmembrane receptor protein serine/threonine kinase signaling pathway;BP:regulation of signal transduction;BP:positive regulation of signal transduction;BP:biological regulation;BP:positive regulation of biological process;BP:regulation of transmembrane receptor protein serine/threonine kinase signaling pathway;BP:regulation of cellular process;BP:biological_process;BP:positive regulation of BMP signaling pathway;BP:regulation of BMP signaling pathway;BP:positive regulation of Wnt signaling pathway;BP:regulation of canonical Wnt signaling pathway;BP:positive regulation of cellular process;CC:extracellular matrix;CC:anchored component of plasma membrane;CC:intrinsic component of plasma membrane;CC:intrinsic component of membrane;CC:anchored component of membrane;CC:cellular anatomical entity;CC:collagen-containing extracellular matrix;CC:external encapsulating structure;CC:cellular_component;	GO:0008589;GO:0090263;GO:0048584;GO:0048583;GO:0023056;GO:0030111;GO:0045880;GO:0023051;GO:0010647;GO:0010646;GO:0050789;GO:0090287;GO:0090100;GO:0009966;GO:0009967;GO:0065007;GO:0048518;GO:0090092;GO:0050794;GO:0008150;GO:0030513;GO:0030510;GO:0030177;GO:0060828;GO:0048522;GO:0031012;GO:0046658;GO:0031226;GO:0031224;GO:0031225;GO:0110165;GO:0062023;GO:0030312;GO:0005575	K02306	DALLY	map04310;ma	Wnt signaling pathway;Hippo signaling pathway - fly	ENOG410XST2	O:Posttranslational modification, protein turnover, chaperones	PF01153.22	Glypican	Glypican	CYT	2	3	60	High
TRINITY_DN37585_c0_g1_i1_orf1	cuticle protein 19.8-like [Ostrinia furnacalis]	0.469451932	-1.09095065	0.001662	down	yes	1.045	2.226	2.305	2.349	2.024	0.841	1.039	1.254	------	------	------	------	------	------	------	------	------	------	------	EXC	1	10	17.7	High
TRINITY_DN4068_c1_g2_i1_orf1	larval cuticle protein LCP-17 [Helicoverpa armigera] >PZC82071.1 hypothetical protein B5X24_HaOG211161 [Helicoverpa armigera] >PZC87412.1 hypothetical protein B5X24_HaOG216859 [Helicoverpa armigera]	3.011462313	1.590464205	1.69E-05	up	yes	2.601	0.8637	0.722	0.924	0.945	2.592	2.613	2.597	MF:molecular_function;MF:structural constituent of cuticle;MF:structural molecule activity;	GO:0003674;GO:0042302;GO:0005198	------	------	------	------	ENOG4110RUZ;EN	S:Function unknown;	PF00379.26	Chitin_bind_4	Insect cuticle protein	CYT	4	64	17	High
TRINITY_DN1230_c1_g1_i5_orf1	uncharacterized protein LOC114353440 [Ostrinia furnacalis]	2.052430887	1.037333642	0.0003795	up	yes	2.153	1.049	1.046	0.898	1.204	2.169	2.224	2.066	------	------	------	------	------	------	ENOG4110OI3;EN	S:Function unknown;	------	------	------	CYT	1	3	35.6	High
TRINITY_DN668_c0_g1_i4_orf1	fatty acid synthase-like isoform X1 [Ostrinia furnacalis]	0.345528455	-1.533123569	5.53E-07	down	yes	0.85	2.46	2.495	2.409	2.477	0.849	0.868	0.833	------	------	K00665	FASN	map04910;ma	Insulin signaling pathway;Alcoholic liver disease;Fatty acid biosynthesis;AMPK signaling pathway	COG3321	Q:Secondary metabolites biosynthesis, transport and catabolism	PF00698.24;PF00109.29;PF08659.13;PF02801.25;PF16197.8;PF00107.29;PF14765.9;PF13602.9;PF00975.23;PF00550.28;PF00106.28	Acyl_transf_1;ketoacyl-synt;KR;Ketoacyl-synt_C;KAsynt_C_assoc;ADH_zinc_N;PS-DH;ADH_zinc_N_2;Thioesterase;PP-binding;adh_short	Acyl transferase domain;Beta-ketoacyl synthase, N-terminal domain;KR domain;Beta-ketoacyl synthase, C-terminal domain;Ketoacyl-synthetase C-terminal extension;Zinc-binding dehydrogenase;Polyketide synthase dehydratase;Zinc-binding dehydrogenase;Thioesterase domain;Phosphopantetheine attachment site;short chain dehydrogenase	CYT	55	29	268.3	High
TRINITY_DN37585_c0_g2_i1_orf1	cuticle protein 19.8-like [Ostrinia furnacalis]	0.409808394	-1.286978559	0.002764	down	yes	0.8983	2.192	2.184	2.528	1.865	0.808	0.944	0.943	MF:molecular_function;MF:structural constituent of cuticle;MF:structural molecule activity;	GO:0003674;GO:0042302;GO:0005198	------	------	------	------	ENOG410YTXG;EN	S:Function unknown;	PF00379.26	Chitin_bind_4	Insect cuticle protein	CYT	1	6	14.7	High
TRINITY_DN111621_c0_g3_i1_orf1	Serine proteinase stubble [Eumeta japonica]	0.419953271	-1.251699289	3.47E-05	down	yes	0.8987	2.14	2.121	2.121	2.177	1.015	0.839	0.842	BP:organic substance metabolic process;BP:organonitrogen compound metabolic process;BP:protein metabolic process;BP:macromolecule metabolic process;BP:biological_process;BP:nitrogen compound metabolic process;BP:metabolic process;BP:proteolysis;BP:primary metabolic process;MF:serine-type endopeptidase activity;MF:endopeptidase activity;MF:hydrolase activity;MF:serine hydrolase activity;MF:catalytic activity, acting on a protein;MF:catalytic activity;MF:molecular_function;MF:peptidase activity;MF:serine-type peptidase activity;	GO:0071704;GO:1901564;GO:0019538;GO:0043170;GO:0008150;GO:0006807;GO:0008152;GO:0006508;GO:0044238;GO:0004252;GO:0004175;GO:0016787;GO:0017171;GO:0140096;GO:0003824;GO:0003674;GO:0008233;GO:0008236	------	------	------	------	COG5640	E:Amino acid transport and metabolism	PF00089.29	Trypsin	Trypsin	CYT	1	4	18.5	Medium
TRINITY_DN3383_c0_g1_i5_orf1	uncharacterized protein LOC114357426 [Ostrinia furnacalis]	0.446920115	-1.161911114	2.08E-06	down	yes	0.9287	2.078	2.105	2.036	2.094	0.961	0.926	0.899	------	------	------	------	------	------	ENOG4110QG2;EI	S:Function unknown;	------	------	------	CYT	4	14	35.5	High
TRINITY_DN3464_c0_g1_i1_orf1	putative mitochondrial aconitate hydratase isoform X1-likeprotein, partial [Cotesia chilonis]	0.391397849	-1.353292266	7.68E-05	down	yes	1.092	2.79	2.905	2.59	2.875	1.126	1.09	1.06	BP:tricarboxylic acid cycle;BP:biological_process;BP:metabolic process;BP:primary metabolic process;CC:membrane-bounded organelle;CC:organelle;CC:mitochondrion;CC:cellular anatomical entity;CC:intracellular membrane-bounded organelle;CC:intracellular organelle;CC:cellular_component;MF:molecular_function;MF:binding;MF:cation binding;MF:metal cluster binding;MF:lyase activity;MF:ion binding;MF:metal ion binding;MF:catalytic activity;MF:citrate dehydratase activity;MF:4 iron, 4 sulfur cluster binding;MF:carbon-oxygen lyase activity;MF:hydro-lyase activity;MF:aconitate hydratase activity;MF:iron-sulfur cluster binding;	GO:0006099;GO:0008150;GO:0008152;GO:0044238;GO:0043227;GO:0043226;GO:0005739;GO:0110165;GO:0043231;GO:0043229;GO:0005575;GO:0003674;GO:0005488;GO:0043169;GO:0051540;GO:0016829;GO:0043167;GO:0046872;GO:0003824;GO:0047780;GO:0051539;GO:0016835;GO:0016836;GO:0003994;GO:0051536	K01681	ACO, acnA	map00020;ma	Citrate cycl;Glyoxylate and dicarboxylate metabolism	COG1048	C:Energy production and conversion	PF00330.23;PF00694.22	Aconitase;Aconitase_C	Aconitase family;Aconitase C-terminal domain	CYT	1	6	89.4	High
TRINITY_DN38230_c0_g1_i4_orf1	hypothetical protein evm_007803 [Chilo suppressalis]	3.859922179	1.948571761	0.003007	up	yes	2.976	0.771	0.75	0.669	0.894	3.151	2.326	3.452	BP:organophosphate catabolic process;BP:nucleotide catabolic process;BP:cellular nitrogen compound metabolic process;BP:nitrogen compound metabolic process;BP:small molecule metabolic process;BP:organic cyclic compound metabolic process;BP:organic cyclic compound catabolic process;BP:nucleobase-containing compound metabolic process;BP:organic substance catabolic process;BP:organic substance metabolic process;BP:cellular catabolic process;BP:cellular process;BP:cellular aromatic compound metabolic process;BP:heterocycle catabolic process;BP:aromatic compound catabolic process;BP:biological_process;BP:metabolic process;BP:nucleobase-containing compound catabolic process;BP:catabolic process;BP:nucleobase-containing small molecule metabolic process;BP:heterocycle metabolic process;BP:primary metabolic process;BP:cellular nitrogen compound catabolic process;BP:cellular metabolic process;BP:phosphate-containing compound metabolic process;BP:nucleoside phosphate catabolic process;BP:phosphorus metabolic process;BP:organophosphate metabolic process;BP:nucleotide metabolic process;BP:nucleoside phosphate metabolic process;CC:cellular anatomical entity;CC:cellular_component;MF:phosphatase activity;MF:nucleotidase activity;MF:phosphoric ester hydrolase activity;MF:molecular_function;MF:nucleotide binding;MF:heterocyclic compound binding;MF:ion binding;MF:nucleoside phosphate binding;MF:metal ion binding;MF:hydrolase activity;MF:cation binding;MF:hydrolase activity, acting on ester bonds;MF:small molecule binding;MF:catalytic activity;MF:organic cyclic compound binding;MF:binding;	GO:0046434;GO:0009166;GO:0034641;GO:0006807;GO:0044281;GO:1901360;GO:1901361;GO:0006139;GO:1901575;GO:0071704;GO:0044248;GO:0009987;GO:0006725;GO:0046700;GO:0019439;GO:0008150;GO:0008152;GO:0034655;GO:0009056;GO:0055086;GO:0046483;GO:0044238;GO:0044270;GO:0044237;GO:0006796;GO:1901292;GO:0006793;GO:0019637;GO:0009117;GO:0006753;GO:0110165;GO:0005575;GO:0016791;GO:0008252;GO:0042578;GO:0003674;GO:0000166;GO:1901363;GO:0043167;GO:1901265;GO:0046872;GO:0016787;GO:0043169;GO:0016788;GO:0036094;GO:0003824;GO:0097159;GO:0005488	------	------	------	------	COG0737	F:Nucleotide transport and metabolism	PF02872.21	5_nucleotid_C	5'-nucleotidase, C-terminal domain	CYT	1	17	12.1	High
TRINITY_DN214_c0_g1_i3_orf1	uncharacterized protein LOC114352813 [Ostrinia furnacalis]	2.698445382	1.432128487	0.0008911	up	yes	2.621	0.9713	0.964	0.943	1.007	2.923	2.285	2.655	------	------	------	------	------	------	ENOG4110S0N;EN	S:Function unknown;	PF06585.14	JHBP	Haemolymph juvenile hormone binding protein	CYT	3	38	19.4	High
TRINITY_DN114834_c0_g1_i1_orf1	uncharacterized protein LOC115444227 [Manduca sexta] >XP_030025790.1 uncharacterized protein LOC115444227 [Manduca sexta] >XP_037296791.1 uncharacterized protein LOC115444227 [Manduca sexta] >XP_037296792.1 uncharacterized protein LOC115444227 [Manduca sexta] >KAG6441350.1 hypothetical protein O3G_MSEX001778 [Manduca sexta]	0.324249605	-1.624823278	3.78E-06	down	yes	0.821	2.532	2.519	2.62	2.458	0.836	0.825	0.802	CC:cellular_component;CC:integral component of membrane;CC:cellular anatomical entity;CC:intrinsic component of membrane;	GO:0005575;GO:0016021;GO:0110165;GO:0031224	------	------	------	------	ENOG410XTAH	S:Function unknown	PF00024.29	PAN_1	PAN domain	CYT	10	19	81.4	High
TRINITY_DN4689_c0_g1_i5_orf1	pericentriolar material 1 protein-like isoform X3 [Ostrinia furnacalis]	0.339551309	-1.558298502	0.001678	down	yes	0.8173	2.407	2.373	2.059	2.789	0.828	0.804	0.82	BP:cellular component assembly;BP:cell projection organization;BP:cell projection assembly;BP:protein localization to cytoskeleton;BP:protein localization;BP:microtubule cytoskeleton organization;BP:plasma membrane bounded cell projection organization;BP:cellular component organization or biogenesis;BP:plasma membrane bounded cell projection assembly;BP:microtubule anchoring at centrosome;BP:protein localization to centrosome;BP:microtubule anchoring at microtubule organizing center;BP:cellular component organization;BP:protein localization to microtubule organizing center;BP:macromolecule localization;BP:microtubule anchoring;BP:cilium assembly;BP:cellular process;BP:biological_process;BP:organelle assembly;BP:localization;BP:cellular localization;BP:organelle organization;BP:microtubule-based process;BP:cytoskeleton organization;BP:cellular macromolecule localization;BP:protein localization to microtubule cytoskeleton;BP:protein localization to organelle;BP:cilium organization;CC:centrosome;CC:microtubule organizing center;CC:cellular anatomical entity;CC:cellular_component;	GO:0022607;GO:0030030;GO:0030031;GO:0044380;GO:0008104;GO:0000226;GO:0120036;GO:0071840;GO:0120031;GO:0034454;GO:0071539;GO:0072393;GO:0016043;GO:1905508;GO:0033036;GO:0034453;GO:0060271;GO:0009987;GO:0008150;GO:0070925;GO:0051179;GO:0051641;GO:0006996;GO:0007017;GO:0007010;GO:0070727;GO:0072698;GO:0033365;GO:0044782;GO:0005813;GO:0005815;GO:0110165;GO:0005575	K16537	PCM1	------	------	ENOG410YNFX	S:Function unknown	PF15717.8	PCM1_C	Pericentriolar material 1 C terminus	CYT	2	1	193.5	High
TRINITY_DN806_c0_g2_i1_orf1	uncharacterized protein LOC114355167 [Ostrinia furnacalis]	0.354946319	-1.494327243	5.06E-06	down	yes	0.9257	2.608	2.529	2.645	2.649	0.952	0.861	0.964	MF:molecular_function;MF:structural constituent of cuticle;MF:structural molecule activity;	GO:0003674;GO:0042302;GO:0005198	------	------	------	------	ENOG410YR66;EN	S:Function unknown;	PF00379.26	Chitin_bind_4	Insect cuticle protein	CYT	1	6	17.8	High
TRINITY_DN12013_c0_g1_i6_orf1	tudor domain-containing protein 7 isoform X3 [Ostrinia furnacalis]	2.072743208	1.051541392	0.0006072	up	yes	2.365	1.141	1.314	0.965	1.143	2.337	2.253	2.504	CC:cytoplasm;CC:cellular_component;CC:cellular anatomical entity;	GO:0005737;GO:0005575;GO:0110165	K18405	TDRD1 4 6 7	------	------	ENOG41104YI;CO	S:Function unknown;	PF00567.27	TUDOR	Tudor domain	CYT	1	7	33.4	High
TRINITY_DN10364_c0_g1_i5_orf1	uncharacterized protein LOC114352615 [Ostrinia furnacalis]	0.443275389	-1.17372483	1.56E-06	down	yes	0.969	2.186	2.142	2.221	2.196	0.996	0.965	0.946	BP:organic substance metabolic process;BP:organonitrogen compound metabolic process;BP:protein metabolic process;BP:nitrogen compound metabolic process;BP:macromolecule metabolic process;BP:biological_process;BP:metabolic process;BP:proteolysis;BP:primary metabolic process;CC:cellular_component;CC:extracellular region;CC:cellular anatomical entity;MF:serine-type endopeptidase activity;MF:endopeptidase activity;MF:hydrolase activity;MF:serine hydrolase activity;MF:catalytic activity, acting on a protein;MF:catalytic activity;MF:molecular_function;MF:peptidase activity;MF:serine-type peptidase activity;	GO:0071704;GO:1901564;GO:0019538;GO:0006807;GO:0043170;GO:0008150;GO:0008152;GO:0006508;GO:0044238;GO:0005575;GO:0005576;GO:0110165;GO:0004252;GO:0004175;GO:0016787;GO:0017171;GO:0140096;GO:0003824;GO:0003674;GO:0008233;GO:0008236	------	------	------	------	COG5640	I:Lipid transport and metabolism	PF00089.29;PF12032.11;PF13365.9	Trypsin;CLIP;Trypsin_2	Trypsin;Regulatory CLIP domain of proteinases;Trypsin-like peptidase domain	CYT	3	6	46.8	High
TRINITY_DN13648_c0_g1_i6_orf1	neurexin-4 [Ostrinia furnacalis]	0.46461825	-1.105882273	2.75E-05	down	yes	0.998	2.148	2.153	2.056	2.235	1.024	0.98	0.99	------	------	K07380	CNTNAP2	map04514	Cell adhesion molecules	COG2091;ENOG4	H:Coenzyme transport and metabolism;S:Function unknown	PF02210.27;PF00054.26;PF00754.28;PF13385.9;PF00008.30	Laminin_G_2;Laminin_G_1;F5_F8_type_C;Laminin_G_3;EGF	Laminin G domain;Laminin G domain;F5/8 type C domain;Concanavalin A-like lectin/glucanases superfamily;EGF-like domain	CYT	5	4	142.6	High
TRINITY_DN15420_c0_g3_i2_orf1	elongation factor 1-alpha 2-like [Galleria mellonella] >XP_031769625.1 elongation factor 1-alpha 2-like [Galleria mellonella]	0.43490566	-1.201225609	5.17E-05	down	yes	0.922	2.12	2.062	2.214	2.084	0.94	0.837	0.989	CC:cytoplasm;CC:cellular anatomical entity;CC:cellular_component;MF:purine ribonucleoside triphosphate binding;MF:molecular_function;MF:binding;MF:nucleic acid binding;MF:ion binding;MF:heterocyclic compound binding;MF:nucleoside phosphate binding;MF:guanyl nucleotide binding;MF:nucleotide binding;MF:guanyl ribonucleotide binding;MF:purine nucleotide binding;MF:GTP binding;MF:hydrolase activity;MF:translation factor activity, RNA binding;MF:GTPase activity;MF:nucleoside-triphosphatase activity;MF:small molecule binding;MF:catalytic activity;MF:translation regulator activity;MF:hydrolase activity, acting on acid anhydrides, in phosphorus-containing anhydrides;MF:translation regulator activity, nucleic acid binding;MF:carbohydrate derivative binding;MF:organic cyclic compound binding;MF:hydrolase activity, acting on acid anhydrides;MF:anion binding;MF:pyrophosphatase activity;MF:purine ribonucleotide binding;MF:translation elongation factor activity;MF:ribonucleotide binding;	GO:0005737;GO:0110165;GO:0005575;GO:0035639;GO:0003674;GO:0005488;GO:0003676;GO:0043167;GO:1901363;GO:1901265;GO:0019001;GO:0000166;GO:0032561;GO:0017076;GO:0005525;GO:0016787;GO:0008135;GO:0003924;GO:0017111;GO:0036094;GO:0003824;GO:0045182;GO:0016818;GO:0090079;GO:0097367;GO:0097159;GO:0016817;GO:0043168;GO:0016462;GO:0032555;GO:0003746;GO:0032553	K03231	EEF1A	map05140;ma	Leishmaniasis;Legionellosis;Nucleocytoplasmic transport	COG5256	S:Function unknown	PF00009.30;PF03143.20;PF03144.28	GTP_EFTU;GTP_EFTU_D3;GTP_EFTU_D2	Elongation factor Tu GTP binding domain;Elongation factor Tu C-terminal domain;Elongation factor Tu domain 2	CYT	3	30	50.6	High
TRINITY_DN18502_c0_g1_i1_orf1	uncharacterized protein LOC114359515 [Ostrinia furnacalis]	0.171817585	-2.541050393	1.68E-05	down	yes	0.5237	3.048	3.119	3.18	2.846	0.497	0.537	0.537	------	------	------	------	------	------	ENOG41117E9	S:Function unknown	------	------	------	CYT	6	15	59.7	High
TRINITY_DN4125_c0_g1_i14_orf1	angiotensin-converting enzyme-like isoform X1 [Ostrinia furnacalis]	0.223740007	-2.160104847	4.82E-05	down	yes	0.6437	2.877	3.012	2.976	2.642	0.641	0.679	0.611	BP:nitrogen compound metabolic process;BP:organic substance metabolic process;BP:proteolysis;BP:biological_process;BP:metabolic process;BP:primary metabolic process;BP:organonitrogen compound metabolic process;BP:protein metabolic process;BP:macromolecule metabolic process;CC:membrane;CC:intrinsic component of membrane;CC:integral component of membrane;CC:cellular anatomical entity;CC:cellular_component;MF:exopeptidase activity;MF:molecular_function;MF:binding;MF:peptidase activity;MF:cation binding;MF:carboxypeptidase activity;MF:ion binding;MF:metallopeptidase activity;MF:metal ion binding;MF:hydrolase activity;MF:peptidyl-dipeptidase activity;MF:catalytic activity, acting on a protein;MF:catalytic activity;	GO:0006807;GO:0071704;GO:0006508;GO:0008150;GO:0008152;GO:0044238;GO:1901564;GO:0019538;GO:0043170;GO:0016020;GO:0031224;GO:0016021;GO:0110165;GO:0005575;GO:0008238;GO:0003674;GO:0005488;GO:0008233;GO:0043169;GO:0004180;GO:0043167;GO:0008237;GO:0046872;GO:0016787;GO:0008241;GO:0140096;GO:0003824	K01283	ACE, CD143	map05142;ma	Chagas disease;Renin-angiotensin system;Renin secretion;Coronavirus disease - COVID-19;Diabetic cardiomyopathy;Hypertrophic cardiomyopathy	ENOG410XPJ3	S:Function unknown	PF01401.21	Peptidase_M2	Angiotensin-converting enzyme	CYT	1	12	74.3	High
TRINITY_DN13973_c0_g1_i6_orf1	27 kDa glycoprotein-like [Ostrinia furnacalis]	0.456730769	-1.13058411	1.82E-05	down	yes	1.235	2.704	2.787	2.592	2.734	1.229	1.204	1.272	------	------	------	------	------	------	ENOG4111PQK	S:Function unknown	PF07165.14	DUF1397	Protein of unknown function	CYT	6	35	29.1	High
TRINITY_DN11970_c0_g1_i4_orf1	myb-like protein AA [Ostrinia furnacalis]	0.287402904	-1.798853453	7.30E-06	down	yes	0.851	2.961	2.887	3.101	2.895	0.841	0.856	0.856	------	------	------	------	------	------	ENOG41117A2	S:Function unknown	------	------	------	CYT	3	5	126.8	High
TRINITY_DN66453_c0_g1_i4_orfp1	TRINITY_DN66453_c0_g1_i4_m.7345 TRINITY_DN66453_c0_g1::TRINITY_DN66453_c0_g1_i4::g.7345 ORF type:internal len:93 (+) score=24.53 TRINITY_DN66453_c0_g1_i4:3-278(+)	2.224926972	1.153757984	4.65E-07	up	yes	2.285	1.027	1.044	1.008	1.029	2.306	2.301	2.249	------	------	------	------	------	------	------	------	------	------	------	CYT	1	21	9.7	High
TRINITY_DN33995_c0_g1_i5_orf1	unnamed protein product [Spodoptera exigua]	3.355987495	1.74673734	3.34E-06	up	yes	2.469	0.7357	0.692	0.781	0.734	2.401	2.468	2.539	CC:cellular_component;CC:integral component of membrane;CC:cellular anatomical entity;CC:intrinsic component of membrane;	GO:0005575;GO:0016021;GO:0110165;GO:0031224	------	------	------	------	ENOG410XPW0	U:Intracellular trafficking, secretion, and vesicular	PF03798.19	TRAM_LAG1_CLN8	TLC domain	CYT	1	4	37.8	High
TRINITY_DN8095_c0_g1_i3_orf1	circadian clock-controlled protein-like [Ostrinia furnacalis]	2.371364653	1.245717528	3.11E-05	up	yes	2.226	0.9387	1.037	0.83	0.949	2.235	2.198	2.245	------	------	------	------	------	------	ENOG41114TE;EN	S:Function unknown;	PF06585.14	JHBP	Haemolymph juvenile hormone binding protein	CYT	4	18	27.1	High

TRINITY_DN147458_c0_g1_i1_orf1	60S ribosomal protein L5, partial [Cotesia chilonis]	0.342266723	-1.54680706	1.09E-05	down	yes	0.8033	2.347	2.323	2.447	2.271	0.821	0.83	0.759	BP:cellular macromolecule biosynthetic process;BP:cellular amide metabolic process;BP:cellular biosynthetic process;BP:organic substance biosynthetic process;BP:cellular nitrogen compound biosynthetic process;BP:cellular macromolecule metabolic process;BP:peptide biosynthetic process;BP:organic substance metabolic process;BP:organonitrogen compound biosynthetic process;BP:macromolecule metabolic process;BP:amide biosynthetic process;BP:cellular process;BP:biosynthetic process;BP:macromolecule biosynthetic process;BP:biological_process;BP:metabolic process;BP:organonitrogen compound metabolic process;BP:primary metabolic process;BP:protein metabolic process;BP:cellular nitrogen compound metabolic process;BP:cellular metabolic process;BP:nitrogen compound metabolic process;BP:peptide metabolic process;BP:translation;CC:intracellular organelle;CC:non-membrane-bounded organelle;CC:membrane-bounded organelle;CC:organelle;CC:cytoplasm;CC:nucleus;CC:cellular anatomical entity;CC:intracellular membrane-bounded organelle;CC:macromolecular complex;CC:ribosome;CC:intracellular non-membrane-bounded organelle;CC:ribonucleoprotein complex;CC:cellular_component;MF:molecular_function;MF:binding;MF:nucleic acid binding;MF:structural molecule activity;MF:heterocyclic compound binding;MF:structural constituent of ribosome;MF:5S rRNA binding;MF:organic cyclic compound binding;MF:rRNA binding;MF:RNA binding;	GO:0034645;GO:0043603;GO:0044249;GO:1901576;GO:0044271;GO:0044260;GO:0043043;GO:0071704;GO:1901566;GO:0043170;GO:0043604;GO:0009987;GO:0009058;GO:0009059;GO:0008150;GO:0008152;GO:1901564;GO:0044238;GO:0019538;GO:0034641;GO:0044237;GO:0006807;GO:0006518;GO:0006412;GO:0043229;GO:0043228;GO:0043227;GO:0043226;GO:0005737;GO:0005634;GO:0110165;GO:0043231;GO:0032991;GO:0005840;GO:0043232;GO:1990904;GO:0005575;GO:0003674;GO:0005488;GO:0003676;GO:0005198;GO:1901363;GO:0003735;GO:0008097;GO:0097159;GO:0019843;GO:0003723	K02932	RP-L5e, RPL5	map03010;ma	Ribosome;Coronavirus disease - COVID-19	COG0256	J:Translation, ribosomal structure and biogenesis	PF17144.7;PF14204.9	Ribosomal_L5e;Ribosomal_L18_c	Ribosomal large subunit proteins 60S L5, and 50S L18;Ribosomal L18 C-terminal region	CYT	1	13	37	High
TRINITY_DN42964_c0_g1_i1_orf1	protein lethal(2)essential for life-like [Galleria mellonella]	0.301034768	-1.731997973	2.82E-05	down	yes	0.7273	2.416	2.447	2.519	2.281	0.729	0.665	0.788	------	------	K09542	CRYAB	map04213;ma	Longevity regulating pathway - multiple species;Protein processing in	ENOG410YERS	O:Posttranslational modification, protein turnover, chaperones	PF00011.24	HSP20	Hsp20/alpha crystallin family	CYT	1	5	23.2	High
TRINITY_DN3255_c0_g1_i1_orf1	uncharacterized protein LOC114351042 [Ostrinia furnacalis]	0.206550761	-2.275431722	1.89E-05	down	yes	0.6653	3.221	3.035	3.37	3.258	0.589	0.664	0.743	------	------	------	------	------	------	ENOG41114RO	S:Function unknown	------	------	------	CYT	1	6	13.9	High
TRINITY_DN206_c0_g1_i11_orf1	A-kinase anchor protein 200-like [Ostrinia furnacalis] >XP_028173114.1 A-kinase anchor protein 200-like [Ostrinia furnacalis] >XP_028173115.1 A-kinase anchor protein 200-like [Ostrinia furnacalis]	0.395658797	-1.337671262	1.56E-05	down	yes	1.039	2.626	2.547	2.697	2.633	1.039	1.12	0.958	------	------	------	------	------	------	ENOG4110RAQ	S:Function unknown	------	------	------	CYT	3	12	67.6	High
TRINITY_DN467_c0_g3_i1_orf1	histone-lysine N-methyltransferase 2B-like, partial [Ostrinia furnacalis]	0.310305823	-1.688237324	6.01E-06	down	yes	0.7407	2.387	2.413	2.42	2.329	0.768	0.798	0.656	MF:molecular_function;MF:structural constituent of cuticle;MF:structural molecule activity;	GO:0003674;GO:0042302;GO:0005198	------	------	------	------	ENOG4111I81	S:Function unknown	PF00379.26	Chitin_bind_4	Insect cuticle protein	CYT	7	19	58.3	High
TRINITY_DN3715_c0_g1_i2_orf1	uncharacterized protein LOC114356437 isoform X1 [Ostrinia furnacalis]	0.247105173	-2.016802882	4.43E-05	down	yes	0.7213	2.919	2.845	3.143	2.77	0.752	0.695	0.717	------	------	------	------	------	------	------	------	PF00041.24	fn3	Fibronectin type III domain	CYT	3	19	19.1	High
TRINITY_DN15755_c0_g1_i1_orf1	cytochrome P450 monooxygenase CYP6AB141 [Ostrinia furnacalis]	2.388675624	1.256210953	2.66E-06	up	yes	2.489	1.042	1.052	1.048	1.025	2.559	2.437	2.47	CC:integral component of membrane;CC:cellular_component;CC:cellular anatomical entity;CC:intrinsic component of membrane;MF:cation binding;MF:monooxygenase activity;MF:heme binding;MF:transition metal ion binding;MF:tetrapyrrole binding;MF:organic cyclic compound binding;MF:oxidoreductase activity, acting on paired donors, with incorporation or reduction of molecular oxygen;MF:ion binding;MF:catalytic activity;MF:molecular_function;MF:binding;MF:iron ion binding;MF:metal ion binding;MF:heterocyclic compound	GO:0016021;GO:0005575;GO:0110165;GO:0031224;GO:0043169;GO:0004497;GO:0020037;GO:0046914;GO:0046906;GO:0097159;GO:0016705;GO:0043167;GO:0003824;GO:0003674;GO:0005488;GO:0005506;GO:0046872;GO:1901363;GO:0016491	K14999	CYP6	------	------	COG2124	Q:Secondary metabolites biosynthesis, transport and catabolism	PF00067.25	p450	Cytochrome P450	CYT	3	28	27.4	High
TRINITY_DN9920_c0_g1_i1_orf1	uncharacterized protein LOC114351526 [Ostrinia furnacalis]	0.474968448	-1.074096417	4.72E-05	down	yes	1.129	2.377	2.37	2.268	2.492	1.136	1.15	1.101	MF:catalytic activity;MF:transferase activity;MF:molecular_function;	GO:0003824;GO:0016740;GO:0003674	K00681	ggt	map00480;ma	Glutathione metabolism;Taurine and hypotaurine metabolism	COG0405	F:Nucleotide transport and metabolism	PF01019.24	G_glu_transpept	Gamma-glutamyltranspeptidase	CYT	5	9	57.9	High
TRINITY_DN311_c0_g1_i8_orfp1	TRINITY_DN311_c0_g1_i8_m.65152 TRINITY_DN311_c0_g1::TRINITY_DN311_c0_g1_i8::g.65152 ORF type:5prime_partial len:138 (+),score=74.30 TRINITY_DN311_c0_g1_i8:2-415(+)	2.104029991	1.073155269	0.0014	up	yes	2.245	1.067	1.036	1.047	1.119	2.518	2.014	2.202	------	------	------	------	------	------	------	------	------	------	------	PLA	1	18	15	High
TRINITY_DN661_c1_g2_i1_orf1	larval/pupal cuticle protein H1C-like [Ostrinia furnacalis]	0.331681124	-1.592131186	2.12E-05	down	yes	0.803	2.421	2.408	2.54	2.314	0.779	0.854	0.776	MF:molecular_function;MF:structural constituent of cuticle;MF:structural molecule activity;	GO:0003674;GO:0042302;GO:0005198	------	------	------	------	ENOG4111EA8;EN	S:Function unknown;	PF00379.26	Chitin_bind_4	Insect cuticle protein	CYT	4	18	25.4	High
TRINITY_DN5074_c0_g1_i7_orf1	zonadhesin-like [Ostrinia furnacalis]	0.436575527	-1.195696836	2.14E-07	down	yes	0.974	2.231	2.21	2.265	2.218	0.972	0.972	0.978	------	------	------	------	------	------	COG4826;ENOG4	O:Posttranslational modification, protein turnover, chaperones;S:Function unknown;S:Function unknown;S:Function unknown;S:Function unknown	PF01826.20	TIL	Trypsin Inhibitor like cysteine rich domain	CYT	4	21	25.8	High
TRINITY_DN113272_c0_g1_i1_orf1	altered inheritance of mitochondria protein 3-like [Ostrinia furnacalis]	0.201397465	-2.31188257	5.47E-05	down	yes	0.6197	3.077	3.34	2.966	2.924	0.621	0.672	0.566	------	------	------	------	------	------	ENOG41113B8	S:Function unknown	------	------	------	PLA	1	5	33	High
TRINITY_DN125521_c0_g2_i1_orf1	seroin transcript 1A2 [Ostrinia nubilalis]	2.745762712	1.457206954	0.0002704	up	yes	2.268	0.826	0.74	0.769	0.969	2.444	2.245	2.116	------	------	------	------	------	------	------	------	------	------	------	CYT	1	35	8.1	High
TRINITY_DN5553_c0_g1_i4_orf1	uncharacterized protein LOC114353828 [Ostrinia furnacalis]	0.478894858	-1.06221915	0.0002178	down	yes	1.248	2.606	2.708	2.464	2.645	1.197	1.4	1.147	BP:positive regulation of ion transmembrane transporter activity;BP:positive regulation of transporter activity;BP:regulation of ion transmembrane transporter activity;BP:regulation of transport;BP:modulation of synaptic transmission;BP:regulation of potassium ion transport;BP:regulation of signaling;BP:positive regulation of transmembrane transport;BP:regulation of ion transmembrane transport;BP:regulation of cell communication;BP:regulation of biological process;BP:positive regulation of potassium ion transport;BP:positive regulation of ion transmembrane transport;BP:rhythmic process;BP:regulation of transporter activity;BP:biological regulation;BP:regulation of transmembrane transport;BP:positive regulation of molecular function;BP:regulation of trans-synaptic signaling;BP:positive regulation of biological process;BP:regulation of molecular function;BP:positive regulation of potassium ion transmembrane transport;BP:multicellular organismal process;BP:positive regulation of transport;BP:regulation of cellular process;BP:positive regulation of ion transport;BP:biological_process;BP:sleep;BP:positive regulation of voltage-gated potassium channel activity;BP:regulation of cation channel activity;BP:regulation of localization;BP:positive regulation of potassium ion transmembrane transporter activity;BP:regulation of metal ion transport;BP:regulation of synaptic transmission, cholinergic;BP:regulation of transmembrane transporter activity;BP:positive regulation of cation transmembrane transport;BP:regulation of cation transmembrane transport;BP:regulation of potassium ion transmembrane transporter activity;BP:regulation of ion transport;BP:positive regulation of cation channel activity;BP:regulation of potassium ion transmembrane transport;BP:positive regulation of cellular process;CC:plasma membrane;CC:intrinsic component of membrane;CC:integral component of membrane;CC:membrane;CC:anchored component of membrane;CC:cellular anatomical entity;CC:cellular_component;	GO:0032414;GO:0032411;GO:0032412;GO:0051049;GO:0050804;GO:0043266;GO:0023051;GO:0034764;GO:0034765;GO:0010646;GO:0050789;GO:0043268;GO:0034767;GO:0048511;GO:0032409;GO:0065007;GO:0034762;GO:0044093;GO:0099177;GO:0048518;GO:0065009;GO:1901381;GO:0032501;GO:0051050;GO:0050794;GO:0043270;GO:0008150;GO:0030431;GO:1903818;GO:2001257;GO:0032879;GO:1901018;GO:0010959;GO:0032222;GO:0022898;GO:1904064;GO:1904062;GO:1901016;GO:0043269;GO:2001259;GO:1901379;GO:0048522;GO:0005886;GO:0031224;GO:0016021;GO:0016020;GO:0031225;GO:0110165;GO:0005575	------	------	------	------	ENOG4111SVR	S:Function unknown	PF17064.8	QVR	Sleepless protein	CYT	1	6	16.5	High
TRINITY_DN4688_c0_g1_i2_orf1	uncharacterized protein LOC114359411 [Ostrinia furnacalis]	0.464831804	-1.105219312	9.33E-06	down	yes	1.216	2.616	2.531	2.678	2.64	1.18	1.258	1.211	------	------	------	------	------	------	ENOG4110SAV	S:Function unknown	------	------	------	CYT	6	10	71.4	High
TRINITY_DN125427_c0_g1_i1_orf1	heat shock protein 19.8 [Chilo suppressalis] >AGM90553.1 HSP19.8 [Chilo suppressalis] >BAE94664.1 small heat shock protein 19.7 [Chilo suppressalis]	0.35922292	-1.477048692	3.46E-06	down	yes	0.9153	2.548	2.54	2.621	2.484	0.881	0.911	0.954	MF:cation binding;MF:structural molecule activity;MF:structural constituent of eye lens;MF:ion binding;MF:molecular_function;MF:binding;MF:metal ion binding;	GO:0043169;GO:0005198;GO:0005212;GO:0043167;GO:0003674;GO:0005488;GO:0046872	K09542	CRYAB	map04213;ma	Longevity regulating pathway - multiple species;Protein processing in	ENOG410YERS	O:Posttranslational modification, protein turnover, chaperones	PF00011.24	HSP20	Hsp20/alpha crystallin family	CYT	3	39	21.2	High
TRINITY_DN13221_c0_g1_i3_orf1	fasciclin-3-like [Ostrinia furnacalis]	0.101792699	-3.296294008	4.89E-09	down	yes	0.3123	3.068	3.076	3.04	3.089	0.31	0.315	0.312	------	------	------	------	------	------	ENOG4111K6M	S:Function unknown	PF08205.15;PF13927.9	C2-set_2;Ig_3	CD80-like C2-set immunoglobulin domain;Immunoglobulin domain	CYT	1	46	19	High
TRINITY_DN2566_c0_g1_i5_orf1	uncharacterized protein LOC114349936 [Ostrinia furnacalis]	0.441501104	-1.17951105	0.0002761	down	yes	1	2.265	2.292	2.344	2.16	0.928	0.893	1.179	------	------	------	------	------	------	ENOG410XZ3K	S:Function unknown	------	------	------	PLA	2	21	12.4	High
TRINITY_DN14009_c0_g1_i1_orf1	proline-rich extensin-like protein EPR1 [Manduca sexta]	0.45054425	-1.150259289	2.85E-05	down	yes	0.952	2.113	2.069	2.113	2.157	1.005	0.856	0.995	------	------	------	------	------	------	ENOG4111PWE	S:Function unknown	PF03103.20	DUF243	Domain of unknown function	CYT	3	12	26.8	High
TRINITY_DN54366_c0_g1_i1_orf1	protein obstructor-E-like [Ostrinia furnacalis]	0.204761905	-2.287980763	4.06E-06	down	yes	0.6493	3.171	3.04	3.29	3.184	0.648	0.65	0.65	CC:cellular_component;CC:extracellular region;CC:cellular anatomical entity;MF:carbohydrate derivative binding;MF:molecular_function;MF:chitin binding;MF:binding;	GO:0005575;GO:0005576;GO:0110165;GO:0097367;GO:0003674;GO:0008061;GO:0005488	------	------	------	------	ENOG4111EK0	S:Function unknown	PF01607.27	CBM_14	Chitin binding Peritrophin-A domain	PLA	6	28	26.9	High
TRINITY_DN97138_c0_g1_i2_orf1	tubulin beta chain-like isoform X2 [Ostrinia furnacalis]	0.499195495	-1.002323181	1.92E-06	down	yes	1.241	2.486	2.491	2.513	2.455	1.201	1.236	1.285	BP:cellular process;BP:biological_process;BP:microtubule-based process;CC:supramolecular fiber;CC:polymeric cytoskeletal fiber;CC:microtubule;CC:supramolecular complex;CC:supramolecular polymer;CC:cellular anatomical entity;CC:cellular_component;MF:structural constituent of cytoskeleton;MF:purine ribonucleoside triphosphate binding;MF:molecular_function;MF:binding;MF:guanyl nucleotide binding;MF:heterocyclic compound binding;MF:nucleoside phosphate binding;MF:anion binding;MF:structural molecule activity;MF:guanyl ribonucleotide binding;MF:purine nucleotide binding;MF:GTP binding;MF:hydrolase activity;MF:nucleotide binding;MF:GTPase activity;MF:nucleoside-triphosphatase activity;MF:small molecule binding;MF:catalytic activity;MF:hydrolase activity, acting on acid anhydrides, in phosphorus-containing anhydrides;MF:ion binding;MF:carbohydrate derivative binding;MF:organic cyclic compound binding;MF:hydrolase activity, acting on acid anhydrides;MF:pyrophosphatase activity;MF:purine ribonucleotide binding;MF:ribonucleotide binding;	GO:0009987;GO:0008150;GO:0007017;GO:0099512;GO:0099513;GO:0005874;GO:0099080;GO:0099081;GO:0110165;GO:0005575;GO:0005200;GO:0035639;GO:0003674;GO:0005488;GO:0019001;GO:1901363;GO:1901265;GO:0043168;GO:0005198;GO:0032561;GO:0017076;GO:0005525;GO:0016787;GO:0000166;GO:0003924;GO:0017111;GO:0036094;GO:0003824;GO:0016818;GO:0043167;GO:0097367;GO:0097159;GO:0016817;GO:0016462;GO:0032555;GO:0032553	K07375	TUBB	map04540;ma	Gap junction;Parkinson disease;Alzheimer disease;Huntington disease;Pathogenic Escherichia coli infection;Amyotrophic lateral sclerosis;Salmonella infection;Phagosome;Pathways of neurodegeneration - multiple diseases;Prion	COG5023	Z:Cytoskeleton	PF00091.28	Tubulin	Tubulin/FtsZ family, GTPase domain	CYT	4	52	31.2	High
TRINITY_DN3952_c0_g1_i3_orf1	protein Skeletor, isoforms D/E-like isoform X1 [Ostrinia furnacalis] >XP_028176405.1 protein Skeletor, isoforms D/E-like isoform X2 [Ostrinia furnacalis] >XP_028176406.1 protein Skeletor, isoforms D/E-like isoform X3 [Ostrinia furnacalis] >XP_028176407.1 protein Skeletor, isoforms D/E-like isoform X4 [Ostrinia furnacalis]	0.397018439	-1.332722083	6.42E-06	down	yes	1.012	2.549	2.638	2.474	2.534	0.99	1.033	1.014	------	------	------	------	------	------	ENOG410XSWZ	U:Intracellular trafficking, secretion, and vesicular transport	PF10517.12;PF03351.20	DM13;DOMON	Electron transfer DM13;DOMON domain	CYT	4	4	162.2	High
TRINITY_DN5770_c0_g1_i4_orf1	rotatin-like [Ostrinia furnacalis]	2.511352419	1.328464496	0.00028	up	yes	2.544	1.013	1.134	1.035	0.87	2.607	2.681	2.344	------	------	------	------	------	------	ENOG4110T3E;EN	S:Function unknown;Q:Secondary metabolites biosynthesis, transport and catabolism;D:Cell cycle control, cell division, chromosome partitioning;U:Intracellular trafficking, secretion, and	------	------	------	CYT	1	1	111.2	Medium
TRINITY_DN14250_c0_g1_i1_orf1	apolipophorins-like [Ostrinia furnacalis]	0.453478261	-1.140894703	9.28E-06	down	yes	1.043	2.3	2.32	2.343	2.237	0.997	1.101	1.031	MF:lipid transporter activity;MF:transporter activity;MF:molecular_function;	GO:0005319;GO:0005215;GO:0003674	------	------	------	------	ENOG41112S0;EN	S:Function unknown;	------	------	------	CYT	2	22	9.7	High
TRINITY_DN28503_c0_g1_i6_orf1	uncharacterized protein LOC114363584 [Ostrinia furnacalis] >AXY94663.1 seroin transcript 3 [Ostrinia nubilalis]	4.49695373	2.16894804	7.76E-05	up	yes	2.731	0.6073	0.656	0.583	0.583	2.717	2.955	2.52	------	------	------	------	------	------	------	------	------	------	------	PLA	5	42	12.8	High
TRINITY_DN31619_c0_g1_i2_orf1	endocuticle structural glycoprotein ABD-4-like [Ostrinia furnacalis]	0.30349076	-1.720275503	0.0005293	down	yes	0.739	2.435	2.45	2.714	2.14	0.757	0.759	0.701	MF:molecular_function;MF:structural constituent of cuticle;MF:structural molecule activity;	GO:0003674;GO:0042302;GO:0005198	------	------	------	------	ENOG4111ZVI	S:Function unknown	PF00379.26	Chitin_bind_4	Insect cuticle protein	CYT	1	7	14.7	High
TRINITY_DN25345_c0_g1_i1_orf1	chromodomain-helicase-DNA-binding protein 1 isoform X3 [Ostrinia furnacalis]	0.31130092	-1.683618255	0.0002376	down	yes	0.7107	2.283	2.244	2.516	2.089	0.697	0.746	0.689	CC:intracellular organelle;CC:membrane-bounded organelle;CC:organelle;CC:nucleus;CC:intracellular membrane-bounded organelle;CC:cellular_component;CC:cellular anatomical entity;MF:purine ribonucleoside triphosphate binding;MF:heterocyclic compound binding;MF:molecular_function;MF:binding;MF:DNA helicase activity;MF:ion binding;MF:nucleoside phosphate binding;MF:anion binding;MF:nucleotide binding;MF:purine nucleotide binding;MF:ATP binding;MF:hydrolase activity;MF:catalytic activity, acting on DNA;MF:helicase activity;MF:small molecule binding;MF:catalytic activity;MF:DNA-dependent ATPase activity;MF:adenyl nucleotide binding;MF:carbohydrate derivative binding;MF:organic cyclic compound binding;MF:adenyl ribonucleotide binding;MF:purine ribonucleotide binding;MF:chromatin binding;MF:ribonucleotide binding;	GO:0043229;GO:0043227;GO:0043226;GO:0005634;GO:0043231;GO:0005575;GO:0110165;GO:0035639;GO:1901363;GO:0003674;GO:0005488;GO:0003678;GO:0043167;GO:1901265;GO:0043168;GO:0000166;GO:0017076;GO:0005524;GO:0016787;GO:0140097;GO:0004386;GO:0036094;GO:0003824;GO:0008094;GO:0030554;GO:0097367;GO:0097159;GO:0032559;GO:0032555;GO:0003682;GO:0032553	K11367	CHD1	------	------	COG0553;ENOG4	U:Intracellular trafficking, secretion, and vesicular transport;L:Replication, recombination and repair	PF00176.26;PF18375.4;PF13907.9;PF00385.27;PF00271.34;PF04851.18	SNF2-rel_dom;CDH1_2_SANT_HL1;DUF4208;Chromo;Helicase_C;ResIII	SNF2-related domain;CDH1/2 SANT-Helical linker 1;Domain of unknown function;Chromo;Helicase conserved C-terminal domain;Type III restriction enzyme, res subunit	CYT	2	1	208.3	High
TRINITY_DN13236_c0_g1_i4_orf1	zonadhesin-like isoform X4 [Ostrinia furnacalis]	3.684402776	1.881430784	4.26E-05	up	yes	3.026	0.8213	0.78	0.868	0.816	2.806	3.167	3.105	------	------	------	------	------	------	COG4826;ENOG4	O:Posttranslational modification, protein turnover, chaperones;S:Function unknown	PF01826.20	TIL	Trypsin Inhibitor like cysteine rich domain	CYT	2	48	10.2	High
TRINITY_DN1226_c0_g1_i11_orfp1	TRINITY_DN1226_c0_g1_i11_m.52385 TRINITY_DN1226_c0_g1::TRINITY_DN1226_c0_g1_i11::g.52385 ORF type:internal len:92 (-),score=5.77 TRINITY_DN1226_c0_g1_i11:2-274(-)	2.155513666	1.10803171	1.89E-05	up	yes	2.287	1.061	1.087	1.058	1.039	2.261	2.383	2.216	------	------	------	------	------	------	------	------	------	------	------	PLA	2	53	9.1	High
TRINITY_DN14532_c0_g1_i1_orf1	pupal cuticle protein-like [Trichoplusia ni]	0.232280431	-2.106060476	3.13E-07	down	yes	0.603	2.596	2.547	2.647	2.595	0.609	0.614	0.586	MF:molecular_function;MF:structural constituent of cuticle;MF:structural molecule activity;	GO:0003674;GO:0042302;GO:0005198	------	------	------	------	ENOG4110QXC;EN	S:Function unknown;	------	------	------	CYT	4	26	25.9	High
TRINITY_DN15327_c2_g1_i2_orf1	protein lethal(2)essential for life-like [Ostrinia furnacalis]	2.950754037	1.561083669	1.93E-06	up	yes	2.211	0.7493	0.765	0.736	0.747	2.215	2.268	2.151	MF:cation binding;MF:structural molecule activity;MF:structural constituent of eye lens;MF:ion binding;MF:molecular_function;MF:binding;MF:metal ion binding;	GO:0043169;GO:0005198;GO:0005212;GO:0043167;GO:0003674;GO:0005488;GO:0046872	K09542	CRYAB	map04213;ma	Longevity regulating pathway - multiple species;Protein processing in	ENOG410YERS	E:Amino acid transport and metabolism	PF00011.24;PF00525.21	HSP20;Crystallin	Hsp20/alpha crystallin family;Alpha crystallin A chain, N terminal	CYT	7	44	22	High
TRINITY_DN10441_c0_g1_i3_orf1	zonadhesin-like [Ostrinia furnacalis]	2.189554201	1.130637163	0.000176	up	yes	1.832	0.8367	0.855	0.858	0.797	1.697	1.859	1.939	------	------	------	------	------	------	COG4826;ENOG4	S:Function unknown;	PF01826.20	TIL	Trypsin Inhibitor like cysteine rich domain	PLA	3	4	119.6	High
TRINITY_DN4123_c0_g1_i1_orf1	uncharacterized protein LOC114355030 [Ostrinia furnacalis]	2.133679123	1.09334323	0.007825	up	yes	1.81	0.8483	0.804	0.748	0.993	1.457	2.048	1.926	------	------	------	------	------	------	------	------	------	------	------	CYT	1	4	15.8	Medium
TRINITY_DN978_c9_g2_i1_orf1	hypothetical protein evm_000959 [Chilo suppressalis]	0.352740385	-1.503321338	2.09E-05	down	yes	0.7337	2.08	2.005	2.175	2.059	0.674	0.763	0.764	------	------	------	------	------	------	ENOG410Z4TG;EN	S:Function unknown;	------	------	------	CYT	1	8	14.8	High
TRINITY_DN34786_c0_g1_i1_orf1	small heat shock protein Hsp29.7 [Ostrinia furnacalis]	0.338751192	-1.561702074	7.32E-06	down	yes	0.7107	2.098	2.081	2.052	2.162	0.739	0.747	0.646	------	------	K09542	CRYAB	map04213;ma	Longevity regulating pathway - multiple species;Protein processing in	ENOG410YERS	O:Posttranslational modification, protein turnover, chaperones	PF00011.24	HSP20	Hsp20/alpha crystallin family	CYT	2	8	28.2	High
TRINITY_DN21170_c0_g1_i5_orf1	twitchin-like [Ostrinia furnacalis]	3.561020036	1.832290553	5.96E-05	up	yes	1.955	0.549	0.512	0.569	0.566	1.801	2.032	2.033	------	------	------	------	------	------	ENOG410XP8U;EN	S:Function unknown;	PF00041.24	fn3	Fibronectin type III domain	CYT	1	52	17	High
TRINITY_DN5848_c0_g1_i6_orf1	brain tumor protein isoform X1 [Ostrinia furnacalis]	0.49295559	-1.020470415	0.006036	down	yes	0.9657	1.959	1.804	2.263	1.811	0.822	0.895	1.18	MF:cation binding;MF:transition metal ion binding;MF:ion binding;MF:molecular_function;MF:binding;MF:metal ion binding;MF:zinc ion binding;	GO:0043169;GO:0046914;GO:0043167;GO:0003674;GO:0005488;GO:0046872;GO:0008270	K11997	TRIM2_3	------	------	ENOG410XQJ4	S:Function unknown	PF01436.24;PF17170.7;PF13445.9;PF00643.27	NHL;DUF5128;zf-RING_UBOX;zf-B_box	NHL repeat;6-bladed beta-propeller;RING-type zinc-finger;B-box zinc finger	CYT	2	2	95.7	High
TRINITY_DN27300_c0_g1_i6_orfp1	TRINITY_DN27300_c0_g1_i6_m.71140 TRINITY_DN27300_c0_g1::TRINITY_DN27300_c0_g1_i6::g.71140 ORF type:internal len:129 (-),score=20.75 TRINITY_DN27300_c0_g1_i6:1-384(-)	3.10364384	1.63396301	2.30E-05	up	yes	2.189	0.7053	0.764	0.718	0.634	2.295	2.149	2.122	------	------	------	------	------	------	------	------	------	------	------	CYT	1	9	14.4	High
TRINITY_DN9560_c0_g1_i5_orf1	uncharacterized protein LOC114357350 [Ostrinia furnacalis]	4.088679245	2.031634889	6.61E-05	up	yes	2.167	0.53	0.629	0.538	0.423	2.071	2.312	2.118	------	------	------	------	------	------	ENOG410XV86;EN	S:Function unknown;	------	------	------	CYT	1	11	27.1	High
TRINITY_DN40434_c0_g1_i2_orf1	deoxyribodipyrimidine photo-lyase [Ostrinia furnacalis]	2.00628719	1.004528135	0.004836	up	yes	1.787	0.8907	0.885	0.825	0.962	1.488	1.998	1.874	BP:nucleic acid metabolic process;BP:cellular nitrogen compound metabolic process;BP:nitrogen compound metabolic process;BP:organic cyclic compound metabolic process;BP:nucleobase-containing compound metabolic process;BP:cellular response to stimulus;BP:cellular macromolecule metabolic process;BP:organic substance metabolic process;BP:DNA repair;BP:cellular process;BP:cellular aromatic compound metabolic process;BP:cellular response to DNA damage stimulus;BP:biological_process;BP:metabolic process;BP:heterocycle metabolic process;BP:primary metabolic process;BP:response to stimulus;BP:response to stress;BP:cellular metabolic process;BP:macromolecule metabolic process;BP:cellular response to stress;BP:DNA metabolic process;MF:nucleic acid binding;MF:molecular_function;MF:binding;MF:DNA photolyase activity;MF:DNA binding;MF:heterocyclic compound binding;MF:lyase activity;MF:catalytic activity, acting on DNA;MF:catalytic activity;MF:deoxyribodipyrimidine photo-lyase activity;MF:organic cyclic compound binding;MF:carbon-carbon lyase activity;	GO:0090304;GO:0034641;GO:0006807;GO:1901360;GO:0006139;GO:0051716;GO:0044260;GO:0071704;GO:0006281;GO:0009987;GO:0006725;GO:0006974;GO:0008150;GO:0008152;GO:0046483;GO:0044238;GO:0050896;GO:0006950;GO:0044237;GO:0043170;GO:0033554;GO:0006259;GO:0003676;GO:0003674;GO:0005488;GO:0003913;GO:0003677;GO:1901363;GO:0016829;GO:0140097;GO:0003824;GO:0003904;GO:0097159;GO:0016830	K01669	phr, PHR1	------	------	COG0415	L:Replication, recombination and repair	PF00875.21	DNA_photolyase	DNA photolyase	CYT	1	1	60.4	Medium
TRINITY_DN22597_c0_g1_i4_orf1	uncharacterized protein LOC114361588 isoform X16 [Ostrinia furnacalis]	2.045851274	1.03270127	0.0004229	up	yes	1.758	0.8593	0.892	0.783	0.903	1.713	1.903	1.658	------	------	K10382	DST	------	------	ENOG410XP41;CC	Z:Cytoskeleton;	PF00435.24	Spectrin	Spectrin repeat	CYT	3	29	12.4	High
TRINITY_DN76633_c0_g1_i1_orfp1	TRINITY_DN76633_c0_g1_i1_m.53394 TRINITY_DN76633_c0_g1::TRINITY_DN76633_c0_g1_i1::g.53394 ORF type:internal len:164 (-),score=26.46,Toxin_2 PF00451.20 0.00022,Toxin_2|PF00451.20|0.00056,Toxin_2|PF00451.20|0.0002,Toxin_2|PF00451.20|5.9e-06,Gamma-thionin|PF00304.21|2,Gamma-thionin|PF00304.21|0.024,Gamma-thionin|PF00304.21|0.027,Gamma-thionin|PF00304.21|0.066,Toxin_38|PF14866.7|0.27,Toxin_38|PF14866.7|0.054,Toxin_38|PF14866.7|0.14,Defensin_2|PF01097.19|1.7,Defensin_2|PF01097.19|0.055,Defensin_2|PF01097.19|1.2,Defensin_2|PF01097.19|0.15 TRINITY_DN76633_c0_g1_i1:1-489(-)	2.156689167	1.108818263	6.79E-05	up	yes	1.949	0.9037	0.899	0.925	0.887	1.93	2.061	1.856	------	------	------	------	------	------	------	------	PF00451.22;PF00304.23	Toxin_2;Gamma-thionin	Scorpion short toxin, BmKK2;Gamma-thionin family	PLA	3	31	19	High
TRINITY_DN804_c0_g1_i7_orf1	hypothetical protein HF086_004695 [Spodoptera exigua] >CAH0695017.1 unnamed protein product [Spodoptera exigua]	2.370552147	1.24522313	3.37E-06	up	yes	1.932	0.815	0.77	0.836	0.839	1.891	1.958	1.947	BP:cellular process;BP:regulation of cellular process;BP:biological_process;BP:signal transduction;BP:biological regulation;BP:intracellular signal transduction;BP:regulation of biological process;MF:molecular_function;MF:enzyme regulator activity;MF:guanyl-nucleotide exchange factor activity;MF:GTPase regulator activity;MF:molecular function regulator;MF:nucleoside-triphosphatase regulator activity;	GO:0009987;GO:0050794;GO:0008150;GO:0007165;GO:0065007;GO:0035556;GO:0050789;GO:0003674;GO:0030234;GO:0005085;GO:0030695;GO:0098772;GO:0060589	K20685	MCF2, ARHGE	------	------	ENOG410XNYV;EN	T:Signal transduction mechanisms;	PF00018.31	SH3_1	SH3 domain	CYT	1	5	12.3	Medium
TRINITY_DN4144_c0_g1_i7_orf1	uncharacterized protein LOC114350172 [Ostrinia furnacalis]	0.419147727	-1.254469288	0.008039	down	yes	0.7377	1.76	2.053	1.377	1.849	0.621	0.801	0.791	CC:cellular_component;CC:integral component of membrane;CC:cellular anatomical entity;CC:intrinsic component of membrane;	GO:0005575;GO:0016021;GO:0110165;GO:0031224	------	------	------	------	ENOG4110KD1	S:Function unknown	PF00100.26	Zona_pellucida	Zona pellucida-like domain	CYT	2	7	42	High
TRINITY_DN2323_c0_g1_i4_orf1	uncharacterized protein LOC114364097 isoform X2 [Ostrinia furnacalis]	0.491593407	-1.024462529	6.08E-05	down	yes	0.8947	1.82	1.75	1.902	1.808	0.843	0.94	0.901	CC:cellular_component;CC:extracellular region;CC:cellular anatomical entity;MF:endopeptidase inhibitor activity;MF:endopeptidase regulator activity;MF:peptidase regulator activity;MF:molecular_function;MF:peptidase inhibitor activity;MF:serine-type endopeptidase inhibitor activity;MF:enzyme inhibitor activity;MF:molecular function regulator;MF:enzyme regulator activity;	GO:0005575;GO:0005576;GO:0110165;GO:0004866;GO:0061135;GO:0061134;GO:0003674;GO:0030414;GO:0004867;GO:0004857;GO:0098772;GO:0030234	------	------	------	------	ENOG410Y6EP	S:Function unknown	PF05375.16	Pacifastin_I	Pacifastin inhibitor	EXC	8	20	53.5	High
TRINITY_DN7735_c1_g1_i1_orf1	cuticular protein CPH [Spodoptera litura]	0.443939394	-1.17156536	0.002264	down	yes	0.9083	2.046	2.284	2.103	1.75	0.821	0.985	0.919	CC:cellular_component;CC:integral component of membrane;CC:cellular anatomical entity;CC:intrinsic component of membrane;	GO:0005575;GO:0016021;GO:0110165;GO:0031224	------	------	------	------	ENOG4110SF2;EN	S:Function unknown;	------	------	------	CYT	1	5	15.2	High
TRINITY_DN72999_c0_g1_i1_orf1	protein obstructor-E-like isoform X1 [Ostrinia furnacalis] >XP_028169319.1 protein obstructor-E-like isoform X2 [Ostrinia furnacalis]	0.272470904	-1.875825915	5.63E-06	down	yes	0.6087	2.234	2.17	2.333	2.2	0.612	0.594	0.62	CC:cellular_component;CC:extracellular region;CC:cellular anatomical entity;MF:carbohydrate derivative binding;MF:molecular_function;MF:chitin binding;MF:binding;	GO:0005575;GO:0005576;GO:0110165;GO:0097367;GO:0003674;GO:0008061;GO:0005488	------	------	------	------	ENOG4111EXD	S:Function unknown	PF01607.27	CBM_14	Chitin binding Peritrophin-A domain	CYT	6	24	32.9	High
TRINITY_DN22513_c0_g1_i4_orf1	DNA-directed RNA polymerase II subunit RPB1 [Ostrinia furnacalis] >XP_028179194.1 DNA-directed RNA polymerase II subunit RPB1 [Ostrinia furnacalis] >XP_028179195.1 DNA-directed RNA polymerase II subunit RPB1 [Ostrinia furnacalis]	2.645120406	1.403333395	0.0006772	up	yes	2.087	0.789	0.784	0.866	0.717	1.829	2.232	2.199	MF:molecular_function;MF:structural constituent of cuticle;MF:structural molecule activity;	GO:0003674;GO:0042302;GO:0005198	------	------	------	------	ENOG4111M49	S:Function unknown	PF00379.26	Chitin_bind_4	Insect cuticle protein	CYT	1	5	40.9	Medium
TRINITY_DN21930_c0_g1_i1_orf1	coactosin-like protein isoform X2 [Trichoplusia ni]	0.482376126	-1.051769588	2.69E-05	down	yes	0.8567	1.776	1.72	1.805	1.804	0.8	0.91	0.86	------	------	------	------	------	------	ENOG4111QQE	S:Function unknown	PF00241.23	Cofilin_ADF	Cofilin/tropomyosin-type actin-binding protein	CYT	1	5	29.8	High
TRINITY_DN1868_c0_g1_i1_orf1	protein obstructor-E isoform X1 [Ostrinia furnacalis]	0.189935662	-2.396417288	3.22E-06	down	yes	0.4133	2.176	2.199	2.244	2.086	0.422	0.396	0.422	------	------	------	------	------	------	ENOG410XQ49	S:Function unknown	PF01607.27	CBM_14	Chitin binding Peritrophin-A domain	CYT	16	68	30.6	High
TRINITY_DN58872_c0_g1_i1_orfp1	cuticle protein 64-like [Pectinophora gossypiella]	0.414285714	-1.271302022	0.0007864	down	yes	0.754	1.82	1.671	2.021	1.769	0.744	0.848	0.67	------	------	------	------	------	------	------	------	------	------	------	CYT	1	9	8.7	High
TRINITY_DN5661_c0_g1_i5_orf1	cytochrome P450 6B7-like [Ostrinia furnacalis]	2.182931401	1.126266794	1.74E-05	up	yes	1.747	0.8003	0.777	0.758	0.866	1.771	1.706	1.764	MF:cation binding;MF:monooxygenase activity;MF:heme binding;MF:transition metal ion binding;MF:tetrapyrrole binding;MF:organic cyclic compound binding;MF:oxidoreductase activity, acting on paired donors, with incorporation or reduction of molecular oxygen;MF:ion binding;MF:catalytic activity;MF:molecular_function;MF:binding;MF:iron ion binding;MF:metal ion binding;MF:heterocyclic compound binding;MF:oxidoreductase activity;	GO:0043169;GO:0004497;GO:0020037;GO:0046914;GO:0046906;GO:0097159;GO:0016705;GO:0043167;GO:0003824;GO:0003674;GO:0005488;GO:0005506;GO:0046872;GO:1901363;GO:0016491	K14999	CYP6	------	------	COG2124	Q:Secondary metabolites biosynthesis, transport and catabolism	PF00067.25	p450	Cytochrome P450	CYT	10	29	60.2	High

TRINITY_DN48590_c0_g1_i1_orf1	acyl-CoA Delta(11) desaturase isoform X1 [Ostrinia furnacalis] >XP_028172999.1 acyl-CoA Delta(11) desaturase isoform X2 [Ostrinia furnacalis] >XP_028173000.1 acyl-CoA Delta(11) desaturase isoform X1 [Ostrinia furnacalis]	0.243857388	-2.035890412	7.22E-06	down	yes	0.5677	2.328	2.331	2.426	2.226	0.584	0.564	0.555	BP:fatty acid biosynthetic process;BP:fatty acid metabolic process;BP:carboxylic acid metabolic process;BP:cellular biosynthetic process;BP:small molecule metabolic process;BP:small molecule biosynthetic process;BP:monocarboxylic acid biosynthetic process;BP:organic substance biosynthetic process;BP:organic substance metabolic process;BP:lipid metabolic process;BP:cellular process;BP:monocarboxylic acid metabolic process;BP:biosynthetic process;BP:biological_process;BP:metabolic process;BP:oxoacid metabolic process;BP:cellular lipid metabolic process;BP:lipid biosynthetic process;BP:primary metabolic process;BP:organic acid metabolic process;BP:carboxylic acid biosynthetic process;BP:organic acid biosynthetic process;BP:cellular metabolic process;CC:intrinsic component of membrane;CC:integral component of membrane;CC:cellular anatomical entity;CC:cellular_component;MF:molecular_function;MF:oxidoreductase activity;MF:oxidoreductase activity, acting on paired donors, with incorporation or reduction of molecular oxygen;MF:catalytic activity;MF:oxidoreductase activity, acting on paired donors, with oxidation of a pair of donors resulting in the reduction of molecular oxygen to two molecules of water;	GO:0006633;GO:0006631;GO:0019752;GO:0044249;GO:0044281;GO:0044283;GO:0072330;GO:1901576;GO:0071704;GO:0006629;GO:0009987;GO:0032787;GO:0009058;GO:0008150;GO:0008152;GO:0043436;GO:0044255;GO:0008610;GO:0044238;GO:0006082;GO:0046394;GO:0016053;GO:0044237;GO:0031224;GO:0016021;GO:0110165;GO:0005575;GO:0003674;GO:0016491;GO:0016705;GO:0003824;GO:0016717	K00507	SCD, desC	map04936;ma	Alcoholic liver disease;AMPK signaling pathway;Biosynthesis of unsaturated fatty acids;PPAR signaling pathway;Longevity regulating pathway - worm	COG1398	J:Translation, ribosomal structure and biogenesis	------	------	------	CYT	4	14	36.9	High
TRINITY_DN9694_c0_g1_i1_orf1	larval cuticle protein LCP-17 [Helicoverpa armigera] >PZC82071.1 hypothetical protein B5X24_HaOG211161 [Helicoverpa armigera] >PZC87412.1 hypothetical protein B5X24_HaOG216859 [Helicoverpa armigera]	2.504923198	1.324766371	3.13E-07	up	yes	1.908	0.7617	0.78	0.735	0.77	1.89	1.927	1.907	MF:molecular_function;MF:structural constituent of cuticle;MF:structural molecule activity;	GO:0003674;GO:0042302;GO:0005198	------	------	------	------	ENOG4110RUZ;EN	S:Function unknown;	PF00379.26	Chitin_bind_4	Insect cuticle protein	CYT	5	82	15.7	High
TRINITY_DN12336_c0_g1_i1_orfp1	TRINITY_DN12336_c0_g1_i1_m.30792 TRINITY_DN12336_c0_g1::TRINITY_DN12336_c0_g1_i1::g.30792 ORF type:internal len:86 (-),score=13.56 TRINITY_DN12336_c0_g1_i1:3-257(-)	2.321307779	1.21493782	4.14E-05	up	yes	2.059	0.887	0.93	0.824	0.907	2.009	2.161	2.007	------	------	------	------	------	------	------	------	------	------	------	CYT	4	51	9.3	High
TRINITY_DN56708_c0_g3_i1_orfp1	TRINITY_DN56708_c0_g3_i1_m.56611 TRINITY_DN56708_c0_g3::TRINITY_DN56708_c0_g3_i1::g.56611 ORF type:internal len:69 (-),score=2.50 TRINITY_DN56708_c0_g3_i1:1-204(-)	2.178599796	1.123401201	0.0002017	up	yes	1.92	0.8813	0.847	0.954	0.843	1.998	1.984	1.778	------	------	------	------	------	------	------	------	------	------	------	CYT	1	18	7.1	High
TRINITY_DN1005_c0_g1_i5_orf1	hypothetical protein evm_008839 [Chilo suppressalis] >CAB3526474.1 unnamed protein product [Chilo suppressalis] >CAH0403802.1 unnamed protein product [Chilo suppressalis]	0.440078918	-1.184165835	5.15E-05	down	yes	0.7807	1.774	1.68	1.785	1.858	0.749	0.799	0.794	BP:nucleic acid metabolic process;BP:cellular nitrogen compound metabolic process;BP:nitrogen compound metabolic process;BP:organic cyclic compound metabolic process;BP:nucleobase-containing compound metabolic process;BP:organic substance metabolic process;BP:primary metabolic process;BP:mRNA 3'-end processing;BP:cellular process;BP:cellular aromatic compound metabolic process;BP:RNA 3'-end processing;BP:biological_process;BP:metabolic process;BP:heterocycle metabolic process;BP:RNA metabolic process;BP:mRNA metabolic process;BP:cellular metabolic process;BP:macromolecule metabolic process;BP:RNA processing;BP:mRNA processing;CC:macromolecular complex;CC:cellular_component;CC:mRNA cleavage factor complex;CC:mRNA cleavage stimulating factor complex;	GO:0090304;GO:0034641;GO:0006807;GO:1901360;GO:0006139;GO:0071704;GO:0044238;GO:0031124;GO:0009987;GO:0006725;GO:0031123;GO:0008150;GO:0008152;GO:0046483;GO:0016070;GO:0016071;GO:0044237;GO:0043170;GO:0006396;GO:0006397;GO:0032991;GO:0005575;GO:0005849;GO:0005848	K14406	CSTF1	map03015	mRNA surveillance pathway	ENOG410XPWJ;EN	A:RNA processing and modification;S:Function unknown	PF00400.35;PF16699.8;PF12894.10;PF20426.1;PF16529.8;PF08662.14	WD40;CSTF1_dimer;ANAPC4_WD40;NBCH_WD40;Ge1_WD40;eIF2A	WD domain, G-beta repeat;Cleavage stimulation factor subunit 1, dimerisation domain;Anaphase-promoting complex subunit 4 WD40 domain;Neurobeachin beta propeller domain;WD40 region of Ge1, enhancer of mRNA-decapping protein;Eukaryotic translation initiation factor eIF2A	CYT	1	6	41.2	High
TRINITY_DN16400_c0_g2_i1_orf1	superoxide dismutase [Cu-Zn]-like isoform X1 [Ostrinia furnacalis]	2.372617659	1.246479633	1.01E-05	up	yes	1.83	0.7713	0.765	0.718	0.831	1.817	1.868	1.804	BP:cellular metabolic process;BP:cellular process;BP:reactive oxygen species metabolic process;BP:superoxide metabolic process;BP:metabolic process;BP:biological_process;MF:oxidoreductase activity, acting on superoxide radicals as acceptor;MF:cation binding;MF:antioxidant activity;MF:ion binding;MF:catalytic activity;MF:molecular_function;MF:binding;MF:superoxide dismutase activity;MF:metal ion binding;MF:oxidoreductase activity;	GO:0044237;GO:0009987;GO:0072593;GO:0006801;GO:0008152;GO:0008150;GO:0016721;GO:0043169;GO:0016209;GO:0043167;GO:0003824;GO:0003674;GO:0005488;GO:0004784;GO:0046872;GO:0016491	K04565	SOD1	map05208;ma	Chemical carcinogenesis - reactive oxygen species;Parkinson disease;Huntington disease;Amyotrophic lateral sclerosis;Longevity regulating pathway - multiple species;Peroxisome;Pathways of neurodegeneration - multiple diseases;Prion	COG2032	Q:Secondary metabolites biosynthesis, transport and catabolism	PF00080.23	Sod_Cu	Copper/zinc superoxide dismutase	CYT	2	10	25.9	High
TRINITY_DN1038_c1_g1_i3_orf1	uncharacterized protein LOC114363583 [Ostrinia furnacalis]	2.052740434	1.037551213	0.0002727	up	yes	1.985	0.967	1.001	0.997	0.903	1.911	2.141	1.902	------	------	------	------	------	------	------	------	------	------	------	CYT	1	11	11	High
TRINITY_DN867_c0_g1_i1_orf1	hemicentin-2-like isoform X1 [Ostrinia furnacalis]	0.440343782	-1.183297802	0.000318	down	yes	0.871	1.978	2.101	1.95	1.884	0.908	0.734	0.971	BP:developmental process;BP:anatomical structure development;BP:biological_process;CC:integral component of membrane;CC:cellular_component;CC:intrinsic component of membrane;CC:cellular anatomical entity;	GO:0032502;GO:0048856;GO:0008150;GO:0016021;GO:0005575;GO:0031224;GO:0110165	K16680	ED	map04391	Hippo signaling pathway - fly	ENOG410ZJSM	S:Function unknown	PF13927.9;PF07679.19;PF13895.9;PF00047.28;PF07686.20;PF08205.15;PF00041.24	Ig_3;I-set;Ig_2;ig;V-set;C2-set_2;fn3	Immunoglobulin domain;Immunoglobulin I-set domain;Immunoglobulin domain;Immunoglobulin domain;Immunoglobulin V-set domain;CD80-like C2-set	CYT	1	1	138.3	High
TRINITY_DN20614_c0_g1_i1_orf1	hypothetical protein evm_003552 [Chilo suppressalis]	0.44249503	-1.176266844	0.01402	down	yes	0.8903	2.012	1.936	1.611	2.49	0.865	0.765	1.041	CC:cytoplasm;CC:cellular_component;CC:cellular anatomical entity;MF:enzyme regulator activity;MF:guanyl-nucleotide exchange factor activity;MF:GTPase regulator activity;MF:molecular_function;MF:molecular function regulator;MF:nucleoside-triphosphatase regulator activity;	GO:0005737;GO:0005575;GO:0110165;GO:0030234;GO:0005085;GO:0030695;GO:0003674;GO:0098772;GO:0060589	K21066	ARHGEF18	map04530	Tight junction	ENOG410XT68;CC	T:Signal transduction mechanisms;	PF00621.23;PF17838.4	RhoGEF;PH_16	RhoGEF domain;PH domain	CYT	2	1	143	High
TRINITY_DN20244_c0_g1_i1_orfp1	uncharacterized protein LOC125235519 [Leguminivora glycinivorella]	0.246378767	-2.021050163	0.001228	down	yes	0.5477	2.223	1.93	2.581	2.157	0.691	0.433	0.519	------	------	------	------	------	------	------	------	------	------	------	CYT	1	7	12.3	High
TRINITY_DN26961_c0_g1_i1_orf1	uncharacterized protein LOC120424957 [Culex pipiens pallens]	3.037821483	1.603037092	0.003818	up	yes	2.008	0.661	0.718	0.642	0.623	1.616	2.383	2.025	CC:sarcomere;CC:myosin complex;CC:macromolecular complex;CC:cellular_component;CC:cellular anatomical entity;MF:purine ribonucleoside triphosphate binding;MF:macromolecular complex binding;MF:binding;MF:ion binding;MF:heterocyclic compound binding;MF:nucleoside phosphate binding;MF:anion binding;MF:nucleotide binding;MF:actin binding;MF:purine nucleotide binding;MF:motor activity;MF:ATP binding;MF:actin filament binding;MF:small molecule binding;MF:cytoskeletal protein binding;MF:carbohydrate derivative binding;MF:organic cyclic compound binding;MF:adenyl nucleotide binding;MF:adenyl ribonucleotide binding;MF:protein binding;MF:purine ribonucleotide binding;MF:molecular_function;MF:ribonucleotide binding;	GO:0030017;GO:0016459;GO:0032991;GO:0005575;GO:0110165;GO:0035639;GO:0044877;GO:0005488;GO:0043167;GO:1901363;GO:1901265;GO:0043168;GO:0000166;GO:0003779;GO:0017076;GO:0003774;GO:0005524;GO:0051015;GO:0036094;GO:0008092;GO:0097367;GO:0097159;GO:0030554;GO:0032559;GO:0005515;GO:0032555;GO:0003674;GO:0032553	K17751	MYH6_7	map04260;ma	Cardiac muscle contraction;Adrenergic signaling in cardiomyocytes;Thyroid hormone signaling pathway;cGMP-PKG signaling pathway;Viral myocarditis;Dilated cardiomyopathy;Hypertrophic cardiomyopathy	COG5022	E:Amino acid transport and metabolism	PF01576.22	Myosin_tail_1	Myosin tail	CYT	5	64	12.3	High
TRINITY_DN2109_c0_g1_i4_orf1	mucin-2-like isoform X2 [Ostrinia furnacalis]	0.353961556	-1.498335416	2.93E-06	down	yes	0.755	2.133	2.072	2.172	2.156	0.717	0.769	0.779	CC:cellular_component;CC:integral component of membrane;CC:cellular anatomical entity;CC:intrinsic component of membrane;	GO:0005575;GO:0016021;GO:0110165;GO:0031224	------	------	------	------	ENOG410Y7ID	S:Function unknown	------	------	------	EXC	3	2	236.3	High
TRINITY_DN19923_c0_g1_i1_orf1	uncharacterized protein LOC114350958 [Ostrinia furnacalis]	0.390276407	-1.357431844	7.38E-05	down	yes	0.7907	2.026	2.055	2.136	1.887	0.801	0.78	0.791	------	------	------	------	------	------	ENOG4111MP7	S:Function unknown	PF15430.9	SVWC	Single domain von Willebrand factor type C	CYT	7	71	16.2	High
TRINITY_DN4068_c0_g2_i4_orf1	larval cuticle protein LCP-17-like precursor [Papilio polytes] >BAM18876.1 cuticular protein PpolCPR2 [Papilio polytes]	3.152822396	1.656643903	9.21E-05	up	yes	2.061	0.6537	0.707	0.695	0.559	1.912	2.147	2.125	MF:molecular_function;MF:structural constituent of cuticle;MF:structural molecule activity;	GO:0003674;GO:0042302;GO:0005198	------	------	------	------	ENOG4110RUZ;EN	S:Function unknown;	PF00379.26	Chitin_bind_4	Insect cuticle protein	CYT	2	56	13.4	High
TRINITY_DN111488_c0_g1_i1_orf1	LOW QUALITY PROTEIN: formin-1-like [Chelonus insularis]	3.009463722	1.589506426	0.00016	up	yes	1.908	0.634	0.694	0.518	0.69	1.809	2.048	1.868	------	------	------	------	------	------	ENOG4111I6P	S:Function unknown	------	------	------	CYT	1	3	22.4	Medium
TRINITY_DN9455_c0_g1_i6_orf1	uncharacterized protein LOC114360866 isoform X4 [Ostrinia furnacalis]	0.361840689	-1.466573447	0.0002049	down	yes	0.6723	1.858	1.878	1.914	1.782	0.602	0.578	0.837	CC:integral component of membrane;CC:cellular_component;CC:extracellular region;CC:intrinsic component of membrane;CC:cellular anatomical entity;MF:enzyme regulator activity;MF:peptidase inhibitor activity;MF:peptidase regulator activity;MF:molecular_function;MF:endopeptidase inhibitor activity;MF:serine-type endopeptidase inhibitor activity;MF:enzyme inhibitor activity;MF:molecular function regulator;MF:endopeptidase regulator activity;	GO:0016021;GO:0005575;GO:0005576;GO:0031224;GO:0110165;GO:0030234;GO:0030414;GO:0061134;GO:0003674;GO:0004866;GO:0004867;GO:0004857;GO:0098772;GO:0061135	------	------	------	------	ENOG410XRYE;EN	S:Function unknown;	PF00086.21;PF00014.26;PF00095.24;PF14625.9;PF02822.17	Thyroglobulin_1;Kunitz_BPTI;WAP;Lustrin_cystein;Antistasin	Thyroglobulin type-1 repeat;Kunitz/Bovine pancreatic trypsin inhibitor domain;WAP-type;Lustrin, cysteine-rich repeated domain;Antistasin family	CYT	1	1	158.2	High
TRINITY_DN7565_c0_g1_i3_orf1	acylphosphatase-2-like [Ostrinia furnacalis]	0.332126697	-1.590194401	0.0004441	down	yes	0.734	2.21	2.064	2.478	2.087	0.794	0.736	0.672	MF:hydrolase activity;MF:hydrolase activity, acting on acid anhydrides, in phosphorus-containing anhydrides;MF:catalytic activity;MF:hydrolase activity, acting on acid anhydrides;MF:pyrophosphatase activity;MF:molecular_function;MF:acylphosphatase activity;	GO:0016787;GO:0016818;GO:0003824;GO:0016817;GO:0016462;GO:0003674;GO:0003998	K01512	acyP	map00620	Pyruvate metabolism	ENOG41126ER	O:Posttranslational modification, protein turnover, chaperones	PF00708.21	Acylphosphatase	Acylphosphatase	CYT	1	9	13.7	High
TRINITY_DN41573_c0_g1_i1_orf1	BRISC and BRCA1-A complex member 2-like [Ostrinia furnacalis]	2.743957606	1.456258192	0.009287	up	yes	2.123	0.7737	0.87	0.826	0.625	1.632	2.147	2.59	BP:regulation of primary metabolic process;BP:regulation of metabolic process;BP:positive regulation of response to stimulus;BP:regulation of response to stimulus;BP:signal transduction;BP:organic cyclic compound metabolic process;BP:cellular response to stimulus;BP:negative regulation of cell cycle;BP:double-strand break repair;BP:biological regulation;BP:positive regulation of DNA metabolic process;BP:regulation of DNA repair;BP:regulation of DNA metabolic process;BP:DNA repair;BP:regulation of macromolecule metabolic process;BP:negative regulation of G2/M transition of mitotic cell cycle;BP:regulation of response to DNA damage stimulus;BP:positive regulation of response to DNA damage stimulus;BP:heterocycle metabolic process;BP:cellular response to stress;BP:positive regulation of metabolic process;BP:nitrogen compound metabolic process;BP:intracellular signal transduction;BP:regulation of biological process;BP:regulation of mitotic cell cycle;BP:cellular macromolecule metabolic process;BP:cellular component organization;BP:cellular component organization or biogenesis;BP:regulation of cellular process;BP:programmed cell death;BP:response to stress;BP:biological_process;BP:metabolic process;BP:negative regulation of cell cycle phase transition;BP:positive regulation of macromolecule metabolic process;BP:regulation of cell cycle phase transition;BP:response to stimulus;BP:regulation of cellular response to stress;BP:positive regulation of DNA repair;BP:response to radiation;BP:cellular nitrogen compound metabolic process;BP:negative regulation of mitotic cell cycle phase transition;BP:regulation of mitotic cell cycle phase transition;BP:mitotic G2/M transition checkpoint;BP:nucleobase-containing compound metabolic process;BP:response to ionizing radiation;BP:regulation of cell cycle process;BP:response to abiotic stimulus;BP:cellular process;BP:cellular aromatic compound metabolic process;BP:cellular response to DNA damage stimulus;BP:positive regulation of biological process;BP:negative regulation of biological process;BP:macromolecule metabolic process;BP:mitotic DNA integrity checkpoint;BP:regulation of response to stress;BP:positive regulation of cellular metabolic process;BP:regulation of cellular metabolic process;BP:mitotic cell cycle process;BP:nucleic acid metabolic process;BP:negative regulation of cell cycle process;BP:mitotic DNA damage checkpoint;BP:cell cycle process;BP:cell death;BP:cell division;BP:chromatin organization;BP:organic substance metabolic process;BP:negative regulation of cell cycle G2/M phase transition;BP:negative regulation of mitotic cell cycle;BP:positive regulation of nucleobase-containing compound metabolic process;BP:regulation of nucleobase-containing compound metabolic process;BP:apoptotic process;BP:regulation of nitrogen compound metabolic process;BP:positive regulation of nitrogen compound metabolic process;BP:regulation of G2/M transition of mitotic cell cycle;BP:signal transduction in response to DNA damage;BP:primary metabolic process;BP:DNA damage checkpoint;BP:DNA integrity checkpoint;BP:cell cycle checkpoint;BP:regulation of cell cycle;BP:cellular metabolic process;BP:regulation of cell cycle G2/M phase transition;BP:DNA metabolic process;BP:mitotic G2 DNA damage checkpoint;BP:positive regulation of cellular	GO:0080090;GO:0019222;GO:0048584;GO:0048583;GO:0007165;GO:1901360;GO:0051716;GO:0045786;GO:0006302;GO:0065007;GO:0051054;GO:0006282;GO:0051052;GO:0006281;GO:0060255;GO:0010972;GO:2001020;GO:2001022;GO:0046483;GO:0033554;GO:0009893;GO:0006807;GO:0035556;GO:0050789;GO:0007346;GO:0044260;GO:0016043;GO:0071840;GO:0050794;GO:0012501;GO:0006950;GO:0008150;GO:0008152;GO:1901988;GO:0010604;GO:1901987;GO:0050896;GO:0080135;GO:0045739;GO:0009314;GO:0034641;GO:1901991;GO:1901990;GO:0044818;GO:0006139;GO:0010212;GO:0010564;GO:0009628;GO:0009987;GO:0006725;GO:0006974;GO:0048518;GO:0048519;GO:0043170;GO:0044774;GO:0080134;GO:0031325;GO:0031323;GO:1903047;GO:0090304;GO:0010948;GO:0044773;GO:0022402;GO:0008219;GO:0051301;GO:0006325;GO:0071704;GO:1902750;GO:0045930;GO:0045935;GO:0019219;GO:0006915;GO:0051171;GO:0051173;GO:0010389;GO:0042770;GO:0044238;GO:0000077;GO:0031570;GO:0000075;GO:0051726;GO:0044237;GO:1902749;GO:0006259;GO:0007095;GO:0048522;GO:0048523;GO:0007093;GO:0070531;GO:0110165;GO:0005737;GO:0032991;GO:0070552;GO:0005575;GO:0031593;GO:0003674;GO:0005488;GO:0140030;GO:0005515	K12173	BRE, BRCC45	map03440	Homologous recombination	ENOG410Y1T7	S:Function unknown	PF06113.15	BRE	Brain and reproductive organ-expressed protein	CYT	1	4	42.2	High
TRINITY_DN14721_c0_g1_i2_orf1	protein masquerade-like isoform X2 [Ostrinia furnacalis]	0.315487572	-1.664344922	0.0002568	down	yes	0.66	2.092	2.27	1.973	2.033	0.603	0.807	0.57	------	------	------	------	------	------	ENOG411279V	S:Function unknown	------	------	------	CYT	1	3	38	High
TRINITY_DN59422_c0_g1_i2_orf1	larval cuticle protein LCP-22-like isoform X2 [Pectinophora gossypiella]	5.529382594	2.467118399	2.36E-06	up	yes	2.23	0.4033	0.393	0.386	0.431	2.21	2.314	2.167	MF:molecular_function;MF:structural constituent of cuticle;MF:structural molecule activity;	GO:0003674;GO:0042302;GO:0005198	------	------	------	------	ENOG4111CO7;EN	S:Function unknown;	PF00379.26	Chitin_bind_4	Insect cuticle protein	CYT	4	38	19.5	High
TRINITY_DN23582_c0_g1_i1_orf1	unnamed protein product [Diatraea saccharalis]	0.410354691	-1.285056649	8.10E-06	down	yes	0.7173	1.748	1.708	1.775	1.762	0.696	0.682	0.774	------	------	------	------	------	------	ENOG4111ASG	S:Function unknown	------	------	------	CYT	1	3	26.5	Medium
TRINITY_DN9820_c0_g1_i1_orf1	endocuticle structural glycoprotein SgAbd-2-like [Ostrinia furnacalis]	4.521525764	2.176809684	3.64E-06	up	yes	2.027	0.4483	0.469	0.404	0.472	1.962	2.094	2.024	MF:molecular_function;MF:structural constituent of cuticle;MF:structural molecule activity;	GO:0003674;GO:0042302;GO:0005198	------	------	------	------	ENOG4111PJ1;EN	S:Function unknown;	PF00379.26	Chitin_bind_4	Insect cuticle protein	CYT	4	56	16	High
TRINITY_DN14922_c0_g3_i2_orf1	probable pseudouridine-5'-phosphatase [Ostrinia furnacalis]	2.165931407	1.114987555	7.09E-05	up	yes	1.522	0.7027	0.665	0.728	0.715	1.465	1.609	1.491	------	------	K17623	PUDP, HDHD	------	------	COG0637	J:Translation, ribosomal structure and biogenesis	PF13419.9;PF00702.29	HAD_2;Hydrolase	Haloacid dehalogenase-like hydrolase;haloacid dehalogenase-like hydrolase	CYT	3	19	25.8	High
TRINITY_DN10057_c0_g2_i1_orf1	cell wall protein DAN4 [Ostrinia furnacalis]	0.277847114	-1.84763684	1.46E-05	down	yes	0.5343	1.923	1.828	1.98	1.96	0.585	0.527	0.491	CC:cellular_component;CC:cellular anatomical entity;CC:extracellular space;MF:endopeptidase inhibitor activity;MF:endopeptidase regulator activity;MF:peptidase regulator activity;MF:molecular_function;MF:peptidase inhibitor activity;MF:serine-type endopeptidase inhibitor activity;MF:enzyme inhibitor activity;MF:molecular function regulator;MF:enzyme regulator activity;	GO:0005575;GO:0110165;GO:0005615;GO:0004866;GO:0061135;GO:0061134;GO:0003674;GO:0030414;GO:0004867;GO:0004857;GO:0098772;GO:0030234	------	------	------	------	COG4826	O:Posttranslational modification, protein turnover, chaperones	PF00079.23	Serpin	Serpin	CYT	1	1	157.8	High
TRINITY_DN650_c0_g1_i3_orf1	chitinase 7 [Glyphodes pyloalis]	0.354676674	-1.495423642	5.20E-06	down	yes	0.6143	1.732	1.759	1.77	1.666	0.631	0.601	0.611	BP:amino sugar catabolic process;BP:nitrogen compound metabolic process;BP:aminoglycan metabolic process;BP:organic substance catabolic process;BP:organic substance metabolic process;BP:chitin metabolic process;BP:chitin catabolic process;BP:biological_process;BP:metabolic process;BP:carbohydrate metabolic process;BP:catabolic process;BP:macromolecule catabolic process;BP:primary metabolic process;BP:organonitrogen compound metabolic process;BP:organonitrogen compound catabolic process;BP:amino sugar metabolic process;BP:carbohydrate derivative catabolic process;BP:carbohydrate derivative metabolic process;BP:macromolecule metabolic process;BP:glucosamine-containing compound catabolic process;BP:glucosamine-containing compound metabolic process;BP:aminoglycan catabolic process;CC:cellular anatomical entity;CC:cellular_component;CC:extracellular region;MF:hydrolase activity, acting on glycosyl bonds;MF:molecular_function;MF:chitin binding;MF:hydrolase activity;MF:catalytic activity;MF:chitinase activity;MF:carbohydrate derivative binding;MF:hydrolase activity, hydrolyzing O-glycosyl compounds;MF:binding;	GO:0046348;GO:0006807;GO:0006022;GO:1901575;GO:0071704;GO:0006030;GO:0006032;GO:0008150;GO:0008152;GO:0005975;GO:0009056;GO:0009057;GO:0044238;GO:1901564;GO:1901565;GO:0006040;GO:1901136;GO:1901135;GO:0043170;GO:1901072;GO:1901071;GO:0006026;GO:0110165;GO:0005575;GO:0005576;GO:0016798;GO:0003674;GO:0008061;GO:0016787;GO:0003824;GO:0004568;GO:0097367;GO:0004553;GO:0005488	K01183	E3.2.1.14	map00520	Amino sugar and nucleotide sugar metabolism	COG3325	S:Function unknown	PF00704.31;PF01607.27	Glyco_hydro_18;CBM_14	Glycosyl hydrolases family 18;Chitin binding Peritrophin-A domain	CYT	7	9	111.3	High
TRINITY_DN38568_c0_g1_i1_orf1	unnamed protein product, partial [Diatraea saccharalis]	2.652439024	1.407319586	0.002235	up	yes	1.74	0.656	0.738	0.646	0.584	1.796	1.965	1.458	------	------	K12567	TTN	map05414;ma	Dilated cardiomyopathy;Hypertrophic cardiomyopathy	ENOG410XQFD;EI	T:Signal transduction mechanisms;S:Function unknown;S:Function unknown;S:Function unknown	PF07679.19;PF13927.9;PF13895.9;PF00047.28	I-set;Ig_3;Ig_2;ig	Immunoglobulin I-set domain;Immunoglobulin domain;Immunoglobulin	CYT	1	4	37.5	High
TRINITY_DN1274_c0_g1_i4_orf1	venom dipeptidyl peptidase 4-like [Ostrinia furnacalis]	0.458887468	-1.123787687	0.002334	down	yes	0.7177	1.564	1.76	1.555	1.378	0.669	0.826	0.658	BP:primary metabolic process;BP:organonitrogen compound metabolic process;BP:protein metabolic process;BP:macromolecule metabolic process;BP:organic substance metabolic process;BP:nitrogen compound metabolic process;BP:biological_process;BP:metabolic process;BP:proteolysis;MF:molecular_function;MF:serine hydrolase activity;MF:hydrolase activity;MF:catalytic activity, acting on a protein;MF:catalytic activity;MF:peptidase activity;MF:serine-type peptidase activity;	GO:0044238;GO:1901564;GO:0019538;GO:0043170;GO:0071704;GO:0006807;GO:0008150;GO:0008152;GO:0006508;GO:0003674;GO:0017171;GO:0016787;GO:0140096;GO:0003824;GO:0008233;GO:0008236	K01278	DPP4, CD26	map04974	Protein digestion and absorption	COG1506	E:Amino acid transport and metabolism	PF00930.24;PF00326.24;PF20434.1	DPPIV_N;Peptidase_S9;BD-FAE	Dipeptidyl peptidase IV;Prolyl oligopeptidase family;BD-FAE	CYT	1	1	83.9	High
TRINITY_DN81181_c0_g1_i6_orfp1	C-type lectin domain family 4 member F [Pieris rapae]	2.025213501	1.018074007	0.0002647	up	yes	1.494	0.7377	0.738	0.807	0.668	1.454	1.439	1.589	MF:molecular_function;MF:carbohydrate binding;MF:binding;	GO:0003674;GO:0030246;GO:0005488	------	------	------	------	------	------	------	------	------	CYT	1	14	9.7	High
TRINITY_DN1267_c0_g2_i10_orf1	secretory phospholipase A2 receptor-like [Ostrinia furnacalis]	2.83853758	1.505147842	2.61E-05	up	yes	1.809	0.6373	0.674	0.588	0.65	1.893	1.803	1.73	MF:molecular_function;MF:carbohydrate binding;MF:binding;	GO:0003674;GO:0030246;GO:0005488	------	------	------	------	ENOG410ZPTE;EN	S:Function unknown;	PF00059.24	Lectin_C	Lectin C-type domain	CYT	2	5	34.1	High
TRINITY_DN10889_c0_g1_i8_orf1	four and a half LIM domains protein 2 isoform X7 [Pectinophora gossypiella]	2.640608683	1.400870521	1.74E-07	up	yes	1.77	0.6703	0.65	0.686	0.675	1.751	1.777	1.782	------	------	------	------	------	------	ENOG410XRFY;EN	S:Function unknown;K:Transcription	PF00412.25	LIM	LIM domain	CYT	19	58	39.3	High
TRINITY_DN6586_c0_g1_i1_orf1	fatty acyl-CoA reductase wat-like isoform X1 [Ostrinia furnacalis]	0.343767786	-1.540493737	0.0001098	down	yes	0.604	1.757	1.738	1.896	1.637	0.59	0.6	0.622	BP:primary metabolic process;BP:lipid metabolic process;BP:fatty acid derivative metabolic process;BP:organic substance metabolic process;BP:biological_process;BP:metabolic process;CC:cellular_component;CC:cellular anatomical entity;CC:integral component of membrane;CC:intrinsic component of membrane;MF:fatty-acyl-CoA reductas;MF:alcohol-forming fatty acyl-CoA reductase activity;MF:catalytic activity;MF:oxidoreductase activity, acting on the aldehyde or oxo group of donors, NAD or NADP as acceptor;MF:oxidoreductase activity, acting on the aldehyde or oxo group of donors;MF:molecular_function;MF:oxidoreductase activity;	GO:0044238;GO:0006629;GO:1901568;GO:0071704;GO:0008150;GO:0008152;GO:0005575;GO:0110165;GO:0016021;GO:0031224;GO:0080019;GO:0102965;GO:0003824;GO:0016620;GO:0016903;GO:0003674;GO:0016491	K13356	FAR	map04146;ma	Peroxisome;Cutin, suberine and wax biosynthesis;Longevity regulating pathway - worm	ENOG410XS7R	O:Posttranslational modification, protein turnover, chaperones	PF07993.15;PF03015.22;PF01370.24	NAD_binding_4;Sterile;Epimerase	Male sterility protein;Male sterility protein;NAD dependent epimerase/dehydratase family	CYT	3	7	71.4	High
TRINITY_DN2722_c0_g1_i1_orf1	troponin C [Pieris rapae] >XP_045490973.1 troponin C-like isoform X1 [Colias croceus] >XP_049866665.1 troponin C-like [Pectinophora gossypiella]	2.455639098	1.296098545	1.55E-06	up	yes	1.633	0.665	0.664	0.666	0.665	1.59	1.661	1.648	MF:cation binding;MF:ion binding;MF:molecular_function;MF:binding;MF:metal ion binding;MF:calcium ion binding;	GO:0043169;GO:0043167;GO:0003674;GO:0005488;GO:0046872;GO:0005509	------	------	------	------	COG5126	S:Function unknown	PF13499.9;PF00036.35;PF13405.9;PF13833.9;PF14658.9;PF13202.9	EF-hand_7;EF-hand_1;EF-hand_6;EF-hand_8;EF-hand_9;EF-hand_5	EF-hand domain pair;EF hand;EF-hand domain;EF-hand domain pair;EF-hand domain;EF hand	CYT	9	58	18.1	High
TRINITY_DN4898_c0_g1_i7_orf1	annulin-like isoform X3 [Ostrinia furnacalis]	0.48204504	-1.052760145	1.32E-05	down	yes	0.792	1.643	1.643	1.696	1.589	0.8	0.806	0.77	BP:primary metabolic process;BP:peptide cross-linking;BP:organonitrogen compound metabolic process;BP:organic substance metabolic process;BP:nitrogen compound metabolic process;BP:macromolecule modification;BP:macromolecule metabolic process;BP:biological_process;BP:protein modification process;BP:metabolic process;BP:protein metabolic process;MF:molecular_function;MF:protein-glutamine gamma-glutamyltransferase activity;MF:catalytic activity, acting on a protein;MF:catalytic activity;MF:transferase activity;MF:transferase activity, transferring acyl groups;MF:transferase activity, transferring amino-acyl groups;	GO:0044238;GO:0018149;GO:1901564;GO:0071704;GO:0006807;GO:0043412;GO:0043170;GO:0008150;GO:0036211;GO:0008152;GO:0019538;GO:0003674;GO:0003810;GO:0140096;GO:0003824;GO:0016740;GO:0016746;GO:0016755	K05619	TGM1	------	------	ENOG410XQEZ	O:Posttranslational modification, protein turnover, chaperones	PF00927.25;PF00868.23;PF01841.22	Transglut_C;Transglut_N;Transglut_core	Transglutaminase family, C-terminal ig like domain;Transglutaminase family;Transglutaminase-like superfamily	CYT	7	9	86.1	High
TRINITY_DN52395_c0_g2_i2_orf1	twitchin isoform X20 [Zerene cesonia]	3.432805928	1.779388298	3.14E-05	up	yes	1.714	0.4993	0.414	0.579	0.505	1.663	1.701	1.777	BP:developmental process;BP:cell differentiation;BP:cellular developmental process;BP:cellular process;BP:biological_process;BP:anatomical structure morphogenesis;	GO:0032502;GO:0030154;GO:0048869;GO:0009987;GO:0008150;GO:0009653	------	------	------	------	ENOG410XP8U;EN	S:Function unknown;	PF07679.19	I-set	Immunoglobulin I-set domain	CYT	1	73	10.1	High

TRINITY_DN4571_c0_g1_i4_orf1	PREDICTED: nuclear factor NF-kappa-B p105 subunit [Microplitis demolitor] >KAG6558391.1 viral ankyrin V1 [Microplitis demolitor]	0.490808824	-1.02676691	0.004032	down	yes	0.801	1.632	1.753	1.779	1.364	0.879	0.774	0.75	BP:developmental process;BP:dephosphorylation;BP:protein dephosphorylation;BP:cell development;BP:nitrogen compound metabolic process;BP:cellular metabolic process;BP:anatomical structure morphogenesis;BP:cellular developmental process;BP:organic substance metabolic process;BP:neuron development;BP:cellular process;BP:macromolecule modification;BP:protein modification process;BP:biological_process;BP:metabolic process;BP:locomotion;BP:primary metabolic process;BP:organonitrogen compound metabolic process;BP:protein metabolic process;BP:anatomical structure development;BP:macromolecule metabolic process;BP:phosphate-containing compound metabolic process;BP:phosphorus metabolic process;MF:phosphatase activity;MF:phosphoric ester hydrolase activity;MF:molecular_function;MF:hydrolase activity;MF:catalytic activity, acting on a protein;MF:hydrolase activity, acting on ester bonds;MF:catalytic activity;MF:protein tyrosine phosphatase activity;MF:phosphoprotein phosphatase activity;	GO:0032502;GO:0016311;GO:0006470;GO:0048468;GO:0006807;GO:0044237;GO:0009653;GO:0048869;GO:0071704;GO:0048666;GO:0009987;GO:0043412;GO:0036211;GO:0008150;GO:0008152;GO:0040011;GO:0044238;GO:1901564;GO:0019538;GO:0048856;GO:0043170;GO:0006796;GO:0006793;GO:0016791;GO:0042578;GO:0003674;GO:0016787;GO:0140096;GO:0016788;GO:0003824;GO:0004725;GO:0004721	K04734	NFKBIA	map05140;ma	Leishmaniasis;Prostate cancer;Chagas disease;IL-17 signaling pathway;Toxoplasmosis;Hepatitis C;Kaposi sarcoma-associated herpesvirus infection;Th17 cell differentiation;Th1 and Th2 cell differentiation;Influenza A;Herpes simplex virus 1 infection;Epstein-Barr virus infection;Viral carcinogenesis;Apoptosis;Human cytomegalovirus infection;Yersinia infection;Legionellosis;Hepatitis B;RIG-I-like receptor signaling pathway;Cytosolic DNA-sensing pathway;Toll-like receptor signaling pathway;NOD-like receptor signaling pathway;Shigellosis;Pathogenic Escherichia coli infection;Toll and Imd signaling pathway;Relaxin	------	------	PF12796.10;PF13637.9;PF13857.9	Ank_2;Ank_4;Ank_5	Ankyrin repeats;Ankyrin repeats;Ankyrin repeats	CYT		1	9	17	High
TRINITY_DN2559_c0_g1_i4_orf1	uricase [Ostrinia furnacalis]	2.088461538	1.062440575	2.05E-05	up	yes	1.629	0.78	0.789	0.749	0.802	1.672	1.65	1.564	BP:cellular catabolic process;BP:cellular nitrogen compound metabolic process;BP:nitrogen compound metabolic process;BP:small molecule metabolic process;BP:small molecule catabolic process;BP:purine-containing compound metabolic process;BP:purine-containing compound catabolic process;BP:organic cyclic compound metabolic process;BP:organic substance catabolic process;BP:urate catabolic process;BP:organic substance metabolic process;BP:organic cyclic compound catabolic process;BP:nucleobase-containing compound metabolic process;BP:cellular process;BP:cellular aromatic compound metabolic process;BP:heterocycle catabolic process;BP:biological_process;BP:metabolic process;BP:catabolic process;BP:nucleobase-containing small molecule metabolic process;BP:heterocycle metabolic process;BP:primary metabolic process;BP:organonitrogen compound metabolic process;BP:cellular nitrogen compound catabolic process;BP:nucleobase metabolic process;BP:cellular metabolic process;BP:aromatic compound catabolic process;BP:purine nucleobase metabolic process;BP:urate metabolic process;BP:organonitrogen compound catabolic process;CC:microbody;CC:membrane-bounded organelle;CC:organelle;CC:peroxisome;CC:cellular anatomical entity;CC:intracellular organelle;CC:intracellular membrane-bounded organelle;CC:cellular_component;MF:molecular_function;MF:oxidoreductase activity;MF:oxidoreductase activity, acting on other nitrogenous compounds as donors;MF:oxidoreductase activity, acting on other nitrogenous compounds as donors, oxygen as acceptor;MF:catalytic activity;MF:urate oxidase activity;	GO:0044248;GO:0034641;GO:0006807;GO:0044281;GO:0044282;GO:0072521;GO:0072523;GO:1901360;GO:1901575;GO:0019628;GO:0071704;GO:1901361;GO:0006139;GO:0009987;GO:0006725;GO:0046700;GO:0008150;GO:0008152;GO:0009056;GO:0055086;GO:0046483;GO:0044238;GO:1901564;GO:0044270;GO:0009112;GO:0044237;GO:0019439;GO:0006144;GO:0046415;GO:1901565;GO:0042579;GO:0043227;GO:0043226;GO:0005777;GO:0110165;GO:0043229;GO:0043231;GO:0005575;GO:0003674;GO:0016491;GO:0016661;GO:0016663;GO:0003824;GO:0004846	K00365	uaZ	map00230;ma	Purine metabolism;Caffeine metabolism	COG3648	Q:Secondary metabolites biosynthesis, transport and catabolism	PF01014.21	Uricase	Uricase	CYT		17	60	38.2	High
TRINITY_DN1329_c0_g1_i5_orf1	neurogenic locus notch homolog protein 3 [Ostrinia furnacalis] >XP_028157678.1 neurogenic locus notch homolog protein 3 [Ostrinia furnacalis]	2.645767076	1.403686057	1.75E-05	up	yes	1.747	0.6603	0.719	0.638	0.624	1.692	1.809	1.741	------	------	------	------	------	------	ENOG410XP6K	L:Replication, recombination and repair	PF12947.10	EGF_3	EGF domain	CYT		13	43	54.1	High
TRINITY_DN11448_c0_g1_i11_orf1	hypothetical protein B5X24_HaOG201808 [Helicoverpa armigera]	2.438841363	1.286195919	1.48E-08	up	yes	1.625	0.6663	0.672	0.656	0.671	1.629	1.629	1.616	------	------	K23353	PDLIM1_2_3_4	------	------	ENOG4111KMK;EI	S:Function unknown;O:Posttranslational modification, protein turnover, chaperones;S:Function unknown	PF15936.8;PF00595.27	DUF4749;PDZ	Domain of unknown function;PDZ domain	CYT		2	54	29.8	High
TRINITY_DN2312_c0_g1_i4_orf1	endoplasmic reticulum-Golgi intermediate compartment protein 3 [Ostrinia furnacalis]	2.140340989	1.097840658	0.0002761	up	yes	1.519	0.7097	0.687	0.722	0.72	1.565	1.604	1.388	CC:organelle membrane;CC:integral component of membrane;CC:membrane;CC:endoplasmic reticulum-Golgi intermediate compartment membrane;CC:cellular_component;CC:intrinsic component of membrane;CC:bounding membrane of organelle;CC:cellular anatomical entity;	GO:0031090;GO:0016021;GO:0016020;GO:0033116;GO:0005575;GO:0031224;GO:0098588;GO:0110165	K20367	ERGIC3, ERV46	------	------	ENOG410XP7X	U:Intracellular trafficking, secretion, and vesicular transport	PF07970.15;PF13850.9	COPIIcoated_ERV;ERGIC_N	Endoplasmic reticulum vesicle transporter;Endoplasmic Reticulum-Golgi Intermediate Compartment	CYT		1	4	43	High
TRINITY_DN7803_c0_g1_i2_orf1	membrane-associated protein Hem [Ostrinia furnacalis]	0.461766449	-1.11476474	1.34E-05	down	yes	0.779	1.687	1.751	1.644	1.667	0.792	0.752	0.793	------	------	K05750	NCKAP1, NAP	map05130;ma	Pathogenic Escherichia coli infection;Regulation of actin cytoskeleton;Salmonella infection	ENOG410XPQI	S:Function unknown	PF09735.12	Nckap1	Nck-associated protein 1	CYT		2	2	129.5	High
TRINITY_DN15865_c0_g1_i1_orf1	carboxylesterase, partial [Ostrinia furnacalis]	0.418118467	-1.258016331	8.37E-06	down	yes	0.72	1.722	1.759	1.733	1.674	0.685	0.765	0.71	MF:hydrolase activity;MF:molecular_function;MF:hydrolase activity, acting on ester bonds;MF:carboxylic ester hydrolase activity;MF:catalytic activity;	GO:0016787;GO:0003674;GO:0016788;GO:0052689;GO:0003824	K01049	ACHE	map00564;ma	Glycerophospholipid metabolism;Cholinergic synapse	COG2272	O:Posttranslational modification, protein turnover, chaperones	PF00135.31;PF20434.1;PF07859.16	COesterase;BD-FAE;Abhydrolase_3	Carboxylesterase family;BD-FAE;alpha/beta hydrolase fold	PLA		4	14	38	High
TRINITY_DN32586_c0_g2_i1_orf1	unnamed protein product [Euphydryas editha]	2.660357518	1.411620139	1.20E-05	up	yes	1.771	0.6657	0.6	0.713	0.684	1.772	1.813	1.729	MF:molecular_function;MF:carbohydrate binding;MF:binding;	GO:0003674;GO:0030246;GO:0005488	------	------	------	------	ENOG410XNMR;E	S:Function unknown;	PF00059.24	Lectin_C	Lectin C-type domain	CYT		4	47	20.5	High
TRINITY_DN7735_c0_g1_i4_orf1	calphotin-like [Ostrinia furnacalis]	7.591911765	2.924463225	0.002154	up	yes	2.065	0.272	0.156	0.339	0.321	2.044	2.505	1.646	------	------	------	------	------	------	ENOG4110RAS;EN	S:Function unknown;	------	------	------	CYT		1	3	22.5	High
TRINITY_DN5568_c0_g2_i2_orf1	carboxypeptidase D isoform X5 [Ostrinia furnacalis]	0.471922111	-1.083379329	0.000169	down	yes	0.7513	1.592	1.708	1.551	1.518	0.718	0.786	0.75	------	------	K01296	CPM	------	------	ENOG410XX0H	S:Function unknown	PF00246.27;PF13620.9	Peptidase_M14;CarboxypepD_reg	Zinc carboxypeptidase;Carboxypeptidase regulatory-like domain	CYT		4	8	53.7	High
TRINITY_DN109931_c0_g1_i1_orf1	hydroxymethylglutaryl-CoA lyase, mitochondrial isoform X1 [Ostrinia furnacalis]	4.820253165	2.26910892	1.68E-05	up	yes	1.904	0.395	0.424	0.379	0.382	1.821	2.021	1.87	MF:hydroxymethylglutaryl-CoA lyase activity;MF:lyase activity;MF:carbon-carbon lyase activity;MF:oxo-acid-lyase activity;MF:catalytic activity;MF:molecular_function;	GO:0004419;GO:0016829;GO:0016830;GO:0016833;GO:0003824;GO:0003674	K01640	HMGCL, hmgL	map04146;ma	Peroxisome;Valine, leucine and isoleucine degradation;Butanoate metabolism	COG0119	E:Amino acid transport and metabolism	------	------	------	CYT		1	15	5.9	High
TRINITY_DN6325_c0_g1_i8_orf1	unnamed protein product [Pieris macdunnoughi]	2.894321767	1.533225318	6.96E-06	up	yes	1.835	0.634	0.657	0.671	0.574	1.861	1.859	1.784	BP:ribonucleoside diphosphate metabolic process;BP:phosphorylation;BP:purine nucleoside triphosphate metabolic process;BP:carboxylic acid metabolic process;BP:cellular nitrogen compound metabolic process;BP:nitrogen compound metabolic process;BP:small molecule metabolic process;BP:cellular aromatic compound metabolic process;BP:purine-containing compound metabolic process;BP:generation of precursor metabolites and energy;BP:pyruvate metabolic process;BP:glycolytic process;BP:organic cyclic compound metabolic process;BP:nucleobase-containing compound metabolic process;BP:organic substance catabolic process;BP:purine nucleoside diphosphate metabolic process;BP:nucleotide phosphorylation;BP:ribonucleotide metabolic process;BP:organic substance metabolic process;BP:ribonucleoside triphosphate metabolic process;BP:nucleoside triphosphate metabolic process;BP:purine ribonucleotide metabolic process;BP:purine ribonucleoside triphosphate metabolic process;BP:ribose phosphate metabolic process;BP:ATP metabolic process;BP:cellular process;BP:ADP metabolic process;BP:monocarboxylic acid metabolic process;BP:biological_process;BP:metabolic process;BP:oxoacid metabolic process;BP:carbohydrate metabolic process;BP:nucleoside diphosphate metabolic process;BP:purine ribonucleoside diphosphate metabolic process;BP:nucleobase-containing small molecule metabolic process;BP:heterocycle metabolic process;BP:primary metabolic process;BP:organonitrogen compound metabolic process;BP:organic acid metabolic process;BP:carbohydrate derivative metabolic process;BP:carbohydrate catabolic process;BP:cellular metabolic process;BP:purine nucleotide metabolic process;BP:phosphate-containing compound metabolic process;BP:nucleoside diphosphate phosphorylation;BP:phosphorus metabolic process;BP:organophosphate metabolic process;BP:nucleotide metabolic process;BP:nucleoside phosphate metabolic process;BP:catabolic process;BP:ATP generation from ADP;MF:molecular_function;MF:lyase activity;MF:carbon-carbon lyase activity;MF:fructose-bisphosphate aldolase	GO:0009185;GO:0016310;GO:0009144;GO:0019752;GO:0034641;GO:0006807;GO:0044281;GO:0006725;GO:0072521;GO:0006091;GO:0006090;GO:0006096;GO:1901360;GO:0006139;GO:1901575;GO:0009135;GO:0046939;GO:0009259;GO:0071704;GO:0009199;GO:0009141;GO:0009150;GO:0009205;GO:0019693;GO:0046034;GO:0009987;GO:0046031;GO:0032787;GO:0008150;GO:0008152;GO:0043436;GO:0005975;GO:0009132;GO:0009179;GO:0055086;GO:0046483;GO:0044238;GO:1901564;GO:0006082;GO:1901135;GO:0016052;GO:0044237;GO:0006163;GO:0006796;GO:0006165;GO:0006793;GO:0019637;GO:0009117;GO:0006753;GO:0009056;GO:0006757;GO:0003674;GO:0016829;GO:0016830;GO:0004332;GO:0016832;GO:0003824	K01623	ALDO	map00010;ma	Glycolysis / Gluconeogenesis;Pentose phosphate pathway;Fructose and mannose metabolism;HIF-1 signaling pathway	COG3588	G:Carbohydrate transport and metabolism	PF00274.22	Glycolytic	Fructose-bisphosphate aldolase class-I	CYT		1	75	42.1	High
TRINITY_DN76529_c0_g1_i1_orfp1	TRINITY_DN76529_c0_g1_i1_m.64079 TRINITY_DN76529_c0_g1::TRINITY_DN76529_c0_g1_i1::g.64079 ORF type:internal len:70 (+) score=14.68 TRINITY_DN76529_c0_g1_i1:3-209(+)	3.739130435	1.902702799	3.65E-05	up	yes	1.806	0.483	0.499	0.469	0.481	1.677	1.889	1.852	------	------	------	------	------	------	------	------	------	------	------	CYT		1	23	7.7	High
TRINITY_DN5954_c0_g1_i2_orf1	myosin-VIIa [Ostrinia furnacalis] >XP_028155907.1 myosin-VIIa [Ostrinia furnacalis]	0.468405276	-1.094170769	0.0001984	down	yes	0.7813	1.668	1.789	1.561	1.653	0.785	0.805	0.754	BP:cell differentiation;BP:anatomical structure morphogenesis;BP:cellular developmental process;BP:developmental process;BP:animal organ morphogenesis;BP:neuron differentiation;BP:cellular process;BP:biological_process;CC:cell periphery;CC:cytoplasm;CC:myosin complex;CC:cellular anatomical entity;CC:plasma membrane bounded cell projection;CC:cell projection;CC:macromolecular complex;CC:cellular_component;MF:purine ribonucleoside triphosphate binding;MF:molecular_function;MF:binding;MF:heterocyclic compound binding;MF:nucleoside phosphate binding;MF:nucleotide binding;MF:actin binding;MF:ion binding;MF:purine nucleotide binding;MF:motor activity;MF:anion binding;MF:small molecule binding;MF:cytoskeletal protein binding;MF:carbohydrate derivative binding;MF:organic cyclic compound binding;MF:adenyl nucleotide binding;MF:adenyl ribonucleotide binding;MF:ATP binding;MF:protein binding;MF:purine ribonucleotide binding;MF:ribonucleotide binding;	GO:0030154;GO:0009653;GO:0048869;GO:0032502;GO:0009887;GO:0030182;GO:0009987;GO:0008150;GO:0071944;GO:0005737;GO:0016459;GO:0110165;GO:0120025;GO:0042995;GO:0032991;GO:0005575;GO:0035639;GO:0003674;GO:0005488;GO:1901363;GO:1901265;GO:0000166;GO:0003779;GO:0043167;GO:0017076;GO:0003774;GO:0043168;GO:0036094;GO:0008092;GO:0097367;GO:0097159;GO:0030554;GO:0032559;GO:0005524;GO:0005515;GO:0032555;GO:0032553	K10359	MYO7A, USH	------	------	COG5022	J:Translation, ribosomal structure and biogenesis	PF00063.24;PF00784.20;PF00373.21;PF00612.30	Myosin_head;MyTH4;FERM_M;IQ	Myosin head;MyTH4 domain;FERM central domain;IQ calmodulin-binding motif	CYT		5	2	249.6	High
TRINITY_DN195_c8_g1_i1_orf1	hypothetical protein evm_009768 [Chilo suppressalis]	2.618590217	1.38879031	0.002632	up	yes	1.772	0.6767	0.654	0.6	0.776	1.532	2.064	1.719	BP:interspecies interaction between organisms;BP:response to biotic stimulus;BP:response to external stimulus;BP:response to external biotic stimulus;BP:response to stimulus;BP:response to bacterium;BP:defense response;BP:response to stress;BP:defense response to other organism;BP:defense response to bacterium;BP:biological_process;BP:response to other organism;CC:cellular_component;CC:extracellular region;CC:cellular anatomical entity;	GO:0044419;GO:0009607;GO:0009605;GO:0043207;GO:0050896;GO:0009617;GO:0006952;GO:0006950;GO:0098542;GO:0042742;GO:0008150;GO:0051707;GO:0005575;GO:0005576;GO:0110165	------	------	------	------	ENOG410ZBQ8	S:Function unknown	PF03769.18	Attacin_C	Attacin, C-terminal region	CYT		1	4	21.6	Medium
TRINITY_DN46372_c0_g1_i1_orf1	unnamed protein product [Chilo suppressalis]	2.551765232	1.351495604	1.74E-05	up	yes	1.713	0.6713	0.671	0.64	0.703	1.651	1.702	1.785	MF:molecular_function;MF:structural constituent of cuticle;MF:structural molecule activity;	GO:0003674;GO:0042302;GO:0005198	------	------	------	------	ENOG4112CT1	S:Function unknown	PF00379.26	Chitin_bind_4	Insect cuticle protein	CYT		1	9	18.1	High
TRINITY_DN8310_c0_g2_i1_orf1	uncharacterized protein LOC116773294 [Danaus plexippus plexippus] >OWR55545.1 hypothetical protein KGM_209260 [Danaus plexippus plexippus]	2.147335423	1.102547564	0.0003907	up	yes	1.37	0.638	0.571	0.677	0.666	1.406	1.446	1.257	------	------	------	------	------	------	ENOG4110R2S;CC	S:Function unknown;	PF08246.15	Inhibitor_I29	Cathepsin propeptide inhibitor domain	CYT		3	46	9.3	High
TRINITY_DN5211_c0_g1_i1_orf1	elongation of very long chain fatty acids protein AAEL008004-like [Ostrinia furnacalis]	0.475121612	-1.073631262	0.0003596	down	yes	0.6837	1.439	1.515	1.308	1.495	0.663	0.678	0.71	BP:fatty acid biosynthetic process;BP:fatty acid metabolic process;BP:carboxylic acid metabolic process;BP:cellular biosynthetic process;BP:small molecule metabolic process;BP:small molecule biosynthetic process;BP:monocarboxylic acid biosynthetic process;BP:organic substance biosynthetic process;BP:organic substance metabolic process;BP:lipid metabolic process;BP:cellular process;BP:monocarboxylic acid metabolic process;BP:biosynthetic process;BP:biological_process;BP:metabolic process;BP:oxoacid metabolic process;BP:cellular lipid metabolic process;BP:lipid biosynthetic process;BP:primary metabolic process;BP:organic acid metabolic process;BP:carboxylic acid biosynthetic process;BP:organic acid biosynthetic process;BP:cellular metabolic process;CC:intrinsic component of membrane;CC:integral component of membrane;CC:cellular anatomical entity;CC:cellular_component;MF:molecular_function;MF:fatty acid elongase activity;MF:transferase activity;MF:transferase activity, transferring acyl groups;MF:transferase activity, transferring acyl groups other than amino-acyl groups;MF:very-long-chain 3-ketoacyl-CoA synthase activity;MF:catalytic activity;MF:fatty acid synthase activity;	GO:0006633;GO:0006631;GO:0019752;GO:0044249;GO:0044281;GO:0044283;GO:0072330;GO:1901576;GO:0071704;GO:0006629;GO:0009987;GO:0032787;GO:0009058;GO:0008150;GO:0008152;GO:0043436;GO:0044255;GO:0008610;GO:0044238;GO:0006082;GO:0046394;GO:0016053;GO:0044237;GO:0031224;GO:0016021;GO:0110165;GO:0005575;GO:0003674;GO:0009922;GO:0016740;GO:0016746;GO:0016747;GO:0102756;GO:0003824;GO:0004312	K10250	ELOVL7	map01040;ma	Biosynthesis of unsaturated fatty acids;Fatty acid elongation	ENOG410XRWT	I:Lipid transport and metabolism	PF01151.21	ELO	GNS1/SUR4 family	PLA		1	4	32.2	High
TRINITY_DN5628_c0_g1_i5_orf1	hypothetical protein O3G_MSEX015036 [Manduca sexta]	2.436644105	1.284895547	2.15E-05	up	yes	1.548	0.6353	0.663	0.629	0.614	1.541	1.616	1.488	MF:cation binding;MF:metal ion binding;MF:molecular_function;MF:binding;MF:ion binding;	GO:0043169;GO:0046872;GO:0003674;GO:0005488;GO:0043167	------	------	------	------	ENOG410Z840;EN	S:Function unknown;O:Posttranslational modification, protein turnover, chaperones	PF00412.25	LIM	LIM domain	CYT		8	55	36.3	High
TRINITY_DN1212_c0_g1_i8_orf1	extensin isoform X5 [Ostrinia furnacalis] >XP_028175473.1 extensin isoform X5 [Ostrinia furnacalis] >XP_028175474.1 extensin isoform X5 [Ostrinia furnacalis]	3.246322768	1.698806448	6.74E-06	up	yes	1.567	0.4827	0.477	0.462	0.509	1.515	1.627	1.56	------	------	------	------	------	------	ENOG4111WKY;EI	S:Function unknown;	PF15936.8	DUF4749	Domain of unknown function	CYT		7	26	44.1	High
TRINITY_DN73224_c0_g4_i2_orf1	PREDICTED: poly(rC)-binding protein 3 isoform X2 [Vollenhovia emeryi]	0.479492916	-1.060418595	0.0004454	down	yes	0.643	1.341	1.327	1.445	1.251	0.691	0.578	0.66	MF:organic cyclic compound binding;MF:molecular_function;MF:binding;MF:nucleic acid binding;MF:heterocyclic compound binding;MF:RNA binding;	GO:0097159;GO:0003674;GO:0005488;GO:0003676;GO:1901363;GO:0003723	K21444	PCBP3_4	------	------	ENOG410XNN8	S:Function unknown	PF00013.32;PF07650.20;PF13083.9;PF16005.8	KH_1;KH_2;KH_4;MOEP19	KH domain;KH domain;KH domain;KH-like RNA-binding domain	CYT		2	5	57.5	High
TRINITY_DN3913_c0_g1_i6_orf1	protein obstructor-E-like [Ostrinia furnacalis]	2.640985598	1.401076434	8.13E-06	up	yes	1.522	0.5763	0.58	0.561	0.588	1.506	1.582	1.477	CC:cellular_component;CC:extracellular region;CC:cellular anatomical entity;MF:carbohydrate derivative binding;MF:molecular_function;MF:chitin binding;MF:binding;	GO:0005575;GO:0005576;GO:0110165;GO:0097367;GO:0003674;GO:0008061;GO:0005488	------	------	------	------	ENOG4111HJZ	S:Function unknown	PF01607.27	CBM_14	Chitin binding Peritrophin-A domain	CYT		9	46	33.8	High
TRINITY_DN5235_c0_g1_i7_orf1	peptidoglycan-recognition protein SA-like [Ostrinia furnacalis]	0.46212976	-1.113630096	8.20E-06	down	yes	0.6553	1.418	1.399	1.469	1.385	0.655	0.66	0.651	BP:glycosaminoglycan metabolic process;BP:nitrogen compound metabolic process;BP:peptidoglycan catabolic process;BP:macromolecule metabolic process;BP:organic substance catabolic process;BP:response to external biotic stimulus;BP:catabolic process;BP:interspecies interaction between organisms;BP:organic substance metabolic process;BP:carbohydrate derivative metabolic process;BP:innate immune response;BP:peptidoglycan metabolic process;BP:defense response;BP:response to stress;BP:biological_process;BP:metabolic process;BP:immune response;BP:organonitrogen compound metabolic process;BP:response to other organism;BP:macromolecule catabolic process;BP:response to biotic stimulus;BP:organonitrogen compound catabolic process;BP:response to external stimulus;BP:carbohydrate derivative catabolic process;BP:response to stimulus;BP:glycosaminoglycan catabolic process;BP:immune system process;BP:aminoglycan metabolic process;BP:defense response to other organism;BP:aminoglycan catabolic process;MF:peptidoglycan binding;MF:N-acetylmuramoyl-L-alanine amidase activity;MF:molecular_function;MF:binding;MF:glycosaminoglycan binding;MF:ion binding;MF:metal ion binding;MF:hydrolase activity;MF:cation binding;MF:transition metal ion binding;MF:catalytic activity;MF:peptidoglycan muralytic activity;MF:carbohydrate derivative binding;MF:hydrolase activity, acting on carbon-nitrogen;MF:hydrolase activity, acting on carbon-nitrogen;MF:zinc ion binding;	GO:0030203;GO:0006807;GO:0009253;GO:0043170;GO:1901575;GO:0043207;GO:0009056;GO:0044419;GO:0071704;GO:1901135;GO:0045087;GO:0002252;GO:0006952;GO:0006950;GO:0008150;GO:0008152;GO:0006955;GO:1901564;GO:0051707;GO:0009057;GO:0009607;GO:1901565;GO:0009605;GO:1901136;GO:0050896;GO:0006027;GO:0002376;GO:0006022;GO:0098542;GO:0006026;GO:0042834;GO:0008745;GO:0003674;GO:0005488;GO:0005539;GO:0043167;GO:0046872;GO:0016787;GO:0043169;GO:0046914;GO:0003824;GO:0061783;GO:0097367;GO:0016811;GO:0016810;GO:0008270	K24096;K014	PGRP-SB;PGF	map04624	Toll and Imd signaling pathway	ENOG4111PAY;EN	O:Posttranslational modification, protein turnover, chaperones;M:Cell wall/membrane/envelope biogenesis;M:Cell wall/membrane/envelope biogenesis	PF01510.28	Amidase_2	N-acetylmuramoyl-L-alanine amidase	CYT		2	16	20.7	High
TRINITY_DN17657_c0_g1_i1_orf1	alcohol dehydrogenase 18, partial [Helicoverpa assulta]	2.289267619	1.194886126	3.07E-05	up	yes	1.491	0.6513	0.663	0.66	0.631	1.568	1.443	1.463	MF:catalytic activity;MF:molecular_function;MF:oxidoreductase activity;	GO:0003824;GO:0003674;GO:0016491	K00069	HPGD	map00590;ma	Arachidonic acid metabolism;Transcriptional misregulation in cancer	COG1028	I:Lipid transport and metabolism	------	------	------	CYT		2	61	5.7	High
TRINITY_DN29969_c0_g1_i5_orf1	twitchin-like [Ostrinia furnacalis]	3.241529622	1.696674757	0.007876	up	yes	1.636	0.5047	0.461	0.478	0.575	1.302	2.069	1.538	------	------	------	------	------	------	ENOG410XQFD;EI	S:Function unknown;	PF00041.24	fn3	Fibronectin type III domain	CYT		1	38	16.3	High
TRINITY_DN73923_c0_g1_i1_orf1	protein obstructor-E-like [Ostrinia furnacalis]	0.146834061	-2.767741426	2.52E-07	down	yes	0.269	1.832	1.849	1.855	1.793	0.257	0.26	0.29	CC:cellular_component;CC:extracellular region;CC:cellular anatomical entity;MF:carbohydrate derivative binding;MF:molecular_function;MF:chitin binding;MF:binding;	GO:0005575;GO:0005576;GO:0110165;GO:0097367;GO:0003674;GO:0008061;GO:0005488	------	------	------	------	ENOG410ZSCN	S:Function unknown	PF01607.27	CBM_14	Chitin binding Peritrophin-A domain	CYT		15	65	26.5	High
TRINITY_DN286_c0_g1_i2_orf1	uncharacterized protein LOC114361329 [Ostrinia furnacalis]	0.436652542	-1.195442355	7.07E-05	down	yes	0.6183	1.416	1.418	1.452	1.378	0.579	0.574	0.702	MF:actin binding;MF:molecular_function;MF:binding;MF:protein binding;MF:cytoskeletal protein binding;	GO:0003779;GO:0003674;GO:0005488;GO:0005515;GO:0008092	K17914	KIF13	------	------	ENOG41102XR	S:Function unknown	------	------	------	CYT		4	3	150.3	High
TRINITY_DN2840_c0_g1_i5_orf1	hypothetical protein evm_002181 [Chilo suppressalis]	0.314156432	-1.670444978	2.60E-06	down	yes	0.486	1.547	1.553	1.578	1.509	0.474	0.462	0.522	------	------	------	------	------	------	ENOG41129M9	S:Function unknown	------	------	------	PLA		3	23	16.5	High
TRINITY_DN35633_c0_g2_i1_orf1	uncharacterized protein LOC114353024 [Ostrinia furnacalis]	0.463335631	-1.109870465	6.87E-06	down	yes	0.6723	1.451	1.419	1.478	1.456	0.644	0.665	0.708	------	------	------	------	------	------	ENOG410YUHN	S:Function unknown	------	------	------	CYT		4	12	45.2	High
TRINITY_DN57856_c0_g2_i1_orf1	cytochrome P450 6B2-like [Ostrinia furnacalis]	2.089067574	1.062859159	0.0001984	up	yes	1.351	0.6467	0.676	0.588	0.676	1.323	1.291	1.44	MF:cation binding;MF:monooxygenase activity;MF:heme binding;MF:transition metal ion binding;MF:tetrapyrrole binding;MF:organic cyclic compound binding;MF:oxidoreductase activity, acting on paired donors, with incorporation or reduction of molecular oxygen;MF:ion binding;MF:catalytic activity;MF:molecular_function;MF:binding;MF:iron ion binding;MF:metal ion binding;MF:heterocyclic compound binding;MF:oxidoreductase activity;	GO:0043169;GO:0004497;GO:0020037;GO:0046914;GO:0046906;GO:0097159;GO:0016705;GO:0043167;GO:0003824;GO:0003674;GO:0005488;GO:0005506;GO:0046872;GO:1901363;GO:0016491	K14999	CYP6	------	------	COG2124	G:Carbohydrate transport and metabolism	PF00067.25	p450	Cytochrome P450	CYT		2	38	7.2	High
TRINITY_DN8651_c0_g1_i18_orf1	glutathione S-transferase theta 2 [Conogethes punctiferalis]	2.54214876	1.346048456	1.85E-06	up	yes	1.538	0.605	0.594	0.602	0.619	1.497	1.562	1.556	CC:cytoplasm;CC:cellular_component;CC:cellular anatomical entity;MF:glutathione transferase activity;MF:catalytic activity;MF:transferase activity, transferring alkyl or ary;MF:transferase activity;MF:molecular_function;	GO:0005737;GO:0005575;GO:0110165;GO:0004364;GO:0003824;GO:0016765;GO:0016740;GO:0003674	K00799	GST, gst	map05207;ma	Chemical carcinogenesis - receptor activation;Glutathione metabolism;Chemical carcinogenesis - DNA adducts;Drug metabolism - other enzymes;Drug metabolism - cytochrome P450;Metabolism of xenobiotics by cytochrome P450;Pathways in cancer;Chemical carcinogenesis - reactive oxygen species;Fluid shear stress and atherosclerosis;Hepatocellular carcinoma;Longevity regulating pathway - worm;Platinum drug	COG0625	O:Posttranslational modification, protein turnover, chaperones	PF00043.28;PF14497.9	GST_C;GST_C_3	Glutathione S-transferase, C-terminal domain;Glutathione S-transferase, C-terminal domain	CYT		2	39	16.8	High
TRINITY_DN122393_c0_g1_i1_orf1	microtubule-associated protein futsch isoform X4 [Ostrinia furnacalis] >XP_028162562.1 microtubule-associated protein futsch isoform X4 [Ostrinia furnacalis]	2.239169236	1.162963571	0.0008001	up	yes	1.38	0.6163	0.535	0.732	0.582	1.381	1.481	1.277	------	------	------	------	------	------	------	------	------	------	------	PLA		2	29	9.5	High
TRINITY_DN99063_c0_g1_i1_orf1	microtubule-associated protein futsch isoform X4 [Ostrinia furnacalis] >XP_028162562.1 microtubule-associated protein futsch isoform X4 [Ostrinia furnacalis]	2.097101449	1.068396655	0.0001476	up	yes	1.447	0.69	0.771	0.682	0.617	1.505	1.429	1.406	------	------	------	------	------	------	ENOG410Y4AT	S:Function unknown	------	------	------	PLA		1	18	9.1	High
TRINITY_DN10479_c0_g1_i6_orf1	unnamed protein product [Chrysodeixis includens]	0.381824417	-1.389018731	8.27E-05	down	yes	0.5567	1.458	1.45	1.369	1.554	0.533	0.58	0.557	------	------	------	------	------	------	ENOG4111SOM;E	S:Function unknown;	------	------	------	CYT		1	4	26.6	High

TRINITY_DN920_c0_g1_i4_orf1	glutathione S-transferase omega 2 [Ostrinia furnacalis]	2.404884923	1.26596786	0.0001117	up	yes	1.536	0.6387	0.711	0.598	0.607	1.576	1.442	1.589	BP:metabolic process;BP:biological_process;BP:organic substance metabolic process;CC:cytoplasm;CC:cellular_component;CC:cellular anatomical entity;MF:glutathione dehydrogenas;MF:glutathione transferase activity;MF:catalytic activity;MF:antioxidant activity;MF:oxidoreductase activity, acting on a sulfur group of donors, quinone or similar compound as acceptor;MF:glutathione disulfide oxidoreductase activity;MF:transferase activity;MF:molecular_function;MF:oxidoreductase activity, acting on a sulfur group of donors;MF:transferase activity, transferring alkyl or ary;MF:oxidoreductase activity;MF:disulfide oxidoreductase activity;	GO:0008152;GO:0008150;GO:0071704;GO:0005737;GO:0005575;GO:0110165;GO:0045174;GO:0004364;GO:0003824;GO:0016209;GO:0016672;GO:0015038;GO:0016740;GO:0003674;GO:0016667;GO:0016765;GO:0016491;GO:0015036	K00310	SE	map00480;ma	Glutathione metabolism;Folate biosynthesis	ENOG410XSIX	O:Posttranslational modification, protein turnover, chaperones	PF13417.9;PF02798.23;PF13409.9;PF13410.9;PF14497.9;PF00043.28	GST_N_3;GST_N;GST_N_2;GST_C_2;GST_C_3;GST_C	Glutathione S-transferase, N-terminal domain;Glutathione S-transferase, N-terminal domain;Glutathione S-transferase, N-terminal domain;Glutathione S-transferase, C-terminal domain;Glutathione S-transferase, C-terminal domain;Glutathione S-transferase, C-terminal domain	CYT	1	11	32.4	High
TRINITY_DN48641_c0_g1_i4_orf1	RNA-binding protein 45-like [Galleria mellonella]	0.423857527	-1.238348688	0.0006581	down	yes	0.6307	1.488	1.367	1.652	1.444	0.577	0.654	0.661	MF:organic cyclic compound binding;MF:molecular_function;MF:binding;MF:nucleic acid binding;MF:heterocyclic compound binding;MF:RNA binding;	GO:0097159;GO:0003674;GO:0005488;GO:0003676;GO:1901363;GO:0003723	K25081	RBM45	------	------	ENOG410XP9Z	S:Function unknown	PF00076.25;PF08777.14	RRM_1;RRM_3	RNA recognition motif;RNA binding	CYT	1	2	53.8	High
TRINITY_DN42337_c0_g1_i6_orf1	cuticle protein 8-like [Leguminivora glycinivorella]	0.318402556	-1.651076178	0.001608	down	yes	0.4983	1.565	1.464	1.822	1.408	0.393	0.534	0.568	MF:molecular_function;MF:structural constituent of cuticle;MF:structural molecule activity;	GO:0003674;GO:0042302;GO:0005198	------	------	------	------	ENOG410YSFI;EN	S:Function unknown;	PF00379.26	Chitin_bind_4	Insect cuticle protein	CYT	1	36	15.3	High
TRINITY_DN31645_c0_g1_i3_orf1	dystonin isoform X43 [Helicoverpa armigera]	2.491763482	1.317167134	0.0003587	up	yes	1.437	0.5767	0.682	0.501	0.547	1.388	1.545	1.378	------	------	K10382	DST	------	------	ENOG410XP41;EN	Z:Cytoskeleton;S:Function unknown;Z:Cytoskeleton	------	------	------	CYT	1	6	15.8	High
TRINITY_DN24322_c0_g1_i4_orf1	unnamed protein product, partial [Brenthis ino]	2.370650802	1.245283169	1.20E-05	up	yes	1.315	0.5547	0.518	0.597	0.549	1.281	1.337	1.326	BP:RNA modification;BP:nucleic acid metabolic process;BP:ncRNA metabolic process;BP:pseudouridine synthesis;BP:organic cyclic compound metabolic process;BP:nucleobase-containing compound metabolic process;BP:organic substance metabolic process;BP:rRNA processing;BP:ncRNA processing;BP:cellular process;BP:cellular aromatic compound metabolic process;BP:macromolecule modification;BP:biological_process;BP:metabolic process;BP:heterocycle metabolic process;BP:RNA metabolic process;BP:primary metabolic process;BP:rRNA metabolic process;BP:cellular nitrogen compound metabolic process;BP:cellular metabolic process;BP:macromolecule metabolic process;BP:nitrogen compound metabolic process;BP:RNA processing;CC:intracellular organelle;CC:non-membrane-bounded organelle;CC:organelle;CC:nucleolus;CC:cellular anatomical entity;CC:macromolecular complex;CC:intracellular non-membrane-bounded organelle;CC:ribonucleoprotein complex;CC:cellular_component;MF:molecular_function;MF:binding;MF:nucleic acid binding;MF:heterocyclic compound binding;MF:organic cyclic compound binding;MF:RNA binding;	GO:0009451;GO:0090304;GO:0034660;GO:0001522;GO:1901360;GO:0006139;GO:0071704;GO:0006364;GO:0034470;GO:0009987;GO:0006725;GO:0043412;GO:0008150;GO:0008152;GO:0046483;GO:0016070;GO:0044238;GO:0016072;GO:0034641;GO:0044237;GO:0043170;GO:0006807;GO:0006396;GO:0043229;GO:0043228;GO:0043226;GO:0005730;GO:0110165;GO:0032991;GO:0043232;GO:1990904;GO:0005575;GO:0003674;GO:0005488;GO:0003676;GO:1901363;GO:0097159;GO:0003723	K11128	GAR1, NOLA1	map03008	Ribosome biogenesis in eukaryotes	COG3277	J:Translation, ribosomal structure and biogenesis	PF04410.17	Gar1	Gar1/Naf1 RNA binding region	CYT	3	20	22.5	High
TRINITY_DN23474_c1_g1_i1_orf1	unnamed protein product [Chrysodeixis includens]	2.648213952	1.405019684	0.0005519	up	yes	1.416	0.5347	0.504	0.605	0.495	1.328	1.343	1.576	------	------	------	------	------	------	ENOG41115FG	S:Function unknown	------	------	------	CYT	1	17	11.4	High
TRINITY_DN27500_c0_g1_i4_orf1	hemicentin-1-like [Ostrinia furnacalis]	5.275754422	2.399377411	1.04E-05	up	yes	1.521	0.2883	0.266	0.297	0.302	1.434	1.564	1.564	BP:developmental process;BP:anatomical structure development;BP:biological_process;CC:integral component of membrane;CC:cellular_component;CC:intrinsic component of membrane;CC:cellular anatomical entity;	GO:0032502;GO:0048856;GO:0008150;GO:0016021;GO:0005575;GO:0031224;GO:0110165	------	------	------	------	ENOG4110SA1;EN	S:Function unknown;	PF13927.9;PF07679.19;PF13895.9;PF00047.28;PF07686.20;PF00041.24	Ig_3;I-set;Ig_2;ig;V-set;fn3	Immunoglobulin domain;Immunoglobulin I-set domain;Immunoglobulin domain;Immunoglobulin domain;Immunoglobulin V-set	CYT	1	2	57.4	High
TRINITY_DN1313_c0_g1_i2_orf1	39S ribosomal protein L40, mitochondrial [Ostrinia furnacalis]	0.313743094	-1.672344392	9.19E-05	down	yes	0.4543	1.448	1.561	1.419	1.363	0.481	0.469	0.413	CC:large ribosomal subunit;CC:organellar large ribosomal subunit;CC:ribonucleoprotein complex;CC:ribosomal subunit;CC:cellular_component;CC:mitochondrial large ribosomal subunit;CC:macromolecular complex;CC:mitochondrial protein complex;	GO:0015934;GO:0000315;GO:1990904;GO:0044391;GO:0005575;GO:0005762;GO:0032991;GO:0098798	K17421	MRPL40	------	------	ENOG4111PQW	S:Function unknown	PF09812.12	MRP-L28	Mitochondrial ribosomal protein L28	CYT	1	11	23.8	High
TRINITY_DN4010_c0_g2_i1_orf1	myophilin [Ostrinia furnacalis]	2.812174276	1.491686004	1.87E-10	up	yes	1.349	0.4797	0.481	0.478	0.48	1.347	1.348	1.353	BP:organelle organization;BP:cytoskeleton organization;BP:actomyosin structure organization;BP:actin filament-based process;BP:cellular process;BP:actin cytoskeleton organization;BP:cellular component organization;BP:biological_process;BP:cellular component organization or biogenesis;MF:cytoskeletal protein binding;MF:actin binding;MF:molecular_function;MF:calmodulin binding;MF:protein binding;MF:binding;	GO:0006996;GO:0007010;GO:0031032;GO:0030029;GO:0009987;GO:0030036;GO:0016043;GO:0008150;GO:0071840;GO:0008092;GO:0003779;GO:0003674;GO:0005516;GO:0005515;GO:0005488	K20526	TAGLN	------	------	COG5199	S:Function unknown	PF00307.34;PF00402.21	CH;Calponin	Calponin homology;Calponin family repeat	CYT	7	47	19.2	High
TRINITY_DN1104_c0_g1_i1_orfp1	TRINITY_DN1104_c0_g1_i1_m.5521 TRINITY_DN1104_c0_g1::TRINITY_DN1104_c0_g1_i1::g.5521 ORF type:5prime_partial len:204 (+),score=39.47 TRINITY_DN1104_c0_g1_i1:3-614(+)	2.019933555	1.014307837	0.01901	up	yes	1.216	0.602	0.527	0.866	0.413	1.045	1.277	1.327	------	------	------	------	------	------	------	------	------	------	------	CYT	1	6	21.2	High
TRINITY_DN46173_c0_g3_i1_orf1	Tropomyosin, partial [Cotesia chilonis]	2.16751358	1.116041032	1.61E-05	up	yes	1.237	0.5707	0.577	0.547	0.588	1.205	1.284	1.221	------	------	K10373	TPM1	map05206;ma	MicroRNAs in cancer;Dilated cardiomyopathy;Adrenergic signaling in cardiomyocytes;Cardiac muscle contraction;Hypertrophic cardiomyopathy	ENOG410XR5K	S:Function unknown	PF00261.23;PF12718.10;PF06009.15;PF07889.15	Tropomyosin;Tropomyosin_1;Laminin_II;DUF1664	Tropomyosin;Tropomyosin like;Laminin Domain II;Protein of unknown function	CYT	12	45	33.2	High
TRINITY_DN1814_c0_g2_i1_orf1	titin-like, partial [Ostrinia furnacalis]	2.151832461	1.105565756	7.25E-06	up	yes	1.233	0.573	0.582	0.588	0.549	1.197	1.255	1.248	------	------	K12567	TTN	map05414;ma	Dilated cardiomyopathy;Hypertrophic cardiomyopathy	ENOG410XQFD;EI	S:Function unknown;	------	------	------	CYT	32	21	230.9	High
TRINITY_DN131603_c0_g1_i4_orfp1	TRINITY_DN131603_c0_g1_i4_m.86149 TRINITY_DN131603_c0_g1::TRINITY_DN131603_c0_g1_i4::g.86149 ORF type:internal len:112 (-),score=8.40 TRINITY_DN131603_c0_g1_i4:2-334(-)	2.417699115	1.273634711	0.002566	up	yes	1.366	0.565	0.749	0.586	0.36	1.289	1.394	1.416	------	------	------	------	------	------	------	------	------	------	------	PLA	1	22	12.9	High
TRINITY_DN31118_c0_g1_i1_orf1	CPR9 [Ostrinia furnacalis]	5.083798883	2.345906958	1.66E-06	up	yes	1.547	0.3043	0.309	0.285	0.319	1.519	1.6	1.521	CC:integral component of membrane;CC:cellular_component;CC:intrinsic component of membrane;CC:cellular anatomical entity;MF:structural molecule activity;MF:molecular_function;MF:structural constituent of cuticle;	GO:0016021;GO:0005575;GO:0031224;GO:0110165;GO:0005198;GO:0003674;GO:0042302	------	------	------	------	ENOG41125BY;EN	S:Function unknown;	PF00379.26	Chitin_bind_4	Insect cuticle protein	EXC	8	78	11.4	High
TRINITY_DN59829_c0_g1_i1_orf1	putative mediator of RNA polymerase II transcription subunit 12 [Ostrinia furnacalis]	0.403997145	-1.307582998	2.26E-06	down	yes	0.566	1.401	1.417	1.416	1.369	0.544	0.564	0.59	------	------	------	------	------	------	ENOG4110RMW	S:Function unknown	------	------	------	CYT	4	10	67.1	High
TRINITY_DN6612_c0_g1_i4_orf1	hypothetical protein O3G_MSEX008151 [Manduca sexta]	2.145587502	1.101372739	6.49E-06	up	yes	1.291	0.6017	0.616	0.58	0.609	1.261	1.284	1.327	CC:cellular_component;CC:integral component of membrane;CC:cellular anatomical entity;CC:intrinsic component of membrane;	GO:0005575;GO:0016021;GO:0110165;GO:0031224	K00019	BDH1, bdhA	map00650	Butanoate metabolism	ENOG410Y7FK	S:Function unknown	PF00106.28;PF13561.9;PF08643.13	adh_short;adh_short_C2;DUF1776	short chain dehydrogenase;Enoyl;Fungal family of unknown function	CYT	8	24	42.7	High
TRINITY_DN9871_c0_g1_i11_orf1	PEST proteolytic signal-containing nuclear protein-like [Ostrinia furnacalis]	3.239145417	1.695613237	3.14E-05	up	yes	1.41	0.4353	0.417	0.471	0.418	1.329	1.477	1.423	BP:nitrogen compound metabolic process;BP:cell cycle;BP:protein modification by small protein conjugation;BP:protein modification by small protein conjugation or removal;BP:organic substance metabolic process;BP:cellular process;BP:macromolecule modification;BP:protein modification process;BP:biological_process;BP:metabolic process;BP:primary metabolic process;BP:organonitrogen compound metabolic process;BP:protein metabolic process;BP:macromolecule metabolic process;BP:protein ubiquitination;CC:intracellular organelle;CC:membrane-bounded organelle;CC:organelle;CC:nucleus;CC:cellular anatomical entity;CC:intracellular membrane-bounded organelle;CC:cellular_component;	GO:0006807;GO:0007049;GO:0032446;GO:0070647;GO:0071704;GO:0009987;GO:0043412;GO:0036211;GO:0008150;GO:0008152;GO:0044238;GO:1901564;GO:0019538;GO:0043170;GO:0016567;GO:0043229;GO:0043227;GO:0043226;GO:0005634;GO:0110165;GO:0043231;GO:0005575	------	------	------	------	ENOG4111S9J	S:Function unknown	PF15473.9	PCNP	PEST, proteolytic signal-containing nuclear protein family	CYT	1	6	24.7	High
TRINITY_DN45949_c0_g1_i1_orf1	uncharacterized protein LOC114355167 [Ostrinia furnacalis]	3.626760563	1.858681503	3.86E-05	up	yes	1.545	0.426	0.423	0.409	0.446	1.435	1.589	1.612	MF:molecular_function;MF:structural constituent of cuticle;MF:structural molecule activity;	GO:0003674;GO:0042302;GO:0005198	------	------	------	------	ENOG410ZET2;EN	O:Posttranslational modification, protein turnover, chaperones;S:Function unknown;S:Function unknown;S:Function unknown	PF00379.26	Chitin_bind_4	Insect cuticle protein	CYT	3	18	22.7	High
TRINITY_DN26254_c0_g1_i1_orf1	hypothetical protein evm_003664 [Chilo suppressalis] >CAB3521132.1 unnamed protein product [Chilo suppressalis] >CAH0398453.1 unnamed protein product [Chilo suppressalis]	4.995287465	2.320567703	3.73E-07	up	yes	1.59	0.3183	0.325	0.304	0.326	1.566	1.577	1.627	MF:molecular_function;MF:structural constituent of cuticle;MF:structural molecule activity;	GO:0003674;GO:0042302;GO:0005198	------	------	------	------	ENOG410ZNDD;E	S:Function unknown;	PF00379.26	Chitin_bind_4	Insect cuticle protein	CYT	3	36	14.7	High
TRINITY_DN30208_c0_g1_i3_orf1	unnamed protein product [Timema cristinae]	3.1	1.632268216	3.44E-08	up	yes	1.426	0.46	0.456	0.461	0.463	1.413	1.441	1.424	------	------	K10374	TPM2	map05414;ma	Dilated cardiomyopathy;Adrenergic signaling in cardiomyocytes;Cardiac muscle contraction;Hypertrophic cardiomyopathy	ENOG410XR5K	Z:Cytoskeleton	PF00261.23;PF12718.10	Tropomyosin;Tropomyosin_1	Tropomyosin;Tropomyosin like	CYT	4	57	7.7	High
TRINITY_DN32687_c0_g1_i2_orf1	protein D2-like isoform X2 [Ostrinia furnacalis] >XP_028164613.1 protein D2-like isoform X2 [Ostrinia furnacalis]	2.231215907	1.157830125	1.64E-06	up	yes	1.268	0.5683	0.575	0.587	0.543	1.264	1.285	1.254	------	------	K06910	PEBP, TFS1	------	------	COG1881	S:Function unknown	------	------	------	CYT	1	66	11.6	High
TRINITY_DN549_c0_g1_i7_orf1	titin-like, partial [Ostrinia furnacalis]	3.581949965	1.840745185	1.86E-05	up	yes	1.532	0.4277	0.395	0.436	0.452	1.51	1.615	1.47	BP:developmental process;BP:cell differentiation;BP:cellular developmental process;BP:cellular process;BP:biological_process;BP:anatomical structure morphogenesis;	GO:0032502;GO:0030154;GO:0048869;GO:0009987;GO:0008150;GO:0009653	K12567	TTN	map05414;ma	Dilated cardiomyopathy;Hypertrophic cardiomyopathy	ENOG410XQFD;EI	S:Function unknown;	------	------	------	CYT	2	4	48.1	High
TRINITY_DN1134_c0_g1_i4_orf1	cytochrome P450 6B5-like [Ostrinia furnacalis]	2.104156558	1.073242051	2.57E-05	up	yes	1.301	0.6183	0.654	0.617	0.584	1.342	1.3	1.26	CC:endoplasmic reticulum membrane;CC:organelle membrane;CC:membrane;CC:cellular anatomical entity;CC:cellular_component;MF:monooxygenase activity;MF:oxidoreductase activity, acting on paired donors, with incorporation or reduction of molecular oxygen;MF:NADPH-hemoprotein reductase activity;MF:molecular_function;MF:binding;MF:heterocyclic compound binding;MF:oxidoreductase activity;MF:cation binding;MF:transition metal ion binding;MF:ion binding;MF:iron ion binding;MF:metal ion binding;MF:heme binding;MF:catalytic activity;MF:tetrapyrrole binding;MF:organic cyclic compound binding;MF:oxidoreductase activity, acting on NA;MF:oxidoreductase activity, acting on NA;	GO:0005789;GO:0031090;GO:0016020;GO:0110165;GO:0005575;GO:0004497;GO:0016705;GO:0003958;GO:0003674;GO:0005488;GO:1901363;GO:0016491;GO:0043169;GO:0046914;GO:0043167;GO:0005506;GO:0046872;GO:0020037;GO:0003824;GO:0046906;GO:0097159;GO:0016651;GO:0016653	K14999	CYP6	------	------	COG2124	S:Function unknown	PF00067.25	p450	Cytochrome P450	CYT	3	16	56.4	High
TRINITY_DN64769_c0_g1_i3_orf1	procollagen-lysine,2-oxoglutarate 5-dioxygenase isoform X2 [Ostrinia furnacalis]	0.45064	-1.149952718	1.30E-06	down	yes	0.5633	1.25	1.271	1.248	1.231	0.548	0.562	0.58	------	------	K13647	PLODN	map00310	Lysine degradation	ENOG410Y4QU	O:Posttranslational modification, protein turnover	------	------	------	CYT	8	13	83.9	High
TRINITY_DN21124_c0_g1_i4_orf1	calsyntenin-1 [Ostrinia furnacalis]	0.364350243	-1.456602141	5.33E-06	down	yes	0.5243	1.439	1.411	1.481	1.425	0.497	0.517	0.559	------	------	K22659	CLSTN1	------	------	ENOG410XT2J	U:Intracellular trafficking, secretion, and vesicular transport	PF19699.2;PF00028.20	CLSTN_C;Cadherin	Calsyntenin C-terminal;Cadherin domain	CYT	10	14	95.1	High
TRINITY_DN4242_c0_g1_i6_orf1	fibrohexamerin-like [Ostrinia furnacalis]	2.237024221	1.161580877	4.12E-05	up	yes	1.293	0.578	0.631	0.587	0.516	1.322	1.271	1.286	CC:cellular_component;CC:extracellular region;CC:cellular anatomical entity;MF:molecular_function;MF:structural molecule activity;	GO:0005575;GO:0005576;GO:0110165;GO:0003674;GO:0005198	------	------	------	------	ENOG4110SS9	S:Function unknown	PF07294.14	Fibroin_P25	Fibroin P25	CYT	3	14	25.5	High
TRINITY_DN3105_c0_g1_i4_orf1	nose resistant to fluoxetine protein 6-like [Ostrinia furnacalis]	2.098607332	1.069432251	0.004574	up	yes	1.311	0.6247	0.611	0.473	0.79	1.464	1.236	1.232	CC:integral component of membrane;CC:cellular_component;CC:intrinsic component of membrane;CC:cellular anatomical entity;MF:catalytic activity;MF:transferase activity;MF:transferase activity, transferring acyl groups;MF:molecular_function;MF:transferase activity, transferring acyl groups other than amino-acyl groups;	GO:0016021;GO:0005575;GO:0031224;GO:0110165;GO:0003824;GO:0016740;GO:0016746;GO:0003674;GO:0016747	------	------	------	------	ENOG410YD1T	S:Function unknown	PF01757.25	Acyl_transf_3	Acyltransferase family	CYT	1	2	81.7	High
TRINITY_DN1029_c0_g1_i1_orfp1	TRINITY_DN1029_c0_g1_i1_m.64408 TRINITY_DN1029_c0_g1::TRINITY_DN1029_c0_g1_i1::g.64408 ORF type:3prime_partial len:55 (+),score=13.47 TRINITY_DN1029_c0_g1_i1:74-235(+)	2.431610942	1.281912416	0.0002397	up	yes	1.36	0.5593	0.668	0.555	0.455	1.384	1.374	1.323	------	------	------	------	------	------	------	------	------	------	------	CYT	1	56	6.1	High
TRINITY_DN3906_c0_g1_i5_orf1	ejaculatory bulb-specific protein 3-like [Ostrinia furnacalis]	2.124125874	1.086869262	2.21E-05	up	yes	1.215	0.572	0.578	0.552	0.586	1.191	1.268	1.187	------	------	------	------	------	------	ENOG4111ATT;EN	S:Function unknown;G:Carbohydrate transport and metabolism	PF03392.16	OS-D	Insect pheromone-binding family, A10/OS-D	CYT	7	44	13.2	High
TRINITY_DN110400_c0_g1_i1_orf1	DNA replication factor Cdt1 [Chelonus insularis]	2.305970149	1.205373837	0.02278	up	yes	1.236	0.536	0.406	0.552	0.65	0.874	1.425	1.41	BP:cellular component assembly;BP:regulation of primary metabolic process;BP:DNA replication preinitiation complex assembly;BP:macromolecular complex subunit organization;BP:regulation of cellular metabolic process;BP:regulation of DNA-dependent DNA replication initiation;BP:cell cycle process;BP:regulation of DNA-dependent DNA replication;BP:cellular component organization or biogenesis;BP:protein-DNA complex subunit organization;BP:regulation of metabolic process;BP:cellular component organization;BP:regulation of biological process;BP:macromolecular complex assembly;BP:regulation of macromolecule metabolic process;BP:biological regulation;BP:protein-DNA complex assembly;BP:regulation of DNA metabolic process;BP:regulation of nucleobase-containing compound metabolic process;BP:cellular process;BP:regulation of cellular process;BP:biological_process;BP:regulation of nitrogen compound metabolic process;BP:regulation of DNA replication;CC:intracellular organelle;CC:membrane-bounded organelle;CC:organelle;CC:nucleus;CC:cellular anatomical entity;CC:intracellular membrane-bounded organelle;CC:cellular_component;MF:binding;MF:nucleic acid binding;MF:DNA binding;MF:organic cyclic compound binding;MF:molecular_function;MF:heterocyclic compound binding;	GO:0022607;GO:0080090;GO:0071163;GO:0043933;GO:0031323;GO:0030174;GO:0022402;GO:0090329;GO:0071840;GO:0071824;GO:0019222;GO:0016043;GO:0050789;GO:0065003;GO:0060255;GO:0065007;GO:0065004;GO:0051052;GO:0019219;GO:0009987;GO:0050794;GO:0008150;GO:0051171;GO:0006275;GO:0043229;GO:0043227;GO:0043226;GO:0005634;GO:0110165;GO:0043231;GO:0005575;GO:0005488;GO:0003676;GO:0003677;GO:0097159;GO:0003674;GO:1901363	K10727	CDT1	------	------	ENOG410XT37	L:Replication, recombination and repair	PF08839.14;PF16679.8	CDT1;CDT1_C	DNA replication factor CDT1 like;DNA replication factor Cdt1 C-terminal domain	CYT	1	1	82.5	High
TRINITY_DN11448_c0_g1_i15_orf1	unnamed protein product [Chilo suppressalis]	2.377962325	1.249725858	3.36E-06	up	yes	1.174	0.4937	0.527	0.486	0.468	1.171	1.186	1.164	------	------	K23353	PDLIM1_2_3_4	------	------	ENOG4111KMK;EI	S:Function unknown;	PF00595.27	PDZ	PDZ domain	CYT	2	52	27	High
TRINITY_DN1455_c0_g1_i8_orf1	troponin T, skeletal muscle isoform X1 [Galleria mellonella]	3.487603306	1.802235951	1.07E-05	up	yes	1.266	0.363	0.348	0.355	0.386	1.214	1.321	1.264	BP:regulation of system process;BP:regulation of muscle contraction;BP:biological regulation;BP:regulation of muscle system process;BP:regulation of multicellular organismal process;BP:biological_process;BP:regulation of biological process;CC:macromolecular complex;CC:cellular_component;CC:troponin complex;	GO:0044057;GO:0006937;GO:0065007;GO:0090257;GO:0051239;GO:0008150;GO:0050789;GO:0032991;GO:0005575;GO:0005861	K12046	TNNT3	------	------	ENOG410XS6A	Z:Cytoskeleton	------	------	------	CYT	5	41	43.1	High
TRINITY_DN4070_c0_g1_i4_orf1	alpha-N-acetylgalactosaminidase isoform X3 [Ostrinia furnacalis]	0.457167091	-1.12920654	1.01E-05	down	yes	0.539	1.179	1.195	1.204	1.139	0.541	0.519	0.557	BP:primary metabolic process;BP:organic substance metabolic process;BP:biological_process;BP:metabolic process;BP:carbohydrate metabolic process;BP:lipid metabolic process;CC:cellular_component;CC:lytic vacuole;CC:intracellular membrane-bounded organelle;CC:vacuole;CC:lysosome;CC:intracellular organelle;CC:membrane-bounded organelle;CC:organelle;CC:cellular anatomical entity;MF:molecular_function;MF:hydrolase activity;MF:hydrolase activity, acting on glycosyl bonds;MF:catalytic activity;MF:hydrolase activity, hydrolyzing O-glycosyl compounds;	GO:0044238;GO:0071704;GO:0008150;GO:0008152;GO:0005975;GO:0006629;GO:0005575;GO:0000323;GO:0043231;GO:0005773;GO:0005764;GO:0043229;GO:0043227;GO:0043226;GO:0110165;GO:0003674;GO:0016787;GO:0016798;GO:0003824;GO:0004553	K01204	NAGA	map00603;ma	Glycosphingolipid biosynthesis - globo and isoglobo series;Lysosome	ENOG410XPF1	O:Posttranslational modification, protein turnover, chaperones	PF16499.8;PF17450.5;PF17801.4	Melibiase_2;Melibiase_2_C;Melibiase_C	Alpha galactosidase A;Alpha galactosidase A C-terminal beta sandwich domain;Alpha galactosidase C-terminal beta sandwich	CYT	5	15	49.7	High
TRINITY_DN1895_c0_g1_i2_orf1	unnamed protein product [Chrysodeixis includens]	2.051505133	1.036682764	5.52E-05	up	yes	1.179	0.5747	0.6	0.572	0.552	1.124	1.184	1.229	BP:secretion;BP:transport;BP:vesicle-mediated transport;BP:export from cell;BP:exocytosis;BP:secretion by cell;BP:biological_process;BP:cellular process;BP:establishment of localization;BP:localization;CC:macromolecular complex;CC:exocyst;CC:cellular_component;CC:tethering complex;	GO:0046903;GO:0006810;GO:0016192;GO:0140352;GO:0006887;GO:0032940;GO:0008150;GO:0009987;GO:0051234;GO:0051179;GO:0032991;GO:0000145;GO:0005575;GO:0099023	K19983	EXOC1, SEC3	------	------	ENOG410XQEE	S:Function unknown	PF09763.12	Sec3_C	Exocyst complex component Sec3	CYT	1	9	23.6	High
TRINITY_DN13119_c0_g1_i4_orf1	endocuticle structural glycoprotein ABD-5-like [Bicyclus anynana]	5.170138889	2.370203037	1.19E-07	up	yes	1.489	0.288	0.266	0.289	0.309	1.478	1.487	1.502	------	------	------	------	------	------	ENOG410YVGC;EI	S:Function unknown;	PF00379.26	Chitin_bind_4	Insect cuticle protein	CYT	3	49	12.3	High
TRINITY_DN116_c1_g1_i8_orf1	uncharacterized protein LOC114350057 isoform X2 [Ostrinia furnacalis]	2.400235895	1.263176201	4.41E-05	up	yes	1.221	0.5087	0.503	0.504	0.519	1.172	1.293	1.197	------	------	------	------	------	------	ENOG4111JGD	S:Function unknown	PF15929.8	Myofilin	Myofilin	CYT	18	58	37.9	High
TRINITY_DN2215_c0_g2_i1_orf1	PREDICTED: larval cuticle protein LCP-22-like [Amyelois transitella]	6.510460251	2.702759537	2.29E-06	up	yes	1.556	0.239	0.264	0.206	0.247	1.543	1.609	1.515	------	------	------	------	------	------	ENOG41119AX;EN	S:Function unknown;	PF00379.26	Chitin_bind_4	Insect cuticle protein	CYT	3	33	16.5	High
TRINITY_DN82628_c0_g1_i2_orf1	ORF type:internal len:148 hit:XP_028162129.1 TRINITY_DN82628_c0_g1_i2:3-446(-)	2.361788618	1.239879848	0.0001573	up	yes	1.162	0.492	0.467	0.439	0.57	1.17	1.205	1.111	------	------	K19326	SYNE1	------	------	------	------	------	------	------	CYT	1	9	16.4	High
TRINITY_DN779_c0_g1_i12_orf1	unnamed protein product [Chilo suppressalis]	2.133666219	1.093334505	0.0002018	up	yes	1.111	0.5207	0.59	0.513	0.459	1.082	1.16	1.09	BP:cellular catabolic process;BP:cellular nitrogen compound metabolic process;BP:nitrogen compound metabolic process;BP:small molecule metabolic process;BP:small molecule catabolic process;BP:purine-containing compound metabolic process;BP:purine-containing compound catabolic process;BP:organic cyclic compound metabolic process;BP:organic cyclic compound catabolic process;BP:nucleobase-containing compound metabolic process;BP:organic substance catabolic process;BP:urate catabolic process;BP:organic substance metabolic process;BP:allantoin metabolic process;BP:cellular process;BP:cellular aromatic compound metabolic process;BP:heterocycle catabolic process;BP:biological_process;BP:metabolic process;BP:catabolic process;BP:nucleobase-containing small molecule metabolic process;BP:heterocycle metabolic process;BP:primary metabolic process;BP:organonitrogen compound metabolic process;BP:cellular nitrogen compound catabolic process;BP:cellular amide metabolic process;BP:nucleobase metabolic process;BP:cellular metabolic process;BP:aromatic compound catabolic process;BP:purine nucleobase metabolic process;BP:urate metabolic process;BP:organonitrogen compound catabolic process;MF:molecular_function;MF:lyase activity;MF:catalytic activity;MF:carbon-carbon lyase activity;MF:carboxy-lyase activity;	GO:0044248;GO:0034641;GO:0006807;GO:0044281;GO:0044282;GO:0072521;GO:0072523;GO:1901360;GO:1901361;GO:0006139;GO:1901575;GO:0019628;GO:0071704;GO:0000255;GO:0009987;GO:0006725;GO:0046700;GO:0008150;GO:0008152;GO:0009056;GO:0055086;GO:0046483;GO:0044238;GO:1901564;GO:0044270;GO:0043603;GO:0009112;GO:0044237;GO:0019439;GO:0006144;GO:0046415;GO:1901565;GO:0003674;GO:0016829;GO:0003824;GO:0016830;GO:0016831	------	------	------	------	ENOG410YZ5M;EI	S:Function unknown;	PF07004.15	SHIPPO-rpt	Sperm-tail PG-rich repeat	CYT	1	16	93.6	High
TRINITY_DN48610_c0_g1_i2_orf1	hypothetical protein evm_002298 [Chilo suppressalis] >CAH0682062.1 unnamed protein product [Chilo suppressalis]	3.12245387	1.642680258	3.85E-05	up	yes	1.303	0.4173	0.435	0.413	0.404	1.26	1.391	1.259	MF:molecular_function;MF:structural constituent of cuticle;MF:structural molecule activity;	GO:0003674;GO:0042302;GO:0005198	------	------	------	------	ENOG410XTK4;EN	S:Function unknown;	PF00379.26	Chitin_bind_4	Insect cuticle protein	CYT	5	41	18.6	High
TRINITY_DN4133_c0_g1_i2_orfp2	unnamed protein product [Spodoptera exigua]	2.150936399	1.104964866	0.0001525	up	yes	1.16	0.5393	0.59	0.516	0.512	1.167	1.22	1.094	------	------	------	------	------	------	------	------	------	------	------	CYT	2	40	6.9	High
TRINITY_DN4695_c0_g1_i3_orf1	glutathione S-transferase epsilon 3 [Ostrinia furnacalis]	2.186182884	1.128414094	1.64E-05	up	yes	1.193	0.5457	0.574	0.535	0.528	1.236	1.163	1.179	MF:catalytic activity;MF:transferase activity;MF:glutathione transferase activity;MF:molecular_function;MF:transferase activity, transferring alkyl or ary;	GO:0003824;GO:0016740;GO:0004364;GO:0003674;GO:0016765	K00799	GST, gst	map05207;ma	Chemical carcinogenesis - receptor activation;Glutathione metabolism;Chemical carcinogenesis - DNA adducts;Drug metabolism - other enzymes;Drug metabolism - cytochrome P450;Metabolism of xenobiotics by cytochrome P450;Pathways in cancer;Chemical carcinogenesis - reactive oxygen species;Fluid shear stress and atherosclerosis;Hepatocellular carcinoma;Longevity regulating pathway - worm;Platinum drug	COG0625	S:Function unknown	PF13417.9;PF02798.23;PF00043.28;PF13409.9;PF14497.9;PF13410.9	GST_N_3;GST_N;GST_C;GST_N_2;GST_C_3;GST_C_2	Glutathione S-transferase, N-terminal domain;Glutathione S-transferase, N-terminal domain;Glutathione S-transferase, C-terminal domain;Glutathione S-transferase, N-terminal domain;Glutathione S-transferase, C-terminal domain;Glutathione S-transferase, C-terminal domain	CYT	2	9	24.8	High
TRINITY_DN1180_c0_g1_i4_orf1	larval cuticle protein LCP-30-like [Ostrinia furnacalis]	6.063518596	2.600155217	1.50E-05	up	yes	1.451	0.2393	0.247	0.225	0.246	1.407	1.546	1.399	MF:molecular_function;MF:structural constituent of cuticle;MF:structural molecule activity;	GO:0003674;GO:0042302;GO:0005198	------	------	------	------	ENOG410YZ4Z;EN	S:Function unknown;	PF00379.26	Chitin_bind_4	Insect cuticle protein	CYT	13	61	31	High
TRINITY_DN41259_c0_g1_i6_orf1	endocuticle structural glycoprotein SgAbd-8 [Ostrinia furnacalis]	0.101804124	-3.296132094	9.71E-06	down	yes	0.158	1.552	1.547	1.639	1.471	0.15	0.144	0.18	MF:molecular_function;MF:structural constituent of cuticle;MF:structural molecule activity;	GO:0003674;GO:0042302;GO:0005198	------	------	------	------	ENOG4111PJ1	S:Function unknown	PF00379.26	Chitin_bind_4	Insect cuticle protein	CYT	7	44	24.7	High
TRINITY_DN1391_c1_g2_i2_orf1	uncharacterized protein LOC119837640 isoform X2 [Zerene cesonia]	2.083838559	1.059243512	1.16E-05	up	yes	1.203	0.5773	0.566	0.558	0.608	1.19	1.238	1.182	------	------	------	------	------	------	ENOG4111R1K;EN	S:Function unknown;	------	------	------	PLA	6	24	35.1	High
TRINITY_DN69334_c0_g1_i1_orf1	PREDICTED: 15-hydroxyprostaglandin dehydrogenase [NAD(+)]-like [Papilio xuthus]	2.470405589	1.304747922	6.48E-05	up	yes	1.273	0.5153	0.493	0.522	0.531	1.276	1.199	1.345	MF:catalytic activity;MF:molecular_function;MF:oxidoreductase activity;	GO:0003824;GO:0003674;GO:0016491	K00069	HPGD	map00590;ma	Arachidonic acid metabolism;Transcriptional misregulation in cancer	COG1028	S:Function unknown	PF00106.28;PF13561.9;PF08659.13	adh_short;adh_short_C2;KR	short chain dehydrogenase;Enoyl;KR domain	CYT	3	11	29.1	High
TRINITY_DN2040_c0_g1_i15_orfp1	TRINITY_DN2040_c0_g1_i15_m.4150 TRINITY_DN2040_c0_g1::TRINITY_DN2040_c0_g1_i15::g.4150 ORF type:complete len:319 (+),score=125.43,Plasmodium_HRP PF05403.12 3.5,Plasmodium_HRP|PF05403.12|1.5 TRINITY_DN2040_c0_g1_i15:118-957(+)	2.529531568	1.338870244	9.06E-05	up	yes	1.242	0.491	0.458	0.459	0.556	1.246	1.299	1.181	------	------	------	------	------	------	------	------	------	------	------	PLA	2	16	30.3	High

TRINITY_DN14217_c0_g1_i1_orf1	putative serine protease K12H4.7 [Ostrinia furnacalis] >XP_028166339.1 putative serine protease K12H4.7 [Ostrinia furnacalis]	0.076923077	-3.700439718	0.01082	down	yes	0.119	1.547	1.769	0.924	1.948	0.14	0.058	0.159	BP:primary metabolic process;BP:organonitrogen compound metabolic process;BP:protein metabolic process;BP:macromolecule metabolic process;BP:organic substance metabolic process;BP:nitrogen compound metabolic process;BP:biological_process;BP:metabolic process;BP:proteolysis;MF:molecular_function;MF:serine hydrolase activity;MF:hydrolase activity;MF:catalytic activity, acting on a protein;MF:catalytic activity;MF:peptidase activity;MF:serine-type peptidase activity;	GO:0044238;GO:1901564;GO:0019538;GO:0043170;GO:0071704;GO:0006807;GO:0008150;GO:0008152;GO:0006508;GO:0003674;GO:0017171;GO:0016787;GO:0140096;GO:0003824;GO:0008233;GO:0008236	------	------	------	------	ENOG410XSGG	S:Function unknown	PF05577.15	Peptidase_S28	Serine carboxypeptidase S28	CYT	1	3	54.9	High
TRINITY_DN57904_c0_g2_i1_orf1	cuticle protein 19 [Plutella xylostella] >CAG9138481.1 unnamed protein product [Plutella xylostella]	0.172836879	-2.532517006	3.21E-05	down	yes	0.2437	1.41	1.356	1.522	1.351	0.246	0.237	0.248	MF:molecular_function;MF:structural constituent of cuticle;MF:structural molecule activity;	GO:0003674;GO:0042302;GO:0005198	------	------	------	------	ENOG410YSFI	S:Function unknown	PF00379.26	Chitin_bind_4	Insect cuticle protein	CYT	1	16	14.1	High
TRINITY_DN592_c0_g1_i6_orf1	PDZ and LIM domain protein Zasp isoform X4 [Pectinophora gossypiella]	2.082080311	1.058025718	3.46E-06	up	yes	1.177	0.5653	0.569	0.555	0.572	1.145	1.195	1.191	------	------	K19867	PDLIM5_6_7	------	------	ENOG410XRD4	S:Function unknown	PF00412.25;PF00595.27;PF15936.8;PF17820.4;PF02163.25;PF13180.9	LIM;PDZ;DUF4749;PDZ_6;Peptidase_M50;PDZ_2	LIM domain;PDZ domain;Domain of unknown function;PDZ domain;Peptidase family M50;PDZ domain	CYT	21	52	68.6	High
TRINITY_DN36061_c0_g4_i2_orf1	putative GPI-anchored protein pfl2 [Ostrinia furnacalis] >XP_028163002.1 putative GPI-anchored protein pfl2 [Ostrinia furnacalis]	0.158361775	-2.658703954	8.94E-06	down	yes	0.232	1.465	1.396	1.496	1.504	0.202	0.283	0.211	CC:cellular_component;CC:extracellular region;CC:cellular anatomical entity;MF:carbohydrate derivative binding;MF:molecular_function;MF:chitin binding;MF:binding;	GO:0005575;GO:0005576;GO:0110165;GO:0097367;GO:0003674;GO:0008061;GO:0005488	------	------	------	------	ENOG4111P12	S:Function unknown	------	------	------	CYT	1	1	91.7	High
TRINITY_DN14019_c0_g1_i5_orf1	hypothetical protein evm_009768 [Chilo suppressalis]	0.400086655	-1.321615586	0.0002093	down	yes	0.4617	1.154	1.063	1.157	1.241	0.491	0.438	0.456	BP:interspecies interaction between organisms;BP:response to biotic stimulus;BP:response to external stimulus;BP:response to external biotic stimulus;BP:response to stimulus;BP:response to bacterium;BP:defense response;BP:response to stress;BP:defense response to other organism;BP:defense response to bacterium;BP:biological_process;BP:response to other organism;CC:cellular_component;CC:extracellular region;CC:cellular anatomical entity;	GO:0044419;GO:0009607;GO:0009605;GO:0043207;GO:0050896;GO:0009617;GO:0006952;GO:0006950;GO:0098542;GO:0042742;GO:0008150;GO:0051707;GO:0005575;GO:0005576;GO:0110165	------	------	------	------	------	------	------	------	------	CYT	1	14	10.8	High
TRINITY_DN1814_c0_g2_i4_orfp1	TRINITY_DN1814_c0_g2_i4_m.63284 TRINITY_DN1814_c0_g2::TRINITY_DN1814_c0_g2_i4::g.63284 ORF type:internal len:258 (-),score=126.31 TRINITY_DN1814_c0_g2_i4:3-773(-)	3.002257336	1.586047642	1.65E-05	up	yes	1.197	0.3987	0.415	0.382	0.399	1.135	1.235	1.22	------	------	------	------	------	------	------	------	------	------	------	CYT	3	34	30.5	High
TRINITY_DN85004_c0_g1_i1_orf1	uncharacterized protein LOC114357684 [Ostrinia furnacalis]	2.04120932	1.029424134	0.0004519	up	yes	1.06	0.5193	0.589	0.519	0.45	1.014	1.121	1.046	------	------	------	------	------	------	------	------	------	------	------	CYT	1	9	18.1	High
TRINITY_DN1073_c0_g1_i3_orf1	carboxylesterase [Loxostege sticticalis]	2.216300244	1.148153338	0.0002531	up	yes	0.998	0.4503	0.44	0.393	0.518	0.995	1.044	0.955	CC:integral component of membrane;CC:cellular_component;CC:intrinsic component of membrane;CC:cellular anatomical entity;MF:hydrolase activity;MF:hydrolase activity, acting on ester bonds;MF:carboxylic ester hydrolase activity;MF:catalytic activity;MF:molecular_function;	GO:0016021;GO:0005575;GO:0031224;GO:0110165;GO:0016787;GO:0016788;GO:0052689;GO:0003824;GO:0003674	------	------	------	------	COG2272;COG05	I:Lipid transport and metabolism;G:Carbohydrate transport and metabolism;T:Signal transduction mechanisms	PF00135.31;PF20434.1;PF07859.16	COesterase;BD-FAE;Abhydrolase_3	Carboxylesterase family;BD-FAE;alpha/beta hydrolase fold	CYT	3	7	68.1	High
TRINITY_DN248_c0_g1_i1_orf1	unnamed protein product [Chilo suppressalis]	2.028813894	1.020636531	4.72E-07	up	yes	1.028	0.5067	0.502	0.505	0.513	1.014	1.027	1.042	BP:phosphorylation;BP:cell differentiation;BP:nitrogen compound metabolic process;BP:anatomical structure morphogenesis;BP:cellular developmental process;BP:organic substance metabolic process;BP:developmental process;BP:protein phosphorylation;BP:cellular process;BP:macromolecule modification;BP:protein modification process;BP:biological_process;BP:metabolic process;BP:primary metabolic process;BP:organonitrogen compound metabolic process;BP:protein metabolic process;BP:cellular metabolic process;BP:macromolecule metabolic process;BP:phosphate-containing compound metabolic process;BP:phosphorus metabolic process;MF:purine ribonucleoside triphosphate binding;MF:molecular_function;MF:binding;MF:heterocyclic compound binding;MF:nucleoside phosphate binding;MF:anion binding;MF:ion binding;MF:nucleotide binding;MF:transferase activity;MF:protein kinase activity;MF:purine nucleotide binding;MF:ATP binding;MF:kinase activity;MF:catalytic activity, acting on a protein;MF:small molecule binding;MF:catalytic activity;MF:transferase activity, transferring phosphorus-containing groups;MF:adenyl nucleotide binding;MF:organic cyclic compound binding;MF:carbohydrate derivative binding;MF:adenyl ribonucleotide binding;MF:purine ribonucleotide binding;MF:phosphotransferase activity, alcohol group as acceptor;MF:ribonucleotide binding;	GO:0016310;GO:0030154;GO:0006807;GO:0009653;GO:0048869;GO:0071704;GO:0032502;GO:0006468;GO:0009987;GO:0043412;GO:0036211;GO:0008150;GO:0008152;GO:0044238;GO:1901564;GO:0019538;GO:0044237;GO:0043170;GO:0006796;GO:0006793;GO:0035639;GO:0003674;GO:0005488;GO:1901363;GO:1901265;GO:0043168;GO:0043167;GO:0000166;GO:0016740;GO:0004672;GO:0017076;GO:0005524;GO:0016301;GO:0140096;GO:0036094;GO:0003824;GO:0016772;GO:0030554;GO:0097159;GO:0097367;GO:0032559;GO:0032555;GO:0016773;GO:0032553	------	------	------	------	ENOG410XQFD;EI	S:Function unknown;	PF00041.24;PF07679.19;PF13927.9;PF13895.9;PF00047.28;PF00069.28;PF07714.20	fn3;I-set;Ig_3;Ig_2;ig;Pkinase;PK_Tyr_Ser-Thr	Fibronectin type III domain;Immunoglobulin I-set domain;Immunoglobulin domain;Immunoglobulin domain;Immunoglobulin domain;Protein kinase domain;Protein tyrosine and serine/threonine kinase	CYT	208	34	1059	High
TRINITY_DN23429_c0_g2_i1_orf1	muscle-specific protein 20 [Zerene cesonia]	2.567718827	1.360487232	8.59E-07	up	yes	1.147	0.4467	0.457	0.434	0.449	1.167	1.126	1.148	------	------	K20526	TAGLN	------	------	COG5199	Z:Cytoskeleton	PF00307.34;PF00402.21	CH;Calponin	Calponin homology;Calponin family repeat	CYT	12	75	20.3	High
TRINITY_DN8915_c0_g1_i3_orf1	filamin-A isoform X1 [Ostrinia furnacalis] >XP_028171553.1 filamin-A isoform X2 [Ostrinia furnacalis] >XP_028171561.1 filamin-A isoform X2 [Ostrinia furnacalis]	2.029674719	1.021248536	2.61E-06	up	yes	1.067	0.5257	0.529	0.531	0.517	1.049	1.06	1.093	BP:organelle organization;BP:cytoskeleton organization;BP:actin filament-based process;BP:cellular process;BP:actin cytoskeleton organization;BP:biological_process;BP:cellular component organization;BP:cellular component organization or biogenesis;MF:molecular_function;MF:cytoskeletal protein binding;MF:actin filament binding;MF:actin binding;MF:protein binding;MF:macromolecular complex binding;MF:binding;	GO:0006996;GO:0007010;GO:0030029;GO:0009987;GO:0030036;GO:0008150;GO:0016043;GO:0071840;GO:0003674;GO:0008092;GO:0051015;GO:0003779;GO:0005515;GO:0044877;GO:0005488	K04437	FLNA	map05205;ma	Proteoglycans in cancer;Focal adhesion;MAPK signaling pathway;Salmonella infection	ENOG410XNS3;C(	S:Function unknown;	PF00630.22	Filamin	Filamin/ABP280 repeat	CYT	37	54	100.3	High
TRINITY_DN6482_c0_g1_i1_orf1	endocuticle structural glycoprotein SgAbd-5-like [Ostrinia furnacalis]	2.019648592	1.014104294	0.0001905	up	yes	1.069	0.5293	0.564	0.486	0.538	1.001	1.103	1.102	MF:molecular_function;MF:structural constituent of cuticle;MF:structural molecule activity;	GO:0003674;GO:0042302;GO:0005198	------	------	------	------	ENOG4111D3H;EI	S:Function unknown;	PF00379.26	Chitin_bind_4	Insect cuticle protein	CYT	3	46	11.3	High
TRINITY_DN416_c0_g1_i1_orf1	unnamed protein product [Diatraea saccharalis]	2.32221523	1.215501692	1.14E-06	up	yes	1.107	0.4767	0.471	0.47	0.489	1.101	1.129	1.09	------	------	K05699	ACTN1_4	map04510;ma	Focal adhesion;Regulation of actin cytoskeleton;Amoebiasis;Systemic lupus erythematosus;Shigellosis;Leukocyte transendothelial migration;Tight junction;Viral	COG5069	S:Function unknown	PF00435.24;PF00307.34;PF08726.13;PF00036.35;PF11971.11;PF13405.9;PF13499.9	Spectrin;CH;EF hand_Ca_insen;EF-hand_1;CAMSAP_CH;EF-hand_6;EF-hand_7	Spectrin repeat;Calponin homology;Ca2+ insensitive EF hand;EF hand;CAMSAP CH domain;EF-hand domain;EF-hand domain pair	CYT	42	55	103.8	High
TRINITY_DN64_c0_g1_i4_orf1	unnamed protein product [Chilo suppressalis]	2.026466522	1.018966342	0.01086	up	yes	1.026	0.5063	0.567	0.613	0.339	1.098	1.112	0.869	BP:nucleobase-containing compound transport;BP:nuclear transport;BP:nuclear export;BP:nucleic acid metabolic process;BP:cellular nitrogen compound metabolic process;BP:nitrogen compound metabolic process;BP:mRNA transport;BP:organic cyclic compound metabolic process;BP:nitrogen compound transport;BP:organic substance metabolic process;BP:organic substance transport;BP:heterocycle metabolic process;BP:RNA export from nucleus;BP:mRNA export from nucleus;BP:cellular process;BP:nucleobase-containing compound metabolic process;BP:transport;BP:cellular aromatic compound metabolic process;BP:nucleocytoplasmic transport;BP:biological_process;BP:metabolic process;BP:establishment of localization in cell;BP:establishment of RNA localization;BP:establishment of localization;BP:primary metabolic process;BP:localization;BP:cellular localization;BP:RNA metabolic process;BP:mRNA metabolic process;BP:RNA transport;BP:intracellular transport;BP:nucleic acid transport;BP:cellular metabolic process;BP:macromolecule metabolic process;BP:RNA processing;BP:mRNA processing;CC:THO complex;CC:macromolecular complex;CC:cellular_component;	GO:0015931;GO:0051169;GO:0051168;GO:0090304;GO:0034641;GO:0006807;GO:0051028;GO:1901360;GO:0071705;GO:0071704;GO:0071702;GO:0046483;GO:0006405;GO:0006406;GO:0009987;GO:0006139;GO:0006810;GO:0006725;GO:0006913;GO:0008150;GO:0008152;GO:0051649;GO:0051236;GO:0051234;GO:0044238;GO:0051179;GO:0051641;GO:0016070;GO:0016071;GO:0050658;GO:0046907;GO:0050657;GO:0044237;GO:0043170;GO:0006396;GO:0006397;GO:0000347;GO:0032991;GO:0005575	K12879	THOC2	map03013;ma	Nucleocytoplasmic transport;Spliceosome	ENOG410XQ2A	S:Function unknown	PF11262.11;PF16134.8;PF11732.11	Tho2;THOC2_N;Thoc2	Transcription factor/nuclear export subunit protein 2;THO complex subunit 2 N-terminus;Transcription- and export-related complex subunit	CYT	1	0	161.7	High
TRINITY_DN8226_c0_g1_i1_orf1	myosin heavy chain, muscle isoform X16 [Helicoverpa armigera]	2.350230415	1.232802204	1.64E-05	up	yes	1.071	0.4557	0.456	0.452	0.459	1.022	1.105	1.085	------	------	K17751	MYH6_7	map04260;ma	Cardiac muscle contraction;Adrenergic signaling in cardiomyocytes;Thyroid hormone signaling pathway;cGMP-PKG signaling pathway;Viral myocarditis;Dilated cardiomyopathy;Hypertrophic cardiomyopathy	COG5022	G:Carbohydrate transport and metabolism	------	------	------	CYT	1	56	6.3	High
TRINITY_DN4408_c6_g1_i1_orf1	polyprotein, partial [Bemisia tabaci]	2.71319797	1.439994318	8.52E-06	up	yes	1.069	0.394	0.429	0.368	0.385	1.097	1.047	1.064	BP:viral RNA genome replication;BP:RNA biosynthetic process;BP:nucleic acid-protein covalent cross-linking;BP:RNA-protein covalent cross-linking;BP:nucleic acid metabolic process;BP:cellular biosynthetic process;BP:cellular nitrogen compound metabolic process;BP:nitrogen compound metabolic process;BP:organic cyclic compound biosynthetic process;BP:organic cyclic compound metabolic process;BP:nucleobase-containing compound metabolic process;BP:cellular macromolecule metabolic process;BP:organic substance metabolic process;BP:nucleic acid-templated transcription;BP:proteolysis;BP:heterocycle biosynthetic process;BP:organic substance biosynthetic process;BP:viral genome replication;BP:cellular aromatic compound metabolic process;BP:cellular process;BP:macromolecule modification;BP:protein modification process;BP:biological_process;BP:metabolic process;BP:aromatic compound biosynthetic process;BP:nucleobase-containing compound biosynthetic process;BP:cellular nitrogen compound biosynthetic process;BP:heterocycle metabolic process;BP:RNA metabolic process;BP:primary metabolic process;BP:transcription, DNA-templated;BP:organonitrogen compound metabolic process;BP:protein metabolic process;BP:biosynthetic process;BP:macromolecule biosynthetic process;BP:macromolecule metabolic process;BP:viral process;BP:DNA metabolic process;BP:cellular metabolic process;BP:DNA biosynthetic process;MF:RNA polymerase activity;MF:molecular_function;MF:binding;MF:nucleic acid binding;MF:RNA-dependent ATPase activity;MF:peptidase activity;MF:cysteine-type peptidase activity;MF:heterocyclic compound binding;MF:transferase activity;MF:hydrolase activity;MF:catalytic activity, acting on RNA;MF:catalytic activity, acting on a protein;MF:helicase activity;MF:catalytic activity;MF:nucleotidyltransferase activity;MF:RNA polymerase activity;MF:transferase activity, transferring phosphorus-containing groups;MF:RNA helicase activity;MF:cysteine-type endopeptidase activity;MF:RNA binding;MF:organic cyclic compound binding;MF:RNA-directed RNA polymerase activity;MF:endopeptidase activity;	GO:0039694;GO:0032774;GO:0018143;GO:0018144;GO:0090304;GO:0044249;GO:0034641;GO:0006807;GO:1901362;GO:1901360;GO:0006139;GO:0044260;GO:0071704;GO:0097659;GO:0006508;GO:0018130;GO:1901576;GO:0019079;GO:0006725;GO:0009987;GO:0043412;GO:0036211;GO:0008150;GO:0008152;GO:0019438;GO:0034654;GO:0044271;GO:0046483;GO:0016070;GO:0044238;GO:0006351;GO:1901564;GO:0019538;GO:0009058;GO:0009059;GO:0043170;GO:0016032;GO:0006259;GO:0044237;GO:0071897;GO:0034062;GO:0003674;GO:0005488;GO:0003676;GO:0008186;GO:0008233;GO:0008234;GO:1901363;GO:0016740;GO:0016787;GO:0140098;GO:0140096;GO:0004386;GO:0003824;GO:0016779;GO:0097747;GO:0016772;GO:0003724;GO:0004197;GO:0003723;GO:0097159;GO:0003968;GO:0004175	------	------	------	------	------	------	PF00680.23	RdRP_1	Viral RNA-dependent RNA polymerase	CYT	6	10	76.2	High
TRINITY_DN248_c0_g1_i12_orf1	twitchin-like [Ostrinia furnacalis]	2.76287681	1.466171237	0.0001049	up	yes	1.164	0.4213	0.41	0.406	0.448	1.072	1.201	1.22	------	------	------	------	------	------	ENOG410YKFP;EI	S:Function unknown;T:Signal transduction mechanisms	PF07679.19;PF13927.9;PF13895.9;PF00047.28;PF07686.20	I-set;Ig_3;Ig_2;ig;V-set	Immunoglobulin I-set domain;Immunoglobulin domain;Immunoglobulin domain;Immunoglobuli	CYT	3	21	226.6	High
TRINITY_DN783_c0_g1_i7_orf1	microtubule-associated protein Jupiter isoform X4 [Helicoverpa armigera]	0.465983225	-1.101650076	0.0001277	down	yes	0.5	1.073	1.148	1.021	1.049	0.513	0.499	0.488	------	------	------	------	------	------	ENOG410Z68G	S:Function unknown	PF17054.8	JUPITER	Microtubule-Associated protein Jupiter	EXC	2	12	24	High
TRINITY_DN279_c0_g1_i10_orf1	RE1-silencing transcription factor-like isoform X1 [Ostrinia furnacalis]	0.417807018	-1.259091371	7.46E-05	down	yes	0.4763	1.14	1.073	1.168	1.179	0.443	0.515	0.471	CC:intracellular membrane-bounded organelle;CC:nucleus;CC:cellular_component;CC:intracellular organelle;CC:membrane-bounded organelle;CC:organelle;CC:cellular anatomical entity;MF:cation binding;MF:transition metal ion binding;MF:ion binding;MF:molecular_function;MF:zinc ion binding;MF:metal ion binding;MF:binding;	GO:0043231;GO:0005634;GO:0005575;GO:0043229;GO:0043227;GO:0043226;GO:0110165;GO:0043169;GO:0046914;GO:0043167;GO:0003674;GO:0008270;GO:0046872;GO:0005488	------	------	------	------	COG5048	O:Posttranslational modification, protein turnover, chaperones	PF00096.29;PF13894.9;PF13465.9;PF07776.18	zf-C2H2;zf-C2H2_4;zf-H2C2_2;zf-AD	Zinc finger, C2H2 type;C2H2-type zinc finger;Zinc-finger double domain;Zinc-finger associated	CYT	1	1	120.9	High
TRINITY_DN2600_c0_g1_i7_orf1	mucin-5AC isoform X2 [Ostrinia furnacalis]	0.393796296	-1.344478552	0.0001942	down	yes	0.4253	1.08	1.096	1.145	1	0.377	0.466	0.433	------	------	------	------	------	------	ENOG4112A7J	S:Function unknown	------	------	------	CYT	9	12	98	High
TRINITY_DN76333_c0_g1_i2_orf1	larval cuticle protein 65Ag1-like [Ostrinia furnacalis]	4.65648855	2.219242431	8.93E-07	up	yes	1.22	0.262	0.274	0.252	0.26	1.194	1.254	1.213	MF:molecular_function;MF:structural constituent of cuticle;MF:structural molecule activity;	GO:0003674;GO:0042302;GO:0005198	------	------	------	------	ENOG410YVGC;EI	S:Function unknown;	PF00379.26	Chitin_bind_4	Insect cuticle protein	CYT	4	43	15.6	High
TRINITY_DN695_c0_g1_i12_orf1	seroin transcript 2A, partial [Ostrinia nubilalis]	2.710843373	1.43874176	0.0004056	up	yes	1.125	0.415	0.361	0.424	0.46	1.141	1.218	1.017	------	------	------	------	------	------	ENOG4110R3H;EI	S:Function unknown;	------	------	------	CYT	1	30	15.1	High
TRINITY_DN1123_c2_g1_i3_orf1	troponin I isoform X8 [Ostrinia furnacalis]	3.638863428	1.863487906	5.70E-06	up	yes	1.191	0.3273	0.299	0.36	0.323	1.15	1.212	1.21	CC:cellular_component;CC:macromolecular complex;CC:troponin complex;	GO:0005575;GO:0032991;GO:0005861	K12044	TNNI3	map04260;ma	Cardiac muscle contraction;Adrenergic signaling in cardiomyocytes;cAMP signaling pathway;Dilated cardiomyopathy;Diabetic cardiomyopathy;Hypertrophic cardiomyopathy	ENOG410Y9IX	Z:Cytoskeleton	PF00992.23	Troponin	Troponin	CYT	3	55	10.3	High
TRINITY_DN31118_c0_g2_i1_orf1	unnamed protein product [Spodoptera exigua]	5.867628341	2.552777493	5.74E-06	up	yes	1.383	0.2357	0.252	0.212	0.243	1.326	1.443	1.379	MF:molecular_function;MF:structural constituent of cuticle;MF:structural molecule activity;	GO:0003674;GO:0042302;GO:0005198	------	------	------	------	ENOG410YVGC;EI	S:Function unknown;	PF00379.26	Chitin_bind_4	Insect cuticle protein	CYT	2	53	12.9	High
TRINITY_DN32601_c0_g1_i2_orf1	uncharacterized protein LOC114363197 [Ostrinia furnacalis]	0.332728843	-1.587581162	2.51E-05	down	yes	0.3853	1.158	1.138	1.165	1.172	0.358	0.346	0.452	------	------	------	------	------	------	ENOG410XRFE	S:Function unknown	PF03022.19	MRJP	Major royal jelly protein	CYT	1	3	48.2	High
TRINITY_DN43328_c0_g1_i1_orf1	tubulin--tyrosine ligase-like protein 12 [Ostrinia furnacalis]	0.4359447	-1.197782954	0.0006227	down	yes	0.473	1.085	1.081	1.068	1.107	0.382	0.446	0.591	------	------	K16609	TTLL12	------	------	ENOG410XQ0A	S:Function unknown	PF03133.18	TTL	Tubulin-tyrosine ligase family	CYT	1	2	71.9	High
TRINITY_DN100_c0_g1_i9_orf1	uncharacterized protein LOC114353052 [Ostrinia furnacalis]	2.027957438	1.020027374	0.0004628	up	yes	0.972	0.4793	0.474	0.475	0.489	0.895	1.056	0.965	------	------	------	------	------	------	ENOG4111OMX	S:Function unknown	------	------	------	CYT	1	9	17.7	High
TRINITY_DN34821_c0_g1_i4_orf1	acetylcholine receptor subunit alpha-L1-like [Ostrinia furnacalis]	0.319290013	-1.647060666	3.87E-05	down	yes	0.313	0.9803	0.945	1.047	0.949	0.321	0.303	0.315	CC:cellular_component;CC:integral component of membrane;CC:cellular anatomical entity;CC:intrinsic component of membrane;MF:signaling receptor activity;MF:molecular transducer activity;MF:inorganic solute uptake transmembrane transporter activity;MF:transporter activity;MF:extracellular ligand-gated ion channel activity;MF:ligand-gated ion channel activity;MF:ion transmembrane transporter activity;MF:transmembrane signaling receptor activity;MF:transmembrane transporter activity;MF:channel activity;MF:molecular_function;MF:gated channel activity;MF:ligand-gated channel activity;MF:passive transmembrane transporter activity;MF:ion channel activity;	GO:0005575;GO:0016021;GO:0110165;GO:0031224;GO:0038023;GO:0060089;GO:0015318;GO:0005215;GO:0005230;GO:0015276;GO:0015075;GO:0004888;GO:0022857;GO:0015267;GO:0003674;GO:0022836;GO:0022834;GO:0022803;GO:0005216	K04803;K048(	CHRNA1;CHR	map04080	Neuroactive ligand-receptor interaction	ENOG410XQGR	S:Function unknown	PF02931.26	Neur_chan_LBD	Neurotransmitter-gated ion-channel ligand binding domain	PLA	1	3	47.7	High
TRINITY_DN389_c0_g1_i2_orf1	uncharacterized protein LOC118068293 isoform X2 [Chelonus insularis]	0.422340426	-1.243521749	1.02E-05	down	yes	0.3573	0.846	0.846	0.838	0.854	0.325	0.383	0.364	------	------	------	------	------	------	ENOG410XX7G	S:Function unknown	PF01607.27;PF00057.21	CBM_14;Ldl_recept_a	Chitin binding Peritrophin-A domain;Low-density lipoprotein receptor domain class A	PLA	4	11	60.2	High
TRINITY_DN76377_c0_g1_i1_orf1	uncharacterized protein LOC111357764, partial [Spodoptera litura]	0.406008052	-1.300419755	2.78E-05	down	yes	0.3933	0.9687	0.958	0.991	0.957	0.367	0.442	0.371	------	------	------	------	------	------	ENOG410Y7Q4;EI	S:Function unknown;	PF03564.18	DUF1759	Protein of unknown function	CYT	1	4	27.4	Medium
TRINITY_DN120439_c1_g1_i1_orf1	myosin heavy chain variant, partial [Bombyx mori]	2.136421807	1.095196515	8.26E-05	up	yes	0.8347	0.3907	0.393	0.369	0.41	0.796	0.88	0.828	CC:cellular_component;CC:myosin complex;CC:macromolecular complex;	GO:0005575;GO:0016459;GO:0032991	K17751	MYH6_7	map04260;ma	Cardiac muscle contraction;Adrenergic signaling in cardiomyocytes;Thyroid hormone signaling pathway;cGMP-PKG signaling pathway;Viral myocarditis;Dilated cardiomyopathy;Hypertrophic cardiomyopathy	COG5022	Z:Cytoskeleton	PF01576.22	Myosin_tail_1	Myosin tail	CYT	7	65	12.3	High
TRINITY_DN4194_c0_g1_i1_orf1	hornerin-like [Ostrinia furnacalis]	0.404317269	-1.30644027	0.002464	down	yes	0.4027	0.996	1.01	1.112	0.866	0.387	0.324	0.497	------	------	K19720;K1972	COL3A;COL5/	map05146;ma	Amoebiasis;Protein digestion and absorption;Relaxin signaling pathway;Diabetic cardiomyopathy;AGE-RAGE signaling pathway in diabetic complications;Platelet activation	ENOG4111IEE;EN(	S:Function unknown;	PF16058.8	Mucin-like	Mucin-like	CYT	2	1	184.6	High
TRINITY_DN195_c4_g1_i1_orf1	beta-1,3-glucan-binding protein 1 [Ostrinia furnacalis]	3.426760093	1.776845193	0.002673	up	yes	1.027	0.2997	0.335	0.292	0.272	1.212	1.031	0.838	BP:immune system process;BP:response to external biotic stimulus;BP:interspecies interaction between organisms;BP:organic substance metabolic process;BP:innate immune response;BP:defense response;BP:response to stress;BP:biological_process;BP:metabolic process;BP:immune response;BP:carbohydrate metabolic process;BP:response to other organism;BP:primary metabolic process;BP:response to biotic stimulus;BP:response to external stimulus;BP:response to stimulus;BP:defense response to other organism;CC:cellular anatomical entity;CC:cellular_component;CC:extracellular region;MF:carbohydrate binding;MF:molecular_function;MF:binding;MF:hydrolase activity;MF:hydrolase activity, acting on glycosyl bonds;MF:catalytic activity;MF:hydrolase activity, hydrolyzing O-glycosyl compounds;	GO:0002376;GO:0043207;GO:0044419;GO:0071704;GO:0045087;GO:0006952;GO:0006950;GO:0008150;GO:0008152;GO:0006955;GO:0005975;GO:0051707;GO:0044238;GO:0009607;GO:0009605;GO:0050896;GO:0098542;GO:0110165;GO:0005575;GO:0005576;GO:0030246;GO:0003674;GO:0005488;GO:0016787;GO:0016798;GO:0003824;GO:0004553	K20692	GNBP3	map04624	Toll and Imd signaling pathway	ENOG410Z83Q;EI	S:Function unknown;	PF15886.8	CBM39	Carbohydrate binding domain	CYT	1	19	14.1	High
TRINITY_DN1173_c1_g1_i10_orf1	hypothetical protein evm_001011 [Chilo suppressalis]	2.819667348	1.49552497	0.0005445	up	yes	0.9663	0.3427	0.32	0.352	0.356	0.884	1.085	0.93	BP:phosphorylation;BP:cell differentiation;BP:nitrogen compound metabolic process;BP:anatomical structure morphogenesis;BP:cellular developmental process;BP:organic substance metabolic process;BP:developmental process;BP:protein phosphorylation;BP:cellular process;BP:macromolecule modification;BP:protein modification process;BP:biological_process;BP:metabolic process;BP:primary metabolic process;BP:organonitrogen compound metabolic process;BP:protein metabolic process;BP:cellular metabolic process;BP:macromolecule metabolic process;BP:phosphate-containing compound metabolic process;BP:phosphorus metabolic process;MF:purine ribonucleoside triphosphate binding;MF:guanyl-nucleotide exchange factor activity;MF:molecular_function;MF:binding;MF:molecular function regulator;MF:heterocyclic compound binding;MF:nucleoside phosphate binding;MF:anion binding;MF:ion binding;MF:nucleotide binding;MF:transferase activity;MF:protein kinase activity;MF:purine nucleotide binding;MF:carbohydrate derivative binding;MF:ATP binding;MF:kinase activity;MF:enzyme regulator activity;MF:catalytic activity, acting on a protein;MF:small molecule binding;MF:GTPase regulator activity;MF:nucleoside-triphosphatase regulator activity;MF:catalytic activity;MF:transferase activity, transferring phosphorus-containing groups;MF:adenyl nucleotide binding;MF:phosphotransferase activity, alcohol group as acceptor;MF:adenyl ribonucleotide binding;MF:purine ribonucleotide binding;MF:organic cyclic compound binding;MF:ribonucleotide binding;	GO:0016310;GO:0030154;GO:0006807;GO:0009653;GO:0048869;GO:0071704;GO:0032502;GO:0006468;GO:0009987;GO:0043412;GO:0036211;GO:0008150;GO:0008152;GO:0044238;GO:1901564;GO:0019538;GO:0044237;GO:0043170;GO:0006796;GO:0006793;GO:0035639;GO:0005085;GO:0003674;GO:0005488;GO:0098772;GO:1901363;GO:1901265;GO:0043168;GO:0043167;GO:0000166;GO:0016740;GO:0004672;GO:0017076;GO:0097367;GO:0005524;GO:0016301;GO:0030234;GO:0140096;GO:0036094;GO:0030695;GO:0060589;GO:0003824;GO:0016772;GO:0030554;GO:0016773;GO:0032559;GO:0032555;GO:0097159;GO:0032553	------	------	------	------	ENOG410XQFD;EI	S:Function unknown;T:Signal transduction mechanisms	PF07679.19;PF13927.9;PF00047.28;PF13895.9;PF00621.23;PF07686.20;PF00169.32	I-set;Ig_3;ig;Ig_2;RhoGEF;V-set;PH	Immunoglobulin I-set domain;Immunoglobulin domain;Immunoglobulin domain;Immunoglobulin domain;RhoGEF domain;Immunoglobulin V-set domain;PH domain	CYT	1	17	169.8	High
TRINITY_DN1123_c2_g1_i5_orf1	troponin I isoform X4 [Leguminivora glycinivorella]	2.323569298	1.216342672	6.43E-05	up	yes	0.8567	0.3687	0.358	0.347	0.401	0.837	0.902	0.831	------	------	K12044	TNNI3	map04260;ma	Cardiac muscle contraction;Adrenergic signaling in cardiomyocytes;cAMP signaling pathway;Dilated cardiomyopathy;Diabetic cardiomyopathy;Hypertrophic cardiomyopathy	ENOG410Y9IX	Z:Cytoskeleton	PF00992.23	Troponin	Troponin	CYT	2	40	16.3	High
TRINITY_DN46216_c0_g3_i1_orf1	unnamed protein product, partial [Brenthis ino]	5.80613736	2.537578703	8.86E-06	up	yes	1.192	0.2053	0.22	0.169	0.227	1.188	1.245	1.144	------	------	------	------	------	------	ENOG410Y7Q4;EI	J:Translation, ribosomal structure and biogenesis;U:Intracellular trafficking, secretion, and vesicular transport;S:Function	PF03564.18	DUF1759	Protein of unknown function	CYT	1	11	9.6	Medium

TRINITY_DN115082_c0_g1_i5_orf1	protein dj-1beta-like isoform X2 [Ostrinia furnacalis]	3.446875	1.785288981	1.46E-05	up	yes	1.103	0.32	0.32	0.322	0.318	1.164	1.082	1.063	------	------	K05687	PARK7	map05012;ma	Parkinson disease;Pathways of neurodegeneration - multiple diseases	COG0693	S:Function unknown	PF01965.27	DJ-1_PfpI	DJ-1/PfpI family	CYT	1	45	11.4 High
TRINITY_DN1123_c2_g1_i4_orf1	troponin I isoform X16 [Ostrinia furnacalis]	3.018748014	1.593950335	2.98E-05	up	yes	0.95	0.3147	0.307	0.297	0.34	0.904	0.998	0.948	CC:cellular_component;CC:macromolecular complex;CC:troponin complex;	GO:0005575;GO:0032991;GO:0005861	K12044	TNNI3	map04260;ma	Cardiac muscle contraction;Adrenergic signaling in cardiomyocytes;cAMP signaling pathway;Dilated cardiomyopathy;Diabetic cardiomyopathy;Hypertrophic cardiomyopathy	ENOG410Y9IX	Z:Cytoskeleton	PF00992.23	Troponin	Troponin	CYT	8	46	24.4 High
TRINITY_DN42461_c0_g1_i4_orf1	obscurin [Ostrinia furnacalis]	3.358088473	1.74764024	3.29E-05	up	yes	1.04	0.3097	0.276	0.336	0.317	1.003	1.101	1.016	BP:phosphorylation;BP:cell differentiation;BP:nitrogen compound metabolic process;BP:anatomical structure morphogenesis;BP:cellular developmental process;BP:organic substance metabolic process;BP:developmental process;BP:protein phosphorylation;BP:cellular process;BP:macromolecule modification;BP:protein modification process;BP:biological_process;BP:metabolic process;BP:primary metabolic process;BP:organonitrogen compound metabolic process;BP:protein metabolic process;BP:cellular metabolic process;BP:macromolecule metabolic process;BP:phosphate-containing compound metabolic process;BP:phosphorus metabolic process;MF:purine ribonucleoside triphosphate binding;MF:guanyl-nucleotide exchange factor activity;MF:molecular_function;MF:binding;MF:molecular function regulator;MF:heterocyclic compound binding;MF:nucleoside phosphate binding;MF:anion binding;MF:ion binding;MF:nucleotide binding;MF:transferase activity;MF:protein kinase activity;MF:purine nucleotide binding;MF:carbohydrate derivative binding;MF:ATP binding;MF:kinase activity;MF:enzyme regulator activity;MF:catalytic activity, acting on a protein;MF:small molecule binding;MF:GTPase regulator activity;MF:nucleoside-triphosphatase regulator activity;MF:catalytic activity;MF:transferase activity, transferring phosphorus-containing groups;MF:adenyl nucleotide binding;MF:phosphotransferase activity, alcohol group as acceptor;MF:adenyl ribonucleotide binding;MF:purine ribonucleotide binding;MF:organic cyclic compound binding;MF:ribonucleotide binding;	GO:0016310;GO:0030154;GO:0006807;GO:0009653;GO:0048869;GO:0071704;GO:0032502;GO:0006468;GO:0009987;GO:0043412;GO:0036211;GO:0008150;GO:0008152;GO:0044238;GO:1901564;GO:0019538;GO:0044237;GO:0043170;GO:0006796;GO:0006793;GO:0035639;GO:0005085;GO:0003674;GO:0005488;GO:0098772;GO:1901363;GO:1901265;GO:0043168;GO:0043167;GO:0000166;GO:0016740;GO:0004672;GO:0017076;GO:0097367;GO:0005524;GO:0016301;GO:0030234;GO:0140096;GO:0036094;GO:0030695;GO:0060589;GO:0003824;GO:0016772;GO:0030554;GO:0016773;GO:0032559;GO:0032555;GO:0097159;GO:0032553	------	------	------	------	ENOG410XQFD;EI	S:Function unknown;	PF07679.19;PF13927.9	I-set;Ig_3	Immunoglobulin I-set domain;Immunoglobulin domain	CYT	1	37	11.1 High
TRINITY_DN38366_c0_g1_i4_orfp1	TRINITY_DN38366_c0_g1_i4_m.10666 TRINITY_DN38366_c0_g1::TRINITY_DN38366_c0_g1_i4::g.10666 ORF type:internal len:143 (+) score=71.68 TRINITY_DN38366_c0_g1_i4:3-428(+)	3.043907095	1.605924326	0.0003918	up	yes	0.9567	0.3143	0.28	0.335	0.328	0.947	1.058	0.865	------	------	------	------	------	------	------	------	------	------	------	CYT	1	8	13.5 High
TRINITY_DN4920_c0_g1_i5_orf1	titin homolog [Ostrinia furnacalis]	2.249125639	1.169364254	2.34E-05	up	yes	0.836	0.3717	0.363	0.385	0.367	0.797	0.858	0.853	------	------	------	------	------	------	ENOG4112AA8	S:Function unknown	------	------	------	CYT	19	26	125.1 High
TRINITY_DN1232_c0_g1_i1_orf1	acanthoscurrin-2-like isoform X1 [Ostrinia furnacalis]	2.088406811	1.062402769	0.0001147	up	yes	0.8953	0.4287	0.411	0.453	0.422	0.908	0.937	0.841	CC:cellular_component;CC:integral component of membrane;CC:cellular anatomical entity;CC:intrinsic component of membrane;	GO:0005575;GO:0016021;GO:0110165;GO:0031224	------	------	------	------	------	------	------	------	------	CYT	2	15	16.6 High
TRINITY_DN31001_c0_g1_i1_orf1	endocuticle structural glycoprotein ABD-5-like [Ostrinia furnacalis]	2.648545401	1.405200239	9.94E-06	up	yes	0.9013	0.3403	0.332	0.371	0.318	0.881	0.924	0.899	MF:molecular_function;MF:structural constituent of cuticle;MF:structural molecule activity;	GO:0003674;GO:0042302;GO:0005198	------	------	------	------	ENOG4111EHD;EN	S:Function unknown;	PF00379.26	Chitin_bind_4	Insect cuticle protein	CYT	3	41	12.2 High
TRINITY_DN29100_c0_g1_i2_orf1	endocuticle structural glycoprotein ABD-5-like [Galleria mellonella]	2.130071876	1.090902113	0.0002088	up	yes	0.9187	0.4313	0.422	0.378	0.494	0.907	0.952	0.897	MF:molecular_function;MF:structural constituent of cuticle;MF:structural molecule activity;	GO:0003674;GO:0042302;GO:0005198	------	------	------	------	ENOG41113X7;EN	S:Function unknown;	PF00379.26	Chitin_bind_4	Insect cuticle protein	CYT	3	61	11.2 High
TRINITY_DN7580_c0_g1_i1_orf1	cytochrome P450 monooxygenase CYP6AB141 [Ostrinia furnacalis]	2.010897287	1.007839393	0.0001196	up	yes	0.8673	0.4313	0.407	0.432	0.455	0.839	0.844	0.919	MF:cation binding;MF:monooxygenase activity;MF:heme binding;MF:transition metal ion binding;MF:tetrapyrrole binding;MF:organic cyclic compound binding;MF:oxidoreductase activity, acting on paired donors, with incorporation or reduction of molecular oxygen;MF:ion binding;MF:catalytic activity;MF:molecular_function;MF:binding;MF:iron ion binding;MF:metal ion binding;MF:heterocyclic compound binding;MF:oxidoreductase activity;	GO:0043169;GO:0004497;GO:0020037;GO:0046914;GO:0046906;GO:0097159;GO:0016705;GO:0043167;GO:0003824;GO:0003674;GO:0005488;GO:0005506;GO:0046872;GO:1901363;GO:0016491	K14999	CYP6	------	------	COG2124	S:Function unknown	PF00067.25	p450	Cytochrome P450	CYT	4	14	52.1 High
TRINITY_DN48097_c0_g1_i1_orf1	unnamed protein product [Homo sapiens]	2.056620799	1.040275813	0.03312	up	yes	0.9117	0.4433	0.419	0.443	0.468	1.069	0.62	1.046	BP:positive regulation of cellular amide metabolic process;BP:positive regulation of RNA binding;BP:regulation of primary metabolic process;BP:regulation of metabolic process;BP:positive regulation of cellular biosynthetic process;BP:macromolecular complex subunit organization;BP:regulation of cellular macromolecule biosynthetic process;BP:positive regulation of cellular metabolic process;BP:regulation of cellular metabolic process;BP:positive regulation of gene expression;BP:formation of cytoplasmic translation initiation complex;BP:regulation of biological process;BP:cellular component organization or biogenesis;BP:positive regulation of metabolic process;BP:ribonucleoprotein complex subunit organization;BP:positive regulation of macromolecule metabolic process;BP:posttranscriptional regulation of gene expression;BP:positive regulation of biosynthetic process;BP:regulation of protein metabolic process;BP:positive regulation of protein metabolic process;BP:cellular component organization;BP:macromolecular complex assembly;BP:ribonucleoprotein complex assembly;BP:biological regulation;BP:positive regulation of molecular function;BP:regulation of cellular biosynthetic process;BP:positive regulation of biological process;BP:regulation of molecular function;BP:positive regulation of translation;BP:regulation of gene expression;BP:cellular component assembly;BP:regulation of macromolecule metabolic process;BP:cellular process;BP:regulation of biosynthetic process;BP:regulation of cellular amide metabolic process;BP:biological_process;BP:regulation of nitrogen compound metabolic process;BP:metabolic process;BP:positive regulation of nitrogen compound metabolic process;BP:regulation of cellular process;BP:regulation of mRNA binding;BP:positive regulation of mRNA binding;BP:positive regulation of binding;BP:regulation of binding;BP:cellular metabolic process;BP:positive regulation of macromolecule biosynthetic process;BP:regulation of macromolecule biosynthetic process;BP:regulation of translation;BP:regulation of RNA binding;BP:translational initiation;BP:positive regulation of cellular process;CC:translation preinitiation complex;CC:eukaryotic translation initiation factor 3 complex;CC:cellular anatomical entity;CC:eukaryotic 48S preinitiation complex;CC:macromolecular complex;CC:ribonucleoprotein complex;CC:cellular_component;CC:eukaryotic 43S preinitiation complex;CC:cytosol;MF:molecular_function;MF:binding;MF:nucleic acid binding;MF:translation initiation factor binding;MF:heterocyclic compound binding;MF:translation factor activity, RNA binding;MF:ribonucleoprotein complex binding;MF:translation regulator activity, nucleic acid binding;MF:ribosome binding;MF:translation regulator activity;MF:organic cyclic compound binding;MF:RNA binding;MF:protein binding;MF:macromolecular complex binding;MF:translation initiation factor activity;	GO:0034250;GO:1905216;GO:0080090;GO:0019222;GO:0031328;GO:0043933;GO:2000112;GO:0031325;GO:0031323;GO:0010628;GO:0001732;GO:0050789;GO:0071840;GO:0009893;GO:0071826;GO:0010604;GO:0010608;GO:0009891;GO:0051246;GO:0051247;GO:0016043;GO:0065003;GO:0022618;GO:0065007;GO:0044093;GO:0031326;GO:0048518;GO:0065009;GO:0045727;GO:0010468;GO:0022607;GO:0060255;GO:0009987;GO:0009889;GO:0034248;GO:0008150;GO:0051171;GO:0008152;GO:0051173;GO:0050794;GO:1902415;GO:1902416;GO:0051099;GO:0051098;GO:0044237;GO:0010557;GO:0010556;GO:0006417;GO:1905214;GO:0006413;GO:0048522;GO:0070993;GO:0005852;GO:0110165;GO:0033290;GO:0032991;GO:1990904;GO:0005575;GO:0016282;GO:0005829;GO:0003674;GO:0005488;GO:0003676;GO:0031369;GO:1901363;GO:0008135;GO:0043021;GO:0090079;GO:0043022;GO:0045182;GO:0097159;GO:0003723;GO:0005515;GO:0044877;GO:0003743	K03252	EIF3C	------	------	ENOG410XRU3	S:Function unknown	PF05470.15;PF01399.30	eIF-3c_N;PCI	Eukaryotic translation initiation factor 3 subunit 8 N-terminus;PCI domain	CYT	1	1	70.4 Medium
TRINITY_DN1982_c0_g1_i17_orf1	unnamed protein product, partial [Iphiclides podalirius]	2.287410926	1.193715565	0.0001287	up	yes	0.963	0.421	0.441	0.395	0.427	0.918	0.94	1.031	CC:cellular_component;CC:integral component of membrane;CC:cellular anatomical entity;CC:intrinsic component of membrane;	GO:0005575;GO:0016021;GO:0110165;GO:0031224	------	------	------	------	COG0526	O:Posttranslational modification, protein turnover, chaperones	PF00085.23;PF13098.9;PF13905.9;PF07449.14	Thioredoxin;Thioredoxin_2;Thioredoxin_8;HyaE	Thioredoxin;Thioredoxin-like domain;Thioredoxin-like;Hydrogenase-1 expression protein HyaE	CYT	6	17	124.3 High
TRINITY_DN13216_c0_g1_i5_orf1	uncharacterized protein LOC114358344 isoform X1 [Ostrinia furnacalis]	0.331100178	-1.594660309	6.42E-05	down	yes	0.3163	0.9553	0.901	0.991	0.974	0.315	0.275	0.359	BP:cellular process;BP:regulation of cellular process;BP:biological regulation;BP:cell surface receptor signaling pathway;BP:biological_process;BP:regulation of biological process;BP:signal transduction;CC:integral component of membrane;CC:cellular_component;CC:cellular anatomical entity;CC:intrinsic component of membrane;MF:G-protein coupled receptor activity;MF:signaling receptor activity;MF:molecular transducer activity;MF:transmembrane signaling receptor activity;MF:molecular_function;	GO:0009987;GO:0050794;GO:0065007;GO:0007166;GO:0008150;GO:0050789;GO:0007165;GO:0016021;GO:0005575;GO:0110165;GO:0031224;GO:0004930;GO:0038023;GO:0060089;GO:0004888;GO:0003674	K08451;K0845	ADGRG2, GPF	------	------	ENOG410XSD2	S:Function unknown	PF00002.27;PF01825.24	7tm_2;GPS	7 transmembrane receptor;GPCR proteolysis site, GPS, motif	CYT	1	1	148.9 Medium
TRINITY_DN27592_c0_g1_i1_orf1	D-arabinitol dehydrogenase 1-like [Ostrinia furnacalis]	4.573053892	2.193157921	0.0005257	up	yes	0.7637	0.167	0.155	0.172	0.174	0.851	0.653	0.787	MF:oxidoreductase activity;MF:cation binding;MF:transition metal ion binding;MF:ion binding;MF:catalytic activity;MF:molecular_function;MF:binding;MF:metal ion binding;MF:zinc ion binding;	GO:0016491;GO:0043169;GO:0046914;GO:0043167;GO:0003824;GO:0003674;GO:0005488;GO:0046872;GO:0008270	K17818	ARD1	map00040	Pentose and glucuronate interconversions	COG1063	E:Amino acid transport and metabolism	PF00107.29	ADH_zinc_N	Zinc-binding dehydrogenase	CYT	1	59	15.1 High
TRINITY_DN99673_c0_g1_i1_orf1	PREDICTED: pistil-specific extensin-like protein isoform X2 [Microplitis demolitor]	2.036939314	1.026402999	0.02241	up	yes	0.772	0.379	0.315	0.408	0.414	0.581	0.797	0.938	CC:intracellular membrane-bounded organelle;CC:nucleus;CC:cellular_component;CC:intracellular organelle;CC:membrane-bounded organelle;CC:organelle;CC:cellular anatomical entity;MF:cation binding;MF:transition metal ion binding;MF:organic cyclic compound binding;MF:heterocyclic compound binding;MF:ion binding;MF:molecular_function;MF:zinc ion binding;MF:nucleic acid binding;MF:metal ion binding;MF:binding;	GO:0043231;GO:0005634;GO:0005575;GO:0043229;GO:0043227;GO:0043226;GO:0110165;GO:0043169;GO:0046914;GO:0097159;GO:1901363;GO:0043167;GO:0003674;GO:0008270;GO:0003676;GO:0046872;GO:0005488	------	------	------	------	ENOG4110YZS	S:Function unknown	PF12874.10;PF12171.11;PF06220.15	zf-met;zf-C2H2_jaz;zf-U1	Zinc-finger of C2H2 type;Zinc-finger double-stranded RNA-binding;U1 zinc finger	PLA	1	4	27.3 Medium
TRINITY_DN67231_c0_g1_i1_orf1	endocuticle structural glycoprotein SgAbd-8-like [Ostrinia furnacalis]	41.09751283	5.360979181	5.40E-06	up	yes	1.041	0.02533	0.024	0.02	0.032	0.996	1.101	1.027	MF:molecular_function;MF:structural constituent of cuticle;MF:structural molecule activity;	GO:0003674;GO:0042302;GO:0005198	------	------	------	------	ENOG4111PJ1;EN	S:Function unknown;	PF00379.26	Chitin_bind_4	Insect cuticle protein	CYT	1	20	13.9 High
TRINITY_DN76815_c0_g1_i3_orf1	5-formyltetrahydrofolate cyclo-ligase [Ostrinia furnacalis]	8.413023446	3.072624365	4.82E-05	up	yes	0.8217	0.09767	0.133	0.079	0.081	0.77	0.808	0.887	MF:purine ribonucleoside triphosphate binding;MF:cyclo-ligase activity;MF:molecular_function;MF:binding;MF:ion binding;MF:heterocyclic compound binding;MF:nucleoside phosphate binding;MF:anion binding;MF:cation binding;MF:nucleotide binding;MF:ligase activity, forming carbon-nitrogen bonds;MF:purine nucleotide binding;MF:metal ion binding;MF:ATP binding;MF:small molecule binding;MF:catalytic activity;MF:ligase activity;MF:5-formyltetrahydrofolate cyclo-ligase activity;MF:adenyl nucleotide binding;MF:carbohydrate derivative binding;MF:organic cyclic compound binding;MF:adenyl ribonucleotide binding;MF:purine ribonucleotide binding;MF:ribonucleotide binding;	GO:0035639;GO:0016882;GO:0003674;GO:0005488;GO:0043167;GO:1901363;GO:1901265;GO:0043168;GO:0043169;GO:0000166;GO:0016879;GO:0017076;GO:0046872;GO:0005524;GO:0036094;GO:0003824;GO:0016874;GO:0030272;GO:0030554;GO:0097367;GO:0097159;GO:0032559;GO:0032555;GO:0032553	K01934	MTHFS	map00670	One carbon pool by folate	COG0212	H:Coenzyme transport and metabolism	PF01812.23	5-FTHF_cyc-lig	5-formyltetrahydrofolate cyclo-ligase family	CYT	1	61	14.2 High
TRINITY_DN18396_c0_g1_i1_orf1	uncharacterized protein LOC114359424 [Ostrinia furnacalis]	2.513333333	1.329602023	3.68E-07	up	yes	0.7163	0.285	0.277	0.286	0.292	0.725	0.717	0.707	MF:molecular_function;MF:structural constituent of cuticle;MF:structural molecule activity;	GO:0003674;GO:0042302;GO:0005198	------	------	------	------	ENOG410YSFI;EN	K:Transcription;S:Function unknown;S:Function unknown;S:Function unknown;S:Function unknown	PF00379.26	Chitin_bind_4	Insect cuticle protein	CYT	1	4	39.7 High
TRINITY_DN116951_c0_g3_i2_orf1	spermine oxidase-like isoform X2 [Ostrinia furnacalis]	2.48951049	1.315862094	1.51E-05	up	yes	0.712	0.286	0.281	0.262	0.315	0.719	0.698	0.719	MF:catalytic activity;MF:molecular_function;MF:oxidoreductase activity;	GO:0003824;GO:0003674;GO:0016491	K12259	SMOX, PAO5	map00330;ma	Arginine and proline metabolism;beta-Alanine metabolism	ENOG4111HZN;C	S:Function unknown;E:Amino acid transport and metabolism;S:Function	PF13450.9;PF01593.27	NAD_binding_8;Amino_oxidase	NA;Flavin containing amine oxidoreductase	CYT	2	38	8.6 High
TRINITY_DN53462_c0_g1_i1_orf1	uncharacterized protein LOC118072968 isoform X1 [Chelonus insularis] >XP_034949073.1 uncharacterized protein LOC118072968 isoform X1 [Chelonus insularis]	2.689841377	1.427521098	0.0002961	up	yes	0.797	0.2963	0.361	0.268	0.26	0.75	0.796	0.845	------	------	------	------	------	------	ENOG41125KI	S:Function unknown	PF15376.9	DUF4603	Domain of unknown function	CYT	1	1	69.1 Medium
TRINITY_DN36817_c0_g1_i1_orf1	uncharacterized protein LOC114357350 [Ostrinia furnacalis]	2.350773994	1.233135843	0.001498	up	yes	0.7593	0.323	0.232	0.385	0.352	0.726	0.823	0.729	MF:translation regulator activity, nucleic acid binding;MF:nucleic acid binding;MF:organic cyclic compound binding;MF:translation factor activity, RNA binding;MF:molecular_function;MF:binding;MF:translation regulator activity;MF:heterocyclic compound binding;MF:translation initiation factor activity;	GO:0090079;GO:0003676;GO:0097159;GO:0008135;GO:0003674;GO:0005488;GO:0045182;GO:1901363;GO:0003743	------	------	------	------	ENOG410XV86;EN	S:Function unknown;	------	------	------	CYT	1	29	7.6 High
TRINITY_DN84357_c0_g1_i1_orf1	4-coumarate--CoA ligase 1-like [Ostrinia furnacalis]	2.350332594	1.232864926	0.0001303	up	yes	0.742	0.3157	0.291	0.351	0.305	0.732	0.708	0.786	MF:catalytic activity;MF:monooxygenase activity;MF:molecular_function;MF:oxidoreductase activity;	GO:0003824;GO:0004497;GO:0003674;GO:0016491	K01904	4CL	map00130	Ubiquinone and other terpenoid-quinone biosynthesis	COG0318;COG03	I:Lipid transport and metabolism;Q:Secondary metabolites biosynthesis, transport and catabolism	PF00501.31;PF13193.9	AMP-binding;AMP-binding_C	AMP-binding enzyme;AMP-binding enzyme C-terminal domain	CYT	1	2	58.8 High
TRINITY_DN82801_c0_g1_i1_orf1	uncharacterized protein LOC114364712 [Ostrinia furnacalis]	0.256719454	-1.96173547	4.80E-06	down	yes	0.1767	0.6883	0.695	0.704	0.666	0.167	0.166	0.197	BP:organic substance metabolic process;BP:carbohydrate metabolic process;BP:primary metabolic process;BP:metabolic process;BP:biological_process;CC:cellular_component;CC:extracellular region;CC:cellular anatomical entity;MF:hydrolase activity;MF:deacetylase activity;MF:carbohydrate derivative binding;MF:hydrolase activity, acting on carbon-nitroge;MF:hydrolase activity, acting on carbon-nitroge;MF:catalytic activity;MF:molecular_function;MF:chitin binding;MF:chitin deacetylase activity;MF:binding;	GO:0071704;GO:0005975;GO:0044238;GO:0008152;GO:0008150;GO:0005575;GO:0005576;GO:0110165;GO:0016787;GO:0019213;GO:0097367;GO:0016810;GO:0016811;GO:0003824;GO:0003674;GO:0008061;GO:0004099;GO:0005488	------	------	------	------	ENOG410XX7G	Z:Cytoskeleton	PF01607.27;PF01522.24;PF00057.21	CBM_14;Polysacc_deac_1;Ldl_recept_a	Chitin binding Peritrophin-A domain;Polysaccharide deacetylase;Low-density lipoprotein receptor domain class A	CYT	10	27	61.3 High
TRINITY_DN19160_c0_g1_i1_orf1	alkyldihydroxyacetonephosphate synthase [Ostrinia furnacalis]	0.227919206	-2.133405591	7.69E-05	down	yes	0.1907	0.8367	0.811	0.88	0.819	0.132	0.197	0.243	------	------	K00803	AGPS, agpS	map00565;ma	Ether lipid metabolism;Peroxisome	COG0277	C:Energy production and conversion	PF02913.22;PF01565.26	FAD-oxidase_C;FAD_binding_4	FAD linked oxidases, C-terminal domain;FAD binding domain	CYT	1	1	69.6 Medium
TRINITY_DN146841_c0_g1_i1_orf1	muscle-specific protein 20 [Temnothorax curvispinosus]	4.443349754	2.151647706	1.38E-05	up	yes	0.902	0.203	0.211	0.236	0.162	0.903	0.93	0.873	------	------	K20526	TAGLN	------	------	COG5199	S:Function unknown	PF00307.34;PF00402.21	CH;Calponin	Calponin homology;Calponin family repeat	CYT	1	10	20.6 High
TRINITY_DN5459_c0_g1_i1_orf1	protein takeout-like isoform X2 [Ostrinia furnacalis]	0.321861502	-1.635488069	0.0002828	down	yes	0.231	0.7177	0.755	0.749	0.649	0.233	0.192	0.268	------	------	------	------	------	------	ENOG4111E83;EN	S:Function unknown;	PF06585.14	JHBP	Haemolymph juvenile hormone binding protein	CYT	1	4	21.2 Medium
TRINITY_DN2186_c0_g1_i17_orf1	paxillin isoform X6 [Leguminivora glycinivorella]	4.392571697	2.135065835	2.76E-06	up	yes	0.9343	0.2127	0.196	0.215	0.227	0.903	0.959	0.941	BP:cell-matrix adhesion;BP:cell-substrate adhesion;BP:cellular process;BP:biological_process;BP:cell adhesion;CC:intracellular organelle;CC:non-membrane-bounded organelle;CC:focal adhesion;CC:organelle;CC:cell junction;CC:cell-substrate junction;CC:cytoskeleton;CC:cellular anatomical entity;CC:cytoplasm;CC:anchoring junction;CC:cell cortex;CC:intracellular non-membrane-bounded organelle;CC:cellular_component;MF:molecular_function;MF:binding;MF:cation binding;MF:ion binding;MF:metal ion binding;	GO:0007160;GO:0031589;GO:0009987;GO:0008150;GO:0007155;GO:0043229;GO:0043228;GO:0005925;GO:0043226;GO:0030054;GO:0030055;GO:0005856;GO:0110165;GO:0005737;GO:0070161;GO:0005938;GO:0043232;GO:0005575;GO:0003674;GO:0005488;GO:0043169;GO:0043167;GO:0046872	K05760	PXN	map05205;ma	Proteoglycans in cancer;Human papillomavirus infection;Viral carcinogenesis;Human cytomegalovirus infection;Focal adhesion;Yersinia infection;Shigellosis;VEGF signaling pathway;Human immunodeficiency virus 1 infection;Leukocyte transendothelial migration;Chemokine signaling pathway;Bacterial invasion of epithelial cells;Regulation of actin	ENOG410XRD4	S:Function unknown	PF00412.25	LIM	LIM domain	CYT	2	65	26.4 High
TRINITY_DN58531_c0_g1_i1_orf1	uncharacterized protein LOC114357371 [Ostrinia furnacalis] >XP_028166768.1 uncharacterized protein LOC114357371 [Ostrinia furnacalis]	0.470398555	-1.088044465	0.006008	down	yes	0.3647	0.7753	0.817	0.658	0.851	0.334	0.299	0.461	------	------	------	------	------	------	ENOG410Y4ER;EN	S:Function unknown;	------	------	------	CYT	1	3	29.7 Medium
TRINITY_DN21035_c0_g1_i14_orf1	mitochondrial amidoxime reducing component 2-like [Ostrinia furnacalis]	3.249111725	1.700045354	9.14E-05	up	yes	0.823	0.2533	0.248	0.244	0.268	0.791	0.785	0.893	MF:anion binding;MF:cation binding;MF:molybdenum ion binding;MF:metal ion binding;MF:transition metal ion binding;MF:organic cyclic compound binding;MF:vitamin binding;MF:vitamin B6 binding;MF:small molecule binding;MF:catalytic activity;MF:transferase activity;MF:molecular_function;MF:pyridoxal phosphate binding;MF:ion binding;MF:heterocyclic compound binding;MF:binding;	GO:0043168;GO:0043169;GO:0030151;GO:0046872;GO:0046914;GO:0097159;GO:0019842;GO:0070279;GO:0036094;GO:0003824;GO:0016740;GO:0003674;GO:0030170;GO:0043167;GO:1901363;GO:0005488	------	------	------	------	COG3217	S:Function unknown	PF03476.19	MOSC_N	MOSC N-terminal beta barrel domain	CYT	1	41	15.6 High
TRINITY_DN6881_c0_g1_i1_orf1	putative protein TPRXL [Ostrinia furnacalis]	4.987865416	2.318422539	1.50E-05	up	yes	0.9043	0.1813	0.186	0.15	0.208	0.861	0.941	0.911	MF:molecular_function;MF:structural constituent of cuticle;MF:structural molecule activity;	GO:0003674;GO:0042302;GO:0005198	------	------	------	------	ENOG410ZF0W;E	S:Function unknown;	PF00379.26	Chitin_bind_4	Insect cuticle protein	CYT	5	23	22.4 High
TRINITY_DN23167_c0_g1_i4_orf1	uncharacterized protein LOC114363065 [Ostrinia furnacalis]	2.182802351	1.126181503	0.001841	up	yes	0.7057	0.3233	0.314	0.336	0.32	0.807	0.637	0.673	BP:organic substance metabolic process;BP:organonitrogen compound metabolic process;BP:protein metabolic process;BP:macromolecule metabolic process;BP:biological_process;BP:nitrogen compound metabolic process;BP:metabolic process;BP:proteolysis;BP:primary metabolic process;MF:serine-type endopeptidase activity;MF:endopeptidase activity;MF:hydrolase activity;MF:serine hydrolase activity;MF:catalytic activity, acting on a protein;MF:catalytic activity;MF:molecular_function;MF:peptidase activity;MF:serine-type peptidase activity;	GO:0071704;GO:1901564;GO:0019538;GO:0043170;GO:0008150;GO:0006807;GO:0008152;GO:0006508;GO:0044238;GO:0004252;GO:0004175;GO:0016787;GO:0017171;GO:0140096;GO:0003824;GO:0003674;GO:0008233;GO:0008236	------	------	------	------	COG5640	O:Posttranslational modification, protein turnover, chaperones	PF18322.4	CLIP_1	Serine protease Clip domain PPAF-2	CYT	7	14	69.4 High
TRINITY_DN96557_c0_g1_i1_orf1	charged multivesicular body protein 4B [Phyllostomus discolor]	2.999572101	1.58475671	1.51E-05	up	yes	0.701	0.2337	0.212	0.253	0.236	0.674	0.706	0.723	BP:protein localization;BP:regulation of metabolic process;BP:anatomical structure homeostasis;BP:membrane organization;BP:plasma membrane organization;BP:establishment of organelle localization;BP:cellular component organization or biogenesis;BP:regulation of spindle assembly;BP:cellular macromolecule localization;BP:regulation of cytoskeleton organization;BP:interspecies interaction between organisms;BP:mitotic metaphase plate congression;BP:late endosome to lysosome transport;BP:negative regulation of biological process;BP:macromolecule localization;BP:protein polymerization;BP:negative regulation of cell death;BP:protein targeting;BP:establishment of protein localization;BP:protein localization to membrane;BP:localization within membrane;BP:mitotic cytokinetic process;BP:interaction with host;BP:organonitrogen compound metabolic process;BP:vesicle-mediated transport;BP:protein metabolic process;BP:regulation of autophagy;BP:negative regulation of organelle organization;BP:negative regulation of autophagy;BP:ubiquitin-independent protein catabolic process via the multivesicular body sorting pathway;BP:regulation of microtubule cytoskeleton organization;BP:regulation of catabolic process;BP:regulation of mitotic spindle assembly;BP:negative regulation of metabolic process;BP:negative regulation of neuron death;BP:protein targeting to membrane;BP:nitrogen compound metabolic process;BP:regulation of biological process;BP:organic substance catabolic process;BP:cellular macromolecule catabolic process;BP:cellular macromolecule metabolic process;BP:intracellular protein transport;BP:cellular component organization;BP:macromolecular complex assembly;BP:biological regulation;BP:multivesicular body sorting pathway;BP:regulation of biological quality;BP:transport;BP:posttranslational protein targeting to membrane;BP:regulation of mitotic spindle organization;BP:protein localization to endoplasmic reticulum;BP:biological_process;BP:metabolic process;BP:regulation of neuron death;BP:regulation of cellular process;BP:plasma membrane repair;BP:proteolysis involved in cellular protein catabolic process;BP:negative regulation of catabolic process;BP:intracellular transport;BP:viral budding via host ESCRT complex;BP:protein targeting to ER;BP:ubiquitin-dependent protein catabolic process;BP:regulation of organelle organization;BP:negative regulation of cellular component organization;BP:regulation of cellular component organization;BP:cellular catabolic process;BP:cellular component assembly;BP:lysosomal transport;BP:regulation of microtubule-based process;BP:establishment of localization;BP:ubiquitin-dependent protein catabolic process via the multivesicular body sorting pathway;BP:regulation of cell cycle process;BP:macromolecule catabolic process;BP:nuclear membrane organization;BP:movement in host;BP:nuclear envelope reassembly;BP:proteolysis;BP:establishment of protein localization to endoplasmic reticulum;BP:maintenance of lens transparency;BP:viral release from host cell;BP:negative regulation of cellular catabolic process;BP:establishment of protein localization to organelle;BP:cellular process;BP:modification-dependent protein catabolic process;BP:regulation of centrosome cycle;BP:metaphase plate congression;BP:tissue homeostasis;BP:regulation of autophagosome assembly;BP:cell	GO:0008104;GO:0019222;GO:0060249;GO:0061024;GO:0007009;GO:0051656;GO:0071840;GO:0090169;GO:0070727;GO:0051493;GO:0044419;GO:0007080;GO:1902774;GO:0048519;GO:0033036;GO:0051258;GO:0060548;GO:0006605;GO:0045184;GO:0072657;GO:0051668;GO:1902410;GO:0051701;GO:1901564;GO:0016192;GO:0019538;GO:0010506;GO:0010639;GO:0010507;GO:0090611;GO:0070507;GO:0009894;GO:1901673;GO:0009892;GO:1901215;GO:0006612;GO:0006807;GO:0050789;GO:1901575;GO:0044265;GO:0044260;GO:0006886;GO:0016043;GO:0065003;GO:0065007;GO:0071985;GO:0065008;GO:0006810;GO:0006620;GO:0060236;GO:0070972;GO:0008150;GO:0008152;GO:1901214;GO:0050794;GO:0001778;GO:0051603;GO:0009895;GO:0046907;GO:0039702;GO:0045047;GO:0006511;GO:0033043;GO:0051129;GO:0051128;GO:0044248;GO:0022607;GO:0007041;GO:0032886;GO:0051234;GO:0043162;GO:0010564;GO:0009057;GO:0071763;GO:0044000;GO:0031468;GO:0006508;GO:0072599;GO:0036438;GO:0019076;GO:0031330;GO:0072594;GO:0009987;GO:0019941;GO:0046605;GO:0051310;GO:0001894;GO:2000785;GO:0044770;GO:0061952;GO:0007034;GO:0032506;GO:1902902;GO:0043170;GO:0033365;GO:0090224;GO:0031329;GO:0043933;GO:0031324;GO:0031323;GO:1903047;GO:0042592;GO:0010824;GO:0044772;GO:0022402;GO:0046755;GO:0043632;GO:0010941;GO:0051303;GO:0046753;GO:0035891;GO:0090148;GO:0044087;GO:0	K12194	CHMP4A_B, S	map03250;ma	Viral life cycle - HIV-1;Endocytosis;Necroptosis	ENOG410YE9I	I:Lipid transport and metabolism	PF03357.24	Snf7	Snf7	CYT	1	14	8 High
TRINITY_DN31118_c1_g1_i1_orf1	endocuticle structural glycoprotein ABD-4-like [Ostrinia furnacalis]	4.981253661	2.31650888	3.00E-05	up	yes	0.8503	0.1707	0.193	0.137	0.182	0.809	0.902	0.84	MF:molecular_function;MF:structural constituent of cuticle;MF:structural molecule activity;	GO:0003674;GO:0042302;GO:0005198	------	------	------	------	ENOG4111PJ1;EN	S:Function unknown;	PF00379.26	Chitin_bind_4	Insect cuticle protein	CYT	1	23	12 High
TRINITY_DN25779_c0_g1_i6_orf1	aldo-keto reductase AKR2E4-like [Ostrinia furnacalis]	0.494062829	-1.017233577	6.60E-05	down	yes	0.3287	0.6653	0.645	0.698	0.653	0.311	0.328	0.347	MF:catalytic activity;MF:molecular_function;MF:oxidoreductase activity;	GO:0003824;GO:0003674;GO:0016491	K00011	AKR1B	map00051;ma	Fructose and mannose metabolism;Pentose and glucuronate interconversions;Glycerolipid metabolism;Folate biosynthesis;Galactose metabolism	COG0656	S:Function unknown	PF00248.24	Aldo_ket_red	Aldo/keto reductase family	CYT	5	23	35.9 High

TRINITY_DN129869_c0_g4_i1_orf1	putative myosin heavy chain, muscle, partial [Cotesia chilonis]	2.134023584	1.09357612	1.65E-06	up	yes	0.742	0.3477	0.35	0.343	0.35	0.725	0.747	0.754	CC:sarcomere;CC:myosin complex;CC:cellular anatomical entity;CC:macromolecular complex;CC:cellular_component;MF:purine ribonucleoside triphosphate binding;MF:molecular_function;MF:binding;MF:ion binding;MF:heterocyclic compound binding;MF:nucleoside phosphate binding;MF:anion binding;MF:nucleotide binding;MF:actin binding;MF:purine nucleotide binding;MF:motor activity;MF:actin filament binding;MF:small molecule binding;MF:adenyl nucleotide binding;MF:carbohydrate derivative binding;MF:organic cyclic compound binding;MF:cytoskeletal protein binding;MF:adenyl ribonucleotide binding;MF:ATP binding;MF:protein binding;MF:purine ribonucleotide binding;MF:macromolecular complex binding;MF:ribonucleotide binding;	GO:0030017;GO:0016459;GO:0110165;GO:0032991;GO:0005575;GO:0035639;GO:0003674;GO:0005488;GO:0043167;GO:1901363;GO:1901265;GO:0043168;GO:0000166;GO:0003779;GO:0017076;GO:0003774;GO:0051015;GO:0036094;GO:0030554;GO:0097367;GO:0097159;GO:0008092;GO:0032559;GO:0005524;GO:0005515;GO:0032555;GO:0044877;GO:0032553	K17751	MYH6_7	map04260;ma	Cardiac muscle contraction;Adrenergic signaling in cardiomyocytes;Thyroid hormone signaling pathway;cGMP-PKG signaling pathway;Viral myocarditis;Dilated cardiomyopathy;Hypertrophic cardiomyopathy	COG5022	Z:Cytoskeleton	PF00063.24;PF01576.22;PF02736.22;PF19220.3	Myosin_head;Myosin_tail_1;Myosin_N;Crescentin	Myosin head;Myosin tail;Myosin N-terminal SH3-like domain;Crescentin protein	CYT	42	22	225.6	High
TRINITY_DN14429_c0_g1_i2_orf1	NADH dehydrogenase [ubiquinone] 1 beta subcomplex subunit 11, mitochondrial [Ostrinia furnacalis]	0.490190205	-1.028586438	0.001334	down	yes	0.3273	0.6677	0.7	0.661	0.642	0.268	0.313	0.401	CC:cellular_component;CC:integral component of membrane;CC:cellular anatomical entity;CC:intrinsic component of membrane;	GO:0005575;GO:0016021;GO:0110165;GO:0031224	K11351	NDUFB11	map04714;ma	Thermogenesis;Oxidative phosphorylation;Chemical carcinogenesis - reactive oxygen species;Parkinson disease;Alzheimer disease;Retrograde endocannabinoid signaling;Huntington disease;Amyotrophic lateral sclerosis;Diabetic cardiomyopathy;Non-alcoholic fatty liver disease;Pathways of neurodegeneration - multiple diseases;Prion	ENOG41120A5	U:Intracellular trafficking, secretion, and vesicular transport	PF10183.12	ESSS	ESSS subunit of NAD	PLA	1	8	16.4	High
TRINITY_DN20957_c0_g1_i1_orf1	adenylate kinase isoenzyme 1 isoform X2 [Ostrinia furnacalis]	2.270501475	1.183010974	5.30E-06	up	yes	0.7697	0.339	0.342	0.333	0.342	0.761	0.753	0.795	------	------	K00939	adk, AK	map00730;ma	Thiamine metabolism;Nucleotide metabolism;Purine metabolism;Biosynthesis of cofactors	COG0563	S:Function unknown	PF00406.25;PF13207.9	ADK;AAA_17	Adenylate kinase;AAA domain	CYT	6	30	26.2	High
TRINITY_DN3235_c0_g1_i1_orf1	SPARC [Trichoplusia ni]	0.328663936	-1.605314938	3.66E-05	down	yes	0.2487	0.7567	0.758	0.795	0.717	0.246	0.23	0.27	CC:extracellular space;CC:cellular_component;CC:cellular anatomical entity;MF:cation binding;MF:ion binding;MF:molecular_function;MF:binding;MF:metal ion binding;MF:calcium ion binding;	GO:0005615;GO:0005575;GO:0110165;GO:0043169;GO:0043167;GO:0003674;GO:0005488;GO:0046872;GO:0005509	K24262	SPARC	------	------	ENOG41101WW	S:Function unknown	PF10591.12	SPARC_Ca_bdg	Secreted protein acidic and rich in cysteine Ca binding region	CYT	6	24	36.9	High
TRINITY_DN19814_c0_g1_i4_orf1	general odorant-binding protein 28a-like [Ostrinia furnacalis]	2.209087551	1.143450597	0.0001996	up	yes	0.598	0.2707	0.296	0.262	0.254	0.555	0.617	0.622	MF:odorant binding;MF:molecular_function;MF:binding;	GO:0005549;GO:0003674;GO:0005488	------	------	------	------	ENOG4110ONN;E	S:Function unknown;	PF01395.25	PBP_GOBP	PBP/GOBP family	CYT	1	5	16.7	High
TRINITY_DN3135_c0_g1_i6_orf1	acanthoscurrin-1-like [Ostrinia furnacalis]	2.54289004	1.346469078	0.0001397	up	yes	0.4417	0.1737	0.145	0.192	0.184	0.461	0.42	0.444	------	------	------	------	------	------	------	------	------	------	------	EXC	1	7	25	High
TRINITY_DN1249_c0_g1_i6_orf1	venom carboxylesterase-6-like [Ostrinia furnacalis]	0.364303178	-1.456788512	0.0002216	down	yes	0.1937	0.5317	0.543	0.519	0.533	0.194	0.238	0.149	------	------	------	------	------	------	COG2272;ENOG4	I:Lipid transport and metabolism;T:Signal transduction mechanisms;G:Carbohydrate	PF00135.31;PF20434.1;PF07859.16	COesterase;BD-FAE;Abhydrolase_3	Carboxylesterase family;BD-FAE;alpha/beta hydrolase fold	PLA	1	6	64.7	High
TRINITY_DN4356_c0_g1_i6_orf1	mulatexin-like [Ostrinia furnacalis]	0.499321267	-1.00195974	0.008413	down	yes	0.2207	0.442	0.461	0.395	0.47	0.181	0.182	0.299	------	------	------	------	------	------	------	------	------	------	------	CYT	1	5	20.1	High
TRINITY_DN47731_c0_g1_i2_orf1	nucleolar GTP-binding protein 2 [Ostrinia furnacalis]	0.347607656	-1.524468239	0.000561	down	yes	0.1453	0.418	0.419	0.412	0.423	0.092	0.18	0.164	CC:organelle;CC:intracellular organelle;CC:non-membrane-bounded organelle;CC:nucleolus;CC:cellular anatomical entity;CC:intracellular non-membrane-bounded organelle;CC:cellular_component;MF:purine ribonucleoside triphosphate binding;MF:molecular_function;MF:binding;MF:ion binding;MF:heterocyclic compound binding;MF:nucleoside phosphate binding;MF:anion binding;MF:nucleotide binding;MF:guanyl ribonucleotide binding;MF:purine nucleotide binding;MF:GTP binding;MF:small molecule binding;MF:carbohydrate derivative binding;MF:organic cyclic compound binding;MF:guanyl nucleotide binding;MF:purine ribonucleotide binding;MF:ribonucleotide binding;	GO:0043226;GO:0043229;GO:0043228;GO:0005730;GO:0110165;GO:0043232;GO:0005575;GO:0035639;GO:0003674;GO:0005488;GO:0043167;GO:1901363;GO:1901265;GO:0043168;GO:0000166;GO:0032561;GO:0017076;GO:0005525;GO:0036094;GO:0097367;GO:0097159;GO:0019001;GO:0032555;GO:0032553	K14537	NUG2, GNL2	map03008	Ribosome biogenesis in eukaryotes	COG1161;ENOG4	S:Function unknown;	PF07572.15	BCNT	Bucentaur or craniofacial development	CYT	1	4	30.3	Medium
TRINITY_DN3504_c0_g1_i4_orfp1	TRINITY_DN3504_c0_g1_i4_m.43930 TRINITY_DN3504_c0_g1::TRINITY_DN3504_c0_g1_i4::g.43930 ORF type:internal len:196 (-),score=84.82 TRINITY_DN3504_c0_g1_i4:3-587(-)	3.034008014	1.601224896	0.00257	up	yes	0.2953	0.09733	0.121	0.053	0.118	0.272	0.334	0.28	------	------	------	------	------	------	------	------	------	------	------	EXC	1	8	21.5	High
TRINITY_DN10940_c0_g1_i10_orfp1	TRINITY_DN10940_c0_g1_i10_m.52163 TRINITY_DN10940_c0_g1::TRINITY_DN10940_c0_g1_i10::g.52163 ORF type:5prime_partial len:248 (-),score=128.24 TRINITY_DN10940_c0_g1_i10:121-864(-)	2.461868146	1.299753495	9.37E-05	up	yes	0.1953	0.07933	0.085	0.085	0.068	0.2	0.186	0.2	------	------	------	------	------	------	------	------	------	------	------	CYT	1	14	22.7	High
TRINITY_DN81803_c0_g2_i1_orf1	cathepsin K-like [Ostrinia furnacalis]	0.498900428	-1.003176189	0.00946	down	yes	0.08167	0.1637	0.159	0.174	0.158	0.081	0.053	0.111	BP:primary metabolic process;BP:organonitrogen compound metabolic process;BP:protein metabolic process;BP:macromolecule metabolic process;BP:organic substance metabolic process;BP:nitrogen compound metabolic process;BP:biological_process;BP:metabolic process;BP:proteolysis;MF:molecular_function;MF:hydrolase activity;MF:catalytic activity;MF:catalytic activity, acting on a protein;MF:peptidase activity;MF:cysteine-type peptidase activity;	GO:0044238;GO:1901564;GO:0019538;GO:0043170;GO:0071704;GO:0006807;GO:0008150;GO:0008152;GO:0006508;GO:0003674;GO:0016787;GO:0003824;GO:0140096;GO:0008233;GO:0008234	------	------	------	------	COG4870	O:Posttranslational modification, protein turnover, chaperones	------	------	------	CYT	1	8	13.3	High
TRINITY_DN22664_c0_g1_i1_orf1	larval cuticle protein LCP-14-like [Ostrinia furnacalis]	3.031470777	1.600017915	5.93E-06	up	yes	0.472	0.1557	0.156	0.148	0.163	0.454	0.481	0.481	MF:molecular_function;MF:structural constituent of cuticle;MF:structural molecule activity;	GO:0003674;GO:0042302;GO:0005198	------	------	------	------	ENOG4111PJ1;EN	S:Function unknown;	PF00379.26	Chitin_bind_4	Insect cuticle protein	CYT	6	52	19.3	High
TRINITY_DN117_c0_g1_i6_orf1	lipase member I-like [Ostrinia furnacalis]	0.41732326	-1.260762763	0.001301	down	yes	0.07733	0.1853	0.192	0.178	0.186	0.059	0.071	0.102	BP:organic substance metabolic process;BP:lipid metabolic process;BP:primary metabolic process;BP:metabolic process;BP:biological_process;CC:cellular_component;CC:extracellular region;CC:cellular anatomical entity;MF:hydrolase activity;MF:hydrolase activity, acting on ester bonds;MF:catalytic activity;MF:molecular_function;MF:lipase activity;	GO:0071704;GO:0006629;GO:0044238;GO:0008152;GO:0008150;GO:0005575;GO:0005576;GO:0110165;GO:0016787;GO:0016788;GO:0003824;GO:0003674;GO:0016298	------	------	------	------	ENOG41116Y2;EN	S:Function unknown;	PF00151.22	Lipase	Lipase	CYT	1	11	42.3	High
TRINITY_DN4959_c0_g1_i1_orf1	pancreatic triacylglycerol lipase-like [Ostrinia furnacalis]	0.281627907	-1.828137795	0.008516	down	yes	0.03633	0.129	0.123	0.135	0.129	0.026	0.01	0.073	------	------	------	------	------	------	ENOG410YGS3;EN	S:Function unknown;	PF00151.22	Lipase	Lipase	CYT	1	7	37.1	High
TRINITY_DN37699_c0_g1_i3_orfp1	TRINITY_DN37699_c0_g1_i3_m.58788 TRINITY_DN37699_c0_g1::TRINITY_DN37699_c0_g1_i3::g.58788 ORF type:internal len:122 (+),score=39.86 TRINITY_DN37699_c0_g1_i3:1-363(+)	2.943150685	1.557361408	0.0001541	up	yes	0.4297	0.146	0.114	0.156	0.168	0.406	0.446	0.437	------	------	------	------	------	------	------	------	------	------	------	CYT	1	12	12.6	High
TRINITY_DN2953_c1_g1_i2_orf1	methionine--tRNA ligase, cytoplasmic isoform X6 [Ostrinia furnacalis]	0.3825	-1.386468347	0.00759	down	yes	0.153	0.4	0.378	0.437	0.385	0.174	0.065	0.22	BP:methionyl-tRNA aminoacylation;BP:carboxylic acid metabolic process;BP:nucleic acid metabolic process;BP:cellular nitrogen compound metabolic process;BP:nitrogen compound metabolic process;BP:small molecule metabolic process;BP:ncRNA metabolic process;BP:organic cyclic compound metabolic process;BP:nucleobase-containing compound metabolic process;BP:cellular amino acid metabolic process;BP:organic substance metabolic process;BP:primary metabolic process;BP:cellular process;BP:cellular aromatic compound metabolic process;BP:biological_process;BP:metabolic process;BP:oxoacid metabolic process;BP:organonitrogen compound metabolic process;BP:heterocycle metabolic process;BP:RNA metabolic process;BP:organic acid metabolic process;BP:cellular metabolic process;BP:macromolecule metabolic process;BP:tRNA metabolic process;BP:amino acid activation;BP:tRNA aminoacylation;BP:tRNA aminoacylation for protein translation;CC:aminoacyl-tRNA synthetase multienzyme complex;CC:catalytic complex;CC:macromolecular complex;CC:cellular_component;MF:purine ribonucleoside triphosphate binding;MF:molecular_function;MF:ion binding;MF:heterocyclic compound binding;MF:anion binding;MF:aminoacyl-tRNA ligase activity;MF:nucleotide binding;MF:nucleoside phosphate binding;MF:purine nucleotide binding;MF:ATP binding;MF:catalytic activity, acting on RNA;MF:small molecule binding;MF:catalytic activity;MF:ligase activity;MF:ligase activity, forming carbon-oxygen bonds;MF:methionine-tRNA ligase activity;MF:adenyl nucleotide binding;MF:carbohydrate derivative binding;MF:organic cyclic compound binding;MF:adenyl ribonucleotide binding;MF:purine ribonucleotide binding;MF:catalytic activity, acting on a tRNA;MF:ribonucleotide binding;MF:binding;	GO:0006431;GO:0019752;GO:0090304;GO:0034641;GO:0006807;GO:0044281;GO:0034660;GO:1901360;GO:0006139;GO:0006520;GO:0071704;GO:0044238;GO:0009987;GO:0006725;GO:0008150;GO:0008152;GO:0043436;GO:1901564;GO:0046483;GO:0016070;GO:0006082;GO:0044237;GO:0043170;GO:0006399;GO:0043038;GO:0043039;GO:0006418;GO:0017101;GO:1902494;GO:0032991;GO:0005575;GO:0035639;GO:0003674;GO:0043167;GO:1901363;GO:0043168;GO:0004812;GO:0000166;GO:1901265;GO:0017076;GO:0005524;GO:0140098;GO:0036094;GO:0003824;GO:0016874;GO:0016875;GO:0004825;GO:0030554;GO:0097367;GO:0097159;GO:0032559;GO:0032555;GO:0140101;GO:0032553;GO:0005488	K01874	MARS, metG	map00450;ma	Selenocompound metabolism;Aminoacyl-tRNA biosynthesis	COG0143	J:Translation, ribosomal structure and biogenesis	PF00458.23	WHEP-TRS	WHEP-TRS domain	CYT	1	43	10.3	High
TRINITY_DN2986_c1_g1_i1_orf1	Troponin C, isoform 1 [Papilio xuthus]	3.913	1.96827511	0.0002458	up	yes	0.3913	0.1	0.103	0.098	0.099	0.363	0.373	0.438	MF:cation binding;MF:ion binding;MF:molecular_function;MF:binding;MF:metal ion binding;MF:calcium ion binding;	GO:0043169;GO:0043167;GO:0003674;GO:0005488;GO:0046872;GO:0005509	------	------	------	------	COG5126	F:Nucleotide transport and metabolism	PF13499.9;PF00036.35;PF14658.9;PF13833.9;PF13405.9;PF13202.9	EF-hand_7;EF-hand_1;EF-hand_9;EF-hand_8;EF-hand_6;EF-hand_5	EF-hand domain pair;EF hand;EF-hand domain;EF-hand domain pair;EF-hand domain;EF hand	CYT	5	22	20.2	High
TRINITY_DN1914_c0_g1_i6_orf1	loricrin-like [Ostrinia furnacalis]	0.439086022	-1.187424489	0.002769	down	yes	0.08167	0.186	0.175	0.211	0.172	0.07	0.074	0.101	------	------	------	------	------	------	ENOG4110QTU;E	S:Function unknown;	------	------	------	CYT	1	15	58.9	High
TRINITY_DN5244_c0_g1_i1_orf1	eukaryotic peptide chain release factor GTP-binding subunit-like [Ostrinia furnacalis]	2.118064516	1.082746534	0.0001796	up	yes	0.3283	0.155	0.159	0.148	0.158	0.313	0.319	0.353	------	------	------	------	------	------	ENOG41116ZG;E	S:Function unknown;	PF03103.20	DUF243	Domain of unknown function	CYT	6	20	48.5	High
TRINITY_DN117_c0_g1_i4_orf1	lipase member I-like [Ostrinia furnacalis]	0.290139988	-1.78517895	0.0008912	down	yes	0.04767	0.1643	0.172	0.142	0.179	0.036	0.048	0.059	BP:organic substance metabolic process;BP:lipid metabolic process;BP:primary metabolic process;BP:metabolic process;BP:biological_process;CC:cellular_component;CC:extracellular region;CC:cellular anatomical entity;MF:hydrolase activity;MF:hydrolase activity, acting on ester bonds;MF:catalytic activity;MF:molecular_function;MF:lipase activity;	GO:0071704;GO:0006629;GO:0044238;GO:0008152;GO:0008150;GO:0005575;GO:0005576;GO:0110165;GO:0016787;GO:0016788;GO:0003824;GO:0003674;GO:0016298	------	------	------	------	ENOG41116Y2;EN	S:Function unknown;	PF00151.22	Lipase	Lipase	CYT	5	82	16.1	High
TRINITY_DN3504_c0_g1_i3_orfp2	TRINITY_DN3504_c0_g1_i3_m.43947 TRINITY_DN3504_c0_g1::TRINITY_DN3504_c0_g1_i3::g.43947 ORF type:5prime_partial len:208 (-),score=77.75 TRINITY_DN3504_c0_g1_i3:185-808(-)	2.664122137	1.413660225	0.0004303	up	yes	0.5933	0.2227	0.225	0.263	0.18	0.634	0.598	0.548	------	------	------	------	------	------	------	------	------	------	------	EXC	1	3	22.4	High
TRINITY_DN5578_c0_g1_i10_orf1	unnamed protein product [Chilo suppressalis]	0.461585181	-1.115331188	0.002278	down	yes	0.152	0.3293	0.362	0.307	0.319	0.117	0.155	0.184	------	------	K23390	BAG6	------	------	ENOG410XS9P	O:Posttranslational modification, protein turnover, chaperones	PF00240.26;PF12057.11;PF11976.11	ubiquitin;BAG6;Rad60-SLD	Ubiquitin family;BCL2-associated athanogene 6;Ubiquitin-2 like Rad60 SUMO-like	CYT	1	4	62.9	High
TRINITY_DN29120_c0_g1_i6_orf1	putative inorganic phosphate cotransporter [Ostrinia furnacalis]	0.451558833	-1.147014127	0.0006916	down	yes	0.1347	0.2983	0.3	0.321	0.274	0.119	0.13	0.155	CC:integral component of membrane;CC:cellular_component;CC:cellular anatomical entity;CC:intrinsic component of membrane;MF:transporter activity;MF:transmembrane transporter activity;MF:molecular_function;	GO:0016021;GO:0005575;GO:0110165;GO:0031224;GO:0005215;GO:0022857;GO:0003674	K08193	SLC17A	------	------	ENOG410XPWC;E	G:Carbohydrate transport and metabolism;	PF07690.19	MFS_1	Major Facilitator Superfamily	CYT	1	4	27.3	High
TRINITY_DN38498_c0_g3_i1_orf1	unnamed protein product [Parnassius apollo]	2.758402029	1.463832741	0.02044	up	yes	0.435	0.1577	0.164	0.154	0.155	0.554	0.453	0.298	------	------	------	------	------	------	ENOG410ZSXY;EN	S:Function unknown;	------	------	------	CYT	1	3	36.4	Medium
