# Supplementary material for: Comparative Proteomics and Metabonomics Analysis of Different Diapause Stages Revealed a New Regulation Mechanism of Diapause in Loxostege sticticalis (Lepidoptera: Pyralidae)
Source: Molecules. 2024 Jul 25;29(15):3472. doi: 10.3390/molecules29153472 (PMC11314584; doi:10.3390/molecules29153472)
Supplement: Supplementary file 1 [file molecules-29-03472-s001.zip › analysis process/proteomic/Functional annotation of Proteins.pdf]

Database	Protein Number	Percentage
NR	5252	0.9786
SubCell-Location	5367	1
GO	3664	0.6827
EggNOG	5103	0.9508
KEGG	3750	0.6987
Pfam	4515	0.8413
Total_anno	5367	1
Total	5367	1
