# Supplementary material for: Comparative Proteomics and Metabonomics Analysis of Different Diapause Stages Revealed a New Regulation Mechanism of Diapause in Loxostege sticticalis (Lepidoptera: Pyralidae)
Source: Molecules. 2024 Jul 25;29(15):3472. doi: 10.3390/molecules29153472 (PMC11314584; doi:10.3390/molecules29153472)
Supplement: Supplementary file 1 [file molecules-29-03472-s001.zip › analysis process/proteomic/Gene Set Enrichment Analysis/Fig. B/CTvsRD.pdf]

Protein set name	Description	Group	Size	ES	NES	NOM p-value	FDR q-value	Rank at MAX	Leading edge
MAP04714	Thermogenesis	RD	97	0.9999998	0.9999998	1	0.5344227	96	97
MAP05208	Chemical carcinogenesis - reactive oxygen species	CT	57	-0.24535544	-0.90902215	0.6666667	0.88646936	39	26
MAP05415	Diabetic cardiomyopathy	CT	57	-0.25830278	-0.9768436	0.51626015	0.9140068	39	26
MAP05020	Prion disease	CT	55	-0.24845512	-0.93112123	0.6161137	0.9407944	39	25
MAP04932	Non-alcoholic fatty liver disease	CT	47	-0.20656392	-0.76039773	0.8589212	0.96387035	15	9
MAP04723	Retrograde endocannabinoid signaling	CT	28	-0.17277257	-0.57372147	0.98349833	0.9878916	11	8
MAP05016	Huntington disease	CT	57	-0.26795936	-1.0086614	0.44036698	1	39	26
MAP05022	Pathways of neurodegeneration - multiple diseases	CT	57	-0.26795936	-1.0014677	0.43925235	1	39	26
MAP05014	Amyotrophic lateral sclerosis	CT	58	-0.20686898	-0.78443646	0.8826291	1	39	26
MAP05010	Alzheimer disease	CT	57	-0.26795936	-0.9967606	0.5064378	1	39	26
MAP00190	Oxidative phosphorylation	CT	60	-0.26634753	-1.0068396	0.42156863	1	39	27
MAP05012	Parkinson disease	CT	56	-0.27990893	-1.026239	0.4187192	1	39	26
