# Supplementary material for: Comparative Proteomics and Metabonomics Analysis of Different Diapause Stages Revealed a New Regulation Mechanism of Diapause in Loxostege sticticalis (Lepidoptera: Pyralidae)
Source: Molecules. 2024 Jul 25;29(15):3472. doi: 10.3390/molecules29153472 (PMC11314584; doi:10.3390/molecules29153472)
Supplement: Supplementary file 1 [file molecules-29-03472-s001.zip › analysis process/proteomic/Gene Set Enrichment Analysis/Fig. B/DvsCT.pdf]

Protein set name	Description	Group	Size	ES	NES	NOM p-value	FDR q-value	Rank at MAX	Leading edge
MAP00190	Oxidative phosphorylation	CT	60	0.4971882	1.7970706	0.002222222	0.015357142	40	31
MAP05020	Prion disease	CT	55	0.41298255	1.4667218	0.025404157	0.08921111	40	27
MAP05012	Parkinson disease	CT	56	0.39940497	1.4354329	0.047169812	0.08991782	40	27
MAP05415	Diabetic cardiomyopathy	CT	57	0.37607637	1.3515995	0.055045873	0.10371271	60	45
MAP05208	Chemical carcinogenesis - reactive oxygen species	CT	57	0.4219787	1.493208	0.019704433	0.11522858	40	28
MAP04932	Non-alcoholic fatty liver disease	CT	47	0.37420163	1.3548102	0.07009346	0.12167621	28	16
MAP05016	Huntington disease	CT	57	0.35848197	1.2579846	0.11058824	0.13077356	40	27
MAP05010	Alzheimer disease	CT	57	0.35848197	1.2709435	0.11034483	0.13757738	40	27
MAP05022	Pathways of neurodegeneration - multiple diseases	CT	57	0.35848197	1.2782998	0.09929078	0.14738369	40	27
MAP04723	Retrograde endocannabinoid signaling	CT	28	0.37392604	1.2153298	0.19067797	0.1559095	12	5
MAP05014	Amyotrophic lateral sclerosis	CT	58	0.3393636	1.1990149	0.18262807	0.15656967	40	27
MAP04714	Thermogenesis	CT	97	0.99999994	0.9999998	1	0.5657482	96	97
