# Supplementary material for: Comparative Proteomics and Metabonomics Analysis of Different Diapause Stages Revealed a New Regulation Mechanism of Diapause in Loxostege sticticalis (Lepidoptera: Pyralidae)
Source: Molecules. 2024 Jul 25;29(15):3472. doi: 10.3390/molecules29153472 (PMC11314584; doi:10.3390/molecules29153472)
Supplement: Supplementary file 1 [file molecules-29-03472-s001.zip › analysis process/proteomic/Gene Set Enrichment Analysis/Fig. B/DvsRD.pdf]

Protein set name	Description	Group	Size	ES	NES	NOM p-value	FDR q-value	Rank at MAX	Leading edge
MAP05016	Huntington disease	RD	57	0.13131541	0.41143638	1	0.99960715	33	18
MAP05208	Chemical carcinogenesis - reactive oxygen species	RD	57	0.20152253	0.6307915	0.95929444	1	33	19
MAP00190	Oxidative phosphorylation	RD	60	0.19727477	0.6219267	0.9733893	1	33	21
MAP04714	Thermogenesis	RD	97	0.99999994	1.0000002	0	1	96	97
MAP05014	Amyotrophic lateral sclerosis	RD	58	0.19096515	0.6057708	0.9781421	1	33	19
MAP04932	Non-alcoholic fatty liver disease	RD	47	0.24837239	0.7799862	0.81051177	1	28	14
MAP05010	Alzheimer disease	RD	57	0.13131541	0.41767454	1	1	33	18
MAP05022	Pathways of neurodegeneration - multiple diseases	RD	57	0.13131541	0.41490254	1	1	33	18
MAP05415	Diabetic cardiomyopathy	RD	57	0.16163436	0.5072585	0.99344695	1	78	52
MAP05012	Parkinson disease	RD	56	0.1554725	0.49271584	0.9972028	1	36	20
MAP05020	Prion disease	RD	55	0.19206133	0.6019362	0.9831461	1	78	51
MAP04723	Retrograde endocannabinoid signaling	RD	28	0.3552874	1.0307028	0.41445428	1	22	7
