# Supplementary material for: Comparative Proteomics and Metabonomics Analysis of Different Diapause Stages Revealed a New Regulation Mechanism of Diapause in Loxostege sticticalis (Lepidoptera: Pyralidae)
Source: Molecules. 2024 Jul 25;29(15):3472. doi: 10.3390/molecules29153472 (PMC11314584; doi:10.3390/molecules29153472)
Supplement: Supplementary file 1 [file molecules-29-03472-s001.zip › analysis process/proteomic/Gene Set Enrichment Analysis/Fig. B/NDvsCT.pdf]

Protein set name	Description	Group	Size	ES	NES	NOM p-value	FDR q-value	Rank at MAX	Leading edge
MAP00190	Oxidative phosphorylation	ND	60	-0.6614489	-2.1635334	0	0	48	41
MAP05022	Pathways of neurodegeneration - multiple diseases	ND	57	-0.5883599	-1.9328731	0	0.00087037	48	38
MAP05016	Huntington disease	ND	57	-0.5883599	-1.9404546	0.001012146	0.001044444	48	38
MAP05014	Amyotrophic lateral sclerosis	ND	58	-0.61294436	-1.968304	0.001006036	0.001055556	48	39
MAP05415	Diabetic cardiomyopathy	ND	57	-0.58631724	-1.902558	0.002014099	0.001152778	48	38
MAP05010	Alzheimer disease	ND	57	-0.5883599	-1.9188994	0.002030457	0.001174603	48	38
MAP05208	Chemical carcinogenesis - reactive oxygen species	ND	57	-0.5868642	-1.883835	0	0.001246914	48	38
MAP05012	Parkinson disease	ND	56	-0.5984287	-1.9715023	0.001012146	0.001407407	48	38
MAP05020	Prion disease	ND	55	-0.6117068	-1.994963	0	0.002111111	48	38
MAP04932	Non-alcoholic fatty liver disease	ND	47	-0.488615	-1.5971253	0.007070707	0.014696659	43	28
MAP04723	Retrograde endocannabinoid signaling	ND	28	-0.48907894	-1.5406426	0.014344262	0.021280834	43	19
MAP04714	Thermogenesis	CT	97	1	0.99999976	1	0.9670001	96	97
