# Supplementary material for: Comparative Proteomics and Metabonomics Analysis of Different Diapause Stages Revealed a New Regulation Mechanism of Diapause in Loxostege sticticalis (Lepidoptera: Pyralidae)
Source: Molecules. 2024 Jul 25;29(15):3472. doi: 10.3390/molecules29153472 (PMC11314584; doi:10.3390/molecules29153472)
Supplement: Supplementary file 1 [file molecules-29-03472-s001.zip › analysis process/proteomic/Gene Set Enrichment Analysis/Fig. B/NDvsD.pdf]

Protein set name	Description	Group	Size	ES	NES	NOM p-value	FDR q-value	Rank at MAX	Leading edge
MAP05022	Pathways of neurodegeneration - multiple diseases	ND	57	-0.6879275	-2.2432148	0	0	57	47
MAP00190	Oxidative phosphorylation	ND	60	-0.8004599	-2.5805542	0	0	57	51
MAP05010	Alzheimer disease	ND	57	-0.6879275	-2.2170215	0	0	57	47
MAP05415	Diabetic cardiomyopathy	ND	57	-0.7213168	-2.3496892	0	0	60	50
MAP05012	Parkinson disease	ND	56	-0.7034179	-2.3075316	0	0	57	49
MAP05208	Chemical carcinogenesis - reactive oxygen species	ND	57	-0.6938943	-2.2541056	0	0	57	47
MAP05014	Amyotrophic lateral sclerosis	ND	58	-0.70975393	-2.3166103	0	0	57	48
MAP05020	Prion disease	ND	55	-0.71802104	-2.3032448	0	0	60	49
MAP05016	Huntington disease	ND	57	-0.6879275	-2.2498915	0	0	57	47
MAP04932	Non-alcoholic fatty liver disease	ND	47	-0.5533356	-1.7775393	0.001007049	0.00192	57	38
MAP04723	Retrograde endocannabinoid signaling	ND	28	-0.5014879	-1.5756046	0.011190234	0.014038376	57	25
MAP04714	Thermogenesis	D	97	1.0000002	1.0000004	0	0.024416668	96	97
