# Supplementary material for: Comparative Proteomics and Metabonomics Analysis of Different Diapause Stages Revealed a New Regulation Mechanism of Diapause in Loxostege sticticalis (Lepidoptera: Pyralidae)
Source: Molecules. 2024 Jul 25;29(15):3472. doi: 10.3390/molecules29153472 (PMC11314584; doi:10.3390/molecules29153472)
Supplement: Supplementary file 1 [file molecules-29-03472-s001.zip › analysis process/proteomic/Gene Set Enrichment Analysis/Fig. B/NDvsPreD.pdf]

Protein set name	Description	Group	Size	ES	NES	NOM p-value	FDR q-value	Rank at MAX	Leading edge
MAP05415	Diabetic cardiomyopathy	ND	57	-0.64112914	-2.1394622	0	0	58	48
MAP05014	Amyotrophic lateral sclerosis	ND	58	-0.66444516	-2.204654	0	0	58	47
MAP05012	Parkinson disease	ND	56	-0.6549208	-2.1877604	0	0	58	46
MAP05020	Prion disease	ND	55	-0.67001253	-2.2770717	0	0	58	46
MAP05208	Chemical carcinogenesis - reactive oxygen species	ND	57	-0.63966286	-2.144971	0	0	58	46
MAP05022	Pathways of neurodegeneration - multiple diseases	ND	57	-0.6441208	-2.1746535	0	0	58	46
MAP05016	Huntington disease	ND	57	-0.6441208	-2.1446857	0	0	58	46
MAP05010	Alzheimer disease	ND	57	-0.6441208	-2.1340718	0	0	58	46
MAP00190	Oxidative phosphorylation	ND	60	-0.74056584	-2.5211117	0	0	58	50
MAP04932	Non-alcoholic fatty liver disease	ND	47	-0.53703135	-1.7965263	0	0.00143	52	34
MAP04723	Retrograde endocannabinoid signaling	ND	28	-0.48452395	-1.5755091	0.02047083	0.01633333	52	24
MAP04714	Thermogenesis	PreD	97	1	1.0000001	0	0.028500002	96	97
