# Supplementary material for: Comparative Proteomics and Metabonomics Analysis of Different Diapause Stages Revealed a New Regulation Mechanism of Diapause in Loxostege sticticalis (Lepidoptera: Pyralidae)
Source: Molecules. 2024 Jul 25;29(15):3472. doi: 10.3390/molecules29153472 (PMC11314584; doi:10.3390/molecules29153472)
Supplement: Supplementary file 1 [file molecules-29-03472-s001.zip › analysis process/proteomic/Gene Set Enrichment Analysis/Fig. B/NDvsRD.pdf]

Protein set name	Description	Group	Size	ES	NES	NOM p-value	FDR q-value	Rank at MAX	Leading edge
MAP05020	Prion disease	ND	55	-0.72639024	-2.34977	0	0	58	48
MAP00190	Oxidative phosphorylation	ND	60	-0.783538	-2.494016	0	0	60	53
MAP05208	Chemical carcinogenesis - reactive oxygen species	ND	57	-0.6977576	-2.2633486	0	0	58	48
MAP05022	Pathways of neurodegeneration - multiple diseases	ND	57	-0.7052654	-2.285599	0	0	58	48
MAP05010	Alzheimer disease	ND	57	-0.7052654	-2.266146	0	0	58	48
MAP05415	Diabetic cardiomyopathy	ND	57	-0.7347132	-2.3921754	0	0	58	49
MAP05016	Huntington disease	ND	57	-0.7052654	-2.2500088	0	0	58	48
MAP05014	Amyotrophic lateral sclerosis	ND	58	-0.6916136	-2.24947	0	0	58	48
MAP05012	Parkinson disease	ND	56	-0.7175543	-2.336482	0	0	58	48
MAP04932	Non-alcoholic fatty liver disease	ND	47	-0.54534155	-1.7694863	0.002012072	0.00141	43	30
MAP04723	Retrograde endocannabinoid signaling	ND	28	-0.48122895	-1.5059316	0.019507186	0.024258632	50	23
MAP04714	Thermogenesis	RD	97	0.99999994	1.0000002	0	0.02916667	96	97
