# Supplementary material for: Comparative Proteomics and Metabonomics Analysis of Different Diapause Stages Revealed a New Regulation Mechanism of Diapause in Loxostege sticticalis (Lepidoptera: Pyralidae)
Source: Molecules. 2024 Jul 25;29(15):3472. doi: 10.3390/molecules29153472 (PMC11314584; doi:10.3390/molecules29153472)
Supplement: Supplementary file 1 [file molecules-29-03472-s001.zip › analysis process/proteomic/Gene Set Enrichment Analysis/Fig. B/PreDvsCT.pdf]

Protein set name	Description	Group	Size	ES	NES	NOM p-value	FDR q-value	Rank at MAX	Leading edge
MAP05022	Pathways of neurodegeneration - multiple diseases	CT	57	0.31867146	1.1037676	0.32170543	0.33780524	10	8
MAP05014	Amyotrophic lateral sclerosis	CT	58	0.30262434	1.0594122	0.3658088	0.37192842	10	8
MAP05208	Chemical carcinogenesis - reactive oxygen species	CT	57	0.31835598	1.1039932	0.28545454	0.37946242	10	8
MAP04932	Non-alcoholic fatty liver disease	CT	47	0.32566768	1.112068	0.29443446	0.41426238	10	7
MAP04714	Thermogenesis	CT	97	0.9999999	1.0000001	0	0.42838824	96	97
MAP05010	Alzheimer disease	CT	57	0.31867146	1.1154072	0.28228784	0.47560617	10	8
MAP04723	Retrograde endocannabinoid signaling	CT	28	0.30921027	0.9914765	0.46880907	0.5592495	10	5
MAP05415	Diabetic cardiomyopathy	CT	57	0.3201471	1.1156918	0.29744527	0.56898457	10	8
MAP05016	Huntington disease	CT	57	0.31867146	1.1217412	0.27124774	0.69237137	10	8
MAP05012	Parkinson disease	CT	56	0.32476175	1.1231948	0.24593128	0.91534424	10	8
MAP05020	Prion disease	CT	55	0.32821426	1.150378	0.21818182	1	10	8
MAP00190	Oxidative phosphorylation	CT	60	0.34950295	1.2125224	0.16698657	1	10	9
