# Supplementary material for: Comparative Proteomics and Metabonomics Analysis of Different Diapause Stages Revealed a New Regulation Mechanism of Diapause in Loxostege sticticalis (Lepidoptera: Pyralidae)
Source: Molecules. 2024 Jul 25;29(15):3472. doi: 10.3390/molecules29153472 (PMC11314584; doi:10.3390/molecules29153472)
Supplement: Supplementary file 1 [file molecules-29-03472-s001.zip › analysis process/proteomic/Gene Set Enrichment Analysis/Fig. B/PreDvsD.pdf]

Protein set name	Description	Group	Size	ES	NES	NOM p-value	FDR q-value	Rank at MAX	Leading edge
MAP00190	Oxidative phosphorylation	PreD	60	-0.4303937	-1.4934328	0.02003643	0.12845555	49	39
MAP05012	Parkinson disease	PreD	56	-0.4018364	-1.4116864	0.042372882	0.12853202	42	31
MAP05014	Amyotrophic lateral sclerosis	PreD	58	-0.3962663	-1.3710241	0.064935066	0.13661487	42	32
MAP05020	Prion disease	PreD	55	-0.4132356	-1.4311773	0.03786575	0.14641051	42	31
MAP05010	Alzheimer disease	PreD	57	-0.3693787	-1.2645333	0.11343805	0.15155853	42	31
MAP05022	Pathways of neurodegeneration - multiple diseases	PreD	57	-0.3693787	-1.2791395	0.11028037	0.15901558	42	31
MAP05016	Huntington disease	PreD	57	-0.3693787	-1.2828693	0.09532374	0.17785963	42	31
MAP05415	Diabetic cardiomyopathy	PreD	57	-0.3715041	-1.2986273	0.110320285	0.18531881	42	31
MAP05208	Chemical carcinogenesis - reactive oxygen species	PreD	57	-0.4297972	-1.5094649	0.012345679	0.21506746	42	32
MAP04932	Non-alcoholic fatty liver disease	PreD	47	-0.3368274	-1.1490043	0.23352166	0.26732814	37	23
MAP04714	Thermogenesis	D	97	1	0.9999999	1	0.57209796	96	97
MAP04723	Retrograde endocannabinoid signaling	PreD	28	-0.2718462	-0.85481626	0.67718	0.7167525	37	14
