# Supplementary material for: Comparative Proteomics and Metabonomics Analysis of Different Diapause Stages Revealed a New Regulation Mechanism of Diapause in Loxostege sticticalis (Lepidoptera: Pyralidae)
Source: Molecules. 2024 Jul 25;29(15):3472. doi: 10.3390/molecules29153472 (PMC11314584; doi:10.3390/molecules29153472)
Supplement: Supplementary file 1 [file molecules-29-03472-s001.zip › analysis process/proteomic/Gene Set Enrichment Analysis/Fig. B/PreDvsRD.pdf]

Protein set nar	Description	Group	Size	ES	NES	NOM p-value	FDR q-value	Rank at MAX	Leading edge
MAP00190	Oxidative phosphorylation	RD	60	0.19011109	0.5910735	0.97357994	0.98116636	5	4
MAP05020	Prion disease	RD	55	0.23634306	0.73690677	0.8771466	1	5	4
MAP05014	Amyotrophic lateral sclerosis	RD	58	0.2903355	0.8985626	0.65789473	1	5	5
MAP04714	Thermogenesis	RD	97	1	1	0	1	96	97
MAP05022	Pathways of neurodegeneration - multiple diseases	RD	57	0.22269456	0.69604796	0.92523366	1	5	4
MAP05415	Diabetic cardiomyopathy	RD	57	0.22706288	0.71596	0.9	1	5	4
MAP05010	Alzheimer disease	RD	57	0.22269456	0.7019738	0.9215425	1	5	4
MAP04932	Non-alcoholic fatty liver disease	RD	47	0.24792045	0.76400906	0.83152175	1	4	3
MAP05012	Parkinson disease	RD	56	0.22809412	0.7279902	0.90142673	1	5	4
MAP04723	Retrograde endocannabinoid signaling	RD	28	0.321082	0.91868603	0.5785007	1	1	2
MAP05016	Huntington disease	RD	57	0.22269456	0.68227506	0.9317585	1	5	4
MAP05208	Chemical carcinogenesis - reactive oxygen species	RD	57	0.21771117	0.6688207	0.95225465	1	5	4
