# Supplementary material for: Comparative Proteomics and Metabonomics Analysis of Different Diapause Stages Revealed a New Regulation Mechanism of Diapause in Loxostege sticticalis (Lepidoptera: Pyralidae)
Source: Molecules. 2024 Jul 25;29(15):3472. doi: 10.3390/molecules29153472 (PMC11314584; doi:10.3390/molecules29153472)
Supplement: Supplementary file 1 [file molecules-29-03472-s001.zip › analysis process/proteomic/Gene Set Enrichment Analysis/Fig.A/CTvsRD.pdf]

Protein set name	Description	Group	Size	ES	NES	NOM p-value	FDR q-value	Rank at MAX	Leading edge
MAP04714	Thermogenesis	RD	97	0.9999998	0.9999998	1	0.529066	96	97
MAP05020	Prion disease	CT	55	-0.24845512	-0.91367036	0.65065503	0.90942883	39	25
MAP05014	Amyotrophic lateral sclerosis	CT	58	-0.20686898	-0.76237524	0.8961039	0.9775174	39	26
MAP04723	Retrograde endocannabinoid signaling	CT	28	-0.17277257	-0.57068664	0.99331105	0.99211454	11	8
MAP04932	Non-alcoholic fatty liver disease	CT	47	-0.20656392	-0.7668343	0.86307055	1	15	9
MAP05010	Alzheimer disease	CT	57	-0.26795936	-0.9966901	0.5020576	1	39	26
MAP05022	Pathways of neurodegeneration - multiple diseases	CT	57	-0.26795936	-1.0240308	0.42986426	1	39	26
MAP05012	Parkinson disease	CT	56	-0.27990893	-1.0353229	0.3846154	1	39	26
MAP00190	Oxidative phosphorylation	CT	60	-0.26634753	-0.9899641	0.5381356	1	39	27
MAP05415	Diabetic cardiomyopathy	CT	57	-0.25830278	-0.96958125	0.5377778	1	39	26
MAP05016	Huntington disease	CT	57	-0.26795936	-1.0016297	0.4857143	1	39	26
MAP05208	Chemical carcinogenesis - reactive oxygen species	CT	57	-0.24535544	-0.92541087	0.6334842	1	39	26
