# Supplementary material for: Comparative Proteomics and Metabonomics Analysis of Different Diapause Stages Revealed a New Regulation Mechanism of Diapause in Loxostege sticticalis (Lepidoptera: Pyralidae)
Source: Molecules. 2024 Jul 25;29(15):3472. doi: 10.3390/molecules29153472 (PMC11314584; doi:10.3390/molecules29153472)
Supplement: Supplementary file 1 [file molecules-29-03472-s001.zip › analysis process/proteomic/Gene Set Enrichment Analysis/Fig.A/DvsCT.pdf]

| Protein set name | Description | Group | Size | ES | NES | NOM p-value | FDR q-value | Rank at MAX | Leading edge |
|---|---|---|---|---|---|---|---|---|---|
| MAP00190 | Oxidative phosphorylation | CT | 60 | 0.4971882 | 1.751744 | 0.002512563 | 0.015133333 | 40 | 31 |
| MAP05012 | Parkinson disease | CT | 56 | 0.39940497 | 1.430966 | 0.023148147 | 0.08741901 | 40 | 27 |
| MAP05020 | Prion disease | CT | 55 | 0.41298255 | 1.463409 | 0.04 | 0.098911084 | 40 | 27 |
| MAP05010 | Alzheimer disease | CT | 57 | 0.35848197 | 1.291832 | 0.09307876 | 0.11865299 | 40 | 27 |
| MAP05016 | Huntington disease | CT | 57 | 0.35848197 | 1.270852 | 0.10526316 | 0.120918 | 40 | 27 |
| MAP05208 | Chemical carcinogenesis - reactive oxygen species | CT | 57 | 0.4219787 | 1.488418 | 0.011494253 | 0.12325954 | 40 | 28 |
| MAP05415 | Diabetic cardiomyopathy | CT | 57 | 0.37607637 | 1.349795 | 0.049261082 | 0.12576573 | 60 | 45 |
| MAP04932 | Non-alcoholic fatty liver disease | CT | 47 | 0.37420163 | 1.296815 | 0.112171836 | 0.13093743 | 28 | 16 |
| MAP04723 | Retrograde endocannabinoid signaling | CT | 28 | 0.37392604 | 1.23866 | 0.17162472 | 0.13429235 | 12 | 5 |
| MAP05014 | Amyotrophic lateral sclerosis | CT | 58 | 0.3393636 | 1.215334 | 0.15801887 | 0.1399705 | 40 | 27 |
| MAP05022 | Pathways of neurodegeneration - multiple disease | CT | 57 | 0.35848197 | 1.300456 | 0.09247312 | 0.14793654 | 40 | 27 |
| MAP04714 | Thermogenesis | CT | 97 | 0.99999994 | 1 | 1 | 0.5690424 | 96 | 97 |
