# Supplementary material for: Comparative Proteomics and Metabonomics Analysis of Different Diapause Stages Revealed a New Regulation Mechanism of Diapause in Loxostege sticticalis (Lepidoptera: Pyralidae)
Source: Molecules. 2024 Jul 25;29(15):3472. doi: 10.3390/molecules29153472 (PMC11314584; doi:10.3390/molecules29153472)
Supplement: Supplementary file 1 [file molecules-29-03472-s001.zip › analysis process/proteomic/Gene Set Enrichment Analysis/Fig.A/NDvsCT.pdf]

Protein set name	Description	Group	Size	ES	NES	NOM p-value	FDR q-value	Rank at MAX	Leading edge
MAP05012	Parkinson disease	ND	56	-0.5984287	-1.9534969	0	0	48	38
MAP05415	Diabetic cardiomyopathy	ND	57	-0.58631724	-1.9066437	0	0	48	38
MAP05014	Amyotrophic lateral sclerosis	ND	58	-0.61294436	-1.9583342	0	0	48	39
MAP00190	Oxidative phosphorylation	ND	60	-0.6614489	-2.1583645	0	0	48	41
MAP05016	Huntington disease	ND	57	-0.5883599	-1.9116352	0	0	48	38
MAP05020	Prion disease	ND	55	-0.6117068	-1.9460838	0	0	48	38
MAP05022	Pathways of neurodegeneration - multiple diseases	ND	57	-0.5883599	-1.9072906	0	0	48	38
MAP05010	Alzheimer disease	ND	57	-0.5883599	-1.8841712	0	0.000111111	48	38
MAP05208	Chemical carcinogenesis - reactive oxygen species	ND	57	-0.5868642	-1.888691	0	0.000125	48	38
MAP04932	Non-alcoholic fatty liver disease	ND	47	-0.488615	-1.6032946	0.009063444	0.010449993	43	28
MAP04723	Retrograde endocannabinoid signaling	ND	28	-0.48907894	-1.5533434	0.015212982	0.015972717	43	19
MAP04714	Thermogenesis	CT	97	1	0.99999976	1	0.9723333	96	97
