# Supplementary material for: Comparative Proteomics and Metabonomics Analysis of Different Diapause Stages Revealed a New Regulation Mechanism of Diapause in Loxostege sticticalis (Lepidoptera: Pyralidae)
Source: Molecules. 2024 Jul 25;29(15):3472. doi: 10.3390/molecules29153472 (PMC11314584; doi:10.3390/molecules29153472)
Supplement: Supplementary file 1 [file molecules-29-03472-s001.zip › analysis process/proteomic/Gene Set Enrichment Analysis/Fig.A/NDvsD.pdf]

| Protein set name | Description | Group | Size | ES | NES | NOM p-value | FDR q-value | Rank at MAX | Leading edge |
|---|---|---|---|---|---|---|---|---|---|
| MAP05022 | Pathways of neurodegeneration - multiple diseases | ND | 57 | 0.6879274 | 2.2370198 | 0 | 0 | 55 | 47 |
| MAP05208 | Chemical carcinogenesis - reactive oxygen species | ND | 57 | 0.6938942 | 2.2316275 | 0 | 0 | 55 | 47 |
| MAP05020 | Prion disease | ND | 55 | 0.7180209 | 2.3383408 | 0 | 0 | 58 | 49 |
| MAP05010 | Alzheimer disease | ND | 57 | 0.6879274 | 2.2515464 | 0 | 0 | 55 | 47 |
| MAP00190 | Oxidative phosphorylation | ND | 60 | 0.8004599 | 2.588476 | 0 | 0 | 55 | 51 |
| MAP05014 | Amyotrophic lateral sclerosis | ND | 58 | 0.70975375 | 2.2931688 | 0 | 0 | 55 | 48 |
| MAP05012 | Parkinson disease | ND | 56 | 0.7034179 | 2.3074138 | 0 | 0 | 55 | 47 |
| MAP05415 | Diabetic cardiomyopathy | ND | 57 | 0.7213168 | 2.3290124 | 0 | 0 | 58 | 50 |
| MAP05016 | Huntington disease | ND | 57 | 0.6879274 | 2.2137492 | 0 | 0 | 55 | 47 |
| MAP04932 | Non-alcoholic fatty liver disease | ND | 47 | 0.55333555 | 1.8022877 | 0 | 0.0011 | 55 | 38 |
| MAP04723 | Retrograde endocannabinoid signaling | ND | 28 | 0.5014879 | 1.5814127 | 0.016243655 | 0.013462799 | 55 | 25 |
| MAP04714 | Thermogenesis | ND | 97 | 1.0000002 | 1.0000004 | 0 | 0.4484924 | 96 | 97 |
