# Supplementary material for: Comparative Proteomics and Metabonomics Analysis of Different Diapause Stages Revealed a New Regulation Mechanism of Diapause in Loxostege sticticalis (Lepidoptera: Pyralidae)
Source: Molecules. 2024 Jul 25;29(15):3472. doi: 10.3390/molecules29153472 (PMC11314584; doi:10.3390/molecules29153472)
Supplement: Supplementary file 1 [file molecules-29-03472-s001.zip › analysis process/proteomic/Gene Set Enrichment Analysis/Fig.A/NDvsRD.pdf]

Protein set name	Description	Group	Size	ES	NES	NOM p-value	FDR q-value	Rank at MAX	Leading edge
MAP05415	Diabetic cardiomyopathy	ND	57	-0.7347132	-2.3678968	0	0	58	49
MAP05012	Parkinson disease	ND	56	-0.7175543	-2.3353314	0	0	58	48
MAP05208	Chemical carcinogenesis - reactive oxygen species	ND	57	-0.6977576	-2.206676	0	0	58	48
MAP05022	Pathways of neurodegeneration - multiple disease	ND	57	-0.7052654	-2.25557	0	0	58	48
MAP05020	Prion disease	ND	55	-0.72639024	-2.345456	0	0	58	48
MAP00190	Oxidative phosphorylation	ND	60	-0.783538	-2.505995	0	0	60	53
MAP05010	Alzheimer disease	ND	57	-0.7052654	-2.2880538	0	0	58	48
MAP05016	Huntington disease	ND	57	-0.7052654	-2.272781	0	0	58	48
MAP05014	Amyotrophic lateral sclerosis	ND	58	-0.6916136	-2.2306857	0	0	58	48
MAP04932	Non-alcoholic fatty liver disease	ND	47	-0.54534155	-1.7581639	0.005040322	0.00242	43	30
MAP04714	Thermogenesis	RD	97	0.99999994	1.0000002	0	0.02	96	97
MAP04723	Retrograde endocannabinoid signaling	ND	28	-0.48122895	-1.508771	0.022289768	0.026413195	50	23
