# Supplementary material for: Comparative Proteomics and Metabonomics Analysis of Different Diapause Stages Revealed a New Regulation Mechanism of Diapause in Loxostege sticticalis (Lepidoptera: Pyralidae)
Source: Molecules. 2024 Jul 25;29(15):3472. doi: 10.3390/molecules29153472 (PMC11314584; doi:10.3390/molecules29153472)
Supplement: Supplementary file 1 [file molecules-29-03472-s001.zip › analysis process/proteomic/Gene Set Enrichment Analysis/Fig.A/PreDvsCT.pdf]

Protein set na	Description	Group	Size	ES	NES	NOM p-value	FDR q-value	Rank at MAX	Leading edge
MAP05016	Huntington disease	CT	57	0.31867146	1.09261	0.3299389	0.35331786	10	8
MAP05014	Amyotrophic lateral sclerosis	CT	58	0.30262434	1.0597043	0.38246268	0.36840668	10	8
MAP05208	Chemical carcinogenesis - reactive oxygen species	CT	57	0.31835598	1.1013987	0.28484848	0.3799527	10	8
MAP04714	Thermogenesis	CT	97	0.9999999	1.0000001	0	0.42803422	96	97
MAP05022	Pathways of neurodegeneration - multiple diseases	CT	57	0.31867146	1.1033878	0.3117338	0.42947242	10	8
MAP05415	Diabetic cardiomyopathy	CT	57	0.3201471	1.1105777	0.29227942	0.47964475	10	8
MAP04932	Non-alcoholic fatty liver disease	CT	47	0.32566768	1.1128951	0.27402136	0.5666985	10	7
MAP04723	Retrograde endocannabinoid signaling	CT	28	0.30921027	0.96024257	0.52681994	0.61015344	10	5
MAP05010	Alzheimer disease	CT	57	0.31867146	1.1185085	0.27724665	0.69348735	10	8
MAP05012	Parkinson disease	CT	56	0.32476175	1.1265157	0.27868852	0.88901174	10	8
MAP00190	Oxidative phosphorylation	CT	60	0.34950295	1.2023335	0.18067978	1	10	9
MAP05020	Prion disease	CT	55	0.32821426	1.1336738	0.23699422	1	10	8
