# Supplementary material for: Comparative Proteomics and Metabonomics Analysis of Different Diapause Stages Revealed a New Regulation Mechanism of Diapause in Loxostege sticticalis (Lepidoptera: Pyralidae)
Source: Molecules. 2024 Jul 25;29(15):3472. doi: 10.3390/molecules29153472 (PMC11314584; doi:10.3390/molecules29153472)
Supplement: Supplementary file 1 [file molecules-29-03472-s001.zip › analysis process/proteomic/Gene Set Enrichment Analysis/Fig.A/PreDvsD.pdf]

Protein set name	Description	Group	Size	ES	NES	NOM p-value	FDR q-value	Rank at MAX	Leading edge
MAP05020	Prion disease	PreD	55	-0.4132356	-1.4393284	0.02434457	0.1290813	42	31
MAP05014	Amyotrophic lateral sclerosis	PreD	58	-0.3962663	-1.3627934	0.06007067	0.13479298	42	32
MAP05012	Parkinson disease	PreD	56	-0.40183643	-1.3897313	0.047368422	0.14383493	42	31
MAP05022	Pathways of neurodegeneration - multiple diseases	PreD	57	-0.36937866	-1.2671019	0.120287254	0.14739682	42	31
MAP00190	Oxidative phosphorylation	PreD	60	-0.4303937	-1.4735453	0.04323308	0.15689419	49	39
MAP05415	Diabetic cardiomyopathy	PreD	57	-0.37150407	-1.2741469	0.11904762	0.15790862	42	31
MAP05010	Alzheimer disease	PreD	57	-0.36937866	-1.275521	0.12633452	0.18001801	42	31
MAP05016	Huntington disease	PreD	57	-0.36937866	-1.284736	0.09318996	0.19746703	42	31
MAP04932	Non-alcoholic fatty liver disease	PreD	47	-0.3368274	-1.1509256	0.2462963	0.25489217	37	23
MAP05208	Chemical carcinogenesis - reactive oxygen species	PreD	57	-0.4297972	-1.4992191	0.02930403	0.2684483	42	32
MAP04714	Thermogenesis	D	97	1	0.9999999	1	0.56323916	96	97
MAP04723	Retrograde endocannabinoid signaling	PreD	28	-0.27184623	-0.850798	0.7111111	0.7382452	37	14
