# Supplementary material for: Comparative Proteomics and Metabonomics Analysis of Different Diapause Stages Revealed a New Regulation Mechanism of Diapause in Loxostege sticticalis (Lepidoptera: Pyralidae)
Source: Molecules. 2024 Jul 25;29(15):3472. doi: 10.3390/molecules29153472 (PMC11314584; doi:10.3390/molecules29153472)
Supplement: Supplementary file 1 [file molecules-29-03472-s001.zip › analysis process/proteomic/Gene Set Enrichment Analysis/Fig.A/PreDvsRD.pdf]

Protein set name	Description	Group	Size	ES	NES	NOM p-value	FDR q-value	Rank at MAX	Leading edge
MAP00190	Oxidative phosphorylation	RD	60	0.19011109	0.586712	0.9737569	0.98156077	5	4
MAP05415	Diabetic cardiomyopathy	RD	57	0.22706288	0.7102508	0.92633015	1	5	4
MAP04714	Thermogenesis	RD	97	1	1	0	1	96	97
MAP05014	Amyotrophic lateral sclerosis	RD	58	0.2903355	0.9082735	0.63143986	1	5	5
MAP05022	Pathways of neurodegeneration - multiple disease	RD	57	0.22269456	0.69683456	0.9238845	1	5	4
MAP04723	Retrograde endocannabinoid signaling	RD	28	0.321082	0.92487866	0.5970803	1	1	2
MAP05016	Huntington disease	RD	57	0.22269456	0.6902505	0.92722374	1	5	4
MAP05020	Prion disease	RD	55	0.23634306	0.7489317	0.8669302	1	5	4
MAP05012	Parkinson disease	RD	56	0.22809412	0.70690435	0.9081225	1	5	4
MAP04932	Non-alcoholic fatty liver disease	RD	47	0.24792045	0.76010376	0.85732985	1	4	3
MAP05010	Alzheimer disease	RD	57	0.22269456	0.69896793	0.9339623	1	5	4
MAP05208	Chemical carcinogenesis - reactive oxygen species	RD	57	0.21771117	0.6758186	0.92970824	1	5	4
