# Supplementary material for: Comparative Proteomics and Metabonomics Analysis of Different Diapause Stages Revealed a New Regulation Mechanism of Diapause in Loxostege sticticalis (Lepidoptera: Pyralidae)
Source: Molecules. 2024 Jul 25;29(15):3472. doi: 10.3390/molecules29153472 (PMC11314584; doi:10.3390/molecules29153472)
Supplement: Supplementary file 1 [file molecules-29-03472-s001.zip › analysis process/proteomic/GO annotations analysis/CTvsD all.pdf]

Term Type	GO Term	GO ID	LCL_vs_ZY_a	LCL_vs_ZY_a	LCL_vs_ZY_all Accession ids
biological_process	immune response-activating signal transduction	GO:0002757	1	1/855	TRINITY_DN2170_c0_g2_i1_orf1
biological_process	activation of innate immune response	GO:0002218	3	3/855	TRINITY_DN1091_c0_g2_i10_orf1;TRINITY_DN2170_c0_g2_i1_orf1;TRINITY_DN5880_c0_g2_i2_orf1
biological_process	cell activation involved in immune response	GO:0002263	1	1/855	TRINITY_DN46409_c0_g1_i1_orf1
biological_process	lymphocyte activation	GO:0046649	1	1/855	TRINITY_DN46409_c0_g1_i1_orf1
biological_process	leukocyte activation involved in immune response	GO:0002366	1	1/855	TRINITY_DN46409_c0_g1_i1_orf1
biological_process	innate immune response	GO:0045087	5	5/855	TRINITY_DN1091_c0_g2_i10_orf1;TRINITY_DN9044_c0_g1_i2_orf1;TRINITY_DN2170_c0_g2_i1_orf1;TRINITY_DN5880_c0_g2_i2_orf1;TRINITY_DN1534_c0_g1_i3_orf1
biological_process	somatic diversification of immune receptors via germline recombination within a single locus	GO:0002562	1	1/855	TRINITY_DN46409_c0_g1_i1_orf1
biological_process	somatic diversification of immunoglobulins	GO:0016445	1	1/855	TRINITY_DN46409_c0_g1_i1_orf1
biological_process	regulation of catalytic activity	GO:0050790	3	3/855	TRINITY_DN46409_c0_g1_i1_orf1;TRINITY_DN8473_c0_g1_i5_orf1;TRINITY_DN140538_c0_g2_i1_orf1
biological_process	positive regulation of molecular function	GO:0044093	3	3/855	TRINITY_DN46409_c0_g1_i1_orf1;TRINITY_DN140538_c0_g2_i1_orf1;TRINITY_DN5553_c0_g1_i4_orf1
biological_process	negative regulation of molecular function	GO:0044092	2	2/855	TRINITY_DN140538_c0_g2_i1_orf1;TRINITY_DN31584_c0_g2_i2_orf1
biological_process	regulation of binding	GO:0051098	1	1/855	TRINITY_DN140538_c0_g2_i1_orf1
biological_process	regulation of transporter activity	GO:0032409	2	2/855	TRINITY_DN31584_c0_g2_i2_orf1;TRINITY_DN5553_c0_g1_i4_orf1
biological_process	regulation of metabolic process	GO:0019222	17	17/855	TRINITY_DN6462_c0_g1_i5_orf1;TRINITY_DN1706_c0_g1_i7_orf1;TRINITY_DN33893_c0_g1_i1_orf1;TRINITY_DN20442_c0_g2_i1_orf1;TRINITY_DN12771_c0_g1_i1_orf1;TRINITY_DN4813_c0_g1_i5_orf1;TRINITY_DN12323_c0_g2_i2_orf1;TRINITY_DN77572_c0_g1_i1_orf1;TRINITY_DN8473_c0_g1_i5_orf1;TRINITY_DN46409_c0_g1_i1_orf1;TRINITY_DN21341_c0_g1_i1_orf1;TRINITY_DN810_c0_g1_i4_orf1;TRINITY_DN31584_c0_g2_i2_orf1;TRINITY_DN4309_c0_g1_i1_orf1;TRINITY_DN3649_c0_g1_i6_orf1;TRINITY_DN140538_c0_g2_i1_orf1;TRINITY_DN2630_c0_g3_i3_orf1
biological_process	regulation of response to stimulus	GO:0048583	8	8/855	TRINITY_DN1091_c0_g2_i10_orf1;TRINITY_DN51938_c0_g3_i1_orf1;TRINITY_DN22572_c0_g1_i1_orf1;TRINITY_DN46409_c0_g1_i1_orf1;TRINITY_DN2170_c0_g2_i1_orf1;TRINITY_DN146119_c0_g1_i1_orf1;TRINITY_DN140538_c0_g2_i1_orf1;TRINITY_DN5880_c0_g2_i2_orf1
biological_process	regulation of cellular process	GO:0050794	28	28/855	TRINITY_DN51938_c0_g3_i1_orf1;TRINITY_DN1706_c0_g1_i7_orf1;TRINITY_DN20442_c0_g2_i1_orf1;TRINITY_DN46409_c0_g1_i1_orf1;TRINITY_DN31584_c0_g2_i2_orf1;TRINITY_DN146119_c0_g1_i1_orf1;TRINITY_DN2770_c0_g2_i4_orf1;TRINITY_DN62729_c0_g1_i13_orf1;TRINITY_DN22572_c0_g1_i1_orf1;TRINITY_DN1008_c0_g1_i2_orf1;TRINITY_DN5553_c0_g1_i4_orf1;TRINITY_DN4142_c0_g1_i5_orf1;TRINITY_DN8473_c0_g1_i5_orf1;TRINITY_DN2630_c0_g3_i3_orf1;TRINITY_DN4628_c0_g1_i1_orf1;TRINITY_DN96739_c0_g1_i1_orf1;TRINITY_DN140538_c0_g2_i1_orf1;TRINITY_DN33893_c0_g1_i1_orf1;TRINITY_DN2170_c0_g2_i1_orf1;TRINITY_DN77572_c0_g1_i1_orf1;TRINITY_DN6462_c0_g1_i5_orf1;TRINITY_DN42854_c0_g3_i2_orf1;TRINITY_DN3649_c0_g1_i6_orf1;TRINITY_DN4309_c0_g1_i1_orf1;TRINITY_DN804_c0_g1_i7_orf1;TRINITY_DN15247_c0_g1_i2_orf1;TRINITY_DN21341_c0_g1_i1_orf1;TRINITY_DN46633_c0_g1_i4_orf1
biological_process	regulation of locomotion	GO:0040012	1	1/855	TRINITY_DN140538_c0_g2_i1_orf1
biological_process	regulation of localization	GO:0032879	4	4/855	TRINITY_DN96739_c0_g1_i1_orf1;TRINITY_DN31584_c0_g2_i2_orf1;TRINITY_DN5553_c0_g1_i4_orf1;TRINITY_DN140538_c0_g2_i1_orf1
biological_process	regulation of multicellular organismal process	GO:0051239	2	2/855	TRINITY_DN46409_c0_g1_i1_orf1;TRINITY_DN96739_c0_g1_i1_orf1
biological_process	regulation of membrane repolarization	GO:0060306	1	1/855	TRINITY_DN31584_c0_g2_i2_orf1
biological_process	regulation of immune system process	GO:0002682	4	4/855	TRINITY_DN1091_c0_g2_i10_orf1;TRINITY_DN46409_c0_g1_i1_orf1;TRINITY_DN2170_c0_g2_i1_orf1;TRINITY_DN5880_c0_g2_i2_orf1
biological_process	positive regulation of biological process	GO:0048518	11	11/855	TRINITY_DN1091_c0_g2_i10_orf1;TRINITY_DN51938_c0_g3_i1_orf1;TRINITY_DN22572_c0_g1_i1_orf1;TRINITY_DN46409_c0_g1_i1_orf1;TRINITY_DN2170_c0_g2_i1_orf1;TRINITY_DN31584_c0_g2_i2_orf1;TRINITY_DN5553_c0_g1_i4_orf1;TRINITY_DN146119_c0_g1_i1_orf1;TRINITY_DN4309_c0_g1_i1_orf1;TRINITY_DN140538_c0_g2_i1_orf1;TRINITY_DN5880_c0_g2_i2_orf1
biological_process	negative regulation of biological process	GO:0048519	9	9/855	TRINITY_DN4813_c0_g1_i5_orf1;TRINITY_DN12771_c0_g1_i1_orf1;TRINITY_DN12323_c0_g2_i2_orf1;TRINITY_DN46409_c0_g1_i1_orf1;TRINITY_DN96739_c0_g1_i1_orf1;TRINITY_DN810_c0_g1_i4_orf1;TRINITY_DN31584_c0_g2_i2_orf1;TRINITY_DN140538_c0_g2_i1_orf1;TRINITY_DN2630_c0_g3_i3_orf1
biological_process	regulation of signaling	GO:0023051	5	5/855	TRINITY_DN22572_c0_g1_i1_orf1;TRINITY_DN51938_c0_g3_i1_orf1;TRINITY_DN140538_c0_g2_i1_orf1;TRINITY_DN5553_c0_g1_i4_orf1;TRINITY_DN146119_c0_g1_i1_orf1
biological_process	regulation of membrane potential	GO:0042391	1	1/855	TRINITY_DN31584_c0_g2_i2_orf1
biological_process	regulation of neurotransmitter levels	GO:0001505	2	2/855	TRINITY_DN17693_c0_g1_i10_orf1;TRINITY_DN1652_c0_g1_i12_orf1
biological_process	homeostatic process	GO:0042592	4	4/855	TRINITY_DN1423_c0_g1_i8_orf1;TRINITY_DN96739_c0_g1_i1_orf1;TRINITY_DN31584_c0_g2_i2_orf1;TRINITY_DN136031_c0_g1_i7_orf1
biological_process	regulation of RNA stability	GO:0043487	1	1/855	TRINITY_DN21341_c0_g1_i1_orf1
biological_process	regulation of protein stability	GO:0031647	2	2/855	TRINITY_DN46409_c0_g1_i1_orf1;TRINITY_DN140538_c0_g2_i1_orf1
biological_process	organonitrogen compound metabolic process	GO:1901564	113	113/855	TRINITY_DN3194_c0_g1_i6_orf1;TRINITY_DN5564_c0_g1_i5_orf1;TRINITY_DN344_c1_g1_i1_orf1;TRINITY_DN1153_c1_g1_i1_orf1;TRINITY_DN140538_c0_g2_i1_orf1;TRINITY_DN31163_c1_g1_i4_orf1;TRINITY_DN14754_c0_g1_i6_orf1;TRINITY_DN73945_c0_g5_i3_orf1;TRINITY_DN48020_c0_g1_i1_orf1;TRINITY_DN4822_c0_g1_i6_orf1;TRINITY_DN1534_c0_g1_i3_orf1;TRINITY_DN18273_c0_g1_i4_orf1;TRINITY_DN1760_c0_g1_i4_orf1;TRINITY_DN84478_c0_g1_i8_orf1;TRINITY_DN20527_c0_g1_i1_orf1;TRINITY_DN143_c0_g3_i1_orf1;TRINITY_DN37165_c0_g1_i4_orf1;TRINITY_DN79734_c0_g2_i3_orf1;TRINITY_DN1380_c0_g1_i5_orf1;TRINITY_DN107288_c0_g1_i2_orf1;TRINITY_DN338_c1_g1_i9_orf1;TRINITY_DN4767_c0_g1_i6_orf1;TRINITY_DN230_c2_g1_i5_orf1;TRINITY_DN143895_c0_g1_i1_orf1;TRINITY_DN13856_c0_g1_i1_orf1;TRINITY_DN12526_c0_g1_i5_orf1;TRINITY_DN100821_c0_g1_i1_orf1;TRINITY_DN28626_c0_g1_i5_orf1;TRINITY_DN1791_c0_g1_i3_orf1;TRINITY_DN8659_c0_g2_i1_orf1;TRINITY_DN97589_c0_g1_i3_orf1;TRINITY_DN19187_c0_g1_i1_orf1;TRINITY_DN1789_c0_g1_i5_orf1;TRINITY_DN42461_c0_g1_i4_orf1;TRINITY_DN11125_c0_g1_i1_orf1;TRINITY_DN48602_c0_g1_i6_orf1;TRINITY_DN277_c1_g1_i1_orf1;TRINITY_DN50787_c0_g2_i2_orf1;TRINITY_DN41_c0_g1_i3_orf1;TRINITY_DN45948_c1_g1_i1_orf1;TRINITY_DN5696_c0_g1_i4_orf1;TRINITY_DN36144_c0_g1_i3_orf1;TRINITY_DN5070_c0_g1_i1_orf1;TRINITY_DN4064_c0_g2_i1_orf1;TRINITY_DN3733_c0_g1_i1_orf1;TRINITY_DN4408_c6_g1_i1_orf1;TRINITY_DN95414_c0_g1_i1_orf1;TRINITY_DN21357_c0_g1_i5_orf1;TRINITY_DN779_c0_g1_i12_orf1;TRINITY_DN4767_c0_g1_i4_orf1;TRINITY_DN3702_c0_g1_i1_orf1;TRINITY_DN147458_c0_g1_i1_orf1;TRINITY_DN52553_c0_g2_i1_orf1;TRINITY_DN1749_c0_g2_i2_orf1;TRINITY_DN5112_c0_g1_i1_orf1;TRINITY_DN36434_c0_g2_i3_orf1;TRINITY_DN30131_c0_g1_i1_orf1;TRINITY_DN6365_c0_g1_i4_orf1;TRINITY_DN620_c0_g1_i4_orf1;TRINITY_DN46409_c0_g1_i1_orf1;TRINITY_DN24318_c0_g1_i1_orf1;TRINITY_DN21619_c0_g1_i1_orf1;TRINITY_DN4817_c0_g1_i4_orf1;TRINITY_DN5310_c2_g1_i2_orf1;TRINITY_DN4571_c0_g1_i4_orf1;TRINITY_DN42646_c0_g2_i1_orf1;TRINITY_DN62729_c0_g1_i13_orf1;TRINITY_DN1266_c2_g1_i1_orf1;TRINITY_DN1706_c0_g1_i7_orf1;TRINITY_DN115210_c0_g4_i1_orf1;TRINITY_DN2803_c4_g1_i1_orf1;TRINITY_DN3343_c0_g2_i1_orf1;TRINITY_DN18388_c0_g1_i6_orf1;TRINITY_DN19866_c0_g1_i4_orf1;TRINITY_DN51813_c0_g1_i1_orf1;TRINITY_DN2673_c2_g1_i2_orf1;TRINITY_DN701_c0_g1_i1_orf1;TRINITY_DN83150_c0_g1_i1_orf1;TRINITY_DN12293_c0_g1_i1_orf1;TRINITY_DN113327_c0_g1_i2_orf1;TRINITY_DN143637_c0_g1_i1_orf1;TRINITY_DN111110_c0_g1_i1_orf1;TRINITY_DN15160_c0_g1_i1_orf1;TRINITY_DN768_c0_g1_i7_orf1;TRINITY_DN6205_c0_g1_i1_orf1;TRINITY_DN3836_c0_g1_i4_orf1;TRINITY_DN146718_c0_g1_i1_orf1;TRINITY_DN8716_c0_g1_i3_orf1;TRINITY_DN753_c0_g1_i4_orf1;TRINITY_DN13651_c0_g1_i2_orf1;TRINITY_DN8949_c0_g1_i2_orf1;TRINITY_DN4822_c0_g1_i9_orf1;TRINITY_DN89483_c0_g1_i1_orf1;TRINITY_DN14874_c0_g1_i6_orf1;TRINITY_DN875_c0_g1_i3_orf1;TRINITY_DN143496_c0_g1_i1_orf1;TRINITY_DN17329_c0_g2_i3_orf1;TRINITY_DN3991_c0_g1_i6_orf1;TRINITY_DN24121_c1_g1_i6_orf1;TRINITY_DN8824_c0_g2_i1_orf1;TRINITY_DN892_c7_g1_i2_orf1;TRINITY_DN15222_c0_g1_i4_orf1;TRINITY_DN3826_c0_g1_i1_orf1;TRINITY_DN747_c0_g1_i1_orf1;TRINITY_DN56690_c0_g1_i4_orf1;TRINITY_DN11798_c0_g2_i1_orf1;TRINITY_DN103118_c0_g1_i4_orf1;TRINITY_DN51498_c0_g1_i1_orf1;TRINITY_DN41086_c0_g1_i4_orf1;TRINITY_DN1592_c0_g1_i1_orf1;TRINITY_DN26824_c0_g1_i1_orf1;TRINITY_DN747_c0_g1_i4_orf1;TRINITY_DN6462_c0_g1_i5_orf1
biological_process	cellular nitrogen compound metabolic process	GO:0034641	71	71/855	TRINITY_DN146718_c0_g1_i1_orf1;TRINITY_DN230_c2_g1_i5_orf1;TRINITY_DN3092_c0_g1_i2_orf1;TRINITY_DN24_c0_g1_i1_orf1;TRINITY_DN5112_c0_g1_i1_orf1;TRINITY_DN15222_c0_g1_i4_orf1;TRINITY_DN115210_c0_g4_i1_orf1;TRINITY_DN40434_c0_g1_i2_orf1;TRINITY_DN8716_c0_g1_i3_orf1;TRINITY_DN27852_c0_g1_i1_orf1;TRINITY_DN3826_c0_g1_i1_orf1;TRINITY_DN6365_c0_g1_i4_orf1;TRINITY_DN620_c0_g1_i4_orf1;TRINITY_DN46409_c0_g1_i1_orf1;TRINITY_DN1978_c0_g1_i4_orf1;TRINITY_DN24318_c0_g1_i1_orf1;TRINITY_DN13651_c0_g1_i2_orf1;TRINITY_DN107288_c0_g1_i2_orf1;TRINITY_DN8949_c0_g1_i2_orf1;TRINITY_DN51934_c0_g2_i1_orf1;TRINITY_DN1153_c1_g1_i1_orf1;TRINITY_DN42646_c0_g2_i1_orf1;TRINITY_DN500_c0_g1_i1_orf1;TRINITY_DN34134_c0_g2_i1_orf1;TRINITY_DN30097_c0_g1_i2_orf1;TRINITY_DN143_c0_g3_i1_orf1;TRINITY_DN12301_c0_g1_i1_orf1;TRINITY_DN48602_c0_g1_i6_orf1;TRINITY_DN30131_c0_g1_i1_orf1;TRINITY_DN810_c0_g1_i4_orf1;TRINITY_DN50787_c0_g2_i2_orf1;TRINITY_DN2201_c0_g1_i1_orf1;TRINITY_DN3862_c0_g1_i7_orf1;TRINITY_DN16978_c0_g1_i1_orf1;TRINITY_DN8824_c0_g2_i1_orf1;TRINITY_DN15900_c0_g1_i6_orf1;TRINITY_DN5670_c0_g1_i2_orf1;TRINITY_DN2749_c0_g2_i3_orf1;TRINITY_DN36144_c0_g1_i3_orf1;TRINITY_DN5070_c0_g1_i1_orf1;TRINITY_DN3062_c0_g1_i1_orf1;TRINITY_DN5686_c0_g1_i4_orf1;TRINITY_DN19866_c0_g1_i4_orf1;TRINITY_DN3991_c0_g1_i6_orf1;TRINITY_DN5238_c0_g1_i2_orf1;TRINITY_DN3733_c0_g1_i1_orf1;TRINITY_DN51813_c0_g1_i1_orf1;TRINITY_DN12323_c0_g2_i2_orf1;TRINITY_DN4408_c6_g1_i1_orf1;TRINITY_DN109733_c0_g1_i1_orf1;TRINITY_DN83150_c0_g1_i1_orf1;TRINITY_DN20527_c0_g1_i1_orf1;TRINITY_DN1116_c0_g1_i6_orf1;TRINITY_DN40945_c0_g1_i1_orf1;TRINITY_DN77318_c0_g2_i1_orf1;TRINITY_DN21357_c0_g1_i5_orf1;TRINITY_DN37165_c0_g1_i4_orf1;TRINITY_DN15160_c0_g1_i1_orf1;TRINITY_DN779_c0_g1_i12_orf1;TRINITY_DN4813_c0_g1_i5_orf1;TRINITY_DN97589_c0_g1_i3_orf1;TRINITY_DN768_c0_g1_i7_orf1;TRINITY_DN51498_c0_g1_i1_orf1;TRINITY_DN21619_c0_g1_i1_orf1;TRINITY_DN79734_c0_g2_i3_orf1;TRINITY_DN147458_c0_g1_i1_orf1;TRINITY_DN2299_c0_g1_i3_orf1;TRINITY_DN26824_c0_g1_i1_orf1;TRINITY_DN13760_c1_g1_i1_orf1;TRINITY_DN89613_c0_g1_i13_orf1;TRINITY_DN2749_c4_g1_i2_orf1
biological_process	cellular lipid metabolic process	GO:0044255	18	18/855	TRINITY_DN22046_c1_g1_i5_orf1;TRINITY_DN27903_c0_g1_i1_orf1;TRINITY_DN41_c0_g1_i3_orf1;TRINITY_DN5055_c0_g1_i12_orf1;TRINITY_DN12526_c0_g1_i5_orf1;TRINITY_DN1999_c0_g1_i9_orf1;TRINITY_DN84478_c0_g1_i8_orf1;TRINITY_DN86833_c0_g3_i1_orf1;TRINITY_DN8964_c0_g1_i4_orf1;TRINITY_DN28626_c0_g1_i5_orf1;TRINITY_DN3991_c0_g1_i6_orf1;TRINITY_DN5841_c0_g1_i2_orf1;TRINITY_DN10430_c0_g1_i4_orf1;TRINITY_DN42759_c0_g2_i1_orf1;TRINITY_DN883_c0_g1_i8_orf1;TRINITY_DN768_c0_g1_i7_orf1;TRINITY_DN2441_c0_g1_i1_orf1;TRINITY_DN4321_c0_g1_i1_orf1
biological_process	generation of precursor metabolites and energy	GO:0006091	4	4/855	TRINITY_DN1791_c0_g1_i3_orf1;TRINITY_DN86149_c0_g1_i1_orf1;TRINITY_DN83150_c0_g1_i1_orf1;TRINITY_DN14073_c0_g1_i1_orf1
biological_process	one-carbon metabolic process	GO:0006730	1	1/855	TRINITY_DN20527_c0_g1_i1_orf1
biological_process	cellular ketone metabolic process	GO:0042180	3	3/855	TRINITY_DN8964_c0_g1_i4_orf1;TRINITY_DN51813_c0_g1_i1_orf1;TRINITY_DN28626_c0_g1_i5_orf1
biological_process	heterocycle metabolic process	GO:0046483	52	52/855	TRINITY_DN230_c2_g1_i5_orf1;TRINITY_DN3092_c0_g1_i2_orf1;TRINITY_DN1978_c0_g1_i4_orf1;TRINITY_DN15222_c0_g1_i4_orf1;TRINITY_DN115210_c0_g4_i1_orf1;TRINITY_DN40434_c0_g1_i2_orf1;TRINITY_DN8716_c0_g1_i3_orf1;TRINITY_DN27852_c0_g1_i1_orf1;TRINITY_DN77318_c0_g2_i1_orf1;TRINITY_DN46409_c0_g1_i1_orf1;TRINITY_DN13760_c1_g1_i1_orf1;TRINITY_DN51934_c0_g2_i1_orf1;TRINITY_DN15900_c0_g1_i6_orf1;TRINITY_DN500_c0_g1_i1_orf1;TRINITY_DN34134_c0_g2_i1_orf1;TRINITY_DN30097_c0_g1_i2_orf1;TRINITY_DN24_c0_g1_i1_orf1;TRINITY_DN48602_c0_g1_i6_orf1;TRINITY_DN2803_c4_g1_i1_orf1;TRINITY_DN810_c0_g1_i4_orf1;TRINITY_DN2201_c0_g1_i1_orf1;TRINITY_DN16978_c0_g1_i1_orf1;TRINITY_DN5670_c0_g1_i2_orf1;TRINITY_DN2749_c0_g2_i3_orf1;TRINITY_DN36144_c0_g1_i3_orf1;TRINITY_DN5070_c0_g1_i1_orf1;TRINITY_DN3062_c0_g1_i1_orf1;TRINITY_DN19866_c0_g1_i4_orf1;TRINITY_DN3991_c0_g1_i6_orf1;TRINITY_DN5238_c0_g1_i2_orf1;TRINITY_DN5686_c0_g1_i4_orf1;TRINITY_DN51813_c0_g1_i1_orf1;TRINITY_DN12323_c0_g2_i2_orf1;TRINITY_DN1760_c0_g1_i4_orf1;TRINITY_DN4408_c6_g1_i1_orf1;TRINITY_DN109733_c0_g1_i1_orf1;TRINITY_DN83150_c0_g1_i1_orf1;TRINITY_DN20527_c0_g1_i1_orf1;TRINITY_DN1116_c0_g1_i6_orf1;TRINITY_DN40945_c0_g1_i1_orf1;TRINITY_DN620_c0_g1_i4_orf1;TRINITY_DN37165_c0_g1_i4_orf1;TRINITY_DN15160_c0_g1_i1_orf1;TRINITY_DN779_c0_g1_i12_orf1;TRINITY_DN4813_c0_g1_i5_orf1;TRINITY_DN12301_c0_g1_i1_orf1;TRINITY_DN51498_c0_g1_i1_orf1;TRINITY_DN107288_c0_g1_i2_orf1;TRINITY_DN2299_c0_g1_i3_orf1;TRINITY_DN3862_c0_g1_i7_orf1;TRINITY_DN89613_c0_g1_i13_orf1;TRINITY_DN2749_c4_g1_i2_orf1
biological_process	cellular carbohydrate metabolic process	GO:0044262	4	4/855	TRINITY_DN3010_c0_g1_i4_orf1;TRINITY_DN11942_c0_g1_i1_orf1;TRINITY_DN52244_c1_g1_i1_orf1;TRINITY_DN95850_c0_g1_i1_orf1
biological_process	sulfur compound metabolic process	GO:0006790	3	3/855	TRINITY_DN3991_c0_g1_i6_orf1;TRINITY_DN85476_c0_g1_i1_orf1;TRINITY_DN6985_c0_g1_i5_orf1
biological_process	phosphorus metabolic process	GO:0006793	24	24/855	TRINITY_DN1749_c0_g2_i2_orf1;TRINITY_DN47151_c0_g1_i1_orf1;TRINITY_DN3534_c0_g1_i2_orf1;TRINITY_DN115210_c0_g4_i1_orf1;TRINITY_DN15222_c0_g1_i4_orf1;TRINITY_DN2770_c0_g2_i4_orf1;TRINITY_DN4571_c0_g1_i4_orf1;TRINITY_DN11942_c0_g1_i1_orf1;TRINITY_DN62729_c0_g1_i13_orf1;TRINITY_DN42461_c0_g1_i4_orf1;TRINITY_DN11125_c0_g1_i1_orf1;TRINITY_DN24_c0_g1_i1_orf1;TRINITY_DN48602_c0_g1_i6_orf1;TRINITY_DN277_c1_g1_i1_orf1;TRINITY_DN73945_c0_g5_i3_orf1;TRINITY_DN36144_c0_g1_i3_orf1;TRINITY_DN5070_c0_g1_i1_orf1;TRINITY_DN12301_c0_g1_i1_orf1;TRINITY_DN3991_c0_g1_i6_orf1;TRINITY_DN51813_c0_g1_i1_orf1;TRINITY_DN83150_c0_g1_i1_orf1;TRINITY_DN143637_c0_g1_i1_orf1;TRINITY_DN1266_c2_g1_i1_orf1;TRINITY_DN52244_c1_g1_i1_orf1
biological_process	cellular aldehyde metabolic process	GO:0006081	2	2/855	TRINITY_DN125150_c0_g1_i1_orf1;TRINITY_DN4596_c0_g1_i14_orf1

biological_process	organic acid metabolic process	GO:0006082	26	26/855	TRINITY_DN5055_c0_g1_i12_orf1;TRINITY_DN115210_c0_g4_i1_orf1;TRINITY_DN8716_c0_g1_i3_orf1;TRINITY_DN5564_c0_g1_i5_orf1;TRINITY_DN100821_c0_g1_i1_orf1;TRINITY_DN4822_c0_g1_i9_orf1;TRINITY_DN89483_c0_g1_i1_orf1;TRINITY_DN19187_c0_g1_i1_orf1;TRINITY_DN27903_c0_g1_i1_orf1;TRINITY_DN31163_c1_g1_i4_orf1;TRINITY_DN2803_c4_g1_i1_orf1;TRINITY_DN42759_c0_g2_i1_orf1;TRINITY_DN10430_c0_g1_i4_orf1;TRINITY_DN15160_c0_g1_i1_orf1;TRINITY_DN4822_c0_g1_i6_orf1;TRINITY_DN1999_c0_g1_i9_orf1;TRINITY_DN3991_c0_g1_i6_orf1;TRINITY_DN51813_c0_g1_i1_orf1;TRINITY_DN1760_c0_g1_i4_orf1;TRINITY_DN83150_c0_g1_i1_orf1;TRINITY_DN12293_c0_g1_i1_orf1;TRINITY_DN20527_c0_g1_i1_orf1;TRINITY_DN620_c0_g1_i4_orf1;TRINITY_DN4321_c0_g1_i1_orf1;TRINITY_DN3836_c0_g1_i4_orf1;TRINITY_DN107288_c0_g1_i2_orf1
biological_process	cellular catabolic process	GO:0044248	17	17/855	TRINITY_DN779_c0_g1_i12_orf1;TRINITY_DN19187_c0_g1_i1_orf1;TRINITY_DN768_c0_g1_i7_orf1;TRINITY_DN22242_c0_g1_i1_orf1;TRINITY_DN143496_c0_g1_i1_orf1;TRINITY_DN5055_c0_g1_i12_orf1;TRINITY_DN24_c0_g1_i1_orf1;TRINITY_DN51813_c0_g1_i1_orf1;TRINITY_DN4822_c0_g1_i6_orf1;TRINITY_DN12323_c0_g2_i2_orf1;TRINITY_DN3836_c0_g1_i4_orf1;TRINITY_DN17693_c0_g1_i10_orf1;TRINITY_DN12293_c0_g1_i1_orf1;TRINITY_DN3010_c0_g1_i4_orf1;TRINITY_DN4822_c0_g1_i9_orf1;TRINITY_DN11172_c0_g1_i4_orf1;TRINITY_DN89483_c0_g1_i1_orf1
biological_process	cellular biosynthetic process	GO:0044249	50	50/855	TRINITY_DN146718_c0_g1_i1_orf1;TRINITY_DN230_c2_g1_i5_orf1;TRINITY_DN5112_c0_g1_i1_orf1;TRINITY_DN22046_c1_g1_i5_orf1;TRINITY_DN115210_c0_g4_i1_orf1;TRINITY_DN13651_c0_g1_i2_orf1;TRINITY_DN27852_c0_g1_i1_orf1;TRINITY_DN6365_c0_g1_i4_orf1;TRINITY_DN5564_c0_g1_i5_orf1;TRINITY_DN24318_c0_g1_i1_orf1;TRINITY_DN28626_c0_g1_i5_orf1;TRINITY_DN21619_c0_g1_i1_orf1;TRINITY_DN41645_c0_g1_i1_orf1;TRINITY_DN8949_c0_g1_i2_orf1;TRINITY_DN95850_c0_g1_i1_orf1;TRINITY_DN97589_c0_g1_i3_orf1;TRINITY_DN1153_c1_g1_i1_orf1;TRINITY_DN27903_c0_g1_i1_orf1;TRINITY_DN21357_c0_g1_i5_orf1;TRINITY_DN883_c0_g1_i8_orf1;TRINITY_DN12301_c0_g1_i1_orf1;TRINITY_DN2803_c4_g1_i1_orf1;TRINITY_DN48602_c0_g1_i6_orf1;TRINITY_DN30131_c0_g1_i1_orf1;TRINITY_DN31163_c1_g1_i4_orf1;TRINITY_DN8964_c0_g1_i4_orf1;TRINITY_DN50787_c0_g2_i2_orf1;TRINITY_DN42759_c0_g2_i1_orf1;TRINITY_DN10430_c0_g1_i4_orf1;TRINITY_DN8824_c0_g2_i1_orf1;TRINITY_DN36144_c0_g1_i3_orf1;TRINITY_DN15222_c0_g1_i4_orf1;TRINITY_DN3826_c0_g1_i1_orf1;TRINITY_DN1999_c0_g1_i9_orf1;TRINITY_DN3991_c0_g1_i6_orf1;TRINITY_DN3733_c0_g1_i1_orf1;TRINITY_DN51813_c0_g1_i1_orf1;TRINITY_DN1760_c0_g1_i4_orf1;TRINITY_DN4408_c6_g1_i1_orf1;TRINITY_DN20527_c0_g1_i1_orf1;TRINITY_DN42646_c0_g2_i1_orf1;TRINITY_DN143_c0_g3_i1_orf1;TRINITY_DN37165_c0_g1_i4_orf1;TRINITY_DN4321_c0_g1_i1_orf1;TRINITY_DN4051_c0_g1_i1_orf1;TRINITY_DN51498_c0_g1_i1_orf1;TRINITY_DN79734_c0_g2_i3_orf1;TRINITY_DN147458_c0_g1_i1_orf1;TRINITY_DN2299_c0_g1_i3_orf1;TRINITY_DN26824_c0_g1_i1_orf1
biological_process	cellular macromolecule metabolic process	GO:0044260	39	39/855	TRINITY_DN146718_c0_g1_i1_orf1;TRINITY_DN3092_c0_g1_i2_orf1;TRINITY_DN5112_c0_g1_i1_orf1;TRINITY_DN30131_c0_g1_i1_orf1;TRINITY_DN40434_c0_g1_i2_orf1;TRINITY_DN21619_c0_g1_i1_orf1;TRINITY_DN27852_c0_g1_i1_orf1;TRINITY_DN6365_c0_g1_i4_orf1;TRINITY_DN77318_c0_g2_i1_orf1;TRINITY_DN46409_c0_g1_i1_orf1;TRINITY_DN24318_c0_g1_i1_orf1;TRINITY_DN51934_c0_g2_i1_orf1;TRINITY_DN41645_c0_g1_i1_orf1;TRINITY_DN8949_c0_g1_i2_orf1;TRINITY_DN97589_c0_g1_i3_orf1;TRINITY_DN26824_c0_g1_i1_orf1;TRINITY_DN143496_c0_g1_i1_orf1;TRINITY_DN143_c0_g3_i1_orf1;TRINITY_DN50787_c0_g2_i2_orf1;TRINITY_DN8824_c0_g2_i1_orf1;TRINITY_DN5670_c0_g1_i2_orf1;TRINITY_DN3826_c0_g1_i1_orf1;TRINITY_DN5686_c0_g1_i4_orf1;TRINITY_DN19866_c0_g1_i4_orf1;TRINITY_DN5238_c0_g1_i2_orf1;TRINITY_DN3733_c0_g1_i1_orf1;TRINITY_DN12323_c0_g2_i2_orf1;TRINITY_DN4408_c6_g1_i1_orf1;TRINITY_DN95414_c0_g1_i1_orf1;TRINITY_DN109733_c0_g1_i1_orf1;TRINITY_DN13651_c0_g1_i2_orf1;TRINITY_DN42646_c0_g2_i1_orf1;TRINITY_DN21357_c0_g1_i5_orf1;TRINITY_DN4051_c0_g1_i1_orf1;TRINITY_DN79734_c0_g2_i3_orf1;TRINITY_DN147458_c0_g1_i1_orf1;TRINITY_DN3862_c0_g1_i7_orf1;TRINITY_DN89613_c0_g1_i13_orf1;TRINITY_DN6462_c0_g1_i5_orf1
biological_process	cellular aromatic compound metabolic process	GO:0006725	57	57/855	TRINITY_DN230_c2_g1_i5_orf1;TRINITY_DN3092_c0_g1_i2_orf1;TRINITY_DN24_c0_g1_i1_orf1;TRINITY_DN15222_c0_g1_i4_orf1;TRINITY_DN115210_c0_g4_i1_orf1;TRINITY_DN40434_c0_g1_i2_orf1;TRINITY_DN8716_c0_g1_i3_orf1;TRINITY_DN27852_c0_g1_i1_orf1;TRINITY_DN4822_c0_g1_i6_orf1;TRINITY_DN77318_c0_g2_i1_orf1;TRINITY_DN46409_c0_g1_i1_orf1;TRINITY_DN1978_c0_g1_i4_orf1;TRINITY_DN13760_c1_g1_i1_orf1;TRINITY_DN51934_c0_g2_i1_orf1;TRINITY_DN4822_c0_g1_i9_orf1;TRINITY_DN15900_c0_g1_i6_orf1;TRINITY_DN19187_c0_g1_i1_orf1;TRINITY_DN500_c0_g1_i1_orf1;TRINITY_DN34134_c0_g2_i1_orf1;TRINITY_DN30097_c0_g1_i2_orf1;TRINITY_DN12301_c0_g1_i1_orf1;TRINITY_DN48602_c0_g1_i6_orf1;TRINITY_DN31163_c1_g1_i4_orf1;TRINITY_DN810_c0_g1_i4_orf1;TRINITY_DN2201_c0_g1_i1_orf1;TRINITY_DN16978_c0_g1_i1_orf1;TRINITY_DN11172_c0_g1_i4_orf1;TRINITY_DN2749_c0_g2_i3_orf1;TRINITY_DN36144_c0_g1_i3_orf1;TRINITY_DN5070_c0_g1_i1_orf1;TRINITY_DN3062_c0_g1_i1_orf1;TRINITY_DN19866_c0_g1_i4_orf1;TRINITY_DN3991_c0_g1_i6_orf1;TRINITY_DN5238_c0_g1_i2_orf1;TRINITY_DN5686_c0_g1_i4_orf1;TRINITY_DN51813_c0_g1_i1_orf1;TRINITY_DN12323_c0_g2_i2_orf1;TRINITY_DN4408_c6_g1_i1_orf1;TRINITY_DN109733_c0_g1_i1_orf1;TRINITY_DN83150_c0_g1_i1_orf1;TRINITY_DN5670_c0_g1_i2_orf1;TRINITY_DN12293_c0_g1_i1_orf1;TRINITY_DN20527_c0_g1_i1_orf1;TRINITY_DN1116_c0_g1_i6_orf1;TRINITY_DN40945_c0_g1_i1_orf1;TRINITY_DN620_c0_g1_i4_orf1;TRINITY_DN37165_c0_g1_i4_orf1;TRINITY_DN15160_c0_g1_i1_orf1;TRINITY_DN779_c0_g1_i12_orf1;TRINITY_DN22242_c0_g1_i1_orf1;TRINITY_DN4813_c0_g1_i5_orf1;TRINITY_DN51498_c0_g1_i1_orf1;TRINITY_DN107288_c0_g1_i2_orf1;TRINITY_DN2299_c0_g1_i3_orf1;TRINITY_DN3862_c0_g1_i7_orf1;TRINITY_DN89613_c0_g1_i13_orf1;TRINITY_DN2749_c4_g1_i2_orf1
biological_process	neurotransmitter metabolic process	GO:0042133	1	1/855	TRINITY_DN17693_c0_g1_i10_orf1
biological_process	cellular modified amino acid metabolic process	GO:0006575	2	2/855	TRINITY_DN20527_c0_g1_i1_orf1;TRINITY_DN1153_c1_g1_i1_orf1
biological_process	secondary metabolite biosynthetic process	GO:0044550	1	1/855	TRINITY_DN31163_c1_g1_i4_orf1
biological_process	small molecule biosynthetic process	GO:0044283	14	14/855	TRINITY_DN230_c2_g1_i5_orf1;TRINITY_DN27903_c0_g1_i1_orf1;TRINITY_DN1999_c0_g1_i9_orf1;TRINITY_DN3991_c0_g1_i6_orf1;TRINITY_DN51813_c0_g1_i1_orf1;TRINITY_DN1760_c0_g1_i4_orf1;TRINITY_DN5564_c0_g1_i5_orf1;TRINITY_DN2803_c4_g1_i1_orf1;TRINITY_DN8964_c0_g1_i4_orf1;TRINITY_DN42759_c0_g2_i1_orf1;TRINITY_DN20527_c0_g1_i1_orf1;TRINITY_DN10430_c0_g1_i4_orf1;TRINITY_DN37165_c0_g1_i4_orf1;TRINITY_DN4321_c0_g1_i1_orf1
biological_process	organic substance biosynthetic process	GO:1901576	51	51/855	TRINITY_DN146718_c0_g1_i1_orf1;TRINITY_DN230_c2_g1_i5_orf1;TRINITY_DN5112_c0_g1_i1_orf1;TRINITY_DN22046_c1_g1_i5_orf1;TRINITY_DN115210_c0_g4_i1_orf1;TRINITY_DN13651_c0_g1_i2_orf1;TRINITY_DN27852_c0_g1_i1_orf1;TRINITY_DN6365_c0_g1_i4_orf1;TRINITY_DN5564_c0_g1_i5_orf1;TRINITY_DN24318_c0_g1_i1_orf1;TRINITY_DN28626_c0_g1_i5_orf1;TRINITY_DN21619_c0_g1_i1_orf1;TRINITY_DN41645_c0_g1_i1_orf1;TRINITY_DN8949_c0_g1_i2_orf1;TRINITY_DN95850_c0_g1_i1_orf1;TRINITY_DN97589_c0_g1_i3_orf1;TRINITY_DN1153_c1_g1_i1_orf1;TRINITY_DN27903_c0_g1_i1_orf1;TRINITY_DN21357_c0_g1_i5_orf1;TRINITY_DN143_c0_g3_i1_orf1;TRINITY_DN12301_c0_g1_i1_orf1;TRINITY_DN2803_c4_g1_i1_orf1;TRINITY_DN48602_c0_g1_i6_orf1;TRINITY_DN30131_c0_g1_i1_orf1;TRINITY_DN31163_c1_g1_i4_orf1;TRINITY_DN8964_c0_g1_i4_orf1;TRINITY_DN50787_c0_g2_i2_orf1;TRINITY_DN42759_c0_g2_i1_orf1;TRINITY_DN2441_c0_g1_i1_orf1;TRINITY_DN10430_c0_g1_i4_orf1;TRINITY_DN8824_c0_g2_i1_orf1;TRINITY_DN36144_c0_g1_i3_orf1;TRINITY_DN15222_c0_g1_i4_orf1;TRINITY_DN3826_c0_g1_i1_orf1;TRINITY_DN1999_c0_g1_i9_orf1;TRINITY_DN3991_c0_g1_i6_orf1;TRINITY_DN3733_c0_g1_i1_orf1;TRINITY_DN51813_c0_g1_i1_orf1;TRINITY_DN1760_c0_g1_i4_orf1;TRINITY_DN4408_c6_g1_i1_orf1;TRINITY_DN20527_c0_g1_i1_orf1;TRINITY_DN42646_c0_g2_i1_orf1;TRINITY_DN883_c0_g1_i8_orf1;TRINITY_DN37165_c0_g1_i4_orf1;TRINITY_DN4321_c0_g1_i1_orf1;TRINITY_DN4051_c0_g1_i1_orf1;TRINITY_DN51498_c0_g1_i1_orf1;TRINITY_DN79734_c0_g2_i3_orf1;TRINITY_DN147458_c0_g1_i1_orf1;TRINITY_DN2299_c0_g1_i3_orf1;TRINITY_DN26824_c0_g1_i1_orf1
biological_process	small molecule catabolic process	GO:0044282	11	11/855	TRINITY_DN779_c0_g1_i12_orf1;TRINITY_DN4822_c0_g1_i6_orf1;TRINITY_DN5055_c0_g1_i12_orf1;TRINITY_DN51813_c0_g1_i1_orf1;TRINITY_DN19187_c0_g1_i1_orf1;TRINITY_DN3836_c0_g1_i4_orf1;TRINITY_DN12293_c0_g1_i1_orf1;TRINITY_DN25997_c1_g2_i4_orf1;TRINITY_DN3010_c0_g1_i4_orf1;TRINITY_DN4822_c0_g1_i9_orf1;TRINITY_DN89483_c0_g1_i1_orf1
biological_process	organic substance catabolic process	GO:1901575	18	18/855	TRINITY_DN779_c0_g1_i12_orf1;TRINITY_DN19187_c0_g1_i1_orf1;TRINITY_DN768_c0_g1_i7_orf1;TRINITY_DN143496_c0_g1_i1_orf1;TRINITY_DN1534_c0_g1_i3_orf1;TRINITY_DN5055_c0_g1_i12_orf1;TRINITY_DN24_c0_g1_i1_orf1;TRINITY_DN51813_c0_g1_i1_orf1;TRINITY_DN4822_c0_g1_i6_orf1;TRINITY_DN12323_c0_g2_i2_orf1;TRINITY_DN3836_c0_g1_i4_orf1;TRINITY_DN83150_c0_g1_i1_orf1;TRINITY_DN12293_c0_g1_i1_orf1;TRINITY_DN4817_c0_g1_i4_orf1;TRINITY_DN25997_c1_g2_i4_orf1;TRINITY_DN3010_c0_g1_i4_orf1;TRINITY_DN4822_c0_g1_i9_orf1;TRINITY_DN89483_c0_g1_i1_orf1
biological_process	vitamin metabolic process	GO:0006766	1	1/855	TRINITY_DN37165_c0_g1_i4_orf1
biological_process	monosaccharide metabolic process	GO:0005996	5	5/855	TRINITY_DN9109_c0_g1_i1_orf1;TRINITY_DN4142_c0_g1_i5_orf1;TRINITY_DN25997_c1_g2_i4_orf1;TRINITY_DN30713_c0_g1_i3_orf1;TRINITY_DN15222_c0_g1_i4_orf1
biological_process	alcohol metabolic process	GO:0006066	4	4/855	TRINITY_DN230_c2_g1_i5_orf1;TRINITY_DN3010_c0_g1_i4_orf1;TRINITY_DN11942_c0_g1_i1_orf1;TRINITY_DN52244_c1_g1_i1_orf1
biological_process	nucleobase-containing small molecule metabolic process	GO:0055086	11	11/855	TRINITY_DN36144_c0_g1_i3_orf1;TRINITY_DN5070_c0_g1_i1_orf1;TRINITY_DN12301_c0_g1_i1_orf1;TRINITY_DN3991_c0_g1_i6_orf1;TRINITY_DN24_c0_g1_i1_orf1;TRINITY_DN51813_c0_g1_i1_orf1;TRINITY_DN48602_c0_g1_i6_orf1;TRINITY_DN115210_c0_g4_i1_orf1;TRINITY_DN15222_c0_g1_i4_orf1;TRINITY_DN83150_c0_g1_i1_orf1;TRINITY_DN779_c0_g1_i12_orf1
biological_process	urate metabolic process	GO:0046415	1	1/855	TRINITY_DN779_c0_g1_i12_orf1
biological_process	macromolecule glycosylation	GO:0043413	2	2/855	TRINITY_DN103118_c0_g1_i4_orf1;TRINITY_DN1789_c0_g1_i5_orf1
biological_process	macromolecule methylation	GO:0043414	2	2/855	TRINITY_DN95414_c0_g1_i1_orf1;TRINITY_DN6462_c0_g1_i5_orf1
biological_process	lipid metabolic process	GO:0006629	20	20/855	TRINITY_DN5055_c0_g1_i12_orf1;TRINITY_DN27903_c0_g1_i1_orf1;TRINITY_DN41_c0_g1_i3_orf1;TRINITY_DN22046_c1_g1_i5_orf1;TRINITY_DN3991_c0_g1_i6_orf1;TRINITY_DN768_c0_g1_i7_orf1;TRINITY_DN1999_c0_g1_i9_orf1;TRINITY_DN86833_c0_g3_i1_orf1;TRINITY_DN84478_c0_g1_i8_orf1;TRINITY_DN12526_c0_g1_i5_orf1;TRINITY_DN5841_c0_g1_i2_orf1;TRINITY_DN8964_c0_g1_i4_orf1;TRINITY_DN28626_c0_g1_i5_orf1;TRINITY_DN2441_c0_g1_i1_orf1;TRINITY_DN10430_c0_g1_i4_orf1;TRINITY_DN3529_c0_g1_i7_orf1;TRINITY_DN42759_c0_g2_i1_orf1;TRINITY_DN883_c0_g1_i8_orf1;TRINITY_DN7861_c0_g1_i5_orf1;TRINITY_DN4321_c0_g1_i1_orf1
biological_process	nucleobase-containing compound metabolic process	GO:0006139	46	46/855	TRINITY_DN3092_c0_g1_i2_orf1;TRINITY_DN1978_c0_g1_i4_orf1;TRINITY_DN15222_c0_g1_i4_orf1;TRINITY_DN115210_c0_g4_i1_orf1;TRINITY_DN40434_c0_g1_i2_orf1;TRINITY_DN8716_c0_g1_i3_orf1;TRINITY_DN27852_c0_g1_i1_orf1;TRINITY_DN77318_c0_g2_i1_orf1;TRINITY_DN46409_c0_g1_i1_orf1;TRINITY_DN13760_c1_g1_i1_orf1;TRINITY_DN51934_c0_g2_i1_orf1;TRINITY_DN15900_c0_g1_i6_orf1;TRINITY_DN500_c0_g1_i1_orf1;TRINITY_DN34134_c0_g2_i1_orf1;TRINITY_DN30097_c0_g1_i2_orf1;TRINITY_DN24_c0_g1_i1_orf1;TRINITY_DN48602_c0_g1_i6_orf1;TRINITY_DN810_c0_g1_i4_orf1;TRINITY_DN2201_c0_g1_i1_orf1;TRINITY_DN16978_c0_g1_i1_orf1;TRINITY_DN5670_c0_g1_i2_orf1;TRINITY_DN2749_c0_g2_i3_orf1;TRINITY_DN36144_c0_g1_i3_orf1;TRINITY_DN5070_c0_g1_i1_orf1;TRINITY_DN3062_c0_g1_i1_orf1;TRINITY_DN19866_c0_g1_i4_orf1;TRINITY_DN3991_c0_g1_i6_orf1;TRINITY_DN5238_c0_g1_i2_orf1;TRINITY_DN5686_c0_g1_i4_orf1;TRINITY_DN51813_c0_g1_i1_orf1;TRINITY_DN12323_c0_g2_i2_orf1;TRINITY_DN4408_c6_g1_i1_orf1;TRINITY_DN109733_c0_g1_i1_orf1;TRINITY_DN83150_c0_g1_i1_orf1;TRINITY_DN1116_c0_g1_i6_orf1;TRINITY_DN40945_c0_g1_i1_orf1;TRINITY_DN620_c0_g1_i4_orf1;TRINITY_DN15160_c0_g1_i1_orf1;TRINITY_DN779_c0_g1_i12_orf1;TRINITY_DN4813_c0_g1_i5_orf1;TRINITY_DN12301_c0_g1_i1_orf1;TRINITY_DN107288_c0_g1_i2_orf1;TRINITY_DN2299_c0_g1_i3_orf1;TRINITY_DN3862_c0_g1_i7_orf1;TRINITY_DN89613_c0_g1_i13_orf1;TRINITY_DN2749_c4_g1_i2_orf1
biological_process	tricarboxylic acid cycle	GO:0006099	1	1/855	TRINITY_DN3464_c0_g1_i1_orf1
biological_process	carbohydrate metabolic process	GO:0005975	21	21/855	TRINITY_DN15222_c0_g1_i4_orf1;TRINITY_DN8703_c0_g1_i2_orf1;TRINITY_DN52244_c1_g1_i1_orf1;TRINITY_DN9109_c0_g1_i1_orf1;TRINITY_DN18918_c0_g1_i3_orf1;TRINITY_DN25997_c1_g2_i4_orf1;TRINITY_DN1785_c0_g1_i5_orf1;TRINITY_DN103118_c0_g1_i4_orf1;TRINITY_DN6108_c0_g1_i5_orf1;TRINITY_DN4142_c0_g1_i5_orf1;TRINITY_DN2170_c0_g2_i1_orf1;TRINITY_DN30713_c0_g1_i3_orf1;TRINITY_DN83150_c0_g1_i1_orf1;TRINITY_DN7534_c0_g1_i15_orf1;TRINITY_DN2894_c0_g2_i3_orf1;TRINITY_DN5852_c0_g1_i6_orf1;TRINITY_DN9044_c0_g1_i2_orf1;TRINITY_DN3010_c0_g1_i4_orf1;TRINITY_DN11942_c0_g1_i1_orf1;TRINITY_DN89483_c0_g1_i1_orf1;TRINITY_DN95850_c0_g1_i1_orf1
biological_process	protein metabolic process	GO:0019538	77	77/855	TRINITY_DN4767_c0_g1_i6_orf1;TRINITY_DN146718_c0_g1_i1_orf1;TRINITY_DN73945_c0_g5_i3_orf1;TRINITY_DN5112_c0_g1_i1_orf1;TRINITY_DN143895_c0_g1_i1_orf1;TRINITY_DN13856_c0_g1_i1_orf1;TRINITY_DN30131_c0_g1_i1_orf1;TRINITY_DN21619_c0_g1_i1_orf1;TRINITY_DN3194_c0_g1_i6_orf1;TRINITY_DN6365_c0_g1_i4_orf1;TRINITY_DN46409_c0_g1_i1_orf1;TRINITY_DN24318_c0_g1_i1_orf1;TRINITY_DN103118_c0_g1_i4_orf1;TRINITY_DN13651_c0_g1_i2_orf1;TRINITY_DN4817_c0_g1_i4_orf1;TRINITY_DN8949_c0_g1_i2_orf1;TRINITY_DN1791_c0_g1_i3_orf1;TRINITY_DN344_c1_g1_i1_orf1;TRINITY_DN5310_c2_g1_i2_orf1;TRINITY_DN97589_c0_g1_i3_orf1;TRINITY_DN4571_c0_g1_i4_orf1;TRINITY_DN52553_c0_g2_i1_orf1;TRINITY_DN62729_c0_g1_i13_orf1;TRINITY_DN143496_c0_g1_i1_orf1;TRINITY_DN17329_c0_g2_i3_orf1;TRINITY_DN42461_c0_g1_i4_orf1;TRINITY_DN143_c0_g3_i1_orf1;TRINITY_DN11125_c0_g1_i1_orf1;TRINITY_DN56690_c0_g1_i4_orf1;TRINITY_DN41086_c0_g1_i4_orf1;TRINITY_DN277_c1_g1_i1_orf1;TRINITY_DN50787_c0_g2_i2_orf1;TRINITY_DN14754_c0_g1_i6_orf1;TRINITY_DN45948_c1_g1_i1_orf1;TRINITY_DN1592_c0_g1_i1_orf1;TRINITY_DN8824_c0_g2_i1_orf1;TRINITY_DN14874_c0_g1_i6_orf1;TRINITY_DN5696_c0_g1_i4_orf1;TRINITY_DN18388_c0_g1_i6_orf1;TRINITY_DN3343_c0_g2_i1_orf1;TRINITY_DN48020_c0_g1_i1_orf1;TRINITY_DN3826_c0_g1_i1_orf1;TRINITY_DN747_c0_g1_i1_orf1;TRINITY_DN875_c0_g1_i3_orf1;TRINITY_DN19866_c0_g1_i4_orf1;TRINITY_DN18273_c0_g1_i4_orf1;TRINITY_DN4064_c0_g2_i1_orf1;TRINITY_DN3733_c0_g1_i1_orf1;TRINITY_DN1706_c0_g1_i7_orf1;TRINITY_DN2673_c2_g1_i2_orf1;TRINITY_DN753_c0_g1_i4_orf1;TRINITY_DN4408_c6_g1_i1_orf1;TRINITY_DN701_c0_g1_i1_orf1;TRINITY_DN95414_c0_g1_i1_orf1;TRINITY_DN113327_c0_g1_i2_orf1;TRINITY_DN42646_c0_g2_i1_orf1;TRINITY_DN21357_c0_g1_i5_orf1;TRINITY_DN140538_c0_g2_i1_orf1;TRINITY_DN111110_c0_g1_i1_orf1;TRINITY_DN36434_c0_g2_i3_orf1;TRINITY_DN8659_c0_g2_i1_orf1;TRINITY_DN1380_c0_g1_i5_orf1;TRINITY_DN1266_c2_g1_i1_orf1;TRINITY_DN1789_c0_g1_i5_orf1;TRINITY_DN4767_c0_g1_i4_orf1;TRINITY_DN143637_c0_g1_i1_orf1;TRINITY_DN24121_c1_g1_i6_orf1;TRINITY_DN6205_c0_g1_i1_orf1;TRINITY_DN892_c7_g1_i2_orf1;TRINITY_DN79734_c0_g2_i3_orf1;TRINITY_DN3702_c0_g1_i1_orf1;TRINITY_DN147458_c0_g1_i1_orf1;TRINITY_DN26824_c0_g1_i1_orf1;TRINITY_DN338_c1_g1_i9_orf1;TRINITY_DN1749_c0_g2_i2_orf1;TRINITY_DN747_c0_g1_i4_orf1;TRINITY_DN6462_c0_g1_i5_orf1

biological_process	cellular amino acid metabolic process	GO:0006520	18	18/855	TRINITY_DN4822_c0_g1_i6_orf1;TRINITY_DN8716_c0_g1_i3_orf1;TRINITY_DN89483_c0_g1_i1_orf1;TRINITY_DN115210_c0_g4_i1_orf1;TRINITY_DN2803_c4_g1_i1_orf1;TRINITY_DN51813_c0_g1_i1_orf1;TRINITY_DN19187_c0_g1_i1_orf1;TRINITY_DN1760_c0_g1_i4_orf1;TRINITY_DN5564_c0_g1_i5_orf1;TRINITY_DN31163_c1_g1_i4_orf1;TRINITY_DN107288_c0_g1_i2_orf1;TRINITY_DN100821_c0_g1_i1_orf1;TRINITY_DN12293_c0_g1_i1_orf1;TRINITY_DN20527_c0_g1_i1_orf1;TRINITY_DN620_c0_g1_i4_orf1;TRINITY_DN3836_c0_g1_i4_orf1;TRINITY_DN4822_c0_g1_i9_orf1;TRINITY_DN15160_c0_g1_i1_orf1
biological_process	organophosphate metabolic process	GO:0019637	11	11/855	TRINITY_DN36144_c0_g1_i3_orf1;TRINITY_DN5070_c0_g1_i1_orf1;TRINITY_DN47151_c0_g1_i1_orf1;TRINITY_DN12301_c0_g1_i1_orf1;TRINITY_DN3991_c0_g1_i6_orf1;TRINITY_DN24_c0_g1_i1_orf1;TRINITY_DN51813_c0_g1_i1_orf1;TRINITY_DN48602_c0_g1_i6_orf1;TRINITY_DN115210_c0_g4_i1_orf1;TRINITY_DN15222_c0_g1_i4_orf1;TRINITY_DN83150_c0_g1_i1_orf1
biological_process	carbohydrate derivative metabolic process	GO:1901135	10	10/855	TRINITY_DN15222_c0_g1_i4_orf1;TRINITY_DN47151_c0_g1_i1_orf1;TRINITY_DN1534_c0_g1_i3_orf1;TRINITY_DN12301_c0_g1_i1_orf1;TRINITY_DN3991_c0_g1_i6_orf1;TRINITY_DN48602_c0_g1_i6_orf1;TRINITY_DN115210_c0_g4_i1_orf1;TRINITY_DN83150_c0_g1_i1_orf1;TRINITY_DN2441_c0_g1_i1_orf1;TRINITY_DN11798_c0_g2_i1_orf1
biological_process	organic hydroxy compound metabolic process	GO:1901615	6	6/855	TRINITY_DN230_c2_g1_i5_orf1;TRINITY_DN52244_c1_g1_i1_orf1;TRINITY_DN31163_c1_g1_i4_orf1;TRINITY_DN3010_c0_g1_i4_orf1;TRINITY_DN11942_c0_g1_i1_orf1;TRINITY_DN37165_c0_g1_i4_orf1
biological_process	organic cyclic compound metabolic process	GO:1901360	57	57/855	TRINITY_DN230_c2_g1_i5_orf1;TRINITY_DN3092_c0_g1_i2_orf1;TRINITY_DN24_c0_g1_i1_orf1;TRINITY_DN15222_c0_g1_i4_orf1;TRINITY_DN115210_c0_g4_i1_orf1;TRINITY_DN40434_c0_g1_i2_orf1;TRINITY_DN8716_c0_g1_i3_orf1;TRINITY_DN27852_c0_g1_i1_orf1;TRINITY_DN4822_c0_g1_i6_orf1;TRINITY_DN77318_c0_g2_i1_orf1;TRINITY_DN46409_c0_g1_i1_orf1;TRINITY_DN1978_c0_g1_i4_orf1;TRINITY_DN13760_c1_g1_i1_orf1;TRINITY_DN51934_c0_g2_i1_orf1;TRINITY_DN4822_c0_g1_i9_orf1;TRINITY_DN15900_c0_g1_i6_orf1;TRINITY_DN19187_c0_g1_i1_orf1;TRINITY_DN500_c0_g1_i1_orf1;TRINITY_DN34134_c0_g2_i1_orf1;TRINITY_DN30097_c0_g1_i2_orf1;TRINITY_DN12301_c0_g1_i1_orf1;TRINITY_DN31163_c1_g1_i4_orf1;TRINITY_DN48602_c0_g1_i6_orf1;TRINITY_DN2803_c4_g1_i1_orf1;TRINITY_DN810_c0_g1_i4_orf1;TRINITY_DN2201_c0_g1_i1_orf1;TRINITY_DN16978_c0_g1_i1_orf1;TRINITY_DN5670_c0_g1_i2_orf1;TRINITY_DN2749_c0_g2_i3_orf1;TRINITY_DN36144_c0_g1_i3_orf1;TRINITY_DN5070_c0_g1_i1_orf1;TRINITY_DN3062_c0_g1_i1_orf1;TRINITY_DN19866_c0_g1_i4_orf1;TRINITY_DN3991_c0_g1_i6_orf1;TRINITY_DN5238_c0_g1_i2_orf1;TRINITY_DN5686_c0_g1_i4_orf1;TRINITY_DN51813_c0_g1_i1_orf1;TRINITY_DN12323_c0_g2_i2_orf1;TRINITY_DN1760_c0_g1_i4_orf1;TRINITY_DN4408_c6_g1_i1_orf1;TRINITY_DN109733_c0_g1_i1_orf1;TRINITY_DN83150_c0_g1_i1_orf1;TRINITY_DN12293_c0_g1_i1_orf1;TRINITY_DN20527_c0_g1_i1_orf1;TRINITY_DN1116_c0_g1_i6_orf1;TRINITY_DN40945_c0_g1_i1_orf1;TRINITY_DN620_c0_g1_i4_orf1;TRINITY_DN37165_c0_g1_i4_orf1;TRINITY_DN15160_c0_g1_i1_orf1;TRINITY_DN779_c0_g1_i12_orf1;TRINITY_DN4813_c0_g1_i5_orf1;TRINITY_DN51498_c0_g1_i1_orf1;TRINITY_DN107288_c0_g1_i2_orf1;TRINITY_DN2299_c0_g1_i3_orf1;TRINITY_DN3862_c0_g1_i7_orf1;TRINITY_DN89613_c0_g1_i13_orf1;TRINITY_DN2749_c4_g1_i2_orf1
biological_process	thioester metabolic process	GO:0035383	1	1/855	TRINITY_DN3991_c0_g1_i6_orf1
biological_process	macromolecule metabolic process	GO:0043170	113	113/855	TRINITY_DN40434_c0_g1_i2_orf1;TRINITY_DN3194_c0_g1_i6_orf1;TRINITY_DN344_c1_g1_i1_orf1;TRINITY_DN34134_c0_g2_i1_orf1;TRINITY_DN14754_c0_g1_i6_orf1;TRINITY_DN73945_c0_g5_i3_orf1;TRINITY_DN5670_c0_g1_i2_orf1;TRINITY_DN48020_c0_g1_i1_orf1;TRINITY_DN1534_c0_g1_i3_orf1;TRINITY_DN18273_c0_g1_i4_orf1;TRINITY_DN1116_c0_g1_i6_orf1;TRINITY_DN77318_c0_g2_i1_orf1;TRINITY_DN143_c0_g3_i1_orf1;TRINITY_DN140538_c0_g2_i1_orf1;TRINITY_DN4813_c0_g1_i5_orf1;TRINITY_DN79734_c0_g2_i3_orf1;TRINITY_DN1380_c0_g1_i5_orf1;TRINITY_DN107288_c0_g1_i2_orf1;TRINITY_DN338_c1_g1_i9_orf1;TRINITY_DN89613_c0_g1_i13_orf1;TRINITY_DN4767_c0_g1_i6_orf1;TRINITY_DN3092_c0_g1_i2_orf1;TRINITY_DN1978_c0_g1_i4_orf1;TRINITY_DN143895_c0_g1_i1_orf1;TRINITY_DN13856_c0_g1_i1_orf1;TRINITY_DN21619_c0_g1_i1_orf1;TRINITY_DN1791_c0_g1_i3_orf1;TRINITY_DN8659_c0_g2_i1_orf1;TRINITY_DN97589_c0_g1_i3_orf1;TRINITY_DN500_c0_g1_i1_orf1;TRINITY_DN1789_c0_g1_i5_orf1;TRINITY_DN41086_c0_g1_i4_orf1;TRINITY_DN11125_c0_g1_i1_orf1;TRINITY_DN277_c1_g1_i1_orf1;TRINITY_DN810_c0_g1_i4_orf1;TRINITY_DN50787_c0_g2_i2_orf1;TRINITY_DN45948_c1_g1_i1_orf1;TRINITY_DN16978_c0_g1_i1_orf1;TRINITY_DN5696_c0_g1_i4_orf1;TRINITY_DN4064_c0_g2_i1_orf1;TRINITY_DN3733_c0_g1_i1_orf1;TRINITY_DN4408_c6_g1_i1_orf1;TRINITY_DN95414_c0_g1_i1_orf1;TRINITY_DN40945_c0_g1_i1_orf1;TRINITY_DN21357_c0_g1_i5_orf1;TRINITY_DN4767_c0_g1_i4_orf1;TRINITY_DN4051_c0_g1_i1_orf1;TRINITY_DN3702_c0_g1_i1_orf1;TRINITY_DN147458_c0_g1_i1_orf1;TRINITY_DN2299_c0_g1_i3_orf1;TRINITY_DN2749_c4_g1_i2_orf1;TRINITY_DN1749_c0_g2_i2_orf1;TRINITY_DN5112_c0_g1_i1_orf1;TRINITY_DN36434_c0_g2_i3_orf1;TRINITY_DN30131_c0_g1_i1_orf1;TRINITY_DN52553_c0_g2_i1_orf1;TRINITY_DN27852_c0_g1_i1_orf1;TRINITY_DN6365_c0_g1_i4_orf1;TRINITY_DN620_c0_g1_i4_orf1;TRINITY_DN46409_c0_g1_i1_orf1;TRINITY_DN24318_c0_g1_i1_orf1;TRINITY_DN51934_c0_g2_i1_orf1;TRINITY_DN4817_c0_g1_i4_orf1;TRINITY_DN41645_c0_g1_i1_orf1;TRINITY_DN5310_c2_g1_i2_orf1;TRINITY_DN4571_c0_g1_i4_orf1;TRINITY_DN11798_c0_g2_i1_orf1;TRINITY_DN62729_c0_g1_i13_orf1;TRINITY_DN1266_c2_g1_i1_orf1;TRINITY_DN1706_c0_g1_i7_orf1;TRINITY_DN3343_c0_g2_i1_orf1;TRINITY_DN18388_c0_g1_i6_orf1;TRINITY_DN2749_c0_g2_i3_orf1;TRINITY_DN3062_c0_g1_i1_orf1;TRINITY_DN19866_c0_g1_i4_orf1;TRINITY_DN5686_c0_g1_i4_orf1;TRINITY_DN2673_c2_g1_i2_orf1;TRINITY_DN701_c0_g1_i1_orf1;TRINITY_DN113327_c0_g1_i2_orf1;TRINITY_DN143637_c0_g1_i1_orf1;TRINITY_DN111110_c0_g1_i1_orf1;TRINITY_DN15160_c0_g1_i1_orf1;TRINITY_DN6205_c0_g1_i1_orf1;TRINITY_DN3862_c0_g1_i7_orf1;TRINITY_DN753_c0_g1_i4_orf1;TRINITY_DN42461_c0_g1_i4_orf1;TRINITY_DN146718_c0_g1_i1_orf1;TRINITY_DN8716_c0_g1_i3_orf1;TRINITY_DN13760_c1_g1_i1_orf1;TRINITY_DN13651_c0_g1_i2_orf1;TRINITY_DN8949_c0_g1_i2_orf1;TRINITY_DN14874_c0_g1_i6_orf1;TRINITY_DN875_c0_g1_i3_orf1;TRINITY_DN143496_c0_g1_i1_orf1;TRINITY_DN17329_c0_g2_i3_orf1;TRINITY_DN2201_c0_g1_i1_orf1;TRINITY_DN24121_c1_g1_i6_orf1;TRINITY_DN8824_c0_g2_i1_orf1;TRINITY_DN892_c7_g1_i2_orf1;TRINITY_DN15900_c0_g1_i6_orf1;TRINITY_DN3826_c0_g1_i1_orf1;TRINITY_DN747_c0_g1_i1_orf1;TRINITY_DN56690_c0_g1_i4_orf1;TRINITY_DN5238_c0_g1_i2_orf1;TRINITY_DN12323_c0_g2_i2_orf1;TRINITY_DN109733_c0_g1_i1_orf1;TRINITY_DN42646_c0_g2_i1_orf1;TRINITY_DN103118_c0_g1_i4_orf1;TRINITY_DN30097_c0_g1_i2_orf1;TRINITY_DN1592_c0_g1_i1_orf1;TRINITY_DN26824_c0_g1_i1_orf1;TRINITY_DN747_c0_g1_i4_orf1;TRINITY_DN6462_c0_g1_i5_orf1
biological_process	melanin metabolic process	GO:0006582	1	1/855	TRINITY_DN31163_c1_g1_i4_orf1
biological_process	pigment biosynthetic process	GO:0046148	1	1/855	TRINITY_DN31163_c1_g1_i4_orf1
biological_process	gamete generation	GO:0007276	1	1/855	TRINITY_DN4813_c0_g1_i5_orf1
biological_process	spermatogenesis	GO:0007283	1	1/855	TRINITY_DN4813_c0_g1_i5_orf1
biological_process	leukocyte activation	GO:0045321	1	1/855	TRINITY_DN46409_c0_g1_i1_orf1
biological_process	secretion by cell	GO:0032940	3	3/855	TRINITY_DN33452_c0_g1_i1_orf1;TRINITY_DN1652_c0_g1_i12_orf1;TRINITY_DN33452_c0_g1_i3_orf1
biological_process	cell-cell adhesion	GO:0098609	1	1/855	TRINITY_DN1008_c0_g1_i2_orf1
biological_process	cellular response to extracellular stimulus	GO:0031668	1	1/855	TRINITY_DN51938_c0_g3_i1_orf1
biological_process	intermediate filament cytoskeleton organization	GO:0045104	1	1/855	TRINITY_DN107962_c0_g1_i1_orf1
biological_process	maintenance of protein location in cell	GO:0032507	1	1/855	TRINITY_DN13783_c0_g4_i2_orf1
biological_process	cellular chemical homeostasis	GO:0055082	4	4/855	TRINITY_DN1423_c0_g1_i8_orf1;TRINITY_DN96739_c0_g1_i1_orf1;TRINITY_DN136031_c0_g1_i7_orf1;TRINITY_DN31584_c0_g2_i2_orf1
biological_process	leukocyte proliferation	GO:0070661	1	1/855	TRINITY_DN46409_c0_g1_i1_orf1
biological_process	mitotic cell cycle process	GO:1903047	1	1/855	TRINITY_DN31119_c0_g1_i1_orf1
biological_process	spindle organization	GO:0007051	1	1/855	TRINITY_DN31119_c0_g1_i1_orf1
biological_process	'de novo' protein folding	GO:0006458	1	1/855	TRINITY_DN46409_c0_g1_i1_orf1
biological_process	protein refolding	GO:0042026	1	1/855	TRINITY_DN46409_c0_g1_i1_orf1
biological_process	cellular macromolecule localization	GO:0070727	10	10/855	TRINITY_DN15339_c0_g1_i6_orf1;TRINITY_DN3209_c0_g1_i1_orf1;TRINITY_DN3747_c1_g1_i3_orf1;TRINITY_DN46409_c0_g1_i1_orf1;TRINITY_DN3450_c0_g1_i3_orf1;TRINITY_DN31584_c0_g2_i2_orf1;TRINITY_DN13783_c0_g4_i2_orf1;TRINITY_DN146119_c0_g1_i1_orf1;TRINITY_DN9931_c0_g1_i1_orf1;TRINITY_DN3209_c0_g2_i6_orf1
biological_process	intracellular transport	GO:0046907	11	11/855	TRINITY_DN578_c0_g1_i5_orf1;TRINITY_DN3209_c0_g1_i1_orf1;TRINITY_DN3747_c1_g1_i3_orf1;TRINITY_DN6231_c0_g1_i6_orf1;TRINITY_DN46409_c0_g1_i1_orf1;TRINITY_DN3450_c0_g1_i3_orf1;TRINITY_DN31584_c0_g2_i2_orf1;TRINITY_DN578_c0_g1_i3_orf1;TRINITY_DN45037_c0_g1_i1_orf1;TRINITY_DN5028_c0_g1_i11_orf1;TRINITY_DN3209_c0_g2_i6_orf1
biological_process	cellular component biogenesis	GO:0044085	2	2/855	TRINITY_DN14391_c1_g1_i2_orf1;TRINITY_DN31225_c0_g1_i1_orf1
biological_process	cellular component organization	GO:0016043	26	26/855	TRINITY_DN1749_c0_g2_i2_orf1;TRINITY_DN51938_c0_g3_i1_orf1;TRINITY_DN8087_c0_g1_i9_orf1;TRINITY_DN20442_c0_g2_i1_orf1;TRINITY_DN46409_c0_g1_i1_orf1;TRINITY_DN3450_c0_g1_i3_orf1;TRINITY_DN34426_c0_g1_i1_orf1;TRINITY_DN146119_c0_g1_i1_orf1;TRINITY_DN3126_c0_g1_i4_orf1;TRINITY_DN71832_c0_g1_i1_orf1;TRINITY_DN107962_c0_g1_i1_orf1;TRINITY_DN11069_c0_g2_i1_orf1;TRINITY_DN12771_c0_g1_i1_orf1;TRINITY_DN2904_c0_g1_i4_orf1;TRINITY_DN1298_c0_g1_i3_orf1;TRINITY_DN6985_c0_g1_i5_orf1;TRINITY_DN96739_c0_g1_i1_orf1;TRINITY_DN109733_c0_g1_i1_orf1;TRINITY_DN31119_c0_g1_i1_orf1;TRINITY_DN36987_c0_g1_i1_orf1;TRINITY_DN85476_c0_g1_i1_orf1;TRINITY_DN140538_c0_g2_i1_orf1;TRINITY_DN27276_c0_g1_i5_orf1;TRINITY_DN6358_c0_g1_i5_orf1;TRINITY_DN42854_c0_g3_i2_orf1;TRINITY_DN3702_c0_g1_i1_orf1
biological_process	cell migration	GO:0016477	2	2/855	TRINITY_DN96739_c0_g1_i1_orf1;TRINITY_DN31584_c0_g2_i2_orf1
biological_process	microtubule cytoskeleton organization	GO:0000226	1	1/855	TRINITY_DN31119_c0_g1_i1_orf1
biological_process	cellular response to chemical stimulus	GO:0070887	2	2/855	TRINITY_DN46409_c0_g1_i1_orf1;TRINITY_DN51938_c0_g3_i1_orf1
biological_process	cellular response to stress	GO:0033554	10	10/855	TRINITY_DN3092_c0_g1_i2_orf1;TRINITY_DN19866_c0_g1_i4_orf1;TRINITY_DN40434_c0_g1_i2_orf1;TRINITY_DN5686_c0_g1_i4_orf1;TRINITY_DN51938_c0_g3_i1_orf1;TRINITY_DN77318_c0_g2_i1_orf1;TRINITY_DN46409_c0_g1_i1_orf1;TRINITY_DN109733_c0_g1_i1_orf1;TRINITY_DN31584_c0_g2_i2_orf1;TRINITY_DN5238_c0_g1_i2_orf1
biological_process	cell surface receptor signaling pathway	GO:0007166	5	5/855	TRINITY_DN4142_c0_g1_i5_orf1;TRINITY_DN1008_c0_g1_i2_orf1;TRINITY_DN2170_c0_g2_i1_orf1;TRINITY_DN15247_c0_g1_i2_orf1;TRINITY_DN51938_c0_g3_i1_orf1
biological_process	immune response-regulating signaling pathway	GO:0002764	2	2/855	TRINITY_DN46409_c0_g1_i1_orf1;TRINITY_DN2170_c0_g2_i1_orf1
biological_process	G protein-coupled receptor signaling pathway	GO:0007186	2	2/855	TRINITY_DN42854_c0_g3_i2_orf1;TRINITY_DN4628_c0_g1_i1_orf1
biological_process	intracellular signal transduction	GO:0035556	3	3/855	TRINITY_DN804_c0_g1_i7_orf1;TRINITY_DN2770_c0_g2_i4_orf1;TRINITY_DN31584_c0_g2_i2_orf1
biological_process	apoptotic signaling pathway	GO:0097190	2	2/855	TRINITY_DN96739_c0_g1_i1_orf1;TRINITY_DN51938_c0_g3_i1_orf1
biological_process	meiotic cell cycle	GO:0051321	1	1/855	TRINITY_DN4813_c0_g1_i5_orf1
biological_process	cell differentiation	GO:0030154	6	6/855	TRINITY_DN4813_c0_g1_i5_orf1;TRINITY_DN42461_c0_g1_i4_orf1;TRINITY_DN4550_c1_g1_i19_orf1;TRINITY_DN23746_c0_g1_i2_orf1;TRINITY_DN96739_c0_g1_i1_orf1;TRINITY_DN140538_c0_g2_i1_orf1
biological_process	cellular component morphogenesis	GO:0032989	1	1/855	TRINITY_DN42854_c0_g3_i2_orf1
biological_process	cell development	GO:0048468	5	5/855	TRINITY_DN1749_c0_g2_i2_orf1;TRINITY_DN8087_c0_g1_i9_orf1;TRINITY_DN71832_c0_g1_i1_orf1;TRINITY_DN36987_c0_g1_i1_orf1;TRINITY_DN4571_c0_g1_i4_orf1
biological_process	protein transmembrane transport	GO:0071806	1	1/855	TRINITY_DN46409_c0_g1_i1_orf1
biological_process	mitochondrial transmembrane transport	GO:1990542	1	1/855	TRINITY_DN46409_c0_g1_i1_orf1
biological_process	ion transmembrane transport	GO:0034220	2	2/855	TRINITY_DN96739_c0_g1_i1_orf1;TRINITY_DN8306_c0_g1_i4_orf1
biological_process	actin cytoskeleton organization	GO:0030036	1	1/855	TRINITY_DN3126_c0_g1_i4_orf1
biological_process	animal organ morphogenesis	GO:0009887	1	1/855	TRINITY_DN23746_c0_g1_i2_orf1
biological_process	cell morphogenesis	GO:0000902	1	1/855	TRINITY_DN34426_c0_g1_i1_orf1
biological_process	system development	GO:0048731	1	1/855	TRINITY_DN42854_c0_g3_i2_orf1
biological_process	hippocampus development	GO:0021766	1	1/855	TRINITY_DN31584_c0_g2_i2_orf1
biological_process	animal organ development	GO:0048513	4	4/855	TRINITY_DN8087_c0_g1_i9_orf1;TRINITY_DN36987_c0_g1_i1_orf1;TRINITY_DN34426_c0_g1_i1_orf1;TRINITY_DN71832_c0_g1_i1_orf1
biological_process	cerebral cortex development	GO:0021987	1	1/855	TRINITY_DN31584_c0_g2_i2_orf1
biological_process	nervous system process	GO:0050877	2	2/855	TRINITY_DN75086_c0_g1_i5_orf1;TRINITY_DN39725_c0_g1_i4_orf1
biological_process	response to bacterium	GO:0009617	5	5/855	TRINITY_DN1091_c0_g2_i10_orf1;TRINITY_DN5880_c0_g2_i2_orf1;TRINITY_DN16840_c1_g1_i1_orf1;TRINITY_DN29190_c0_g1_i4_orf1;TRINITY_DN14904_c0_g1_i1_orf1
biological_process	defense response to other organism	GO:0098542	8	8/855	TRINITY_DN14904_c0_g1_i1_orf1;TRINITY_DN1534_c0_g1_i3_orf1;TRINITY_DN16840_c1_g1_i1_orf1;TRINITY_DN1091_c0_g2_i10_orf1;TRINITY_DN2170_c0_g2_i1_orf1;TRINITY_DN9044_c0_g1_i2_orf1;TRINITY_DN29190_c0_g1_i4_orf1;TRINITY_DN5880_c0_g2_i2_orf1
biological_process	biological process involved in interaction with symbiont	GO:0051702	1	1/855	TRINITY_DN46409_c0_g1_i1_orf1
biological_process	lipid storage	GO:0019915	1	1/855	TRINITY_DN11069_c0_g2_i1_orf1
biological_process	maintenance of protein location	GO:0045185	1	1/855	TRINITY_DN13783_c0_g4_i2_orf1
biological_process	maintenance of location in cell	GO:0051651	1	1/855	TRINITY_DN13783_c0_g4_i2_orf1
biological_process	establishment of protein localization	GO:0045184	10	10/855	TRINITY_DN15339_c0_g1_i6_orf1;TRINITY_DN3209_c0_g1_i1_orf1;TRINITY_DN3747_c1_g1_i3_orf1;TRINITY_DN46409_c0_g1_i1_orf1;TRINITY_DN3450_c0_g1_i3_orf1;TRINITY_DN31584_c0_g2_i2_orf1;TRINITY_DN13783_c0_g4_i2_orf1;TRINITY_DN146119_c0_g1_i1_orf1;TRINITY_DN9931_c0_g1_i1_orf1;TRINITY_DN3209_c0_g2_i6_orf1

biological_process	establishment of localization in cell	GO:0051649	14	14/855	TRINITY_DN578_c0_g1_i5_orf1;TRINITY_DN3209_c0_g1_i1_orf1;TRINITY_DN3747_c1_g1_i3_orf1;TRINITY_DN6231_c0_g1_i6_orf1;TRINITY_DN46409_c0_g1_i1_orf1;TRINITY_DN3450_c0_g1_i3_orf1;TRINITY_DN1652_c0_g1_i12_orf1;TRINITY_DN31584_c0_g2_i2_orf1;TRINITY_DN578_c0_g1_i3_orf1;TRINITY_DN45037_c0_g1_i1_orf1;TRINITY_DN29144_c0_g3_i1_orf1;TRINITY_DN42185_c0_g1_i7_orf1;TRINITY_DN5028_c0_g1_i11_orf1;TRINITY_DN3209_c0_g2_i6_orf1
biological_process	establishment of RNA localization	GO:0051236	1	1/855	TRINITY_DN146119_c0_g1_i1_orf1
biological_process	transport	GO:0006810	34	34/855	TRINITY_DN81488_c0_g1_i1_orf1;TRINITY_DN13923_c0_g2_i1_orf1;TRINITY_DN3209_c0_g1_i1_orf1;TRINITY_DN3747_c1_g1_i3_orf1;TRINITY_DN6231_c0_g1_i6_orf1;TRINITY_DN33452_c0_g1_i3_orf1;TRINITY_DN46409_c0_g1_i1_orf1;TRINITY_DN3450_c0_g1_i3_orf1;TRINITY_DN31584_c0_g2_i2_orf1;TRINITY_DN578_c0_g1_i3_orf1;TRINITY_DN146119_c0_g1_i1_orf1;TRINITY_DN9239_c0_g1_i1_orf1;TRINITY_DN47389_c0_g1_i2_orf1;TRINITY_DN3219_c0_g1_i6_orf1;TRINITY_DN9239_c0_g2_i2_orf1;TRINITY_DN45037_c0_g1_i1_orf1;TRINITY_DN29144_c0_g3_i1_orf1;TRINITY_DN15339_c0_g1_i6_orf1;TRINITY_DN9354_c0_g1_i7_orf1;TRINITY_DN1423_c0_g1_i8_orf1;TRINITY_DN136031_c0_g1_i7_orf1;TRINITY_DN8766_c0_g1_i1_orf1;TRINITY_DN96739_c0_g1_i1_orf1;TRINITY_DN12286_c1_g1_i2_orf1;TRINITY_DN13783_c0_g4_i2_orf1;TRINITY_DN42185_c0_g1_i7_orf1;TRINITY_DN3209_c0_g2_i6_orf1;TRINITY_DN578_c0_g1_i5_orf1;TRINITY_DN1407_c0_g1_i5_orf1;TRINITY_DN8306_c0_g1_i4_orf1;TRINITY_DN33452_c0_g1_i1_orf1;TRINITY_DN1652_c0_g1_i12_orf1;TRINITY_DN9931_c0_g1_i1_orf1;TRINITY_DN5028_c0_g1_i11_orf1
biological_process	viral RNA genome replication	GO:0039694	1	1/855	TRINITY_DN4408_c6_g1_i1_orf1
biological_process	response to external biotic stimulus	GO:0043207	14	14/855	TRINITY_DN14904_c0_g1_i1_orf1;TRINITY_DN1534_c0_g1_i3_orf1;TRINITY_DN109503_c0_g1_i4_orf1;TRINITY_DN59429_c0_g1_i6_orf1;TRINITY_DN16840_c1_g1_i1_orf1;TRINITY_DN3166_c1_g1_i6_orf1;TRINITY_DN1091_c0_g2_i10_orf1;TRINITY_DN2170_c0_g2_i1_orf1;TRINITY_DN2407_c0_g1_i2_orf1;TRINITY_DN9044_c0_g1_i2_orf1;TRINITY_DN86772_c0_g1_i3_orfp1;TRINITY_DN29190_c0_g1_i4_orf1;TRINITY_DN4802_c0_g1_i4_orf1;TRINITY_DN5880_c0_g2_i2_orf1
biological_process	detection of biotic stimulus	GO:0009595	2	2/855	TRINITY_DN1091_c0_g2_i10_orf1;TRINITY_DN5880_c0_g2_i2_orf1
biological_process	response to extracellular stimulus	GO:0009991	1	1/855	TRINITY_DN51938_c0_g3_i1_orf1
biological_process	cellular response to external stimulus	GO:0071496	1	1/855	TRINITY_DN51938_c0_g3_i1_orf1
biological_process	cellular response to endogenous stimulus	GO:0071495	1	1/855	TRINITY_DN51938_c0_g3_i1_orf1
biological_process	response to transforming growth factor beta	GO:0071559	1	1/855	TRINITY_DN51938_c0_g3_i1_orf1
biological_process	response to hormone	GO:0009725	1	1/855	TRINITY_DN51938_c0_g3_i1_orf1
biological_process	response to hypoxia	GO:0001666	2	2/855	TRINITY_DN51938_c0_g3_i1_orf1;TRINITY_DN140538_c0_g2_i1_orf1
biological_process	response to topologically incorrect protein	GO:0035966	1	1/855	TRINITY_DN46409_c0_g1_i1_orf1
biological_process	response to ischemia	GO:0002931	1	1/855	TRINITY_DN51938_c0_g3_i1_orf1
biological_process	response to cold	GO:0009409	1	1/855	TRINITY_DN46409_c0_g1_i1_orf1
biological_process	response to heat	GO:0009408	1	1/855	TRINITY_DN31584_c0_g2_i2_orf1
biological_process	defense response	GO:0006952	14	14/855	TRINITY_DN14904_c0_g1_i1_orf1;TRINITY_DN1534_c0_g1_i3_orf1;TRINITY_DN59429_c0_g1_i6_orf1;TRINITY_DN16840_c1_g1_i1_orf1;TRINITY_DN3166_c1_g1_i6_orf1;TRINITY_DN1091_c0_g2_i10_orf1;TRINITY_DN31163_c1_g1_i4_orf1;TRINITY_DN2407_c0_g1_i2_orf1;TRINITY_DN2170_c0_g2_i1_orf1;TRINITY_DN9044_c0_g1_i2_orf1;TRINITY_DN86772_c0_g1_i3_orfp1;TRINITY_DN29190_c0_g1_i4_orf1;TRINITY_DN4802_c0_g1_i4_orf1;TRINITY_DN5880_c0_g2_i2_orf1
biological_process	response to hyperoxia	GO:0055093	1	1/855	TRINITY_DN51938_c0_g3_i1_orf1
biological_process	response to oxidative stress	GO:0006979	3	3/855	TRINITY_DN80660_c0_g1_i1_orf1;TRINITY_DN1622_c0_g1_i6_orf1;TRINITY_DN21420_c0_g1_i2_orf1
biological_process	response to oxygen-containing compound	GO:1901700	1	1/855	TRINITY_DN51938_c0_g3_i1_orf1
biological_process	response to nutrient	GO:0007584	1	1/855	TRINITY_DN51938_c0_g3_i1_orf1
biological_process	response to organic substance	GO:0010033	4	4/855	TRINITY_DN1091_c0_g2_i10_orf1;TRINITY_DN46409_c0_g1_i1_orf1;TRINITY_DN51938_c0_g3_i1_orf1;TRINITY_DN5880_c0_g2_i2_orf1
biological_process	response to temperature stimulus	GO:0009266	2	2/855	TRINITY_DN46409_c0_g1_i1_orf1;TRINITY_DN31584_c0_g2_i2_orf1
biological_process	response to oxygen levels	GO:0070482	2	2/855	TRINITY_DN51938_c0_g3_i1_orf1;TRINITY_DN140538_c0_g2_i1_orf1
biological_process	detection of chemical stimulus	GO:0009593	2	2/855	TRINITY_DN1091_c0_g2_i10_orf1;TRINITY_DN5880_c0_g2_i2_orf1
cellular_component	nucleosome	GO:0000786	3	3/855	TRINITY_DN20442_c0_g2_i1_orf1;TRINITY_DN96801_c0_g1_i1_orf1;TRINITY_DN6358_c0_g1_i5_orf1
cellular_component	SWI/SNF superfamily-type complex	GO:0070603	1	1/855	TRINITY_DN3649_c0_g1_i6_orf1
cellular_component	transcription elongation factor complex	GO:0008023	1	1/855	TRINITY_DN5686_c0_g1_i4_orf1
cellular_component	spliceosomal complex	GO:0005681	2	2/855	TRINITY_DN30097_c0_g1_i2_orf1;TRINITY_DN27276_c0_g1_i5_orf1
cellular_component	BRISC complex	GO:0070552	1	1/855	TRINITY_DN19866_c0_g1_i4_orf1
cellular_component	BRCA1-A complex	GO:0070531	1	1/855	TRINITY_DN19866_c0_g1_i4_orf1
cellular_component	nuclear pore	GO:0005643	3	3/855	TRINITY_DN10415_c0_g1_i5_orf1;TRINITY_DN15339_c0_g1_i6_orf1;TRINITY_DN146119_c0_g1_i1_orf1
cellular_component	transmembrane transporter complex	GO:1902495	1	1/855	TRINITY_DN20558_c0_g1_i2_orf1
cellular_component	dynein complex	GO:0030286	1	1/855	TRINITY_DN4257_c0_g1_i2_orf1
cellular_component	catalytic step 2 spliceosome	GO:0071013	1	1/855	TRINITY_DN30097_c0_g1_i2_orf1
cellular_component	proteasome core complex	GO:0005839	1	1/855	TRINITY_DN113327_c0_g1_i2_orf1
cellular_component	cytochrome complex	GO:0070069	1	1/855	TRINITY_DN14073_c0_g1_i1_orf1
cellular_component	transferase complex	GO:1990234	3	3/855	TRINITY_DN89613_c0_g1_i13_orf1;TRINITY_DN143496_c0_g1_i1_orf1;TRINITY_DN2299_c0_g1_i3_orf1
cellular_component	aminoacyl-tRNA synthetase multienzyme complex	GO:0017101	2	2/855	TRINITY_DN22572_c0_g1_i1_orf1;TRINITY_DN107288_c0_g1_i2_orf1
cellular_component	ATPase complex	GO:1904949	1	1/855	TRINITY_DN3649_c0_g1_i6_orf1
cellular_component	lipopolysaccharide receptor complex	GO:0046696	1	1/855	TRINITY_DN46409_c0_g1_i1_orf1
cellular_component	Tapasin-ERp57 complex	GO:0061779	1	1/855	TRINITY_DN51938_c0_g3_i1_orf1
cellular_component	MHC class I peptide loading complex	GO:0042824	1	1/855	TRINITY_DN51938_c0_g3_i1_orf1
cellular_component	TAP complex	GO:0042825	1	1/855	TRINITY_DN51938_c0_g3_i1_orf1
cellular_component	respiratory chain complex	GO:0098803	1	1/855	TRINITY_DN14073_c0_g1_i1_orf1
cellular_component	inner mitochondrial membrane protein complex	GO:0098800	2	2/855	TRINITY_DN15222_c0_g1_i4_orf1;TRINITY_DN14073_c0_g1_i1_orf1
cellular_component	membrane coat	GO:0030117	3	3/855	TRINITY_DN3209_c0_g1_i1_orf1;TRINITY_DN3209_c0_g2_i6_orf1;TRINITY_DN146119_c0_g1_i1_orf1
cellular_component	proton-transporting two-sector ATPase complex, proton-transporting domain	GO:0033177	1	1/855	TRINITY_DN15222_c0_g1_i4_orf1
cellular_component	mitochondrial large ribosomal subunit	GO:0005762	1	1/855	TRINITY_DN1313_c0_g1_i2_orf1
cellular_component	TRAPP complex	GO:0030008	1	1/855	TRINITY_DN45037_c0_g1_i1_orf1
cellular_component	ribosomal subunit	GO:0044391	9	9/855	TRINITY_DN3534_c0_g1_i2_orf1;TRINITY_DN79734_c0_g2_i3_orf1;TRINITY_DN1313_c0_g1_i2_orf1;TRINITY_DN4051_c0_g1_i1_orf1;TRINITY_DN50787_c0_g2_i2_orf1;TRINITY_DN13651_c0_g1_i2_orf1;TRINITY_DN41645_c0_g1_i1_orf1;TRINITY_DN42646_c0_g2_i1_orf1;TRINITY_DN8949_c0_g1_i2_orf1
cellular_component	DNA polymerase complex	GO:0042575	1	1/855	TRINITY_DN89613_c0_g1_i13_orf1
cellular_component	ubiquitin ligase complex	GO:0000151	1	1/855	TRINITY_DN143496_c0_g1_i1_orf1
cellular_component	RNA polymerase complex	GO:0030880	1	1/855	TRINITY_DN2299_c0_g1_i3_orf1
cellular_component	organelle lumen	GO:0043233	5	5/855	TRINITY_DN42854_c0_g3_i2_orf1;TRINITY_DN46409_c0_g1_i1_orf1;TRINITY_DN51938_c0_g3_i1_orf1;TRINITY_DN1791_c0_g1_i3_orf1;TRINITY_DN2299_c0_g1_i3_orf1
cellular_component	chromosome, telomeric region	GO:0000781	1	1/855	TRINITY_DN12771_c0_g1_i1_orf1
cellular_component	anchored component of membrane	GO:0031225	2	2/855	TRINITY_DN5553_c0_g1_i4_orf1;TRINITY_DN56690_c0_g1_i4_orf1
cellular_component	integral component of membrane	GO:0016021	120	120/855	TRINITY_DN129226_c0_g1_i2_orf1;TRINITY_DN13563_c0_g1_i1_orf1;TRINITY_DN9608_c0_g1_i3_orf1;TRINITY_DN13923_c0_g2_i1_orf1;TRINITY_DN1239_c0_g1_i3_orf1;TRINITY_DN3194_c0_g1_i6_orf1;TRINITY_DN52761_c0_g1_i2_orf1;TRINITY_DN2109_c0_g1_i4_orf1;TRINITY_DN72816_c0_g1_i2_orf1;TRINITY_DN34426_c0_g1_i1_orf1;TRINITY_DN3949_c1_g1_i1_orf1;TRINITY_DN1672_c0_g1_i6_orf1;TRINITY_DN11069_c0_g2_i1_orf1;TRINITY_DN8964_c0_g1_i4_orf1;TRINITY_DN48020_c0_g1_i1_orf1;TRINITY_DN15755_c0_g1_i1_orf1;TRINITY_DN41708_c0_g1_i1_orf1;TRINITY_DN33995_c0_g1_i5_orf1;TRINITY_DN18338_c0_g1_i6_orf1;TRINITY_DN20558_c0_g1_i2_orf1;TRINITY_DN15157_c0_g1_i1_orf1;TRINITY_DN17505_c0_g1_i15_orf1;TRINITY_DN32479_c0_g1_i8_orf1;TRINITY_DN3177_c0_g1_i1_orf1;TRINITY_DN3276_c0_g1_i4_orf1;TRINITY_DN3529_c0_g1_i7_orf1;TRINITY_DN883_c0_g1_i8_orf1;TRINITY_DN2343_c1_g1_i2_orf1;TRINITY_DN29879_c0_g1_i3_orf1;TRINITY_DN10231_c0_g2_i1_orf1;TRINITY_DN4596_c0_g1_i14_orf1;TRINITY_DN14937_c0_g1_i7_orf1;TRINITY_DN2343_c1_g1_i8_orf1;TRINITY_DN4116_c0_g1_i3_orf1;TRINITY_DN5852_c0_g1_i6_orf1;TRINITY_DN52761_c0_g2_i1_orf1;TRINITY_DN1749_c0_g2_i2_orf1;TRINITY_DN13856_c0_g1_i1_orf1;TRINITY_DN25783_c0_g1_i2_orf1;TRINITY_DN75086_c0_g1_i5_orf1;TRINITY_DN28626_c0_g1_i5_orf1;TRINITY_DN5174_c0_g3_i1_orf1;TRINITY_DN1789_c0_g1_i5_orf1;TRINITY_DN483_c0_g1_i6_orf1;TRINITY_DN48602_c0_g1_i6_orf1;TRINITY_DN1402_c1_g1_i6_orf1;TRINITY_DN1008_c0_g1_i2_orf1;TRINITY_DN5991_c0_g1_i6_orf1;TRINITY_DN810_c0_g1_i4_orf1;TRINITY_DN3647_c2_g1_i3_orf1;TRINITY_DN2441_c0_g1_i1_orf1;TRINITY_DN1999_c0_g1_i9_orf1;TRINITY_DN15318_c0_g1_i1_orf1;TRINITY_DN4064_c0_g2_i1_orf1;TRINITY_DN5408_c0_g1_i5_orf1;TRINITY_DN867_c0_g1_i1_orf1;TRINITY_DN40945_c0_g1_i1_orf1;TRINITY_DN7861_c0_g1_i5_orf1;TRINITY_DN3209_c0_g2_i6_orf1;TRINITY_DN22_c0_g1_i3_orf1;TRINITY_DN23978_c0_g1_i2_orf1;TRINITY_DN28592_c0_g1_i2_orf1;TRINITY_DN1652_c0_g1_i12_orf1;TRINITY_DN9931_c0_g1_i1_orf1;TRINITY_DN120500_c0_g1_i1_orf1;TRINITY_DN15903_c0_g1_i2_orf1;TRINITY_DN3209_c0_g1_i1_orf1;TRINITY_DN4497_c0_g1_i4_orf1;TRINITY_DN24873_c0_g1_i4_orf1;TRINITY_DN14262_c0_g1_i5_orf1;TRINITY_DN79803_c0_g1_i7_orf1;TRINITY_DN3616_c0_g2_i2_orf1;TRINITY_DN27903_c0_g1_i1_orf1;TRINITY_DN46090_c0_g3_i1_orf1;TRINITY_DN1622_c0_g1_i6_orf1;TRINITY_DN3343_c0_g2_i1_orf1;TRINITY_DN5553_c0_g1_i4_orf1;TRINITY_DN11566_c0_g1_i6_orf1;TRINITY_DN1882_c0_g1_i4_orf1;TRINITY_DN15597_c0_g1_i1_orf1;TRINITY_DN19303_c0_g1_i5_orf1;TRINITY_DN23783_c0_g2_i1_orf1;TRINITY_DN12508_c0_g1_i1_orf1;TRINITY_DN32780_c0_g1_i2_orf1;TRINITY_DN4321_c0_g1_i1_orf1;TRINITY_DN31327_c0_g2_i1_orf1;TRINITY_DN6612_c0_g1_i4_orf1;TRINITY_DN42854_c0_g3_i2_orf1;TRINITY_DN14073_c0_g1_i1_orf1;TRINITY_DN20680_c0_g1_i5_orf1;TRINITY_DN14597_c0_g1_i5_orf1;TRINITY_DN43369_c0_g2_i1_orf1;TRINITY_DN36324_c0_g1_i12_orf1;TRINITY_DN2392_c0_g2_i1_orf1;TRINITY_DN753_c0_g1_i4_orf1;TRINITY_DN5841_c0_g1_i2_orf1;TRINITY_DN448_c0_g1_i20_orf1;TRINITY_DN14874_c0_g1_i6_orf1;TRINITY_DN6140_c0_g3_i3_orf1;TRINITY_DN9457_c0_g1_i9_orf1;TRINITY_DN20984_c0_g1_i4_orf1;TRINITY_DN452_c0_g1_i4_orf1;TRINITY_DN11172_c0_g1_i4_orf1;TRINITY_DN39725_c0_g1_i4_orf1;TRINITY_DN9354_c0_g1_i7_orf1;TRINITY_DN4394_c0_g2_i1_orf1;TRINITY_DN1012_c0_g1_i2_orf1;TRINITY_DN1292_c0_g1_i3_orf1;TRINITY_DN69049_c0_g2_i1_orf1;TRINITY_DN12286_c1_g1_i2_orf1;TRINITY_DN13783_c0_g4_i2_orf1;TRINITY_DN27500_c0_g1_i4_orf1;TRINITY_DN7868_c0_g1_i8_orf1;TRINITY_DN31390_c0_g1_i2_orf1;TRINITY_DN6545_c0_g1_i6_orf1;TRINITY_DN8306_c0_g1_i4_orf1;TRINITY_DN3616_c0_g1_i4_orf1;TRINITY_DN103118_c0_g1_i4_orf1;TRINITY_DN15247_c0_g1_i2_orf1;TRINITY_DN91946_c0_g1_i1_orf1
cellular_component	intrinsic component of organelle membrane	GO:0031300	2	2/855	TRINITY_DN8964_c0_g1_i4_orf1;TRINITY_DN483_c0_g1_i6_orf1
cellular_component	intracellular organelle	GO:0043229	75	75/855	TRINITY_DN146718_c0_g1_i1_orf1;TRINITY_DN8703_c0_g1_i2_orf1;TRINITY_DN6556_c0_g1_i7_orf1;TRINITY_DN5112_c0_g1_i1_orf1;TRINITY_DN24318_c0_g1_i1_orf1;TRINITY_DN5055_c0_g1_i12_orf1;TRINITY_DN30131_c0_g1_i1_orf1;TRINITY_DN5670_c0_g1_i2_orf1;TRINITY_DN146119_c0_g1_i1_orf1;TRINITY_DN3092_c0_g1_i2_orf1;TRINITY_DN6365_c0_g1_i4_orf1;TRINITY_DN14391_c1_g1_i2_orf1;TRINITY_DN46409_c0_g1_i1_orf1;TRINITY_DN25783_c0_g1_i2_orf1;TRINITY_DN13760_c1_g1_i1_orf1;TRINITY_DN31584_c0_g2_i2_orf1;TRINITY_DN18933_c0_g1_i3_orf1;TRINITY_DN2168_c0_g1_i2_orf1;TRINITY_DN96801_c0_g1_i1_orf1;TRINITY_DN5962_c0_g1_i1_orf1;TRINITY_DN30663_c0_g1_i1_orf1;TRINITY_DN18804_c0_g1_i5_orf1;TRINITY_DN97589_c0_g1_i3_orf1;TRINITY_DN107962_c0_g1_i1_orf1;TRINITY_DN51938_c0_g3_i1_orf1;TRINITY_DN3702_c0_g1_i1_orf1;TRINITY_DN500_c0_g1_i1_orf1;TRINITY_DN30233_c0_g1_i2_orf1;TRINITY_DN12771_c0_g1_i1_orf1;TRINITY_DN1706_c0_g1_i7_orf1;TRINITY_DN23746_c0_g1_i2_orf1;TRINITY_DN12526_c0_g1_i5_orf1;TRINITY_DN4793_c0_g1_i7_orf1;TRINITY_DN3219_c0_g1_i6_orf1;TRINITY_DN50787_c0_g2_i2_orf1;TRINITY_DN41_c0_g1_i3_orf1;TRINITY_DN45037_c0_g1_i1_orf1;TRINITY_DN4142_c0_g1_i5_orf1;TRINITY_DN279_c0_g1_i10_orf1;TRINITY_DN8824_c0_g2_i1_orf1;TRINITY_DN3464_c0_g1_i1_orf1;TRINITY_DN26789_c0_g1_i2_orf1;TRINITY_DN147596_c0_g1_i1_orf1;TRINITY_DN143_c0_g3_i1_orf1;TRINITY_DN3826_c0_g1_i1_orf1;TRINITY_DN22009_c0_g1_i1_orf1;TRINITY_DN31225_c0_g1_i1_orf1;TRINITY_DN47114_c0_g1_i5_orf1;TRINITY_DN3733_c0_g1_i1_orf1;TRINITY_DN106534_c0_g1_i1_orf1;TRINITY_DN14274_c0_g1_i3_orf1;TRINITY_DN84478_c0_g1_i8_orf1;TRINITY_DN96739_c0_g1_i1_orf1;TRINITY_DN31119_c0_g1_i1_orf1;TRINITY_DN113327_c0_g1_i2_orf1;TRINITY_DN21357_c0_g1_i5_orf1;TRINITY_DN140538_c0_g2_i1_orf1;TRINITY_DN111110_c0_g1_i1_orf1;TRINITY_DN7909_c0_g2_i1_orf1;TRINITY_DN3909_c0_g2_i2_orf1;TRINITY_DN6358_c0_g1_i5_orf1;TRINITY_DN33893_c0_g1_i1_orf1;TRINITY_DN86149_c0_g1_i1_orf1;TRINITY_DN21619_c0_g1_i1_orf1;TRINITY_DN6462_c0_g1_i5_orf1;TRINITY_DN42854_c0_g3_i2_orf1;TRINITY_DN16123_c0_g1_i1_orf1;TRINITY_DN5578_c0_g1_i4_orf1;TRINITY_DN147458_c0_g1_i1_orf1;TRINITY_DN47389_c0_g1_i2_orf1;TRINITY_DN26824_c0_g1_i1_orf1;TRINITY_DN25997_c1_g2_i4_orf1;TRINITY_DN54612_c0_g1_i3_orf1;TRINITY_DN3062_c0_g1_i1_orf1;TRINITY_DN1298_c0_g1_i3_orf1

cellular_component	non-membrane-bounded organelle	GO:0043228	28	28/855	TRINITY_DN146718_c0_g1_i1_orf1;TRINITY_DN5112_c0_g1_i1_orf1;TRINITY_DN30131_c0_g1_i1_orf1;TRINITY_DN3826_c0_g1_i1_orf1;TRINITY_DN6365_c0_g1_i4_orf1;TRINITY_DN24318_c0_g1_i1_orf1;TRINITY_DN21619_c0_g1_i1_orf1;TRINITY_DN97589_c0_g1_i3_orf1;TRINITY_DN107962_c0_g1_i1_orf1;TRINITY_DN143_c0_g3_i1_orf1;TRINITY_DN1298_c0_g1_i3_orf1;TRINITY_DN50787_c0_g2_i2_orf1;TRINITY_DN8824_c0_g2_i1_orf1;TRINITY_DN147596_c0_g1_i1_orf1;TRINITY_DN3062_c0_g1_i1_orf1;TRINITY_DN47114_c0_g1_i5_orf1;TRINITY_DN3733_c0_g1_i1_orf1;TRINITY_DN3909_c0_g2_i2_orf1;TRINITY_DN31119_c0_g1_i1_orf1;TRINITY_DN21357_c0_g1_i5_orf1;TRINITY_DN23746_c0_g1_i2_orf1;TRINITY_DN14391_c1_g1_i2_orf1;TRINITY_DN16123_c0_g1_i1_orf1;TRINITY_DN31225_c0_g1_i1_orf1;TRINITY_DN147458_c0_g1_i1_orf1;TRINITY_DN26824_c0_g1_i1_orf1;TRINITY_DN30233_c0_g1_i2_orf1;TRINITY_DN6462_c0_g1_i5_orf1
cellular_component	membrane-bounded organelle	GO:0043227	50	50/855	TRINITY_DN8703_c0_g1_i2_orf1;TRINITY_DN6556_c0_g1_i7_orf1;TRINITY_DN5055_c0_g1_i12_orf1;TRINITY_DN12526_c0_g1_i5_orf1;TRINITY_DN5670_c0_g1_i2_orf1;TRINITY_DN146119_c0_g1_i1_orf1;TRINITY_DN3092_c0_g1_i2_orf1;TRINITY_DN6365_c0_g1_i4_orf1;TRINITY_DN46409_c0_g1_i1_orf1;TRINITY_DN13760_c1_g1_i1_orf1;TRINITY_DN31584_c0_g2_i2_orf1;TRINITY_DN18933_c0_g1_i3_orf1;TRINITY_DN2168_c0_g1_i2_orf1;TRINITY_DN96801_c0_g1_i1_orf1;TRINITY_DN5962_c0_g1_i1_orf1;TRINITY_DN30663_c0_g1_i1_orf1;TRINITY_DN18804_c0_g1_i5_orf1;TRINITY_DN51938_c0_g3_i1_orf1;TRINITY_DN47389_c0_g1_i2_orf1;TRINITY_DN500_c0_g1_i1_orf1;TRINITY_DN12771_c0_g1_i1_orf1;TRINITY_DN1706_c0_g1_i7_orf1;TRINITY_DN4793_c0_g1_i7_orf1;TRINITY_DN3219_c0_g1_i6_orf1;TRINITY_DN50787_c0_g2_i2_orf1;TRINITY_DN41_c0_g1_i3_orf1;TRINITY_DN45037_c0_g1_i1_orf1;TRINITY_DN4142_c0_g1_i5_orf1;TRINITY_DN279_c0_g1_i10_orf1;TRINITY_DN3464_c0_g1_i1_orf1;TRINITY_DN26789_c0_g1_i2_orf1;TRINITY_DN22009_c0_g1_i1_orf1;TRINITY_DN106534_c0_g1_i1_orf1;TRINITY_DN84478_c0_g1_i8_orf1;TRINITY_DN96739_c0_g1_i1_orf1;TRINITY_DN113327_c0_g1_i2_orf1;TRINITY_DN143_c0_g3_i1_orf1;TRINITY_DN140538_c0_g2_i1_orf1;TRINITY_DN111110_c0_g1_i1_orf1;TRINITY_DN7909_c0_g2_i1_orf1;TRINITY_DN6358_c0_g1_i5_orf1;TRINITY_DN33893_c0_g1_i1_orf1;TRINITY_DN86149_c0_g1_i1_orf1;TRINITY_DN42854_c0_g3_i2_orf1;TRINITY_DN5578_c0_g1_i4_orf1;TRINITY_DN147458_c0_g1_i1_orf1;TRINITY_DN3702_c0_g1_i1_orf1;TRINITY_DN25997_c1_g2_i4_orf1;TRINITY_DN54612_c0_g1_i3_orf1;TRINITY_DN6462_c0_g1_i5_orf1
cellular_component	extracellular organelle	GO:0043230	3	3/855	TRINITY_DN42854_c0_g3_i2_orf1;TRINITY_DN46409_c0_g1_i1_orf1;TRINITY_DN96739_c0_g1_i1_orf1
cellular_component	organelle membrane	GO:0031090	30	30/855	TRINITY_DN9608_c0_g1_i3_orf1;TRINITY_DN13923_c0_g2_i1_orf1;TRINITY_DN3209_c0_g1_i1_orf1;TRINITY_DN36324_c0_g1_i12_orf1;TRINITY_DN46409_c0_g1_i1_orf1;TRINITY_DN3450_c0_g1_i3_orf1;TRINITY_DN14262_c0_g1_i5_orf1;TRINITY_DN1789_c0_g1_i5_orf1;TRINITY_DN11069_c0_g2_i1_orf1;TRINITY_DN20984_c0_g1_i4_orf1;TRINITY_DN13186_c0_g1_i1_orf1;TRINITY_DN11172_c0_g1_i4_orf1;TRINITY_DN95558_c0_g3_i1_orf1;TRINITY_DN448_c0_g1_i20_orf1;TRINITY_DN13783_c0_g4_i2_orf1;TRINITY_DN19303_c0_g1_i5_orf1;TRINITY_DN5439_c0_g1_i2_orf1;TRINITY_DN96739_c0_g1_i1_orf1;TRINITY_DN12286_c1_g1_i2_orf1;TRINITY_DN52887_c0_g1_i1_orf1;TRINITY_DN883_c0_g1_i8_orf1;TRINITY_DN32780_c0_g1_i2_orf1;TRINITY_DN3209_c0_g2_i6_orf1;TRINITY_DN22242_c0_g1_i1_orf1;TRINITY_DN24873_c0_g1_i4_orf1;TRINITY_DN103118_c0_g1_i4_orf1;TRINITY_DN42854_c0_g3_i2_orf1;TRINITY_DN72816_c0_g1_i2_orf1;TRINITY_DN1652_c0_g1_i12_orf1;TRINITY_DN9931_c0_g1_i1_orf1
cellular_component	outer membrane	GO:0019867	1	1/855	TRINITY_DN142657_c0_g1_i1_orf1
cellular_component	plasma membrane	GO:0005886	10	10/855	TRINITY_DN51938_c0_g3_i1_orf1;TRINITY_DN23926_c0_g1_i4_orf1;TRINITY_DN39725_c0_g1_i4_orf1;TRINITY_DN1012_c0_g1_i2_orf1;TRINITY_DN42854_c0_g3_i2_orf1;TRINITY_DN46409_c0_g1_i1_orf1;TRINITY_DN75086_c0_g1_i5_orf1;TRINITY_DN31584_c0_g2_i2_orf1;TRINITY_DN5553_c0_g1_i4_orf1;TRINITY_DN32780_c0_g1_i2_orf1
cellular_component	plasma membrane region	GO:0098590	2	2/855	TRINITY_DN46409_c0_g1_i1_orf1;TRINITY_DN51938_c0_g3_i1_orf1
cellular_component	extracellular matrix	GO:0031012	2	2/855	TRINITY_DN35147_c0_g1_i1_orf1;TRINITY_DN96739_c0_g1_i1_orf1
cellular_component	synapse	GO:0045202	2	2/855	TRINITY_DN17693_c0_g1_i10_orf1;TRINITY_DN140538_c0_g2_i1_orf1
cellular_component	anchoring junction	GO:0070161	4	4/855	TRINITY_DN96739_c0_g1_i1_orf1;TRINITY_DN17693_c0_g1_i10_orf1;TRINITY_DN1652_c0_g1_i12_orf1;TRINITY_DN23746_c0_g1_i2_orf1
cellular_component	Golgi apparatus subcompartment	GO:0098791	1	1/855	TRINITY_DN4401_c0_g2_i1_orf1
cellular_component	nuclear speck	GO:0016607	1	1/855	TRINITY_DN140538_c0_g2_i1_orf1
cellular_component	cytosolic region	GO:0099522	1	1/855	TRINITY_DN140538_c0_g2_i1_orf1
cellular_component	ciliary basal body	GO:0036064	1	1/855	TRINITY_DN140538_c0_g2_i1_orf1
cellular_component	ribonucleoprotein granule	GO:0035770	1	1/855	TRINITY_DN1298_c0_g1_i3_orf1
molecular_function	translation factor activity, RNA binding	GO:0008135	6	6/855	TRINITY_DN126648_c0_g1_i1_orf1;TRINITY_DN21609_c0_g2_i1_orf1;TRINITY_DN22572_c0_g1_i1_orf1;TRINITY_DN4309_c0_g1_i1_orf1;TRINITY_DN29521_c0_g1_i1_orf1;TRINITY_DN2630_c0_g3_i3_orf1
molecular_function	transcription coactivator activity	GO:0003713	1	1/855	TRINITY_DN77572_c0_g1_i1_orf1
molecular_function	RNA helicase activity	GO:0003724	3	3/855	TRINITY_DN4408_c6_g1_i1_orf1;TRINITY_DN2904_c0_g1_i4_orf1;TRINITY_DN14274_c0_g1_i3_orf1
molecular_function	DNA helicase activity	GO:0003678	1	1/855	TRINITY_DN109733_c0_g1_i1_orf1
molecular_function	DNA clamp loader activity	GO:0003689	1	1/855	TRINITY_DN3092_c0_g1_i2_orf1
molecular_function	long-chain fatty acid-CoA ligase activity	GO:0004467	1	1/855	TRINITY_DN2193_c0_g1_i7_orf1
molecular_function	ABC-type transporter activity	GO:0140359	3	3/855	TRINITY_DN13563_c0_g1_i1_orf1;TRINITY_DN14937_c0_g1_i7_orf1;TRINITY_DN31327_c0_g2_i1_orf1
molecular_function	nuclear export signal receptor activity	GO:0005049	1	1/855	TRINITY_DN3747_c1_g1_i3_orf1
molecular_function	ATP-dependent FeS chaperone activity	GO:0140663	1	1/855	TRINITY_DN85476_c0_g1_i1_orf1
molecular_function	peroxiredoxin activity	GO:0051920	1	1/855	TRINITY_DN69236_c0_g1_i1_orf1
molecular_function	glutathione peroxidase activity	GO:0004602	3	3/855	TRINITY_DN80660_c0_g1_i1_orf1;TRINITY_DN1622_c0_g1_i6_orf1;TRINITY_DN21420_c0_g1_i2_orf1
molecular_function	passive transmembrane transporter activity	GO:0022803	4	4/855	TRINITY_DN11566_c0_g1_i6_orf1;TRINITY_DN96739_c0_g1_i1_orf1;TRINITY_DN18338_c0_g1_i6_orf1;TRINITY_DN20558_c0_g1_i2_orf1
molecular_function	active transmembrane transporter activity	GO:0022804	3	3/855	TRINITY_DN13563_c0_g1_i1_orf1;TRINITY_DN14937_c0_g1_i7_orf1;TRINITY_DN31327_c0_g2_i1_orf1
molecular_function	ion transmembrane transporter activity	GO:0015075	8	8/855	TRINITY_DN15222_c0_g1_i4_orf1;TRINITY_DN13923_c0_g2_i1_orf1;TRINITY_DN9354_c0_g1_i7_orf1;TRINITY_DN20558_c0_g1_i2_orf1;TRINITY_DN96739_c0_g1_i1_orf1;TRINITY_DN12286_c1_g1_i2_orf1;TRINITY_DN91946_c0_g1_i1_orf1;TRINITY_DN1882_c0_g1_i4_orf1
molecular_function	inorganic molecular entity transmembrane transporter activity	GO:0015318	6	6/855	TRINITY_DN15222_c0_g1_i4_orf1;TRINITY_DN1882_c0_g1_i4_orf1;TRINITY_DN9354_c0_g1_i7_orf1;TRINITY_DN20558_c0_g1_i2_orf1;TRINITY_DN96739_c0_g1_i1_orf1;TRINITY_DN91946_c0_g1_i1_orf1
molecular_function	ion channel regulator activity	GO:0099106	1	1/855	TRINITY_DN31584_c0_g2_i2_orf1
molecular_function	nucleoside-triphosphatase regulator activity	GO:0060589	3	3/855	TRINITY_DN42461_c0_g1_i4_orf1;TRINITY_DN804_c0_g1_i7_orf1;TRINITY_DN21609_c0_g2_i1_orf1
molecular_function	peptidase regulator activity	GO:0061134	4	4/855	TRINITY_DN3609_c0_g1_i6_orf1;TRINITY_DN42854_c0_g3_i2_orf1;TRINITY_DN2271_c0_g1_i12_orf1;TRINITY_DN45948_c1_g1_i1_orf1
molecular_function	enzyme inhibitor activity	GO:0004857	4	4/855	TRINITY_DN3609_c0_g1_i6_orf1;TRINITY_DN42854_c0_g3_i2_orf1;TRINITY_DN2271_c0_g1_i12_orf1;TRINITY_DN45948_c1_g1_i1_orf1
molecular_function	nucleic acid binding	GO:0003676	57	57/855	TRINITY_DN129226_c0_g1_i2_orf1;TRINITY_DN3092_c0_g1_i2_orf1;TRINITY_DN1978_c0_g1_i4_orf1;TRINITY_DN3301_c0_g1_i2_orf1;TRINITY_DN40434_c0_g1_i2_orf1;TRINITY_DN8716_c0_g1_i3_orf1;TRINITY_DN8008_c0_g1_i6_orf1;TRINITY_DN620_c0_g1_i4_orf1;TRINITY_DN46409_c0_g1_i1_orf1;TRINITY_DN4262_c0_g1_i16_orf1;TRINITY_DN51934_c0_g2_i1_orf1;TRINITY_DN19651_c0_g1_i1_orf1;TRINITY_DN5962_c0_g1_i1_orf1;TRINITY_DN18804_c0_g1_i5_orf1;TRINITY_DN22572_c0_g1_i1_orf1;TRINITY_DN500_c0_g1_i1_orf1;TRINITY_DN34134_c0_g2_i1_orf1;TRINITY_DN126648_c0_g1_i1_orf1;TRINITY_DN96801_c0_g1_i1_orf1;TRINITY_DN2904_c0_g1_i4_orf1;TRINITY_DN16965_c0_g2_i1_orf1;TRINITY_DN1298_c0_g1_i3_orf1;TRINITY_DN810_c0_g1_i4_orf1;TRINITY_DN50787_c0_g2_i2_orf1;TRINITY_DN20442_c0_g2_i1_orf1;TRINITY_DN29521_c0_g1_i1_orf1;TRINITY_DN5670_c0_g1_i2_orf1;TRINITY_DN2630_c0_g3_i3_orf1;TRINITY_DN147596_c0_g1_i1_orf1;TRINITY_DN5238_c0_g1_i2_orf1;TRINITY_DN3733_c0_g1_i1_orf1;TRINITY_DN12323_c0_g2_i2_orf1;TRINITY_DN14274_c0_g1_i3_orf1;TRINITY_DN21609_c0_g2_i1_orf1;TRINITY_DN4408_c6_g1_i1_orf1;TRINITY_DN16978_c0_g1_i1_orf1;TRINITY_DN1116_c0_g1_i6_orf1;TRINITY_DN40945_c0_g1_i1_orf1;TRINITY_DN42646_c0_g2_i1_orf1;TRINITY_DN15160_c0_g1_i1_orf1;TRINITY_DN2749_c0_g2_i3_orf1;TRINITY_DN6358_c0_g1_i5_orf1;TRINITY_DN2676_c0_g1_i2_orf1;TRINITY_DN33893_c0_g1_i1_orf1;TRINITY_DN251_c0_g1_i2_orf1;TRINITY_DN36496_c0_g1_i1_orf1;TRINITY_DN4813_c0_g1_i5_orf1;TRINITY_DN5210_c0_g1_i3_orf1;TRINITY_DN42854_c0_g3_i2_orf1;TRINITY_DN147458_c0_g1_i1_orf1;TRINITY_DN23004_c0_g1_i1_orf1;TRINITY_DN2299_c0_g1_i3_orf1;TRINITY_DN3862_c0_g1_i7_orf1;TRINITY_DN4309_c0_g1_i1_orf1;TRINITY_DN89613_c0_g1_i13_orf1;TRINITY_DN430_c0_g1_i5_orf1;TRINITY_DN2749_c4_g1_i2_orf1
molecular_function	nucleoside phosphate binding	GO:1901265	67	67/855	TRINITY_DN5422_c0_g1_i1_orf1;TRINITY_DN3092_c0_g1_i2_orf1;TRINITY_DN2904_c0_g1_i4_orf1;TRINITY_DN47151_c0_g1_i1_orf1;TRINITY_DN2772_c0_g1_i3_orf1;TRINITY_DN115210_c0_g4_i1_orf1;TRINITY_DN8716_c0_g1_i3_orf1;TRINITY_DN13563_c0_g1_i1_orf1;TRINITY_DN620_c0_g1_i4_orf1;TRINITY_DN52761_c0_g1_i2_orf1;TRINITY_DN14937_c0_g1_i7_orf1;TRINITY_DN76815_c0_g1_i3_orf1;TRINITY_DN100821_c0_g1_i1_orf1;TRINITY_DN2770_c0_g2_i4_orf1;TRINITY_DN8659_c0_g2_i1_orf1;TRINITY_DN85476_c0_g1_i1_orf1;TRINITY_DN4744_c0_g1_i7_orf1;TRINITY_DN11942_c0_g1_i1_orf1;TRINITY_DN62729_c0_g1_i13_orf1;TRINITY_DN5161_c0_g1_i5_orf1;TRINITY_DN3131_c0_g1_i5_orf1;TRINITY_DN42461_c0_g1_i4_orf1;TRINITY_DN277_c1_g1_i1_orf1;TRINITY_DN5055_c0_g1_i12_orf1;TRINITY_DN46409_c0_g1_i1_orf1;TRINITY_DN46090_c0_g3_i1_orf1;TRINITY_DN20984_c0_g1_i4_orf1;TRINITY_DN7388_c0_g1_i7_orf1;TRINITY_DN810_c0_g1_i4_orf1;TRINITY_DN3343_c0_g2_i1_orf1;TRINITY_DN29144_c0_g3_i1_orf1;TRINITY_DN107288_c0_g1_i2_orf1;TRINITY_DN73945_c0_g5_i3_orf1;TRINITY_DN15160_c0_g1_i1_orf1;TRINITY_DN24_c0_g1_i1_orf1;TRINITY_DN1125_c0_g1_i4_orf1;TRINITY_DN4628_c0_g1_i1_orf1;TRINITY_DN4794_c1_g1_i9_orf1;TRINITY_DN5070_c0_g1_i1_orf1;TRINITY_DN12301_c0_g1_i1_orf1;TRINITY_DN3991_c0_g1_i6_orf1;TRINITY_DN14274_c0_g1_i3_orf1;TRINITY_DN96739_c0_g1_i1_orf1;TRINITY_DN2193_c0_g1_i7_orf1;TRINITY_DN20527_c0_g1_i1_orf1;TRINITY_DN42185_c0_g1_i7_orf1;TRINITY_DN143637_c0_g1_i1_orf1;TRINITY_DN1266_c2_g1_i1_orf1;TRINITY_DN37165_c0_g1_i4_orf1;TRINITY_DN111110_c0_g1_i1_orf1;TRINITY_DN41697_c0_g1_i1_orf1;TRINITY_DN52244_c1_g1_i1_orf1;TRINITY_DN7909_c0_g2_i1_orf1;TRINITY_DN8979_c0_g1_i5_orf1;TRINITY_DN31327_c0_g2_i1_orf1;TRINITY_DN2146_c0_g2_i1_orf1;TRINITY_DN26789_c0_g1_i2_orf1;TRINITY_DN126648_c0_g1_i1_orf1;TRINITY_DN9109_c0_g1_i1_orf1;TRINITY_DN31225_c0_g1_i1_orf1;TRINITY_DN70236_c0_g1_i1_orf1;TRINITY_DN38424_c0_g1_i1_orf1;TRINITY_DN25997_c1_g2_i4_orf1;TRINITY_DN54612_c0_g1_i3_orf1;TRINITY_DN11125_c0_g1_i1_orf1;TRINITY_DN52761_c0_g2_i1_orf1;TRINITY_DN1209_c0_g1_i9_orf1
molecular_function	L-ascorbic acid binding	GO:0031418	1	1/855	TRINITY_DN43293_c0_g1_i2_orf1
molecular_function	tetrapyrrole binding	GO:0046906	27	27/855	TRINITY_DN43369_c0_g2_i1_orf1;TRINITY_DN120500_c0_g1_i1_orf1;TRINITY_DN9608_c0_g1_i3_orf1;TRINITY_DN3949_c1_g1_i1_orf1;TRINITY_DN23564_c0_g1_i7_orf1;TRINITY_DN4497_c0_g1_i4_orf1;TRINITY_DN7580_c0_g1_i1_orf1;TRINITY_DN2392_c0_g2_i1_orf1;TRINITY_DN24873_c0_g1_i4_orf1;TRINITY_DN14262_c0_g1_i5_orf1;TRINITY_DN3732_c0_g1_i2_orf1;TRINITY_DN829_c0_g1_i8_orf1;TRINITY_DN3732_c1_g1_i5_orf1;TRINITY_DN95558_c0_g3_i1_orf1;TRINITY_DN15755_c0_g1_i1_orf1;TRINITY_DN448_c0_g1_i20_orf1;TRINITY_DN27045_c0_g1_i1_orf1;TRINITY_DN57856_c0_g2_i1_orf1;TRINITY_DN5439_c0_g1_i2_orf1;TRINITY_DN23783_c0_g2_i1_orf1;TRINITY_DN52887_c0_g1_i1_orf1;TRINITY_DN30037_c0_g1_i5_orf1;TRINITY_DN7861_c0_g1_i5_orf1;TRINITY_DN5126_c0_g2_i1_orf1;TRINITY_DN2676_c0_g1_i2_orf1;TRINITY_DN5661_c0_g1_i5_orf1;TRINITY_DN2442_c0_g1_i6_orf1
molecular_function	vitamin B6 binding	GO:0070279	4	4/855	TRINITY_DN2803_c4_g1_i1_orf1;TRINITY_DN51813_c0_g1_i1_orf1;TRINITY_DN14935_c0_g1_i1_orf1;TRINITY_DN21035_c0_g1_i14_orf1
molecular_function	histone binding	GO:0042393	1	1/855	TRINITY_DN12771_c0_g1_i1_orf1
molecular_function	identical protein binding	GO:0042802	4	4/855	TRINITY_DN42854_c0_g3_i2_orf1;TRINITY_DN51938_c0_g3_i1_orf1;TRINITY_DN96739_c0_g1_i1_orf1;TRINITY_DN31584_c0_g2_i2_orf1
molecular_function	p53 binding	GO:0002039	1	1/855	TRINITY_DN46409_c0_g1_i1_orf1
molecular_function	apolipoprotein binding	GO:0034185	1	1/855	TRINITY_DN46409_c0_g1_i1_orf1
molecular_function	enzyme binding	GO:0019899	6	6/855	TRINITY_DN143496_c0_g1_i1_orf1;TRINITY_DN8473_c0_g1_i5_orf1;TRINITY_DN46409_c0_g1_i1_orf1;TRINITY_DN31584_c0_g2_i2_orf1;TRINITY_DN3747_c1_g1_i3_orf1;TRINITY_DN140538_c0_g2_i1_orf1
molecular_function	chaperone binding	GO:0051087	1	1/855	TRINITY_DN46409_c0_g1_i1_orf1
molecular_function	unfolded protein binding	GO:0051082	1	1/855	TRINITY_DN46409_c0_g1_i1_orf1
molecular_function	calmodulin binding	GO:0005516	1	1/855	TRINITY_DN3126_c0_g1_i4_orf1
molecular_function	beta-catenin binding	GO:0008013	1	1/855	TRINITY_DN140538_c0_g2_i1_orf1
molecular_function	translation initiation factor binding	GO:0031369	2	2/855	TRINITY_DN21609_c0_g2_i1_orf1;TRINITY_DN2630_c0_g3_i3_orf1
molecular_function	signaling receptor binding	GO:0005102	2	2/855	TRINITY_DN51938_c0_g3_i1_orf1;TRINITY_DN4628_c0_g1_i1_orf1
molecular_function	cytoskeletal protein binding	GO:0008092	6	6/855	TRINITY_DN107962_c0_g1_i1_orf1;TRINITY_DN22824_c0_g1_i4_orf1;TRINITY_DN23746_c0_g1_i2_orf1;TRINITY_DN96739_c0_g1_i1_orf1;TRINITY_DN3126_c0_g1_i4_orf1;TRINITY_DN140538_c0_g2_i1_orf1
molecular_function	protein dimerization activity	GO:0046983	2	2/855	TRINITY_DN96801_c0_g1_i1_orf1;TRINITY_DN31584_c0_g2_i2_orf1
molecular_function	phosphoprotein binding	GO:0051219	2	2/855	TRINITY_DN140538_c0_g2_i1_orf1;TRINITY_DN31584_c0_g2_i2_orf1
molecular_function	protein domain specific binding	GO:0019904	1	1/855	TRINITY_DN31584_c0_g2_i2_orf1
molecular_function	calcium-dependent protein binding	GO:0048306	1	1/855	TRINITY_DN96739_c0_g1_i1_orf1
molecular_function	GTPase activating protein binding	GO:0032794	1	1/855	TRINITY_DN140538_c0_g2_i1_orf1
molecular_function	transmembrane transporter binding	GO:0044325	1	1/855	TRINITY_DN31584_c0_g2_i2_orf1

molecular_function	scaffold protein binding	GO:0097110	1	1/855	TRINITY_DN31584_c0_g2_i2_orf1
molecular_function	chitin binding	GO:0008061	1	1/855	TRINITY_DN3759_c0_g1_i1_orf1
molecular_function	lipopolysaccharide binding	GO:0001530	2	2/855	TRINITY_DN46409_c0_g1_i1_orf1;TRINITY_DN2170_c0_g2_i1_orf1
molecular_function	lipoteichoic acid binding	GO:0070891	1	1/855	TRINITY_DN2170_c0_g2_i1_orf1
molecular_function	ribonucleotide binding	GO:0032553	51	51/855	TRINITY_DN3092_c0_g1_i2_orf1;TRINITY_DN47151_c0_g1_i1_orf1;TRINITY_DN2772_c0_g1_i3_orf1;TRINITY_DN115210_c0_g4_i1_orf1;TRINITY_DN8716_c0_g1_i3_orf1;TRINITY_DN13563_c0_g1_i1_orf1;TRINITY_DN620_c0_g1_i4_orf1;TRINITY_DN46409_c0_g1_i1_orf1;TRINITY_DN14937_c0_g1_i7_orf1;TRINITY_DN76815_c0_g1_i3_orf1;TRINITY_DN277_c1_g1_i1_orf1;TRINITY_DN2770_c0_g2_i4_orf1;TRINITY_DN8659_c0_g2_i1_orf1;TRINITY_DN85476_c0_g1_i1_orf1;TRINITY_DN11942_c0_g1_i1_orf1;TRINITY_DN62729_c0_g1_i13_orf1;TRINITY_DN42461_c0_g1_i4_orf1;TRINITY_DN11125_c0_g1_i1_orf1;TRINITY_DN2904_c0_g1_i4_orf1;TRINITY_DN46090_c0_g3_i1_orf1;TRINITY_DN20984_c0_g1_i4_orf1;TRINITY_DN7388_c0_g1_i7_orf1;TRINITY_DN810_c0_g1_i4_orf1;TRINITY_DN3343_c0_g2_i1_orf1;TRINITY_DN29144_c0_g3_i1_orf1;TRINITY_DN73945_c0_g5_i3_orf1;TRINITY_DN15160_c0_g1_i1_orf1;TRINITY_DN5070_c0_g1_i1_orf1;TRINITY_DN12301_c0_g1_i1_orf1;TRINITY_DN3991_c0_g1_i6_orf1;TRINITY_DN14274_c0_g1_i3_orf1;TRINITY_DN96739_c0_g1_i1_orf1;TRINITY_DN2193_c0_g1_i7_orf1;TRINITY_DN52761_c0_g1_i2_orf1;TRINITY_DN42185_c0_g1_i7_orf1;TRINITY_DN143637_c0_g1_i1_orf1;TRINITY_DN1266_c2_g1_i1_orf1;TRINITY_DN37165_c0_g1_i4_orf1;TRINITY_DN41697_c0_g1_i1_orf1;TRINITY_DN52244_c1_g1_i1_orf1;TRINITY_DN8979_c0_g1_i5_orf1;TRINITY_DN31327_c0_g2_i1_orf1;TRINITY_DN2146_c0_g2_i1_orf1;TRINITY_DN126648_c0_g1_i1_orf1;TRINITY_DN9109_c0_g1_i1_orf1;TRINITY_DN31225_c0_g1_i1_orf1;TRINITY_DN107288_c0_g1_i2_orf1;TRINITY_DN25997_c1_g2_i4_orf1;TRINITY_DN54612_c0_g1_i3_orf1;TRINITY_DN52761_c0_g2_i1_orf1;TRINITY_DN100821_c0_g1_i1_orf1
molecular_function	glycosaminoglycan binding	GO:0005539	3	3/855	TRINITY_DN42854_c0_g3_i2_orf1;TRINITY_DN96739_c0_g1_i1_orf1;TRINITY_DN1534_c0_g1_i3_orf1
molecular_function	heparin binding	GO:0008201	1	1/855	TRINITY_DN42854_c0_g3_i2_orf1
molecular_function	acyl-CoA binding	GO:0120227	1	1/855	TRINITY_DN13563_c0_g1_i1_orf1
molecular_function	chondroitin sulfate binding	GO:0035374	1	1/855	TRINITY_DN96739_c0_g1_i1_orf1
molecular_function	peptide binding	GO:0042277	1	1/855	TRINITY_DN13783_c0_g4_i2_orf1
molecular_function	phosphopantetheine binding	GO:0031177	1	1/855	TRINITY_DN10430_c0_g1_i4_orf1
molecular_function	cation binding	GO:0043169	113	113/855	TRINITY_DN129226_c0_g1_i2_orf1;TRINITY_DN9608_c0_g1_i3_orf1;TRINITY_DN3949_c1_g1_i1_orf1;TRINITY_DN3301_c0_g1_i2_orf1;TRINITY_DN3194_c0_g1_i6_orf1;TRINITY_DN7580_c0_g1_i1_orf1;TRINITY_DN43293_c0_g1_i2_orf1;TRINITY_DN19651_c0_g1_i1_orf1;TRINITY_DN18804_c0_g1_i5_orf1;TRINITY_DN47842_c0_g1_i1_orf1;TRINITY_DN1153_c1_g1_i1_orf1;TRINITY_DN8621_c0_g1_i4_orf1;TRINITY_DN1492_c0_g1_i4_orf1;TRINITY_DN3732_c1_g1_i5_orf1;TRINITY_DN31163_c1_g1_i4_orf1;TRINITY_DN3235_c0_g1_i1_orf1;TRINITY_DN78492_c0_g1_i1_orf1;TRINITY_DN4321_c0_g1_i1_orf1;TRINITY_DN3464_c0_g1_i1_orf1;TRINITY_DN95558_c0_g3_i1_orf1;TRINITY_DN15755_c0_g1_i1_orf1;TRINITY_DN4822_c0_g1_i6_orf1;TRINITY_DN7740_c0_g1_i2_orf1;TRINITY_DN1534_c0_g1_i3_orf1;TRINITY_DN3991_c0_g1_i6_orf1;TRINITY_DN1999_c0_g1_i9_orf1;TRINITY_DN5439_c0_g1_i2_orf1;TRINITY_DN3276_c0_g1_i4_orf1;TRINITY_DN7909_c0_g2_i1_orf1;TRINITY_DN4813_c0_g1_i5_orf1;TRINITY_DN5210_c0_g1_i3_orf1;TRINITY_DN2338_c0_g2_i1_orf1;TRINITY_DN230_c2_g1_i5_orf1;TRINITY_DN115210_c0_g4_i1_orf1;TRINITY_DN20442_c0_g2_i1_orf1;TRINITY_DN78686_c0_g1_i1_orf1;TRINITY_DN27592_c0_g1_i1_orf1;TRINITY_DN3010_c0_g1_i4_orf1;TRINITY_DN19187_c0_g1_i1_orf1;TRINITY_DN56690_c0_g1_i4_orf1;TRINITY_DN30713_c0_g1_i3_orf1;TRINITY_DN50787_c0_g2_i2_orf1;TRINITY_DN6602_c0_g1_i4_orf1;TRINITY_DN6415_c0_g1_i1_orf1;TRINITY_DN12301_c0_g1_i1_orf1;TRINITY_DN1423_c0_g1_i8_orf1;TRINITY_DN3733_c0_g1_i1_orf1;TRINITY_DN136031_c0_g1_i7_orf1;TRINITY_DN1266_c2_g1_i1_orf1;TRINITY_DN7861_c0_g1_i5_orf1;TRINITY_DN3702_c0_g1_i1_orf1;TRINITY_DN1652_c0_g1_i12_orf1;TRINITY_DN2299_c0_g1_i3_orf1;TRINITY_DN25997_c1_g2_i4_orf1;TRINITY_DN1209_c0_g1_i9_orf1;TRINITY_DN120500_c0_g1_i1_orf1;TRINITY_DN10672_c0_g1_i3_orf1;TRINITY_DN30037_c0_g1_i5_orf1;TRINITY_DN4497_c0_g1_i4_orf1;TRINITY_DN24873_c0_g1_i4_orf1;TRINITY_DN14262_c0_g1_i5_orf1;TRINITY_DN24_c0_g1_i1_orf1;TRINITY_DN829_c0_g1_i8_orf1;TRINITY_DN23564_c0_g1_i7_orf1;TRINITY_DN4793_c0_g1_i7_orf1;TRINITY_DN276_c0_g1_i2_orf1;TRINITY_DN3343_c0_g2_i1_orf1;TRINITY_DN27833_c0_g2_i1_orf1;TRINITY_DN6415_c0_g2_i1_orf1;TRINITY_DN8771_c0_g2_i1_orf1;TRINITY_DN19866_c0_g1_i4_orf1;TRINITY_DN27045_c0_g1_i1_orf1;TRINITY_DN2673_c2_g1_i2_orf1;TRINITY_DN23783_c0_g2_i1_orf1;TRINITY_DN31348_c0_g1_i1_orf1;TRINITY_DN111110_c0_g1_i1_orf1;TRINITY_DN31609_c0_g1_i3_orf1;TRINITY_DN5126_c0_g2_i1_orf1;TRINITY_DN5661_c0_g1_i5_orf1;TRINITY_DN2472_c0_g1_i6_orf1;TRINITY_DN42854_c0_g3_i2_orf1;TRINITY_DN70236_c0_g1_i1_orf1;TRINITY_DN43369_c0_g2_i1_orf1;TRINITY_DN48020_c0_g1_i1_orf1;TRINITY_DN6556_c0_g1_i7_orf1;TRINITY_DN47151_c0_g1_i1_orf1;TRINITY_DN8087_c0_g1_i9_orf1;TRINITY_DN2986_c1_g1_i1_orf1;TRINITY_DN2392_c0_g2_i1_orf1;TRINITY_DN76815_c0_g1_i3_orf1;TRINITY_DN448_c0_g1_i20_orf1;TRINITY_DN4822_c0_g1_i9_orf1;TRINITY_DN89483_c0_g1_i1_orf1;TRINITY_DN14874_c0_g1_i6_orf1;TRINITY_DN21035_c0_g1_i14_orf1;TRINITY_DN3732_c0_g1_i2_orf1;TRINITY_DN279_c0_g1_i10_orf1;TRINITY_DN15222_c0_g1_i4_orf1;TRINITY_DN22824_c0_g1_i4_orf1;TRINITY_DN19303_c0_g1_i5_orf1;TRINITY_DN5238_c0_g1_i2_orf1;TRINITY_DN57856_c0_g2_i1_orf1;TRINITY_DN69049_c0_g2_i1_orf1;TRINITY_DN52887_c0_g1_i1_orf1;TRINITY_DN140_c0_g1_i5_orf1;TRINITY_DN41697_c0_g1_i1_orf1;TRINITY_DN810_c0_g1_i4_orf1;TRINITY_DN430_c0_g1_i5_orf1;TRINITY_DN2676_c0_g1_i2_orf1;TRINITY_DN96739_c0_g1_i1_orf1;TRINITY_DN2442_c0_g1_i6_orf1;TRINITY_DN51498_c0_g1_i1_orf1;TRINITY_DN11820_c0_g1_i1_orf1
molecular_function	anion binding	GO:0043168	65	65/855	TRINITY_DN3092_c0_g1_i2_orf1;TRINITY_DN2904_c0_g1_i4_orf1;TRINITY_DN47151_c0_g1_i1_orf1;TRINITY_DN2772_c0_g1_i3_orf1;TRINITY_DN115210_c0_g4_i1_orf1;TRINITY_DN8716_c0_g1_i3_orf1;TRINITY_DN13563_c0_g1_i1_orf1;TRINITY_DN620_c0_g1_i4_orf1;TRINITY_DN46409_c0_g1_i1_orf1;TRINITY_DN14937_c0_g1_i7_orf1;TRINITY_DN76815_c0_g1_i3_orf1;TRINITY_DN43293_c0_g1_i2_orf1;TRINITY_DN100821_c0_g1_i1_orf1;TRINITY_DN2770_c0_g2_i4_orf1;TRINITY_DN8659_c0_g2_i1_orf1;TRINITY_DN85476_c0_g1_i1_orf1;TRINITY_DN4744_c0_g1_i7_orf1;TRINITY_DN20984_c0_g1_i4_orf1;TRINITY_DN11942_c0_g1_i1_orf1;TRINITY_DN62729_c0_g1_i13_orf1;TRINITY_DN3131_c0_g1_i5_orf1;TRINITY_DN42461_c0_g1_i4_orf1;TRINITY_DN277_c1_g1_i1_orf1;TRINITY_DN5055_c0_g1_i12_orf1;TRINITY_DN46090_c0_g3_i1_orf1;TRINITY_DN21035_c0_g1_i14_orf1;TRINITY_DN2803_c4_g1_i1_orf1;TRINITY_DN7388_c0_g1_i7_orf1;TRINITY_DN810_c0_g1_i4_orf1;TRINITY_DN3343_c0_g2_i1_orf1;TRINITY_DN29144_c0_g3_i1_orf1;TRINITY_DN107288_c0_g1_i2_orf1;TRINITY_DN73945_c0_g5_i3_orf1;TRINITY_DN15160_c0_g1_i1_orf1;TRINITY_DN1125_c0_g1_i4_orf1;TRINITY_DN5070_c0_g1_i1_orf1;TRINITY_DN12301_c0_g1_i1_orf1;TRINITY_DN3991_c0_g1_i6_orf1;TRINITY_DN14935_c0_g1_i1_orf1;TRINITY_DN51813_c0_g1_i1_orf1;TRINITY_DN14274_c0_g1_i3_orf1;TRINITY_DN96739_c0_g1_i1_orf1;TRINITY_DN2193_c0_g1_i7_orf1;TRINITY_DN52761_c0_g1_i2_orf1;TRINITY_DN42185_c0_g1_i7_orf1;TRINITY_DN143637_c0_g1_i1_orf1;TRINITY_DN1266_c2_g1_i1_orf1;TRINITY_DN37165_c0_g1_i4_orf1;TRINITY_DN111110_c0_g1_i1_orf1;TRINITY_DN41697_c0_g1_i1_orf1;TRINITY_DN52244_c1_g1_i1_orf1;TRINITY_DN7909_c0_g2_i1_orf1;TRINITY_DN8979_c0_g1_i5_orf1;TRINITY_DN31327_c0_g2_i1_orf1;TRINITY_DN2146_c0_g2_i1_orf1;TRINITY_DN26789_c0_g1_i2_orf1;TRINITY_DN126648_c0_g1_i1_orf1;TRINITY_DN9109_c0_g1_i1_orf1;TRINITY_DN31225_c0_g1_i1_orf1;TRINITY_DN70236_c0_g1_i1_orf1;TRINITY_DN38424_c0_g1_i1_orf1;TRINITY_DN25997_c1_g2_i4_orf1;TRINITY_DN54612_c0_g1_i3_orf1;TRINITY_DN11125_c0_g1_i1_orf1;TRINITY_DN52761_c0_g2_i1_orf1
molecular_function	phospholipid binding	GO:0005543	4	4/855	TRINITY_DN1652_c0_g1_i12_orf1;TRINITY_DN96739_c0_g1_i1_orf1;TRINITY_DN2770_c0_g2_i4_orf1;TRINITY_DN143637_c0_g1_i1_orf1
molecular_function	steroid binding	GO:0005496	1	1/855	TRINITY_DN96739_c0_g1_i1_orf1
molecular_function	fatty acid derivative binding	GO:1901567	1	1/855	TRINITY_DN13563_c0_g1_i1_orf1
molecular_function	oxygen binding	GO:0019825	1	1/855	TRINITY_DN30037_c0_g1_i5_orf1
molecular_function	monosaccharide binding	GO:0048029	1	1/855	TRINITY_DN43293_c0_g1_i2_orf1
molecular_function	organic acid binding	GO:0043177	2	2/855	TRINITY_DN115210_c0_g4_i1_orf1;TRINITY_DN43293_c0_g1_i2_orf1
molecular_function	alcohol binding	GO:0043178	1	1/855	TRINITY_DN96739_c0_g1_i1_orf1
molecular_function	vitamin binding	GO:0019842	6	6/855	TRINITY_DN14935_c0_g1_i1_orf1;TRINITY_DN51813_c0_g1_i1_orf1;TRINITY_DN21035_c0_g1_i14_orf1;TRINITY_DN2803_c4_g1_i1_orf1;TRINITY_DN43293_c0_g1_i2_orf1;TRINITY_DN10430_c0_g1_i4_orf1
molecular_function	nucleotide binding	GO:0000166	67	67/855	TRINITY_DN5422_c0_g1_i1_orf1;TRINITY_DN3092_c0_g1_i2_orf1;TRINITY_DN2904_c0_g1_i4_orf1;TRINITY_DN47151_c0_g1_i1_orf1;TRINITY_DN2772_c0_g1_i3_orf1;TRINITY_DN115210_c0_g4_i1_orf1;TRINITY_DN8716_c0_g1_i3_orf1;TRINITY_DN13563_c0_g1_i1_orf1;TRINITY_DN620_c0_g1_i4_orf1;TRINITY_DN52761_c0_g1_i2_orf1;TRINITY_DN14937_c0_g1_i7_orf1;TRINITY_DN76815_c0_g1_i3_orf1;TRINITY_DN100821_c0_g1_i1_orf1;TRINITY_DN2770_c0_g2_i4_orf1;TRINITY_DN8659_c0_g2_i1_orf1;TRINITY_DN85476_c0_g1_i1_orf1;TRINITY_DN4744_c0_g1_i7_orf1;TRINITY_DN11942_c0_g1_i1_orf1;TRINITY_DN62729_c0_g1_i13_orf1;TRINITY_DN5161_c0_g1_i5_orf1;TRINITY_DN3131_c0_g1_i5_orf1;TRINITY_DN42461_c0_g1_i4_orf1;TRINITY_DN277_c1_g1_i1_orf1;TRINITY_DN5055_c0_g1_i12_orf1;TRINITY_DN46409_c0_g1_i1_orf1;TRINITY_DN46090_c0_g3_i1_orf1;TRINITY_DN20984_c0_g1_i4_orf1;TRINITY_DN7388_c0_g1_i7_orf1;TRINITY_DN810_c0_g1_i4_orf1;TRINITY_DN3343_c0_g2_i1_orf1;TRINITY_DN29144_c0_g3_i1_orf1;TRINITY_DN107288_c0_g1_i2_orf1;TRINITY_DN73945_c0_g5_i3_orf1;TRINITY_DN15160_c0_g1_i1_orf1;TRINITY_DN24_c0_g1_i1_orf1;TRINITY_DN1125_c0_g1_i4_orf1;TRINITY_DN4628_c0_g1_i1_orf1;TRINITY_DN4794_c1_g1_i9_orf1;TRINITY_DN5070_c0_g1_i1_orf1;TRINITY_DN12301_c0_g1_i1_orf1;TRINITY_DN3991_c0_g1_i6_orf1;TRINITY_DN14274_c0_g1_i3_orf1;TRINITY_DN96739_c0_g1_i1_orf1;TRINITY_DN2193_c0_g1_i7_orf1;TRINITY_DN20527_c0_g1_i1_orf1;TRINITY_DN42185_c0_g1_i7_orf1;TRINITY_DN143637_c0_g1_i1_orf1;TRINITY_DN1266_c2_g1_i1_orf1;TRINITY_DN37165_c0_g1_i4_orf1;TRINITY_DN111110_c0_g1_i1_orf1;TRINITY_DN41697_c0_g1_i1_orf1;TRINITY_DN52244_c1_g1_i1_orf1;TRINITY_DN7909_c0_g2_i1_orf1;TRINITY_DN8979_c0_g1_i5_orf1;TRINITY_DN31327_c0_g2_i1_orf1;TRINITY_DN2146_c0_g2_i1_orf1;TRINITY_DN26789_c0_g1_i2_orf1;TRINITY_DN126648_c0_g1_i1_orf1;TRINITY_DN9109_c0_g1_i1_orf1;TRINITY_DN31225_c0_g1_i1_orf1;TRINITY_DN70236_c0_g1_i1_orf1;TRINITY_DN38424_c0_g1_i1_orf1;TRINITY_DN25997_c1_g2_i4_orf1;TRINITY_DN54612_c0_g1_i3_orf1;TRINITY_DN11125_c0_g1_i1_orf1;TRINITY_DN52761_c0_g2_i1_orf1;TRINITY_DN1209_c0_g1_i9_orf1
molecular_function	polysaccharide binding	GO:0030247	1	1/855	TRINITY_DN2170_c0_g2_i1_orf1
molecular_function	phosphatidylserine binding	GO:0001786	1	1/855	TRINITY_DN96739_c0_g1_i1_orf1
molecular_function	ribonucleoprotein complex binding	GO:0043021	2	2/855	TRINITY_DN4309_c0_g1_i1_orf1;TRINITY_DN3534_c0_g1_i2_orf1
molecular_function	G-protein beta/gamma-subunit complex binding	GO:0031683	1	1/855	TRINITY_DN4628_c0_g1_i1_orf1
molecular_function	protein-lipid complex binding	GO:0071814	1	1/855	TRINITY_DN46409_c0_g1_i1_orf1
molecular_function	actin filament binding	GO:0051015	2	2/855	TRINITY_DN96739_c0_g1_i1_orf1;TRINITY_DN22824_c0_g1_i4_orf1
molecular_function	iron-sulfur cluster binding	GO:0051536	3	3/855	TRINITY_DN85476_c0_g1_i1_orf1;TRINITY_DN3464_c0_g1_i1_orf1;TRINITY_DN7909_c0_g2_i1_orf1
molecular_function	transmembrane signaling receptor activity	GO:0004888	3	3/855	TRINITY_DN15247_c0_g1_i2_orf1;TRINITY_DN46090_c0_g3_i1_orf1;TRINITY_DN19662_c4_g1_i1_orf1
molecular_function	pattern recognition receptor activity	GO:0038187	1	1/855	TRINITY_DN2170_c0_g2_i1_orf1
molecular_function	ligase activity, forming carbon-oxygen bonds	GO:0016875	4	4/855	TRINITY_DN15160_c0_g1_i1_orf1;TRINITY_DN8716_c0_g1_i3_orf1;TRINITY_DN107288_c0_g1_i2_orf1;TRINITY_DN620_c0_g1_i4_orf1
molecular_function	ligase activity, forming carbon-sulfur bonds	GO:0016877	2	2/855	TRINITY_DN8659_c0_g2_i1_orf1;TRINITY_DN2193_c0_g1_i7_orf1
molecular_function	ligase activity, forming carbon-nitrogen bonds	GO:0016879	5	5/855	TRINITY_DN115210_c0_g4_i1_orf1;TRINITY_DN36144_c0_g1_i3_orf1;TRINITY_DN76815_c0_g1_i3_orf1;TRINITY_DN100821_c0_g1_i1_orf1;TRINITY_DN41697_c0_g1_i1_orf1
molecular_function	ligase activity, forming carbon-carbon bonds	GO:0016885	1	1/855	TRINITY_DN3991_c0_g1_i6_orf1
molecular_function	phosphorus-oxygen lyase activity	GO:0016849	2	2/855	TRINITY_DN11942_c0_g1_i1_orf1;TRINITY_DN52244_c1_g1_i1_orf1
molecular_function	carbon-oxygen lyase activity	GO:0016835	7	7/855	TRINITY_DN230_c2_g1_i5_orf1;TRINITY_DN5070_c0_g1_i1_orf1;TRINITY_DN51498_c0_g1_i1_orf1;TRINITY_DN42759_c0_g2_i1_orf1;TRINITY_DN10430_c0_g1_i4_orf1;TRINITY_DN3464_c0_g1_i1_orf1;TRINITY_DN89483_c0_g1_i1_orf1
molecular_function	carbon-carbon lyase activity	GO:0016830	3	3/855	TRINITY_DN779_c0_g1_i12_orf1;TRINITY_DN40434_c0_g1_i2_orf1;TRINITY_DN83150_c0_g1_i1_orf1
molecular_function	protein-malonyllysine demalonylase activity	GO:0036054	1	1/855	TRINITY_DN111110_c0_g1_i1_orf1
molecular_function	peptide-lysine-N-acetyltransferase activity	GO:0061733	2	2/855	TRINITY_DN20442_c0_g2_i1_orf1;TRINITY_DN12771_c0_g1_i1_orf1
molecular_function	palmitoyl-(protein) hydrolase activity	GO:0008474	1	1/855	TRINITY_DN4817_c0_g1_i4_orf1
molecular_function	protein-disulfide reductase activity	GO:0015035	1	1/855	TRINITY_DN51938_c0_g3_i1_orf1
molecular_function	protein-glutaryllysine deglutarylase activity	GO:0061697	1	1/855	TRINITY_DN111110_c0_g1_i1_orf1
molecular_function	protein methyltransferase activity	GO:0008276	3	3/855	TRINITY_DN95414_c0_g1_i1_orf1;TRINITY_DN6462_c0_g1_i5_orf1;TRINITY_DN2168_c0_g1_i2_orf1
molecular_function	phosphoprotein phosphatase activity	GO:0004721	2	2/855	TRINITY_DN1749_c0_g2_i2_orf1;TRINITY_DN4571_c0_g1_i4_orf1
molecular_function	ubiquitin-like protein transferase activity	GO:0019787	2	2/855	TRINITY_DN11820_c0_g1_i1_orf1;TRINITY_DN1380_c0_g1_i5_orf1
molecular_function	aminoacyltransferase activity	GO:0016755	1	1/855	TRINITY_DN52553_c0_g2_i1_orf1
molecular_function	protein lysine deacetylase activity	GO:0033558	2	2/855	TRINITY_DN70236_c0_g1_i1_orf1;TRINITY_DN111110_c0_g1_i1_orf1
molecular_function	protein kinase activity	GO:0004672	9	9/855	TRINITY_DN62729_c0_g1_i13_orf1;TRINITY_DN42461_c0_g1_i4_orf1;TRINITY_DN11125_c0_g1_i1_orf1;TRINITY_DN46090_c0_g3_i1_orf1;TRINITY_DN277_c1_g1_i1_orf1;TRINITY_DN19662_c4_g1_i1_orf1;TRINITY_DN73945_c0_g5_i3_orf1;TRINITY_DN143637_c0_g1_i1_orf1;TRINITY_DN1266_c2_g1_i1_orf1

molecular_function	peptidase activity	GO:0008233	40	40/855	TRINITY_DN4767_c0_g1_i6_orf1;TRINITY_DN51938_c0_g3_i1_orf1;TRINITY_DN143895_c0_g1_i1_orf1;TRINITY_DN13856_c0_g1_i1_orf1;TRINITY_DN3194_c0_g1_i6_orf1;TRINITY_DN753_c0_g1_i4_orf1;TRINITY_DN6122_c0_g1_i6_orf1;TRINITY_DN19651_c0_g1_i1_orf1;TRINITY_DN344_c1_g1_i1_orf1;TRINITY_DN5310_c2_g1_i2_orf1;TRINITY_DN17329_c0_g2_i3_orf1;TRINITY_DN41086_c0_g1_i4_orf1;TRINITY_DN8621_c0_g1_i4_orf1;TRINITY_DN36434_c0_g2_i3_orf1;TRINITY_DN3343_c0_g2_i1_orf1;TRINITY_DN14754_c0_g1_i6_orf1;TRINITY_DN45948_c1_g1_i1_orf1;TRINITY_DN1592_c0_g1_i1_orf1;TRINITY_DN14874_c0_g1_i6_orf1;TRINITY_DN5696_c0_g1_i4_orf1;TRINITY_DN18388_c0_g1_i6_orf1;TRINITY_DN48020_c0_g1_i1_orf1;TRINITY_DN747_c0_g1_i1_orf1;TRINITY_DN875_c0_g1_i3_orf1;TRINITY_DN19866_c0_g1_i4_orf1;TRINITY_DN18273_c0_g1_i4_orf1;TRINITY_DN4064_c0_g2_i1_orf1;TRINITY_DN2673_c2_g1_i2_orf1;TRINITY_DN4408_c6_g1_i1_orf1;TRINITY_DN701_c0_g1_i1_orf1;TRINITY_DN113327_c0_g1_i2_orf1;TRINITY_DN4767_c0_g1_i4_orf1;TRINITY_DN69049_c0_g2_i1_orf1;TRINITY_DN6205_c0_g1_i1_orf1;TRINITY_DN892_c7_g1_i2_orf1;TRINITY_DN3702_c0_g1_i1_orf1;TRINITY_DN24121_c1_g1_i6_orf1;TRINITY_DN56690_c0_g1_i4_orf1;TRINITY_DN338_c1_g1_i9_orf1;TRINITY_DN747_c0_g1_i4_orf1
molecular_function	protein disulfide isomerase activity	GO:0003756	1	1/855	TRINITY_DN51938_c0_g3_i1_orf1
molecular_function	peptidyl-prolyl cis-trans isomerase activity	GO:0003755	1	1/855	TRINITY_DN140538_c0_g2_i1_orf1
molecular_function	ubiquitin-like modifier activating enzyme activity	GO:0008641	1	1/855	TRINITY_DN8659_c0_g2_i1_orf1
molecular_function	peptide-O-fucosyltransferase activity	GO:0046922	1	1/855	TRINITY_DN4142_c0_g1_i5_orf1
molecular_function	hydrolase activity, acting on glycosyl bonds	GO:0016798	11	11/855	TRINITY_DN15222_c0_g1_i4_orf1;TRINITY_DN8703_c0_g1_i2_orf1;TRINITY_DN467_c3_g1_i5_orf1;TRINITY_DN18918_c0_g1_i3_orf1;TRINITY_DN1785_c0_g1_i5_orf1;TRINITY_DN103118_c0_g1_i4_orf1;TRINITY_DN6108_c0_g1_i5_orf1;TRINITY_DN2170_c0_g2_i1_orf1;TRINITY_DN2894_c0_g2_i3_orf1;TRINITY_DN5852_c0_g1_i6_orf1;TRINITY_DN9044_c0_g1_i2_orf1
molecular_function	hydrolase activity, acting on acid carbon-carbon bonds	GO:0016822	2	2/855	TRINITY_DN19187_c0_g1_i1_orf1;TRINITY_DN51813_c0_g1_i1_orf1
molecular_function	hydrolase activity, acting on carbon-nitrogen (but not peptide) bonds	GO:0016810	6	6/855	TRINITY_DN5422_c0_g1_i1_orf1;TRINITY_DN1534_c0_g1_i3_orf1;TRINITY_DN115210_c0_g4_i1_orf1;TRINITY_DN768_c0_g1_i7_orf1;TRINITY_DN70236_c0_g1_i1_orf1;TRINITY_DN111110_c0_g1_i1_orf1
molecular_function	hydrolase activity, acting on acid anhydrides	GO:0016817	13	13/855	TRINITY_DN5422_c0_g1_i1_orf1;TRINITY_DN3092_c0_g1_i2_orf1;TRINITY_DN8979_c0_g1_i5_orf1;TRINITY_DN11069_c0_g2_i1_orf1;TRINITY_DN29144_c0_g3_i1_orf1;TRINITY_DN46409_c0_g1_i1_orf1;TRINITY_DN7388_c0_g1_i7_orf1;TRINITY_DN3343_c0_g2_i1_orf1;TRINITY_DN52761_c0_g1_i2_orf1;TRINITY_DN42185_c0_g1_i7_orf1;TRINITY_DN4628_c0_g1_i1_orf1;TRINITY_DN52761_c0_g2_i1_orf1;TRINITY_DN100821_c0_g1_i1_orf1
molecular_function	serine hydrolase activity	GO:0017171	15	15/855	TRINITY_DN747_c0_g1_i1_orf1;TRINITY_DN36434_c0_g2_i3_orf1;TRINITY_DN18273_c0_g1_i4_orf1;TRINITY_DN5696_c0_g1_i4_orf1;TRINITY_DN1592_c0_g1_i1_orf1;TRINITY_DN6205_c0_g1_i1_orf1;TRINITY_DN24121_c1_g1_i6_orf1;TRINITY_DN41086_c0_g1_i4_orf1;TRINITY_DN701_c0_g1_i1_orf1;TRINITY_DN753_c0_g1_i4_orf1;TRINITY_DN338_c1_g1_i9_orf1;TRINITY_DN747_c0_g1_i4_orf1;TRINITY_DN344_c1_g1_i1_orf1;TRINITY_DN18388_c0_g1_i6_orf1;TRINITY_DN5310_c2_g1_i2_orf1
molecular_function	hydrolase activity, acting on ester bonds	GO:0016788	30	30/855	TRINITY_DN1749_c0_g2_i2_orf1;TRINITY_DN2749_c4_g1_i2_orf1;TRINITY_DN1978_c0_g1_i4_orf1;TRINITY_DN2772_c0_g1_i3_orf1;TRINITY_DN8771_c0_g2_i1_orf1;TRINITY_DN4817_c0_g1_i4_orf1;TRINITY_DN95850_c0_g1_i1_orf1;TRINITY_DN4571_c0_g1_i4_orf1;TRINITY_DN53294_c0_g1_i1_orf1;TRINITY_DN34465_c0_g1_i1_orf1;TRINITY_DN2798_c0_g1_i5_orf1;TRINITY_DN24_c0_g1_i1_orf1;TRINITY_DN810_c0_g1_i4_orf1;TRINITY_DN42759_c0_g2_i1_orf1;TRINITY_DN10430_c0_g1_i4_orf1;TRINITY_DN2749_c0_g2_i3_orf1;TRINITY_DN17693_c0_g1_i10_orf1;TRINITY_DN81926_c0_g1_i1_orf1;TRINITY_DN4394_c0_g2_i1_orf1;TRINITY_DN5238_c0_g1_i2_orf1;TRINITY_DN1116_c0_g1_i6_orf1;TRINITY_DN40945_c0_g1_i1_orf1;TRINITY_DN11798_c0_g2_i1_orf1;TRINITY_DN4813_c0_g1_i5_orf1;TRINITY_DN64403_c0_g2_i1_orf1;TRINITY_DN23004_c0_g1_i1_orf1;TRINITY_DN3862_c0_g1_i7_orf1;TRINITY_DN9931_c0_g1_i1_orf1;TRINITY_DN10662_c0_g1_i4_orf1;TRINITY_DN18128_c0_g1_i4_orf1
molecular_function	deacetylase activity	GO:0019213	2	2/855	TRINITY_DN70236_c0_g1_i1_orf1;TRINITY_DN111110_c0_g1_i1_orf1
molecular_function	palmitoyl hydrolase activity	GO:0098599	1	1/855	TRINITY_DN4817_c0_g1_i4_orf1
molecular_function	hydrolase activity, acting on ether bonds	GO:0016801	2	2/855	TRINITY_DN11172_c0_g1_i4_orf1;TRINITY_DN22242_c0_g1_i1_orf1
molecular_function	FAD-AMP lyase (cyclizing) activity	GO:0034012	2	2/855	TRINITY_DN11942_c0_g1_i1_orf1;TRINITY_DN52244_c1_g1_i1_orf1
molecular_function	oxidoreductase activity, acting on CH-OH group of donors	GO:0016614	12	12/855	TRINITY_DN4794_c1_g1_i9_orf1;TRINITY_DN42759_c0_g2_i1_orf1;TRINITY_DN5161_c0_g1_i5_orf1;TRINITY_DN29018_c0_g1_i4_orf1;TRINITY_DN20658_c0_g2_i3_orf1;TRINITY_DN4793_c0_g1_i7_orf1;TRINITY_DN12193_c0_g1_i6_orf1;TRINITY_DN28626_c0_g1_i5_orf1;TRINITY_DN38424_c0_g1_i1_orf1;TRINITY_DN10430_c0_g1_i4_orf1;TRINITY_DN1209_c0_g1_i9_orf1;TRINITY_DN31609_c0_g1_i3_orf1
molecular_function	oxidoreductase activity, acting on the aldehyde or oxo group of donors	GO:0016903	3	3/855	TRINITY_DN3529_c0_g1_i7_orf1;TRINITY_DN3836_c0_g1_i4_orf1;TRINITY_DN4596_c0_g1_i14_orf1
molecular_function	oxidoreductase activity, acting on the CH-NH group of donors	GO:0016645	2	2/855	TRINITY_DN20527_c0_g1_i1_orf1;TRINITY_DN1760_c0_g1_i4_orf1
molecular_function	dioxygenase activity	GO:0051213	5	5/855	TRINITY_DN1153_c1_g1_i1_orf1;TRINITY_DN4822_c0_g1_i9_orf1;TRINITY_DN43293_c0_g1_i2_orf1;TRINITY_DN4822_c0_g1_i6_orf1;TRINITY_DN57900_c0_g1_i2_orf1
molecular_function	oxidoreductase activity, acting on paired donors, with incorporation or reduction of molecular	GO:0016705	30	30/855	TRINITY_DN43369_c0_g2_i1_orf1;TRINITY_DN120500_c0_g1_i1_orf1;TRINITY_DN9608_c0_g1_i3_orf1;TRINITY_DN3949_c1_g1_i1_orf1;TRINITY_DN23564_c0_g1_i7_orf1;TRINITY_DN4497_c0_g1_i4_orf1;TRINITY_DN7580_c0_g1_i1_orf1;TRINITY_DN2392_c0_g2_i1_orf1;TRINITY_DN24873_c0_g1_i4_orf1;TRINITY_DN43293_c0_g1_i2_orf1;TRINITY_DN14262_c0_g1_i5_orf1;TRINITY_DN27903_c0_g1_i1_orf1;TRINITY_DN3131_c0_g1_i5_orf1;TRINITY_DN3732_c0_g1_i2_orf1;TRINITY_DN829_c0_g1_i8_orf1;TRINITY_DN3732_c1_g1_i5_orf1;TRINITY_DN31163_c1_g1_i4_orf1;TRINITY_DN95558_c0_g3_i1_orf1;TRINITY_DN15755_c0_g1_i1_orf1;TRINITY_DN448_c0_g1_i20_orf1;TRINITY_DN1999_c0_g1_i9_orf1;TRINITY_DN27045_c0_g1_i1_orf1;TRINITY_DN57856_c0_g2_i1_orf1;TRINITY_DN5439_c0_g1_i2_orf1;TRINITY_DN52887_c0_g1_i1_orf1;TRINITY_DN4321_c0_g1_i1_orf1;TRINITY_DN5126_c0_g2_i1_orf1;TRINITY_DN2676_c0_g1_i2_orf1;TRINITY_DN5661_c0_g1_i5_orf1;TRINITY_DN2442_c0_g1_i6_orf1
molecular_function	oxidoreductase activity, acting on single donors with incorporation of molecular oxygen	GO:0016701	3	3/855	TRINITY_DN4822_c0_g1_i6_orf1;TRINITY_DN4822_c0_g1_i9_orf1;TRINITY_DN3010_c0_g1_i4_orf1
molecular_function	oxidoreductase activity, acting on the CH-CH group of donors	GO:0016627	7	7/855	TRINITY_DN5055_c0_g1_i12_orf1;TRINITY_DN29018_c0_g1_i4_orf1;TRINITY_DN20658_c0_g2_i3_orf1;TRINITY_DN42759_c0_g2_i1_orf1;TRINITY_DN10430_c0_g1_i4_orf1;TRINITY_DN1125_c0_g1_i4_orf1;TRINITY_DN4744_c0_g1_i7_orf1
molecular_function	oxidoreductase activity, acting on NAD(P)H	GO:0016651	4	4/855	TRINITY_DN20984_c0_g1_i4_orf1;TRINITY_DN4497_c0_g1_i4_orf1;TRINITY_DN8306_c0_g1_i4_orf1;TRINITY_DN52887_c0_g1_i1_orf1
molecular_function	oxidoreductase activity, acting on metal ions	GO:0016722	2	2/855	TRINITY_DN1423_c0_g1_i8_orf1;TRINITY_DN136031_c0_g1_i7_orf1
molecular_function	oxidoreductase activity, acting on peroxide as acceptor	GO:0016684	4	4/855	TRINITY_DN80660_c0_g1_i1_orf1;TRINITY_DN1622_c0_g1_i6_orf1;TRINITY_DN21420_c0_g1_i2_orf1;TRINITY_DN69236_c0_g1_i1_orf1
molecular_function	monooxygenase activity	GO:0004497	31	31/855	TRINITY_DN43369_c0_g2_i1_orf1;TRINITY_DN120500_c0_g1_i1_orf1;TRINITY_DN9608_c0_g1_i3_orf1;TRINITY_DN3949_c1_g1_i1_orf1;TRINITY_DN84357_c0_g1_i1_orf1;TRINITY_DN23564_c0_g1_i7_orf1;TRINITY_DN4497_c0_g1_i4_orf1;TRINITY_DN7580_c0_g1_i1_orf1;TRINITY_DN2392_c0_g2_i1_orf1;TRINITY_DN24873_c0_g1_i4_orf1;TRINITY_DN14262_c0_g1_i5_orf1;TRINITY_DN3010_c0_g1_i4_orf1;TRINITY_DN3131_c0_g1_i5_orf1;TRINITY_DN3732_c0_g1_i2_orf1;TRINITY_DN829_c0_g1_i8_orf1;TRINITY_DN3732_c1_g1_i5_orf1;TRINITY_DN31163_c1_g1_i4_orf1;TRINITY_DN95558_c0_g3_i1_orf1;TRINITY_DN15755_c0_g1_i1_orf1;TRINITY_DN448_c0_g1_i20_orf1;TRINITY_DN27045_c0_g1_i1_orf1;TRINITY_DN57856_c0_g2_i1_orf1;TRINITY_DN5439_c0_g1_i2_orf1;TRINITY_DN52887_c0_g1_i1_orf1;TRINITY_DN5126_c0_g2_i1_orf1;TRINITY_DN2676_c0_g1_i2_orf1;TRINITY_DN5661_c0_g1_i5_orf1;TRINITY_DN2442_c0_g1_i6_orf1;TRINITY_DN2338_c0_g2_i1_orf1;TRINITY_DN2338_c0_g1_i3_orf1;TRINITY_DN31584_c0_g2_i2_orf1
molecular_function	oxidoreductase activity, acting on the CH-NH2 group of donors	GO:0016638	1	1/855	TRINITY_DN37165_c0_g1_i4_orf1
molecular_function	oxidoreductase activity, acting on a sulfur group of donors	GO:0016667	4	4/855	TRINITY_DN920_c0_g1_i6_orf1;TRINITY_DN920_c0_g1_i4_orf1;TRINITY_DN51938_c0_g3_i1_orf1;TRINITY_DN1491_c0_g1_i4_orf1
molecular_function	lysozyme activity	GO:0003796	1	1/855	TRINITY_DN467_c3_g1_i5_orf1
molecular_function	N-acetylmuramoyl-L-alanine amidase activity	GO:0008745	1	1/855	TRINITY_DN1534_c0_g1_i3_orf1
molecular_function	intramolecular transferase activity	GO:0016866	1	1/855	TRINITY_DN30713_c0_g1_i3_orf1
molecular_function	intramolecular oxidoreductase activity	GO:0016860	1	1/855	TRINITY_DN51938_c0_g3_i1_orf1
molecular_function	cis-trans isomerase activity	GO:0016859	2	2/855	TRINITY_DN140538_c0_g2_i1_orf1;TRINITY_DN12293_c0_g1_i1_orf1
molecular_function	catalytic activity, acting on RNA	GO:0140098	13	13/855	TRINITY_DN8716_c0_g1_i3_orf1;TRINITY_DN34465_c0_g1_i1_orf1;TRINITY_DN4813_c0_g1_i5_orf1;TRINITY_DN2904_c0_g1_i4_orf1;TRINITY_DN14274_c0_g1_i3_orf1;TRINITY_DN620_c0_g1_i4_orf1;TRINITY_DN4408_c6_g1_i1_orf1;TRINITY_DN810_c0_g1_i4_orf1;TRINITY_DN23004_c0_g1_i1_orf1;TRINITY_DN2299_c0_g1_i3_orf1;TRINITY_DN107288_c0_g1_i2_orf1;TRINITY_DN5962_c0_g1_i1_orf1;TRINITY_DN15160_c0_g1_i1_orf1
molecular_function	catalytic activity, acting on DNA	GO:0140097	4	4/855	TRINITY_DN3092_c0_g1_i2_orf1;TRINITY_DN89613_c0_g1_i13_orf1;TRINITY_DN109733_c0_g1_i1_orf1;TRINITY_DN40434_c0_g1_i2_orf1
molecular_function	helicase activity	GO:0004386	6	6/855	TRINITY_DN6556_c0_g1_i7_orf1;TRINITY_DN2904_c0_g1_i4_orf1;TRINITY_DN14274_c0_g1_i3_orf1;TRINITY_DN4408_c6_g1_i1_orf1;TRINITY_DN109733_c0_g1_i1_orf1;TRINITY_DN810_c0_g1_i4_orf1
molecular_function	transferase activity, transferring alkyl or aryl (other than methyl) groups	GO:0016765	13	13/855	TRINITY_DN920_c0_g1_i4_orf1;TRINITY_DN12134_c0_g1_i4_orf1;TRINITY_DN22_c0_g1_i3_orf1;TRINITY_DN3929_c0_g3_i3_orf1;TRINITY_DN128231_c0_g1_i5_orf1;TRINITY_DN22046_c1_g1_i5_orf1;TRINITY_DN15597_c0_g1_i1_orf1;TRINITY_DN8651_c0_g1_i18_orf1;TRINITY_DN8964_c0_g1_i4_orf1;TRINITY_DN8651_c0_g1_i16_orf1;TRINITY_DN3332_c0_g1_i9_orf1;TRINITY_DN225_c0_g1_i6_orf1;TRINITY_DN920_c0_g1_i6_orf1
molecular_function	transferase activity, transferring nitrogenous groups	GO:0016769	3	3/855	TRINITY_DN6908_c0_g1_i3_orf1;TRINITY_DN2803_c4_g1_i1_orf1;TRINITY_DN5564_c0_g1_i5_orf1
molecular_function	glycosyltransferase activity	GO:0016757	7	7/855	TRINITY_DN31390_c0_g1_i2_orf1;TRINITY_DN15157_c0_g1_i1_orf1;TRINITY_DN48602_c0_g1_i6_orf1;TRINITY_DN12508_c0_g1_i1_orf1;TRINITY_DN28592_c0_g1_i2_orf1;TRINITY_DN4142_c0_g1_i5_orf1;TRINITY_DN14597_c0_g1_i5_orf1
molecular_function	transferase activity, transferring phosphorus-containing groups	GO:0016772	23	23/855	TRINITY_DN56998_c0_g1_i2_orf1;TRINITY_DN47151_c0_g1_i1_orf1;TRINITY_DN3534_c0_g1_i2_orf1;TRINITY_DN2770_c0_g2_i4_orf1;TRINITY_DN11942_c0_g1_i1_orf1;TRINITY_DN62729_c0_g1_i13_orf1;TRINITY_DN42461_c0_g1_i4_orf1;TRINITY_DN11125_c0_g1_i1_orf1;TRINITY_DN46090_c0_g3_i1_orf1;TRINITY_DN277_c1_g1_i1_orf1;TRINITY_DN19662_c4_g1_i1_orf1;TRINITY_DN73945_c0_g5_i3_orf1;TRINITY_DN12301_c0_g1_i1_orf1;TRINITY_DN12323_c0_g2_i2_orf1;TRINITY_DN4408_c6_g1_i1_orf1;TRINITY_DN36632_c0_g1_i1_orf1;TRINITY_DN143637_c0_g1_i1_orf1;TRINITY_DN1266_c2_g1_i1_orf1;TRINITY_DN52244_c1_g1_i1_orf1;TRINITY_DN9109_c0_g1_i1_orf1;TRINITY_DN2299_c0_g1_i3_orf1;TRINITY_DN25997_c1_g2_i4_orf1;TRINITY_DN89613_c0_g1_i13_orf1
molecular_function	transferase activity, transferring one-carbon groups	GO:0016741	7	7/855	TRINITY_DN115210_c0_g4_i1_orf1;TRINITY_DN6462_c0_g1_i5_orf1;TRINITY_DN77318_c0_g2_i1_orf1;TRINITY_DN95414_c0_g1_i1_orf1;TRINITY_DN2168_c0_g1_i2_orf1;TRINITY_DN31431_c0_g1_i1_orf1;TRINITY_DN5962_c0_g1_i1_orf1
molecular_function	acyltransferase activity	GO:0016746	11	11/855	TRINITY_DN52553_c0_g2_i1_orf1;TRINITY_DN47389_c0_g1_i2_orf1;TRINITY_DN86833_c0_g3_i1_orf1;TRINITY_DN20442_c0_g2_i1_orf1;TRINITY_DN12771_c0_g1_i1_orf1;TRINITY_DN24142_c0_g1_i1_orf1;TRINITY_DN3219_c0_g1_i6_orf1;TRINITY_DN42759_c0_g2_i1_orf1;TRINITY_DN5841_c0_g1_i2_orf1;TRINITY_DN10430_c0_g1_i4_orf1;TRINITY_DN883_c0_g1_i8_orf1
