# Supplementary material for: Comparative Proteomics and Metabonomics Analysis of Different Diapause Stages Revealed a New Regulation Mechanism of Diapause in Loxostege sticticalis (Lepidoptera: Pyralidae)
Source: Molecules. 2024 Jul 25;29(15):3472. doi: 10.3390/molecules29153472 (PMC11314584; doi:10.3390/molecules29153472)
Supplement: Supplementary file 1 [file molecules-29-03472-s001.zip › analysis process/proteomic/GO annotations analysis/CTvsPreD all.pdf]

Term Type	GO Term	GO ID	LCL_vs_ZYQ_all num	LCL_vs_ZYQ_all p	LCL_vs_ZYQ_all Accession ids
biological_process	immune response-activating signal transduction	GO:0002757	1	1/1363	TRINITY_DN2170_c0_g2_i1_orf1
biological_process	activation of innate immune response	GO:0002218	3	3/1363	TRINITY_DN8685_c0_g1_i5_orf1;TRINITY_DN2170_c0_g2_i1_orf1;TRINITY_DN5880_c0_g2_i2_orf1
biological_process	cell activation involved in immune response	GO:0002263	1	1/1363	TRINITY_DN46409_c0_g1_i1_orf1
biological_process	lymphocyte activation	GO:0046649	1	1/1363	TRINITY_DN46409_c0_g1_i1_orf1
biological_process	leukocyte activation involved in immune response	GO:0002366	1	1/1363	TRINITY_DN46409_c0_g1_i1_orf1
biological_process	innate immune response	GO:0045087	11	11/1363	TRINITY_DN827_c1_g1_i1_orf1;TRINITY_DN1534_c0_g1_i3_orf1;TRINITY_DN8685_c0_g1_i5_orf1;TRINITY_DN195_c4_g1_i1_orf1;TRINITY_DN5880_c0_g2_i2_orf1;TRINITY_DN2170_c0_g2_i1_orf1;TRINITY_DN670_c0_g1_i15_orf1;TRINITY_DN1666_c0_g1_i2_orf1;TRINITY_DN9044_c0_g1_i2_orf1;TRINITY_DN2848_c0_g1_i2_orf1;TRINITY_DN9724_c0_g1_i4_orf1
biological_process	humoral immune response	GO:0006959	1	1/1363	TRINITY_DN2848_c0_g1_i2_orf1
biological_process	somatic diversification of immune receptors via germline recombination within a single locus	GO:0002562	1	1/1363	TRINITY_DN46409_c0_g1_i1_orf1
biological_process	somatic diversification of immunoglobulins	GO:0016445	1	1/1363	TRINITY_DN46409_c0_g1_i1_orf1
biological_process	regulation of catalytic activity	GO:0050790	5	5/1363	TRINITY_DN1328_c0_g1_i6_orf1;TRINITY_DN46409_c0_g1_i1_orf1;TRINITY_DN2848_c0_g1_i2_orf1;TRINITY_DN140538_c0_g2_i1_orf1;TRINITY_DN50074_c0_g1_i1_orf1
biological_process	positive regulation of molecular function	GO:0044093	5	5/1363	TRINITY_DN46409_c0_g1_i1_orf1;TRINITY_DN50074_c0_g1_i1_orf1;TRINITY_DN5406_c0_g2_i1_orf1;TRINITY_DN5553_c0_g1_i4_orf1;TRINITY_DN140538_c0_g2_i1_orf1
biological_process	negative regulation of molecular function	GO:0044092	4	4/1363	TRINITY_DN1328_c0_g1_i6_orf1;TRINITY_DN2848_c0_g1_i2_orf1;TRINITY_DN140538_c0_g2_i1_orf1;TRINITY_DN31584_c0_g2_i2_orf1
biological_process	regulation of binding	GO:0051098	1	1/1363	TRINITY_DN140538_c0_g2_i1_orf1
biological_process	regulation of transporter activity	GO:0032409	3	3/1363	TRINITY_DN5406_c0_g2_i1_orf1;TRINITY_DN5553_c0_g1_i4_orf1;TRINITY_DN31584_c0_g2_i2_orf1
biological_process	regulation of metabolic process	GO:0019222	25	25/1363	TRINITY_DN59804_c0_g1_i1_orf1;TRINITY_DN19260_c0_g1_i5_orf1;TRINITY_DN1706_c0_g1_i7_orf1;TRINITY_DN20442_c0_g2_i1_orf1;TRINITY_DN46409_c0_g1_i1_orf1;TRINITY_DN31584_c0_g2_i2_orf1;TRINITY_DN44407_c0_g4_i2_orf1;TRINITY_DN21150_c0_g1_i4_orf1;TRINITY_DN142442_c0_g1_i1_orf1;TRINITY_DN1328_c0_g1_i6_orf1;TRINITY_DN50074_c0_g1_i1_orf1;TRINITY_DN20009_c0_g1_i1_orf1;TRINITY_DN9510_c0_g2_i1_orf1;TRINITY_DN1710_c0_g2_i2_orf1;TRINITY_DN50085_c0_g1_i1_orf1;TRINITY_DN8107_c0_g1_i1_orf1;TRINITY_DN140538_c0_g2_i1_orf1;TRINITY_DN96557_c0_g1_i1_orf1;TRINITY_DN33893_c0_g1_i1_orf1;TRINITY_DN31342_c2_g2_i1_orf1;TRINITY_DN77572_c0_g1_i1_orf1;TRINITY_DN6462_c0_g1_i5_orf1;TRINITY_DN3649_c0_g1_i6_orf1;TRINITY_DN2848_c0_g1_i2_orf1;TRINITY_DN21341_c0_g1_i1_orf1
biological_process	regulation of response to stimulus	GO:0048583	10	10/1363	TRINITY_DN24323_c0_g1_i3_orf1;TRINITY_DN7316_c0_g2_i1_orf1;TRINITY_DN8685_c0_g1_i5_orf1;TRINITY_DN22572_c0_g1_i1_orf1;TRINITY_DN46409_c0_g1_i1_orf1;TRINITY_DN2170_c0_g2_i1_orf1;TRINITY_DN670_c0_g1_i15_orf1;TRINITY_DN2848_c0_g1_i2_orf1;TRINITY_DN140538_c0_g2_i1_orf1;TRINITY_DN5880_c0_g2_i2_orf1
biological_process	regulation of cellular process	GO:0050794	42	42/1363	TRINITY_DN59804_c0_g1_i1_orf1;TRINITY_DN1706_c0_g1_i7_orf1;TRINITY_DN20442_c0_g2_i1_orf1;TRINITY_DN4676_c0_g1_i16_orf1;TRINITY_DN46409_c0_g1_i1_orf1;TRINITY_DN670_c0_g1_i15_orf1;TRINITY_DN4742_c0_g1_i1_orf1;TRINITY_DN10455_c0_g1_i2_orf1;TRINITY_DN2770_c0_g2_i4_orf1;TRINITY_DN44407_c0_g4_i2_orf1;TRINITY_DN54477_c0_g1_i1_orf1;TRINITY_DN5406_c0_g2_i1_orf1;TRINITY_DN21150_c0_g1_i4_orf1;TRINITY_DN7316_c0_g2_i1_orf1;TRINITY_DN142442_c0_g1_i1_orf1;TRINITY_DN4798_c0_g1_i3_orf1;TRINITY_DN22572_c0_g1_i1_orf1;TRINITY_DN1008_c0_g1_i2_orf1;TRINITY_DN6462_c0_g1_i5_orf1;TRINITY_DN5553_c0_g1_i4_orf1;TRINITY_DN20009_c0_g1_i1_orf1;TRINITY_DN9510_c0_g2_i1_orf1;TRINITY_DN24323_c0_g1_i3_orf1;TRINITY_DN1710_c0_g2_i2_orf1;TRINITY_DN50085_c0_g1_i1_orf1;TRINITY_DN15478_c0_g1_i1_orf1;TRINITY_DN2170_c0_g2_i1_orf1;TRINITY_DN140538_c0_g2_i1_orf1;TRINITY_DN9724_c0_g1_i4_orf1;TRINITY_DN96557_c0_g1_i1_orf1;TRINITY_DN33893_c0_g1_i1_orf1;TRINITY_DN15247_c0_g1_i2_orf1;TRINITY_DN80424_c0_g1_i1_orf1;TRINITY_DN96739_c0_g1_i1_orf1;TRINITY_DN77572_c0_g1_i1_orf1;TRINITY_DN23020_c0_g1_i1_orf1;TRINITY_DN42854_c0_g3_i2_orf1;TRINITY_DN3649_c0_g1_i6_orf1;TRINITY_DN804_c0_g1_i7_orf1;TRINITY_DN2848_c0_g1_i2_orf1;TRINITY_DN21341_c0_g1_i1_orf1;TRINITY_DN31584_c0_g2_i2_orf1
biological_process	regulation of locomotion	GO:0040012	1	1/1363	TRINITY_DN140538_c0_g2_i1_orf1
biological_process	regulation of localization	GO:0032879	5	5/1363	TRINITY_DN140538_c0_g2_i1_orf1;TRINITY_DN96739_c0_g1_i1_orf1;TRINITY_DN5406_c0_g2_i1_orf1;TRINITY_DN5553_c0_g1_i4_orf1;TRINITY_DN31584_c0_g2_i2_orf1
biological_process	regulation of multicellular organismal process	GO:0051239	4	4/1363	TRINITY_DN1455_c0_g1_i8_orf1;TRINITY_DN96739_c0_g1_i1_orf1;TRINITY_DN2848_c0_g1_i2_orf1;TRINITY_DN46409_c0_g1_i1_orf1
biological_process	regulation of membrane repolarization	GO:0060306	1	1/1363	TRINITY_DN31584_c0_g2_i2_orf1
biological_process	regulation of immune system process	GO:0002682	4	4/1363	TRINITY_DN8685_c0_g1_i5_orf1;TRINITY_DN46409_c0_g1_i1_orf1;TRINITY_DN2170_c0_g2_i1_orf1;TRINITY_DN5880_c0_g2_i2_orf1
biological_process	positive regulation of biological process	GO:0048518	15	15/1363	TRINITY_DN1710_c0_g2_i2_orf1;TRINITY_DN5553_c0_g1_i4_orf1;TRINITY_DN5406_c0_g2_i1_orf1;TRINITY_DN8685_c0_g1_i5_orf1;TRINITY_DN140538_c0_g2_i1_orf1;TRINITY_DN9510_c0_g2_i1_orf1;TRINITY_DN22572_c0_g1_i1_orf1;TRINITY_DN46409_c0_g1_i1_orf1;TRINITY_DN2170_c0_g2_i1_orf1;TRINITY_DN31584_c0_g2_i2_orf1;TRINITY_DN50074_c0_g1_i1_orf1;TRINITY_DN20009_c0_g1_i1_orf1;TRINITY_DN2848_c0_g1_i2_orf1;TRINITY_DN44407_c0_g4_i2_orf1;TRINITY_DN5880_c0_g2_i2_orf1
biological_process	negative regulation of biological process	GO:0048519	10	10/1363	TRINITY_DN59804_c0_g1_i1_orf1;TRINITY_DN96557_c0_g1_i1_orf1;TRINITY_DN1328_c0_g1_i6_orf1;TRINITY_DN46409_c0_g1_i1_orf1;TRINITY_DN96739_c0_g1_i1_orf1;TRINITY_DN31584_c0_g2_i2_orf1;TRINITY_DN20009_c0_g1_i1_orf1;TRINITY_DN8107_c0_g1_i1_orf1;TRINITY_DN2848_c0_g1_i2_orf1;TRINITY_DN140538_c0_g2_i1_orf1
biological_process	regulation of signaling	GO:0023051	7	7/1363	TRINITY_DN24323_c0_g1_i3_orf1;TRINITY_DN5406_c0_g2_i1_orf1;TRINITY_DN7316_c0_g2_i1_orf1;TRINITY_DN22572_c0_g1_i1_orf1;TRINITY_DN5553_c0_g1_i4_orf1;TRINITY_DN2848_c0_g1_i2_orf1;TRINITY_DN140538_c0_g2_i1_orf1
biological_process	regulation of membrane potential	GO:0042391	1	1/1363	TRINITY_DN31584_c0_g2_i2_orf1
biological_process	regulation of neurotransmitter levels	GO:0001505	2	2/1363	TRINITY_DN17693_c0_g1_i10_orf1;TRINITY_DN82017_c0_g1_i5_orf1
biological_process	homeostatic process	GO:0042592	9	9/1363	TRINITY_DN96557_c0_g1_i1_orf1;TRINITY_DN46625_c0_g1_i1_orf1;TRINITY_DN65681_c0_g1_i1_orf1;TRINITY_DN1423_c0_g1_i4_orf1;TRINITY_DN1423_c0_g1_i8_orf1;TRINITY_DN4469_c0_g1_i2_orf1;TRINITY_DN136031_c0_g1_i7_orf1;TRINITY_DN96739_c0_g1_i1_orf1;TRINITY_DN31584_c0_g2_i2_orf1
biological_process	regulation of anatomical structure size	GO:0090066	3	3/1363	TRINITY_DN10455_c0_g1_i2_orf1;TRINITY_DN80424_c0_g1_i1_orf1;TRINITY_DN23020_c0_g1_i1_orf1
biological_process	regulation of RNA stability	GO:0043487	2	2/1363	TRINITY_DN20009_c0_g1_i1_orf1;TRINITY_DN21341_c0_g1_i1_orf1
biological_process	regulation of protein stability	GO:0031647	3	3/1363	TRINITY_DN46409_c0_g1_i1_orf1;TRINITY_DN2848_c0_g1_i2_orf1;TRINITY_DN140538_c0_g2_i1_orf1
biological_process	organonitrogen compound metabolic process	GO:1901564	192	192/1363	TRINITY_DN42856_c0_g1_i1_orf1;TRINITY_DN827_c1_g1_i1_orf1;TRINITY_DN98242_c0_g1_i1_orf1;TRINITY_DN27771_c0_g1_i1_orf1;TRINITY_DN5564_c0_g1_i5_orf1;TRINITY_DN4121_c0_g1_i1_orf1;TRINITY_DN5873_c0_g4_i1_orf1;TRINITY_DN344_c1_g1_i1_orf1;TRINITY_DN54477_c0_g1_i1_orf1;TRINITY_DN11065_c0_g2_i1_orf1;TRINITY_DN6325_c0_g1_i8_orf1;TRINITY_DN2682_c0_g1_i4_orf1;TRINITY_DN30300_c0_g2_i1_orf1;TRINITY_DN2442_c0_g1_i2_orf1;TRINITY_DN142442_c0_g1_i1_orf1;TRINITY_DN4798_c0_g1_i3_orf1;TRINITY_DN30131_c0_g1_i1_orf1;TRINITY_DN31163_c1_g1_i4_orf1;TRINITY_DN277_c1_g1_i1_orf1;TRINITY_DN14754_c0_g1_i6_orf1;TRINITY_DN2954_c0_g1_i1_orf1;TRINITY_DN14774_c0_g1_i4_orf1;TRINITY_DN244_c1_g1_i5_orf1;TRINITY_DN4822_c0_g1_i6_orf1;TRINITY_DN1534_c0_g1_i3_orf1;TRINITY_DN95056_c0_g2_i2_orf1;TRINITY_DN18273_c0_g1_i4_orf1;TRINITY_DN59885_c0_g1_i3_orf1;TRINITY_DN84478_c0_g1_i8_orf1;TRINITY_DN6205_c0_g1_i8_orf1;TRINITY_DN817_c0_g1_i3_orf1;TRINITY_DN137_c0_g1_i1_orf1;TRINITY_DN143_c0_g3_i1_orf1;TRINITY_DN140538_c0_g2_i1_orf1;TRINITY_DN17376_c0_g1_i2_orf1;TRINITY_DN3483_c0_g1_i5_orf1;TRINITY_DN22674_c0_g1_i2_orf1;TRINITY_DN79734_c0_g2_i3_orf1;TRINITY_DN46715_c0_g1_i1_orf1;TRINITY_DN21971_c0_g1_i4_orf1;TRINITY_DN46202_c0_g1_i1_orf1;TRINITY_DN121893_c0_g1_i1_orf1;TRINITY_DN2848_c0_g1_i2_orf1;TRINITY_DN4767_c0_g1_i6_orf1;TRINITY_DN230_c2_g1_i5_orf1;TRINITY_DN6199_c2_g1_i3_orf1;TRINITY_DN18249_c0_g1_i1_orf1;TRINITY_DN143895_c0_g1_i1_orf1;TRINITY_DN1706_c0_g1_i7_orf1;TRINITY_DN12526_c0_g1_i5_orf1;TRINITY_DN144956_c0_g1_i1_orf1;TRINITY_DN10824_c0_g1_i3_orf1;TRINITY_DN100821_c0_g1_i1_orf1;TRINITY_DN2719_c1_g1_i6_orf1;TRINITY_DN4189_c0_g2_i1_orf1;TRINITY_DN1791_c0_g1_i3_orf1;TRINITY_DN1272_c1_g1_i4_orf1;TRINITY_DN53760_c0_g1_i1_orf1;TRINITY_DN97589_c0_g1_i3_orf1;TRINITY_DN19187_c0_g1_i1_orf1;TRINITY_DN9871_c0_g1_i11_orf1;TRINITY_DN42461_c0_g1_i4_orf1;TRINITY_DN46409_c0_g1_i1_orf1;TRINITY_DN7464_c0_g1_i14_orf1;TRINITY_DN50787_c0_g2_i2_orf1;TRINITY_DN45948_c1_g1_i1_orf1;TRINITY_DN5696_c0_g1_i4_orf1;TRINITY_DN36144_c0_g1_i3_orf1;TRINITY_DN71863_c0_g1_i2_orf1;TRINITY_DN799_c0_g1_i7_orf1;TRINITY_DN3324_c0_g1_i3_orf1;TRINITY_DN3733_c0_g1_i1_orf1;TRINITY_DN13732_c0_g2_i3_orf1;TRINITY_DN7957_c0_g1_i5_orf1;TRINITY_DN95414_c0_g1_i1_orf1;TRINITY_DN3472_c0_g1_i6_orf1;TRINITY_DN376_c1_g1_i1_orf1;TRINITY_DN21357_c0_g1_i5_orf1;TRINITY_DN1954_c0_g1_i4_orf1;TRINITY_DN7991_c0_g1_i9_orf1;TRINITY_DN58207_c0_g1_i1_orf1;TRINITY_DN4767_c0_g1_i4_orf1;TRINITY_DN4408_c6_g1_i1_orf1;TRINITY_DN13233_c0_g1_i3_orf1;TRINITY_DN10831_c1_g1_i1_orf1;TRINITY_DN40015_c0_g1_i2_orf1;TRINITY_DN14874_c0_g1_i6_orf1;TRINITY_DN17326_c0_g1_i5_orf1;TRINITY_DN21984_c0_g1_i6_orf1;TRINITY_DN147458_c0_g1_i1_orf1;TRINITY_DN12397_c0_g1_i1_orf1;TRINITY_DN51658_c0_g1_i1_orf1;TRINITY_DN143852_c0_g1_i1_orf1;TRINITY_DN21251_c1_g1_i1_orf1;TRINITY_DN1749_c0_g2_i2_orf1;TRINITY_DN5112_c0_g1_i1_orf1;TRINITY_DN92153_c0_g2_i2_orf1;TRINITY_DN10766_c0_g1_i1_orf1;TRINITY_DN18159_c0_g1_i6_orf1;TRINITY_DN620_c0_g1_i4_orf1;TRINITY_DN67026_c0_g1_i6_orf1;TRINITY_DN27035_c0_g1_i1_orf1;TRINITY_DN1575_c0_g1_i7_orf1;TRINITY_DN24318_c0_g1_i1_orf1;TRINITY_DN670_c0_g1_i15_orf1;TRINITY_DN4742_c0_g1_i1_orf1;TRINITY_DN4817_c0_g1_i4_orf1;TRINITY_DN5009_c0_g1_i2_orf1;TRINITY_DN753_c0_g1_i4_orf1;TRINITY_DN57111_c0_g1_i1_orf1;TRINITY_DN7212_c0_g1_i4_orf1;TRINITY_DN140_c1_g1_i2_orf1;TRINITY_DN28729_c0_g1_i9_orf1;TRINITY_DN61222_c0_g1_i1_orf1;TRINITY_DN40345_c0_g1_i6_orf1;TRINITY_DN34479_c0_g1_i2_orf1;TRINITY_DN2885_c1_g1_i2_orf1;TRINITY_DN45924_c0_g1_i14_orf1;TRINITY_DN2559_c0_g1_i4_orf1;TRINITY_DN130778_c0_g1_i1_orf1;TRINITY_DN30027_c0_g1_i1_orf1;TRINITY_DN82324_c0_g1_i4_orf1;TRINITY_DN147676_c0_g1_i1_orf1;TRINITY_DN19866_c0_g1_i4_orf1;TRINITY_DN825_c23_g1_i5_orf1;TRINITY_DN84322_c0_g2_i1_orf1;TRINITY_DN51813_c0_g1_i1_orf1;TRINITY_DN2673_c2_g1_i2_orf1;TRINITY_DN1308_c0_g1_i4_orf1;TRINITY_DN23167_c0_g1_i4_orf1;TRINITY_DN631_c0_g1_i6_orf1;TRINITY_DN113327_c0_g1_i2_orf1;TRINITY_DN111110_c0_g1_i1_orf1;TRINITY_DN5811_c0_g1_i4_orf1;TRINITY_DN96557_c0_g1_i1_orf1;TRINITY_DN37856_c0_g1_i5_orf1;TRINITY_DN28299_c0_g1_i1_orf1;TRINITY_DN6470_c0_g3_i2_orf1;TRINITY_DN1287_c0_g1_i5_orf1;TRINITY_DN1957_c0_g1_i4_orf1;TRINITY_DN24121_c1_g1_i6_orf1;TRINITY_DN10548_c0_g2_i1_orf1;TRINITY_DN49047_c0_g1_i2_orf1;TRINITY_DN14464_c0_g1_i1_orf1;TRINITY_DN9979_c0_g1_i1_orf1;TRINITY_DN16939_c0_g1_i4_orf1;TRINITY_DN59804_c0_g1_i1_orf1;TRINITY_DN140423_c0_g1_i2_orf1;TRINITY_DN40650_c0_g1_i1_orf1;TRINITY_DN5031_c0_g1_i1_orf1;TRINITY_DN7316_c0_g2_i1_orf1;TRINITY_DN7613_c1_g2_i1_orf1;TRINITY_DN36893_c0_g1_i1_orf1;TRINITY_DN801_c0_g1_i2_orf1;TRINITY_DN38562_c0_g1_i3_orf1;TRINITY_DN41166_c0_g1_i1_orf1;TRINITY_DN18593_c0_g1_i1_orf1;TRINITY_DN25423_c0_g1_i1_orf1;TRINITY_DN13651_c0_g1_i2_orf1;TRINITY_DN8949_c0_g1_i2_orf1;TRINITY_DN4822_c0_g1_i9_orf1;TRINITY_DN27958_c0_g1_i1_orf1;TRINITY_DN4030_c0_g2_i1_orf1;TRINITY_DN143496_c0_g1_i1_orf1;TRINITY_DN17329_c0_g2_i3_orf1;TRINITY_DN7647_c0_g1_i4_orf1;TRINITY_DN3702_c0_g1_i1_orf1;TRINITY_DN15265_c0_g1_i1_orf1;TRINITY_DN4494_c0_g1_i1_orf1;TRINITY_DN8824_c0_g2_i1_orf1;TRINITY_DN152_c0_g1_i4_orf1;TRINITY_DN892_c7_g1_i2_orf1;TRINITY_DN18782_c0_g1_i4_orf1;TRINITY_DN3826_c0_g1_i1_orf1;TRINITY_DN17913_c0_g1_i8_orf1;TRINITY_DN10234_c0_g1_i1_orf1;TRINITY_DN15478_c0_g1_i1_orf1;TRINITY_DN80134_c0_g1_i1_orf1;TRINITY_DN34399_c0_g1_i1_orf1;TRINITY_DN5064_c0_g1_i4_orf1;TRINITY_DN42646_c0_g2_i1_orf1;TRINITY_DN26130_c0_g1_i1_orf1;TRINITY_DN48619_c0_g1_i1_orf1;TRINITY_DN103118_c0_g1_i4_orf1;TRINITY_DN9862_c0_g2_i1_orf1;TRINITY_DN1592_c0_g1_i1_orf1;TRINITY_DN1084_c0_g1_i2_orf1;TRINITY_DN19251_c0_g1_i8_orf1;TRINITY_DN26824_c0_g1_i1_orf1;TRINITY_DN5697_c0_g1_i1_orf1;TRINITY_DN6462_c0_g1_i5_orf1;TRINITY_DN3393_c0_g2_i1_orf1
biological_process	cellular nitrogen compound metabolic process	GO:0034641	142	142/1363	TRINITY_DN38230_c0_g1_i4_orf1;TRINITY_DN40434_c0_g1_i2_orf1;TRINITY_DN27771_c0_g1_i1_orf1;TRINITY_DN137_c0_g1_i1_orf1;TRINITY_DN1116_c0_g1_i6_orf1;TRINITY_DN5873_c0_g4_i1_orf1;TRINITY_DN2054_c0_g1_i1_orf1;TRINITY_DN11065_c0_g2_i1_orf1;TRINITY_DN58207_c0_g1_i1_orf1;TRINITY_DN30300_c0_g2_i1_orf1;TRINITY_DN142442_c0_g1_i1_orf1;TRINITY_DN30131_c0_g1_i1_orf1;TRINITY_DN7957_c0_g1_i5_orf1;TRINITY_DN8107_c0_g1_i1_orf1;TRINITY_DN5670_c0_g1_i2_orf1;TRINITY_DN95056_c0_g2_i2_orf1;TRINITY_DN47123_c0_g1_i1_orf1;TRINITY_DN123184_c0_g1_i1_orf1;TRINITY_DN2110_c0_g1_i3_orf1;TRINITY_DN10234_c0_g1_i1_orf1;TRINITY_DN817_c0_g1_i3_orf1;TRINITY_DN77318_c0_g2_i1_orf1;TRINITY_DN143_c0_g3_i1_orf1;TRINITY_DN8824_c0_g2_i1_orf1;TRINITY_DN20499_c0_g3_i1_orf1;TRINITY_DN46409_c0_g1_i1_orf1;TRINITY_DN79734_c0_g2_i3_orf1;TRINITY_DN6235_c0_g1_i5_orf1;TRINITY_DN121893_c0_g1_i1_orf1;TRINITY_DN2848_c0_g1_i2_orf1;TRINITY_DN1957_c0_g1_i4_orf1;TRINITY_DN230_c2_g1_i5_orf1;TRINITY_DN3092_c0_g1_i2_orf1;TRINITY_DN1978_c0_g1_i4_orf1;TRINITY_DN17271_c0_g1_i1_orf1;TRINITY_DN140212_c0_g1_i1_orf1;TRINITY_DN144956_c0_g1_i1_orf1;TRINITY_DN37532_c0_g1_i1_orf1;TRINITY_DN51968_c0_g1_i1_orf1;TRINITY_DN20238_c0_g1_i7_orf1;TRINITY_DN97589_c0_g1_i3_orf1;TRINITY_DN1091_c0_g1_i1_orf1;TRINITY_DN115658_c0_g1_i1_orf1;TRINITY_DN47575_c0_g1_i1_orf1;TRINITY_DN7464_c0_g1_i14_orf1;TRINITY_DN50787_c0_g2_i2_orf1;TRINITY_DN2769_c0_g1_i1_orf1;TRINITY_DN41664_c0_g1_i4_orf1;TRINITY_DN1091_c0_g3_i1_orf1;TRINITY_DN36144_c0_g1_i3_orf1;TRINITY_DN799_c0_g1_i7_orf1;TRINITY_DN3324_c0_g1_i3_orf1;TRINITY_DN12301_c0_g1_i1_orf1;TRINITY_DN3733_c0_g1_i1_orf1;TRINITY_DN13732_c0_g2_i3_orf1;TRINITY_DN4408_c6_g1_i1_orf1;TRINITY_DN51568_c0_g1_i1_orf1;TRINITY_DN3472_c0_g1_i6_orf1;TRINITY_DN40945_c0_g1_i1_orf1;TRINITY_DN21357_c0_g1_i5_orf1;TRINITY_DN7991_c0_g1_i9_orf1;TRINITY_DN89613_c0_g1_i13_orf1;TRINITY_DN2682_c0_g1_i4_orf1;TRINITY_DN3847_c1_g1_i1_orf1;TRINITY_DN13233_c0_g1_i3_orf1;TRINITY_DN10831_c1_g1_i1_orf1;TRINITY_DN40015_c0_g1_i2_orf1;TRINITY_DN147458_c0_g1_i1_orf1;TRINITY_DN12397_c0_g1_i1_orf1;TRINITY_DN2299_c0_g1_i3_orf1;TRINITY_DN5064_c0_g1_i4_orf1;TRINITY_DN143852_c0_g1_i1_orf1;TRINITY_DN5029_c0_g1_i1_orf1;TRINITY_DN2749_c4_g1_i2_orf1;TRINITY_DN21251_c1_g1_i1_orf1;TRINITY_DN5112_c0_g1_i1_orf1;TRINITY_DN6325_c0_g1_i8_orf1;TRINITY_DN2749_c0_g1_i4_orf1;TRINITY_DN620_c0_g1_i4_orf1;TRINITY_DN27035_c0_g1_i1_orf1;TRINITY_DN1575_c0_g1_i7_orf1;TRINITY_DN24318_c0_g1_i1_orf1;TRINITY_DN2718_c0_g1_i6_orf1;TRINITY_DN18249_c0_g1_i1_orf1;TRINITY_DN7212_c0_g1_i4_orf1;TRINITY_DN61222_c0_g1_i1_orf1;TRINITY_DN40345_c0_g1_i6_orf1;TRINITY_DN24_c0_g1_i1_orf1;TRINITY_DN2559_c0_g1_i4_orf1;TRINITY_DN98242_c0_g1_i1_orf1;TRINITY_DN30027_c0_g1_i1_orf1;TRINITY_DN82324_c0_g1_i4_orf1;TRINITY_DN4135_c0_g1_i5_orf1;TRINITY_DN2749_c0_g2_i3_orf1;TRINITY_DN3062_c0_g1_i1_orf1;TRINITY_DN19866_c0_g1_i4_orf1;TRINITY_DN825_c23_g1_i5_orf1;TRINITY_DN84322_c0_g2_i1_orf1;TRINITY_DN51813_c0_g1_i1_orf1;TRINITY_DN631_c0_g1_i6_orf1;TRINITY_DN111_c0_g2_i2_orf1;TRINITY_DN5811_c0_g1_i4_orf1;TRINITY_DN37856_c0_g1_i5_orf1;TRINITY_DN28299_c0_g1_i1_orf1;TRINITY_DN21971_c0_g1_i4_orf1;TRINITY_DN2647_c0_g1_i3_orf1;TRINITY_DN10548_c0_g2_i1_orf1;TRINITY_DN5031_c0_g1_i1_orf1;TRINITY_DN9979_c0_g1_i1_orf1;TRINITY_DN16939_c0_g1_i4_orf1;TRINITY_DN59804_c0_g1_i1_orf1;TRINITY_DN40650_c0_g1_i1_orf1;TRINITY_DN2971_c0_g1_i1_orf1;TRINITY_DN5507_c0_g1_i1_orf1;TRINITY_DN7613_c1_g2_i1_orf1;TRINITY_DN36893_c0_g1_i1_orf1;TRINITY_DN4835_c0_g1_i2_orf1;TRINITY_DN38562_c0_g1_i3_orf1;TRINITY_DN25423_c0_g1_i1_orf1;TRINITY_DN13651_c0_g1_i2_orf1;TRINITY_DN8949_c0_g1_i2_orf1;TRINITY_DN104507_c0_g1_i2_orf1;TRINITY_DN27958_c0_g1_i1_orf1;TRINITY_DN5009_c0_g1_i2_orf1;TRINITY_DN147676_c0_g1_i1_orf1;TRINITY_DN2367_c1_g1_i20_orf1;TRINITY_DN17045_c0_g2_i3_orf1;TRINITY_DN18782_c0_g1_i4_orf1;TRINITY_DN3826_c0_g1_i1_orf1;TRINITY_DN5238_c0_g1_i2_orf1;TRINITY_DN19251_c0_g1_i8_orf1;TRINITY_DN13055_c0_g1_i5_orf1;TRINITY_DN42646_c0_g2_i1_orf1;TRINITY_DN45924_c0_g1_i14_orf1;TRINITY_DN48619_c0_g1_i1_orf1;TRINITY_DN346_c0_g1_i7_orf1;TRINITY_DN18593_c0_g1_i1_orf1;TRINITY_DN9862_c0_g2_i1_orf1;TRINITY_DN1084_c0_g1_i2_orf1;TRINITY_DN30950_c0_g1_i13_orf1;TRINITY_DN26824_c0_g1_i1_orf1;TRINITY_DN3393_c0_g2_i1_orf1
biological_process	cellular lipid metabolic process	GO:0044255	11	11/1363	TRINITY_DN45220_c0_g1_i1_orf1;TRINITY_DN2618_c0_g1_i3_orf1;TRINITY_DN5055_c0_g1_i12_orf1;TRINITY_DN12526_c0_g1_i5_orf1;TRINITY_DN44110_c0_g1_i4_orf1;TRINITY_DN3588_c0_g1_i4_orf1;TRINITY_DN84478_c0_g1_i8_orf1;TRINITY_DN10900_c0_g1_i7_orf1;TRINITY_DN8964_c0_g1_i4_orf1;TRINITY_DN5841_c0_g1_i2_orf1;TRINITY_DN5697_c0_g1_i1_orf1
biological_process	generation of precursor metabolites and energy	GO:0006091	8	8/1363	TRINITY_DN6325_c0_g1_i8_orf1;TRINITY_DN2848_c0_g1_i2_orf1;TRINITY_DN27035_c0_g1_i1_orf1;TRINITY_DN4270_c0_g1_i1_orf1;TRINITY_DN14073_c0_g1_i1_orf1;TRINITY_DN1791_c0_g1_i3_orf1;TRINITY_DN140613_c0_g1_i1_orf1;TRINITY_DN9979_c0_g1
biological_process	one-carbon metabolic process	GO:0006730	4	4/1363	TRINITY_DN98313_c0_g1_i1_orf1;TRINITY_DN244_c1_g1_i5_orf1;TRINITY_DN92153_c0_g2_i2_orf1;TRINITY_DN631_c0_g1_i6_orf1
biological_process	cellular ketone metabolic process	GO:0042180	2	2/1363	TRINITY_DN8964_c0_g1_i4_orf1;TRINITY_DN51813_c0_g1_i1_orf1
biological_process	heterocycle metabolic process	GO:0046483	90	90/1363	TRINITY_DN38230_c0_g1_i4_orf1;TRINITY_DN40434_c0_g1_i2_orf1;TRINITY_DN27771_c0_g1_i1_orf1;TRINITY_DN2054_c0_g1_i1_orf1;TRINITY_DN6325_c0_g1_i8_orf1;TRINITY_DN8107_c0_g1_i1_orf1;TRINITY_DN5670_c0_g1_i2_orf1;TRINITY_DN47123_c0_g1_i1_orf1;TRINITY_DN123184_c0_g1_i1_orf1;TRINITY_DN2769_c0_g1_i1_orf1;TRINITY_DN1116_c0_g1_i6_orf1;TRINITY_DN817_c0_g1_i3_orf1;TRINITY_DN620_c0_g1_i4_orf1;TRINITY_DN41664_c0_g1_i4_orf1;TRINITY_DN20499_c0_g3_i1_orf1;TRINITY_DN46409_c0_g1_i1_orf1;TRINITY_DN6235_c0_g1_i5_orf1;TRINITY_DN89613_c0_g1_i13_orf1;TRINITY_DN2848_c0_g1_i2_orf1;TRINITY_DN1957_c0_g1_i4_orf1;TRINITY_DN230_c2_g1_i5_orf1;TRINITY_DN3092_c0_g1_i2_orf1;TRINITY_DN1978_c0_g1_i4_orf1;TRINITY_DN17271_c0_g1_i1_orf1;TRINITY_DN140212_c0_g1_i1_orf1;TRINITY_DN2719_c1_g1_i6_orf1;TRINITY_DN51968_c0_g1_i1_orf1;TRINITY_DN20238_c0_g1_i7_orf1;TRINITY_DN115658_c0_g1_i1_orf1;TRINITY_DN47575_c0_g1_i1_orf1;TRINITY_DN244_c1_g1_i5_orf1;TRINITY_DN2110_c0_g1_i3_orf1;TRINITY_DN5507_c0_g1_i1_orf1;TRINITY_DN1091_c0_g3_i1_orf1;TRINITY_DN36144_c0_g1_i3_orf1;TRINITY_DN3324_c0_g1_i3_orf1;TRINITY_DN12301_c0_g1_i1_orf1;TRINITY_DN13732_c0_g2_i3_orf1;TRINITY_DN4408_c6_g1_i1_orf1;TRINITY_DN51568_c0_g1_i1_orf1;TRINITY_DN3472_c0_g1_i6_orf1;TRINITY_DN40945_c0_g1_i1_orf1;TRINITY_DN5811_c0_g1_i4_orf1;TRINITY_DN3847_c1_g1_i1_orf1;TRINITY_DN1091_c0_g1_i1_orf1;TRINITY_DN2299_c0_g1_i3_orf1;TRINITY_DN5029_c0_g1_i1_orf1;TRINITY_DN2749_c4_g1_i2_orf1;TRINITY_DN92153_c0_g2_i2_orf1;TRINITY_DN2749_c0_g1_i4_orf1;TRINITY_DN77318_c0_g2_i1_orf1;TRINITY_DN27035_c0_g1_i1_orf1;TRINITY_DN1575_c0_g1_i7_orf1;TRINITY_DN2718_c0_g1_i6_orf1;TRINITY_DN24_c0_g1_i1_orf1;TRINITY_DN2559_c0_g1_i4_orf1;TRINITY_DN98242_c0_g1_i1_orf1;TRINITY_DN4135_c0_g1_i5_orf1;TRINITY_DN2749_c0_g2_i3_orf1;TRINITY_DN4835_c0_g1_i2_orf1;TRINITY_DN19866_c0_g1_i4_orf1;TRINITY_DN825_c23_g1_i5_orf1;TRINITY_DN84322_c0_g2_i1_orf1;TRINITY_DN51813_c0_g1_i1_orf1;TRINITY_DN631_c0_g1_i6_orf1;TRINITY_DN111_c0_g2_i2_orf1;TRINITY_DN28299_c0_g1_i1_orf1;TRINITY_DN2647_c0_g1_i3_orf1;TRINITY_DN10548_c0_g2_i1_orf1;TRINITY_DN9979_c0_g1_i1_orf1;TRINITY_DN59804_c0_g1_i1_orf1;TRINITY_DN2971_c0_g1_i1_orf1;TRINITY_DN37532_c0_g1_i1_orf1;TRINITY_DN3062_c0_g1_i1_orf1;TRINITY_DN38562_c0_g1_i3_orf1;TRINITY_DN25423_c0_g1_i1_orf1;TRINITY_DN104507_c0_g1_i2_orf1;TRINITY_DN27958_c0_g1_i1_orf1;TRINITY_DN2367_c1_g1_i20_orf1;TRINITY_DN17045_c0_g2_i3_orf1;TRINITY_DN1084_c0_g1_i2_orf1;TRINITY_DN5238_c0_g1_i2_orf1;TRINITY_DN19251_c0_g1_i8_orf1;TRINITY_DN13055_c0_g1_i5_orf1;TRINITY_DN80134_c0_g1_i1_orf1;TRINITY_DN45924_c0_g1_i14_orf1;TRINITY_DN48619_c0_g1_i1_orf1;TRINITY_DN346_c0_g1_i7_orf1;TRINITY_DN18782_c0_g1_i4_orf1;TRINITY_DN30950_c0_g1_i13_orf1

biological_process	reactive oxygen species metabolic process	GO:0072593	2	2/1363	TRINITY_DN8637_c0_g1_i1_orf1;TRINITY_DN16400_c0_g2_i1_orf1
biological_process	cellular carbohydrate metabolic process	GO:0044262	7	7/1363	TRINITY_DN52244_c1_g1_i1_orf1;TRINITY_DN36788_c0_g1_i2_orf1;TRINITY_DN1707_c0_g1_i1_orf1;TRINITY_DN3010_c0_g1_i4_orf1;TRINITY_DN11942_c0_g1_i1_orf1;TRINITY_DN140613_c0_g1_i1_orf1;TRINITY_DN95850_c0_g1_i1_orf1
biological_process	sulfur compound metabolic process	GO:0006790	9	9/1363	TRINITY_DN92153_c0_g2_i2_orf1;TRINITY_DN7170_c0_g1_i11_orf1;TRINITY_DN1285_c0_g1_i6_orf1;TRINITY_DN38562_c0_g1_i3_orf1;TRINITY_DN34399_c0_g1_i1_orf1;TRINITY_DN35002_c0_g2_i2_orf1;TRINITY_DN1084_c0_g1_i2_orf1;TRINITY_DN19251_c0_g1_i8_orf1;TRINITY_DN85476_c0_g1_i1_orf1
biological_process	phosphorus metabolic process	GO:0006793	45	45/1363	TRINITY_DN1749_c0_g2_i2_orf1;TRINITY_DN140423_c0_g1_i2_orf1;TRINITY_DN44110_c0_g1_i4_orf1;TRINITY_DN47151_c0_g1_i1_orf1;TRINITY_DN38230_c0_g1_i4_orf1;TRINITY_DN6325_c0_g1_i8_orf1;TRINITY_DN27035_c0_g1_i1_orf1;TRINITY_DN1575_c0_g1_i7_orf1;TRINITY_DN41166_c0_g1_i1_orf1;TRINITY_DN670_c0_g1_i15_orf1;TRINITY_DN2719_c1_g1_i6_orf1;TRINITY_DN2770_c0_g2_i4_orf1;TRINITY_DN54477_c0_g1_i1_orf1;TRINITY_DN11942_c0_g1_i1_orf1;TRINITY_DN28729_c0_g1_i9_orf1;TRINITY_DN42461_c0_g1_i4_orf1;TRINITY_DN24_c0_g1_i1_orf1;TRINITY_DN1954_c0_g1_i4_orf1;TRINITY_DN277_c1_g1_i1_orf1;TRINITY_DN59885_c0_g1_i3_orf1;TRINITY_DN2110_c0_g1_i3_orf1;TRINITY_DN152_c0_g1_i4_orf1;TRINITY_DN1084_c0_g1_i2_orf1;TRINITY_DN36144_c0_g1_i3_orf1;TRINITY_DN12301_c0_g1_i1_orf1;TRINITY_DN51813_c0_g1_i1_orf1;TRINITY_DN15478_c0_g1_i1_orf1;TRINITY_DN80134_c0_g1_i1_orf1;TRINITY_DN28299_c0_g1_i1_orf1;TRINITY_DN4798_c0_g1_i3_orf1;TRINITY_DN52244_c1_g1_i1_orf1;TRINITY_DN5811_c0_g1_i4_orf1;TRINITY_DN45924_c0_g1_i14_orf1;TRINITY_DN2618_c0_g1_i3_orf1;TRINITY_DN1957_c0_g1_i4_orf1;TRINITY_DN18782_c0_g1_i4_orf1;TRINITY_DN4929_c1_g2_i5_orf1;TRINITY_DN10548_c0_g2_i1_orf1;TRINITY_DN4742_c0_g1_i1_orf1;TRINITY_DN46715_c0_g1_i1_orf1;TRINITY_DN19251_c0_g1_i8_orf1;TRINITY_DN5697_c0_g1_i1_orf1;TRINITY_DN5029_c0_g1_i1_orf1;TRINITY_DN2848_c0_g1_i2_orf1;TRINITY_DN9979_c0_g1_i1_orf1
biological_process	cellular aldehyde metabolic process	GO:0006081	1	1/1363	TRINITY_DN36788_c0_g1_i2_orf1
biological_process	organic acid metabolic process	GO:0006082	34	34/1363	TRINITY_DN42856_c0_g1_i1_orf1;TRINITY_DN6199_c2_g1_i3_orf1;TRINITY_DN92153_c0_g2_i2_orf1;TRINITY_DN5055_c0_g1_i12_orf1;TRINITY_DN27771_c0_g1_i1_orf1;TRINITY_DN5564_c0_g1_i5_orf1;TRINITY_DN27035_c0_g1_i1_orf1;TRINITY_DN11159_c0_g1_i5_orf1;TRINITY_DN100821_c0_g1_i1_orf1;TRINITY_DN2719_c1_g1_i6_orf1;TRINITY_DN4822_c0_g1_i9_orf1;TRINITY_DN19187_c0_g1_i1_orf1;TRINITY_DN45220_c0_g1_i1_orf1;TRINITY_DN36788_c0_g1_i2_orf1;TRINITY_DN3588_c0_g1_i4_orf1;TRINITY_DN17913_c0_g1_i8_orf1;TRINITY_DN31163_c1_g1_i4_orf1;TRINITY_DN4822_c0_g1_i6_orf1;TRINITY_DN10900_c0_g1_i7_orf1;TRINITY_DN825_c23_g1_i5_orf1;TRINITY_DN84322_c0_g2_i1_orf1;TRINITY_DN51813_c0_g1_i1_orf1;TRINITY_DN34399_c0_g1_i1_orf1;TRINITY_DN631_c0_g1_i6_orf1;TRINITY_DN817_c0_g1_i3_orf1;TRINITY_DN620_c0_g1_i4_orf1;TRINITY_DN48619_c0_g1_i1_orf1;TRINITY_DN6325_c0_g1_i8_orf1;TRINITY_DN2684_c0_g2_i3_orf1;TRINITY_DN17326_c0_g1_i5_orf1;TRINITY_DN1084_c0_g1_i2_orf1;TRINITY_DN14464_c0_g1_i1_orf1;TRINITY_DN2848_c0_g1_i2_orf1;TRINITY_DN9979_c0_g1_i1_orf1
biological_process	cellular catabolic process	GO:0044248	31	31/1363	TRINITY_DN42856_c0_g1_i1_orf1;TRINITY_DN6199_c2_g1_i3_orf1;TRINITY_DN38230_c0_g1_i4_orf1;TRINITY_DN5055_c0_g1_i12_orf1;TRINITY_DN25423_c0_g1_i1_orf1;TRINITY_DN3010_c0_g1_i4_orf1;TRINITY_DN4822_c0_g1_i9_orf1;TRINITY_DN1272_c1_g1_i4_orf1;TRINITY_DN27958_c0_g1_i1_orf1;TRINITY_DN19187_c0_g1_i1_orf1;TRINITY_DN45220_c0_g1_i1_orf1;TRINITY_DN143496_c0_g1_i1_orf1;TRINITY_DN24_c0_g1_i1_orf1;TRINITY_DN3588_c0_g1_i4_orf1;TRINITY_DN2559_c0_g1_i4_orf1;TRINITY_DN98242_c0_g1_i1_orf1;TRINITY_DN82017_c0_g1_i5_orf1;TRINITY_DN8107_c0_g1_i1_orf1;TRINITY_DN11172_c0_g1_i4_orf1;TRINITY_DN4822_c0_g1_i6_orf1;TRINITY_DN17693_c0_g1_i10_orf1;TRINITY_DN17913_c0_g1_i8_orf1;TRINITY_DN15265_c0_g1_i1_orf1;TRINITY_DN51813_c0_g1_i1_orf1;TRINITY_DN631_c0_g1_i6_orf1;TRINITY_DN1707_c0_g1_i1_orf1;TRINITY_DN4121_c0_g1_i1_orf1;TRINITY_DN96557_c0_g1_i1_orf1;TRINITY_DN20499_c0_g3_i1_orf1;TRINITY_DN113353_c0_g1_i1_orf1;TRINITY_DN51658_c0_g1_i1_orf1
biological_process	cellular biosynthetic process	GO:0044249	86	86/1363	TRINITY_DN5564_c0_g1_i5_orf1;TRINITY_DN5873_c0_g4_i1_orf1;TRINITY_DN95850_c0_g1_i1_orf1;TRINITY_DN11065_c0_g2_i1_orf1;TRINITY_DN2682_c0_g1_i4_orf1;TRINITY_DN30300_c0_g2_i1_orf1;TRINITY_DN142442_c0_g1_i1_orf1;TRINITY_DN31163_c1_g1_i4_orf1;TRINITY_DN8964_c0_g1_i4_orf1;TRINITY_DN95056_c0_g2_i2_orf1;TRINITY_DN28299_c0_g1_i1_orf1;TRINITY_DN137_c0_g1_i1_orf1;TRINITY_DN143_c0_g3_i1_orf1;TRINITY_DN79734_c0_g2_i3_orf1;TRINITY_DN121893_c0_g1_i1_orf1;TRINITY_DN230_c2_g1_i5_orf1;TRINITY_DN18249_c0_g1_i1_orf1;TRINITY_DN144956_c0_g1_i1_orf1;TRINITY_DN2719_c1_g1_i6_orf1;TRINITY_DN97589_c0_g1_i3_orf1;TRINITY_DN115658_c0_g1_i1_orf1;TRINITY_DN7464_c0_g1_i14_orf1;TRINITY_DN50787_c0_g2_i2_orf1;TRINITY_DN2110_c0_g1_i3_orf1;TRINITY_DN36144_c0_g1_i3_orf1;TRINITY_DN799_c0_g1_i7_orf1;TRINITY_DN12301_c0_g1_i1_orf1;TRINITY_DN10900_c0_g1_i7_orf1;TRINITY_DN3733_c0_g1_i1_orf1;TRINITY_DN13732_c0_g2_i3_orf1;TRINITY_DN4408_c6_g1_i1_orf1;TRINITY_DN3472_c0_g1_i6_orf1;TRINITY_DN21357_c0_g1_i5_orf1;TRINITY_DN7991_c0_g1_i9_orf1;TRINITY_DN5811_c0_g1_i4_orf1;TRINITY_DN13233_c0_g1_i3_orf1;TRINITY_DN10831_c1_g1_i1_orf1;TRINITY_DN147458_c0_g1_i1_orf1;TRINITY_DN12397_c0_g1_i1_orf1;TRINITY_DN2299_c0_g1_i3_orf1;TRINITY_DN5064_c0_g1_i4_orf1;TRINITY_DN143852_c0_g1_i1_orf1;TRINITY_DN5029_c0_g1_i1_orf1;TRINITY_DN21251_c1_g1_i1_orf1;TRINITY_DN5112_c0_g1_i1_orf1;TRINITY_DN30131_c0_g1_i1_orf1;TRINITY_DN1575_c0_g1_i7_orf1;TRINITY_DN24318_c0_g1_i1_orf1;TRINITY_DN5031_c0_g1_i1_orf1;TRINITY_DN41645_c0_g1_i1_orf1;TRINITY_DN61222_c0_g1_i1_orf1;TRINITY_DN40345_c0_g1_i6_orf1;TRINITY_DN7957_c0_g1_i5_orf1;TRINITY_DN30027_c0_g1_i1_orf1;TRINITY_DN147676_c0_g1_i1_orf1;TRINITY_DN51813_c0_g1_i1_orf1;TRINITY_DN37856_c0_g1_i5_orf1;TRINITY_DN10234_c0_g1_i1_orf1;TRINITY_DN21971_c0_g1_i4_orf1;TRINITY_DN1957_c0_g1_i4_orf1;TRINITY_DN10548_c0_g2_i1_orf1;TRINITY_DN40015_c0_g1_i2_orf1;TRINITY_DN16939_c0_g1_i4_orf1;TRINITY_DN40650_c0_g1_i1_orf1;TRINITY_DN5009_c0_g1_i2_orf1;TRINITY_DN37532_c0_g1_i1_orf1;TRINITY_DN7613_c1_g2_i1_orf1;TRINITY_DN36893_c0_g1_i1_orf1;TRINITY_DN13651_c0_g1_i2_orf1;TRINITY_DN8949_c0_g1_i2_orf1;TRINITY_DN8824_c0_g2_i1_orf1;TRINITY_DN1084_c0_g1_i2_orf1;TRINITY_DN3826_c0_g1_i1_orf1;TRINITY_DN80134_c0_g1_i1_orf1;TRINITY_DN34399_c0_g1_i1_orf1;TRINITY_DN42646_c0_g2_i1_orf1;TRINITY_DN45924_c0_g1_i14_orf1;TRINITY_DN82324_c0_g1_i4_orf1;TRINITY_DN2618_c0_g1_i3_orf1;TRINITY_DN58207_c0_g1_i1_orf1;TRINITY_DN18593_c0_g1_i1_orf1;TRINITY_DN9862_c0_g2_i1_orf1;TRINITY_DN18782_c0_g1_i4_orf1;TRINITY_DN26824_c0_g1_i1_orf1;TRINITY_DN5697_c0_g1_i1_orf1;TRINITY_DN3393_c0_g2_i1_orf1
biological_process	cellular macromolecule metabolic process	GO:0044260	93	93/1363	TRINITY_DN40434_c0_g1_i2_orf1;TRINITY_DN137_c0_g1_i1_orf1;TRINITY_DN5873_c0_g4_i1_orf1;TRINITY_DN2054_c0_g1_i1_orf1;TRINITY_DN11065_c0_g2_i1_orf1;TRINITY_DN2682_c0_g1_i4_orf1;TRINITY_DN30300_c0_g2_i1_orf1;TRINITY_DN142442_c0_g1_i1_orf1;TRINITY_DN8107_c0_g1_i1_orf1;TRINITY_DN5670_c0_g1_i2_orf1;TRINITY_DN95056_c0_g2_i2_orf1;TRINITY_DN123184_c0_g1_i1_orf1;TRINITY_DN143_c0_g3_i1_orf1;TRINITY_DN17045_c0_g2_i3_orf1;TRINITY_DN20499_c0_g3_i1_orf1;TRINITY_DN22674_c0_g1_i2_orf1;TRINITY_DN79734_c0_g2_i3_orf1;TRINITY_DN121893_c0_g1_i1_orf1;TRINITY_DN3092_c0_g1_i2_orf1;TRINITY_DN17271_c0_g1_i1_orf1;TRINITY_DN140212_c0_g1_i1_orf1;TRINITY_DN144956_c0_g1_i1_orf1;TRINITY_DN20238_c0_g1_i7_orf1;TRINITY_DN1272_c1_g1_i4_orf1;TRINITY_DN97589_c0_g1_i3_orf1;TRINITY_DN18249_c0_g1_i1_orf1;TRINITY_DN115658_c0_g1_i1_orf1;TRINITY_DN7464_c0_g1_i14_orf1;TRINITY_DN50787_c0_g2_i2_orf1;TRINITY_DN41664_c0_g1_i4_orf1;TRINITY_DN1091_c0_g3_i1_orf1;TRINITY_DN5238_c0_g1_i2_orf1;TRINITY_DN799_c0_g1_i7_orf1;TRINITY_DN3733_c0_g1_i1_orf1;TRINITY_DN13732_c0_g2_i3_orf1;TRINITY_DN4408_c6_g1_i1_orf1;TRINITY_DN95414_c0_g1_i1_orf1;TRINITY_DN21357_c0_g1_i5_orf1;TRINITY_DN7991_c0_g1_i9_orf1;TRINITY_DN89613_c0_g1_i13_orf1;TRINITY_DN58207_c0_g1_i1_orf1;TRINITY_DN13233_c0_g1_i3_orf1;TRINITY_DN10831_c1_g1_i1_orf1;TRINITY_DN1091_c0_g1_i1_orf1;TRINITY_DN147458_c0_g1_i1_orf1;TRINITY_DN12397_c0_g1_i1_orf1;TRINITY_DN5064_c0_g1_i4_orf1;TRINITY_DN143852_c0_g1_i1_orf1;TRINITY_DN21251_c1_g1_i1_orf1;TRINITY_DN5112_c0_g1_i1_orf1;TRINITY_DN30131_c0_g1_i1_orf1;TRINITY_DN77318_c0_g2_i1_orf1;TRINITY_DN46409_c0_g1_i1_orf1;TRINITY_DN24318_c0_g1_i1_orf1;TRINITY_DN5009_c0_g1_i2_orf1;TRINITY_DN41645_c0_g1_i1_orf1;TRINITY_DN61222_c0_g1_i1_orf1;TRINITY_DN40345_c0_g1_i6_orf1;TRINITY_DN30027_c0_g1_i1_orf1;TRINITY_DN147676_c0_g1_i1_orf1;TRINITY_DN4835_c0_g1_i2_orf1;TRINITY_DN19866_c0_g1_i4_orf1;TRINITY_DN15265_c0_g1_i1_orf1;TRINITY_DN4121_c0_g1_i1_orf1;TRINITY_DN96557_c0_g1_i1_orf1;TRINITY_DN10234_c0_g1_i1_orf1;TRINITY_DN21971_c0_g1_i4_orf1;TRINITY_DN2647_c0_g1_i3_orf1;TRINITY_DN40015_c0_g1_i2_orf1;TRINITY_DN16939_c0_g1_i4_orf1;TRINITY_DN59804_c0_g1_i1_orf1;TRINITY_DN40650_c0_g1_i1_orf1;TRINITY_DN2971_c0_g1_i1_orf1;TRINITY_DN7613_c1_g2_i1_orf1;TRINITY_DN36893_c0_g1_i1_orf1;TRINITY_DN13651_c0_g1_i2_orf1;TRINITY_DN8949_c0_g1_i2_orf1;TRINITY_DN104507_c0_g1_i2_orf1;TRINITY_DN27958_c0_g1_i1_orf1;TRINITY_DN143496_c0_g1_i1_orf1;TRINITY_DN8824_c0_g2_i1_orf1;TRINITY_DN140613_c0_g1_i1_orf1;TRINITY_DN3826_c0_g1_i1_orf1;TRINITY_DN5031_c0_g1_i1_orf1;TRINITY_DN51658_c0_g1_i1_orf1;TRINITY_DN42646_c0_g2_i1_orf1;TRINITY_DN82324_c0_g1_i4_orf1;TRINITY_DN346_c0_g1_i7_orf1;TRINITY_DN18593_c0_g1_i1_orf1;TRINITY_DN9862_c0_g2_i1_orf1;TRINITY_DN26824_c0_g1_i1_orf1;TRINITY_DN6462_c0_g1_i5_orf1;TRINITY_DN3393_c0_g2_i1_orf1
biological_process	cellular aromatic compound metabolic process	GO:0006725	94	94/1363	TRINITY_DN38230_c0_g1_i4_orf1;TRINITY_DN40434_c0_g1_i2_orf1;TRINITY_DN27771_c0_g1_i1_orf1;TRINITY_DN2054_c0_g1_i1_orf1;TRINITY_DN6325_c0_g1_i8_orf1;TRINITY_DN31163_c1_g1_i4_orf1;TRINITY_DN8107_c0_g1_i1_orf1;TRINITY_DN5670_c0_g1_i2_orf1;TRINITY_DN4822_c0_g1_i6_orf1;TRINITY_DN47123_c0_g1_i1_orf1;TRINITY_DN123184_c0_g1_i1_orf1;TRINITY_DN2769_c0_g1_i1_orf1;TRINITY_DN1116_c0_g1_i6_orf1;TRINITY_DN817_c0_g1_i3_orf1;TRINITY_DN620_c0_g1_i4_orf1;TRINITY_DN41664_c0_g1_i4_orf1;TRINITY_DN20499_c0_g3_i1_orf1;TRINITY_DN46409_c0_g1_i1_orf1;TRINITY_DN6235_c0_g1_i5_orf1;TRINITY_DN89613_c0_g1_i13_orf1;TRINITY_DN2848_c0_g1_i2_orf1;TRINITY_DN1957_c0_g1_i4_orf1;TRINITY_DN230_c2_g1_i5_orf1;TRINITY_DN3092_c0_g1_i2_orf1;TRINITY_DN1978_c0_g1_i4_orf1;TRINITY_DN17271_c0_g1_i1_orf1;TRINITY_DN140212_c0_g1_i1_orf1;TRINITY_DN37532_c0_g1_i1_orf1;TRINITY_DN51968_c0_g1_i1_orf1;TRINITY_DN20238_c0_g1_i7_orf1;TRINITY_DN19187_c0_g1_i1_orf1;TRINITY_DN115658_c0_g1_i1_orf1;TRINITY_DN47575_c0_g1_i1_orf1;TRINITY_DN244_c1_g1_i5_orf1;TRINITY_DN2110_c0_g1_i3_orf1;TRINITY_DN5507_c0_g1_i1_orf1;TRINITY_DN1091_c0_g3_i1_orf1;TRINITY_DN36144_c0_g1_i3_orf1;TRINITY_DN3324_c0_g1_i3_orf1;TRINITY_DN12301_c0_g1_i1_orf1;TRINITY_DN13732_c0_g2_i3_orf1;TRINITY_DN4408_c6_g1_i1_orf1;TRINITY_DN51568_c0_g1_i1_orf1;TRINITY_DN3472_c0_g1_i6_orf1;TRINITY_DN40945_c0_g1_i1_orf1;TRINITY_DN9724_c0_g1_i4_orf1;TRINITY_DN5811_c0_g1_i4_orf1;TRINITY_DN3847_c1_g1_i1_orf1;TRINITY_DN1091_c0_g1_i1_orf1;TRINITY_DN2299_c0_g1_i3_orf1;TRINITY_DN5029_c0_g1_i1_orf1;TRINITY_DN2749_c4_g1_i2_orf1;TRINITY_DN92153_c0_g2_i2_orf1;TRINITY_DN2749_c0_g1_i4_orf1;TRINITY_DN77318_c0_g2_i1_orf1;TRINITY_DN27035_c0_g1_i1_orf1;TRINITY_DN1575_c0_g1_i7_orf1;TRINITY_DN2718_c0_g1_i6_orf1;TRINITY_DN24_c0_g1_i1_orf1;TRINITY_DN2559_c0_g1_i4_orf1;TRINITY_DN98242_c0_g1_i1_orf1;TRINITY_DN4135_c0_g1_i5_orf1;TRINITY_DN2749_c0_g2_i3_orf1;TRINITY_DN3062_c0_g1_i1_orf1;TRINITY_DN19866_c0_g1_i4_orf1;TRINITY_DN825_c23_g1_i5_orf1;TRINITY_DN84322_c0_g2_i1_orf1;TRINITY_DN51813_c0_g1_i1_orf1;TRINITY_DN631_c0_g1_i6_orf1;TRINITY_DN111_c0_g2_i2_orf1;TRINITY_DN28299_c0_g1_i1_orf1;TRINITY_DN2647_c0_g1_i3_orf1;TRINITY_DN10548_c0_g2_i1_orf1;TRINITY_DN9979_c0_g1_i1_orf1;TRINITY_DN59804_c0_g1_i1_orf1;TRINITY_DN2971_c0_g1_i1_orf1;TRINITY_DN4835_c0_g1_i2_orf1;TRINITY_DN38562_c0_g1_i3_orf1;TRINITY_DN4822_c0_g1_i9_orf1;TRINITY_DN104507_c0_g1_i2_orf1;TRINITY_DN27958_c0_g1_i1_orf1;TRINITY_DN2367_c1_g1_i20_orf1;TRINITY_DN17045_c0_g2_i3_orf1;TRINITY_DN11172_c0_g1_i4_orf1;TRINITY_DN1084_c0_g1_i2_orf1;TRINITY_DN17913_c0_g1_i8_orf1;TRINITY_DN5238_c0_g1_i2_orf1;TRINITY_DN19251_c0_g1_i8_orf1;TRINITY_DN13055_c0_g1_i5_orf1;TRINITY_DN45924_c0_g1_i14_orf1;TRINITY_DN48619_c0_g1_i1_orf1;TRINITY_DN346_c0_g1_i7_orf1;TRINITY_DN18782_c0_g1_i4_orf1;TRINITY_DN30950_c0_g1_i13_orf1
biological_process	cellular metabolic compound salvage	GO:0043094	2	2/1363	TRINITY_DN1575_c0_g1_i7_orf1;TRINITY_DN5811_c0_g1_i4_orf1
biological_process	neurotransmitter metabolic process	GO:0042133	2	2/1363	TRINITY_DN17693_c0_g1_i10_orf1;TRINITY_DN82017_c0_g1_i5_orf1
biological_process	cellular modified amino acid metabolic process	GO:0006575	5	5/1363	TRINITY_DN631_c0_g1_i6_orf1;TRINITY_DN41166_c0_g1_i1_orf1;TRINITY_DN38562_c0_g1_i3_orf1;TRINITY_DN92153_c0_g2_i2_orf1;TRINITY_DN244_c1_g1_i5_orf1
biological_process	secondary metabolite biosynthetic process	GO:0044550	1	1/1363	TRINITY_DN31163_c1_g1_i4_orf1
biological_process	small molecule biosynthetic process	GO:0044283	11	11/1363	TRINITY_DN230_c2_g1_i5_orf1;TRINITY_DN8964_c0_g1_i4_orf1;TRINITY_DN10900_c0_g1_i7_orf1;TRINITY_DN51813_c0_g1_i1_orf1;TRINITY_DN5564_c0_g1_i5_orf1;TRINITY_DN34399_c0_g1_i1_orf1;TRINITY_DN27035_c0_g1_i1_orf1;TRINITY_DN1575_c0_g1_i7_orf1;TRINITY_DN18782_c0_g1_i4_orf1;TRINITY_DN2719_c1_g1_i6_orf1;TRINITY_DN3472_c0_g1_i6_orf1
biological_process	organic substance biosynthetic process	GO:1901576	89	89/1363	TRINITY_DN5564_c0_g1_i5_orf1;TRINITY_DN5873_c0_g4_i1_orf1;TRINITY_DN95850_c0_g1_i1_orf1;TRINITY_DN11065_c0_g2_i1_orf1;TRINITY_DN2682_c0_g1_i4_orf1;TRINITY_DN30300_c0_g2_i1_orf1;TRINITY_DN142442_c0_g1_i1_orf1;TRINITY_DN31163_c1_g1_i4_orf1;TRINITY_DN7957_c0_g1_i5_orf1;TRINITY_DN95056_c0_g2_i2_orf1;TRINITY_DN28299_c0_g1_i1_orf1;TRINITY_DN137_c0_g1_i1_orf1;TRINITY_DN143_c0_g3_i1_orf1;TRINITY_DN10785_c0_g1_i4_orf1;TRINITY_DN79734_c0_g2_i3_orf1;TRINITY_DN121893_c0_g1_i1_orf1;TRINITY_DN230_c2_g1_i5_orf1;TRINITY_DN18249_c0_g1_i1_orf1;TRINITY_DN144956_c0_g1_i1_orf1;TRINITY_DN2719_c1_g1_i6_orf1;TRINITY_DN97589_c0_g1_i3_orf1;TRINITY_DN115658_c0_g1_i1_orf1;TRINITY_DN7464_c0_g1_i14_orf1;TRINITY_DN50787_c0_g2_i2_orf1;TRINITY_DN2110_c0_g1_i3_orf1;TRINITY_DN36144_c0_g1_i3_orf1;TRINITY_DN799_c0_g1_i7_orf1;TRINITY_DN12301_c0_g1_i1_orf1;TRINITY_DN10900_c0_g1_i7_orf1;TRINITY_DN3733_c0_g1_i1_orf1;TRINITY_DN13732_c0_g2_i3_orf1;TRINITY_DN4408_c6_g1_i1_orf1;TRINITY_DN3472_c0_g1_i6_orf1;TRINITY_DN21357_c0_g1_i5_orf1;TRINITY_DN7991_c0_g1_i9_orf1;TRINITY_DN5811_c0_g1_i4_orf1;TRINITY_DN13233_c0_g1_i3_orf1;TRINITY_DN10831_c1_g1_i1_orf1;TRINITY_DN147458_c0_g1_i1_orf1;TRINITY_DN12397_c0_g1_i1_orf1;TRINITY_DN2299_c0_g1_i3_orf1;TRINITY_DN5064_c0_g1_i4_orf1;TRINITY_DN143852_c0_g1_i1_orf1;TRINITY_DN5029_c0_g1_i1_orf1;TRINITY_DN21251_c1_g1_i1_orf1;TRINITY_DN5112_c0_g1_i1_orf1;TRINITY_DN30131_c0_g1_i1_orf1;TRINITY_DN27035_c0_g1_i1_orf1;TRINITY_DN1575_c0_g1_i7_orf1;TRINITY_DN24318_c0_g1_i1_orf1;TRINITY_DN5031_c0_g1_i1_orf1;TRINITY_DN41645_c0_g1_i1_orf1;TRINITY_DN61222_c0_g1_i1_orf1;TRINITY_DN40345_c0_g1_i6_orf1;TRINITY_DN8964_c0_g1_i4_orf1;TRINITY_DN30027_c0_g1_i1_orf1;TRINITY_DN147676_c0_g1_i1_orf1;TRINITY_DN51813_c0_g1_i1_orf1;TRINITY_DN37856_c0_g1_i5_orf1;TRINITY_DN10234_c0_g1_i1_orf1;TRINITY_DN21971_c0_g1_i4_orf1;TRINITY_DN1957_c0_g1_i4_orf1;TRINITY_DN10548_c0_g2_i1_orf1;TRINITY_DN40015_c0_g1_i2_orf1;TRINITY_DN16939_c0_g1_i4_orf1;TRINITY_DN40650_c0_g1_i1_orf1;TRINITY_DN5009_c0_g1_i2_orf1;TRINITY_DN37532_c0_g1_i1_orf1;TRINITY_DN7613_c1_g2_i1_orf1;TRINITY_DN36893_c0_g1_i1_orf1;TRINITY_DN41166_c0_g1_i1_orf1;TRINITY_DN13651_c0_g1_i2_orf1;TRINITY_DN8949_c0_g1_i2_orf1;TRINITY_DN8824_c0_g2_i1_orf1;TRINITY_DN1084_c0_g1_i2_orf1;TRINITY_DN3826_c0_g1_i1_orf1;TRINITY_DN80134_c0_g1_i1_orf1;TRINITY_DN34399_c0_g1_i1_orf1;TRINITY_DN42646_c0_g2_i1_orf1;TRINITY_DN45924_c0_g1_i14_orf1;TRINITY_DN82324_c0_g1_i4_orf1;TRINITY_DN2618_c0_g1_i3_orf1;TRINITY_DN58207_c0_g1_i1_orf1;TRINITY_DN18593_c0_g1_i1_orf1;TRINITY_DN9862_c0_g2_i1_orf1;TRINITY_DN18782_c0_g1_i4_orf1;TRINITY_DN26824_c0_g1_i1_orf1;TRINITY_DN5697_c0_g1_i1_orf1;TRINITY_DN3393_c0_g2_i1_orf1
biological_process	small molecule catabolic process	GO:0044282	15	15/1363	TRINITY_DN42856_c0_g1_i1_orf1;TRINITY_DN6199_c2_g1_i3_orf1;TRINITY_DN4822_c0_g1_i6_orf1;TRINITY_DN45220_c0_g1_i1_orf1;TRINITY_DN5055_c0_g1_i12_orf1;TRINITY_DN17913_c0_g1_i8_orf1;TRINITY_DN51813_c0_g1_i1_orf1;TRINITY_DN3588_c0_g1_i4_orf1;TRINITY_DN19187_c0_g1_i1_orf1;TRINITY_DN2559_c0_g1_i4_orf1;TRINITY_DN98242_c0_g1_i1_orf1;TRINITY_DN631_c0_g1_i6_orf1;TRINITY_DN1707_c0_g1_i1_orf1;TRINITY_DN3010_c0_g1_i4_orf1;TRINITY_DN4822_c0_g1_i9_orf1
biological_process	organic substance catabolic process	GO:1901575	38	38/1363	TRINITY_DN42856_c0_g1_i1_orf1;TRINITY_DN6199_c2_g1_i3_orf1;TRINITY_DN38230_c0_g1_i4_orf1;TRINITY_DN827_c1_g1_i1_orf1;TRINITY_DN5055_c0_g1_i12_orf1;TRINITY_DN34479_c0_g1_i2_orf1;TRINITY_DN10824_c0_g1_i3_orf1;TRINITY_DN27035_c0_g1_i1_orf1;TRINITY_DN25423_c0_g1_i1_orf1;TRINITY_DN4817_c0_g1_i4_orf1;TRINITY_DN3010_c0_g1_i4_orf1;TRINITY_DN4822_c0_g1_i9_orf1;TRINITY_DN1272_c1_g1_i4_orf1;TRINITY_DN27958_c0_g1_i1_orf1;TRINITY_DN19187_c0_g1_i1_orf1;TRINITY_DN6325_c0_g1_i8_orf1;TRINITY_DN143496_c0_g1_i1_orf1;TRINITY_DN24_c0_g1_i1_orf1;TRINITY_DN3588_c0_g1_i4_orf1;TRINITY_DN2559_c0_g1_i4_orf1;TRINITY_DN98242_c0_g1_i1_orf1;TRINITY_DN8107_c0_g1_i1_orf1;TRINITY_DN4822_c0_g1_i6_orf1;TRINITY_DN1534_c0_g1_i3_orf1;TRINITY_DN17913_c0_g1_i8_orf1;TRINITY_DN15265_c0_g1_i1_orf1;TRINITY_DN51813_c0_g1_i1_orf1;TRINITY_DN631_c0_g1_i6_orf1;TRINITY_DN1707_c0_g1_i1_orf1;TRINITY_DN4121_c0_g1_i1_orf1;TRINITY_DN45220_c0_g1_i1_orf1;TRINITY_DN96557_c0_g1_i1_orf1;TRINITY_DN20499_c0_g3_i1_orf1;TRINITY_DN1287_c0_g1_i5_orf1;TRINITY_DN49047_c0_g1_i2_orf1;TRINITY_DN51658_c0_g1_i1_orf1;TRINITY_DN2848_c0_g1_i2_orf1;TRINITY_DN9979_c0_g1_i1_orf1
biological_process	vitamin metabolic process	GO:0006766	1	1/1363	TRINITY_DN18782_c0_g1_i4_orf1
biological_process	monosaccharide metabolic process	GO:0005996	6	6/1363	TRINITY_DN12545_c0_g1_i7_orf1;TRINITY_DN18650_c0_g1_i1_orf1;TRINITY_DN27035_c0_g1_i1_orf1;TRINITY_DN30713_c0_g1_i3_orf1;TRINITY_DN45530_c0_g1_i1_orf1;TRINITY_DN2848_c0_g1_i2_orf1
biological_process	alcohol metabolic process	GO:0006066	7	7/1363	TRINITY_DN230_c2_g1_i5_orf1;TRINITY_DN52244_c1_g1_i1_orf1;TRINITY_DN36788_c0_g1_i2_orf1;TRINITY_DN3472_c0_g1_i6_orf1;TRINITY_DN1707_c0_g1_i1_orf1;TRINITY_DN3010_c0_g1_i4_orf1;TRINITY_DN11942_c0_g1_i1_orf1
biological_process	nucleobase-containing small molecule metabolic process	GO:0055086	23	23/1363	TRINITY_DN38230_c0_g1_i4_orf1;TRINITY_DN6325_c0_g1_i8_orf1;TRINITY_DN27035_c0_g1_i1_orf1;TRINITY_DN1575_c0_g1_i7_orf1;TRINITY_DN24_c0_g1_i1_orf1;TRINITY_DN2559_c0_g1_i4_orf1;TRINITY_DN98242_c0_g1_i1_orf1;TRINITY_DN2110_c0_g1_i3_orf1;TRINITY_DN1084_c0_g1_i2_orf1;TRINITY_DN36144_c0_g1_i3_orf1;TRINITY_DN3324_c0_g1_i3_orf1;TRINITY_DN12301_c0_g1_i1_orf1;TRINITY_DN51813_c0_g1_i1_orf1;TRINITY_DN45924_c0_g1_i14_orf1;TRINITY_DN28299_c0_g1_i1_orf1;TRINITY_DN5811_c0_g1_i4_orf1;TRINITY_DN1957_c0_g1_i4_orf1;TRINITY_DN10548_c0_g2_i1_orf1;TRINITY_DN18782_c0_g1_i4_orf1;TRINITY_DN19251_c0_g1_i8_orf1;TRINITY_DN5029_c0_g1_i1_orf1;TRINITY_DN2848_c0_g1_i2_orf1;TRINITY_DN9979_c0_g1_i1_orf1
biological_process	urate metabolic process	GO:0046415	1	1/1363	TRINITY_DN2559_c0_g1_i4_orf1
biological_process	macromolecule glycosylation	GO:0043413	2	2/1363	TRINITY_DN103118_c0_g1_i4_orf1;TRINITY_DN53760_c0_g1_i1_orf1
biological_process	macromolecule methylation	GO:0043414	4	4/1363	TRINITY_DN22674_c0_g1_i2_orf1;TRINITY_DN41664_c0_g1_i4_orf1;TRINITY_DN95414_c0_g1_i1_orf1;TRINITY_DN6462_c0_g1_i5_orf1
biological_process	molybdopterin cofactor metabolic process	GO:0043545	1	1/1363	TRINITY_DN80134_c0_g1_i1_orf1
biological_process	lipid metabolic process	GO:0006629	25	25/1363	TRINITY_DN5055_c0_g1_i12_orf1;TRINITY_DN12526_c0_g1_i5_orf1;TRINITY_DN5841_c0_g1_i2_orf1;TRINITY_DN3784_c0_g1_i1_orf1;TRINITY_DN45220_c0_g1_i1_orf1;TRINITY_DN44110_c0_g1_i4_orf1;TRINITY_DN3588_c0_g1_i4_orf1;TRINITY_DN44658_c0_g1_i2_orf1;TRINITY_DN8964_c0_g1_i4_orf1;TRINITY_DN117_c0_g1_i6_orf1;TRINITY_DN10785_c0_g1_i4_orf1;TRINITY_DN12024_c0_g2_i2_orf1;TRINITY_DN10900_c0_g1_i7_orf1;TRINITY_DN84478_c0_g1_i8_orf1;TRINITY_DN4070_c0_g1_i4_orf1;TRINITY_DN3529_c0_g1_i7_orf1;TRINITY_DN12806_c0_g2_i1_orf1;TRINITY_DN7861_c0_g1_i5_orf1;TRINITY_DN117_c0_g1_i4_orf1;TRINITY_DN2618_c0_g1_i3_orf1;TRINITY_DN1293_c0_g1_i4_orf1;TRINITY_DN11886_c0_g1_i1_orf1;TRINITY_DN1293_c1_g1_i4_orf1;TRINITY_DN1084_c0_g1_i2_orf1;TRINITY_DN5697_c0_g1_i1_orf1
biological_process	nucleobase-containing compound metabolic process	GO:0006139	82	82/1363	TRINITY_DN59804_c0_g1_i1_orf1;TRINITY_DN3092_c0_g1_i2_orf1;TRINITY_DN24_c0_g1_i1_orf1;TRINITY_DN17271_c0_g1_i1_orf1;TRINITY_DN38230_c0_g1_i4_orf1;TRINITY_DN6325_c0_g1_i8_orf1;TRINITY_DN5507_c0_g1_i1_orf1;TRINITY_DN2749_c0_g1_i4_orf1;TRINITY_DN140212_c0_g1_i1_orf1;TRINITY_DN40434_c0_g1_i2_orf1;TRINITY_DN2110_c0_g1_i3_orf1;TRINITY_DN4835_c0_g1_i2_orf1;TRINITY_DN38562_c0_g1_i3_orf1;TRINITY_DN77318_c0_g2_i1_orf1;TRINITY_DN5238_c0_g1_i2_orf1;TRINITY_DN27035_c0_g1_i1_orf1;TRINITY_DN51813_c0_g1_i1_orf1;TRINITY_DN1978_c0_g1_i4_orf1;TRINITY_DN37532_c0_g1_i1_orf1;TRINITY_DN51968_c0_g1_i1_orf1;TRINITY_DN2718_c0_g1_i6_orf1;TRINITY_DN20238_c0_g1_i7_orf1;TRINITY_DN2971_c0_g1_i1_orf1;TRINITY_DN40945_c0_g1_i1_orf1;TRINITY_DN2054_c0_g1_i1_orf1;TRINITY_DN27958_c0_g1_i1_orf1;TRINITY_DN5670_c0_g1_i2_orf1;TRINITY_DN45924_c0_g1_i14_orf1;TRINITY_DN115658_c0_g1_i1_orf1;TRINITY_DN30950_c0_g1_i13_orf1;TRINITY_DN47575_c0_g1_i1_orf1;TRINITY_DN12301_c0_g1_i1_orf1;TRINITY_DN111_c0_g2_i2_orf1;TRINITY_DN2559_c0_g1_i4_orf1;TRINITY_DN98242_c0_g1_i1_orf1;TRINITY_DN41664_c0_g1_i4_orf1;TRINITY_DN2367_c1_g1_i20_orf1;TRINITY_DN8107_c0_g1_i1_orf1;TRINITY_DN17045_c0_g2_i3_orf1;TRINITY_DN19251_c0_g1_i8_orf1;TRINITY_DN1091_c0_g3_i1_orf1;TRINITY_DN2647_c0_g1_i3_orf1;TRINITY_DN2749_c0_g2_i3_orf1;TRINITY_DN18782_c0_g1_i4_orf1;TRINITY_DN36144_c0_g1_i3_orf1;TRINITY_DN3062_c0_g1_i1_orf1;TRINITY_DN3324_c0_g1_i3_orf1;TRINITY_DN19866_c0_g1_i4_orf1;TRINITY_DN825_c23_g1_i5_orf1;TRINITY_DN84322_c0_g2_i1_orf1;TRINITY_DN6235_c0_g1_i5_orf1;TRINITY_DN13055_c0_g1_i5_orf1;TRINITY_DN13732_c0_g2_i3_orf1;TRINITY_DN4408_c6_g1_i1_orf1;TRINITY_DN51568_c0_g1_i1_orf1;TRINITY_DN123184_c0_g1_i1_orf1;TRINITY_DN27771_c0_g1_i1_orf1;TRINITY_DN2769_c0_g1_i1_orf1;TRINITY_DN104507_c0_g1_i2_orf1;TRINITY_DN1116_c0_g1_i6_orf1;TRINITY_DN817_c0_g1_i3_orf1;TRINITY_DN47123_c0_g1_i1_orf1;TRINITY_DN4135_c0_g1_i5_orf1;TRINITY_DN48619_c0_g1_i1_orf1;TRINITY_DN89613_c0_g1_i13_orf1;TRINITY_DN28299_c0_g1_i1_orf1;TRINITY_DN5811_c0_g1_i4_orf1;TRINITY_DN3847_c1_g1_i1_orf1;TRINITY_DN46409_c0_g1_i1_orf1;TRINITY_DN1957_c0_g1_i4_orf1;TRINITY_DN346_c0_g1_i7_orf1;TRINITY_DN1091_c0_g1_i1_orf1;TRINITY_DN20499_c0_g3_i1_orf1;TRINITY_DN620_c0_g1_i4_orf1;TRINITY_DN1575_c0_g1_i7_orf1;TRINITY_DN1084_c0_g1_i2_orf1;TRINITY_DN2299_c0_g1_i3_orf1;TRINITY_DN5029_c0_g1_i1_orf1;TRINITY_DN2848_c0_g1_i2_orf1;TRINITY_DN9979_c0_g1_i1_orf1;TRINITY_DN2749_c4_g1_i2_orf1;TRINITY_DN10548_c0_g2_i1_orf1

biological_process	tricarboxylic acid cycle	GO:0006099	3	3/1363	TRINITY_DN36788_c0_g1_i2_orf1;TRINITY_DN3464_c0_g1_i1_orf1;TRINITY_DN19251_c0_g1_i8_orf1
biological_process	carbohydrate metabolic process	GO:0005975	39	39/1363	TRINITY_DN14235_c0_g1_i1_orf1;TRINITY_DN8703_c0_g1_i2_orf1;TRINITY_DN6325_c0_g1_i8_orf1;TRINITY_DN18030_c0_g1_i1_orf1;TRINITY_DN10824_c0_g1_i3_orf1;TRINITY_DN48410_c0_g1_i1_orf1;TRINITY_DN6108_c0_g1_i5_orf1;TRINITY_DN27035_c0_g1_i1_orf1;TRINITY_DN14409_c0_g1_i1_orf1;TRINITY_DN45530_c0_g1_i1_orf1;TRINITY_DN1196_c0_g1_i5_orf1;TRINITY_DN3010_c0_g1_i4_orf1;TRINITY_DN11942_c0_g1_i1_orf1;TRINITY_DN95850_c0_g1_i1_orf1;TRINITY_DN9044_c0_g1_i1_orf1;TRINITY_DN12545_c0_g1_i7_orf1;TRINITY_DN36788_c0_g1_i2_orf1;TRINITY_DN28741_c0_g1_i3_orf1;TRINITY_DN30713_c0_g1_i3_orf1;TRINITY_DN2894_c0_g2_i3_orf1;TRINITY_DN3322_c0_g1_i2_orf1;TRINITY_DN140613_c0_g1_i1_orf1;TRINITY_DN1785_c0_g1_i5_orf1;TRINITY_DN18918_c0_g1_i3_orf1;TRINITY_DN195_c4_g1_i1_orf1;TRINITY_DN2170_c0_g2_i1_orf1;TRINITY_DN4070_c0_g1_i4_orf1;TRINITY_DN7534_c0_g1_i15_orf1;TRINITY_DN1707_c0_g1_i1_orf1;TRINITY_DN9044_c0_g1_i2_orf1;TRINITY_DN1732_c0_g1_i15_orf1;TRINITY_DN52244_c1_g1_i1_orf1;TRINITY_DN1732_c0_g1_i17_orf1;TRINITY_DN1287_c0_g1_i5_orf1;TRINITY_DN82801_c0_g1_i1_orf1;TRINITY_DN103118_c0_g1_i4_orf1;TRINITY_DN664_c0_g1_i18_orf1;TRINITY_DN2848_c0_g1_i2_orf1;TRINITY_DN9979_c0_g1_i1_orf1
biological_process	protein metabolic process	GO:0019538	138	138/1363	TRINITY_DN137_c0_g1_i1_orf1;TRINITY_DN4121_c0_g1_i1_orf1;TRINITY_DN5873_c0_g4_i1_orf1;TRINITY_DN344_c1_g1_i1_orf1;TRINITY_DN54477_c0_g1_i1_orf1;TRINITY_DN11065_c0_g2_i1_orf1;TRINITY_DN2682_c0_g1_i4_orf1;TRINITY_DN30300_c0_g2_i1_orf1;TRINITY_DN2442_c0_g1_i2_orf1;TRINITY_DN142442_c0_g1_i1_orf1;TRINITY_DN4798_c0_g1_i3_orf1;TRINITY_DN30131_c0_g1_i1_orf1;TRINITY_DN2885_c1_g1_i2_orf1;TRINITY_DN7464_c0_g1_i14_orf1;TRINITY_DN14754_c0_g1_i6_orf1;TRINITY_DN2954_c0_g1_i1_orf1;TRINITY_DN14774_c0_g1_i4_orf1;TRINITY_DN95056_c0_g2_i2_orf1;TRINITY_DN18273_c0_g1_i4_orf1;TRINITY_DN59885_c0_g1_i3_orf1;TRINITY_DN6205_c0_g1_i8_orf1;TRINITY_DN143_c0_g3_i1_orf1;TRINITY_DN140538_c0_g2_i1_orf1;TRINITY_DN17376_c0_g1_i2_orf1;TRINITY_DN46409_c0_g1_i1_orf1;TRINITY_DN22674_c0_g1_i2_orf1;TRINITY_DN79734_c0_g2_i3_orf1;TRINITY_DN46715_c0_g1_i1_orf1;TRINITY_DN46202_c0_g1_i1_orf1;TRINITY_DN121893_c0_g1_i1_orf1;TRINITY_DN2848_c0_g1_i2_orf1;TRINITY_DN4767_c0_g1_i6_orf1;TRINITY_DN18249_c0_g1_i1_orf1;TRINITY_DN143895_c0_g1_i1_orf1;TRINITY_DN1706_c0_g1_i7_orf1;TRINITY_DN144956_c0_g1_i1_orf1;TRINITY_DN4189_c0_g2_i1_orf1;TRINITY_DN1791_c0_g1_i3_orf1;TRINITY_DN1272_c1_g1_i4_orf1;TRINITY_DN53760_c0_g1_i1_orf1;TRINITY_DN97589_c0_g1_i3_orf1;TRINITY_DN9871_c0_g1_i11_orf1;TRINITY_DN42461_c0_g1_i4_orf1;TRINITY_DN277_c1_g1_i1_orf1;TRINITY_DN50787_c0_g2_i2_orf1;TRINITY_DN45948_c1_g1_i1_orf1;TRINITY_DN5696_c0_g1_i4_orf1;TRINITY_DN71863_c0_g1_i2_orf1;TRINITY_DN799_c0_g1_i7_orf1;TRINITY_DN3733_c0_g1_i1_orf1;TRINITY_DN13732_c0_g2_i3_orf1;TRINITY_DN4408_c6_g1_i1_orf1;TRINITY_DN21984_c0_g1_i6_orf1;TRINITY_DN376_c1_g1_i1_orf1;TRINITY_DN21357_c0_g1_i5_orf1;TRINITY_DN1954_c0_g1_i4_orf1;TRINITY_DN7991_c0_g1_i9_orf1;TRINITY_DN58207_c0_g1_i1_orf1;TRINITY_DN4767_c0_g1_i4_orf1;TRINITY_DN13233_c0_g1_i3_orf1;TRINITY_DN10831_c1_g1_i1_orf1;TRINITY_DN95414_c0_g1_i1_orf1;TRINITY_DN4494_c0_g1_i1_orf1;TRINITY_DN12397_c0_g1_i1_orf1;TRINITY_DN5064_c0_g1_i4_orf1;TRINITY_DN143852_c0_g1_i1_orf1;TRINITY_DN21251_c1_g1_i1_orf1;TRINITY_DN1749_c0_g2_i2_orf1;TRINITY_DN5112_c0_g1_i1_orf1;TRINITY_DN10766_c0_g1_i1_orf1;TRINITY_DN18159_c0_g1_i6_orf1;TRINITY_DN67026_c0_g1_i6_orf1;TRINITY_DN3483_c0_g1_i5_orf1;TRINITY_DN24318_c0_g1_i1_orf1;TRINITY_DN670_c0_g1_i15_orf1;TRINITY_DN4742_c0_g1_i1_orf1;TRINITY_DN4817_c0_g1_i4_orf1;TRINITY_DN5009_c0_g1_i2_orf1;TRINITY_DN57111_c0_g1_i1_orf1;TRINITY_DN140_c1_g1_i2_orf1;TRINITY_DN28729_c0_g1_i9_orf1;TRINITY_DN61222_c0_g1_i1_orf1;TRINITY_DN40345_c0_g1_i6_orf1;TRINITY_DN34479_c0_g1_i2_orf1;TRINITY_DN130778_c0_g1_i1_orf1;TRINITY_DN30027_c0_g1_i1_orf1;TRINITY_DN147676_c0_g1_i1_orf1;TRINITY_DN19866_c0_g1_i4_orf1;TRINITY_DN15265_c0_g1_i1_orf1;TRINITY_DN2673_c2_g1_i2_orf1;TRINITY_DN1308_c0_g1_i4_orf1;TRINITY_DN23167_c0_g1_i4_orf1;TRINITY_DN113327_c0_g1_i2_orf1;TRINITY_DN111110_c0_g1_i1_orf1;TRINITY_DN96557_c0_g1_i1_orf1;TRINITY_DN10234_c0_g1_i1_orf1;TRINITY_DN6470_c0_g3_i2_orf1;TRINITY_DN21971_c0_g1_i4_orf1;TRINITY_DN24121_c1_g1_i6_orf1;TRINITY_DN49047_c0_g1_i2_orf1;TRINITY_DN40015_c0_g1_i2_orf1;TRINITY_DN16939_c0_g1_i4_orf1;TRINITY_DN59804_c0_g1_i1_orf1;TRINITY_DN140423_c0_g1_i2_orf1;TRINITY_DN40650_c0_g1_i1_orf1;TRINITY_DN5031_c0_g1_i1_orf1;TRINITY_DN7316_c0_g2_i1_orf1;TRINITY_DN7613_c1_g2_i1_orf1;TRINITY_DN36893_c0_g1_i1_orf1;TRINITY_DN801_c0_g1_i2_orf1;TRINITY_DN753_c0_g1_i4_orf1;TRINITY_DN18593_c0_g1_i1_orf1;TRINITY_DN13651_c0_g1_i2_orf1;TRINITY_DN8949_c0_g1_i2_orf1;TRINITY_DN27958_c0_g1_i1_orf1;TRINITY_DN4030_c0_g2_i1_orf1;TRINITY_DN143496_c0_g1_i1_orf1;TRINITY_DN17329_c0_g2_i3_orf1;TRINITY_DN7647_c0_g1_i4_orf1;TRINITY_DN3702_c0_g1_i1_orf1;TRINITY_DN147458_c0_g1_i1_orf1;TRINITY_DN8824_c0_g2_i1_orf1;TRINITY_DN14874_c0_g1_i6_orf1;TRINITY_DN892_c7_g1_i2_orf1;TRINITY_DN3826_c0_g1_i1_orf1;TRINITY_DN15478_c0_g1_i1_orf1;TRINITY_DN51658_c0_g1_i1_orf1;TRINITY_DN42646_c0_g2_i1_orf1;TRINITY_DN5697_c0_g1_i1_orf1;TRINITY_DN82324_c0_g1_i4_orf1;TRINITY_DN152_c0_g1_i4_orf1;TRINITY_DN103118_c0_g1_i4_orf1;TRINITY_DN9862_c0_g2_i1_orf1;TRINITY_DN1592_c0_g1_i1_orf1;TRINITY_DN26824_c0_g1_i1_orf1;TRINITY_DN26130_c0_g1_i1_orf1;TRINITY_DN6462_c0_g1_i5_orf1;TRINITY_DN3393_c0_g2_i1_orf1
biological_process	cellular amino acid metabolic process	GO:0006520	21	21/1363	TRINITY_DN42856_c0_g1_i1_orf1;TRINITY_DN4822_c0_g1_i6_orf1;TRINITY_DN4822_c0_g1_i9_orf1;TRINITY_DN92153_c0_g2_i2_orf1;TRINITY_DN17913_c0_g1_i8_orf1;TRINITY_DN825_c23_g1_i5_orf1;TRINITY_DN84322_c0_g2_i1_orf1;TRINITY_DN51813_c0_g1_i1_orf1;TRINITY_DN6199_c2_g1_i3_orf1;TRINITY_DN27771_c0_g1_i1_orf1;TRINITY_DN5564_c0_g1_i5_orf1;TRINITY_DN34399_c0_g1_i1_orf1;TRINITY_DN17326_c0_g1_i5_orf1;TRINITY_DN31163_c1_g1_i4_orf1;TRINITY_DN100821_c0_g1_i1_orf1;TRINITY_DN2719_c1_g1_i6_orf1;TRINITY_DN19187_c0_g1_i1_orf1;TRINITY_DN14464_c0_g1_i1_orf1;TRINITY_DN817_c0_g1_i3_orf1;TRINITY_DN620_c0_g1_i4_orf1;TRINITY_DN48619_c0_g1_i1_orf1
biological_process	organophosphate metabolic process	GO:0019637	26	26/1363	TRINITY_DN24_c0_g1_i1_orf1;TRINITY_DN47151_c0_g1_i1_orf1;TRINITY_DN38230_c0_g1_i4_orf1;TRINITY_DN6325_c0_g1_i8_orf1;TRINITY_DN27035_c0_g1_i1_orf1;TRINITY_DN1575_c0_g1_i7_orf1;TRINITY_DN41166_c0_g1_i1_orf1;TRINITY_DN44110_c0_g1_i4_orf1;TRINITY_DN2110_c0_g1_i3_orf1;TRINITY_DN18782_c0_g1_i4_orf1;TRINITY_DN36144_c0_g1_i3_orf1;TRINITY_DN12301_c0_g1_i1_orf1;TRINITY_DN51813_c0_g1_i1_orf1;TRINITY_DN80134_c0_g1_i1_orf1;TRINITY_DN45924_c0_g1_i14_orf1;TRINITY_DN28299_c0_g1_i1_orf1;TRINITY_DN5811_c0_g1_i4_orf1;TRINITY_DN2618_c0_g1_i3_orf1;TRINITY_DN1957_c0_g1_i4_orf1;TRINITY_DN10548_c0_g2_i1_orf1;TRINITY_DN1084_c0_g1_i2_orf1;TRINITY_DN19251_c0_g1_i8_orf1;TRINITY_DN5697_c0_g1_i1_orf1;TRINITY_DN5029_c0_g1_i1_orf1;TRINITY_DN2848_c0_g1_i2_orf1;TRINITY_DN9979_c0_g1_i1_orf1
biological_process	carbohydrate derivative metabolic process	GO:1901135	24	24/1363	TRINITY_DN47151_c0_g1_i1_orf1;TRINITY_DN6325_c0_g1_i8_orf1;TRINITY_DN10824_c0_g1_i3_orf1;TRINITY_DN27035_c0_g1_i1_orf1;TRINITY_DN1575_c0_g1_i7_orf1;TRINITY_DN1196_c0_g1_i5_orf1;TRINITY_DN827_c1_g1_i1_orf1;TRINITY_DN98242_c0_g1_i1_orf1;TRINITY_DN2110_c0_g1_i3_orf1;TRINITY_DN1084_c0_g1_i2_orf1;TRINITY_DN3324_c0_g1_i3_orf1;TRINITY_DN1534_c0_g1_i3_orf1;TRINITY_DN12301_c0_g1_i1_orf1;TRINITY_DN45924_c0_g1_i14_orf1;TRINITY_DN28299_c0_g1_i1_orf1;TRINITY_DN5811_c0_g1_i4_orf1;TRINITY_DN1287_c0_g1_i5_orf1;TRINITY_DN10548_c0_g2_i1_orf1;TRINITY_DN18782_c0_g1_i4_orf1;TRINITY_DN19251_c0_g1_i8_orf1;TRINITY_DN5697_c0_g1_i1_orf1;TRINITY_DN5029_c0_g1_i1_orf1;TRINITY_DN2848_c0_g1_i2_orf1;TRINITY_DN9979_c0_g1_i1_orf1
biological_process	organic hydroxy compound metabolic process	GO:1901615	9	9/1363	TRINITY_DN230_c2_g1_i5_orf1;TRINITY_DN52244_c1_g1_i1_orf1;TRINITY_DN36788_c0_g1_i2_orf1;TRINITY_DN1707_c0_g1_i1_orf1;TRINITY_DN31163_c1_g1_i4_orf1;TRINITY_DN3472_c0_g1_i6_orf1;TRINITY_DN3010_c0_g1_i4_orf1;TRINITY_DN11942_c0_g1_i1_orf1;TRINITY_DN9724_c0_g1_i4_orf1
biological_process	organic cyclic compound metabolic process	GO:1901360	96	96/1363	TRINITY_DN38230_c0_g1_i4_orf1;TRINITY_DN40434_c0_g1_i2_orf1;TRINITY_DN27771_c0_g1_i1_orf1;TRINITY_DN2054_c0_g1_i1_orf1;TRINITY_DN6325_c0_g1_i8_orf1;TRINITY_DN31163_c1_g1_i4_orf1;TRINITY_DN8107_c0_g1_i1_orf1;TRINITY_DN5670_c0_g1_i2_orf1;TRINITY_DN4822_c0_g1_i6_orf1;TRINITY_DN47123_c0_g1_i1_orf1;TRINITY_DN123184_c0_g1_i1_orf1;TRINITY_DN2769_c0_g1_i1_orf1;TRINITY_DN1116_c0_g1_i6_orf1;TRINITY_DN817_c0_g1_i3_orf1;TRINITY_DN620_c0_g1_i4_orf1;TRINITY_DN41664_c0_g1_i4_orf1;TRINITY_DN20499_c0_g3_i1_orf1;TRINITY_DN46409_c0_g1_i1_orf1;TRINITY_DN6235_c0_g1_i5_orf1;TRINITY_DN89613_c0_g1_i13_orf1;TRINITY_DN2848_c0_g1_i2_orf1;TRINITY_DN1957_c0_g1_i4_orf1;TRINITY_DN230_c2_g1_i5_orf1;TRINITY_DN3092_c0_g1_i2_orf1;TRINITY_DN1978_c0_g1_i4_orf1;TRINITY_DN17271_c0_g1_i1_orf1;TRINITY_DN140212_c0_g1_i1_orf1;TRINITY_DN2719_c1_g1_i6_orf1;TRINITY_DN51968_c0_g1_i1_orf1;TRINITY_DN20238_c0_g1_i7_orf1;TRINITY_DN19187_c0_g1_i1_orf1;TRINITY_DN115658_c0_g1_i1_orf1;TRINITY_DN47575_c0_g1_i1_orf1;TRINITY_DN244_c1_g1_i5_orf1;TRINITY_DN2110_c0_g1_i3_orf1;TRINITY_DN5507_c0_g1_i1_orf1;TRINITY_DN1091_c0_g3_i1_orf1;TRINITY_DN36144_c0_g1_i3_orf1;TRINITY_DN3324_c0_g1_i3_orf1;TRINITY_DN12301_c0_g1_i1_orf1;TRINITY_DN13732_c0_g2_i3_orf1;TRINITY_DN4408_c6_g1_i1_orf1;TRINITY_DN51568_c0_g1_i1_orf1;TRINITY_DN3472_c0_g1_i6_orf1;TRINITY_DN40945_c0_g1_i1_orf1;TRINITY_DN9724_c0_g1_i4_orf1;TRINITY_DN5811_c0_g1_i4_orf1;TRINITY_DN3847_c1_g1_i1_orf1;TRINITY_DN1091_c0_g1_i1_orf1;TRINITY_DN2299_c0_g1_i3_orf1;TRINITY_DN5029_c0_g1_i1_orf1;TRINITY_DN2749_c4_g1_i2_orf1;TRINITY_DN92153_c0_g2_i2_orf1;TRINITY_DN2749_c0_g1_i4_orf1;TRINITY_DN77318_c0_g2_i1_orf1;TRINITY_DN27035_c0_g1_i1_orf1;TRINITY_DN1575_c0_g1_i7_orf1;TRINITY_DN2718_c0_g1_i6_orf1;TRINITY_DN24_c0_g1_i1_orf1;TRINITY_DN2559_c0_g1_i4_orf1;TRINITY_DN98242_c0_g1_i1_orf1;TRINITY_DN4135_c0_g1_i5_orf1;TRINITY_DN2749_c0_g2_i3_orf1;TRINITY_DN3062_c0_g1_i1_orf1;TRINITY_DN19866_c0_g1_i4_orf1;TRINITY_DN825_c23_g1_i5_orf1;TRINITY_DN84322_c0_g2_i1_orf1;TRINITY_DN51813_c0_g1_i1_orf1;TRINITY_DN631_c0_g1_i6_orf1;TRINITY_DN111_c0_g2_i2_orf1;TRINITY_DN28299_c0_g1_i1_orf1;TRINITY_DN2647_c0_g1_i3_orf1;TRINITY_DN10548_c0_g2_i1_orf1;TRINITY_DN9979_c0_g1_i1_orf1;TRINITY_DN59804_c0_g1_i1_orf1;TRINITY_DN2971_c0_g1_i1_orf1;TRINITY_DN37532_c0_g1_i1_orf1;TRINITY_DN4835_c0_g1_i2_orf1;TRINITY_DN38562_c0_g1_i3_orf1;TRINITY_DN25423_c0_g1_i1_orf1;TRINITY_DN4822_c0_g1_i9_orf1;TRINITY_DN104507_c0_g1_i2_orf1;TRINITY_DN27958_c0_g1_i1_orf1;TRINITY_DN2367_c1_g1_i20_orf1;TRINITY_DN17045_c0_g2_i3_orf1;TRINITY_DN1084_c0_g1_i2_orf1;TRINITY_DN17913_c0_g1_i8_orf1;TRINITY_DN5238_c0_g1_i2_orf1;TRINITY_DN19251_c0_g1_i8_orf1;TRINITY_DN13055_c0_g1_i5_orf1;TRINITY_DN80134_c0_g1_i1_orf1;TRINITY_DN45924_c0_g1_i14_orf1;TRINITY_DN48619_c0_g1_i1_orf1;TRINITY_DN346_c0_g1_i7_orf1;TRINITY_DN18782_c0_g1_i4_orf1;TRINITY_DN30950_c0_g1_i13_orf1
biological_process	thioester metabolic process	GO:0035383	2	2/1363	TRINITY_DN1084_c0_g1_i2_orf1;TRINITY_DN19251_c0_g1_i8_orf1
biological_process	macromolecule metabolic process	GO:0043170	197	197/1363	TRINITY_DN827_c1_g1_i1_orf1;TRINITY_DN40434_c0_g1_i2_orf1;TRINITY_DN27771_c0_g1_i1_orf1;TRINITY_DN137_c0_g1_i1_orf1;TRINITY_DN4121_c0_g1_i1_orf1;TRINITY_DN5873_c0_g4_i1_orf1;TRINITY_DN344_c1_g1_i1_orf1;TRINITY_DN2054_c0_g1_i1_orf1;TRINITY_DN54477_c0_g1_i1_orf1;TRINITY_DN11065_c0_g2_i1_orf1;TRINITY_DN58207_c0_g1_i1_orf1;TRINITY_DN30300_c0_g2_i1_orf1;TRINITY_DN2442_c0_g1_i2_orf1;TRINITY_DN142442_c0_g1_i1_orf1;TRINITY_DN111_c0_g2_i2_orf1;TRINITY_DN2749_c0_g1_i4_orf1;TRINITY_DN2885_c1_g1_i2_orf1;TRINITY_DN7464_c0_g1_i14_orf1;TRINITY_DN14754_c0_g1_i6_orf1;TRINITY_DN8107_c0_g1_i1_orf1;TRINITY_DN2954_c0_g1_i1_orf1;TRINITY_DN5670_c0_g1_i2_orf1;TRINITY_DN14774_c0_g1_i4_orf1;TRINITY_DN1534_c0_g1_i3_orf1;TRINITY_DN95056_c0_g2_i2_orf1;TRINITY_DN18273_c0_g1_i4_orf1;TRINITY_DN45948_c1_g1_i1_orf1;TRINITY_DN47123_c0_g1_i1_orf1;TRINITY_DN123184_c0_g1_i1_orf1;TRINITY_DN6205_c0_g1_i8_orf1;TRINITY_DN817_c0_g1_i3_orf1;TRINITY_DN77318_c0_g2_i1_orf1;TRINITY_DN143_c0_g3_i1_orf1;TRINITY_DN1091_c0_g1_i1_orf1;TRINITY_DN17376_c0_g1_i2_orf1;TRINITY_DN20499_c0_g3_i1_orf1;TRINITY_DN3483_c0_g1_i5_orf1;TRINITY_DN22674_c0_g1_i2_orf1;TRINITY_DN5696_c0_g1_i4_orf1;TRINITY_DN79734_c0_g2_i3_orf1;TRINITY_DN46715_c0_g1_i1_orf1;TRINITY_DN6235_c0_g1_i5_orf1;TRINITY_DN46202_c0_g1_i1_orf1;TRINITY_DN121893_c0_g1_i1_orf1;TRINITY_DN2848_c0_g1_i2_orf1;TRINITY_DN4767_c0_g1_i6_orf1;TRINITY_DN3092_c0_g1_i2_orf1;TRINITY_DN1978_c0_g1_i4_orf1;TRINITY_DN17271_c0_g1_i1_orf1;TRINITY_DN143895_c0_g1_i1_orf1;TRINITY_DN1116_c0_g1_i6_orf1;TRINITY_DN1706_c0_g1_i7_orf1;TRINITY_DN140212_c0_g1_i1_orf1;TRINITY_DN144956_c0_g1_i1_orf1;TRINITY_DN10824_c0_g1_i3_orf1;TRINITY_DN4817_c0_g1_i4_orf1;TRINITY_DN37532_c0_g1_i1_orf1;TRINITY_DN51968_c0_g1_i1_orf1;TRINITY_DN4189_c0_g2_i1_orf1;TRINITY_DN20238_c0_g1_i7_orf1;TRINITY_DN1791_c0_g1_i3_orf1;TRINITY_DN1272_c1_g1_i4_orf1;TRINITY_DN53760_c0_g1_i1_orf1;TRINITY_DN97589_c0_g1_i3_orf1;TRINITY_DN18249_c0_g1_i1_orf1;TRINITY_DN9871_c0_g1_i11_orf1;TRINITY_DN115658_c0_g1_i1_orf1;TRINITY_DN51568_c0_g1_i1_orf1;TRINITY_DN42461_c0_g1_i4_orf1;TRINITY_DN47575_c0_g1_i1_orf1;TRINITY_DN277_c1_g1_i1_orf1;TRINITY_DN50787_c0_g2_i2_orf1;TRINITY_DN59885_c0_g1_i3_orf1;TRINITY_DN2769_c0_g1_i1_orf1;TRINITY_DN41664_c0_g1_i4_orf1;TRINITY_DN1091_c0_g3_i1_orf1;TRINITY_DN21971_c0_g1_i4_orf1;TRINITY_DN71863_c0_g1_i2_orf1;TRINITY_DN799_c0_g1_i7_orf1;TRINITY_DN3733_c0_g1_i1_orf1;TRINITY_DN13732_c0_g2_i3_orf1;TRINITY_DN4408_c6_g1_i1_orf1;TRINITY_DN95414_c0_g1_i1_orf1;TRINITY_DN40945_c0_g1_i1_orf1;TRINITY_DN376_c1_g1_i1_orf1;TRINITY_DN21357_c0_g1_i5_orf1;TRINITY_DN1954_c0_g1_i4_orf1;TRINITY_DN7991_c0_g1_i9_orf1;TRINITY_DN89613_c0_g1_i13_orf1;TRINITY_DN2682_c0_g1_i4_orf1;TRINITY_DN3847_c1_g1_i1_orf1;TRINITY_DN13233_c0_g1_i3_orf1;TRINITY_DN10831_c1_g1_i1_orf1;TRINITY_DN21984_c0_g1_i6_orf1;TRINITY_DN147458_c0_g1_i1_orf1;TRINITY_DN12397_c0_g1_i1_orf1;TRINITY_DN2299_c0_g1_i3_orf1;TRINITY_DN51658_c0_g1_i1_orf1;TRINITY_DN143852_c0_g1_i1_orf1;TRINITY_DN2749_c4_g1_i2_orf1;TRINITY_DN21251_c1_g1_i1_orf1;TRINITY_DN1749_c0_g2_i2_orf1;TRINITY_DN5112_c0_g1_i1_orf1;TRINITY_DN10766_c0_g1_i1_orf1;TRINITY_DN18159_c0_g1_i6_orf1;TRINITY_DN140538_c0_g2_i1_orf1;TRINITY_DN620_c0_g1_i4_orf1;TRINITY_DN67026_c0_g1_i6_orf1;TRINITY_DN46409_c0_g1_i1_orf1;TRINITY_DN24318_c0_g1_i1_orf1;TRINITY_DN670_c0_g1_i15_orf1;TRINITY_DN4742_c0_g1_i1_orf1;TRINITY_DN2718_c0_g1_i6_orf1;TRINITY_DN5031_c0_g1_i1_orf1;TRINITY_DN41645_c0_g1_i1_orf1;TRINITY_DN57111_c0_g1_i1_orf1;TRINITY_DN140_c1_g1_i2_orf1;TRINITY_DN28729_c0_g1_i9_orf1;TRINITY_DN61222_c0_g1_i1_orf1;TRINITY_DN40345_c0_g1_i6_orf1;TRINITY_DN7316_c0_g2_i1_orf1;TRINITY_DN34479_c0_g1_i2_orf1;TRINITY_DN130778_c0_g1_i1_orf1;TRINITY_DN30027_c0_g1_i1_orf1;TRINITY_DN82324_c0_g1_i4_orf1;TRINITY_DN147676_c0_g1_i1_orf1;TRINITY_DN2749_c0_g2_i3_orf1;TRINITY_DN3062_c0_g1_i1_orf1;TRINITY_DN19866_c0_g1_i4_orf1;TRINITY_DN825_c23_g1_i5_orf1;TRINITY_DN84322_c0_g2_i1_orf1;TRINITY_DN2673_c2_g1_i2_orf1;TRINITY_DN1308_c0_g1_i4_orf1;TRINITY_DN23167_c0_g1_i4_orf1;TRINITY_DN113327_c0_g1_i2_orf1;TRINITY_DN4135_c0_g1_i5_orf1;TRINITY_DN111110_c0_g1_i1_orf1;TRINITY_DN96557_c0_g1_i1_orf1;TRINITY_DN10234_c0_g1_i1_orf1;TRINITY_DN4767_c0_g1_i4_orf1;TRINITY_DN6470_c0_g3_i2_orf1;TRINITY_DN1287_c0_g1_i5_orf1;TRINITY_DN2647_c0_g1_i3_orf1;TRINITY_DN24121_c1_g1_i6_orf1;TRINITY_DN8824_c0_g2_i1_orf1;TRINITY_DN49047_c0_g1_i2_orf1;TRINITY_DN40015_c0_g1_i2_orf1;TRINITY_DN16939_c0_g1_i4_orf1;TRINITY_DN59804_c0_g1_i1_orf1;TRINITY_DN140423_c0_g1_i2_orf1;TRINITY_DN40650_c0_g1_i1_orf1;TRINITY_DN2971_c0_g1_i1_orf1;TRINITY_DN5507_c0_g1_i1_orf1;TRINITY_DN7613_c1_g2_i1_orf1;TRINITY_DN36893_c0_g1_i1_orf1;TRINITY_DN801_c0_g1_i2_orf1;TRINITY_DN4835_c0_g1_i2_orf1;TRINITY_DN38562_c0_g1_i3_orf1;TRINITY_DN753_c0_g1_i4_orf1;TRINITY_DN18593_c0_g1_i1_orf1;TRINITY_DN13651_c0_g1_i2_orf1;TRINITY_DN8949_c0_g1_i2_orf1;TRINITY_DN104507_c0_g1_i2_orf1;TRINITY_DN4798_c0_g1_i3_orf1;TRINITY_DN27958_c0_g1_i1_orf1;TRINITY_DN4030_c0_g2_i1_orf1;TRINITY_DN143496_c0_g1_i1_orf1;TRINITY_DN17329_c0_g2_i3_orf1;TRINITY_DN7647_c0_g1_i4_orf1;TRINITY_DN5009_c0_g1_i2_orf1;TRINITY_DN3702_c0_g1_i1_orf1;TRINITY_DN15265_c0_g1_i1_orf1;TRINITY_DN2367_c1_g1_i20_orf1;TRINITY_DN4494_c0_g1_i1_orf1;TRINITY_DN17045_c0_g2_i3_orf1;TRINITY_DN14874_c0_g1_i6_orf1;TRINITY_DN140613_c0_g1_i1_orf1;TRINITY_DN892_c7_g1_i2_orf1;TRINITY_DN3826_c0_g1_i1_orf1;TRINITY_DN5238_c0_g1_i2_orf1;TRINITY_DN13055_c0_g1_i5_orf1;TRINITY_DN15478_c0_g1_i1_orf1;TRINITY_DN30131_c0_g1_i1_orf1;TRINITY_DN5064_c0_g1_i4_orf1;TRINITY_DN42646_c0_g2_i1_orf1;TRINITY_DN26130_c0_g1_i1_orf1;TRINITY_DN48619_c0_g1_i1_orf1;TRINITY_DN346_c0_g1_i7_orf1;TRINITY_DN152_c0_g1_i4_orf1;TRINITY_DN103118_c0_g1_i4_orf1;TRINITY_DN9862_c0_g2_i1_orf1;TRINITY_DN1592_c0_g1_i1_orf1;TRINITY_DN30950_c0_g1_i13_orf1;TRINITY_DN26824_c0_g1_i1_orf1;TRINITY_DN5697_c0_g1_i1_orf1;TRINITY_DN6462_c0_g1_i5_orf1;TRINITY_DN3393_c0_g2_i1_orf1
biological_process	melanin metabolic process	GO:0006582	2	2/1363	TRINITY_DN31163_c1_g1_i4_orf1;TRINITY_DN9724_c0_g1_i4_orf1
biological_process	pigment biosynthetic process	GO:0046148	1	1/1363	TRINITY_DN31163_c1_g1_i4_orf1
biological_process	ovarian follicle cell development	GO:0030707	1	1/1363	TRINITY_DN9724_c0_g1_i4_orf1
biological_process	killing of cells of another organism	GO:0031640	1	1/1363	TRINITY_DN2848_c0_g1_i2_orf1
biological_process	leukocyte activation	GO:0045321	1	1/1363	TRINITY_DN46409_c0_g1_i1_orf1
biological_process	neuron death	GO:0070997	1	1/1363	TRINITY_DN2848_c0_g1_i2_orf1
biological_process	programmed cell death	GO:0012501	3	3/1363	TRINITY_DN2848_c0_g1_i2_orf1;TRINITY_DN2655_c0_g2_i1_orf1;TRINITY_DN50074_c0_g1_i1_orf1
biological_process	autophagy	GO:0006914	1	1/1363	TRINITY_DN113353_c0_g1_i1_orf1
biological_process	secretion by cell	GO:0032940	3	3/1363	TRINITY_DN33452_c0_g1_i1_orf1;TRINITY_DN1895_c0_g1_i2_orf1;TRINITY_DN33452_c0_g1_i3_orf1
biological_process	cell-substrate adhesion	GO:0031589	2	2/1363	TRINITY_DN2186_c0_g1_i17_orf1;TRINITY_DN2919_c0_g1_i5_orf1
biological_process	cell-cell adhesion	GO:0098609	1	1/1363	TRINITY_DN1008_c0_g1_i2_orf1
biological_process	cellular response to extracellular stimulus	GO:0031668	4	4/1363	TRINITY_DN140212_c0_g1_i1_orf1;TRINITY_DN1091_c0_g1_i1_orf1;TRINITY_DN1091_c0_g3_i1_orf1;TRINITY_DN2054_c0_g1_i1_orf1
biological_process	intermediate filament cytoskeleton organization	GO:0045104	2	2/1363	TRINITY_DN20009_c0_g1_i1_orf1;TRINITY_DN77480_c0_g1_i2_orf1
biological_process	cellular chemical homeostasis	GO:0055082	7	7/1363	TRINITY_DN46625_c0_g1_i1_orf1;TRINITY_DN65681_c0_g1_i1_orf1;TRINITY_DN1423_c0_g1_i4_orf1;TRINITY_DN1423_c0_g1_i8_orf1;TRINITY_DN136031_c0_g1_i7_orf1;TRINITY_DN96739_c0_g1_i1_orf1;TRINITY_DN31584_c0_g2_i2_orf1
biological_process	leukocyte proliferation	GO:0070661	1	1/1363	TRINITY_DN46409_c0_g1_i1_orf1
biological_process	mitotic cell cycle process	GO:1903047	4	4/1363	TRINITY_DN96557_c0_g1_i1_orf1;TRINITY_DN20238_c0_g1_i7_orf1;TRINITY_DN235_c0_g3_i1_orf1;TRINITY_DN31119_c0_g1_i1_orf1
biological_process	cell cycle phase transition	GO:0044770	1	1/1363	TRINITY_DN96557_c0_g1_i1_orf1
biological_process	spindle organization	GO:0007051	2	2/1363	TRINITY_DN25960_c0_g1_i1_orf1;TRINITY_DN31119_c0_g1_i1_orf1
biological_process	cytokinesis	GO:0000910	2	2/1363	TRINITY_DN20238_c0_g1_i7_orf1;TRINITY_DN235_c0_g3_i1_orf1
biological_process	cytokinetic process	GO:0032506	1	1/1363	TRINITY_DN96557_c0_g1_i1_orf1
biological_process	'de novo' protein folding	GO:0006458	1	1/1363	TRINITY_DN46409_c0_g1_i1_orf1
biological_process	protein refolding	GO:0042026	1	1/1363	TRINITY_DN46409_c0_g1_i1_orf1
biological_process	post-chaperonin tubulin folding pathway	GO:0007023	2	2/1363	TRINITY_DN1054_c0_g1_i8_orf1;TRINITY_DN104297_c0_g1_i1_orf1
biological_process	cellular macromolecule localization	GO:0070727	22	22/1363	TRINITY_DN3209_c0_g1_i1_orf1;TRINITY_DN46409_c0_g1_i1_orf1;TRINITY_DN31584_c0_g2_i2_orf1;TRINITY_DN14677_c0_g2_i3_orf1;TRINITY_DN36230_c0_g1_i1_orf1;TRINITY_DN65299_c0_g4_i1_orf1;TRINITY_DN13118_c0_g1_i6_orf1;TRINITY_DN1447_c0_g1_i5_orf1;TRINITY_DN3698_c0_g1_i4_orf1;TRINITY_DN6680_c0_g1_i1_orf1;TRINITY_DN146236_c0_g1_i1_orf1;TRINITY_DN8405_c0_g1_i4_orf1;TRINITY_DN486_c0_g1_i5_orf1;TRINITY_DN106476_c0_g1_i3_orf1;TRINITY_DN2879_c0_g1_i4_orf1;TRINITY_DN96557_c0_g1_i1_orf1;TRINITY_DN2172_c0_g2_i8_orf1;TRINITY_DN5630_c4_g1_i2_orf1;TRINITY_DN12777_c0_g1_i5_orf1;TRINITY_DN82_c0_g1_i1_orf1;TRINITY_DN4207_c0_g1_i1_orf1;TRINITY_DN11693_c0_g1_i6_orf1
biological_process	intracellular transport	GO:0046907	26	26/1363	TRINITY_DN960_c1_g1_i6_orf1;TRINITY_DN54586_c1_g1_i1_orf1;TRINITY_DN3209_c0_g1_i1_orf1;TRINITY_DN35377_c0_g1_i3_orf1;TRINITY_DN3821_c1_g1_i7_orf1;TRINITY_DN6231_c0_g1_i6_orf1;TRINITY_DN46409_c0_g1_i1_orf1;TRINITY_DN31584_c0_g2_i2_orf1;TRINITY_DN578_c0_g1_i3_orf1;TRINITY_DN12432_c0_g1_i2_orf1;TRINITY_DN14677_c0_g2_i3_orf1;TRINITY_DN855_c0_g1_i5_orf1;TRINITY_DN36230_c0_g1_i1_orf1;TRINITY_DN45037_c0_g1_i1_orf1;TRINITY_DN13118_c0_g1_i6_orf1;TRINITY_DN1447_c0_g1_i5_orf1;TRINITY_DN8405_c0_g1_i4_orf1;TRINITY_DN486_c0_g1_i5_orf1;TRINITY_DN106476_c0_g1_i3_orf1;TRINITY_DN578_c0_g1_i5_orf1;TRINITY_DN96557_c0_g1_i1_orf1;TRINITY_DN6535_c0_g1_i3_orf1;TRINITY_DN12777_c0_g1_i5_orf1;TRINITY_DN5558_c0_g1_i4_orf1;TRINITY_DN82_c0_g1_i1_orf1;TRINITY_DN4207_c0_g1_i1_orf1
biological_process	localization within membrane	GO:0051668	1	1/1363	TRINITY_DN96557_c0_g1_i1_orf1

biological_process	cellular component biogenesis	GO:0044085	4	4/1363	TRINITY_DN102260_c0_g1_i1_orf1;TRINITY_DN14391_c1_g1_i2_orf1;TRINITY_DN31225_c0_g1_i1_orf1;TRINITY_DN9101_c0_g2_i1_orf1
biological_process	cellular component organization	GO:0016043	53	53/1363	TRINITY_DN1749_c0_g2_i2_orf1;TRINITY_DN59804_c0_g1_i1_orf1;TRINITY_DN25960_c0_g1_i1_orf1;TRINITY_DN20442_c0_g2_i1_orf1;TRINITY_DN18009_c0_g1_i1_orf1;TRINITY_DN46409_c0_g1_i1_orf1;TRINITY_DN89083_c0_g1_i1_orf1;TRINITY_DN34426_c0_g1_i1_orf1;TRINITY_DN104596_c0_g1_i1_orf1;TRINITY_DN27751_c0_g2_i1_orf1;TRINITY_DN4237_c1_g1_i5_orf1;TRINITY_DN71832_c0_g1_i1_orf1;TRINITY_DN4798_c0_g1_i3_orf1;TRINITY_DN1054_c0_g1_i8_orf1;TRINITY_DN43412_c0_g1_i2_orf1;TRINITY_DN4010_c0_g2_i1_orf1;TRINITY_DN40911_c0_g1_i1_orf1;TRINITY_DN101682_c0_g1_i1_orf1;TRINITY_DN11069_c0_g2_i1_orf1;TRINITY_DN104297_c0_g1_i1_orf1;TRINITY_DN142442_c0_g1_i1_orf1;TRINITY_DN4108_c0_g1_i6_orf1;TRINITY_DN1298_c0_g1_i3_orf1;TRINITY_DN20009_c0_g1_i1_orf1;TRINITY_DN17045_c0_g2_i3_orf1;TRINITY_DN152_c0_g1_i4_orf1;TRINITY_DN30273_c1_g1_i1_orf1;TRINITY_DN50085_c0_g1_i1_orf1;TRINITY_DN841_c0_g1_i8_orf1;TRINITY_DN146236_c0_g1_i1_orf1;TRINITY_DN37986_c0_g1_i2_orf1;TRINITY_DN235_c0_g3_i1_orf1;TRINITY_DN77480_c0_g1_i2_orf1;TRINITY_DN85476_c0_g1_i1_orf1;TRINITY_DN140538_c0_g2_i1_orf1;TRINITY_DN96557_c0_g1_i1_orf1;TRINITY_DN110231_c0_g1_i1_orf1;TRINITY_DN1860_c0_g1_i1_orf1;TRINITY_DN27276_c0_g1_i5_orf1;TRINITY_DN6358_c0_g1_i5_orf1;TRINITY_DN3847_c1_g1_i1_orf1;TRINITY_DN3459_c0_g1_i4_orf1;TRINITY_DN96739_c0_g1_i1_orf1;TRINITY_DN23020_c0_g1_i1_orf1;TRINITY_DN42854_c0_g3_i2_orf1;TRINITY_DN7647_c0_g1_i4_orf1;TRINITY_DN3702_c0_g1_i1_orf1;TRINITY_DN10455_c0_g1_i2_orf1;TRINITY_DN24266_c0_g2_i2_orf1;TRINITY_DN23502_c0_g1_i1_orf1;TRINITY_DN2848_c0_g1_i2_orf1;TRINITY_DN80424_c0_g1_i1_orf1;TRINITY_DN31119_c0_g1_i1_orf1
biological_process	cell migration	GO:0016477	4	4/1363	TRINITY_DN110231_c0_g1_i1_orf1;TRINITY_DN96739_c0_g1_i1_orf1;TRINITY_DN31584_c0_g2_i2_orf1;TRINITY_DN9724_c0_g1_i4_orf1
biological_process	microtubule-based movement	GO:0007018	1	1/1363	TRINITY_DN14298_c0_g3_i1_orf1
biological_process	microtubule cytoskeleton organization	GO:0000226	3	3/1363	TRINITY_DN2848_c0_g1_i2_orf1;TRINITY_DN25960_c0_g1_i1_orf1;TRINITY_DN31119_c0_g1_i1_orf1
biological_process	cellular response to chemical stimulus	GO:0070887	3	3/1363	TRINITY_DN46409_c0_g1_i1_orf1;TRINITY_DN2848_c0_g1_i2_orf1;TRINITY_DN20009_c0_g1_i1_orf1
biological_process	cellular response to stress	GO:0033554	21	21/1363	TRINITY_DN59804_c0_g1_i1_orf1;TRINITY_DN17271_c0_g1_i1_orf1;TRINITY_DN3092_c0_g1_i2_orf1;TRINITY_DN2971_c0_g1_i1_orf1;TRINITY_DN19866_c0_g1_i4_orf1;TRINITY_DN140212_c0_g1_i1_orf1;TRINITY_DN40434_c0_g1_i2_orf1;TRINITY_DN346_c0_g1_i7_orf1;TRINITY_DN77318_c0_g2_i1_orf1;TRINITY_DN5238_c0_g1_i2_orf1;TRINITY_DN46409_c0_g1_i1_orf1;TRINITY_DN123184_c0_g1_i1_orf1;TRINITY_DN31584_c0_g2_i2_orf1;TRINITY_DN2647_c0_g1_i3_orf1;TRINITY_DN51658_c0_g1_i1_orf1;TRINITY_DN20238_c0_g1_i7_orf1;TRINITY_DN1091_c0_g1_i1_orf1;TRINITY_DN1091_c0_g3_i1_orf1;TRINITY_DN104507_c0_g1_i2_orf1;TRINITY_DN2054_c0_g1_i1_orf1;TRINITY_DN27958_c0_g1_i1_orf1
biological_process	cellular response to biotic stimulus	GO:0071216	1	1/1363	TRINITY_DN20009_c0_g1_i1_orf1
biological_process	establishment or maintenance of cytoskeleton polarity	GO:0030952	1	1/1363	TRINITY_DN25960_c0_g1_i1_orf1
biological_process	cell surface receptor signaling pathway	GO:0007166	3	3/1363	TRINITY_DN1008_c0_g1_i2_orf1;TRINITY_DN2170_c0_g2_i1_orf1;TRINITY_DN15247_c0_g1_i2_orf1
biological_process	immune response-regulating signaling pathway	GO:0002764	2	2/1363	TRINITY_DN46409_c0_g1_i1_orf1;TRINITY_DN2170_c0_g2_i1_orf1
biological_process	SMAD protein signal transduction	GO:0060395	1	1/1363	TRINITY_DN20009_c0_g1_i1_orf1
biological_process	G protein-coupled receptor signaling pathway	GO:0007186	1	1/1363	TRINITY_DN42854_c0_g3_i2_orf1
biological_process	intracellular signal transduction	GO:0035556	6	6/1363	TRINITY_DN54477_c0_g1_i1_orf1;TRINITY_DN15478_c0_g1_i1_orf1;TRINITY_DN31584_c0_g2_i2_orf1;TRINITY_DN804_c0_g1_i7_orf1;TRINITY_DN2770_c0_g2_i4_orf1;TRINITY_DN9724_c0_g1_i4_orf1
biological_process	apoptotic signaling pathway	GO:0097190	1	1/1363	TRINITY_DN96739_c0_g1_i1_orf1
biological_process	meiotic cell cycle	GO:0051321	1	1/1363	TRINITY_DN123184_c0_g1_i1_orf1
biological_process	cell differentiation	GO:0030154	11	11/1363	TRINITY_DN140423_c0_g1_i2_orf1;TRINITY_DN52395_c0_g2_i2_orf1;TRINITY_DN50725_c0_g1_i6_orf1;TRINITY_DN42461_c0_g1_i4_orf1;TRINITY_DN4550_c1_g1_i19_orf1;TRINITY_DN23746_c0_g1_i2_orf1;TRINITY_DN96739_c0_g1_i1_orf1;TRINITY_DN26790_c0_g1_i3_orf1;TRINITY_DN20009_c0_g1_i1_orf1;TRINITY_DN3158_c0_g1_i5_orf1;TRINITY_DN140538_c0_g2_i1_orf1
biological_process	cellular component morphogenesis	GO:0032989	1	1/1363	TRINITY_DN42854_c0_g3_i2_orf1
biological_process	cell development	GO:0048468	8	8/1363	TRINITY_DN1749_c0_g2_i2_orf1;TRINITY_DN1710_c0_g2_i2_orf1;TRINITY_DN101682_c0_g1_i1_orf1;TRINITY_DN104596_c0_g1_i1_orf1;TRINITY_DN20009_c0_g1_i1_orf1;TRINITY_DN152_c0_g1_i4_orf1;TRINITY_DN71832_c0_g1_i1_orf1;TRINITY_DN9724_c0_g
biological_process	cell maturation	GO:0048469	1	1/1363	TRINITY_DN1272_c1_g1_i4_orf1
biological_process	protein transmembrane transport	GO:0071806	3	3/1363	TRINITY_DN46409_c0_g1_i1_orf1;TRINITY_DN4207_c0_g1_i1_orf1;TRINITY_DN106476_c0_g1_i3_orf1
biological_process	mitochondrial transmembrane transport	GO:1990542	3	3/1363	TRINITY_DN46409_c0_g1_i1_orf1;TRINITY_DN4207_c0_g1_i1_orf1;TRINITY_DN106476_c0_g1_i3_orf1
biological_process	ion transmembrane transport	GO:0034220	2	2/1363	TRINITY_DN96739_c0_g1_i1_orf1;TRINITY_DN8306_c0_g1_i4_orf1
biological_process	actin cytoskeleton organization	GO:0030036	2	2/1363	TRINITY_DN4010_c0_g2_i1_orf1;TRINITY_DN235_c0_g3_i1_orf1
biological_process	anatomical structure maturation	GO:0071695	1	1/1363	TRINITY_DN1272_c1_g1_i4_orf1
biological_process	cellular component assembly involved in morphogenesis	GO:0010927	1	1/1363	TRINITY_DN235_c0_g3_i1_orf1
biological_process	embryonic morphogenesis	GO:0048598	1	1/1363	TRINITY_DN142442_c0_g1_i1_orf1
biological_process	animal organ morphogenesis	GO:0009887	1	1/1363	TRINITY_DN23746_c0_g1_i2_orf1
biological_process	cell morphogenesis	GO:0000902	1	1/1363	TRINITY_DN34426_c0_g1_i1_orf1
biological_process	system development	GO:0048731	3	3/1363	TRINITY_DN42854_c0_g3_i2_orf1;TRINITY_DN1710_c0_g2_i2_orf1;TRINITY_DN142442_c0_g1_i1_orf1
biological_process	hippocampus development	GO:0021766	1	1/1363	TRINITY_DN31584_c0_g2_i2_orf1
biological_process	animal organ development	GO:0048513	6	6/1363	TRINITY_DN8044_c0_g1_i2_orf1;TRINITY_DN101682_c0_g1_i1_orf1;TRINITY_DN26790_c0_g1_i3_orf1;TRINITY_DN34426_c0_g1_i1_orf1;TRINITY_DN104596_c0_g1_i1_orf1;TRINITY_DN71832_c0_g1_i1_orf1
biological_process	muscle structure development	GO:0061061	2	2/1363	TRINITY_DN26790_c0_g1_i3_orf1;TRINITY_DN8044_c0_g1_i2_orf1
biological_process	cerebral cortex development	GO:0021987	1	1/1363	TRINITY_DN31584_c0_g2_i2_orf1
biological_process	tissue development	GO:0009888	1	1/1363	TRINITY_DN142442_c0_g1_i1_orf1
biological_process	nervous system process	GO:0050877	1	1/1363	TRINITY_DN142442_c0_g1_i1_orf1
biological_process	envenomation resulting in modulation of process in another organism	GO:0035738	1	1/1363	TRINITY_DN1215_c0_g1_i2_orf1
biological_process	response to bacterium	GO:0009617	7	7/1363	TRINITY_DN14904_c0_g1_i1_orf1;TRINITY_DN8685_c0_g1_i5_orf1;TRINITY_DN14019_c0_g1_i5_orf1;TRINITY_DN16840_c1_g1_i1_orf1;TRINITY_DN195_c8_g1_i1_orf1;TRINITY_DN1666_c0_g1_i2_orf1;TRINITY_DN5880_c0_g2_i2_orf1
biological_process	response to fungus	GO:0009620	1	1/1363	TRINITY_DN2848_c0_g1_i2_orf1
biological_process	defense response to other organism	GO:0098542	15	15/1363	TRINITY_DN14904_c0_g1_i1_orf1;TRINITY_DN1534_c0_g1_i3_orf1;TRINITY_DN827_c1_g1_i1_orf1;TRINITY_DN8685_c0_g1_i5_orf1;TRINITY_DN14019_c0_g1_i5_orf1;TRINITY_DN16840_c1_g1_i1_orf1;TRINITY_DN195_c8_g1_i1_orf1;TRINITY_DN195_c4_g1_i1_orf1;TRINITY_DN5880_c0_g2_i2_orf1;TRINITY_DN2170_c0_g2_i1_orf1;TRINITY_DN670_c0_g1_i15_orf1;TRINITY_DN1666_c0_g1_i2_orf1;TRINITY_DN9044_c0_g1_i2_orf1;TRINITY_DN2848_c0_g1_i2_orf1;TRINITY_DN9724_c0_g1_i4_orf1
biological_process	biological process involved in interaction with symbiont	GO:0051702	2	2/1363	TRINITY_DN46409_c0_g1_i1_orf1;TRINITY_DN2848_c0_g1_i2_orf1
biological_process	biological process involved in interaction with host	GO:0051701	1	1/1363	TRINITY_DN96557_c0_g1_i1_orf1
biological_process	establishment of organelle localization	GO:0051656	1	1/1363	TRINITY_DN96557_c0_g1_i1_orf1
biological_process	chromosome localization	GO:0050000	1	1/1363	TRINITY_DN96557_c0_g1_i1_orf1
biological_process	lipid storage	GO:0019915	1	1/1363	TRINITY_DN11069_c0_g2_i1_orf1
biological_process	establishment of protein localization	GO:0045184	22	22/1363	TRINITY_DN3209_c0_g1_i1_orf1;TRINITY_DN46409_c0_g1_i1_orf1;TRINITY_DN31584_c0_g2_i2_orf1;TRINITY_DN14677_c0_g2_i3_orf1;TRINITY_DN36230_c0_g1_i1_orf1;TRINITY_DN65299_c0_g4_i1_orf1;TRINITY_DN13118_c0_g1_i6_orf1;TRINITY_DN1447_c0_g1_i5_orf1;TRINITY_DN3698_c0_g1_i4_orf1;TRINITY_DN6680_c0_g1_i1_orf1;TRINITY_DN146236_c0_g1_i1_orf1;TRINITY_DN8405_c0_g1_i4_orf1;TRINITY_DN486_c0_g1_i5_orf1;TRINITY_DN106476_c0_g1_i3_orf1;TRINITY_DN2879_c0_g1_i4_orf1;TRINITY_DN96557_c0_g1_i1_orf1;TRINITY_DN2172_c0_g2_i8_orf1;TRINITY_DN5630_c4_g1_i2_orf1;TRINITY_DN12777_c0_g1_i5_orf1;TRINITY_DN82_c0_g1_i1_orf1;TRINITY_DN4207_c0_g1_i1_orf1;TRINITY_DN11693_c0_g1_i6_orf1
biological_process	establishment of localization in cell	GO:0051649	28	28/1363	TRINITY_DN960_c1_g1_i6_orf1;TRINITY_DN54586_c1_g1_i1_orf1;TRINITY_DN3209_c0_g1_i1_orf1;TRINITY_DN35377_c0_g1_i3_orf1;TRINITY_DN3821_c1_g1_i7_orf1;TRINITY_DN6231_c0_g1_i6_orf1;TRINITY_DN46409_c0_g1_i1_orf1;TRINITY_DN31584_c0_g2_i2_orf1;TRINITY_DN578_c0_g1_i3_orf1;TRINITY_DN12432_c0_g1_i2_orf1;TRINITY_DN14677_c0_g2_i3_orf1;TRINITY_DN486_c0_g1_i5_orf1;TRINITY_DN855_c0_g1_i5_orf1;TRINITY_DN36230_c0_g1_i1_orf1;TRINITY_DN45037_c0_g1_i1_orf1;TRINITY_DN29144_c0_g3_i1_orf1;TRINITY_DN13118_c0_g1_i6_orf1;TRINITY_DN1447_c0_g1_i5_orf1;TRINITY_DN8405_c0_g1_i4_orf1;TRINITY_DN42185_c0_g1_i7_orf1;TRINITY_DN106476_c0_g1_i3_orf1;TRINITY_DN578_c0_g1_i5_orf1;TRINITY_DN96557_c0_g1_i1_orf1;TRINITY_DN6535_c0_g1_i3_orf1;TRINITY_DN12777_c0_g1_i5_orf1;TRINITY_DN5558_c0_g1_i4_orf1;TRINITY_DN82_c0_g1_i1_orf1;TRINITY_DN4207_c0_g1_i1_orf1
biological_process	establishment of RNA localization	GO:0051236	3	3/1363	TRINITY_DN6535_c0_g1_i3_orf1;TRINITY_DN2879_c0_g1_i4_orf1;TRINITY_DN6680_c0_g1_i1_orf1
biological_process	transport	GO:0006810	59	59/1363	TRINITY_DN960_c1_g1_i6_orf1;TRINITY_DN65681_c0_g1_i1_orf1;TRINITY_DN13923_c0_g2_i1_orf1;TRINITY_DN13118_c0_g1_i6_orf1;TRINITY_DN3209_c0_g1_i1_orf1;TRINITY_DN35377_c0_g1_i3_orf1;TRINITY_DN3821_c1_g1_i7_orf1;TRINITY_DN6231_c0_g1_i6_orf1;TRINITY_DN33452_c0_g1_i3_orf1;TRINITY_DN46409_c0_g1_i1_orf1;TRINITY_DN31584_c0_g2_i2_orf1;TRINITY_DN578_c0_g1_i3_orf1;TRINITY_DN12432_c0_g1_i2_orf1;TRINITY_DN14677_c0_g2_i3_orf1;TRINITY_DN1895_c0_g1_i2_orf1;TRINITY_DN9239_c0_g1_i1_orf1;TRINITY_DN19521_c0_g1_i1_orf1;TRINITY_DN1423_c0_g1_i4_orf1;TRINITY_DN54586_c1_g1_i1_orf1;TRINITY_DN3219_c0_g1_i6_orf1;TRINITY_DN36230_c0_g1_i1_orf1;TRINITY_DN2879_c0_g1_i4_orf1;TRINITY_DN45037_c0_g1_i1_orf1;TRINITY_DN29144_c0_g3_i1_orf1;TRINITY_DN9239_c0_g2_i2_orf1;TRINITY_DN5666_c0_g1_i2_orf1;TRINITY_DN61711_c0_g1_i1_orf1;TRINITY_DN46625_c0_g1_i1_orf1;TRINITY_DN29934_c0_g1_i6_orf1;TRINITY_DN1447_c0_g1_i5_orf1;TRINITY_DN3698_c0_g1_i4_orf1;TRINITY_DN1423_c0_g1_i8_orf1;TRINITY_DN136031_c0_g1_i7_orf1;TRINITY_DN6680_c0_g1_i1_orf1;TRINITY_DN146236_c0_g1_i1_orf1;TRINITY_DN96739_c0_g1_i1_orf1;TRINITY_DN12286_c1_g1_i2_orf1;TRINITY_DN8405_c0_g1_i4_orf1;TRINITY_DN42185_c0_g1_i7_orf1;TRINITY_DN3132_c0_g1_i10_orf1;TRINITY_DN486_c0_g1_i5_orf1;TRINITY_DN106476_c0_g1_i3_orf1;TRINITY_DN65299_c0_g4_i1_orf1;TRINITY_DN8306_c0_g1_i4_orf1;TRINITY_DN578_c0_g1_i5_orf1;TRINITY_DN96557_c0_g1_i1_orf1;TRINITY_DN6535_c0_g1_i3_orf1;TRINITY_DN855_c0_g1_i5_orf1;TRINITY_DN2172_c0_g2_i8_orf1;TRINITY_DN33452_c0_g1_i1_orf1;TRINITY_DN5630_c4_g1_i2_orf1;TRINITY_DN12777_c0_g1_i5_orf1;TRINITY_DN81488_c0_g1_i1_orf1;TRINITY_DN5558_c0_g1_i4_orf1;TRINITY_DN82_c0_g1_i1_orf1;TRINITY_DN113353_c0_g1_i1_orf1;TRINITY_DN5064_c0_g1_i4_orf1;TRINITY_DN4207_c0_g1_i1_orf1;TRINITY_DN11693_c0_g1_i6_orf1
biological_process	non-lytic viral release	GO:0046753	1	1/1363	TRINITY_DN96557_c0_g1_i1_orf1
biological_process	viral RNA genome replication	GO:0039694	1	1/1363	TRINITY_DN4408_c6_g1_i1_orf1
biological_process	viral budding via host ESCRT complex	GO:0039702	1	1/1363	TRINITY_DN96557_c0_g1_i1_orf1
biological_process	viral budding from plasma membrane	GO:0046761	1	1/1363	TRINITY_DN96557_c0_g1_i1_orf1
biological_process	response to external biotic stimulus	GO:0043207	21	21/1363	TRINITY_DN14904_c0_g1_i1_orf1;TRINITY_DN1534_c0_g1_i3_orf1;TRINITY_DN827_c1_g1_i1_orf1;TRINITY_DN8685_c0_g1_i5_orf1;TRINITY_DN14019_c0_g1_i5_orf1;TRINITY_DN16840_c1_g1_i1_orf1;TRINITY_DN195_c8_g1_i1_orf1;TRINITY_DN20009_c0_g1_i1_orf1;TRINITY_DN5880_c0_g2_i2_orf1;TRINITY_DN195_c4_g1_i1_orf1;TRINITY_DN9724_c0_g1_i4_orf1;TRINITY_DN109503_c0_g1_i4_orf1;TRINITY_DN2170_c0_g2_i1_orf1;TRINITY_DN2407_c0_g1_i2_orf1;TRINITY_DN670_c0_g1_i15_orf1;TRINITY_DN9044_c0_g1_i2_orf1;TRINITY_DN1666_c0_g1_i2_orf1;TRINITY_DN86772_c0_g1_i3_orfp1;TRINITY_DN2848_c0_g1_i2_orf1;TRINITY_DN4802_c0_g1_i4_orf1;TRINITY_DN66287_c0_g1_i1_orfp1
biological_process	detection of biotic stimulus	GO:0009595	2	2/1363	TRINITY_DN8685_c0_g1_i5_orf1;TRINITY_DN5880_c0_g2_i2_orf1
biological_process	response to extracellular stimulus	GO:0009991	4	4/1363	TRINITY_DN140212_c0_g1_i1_orf1;TRINITY_DN1091_c0_g1_i1_orf1;TRINITY_DN1091_c0_g3_i1_orf1;TRINITY_DN2054_c0_g1_i1_orf1
biological_process	cellular response to external stimulus	GO:0071496	4	4/1363	TRINITY_DN140212_c0_g1_i1_orf1;TRINITY_DN1091_c0_g1_i1_orf1;TRINITY_DN1091_c0_g3_i1_orf1;TRINITY_DN2054_c0_g1_i1_orf1
biological_process	cellular response to endogenous stimulus	GO:0071495	1	1/1363	TRINITY_DN20009_c0_g1_i1_orf1
biological_process	response to hypoxia	GO:0001666	1	1/1363	TRINITY_DN140538_c0_g2_i1_orf1
biological_process	response to topologically incorrect protein	GO:0035966	1	1/1363	TRINITY_DN46409_c0_g1_i1_orf1
biological_process	response to cold	GO:0009409	1	1/1363	TRINITY_DN46409_c0_g1_i1_orf1
biological_process	response to heat	GO:0009408	1	1/1363	TRINITY_DN31584_c0_g2_i2_orf1
biological_process	defense response	GO:0006952	20	20/1363	TRINITY_DN14904_c0_g1_i1_orf1;TRINITY_DN1534_c0_g1_i3_orf1;TRINITY_DN827_c1_g1_i1_orf1;TRINITY_DN8685_c0_g1_i5_orf1;TRINITY_DN14019_c0_g1_i5_orf1;TRINITY_DN16840_c1_g1_i1_orf1;TRINITY_DN195_c8_g1_i1_orf1;TRINITY_DN2170_c0_g2_i1_orf1;TRINITY_DN195_c4_g1_i1_orf1;TRINITY_DN9724_c0_g1_i4_orf1;TRINITY_DN5880_c0_g2_i2_orf1;TRINITY_DN31163_c1_g1_i4_orf1;TRINITY_DN2407_c0_g1_i2_orf1;TRINITY_DN670_c0_g1_i15_orf1;TRINITY_DN9044_c0_g1_i2_orf1;TRINITY_DN1666_c0_g1_i2_orf1;TRINITY_DN86772_c0_g1_i3_orfp1;TRINITY_DN2848_c0_g1_i2_orf1;TRINITY_DN4802_c0_g1_i4_orf1;TRINITY_DN66287_c0_g1_i1_orfp1
biological_process	response to oxidative stress	GO:0006979	3	3/1363	TRINITY_DN21420_c0_g1_i2_orf1;TRINITY_DN1622_c0_g1_i6_orf1;TRINITY_DN3321_c0_g1_i3_orf1
biological_process	response to oxygen-containing compound	GO:1901700	1	1/1363	TRINITY_DN20009_c0_g1_i1_orf1
biological_process	response to nitrogen compound	GO:1901698	2	2/1363	TRINITY_DN20009_c0_g1_i1_orf1;TRINITY_DN51658_c0_g1_i1_orf1
biological_process	response to organic substance	GO:0010033	7	7/1363	TRINITY_DN8685_c0_g1_i5_orf1;TRINITY_DN46409_c0_g1_i1_orf1;TRINITY_DN20009_c0_g1_i1_orf1;TRINITY_DN51658_c0_g1_i1_orf1;TRINITY_DN2227_c0_g1_i5_orf1;TRINITY_DN2848_c0_g1_i2_orf1;TRINITY_DN5880_c0_g2_i2_orf1
biological_process	response to temperature stimulus	GO:0009266	2	2/1363	TRINITY_DN46409_c0_g1_i1_orf1;TRINITY_DN31584_c0_g2_i2_orf1
biological_process	response to oxygen levels	GO:0070482	1	1/1363	TRINITY_DN140538_c0_g2_i1_orf1
biological_process	detection of chemical stimulus	GO:0009593	2	2/1363	TRINITY_DN8685_c0_g1_i5_orf1;TRINITY_DN5880_c0_g2_i2_orf1
cellular_component	nucleosome	GO:0000786	3	3/1363	TRINITY_DN20442_c0_g2_i1_orf1;TRINITY_DN96801_c0_g1_i1_orf1;TRINITY_DN6358_c0_g1_i5_orf1
cellular_component	Mre11 complex	GO:0030870	1	1/1363	TRINITY_DN123184_c0_g1_i1_orf1
cellular_component	mRNA cleavage factor complex	GO:0005849	1	1/1363	TRINITY_DN2718_c0_g1_i6_orf1
cellular_component	SWI/SNF superfamily-type complex	GO:0070603	1	1/1363	TRINITY_DN3649_c0_g1_i6_orf1
cellular_component	integrator complex	GO:0032039	1	1/1363	TRINITY_DN5008_c0_g1_i1_orf1
cellular_component	U2AF complex	GO:0089701	1	1/1363	TRINITY_DN51968_c0_g1_i1_orf1
cellular_component	spliceosomal complex	GO:0005681	9	9/1363	TRINITY_DN27276_c0_g1_i5_orf1;TRINITY_DN51568_c0_g1_i1_orf1;TRINITY_DN3459_c0_g1_i4_orf1;TRINITY_DN47575_c0_g1_i1_orf1;TRINITY_DN13055_c0_g1_i5_orf1;TRINITY_DN43412_c0_g1_i2_orf1;TRINITY_DN142652_c0_g1_i1_orf1;TRINITY_DN4135_c0_g1_i5_orf1;TRINITY_DN23502_c0_g1_i1_orf1
cellular_component	BRISC complex	GO:0070552	1	1/1363	TRINITY_DN19866_c0_g1_i4_orf1
cellular_component	histone acetyltransferase complex	GO:0000123	1	1/1363	TRINITY_DN59804_c0_g1_i1_orf1

cellular_component	exon-exon junction complex	GO:0035145	1	1/1363	TRINITY_DN5507_c0_g1_i1_orf1
cellular_component	small nuclear ribonucleoprotein complex	GO:0030532	2	2/1363	TRINITY_DN4135_c0_g1_i5_orf1;TRINITY_DN43412_c0_g1_i2_orf1
cellular_component	BRCA1-A complex	GO:0070531	1	1/1363	TRINITY_DN19866_c0_g1_i4_orf1
cellular_component	RNA polymerase II transcription regulator complex	GO:0090575	1	1/1363	TRINITY_DN346_c0_g1_i7_orf1
cellular_component	carboxy-terminal domain protein kinase complex	GO:0032806	1	1/1363	TRINITY_DN346_c0_g1_i7_orf1
cellular_component	nuclear pore outer ring	GO:0031080	1	1/1363	TRINITY_DN6680_c0_g1_i1_orf1
cellular_component	nuclear pore	GO:0005643	2	2/1363	TRINITY_DN96557_c0_g1_i1_orf1;TRINITY_DN2879_c0_g1_i4_orf1
cellular_component	ESCRT III complex	GO:0000815	1	1/1363	TRINITY_DN96557_c0_g1_i1_orf1
cellular_component	ESCRT I complex	GO:0000813	1	1/1363	TRINITY_DN4013_c0_g1_i4_orf1
cellular_component	transmembrane transporter complex	GO:1902495	5	5/1363	TRINITY_DN16408_c0_g1_i1_orf1;TRINITY_DN19521_c0_g1_i1_orf1;TRINITY_DN4270_c0_g1_i1_orf1;TRINITY_DN29934_c0_g1_i6_orf1;TRINITY_DN20558_c0_g1_i2_orf1
cellular_component	proteasome core complex	GO:0005839	1	1/1363	TRINITY_DN113327_c0_g1_i2_orf1
cellular_component	cytochrome complex	GO:0070069	2	2/1363	TRINITY_DN4270_c0_g1_i1_orf1;TRINITY_DN14073_c0_g1_i1_orf1
cellular_component	oxidoreductase complex	GO:1990204	2	2/1363	TRINITY_DN6199_c2_g1_i3_orf1;TRINITY_DN4270_c0_g1_i1_orf1
cellular_component	transferase complex	GO:1990234	8	8/1363	TRINITY_DN59804_c0_g1_i1_orf1;TRINITY_DN143496_c0_g1_i1_orf1;TRINITY_DN15265_c0_g1_i1_orf1;TRINITY_DN346_c0_g1_i7_orf1;TRINITY_DN12594_c0_g1_i1_orf1;TRINITY_DN2299_c0_g1_i3_orf1;TRINITY_DN51658_c0_g1_i1_orf1;TRINITY_DN89613_c0
cellular_component	peptidase complex	GO:1905368	5	5/1363	TRINITY_DN6684_c0_g1_i4_orf1;TRINITY_DN34479_c0_g1_i2_orf1;TRINITY_DN49047_c0_g1_i2_orf1;TRINITY_DN19260_c0_g1_i5_orf1;TRINITY_DN32359_c0_g2_i1_orf1
cellular_component	aminoacyl-tRNA synthetase multienzyme complex	GO:0017101	3	3/1363	TRINITY_DN22572_c0_g1_i1_orf1;TRINITY_DN825_c23_g1_i5_orf1;TRINITY_DN5857_c0_g1_i13_orf1
cellular_component	ATPase complex	GO:1904949	1	1/1363	TRINITY_DN3649_c0_g1_i6_orf1
cellular_component	lipopolysaccharide receptor complex	GO:0046696	1	1/1363	TRINITY_DN46409_c0_g1_i1_orf1
cellular_component	plasma membrane protein complex	GO:0098797	5	5/1363	TRINITY_DN16408_c0_g1_i1_orf1;TRINITY_DN19521_c0_g1_i1_orf1;TRINITY_DN12777_c0_g1_i5_orf1;TRINITY_DN29934_c0_g1_i6_orf1;TRINITY_DN8405_c0_g1_i4_orf1
cellular_component	ER ubiquitin ligase complex	GO:0000835	1	1/1363	TRINITY_DN51658_c0_g1_i1_orf1
cellular_component	retromer complex	GO:0030904	1	1/1363	TRINITY_DN36230_c0_g1_i1_orf1
cellular_component	EMC complex	GO:0072546	2	2/1363	TRINITY_DN3838_c0_g1_i8_orf1;TRINITY_DN9002_c0_g1_i1_orf1
cellular_component	clathrin complex	GO:0071439	1	1/1363	TRINITY_DN8405_c0_g1_i4_orf1
cellular_component	respiratory chain complex	GO:0098803	2	2/1363	TRINITY_DN4270_c0_g1_i1_orf1;TRINITY_DN14073_c0_g1_i1_orf1
cellular_component	inner mitochondrial membrane protein complex	GO:0098800	3	3/1363	TRINITY_DN4207_c0_g1_i1_orf1;TRINITY_DN4270_c0_g1_i1_orf1;TRINITY_DN14073_c0_g1_i1_orf1
cellular_component	membrane coat	GO:0030117	6	6/1363	TRINITY_DN96557_c0_g1_i1_orf1;TRINITY_DN1447_c0_g1_i5_orf1;TRINITY_DN3209_c0_g1_i1_orf1;TRINITY_DN12777_c0_g1_i5_orf1;TRINITY_DN8405_c0_g1_i4_orf1;TRINITY_DN14677_c0_g2_i3_orf1
cellular_component	AP-type membrane coat adaptor complex	GO:0030119	2	2/1363	TRINITY_DN13118_c0_g1_i6_orf1;TRINITY_DN486_c0_g1_i5_orf1
cellular_component	mitochondrial large ribosomal subunit	GO:0005762	1	1/1363	TRINITY_DN1313_c0_g1_i2_orf1
cellular_component	exocyst	GO:0000145	1	1/1363	TRINITY_DN1895_c0_g1_i2_orf1
cellular_component	TRAPP complex	GO:0030008	1	1/1363	TRINITY_DN45037_c0_g1_i1_orf1
cellular_component	polysome	GO:0005844	1	1/1363	TRINITY_DN20009_c0_g1_i1_orf1
cellular_component	translation preinitiation complex	GO:0070993	4	4/1363	TRINITY_DN17045_c0_g2_i3_orf1;TRINITY_DN4237_c1_g1_i5_orf1;TRINITY_DN50085_c0_g1_i1_orf1;TRINITY_DN27751_c0_g2_i1_orf1
cellular_component	translation initiation complex	GO:0070992	1	1/1363	TRINITY_DN142442_c0_g1_i1_orf1
cellular_component	ribosomal subunit	GO:0044391	19	19/1363	TRINITY_DN441_c0_g2_i1_orf1;TRINITY_DN121893_c0_g1_i1_orf1;TRINITY_DN9101_c0_g2_i1_orf1;TRINITY_DN2682_c0_g1_i4_orf1;TRINITY_DN95056_c0_g2_i2_orf1;TRINITY_DN7613_c1_g2_i1_orf1;TRINITY_DN30300_c0_g2_i1_orf1;TRINITY_DN36893_c0_g1_i1_orf1;TRINITY_DN79734_c0_g2_i3_orf1;TRINITY_DN1313_c0_g1_i2_orf1;TRINITY_DN142442_c0_g1_i1_orf1;TRINITY_DN137_c0_g1_i1_orf1;TRINITY_DN13732_c0_g2_i3_orf1;TRINITY_DN50787_c0_g2_i2_orf1;TRINITY_DN13651_c0_g1_i2_orf1;TRINITY_DN41645_c0_g1_i1_orf1;TRINITY_DN42646_c0_g2_i1_orf1;TRINITY_DN8949_c0_g1_i2_orf1;TRINITY_DN82324_c0_g1_i4_orf1
cellular_component	mRNA cap binding complex	GO:0005845	1	1/1363	TRINITY_DN41664_c0_g1_i4_orf1
cellular_component	proteasome complex	GO:0000502	5	5/1363	TRINITY_DN6684_c0_g1_i4_orf1;TRINITY_DN34479_c0_g1_i2_orf1;TRINITY_DN49047_c0_g1_i2_orf1;TRINITY_DN19260_c0_g1_i5_orf1;TRINITY_DN32359_c0_g2_i1_orf1
cellular_component	DNA polymerase complex	GO:0042575	1	1/1363	TRINITY_DN89613_c0_g1_i13_orf1
cellular_component	ubiquitin ligase complex	GO:0000151	3	3/1363	TRINITY_DN15265_c0_g1_i1_orf1;TRINITY_DN143496_c0_g1_i1_orf1;TRINITY_DN51658_c0_g1_i1_orf1
cellular_component	RNA polymerase complex	GO:0030880	2	2/1363	TRINITY_DN12594_c0_g1_i1_orf1;TRINITY_DN2299_c0_g1_i3_orf1
cellular_component	protein acetyltransferase complex	GO:0031248	1	1/1363	TRINITY_DN59804_c0_g1_i1_orf1
cellular_component	organelle lumen	GO:0043233	9	9/1363	TRINITY_DN68401_c1_g1_i1_orf1;TRINITY_DN17271_c0_g1_i1_orf1;TRINITY_DN4424_c0_g1_i1_orf1;TRINITY_DN42854_c0_g3_i2_orf1;TRINITY_DN46409_c0_g1_i1_orf1;TRINITY_DN2299_c0_g1_i3_orf1;TRINITY_DN3037_c0_g1_i1_orf1;TRINITY_DN1791_c0_g1_i3_orf1;TRINITY_DN146264_c0_g1_i1_orf1
cellular_component	cell cortex	GO:0005938	1	1/1363	TRINITY_DN2186_c0_g1_i17_orf1
cellular_component	heterochromatin	GO:0000792	1	1/1363	TRINITY_DN24266_c0_g2_i2_orf1
cellular_component	anchored component of membrane	GO:0031225	2	2/1363	TRINITY_DN5406_c0_g2_i1_orf1;TRINITY_DN5553_c0_g1_i4_orf1
cellular_component	integral component of membrane	GO:0016021	154	154/1363	TRINITY_DN13563_c0_g1_i1_orf1;TRINITY_DN9608_c0_g1_i3_orf1;TRINITY_DN13923_c0_g2_i1_orf1;TRINITY_DN827_c1_g1_i1_orf1;TRINITY_DN79868_c0_g1_i1_orf1;TRINITY_DN12227_c0_g2_i3_orf1;TRINITY_DN1239_c0_g1_i3_orf1;TRINITY_DN496_c0_g1_i7_orf1;TRINITY_DN2109_c0_g1_i4_orf1;TRINITY_DN72816_c0_g1_i2_orf1;TRINITY_DN34426_c0_g1_i1_orf1;TRINITY_DN11069_c0_g2_i1_orf1;TRINITY_DN1622_c0_g1_i6_orf1;TRINITY_DN104297_c0_g1_i1_orf1;TRINITY_DN4469_c0_g1_i2_orf1;TRINITY_DN101682_c0_g1_i1_orf1;TRINITY_DN3105_c0_g1_i4_orf1;TRINITY_DN8964_c0_g1_i4_orf1;TRINITY_DN1099_c1_g1_i2_orf1;TRINITY_DN2855_c0_g1_i6_orf1;TRINITY_DN3209_c0_g1_i1_orf1;TRINITY_DN4076_c1_g2_i2_orf1;TRINITY_DN61711_c0_g1_i1_orf1;TRINITY_DN15755_c0_g1_i1_orf1;TRINITY_DN33995_c0_g1_i5_orf1;TRINITY_DN20558_c0_g1_i2_orf1;TRINITY_DN3738_c0_g1_i5_orf1;TRINITY_DN3821_c1_g1_i7_orf1;TRINITY_DN32479_c0_g1_i8_orf1;TRINITY_DN52761_c0_g1_i2_orf1;TRINITY_DN3529_c0_g1_i7_orf1;TRINITY_DN2346_c0_g2_i1_orf1;TRINITY_DN3158_c0_g1_i5_orf1;TRINITY_DN919_c0_g1_i7_orf1;TRINITY_DN10785_c0_g1_i4_orf1;TRINITY_DN2343_c1_g1_i2_orf1;TRINITY_DN10231_c0_g2_i1_orf1;TRINITY_DN11670_c0_g1_i1_orf1;TRINITY_DN4116_c0_g1_i3_orf1;TRINITY_DN52761_c0_g2_i1_orf1;TRINITY_DN1749_c0_g2_i2_orf1;TRINITY_DN15607_c0_g1_i6_orf1;TRINITY_DN8555_c0_g1_i1_orf1;TRINITY_DN25251_c0_g2_i1_orf1;TRINITY_DN8136_c0_g1_i1_orf1;TRINITY_DN957_c0_g1_i18_orf1;TRINITY_DN53760_c0_g1_i1_orf1;TRINITY_DN1054_c0_g1_i8_orf1;TRINITY_DN5153_c1_g1_i1_orf1;TRINITY_DN2326_c0_g1_i1_orf1;TRINITY_DN1008_c0_g1_i2_orf1;TRINITY_DN5991_c0_g1_i6_orf1;TRINITY_DN7464_c0_g1_i14_orf1;TRINITY_DN7336_c0_g1_i13_orf1;TRINITY_DN3227_c0_g1_i5_orf1;TRINITY_DN34821_c0_g1_i4_orf1;TRINITY_DN15318_c0_g1_i1_orf1;TRINITY_DN1720_c0_g1_i3_orf1;TRINITY_DN5408_c0_g1_i5_orf1;TRINITY_DN9243_c0_g1_i4_orf1;TRINITY_DN10650_c0_g1_i1_orf1;TRINITY_DN62707_c0_g1_i1_orf1;TRINITY_DN642_c0_g1_i6_orf1;TRINITY_DN40945_c0_g1_i1_orf1;TRINITY_DN140_c1_g1_i2_orf1;TRINITY_DN30509_c0_g1_i9_orf1;TRINITY_DN23978_c0_g1_i2_orf1;TRINITY_DN2172_c0_g2_i8_orf1;TRINITY_DN28592_c0_g1_i2_orf1;TRINITY_DN11886_c0_g1_i1_orf1;TRINITY_DN9090_c0_g1_i9_orf1;TRINITY_DN6381_c0_g1_i2_orf1;TRINITY_DN51658_c0_g1_i1_orf1;TRINITY_DN49786_c0_g1_i1_orf1;TRINITY_DN98091_c0_g1_i3_orf1;TRINITY_DN7570_c0_g1_i18_orf1;TRINITY_DN6992_c0_g1_i6_orf1;TRINITY_DN18159_c0_g1_i6_orf1;TRINITY_DN1232_c0_g1_i1_orf1;TRINITY_DN24873_c0_g1_i4_orf1;TRINITY_DN2343_c1_g1_i12_orf1;TRINITY_DN79803_c0_g1_i7_orf1;TRINITY_DN7868_c0_g1_i8_orf1;TRINITY_DN46090_c0_g3_i1_orf1;TRINITY_DN7861_c0_g1_i5_orf1;TRINITY_DN27500_c0_g1_i4_orf1;TRINITY_DN5553_c0_g1_i4_orf1;TRINITY_DN1982_c0_g1_i24_orf1;TRINITY_DN42177_c0_g1_i4_orf1;TRINITY_DN48713_c0_g1_i1_orf1;TRINITY_DN20356_c0_g1_i5_orf1;TRINITY_DN38225_c0_g2_i1_orf1;TRINITY_DN1882_c0_g1_i4_orf1;TRINITY_DN19303_c0_g1_i5_orf1;TRINITY_DN4820_c0_g1_i1_orf1;TRINITY_DN60792_c0_g1_i2_orf1;TRINITY_DN12508_c0_g1_i1_orf1;TRINITY_DN32780_c0_g1_i2_orf1;TRINITY_DN14611_c0_g1_i5_orf1;TRINITY_DN6685_c0_g1_i8_orf1;TRINITY_DN31327_c0_g2_i1_orf1;TRINITY_DN6612_c0_g1_i4_orf1;TRINITY_DN5630_c4_g1_i2_orf1;TRINITY_DN1293_c0_g1_i4_orf1;TRINITY_DN42854_c0_g3_i2_orf1;TRINITY_DN1293_c1_g1_i4_orf1;TRINITY_DN14073_c0_g1_i1_orf1;TRINITY_DN18136_c0_g1_i1_orf1;TRINITY_DN4207_c0_g1_i1_orf1;TRINITY_DN2356_c2_g1_i6_orf1;TRINITY_DN2880_c0_g1_i2_orf1;TRINITY_DN17907_c0_g1_i13_orf1;TRINITY_DN8083_c0_g1_i1_orf1;TRINITY_DN54586_c1_g1_i1_orf1;TRINITY_DN36324_c0_g1_i12_orf1;TRINITY_DN35099_c0_g1_i1_orf1;TRINITY_DN230_c1_g1_i3_orf1;TRINITY_DN2392_c0_g2_i1_orf1;TRINITY_DN753_c0_g1_i4_orf1;TRINITY_DN14409_c0_g1_i1_orf1;TRINITY_DN5841_c0_g1_i2_orf1;TRINITY_DN14874_c0_g1_i6_orf1;TRINITY_DN38720_c0_g1_i3_orf1;TRINITY_DN3949_c0_g1_i1_orf1;TRINITY_DN44110_c0_g1_i4_orf1;TRINITY_DN17394_c0_g1_i1_orf1;TRINITY_DN9457_c0_g1_i9_orf1;TRINITY_DN20984_c0_g1_i4_orf1;TRINITY_DN27491_c0_g1_i1_orf1;TRINITY_DN452_c0_g1_i4_orf1;TRINITY_DN11172_c0_g1_i4_orf1;TRINITY_DN4394_c0_g2_i1_orf1;TRINITY_DN1012_c0_g1_i2_orf1;TRINITY_DN1960_c5_g1_i3_orf1;TRINITY_DN4443_c0_g1_i4_orf1;TRINITY_DN12286_c1_g1_i2_orf1;TRINITY_DN5064_c0_g1_i4_orf1;TRINITY_DN351_c14_g1_i2_orf1;TRINITY_DN8454_c0_g1_i4_orf1;TRINITY_DN26130_c0_g1_i1_orf1;TRINITY_DN2967_c0_g1_i4_orf1;TRINITY_DN8306_c0_g1_i4_orf1;TRINITY_DN2618_c0_g1_i3_orf1;TRINITY_DN325_c0_g1_i15_orf1;TRINITY_DN103118_c0_g1_i4_orf1;TRINITY_DN5558_c0_g1_i4_orf1;TRINITY_DN44658_c0_g1_i2_orf1;TRINITY_DN3260_c0_g1_i6_orf1;TRINITY_DN113353_c0_g1_i1_orf1;TRINITY_DN3616_c0_g1_i4_orf1;TRINITY_DN5697_c0_g1_i1_orf1;TRINITY_DN15247_c0_g1_i2_orf1;TRINITY_DN4782_c0_g1_i1_orf1;TRINITY_DN91946_c0_g1_i1_orf1
cellular_component	intrinsic component of organelle membrane	GO:0031300	2	2/1363	TRINITY_DN8964_c0_g1_i4_orf1;TRINITY_DN2172_c0_g2_i8_orf1
cellular_component	external side of plasma membrane	GO:0009897	1	1/1363	TRINITY_DN20339_c0_g1_i3_orf1
cellular_component	cytoplasmic side of membrane	GO:0098562	1	1/1363	TRINITY_DN96557_c0_g1_i1_orf1
cellular_component	intracellular organelle	GO:0043229	157	157/1363	TRINITY_DN42856_c0_g1_i1_orf1;TRINITY_DN25960_c0_g1_i1_orf1;TRINITY_DN4380_c0_g1_i9_orf1;TRINITY_DN5670_c0_g1_i2_orf1;TRINITY_DN137_c0_g1_i1_orf1;TRINITY_DN40176_c0_g1_i1_orf1;TRINITY_DN5873_c0_g4_i1_orf1;TRINITY_DN54477_c0_g1_i1_orf1;TRINITY_DN11065_c0_g2_i1_orf1;TRINITY_DN58207_c0_g1_i1_orf1;TRINITY_DN142442_c0_g1_i1_orf1;TRINITY_DN140538_c0_g2_i1_orf1;TRINITY_DN19810_c1_g1_i7_orf1;TRINITY_DN50074_c0_g1_i1_orf1;TRINITY_DN8107_c0_g1_i1_orf1;TRINITY_DN4711_c0_g1_i2_orf1;TRINITY_DN3464_c0_g1_i1_orf1;TRINITY_DN26789_c0_g1_i2_orf1;TRINITY_DN96557_c0_g1_i1_orf1;TRINITY_DN11596_c0_g1_i1_orf1;TRINITY_DN95056_c0_g2_i2_orf1;TRINITY_DN3698_c0_g1_i4_orf1;TRINITY_DN364_c1_g1_i2_orf1;TRINITY_DN18148_c0_g2_i1_orf1;TRINITY_DN84478_c0_g1_i8_orf1;TRINITY_DN235_c0_g3_i1_orf1;TRINITY_DN77480_c0_g1_i2_orf1;TRINITY_DN4929_c0_g1_i1_orf1;TRINITY_DN143_c0_g3_i1_orf1;TRINITY_DN23746_c0_g1_i2_orf1;TRINITY_DN110231_c0_g1_i1_orf1;TRINITY_DN14347_c0_g1_i1_orf1;TRINITY_DN7909_c0_g2_i1_orf1;TRINITY_DN33893_c0_g1_i1_orf1;TRINITY_DN14391_c1_g1_i2_orf1;TRINITY_DN31225_c0_g1_i1_orf1;TRINITY_DN121893_c0_g1_i1_orf1;TRINITY_DN2848_c0_g1_i2_orf1;TRINITY_DN6199_c2_g1_i3_orf1;TRINITY_DN18249_c0_g1_i1_orf1;TRINITY_DN1706_c0_g1_i7_orf1;TRINITY_DN12526_c0_g1_i5_orf1;TRINITY_DN144956_c0_g1_i1_orf1;TRINITY_DN18009_c0_g1_i1_orf1;TRINITY_DN799_c0_g1_i7_orf1;TRINITY_DN18933_c0_g1_i3_orf1;TRINITY_DN2719_c1_g1_i6_orf1;TRINITY_DN97589_c0_g1_i3_orf1;TRINITY_DN9871_c0_g1_i11_orf1;TRINITY_DN4381_c0_g2_i1_orf1;TRINITY_DN60821_c0_g1_i1_orf1;TRINITY_DN3588_c0_g1_i4_orf1;TRINITY_DN7464_c0_g1_i14_orf1;TRINITY_DN50787_c0_g2_i2_orf1;TRINITY_DN45037_c0_g1_i1_orf1;TRINITY_DN937_c0_g1_i2_orf1;TRINITY_DN2621_c0_g1_i1_orf1;TRINITY_DN23534_c0_g2_i2_orf1;TRINITY_DN3733_c0_g1_i1_orf1;TRINITY_DN106534_c0_g1_i1_orf1;TRINITY_DN14274_c0_g1_i3_orf1;TRINITY_DN4938_c0_g1_i13_orf1;TRINITY_DN18922_c0_g1_i1_orf1;TRINITY_DN40911_c0_g1_i1_orf1;TRINITY_DN22272_c0_g1_i1_orf1;TRINITY_DN11263_c0_g1_i5_orf1;TRINITY_DN21357_c0_g1_i5_orf1;TRINITY_DN3335_c0_g1_i1_orf1;TRINITY_DN7991_c0_g1_i9_orf1;TRINITY_DN6358_c0_g1_i5_orf1;TRINITY_DN3847_c1_g1_i1_orf1;TRINITY_DN13233_c0_g1_i3_orf1;TRINITY_DN10831_c1_g1_i1_orf1;TRINITY_DN23020_c0_g1_i1_orf1;TRINITY_DN3702_c0_g1_i1_orf1;TRINITY_DN147458_c0_g1_i1_orf1;TRINITY_DN12397_c0_g1_i1_orf1;TRINITY_DN3860_c0_g1_i5_orf1;TRINITY_DN5064_c0_g1_i4_orf1;TRINITY_DN143852_c0_g1_i1_orf1;TRINITY_DN31119_c0_g1_i1_orf1;TRINITY_DN21251_c1_g1_i1_orf1;TRINITY_DN8703_c0_g1_i2_orf1;TRINITY_DN5112_c0_g1_i1_orf1;TRINITY_DN5055_c0_g1_i12_orf1;TRINITY_DN30131_c0_g1_i1_orf1;TRINITY_DN26251_c0_g1_i1_orf1;TRINITY_DN3092_c0_g1_i2_orf1;TRINITY_DN2186_c0_g1_i17_orf1;TRINITY_DN46409_c0_g1_i1_orf1;TRINITY_DN89083_c0_g1_i1_orf1;TRINITY_DN24318_c0_g1_i1_orf1;TRINITY_DN31584_c0_g2_i2_orf1;TRINITY_DN102260_c0_g1_i1_orf1;TRINITY_DN2971_c0_g1_i1_orf1;TRINITY_DN33248_c0_g1_i1_orf1;TRINITY_DN34830_c0_g1_i1_orf1;TRINITY_DN61222_c0_g1_i1_orf1;TRINITY_DN40345_c0_g1_i6_orf1;TRINITY_DN34479_c0_g1_i2_orf1;TRINITY_DN12594_c0_g1_i1_orf1;TRINITY_DN2559_c0_g1_i4_orf1;TRINITY_DN4793_c0_g1_i7_orf1;TRINITY_DN3219_c0_g1_i6_orf1;TRINITY_DN30027_c0_g1_i1_orf1;TRINITY_DN20009_c0_g1_i1_orf1;TRINITY_DN147676_c0_g1_i1_orf1;TRINITY_DN3062_c0_g1_i1_orf1;TRINITY_DN3063_c0_g1_i5_orf1;TRINITY_DN166_c0_g1_i4_orf1;TRINITY_DN44288_c0_g1_i2_orf1;TRINITY_DN96739_c0_g1_i1_orf1;TRINITY_DN65299_c0_g4_i1_orf1;TRINITY_DN113327_c0_g1_i2_orf1;TRINITY_DN111_c0_g2_i2_orf1;TRINITY_DN111110_c0_g1_i1_orf1;TRINITY_DN62_c1_g1_i3_orf1;TRINITY_DN2954_c0_g1_i1_orf1;TRINITY_DN10234_c0_g1_i1_orf1;TRINITY_DN21971_c0_g1_i4_orf1;TRINITY_DN2647_c0_g1_i3_orf1;TRINITY_DN42854_c0_g3_i2_orf1;TRINITY_DN40015_c0_g1_i2_orf1;TRINITY_DN16939_c0_g1_i4_orf1;TRINITY_DN40650_c0_g1_i1_orf1;TRINITY_DN5031_c0_g1_i1_orf1;TRINITY_DN37532_c0_g1_i1_orf1;TRINITY_DN8044_c0_g1_i2_orf1;TRINITY_DN59291_c0_g1_i1_orf1;TRINITY_DN96801_c0_g1_i1_orf1;TRINITY_DN4056_c0_g1_i8_orf1;TRINITY_DN1772_c1_g3_i1_orf1;TRINITY_DN50725_c0_g1_i6_orf1;TRINITY_DN7647_c0_g1_i4_orf1;TRINITY_DN5009_c0_g1_i2_orf1;TRINITY_DN9383_c0_g1_i3_orf1;TRINITY_DN104507_c0_g1_i2_orf1;TRINITY_DN1298_c0_g1_i3_orf1;TRINITY_DN279_c0_g1_i10_orf1;TRINITY_DN8824_c0_g2_i1_orf1;TRINITY_DN3614_c0_g2_i1_orf1;TRINITY_DN9510_c0_g2_i1_orf1;TRINITY_DN3826_c0_g1_i1_orf1;TRINITY_DN47114_c0_g1_i5_orf1;TRINITY_DN3909_c0_g2_i2_orf1;TRINITY_DN24266_c0_g2_i2_orf1;TRINITY_DN4070_c0_g1_i4_orf1;TRINITY_DN351_c14_g1_i2_orf1;TRINITY_DN30233_c0_g1_i2_orf1;TRINITY_DN18593_c0_g1_i1_orf1;TRINITY_DN5558_c0_g1_i4_orf1;TRINITY_DN9862_c0_g2_i1_orf1;TRINITY_DN19251_c0_g1_i8_orf1;TRINITY_DN26824_c0_g1_i1_orf1;TRINITY_DN7241_c0_g2_i2_orf1;TRINITY_DN6462_c0_g1_i5_orf1;TRINITY_DN3393_c0_g2_i1_orf1
cellular_component	non-membrane-bounded organelle	GO:0043228	73	73/1363	TRINITY_DN21251_c1_g1_i1_orf1;TRINITY_DN1298_c0_g1_i3_orf1;TRINITY_DN40650_c0_g1_i1_orf1;TRINITY_DN18249_c0_g1_i1_orf1;TRINITY_DN30131_c0_g1_i1_orf1;TRINITY_DN144956_c0_g1_i1_orf1;TRINITY_DN3826_c0_g1_i1_orf1;TRINITY_DN8044_c0_g1_i2_orf1;TRINITY_DN137_c0_g1_i1_orf1;TRINITY_DN2186_c0_g1_i17_orf1;TRINITY_DN30233_c0_g1_i2_orf1;TRINITY_DN9862_c0_g2_i1_orf1;TRINITY_DN24318_c0_g1_i1_orf1;TRINITY_DN9383_c0_g1_i3_orf1;TRINITY_DN102260_c0_g1_i1_orf1;TRINITY_DN25960_c0_g1_i1_orf1;TRINITY_DN5873_c0_g4_i1_orf1;TRINITY_DN4056_c0_g1_i8_orf1;TRINITY_DN97589_c0_g1_i3_orf1;TRINITY_DN54477_c0_g1_i1_orf1;TRINITY_DN11065_c0_g2_i1_orf1;TRINITY_DN61222_c0_g1_i1_orf1;TRINITY_DN58207_c0_g1_i1_orf1;TRINITY_DN60821_c0_g1_i1_orf1;TRINITY_DN21357_c0_g1_i5_orf1;TRINITY_DN5009_c0_g1_i2_orf1;TRINITY_DN6462_c0_g1_i5_orf1;TRINITY_DN142442_c0_g1_i1_orf1;TRINITY_DN7464_c0_g1_i14_orf1;TRINITY_DN30027_c0_g1_i1_orf1;TRINITY_DN50787_c0_g2_i2_orf1;TRINITY_DN364_c1_g1_i2_orf1;TRINITY_DN20009_c0_g1_i1_orf1;TRINITY_DN147676_c0_g1_i1_orf1;TRINITY_DN7991_c0_g1_i9_orf1;TRINITY_DN3062_c0_g1_i1_orf1;TRINITY_DN799_c0_g1_i7_orf1;TRINITY_DN5112_c0_g1_i1_orf1;TRINITY_DN95056_c0_g2_i2_orf1;TRINITY_DN47114_c0_g1_i5_orf1;TRINITY_DN5031_c0_g1_i1_orf1;TRINITY_DN3733_c0_g1_i1_orf1;TRINITY_DN77480_c0_g1_i2_orf1;TRINITY_DN3909_c0_g2_i2_orf1;TRINITY_DN235_c0_g3_i1_orf1;TRINITY_DN31119_c0_g1_i1_orf1;TRINITY_DN22272_c0_g1_i1_orf1;TRINITY_DN143_c0_g3_i1_orf1;TRINITY_DN23746_c0_g1_i2_orf1;TRINITY_DN96557_c0_g1_i1_orf1;TRINITY_DN110231_c0_g1_i1_orf1;TRINITY_DN7241_c0_g2_i2_orf1;TRINITY_DN10234_c0_g1_i1_orf1;TRINITY_DN50725_c0_g1_i6_orf1;TRINITY_DN3847_c1_g1_i1_orf1;TRINITY_DN14391_c1_g1_i2_orf1;TRINITY_DN21971_c0_g1_i4_orf1;TRINITY_DN10831_c1_g1_i1_orf1;TRINITY_DN18593_c0_g1_i1_orf1;TRINITY_DN8824_c0_g2_i1_orf1;TRINITY_DN13233_c0_g1_i3_orf1;TRINITY_DN31225_c0_g1_i1_orf1;TRINITY_DN147458_c0_g1_i1_orf1;TRINITY_DN12397_c0_g1_i1_orf1;TRINITY_DN40345_c0_g1_i6_orf1;TRINITY_DN26824_c0_g1_i1_orf1;TRINITY_DN5064_c0_g1_i4_orf1;TRINITY_DN143852_c0_g1_i1_orf1;TRINITY_DN121893_c0_g1_i1_orf1;TRINITY_DN2848_c0_g1_i2_orf1;TRINITY_DN40015_c0_g1_i2_orf1;TRINITY_DN3393_c0_g2_i1_orf1;TRINITY_DN16939_c0_g1_i4_orf1

cellular_component	membrane-bounded organelle	GO:0043227	86	86/1363	TRINITY_DN42856_c0_g1_i1_orf1;TRINITY_DN5670_c0_g1_i2_orf1;TRINITY_DN40176_c0_g1_i1_orf1;TRINITY_DN30300_c0_g2_i1_orf1;TRINITY_DN65299_c0_g4_i1_orf1;TRINITY_DN50074_c0_g1_i1_orf1;TRINITY_DN8107_c0_g1_i1_orf1;TRINITY_DN4711_c0_g1_i2_orf1;TRINITY_DN3464_c0_g1_i1_orf1;TRINITY_DN26789_c0_g1_i2_orf1;TRINITY_DN3698_c0_g1_i4_orf1;TRINITY_DN18148_c0_g2_i1_orf1;TRINITY_DN84478_c0_g1_i8_orf1;TRINITY_DN4929_c0_g1_i1_orf1;TRINITY_DN143_c0_g3_i1_orf1;TRINITY_DN140538_c0_g2_i1_orf1;TRINITY_DN14347_c0_g1_i1_orf1;TRINITY_DN7909_c0_g2_i1_orf1;TRINITY_DN33893_c0_g1_i1_orf1;TRINITY_DN2848_c0_g1_i2_orf1;TRINITY_DN6199_c2_g1_i3_orf1;TRINITY_DN1706_c0_g1_i7_orf1;TRINITY_DN12526_c0_g1_i5_orf1;TRINITY_DN18009_c0_g1_i1_orf1;TRINITY_DN18933_c0_g1_i3_orf1;TRINITY_DN9871_c0_g1_i11_orf1;TRINITY_DN3588_c0_g1_i4_orf1;TRINITY_DN50787_c0_g2_i2_orf1;TRINITY_DN45037_c0_g1_i1_orf1;TRINITY_DN937_c0_g1_i2_orf1;TRINITY_DN2621_c0_g1_i1_orf1;TRINITY_DN23534_c0_g2_i2_orf1;TRINITY_DN106534_c0_g1_i1_orf1;TRINITY_DN4938_c0_g1_i13_orf1;TRINITY_DN18922_c0_g1_i1_orf1;TRINITY_DN40911_c0_g1_i1_orf1;TRINITY_DN22272_c0_g1_i1_orf1;TRINITY_DN6358_c0_g1_i5_orf1;TRINITY_DN23020_c0_g1_i1_orf1;TRINITY_DN3702_c0_g1_i1_orf1;TRINITY_DN147458_c0_g1_i1_orf1;TRINITY_DN3860_c0_g1_i5_orf1;TRINITY_DN8703_c0_g1_i2_orf1;TRINITY_DN5055_c0_g1_i12_orf1;TRINITY_DN26251_c0_g1_i1_orf1;TRINITY_DN3092_c0_g1_i2_orf1;TRINITY_DN46409_c0_g1_i1_orf1;TRINITY_DN89083_c0_g1_i1_orf1;TRINITY_DN31584_c0_g2_i2_orf1;TRINITY_DN33248_c0_g1_i1_orf1;TRINITY_DN34830_c0_g1_i1_orf1;TRINITY_DN3335_c0_g1_i1_orf1;TRINITY_DN34479_c0_g1_i2_orf1;TRINITY_DN12594_c0_g1_i1_orf1;TRINITY_DN2559_c0_g1_i4_orf1;TRINITY_DN4793_c0_g1_i7_orf1;TRINITY_DN3219_c0_g1_i6_orf1;TRINITY_DN20009_c0_g1_i1_orf1;TRINITY_DN3063_c0_g1_i5_orf1;TRINITY_DN166_c0_g1_i4_orf1;TRINITY_DN44288_c0_g1_i2_orf1;TRINITY_DN96739_c0_g1_i1_orf1;TRINITY_DN113327_c0_g1_i2_orf1;TRINITY_DN111_c0_g2_i2_orf1;TRINITY_DN19810_c1_g1_i7_orf1;TRINITY_DN62_c1_g1_i3_orf1;TRINITY_DN2954_c0_g1_i1_orf1;TRINITY_DN111110_c0_g1_i1_orf1;TRINITY_DN2647_c0_g1_i3_orf1;TRINITY_DN42854_c0_g3_i2_orf1;TRINITY_DN2971_c0_g1_i1_orf1;TRINITY_DN2719_c1_g1_i6_orf1;TRINITY_DN96801_c0_g1_i1_orf1;TRINITY_DN104507_c0_g1_i2_orf1;TRINITY_DN1772_c1_g3_i1_orf1;TRINITY_DN7647_c0_g1_i4_orf1;TRINITY_DN279_c0_g1_i10_orf1;TRINITY_DN3614_c0_g2_i1_orf1;TRINITY_DN9510_c0_g2_i1_orf1;TRINITY_DN4070_c0_g1_i4_orf1;TRINITY_DN351_c14_g1_i2_orf1;TRINITY_DN5558_c0_g1_i4_orf1;TRINITY_DN19251_c0_g1_i8_orf1;TRINITY_DN24266_c0_g2_i2_orf1;TRINITY_DN6462_c0_g1_i5_orf1;TRINITY_DN[illegible]
cellular_component	postsynaptic specialization	GO:0099572	1	1/1363	TRINITY_DN142442_c0_g1_i1_orf1
cellular_component	extracellular organelle	GO:0043230	6	6/1363	TRINITY_DN30300_c0_g2_i1_orf1;TRINITY_DN42854_c0_g3_i2_orf1;TRINITY_DN46409_c0_g1_i1_orf1;TRINITY_DN96739_c0_g1_i1_orf1;TRINITY_DN20009_c0_g1_i1_orf1;TRINITY_DN2848_c0_g1_i2_orf1
cellular_component	striated muscle thin filament	GO:0005865	1	1/1363	TRINITY_DN235_c0_g3_i1_orf1
cellular_component	organelle membrane	GO:0031090	39	39/1363	TRINITY_DN9608_c0_g1_i3_orf1;TRINITY_DN13923_c0_g2_i1_orf1;TRINITY_DN3209_c0_g1_i1_orf1;TRINITY_DN36324_c0_g1_i12_orf1;TRINITY_DN3821_c1_g1_i7_orf1;TRINITY_DN24873_c0_g1_i4_orf1;TRINITY_DN72816_c0_g1_i2_orf1;TRINITY_DN53760_c0_g1_i1_orf1;TRINITY_DN38720_c0_g1_i3_orf1;TRINITY_DN8454_c0_g1_i4_orf1;TRINITY_DN57765_c0_g1_i1_orf1;TRINITY_DN11069_c0_g2_i1_orf1;TRINITY_DN106476_c0_g1_i3_orf1;TRINITY_DN20984_c0_g1_i4_orf1;TRINITY_DN9316_c0_g3_i1_orf1;TRINITY_DN5432_c1_g1_i3_orf1;TRINITY_DN11172_c0_g1_i4_orf1;TRINITY_DN7336_c0_g1_i13_orf1;TRINITY_DN1447_c0_g1_i5_orf1;TRINITY_DN19303_c0_g1_i5_orf1;TRINITY_DN1960_c5_g1_i3_orf1;TRINITY_DN4443_c0_g1_i4_orf1;TRINITY_DN96739_c0_g1_i1_orf1;TRINITY_DN12286_c1_g1_i2_orf1;TRINITY_DN40911_c0_g1_i1_orf1;TRINITY_DN642_c0_g1_i6_orf1;TRINITY_DN6680_c0_g1_i1_orf1;TRINITY_DN32780_c0_g1_i2_orf1;TRINITY_DN2879_c0_g1_i4_orf1;TRINITY_DN96557_c0_g1_i1_orf1;TRINITY_DN46409_c0_g1_i1_orf1;TRINITY_DN5630_c4_g1_i2_orf1;TRINITY_DN103118_c0_g1_i4_orf1;TRINITY_DN42854_c0_g3_i2_orf1;TRINITY_DN5558_c0_g1_i4_orf1;TRINITY_DN5064_c0_g1_i4_orf1;TRINITY_DN5697_c0_g1_i1_orf1;TRINITY_DN2848_c0_g1_i2_orf1;TRINITY_DN9002_c0_g1_i1_orf1
cellular_component	outer membrane	GO:0019867	1	1/1363	TRINITY_DN142657_c0_g1_i1_orf1
cellular_component	plasma membrane	GO:0005886	15	15/1363	TRINITY_DN54477_c0_g1_i1_orf1;TRINITY_DN23926_c0_g1_i4_orf1;TRINITY_DN5406_c0_g2_i1_orf1;TRINITY_DN7570_c0_g1_i18_orf1;TRINITY_DN11670_c0_g1_i1_orf1;TRINITY_DN1012_c0_g1_i2_orf1;TRINITY_DN2848_c0_g1_i2_orf1;TRINITY_DN42854_c0_g3_i2_orf1;TRINITY_DN46409_c0_g1_i1_orf1;TRINITY_DN31584_c0_g2_i2_orf1;TRINITY_DN5553_c0_g1_i4_orf1;TRINITY_DN5064_c0_g1_i4_orf1;TRINITY_DN20009_c0_g1_i1_orf1;TRINITY_DN140613_c0_g1_i1_orf1;TRINITY_DN32780_c0_g1_i2_orf1
cellular_component	plasma membrane region	GO:0098590	2	2/1363	TRINITY_DN46409_c0_g1_i1_orf1;TRINITY_DN486_c0_g1_i5_orf1
cellular_component	phagophore assembly site membrane	GO:0034045	1	1/1363	TRINITY_DN113353_c0_g1_i1_orf1
cellular_component	extracellular matrix	GO:0031012	3	3/1363	TRINITY_DN96739_c0_g1_i1_orf1;TRINITY_DN376_c1_g1_i1_orf1;TRINITY_DN2919_c0_g1_i5_orf1
cellular_component	synapse	GO:0045202	4	4/1363	TRINITY_DN17693_c0_g1_i10_orf1;TRINITY_DN82017_c0_g1_i5_orf1;TRINITY_DN140538_c0_g2_i1_orf1;TRINITY_DN142442_c0_g1_i1_orf1
cellular_component	anchoring junction	GO:0070161	9	9/1363	TRINITY_DN17693_c0_g1_i10_orf1;TRINITY_DN30300_c0_g2_i1_orf1;TRINITY_DN142442_c0_g1_i1_orf1;TRINITY_DN2186_c0_g1_i17_orf1;TRINITY_DN96739_c0_g1_i1_orf1;TRINITY_DN9383_c0_g1_i3_orf1;TRINITY_DN364_c1_g1_i2_orf1;TRINITY_DN20009_c0_g1_i1_orf1;TRINITY_DN23746_c0_g1_i2_orf1
cellular_component	Golgi apparatus subcompartment	GO:0098791	1	1/1363	TRINITY_DN9243_c0_g1_i4_orf1
cellular_component	plasma membrane bounded cell projection	GO:0120025	3	3/1363	TRINITY_DN20009_c0_g1_i1_orf1;TRINITY_DN9383_c0_g1_i3_orf1;TRINITY_DN364_c1_g1_i2_orf1
cellular_component	Cajal body	GO:0015030	1	1/1363	TRINITY_DN8044_c0_g1_i2_orf1
cellular_component	nuclear speck	GO:0016607	2	2/1363	TRINITY_DN140538_c0_g2_i1_orf1;TRINITY_DN47123_c0_g1_i1_orf1
cellular_component	cytosolic region	GO:0099522	1	1/1363	TRINITY_DN140538_c0_g2_i1_orf1
cellular_component	ciliary basal body	GO:0036064	1	1/1363	TRINITY_DN140538_c0_g2_i1_orf1
cellular_component	kinetochore	GO:0000776	1	1/1363	TRINITY_DN96557_c0_g1_i1_orf1
cellular_component	ribonucleoprotein granule	GO:0035770	2	2/1363	TRINITY_DN1298_c0_g1_i3_orf1;TRINITY_DN50725_c0_g1_i6_orf1
cellular_component	supramolecular polymer	GO:0099081	6	6/1363	TRINITY_DN96557_c0_g1_i1_orf1;TRINITY_DN36612_c0_g1_i1_orf1;TRINITY_DN235_c0_g3_i1_orf1;TRINITY_DN46202_c0_g1_i1_orf1;TRINITY_DN14298_c0_g3_i1_orf1;TRINITY_DN20009_c0_g1_i1_orf1
molecular_function	translation factor activity, RNA binding	GO:0008135	23	23/1363	TRINITY_DN33967_c0_g1_i1_orf1;TRINITY_DN2716_c0_g2_i1_orf1;TRINITY_DN33248_c0_g1_i1_orf1;TRINITY_DN4237_c1_g1_i5_orf1;TRINITY_DN44407_c0_g4_i2_orf1;TRINITY_DN15362_c0_g1_i1_orf1;TRINITY_DN136906_c0_g1_i1_orf1;TRINITY_DN14498_c0_g1_i1_orf1;TRINITY_DN4381_c0_g2_i1_orf1;TRINITY_DN48096_c0_g2_i2_orf1;TRINITY_DN27751_c0_g2_i1_orf1;TRINITY_DN22572_c0_g1_i1_orf1;TRINITY_DN29521_c0_g1_i1_orf1;TRINITY_DN17045_c0_g2_i3_orf1;TRINITY_DN50085_c0_g1_i1_orf1;TRINITY_DN9164_c0_g1_i3_orf1;TRINITY_DN359_c0_g1_i5_orf1;TRINITY_DN28039_c0_g1_i1_orf1;TRINITY_DN11612_c0_g2_i1_orf1;TRINITY_DN19659_c1_g1_i1_orf1;TRINITY_DN21609_c0_g2_i1_orf1;TRINITY_DN126648_c0_g1_i1_orf1;TRINITY_DN21000_c0_g1_i1_orf1
molecular_function	transcription coactivator activity	GO:0003713	2	2/1363	TRINITY_DN9510_c0_g2_i1_orf1;TRINITY_DN77572_c0_g1_i1_orf1
molecular_function	RNA helicase activity	GO:0003724	9	9/1363	TRINITY_DN20499_c0_g3_i1_orf1;TRINITY_DN11596_c0_g1_i1_orf1;TRINITY_DN4381_c0_g2_i1_orf1;TRINITY_DN44288_c0_g1_i2_orf1;TRINITY_DN4380_c0_g1_i9_orf1;TRINITY_DN14274_c0_g1_i3_orf1;TRINITY_DN4408_c6_g1_i1_orf1;TRINITY_DN59291_c0_g1_i1_orf1;TRINITY_DN7213_c0_g1_i2_orf1
molecular_function	DNA helicase activity	GO:0003678	1	1/1363	TRINITY_DN2971_c0_g1_i1_orf1
molecular_function	DNA clamp loader activity	GO:0003689	1	1/1363	TRINITY_DN3092_c0_g1_i2_orf1
molecular_function	long-chain fatty acid-CoA ligase activity	GO:0004467	1	1/1363	TRINITY_DN2193_c0_g1_i7_orf1
molecular_function	ABC-type transporter activity	GO:0140359	4	4/1363	TRINITY_DN16408_c0_g1_i1_orf1;TRINITY_DN13563_c0_g1_i1_orf1;TRINITY_DN60792_c0_g1_i2_orf1;TRINITY_DN31327_c0_g2_i1_orf1
molecular_function	P-type transmembrane transporter activity	GO:0140358	3	3/1363	TRINITY_DN7336_c0_g1_i13_orf1;TRINITY_DN7570_c0_g1_i18_orf1;TRINITY_DN9243_c0_g1_i4_orf1
molecular_function	ATPase-coupled cation transmembrane transporter activity	GO:0019829	3	3/1363	TRINITY_DN7336_c0_g1_i13_orf1;TRINITY_DN7570_c0_g1_i18_orf1;TRINITY_DN9243_c0_g1_i4_orf1
molecular_function	SNAP receptor activity	GO:0005484	1	1/1363	TRINITY_DN5558_c0_g1_i4_orf1
molecular_function	ATP-dependent FeS chaperone activity	GO:0140663	1	1/1363	TRINITY_DN85476_c0_g1_i1_orf1
molecular_function	lactoperoxidase activity	GO:0140825	1	1/1363	TRINITY_DN3321_c0_g1_i3_orf1
molecular_function	peroxiredoxin activity	GO:0051920	3	3/1363	TRINITY_DN2542_c0_g2_i1_orf1;TRINITY_DN7778_c0_g1_i1_orf1;TRINITY_DN69236_c0_g1_i1_orf1
molecular_function	glutathione peroxidase activity	GO:0004602	2	2/1363	TRINITY_DN1622_c0_g1_i6_orf1;TRINITY_DN21420_c0_g1_i2_orf1
molecular_function	passive transmembrane transporter activity	GO:0022803	4	4/1363	TRINITY_DN957_c0_g1_i18_orf1;TRINITY_DN96739_c0_g1_i1_orf1;TRINITY_DN34821_c0_g1_i4_orf1;TRINITY_DN20558_c0_g1_i2_orf1
molecular_function	active transmembrane transporter activity	GO:0022804	8	8/1363	TRINITY_DN7336_c0_g1_i13_orf1;TRINITY_DN13563_c0_g1_i1_orf1;TRINITY_DN31327_c0_g2_i1_orf1;TRINITY_DN7570_c0_g1_i18_orf1;TRINITY_DN9243_c0_g1_i4_orf1;TRINITY_DN60792_c0_g1_i2_orf1;TRINITY_DN16408_c0_g1_i1_orf1;TRINITY_DN6381_c0
molecular_function	ion transmembrane transporter activity	GO:0015075	12	12/1363	TRINITY_DN7336_c0_g1_i13_orf1;TRINITY_DN13923_c0_g2_i1_orf1;TRINITY_DN20558_c0_g1_i2_orf1;TRINITY_DN7570_c0_g1_i18_orf1;TRINITY_DN9243_c0_g1_i4_orf1;TRINITY_DN96739_c0_g1_i1_orf1;TRINITY_DN12286_c1_g1_i2_orf1;TRINITY_DN34821_c0_g1_i4_orf1;TRINITY_DN957_c0_g1_i18_orf1;TRINITY_DN91946_c0_g1_i1_orf1;TRINITY_DN6381_c0_g1_i2_orf1;TRINITY_DN1882_c0_g1_i4_orf1
molecular_function	inorganic molecular entity transmembrane transporter activity	GO:0015318	10	10/1363	TRINITY_DN7336_c0_g1_i13_orf1;TRINITY_DN34821_c0_g1_i4_orf1;TRINITY_DN20558_c0_g1_i2_orf1;TRINITY_DN7570_c0_g1_i18_orf1;TRINITY_DN9243_c0_g1_i4_orf1;TRINITY_DN96739_c0_g1_i1_orf1;TRINITY_DN957_c0_g1_i18_orf1;TRINITY_DN91946_c0_g1_i1_orf1;TRINITY_DN6381_c0_g1_i2_orf1;TRINITY_DN1882_c0_g1_i4_orf1
molecular_function	ion channel regulator activity	GO:0099106	1	1/1363	TRINITY_DN31584_c0_g2_i2_orf1
molecular_function	kinase regulator activity	GO:0019207	1	1/1363	TRINITY_DN346_c0_g1_i7_orf1
molecular_function	phosphatase regulator activity	GO:0019208	1	1/1363	TRINITY_DN12087_c0_g1_i2_orf1
molecular_function	nucleoside-triphosphatase regulator activity	GO:0060589	6	6/1363	TRINITY_DN1054_c0_g1_i8_orf1;TRINITY_DN42461_c0_g1_i4_orf1;TRINITY_DN21609_c0_g2_i1_orf1;TRINITY_DN104297_c0_g1_i1_orf1;TRINITY_DN27491_c0_g1_i1_orf1;TRINITY_DN804_c0_g1_i7_orf1
molecular_function	peptidase regulator activity	GO:0061134	12	12/1363	TRINITY_DN42854_c0_g3_i2_orf1;TRINITY_DN16234_c0_g2_i3_orf1;TRINITY_DN4314_c0_g1_i9_orf1;TRINITY_DN1215_c0_g1_i2_orf1;TRINITY_DN8780_c0_g1_i3_orf1;TRINITY_DN1328_c0_g1_i6_orf1;TRINITY_DN2097_c1_g2_i2_orf1;TRINITY_DN399_c3_g2_i6_orf1;TRINITY_DN45948_c1_g1_i1_orf1;TRINITY_DN3055_c0_g1_i9_orf1;TRINITY_DN2848_c0_g1_i2_orf1;TRINITY_DN1540_c0_g1_i7_orf1
molecular_function	enzyme activator activity	GO:0008047	3	3/1363	TRINITY_DN1054_c0_g1_i8_orf1;TRINITY_DN346_c0_g1_i7_orf1;TRINITY_DN104297_c0_g1_i1_orf1
molecular_function	enzyme inhibitor activity	GO:0004857	13	13/1363	TRINITY_DN42854_c0_g3_i2_orf1;TRINITY_DN16234_c0_g2_i3_orf1;TRINITY_DN4314_c0_g1_i9_orf1;TRINITY_DN12087_c0_g1_i2_orf1;TRINITY_DN1215_c0_g1_i2_orf1;TRINITY_DN8780_c0_g1_i3_orf1;TRINITY_DN1328_c0_g1_i6_orf1;TRINITY_DN2097_c1_g2_i2_orf1;TRINITY_DN399_c3_g2_i6_orf1;TRINITY_DN45948_c1_g1_i1_orf1;TRINITY_DN3055_c0_g1_i9_orf1;TRINITY_DN2848_c0_g1_i2_orf1;TRINITY_DN1540_c0_g1_i7_orf1
molecular_function	signaling receptor activator activity	GO:0030546	2	2/1363	TRINITY_DN18650_c0_g1_i1_orf1;TRINITY_DN2227_c0_g1_i5_orf1
molecular_function	nucleic acid binding	GO:0003676	126	126/1363	TRINITY_DN4380_c0_g1_i9_orf1;TRINITY_DN3301_c0_g1_i2_orf1;TRINITY_DN40434_c0_g1_i2_orf1;TRINITY_DN12227_c0_g2_i3_orf1;TRINITY_DN137_c0_g1_i1_orf1;TRINITY_DN4262_c0_g1_i16_orf1;TRINITY_DN1116_c0_g1_i6_orf1;TRINITY_DN7213_c0_g1_i2_orf1;TRINITY_DN4237_c1_g1_i5_orf1;TRINITY_DN359_c0_g1_i5_orf1;TRINITY_DN16965_c0_g2_i1_orf1;TRINITY_DN2682_c0_g1_i4_orf1;TRINITY_DN142442_c0_g1_i1_orf1;TRINITY_DN24689_c0_g1_i1_orf1;TRINITY_DN29521_c0_g1_i1_orf1;TRINITY_DN5670_c0_g1_i2_orf1;TRINITY_DN1710_c0_g2_i2_orf1;TRINITY_DN11596_c0_g1_i1_orf1;TRINITY_DN95056_c0_g2_i2_orf1;TRINITY_DN9164_c0_g1_i3_orf1;TRINITY_DN9101_c0_g2_i1_orf1;TRINITY_DN4929_c0_g1_i1_orf1;TRINITY_DN817_c0_g1_i3_orf1;TRINITY_DN19659_c1_g1_i1_orf1;TRINITY_DN48641_c0_g1_i4_orf1;TRINITY_DN20499_c0_g3_i1_orf1;TRINITY_DN227_c0_g1_i1_orf1;TRINITY_DN33893_c0_g1_i1_orf1;TRINITY_DN251_c0_g1_i2_orf1;TRINITY_DN6235_c0_g1_i5_orf1;TRINITY_DN10558_c0_g1_i4_orf1;TRINITY_DN89613_c0_g1_i13_orf1;TRINITY_DN3092_c0_g1_i2_orf1;TRINITY_DN1978_c0_g1_i4_orf1;TRINITY_DN17271_c0_g1_i1_orf1;TRINITY_DN20442_c0_g2_i1_orf1;TRINITY_DN144956_c0_g1_i1_orf1;TRINITY_DN3712_c0_g1_i1_orf1;TRINITY_DN18242_c0_g1_i3_orf1;TRINITY_DN37532_c0_g1_i1_orf1;TRINITY_DN51968_c0_g1_i1_orf1;TRINITY_DN4381_c0_g2_i1_orf1;TRINITY_DN47575_c0_g1_i1_orf1;TRINITY_DN27751_c0_g2_i1_orf1;TRINITY_DN14498_c0_g1_i1_orf1;TRINITY_DN7464_c0_g1_i14_orf1;TRINITY_DN50787_c0_g2_i2_orf1;TRINITY_DN2769_c0_g1_i1_orf1;TRINITY_DN41664_c0_g1_i4_orf1;TRINITY_DN6532_c2_g1_i1_orf1;TRINITY_DN799_c0_g1_i7_orf1;TRINITY_DN50085_c0_g1_i1_orf1;TRINITY_DN3733_c0_g1_i1_orf1;TRINITY_DN14274_c0_g1_i3_orf1;TRINITY_DN28039_c0_g1_i1_orf1;TRINITY_DN4408_c6_g1_i1_orf1;TRINITY_DN51568_c0_g1_i1_orf1;TRINITY_DN104597_c0_g1_i2_orf1;TRINITY_DN40945_c0_g1_i1_orf1;TRINITY_DN23502_c0_g1_i1_orf1;TRINITY_DN6358_c0_g1_i5_orf1;TRINITY_DN3847_c1_g1_i1_orf1;TRINITY_DN3459_c0_g1_i4_orf1;TRINITY_DN147458_c0_g1_i1_orf1;TRINITY_DN23004_c0_g1_i1_orf1;TRINITY_DN2299_c0_g1_i3_orf1;TRINITY_DN2749_c4_g1_i2_orf1;TRINITY_DN21000_c0_g1_i1_orf1;TRINITY_DN257_c0_g1_i7_orf1;TRINITY_DN8008_c0_g1_i6_orf1;TRINITY_DN2749_c0_g1_i4_orf1;TRINITY_DN26251_c0_g1_i1_orf1;TRINITY_DN620_c0_g1_i4_orf1;TRINITY_DN2117_c0_g1_i1_orf1;TRINITY_DN46409_c0_g1_i1_orf1;TRINITY_DN2718_c0_g1_i6_orf1;TRINITY_DN33248_c0_g1_i1_orf1;TRINITY_DN12594_c0_g1_i1_orf1;TRINITY_DN15362_c0_g1_i1_orf1;TRINITY_DN136906_c0_g1_i1_orf1;TRINITY_DN36494_c0_g1_i1_orf1;TRINITY_DN3335_c0_g1_i1_orf1;TRINITY_DN48096_c0_g2_i2_orf1;TRINITY_DN43412_c0_g1_i2_orf1;TRINITY_DN33967_c0_g1_i1_orf1;TRINITY_DN142652_c0_g1_i1_orf1;TRINITY_DN2879_c0_g1_i4_orf1;TRINITY_DN20009_c0_g1_i1_orf1;TRINITY_DN1066_c0_g1_i8_orf1;TRINITY_DN2749_c0_g2_i3_orf1;TRINITY_DN84322_c0_g2_i1_orf1;TRINITY_DN4820_c0_g1_i1_orf1;TRINITY_DN44288_c0_g1_i2_orf1;TRINITY_DN20339_c0_g1_i3_orf1;TRINITY_DN111_c0_g2_i2_orf1;TRINITY_DN19810_c1_g1_i7_orf1;TRINITY_DN1860_c0_g1_i1_orf1;TRINITY_DN10234_c0_g1_i1_orf1;TRINITY_DN2647_c0_g1_i3_orf1;TRINITY_DN42854_c0_g3_i2_orf1;TRINITY_DN7613_c1_g2_i1_orf1;TRINITY_DN36893_c0_g1_i1_orf1;TRINITY_DN2716_c0_g2_i1_orf1;TRINITY_DN59291_c0_g1_i1_orf1;TRINITY_DN271_c0_g2_i6_orf1;TRINITY_DN96801_c0_g1_i1_orf1;TRINITY_DN4056_c0_g1_i8_orf1;TRINITY_DN27958_c0_g1_i1_orf1;TRINITY_DN21150_c0_g1_i4_orf1;TRINITY_DN27_c0_g1_i1_orf1;TRINITY_DN104507_c0_g1_i2_orf1;TRINITY_DN22572_c0_g1_i1_orf1;TRINITY_DN1298_c0_g1_i3_orf1;TRINITY_DN2367_c1_g1_i20_orf1;TRINITY_DN17045_c0_g2_i3_orf1;TRINITY_DN9510_c0_g2_i1_orf1;TRINITY_DN7112_c0_g1_i1_orf1;TRINITY_DN5238_c0_g1_i2_orf1;TRINITY_DN11612_c0_g2_i1_orf1;TRINITY_DN42646_c0_g2_i1_orf1;TRINITY_DN48619_c0_g1_i1_orf1;TRINITY_DN430_c0_g1_i5_orf1;TRINITY_DN21609_c0_g2_i1_orf1;TRINITY_DN126648_c0_g1_i1_orf1;TRINITY_DN9863_c0_g2_i1_orf1;TRINITY_DN44407_c0_g4_i2_orf1
molecular_function	nucleoside phosphate binding	GO:1901265	116	116/1363	TRINITY_DN98313_c0_g1_i1_orf1;TRINITY_DN13563_c0_g1_i1_orf1;TRINITY_DN38230_c0_g1_i4_orf1;TRINITY_DN4380_c0_g1_i9_orf1;TRINITY_DN27771_c0_g1_i1_orf1;TRINITY_DN33801_c0_g1_i1_orf1;TRINITY_DN359_c0_g1_i5_orf1;TRINITY_DN54477_c0_g1_i1_orf1;TRINITY_DN4711_c0_g1_i2_orf1;TRINITY_DN26789_c0_g1_i2_orf1;TRINITY_DN5422_c0_g1_i1_orf1;TRINITY_DN11596_c0_g1_i1_orf1;TRINITY_DN235_c0_g3_i1_orf1;TRINITY_DN52761_c0_g1_i2_orf1;TRINITY_DN817_c0_g1_i3_orf1;TRINITY_DN4798_c0_g1_i3_orf1;TRINITY_DN20499_c0_g3_i1_orf1;TRINITY_DN7909_c0_g2_i1_orf1;TRINITY_DN987_c0_g1_i3_orf1;TRINITY_DN4501_c0_g1_i3_orf1;TRINITY_DN31225_c0_g1_i1_orf1;TRINITY_DN46715_c0_g1_i1_orf1;TRINITY_DN2848_c0_g1_i2_orf1;TRINITY_DN52761_c0_g2_i1_orf1;TRINITY_DN7336_c0_g1_i13_orf1;TRINITY_DN3092_c0_g1_i2_orf1;TRINITY_DN2772_c0_g1_i3_orf1;TRINITY_DN100821_c0_g1_i1_orf1;TRINITY_DN2719_c1_g1_i6_orf1;TRINITY_DN7213_c0_g1_i2_orf1;TRINITY_DN11942_c0_g1_i1_orf1;TRINITY_DN1604_c0_g1_i4_orf1;TRINITY_DN4381_c0_g2_i1_orf1;TRINITY_DN60821_c0_g1_i1_orf1;TRINITY_DN42461_c0_g1_i4_orf1;TRINITY_DN3588_c0_g1_i4_orf1;TRINITY_DN277_c1_g1_i1_orf1;TRINITY_DN244_c1_g1_i5_orf1;TRINITY_DN2110_c0_g1_i3_orf1;TRINITY_DN2621_c0_g1_i1_orf1;TRINITY_DN3805_c0_g1_i2_orf1;TRINITY_DN12301_c0_g1_i1_orf1;TRINITY_DN38424_c0_g1_i1_orf1;TRINITY_DN14274_c0_g1_i3_orf1;TRINITY_DN28039_c0_g1_i1_orf1;TRINITY_DN9243_c0_g1_i4_orf1;TRINITY_DN42185_c0_g1_i7_orf1;TRINITY_DN1954_c0_g1_i4_orf1;TRINITY_DN9724_c0_g1_i4_orf1;TRINITY_DN5811_c0_g1_i4_orf1;TRINITY_DN16408_c0_g1_i1_orf1;TRINITY_DN3428_c0_g1_i1_orf1;TRINITY_DN5029_c0_g1_i1_orf1;TRINITY_DN4501_c0_g2_i1_orf1;TRINITY_DN21000_c0_g1_i1_orf1;TRINITY_DN5055_c0_g1_i12_orf1;TRINITY_DN7570_c0_g1_i18_orf1;TRINITY_DN620_c0_g1_i4_orf1;TRINITY_DN46409_c0_g1_i1_orf1;TRINITY_DN11178_c0_g1_i1_orf1;TRINITY_DN670_c0_g1_i15_orf1;TRINITY_DN4742_c0_g1_i1_orf1;TRINITY_DN2770_c0_g2_i4_orf1;TRINITY_DN136906_c0_g1_i1_orf1;TRINITY_DN28729_c0_g1_i9_orf1;TRINITY_DN36788_c0_g1_i2_orf1;TRINITY_DN24_c0_g1_i1_orf1;TRINITY_DN34479_c0_g1_i2_orf1;TRINITY_DN46090_c0_g3_i1_orf1;TRINITY_DN29144_c0_g3_i1_orf1;TRINITY_DN4835_c0_g1_i2_orf1;TRINITY_DN825_c23_g1_i5_orf1;TRINITY_DN84322_c0_g2_i1_orf1;TRINITY_DN146236_c0_g1_i1_orf1;TRINITY_DN96739_c0_g1_i1_orf1;TRINITY_DN60792_c0_g1_i2_orf1;TRINITY_DN2193_c0_g1_i7_orf1;TRINITY_DN659_c0_g2_i1_orf1;TRINITY_DN85476_c0_g1_i1_orf1;TRINITY_DN111110_c0_g1_i1_orf1;TRINITY_DN62_c1_g1_i3_orf1;TRINITY_DN52244_c1_g1_i1_orf1;TRINITY_DN31327_c0_g2_i1_orf1;TRINITY_DN49047_c0_g1_i2_orf1;TRINITY_DN70236_c0_g1_i1_orf1;TRINITY_DN2146_c0_g2_i1_orf1;TRINITY_DN14464_c0_g1_i1_orf1;TRINITY_DN140423_c0_g1_i2_orf1;TRINITY_DN47151_c0_g1_i1_orf1;TRINITY_DN33967_c0_g1_i1_orf1;TRINITY_DN76815_c0_g1_i3_orf1;TRINITY_DN41166_c0_g1_i1_orf1;TRINITY_DN59291_c0_g1_i1_orf1;TRINITY_DN119893_c0_g2_i3_orf1;TRINITY_DN4056_c0_g1_i8_orf1;TRINITY_DN1285_c0_g1_i6_orf1;TRINITY_DN20984_c0_g1_i4_orf1;TRINITY_DN1084_c0_g1_i2_orf1;TRINITY_DN2432_c0_g1_i1_orf1;TRINITY_DN13055_c0_g1_i5_orf1;TRINITY_DN15478_c0_g1_i1_orf1;TRINITY_DN80134_c0_g1_i1_orf1;TRINITY_DN11612_c0_g2_i1_orf1;TRINITY_DN45924_c0_g1_i14_orf1;TRINITY_DN48619_c0_g1_i1_orf1;TRINITY_DN41697_c0_g1_i1_orf1;TRINITY_DN68401_c1_g1_i1_orf1;TRINITY_DN44288_c0_g1_i2_orf1;TRINITY_DN3131_c0_g1_i5_orf1;TRINITY_DN126648_c0_g1_i1_orf1;TRINITY_DN18782_c0_g1_i4_orf1;TRINITY_DN19251_c0_g1_i8_orf1;TRINITY_DN14298_c0_g3_i1_orf1;TRINITY_DN1209_c0_g1_i9_orf1;TRINITY_DN4929_c1_g2_i5_orf1;TRINITY_DN1665_c1_g1_i2_orf1
molecular_function	tetrapyrrole binding	GO:0046906	23	23/1363	TRINITY_DN8985_c0_g1_i4_orf1;TRINITY_DN9608_c0_g1_i3_orf1;TRINITY_DN23564_c0_g1_i7_orf1;TRINITY_DN7580_c0_g1_i1_orf1;TRINITY_DN2392_c0_g2_i1_orf1;TRINITY_DN24873_c0_g1_i4_orf1;TRINITY_DN35002_c0_g2_i2_orf1;TRINITY_DN3732_c0_g1_i6_orf1;TRINITY_DN57765_c0_g1_i1_orf1;TRINITY_DN3949_c0_g1_i1_orf1;TRINITY_DN3732_c1_g1_i5_orf1;TRINITY_DN1363_c0_g1_i11_orf1;TRINITY_DN9316_c0_g3_i1_orf1;TRINITY_DN50743_c0_g1_i1_orf1;TRINITY_DN15755_c0_g1_i1_orf1;TRINITY_DN7170_c0_g1_i11_orf1;TRINITY_DN1960_c5_g1_i3_orf1;TRINITY_DN57856_c0_g2_i1_orf1;TRINITY_DN30037_c0_g1_i5_orf1;TRINITY_DN7861_c0_g1_i5_orf1;TRINITY_DN5126_c0_g1_i3_orf1;TRINITY_DN5661_c0_g1_i5_orf1;TRINITY_DN3321_c0_g1_i3_orf1
molecular_function	vitamin B6 binding	GO:0070279	4	4/1363	TRINITY_DN11159_c0_g1_i5_orf1;TRINITY_DN51813_c0_g1_i1_orf1;TRINITY_DN2684_c0_g2_i3_orf1;TRINITY_DN21035_c0_g1_i14_orf1

molecular_function	identical protein binding	GO:0042802	6	6/1363	TRINITY_DN96557_c0_g1_i1_orf1;TRINITY_DN42854_c0_g3_i2_orf1;TRINITY_DN96739_c0_g1_i1_orf1;TRINITY_DN31584_c0_g2_i2_orf1;TRINITY_DN20009_c0_g1_i1_orf1;TRINITY_DN2848_c0_g1_i2_orf1
molecular_function	p53 binding	GO:0002039	1	1/1363	TRINITY_DN46409_c0_g1_i1_orf1
molecular_function	apolipoprotein binding	GO:0034185	1	1/1363	TRINITY_DN46409_c0_g1_i1_orf1
molecular_function	enzyme binding	GO:0019899	7	7/1363	TRINITY_DN54477_c0_g1_i1_orf1;TRINITY_DN143496_c0_g1_i1_orf1;TRINITY_DN15265_c0_g1_i1_orf1;TRINITY_DN46409_c0_g1_i1_orf1;TRINITY_DN80424_c0_g1_i1_orf1;TRINITY_DN31584_c0_g2_i2_orf1;TRINITY_DN140538_c0_g2_i1_orf1
molecular_function	chaperone binding	GO:0051087	2	2/1363	TRINITY_DN46409_c0_g1_i1_orf1;TRINITY_DN106476_c0_g1_i3_orf1
molecular_function	unfolded protein binding	GO:0051082	7	7/1363	TRINITY_DN7674_c0_g1_i2_orf1;TRINITY_DN3805_c0_g1_i2_orf1;TRINITY_DN16128_c0_g1_i5_orf1;TRINITY_DN4424_c0_g1_i1_orf1;TRINITY_DN46409_c0_g1_i1_orf1;TRINITY_DN351_c14_g1_i2_orf1;TRINITY_DN33801_c0_g1_i1_orf1
molecular_function	calmodulin binding	GO:0005516	3	3/1363	TRINITY_DN4010_c0_g2_i1_orf1;TRINITY_DN140613_c0_g1_i1_orf1;TRINITY_DN32022_c0_g1_i1_orf1
molecular_function	heat shock protein binding	GO:0031072	1	1/1363	TRINITY_DN79083_c0_g1_i2_orf1
molecular_function	beta-catenin binding	GO:0008013	1	1/1363	TRINITY_DN140538_c0_g2_i1_orf1
molecular_function	modification-dependent protein binding	GO:0140030	1	1/1363	TRINITY_DN27958_c0_g1_i1_orf1
molecular_function	translation initiation factor binding	GO:0031369	2	2/1363	TRINITY_DN21609_c0_g2_i1_orf1;TRINITY_DN50085_c0_g1_i1_orf1
molecular_function	signaling receptor binding	GO:0005102	2	2/1363	TRINITY_DN18650_c0_g1_i1_orf1;TRINITY_DN2227_c0_g1_i5_orf1
molecular_function	cytoskeletal protein binding	GO:0008092	19	19/1363	TRINITY_DN1054_c0_g1_i8_orf1;TRINITY_DN110231_c0_g1_i1_orf1;TRINITY_DN4010_c0_g2_i1_orf1;TRINITY_DN25960_c0_g1_i1_orf1;TRINITY_DN22824_c0_g1_i4_orf1;TRINITY_DN2848_c0_g1_i2_orf1;TRINITY_DN8406_c0_g1_i2_orf1;TRINITY_DN104297_c0_g1_i1_orf1;TRINITY_DN9383_c0_g1_i3_orf1;TRINITY_DN23020_c0_g1_i1_orf1;TRINITY_DN96739_c0_g1_i1_orf1;TRINITY_DN10455_c0_g1_i2_orf1;TRINITY_DN77480_c0_g1_i2_orf1;TRINITY_DN364_c1_g1_i2_orf1;TRINITY_DN14298_c0_g3_i1_orf1;TRINITY_DN841_c0_g1_i8_orf1;TRINITY_DN23746_c0_g1_i2_orf1;TRINITY_DN364_c0_g1_i2_orf1;TRINITY_DN140538_c0_g2_i1_orf1
molecular_function	protein dimerization activity	GO:0046983	6	6/1363	TRINITY_DN96557_c0_g1_i1_orf1;TRINITY_DN8646_c0_g1_i2_orf1;TRINITY_DN12594_c0_g1_i1_orf1;TRINITY_DN31584_c0_g2_i2_orf1;TRINITY_DN96801_c0_g1_i1_orf1;TRINITY_DN14611_c0_g1_i5_orf1
molecular_function	phosphoprotein binding	GO:0051219	2	2/1363	TRINITY_DN140538_c0_g2_i1_orf1;TRINITY_DN31584_c0_g2_i2_orf1
molecular_function	protein domain specific binding	GO:0019904	3	3/1363	TRINITY_DN20009_c0_g1_i1_orf1;TRINITY_DN2848_c0_g1_i2_orf1;TRINITY_DN31584_c0_g2_i2_orf1
molecular_function	clathrin binding	GO:0030276	2	2/1363	TRINITY_DN8405_c0_g1_i4_orf1;TRINITY_DN13118_c0_g1_i6_orf1
molecular_function	ubiquitin-like protein binding	GO:0032182	2	2/1363	TRINITY_DN65299_c0_g4_i1_orf1;TRINITY_DN27958_c0_g1_i1_orf1
molecular_function	calcium-dependent protein binding	GO:0048306	1	1/1363	TRINITY_DN96739_c0_g1_i1_orf1
molecular_function	GTPase activating protein binding	GO:0032794	1	1/1363	TRINITY_DN140538_c0_g2_i1_orf1
molecular_function	transmembrane transporter binding	GO:0044325	1	1/1363	TRINITY_DN31584_c0_g2_i2_orf1
molecular_function	scaffold protein binding	GO:0097110	2	2/1363	TRINITY_DN20009_c0_g1_i1_orf1;TRINITY_DN31584_c0_g2_i2_orf1
molecular_function	chitin binding	GO:0008061	8	8/1363	TRINITY_DN77642_c0_g1_i1_orf1;TRINITY_DN1287_c0_g1_i5_orf1;TRINITY_DN10824_c0_g1_i3_orf1;TRINITY_DN82801_c0_g1_i1_orf1;TRINITY_DN2958_c0_g1_i2_orf1;TRINITY_DN664_c0_g1_i18_orf1;TRINITY_DN619_c0_g1_i1_orf1;TRINITY_DN3759_c0_g1_i1
molecular_function	lipopolysaccharide binding	GO:0001530	2	2/1363	TRINITY_DN46409_c0_g1_i1_orf1;TRINITY_DN2170_c0_g2_i1_orf1
molecular_function	lipoteichoic acid binding	GO:0070891	1	1/1363	TRINITY_DN2170_c0_g2_i1_orf1
molecular_function	ribonucleotide binding	GO:0032553	99	99/1363	TRINITY_DN98313_c0_g1_i1_orf1;TRINITY_DN13563_c0_g1_i1_orf1;TRINITY_DN4380_c0_g1_i9_orf1;TRINITY_DN27771_c0_g1_i1_orf1;TRINITY_DN33801_c0_g1_i1_orf1;TRINITY_DN359_c0_g1_i5_orf1;TRINITY_DN54477_c0_g1_i1_orf1;TRINITY_DN11596_c0_g1_i1_orf1;TRINITY_DN235_c0_g3_i1_orf1;TRINITY_DN52761_c0_g1_i2_orf1;TRINITY_DN817_c0_g1_i3_orf1;TRINITY_DN4798_c0_g1_i3_orf1;TRINITY_DN20499_c0_g3_i1_orf1;TRINITY_DN987_c0_g1_i3_orf1;TRINITY_DN4501_c0_g1_i3_orf1;TRINITY_DN31225_c0_g1_i1_orf1;TRINITY_DN46715_c0_g1_i1_orf1;TRINITY_DN52761_c0_g2_i1_orf1;TRINITY_DN2621_c0_g1_i1_orf1;TRINITY_DN3092_c0_g1_i2_orf1;TRINITY_DN2772_c0_g1_i3_orf1;TRINITY_DN100821_c0_g1_i1_orf1;TRINITY_DN2719_c1_g1_i6_orf1;TRINITY_DN7213_c0_g1_i2_orf1;TRINITY_DN11942_c0_g1_i1_orf1;TRINITY_DN1604_c0_g1_i4_orf1;TRINITY_DN4381_c0_g2_i1_orf1;TRINITY_DN60821_c0_g1_i1_orf1;TRINITY_DN42461_c0_g1_i4_orf1;TRINITY_DN277_c1_g1_i1_orf1;TRINITY_DN244_c1_g1_i5_orf1;TRINITY_DN2110_c0_g1_i3_orf1;TRINITY_DN7336_c0_g1_i13_orf1;TRINITY_DN3805_c0_g1_i2_orf1;TRINITY_DN12301_c0_g1_i1_orf1;TRINITY_DN14274_c0_g1_i3_orf1;TRINITY_DN28039_c0_g1_i1_orf1;TRINITY_DN9243_c0_g1_i4_orf1;TRINITY_DN42185_c0_g1_i7_orf1;TRINITY_DN1954_c0_g1_i4_orf1;TRINITY_DN9724_c0_g1_i4_orf1;TRINITY_DN5811_c0_g1_i4_orf1;TRINITY_DN119893_c0_g2_i3_orf1;TRINITY_DN3428_c0_g1_i1_orf1;TRINITY_DN5029_c0_g1_i1_orf1;TRINITY_DN4501_c0_g2_i1_orf1;TRINITY_DN21000_c0_g1_i1_orf1;TRINITY_DN7570_c0_g1_i18_orf1;TRINITY_DN620_c0_g1_i4_orf1;TRINITY_DN46409_c0_g1_i1_orf1;TRINITY_DN11178_c0_g1_i1_orf1;TRINITY_DN670_c0_g1_i15_orf1;TRINITY_DN4742_c0_g1_i1_orf1;TRINITY_DN2770_c0_g2_i4_orf1;TRINITY_DN136906_c0_g1_i1_orf1;TRINITY_DN28729_c0_g1_i9_orf1;TRINITY_DN34479_c0_g1_i2_orf1;TRINITY_DN46090_c0_g3_i1_orf1;TRINITY_DN29144_c0_g3_i1_orf1;TRINITY_DN4835_c0_g1_i2_orf1;TRINITY_DN825_c23_g1_i5_orf1;TRINITY_DN84322_c0_g2_i1_orf1;TRINITY_DN146236_c0_g1_i1_orf1;TRINITY_DN96739_c0_g1_i1_orf1;TRINITY_DN60792_c0_g1_i2_orf1;TRINITY_DN2193_c0_g1_i7_orf1;TRINITY_DN85476_c0_g1_i1_orf1;TRINITY_DN52244_c1_g1_i1_orf1;TRINITY_DN31327_c0_g2_i1_orf1;TRINITY_DN16408_c0_g1_i1_orf1;TRINITY_DN49047_c0_g1_i2_orf1;TRINITY_DN2146_c0_g2_i1_orf1;TRINITY_DN14464_c0_g1_i1_orf1;TRINITY_DN140423_c0_g1_i2_orf1;TRINITY_DN47151_c0_g1_i1_orf1;TRINITY_DN33967_c0_g1_i1_orf1;TRINITY_DN76815_c0_g1_i3_orf1;TRINITY_DN41166_c0_g1_i1_orf1;TRINITY_DN59291_c0_g1_i1_orf1;TRINITY_DN4056_c0_g1_i8_orf1;TRINITY_DN1285_c0_g1_i6_orf1;TRINITY_DN20984_c0_g1_i4_orf1;TRINITY_DN1084_c0_g1_i2_orf1;TRINITY_DN2432_c0_g1_i1_orf1;TRINITY_DN13055_c0_g1_i5_orf1;TRINITY_DN15478_c0_g1_i1_orf1;TRINITY_DN80134_c0_g1_i1_orf1;TRINITY_DN11612_c0_g2_i1_orf1;TRINITY_DN45924_c0_g1_i14_orf1;TRINITY_DN48619_c0_g1_i1_orf1;TRINITY_DN41697_c0_g1_i1_orf1;TRINITY_DN68401_c1_g1_i1_orf1;TRINITY_DN44288_c0_g1_i2_orf1;TRINITY_DN126648_c0_g1_i1_orf1;TRINITY_DN18782_c0_g1_i4_orf1;TRINITY_DN19251_c0_g1_i8_orf1;TRINITY_DN14298_c0_g3_i1_orf1;TRINITY_DN4929_c1_g2_i5_orf1;TRINITY_DN1665_c1_g1_i2_orf1
molecular_function	glycosaminoglycan binding	GO:0005539	3	3/1363	TRINITY_DN42854_c0_g3_i2_orf1;TRINITY_DN96739_c0_g1_i1_orf1;TRINITY_DN1534_c0_g1_i3_orf1
molecular_function	heparin binding	GO:0008201	1	1/1363	TRINITY_DN42854_c0_g3_i2_orf1
molecular_function	acyl-CoA binding	GO:0120227	1	1/1363	TRINITY_DN13563_c0_g1_i1_orf1
molecular_function	chondroitin sulfate binding	GO:0035374	1	1/1363	TRINITY_DN96739_c0_g1_i1_orf1
molecular_function	cation binding	GO:0043169	171	171/1363	TRINITY_DN276_c0_g1_i1_orf1;TRINITY_DN9608_c0_g1_i3_orf1;TRINITY_DN38230_c0_g1_i4_orf1;TRINITY_DN827_c1_g1_i1_orf1;TRINITY_DN3301_c0_g1_i2_orf1;TRINITY_DN7580_c0_g1_i1_orf1;TRINITY_DN35002_c0_g2_i2_orf1;TRINITY_DN3732_c0_g1_i6_orf1;TRINITY_DN40176_c0_g1_i1_orf1;TRINITY_DN51342_c0_g1_i7_orf1;TRINITY_DN2694_c0_g1_i3_orf1;TRINITY_DN47842_c0_g1_i1_orf1;TRINITY_DN12545_c0_g1_i7_orf1;TRINITY_DN8621_c0_g1_i4_orf1;TRINITY_DN1492_c0_g1_i4_orf1;TRINITY_DN24689_c0_g1_i1_orf1;TRINITY_DN22928_c0_g1_i6_orf1;TRINITY_DN3732_c1_g1_i5_orf1;TRINITY_DN31163_c1_g1_i4_orf1;TRINITY_DN3433_c0_g1_i6_orf1;TRINITY_DN8107_c0_g1_i1_orf1;TRINITY_DN4711_c0_g1_i2_orf1;TRINITY_DN3464_c0_g1_i1_orf1;TRINITY_DN14774_c0_g1_i4_orf1;TRINITY_DN15755_c0_g1_i1_orf1;TRINITY_DN4822_c0_g1_i6_orf1;TRINITY_DN1534_c0_g1_i3_orf1;TRINITY_DN123184_c0_g1_i1_orf1;TRINITY_DN817_c0_g1_i3_orf1;TRINITY_DN10785_c0_g1_i4_orf1;TRINITY_DN2117_c0_g1_i1_orf1;TRINITY_DN14347_c0_g1_i1_orf1;TRINITY_DN7909_c0_g2_i1_orf1;TRINITY_DN227_c0_g1_i1_orf1;TRINITY_DN987_c0_g1_i3_orf1;TRINITY_DN2919_c0_g1_i5_orf1;TRINITY_DN4501_c0_g1_i3_orf1;TRINITY_DN2338_c0_g2_i1_orf1;TRINITY_DN1957_c0_g1_i4_orf1;TRINITY_DN230_c2_g1_i5_orf1;TRINITY_DN65681_c0_g1_i1_orf1;TRINITY_DN20442_c0_g2_i1_orf1;TRINITY_DN4367_c0_g1_i1_orf1;TRINITY_DN18242_c0_g1_i3_orf1;TRINITY_DN37532_c0_g1_i1_orf1;TRINITY_DN22018_c0_g1_i3_orf1;TRINITY_DN4189_c0_g2_i1_orf1;TRINITY_DN20238_c0_g1_i7_orf1;TRINITY_DN3010_c0_g1_i4_orf1;TRINITY_DN1272_c1_g1_i4_orf1;TRINITY_DN19187_c0_g1_i1_orf1;TRINITY_DN1423_c0_g1_i4_orf1;TRINITY_DN47575_c0_g1_i1_orf1;TRINITY_DN3275_c0_g2_i3_orf1;TRINITY_DN30713_c0_g1_i3_orf1;TRINITY_DN50787_c0_g2_i2_orf1;TRINITY_DN9316_c0_g3_i1_orf1;TRINITY_DN2110_c0_g1_i3_orf1;TRINITY_DN6602_c0_g1_i4_orf1;TRINITY_DN6415_c0_g1_i1_orf1;TRINITY_DN8241_c0_g1_i3_orf1;TRINITY_DN12301_c0_g1_i1_orf1;TRINITY_DN1083_c0_g1_i4_orf1;TRINITY_DN1423_c0_g1_i8_orf1;TRINITY_DN3733_c0_g1_i1_orf1;TRINITY_DN136031_c0_g1_i7_orf1;TRINITY_DN9243_c0_g1_i4_orf1;TRINITY_DN18922_c0_g1_i1_orf1;TRINITY_DN51568_c0_g1_i1_orf1;TRINITY_DN80134_c0_g1_i1_orf1;TRINITY_DN104597_c0_g1_i2_orf1;TRINITY_DN376_c1_g1_i1_orf1;TRINITY_DN7861_c0_g1_i5_orf1;TRINITY_DN46778_c0_g1_i2_orf1;TRINITY_DN48410_c0_g1_i1_orf1;TRINITY_DN3702_c0_g1_i1_orf1;TRINITY_DN103107_c0_g1_i2_orf1;TRINITY_DN2299_c0_g1_i3_orf1;TRINITY_DN51658_c0_g1_i1_orf1;TRINITY_DN5029_c0_g1_i1_orf1;TRINITY_DN4501_c0_g2_i1_orf1;TRINITY_DN125427_c0_g1_i1_orf1;TRINITY_DN8985_c0_g1_i4_orf1;TRINITY_DN1209_c0_g1_i9_orf1;TRINITY_DN257_c0_g1_i7_orf1;TRINITY_DN10672_c0_g1_i3_orf1;TRINITY_DN7570_c0_g1_i18_orf1;TRINITY_DN1363_c0_g1_i11_orf1;TRINITY_DN18159_c0_g1_i6_orf1;TRINITY_DN16400_c0_g2_i1_orf1;TRINITY_DN2186_c0_g1_i17_orf1;TRINITY_DN24873_c0_g1_i4_orf1;TRINITY_DN89083_c0_g1_i1_orf1;TRINITY_DN47151_c0_g1_i1_orf1;TRINITY_DN7212_c0_g1_i4_orf1;TRINITY_DN30037_c0_g1_i5_orf1;TRINITY_DN36788_c0_g1_i2_orf1;TRINITY_DN24_c0_g1_i1_orf1;TRINITY_DN4793_c0_g1_i7_orf1;TRINITY_DN43412_c0_g1_i2_orf1;TRINITY_DN4565_c0_g2_i1_orf1;TRINITY_DN142652_c0_g1_i1_orf1;TRINITY_DN276_c0_g1_i2_orf1;TRINITY_DN3275_c0_g1_i4_orf1;TRINITY_DN5432_c1_g1_i3_orf1;TRINITY_DN50743_c0_g1_i1_orf1;TRINITY_DN8771_c0_g2_i1_orf1;TRINITY_DN7170_c0_g1_i11_orf1;TRINITY_DN19866_c0_g1_i4_orf1;TRINITY_DN84322_c0_g2_i1_orf1;TRINITY_DN19303_c0_g1_i5_orf1;TRINITY_DN2338_c0_g2_i2_orf1;TRINITY_DN2673_c2_g1_i2_orf1;TRINITY_DN146236_c0_g1_i1_orf1;TRINITY_DN96739_c0_g1_i1_orf1;TRINITY_DN1707_c0_g1_i1_orf1;TRINITY_DN31348_c0_g1_i1_orf1;TRINITY_DN101682_c0_g1_i1_orf1;TRINITY_DN111110_c0_g1_i1_orf1;TRINITY_DN5126_c0_g1_i3_orf1;TRINITY_DN1860_c0_g1_i1_orf1;TRINITY_DN5661_c0_g1_i5_orf1;TRINITY_DN2647_c0_g1_i3_orf1;TRINITY_DN2472_c0_g1_i6_orf1;TRINITY_DN42854_c0_g3_i2_orf1;TRINITY_DN8637_c0_g1_i1_orf1;TRINITY_DN70236_c0_g1_i1_orf1;TRINITY_DN892_c0_g1_i9_orf1;TRINITY_DN61536_c0_g3_i1_orf1;TRINITY_DN1194_c0_g1_i5_orf1;TRINITY_DN78492_c0_g1_i1_orf1;TRINITY_DN2971_c0_g1_i1_orf1;TRINITY_DN46625_c0_g1_i1_orf1;TRINITY_DN23564_c0_g1_i7_orf1;TRINITY_DN51968_c0_g1_i1_orf1;TRINITY_DN2986_c1_g1_i1_orf1;TRINITY_DN4424_c0_g1_i1_orf1;TRINITY_DN2392_c0_g2_i1_orf1;TRINITY_DN76815_c0_g1_i3_orf1;TRINITY_DN2342_c0_g1_i1_orf1;TRINITY_DN25423_c0_g1_i1_orf1;TRINITY_DN27592_c0_g1_i1_orf1;TRINITY_DN4822_c0_g1_i9_orf1;TRINITY_DN4056_c0_g1_i8_orf1;TRINITY_DN14874_c0_g1_i6_orf1;TRINITY_DN57765_c0_g1_i1_orf1;TRINITY_DN7647_c0_g1_i4_orf1;TRINITY_DN3949_c0_g1_i1_orf1;TRINITY_DN21035_c0_g1_i14_orf1;TRINITY_DN14670_c0_g1_i1_orf1;TRINITY_DN12024_c0_g2_i2_orf1;TRINITY_DN1884_c0_g2_i2_orf1;TRINITY_DN279_c0_g1_i10_orf1;TRINITY_DN37307_c0_g1_i4_orf1;TRINITY_DN22824_c0_g1_i4_orf1;TRINITY_DN17913_c0_g1_i8_orf1;TRINITY_DN5238_c0_g1_i2_orf1;TRINITY_DN1960_c5_g1_i3_orf1;TRINITY_DN57856_c0_g2_i1_orf1;TRINITY_DN140_c0_g1_i5_orf1;TRINITY_DN351_c14_g1_i2_orf1;TRINITY_DN45924_c0_g1_i14_orf1;TRINITY_DN21533_c0_g1_i7_orf1;TRINITY_DN41697_c0_g1_i1_orf1;TRINITY_DN430_c0_g1_i5_orf1;TRINITY_DN346_c0_g1_i7_orf1;TRINITY_DN14409_c0_g1_i1_orf1;TRINITY_DN11820_c0_g1_i1_orf1;TRINITY_DN1084_c0_g1_i2_orf1;TRINITY_DN19251_c0_g1_i8_orf1;TRINITY_DN26130_c0_g1_i1_orf1
molecular_function	anion binding	GO:0043168	114	114/1363	TRINITY_DN98313_c0_g1_i1_orf1;TRINITY_DN13563_c0_g1_i1_orf1;TRINITY_DN4380_c0_g1_i9_orf1;TRINITY_DN27771_c0_g1_i1_orf1;TRINITY_DN11159_c0_g1_i5_orf1;TRINITY_DN33801_c0_g1_i1_orf1;TRINITY_DN359_c0_g1_i5_orf1;TRINITY_DN54477_c0_g1_i1_orf1;TRINITY_DN4711_c0_g1_i2_orf1;TRINITY_DN26789_c0_g1_i2_orf1;TRINITY_DN11596_c0_g1_i1_orf1;TRINITY_DN235_c0_g3_i1_orf1;TRINITY_DN52761_c0_g1_i2_orf1;TRINITY_DN817_c0_g1_i3_orf1;TRINITY_DN4798_c0_g1_i3_orf1;TRINITY_DN20499_c0_g3_i1_orf1;TRINITY_DN7909_c0_g2_i1_orf1;TRINITY_DN987_c0_g1_i3_orf1;TRINITY_DN4501_c0_g1_i3_orf1;TRINITY_DN31225_c0_g1_i1_orf1;TRINITY_DN46715_c0_g1_i1_orf1;TRINITY_DN52761_c0_g2_i1_orf1;TRINITY_DN2621_c0_g1_i1_orf1;TRINITY_DN3092_c0_g1_i2_orf1;TRINITY_DN2772_c0_g1_i3_orf1;TRINITY_DN100821_c0_g1_i1_orf1;TRINITY_DN2719_c1_g1_i6_orf1;TRINITY_DN7213_c0_g1_i2_orf1;TRINITY_DN11942_c0_g1_i1_orf1;TRINITY_DN1604_c0_g1_i4_orf1;TRINITY_DN4381_c0_g2_i1_orf1;TRINITY_DN60821_c0_g1_i1_orf1;TRINITY_DN42461_c0_g1_i4_orf1;TRINITY_DN3588_c0_g1_i4_orf1;TRINITY_DN277_c1_g1_i1_orf1;TRINITY_DN244_c1_g1_i5_orf1;TRINITY_DN2110_c0_g1_i3_orf1;TRINITY_DN7336_c0_g1_i13_orf1;TRINITY_DN3805_c0_g1_i2_orf1;TRINITY_DN12301_c0_g1_i1_orf1;TRINITY_DN38424_c0_g1_i1_orf1;TRINITY_DN14274_c0_g1_i3_orf1;TRINITY_DN28039_c0_g1_i1_orf1;TRINITY_DN9243_c0_g1_i4_orf1;TRINITY_DN42185_c0_g1_i7_orf1;TRINITY_DN1954_c0_g1_i4_orf1;TRINITY_DN9724_c0_g1_i4_orf1;TRINITY_DN5811_c0_g1_i4_orf1;TRINITY_DN119893_c0_g2_i3_orf1;TRINITY_DN3428_c0_g1_i1_orf1;TRINITY_DN2684_c0_g2_i3_orf1;TRINITY_DN5029_c0_g1_i1_orf1;TRINITY_DN4501_c0_g2_i1_orf1;TRINITY_DN21000_c0_g1_i1_orf1;TRINITY_DN5055_c0_g1_i12_orf1;TRINITY_DN7570_c0_g1_i18_orf1;TRINITY_DN620_c0_g1_i4_orf1;TRINITY_DN46409_c0_g1_i1_orf1;TRINITY_DN11178_c0_g1_i1_orf1;TRINITY_DN670_c0_g1_i15_orf1;TRINITY_DN4742_c0_g1_i1_orf1;TRINITY_DN2770_c0_g2_i4_orf1;TRINITY_DN136906_c0_g1_i1_orf1;TRINITY_DN28729_c0_g1_i9_orf1;TRINITY_DN34479_c0_g1_i2_orf1;TRINITY_DN46090_c0_g3_i1_orf1;TRINITY_DN29144_c0_g3_i1_orf1;TRINITY_DN4835_c0_g1_i2_orf1;TRINITY_DN825_c23_g1_i5_orf1;TRINITY_DN84322_c0_g2_i1_orf1;TRINITY_DN51813_c0_g1_i1_orf1;TRINITY_DN146236_c0_g1_i1_orf1;TRINITY_DN96739_c0_g1_i1_orf1;TRINITY_DN60792_c0_g1_i2_orf1;TRINITY_DN2193_c0_g1_i7_orf1;TRINITY_DN659_c0_g2_i1_orf1;TRINITY_DN85476_c0_g1_i1_orf1;TRINITY_DN111110_c0_g1_i1_orf1;TRINITY_DN62_c1_g1_i3_orf1;TRINITY_DN52244_c1_g1_i1_orf1;TRINITY_DN31327_c0_g2_i1_orf1;TRINITY_DN16408_c0_g1_i1_orf1;TRINITY_DN49047_c0_g1_i2_orf1;TRINITY_DN70236_c0_g1_i1_orf1;TRINITY_DN2146_c0_g2_i1_orf1;TRINITY_DN14464_c0_g1_i1_orf1;TRINITY_DN140423_c0_g1_i2_orf1;TRINITY_DN47151_c0_g1_i1_orf1;TRINITY_DN33967_c0_g1_i1_orf1;TRINITY_DN76815_c0_g1_i3_orf1;TRINITY_DN41166_c0_g1_i1_orf1;TRINITY_DN59291_c0_g1_i1_orf1;TRINITY_DN4056_c0_g1_i8_orf1;TRINITY_DN1285_c0_g1_i6_orf1;TRINITY_DN21035_c0_g1_i14_orf1;TRINITY_DN20984_c0_g1_i4_orf1;TRINITY_DN1084_c0_g1_i2_orf1;TRINITY_DN2432_c0_g1_i1_orf1;TRINITY_DN13055_c0_g1_i5_orf1;TRINITY_DN15478_c0_g1_i1_orf1;TRINITY_DN80134_c0_g1_i1_orf1;TRINITY_DN11612_c0_g2_i1_orf1;TRINITY_DN45924_c0_g1_i14_orf1;TRINITY_DN48619_c0_g1_i1_orf1;TRINITY_DN41697_c0_g1_i1_orf1;TRINITY_DN68401_c1_g1_i1_orf1;TRINITY_DN44288_c0_g1_i2_orf1;TRINITY_DN3131_c0_g1_i5_orf1;TRINITY_DN126648_c0_g1_i1_orf1;TRINITY_DN18782_c0_g1_i4_orf1;TRINITY_DN19251_c0_g1_i8_orf1;TRINITY_DN14298_c0_g3_i1_orf1;TRINITY_DN4929_c1_g2_i5_orf1;TRINITY_DN1665_c1_g1_i2_orf1
molecular_function	phospholipid binding	GO:0005543	9	9/1363	TRINITY_DN96739_c0_g1_i1_orf1;TRINITY_DN36230_c0_g1_i1_orf1;TRINITY_DN65299_c0_g4_i1_orf1;TRINITY_DN12432_c0_g1_i2_orf1;TRINITY_DN20238_c0_g1_i7_orf1;TRINITY_DN119265_c0_g2_i1_orf1;TRINITY_DN2770_c0_g2_i4_orf1;TRINITY_DN21533_c0_g1_i7_orf1;TRINITY_DN11693_c0_g1_i6_orf1
molecular_function	steroid binding	GO:0005496	1	1/1363	TRINITY_DN96739_c0_g1_i1_orf1
molecular_function	fatty acid derivative binding	GO:1901567	1	1/1363	TRINITY_DN13563_c0_g1_i1_orf1
molecular_function	oxygen binding	GO:0019825	1	1/1363	TRINITY_DN30037_c0_g1_i5_orf1
molecular_function	alcohol binding	GO:0043178	1	1/1363	TRINITY_DN96739_c0_g1_i1_orf1
molecular_function	vitamin binding	GO:0019842	4	4/1363	TRINITY_DN11159_c0_g1_i5_orf1;TRINITY_DN51813_c0_g1_i1_orf1;TRINITY_DN2684_c0_g2_i3_orf1;TRINITY_DN21035_c0_g1_i14_orf1
molecular_function	nucleotide binding	GO:0000166	116	116/1363	TRINITY_DN98313_c0_g1_i1_orf1;TRINITY_DN13563_c0_g1_i1_orf1;TRINITY_DN38230_c0_g1_i4_orf1;TRINITY_DN4380_c0_g1_i9_orf1;TRINITY_DN27771_c0_g1_i1_orf1;TRINITY_DN33801_c0_g1_i1_orf1;TRINITY_DN359_c0_g1_i5_orf1;TRINITY_DN54477_c0_g1_i1_orf1;TRINITY_DN4711_c0_g1_i2_orf1;TRINITY_DN26789_c0_g1_i2_orf1;TRINITY_DN5422_c0_g1_i1_orf1;TRINITY_DN11596_c0_g1_i1_orf1;TRINITY_DN235_c0_g3_i1_orf1;TRINITY_DN52761_c0_g1_i2_orf1;TRINITY_DN817_c0_g1_i3_orf1;TRINITY_DN4798_c0_g1_i3_orf1;TRINITY_DN20499_c0_g3_i1_orf1;TRINITY_DN7909_c0_g2_i1_orf1;TRINITY_DN987_c0_g1_i3_orf1;TRINITY_DN4501_c0_g1_i3_orf1;TRINITY_DN31225_c0_g1_i1_orf1;TRINITY_DN46715_c0_g1_i1_orf1;TRINITY_DN2848_c0_g1_i2_orf1;TRINITY_DN52761_c0_g2_i1_orf1;TRINITY_DN7336_c0_g1_i13_orf1;TRINITY_DN3092_c0_g1_i2_orf1;TRINITY_DN2772_c0_g1_i3_orf1;TRINITY_DN100821_c0_g1_i1_orf1;TRINITY_DN2719_c1_g1_i6_orf1;TRINITY_DN7213_c0_g1_i2_orf1;TRINITY_DN11942_c0_g1_i1_orf1;TRINITY_DN1604_c0_g1_i4_orf1;TRINITY_DN4381_c0_g2_i1_orf1;TRINITY_DN60821_c0_g1_i1_orf1;TRINITY_DN42461_c0_g1_i4_orf1;TRINITY_DN3588_c0_g1_i4_orf1;TRINITY_DN277_c1_g1_i1_orf1;TRINITY_DN244_c1_g1_i5_orf1;TRINITY_DN2110_c0_g1_i3_orf1;TRINITY_DN2621_c0_g1_i1_orf1;TRINITY_DN3805_c0_g1_i2_orf1;TRINITY_DN12301_c0_g1_i1_orf1;TRINITY_DN38424_c0_g1_i1_orf1;TRINITY_DN14274_c0_g1_i3_orf1;TRINITY_DN28039_c0_g1_i1_orf1;TRINITY_DN9243_c0_g1_i4_orf1;TRINITY_DN42185_c0_g1_i7_orf1;TRINITY_DN1954_c0_g1_i4_orf1;TRINITY_DN9724_c0_g1_i4_orf1;TRINITY_DN5811_c0_g1_i4_orf1;TRINITY_DN16408_c0_g1_i1_orf1;TRINITY_DN3428_c0_g1_i1_orf1;TRINITY_DN5029_c0_g1_i1_orf1;TRINITY_DN4501_c0_g2_i1_orf1;TRINITY_DN21000_c0_g1_i1_orf1;TRINITY_DN5055_c0_g1_i12_orf1;TRINITY_DN7570_c0_g1_i18_orf1;TRINITY_DN620_c0_g1_i4_orf1;TRINITY_DN46409_c0_g1_i1_orf1;TRINITY_DN11178_c0_g1_i1_orf1;TRINITY_DN670_c0_g1_i15_orf1;TRINITY_DN4742_c0_g1_i1_orf1;TRINITY_DN2770_c0_g2_i4_orf1;TRINITY_DN136906_c0_g1_i1_orf1;TRINITY_DN28729_c0_g1_i9_orf1;TRINITY_DN36788_c0_g1_i2_orf1;TRINITY_DN24_c0_g1_i1_orf1;TRINITY_DN34479_c0_g1_i2_orf1;TRINITY_DN46090_c0_g3_i1_orf1;TRINITY_DN29144_c0_g3_i1_orf1;TRINITY_DN4835_c0_g1_i2_orf1;TRINITY_DN825_c23_g1_i5_orf1;TRINITY_DN84322_c0_g2_i1_orf1;TRINITY_DN146236_c0_g1_i1_orf1;TRINITY_DN96739_c0_g1_i1_orf1;TRINITY_DN60792_c0_g1_i2_orf1;TRINITY_DN2193_c0_g1_i7_orf1;TRINITY_DN659_c0_g2_i1_orf1;TRINITY_DN85476_c0_g1_i1_orf1;TRINITY_DN111110_c0_g1_i1_orf1;TRINITY_DN62_c1_g1_i3_orf1;TRINITY_DN52244_c1_g1_i1_orf1;TRINITY_DN31327_c0_g2_i1_orf1;TRINITY_DN49047_c0_g1_i2_orf1;TRINITY_DN70236_c0_g1_i1_orf1;TRINITY_DN2146_c0_g2_i1_orf1;TRINITY_DN14464_c0_g1_i1_orf1;TRINITY_DN140423_c0_g1_i2_orf1;TRINITY_DN47151_c0_g1_i1_orf1;TRINITY_DN33967_c0_g1_i1_orf1;TRINITY_DN76815_c0_g1_i3_orf1;TRINITY_DN41166_c0_g1_i1_orf1;TRINITY_DN59291_c0_g1_i1_orf1;TRINITY_DN119893_c0_g2_i3_orf1;TRINITY_DN4056_c0_g1_i8_orf1;TRINITY_DN1285_c0_g1_i6_orf1;TRINITY_DN20984_c0_g1_i4_orf1;TRINITY_DN1084_c0_g1_i2_orf1;TRINITY_DN2432_c0_g1_i1_orf1;TRINITY_DN13055_c0_g1_i5_orf1;TRINITY_DN15478_c0_g1_i1_orf1;TRINITY_DN80134_c0_g1_i1_orf1;TRINITY_DN11612_c0_g2_i1_orf1;TRINITY_DN45924_c0_g1_i14_orf1;TRINITY_DN48619_c0_g1_i1_orf1;TRINITY_DN41697_c0_g1_i1_orf1;TRINITY_DN68401_c1_g1_i1_orf1;TRINITY_DN44288_c0_g1_i2_orf1;TRINITY_DN3131_c0_g1_i5_orf1;TRINITY_DN126648_c0_g1_i1_orf1;TRINITY_DN18782_c0_g1_i4_orf1;TRINITY_DN19251_c0_g1_i8_orf1;TRINITY_DN14298_c0_g3_i1_orf1;TRINITY_DN1209_c0_g1_i9_orf1;TRINITY_DN4929_c1_g2_i5_orf1;TRINITY_DN1665_c1_g1_i2_orf1
molecular_function	polysaccharide binding	GO:0030247	1	1/1363	TRINITY_DN2170_c0_g2_i1_orf1
molecular_function	phosphatidylserine binding	GO:0001786	1	1/1363	TRINITY_DN96739_c0_g1_i1_orf1

molecular_function	ribonucleoprotein complex binding	GO:0043021	2	2/1363	TRINITY_DN44407_c0_g4_i2_orf1;TRINITY_DN441_c0_g2_i1_orf1
molecular_function	protein-lipid complex binding	GO:0071814	1	1/1363	TRINITY_DN46409_c0_g1_i1_orf1
molecular_function	intermediate filament binding	GO:0019215	1	1/1363	TRINITY_DN20009_c0_g1_i1_orf1
molecular_function	actin filament binding	GO:0051015	5	5/1363	TRINITY_DN110231_c0_g1_i1_orf1;TRINITY_DN96739_c0_g1_i1_orf1;TRINITY_DN364_c0_g1_i2_orf1;TRINITY_DN9383_c0_g1_i3_orf1;TRINITY_DN22824_c0_g1_i4_orf1
molecular_function	iron-sulfur cluster binding	GO:0051536	6	6/1363	TRINITY_DN7909_c0_g2_i1_orf1;TRINITY_DN49221_c0_g1_i1_orf1;TRINITY_DN85476_c0_g1_i1_orf1;TRINITY_DN4711_c0_g1_i2_orf1;TRINITY_DN3464_c0_g1_i1_orf1;TRINITY_DN5432_c1_g1_i3_orf1
molecular_function	transmembrane signaling receptor activity	GO:0004888	4	4/1363	TRINITY_DN15247_c0_g1_i2_orf1;TRINITY_DN46090_c0_g3_i1_orf1;TRINITY_DN19662_c4_g1_i1_orf1;TRINITY_DN34821_c0_g1_i4_orf1
molecular_function	pattern recognition receptor activity	GO:0038187	1	1/1363	TRINITY_DN2170_c0_g2_i1_orf1
molecular_function	ligase activity, forming carbon-oxygen bonds	GO:0016875	6	6/1363	TRINITY_DN825_c23_g1_i5_orf1;TRINITY_DN84322_c0_g2_i1_orf1;TRINITY_DN27771_c0_g1_i1_orf1;TRINITY_DN620_c0_g1_i4_orf1;TRINITY_DN817_c0_g1_i3_orf1;TRINITY_DN48619_c0_g1_i1_orf1
molecular_function	ligase activity, forming carbon-sulfur bonds	GO:0016877	3	3/1363	TRINITY_DN22928_c0_g1_i6_orf1;TRINITY_DN2193_c0_g1_i7_orf1;TRINITY_DN19251_c0_g1_i8_orf1
molecular_function	ligase activity, forming carbon-nitrogen bonds	GO:0016879	9	9/1363	TRINITY_DN98313_c0_g1_i1_orf1;TRINITY_DN36144_c0_g1_i3_orf1;TRINITY_DN987_c0_g1_i3_orf1;TRINITY_DN76815_c0_g1_i3_orf1;TRINITY_DN100821_c0_g1_i1_orf1;TRINITY_DN14464_c0_g1_i1_orf1;TRINITY_DN41697_c0_g1_i1_orf1;TRINITY_DN45924_c0_g1_i14_orf1;TRINITY_DN244_c1_g1_i5_orf1
molecular_function	carbon-nitrogen lyase activity	GO:0016840	2	2/1363	TRINITY_DN16868_c0_g2_i1_orf1;TRINITY_DN28299_c0_g1_i1_orf1
molecular_function	phosphorus-oxygen lyase activity	GO:0016849	3	3/1363	TRINITY_DN3712_c0_g1_i1_orf1;TRINITY_DN11942_c0_g1_i1_orf1;TRINITY_DN52244_c1_g1_i1_orf1
molecular_function	carbon-oxygen lyase activity	GO:0016835	3	3/1363	TRINITY_DN10900_c0_g1_i7_orf1;TRINITY_DN230_c2_g1_i5_orf1;TRINITY_DN3464_c0_g1_i1_orf1
molecular_function	carbon-carbon lyase activity	GO:0016830	6	6/1363	TRINITY_DN6325_c0_g1_i8_orf1;TRINITY_DN40434_c0_g1_i2_orf1;TRINITY_DN109931_c0_g1_i1_orf1;TRINITY_DN2684_c0_g2_i3_orf1;TRINITY_DN10548_c0_g2_i1_orf1;TRINITY_DN11159_c0_g1_i5_orf1
molecular_function	protein-malonyllysine demalonylase activity	GO:0036054	1	1/1363	TRINITY_DN111110_c0_g1_i1_orf1
molecular_function	peptide-lysine-N-acetyltransferase activity	GO:0061733	2	2/1363	TRINITY_DN20442_c0_g2_i1_orf1;TRINITY_DN46202_c0_g1_i1_orf1
molecular_function	palmitoyl-(protein) hydrolase activity	GO:0008474	1	1/1363	TRINITY_DN4817_c0_g1_i4_orf1
molecular_function	protein-disulfide reductase activity	GO:0015035	1	1/1363	TRINITY_DN24689_c0_g1_i1_orf1
molecular_function	protein-glutaryllysine deglutarylase activity	GO:0061697	1	1/1363	TRINITY_DN111110_c0_g1_i1_orf1
molecular_function	protein methyltransferase activity	GO:0008276	3	3/1363	TRINITY_DN22674_c0_g1_i2_orf1;TRINITY_DN95414_c0_g1_i1_orf1;TRINITY_DN6462_c0_g1_i5_orf1
molecular_function	phosphoprotein phosphatase activity	GO:0004721	4	4/1363	TRINITY_DN1749_c0_g2_i2_orf1;TRINITY_DN152_c0_g1_i4_orf1;TRINITY_DN34830_c0_g1_i1_orf1;TRINITY_DN59885_c0_g1_i3_orf1
molecular_function	peptidyl-cysteine S-nitrosylase activity	GO:0035605	1	1/1363	TRINITY_DN2848_c0_g1_i2_orf1
molecular_function	ubiquitin-like protein transferase activity	GO:0019787	7	7/1363	TRINITY_DN24323_c0_g1_i3_orf1;TRINITY_DN17376_c0_g1_i2_orf1;TRINITY_DN7316_c0_g2_i1_orf1;TRINITY_DN7647_c0_g1_i4_orf1;TRINITY_DN11820_c0_g1_i1_orf1;TRINITY_DN51658_c0_g1_i1_orf1;TRINITY_DN1272_c1_g1_i4_orf1
molecular_function	protein lysine deacetylase activity	GO:0033558	2	2/1363	TRINITY_DN70236_c0_g1_i1_orf1;TRINITY_DN111110_c0_g1_i1_orf1
molecular_function	protein kinase activity	GO:0004672	13	13/1363	TRINITY_DN54477_c0_g1_i1_orf1;TRINITY_DN140423_c0_g1_i2_orf1;TRINITY_DN28729_c0_g1_i9_orf1;TRINITY_DN19662_c4_g1_i1_orf1;TRINITY_DN42461_c0_g1_i4_orf1;TRINITY_DN46090_c0_g3_i1_orf1;TRINITY_DN4798_c0_g1_i3_orf1;TRINITY_DN277_c1_g1_i1_orf1;TRINITY_DN670_c0_g1_i15_orf1;TRINITY_DN4742_c0_g1_i1_orf1;TRINITY_DN15478_c0_g1_i1_orf1;TRINITY_DN1954_c0_g1_i4_orf1;TRINITY_DN46715_c0_g1_i1_orf1
molecular_function	peptidase activity	GO:0008233	46	46/1363	TRINITY_DN4767_c0_g1_i6_orf1;TRINITY_DN143895_c0_g1_i1_orf1;TRINITY_DN10766_c0_g1_i1_orf1;TRINITY_DN801_c0_g1_i2_orf1;TRINITY_DN18159_c0_g1_i6_orf1;TRINITY_DN67026_c0_g1_i6_orf1;TRINITY_DN3483_c0_g1_i5_orf1;TRINITY_DN753_c0_g1_i4_orf1;TRINITY_DN113327_c0_g1_i2_orf1;TRINITY_DN4189_c0_g2_i1_orf1;TRINITY_DN6122_c0_g1_i6_orf1;TRINITY_DN344_c1_g1_i1_orf1;TRINITY_DN57111_c0_g1_i1_orf1;TRINITY_DN9836_c0_g1_i2_orf1;TRINITY_DN14874_c0_g1_i6_orf1;TRINITY_DN376_c1_g1_i1_orf1;TRINITY_DN4030_c0_g2_i1_orf1;TRINITY_DN17329_c0_g2_i3_orf1;TRINITY_DN8621_c0_g1_i4_orf1;TRINITY_DN2442_c0_g1_i2_orf1;TRINITY_DN34479_c0_g1_i2_orf1;TRINITY_DN1592_c0_g1_i1_orf1;TRINITY_DN2885_c1_g1_i2_orf1;TRINITY_DN49047_c0_g1_i2_orf1;TRINITY_DN14754_c0_g1_i6_orf1;TRINITY_DN45948_c1_g1_i1_orf1;TRINITY_DN24121_c1_g1_i6_orf1;TRINITY_DN5696_c0_g1_i4_orf1;TRINITY_DN892_c7_g1_i2_orf1;TRINITY_DN14774_c0_g1_i4_orf1;TRINITY_DN71863_c0_g1_i2_orf1;TRINITY_DN19866_c0_g1_i4_orf1;TRINITY_DN18273_c0_g1_i4_orf1;TRINITY_DN2673_c2_g1_i2_orf1;TRINITY_DN1308_c0_g1_i4_orf1;TRINITY_DN4408_c6_g1_i1_orf1;TRINITY_DN23167_c0_g1_i4_orf1;TRINITY_DN21984_c0_g1_i6_orf1;TRINITY_DN6205_c0_g1_i8_orf1;TRINITY_DN140_c1_g1_i2_orf1;TRINITY_DN4121_c0_g1_i1_orf1;TRINITY_DN4767_c0_g1_i4_orf1;TRINITY_DN6470_c0_g3_i2_orf1;TRINITY_DN3702_c0_g1_i1_orf1;TRINITY_DN4494_c0_g1_i1_orf1;TRINITY_DN26130_c0_g1_i1_orf1
molecular_function	peptidyl-prolyl cis-trans isomerase activity	GO:0003755	2	2/1363	TRINITY_DN2807_c0_g1_i4_orf1;TRINITY_DN140538_c0_g2_i1_orf1
molecular_function	protein demethylase activity	GO:0140457	1	1/1363	TRINITY_DN89083_c0_g1_i1_orf1
molecular_function	ubiquitin-like modifier activating enzyme activity	GO:0008641	1	1/1363	TRINITY_DN22928_c0_g1_i6_orf1
molecular_function	microtubule plus end polymerase	GO:0061863	1	1/1363	TRINITY_DN25960_c0_g1_i1_orf1
molecular_function	hydrolase activity, acting on glycosyl bonds	GO:0016798	22	22/1363	TRINITY_DN14235_c0_g1_i1_orf1;TRINITY_DN8703_c0_g1_i2_orf1;TRINITY_DN18918_c0_g1_i3_orf1;TRINITY_DN6108_c0_g1_i5_orf1;TRINITY_DN14409_c0_g1_i1_orf1;TRINITY_DN9044_c0_g1_i1_orf1;TRINITY_DN467_c3_g1_i5_orf1;TRINITY_DN28741_c0_g1_i3_orf1;TRINITY_DN3322_c0_g1_i2_orf1;TRINITY_DN2894_c0_g2_i3_orf1;TRINITY_DN1785_c0_g1_i5_orf1;TRINITY_DN10824_c0_g1_i3_orf1;TRINITY_DN195_c4_g1_i1_orf1;TRINITY_DN2170_c0_g2_i1_orf1;TRINITY_DN4070_c0_g1_i4_orf1;TRINITY_DN9044_c0_g1_i2_orf1;TRINITY_DN1732_c0_g1_i15_orf1;TRINITY_DN54410_c0_g2_i1_orf1;TRINITY_DN1732_c0_g1_i17_orf1;TRINITY_DN1287_c0_g1_i5_orf1;TRINITY_DN48410_c0_g1_i1_orf1;TRINITY_DN103118_c0_g1_i4_orf1
molecular_function	hydrolase activity, acting on acid carbon-carbon bonds	GO:0016822	3	3/1363	TRINITY_DN17913_c0_g1_i8_orf1;TRINITY_DN19187_c0_g1_i1_orf1;TRINITY_DN51813_c0_g1_i1_orf1
molecular_function	hydrolase activity, acting on carbon-nitrogen (but not peptide) bonds	GO:0016810	11	11/1363	TRINITY_DN5422_c0_g1_i1_orf1;TRINITY_DN827_c1_g1_i1_orf1;TRINITY_DN1534_c0_g1_i3_orf1;TRINITY_DN82801_c0_g1_i1_orf1;TRINITY_DN17326_c0_g1_i5_orf1;TRINITY_DN98242_c0_g1_i1_orf1;TRINITY_DN70236_c0_g1_i1_orf1;TRINITY_DN244_c1_g1_i5_orf1;TRINITY_DN25423_c0_g1_i1_orf1;TRINITY_DN2835_c0_g1_i6_orf1;TRINITY_DN111110_c0_g1_i1_orf1
molecular_function	hydrolase activity, acting on acid anhydrides	GO:0016817	32	32/1363	TRINITY_DN7336_c0_g1_i13_orf1;TRINITY_DN3092_c0_g1_i2_orf1;TRINITY_DN33967_c0_g1_i1_orf1;TRINITY_DN140212_c0_g1_i1_orf1;TRINITY_DN46409_c0_g1_i1_orf1;TRINITY_DN100821_c0_g1_i1_orf1;TRINITY_DN33801_c0_g1_i1_orf1;TRINITY_DN2054_c0_g1_i1_orf1;TRINITY_DN136906_c0_g1_i1_orf1;TRINITY_DN11612_c0_g2_i1_orf1;TRINITY_DN11069_c0_g2_i1_orf1;TRINITY_DN34479_c0_g1_i2_orf1;TRINITY_DN7570_c0_g1_i18_orf1;TRINITY_DN29144_c0_g3_i1_orf1;TRINITY_DN1091_c0_g1_i1_orf1;TRINITY_DN1091_c0_g3_i1_orf1;TRINITY_DN5422_c0_g1_i1_orf1;TRINITY_DN3805_c0_g1_i2_orf1;TRINITY_DN13055_c0_g1_i5_orf1;TRINITY_DN9243_c0_g1_i4_orf1;TRINITY_DN28039_c0_g1_i1_orf1;TRINITY_DN146236_c0_g1_i1_orf1;TRINITY_DN52761_c0_g1_i2_orf1;TRINITY_DN42185_c0_g1_i7_orf1;TRINITY_DN9724_c0_g1_i4_orf1;TRINITY_DN7565_c0_g1_i3_orf1;TRINITY_DN49047_c0_g1_i2_orf1;TRINITY_DN14464_c0_g1_i1_orf1;TRINITY_DN1665_c1_g1_i2_orf1;TRINITY_DN2621_c0_g1_i1_orf1;TRINITY_DN52761_c0_g2_i1_orf1;TRINITY_DN[illegible]
molecular_function	serine hydrolase activity	GO:0017171	19	19/1363	TRINITY_DN1308_c0_g1_i4_orf1;TRINITY_DN71863_c0_g1_i2_orf1;TRINITY_DN24121_c1_g1_i6_orf1;TRINITY_DN4030_c0_g2_i1_orf1;TRINITY_DN3483_c0_g1_i5_orf1;TRINITY_DN18273_c0_g1_i4_orf1;TRINITY_DN344_c1_g1_i1_orf1;TRINITY_DN6470_c0_g3_i2_orf1;TRINITY_DN1592_c0_g1_i1_orf1;TRINITY_DN67026_c0_g1_i6_orf1;TRINITY_DN10766_c0_g1_i1_orf1;TRINITY_DN23167_c0_g1_i4_orf1;TRINITY_DN21984_c0_g1_i6_orf1;TRINITY_DN4494_c0_g1_i1_orf1;TRINITY_DN6205_c0_g1_i8_orf1;TRINITY_DN753_c0_g1_i4_orf1;TRINITY_DN140_c1_g1_i2_orf1;TRINITY_DN5696_c0_g1_i4_orf1;TRINITY_DN57111_c0_g1_i1_orf1
molecular_function	hydrolase activity, acting on ester bonds	GO:0016788	51	51/1363	TRINITY_DN1749_c0_g2_i2_orf1;TRINITY_DN2749_c4_g1_i2_orf1;TRINITY_DN1978_c0_g1_i4_orf1;TRINITY_DN38230_c0_g1_i4_orf1;TRINITY_DN2772_c0_g1_i3_orf1;TRINITY_DN2749_c0_g1_i4_orf1;TRINITY_DN12227_c0_g2_i3_orf1;TRINITY_DN38562_c0_g1_i3_orf1;TRINITY_DN17693_c0_g1_i10_orf1;TRINITY_DN3712_c0_g1_i1_orf1;TRINITY_DN4817_c0_g1_i4_orf1;TRINITY_DN1330_c0_g1_i1_orf1;TRINITY_DN95850_c0_g1_i1_orf1;TRINITY_DN3784_c0_g1_i1_orf1;TRINITY_DN53294_c0_g1_i1_orf1;TRINITY_DN1841_c0_g1_i2_orf1;TRINITY_DN24_c0_g1_i1_orf1;TRINITY_DN34830_c0_g1_i1_orf1;TRINITY_DN4394_c0_g2_i1_orf1;TRINITY_DN4565_c0_g2_i1_orf1;TRINITY_DN82017_c0_g1_i5_orf1;TRINITY_DN12024_c0_g2_i2_orf1;TRINITY_DN1884_c0_g2_i2_orf1;TRINITY_DN59885_c0_g1_i3_orf1;TRINITY_DN2367_c1_g1_i20_orf1;TRINITY_DN8107_c0_g1_i1_orf1;TRINITY_DN152_c0_g1_i4_orf1;TRINITY_DN616_c1_g1_i6_orf1;TRINITY_DN2749_c0_g2_i3_orf1;TRINITY_DN1249_c0_g1_i10_orf1;TRINITY_DN8771_c0_g2_i1_orf1;TRINITY_DN117_c0_g1_i6_orf1;TRINITY_DN10900_c0_g1_i7_orf1;TRINITY_DN5238_c0_g1_i2_orf1;TRINITY_DN42705_c0_g1_i3_orf1;TRINITY_DN18922_c0_g1_i1_orf1;TRINITY_DN123184_c0_g1_i1_orf1;TRINITY_DN1116_c0_g1_i6_orf1;TRINITY_DN40945_c0_g1_i1_orf1;TRINITY_DN12806_c0_g2_i1_orf1;TRINITY_DN30509_c0_g1_i9_orf1;TRINITY_DN117_c0_g1_i4_orf1;TRINITY_DN1557_c0_g1_i9_orf1;TRINITY_DN227_c0_g1_i1_orf1;TRINITY_DN2647_c0_g1_i3_orf1;TRINITY_DN48713_c0_g1_i1_orf1;TRINITY_DN44658_c0_g1_i2_orf1;TRINITY_DN23004_c0_g1_i1_orf1;TRINITY_DN11886_c0_g1_i1_orf1;TRINITY_DN10662_c0_g1_i4_orf1;TRINITY_DN18128_c0_g1_i4_orf1
molecular_function	deacetylase activity	GO:0019213	3	3/1363	TRINITY_DN70236_c0_g1_i1_orf1;TRINITY_DN82801_c0_g1_i1_orf1;TRINITY_DN111110_c0_g1_i1_orf1
molecular_function	deaminase activity	GO:0019239	2	2/1363	TRINITY_DN1196_c0_g1_i5_orf1;TRINITY_DN98242_c0_g1_i1_orf1
molecular_function	palmitoyl hydrolase activity	GO:0098599	1	1/1363	TRINITY_DN4817_c0_g1_i4_orf1
molecular_function	hydrolase activity, acting on ether bonds	GO:0016801	1	1/1363	TRINITY_DN11172_c0_g1_i4_orf1
molecular_function	FAD-AMP lyase (cyclizing) activity	GO:0034012	2	2/1363	TRINITY_DN11942_c0_g1_i1_orf1;TRINITY_DN52244_c1_g1_i1_orf1
molecular_function	oxidoreductase activity, acting on CH-OH group of donors	GO:0016614	10	10/1363	TRINITY_DN10900_c0_g1_i7_orf1;TRINITY_DN36788_c0_g1_i2_orf1;TRINITY_DN29018_c0_g1_i4_orf1;TRINITY_DN4793_c0_g1_i7_orf1;TRINITY_DN38424_c0_g1_i1_orf1;TRINITY_DN12193_c0_g1_i6_orf1;TRINITY_DN3053_c0_g1_i2_orf1;TRINITY_DN3472_c0_g1_i6_orf1;TRINITY_DN77830_c0_g2_i2_orf1;TRINITY_DN1209_c0_g1_i9_orf1
molecular_function	oxidoreductase activity, acting on the aldehyde or oxo group of donors	GO:0016903	10	10/1363	TRINITY_DN7075_c0_g2_i1_orf1;TRINITY_DN1293_c0_g1_i4_orf1;TRINITY_DN81031_c0_g1_i1_orf1;TRINITY_DN1293_c1_g1_i4_orf1;TRINITY_DN11826_c0_g1_i4_orf1;TRINITY_DN64772_c0_g1_i1_orf1;TRINITY_DN2719_c1_g1_i6_orf1;TRINITY_DN631_c0_g1_i6_orf1;TRINITY_DN3529_c0_g1_i7_orf1;TRINITY_DN2848_c0_g1_i2_orf1
molecular_function	oxidoreductase activity, acting on the CH-NH group of donors	GO:0016645	4	4/1363	TRINITY_DN5432_c1_g1_i3_orf1;TRINITY_DN244_c1_g1_i5_orf1;TRINITY_DN92153_c0_g2_i2_orf1;TRINITY_DN631_c0_g1_i6_orf1
molecular_function	dioxygenase activity	GO:0051213	4	4/1363	TRINITY_DN4822_c0_g1_i6_orf1;TRINITY_DN4822_c0_g1_i9_orf1;TRINITY_DN89083_c0_g1_i1_orf1;TRINITY_DN38562_c0_g1_i3_orf1
molecular_function	electron transfer activity	GO:0009055	1	1/1363	TRINITY_DN5432_c1_g1_i3_orf1
molecular_function	oxidoreductase activity, acting on paired donors, with incorporation or reduction of molecular oxygen	GO:0016705	22	22/1363	TRINITY_DN8985_c0_g1_i4_orf1;TRINITY_DN9608_c0_g1_i3_orf1;TRINITY_DN23564_c0_g1_i7_orf1;TRINITY_DN7580_c0_g1_i1_orf1;TRINITY_DN2392_c0_g2_i1_orf1;TRINITY_DN24873_c0_g1_i4_orf1;TRINITY_DN89083_c0_g1_i1_orf1;TRINITY_DN3732_c0_g1_i6_orf1;TRINITY_DN7212_c0_g1_i4_orf1;TRINITY_DN57765_c0_g1_i1_orf1;TRINITY_DN3131_c0_g1_i5_orf1;TRINITY_DN3949_c0_g1_i1_orf1;TRINITY_DN31163_c1_g1_i4_orf1;TRINITY_DN3732_c1_g1_i5_orf1;TRINITY_DN1363_c0_g1_i11_orf1;TRINITY_DN9316_c0_g3_i1_orf1;TRINITY_DN50743_c0_g1_i1_orf1;TRINITY_DN15755_c0_g1_i1_orf1;TRINITY_DN1960_c5_g1_i3_orf1;TRINITY_DN57856_c0_g2_i1_orf1;TRINITY_DN5126_c0_g1_i3_orf1;TRINITY_DN5661_c0_g1_i5_orf1
molecular_function	oxidoreductase activity, acting on single donors with incorporation of molecular oxygen	GO:0016701	5	5/1363	TRINITY_DN1707_c0_g1_i1_orf1;TRINITY_DN4822_c0_g1_i6_orf1;TRINITY_DN4822_c0_g1_i9_orf1;TRINITY_DN3010_c0_g1_i4_orf1;TRINITY_DN38562_c0_g1_i3_orf1
molecular_function	oxidoreductase activity, acting on the CH-CH group of donors	GO:0016627	8	8/1363	TRINITY_DN659_c0_g2_i1_orf1;TRINITY_DN5055_c0_g1_i12_orf1;TRINITY_DN10900_c0_g1_i7_orf1;TRINITY_DN29018_c0_g1_i4_orf1;TRINITY_DN3588_c0_g1_i4_orf1;TRINITY_DN3053_c0_g1_i2_orf1;TRINITY_DN3478_c0_g1_i10_orf1;TRINITY_DN77830_c0_g2
molecular_function	oxidoreductase activity, acting on NAD(P)H	GO:0016651	3	3/1363	TRINITY_DN49221_c0_g1_i1_orf1;TRINITY_DN20984_c0_g1_i4_orf1;TRINITY_DN8306_c0_g1_i4_orf1
molecular_function	oxidoreductase activity, acting on superoxide radicals as acceptor	GO:0016721	4	4/1363	TRINITY_DN103107_c0_g1_i2_orf1;TRINITY_DN37307_c0_g1_i4_orf1;TRINITY_DN8637_c0_g1_i1_orf1;TRINITY_DN16400_c0_g2_i1_orf1
molecular_function	oxidoreductase activity, acting on metal ions	GO:0016722	5	5/1363	TRINITY_DN46625_c0_g1_i1_orf1;TRINITY_DN1423_c0_g1_i8_orf1;TRINITY_DN65681_c0_g1_i1_orf1;TRINITY_DN136031_c0_g1_i7_orf1;TRINITY_DN1423_c0_g1_i4_orf1
molecular_function	oxidoreductase activity, acting on CH or CH2 groups	GO:0016725	1	1/1363	TRINITY_DN4835_c0_g1_i2_orf1
molecular_function	oxidoreductase activity, acting on peroxide as acceptor	GO:0016684	6	6/1363	TRINITY_DN7778_c0_g1_i1_orf1;TRINITY_DN1622_c0_g1_i6_orf1;TRINITY_DN3321_c0_g1_i3_orf1;TRINITY_DN69236_c0_g1_i1_orf1;TRINITY_DN2542_c0_g2_i1_orf1;TRINITY_DN21420_c0_g1_i2_orf1
molecular_function	monooxygenase activity	GO:0004497	29	29/1363	TRINITY_DN8985_c0_g1_i4_orf1;TRINITY_DN9608_c0_g1_i3_orf1;TRINITY_DN84357_c0_g1_i1_orf1;TRINITY_DN23564_c0_g1_i7_orf1;TRINITY_DN7580_c0_g1_i1_orf1;TRINITY_DN2392_c0_g2_i1_orf1;TRINITY_DN24873_c0_g1_i4_orf1;TRINITY_DN3732_c0_g1_i6_orf1;TRINITY_DN31584_c0_g2_i2_orf1;TRINITY_DN3010_c0_g1_i4_orf1;TRINITY_DN51342_c0_g1_i7_orf1;TRINITY_DN7212_c0_g1_i4_orf1;TRINITY_DN57765_c0_g1_i1_orf1;TRINITY_DN3131_c0_g1_i5_orf1;TRINITY_DN3949_c0_g1_i1_orf1;TRINITY_DN31163_c1_g1_i4_orf1;TRINITY_DN3732_c1_g1_i5_orf1;TRINITY_DN1363_c0_g1_i11_orf1;TRINITY_DN9316_c0_g3_i1_orf1;TRINITY_DN50743_c0_g1_i1_orf1;TRINITY_DN15755_c0_g1_i1_orf1;TRINITY_DN2338_c0_g2_i2_orf1;TRINITY_DN1960_c5_g1_i3_orf1;TRINITY_DN57856_c0_g2_i1_orf1;TRINITY_DN1707_c0_g1_i1_orf1;TRINITY_DN5126_c0_g1_i3_orf1;TRINITY_DN5661_c0_g1_i5_orf1;TRINITY_DN2338_c0_g2_i1_orf1;TRINITY_DN2338_c0_g1_i3_orf1
molecular_function	oxidoreductase activity, acting on the CH-NH2 group of donors	GO:0016638	1	1/1363	TRINITY_DN42856_c0_g1_i1_orf1
molecular_function	oxidoreductase activity, acting on other nitrogenous compounds as donors	GO:0016661	1	1/1363	TRINITY_DN2559_c0_g1_i4_orf1
molecular_function	oxidoreductase activity, acting on a sulfur group of donors	GO:0016667	6	6/1363	TRINITY_DN920_c0_g1_i4_orf1;TRINITY_DN4390_c0_g1_i4_orf1;TRINITY_DN7170_c0_g1_i11_orf1;TRINITY_DN24689_c0_g1_i1_orf1;TRINITY_DN920_c0_g1_i6_orf1;TRINITY_DN1491_c0_g1_i4_orf1
molecular_function	lysozyme activity	GO:0003796	2	2/1363	TRINITY_DN54410_c0_g2_i1_orf1;TRINITY_DN467_c3_g1_i5_orf1
molecular_function	N-acetylmuramoyl-L-alanine amidase activity	GO:0008745	2	2/1363	TRINITY_DN1534_c0_g1_i3_orf1;TRINITY_DN827_c1_g1_i1_orf1
molecular_function	intramolecular transferase activity	GO:0016866	3	3/1363	TRINITY_DN2769_c0_g1_i1_orf1;TRINITY_DN30713_c0_g1_i3_orf1;TRINITY_DN12545_c0_g1_i7_orf1
molecular_function	intramolecular oxidoreductase activity	GO:0016860	2	2/1363	TRINITY_DN1196_c0_g1_i5_orf1;TRINITY_DN27035_c0_g1_i1_orf1
molecular_function	racemase and epimerase activity	GO:0016854	1	1/1363	TRINITY_DN45530_c0_g1_i1_orf1
molecular_function	cis-trans isomerase activity	GO:0016859	2	2/1363	TRINITY_DN2807_c0_g1_i4_orf1;TRINITY_DN140538_c0_g2_i1_orf1
molecular_function	catalytic activity, acting on RNA	GO:0140098	20	20/1363	TRINITY_DN20499_c0_g3_i1_orf1;TRINITY_DN4381_c0_g2_i1_orf1;TRINITY_DN44288_c0_g1_i2_orf1;TRINITY_DN4380_c0_g1_i9_orf1;TRINITY_DN825_c23_g1_i5_orf1;TRINITY_DN84322_c0_g2_i1_orf1;TRINITY_DN27771_c0_g1_i1_orf1;TRINITY_DN620_c0_g1_i4_orf1;TRINITY_DN11596_c0_g1_i1_orf1;TRINITY_DN4408_c6_g1_i1_orf1;TRINITY_DN18922_c0_g1_i1_orf1;TRINITY_DN3712_c0_g1_i1_orf1;TRINITY_DN14274_c0_g1_i3_orf1;TRINITY_DN2299_c0_g1_i3_orf1;TRINITY_DN59291_c0_g1_i1_orf1;TRINITY_DN7213_c0_g1_i2_orf1;TRINITY_DN817_c0_g1_i3_orf1;TRINITY_DN12594_c0_g1_i1_orf1;TRINITY_DN48619_c0_g1_i1_orf1;TRINITY_DN23004_c0_g1_i1_orf1
molecular_function	catalytic activity, acting on DNA	GO:0140097	5	5/1363	TRINITY_DN123184_c0_g1_i1_orf1;TRINITY_DN3092_c0_g1_i2_orf1;TRINITY_DN89613_c0_g1_i13_orf1;TRINITY_DN2971_c0_g1_i1_orf1;TRINITY_DN40434_c0_g1_i2_orf1
molecular_function	helicase activity	GO:0004386	10	10/1363	TRINITY_DN20499_c0_g3_i1_orf1;TRINITY_DN11596_c0_g1_i1_orf1;TRINITY_DN4381_c0_g2_i1_orf1;TRINITY_DN44288_c0_g1_i2_orf1;TRINITY_DN4380_c0_g1_i9_orf1;TRINITY_DN14274_c0_g1_i3_orf1;TRINITY_DN4408_c6_g1_i1_orf1;TRINITY_DN7213_c0_g1_i2_orf1;TRINITY_DN59291_c0_g1_i1_orf1;TRINITY_DN2971_c0_g1_i1_orf1
molecular_function	transferase activity, transferring alkyl or aryl (other than methyl) groups	GO:0016765	14	14/1363	TRINITY_DN920_c0_g1_i4_orf1;TRINITY_DN10222_c0_g1_i2_orf1;TRINITY_DN12134_c0_g1_i4_orf1;TRINITY_DN1305_c0_g1_i6_orf1;TRINITY_DN3929_c0_g3_i3_orf1;TRINITY_DN128231_c0_g1_i5_orf1;TRINITY_DN37856_c0_g1_i5_orf1;TRINITY_DN23732_c0_g1_i1_orf1;TRINITY_DN8651_c0_g1_i18_orf1;TRINITY_DN82320_c0_g1_i2_orf1;TRINITY_DN4695_c0_g1_i3_orf1;TRINITY_DN62707_c0_g1_i1_orf1;TRINITY_DN8964_c0_g1_i4_orf1;TRINITY_DN920_c0_g1_i6_orf1
molecular_function	transferase activity, transferring nitrogenous groups	GO:0016769	2	2/1363	TRINITY_DN2848_c0_g1_i2_orf1;TRINITY_DN5564_c0_g1_i5_orf1
molecular_function	glycosyltransferase activity	GO:0016757	11	11/1363	TRINITY_DN49786_c0_g1_i1_orf1;TRINITY_DN2967_c0_g1_i4_orf1;TRINITY_DN79868_c0_g1_i1_orf1;TRINITY_DN6992_c0_g1_i6_orf1;TRINITY_DN25251_c0_g2_i1_orf1;TRINITY_DN10548_c0_g2_i1_orf1;TRINITY_DN1575_c0_g1_i7_orf1;TRINITY_DN12508_c0_g1_i1_orf1;TRINITY_DN28592_c0_g1_i2_orf1;TRINITY_DN98091_c0_g1_i3_orf1;TRINITY_DN53760_c0_g1_i1_orf1
molecular_function	molybdopterin molybdotransferase activity	GO:0061599	1	1/1363	TRINITY_DN80134_c0_g1_i1_orf1

molecular_function	transferase activity, transferring phosphorus-containing groups	GO:0016772	37	37/1363	TRINITY_DN140423_c0_g1_i2_orf1;TRINITY_DN47151_c0_g1_i1_orf1;TRINITY_DN2719_c1_g1_i6_orf1;TRINITY_DN41166_c0_g1_i1_orf1;TRINITY_DN670_c0_g1_i15_orf1;TRINITY_DN4742_c0_g1_i1_orf1;TRINITY_DN11942_c0_g1_i1_orf1;TRINITY_DN4056_c0_g1_i8_orf1;TRINITY_DN54477_c0_g1_i1_orf1;TRINITY_DN2770_c0_g2_i4_orf1;TRINITY_DN28729_c0_g1_i9_orf1;TRINITY_DN42461_c0_g1_i4_orf1;TRINITY_DN1285_c0_g1_i6_orf1;TRINITY_DN46090_c0_g3_i1_orf1;TRINITY_DN4798_c0_g1_i3_orf1;TRINITY_DN277_c1_g1_i1_orf1;TRINITY_DN19662_c4_g1_i1_orf1;TRINITY_DN2110_c0_g1_i3_orf1;TRINITY_DN18782_c0_g1_i4_orf1;TRINITY_DN12594_c0_g1_i1_orf1;TRINITY_DN12301_c0_g1_i1_orf1;TRINITY_DN15478_c0_g1_i1_orf1;TRINITY_DN80134_c0_g1_i1_orf1;TRINITY_DN4408_c6_g1_i1_orf1;TRINITY_DN36632_c0_g1_i1_orf1;TRINITY_DN1954_c0_g1_i4_orf1;TRINITY_DN89613_c0_g1_i13_orf1;TRINITY_DN52244_c1_g1_i1_orf1;TRINITY_DN5811_c0_g1_i4_orf1;TRINITY_DN2618_c0_g1_i3_orf1;TRINITY_DN1957_c0_g1_i4_orf1;TRINITY_DN4929_c1_g2_i5_orf1;TRINITY_DN46715_c0_g1_i1_orf1;TRINITY_DN2299_c0_g1_i3_orf1;TRINITY_DN5697_c0_g1_i1_orf1;TRINITY_DN5029_c0_g1_i1_orf1;TRINITY_DN9979_c0_g1_i1_orf1
molecular_function	transferase activity, transferring one-carbon groups	GO:0016741	9	9/1363	TRINITY_DN6462_c0_g1_i5_orf1;TRINITY_DN22674_c0_g1_i2_orf1;TRINITY_DN5748_c0_g1_i6_orf1;TRINITY_DN6235_c0_g1_i5_orf1;TRINITY_DN77318_c0_g2_i1_orf1;TRINITY_DN95414_c0_g1_i1_orf1;TRINITY_DN631_c0_g1_i6_orf1;TRINITY_DN31431_c0_g1_i1_orf1;TRINITY_DN2457_c0_g1_i8_orf1
molecular_function	acyltransferase activity	GO:0016746	10	10/1363	TRINITY_DN5153_c1_g1_i1_orf1;TRINITY_DN10900_c0_g1_i7_orf1;TRINITY_DN20442_c0_g2_i1_orf1;TRINITY_DN2346_c0_g2_i1_orf1;TRINITY_DN3105_c0_g1_i4_orf1;TRINITY_DN3219_c0_g1_i6_orf1;TRINITY_DN46202_c0_g1_i1_orf1;TRINITY_DN1084_c0_g1_i2_orf1;TRINITY_DN5841_c0_g1_i2_orf1;TRINITY_DN24142_c0_g1_i1_orf1
