# Supplementary material for: Comparative Proteomics and Metabonomics Analysis of Different Diapause Stages Revealed a New Regulation Mechanism of Diapause in Loxostege sticticalis (Lepidoptera: Pyralidae)
Source: Molecules. 2024 Jul 25;29(15):3472. doi: 10.3390/molecules29153472 (PMC11314584; doi:10.3390/molecules29153472)
Supplement: Supplementary file 1 [file molecules-29-03472-s001.zip › analysis process/proteomic/GO annotations analysis/DvsND all.pdf]

Term Type	GO Term	GO ID	ZY_vs_CK_all num	ZY_vs_CK_all percent	ZY_vs_CK_all Accession ids
biological_process	immune response-activating signal transduction	GO:0002757	2	2/3516	TRINITY_DN2170_c0_g2_i1_orf1;TRINITY_DN2170_c1_g1_i3_orf1
biological_process	activation of innate immune response	GO:0002218	4	4/3516	TRINITY_DN8685_c0_g1_i5_orf1;TRINITY_DN2170_c0_g2_i1_orf1;TRINITY_DN2170_c1_g1_i3_orf1;TRINITY_DN5880_c0_g2_i2_orf1
biological_process	cell activation involved in immune response	GO:0002263	1	1/3516	TRINITY_DN46409_c0_g1_i1_orf1
biological_process	lymphocyte activation	GO:0046649	1	1/3516	TRINITY_DN46409_c0_g1_i1_orf1
biological_process	leukocyte activation involved in immune response	GO:0002366	1	1/3516	TRINITY_DN46409_c0_g1_i1_orf1
biological_process	innate immune response	GO:0045087	17	17/3516	TRINITY_DN827_c1_g1_i1_orf1;TRINITY_DN21545_c0_g1_i2_orf1;TRINITY_DN479_c6_g1_i2_orf1;TRINITY_DN8685_c0_g1_i5_orf1;TRINITY_DN1444_c1_g1_i5_orf1;TRINITY_DN1534_c0_g1_i3_orf1;TRINITY_DN5235_c0_g1_i7_orf1;TRINITY_DN195_c4_g1_i1_orf1;TRINITY_DN6098_c1_g1_i5_orf1;TRINITY_DN2170_c0_g2_i1_orf1;TRINITY_DN2170_c1_g1_i3_orf1;TRINITY_DN15706_c0_g2_i5_orf1;TRINITY_DN1666_c0_g1_i2_orf1;TRINITY_DN2170_c4_g1_i2_orf1;TRINITY_DN9044_c0_g1_i2_orf1;TRINITY_DN2848_c0_g1_i2_orf1;TRINITY_DN5880_c0_g2_i2_orf1
biological_process	humoral immune response	GO:0006959	3	3/3516	TRINITY_DN5667_c0_g1_i4_orf1;TRINITY_DN2848_c0_g1_i2_orf1;TRINITY_DN2848_c0_g1_i1_orf1
biological_process	somatic diversification of immune receptors via germline recombination within a single locus	GO:0002562	1	1/3516	TRINITY_DN46409_c0_g1_i1_orf1
biological_process	somatic diversification of immunoglobulins	GO:0016445	1	1/3516	TRINITY_DN46409_c0_g1_i1_orf1
biological_process	regulation of catalytic activity	GO:0050790	20	20/3516	TRINITY_DN11076_c0_g2_i1_orf1;TRINITY_DN111985_c0_g1_i1_orf1;TRINITY_DN13999_c0_g1_i4_orf1;TRINITY_DN8473_c0_g1_i6_orf1;TRINITY_DN2943_c2_g2_i1_orf1;TRINITY_DN518_c0_g1_i1_orf1;TRINITY_DN130075_c1_g2_i1_orf1;TRINITY_DN140538_c0_g2_i1_orf1;TRINITY_DN802_c0_g1_i2_orf1;TRINITY_DN1328_c0_g1_i6_orf1;TRINITY_DN46409_c0_g1_i1_orf1;TRINITY_DN2848_c0_g1_i1_orf1;TRINITY_DN50074_c0_g1_i1_orf1;TRINITY_DN46022_c0_g1_i1_orf1;TRINITY_DN975_c0_g1_i1_orf1;TRINITY_DN147475_c0_g1_i1_orf1;TRINITY_DN2848_c0_g1_i2_orf1;TRINITY_DN55148_c0_g1_i1_orf1;TRINITY_DN8473_c0_g1_i5_orf1;TRINITY_DN28661_c0_g1_i1_orf1
biological_process	positive regulation of molecular function	GO:0044093	11	11/3516	TRINITY_DN111985_c0_g1_i1_orf1;TRINITY_DN5406_c0_g2_i1_orf1;TRINITY_DN802_c0_g1_i2_orf1;TRINITY_DN46409_c0_g1_i1_orf1;TRINITY_DN1352_c0_g1_i5_orf1;TRINITY_DN55148_c0_g1_i1_orf1;TRINITY_DN5553_c0_g1_i4_orf1;TRINITY_DN2175_c0_g1_i4_orf1;TRINITY_DN46022_c0_g1_i1_orf1;TRINITY_DN50074_c0_g1_i1_orf1;TRINITY_DN140538_c0_g2_i1_orf1
biological_process	negative regulation of molecular function	GO:0044092	8	8/3516	TRINITY_DN5442_c0_g1_i4_orf1;TRINITY_DN13999_c0_g1_i4_orf1;TRINITY_DN130075_c1_g2_i1_orf1;TRINITY_DN55148_c0_g1_i1_orf1;TRINITY_DN1328_c0_g1_i6_orf1;TRINITY_DN2848_c0_g1_i1_orf1;TRINITY_DN2848_c0_g1_i2_orf1;TRINITY_DN140538_c0_g2_i1_orf1
biological_process	regulation of binding	GO:0051098	3	3/3516	TRINITY_DN147475_c0_g1_i1_orf1;TRINITY_DN55148_c0_g1_i1_orf1;TRINITY_DN140538_c0_g2_i1_orf1
biological_process	regulation of ATP-dependent activity	GO:0043462	1	1/3516	TRINITY_DN5442_c0_g1_i4_orf1
biological_process	regulation of transporter activity	GO:0032409	5	5/3516	TRINITY_DN111985_c0_g1_i1_orf1;TRINITY_DN1352_c0_g1_i5_orf1;TRINITY_DN5406_c0_g2_i1_orf1;TRINITY_DN5553_c0_g1_i4_orf1;TRINITY_DN2175_c0_g1_i4_orf1
biological_process	regulation of metabolic process	GO:0019222	82	82/3516	TRINITY_DN3649_c0_g1_i6_orf1;TRINITY_DN21214_c0_g2_i1_orf1;TRINITY_DN13999_c0_g1_i4_orf1;TRINITY_DN19260_c0_g1_i5_orf1;TRINITY_DN288_c0_g1_i9_orf1;TRINITY_DN8702_c0_g1_i1_orf1;TRINITY_DN1706_c0_g1_i7_orf1;TRINITY_DN5207_c0_g2_i3_orf1;TRINITY_DN20442_c0_g2_i1_orf1;TRINITY_DN23360_c0_g1_i3_orf1;TRINITY_DN130075_c1_g2_i1_orf1;TRINITY_DN1710_c0_g1_i1_orf1;TRINITY_DN46409_c0_g1_i1_orf1;TRINITY_DN28981_c0_g1_i1_orf1;TRINITY_DN29402_c0_g1_i1_orf1;TRINITY_DN20009_c0_g1_i1_orf1;TRINITY_DN9938_c0_g2_i1_orf1;TRINITY_DN14701_c0_g1_i2_orf1;TRINITY_DN147475_c0_g1_i1_orf1;TRINITY_DN15448_c0_g1_i1_orf1;TRINITY_DN44407_c0_g4_i2_orf1;TRINITY_DN18538_c0_g3_i1_orf1;TRINITY_DN1191_c0_g1_i4_orf1;TRINITY_DN5262_c0_g1_i7_orf1;TRINITY_DN10636_c0_g1_i1_orf1;TRINITY_DN21150_c0_g1_i4_orf1;TRINITY_DN3673_c0_g1_i10_orf1;TRINITY_DN21341_c0_g1_i4_orf1;TRINITY_DN29707_c0_g1_i2_orf1;TRINITY_DN6462_c0_g1_i5_orf1;TRINITY_DN975_c0_g1_i1_orf1;TRINITY_DN142442_c0_g1_i1_orf1;TRINITY_DN10886_c0_g2_i4_orf1;TRINITY_DN1328_c0_g1_i6_orf1;TRINITY_DN3457_c0_g1_i4_orf1;TRINITY_DN41602_c0_g3_i1_orf1;TRINITY_DN19286_c0_g1_i1_orf1;TRINITY_DN2848_c0_g1_i1_orf1;TRINITY_DN50074_c0_g1_i1_orf1;TRINITY_DN5562_c1_g1_i3_orf1;TRINITY_DN810_c0_g1_i4_orf1;TRINITY_DN18681_c0_g1_i7_orf1;TRINITY_DN8473_c0_g1_i5_orf1;TRINITY_DN2630_c0_g3_i3_orf1;TRINITY_DN22430_c0_g3_i1_orf1;TRINITY_DN1639_c0_g2_i2_orf1;TRINITY_DN5562_c1_g2_i1_orf1;TRINITY_DN17655_c0_g1_i1_orf1;TRINITY_DN5562_c0_g1_i3_orf1;TRINITY_DN50085_c0_g1_i1_orf1;TRINITY_DN44877_c0_g1_i2_orf1;TRINITY_DN7289_c0_g1_i1_orf1;TRINITY_DN1198_c0_g1_i1_orf1;TRINITY_DN33926_c0_g1_i1_orf1;TRINITY_DN8986_c0_g1_i1_orf1;TRINITY_DN31851_c0_g1_i2_orf1;TRINITY_DN18036_c0_g1_i7_orf1;TRINITY_DN5105_c0_g1_i10_orf1;TRINITY_DN3366_c0_g1_i6_orf1;TRINITY_DN8473_c0_g1_i6_orf1;TRINITY_DN66596_c0_g1_i1_orf1;TRINITY_DN55148_c0_g1_i1_orf1;TRINITY_DN140538_c0_g2_i1_orf1;TRINITY_DN12323_c0_g2_i2_orf1;TRINITY_DN96557_c0_g1_i1_orf1;TRINITY_DN6125_c0_g1_i2_orf1;TRINITY_DN2943_c2_g2_i1_orf1;TRINITY_DN4813_c0_g1_i5_orf1;TRINITY_DN9542_c0_g1_i4_orf1;TRINITY_DN2848_c0_g1_i2_orf1;TRINITY_DN67649_c0_g1_i1_orf1;TRINITY_DN2802_c0_g1_i1_orf1;TRINITY_DN2802_c1_g1_i1_orf1;TRINITY_DN9510_c0_g2_i1_orf1;TRINITY_DN4014_c0_g1_i1_orf1;TRINITY_DN111985_c0_g1_i1_orf1;TRINITY_DN31585_c0_g1_i1_orf1;TRINITY_DN34689_c0_g1_i4_orf1;TRINITY_DN46022_c0_g1_i1_orf1;TRINITY_DN4309_c0_g1_i1_orf1;TRINITY_DN4950_c0_g1_i2_orf1;TRINITY_DN12771_c0_g1_i1_orf1

biological_process	regulation of response to stimulus	GO:0048583	27	27/3516	TRINITY_DN51938_c0_g3_i1_orf1;TRINITY_DN8685_c0_g1_i5_orf1;TRINITY_DN130075_c1_g2_i1_orf1;TRINITY_DN46409_c0_g1_i1_orf1;TRINITY_DN12320_c0_g1_i1_orf1;TRINITY_DN146119_c0_g1_i1_orf1;TRINITY_DN34745_c0_g2_i1_orf1;TRINITY_DN15448_c0_g1_i1_orf1;TRINITY_DN9119_c0_g1_i3_orf1;TRINITY_DN21545_c0_g1_i2_orf1;TRINITY_DN479_c6_g1_i2_orf1;TRINITY_DN9711_c0_g1_i10_orf1;TRINITY_DN55148_c0_g1_i1_orf1;TRINITY_DN22572_c0_g1_i1_orf1;TRINITY_DN2848_c0_g1_i1_orf1;TRINITY_DN1612_c0_g1_i3_orf1;TRINITY_DN22430_c0_g3_i1_orf1;TRINITY_DN111985_c0_g1_i1_orf1;TRINITY_DN17655_c0_g1_i1_orf1;TRINITY_DN2170_c0_g2_i1_orf1;TRINITY_DN140538_c0_g2_i1_orf1;TRINITY_DN2943_c2_g2_i1_orf1;TRINITY_DN4464_c0_g2_i1_orf1;TRINITY_DN147475_c0_g1_i1_orf1;TRINITY_DN2170_c1_g1_i3_orf1;TRINITY_DN2848_c0_g1_i2_orf1;TRINITY_DN5880_c0_g2_i2_orf1
biological_process	regulation of developmental process	GO:0050793	3	3/3516	TRINITY_DN41602_c0_g3_i1_orf1;TRINITY_DN7493_c0_g1_i1_orf1;TRINITY_DN20133_c0_g1_i1_orf1
biological_process	regulation of cellular process	GO:0050794	129	129/3516	TRINITY_DN5207_c0_g2_i3_orf1;TRINITY_DN9711_c0_g1_i10_orf1;TRINITY_DN44407_c0_g4_i2_orf1;TRINITY_DN5262_c0_g1_i7_orf1;TRINITY_DN21545_c0_g1_i2_orf1;TRINITY_DN1008_c0_g1_i2_orf1;TRINITY_DN2983_c0_g1_i6_orf1;TRINITY_DN140538_c0_g2_i1_orf1;TRINITY_DN3457_c0_g1_i4_orf1;TRINITY_DN19286_c0_g1_i1_orf1;TRINITY_DN2848_c0_g1_i1_orf1;TRINITY_DN1612_c0_g1_i3_orf1;TRINITY_DN18681_c0_g1_i7_orf1;TRINITY_DN1639_c0_g2_i2_orf1;TRINITY_DN2202_c0_g1_i9_orf1;TRINITY_DN17655_c0_g1_i1_orf1;TRINITY_DN2170_c0_g2_i1_orf1;TRINITY_DN66596_c0_g1_i1_orf1;TRINITY_DN4798_c0_g1_i3_orf1;TRINITY_DN2943_c2_g2_i1_orf1;TRINITY_DN2947_c0_g1_i4_orf1;TRINITY_DN7102_c0_g1_i5_orf1;TRINITY_DN9146_c0_g1_i1_orf1;TRINITY_DN2802_c1_g1_i1_orf1;TRINITY_DN48536_c0_g1_i3_orf1;TRINITY_DN4014_c0_g1_i1_orf1;TRINITY_DN2170_c1_g1_i3_orf1;TRINITY_DN2848_c0_g1_i2_orf1;TRINITY_DN13216_c0_g1_i5_orf1;TRINITY_DN3755_c0_g1_i3_orf1;TRINITY_DN51938_c0_g3_i1_orf1;TRINITY_DN91198_c0_g2_i1_orf1;TRINITY_DN8702_c0_g1_i1_orf1;TRINITY_DN1706_c0_g1_i7_orf1;TRINITY_DN20442_c0_g2_i1_orf1;TRINITY_DN23360_c0_g1_i3_orf1;TRINITY_DN52649_c0_g1_i6_orf1;TRINITY_DN492_c0_g1_i4_orf1;TRINITY_DN2623_c0_g1_i3_orf1;TRINITY_DN146119_c0_g1_i1_orf1;TRINITY_DN34745_c0_g2_i1_orf1;TRINITY_DN9119_c0_g1_i3_orf1;TRINITY_DN5406_c0_g2_i1_orf1;TRINITY_DN288_c0_g1_i9_orf1;TRINITY_DN29707_c0_g1_i2_orf1;TRINITY_DN3673_c0_g1_i10_orf1;TRINITY_DN13259_c0_g1_i2_orf1;TRINITY_DN8473_c0_g1_i5_orf1;TRINITY_DN111985_c0_g1_i1_orf1;TRINITY_DN21341_c0_g1_i4_orf1;TRINITY_DN32700_c0_g1_i2_orf1;TRINITY_DN50085_c0_g1_i1_orf1;TRINITY_DN8986_c0_g1_i1_orf1;TRINITY_DN5105_c0_g1_i10_orf1;TRINITY_DN3366_c0_g1_i6_orf1;TRINITY_DN4439_c0_g1_i2_orf1;TRINITY_DN7493_c0_g1_i1_orf1;TRINITY_DN1738_c0_g1_i5_orf1;TRINITY_DN3649_c0_g1_i6_orf1;TRINITY_DN2270_c0_g2_i1_orf1;TRINITY_DN10287_c0_g1_i1_orf1;TRINITY_DN13999_c0_g1_i4_orf1;TRINITY_DN21214_c0_g2_i1_orf1;TRINITY_DN376_c0_g1_i1_orf1;TRINITY_DN46409_c0_g1_i1_orf1;TRINITY_DN40211_c0_g1_i1_orf1;TRINITY_DN142442_c0_g1_i1_orf1;TRINITY_DN5182_c0_g1_i5_orf1;TRINITY_DN31851_c0_g1_i2_orf1;TRINITY_DN14987_c0_g1_i3_orf1;TRINITY_DN6426_c0_g1_i2_orf1;TRINITY_DN22430_c0_g3_i1_orf1;TRINITY_DN5553_c0_g1_i4_orf1;TRINITY_DN20009_c0_g1_i1_orf1;TRINITY_DN2630_c0_g3_i3_orf1;TRINITY_DN5562_c1_g2_i1_orf1;TRINITY_DN5562_c0_g1_i3_orf1;TRINITY_DN10636_c0_g1_i1_orf1;TRINITY_DN3418_c0_g1_i3_orf1;TRINITY_DN5562_c1_g1_i3_orf1;TRINITY_DN1198_c0_g1_i1_orf1;TRINITY_DN2793_c0_g2_i1_orf1;TRINITY_DN17838_c0_g1_i4_orf1;TRINITY_DN96739_c0_g1_i1_orf1;TRINITY_DN18036_c0_g1_i7_orf1;TRINITY_DN6125_c0_g1_i2_orf1;TRINITY_DN96557_c0_g1_i1_orf1;TRINITY_DN2802_c0_g1_i1_orf1;TRINITY_DN18696_c0_g1_i1_orf1;TRINITY_DN802_c0_g1_i2_orf1;TRINITY_DN42854_c0_g3_i2_orf1;TRINITY_DN10455_c0_g1_i2_orf1;TRINITY_DN37336_c1_g1_i1_orf1;TRINITY_DN46633_c0_g1_i4_orf1;TRINITY_DN79000_c1_g1_i1_orf1;TRINITY_DN67649_c0_g1_i1_orf1;TRINITY_DN28981_c0_g1_i1_orf1;TRINITY_DN4159_c1_g1_i1_orf1;TRINITY_DN1710_c0_g1_i1_orf1;TRINITY_DN9965_c0_g1_i1_orf1;TRINITY_DN15706_c0_g2_i5_orf1;TRINITY_DN9938_c0_g2_i1_orf1;TRINITY_DN1405_c0_g1_i1_orf1;TRINITY_DN15448_c0_g1_i1_orf1;TRINITY_DN55148_c0_g1_i1_orf1;TRINITY_DN8473_c0_g1_i6_orf1;TRINITY_DN21150_c0_g1_i4_orf1;TRINITY_DN975_c0_g1_i1_orf1;TRINITY_DN33926_c0_g1_i1_orf1;TRINITY_DN22572_c0_g1_i1_orf1;TRINITY_DN1352_c0_g1_i5_orf1;TRINITY_DN41602_c0_g3_i1_orf1;TRINITY_DN12320_c0_g1_i1_orf1;TRINITY_DN9510_c0_g2_i1_orf1;TRINITY_DN4628_c0_g1_i1_orf1;TRINITY_DN31585_c0_g1_i1_orf1;TRINITY_DN2175_c0_g1_i4_orf1;TRINITY_DN15478_c0_g1_i1_orf1;TRINITY_DN1498_c0_g1_i2_orf1;TRINITY_DN130075_c1_g2_i1_orf1;TRINITY_DN804_c0_g1_i7_orf1;TRINITY_DN9
biological_process	regulation of locomotion	GO:0040012	3	3/3516	TRINITY_DN147475_c0_g1_i1_orf1;TRINITY_DN140538_c0_g2_i1_orf1;TRINITY_DN20133_c0_g1_i1_orf1
biological_process	regulation of localization	GO:0032879	8	8/3516	TRINITY_DN111985_c0_g1_i1_orf1;TRINITY_DN5406_c0_g2_i1_orf1;TRINITY_DN14987_c0_g1_i3_orf1;TRINITY_DN96739_c0_g1_i1_orf1;TRINITY_DN1352_c0_g1_i5_orf1;TRINITY_DN5553_c0_g1_i4_orf1;TRINITY_DN2175_c0_g1_i4_orf1;TRINITY_DN140538_c0_g2_i1_orf1
biological_process	regulation of multicellular organismal process	GO:0051239	8	8/3516	TRINITY_DN1455_c0_g1_i4_orf1;TRINITY_DN7493_c0_g1_i1_orf1;TRINITY_DN147475_c0_g1_i1_orf1;TRINITY_DN46409_c0_g1_i1_orf1;TRINITY_DN96739_c0_g1_i1_orf1;TRINITY_DN41602_c0_g3_i1_orf1;TRINITY_DN2848_c0_g1_i1_orf1;TRINITY_DN2848_c0_g1_i2_orf1
biological_process	regulation of immune system process	GO:0002682	8	8/3516	TRINITY_DN111985_c0_g1_i1_orf1;TRINITY_DN21545_c0_g1_i2_orf1;TRINITY_DN479_c6_g1_i2_orf1;TRINITY_DN8685_c0_g1_i5_orf1;TRINITY_DN46409_c0_g1_i1_orf1;TRINITY_DN2170_c0_g2_i1_orf1;TRINITY_DN2170_c1_g1_i3_orf1;TRINITY_DN5880_c0_g2_i2_orf1
biological_process	positive regulation of biological process	GO:0048518	37	37/3516	TRINITY_DN9119_c0_g1_i3_orf1;TRINITY_DN21214_c0_g2_i1_orf1;TRINITY_DN51938_c0_g3_i1_orf1;TRINITY_DN15706_c0_g2_i5_orf1;TRINITY_DN8685_c0_g1_i5_orf1;TRINITY_DN130075_c1_g2_i1_orf1;TRINITY_DN1710_c0_g1_i1_orf1;TRINITY_DN46409_c0_g1_i1_orf1;TRINITY_DN288_c0_g1_i9_orf1;TRINITY_DN146119_c0_g1_i1_orf1;TRINITY_DN15448_c0_g1_i1_orf1;TRINITY_DN44407_c0_g4_i2_orf1;TRINITY_DN5406_c0_g2_i1_orf1;TRINITY_DN55148_c0_g1_i1_orf1;TRINITY_DN22572_c0_g1_i1_orf1;TRINITY_DN3457_c0_g1_i4_orf1;TRINITY_DN1352_c0_g1_i5_orf1;TRINITY_DN2848_c0_g1_i1_orf1;TRINITY_DN50074_c0_g1_i1_orf1;TRINITY_DN20009_c0_g1_i1_orf1;TRINITY_DN9510_c0_g2_i1_orf1;TRINITY_DN22430_c0_g3_i1_orf1;TRINITY_DN1639_c0_g2_i2_orf1;TRINITY_DN17655_c0_g1_i1_orf1;TRINITY_DN5553_c0_g1_i4_orf1;TRINITY_DN2175_c0_g1_i4_orf1;TRINITY_DN33926_c0_g1_i1_orf1;TRINITY_DN2170_c0_g2_i1_orf1;TRINITY_DN140538_c0_g2_i1_orf1;TRINITY_DN111985_c0_g1_i1_orf1;TRINITY_DN7493_c0_g1_i1_orf1;TRINITY_DN147475_c0_g1_i1_orf1;TRINITY_DN2170_c1_g1_i3_orf1;TRINITY_DN20133_c0_g1_i1_orf1;TRINITY_DN4309_c0_g1_i1_orf1;TRINITY_DN2848_c0_g1_i2_orf1;TRINITY_DN5880_c0_g2_i2_orf1

biological_process	negative regulation of biological process	GO:0048519	40	40/3516	TRINITY_DN21214_c0_g2_i1_orf1;TRINITY_DN13999_c0_g1_i4_orf1;TRINITY_DN8702_c0_g1_i1_orf1;TRINITY_DN52649_c0_g1_i6_orf1;TRINITY_DN130075_c1_g2_i1_orf1;TRINITY_DN46409_c0_g1_i1_orf1;TRINITY_DN29402_c0_g1_i1_orf1;TRINITY_DN4159_c1_g1_i1_orf1;TRINITY_DN14701_c0_g1_i2_orf1;TRINITY_DN55148_c0_g1_i1_orf1;TRINITY_DN21545_c0_g1_i2_orf1;TRINITY_DN12771_c0_g1_i1_orf1;TRINITY_DN10886_c0_g2_i4_orf1;TRINITY_DN1328_c0_g1_i6_orf1;TRINITY_DN3673_c0_g1_i10_orf1;TRINITY_DN19286_c0_g1_i1_orf1;TRINITY_DN2848_c0_g1_i1_orf1;TRINITY_DN810_c0_g1_i4_orf1;TRINITY_DN13259_c0_g1_i2_orf1;TRINITY_DN2630_c0_g3_i3_orf1;TRINITY_DN1639_c0_g2_i2_orf1;TRINITY_DN18538_c0_g3_i1_orf1;TRINITY_DN44877_c0_g1_i2_orf1;TRINITY_DN7289_c0_g1_i1_orf1;TRINITY_DN1198_c0_g1_i1_orf1;TRINITY_DN12323_c0_g2_i2_orf1;TRINITY_DN96739_c0_g1_i1_orf1;TRINITY_DN20009_c0_g1_i1_orf1;TRINITY_DN66596_c0_g1_i1_orf1;TRINITY_DN140538_c0_g2_i1_orf1;TRINITY_DN96557_c0_g1_i1_orf1;TRINITY_DN4813_c0_g1_i5_orf1;TRINITY_DN111985_c0_g1_i1_orf1;TRINITY_DN7493_c0_g1_i1_orf1;TRINITY_DN147475_c0_g1_i1_orf1;TRINITY_DN34689_c0_g1_i4_orf1;TRINITY_DN20133_c0_g1_i1_orf1;TRINITY_DN46022_c0_g1_i1_orf1;TRINITY_DN10287_c0_g1_i1_orf1;TRINITY_DN2848_c0_g1_i2_orf1
biological_process	regulation of signaling	GO:0023051	24	24/3516	TRINITY_DN51938_c0_g3_i1_orf1;TRINITY_DN130075_c1_g2_i1_orf1;TRINITY_DN146119_c0_g1_i1_orf1;TRINITY_DN34745_c0_g2_i1_orf1;TRINITY_DN15448_c0_g1_i1_orf1;TRINITY_DN55148_c0_g1_i1_orf1;TRINITY_DN5406_c0_g2_i1_orf1;TRINITY_DN21545_c0_g1_i2_orf1;TRINITY_DN9711_c0_g1_i10_orf1;TRINITY_DN9119_c0_g1_i3_orf1;TRINITY_DN22572_c0_g1_i1_orf1;TRINITY_DN1352_c0_g1_i5_orf1;TRINITY_DN22430_c0_g3_i1_orf1;TRINITY_DN5553_c0_g1_i4_orf1;TRINITY_DN1612_c0_g1_i3_orf1;TRINITY_DN2848_c0_g1_i1_orf1;TRINITY_DN111985_c0_g1_i1_orf1;TRINITY_DN140538_c0_g2_i1_orf1;TRINITY_DN2943_c2_g2_i1_orf1;TRINITY_DN4464_c0_g2_i1_orf1;TRINITY_DN147475_c0_g1_i1_orf1;TRINITY_DN12320_c0_g1_i1_orf1;TRINITY_DN2175_c0_g1_i4_orf1;TRINITY_DN2848_c0_g1_i2_orf1
biological_process	regulation of growth	GO:0040008	1	1/3516	TRINITY_DN59965_c0_g4_i1_orf1
biological_process	regulation of membrane potential	GO:0042391	1	1/3516	TRINITY_DN111985_c0_g1_i1_orf1
biological_process	regulation of neurotransmitter levels	GO:0001505	4	4/3516	TRINITY_DN111985_c0_g1_i1_orf1;TRINITY_DN49527_c0_g1_i1_orf1;TRINITY_DN2047_c0_g1_i1_orf1;TRINITY_DN14565_c0_g1_i11_orf1
biological_process	regulation of body fluid levels	GO:0050878	1	1/3516	TRINITY_DN4016_c0_g1_i1_orf1
biological_process	homeostatic process	GO:0042592	19	19/3516	TRINITY_DN111985_c0_g1_i1_orf1;TRINITY_DN46625_c0_g1_i1_orf1;TRINITY_DN65681_c0_g1_i1_orf1;TRINITY_DN1423_c0_g1_i4_orf1;TRINITY_DN96557_c0_g1_i1_orf1;TRINITY_DN1423_c0_g1_i8_orf1;TRINITY_DN20133_c0_g1_i1_orf1;TRINITY_DN4469_c0_g1_i2_orf1;TRINITY_DN96739_c0_g1_i1_orf1;TRINITY_DN136031_c0_g1_i7_orf1;TRINITY_DN376_c0_g1_i1_orf1;TRINITY_DN3461_c0_g1_i1_orf1;TRINITY_DN9965_c0_g1_i1_orf1;TRINITY_DN3434_c0_g1_i1_orf1;TRINITY_DN15812_c0_g1_i2_orf1;TRINITY_DN22430_c0_g3_i1_orf1;TRINITY_DN7405_c0_g1_i3_orf1;TRINITY_DN44256_c0_g1_i1_orf1;TRINITY_DN15448_c0_g1_i1_orf1
biological_process	regulation of anatomical structure size	GO:0090066	5	5/3516	TRINITY_DN37336_c1_g1_i1_orf1;TRINITY_DN4439_c0_g1_i2_orf1;TRINITY_DN52649_c0_g1_i6_orf1;TRINITY_DN10455_c0_g1_i2_orf1;TRINITY_DN4159_c1_g1_i1_orf1
biological_process	regulation of translational fidelity	GO:0006450	2	2/3516	TRINITY_DN11215_c0_g1_i1_orf1;TRINITY_DN33926_c0_g1_i1_orf1
biological_process	regulation of hormone levels	GO:0010817	1	1/3516	TRINITY_DN111985_c0_g1_i1_orf1
biological_process	regulation of RNA stability	GO:0043487	3	3/3516	TRINITY_DN20009_c0_g1_i1_orf1;TRINITY_DN21341_c0_g1_i4_orf1;TRINITY_DN5262_c0_g1_i7_orf1
biological_process	regulation of protein stability	GO:0031647	7	7/3516	TRINITY_DN111985_c0_g1_i1_orf1;TRINITY_DN130075_c1_g2_i1_orf1;TRINITY_DN55148_c0_g1_i1_orf1;TRINITY_DN46409_c0_g1_i1_orf1;TRINITY_DN2848_c0_g1_i1_orf1;TRINITY_DN2848_c0_g1_i2_orf1;TRINITY_DN140538_c0_g2_i1_orf1
biological_process	NADH regeneration	GO:0006735	2	2/3516	TRINITY_DN2848_c0_g1_i1_orf1;TRINITY_DN20133_c0_g1_i1_orf1

biological_process	organonitrogen compound metabolic process	GO:1901564	526	526/3516	TRINITY_DN57074_c0_g2_i1_orf1;TRINITY_DN42856_c0_g1_i1_orf1;TRINITY_DN3194_c0_g1_i6_orf1;TRINITY_DN11492_c0_g1_i8_orf1;TRINITY_DN2818_c0_g1_i2_orf1;TRINITY_DN1533_c0_g2_i1_orf1;TRINITY_DN35763_c0_g1_i2_orf1;TRINITY_DN8019_c0_g1_i4_orf1;TRINITY_DN117362_c0_g1_i5_orf1;TRINITY_DN2069_c1_g1_i8_orf1;TRINITY_DN2274_c0_g1_i6_orf1;TRINITY_DN30300_c0_g2_i1_orf1;TRINITY_DN2983_c0_g1_i6_orf1;TRINITY_DN31163_c1_g1_i4_orf1;TRINITY_DN31611_c0_g1_i2_orf1;TRINITY_DN11013_c0_g1_i3_orf1;TRINITY_DN16343_c0_g1_i6_orf1;TRINITY_DN14217_c0_g1_i1_orf1;TRINITY_DN48020_c0_g1_i1_orf1;TRINITY_DN2861_c0_g2_i1_orf1;TRINITY_DN14953_c0_g1_i5_orf1;TRINITY_DN3991_c0_g1_i6_orf1;TRINITY_DN2794_c1_g1_i8_orf1;TRINITY_DN84478_c0_g1_i8_orf1;TRINITY_DN1827_c0_g1_i4_orf1;TRINITY_DN817_c0_g1_i3_orf1;TRINITY_DN2559_c0_g1_i4_orf1;TRINITY_DN25896_c0_g1_i6_orf1;TRINITY_DN22674_c0_g1_i2_orf1;TRINITY_DN8908_c0_g1_i1_orf1;TRINITY_DN6059_c0_g1_i1_orf1;TRINITY_DN107261_c0_g1_i1_orf1;TRINITY_DN79734_c0_g2_i3_orf1;TRINITY_DN123396_c0_g1_i1_orf1;TRINITY_DN3755_c0_g1_i3_orf1;TRINITY_DN6199_c2_g1_i3_orf1;TRINITY_DN143895_c0_g1_i1_orf1;TRINITY_DN2627_c0_g1_i2_orf1;TRINITY_DN2038_c0_g1_i2_orf1;TRINITY_DN19829_c0_g2_i1_orf1;TRINITY_DN18230_c1_g2_i1_orf1;TRINITY_DN1965_c0_g1_i7_orf1;TRINITY_DN29038_c0_g2_i1_orf1;TRINITY_DN4125_c1_g1_i5_orf1;TRINITY_DN7583_c0_g1_i1_orf1;TRINITY_DN1607_c0_g1_i16_orf1;TRINITY_DN7464_c0_g1_i14_orf1;TRINITY_DN66302_c0_g1_i1_orf1;TRINITY_DN5696_c0_g1_i4_orf1;TRINITY_DN3733_c0_g1_i1_orf1;TRINITY_DN5444_c0_g2_i1_orf1;TRINITY_DN95414_c0_g1_i1_orf1;TRINITY_DN27033_c1_g1_i3_orfp1;TRINITY_DN376_c1_g1_i1_orf1;TRINITY_DN1494_c0_g1_i3_orf1;TRINITY_DN9062_c0_g2_i3_orf1;TRINITY_DN4767_c0_g1_i4_orf1;TRINITY_DN7776_c0_g1_i5_orf1;TRINITY_DN40015_c0_g1_i2_orf1;TRINITY_DN147458_c0_g1_i1_orf1;TRINITY_DN1068_c0_g1_i3_orf1;TRINITY_DN21251_c1_g1_i1_orf1;TRINITY_DN5112_c0_g1_i1_orf1;TRINITY_DN92153_c0_g2_i2_orf1;TRINITY_DN25534_c0_g1_i1_orf1;TRINITY_DN10766_c0_g1_i1_orf1;TRINITY_DN620_c0_g1_i4_orf1;TRINITY_DN2026_c0_g1_i4_orf1;TRINITY_DN5182_c0_g1_i5_orf1;TRINITY_DN28577_c0_g1_i6_orf1;TRINITY_DN4016_c0_g1_i1_orf1;TRINITY_DN542_c0_g2_i1_orf1;TRINITY_DN3975_c0_g1_i10_orf1;TRINITY_DN43792_c0_g1_i1_orf1;TRINITY_DN2803_c4_g1_i1_orf1;TRINITY_DN98242_c0_g1_i1_orf1;TRINITY_DN18388_c0_g1_i6_orf1;TRINITY_DN4449_c0_g2_i1_orf1;TRINITY_DN2579_c0_g1_i7_orf1;TRINITY_DN36893_c0_g1_i1_orf1;TRINITY_DN18172_c0_g1_i6_orf1;TRINITY_DN10455_c0_g2_i1_orf1;TRINITY_DN17838_c0_g1_i4_orf1;TRINITY_DN2515_c0_g1_i6_orf1;TRINITY_DN1173_c1_g1_i9_orf1;TRINITY_DN3401_c0_g1_i1_orf1;TRINITY_DN42506_c0_g1_i1_orf1;TRINITY_DN3971_c0_g1_i1_orf1;TRINITY_DN1287_c0_g1_i5_orf1;TRINITY_DN1957_c0_g1_i4_orf1;TRINITY_DN29034_c0_g1_i1_orf1;TRINITY_DN9874_c0_g1_i7_orf1;TRINITY_DN69697_c0_g1_i1_orf1;TRINITY_DN10385_c0_g1_i5_orf1;TRINITY_DN4944_c0_g1_i2_orf1;TRINITY_DN41997_c0_g1_i2_orf1;TRINITY_DN5031_c0_g1_i1_orf1;TRINITY_DN461_c0_g1_i5_orf1;TRINITY_DN44792_c0_g1_i1_orf1;TRINITY_DN12973_c0_g1_i1_orf1;TRINITY_DN24970_c0_g1_i4_orf1;TRINITY_DN24539_c0_g1_i4_orf1;TRINITY_DN8949_c0_g1_i2_orf1;TRINITY_DN55148_c0_g1_i1_orf1;TRINITY_DN142588_c0_g1_i1_orf1;TRINITY_DN975_c0_g1_i1_orf1;TRINITY_DN3702_c0_g1_i1_orf1;TRINITY_DN10994_c0_g1_i4_orf1;TRINITY_DN19115_c0_g1_i1_orf1;TRINITY_DN1080_c0_g1_i1_orf1;TRINITY_DN2593_c0_g3_i1_orf1;TRINITY_DN2593_c0_g1_i1_orf1;TRINITY_DN334_c0_g1_i3_orf1;TRINITY_DN17913_c0_g1_i8_orf1;TRINITY_DN58413_c0_g1_i4_orf1;TRINITY_DN46132_c0_g2_i2_orf1;TRINITY_DN6669_c0_g1_i3_orf1;TRINITY_DN81248_c0_g1_i1_orf1;TRINITY_DN11798_c0_g2_i1_orf1;TRINITY_DN48619_c0_g1_i1_orf1;TRINITY_DN2338_c0_g1_i5_orf1;TRINITY_D
biological_process	cellular nitrogen compound metabolic process	GO:0034641	377	377/3516	TRINITY_DN57074_c0_g2_i1_orf1;TRINITY_DN38230_c0_g1_i4_orf1;TRINITY_DN125565_c1_g1_i1_orf1;TRINITY_DN40434_c0_g1_i2_orf1;TRINITY_DN1827_c0_g1_i4_orf1;TRINITY_DN2054_c0_g1_i1_orf1;TRINITY_DN22941_c0_g1_i1_orf1;TRINITY_DN30300_c0_g2_i1_orf1;TRINITY_DN31611_c0_g1_i2_orf1;TRINITY_DN11013_c0_g1_i3_orf1;TRINITY_DN18404_c0_g1_i5_orf1;TRINITY_DN3991_c0_g1_i6_orf1;TRINITY_DN2794_c1_g1_i8_orf1;TRINITY_DN123184_c0_g1_i1_orf1;TRINITY_DN817_c0_g1_i3_orf1;TRINITY_DN25896_c0_g1_i6_orf1;TRINITY_DN8908_c0_g1_i1_orf1;TRINITY_DN107261_c0_g1_i1_orf1;TRINITY_DN79734_c0_g2_i3_orf1;TRINITY_DN2647_c0_g1_i3_orf1;TRINITY_DN5603_c0_g1_i1_orf1;TRINITY_DN4300_c0_g1_i5_orf1;TRINITY_DN2038_c0_g1_i2_orf1;TRINITY_DN19829_c0_g2_i1_orf1;TRINITY_DN18728_c0_g1_i2_orf1;TRINITY_DN1965_c0_g1_i7_orf1;TRINITY_DN6248_c0_g1_i1_orf1;TRINITY_DN29038_c0_g2_i1_orf1;TRINITY_DN5873_c0_g4_i1_orf1;TRINITY_DN115658_c0_g1_i1_orf1;TRINITY_DN47575_c0_g1_i1_orf1;TRINITY_DN47666_c0_g1_i4_orf1;TRINITY_DN7583_c0_g1_i1_orf1;TRINITY_DN1607_c0_g1_i16_orf1;TRINITY_DN7464_c0_g1_i14_orf1;TRINITY_DN810_c0_g1_i4_orf1;TRINITY_DN18863_c0_g1_i3_orf1;TRINITY_DN3733_c0_g1_i1_orf1;TRINITY_DN74037_c0_g5_i1_orf1;TRINITY_DN147458_c0_g1_i1_orf1;TRINITY_DN21251_c1_g1_i1_orf1;TRINITY_DN5112_c0_g1_i1_orf1;TRINITY_DN1750_c1_g1_i5_orf1;TRINITY_DN77318_c0_g2_i1_orf1;TRINITY_DN2026_c0_g1_i4_orf1;TRINITY_DN19262_c0_g1_i1_orf1;TRINITY_DN33346_c0_g1_i1_orf1;TRINITY_DN1554_c0_g1_i9_orf1;TRINITY_DN4016_c0_g1_i1_orf1;TRINITY_DN934_c2_g1_i7_orf1;TRINITY_DN43792_c0_g1_i1_orf1;TRINITY_DN98242_c0_g1_i1_orf1;TRINITY_DN4135_c0_g1_i5_orf1;TRINITY_DN6660_c0_g1_i5_orf1;TRINITY_DN3401_c0_g1_i1_orf1;TRINITY_DN42506_c0_g1_i1_orf1;TRINITY_DN34509_c0_g1_i1_orf1;TRINITY_DN1957_c0_g1_i4_orf1;TRINITY_DN9874_c0_g1_i7_orf1;TRINITY_DN31663_c0_g1_i2_orf1;TRINITY_DN4944_c0_g1_i2_orf1;TRINITY_DN41997_c0_g1_i2_orf1;TRINITY_DN5031_c0_g1_i1_orf1;TRINITY_DN44792_c0_g1_i1_orf1;TRINITY_DN36893_c0_g1_i1_orf1;TRINITY_DN8949_c0_g1_i2_orf1;TRINITY_DN55148_c0_g1_i1_orf1;TRINITY_DN71840_c0_g1_i1_orf1;TRINITY_DN975_c0_g1_i1_orf1;TRINITY_DN39490_c0_g1_i1_orf1;TRINITY_DN19115_c0_g1_i1_orf1;TRINITY_DN17045_c0_g2_i3_orf1;TRINITY_DN4151_c1_g1_i4_orf1;TRINITY_DN81248_c0_g1_i1_orf1;TRINITY_DN82324_c0_g1_i4_orf1;TRINITY_DN8717_c0_g1_i5_orf1;TRINITY_DN21539_c0_g1_i1_orf1;TRINITY_DN9542_c0_g1_i4_orf1;TRINITY_DN31253_c0_g1_i2_orf1;TRINITY_DN346_c0_g1_i7_orf1;TRINITY_DN68725_c0_g1_i1_orf1;TRINITY_DN1084_c0_g1_i2_orf1;TRINITY_DN26824_c0_g1_i1_orf1;TRINITY_DN16749_c0_g1_i1_orf1;TRINITY_DN8430_c0_g1_i1_orf1;TRINITY_DN56910_c0_g2_i1_orf1;TRINITY_DN2738_c1_g1_i3_orf1;TRINITY_DN34134_c0_g2_i1_orf1;TRINITY_DN1216_c0_g1_i4_orf1;TRINITY_DN23616_c0_g1_i4_orf1;TRINITY_DN2953_c1_g1_i10_orf1;TRINITY_DN7957_c0_g1_i5_orf1;TRINITY_DN2848_c0_g1_i1_orf1;TRINITY_DN23432_c0_g1_i1_orf1;TRINITY_DN74889_c0_g1_i1_orf1;TRINITY_DN15370_c0_g1_i4_orf1;TRINITY_DN38075_c0_g1_i1_orf1;TRINITY_DN145647_c0_g1_i1_orf1;TRINITY_DN37165_c0_g1_i4_orf1;TRINITY_DN24310_c0_g1_i2_orf1;TRINITY_DN18860_c0_g1_i1_orf1;TRINITY_DN6235_c0_g1_i5_orf1;TRINITY_DN3092_c0_g1_i2_orf1;TRINITY_DN5756_c0_g1_i4_orf1;TRINITY_DN23360_c0_g1_i3_orf1;TRINITY_DN7405_c0_g1_i3_orf1;TRINITY_DN33926_c0_g1_i1_orf1;TRINITY_DN30097_c0_g1_i2_orf1;TRINITY_DN25582_c0_g1_i3_orf1;TRINITY_DN50787_c0_g2_i2_orf1;TRINITY_DN2769_c0_g1_i1_orf1;TRINITY_DN1091_c0_g3_i1_orf1;TRINITY_DN4145_c0_g1_i1_orf1;TRINITY_DN36144_c0_g1_i3_orf1;TRINITY_DN131662_c0_g1_i4_orf1;TRINITY_DN1616_c0_g1_i3_orf1;TRINITY_DN12301_c0_g1_i1_orf1;TRINITY_DN7289_c0_g1_i1_orf1;TRINITY_DN19829_c0_g1_i1_orf1;TRINITY_DN21357_c0_g1_i5_orf1;TRINITY_DN30638_c0_g1_i1_orf
biological_process	nitrogen cycle metabolic process	GO:0071941	3	3/3516	TRINITY_DN24723_c2_g1_i1_orf1;TRINITY_DN17031_c0_g1_i1_orf1;TRINITY_DN28221_c0_g2_i1_orf1

biological_process	cellular lipid metabolic process	GO:0044255	52	52/3516	TRINITY_DN48590_c0_g1_i1_orf1;TRINITY_DN8173_c0_g1_i3_orf1;TRINITY_DN25733_c0_g1_i3_orf1;TRINITY_DN10722_c0_g3_i1_orf1;TRINITY_DN5055_c0_g1_i12_orf1;TRINITY_DN12526_c0_g1_i5_orf1;TRINITY_DN10399_c0_g1_i2_orf1;TRINITY_DN76283_c0_g6_i1_orf1;TRINITY_DN86833_c0_g3_i1_orf1;TRINITY_DN64627_c0_g1_i1_orf1;TRINITY_DN5841_c0_g1_i2_orf1;TRINITY_DN14306_c0_g1_i1_orf1;TRINITY_DN11076_c0_g2_i1_orf1;TRINITY_DN5092_c0_g1_i2_orf1;TRINITY_DN9718_c0_g1_i7_orf1;TRINITY_DN45220_c0_g1_i1_orf1;TRINITY_DN44110_c0_g1_i4_orf1;TRINITY_DN5211_c0_g1_i1_orf1;TRINITY_DN41_c0_g1_i3_orf1;TRINITY_DN43656_c0_g1_i1_orf1;TRINITY_DN10066_c0_g2_i2_orf1;TRINITY_DN7134_c0_g1_i1_orf1;TRINITY_DN3588_c0_g1_i1_orf1;TRINITY_DN59335_c0_g1_i2_orf1;TRINITY_DN40197_c0_g1_i1_orf1;TRINITY_DN2441_c0_g1_i1_orf1;TRINITY_DN10430_c0_g1_i4_orf1;TRINITY_DN10742_c0_g1_i4_orf1;TRINITY_DN19122_c0_g1_i7_orf1;TRINITY_DN3175_c0_g1_i7_orf1;TRINITY_DN127151_c0_g1_i1_orf1;TRINITY_DN3991_c0_g1_i6_orf1;TRINITY_DN42759_c0_g3_i1_orf1;TRINITY_DN84478_c0_g1_i8_orf1;TRINITY_DN42759_c0_g2_i1_orf1;TRINITY_DN76283_c0_g2_i1_orf1;TRINITY_DN659_c0_g1_i3_orf1;TRINITY_DN5512_c0_g1_i8_orf1;TRINITY_DN883_c0_g1_i8_orf1;TRINITY_DN4321_c0_g1_i1_orf1;TRINITY_DN22046_c1_g1_i5_orf1;TRINITY_DN2618_c0_g1_i3_orf1;TRINITY_DN25896_c0_g1_i6_orf1;TRINITY_DN357_c0_g1_i8_orf1;TRINITY_DN33178_c0_g1_i1_orf1;TRINITY_DN768_c0_g1_i7_orf1;TRINITY_DN3551_c0_g1_i4_orf1;TRINITY_DN115498_c0_g1_i1_orf1;TRINITY_DN68725_c0_g1_i1_orf1;TRINITY_DN5697_c0_g1_i1_orf1;TRINITY_DN905_c0_g1_i4_orf1;TRINITY_DN10900_c0_g1_i7_orf1
biological_process	generation of precursor metabolites and energy	GO:0006091	39	39/3516	TRINITY_DN5417_c0_g1_i1_orf1;TRINITY_DN19000_c0_g1_i4_orf1;TRINITY_DN14967_c0_g2_i1_orf1;TRINITY_DN108051_c0_g1_i2_orf1;TRINITY_DN1827_c0_g1_i4_orf1;TRINITY_DN59965_c0_g4_i1_orf1;TRINITY_DN60787_c0_g1_i5_orf1;TRINITY_DN27035_c0_g1_i1_orf1;TRINITY_DN812_c2_g1_i1_orf1;TRINITY_DN9558_c0_g1_i2_orf1;TRINITY_DN7405_c0_g1_i3_orf1;TRINITY_DN1791_c0_g1_i3_orf1;TRINITY_DN5867_c0_g1_i1_orf1;TRINITY_DN1201_c0_g1_i4_orf1;TRINITY_DN49038_c0_g4_i1_orf1;TRINITY_DN9286_c0_g1_i2_orf1;TRINITY_DN11817_c0_g1_i4_orf1;TRINITY_DN16830_c0_g1_i5_orf1;TRINITY_DN1132_c0_g1_i5_orf1;TRINITY_DN31611_c0_g1_i2_orf1;TRINITY_DN2848_c0_g1_i1_orf1;TRINITY_DN76036_c0_g1_i1_orf1;TRINITY_DN29873_c0_g1_i1_orf1;TRINITY_DN1422_c0_g1_i4_orf1;TRINITY_DN83150_c0_g1_i1_orf1;TRINITY_DN6325_c0_g1_i9_orf1;TRINITY_DN26010_c0_g1_i2_orf1;TRINITY_DN24310_c0_g1_i2_orf1;TRINITY_DN136028_c0_g2_i1_orf1;TRINITY_DN86149_c0_g1_i1_orf1;TRINITY_DN95665_c0_g1_i1_orf1;TRINITY_DN9536_c0_g1_i4_orf1;TRINITY_DN14073_c0_g1_i1_orf1;TRINITY_DN5111_c0_g1_i2_orf1;TRINITY_DN20133_c0_g1_i1_orf1;TRINITY_DN6535_c0_g2_i1_orf1;TRINITY_DN2848_c0_g1_i2_orf1;TRINITY_DN4954_c0_g1_i5_orf1;TRINITY_DN679_c0_g1_i2_orf1
biological_process	one-carbon metabolic process	GO:0006730	12	12/3516	TRINITY_DN98313_c0_g1_i1_orf1;TRINITY_DN63536_c0_g1_i1_orf1;TRINITY_DN92153_c0_g2_i2_orf1;TRINITY_DN130051_c0_g1_i1_orf1;TRINITY_DN3263_c0_g1_i2_orf1;TRINITY_DN1578_c0_g3_i1_orf1;TRINITY_DN38506_c0_g1_i4_orf1;TRINITY_DN7512_c0_g1_i1_orf1;TRINITY_DN244_c1_g1_i5_orf1;TRINITY_DN631_c0_g1_i6_orf1;TRINITY_DN5768_c0_g1_i2_orf1;TRINITY_DN14107_c0_g1_i4_orf1
biological_process	cellular ketone metabolic process	GO:0042180	10	10/3516	TRINITY_DN18291_c0_g1_i1_orf1;TRINITY_DN87170_c0_g1_i3_orf1;TRINITY_DN13941_c0_g1_i6_orf1;TRINITY_DN51813_c0_g1_i1_orf1;TRINITY_DN6027_c0_g1_i13_orf1;TRINITY_DN135781_c0_g1_i1_orf1;TRINITY_DN6638_c0_g1_i1_orf1;TRINITY_DN20133_c0_g1_i1_orf1;TRINITY_DN27885_c0_g1_i3_orf1;TRINITY_DN36592_c0_g1_i1_orf1
biological_process	heterocycle metabolic process	GO:0046483	273	273/3516	TRINITY_DN38230_c0_g1_i4_orf1;TRINITY_DN22941_c0_g1_i1_orf1;TRINITY_DN86090_c0_g1_i1_orf1;TRINITY_DN40434_c0_g1_i2_orf1;TRINITY_DN130051_c0_g1_i1_orf1;TRINITY_DN15836_c0_g1_i1_orf1;TRINITY_DN1827_c0_g1_i4_orf1;TRINITY_DN1316_c0_g1_i1_orf1;TRINITY_DN60787_c0_g1_i5_orf1;TRINITY_DN1354_c0_g1_i6_orf1;TRINITY_DN47666_c0_g1_i4_orf1;TRINITY_DN56910_c0_g2_i1_orf1;TRINITY_DN35669_c0_g1_i1_orf1;TRINITY_DN2054_c0_g1_i1_orf1;TRINITY_DN51934_c0_g2_i1_orf1;TRINITY_DN124950_c0_g2_i1_orf1;TRINITY_DN2738_c1_g1_i3_orf1;TRINITY_DN20499_c0_g3_i1_orf1;TRINITY_DN125565_c1_g1_i1_orf1;TRINITY_DN34134_c0_g2_i1_orf1;TRINITY_DN1344_c0_g1_i1_orf1;TRINITY_DN1366_c0_g1_i5_orf1;TRINITY_DN1216_c0_g1_i4_orf1;TRINITY_DN25582_c0_g1_i3_orf1;TRINITY_DN44877_c0_g1_i2_orf1;TRINITY_DN23616_c0_g1_i4_orf1;TRINITY_DN2953_c1_g1_i10_orf1;TRINITY_DN31611_c0_g1_i2_orf1;TRINITY_DN2848_c0_g1_i1_orf1;TRINITY_DN16933_c0_g1_i10_orf1;TRINITY_DN23432_c0_g1_i1_orf1;TRINITY_DN18404_c0_g1_i5_orf1;TRINITY_DN8430_c0_g1_i1_orf1;TRINITY_DN9207_c0_g1_i1_orf1;TRINITY_DN6587_c0_g1_i3_orf1;TRINITY_DN18291_c0_g1_i1_orf1;TRINITY_DN1393_c0_g1_i2_orf1;TRINITY_DN5507_c0_g1_i1_orf1;TRINITY_DN3991_c0_g1_i6_orf1;TRINITY_DN29873_c0_g1_i1_orf1;TRINITY_DN17738_c0_g1_i2_orf1;TRINITY_DN15370_c0_g1_i4_orf1;TRINITY_DN1760_c0_g1_i4_orf1;TRINITY_DN47123_c0_g1_i1_orf1;TRINITY_DN18538_c0_g3_i1_orf1;TRINITY_DN123184_c0_g1_i1_orf1;TRINITY_DN26805_c0_g2_i3_orf1;TRINITY_DN2110_c0_g1_i3_orf1;TRINITY_DN1116_c0_g1_i6_orf1;TRINITY_DN817_c0_g1_i3_orf1;TRINITY_DN1066_c0_g1_i4_orf1;TRINITY_DN145647_c0_g1_i1_orf1;TRINITY_DN37165_c0_g1_i4_orf1;TRINITY_DN9094_c0_g1_i1_orf1;TRINITY_DN5952_c0_g1_i6_orf1;TRINITY_DN2224_c0_g1_i1_orf1;TRINITY_DN3822_c0_g1_i7_orf1;TRINITY_DN24310_c0_g1_i2_orf1;TRINITY_DN46409_c0_g1_i1_orf1;TRINITY_DN15040_c0_g4_i1_orf1;TRINITY_DN8908_c0_g1_i1_orf1;TRINITY_DN4813_c0_g1_i5_orf1;TRINITY_DN58636_c0_g1_i1_orf1;TRINITY_DN107261_c0_g1_i1_orf1;TRINITY_DN9156_c0_g1_i1_orf1;TRINITY_DN107288_c0_g1_i2_orf1;TRINITY_DN18860_c0_g1_i1_orf1;TRINITY_DN6235_c0_g1_i5_orf1;TRINITY_DN7808_c0_g1_i1_orf1;TRINITY_DN89613_c0_g1_i13_orf1;TRINITY_DN2848_c0_g1_i2_orf1;TRINITY_DN2647_c0_g1_i3_orf1;TRINITY_DN5603_c0_g1_i1_orf1;TRINITY_DN3092_c0_g1_i2_orf1;TRINITY_DN1978_c0_g1_i4_orf1;TRINITY_DN4300_c0_g1_i5_orf1;TRINITY_DN6313_c0_g1_i4_orf1;TRINITY_DN2038_c0_g1_i2_orf1;TRINITY_DN45271_c0_g1_i1_orf1;TRINITY_DN3082_c1_g1_i7_orf1;TRINITY_DN59965_c0_g4_i1_orf1;TRINITY_DN26649_c0_g1_i2_orf1;TRINITY_DN18728_c0_g1_i2_orf1;TRINITY_DN29402_c0_g1_i1_orf1;TRINITY_DN37532_c0_g1_i1_orf1;TRINITY_DN4710_c0_g1_i1_orf1;TRINITY_DN1965_c0_g1_i7_orf1;TRINITY_DN7405_c0_g1_i3_orf1;TRINITY_DN29038_c0_g2_i1_orf1;TRINITY_DN11639_c0_g1_i1_orf1;TRINITY_DN15900_c0_g1_i6_orf1;TRINITY_DN14967_c0_g2_i1_orf1;TRINITY_DN115658_c0_g1_i1_orf1;TRINITY_DN10548_c0_g2_i1_orf1;TRINITY_DN30097_c0_g1_i2_orf1;TRINITY_DN47575_c0_g1_i1_orf1;TRINITY_DN51968_c0_g1_i1_orf1;TRINITY_DN8625_c0_g1_i1_orf1;TRINITY_DN779_c0_g1_i3_orf1;TRINITY_DN1607_c0_g1_i16_orf1;TRINITY_DN810_c0_g1_i4_orf1;TRINITY_DN244_c1_g1_i5_orf1;TRINITY_DN18863_c0_g1_i3_orf1;TRINITY_DN2769_c0_g1_i1_orf1;TRINITY_DN41664_c0_g1_i4_orf1;TRINITY_DN1091_c0_g3_i1_orf1;TRINITY_DN23714_c0_g1_i4_orf1;TRINITY_DN4145_c0_g1_i1_orf1;TRINITY_DN36144_c0_g1_i3_orf1;TRINITY_DN12527_c0_g1_i4_orf1;TRINITY_DN131662_c0_g1_i4_orf1;TRINITY_DN1616_c0_g1_i3_orf1;TRINITY_DN117844_c0_g1_i1_orf1;TRINITY_DN12301_c0_g1_i1_orf1;TRINITY_DN2953_c1_g1_i11_orf1;TRINITY_DN6642_c0_g1_i2_orf1;TRINITY_DN7289_c0_g1_i1_orf1;TRINITY_DN12323_c0_g2_i2_orf1;TRINITY_DN46022_c0_g1_i1_orf1;TRINITY_DN51568_c0_g1_i1_orf1;TRINITY_DN1277_c4_g1_i5_
biological_process	reactive oxygen species metabolic process	GO:0072593	5	5/3516	TRINITY_DN111985_c0_g1_i1_orf1;TRINITY_DN8637_c0_g1_i1_orf1;TRINITY_DN285_c0_g1_i4_orf1;TRINITY_DN16400_c0_g2_i1_orf1;TRINITY_DN6580_c0_g1_i4_orf1

biological_process	cellular carbohydrate metabolic process	GO:0044262	14	14/3516	TRINITY_DN618_c0_g1_i3_orf1;TRINITY_DN52244_c1_g1_i1_orf1;TRINITY_DN49038_c0_g4_i1_orf1;TRINITY_DN10722_c0_g3_i1_orf1;TRINITY_DN1034_c0_g1_i4_orf1;TRINITY_DN36788_c0_g1_i2_orf1;TRINITY_DN98723_c1_g1_i1_orf1;TRINITY_DN11817_c0_g1_i4_orf1;TRINITY_DN812_c2_g1_i1_orf1;TRINITY_DN29369_c0_g1_i1_orf1;TRINITY_DN1707_c0_g1_i1_orf1;TRINITY_DN11657_c0_g1_i2_orf1;TRINITY_DN4954_c0_g1_i5_orf1;TRINITY_DN95850_c0_g1_i1_orf1
biological_process	sulfur compound metabolic process	GO:0006790	24	24/3516	TRINITY_DN14920_c0_g1_i1_orf1;TRINITY_DN92153_c0_g2_i2_orf1;TRINITY_DN130051_c0_g1_i1_orf1;TRINITY_DN5497_c0_g1_i6_orf1;TRINITY_DN38562_c0_g1_i3_orf1;TRINITY_DN33183_c0_g1_i4_orf1;TRINITY_DN35763_c0_g1_i2_orf1;TRINITY_DN11948_c0_g1_i8_orf1;TRINITY_DN144807_c0_g1_i1_orf1;TRINITY_DN1285_c0_g1_i6_orf1;TRINITY_DN1578_c0_g3_i1_orf1;TRINITY_DN27848_c0_g1_i2_orf1;TRINITY_DN3991_c0_g1_i6_orf1;TRINITY_DN54134_c0_g1_i1_orf1;TRINITY_DN6313_c0_g1_i4_orf1;TRINITY_DN117844_c0_g1_i1_orf1;TRINITY_DN19727_c0_g1_i7_orf1;TRINITY_DN34399_c0_g1_i1_orf1;TRINITY_DN85476_c0_g1_i1_orf1;TRINITY_DN7512_c0_g1_i1_orf1;TRINITY_DN68725_c0_g1_i1_orf1;TRINITY_DN1084_c0_g1_i2_orf1;TRINITY_DN18558_c0_g1_i7_orf1;TRINITY_DN7808_c0_g1_i1_orf1
biological_process	phosphorus metabolic process	GO:0006793	145	145/3516	TRINITY_DN38230_c0_g1_i4_orf1;TRINITY_DN10722_c0_g3_i1_orf1;TRINITY_DN86090_c0_g1_i1_orf1;TRINITY_DN39404_c0_g1_i7_orf1;TRINITY_DN26805_c0_g2_i3_orf1;TRINITY_DN60787_c0_g1_i5_orf1;TRINITY_DN2738_c1_g1_i3_orf1;TRINITY_DN21545_c0_g1_i2_orf1;TRINITY_DN96170_c0_g1_i1_orf1;TRINITY_DN3534_c0_g1_i2_orf1;TRINITY_DN1216_c0_g1_i4_orf1;TRINITY_DN70485_c0_g1_i2_orf1;TRINITY_DN2983_c0_g1_i6_orf1;TRINITY_DN143509_c0_g1_i1_orf1;TRINITY_DN2848_c0_g1_i1_orf1;TRINITY_DN16933_c0_g1_i10_orf1;TRINITY_DN73945_c0_g5_i3_orf1;TRINITY_DN9555_c0_g1_i1_orf1;TRINITY_DN10742_c0_g1_i4_orf1;TRINITY_DN1334_c0_g1_i2_orf1;TRINITY_DN3991_c0_g1_i6_orf1;TRINITY_DN29873_c0_g1_i1_orf1;TRINITY_DN1827_c0_g1_i4_orf1;TRINITY_DN6185_c0_g1_i12_orf1;TRINITY_DN28299_c0_g1_i1_orf1;TRINITY_DN6436_c0_g1_i1_orf1;TRINITY_DN4798_c0_g1_i3_orf1;TRINITY_DN5952_c0_g1_i6_orf1;TRINITY_DN3822_c0_g1_i7_orf1;TRINITY_DN24310_c0_g1_i2_orf1;TRINITY_DN9156_c0_g1_i1_orf1;TRINITY_DN8908_c0_g1_i1_orf1;TRINITY_DN107261_c0_g1_i1_orf1;TRINITY_DN7808_c0_g1_i1_orf1;TRINITY_DN2848_c0_g1_i2_orf1;TRINITY_DN70_c2_g1_i1_orf1;TRINITY_DN25733_c0_g1_i3_orf1;TRINITY_DN39813_c0_g1_i1_orf1;TRINITY_DN16905_c0_g1_i1_orf1;TRINITY_DN96170_c0_g2_i1_orf1;TRINITY_DN59965_c0_g4_i1_orf1;TRINITY_DN1965_c0_g1_i7_orf1;TRINITY_DN7405_c0_g1_i3_orf1;TRINITY_DN37923_c0_g1_i1_orf1;TRINITY_DN29038_c0_g2_i1_orf1;TRINITY_DN1201_c0_g1_i4_orf1;TRINITY_DN1034_c0_g1_i4_orf1;TRINITY_DN12301_c0_g1_i1_orf1;TRINITY_DN10066_c0_g2_i2_orf1;TRINITY_DN116972_c0_g1_i1_orf1;TRINITY_DN2812_c0_g1_i5_orf1;TRINITY_DN21126_c0_g1_i1_orf1;TRINITY_DN59885_c0_g1_i3_orf1;TRINITY_DN2110_c0_g1_i3_orf1;TRINITY_DN36144_c0_g1_i3_orf1;TRINITY_DN32700_c0_g1_i2_orf1;TRINITY_DN117844_c0_g1_i1_orf1;TRINITY_DN3312_c0_g1_i10_orf1;TRINITY_DN1277_c4_g1_i5_orf1;TRINITY_DN5525_c0_g1_i4_orf1;TRINITY_DN1154_c0_g1_i1_orf1;TRINITY_DN6325_c0_g1_i9_orf1;TRINITY_DN5281_c0_g2_i3_orf1;TRINITY_DN1266_c2_g1_i1_orf1;TRINITY_DN71465_c0_g1_i1_orf1;TRINITY_DN1173_c1_g1_i10_orf1;TRINITY_DN16487_c0_g1_i1_orf1;TRINITY_DN57536_c0_g1_i14_orf1;TRINITY_DN5029_c0_g1_i1_orf1;TRINITY_DN1173_c0_g1_i12_orf1;TRINITY_DN82008_c0_g1_i1_orf1;TRINITY_DN1366_c0_g1_i5_orf1;TRINITY_DN35991_c0_g1_i2_orf1;TRINITY_DN27035_c0_g1_i1_orf1;TRINITY_DN15222_c0_g1_i4_orf1;TRINITY_DN1575_c0_g1_i7_orf1;TRINITY_DN1552_c0_g1_i3_orf1;TRINITY_DN98538_c0_g1_i1_orf1;TRINITY_DN26649_c0_g1_i2_orf1;TRINITY_DN70409_c0_g1_i3_orf1;TRINITY_DN105749_c0_g1_i1_orf1;TRINITY_DN6876_c0_g2_i1_orf1;TRINITY_DN28729_c0_g1_i9_orf1;TRINITY_DN49038_c0_g4_i1_orf1;TRINITY_DN19261_c0_g1_i3_orf1;TRINITY_DN24_c0_g1_i1_orf1;TRINITY_DN43656_c0_g1_i1_orf1;TRINITY_DN13160_c0_g1_i1_orf1;TRINITY_DN31611_c0_g1_i2_orf1;TRINITY_DN21181_c0_g1_i6_orf1;TRINITY_DN7688_c0_g1_i10_orf1;TRINITY_DN26293_c0_g1_i4_orf1;TRINITY_DN4449_c0_g2_i1_orf1;TRINITY_DN14477_c0_g1_i12_orf1;TRINITY_DN19727_c0_g1_i7_orf1;TRINITY_DN51813_c0_g1_i1_orf1;TRINITY_DN8012_c0_g1_i3_orf1;TRINITY_DN83150_c0_g1_i1_orf1;TRINITY_DN7688_c0_g1_i2_orf1;TRINITY_DN1173_c1_g1_i9_orf1;TRINITY_DN143637_c0_g1_i1_orf1;TRINITY_DN23432_c0_g1_i1_orf1;TRINITY_DN52244_c1_g1_i1_orf1;TRINITY_DN1957_c0_g1_i4_orf1;TRINITY_DN10548_c0_g2_i1_orf1;TRINITY_DN6813_c1_g1_i1_orf1;TRINITY_DN40562_c0_g2_i1_orf1;TRINITY_DN33178_c0_g1_i1_orf1;TRINITY_DN47151_c0_g1_i1_orf1;TRINITY_DN6587_c0_g1_i3_orf1;TRINITY_DN14967_c0_g2_i1_orf1;TRINITY_DN24539_c0_g1_i4_orf1;TRINITY_DN1494_c0_g1_i3_orf1;TRINITY_DN19122_c0_g1_i7_orf1;TRINITY_DN41166_c0_g1_i1_orf1;TRINITY_DN1405_c0_g1_i1_orf1;TRINITY_DN1741_c0_g1_i5_orf1;TRINITY_DN618_c0_g1_i3_orf1;TRINITY_DN1494_c0_g2_i1_orf1;TRINITY_DN10680_c0_g1_i5_orf1;TRINITY_DN67716_c
biological_process	cellular aldehyde metabolic process	GO:0006081	10	10/3516	TRINITY_DN111985_c0_g1_i1_orf1;TRINITY_DN18291_c0_g1_i1_orf1;TRINITY_DN36788_c0_g1_i2_orf1;TRINITY_DN4596_c0_g1_i14_orf1;TRINITY_DN29369_c0_g1_i1_orf1;TRINITY_DN59965_c0_g4_i1_orf1;TRINITY_DN125150_c0_g1_i1_orf1;TRINITY_DN3758_c0_g1_i2_orf1;TRINITY_DN20133_c0_g1_i1_orf1;TRINITY_DN9555_c0_g1_i1_orf1

biological_process	organic acid metabolic process	GO:0006082	142 142/3516	TRINITY_DN42856_c0_g1_i1_orf1;TRINITY_DN2065_c1_g2_i1_orf1;TRINITY_DN146126_c0_g1_i1_orf1;TRINITY_DN14565_c0_g1_i11_orf1;TRINITY_DN130051_c0_g1_i1_orf1;TRINITY_DN659_c0_g1_i3_orf1;TRINITY_DN27771_c0_g1_i1_orf1;TRINITY_DN35763_c0_g1_i2_orf1;TRINITY_DN12474_c0_g1_i6_orf1;TRINITY_DN24723_c2_g1_i1_orf1;TRINITY_DN27771_c0_g2_i1_orf1;TRINITY_DN2953_c1_g1_i10_orf1;TRINITY_DN31611_c0_g1_i2_orf1;TRINITY_DN2848_c0_g1_i1_orf1;TRINITY_DN59335_c0_g1_i2_orf1;TRINITY_DN11013_c0_g1_i3_orf1;TRINITY_DN4321_c0_g1_i1_orf1;TRINITY_DN6587_c0_g1_i3_orf1;TRINITY_DN4822_c0_g1_i6_orf1;TRINITY_DN18291_c0_g1_i1_orf1;TRINITY_DN17326_c0_g1_i8_orf1;TRINITY_DN3859_c0_g1_i5_orf1;TRINITY_DN29873_c0_g1_i1_orf1;TRINITY_DN1760_c0_g1_i4_orf1;TRINITY_DN1827_c0_g1_i4_orf1;TRINITY_DN817_c0_g1_i3_orf1;TRINITY_DN2224_c0_g1_i1_orf1;TRINITY_DN357_c0_g1_i8_orf1;TRINITY_DN115498_c0_g1_i1_orf1;TRINITY_DN107288_c0_g1_i2_orf1;TRINITY_DN7808_c0_g1_i1_orf1;TRINITY_DN2848_c0_g1_i2_orf1;TRINITY_DN6199_c2_g1_i3_orf1;TRINITY_DN2038_c0_g1_i2_orf1;TRINITY_DN5497_c0_g1_i6_orf1;TRINITY_DN18230_c1_g2_i1_orf1;TRINITY_DN100821_c0_g1_i1_orf1;TRINITY_DN1965_c0_g1_i7_orf1;TRINITY_DN7405_c0_g1_i3_orf1;TRINITY_DN30224_c0_g1_i1_orf1;TRINITY_DN11639_c0_g1_i1_orf1;TRINITY_DN1201_c0_g1_i4_orf1;TRINITY_DN4451_c0_g2_i4_orf1;TRINITY_DN19187_c0_g1_i1_orf1;TRINITY_DN45220_c0_g1_i1_orf1;TRINITY_DN10264_c1_g1_i5_orf1;TRINITY_DN5211_c0_g1_i1_orf1;TRINITY_DN27848_c0_g1_i2_orf1;TRINITY_DN1607_c0_g1_i16_orf1;TRINITY_DN42759_c0_g3_i1_orf1;TRINITY_DN10430_c0_g1_i4_orf1;TRINITY_DN123396_c0_g1_i1_orf1;TRINITY_DN111985_c0_g1_i1_orf1;TRINITY_DN20796_c0_g1_i4_orf1;TRINITY_DN117844_c0_g1_i1_orf1;TRINITY_DN43431_c0_g1_i1_orf1;TRINITY_DN10900_c0_g1_i7_orf1;TRINITY_DN6325_c0_g1_i9_orf1;TRINITY_DN511_c0_g2_i1_orf1;TRINITY_DN4451_c0_g1_i1_orf1;TRINITY_DN30638_c0_g1_i1_orf1;TRINITY_DN905_c0_g1_i4_orf1;TRINITY_DN11159_c0_g2_i1_orf1;TRINITY_DN2684_c0_g2_i3_orf1;TRINITY_DN17326_c0_g1_i5_orf1;TRINITY_DN1068_c0_g1_i3_orf1;TRINITY_DN48590_c0_g1_i1_orf1;TRINITY_DN8173_c0_g1_i3_orf1;TRINITY_DN92153_c0_g2_i2_orf1;TRINITY_DN5055_c0_g1_i12_orf1;TRINITY_DN620_c0_g1_i4_orf1;TRINITY_DN27035_c0_g1_i1_orf1;TRINITY_DN64627_c0_g1_i1_orf1;TRINITY_DN28577_c0_g1_i6_orf1;TRINITY_DN81719_c0_g1_i1_orf1;TRINITY_DN11076_c0_g2_i1_orf1;TRINITY_DN2953_c1_g1_i11_orf1;TRINITY_DN1824_c0_g2_i2_orf1;TRINITY_DN49038_c0_g4_i1_orf1;TRINITY_DN11948_c0_g1_i8_orf1;TRINITY_DN64810_c0_g1_i1_orf1;TRINITY_DN36788_c0_g1_i2_orf1;TRINITY_DN28221_c0_g2_i1_orf1;TRINITY_DN2803_c4_g1_i1_orf1;TRINITY_DN5129_c0_g3_i3_orf1;TRINITY_DN42759_c0_g2_i1_orf1;TRINITY_DN5756_c0_g1_i4_orf1;TRINITY_DN76283_c0_g2_i1_orf1;TRINITY_DN26293_c0_g1_i4_orf1;TRINITY_DN8716_c0_g1_i3_orf1;TRINITY_DN3175_c0_g1_i7_orf1;TRINITY_DN127151_c0_g1_i1_orf1;TRINITY_DN1375_c0_g1_i5_orf1;TRINITY_DN19727_c0_g1_i7_orf1;TRINITY_DN84322_c0_g2_i1_orf1;TRINITY_DN13941_c0_g1_i6_orf1;TRINITY_DN51813_c0_g1_i1_orf1;TRINITY_DN76283_c0_g6_i1_orf1;TRINITY_DN8598_c0_g1_i2_orf1;TRINITY_DN17031_c0_g1_i1_orf1;TRINITY_DN83150_c0_g1_i1_orf1;TRINITY_DN631_c0_g1_i6_orf1;TRINITY_DN15160_c0_g1_i1_orf1;TRINITY_DN3971_c0_g1_i1_orf1;TRINITY_DN3263_c0_g1_i2_orf1;TRINITY_DN3551_c0_g1_i4_orf1;TRINITY_DN3836_c0_g1_i4_orf1;TRINITY_DN21506_c0_g1_i4_orf1;TRINITY_DN53807_c0_g2_i1_orf1;TRINITY_DN29369_c0_g1_i1_orf1;TRINITY_DN14464_c0_g1_i1_orf1;TRINITY_DN4944_c0_g1_i2_orf1;TRINITY_DN5266_c0_g1_i1_orf1;TRINITY_DN57918_c0_g1_i1_orf1;TRINITY_DN14967_c0_g2_i1_orf1;TRINITY_DN24970_c0_g1_i4_orf1;TRINITY_DN1494_c0_g1_i3_orf1;TRINITY_DN1494_c0_g2_i1_orf1;TRINITY_DN89483_c0_g1_i1_orf1;TRINITY_DN42738_c0_g1_i1_orf1;TRINITY_DN144807_c0
biological_process	cellular catabolic process	GO:0044248	97 97/3516	TRINITY_DN29017_c0_g1_i4_orf1;TRINITY_DN42856_c0_g1_i1_orf1;TRINITY_DN2065_c1_g2_i1_orf1;TRINITY_DN38230_c0_g1_i4_orf1;TRINITY_DN14565_c0_g1_i11_orf1;TRINITY_DN181_c0_g1_i3_orf1;TRINITY_DN2818_c0_g1_i2_orf1;TRINITY_DN1757_c0_g1_i4_orf1;TRINITY_DN21545_c0_g1_i2_orf1;TRINITY_DN5092_c0_g1_i2_orf1;TRINITY_DN59335_c0_g1_i2_orf1;TRINITY_DN16933_c0_g1_i10_orf1;TRINITY_DN2861_c0_g2_i1_orf1;TRINITY_DN4822_c0_g1_i6_orf1;TRINITY_DN18291_c0_g1_i1_orf1;TRINITY_DN285_c0_g1_i4_orf1;TRINITY_DN2181_c1_g1_i8_orf1;TRINITY_DN20499_c0_g3_i1_orf1;TRINITY_DN357_c0_g1_i8_orf1;TRINITY_DN8908_c0_g1_i1_orf1;TRINITY_DN8037_c0_g2_i1_orf1;TRINITY_DN2947_c0_g1_i4_orf1;TRINITY_DN34689_c0_g1_i4_orf1;TRINITY_DN11172_c1_g1_i1_orf1;TRINITY_DN53807_c0_g2_i1_orf1;TRINITY_DN4145_c0_g1_i1_orf1;TRINITY_DN4954_c0_g1_i5_orf1;TRINITY_DN6199_c2_g1_i3_orf1;TRINITY_DN18230_c1_g2_i1_orf1;TRINITY_DN29402_c0_g1_i1_orf1;TRINITY_DN37923_c0_g1_i1_orf1;TRINITY_DN4451_c0_g2_i4_orf1;TRINITY_DN19187_c0_g1_i1_orf1;TRINITY_DN45220_c0_g1_i1_orf1;TRINITY_DN1034_c0_g1_i4_orf1;TRINITY_DN19821_c0_g2_i4_orf1;TRINITY_DN779_c0_g1_i3_orf1;TRINITY_DN21126_c0_g1_i1_orf1;TRINITY_DN123396_c0_g1_i1_orf1;TRINITY_DN111985_c0_g1_i1_orf1;TRINITY_DN43431_c0_g1_i1_orf1;TRINITY_DN12323_c0_g2_i2_orf1;TRINITY_DN4451_c0_g1_i1_orf1;TRINITY_DN779_c0_g1_i12_orf1;TRINITY_DN9062_c0_g2_i3_orf1;TRINITY_DN10229_c0_g1_i6_orf1;TRINITY_DN22441_c0_g1_i1_orf1;TRINITY_DN5055_c0_g1_i12_orf1;TRINITY_DN22242_c0_g2_i1_orf1;TRINITY_DN48536_c0_g1_i3_orf1;TRINITY_DN10429_c0_g1_i2_orf1;TRINITY_DN28577_c0_g1_i6_orf1;TRINITY_DN3758_c0_g1_i2_orf1;TRINITY_DN24_c0_g1_i1_orf1;TRINITY_DN2559_c0_g1_i4_orf1;TRINITY_DN98242_c0_g1_i1_orf1;TRINITY_DN8926_c0_g1_i4_orf1;TRINITY_DN13941_c0_g1_i6_orf1;TRINITY_DN17726_c0_g1_i1_orf1;TRINITY_DN18538_c0_g3_i1_orf1;TRINITY_DN19727_c0_g1_i7_orf1;TRINITY_DN10455_c0_g2_i1_orf1;TRINITY_DN51813_c0_g1_i1_orf1;TRINITY_DN8012_c0_g1_i3_orf1;TRINITY_DN631_c0_g1_i6_orf1;TRINITY_DN1707_c0_g1_i1_orf1;TRINITY_DN4121_c0_g1_i1_orf1;TRINITY_DN44877_c0_g1_i2_orf1;TRINITY_DN96557_c0_g1_i1_orf1;TRINITY_DN9376_c1_g1_i3_orf1;TRINITY_DN768_c0_g1_i7_orf1;TRINITY_DN3551_c0_g1_i4_orf1;TRINITY_DN3836_c0_g1_i4_orf1;TRINITY_DN46022_c0_g1_i1_orf1;TRINITY_DN38180_c0_g1_i3_orf1;TRINITY_DN461_c0_g1_i5_orf1;TRINITY_DN1494_c0_g1_i3_orf1;TRINITY_DN1494_c0_g2_i1_orf1;TRINITY_DN89483_c0_g1_i1_orf1;TRINITY_DN146138_c0_g1_i1_orf1;TRINITY_DN87170_c0_g1_i3_orf1;TRINITY_DN3588_c0_g1_i1_orf1;TRINITY_DN11172_c0_g1_i4_orf1;TRINITY_DN17913_c0_g1_i8_orf1;TRINITY_DN58413_c0_g1_i4_orf1;TRINITY_DN57798_c0_g1_i1_orf1;TRINITY_DN46132_c0_g2_i2_orf1;TRINITY_DN5001_c0_g1_i4_orf1;TRINITY_DN37366_c0_g1_i7_orf1;TRINITY_DN5512_c0_g1_i8_orf1;TRINITY_DN2047_c0_g1_i1_orf1;TRINITY_DN22242_c0_g1_i1_orf1;TRINITY_DN6580_c0_g1_i4_orf1;TRINITY_DN135188_c0_g1_i2_orf1;TRINITY_DN195_c0_g3_i6_orf1;TRINITY_DN113353_c0_g1_i1_orf1;TRINITY_DN18230_c1_g1_i1_orf1

biological_process	cellular biosynthetic process	GO:0044249	247	247/3516	TRINITY_DN57074_c0_g2_i1_orf1;TRINITY_DN21251_c1_g1_i1_orf1;TRINITY_DN10722_c0_g3_i1_orf1;TRINITY_DN86090_c0_g1_i1_orf1;TRINITY_DN10399_c0_g1_i2_orf1;TRINITY_DN659_c0_g1_i3_orf1;TRINITY_DN26805_c0_g2_i3_orf1;TRINITY_DN137_c0_g1_i1_orf1;TRINITY_DN8625_c0_g1_i1_orf1;TRINITY_DN135_c0_g1_i1_orf1;TRINITY_DN35763_c0_g1_i2_orf1;TRINITY_DN27885_c0_g1_i3_orf1;TRINITY_DN5873_c0_g4_i1_orf1;TRINITY_DN40650_c0_g1_i1_orf1;TRINITY_DN95850_c0_g1_i1_orf1;TRINITY_DN124950_c0_g2_i1_orf1;TRINITY_DN2738_c1_g1_i3_orf1;TRINITY_DN36592_c0_g1_i1_orf1;TRINITY_DN11065_c0_g2_i1_orf1;TRINITY_DN2682_c0_g1_i4_orf1;TRINITY_DN15836_c0_g1_i1_orf1;TRINITY_DN30300_c0_g2_i1_orf1;TRINITY_DN1216_c0_g1_i4_orf1;TRINITY_DN5976_c0_g1_i1_orf1;TRINITY_DN142442_c0_g1_i1_orf1;TRINITY_DN3985_c0_g2_i1_orf1;TRINITY_DN24723_c2_g1_i1_orf1;TRINITY_DN31163_c1_g1_i4_orf1;TRINITY_DN7957_c0_g1_i5_orf1;TRINITY_DN29448_c0_g1_i1_orf1;TRINITY_DN11013_c0_g1_i3_orf1;TRINITY_DN9555_c0_g1_i1_orf1;TRINITY_DN50787_c0_g2_i2_orf1;TRINITY_DN10742_c0_g1_i4_orf1;TRINITY_DN19122_c0_g1_i7_orf1;TRINITY_DN11825_c0_g1_i4_orf1;TRINITY_DN1393_c0_g1_i2_orf1;TRINITY_DN74889_c0_g1_i1_orf1;TRINITY_DN10070_c0_g1_i1_orf1;TRINITY_DN1760_c0_g1_i4_orf1;TRINITY_DN1509_c0_g1_i1_orf1;TRINITY_DN2110_c0_g1_i3_orf1;TRINITY_DN38075_c0_g1_i1_orf1;TRINITY_DN58207_c0_g1_i1_orf1;TRINITY_DN883_c0_g1_i8_orf1;TRINITY_DN37165_c0_g1_i4_orf1;TRINITY_DN3822_c0_g1_i7_orf1;TRINITY_DN5952_c0_g1_i6_orf1;TRINITY_DN25896_c0_g1_i6_orf1;TRINITY_DN9156_c0_g1_i1_orf1;TRINITY_DN55148_c0_g1_i1_orf1;TRINITY_DN107261_c0_g1_i1_orf1;TRINITY_DN2265_c0_g1_i5_orf1;TRINITY_DN1575_c0_g1_i7_orf1;TRINITY_DN34509_c0_g1_i1_orf1;TRINITY_DN18869_c0_g1_i1_orf1;TRINITY_DN7808_c0_g1_i1_orf1;TRINITY_DN121893_c0_g1_i1_orf1;TRINITY_DN111985_c0_g1_i1_orf1;TRINITY_DN42738_c0_g1_i1_orf1;TRINITY_DN18249_c0_g1_i1_orf1;TRINITY_DN15222_c0_g1_i4_orf1;TRINITY_DN37830_c0_g1_i1_orf1;TRINITY_DN144956_c0_g1_i1_orf1;TRINITY_DN98538_c0_g1_i1_orf1;TRINITY_DN19829_c0_g2_i1_orf1;TRINITY_DN5497_c0_g1_i6_orf1;TRINITY_DN59965_c0_g4_i1_orf1;TRINITY_DN33183_c0_g1_i4_orf1;TRINITY_DN37532_c0_g1_i1_orf1;TRINITY_DN1965_c0_g1_i7_orf1;TRINITY_DN9874_c0_g1_i7_orf1;TRINITY_DN29038_c0_g2_i1_orf1;TRINITY_DN14306_c0_g1_i1_orf1;TRINITY_DN93566_c0_g2_i1_orf1;TRINITY_DN31253_c0_g1_i2_orf1;TRINITY_DN115658_c0_g1_i1_orf1;TRINITY_DN79734_c0_g2_i3_orf1;TRINITY_DN29707_c0_g1_i2_orf1;TRINITY_DN5211_c0_g1_i1_orf1;TRINITY_DN7512_c0_g1_i1_orf1;TRINITY_DN1578_c0_g3_i1_orf1;TRINITY_DN7583_c0_g1_i1_orf1;TRINITY_DN25582_c0_g1_i3_orf1;TRINITY_DN7464_c0_g1_i14_orf1;TRINITY_DN9207_c0_g1_i1_orf1;TRINITY_DN42759_c0_g3_i1_orf1;TRINITY_DN10430_c0_g1_i4_orf1;TRINITY_DN36144_c0_g1_i3_orf1;TRINITY_DN11076_c0_g2_i1_orf1;TRINITY_DN12527_c0_g1_i4_orf1;TRINITY_DN799_c0_g1_i7_orf1;TRINITY_DN117844_c0_g1_i1_orf1;TRINITY_DN12301_c0_g1_i1_orf1;TRINITY_DN10900_c0_g1_i7_orf1;TRINITY_DN23714_c0_g1_i4_orf1;TRINITY_DN3733_c0_g1_i1_orf1;TRINITY_DN49936_c0_g2_i1_orf1;TRINITY_DN8986_c0_g1_i1_orf1;TRINITY_DN3814_c1_g1_i1_orf1;TRINITY_DN1277_c4_g1_i5_orf1;TRINITY_DN5525_c0_g1_i4_orf1;TRINITY_DN4707_c0_g1_i1_orf1;TRINITY_DN97680_c0_g1_i1_orf1;TRINITY_DN19829_c0_g1_i1_orf1;TRINITY_DN21357_c0_g1_i5_orf1;TRINITY_DN7991_c0_g1_i9_orf1;TRINITY_DN4321_c0_g1_i1_orf1;TRINITY_DN11948_c0_g1_i8_orf1;TRINITY_DN22175_c0_g1_i1_orf1;TRINITY_DN13233_c0_g1_i3_orf1;TRINITY_DN31520_c1_g1_i1_orf1;TRINITY_DN10831_c1_g1_i1_orf1;TRINITY_DN41997_c0_g1_i2_orf1;TRINITY_DN147458_c0_g1_i1_orf1;TRINITY_DN2299_c0_g1_i3_orf1;TRINITY_DN5064_c0_g1_i4_orf1;TRINITY_DN143852_c0_g1_i1_orf1;TRINITY_DN5029_c0_g1_i1_orf1;TRINITY_DN812_c2_g1_i1_orf1;TRINITY_DN4859
biological_process	cellular macromolecule metabolic process	GO:0044260	191	191/3516	TRINITY_DN57074_c0_g2_i1_orf1;TRINITY_DN125565_c1_g1_i1_orf1;TRINITY_DN40434_c0_g1_i2_orf1;TRINITY_DN137_c0_g1_i1_orf1;TRINITY_DN181_c0_g1_i3_orf1;TRINITY_DN2818_c0_g1_i2_orf1;TRINITY_DN55148_c0_g1_i1_orf1;TRINITY_DN135_c0_g1_i1_orf1;TRINITY_DN1757_c0_g1_i4_orf1;TRINITY_DN5873_c0_g4_i1_orf1;TRINITY_DN2054_c0_g1_i1_orf1;TRINITY_DN11065_c0_g2_i1_orf1;TRINITY_DN15040_c0_g4_i1_orf1;TRINITY_DN2682_c0_g1_i4_orf1;TRINITY_DN1344_c0_g1_i1_orf1;TRINITY_DN30300_c0_g2_i1_orf1;TRINITY_DN142442_c0_g1_i1_orf1;TRINITY_DN11817_c0_g1_i4_orf1;TRINITY_DN3814_c1_g1_i1_orf1;TRINITY_DN29448_c0_g1_i1_orf1;TRINITY_DN42506_c0_g1_i1_orf1;TRINITY_DN2861_c0_g2_i1_orf1;TRINITY_DN11825_c0_g1_i4_orf1;TRINITY_DN1393_c0_g1_i2_orf1;TRINITY_DN74889_c0_g1_i1_orf1;TRINITY_DN10070_c0_g1_i1_orf1;TRINITY_DN15370_c0_g1_i4_orf1;TRINITY_DN9874_c0_g1_i7_orf1;TRINITY_DN1509_c0_g1_i1_orf1;TRINITY_DN38075_c0_g1_i1_orf1;TRINITY_DN143_c0_g3_i1_orf1;TRINITY_DN3985_c0_g2_i1_orf1;TRINITY_DN20499_c0_g3_i1_orf1;TRINITY_DN2265_c0_g1_i5_orf1;TRINITY_DN48536_c0_g1_i3_orf1;TRINITY_DN21619_c0_g1_i1_orf1;TRINITY_DN79734_c0_g2_i3_orf1;TRINITY_DN24318_c0_g1_i1_orf1;TRINITY_DN121893_c0_g1_i1_orf1;TRINITY_DN14313_c0_g1_i1_orf1;TRINITY_DN4954_c0_g1_i5_orf1;TRINITY_DN10429_c0_g1_i2_orf1;TRINITY_DN3092_c0_g1_i2_orf1;TRINITY_DN17271_c0_g1_i1_orf1;TRINITY_DN140212_c0_g1_i1_orf1;TRINITY_DN144956_c0_g1_i1_orf1;TRINITY_DN45271_c0_g1_i1_orf1;TRINITY_DN19829_c0_g2_i1_orf1;TRINITY_DN130075_c1_g2_i1_orf1;TRINITY_DN29402_c0_g1_i1_orf1;TRINITY_DN6248_c0_g1_i1_orf1;TRINITY_DN37923_c0_g1_i1_orf1;TRINITY_DN33926_c0_g1_i1_orf1;TRINITY_DN93566_c0_g2_i1_orf1;TRINITY_DN57798_c0_g1_i1_orf1;TRINITY_DN31253_c0_g1_i2_orf1;TRINITY_DN115658_c0_g1_i1_orf1;TRINITY_DN2930_c0_g1_i8_orf1;TRINITY_DN7583_c0_g1_i1_orf1;TRINITY_DN7464_c0_g1_i14_orf1;TRINITY_DN50787_c0_g2_i2_orf1;TRINITY_DN14953_c0_g1_i5_orf1;TRINITY_DN41664_c0_g1_i4_orf1;TRINITY_DN1091_c0_g3_i1_orf1;TRINITY_DN799_c0_g1_i7_orf1;TRINITY_DN6642_c0_g1_i2_orf1;TRINITY_DN3733_c0_g1_i1_orf1;TRINITY_DN49936_c0_g2_i1_orf1;TRINITY_DN8986_c0_g1_i1_orf1;TRINITY_DN46022_c0_g1_i1_orf1;TRINITY_DN95414_c0_g1_i1_orf1;TRINITY_DN97680_c0_g1_i1_orf1;TRINITY_DN19829_c0_g1_i1_orf1;TRINITY_DN21357_c0_g1_i5_orf1;TRINITY_DN46132_c0_g2_i2_orf1;TRINITY_DN9062_c0_g2_i3_orf1;TRINITY_DN89613_c0_g1_i13_orf1;TRINITY_DN58207_c0_g1_i1_orf1;TRINITY_DN22175_c0_g1_i1_orf1;TRINITY_DN13233_c0_g1_i3_orf1;TRINITY_DN10831_c1_g1_i1_orf1;TRINITY_DN41997_c0_g1_i2_orf1;TRINITY_DN147458_c0_g1_i1_orf1;TRINITY_DN4908_c1_g1_i5_orf1;TRINITY_DN109733_c0_g1_i1_orf1;TRINITY_DN10287_c0_g1_i1_orf1;TRINITY_DN145666_c0_g1_i1_orf1;TRINITY_DN812_c2_g1_i1_orf1;TRINITY_DN21251_c1_g1_i1_orf1;TRINITY_DN5112_c0_g1_i1_orf1;TRINITY_DN30131_c0_g1_i1_orf1;TRINITY_DN1750_c1_g1_i5_orf1;TRINITY_DN27852_c0_g1_i1_orf1;TRINITY_DN19942_c0_g1_i2_orf1;TRINITY_DN77318_c0_g2_i1_orf1;TRINITY_DN46409_c0_g1_i1_orf1;TRINITY_DN2026_c0_g1_i4_orf1;TRINITY_DN40650_c0_g1_i1_orf1;TRINITY_DN14734_c0_g1_i2_orf1;TRINITY_DN2084_c0_g1_i1_orf1;TRINITY_DN51934_c0_g2_i1_orf1;TRINITY_DN4300_c0_g1_i5_orf1;TRINITY_DN41645_c0_g1_i1_orf1;TRINITY_DN19262_c0_g1_i1_orf1;TRINITY_DN17045_c0_g2_i3_orf1;TRINITY_DN61222_c0_g1_i1_orf1;TRINITY_DN4016_c0_g1_i1_orf1;TRINITY_DN19261_c0_g1_i3_orf1;TRINITY_DN934_c2_g1_i7_orf1;TRINITY_DN40345_c0_g1_i6_orf1;TRINITY_DN43792_c0_g1_i1_orf1;TRINITY_DN11297_c0_g1_i1_orf1;TRINITY_DN30027_c0_g1_i1_orf1;TRINITY_DN82324_c0_g1_i4_orf1;TRINITY_DN452_c1_g1_i3_orf1;TRINITY_DN34689_c0_g1_i4_orf1;TRINITY_DN147676_c0_g1_i1_orf1;TRINITY_DN23360_c0_g1_i3_orf1;TRINITY_DN18538_c0_g3_i1_orf1;TRINITY_DN4835_c0_g1_i2_orf1;TRINITY_D

biological_process	cellular aromatic compound metabolic process	GO:0006725	280	280/3516	TRINITY_DN38230_c0_g1_i4_orf1;TRINITY_DN22941_c0_g1_i1_orf1;TRINITY_DN86090_c0_g1_i1_orf1;TRINITY_DN40434_c0_g1_i2_orf1;TRINITY_DN130051_c0_g1_i1_orf1;TRINITY_DN15836_c0_g1_i1_orf1;TRINITY_DN1827_c0_g1_i4_orf1;TRINITY_DN1316_c0_g1_i1_orf1;TRINITY_DN60787_c0_g1_i5_orf1;TRINITY_DN1354_c0_g1_i6_orf1;TRINITY_DN47666_c0_g1_i4_orf1;TRINITY_DN56910_c0_g2_i1_orf1;TRINITY_DN35669_c0_g1_i1_orf1;TRINITY_DN2054_c0_g1_i1_orf1;TRINITY_DN124950_c0_g2_i1_orf1;TRINITY_DN2738_c1_g1_i3_orf1;TRINITY_DN14967_c0_g2_i1_orf1;TRINITY_DN125565_c1_g1_i1_orf1;TRINITY_DN34134_c0_g2_i1_orf1;TRINITY_DN1344_c0_g1_i1_orf1;TRINITY_DN1366_c0_g1_i5_orf1;TRINITY_DN1216_c0_g1_i4_orf1;TRINITY_DN25582_c0_g1_i3_orf1;TRINITY_DN44877_c0_g1_i2_orf1;TRINITY_DN23616_c0_g1_i4_orf1;TRINITY_DN31163_c1_g1_i4_orf1;TRINITY_DN31611_c0_g1_i2_orf1;TRINITY_DN2848_c0_g1_i1_orf1;TRINITY_DN16933_c0_g1_i10_orf1;TRINITY_DN23432_c0_g1_i1_orf1;TRINITY_DN18404_c0_g1_i5_orf1;TRINITY_DN8430_c0_g1_i1_orf1;TRINITY_DN9207_c0_g1_i1_orf1;TRINITY_DN6587_c0_g1_i3_orf1;TRINITY_DN4822_c0_g1_i6_orf1;TRINITY_DN18291_c0_g1_i1_orf1;TRINITY_DN1393_c0_g1_i2_orf1;TRINITY_DN5507_c0_g1_i1_orf1;TRINITY_DN3991_c0_g1_i6_orf1;TRINITY_DN29873_c0_g1_i1_orf1;TRINITY_DN17738_c0_g1_i2_orf1;TRINITY_DN15370_c0_g1_i4_orf1;TRINITY_DN47123_c0_g1_i1_orf1;TRINITY_DN18538_c0_g3_i1_orf1;TRINITY_DN123184_c0_g1_i1_orf1;TRINITY_DN26805_c0_g2_i3_orf1;TRINITY_DN2110_c0_g1_i3_orf1;TRINITY_DN1116_c0_g1_i6_orf1;TRINITY_DN817_c0_g1_i3_orf1;TRINITY_DN1066_c0_g1_i4_orf1;TRINITY_DN145647_c0_g1_i1_orf1;TRINITY_DN37165_c0_g1_i4_orf1;TRINITY_DN9094_c0_g1_i1_orf1;TRINITY_DN5952_c0_g1_i6_orf1;TRINITY_DN2224_c0_g1_i1_orf1;TRINITY_DN3822_c0_g1_i7_orf1;TRINITY_DN24310_c0_g1_i2_orf1;TRINITY_DN46409_c0_g1_i1_orf1;TRINITY_DN15040_c0_g4_i1_orf1;TRINITY_DN8908_c0_g1_i1_orf1;TRINITY_DN4813_c0_g1_i5_orf1;TRINITY_DN58636_c0_g1_i1_orf1;TRINITY_DN107261_c0_g1_i1_orf1;TRINITY_DN9156_c0_g1_i1_orf1;TRINITY_DN107288_c0_g1_i2_orf1;TRINITY_DN18860_c0_g1_i1_orf1;TRINITY_DN6235_c0_g1_i5_orf1;TRINITY_DN11172_c1_g1_i1_orf1;TRINITY_DN7808_c0_g1_i1_orf1;TRINITY_DN89613_c0_g1_i13_orf1;TRINITY_DN2848_c0_g1_i2_orf1;TRINITY_DN2647_c0_g1_i3_orf1;TRINITY_DN5603_c0_g1_i1_orf1;TRINITY_DN3092_c0_g1_i2_orf1;TRINITY_DN1978_c0_g1_i4_orf1;TRINITY_DN4300_c0_g1_i5_orf1;TRINITY_DN6313_c0_g1_i4_orf1;TRINITY_DN2038_c0_g1_i2_orf1;TRINITY_DN45271_c0_g1_i1_orf1;TRINITY_DN3082_c1_g1_i7_orf1;TRINITY_DN59965_c0_g4_i1_orf1;TRINITY_DN26649_c0_g1_i2_orf1;TRINITY_DN37366_c0_g1_i7_orf1;TRINITY_DN18728_c0_g1_i2_orf1;TRINITY_DN29402_c0_g1_i1_orf1;TRINITY_DN37532_c0_g1_i1_orf1;TRINITY_DN4710_c0_g1_i1_orf1;TRINITY_DN1965_c0_g1_i7_orf1;TRINITY_DN7405_c0_g1_i3_orf1;TRINITY_DN29038_c0_g2_i1_orf1;TRINITY_DN11639_c0_g1_i1_orf1;TRINITY_DN15900_c0_g1_i6_orf1;TRINITY_DN19187_c0_g1_i1_orf1;TRINITY_DN115658_c0_g1_i1_orf1;TRINITY_DN22242_c0_g1_i1_orf1;TRINITY_DN10548_c0_g2_i1_orf1;TRINITY_DN30097_c0_g1_i2_orf1;TRINITY_DN47575_c0_g1_i1_orf1;TRINITY_DN51968_c0_g1_i1_orf1;TRINITY_DN8625_c0_g1_i1_orf1;TRINITY_DN779_c0_g1_i3_orf1;TRINITY_DN1607_c0_g1_i16_orf1;TRINITY_DN810_c0_g1_i4_orf1;TRINITY_DN244_c1_g1_i5_orf1;TRINITY_DN18863_c0_g1_i3_orf1;TRINITY_DN2769_c0_g1_i1_orf1;TRINITY_DN41664_c0_g1_i4_orf1;TRINITY_DN51813_c0_g1_i1_orf1;TRINITY_DN1091_c0_g3_i1_orf1;TRINITY_DN23714_c0_g1_i4_orf1;TRINITY_DN36144_c0_g1_i3_orf1;TRINITY_DN12527_c0_g1_i4_orf1;TRINITY_DN131662_c0_g1_i4_orf1;TRINITY_DN1616_c0_g1_i3_orf1;TRINITY_DN117844_c0_g1_i1_orf1;TRINITY_DN12301_c0_g1_i1_orf1;TRINITY_DN2953_c1_g1_i11_orf1;TRINITY_DN6642_c0_g1_i2_orf1;TRINITY_DN7289_c0_g1_i1_orf1;TRINITY_DN12323_c0_g2_i2_orf1;TRINITY_DN46022_c0_g
biological_process	cellular metabolic compound salvage	GO:0043094	7	7/3516	TRINITY_DN2738_c1_g1_i3_orf1;TRINITY_DN144807_c0_g1_i1_orf1;TRINITY_DN5497_c0_g1_i6_orf1;TRINITY_DN1575_c0_g1_i7_orf1;TRINITY_DN35763_c0_g1_i2_orf1;TRINITY_DN9555_c0_g1_i1_orf1;TRINITY_DN140669_c0_g1_i1_orf1
biological_process	neurotransmitter metabolic process	GO:0042133	2	2/3516	TRINITY_DN2047_c0_g1_i1_orf1;TRINITY_DN14565_c0_g1_i11_orf1
biological_process	cellular modified amino acid metabolic process	GO:0006575	10	10/3516	TRINITY_DN92153_c0_g2_i2_orf1;TRINITY_DN33183_c0_g1_i4_orf1;TRINITY_DN130051_c0_g1_i1_orf1;TRINITY_DN3263_c0_g1_i2_orf1;TRINITY_DN38562_c0_g1_i3_orf1;TRINITY_DN38506_c0_g1_i4_orf1;TRINITY_DN41166_c0_g1_i1_orf1;TRINITY_DN244_c1_g1_i5_orf1;TRINITY_DN631_c0_g1_i6_orf1;TRINITY_DN1277_c4_g1_i5_orf1
biological_process	translational initiation	GO:0006413	1	1/3516	TRINITY_DN1572_c0_g1_i6_orf1
biological_process	prenylation	GO:0097354	1	1/3516	TRINITY_DN5182_c0_g1_i5_orf1
biological_process	secondary metabolite biosynthetic process	GO:0044550	3	3/3516	TRINITY_DN31163_c1_g1_i4_orf1;TRINITY_DN2338_c0_g1_i5_orf1;TRINITY_DN13941_c0_g1_i6_orf1
biological_process	small molecule biosynthetic process	GO:0044283	59	59/3516	TRINITY_DN48590_c0_g1_i1_orf1;TRINITY_DN230_c2_g1_i5_orf1;TRINITY_DN8173_c0_g1_i3_orf1;TRINITY_DN10722_c0_g3_i1_orf1;TRINITY_DN24970_c0_g1_i4_orf1;TRINITY_DN5497_c0_g1_i6_orf1;TRINITY_DN27035_c0_g1_i1_orf1;TRINITY_DN1575_c0_g1_i7_orf1;TRINITY_DN35763_c0_g1_i2_orf1;TRINITY_DN27885_c0_g1_i3_orf1;TRINITY_DN1494_c0_g2_i1_orf1;TRINITY_DN14306_c0_g1_i1_orf1;TRINITY_DN140669_c0_g1_i1_orf1;TRINITY_DN11076_c0_g2_i1_orf1;TRINITY_DN1824_c0_g2_i2_orf1;TRINITY_DN42738_c0_g1_i1_orf1;TRINITY_DN11948_c0_g1_i8_orf1;TRINITY_DN144807_c0_g1_i1_orf1;TRINITY_DN905_c0_g1_i4_orf1;TRINITY_DN5211_c0_g1_i1_orf1;TRINITY_DN2803_c4_g1_i1_orf1;TRINITY_DN6027_c0_g1_i13_orf1;TRINITY_DN24723_c2_g1_i1_orf1;TRINITY_DN3991_c0_g1_i6_orf1;TRINITY_DN28221_c0_g2_i1_orf1;TRINITY_DN31611_c0_g1_i2_orf1;TRINITY_DN2848_c0_g1_i1_orf1;TRINITY_DN42759_c0_g2_i1_orf1;TRINITY_DN42759_c0_g3_i1_orf1;TRINITY_DN10430_c0_g1_i4_orf1;TRINITY_DN9555_c0_g1_i1_orf1;TRINITY_DN111985_c0_g1_i1_orf1;TRINITY_DN1201_c0_g1_i4_orf1;TRINITY_DN1334_c0_g1_i2_orf1;TRINITY_DN127151_c0_g1_i1_orf1;TRINITY_DN27848_c0_g1_i2_orf1;TRINITY_DN51813_c0_g1_i1_orf1;TRINITY_DN1760_c0_g1_i4_orf1;TRINITY_DN76283_c0_g6_i1_orf1;TRINITY_DN34399_c0_g1_i1_orf1;TRINITY_DN2570_c0_g1_i1_orf1;TRINITY_DN6638_c0_g1_i1_orf1;TRINITY_DN1277_c4_g1_i5_orf1;TRINITY_DN6669_c0_g1_i3_orf1;TRINITY_DN76283_c0_g2_i1_orf1;TRINITY_DN659_c0_g1_i3_orf1;TRINITY_DN36592_c0_g1_i1_orf1;TRINITY_DN511_c0_g2_i1_orf1;TRINITY_DN37165_c0_g1_i4_orf1;TRINITY_DN4321_c0_g1_i1_orf1;TRINITY_DN26293_c0_g1_i4_orf1;TRINITY_DN1494_c0_g1_i3_orf1;TRINITY_DN3263_c0_g1_i2_orf1;TRINITY_DN130051_c0_g1_i1_orf1;TRINITY_DN115498_c0_g1_i1_orf1;TRINITY_DN18782_c0_g1_i4_orf1;TRINITY_DN20133_c0_g1_i1_orf1;TRINITY_DN1161_c0_g1_i2_orf1;TRINITY_DN10900_c0_g1_i7_orf1
biological_process	peptidyl-lysine modification to peptidyl-hypusine	GO:0008612	1	1/3516	TRINITY_DN8019_c0_g1_i4_orf1

biological_process	organic substance biosynthetic process	GO:1901576	259	259/3516	TRINITY_DN57074_c0_g2_i1_orf1;TRINITY_DN21251_c1_g1_i1_orf1;TRINITY_DN10722_c0_g3_i1_orf1;TRINITY_DN86090_c0_g1_i1_orf1;TRINITY_DN10399_c0_g1_i2_orf1;TRINITY_DN659_c0_g1_i3_orf1;TRINITY_DN26805_c0_g2_i3_orf1;TRINITY_DN137_c0_g1_i1_orf1;TRINITY_DN1578_c0_g3_i1_orf1;TRINITY_DN135_c0_g1_i1_orf1;TRINITY_DN35763_c0_g1_i2_orf1;TRINITY_DN27885_c0_g1_i3_orf1;TRINITY_DN5873_c0_g4_i1_orf1;TRINITY_DN40650_c0_g1_i1_orf1;TRINITY_DN95850_c0_g1_i1_orf1;TRINITY_DN124950_c0_g2_i1_orf1;TRINITY_DN2738_c1_g1_i3_orf1;TRINITY_DN36592_c0_g1_i1_orf1;TRINITY_DN11065_c0_g2_i1_orf1;TRINITY_DN2682_c0_g1_i4_orf1;TRINITY_DN15836_c0_g1_i1_orf1;TRINITY_DN30300_c0_g2_i1_orf1;TRINITY_DN1216_c0_g1_i4_orf1;TRINITY_DN5976_c0_g1_i1_orf1;TRINITY_DN142442_c0_g1_i1_orf1;TRINITY_DN37165_c0_g1_i4_orf1;TRINITY_DN24723_c2_g1_i1_orf1;TRINITY_DN31163_c1_g1_i4_orf1;TRINITY_DN31611_c0_g1_i2_orf1;TRINITY_DN2848_c0_g1_i1_orf1;TRINITY_DN29448_c0_g1_i1_orf1;TRINITY_DN11013_c0_g1_i3_orf1;TRINITY_DN9555_c0_g1_i1_orf1;TRINITY_DN50787_c0_g2_i2_orf1;TRINITY_DN10742_c0_g1_i4_orf1;TRINITY_DN19122_c0_g1_i7_orf1;TRINITY_DN11825_c0_g1_i4_orf1;TRINITY_DN1393_c0_g1_i2_orf1;TRINITY_DN74889_c0_g1_i1_orf1;TRINITY_DN10070_c0_g1_i1_orf1;TRINITY_DN40197_c0_g1_i1_orf1;TRINITY_DN1760_c0_g1_i4_orf1;TRINITY_DN1509_c0_g1_i1_orf1;TRINITY_DN2110_c0_g1_i3_orf1;TRINITY_DN38075_c0_g1_i1_orf1;TRINITY_DN883_c0_g1_i8_orf1;TRINITY_DN3985_c0_g2_i1_orf1;TRINITY_DN10785_c0_g1_i4_orf1;TRINITY_DN3822_c0_g1_i7_orf1;TRINITY_DN5952_c0_g1_i6_orf1;TRINITY_DN25896_c0_g1_i6_orf1;TRINITY_DN2265_c0_g1_i5_orf1;TRINITY_DN55148_c0_g1_i1_orf1;TRINITY_DN107261_c0_g1_i1_orf1;TRINITY_DN79734_c0_g2_i3_orf1;TRINITY_DN1575_c0_g1_i7_orf1;TRINITY_DN34509_c0_g1_i1_orf1;TRINITY_DN18869_c0_g1_i1_orf1;TRINITY_DN7808_c0_g1_i1_orf1;TRINITY_DN1161_c0_g1_i2_orf1;TRINITY_DN111985_c0_g1_i1_orf1;TRINITY_DN42738_c0_g1_i1_orf1;TRINITY_DN18249_c0_g1_i1_orf1;TRINITY_DN15222_c0_g1_i4_orf1;TRINITY_DN37830_c0_g1_i1_orf1;TRINITY_DN144956_c0_g1_i1_orf1;TRINITY_DN98538_c0_g1_i1_orf1;TRINITY_DN19829_c0_g2_i1_orf1;TRINITY_DN5497_c0_g1_i6_orf1;TRINITY_DN59965_c0_g4_i1_orf1;TRINITY_DN33183_c0_g1_i4_orf1;TRINITY_DN37532_c0_g1_i1_orf1;TRINITY_DN1965_c0_g1_i7_orf1;TRINITY_DN9874_c0_g1_i7_orf1;TRINITY_DN29038_c0_g2_i1_orf1;TRINITY_DN14306_c0_g1_i1_orf1;TRINITY_DN93566_c0_g2_i1_orf1;TRINITY_DN31253_c0_g1_i2_orf1;TRINITY_DN115658_c0_g1_i1_orf1;TRINITY_DN9156_c0_g1_i1_orf1;TRINITY_DN5211_c0_g1_i1_orf1;TRINITY_DN7512_c0_g1_i1_orf1;TRINITY_DN8625_c0_g1_i1_orf1;TRINITY_DN7583_c0_g1_i1_orf1;TRINITY_DN25582_c0_g1_i3_orf1;TRINITY_DN7464_c0_g1_i14_orf1;TRINITY_DN9207_c0_g1_i1_orf1;TRINITY_DN42759_c0_g3_i1_orf1;TRINITY_DN2441_c0_g1_i1_orf1;TRINITY_DN10430_c0_g1_i4_orf1;TRINITY_DN36144_c0_g1_i3_orf1;TRINITY_DN11076_c0_g2_i1_orf1;TRINITY_DN12527_c0_g1_i4_orf1;TRINITY_DN799_c0_g1_i7_orf1;TRINITY_DN117844_c0_g1_i1_orf1;TRINITY_DN12301_c0_g1_i1_orf1;TRINITY_DN10900_c0_g1_i7_orf1;TRINITY_DN23714_c0_g1_i4_orf1;TRINITY_DN3733_c0_g1_i1_orf1;TRINITY_DN49936_c0_g2_i1_orf1;TRINITY_DN8986_c0_g1_i1_orf1;TRINITY_DN3814_c1_g1_i1_orf1;TRINITY_DN1277_c4_g1_i5_orf1;TRINITY_DN5525_c0_g1_i4_orf1;TRINITY_DN4707_c0_g1_i1_orf1;TRINITY_DN97680_c0_g1_i1_orf1;TRINITY_DN19829_c0_g1_i1_orf1;TRINITY_DN511_c0_g2_i1_orf1;TRINITY_DN7991_c0_g1_i9_orf1;TRINITY_DN4321_c0_g1_i1_orf1;TRINITY_DN121893_c0_g1_i1_orf1;TRINITY_DN58207_c0_g1_i1_orf1;TRINITY_DN22175_c0_g1_i1_orf1;TRINITY_DN13233_c0_g1_i3_orf1;TRINITY_DN31520_c1_g1_i1_orf1;TRINITY_DN10831_c1_g1_i1_orf1;TRINITY_DN41997_c0_g1_i2_orf1;TRINITY_DN147458_c0_g1_i1_orf1;TRINITY_DN2299_c0_g1_i3_orf1;TRINITY_DN5064_c0_g1_i4_orf1;TRINITY_DN143852
biological_process	small molecule catabolic process	GO:0044282	40	40/3516	TRINITY_DN42856_c0_g1_i1_orf1;TRINITY_DN6199_c2_g1_i3_orf1;TRINITY_DN5055_c0_g1_i12_orf1;TRINITY_DN2065_c1_g2_i1_orf1;TRINITY_DN18230_c1_g2_i1_orf1;TRINITY_DN8037_c0_g2_i1_orf1;TRINITY_DN28577_c0_g1_i6_orf1;TRINITY_DN89483_c0_g1_i1_orf1;TRINITY_DN4451_c0_g2_i4_orf1;TRINITY_DN19187_c0_g1_i1_orf1;TRINITY_DN3758_c0_g1_i2_orf1;TRINITY_DN45220_c0_g1_i1_orf1;TRINITY_DN1034_c0_g1_i4_orf1;TRINITY_DN779_c0_g1_i3_orf1;TRINITY_DN87170_c0_g1_i3_orf1;TRINITY_DN5092_c0_g1_i2_orf1;TRINITY_DN2559_c0_g1_i4_orf1;TRINITY_DN98242_c0_g1_i1_orf1;TRINITY_DN2848_c0_g1_i1_orf1;TRINITY_DN59335_c0_g1_i2_orf1;TRINITY_DN13941_c0_g1_i6_orf1;TRINITY_DN123396_c0_g1_i1_orf1;TRINITY_DN4822_c0_g1_i6_orf1;TRINITY_DN18291_c0_g1_i1_orf1;TRINITY_DN43431_c0_g1_i1_orf1;TRINITY_DN17913_c0_g1_i8_orf1;TRINITY_DN51813_c0_g1_i1_orf1;TRINITY_DN3588_c0_g1_i1_orf1;TRINITY_DN631_c0_g1_i6_orf1;TRINITY_DN1707_c0_g1_i1_orf1;TRINITY_DN19727_c0_g1_i7_orf1;TRINITY_DN5512_c0_g1_i8_orf1;TRINITY_DN4451_c0_g1_i1_orf1;TRINITY_DN779_c0_g1_i12_orf1;TRINITY_DN357_c0_g1_i8_orf1;TRINITY_DN3551_c0_g1_i4_orf1;TRINITY_DN3836_c0_g1_i4_orf1;TRINITY_DN20133_c0_g1_i1_orf1;TRINITY_DN53807_c0_g2_i1_orf1;TRINITY_DN18230_c1_g1_i1_orf1

biological_process	organic substance catabolic process	GO:1901575	103	103/3516	TRINITY_DN42856_c0_g1_i1_orf1;TRINITY_DN2065_c1_g2_i1_orf1;TRINITY_DN38230_c0_g1_i4_orf1;TRINITY_DN827_c1_g1_i1_orf1;TRINITY_DN1827_c0_g1_i4_orf1;TRINITY_DN181_c0_g1_i3_orf1;TRINITY_DN2818_c0_g1_i2_orf1;TRINITY_DN1757_c0_g1_i4_orf1;TRINITY_DN21545_c0_g1_i2_orf1;TRINITY_DN5092_c0_g1_i2_orf1;TRINITY_DN31611_c0_g1_i2_orf1;TRINITY_DN2848_c0_g1_i1_orf1;TRINITY_DN59335_c0_g1_i2_orf1;TRINITY_DN16933_c0_g1_i10_orf1;TRINITY_DN2861_c0_g2_i1_orf1;TRINITY_DN4822_c0_g1_i6_orf1;TRINITY_DN18291_c0_g1_i1_orf1;TRINITY_DN1534_c0_g1_i3_orf1;TRINITY_DN29873_c0_g1_i1_orf1;TRINITY_DN20499_c0_g3_i1_orf1;TRINITY_DN357_c0_g1_i8_orf1;TRINITY_DN8908_c0_g1_i1_orf1;TRINITY_DN9717_c0_g2_i1_orf1;TRINITY_DN8037_c0_g2_i1_orf1;TRINITY_DN53807_c0_g2_i1_orf1;TRINITY_DN4145_c0_g1_i1_orf1;TRINITY_DN2848_c0_g1_i2_orf1;TRINITY_DN4954_c0_g1_i5_orf1;TRINITY_DN6199_c2_g1_i3_orf1;TRINITY_DN18230_c1_g2_i1_orf1;TRINITY_DN29402_c0_g1_i1_orf1;TRINITY_DN7405_c0_g1_i3_orf1;TRINITY_DN37923_c0_g1_i1_orf1;TRINITY_DN1201_c0_g1_i4_orf1;TRINITY_DN4451_c0_g2_i4_orf1;TRINITY_DN19187_c0_g1_i1_orf1;TRINITY_DN45220_c0_g1_i1_orf1;TRINITY_DN1034_c0_g1_i4_orf1;TRINITY_DN779_c0_g1_i3_orf1;TRINITY_DN123396_c0_g1_i1_orf1;TRINITY_DN17913_c0_g1_i8_orf1;TRINITY_DN43431_c0_g1_i1_orf1;TRINITY_DN12323_c0_g2_i2_orf1;TRINITY_DN6325_c0_g1_i9_orf1;TRINITY_DN4451_c0_g1_i1_orf1;TRINITY_DN779_c0_g1_i12_orf1;TRINITY_DN9062_c0_g2_i3_orf1;TRINITY_DN145227_c0_g1_i1_orf1;TRINITY_DN5235_c0_g1_i7_orf1;TRINITY_DN650_c0_g1_i3_orf1;TRINITY_DN5055_c0_g1_i12_orf1;TRINITY_DN27035_c0_g1_i1_orf1;TRINITY_DN48536_c0_g1_i3_orf1;TRINITY_DN10429_c0_g1_i2_orf1;TRINITY_DN4817_c0_g1_i4_orf1;TRINITY_DN28577_c0_g1_i6_orf1;TRINITY_DN3758_c0_g1_i2_orf1;TRINITY_DN542_c0_g2_i1_orf1;TRINITY_DN24_c0_g1_i1_orf1;TRINITY_DN34479_c0_g1_i2_orf1;TRINITY_DN2559_c0_g1_i4_orf1;TRINITY_DN98242_c0_g1_i1_orf1;TRINITY_DN13941_c0_g1_i6_orf1;TRINITY_DN34689_c0_g1_i4_orf1;TRINITY_DN18538_c0_g3_i1_orf1;TRINITY_DN19727_c0_g1_i7_orf1;TRINITY_DN10455_c0_g2_i1_orf1;TRINITY_DN51813_c0_g1_i1_orf1;TRINITY_DN8012_c0_g1_i3_orf1;TRINITY_DN2515_c0_g1_i6_orf1;TRINITY_DN83150_c0_g1_i1_orf1;TRINITY_DN631_c0_g1_i6_orf1;TRINITY_DN1707_c0_g1_i1_orf1;TRINITY_DN4121_c0_g1_i1_orf1;TRINITY_DN44877_c0_g1_i2_orf1;TRINITY_DN96557_c0_g1_i1_orf1;TRINITY_DN1287_c0_g1_i5_orf1;TRINITY_DN768_c0_g1_i7_orf1;TRINITY_DN3551_c0_g1_i4_orf1;TRINITY_DN3836_c0_g1_i4_orf1;TRINITY_DN49047_c0_g1_i2_orf1;TRINITY_DN46022_c0_g1_i1_orf1;TRINITY_DN38180_c0_g1_i3_orf1;TRINITY_DN461_c0_g1_i5_orf1;TRINITY_DN14967_c0_g2_i1_orf1;TRINITY_DN1494_c0_g1_i3_orf1;TRINITY_DN1494_c0_g2_i1_orf1;TRINITY_DN89483_c0_g1_i1_orf1;TRINITY_DN87170_c0_g1_i3_orf1;TRINITY_DN17726_c0_g1_i1_orf1;TRINITY_DN3588_c0_g1_i1_orf1;TRINITY_DN542_c0_g1_i4_orf1;TRINITY_DN146138_c0_g1_i1_orf1;TRINITY_DN58413_c0_g1_i4_orf1;TRINITY_DN57798_c0_g1_i1_orf1;TRINITY_DN46132_c0_g2_i2_orf1;TRINITY_DN5001_c0_g1_i4_orf1;TRINITY_DN5512_c0_g1_i8_orf1;TRINITY_DN21555_c0_g1_i4_orf1;TRINITY_DN135188_c0_g1_i2_orf1;TRINITY_DN195_c0_g3_i6_orf1;TRINITY_DN20133_c0_g1_i1_orf1;TRINITY_DN18230_c1_g1_i1_orf1
biological_process	formaldehyde metabolic process	GO:0046292	1	1/3516	TRINITY_DN3758_c0_g1_i2_orf1
biological_process	vitamin metabolic process	GO:0006766	4	4/3516	TRINITY_DN135781_c0_g1_i1_orf1;TRINITY_DN9555_c0_g1_i1_orf1;TRINITY_DN18782_c0_g1_i4_orf1;TRINITY_DN37165_c0_g1_i4_orf1
biological_process	urea metabolic process	GO:0019627	3	3/3516	TRINITY_DN24723_c2_g1_i1_orf1;TRINITY_DN17031_c0_g1_i1_orf1;TRINITY_DN28221_c0_g2_i1_orf1
biological_process	monosaccharide metabolic process	GO:0005996	24	24/3516	TRINITY_DN120089_c0_g1_i1_orf1;TRINITY_DN14967_c0_g2_i1_orf1;TRINITY_DN18650_c0_g1_i1_orf1;TRINITY_DN27035_c0_g1_i1_orf1;TRINITY_DN7405_c0_g1_i3_orf1;TRINITY_DN31967_c0_g1_i5_orf1;TRINITY_DN1201_c0_g1_i4_orf1;TRINITY_DN12545_c0_g1_i7_orf1;TRINITY_DN9109_c0_g1_i1_orf1;TRINITY_DN31611_c0_g1_i2_orf1;TRINITY_DN2848_c0_g1_i1_orf1;TRINITY_DN15222_c0_g1_i4_orf1;TRINITY_DN1334_c0_g1_i2_orf1;TRINITY_DN29873_c0_g1_i1_orf1;TRINITY_DN2570_c0_g1_i1_orf1;TRINITY_DN45530_c0_g1_i1_orf1;TRINITY_DN511_c0_g2_i1_orf1;TRINITY_DN2516_c0_g2_i10_orf1;TRINITY_DN24310_c0_g1_i2_orf1;TRINITY_DN19261_c0_g1_i3_orf1;TRINITY_DN20133_c0_g1_i1_orf1;TRINITY_DN1161_c0_g1_i2_orf1;TRINITY_DN2848_c0_g1_i2_orf1;TRINITY_DN1353_c0_g1_i1_orf1
biological_process	alcohol metabolic process	GO:0006066	11	11/3516	TRINITY_DN111985_c0_g1_i1_orf1;TRINITY_DN230_c2_g1_i5_orf1;TRINITY_DN52244_c1_g1_i1_orf1;TRINITY_DN1034_c0_g1_i4_orf1;TRINITY_DN10722_c0_g3_i1_orf1;TRINITY_DN36788_c0_g1_i2_orf1;TRINITY_DN9286_c0_g1_i2_orf1;TRINITY_DN1277_c4_g1_i5_orf1;TRINITY_DN1707_c0_g1_i1_orf1;TRINITY_DN618_c0_g1_i3_orf1;TRINITY_DN70_c2_g1_i1_orf1

biological_process	nucleobase-containing small molecule metabolic process	GO:0055086	79	79/3516	TRINITY_DN24_c0_g1_i1_orf1;TRINITY_DN82008_c0_g1_i1_orf1;TRINITY_DN38230_c0_g1_i4_orf1;TRINITY_DN86090_c0_g1_i1_orf1;TRINITY_DN14967_c0_g2_i1_orf1;TRINITY_DN98242_c0_g1_i1_orf1;TRINITY_DN1827_c0_g1_i4_orf1;TRINITY_DN59965_c0_g4_i1_orf1;TRINITY_DN60787_c0_g1_i5_orf1;TRINITY_DN27035_c0_g1_i1_orf1;TRINITY_DN8037_c0_g2_i1_orf1;TRINITY_DN1575_c0_g1_i7_orf1;TRINITY_DN39813_c0_g1_i1_orf1;TRINITY_DN98538_c0_g1_i1_orf1;TRINITY_DN1965_c0_g1_i7_orf1;TRINITY_DN7405_c0_g1_i3_orf1;TRINITY_DN1494_c0_g2_i1_orf1;TRINITY_DN29038_c0_g2_i1_orf1;TRINITY_DN6313_c0_g1_i4_orf1;TRINITY_DN140669_c0_g1_i1_orf1;TRINITY_DN1201_c0_g1_i4_orf1;TRINITY_DN21981_c0_g1_i8_orf1;TRINITY_DN2738_c1_g1_i3_orf1;TRINITY_DN125_c0_g1_i2_orf1;TRINITY_DN49038_c0_g4_i1_orf1;TRINITY_DN21545_c0_g1_i2_orf1;TRINITY_DN19261_c0_g1_i3_orf1;TRINITY_DN779_c0_g1_i3_orf1;TRINITY_DN1366_c0_g1_i5_orf1;TRINITY_DN1216_c0_g1_i4_orf1;TRINITY_DN8625_c0_g1_i1_orf1;TRINITY_DN45924_c0_g1_i14_orf1;TRINITY_DN19727_c0_g1_i7_orf1;TRINITY_DN2559_c0_g1_i4_orf1;TRINITY_DN31611_c0_g1_i2_orf1;TRINITY_DN2848_c0_g1_i1_orf1;TRINITY_DN14477_c0_g1_i12_orf1;TRINITY_DN19115_c0_g1_i1_orf1;TRINITY_DN16933_c0_g1_i10_orf1;TRINITY_DN23432_c0_g1_i1_orf1;TRINITY_DN26293_c0_g1_i4_orf1;TRINITY_DN18782_c0_g1_i4_orf1;TRINITY_DN36144_c0_g1_i3_orf1;TRINITY_DN6587_c0_g1_i3_orf1;TRINITY_DN38180_c0_g1_i3_orf1;TRINITY_DN117844_c0_g1_i1_orf1;TRINITY_DN12301_c0_g1_i1_orf1;TRINITY_DN3991_c0_g1_i6_orf1;TRINITY_DN29873_c0_g1_i1_orf1;TRINITY_DN51813_c0_g1_i1_orf1;TRINITY_DN28299_c0_g1_i1_orf1;TRINITY_DN26649_c0_g1_i2_orf1;TRINITY_DN15222_c0_g1_i4_orf1;TRINITY_DN57536_c0_g1_i14_orf1;TRINITY_DN26805_c0_g2_i3_orf1;TRINITY_DN2110_c0_g1_i3_orf1;TRINITY_DN6325_c0_g1_i9_orf1;TRINITY_DN83150_c0_g1_i1_orf1;TRINITY_DN11013_c0_g1_i3_orf1;TRINITY_DN17559_c0_g1_i4_orf1;TRINITY_DN5952_c0_g1_i6_orf1;TRINITY_DN779_c0_g1_i12_orf1;TRINITY_DN8012_c0_g1_i3_orf1;TRINITY_DN3822_c0_g1_i7_orf1;TRINITY_DN24310_c0_g1_i2_orf1;TRINITY_DN1494_c0_g1_i3_orf1;TRINITY_DN9156_c0_g1_i1_orf1;TRINITY_DN8908_c0_g1_i1_orf1;TRINITY_DN107261_c0_g1_i1_orf1;TRINITY_DN10548_c0_g2_i1_orf1;TRINITY_DN6813_c1_g1_i1_orf1;TRINITY_DN68725_c0_g1_i1_orf1;TRINITY_DN1084_c0_g1_i2_orf1;TRINITY_DN20133_c0_g1_i1_orf1;TRINITY_DN7808_c0_g1_i1_orf1;TRINITY_DN5029_c0_g1_i1_orf1;TRINITY_DN2848_c0_g1_i2_orf1;TRINITY_DN1957_c0_g1_i4_orf1;TRINITY_DN5525_c0_g1_i4_orf1
biological_process	urate metabolic process	GO:0046415	4	4/3516	TRINITY_DN779_c0_g1_i12_orf1;TRINITY_DN2559_c0_g1_i4_orf1;TRINITY_DN8037_c0_g2_i1_orf1;TRINITY_DN779_c0_g1_i3_orf1
biological_process	macromolecule glycosylation	GO:0043413	4	4/3516	TRINITY_DN332_c0_g1_i6_orf1;TRINITY_DN24024_c0_g1_i1_orf1;TRINITY_DN31676_c0_g1_i4_orf1;TRINITY_DN10058_c0_g1_i1_orf1
biological_process	protein demethylation	GO:0006482	1	1/3516	TRINITY_DN10336_c0_g1_i9_orf1
biological_process	macromolecule methylation	GO:0043414	13	13/3516	TRINITY_DN2930_c0_g1_i8_orf1;TRINITY_DN1344_c0_g1_i1_orf1;TRINITY_DN22674_c0_g1_i2_orf1;TRINITY_DN20749_c0_g1_i3_orf1;TRINITY_DN4151_c1_g1_i4_orf1;TRINITY_DN3028_c0_g1_i1_orf1;TRINITY_DN95414_c0_g1_i1_orf1;TRINITY_DN14734_c0_g1_i2_orf1;TRINITY_DN14953_c0_g1_i5_orf1;TRINITY_DN41664_c0_g1_i4_orf1;TRINITY_DN14313_c0_g1_i1_orf1;TRINITY_DN6462_c0_g1_i5_orf1;TRINITY_DN19262_c0_g1_i1_orf1
biological_process	molybdopterin cofactor metabolic process	GO:0043545	1	1/3516	TRINITY_DN1741_c0_g1_i5_orf1
biological_process	lipid metabolic process	GO:0006629	75	75/3516	TRINITY_DN48590_c0_g1_i1_orf1;TRINITY_DN117_c0_g1_i5_orf1;TRINITY_DN659_c0_g1_i3_orf1;TRINITY_DN44110_c0_g1_i4_orf1;TRINITY_DN25733_c0_g1_i3_orf1;TRINITY_DN10722_c0_g3_i1_orf1;TRINITY_DN22046_c1_g1_i5_orf1;TRINITY_DN12526_c0_g1_i5_orf1;TRINITY_DN10399_c0_g1_i2_orf1;TRINITY_DN76283_c0_g6_i1_orf1;TRINITY_DN86833_c0_g3_i1_orf1;TRINITY_DN6586_c0_g1_i1_orf1;TRINITY_DN115498_c0_g1_i1_orf1;TRINITY_DN3175_c0_g1_i7_orf1;TRINITY_DN5841_c0_g1_i2_orf1;TRINITY_DN14306_c0_g1_i1_orf1;TRINITY_DN43656_c0_g1_i1_orf1;TRINITY_DN11076_c0_g2_i1_orf1;TRINITY_DN3784_c0_g1_i1_orf1;TRINITY_DN9718_c0_g1_i7_orf1;TRINITY_DN127151_c0_g1_i1_orf1;TRINITY_DN45220_c0_g1_i1_orf1;TRINITY_DN29440_c1_g1_i4_orf1;TRINITY_DN12806_c0_g2_i1_orf1;TRINITY_DN5055_c0_g1_i12_orf1;TRINITY_DN5211_c0_g1_i1_orf1;TRINITY_DN7134_c0_g1_i1_orf1;TRINITY_DN5092_c0_g1_i2_orf1;TRINITY_DN10066_c0_g2_i2_orf1;TRINITY_DN1038_c0_g1_i4_orf1;TRINITY_DN3588_c0_g1_i1_orf1;TRINITY_DN117_c0_g1_i6_orf1;TRINITY_DN117_c0_g1_i4_orf1;TRINITY_DN59335_c0_g1_i2_orf1;TRINITY_DN42759_c0_g3_i1_orf1;TRINITY_DN2441_c0_g1_i1_orf1;TRINITY_DN10430_c0_g1_i4_orf1;TRINITY_DN8173_c0_g1_i3_orf1;TRINITY_DN49508_c0_g2_i8_orf1;TRINITY_DN1109_c0_g1_i6_orf1;TRINITY_DN12024_c0_g2_i2_orf1;TRINITY_DN10742_c0_g1_i4_orf1;TRINITY_DN2668_c0_g1_i7_orf1;TRINITY_DN19122_c0_g1_i7_orf1;TRINITY_DN41_c0_g1_i3_orf1;TRINITY_DN117844_c0_g1_i1_orf1;TRINITY_DN3991_c0_g1_i6_orf1;TRINITY_DN40197_c0_g1_i1_orf1;TRINITY_DN84478_c0_g1_i8_orf1;TRINITY_DN21494_c0_g1_i2_orf1;TRINITY_DN42759_c0_g2_i1_orf1;TRINITY_DN4070_c0_g1_i4_orf1;TRINITY_DN76283_c0_g2_i1_orf1;TRINITY_DN2668_c0_g1_i6_orf1;TRINITY_DN5512_c0_g1_i8_orf1;TRINITY_DN883_c0_g1_i8_orf1;TRINITY_DN7861_c0_g1_i5_orf1;TRINITY_DN10785_c0_g1_i4_orf1;TRINITY_DN4321_c0_g1_i1_orf1;TRINITY_DN12024_c0_g1_i4_orf1;TRINITY_DN3545_c0_g1_i6_orf1;TRINITY_DN2618_c0_g1_i3_orf1;TRINITY_DN25896_c0_g1_i6_orf1;TRINITY_DN357_c0_g1_i8_orf1;TRINITY_DN33178_c0_g1_i1_orf1;TRINITY_DN768_c0_g1_i7_orf1;TRINITY_DN1293_c0_g1_i4_orf1;TRINITY_DN3551_c0_g1_i4_orf1;TRINITY_DN11886_c0_g1_i1_orf1;TRINITY_DN64627_c0_g1_i1_orf1;TRINITY_DN68725_c0_g1_i1_orf1;TRINITY_DN1084_c0_g1_i2_orf1;TRINITY_DN5697_c0_g1_i1_orf1;TRINITY_DN905_c0_g1_i4_orf1;TRINITY_DN10900_c0_g1_i7_orf1

biological_process	nucleobase-containing compound metabolic process	GO:0006139	252	252/3516	TRINITY_DN38230_c0_g1_i4_orf1;TRINITY_DN22941_c0_g1_i1_orf1;TRINITY_DN86090_c0_g1_i1_orf1;TRINITY_DN8430_c0_g1_i1_orf1;TRINITY_DN15836_c0_g1_i1_orf1;TRINITY_DN1827_c0_g1_i4_orf1;TRINITY_DN1316_c0_g1_i1_orf1;TRINITY_DN60787_c0_g1_i5_orf1;TRINITY_DN1354_c0_g1_i6_orf1;TRINITY_DN47666_c0_g1_i4_orf1;TRINITY_DN56910_c0_g2_i1_orf1;TRINITY_DN35669_c0_g1_i1_orf1;TRINITY_DN2054_c0_g1_i1_orf1;TRINITY_DN124950_c0_g2_i1_orf1;TRINITY_DN2738_c1_g1_i3_orf1;TRINITY_DN20499_c0_g3_i1_orf1;TRINITY_DN125565_c1_g1_i1_orf1;TRINITY_DN34134_c0_g2_i1_orf1;TRINITY_DN1344_c0_g1_i1_orf1;TRINITY_DN1366_c0_g1_i5_orf1;TRINITY_DN1216_c0_g1_i4_orf1;TRINITY_DN44877_c0_g1_i2_orf1;TRINITY_DN23616_c0_g1_i4_orf1;TRINITY_DN2953_c1_g1_i10_orf1;TRINITY_DN31611_c0_g1_i2_orf1;TRINITY_DN2848_c0_g1_i1_orf1;TRINITY_DN16933_c0_g1_i10_orf1;TRINITY_DN23432_c0_g1_i1_orf1;TRINITY_DN18404_c0_g1_i5_orf1;TRINITY_DN40434_c0_g1_i2_orf1;TRINITY_DN9207_c0_g1_i1_orf1;TRINITY_DN6587_c0_g1_i3_orf1;TRINITY_DN18291_c0_g1_i1_orf1;TRINITY_DN1393_c0_g1_i2_orf1;TRINITY_DN3991_c0_g1_i6_orf1;TRINITY_DN29873_c0_g1_i1_orf1;TRINITY_DN17738_c0_g1_i2_orf1;TRINITY_DN15370_c0_g1_i4_orf1;TRINITY_DN47123_c0_g1_i1_orf1;TRINITY_DN18538_c0_g3_i1_orf1;TRINITY_DN123184_c0_g1_i1_orf1;TRINITY_DN26805_c0_g2_i3_orf1;TRINITY_DN2110_c0_g1_i3_orf1;TRINITY_DN1116_c0_g1_i6_orf1;TRINITY_DN817_c0_g1_i3_orf1;TRINITY_DN1066_c0_g1_i4_orf1;TRINITY_DN145647_c0_g1_i1_orf1;TRINITY_DN5507_c0_g1_i1_orf1;TRINITY_DN9094_c0_g1_i1_orf1;TRINITY_DN5952_c0_g1_i6_orf1;TRINITY_DN2224_c0_g1_i1_orf1;TRINITY_DN3822_c0_g1_i7_orf1;TRINITY_DN24310_c0_g1_i2_orf1;TRINITY_DN46409_c0_g1_i1_orf1;TRINITY_DN15040_c0_g4_i1_orf1;TRINITY_DN8908_c0_g1_i1_orf1;TRINITY_DN4813_c0_g1_i5_orf1;TRINITY_DN58636_c0_g1_i1_orf1;TRINITY_DN107261_c0_g1_i1_orf1;TRINITY_DN9156_c0_g1_i1_orf1;TRINITY_DN107288_c0_g1_i2_orf1;TRINITY_DN18860_c0_g1_i1_orf1;TRINITY_DN6235_c0_g1_i5_orf1;TRINITY_DN7808_c0_g1_i1_orf1;TRINITY_DN89613_c0_g1_i13_orf1;TRINITY_DN2848_c0_g1_i2_orf1;TRINITY_DN2647_c0_g1_i3_orf1;TRINITY_DN5603_c0_g1_i1_orf1;TRINITY_DN3092_c0_g1_i2_orf1;TRINITY_DN1978_c0_g1_i4_orf1;TRINITY_DN4300_c0_g1_i5_orf1;TRINITY_DN6313_c0_g1_i4_orf1;TRINITY_DN2038_c0_g1_i2_orf1;TRINITY_DN45271_c0_g1_i1_orf1;TRINITY_DN3082_c1_g1_i7_orf1;TRINITY_DN59965_c0_g4_i1_orf1;TRINITY_DN26649_c0_g1_i2_orf1;TRINITY_DN18728_c0_g1_i2_orf1;TRINITY_DN29402_c0_g1_i1_orf1;TRINITY_DN37532_c0_g1_i1_orf1;TRINITY_DN4710_c0_g1_i1_orf1;TRINITY_DN1965_c0_g1_i7_orf1;TRINITY_DN7405_c0_g1_i3_orf1;TRINITY_DN29038_c0_g2_i1_orf1;TRINITY_DN11639_c0_g1_i1_orf1;TRINITY_DN15900_c0_g1_i6_orf1;TRINITY_DN14967_c0_g2_i1_orf1;TRINITY_DN115658_c0_g1_i1_orf1;TRINITY_DN10548_c0_g2_i1_orf1;TRINITY_DN30097_c0_g1_i2_orf1;TRINITY_DN47575_c0_g1_i1_orf1;TRINITY_DN51968_c0_g1_i1_orf1;TRINITY_DN8625_c0_g1_i1_orf1;TRINITY_DN779_c0_g1_i3_orf1;TRINITY_DN1607_c0_g1_i16_orf1;TRINITY_DN810_c0_g1_i4_orf1;TRINITY_DN56270_c0_g1_i1_orf1;TRINITY_DN18863_c0_g1_i3_orf1;TRINITY_DN2769_c0_g1_i1_orf1;TRINITY_DN41664_c0_g1_i4_orf1;TRINITY_DN1091_c0_g3_i1_orf1;TRINITY_DN23714_c0_g1_i4_orf1;TRINITY_DN36144_c0_g1_i3_orf1;TRINITY_DN12527_c0_g1_i4_orf1;TRINITY_DN131662_c0_g1_i4_orf1;TRINITY_DN1616_c0_g1_i3_orf1;TRINITY_DN117844_c0_g1_i1_orf1;TRINITY_DN12301_c0_g1_i1_orf1;TRINITY_DN2953_c1_g1_i11_orf1;TRINITY_DN6642_c0_g1_i2_orf1;TRINITY_DN7289_c0_g1_i1_orf1;TRINITY_DN12323_c0_g2_i2_orf1;TRINITY_DN46022_c0_g1_i1_orf1;TRINITY_DN51568_c0_g1_i1_orf1;TRINITY_DN5525_c0_g1_i4_orf1;TRINITY_DN5105_c0_g1_i10_orf1;TRINITY_DN6325_c0_g1_i9_orf1;TRINITY_DN40945_c0_g1_i1_orf1;TRINITY_DN140212_c0_g1_i1_orf1;TRINITY_DN30638_c0_g1_i1_orf1;TRINITY_DN56110
biological_process	tricarboxylic acid cycle	GO:0006099	15	15/3516	TRINITY_DN5266_c0_g1_i1_orf1;TRINITY_DN146126_c0_g1_i1_orf1;TRINITY_DN10379_c0_g1_i3_orf1;TRINITY_DN2103_c0_g1_i1_orf1;TRINITY_DN19727_c0_g1_i7_orf1;TRINITY_DN36788_c0_g1_i2_orf1;TRINITY_DN29369_c0_g1_i1_orf1;TRINITY_DN27641_c0_g1_i1_orf1;TRINITY_DN5925_c0_g1_i5_orf1;TRINITY_DN21492_c0_g1_i1_orf1;TRINITY_DN1132_c0_g1_i5_orf1;TRINITY_DN3959_c1_g2_i1_orf1;TRINITY_DN2594_c0_g2_i4_orf1;TRINITY_DN15882_c0_g1_i1_orf1;TRINITY_DN5129_c0_g3_i3_orf1
biological_process	carbohydrate metabolic process	GO:0005975	96	96/3516	TRINITY_DN6510_c1_g1_i1_orf1;TRINITY_DN14235_c0_g1_i1_orf1;TRINITY_DN10722_c0_g3_i1_orf1;TRINITY_DN18650_c0_g1_i1_orf1;TRINITY_DN1827_c0_g1_i4_orf1;TRINITY_DN60787_c0_g1_i5_orf1;TRINITY_DN2170_c4_g1_i2_orf1;TRINITY_DN31967_c0_g1_i5_orf1;TRINITY_DN95850_c0_g1_i1_orf1;TRINITY_DN12545_c0_g1_i7_orf1;TRINITY_DN7183_c0_g1_i2_orf1;TRINITY_DN11817_c0_g1_i4_orf1;TRINITY_DN31611_c0_g1_i2_orf1;TRINITY_DN3322_c0_g1_i2_orf1;TRINITY_DN2848_c0_g1_i1_orf1;TRINITY_DN67623_c0_g1_i1_orf1;TRINITY_DN1334_c0_g1_i2_orf1;TRINITY_DN29873_c0_g1_i1_orf1;TRINITY_DN2170_c0_g2_i1_orf1;TRINITY_DN9044_c0_g1_i2_orf1;TRINITY_DN24310_c0_g1_i2_orf1;TRINITY_DN5952_c0_g1_i6_orf1;TRINITY_DN82801_c0_g1_i1_orf1;TRINITY_DN2170_c1_g1_i3_orf1;TRINITY_DN1161_c0_g1_i2_orf1;TRINITY_DN2848_c0_g1_i2_orf1;TRINITY_DN4954_c0_g1_i5_orf1;TRINITY_DN1353_c0_g1_i1_orf1;TRINITY_DN120089_c0_g1_i1_orf1;TRINITY_DN6108_c0_g1_i5_orf1;TRINITY_DN7405_c0_g1_i3_orf1;TRINITY_DN43391_c0_g1_i5_orf1;TRINITY_DN9044_c0_g1_i1_orf1;TRINITY_DN5488_c0_g1_i5_orf1;TRINITY_DN1034_c0_g1_i4_orf1;TRINITY_DN53167_c0_g1_i3_orf1;TRINITY_DN361_c0_g1_i5_orf1;TRINITY_DN2205_c0_g1_i3_orf1;TRINITY_DN23586_c0_g1_i3_orf1;TRINITY_DN2894_c0_g2_i3_orf1;TRINITY_DN7228_c0_g1_i6_orf1;TRINITY_DN9000_c0_g2_i1_orf1;TRINITY_DN26688_c0_g1_i2_orf1;TRINITY_DN6472_c0_g1_i5_orf1;TRINITY_DN6325_c0_g1_i9_orf1;TRINITY_DN511_c0_g2_i1_orf1;TRINITY_DN7828_c0_g1_i2_orf1;TRINITY_DN1732_c0_g1_i17_orf1;TRINITY_DN48410_c0_g1_i1_orf1;TRINITY_DN542_c0_g2_i1_orf1;TRINITY_DN48410_c0_g2_i1_orf1;TRINITY_DN2894_c0_g3_i1_orf1;TRINITY_DN650_c0_g1_i3_orf1;TRINITY_DN8703_c0_g1_i2_orf1;TRINITY_DN2474_c0_g1_i5_orf1;TRINITY_DN27035_c0_g1_i1_orf1;TRINITY_DN11657_c0_g1_i2_orf1;TRINITY_DN49038_c0_g4_i1_orf1;TRINITY_DN19261_c0_g1_i3_orf1;TRINITY_DN9109_c0_g1_i1_orf1;TRINITY_DN36788_c0_g1_i2_orf1;TRINITY_DN48237_c0_g1_i5_orf1;TRINITY_DN2894_c0_g1_i2_orf1;TRINITY_DN18918_c0_g1_i2_orf1;TRINITY_DN2515_c0_g1_i6_orf1;TRINITY_DN83150_c0_g1_i1_orf1;TRINITY_DN1707_c0_g1_i1_orf1;TRINITY_DN2516_c0_g2_i10_orf1;TRINITY_DN670_c0_g1_i3_orf1;TRINITY_DN52244_c1_g1_i1_orf1;TRINITY_DN1287_c0_g1_i5_orf1;TRINITY_DN29369_c0_g1_i1_orf1;TRINITY_DN3476_c0_g1_i5_orf1;TRINITY_DN25492_c0_g1_i1_orf1;TRINITY_DN14967_c0_g2_i1_orf1;TRINITY_DN18918_c0_g1_i3_orf1;TRINITY_DN2425_c0_g1_i3_orf1;TRINITY_DN618_c0_g1_i3_orf1;TRINITY_DN89483_c0_g1_i1_orf1;TRINITY_DN479_c6_g1_i2_orf1;TRINITY_DN542_c0_g1_i4_orf1;TRINITY_DN1201_c0_g1_i4_orf1;TRINITY_DN15222_c0_g1_i4_orf1;TRINITY_DN664_c0_g1_i18_orf1;TRINITY_DN195_c4_g1_i1_orf1;TRINITY_DN2570_c0_g1_i1_orf1;TRINITY_DN4070_c0_g1_i4_orf1;TRINITY_DN7534_c0_g1_i15_orf1;TRINITY_DN45530_c0_g1_i1_orf1;TRINITY_DN143603_c0_g1_i1_orf1;TRINITY_DN1732_c0_g1_i15_orf1;TRINITY_DN21555_c0_g1_i4_orf1;TRINITY_DN98723_c1_g1_i1_orf1;TRINITY_DN53167_c0_g1_i2_orf1;TRINITY_DN20133_c0_g1_i1_orf1;TRINITY_DN812_c2_g1_i1_orf1

biological_process	protein metabolic process	GO:0019538	352 352/3516	[illegible]TRINITY_DN57074_c0_g2_i1_orf1;TRINITY_DN3194_c0_g1_i6_orf1;TRINITY_DN11492_c0_g1_i8_orf1;TRINITY_DN2818_c0_g1_i2_orf1;TRINITY_DN1533_c0_g2_i1_orf1;TRINITY_DN8019_c0_g1_i4_orf1;TRINITY_DN117362_c0_g1_i5_orf1;TRINITY_DN2069_c1_g1_i8_orf1;TRINITY_DN3401_c0_g1_i1_orf1;TRINITY_DN30300_c0_g2_i1_orf1;TRINITY_DN2983_c0_g1_i6_orf1;TRINITY_DN16343_c0_g1_i6_orf1;TRINITY_DN14217_c0_g1_i1_orf1;TRINITY_DN48020_c0_g1_i1_orf1;TRINITY_DN2861_c0_g2_i1_orf1;TRINITY_DN2794_c1_g1_i8_orf1;TRINITY_DN22674_c0_g1_i2_orf1;TRINITY_DN33883_c0_g1_i1_orf1;TRINITY_DN79734_c0_g2_i3_orf1;TRINITY_DN3755_c0_g1_i3_orf1;TRINITY_DN143895_c0_g1_i1_orf1;TRINITY_DN2627_c0_g1_i2_orf1;TRINITY_DN19829_c0_g2_i1_orf1;TRINITY_DN4189_c0_g2_i1_orf1;TRINITY_DN7583_c0_g1_i1_orf1;TRINITY_DN7464_c0_g1_i14_orf1;TRINITY_DN66302_c0_g1_i1_orf1;TRINITY_DN5696_c0_g1_i4_orf1;TRINITY_DN3733_c0_g1_i1_orf1;TRINITY_DN5444_c0_g2_i1_orf1;TRINITY_DN95414_c0_g1_i1_orf1;TRINITY_DN27033_c1_g1_i3_orfp1;TRINITY_DN376_c1_g1_i1_orf1;TRINITY_DN9062_c0_g2_i3_orf1;TRINITY_DN4767_c0_g1_i4_orf1;TRINITY_DN7776_c0_g1_i5_orf1;TRINITY_DN1459_c1_g1_i1_orf1;TRINITY_DN147458_c0_g1_i1_orf1;TRINITY_DN21251_c1_g1_i1_orf1;TRINITY_DN5112_c0_g1_i1_orf1;TRINITY_DN36434_c0_g2_i3_orf1;TRINITY_DN10766_c0_g1_i1_orf1;TRINITY_DN2026_c0_g1_i4_orf1;TRINITY_DN5182_c0_g1_i5_orf1;TRINITY_DN4016_c0_g1_i1_orf1;TRINITY_DN934_c2_g1_i7_orf1;TRINITY_DN3975_c0_g1_i10_orf1;TRINITY_DN43792_c0_g1_i1_orf1;TRINITY_DN18388_c0_g1_i6_orf1;TRINITY_DN4449_c0_g2_i1_orf1;TRINITY_DN2579_c0_g1_i7_orf1;TRINITY_DN18172_c0_g1_i6_orf1;TRINITY_DN10455_c0_g2_i1_orf1;TRINITY_DN17838_c0_g1_i4_orf1;TRINITY_DN1173_c1_g1_i9_orf1;TRINITY_DN2274_c0_g1_i6_orf1;TRINITY_DN42506_c0_g1_i1_orf1;TRINITY_DN29034_c0_g1_i1_orf1;TRINITY_DN9874_c0_g1_i7_orf1;TRINITY_DN69697_c0_g1_i1_orf1;TRINITY_DN10385_c0_g1_i5_orf1;TRINITY_DN41997_c0_g1_i2_orf1;TRINITY_DN5031_c0_g1_i1_orf1;TRINITY_DN461_c0_g1_i5_orf1;TRINITY_DN44792_c0_g1_i1_orf1;TRINITY_DN12973_c0_g1_i1_orf1;TRINITY_DN36893_c0_g1_i1_orf1;TRINITY_DN24539_c0_g1_i4_orf1;TRINITY_DN8949_c0_g1_i2_orf1;TRINITY_DN55148_c0_g1_i1_orf1;TRINITY_DN142588_c0_g1_i1_orf1;TRINITY_DN975_c0_g1_i1_orf1;TRINITY_DN10336_c0_g1_i9_orf1;TRINITY_DN10994_c0_g1_i4_orf1;TRINITY_DN1080_c0_g1_i1_orf1;TRINITY_DN2593_c0_g3_i1_orf1;TRINITY_DN2593_c0_g1_i1_orf1;TRINITY_DN334_c0_g1_i3_orf1;TRINITY_DN96_c0_g1_i1_orf1;TRINITY_DN46132_c0_g2_i2_orf1;TRINITY_DN81248_c0_g1_i1_orf1;TRINITY_DN82324_c0_g1_i4_orf1;TRINITY_DN36262_c0_g1_i1_orf1;TRINITY_DN30_c0_g1_i6_orf1;TRINITY_DN147475_c0_g1_i1_orf1;TRINITY_DN135188_c0_g1_i2_orf1;TRINITY_DN24121_c1_g1_i6_orf1;TRINITY_DN4125_c0_g1_i6_orf1;TRINITY_DN16749_c0_g1_i1_orf1;TRINITY_DN8480_c0_g1_i1_orf1;TRINITY_DN130051_c0_g1_i1_orf1;TRINITY_DN181_c0_g1_i3_orf1;TRINITY_DN1757_c0_g1_i4_orf1;TRINITY_DN344_c1_g1_i1_orf1;TRINITY_DN119291_c0_g1_i1_orf1;TRINITY_DN2682_c0_g1_i4_orf1;TRINITY_DN3499_c0_g1_i8_orf1;TRINITY_DN2442_c0_g1_i2_orf1;TRINITY_DN3861_c0_g3_i2_orf1;TRINITY_DN16258_c0_g1_i2_orf1;TRINITY_DN2848_c0_g1_i1_orf1;TRINITY_DN14754_c0_g1_i6_orf1;TRINITY_DN2954_c0_g1_i1_orf1;TRINITY_DN74889_c0_g1_i1_orf1;TRINITY_DN4125_c0_g1_i14_orf1;TRINITY_DN2178_c0_g1_i1_orf1;TRINITY_DN135_c0_g1_i1_orf1;TRINITY_DN6436_c0_g1_i1_orf1;TRINITY_DN3985_c0_g2_i1_orf1;TRINITY_DN17376_c0_g1_i2_orf1;TRINITY_DN40_c0_g2_i1_orf1;TRINITY_DN9717_c0_g2_i1_orf1;TRINITY_DN18860_c0_g1_i1_orf1;TRINITY_DN338_c1_g1_i9_orf1;TRINITY_DN10429_c0_g1_i2_orf1;TRINITY_DN4767_c0_g1_i6_orf1;TRINITY_DN23360_c0_g1_i3_orf1;TRINITY_DN21619_c0_g1_i1_orf1;TRINITY_DN45633_c0_g1_i1_orf1;TRINITY_DN37923_c0_g1_i1_orf1;TRINITY_DN33926_c0_g1_i1_orf1;TRINITY_DN501[illegible]
biological_process	cellular amino acid metabolic process	GO:0006520	85 85/3516	TRINITY_DN42856_c0_g1_i1_orf1;TRINITY_DN2065_c1_g2_i1_orf1;TRINITY_DN57918_c0_g1_i1_orf1;TRINITY_DN92153_c0_g2_i2_orf1;TRINITY_DN14565_c0_g1_i11_orf1;TRINITY_DN5756_c0_g1_i4_orf1;TRINITY_DN6587_c0_g1_i3_orf1;TRINITY_DN130051_c0_g1_i1_orf1;TRINITY_DN17326_c0_g1_i8_orf1;TRINITY_DN6199_c2_g1_i3_orf1;TRINITY_DN27771_c0_g1_i1_orf1;TRINITY_DN620_c0_g1_i4_orf1;TRINITY_DN18230_c1_g2_i1_orf1;TRINITY_DN1494_c0_g1_i3_orf1;TRINITY_DN144807_c0_g1_i1_orf1;TRINITY_DN100821_c0_g1_i1_orf1;TRINITY_DN1965_c0_g1_i7_orf1;TRINITY_DN35763_c0_g1_i2_orf1;TRINITY_DN28577_c0_g1_i6_orf1;TRINITY_DN81719_c0_g1_i1_orf1;TRINITY_DN30224_c0_g1_i1_orf1;TRINITY_DN11639_c0_g1_i1_orf1;TRINITY_DN12474_c0_g1_i6_orf1;TRINITY_DN19187_c0_g1_i1_orf1;TRINITY_DN1824_c0_g2_i2_orf1;TRINITY_DN8716_c0_g1_i3_orf1;TRINITY_DN11948_c0_g1_i8_orf1;TRINITY_DN64810_c0_g1_i1_orf1;TRINITY_DN10264_c1_g1_i5_orf1;TRINITY_DN43431_c0_g1_i1_orf1;TRINITY_DN21539_c0_g1_i1_orf1;TRINITY_DN1607_c0_g1_i16_orf1;TRINITY_DN24723_c2_g1_i1_orf1;TRINITY_DN27848_c0_g1_i2_orf1;TRINITY_DN28221_c0_g2_i1_orf1;TRINITY_DN21506_c0_g1_i4_orf1;TRINITY_DN89483_c0_g1_i1_orf1;TRINITY_DN2953_c1_g1_i2_orf1;TRINITY_DN2038_c0_g1_i2_orf1;TRINITY_DN11013_c0_g1_i3_orf1;TRINITY_DN2338_c0_g1_i5_orf1;TRINITY_DN2953_c1_g1_i11_orf1;TRINITY_DN123396_c0_g1_i1_orf1;TRINITY_DN20796_c0_g1_i4_orf1;TRINITY_DN4822_c0_g1_i6_orf1;TRINITY_DN24970_c0_g1_i4_orf1;TRINITY_DN17913_c0_g1_i8_orf1;TRINITY_DN42738_c0_g1_i1_orf1;TRINITY_DN4451_c0_g2_i4_orf1;TRINITY_DN3859_c0_g1_i5_orf1;TRINITY_DN5218_c0_g1_i4_orf1;TRINITY_DN84322_c0_g2_i1_orf1;TRINITY_DN13941_c0_g1_i6_orf1;TRINITY_DN51813_c0_g1_i1_orf1;TRINITY_DN1760_c0_g1_i4_orf1;TRINITY_DN5497_c0_g1_i6_orf1;TRINITY_DN8598_c0_g1_i2_orf1;TRINITY_DN48619_c0_g1_i1_orf1;TRINITY_DN17031_c0_g1_i1_orf1;TRINITY_DN6669_c0_g1_i3_orf1;TRINITY_DN2803_c4_g1_i1_orf1;TRINITY_DN817_c0_g1_i3_orf1;TRINITY_DN4451_c0_g1_i1_orf1;TRINITY_DN30638_c0_g1_i1_orf1;TRINITY_DN15160_c0_g1_i1_orf1;TRINITY_DN2224_c0_g1_i1_orf1;TRINITY_DN31163_c1_g1_i4_orf1;TRINITY_DN34399_c0_g1_i1_orf1;TRINITY_DN27771_c0_g2_i1_orf1;TRINITY_DN1494_c0_g2_i1_orf1;TRINITY_DN3971_c0_g1_i1_orf1;TRINITY_DN11383_c0_g2_i4_orf1;TRINITY_DN3263_c0_g1_i2_orf1;TRINITY_DN1375_c0_g1_i5_orf1;TRINITY_DN17326_c0_g1_i5_orf1;TRINITY_DN19727_c0_g1_i7_orf1;TRINITY_DN2953_c1_g1_i10_orf1;TRINITY_DN107288_c0_g1_i2_orf1;TRINITY_DN87170_c0_g1_i3_orf1;TRINITY_DN14464_c0_g1_i1_orf1;TRINITY_DN53807_c0_g2_i1_orf1;TRINITY_DN4944_c0_g1_i2_orf1;TRINITY_DN1068_c0_g1_i3_orf1;TRINITY_DN3836_c0_g1_i4_orf1;TRINITY_DN18230_c1_g1_i1_orf1

biological_process	organophosphate metabolic process	GO:0019637	85	85/3516	TRINITY_DN143509_c0_g1_i1_orf1;TRINITY_DN24_c0_g1_i1_orf1;TRINITY_DN25733_c0_g1_i3_orf1;TRINITY_DN38230_c0_g1_i4_orf1;TRINITY_DN10722_c0_g3_i1_orf1;TRINITY_DN86090_c0_g1_i1_orf1;TRINITY_DN1034_c0_g1_i4_orf1;TRINITY_DN6587_c0_g1_i3_orf1;TRINITY_DN98538_c0_g1_i1_orf1;TRINITY_DN5697_c0_g1_i1_orf1;TRINITY_DN1827_c0_g1_i4_orf1;TRINITY_DN59965_c0_g4_i1_orf1;TRINITY_DN60787_c0_g1_i5_orf1;TRINITY_DN27035_c0_g1_i1_orf1;TRINITY_DN15222_c0_g1_i4_orf1;TRINITY_DN1575_c0_g1_i7_orf1;TRINITY_DN41166_c0_g1_i1_orf1;TRINITY_DN39813_c0_g1_i1_orf1;TRINITY_DN1366_c0_g1_i5_orf1;TRINITY_DN1965_c0_g1_i7_orf1;TRINITY_DN7405_c0_g1_i3_orf1;TRINITY_DN1741_c0_g1_i5_orf1;TRINITY_DN1494_c0_g2_i1_orf1;TRINITY_DN29038_c0_g2_i1_orf1;TRINITY_DN6313_c0_g1_i4_orf1;TRINITY_DN1201_c0_g1_i4_orf1;TRINITY_DN21981_c0_g1_i8_orf1;TRINITY_DN2738_c1_g1_i3_orf1;TRINITY_DN49038_c0_g4_i1_orf1;TRINITY_DN21545_c0_g1_i2_orf1;TRINITY_DN82008_c0_g1_i1_orf1;TRINITY_DN3312_c0_g1_i10_orf1;TRINITY_DN1216_c0_g1_i4_orf1;TRINITY_DN43656_c0_g1_i1_orf1;TRINITY_DN10066_c0_g2_i2_orf1;TRINITY_DN7134_c0_g1_i1_orf1;TRINITY_DN31611_c0_g1_i2_orf1;TRINITY_DN2848_c0_g1_i1_orf1;TRINITY_DN14477_c0_g1_i12_orf1;TRINITY_DN19115_c0_g1_i1_orf1;TRINITY_DN16933_c0_g1_i10_orf1;TRINITY_DN23432_c0_g1_i1_orf1;TRINITY_DN9555_c0_g1_i1_orf1;TRINITY_DN14967_c0_g2_i1_orf1;TRINITY_DN47151_c0_g1_i1_orf1;TRINITY_DN1084_c0_g1_i2_orf1;TRINITY_DN36144_c0_g1_i3_orf1;TRINITY_DN10742_c0_g1_i4_orf1;TRINITY_DN19122_c0_g1_i7_orf1;TRINITY_DN117844_c0_g1_i1_orf1;TRINITY_DN12301_c0_g1_i1_orf1;TRINITY_DN3991_c0_g1_i6_orf1;TRINITY_DN29873_c0_g1_i1_orf1;TRINITY_DN51813_c0_g1_i1_orf1;TRINITY_DN44110_c0_g1_i4_orf1;TRINITY_DN28299_c0_g1_i1_orf1;TRINITY_DN26649_c0_g1_i2_orf1;TRINITY_DN57536_c0_g1_i14_orf1;TRINITY_DN1277_c4_g1_i5_orf1;TRINITY_DN26805_c0_g2_i3_orf1;TRINITY_DN2110_c0_g1_i3_orf1;TRINITY_DN6325_c0_g1_i9_orf1;TRINITY_DN19727_c0_g1_i7_orf1;TRINITY_DN45924_c0_g1_i14_orf1;TRINITY_DN8012_c0_g1_i3_orf1;TRINITY_DN3822_c0_g1_i7_orf1;TRINITY_DN24310_c0_g1_i2_orf1;TRINITY_DN2618_c0_g1_i3_orf1;TRINITY_DN1494_c0_g1_i3_orf1;TRINITY_DN9156_c0_g1_i1_orf1;TRINITY_DN33178_c0_g1_i1_orf1;TRINITY_DN8908_c0_g1_i1_orf1;TRINITY_DN18782_c0_g1_i4_orf1;TRINITY_DN107261_c0_g1_i1_orf1;TRINITY_DN10548_c0_g2_i1_orf1;TRINITY_DN6813_c1_g1_i1_orf1;TRINITY_DN68725_c0_g1_i1_orf1;TRINITY_DN116972_c0_g1_i1_orf1;TRINITY_DN20133_c0_g1_i1_orf1;TRINITY_DN7808_c0_g1_i1_orf1;TRINITY_DN5029_c0_g1_i1_orf1;TRINITY_DN2848_c0_g1_i2_orf1;TRINITY_DN1957_c0_g1_i4_orf1;TRINITY_DN70_c2_g1_i1_orf1;TRINITY_DN83150_c0_g1_i1_orf1
biological_process	carbohydrate derivative metabolic process	GO:1901135	83	83/3516	TRINITY_DN143509_c0_g1_i1_orf1;TRINITY_DN47151_c0_g1_i1_orf1;TRINITY_DN827_c1_g1_i1_orf1;TRINITY_DN86090_c0_g1_i1_orf1;TRINITY_DN14967_c0_g2_i1_orf1;TRINITY_DN98242_c0_g1_i1_orf1;TRINITY_DN98538_c0_g1_i1_orf1;TRINITY_DN5697_c0_g1_i1_orf1;TRINITY_DN1827_c0_g1_i4_orf1;TRINITY_DN59965_c0_g4_i1_orf1;TRINITY_DN60787_c0_g1_i5_orf1;TRINITY_DN27035_c0_g1_i1_orf1;TRINITY_DN15222_c0_g1_i4_orf1;TRINITY_DN1575_c0_g1_i7_orf1;TRINITY_DN39813_c0_g1_i1_orf1;TRINITY_DN1366_c0_g1_i5_orf1;TRINITY_DN1965_c0_g1_i7_orf1;TRINITY_DN7405_c0_g1_i3_orf1;TRINITY_DN1494_c0_g2_i1_orf1;TRINITY_DN29038_c0_g2_i1_orf1;TRINITY_DN6313_c0_g1_i4_orf1;TRINITY_DN140669_c0_g1_i1_orf1;TRINITY_DN1201_c0_g1_i4_orf1;TRINITY_DN21981_c0_g1_i8_orf1;TRINITY_DN2738_c1_g1_i3_orf1;TRINITY_DN125_c0_g1_i2_orf1;TRINITY_DN117844_c0_g1_i1_orf1;TRINITY_DN1034_c0_g1_i4_orf1;TRINITY_DN19261_c0_g1_i3_orf1;TRINITY_DN542_c0_g2_i1_orf1;TRINITY_DN12301_c0_g1_i1_orf1;TRINITY_DN1216_c0_g1_i4_orf1;TRINITY_DN8625_c0_g1_i1_orf1;TRINITY_DN43656_c0_g1_i1_orf1;TRINITY_DN19727_c0_g1_i7_orf1;TRINITY_DN31611_c0_g1_i2_orf1;TRINITY_DN2848_c0_g1_i1_orf1;TRINITY_DN83150_c0_g1_i1_orf1;TRINITY_DN19115_c0_g1_i1_orf1;TRINITY_DN2441_c0_g1_i1_orf1;TRINITY_DN16933_c0_g1_i10_orf1;TRINITY_DN11013_c0_g1_i3_orf1;TRINITY_DN26293_c0_g1_i4_orf1;TRINITY_DN1084_c0_g1_i2_orf1;TRINITY_DN6587_c0_g1_i3_orf1;TRINITY_DN49038_c0_g4_i1_orf1;TRINITY_DN1534_c0_g1_i3_orf1;TRINITY_DN3312_c0_g1_i10_orf1;TRINITY_DN3991_c0_g1_i6_orf1;TRINITY_DN29873_c0_g1_i1_orf1;TRINITY_DN40197_c0_g1_i1_orf1;TRINITY_DN650_c0_g1_i3_orf1;TRINITY_DN26649_c0_g1_i2_orf1;TRINITY_DN542_c0_g1_i4_orf1;TRINITY_DN2515_c0_g1_i6_orf1;TRINITY_DN26805_c0_g2_i3_orf1;TRINITY_DN2110_c0_g1_i3_orf1;TRINITY_DN6325_c0_g1_i9_orf1;TRINITY_DN11798_c0_g2_i1_orf1;TRINITY_DN45924_c0_g1_i14_orf1;TRINITY_DN17559_c0_g1_i4_orf1;TRINITY_DN5952_c0_g1_i6_orf1;TRINITY_DN28299_c0_g1_i1_orf1;TRINITY_DN21555_c0_g1_i4_orf1;TRINITY_DN3822_c0_g1_i7_orf1;TRINITY_DN24310_c0_g1_i2_orf1;TRINITY_DN1494_c0_g1_i3_orf1;TRINITY_DN9156_c0_g1_i1_orf1;TRINITY_DN1287_c0_g1_i5_orf1;TRINITY_DN8908_c0_g1_i1_orf1;TRINITY_DN18782_c0_g1_i4_orf1;TRINITY_DN107261_c0_g1_i1_orf1;TRINITY_DN10548_c0_g2_i1_orf1;TRINITY_DN6813_c1_g1_i1_orf1;TRINITY_DN57536_c0_g1_i14_orf1;TRINITY_DN68725_c0_g1_i1_orf1;TRINITY_DN116972_c0_g1_i1_orf1;TRINITY_DN20133_c0_g1_i1_orf1;TRINITY_DN5235_c0_g1_i7_orf1;TRINITY_DN7808_c0_g1_i1_orf1;TRINITY_DN5029_c0_g1_i1_orf1;TRINITY_DN2848_c0_g1_i2_orf1;TRINITY_DN5525_c0_g1_i4_orf1
biological_process	organic hydroxy compound metabolic process	GO:1901615	19	19/3516	TRINITY_DN111985_c0_g1_i1_orf1;TRINITY_DN230_c2_g1_i5_orf1;TRINITY_DN9555_c0_g1_i1_orf1;TRINITY_DN52244_c1_g1_i1_orf1;TRINITY_DN1034_c0_g1_i4_orf1;TRINITY_DN10722_c0_g3_i1_orf1;TRINITY_DN36788_c0_g1_i2_orf1;TRINITY_DN9286_c0_g1_i2_orf1;TRINITY_DN31163_c1_g1_i4_orf1;TRINITY_DN18291_c0_g1_i1_orf1;TRINITY_DN1277_c4_g1_i5_orf1;TRINITY_DN15706_c0_g2_i5_orf1;TRINITY_DN1707_c0_g1_i1_orf1;TRINITY_DN68725_c0_g1_i1_orf1;TRINITY_DN618_c0_g1_i3_orf1;TRINITY_DN81719_c0_g1_i1_orf1;TRINITY_DN37165_c0_g1_i4_orf1;TRINITY_DN70_c2_g1_i1_orf1;TRINITY_DN2338_c0_g1_i5_orf1

biological_process	organic cyclic compound metabolic process	GO:1901360	280 280/3516	TRINITY_DN38230_c0_g1_i4_orf1;TRINITY_DN22941_c0_g1_i1_orf1;TRINITY_DN86090_c0_g1_i1_orf1;TRINITY_DN40434_c0_g1_i2_orf1;TRINITY_DN130051_c0_g1_i1_orf1;TRINITY_DN15836_c0_g1_i1_orf1;TRINITY_DN1827_c0_g1_i4_orf1;TRINITY_DN1316_c0_g1_i1_orf1;TRINITY_DN60787_c0_g1_i5_orf1;TRINITY_DN1354_c0_g1_i6_orf1;TRINITY_DN47666_c0_g1_i4_orf1;TRINITY_DN56910_c0_g2_i1_orf1;TRINITY_DN35669_c0_g1_i1_orf1;TRINITY_DN2054_c0_g1_i1_orf1;TRINITY_DN51934_c0_g2_i1_orf1;TRINITY_DN124950_c0_g2_i1_orf1;TRINITY_DN2738_c1_g1_i3_orf1;TRINITY_DN14967_c0_g2_i1_orf1;TRINITY_DN125565_c1_g1_i1_orf1;TRINITY_DN34134_c0_g2_i1_orf1;TRINITY_DN1344_c0_g1_i1_orf1;TRINITY_DN1366_c0_g1_i5_orf1;TRINITY_DN1216_c0_g1_i4_orf1;TRINITY_DN25582_c0_g1_i3_orf1;TRINITY_DN44877_c0_g1_i2_orf1;TRINITY_DN23616_c0_g1_i4_orf1;TRINITY_DN31163_c1_g1_i4_orf1;TRINITY_DN31611_c0_g1_i2_orf1;TRINITY_DN2848_c0_g1_i1_orf1;TRINITY_DN16933_c0_g1_i10_orf1;TRINITY_DN23432_c0_g1_i1_orf1;TRINITY_DN18404_c0_g1_i5_orf1;TRINITY_DN8430_c0_g1_i1_orf1;TRINITY_DN9207_c0_g1_i1_orf1;TRINITY_DN6587_c0_g1_i3_orf1;TRINITY_DN4822_c0_g1_i6_orf1;TRINITY_DN18291_c0_g1_i1_orf1;TRINITY_DN1393_c0_g1_i2_orf1;TRINITY_DN5507_c0_g1_i1_orf1;TRINITY_DN3991_c0_g1_i6_orf1;TRINITY_DN29873_c0_g1_i1_orf1;TRINITY_DN17738_c0_g1_i2_orf1;TRINITY_DN15370_c0_g1_i4_orf1;TRINITY_DN1760_c0_g1_i4_orf1;TRINITY_DN47123_c0_g1_i1_orf1;TRINITY_DN18538_c0_g3_i1_orf1;TRINITY_DN123184_c0_g1_i1_orf1;TRINITY_DN26805_c0_g2_i3_orf1;TRINITY_DN2110_c0_g1_i3_orf1;TRINITY_DN1116_c0_g1_i6_orf1;TRINITY_DN817_c0_g1_i3_orf1;TRINITY_DN1066_c0_g1_i4_orf1;TRINITY_DN145647_c0_g1_i1_orf1;TRINITY_DN37165_c0_g1_i4_orf1;TRINITY_DN9094_c0_g1_i1_orf1;TRINITY_DN5952_c0_g1_i6_orf1;TRINITY_DN2224_c0_g1_i1_orf1;TRINITY_DN3822_c0_g1_i7_orf1;TRINITY_DN24310_c0_g1_i2_orf1;TRINITY_DN46409_c0_g1_i1_orf1;TRINITY_DN15040_c0_g4_i1_orf1;TRINITY_DN8908_c0_g1_i1_orf1;TRINITY_DN4813_c0_g1_i5_orf1;TRINITY_DN58636_c0_g1_i1_orf1;TRINITY_DN107261_c0_g1_i1_orf1;TRINITY_DN9156_c0_g1_i1_orf1;TRINITY_DN107288_c0_g1_i2_orf1;TRINITY_DN18860_c0_g1_i1_orf1;TRINITY_DN6235_c0_g1_i5_orf1;TRINITY_DN7808_c0_g1_i1_orf1;TRINITY_DN89613_c0_g1_i13_orf1;TRINITY_DN2848_c0_g1_i2_orf1;TRINITY_DN2647_c0_g1_i3_orf1;TRINITY_DN5603_c0_g1_i1_orf1;TRINITY_DN3092_c0_g1_i2_orf1;TRINITY_DN1978_c0_g1_i4_orf1;TRINITY_DN4300_c0_g1_i5_orf1;TRINITY_DN6313_c0_g1_i4_orf1;TRINITY_DN2038_c0_g1_i2_orf1;TRINITY_DN45271_c0_g1_i1_orf1;TRINITY_DN3082_c1_g1_i7_orf1;TRINITY_DN59965_c0_g4_i1_orf1;TRINITY_DN26649_c0_g1_i2_orf1;TRINITY_DN18728_c0_g1_i2_orf1;TRINITY_DN29402_c0_g1_i1_orf1;TRINITY_DN37532_c0_g1_i1_orf1;TRINITY_DN4710_c0_g1_i1_orf1;TRINITY_DN1965_c0_g1_i7_orf1;TRINITY_DN7405_c0_g1_i3_orf1;TRINITY_DN29038_c0_g2_i1_orf1;TRINITY_DN11639_c0_g1_i1_orf1;TRINITY_DN15900_c0_g1_i6_orf1;TRINITY_DN19187_c0_g1_i1_orf1;TRINITY_DN115658_c0_g1_i1_orf1;TRINITY_DN10548_c0_g2_i1_orf1;TRINITY_DN30097_c0_g1_i2_orf1;TRINITY_DN47575_c0_g1_i1_orf1;TRINITY_DN51968_c0_g1_i1_orf1;TRINITY_DN8625_c0_g1_i1_orf1;TRINITY_DN779_c0_g1_i3_orf1;TRINITY_DN1607_c0_g1_i16_orf1;TRINITY_DN810_c0_g1_i4_orf1;TRINITY_DN244_c1_g1_i5_orf1;TRINITY_DN18863_c0_g1_i3_orf1;TRINITY_DN2769_c0_g1_i1_orf1;TRINITY_DN41664_c0_g1_i4_orf1;TRINITY_DN1091_c0_g3_i1_orf1;TRINITY_DN23714_c0_g1_i4_orf1;TRINITY_DN4145_c0_g1_i1_orf1;TRINITY_DN36144_c0_g1_i3_orf1;TRINITY_DN12527_c0_g1_i4_orf1;TRINITY_DN131662_c0_g1_i4_orf1;TRINITY_DN1616_c0_g1_i3_orf1;TRINITY_DN117844_c0_g1_i1_orf1;TRINITY_DN12301_c0_g1_i1_orf1;TRINITY_DN2953_c1_g1_i11_orf1;TRINITY_DN6642_c0_g1_i2_orf1;TRINITY_DN7289_c0_g1_i1_orf1;TRINITY_DN12323_c0_g2_i2_orf1;TRINITY_DN46022_c0_g1_i1_orf1;TRINITY_DN51568_c0_g1_i
biological_process	thioester metabolic process	GO:0035383	7 7/3516	TRINITY_DN6313_c0_g1_i4_orf1;TRINITY_DN117844_c0_g1_i1_orf1;TRINITY_DN19727_c0_g1_i7_orf1;TRINITY_DN3991_c0_g1_i6_orf1;TRINITY_DN68725_c0_g1_i1_orf1;TRINITY_DN1084_c0_g1_i2_orf1;TRINITY_DN7808_c0_g1_i1_orf1
biological_process	macromolecule metabolic process	GO:0043170	538 538/3516	TRINITY_DN57074_c0_g2_i1_orf1;TRINITY_DN22941_c0_g1_i1_orf1;TRINITY_DN40434_c0_g1_i2_orf1;TRINITY_DN3194_c0_g1_i6_orf1;TRINITY_DN11492_c0_g1_i8_orf1;TRINITY_DN2818_c0_g1_i2_orf1;TRINITY_DN1533_c0_g2_i1_orf1;TRINITY_DN8019_c0_g1_i4_orf1;TRINITY_DN117362_c0_g1_i5_orf1;TRINITY_DN2054_c0_g1_i1_orf1;TRINITY_DN3401_c0_g1_i1_orf1;TRINITY_DN125565_c1_g1_i1_orf1;TRINITY_DN30300_c0_g2_i1_orf1;TRINITY_DN2983_c0_g1_i6_orf1;TRINITY_DN18404_c0_g1_i5_orf1;TRINITY_DN16343_c0_g1_i6_orf1;TRINITY_DN14217_c0_g1_i1_orf1;TRINITY_DN48020_c0_g1_i1_orf1;TRINITY_DN2861_c0_g2_i1_orf1;TRINITY_DN2794_c1_g1_i8_orf1;TRINITY_DN123184_c0_g1_i1_orf1;TRINITY_DN817_c0_g1_i3_orf1;TRINITY_DN6299_c0_g1_i1_orf1;TRINITY_DN22674_c0_g1_i2_orf1;TRINITY_DN33883_c0_g1_i1_orf1;TRINITY_DN79734_c0_g2_i3_orf1;TRINITY_DN34509_c0_g1_i1_orf1;TRINITY_DN5603_c0_g1_i1_orf1;TRINITY_DN4300_c0_g1_i5_orf1;TRINITY_DN143895_c0_g1_i1_orf1;TRINITY_DN2627_c0_g1_i2_orf1;TRINITY_DN2038_c0_g1_i2_orf1;TRINITY_DN19829_c0_g2_i1_orf1;TRINITY_DN71863_c0_g1_i2_orf1;TRINITY_DN18728_c0_g1_i2_orf1;TRINITY_DN4189_c0_g2_i1_orf1;TRINITY_DN6248_c0_g1_i1_orf1;TRINITY_DN5873_c0_g4_i1_orf1;TRINITY_DN115658_c0_g1_i1_orf1;TRINITY_DN47575_c0_g1_i1_orf1;TRINITY_DN47666_c0_g1_i4_orf1;TRINITY_DN7583_c0_g1_i1_orf1;TRINITY_DN1607_c0_g1_i16_orf1;TRINITY_DN7464_c0_g1_i14_orf1;TRINITY_DN810_c0_g1_i4_orf1;TRINITY_DN2069_c1_g1_i8_orf1;TRINITY_DN18863_c0_g1_i3_orf1;TRINITY_DN66302_c0_g1_i1_orf1;TRINITY_DN5696_c0_g1_i4_orf1;TRINITY_DN3733_c0_g1_i1_orf1;TRINITY_DN5444_c0_g2_i1_orf1;TRINITY_DN95414_c0_g1_i1_orf1;TRINITY_DN27033_c1_g1_i3_orfp1;TRINITY_DN376_c1_g1_i1_orf1;TRINITY_DN83295_c0_g1_i3_orf1;TRINITY_DN9062_c0_g2_i3_orf1;TRINITY_DN4767_c0_g1_i4_orf1;TRINITY_DN74037_c0_g5_i1_orf1;TRINITY_DN7776_c0_g1_i5_orf1;TRINITY_DN40015_c0_g1_i2_orf1;TRINITY_DN147458_c0_g1_i1_orf1;TRINITY_DN812_c2_g1_i1_orf1;TRINITY_DN21251_c1_g1_i1_orf1;TRINITY_DN5112_c0_g1_i1_orf1;TRINITY_DN25534_c0_g1_i1_orf1;TRINITY_DN10766_c0_g1_i1_orf1;TRINITY_DN1750_c1_g1_i5_orf1;TRINITY_DN620_c0_g1_i4_orf1;TRINITY_DN2026_c0_g1_i4_orf1;TRINITY_DN5182_c0_g1_i5_orf1;TRINITY_DN19262_c0_g1_i1_orf1;TRINITY_DN33346_c0_g1_i1_orf1;TRINITY_DN1554_c0_g1_i9_orf1;TRINITY_DN4016_c0_g1_i1_orf1;TRINITY_DN542_c0_g2_i1_orf1;TRINITY_DN3975_c0_g1_i10_orf1;TRINITY_DN43792_c0_g1_i1_orf1;TRINITY_DN4135_c0_g1_i5_orf1;TRINITY_DN18388_c0_g1_i6_orf1;TRINITY_DN4449_c0_g2_i1_orf1;TRINITY_DN2579_c0_g1_i7_orf1;TRINITY_DN18172_c0_g1_i6_orf1;TRINITY_DN10455_c0_g2_i1_orf1;TRINITY_DN6660_c0_g1_i5_orf1;TRINITY_DN17838_c0_g1_i4_orf1;TRINITY_DN2515_c0_g1_i6_orf1;TRINITY_DN1173_c1_g1_i9_orf1;TRINITY_DN2274_c0_g1_i6_orf1;TRINITY_DN42506_c0_g1_i1_orf1;TRINITY_DN1287_c0_g1_i5_orf1;TRINITY_DN2647_c0_g1_i3_orf1;TRINITY_DN29034_c0_g1_i1_orf1;TRINITY_DN9874_c0_g1_i7_orf1;TRINITY_DN69697_c0_g1_i1_orf1;TRINITY_DN10385_c0_g1_i5_orf1;TRINITY_DN4944_c0_g1_i2_orf1;TRINITY_DN41997_c0_g1_i2_orf1;TRINITY_DN2265_c0_g2_i1_orf1;TRINITY_DN5031_c0_g1_i1_orf1;TRINITY_DN461_c0_g1_i5_orf1;TRINITY_DN44792_c0_g1_i1_orf1;TRINITY_DN12973_c0_g1_i1_orf1;TRINITY_DN36893_c0_g1_i1_orf1;TRINITY_DN24539_c0_g1_i4_orf1;TRINITY_DN8949_c0_g1_i2_orf1;TRINITY_DN55148_c0_g1_i1_orf1;TRINITY_DN71840_c0_g1_i1_orf1;TRINITY_DN142588_c0_g1_i1_orf1;TRINITY_DN975_c0_g1_i1_orf1;TRINITY_DN3702_c0_g1_i1_orf1;TRINITY_DN39490_c0_g1_i1_orf1;TRINITY_DN10994_c0_g1_i4_orf1;TRINITY_DN1080_c0_g1_i1_orf1;TRINITY_DN17045_c0_g2_i3_orf1;TRINITY_DN2593_c0_g3_i1_orf1;TRINITY_DN2593_c0_g1_i1_orf1;TRINITY_DN334_c0_g1_i3_orf1;TRINITY_DN58413_c0_g1_i4_orf1;TRINITY_DN4151_c1_g1_i4_orf1;TRINITY_DN46132_c0_g2_i2_orf1;TRINITY_DN81248_c0_g1_i1_orf1;TRINITY_DN
biological_process	S-adenosylmethionine metabolic process	GO:0046500	2 2/3516	TRINITY_DN7512_c0_g1_i1_orf1;TRINITY_DN1578_c0_g3_i1_orf1
biological_process	pigment metabolic process involved in	GO:0043474	1 1/3516	TRINITY_DN13941_c0_g1_i6_orf1
biological_process	melanin metabolic process	GO:0006582	3 3/3516	TRINITY_DN31163_c1_g1_i4_orf1;TRINITY_DN15706_c0_g2_i5_orf1;TRINITY_DN2338_c0_g1_i5_orf1
biological_process	pigment biosynthetic process	GO:0046148	5 5/3516	TRINITY_DN31163_c1_g1_i4_orf1;TRINITY_DN5559_c0_g1_i1_orf1;TRINITY_DN6563_c0_g1_i1_orf1;TRINITY_DN2338_c0_g1_i5_orf1;TRINITY_DN13941_c0_g1_i6_orf1
biological_process	heme metabolic process	GO:0042168	2 2/3516	TRINITY_DN5559_c0_g1_i1_orf1;TRINITY_DN6563_c0_g1_i1_orf1

biological_process	ocellus pigment metabolic process	GO:0046158	1	1/3516	TRINITY_DN13941_c0_g1_i6_orf1
biological_process	single fertilization	GO:0007338	1	1/3516	TRINITY_DN111985_c0_g1_i1_orf1
biological_process	gamete generation	GO:0007276	4	4/3516	TRINITY_DN31216_c0_g1_i2_orf1;TRINITY_DN10886_c0_g2_i4_orf1;TRINITY_DN2652_c0_g2_i1_orf1;TRINITY_DN4813_c0_g1_i5_orf1
biological_process	germ cell development	GO:0007281	2	2/3516	TRINITY_DN31216_c0_g1_i2_orf1;TRINITY_DN2652_c0_g2_i1_orf1
biological_process	ovarian follicle cell development	GO:0030707	1	1/3516	TRINITY_DN15706_c0_g2_i5_orf1
biological_process	bicoid mRNA localization	GO:0045450	1	1/3516	TRINITY_DN21123_c0_g1_i1_orf1
biological_process	spermatogenesis	GO:0007283	2	2/3516	TRINITY_DN10886_c0_g2_i4_orf1;TRINITY_DN4813_c0_g1_i5_orf1
biological_process	binding of sperm to zona pellucida	GO:0007339	1	1/3516	TRINITY_DN20133_c0_g1_i1_orf1
biological_process	female pregnancy	GO:0007565	1	1/3516	TRINITY_DN2848_c0_g1_i1_orf1
biological_process	killing of cells of another organism	GO:0031640	4	4/3516	TRINITY_DN5667_c0_g1_i4_orf1;TRINITY_DN6098_c1_g1_i5_orf1;TRINITY_DN2848_c0_g1_i2_orf1;TRINITY_DN2848_c0_g1_i1_orf1
biological_process	leukocyte activation	GO:0045321	1	1/3516	TRINITY_DN46409_c0_g1_i1_orf1
biological_process	neuron death	GO:0070997	2	2/3516	TRINITY_DN2848_c0_g1_i2_orf1;TRINITY_DN2848_c0_g1_i1_orf1
biological_process	programmed cell death	GO:0012501	8	8/3516	TRINITY_DN14967_c0_g2_i1_orf1;TRINITY_DN2848_c0_g1_i2_orf1;TRINITY_DN2848_c0_g1_i1_orf1;TRINITY_DN1738_c0_g1_i5_orf1;TRINITY_DN17215_c0_g1_i4_orf1;TRINITY_DN50074_c0_g1_i1_orf1;TRINITY_DN18912_c1_g1_i1_orf1;TRINITY_DN108122_c0_g1_i9_orf1
biological_process	autophagy	GO:0006914	10	10/3516	TRINITY_DN29017_c0_g1_i4_orf1;TRINITY_DN111985_c0_g1_i1_orf1;TRINITY_DN9376_c1_g1_i3_orf1;TRINITY_DN10229_c0_g1_i6_orf1;TRINITY_DN2947_c0_g1_i4_orf1;TRINITY_DN19821_c0_g2_i4_orf1;TRINITY_DN113353_c0_g1_i1_orf1;TRINITY_DN21126_c0_g1_i1_orf1;TRINITY_DN22441_c0_g1_i1_orf1;TRINITY_DN8926_c0_g1_i4_orf1
biological_process	secretion by cell	GO:0032940	6	6/3516	TRINITY_DN111985_c0_g1_i1_orf1;TRINITY_DN61777_c0_g1_i4_orf1;TRINITY_DN121_c0_g1_i9_orf1;TRINITY_DN33452_c0_g1_i1_orf1;TRINITY_DN49527_c0_g1_i1_orf1;TRINITY_DN33452_c0_g1_i3_orf1
biological_process	cell-substrate adhesion	GO:0031589	3	3/3516	TRINITY_DN2186_c0_g1_i17_orf1;TRINITY_DN6698_c0_g2_i1_orf1;TRINITY_DN2919_c0_g1_i5_orf1
biological_process	cell-cell adhesion	GO:0098609	3	3/3516	TRINITY_DN1008_c0_g1_i2_orf1;TRINITY_DN10070_c0_g1_i1_orf1;TRINITY_DN2270_c0_g2_i1_orf1
biological_process	cellular response to extracellular stimulus	GO:0031668	4	4/3516	TRINITY_DN140212_c0_g1_i1_orf1;TRINITY_DN51938_c0_g3_i1_orf1;TRINITY_DN1091_c0_g3_i1_orf1;TRINITY_DN2054_c0_g1_i1_orf1
biological_process	intermediate filament cytoskeleton organization	GO:0045104	4	4/3516	TRINITY_DN34166_c0_g1_i1_orf1;TRINITY_DN59852_c0_g1_i1_orf1;TRINITY_DN97097_c0_g1_i4_orf1;TRINITY_DN20009_c0_g1_i1_orf1
biological_process	maintenance of protein location in cell	GO:0032507	2	2/3516	TRINITY_DN245_c0_g1_i4_orf1;TRINITY_DN13783_c0_g4_i2_orf1
biological_process	muscle cell cellular homeostasis	GO:0046716	1	1/3516	TRINITY_DN20133_c0_g1_i1_orf1
biological_process	cell redox homeostasis	GO:0045454	2	2/3516	TRINITY_DN376_c0_g1_i1_orf1;TRINITY_DN9965_c0_g1_i1_orf1
biological_process	cellular chemical homeostasis	GO:0055082	11	11/3516	TRINITY_DN46625_c0_g1_i1_orf1;TRINITY_DN65681_c0_g1_i1_orf1;TRINITY_DN1423_c0_g1_i4_orf1;TRINITY_DN1423_c0_g1_i8_orf1;TRINITY_DN136031_c0_g1_i7_orf1;TRINITY_DN3461_c0_g1_i1_orf1;TRINITY_DN96739_c0_g1_i1_orf1;TRINITY_DN3434_c0_g1_i1_orf1;TRINITY_DN22430_c0_g3_i1_orf1;TRINITY_DN7405_c0_g1_i3_orf1;TRINITY_DN44256_c0_g1_i1_orf1
biological_process	leukocyte proliferation	GO:0070661	1	1/3516	TRINITY_DN46409_c0_g1_i1_orf1
biological_process	mitotic cell cycle process	GO:1903047	6	6/3516	TRINITY_DN96557_c0_g1_i1_orf1;TRINITY_DN7493_c0_g1_i1_orf1;TRINITY_DN235_c0_g3_i1_orf1;TRINITY_DN31119_c0_g1_i1_orf1;TRINITY_DN10287_c0_g1_i1_orf1;TRINITY_DN13259_c0_g1_i2_orf1
biological_process	cell cycle phase transition	GO:0044770	1	1/3516	TRINITY_DN96557_c0_g1_i1_orf1
biological_process	spindle organization	GO:0007051	1	1/3516	TRINITY_DN31119_c0_g1_i1_orf1
biological_process	spindle localization	GO:0051653	1	1/3516	TRINITY_DN7493_c0_g1_i1_orf1
biological_process	G1 to G0 transition	GO:0070314	1	1/3516	TRINITY_DN130075_c1_g2_i1_orf1
biological_process	cytokinesis	GO:0000910	2	2/3516	TRINITY_DN235_c0_g3_i1_orf1;TRINITY_DN7493_c0_g1_i1_orf1
biological_process	sister chromatid cohesion	GO:0007062	1	1/3516	TRINITY_DN2638_c0_g1_i7_orf1
biological_process	cytokinetic process	GO:0032506	1	1/3516	TRINITY_DN96557_c0_g1_i1_orf1
biological_process	chaperone-mediated protein folding	GO:0061077	2	2/3516	TRINITY_DN21214_c0_g2_i1_orf1;TRINITY_DN34056_c0_g1_i4_orf1
biological_process	protein folding in endoplasmic reticulum	GO:0034975	1	1/3516	TRINITY_DN5169_c0_g1_i5_orf1
biological_process	'de novo' protein folding	GO:0006458	3	3/3516	TRINITY_DN11215_c0_g1_i1_orf1;TRINITY_DN21214_c0_g2_i1_orf1;TRINITY_DN46409_c0_g1_i1_orf1
biological_process	protein refolding	GO:0042026	4	4/3516	TRINITY_DN21214_c0_g2_i1_orf1;TRINITY_DN45598_c0_g1_i2_orf1;TRINITY_DN18031_c0_g1_i1_orf1;TRINITY_DN46409_c0_g1_i1_orf1
biological_process	post-chaperonin tubulin folding pathway	GO:0007023	2	2/3516	TRINITY_DN1054_c0_g1_i8_orf1;TRINITY_DN104297_c0_g1_i1_orf1

biological_process	cellular macromolecule localization	GO:0070727	71	71/3516	TRINITY_DN29017_c0_g1_i4_orf1;TRINITY_DN245_c0_g1_i4_orf1;TRINITY_DN44219_c0_g1_i1_orf1;TRINITY_DN4790_c0_g1_i6_orf1;TRINITY_DN38835_c0_g2_i1_orf1;TRINITY_DN5182_c0_g1_i5_orf1;TRINITY_DN15811_c0_g1_i7_orf1;TRINITY_DN46409_c0_g1_i1_orf1;TRINITY_DN3450_c0_g1_i3_orf1;TRINITY_DN6243_c0_g1_i5_orf1;TRINITY_DN55148_c0_g1_i1_orf1;TRINITY_DN3747_c1_g1_i3_orf1;TRINITY_DN383_c0_g1_i1_orf1;TRINITY_DN14677_c0_g2_i3_orf1;TRINITY_DN3664_c0_g1_i8_orf1;TRINITY_DN327_c1_g1_i4_orf1;TRINITY_DN34159_c0_g2_i1_orf1;TRINITY_DN27721_c1_g1_i2_orf1;TRINITY_DN41842_c0_g1_i2_orf1;TRINITY_DN72859_c0_g1_i1_orf1;TRINITY_DN5982_c0_g1_i3_orf1;TRINITY_DN1447_c0_g1_i5_orf1;TRINITY_DN49527_c0_g1_i1_orf1;TRINITY_DN2172_c0_g2_i8_orf1;TRINITY_DN42903_c0_g1_i4_orf1;TRINITY_DN146758_c0_g1_i1_orf1;TRINITY_DN2879_c0_g1_i4_orf1;TRINITY_DN19286_c0_g1_i1_orf1;TRINITY_DN472_c1_g1_i3_orf1;TRINITY_DN3513_c0_g1_i5_orf1;TRINITY_DN2238_c0_g2_i1_orf1;TRINITY_DN3299_c0_g1_i2_orf1;TRINITY_DN111985_c0_g1_i1_orf1;TRINITY_DN61777_c0_g1_i4_orf1;TRINITY_DN3562_c0_g1_i4_orf1;TRINITY_DN740_c0_g1_i1_orf1;TRINITY_DN1437_c0_g1_i6_orf1;TRINITY_DN25681_c0_g1_i5_orf1;TRINITY_DN2286_c2_g1_i1_orf1;TRINITY_DN124300_c0_g1_i2_orf1;TRINITY_DN1384_c0_g1_i5_orf1;TRINITY_DN48460_c0_g1_i1_orf1;TRINITY_DN1901_c0_g1_i6_orf1;TRINITY_DN13783_c0_g4_i2_orf1;TRINITY_DN959_c0_g1_i7_orf1;TRINITY_DN2181_c1_g1_i8_orf1;TRINITY_DN486_c0_g1_i5_orf1;TRINITY_DN13944_c0_g1_i1_orf1;TRINITY_DN106476_c0_g1_i3_orf1;TRINITY_DN65299_c0_g4_i1_orf1;TRINITY_DN3209_c0_g2_i6_orf1;TRINITY_DN96557_c0_g1_i1_orf1;TRINITY_DN5383_c0_g1_i4_orf1;TRINITY_DN9741_c0_g1_i3_orf1;TRINITY_DN6680_c0_g1_i1_orf1;TRINITY_DN25896_c0_g1_i6_orf1;TRINITY_DN92232_c0_g1_i1_orf1;TRINITY_DN5630_c4_g1_i2_orf1;TRINITY_DN13139_c0_g1_i1_orf1;TRINITY_DN4859_c0_g1_i5_orf1;TRINITY_DN147427_c0_g1_i1_orf1;TRINITY_DN25210_c0_g1_i1_orf1;TRINITY_DN50875_c0_g1_i3_orf1;TRINITY_DN4304_c0_g1_i3_orf1;TRINITY_DN21123_c0_g1_i1_orf1;TRINITY_DN12320_c0_g1_i1_orf1;TRINITY_DN4394_c0_g1_i4_orf1;TRINITY_DN9931_c0_g1_i1_orf1;TRINITY_DN38835_c0_g3_i1_orf1;TRINITY_DN146119_c0_g1_i1_orf1;TRINITY_DN59042_c1_g1_i1_orf1
biological_process	intracellular transport	GO:0046907	58	58/3516	TRINITY_DN3835_c0_g1_i3_orf1;TRINITY_DN21214_c0_g2_i1_orf1;TRINITY_DN31751_c0_g1_i5_orf1;TRINITY_DN10396_c0_g1_i1_orf1;TRINITY_DN960_c1_g1_i6_orf1;TRINITY_DN4770_c0_g1_i4_orf1;TRINITY_DN5182_c0_g1_i5_orf1;TRINITY_DN15811_c0_g1_i7_orf1;TRINITY_DN3513_c0_g1_i5_orf1;TRINITY_DN6231_c0_g1_i6_orf1;TRINITY_DN4859_c0_g1_i5_orf1;TRINITY_DN46409_c0_g1_i1_orf1;TRINITY_DN3450_c0_g1_i3_orf1;TRINITY_DN25210_c0_g1_i1_orf1;TRINITY_DN3747_c1_g1_i3_orf1;TRINITY_DN14677_c0_g2_i3_orf1;TRINITY_DN15448_c0_g1_i1_orf1;TRINITY_DN3664_c0_g1_i8_orf1;TRINITY_DN327_c1_g1_i4_orf1;TRINITY_DN3835_c0_g1_i4_orf1;TRINITY_DN34159_c0_g2_i1_orf1;TRINITY_DN41842_c0_g1_i2_orf1;TRINITY_DN13139_c0_g1_i1_orf1;TRINITY_DN5982_c0_g1_i3_orf1;TRINITY_DN740_c0_g1_i1_orf1;TRINITY_DN1447_c0_g1_i5_orf1;TRINITY_DN55148_c0_g1_i1_orf1;TRINITY_DN72859_c0_g1_i1_orf1;TRINITY_DN146758_c0_g1_i1_orf1;TRINITY_DN45037_c0_g1_i1_orf1;TRINITY_DN19286_c0_g1_i1_orf1;TRINITY_DN3209_c0_g2_i6_orf1;TRINITY_DN14673_c0_g1_i3_orf1;TRINITY_DN96557_c0_g1_i1_orf1;TRINITY_DN3821_c1_g1_i7_orf1;TRINITY_DN1437_c0_g1_i6_orf1;TRINITY_DN25681_c0_g1_i5_orf1;TRINITY_DN124300_c0_g1_i2_orf1;TRINITY_DN1384_c0_g1_i5_orf1;TRINITY_DN48460_c0_g1_i1_orf1;TRINITY_DN1245_c0_g1_i4_orf1;TRINITY_DN1901_c0_g1_i6_orf1;TRINITY_DN35377_c0_g1_i3_orf1;TRINITY_DN486_c0_g1_i5_orf1;TRINITY_DN27721_c1_g1_i2_orf1;TRINITY_DN106476_c0_g1_i3_orf1;TRINITY_DN10195_c0_g1_i8_orf1;TRINITY_DN578_c0_g1_i5_orf1;TRINITY_DN6535_c0_g1_i3_orf1;TRINITY_DN5383_c0_g1_i4_orf1;TRINITY_DN54586_c1_g1_i1_orf1;TRINITY_DN4814_c0_g1_i6_orf1;TRINITY_DN147427_c0_g1_i1_orf1;TRINITY_DN5558_c0_g1_i4_orf1;TRINITY_DN4394_c0_g1_i4_orf1;TRINITY_DN942_c0_g1_i1_orf1;TRINITY_DN92232_c0_g1_i1_orf1;TRINITY_DN59042_c1_g1_i1_orf1
biological_process	localization within membrane	GO:0051668	6	6/3516	TRINITY_DN96557_c0_g1_i1_orf1;TRINITY_DN61777_c0_g1_i4_orf1;TRINITY_DN15811_c0_g1_i7_orf1;TRINITY_DN48460_c0_g1_i1_orf1;TRINITY_DN19286_c0_g1_i1_orf1;TRINITY_DN959_c0_g1_i7_orf1
biological_process	cellular component biogenesis	GO:0044085	15	15/3516	TRINITY_DN14313_c0_g1_i1_orf1;TRINITY_DN4016_c0_g1_i1_orf1;TRINITY_DN41179_c0_g1_i1_orf1;TRINITY_DN13496_c0_g1_i7_orf1;TRINITY_DN4956_c0_g1_i6_orf1;TRINITY_DN9101_c0_g2_i1_orf1;TRINITY_DN31225_c0_g1_i1_orf1;TRINITY_DN3292_c2_g1_i4_orf1;TRINITY_DN7573_c0_g2_i1_orf1;TRINITY_DN102260_c0_g1_i1_orf1;TRINITY_DN17299_c0_g1_i4_orf1;TRINITY_DN92232_c0_g1_i1_orf1;TRINITY_DN55148_c0_g1_i1_orf1;TRINITY_DN8676_c0_g1_i1_orf1;TRINITY_DN6239_c0_g1_i1_orf1

biological_process	cellular component organization	GO:0016043	137	137/3516	TRINITY_DN14920_c0_g1_i1_orf1;TRINITY_DN125565_c1_g1_i1_orf1;TRINITY_DN2304_c0_g1_i4_orf1;TRINITY_DN1497_c0_g2_i6_orf1;TRINITY_DN39404_c0_g1_i7_orf1;TRINITY_DN11194_c0_g1_i4_orf1;TRINITY_DN3450_c0_g1_i3_orf1;TRINITY_DN104596_c0_g1_i1_orf1;TRINITY_DN35669_c0_g1_i1_orf1;TRINITY_DN4237_c1_g1_i5_orf1;TRINITY_DN91877_c0_g1_i1_orf1;TRINITY_DN101922_c0_g1_i1_orf1;TRINITY_DN35245_c0_g1_i1_orf1;TRINITY_DN2904_c0_g1_i4_orf1;TRINITY_DN104297_c0_g1_i1_orf1;TRINITY_DN70485_c0_g1_i2_orf1;TRINITY_DN142442_c0_g1_i1_orf1;TRINITY_DN140538_c0_g2_i1_orf1;TRINITY_DN59852_c0_g1_i1_orf1;TRINITY_DN1298_c0_g1_i3_orf1;TRINITY_DN2638_c0_g1_i7_orf1;TRINITY_DN2848_c0_g1_i1_orf1;TRINITY_DN1639_c0_g2_i2_orf1;TRINITY_DN15811_c0_g1_i7_orf1;TRINITY_DN12442_c0_g1_i4_orf1;TRINITY_DN43505_c0_g1_i1_orf1;TRINITY_DN698_c0_g1_i5_orf1;TRINITY_DN4798_c0_g1_i3_orf1;TRINITY_DN110231_c0_g1_i1_orf1;TRINITY_DN662_c0_g1_i1_orf1;TRINITY_DN3513_c0_g1_i5_orf1;TRINITY_DN3461_c0_g1_i1_orf1;TRINITY_DN53684_c0_g1_i1_orf1;TRINITY_DN23502_c0_g1_i1_orf1;TRINITY_DN4950_c0_g1_i2_orf1;TRINITY_DN19092_c0_g1_i2_orf1;TRINITY_DN51938_c0_g3_i1_orf1;TRINITY_DN11464_c0_g1_i3_orf1;TRINITY_DN19980_c0_g1_i4_orf1;TRINITY_DN20442_c0_g2_i1_orf1;TRINITY_DN52649_c0_g1_i6_orf1;TRINITY_DN130075_c1_g2_i1_orf1;TRINITY_DN146119_c0_g1_i1_orf1;TRINITY_DN23790_c0_g1_i1_orf1;TRINITY_DN6248_c0_g1_i1_orf1;TRINITY_DN71832_c0_g1_i1_orf1;TRINITY_DN1054_c0_g1_i8_orf1;TRINITY_DN42854_c0_g3_i2_orf1;TRINITY_DN298_c0_g1_i4_orf1;TRINITY_DN30150_c0_g1_i7_orf1;TRINITY_DN4108_c0_g1_i6_orf1;TRINITY_DN28622_c0_g1_i1_orf1;TRINITY_DN111985_c0_g1_i1_orf1;TRINITY_DN50085_c0_g1_i1_orf1;TRINITY_DN6642_c0_g1_i2_orf1;TRINITY_DN315_c0_g1_i1_orf1;TRINITY_DN37986_c0_g1_i2_orf1;TRINITY_DN31119_c0_g1_i1_orf1;TRINITY_DN31310_c0_g1_i1_orf1;TRINITY_DN40508_c0_g1_i1_orf1;TRINITY_DN3366_c0_g1_i6_orf1;TRINITY_DN4439_c0_g1_i2_orf1;TRINITY_DN3847_c1_g1_i1_orf1;TRINITY_DN2848_c0_g1_i2_orf1;TRINITY_DN7493_c0_g1_i1_orf1;TRINITY_DN11069_c0_g2_i1_orf1;TRINITY_DN3702_c0_g1_i1_orf1;TRINITY_DN4908_c1_g1_i5_orf1;TRINITY_DN13371_c0_g1_i4_orf1;TRINITY_DN40911_c0_g1_i1_orf1;TRINITY_DN46409_c0_g1_i1_orf1;TRINITY_DN89083_c0_g1_i1_orf1;TRINITY_DN5458_c1_g1_i9_orf1;TRINITY_DN10429_c0_g1_i2_orf1;TRINITY_DN70409_c0_g1_i3_orf1;TRINITY_DN4016_c0_g1_i1_orf1;TRINITY_DN14987_c0_g1_i3_orf1;TRINITY_DN12771_c0_g1_i1_orf1;TRINITY_DN43412_c0_g1_i2_orf1;TRINITY_DN452_c1_g1_i3_orf1;TRINITY_DN20009_c0_g1_i1_orf1;TRINITY_DN54134_c0_g1_i1_orf1;TRINITY_DN73_c0_g1_i6_orf1;TRINITY_DN1572_c0_g1_i6_orf1;TRINITY_DN124300_c0_g1_i2_orf1;TRINITY_DN96739_c0_g1_i1_orf1;TRINITY_DN25976_c0_g1_i4_orf1;TRINITY_DN85476_c0_g1_i1_orf1;TRINITY_DN101682_c0_g1_i1_orf1;TRINITY_DN96557_c0_g1_i1_orf1;TRINITY_DN18869_c0_g1_i1_orf1;TRINITY_DN9376_c1_g1_i3_orf1;TRINITY_DN33619_c0_g1_i1_orf1;TRINITY_DN11327_c0_g1_i1_orf1;TRINITY_DN14904_c1_g2_i2_orf1;TRINITY_DN34166_c0_g1_i1_orf1;TRINITY_DN164_c0_g1_i11_orf1;TRINITY_DN10455_c0_g1_i2_orf1;TRINITY_DN4842_c0_g1_i5_orf1;TRINITY_DN10385_c0_g1_i5_orf1;TRINITY_DN37336_c1_g1_i1_orf1;TRINITY_DN92232_c0_g1_i1_orf1;TRINITY_DN6071_c0_g1_i1_orf1;TRINITY_DN54336_c0_g1_i1_orf1;TRINITY_DN8087_c0_g1_i9_orf1;TRINITY_DN72369_c0_g1_i1_orf1;TRINITY_DN11215_c0_g1_i1_orf1;TRINITY_DN57202_c0_g1_i1_orf1;TRINITY_DN4159_c1_g1_i1_orf1;TRINITY_DN15448_c0_g1_i1_orf1;TRINITY_DN55148_c0_g1_i1_orf1;TRINITY_DN38540_c0_g1_i1_orf1;TRINITY_DN10636_c0_g1_i1_orf1;TRINITY_DN7647_c0_g1_i4_orf1;TRINITY_DN19092_c2_g1_i1_orf1;TRINITY_DN17045_c0_g2_i3_orf1;TRINITY_DN152_c0_g1_i4_orf1;TRINITY_DN115_c0_g1_i6_orf1;TRINITY_DN35635_c0_g1_i1_orf1;TRINITY_DN5678_c0_g2_i3_orf1;TRINITY_DN170
biological_process	cell migration	GO:0016477	3	3/3516	TRINITY_DN110231_c0_g1_i1_orf1;TRINITY_DN96739_c0_g1_i1_orf1;TRINITY_DN15706_c0_g2_i5_orf1
biological_process	cilium or flagellum-dependent cell motility	GO:0001539	1	1/3516	TRINITY_DN26243_c0_g1_i2_orf1
biological_process	microtubule-based movement	GO:0007018	3	3/3516	TRINITY_DN14298_c0_g3_i1_orf1;TRINITY_DN26243_c0_g1_i2_orf1;TRINITY_DN14298_c0_g1_i1_orf1
biological_process	microtubule cytoskeleton organization	GO:0000226	6	6/3516	TRINITY_DN2848_c0_g1_i2_orf1;TRINITY_DN7493_c0_g1_i1_orf1;TRINITY_DN315_c0_g1_i1_orf1;TRINITY_DN31119_c0_g1_i1_orf1;TRINITY_DN2745_c0_g1_i4_orf1;TRINITY_DN2848_c0_g1_i1_orf1
biological_process	cellular response to chemical stimulus	GO:0070887	13	13/3516	TRINITY_DN111985_c0_g1_i1_orf1;TRINITY_DN21214_c0_g2_i1_orf1;TRINITY_DN51938_c0_g3_i1_orf1;TRINITY_DN4016_c0_g1_i1_orf1;TRINITY_DN315_c0_g1_i1_orf1;TRINITY_DN2848_c0_g1_i2_orf1;TRINITY_DN975_c0_g1_i1_orf1;TRINITY_DN130075_c1_g2_i1_orf1;TRINITY_DN46409_c0_g1_i1_orf1;TRINITY_DN10429_c0_g1_i2_orf1;TRINITY_DN20009_c0_g1_i1_orf1;TRINITY_DN15448_c0_g1_i1_orf1;TRINITY_DN2848_c0_g1_i1_orf1
biological_process	cellular response to stress	GO:0033554	31	31/3516	TRINITY_DN21214_c0_g2_i1_orf1;TRINITY_DN3092_c0_g1_i2_orf1;TRINITY_DN17271_c0_g1_i1_orf1;TRINITY_DN125565_c1_g1_i1_orf1;TRINITY_DN140212_c0_g1_i1_orf1;TRINITY_DN40434_c0_g1_i2_orf1;TRINITY_DN45271_c0_g1_i1_orf1;TRINITY_DN51938_c0_g3_i1_orf1;TRINITY_DN77318_c0_g2_i1_orf1;TRINITY_DN46409_c0_g1_i1_orf1;TRINITY_DN48536_c0_g1_i3_orf1;TRINITY_DN10429_c0_g1_i2_orf1;TRINITY_DN104507_c0_g1_i2_orf1;TRINITY_DN2054_c0_g1_i1_orf1;TRINITY_DN452_c1_g1_i3_orf1;TRINITY_DN17726_c0_g1_i1_orf1;TRINITY_DN1091_c0_g3_i1_orf1;TRINITY_DN111985_c0_g1_i1_orf1;TRINITY_DN6503_c0_g1_i8_orf1;TRINITY_DN5238_c0_g1_i2_orf1;TRINITY_DN5686_c0_g1_i4_orf1;TRINITY_DN6642_c0_g1_i2_orf1;TRINITY_DN123184_c0_g1_i1_orf1;TRINITY_DN6185_c0_g1_i12_orf1;TRINITY_DN14487_c0_g1_i4_orf1;TRINITY_DN9062_c0_g2_i3_orf1;TRINITY_DN2647_c0_g1_i3_orf1;TRINITY_DN346_c0_g1_i7_orf1;TRINITY_DN4429_c0_g1_i5_orf1;TRINITY_DN109733_c0_g1_i1_orf1;TRINITY_DN10287_c0_g1_i1_orf1
biological_process	cellular response to biotic stimulus	GO:0071216	1	1/3516	TRINITY_DN20009_c0_g1_i1_orf1
biological_process	developmental cell growth	GO:0048588	1	1/3516	TRINITY_DN501_c1_g1_i1_orf1
biological_process	cell surface receptor signaling pathway	GO:0007166	12	12/3516	TRINITY_DN2202_c0_g1_i9_orf1;TRINITY_DN492_c0_g1_i4_orf1;TRINITY_DN91198_c0_g2_i1_orf1;TRINITY_DN3418_c0_g1_i3_orf1;TRINITY_DN51938_c0_g3_i1_orf1;TRINITY_DN147475_c0_g1_i1_orf1;TRINITY_DN1008_c0_g1_i2_orf1;TRINITY_DN2170_c0_g2_i1_orf1;TRINITY_DN2170_c1_g1_i3_orf1;TRINITY_DN13216_c0_g1_i5_orf1;TRINITY_DN2270_c0_g2_i1_orf1;TRINITY_DN15247_c0_g1_i2_orf1
biological_process	hormone-mediated signaling pathway	GO:0009755	1	1/3516	TRINITY_DN147475_c0_g1_i1_orf1
biological_process	endoplasmic reticulum unfolded protein response	GO:0030968	1	1/3516	TRINITY_DN48536_c0_g1_i3_orf1
biological_process	immune response-regulating signaling pathway	GO:0002764	3	3/3516	TRINITY_DN46409_c0_g1_i1_orf1;TRINITY_DN2170_c0_g2_i1_orf1;TRINITY_DN2170_c1_g1_i3_orf1
biological_process	SMAD protein signal transduction	GO:0060395	1	1/3516	TRINITY_DN20009_c0_g1_i1_orf1
biological_process	G protein-coupled receptor signaling pathway	GO:0007186	3	3/3516	TRINITY_DN42854_c0_g3_i2_orf1;TRINITY_DN11245_c0_g1_i2_orf1;TRINITY_DN4628_c0_g1_i1_orf1
biological_process	intracellular signal transduction	GO:0035556	18	18/3516	TRINITY_DN17838_c0_g1_i4_orf1;TRINITY_DN802_c0_g1_i2_orf1;TRINITY_DN79000_c1_g1_i1_orf1;TRINITY_DN2947_c0_g1_i4_orf1;TRINITY_DN18696_c0_g1_i1_orf1;TRINITY_DN2793_c0_g2_i1_orf1;TRINITY_DN2983_c0_g1_i6_orf1;TRINITY_DN15478_c0_g1_i1_orf1;TRINITY_DN2623_c0_g1_i3_orf1;TRINITY_DN6426_c0_g1_i2_orf1;TRINITY_DN7493_c0_g1_i1_orf1;TRINITY_DN2848_c0_g1_i1_orf1;TRINITY_DN15706_c0_g2_i5_orf1;TRINITY_DN5182_c0_g1_i5_orf1;TRINITY_DN10287_c0_g1_i1_orf1;TRINITY_DN13259_c0_g1_i2_orf1;TRINITY_DN804_c0_g1_i7_orf1;TRINITY_DN32700_c0_g1_i2_orf1
biological_process	apoptotic signaling pathway	GO:0097190	2	2/3516	TRINITY_DN96739_c0_g1_i1_orf1;TRINITY_DN51938_c0_g3_i1_orf1
biological_process	cellular detoxification of aldehyde	GO:0110095	2	2/3516	TRINITY_DN111985_c0_g1_i1_orf1;TRINITY_DN3758_c0_g1_i2_orf1

biological_process	meiotic cell cycle	GO:0051321	4	4/3516	TRINITY_DN45271_c0_g1_i1_orf1;TRINITY_DN123184_c0_g1_i1_orf1;TRINITY_DN10886_c0_g2_i4_orf1;TRINITY_DN4813_c0_g1_i5_orf1
biological_process	mitotic cell cycle	GO:0000278	2	2/3516	TRINITY_DN315_c0_g1_i1_orf1;TRINITY_DN2745_c0_g1_i4_orf1
biological_process	cell differentiation	GO:0030154	18	18/3516	TRINITY_DN1173_c0_g1_i12_orf1;TRINITY_DN928_c0_g1_i3_orf1;TRINITY_DN4813_c0_g1_i5_orf1;TRINITY_DN50725_c0_g1_i6_orf1;TRINITY_DN5954_c0_g1_i2_orf1;TRINITY_DN1173_c1_g1_i10_orf1;TRINITY_DN23746_c0_g1_i2_orf1;TRINITY_DN10886_c0_g2_i4_orf1;TRINITY_DN1173_c1_g1_i9_orf1;TRINITY_DN96739_c0_g1_i1_orf1;TRINITY_DN11388_c0_g1_i4_orf1;TRINITY_DN741_c0_g1_i10_orf1;TRINITY_DN655_c0_g1_i3_orf1;TRINITY_DN928_c0_g2_i1_orf1;TRINITY_DN20009_c0_g1_i1_orf1;TRINITY_DN467_c9_g1_i2_orf1;TRINITY_DN140538_c0_g2_i1_orf1;TRINITY_DN15244_c0_g1_i5_orf1
biological_process	cellular component morphogenesis	GO:0032989	1	1/3516	TRINITY_DN42854_c0_g3_i2_orf1
biological_process	cell development	GO:0048468	20	20/3516	TRINITY_DN61777_c0_g1_i4_orf1;TRINITY_DN54336_c0_g1_i1_orf1;TRINITY_DN25976_c0_g1_i4_orf1;TRINITY_DN288_c0_g1_i9_orf1;TRINITY_DN101682_c0_g1_i1_orf1;TRINITY_DN8087_c0_g1_i9_orf1;TRINITY_DN70409_c0_g1_i3_orf1;TRINITY_DN15706_c0_g2_i5_orf1;TRINITY_DN39404_c0_g1_i7_orf1;TRINITY_DN71832_c0_g1_i1_orf1;TRINITY_DN1710_c0_g1_i1_orf1;TRINITY_DN104596_c0_g1_i1_orf1;TRINITY_DN237_c1_g1_i1_orf1;TRINITY_DN31216_c0_g1_i2_orf1;TRINITY_DN20009_c0_g1_i1_orf1;TRINITY_DN19980_c0_g1_i4_orf1;TRINITY_DN152_c0_g1_i4_orf1;TRINITY_DN2652_c0_g2_i1_orf1;TRINITY_DN20710_c0_g2_i2_orf1;TRINITY_DN31310_c0_g1_i1_orf1
biological_process	protein transmembrane transport	GO:0071806	5	5/3516	TRINITY_DN46409_c0_g1_i1_orf1;TRINITY_DN146758_c0_g1_i1_orf1;TRINITY_DN1901_c0_g1_i6_orf1;TRINITY_DN327_c1_g1_i4_orf1;TRINITY_DN106476_c0_g1_i3_orf1
biological_process	mitochondrial transmembrane transport	GO:1990542	8	8/3516	TRINITY_DN760_c1_g2_i6_orf1;TRINITY_DN2267_c0_g1_i1_orf1;TRINITY_DN46409_c0_g1_i1_orf1;TRINITY_DN146758_c0_g1_i1_orf1;TRINITY_DN1901_c0_g1_i6_orf1;TRINITY_DN44256_c0_g1_i1_orf1;TRINITY_DN106476_c0_g1_i3_orf1;TRINITY_DN327_c1_g1_i4_orf1
biological_process	ion transmembrane transport	GO:0034220	7	7/3516	TRINITY_DN760_c1_g2_i6_orf1;TRINITY_DN1661_c0_g1_i1_orf1;TRINITY_DN2267_c0_g1_i1_orf1;TRINITY_DN96739_c0_g1_i1_orf1;TRINITY_DN22430_c0_g3_i1_orf1;TRINITY_DN19115_c0_g1_i1_orf1;TRINITY_DN44256_c0_g1_i1_orf1
biological_process	purine-containing compound transmembrane transport	GO:0072530	1	1/3516	TRINITY_DN760_c1_g2_i6_orf1
biological_process	nucleotide transmembrane transport	GO:1901679	1	1/3516	TRINITY_DN760_c1_g2_i6_orf1
biological_process	cell-cell recognition	GO:0009988	1	1/3516	TRINITY_DN20133_c0_g1_i1_orf1
biological_process	actin cytoskeleton organization	GO:0030036	6	6/3516	TRINITY_DN30150_c0_g1_i7_orf1;TRINITY_DN7493_c0_g1_i1_orf1;TRINITY_DN235_c0_g3_i1_orf1;TRINITY_DN4159_c1_g1_i1_orf1;TRINITY_DN23790_c0_g1_i1_orf1;TRINITY_DN92232_c0_g1_i1_orf1
biological_process	actin filament severing	GO:0051014	2	2/3516	TRINITY_DN30150_c0_g1_i7_orf1;TRINITY_DN7493_c0_g1_i1_orf1
biological_process	cellular component assembly involved in morphogenesis	GO:0010927	1	1/3516	TRINITY_DN235_c0_g3_i1_orf1
biological_process	tube morphogenesis	GO:0035239	2	2/3516	TRINITY_DN1639_c0_g2_i2_orf1;TRINITY_DN147475_c0_g1_i1_orf1
biological_process	embryonic morphogenesis	GO:0048598	1	1/3516	TRINITY_DN142442_c0_g1_i1_orf1
biological_process	tissue morphogenesis	GO:0048729	2	2/3516	TRINITY_DN237_c1_g1_i1_orf1;TRINITY_DN147475_c0_g1_i1_orf1
biological_process	animal organ morphogenesis	GO:0009887	6	6/3516	TRINITY_DN1639_c0_g2_i2_orf1;TRINITY_DN5954_c0_g1_i2_orf1;TRINITY_DN467_c9_g1_i2_orf1;TRINITY_DN741_c0_g1_i10_orf1;TRINITY_DN655_c0_g1_i3_orf1;TRINITY_DN23746_c0_g1_i2_orf1
biological_process	system development	GO:0048731	6	6/3516	TRINITY_DN61777_c0_g1_i4_orf1;TRINITY_DN1710_c0_g1_i1_orf1;TRINITY_DN142442_c0_g1_i1_orf1;TRINITY_DN42854_c0_g3_i2_orf1;TRINITY_DN288_c0_g1_i9_orf1;TRINITY_DN20710_c0_g2_i2_orf1
biological_process	multicellular organism development	GO:0007275	1	1/3516	TRINITY_DN1639_c0_g2_i2_orf1
biological_process	animal organ development	GO:0048513	12	12/3516	TRINITY_DN1639_c0_g2_i2_orf1;TRINITY_DN54336_c0_g1_i1_orf1;TRINITY_DN25976_c0_g1_i4_orf1;TRINITY_DN91198_c0_g2_i1_orf1;TRINITY_DN8087_c0_g1_i9_orf1;TRINITY_DN104596_c0_g1_i1_orf1;TRINITY_DN101682_c0_g1_i1_orf1;TRINITY_DN31310_c0_g1_i1_orf1;TRINITY_DN5458_c1_g1_i9_orf1;TRINITY_DN19980_c0_g1_i4_orf1;TRINITY_DN237_c1_g1_i1_orf1;TRINITY_DN71832_c0_g1_i1_orf1
biological_process	muscle structure development	GO:0061061	1	1/3516	TRINITY_DN5458_c1_g1_i9_orf1
biological_process	tissue development	GO:0009888	1	1/3516	TRINITY_DN142442_c0_g1_i1_orf1
biological_process	embryo development	GO:0009790	1	1/3516	TRINITY_DN1639_c0_g2_i2_orf1
biological_process	nervous system process	GO:0050877	7	7/3516	TRINITY_DN39725_c0_g1_i4_orf1;TRINITY_DN501_c1_g1_i1_orf1;TRINITY_DN14460_c0_g1_i6_orf1;TRINITY_DN142442_c0_g1_i1_orf1;TRINITY_DN75086_c0_g1_i5_orf1;TRINITY_DN26337_c0_g1_i3_orf1;TRINITY_DN12256_c0_g1_i1_orf1
biological_process	muscle system process	GO:0003012	1	1/3516	TRINITY_DN20133_c0_g1_i1_orf1
biological_process	regionalization	GO:0003002	1	1/3516	TRINITY_DN1639_c0_g2_i2_orf1
biological_process	transmission of nerve impulse	GO:0019226	1	1/3516	TRINITY_DN501_c1_g1_i1_orf1
biological_process	adult behavior	GO:0030534	1	1/3516	TRINITY_DN111985_c0_g1_i1_orf1
biological_process	locomotory behavior	GO:0007626	1	1/3516	TRINITY_DN111985_c0_g1_i1_orf1
biological_process	envenomation resulting in modulation of process in another organism	GO:0035738	1	1/3516	TRINITY_DN1215_c0_g1_i2_orf1
biological_process	response to virus	GO:0009615	1	1/3516	TRINITY_DN7493_c0_g1_i1_orf1
biological_process	response to bacterium	GO:0009617	10	10/3516	TRINITY_DN1444_c1_g1_i5_orf1;TRINITY_DN14904_c0_g1_i1_orf1;TRINITY_DN479_c6_g1_i2_orf1;TRINITY_DN8685_c0_g1_i5_orf1;TRINITY_DN16840_c1_g1_i1_orf1;TRINITY_DN195_c8_g1_i1_orf1;TRINITY_DN21856_c0_g1_i1_orf1;TRINITY_DN1666_c0_g1_i2_orf1;TRINITY_DN29190_c0_g1_i4_orf1;TRINITY_DN5880_c0_g2_i2_orf1
biological_process	response to host	GO:0075136	1	1/3516	TRINITY_DN3159_c0_g1_i4_orf1
biological_process	response to defenses of other organism	GO:0052173	1	1/3516	TRINITY_DN3159_c0_g1_i4_orf1
biological_process	response to fungus	GO:0009620	4	4/3516	TRINITY_DN5667_c0_g1_i4_orf1;TRINITY_DN6098_c1_g1_i5_orf1;TRINITY_DN2848_c0_g1_i2_orf1;TRINITY_DN2848_c0_g1_i1_orf1

biological_process	defense response to other organism	GO:0098542	24	24/3516	TRINITY_DN195_c8_g1_i1_orf1;TRINITY_DN827_c1_g1_i1_orf1;TRINITY_DN8685_c0_g1_i5_orf1;TRINITY_DN16840_c1_g1_i1_orf1;TRINITY_DN15706_c0_g2_i5_orf1;TRINITY_DN1666_c0_g1_i2_orf1;TRINITY_DN29190_c0_g1_i4_orf1;TRINITY_DN21545_c0_g1_i2_orf1;TRINITY_DN479_c6_g1_i2_orf1;TRINITY_DN2848_c0_g1_i1_orf1;TRINITY_DN1534_c0_g1_i3_orf1;TRINITY_DN195_c4_g1_i1_orf1;TRINITY_DN6098_c1_g1_i5_orf1;TRINITY_DN2170_c0_g2_i1_orf1;TRINITY_DN21856_c0_g1_i1_orf1;TRINITY_DN2170_c4_g1_i2_orf1;TRINITY_DN9044_c0_g1_i2_orf1;TRINITY_DN14904_c0_g1_i1_orf1;TRINITY_DN5667_c0_g1_i4_orf1;TRINITY_DN1444_c1_g1_i5_orf1;TRINITY_DN2170_c1_g1_i3_orf1;TRINITY_DN5235_c0_g1_i7_orf1;TRINITY_DN2848_c0_g1_i2_orf1;TRINITY_DN5880_c0_g2_i2_orf1
biological_process	biological process involved in interaction with symbiont	GO:0051702	5	5/3516	TRINITY_DN46409_c0_g1_i1_orf1;TRINITY_DN2848_c0_g1_i2_orf1;TRINITY_DN2848_c0_g1_i1_orf1;TRINITY_DN7493_c0_g1_i1_orf1;TRINITY_DN975_c0_g1_i1_orf1
biological_process	biological process involved in interaction with host	GO:0051701	2	2/3516	TRINITY_DN96557_c0_g1_i1_orf1;TRINITY_DN3159_c0_g1_i4_orf1
biological_process	ribosomal subunit export from nucleus	GO:0000054	1	1/3516	TRINITY_DN92232_c0_g1_i1_orf1
biological_process	establishment of organelle localization	GO:0051656	3	3/3516	TRINITY_DN96557_c0_g1_i1_orf1;TRINITY_DN92232_c0_g1_i1_orf1;TRINITY_DN7493_c0_g1_i1_orf1
biological_process	chromosome localization	GO:0050000	1	1/3516	TRINITY_DN96557_c0_g1_i1_orf1
biological_process	ribosome localization	GO:0033750	1	1/3516	TRINITY_DN92232_c0_g1_i1_orf1
biological_process	lipid storage	GO:0019915	1	1/3516	TRINITY_DN11069_c0_g2_i1_orf1
biological_process	maintenance of protein location	GO:0045185	2	2/3516	TRINITY_DN245_c0_g1_i4_orf1;TRINITY_DN13783_c0_g4_i2_orf1
biological_process	maintenance of location in cell	GO:0051651	2	2/3516	TRINITY_DN245_c0_g1_i4_orf1;TRINITY_DN13783_c0_g4_i2_orf1
biological_process	establishment of protein localization	GO:0045184	69	69/3516	TRINITY_DN29017_c0_g1_i4_orf1;TRINITY_DN111985_c0_g1_i1_orf1;TRINITY_DN44219_c0_g1_i1_orf1;TRINITY_DN4790_c0_g1_i6_orf1;TRINITY_DN38835_c0_g2_i1_orf1;TRINITY_DN146119_c0_g1_i1_orf1;TRINITY_DN15811_c0_g1_i7_orf1;TRINITY_DN46409_c0_g1_i1_orf1;TRINITY_DN3450_c0_g1_i3_orf1;TRINITY_DN6243_c0_g1_i5_orf1;TRINITY_DN55148_c0_g1_i1_orf1;TRINITY_DN3747_c1_g1_i3_orf1;TRINITY_DN383_c0_g1_i1_orf1;TRINITY_DN14677_c0_g2_i3_orf1;TRINITY_DN3664_c0_g1_i8_orf1;TRINITY_DN327_c1_g1_i4_orf1;TRINITY_DN34159_c0_g2_i1_orf1;TRINITY_DN27721_c1_g1_i2_orf1;TRINITY_DN41842_c0_g1_i2_orf1;TRINITY_DN72859_c0_g1_i1_orf1;TRINITY_DN5982_c0_g1_i3_orf1;TRINITY_DN1447_c0_g1_i5_orf1;TRINITY_DN49527_c0_g1_i1_orf1;TRINITY_DN2172_c0_g2_i8_orf1;TRINITY_DN42903_c0_g1_i4_orf1;TRINITY_DN146758_c0_g1_i1_orf1;TRINITY_DN2879_c0_g1_i4_orf1;TRINITY_DN19286_c0_g1_i1_orf1;TRINITY_DN472_c1_g1_i3_orf1;TRINITY_DN3513_c0_g1_i5_orf1;TRINITY_DN2238_c0_g2_i1_orf1;TRINITY_DN3299_c0_g1_i2_orf1;TRINITY_DN5182_c0_g1_i5_orf1;TRINITY_DN61777_c0_g1_i4_orf1;TRINITY_DN740_c0_g1_i1_orf1;TRINITY_DN1437_c0_g1_i6_orf1;TRINITY_DN25681_c0_g1_i5_orf1;TRINITY_DN2286_c2_g1_i1_orf1;TRINITY_DN124300_c0_g1_i2_orf1;TRINITY_DN1384_c0_g1_i5_orf1;TRINITY_DN48460_c0_g1_i1_orf1;TRINITY_DN1901_c0_g1_i6_orf1;TRINITY_DN13783_c0_g4_i2_orf1;TRINITY_DN959_c0_g1_i7_orf1;TRINITY_DN2181_c1_g1_i8_orf1;TRINITY_DN486_c0_g1_i5_orf1;TRINITY_DN13944_c0_g1_i1_orf1;TRINITY_DN106476_c0_g1_i3_orf1;TRINITY_DN65299_c0_g4_i1_orf1;TRINITY_DN3209_c0_g2_i6_orf1;TRINITY_DN96557_c0_g1_i1_orf1;TRINITY_DN245_c0_g1_i4_orf1;TRINITY_DN5383_c0_g1_i4_orf1;TRINITY_DN9741_c0_g1_i3_orf1;TRINITY_DN6680_c0_g1_i1_orf1;TRINITY_DN25896_c0_g1_i6_orf1;TRINITY_DN5630_c4_g1_i2_orf1;TRINITY_DN13139_c0_g1_i1_orf1;TRINITY_DN4859_c0_g1_i5_orf1;TRINITY_DN147427_c0_g1_i1_orf1;TRINITY_DN25210_c0_g1_i1_orf1;TRINITY_DN50875_c0_g1_i3_orf1;TRINITY_DN4304_c0_g1_i3_orf1;TRINITY_DN12320_c0_g1_i1_orf1;TRINITY_DN4394_c0_g1_i4_orf1;TRINITY_DN9931_c0_g1_i1_orf1;TRINITY_DN38835_c0_g3_i1_orf1;TRINITY_DN92232_c0_g1_i1_orf1;TRINITY_DN59042_c1_g1_i1_orf1
biological_process	establishment of localization in cell	GO:0051649	64	64/3516	TRINITY_DN3835_c0_g1_i3_orf1;TRINITY_DN21214_c0_g2_i1_orf1;TRINITY_DN31751_c0_g1_i5_orf1;TRINITY_DN10396_c0_g1_i1_orf1;TRINITY_DN4790_c0_g1_i6_orf1;TRINITY_DN960_c1_g1_i6_orf1;TRINITY_DN4770_c0_g1_i4_orf1;TRINITY_DN5182_c0_g1_i5_orf1;TRINITY_DN15811_c0_g1_i7_orf1;TRINITY_DN3513_c0_g1_i5_orf1;TRINITY_DN6231_c0_g1_i6_orf1;TRINITY_DN4859_c0_g1_i5_orf1;TRINITY_DN46409_c0_g1_i1_orf1;TRINITY_DN3450_c0_g1_i3_orf1;TRINITY_DN7493_c0_g1_i1_orf1;TRINITY_DN3747_c1_g1_i3_orf1;TRINITY_DN14677_c0_g2_i3_orf1;TRINITY_DN15448_c0_g1_i1_orf1;TRINITY_DN3664_c0_g1_i8_orf1;TRINITY_DN327_c1_g1_i4_orf1;TRINITY_DN3835_c0_g1_i4_orf1;TRINITY_DN34159_c0_g2_i1_orf1;TRINITY_DN41842_c0_g1_i2_orf1;TRINITY_DN13139_c0_g1_i1_orf1;TRINITY_DN5982_c0_g1_i3_orf1;TRINITY_DN740_c0_g1_i1_orf1;TRINITY_DN1447_c0_g1_i5_orf1;TRINITY_DN49527_c0_g1_i1_orf1;TRINITY_DN55148_c0_g1_i1_orf1;TRINITY_DN72859_c0_g1_i1_orf1;TRINITY_DN146758_c0_g1_i1_orf1;TRINITY_DN45037_c0_g1_i1_orf1;TRINITY_DN29144_c0_g3_i1_orf1;TRINITY_DN19286_c0_g1_i1_orf1;TRINITY_DN3209_c0_g2_i6_orf1;TRINITY_DN14673_c0_g1_i3_orf1;TRINITY_DN111985_c0_g1_i1_orf1;TRINITY_DN96557_c0_g1_i1_orf1;TRINITY_DN3821_c1_g1_i7_orf1;TRINITY_DN1437_c0_g1_i6_orf1;TRINITY_DN25681_c0_g1_i5_orf1;TRINITY_DN25210_c0_g1_i1_orf1;TRINITY_DN124300_c0_g1_i2_orf1;TRINITY_DN1384_c0_g1_i5_orf1;TRINITY_DN48460_c0_g1_i1_orf1;TRINITY_DN1245_c0_g1_i4_orf1;TRINITY_DN1901_c0_g1_i6_orf1;TRINITY_DN35377_c0_g1_i3_orf1;TRINITY_DN486_c0_g1_i5_orf1;TRINITY_DN27721_c1_g1_i2_orf1;TRINITY_DN106476_c0_g1_i3_orf1;TRINITY_DN10195_c0_g1_i8_orf1;TRINITY_DN578_c0_g1_i5_orf1;TRINITY_DN6535_c0_g1_i3_orf1;TRINITY_DN5383_c0_g1_i4_orf1;TRINITY_DN54586_c1_g1_i1_orf1;TRINITY_DN92232_c0_g1_i1_orf1;TRINITY_DN4814_c0_g1_i6_orf1;TRINITY_DN147427_c0_g1_i1_orf1;TRINITY_DN5558_c0_g1_i4_orf1;TRINITY_DN4394_c0_g1_i4_orf1;TRINITY_DN942_c0_g1_i1_orf1;TRINITY_DN33178_c0_g1_i1_orf1;TRINITY_DN59042_c1_g1_i1_orf1
biological_process	establishment of RNA localization	GO:0051236	7	7/3516	TRINITY_DN6535_c0_g1_i3_orf1;TRINITY_DN31751_c0_g1_i5_orf1;TRINITY_DN6680_c0_g1_i1_orf1;TRINITY_DN146119_c0_g1_i1_orf1;TRINITY_DN1245_c0_g1_i4_orf1;TRINITY_DN59042_c1_g1_i1_orf1;TRINITY_DN2879_c0_g1_i4_orf1

biological_process	transport	GO:0006810	151 151/3516	TRINITY_DN29017_c0_g1_i4_orf1;TRINITY_DN9356_c0_g1_i1_orf1;TRINITY_DN105574_c0_g1_i1_orf1;TRINITY_DN121_c0_g1_i9_orf1;TRINITY_DN1497_c0_g2_i6_orf1;TRINITY_DN25681_c0_g1_i5_orf1;TRINITY_DN21872_c0_g1_i2_orf1;TRINITY_DN3450_c0_g1_i3_orf1;TRINITY_DN3747_c1_g1_i3_orf1;TRINITY_DN383_c0_g1_i1_orf1;TRINITY_DN44256_c0_g1_i1_orf1;TRINITY_DN14677_c0_g2_i3_orf1;TRINITY_DN13923_c0_g2_i1_orf1;TRINITY_DN34159_c0_g2_i1_orf1;TRINITY_DN19521_c0_g1_i1_orf1;TRINITY_DN49527_c0_g1_i1_orf1;TRINITY_DN42903_c0_g1_i4_orf1;TRINITY_DN3835_c0_g1_i3_orf1;TRINITY_DN146758_c0_g1_i1_orf1;TRINITY_DN7590_c0_g1_i4_orf1;TRINITY_DN61711_c0_g1_i1_orf1;TRINITY_DN46625_c0_g1_i1_orf1;TRINITY_DN4810_c0_g1_i3_orf1;TRINITY_DN22944_c0_g3_i1_orf1;TRINITY_DN1437_c0_g1_i6_orf1;TRINITY_DN15811_c0_g1_i7_orf1;TRINITY_DN2267_c0_g1_i1_orf1;TRINITY_DN8766_c0_g1_i1_orf1;TRINITY_DN2181_c1_g1_i8_orf1;TRINITY_DN9239_c0_g1_i1_orf1;TRINITY_DN10195_c0_g1_i8_orf1;TRINITY_DN51766_c0_g1_i2_orf1;TRINITY_DN5383_c0_g1_i4_orf1;TRINITY_DN25896_c0_g1_i6_orf1;TRINITY_DN37654_c0_g1_i5_orf1;TRINITY_DN4814_c0_g1_i6_orf1;TRINITY_DN147427_c0_g1_i1_orf1;TRINITY_DN4394_c0_g1_i4_orf1;TRINITY_DN7633_c0_g1_i1_orf1;TRINITY_DN63561_c1_g1_i2_orf1;TRINITY_DN74020_c0_g1_i2_orf1;TRINITY_DN65681_c0_g1_i1_orf1;TRINITY_DN10396_c0_g1_i1_orf1;TRINITY_DN198_c2_g1_i2_orf1;TRINITY_DN6231_c0_g1_i6_orf1;TRINITY_DN146119_c0_g1_i1_orf1;TRINITY_DN9354_c0_g1_i7_orf1;TRINITY_DN1423_c0_g1_i4_orf1;TRINITY_DN13139_c0_g1_i1_orf1;TRINITY_DN5982_c0_g1_i3_orf1;TRINITY_DN1447_c0_g1_i5_orf1;TRINITY_DN45037_c0_g1_i1_orf1;TRINITY_DN111985_c0_g1_i1_orf1;TRINITY_DN29934_c0_g1_i6_orf1;TRINITY_DN3299_c0_g1_i2_orf1;TRINITY_DN1423_c0_g1_i8_orf1;TRINITY_DN2286_c2_g1_i1_orf1;TRINITY_DN136031_c0_g1_i7_orf1;TRINITY_DN1245_c0_g1_i4_orf1;TRINITY_DN741_c0_g1_i10_orf1;TRINITY_DN13944_c0_g1_i1_orf1;TRINITY_DN3209_c0_g2_i6_orf1;TRINITY_DN578_c0_g1_i5_orf1;TRINITY_DN1407_c0_g1_i5_orf1;TRINITY_DN2172_c0_g2_i8_orf1;TRINITY_DN33178_c0_g1_i1_orf1;TRINITY_DN12320_c0_g1_i1_orf1;TRINITY_DN9931_c0_g1_i1_orf1;TRINITY_DN960_c1_g1_i6_orf1;TRINITY_DN31751_c0_g1_i5_orf1;TRINITY_DN44219_c0_g1_i1_orf1;TRINITY_DN81488_c0_g1_i1_orf1;TRINITY_DN38835_c0_g2_i1_orf1;TRINITY_DN3821_c1_g1_i7_orf1;TRINITY_DN21214_c0_g2_i1_orf1;TRINITY_DN33452_c0_g1_i3_orf1;TRINITY_DN46409_c0_g1_i1_orf1;TRINITY_DN6243_c0_g1_i5_orf1;TRINITY_DN5182_c0_g1_i5_orf1;TRINITY_DN3664_c0_g1_i8_orf1;TRINITY_DN47389_c0_g1_i2_orf1;TRINITY_DN4016_c0_g1_i1_orf1;TRINITY_DN54586_c1_g1_i1_orf1;TRINITY_DN3219_c0_g1_i6_orf1;TRINITY_DN22430_c0_g3_i1_orf1;TRINITY_DN29144_c0_g3_i1_orf1;TRINITY_DN9239_c0_g2_i2_orf1;TRINITY_DN472_c1_g1_i3_orf1;TRINITY_DN5312_c4_g1_i2_orf1;TRINITY_DN2238_c0_g2_i1_orf1;TRINITY_DN6247_c0_g1_i2_orf1;TRINITY_DN124300_c0_g1_i2_orf1;TRINITY_DN1384_c0_g1_i5_orf1;TRINITY_DN48460_c0_g1_i1_orf1;TRINITY_DN96739_c0_g1_i1_orf1;TRINITY_DN13901_c0_g1_i4_orf1;TRINITY_DN86956_c0_g5_i1_orf1;TRINITY_DN486_c0_g1_i5_orf1;TRINITY_DN106476_c0_g1_i3_orf1;TRINITY_DN65299_c0_g4_i1_orf1;TRINITY_DN96557_c0_g1_i1_orf1;TRINITY_DN6535_c0_g1_i3_orf1;TRINITY_DN5630_c4_g1_i2_orf1;TRINITY_DN45446_c0_g1_i2_orf1;TRINITY_DN110402_c0_g2_i1_orf1;TRINITY_DN38835_c0_g3_i1_orf1;TRINITY_DN92232_c0_g1_i1_orf1;TRINITY_DN59042_c1_g1_i1_orf1;TRINITY_DN245_c0_g1_i4_orf1;TRINITY_DN28759_c0_g1_i1_orf1;TRINITY_DN3835_c0_g1_i4_orf1;TRINITY_DN4790_c0_g1_i6_orf1;TRINITY_DN61777_c0_g1_i4_orf1;TRINITY_DN4770_c0_g1_i4_orf1;TRINITY_DN33272_c0_g1_i5_orf1;TRINITY_DN15448_c0_g1_i1_orf1;TRINITY_DN55148_c0_g1_i1_orf1;TRINITY_DN327_c1_g1_i4_orf1;TRINITY_DN12885_c0_g1_i1_orf1;TRINITY_DN7407_c0_g1_i9_orf1;TRINITY_DN50875_c0_g1_i3_orf1;TRI
biological_process	RNA localization	GO:0006403	1 1/3516	TRINITY_DN21123_c0_g1_i1_orf1
biological_process	non-lytic viral release	GO:0046753	1 1/3516	TRINITY_DN96557_c0_g1_i1_orf1
biological_process	viral budding via host ESCRT complex	GO:0039702	1 1/3516	TRINITY_DN96557_c0_g1_i1_orf1
biological_process	viral budding from plasma membrane	GO:0046761	1 1/3516	TRINITY_DN96557_c0_g1_i1_orf1
biological_process	detoxification of mercury ion	GO:0050787	1 1/3516	TRINITY_DN111985_c0_g1_i1_orf1
biological_process	detoxification of copper ion	GO:0010273	1 1/3516	TRINITY_DN111985_c0_g1_i1_orf1
biological_process	response to external biotic stimulus	GO:0043207	35 35/3516	TRINITY_DN1444_c1_g1_i5_orf1;TRINITY_DN827_c1_g1_i1_orf1;TRINITY_DN8685_c0_g1_i5_orf1;TRINITY_DN16840_c1_g1_i1_orf1;TRINITY_DN3159_c0_g1_i4_orf1;TRINITY_DN2407_c0_g1_i2_orf1;TRINITY_DN15706_c0_g2_i5_orf1;TRINITY_DN1666_c0_g1_i2_orf1;TRINITY_DN29190_c0_g1_i4_orf1;TRINITY_DN21545_c0_g1_i2_orf1;TRINITY_DN479_c6_g1_i2_orf1;TRINITY_DN3166_c1_g1_i6_orf1;TRINITY_DN2407_c0_g1_i6_orf1;TRINITY_DN2848_c0_g1_i1_orf1;TRINITY_DN20009_c0_g1_i1_orf1;TRINITY_DN1534_c0_g1_i3_orf1;TRINITY_DN195_c4_g1_i1_orf1;TRINITY_DN6098_c1_g1_i5_orf1;TRINITY_DN2170_c0_g2_i1_orf1;TRINITY_DN21856_c0_g1_i1_orf1;TRINITY_DN86772_c0_g1_i3_orfp1;TRINITY_DN2170_c4_g1_i2_orf1;TRINITY_DN9044_c0_g1_i2_orf1;TRINITY_DN4748_c0_g1_i5_orf1;TRINITY_DN12534_c0_g1_i4_orf1;TRINITY_DN14904_c0_g1_i1_orf1;TRINITY_DN5667_c0_g1_i4_orf1;TRINITY_DN4802_c0_g1_i4_orf1;TRINITY_DN195_c8_g1_i1_orf1;TRINITY_DN7493_c0_g1_i1_orf1;TRINITY_DN59429_c0_g1_i6_orf1;TRINITY_DN2170_c1_g1_i3_orf1;TRINITY_DN5235_c0_g1_i7_orf1;TRINITY_DN2848_c0_g1_i2_orf1;TRINITY_DN5880_c0_g2_i2_orf1
biological_process	detection of biotic stimulus	GO:0009595	2 2/3516	TRINITY_DN8685_c0_g1_i5_orf1;TRINITY_DN5880_c0_g2_i2_orf1
biological_process	response to extracellular stimulus	GO:0009991	5 5/3516	TRINITY_DN140212_c0_g1_i1_orf1;TRINITY_DN51938_c0_g3_i1_orf1;TRINITY_DN1091_c0_g3_i1_orf1;TRINITY_DN143603_c0_g1_i1_orf1;TRINITY_DN2054_c0_g1_i1_orf1
biological_process	cellular response to external stimulus	GO:0071496	4 4/3516	TRINITY_DN140212_c0_g1_i1_orf1;TRINITY_DN51938_c0_g3_i1_orf1;TRINITY_DN1091_c0_g3_i1_orf1;TRINITY_DN2054_c0_g1_i1_orf1
biological_process	cellular response to endogenous stimulus	GO:0071495	6 6/3516	TRINITY_DN51938_c0_g3_i1_orf1;TRINITY_DN4016_c0_g1_i1_orf1;TRINITY_DN975_c0_g1_i1_orf1;TRINITY_DN130075_c1_g2_i1_orf1;TRINITY_DN20009_c0_g1_i1_orf1;TRINITY_DN15448_c0_g1_i1_orf1
biological_process	response to epidermal growth factor	GO:0070849	1 1/3516	TRINITY_DN975_c0_g1_i1_orf1
biological_process	response to transforming growth factor beta	GO:0071559	1 1/3516	TRINITY_DN51938_c0_g3_i1_orf1
biological_process	response to hormone	GO:0009725	1 1/3516	TRINITY_DN51938_c0_g3_i1_orf1
biological_process	response to hypoxia	GO:0001666	2 2/3516	TRINITY_DN51938_c0_g3_i1_orf1;TRINITY_DN140538_c0_g2_i1_orf1
biological_process	response to wounding	GO:0009611	1 1/3516	TRINITY_DN21545_c0_g1_i2_orf1
biological_process	response to topologically incorrect protein	GO:0035966	2 2/3516	TRINITY_DN21214_c0_g2_i1_orf1;TRINITY_DN46409_c0_g1_i1_orf1
biological_process	response to ischemia	GO:0002931	1 1/3516	TRINITY_DN51938_c0_g3_i1_orf1
biological_process	response to cold	GO:0009409	1 1/3516	TRINITY_DN46409_c0_g1_i1_orf1
biological_process	response to heat	GO:0009408	3 3/3516	TRINITY_DN12964_c0_g1_i1_orf1;TRINITY_DN15959_c0_g1_i1_orf1;TRINITY_DN5648_c0_g1_i5_orf1

biological_process	defense response	GO:0006952	34 34/3516	TRINITY_DN1444_c1_g1_i5_orf1;TRINITY_DN827_c1_g1_i1_orf1;TRINITY_DN8685_c0_g1_i5_orf1;TRINITY_DN16840_c1_g1_i1_orf1;TRINITY_DN2407_c0_g1_i2_orf1;TRINITY_DN15706_c0_g2_i5_orf1;TRINITY_DN1666_c0_g1_i2_orf1;TRINITY_DN29190_c0_g1_i4_orf1;TRINITY_DN12534_c0_g1_i4_orf1;TRINITY_DN479_c6_g1_i2_orf1;TRINITY_DN3166_c1_g1_i6_orf1;TRINITY_DN2407_c0_g1_i6_orf1;TRINITY_DN31163_c1_g1_i4_orf1;TRINITY_DN2848_c0_g1_i1_orf1;TRINITY_DN86772_c0_g1_i3_orfp1;TRINITY_DN1534_c0_g1_i3_orf1;TRINITY_DN195_c4_g1_i1_orf1;TRINITY_DN6098_c1_g1_i5_orf1;TRINITY_DN2170_c0_g2_i1_orf1;TRINITY_DN21856_c0_g1_i1_orf1;TRINITY_DN2170_c4_g1_i2_orf1;TRINITY_DN9044_c0_g1_i2_orf1;TRINITY_DN2338_c0_g1_i5_orf1;TRINITY_DN4748_c0_g1_i5_orf1;TRINITY_DN21545_c0_g1_i2_orf1;TRINITY_DN14904_c0_g1_i1_orf1;TRINITY_DN5667_c0_g1_i4_orf1;TRINITY_DN4802_c0_g1_i4_orf1;TRINITY_DN195_c8_g1_i1_orf1;TRINITY_DN59429_c0_g1_i6_orf1;TRINITY_DN2170_c1_g1_i3_orf1;TRINITY_DN5235_c0_g1_i7_orf1;TRINITY_DN2848_c0_g1_i2_orf1;TRINITY_DN5880_c0_g2_i2_orf1
biological_process	response to hyperoxia	GO:0055093	1 1/3516	TRINITY_DN51938_c0_g3_i1_orf1
biological_process	response to oxidative stress	GO:0006979	14 14/3516	TRINITY_DN111985_c0_g1_i1_orf1;TRINITY_DN6580_c0_g1_i4_orf1;TRINITY_DN12514_c0_g2_i1_orf1;TRINITY_DN1622_c0_g1_i6_orf1;TRINITY_DN51252_c0_g2_i1_orf1;TRINITY_DN2207_c0_g1_i6_orf1;TRINITY_DN80660_c0_g1_i1_orf1;TRINITY_DN5933_c0_g1_i1_orf1;TRINITY_DN285_c0_g1_i4_orf1;TRINITY_DN10429_c0_g1_i2_orf1;TRINITY_DN54387_c0_g1_i1_orf1;TRINITY_DN3321_c0_g1_i3_orf1;TRINITY_DN21420_c0_g1_i2_orf1;TRINITY_DN2652_c0_g2_i1_orf1
biological_process	response to antibiotic	GO:0046677	1 1/3516	TRINITY_DN130075_c1_g2_i1_orf1
biological_process	response to oxygen-containing compound	GO:1901700	6 6/3516	TRINITY_DN111985_c0_g1_i1_orf1;TRINITY_DN51938_c0_g3_i1_orf1;TRINITY_DN4016_c0_g1_i1_orf1;TRINITY_DN130075_c1_g2_i1_orf1;TRINITY_DN20009_c0_g1_i1_orf1;TRINITY_DN15448_c0_g1_i1_orf1
biological_process	response to nitrogen compound	GO:1901698	8 8/3516	TRINITY_DN9062_c0_g2_i3_orf1;TRINITY_DN4016_c0_g1_i1_orf1;TRINITY_DN130075_c1_g2_i1_orf1;TRINITY_DN48536_c0_g1_i3_orf1;TRINITY_DN2848_c0_g1_i1_orf1;TRINITY_DN20009_c0_g1_i1_orf1;TRINITY_DN17726_c0_g1_i1_orf1;TRINITY_DN15448_c0_g1_i1_orf1
biological_process	response to nutrient	GO:0007584	1 1/3516	TRINITY_DN51938_c0_g3_i1_orf1
biological_process	response to acid chemical	GO:0001101	1 1/3516	TRINITY_DN15448_c0_g1_i1_orf1
biological_process	response to inorganic substance	GO:0010035	3 3/3516	TRINITY_DN111985_c0_g1_i1_orf1;TRINITY_DN2848_c0_g1_i1_orf1;TRINITY_DN4016_c0_g1_i1_orf1
biological_process	response to organic substance	GO:0010033	19 19/3516	TRINITY_DN111985_c0_g1_i1_orf1;TRINITY_DN21214_c0_g2_i1_orf1;TRINITY_DN51938_c0_g3_i1_orf1;TRINITY_DN4016_c0_g1_i1_orf1;TRINITY_DN315_c0_g1_i1_orf1;TRINITY_DN8685_c0_g1_i5_orf1;TRINITY_DN2848_c0_g1_i2_orf1;TRINITY_DN9062_c0_g2_i3_orf1;TRINITY_DN18218_c0_g1_i7_orf1;TRINITY_DN130075_c1_g2_i1_orf1;TRINITY_DN975_c0_g1_i1_orf1;TRINITY_DN46409_c0_g1_i1_orf1;TRINITY_DN48536_c0_g1_i3_orf1;TRINITY_DN2848_c0_g1_i1_orf1;TRINITY_DN17726_c0_g1_i1_orf1;TRINITY_DN20009_c0_g1_i1_orf1;TRINITY_DN2227_c0_g1_i5_orf1;TRINITY_DN15448_c0_g1_i1_orf1;TRINITY_DN5880_c0_g2_i2_orf1
biological_process	response to temperature stimulus	GO:0009266	4 4/3516	TRINITY_DN46409_c0_g1_i1_orf1;TRINITY_DN12964_c0_g1_i1_orf1;TRINITY_DN15959_c0_g1_i1_orf1;TRINITY_DN5648_c0_g1_i5_orf1
biological_process	response to oxygen levels	GO:0070482	2 2/3516	TRINITY_DN51938_c0_g3_i1_orf1;TRINITY_DN140538_c0_g2_i1_orf1
biological_process	detection of chemical stimulus	GO:0009593	2 2/3516	TRINITY_DN8685_c0_g1_i5_orf1;TRINITY_DN5880_c0_g2_i2_orf1
cellular_component	nucleosome	GO:0000786	5 5/3516	TRINITY_DN96801_c0_g1_i1_orf1;TRINITY_DN20442_c0_g2_i1_orf1;TRINITY_DN24917_c0_g2_i1_orf1;TRINITY_DN5458_c1_g1_i9_orf1;TRINITY_DN3325_c0_g1_i1_orf1
cellular_component	cohesin complex	GO:0008278	1 1/3516	TRINITY_DN2638_c0_g1_i7_orf1
cellular_component	Mre11 complex	GO:0030870	3 3/3516	TRINITY_DN10287_c0_g1_i1_orf1;TRINITY_DN45271_c0_g1_i1_orf1;TRINITY_DN123184_c0_g1_i1_orf1
cellular_component	mRNA cleavage factor complex	GO:0005849	2 2/3516	TRINITY_DN2718_c0_g1_i6_orf1;TRINITY_DN2859_c0_g1_i7_orf1
cellular_component	SWI/SNF superfamily-type complex	GO:0070603	3 3/3516	TRINITY_DN452_c1_g1_i3_orf1;TRINITY_DN11327_c0_g1_i1_orf1;TRINITY_DN3649_c0_g1_i6_orf1
cellular_component	U2AF complex	GO:0089701	1 1/3516	TRINITY_DN51968_c0_g1_i1_orf1
cellular_component	histone deacetylase complex	GO:0000118	1 1/3516	TRINITY_DN10636_c0_g1_i1_orf1
cellular_component	transcription elongation factor complex	GO:0008023	2 2/3516	TRINITY_DN44792_c0_g1_i1_orf1;TRINITY_DN5686_c0_g1_i4_orf1
cellular_component	PcG protein complex	GO:0031519	1 1/3516	TRINITY_DN1639_c0_g2_i2_orf1
cellular_component	spliceosomal complex	GO:0005681	25 25/3516	TRINITY_DN22941_c0_g1_i1_orf1;TRINITY_DN57202_c0_g1_i1_orf1;TRINITY_DN29402_c0_g1_i1_orf1;TRINITY_DN33346_c0_g1_i1_orf1;TRINITY_DN1554_c0_g1_i9_orf1;TRINITY_DN30097_c0_g1_i2_orf1;TRINITY_DN47575_c0_g1_i1_orf1;TRINITY_DN47666_c0_g1_i4_orf1;TRINITY_DN43412_c0_g1_i2_orf1;TRINITY_DN5767_c0_g1_i4_orf1;TRINITY_DN142652_c0_g1_i1_orf1;TRINITY_DN20215_c0_g2_i1_orf1;TRINITY_DN18863_c0_g1_i3_orf1;TRINITY_DN4135_c0_g1_i5_orf1;TRINITY_DN131662_c0_g1_i4_orf1;TRINITY_DN13055_c0_g1_i5_orf1;TRINITY_DN51568_c0_g1_i1_orf1;TRINITY_DN698_c0_g1_i5_orf1;TRINITY_DN145647_c0_g1_i1_orf1;TRINITY_DN44877_c0_g1_i2_orf1;TRINITY_DN14487_c0_g1_i4_orf1;TRINITY_DN8717_c0_g1_i5_orf1;TRINITY_DN31663_c0_g1_i2_orf1;TRINITY_DN107035_c0_g1_i1_orf1;TRINITY_DN23502_c0_g1_i1_orf1
cellular_component	BRISC complex	GO:0070552	1 1/3516	TRINITY_DN17655_c0_g1_i1_orf1
cellular_component	nuclear DNA-directed RNA polymerase complex	GO:0055029	5 5/3516	TRINITY_DN10658_c0_g1_i1_orf1;TRINITY_DN12527_c0_g1_i4_orf1;TRINITY_DN4707_c0_g1_i1_orf1;TRINITY_DN31520_c1_g1_i1_orf1;TRINITY_DN9207_c0_g1_i1_orf1
cellular_component	histone acetyltransferase complex	GO:0000123	2 2/3516	TRINITY_DN452_c1_g1_i3_orf1;TRINITY_DN10636_c0_g1_i1_orf1
cellular_component	exon-exon junction complex	GO:0035145	1 1/3516	TRINITY_DN5507_c0_g1_i1_orf1
cellular_component	small nuclear ribonucleoprotein complex	GO:0030532	10 10/3516	TRINITY_DN38540_c0_g1_i1_orf1;TRINITY_DN33346_c0_g1_i1_orf1;TRINITY_DN298_c0_g1_i4_orf1;TRINITY_DN1616_c0_g1_i3_orf1;TRINITY_DN31663_c0_g1_i2_orf1;TRINITY_DN47666_c0_g1_i4_orf1;TRINITY_DN43412_c0_g1_i2_orf1;TRINITY_DN57202_c0_g1_i1_orf1;TRINITY_DN5834_c0_g1_i2_orf1;TRINITY_DN4135_c0_g1_i5_orf1
cellular_component	nuclear ubiquitin ligase complex	GO:0000152	1 1/3516	TRINITY_DN146493_c0_g1_i1_orf1
cellular_component	BRCA1-A complex	GO:0070531	1 1/3516	TRINITY_DN17655_c0_g1_i1_orf1
cellular_component	RNA polymerase II transcription regulator complex	GO:0090575	2 2/3516	TRINITY_DN34509_c0_g1_i1_orf1;TRINITY_DN346_c0_g1_i7_orf1
cellular_component	carboxy-terminal domain protein kinase complex	GO:0032806	1 1/3516	TRINITY_DN346_c0_g1_i7_orf1
cellular_component	THO complex	GO:0000347	1 1/3516	TRINITY_DN133760_c0_g1_i1_orf1

cellular_component	nuclear pore outer ring	GO:0031080	1	1/3516	TRINITY_DN6680_c0_g1_i1_orf1
cellular_component	nuclear pore	GO:0005643	6	6/3516	TRINITY_DN96557_c0_g1_i1_orf1;TRINITY_DN1437_c0_g1_i6_orf1;TRINITY_DN146119_c0_g1_i1_orf1;TRINITY_DN1268_c0_g1_i1_orf1;TRINITY_DN59042_c1_g1_i1_orf1;TRINITY_DN2879_c0_g1_i4_orf1
cellular_component	ESCRT III complex	GO:0000815	1	1/3516	TRINITY_DN96557_c0_g1_i1_orf1
cellular_component	ESCRT I complex	GO:0000813	1	1/3516	TRINITY_DN2181_c1_g1_i8_orf1
cellular_component	mitochondrial intermembrane space protein transporter complex	GO:0042719	1	1/3516	TRINITY_DN15811_c0_g1_i7_orf1
cellular_component	transmembrane transporter complex	GO:1902495	14	14/3516	TRINITY_DN5417_c0_g1_i1_orf1;TRINITY_DN20346_c0_g1_i1_orf1;TRINITY_DN19521_c0_g1_i1_orf1;TRINITY_DN9558_c0_g1_i2_orf1;TRINITY_DN29934_c0_g1_i6_orf1;TRINITY_DN20558_c0_g1_i2_orf1;TRINITY_DN108051_c0_g1_i2_orf1;TRINITY_DN7626_c0_g1_i1_orf1;TRINITY_DN391_c1_g2_i1_orf1;TRINITY_DN45227_c0_g1_i3_orf1;TRINITY_DN16408_c0_g1_i1_orf1;TRINITY_DN44256_c0_g1_i1_orf1;TRINITY_DN26010_c0_g1_i2_orf1;TRINITY_DN679_c0_g1_i2_orf1
cellular_component	dynein complex	GO:0030286	2	2/3516	TRINITY_DN107_c0_g1_i1_orf1;TRINITY_DN26243_c0_g1_i2_orf1
cellular_component	catalytic step 2 spliceosome	GO:0071013	1	1/3516	TRINITY_DN30097_c0_g1_i2_orf1
cellular_component	fatty acid beta-oxidation multienzyme complex	GO:0036125	1	1/3516	TRINITY_DN357_c0_g1_i8_orf1
cellular_component	proteasome core complex	GO:0005839	2	2/3516	TRINITY_DN9717_c0_g2_i1_orf1;TRINITY_DN443_c0_g1_i2_orf1
cellular_component	cytochrome complex	GO:0070069	8	8/3516	TRINITY_DN3749_c0_g1_i1_orf1;TRINITY_DN136028_c0_g2_i1_orf1;TRINITY_DN76036_c0_g1_i1_orf1;TRINITY_DN14073_c0_g1_i1_orf1;TRINITY_DN5111_c0_g1_i2_orf1;TRINITY_DN26010_c0_g1_i2_orf1;TRINITY_DN95665_c0_g1_i1_orf1;TRINITY_DN679_c0_g1_i2_orf1
cellular_component	phosphatase complex	GO:1903293	1	1/3516	TRINITY_DN2257_c0_g1_i4_orf1
cellular_component	mitochondrial processing peptidase complex	GO:0017087	1	1/3516	TRINITY_DN141462_c0_g1_i1_orf1
cellular_component	oxidoreductase complex	GO:1990204	15	15/3516	TRINITY_DN5417_c0_g1_i1_orf1;TRINITY_DN20346_c0_g1_i1_orf1;TRINITY_DN6199_c2_g1_i3_orf1;TRINITY_DN9558_c0_g1_i2_orf1;TRINITY_DN82008_c0_g1_i1_orf1;TRINITY_DN3312_c0_g1_i10_orf1;TRINITY_DN19727_c0_g1_i7_orf1;TRINITY_DN108051_c0_g1_i2_orf1;TRINITY_DN7626_c0_g1_i1_orf1;TRINITY_DN391_c1_g2_i1_orf1;TRINITY_DN45227_c0_g1_i3_orf1;TRINITY_DN3959_c1_g2_i1_orf1;TRINITY_DN2594_c0_g2_i4_orf1;TRINITY_DN26010_c0_g1_i2_orf1;TRINITY_DN679_c0_g1_i2_orf1
cellular_component	tricarboxylic acid cycle enzyme complex	GO:0045239	3	3/3516	TRINITY_DN19727_c0_g1_i7_orf1;TRINITY_DN3959_c1_g2_i1_orf1;TRINITY_DN2594_c0_g2_i4_orf1
cellular_component	endonuclease complex	GO:1905348	1	1/3516	TRINITY_DN9094_c0_g1_i1_orf1
cellular_component	transferase complex	GO:1990234	31	31/3516	TRINITY_DN461_c0_g1_i5_orf1;TRINITY_DN13174_c0_g1_i4_orf1;TRINITY_DN9094_c0_g1_i1_orf1;TRINITY_DN1757_c0_g1_i4_orf1;TRINITY_DN24024_c0_g1_i1_orf1;TRINITY_DN5182_c0_g1_i5_orf1;TRINITY_DN146493_c0_g1_i1_orf1;TRINITY_DN15040_c0_g4_i1_orf1;TRINITY_DN15836_c0_g1_i1_orf1;TRINITY_DN70485_c0_g1_i2_orf1;TRINITY_DN9207_c0_g1_i1_orf1;TRINITY_DN452_c1_g1_i3_orf1;TRINITY_DN17726_c0_g1_i1_orf1;TRINITY_DN18538_c0_g3_i1_orf1;TRINITY_DN12527_c0_g1_i4_orf1;TRINITY_DN10636_c0_g1_i1_orf1;TRINITY_DN19727_c0_g1_i7_orf1;TRINITY_DN23714_c0_g1_i4_orf1;TRINITY_DN10058_c0_g1_i1_orf1;TRINITY_DN4707_c0_g1_i1_orf1;TRINITY_DN110534_c0_g1_i3_orf1;TRINITY_DN9062_c0_g2_i3_orf1;TRINITY_DN74037_c0_g5_i1_orf1;TRINITY_DN31520_c1_g1_i1_orf1;TRINITY_DN346_c0_g1_i7_orf1;TRINITY_DN147475_c0_g1_i1_orf1;TRINITY_DN879_c0_g1_i2_orf1;TRINITY_DN2299_c0_g1_i3_orf1;TRINITY_DN2064_c1_g1_i1_orf1;TRINITY_DN89613_c0_g1_i13_orf1;TRINITY_DN10658_c0_g1_i1_orf1
cellular_component	peptidase complex	GO:1905368	7	7/3516	TRINITY_DN5775_c0_g1_i1_orf1;TRINITY_DN19260_c0_g1_i5_orf1;TRINITY_DN34479_c0_g1_i2_orf1;TRINITY_DN145227_c0_g1_i1_orf1;TRINITY_DN49047_c0_g1_i2_orf1;TRINITY_DN2591_c0_g1_i4_orf1;TRINITY_DN32359_c0_g2_i1_orf1
cellular_component	aminoacyl-tRNA synthetase multienzyme complex	GO:0017101	6	6/3516	TRINITY_DN2953_c1_g1_i11_orf1;TRINITY_DN5857_c0_g1_i13_orf1;TRINITY_DN22572_c0_g1_i1_orf1;TRINITY_DN2953_c1_g1_i10_orf1;TRINITY_DN107288_c0_g1_i2_orf1;TRINITY_DN2953_c1_g1_i2_orf1
cellular_component	elongator holoenzyme complex	GO:0033588	3	3/3516	TRINITY_DN1354_c0_g1_i6_orf1;TRINITY_DN56270_c0_g1_i1_orf1;TRINITY_DN38650_c0_g1_i2_orf1
cellular_component	ATPase complex	GO:1904949	3	3/3516	TRINITY_DN452_c1_g1_i3_orf1;TRINITY_DN11327_c0_g1_i1_orf1;TRINITY_DN3649_c0_g1_i6_orf1
cellular_component	exoribonuclease complex	GO:1905354	1	1/3516	TRINITY_DN9094_c0_g1_i1_orf1
cellular_component	dystrophin-associated glycoprotein complex	GO:0016010	1	1/3516	TRINITY_DN7128_c0_g1_i7_orf1
cellular_component	eukaryotic translation initiation factor 3 complex, eIF3m	GO:0071541	1	1/3516	TRINITY_DN53684_c0_g1_i1_orf1
cellular_component	HOPS complex	GO:0030897	1	1/3516	TRINITY_DN3513_c0_g1_i5_orf1
cellular_component	Ragulator complex	GO:0071986	1	1/3516	TRINITY_DN15448_c0_g1_i1_orf1
cellular_component	lipopolysaccharide receptor complex	GO:0046696	1	1/3516	TRINITY_DN46409_c0_g1_i1_orf1
cellular_component	oligosaccharyltransferase complex	GO:0008250	2	2/3516	TRINITY_DN24024_c0_g1_i1_orf1;TRINITY_DN10058_c0_g1_i1_orf1
cellular_component	plasma membrane protein complex	GO:0098797	5	5/3516	TRINITY_DN16408_c0_g1_i1_orf1;TRINITY_DN19521_c0_g1_i1_orf1;TRINITY_DN49527_c0_g1_i1_orf1;TRINITY_DN29934_c0_g1_i6_orf1;TRINITY_DN7128_c0_g1_i7_orf1
cellular_component	outer mitochondrial membrane protein complex	GO:0098799	3	3/3516	TRINITY_DN9741_c0_g1_i3_orf1;TRINITY_DN27721_c1_g1_i2_orf1;TRINITY_DN3299_c0_g1_i2_orf1
cellular_component	Tapasin-ERp57 complex	GO:0061779	1	1/3516	TRINITY_DN51938_c0_g3_i1_orf1
cellular_component	retromer, cargo-selective complex	GO:0030906	1	1/3516	TRINITY_DN5383_c0_g1_i4_orf1
cellular_component	proton-transporting two-sector ATPase complex, catalytic domain	GO:0033178	10	10/3516	TRINITY_DN45000_c0_g1_i5_orf1;TRINITY_DN1366_c0_g1_i5_orf1;TRINITY_DN4434_c0_g1_i7_orf1;TRINITY_DN2300_c0_g1_i1_orf1;TRINITY_DN1044_c0_g1_i2_orf1;TRINITY_DN96080_c0_g2_i1_orf1;TRINITY_DN9715_c0_g1_i1_orf1;TRINITY_DN17351_c0_g1_i3_orf1;TRINITY_DN80560_c0_g1_i1_orf1;TRINITY_DN700_c0_g1_i3_orf1
cellular_component	EMC complex	GO:0072546	4	4/3516	TRINITY_DN3838_c0_g1_i8_orf1;TRINITY_DN17828_c0_g1_i1_orf1;TRINITY_DN16886_c0_g1_i4_orf1;TRINITY_DN9002_c0_g1_i1_orf1
cellular_component	MHC class I peptide loading complex	GO:0042824	1	1/3516	TRINITY_DN51938_c0_g3_i1_orf1
cellular_component	TAP complex	GO:0042825	1	1/3516	TRINITY_DN51938_c0_g3_i1_orf1

cellular_component	NADH dehydrogenase complex	GO:0030964	7	7/3516	TRINITY_DN5417_c0_g1_i1_orf1;TRINITY_DN20346_c0_g1_i1_orf1;TRINITY_DN9558_c0_g1_i2_orf1;TRINITY_DN108051_c0_g1_i2_orf1;TRINITY_DN7626_c0_g1_i1_orf1;TRINITY_DN391_c1_g2_i1_orf1;TRINITY_DN45227_c0_g1_i3_orf1
cellular_component	respiratory chain complex	GO:0098803	15	15/3516	TRINITY_DN3749_c0_g1_i1_orf1;TRINITY_DN20346_c0_g1_i1_orf1;TRINITY_DN9558_c0_g1_i2_orf1;TRINITY_DN136028_c0_g2_i1_orf1;TRINITY_DN76036_c0_g1_i1_orf1;TRINITY_DN108051_c0_g1_i2_orf1;TRINITY_DN5417_c0_g1_i1_orf1;TRINITY_DN7626_c0_g1_i1_orf1;TRINITY_DN391_c1_g2_i1_orf1;TRINITY_DN45227_c0_g1_i3_orf1;TRINITY_DN14073_c0_g1_i1_orf1;TRINITY_DN5111_c0_g1_i2_orf1;TRINITY_DN26010_c0_g1_i2_orf1;TRINITY_DN95665_c0_g1_i1_orf1;TRINITY_DN679_c0_g1_i2_orf1
cellular_component	inner mitochondrial membrane protein complex	GO:0098800	24	24/3516	TRINITY_DN5417_c0_g1_i1_orf1;TRINITY_DN9558_c0_g1_i2_orf1;TRINITY_DN86090_c0_g1_i1_orf1;TRINITY_DN108051_c0_g1_i2_orf1;TRINITY_DN98538_c0_g1_i1_orf1;TRINITY_DN26649_c0_g1_i2_orf1;TRINITY_DN44256_c0_g1_i1_orf1;TRINITY_DN45227_c0_g1_i3_orf1;TRINITY_DN146758_c0_g1_i1_orf1;TRINITY_DN3454_c0_g1_i1_orf1;TRINITY_DN15222_c0_g1_i4_orf1;TRINITY_DN24325_c0_g1_i12_orf1;TRINITY_DN76036_c0_g1_i1_orf1;TRINITY_DN28152_c0_g1_i1_orf1;TRINITY_DN44219_c0_g1_i1_orf1;TRINITY_DN26010_c0_g1_i2_orf1;TRINITY_DN20346_c0_g1_i1_orf1;TRINITY_DN136028_c0_g2_i1_orf1;TRINITY_DN391_c1_g2_i1_orf1;TRINITY_DN107261_c0_g1_i1_orf1;TRINITY_DN14073_c0_g1_i1_orf1;TRINITY_DN5111_c0_g1_i2_orf1;TRINITY_DN95665_c0_g1_i1_orf1;TRINITY_DN679_c0_g1_i2_orf1
cellular_component	membrane coat	GO:0030117	8	8/3516	TRINITY_DN96557_c0_g1_i1_orf1;TRINITY_DN1447_c0_g1_i5_orf1;TRINITY_DN5982_c0_g1_i3_orf1;TRINITY_DN2286_c2_g1_i1_orf1;TRINITY_DN124300_c0_g1_i2_orf1;TRINITY_DN146119_c0_g1_i1_orf1;TRINITY_DN14677_c0_g2_i3_orf1;TRINITY_DN3209_c0_g2_i6_orf1
cellular_component	AP-type membrane coat adaptor complex	GO:0030119	3	3/3516	TRINITY_DN72859_c0_g1_i1_orf1;TRINITY_DN486_c0_g1_i5_orf1;TRINITY_DN13139_c0_g1_i1_orf1
cellular_component	proton-transporting two-sector ATPase complex	GO:0016469	1	1/3516	TRINITY_DN22430_c0_g3_i1_orf1
cellular_component	proton-transporting two-sector ATPase complex, proton-transporting domain	GO:0033177	14	14/3516	TRINITY_DN98538_c0_g1_i1_orf1;TRINITY_DN15222_c0_g1_i4_orf1;TRINITY_DN79210_c0_g1_i1_orf1;TRINITY_DN6221_c0_g1_i5_orf1;TRINITY_DN86090_c0_g1_i1_orf1;TRINITY_DN21722_c0_g1_i3_orf1;TRINITY_DN47605_c0_g2_i1_orf1;TRINITY_DN107261_c0_g1_i1_orf1;TRINITY_DN26649_c0_g1_i2_orf1;TRINITY_DN22430_c0_g3_i1_orf1;TRINITY_DN19115_c0_g1_i1_orf1;TRINITY_DN10458_c0_g1_i1_orf1;TRINITY_DN10637_c0_g1_i4_orf1;TRINITY_DN29038_c0_g2_i1_orf1
cellular_component	mitochondrial tricarboxylic acid cycle enzyme	GO:0030062	2	2/3516	TRINITY_DN3959_c1_g2_i1_orf1;TRINITY_DN2594_c0_g2_i4_orf1
cellular_component	mitochondrial large ribosomal subunit	GO:0005762	3	3/3516	TRINITY_DN97680_c0_g1_i1_orf1;TRINITY_DN1313_c0_g1_i2_orf1;TRINITY_DN43611_c0_g1_i1_orf1
cellular_component	mitochondrial small ribosomal subunit	GO:0005763	1	1/3516	TRINITY_DN10007_c0_g1_i1_orf1
cellular_component	mitochondrial fatty acid beta-oxidation multienzyme complex	GO:0016507	1	1/3516	TRINITY_DN357_c0_g1_i8_orf1
cellular_component	Golgi transport complex	GO:0017119	1	1/3516	TRINITY_DN50875_c0_g1_i3_orf1
cellular_component	exocyst	GO:0000145	1	1/3516	TRINITY_DN61777_c0_g1_i4_orf1
cellular_component	CORVET complex	GO:0033263	1	1/3516	TRINITY_DN3513_c0_g1_i5_orf1
cellular_component	TRAPP complex	GO:0030008	1	1/3516	TRINITY_DN45037_c0_g1_i1_orf1
cellular_component	dynactin complex	GO:0005869	1	1/3516	TRINITY_DN8561_c0_g4_i1_orf1
cellular_component	kinesin complex	GO:0005871	1	1/3516	TRINITY_DN4808_c0_g1_i3_orf1
cellular_component	sno(s)RNA-containing ribonucleoprotein complex	GO:0005732	2	2/3516	TRINITY_DN13496_c0_g1_i7_orf1;TRINITY_DN7573_c0_g2_i1_orf1
cellular_component	polysome	GO:0005844	1	1/3516	TRINITY_DN20009_c0_g1_i1_orf1
cellular_component	translation preinitiation complex	GO:0070993	11	11/3516	TRINITY_DN19092_c0_g1_i2_orf1;TRINITY_DN50085_c0_g1_i1_orf1;TRINITY_DN1572_c0_g1_i6_orf1;TRINITY_DN3878_c0_g1_i4_orf1;TRINITY_DN17049_c0_g1_i6_orf1;TRINITY_DN27751_c0_g2_i1_orf1;TRINITY_DN53684_c0_g1_i1_orf1;TRINITY_DN33619_c0_g1_i1_orf1;TRINITY_DN3366_c0_g1_i6_orf1;TRINITY_DN17045_c0_g2_i3_orf1;TRINITY_DN4237_c1_g1_i5_orf1
cellular_component	translation initiation complex	GO:0070992	1	1/3516	TRINITY_DN142442_c0_g1_i1_orf1
cellular_component	RNAi effector complex	GO:0031332	1	1/3516	TRINITY_DN14701_c0_g1_i2_orf1
cellular_component	signal recognition particle	GO:0048500	2	2/3516	TRINITY_DN48460_c0_g1_i1_orf1;TRINITY_DN19286_c0_g1_i1_orf1
cellular_component	preribosome	GO:0030684	6	6/3516	TRINITY_DN8430_c0_g1_i1_orf1;TRINITY_DN3082_c1_g1_i7_orf1;TRINITY_DN7573_c0_g2_i1_orf1;TRINITY_DN1066_c0_g1_i4_orf1;TRINITY_DN13496_c0_g1_i7_orf1;TRINITY_DN56110_c0_g1_i1_orf1
cellular_component	ribosomal subunit	GO:0044391	40	40/3516	TRINITY_DN3534_c0_g1_i2_orf1;TRINITY_DN7613_c1_g2_i1_orf1;TRINITY_DN36893_c0_g1_i1_orf1;TRINITY_DN10007_c0_g1_i1_orf1;TRINITY_DN130075_c1_g2_i1_orf1;TRINITY_DN19942_c0_g1_i2_orf1;TRINITY_DN137_c0_g1_i1_orf1;TRINITY_DN142442_c0_g1_i1_orf1;TRINITY_DN13651_c0_g1_i2_orf1;TRINITY_DN41645_c0_g1_i1_orf1;TRINITY_DN33926_c0_g1_i1_orf1;TRINITY_DN71840_c0_g1_i1_orf1;TRINITY_DN97680_c0_g1_i1_orf1;TRINITY_DN4016_c0_g1_i1_orf1;TRINITY_DN30300_c0_g2_i1_orf1;TRINITY_DN43792_c0_g1_i1_orf1;TRINITY_DN11297_c0_g1_i1_orf1;TRINITY_DN55148_c0_g1_i1_orf1;TRINITY_DN15234_c0_g1_i3_orf1;TRINITY_DN50787_c0_g2_i2_orf1;TRINITY_DN11825_c0_g1_i4_orf1;TRINITY_DN36701_c0_g1_i4_orf1;TRINITY_DN10070_c0_g1_i1_orf1;TRINITY_DN9101_c0_g2_i1_orf1;TRINITY_DN38075_c0_g1_i1_orf1;TRINITY_DN42646_c0_g2_i1_orf1;TRINITY_DN82324_c0_g1_i4_orf1;TRINITY_DN43611_c0_g1_i1_orf1;TRINITY_DN18869_c0_g1_i1_orf1;TRINITY_DN2682_c0_g1_i4_orf1;TRINITY_DN15380_c0_g1_i1_orf1;TRINITY_DN1313_c0_g1_i2_orf1;TRINITY_DN441_c0_g2_i1_orf1;TRINITY_DN9874_c0_g1_i7_orf1;TRINITY_DN79734_c0_g2_i3_orf1;TRINITY_DN17215_c0_g1_i4_orf1;TRINITY_DN64510_c0_g1_i1_orf1;TRINITY_DN121893_c0_g1_i1_orf1;TRINITY_DN754_c1_g1_i6_orf1;TRINITY_DN8949_c0_g1_i2_orf1
cellular_component	mRNA cap binding complex	GO:0005845	1	1/3516	TRINITY_DN41664_c0_g1_i4_orf1
cellular_component	nuclear cap binding complex	GO:0005846	1	1/3516	TRINITY_DN7289_c0_g1_i1_orf1
cellular_component	proteasome complex	GO:0000502	7	7/3516	TRINITY_DN5775_c0_g1_i1_orf1;TRINITY_DN19260_c0_g1_i5_orf1;TRINITY_DN34479_c0_g1_i2_orf1;TRINITY_DN145227_c0_g1_i1_orf1;TRINITY_DN49047_c0_g1_i2_orf1;TRINITY_DN32359_c0_g2_i1_orf1;TRINITY_DN2591_c0_g1_i4_orf1
cellular_component	DNA polymerase complex	GO:0042575	6	6/3516	TRINITY_DN18538_c0_g3_i1_orf1;TRINITY_DN15040_c0_g4_i1_orf1;TRINITY_DN74037_c0_g5_i1_orf1;TRINITY_DN70485_c0_g1_i2_orf1;TRINITY_DN110534_c0_g1_i3_orf1;TRINITY_DN89613_c0_g1_i13_orf1

cellular_component	chaperone complex	GO:0101031	2	2/3516	TRINITY_DN5262_c0_g1_i7_orf1;TRINITY_DN1725_c0_g1_i7_orf1
cellular_component	ubiquitin ligase complex	GO:0000151	5	5/3516	TRINITY_DN1757_c0_g1_i4_orf1;TRINITY_DN9062_c0_g2_i3_orf1;TRINITY_DN17726_c0_g1_i1_orf1;TRINITY_DN146493_c0_g1_i1_orf1;TRINITY_DN461_c0_g1_i5_orf1
cellular_component	TOR complex	GO:0038201	2	2/3516	TRINITY_DN105749_c0_g1_i1_orf1;TRINITY_DN40191_c2_g1_i1_orf1
cellular_component	CCR4-NOT complex	GO:0030014	2	2/3516	TRINITY_DN88539_c0_g2_i1_orf1;TRINITY_DN66596_c0_g1_i1_orf1
cellular_component	CCR4-NOT core complex	GO:0030015	1	1/3516	TRINITY_DN41602_c0_g3_i1_orf1
cellular_component	guanyl-nucleotide exchange factor complex	GO:0032045	2	2/3516	TRINITY_DN34159_c0_g2_i1_orf1;TRINITY_DN15448_c0_g1_i1_orf1
cellular_component	RNA polymerase complex	GO:0030880	9	9/3516	TRINITY_DN12527_c0_g1_i4_orf1;TRINITY_DN15836_c0_g1_i1_orf1;TRINITY_DN10658_c0_g1_i1_orf1;TRINITY_DN4707_c0_g1_i1_orf1;TRINITY_DN879_c0_g1_i2_orf1;TRINITY_DN2299_c0_g1_i3_orf1;TRINITY_DN23714_c0_g1_i4_orf1;TRINITY_DN31520_c1_g1_i1_orf1;TRINITY_DN9207_c0_g1_i1_orf1
cellular_component	protein acetyltransferase complex	GO:0031248	4	4/3516	TRINITY_DN452_c1_g1_i3_orf1;TRINITY_DN13174_c0_g1_i4_orf1;TRINITY_DN2064_c1_g1_i1_orf1;TRINITY_DN10636_c0_g1_i1_orf1
cellular_component	protein kinase CK2 complex	GO:0005956	1	1/3516	TRINITY_DN147475_c0_g1_i1_orf1
cellular_component	actin rod	GO:0031002	1	1/3516	TRINITY_DN7493_c0_g1_i1_orf1
cellular_component	organelle lumen	GO:0043233	29	29/3516	TRINITY_DN5417_c0_g1_i1_orf1;TRINITY_DN14920_c0_g1_i1_orf1;TRINITY_DN51938_c0_g3_i1_orf1;TRINITY_DN17271_c0_g1_i1_orf1;TRINITY_DN12973_c0_g1_i1_orf1;TRINITY_DN46409_c0_g1_i1_orf1;TRINITY_DN10429_c0_g1_i2_orf1;TRINITY_DN1791_c0_g1_i3_orf1;TRINITY_DN146264_c0_g1_i1_orf1;TRINITY_DN49265_c0_g3_i2_orf1;TRINITY_DN95850_c0_g4_i3_orf1;TRINITY_DN975_c0_g1_i1_orf1;TRINITY_DN2299_c0_g1_i3_orf1;TRINITY_DN7579_c1_g3_i1_orf1;TRINITY_DN2848_c0_g1_i1_orf1;TRINITY_DN2238_c0_g2_i1_orf1;TRINITY_DN21715_c0_g1_i1_orf1;TRINITY_DN111985_c0_g1_i1_orf1;TRINITY_DN21909_c0_g1_i1_orf1;TRINITY_DN5122_c0_g1_i3_orf1;TRINITY_DN825_c2_g1_i5_orf1;TRINITY_DN24751_c0_g1_i1_orf1;TRINITY_DN5129_c0_g3_i3_orf1;TRINITY_DN21539_c0_g1_i1_orf1;TRINITY_DN9156_c0_g1_i1_orf1;TRINITY_DN4842_c0_g1_i5_orf1;TRINITY_DN147475_c0_g1_i1_orf1;TRINITY_DN42854_c0_g3_i2_orf1;TRINITY_DN20133_c0_g1_i1_orf1
cellular_component	polytene chromosome band	GO:0005704	1	1/3516	TRINITY_DN5458_c1_g1_i9_orf1
cellular_component	chromosome, telomeric region	GO:0000781	1	1/3516	TRINITY_DN12771_c0_g1_i1_orf1
cellular_component	cell cortex	GO:0005938	1	1/3516	TRINITY_DN2186_c0_g1_i17_orf1
cellular_component	extrinsic component of organelle membrane	GO:0031312	3	3/3516	TRINITY_DN6638_c0_g1_i1_orf1;TRINITY_DN36592_c0_g1_i1_orf1;TRINITY_DN6027_c0_g1_i13_orf1
cellular_component	heterochromatin	GO:0000792	2	2/3516	TRINITY_DN24266_c0_g2_i2_orf1;TRINITY_DN20133_c0_g1_i1_orf1
cellular_component	intrinsic component of plasma membrane	GO:0031226	2	2/3516	TRINITY_DN11569_c0_g1_i1_orf1;TRINITY_DN4464_c0_g2_i1_orf1
cellular_component	anchored component of membrane	GO:0031225	6	6/3516	TRINITY_DN5406_c0_g2_i1_orf1;TRINITY_DN4464_c0_g2_i1_orf1;TRINITY_DN1352_c0_g1_i5_orf1;TRINITY_DN5553_c0_g1_i4_orf1;TRINITY_DN2175_c0_g1_i4_orf1;TRINITY_DN932_c0_g1_i4_orf1
cellular_component	integral component of membrane	GO:0016021	452	452/3516	TRINITY_DN12227_c0_g2_i3_orf1;TRINITY_DN5597_c0_g1_i2_orf1;TRINITY_DN3194_c0_g1_i6_orf1;TRINITY_DN11492_c0_g1_i8_orf1;TRINITY_DN63943_c0_g1_i5_orf1;TRINITY_DN101922_c0_g1_i1_orf1;TRINITY_DN7414_c0_g1_i1_orf1;TRINITY_DN14826_c0_g1_i1_orf1;TRINITY_DN16343_c0_g1_i6_orf1;TRINITY_DN3196_c0_g1_i1_orf1;TRINITY_DN48020_c0_g1_i1_orf1;TRINITY_DN15755_c0_g1_i1_orf1;TRINITY_DN18338_c0_g1_i6_orf1;TRINITY_DN71917_c0_g3_i1_orf1;TRINITY_DN5337_c0_g1_i6_orf1;TRINITY_DN15157_c0_g1_i1_orf1;TRINITY_DN17505_c0_g1_i15_orf1;TRINITY_DN3276_c0_g1_i4_orf1;TRINITY_DN30932_c0_g1_i2_orf1;TRINITY_DN2343_c1_g1_i2_orf1;TRINITY_DN51766_c0_g1_i2_orf1;TRINITY_DN16482_c0_g1_i6_orf1;TRINITY_DN14937_c0_g1_i7_orf1;TRINITY_DN11172_c1_g1_i1_orf1;TRINITY_DN52761_c0_g2_i1_orf1;TRINITY_DN25733_c0_g1_i3_orf1;TRINITY_DN30704_c0_g1_i1_orf1;TRINITY_DN2627_c0_g1_i2_orf1;TRINITY_DN198_c2_g1_i2_orf1;TRINITY_DN24043_c0_g1_i1_orf1;TRINITY_DN1664_c0_g1_i4_orf1;TRINITY_DN5046_c0_g3_i1_orf1;TRINITY_DN29038_c0_g2_i1_orf1;TRINITY_DN2177_c0_g1_i1_orf1;TRINITY_DN625_c9_g1_i7_orf1;TRINITY_DN22678_c0_g1_i4_orf1;TRINITY_DN7464_c0_g1_i14_orf1;TRINITY_DN810_c0_g1_i4_orf1;TRINITY_DN66302_c0_g1_i1_orf1;TRINITY_DN8838_c0_g1_i1_orf1;TRINITY_DN15607_c0_g1_i6_orf1;TRINITY_DN1786_c0_g1_i11_orf1;TRINITY_DN12666_c0_g1_i2_orf1;TRINITY_DN19917_c0_g1_i1_orf1;TRINITY_DN7861_c0_g1_i5_orf1;TRINITY_DN17651_c0_g1_i2_orf1;TRINITY_DN867_c0_g1_i1_orf1;TRINITY_DN5908_c0_g1_i2_orf1;TRINITY_DN1750_c1_g1_i5_orf1;TRINITY_DN4497_c0_g1_i4_orf1;TRINITY_DN17907_c0_g1_i13_orf1;TRINITY_DN24873_c0_g1_i4_orf1;TRINITY_DN928_c0_g1_i3_orf1;TRINITY_DN19135_c0_g1_i1_orf1;TRINITY_DN46090_c0_g3_i1_orf1;TRINITY_DN1348_c0_g1_i1_orf1;TRINITY_DN14046_c0_g1_i1_orf1;TRINITY_DN13411_c0_g1_i4_orf1;TRINITY_DN117042_c0_g1_i2_orf1;TRINITY_DN48713_c0_g1_i1_orf1;TRINITY_DN2579_c0_g1_i7_orf1;TRINITY_DN10379_c0_g1_i3_orf1;TRINITY_DN2876_c0_g1_i3_orf1;TRINITY_DN10530_c0_g1_i1_orf1;TRINITY_DN1617_c0_g1_i5_orf1;TRINITY_DN79210_c0_g1_i1_orf1;TRINITY_DN4144_c0_g1_i7_orf1;TRINITY_DN22175_c0_g1_i1_orf1;TRINITY_DN1293_c0_g1_i4_orf1;TRINITY_DN14073_c0_g1_i1_orf1;TRINITY_DN5111_c0_g1_i2_orf1;TRINITY_DN9455_c0_g1_i6_orf1;TRINITY_DN642_c0_g1_i6_orf1;TRINITY_DN2880_c0_g1_i2_orf1;TRINITY_DN54586_c1_g1_i1_orf1;TRINITY_DN33272_c0_g1_i5_orf1;TRINITY_DN7128_c0_g1_i7_orf1;TRINITY_DN448_c0_g1_i20_orf1;TRINITY_DN17394_c0_g1_i1_orf1;TRINITY_DN1672_c0_g1_i6_orf1;TRINITY_DN9316_c1_g1_i1_orf1;TRINITY_DN2794_c1_g1_i8_orf1;TRINITY_DN19748_c0_g1_i4_orf1;TRINITY_DN2267_c0_g1_i1_orf1;TRINITY_DN14168_c0_g1_i1_orf1;TRINITY_DN1292_c0_g1_i3_orf1;TRINITY_DN1960_c5_g1_i3_orf1;TRINITY_DN69049_c0_g2_i1_orf1;TRINITY_DN13783_c0_g4_i2_orf1;TRINITY_DN91_c0_g1_i9_orf1;TRINITY_DN7867_c0_g1_i1_orf1;TRINITY_DN760_c1_g2_i6_orf1;TRINITY_DN4125_c0_g1_i6_orf1;TRINITY_DN6710_c0_g1_i6_orf1;TRINITY_DN1803_c0_g1_i3_orf1;TRINITY_DN6510_c1_g1_i1_orf1;TRINITY_DN38435_c0_g1_i1_orf1;TRINITY_DN9608_c0_g1_i3_orf1;TRINITY_DN13923_c0_g2_i1_orf1;TRINITY_DN827_c1_g1_i1_orf1;TRINITY_DN79868_c0_g1_i1_orf1;TRINITY_DN130051_c0_g1_i1_orf1;TRINITY_DN1239_c0_g1_i3_orf1;TRINITY_DN496_c0_g1_i7_orf1;TRINITY_DN9615_c0_g1_i1_orf1;TRINITY_DN2109_c0_g1_i4_orf1;TRINITY_DN108819_c0_g1_i1_orf1;TRINITY_DN61_c0_g2_i3_orf1;TRINITY_DN4469_c0_g1_i2_orf1;TRINITY_DN16122_c0_g1_i4_orf1;TRINITY_DN23432_c0_g1_i1_orf1;TRINITY_DN7590_c0_g1_i4_orf1;TRINITY_DN585_c0_g1_i12_orf1;TRINITY_DN61711_c0_g1_i1_orf1;TRINITY_DN10774_c0_g2_i3_orf1;TRINITY_DN146364_c0_g1_i1_orf1;TRINITY_DN22944_c0_g3_i1_orf1;TRINITY_DN19115_c0_g1_i1_orf1;TRINITY_DN4125_c0_g1_i14_orf1;TRINITY_DN3355_c0_g2_i4_orf1;TRINITY_DN29879_c0_g1_i3_orf1;TRINITY_D
cellular_component	intrinsic component of organelle membrane	GO:0031300	4	4/3516	TRINITY_DN19122_c0_g1_i7_orf1;TRINITY_DN91_c0_g1_i9_orf1;TRINITY_DN483_c0_g1_i6_orf1;TRINITY_DN2172_c0_g2_i8_orf1
cellular_component	external side of plasma membrane	GO:0009897	1	1/3516	TRINITY_DN20339_c0_g1_i3_orf1
cellular_component	cytoplasmic side of membrane	GO:0098562	2	2/3516	TRINITY_DN96557_c0_g1_i1_orf1;TRINITY_DN975_c0_g1_i1_orf1

cellular_component	intracellular organelle	GO:0043229	393 393/3516	TRINITY_DN57074_c0_g2_i1_orf1;TRINITY_DN42856_c0_g1_i1_orf1;TRINITY_DN5019_c0_g1_i2_orf1;TRINITY_DN1497_c0_g2_i6_orf1;TRINITY_DN2818_c0_g1_i2_orf1;TRINITY_DN879_c0_g1_i2_orf1;TRINITY_DN101922_c0_g1_i1_orf1;TRINITY_DN5092_c0_g1_i2_orf1;TRINITY_DN19810_c1_g1_i7_orf1;TRINITY_DN59335_c0_g1_i2_orf1;TRINITY_DN26789_c0_g1_i2_orf1;TRINITY_DN1639_c0_g2_i2_orf1;TRINITY_DN2861_c0_g2_i1_orf1;TRINITY_DN535_c3_g2_i1_orf1;TRINITY_DN84478_c0_g1_i8_orf1;TRINITY_DN21492_c0_g1_i1_orf1;TRINITY_DN235_c0_g3_i1_orf1;TRINITY_DN30932_c0_g1_i2_orf1;TRINITY_DN110231_c0_g1_i1_orf1;TRINITY_DN25896_c0_g1_i6_orf1;TRINITY_DN2802_c0_g1_i1_orf1;TRINITY_DN16174_c0_g1_i2_orf1;TRINITY_DN5603_c0_g1_i1_orf1;TRINITY_DN6199_c2_g1_i3_orf1;TRINITY_DN364_c0_g2_i1_orf1;TRINITY_DN52649_c0_g1_i6_orf1;TRINITY_DN19829_c0_g2_i1_orf1;TRINITY_DN18230_c1_g2_i1_orf1;TRINITY_DN146119_c0_g1_i1_orf1;TRINITY_DN56121_c0_g1_i4_orf1;TRINITY_DN23790_c0_g1_i1_orf1;TRINITY_DN5873_c0_g4_i1_orf1;TRINITY_DN9790_c0_g1_i4_orf1;TRINITY_DN7583_c0_g1_i1_orf1;TRINITY_DN19920_c1_g1_i2_orf1;TRINITY_DN7464_c0_g1_i14_orf1;TRINITY_DN45037_c0_g1_i1_orf1;TRINITY_DN3733_c0_g1_i1_orf1;TRINITY_DN9085_c0_g1_i1_orf1;TRINITY_DN4938_c0_g1_i13_orf1;TRINITY_DN71465_c0_g1_i1_orf1;TRINITY_DN7573_c0_g2_i1_orf1;TRINITY_DN147458_c0_g1_i1_orf1;TRINITY_DN21251_c1_g1_i1_orf1;TRINITY_DN8703_c0_g1_i2_orf1;TRINITY_DN5112_c0_g1_i1_orf1;TRINITY_DN5055_c0_g1_i12_orf1;TRINITY_DN26251_c0_g1_i1_orf1;TRINITY_DN15373_c0_g1_i2_orf1;TRINITY_DN2186_c0_g1_i17_orf1;TRINITY_DN2026_c0_g1_i4_orf1;TRINITY_DN2168_c0_g1_i2_orf1;TRINITY_DN19262_c0_g1_i1_orf1;TRINITY_DN19829_c0_g1_i1_orf1;TRINITY_DN19135_c0_g1_i1_orf1;TRINITY_DN4016_c0_g1_i1_orf1;TRINITY_DN934_c2_g1_i7_orf1;TRINITY_DN12771_c0_g1_i1_orf1;TRINITY_DN3219_c0_g1_i6_orf1;TRINITY_DN38341_c0_g2_i2_orf1;TRINITY_DN2710_c0_g1_i4_orf1;TRINITY_DN56110_c0_g1_i1_orf1;TRINITY_DN12576_c0_g1_i2_orf1;TRINITY_DN27960_c0_g1_i1_orf1;TRINITY_DN3401_c0_g1_i1_orf1;TRINITY_DN65299_c0_g4_i1_orf1;TRINITY_DN42506_c0_g1_i1_orf1;TRINITY_DN2647_c0_g1_i3_orf1;TRINITY_DN6248_c0_g1_i1_orf1;TRINITY_DN10385_c0_g1_i5_orf1;TRINITY_DN41997_c0_g1_i2_orf1;TRINITY_DN33038_c0_g1_i1_orf1;TRINITY_DN2265_c0_g2_i1_orf1;TRINITY_DN5031_c0_g1_i1_orf1;TRINITY_DN1952_c0_g1_i2_orf1;TRINITY_DN147691_c0_g1_i1_orf1;TRINITY_DN10831_c1_g1_i1_orf1;TRINITY_DN72369_c0_g1_i1_orf1;TRINITY_DN7128_c0_g1_i7_orf1;TRINITY_DN90289_c0_g1_i5_orf1;TRINITY_DN55148_c0_g1_i1_orf1;TRINITY_DN13711_c0_g1_i1_orf1;TRINITY_DN15845_c0_g1_i1_orf1;TRINITY_DN975_c0_g1_i1_orf1;TRINITY_DN9383_c0_g1_i3_orf1;TRINITY_DN4056_c0_g1_i8_orf1;TRINITY_DN108819_c0_g1_i1_orf1;TRINITY_DN3588_c0_g1_i1_orf1;TRINITY_DN10994_c0_g1_i4_orf1;TRINITY_DN19115_c0_g1_i1_orf1;TRINITY_DN3614_c0_g2_i1_orf1;TRINITY_DN9510_c0_g2_i1_orf1;TRINITY_DN81248_c0_g1_i1_orf1;TRINITY_DN17825_c1_g1_i1_orf1;TRINITY_DN9542_c0_g1_i4_orf1;TRINITY_DN31253_c0_g1_i2_orf1;TRINITY_DN147475_c0_g1_i1_orf1;TRINITY_DN26824_c0_g1_i1_orf1;TRINITY_DN10745_c0_g1_i14_orf1;TRINITY_DN16749_c0_g1_i1_orf1;TRINITY_DN18230_c1_g1_i1_orf1;TRINITY_DN8430_c0_g1_i1_orf1;TRINITY_DN13496_c0_g1_i7_orf1;TRINITY_DN11194_c0_g1_i4_orf1;TRINITY_DN56910_c0_g2_i1_orf1;TRINITY_DN27885_c0_g1_i3_orf1;TRINITY_DN8676_c0_g1_i1_orf1;TRINITY_DN23746_c0_g1_i2_orf1;TRINITY_DN3457_c0_g1_i4_orf1;TRINITY_DN2638_c0_g1_i7_orf1;TRINITY_DN2848_c0_g1_i1_orf1;TRINITY_DN2954_c0_g1_i1_orf1;TRINITY_DN4810_c0_g1_i3_orf1;TRINITY_DN5442_c0_g1_i4_orf1;TRINITY_DN74889_c0_g1_i1_orf1;TRINITY_DN50571_c1_g1_i1_orf1;TRINITY_DN12442_c0_g1_i4_orf1;TRINITY_DN135_c0_g1_i1_orf1;TRINITY_DN66596_c0_g1_i1_orf1;TRINITY_DN3985_c0_g2_i1_orf1;TRINITY_DN9717_c0_g2_i1
cellular_component	non-membrane-bounded organelle	GO:0043228	164 164/3516	TRINITY_DN57074_c0_g2_i1_orf1;TRINITY_DN47731_c0_g1_i2_orf1;TRINITY_DN8430_c0_g1_i1_orf1;TRINITY_DN4956_c0_g1_i6_orf1;TRINITY_DN137_c0_g1_i1_orf1;TRINITY_DN5873_c0_g4_i1_orf1;TRINITY_DN8676_c0_g1_i1_orf1;TRINITY_DN11065_c0_g2_i1_orf1;TRINITY_DN58207_c0_g1_i1_orf1;TRINITY_DN15836_c0_g1_i1_orf1;TRINITY_DN101922_c0_g1_i1_orf1;TRINITY_DN142442_c0_g1_i1_orf1;TRINITY_DN3985_c0_g2_i1_orf1;TRINITY_DN23616_c0_g1_i4_orf1;TRINITY_DN3814_c1_g1_i1_orf1;TRINITY_DN2848_c0_g1_i1_orf1;TRINITY_DN29448_c0_g1_i1_orf1;TRINITY_DN1639_c0_g2_i2_orf1;TRINITY_DN97097_c0_g1_i4_orf1;TRINITY_DN74889_c0_g1_i1_orf1;TRINITY_DN766_c0_g1_i1_orf1;TRINITY_DN50571_c1_g1_i1_orf1;TRINITY_DN1509_c0_g1_i1_orf1;TRINITY_DN135_c0_g1_i1_orf1;TRINITY_DN143_c0_g3_i1_orf1;TRINITY_DN23746_c0_g1_i2_orf1;TRINITY_DN110231_c0_g1_i1_orf1;TRINITY_DN33883_c0_g1_i1_orf1;TRINITY_DN2026_c0_g1_i4_orf1;TRINITY_DN31225_c0_g1_i1_orf1;TRINITY_DN18860_c0_g1_i1_orf1;TRINITY_DN5458_c1_g1_i9_orf1;TRINITY_DN121893_c0_g1_i1_orf1;TRINITY_DN14313_c0_g1_i1_orf1;TRINITY_DN18249_c0_g1_i1_orf1;TRINITY_DN23360_c0_g1_i3_orf1;TRINITY_DN52649_c0_g1_i6_orf1;TRINITY_DN130075_c1_g2_i1_orf1;TRINITY_DN31433_c0_g1_i1_orf1;TRINITY_DN29402_c0_g1_i1_orf1;TRINITY_DN56121_c0_g1_i4_orf1;TRINITY_DN23790_c0_g1_i1_orf1;TRINITY_DN6248_c0_g1_i1_orf1;TRINITY_DN93566_c0_g2_i1_orf1;TRINITY_DN60821_c0_g1_i1_orf1;TRINITY_DN53810_c0_g1_i1_orf1;TRINITY_DN7583_c0_g1_i1_orf1;TRINITY_DN7464_c0_g1_i14_orf1;TRINITY_DN50787_c0_g2_i2_orf1;TRINITY_DN364_c1_g1_i2_orf1;TRINITY_DN799_c0_g1_i7_orf1;TRINITY_DN3733_c0_g1_i1_orf1;TRINITY_DN49936_c0_g2_i1_orf1;TRINITY_DN315_c0_g1_i1_orf1;TRINITY_DN741_c0_g1_i10_orf1;TRINITY_DN655_c0_g1_i3_orf1;TRINITY_DN40508_c0_g1_i1_orf1;TRINITY_DN147691_c0_g1_i1_orf1;TRINITY_DN21357_c0_g1_i5_orf1;TRINITY_DN56110_c0_g1_i1_orf1;TRINITY_DN7991_c0_g1_i9_orf1;TRINITY_DN31119_c0_g1_i1_orf1;TRINITY_DN3847_c1_g1_i1_orf1;TRINITY_DN13233_c0_g1_i3_orf1;TRINITY_DN2848_c0_g1_i2_orf1;TRINITY_DN10658_c0_g1_i1_orf1;TRINITY_DN10831_c1_g1_i1_orf1;TRINITY_DN7573_c0_g2_i1_orf1;TRINITY_DN41997_c0_g1_i2_orf1;TRINITY_DN147458_c0_g1_i1_orf1;TRINITY_DN17215_c0_g1_i4_orf1;TRINITY_DN5064_c0_g1_i4_orf1;TRINITY_DN143852_c0_g1_i1_orf1;TRINITY_DN16939_c0_g1_i4_orf1;TRINITY_DN6239_c0_g1_i1_orf1;TRINITY_DN21251_c1_g1_i1_orf1;TRINITY_DN5112_c0_g1_i1_orf1;TRINITY_DN30131_c0_g1_i1_orf1;TRINITY_DN1066_c0_g1_i4_orf1;TRINITY_DN2186_c0_g1_i17_orf1;TRINITY_DN58636_c0_g1_i1_orf1;TRINITY_DN24318_c0_g1_i1_orf1;TRINITY_DN2084_c0_g1_i1_orf1;TRINITY_DN21619_c0_g1_i1_orf1;TRINITY_DN102260_c0_g1_i1_orf1;TRINITY_DN5009_c0_g1_i2_orf1;TRINITY_DN19186_c0_g1_i1_orf1;TRINITY_DN61222_c0_g1_i1_orf1;TRINITY_DN4016_c0_g1_i1_orf1;TRINITY_DN934_c2_g1_i7_orf1;TRINITY_DN40345_c0_g1_i6_orf1;TRINITY_DN467_c9_g1_i2_orf1;TRINITY_DN30027_c0_g1_i1_orf1;TRINITY_DN20009_c0_g1_i1_orf1;TRINITY_DN147676_c0_g1_i1_orf1;TRINITY_DN144956_c0_g1_i1_orf1;TRINITY_DN3062_c0_g1_i1_orf1;TRINITY_DN12576_c0_g1_i2_orf1;TRINITY_DN8079_c0_g1_i2_orf1;TRINITY_DN17299_c0_g1_i4_orf1;TRINITY_DN3401_c0_g1_i1_orf1;TRINITY_DN52861_c0_g1_i1_orf1;TRINITY_DN42506_c0_g1_i1_orf1;TRINITY_DN96557_c0_g1_i1_orf1;TRINITY_DN9146_c0_g1_i1_orf1;TRINITY_DN21971_c0_g1_i4_orf1;TRINITY_DN15380_c0_g1_i1_orf1;TRINITY_DN2258_c0_g2_i1_orf1;TRINITY_DN34166_c0_g1_i1_orf1;TRINITY_DN4429_c0_g1_i5_orf1;TRINITY_DN879_c0_g1_i2_orf1;TRINITY_DN31253_c0_g1_i2_orf1;TRINITY_DN92232_c0_g1_i1_orf1;TRINITY_DN40015_c0_g1_i2_orf1;TRINITY_DN145666_c0_g1_i1_orf1;TRINITY_DN146718_c0_g1_i1_orf1;TRINITY_DN33038_c0_g1_i1_orf1;TRINITY_DN15965_c0_g1_i1_orf1;TRINITY_DN40650_c0_g1_i1_orf1;TRINITY_DN5031_c0_g1_i1_orf1;TRINITY_DN364_c0_g

cellular_component	membrane-bounded organelle	GO:0043227	240	240/3516	TRINITY_DN29017_c0_g1_i4_orf1;TRINITY_DN42856_c0_g1_i1_orf1;TRINITY_DN5019_c0_g1_i2_orf1;TRINITY_DN1497_c0_g2_i6_orf1;TRINITY_DN5925_c0_g1_i5_orf1;TRINITY_DN11194_c0_g1_i4_orf1;TRINITY_DN2818_c0_g1_i2_orf1;TRINITY_DN56910_c0_g2_i1_orf1;TRINITY_DN3292_c2_g1_i4_orf1;TRINITY_DN27885_c0_g1_i3_orf1;TRINITY_DN35669_c0_g1_i1_orf1;TRINITY_DN30932_c0_g1_i2_orf1;TRINITY_DN30663_c0_g1_i1_orf1;TRINITY_DN14967_c0_g2_i1_orf1;TRINITY_DN101922_c0_g1_i1_orf1;TRINITY_DN30300_c0_g2_i1_orf1;TRINITY_DN140538_c0_g2_i1_orf1;TRINITY_DN5092_c0_g1_i2_orf1;TRINITY_DN3457_c0_g1_i4_orf1;TRINITY_DN41602_c0_g3_i1_orf1;TRINITY_DN2638_c0_g1_i7_orf1;TRINITY_DN2848_c0_g1_i1_orf1;TRINITY_DN59335_c0_g1_i2_orf1;TRINITY_DN2954_c0_g1_i1_orf1;TRINITY_DN26789_c0_g1_i2_orf1;TRINITY_DN443_c0_g1_i2_orf1;TRINITY_DN7047_c0_g1_i1_orf1;TRINITY_DN2861_c0_g2_i1_orf1;TRINITY_DN4810_c0_g1_i3_orf1;TRINITY_DN3325_c0_g1_i1_orf1;TRINITY_DN74889_c0_g1_i1_orf1;TRINITY_DN535_c3_g2_i1_orf1;TRINITY_DN15370_c0_g1_i4_orf1;TRINITY_DN84478_c0_g1_i8_orf1;TRINITY_DN21492_c0_g1_i1_orf1;TRINITY_DN66596_c0_g1_i1_orf1;TRINITY_DN143_c0_g3_i1_orf1;TRINITY_DN3985_c0_g2_i1_orf1;TRINITY_DN5442_c0_g1_i4_orf1;TRINITY_DN7909_c0_g2_i1_orf1;TRINITY_DN25896_c0_g1_i6_orf1;TRINITY_DN2265_c0_g1_i5_orf1;TRINITY_DN9717_c0_g2_i1_orf1;TRINITY_DN48536_c0_g1_i3_orf1;TRINITY_DN16174_c0_g1_i2_orf1;TRINITY_DN17312_c0_g1_i1_orf1;TRINITY_DN53807_c0_g2_i1_orf1;TRINITY_DN2848_c0_g1_i2_orf1;TRINITY_DN5603_c0_g1_i1_orf1;TRINITY_DN6199_c2_g1_i3_orf1;TRINITY_DN1706_c0_g1_i7_orf1;TRINITY_DN12526_c0_g1_i5_orf1;TRINITY_DN2802_c1_g1_i1_orf1;TRINITY_DN23360_c0_g1_i3_orf1;TRINITY_DN19829_c0_g2_i1_orf1;TRINITY_DN5497_c0_g1_i6_orf1;TRINITY_DN59965_c0_g4_i1_orf1;TRINITY_DN18230_c1_g2_i1_orf1;TRINITY_DN383_c0_g1_i1_orf1;TRINITY_DN95850_c0_g4_i3_orf1;TRINITY_DN18933_c0_g1_i3_orf1;TRINITY_DN146119_c0_g1_i1_orf1;TRINITY_DN17864_c0_g1_i1_orf1;TRINITY_DN40704_c0_g1_i2_orf1;TRINITY_DN500_c0_g1_i1_orf1;TRINITY_DN41842_c0_g1_i2_orf1;TRINITY_DN4747_c0_g1_i4_orf1;TRINITY_DN740_c0_g1_i1_orf1;TRINITY_DN53810_c0_g1_i1_orf1;TRINITY_DN9790_c0_g1_i4_orf1;TRINITY_DN50787_c0_g2_i2_orf1;TRINITY_DN41_c0_g1_i3_orf1;TRINITY_DN45037_c0_g1_i1_orf1;TRINITY_DN3562_c0_g1_i4_orf1;TRINITY_DN937_c0_g1_i2_orf1;TRINITY_DN3251_c0_g1_i6_orf1;TRINITY_DN111985_c0_g1_i1_orf1;TRINITY_DN141_c0_g1_i1_orf1;TRINITY_DN5458_c1_g1_i9_orf1;TRINITY_DN3312_c0_g1_i10_orf1;TRINITY_DN106534_c0_g1_i1_orf1;TRINITY_DN6556_c0_g1_i7_orf1;TRINITY_DN9085_c0_g1_i1_orf1;TRINITY_DN4938_c0_g1_i13_orf1;TRINITY_DN17738_c0_g1_i2_orf1;TRINITY_DN53400_c0_g1_i1_orf1;TRINITY_DN40911_c0_g1_i1_orf1;TRINITY_DN5105_c0_g1_i10_orf1;TRINITY_DN52893_c0_g1_i1_orf1;TRINITY_DN13944_c0_g1_i1_orf1;TRINITY_DN7808_c0_g1_i1_orf1;TRINITY_DN3335_c0_g1_i1_orf1;TRINITY_DN71465_c0_g1_i1_orf1;TRINITY_DN7289_c0_g1_i1_orf1;TRINITY_DN110460_c0_g2_i1_orf1;TRINITY_DN24917_c0_g2_i1_orf1;TRINITY_DN86149_c0_g1_i1_orf1;TRINITY_DN649_c1_g1_i13_orf1;TRINITY_DN80547_c0_g1_i5_orf1;TRINITY_DN3702_c0_g1_i1_orf1;TRINITY_DN147458_c0_g1_i1_orf1;TRINITY_DN3860_c0_g1_i5_orf1;TRINITY_DN38650_c0_g1_i2_orf1;TRINITY_DN13371_c0_g1_i4_orf1;TRINITY_DN8703_c0_g1_i2_orf1;TRINITY_DN31851_c0_g1_i2_orf1;TRINITY_DN5055_c0_g1_i12_orf1;TRINITY_DN26251_c0_g1_i1_orf1;TRINITY_DN3092_c0_g1_i2_orf1;TRINITY_DN46409_c0_g1_i1_orf1;TRINITY_DN89083_c0_g1_i1_orf1;TRINITY_DN129_c0_g1_i6_orf1;TRINITY_DN19810_c1_g1_i7_orf1;TRINITY_DN2168_c0_g1_i2_orf1;TRINITY_DN7739_c0_g1_i2_orf1;TRINITY_DN3057_c0_g2_i1_orf1;TRINITY_DN33248_c0_g1_i1_orf1;TRINITY_DN1322_c0_g1_i4_orf1;TRINITY_DN3664_c0_g1_i8_orf1;TRINITY_DN19262_c0_g1_i1_orf1;TRINITY_DN5081_c0_g1_i5_orf1;TRINITY
cellular_component	postsynaptic specialization	GO:0099572	3	3/3516	TRINITY_DN4016_c0_g1_i1_orf1;TRINITY_DN142442_c0_g1_i1_orf1;TRINITY_DN130075_c1_g2_i1_orf1
cellular_component	extracellular organelle	GO:0043230	15	15/3516	TRINITY_DN4016_c0_g1_i1_orf1;TRINITY_DN30300_c0_g2_i1_orf1;TRINITY_DN975_c0_g1_i1_orf1;TRINITY_DN7493_c0_g1_i1_orf1;TRINITY_DN147475_c0_g1_i1_orf1;TRINITY_DN42854_c0_g3_i2_orf1;TRINITY_DN46409_c0_g1_i1_orf1;TRINITY_DN96739_c0_g1_i1_orf1;TRINITY_DN22430_c0_g3_i1_orf1;TRINITY_DN20133_c0_g1_i1_orf1;TRINITY_DN20009_c0_g1_i1_orf1;TRINITY_DN59965_c0_g4_i1_orf1;TRINITY_DN2848_c0_g1_i2_orf1;TRINITY_DN55148_c0_g1_i1_orf1;TRINITY_DN10070_c0_g1_i1_orf1
cellular_component	striated muscle thin filament	GO:0005865	1	1/3516	TRINITY_DN235_c0_g3_i1_orf1
cellular_component	neuronal cell body	GO:0043025	1	1/3516	TRINITY_DN10070_c0_g1_i1_orf1
cellular_component	organelle membrane	GO:0031090	113	113/3516	TRINITY_DN9608_c0_g1_i3_orf1;TRINITY_DN13923_c0_g2_i1_orf1;TRINITY_DN2312_c0_g1_i4_orf1;TRINITY_DN11392_c0_g1_i4_orf1;TRINITY_DN10476_c0_g1_i1_orf1;TRINITY_DN3461_c0_g1_i1_orf1;TRINITY_DN72816_c0_g1_i2_orf1;TRINITY_DN5169_c0_g1_i5_orf1;TRINITY_DN5867_c0_g1_i1_orf1;TRINITY_DN8511_c0_g1_i1_orf1;TRINITY_DN11069_c0_g2_i1_orf1;TRINITY_DN1134_c0_g1_i4_orf1;TRINITY_DN106476_c0_g1_i3_orf1;TRINITY_DN1132_c0_g1_i5_orf1;TRINITY_DN2848_c0_g1_i1_orf1;TRINITY_DN95558_c0_g3_i1_orf1;TRINITY_DN19122_c0_g1_i7_orf1;TRINITY_DN448_c0_g1_i20_orf1;TRINITY_DN16084_c0_g1_i4_orf1;TRINITY_DN2267_c0_g1_i1_orf1;TRINITY_DN5439_c0_g1_i2_orf1;TRINITY_DN27641_c0_g1_i1_orf1;TRINITY_DN10745_c0_g1_i14_orf1;TRINITY_DN24528_c0_g1_i1_orf1;TRINITY_DN2181_c1_g1_i8_orf1;TRINITY_DN883_c0_g1_i8_orf1;TRINITY_DN24751_c0_g1_i1_orf1;TRINITY_DN46409_c0_g1_i1_orf1;TRINITY_DN4814_c0_g1_i6_orf1;TRINITY_DN3450_c0_g1_i3_orf1;TRINITY_DN4394_c0_g1_i4_orf1;TRINITY_DN11172_c1_g1_i1_orf1;TRINITY_DN2848_c0_g1_i2_orf1;TRINITY_DN19000_c0_g1_i4_orf1;TRINITY_DN59965_c0_g4_i1_orf1;TRINITY_DN24043_c0_g1_i1_orf1;TRINITY_DN29038_c0_g2_i1_orf1;TRINITY_DN22242_c0_g1_i1_orf1;TRINITY_DN5982_c0_g1_i3_orf1;TRINITY_DN135781_c0_g1_i1_orf1;TRINITY_DN9316_c0_g3_i1_orf1;TRINITY_DN7336_c0_g1_i13_orf1;TRINITY_DN154_c0_g1_i4_orf1;TRINITY_DN2286_c2_g1_i1_orf1;TRINITY_DN40911_c0_g1_i1_orf1;TRINITY_DN6680_c0_g1_i1_orf1;TRINITY_DN3209_c0_g2_i6_orf1;TRINITY_DN83005_c0_g1_i1_orf1;TRINITY_DN5064_c0_g1_i4_orf1;TRINITY_DN9002_c0_g1_i1_orf1;TRINITY_DN22044_c0_g2_i1_orf1;TRINITY_DN8173_c0_g1_i3_orf1;TRINITY_DN13783_c0_g4_i2_orf1;TRINITY_DN22242_c0_g2_i1_orf1;TRINITY_DN3821_c1_g1_i7_orf1;TRINITY_DN12823_c0_g1_i1_orf1;TRINITY_DN24873_c0_g1_i4_orf1;TRINITY_DN6243_c0_g1_i5_orf1;TRINITY_DN14262_c0_g1_i5_orf1;TRINITY_DN3619_c0_g2_i1_orf1;TRINITY_DN31851_c0_g1_i2_orf1;TRINITY_DN4538_c0_g1_i4_orf1;TRINITY_DN43656_c0_g1_i1_orf1;TRINITY_DN1348_c0_g1_i1_orf1;TRINITY_DN22430_c0_g3_i1_orf1;TRINITY_DN2238_c0_g2_i1_orf1;TRINITY_DN10379_c0_g1_i3_orf1;TRINITY_DN5122_c0_g1_i3_orf1;TRINITY_DN124300_c0_g1_i2_orf1;TRINITY_DN1384_c0_g1_i5_orf1;TRINITY_DN96739_c0_g1_i1_orf1;TRINITY_DN32780_c0_g1_i2_orf1;TRINITY_DN96557_c0_g1_i1_orf1;TRINITY_DN79210_c0_g1_i1_orf1;TRINITY_DN5630_c4_g1_i2_orf1;TRINITY_DN42854_c0_g3_i2_orf1;TRINITY_DN164_c0_g1_i11_orf1;TRINITY_DN3434_c0_g1_i1_orf1;TRINITY_DN3562_c0_g1_i4_orf1;TRINITY_DN642_c0_g1_i6_orf1;TRINITY_DN6535_c0_g2_i1_orf1;TRINITY_DN245_c0_g1_i4_orf1;TRINITY_DN5559_c0_g1_i1_orf1;TRINITY_DN31676_c0_g1_i4_orf1;TRINITY_DN4790_c0_g1_i6_orf1;TRINITY_DN13186_c0_g1_i1_orf1;TRINITY_DN15448_c0_g1_i1_orf1;TRINITY_DN327_c1_g1_i4_orf1;TRINITY_DN35635_c0_g1_i1_orf1;TRINITY_DN57765_c0_g1_i1_orf1;TRINITY_DN1280_c0_g1_i1_orf1;TRINITY_DN21981_c0_g1_i8_orf1;TRINITY_DN20984_c0_g1_i4_orf1;TRINITY_DN19092_c2_g1_i1_orf1;TRINITY_DN40197_c0_g1_i1_orf1;TRINITY_DN2879_c0_g1_i4_orf1;TRINITY_DN3513_c0_g1_i5_orf1;TRINITY_DN11172_c0_g1_i4_orf1;TRINITY_DN1447_c0_g1_i5_orf1;TRINITY_DN2264_c0_g1_i1_orf1;TRINITY_DN1960_c5_g1_i3_orf1;TRINITY_DN4443_c0_g1_i4_orf1;TRINITY_DN12286_c1_g1_i2_orf1;TRINITY_DN52887_c0_g1_i1_orf1;TRINITY_DN9931_c0_g1_i1_orf1;TRINITY_DN37366_c0_g1_i7_orf1;TRINITY_DN5697_c0_g1_i1_orf1;TRINITY_DN3134_c0_g1_i1_orf1;TRINITY_DN760_c1_g2_i6_orf1;TRINITY_DN7626_c0_g1_i1_orf1;TRINITY_DN25210_c0_g1_i1_orf1;TRINITY_DN5558_c0_g1_i4_orf1;TRINITY_DN8747_c0_g1_i2_orf1

cellular_component	outer membrane	GO:0019867	6	6/3516	TRINITY_DN25210_c0_g1_i1_orf1;TRINITY_DN164_c0_g1_i11_orf1;TRINITY_DN19092_c2_g1_i1_orf1;TRINITY_DN142657_c0_g1_i1_orf1;TRINITY_DN3619_c0_g2_i1_orf1;TRINITY_DN327_c1_g1_i4_orf1
cellular_component	plasma membrane	GO:0005886	43	43/3516	TRINITY_DN28759_c0_g1_i1_orf1;TRINITY_DN51938_c0_g3_i1_orf1;TRINITY_DN23926_c0_g1_i4_orf1;TRINITY_DN21214_c0_g2_i1_orf1;TRINITY_DN198_c2_g1_i2_orf1;TRINITY_DN7128_c0_g1_i7_orf1;TRINITY_DN46409_c0_g1_i1_orf1;TRINITY_DN75086_c0_g1_i5_orf1;TRINITY_DN15706_c0_g2_i5_orf1;TRINITY_DN5064_c0_g1_i4_orf1;TRINITY_DN745_c5_g1_i2_orf1;TRINITY_DN20710_c0_g2_i2_orf1;TRINITY_DN39725_c0_g1_i4_orf1;TRINITY_DN2735_c0_g1_i4_orf1;TRINITY_DN5406_c0_g2_i1_orf1;TRINITY_DN975_c0_g1_i1_orf1;TRINITY_DN1352_c0_g1_i5_orf1;TRINITY_DN22430_c0_g3_i1_orf1;TRINITY_DN5553_c0_g1_i4_orf1;TRINITY_DN20009_c0_g1_i1_orf1;TRINITY_DN7590_c0_g1_i4_orf1;TRINITY_DN115_c0_g1_i6_orf1;TRINITY_DN2848_c0_g1_i1_orf1;TRINITY_DN111985_c0_g1_i1_orf1;TRINITY_DN492_c0_g1_i4_orf1;TRINITY_DN6247_c0_g1_i2_orf1;TRINITY_DN10070_c0_g1_i1_orf1;TRINITY_DN2175_c0_g1_i4_orf1;TRINITY_DN1012_c0_g1_i2_orf1;TRINITY_DN10581_c0_g1_i5_orf1;TRINITY_DN26337_c0_g1_i3_orf1;TRINITY_DN655_c0_g1_i3_orf1;TRINITY_DN4694_c0_g1_i6_orf1;TRINITY_DN32780_c0_g1_i2_orf1;TRINITY_DN12256_c0_g1_i1_orf1;TRINITY_DN14460_c0_g1_i6_orf1;TRINITY_DN11670_c0_g1_i1_orf1;TRINITY_DN42854_c0_g3_i2_orf1;TRINITY_DN2947_c0_g1_i4_orf1;TRINITY_DN57348_c0_g1_i4_orf1;TRINITY_DN20133_c0_g1_i1_orf1;TRINITY_DN932_c0_g1_i4_orf1;TRINITY_DN2848_c0_g1_i2_orf1
cellular_component	membrane microdomain	GO:0098857	2	2/3516	TRINITY_DN111985_c0_g1_i1_orf1;TRINITY_DN315_c0_g1_i1_orf1
cellular_component	plasma membrane region	GO:0098590	10	10/3516	TRINITY_DN51938_c0_g3_i1_orf1;TRINITY_DN2257_c0_g1_i4_orf1;TRINITY_DN975_c0_g1_i1_orf1;TRINITY_DN7493_c0_g1_i1_orf1;TRINITY_DN49527_c0_g1_i1_orf1;TRINITY_DN46409_c0_g1_i1_orf1;TRINITY_DN10745_c0_g1_i14_orf1;TRINITY_DN10796_c0_g2_i1_orf1;TRINITY_DN486_c0_g1_i5_orf1;TRINITY_DN7128_c0_g1_i7_orf1
cellular_component	phagophore assembly site membrane	GO:0034045	2	2/3516	TRINITY_DN113353_c0_g1_i1_orf1;TRINITY_DN10229_c0_g1_i6_orf1
cellular_component	dendritic spine	GO:0043197	1	1/3516	TRINITY_DN802_c0_g1_i2_orf1
cellular_component	extracellular matrix	GO:0031012	7	7/3516	TRINITY_DN12960_c0_g1_i1_orf1;TRINITY_DN35147_c0_g1_i1_orf1;TRINITY_DN4464_c0_g2_i1_orf1;TRINITY_DN96739_c0_g1_i1_orf1;TRINITY_DN2919_c0_g1_i5_orf1;TRINITY_DN376_c1_g1_i1_orf1;TRINITY_DN10070_c0_g1_i1_orf1
cellular_component	egg chorion	GO:0042600	3	3/3516	TRINITY_DN12514_c0_g2_i1_orf1;TRINITY_DN5933_c0_g1_i1_orf1;TRINITY_DN51252_c0_g2_i1_orf1
cellular_component	synapse	GO:0045202	6	6/3516	TRINITY_DN4016_c0_g1_i1_orf1;TRINITY_DN142442_c0_g1_i1_orf1;TRINITY_DN2047_c0_g1_i1_orf1;TRINITY_DN140538_c0_g2_i1_orf1;TRINITY_DN10070_c0_g1_i1_orf1;TRINITY_DN2848_c0_g1_i1_orf1
cellular_component	anchoring junction	GO:0070161	21	21/3516	TRINITY_DN111985_c0_g1_i1_orf1;TRINITY_DN28759_c0_g1_i1_orf1;TRINITY_DN741_c0_g1_i10_orf1;TRINITY_DN7590_c0_g1_i4_orf1;TRINITY_DN364_c0_g2_i1_orf1;TRINITY_DN4016_c0_g1_i1_orf1;TRINITY_DN30300_c0_g2_i1_orf1;TRINITY_DN655_c0_g1_i3_orf1;TRINITY_DN9383_c0_g1_i3_orf1;TRINITY_DN7128_c0_g1_i7_orf1;TRINITY_DN2186_c0_g1_i17_orf1;TRINITY_DN7493_c0_g1_i1_orf1;TRINITY_DN96739_c0_g1_i1_orf1;TRINITY_DN802_c0_g1_i2_orf1;TRINITY_DN142442_c0_g1_i1_orf1;TRINITY_DN6247_c0_g1_i2_orf1;TRINITY_DN364_c1_g1_i2_orf1;TRINITY_DN20009_c0_g1_i1_orf1;TRINITY_DN467_c9_g1_i2_orf1;TRINITY_DN23746_c0_g1_i2_orf1;TRINITY_DN22430_c0_g3_i1_orf1
cellular_component	ruffle membrane	GO:0032587	2	2/3516	TRINITY_DN975_c0_g1_i1_orf1;TRINITY_DN7493_c0_g1_i1_orf1
cellular_component	lamellipodium membrane	GO:0031258	1	1/3516	TRINITY_DN7493_c0_g1_i1_orf1
cellular_component	plasma membrane bounded cell projection	GO:0120025	12	12/3516	TRINITY_DN111985_c0_g1_i1_orf1;TRINITY_DN364_c0_g2_i1_orf1;TRINITY_DN4016_c0_g1_i1_orf1;TRINITY_DN5954_c0_g1_i2_orf1;TRINITY_DN101922_c0_g1_i1_orf1;TRINITY_DN9383_c0_g1_i3_orf1;TRINITY_DN802_c0_g1_i2_orf1;TRINITY_DN7493_c0_g1_i1_orf1;TRINITY_DN364_c1_g1_i2_orf1;TRINITY_DN20009_c0_g1_i1_orf1;TRINITY_DN81719_c0_g1_i1_orf1;TRINITY_DN741_c0_g1_i10_orf1
cellular_component	growth cone	GO:0030426	1	1/3516	TRINITY_DN7493_c0_g1_i1_orf1
cellular_component	nuclear speck	GO:0016607	3	3/3516	TRINITY_DN140538_c0_g2_i1_orf1;TRINITY_DN19413_c0_g1_i2_orf1;TRINITY_DN47123_c0_g1_i1_orf1
cellular_component	cytosolic region	GO:0099522	1	1/3516	TRINITY_DN140538_c0_g2_i1_orf1
cellular_component	ciliary basal body	GO:0036064	1	1/3516	TRINITY_DN140538_c0_g2_i1_orf1
cellular_component	centrosome	GO:0005813	1	1/3516	TRINITY_DN21214_c0_g2_i1_orf1
cellular_component	kinetochore	GO:0000776	1	1/3516	TRINITY_DN96557_c0_g1_i1_orf1
cellular_component	ribonucleoprotein granule	GO:0035770	5	5/3516	TRINITY_DN4016_c0_g1_i1_orf1;TRINITY_DN1298_c0_g1_i3_orf1;TRINITY_DN50725_c0_g1_i6_orf1;TRINITY_DN12576_c0_g1_i2_orf1;TRINITY_DN29402_c0_g1_i1_orf1
cellular_component	supramolecular polymer	GO:0099081	16	16/3516	TRINITY_DN96557_c0_g1_i1_orf1;TRINITY_DN63561_c1_g1_i2_orf1;TRINITY_DN107_c0_g1_i1_orf1;TRINITY_DN4808_c0_g1_i3_orf1;TRINITY_DN14298_c0_g1_i1_orf1;TRINITY_DN280_c0_g1_i12_orf1;TRINITY_DN350_c0_g1_i5_orf1;TRINITY_DN26243_c0_g1_i2_orf1;TRINITY_DN315_c0_g1_i1_orf1;TRINITY_DN235_c0_g3_i1_orf1;TRINITY_DN2745_c0_g1_i2_orf1;TRINITY_DN97138_c0_g1_i2_orf1;TRINITY_DN14298_c0_g3_i1_orf1;TRINITY_DN20009_c0_g1_i1_orf1;TRINITY_DN10521_c0_g1_i7_orf1;TRINITY_DN2745_c0_g1_i4_orf1
molecular_function	mRNA regulatory element binding translation repressor activity	GO:0000900	1	1/3516	TRINITY_DN3673_c0_g1_i10_orf1

molecular_function	translation factor activity, RNA binding	GO:0008135	52	52/3516	TRINITY_DN2265_c0_g2_i1_orf1;TRINITY_DN1771_c0_g2_i1_orf1;TRINITY_DN33967_c0_g1_i1_orf1;TRINITY_DN11612_c0_g3_i1_orf1;TRINITY_DN31503_c0_g1_i4_orf1;TRINITY_DN9575_c0_g1_i1_orf1;TRINITY_DN975_c0_g1_i1_orf1;TRINITY_DN38412_c0_g1_i1_orf1;TRINITY_DN33249_c0_g1_i1_orf1;TRINITY_DN2716_c0_g2_i1_orf1;TRINITY_DN33248_c0_g1_i1_orf1;TRINITY_DN4237_c1_g1_i5_orf1;TRINITY_DN44407_c0_g4_i2_orf1;TRINITY_DN15362_c0_g1_i1_orf1;TRINITY_DN136906_c0_g1_i1_orf1;TRINITY_DN22572_c0_g1_i1_orf1;TRINITY_DN4381_c0_g2_i1_orf1;TRINITY_DN3878_c0_g1_i4_orf1;TRINITY_DN48096_c0_g2_i2_orf1;TRINITY_DN147517_c0_g1_i1_orf1;TRINITY_DN27751_c0_g2_i1_orf1;TRINITY_DN14498_c0_g1_i1_orf1;TRINITY_DN29521_c0_g1_i1_orf1;TRINITY_DN17045_c0_g2_i3_orf1;TRINITY_DN2630_c0_g3_i3_orf1;TRINITY_DN36817_c0_g1_i1_orf1;TRINITY_DN19092_c0_g1_i2_orf1;TRINITY_DN53684_c0_g1_i1_orf1;TRINITY_DN50085_c0_g1_i1_orf1;TRINITY_DN1572_c0_g1_i6_orf1;TRINITY_DN9164_c0_g1_i3_orf1;TRINITY_DN17049_c0_g1_i6_orf1;TRINITY_DN53311_c0_g2_i1_orf1;TRINITY_DN25136_c0_g1_i1_orf1;TRINITY_DN9498_c0_g1_i3_orf1;TRINITY_DN4309_c0_g1_i1_orf1;TRINITY_DN3366_c0_g1_i6_orf1;TRINITY_DN94625_c0_g1_i1_orf1;TRINITY_DN19659_c1_g1_i1_orf1;TRINITY_DN24317_c0_g1_i7_orf1;TRINITY_DN31232_c1_g1_i9_orf1;TRINITY_DN5086_c0_g1_i1_orf1;TRINITY_DN2265_c0_g1_i5_orf1;TRINITY_DN34509_c0_g1_i1_orf1;TRINITY_DN21609_c0_g2_i1_orf1;TRINITY_DN1074_c0_g1_i7_orf1;TRINITY_DN32822_c0_g1_i1_orf1;TRINITY_DN126648_c0_g1_i1_orf1;TRINITY_DN33619_c0_g1_i1_orf1;TRINITY_DN10871_c0_g1_i3_orf1;TRINITY_DN21000_c0_g1_i1_orf1;TRINITY_DN6239_c0_g1_i1_orf1
molecular_function	transcription corepressor activity	GO:0003714	3	3/3516	TRINITY_DN21214_c0_g2_i1_orf1;TRINITY_DN34726_c0_g2_i1_orf1;TRINITY_DN1921_c1_g1_i5_orf1
molecular_function	transcription coactivator activity	GO:0003713	1	1/3516	TRINITY_DN9510_c0_g2_i1_orf1
molecular_function	DNA-binding transcription factor activity, RNA polymerase II-specific	GO:0000981	1	1/3516	TRINITY_DN1926_c0_g1_i5_orf1
molecular_function	DNA-binding transcription repressor activity	GO:0001217	1	1/3516	TRINITY_DN1926_c0_g1_i5_orf1
molecular_function	RNA helicase activity	GO:0003724	18	18/3516	TRINITY_DN2904_c0_g1_i4_orf1;TRINITY_DN20499_c0_g3_i1_orf1;TRINITY_DN15845_c0_g1_i1_orf1;TRINITY_DN4381_c0_g2_i1_orf1;TRINITY_DN44288_c0_g1_i2_orf1;TRINITY_DN4380_c0_g1_i9_orf1;TRINITY_DN4950_c0_g1_i2_orf1;TRINITY_DN31503_c0_g1_i4_orf1;TRINITY_DN13094_c0_g1_i1_orf1;TRINITY_DN19920_c1_g1_i2_orf1;TRINITY_DN16174_c0_g1_i2_orf1;TRINITY_DN26168_c0_g1_i1_orf1;TRINITY_DN12495_c0_g1_i2_orf1;TRINITY_DN8980_c0_g1_i2_orf1;TRINITY_DN59291_c0_g1_i1_orf1;TRINITY_DN7213_c0_g1_i2_orf1;TRINITY_DN2709_c0_g1_i4_orf1;TRINITY_DN2535_c0_g1_i4_orf1
molecular_function	minus-end-directed microtubule motor activity	GO:0008569	1	1/3516	TRINITY_DN26243_c0_g1_i2_orf1
molecular_function	DNA helicase activity	GO:0003678	7	7/3516	TRINITY_DN25345_c0_g1_i1_orf1;TRINITY_DN125565_c1_g1_i1_orf1;TRINITY_DN6642_c0_g1_i2_orf1;TRINITY_DN15370_c0_g1_i4_orf1;TRINITY_DN109733_c0_g1_i1_orf1;TRINITY_DN452_c1_g1_i3_orf1;TRINITY_DN3057_c0_g2_i1_orf1
molecular_function	ATP-dependent chromatin remodeler activity	GO:0140658	3	3/3516	TRINITY_DN3057_c0_g2_i1_orf1;TRINITY_DN25345_c0_g1_i1_orf1;TRINITY_DN12820_c0_g1_i1_orf1
molecular_function	DNA topoisomerase type II (double strand cut, ATP-hydrolyzing) activity	GO:0003918	1	1/3516	TRINITY_DN4908_c1_g1_i5_orf1
molecular_function	DNA clamp loader activity	GO:0003689	1	1/3516	TRINITY_DN3092_c0_g1_i2_orf1
molecular_function	long-chain fatty acid-CoA ligase activity	GO:0004467	1	1/3516	TRINITY_DN2193_c0_g1_i7_orf1
molecular_function	ABC-type transporter activity	GO:0140359	12	12/3516	TRINITY_DN13563_c0_g1_i1_orf1;TRINITY_DN1786_c0_g1_i11_orf1;TRINITY_DN5908_c0_g1_i2_orf1;TRINITY_DN4911_c0_g1_i6_orf1;TRINITY_DN31327_c0_g2_i1_orf1;TRINITY_DN16408_c0_g1_i1_orf1;TRINITY_DN157_c0_g1_i4_orf1;TRINITY_DN14937_c0_g1_i7_orf1;TRINITY_DN60792_c0_g1_i2_orf1;TRINITY_DN2826_c0_g1_i7_orf1;TRINITY_DN37218_c0_g1_i12_orf1;TRINITY_DN2706_c0_g1_i3_orf1
molecular_function	P-type transmembrane transporter activity	GO:0140358	2	2/3516	TRINITY_DN7336_c0_g1_i13_orf1;TRINITY_DN4977_c0_g1_i2_orf1
molecular_function	ATPase-coupled cation transmembrane transporter activity	GO:0019829	18	18/3516	TRINITY_DN7336_c0_g1_i13_orf1;TRINITY_DN25975_c0_g3_i2_orf1;TRINITY_DN47605_c0_g2_i1_orf1;TRINITY_DN6221_c0_g1_i5_orf1;TRINITY_DN79210_c0_g1_i1_orf1;TRINITY_DN4434_c0_g1_i7_orf1;TRINITY_DN21722_c0_g1_i3_orf1;TRINITY_DN1366_c0_g1_i5_orf1;TRINITY_DN4977_c0_g1_i2_orf1;TRINITY_DN2300_c0_g1_i1_orf1;TRINITY_DN45000_c0_g1_i5_orf1;TRINITY_DN22430_c0_g3_i1_orf1;TRINITY_DN1044_c0_g1_i2_orf1;TRINITY_DN9715_c0_g1_i1_orf1;TRINITY_DN10458_c0_g1_i1_orf1;TRINITY_DN17351_c0_g1_i3_orf1;TRINITY_DN10637_c0_g1_i4_orf1;TRINITY_DN700_c0_g1_i3_orf1
molecular_function	ATPase-coupled ion transmembrane transporter activity	GO:0042625	16	16/3516	TRINITY_DN25975_c0_g3_i2_orf1;TRINITY_DN47605_c0_g2_i1_orf1;TRINITY_DN6221_c0_g1_i5_orf1;TRINITY_DN79210_c0_g1_i1_orf1;TRINITY_DN4434_c0_g1_i7_orf1;TRINITY_DN21722_c0_g1_i3_orf1;TRINITY_DN1366_c0_g1_i5_orf1;TRINITY_DN2300_c0_g1_i1_orf1;TRINITY_DN45000_c0_g1_i5_orf1;TRINITY_DN22430_c0_g3_i1_orf1;TRINITY_DN1044_c0_g1_i2_orf1;TRINITY_DN9715_c0_g1_i1_orf1;TRINITY_DN10458_c0_g1_i1_orf1;TRINITY_DN17351_c0_g1_i3_orf1;TRINITY_DN10637_c0_g1_i4_orf1;TRINITY_DN700_c0_g1_i3_orf1
molecular_function	signaling adaptor activity	GO:0035591	1	1/3516	TRINITY_DN21545_c0_g1_i2_orf1
molecular_function	cytoskeletal anchor activity	GO:0008093	2	2/3516	TRINITY_DN21559_c0_g2_i1_orf1;TRINITY_DN21559_c0_g1_i2_orf1
molecular_function	SNAP receptor activity	GO:0005484	2	2/3516	TRINITY_DN383_c0_g1_i1_orf1;TRINITY_DN5558_c0_g1_i4_orf1
molecular_function	very-low-density lipoprotein particle receptor	GO:0030229	1	1/3516	TRINITY_DN585_c0_g1_i12_orf1
molecular_function	nuclear export signal receptor activity	GO:0005049	1	1/3516	TRINITY_DN3747_c1_g1_i3_orf1
molecular_function	nuclear import signal receptor activity	GO:0061608	1	1/3516	TRINITY_DN147427_c0_g1_i1_orf1
molecular_function	membrane insertase activity	GO:0032977	1	1/3516	TRINITY_DN64759_c0_g1_i1_orf1
molecular_function	copper chaperone activity	GO:0016531	2	2/3516	TRINITY_DN825_c2_g1_i5_orf1;TRINITY_DN3461_c0_g1_i1_orf1
molecular_function	ATP-dependent FeS chaperone activity	GO:0140663	2	2/3516	TRINITY_DN85476_c0_g1_i1_orf1;TRINITY_DN18558_c0_g1_i7_orf1
molecular_function	lactoperoxidase activity	GO:0140825	1	1/3516	TRINITY_DN3321_c0_g1_i3_orf1
molecular_function	peroxiredoxin activity	GO:0051920	5	5/3516	TRINITY_DN111985_c0_g1_i1_orf1;TRINITY_DN2542_c0_g2_i1_orf1;TRINITY_DN7579_c1_g3_i1_orf1;TRINITY_DN791_c0_g1_i2_orf1;TRINITY_DN69236_c0_g1_i1_orf1
molecular_function	catalase activity	GO:0004096	3	3/3516	TRINITY_DN54387_c0_g1_i1_orf1;TRINITY_DN285_c0_g1_i4_orf1;TRINITY_DN6580_c0_g1_i4_orf1
molecular_function	glutathione peroxidase activity	GO:0004602	3	3/3516	TRINITY_DN80660_c0_g1_i1_orf1;TRINITY_DN1622_c0_g1_i6_orf1;TRINITY_DN21420_c0_g1_i2_orf1

molecular_function	phospholipid transporter activity	GO:0005548	6	6/3516	TRINITY_DN101658_c0_g1_i1_orf1;TRINITY_DN2160_c0_g1_i13_orf1;TRINITY_DN72369_c0_g1_i1_orf1;TRINITY_DN15896_c0_g1_i4_orf1;TRINITY_DN42310_c0_g1_i1_orf1;TRINITY_DN79657_c0_g1_i1_orf1
molecular_function	protein transmembrane transporter activity	GO:0008320	1	1/3516	TRINITY_DN327_c1_g1_i4_orf1
molecular_function	organophosphate ester transmembrane transporter activity	GO:0015605	1	1/3516	TRINITY_DN760_c1_g2_i6_orf1
molecular_function	neurotransmitter transmembrane transporter	GO:0005326	1	1/3516	TRINITY_DN501_c1_g1_i1_orf1
molecular_function	sulfur compound transmembrane transporter	GO:1901682	1	1/3516	TRINITY_DN21331_c0_g1_i6_orf1
molecular_function	carbohydrate transmembrane transporter activity	GO:0015144	4	4/3516	TRINITY_DN2735_c0_g1_i4_orf1;TRINITY_DN745_c5_g1_i2_orf1;TRINITY_DN57348_c0_g1_i4_orf1;TRINITY_DN10581_c0_g1_i5_orf1
molecular_function	passive transmembrane transporter activity	GO:0022803	13	13/3516	TRINITY_DN18338_c0_g1_i6_orf1;TRINITY_DN34821_c0_g1_i4_orf1;TRINITY_DN11569_c0_g1_i1_orf1;TRINITY_DN20558_c0_g1_i2_orf1;TRINITY_DN4434_c0_g1_i7_orf1;TRINITY_DN96739_c0_g1_i1_orf1;TRINITY_DN2300_c0_g1_i1_orf1;TRINITY_DN45000_c0_g1_i5_orf1;TRINITY_DN96080_c0_g2_i1_orf1;TRINITY_DN22430_c0_g3_i1_orf1;TRINITY_DN83005_c0_g1_i1_orf1;TRINITY_DN7787_c0_g1_i1_orf1;TRINITY_DN80560_c0_g1_i1_orf1
molecular_function	active transmembrane transporter activity	GO:0022804	44	44/3516	TRINITY_DN13563_c0_g1_i1_orf1;TRINITY_DN5908_c0_g1_i2_orf1;TRINITY_DN1366_c0_g1_i5_orf1;TRINITY_DN4977_c0_g1_i2_orf1;TRINITY_DN2300_c0_g1_i1_orf1;TRINITY_DN29_c0_g1_i4_orf1;TRINITY_DN14937_c0_g1_i7_orf1;TRINITY_DN76036_c0_g1_i1_orf1;TRINITY_DN4911_c0_g1_i6_orf1;TRINITY_DN501_c1_g1_i1_orf1;TRINITY_DN1044_c0_g1_i2_orf1;TRINITY_DN1422_c0_g1_i4_orf1;TRINITY_DN9715_c0_g1_i1_orf1;TRINITY_DN20279_c0_g1_i1_orf1;TRINITY_DN2267_c0_g1_i1_orf1;TRINITY_DN22430_c0_g3_i1_orf1;TRINITY_DN7336_c0_g1_i13_orf1;TRINITY_DN1786_c0_g1_i11_orf1;TRINITY_DN21331_c0_g1_i6_orf1;TRINITY_DN4434_c0_g1_i7_orf1;TRINITY_DN21722_c0_g1_i3_orf1;TRINITY_DN47605_c0_g2_i1_orf1;TRINITY_DN12666_c0_g1_i2_orf1;TRINITY_DN60792_c0_g1_i2_orf1;TRINITY_DN2826_c0_g1_i7_orf1;TRINITY_DN17351_c0_g1_i3_orf1;TRINITY_DN37218_c0_g1_i12_orf1;TRINITY_DN10637_c0_g1_i4_orf1;TRINITY_DN700_c0_g1_i3_orf1;TRINITY_DN2706_c0_g1_i3_orf1;TRINITY_DN25975_c0_g3_i2_orf1;TRINITY_DN6221_c0_g1_i5_orf1;TRINITY_DN31327_c0_g2_i1_orf1;TRINITY_DN760_c1_g2_i6_orf1;TRINITY_DN16408_c0_g1_i1_orf1;TRINITY_DN79210_c0_g1_i1_orf1;TRINITY_DN7626_c0_g1_i1_orf1;TRINITY_DN4040_c0_g1_i10_orf1;TRINITY_DN26186_c0_g1_i7_orf1;TRINITY_DN45000_c0_g1_i5_orf1;TRINITY_DN157_c0_g1_i4_orf1;TRINITY_DN10458_c0_g1_i1_orf1;TRINITY_DN10030_c0_g1_i2_orf1;TRINITY_DN6381_c0_g1_i2_orf1
molecular_function	macromolecule transmembrane transporter activity	GO:0022884	1	1/3516	TRINITY_DN327_c1_g1_i4_orf1
molecular_function	carbohydrate derivative transmembrane transporter activity	GO:1901505	1	1/3516	TRINITY_DN760_c1_g2_i6_orf1
molecular_function	nucleobase-containing compound transmembrane transporter activity	GO:0015932	1	1/3516	TRINITY_DN760_c1_g2_i6_orf1
molecular_function	ion transmembrane transporter activity	GO:0015075	44	44/3516	TRINITY_DN13923_c0_g2_i1_orf1;TRINITY_DN1366_c0_g1_i5_orf1;TRINITY_DN4977_c0_g1_i2_orf1;TRINITY_DN2300_c0_g1_i1_orf1;TRINITY_DN80560_c0_g1_i1_orf1;TRINITY_DN4040_c0_g1_i10_orf1;TRINITY_DN26429_c0_g1_i4_orf1;TRINITY_DN98538_c0_g1_i1_orf1;TRINITY_DN26649_c0_g1_i2_orf1;TRINITY_DN29038_c0_g2_i1_orf1;TRINITY_DN1882_c0_g1_i4_orf1;TRINITY_DN76036_c0_g1_i1_orf1;TRINITY_DN11569_c0_g1_i1_orf1;TRINITY_DN501_c1_g1_i1_orf1;TRINITY_DN86090_c0_g1_i1_orf1;TRINITY_DN1044_c0_g1_i2_orf1;TRINITY_DN34821_c0_g1_i4_orf1;TRINITY_DN22430_c0_g3_i1_orf1;TRINITY_DN9715_c0_g1_i1_orf1;TRINITY_DN7336_c0_g1_i13_orf1;TRINITY_DN15222_c0_g1_i4_orf1;TRINITY_DN21331_c0_g1_i6_orf1;TRINITY_DN9354_c0_g1_i7_orf1;TRINITY_DN20558_c0_g1_i2_orf1;TRINITY_DN4434_c0_g1_i7_orf1;TRINITY_DN21722_c0_g1_i3_orf1;TRINITY_DN47605_c0_g2_i1_orf1;TRINITY_DN96739_c0_g1_i1_orf1;TRINITY_DN12286_c1_g1_i2_orf1;TRINITY_DN17351_c0_g1_i3_orf1;TRINITY_DN10637_c0_g1_i4_orf1;TRINITY_DN700_c0_g1_i3_orf1;TRINITY_DN25975_c0_g3_i2_orf1;TRINITY_DN6221_c0_g1_i5_orf1;TRINITY_DN79210_c0_g1_i1_orf1;TRINITY_DN760_c1_g2_i6_orf1;TRINITY_DN107261_c0_g1_i1_orf1;TRINITY_DN45000_c0_g1_i5_orf1;TRINITY_DN96080_c0_g2_i1_orf1;TRINITY_DN83005_c0_g1_i1_orf1;TRINITY_DN10458_c0_g1_i1_orf1;TRINITY_DN7787_c0_g1_i1_orf1;TRINITY_DN6381_c0_g1_i2_orf1;TRINITY_DN91946_c0_g1_i1_orf1
molecular_function	inorganic molecular entity transmembrane transporter activity	GO:0015318	41	41/3516	TRINITY_DN1366_c0_g1_i5_orf1;TRINITY_DN4977_c0_g1_i2_orf1;TRINITY_DN2300_c0_g1_i1_orf1;TRINITY_DN80560_c0_g1_i1_orf1;TRINITY_DN4040_c0_g1_i10_orf1;TRINITY_DN98538_c0_g1_i1_orf1;TRINITY_DN26649_c0_g1_i2_orf1;TRINITY_DN29038_c0_g2_i1_orf1;TRINITY_DN34821_c0_g1_i4_orf1;TRINITY_DN76036_c0_g1_i1_orf1;TRINITY_DN11569_c0_g1_i1_orf1;TRINITY_DN501_c1_g1_i1_orf1;TRINITY_DN86090_c0_g1_i1_orf1;TRINITY_DN1044_c0_g1_i2_orf1;TRINITY_DN22430_c0_g3_i1_orf1;TRINITY_DN2267_c0_g1_i1_orf1;TRINITY_DN21331_c0_g1_i6_orf1;TRINITY_DN9715_c0_g1_i1_orf1;TRINITY_DN7336_c0_g1_i13_orf1;TRINITY_DN15222_c0_g1_i4_orf1;TRINITY_DN1882_c0_g1_i4_orf1;TRINITY_DN9354_c0_g1_i7_orf1;TRINITY_DN20558_c0_g1_i2_orf1;TRINITY_DN4434_c0_g1_i7_orf1;TRINITY_DN21722_c0_g1_i3_orf1;TRINITY_DN47605_c0_g2_i1_orf1;TRINITY_DN96739_c0_g1_i1_orf1;TRINITY_DN17351_c0_g1_i3_orf1;TRINITY_DN10637_c0_g1_i4_orf1;TRINITY_DN700_c0_g1_i3_orf1;TRINITY_DN25975_c0_g3_i2_orf1;TRINITY_DN6221_c0_g1_i5_orf1;TRINITY_DN79210_c0_g1_i1_orf1;TRINITY_DN107261_c0_g1_i1_orf1;TRINITY_DN45000_c0_g1_i5_orf1;TRINITY_DN96080_c0_g2_i1_orf1;TRINITY_DN83005_c0_g1_i1_orf1;TRINITY_DN10458_c0_g1_i1_orf1;TRINITY_DN7787_c0_g1_i1_orf1;TRINITY_DN6381_c0_g1_i2_orf1;TRINITY_DN91946_c0_g1_i1_orf1
molecular_function	channel inhibitor activity	GO:0016248	3	3/3516	TRINITY_DN5667_c0_g1_i4_orf1;TRINITY_DN6098_c1_g1_i5_orf1;TRINITY_DN4748_c0_g1_i5_orf1
molecular_function	ATPase inhibitor activity	GO:0042030	1	1/3516	TRINITY_DN5442_c0_g1_i4_orf1
molecular_function	ion channel regulator activity	GO:0099106	5	5/3516	TRINITY_DN5667_c0_g1_i4_orf1;TRINITY_DN6098_c1_g1_i5_orf1;TRINITY_DN4748_c0_g1_i5_orf1;TRINITY_DN10994_c0_g1_i4_orf1;TRINITY_DN5312_c4_g1_i2_orf1
molecular_function	ubiquitin-protein transferase regulator activity	GO:0055106	2	2/3516	TRINITY_DN130075_c1_g2_i1_orf1;TRINITY_DN55148_c0_g1_i1_orf1
molecular_function	kinase regulator activity	GO:0019207	3	3/3516	TRINITY_DN4014_c0_g1_i1_orf1;TRINITY_DN346_c0_g1_i7_orf1;TRINITY_DN147475_c0_g1_i1_orf1
molecular_function	phosphatase regulator activity	GO:0019208	4	4/3516	TRINITY_DN14524_c0_g1_i1_orf1;TRINITY_DN2943_c2_g2_i1_orf1;TRINITY_DN400_c0_g1_i1_orf1;TRINITY_DN13999_c0_g1_i4_orf1

molecular_function	nucleoside-triphosphatase regulator activity	GO:0060589	21	21/3516	TRINITY_DN1173_c0_g1_i12_orf1;TRINITY_DN1054_c0_g1_i8_orf1;TRINITY_DN1498_c0_g1_i2_orf1;TRINITY_DN21609_c0_g2_i1_orf1;TRINITY_DN42738_c0_g1_i1_orf1;TRINITY_DN138086_c0_g1_i1_orf1;TRINITY_DN18696_c0_g1_i1_orf1;TRINITY_DN104297_c0_g1_i1_orf1;TRINITY_DN518_c0_g1_i1_orf1;TRINITY_DN493_c0_g1_i4_orf1;TRINITY_DN69170_c0_g2_i1_orf1;TRINITY_DN9248_c0_g1_i10_orf1;TRINITY_DN802_c0_g1_i2_orf1;TRINITY_DN2623_c0_g1_i3_orf1;TRINITY_DN42903_c0_g1_i4_orf1;TRINITY_DN12320_c0_g1_i1_orf1;TRINITY_DN5182_c0_g1_i5_orf1;TRINITY_DN1173_c1_g1_i9_orf1;TRINITY_DN804_c0_g1_i7_orf1;TRINITY_DN15753_c0_g1_i1_orf1;TRINITY_DN1173_c1_g1_i10_orf1
molecular_function	peptidase regulator activity	GO:0061134	37	37/3516	TRINITY_DN122321_c0_g1_i1_orf1;TRINITY_DN4314_c0_g1_i9_orf1;TRINITY_DN8258_c0_g1_i3_orf1;TRINITY_DN1986_c0_g1_i1_orf1;TRINITY_DN2271_c0_g1_i12_orf1;TRINITY_DN834_c0_g1_i1_orf1;TRINITY_DN1079_c0_g1_i4_orf1;TRINITY_DN7776_c0_g1_i9_orf1;TRINITY_DN69697_c0_g1_i1_orf1;TRINITY_DN4235_c0_g1_i2_orf1;TRINITY_DN18196_c0_g1_i4_orf1;TRINITY_DN7776_c0_g1_i1_orf1;TRINITY_DN1328_c0_g1_i6_orf1;TRINITY_DN2848_c0_g1_i1_orf1;TRINITY_DN10994_c0_g1_i4_orf1;TRINITY_DN45948_c1_g1_i1_orf1;TRINITY_DN77425_c0_g1_i2_orf1;TRINITY_DN3609_c0_g1_i6_orf1;TRINITY_DN8780_c0_g1_i3_orf1;TRINITY_DN16234_c0_g2_i3_orf1;TRINITY_DN2097_c1_g2_i2_orf1;TRINITY_DN399_c3_g2_i6_orf1;TRINITY_DN71308_c0_g1_i4_orf1;TRINITY_DN1540_c0_g1_i7_orf1;TRINITY_DN42854_c0_g3_i2_orf1;TRINITY_DN7776_c0_g1_i5_orf1;TRINITY_DN1444_c1_g1_i5_orf1;TRINITY_DN1215_c0_g1_i2_orf1;TRINITY_DN7539_c0_g1_i2_orf1;TRINITY_DN10057_c0_g2_i1_orf1;TRINITY_DN1540_c0_g1_i9_orf1;TRINITY_DN135188_c0_g1_i2_orf1;TRINITY_DN9455_c0_g1_i6_orf1;TRINITY_DN1540_c0_g1_i14_orf1;TRINITY_DN712_c0_g2_i1_orf1;TRINITY_DN121047_c0_g1_i3_orf1;TRINITY_DN2848_c0_g1_i2_orf1
molecular_function	enzyme activator activity	GO:0008047	18	18/3516	TRINITY_DN1054_c0_g1_i8_orf1;TRINITY_DN1498_c0_g1_i2_orf1;TRINITY_DN346_c0_g1_i7_orf1;TRINITY_DN42738_c0_g1_i1_orf1;TRINITY_DN67649_c0_g1_i1_orf1;TRINITY_DN138086_c0_g1_i1_orf1;TRINITY_DN9542_c0_g1_i4_orf1;TRINITY_DN104297_c0_g1_i1_orf1;TRINITY_DN518_c0_g1_i1_orf1;TRINITY_DN493_c0_g1_i4_orf1;TRINITY_DN69170_c0_g2_i1_orf1;TRINITY_DN9248_c0_g1_i10_orf1;TRINITY_DN802_c0_g1_i2_orf1;TRINITY_DN42903_c0_g1_i4_orf1;TRINITY_DN18696_c0_g1_i1_orf1;TRINITY_DN400_c0_g1_i1_orf1;TRINITY_DN5182_c0_g1_i5_orf1;TRINITY_DN46022_c0_g1_i1_orf1
molecular_function	enzyme inhibitor activity	GO:0004857	41	41/3516	TRINITY_DN13999_c0_g1_i4_orf1;TRINITY_DN122321_c0_g1_i1_orf1;TRINITY_DN4314_c0_g1_i9_orf1;TRINITY_DN8258_c0_g1_i3_orf1;TRINITY_DN130075_c1_g2_i1_orf1;TRINITY_DN1986_c0_g1_i1_orf1;TRINITY_DN2271_c0_g1_i12_orf1;TRINITY_DN55148_c0_g1_i1_orf1;TRINITY_DN834_c0_g1_i1_orf1;TRINITY_DN1079_c0_g1_i4_orf1;TRINITY_DN7776_c0_g1_i9_orf1;TRINITY_DN69697_c0_g1_i1_orf1;TRINITY_DN4235_c0_g1_i2_orf1;TRINITY_DN18196_c0_g1_i4_orf1;TRINITY_DN7776_c0_g1_i1_orf1;TRINITY_DN1328_c0_g1_i6_orf1;TRINITY_DN2848_c0_g1_i1_orf1;TRINITY_DN10994_c0_g1_i4_orf1;TRINITY_DN45948_c1_g1_i1_orf1;TRINITY_DN77425_c0_g1_i2_orf1;TRINITY_DN3609_c0_g1_i6_orf1;TRINITY_DN399_c3_g2_i6_orf1;TRINITY_DN16234_c0_g2_i3_orf1;TRINITY_DN2097_c1_g2_i2_orf1;TRINITY_DN8780_c0_g1_i3_orf1;TRINITY_DN71308_c0_g1_i4_orf1;TRINITY_DN1540_c0_g1_i7_orf1;TRINITY_DN42854_c0_g3_i2_orf1;TRINITY_DN2943_c2_g2_i1_orf1;TRINITY_DN7776_c0_g1_i5_orf1;TRINITY_DN1444_c1_g1_i5_orf1;TRINITY_DN1215_c0_g1_i2_orf1;TRINITY_DN7539_c0_g1_i2_orf1;TRINITY_DN10057_c0_g2_i1_orf1;TRINITY_DN1540_c0_g1_i9_orf1;TRINITY_DN135188_c0_g1_i2_orf1;TRINITY_DN9455_c0_g1_i6_orf1;TRINITY_DN1540_c0_g1_i14_orf1;TRINITY_DN712_c0_g2_i1_orf1;TRINITY_DN121047_c0_g1_i3_orf1;TRINITY_DN2848_c0_g1_i2_orf1
molecular_function	signaling receptor activator activity	GO:0030546	5	5/3516	TRINITY_DN42903_c0_g1_i4_orf1;TRINITY_DN18650_c0_g1_i1_orf1;TRINITY_DN18218_c0_g1_i7_orf1;TRINITY_DN2227_c0_g1_i5_orf1;TRINITY_DN141738_c0_g1_i1_orf1
molecular_function	signaling receptor inhibitor activity	GO:0030547	1	1/3516	TRINITY_DN108433_c0_g1_i1_orf1

molecular_function	nucleic acid binding	GO:0003676	307	307/3516	TRINITY_DN129226_c0_g1_i2_orf1;TRINITY_DN6380_c0_g1_i1_orf1;TRINITY_DN9498_c0_g1_i3_orf1;TRINITY_DN1856_c0_g1_i3_orf1;TRINITY_DN4380_c0_g1_i9_orf1;TRINITY_DN3301_c0_g1_i2_orf1;TRINITY_DN40434_c0_g1_i2_orf1;TRINITY_DN12227_c0_g2_i3_orf1;TRINITY_DN9575_c0_g1_i1_orf1;TRINITY_DN975_c0_g1_i1_orf1;TRINITY_DN38412_c0_g1_i1_orf1;TRINITY_DN4262_c0_g1_i16_orf1;TRINITY_DN21123_c0_g1_i1_orf1;TRINITY_DN1116_c0_g1_i6_orf1;TRINITY_DN7213_c0_g1_i2_orf1;TRINITY_DN35669_c0_g1_i1_orf1;TRINITY_DN19651_c0_g1_i1_orf1;TRINITY_DN44407_c0_g4_i2_orf1;TRINITY_DN51934_c0_g2_i1_orf1;TRINITY_DN16965_c0_g2_i1_orf1;TRINITY_DN100885_c0_g2_i1_orfp1;TRINITY_DN15040_c0_g4_i1_orf1;TRINITY_DN34134_c0_g2_i1_orf1;TRINITY_DN15388_c0_g1_i5_orf1;TRINITY_DN35245_c0_g1_i1_orf1;TRINITY_DN2904_c0_g1_i4_orf1;TRINITY_DN70485_c0_g1_i2_orf1;TRINITY_DN142442_c0_g1_i1_orf1;TRINITY_DN3985_c0_g2_i1_orf1;TRINITY_DN3457_c0_g1_i4_orf1;TRINITY_DN1298_c0_g1_i3_orf1;TRINITY_DN19286_c0_g1_i1_orf1;TRINITY_DN5962_c0_g1_i1_orf1;TRINITY_DN2304_c0_g1_i4_orf1;TRINITY_DN29521_c0_g1_i1_orf1;TRINITY_DN18681_c0_g1_i7_orf1;TRINITY_DN15160_c0_g1_i1_orf1;TRINITY_DN9637_c0_g1_i14_orf1;TRINITY_DN36817_c0_g1_i1_orf1;TRINITY_DN1639_c0_g2_i2_orf1;TRINITY_DN17271_c0_g1_i1_orf1;TRINITY_DN3325_c0_g1_i1_orf1;TRINITY_DN10070_c0_g1_i1_orf1;TRINITY_DN9164_c0_g1_i3_orf1;TRINITY_DN25136_c0_g1_i1_orf1;TRINITY_DN9101_c0_g2_i1_orf1;TRINITY_DN9874_c0_g1_i7_orf1;TRINITY_DN129207_c0_g1_i1_orf1;TRINITY_DN53400_c0_g1_i1_orf1;TRINITY_DN38075_c0_g1_i1_orf1;TRINITY_DN817_c0_g1_i3_orf1;TRINITY_DN137_c0_g1_i1_orf1;TRINITY_DN145647_c0_g1_i1_orf1;TRINITY_DN24317_c0_g1_i7_orf1;TRINITY_DN5442_c0_g1_i4_orf1;TRINITY_DN9410_c0_g1_i4_orf1;TRINITY_DN20499_c0_g3_i1_orf1;TRINITY_DN227_c0_g1_i1_orf1;TRINITY_DN2265_c0_g1_i5_orf1;TRINITY_DN33883_c0_g1_i1_orf1;TRINITY_DN2802_c1_g1_i1_orf1;TRINITY_DN33346_c0_g1_i1_orf1;TRINITY_DN32822_c0_g1_i1_orf1;TRINITY_DN53684_c0_g1_i1_orf1;TRINITY_DN33249_c0_g1_i1_orf1;TRINITY_DN6235_c0_g1_i5_orf1;TRINITY_DN1245_c0_g1_i4_orf1;TRINITY_DN5458_c1_g1_i9_orf1;TRINITY_DN10558_c0_g1_i4_orf1;TRINITY_DN89613_c0_g1_i13_orf1;TRINITY_DN14313_c0_g1_i1_orf1;TRINITY_DN13347_c0_g1_i1_orf1;TRINITY_DN5603_c0_g1_i1_orf1;TRINITY_DN3092_c0_g1_i2_orf1;TRINITY_DN1978_c0_g1_i4_orf1;TRINITY_DN1771_c0_g2_i1_orf1;TRINITY_DN20442_c0_g2_i1_orf1;TRINITY_DN144956_c0_g1_i1_orf1;TRINITY_DN31503_c0_g1_i4_orf1;TRINITY_DN47723_c0_g1_i1_orf1;TRINITY_DN1074_c0_g1_i7_orf1;TRINITY_DN131662_c0_g1_i4_orf1;TRINITY_DN3712_c0_g1_i1_orf1;TRINITY_DN18242_c0_g1_i3_orf1;TRINITY_DN12495_c0_g1_i2_orf1;TRINITY_DN3057_c0_g2_i1_orf1;TRINITY_DN8224_c0_g1_i7_orf1;TRINITY_DN19687_c0_g1_i1_orf1;TRINITY_DN6248_c0_g1_i1_orf1;TRINITY_DN33926_c0_g1_i1_orf1;TRINITY_DN1870_c0_g1_i6_orf1;TRINITY_DN298_c0_g1_i4_orf1;TRINITY_DN17738_c0_g1_i2_orf1;TRINITY_DN4381_c0_g2_i1_orf1;TRINITY_DN4955_c0_g1_i2_orf1;TRINITY_DN4237_c1_g1_i5_orf1;TRINITY_DN47575_c0_g1_i1_orf1;TRINITY_DN51968_c0_g1_i1_orf1;TRINITY_DN27751_c0_g2_i1_orf1;TRINITY_DN7583_c0_g1_i1_orf1;TRINITY_DN19920_c1_g1_i2_orf1;TRINITY_DN1607_c0_g1_i16_orf1;TRINITY_DN7464_c0_g1_i14_orf1;TRINITY_DN810_c0_g1_i4_orf1;TRINITY_DN50787_c0_g2_i2_orf1;TRINITY_DN41664_c0_g1_i4_orf1;TRINITY_DN18863_c0_g1_i3_orf1;TRINITY_DN2769_c0_g1_i1_orf1;TRINITY_DN48641_c0_g1_i4_orf1;TRINITY_DN14498_c0_g1_i1_orf1;TRINITY_DN5238_c0_g1_i2_orf1;TRINITY_DN26168_c0_g1_i1_orf1;TRINITY_DN19092_c0_g1_i2_orf1;TRINITY_DN6532_c2_g1_i1_orf1;TRINITY_DN12527_c0_g1_i4_orf1;TRINITY_DN1786_c0_g1_i11_orf1;TRINITY_DN50085_c0_g1_i1_orf1;TRINITY_DN124950_c0_g2_i1_orf1;TRINITY_DN23714_c0_g1_i4_orf1;TRINITY_DN3733_c0_g1_i1_orf1;TRINITY_DN49936_c0_
molecular_function	nucleoside phosphate binding	GO:1901265	342	342/3516	TRINITY_DN98313_c0_g1_i1_orf1;TRINITY_DN20776_c0_g1_i3_orf1;TRINITY_DN38230_c0_g1_i4_orf1;TRINITY_DN125565_c1_g1_i1_orf1;TRINITY_DN5262_c0_g1_i7_orf1;TRINITY_DN2983_c0_g1_i6_orf1;TRINITY_DN5092_c0_g1_i2_orf1;TRINITY_DN45598_c0_g1_i2_orf1;TRINITY_DN143509_c0_g1_i1_orf1;TRINITY_DN59335_c0_g1_i2_orf1;TRINITY_DN26789_c0_g1_i2_orf1;TRINITY_DN28622_c0_g1_i1_orf1;TRINITY_DN14298_c0_g1_i1_orf1;TRINITY_DN3991_c0_g1_i6_orf1;TRINITY_DN29873_c0_g1_i1_orf1;TRINITY_DN235_c0_g3_i1_orf1;TRINITY_DN30932_c0_g1_i2_orf1;TRINITY_DN357_c0_g1_i8_orf1;TRINITY_DN16174_c0_g1_i2_orf1;TRINITY_DN52761_c0_g2_i1_orf1;TRINITY_DN4300_c0_g1_i5_orf1;TRINITY_DN46367_c0_g1_i2_orf1;TRINITY_DN2038_c0_g1_i2_orf1;TRINITY_DN7161_c0_g1_i7_orf1;TRINITY_DN825_c8_g1_i5_orf1;TRINITY_DN12495_c0_g1_i2_orf1;TRINITY_DN1965_c0_g1_i7_orf1;TRINITY_DN6044_c0_g1_i4_orf1;TRINITY_DN19920_c1_g1_i2_orf1;TRINITY_DN1607_c0_g1_i16_orf1;TRINITY_DN810_c0_g1_i4_orf1;TRINITY_DN1786_c0_g1_i11_orf1;TRINITY_DN71465_c0_g1_i1_orf1;TRINITY_DN5908_c0_g1_i2_orf1;TRINITY_DN5055_c0_g1_i12_orf1;TRINITY_DN620_c0_g1_i4_orf1;TRINITY_DN8980_c0_g1_i2_orf1;TRINITY_DN6063_c1_g2_i1_orf1;TRINITY_DN36788_c0_g1_i2_orf1;TRINITY_DN46090_c0_g3_i1_orf1;TRINITY_DN16673_c0_g1_i1_orf1;TRINITY_DN38341_c0_g2_i2_orf1;TRINITY_DN32769_c1_g1_i5_orf1;TRINITY_DN4449_c0_g2_i1_orf1;TRINITY_DN36899_c0_g1_i1_orf1;TRINITY_DN17838_c0_g1_i4_orf1;TRINITY_DN1173_c1_g1_i9_orf1;TRINITY_DN1617_c0_g1_i5_orf1;TRINITY_DN2146_c0_g2_i1_orf1;TRINITY_DN4944_c0_g1_i2_orf1;TRINITY_DN5740_c0_g1_i4_orf1;TRINITY_DN2265_c0_g2_i1_orf1;TRINITY_DN76815_c0_g1_i3_orf1;TRINITY_DN4056_c0_g1_i8_orf1;TRINITY_DN2803_c2_g1_i8_orf1;TRINITY_DN15845_c0_g1_i1_orf1;TRINITY_DN1285_c0_g1_i6_orf1;TRINITY_DN17861_c0_g1_i5_orf1;TRINITY_DN5169_c0_g1_i5_orf1;TRINITY_DN3588_c0_g1_i1_orf1;TRINITY_DN39490_c0_g1_i1_orf1;TRINITY_DN23946_c0_g1_i1_orf1;TRINITY_DN4908_c1_g1_i5_orf1;TRINITY_DN2570_c0_g1_i1_orf1;TRINITY_DN6669_c0_g1_i3_orf1;TRINITY_DN280_c0_g1_i12_orf1;TRINITY_DN48619_c0_g1_i1_orf1;TRINITY_DN63719_c0_g1_i5_orf1;TRINITY_DN21539_c0_g1_i1_orf1;TRINITY_DN9542_c0_g1_i4_orf1;TRINITY_DN30_c0_g1_i6_orf1;TRINITY_DN7626_c0_g1_i1_orf1;TRINITY_DN1084_c0_g1_i2_orf1;TRINITY_DN4779_c0_g1_i5_orf1;TRINITY_DN6771_c0_g2_i1_orf1;TRINITY_DN41311_c0_g2_i3_orf1;TRINITY_DN975_c0_g1_i1_orf1;TRINITY_DN11194_c0_g1_i4_orf1;TRINITY_DN15959_c0_g1_i1_orf1;TRINITY_DN28875_c0_g1_i1_orf1;TRINITY_DN7213_c0_g1_i2_orf1;TRINITY_DN817_c0_g1_i3_orf1;TRINITY_DN25341_c0_g1_i1_orf1;TRINITY_DN2738_c1_g1_i3_orf1;TRINITY_DN2904_c0_g1_i4_orf1;TRINITY_DN28221_c0_g2_i1_orf1;TRINITY_DN7388_c0_g1_i7_orf1;TRINITY_DN122786_c0_g2_i1_orf1;TRINITY_DN2848_c0_g1_i1_orf1;TRINITY_DN23432_c0_g1_i1_orf1;TRINITY_DN10774_c0_g2_i3_orf1;TRINITY_DN1334_c0_g1_i2_orf1;TRINITY_DN4434_c0_g1_i7_orf1;TRINITY_DN15370_c0_g1_i4_orf1;TRINITY_DN5354_c0_g1_i4_orf1;TRINITY_DN12442_c0_g1_i4_orf1;TRINITY_DN6436_c0_g1_i1_orf1;TRINITY_DN37165_c0_g1_i4_orf1;TRINITY_DN662_c0_g1_i1_orf1;TRINITY_DN24310_c0_g1_i2_orf1;TRINITY_DN44119_c0_g1_i1_orf1;TRINITY_DN20007_c0_g1_i1_orf1;TRINITY_DN63561_c1_g1_i2_orf1;TRINITY_DN3092_c0_g1_i2_orf1;TRINITY_DN39813_c0_g1_i1_orf1;TRINITY_DN2772_c0_g1_i3_orf1;TRINITY_DN5756_c0_g1_i4_orf1;TRINITY_DN31503_c0_g1_i4_orf1;TRINITY_DN157_c0_g1_i4_orf1;TRINITY_DN100821_c0_g1_i1_orf1;TRINITY_DN7405_c0_g1_i3_orf1;TRINITY_DN1034_c0_g1_i4_orf1;TRINITY_DN1725_c0_g1_i7_orf1;TRINITY_DN12301_c0_g1_i1_orf1;TRINITY_DN48554_c0_g1_i1_orf1;TRINITY_DN511_c0_g2_i1_orf1;TRINITY_DN30638_c0_g1_i1_orf1;TRINITY_DN2535_c0_g1_i4_orf1;TRINITY_DN16408_c0_g1_i1_orf1;TRINITY_DN70_c2_g1_i1_orf1;TRINITY_DN21000_c0_g1_i1_orf1;TRINITY_DN12064_c0_
molecular_function	L-ascorbic acid binding	GO:0031418	1	1/3516	TRINITY_DN43293_c0_g1_i2_orf1
molecular_function	thiamine pyrophosphate binding	GO:0030976	2	2/3516	TRINITY_DN4861_c0_g1_i7_orf1;TRINITY_DN59965_c0_g4_i1_orf1

molecular_function	tetrapyrrole binding	GO:0046906	56	56/3516	TRINITY_DN43369_c0_g2_i1_orf1;TRINITY_DN8985_c0_g1_i4_orf1;TRINITY_DN8173_c0_g1_i3_orf1;TRINITY_DN9608_c0_g1_i3_orf1;TRINITY_DN3949_c1_g1_i1_orf1;TRINITY_DN30704_c0_g1_i1_orf1;TRINITY_DN120500_c0_g1_i1_orf1;TRINITY_DN23564_c0_g1_i7_orf1;TRINITY_DN4497_c2_g1_i3_orf1;TRINITY_DN4497_c0_g1_i4_orf1;TRINITY_DN2392_c0_g2_i1_orf1;TRINITY_DN24873_c0_g1_i4_orf1;TRINITY_DN23398_c0_g1_i1_orf1;TRINITY_DN5933_c0_g1_i1_orf1;TRINITY_DN14262_c0_g1_i5_orf1;TRINITY_DN24043_c0_g1_i1_orf1;TRINITY_DN625_c9_g1_i7_orf1;TRINITY_DN1664_c0_g1_i4_orf1;TRINITY_DN49265_c0_g3_i2_orf1;TRINITY_DN22604_c0_g1_i3_orf1;TRINITY_DN57765_c0_g1_i1_orf1;TRINITY_DN3949_c0_g1_i1_orf1;TRINITY_DN1134_c0_g1_i4_orf1;TRINITY_DN7861_c0_g1_i5_orf1;TRINITY_DN829_c0_g1_i8_orf1;TRINITY_DN3732_c1_g1_i5_orf1;TRINITY_DN1363_c0_g1_i11_orf1;TRINITY_DN16122_c0_g1_i4_orf1;TRINITY_DN13941_c0_g1_i6_orf1;TRINITY_DN9316_c0_g3_i1_orf1;TRINITY_DN50743_c0_g1_i1_orf1;TRINITY_DN95558_c0_g3_i1_orf1;TRINITY_DN15755_c0_g1_i1_orf1;TRINITY_DN448_c0_g1_i20_orf1;TRINITY_DN6351_c0_g1_i4_orf1;TRINITY_DN109144_c0_g1_i5_orf1;TRINITY_DN2264_c0_g1_i1_orf1;TRINITY_DN1960_c5_g1_i3_orf1;TRINITY_DN57856_c0_g2_i1_orf1;TRINITY_DN5439_c0_g1_i2_orf1;TRINITY_DN23783_c0_g2_i1_orf1;TRINITY_DN51252_c0_g2_i1_orf1;TRINITY_DN52887_c0_g1_i1_orf1;TRINITY_DN30037_c0_g1_i5_orf1;TRINITY_DN9647_c0_g1_i1_orf1;TRINITY_DN5126_c0_g1_i3_orf1;TRINITY_DN5126_c0_g2_i1_orf1;TRINITY_DN6580_c0_g1_i4_orf1;TRINITY_DN12514_c0_g2_i1_orf1;TRINITY_DN2652_c0_g2_i1_orf1;TRINITY_DN2442_c0_g1_i6_orf1;TRINITY_DN54387_c0_g1_i1_orf1;TRINITY_DN3321_c0_g1_i3_orf1;TRINITY_DN49742_c0_g1_i4_orf1;TRINITY_DN5661_c0_g1_i5_orf1;TRINITY_DN285_c0_g1_i4_orf1
molecular_function	molybdopterin cofactor binding	GO:0043546	1	1/3516	TRINITY_DN129_c0_g1_i6_orf1
molecular_function	vitamin B6 binding	GO:0070279	15	15/3516	TRINITY_DN12474_c0_g1_i6_orf1;TRINITY_DN2065_c1_g2_i1_orf1;TRINITY_DN2688_c0_g2_i1_orf1;TRINITY_DN11948_c0_g1_i8_orf1;TRINITY_DN14565_c0_g1_i11_orf1;TRINITY_DN11159_c0_g2_i1_orf1;TRINITY_DN3263_c0_g1_i2_orf1;TRINITY_DN51813_c0_g1_i1_orf1;TRINITY_DN36699_c0_g1_i3_orf1;TRINITY_DN21035_c0_g1_i14_orf1;TRINITY_DN11817_c0_g1_i4_orf1;TRINITY_DN2803_c4_g1_i1_orf1;TRINITY_DN2684_c0_g2_i3_orf1;TRINITY_DN2688_c0_g1_i3_orf1;TRINITY_DN1068_c0_g1_i3_orf1
molecular_function	laminin binding	GO:0043236	1	1/3516	TRINITY_DN10070_c0_g1_i1_orf1
molecular_function	histone binding	GO:0042393	1	1/3516	TRINITY_DN12771_c0_g1_i1_orf1
molecular_function	identical protein binding	GO:0042802	13	13/3516	TRINITY_DN96557_c0_g1_i1_orf1;TRINITY_DN1639_c0_g2_i2_orf1;TRINITY_DN51938_c0_g3_i1_orf1;TRINITY_DN147475_c0_g1_i1_orf1;TRINITY_DN42854_c0_g3_i2_orf1;TRINITY_DN96739_c0_g1_i1_orf1;TRINITY_DN21123_c0_g1_i1_orf1;TRINITY_DN2848_c0_g1_i1_orf1;TRINITY_DN20133_c0_g1_i1_orf1;TRINITY_DN20009_c0_g1_i1_orf1;TRINITY_DN7787_c0_g1_i1_orf1;TRINITY_DN59965_c0_g4_i1_orf1;TRINITY_DN2848_c0_g1_i2_orf1
molecular_function	p53 binding	GO:0002039	1	1/3516	TRINITY_DN46409_c0_g1_i1_orf1
molecular_function	apolipoprotein binding	GO:0034185	1	1/3516	TRINITY_DN46409_c0_g1_i1_orf1
molecular_function	enzyme binding	GO:0019899	23	23/3516	TRINITY_DN21214_c0_g2_i1_orf1;TRINITY_DN461_c0_g1_i5_orf1;TRINITY_DN130075_c1_g2_i1_orf1;TRINITY_DN46409_c0_g1_i1_orf1;TRINITY_DN3747_c1_g1_i3_orf1;TRINITY_DN7739_c0_g1_i2_orf1;TRINITY_DN17864_c0_g1_i1_orf1;TRINITY_DN55148_c0_g1_i1_orf1;TRINITY_DN8473_c0_g1_i6_orf1;TRINITY_DN975_c0_g1_i1_orf1;TRINITY_DN22430_c0_g3_i1_orf1;TRINITY_DN8473_c0_g1_i5_orf1;TRINITY_DN2848_c0_g1_i1_orf1;TRINITY_DN41736_c0_g2_i1_orf1;TRINITY_DN315_c0_g1_i1_orf1;TRINITY_DN2532_c0_g3_i1_orf1;TRINITY_DN4439_c0_g1_i2_orf1;TRINITY_DN140538_c0_g2_i1_orf1;TRINITY_DN33183_c0_g1_i4_orf1;TRINITY_DN1532_c0_g1_i6_orf1;TRINITY_DN4859_c0_g1_i5_orf1;TRINITY_DN7493_c0_g1_i1_orf1;TRINITY_DN20133_c0_g1_i1_orf1
molecular_function	SNARE binding	GO:0000149	1	1/3516	TRINITY_DN38301_c0_g1_i2_orf1
molecular_function	chaperone binding	GO:0051087	2	2/3516	TRINITY_DN46409_c0_g1_i1_orf1;TRINITY_DN106476_c0_g1_i3_orf1
molecular_function	unfolded protein binding	GO:0051082	25	25/3516	TRINITY_DN21214_c0_g2_i1_orf1;TRINITY_DN20776_c0_g1_i3_orf1;TRINITY_DN46367_c0_g1_i2_orf1;TRINITY_DN6771_c0_g2_i1_orf1;TRINITY_DN2993_c0_g1_i4_orf1;TRINITY_DN46409_c0_g1_i1_orf1;TRINITY_DN15959_c0_g1_i1_orf1;TRINITY_DN5648_c0_g1_i5_orf1;TRINITY_DN37141_c0_g1_i2_orf1;TRINITY_DN33801_c0_g1_i1_orf1;TRINITY_DN6671_c0_g1_i6_orf1;TRINITY_DN25341_c0_g1_i1_orf1;TRINITY_DN5262_c0_g1_i7_orf1;TRINITY_DN95850_c0_g4_i3_orf1;TRINITY_DN7942_c0_g1_i1_orf1;TRINITY_DN16128_c0_g1_i5_orf1;TRINITY_DN10257_c0_g1_i2_orf1;TRINITY_DN1725_c0_g1_i7_orf1;TRINITY_DN2927_c0_g1_i6_orf1;TRINITY_DN32487_c0_g1_i1_orf1;TRINITY_DN12964_c0_g1_i1_orf1;TRINITY_DN7674_c0_g1_i2_orf1;TRINITY_DN139438_c0_g1_i1_orf1;TRINITY_DN7464_c1_g1_i1_orf1;TRINITY_DN4779_c0_g1_i5_orf1
molecular_function	calmodulin binding	GO:0005516	3	3/3516	TRINITY_DN6642_c0_g1_i2_orf1;TRINITY_DN975_c0_g1_i1_orf1;TRINITY_DN32022_c0_g1_i1_orf1
molecular_function	misfolded protein binding	GO:0051787	1	1/3516	TRINITY_DN21214_c0_g2_i1_orf1
molecular_function	heat shock protein binding	GO:0031072	6	6/3516	TRINITY_DN21214_c0_g2_i1_orf1;TRINITY_DN43355_c0_g1_i1_orf1;TRINITY_DN12964_c0_g1_i1_orf1;TRINITY_DN11215_c0_g1_i1_orf1;TRINITY_DN15959_c0_g1_i1_orf1;TRINITY_DN5648_c0_g1_i5_orf1
molecular_function	transcription factor binding	GO:0008134	3	3/3516	TRINITY_DN6071_c0_g1_i1_orf1;TRINITY_DN130075_c1_g2_i1_orf1;TRINITY_DN147475_c0_g1_i1_orf1
molecular_function	beta-catenin binding	GO:0008013	1	1/3516	TRINITY_DN140538_c0_g2_i1_orf1
molecular_function	translation initiation factor binding	GO:0031369	4	4/3516	TRINITY_DN33619_c0_g1_i1_orf1;TRINITY_DN21609_c0_g2_i1_orf1;TRINITY_DN2630_c0_g3_i3_orf1;TRINITY_DN50085_c0_g1_i1_orf1
molecular_function	signaling receptor binding	GO:0005102	9	9/3516	TRINITY_DN51938_c0_g3_i1_orf1;TRINITY_DN18650_c0_g1_i1_orf1;TRINITY_DN18218_c0_g1_i7_orf1;TRINITY_DN147475_c0_g1_i1_orf1;TRINITY_DN108433_c0_g1_i1_orf1;TRINITY_DN42903_c0_g1_i4_orf1;TRINITY_DN141738_c0_g1_i1_orf1;TRINITY_DN2227_c0_g1_i5_orf1;TRINITY_DN4628_c0_g1_i1_orf1

molecular_function	cytoskeletal protein binding	GO:0008092	46	46/3516	TRINITY_DN11464_c0_g1_i3_orf1;TRINITY_DN8406_c0_g1_i4_orf1;TRINITY_DN350_c0_g1_i4_orf1;TRINITY_DN52649_c0_g1_i6_orf1;TRINITY_DN23746_c0_g1_i2_orf1;TRINITY_DN350_c0_g1_i5_orf1;TRINITY_DN4159_c1_g1_i1_orf1;TRINITY_DN23790_c0_g1_i1_orf1;TRINITY_DN9119_c0_g1_i3_orf1;TRINITY_DN1054_c0_g1_i8_orf1;TRINITY_DN129869_c0_g4_i1_orf1;TRINITY_DN364_c0_g2_i1_orf1;TRINITY_DN5954_c0_g1_i2_orf1;TRINITY_DN30150_c0_g1_i7_orf1;TRINITY_DN104297_c0_g1_i1_orf1;TRINITY_DN9383_c0_g1_i3_orf1;TRINITY_DN467_c9_g1_i2_orf1;TRINITY_DN59852_c0_g1_i1_orf1;TRINITY_DN2848_c0_g1_i1_orf1;TRINITY_DN28622_c0_g1_i1_orf1;TRINITY_DN364_c1_g1_i2_orf1;TRINITY_DN26961_c0_g1_i1_orf1;TRINITY_DN2848_c0_g1_i2_orf1;TRINITY_DN14298_c0_g1_i1_orf1;TRINITY_DN22824_c0_g1_i4_orf1;TRINITY_DN41736_c0_g2_i1_orf1;TRINITY_DN16673_c0_g1_i1_orf1;TRINITY_DN96739_c0_g1_i1_orf1;TRINITY_DN4731_c0_g2_i1_orf1;TRINITY_DN741_c0_g1_i10_orf1;TRINITY_DN655_c0_g1_i3_orf1;TRINITY_DN21451_c0_g1_i3_orf1;TRINITY_DN286_c0_g1_i2_orf1;TRINITY_DN140538_c0_g2_i1_orf1;TRINITY_DN110231_c0_g1_i1_orf1;TRINITY_DN662_c0_g1_i1_orf1;TRINITY_DN104663_c1_g1_i2_orf1;TRINITY_DN9146_c0_g1_i1_orf1;TRINITY_DN5740_c0_g1_i4_orf1;TRINITY_DN7493_c0_g1_i1_orf1;TRINITY_DN10455_c0_g1_i2_orf1;TRINITY_DN20133_c0_g1_i1_orf1;TRINITY_DN14298_c0_g3_i1_orf1;TRINITY_DN97097_c0_g1_i4_orf1;TRINITY_DN37336_c1_g1_i1_orf1;TRINITY_DN364_c0_g1_i2_orf1
molecular_function	basal transcription machinery binding	GO:0001098	1	1/3516	TRINITY_DN1532_c0_g1_i6_orf1
molecular_function	protein dimerization activity	GO:0046983	13	13/3516	TRINITY_DN1639_c0_g2_i2_orf1;TRINITY_DN96557_c0_g1_i1_orf1;TRINITY_DN14301_c0_g1_i1_orf1;TRINITY_DN3325_c0_g1_i1_orf1;TRINITY_DN24917_c0_g2_i1_orf1;TRINITY_DN34115_c0_g1_i1_orf1;TRINITY_DN59965_c0_g4_i1_orf1;TRINITY_DN3457_c0_g1_i4_orf1;TRINITY_DN21123_c0_g1_i1_orf1;TRINITY_DN5458_c1_g1_i9_orf1;TRINITY_DN6162_c1_g1_i1_orf1;TRINITY_DN96801_c0_g1_i1_orf1;TRINITY_DN14611_c0_g1_i5_orf1
molecular_function	cytokine binding	GO:0019955	1	1/3516	TRINITY_DN111985_c0_g1_i1_orf1
molecular_function	phosphoprotein binding	GO:0051219	1	1/3516	TRINITY_DN140538_c0_g2_i1_orf1
molecular_function	protein domain specific binding	GO:0019904	8	8/3516	TRINITY_DN1639_c0_g2_i2_orf1;TRINITY_DN21214_c0_g2_i1_orf1;TRINITY_DN2848_c0_g1_i2_orf1;TRINITY_DN49527_c0_g1_i1_orf1;TRINITY_DN147475_c0_g1_i1_orf1;TRINITY_DN2848_c0_g1_i1_orf1;TRINITY_DN20009_c0_g1_i1_orf1;TRINITY_DN18912_c1_g1_i1_orf1
molecular_function	clathrin binding	GO:0030276	2	2/3516	TRINITY_DN1497_c0_g2_i6_orf1;TRINITY_DN741_c0_g1_i10_orf1
molecular_function	ubiquitin-like protein binding	GO:0032182	3	3/3516	TRINITY_DN4304_c0_g1_i3_orf1;TRINITY_DN45633_c0_g1_i1_orf1;TRINITY_DN65299_c0_g4_i1_orf1
molecular_function	cell adhesion molecule binding	GO:0050839	4	4/3516	TRINITY_DN111985_c0_g1_i1_orf1;TRINITY_DN492_c0_g1_i4_orf1;TRINITY_DN10070_c0_g1_i1_orf1;TRINITY_DN20133_c0_g1_i1_orf1
molecular_function	S100 protein binding	GO:0044548	1	1/3516	TRINITY_DN41736_c0_g2_i1_orf1
molecular_function	calcium-dependent protein binding	GO:0048306	1	1/3516	TRINITY_DN96739_c0_g1_i1_orf1
molecular_function	GTPase activating protein binding	GO:0032794	1	1/3516	TRINITY_DN140538_c0_g2_i1_orf1
molecular_function	scaffold protein binding	GO:0097110	1	1/3516	TRINITY_DN20009_c0_g1_i1_orf1
molecular_function	chitin binding	GO:0008061	17	17/3516	TRINITY_DN26301_c0_g1_i1_orf1;TRINITY_DN21555_c0_g1_i4_orf1;TRINITY_DN2205_c0_g1_i3_orf1;TRINITY_DN9000_c0_g2_i1_orf1;TRINITY_DN17003_c0_g1_i1_orf1;TRINITY_DN3913_c0_g1_i6_orf1;TRINITY_DN664_c0_g1_i18_orf1;TRINITY_DN619_c0_g1_i1_orf1;TRINITY_DN82801_c0_g1_i1_orf1;TRINITY_DN73923_c0_g1_i1_orf1;TRINITY_DN1287_c0_g1_i5_orf1;TRINITY_DN72999_c0_g1_i1_orf1;TRINITY_DN36061_c0_g4_i2_orf1;TRINITY_DN650_c0_g1_i3_orf1;TRINITY_DN54366_c0_g1_i1_orf1;TRINITY_DN6418_c0_g1_i28_orf1;TRINITY_DN3759_c0_g1_i1_orf1
molecular_function	lipopolysaccharide binding	GO:0001530	2	2/3516	TRINITY_DN46409_c0_g1_i1_orf1;TRINITY_DN2170_c0_g2_i1_orf1
molecular_function	lipoteichoic acid binding	GO:0070891	1	1/3516	TRINITY_DN2170_c0_g2_i1_orf1

molecular_function	ribonucleotide binding	GO:0032553	283	283/3516	TRINITY_DN98313_c0_g1_i1_orf1;TRINITY_DN47731_c0_g1_i2_orf1;TRINITY_DN20776_c0_g1_i3_orf1;TRINITY_DN125565_c1_g1_i1_orf1;TRINITY_DN4380_c0_g1_i9_orf1;TRINITY_DN6771_c0_g2_i1_orf1;TRINITY_DN2993_c0_g1_i4_orf1;TRINITY_DN41311_c0_g2_i3_orf1;TRINITY_DN4956_c0_g1_i6_orf1;TRINITY_DN17861_c0_g1_i5_orf1;TRINITY_DN11194_c0_g1_i4_orf1;TRINITY_DN14937_c0_g1_i7_orf1;TRINITY_DN15959_c0_g1_i1_orf1;TRINITY_DN6044_c0_g1_i4_orf1;TRINITY_DN28875_c0_g1_i1_orf1;TRINITY_DN7213_c0_g1_i2_orf1;TRINITY_DN6436_c0_g1_i1_orf1;TRINITY_DN31967_c0_g1_i5_orf1;TRINITY_DN25341_c0_g1_i1_orf1;TRINITY_DN2738_c1_g1_i3_orf1;TRINITY_DN5262_c0_g1_i7_orf1;TRINITY_DN33705_c0_g1_i1_orf1;TRINITY_DN96170_c0_g1_i1_orf1;TRINITY_DN2904_c0_g1_i4_orf1;TRINITY_DN70485_c0_g1_i2_orf1;TRINITY_DN2983_c0_g1_i6_orf1;TRINITY_DN4798_c0_g1_i3_orf1;TRINITY_DN24723_c2_g1_i1_orf1;TRINITY_DN27771_c0_g2_i1_orf1;TRINITY_DN45598_c0_g1_i2_orf1;TRINITY_DN143509_c0_g1_i1_orf1;TRINITY_DN2638_c0_g1_i7_orf1;TRINITY_DN2265_c0_g1_i5_orf1;TRINITY_DN73945_c0_g5_i3_orf1;TRINITY_DN80560_c0_g1_i1_orf1;TRINITY_DN47151_c0_g1_i1_orf1;TRINITY_DN10774_c0_g2_i3_orf1;TRINITY_DN6587_c0_g1_i3_orf1;TRINITY_DN1334_c0_g1_i2_orf1;TRINITY_DN1725_c0_g1_i7_orf1;TRINITY_DN14298_c0_g1_i1_orf1;TRINITY_DN4434_c0_g1_i7_orf1;TRINITY_DN15370_c0_g1_i4_orf1;TRINITY_DN9575_c0_g1_i1_orf1;TRINITY_DN235_c0_g3_i1_orf1;TRINITY_DN27771_c0_g1_i1_orf1;TRINITY_DN6185_c0_g1_i12_orf1;TRINITY_DN52761_c0_g1_i2_orf1;TRINITY_DN24693_c1_g1_i1_orf1;TRINITY_DN817_c0_g1_i3_orf1;TRINITY_DN24164_c0_g1_i1_orf1;TRINITY_DN37165_c0_g1_i4_orf1;TRINITY_DN2224_c0_g1_i1_orf1;TRINITY_DN662_c0_g1_i1_orf1;TRINITY_DN139438_c0_g1_i1_orf1;TRINITY_DN3822_c0_g1_i7_orf1;TRINITY_DN46409_c0_g1_i1_orf1;TRINITY_DN2947_c0_g1_i4_orf1;TRINITY_DN26961_c0_g1_i1_orf1;TRINITY_DN7247_c0_g1_i7_orf1;TRINITY_DN987_c0_g1_i3_orf1;TRINITY_DN29956_c1_g1_i1_orf1;TRINITY_DN33249_c0_g1_i1_orf1;TRINITY_DN16174_c0_g1_i2_orf1;TRINITY_DN2173_c0_g1_i1_orf1;TRINITY_DN452_c1_g1_i3_orf1;TRINITY_DN20007_c0_g1_i1_orf1;TRINITY_DN4950_c0_g1_i2_orf1;TRINITY_DN52761_c0_g2_i1_orf1;TRINITY_DN164_c0_g1_i11_orf1;TRINITY_DN63561_c1_g1_i2_orf1;TRINITY_DN3092_c0_g1_i2_orf1;TRINITY_DN4300_c0_g1_i5_orf1;TRINITY_DN1552_c0_g1_i3_orf1;TRINITY_DN46367_c0_g1_i2_orf1;TRINITY_DN2038_c0_g1_i2_orf1;TRINITY_DN31503_c0_g1_i4_orf1;TRINITY_DN4977_c0_g1_i2_orf1;TRINITY_DN7161_c0_g1_i7_orf1;TRINITY_DN7464_c1_g1_i1_orf1;TRINITY_DN1366_c0_g1_i5_orf1;TRINITY_DN157_c0_g1_i4_orf1;TRINITY_DN825_c8_g1_i5_orf1;TRINITY_DN100821_c0_g1_i1_orf1;TRINITY_DN12495_c0_g1_i2_orf1;TRINITY_DN33801_c0_g1_i1_orf1;TRINITY_DN1965_c0_g1_i7_orf1;TRINITY_DN7405_c0_g1_i3_orf1;TRINITY_DN30224_c0_g1_i1_orf1;TRINITY_DN11639_c0_g1_i1_orf1;TRINITY_DN17935_c0_g1_i1_orf1;TRINITY_DN107288_c0_g1_i2_orf1;TRINITY_DN1034_c0_g1_i4_orf1;TRINITY_DN60821_c0_g1_i1_orf1;TRINITY_DN9156_c0_g1_i1_orf1;TRINITY_DN740_c0_g1_i1_orf1;TRINITY_DN6813_c1_g1_i1_orf1;TRINITY_DN1578_c0_g3_i1_orf1;TRINITY_DN19920_c1_g1_i2_orf1;TRINITY_DN1607_c0_g1_i16_orf1;TRINITY_DN28221_c0_g2_i1_orf1;TRINITY_DN810_c0_g1_i4_orf1;TRINITY_DN244_c1_g1_i5_orf1;TRINITY_DN21126_c0_g1_i1_orf1;TRINITY_DN2110_c0_g1_i3_orf1;TRINITY_DN2202_c0_g1_i9_orf1;TRINITY_DN2927_c0_g1_i6_orf1;TRINITY_DN15882_c0_g1_i1_orf1;TRINITY_DN7336_c0_g1_i13_orf1;TRINITY_DN1786_c0_g1_i11_orf1;TRINITY_DN32700_c0_g1_i2_orf1;TRINITY_DN117844_c0_g1_i1_orf1;TRINITY_DN12301_c0_g1_i1_orf1;TRINITY_DN31225_c0_g1_i1_orf1;TRINITY_DN13563_c0_g1_i1_orf1;TRINITY_DN38506_c0_g1_i4_orf1;TRINITY_DN8986_c0_g1_i1_orf1;TRINITY_DN315_c0_g1_i1_orf1;TRINITY_DN2826_c0_g1_i7_orf1;TRINITY_DN16816_c0_g1_i1_orf1;TRINITY_DN5281_c0_g2_i3_orf1;TRINITY_DN5
molecular_function	glycosaminoglycan binding	GO:0005539	5	5/3516	TRINITY_DN108433_c0_g1_i1_orf1;TRINITY_DN42854_c0_g3_i2_orf1;TRINITY_DN96739_c0_g1_i1_orf1;TRINITY_DN5235_c0_g1_i7_orf1;TRINITY_DN1534_c0_g1_i3_orf1
molecular_function	heparin binding	GO:0008201	2	2/3516	TRINITY_DN42854_c0_g3_i2_orf1;TRINITY_DN108433_c0_g1_i1_orf1
molecular_function	acyl-CoA binding	GO:0120227	3	3/3516	TRINITY_DN13563_c0_g1_i1_orf1;TRINITY_DN17861_c0_g1_i5_orf1;TRINITY_DN6044_c0_g1_i4_orf1
molecular_function	chondroitin sulfate binding	GO:0035374	1	1/3516	TRINITY_DN96739_c0_g1_i1_orf1
molecular_function	peptide binding	GO:0042277	3	3/3516	TRINITY_DN245_c0_g1_i4_orf1;TRINITY_DN4016_c0_g1_i1_orf1;TRINITY_DN13783_c0_g4_i2_orf1
molecular_function	phosphopantetheine binding	GO:0031177	1	1/3516	TRINITY_DN10430_c0_g1_i4_orf1
molecular_function	cation binding	GO:0043169	397	397/3516	TRINITY_DN38230_c0_g1_i4_orf1;TRINITY_DN3194_c0_g1_i6_orf1;TRINITY_DN35763_c0_g1_i2_orf1;TRINITY_DN8019_c0_g1_i4_orf1;TRINITY_DN9711_c0_g1_i10_orf1;TRINITY_DN1134_c0_g1_i4_orf1;TRINITY_DN59852_c0_g1_i1_orf1;TRINITY_DN31163_c1_g1_i4_orf1;TRINITY_DN143509_c0_g1_i1_orf1;TRINITY_DN3235_c0_g1_i1_orf1;TRINITY_DN16343_c0_g1_i6_orf1;TRINITY_DN48020_c0_g1_i1_orf1;TRINITY_DN15755_c0_g1_i1_orf1;TRINITY_DN448_c0_g1_i20_orf1;TRINITY_DN3991_c0_g1_i6_orf1;TRINITY_DN2794_c1_g1_i8_orf1;TRINITY_DN123184_c0_g1_i1_orf1;TRINITY_DN3276_c0_g1_i4_orf1;TRINITY_DN817_c0_g1_i3_orf1;TRINITY_DN25896_c0_g1_i6_orf1;TRINITY_DN2627_c0_g2_i1_orf1;TRINITY_DN5190_c0_g3_i1_orf1;TRINITY_DN1161_c0_g1_i2_orf1;TRINITY_DN1957_c0_g1_i4_orf1;TRINITY_DN70_c2_g1_i1_orf1;TRINITY_DN30704_c0_g1_i1_orf1;TRINITY_DN2627_c0_g1_i2_orf1;TRINITY_DN47723_c0_g1_i1_orf1;TRINITY_DN24043_c0_g1_i1_orf1;TRINITY_DN4189_c0_g2_i1_orf1;TRINITY_DN1664_c0_g1_i4_orf1;TRINITY_DN117844_c0_g1_i1_orf1;TRINITY_DN47575_c0_g1_i1_orf1;TRINITY_DN12301_c0_g1_i1_orf1;TRINITY_DN1578_c0_g3_i1_orf1;TRINITY_DN810_c0_g1_i4_orf1;TRINITY_DN20294_c0_g2_i1_orf1;TRINITY_DN66302_c0_g1_i1_orf1;TRINITY_DN7655_c0_g1_i3_orf1;TRINITY_DN15327_c2_g1_i2_orf1;TRINITY_DN3733_c0_g1_i1_orf1;TRINITY_DN12024_c0_g1_i4_orf1;TRINITY_DN18300_c0_g1_i17_orf1;TRINITY_DN27033_c1_g1_i3_orfp1;TRINITY_DN376_c1_g1_i1_orf1;TRINITY_DN7861_c0_g1_i5_orf1;TRINITY_DN83295_c0_g1_i3_orf1;TRINITY_DN1206_c0_g1_i6_orf1;TRINITY_DN812_c2_g1_i1_orf1;TRINITY_DN10672_c0_g1_i3_orf1;TRINITY_DN22604_c0_g1_i3_orf1;TRINITY_DN109144_c0_g1_i5_orf1;TRINITY_DN2186_c0_g1_i17_orf1;TRINITY_DN24873_c0_g1_i4_orf1;TRINITY_DN89083_c0_g1_i1_orf1;TRINITY_DN73900_c0_g1_i1_orf1;TRINITY_DN1952_c0_g1_i2_orf1;TRINITY_DN3732_c1_g1_i5_orf1;TRINITY_DN934_c2_g1_i7_orf1;TRINITY_DN36788_c0_g1_i2_orf1;TRINITY_DN8087_c0_g1_i9_orf1;TRINITY_DN21533_c0_g1_i4_orf1;TRINITY_DN15812_c0_g1_i2_orf1;TRINITY_DN2579_c0_g1_i7_orf1;TRINITY_DN8771_c0_g2_i1_orf1;TRINITY_DN13098_c2_g1_i2_orf1;TRINITY_DN2274_c0_g1_i6_orf1;TRINITY_DN5126_c0_g1_i3_orf1;TRINITY_DN5661_c0_g1_i5_orf1;TRINITY_DN2647_c0_g1_i3_orf1;TRINITY_DN10385_c0_g1_i5_orf1;TRINITY_DN79000_c1_g1_i1_orf1;TRINITY_DN23564_c0_g1_i7_orf1;TRINITY_DN12973_c0_g1_i1_orf1;TRINITY_DN33272_c0_g1_i5_orf1;TRINITY_DN7128_c0_g1_i7_orf1;TRINITY_DN76815_c0_g1_i3_orf1;TRINITY_DN4998_c0_g1_i21_orf1;TRINITY_DN4056_c0_g1_i8_orf1;TRINITY_DN13711_c0_g1_i1_orf1;TRINITY_DN54387_c0_g1_i1_orf1;TRINITY_DN1884_c0_g2_i2_orf1;TRINITY_DN132043_c0_g1_i1_orf1;TRINITY_DN2593_c0_g3_i1_orf1;TRINITY_DN4908_c1_g1_i5_orf1;TRINITY_DN2593_c0_g1_i1_orf1;TRINITY_DN17913_c0_g1_i8_orf1;TRINITY_DN58413_c0_g1_i4_orf1;TRINITY_DN2264_c0_g1_i1_orf1;TRINITY_DN1960_c5_g1_i3_orf1;TRINITY_DN2570_c0_g1_i1_orf1;TRINITY_DN49265_c0_g3_i2_orf1;TRINITY_DN48410_c0_g2_i1_orf1;TRINITY_DN2338_c0_g1_i5_orf1;TRINITY_DN136028_c0_g2_i1_orf1;TRINITY_DN9542_c0_g1_i4_orf1;TRINITY_DN6299_c0_g1_i1_orf1;TRINITY_DN346_c0_g1_i7_orf1;TRINITY_DN7626_c0_g1_i1_orf1;TRINITY_DN147475_c0_g1_i1_orf1;TRINITY_DN11820_c0_g1_i1_orf1;TRINITY_DN195_c0_g3_i6_orf1;TRINITY_DN1084_c0_g1_i2_orf1;TRINITY_DN4125_c0_g1_i6_orf1;TRINITY_DN129226_c0_g1_i2_orf1;TRINITY_DN9608_c0_g1_i3_orf1;TRINITY_DN827_c1_g1_i1_orf1;TRINITY_DN3301_c0_g1_i2_orf1;TRINITY_DN130051_c0_g1_i1_orf1;TRINITY_DN15959_c0_g1_i1_orf1;TRINITY_DN6027_c0_g1_i13_orf1;TRINITY_DN1231_c0_g1_i4_orf1;TRINITY_DN3861_c0_g3_i2_orf1;TRINITY_DN16122_c0_g1_i4_orf1;TRINITY_DN3433_c0_g1_i6_orf1;TRINITY_DN23432_c0_g1_i1_orf1;TRINITY_DN585_c0_g1_i12_orf1;TRINITY_DN95558_c0_g3_i1_orf1;TRINITY_DN46625_c0_g1_i1_orf1;TRINITY_DN1334_c0_g1_i2_orf1;TRINITY_DN7740_c0_g1_i2_orf1;TRINITY_DN

molecular_function	anion binding	GO:0043168	328	328/3516	TRINITY_DN98313_c0_g1_i1_orf1;TRINITY_DN129_c0_g1_i6_orf1;TRINITY_DN20776_c0_g1_i3_orf1;TRINITY_DN987_c0_g1_i3_orf1;TRINITY_DN125565_c1_g1_i1_orf1;TRINITY_DN47731_c0_g1_i2_orf1;TRINITY_DN4380_c0_g1_i9_orf1;TRINITY_DN6771_c0_g2_i1_orf1;TRINITY_DN2993_c0_g1_i4_orf1;TRINITY_DN2065_c1_g2_i1_orf1;TRINITY_DN4956_c0_g1_i6_orf1;TRINITY_DN28622_c0_g1_i1_orf1;TRINITY_DN11194_c0_g1_i4_orf1;TRINITY_DN14937_c0_g1_i7_orf1;TRINITY_DN15959_c0_g1_i1_orf1;TRINITY_DN43293_c0_g1_i2_orf1;TRINITY_DN21035_c0_g1_i14_orf1;TRINITY_DN28875_c0_g1_i1_orf1;TRINITY_DN2688_c0_g1_i3_orf1;TRINITY_DN26243_c0_g1_i2_orf1;TRINITY_DN30932_c0_g1_i2_orf1;TRINITY_DN31967_c0_g1_i5_orf1;TRINITY_DN3859_c0_g1_i5_orf1;TRINITY_DN12474_c0_g1_i6_orf1;TRINITY_DN2738_c1_g1_i3_orf1;TRINITY_DN5262_c0_g1_i7_orf1;TRINITY_DN1023_c1_g1_i1_orf1;TRINITY_DN96170_c0_g1_i1_orf1;TRINITY_DN452_c1_g1_i3_orf1;TRINITY_DN2904_c0_g1_i4_orf1;TRINITY_DN70485_c0_g1_i2_orf1;TRINITY_DN2983_c0_g1_i6_orf1;TRINITY_DN11817_c0_g1_i4_orf1;TRINITY_DN5092_c0_g1_i2_orf1;TRINITY_DN27771_c0_g2_i1_orf1;TRINITY_DN45598_c0_g1_i2_orf1;TRINITY_DN143509_c0_g1_i1_orf1;TRINITY_DN122786_c0_g2_i1_orf1;TRINITY_DN59335_c0_g1_i2_orf1;TRINITY_DN2265_c0_g1_i5_orf1;TRINITY_DN73945_c0_g5_i3_orf1;TRINITY_DN80560_c0_g1_i1_orf1;TRINITY_DN26789_c0_g1_i2_orf1;TRINITY_DN47151_c0_g1_i1_orf1;TRINITY_DN2688_c0_g2_i1_orf1;TRINITY_DN10774_c0_g2_i3_orf1;TRINITY_DN6587_c0_g1_i3_orf1;TRINITY_DN1334_c0_g1_i2_orf1;TRINITY_DN1725_c0_g1_i7_orf1;TRINITY_DN14298_c0_g1_i1_orf1;TRINITY_DN3991_c0_g1_i6_orf1;TRINITY_DN24723_c2_g1_i1_orf1;TRINITY_DN15370_c0_g1_i4_orf1;TRINITY_DN41311_c0_g2_i3_orf1;TRINITY_DN1132_c0_g1_i5_orf1;TRINITY_DN235_c0_g3_i1_orf1;TRINITY_DN1068_c0_g1_i3_orf1;TRINITY_DN6185_c0_g1_i12_orf1;TRINITY_DN52761_c0_g1_i2_orf1;TRINITY_DN24693_c1_g1_i1_orf1;TRINITY_DN817_c0_g1_i3_orf1;TRINITY_DN24164_c0_g1_i1_orf1;TRINITY_DN37165_c0_g1_i4_orf1;TRINITY_DN2224_c0_g1_i1_orf1;TRINITY_DN662_c0_g1_i1_orf1;TRINITY_DN139438_c0_g1_i1_orf1;TRINITY_DN3822_c0_g1_i7_orf1;TRINITY_DN7909_c0_g2_i1_orf1;TRINITY_DN46409_c0_g1_i1_orf1;TRINITY_DN2947_c0_g1_i4_orf1;TRINITY_DN26961_c0_g1_i1_orf1;TRINITY_DN7247_c0_g1_i7_orf1;TRINITY_DN357_c0_g1_i8_orf1;TRINITY_DN29956_c1_g1_i1_orf1;TRINITY_DN33249_c0_g1_i1_orf1;TRINITY_DN16174_c0_g1_i2_orf1;TRINITY_DN2173_c0_g1_i1_orf1;TRINITY_DN52761_c0_g2_i1_orf1;TRINITY_DN11178_c0_g1_i1_orf1;TRINITY_DN20007_c0_g1_i1_orf1;TRINITY_DN4950_c0_g1_i2_orf1;TRINITY_DN70_c2_g1_i1_orf1;TRINITY_DN164_c0_g1_i11_orf1;TRINITY_DN63561_c1_g1_i2_orf1;TRINITY_DN3092_c0_g1_i2_orf1;TRINITY_DN4300_c0_g1_i5_orf1;TRINITY_DN4501_c0_g1_i3_orf1;TRINITY_DN46367_c0_g1_i2_orf1;TRINITY_DN2038_c0_g1_i2_orf1;TRINITY_DN31503_c0_g1_i4_orf1;TRINITY_DN4977_c0_g1_i2_orf1;TRINITY_DN7161_c0_g1_i7_orf1;TRINITY_DN59965_c0_g4_i1_orf1;TRINITY_DN1366_c0_g1_i5_orf1;TRINITY_DN157_c0_g1_i4_orf1;TRINITY_DN825_c8_g1_i5_orf1;TRINITY_DN100821_c0_g1_i1_orf1;TRINITY_DN12495_c0_g1_i2_orf1;TRINITY_DN7213_c0_g1_i2_orf1;TRINITY_DN1965_c0_g1_i7_orf1;TRINITY_DN7405_c0_g1_i3_orf1;TRINITY_DN30224_c0_g1_i1_orf1;TRINITY_DN11639_c0_g1_i1_orf1;TRINITY_DN17935_c0_g1_i1_orf1;TRINITY_DN107288_c0_g1_i2_orf1;TRINITY_DN1034_c0_g1_i4_orf1;TRINITY_DN60821_c0_g1_i1_orf1;TRINITY_DN9156_c0_g1_i1_orf1;TRINITY_DN740_c0_g1_i1_orf1;TRINITY_DN9575_c0_g1_i1_orf1;TRINITY_DN6813_c1_g1_i1_orf1;TRINITY_DN1578_c0_g3_i1_orf1;TRINITY_DN19920_c1_g1_i2_orf1;TRINITY_DN1607_c0_g1_i16_orf1;TRINITY_DN28221_c0_g2_i1_orf1;TRINITY_DN810_c0_g1_i4_orf1;TRINITY_DN244_c1_g1_i5_orf1;TRINITY_DN21126_c0_g1_i1_orf1;TRINITY_DN2110_c0_g1_i3_orf1;TRINITY_DN2202_c0_g1_i9_orf1;TRINITY_DN2927_c0_g1_i6_orf1;TRINITY_DN158
molecular_function	phospholipid binding	GO:0005543	9	9/3516	TRINITY_DN1497_c0_g2_i6_orf1;TRINITY_DN4304_c0_g1_i3_orf1;TRINITY_DN7493_c0_g1_i1_orf1;TRINITY_DN96739_c0_g1_i1_orf1;TRINITY_DN21533_c0_g1_i4_orf1;TRINITY_DN6243_c0_g1_i5_orf1;TRINITY_DN65299_c0_g4_i1_orf1;TRINITY_DN143637_c0_g1_i1_orf1;TRINITY_DN21533_c0_g1_i7_orf1
molecular_function	steroid binding	GO:0005496	1	1/3516	TRINITY_DN96739_c0_g1_i1_orf1
molecular_function	fatty acid derivative binding	GO:1901567	3	3/3516	TRINITY_DN13563_c0_g1_i1_orf1;TRINITY_DN17861_c0_g1_i5_orf1;TRINITY_DN6044_c0_g1_i4_orf1
molecular_function	oxygen binding	GO:0019825	3	3/3516	TRINITY_DN111985_c0_g1_i1_orf1;TRINITY_DN49742_c0_g1_i4_orf1;TRINITY_DN30037_c0_g1_i5_orf1
molecular_function	monosaccharide binding	GO:0048029	5	5/3516	TRINITY_DN3835_c0_g1_i3_orf1;TRINITY_DN7405_c0_g1_i3_orf1;TRINITY_DN43293_c0_g1_i2_orf1;TRINITY_DN3835_c0_g1_i4_orf1;TRINITY_DN20133_c0_g1_i1_orf1
molecular_function	organic acid binding	GO:0043177	1	1/3516	TRINITY_DN43293_c0_g1_i2_orf1
molecular_function	alcohol binding	GO:0043178	1	1/3516	TRINITY_DN96739_c0_g1_i1_orf1
molecular_function	vitamin binding	GO:0019842	19	19/3516	TRINITY_DN12474_c0_g1_i6_orf1;TRINITY_DN2065_c1_g2_i1_orf1;TRINITY_DN2688_c0_g2_i1_orf1;TRINITY_DN11948_c0_g1_i8_orf1;TRINITY_DN14565_c0_g1_i11_orf1;TRINITY_DN11159_c0_g2_i1_orf1;TRINITY_DN3263_c0_g1_i2_orf1;TRINITY_DN51813_c0_g1_i1_orf1;TRINITY_DN36699_c0_g1_i3_orf1;TRINITY_DN21035_c0_g1_i14_orf1;TRINITY_DN59965_c0_g4_i1_orf1;TRINITY_DN2803_c4_g1_i1_orf1;TRINITY_DN43293_c0_g1_i2_orf1;TRINITY_DN2684_c0_g2_i3_orf1;TRINITY_DN10430_c0_g1_i4_orf1;TRINITY_DN2688_c0_g1_i3_orf1;TRINITY_DN4861_c0_g1_i7_orf1;TRINITY_DN11817_c0_g1_i4_orf1;TRINITY_DN1068_c0_g1_i3_orf1

molecular_function	nucleotide binding	GO:0000166	342	342/3516	TRINITY_DN98313_c0_g1_i1_orf1;TRINITY_DN20776_c0_g1_i3_orf1;TRINITY_DN38230_c0_g1_i4_orf1;TRINITY_DN125565_c1_g1_i1_orf1;TRINITY_DN5262_c0_g1_i7_orf1;TRINITY_DN2983_c0_g1_i6_orf1;TRINITY_DN5092_c0_g1_i2_orf1;TRINITY_DN45598_c0_g1_i2_orf1;TRINITY_DN143509_c0_g1_i1_orf1;TRINITY_DN59335_c0_g1_i2_orf1;TRINITY_DN26789_c0_g1_i2_orf1;TRINITY_DN28622_c0_g1_i1_orf1;TRINITY_DN14298_c0_g1_i1_orf1;TRINITY_DN3991_c0_g1_i6_orf1;TRINITY_DN29873_c0_g1_i1_orf1;TRINITY_DN235_c0_g3_i1_orf1;TRINITY_DN30932_c0_g1_i2_orf1;TRINITY_DN357_c0_g1_i8_orf1;TRINITY_DN16174_c0_g1_i2_orf1;TRINITY_DN52761_c0_g2_i1_orf1;TRINITY_DN4300_c0_g1_i5_orf1;TRINITY_DN46367_c0_g1_i2_orf1;TRINITY_DN2038_c0_g1_i2_orf1;TRINITY_DN7161_c0_g1_i7_orf1;TRINITY_DN825_c8_g1_i5_orf1;TRINITY_DN12495_c0_g1_i2_orf1;TRINITY_DN1965_c0_g1_i7_orf1;TRINITY_DN6044_c0_g1_i4_orf1;TRINITY_DN19920_c1_g1_i2_orf1;TRINITY_DN1607_c0_g1_i16_orf1;TRINITY_DN810_c0_g1_i4_orf1;TRINITY_DN1786_c0_g1_i11_orf1;TRINITY_DN71465_c0_g1_i1_orf1;TRINITY_DN5908_c0_g1_i2_orf1;TRINITY_DN5055_c0_g1_i12_orf1;TRINITY_DN620_c0_g1_i4_orf1;TRINITY_DN8980_c0_g1_i2_orf1;TRINITY_DN6063_c1_g2_i1_orf1;TRINITY_DN36788_c0_g1_i2_orf1;TRINITY_DN46090_c0_g3_i1_orf1;TRINITY_DN16673_c0_g1_i1_orf1;TRINITY_DN38341_c0_g2_i2_orf1;TRINITY_DN32769_c1_g1_i5_orf1;TRINITY_DN4449_c0_g2_i1_orf1;TRINITY_DN36899_c0_g1_i1_orf1;TRINITY_DN17838_c0_g1_i4_orf1;TRINITY_DN1173_c1_g1_i9_orf1;TRINITY_DN1617_c0_g1_i5_orf1;TRINITY_DN2146_c0_g2_i1_orf1;TRINITY_DN4944_c0_g1_i2_orf1;TRINITY_DN5740_c0_g1_i4_orf1;TRINITY_DN2265_c0_g2_i1_orf1;TRINITY_DN76815_c0_g1_i3_orf1;TRINITY_DN4056_c0_g1_i8_orf1;TRINITY_DN2803_c2_g1_i8_orf1;TRINITY_DN15845_c0_g1_i1_orf1;TRINITY_DN1285_c0_g1_i6_orf1;TRINITY_DN17861_c0_g1_i5_orf1;TRINITY_DN5169_c0_g1_i5_orf1;TRINITY_DN3588_c0_g1_i1_orf1;TRINITY_DN39490_c0_g1_i1_orf1;TRINITY_DN23946_c0_g1_i1_orf1;TRINITY_DN4908_c1_g1_i5_orf1;TRINITY_DN2570_c0_g1_i1_orf1;TRINITY_DN6669_c0_g1_i3_orf1;TRINITY_DN280_c0_g1_i12_orf1;TRINITY_DN48619_c0_g1_i1_orf1;TRINITY_DN63719_c0_g1_i5_orf1;TRINITY_DN21539_c0_g1_i1_orf1;TRINITY_DN9542_c0_g1_i4_orf1;TRINITY_DN30_c0_g1_i6_orf1;TRINITY_DN7626_c0_g1_i1_orf1;TRINITY_DN1084_c0_g1_i2_orf1;TRINITY_DN4779_c0_g1_i5_orf1;TRINITY_DN6771_c0_g2_i1_orf1;TRINITY_DN41311_c0_g2_i3_orf1;TRINITY_DN975_c0_g1_i1_orf1;TRINITY_DN11194_c0_g1_i4_orf1;TRINITY_DN15959_c0_g1_i1_orf1;TRINITY_DN28875_c0_g1_i1_orf1;TRINITY_DN7213_c0_g1_i2_orf1;TRINITY_DN817_c0_g1_i3_orf1;TRINITY_DN25341_c0_g1_i1_orf1;TRINITY_DN2738_c1_g1_i3_orf1;TRINITY_DN2904_c0_g1_i4_orf1;TRINITY_DN28221_c0_g2_i1_orf1;TRINITY_DN7388_c0_g1_i7_orf1;TRINITY_DN122786_c0_g2_i1_orf1;TRINITY_DN2848_c0_g1_i1_orf1;TRINITY_DN23432_c0_g1_i1_orf1;TRINITY_DN10774_c0_g2_i3_orf1;TRINITY_DN1334_c0_g1_i2_orf1;TRINITY_DN4434_c0_g1_i7_orf1;TRINITY_DN15370_c0_g1_i4_orf1;TRINITY_DN5354_c0_g1_i4_orf1;TRINITY_DN12442_c0_g1_i4_orf1;TRINITY_DN6436_c0_g1_i1_orf1;TRINITY_DN37165_c0_g1_i4_orf1;TRINITY_DN662_c0_g1_i1_orf1;TRINITY_DN24310_c0_g1_i2_orf1;TRINITY_DN44119_c0_g1_i1_orf1;TRINITY_DN20007_c0_g1_i1_orf1;TRINITY_DN63561_c1_g1_i2_orf1;TRINITY_DN3092_c0_g1_i2_orf1;TRINITY_DN39813_c0_g1_i1_orf1;TRINITY_DN2772_c0_g1_i3_orf1;TRINITY_DN5756_c0_g1_i4_orf1;TRINITY_DN31503_c0_g1_i4_orf1;TRINITY_DN157_c0_g1_i4_orf1;TRINITY_DN100821_c0_g1_i1_orf1;TRINITY_DN7405_c0_g1_i3_orf1;TRINITY_DN1034_c0_g1_i4_orf1;TRINITY_DN1725_c0_g1_i7_orf1;TRINITY_DN12301_c0_g1_i1_orf1;TRINITY_DN48554_c0_g1_i1_orf1;TRINITY_DN511_c0_g2_i1_orf1;TRINITY_DN30638_c0_g1_i1_orf1;TRINITY_DN2535_c0_g1_i4_orf1;TRINITY_DN16408_c0_g1_i1_orf1;TRINITY_DN70_c2_g1_i1_orf1;TRINITY_DN21000_c0_g1_i1_orf1;TRINITY_DN12064_c0
molecular_function	polysaccharide binding	GO:0030247	1	1/3516	TRINITY_DN2170_c0_g2_i1_orf1
molecular_function	phosphatidylserine binding	GO:0001786	1	1/3516	TRINITY_DN96739_c0_g1_i1_orf1
molecular_function	ribonucleoprotein complex binding	GO:0043021	12	12/3516	TRINITY_DN3534_c0_g1_i2_orf1;TRINITY_DN23360_c0_g1_i3_orf1;TRINITY_DN25896_c0_g1_i6_orf1;TRINITY_DN441_c0_g2_i1_orf1;TRINITY_DN1066_c0_g1_i4_orf1;TRINITY_DN11215_c0_g1_i1_orf1;TRINITY_DN3366_c0_g1_i6_orf1;TRINITY_DN4309_c0_g1_i1_orf1;TRINITY_DN6239_c0_g1_i1_orf1;TRINITY_DN44407_c0_g4_i2_orf1;TRINITY_DN10070_c0_g1_i1_orf1;TRINITY_DN56110_c0_g1_i1_orf1
molecular_function	G-protein beta/gamma-subunit complex binding	GO:0031683	1	1/3516	TRINITY_DN4628_c0_g1_i1_orf1
molecular_function	protein-lipid complex binding	GO:0071814	1	1/3516	TRINITY_DN46409_c0_g1_i1_orf1
molecular_function	intermediate filament binding	GO:0019215	1	1/3516	TRINITY_DN20009_c0_g1_i1_orf1
molecular_function	dynein complex binding	GO:0070840	2	2/3516	TRINITY_DN21559_c0_g2_i1_orf1;TRINITY_DN21559_c0_g1_i2_orf1
molecular_function	proteasome binding	GO:0070628	1	1/3516	TRINITY_DN135188_c0_g1_i2_orf1
molecular_function	actin filament binding	GO:0051015	14	14/3516	TRINITY_DN110231_c0_g1_i1_orf1;TRINITY_DN104663_c1_g1_i2_orf1;TRINITY_DN364_c0_g2_i1_orf1;TRINITY_DN129869_c0_g4_i1_orf1;TRINITY_DN30150_c0_g1_i7_orf1;TRINITY_DN22824_c0_g1_i4_orf1;TRINITY_DN7493_c0_g1_i1_orf1;TRINITY_DN364_c0_g1_i2_orf1;TRINITY_DN96739_c0_g1_i1_orf1;TRINITY_DN4731_c0_g2_i1_orf1;TRINITY_DN9383_c0_g1_i3_orf1;TRINITY_DN21451_c0_g1_i3_orf1;TRINITY_DN26961_c0_g1_i1_orf1;TRINITY_DN741_c0_g1_i10_orf1
molecular_function	iron-sulfur cluster binding	GO:0051536	21	21/3516	TRINITY_DN14920_c0_g1_i1_orf1;TRINITY_DN129_c0_g1_i6_orf1;TRINITY_DN7909_c0_g2_i1_orf1;TRINITY_DN10030_c0_g1_i2_orf1;TRINITY_DN49221_c0_g1_i1_orf1;TRINITY_DN96566_c0_g1_i1_orf1;TRINITY_DN2103_c0_g1_i1_orf1;TRINITY_DN7626_c0_g1_i1_orf1;TRINITY_DN27641_c0_g1_i1_orf1;TRINITY_DN57536_c0_g1_i14_orf1;TRINITY_DN9536_c0_g1_i4_orf1;TRINITY_DN16830_c0_g1_i5_orf1;TRINITY_DN1494_c0_g1_i3_orf1;TRINITY_DN10716_c1_g1_i1_orf1;TRINITY_DN1422_c0_g1_i4_orf1;TRINITY_DN48638_c0_g1_i5_orf1;TRINITY_DN18558_c0_g1_i7_orf1;TRINITY_DN85476_c0_g1_i1_orf1;TRINITY_DN1494_c0_g2_i1_orf1;TRINITY_DN31851_c0_g1_i2_orf1;TRINITY_DN54134_c0_g1_i1_orf1
molecular_function	virus receptor activity	GO:0001618	1	1/3516	TRINITY_DN10070_c0_g1_i1_orf1
molecular_function	transmembrane signaling receptor activity	GO:0004888	11	11/3516	TRINITY_DN16905_c0_g1_i1_orf1;TRINITY_DN8953_c0_g1_i4_orf1;TRINITY_DN501_c1_g1_i1_orf1;TRINITY_DN3418_c0_g1_i3_orf1;TRINITY_DN46090_c0_g3_i1_orf1;TRINITY_DN13216_c0_g1_i5_orf1;TRINITY_DN19662_c4_g1_i1_orf1;TRINITY_DN2202_c0_g1_i9_orf1;TRINITY_DN15247_c0_g1_i2_orf1;TRINITY_DN20710_c0_g2_i2_orf1;TRINITY_DN34821_c0_g1_i4_orf1
molecular_function	nuclear steroid receptor activity	GO:0003707	1	1/3516	TRINITY_DN938_c0_g1_i7_orf1
molecular_function	pattern recognition receptor activity	GO:0038187	2	2/3516	TRINITY_DN2170_c0_g2_i1_orf1;TRINITY_DN2170_c1_g1_i3_orf1
molecular_function	ligase activity, forming carbon-oxygen bonds	GO:0016875	25	25/3516	TRINITY_DN57918_c0_g1_i1_orf1;TRINITY_DN2038_c0_g1_i2_orf1;TRINITY_DN8716_c0_g1_i3_orf1;TRINITY_DN27771_c0_g1_i1_orf1;TRINITY_DN620_c0_g1_i4_orf1;TRINITY_DN30224_c0_g1_i1_orf1;TRINITY_DN11639_c0_g1_i1_orf1;TRINITY_DN2953_c1_g1_i11_orf1;TRINITY_DN64810_c0_g1_i1_orf1;TRINITY_DN2953_c1_g1_i10_orf1;TRINITY_DN27771_c0_g2_i1_orf1;TRINITY_DN1607_c0_g1_i16_orf1;TRINITY_DN48619_c0_g1_i1_orf1;TRINITY_DN2953_c1_g1_i2_orf1;TRINITY_DN5756_c0_g1_i4_orf1;TRINITY_DN84322_c0_g2_i1_orf1;TRINITY_DN8598_c0_g1_i2_orf1;TRINITY_DN817_c0_g1_i3_orf1;TRINITY_DN30638_c0_g1_i1_orf1;TRINITY_DN15160_c0_g1_i1_orf1;TRINITY_DN2224_c0_g1_i1_orf1;TRINITY_DN21539_c0_g1_i1_orf1;TRINITY_DN5218_c0_g1_i4_orf1;TRINITY_DN107288_c0_g1_i2_orf1;TRINITY_DN4944_c0_g1_i2_orf1

molecular_function	ligase activity, forming carbon-sulfur bonds	GO:0016877	5	5/3516	TRINITY_DN22928_c0_g1_i6_orf1;TRINITY_DN15882_c0_g1_i1_orf1;TRINITY_DN8659_c0_g1_i1_orf1;TRINITY_DN2193_c0_g1_i7_orf1;TRINITY_DN37729_c0_g1_i8_orf1
molecular_function	ligase activity, forming carbon-nitrogen bonds	GO:0016879	17	17/3516	TRINITY_DN98313_c0_g1_i1_orf1;TRINITY_DN36144_c0_g1_i3_orf1;TRINITY_DN3822_c0_g1_i7_orf1;TRINITY_DN42738_c0_g1_i1_orf1;TRINITY_DN6587_c0_g1_i3_orf1;TRINITY_DN987_c0_g1_i3_orf1;TRINITY_DN24723_c2_g1_i1_orf1;TRINITY_DN38506_c0_g1_i4_orf1;TRINITY_DN28221_c0_g2_i1_orf1;TRINITY_DN76815_c0_g1_i3_orf1;TRINITY_DN100821_c0_g1_i1_orf1;TRINITY_DN6669_c0_g1_i3_orf1;TRINITY_DN1965_c0_g1_i7_orf1;TRINITY_DN14464_c0_g1_i1_orf1;TRINITY_DN41697_c0_g1_i1_orf1;TRINITY_DN45924_c0_g1_i14_orf1;TRINITY_DN244_c1_g1_i5_orf1
molecular_function	proton-transporting ATP synthase activity, rotational mechanism	GO:0046933	7	7/3516	TRINITY_DN80560_c0_g1_i1_orf1;TRINITY_DN4434_c0_g1_i7_orf1;TRINITY_DN2300_c0_g1_i1_orf1;TRINITY_DN45000_c0_g1_i5_orf1;TRINITY_DN96080_c0_g2_i1_orf1;TRINITY_DN22430_c0_g3_i1_orf1;TRINITY_DN83005_c0_g1_i1_orf1
molecular_function	ligase activity, forming carbon-carbon bonds	GO:0016885	3	3/3516	TRINITY_DN3991_c0_g1_i6_orf1;TRINITY_DN2570_c0_g1_i1_orf1;TRINITY_DN511_c0_g2_i1_orf1
molecular_function	ligase activity, forming phosphoric ester bonds	GO:0016886	1	1/3516	TRINITY_DN39490_c0_g1_i1_orf1
molecular_function	ferrochelatase activity	GO:0004325	1	1/3516	TRINITY_DN5559_c0_g1_i1_orf1
molecular_function	carbon-sulfur lyase activity	GO:0016846	2	2/3516	TRINITY_DN12003_c0_g2_i1_orf1;TRINITY_DN11948_c0_g1_i8_orf1
molecular_function	carbon-nitrogen lyase activity	GO:0016840	3	3/3516	TRINITY_DN16868_c0_g2_i1_orf1;TRINITY_DN1716_c0_g1_i14_orf1;TRINITY_DN28299_c0_g1_i1_orf1
molecular_function	phosphorus-oxygen lyase activity	GO:0016849	4	4/3516	TRINITY_DN10774_c0_g2_i3_orf1;TRINITY_DN3712_c0_g1_i1_orf1;TRINITY_DN618_c0_g1_i3_orf1;TRINITY_DN52244_c1_g1_i1_orf1
molecular_function	carbon-oxygen lyase activity	GO:0016835	17	17/3516	TRINITY_DN111985_c0_g1_i1_orf1;TRINITY_DN230_c2_g1_i5_orf1;TRINITY_DN82810_c0_g1_i1_orf1;TRINITY_DN2103_c0_g1_i1_orf1;TRINITY_DN27848_c0_g1_i2_orf1;TRINITY_DN10430_c0_g1_i4_orf1;TRINITY_DN17559_c0_g1_i4_orf1;TRINITY_DN51498_c0_g1_i1_orf1;TRINITY_DN357_c0_g1_i8_orf1;TRINITY_DN10900_c0_g1_i7_orf1;TRINITY_DN73900_c0_g1_i1_orf1;TRINITY_DN42759_c0_g2_i1_orf1;TRINITY_DN42759_c0_g3_i1_orf1;TRINITY_DN2825_c0_g1_i3_orf1;TRINITY_DN35763_c0_g1_i2_orf1;TRINITY_DN143603_c0_g1_i1_orf1;TRINITY_DN89483_c0_g1_i1_orf1
molecular_function	carbon-carbon lyase activity	GO:0016830	18	18/3516	TRINITY_DN779_c0_g1_i12_orf1;TRINITY_DN12474_c0_g1_i6_orf1;TRINITY_DN1334_c0_g1_i2_orf1;TRINITY_DN1045_c0_g1_i6_orf1;TRINITY_DN19261_c0_g1_i3_orf1;TRINITY_DN11159_c0_g2_i1_orf1;TRINITY_DN40434_c0_g1_i2_orf1;TRINITY_DN3822_c0_g1_i7_orf1;TRINITY_DN109931_c0_g1_i1_orf1;TRINITY_DN25582_c0_g1_i3_orf1;TRINITY_DN2684_c0_g2_i3_orf1;TRINITY_DN779_c0_g1_i3_orf1;TRINITY_DN8037_c0_g2_i1_orf1;TRINITY_DN19122_c0_g1_i7_orf1;TRINITY_DN83150_c0_g1_i1_orf1;TRINITY_DN20133_c0_g1_i1_orf1;TRINITY_DN6325_c0_g1_i9_orf1;TRINITY_DN10548_c0_g2_i1_orf1
molecular_function	protein methylesterase activity	GO:0051723	1	1/3516	TRINITY_DN10336_c0_g1_i9_orf1
molecular_function	peptide-lysine-N-acetyltransferase activity	GO:0061733	3	3/3516	TRINITY_DN20442_c0_g2_i1_orf1;TRINITY_DN12771_c0_g1_i1_orf1;TRINITY_DN51737_c0_g1_i3_orf1
molecular_function	palmitoyl-(protein) hydrolase activity	GO:0008474	1	1/3516	TRINITY_DN4817_c0_g1_i4_orf1
molecular_function	protein-disulfide reductase activity	GO:0015035	7	7/3516	TRINITY_DN51938_c0_g3_i1_orf1;TRINITY_DN79673_c0_g1_i1_orf1;TRINITY_DN5169_c0_g1_i5_orf1;TRINITY_DN376_c0_g1_i1_orf1;TRINITY_DN14306_c0_g1_i1_orf1;TRINITY_DN21715_c0_g1_i1_orf1;TRINITY_DN1901_c0_g1_i6_orf1
molecular_function	protein methyltransferase activity	GO:0008276	8	8/3516	TRINITY_DN2930_c0_g1_i8_orf1;TRINITY_DN22674_c0_g1_i2_orf1;TRINITY_DN95414_c0_g1_i1_orf1;TRINITY_DN14734_c0_g1_i2_orf1;TRINITY_DN14953_c0_g1_i5_orf1;TRINITY_DN2168_c0_g1_i2_orf1;TRINITY_DN20749_c0_g1_i3_orf1;TRINITY_DN6462_c0_g1_i5_orf1
molecular_function	phosphoprotein phosphatase activity	GO:0004721	9	9/3516	TRINITY_DN2257_c0_g1_i4_orf1;TRINITY_DN39404_c0_g1_i7_orf1;TRINITY_DN24539_c0_g1_i4_orf1;TRINITY_DN7134_c0_g1_i1_orf1;TRINITY_DN40562_c0_g2_i1_orf1;TRINITY_DN59885_c0_g1_i3_orf1;TRINITY_DN70409_c0_g1_i3_orf1;TRINITY_DN152_c0_g1_i4_orf1;TRINITY_DN6876_c0_g2_i1_orf1
molecular_function	peptidyl-cysteine S-nitrosylase activity	GO:0035605	2	2/3516	TRINITY_DN2848_c0_g1_i2_orf1;TRINITY_DN2848_c0_g1_i1_orf1
molecular_function	protein-cysteine S-acyltransferase activity	GO:0019707	1	1/3516	TRINITY_DN3628_c0_g1_i5_orf1
molecular_function	deoxyhypusine monooxygenase activity	GO:0019135	1	1/3516	TRINITY_DN8019_c0_g1_i4_orf1
molecular_function	ubiquitin-like protein transferase activity	GO:0019787	12	12/3516	TRINITY_DN88539_c0_g2_i1_orf1;TRINITY_DN9062_c0_g2_i3_orf1;TRINITY_DN17376_c0_g1_i2_orf1;TRINITY_DN7647_c0_g1_i4_orf1;TRINITY_DN1738_c0_g1_i5_orf1;TRINITY_DN146138_c0_g1_i1_orf1;TRINITY_DN11820_c0_g1_i1_orf1;TRINITY_DN1380_c0_g1_i5_orf1;TRINITY_DN17726_c0_g1_i1_orf1;TRINITY_DN9132_c0_g1_i5_orf1;TRINITY_DN7655_c0_g1_i3_orf1;TRINITY_DN14487_c0_g1_i4_orf1
molecular_function	aminoacyltransferase activity	GO:0016755	1	1/3516	TRINITY_DN4898_c0_g1_i7_orf1
molecular_function	protein lysine deacetylase activity	GO:0033558	1	1/3516	TRINITY_DN10385_c0_g1_i5_orf1
molecular_function	protein kinase activity	GO:0004672	37	37/3516	TRINITY_DN1173_c0_g1_i12_orf1;TRINITY_DN16905_c0_g1_i1_orf1;TRINITY_DN70485_c0_g1_i2_orf1;TRINITY_DN35991_c0_g1_i2_orf1;TRINITY_DN1552_c0_g1_i3_orf1;TRINITY_DN1405_c0_g1_i1_orf1;TRINITY_DN105749_c0_g1_i1_orf1;TRINITY_DN6436_c0_g1_i1_orf1;TRINITY_DN32700_c0_g1_i2_orf1;TRINITY_DN10680_c0_g1_i5_orf1;TRINITY_DN28729_c0_g1_i9_orf1;TRINITY_DN3418_c0_g1_i3_orf1;TRINITY_DN46090_c0_g3_i1_orf1;TRINITY_DN2983_c0_g1_i6_orf1;TRINITY_DN13160_c0_g1_i1_orf1;TRINITY_DN21181_c0_g1_i6_orf1;TRINITY_DN73945_c0_g5_i3_orf1;TRINITY_DN10774_c0_g2_i3_orf1;TRINITY_DN2202_c0_g1_i9_orf1;TRINITY_DN19662_c4_g1_i1_orf1;TRINITY_DN30154_c0_g1_i1_orf1;TRINITY_DN15478_c0_g1_i1_orf1;TRINITY_DN21126_c0_g1_i1_orf1;TRINITY_DN17838_c0_g1_i4_orf1;TRINITY_DN1673_c0_g1_i2_orf1;TRINITY_DN6185_c0_g1_i12_orf1;TRINITY_DN1173_c1_g1_i9_orf1;TRINITY_DN5281_c0_g2_i3_orf1;TRINITY_DN143637_c0_g1_i1_orf1;TRINITY_DN1266_c2_g1_i1_orf1;TRINITY_DN4798_c0_g1_i3_orf1;TRINITY_DN1173_c1_g1_i10_orf1;TRINITY_DN30_c0_g1_i6_orf1;TRINITY_DN4449_c0_g2_i1_orf1;TRINITY_DN147475_c0_g1_i1_orf1;TRINITY_DN29956_c1_g1_i1_orf1;TRINITY_DN3515_c0_g1_i3_orf1

molecular_function	peptidase activity	GO:0008233	171	171/3516	TRINITY_DN29017_c0_g1_i4_orf1;TRINITY_DN130051_c0_g1_i1_orf1;TRINITY_DN8076_c0_g1_i5_orf1;TRINITY_DN3194_c0_g1_i6_orf1;TRINITY_DN11492_c0_g1_i8_orf1;TRINITY_DN181_c0_g1_i3_orf1;TRINITY_DN1533_c0_g2_i1_orf1;TRINITY_DN11928_c0_g1_i3_orf1;TRINITY_DN2563_c0_g1_i4_orf1;TRINITY_DN344_c1_g1_i1_orf1;TRINITY_DN117362_c0_g1_i5_orf1;TRINITY_DN2069_c1_g1_i8_orf1;TRINITY_DN83295_c0_g1_i3_orf1;TRINITY_DN3499_c0_g1_i8_orf1;TRINITY_DN8621_c0_g1_i4_orf1;TRINITY_DN2442_c0_g1_i2_orf1;TRINITY_DN36434_c0_g2_i3_orf1;TRINITY_DN3861_c0_g3_i2_orf1;TRINITY_DN16258_c0_g1_i2_orf1;TRINITY_DN2885_c1_g1_i2_orf1;TRINITY_DN14754_c0_g1_i6_orf1;TRINITY_DN27033_c1_g1_i3_orfp1;TRINITY_DN16343_c0_g1_i6_orf1;TRINITY_DN14217_c0_g1_i1_orf1;TRINITY_DN443_c0_g1_i2_orf1;TRINITY_DN48020_c0_g1_i1_orf1;TRINITY_DN71917_c0_g3_i1_orf1;TRINITY_DN18273_c0_g1_i4_orf1;TRINITY_DN2794_c1_g1_i8_orf1;TRINITY_DN4125_c0_g1_i14_orf1;TRINITY_DN2178_c0_g1_i1_orf1;TRINITY_DN391_c0_g1_i4_orf1;TRINITY_DN67026_c0_g1_i6_orf1;TRINITY_DN40_c0_g2_i1_orf1;TRINITY_DN28428_c0_g1_i2_orf1;TRINITY_DN892_c7_g1_i2_orf1;TRINITY_DN932_c0_g1_i4_orf1;TRINITY_DN4767_c0_g1_i6_orf1;TRINITY_DN3755_c0_g1_i3_orf1;TRINITY_DN51938_c0_g3_i1_orf1;TRINITY_DN334_c0_g1_i2_orf1;TRINITY_DN143895_c0_g1_i1_orf1;TRINITY_DN2627_c0_g1_i2_orf1;TRINITY_DN45633_c0_g1_i1_orf1;TRINITY_DN10090_c0_g1_i1_orf1;TRINITY_DN40_c0_g1_i3_orf1;TRINITY_DN6205_c0_g1_i1_orf1;TRINITY_DN4189_c0_g2_i1_orf1;TRINITY_DN9836_c0_g1_i2_orf1;TRINITY_DN57798_c0_g1_i1_orf1;TRINITY_DN1310_c0_g1_i4_orf1;TRINITY_DN5012_c0_g1_i6_orf1;TRINITY_DN72541_c0_g1_i2_orf1;TRINITY_DN1947_c0_g1_i6_orf1;TRINITY_DN41086_c0_g1_i4_orf1;TRINITY_DN19651_c0_g1_i1_orf1;TRINITY_DN41952_c0_g1_i1_orf1;TRINITY_DN4476_c0_g1_i5_orf1;TRINITY_DN45948_c1_g1_i1_orf1;TRINITY_DN66302_c0_g1_i1_orf1;TRINITY_DN5696_c0_g1_i4_orf1;TRINITY_DN2040_c0_g1_i6_orf1;TRINITY_DN71863_c0_g1_i2_orf1;TRINITY_DN29034_c0_g1_i2_orf1;TRINITY_DN4125_c1_g1_i5_orf1;TRINITY_DN1703_c0_g1_i6_orf1;TRINITY_DN4064_c0_g2_i1_orf1;TRINITY_DN5444_c0_g2_i1_orf1;TRINITY_DN21984_c0_g1_i6_orf1;TRINITY_DN338_c1_g1_i9_orf1;TRINITY_DN6423_c0_g1_i6_orf1;TRINITY_DN140_c1_g1_i2_orf1;TRINITY_DN3474_c1_g2_i7_orf1;TRINITY_DN4767_c0_g1_i4_orf1;TRINITY_DN7776_c0_g1_i5_orf1;TRINITY_DN10403_c0_g1_i1_orf1;TRINITY_DN145227_c0_g1_i1_orf1;TRINITY_DN1459_c1_g1_i1_orf1;TRINITY_DN3702_c0_g1_i1_orf1;TRINITY_DN4494_c0_g1_i1_orf1;TRINITY_DN21218_c0_g1_i4_orf1;TRINITY_DN25534_c0_g1_i1_orf1;TRINITY_DN10766_c0_g1_i1_orf1;TRINITY_DN554_c0_g1_i1_orf1;TRINITY_DN1421_c0_g1_i1_orf1;TRINITY_DN18159_c0_g1_i6_orf1;TRINITY_DN36538_c0_g1_i2_orf1;TRINITY_DN74654_c0_g1_i4_orf1;TRINITY_DN9991_c0_g1_i4_orf1;TRINITY_DN3483_c0_g1_i5_orf1;TRINITY_DN69049_c0_g1_i2_orf1;TRINITY_DN10429_c0_g1_i2_orf1;TRINITY_DN8621_c0_g1_i5_orf1;TRINITY_DN344_c0_g1_i1_orf1;TRINITY_DN6122_c0_g1_i6_orf1;TRINITY_DN7325_c0_g1_i1_orf1;TRINITY_DN57111_c0_g1_i1_orf1;TRINITY_DN5310_c2_g1_i2_orf1;TRINITY_DN6423_c0_g1_i5_orf1;TRINITY_DN376_c1_g1_i1_orf1;TRINITY_DN1404_c0_g1_i6_orf1;TRINITY_DN3975_c0_g1_i10_orf1;TRINITY_DN34479_c0_g1_i2_orf1;TRINITY_DN52768_c0_g1_i1_orf1;TRINITY_DN3343_c0_g2_i1_orf1;TRINITY_DN18388_c0_g1_i6_orf1;TRINITY_DN29414_c1_g2_i1_orf1;TRINITY_DN428_c0_g1_i8_orf1;TRINITY_DN2579_c0_g1_i7_orf1;TRINITY_DN7754_c0_g1_i2_orf1;TRINITY_DN18172_c0_g1_i6_orf1;TRINITY_DN36262_c0_g1_i1_orf1;TRINITY_DN701_c0_g1_i1_orf1;TRINITY_DN28661_c0_g1_i1_orf1;TRINITY_DN4228_c0_g1_i5_orf1;TRINITY_DN4121_c0_g1_i1_orf1;TRINITY_DN2274_c0_g1_i6_orf1;TRINITY_DN1337_c0_g2_i1_orf1;TRINITY_DN6059_c0_g1_i1_orf1;TRINITY_DN29034_c0_g1_i1_orf1;TRINITY_DN69697_c0_g1_i1_orf1;TRINITY_DN6813_c1_g1_i1_orf1;TRIN
molecular_function	protein disulfide isomerase activity	GO:0003756	3	3/3516	TRINITY_DN51938_c0_g3_i1_orf1;TRINITY_DN21715_c0_g1_i1_orf1;TRINITY_DN1831_c0_g1_i3_orf1
molecular_function	peptidyl-prolyl cis-trans isomerase activity	GO:0003755	10	10/3516	TRINITY_DN3773_c0_g1_i4_orf1;TRINITY_DN119291_c0_g1_i1_orf1;TRINITY_DN142588_c0_g1_i1_orf1;TRINITY_DN1888_c0_g2_i1_orf1;TRINITY_DN14372_c0_g2_i1_orf1;TRINITY_DN54275_c0_g1_i4_orf1;TRINITY_DN2807_c0_g1_i4_orf1;TRINITY_DN34056_c0_g1_i4_orf1;TRINITY_DN140538_c0_g2_i1_orf1;TRINITY_DN1294_c0_g1_i3_orf1
molecular_function	protein demethylase activity	GO:0140457	1	1/3516	TRINITY_DN89083_c0_g1_i1_orf1
molecular_function	ubiquitin-like modifier activating enzyme activity	GO:0008641	3	3/3516	TRINITY_DN22928_c0_g1_i6_orf1;TRINITY_DN8659_c0_g1_i1_orf1;TRINITY_DN37729_c0_g1_i8_orf1
molecular_function	dihydrolipoyllysine-residue succinyltransferase	GO:0004149	1	1/3516	TRINITY_DN19727_c0_g1_i7_orf1
molecular_function	hydrolase activity, acting on glycosyl bonds	GO:0016798	56	56/3516	TRINITY_DN6510_c1_g1_i1_orf1;TRINITY_DN14235_c0_g1_i1_orf1;TRINITY_DN8703_c0_g1_i2_orf1;TRINITY_DN3476_c0_g1_i5_orf1;TRINITY_DN25492_c0_g1_i1_orf1;TRINITY_DN53167_c0_g1_i3_orf1;TRINITY_DN18918_c0_g1_i3_orf1;TRINITY_DN467_c3_g1_i5_orf1;TRINITY_DN6108_c0_g1_i5_orf1;TRINITY_DN9044_c0_g1_i2_orf1;TRINITY_DN43391_c0_g1_i5_orf1;TRINITY_DN11657_c0_g1_i2_orf1;TRINITY_DN9044_c0_g1_i1_orf1;TRINITY_DN5488_c0_g1_i5_orf1;TRINITY_DN21545_c0_g1_i2_orf1;TRINITY_DN479_c6_g1_i2_orf1;TRINITY_DN6074_c0_g1_i1_orf1;TRINITY_DN7183_c0_g1_i2_orf1;TRINITY_DN361_c0_g1_i5_orf1;TRINITY_DN48237_c0_g1_i5_orf1;TRINITY_DN23586_c0_g1_i3_orf1;TRINITY_DN2894_c0_g2_i3_orf1;TRINITY_DN542_c0_g1_i4_orf1;TRINITY_DN3322_c0_g1_i2_orf1;TRINITY_DN7228_c0_g1_i6_orf1;TRINITY_DN15222_c0_g1_i4_orf1;TRINITY_DN67623_c0_g1_i1_orf1;TRINITY_DN2894_c0_g1_i2_orf1;TRINITY_DN18918_c0_g1_i2_orf1;TRINITY_DN195_c4_g1_i1_orf1;TRINITY_DN2170_c0_g2_i1_orf1;TRINITY_DN2515_c0_g1_i6_orf1;TRINITY_DN4070_c0_g1_i4_orf1;TRINITY_DN6472_c0_g1_i5_orf1;TRINITY_DN2170_c4_g1_i2_orf1;TRINITY_DN2205_c0_g1_i3_orf1;TRINITY_DN143603_c0_g1_i1_orf1;TRINITY_DN1732_c0_g1_i15_orf1;TRINITY_DN2516_c0_g2_i10_orf1;TRINITY_DN1098_c1_g1_i4_orf1;TRINITY_DN670_c0_g1_i3_orf1;TRINITY_DN26688_c0_g1_i2_orf1;TRINITY_DN21555_c0_g1_i4_orf1;TRINITY_DN7828_c0_g1_i2_orf1;TRINITY_DN1732_c0_g1_i17_orf1;TRINITY_DN25896_c0_g1_i6_orf1;TRINITY_DN1287_c0_g1_i5_orf1;TRINITY_DN98723_c1_g1_i1_orf1;TRINITY_DN53167_c0_g1_i2_orf1;TRINITY_DN48410_c0_g1_i1_orf1;TRINITY_DN2170_c1_g1_i3_orf1;TRINITY_DN48410_c0_g2_i1_orf1;TRINITY_DN812_c2_g1_i1_orf1;TRINITY_DN2894_c0_g3_i1_orf1;TRINITY_DN4954_c0_g1_i5_orf1;TRINITY_DN650_c0_g1_i3_orf1
molecular_function	hydrolase activity, acting on acid carbon-carbon bonds	GO:0016822	3	3/3516	TRINITY_DN17913_c0_g1_i8_orf1;TRINITY_DN19187_c0_g1_i1_orf1;TRINITY_DN51813_c0_g1_i1_orf1
molecular_function	hydrolase activity, acting on carbon-nitrogen (but not peptide) bonds	GO:0016810	27	27/3516	TRINITY_DN827_c1_g1_i1_orf1;TRINITY_DN1534_c0_g1_i3_orf1;TRINITY_DN542_c0_g2_i1_orf1;TRINITY_DN87170_c0_g1_i3_orf1;TRINITY_DN1216_c0_g1_i4_orf1;TRINITY_DN38506_c0_g1_i4_orf1;TRINITY_DN17031_c0_g1_i1_orf1;TRINITY_DN98242_c0_g1_i1_orf1;TRINITY_DN17172_c0_g1_i5_orf1;TRINITY_DN244_c1_g1_i5_orf1;TRINITY_DN13660_c0_g1_i1_orf1;TRINITY_DN542_c0_g1_i4_orf1;TRINITY_DN14107_c0_g1_i4_orf1;TRINITY_DN4145_c0_g1_i1_orf1;TRINITY_DN17326_c0_g1_i8_orf1;TRINITY_DN1375_c0_g1_i5_orf1;TRINITY_DN5235_c0_g1_i7_orf1;TRINITY_DN1277_c4_g1_i5_orf1;TRINITY_DN3971_c0_g1_i1_orf1;TRINITY_DN8674_c0_g2_i1_orf1;TRINITY_DN768_c0_g1_i7_orf1;TRINITY_DN82801_c0_g1_i1_orf1;TRINITY_DN17326_c0_g1_i5_orf1;TRINITY_DN2835_c0_g1_i6_orf1;TRINITY_DN10385_c0_g1_i5_orf1;TRINITY_DN38180_c0_g1_i3_orf1;TRINITY_DN11383_c0_g2_i4_orf1

molecular_function	hydrolase activity, acting on acid anhydrides	GO:0016817	90	90/3516	TRINITY_DN20776_c0_g1_i3_orf1;TRINITY_DN125565_c1_g1_i1_orf1;TRINITY_DN6771_c0_g2_i1_orf1;TRINITY_DN41311_c0_g2_i3_orf1;TRINITY_DN11194_c0_g1_i4_orf1;TRINITY_DN33249_c0_g1_i1_orf1;TRINITY_DN28875_c0_g1_i1_orf1;TRINITY_DN33801_c0_g1_i1_orf1;TRINITY_DN2054_c0_g1_i1_orf1;TRINITY_DN25341_c0_g1_i1_orf1;TRINITY_DN5262_c0_g1_i7_orf1;TRINITY_DN1023_c1_g1_i1_orf1;TRINITY_DN11069_c0_g2_i1_orf1;TRINITY_DN7388_c0_g1_i7_orf1;TRINITY_DN2638_c0_g1_i7_orf1;TRINITY_DN12582_c0_g1_i5_orf1;TRINITY_DN32487_c0_g1_i1_orf1;TRINITY_DN15370_c0_g1_i4_orf1;TRINITY_DN452_c1_g1_i3_orf1;TRINITY_DN12442_c0_g1_i4_orf1;TRINITY_DN52761_c0_g1_i2_orf1;TRINITY_DN24164_c0_g1_i1_orf1;TRINITY_DN139438_c0_g1_i1_orf1;TRINITY_DN44119_c0_g1_i1_orf1;TRINITY_DN2265_c0_g1_i5_orf1;TRINITY_DN2947_c0_g1_i4_orf1;TRINITY_DN16174_c0_g1_i2_orf1;TRINITY_DN121047_c0_g1_i3_orf1;TRINITY_DN52761_c0_g2_i1_orf1;TRINITY_DN63561_c1_g1_i2_orf1;TRINITY_DN3092_c0_g1_i2_orf1;TRINITY_DN46367_c0_g1_i2_orf1;TRINITY_DN140212_c0_g1_i1_orf1;TRINITY_DN4977_c0_g1_i2_orf1;TRINITY_DN100821_c0_g1_i1_orf1;TRINITY_DN9575_c0_g1_i1_orf1;TRINITY_DN19920_c1_g1_i2_orf1;TRINITY_DN1725_c0_g1_i7_orf1;TRINITY_DN1091_c0_g3_i1_orf1;TRINITY_DN2927_c0_g1_i6_orf1;TRINITY_DN6642_c0_g1_i2_orf1;TRINITY_DN8986_c0_g1_i1_orf1;TRINITY_DN315_c0_g1_i1_orf1;TRINITY_DN71465_c0_g1_i1_orf1;TRINITY_DN31232_c1_g1_i9_orf1;TRINITY_DN33705_c0_g1_i1_orf1;TRINITY_DN7336_c0_g1_i13_orf1;TRINITY_DN145227_c0_g1_i1_orf1;TRINITY_DN21000_c0_g1_i1_orf1;TRINITY_DN12064_c0_g2_i1_orf1;TRINITY_DN21214_c0_g2_i1_orf1;TRINITY_DN11612_c0_g3_i1_orf1;TRINITY_DN46409_c0_g1_i1_orf1;TRINITY_DN10429_c0_g1_i2_orf1;TRINITY_DN3664_c0_g1_i8_orf1;TRINITY_DN136906_c0_g1_i1_orf1;TRINITY_DN6426_c0_g1_i2_orf1;TRINITY_DN34479_c0_g1_i2_orf1;TRINITY_DN3343_c0_g2_i1_orf1;TRINITY_DN32769_c1_g1_i5_orf1;TRINITY_DN4762_c0_g1_i2_orf1;TRINITY_DN2793_c0_g2_i1_orf1;TRINITY_DN48460_c0_g1_i1_orf1;TRINITY_DN29144_c0_g3_i1_orf1;TRINITY_DN97138_c0_g1_i2_orf1;TRINITY_DN8979_c0_g1_i5_orf1;TRINITY_DN45097_c0_g1_i5_orf1;TRINITY_DN164_c0_g1_i11_orf1;TRINITY_DN49047_c0_g1_i2_orf1;TRINITY_DN6855_c1_g1_i3_orf1;TRINITY_DN14464_c0_g1_i1_orf1;TRINITY_DN2265_c0_g2_i1_orf1;TRINITY_DN4790_c0_g1_i6_orf1;TRINITY_DN33967_c0_g1_i1_orf1;TRINITY_DN15706_c0_g2_i5_orf1;TRINITY_DN3343_c0_g1_i4_orf1;TRINITY_DN10521_c0_g1_i7_orf1;TRINITY_DN67716_c0_g1_i1_orf1;TRINITY_DN975_c0_g1_i1_orf1;TRINITY_DN26243_c0_g1_i2_orf1;TRINITY_DN4628_c0_g1_i1_orf1;TRINITY_DN740_c0_g1_i1_orf1;TRINITY_DN13055_c0_g1_i5_orf1;TRINITY_DN280_c0_g1_i12_orf1;TRINITY_DN94625_c0_g1_i1_orf1;TRINITY_DN108122_c0_g1_i9_orf1;TRINITY_DN63719_c0_g1_i5_orf1;TRINITY_DN7464_c1_g1_i1_orf1;TRINITY_DN4779_c0_g1_i5_orf1;TRINITY_DN1665_c1_g1_i2_orf1
molecular_function	serine hydrolase activity	GO:0017171	79	79/3516	TRINITY_DN43420_c0_g2_i1_orf1;TRINITY_DN334_c0_g1_i2_orf1;TRINITY_DN29034_c0_g1_i2_orf1;TRINITY_DN36434_c0_g2_i3_orf1;TRINITY_DN10766_c0_g1_i1_orf1;TRINITY_DN130051_c0_g1_i1_orf1;TRINITY_DN8076_c0_g1_i5_orf1;TRINITY_DN11492_c0_g1_i8_orf1;TRINITY_DN74654_c0_g1_i4_orf1;TRINITY_DN10090_c0_g1_i1_orf1;TRINITY_DN3483_c0_g1_i5_orf1;TRINITY_DN40_c0_g1_i3_orf1;TRINITY_DN1533_c0_g2_i1_orf1;TRINITY_DN10429_c0_g1_i2_orf1;TRINITY_DN344_c0_g1_i1_orf1;TRINITY_DN334_c0_g1_i4_orf1;TRINITY_DN2563_c0_g1_i4_orf1;TRINITY_DN344_c1_g1_i1_orf1;TRINITY_DN117362_c0_g1_i5_orf1;TRINITY_DN5310_c2_g1_i2_orf1;TRINITY_DN6423_c0_g1_i5_orf1;TRINITY_DN10071_c0_g1_i2_orf1;TRINITY_DN1310_c0_g1_i4_orf1;TRINITY_DN5012_c0_g1_i6_orf1;TRINITY_DN1404_c0_g1_i6_orf1;TRINITY_DN805_c0_g1_i5_orf1;TRINITY_DN3499_c0_g1_i8_orf1;TRINITY_DN41086_c0_g1_i4_orf1;TRINITY_DN3975_c0_g1_i10_orf1;TRINITY_DN25534_c0_g1_i1_orf1;TRINITY_DN83327_c0_g1_i1_orf1;TRINITY_DN57111_c0_g1_i1_orf1;TRINITY_DN23570_c0_g1_i2_orf1;TRINITY_DN16258_c0_g1_i2_orf1;TRINITY_DN4476_c0_g1_i5_orf1;TRINITY_DN29034_c0_g1_i1_orf1;TRINITY_DN334_c0_g1_i3_orf1;TRINITY_DN5696_c0_g1_i4_orf1;TRINITY_DN18388_c0_g1_i6_orf1;TRINITY_DN29414_c1_g2_i1_orf1;TRINITY_DN2040_c0_g1_i6_orf1;TRINITY_DN71863_c0_g1_i2_orf1;TRINITY_DN4631_c0_g1_i7_orf1;TRINITY_DN747_c0_g1_i1_orf1;TRINITY_DN36262_c0_g1_i1_orf1;TRINITY_DN18273_c0_g1_i4_orf1;TRINITY_DN67026_c0_g1_i6_orf1;TRINITY_DN7325_c0_g1_i1_orf1;TRINITY_DN96_c0_g1_i1_orf1;TRINITY_DN371_c0_g1_i6_orf1;TRINITY_DN5444_c0_g2_i1_orf1;TRINITY_DN753_c0_g1_i4_orf1;TRINITY_DN701_c0_g1_i1_orf1;TRINITY_DN21984_c0_g1_i6_orf1;TRINITY_DN334_c0_g1_i1_orf1;TRINITY_DN4228_c0_g1_i5_orf1;TRINITY_DN6423_c0_g1_i6_orf1;TRINITY_DN23167_c0_g2_i1_orf1;TRINITY_DN140_c1_g1_i2_orf1;TRINITY_DN391_c0_g1_i4_orf1;TRINITY_DN4030_c0_g2_i1_orf1;TRINITY_DN9991_c0_g1_i4_orf1;TRINITY_DN4026_c0_g1_i4_orf1;TRINITY_DN40_c0_g2_i1_orf1;TRINITY_DN6059_c0_g1_i1_orf1;TRINITY_DN10403_c0_g1_i1_orf1;TRINITY_DN23167_c0_g1_i4_orf1;TRINITY_DN6205_c0_g1_i1_orf1;TRINITY_DN14217_c0_g1_i1_orf1;TRINITY_DN24121_c1_g1_i6_orf1;TRINITY_DN1592_c0_g1_i1_orf1;TRINITY_DN747_c0_g2_i1_orf1;TRINITY_DN4494_c0_g1_i1_orf1;TRINITY_DN1421_c0_g1_i1_orf1;TRINITY_DN8480_c0_g1_i1_orf1;TRINITY_DN338_c1_g1_i9_orf1;TRINITY_DN747_c0_g1_i4_orf1;TRINITY_DN428_c0_g1_i8_orf1;TRINITY_DN13686_c0_g2_i1_orf1

molecular_function	hydrolase activity, acting on ester bonds	GO:0016788	123	123/3516	TRINITY_DN38230_c0_g1_i4_orf1;TRINITY_DN11117_c0_g1_i1_orf1;TRINITY_DN12227_c0_g2_i3_orf1;TRINITY_DN39404_c0_g1_i7_orf1;TRINITY_DN1073_c0_g1_i4_orf1;TRINITY_DN1073_c0_g1_i1_orf1;TRINITY_DN9711_c0_g1_i10_orf1;TRINITY_DN95850_c0_g1_i1_orf1;TRINITY_DN3784_c0_g1_i1_orf1;TRINITY_DN70485_c0_g1_i2_orf1;TRINITY_DN117_c0_g1_i6_orf1;TRINITY_DN23432_c0_g1_i1_orf1;TRINITY_DN616_c1_g1_i6_orf1;TRINITY_DN18291_c0_g1_i1_orf1;TRINITY_DN17437_c0_g1_i1_orf1;TRINITY_DN42759_c0_g3_i1_orf1;TRINITY_DN21494_c0_g1_i2_orf1;TRINITY_DN123184_c0_g1_i1_orf1;TRINITY_DN2668_c0_g1_i6_orf1;TRINITY_DN1116_c0_g1_i6_orf1;TRINITY_DN446_c0_g1_i20_orf1;TRINITY_DN4813_c0_g1_i5_orf1;TRINITY_DN1978_c0_g1_i4_orf1;TRINITY_DN25896_c0_g1_i6_orf1;TRINITY_DN2627_c0_g2_i1_orf1;TRINITY_DN1161_c0_g1_i2_orf1;TRINITY_DN69535_c0_g1_i2_orf1;TRINITY_DN25733_c0_g1_i3_orf1;TRINITY_DN2772_c0_g1_i3_orf1;TRINITY_DN5756_c0_g1_i4_orf1;TRINITY_DN45271_c0_g1_i1_orf1;TRINITY_DN76283_c0_g6_i1_orf1;TRINITY_DN3712_c0_g1_i1_orf1;TRINITY_DN121650_c0_g1_i1_orf1;TRINITY_DN4710_c0_g1_i1_orf1;TRINITY_DN14701_c0_g1_i2_orf1;TRINITY_DN1330_c0_g1_i1_orf1;TRINITY_DN1841_c0_g1_i2_orf1;TRINITY_DN34465_c0_g1_i1_orf1;TRINITY_DN5919_c0_g1_i4_orf1;TRINITY_DN10066_c0_g2_i2_orf1;TRINITY_DN2812_c0_g1_i5_orf1;TRINITY_DN59885_c0_g1_i3_orf1;TRINITY_DN10430_c0_g1_i4_orf1;TRINITY_DN117707_c0_g1_i3_orf1;TRINITY_DN1249_c0_g1_i10_orf1;TRINITY_DN10900_c0_g1_i7_orf1;TRINITY_DN12024_c0_g1_i4_orf1;TRINITY_DN9094_c0_g1_i1_orf1;TRINITY_DN27033_c1_g1_i3_orfp1;TRINITY_DN40945_c0_g1_i1_orf1;TRINITY_DN72707_c0_g1_i1_orf1;TRINITY_DN12806_c0_g2_i1_orf1;TRINITY_DN1557_c0_g1_i9_orf1;TRINITY_DN74037_c0_g5_i1_orf1;TRINITY_DN10336_c0_g1_i9_orf1;TRINITY_DN23004_c0_g1_i1_orf1;TRINITY_DN9931_c0_g1_i1_orf1;TRINITY_DN101325_c0_g1_i4_orf1;TRINITY_DN10662_c0_g1_i4_orf1;TRINITY_DN2749_c0_g1_i4_orf1;TRINITY_DN171_c0_g1_i1_orf1;TRINITY_DN2668_c0_g1_i7_orf1;TRINITY_DN29440_c1_g1_i4_orf1;TRINITY_DN85319_c0_g1_i1_orf1;TRINITY_DN4817_c0_g1_i4_orf1;TRINITY_DN70409_c0_g1_i3_orf1;TRINITY_DN29291_c0_g1_i1_orf1;TRINITY_DN6876_c0_g2_i1_orf1;TRINITY_DN18909_c0_g1_i8_orf1;TRINITY_DN3758_c0_g1_i2_orf1;TRINITY_DN4276_c0_g1_i11_orf1;TRINITY_DN24_c0_g1_i1_orf1;TRINITY_DN4565_c0_g2_i1_orf1;TRINITY_DN2456_c0_g1_i2_orf1;TRINITY_DN42759_c0_g2_i1_orf1;TRINITY_DN41179_c0_g1_i1_orf1;TRINITY_DN48713_c0_g1_i1_orf1;TRINITY_DN18538_c0_g3_i1_orf1;TRINITY_DN8771_c0_g2_i1_orf1;TRINITY_DN10644_c0_g1_i2_orf1;TRINITY_DN13330_c0_g1_i4_orf1;TRINITY_DN112120_c0_g1_i1_orf1;TRINITY_DN19293_c0_g1_i4_orf1;TRINITY_DN3598_c0_g1_i1_orf1;TRINITY_DN117_c0_g1_i4_orf1;TRINITY_DN2647_c0_g1_i3_orf1;TRINITY_DN6087_c0_g1_i7_orf1;TRINITY_DN40562_c0_g2_i1_orf1;TRINITY_DN26168_c0_g1_i1_orf1;TRINITY_DN24539_c0_g1_i4_orf1;TRINITY_DN3862_c0_g1_i7_orf1;TRINITY_DN33178_c0_g1_i1_orf1;TRINITY_DN117_c0_g1_i5_orf1;TRINITY_DN72934_c0_g1_i1_orf1;TRINITY_DN1952_c0_g1_i2_orf1;TRINITY_DN38562_c0_g1_i3_orf1;TRINITY_DN81926_c0_g1_i1_orf1;TRINITY_DN144807_c0_g1_i1_orf1;TRINITY_DN69713_c0_g1_i1_orf1;TRINITY_DN9316_c1_g1_i1_orf1;TRINITY_DN7134_c0_g1_i1_orf1;TRINITY_DN12024_c0_g2_i2_orf1;TRINITY_DN1884_c0_g2_i2_orf1;TRINITY_DN152_c0_g1_i4_orf1;TRINITY_DN38783_c0_g1_i1_orf1;TRINITY_DN2257_c0_g1_i4_orf1;TRINITY_DN227_c0_g1_i1_orf1;TRINITY_DN4394_c0_g2_i1_orf1;TRINITY_DN5238_c0_g1_i2_orf1;TRINITY_DN42705_c0_g1_i3_orf1;TRINITY_DN34432_c0_g1_i1_orf1;TRINITY_DN11798_c0_g2_i1_orf1;TRINITY_DN44517_c0_g1_i4_orf1;TRINITY_DN2047_c0_g1_i1_orf1;TRINITY_DN810_c0_g1_i4_orf1;TRINITY_DN26408_c0_g1_i7_orf1;TRINITY_DN4565_c0_g1_i3_orf1;TRINITY_DN64403_c0_g2_i1_orf1;TRINITY_DN1109_c0_g1_i6_orf1;TRINITY_DN2798_c0_g1_i5_orf1;TRI
molecular_function	deacetylase activity	GO:0019213	4	4/3516	TRINITY_DN542_c0_g2_i1_orf1;TRINITY_DN542_c0_g1_i4_orf1;TRINITY_DN82801_c0_g1_i1_orf1;TRINITY_DN10385_c0_g1_i5_orf1
molecular_function	deaminase activity	GO:0019239	2	2/3516	TRINITY_DN38180_c0_g1_i3_orf1;TRINITY_DN98242_c0_g1_i1_orf1
molecular_function	palmitoyl hydrolase activity	GO:0098599	1	1/3516	TRINITY_DN4817_c0_g1_i4_orf1
molecular_function	hydrolase activity, acting on ether bonds	GO:0016801	8	8/3516	TRINITY_DN22242_c0_g1_i1_orf1;TRINITY_DN22242_c0_g2_i1_orf1;TRINITY_DN63536_c0_g1_i1_orf1;TRINITY_DN5768_c0_g1_i2_orf1;TRINITY_DN11172_c1_g1_i1_orf1;TRINITY_DN39200_c0_g1_i5_orf1;TRINITY_DN37366_c0_g1_i7_orf1;TRINITY_DN11172_c0_g1_i4_orf1
molecular_function	FAD-AMP lyase (cyclizing) activity	GO:0034012	2	2/3516	TRINITY_DN618_c0_g1_i3_orf1;TRINITY_DN52244_c1_g1_i1_orf1
molecular_function	guanylate cyclase activity	GO:0004383	1	1/3516	TRINITY_DN10774_c0_g2_i3_orf1
molecular_function	RNA-3'-phosphate cyclase activity	GO:0003963	1	1/3516	TRINITY_DN39490_c0_g1_i1_orf1
molecular_function	oxidoreductase activity, acting on CH-OH group of donors	GO:0016614	35	35/3516	TRINITY_DN5266_c0_g1_i1_orf1;TRINITY_DN83948_c0_g1_i3_orf1;TRINITY_DN146126_c0_g1_i1_orf1;TRINITY_DN20658_c0_g2_i3_orf1;TRINITY_DN64627_c0_g1_i1_orf1;TRINITY_DN1921_c1_g1_i5_orf1;TRINITY_DN4451_c0_g2_i4_orf1;TRINITY_DN49038_c0_g4_i1_orf1;TRINITY_DN36788_c0_g1_i2_orf1;TRINITY_DN29018_c0_g1_i4_orf1;TRINITY_DN9286_c0_g1_i2_orf1;TRINITY_DN135781_c0_g1_i1_orf1;TRINITY_DN4793_c0_g1_i7_orf1;TRINITY_DN122786_c0_g2_i1_orf1;TRINITY_DN42759_c0_g2_i1_orf1;TRINITY_DN42759_c0_g3_i1_orf1;TRINITY_DN10430_c0_g1_i4_orf1;TRINITY_DN40281_c0_g2_i1_orf1;TRINITY_DN26293_c0_g1_i4_orf1;TRINITY_DN3175_c0_g1_i7_orf1;TRINITY_DN9437_c0_g1_i1_orf1;TRINITY_DN3312_c0_g1_i10_orf1;TRINITY_DN10900_c0_g1_i7_orf1;TRINITY_DN3959_c1_g2_i1_orf1;TRINITY_DN38424_c0_g1_i1_orf1;TRINITY_DN36899_c0_g1_i1_orf1;TRINITY_DN5354_c0_g1_i4_orf1;TRINITY_DN2594_c0_g2_i4_orf1;TRINITY_DN4451_c0_g1_i1_orf1;TRINITY_DN31609_c0_g1_i3_orf1;TRINITY_DN1206_c0_g1_i6_orf1;TRINITY_DN24310_c0_g1_i2_orf1;TRINITY_DN357_c0_g1_i8_orf1;TRINITY_DN3053_c0_g1_i2_orf1;TRINITY_DN1209_c0_g1_i9_orf1
molecular_function	oxidoreductase activity, acting on the aldehyde or oxo group of donors	GO:0016903	25	25/3516	TRINITY_DN7075_c0_g2_i1_orf1;TRINITY_DN1103_c0_g1_i12_orf1;TRINITY_DN14967_c0_g2_i1_orf1;TRINITY_DN81031_c0_g1_i1_orf1;TRINITY_DN6586_c0_g1_i1_orf1;TRINITY_DN64772_c0_g1_i1_orf1;TRINITY_DN7335_c0_g1_i1_orf1;TRINITY_DN28577_c0_g1_i6_orf1;TRINITY_DN11826_c0_g1_i4_orf1;TRINITY_DN108818_c0_g1_i5_orf1;TRINITY_DN49508_c0_g2_i8_orf1;TRINITY_DN2848_c0_g1_i1_orf1;TRINITY_DN6313_c0_g1_i4_orf1;TRINITY_DN40126_c0_g1_i1_orf1;TRINITY_DN29873_c0_g1_i1_orf1;TRINITY_DN631_c0_g1_i6_orf1;TRINITY_DN64892_c0_g1_i1_orf1;TRINITY_DN4596_c0_g1_i14_orf1;TRINITY_DN15382_c0_g1_i3_orf1;TRINITY_DN1293_c0_g1_i4_orf1;TRINITY_DN3836_c0_g1_i4_orf1;TRINITY_DN40126_c0_g2_i1_orf1;TRINITY_DN7808_c0_g1_i1_orf1;TRINITY_DN123396_c0_g1_i1_orf1;TRINITY_DN2848_c0_g1_i2_orf1
molecular_function	oxidoreductase activity, acting on a heme group of donors	GO:0016675	1	1/3516	TRINITY_DN76036_c0_g1_i1_orf1
molecular_function	oxidoreductase activity, acting on the CH-NH group of donors	GO:0016645	8	8/3516	TRINITY_DN24970_c0_g1_i4_orf1;TRINITY_DN92153_c0_g2_i2_orf1;TRINITY_DN130051_c0_g1_i1_orf1;TRINITY_DN1760_c0_g1_i4_orf1;TRINITY_DN38506_c0_g1_i4_orf1;TRINITY_DN244_c1_g1_i5_orf1;TRINITY_DN631_c0_g1_i6_orf1;TRINITY_DN14107_c0_g1_i4_orf1
molecular_function	dioxygenase activity	GO:0051213	8	8/3516	TRINITY_DN4822_c0_g1_i6_orf1;TRINITY_DN5497_c0_g1_i6_orf1;TRINITY_DN38562_c0_g1_i3_orf1;TRINITY_DN44083_c0_g1_i2_orf1;TRINITY_DN89083_c0_g1_i1_orf1;TRINITY_DN43293_c0_g1_i2_orf1;TRINITY_DN57900_c0_g1_i2_orf1;TRINITY_DN13941_c0_g1_i6_orf1

molecular_function	electron transfer activity	GO:0009055	9	9/3516	TRINITY_DN14920_c0_g1_i1_orf1;TRINITY_DN49265_c0_g3_i2_orf1;TRINITY_DN10030_c0_g1_i2_orf1;TRINITY_DN76036_c0_g1_i1_orf1;TRINITY_DN7626_c0_g1_i1_orf1;TRINITY_DN27641_c0_g1_i1_orf1;TRINITY_DN1422_c0_g1_i4_orf1;TRINITY_DN20279_c0_g1_i1_orf1;TRINITY_DN24043_c0_g1_i1_orf1
molecular_function	oxidoreductase activity, acting on paired donors, with incorporation or reduction of molecular oxygen	GO:0016705	54	54/3516	TRINITY_DN43369_c0_g2_i1_orf1;TRINITY_DN8985_c0_g1_i4_orf1;TRINITY_DN9608_c0_g1_i3_orf1;TRINITY_DN3949_c1_g1_i1_orf1;TRINITY_DN30704_c0_g1_i1_orf1;TRINITY_DN120500_c0_g1_i1_orf1;TRINITY_DN23564_c0_g1_i7_orf1;TRINITY_DN109144_c0_g1_i5_orf1;TRINITY_DN64126_c0_g1_i1_orf1;TRINITY_DN2392_c0_g2_i1_orf1;TRINITY_DN24873_c0_g1_i4_orf1;TRINITY_DN89083_c0_g1_i1_orf1;TRINITY_DN43293_c0_g1_i2_orf1;TRINITY_DN14262_c0_g1_i5_orf1;TRINITY_DN4998_c0_g1_i21_orf1;TRINITY_DN625_c9_g1_i7_orf1;TRINITY_DN8019_c0_g1_i4_orf1;TRINITY_DN1664_c0_g1_i4_orf1;TRINITY_DN81719_c0_g1_i1_orf1;TRINITY_DN48590_c0_g1_i1_orf1;TRINITY_DN7212_c0_g1_i4_orf1;TRINITY_DN22604_c0_g1_i3_orf1;TRINITY_DN57765_c0_g1_i1_orf1;TRINITY_DN3949_c0_g1_i1_orf1;TRINITY_DN1134_c0_g1_i4_orf1;TRINITY_DN1363_c0_g1_i11_orf1;TRINITY_DN6027_c0_g1_i13_orf1;TRINITY_DN23398_c0_g1_i1_orf1;TRINITY_DN829_c0_g1_i8_orf1;TRINITY_DN3732_c1_g1_i5_orf1;TRINITY_DN31163_c1_g1_i4_orf1;TRINITY_DN16122_c0_g1_i4_orf1;TRINITY_DN9316_c0_g3_i1_orf1;TRINITY_DN50743_c0_g1_i1_orf1;TRINITY_DN95558_c0_g3_i1_orf1;TRINITY_DN15755_c0_g1_i1_orf1;TRINITY_DN448_c0_g1_i20_orf1;TRINITY_DN6351_c0_g1_i4_orf1;TRINITY_DN4497_c0_g1_i4_orf1;TRINITY_DN2264_c0_g1_i1_orf1;TRINITY_DN1960_c5_g1_i3_orf1;TRINITY_DN57856_c0_g2_i1_orf1;TRINITY_DN5439_c0_g1_i2_orf1;TRINITY_DN52887_c0_g1_i1_orf1;TRINITY_DN4497_c2_g1_i3_orf1;TRINITY_DN9647_c0_g1_i1_orf1;TRINITY_DN5126_c0_g1_i3_orf1;TRINITY_DN5126_c0_g2_i1_orf1;TRINITY_DN4321_c0_g1_i1_orf1;TRINITY_DN5661_c0_g1_i5_orf1;TRINITY_DN2442_c0_g1_i6_orf1;TRINITY_DN82944_c0_g1_i4_orf1;TRINITY_DN2338_c0_g1_i5_orf1;TRINITY_DN905_c0_g1_i4_orf1
molecular_function	oxidoreductase activity, acting on single donors with incorporation of molecular oxygen	GO:0016701	5	5/3516	TRINITY_DN1707_c0_g1_i1_orf1;TRINITY_DN4822_c0_g1_i6_orf1;TRINITY_DN5497_c0_g1_i6_orf1;TRINITY_DN38562_c0_g1_i3_orf1;TRINITY_DN13941_c0_g1_i6_orf1
molecular_function	oxidoreductase activity, acting on the CH-CH group of donors	GO:0016627	22	22/3516	TRINITY_DN5055_c0_g1_i12_orf1;TRINITY_DN20658_c0_g2_i3_orf1;TRINITY_DN1494_c0_g1_i3_orf1;TRINITY_DN1494_c0_g2_i1_orf1;TRINITY_DN30932_c0_g1_i2_orf1;TRINITY_DN21981_c0_g1_i8_orf1;TRINITY_DN6063_c1_g2_i1_orf1;TRINITY_DN29018_c0_g1_i4_orf1;TRINITY_DN5092_c0_g1_i2_orf1;TRINITY_DN1132_c0_g1_i5_orf1;TRINITY_DN3588_c0_g1_i1_orf1;TRINITY_DN38341_c0_g2_i2_orf1;TRINITY_DN59335_c0_g1_i2_orf1;TRINITY_DN42759_c0_g3_i1_orf1;TRINITY_DN10430_c0_g1_i4_orf1;TRINITY_DN42759_c0_g2_i1_orf1;TRINITY_DN10900_c0_g1_i7_orf1;TRINITY_DN27641_c0_g1_i1_orf1;TRINITY_DN143603_c0_g1_i1_orf1;TRINITY_DN25542_c0_g1_i1_orf1;TRINITY_DN3053_c0_g1_i2_orf1;TRINITY_DN3478_c0_g1_i10_orf1
molecular_function	oxidoreductase activity, acting on NAD(P)H	GO:0016651	15	15/3516	TRINITY_DN20279_c0_g1_i1_orf1;TRINITY_DN10030_c0_g1_i2_orf1;TRINITY_DN49221_c0_g1_i1_orf1;TRINITY_DN1661_c0_g1_i1_orf1;TRINITY_DN1134_c0_g1_i4_orf1;TRINITY_DN7626_c0_g1_i1_orf1;TRINITY_DN20984_c0_g1_i4_orf1;TRINITY_DN391_c5_g1_i1_orf1;TRINITY_DN33430_c0_g1_i5_orf1;TRINITY_DN22678_c0_g1_i4_orf1;TRINITY_DN1422_c0_g1_i4_orf1;TRINITY_DN4497_c0_g1_i4_orf1;TRINITY_DN52887_c0_g1_i1_orf1;TRINITY_DN96566_c0_g1_i1_orf1;TRINITY_DN6563_c0_g1_i1_orf1
molecular_function	fatty acid alpha-hydroxylase activity	GO:0080132	1	1/3516	TRINITY_DN8173_c0_g1_i3_orf1
molecular_function	oxidoreductase activity, acting on superoxide radicals as acceptor	GO:0016721	5	5/3516	TRINITY_DN14967_c0_g2_i1_orf1;TRINITY_DN37307_c0_g1_i4_orf1;TRINITY_DN8637_c0_g1_i1_orf1;TRINITY_DN16400_c0_g2_i1_orf1;TRINITY_DN1024_c0_g4_i1_orf1
molecular_function	oxidoreductase activity, acting on metal ions	GO:0016722	5	5/3516	TRINITY_DN46625_c0_g1_i1_orf1;TRINITY_DN1423_c0_g1_i8_orf1;TRINITY_DN65681_c0_g1_i1_orf1;TRINITY_DN136031_c0_g1_i7_orf1;TRINITY_DN1423_c0_g1_i4_orf1
molecular_function	oxidoreductase activity, acting on CH or CH2 groups	GO:0016725	3	3/3516	TRINITY_DN135781_c0_g1_i1_orf1;TRINITY_DN4835_c0_g1_i2_orf1;TRINITY_DN129_c0_g1_i6_orf1
molecular_function	oxidoreductase activity, acting on peroxide as acceptor	GO:0016684	16	16/3516	TRINITY_DN111985_c0_g1_i1_orf1;TRINITY_DN6580_c0_g1_i4_orf1;TRINITY_DN12514_c0_g2_i1_orf1;TRINITY_DN1622_c0_g1_i6_orf1;TRINITY_DN21420_c0_g1_i2_orf1;TRINITY_DN7579_c1_g3_i1_orf1;TRINITY_DN3321_c0_g1_i3_orf1;TRINITY_DN51252_c0_g2_i1_orf1;TRINITY_DN80660_c0_g1_i1_orf1;TRINITY_DN5933_c0_g1_i1_orf1;TRINITY_DN285_c0_g1_i4_orf1;TRINITY_DN69236_c0_g1_i1_orf1;TRINITY_DN54387_c0_g1_i1_orf1;TRINITY_DN2542_c0_g2_i1_orf1;TRINITY_DN791_c0_g1_i2_orf1;TRINITY_DN2652_c0_g2_i1_orf1
molecular_function	monooxygenase activity	GO:0004497	54	54/3516	TRINITY_DN43369_c0_g2_i1_orf1;TRINITY_DN8985_c0_g1_i4_orf1;TRINITY_DN9608_c0_g1_i3_orf1;TRINITY_DN3949_c1_g1_i1_orf1;TRINITY_DN30704_c0_g1_i1_orf1;TRINITY_DN120500_c0_g1_i1_orf1;TRINITY_DN23564_c0_g1_i7_orf1;TRINITY_DN4497_c0_g1_i4_orf1;TRINITY_DN64126_c0_g1_i1_orf1;TRINITY_DN2392_c0_g2_i1_orf1;TRINITY_DN24873_c0_g1_i4_orf1;TRINITY_DN14262_c0_g1_i5_orf1;TRINITY_DN4998_c0_g1_i21_orf1;TRINITY_DN625_c9_g1_i7_orf1;TRINITY_DN8019_c0_g1_i4_orf1;TRINITY_DN1664_c0_g1_i4_orf1;TRINITY_DN81719_c0_g1_i1_orf1;TRINITY_DN7212_c0_g1_i4_orf1;TRINITY_DN22604_c0_g1_i3_orf1;TRINITY_DN57765_c0_g1_i1_orf1;TRINITY_DN3949_c0_g1_i1_orf1;TRINITY_DN1134_c0_g1_i4_orf1;TRINITY_DN1363_c0_g1_i11_orf1;TRINITY_DN6027_c0_g1_i13_orf1;TRINITY_DN23398_c0_g1_i1_orf1;TRINITY_DN829_c0_g1_i8_orf1;TRINITY_DN3732_c1_g1_i5_orf1;TRINITY_DN31163_c1_g1_i4_orf1;TRINITY_DN16122_c0_g1_i4_orf1;TRINITY_DN9316_c0_g3_i1_orf1;TRINITY_DN50743_c0_g1_i1_orf1;TRINITY_DN95558_c0_g3_i1_orf1;TRINITY_DN15755_c0_g1_i1_orf1;TRINITY_DN448_c0_g1_i20_orf1;TRINITY_DN6351_c0_g1_i4_orf1;TRINITY_DN109144_c0_g1_i5_orf1;TRINITY_DN2264_c0_g1_i1_orf1;TRINITY_DN1960_c5_g1_i3_orf1;TRINITY_DN2338_c0_g2_i2_orf1;TRINITY_DN57856_c0_g2_i1_orf1;TRINITY_DN5439_c0_g1_i2_orf1;TRINITY_DN52887_c0_g1_i1_orf1;TRINITY_DN4497_c2_g1_i3_orf1;TRINITY_DN1707_c0_g1_i1_orf1;TRINITY_DN9647_c0_g1_i1_orf1;TRINITY_DN84357_c0_g1_i1_orf1;TRINITY_DN5126_c0_g1_i3_orf1;TRINITY_DN5126_c0_g2_i1_orf1;TRINITY_DN1465_c0_g2_i1_orf1;TRINITY_DN109540_c0_g1_i3_orf1;TRINITY_DN5661_c0_g1_i5_orf1;TRINITY_DN2442_c0_g1_i6_orf1;TRINITY_DN82944_c0_g1_i4_orf1;TRINITY_DN2338_c0_g1_i5_orf1
molecular_function	oxidoreductase activity, acting on the CH-NH2 group of donors	GO:0016638	7	7/3516	TRINITY_DN42856_c0_g1_i1_orf1;TRINITY_DN43431_c0_g1_i1_orf1;TRINITY_DN3859_c0_g1_i5_orf1;TRINITY_DN18230_c1_g2_i1_orf1;TRINITY_DN21506_c0_g1_i4_orf1;TRINITY_DN37165_c0_g1_i4_orf1;TRINITY_DN18230_c1_g1_i1_orf1
molecular_function	oxidoreductase activity, acting on other nitrogenous compounds as donors	GO:0016661	2	2/3516	TRINITY_DN2559_c0_g1_i4_orf1;TRINITY_DN82008_c0_g1_i1_orf1

molecular_function	oxidoreductase activity, acting on a sulfur group of donors	GO:0016667	13	13/3516	TRINITY_DN81715_c0_g1_i1_orf1;TRINITY_DN51938_c0_g3_i1_orf1;TRINITY_DN21715_c0_g1_i1_orf1;TRINITY_DN2430_c0_g1_i1_orf1;TRINITY_DN79673_c0_g1_i1_orf1;TRINITY_DN5169_c0_g1_i5_orf1;TRINITY_DN376_c0_g1_i1_orf1;TRINITY_DN2207_c0_g1_i6_orf1;TRINITY_DN9965_c0_g1_i1_orf1;TRINITY_DN5107_c0_g1_i4_orf1;TRINITY_DN920_c0_g1_i6_orf1;TRINITY_DN14306_c0_g1_i1_orf1;TRINITY_DN1901_c0_g1_i6_orf1
molecular_function	lysozyme activity	GO:0003796	2	2/3516	TRINITY_DN467_c3_g1_i5_orf1;TRINITY_DN1098_c1_g1_i4_orf1
molecular_function	N-acetylmuramoyl-L-alanine amidase activity	GO:0008745	3	3/3516	TRINITY_DN5235_c0_g1_i7_orf1;TRINITY_DN1534_c0_g1_i3_orf1;TRINITY_DN827_c1_g1_i1_orf1
molecular_function	intramolecular lyase activity	GO:0016872	1	1/3516	TRINITY_DN10722_c0_g3_i1_orf1
molecular_function	DNA topoisomerase activity	GO:0003916	2	2/3516	TRINITY_DN4908_c1_g1_i5_orf1;TRINITY_DN6248_c0_g1_i1_orf1
molecular_function	intramolecular transferase activity	GO:0016866	8	8/3516	TRINITY_DN5952_c0_g1_i6_orf1;TRINITY_DN2283_c0_g2_i1_orf1;TRINITY_DN12545_c0_g1_i7_orf1;TRINITY_DN1827_c0_g1_i4_orf1;TRINITY_DN4955_c0_g1_i2_orf1;TRINITY_DN31303_c0_g1_i4_orf1;TRINITY_DN2769_c0_g1_i1_orf1;TRINITY_DN120089_c0_g1_i1_orf1
molecular_function	intramolecular oxidoreductase activity	GO:0016860	8	8/3516	TRINITY_DN1201_c0_g1_i4_orf1;TRINITY_DN51938_c0_g3_i1_orf1;TRINITY_DN144807_c0_g1_i1_orf1;TRINITY_DN27035_c0_g1_i1_orf1;TRINITY_DN31611_c0_g1_i2_orf1;TRINITY_DN21715_c0_g1_i1_orf1;TRINITY_DN1831_c0_g1_i3_orf1;TRINITY_DN14306_c0_g1_i1_orf1
molecular_function	racemase and epimerase activity	GO:0016854	3	3/3516	TRINITY_DN9542_c0_g1_i4_orf1;TRINITY_DN45530_c0_g1_i1_orf1;TRINITY_DN1353_c0_g1_i1_orf1
molecular_function	cis-trans isomerase activity	GO:0016859	10	10/3516	TRINITY_DN3773_c0_g1_i4_orf1;TRINITY_DN119291_c0_g1_i1_orf1;TRINITY_DN142588_c0_g1_i1_orf1;TRINITY_DN1888_c0_g2_i1_orf1;TRINITY_DN14372_c0_g2_i1_orf1;TRINITY_DN54275_c0_g1_i4_orf1;TRINITY_DN2807_c0_g1_i4_orf1;TRINITY_DN34056_c0_g1_i4_orf1;TRINITY_DN140538_c0_g2_i1_orf1;TRINITY_DN1294_c0_g1_i3_orf1
molecular_function	catalytic activity, acting on RNA	GO:0140098	71	71/3516	TRINITY_DN8980_c0_g1_i2_orf1;TRINITY_DN57918_c0_g1_i1_orf1;TRINITY_DN4380_c0_g1_i9_orf1;TRINITY_DN5756_c0_g1_i4_orf1;TRINITY_DN31503_c0_g1_i4_orf1;TRINITY_DN27771_c0_g1_i1_orf1;TRINITY_DN620_c0_g1_i4_orf1;TRINITY_DN48619_c0_g1_i1_orf1;TRINITY_DN59291_c0_g1_i1_orf1;TRINITY_DN3712_c0_g1_i1_orf1;TRINITY_DN25542_c0_g1_i1_orf1;TRINITY_DN12495_c0_g1_i2_orf1;TRINITY_DN4710_c0_g1_i1_orf1;TRINITY_DN14701_c0_g1_i2_orf1;TRINITY_DN7213_c0_g1_i2_orf1;TRINITY_DN30224_c0_g1_i1_orf1;TRINITY_DN11639_c0_g1_i1_orf1;TRINITY_DN2953_c1_g1_i11_orf1;TRINITY_DN39490_c0_g1_i1_orf1;TRINITY_DN15845_c0_g1_i1_orf1;TRINITY_DN34465_c0_g1_i1_orf1;TRINITY_DN1344_c0_g1_i1_orf1;TRINITY_DN3028_c0_g1_i1_orf1;TRINITY_DN2904_c0_g1_i4_orf1;TRINITY_DN70485_c0_g1_i2_orf1;TRINITY_DN5962_c0_g1_i1_orf1;TRINITY_DN143603_c0_g1_i1_orf1;TRINITY_DN27771_c0_g2_i1_orf1;TRINITY_DN2953_c1_g1_i10_orf1;TRINITY_DN19807_c0_g1_i1_orf1;TRINITY_DN810_c0_g1_i4_orf1;TRINITY_DN9207_c0_g1_i1_orf1;TRINITY_DN2953_c1_g1_i2_orf1;TRINITY_DN2038_c0_g1_i2_orf1;TRINITY_DN16174_c0_g1_i2_orf1;TRINITY_DN15160_c0_g1_i1_orf1;TRINITY_DN41179_c0_g1_i1_orf1;TRINITY_DN84322_c0_g2_i1_orf1;TRINITY_DN9316_c1_g1_i1_orf1;TRINITY_DN26168_c0_g1_i1_orf1;TRINITY_DN18538_c0_g3_i1_orf1;TRINITY_DN8716_c0_g1_i3_orf1;TRINITY_DN23714_c0_g1_i4_orf1;TRINITY_DN19920_c1_g1_i2_orf1;TRINITY_DN13094_c0_g1_i1_orf1;TRINITY_DN34432_c0_g1_i1_orf1;TRINITY_DN8598_c0_g1_i2_orf1;TRINITY_DN44288_c0_g1_i2_orf1;TRINITY_DN4707_c0_g1_i1_orf1;TRINITY_DN27033_c1_g1_i3_orfp1;TRINITY_DN817_c0_g1_i3_orf1;TRINITY_DN30638_c0_g1_i1_orf1;TRINITY_DN2535_c0_g1_i4_orf1;TRINITY_DN2224_c0_g1_i1_orf1;TRINITY_DN446_c0_g1_i20_orf1;TRINITY_DN20499_c0_g3_i1_orf1;TRINITY_DN4381_c0_g2_i1_orf1;TRINITY_DN21539_c0_g1_i1_orf1;TRINITY_DN4813_c0_g1_i5_orf1;TRINITY_DN2709_c0_g1_i4_orf1;TRINITY_DN31520_c1_g1_i1_orf1;TRINITY_DN1532_c0_g1_i6_orf1;TRINITY_DN5218_c0_g1_i4_orf1;TRINITY_DN64810_c0_g1_i1_orf1;TRINITY_DN107288_c0_g1_i2_orf1;TRINITY_DN23004_c0_g1_i1_orf1;TRINITY_DN2299_c0_g1_i3_orf1;TRINITY_DN17312_c0_g1_i1_orf1;TRINITY_DN4944_c0_g1_i2_orf1;TRINITY_DN4950_c0_g1_i2_orf1;TRINITY_DN1607_c0_g1_i16_orf1
molecular_function	catalytic activity, acting on DNA	GO:0140097	20	20/3516	TRINITY_DN123184_c0_g1_i1_orf1;TRINITY_DN18538_c0_g3_i1_orf1;TRINITY_DN3092_c0_g1_i2_orf1;TRINITY_DN89613_c0_g1_i13_orf1;TRINITY_DN15040_c0_g4_i1_orf1;TRINITY_DN125565_c1_g1_i1_orf1;TRINITY_DN74037_c0_g5_i1_orf1;TRINITY_DN40434_c0_g1_i2_orf1;TRINITY_DN45271_c0_g1_i1_orf1;TRINITY_DN6642_c0_g1_i2_orf1;TRINITY_DN70485_c0_g1_i2_orf1;TRINITY_DN12820_c0_g1_i1_orf1;TRINITY_DN109733_c0_g1_i1_orf1;TRINITY_DN4908_c1_g1_i5_orf1;TRINITY_DN110534_c0_g1_i3_orf1;TRINITY_DN452_c1_g1_i3_orf1;TRINITY_DN3057_c0_g2_i1_orf1;TRINITY_DN6248_c0_g1_i1_orf1;TRINITY_DN15370_c0_g1_i4_orf1;TRINITY_DN25345_c0_g1_i1_orf1
molecular_function	helicase activity	GO:0004386	28	28/3516	TRINITY_DN6556_c0_g1_i7_orf1;TRINITY_DN125565_c1_g1_i1_orf1;TRINITY_DN4380_c0_g1_i9_orf1;TRINITY_DN31503_c0_g1_i4_orf1;TRINITY_DN3057_c0_g2_i1_orf1;TRINITY_DN12495_c0_g1_i2_orf1;TRINITY_DN8980_c0_g1_i2_orf1;TRINITY_DN59291_c0_g1_i1_orf1;TRINITY_DN7213_c0_g1_i2_orf1;TRINITY_DN10886_c0_g2_i4_orf1;TRINITY_DN15845_c0_g1_i1_orf1;TRINITY_DN4381_c0_g2_i1_orf1;TRINITY_DN2904_c0_g1_i4_orf1;TRINITY_DN19920_c1_g1_i2_orf1;TRINITY_DN810_c0_g1_i4_orf1;TRINITY_DN452_c1_g1_i3_orf1;TRINITY_DN25345_c0_g1_i1_orf1;TRINITY_DN6642_c0_g1_i2_orf1;TRINITY_DN13094_c0_g1_i1_orf1;TRINITY_DN15370_c0_g1_i4_orf1;TRINITY_DN44288_c0_g1_i2_orf1;TRINITY_DN109733_c0_g1_i1_orf1;TRINITY_DN2535_c0_g1_i4_orf1;TRINITY_DN20499_c0_g3_i1_orf1;TRINITY_DN2709_c0_g1_i4_orf1;TRINITY_DN16174_c0_g1_i2_orf1;TRINITY_DN26168_c0_g1_i1_orf1;TRINITY_DN4950_c0_g1_i2_orf1
molecular_function	transferase activity, transferring alkyl or aryl (other than methyl) groups	GO:0016765	32	32/3516	TRINITY_DN9506_c0_g1_i2_orf1;TRINITY_DN22046_c1_g1_i5_orf1;TRINITY_DN4695_c0_g1_i4_orf1;TRINITY_DN82320_c0_g1_i2_orf1;TRINITY_DN10399_c0_g1_i2_orf1;TRINITY_DN57462_c0_g1_i1_orf1;TRINITY_DN225_c0_g1_i6_orf1;TRINITY_DN8854_c0_g1_i2_orf1;TRINITY_DN12134_c0_g1_i4_orf1;TRINITY_DN128231_c0_g1_i5_orf1;TRINITY_DN8651_c0_g1_i18_orf1;TRINITY_DN3332_c0_g1_i2_orf1;TRINITY_DN2430_c0_g1_i1_orf1;TRINITY_DN4695_c0_g1_i3_orf1;TRINITY_DN1578_c0_g3_i1_orf1;TRINITY_DN53136_c0_g1_i1_orf1;TRINITY_DN1305_c0_g1_i6_orf1;TRINITY_DN3929_c0_g3_i3_orf1;TRINITY_DN15597_c0_g1_i1_orf1;TRINITY_DN62707_c0_g1_i1_orf1;TRINITY_DN3332_c0_g1_i11_orf1;TRINITY_DN8651_c0_g1_i16_orf1;TRINITY_DN8640_c0_g1_i4_orf1;TRINITY_DN920_c0_g1_i6_orf1;TRINITY_DN9234_c0_g1_i5_orf1;TRINITY_DN10222_c0_g1_i2_orf1;TRINITY_DN37856_c0_g1_i5_orf1;TRINITY_DN20682_c0_g2_i1_orf1;TRINITY_DN2255_c0_g1_i1_orf1;TRINITY_DN7512_c0_g1_i1_orf1;TRINITY_DN48548_c0_g1_i1_orf1;TRINITY_DN29707_c0_g1_i2_orf1

molecular_function	transferase activity, transferring nitrogenous groups	GO:0016769	10 10/3516	TRINITY_DN1824_c0_g2_i2_orf1;TRINITY_DN14565_c0_g1_i11_orf1;TRINITY_DN2848_c0_g1_i2_orf1;TRINITY_DN6908_c0_g1_i3_orf1;TRINITY_DN2803_c4_g1_i1_orf1;TRINITY_DN6908_c0_g1_i1_orf1;TRINITY_DN2848_c0_g1_i1_orf1;TRINITY_DN11013_c0_g1_i3_orf1;TRINITY_DN1068_c0_g1_i3_orf1;TRINITY_DN53807_c0_g2_i1_orf1
molecular_function	transferase activity, transferring sulphur-containing groups	GO:0016782	3 3/3516	TRINITY_DN6693_c0_g1_i1_orf1;TRINITY_DN9059_c0_g1_i1_orf1;TRINITY_DN874_c2_g1_i1_orf1
molecular_function	glycosyltransferase activity	GO:0016757	28 28/3516	TRINITY_DN38435_c0_g1_i1_orf1;TRINITY_DN31676_c0_g1_i4_orf1;TRINITY_DN79868_c0_g1_i1_orf1;TRINITY_DN11392_c0_g1_i4_orf1;TRINITY_DN9079_c1_g1_i1_orf1;TRINITY_DN1575_c0_g1_i7_orf1;TRINITY_DN140669_c0_g1_i1_orf1;TRINITY_DN2483_c0_g1_i1_orf1;TRINITY_DN125_c0_g1_i2_orf1;TRINITY_DN10548_c0_g2_i1_orf1;TRINITY_DN11817_c0_g1_i4_orf1;TRINITY_DN40197_c0_g1_i1_orf1;TRINITY_DN16933_c0_g1_i10_orf1;TRINITY_DN3355_c0_g1_i1_orf1;TRINITY_DN9079_c0_g1_i5_orf1;TRINITY_DN15157_c0_g1_i1_orf1;TRINITY_DN57536_c0_g1_i14_orf1;TRINITY_DN3355_c0_g2_i4_orf1;TRINITY_DN14018_c0_g1_i4_orf1;TRINITY_DN2967_c0_g1_i4_orf1;TRINITY_DN28592_c0_g1_i2_orf1;TRINITY_DN8908_c0_g1_i1_orf1;TRINITY_DN332_c0_g1_i6_orf1;TRINITY_DN5813_c0_g1_i9_orf1;TRINITY_DN14597_c0_g1_i5_orf1;TRINITY_DN812_c2_g1_i1_orf1;TRINITY_DN4954_c0_g1_i5_orf1;TRINITY_DN98091_c0_g1_i3_orf1
molecular_function	transketolase or transaldolase activity	GO:0016744	3 3/3516	TRINITY_DN14967_c0_g2_i1_orf1;TRINITY_DN60787_c0_g1_i5_orf1;TRINITY_DN59965_c0_g4_i1_orf1
molecular_function	molybdopterin molybdotransferase activity	GO:0061599	1 1/3516	TRINITY_DN1741_c0_g1_i5_orf1
molecular_function	transferase activity, transferring phosphorus-containing groups	GO:0016772	97 97/3516	TRINITY_DN31967_c0_g1_i5_orf1;TRINITY_DN2738_c1_g1_i3_orf1;TRINITY_DN15040_c0_g4_i1_orf1;TRINITY_DN70485_c0_g1_i2_orf1;TRINITY_DN2983_c0_g1_i6_orf1;TRINITY_DN143509_c0_g1_i1_orf1;TRINITY_DN7688_c0_g1_i2_orf1;TRINITY_DN73945_c0_g5_i3_orf1;TRINITY_DN9555_c0_g1_i1_orf1;TRINITY_DN10774_c0_g2_i3_orf1;TRINITY_DN2202_c0_g1_i9_orf1;TRINITY_DN1334_c0_g1_i2_orf1;TRINITY_DN6185_c0_g1_i12_orf1;TRINITY_DN6436_c0_g1_i1_orf1;TRINITY_DN4798_c0_g1_i3_orf1;TRINITY_DN9156_c0_g1_i1_orf1;TRINITY_DN89613_c0_g1_i13_orf1;TRINITY_DN70_c2_g1_i1_orf1;TRINITY_DN56998_c0_g1_i2_orf1;TRINITY_DN39813_c0_g1_i1_orf1;TRINITY_DN96170_c0_g2_i1_orf1;TRINITY_DN21278_c0_g2_i2_orf1;TRINITY_DN7405_c0_g1_i3_orf1;TRINITY_DN37923_c0_g1_i1_orf1;TRINITY_DN1034_c0_g1_i4_orf1;TRINITY_DN96170_c0_g1_i1_orf1;TRINITY_DN116972_c0_g1_i1_orf1;TRINITY_DN9207_c0_g1_i1_orf1;TRINITY_DN21126_c0_g1_i1_orf1;TRINITY_DN2110_c0_g1_i3_orf1;TRINITY_DN32700_c0_g1_i2_orf1;TRINITY_DN12301_c0_g1_i1_orf1;TRINITY_DN23714_c0_g1_i4_orf1;TRINITY_DN10742_c0_g1_i4_orf1;TRINITY_DN12323_c0_g2_i2_orf1;TRINITY_DN36632_c0_g1_i1_orf1;TRINITY_DN4707_c0_g1_i1_orf1;TRINITY_DN5281_c0_g2_i3_orf1;TRINITY_DN1266_c2_g1_i1_orf1;TRINITY_DN71465_c0_g1_i1_orf1;TRINITY_DN1173_c1_g1_i10_orf1;TRINITY_DN31520_c1_g1_i1_orf1;TRINITY_DN2299_c0_g1_i3_orf1;TRINITY_DN5029_c0_g1_i1_orf1;TRINITY_DN1173_c0_g1_i12_orf1;TRINITY_DN1154_c0_g1_i1_orf1;TRINITY_DN3534_c0_g1_i2_orf1;TRINITY_DN35991_c0_g1_i2_orf1;TRINITY_DN1552_c0_g1_i3_orf1;TRINITY_DN105749_c0_g1_i1_orf1;TRINITY_DN8261_c0_g1_i1_orf1;TRINITY_DN28729_c0_g1_i9_orf1;TRINITY_DN9109_c0_g1_i1_orf1;TRINITY_DN2082_c0_g1_i2_orf1;TRINITY_DN46090_c0_g3_i1_orf1;TRINITY_DN43656_c0_g1_i1_orf1;TRINITY_DN13160_c0_g1_i1_orf1;TRINITY_DN14477_c0_g1_i12_orf1;TRINITY_DN7688_c0_g1_i10_orf1;TRINITY_DN4449_c0_g2_i1_orf1;TRINITY_DN19662_c4_g1_i1_orf1;TRINITY_DN3418_c0_g1_i3_orf1;TRINITY_DN17838_c0_g1_i4_orf1;TRINITY_DN1173_c1_g1_i9_orf1;TRINITY_DN143637_c0_g1_i1_orf1;TRINITY_DN52244_c1_g1_i1_orf1;TRINITY_DN1957_c0_g1_i4_orf1;TRINITY_DN74037_c0_g5_i1_orf1;TRINITY_DN6813_c1_g1_i1_orf1;TRINITY_DN21181_c0_g1_i6_orf1;TRINITY_DN16487_c0_g1_i1_orf1;TRINITY_DN47151_c0_g1_i1_orf1;TRINITY_DN18538_c0_g3_i1_orf1;TRINITY_DN14967_c0_g2_i1_orf1;TRINITY_DN41166_c0_g1_i1_orf1;TRINITY_DN1405_c0_g1_i1_orf1;TRINITY_DN1741_c0_g1_i5_orf1;TRINITY_DN618_c0_g1_i3_orf1;TRINITY_DN4056_c0_g1_i8_orf1;TRINITY_DN10680_c0_g1_i5_orf1;TRINITY_DN1285_c0_g1_i6_orf1;TRINITY_DN19807_c0_g1_i1_orf1;TRINITY_DN40197_c0_g1_i1_orf1;TRINITY_DN7247_c0_g1_i7_orf1;TRINITY_DN16905_c0_g1_i1_orf1;TRINITY_DN30154_c0_g1_i1_orf1;TRINITY_DN15478_c0_g1_i1_orf1;TRINITY_DN1673_c0_g1_i2_orf1;TRINITY_DN110534_c0_g1_i3_orf1;TRINITY_DN2618_c0_g1_i3_orf1;TRINITY_DN30_c0_g1_i6_orf1;TRINITY_DN147475_c0_g1_i1_orf1;TRINITY_DN18782_c0_g1_i4_orf1;TRINITY_DN5697_c0_g1_i1_orf1;TRINITY_DN29956_c1_g1_i1_orf1;TRINITY_DN4929_c1_g2_i5_orf1;TRINITY_DN3515_c0_g1_i3_orf1
molecular_function	transferase activity, transferring one-carbon groups	GO:0016741	31 31/3516	TRINITY_DN2168_c0_g1_i2_orf1;TRINITY_DN130051_c0_g1_i1_orf1;TRINITY_DN77318_c0_g2_i1_orf1;TRINITY_DN17312_c0_g1_i1_orf1;TRINITY_DN5748_c0_g1_i5_orf1;TRINITY_DN56910_c0_g2_i1_orf1;TRINITY_DN2457_c0_g1_i8_orf1;TRINITY_DN5962_c0_g1_i1_orf1;TRINITY_DN15338_c0_g1_i7_orf1;TRINITY_DN2930_c0_g1_i8_orf1;TRINITY_DN1344_c0_g1_i1_orf1;TRINITY_DN3028_c0_g1_i1_orf1;TRINITY_DN1216_c0_g1_i4_orf1;TRINITY_DN33953_c0_g1_i4_orf1;TRINITY_DN14953_c0_g1_i5_orf1;TRINITY_DN5748_c0_g1_i6_orf1;TRINITY_DN4151_c1_g1_i4_orf1;TRINITY_DN95414_c0_g1_i1_orf1;TRINITY_DN631_c0_g1_i6_orf1;TRINITY_DN31431_c0_g1_i1_orf1;TRINITY_DN36592_c0_g1_i1_orf1;TRINITY_DN20749_c0_g1_i3_orf1;TRINITY_DN22674_c0_g1_i2_orf1;TRINITY_DN1532_c0_g1_i6_orf1;TRINITY_DN3263_c0_g1_i2_orf1;TRINITY_DN2114_c0_g1_i5_orf1;TRINITY_DN6235_c0_g1_i5_orf1;TRINITY_DN14734_c0_g1_i2_orf1;TRINITY_DN53807_c0_g2_i1_orf1;TRINITY_DN14313_c0_g1_i1_orf1;TRINITY_DN6462_c0_g1_i5_orf1

molecular_function	acyltransferase activity	GO:0016746	44	44/3516	TRINITY_DN2065_c1_g2_i1_orf1;TRINITY_DN86833_c0_g3_i1_orf1;TRINITY_DN20442_c0_g2_i1_orf1;TRINITY_DN76283_c0_g6_i1_orf1;TRINITY_DN5841_c0_g1_i2_orf1;TRINITY_DN20710_c0_g2_i2_orf1;TRINITY_DN9718_c0_g1_i7_orf1;TRINITY_DN47389_c0_g1_i2_orf1;TRINITY_DN127151_c0_g1_i1_orf1;TRINITY_DN3022_c0_g1_i1_orf1;TRINITY_DN4538_c0_g1_i4_orf1;TRINITY_DN12771_c0_g1_i1_orf1;TRINITY_DN5211_c0_g1_i1_orf1;TRINITY_DN3628_c0_g1_i5_orf1;TRINITY_DN10900_c0_g1_i7_orf1;TRINITY_DN3219_c0_g1_i6_orf1;TRINITY_DN2365_c0_g1_i6_orf1;TRINITY_DN12497_c0_g1_i1_orf1;TRINITY_DN42759_c0_g2_i1_orf1;TRINITY_DN51737_c0_g1_i3_orf1;TRINITY_DN12133_c0_g2_i1_orf1;TRINITY_DN101358_c0_g2_i1_orf1;TRINITY_DN6871_c0_g1_i3_orf1;TRINITY_DN117844_c0_g1_i1_orf1;TRINITY_DN3179_c0_g1_i1_orf1;TRINITY_DN19727_c0_g1_i7_orf1;TRINITY_DN42759_c0_g3_i1_orf1;TRINITY_DN5525_c0_g1_i4_orf1;TRINITY_DN10430_c0_g1_i4_orf1;TRINITY_DN17299_c0_g1_i4_orf1;TRINITY_DN883_c0_g1_i8_orf1;TRINITY_DN76283_c0_g2_i1_orf1;TRINITY_DN5129_c0_g3_i3_orf1;TRINITY_DN5153_c1_g1_i1_orf1;TRINITY_DN1081_c0_g1_i7_orf1;TRINITY_DN22956_c0_g1_i1_orf1;TRINITY_DN3551_c0_g1_i4_orf1;TRINITY_DN24142_c0_g1_i1_orf1;TRINITY_DN68725_c0_g1_i1_orf1;TRINITY_DN1084_c0_g1_i2_orf1;TRINITY_DN4898_c0_g1_i7_orf1;TRINITY_DN29369_c0_g1_i1_orf1;TRINITY_DN2064_c1_g1_i1_orf1;TRINITY_DN3545_c0_g1_i6_orf1
