# Supplementary material for: Comparative Proteomics and Metabonomics Analysis of Different Diapause Stages Revealed a New Regulation Mechanism of Diapause in Loxostege sticticalis (Lepidoptera: Pyralidae)
Source: Molecules. 2024 Jul 25;29(15):3472. doi: 10.3390/molecules29153472 (PMC11314584; doi:10.3390/molecules29153472)
Supplement: Supplementary file 1 [file molecules-29-03472-s001.zip › analysis process/proteomic/GO annotations analysis/DvsPreD all.pdf]

Term Type	GO Term	GO ID	ZY_vs_ZYQ_all num	ZY_vs_ZYQ_all percent	ZY_vs_ZYQ_all Accession ids
biological_process	activation of innate immune response	GO:0002218	3	3/1043	TRINITY_DN8685_c0_g1_i5_orf1;TRINITY_DN1091_c0_g2_i10_orf1;TRINITY_DN5880_c0_g2_i2_orf1
biological_process	innate immune response	GO:0045087	8	8/1043	TRINITY_DN827_c1_g1_i1_orf1;TRINITY_DN1534_c0_g1_i3_orf1;TRINITY_DN8685_c0_g1_i5_orf1;TRINITY_DN195_c4_g1_i1_orf1;TRINITY_DN1091_c0_g2_i10_orf1;TRINITY_DN1666_c0_g1_i2_orf1;TRINITY_DN9044_c0_g1_i2_orf1;TRINITY_DN5880_c0_g2_i2_orf1
biological_process	regulation of catalytic activity	GO:0050790	3	3/1043	TRINITY_DN27021_c0_g1_i1_orf1;TRINITY_DN55148_c0_g1_i1_orf1;TRINITY_DN975_c0_g1_i1_orf1
biological_process	positive regulation of molecular function	GO:0044093	4	4/1043	TRINITY_DN55148_c0_g1_i1_orf1;TRINITY_DN27021_c0_g1_i1_orf1;TRINITY_DN5406_c0_g2_i1_orf1;TRINITY_DN2175_c0_g1_i4_orf1
biological_process	negative regulation of molecular function	GO:0044092	2	2/1043	TRINITY_DN55148_c0_g1_i1_orf1;TRINITY_DN31584_c0_g2_i2_orf1
biological_process	regulation of binding	GO:0051098	1	1/1043	TRINITY_DN55148_c0_g1_i1_orf1
biological_process	regulation of transporter activity	GO:0032409	3	3/1043	TRINITY_DN31584_c0_g2_i2_orf1;TRINITY_DN5406_c0_g2_i1_orf1;TRINITY_DN2175_c0_g1_i4_orf1
biological_process	regulation of metabolic process	GO:0019222	15	15/1043	TRINITY_DN17655_c0_g1_i1_orf1;TRINITY_DN19260_c0_g1_i5_orf1;TRINITY_DN20442_c0_g2_i1_orf1;TRINITY_DN31342_c2_g2_i1_orf1;TRINITY_DN2802_c1_g1_i1_orf1;TRINITY_DN1710_c0_g2_i2_orf1;TRINITY_DN142442_c0_g1_i1_orf1;TRINITY_DN55148_c0_g1_i1_orf1;TRINITY_DN4014_c0_g1_i1_orf1;TRINITY_DN810_c0_g1_i4_orf1;TRINITY_DN31584_c0_g2_i2_orf1;TRINITY_DN52_c0_g1_i4_orf1;TRINITY_DN975_c0_g1_i1_orf1;TRINITY_DN31851_c0_g1_i2_orf1;TRINITY_DN9510_c0_g2_i1_orf1
biological_process	regulation of response to stimulus	GO:0048583	7	7/1043	TRINITY_DN51938_c0_g3_i1_orf1;TRINITY_DN7316_c0_g2_i1_orf1;TRINITY_DN8685_c0_g1_i5_orf1;TRINITY_DN17655_c0_g1_i1_orf1;TRINITY_DN1091_c0_g2_i10_orf1;TRINITY_DN55148_c0_g1_i1_orf1;TRINITY_DN5880_c0_g2_i2_orf1
biological_process	regulation of developmental process	GO:0050793	1	1/1043	TRINITY_DN52_c0_g1_i4_orf1
biological_process	regulation of cellular process	GO:0050794	21	21/1043	TRINITY_DN1710_c0_g2_i2_orf1;TRINITY_DN31851_c0_g1_i2_orf1;TRINITY_DN5406_c0_g2_i1_orf1;TRINITY_DN7316_c0_g2_i1_orf1;TRINITY_DN17655_c0_g1_i1_orf1;TRINITY_DN20442_c0_g2_i1_orf1;TRINITY_DN2175_c0_g1_i4_orf1;TRINITY_DN2802_c1_g1_i1_orf1;TRINITY_DN51938_c0_g3_i1_orf1;TRINITY_DN142442_c0_g1_i1_orf1;TRINITY_DN4676_c0_g1_i16_orf1;TRINITY_DN4014_c0_g1_i1_orf1;TRINITY_DN804_c0_g1_i7_orf1;TRINITY_DN10455_c0_g1_i2_orf1;TRINITY_DN31584_c0_g2_i2_orf1;TRINITY_DN52_c0_g1_i4_orf1;TRINITY_DN55148_c0_g1_i1_orf1;TRINITY_DN9510_c0_g2_i1_orf1;TRINITY_DN975_c0_g1_i1_orf1;TRINITY_DN4798_c0_g1_i3_orf1;TRINITY_DN80424_c0_g1_i1_orf1
biological_process	regulation of localization	GO:0032879	3	3/1043	TRINITY_DN2175_c0_g1_i4_orf1;TRINITY_DN5406_c0_g2_i1_orf1;TRINITY_DN31584_c0_g2_i2_orf1
biological_process	regulation of multicellular organismal process	GO:0051239	1	1/1043	TRINITY_DN52_c0_g1_i4_orf1
biological_process	regulation of membrane repolarization	GO:0060306	1	1/1043	TRINITY_DN31584_c0_g2_i2_orf1
biological_process	regulation of immune system process	GO:0002682	3	3/1043	TRINITY_DN8685_c0_g1_i5_orf1;TRINITY_DN1091_c0_g2_i10_orf1;TRINITY_DN5880_c0_g2_i2_orf1
biological_process	positive regulation of biological process	GO:0048518	11	11/1043	TRINITY_DN51938_c0_g3_i1_orf1;TRINITY_DN5406_c0_g2_i1_orf1;TRINITY_DN8685_c0_g1_i5_orf1;TRINITY_DN17655_c0_g1_i1_orf1;TRINITY_DN9510_c0_g2_i1_orf1;TRINITY_DN1091_c0_g2_i10_orf1;TRINITY_DN1710_c0_g2_i2_orf1;TRINITY_DN31584_c0_g2_i2_orf1;TRINITY_DN2175_c0_g1_i4_orf1;TRINITY_DN55148_c0_g1_i1_orf1;TRINITY_DN5880_c0_g2_i2_orf1
biological_process	negative regulation of biological process	GO:0048519	3	3/1043	TRINITY_DN810_c0_g1_i4_orf1;TRINITY_DN55148_c0_g1_i1_orf1;TRINITY_DN31584_c0_g2_i2_orf1
biological_process	regulation of signaling	GO:0023051	5	5/1043	TRINITY_DN55148_c0_g1_i1_orf1;TRINITY_DN51938_c0_g3_i1_orf1;TRINITY_DN2175_c0_g1_i4_orf1;TRINITY_DN5406_c0_g2_i1_orf1;TRINITY_DN7316_c0_g2_i1_orf1
biological_process	regulation of membrane potential	GO:0042391	1	1/1043	TRINITY_DN31584_c0_g2_i2_orf1
biological_process	regulation of neurotransmitter levels	GO:0001505	2	2/1043	TRINITY_DN82017_c0_g1_i5_orf1;TRINITY_DN14565_c0_g1_i11_orf1
biological_process	homeostatic process	GO:0042592	3	3/1043	TRINITY_DN65681_c0_g1_i1_orf1;TRINITY_DN31584_c0_g2_i2_orf1;TRINITY_DN136031_c0_g1_i7_orf1
biological_process	regulation of anatomical structure size	GO:0090066	2	2/1043	TRINITY_DN10455_c0_g1_i2_orf1;TRINITY_DN80424_c0_g1_i1_orf1
biological_process	regulation of protein stability	GO:0031647	1	1/1043	TRINITY_DN55148_c0_g1_i1_orf1
biological_process	organonitrogen compound metabolic process	GO:1901564	130	130/1043	TRINITY_DN827_c1_g1_i1_orf1;TRINITY_DN14565_c0_g1_i11_orf1;TRINITY_DN4360_c0_g1_i4_orf1;TRINITY_DN3194_c0_g1_i6_orf1;TRINITY_DN137_c0_g1_i1_orf1;TRINITY_DN863_c0_g1_i6_orf1;TRINITY_DN56164_c0_g1_i1_orf1;TRINITY_DN2069_c1_g1_i8_orf1;TRINITY_DN119291_c0_g1_i1_orf1;TRINITY_DN1153_c1_g1_i1_orf1;TRINITY_DN6325_c0_g1_i8_orf1;TRINITY_DN1216_c0_g1_i4_orf1;TRINITY_DN8603_c0_g1_i1_orf1;TRINITY_DN2953_c1_g1_i10_orf1;TRINITY_DN7957_c0_g1_i5_orf1;TRINITY_DN14754_c0_g1_i6_orf1;TRINITY_DN3975_c0_g1_i7_orf1;TRINITY_DN14774_c0_g1_i4_orf1;TRINITY_DN48020_c0_g1_i1_orf1;TRINITY_DN4822_c0_g1_i6_orf1;TRINITY_DN1534_c0_g1_i3_orf1;TRINITY_DN74889_c0_g1_i1_orf1;TRINITY_DN1760_c0_g1_i4_orf1;TRINITY_DN84478_c0_g1_i8_orf1;TRINITY_DN20527_c0_g1_i1_orf1;TRINITY_DN6205_c0_g1_i8_orf1;TRINITY_DN4798_c0_g1_i3_orf1;TRINITY_DN9991_c0_g1_i4_orf1;TRINITY_DN3483_c0_g1_i5_orf1;TRINITY_DN22674_c0_g1_i2_orf1;TRINITY_DN21619_c0_g1_i1_orf1;TRINITY_DN9156_c0_g1_i1_orf1;TRINITY_DN1380_c0_g1_i5_orf1;TRINITY_DN107288_c0_g1_i2_orf1;TRINITY_DN46715_c0_g1_i1_orf1;TRINITY_DN4767_c0_g1_i6_orf1;TRINITY_DN230_c2_g1_i5_orf1;TRINITY_DN6199_c2_g1_i3_orf1;TRINITY_DN143895_c0_g1_i1_orf1;TRINITY_DN12526_c0_g1_i5_orf1;TRINITY_DN10824_c0_g1_i3_orf1;TRINITY_DN5497_c0_g1_i6_orf1;TRINITY_DN2719_c1_g1_i6_orf1;TRINITY_DN8659_c0_g2_i1_orf1;TRINITY_DN1310_c0_g1_i4_orf1;TRINITY_DN9871_c0_g1_i11_orf1;TRINITY_DN1789_c0_g1_i5_orf1;TRINITY_DN48602_c0_g1_i6_orf1;TRINITY_DN244_c1_g1_i5_orf1;TRINITY_DN59885_c0_g1_i3_orf1;TRINITY_DN66302_c0_g1_i1_orf1;TRINITY_DN71863_c0_g1_i2_orf1;TRINITY_DN3324_c0_g1_i3_orf1;TRINITY_DN9794_c0_g2_i8_orf1;TRINITY_DN4064_c0_g2_i1_orf1;TRINITY_DN49936_c0_g2_i1_orf1;TRINITY_DN21984_c0_g1_i6_orf1;TRINITY_DN27033_c1_g1_i3_orfp1;TRINITY_DN140_c1_g1_i2_orf1;TRINITY_DN1266_c2_g1_i1_orf1;TRINITY_DN779_c0_g1_i12_orf1;TRINITY_DN5811_c0_g1_i4_orf1;TRINITY_DN1173_c1_g1_i10_orf1;TRINITY_DN17326_c0_g1_i5_orf1;TRINITY_DN4494_c0_g1_i1_orf1;TRINITY_DN51658_c0_g1_i1_orf1;TRINITY_DN650_c0_g1_i3_orf1;TRINITY_DN92153_c0_g2_i2_orf1;TRINITY_DN36434_c0_g2_i3_orf1;TRINITY_DN67026_c0_g1_i6_orf1;TRINITY_DN27035_c0_g1_i1_orf1;TRINITY_DN8037_c0_g2_i1_orf1;TRINITY_DN142442_c0_g1_i1_orf1;TRINITY_DN6325_c0_g1_i9_orf1;TRINITY_DN4817_c0_g1_i4_orf1;TRINITY_DN57111_c0_g1_i1_orf1;TRINITY_DN5310_c2_g1_i2_orf1;TRINITY_DN7212_c0_g1_i4_orf1;TRINITY_DN1824_c0_g2_i2_orf1;TRINITY_DN542_c0_g2_i1_orf1;TRINITY_DN34479_c0_g1_i2_orf1;TRINITY_DN2885_c1_g1_i2_orf1;TRINITY_DN2559_c0_g1_i4_orf1;TRINITY_DN98242_c0_g1_i1_orf1;TRINITY_DN18172_c0_g1_i6_orf1;TRINITY_DN825_c23_g1_i5_orf1;TRINITY_DN1308_c0_g1_i4_orf1;TRINITY_DN23167_c0_g1_i4_orf1;TRINITY_DN83150_c0_g1_i1_orf1;TRINITY_DN12293_c0_g1_i1_orf1;TRINITY_DN15160_c0_g1_i1_orf1;TRINITY_DN6470_c0_g3_i2_orf1;TRINITY_DN1287_c0_g1_i5_orf1;TRINITY_DN1957_c0_g1_i4_orf1;TRINITY_DN6205_c0_g1_i1_orf1;TRINITY_DN49047_c0_g1_i2_orf1;TRINITY_DN38180_c0_g1_i3_orf1;TRINITY_DN9979_c0_g1_i1_orf1;TRINITY_DN7316_c0_g2_i1_orf1;TRINITY_DN36893_c0_g1_i1_orf1;TRINITY_DN801_c0_g1_i2_orf1;TRINITY_DN13686_c0_g2_i1_orf1;TRINITY_DN4822_c0_g1_i9_orf1;TRINITY_DN55148_c0_g1_i1_orf1;TRINITY_DN89483_c0_g1_i1_orf1;TRINITY_DN14874_c0_g1_i6_orf1;TRINITY_DN3119_c0_g1_i7_orf1;TRINITY_DN4030_c0_g2_i1_orf1;TRINITY_DN7647_c0_g1_i4_orf1;TRINITY_DN87170_c0_g1_i3_orf1;TRINITY_DN975_c0_g1_i1_orf1;TRINITY_DN3991_c0_g1_i6_orf1;TRINITY_DN18782_c0_g1_i4_orf1;TRINITY_DN2593_c0_g1_i1_orf1;TRINITY_DN15222_c0_g1_i4_orf1;TRINITY_DN323_c0_g2_i5_orf1;TRINITY_DN56690_c0_g1_i4_orf1;TRINITY_DN46132_c0_g2_i2_orf1;TRINITY_DN5001_c0_g1_i4_orf1;TRINITY_DN11798_c0_g2_i1_orf1;TRINITY_DN82324_c0_g1_i4_orf1;TRINITY_DN21555_c0_g1_i4_orf1;TRINITY_DN11383_c0_g2_i4_orf1;TRINITY_DN115210_c0_g4_i1_orf1;TRINITY_DN701_c0_g1_i1_orf1;TRINITY_DN51498_c0_g1_i1_orf1;TRINITY_DN22797_c0_g1_i5_orf1;TRINITY_DN5697_c0_g1_i1_orf1;TRINITY
biological_process	cellular nitrogen compound metabolic process	GO:0034641	76	76/1043	TRINITY_DN230_c2_g1_i5_orf1;TRINITY_DN51968_c0_g1_i1_orf1;TRINITY_DN6325_c0_g1_i8_orf1;TRINITY_DN2749_c0_g1_i4_orf1;TRINITY_DN4360_c0_g1_i4_orf1;TRINITY_DN40434_c0_g1_i2_orf1;TRINITY_DN36893_c0_g1_i1_orf1;TRINITY_DN137_c0_g1_i1_orf1;TRINITY_DN27035_c0_g1_i1_orf1;TRINITY_DN8037_c0_g2_i1_orf1;TRINITY_DN13760_c1_g1_i1_orf1;TRINITY_DN142442_c0_g1_i1_orf1;TRINITY_DN1116_c0_g1_i6_orf1;TRINITY_DN20238_c0_g1_i7_orf1;TRINITY_DN55148_c0_g1_i1_orf1;TRINITY_DN2054_c0_g1_i1_orf1;TRINITY_DN3324_c0_g1_i3_orf1;TRINITY_DN24322_c0_g1_i4_orf1;TRINITY_DN33346_c0_g1_i1_orf1;TRINITY_DN1153_c1_g1_i1_orf1;TRINITY_DN1091_c0_g1_i1_orf1;TRINITY_DN115658_c0_g1_i1_orf1;TRINITY_DN2559_c0_g1_i4_orf1;TRINITY_DN87170_c0_g1_i3_orf1;TRINITY_DN1216_c0_g1_i4_orf1;TRINITY_DN975_c0_g1_i1_orf1;TRINITY_DN8603_c0_g1_i1_orf1;TRINITY_DN48602_c0_g1_i6_orf1;TRINITY_DN74889_c0_g1_i1_orf1;TRINITY_DN2953_c1_g1_i10_orf1;TRINITY_DN98242_c0_g1_i1_orf1;TRINITY_DN810_c0_g1_i4_orf1;TRINITY_DN9207_c0_g1_i1_orf1;TRINITY_DN2201_c0_g1_i1_orf1;TRINITY_DN140212_c0_g1_i1_orf1;TRINITY_DN2110_c0_g1_i3_orf1;TRINITY_DN41664_c0_g1_i4_orf1;TRINITY_DN1091_c0_g3_i1_orf1;TRINITY_DN1957_c0_g1_i4_orf1;TRINITY_DN2749_c0_g2_i3_orf1;TRINITY_DN9794_c0_g2_i8_orf1;TRINITY_DN15222_c0_g1_i4_orf1;TRINITY_DN4835_c0_g1_i2_orf1;TRINITY_DN19687_c0_g1_i1_orf1;TRINITY_DN323_c0_g2_i5_orf1;TRINITY_DN124950_c0_g2_i1_orf1;TRINITY_DN3991_c0_g1_i6_orf1;TRINITY_DN825_c23_g1_i5_orf1;TRINITY_DN5238_c0_g1_i2_orf1;TRINITY_DN49936_c0_g2_i1_orf1;TRINITY_DN7212_c0_g1_i4_orf1;TRINITY_DN7957_c0_g1_i5_orf1;TRINITY_DN83150_c0_g1_i1_orf1;TRINITY_DN20527_c0_g1_i1_orf1;TRINITY_DN110534_c0_g1_i3_orf1;TRINITY_DN5507_c0_g1_i1_orf1;TRINITY_DN82324_c0_g1_i4_orf1;TRINITY_DN15160_c0_g1_i1_orf1;TRINITY_DN779_c0_g1_i12_orf1;TRINITY_DN8717_c0_g1_i5_orf1;TRINITY_DN5811_c0_g1_i4_orf1;TRINITY_DN9156_c0_g1_i1_orf1;TRINITY_DN41179_c0_g1_i1_orf1;TRINITY_DN2647_c0_g1_i3_orf1;TRINITY_DN115210_c0_g4_i1_orf1;TRINITY_DN51498_c0_g1_i1_orf1;TRINITY_DN21619_c0_g1_i1_orf1;TRINITY_DN6325_c0_g1_i9_orf1;TRINITY_DN107288_c0_g1_i2_orf1;TRINITY_DN18782_c0_g1_i4_orf1;TRINITY_DN3651_c0_g1_i5_orf1;TRINITY_DN2299_c0_g1_i3_orf1;TRINITY_DN38180_c0_g1_i3_orf1;TRINITY_DN5029_c0_g1_i1_orf1;TRINITY_DN9979_c0_g1_i1_orf1;TRINITY_DN2749_c4_g1_i2_orf1
biological_process	cellular lipid metabolic process	GO:0044255	15	15/1043	TRINITY_DN22046_c1_g1_i5_orf1;TRINITY_DN27903_c0_g1_i1_orf1;TRINITY_DN45220_c0_g1_i1_orf1;TRINITY_DN2618_c0_g1_i3_orf1;TRINITY_DN1999_c0_g1_i9_orf1;TRINITY_DN12526_c0_g1_i5_orf1;TRINITY_DN44110_c0_g1_i4_orf1;TRINITY_DN3588_c0_g1_i4_orf1;TRINITY_DN84478_c0_g1_i8_orf1;TRINITY_DN3991_c0_g1_i6_orf1;TRINITY_DN42759_c0_g2_i1_orf1;TRINITY_DN76283_c0_g2_i1_orf1;TRINITY_DN5697_c0_g1_i1_orf1;TRINITY_DN10900_c0_g1_i7_orf1;TRINITY_DN4321_c0_g1_i1_orf1
biological_process	generation of precursor metabolites and energy	GO:0006091	8	8/1043	TRINITY_DN6325_c0_g1_i8_orf1;TRINITY_DN76036_c0_g1_i1_orf1;TRINITY_DN4360_c0_g1_i4_orf1;TRINITY_DN9286_c0_g1_i2_orf1;TRINITY_DN27035_c0_g1_i1_orf1;TRINITY_DN83150_c0_g1_i1_orf1;TRINITY_DN6325_c0_g1_i9_orf1;TRINITY_DN9979_c0_g1_i1_orf1
biological_process	one-carbon metabolic process	GO:0006730	3	3/1043	TRINITY_DN20527_c0_g1_i1_orf1;TRINITY_DN244_c1_g1_i5_orf1;TRINITY_DN92153_c0_g2_i2_orf1
biological_process	cellular ketone metabolic process	GO:0042180	2	2/1043	TRINITY_DN87170_c0_g1_i3_orf1;TRINITY_DN6638_c0_g1_i1_orf1
biological_process	heterocycle metabolic process	GO:0046483	69	69/1043	TRINITY_DN230_c2_g1_i5_orf1;TRINITY_DN92153_c0_g2_i2_orf1;TRINITY_DN5507_c0_g1_i1_orf1;TRINITY_DN2749_c0_g1_i4_orf1;TRINITY_DN4360_c0_g1_i4_orf1;TRINITY_DN40434_c0_g1_i2_orf1;TRINITY_DN51968_c0_g1_i1_orf1;TRINITY_DN27035_c0_g1_i1_orf1;TRINITY_DN8037_c0_g2_i1_orf1;TRINITY_DN13760_c1_g1_i1_orf1;TRINITY_DN2719_c1_g1_i6_orf1;TRINITY_DN110534_c0_g1_i3_orf1;TRINITY_DN20238_c0_g1_i7_orf1;TRINITY_DN55148_c0_g1_i1_orf1;TRINITY_DN2054_c0_g1_i1_orf1;TRINITY_DN3324_c0_g1_i3_orf1;TRINITY_DN24322_c0_g1_i4_orf1;TRINITY_DN33346_c0_g1_i1_orf1;TRINITY_DN115210_c0_g4_i1_orf1;TRINITY_DN115658_c0_g1_i1_orf1;TRINITY_DN87170_c0_g1_i3_orf1;TRINITY_DN1216_c0_g1_i4_orf1;TRINITY_DN8603_c0_g1_i1_orf1;TRINITY_DN48602_c0_g1_i6_orf1;TRINITY_DN2559_c0_g1_i4_orf1;TRINITY_DN2953_c1_g1_i10_orf1;TRINITY_DN98242_c0_g1_i1_orf1;TRINITY_DN810_c0_g1_i4_orf1;TRINITY_DN244_c1_g1_i5_orf1;TRINITY_DN2201_c0_g1_i1_orf1;TRINITY_DN140212_c0_g1_i1_orf1;TRINITY_DN2110_c0_g1_i3_orf1;TRINITY_DN1091_c0_g1_i1_orf1;TRINITY_DN1091_c0_g3_i1_orf1;TRINITY_DN2647_c0_g1_i3_orf1;TRINITY_DN2749_c0_g2_i3_orf1;TRINITY_DN9207_c0_g1_i1_orf1;TRINITY_DN15222_c0_g1_i4_orf1;TRINITY_DN4835_c0_g1_i2_orf1;TRINITY_DN19687_c0_g1_i1_orf1;TRINITY_DN323_c0_g2_i5_orf1;TRINITY_DN124950_c0_g2_i1_orf1;TRINITY_DN3991_c0_g1_i6_orf1;TRINITY_DN825_c23_g1_i5_orf1;TRINITY_DN5238_c0_g1_i2_orf1;TRINITY_DN1760_c0_g1_i4_orf1;TRINITY_DN83150_c0_g1_i1_orf1;TRINITY_DN20527_c0_g1_i1_orf1;TRINITY_DN1116_c0_g1_i6_orf1;TRINITY_DN41664_c0_g1_i4_orf1;TRINITY_DN15160_c0_g1_i1_orf1;TRINITY_DN779_c0_g1_i12_orf1;TRINITY_DN6325_c0_g1_i8_orf1;TRINITY_DN8717_c0_g1_i5_orf1;TRINITY_DN5811_c0_g1_i4_orf1;TRINITY_DN9156_c0_g1_i1_orf1;TRINITY_DN41179_c0_g1_i1_orf1;TRINITY_DN1957_c0_g1_i4_orf1;TRINITY_DN51498_c0_g1_i1_orf1;TRINITY_DN6325_c0_g1_i9_orf1;TRINITY_DN107288_c0_g1_i2_orf1;TRINITY_DN18782_c0_g1_i4_orf1;TRINITY_DN3651_c0_g1_i5_orf1;TRINITY_DN2299_c0_g1_i3_orf1;TRINITY_DN9794_c0_g2_i8_orf1;TRINITY_DN38180_c0_g1_i3_orf1;TRINITY_DN5029_c0_g1_i1_orf1;TRINITY_DN9979_c0_g1_i1_orf1;TRINITY_DN2749_c4_g1_i2_orf1
biological_process	reactive oxygen species metabolic process	GO:0072593	4	4/1043	TRINITY_DN8637_c0_g1_i1_orf1;TRINITY_DN285_c0_g1_i4_orf1;TRINITY_DN16400_c0_g2_i1_orf1;TRINITY_DN6580_c0_g1_i4_orf1
biological_process	cellular carbohydrate metabolic process	GO:0044262	1	1/1043	TRINITY_DN52244_c1_g1_i1_orf1

biological_process	sulfur compound metabolic process	GO:0006790	6	6/1043	TRINITY_DN92153_c0_g2_i2_orf1;TRINITY_DN9794_c0_g2_i8_orf1;TRINITY_DN5497_c0_g1_i6_orf1;TRINITY_DN3991_c0_g1_i6_orf1;TRINITY_DN35002_c0_g2_i2_orf1;TRINITY_DN49872_c0_g1_i2_orf1
biological_process	phosphorus metabolic process	GO:0006793	36	36/1043	TRINITY_DN6325_c0_g1_i8_orf1;TRINITY_DN115210_c0_g4_i1_orf1;TRINITY_DN4360_c0_g1_i4_orf1;TRINITY_DN96170_c0_g2_i1_orf1;TRINITY_DN27035_c0_g1_i1_orf1;TRINITY_DN2719_c1_g1_i6_orf1;TRINITY_DN3119_c0_g1_i7_orf1;TRINITY_DN44110_c0_g1_i4_orf1;TRINITY_DN1216_c0_g1_i4_orf1;TRINITY_DN8603_c0_g1_i1_orf1;TRINITY_DN48602_c0_g1_i6_orf1;TRINITY_DN3991_c0_g1_i6_orf1;TRINITY_DN59885_c0_g1_i3_orf1;TRINITY_DN2110_c0_g1_i3_orf1;TRINITY_DN18782_c0_g1_i4_orf1;TRINITY_DN15222_c0_g1_i4_orf1;TRINITY_DN9794_c0_g2_i8_orf1;TRINITY_DN11015_c0_g1_i8_orf1;TRINITY_DN83150_c0_g1_i1_orf1;TRINITY_DN1154_c0_g1_i1_orf1;TRINITY_DN6325_c0_g1_i9_orf1;TRINITY_DN4929_c1_g2_i5_orf1;TRINITY_DN1266_c2_g1_i1_orf1;TRINITY_DN4798_c0_g1_i3_orf1;TRINITY_DN52244_c1_g1_i1_orf1;TRINITY_DN5811_c0_g1_i4_orf1;TRINITY_DN2618_c0_g1_i3_orf1;TRINITY_DN1173_c1_g1_i10_orf1;TRINITY_DN9156_c0_g1_i1_orf1;TRINITY_DN16487_c0_g1_i1_orf1;TRINITY_DN9979_c0_g1_i1_orf1;TRINITY_DN46715_c0_g1_i1_orf1;TRINITY_DN5697_c0_g1_i1_orf1;TRINITY_DN5029_c0_g1_i1_orf1;TRINITY_DN29956_c1_g1_i1_orf1;TRINITY_DN1957_c0_g1_i4_orf1
biological_process	organic acid metabolic process	GO:0006082	40	40/1043	TRINITY_DN6199_c2_g1_i3_orf1;TRINITY_DN92153_c0_g2_i2_orf1;TRINITY_DN14565_c0_g1_i11_orf1;TRINITY_DN115210_c0_g4_i1_orf1;TRINITY_DN4360_c0_g1_i4_orf1;TRINITY_DN5497_c0_g1_i6_orf1;TRINITY_DN863_c0_g1_i6_orf1;TRINITY_DN27035_c0_g1_i1_orf1;TRINITY_DN11159_c0_g1_i5_orf1;TRINITY_DN2719_c1_g1_i6_orf1;TRINITY_DN1264_c0_g1_i2_orf1;TRINITY_DN4822_c0_g1_i9_orf1;TRINITY_DN89483_c0_g1_i1_orf1;TRINITY_DN27903_c0_g1_i1_orf1;TRINITY_DN1824_c0_g2_i2_orf1;TRINITY_DN45220_c0_g1_i1_orf1;TRINITY_DN87170_c0_g1_i3_orf1;TRINITY_DN3588_c0_g1_i4_orf1;TRINITY_DN9794_c0_g2_i8_orf1;TRINITY_DN2953_c1_g1_i10_orf1;TRINITY_DN42759_c0_g2_i1_orf1;TRINITY_DN3991_c0_g1_i6_orf1;TRINITY_DN76283_c0_g2_i1_orf1;TRINITY_DN4321_c0_g1_i1_orf1;TRINITY_DN4822_c0_g1_i6_orf1;TRINITY_DN1999_c0_g1_i9_orf1;TRINITY_DN10900_c0_g1_i7_orf1;TRINITY_DN825_c23_g1_i5_orf1;TRINITY_DN1760_c0_g1_i4_orf1;TRINITY_DN83150_c0_g1_i1_orf1;TRINITY_DN12293_c0_g1_i1_orf1;TRINITY_DN20527_c0_g1_i1_orf1;TRINITY_DN6325_c0_g1_i9_orf1;TRINITY_DN15160_c0_g1_i1_orf1;TRINITY_DN6325_c0_g1_i8_orf1;TRINITY_DN2684_c0_g2_i3_orf1;TRINITY_DN17326_c0_g1_i5_orf1;TRINITY_DN107288_c0_g1_i2_orf1;TRINITY_DN11383_c0_g2_i4_orf1;TRINITY_DN9979_c0_g1_i1_orf1
biological_process	cellular catabolic process	GO:0044248	22	22/1043	TRINITY_DN6199_c2_g1_i3_orf1;TRINITY_DN14565_c0_g1_i11_orf1;TRINITY_DN863_c0_g1_i6_orf1;TRINITY_DN8037_c0_g2_i1_orf1;TRINITY_DN4822_c0_g1_i9_orf1;TRINITY_DN89483_c0_g1_i1_orf1;TRINITY_DN45220_c0_g1_i1_orf1;TRINITY_DN87170_c0_g1_i3_orf1;TRINITY_DN3588_c0_g1_i4_orf1;TRINITY_DN2559_c0_g1_i4_orf1;TRINITY_DN98242_c0_g1_i1_orf1;TRINITY_DN82017_c0_g1_i5_orf1;TRINITY_DN51658_c0_g1_i1_orf1;TRINITY_DN11172_c0_g1_i4_orf1;TRINITY_DN4822_c0_g1_i6_orf1;TRINITY_DN46132_c0_g2_i2_orf1;TRINITY_DN5001_c0_g1_i4_orf1;TRINITY_DN12293_c0_g1_i1_orf1;TRINITY_DN779_c0_g1_i12_orf1;TRINITY_DN6580_c0_g1_i4_orf1;TRINITY_DN285_c0_g1_i4_orf1;TRINITY_DN38180_c0_g1_i3_orf1
biological_process	cellular biosynthetic process	GO:0044249	47	47/1043	TRINITY_DN230_c2_g1_i5_orf1;TRINITY_DN22046_c1_g1_i5_orf1;TRINITY_DN115210_c0_g4_i1_orf1;TRINITY_DN36893_c0_g1_i1_orf1;TRINITY_DN5497_c0_g1_i6_orf1;TRINITY_DN137_c0_g1_i1_orf1;TRINITY_DN8603_c0_g1_i1_orf1;TRINITY_DN2719_c1_g1_i6_orf1;TRINITY_DN21619_c0_g1_i1_orf1;TRINITY_DN55148_c0_g1_i1_orf1;TRINITY_DN124950_c0_g2_i1_orf1;TRINITY_DN1153_c1_g1_i1_orf1;TRINITY_DN27903_c0_g1_i1_orf1;TRINITY_DN1824_c0_g2_i2_orf1;TRINITY_DN115658_c0_g1_i1_orf1;TRINITY_DN1216_c0_g1_i4_orf1;TRINITY_DN975_c0_g1_i1_orf1;TRINITY_DN142442_c0_g1_i1_orf1;TRINITY_DN48602_c0_g1_i6_orf1;TRINITY_DN10900_c0_g1_i7_orf1;TRINITY_DN6638_c0_g1_i1_orf1;TRINITY_DN7957_c0_g1_i5_orf1;TRINITY_DN9207_c0_g1_i1_orf1;TRINITY_DN9794_c0_g2_i8_orf1;TRINITY_DN3991_c0_g1_i6_orf1;TRINITY_DN2110_c0_g1_i3_orf1;TRINITY_DN15222_c0_g1_i4_orf1;TRINITY_DN323_c0_g2_i5_orf1;TRINITY_DN1999_c0_g1_i9_orf1;TRINITY_DN74889_c0_g1_i1_orf1;TRINITY_DN49936_c0_g2_i1_orf1;TRINITY_DN1760_c0_g1_i4_orf1;TRINITY_DN42759_c0_g2_i1_orf1;TRINITY_DN76283_c0_g2_i1_orf1;TRINITY_DN20527_c0_g1_i1_orf1;TRINITY_DN82324_c0_g1_i4_orf1;TRINITY_DN4321_c0_g1_i1_orf1;TRINITY_DN5811_c0_g1_i4_orf1;TRINITY_DN2618_c0_g1_i3_orf1;TRINITY_DN2265_c0_g1_i5_orf1;TRINITY_DN1957_c0_g1_i4_orf1;TRINITY_DN51498_c0_g1_i1_orf1;TRINITY_DN9156_c0_g1_i1_orf1;TRINITY_DN18782_c0_g1_i4_orf1;TRINITY_DN2299_c0_g1_i3_orf1;TRINITY_DN5697_c0_g1_i1_orf1;TRINITY_DN5029_c0_g1_i1_orf1
biological_process	cellular macromolecule metabolic process	GO:0044260	26	26/1043	TRINITY_DN140212_c0_g1_i1_orf1;TRINITY_DN40434_c0_g1_i2_orf1;TRINITY_DN36893_c0_g1_i1_orf1;TRINITY_DN137_c0_g1_i1_orf1;TRINITY_DN21619_c0_g1_i1_orf1;TRINITY_DN20238_c0_g1_i7_orf1;TRINITY_DN55148_c0_g1_i1_orf1;TRINITY_DN2054_c0_g1_i1_orf1;TRINITY_DN115658_c0_g1_i1_orf1;TRINITY_DN975_c0_g1_i1_orf1;TRINITY_DN142442_c0_g1_i1_orf1;TRINITY_DN41664_c0_g1_i4_orf1;TRINITY_DN1091_c0_g3_i1_orf1;TRINITY_DN4835_c0_g1_i2_orf1;TRINITY_DN74889_c0_g1_i1_orf1;TRINITY_DN5238_c0_g1_i2_orf1;TRINITY_DN49936_c0_g2_i1_orf1;TRINITY_DN46132_c0_g2_i2_orf1;TRINITY_DN5001_c0_g1_i4_orf1;TRINITY_DN110534_c0_g1_i3_orf1;TRINITY_DN1091_c0_g1_i1_orf1;TRINITY_DN82324_c0_g1_i4_orf1;TRINITY_DN2265_c0_g1_i5_orf1;TRINITY_DN2647_c0_g1_i3_orf1;TRINITY_DN22674_c0_g1_i2_orf1;TRINITY_DN51
biological_process	cellular aromatic compound metabolic process	GO:0006725	72	72/1043	TRINITY_DN230_c2_g1_i5_orf1;TRINITY_DN92153_c0_g2_i2_orf1;TRINITY_DN5507_c0_g1_i1_orf1;TRINITY_DN2749_c0_g1_i4_orf1;TRINITY_DN4360_c0_g1_i4_orf1;TRINITY_DN40434_c0_g1_i2_orf1;TRINITY_DN51968_c0_g1_i1_orf1;TRINITY_DN4835_c0_g1_i2_orf1;TRINITY_DN863_c0_g1_i6_orf1;TRINITY_DN27035_c0_g1_i1_orf1;TRINITY_DN8037_c0_g2_i1_orf1;TRINITY_DN13760_c1_g1_i1_orf1;TRINITY_DN110534_c0_g1_i3_orf1;TRINITY_DN20238_c0_g1_i7_orf1;TRINITY_DN51498_c0_g1_i1_orf1;TRINITY_DN55148_c0_g1_i1_orf1;TRINITY_DN2054_c0_g1_i1_orf1;TRINITY_DN3324_c0_g1_i3_orf1;TRINITY_DN24322_c0_g1_i4_orf1;TRINITY_DN33346_c0_g1_i1_orf1;TRINITY_DN115210_c0_g4_i1_orf1;TRINITY_DN4822_c0_g1_i9_orf1;TRINITY_DN115658_c0_g1_i1_orf1;TRINITY_DN87170_c0_g1_i3_orf1;TRINITY_DN1216_c0_g1_i4_orf1;TRINITY_DN8603_c0_g1_i1_orf1;TRINITY_DN48602_c0_g1_i6_orf1;TRINITY_DN2559_c0_g1_i4_orf1;TRINITY_DN2953_c1_g1_i10_orf1;TRINITY_DN98242_c0_g1_i1_orf1;TRINITY_DN810_c0_g1_i4_orf1;TRINITY_DN244_c1_g1_i5_orf1;TRINITY_DN2201_c0_g1_i1_orf1;TRINITY_DN140212_c0_g1_i1_orf1;TRINITY_DN2110_c0_g1_i3_orf1;TRINITY_DN1091_c0_g1_i1_orf1;TRINITY_DN1091_c0_g3_i1_orf1;TRINITY_DN2647_c0_g1_i3_orf1;TRINITY_DN2749_c0_g2_i3_orf1;TRINITY_DN9207_c0_g1_i1_orf1;TRINITY_DN15222_c0_g1_i4_orf1;TRINITY_DN4822_c0_g1_i6_orf1;TRINITY_DN19687_c0_g1_i1_orf1;TRINITY_DN323_c0_g2_i5_orf1;TRINITY_DN124950_c0_g2_i1_orf1;TRINITY_DN3991_c0_g1_i6_orf1;TRINITY_DN825_c23_g1_i5_orf1;TRINITY_DN5238_c0_g1_i2_orf1;TRINITY_DN83150_c0_g1_i1_orf1;TRINITY_DN12293_c0_g1_i1_orf1;TRINITY_DN20527_c0_g1_i1_orf1;TRINITY_DN1116_c0_g1_i6_orf1;TRINITY_DN41664_c0_g1_i4_orf1;TRINITY_DN15160_c0_g1_i1_orf1;TRINITY_DN779_c0_g1_i12_orf1;TRINITY_DN6325_c0_g1_i8_orf1;TRINITY_DN8717_c0_g1_i5_orf1;TRINITY_DN5811_c0_g1_i4_orf1;TRINITY_DN9156_c0_g1_i1_orf1;TRINITY_DN41179_c0_g1_i1_orf1;TRINITY_DN1957_c0_g1_i4_orf1;TRINITY_DN11172_c0_g1_i4_orf1;TRINITY_DN6325_c0_g1_i9_orf1;TRINITY_DN107288_c0_g1_i2_orf1;TRINITY_DN18782_c0_g1_i4_orf1;TRINITY_DN3651_c0_g1_i5_orf1;TRINITY_DN2299_c0_g1_i3_orf1;TRINITY_DN9794_c0_g2_i8_orf1;TRINITY_DN38180_c0_g1_i3_orf1;TRINITY_DN5029_c0_g1_i1_orf1;TRINITY_DN9979
biological_process	cellular metabolic compound salvage	GO:0043094	3	3/1043	TRINITY_DN5811_c0_g1_i4_orf1;TRINITY_DN8603_c0_g1_i1_orf1;TRINITY_DN5497_c0_g1_i6_orf1
biological_process	neurotransmitter metabolic process	GO:0042133	2	2/1043	TRINITY_DN82017_c0_g1_i5_orf1;TRINITY_DN14565_c0_g1_i11_orf1
biological_process	cellular modified amino acid metabolic process	GO:0006575	4	4/1043	TRINITY_DN20527_c0_g1_i1_orf1;TRINITY_DN1153_c1_g1_i1_orf1;TRINITY_DN244_c1_g1_i5_orf1;TRINITY_DN92153_c0_g2_i2_orf1
biological_process	small molecule biosynthetic process	GO:0044283	18	18/1043	TRINITY_DN230_c2_g1_i5_orf1;TRINITY_DN27903_c0_g1_i1_orf1;TRINITY_DN1824_c0_g2_i2_orf1;TRINITY_DN42759_c0_g2_i1_orf1;TRINITY_DN1999_c0_g1_i9_orf1;TRINITY_DN3991_c0_g1_i6_orf1;TRINITY_DN4360_c0_g1_i4_orf1;TRINITY_DN18782_c0_g1_i4_orf1;TRINITY_DN5497_c0_g1_i6_orf1;TRINITY_DN8603_c0_g1_i1_orf1;TRINITY_DN27035_c0_g1_i1_orf1;TRINITY_DN6638_c0_g1_i1_orf1;TRINITY_DN1760_c0_g1_i4_orf1;TRINITY_DN2719_c1_g1_i6_orf1;TRINITY_DN20527_c0_g1_i1_orf1;TRINITY_DN76283_c0_g2_i1_orf1;TRINITY_DN10900_c0_g1_i7_orf1;TRINITY_DN4321_c0_g1_i1_orf1
biological_process	organic substance biosynthetic process	GO:1901576	49	49/1043	TRINITY_DN230_c2_g1_i5_orf1;TRINITY_DN22046_c1_g1_i5_orf1;TRINITY_DN115210_c0_g4_i1_orf1;TRINITY_DN4360_c0_g1_i4_orf1;TRINITY_DN36893_c0_g1_i1_orf1;TRINITY_DN5497_c0_g1_i6_orf1;TRINITY_DN137_c0_g1_i1_orf1;TRINITY_DN27035_c0_g1_i1_orf1;TRINITY_DN8603_c0_g1_i1_orf1;TRINITY_DN2719_c1_g1_i6_orf1;TRINITY_DN21619_c0_g1_i1_orf1;TRINITY_DN55148_c0_g1_i1_orf1;TRINITY_DN124950_c0_g2_i1_orf1;TRINITY_DN1153_c1_g1_i1_orf1;TRINITY_DN27903_c0_g1_i1_orf1;TRINITY_DN1824_c0_g2_i2_orf1;TRINITY_DN115658_c0_g1_i1_orf1;TRINITY_DN1216_c0_g1_i4_orf1;TRINITY_DN975_c0_g1_i1_orf1;TRINITY_DN142442_c0_g1_i1_orf1;TRINITY_DN48602_c0_g1_i6_orf1;TRINITY_DN74889_c0_g1_i1_orf1;TRINITY_DN6638_c0_g1_i1_orf1;TRINITY_DN7957_c0_g1_i5_orf1;TRINITY_DN9207_c0_g1_i1_orf1;TRINITY_DN9794_c0_g2_i8_orf1;TRINITY_DN2110_c0_g1_i3_orf1;TRINITY_DN15222_c0_g1_i4_orf1;TRINITY_DN323_c0_g2_i5_orf1;TRINITY_DN1999_c0_g1_i9_orf1;TRINITY_DN3991_c0_g1_i6_orf1;TRINITY_DN49936_c0_g2_i1_orf1;TRINITY_DN1760_c0_g1_i4_orf1;TRINITY_DN42759_c0_g2_i1_orf1;TRINITY_DN76283_c0_g2_i1_orf1;TRINITY_DN20527_c0_g1_i1_orf1;TRINITY_DN82324_c0_g1_i4_orf1;TRINITY_DN4321_c0_g1_i1_orf1;TRINITY_DN5811_c0_g1_i4_orf1;TRINITY_DN2618_c0_g1_i3_orf1;TRINITY_DN9156_c0_g1_i1_orf1;TRINITY_DN1957_c0_g1_i4_orf1;TRINITY_DN51498_c0_g1_i1_orf1;TRINITY_DN2265_c0_g1_i5_orf1;TRINITY_DN18782_c0_g1_i4_orf1;TRINITY_DN2299_c0_g1_i3_orf1;TRINITY_DN5697_c0_g1_i1_orf1;TRINITY_DN5029_c0_g1_i1_orf1;TRINITY_DN10900_c0_g1_i7_orf1
biological_process	small molecule catabolic process	GO:0044282	14	14/1043	TRINITY_DN779_c0_g1_i12_orf1;TRINITY_DN6199_c2_g1_i3_orf1;TRINITY_DN45220_c0_g1_i1_orf1;TRINITY_DN87170_c0_g1_i3_orf1;TRINITY_DN3588_c0_g1_i4_orf1;TRINITY_DN4822_c0_g1_i6_orf1;TRINITY_DN863_c0_g1_i6_orf1;TRINITY_DN2559_c0_g1_i4_orf1;TRINITY_DN8037_c0_g2_i1_orf1;TRINITY_DN98242_c0_g1_i1_orf1;TRINITY_DN12293_c0_g1_i1_orf1;TRINITY_DN25997_c1_g2_i4_orf1;TRINITY_DN4822_c0_g1_i9_orf1;TRINITY_DN89483_c0_g1_i1_orf1
biological_process	organic substance catabolic process	GO:1901575	34	34/1043	TRINITY_DN6199_c2_g1_i3_orf1;TRINITY_DN827_c1_g1_i1_orf1;TRINITY_DN4360_c0_g1_i4_orf1;TRINITY_DN10824_c0_g1_i3_orf1;TRINITY_DN863_c0_g1_i6_orf1;TRINITY_DN27035_c0_g1_i1_orf1;TRINITY_DN8037_c0_g2_i1_orf1;TRINITY_DN4817_c0_g1_i4_orf1;TRINITY_DN4822_c0_g1_i9_orf1;TRINITY_DN89483_c0_g1_i1_orf1;TRINITY_DN6325_c0_g1_i8_orf1;TRINITY_DN542_c0_g2_i1_orf1;TRINITY_DN87170_c0_g1_i3_orf1;TRINITY_DN34479_c0_g1_i2_orf1;TRINITY_DN2559_c0_g1_i4_orf1;TRINITY_DN98242_c0_g1_i1_orf1;TRINITY_DN51658_c0_g1_i1_orf1;TRINITY_DN4822_c0_g1_i6_orf1;TRINITY_DN1534_c0_g1_i3_orf1;TRINITY_DN21555_c0_g1_i4_orf1;TRINITY_DN46132_c0_g2_i2_orf1;TRINITY_DN83150_c0_g1_i1_orf1;TRINITY_DN5001_c0_g1_i4_orf1;TRINITY_DN12293_c0_g1_i1_orf1;TRINITY_DN6325_c0_g1_i9_orf1;TRINITY_DN45220_c0_g1_i1_orf1;TRINITY_DN779_c0_g1_i12_orf1;TRINITY_DN3588_c0_g1_i4_orf1;TRINITY_DN1287_c0_g1_i5_orf1;TRINITY_DN49047_c0_g1_i2_orf1;TRINITY_DN25997_c1_g2_i4_orf1;TRINITY_DN38180_c0_g1_i3_orf1;TRINITY_DN9979_c0_g1_i1_orf1;TRINITY_DN650_c0_g1_i3_orf1
biological_process	vitamin metabolic process	GO:0006766	1	1/1043	TRINITY_DN18782_c0_g1_i4_orf1
biological_process	monosaccharide metabolic process	GO:0005996	7	7/1043	TRINITY_DN15222_c0_g1_i4_orf1;TRINITY_DN9109_c0_g1_i1_orf1;TRINITY_DN4360_c0_g1_i4_orf1;TRINITY_DN18650_c0_g1_i1_orf1;TRINITY_DN4247_c0_g1_i4_orf1;TRINITY_DN27035_c0_g1_i1_orf1;TRINITY_DN25997_c1_g2_i4_orf1
biological_process	alcohol metabolic process	GO:0006066	3	3/1043	TRINITY_DN230_c2_g1_i5_orf1;TRINITY_DN52244_c1_g1_i1_orf1;TRINITY_DN9286_c0_g1_i2_orf1
biological_process	nucleobase-containing small molecule metaboli	GO:0055086	25	25/1043	TRINITY_DN6325_c0_g1_i8_orf1;TRINITY_DN115210_c0_g4_i1_orf1;TRINITY_DN4360_c0_g1_i4_orf1;TRINITY_DN27035_c0_g1_i1_orf1;TRINITY_DN8037_c0_g2_i1_orf1;TRINITY_DN2559_c0_g1_i4_orf1;TRINITY_DN1216_c0_g1_i4_orf1;TRINITY_DN8603_c0_g1_i1_orf1;TRINITY_DN48602_c0_g1_i6_orf1;TRINITY_DN9794_c0_g2_i8_orf1;TRINITY_DN98242_c0_g1_i1_orf1;TRINITY_DN2110_c0_g1_i3_orf1;TRINITY_DN15222_c0_g1_i4_orf1;TRINITY_DN3324_c0_g1_i3_orf1;TRINITY_DN3991_c0_g1_i6_orf1;TRINITY_DN83150_c0_g1_i1_orf1;TRINITY_DN6325_c0_g1_i9_orf1;TRINITY_DN779_c0_g1_i12_orf1;TRINITY_DN5811_c0_g1_i4_orf1;TRINITY_DN9156_c0_g1_i1_orf1;TRINITY_DN1957_c0_g1_i4_orf1;TRINITY_DN18782_c0_g1_i4_orf1;TRINITY_DN38180_c0_g1_i3_orf1;TRINITY_DN5029_c0_g1_i1_orf1;TRINITY_DN9979_c0_g1_i1_orf1
biological_process	urate metabolic process	GO:0046415	3	3/1043	TRINITY_DN779_c0_g1_i12_orf1;TRINITY_DN2559_c0_g1_i4_orf1;TRINITY_DN8037_c0_g2_i1_orf1
biological_process	macromolecule glycosylation	GO:0043413	2	2/1043	TRINITY_DN56164_c0_g1_i1_orf1;TRINITY_DN1789_c0_g1_i5_orf1
biological_process	macromolecule methylation	GO:0043414	2	2/1043	TRINITY_DN22674_c0_g1_i2_orf1;TRINITY_DN41664_c0_g1_i4_orf1
biological_process	lipid metabolic process	GO:0006629	18	18/1043	TRINITY_DN27903_c0_g1_i1_orf1;TRINITY_DN45220_c0_g1_i1_orf1;TRINITY_DN2618_c0_g1_i3_orf1;TRINITY_DN22046_c1_g1_i5_orf1;TRINITY_DN3991_c0_g1_i6_orf1;TRINITY_DN1999_c0_g1_i9_orf1;TRINITY_DN3588_c0_g1_i4_orf1;TRINITY_DN44110_c0_g1_i4_orf1;TRINITY_DN84478_c0_g1_i8_orf1;TRINITY_DN12526_c0_g1_i5_orf1;TRINITY_DN1293_c1_g1_i4_orf1;TRINITY_DN42759_c0_g2_i1_orf1;TRINITY_DN76283_c0_g2_i1_orf1;TRINITY_DN5697_c0_g1_i1_orf1;TRINITY_DN10900_c0_g1_i7_orf1;TRINITY_DN7861_c0_g1_i5_orf1;TRINITY_DN117_c0_g1_i4_orf1;TRINITY_DN4321_c0_g1_i1_orf1

biological_process	nucleobase-containing compound metabolic pr	GO:0006139	60	60/1043	TRINITY_DN6325_c0_g1_i8_orf1;TRINITY_DN5507_c0_g1_i1_orf1;TRINITY_DN2749_c0_g1_i4_orf1;TRINITY_DN4360_c0_g1_i4_orf1;TRINITY_DN40434_c0_g1_i2_orf1;TRINITY_DN51968_c0_g1_i1_orf1;TRINITY_DN27035_c0_g1_i1_orf1;TRINITY_DN8037_c0_g2_i1_orf1;TRINITY_DN13760_c1_g1_i1_orf1;TRINITY_DN110534_c0_g1_i3_orf1;TRINITY_DN20238_c0_g1_i7_orf1;TRINITY_DN55148_c0_g1_i1_orf1;TRINITY_DN2054_c0_g1_i1_orf1;TRINITY_DN24322_c0_g1_i4_orf1;TRINITY_DN33346_c0_g1_i1_orf1;TRINITY_DN115210_c0_g4_i1_orf1;TRINITY_DN115658_c0_g1_i1_orf1;TRINITY_DN1216_c0_g1_i4_orf1;TRINITY_DN8603_c0_g1_i1_orf1;TRINITY_DN48602_c0_g1_i6_orf1;TRINITY_DN2559_c0_g1_i4_orf1;TRINITY_DN2953_c1_g1_i10_orf1;TRINITY_DN98242_c0_g1_i1_orf1;TRINITY_DN810_c0_g1_i4_orf1;TRINITY_DN9207_c0_g1_i1_orf1;TRINITY_DN2201_c0_g1_i1_orf1;TRINITY_DN140212_c0_g1_i1_orf1;TRINITY_DN2110_c0_g1_i3_orf1;TRINITY_DN1091_c0_g1_i1_orf1;TRINITY_DN1091_c0_g3_i1_orf1;TRINITY_DN2647_c0_g1_i3_orf1;TRINITY_DN2749_c0_g2_i3_orf1;TRINITY_DN9794_c0_g2_i8_orf1;TRINITY_DN15222_c0_g1_i4_orf1;TRINITY_DN4835_c0_g1_i2_orf1;TRINITY_DN19687_c0_g1_i1_orf1;TRINITY_DN3324_c0_g1_i3_orf1;TRINITY_DN124950_c0_g2_i1_orf1;TRINITY_DN3991_c0_g1_i6_orf1;TRINITY_DN825_c23_g1_i5_orf1;TRINITY_DN5238_c0_g1_i2_orf1;TRINITY_DN83150_c0_g1_i1_orf1;TRINITY_DN1116_c0_g1_i6_orf1;TRINITY_DN41664_c0_g1_i4_orf1;TRINITY_DN15160_c0_g1_i1_orf1;TRINITY_DN779_c0_g1_i12_orf1;TRINITY_DN8717_c0_g1_i5_orf1;TRINITY_DN5811_c0_g1_i4_orf1;TRINITY_DN9156_c0_g1_i1_orf1;TRINITY_DN41179_c0_g1_i1_orf1;TRINITY_DN1957_c0_g1_i4_orf1;TRINITY_DN6325_c0_g1_i9_orf1;TRINITY_DN107288_c0_g1_i2_orf1;TRINITY_DN18782_c0_g1_i4_orf1;TRINITY_DN3651_c0_g1_i5_orf1;TRINITY_DN2299_c0_g1_i3_orf1;TRINITY_DN38180_c0_g1_i3_orf1;TRINITY_DN5029_c0_g1_i1_orf1;TRINITY_DN9979_c0_g1_i1_orf1;TRINITY_DN2749_c4_g1_i2_orf1
biological_process	tricarboxylic acid cycle	GO:0006099	1	1/1043	TRINITY_DN5925_c0_g1_i5_orf1
biological_process	carbohydrate metabolic process	GO:0005975	32	32/1043	TRINITY_DN14235_c0_g1_i1_orf1;TRINITY_DN6325_c0_g1_i8_orf1;TRINITY_DN4360_c0_g1_i4_orf1;TRINITY_DN18650_c0_g1_i1_orf1;TRINITY_DN10824_c0_g1_i3_orf1;TRINITY_DN4247_c0_g1_i4_orf1;TRINITY_DN48410_c0_g1_i1_orf1;TRINITY_DN27035_c0_g1_i1_orf1;TRINITY_DN9044_c0_g1_i2_orf1;TRINITY_DN89483_c0_g1_i1_orf1;TRINITY_DN9044_c0_g1_i1_orf1;TRINITY_DN2205_c0_g1_i3_orf1;TRINITY_DN9109_c0_g1_i1_orf1;TRINITY_DN28741_c0_g1_i3_orf1;TRINITY_DN2894_c0_g2_i3_orf1;TRINITY_DN15222_c0_g1_i4_orf1;TRINITY_DN26994_c1_g1_i6_orf1;TRINITY_DN195_c4_g1_i1_orf1;TRINITY_DN83150_c0_g1_i1_orf1;TRINITY_DN6325_c0_g1_i9_orf1;TRINITY_DN1732_c0_g1_i15_orf1;TRINITY_DN21555_c0_g1_i4_orf1;TRINITY_DN52244_c1_g1_i1_orf1;TRINITY_DN1732_c0_g1_i17_orf1;TRINITY_DN1287_c0_g1_i5_orf1;TRINITY_DN82801_c0_g1_i1_orf1;TRINITY_DN542_c0_g2_i1_orf1;TRINITY_DN664_c0_g1_i18_orf1;TRINITY_DN5852_c0_g1_i6_orf1;TRINITY_DN25997_c1_g2_i4_orf1;TRINITY_DN9979_c0_g1_i1_orf1;TRINITY_DN650_c0_g1_i3_orf1
biological_process	protein metabolic process	GO:0019538	70	70/1043	TRINITY_DN4767_c0_g1_i6_orf1;TRINITY_DN2593_c0_g1_i1_orf1;TRINITY_DN14874_c0_g1_i6_orf1;TRINITY_DN143895_c0_g1_i1_orf1;TRINITY_DN36434_c0_g2_i3_orf1;TRINITY_DN36893_c0_g1_i1_orf1;TRINITY_DN801_c0_g1_i2_orf1;TRINITY_DN3194_c0_g1_i6_orf1;TRINITY_DN137_c0_g1_i1_orf1;TRINITY_DN9991_c0_g1_i4_orf1;TRINITY_DN3483_c0_g1_i5_orf1;TRINITY_DN13686_c0_g2_i1_orf1;TRINITY_DN27033_c1_g1_i3_orfp1;TRINITY_DN4817_c0_g1_i4_orf1;TRINITY_DN56164_c0_g1_i1_orf1;TRINITY_DN8659_c0_g2_i1_orf1;TRINITY_DN55148_c0_g1_i1_orf1;TRINITY_DN2069_c1_g1_i8_orf1;TRINITY_DN5310_c2_g1_i2_orf1;TRINITY_DN119291_c0_g1_i1_orf1;TRINITY_DN3119_c0_g1_i7_orf1;TRINITY_DN9871_c0_g1_i11_orf1;TRINITY_DN4030_c0_g2_i1_orf1;TRINITY_DN21984_c0_g1_i6_orf1;TRINITY_DN7647_c0_g1_i4_orf1;TRINITY_DN7316_c0_g2_i1_orf1;TRINITY_DN34479_c0_g1_i2_orf1;TRINITY_DN975_c0_g1_i1_orf1;TRINITY_DN142442_c0_g1_i1_orf1;TRINITY_DN57111_c0_g1_i1_orf1;TRINITY_DN2885_c1_g1_i2_orf1;TRINITY_DN49047_c0_g1_i2_orf1;TRINITY_DN14754_c0_g1_i6_orf1;TRINITY_DN59885_c0_g1_i3_orf1;TRINITY_DN66302_c0_g1_i1_orf1;TRINITY_DN3975_c0_g1_i7_orf1;TRINITY_DN14774_c0_g1_i4_orf1;TRINITY_DN46715_c0_g1_i1_orf1;TRINITY_DN48020_c0_g1_i1_orf1;TRINITY_DN71863_c0_g1_i2_orf1;TRINITY_DN18172_c0_g1_i6_orf1;TRINITY_DN56690_c0_g1_i4_orf1;TRINITY_DN74889_c0_g1_i1_orf1;TRINITY_DN4064_c0_g2_i1_orf1;TRINITY_DN49936_c0_g2_i1_orf1;TRINITY_DN1308_c0_g1_i4_orf1;TRINITY_DN23167_c0_g1_i4_orf1;TRINITY_DN1310_c0_g1_i4_orf1;TRINITY_DN46132_c0_g2_i2_orf1;TRINITY_DN5001_c0_g1_i4_orf1;TRINITY_DN6205_c0_g1_i8_orf1;TRINITY_DN140_c1_g1_i2_orf1;TRINITY_DN1266_c2_g1_i1_orf1;TRINITY_DN4798_c0_g1_i3_orf1;TRINITY_DN82324_c0_g1_i4_orf1;TRINITY_DN67026_c0_g1_i6_orf1;TRINITY_DN1789_c0_g1_i5_orf1;TRINITY_DN6470_c0_g3_i2_orf1;TRINITY_DN22674_c0_g1_i2_orf1;TRINITY_DN1173_c1_g1_i10_orf1;TRINITY_DN701_c0_g1_i1_orf1;TRINITY_DN6205_c0_g1_i1_orf1;TRINITY_DN21619_c0_g1_i1_orf1;TRINITY_DN1380_c0_g1_i5_orf1;TRINITY_DN4494_c0_g1_i1_orf1;TRINITY_DN22797_c0_g1_i5_orf1;TRINITY_DN51658_c0_g1_i1_orf1;TRINITY_DN5697_c0_g1_i1_orf1;TRINITY_DN747_c0_g1_i4_orf1;TRINITY_DN29956_c1_g1_i1_orf1
biological_process	cellular amino acid metabolic process	GO:0006520	21	21/1043	TRINITY_DN87170_c0_g1_i3_orf1;TRINITY_DN4822_c0_g1_i6_orf1;TRINITY_DN1824_c0_g2_i2_orf1;TRINITY_DN92153_c0_g2_i2_orf1;TRINITY_DN14565_c0_g1_i11_orf1;TRINITY_DN115210_c0_g4_i1_orf1;TRINITY_DN825_c23_g1_i5_orf1;TRINITY_DN6199_c2_g1_i3_orf1;TRINITY_DN1760_c0_g1_i4_orf1;TRINITY_DN5497_c0_g1_i6_orf1;TRINITY_DN863_c0_g1_i6_orf1;TRINITY_DN17326_c0_g1_i5_orf1;TRINITY_DN2953_c1_g1_i10_orf1;TRINITY_DN107288_c0_g1_i2_orf1;TRINITY_DN2719_c1_g1_i6_orf1;TRINITY_DN12293_c0_g1_i1_orf1;TRINITY_DN20527_c0_g1_i1_orf1;TRINITY_DN4822_c0_g1_i9_orf1;TRINITY_DN11383_c0_g2_i4_orf1;TRINITY_DN89483_c0_g1_i1_orf1;TRINITY_DN15160_c0_g1_i1_orf1
biological_process	organophosphate metabolic process	GO:0019637	22	22/1043	TRINITY_DN6325_c0_g1_i8_orf1;TRINITY_DN115210_c0_g4_i1_orf1;TRINITY_DN4360_c0_g1_i4_orf1;TRINITY_DN27035_c0_g1_i1_orf1;TRINITY_DN44110_c0_g1_i4_orf1;TRINITY_DN1216_c0_g1_i4_orf1;TRINITY_DN8603_c0_g1_i1_orf1;TRINITY_DN48602_c0_g1_i6_orf1;TRINITY_DN9794_c0_g2_i8_orf1;TRINITY_DN2110_c0_g1_i3_orf1;TRINITY_DN15222_c0_g1_i4_orf1;TRINITY_DN3991_c0_g1_i6_orf1;TRINITY_DN83150_c0_g1_i1_orf1;TRINITY_DN6325_c0_g1_i9_orf1;TRINITY_DN5811_c0_g1_i4_orf1;TRINITY_DN2618_c0_g1_i3_orf1;TRINITY_DN9156_c0_g1_i1_orf1;TRINITY_DN1957_c0_g1_i4_orf1;TRINITY_DN18782_c0_g1_i4_orf1;TRINITY_DN5697_c0_g1_i1_orf1;TRINITY_DN5029_c0_g1_i1_orf1;TRINITY_DN9979_c0_g1_i1_orf1
biological_process	carbohydrate derivative metabolic process	GO:1901135	29	29/1043	TRINITY_DN6325_c0_g1_i8_orf1;TRINITY_DN115210_c0_g4_i1_orf1;TRINITY_DN4360_c0_g1_i4_orf1;TRINITY_DN10824_c0_g1_i3_orf1;TRINITY_DN27035_c0_g1_i1_orf1;TRINITY_DN827_c1_g1_i1_orf1;TRINITY_DN542_c0_g2_i1_orf1;TRINITY_DN1216_c0_g1_i4_orf1;TRINITY_DN8603_c0_g1_i1_orf1;TRINITY_DN48602_c0_g1_i6_orf1;TRINITY_DN9794_c0_g2_i8_orf1;TRINITY_DN98242_c0_g1_i1_orf1;TRINITY_DN2110_c0_g1_i3_orf1;TRINITY_DN15222_c0_g1_i4_orf1;TRINITY_DN3324_c0_g1_i3_orf1;TRINITY_DN1534_c0_g1_i3_orf1;TRINITY_DN3991_c0_g1_i6_orf1;TRINITY_DN83150_c0_g1_i1_orf1;TRINITY_DN6325_c0_g1_i9_orf1;TRINITY_DN11798_c0_g2_i1_orf1;TRINITY_DN21555_c0_g1_i4_orf1;TRINITY_DN5811_c0_g1_i4_orf1;TRINITY_DN9156_c0_g1_i1_orf1;TRINITY_DN1287_c0_g1_i5_orf1;TRINITY_DN18782_c0_g1_i4_orf1;TRINITY_DN5697_c0_g1_i1_orf1;TRINITY_DN5029_c0_g1_i1_orf1;TRINITY_DN9979_c0_g1_i1_orf1;TRINITY_DN650_c0_g1_i3_orf1
biological_process	organic hydroxy compound metabolic process	GO:1901615	3	3/1043	TRINITY_DN230_c2_g1_i5_orf1;TRINITY_DN52244_c1_g1_i1_orf1;TRINITY_DN9286_c0_g1_i2_orf1
biological_process	organic cyclic compound metabolic process	GO:1901360	73	73/1043	TRINITY_DN230_c2_g1_i5_orf1;TRINITY_DN92153_c0_g2_i2_orf1;TRINITY_DN5507_c0_g1_i1_orf1;TRINITY_DN2749_c0_g1_i4_orf1;TRINITY_DN4360_c0_g1_i4_orf1;TRINITY_DN40434_c0_g1_i2_orf1;TRINITY_DN51968_c0_g1_i1_orf1;TRINITY_DN4835_c0_g1_i2_orf1;TRINITY_DN863_c0_g1_i6_orf1;TRINITY_DN27035_c0_g1_i1_orf1;TRINITY_DN8037_c0_g2_i1_orf1;TRINITY_DN13760_c1_g1_i1_orf1;TRINITY_DN2719_c1_g1_i6_orf1;TRINITY_DN110534_c0_g1_i3_orf1;TRINITY_DN20238_c0_g1_i7_orf1;TRINITY_DN4822_c0_g1_i9_orf1;TRINITY_DN55148_c0_g1_i1_orf1;TRINITY_DN2054_c0_g1_i1_orf1;TRINITY_DN3324_c0_g1_i3_orf1;TRINITY_DN24322_c0_g1_i4_orf1;TRINITY_DN33346_c0_g1_i1_orf1;TRINITY_DN115210_c0_g4_i1_orf1;TRINITY_DN115658_c0_g1_i1_orf1;TRINITY_DN87170_c0_g1_i3_orf1;TRINITY_DN1216_c0_g1_i4_orf1;TRINITY_DN8603_c0_g1_i1_orf1;TRINITY_DN48602_c0_g1_i6_orf1;TRINITY_DN2559_c0_g1_i4_orf1;TRINITY_DN2953_c1_g1_i10_orf1;TRINITY_DN98242_c0_g1_i1_orf1;TRINITY_DN810_c0_g1_i4_orf1;TRINITY_DN244_c1_g1_i5_orf1;TRINITY_DN2201_c0_g1_i1_orf1;TRINITY_DN140212_c0_g1_i1_orf1;TRINITY_DN2110_c0_g1_i3_orf1;TRINITY_DN1091_c0_g1_i1_orf1;TRINITY_DN1091_c0_g3_i1_orf1;TRINITY_DN2647_c0_g1_i3_orf1;TRINITY_DN2749_c0_g2_i3_orf1;TRINITY_DN9207_c0_g1_i1_orf1;TRINITY_DN15222_c0_g1_i4_orf1;TRINITY_DN4822_c0_g1_i6_orf1;TRINITY_DN19687_c0_g1_i1_orf1;TRINITY_DN323_c0_g2_i5_orf1;TRINITY_DN124950_c0_g2_i1_orf1;TRINITY_DN3991_c0_g1_i6_orf1;TRINITY_DN825_c23_g1_i5_orf1;TRINITY_DN5238_c0_g1_i2_orf1;TRINITY_DN1760_c0_g1_i4_orf1;TRINITY_DN83150_c0_g1_i1_orf1;TRINITY_DN12293_c0_g1_i1_orf1;TRINITY_DN20527_c0_g1_i1_orf1;TRINITY_DN1116_c0_g1_i6_orf1;TRINITY_DN41664_c0_g1_i4_orf1;TRINITY_DN15160_c0_g1_i1_orf1;TRINITY_DN779_c0_g1_i12_orf1;TRINITY_DN6325_c0_g1_i8_orf1;TRINITY_DN8717_c0_g1_i5_orf1;TRINITY_DN5811_c0_g1_i4_orf1;TRINITY_DN9156_c0_g1_i1_orf1;TRINITY_DN41179_c0_g1_i1_orf1;TRINITY_DN1957_c0_g1_i4_orf1;TRINITY_DN51498_c0_g1_i1_orf1;TRINITY_DN6325_c0_g1_i9_orf1;TRINITY_DN107288_c0_g1_i2_orf1;TRINITY_DN18782_c0_g1_i4_orf1;TRINITY_DN3651_c0_g1_i5_orf1;TRINITY_DN2299_c0_g1_i3_orf1;TRINITY_DN9794_c0_g2_i8_orf1;TRINITY_DN38180_c0_g1_i3_orf1;TRINITY_DN5029_c0_g1_i1_orf1;TRINITY_DN9979_c0_g1_i1_orf1;TRINITY_DN2749_c4_g1_i2_orf1
biological_process	thioester metabolic process	GO:0035383	2	2/1043	TRINITY_DN9794_c0_g2_i8_orf1;TRINITY_DN3991_c0_g1_i6_orf1
biological_process	macromolecule metabolic process	GO:0043170	113	113/1043	TRINITY_DN827_c1_g1_i1_orf1;TRINITY_DN40434_c0_g1_i2_orf1;TRINITY_DN3194_c0_g1_i6_orf1;TRINITY_DN137_c0_g1_i1_orf1;TRINITY_DN6205_c0_g1_i8_orf1;TRINITY_DN56164_c0_g1_i1_orf1;TRINITY_DN2054_c0_g1_i1_orf1;TRINITY_DN119291_c0_g1_i1_orf1;TRINITY_DN124950_c0_g2_i1_orf1;TRINITY_DN142442_c0_g1_i1_orf1;TRINITY_DN2885_c1_g1_i2_orf1;TRINITY_DN14754_c0_g1_i6_orf1;TRINITY_DN3975_c0_g1_i7_orf1;TRINITY_DN14774_c0_g1_i4_orf1;TRINITY_DN9207_c0_g1_i1_orf1;TRINITY_DN48020_c0_g1_i1_orf1;TRINITY_DN1534_c0_g1_i3_orf1;TRINITY_DN5507_c0_g1_i1_orf1;TRINITY_DN74889_c0_g1_i1_orf1;TRINITY_DN1116_c0_g1_i6_orf1;TRINITY_DN4798_c0_g1_i3_orf1;TRINITY_DN9991_c0_g1_i4_orf1;TRINITY_DN22674_c0_g1_i2_orf1;TRINITY_DN2265_c0_g1_i5_orf1;TRINITY_DN1380_c0_g1_i5_orf1;TRINITY_DN107288_c0_g1_i2_orf1;TRINITY_DN46715_c0_g1_i1_orf1;TRINITY_DN4767_c0_g1_i6_orf1;TRINITY_DN143895_c0_g1_i1_orf1;TRINITY_DN140212_c0_g1_i1_orf1;TRINITY_DN10824_c0_g1_i3_orf1;TRINITY_DN51968_c0_g1_i1_orf1;TRINITY_DN20238_c0_g1_i7_orf1;TRINITY_DN8659_c0_g2_i1_orf1;TRINITY_DN1310_c0_g1_i4_orf1;TRINITY_DN9871_c0_g1_i11_orf1;TRINITY_DN115658_c0_g1_i1_orf1;TRINITY_DN1789_c0_g1_i5_orf1;TRINITY_DN810_c0_g1_i4_orf1;TRINITY_DN2069_c1_g1_i8_orf1;TRINITY_DN59885_c0_g1_i3_orf1;TRINITY_DN41664_c0_g1_i4_orf1;TRINITY_DN66302_c0_g1_i1_orf1;TRINITY_DN1091_c0_g3_i1_orf1;TRINITY_DN71863_c0_g1_i2_orf1;TRINITY_DN4064_c0_g2_i1_orf1;TRINITY_DN49936_c0_g2_i1_orf1;TRINITY_DN21984_c0_g1_i6_orf1;TRINITY_DN27033_c1_g1_i3_orfp1;TRINITY_DN140_c1_g1_i2_orf1;TRINITY_DN1266_c2_g1_i1_orf1;TRINITY_DN1173_c1_g1_i10_orf1;TRINITY_DN4494_c0_g1_i1_orf1;TRINITY_DN2299_c0_g1_i3_orf1;TRINITY_DN51658_c0_g1_i1_orf1;TRINITY_DN2749_c4_g1_i2_orf1;TRINITY_DN650_c0_g1_i3_orf1;TRINITY_DN36434_c0_g2_i3_orf1;TRINITY_DN2749_c0_g1_i4_orf1;TRINITY_DN67026_c0_g1_i6_orf1;TRINITY_DN3483_c0_g1_i5_orf1;TRINITY_DN21619_c0_g1_i1_orf1;TRINITY_DN4817_c0_g1_i4_orf1;TRINITY_DN57111_c0_g1_i1_orf1;TRINITY_DN5310_c2_g1_i2_orf1;TRINITY_DN33346_c0_g1_i1_orf1;TRINITY_DN542_c0_g2_i1_orf1;TRINITY_DN34479_c0_g1_i2_orf1;TRINITY_DN2953_c1_g1_i10_orf1;TRINITY_DN41179_c0_g1_i1_orf1;TRINITY_DN2749_c0_g2_i3_orf1;TRINITY_DN4835_c0_g1_i2_orf1;TRINITY_DN18172_c0_g1_i6_orf1;TRINITY_DN24322_c0_g1_i4_orf1;TRINITY_DN825_c23_g1_i5_orf1;TRINITY_DN1308_c0_g1_i4_orf1;TRINITY_DN23167_c0_g1_i4_orf1;TRINITY_DN15160_c0_g1_i1_orf1;TRINITY_DN6470_c0_g3_i2_orf1;TRINITY_DN1287_c0_g1_i5_orf1;TRINITY_DN2647_c0_g1_i3_orf1;TRINITY_DN6205_c0_g1_i1_orf1;TRINITY_DN49047_c0_g1_i2_orf1;TRINITY_DN3651_c0_g1_i5_orf1;TRINITY_DN7316_c0_g2_i1_orf1;TRINITY_DN36893_c0_g1_i1_orf1;TRINITY_DN801_c0_g1_i2_orf1;TRINITY_DN13760_c1_g1_i1_orf1;TRINITY_DN13686_c0_g2_i1_orf1;TRINITY_DN19687_c0_g1_i1_orf1;TRINITY_DN55148_c0_g1_i1_orf1;TRINITY_DN14874_c0_g1_i6_orf1;TRINITY_DN3119_c0_g1_i7_orf1;TRINITY_DN4030_c0_g2_i1_orf1;TRINITY_DN7647_c0_g1_i4_orf1;TRINITY_DN975_c0_g1_i1_orf1;TRINITY_DN2201_c0_g1_i1_orf1;TRINITY_DN1091_c0_g1_i1_orf1;TRINITY_DN2593_c0_g1_i1_orf1;TRINITY_DN56690_c0_g1_i4_orf1;TRINITY_DN5238_c0_g1_i2_orf1;TRINITY_DN46132_c0_g2_i2_orf1;TRINITY_DN5001_c0_g1_i4_orf1;TRINITY_DN110534_c0_g1_i3_orf1;TRINITY_DN11798_c0_g2_i1_orf1;TRINITY_DN82324_c0_g1_i4_orf1;TRINITY_DN21555_c0_g1_i4_orf1;TRINITY_DN8717_c0_g1_i5_orf1;TRINITY_DN701_c0_g1_i1_orf1;TRINITY_DN22797_c0_g1_i5_orf1;TRINITY_DN5697_c0_g1_i1_orf1;TRINITY_DN747_c0_g1_i4_orf1;TRINITY_DN29956_c1_g1_i1_orf1
biological_process	programmed cell death	GO:0012501	1	1/1043	TRINITY_DN87603_c0_g2_i1_orf1
biological_process	secretion by cell	GO:0032940	2	2/1043	TRINITY_DN33452_c0_g1_i1_orf1;TRINITY_DN33452_c0_g1_i3_orf1
biological_process	cell-substrate adhesion	GO:0031589	2	2/1043	TRINITY_DN2186_c0_g1_i17_orf1;TRINITY_DN2919_c0_g1_i5_orf1
biological_process	cell-cell adhesion	GO:0098609	1	1/1043	TRINITY_DN3469_c0_g1_i4_orf1
biological_process	cellular response to extracellular stimulus	GO:0031668	5	5/1043	TRINITY_DN140212_c0_g1_i1_orf1;TRINITY_DN1091_c0_g1_i1_orf1;TRINITY_DN1091_c0_g3_i1_orf1;TRINITY_DN51938_c0_g3_i1_orf1;TRINITY_DN2054_c0_g1_i1_orf1
biological_process	vesicle tethering involved in exocytosis	GO:0090522	1	1/1043	TRINITY_DN16316_c0_g1_i7_orf1
biological_process	cellular chemical homeostasis	GO:0055082	3	3/1043	TRINITY_DN65681_c0_g1_i1_orf1;TRINITY_DN136031_c0_g1_i7_orf1;TRINITY_DN31584_c0_g2_i2_orf1
biological_process	mitotic cell cycle process	GO:1903047	1	1/1043	TRINITY_DN20238_c0_g1_i7_orf1
biological_process	cytokinesis	GO:0000910	1	1/1043	TRINITY_DN20238_c0_g1_i7_orf1
biological_process	post-chaperonin tubulin folding pathway	GO:0007023	1	1/1043	TRINITY_DN104297_c0_g1_i1_orf1
biological_process	cellular macromolecule localization	GO:0070727	13	13/1043	TRINITY_DN3299_c0_g1_i2_orf1;TRINITY_DN38835_c0_g2_i1_orf1;TRINITY_DN12777_c0_g1_i5_orf1;TRINITY_DN55148_c0_g1_i1_orf1;TRINITY_DN146236_c0_g1_i1_orf1;TRINITY_DN31584_c0_g2_i2_orf1;TRINITY_DN16316_c0_g1_i7_orf1;TRINITY_DN14677_c0_g2_i3_orf1;TRINITY_DN6680_c0_g1_i1_orf1;TRINITY_DN4207_c0_g1_i1_orf1;TRINITY_DN5630_c4_g1_i2_orf1;TRINITY_DN65299_c0_g4_i1_orf1;TRINITY_DN3209_c0_

biological_process	intracellular transport	GO:0046907	12	12/1043	TRINITY_DN3835_c0_g1_i4_orf1;TRINITY_DN54586_c1_g1_i1_orf1;TRINITY_DN12777_c0_g1_i5_orf1;TRINITY_DN4814_c0_g1_i6_orf1;TRINITY_DN55148_c0_g1_i1_orf1;TRINITY_DN31584_c0_g2_i2_orf1;TRINITY_DN12432_c0_g1_i2_orf1;TRINITY_DN1245_c0_g1_i4_orf1;TRINITY_DN16316_c0_g1_i7_orf1;TRINITY_DN14677_c0_g2_i3_orf1;TRINITY_DN4207_c0_g1_i1_orf1;TRINITY_DN3209_c0_g2_i6_orf1
biological_process	cellular component biogenesis	GO:0044085	5	5/1043	TRINITY_DN102260_c0_g1_i1_orf1;TRINITY_DN3618_c0_g1_i4_orf1;TRINITY_DN3292_c2_g1_i4_orf1;TRINITY_DN41179_c0_g1_i1_orf1;TRINITY_DN55148_c0_g1_i1_orf1
biological_process	cellular component organization	GO:0016043	29	29/1043	TRINITY_DN6071_c0_g1_i1_orf1;TRINITY_DN51938_c0_g3_i1_orf1;TRINITY_DN20442_c0_g2_i1_orf1;TRINITY_DN1497_c0_g2_i6_orf1;TRINITY_DN1860_c0_g1_i2_orf1;TRINITY_DN89083_c0_g1_i1_orf1;TRINITY_DN3126_c0_g1_i4_orf1;TRINITY_DN55148_c0_g1_i1_orf1;TRINITY_DN35635_c0_g1_i1_orf1;TRINITY_DN4010_c0_g2_i1_orf1;TRINITY_DN91877_c0_g1_i1_orf1;TRINITY_DN7647_c0_g1_i4_orf1;TRINITY_DN104297_c0_g1_i1_orf1;TRINITY_DN142442_c0_g1_i1_orf1;TRINITY_DN43412_c0_g1_i2_orf1;TRINITY_DN37418_c0_g1_i4_orf1;TRINITY_DN2304_c0_g1_i4_orf1;TRINITY_DN16316_c0_g1_i7_orf1;TRINITY_DN7794_c0_g1_i1_orf1;TRINITY_DN5678_c0_g2_i3_orf1;TRINITY_DN49872_c0_g1_i2_orf1;TRINITY_DN146236_c0_g1_i1_orf1;TRINITY_DN40911_c0_g1_i1_orf1;TRINITY_DN4798_c0_g1_i3_orf1;TRINITY_DN110231_c0_g1_i1_orf1;TRINITY_DN10455_c0_g1_i2_orf1;TRINITY_DN24266_c0_g2_i2_orf1;TRINITY_DN23502_c0_g1_i1_orf1;TRINITY_DN80424_c0_g1_i1_orf1
biological_process	cell migration	GO:0016477	2	2/1043	TRINITY_DN110231_c0_g1_i1_orf1;TRINITY_DN31584_c0_g2_i2_orf1
biological_process	cellular response to chemical stimulus	GO:0070887	3	3/1043	TRINITY_DN51938_c0_g3_i1_orf1;TRINITY_DN87603_c0_g2_i1_orf1;TRINITY_DN975_c0_g1_i1_orf1
biological_process	cellular response to stress	GO:0033554	13	13/1043	TRINITY_DN51938_c0_g3_i1_orf1;TRINITY_DN6503_c0_g1_i8_orf1;TRINITY_DN87603_c0_g2_i1_orf1;TRINITY_DN140212_c0_g1_i1_orf1;TRINITY_DN40434_c0_g1_i2_orf1;TRINITY_DN31584_c0_g2_i2_orf1;TRINITY_DN2647_c0_g1_i3_orf1;TRINITY_DN51658_c0_g1_i1_orf1;TRINITY_DN20238_c0_g1_i7_orf1;TRINITY_DN1091_c0_g1_i1_orf1;TRINITY_DN1091_c0_g3_i1_orf1;TRINITY_DN5238_c0_g1_i2_orf1;TRINITY_DN2054_c0_g
biological_process	cell surface receptor signaling pathway	GO:0007166	1	1/1043	TRINITY_DN51938_c0_g3_i1_orf1
biological_process	intracellular signal transduction	GO:0035556	2	2/1043	TRINITY_DN804_c0_g1_i7_orf1;TRINITY_DN31584_c0_g2_i2_orf1
biological_process	apoptotic signaling pathway	GO:0097190	1	1/1043	TRINITY_DN51938_c0_g3_i1_orf1
biological_process	cell differentiation	GO:0030154	4	4/1043	TRINITY_DN928_c0_g1_i3_orf1;TRINITY_DN52395_c0_g2_i2_orf1;TRINITY_DN50725_c0_g1_i6_orf1;TRINITY_DN1173_c1_g1_i10_orf1
biological_process	cell development	GO:0048468	1	1/1043	TRINITY_DN1710_c0_g2_i2_orf1
biological_process	protein transmembrane transport	GO:0071806	1	1/1043	TRINITY_DN4207_c0_g1_i1_orf1
biological_process	mitochondrial transmembrane transport	GO:1990542	1	1/1043	TRINITY_DN4207_c0_g1_i1_orf1
biological_process	actin cytoskeleton organization	GO:0030036	2	2/1043	TRINITY_DN3126_c0_g1_i4_orf1;TRINITY_DN4010_c0_g2_i1_orf1
biological_process	embryonic morphogenesis	GO:0048598	1	1/1043	TRINITY_DN142442_c0_g1_i1_orf1
biological_process	cell morphogenesis	GO:0000902	1	1/1043	TRINITY_DN7794_c0_g1_i1_orf1
biological_process	system development	GO:0048731	2	2/1043	TRINITY_DN1710_c0_g2_i2_orf1;TRINITY_DN142442_c0_g1_i1_orf1
biological_process	hippocampus development	GO:0021766	1	1/1043	TRINITY_DN31584_c0_g2_i2_orf1
biological_process	animal organ development	GO:0048513	1	1/1043	TRINITY_DN7794_c0_g1_i1_orf1
biological_process	cerebral cortex development	GO:0021987	1	1/1043	TRINITY_DN31584_c0_g2_i2_orf1
biological_process	tissue development	GO:0009888	1	1/1043	TRINITY_DN142442_c0_g1_i1_orf1
biological_process	nervous system process	GO:0050877	2	2/1043	TRINITY_DN26337_c0_g1_i3_orf1;TRINITY_DN142442_c0_g1_i1_orf1
biological_process	envenomation resulting in modulation of proces	GO:0035738	1	1/1043	TRINITY_DN1215_c0_g1_i2_orf1
biological_process	response to bacterium	GO:0009617	8	8/1043	TRINITY_DN14904_c0_g1_i1_orf1;TRINITY_DN8685_c0_g1_i5_orf1;TRINITY_DN16840_c1_g1_i1_orf1;TRINITY_DN195_c8_g1_i1_orf1;TRINITY_DN1091_c0_g2_i10_orf1;TRINITY_DN1666_c0_g1_i2_orf1;TRINITY_DN29190_c0_g1_i4_orf1;TRINITY_DN5880_c0_g2_i2_orf1
biological_process	defense response to other organism	GO:0098542	12	12/1043	TRINITY_DN14904_c0_g1_i1_orf1;TRINITY_DN1534_c0_g1_i3_orf1;TRINITY_DN827_c1_g1_i1_orf1;TRINITY_DN8685_c0_g1_i5_orf1;TRINITY_DN16840_c1_g1_i1_orf1;TRINITY_DN195_c8_g1_i1_orf1;TRINITY_DN195_c4_g1_i1_orf1;TRINITY_DN1091_c0_g2_i10_orf1;TRINITY_DN1666_c0_g1_i2_orf1;TRINITY_DN9044_c0_g1_i2_orf1;TRINITY_DN29190_c0_g1_i4_orf1;TRINITY_DN5880_c0_g2_i2_orf1
biological_process	biological process involved in interaction with sy	GO:0051702	1	1/1043	TRINITY_DN975_c0_g1_i1_orf1
biological_process	establishment of protein localization	GO:0045184	13	13/1043	TRINITY_DN3299_c0_g1_i2_orf1;TRINITY_DN38835_c0_g2_i1_orf1;TRINITY_DN12777_c0_g1_i5_orf1;TRINITY_DN55148_c0_g1_i1_orf1;TRINITY_DN146236_c0_g1_i1_orf1;TRINITY_DN31584_c0_g2_i2_orf1;TRINITY_DN16316_c0_g1_i7_orf1;TRINITY_DN14677_c0_g2_i3_orf1;TRINITY_DN6680_c0_g1_i1_orf1;TRINITY_DN4207_c0_g1_i1_orf1;TRINITY_DN5630_c4_g1_i2_orf1;TRINITY_DN65299_c0_g4_i1_orf1;TRINITY_DN3209_c0
biological_process	establishment of localization in cell	GO:0051649	12	12/1043	TRINITY_DN3835_c0_g1_i4_orf1;TRINITY_DN54586_c1_g1_i1_orf1;TRINITY_DN12777_c0_g1_i5_orf1;TRINITY_DN4814_c0_g1_i6_orf1;TRINITY_DN55148_c0_g1_i1_orf1;TRINITY_DN31584_c0_g2_i2_orf1;TRINITY_DN12432_c0_g1_i2_orf1;TRINITY_DN1245_c0_g1_i4_orf1;TRINITY_DN16316_c0_g1_i7_orf1;TRINITY_DN14677_c0_g2_i3_orf1;TRINITY_DN4207_c0_g1_i1_orf1;TRINITY_DN3209_c0_g2_i6_orf1
biological_process	establishment of RNA localization	GO:0051236	2	2/1043	TRINITY_DN1245_c0_g1_i4_orf1;TRINITY_DN6680_c0_g1_i1_orf1
biological_process	transport	GO:0006810	28	28/1043	TRINITY_DN74020_c0_g1_i2_orf1;TRINITY_DN65681_c0_g1_i1_orf1;TRINITY_DN3835_c0_g1_i4_orf1;TRINITY_DN54586_c1_g1_i1_orf1;TRINITY_DN1497_c0_g2_i6_orf1;TRINITY_DN33452_c0_g1_i3_orf1;TRINITY_DN31584_c0_g2_i2_orf1;TRINITY_DN12432_c0_g1_i2_orf1;TRINITY_DN14677_c0_g2_i3_orf1;TRINITY_DN55148_c0_g1_i1_orf1;TRINITY_DN47389_c0_g1_i2_orf1;TRINITY_DN16316_c0_g1_i7_orf1;TRINITY_DN7590_c0_g1_i4_orf1;TRINITY_DN38835_c0_g2_i1_orf1;TRINITY_DN29934_c0_g1_i6_orf1;TRINITY_DN3299_c0_g1_i2_orf1;TRINITY_DN136031_c0_g1_i7_orf1;TRINITY_DN6680_c0_g1_i1_orf1;TRINITY_DN146236_c0_g1_i1_orf1;TRINITY_DN65299_c0_g4_i1_orf1;TRINITY_DN3209_c0_g2_i6_orf1;TRINITY_DN33452_c0_g1_i1_orf1;TRINITY_DN5630_c4_g1_i2_orf1;TRINITY_DN12777_c0_g1_i5_orf1;TRINITY_DN4814_c0_g1_i6_orf1;TRINITY_DN2675_c0_g1_i1_orf1;TRINITY_DN1245_c0_g1_i4_orf1;TRINITY_DN4207_c0_g1_i1_orf1
biological_process	response to external biotic stimulus	GO:0043207	18	18/1043	TRINITY_DN14904_c0_g1_i1_orf1;TRINITY_DN1534_c0_g1_i3_orf1;TRINITY_DN827_c1_g1_i1_orf1;TRINITY_DN8685_c0_g1_i5_orf1;TRINITY_DN16840_c1_g1_i1_orf1;TRINITY_DN195_c8_g1_i1_orf1;TRINITY_DN3166_c1_g1_i6_orf1;TRINITY_DN195_c4_g1_i1_orf1;TRINITY_DN1091_c0_g2_i10_orf1;TRINITY_DN109503_c0_g1_i4_orf1;TRINITY_DN59429_c0_g1_i6_orf1;TRINITY_DN2407_c0_g1_i2_orf1;TRINITY_DN5880_c0_g2_i2_orf1;TRINITY_DN1666_c0_g1_i2_orf1;TRINITY_DN9044_c0_g1_i2_orf1;TRINITY_DN29190_c0_g1_i4_orf1;TRINITY_DN4802_c0_g1_i4_orf1;TRINITY_DN66287_c0_g1_i1_orfp1
biological_process	detection of biotic stimulus	GO:0009595	3	3/1043	TRINITY_DN1091_c0_g2_i10_orf1;TRINITY_DN8685_c0_g1_i5_orf1;TRINITY_DN5880_c0_g2_i2_orf1
biological_process	response to extracellular stimulus	GO:0009991	5	5/1043	TRINITY_DN140212_c0_g1_i1_orf1;TRINITY_DN51938_c0_g3_i1_orf1;TRINITY_DN1091_c0_g3_i1_orf1;TRINITY_DN1091_c0_g1_i1_orf1;TRINITY_DN2054_c0_g1_i1_orf1
biological_process	cellular response to external stimulus	GO:0071496	5	5/1043	TRINITY_DN140212_c0_g1_i1_orf1;TRINITY_DN1091_c0_g1_i1_orf1;TRINITY_DN1091_c0_g3_i1_orf1;TRINITY_DN51938_c0_g3_i1_orf1;TRINITY_DN2054_c0_g1_i1_orf1
biological_process	cellular response to endogenous stimulus	GO:0071495	2	2/1043	TRINITY_DN51938_c0_g3_i1_orf1;TRINITY_DN975_c0_g1_i1_orf1
biological_process	response to epidermal growth factor	GO:0070849	1	1/1043	TRINITY_DN975_c0_g1_i1_orf1
biological_process	response to transforming growth factor beta	GO:0071559	1	1/1043	TRINITY_DN51938_c0_g3_i1_orf1
biological_process	response to hormone	GO:0009725	1	1/1043	TRINITY_DN51938_c0_g3_i1_orf1
biological_process	response to hypoxia	GO:0001666	1	1/1043	TRINITY_DN51938_c0_g3_i1_orf1
biological_process	response to ischemia	GO:0002931	1	1/1043	TRINITY_DN51938_c0_g3_i1_orf1
biological_process	response to heat	GO:0009408	1	1/1043	TRINITY_DN31584_c0_g2_i2_orf1
biological_process	defense response	GO:0006952	17	17/1043	TRINITY_DN14904_c0_g1_i1_orf1;TRINITY_DN1534_c0_g1_i3_orf1;TRINITY_DN827_c1_g1_i1_orf1;TRINITY_DN8685_c0_g1_i5_orf1;TRINITY_DN16840_c1_g1_i1_orf1;TRINITY_DN195_c8_g1_i1_orf1;TRINITY_DN3166_c1_g1_i6_orf1;TRINITY_DN195_c4_g1_i1_orf1;TRINITY_DN1091_c0_g2_i10_orf1;TRINITY_DN59429_c0_g1_i6_orf1;TRINITY_DN2407_c0_g1_i2_orf1;TRINITY_DN5880_c0_g2_i2_orf1;TRINITY_DN1666_c0_g1_i2_orf1;TRINITY_DN9044_c0_g1_i2_orf1;TRINITY_DN29190_c0_g1_i4_orf1;TRINITY_DN4802_c0_g1_i4_orf1;TRINITY_DN66287_c0_g1_i1_orfp1
biological_process	response to hyperoxia	GO:0055093	1	1/1043	TRINITY_DN51938_c0_g3_i1_orf1
biological_process	response to oxidative stress	GO:0006979	4	4/1043	TRINITY_DN54387_c0_g1_i1_orf1;TRINITY_DN285_c0_g1_i4_orf1;TRINITY_DN87603_c0_g2_i1_orf1;TRINITY_DN6580_c0_g1_i4_orf1
biological_process	response to oxygen-containing compound	GO:1901700	2	2/1043	TRINITY_DN51938_c0_g3_i1_orf1;TRINITY_DN87603_c0_g2_i1_orf1
biological_process	response to nitrogen compound	GO:1901698	1	1/1043	TRINITY_DN51658_c0_g1_i1_orf1
biological_process	response to nutrient	GO:0007584	1	1/1043	TRINITY_DN51938_c0_g3_i1_orf1
biological_process	response to inorganic substance	GO:0010035	1	1/1043	TRINITY_DN87603_c0_g2_i1_orf1
biological_process	response to organic substance	GO:0010033	7	7/1043	TRINITY_DN51938_c0_g3_i1_orf1;TRINITY_DN8685_c0_g1_i5_orf1;TRINITY_DN18218_c0_g1_i7_orf1;TRINITY_DN975_c0_g1_i1_orf1;TRINITY_DN1091_c0_g2_i10_orf1;TRINITY_DN51658_c0_g1_i1_orf1;TRINITY_DN5880_c0_g2_i2_orf1
biological_process	response to temperature stimulus	GO:0009266	1	1/1043	TRINITY_DN31584_c0_g2_i2_orf1
biological_process	response to oxygen levels	GO:0070482	1	1/1043	TRINITY_DN51938_c0_g3_i1_orf1
biological_process	detection of chemical stimulus	GO:0009593	3	3/1043	TRINITY_DN1091_c0_g2_i10_orf1;TRINITY_DN8685_c0_g1_i5_orf1;TRINITY_DN5880_c0_g2_i2_orf1
cellular_component	nucleosome	GO:0000786	1	1/1043	TRINITY_DN20442_c0_g2_i1_orf1
cellular_component	U2AF complex	GO:0089701	1	1/1043	TRINITY_DN51968_c0_g1_i1_orf1
cellular_component	spliceosomal complex	GO:0005681	4	4/1043	TRINITY_DN33346_c0_g1_i1_orf1;TRINITY_DN23502_c0_g1_i1_orf1;TRINITY_DN8717_c0_g1_i5_orf1;TRINITY_DN43412_c0_g1_i2_orf1
cellular_component	BRISC complex	GO:0070552	1	1/1043	TRINITY_DN17655_c0_g1_i1_orf1
cellular_component	nuclear DNA-directed RNA polymerase comple:	GO:0055029	1	1/1043	TRINITY_DN9207_c0_g1_i1_orf1
cellular_component	exon-exon junction complex	GO:0035145	1	1/1043	TRINITY_DN5507_c0_g1_i1_orf1
cellular_component	small nuclear ribonucleoprotein complex	GO:0030532	2	2/1043	TRINITY_DN33346_c0_g1_i1_orf1;TRINITY_DN43412_c0_g1_i2_orf1
cellular_component	BRCA1-A complex	GO:0070531	1	1/1043	TRINITY_DN17655_c0_g1_i1_orf1
cellular_component	nuclear pore outer ring	GO:0031080	1	1/1043	TRINITY_DN6680_c0_g1_i1_orf1
cellular_component	transmembrane transporter complex	GO:1902495	2	2/1043	TRINITY_DN29934_c0_g1_i6_orf1;TRINITY_DN20558_c0_g1_i2_orf1
cellular_component	cytochrome complex	GO:0070069	1	1/1043	TRINITY_DN76036_c0_g1_i1_orf1
cellular_component	oxidoreductase complex	GO:1990204	1	1/1043	TRINITY_DN6199_c2_g1_i3_orf1
cellular_component	transferase complex	GO:1990234	4	4/1043	TRINITY_DN110534_c0_g1_i3_orf1;TRINITY_DN51658_c0_g1_i1_orf1;TRINITY_DN9207_c0_g1_i1_orf1;TRINITY_DN2299_c0_g1_i3_orf1

cellular_component	peptidase complex	GO:1905368	6	6/1043	TRINITY_DN19260_c0_g1_i5_orf1;TRINITY_DN34479_c0_g1_i2_orf1;TRINITY_DN6684_c0_g1_i4_orf1;TRINITY_DN49047_c0_g1_i2_orf1;TRINITY_DN32359_c0_g2_i1_orf1;TRINITY_DN2058_c0_g1_i2_orf1
cellular_component	aminoacyl-tRNA synthetase multienzyme compl	GO:0017101	3	3/1043	TRINITY_DN825_c23_g1_i5_orf1;TRINITY_DN2953_c1_g1_i10_orf1;TRINITY_DN107288_c0_g1_i2_orf1
cellular_component	plasma membrane protein complex	GO:0098797	2	2/1043	TRINITY_DN12777_c0_g1_i5_orf1;TRINITY_DN29934_c0_g1_i6_orf1
cellular_component	outer mitochondrial membrane protein complex	GO:0098799	1	1/1043	TRINITY_DN3299_c0_g1_i2_orf1
cellular_component	Tapasin-ERp57 complex	GO:0061779	1	1/1043	TRINITY_DN51938_c0_g3_i1_orf1
cellular_component	ER ubiquitin ligase complex	GO:0000835	1	1/1043	TRINITY_DN51658_c0_g1_i1_orf1
cellular_component	MHC class I peptide loading complex	GO:0042824	1	1/1043	TRINITY_DN51938_c0_g3_i1_orf1
cellular_component	TAP complex	GO:0042825	1	1/1043	TRINITY_DN51938_c0_g3_i1_orf1
cellular_component	respiratory chain complex	GO:0098803	1	1/1043	TRINITY_DN76036_c0_g1_i1_orf1
cellular_component	inner mitochondrial membrane protein complex	GO:0098800	3	3/1043	TRINITY_DN76036_c0_g1_i1_orf1;TRINITY_DN15222_c0_g1_i4_orf1;TRINITY_DN4207_c0_g1_i1_orf1
cellular_component	membrane coat	GO:0030117	3	3/1043	TRINITY_DN14677_c0_g2_i3_orf1;TRINITY_DN12777_c0_g1_i5_orf1;TRINITY_DN3209_c0_g2_i6_orf1
cellular_component	proton-transporting two-sector ATPase comple	GO:0033177	1	1/1043	TRINITY_DN15222_c0_g1_i4_orf1
cellular_component	exocyst	GO:0000145	1	1/1043	TRINITY_DN16316_c0_g1_i7_orf1
cellular_component	translation initiation complex	GO:0070992	1	1/1043	TRINITY_DN142442_c0_g1_i1_orf1
cellular_component	ribosomal subunit	GO:0044391	6	6/1043	TRINITY_DN87603_c0_g2_i1_orf1;TRINITY_DN36893_c0_g1_i1_orf1;TRINITY_DN142442_c0_g1_i1_orf1;TRINITY_DN137_c0_g1_i1_orf1;TRINITY_DN55148_c0_g1_i1_orf1;TRINITY_DN82324_c0_g1_i4_orf1
cellular_component	mRNA cap binding complex	GO:0005845	1	1/1043	TRINITY_DN41664_c0_g1_i4_orf1
cellular_component	proteasome complex	GO:0000502	6	6/1043	TRINITY_DN19260_c0_g1_i5_orf1;TRINITY_DN34479_c0_g1_i2_orf1;TRINITY_DN6684_c0_g1_i4_orf1;TRINITY_DN49047_c0_g1_i2_orf1;TRINITY_DN32359_c0_g2_i1_orf1;TRINITY_DN2058_c0_g1_i2_orf1
cellular_component	DNA polymerase complex	GO:0042575	1	1/1043	TRINITY_DN110534_c0_g1_i3_orf1
cellular_component	ubiquitin ligase complex	GO:0000151	1	1/1043	TRINITY_DN51658_c0_g1_i1_orf1
cellular_component	CCR4-NOT complex	GO:0030014	1	1/1043	TRINITY_DN88539_c0_g2_i1_orf1
cellular_component	RNA polymerase complex	GO:0030880	2	2/1043	TRINITY_DN9207_c0_g1_i1_orf1;TRINITY_DN2299_c0_g1_i3_orf1
cellular_component	CIA complex	GO:0097361	1	1/1043	TRINITY_DN49872_c0_g1_i2_orf1
cellular_component	organelle lumen	GO:0043233	5	5/1043	TRINITY_DN9156_c0_g1_i1_orf1;TRINITY_DN51938_c0_g3_i1_orf1;TRINITY_DN49265_c0_g3_i2_orf1;TRINITY_DN975_c0_g1_i1_orf1;TRINITY_DN2299_c0_g1_i3_orf1
cellular_component	cell cortex	GO:0005938	1	1/1043	TRINITY_DN2186_c0_g1_i17_orf1
cellular_component	extrinsic component of organelle membrane	GO:0031312	1	1/1043	TRINITY_DN6638_c0_g1_i1_orf1
cellular_component	heterochromatin	GO:0000792	1	1/1043	TRINITY_DN24266_c0_g2_i2_orf1
cellular_component	anchored component of membrane	GO:0031225	3	3/1043	TRINITY_DN2175_c0_g1_i4_orf1;TRINITY_DN5406_c0_g2_i1_orf1;TRINITY_DN56690_c0_g1_i4_orf1
cellular_component	integral component of membrane	GO:0016021	129	129/1043	TRINITY_DN9608_c0_g1_i3_orf1;TRINITY_DN3949_c1_g1_i1_orf1;TRINITY_DN827_c1_g1_i1_orf1;TRINITY_DN12227_c0_g2_i3_orf1;TRINITY_DN3194_c0_g1_i6_orf1;TRINITY_DN2967_c0_g1_i7_orf1;TRINITY_DN14937_c0_g1_i7_orf1;TRINITY_DN43369_c0_g2_i1_orf1;TRINITY_DN33705_c0_g1_i1_orf1;TRINITY_DN8030_c0_g1_i2_orf1;TRINITY_DN29633_c0_g1_i8_orf1;TRINITY_DN130439_c0_g1_i1_orf1;TRINITY_DN3105_c0_g1_i4_orf1;TRINITY_DN16122_c0_g1_i4_orf1;TRINITY_DN7590_c0_g1_i4_orf1;TRINITY_DN3196_c0_g1_i1_orf1;TRINITY_DN48020_c0_g1_i1_orf1;TRINITY_DN146364_c0_g1_i1_orf1;TRINITY_DN41708_c0_g1_i1_orf1;TRINITY_DN33995_c0_g1_i5_orf1;TRINITY_DN448_c0_g1_i20_orf1;TRINITY_DN20558_c0_g1_i2_orf1;TRINITY_DN5337_c0_g1_i6_orf1;TRINITY_DN6992_c0_g1_i6_orf1;TRINITY_DN17505_c0_g1_i15_orf1;TRINITY_DN3355_c0_g2_i4_orf1;TRINITY_DN919_c0_g1_i7_orf1;TRINITY_DN29879_c0_g1_i3_orf1;TRINITY_DN4814_c0_g1_i6_orf1;TRINITY_DN5852_c0_g1_i6_orf1;TRINITY_DN6381_c0_g1_i2_orf1;TRINITY_DN49786_c0_g1_i1_orf1;TRINITY_DN25251_c0_g2_i1_orf1;TRINITY_DN96170_c0_g2_i1_orf1;TRINITY_DN9079_c1_g1_i1_orf1;TRINITY_DN8136_c0_g1_i1_orf1;TRINITY_DN957_c0_g1_i18_orf1;TRINITY_DN5174_c0_g3_i1_orf1;TRINITY_DN1789_c0_g1_i5_orf1;TRINITY_DN483_c0_g1_i6_orf1;TRINITY_DN48602_c0_g1_i6_orf1;TRINITY_DN172_c8_g2_i1_orf1;TRINITY_DN5991_c0_g1_i6_orf1;TRINITY_DN810_c0_g1_i4_orf1;TRINITY_DN9207_c0_g1_i1_orf1;TRINITY_DN66302_c0_g1_i1_orf1;TRINITY_DN15607_c0_g1_i6_orf1;TRINITY_DN3469_c0_g1_i4_orf1;TRINITY_DN34821_c0_g1_i4_orf1;TRINITY_DN1999_c0_g1_i9_orf1;TRINITY_DN4064_c0_g2_i1_orf1;TRINITY_DN1720_c0_g1_i3_orf1;TRINITY_DN5408_c0_g1_i5_orf1;TRINITY_DN62707_c0_g1_i1_orf1;TRINITY_DN2826_c0_g1_i7_orf1;TRINITY_DN140_c1_g1_i2_orf1;TRINITY_DN7861_c0_g1_i5_orf1;TRINITY_DN3209_c0_g2_i6_orf1;TRINITY_DN4321_c0_g1_i1_orf1;TRINITY_DN22_c0_g1_i3_orf1;TRINITY_DN23978_c0_g1_i2_orf1;TRINITY_DN2675_c0_g1_i1_orf1;TRINITY_DN51658_c0_g1_i1_orf1;TRINITY_DN11069_c0_g1_i6_orf1;TRINITY_DN120500_c0_g1_i1_orf1;TRINITY_DN7570_c0_g1_i18_orf1;TRINITY_DN38835_c0_g2_i1_orf1;TRINITY_DN4497_c0_g1_i4_orf1;TRINITY_DN2343_c1_g1_i12_orf1;TRINITY_DN14262_c0_g1_i5_orf1;TRINITY_DN35757_c0_g1_i1_orf1;TRINITY_DN31851_c0_g1_i2_orf1;TRINITY_DN928_c0_g1_i3_orf1;TRINITY_DN27903_c0_g1_i1_orf1;TRINITY_DN23398_c0_g1_i1_orf1;TRINITY_DN27500_c0_g1_i4_orf1;TRINITY_DN35277_c0_g1_i1_orf1;TRINITY_DN11566_c0_g1_i6_orf1;TRINITY_DN14679_c0_g1_i1_orf1;TRINITY_DN10484_c0_g1_i8_orf1;TRINITY_DN15597_c0_g1_i1_orf1;TRINITY_DN19303_c0_g1_i5_orf1;TRINITY_DN20356_c0_g1_i5_orf1;TRINITY_DN23783_c0_g2_i1_orf1;TRINITY_DN4497_c2_g1_i3_orf1;TRINITY_DN14611_c0_g1_i5_orf1;TRINITY_DN23229_c0_g1_i2_orf1;TRINITY_DN1617_c0_g1_i5_orf1;TRINITY_DN3616_c0_g1_i4_orf1;TRINITY_DN6612_c0_g1_i4_orf1;TRINITY_DN5630_c4_g1_i2_orf1;TRINITY_DN104297_c0_g1_i1_orf1;TRINITY_DN1293_c1_g1_i4_orf1;TRINITY_DN18136_c0_g1_i1_orf1;TRINITY_DN4207_c0_g1_i1_orf1;TRINITY_DN2356_c2_g1_i6_orf1;TRINITY_DN2880_c0_g1_i2_orf1;TRINITY_DN8083_c0_g1_i1_orf1;TRINITY_DN3835_c0_g1_i4_orf1;TRINITY_DN54586_c1_g1_i1_orf1;TRINITY_DN38225_c0_g2_i1_orf1;TRINITY_DN230_c1_g1_i3_orf1;TRINITY_DN2392_c0_g2_i1_orf1;TRINITY_DN14874_c0_g1_i6_orf1;TRINITY_DN6140_c0_g3_i3_orf1;TRINITY_DN44110_c0_g1_i4_orf1;TRINITY_DN17394_c0_g1_i1_orf1;TRINITY_DN1672_c0_g1_i6_orf1;TRINITY_DN15157_c0_g1_i1_orf1;TRINITY_DN1362_c0_g1_i4_orf1;TRINITY_DN11172_c0_g1_i4_orf1;TRINITY_DN1073_c0_g1_i3_orf1;TRINITY_DN76036_c0_g1_i1_orf1;TRINITY_DN5191_c0_g2_i1_orf1;TRINITY_DN1292_c0_g1_i3_orf1;TRINITY_DN1960_c5_g1_i3_orf1;TRINITY_DN4443_c0_g1_i4_orf1;TRINITY_DN69049_c0_g2_i1_orf1;TRINITY_DN26337_c0_g1_i3_orf1;TRINITY_DN30509_c0_g1_i9_orf1;TRINITY_DN2967_c0_g1_i4_orf1;TRINITY_DN31390_c0_g1_i2_orf1;TRINITY_DN5153_c1_g1_i1_orf1;TRINITY_DN2618_c0_g1_i3_orf1;TRINITY_DN325_c0_g1_i15_orf1;TRINITY_DN27745_c0_g1_i6_orf1;TRINITY_DN585_c0_g1_i5_orf1;TRINITY_DN2175_c0_g1_i4_orf1;TRINITY_DN
cellular_component	intrinsic component of organelle membrane	GO:0031300	1	1/1043	TRINITY_DN483_c0_g1_i6_orf1
cellular_component	cytoplasmic side of membrane	GO:0098562	1	1/1043	TRINITY_DN975_c0_g1_i1_orf1
cellular_component	intracellular organelle	GO:0043229	70	70/1043	TRINITY_DN30673_c0_g1_i5_orf1;TRINITY_DN6199_c2_g1_i3_orf1;TRINITY_DN129_c0_g1_i6_orf1;TRINITY_DN12526_c0_g1_i5_orf1;TRINITY_DN34479_c0_g1_i2_orf1;TRINITY_DN1497_c0_g2_i6_orf1;TRINITY_DN2058_c0_g1_i2_orf1;TRINITY_DN5497_c0_g1_i6_orf1;TRINITY_DN5925_c0_g1_i5_orf1;TRINITY_DN137_c0_g1_i1_orf1;TRINITY_DN13760_c1_g1_i1_orf1;TRINITY_DN102260_c0_g1_i1_orf1;TRINITY_DN89083_c0_g1_i1_orf1;TRINITY_DN6556_c0_g1_i7_orf1;TRINITY_DN17312_c0_g1_i1_orf1;TRINITY_DN9383_c0_g1_i3_orf1;TRINITY_DN2719_c1_g1_i6_orf1;TRINITY_DN21619_c0_g1_i1_orf1;TRINITY_DN40176_c0_g1_i1_orf1;TRINITY_DN1865_c1_g1_i3_orf1;TRINITY_DN31851_c0_g1_i2_orf1;TRINITY_DN24322_c0_g1_i4_orf1;TRINITY_DN9871_c0_g1_i11_orf1;TRINITY_DN3335_c0_g1_i1_orf1;TRINITY_DN50725_c0_g1_i6_orf1;TRINITY_DN60821_c0_g1_i1_orf1;TRINITY_DN7647_c0_g1_i4_orf1;TRINITY_DN3588_c0_g1_i4_orf1;TRINITY_DN975_c0_g1_i1_orf1;TRINITY_DN8603_c0_g1_i1_orf1;TRINITY_DN55148_c0_g1_i1_orf1;TRINITY_DN2559_c0_g1_i4_orf1;TRINITY_DN37418_c0_g1_i4_orf1;TRINITY_DN44633_c0_g1_i4_orf1;TRINITY_DN51938_c0_g3_i1_orf1;TRINITY_DN364_c1_g1_i2_orf1;TRINITY_DN4056_c0_g1_i8_orf1;TRINITY_DN3614_c0_g2_i1_orf1;TRINITY_DN9510_c0_g2_i1_orf1;TRINITY_DN7022_c0_g1_i7_orf1;TRINITY_DN24266_c0_g2_i2_orf1;TRINITY_DN74889_c0_g1_i1_orf1;TRINITY_DN166_c0_g1_i4_orf1;TRINITY_DN49936_c0_g2_i1_orf1;TRINITY_DN142442_c0_g1_i1_orf1;TRINITY_DN4938_c0_g1_i13_orf1;TRINITY_DN84478_c0_g1_i8_orf1;TRINITY_DN44288_c0_g1_i2_orf1;TRINITY_DN40911_c0_g1_i1_orf1;TRINITY_DN22272_c0_g1_i1_orf1;TRINITY_DN5664_c0_g1_i1_orf1;TRINITY_DN4929_c0_g1_i1_orf1;TRINITY_DN11263_c0_g1_i5_orf1;TRINITY_DN65299_c0_g4_i1_orf1;TRINITY_DN3618_c0_g1_i4_orf1;TRINITY_DN110231_c0_g1_i1_orf1;TRINITY_DN87603_c0_g2_i1_orf1;TRINITY_DN2265_c0_g1_i5_orf1;TRINITY_DN41179_c0_g1_i1_orf1;TRINITY_DN2647_c0_g1_i3_orf1;TRINITY_DN2802_c1_g1_i1_orf1;TRINITY_DN2186_c0_g1_i17_orf1;TRINITY_DN3292_c2_g1_i4_orf1;TRINITY_DN5962_c0_g1_i1_orf1;TRINITY_DN3651_c0_g1_i5_orf1;TRINITY_DN47389_c0_g1_i2_orf1;TRINITY_DN3860_c0_g1_i5_orf1;TRINITY_DN25997_c1_g2_i4_orf1;TRINITY_DN7241_c0_g2_i2_orf1;TRINITY_DN31584_c0_g2_i2_orf1
cellular_component	non-membrane-bounded organelle	GO:0043228	20	20/1043	TRINITY_DN3618_c0_g1_i4_orf1;TRINITY_DN110231_c0_g1_i1_orf1;TRINITY_DN4056_c0_g1_i8_orf1;TRINITY_DN87603_c0_g2_i1_orf1;TRINITY_DN50725_c0_g1_i6_orf1;TRINITY_DN60821_c0_g1_i1_orf1;TRINITY_DN74889_c0_g1_i1_orf1;TRINITY_DN22272_c0_g1_i1_orf1;TRINITY_DN49936_c0_g2_i1_orf1;TRINITY_DN975_c0_g1_i1_orf1;TRINITY_DN142442_c0_g1_i1_orf1;TRINITY_DN137_c0_g1_i1_orf1;TRINITY_DN21619_c0_g1_i1_orf1;TRINITY_DN24322_c0_g1_i4_orf1;TRINITY_DN2186_c0_g1_i17_orf1;TRINITY_DN9383_c0_g1_i3_orf1;TRINITY_DN364_c1_g1_i2_orf1;TRINITY_DN102260_c0_g1_i1_orf1;TRINITY_DN7241_c0_g2_i2_orf1;TRINITY_DN55148_c0_g1_i1_orf1
cellular_component	membrane-bounded organelle	GO:0043227	53	53/1043	TRINITY_DN30673_c0_g1_i5_orf1;TRINITY_DN6199_c2_g1_i3_orf1;TRINITY_DN129_c0_g1_i6_orf1;TRINITY_DN12526_c0_g1_i5_orf1;TRINITY_DN34479_c0_g1_i2_orf1;TRINITY_DN1497_c0_g2_i6_orf1;TRINITY_DN2058_c0_g1_i2_orf1;TRINITY_DN5497_c0_g1_i6_orf1;TRINITY_DN5925_c0_g1_i5_orf1;TRINITY_DN13760_c1_g1_i1_orf1;TRINITY_DN89083_c0_g1_i1_orf1;TRINITY_DN6556_c0_g1_i7_orf1;TRINITY_DN17312_c0_g1_i1_orf1;TRINITY_DN31584_c0_g2_i2_orf1;TRINITY_DN2719_c1_g1_i6_orf1;TRINITY_DN40176_c0_g1_i1_orf1;TRINITY_DN1865_c1_g1_i3_orf1;TRINITY_DN55148_c0_g1_i1_orf1;TRINITY_DN7022_c0_g1_i7_orf1;TRINITY_DN9871_c0_g1_i11_orf1;TRINITY_DN3335_c0_g1_i1_orf1;TRINITY_DN7647_c0_g1_i4_orf1;TRINITY_DN3588_c0_g1_i4_orf1;TRINITY_DN975_c0_g1_i1_orf1;TRINITY_DN8603_c0_g1_i1_orf1;TRINITY_DN31851_c0_g1_i2_orf1;TRINITY_DN2559_c0_g1_i4_orf1;TRINITY_DN37418_c0_g1_i4_orf1;TRINITY_DN44633_c0_g1_i4_orf1;TRINITY_DN51938_c0_g3_i1_orf1;TRINITY_DN3614_c0_g2_i1_orf1;TRINITY_DN9510_c0_g2_i1_orf1;TRINITY_DN24266_c0_g2_i2_orf1;TRINITY_DN74889_c0_g1_i1_orf1;TRINITY_DN166_c0_g1_i4_orf1;TRINITY_DN4938_c0_g1_i13_orf1;TRINITY_DN84478_c0_g1_i8_orf1;TRINITY_DN44288_c0_g1_i2_orf1;TRINITY_DN40911_c0_g1_i1_orf1;TRINITY_DN22272_c0_g1_i1_orf1;TRINITY_DN5664_c0_g1_i1_orf1;TRINITY_DN4929_c0_g1_i1_orf1;TRINITY_DN65299_c0_g4_i1_orf1;TRINITY_DN2265_c0_g1_i5_orf1;TRINITY_DN41179_c0_g1_i1_orf1;TRINITY_DN2647_c0_g1_i3_orf1;TRINITY_DN2802_c1_g1_i1_orf1;TRINITY_DN3292_c2_g1_i4_orf1;TRINITY_DN5962_c0_g1_i1_orf1;TRINITY_DN3651_c0_g1_i5_orf1;TRINITY_DN47389_c0_g1_i2_orf1;TRINITY_DN3860_c0_g1_i5_orf1;TRINITY_DN25997_c1_g2_i4_orf1
cellular_component	postsynaptic specialization	GO:0099572	1	1/1043	TRINITY_DN142442_c0_g1_i1_orf1
cellular_component	extracellular organelle	GO:0043230	2	2/1043	TRINITY_DN975_c0_g1_i1_orf1;TRINITY_DN55148_c0_g1_i1_orf1
cellular_component	organelle membrane	GO:0031090	25	25/1043	TRINITY_DN9608_c0_g1_i3_orf1;TRINITY_DN14262_c0_g1_i5_orf1;TRINITY_DN56164_c0_g1_i1_orf1;TRINITY_DN31851_c0_g1_i2_orf1;TRINITY_DN35635_c0_g1_i1_orf1;TRINITY_DN57765_c0_g1_i1_orf1;TRINITY_DN37418_c0_g1_i4_orf1;TRINITY_DN1362_c0_g1_i4_orf1;TRINITY_DN5432_c1_g1_i3_orf1;TRINITY_DN11172_c0_g1_i4_orf1;TRINITY_DN448_c0_g1_i20_orf1;TRINITY_DN19303_c0_g1_i5_orf1;TRINITY_DN2264_c0_g1_i1_orf1;TRINITY_DN1960_c5_g1_i3_orf1;TRINITY_DN1789_c0_g1_i5_orf1;TRINITY_DN5439_c0_g1_i2_orf1;TRINITY_DN40911_c0_g1_i1_orf1;TRINITY_DN52887_c0_g1_i1_orf1;TRINITY_DN4443_c0_g1_i4_orf1;TRINITY_DN3209_c0_g2_i6_orf1;TRINITY_DN87603_c0_g2_i1_orf1;TRINITY_DN6680_c0_g1_i1_orf1;TRINITY_DN5630_c4_g1_i2_orf1;TRINITY_DN4814_c0_g1_i6_orf1;TRINITY_DN5697_c0_g1_i1_orf1
cellular_component	outer membrane	GO:0019867	1	1/1043	TRINITY_DN142657_c0_g1_i1_orf1
cellular_component	plasma membrane	GO:0005886	9	9/1043	TRINITY_DN3469_c0_g1_i4_orf1;TRINITY_DN5406_c0_g2_i1_orf1;TRINITY_DN7570_c0_g1_i18_orf1;TRINITY_DN51938_c0_g3_i1_orf1;TRINITY_DN975_c0_g1_i1_orf1;TRINITY_DN31584_c0_g2_i2_orf1;TRINITY_DN2175_c0_g1_i4_orf1;TRINITY_DN7590_c0_g1_i4_orf1;TRINITY_DN26337_c0_g1_i3_orf1
cellular_component	plasma membrane region	GO:0098590	2	2/1043	TRINITY_DN51938_c0_g3_i1_orf1;TRINITY_DN975_c0_g1_i1_orf1
cellular_component	extracellular matrix	GO:0031012	2	2/1043	TRINITY_DN35147_c0_g1_i1_orf1;TRINITY_DN2919_c0_g1_i5_orf1

cellular_component	synapse	GO:0045202	2	2/1043	TRINITY_DN82017_c0_g1_i5_orf1;TRINITY_DN142442_c0_g1_i1_orf1
cellular_component	anchoring junction	GO:0070161	5	5/1043	TRINITY_DN2186_c0_g1_i17_orf1;TRINITY_DN7590_c0_g1_i4_orf1;TRINITY_DN142442_c0_g1_i1_orf1;TRINITY_DN9383_c0_g1_i3_orf1;TRINITY_DN364_c1_g1_i2_orf1
cellular_component	Golgi apparatus subcompartment	GO:0098791	1	1/1043	TRINITY_DN4401_c0_g2_i1_orf1
cellular_component	ruffle membrane	GO:0032587	1	1/1043	TRINITY_DN975_c0_g1_i1_orf1
cellular_component	plasma membrane bounded cell projection	GO:0120025	2	2/1043	TRINITY_DN9383_c0_g1_i3_orf1;TRINITY_DN364_c1_g1_i2_orf1
cellular_component	ribonucleoprotein granule	GO:0035770	1	1/1043	TRINITY_DN50725_c0_g1_i6_orf1
cellular_component	supramolecular polymer	GO:0099081	2	2/1043	TRINITY_DN350_c0_g1_i5_orf1;TRINITY_DN2745_c0_g1_i2_orf1
molecular_function	translation factor activity, RNA binding	GO:0008135	9	9/1043	TRINITY_DN10871_c0_g2_i1_orf1;TRINITY_DN5086_c0_g1_i1_orf1;TRINITY_DN2265_c0_g1_i5_orf1;TRINITY_DN15420_c0_g3_i2_orf1;TRINITY_DN975_c0_g1_i1_orf1;TRINITY_DN28039_c0_g1_i1_orf1;TRINITY_DN11612_c0_g2_i1_orf1;TRINITY_DN19659_c1_g1_i1_orf1;TRINITY_DN21000_c0_g1_i1_orf1
molecular_function	transcription coactivator activity	GO:0003713	1	1/1043	TRINITY_DN9510_c0_g2_i1_orf1
molecular_function	RNA helicase activity	GO:0003724	1	1/1043	TRINITY_DN44288_c0_g1_i2_orf1
molecular_function	ABC-type transporter activity	GO:0140359	2	2/1043	TRINITY_DN14937_c0_g1_i7_orf1;TRINITY_DN2826_c0_g1_i7_orf1
molecular_function	P-type transmembrane transporter activity	GO:0140358	1	1/1043	TRINITY_DN7570_c0_g1_i18_orf1
molecular_function	ATPase-coupled cation transmembrane transpo	GO:0019829	1	1/1043	TRINITY_DN7570_c0_g1_i18_orf1
molecular_function	very-low-density lipoprotein particle receptor a	GO:0030229	1	1/1043	TRINITY_DN585_c0_g1_i5_orf1
molecular_function	peroxiredoxin activity	GO:0051920	1	1/1043	TRINITY_DN2542_c0_g2_i1_orf1
molecular_function	catalase activity	GO:0004096	3	3/1043	TRINITY_DN54387_c0_g1_i1_orf1;TRINITY_DN285_c0_g1_i4_orf1;TRINITY_DN6580_c0_g1_i4_orf1
molecular_function	lipid transfer activity	GO:0120013	1	1/1043	TRINITY_DN64196_c0_g1_i2_orf1
molecular_function	passive transmembrane transporter activity	GO:0022803	4	4/1043	TRINITY_DN11566_c0_g1_i6_orf1;TRINITY_DN957_c0_g1_i18_orf1;TRINITY_DN34821_c0_g1_i4_orf1;TRINITY_DN20558_c0_g1_i2_orf1
molecular_function	active transmembrane transporter activity	GO:0022804	5	5/1043	TRINITY_DN76036_c0_g1_i1_orf1;TRINITY_DN7570_c0_g1_i18_orf1;TRINITY_DN14937_c0_g1_i7_orf1;TRINITY_DN6381_c0_g1_i2_orf1;TRINITY_DN2826_c0_g1_i7_orf1
molecular_function	ion transmembrane transporter activity	GO:0015075	7	7/1043	TRINITY_DN15222_c0_g1_i4_orf1;TRINITY_DN34821_c0_g1_i4_orf1;TRINITY_DN20558_c0_g1_i2_orf1;TRINITY_DN76036_c0_g1_i1_orf1;TRINITY_DN7570_c0_g1_i18_orf1;TRINITY_DN957_c0_g1_i18_orf1;TRINITY_DN6381_c0_g1_i2_orf1
molecular_function	inorganic molecular entity transmembrane trans	GO:0015318	7	7/1043	TRINITY_DN15222_c0_g1_i4_orf1;TRINITY_DN34821_c0_g1_i4_orf1;TRINITY_DN20558_c0_g1_i2_orf1;TRINITY_DN76036_c0_g1_i1_orf1;TRINITY_DN7570_c0_g1_i18_orf1;TRINITY_DN957_c0_g1_i18_orf1;TRINITY_DN6381_c0_g1_i2_orf1
molecular_function	ion channel regulator activity	GO:0099106	1	1/1043	TRINITY_DN31584_c0_g2_i2_orf1
molecular_function	ubiquitin-protein transferase regulator activity	GO:0055106	1	1/1043	TRINITY_DN55148_c0_g1_i1_orf1
molecular_function	kinase regulator activity	GO:0019207	1	1/1043	TRINITY_DN4014_c0_g1_i1_orf1
molecular_function	nucleoside-triphosphatase regulator activity	GO:0060589	4	4/1043	TRINITY_DN27021_c0_g1_i1_orf1;TRINITY_DN104297_c0_g1_i1_orf1;TRINITY_DN804_c0_g1_i7_orf1;TRINITY_DN1173_c1_g1_i10_orf1
molecular_function	peptidase regulator activity	GO:0061134	8	8/1043	TRINITY_DN16234_c0_g2_i3_orf1;TRINITY_DN3609_c0_g1_i6_orf1;TRINITY_DN1215_c0_g1_i2_orf1;TRINITY_DN7539_c0_g1_i2_orf1;TRINITY_DN1540_c0_g1_i9_orf1;TRINITY_DN2097_c1_g2_i2_orf1;TRINITY_DN1540_c0_g1_i14_orf1;TRINITY_DN1540_c0_g1_i7_orf1
molecular_function	enzyme activator activity	GO:0008047	2	2/1043	TRINITY_DN27021_c0_g1_i1_orf1;TRINITY_DN104297_c0_g1_i1_orf1
molecular_function	enzyme inhibitor activity	GO:0004857	9	9/1043	TRINITY_DN16234_c0_g2_i3_orf1;TRINITY_DN3609_c0_g1_i6_orf1;TRINITY_DN1215_c0_g1_i2_orf1;TRINITY_DN7539_c0_g1_i2_orf1;TRINITY_DN1540_c0_g1_i9_orf1;TRINITY_DN2097_c1_g2_i2_orf1;TRINITY_DN1540_c0_g1_i14_orf1;TRINITY_DN55148_c0_g1_i1_orf1;TRINITY_DN1540_c0_g1_i7_orf1
molecular_function	signaling receptor activator activity	GO:0030546	2	2/1043	TRINITY_DN18650_c0_g1_i1_orf1;TRINITY_DN18218_c0_g1_i7_orf1
molecular_function	nucleic acid binding	GO:0003676	67	67/1043	TRINITY_DN1865_c1_g1_i3_orf1;TRINITY_DN51968_c0_g1_i1_orf1;TRINITY_DN23502_c0_g1_i1_orf1;TRINITY_DN1116_c0_g1_i6_orf1;TRINITY_DN512_c1_g1_i4_orf1;TRINITY_DN20442_c0_g2_i1_orf1;TRINITY_DN40434_c0_g1_i2_orf1;TRINITY_DN36893_c0_g1_i1_orf1;TRINITY_DN8008_c0_g1_i6_orf1;TRINITY_DN137_c0_g1_i1_orf1;TRINITY_DN2117_c0_g1_i1_orf1;TRINITY_DN17312_c0_g1_i1_orf1;TRINITY_DN18242_c0_g1_i3_orf1;TRINITY_DN4929_c0_g1_i1_orf1;TRINITY_DN19687_c0_g1_i1_orf1;TRINITY_DN24689_c0_g1_i1_orf1;TRINITY_DN4056_c0_g1_i8_orf1;TRINITY_DN88539_c0_g2_i1_orf1;TRINITY_DN43412_c0_g1_i2_orf1;TRINITY_DN19659_c1_g1_i1_orf1;TRINITY_DN3335_c0_g1_i1_orf1;TRINITY_DN5086_c0_g1_i1_orf1;TRINITY_DN271_c0_g2_i6_orf1;TRINITY_DN975_c0_g1_i1_orf1;TRINITY_DN142442_c0_g1_i1_orf1;TRINITY_DN55148_c0_g1_i1_orf1;TRINITY_DN2749_c0_g1_i4_orf1;TRINITY_DN810_c0_g1_i4_orf1;TRINITY_DN9207_c0_g1_i1_orf1;TRINITY_DN3127_c0_g1_i9_orf1;TRINITY_DN2304_c0_g1_i4_orf1;TRINITY_DN48641_c0_g1_i4_orf1;TRINITY_DN1066_c0_g1_i8_orf1;TRINITY_DN9510_c0_g2_i1_orf1;TRINITY_DN2749_c0_g2_i3_orf1;TRINITY_DN1710_c0_g2_i2_orf1;TRINITY_DN12227_c0_g2_i3_orf1;TRINITY_DN7112_c0_g1_i1_orf1;TRINITY_DN124950_c0_g2_i1_orf1;TRINITY_DN24322_c0_g1_i4_orf1;TRINITY_DN5238_c0_g1_i2_orf1;TRINITY_DN49936_c0_g2_i1_orf1;TRINITY_DN15420_c0_g3_i2_orf1;TRINITY_DN28039_c0_g1_i1_orf1;TRINITY_DN44288_c0_g1_i2_orf1;TRINITY_DN110534_c0_g1_i3_orf1;TRINITY_DN11612_c0_g2_i1_orf1;TRINITY_DN27033_c1_g1_i3_orfp1;TRINITY_DN1860_c0_g1_i2_orf1;TRINITY_DN41664_c0_g1_i4_orf1;TRINITY_DN15160_c0_g1_i1_orf1;TRINITY_DN8717_c0_g1_i5_orf1;TRINITY_DN87603_c0_g2_i1_orf1;TRINITY_DN2265_c0_g1_i5_orf1;TRINITY_DN36496_c0_g1_i1_orf1;TRINITY_DN2802_c1_g1_i1_orf1;TRINITY_DN33346_c0_g1_i1_orf1;TRINITY_DN5210_c0_g1_i3_orf1;TRINITY_DN10871_c0_g2_i1_orf1;TRINITY_DN5962_c0_g1_i1_orf1;TRINITY_DN2299_c0_g1_i3_orf1;TRINITY_DN1245_c0_g1_i4_orf1;TRINITY_DN3712_c0_g1_i1_orf1;TRINITY_DN10558_c0_g1_i4_orf1;TRINITY_DN2647_c0_g1_i3_orf1;TRINITY_DN2749_c4_g1_i2_orf1;TRINITY_DN21000_c0_g1_i1_orf1
molecular_function	nucleoside phosphate binding	GO:1901265	63	63/1043	TRINITY_DN33430_c0_g1_i5_orf1;TRINITY_DN129_c0_g1_i6_orf1;TRINITY_DN115210_c0_g4_i1_orf1;TRINITY_DN6771_c0_g2_i1_orf1;TRINITY_DN5099_c0_g1_i3_orf1;TRINITY_DN4247_c0_g1_i4_orf1;TRINITY_DN96170_c0_g2_i1_orf1;TRINITY_DN20527_c0_g1_i1_orf1;TRINITY_DN14937_c0_g1_i7_orf1;TRINITY_DN76815_c0_g1_i3_orf1;TRINITY_DN2719_c1_g1_i6_orf1;TRINITY_DN34479_c0_g1_i2_orf1;TRINITY_DN8659_c0_g2_i1_orf1;TRINITY_DN4056_c0_g1_i8_orf1;TRINITY_DN1604_c0_g1_i4_orf1;TRINITY_DN33705_c0_g1_i1_orf1;TRINITY_DN60821_c0_g1_i1_orf1;TRINITY_DN9109_c0_g1_i1_orf1;TRINITY_DN9156_c0_g1_i1_orf1;TRINITY_DN3588_c0_g1_i4_orf1;TRINITY_DN975_c0_g1_i1_orf1;TRINITY_DN8603_c0_g1_i1_orf1;TRINITY_DN9794_c0_g2_i8_orf1;TRINITY_DN2953_c1_g1_i10_orf1;TRINITY_DN7388_c0_g1_i7_orf1;TRINITY_DN810_c0_g1_i4_orf1;TRINITY_DN244_c1_g1_i5_orf1;TRINITY_DN7570_c0_g1_i18_orf1;TRINITY_DN2110_c0_g1_i3_orf1;TRINITY_DN15160_c0_g1_i1_orf1;TRINITY_DN46715_c0_g1_i1_orf1;TRINITY_DN4835_c0_g1_i2_orf1;TRINITY_DN3991_c0_g1_i6_orf1;TRINITY_DN825_c23_g1_i5_orf1;TRINITY_DN38424_c0_g1_i1_orf1;TRINITY_DN15420_c0_g3_i2_orf1;TRINITY_DN28039_c0_g1_i1_orf1;TRINITY_DN146236_c0_g1_i1_orf1;TRINITY_DN2745_c0_g1_i2_orf1;TRINITY_DN2826_c0_g1_i7_orf1;TRINITY_DN11612_c0_g2_i1_orf1;TRINITY_DN1266_c2_g1_i1_orf1;TRINITY_DN4798_c0_g1_i3_orf1;TRINITY_DN44288_c0_g1_i2_orf1;TRINITY_DN41697_c0_g1_i1_orf1;TRINITY_DN1617_c0_g1_i5_orf1;TRINITY_DN52244_c1_g1_i1_orf1;TRINITY_DN5811_c0_g1_i4_orf1;TRINITY_DN11015_c0_g1_i8_orf1;TRINITY_DN987_c0_g1_i3_orf1;TRINITY_DN2146_c0_g2_i1_orf1;TRINITY_DN1173_c1_g1_i10_orf1;TRINITY_DN2265_c0_g1_i5_orf1;TRINITY_DN29956_c1_g1_i1_orf1;TRINITY_DN49047_c0_g1_i2_orf1;TRINITY_DN107288_c0_g1_i2_orf1;TRINITY_DN18782_c0_g1_i4_orf1;TRINITY_DN119893_c0_g2_i3_orf1;TRINITY_DN25997_c1_g2_i4_orf1;TRINITY_DN5029_c0_g1_i1_orf1;TRINITY_DN4501_c0_g2_i1_orf1;TRINITY_DN4929_c1_g2_i5_orf1;TRINITY_DN21000_c0_g1_i1_orf1
molecular_function	tetrapyrrole binding	GO:0046906	31	31/1043	TRINITY_DN43369_c0_g2_i1_orf1;TRINITY_DN8985_c0_g1_i4_orf1;TRINITY_DN9608_c0_g1_i3_orf1;TRINITY_DN3949_c1_g1_i1_orf1;TRINITY_DN120500_c0_g1_i1_orf1;TRINITY_DN4497_c2_g1_i3_orf1;TRINITY_DN4497_c0_g1_i4_orf1;TRINITY_DN2392_c0_g2_i1_orf1;TRINITY_DN35002_c0_g2_i2_orf1;TRINITY_DN14262_c0_g1_i5_orf1;TRINITY_DN49265_c0_g3_i2_orf1;TRINITY_DN57765_c0_g1_i1_orf1;TRINITY_DN3732_c0_g1_i2_orf1;TRINITY_DN829_c0_g1_i8_orf1;TRINITY_DN16122_c0_g1_i4_orf1;TRINITY_DN50743_c0_g1_i1_orf1;TRINITY_DN448_c0_g1_i20_orf1;TRINITY_DN27045_c0_g1_i1_orf1;TRINITY_DN2264_c0_g1_i1_orf1;TRINITY_DN1960_c5_g1_i3_orf1;TRINITY_DN5439_c0_g1_i2_orf1;TRINITY_DN23783_c0_g2_i1_orf1;TRINITY_DN285_c0_g1_i4_orf1;TRINITY_DN52887_c0_g1_i1_orf1;TRINITY_DN30037_c0_g1_i5_orf1;TRINITY_DN7861_c0_g1_i5_orf1;TRINITY_DN5126_c0_g1_i3_orf1;TRINITY_DN5126_c0_g2_i1_orf1;TRINITY_DN6580_c0_g1_i4_orf1;TRINITY_DN54387_c0_g1_i1_orf1;TRINITY_DN23398_c0_g1_i1_orf1
molecular_function	molybdopterin cofactor binding	GO:0043546	1	1/1043	TRINITY_DN129_c0_g1_i6_orf1
molecular_function	vitamin B6 binding	GO:0070279	6	6/1043	TRINITY_DN14565_c0_g1_i11_orf1;TRINITY_DN14935_c0_g1_i1_orf1;TRINITY_DN2684_c0_g2_i3_orf1;TRINITY_DN11159_c0_g1_i5_orf1;TRINITY_DN21035_c0_g1_i14_orf1;TRINITY_DN2688_c0_g1_i3_orf1
molecular_function	identical protein binding	GO:0042802	2	2/1043	TRINITY_DN51938_c0_g3_i1_orf1;TRINITY_DN31584_c0_g2_i2_orf1
molecular_function	enzyme binding	GO:0019899	4	4/1043	TRINITY_DN55148_c0_g1_i1_orf1;TRINITY_DN80424_c0_g1_i1_orf1;TRINITY_DN31584_c0_g2_i2_orf1;TRINITY_DN975_c0_g1_i1_orf1
molecular_function	unfolded protein binding	GO:0051082	1	1/1043	TRINITY_DN6771_c0_g2_i1_orf1
molecular_function	calmodulin binding	GO:0005516	3	3/1043	TRINITY_DN3126_c0_g1_i4_orf1;TRINITY_DN4010_c0_g2_i1_orf1;TRINITY_DN975_c0_g1_i1_orf1
molecular_function	heat shock protein binding	GO:0031072	1	1/1043	TRINITY_DN79083_c0_g1_i2_orf1
molecular_function	transcription factor binding	GO:0008134	1	1/1043	TRINITY_DN6071_c0_g1_i1_orf1
molecular_function	signaling receptor binding	GO:0005102	3	3/1043	TRINITY_DN51938_c0_g3_i1_orf1;TRINITY_DN18218_c0_g1_i7_orf1;TRINITY_DN18650_c0_g1_i1_orf1
molecular_function	cytoskeletal protein binding	GO:0008092	11	11/1043	TRINITY_DN110231_c0_g1_i1_orf1;TRINITY_DN4010_c0_g2_i1_orf1;TRINITY_DN22824_c0_g1_i4_orf1;TRINITY_DN104297_c0_g1_i1_orf1;TRINITY_DN9383_c0_g1_i3_orf1;TRINITY_DN4731_c0_g2_i1_orf1;TRINITY_DN350_c0_g1_i5_orf1;TRINITY_DN364_c1_g1_i2_orf1;TRINITY_DN3126_c0_g1_i4_orf1;TRINITY_DN10455_c0_g1_i2_orf1;TRINITY_DN364_c0_g1_i2_orf1
molecular_function	protein dimerization activity	GO:0046983	2	2/1043	TRINITY_DN31584_c0_g2_i2_orf1;TRINITY_DN14611_c0_g1_i5_orf1
molecular_function	phosphoprotein binding	GO:0051219	1	1/1043	TRINITY_DN31584_c0_g2_i2_orf1
molecular_function	protein domain specific binding	GO:0019904	1	1/1043	TRINITY_DN31584_c0_g2_i2_orf1
molecular_function	clathrin binding	GO:0030276	1	1/1043	TRINITY_DN1497_c0_g2_i6_orf1
molecular_function	ubiquitin-like protein binding	GO:0032182	1	1/1043	TRINITY_DN65299_c0_g4_i1_orf1
molecular_function	transmembrane transporter binding	GO:0044325	1	1/1043	TRINITY_DN31584_c0_g2_i2_orf1
molecular_function	scaffold protein binding	GO:0097110	1	1/1043	TRINITY_DN31584_c0_g2_i2_orf1
molecular_function	chitin binding	GO:0008061	9	9/1043	TRINITY_DN77642_c0_g1_i1_orf1;TRINITY_DN21555_c0_g1_i4_orf1;TRINITY_DN2205_c0_g1_i3_orf1;TRINITY_DN1287_c0_g1_i5_orf1;TRINITY_DN10824_c0_g1_i3_orf1;TRINITY_DN82801_c0_g1_i1_orf1;TRINITY_DN2958_c0_g1_i2_orf1;TRINITY_DN664_c0_g1_i18_orf1;TRINITY_DN650_c0_g1_i3_orf1

molecular_function	ribonucleotide binding	GO:0032553	57	57/1043	TRINITY_DN115210_c0_g4_i1_orf1;TRINITY_DN6771_c0_g2_i1_orf1;TRINITY_DN4247_c0_g1_i4_orf1;TRINITY_DN96170_c0_g2_i1_orf1;TRINITY_DN14937_c0_g1_i7_orf1;TRINITY_DN76815_c0_g1_i3_orf1;TRINITY_DN2719_c1_g1_i6_orf1;TRINITY_DN8659_c0_g2_i1_orf1;TRINITY_DN4056_c0_g1_i8_orf1;TRINITY_DN1604_c0_g1_i4_orf1;TRINITY_DN33705_c0_g1_i1_orf1;TRINITY_DN60821_c0_g1_i1_orf1;TRINITY_DN9109_c0_g1_i1_orf1;TRINITY_DN9156_c0_g1_i1_orf1;TRINITY_DN34479_c0_g1_i2_orf1;TRINITY_DN975_c0_g1_i1_orf1;TRINITY_DN8603_c0_g1_i1_orf1;TRINITY_DN9794_c0_g2_i8_orf1;TRINITY_DN2953_c1_g1_i10_orf1;TRINITY_DN7388_c0_g1_i7_orf1;TRINITY_DN810_c0_g1_i4_orf1;TRINITY_DN244_c1_g1_i5_orf1;TRINITY_DN7570_c0_g1_i18_orf1;TRINITY_DN2110_c0_g1_i3_orf1;TRINITY_DN15160_c0_g1_i1_orf1;TRINITY_DN46715_c0_g1_i1_orf1;TRINITY_DN4835_c0_g1_i2_orf1;TRINITY_DN3991_c0_g1_i6_orf1;TRINITY_DN825_c23_g1_i5_orf1;TRINITY_DN15420_c0_g3_i2_orf1;TRINITY_DN28039_c0_g1_i1_orf1;TRINITY_DN146236_c0_g1_i1_orf1;TRINITY_DN2745_c0_g1_i2_orf1;TRINITY_DN2826_c0_g1_i7_orf1;TRINITY_DN11612_c0_g2_i1_orf1;TRINITY_DN1266_c2_g1_i1_orf1;TRINITY_DN4798_c0_g1_i3_orf1;TRINITY_DN44288_c0_g1_i2_orf1;TRINITY_DN41697_c0_g1_i1_orf1;TRINITY_DN1617_c0_g1_i5_orf1;TRINITY_DN52244_c1_g1_i1_orf1;TRINITY_DN5811_c0_g1_i4_orf1;TRINITY_DN11015_c0_g1_i8_orf1;TRINITY_DN987_c0_g1_i3_orf1;TRINITY_DN2146_c0_g2_i1_orf1;TRINITY_DN1173_c1_g1_i10_orf1;TRINITY_DN2265_c0_g1_i5_orf1;TRINITY_DN29956_c1_g1_i1_orf1;TRINITY_DN49047_c0_g1_i2_orf1;TRINITY_DN107288_c0_g1_i2_orf1;TRINITY_DN18782_c0_g1_i4_orf1;TRINITY_DN119893_c0_g2_i3_orf1;TRINITY_DN25997_c1_g2_i4_orf1;TRINITY_DN5029_c0_g1_i1_orf1;TRINITY_DN4501_c0_g2_i1_orf1;TRINITY_DN4929_c1_g2_i5_orf1;TRINITY_DN21000_c0_g1_i1_orf1
molecular_function	glycosaminoglycan binding	GO:0005539	1	1/1043	TRINITY_DN1534_c0_g1_i3_orf1
molecular_function	cation binding	GO:0043169	147	147/1043	TRINITY_DN276_c0_g1_i1_orf1;TRINITY_DN9608_c0_g1_i3_orf1;TRINITY_DN3949_c1_g1_i1_orf1;TRINITY_DN827_c1_g1_i1_orf1;TRINITY_DN3194_c0_g1_i6_orf1;TRINITY_DN863_c0_g1_i6_orf1;TRINITY_DN35002_c0_g2_i2_orf1;TRINITY_DN49204_c0_g1_i1_orf1;TRINITY_DN40176_c0_g1_i1_orf1;TRINITY_DN2688_c0_g1_i3_orf1;TRINITY_DN56164_c0_g1_i1_orf1;TRINITY_DN3275_c0_g2_i3_orf1;TRINITY_DN47842_c0_g1_i1_orf1;TRINITY_DN1153_c1_g1_i1_orf1;TRINITY_DN125427_c0_g1_i1_orf1;TRINITY_DN24689_c0_g1_i1_orf1;TRINITY_DN16122_c0_g1_i4_orf1;TRINITY_DN27833_c0_g2_i1_orf1;TRINITY_DN3433_c0_g1_i6_orf1;TRINITY_DN31609_c0_g1_i3_orf1;TRINITY_DN14774_c0_g1_i4_orf1;TRINITY_DN48020_c0_g1_i1_orf1;TRINITY_DN4822_c0_g1_i6_orf1;TRINITY_DN448_c0_g1_i20_orf1;TRINITY_DN1534_c0_g1_i3_orf1;TRINITY_DN3991_c0_g1_i6_orf1;TRINITY_DN5439_c0_g1_i2_orf1;TRINITY_DN285_c0_g1_i4_orf1;TRINITY_DN12003_c0_g1_i1_orf1;TRINITY_DN2694_c0_g1_i3_orf1;TRINITY_DN2117_c0_g1_i1_orf1;TRINITY_DN987_c0_g1_i3_orf1;TRINITY_DN2627_c0_g2_i1_orf1;TRINITY_DN2919_c0_g1_i5_orf1;TRINITY_DN5210_c0_g1_i3_orf1;TRINITY_DN2647_c0_g1_i3_orf1;TRINITY_DN230_c2_g1_i5_orf1;TRINITY_DN65681_c0_g1_i1_orf1;TRINITY_DN115210_c0_g4_i1_orf1;TRINITY_DN20442_c0_g2_i1_orf1;TRINITY_DN5497_c0_g1_i6_orf1;TRINITY_DN4367_c0_g1_i1_orf1;TRINITY_DN18242_c0_g1_i3_orf1;TRINITY_DN22018_c0_g1_i3_orf1;TRINITY_DN20238_c0_g1_i7_orf1;TRINITY_DN56690_c0_g1_i4_orf1;TRINITY_DN810_c0_g1_i4_orf1;TRINITY_DN9207_c0_g1_i1_orf1;TRINITY_DN2110_c0_g1_i3_orf1;TRINITY_DN66302_c0_g1_i1_orf1;TRINITY_DN3469_c0_g1_i4_orf1;TRINITY_DN8241_c0_g1_i3_orf1;TRINITY_DN1999_c0_g1_i9_orf1;TRINITY_DN9794_c0_g2_i8_orf1;TRINITY_DN6415_c0_g1_i1_orf1;TRINITY_DN136031_c0_g1_i7_orf1;TRINITY_DN1154_c0_g1_i1_orf1;TRINITY_DN5664_c0_g1_i1_orf1;TRINITY_DN27033_c1_g1_i3_orfp1;TRINITY_DN1266_c2_g1_i1_orf1;TRINITY_DN7861_c0_g1_i5_orf1;TRINITY_DN4321_c0_g1_i1_orf1;TRINITY_DN48410_c0_g1_i1_orf1;TRINITY_DN54387_c0_g1_i1_orf1;TRINITY_DN103107_c0_g1_i2_orf1;TRINITY_DN2299_c0_g1_i3_orf1;TRINITY_DN51658_c0_g1_i1_orf1;TRINITY_DN5029_c0_g1_i1_orf1;TRINITY_DN4501_c0_g2_i1_orf1;TRINITY_DN8985_c0_g1_i4_orf1;TRINITY_DN120500_c0_g1_i1_orf1;TRINITY_DN5001_c0_g1_i4_orf1;TRINITY_DN7570_c0_g1_i18_orf1;TRINITY_DN30037_c0_g1_i5_orf1;TRINITY_DN4497_c0_g1_i4_orf1;TRINITY_DN16400_c0_g2_i1_orf1;TRINITY_DN2186_c0_g1_i17_orf1;TRINITY_DN89083_c0_g1_i1_orf1;TRINITY_DN6556_c0_g1_i7_orf1;TRINITY_DN14262_c0_g1_i5_orf1;TRINITY_DN31851_c0_g1_i2_orf1;TRINITY_DN7212_c0_g1_i4_orf1;TRINITY_DN542_c0_g2_i1_orf1;TRINITY_DN43412_c0_g1_i2_orf1;TRINITY_DN829_c0_g1_i8_orf1;TRINITY_DN4565_c0_g2_i1_orf1;TRINITY_DN276_c0_g1_i2_orf1;TRINITY_DN48638_c0_g1_i5_orf1;TRINITY_DN5432_c1_g1_i3_orf1;TRINITY_DN50743_c0_g1_i1_orf1;TRINITY_DN6415_c0_g2_i1_orf1;TRINITY_DN1989_c0_g1_i1_orf1;TRINITY_DN27045_c0_g1_i1_orf1;TRINITY_DN146236_c0_g1_i1_orf1;TRINITY_DN23783_c0_g2_i1_orf1;TRINITY_DN4497_c2_g1_i3_orf1;TRINITY_DN5126_c0_g1_i3_orf1;TRINITY_DN5126_c0_g2_i1_orf1;TRINITY_DN41179_c0_g1_i1_orf1;TRINITY_DN1957_c0_g1_i4_orf1;TRINITY_DN8637_c0_g1_i1_orf1;TRINITY_DN3651_c0_g1_i5_orf1;TRINITY_DN61536_c0_g3_i1_orf1;TRINITY_DN38180_c0_g1_i3_orf1;TRINITY_DN1194_c0_g1_i5_orf1;TRINITY_DN43369_c0_g2_i1_orf1;TRINITY_DN129_c0_g1_i6_orf1;TRINITY_DN51968_c0_g1_i1_orf1;TRINITY_DN2986_c1_g1_i1_orf1;TRINITY_DN1860_c0_g1_i2_orf1;TRINITY_DN2392_c0_g2_i1_orf1;TRINITY_DN23398_c0_g1_i1_orf1;TRINITY_DN76815_c0_g1_i3_orf1;TRINITY_DN2342_c0_g1_i1_orf1;TRINITY_DN27592_c0_g1_i1_orf1;TRINITY_DN4822_c0_g1_i9_orf1;TRINITY_DN4056_c0_g1_i8_orf1;TRINITY_DN89483_c0_g1_i1_orf1;TRINITY_DN14874_c0_g1_i6_orf1;TRINITY_DN88539_c0_g2_i1_orf1;TRINITY_DN49265_c0_g3_i2_orf1;TRINITY_DN5848_c0_g1_i6_orf1;TRINITY_DN57765_c0_g1_i1_orf1;TRINITY_DN7647_c0_g1_i4_orf1;TRINITY_DN9286_c0_g1_i2_orf1;TRINITY_DN21035_c0_g1_i14_orf1;TRINITY_DN3732_c0_g1_i2_orf1;TRINITY_DN14670_c0_g1_i1_orf1;TRINITY_DN1884_c0_g2_i2_orf1;TRINITY_DN38783_c0_g1_i1_orf1;TRINITY_DN2593_c0_g1_i1_orf1;TRINITY_DN15222_c0_g1_i4_orf1;TRINITY_DN37307_c0_g1_i4_orf1;TRINITY_DN22824_c0_g1_i4_orf1;TRINITY_DN19303_c0_g1_i5_orf1;TRINITY_DN5238_c0_g1_i2_orf1;TRINITY_DN2264_c0_g1_i1_orf1;TRINITY_DN1960_c5_g1_i3_orf1;TRINITY_DN69049_c0_g2_i1_orf1;TRINITY_DN52887_c0_g1_i1_orf1;TRINITY_DN25997_c1_g2_i4_orf1;TRINITY_DN110534_c0_g1_i3_orf1;TRINITY_DN21533_c0_g1_i7_orf1;TRINITY_DN41697_c0_g1_i1_orf1;TRINITY_DN6580_c0_g1_i4_orf1;TRINITY_DN51498_c0_g1_i1_orf1;TRINITY_DN585_c0_g1_i5_orf1
molecular_function	anion binding	GO:0043168	66	66/1043	TRINITY_DN129_c0_g1_i6_orf1;TRINITY_DN14565_c0_g1_i11_orf1;TRINITY_DN115210_c0_g4_i1_orf1;TRINITY_DN6771_c0_g2_i1_orf1;TRINITY_DN4247_c0_g1_i4_orf1;TRINITY_DN96170_c0_g2_i1_orf1;TRINITY_DN14937_c0_g1_i7_orf1;TRINITY_DN76815_c0_g1_i3_orf1;TRINITY_DN21035_c0_g1_i14_orf1;TRINITY_DN2719_c1_g1_i6_orf1;TRINITY_DN2688_c0_g1_i3_orf1;TRINITY_DN8659_c0_g2_i1_orf1;TRINITY_DN4056_c0_g1_i8_orf1;TRINITY_DN1604_c0_g1_i4_orf1;TRINITY_DN33705_c0_g1_i1_orf1;TRINITY_DN60821_c0_g1_i1_orf1;TRINITY_DN9109_c0_g1_i1_orf1;TRINITY_DN9156_c0_g1_i1_orf1;TRINITY_DN3588_c0_g1_i4_orf1;TRINITY_DN975_c0_g1_i1_orf1;TRINITY_DN8603_c0_g1_i1_orf1;TRINITY_DN9794_c0_g2_i8_orf1;TRINITY_DN2953_c1_g1_i10_orf1;TRINITY_DN7388_c0_g1_i7_orf1;TRINITY_DN810_c0_g1_i4_orf1;TRINITY_DN244_c1_g1_i5_orf1;TRINITY_DN7570_c0_g1_i18_orf1;TRINITY_DN2110_c0_g1_i3_orf1;TRINITY_DN49047_c0_g1_i2_orf1;TRINITY_DN15160_c0_g1_i1_orf1;TRINITY_DN46715_c0_g1_i1_orf1;TRINITY_DN4835_c0_g1_i2_orf1;TRINITY_DN3991_c0_g1_i6_orf1;TRINITY_DN825_c23_g1_i5_orf1;TRINITY_DN14935_c0_g1_i1_orf1;TRINITY_DN38424_c0_g1_i1_orf1;TRINITY_DN15420_c0_g3_i2_orf1;TRINITY_DN28039_c0_g1_i1_orf1;TRINITY_DN146236_c0_g1_i1_orf1;TRINITY_DN2745_c0_g1_i2_orf1;TRINITY_DN2826_c0_g1_i7_orf1;TRINITY_DN11612_c0_g2_i1_orf1;TRINITY_DN34479_c0_g1_i2_orf1;TRINITY_DN1266_c2_g1_i1_orf1;TRINITY_DN4798_c0_g1_i3_orf1;TRINITY_DN44288_c0_g1_i2_orf1;TRINITY_DN41697_c0_g1_i1_orf1;TRINITY_DN1617_c0_g1_i5_orf1;TRINITY_DN52244_c1_g1_i1_orf1;TRINITY_DN5811_c0_g1_i4_orf1;TRINITY_DN11015_c0_g1_i8_orf1;TRINITY_DN987_c0_g1_i3_orf1;TRINITY_DN2146_c0_g2_i1_orf1;TRINITY_DN1173_c1_g1_i10_orf1;TRINITY_DN2684_c0_g2_i3_orf1;TRINITY_DN2265_c0_g1_i5_orf1;TRINITY_DN29956_c1_g1_i1_orf1;TRINITY_DN11159_c0_g1_i5_orf1;TRINITY_DN107288_c0_g1_i2_orf1;TRINITY_DN18782_c0_g1_i4_orf1;TRINITY_DN119893_c0_g2_i3_orf1;TRINITY_DN25997_c1_g2_i4_orf1;TRINITY_DN5029_c0_g1_i1_orf1;TRINITY_DN4501_c0_g2_i1_orf1;TRINITY_DN4929_c1_g2_i5_orf1;TRINITY_DN21000_c0_g1_i1_orf1
molecular_function	phospholipid binding	GO:0005543	6	6/1043	TRINITY_DN1497_c0_g2_i6_orf1;TRINITY_DN21533_c0_g1_i7_orf1;TRINITY_DN12432_c0_g1_i2_orf1;TRINITY_DN20238_c0_g1_i7_orf1;TRINITY_DN119265_c0_g2_i1_orf1;TRINITY_DN65299_c0_g4_i1_orf1
molecular_function	oxygen binding	GO:0019825	1	1/1043	TRINITY_DN30037_c0_g1_i5_orf1
molecular_function	monosaccharide binding	GO:0048029	1	1/1043	TRINITY_DN3835_c0_g1_i4_orf1
molecular_function	organic acid binding	GO:0043177	1	1/1043	TRINITY_DN115210_c0_g4_i1_orf1
molecular_function	vitamin binding	GO:0019842	6	6/1043	TRINITY_DN14565_c0_g1_i11_orf1;TRINITY_DN14935_c0_g1_i1_orf1;TRINITY_DN2684_c0_g2_i3_orf1;TRINITY_DN11159_c0_g1_i5_orf1;TRINITY_DN21035_c0_g1_i14_orf1;TRINITY_DN2688_c0_g1_i3_orf1
molecular_function	nucleotide binding	GO:0000166	63	63/1043	TRINITY_DN33430_c0_g1_i5_orf1;TRINITY_DN129_c0_g1_i6_orf1;TRINITY_DN115210_c0_g4_i1_orf1;TRINITY_DN6771_c0_g2_i1_orf1;TRINITY_DN5099_c0_g1_i3_orf1;TRINITY_DN4247_c0_g1_i4_orf1;TRINITY_DN96170_c0_g2_i1_orf1;TRINITY_DN20527_c0_g1_i1_orf1;TRINITY_DN14937_c0_g1_i7_orf1;TRINITY_DN76815_c0_g1_i3_orf1;TRINITY_DN2719_c1_g1_i6_orf1;TRINITY_DN34479_c0_g1_i2_orf1;TRINITY_DN8659_c0_g2_i1_orf1;TRINITY_DN4056_c0_g1_i8_orf1;TRINITY_DN1604_c0_g1_i4_orf1;TRINITY_DN33705_c0_g1_i1_orf1;TRINITY_DN60821_c0_g1_i1_orf1;TRINITY_DN9109_c0_g1_i1_orf1;TRINITY_DN9156_c0_g1_i1_orf1;TRINITY_DN3588_c0_g1_i4_orf1;TRINITY_DN975_c0_g1_i1_orf1;TRINITY_DN8603_c0_g1_i1_orf1;TRINITY_DN9794_c0_g2_i8_orf1;TRINITY_DN2953_c1_g1_i10_orf1;TRINITY_DN7388_c0_g1_i7_orf1;TRINITY_DN810_c0_g1_i4_orf1;TRINITY_DN244_c1_g1_i5_orf1;TRINITY_DN7570_c0_g1_i18_orf1;TRINITY_DN2110_c0_g1_i3_orf1;TRINITY_DN15160_c0_g1_i1_orf1;TRINITY_DN46715_c0_g1_i1_orf1;TRINITY_DN4835_c0_g1_i2_orf1;TRINITY_DN3991_c0_g1_i6_orf1;TRINITY_DN825_c23_g1_i5_orf1;TRINITY_DN38424_c0_g1_i1_orf1;TRINITY_DN15420_c0_g3_i2_orf1;TRINITY_DN28039_c0_g1_i1_orf1;TRINITY_DN146236_c0_g1_i1_orf1;TRINITY_DN2745_c0_g1_i2_orf1;TRINITY_DN2826_c0_g1_i7_orf1;TRINITY_DN11612_c0_g2_i1_orf1;TRINITY_DN1266_c2_g1_i1_orf1;TRINITY_DN4798_c0_g1_i3_orf1;TRINITY_DN44288_c0_g1_i2_orf1;TRINITY_DN41697_c0_g1_i1_orf1;TRINITY_DN1617_c0_g1_i5_orf1;TRINITY_DN52244_c1_g1_i1_orf1;TRINITY_DN5811_c0_g1_i4_orf1;TRINITY_DN11015_c0_g1_i8_orf1;TRINITY_DN987_c0_g1_i3_orf1;TRINITY_DN2146_c0_g2_i1_orf1;TRINITY_DN1173_c1_g1_i10_orf1;TRINITY_DN2265_c0_g1_i5_orf1;TRINITY_DN29956_c1_g1_i1_orf1;TRINITY_DN49047_c0_g1_i2_orf1;TRINITY_DN107288_c0_g1_i2_orf1;TRINITY_DN18782_c0_g1_i4_orf1;TRINITY_DN119893_c0_g2_i3_orf1;TRINITY_DN25997_c1_g2_i4_orf1;TRINITY_DN5029_c0_g1_i1_orf1;TRINITY_DN4501_c0_g2_i1_orf1;TRINITY_DN4929_c1_g2_i5_orf1;TRINITY_DN21000_c0_g1_i1_orf1
molecular_function	actin filament binding	GO:0051015	5	5/1043	TRINITY_DN110231_c0_g1_i1_orf1;TRINITY_DN4731_c0_g2_i1_orf1;TRINITY_DN364_c0_g1_i2_orf1;TRINITY_DN9383_c0_g1_i3_orf1;TRINITY_DN22824_c0_g1_i4_orf1
molecular_function	iron-sulfur cluster binding	GO:0051536	6	6/1043	TRINITY_DN129_c0_g1_i6_orf1;TRINITY_DN49221_c0_g1_i1_orf1;TRINITY_DN48638_c0_g1_i5_orf1;TRINITY_DN5664_c0_g1_i1_orf1;TRINITY_DN5432_c1_g1_i3_orf1;TRINITY_DN31851_c0_g1_i2_orf1
molecular_function	transmembrane signaling receptor activity	GO:0004888	1	1/1043	TRINITY_DN34821_c0_g1_i4_orf1
molecular_function	ligase activity, forming carbon-oxygen bonds	GO:0016875	4	4/1043	TRINITY_DN825_c23_g1_i5_orf1;TRINITY_DN2953_c1_g1_i10_orf1;TRINITY_DN107288_c0_g1_i2_orf1;TRINITY_DN15160_c0_g1_i1_orf1
molecular_function	ligase activity, forming carbon-sulfur bonds	GO:0016877	2	2/1043	TRINITY_DN9794_c0_g2_i8_orf1;TRINITY_DN8659_c0_g2_i1_orf1
molecular_function	ligase activity, forming carbon-nitrogen bonds	GO:0016879	5	5/1043	TRINITY_DN115210_c0_g4_i1_orf1;TRINITY_DN987_c0_g1_i3_orf1;TRINITY_DN76815_c0_g1_i3_orf1;TRINITY_DN244_c1_g1_i5_orf1;TRINITY_DN41697_c0_g1_i1_orf1
molecular_function	ligase activity, forming carbon-carbon bonds	GO:0016885	1	1/1043	TRINITY_DN3991_c0_g1_i6_orf1
molecular_function	carbon-sulfur lyase activity	GO:0016846	1	1/1043	TRINITY_DN12003_c0_g1_i1_orf1
molecular_function	carbon-nitrogen lyase activity	GO:0016840	1	1/1043	TRINITY_DN16868_c0_g2_i1_orf1
molecular_function	phosphorus-oxygen lyase activity	GO:0016849	2	2/1043	TRINITY_DN3712_c0_g1_i1_orf1;TRINITY_DN52244_c1_g1_i1_orf1
molecular_function	carbon-oxygen lyase activity	GO:0016835	6	6/1043	TRINITY_DN230_c2_g1_i5_orf1;TRINITY_DN87603_c0_g2_i1_orf1;TRINITY_DN10900_c0_g1_i7_orf1;TRINITY_DN51498_c0_g1_i1_orf1;TRINITY_DN42759_c0_g2_i1_orf1;TRINITY_DN89483_c0_g1_i1_orf1
molecular_function	carbon-carbon lyase activity	GO:0016830	8	8/1043	TRINITY_DN779_c0_g1_i12_orf1;TRINITY_DN6325_c0_g1_i8_orf1;TRINITY_DN40434_c0_g1_i2_orf1;TRINITY_DN2684_c0_g2_i3_orf1;TRINITY_DN8037_c0_g2_i1_orf1;TRINITY_DN11159_c0_g1_i5_orf1;TRINITY_DN83150_c0_g1_i1_orf1;TRINITY_DN6325_c0_g1_i9_orf1
molecular_function	peptide-lysine-N-acetyltransferase activity	GO:0061733	1	1/1043	TRINITY_DN20442_c0_g2_i1_orf1
molecular_function	palmitoyl-(protein) hydrolase activity	GO:0008474	1	1/1043	TRINITY_DN4817_c0_g1_i4_orf1
molecular_function	protein-disulfide reductase activity	GO:0015035	2	2/1043	TRINITY_DN51938_c0_g3_i1_orf1;TRINITY_DN24689_c0_g1_i1_orf1
molecular_function	protein methyltransferase activity	GO:0008276	1	1/1043	TRINITY_DN22674_c0_g1_i2_orf1
molecular_function	phosphoprotein phosphatase activity	GO:0004721	2	2/1043	TRINITY_DN3119_c0_g1_i7_orf1;TRINITY_DN59885_c0_g1_i3_orf1
molecular_function	ubiquitin-like protein transferase activity	GO:0019787	5	5/1043	TRINITY_DN88539_c0_g2_i1_orf1;TRINITY_DN7647_c0_g1_i4_orf1;TRINITY_DN51658_c0_g1_i1_orf1;TRINITY_DN1380_c0_g1_i5_orf1;TRINITY_DN7316_c0_g2_i1_orf1
molecular_function	protein kinase activity	GO:0004672	5	5/1043	TRINITY_DN46715_c0_g1_i1_orf1;TRINITY_DN29956_c1_g1_i1_orf1;TRINITY_DN1266_c2_g1_i1_orf1;TRINITY_DN4798_c0_g1_i3_orf1;TRINITY_DN1173_c1_g1_i10_orf1

molecular_function	peptidase activity	GO:0008233	46	46/1043	TRINITY_DN4767_c0_g1_i6_orf1;TRINITY_DN2593_c0_g1_i1_orf1;TRINITY_DN51938_c0_g3_i1_orf1;TRINITY_DN143895_c0_g1_i1_orf1;TRINITY_DN36434_c0_g2_i3_orf1;TRINITY_DN554_c0_g1_i1_orf1;TRINITY_DN801_c0_g1_i2_orf1;TRINITY_DN3194_c0_g1_i6_orf1;TRINITY_DN67026_c0_g1_i6_orf1;TRINITY_DN3483_c0_g1_i5_orf1;TRINITY_DN13686_c0_g2_i1_orf1;TRINITY_DN27033_c1_g1_i3_orfp1;TRINITY_DN57111_c0_g1_i1_orf1;TRINITY_DN2069_c1_g1_i8_orf1;TRINITY_DN5310_c2_g1_i2_orf1;TRINITY_DN1310_c0_g1_i4_orf1;TRINITY_DN14874_c0_g1_i6_orf1;TRINITY_DN4030_c0_g2_i1_orf1;TRINITY_DN34479_c0_g1_i2_orf1;TRINITY_DN2885_c1_g1_i2_orf1;TRINITY_DN9836_c0_g1_i2_orf1;TRINITY_DN14754_c0_g1_i6_orf1;TRINITY_DN1030_c0_g1_i6_orf1;TRINITY_DN66302_c0_g1_i1_orf1;TRINITY_DN3975_c0_g1_i7_orf1;TRINITY_DN14774_c0_g1_i4_orf1;TRINITY_DN48020_c0_g1_i1_orf1;TRINITY_DN71863_c0_g1_i2_orf1;TRINITY_DN18172_c0_g1_i6_orf1;TRINITY_DN56690_c0_g1_i4_orf1;TRINITY_DN4064_c0_g2_i1_orf1;TRINITY_DN1308_c0_g1_i4_orf1;TRINITY_DN69049_c0_g2_i1_orf1;TRINITY_DN701_c0_g1_i1_orf1;TRINITY_DN21984_c0_g1_i6_orf1;TRINITY_DN46132_c0_g2_i2_orf1;TRINITY_DN6205_c0_g1_i8_orf1;TRINITY_DN140_c1_g1_i2_orf1;TRINITY_DN9991_c0_g1_i4_orf1;TRINITY_DN6470_c0_g3_i2_orf1;TRINITY_DN23167_c0_g1_i4_orf1;TRINITY_DN6205_c0_g1_i1_orf1;TRINITY_DN49047_c0_g1_i2_orf1;TRINITY_DN4494_c0_g1_i1_orf1;TRINITY_DN22797_c0_g1_i5_orf
molecular_function	protein disulfide isomerase activity	GO:0003756	1	1/1043	TRINITY_DN51938_c0_g3_i1_orf1
molecular_function	peptidyl-prolyl cis-trans isomerase activity	GO:0003755	1	1/1043	TRINITY_DN119291_c0_g1_i1_orf1
molecular_function	protein demethylase activity	GO:0140457	1	1/1043	TRINITY_DN89083_c0_g1_i1_orf1
molecular_function	ubiquitin-like modifier activating enzyme activity	GO:0008641	1	1/1043	TRINITY_DN8659_c0_g2_i1_orf1
molecular_function	hydrolase activity, acting on glycosyl bonds	GO:0016798	18	18/1043	TRINITY_DN9044_c0_g1_i1_orf1;TRINITY_DN14235_c0_g1_i1_orf1;TRINITY_DN21555_c0_g1_i4_orf1;TRINITY_DN2205_c0_g1_i3_orf1;TRINITY_DN1732_c0_g1_i17_orf1;TRINITY_DN15222_c0_g1_i4_orf1;TRINITY_DN1287_c0_g1_i5_orf1;TRINITY_DN10824_c0_g1_i3_orf1;TRINITY_DN28741_c0_g1_i3_orf1;TRINITY_DN467_c3_g1_i5_orf1;TRINITY_DN195_c4_g1_i1_orf1;TRINITY_DN48410_c0_g1_i1_orf1;TRINITY_DN2894_c0_g2_i3_orf1;TRINITY_DN5852_c0_g1_i6_orf1;TRINITY_DN9044_c0_g1_i2_orf1;TRINITY_DN1098_c1_g1_i4_orf1;TRINITY_DN1732_c0_g1_i15_orf1;TRINITY_DN650_c0_g1_i3_orf1
molecular_function	hydrolase activity, acting on carbon-nitrogen (b	GO:0016810	13	13/1043	TRINITY_DN1534_c0_g1_i3_orf1;TRINITY_DN827_c1_g1_i1_orf1;TRINITY_DN17326_c0_g1_i5_orf1;TRINITY_DN115210_c0_g4_i1_orf1;TRINITY_DN87170_c0_g1_i3_orf1;TRINITY_DN1216_c0_g1_i4_orf1;TRINITY_DN82801_c0_g1_i1_orf1;TRINITY_DN542_c0_g2_i1_orf1;TRINITY_DN98242_c0_g1_i1_orf1;TRINITY_DN244_c1_g1_i5_orf1;TRINITY_DN2835_c0_g1_i6_orf1;TRINITY_DN38180_c0_g1_i3_orf1;TRINITY_DN11383_c0_g2_i4_
molecular_function	hydrolase activity, acting on acid anhydrides	GO:0016817	18	18/1043	TRINITY_DN7565_c0_g1_i3_orf1;TRINITY_DN33705_c0_g1_i1_orf1;TRINITY_DN2265_c0_g1_i5_orf1;TRINITY_DN6771_c0_g2_i1_orf1;TRINITY_DN34479_c0_g1_i2_orf1;TRINITY_DN975_c0_g1_i1_orf1;TRINITY_DN15420_c0_g3_i2_orf1;TRINITY_DN28039_c0_g1_i1_orf1;TRINITY_DN146236_c0_g1_i1_orf1;TRINITY_DN7388_c0_g1_i7_orf1;TRINITY_DN140212_c0_g1_i1_orf1;TRINITY_DN11612_c0_g2_i1_orf1;TRINITY_DN7570_c0_g1_i18_orf1;TRINITY_DN49047_c0_g1_i2_orf1;TRINITY_DN1091_c0_g3_i1_orf1;TRINITY_DN1091_c0_g1_i1_orf1;TRINITY_DN2054_c0_g1_i1_orf1;TRINITY_DN21000_c0_g1_i1_orf1
molecular_function	serine hydrolase activity	GO:0017171	22	22/1043	TRINITY_DN36434_c0_g2_i3_orf1;TRINITY_DN67026_c0_g1_i6_orf1;TRINITY_DN3483_c0_g1_i5_orf1;TRINITY_DN13686_c0_g2_i1_orf1;TRINITY_DN57111_c0_g1_i1_orf1;TRINITY_DN5310_c2_g1_i2_orf1;TRINITY_DN1310_c0_g1_i4_orf1;TRINITY_DN4030_c0_g2_i1_orf1;TRINITY_DN3975_c0_g1_i7_orf1;TRINITY_DN71863_c0_g1_i2_orf1;TRINITY_DN1308_c0_g1_i4_orf1;TRINITY_DN701_c0_g1_i1_orf1;TRINITY_DN21984_c0_g1_i6_orf1;TRINITY_DN6205_c0_g1_i8_orf1;TRINITY_DN140_c1_g1_i2_orf1;TRINITY_DN9991_c0_g1_i4_orf1;TRINITY_DN6470_c0_g3_i2_orf1;TRINITY_DN23167_c0_g1_i4_orf1;TRINITY_DN6205_c0_g1_i1_orf1;TRINITY_DN4494_c0_g1_i1_orf1;TRINITY_DN22797_c0_g1_i5_orf1;TRINITY_DN747_c0_g1_i4_orf1
molecular_function	hydrolase activity, acting on ester bonds	GO:0016788	41	41/1043	TRINITY_DN1865_c1_g1_i3_orf1;TRINITY_DN2749_c0_g1_i4_orf1;TRINITY_DN171_c0_g1_i1_orf1;TRINITY_DN12227_c0_g2_i3_orf1;TRINITY_DN3712_c0_g1_i1_orf1;TRINITY_DN27033_c1_g1_i3_orfp1;TRINITY_DN4817_c0_g1_i4_orf1;TRINITY_DN18909_c0_g1_i8_orf1;TRINITY_DN3119_c0_g1_i7_orf1;TRINITY_DN1841_c0_g1_i2_orf1;TRINITY_DN34465_c0_g1_i1_orf1;TRINITY_DN69713_c0_g1_i1_orf1;TRINITY_DN4565_c0_g2_i1_orf1;TRINITY_DN82017_c0_g1_i5_orf1;TRINITY_DN42759_c0_g2_i1_orf1;TRINITY_DN59885_c0_g1_i3_orf1;TRINITY_DN810_c0_g1_i4_orf1;TRINITY_DN41179_c0_g1_i1_orf1;TRINITY_DN616_c1_g1_i6_orf1;TRINITY_DN2749_c0_g2_i3_orf1;TRINITY_DN1249_c0_g1_i10_orf1;TRINITY_DN1073_c0_g1_i3_orf1;TRINITY_DN1884_c0_g2_i2_orf1;TRINITY_DN13330_c0_g1_i4_orf1;TRINITY_DN10900_c0_g1_i7_orf1;TRINITY_DN38783_c0_g1_i1_orf1;TRINITY_DN5238_c0_g1_i2_orf1;TRINITY_DN42705_c0_g1_i3_orf1;TRINITY_DN18909_c0_g1_i6_orf1;TRINITY_DN19293_c0_g1_i4_orf1;TRINITY_DN935_c0_g1_i3_orf1;TRINITY_DN1116_c0_g1_i6_orf1;TRINITY_DN11798_c0_g2_i1_orf1;TRINITY_DN30509_c0_g1_i9_orf1;TRINITY_DN117_c0_g1_i4_orf1;TRINITY_DN2647_c0_g1_i3_orf1;TRINITY_DN2627_c0_g2_i1_orf1;TRINITY_DN4276_c0_g1_i6_orf1;TRINITY_DN64403_c0_g2_i1_orf1;TRINITY_DN2749_c4_g1_i2_orf1;TRINITY_DN18128_c0_g1_i4_orf1
molecular_function	deacetylase activity	GO:0019213	2	2/1043	TRINITY_DN542_c0_g2_i1_orf1;TRINITY_DN82801_c0_g1_i1_orf1
molecular_function	deaminase activity	GO:0019239	2	2/1043	TRINITY_DN38180_c0_g1_i3_orf1;TRINITY_DN98242_c0_g1_i1_orf1
molecular_function	palmitoyl hydrolase activity	GO:0098599	1	1/1043	TRINITY_DN4817_c0_g1_i4_orf1
molecular_function	hydrolase activity, acting on ether bonds	GO:0016801	1	1/1043	TRINITY_DN11172_c0_g1_i4_orf1
molecular_function	FAD-AMP lyase (cyclizing) activity	GO:0034012	1	1/1043	TRINITY_DN52244_c1_g1_i1_orf1
molecular_function	oxidoreductase activity, acting on CH-OH group	GO:0016614	8	8/1043	TRINITY_DN10900_c0_g1_i7_orf1;TRINITY_DN9286_c0_g1_i2_orf1;TRINITY_DN20658_c0_g2_i3_orf1;TRINITY_DN42759_c0_g2_i1_orf1;TRINITY_DN38424_c0_g1_i1_orf1;TRINITY_DN1264_c0_g1_i2_orf1;TRINITY_DN77830_c0_g2_i2_orf1;TRINITY_DN31609_c0_g1_i3_orf1
molecular_function	oxidoreductase activity, acting on the aldehyde	GO:0016903	10	10/1043	TRINITY_DN7075_c0_g2_i1_orf1;TRINITY_DN1103_c0_g1_i12_orf1;TRINITY_DN6291_c0_g1_i4_orf1;TRINITY_DN81031_c0_g1_i1_orf1;TRINITY_DN1293_c1_g1_i4_orf1;TRINITY_DN11826_c0_g1_i4_orf1;TRINITY_DN64772_c0_g1_i1_orf1;TRINITY_DN2719_c1_g1_i6_orf1;TRINITY_DN28366_c0_g1_i1_orf1;TRINITY_DN64892_c0_g1_i1_orf1
molecular_function	oxidoreductase activity, acting on a heme group	GO:0016675	1	1/1043	TRINITY_DN76036_c0_g1_i1_orf1
molecular_function	oxidoreductase activity, acting on the CH-NH gr	GO:0016645	5	5/1043	TRINITY_DN20527_c0_g1_i1_orf1;TRINITY_DN5432_c1_g1_i3_orf1;TRINITY_DN1760_c0_g1_i4_orf1;TRINITY_DN92153_c0_g2_i2_orf1;TRINITY_DN244_c1_g1_i5_orf1
molecular_function	dioxygenase activity	GO:0051213	6	6/1043	TRINITY_DN1153_c1_g1_i1_orf1;TRINITY_DN4822_c0_g1_i6_orf1;TRINITY_DN5497_c0_g1_i6_orf1;TRINITY_DN89083_c0_g1_i1_orf1;TRINITY_DN57900_c0_g1_i2_orf1;TRINITY_DN4822_c0_g1_i9_orf1
molecular_function	electron transfer activity	GO:0009055	3	3/1043	TRINITY_DN76036_c0_g1_i1_orf1;TRINITY_DN5432_c1_g1_i3_orf1;TRINITY_DN49265_c0_g3_i2_orf1
molecular_function	oxidoreductase activity, acting on paired donors	GO:0016705	29	29/1043	TRINITY_DN43369_c0_g2_i1_orf1;TRINITY_DN8985_c0_g1_i4_orf1;TRINITY_DN9608_c0_g1_i3_orf1;TRINITY_DN3949_c1_g1_i1_orf1;TRINITY_DN120500_c0_g1_i1_orf1;TRINITY_DN4497_c0_g1_i4_orf1;TRINITY_DN863_c0_g1_i6_orf1;TRINITY_DN89083_c0_g1_i1_orf1;TRINITY_DN14262_c0_g1_i5_orf1;TRINITY_DN7212_c0_g1_i4_orf1;TRINITY_DN27903_c0_g1_i1_orf1;TRINITY_DN57765_c0_g1_i1_orf1;TRINITY_DN3732_c0_g1_i2_orf1;TRINITY_DN829_c0_g1_i8_orf1;TRINITY_DN16122_c0_g1_i4_orf1;TRINITY_DN50743_c0_g1_i1_orf1;TRINITY_DN448_c0_g1_i20_orf1;TRINITY_DN1999_c0_g1_i9_orf1;TRINITY_DN27045_c0_g1_i1_orf1;TRINITY_DN2264_c0_g1_i1_orf1;TRINITY_DN1960_c5_g1_i3_orf1;TRINITY_DN5439_c0_g1_i2_orf1;TRINITY_DN52887_c0_g1_i1_orf1;TRINITY_DN4497_c2_g1_i3_orf1;TRINITY_DN23398_c0_g1_i1_orf1;TRINITY_DN5126_c0_g1_i3_orf1;TRINITY_DN2392_c0_g2_i1_orf1;TRINITY_DN5126_c0_g2_i1_orf1;TRINITY_DN4321_c0_g1_i1_orf1
molecular_function	oxidoreductase activity, acting on single donors	GO:0016701	3	3/1043	TRINITY_DN4822_c0_g1_i6_orf1;TRINITY_DN4822_c0_g1_i9_orf1;TRINITY_DN5497_c0_g1_i6_orf1
molecular_function	oxidoreductase activity, acting on the CH-CH gr	GO:0016627	6	6/1043	TRINITY_DN323_c0_g2_i5_orf1;TRINITY_DN10900_c0_g1_i7_orf1;TRINITY_DN3588_c0_g1_i4_orf1;TRINITY_DN20658_c0_g2_i3_orf1;TRINITY_DN42759_c0_g2_i1_orf1;TRINITY_DN77830_c0_g2_i2_orf1
molecular_function	oxidoreductase activity, acting on NAD(P)H	GO:0016651	4	4/1043	TRINITY_DN49221_c0_g1_i1_orf1;TRINITY_DN33430_c0_g1_i5_orf1;TRINITY_DN4497_c0_g1_i4_orf1;TRINITY_DN52887_c0_g1_i1_orf1
molecular_function	oxidoreductase activity, acting on superoxide ra	GO:0016721	4	4/1043	TRINITY_DN103107_c0_g1_i2_orf1;TRINITY_DN37307_c0_g1_i4_orf1;TRINITY_DN8637_c0_g1_i1_orf1;TRINITY_DN16400_c0_g2_i1_orf1
molecular_function	oxidoreductase activity, acting on metal ions	GO:0016722	2	2/1043	TRINITY_DN65681_c0_g1_i1_orf1;TRINITY_DN136031_c0_g1_i7_orf1
molecular_function	oxidoreductase activity, acting on CH or CH2 gr	GO:0016725	2	2/1043	TRINITY_DN4835_c0_g1_i2_orf1;TRINITY_DN129_c0_g1_i6_orf1
molecular_function	oxidoreductase activity, acting on peroxide as a	GO:0016684	4	4/1043	TRINITY_DN2542_c0_g2_i1_orf1;TRINITY_DN54387_c0_g1_i1_orf1;TRINITY_DN285_c0_g1_i4_orf1;TRINITY_DN6580_c0_g1_i4_orf1
molecular_function	monooxygenase activity	GO:0004497	28	28/1043	TRINITY_DN43369_c0_g2_i1_orf1;TRINITY_DN8985_c0_g1_i4_orf1;TRINITY_DN9608_c0_g1_i3_orf1;TRINITY_DN3949_c1_g1_i1_orf1;TRINITY_DN120500_c0_g1_i1_orf1;TRINITY_DN4497_c0_g1_i4_orf1;TRINITY_DN2392_c0_g2_i1_orf1;TRINITY_DN14262_c0_g1_i5_orf1;TRINITY_DN7212_c0_g1_i4_orf1;TRINITY_DN57765_c0_g1_i1_orf1;TRINITY_DN3732_c0_g1_i2_orf1;TRINITY_DN829_c0_g1_i8_orf1;TRINITY_DN16122_c0_g1_i4_orf1;TRINITY_DN50743_c0_g1_i1_orf1;TRINITY_DN84357_c0_g1_i1_orf1;TRINITY_DN448_c0_g1_i20_orf1;TRINITY_DN27045_c0_g1_i1_orf1;TRINITY_DN2264_c0_g1_i1_orf1;TRINITY_DN1960_c5_g1_i3_orf1;TRINITY_DN5439_c0_g1_i2_orf1;TRINITY_DN52887_c0_g1_i1_orf1;TRINITY_DN4497_c2_g1_i3_orf1;TRINITY_DN23398_c0_g1_i1_orf1;TRINITY_DN5126_c0_g1_i3_orf1;TRINITY_DN863_c0_g1_i6_orf1;TRINITY_DN5126_c0_g2_i1_orf1;TRINITY_DN2338_c0_g1_i3_orf1;TRINITY_DN31584_c0_g2_i2_orf1
molecular_function	oxidoreductase activity, acting on other nitroger	GO:0016661	1	1/1043	TRINITY_DN2559_c0_g1_i4_orf1
molecular_function	oxidoreductase activity, acting on a sulfur group	GO:0016667	5	5/1043	TRINITY_DN920_c0_g1_i6_orf1;TRINITY_DN920_c0_g1_i4_orf1;TRINITY_DN51938_c0_g3_i1_orf1;TRINITY_DN1491_c0_g1_i4_orf1;TRINITY_DN24689_c0_g1_i1_orf1
molecular_function	lysozyme activity	GO:0003796	2	2/1043	TRINITY_DN467_c3_g1_i5_orf1;TRINITY_DN1098_c1_g1_i4_orf1
molecular_function	N-acetylmuramoyl-L-alanine amidase activity	GO:0008745	2	2/1043	TRINITY_DN1534_c0_g1_i3_orf1;TRINITY_DN827_c1_g1_i1_orf1
molecular_function	intramolecular oxidoreductase activity	GO:0016860	3	3/1043	TRINITY_DN4360_c0_g1_i4_orf1;TRINITY_DN51938_c0_g3_i1_orf1;TRINITY_DN27035_c0_g1_i1_orf1
molecular_function	cis-trans isomerase activity	GO:0016859	2	2/1043	TRINITY_DN119291_c0_g1_i1_orf1;TRINITY_DN12293_c0_g1_i1_orf1
molecular_function	catalytic activity, acting on RNA	GO:0140098	15	15/1043	TRINITY_DN1865_c1_g1_i3_orf1;TRINITY_DN34465_c0_g1_i1_orf1;TRINITY_DN44288_c0_g1_i2_orf1;TRINITY_DN825_c23_g1_i5_orf1;TRINITY_DN2299_c0_g1_i3_orf1;TRINITY_DN107288_c0_g1_i2_orf1;TRINITY_DN2953_c1_g1_i10_orf1;TRINITY_DN3712_c0_g1_i1_orf1;TRINITY_DN9207_c0_g1_i1_orf1;TRINITY_DN17312_c0_g1_i1_orf1;TRINITY_DN27033_c1_g1_i3_orfp1;TRINITY_DN810_c0_g1_i4_orf1;TRINITY_DN41179_c0_g1_i1_orf1;TRINITY_DN5962_c0_g1_i1_orf1;TRINITY_DN15160_c0_g1_i1_orf1
molecular_function	catalytic activity, acting on DNA	GO:0140097	3	3/1043	TRINITY_DN110534_c0_g1_i3_orf1;TRINITY_DN40434_c0_g1_i2_orf1;TRINITY_DN87603_c0_g2_i1_orf1
molecular_function	helicase activity	GO:0004386	3	3/1043	TRINITY_DN44288_c0_g1_i2_orf1;TRINITY_DN6556_c0_g1_i7_orf1;TRINITY_DN810_c0_g1_i4_orf1
molecular_function	transferase activity, transferring alkyl or aryl (oth	GO:0016765	14	14/1043	TRINITY_DN920_c0_g1_i4_orf1;TRINITY_DN1305_c0_g1_i6_orf1;TRINITY_DN3929_c0_g3_i3_orf1;TRINITY_DN128231_c0_g1_i5_orf1;TRINITY_DN22046_c1_g1_i5_orf1;TRINITY_DN15597_c0_g1_i1_orf1;TRINITY_DN82320_c0_g1_i2_orf1;TRINITY_DN4695_c0_g1_i3_orf1;TRINITY_DN57462_c0_g1_i1_orf1;TRINITY_DN22_c0_g1_i3_orf1;TRINITY_DN8651_c0_g1_i16_orf1;TRINITY_DN62707_c0_g1_i1_orf1;TRINITY_DN225_c0_g1_i6_orf1;TRINITY_DN920_c0_g1_i6_orf1
molecular_function	transferase activity, transferring nitrogenous gro	GO:0016769	4	4/1043	TRINITY_DN6908_c0_g1_i1_orf1;TRINITY_DN6908_c0_g1_i3_orf1;TRINITY_DN1824_c0_g2_i2_orf1;TRINITY_DN14565_c0_g1_i11_orf1
molecular_function	transferase activity, transferring sulphur-contain	GO:0016782	1	1/1043	TRINITY_DN16516_c0_g1_i1_orf1
molecular_function	glycosyltransferase activity	GO:0016757	11	11/1043	TRINITY_DN49786_c0_g1_i1_orf1;TRINITY_DN2967_c0_g1_i4_orf1;TRINITY_DN31390_c0_g1_i2_orf1;TRINITY_DN6992_c0_g1_i6_orf1;TRINITY_DN3355_c0_g2_i4_orf1;TRINITY_DN25251_c0_g2_i1_orf1;TRINITY_DN48602_c0_g1_i6_orf1;TRINITY_DN15157_c0_g1_i1_orf1;TRINITY_DN2967_c0_g1_i7_orf1;TRINITY_DN56164_c0_g1_i1_orf1;TRINITY_DN9079_c1_g1_i1_orf1
molecular_function	transferase activity, transferring phosphorus-cor	GO:0016772	30	30/1043	TRINITY_DN4247_c0_g1_i4_orf1;TRINITY_DN96170_c0_g2_i1_orf1;TRINITY_DN2719_c1_g1_i6_orf1;TRINITY_DN4056_c0_g1_i8_orf1;TRINITY_DN9109_c0_g1_i1_orf1;TRINITY_DN8603_c0_g1_i1_orf1;TRINITY_DN9207_c0_g1_i1_orf1;TRINITY_DN2110_c0_g1_i3_orf1;TRINITY_DN1957_c0_g1_i4_orf1;TRINITY_DN18782_c0_g1_i4_orf1;TRINITY_DN26994_c1_g1_i6_orf1;TRINITY_DN11015_c0_g1_i8_orf1;TRINITY_DN1154_c0_g1_i1_orf1;TRINITY_DN110534_c0_g1_i3_orf1;TRINITY_DN1266_c2_g1_i1_orf1;TRINITY_DN4798_c0_g1_i3_orf1;TRINITY_DN52244_c1_g1_i1_orf1;TRINITY_DN5811_c0_g1_i4_orf1;TRINITY_DN2618_c0_g1_i3_orf1;TRINITY_DN1173_c1_g1_i10_orf1;TRINITY_DN9156_c0_g1_i1_orf1;TRINITY_DN16487_c0_g1_i1_orf1;TRINITY_DN4929_c1_g2_i5_orf1;TRINITY_DN46715_c0_g1_i1_orf1;TRINITY_DN2299_c0_g1_i3_orf1;TRINITY_DN25997_c1_g2_i4_orf1;TRINITY_DN5697_c0_g1_i1_orf1;TRINITY_DN5029_c0_g1_i1_orf1;TRINITY_DN29956_c1_g1_i1_orf1;TRINITY_DN9979_c0_g1_i1_orf1

molecular_function	transferase activity, transferring one-carbon gro	GO:0016741	7	7/1043	TRINITY_DN115210_c0_g4_i1_orf1;TRINITY_DN5748_c0_g1_i6_orf1;TRINITY_DN1216_c0_g1_i4_orf1;TRINITY_DN22674_c0_g1_i2_orf1;TRINITY_DN17312_c0_g1_i1_orf1;TRINITY_DN2457_c0_g1_i8_orf1;TRINITY_DN5962_c0_g1_i1_orf1
molecular_function	acyltransferase activity	GO:0016746	10	10/1043	TRINITY_DN47389_c0_g1_i2_orf1;TRINITY_DN3651_c0_g1_i5_orf1;TRINITY_DN5153_c1_g1_i1_orf1;TRINITY_DN1081_c0_g1_i7_orf1;TRINITY_DN10900_c0_g1_i7_orf1;TRINITY_DN20442_c0_g2_i1_orf1;TRINITY_DN3105_c0_g1_i4_orf1;TRINITY_DN42759_c0_g2_i1_orf1;TRINITY_DN1362_c0_g1_i4_orf1;TRINITY_DN76283_c0_g2_i1_orf1
