# Supplementary material for: Comparative Proteomics and Metabonomics Analysis of Different Diapause Stages Revealed a New Regulation Mechanism of Diapause in Loxostege sticticalis (Lepidoptera: Pyralidae)
Source: Molecules. 2024 Jul 25;29(15):3472. doi: 10.3390/molecules29153472 (PMC11314584; doi:10.3390/molecules29153472)
Supplement: Supplementary file 1 [file molecules-29-03472-s001.zip › analysis process/proteomic/GO annotations analysis/RDvsCT all.pdf]

Term Type	GO Term	GO ID	JCZY_vs_LCL_all num	JCZY_vs_LCL_all percent	JCZY vs LCL all Accession ids
biological_process	immune response-activating signal transduction	GO:0002757	2	2/2360	TRINITY_DN2170_c0_g2_i1_orf1;TRINITY_DN2170_c1_g1_i3_orf1
biological_process	activation of innate immune response	GO:0002218	5	5/2360	TRINITY_DN8685_c0_g1_i5_orf1;TRINITY_DN1091_c0_g2_i10_orf1;TRINITY_DN2170_c0_g2_i1_orf1;TRINITY_DN2170_c1_g1_i3_orf1;TRINITY_DN5880_c0_g2_i2_orf1
biological_process	innate immune response	GO:0045087	12	12/2360	TRINITY_DN1534_c0_g1_i3_orf1;TRINITY_DN479_c6_g1_i2_orf1;TRINITY_DN8685_c0_g1_i5_orf1;TRINITY_DN1444_c1_g1_i5_orf1;TRINITY_DN1091_c0_g2_i10_orf1;TRINITY_DN6098_c1_g1_i5_orf1;TRINITY_DN2170_c0_g2_i1_orf1;TRINITY_DN2170_c1_g1_i3_orf1;TRINITY_DN15706_c0_g2_i5_orf1;TRINITY_DN1666_c0_g1_i2_orf1;TRINITY_DN5235_c0_g1_i7_orf1;TRINITY_DN5880_c0_g2_i2_orf1
biological_process	regulation of catalytic activity	GO:0050790	10	10/2360	TRINITY_DN1475_c0_g1_i6_orf1;TRINITY_DN8473_c0_g1_i6_orf1;TRINITY_DN518_c0_g1_i1_orf1;TRINITY_DN802_c0_g1_i2_orf1;TRINITY_DN1328_c0_g1_i6_orf1;TRINITY_DN28661_c0_g1_i1_orf1;TRINITY_DN50074_c0_g1_i1_orf1;TRINITY_DN46022_c0_g1_i1_orf1;TRINITY_DN147475_c0_g1_i1_orf1;TRINITY_DN140538_c0_g2_i1_orf1
biological_process	positive regulation of molecular function	GO:0044093	10	10/2360	TRINITY_DN5406_c0_g2_i1_orf1;TRINITY_DN9475_c0_g1_i6_orf1;TRINITY_DN802_c0_g1_i2_orf1;TRINITY_DN1352_c0_g1_i5_orf1;TRINITY_DN5553_c0_g1_i4_orf1;TRINITY_DN2175_c0_g1_i4_orf1;TRINITY_DN46022_c0_g1_i1_orf1;TRINITY_DN48097_c0_g1_i1_orf1;TRINITY_DN50074_c0_g1_i1_orf1;TRINITY_DN140538_c0_g2_i1_orf1
biological_process	negative regulation of molecular function	GO:0044092	3	3/2360	TRINITY_DN1328_c0_g1_i6_orf1;TRINITY_DN5442_c0_g1_i4_orf1;TRINITY_DN140538_c0_g2_i1_orf1
biological_process	regulation of binding	GO:0051098	3	3/2360	TRINITY_DN48097_c0_g1_i1_orf1;TRINITY_DN140538_c0_g2_i1_orf1;TRINITY_DN147475_c0_g1_i1_orf1
biological_process	regulation of ATP-dependent activity	GO:0043462	1	1/2360	TRINITY_DN5442_c0_g1_i4_orf1
biological_process	regulation of transporter activity	GO:0032409	5	5/2360	TRINITY_DN9475_c0_g1_i6_orf1;TRINITY_DN1352_c0_g1_i5_orf1;TRINITY_DN5406_c0_g2_i1_orf1;TRINITY_DN5553_c0_g1_i4_orf1;TRINITY_DN2175_c0_g1_i4_orf1
biological_process	regulation of metabolic process	GO:0019222	49	49/2360	TRINITY_DN19260_c0_g1_i5_orf1;TRINITY_DN8702_c0_g1_i1_orf1;TRINITY_DN1706_c0_g1_i7_orf1;TRINITY_DN20442_c0_g2_i1_orf1;TRINITY_DN23360_c0_g1_i3_orf1;TRINITY_DN1710_c0_g2_i2_orf1;TRINITY_DN288_c0_g1_i9_orf1;TRINITY_DN9938_c0_g2_i1_orf1;TRINITY_DN48097_c0_g1_i1_orf1;TRINITY_DN44407_c0_g4_i2_orf1;TRINITY_DN1475_c0_g1_i6_orf1;TRINITY_DN5262_c0_g1_i7_orf1;TRINITY_DN67649_c0_g1_i1_orf1;TRINITY_DN21150_c0_g1_i4_orf1;TRINITY_DN12771_c0_g1_i1_orf1;TRINITY_DN142442_c0_g1_i1_orf1;TRINITY_DN44877_c0_g1_i2_orf1;TRINITY_DN1328_c0_g1_i6_orf1;TRINITY_DN3457_c0_g1_i4_orf1;TRINITY_DN50074_c0_g1_i1_orf1;TRINITY_DN34689_c0_g1_i4_orf1;TRINITY_DN18681_c0_g1_i7_orf1;TRINITY_DN1639_c0_g2_i2_orf1;TRINITY_DN17655_c0_g1_i1_orf1;TRINITY_DN5562_c0_g1_i3_orf1;TRINITY_DN50085_c0_g1_i1_orf1;TRINITY_DN15256_c0_g1_i8_orf1;TRINITY_DN18036_c0_g1_i7_orf1;TRINITY_DN3366_c0_g1_i6_orf1;TRINITY_DN8473_c0_g1_i6_orf1;TRINITY_DN140538_c0_g2_i1_orf1;TRINITY_DN72_c0_g1_i16_orf1;TRINITY_DN96557_c0_g1_i1_orf1;TRINITY_DN45449_c0_g1_i1_orf1;TRINITY_DN4813_c0_g1_i5_orf1;TRINITY_DN33893_c0_g1_i1_orf1;TRINITY_DN13384_c0_g1_i1_orf1;TRINITY_DN31342_c2_g2_i1_orf1;TRINITY_DN2802_c0_g1_i1_orf1;TRINITY_DN2802_c1_g1_i1_orf1;TRINITY_DN77572_c0_g1_i1_orf1;TRINITY_DN147475_c0_g1_i1_orf1;TRINITY_DN3649_c0_g1_i6_orf1;TRINITY_DN31585_c0_g1_i1_orf1;TRINITY_DN46022_c0_g1_i1_orf1;TRINITY_DN18563_c2_g1_i1_orf1;TRINITY_DN4950_c0_g1_i2_orf1;TRINITY_DN21341_c0_g1_i1_orf1;TRINITY_DN9517_c0_g1_i7_orf1
biological_process	regulation of response to stimulus	GO:0048583	19	19/2360	TRINITY_DN24323_c0_g1_i3_orf1;TRINITY_DN1091_c0_g2_i10_orf1;TRINITY_DN17655_c0_g1_i1_orf1;TRINITY_DN7316_c0_g2_i1_orf1;TRINITY_DN8685_c0_g1_i5_orf1;TRINITY_DN479_c6_g1_i2_orf1;TRINITY_DN4464_c0_g2_i1_orf1;TRINITY_DN147475_c0_g1_i1_orf1;TRINITY_DN22572_c0_g1_i1_orf1;TRINITY_DN2170_c0_g2_i1_orf1;TRINITY_DN2170_c1_g1_i3_orf1;TRINITY_DN48983_c0_g1_i2_orf1;TRINITY_DN146119_c0_g1_i1_orf1;TRINITY_DN34745_c0_g2_i1_orf1;TRINITY_DN12320_c0_g1_i1_orf1;TRINITY_DN3833_c0_g1_i4_orf1;TRINITY_DN140538_c0_g2_i1_orf1;TRINITY_DN5880_c0_g2_i2_orf1;TRINITY_DN21367_c0_g1_i1_orf1
biological_process	regulation of developmental process	GO:0050793	1	1/2360	TRINITY_DN3887_c0_g1_i1_orf1
biological_process	regulation of cellular process	GO:0050794	84	84/2360	TRINITY_DN77572_c0_g1_i1_orf1;TRINITY_DN24323_c0_g1_i3_orf1;TRINITY_DN2942_c0_g1_i6_orf1;TRINITY_DN4159_c1_g1_i1_orf1;TRINITY_DN79000_c1_g1_i1_orf1;TRINITY_DN8702_c0_g1_i1_orf1;TRINITY_DN1706_c0_g1_i7_orf1;TRINITY_DN38371_c0_g1_i7_orf1;TRINITY_DN20442_c0_g2_i1_orf1;TRINITY_DN23360_c0_g1_i3_orf1;TRINITY_DN146119_c0_g1_i1_orf1;TRINITY_DN17655_c0_g1_i1_orf1;TRINITY_DN4676_c0_g1_i16_orf1;TRINITY_DN2623_c0_g1_i3_orf1;TRINITY_DN11245_c0_g1_i2_orf1;TRINITY_DN48983_c0_g1_i2_orf1;TRINITY_DN15706_c0_g2_i5_orf1;TRINITY_DN9938_c0_g2_i1_orf1;TRINITY_DN34745_c0_g2_i1_orf1;TRINITY_DN10455_c0_g1_i2_orf1;TRINITY_DN48097_c0_g1_i1_orf1;TRINITY_DN23020_c0_g1_i1_orf1;TRINITY_DN44407_c0_g4_i2_orf1;TRINITY_DN80424_c0_g1_i1_orf1;TRINITY_DN1475_c0_g1_i6_orf1;TRINITY_DN5262_c0_g1_i7_orf1;TRINITY_DN5406_c0_g2_i1_orf1;TRINITY_DN288_c0_g1_i9_orf1;TRINITY_DN14987_c0_g1_i3_orf1;TRINITY_DN8692_c0_g1_i2_orf1;TRINITY_DN7316_c0_g2_i1_orf1;TRINITY_DN142442_c0_g1_i1_orf1;TRINITY_DN4798_c0_g1_i3_orf1;TRINITY_DN1405_c0_g1_i1_orf1;TRINITY_DN3457_c0_g1_i4_orf1;TRINITY_DN1352_c0_g1_i5_orf1;TRINITY_DN33418_c0_g1_i1_orf1;TRINITY_DN5553_c0_g1_i4_orf1;TRINITY_DN9475_c0_g1_i6_orf1;TRINITY_DN3649_c0_g1_i6_orf1;TRINITY_DN18681_c0_g1_i7_orf1;TRINITY_DN1639_c0_g2_i2_orf1;TRINITY_DN1710_c0_g2_i2_orf1;TRINITY_DN5562_c0_g1_i3_orf1;TRINITY_DN32700_c0_g1_i2_orf1;TRINITY_DN50085_c0_g1_i1_orf1;TRINITY_DN22572_c0_g1_i1_orf1;TRINITY_DN15478_c0_g1_i1_orf1;TRINITY_DN13216_c0_g1_i5_orf1;TRINITY_DN15256_c0_g1_i8_orf1;TRINITY_DN5182_c0_g1_i5_orf1;TRINITY_DN2170_c0_g2_i1_orf1;TRINITY_DN46633_c0_g1_i4_orf1;TRINITY_DN21367_c0_g1_i1_orf1;TRINITY_DN18036_c0_g1_i7_orf1;TRINITY_DN147475_c0_g1_i1_orf1;TRINITY_DN3366_c0_g1_i6_orf1;TRINITY_DN62729_c0_g1_i13_orf1;TRINITY_DN4439_c0_g1_i2_orf1;TRINITY_DN140538_c0_g2_i1_orf1;TRINITY_DN72_c0_g1_i16_orf1;TRINITY_DN96557_c0_g1_i1_orf1;TRINITY_DN45449_c0_g1_i1_orf1;TRINITY_DN21150_c0_g1_i4_orf1;TRINITY_DN8473_c0_g1_i6_orf1;TRINITY_DN2802_c0_g1_i1_orf1;TRINITY_DN33893_c0_g1_i1_orf1;TRINITY_DN15247_c0_g1_i2_orf1;TRINITY_DN67649_c0_g1_i1_orf1;TRINITY_DN9146_c0_g1_i1_orf1;TRINITY_DN2802_c1_g1_i1_orf1;TRINITY_DN96739_c0_g1_i1_orf1;TRINITY_DN4464_c0_g2_i1_orf1;TRINITY_DN802_c0_g1_i2_orf1;TRINITY_DN2170_c1_g1_i3_orf1;TRINITY_DN31585_c0_g1_i1_orf1;TRINITY_DN12320_c0_g1_i1_orf1;TRINITY_DN2175_c0_g1_i4_orf1;TRINITY_DN18563_c2_g1_i1_orf1;TRINITY_DN804_c0_g1_i7_orf1;TRINITY_DN3833_c0_g1_i4_orf1;TRINITY_DN16011_c0_g1_i3_orf1;TRINITY_DN21341_c0_g1_i1_orf1;TRINITY_DN9517_c0_g1_i7_orf1
biological_process	regulation of locomotion	GO:0040012	2	2/2360	TRINITY_DN140538_c0_g2_i1_orf1;TRINITY_DN147475_c0_g1_i1_orf1
biological_process	regulation of localization	GO:0032879	8	8/2360	TRINITY_DN5406_c0_g2_i1_orf1;TRINITY_DN14987_c0_g1_i3_orf1;TRINITY_DN9475_c0_g1_i6_orf1;TRINITY_DN96739_c0_g1_i1_orf1;TRINITY_DN1352_c0_g1_i5_orf1;TRINITY_DN5553_c0_g1_i4_orf1;TRINITY_DN2175_c0_g1_i4_orf1;TRINITY_DN140538_c0_g2_i1_orf1
biological_process	regulation of multicellular organismal process	GO:0051239	4	4/2360	TRINITY_DN1455_c0_g1_i8_orf1;TRINITY_DN96739_c0_g1_i1_orf1;TRINITY_DN1455_c0_g1_i4_orf1;TRINITY_DN147475_c0_g1_i1_orf1
biological_process	regulation of immune system process	GO:0002682	6	6/2360	TRINITY_DN479_c6_g1_i2_orf1;TRINITY_DN8685_c0_g1_i5_orf1;TRINITY_DN1091_c0_g2_i10_orf1;TRINITY_DN2170_c0_g2_i1_orf1;TRINITY_DN2170_c1_g1_i3_orf1;TRINITY_DN5880_c0_g2_i2_orf1
biological_process	positive regulation of biological process	GO:0048518	25	25/2360	TRINITY_DN288_c0_g1_i9_orf1;TRINITY_DN8685_c0_g1_i5_orf1;TRINITY_DN9475_c0_g1_i6_orf1;TRINITY_DN1710_c0_g2_i2_orf1;TRINITY_DN15706_c0_g2_i5_orf1;TRINITY_DN146119_c0_g1_i1_orf1;TRINITY_DN48097_c0_g1_i1_orf1;TRINITY_DN44407_c0_g4_i2_orf1;TRINITY_DN5406_c0_g2_i1_orf1;TRINITY_DN22572_c0_g1_i1_orf1;TRINITY_DN3457_c0_g1_i4_orf1;TRINITY_DN1352_c0_g1_i5_orf1;TRINITY_DN5553_c0_g1_i4_orf1;TRINITY_DN1639_c0_g2_i2_orf1;TRINITY_DN17655_c0_g1_i1_orf1;TRINITY_DN50074_c0_g1_i1_orf1;TRINITY_DN1091_c0_g2_i10_orf1;TRINITY_DN2170_c0_g2_i1_orf1;TRINITY_DN21367_c0_g1_i1_orf1;TRINITY_DN140538_c0_g2_i1_orf1;TRINITY_DN147475_c0_g1_i1_orf1;TRINITY_DN2170_c1_g1_i3_orf1;TRINITY_DN2175_c0_g1_i4_orf1;TRINITY_DN3833_c0_g1_i4_orf1;TRINITY_DN5880_c0_g2_i2_orf1
biological_process	negative regulation of biological process	GO:0048519	14	14/2360	TRINITY_DN1639_c0_g2_i2_orf1;TRINITY_DN96557_c0_g1_i1_orf1;TRINITY_DN8702_c0_g1_i1_orf1;TRINITY_DN4813_c0_g1_i5_orf1;TRINITY_DN12771_c0_g1_i1_orf1;TRINITY_DN44877_c0_g1_i2_orf1;TRINITY_DN1328_c0_g1_i6_orf1;TRINITY_DN96739_c0_g1_i1_orf1;TRINITY_DN4159_c1_g1_i1_orf1;TRINITY_DN46022_c0_g1_i1_orf1;TRINITY_DN34689_c0_g1_i4_orf1;TRINITY_DN147475_c0_g1_i1_orf1;TRINITY_DN140538_c0_g2_i1_orf1;TRINITY_DN9517_c0_g1_i7_orf1
biological_process	regulation of signaling	GO:0023051	17	17/2360	TRINITY_DN24323_c0_g1_i3_orf1;TRINITY_DN5406_c0_g2_i1_orf1;TRINITY_DN7316_c0_g2_i1_orf1;TRINITY_DN9475_c0_g1_i6_orf1;TRINITY_DN2175_c0_g1_i4_orf1;TRINITY_DN4464_c0_g2_i1_orf1;TRINITY_DN147475_c0_g1_i1_orf1;TRINITY_DN22572_c0_g1_i1_orf1;TRINITY_DN1352_c0_g1_i5_orf1;TRINITY_DN12320_c0_g1_i1_orf1;TRINITY_DN48983_c0_g1_i2_orf1;TRINITY_DN5553_c0_g1_i4_orf1;TRINITY_DN146119_c0_g1_i1_orf1;TRINITY_DN34745_c0_g2_i1_orf1;TRINITY_DN3833_c0_g1_i4_orf1;TRINITY_DN140538_c0_g2_i1_orf1;TRINITY_DN21367_c0_g1_i1_orf1
biological_process	regulation of neurotransmitter levels	GO:0001505	2	2/2360	TRINITY_DN17693_c0_g1_i10_orf1;TRINITY_DN14565_c0_g1_i11_orf1
biological_process	regulation of body fluid levels	GO:0050878	1	1/2360	TRINITY_DN4016_c0_g1_i1_orf1
biological_process	homeostatic process	GO:0042592	10	10/2360	TRINITY_DN96557_c0_g1_i1_orf1;TRINITY_DN46625_c0_g1_i1_orf1;TRINITY_DN65681_c0_g1_i1_orf1;TRINITY_DN1423_c0_g1_i4_orf1;TRINITY_DN2433_c0_g1_i3_orf1;TRINITY_DN1423_c0_g1_i8_orf1;TRINITY_DN3461_c0_g1_i1_orf1;TRINITY_DN96739_c0_g1_i1_orf1;TRINITY_DN15812_c0_g1_i2_orf1;TRINITY_DN44256_c0_g1_i1_orf1
biological_process	regulation of anatomical structure size	GO:0090066	5	5/2360	TRINITY_DN10455_c0_g1_i2_orf1;TRINITY_DN4439_c0_g1_i2_orf1;TRINITY_DN4159_c1_g1_i1_orf1;TRINITY_DN80424_c0_g1_i1_orf1;TRINITY_DN23020_c0_g1_i1_orf1
biological_process	regulation of RNA stability	GO:0043487	2	2/2360	TRINITY_DN5262_c0_g1_i7_orf1;TRINITY_DN21341_c0_g1_i1_orf1
biological_process	regulation of protein stability	GO:0031647	1	1/2360	TRINITY_DN140538_c0_g2_i1_orf1

biological_process	organonitrogen compound metabolic process	GO:1901564	283	283/2360	TRINITY_DN24469_c0_g2_i2_orf1;TRINITY_DN2065_c1_g2_i1_orf1;TRINITY_DN10364_c0_g1_i5_orf1;TRINITY_DN14565_c0_g1_i11_orf1;TRINITY_DN98242_c0_g1_i1_orf1;TRINITY_DN39404_c0_g1_i7_orf1;TRINITY_DN3194_c0_g1_i6_orf1;TRINITY_DN1827_c0_g1_i4_orf1;TRINITY_DN5564_c0_g1_i5_orf1;TRINITY_DN863_c0_g1_i6_orf1;TRINITY_DN181_c0_g1_i3_orf1;TRINITY_DN71610_c0_g1_i1_orf1;TRINITY_DN1533_c0_g2_i1_orf1;TRINITY_DN48983_c0_g1_i2_orf1;TRINITY_DN28661_c0_g1_i1_orf1;TRINITY_DN135_c0_g1_i1_orf1;TRINITY_DN35763_c0_g1_i2_orf1;TRINITY_DN344_c1_g1_i1_orf1;TRINITY_DN2069_c1_g1_i8_orf1;TRINITY_DN2738_c1_g1_i3_orf1;TRINITY_DN4217_c0_g1_i2_orf1;TRINITY_DN38431_c0_g1_i1_orf1;TRINITY_DN33146_c0_g1_i1_orf1;TRINITY_DN30300_c0_g2_i1_orf1;TRINITY_DN2442_c0_g1_i2_orf1;TRINITY_DN8659_c0_g1_i1_orf1;TRINITY_DN1216_c0_g1_i4_orf1;TRINITY_DN70485_c0_g1_i2_orf1;TRINITY_DN142442_c0_g1_i1_orf1;TRINITY_DN37165_c0_g1_i4_orf1;TRINITY_DN24723_c2_g1_i1_orf1;TRINITY_DN16258_c0_g1_i2_orf1;TRINITY_DN31163_c1_g1_i4_orf1;TRINITY_DN14754_c0_g1_i6_orf1;TRINITY_DN29448_c0_g1_i1_orf1;TRINITY_DN11013_c0_g1_i3_orf1;TRINITY_DN15160_c0_g1_i1_orf1;TRINITY_DN14774_c0_g1_i4_orf1;TRINITY_DN6587_c0_g1_i3_orf1;TRINITY_DN4822_c0_g1_i6_orf1;TRINITY_DN21570_c0_g1_i1_orf1;TRINITY_DN2953_c1_g1_i2_orf1;TRINITY_DN17326_c0_g1_i8_orf1;TRINITY_DN95056_c0_g2_i2_orf1;TRINITY_DN3991_c0_g1_i6_orf1;TRINITY_DN2794_c1_g1_i8_orf1;TRINITY_DN17759_c0_g1_i5_orf1;TRINITY_DN3800_c0_g1_i7_orf1;TRINITY_DN84478_c0_g1_i8_orf1;TRINITY_DN4886_c0_g1_i6_orf1;TRINITY_DN27771_c0_g1_i1_orf1;TRINITY_DN6185_c0_g1_i12_orf1;TRINITY_DN6205_c0_g1_i8_orf1;TRINITY_DN6436_c0_g1_i1_orf1;TRINITY_DN140538_c0_g2_i1_orf1;TRINITY_DN391_c0_g1_i4_orf1;TRINITY_DN7134_c0_g1_i1_orf1;TRINITY_DN26195_c0_g1_i6_orf1;TRINITY_DN2224_c0_g1_i1_orf1;TRINITY_DN10769_c0_g1_i1_orf1;TRINITY_DN6563_c0_g1_i1_orf1;TRINITY_DN23167_c0_g2_i1_orf1;TRINITY_DN15591_c0_g1_i3_orf1;TRINITY_DN107261_c0_g1_i1_orf1;TRINITY_DN19187_c0_g1_i1_orf1;TRINITY_DN1380_c0_g1_i5_orf1;TRINITY_DN107288_c0_g1_i2_orf1;TRINITY_DN46715_c0_g1_i1_orf1;TRINITY_DN7808_c0_g1_i1_orf1;TRINITY_DN1749_c0_g2_i2_orf1;TRINITY_DN1552_c0_g1_i3_orf1;TRINITY_DN1706_c0_g1_i7_orf1;TRINITY_DN12526_c0_g1_i5_orf1;TRINITY_DN23360_c0_g1_i3_orf1;TRINITY_DN10824_c0_g1_i3_orf1;TRINITY_DN18222_c0_g1_i5_orf1;TRINITY_DN14398_c0_g1_i4_orf1;TRINITY_DN29034_c0_g1_i2_orf1;TRINITY_DN100821_c0_g1_i1_orf1;TRINITY_DN38562_c0_g1_i3_orf1;TRINITY_DN19537_c0_g1_i1_orf1;TRINITY_DN4189_c0_g2_i1_orf1;TRINITY_DN37923_c0_g1_i1_orf1;TRINITY_DN1272_c1_g1_i4_orf1;TRINITY_DN53760_c0_g1_i1_orf1;TRINITY_DN26947_c0_g1_i1_orf1;TRINITY_DN4451_c0_g2_i4_orf1;TRINITY_DN57798_c0_g1_i1_orf1;TRINITY_DN9871_c0_g1_i11_orf1;TRINITY_DN4125_c1_g1_i5_orf1;TRINITY_DN1789_c0_g1_i5_orf1;TRINITY_DN10548_c0_g2_i1_orf1;TRINITY_DN42461_c0_g1_i4_orf1;TRINITY_DN1274_c0_g1_i4_orf1;TRINITY_DN11125_c0_g1_i1_orf1;TRINITY_DN6813_c1_g1_i1_orf1;TRINITY_DN21719_c0_g1_i2_orf1;TRINITY_DN7583_c0_g1_i1_orf1;TRINITY_DN779_c0_g1_i3_orf1;TRINITY_DN1607_c0_g1_i16_orf1;TRINITY_DN277_c1_g1_i1_orf1;TRINITY_DN50787_c0_g2_i2_orf1;TRINITY_DN41_c0_g1_i3_orf1;TRINITY_DN45948_c1_g1_i1_orf1;TRINITY_DN36434_c0_g2_i3_orf1;TRINITY_DN12757_c0_g1_i1_orf1;TRINITY_DN140669_c0_g1_i1_orf1;TRINITY_DN875_c0_g1_i3_orf1;TRINITY_DN4145_c0_g1_i1_orf1;TRINITY_DN36144_c0_g1_i3_orf1;TRINITY_DN6423_c0_g1_i5_orf1;TRINITY_DN71863_c0_g1_i2_orf1;TRINITY_DN9591_c0_g1_i1_orf1;TRINITY_DN32700_c0_g1_i2_orf1;TRINITY_DN117844_c0_g1_i1_orf1;TRINITY_DN43431_c0_g1_i1_orf1;TRINITY_DN96_c0_g1_i1_orf1;TRINITY_DN3073_c0_g1_i7_orf1;TRINITY_DN3733_c0_g1_i1_orf1;TRINITY_DN5444_c0_g2_i1_orf1;TRINITY_DN4408_c6_g1_i1_orf1;TRINITY_DN52553_c0_g2_i1_orf1;TRINITY_DN3472_c0_g1_i6_orf1;TRINITY_DN6423_c0_g1_i6_orf1;TRINITY_DN140_c1_g1_i2_orf1;TRINITY_DN1266_c2_g1_i1_orf1;TRINITY_DN1718_c6_g1_i4_orf1;TRINITY_DN83295_c0_g1_i3_orf1;TRINITY_DN3773_c0_g1_i4_orf1;TRINITY_DN9062_c0_g2_i3_orf1;TRINITY_DN11948_c0_g1_i8_orf1;TRINITY_DN1262_c0_g1_i2_orf1;TRINITY_DN1528_c0_g1_i4_orf1;TRINITY_DN7776_c0_g1_i5_orf1;TRINITY_DN3499_c0_g1_i8_orf1;TRINITY_DN10403_c0_g1_i1_orf1;TRINITY_DN1459_c1_g1_i1_orf1;TRINITY_DN15136_c0_g1_i2_orf1;TRINITY_DN147458_c0_g1_i1_orf1;TRINITY_DN21218_c0_g1_i4_orf1;TRINITY_DN12397_c0_g1_i1_orf1;TRINITY_DN8692_c0_g1_i2_orf1;TRINITY_DN5235_c0_g1_i7_orf1;TRINITY_DN5064_c0_g1_i4_orf1;TRINITY_DN1068_c0_g1_i3_orf1;TRINITY_DN650_c0_g1_i3_orf1;TRINITY_DN152_c0_g1_i4_orf1;TRINITY_DN5112_c0_g1_i1_orf1;TRINITY_DN92153_c0_g2_i2_orf1;TRINITY_DN25534_c0_g1_i1_orf1;TRINITY_DN10766_c0_g1_i1_orf1;TRINITY_DN2043_c0_g1_i11_orf1;TRINITY_DN4798_c0_g1_i3_orf1;TRINITY_DN6365_c0_g1_i4_orf1;TRINITY_DN127056_c0_g1_i1_orf1;TRINITY_DN46202_c0_g1_i1_orf1;TRINITY_DN39813_c0_g1_i1_orf1;TRINITY_DN5182_c0_g1_i5_orf1;TRINITY_DN4817_c0_g1_i4_orf1;TRINITY_DN81803_c0_g2_i1_orf1;TRINITY_DN70409_c0_g1_i3_orf1;TRINITY_DN57111_c0_g1_i1_orf1;TRINITY_DN5310_c2_g1_i2_orf1;TRINITY_DN2040_c0_g1_i6_orf1;TRINITY_DN4571_c0_g1_i4_orf1;TRINITY_DN7212_c0_g1_i4_orf1;TRINITY_DN125_c0_g1_i2_orf1;TRINITY_DN62729_c0_g1_i13_orf1;TRINITY_DN49038_c0_g4_i1_orf1;TRINITY_DN4016_c0_g1_i1_orf1;TRINITY_DN38506_c0_g1_i4_orf1;TRINITY_DN934_c2_g1_i7_orf1;TRINITY_DN34479_c0_g1_i2_orf1;TRINITY_DN2953_c1_g1_i10_orf1;TRINITY_DN1405_c0_g1_i1_orf1;TRINITY_DN2559_c0_g1_i4_orf1;TRINITY_DN2803_c4_g1_i1_orf1;TRINITY_DN130778_c0_g1_i1_orf1;TRINITY_DN3343_c0_g2_i1_orf1;TRINITY_DN779_c0_g1_i12_orf1;TRINITY_DN2885_c1_g1_i2_orf1;TRINITY_DN18172_c0_g1_i6_orf1;TRINITY_DN19866_c0_g1_i4_orf1;TRINITY_DN84322_c0_g2_i1_orf1;[illegible]
biological_process	cellular nitrogen compound metabolic process	GO:0034641	184	184/2360	TRINITY_DN38230_c0_g1_i4_orf1;TRINITY_DN5670_c0_g1_i2_orf1;TRINITY_DN13350_c0_g1_i4_orf1;TRINITY_DN1827_c0_g1_i4_orf1;TRINITY_DN60787_c0_g1_i5_orf1;TRINITY_DN7122_c0_g1_i1_orf1;TRINITY_DN35669_c0_g1_i1_orf1;TRINITY_DN2054_c0_g1_i1_orf1;TRINITY_DN124950_c0_g2_i1_orf1;TRINITY_DN2738_c1_g1_i3_orf1;TRINITY_DN15040_c0_g4_i1_orf1;TRINITY_DN34134_c0_g2_i1_orf1;TRINITY_DN33146_c0_g1_i1_orf1;TRINITY_DN30300_c0_g2_i1_orf1;TRINITY_DN1216_c0_g1_i4_orf1;TRINITY_DN142442_c0_g1_i1_orf1;TRINITY_DN44877_c0_g1_i2_orf1;TRINITY_DN24723_c2_g1_i1_orf1;TRINITY_DN2953_c1_g1_i10_orf1;TRINITY_DN29448_c0_g1_i1_orf1;TRINITY_DN11013_c0_g1_i3_orf1;TRINITY_DN15160_c0_g1_i1_orf1;TRINITY_DN6587_c0_g1_i3_orf1;TRINITY_DN21570_c0_g1_i1_orf1;TRINITY_DN2953_c1_g1_i2_orf1;TRINITY_DN1393_c0_g1_i2_orf1;TRINITY_DN95056_c0_g2_i2_orf1;TRINITY_DN74889_c0_g1_i1_orf1;TRINITY_DN2794_c1_g1_i8_orf1;TRINITY_DN3800_c0_g1_i7_orf1;TRINITY_DN47123_c0_g1_i1_orf1;TRINITY_DN15256_c0_g1_i8_orf1;TRINITY_DN123184_c0_g1_i1_orf1;TRINITY_DN27771_c0_g1_i1_orf1;TRINITY_DN2769_c0_g1_i1_orf1;TRINITY_DN135_c0_g1_i1_orf1;TRINITY_DN37165_c0_g1_i4_orf1;TRINITY_DN26195_c0_g1_i6_orf1;TRINITY_DN2224_c0_g1_i1_orf1;TRINITY_DN2401_c0_g2_i1_orf1;TRINITY_DN20499_c0_g3_i1_orf1;TRINITY_DN5952_c0_g1_i6_orf1;TRINITY_DN4813_c0_g1_i5_orf1;TRINITY_DN107261_c0_g1_i1_orf1;TRINITY_DN15136_c0_g1_i2_orf1;TRINITY_DN107288_c0_g1_i2_orf1;TRINITY_DN7808_c0_g1_i1_orf1;TRINITY_DN56993_c0_g1_i4_orf1;TRINITY_DN14313_c0_g1_i1_orf1;TRINITY_DN2647_c0_g1_i3_orf1;TRINITY_DN3092_c0_g1_i2_orf1;TRINITY_DN1978_c0_g1_i4_orf1;TRINITY_DN17271_c0_g1_i1_orf1;TRINITY_DN115210_c0_g4_i1_orf1;TRINITY_DN23360_c0_g1_i3_orf1;TRINITY_DN45271_c0_g1_i1_orf1;TRINITY_DN18222_c0_g1_i5_orf1;TRINITY_DN14398_c0_g1_i4_orf1;TRINITY_DN1005_c0_g1_i5_orf1;TRINITY_DN37532_c0_g1_i1_orf1;TRINITY_DN51968_c0_g1_i1_orf1;TRINITY_DN15900_c0_g1_i6_orf1;TRINITY_DN81258_c0_g1_i2_orf1;TRINITY_DN21567_c0_g1_i7_orf1;TRINITY_DN30097_c0_g1_i2_orf1;TRINITY_DN1005_c0_g2_i1_orf1;TRINITY_DN8625_c0_g1_i1_orf1;TRINITY_DN7583_c0_g1_i1_orf1;TRINITY_DN779_c0_g1_i3_orf1;TRINITY_DN1607_c0_g1_i16_orf1;TRINITY_DN50787_c0_g2_i2_orf1;TRINITY_DN16978_c0_g1_i1_orf1;TRINITY_DN41664_c0_g1_i4_orf1;TRINITY_DN1091_c0_g3_i1_orf1;TRINITY_DN19034_c0_g1_i1_orf1;TRINITY_DN4145_c0_g1_i1_orf1;TRINITY_DN36144_c0_g1_i3_orf1;TRINITY_DN9591_c0_g1_i1_orf1;TRINITY_DN1616_c0_g1_i3_orf1;TRINITY_DN117844_c0_g1_i1_orf1;TRINITY_DN3733_c0_g1_i1_orf1;TRINITY_DN6642_c0_g1_i2_orf1;TRINITY_DN7212_c0_g1_i4_orf1;TRINITY_DN4408_c6_g1_i1_orf1;TRINITY_DN51568_c0_g1_i1_orf1;TRINITY_DN4707_c0_g1_i1_orf1;TRINITY_DN3472_c0_g1_i6_orf1;TRINITY_DN40945_c0_g1_i1_orf1;TRINITY_DN1718_c6_g1_i4_orf1;TRINITY_DN779_c0_g1_i12_orf1;TRINITY_DN31663_c0_g1_i2_orf1;TRINITY_DN139537_c0_g1_i1_orf1;TRINITY_DN1344_c0_g1_i1_orf1;TRINITY_DN107035_c0_g1_i1_orf1;TRINITY_DN147458_c0_g1_i1_orf1;TRINITY_DN4908_c1_g1_i5_orf1;TRINITY_DN12397_c0_g1_i1_orf1;TRINITY_DN5064_c0_g1_i4_orf1;TRINITY_DN291_c0_g1_i2_orf1;TRINITY_DN2749_c4_g1_i2_orf1;TRINITY_DN5112_c0_g1_i1_orf1;TRINITY_DN6325_c0_g1_i8_orf1;TRINITY_DN2749_c0_g1_i4_orf1;TRINITY_DN22951_c0_g1_i1_orf1;TRINITY_DN1750_c1_g1_i5_orf1;TRINITY_DN6365_c0_g1_i4_orf1;TRINITY_DN1768_c0_g1_i2_orf1;TRINITY_DN127056_c0_g1_i1_orf1;TRINITY_DN39813_c0_g1_i1_orf1;TRINITY_DN2718_c0_g1_i6_orf1;TRINITY_DN57918_c0_g1_i1_orf1;TRINITY_DN6563_c0_g1_i1_orf1;TRINITY_DN56270_c0_g1_i1_orf1;TRINITY_DN140669_c0_g1_i1_orf1;TRINITY_DN33346_c0_g1_i1_orf1;TRINITY_DN1554_c0_g1_i9_orf1;TRINITY_DN125_c0_g1_i2_orf1;TRINITY_DN49038_c0_g4_i1_orf1;TRINITY_DN4016_c0_g1_i1_orf1;TRINITY_DN934_c2_g1_i7_orf1;TRINITY_DN2559_c0_g1_i4_orf1;TRINITY_DN98242_c0_g1_i1_orf1;TRINITY_DN14477_c0_g1_i12_orf1;TRINITY_DN452_c1_g1_i3_orf1;TRINITY_DN34689_c0_g1_i4_orf1;TRINITY_DN4135_c0_g1_i5_orf1;TRINITY_DN26293_c0_g1_i4_orf1;TRINITY_DN3062_c0_g1_i1_orf1;TRINITY_DN7341_c0_g1_i8_orf1;TRINITY_DN19866_c0_g1_i4_orf1;TRINITY_DN24322_c0_g1_i4_orf1;TRINITY_DN84322_c0_g2_i1_orf1;TRINITY_DN5686_c0_g1_i4_orf1;TRINITY_DN53311_c0_g2_i1_orf1;TRINITY_DN3082_c1_g1_i7_orf1;TRINITY_DN21367_c0_g1_i1_orf1;TRINITY_DN38274_c0_g1_i1_orf1;TRINITY_DN111_c0_g2_i2_orf1;TRINITY_DN14487_c0_g1_i4_orf1;TRINITY_DN23042_c0_g1_i1_orf1;TRINITY_DN28299_c0_g1_i1_orf1;TRINITY_DN21971_c0_g1_i4_orf1;TRINITY_DN1957_c0_g1_i4_orf1;TRINITY_DN120144_c0_g1_i1_orf1;TRINITY_DN6248_c0_g1_i1_orf1;TRINITY_DN10548_c0_g2_i1_orf1;TRINITY_DN6813_c1_g1_i1_orf1;TRINITY_DN46022_c0_g1_i1_orf1;TRINITY_DN1084_c0_g2_i2_orf1;TRINITY_DN2971_c0_g1_i1_orf1;TRINITY_DN67649_c0_g1_i1_orf1;TRINITY_DN5507_c0_g1_i1_orf1;TRINITY_DN4835_c0_g1_i2_orf1;TRINITY_DN38562_c0_g1_i3_orf1;TRINITY_DN3482_c0_g2_i1_orf1;TRINITY_DN13760_c1_g1_i1_orf1;TRINITY_DN13651_c0_g1_i2_orf1;TRINITY_DN104507_c0_g1_i2_orf1;TRINITY_DN1045_c0_g1_i6_orf1;TRINITY_DN87170_c0_g1_i3_orf1;TRINITY_DN5200_c0_g1_i2_orf1;TRINITY_DN3991_c0_g1_i6_orf1;TRINITY_DN34676_c1_g1_i3_orf1;TRINITY_DN2201_c0_g1_i1_orf1;TRINITY_DN29873_c0_g1_i1_orf1;TRINITY_DN2367_c1_g1_i20_orf1;TRINITY_DN1091_c0_g1_i1_orf1;TRINITY_DN18782_c0_g1_i4_orf1;TRINITY_DN15222_c0_g1_i4_orf1;TRINITY_DN3826_c0_g1_i1_orf1;TRINITY_DN23343_c0_g1_i9_orf1;TRINITY_DN5238_c0_g1_i2_orf1;TRINITY_DN19251_c0_g1_i8_orf1;TRINITY_DN13055_c0_g1_i5_orf1;TRINITY_DN34432_c0_g1_i1_orf1;TRINITY_DN110534_c0_g1_i3_orf1;TRINITY_DN5757_c0_g1_i1_orf1;TRINITY_DN10070_c0_g1_i1_orf1;TRINITY_DN26947_c0_g1_i1_orf1;TRINITY_DN21539_c0_g1_i1_orf1;TRINITY_DN346_c0_g1_i7_orf1;TRINITY_DN1084_c0_g1_i2_orf1;TRINITY_DN20950_c0_g1_i12_orf1;TRINITY_DN26824_c0_g1_i1_orf1
biological_process	nitrogen cycle metabolic process	GO:0071941	1	1/2360	TRINITY_DN24723_c2_g1_i1_orf1
biological_process	cellular lipid metabolic process	GO:0044255	25	25/2360	TRINITY_DN48590_c0_g1_i1_orf1;TRINITY_DN10722_c0_g3_i1_orf1;TRINITY_DN5055_c0_g1_i12_orf1;TRINITY_DN12526_c0_g1_i5_orf1;TRINITY_DN5841_c0_g1_i2_orf1;TRINITY_DN72017_c0_g1_i1_orf1;TRINITY_DN45220_c0_g1_i1_orf1;TRINITY_DN5211_c0_g1_i1_orf1;TRINITY_DN86833_c0_g3_i1_orf1;TRINITY_DN7134_c0_g1_i1_orf1;TRINITY_DN8964_c0_g1_i4_orf1;TRINITY_DN41_c0_g1_i3_orf1;TRINITY_DN40197_c0_g1_i1_orf1;TRINITY_DN76283_c0_g2_i1_orf1;TRINITY_DN9028_c0_g1_i5_orf1;TRINITY_DN42759_c0_g3_i1_orf1;TRINITY_DN21570_c0_g1_i1_orf1;TRINITY_DN3175_c0_g1_i7_orf1;TRINITY_DN1999_c0_g1_i9_orf1;TRINITY_DN3991_c0_g1_i6_orf1;TRINITY_DN38644_c0_g1_i1_orf1;TRINITY_DN84478_c0_g1_i8_orf1;TRINITY_DN10430_c0_g1_i4_orf1;TRINITY_DN2618_c0_g1_i3_orf1;TRINITY_DN5697_c0_g1_i1_orf1
biological_process	generation of precursor metabolites and energy	GO:0006091	13	13/2360	TRINITY_DN60787_c0_g1_i5_orf1;TRINITY_DN49038_c0_g4_i1_orf1;TRINITY_DN6325_c0_g1_i8_orf1;TRINITY_DN136028_c0_g2_i1_orf1;TRINITY_DN29873_c0_g1_i1_orf1;TRINITY_DN9286_c0_g1_i2_orf1;TRINITY_DN1827_c0_g1_i4_orf1;TRINITY_DN18222_c0_g1_i5_orf1;TRINITY_DN9536_c0_g1_i4_orf1;TRINITY_DN5111_c0_g1_i2_orf1;TRINITY_DN11817_c0_g1_i4_orf1;TRINITY_DN5867_c0_g1_i1_orf1;TRINITY_DN812_c2_g1_i1_orf1
biological_process	one-carbon metabolic process	GO:0006730	5	5/2360	TRINITY_DN5768_c0_g1_i2_orf1;TRINITY_DN38506_c0_g1_i4_orf1;TRINITY_DN7512_c0_g1_i1_orf1;TRINITY_DN1578_c0_g3_i1_orf1;TRINITY_DN92153_c0_g2_i2_orf1
biological_process	cellular ketone metabolic process	GO:0042180	4	4/2360	TRINITY_DN87170_c0_g1_i3_orf1;TRINITY_DN27885_c0_g1_i3_orf1;TRINITY_DN8964_c0_g1_i4_orf1;TRINITY_DN6027_c0_g1_i13_orf1

biological_process	heterocycle metabolic process	GO:0046483	158	158/2360	TRINITY_DN38230_c0_g1_i4_orf1;TRINITY_DN5670_c0_g1_i2_orf1;TRINITY_DN13350_c0_g1_i4_orf1;TRINITY_DN1827_c0_g1_i4_orf1;TRINITY_DN60787_c0_g1_i5_orf1;TRINITY_DN7122_c0_g1_i1_orf1;TRINITY_DN35669_c0_g1_i1_orf1;TRINITY_DN2054_c0_g1_i1_orf1;TRINITY_DN124950_c0_g2_i1_orf1;TRINITY_DN2738_c1_g1_i3_orf1;TRINITY_DN15040_c0_g4_i1_orf1;TRINITY_DN34134_c0_g2_i1_orf1;TRINITY_DN33146_c0_g1_i1_orf1;TRINITY_DN1768_c0_g1_i2_orf1;TRINITY_DN44877_c0_g1_i2_orf1;TRINITY_DN2953_c1_g1_i10_orf1;TRINITY_DN11013_c0_g1_i3_orf1;TRINITY_DN15160_c0_g1_i1_orf1;TRINITY_DN6587_c0_g1_i3_orf1;TRINITY_DN2953_c1_g1_i2_orf1;TRINITY_DN1393_c0_g1_i2_orf1;TRINITY_DN5507_c0_g1_i1_orf1;TRINITY_DN3991_c0_g1_i6_orf1;TRINITY_DN29873_c0_g1_i1_orf1;TRINITY_DN3800_c0_g1_i7_orf1;TRINITY_DN47123_c0_g1_i1_orf1;TRINITY_DN15256_c0_g1_i8_orf1;TRINITY_DN123184_c0_g1_i1_orf1;TRINITY_DN27771_c0_g1_i1_orf1;TRINITY_DN2769_c0_g1_i1_orf1;TRINITY_DN37165_c0_g1_i4_orf1;TRINITY_DN26195_c0_g1_i6_orf1;TRINITY_DN2224_c0_g1_i1_orf1;TRINITY_DN2401_c0_g2_i1_orf1;TRINITY_DN20499_c0_g3_i1_orf1;TRINITY_DN5952_c0_g1_i6_orf1;TRINITY_DN4813_c0_g1_i5_orf1;TRINITY_DN107261_c0_g1_i1_orf1;TRINITY_DN15136_c0_g1_i2_orf1;TRINITY_DN107288_c0_g1_i2_orf1;TRINITY_DN7808_c0_g1_i1_orf1;TRINITY_DN56993_c0_g1_i4_orf1;TRINITY_DN14313_c0_g1_i1_orf1;TRINITY_DN1957_c0_g1_i4_orf1;TRINITY_DN3092_c0_g1_i2_orf1;TRINITY_DN1978_c0_g1_i4_orf1;TRINITY_DN17271_c0_g1_i1_orf1;TRINITY_DN115210_c0_g4_i1_orf1;TRINITY_DN45271_c0_g1_i1_orf1;TRINITY_DN18222_c0_g1_i5_orf1;TRINITY_DN1005_c0_g1_i5_orf1;TRINITY_DN37532_c0_g1_i1_orf1;TRINITY_DN51968_c0_g1_i1_orf1;TRINITY_DN15900_c0_g1_i6_orf1;TRINITY_DN81258_c0_g1_i2_orf1;TRINITY_DN21567_c0_g1_i7_orf1;TRINITY_DN30097_c0_g1_i2_orf1;TRINITY_DN1005_c0_g2_i1_orf1;TRINITY_DN8625_c0_g1_i1_orf1;TRINITY_DN779_c0_g1_i3_orf1;TRINITY_DN1607_c0_g1_i16_orf1;TRINITY_DN16978_c0_g1_i1_orf1;TRINITY_DN41664_c0_g1_i4_orf1;TRINITY_DN1091_c0_g3_i1_orf1;TRINITY_DN19034_c0_g1_i1_orf1;TRINITY_DN4145_c0_g1_i1_orf1;TRINITY_DN36144_c0_g1_i3_orf1;TRINITY_DN1616_c0_g1_i3_orf1;TRINITY_DN117844_c0_g1_i1_orf1;TRINITY_DN6642_c0_g1_i2_orf1;TRINITY_DN4408_c6_g1_i1_orf1;TRINITY_DN51568_c0_g1_i1_orf1;TRINITY_DN4707_c0_g1_i1_orf1;TRINITY_DN3472_c0_g1_i6_orf1;TRINITY_DN40945_c0_g1_i1_orf1;TRINITY_DN1718_c6_g1_i4_orf1;TRINITY_DN779_c0_g1_i12_orf1;TRINITY_DN31663_c0_g1_i2_orf1;TRINITY_DN139537_c0_g1_i1_orf1;TRINITY_DN1344_c0_g1_i1_orf1;TRINITY_DN107035_c0_g1_i1_orf1;TRINITY_DN4908_c1_g1_i5_orf1;TRINITY_DN291_c0_g1_i2_orf1;TRINITY_DN2749_c4_g1_i2_orf1;TRINITY_DN92153_c0_g2_i2_orf1;TRINITY_DN2749_c0_g1_i4_orf1;TRINITY_DN22951_c0_g1_i1_orf1;TRINITY_DN1750_c1_g1_i5_orf1;TRINITY_DN127056_c0_g1_i1_orf1;TRINITY_DN39813_c0_g1_i1_orf1;TRINITY_DN2718_c0_g1_i6_orf1;TRINITY_DN57918_c0_g1_i1_orf1;TRINITY_DN6563_c0_g1_i1_orf1;TRINITY_DN56270_c0_g1_i1_orf1;TRINITY_DN140669_c0_g1_i1_orf1;TRINITY_DN33346_c0_g1_i1_orf1;TRINITY_DN1554_c0_g1_i9_orf1;TRINITY_DN125_c0_g1_i2_orf1;TRINITY_DN49038_c0_g4_i1_orf1;TRINITY_DN38506_c0_g1_i4_orf1;TRINITY_DN2559_c0_g1_i4_orf1;TRINITY_DN2803_c4_g1_i1_orf1;TRINITY_DN98242_c0_g1_i1_orf1;TRINITY_DN14477_c0_g1_i12_orf1;TRINITY_DN452_c1_g1_i3_orf1;TRINITY_DN34689_c0_g1_i4_orf1;TRINITY_DN4135_c0_g1_i5_orf1;TRINITY_DN26293_c0_g1_i4_orf1;TRINITY_DN3062_c0_g1_i1_orf1;TRINITY_DN7341_c0_g1_i8_orf1;TRINITY_DN19866_c0_g1_i4_orf1;TRINITY_DN24322_c0_g1_i4_orf1;TRINITY_DN84322_c0_g2_i1_orf1;TRINITY_DN5686_c0_g1_i4_orf1;TRINITY_DN53311_c0_g2_i1_orf1;TRINITY_DN3082_c1_g1_i7_orf1;TRINITY_DN21367_c0_g1_i1_orf1;TRINITY_DN38274_c0_g1_i1_orf1;TRINITY_DN111_c0_g2_i2_orf1;TRINITY_DN14487_c0_g1_i4_orf1;TRINITY_DN23042_c0_g1_i1_orf1;TRINITY_DN28299_c0_g1_i1_orf1;TRINITY_DN2647_c0_g1_i3_orf1;TRINITY_DN120144_c0_g1_i1_orf1;TRINITY_DN6248_c0_g1_i1_orf1;TRINITY_DN10548_c0_g2_i1_orf1;TRINITY_DN6813_c1_g1_i1_orf1;TRINITY_DN46022_c0_g1_i1_orf1;TRINITY_DN1084_c0_g2_i2_orf1;TRINITY_DN1216_c0_g1_i4_orf1;TRINITY_DN2971_c0_g1_i1_orf1;TRINITY_DN67649_c0_g1_i1_orf1;TRINITY_DN24970_c0_g1_i4_orf1;TRINITY_DN4835_c0_g1_i2_orf1;TRINITY_DN38562_c0_g1_i3_orf1;TRINITY_DN3482_c0_g2_i1_orf1;TRINITY_DN13760_c1_g1_i1_orf1;TRINITY_DN104507_c0_g1_i2_orf1;TRINITY_DN87170_c0_g1_i3_orf1;TRINITY_DN5200_c0_g1_i2_orf1;TRINITY_DN34676_c1_g1_i3_orf1;TRINITY_DN2201_c0_g1_i1_orf1;TRINITY_DN2367_c1_g1_i20_orf1;TRINITY_DN1091_c0_g1_i1_orf1;TRINITY_DN18782_c0_g1_i4_orf1;TRINITY_DN15222_c0_g1_i4_orf1;TRINITY_DN23343_c0_g1_i9_orf1;TRINITY_DN5238_c0_g1_i2_orf1;TRINITY_DN19251_c0_g1_i8_orf1;TRINITY_DN13055_c0_g1_i5_orf1;TRINITY_DN34432_c0_g1_i1_orf1;TRINITY_DN110534_c0_g1_i3_orf1;TRINITY_DN5757_c0_g1_i1_orf1;TRINITY_DN6325_c0_g1_i8_orf1;TRINITY_DN21539_c0_g1_i1_orf1;TRINITY_DN346_c0_g1_i7_orf1;TRINITY_DN1084_c0_g1_i2_orf1;TRINITY_DN30950_c0_g1_i13_orf1
biological_process	reactive oxygen species metabolic process	GO:0072593	2	2/2360	TRINITY_DN285_c0_g1_i4_orf1;TRINITY_DN16400_c0_g2_i1_orf1
biological_process	cellular carbohydrate metabolic process	GO:0044262	12	12/2360	TRINITY_DN618_c0_g1_i3_orf1;TRINITY_DN49038_c0_g4_i1_orf1;TRINITY_DN10722_c0_g3_i1_orf1;TRINITY_DN1034_c0_g1_i4_orf1;TRINITY_DN36788_c0_g1_i2_orf1;TRINITY_DN38644_c0_g1_i1_orf1;TRINITY_DN11817_c0_g1_i4_orf1;TRINITY_DN1707_c0_g1_i1_orf1;TRINITY_DN812_c2_g1_i1_orf1;TRINITY_DN11657_c0_g1_i2_orf1;TRINITY_DN11942_c0_g1_i1_orf1;TRINITY_DN95850_c0_g1_i1_orf1
biological_process	sulfur compound metabolic process	GO:0006790	17	17/2360	TRINITY_DN14920_c0_g1_i1_orf1;TRINITY_DN92153_c0_g2_i2_orf1;TRINITY_DN117844_c0_g1_i1_orf1;TRINITY_DN11948_c0_g1_i8_orf1;TRINITY_DN3991_c0_g1_i6_orf1;TRINITY_DN1285_c0_g1_i6_orf1;TRINITY_DN19251_c0_g1_i8_orf1;TRINITY_DN38562_c0_g1_i3_orf1;TRINITY_DN7512_c0_g1_i1_orf1;TRINITY_DN15136_c0_g1_i2_orf1;TRINITY_DN1578_c0_g3_i1_orf1;TRINITY_DN49872_c0_g1_i2_orf1;TRINITY_DN35763_c0_g1_i2_orf1;TRINITY_DN7808_c0_g1_i1_orf1;TRINITY_DN3073_c0_g1_i7_orf1;TRINITY_DN1084_c0_g2_i2_orf1;TRINITY_DN1084_c0_g1_i2_orf1
biological_process	phosphorus metabolic process	GO:0006793	84	84/2360	TRINITY_DN38482_c0_g1_i4_orf1;TRINITY_DN1749_c0_g2_i2_orf1;TRINITY_DN14477_c0_g1_i12_orf1;TRINITY_DN47151_c0_g1_i1_orf1;TRINITY_DN38230_c0_g1_i4_orf1;TRINITY_DN10722_c0_g3_i1_orf1;TRINITY_DN15222_c0_g1_i4_orf1;TRINITY_DN115210_c0_g4_i1_orf1;TRINITY_DN39404_c0_g1_i7_orf1;TRINITY_DN18222_c0_g1_i5_orf1;TRINITY_DN1827_c0_g1_i4_orf1;TRINITY_DN60787_c0_g1_i5_orf1;TRINITY_DN62557_c0_g1_i1_orf1;TRINITY_DN277_c1_g1_i1_orf1;TRINITY_DN1552_c0_g1_i3_orf1;TRINITY_DN1405_c0_g1_i1_orf1;TRINITY_DN70409_c0_g1_i3_orf1;TRINITY_DN11942_c0_g1_i1_orf1;TRINITY_DN37923_c0_g1_i1_orf1;TRINITY_DN248_c0_g1_i1_orf1;TRINITY_DN1475_c0_g1_i6_orf1;TRINITY_DN2738_c1_g1_i3_orf1;TRINITY_DN618_c0_g1_i3_orf1;TRINITY_DN4217_c0_g1_i2_orf1;TRINITY_DN6325_c0_g1_i8_orf1;TRINITY_DN1034_c0_g1_i4_orf1;TRINITY_DN33146_c0_g1_i1_orf1;TRINITY_DN42461_c0_g1_i4_orf1;TRINITY_DN1173_c0_g1_i11_orf1;TRINITY_DN11125_c0_g1_i1_orf1;TRINITY_DN30154_c0_g1_i1_orf1;TRINITY_DN1216_c0_g1_i4_orf1;TRINITY_DN70485_c0_g1_i2_orf1;TRINITY_DN1718_c6_g1_i4_orf1;TRINITY_DN7134_c0_g1_i1_orf1;TRINITY_DN143509_c0_g1_i1_orf1;TRINITY_DN2812_c0_g1_i5_orf1;TRINITY_DN4320_c0_g1_i1_orf1;TRINITY_DN39813_c0_g1_i1_orf1;TRINITY_DN11013_c0_g1_i3_orf1;TRINITY_DN26293_c0_g1_i4_orf1;TRINITY_DN1084_c0_g1_i2_orf1;TRINITY_DN36144_c0_g1_i3_orf1;TRINITY_DN6587_c0_g1_i3_orf1;TRINITY_DN32700_c0_g1_i2_orf1;TRINITY_DN117844_c0_g1_i1_orf1;TRINITY_DN4571_c0_g1_i4_orf1;TRINITY_DN3991_c0_g1_i6_orf1;TRINITY_DN29873_c0_g1_i1_orf1;TRINITY_DN38644_c0_g1_i1_orf1;TRINITY_DN3800_c0_g1_i7_orf1;TRINITY_DN116972_c0_g1_i1_orf1;TRINITY_DN15478_c0_g1_i1_orf1;TRINITY_DN3119_c0_g1_i7_orf1;TRINITY_DN24539_c0_g1_i4_orf1;TRINITY_DN6185_c0_g1_i12_orf1;TRINITY_DN62729_c0_g1_i13_orf1;TRINITY_DN6436_c0_g1_i1_orf1;TRINITY_DN1266_c2_g1_i1_orf1;TRINITY_DN4798_c0_g1_i3_orf1;TRINITY_DN49038_c0_g4_i1_orf1;TRINITY_DN26195_c0_g1_i6_orf1;TRINITY_DN127056_c0_g1_i1_orf1;TRINITY_DN152_c0_g1_i4_orf1;TRINITY_DN28299_c0_g1_i1_orf1;TRINITY_DN5952_c0_g1_i6_orf1;TRINITY_DN2618_c0_g1_i3_orf1;TRINITY_DN1173_c1_g1_i10_orf1;TRINITY_DN30_c0_g1_i6_orf1;TRINITY_DN1957_c0_g1_i4_orf1;TRINITY_DN18782_c0_g1_i4_orf1;TRINITY_DN15591_c0_g1_i3_orf1;TRINITY_DN107261_c0_g1_i1_orf1;TRINITY_DN147475_c0_g1_i1_orf1;TRINITY_DN10548_c0_g2_i1_orf1;TRINITY_DN6813_c1_g1_i1_orf1;TRINITY_DN15136_c0_g1_i2_orf1;TRINITY_DN40562_c0_g2_i1_orf1;TRINITY_DN46715_c0_g1_i1_orf1;TRINITY_DN19251_c0_g1_i8_orf1;TRINITY_DN7808_c0_g1_i1_orf1;TRINITY_DN5697_c0_g1_i1_orf1;TRINITY_DN4929_c1_g2_i5_orf1;TRINITY_DN1084_c0_g2_i2_orf1
biological_process	cellular aldehyde metabolic process	GO:0006081	5	5/2360	TRINITY_DN36788_c0_g1_i2_orf1;TRINITY_DN4596_c0_g1_i14_orf1;TRINITY_DN3758_c0_g1_i2_orf1;TRINITY_DN14755_c0_g1_i4_orf1;TRINITY_DN125150_c0_g1_i1_orf1
biological_process	organic acid metabolic process	GO:0006082	69	69/2360	TRINITY_DN48590_c0_g1_i1_orf1;TRINITY_DN2065_c1_g2_i1_orf1;TRINITY_DN57918_c0_g1_i1_orf1;TRINITY_DN146126_c0_g1_i1_orf1;TRINITY_DN92153_c0_g2_i2_orf1;TRINITY_DN14565_c0_g1_i11_orf1;TRINITY_DN115210_c0_g4_i1_orf1;TRINITY_DN24970_c0_g1_i4_orf1;TRINITY_DN18222_c0_g1_i5_orf1;TRINITY_DN1827_c0_g1_i4_orf1;TRINITY_DN5564_c0_g1_i5_orf1;TRINITY_DN863_c0_g1_i6_orf1;TRINITY_DN117844_c0_g1_i1_orf1;TRINITY_DN100821_c0_g1_i1_orf1;TRINITY_DN35763_c0_g1_i2_orf1;TRINITY_DN4822_c0_g1_i9_orf1;TRINITY_DN6587_c0_g1_i3_orf1;TRINITY_DN72017_c0_g1_i1_orf1;TRINITY_DN4451_c0_g2_i4_orf1;TRINITY_DN19187_c0_g1_i1_orf1;TRINITY_DN2890_c0_g1_i2_orf1;TRINITY_DN45220_c0_g1_i1_orf1;TRINITY_DN11948_c0_g1_i8_orf1;TRINITY_DN36788_c0_g1_i2_orf1;TRINITY_DN5055_c0_g1_i12_orf1;TRINITY_DN5211_c0_g1_i1_orf1;TRINITY_DN1607_c0_g1_i16_orf1;TRINITY_DN24723_c2_g1_i1_orf1;TRINITY_DN3991_c0_g1_i6_orf1;TRINITY_DN2953_c1_g1_i10_orf1;TRINITY_DN89483_c0_g1_i1_orf1;TRINITY_DN2953_c1_g1_i2_orf1;TRINITY_DN42759_c0_g3_i1_orf1;TRINITY_DN10430_c0_g1_i4_orf1;TRINITY_DN11013_c0_g1_i3_orf1;TRINITY_DN26293_c0_g1_i4_orf1;TRINITY_DN84322_c0_g2_i1_orf1;TRINITY_DN20796_c0_g1_i4_orf1;TRINITY_DN4822_c0_g1_i6_orf1;TRINITY_DN3175_c0_g1_i7_orf1;TRINITY_DN49038_c0_g4_i1_orf1;TRINITY_DN17326_c0_g1_i8_orf1;TRINITY_DN43431_c0_g1_i1_orf1;TRINITY_DN3859_c0_g1_i5_orf1;TRINITY_DN29873_c0_g1_i1_orf1;TRINITY_DN3073_c0_g1_i7_orf1;TRINITY_DN1999_c0_g1_i9_orf1;TRINITY_DN27771_c0_g1_i1_orf1;TRINITY_DN76283_c0_g2_i1_orf1;TRINITY_DN2803_c4_g1_i1_orf1;TRINITY_DN1264_c0_g1_i2_orf1;TRINITY_DN511_c0_g2_i1_orf1;TRINITY_DN5266_c0_g1_i1_orf1;TRINITY_DN15160_c0_g1_i1_orf1;TRINITY_DN2224_c0_g1_i1_orf1;TRINITY_DN6325_c0_g1_i8_orf1;TRINITY_DN31163_c1_g1_i4_orf1;TRINITY_DN21539_c0_g1_i1_orf1;TRINITY_DN1262_c0_g1_i2_orf1;TRINITY_DN11383_c0_g2_i4_orf1;TRINITY_DN2684_c0_g2_i3_orf1;TRINITY_DN15136_c0_g1_i2_orf1;TRINITY_DN107288_c0_g1_i2_orf1;TRINITY_DN1084_c0_g1_i2_orf1;TRINITY_DN2338_c0_g1_i5_orf1;TRINITY_DN87170_c0_g1_i3_orf1;TRINITY_DN7808_c0_g1_i1_orf1;TRINITY_DN1068_c0_g1_i3_orf1;TRINITY_DN1084_c0_g2_i2_orf1
biological_process	cellular catabolic process	GO:0044248	46	46/2360	TRINITY_DN2065_c1_g2_i1_orf1;TRINITY_DN38230_c0_g1_i4_orf1;TRINITY_DN14565_c0_g1_i11_orf1;TRINITY_DN863_c0_g1_i6_orf1;TRINITY_DN181_c0_g1_i3_orf1;TRINITY_DN48983_c0_g1_i2_orf1;TRINITY_DN4822_c0_g1_i9_orf1;TRINITY_DN37923_c0_g1_i1_orf1;TRINITY_DN1272_c1_g1_i4_orf1;TRINITY_DN12757_c0_g1_i1_orf1;TRINITY_DN72017_c0_g1_i1_orf1;TRINITY_DN4451_c0_g2_i4_orf1;TRINITY_DN19187_c0_g1_i1_orf1;TRINITY_DN3758_c0_g1_i2_orf1;TRINITY_DN45220_c0_g1_i1_orf1;TRINITY_DN1034_c0_g1_i4_orf1;TRINITY_DN2559_c0_g1_i4_orf1;TRINITY_DN87170_c0_g1_i3_orf1;TRINITY_DN5055_c0_g1_i12_orf1;TRINITY_DN779_c0_g1_i3_orf1;TRINITY_DN98242_c0_g1_i1_orf1;TRINITY_DN5531_c0_g3_i3_orf1;TRINITY_DN34689_c0_g1_i4_orf1;TRINITY_DN4145_c0_g1_i1_orf1;TRINITY_DN96557_c0_g1_i1_orf1;TRINITY_DN4822_c0_g1_i6_orf1;TRINITY_DN17693_c0_g1_i10_orf1;TRINITY_DN89483_c0_g1_i1_orf1;TRINITY_DN43431_c0_g1_i1_orf1;TRINITY_DN57798_c0_g1_i1_orf1;TRINITY_DN2890_c0_g1_i2_orf1;TRINITY_DN285_c0_g1_i4_orf1;TRINITY_DN5001_c0_g1_i4_orf1;TRINITY_DN1707_c0_g1_i1_orf1;TRINITY_DN9980_c0_g1_i1_orf1;TRINITY_DN44877_c0_g1_i2_orf1;TRINITY_DN779_c0_g1_i12_orf1;TRINITY_DN9062_c0_g2_i3_orf1;TRINITY_DN10769_c0_g1_i1_orf1;TRINITY_DN20499_c0_g3_i1_orf1;TRINITY_DN143496_c0_g1_i1_orf1;TRINITY_DN1262_c0_g1_i2_orf1;TRINITY_DN135188_c0_g1_i2_orf1;TRINITY_DN15136_c0_g1_i2_orf1;TRINITY_DN28989_c0_g1_i7_orf1;TRINITY_DN46022_c0_g1_i1_orf1

biological_process	cellular biosynthetic process	GO:0044249	103	103/2360	TRINITY_DN10722_c0_g3_i1_orf1;TRINITY_DN5564_c0_g1_i5_orf1;TRINITY_DN8625_c0_g1_i1_orf1;TRINITY_DN35763_c0_g1_i2_orf1;TRINITY_DN27885_c0_g1_i3_orf1;TRINITY_DN95850_c0_g1_i1_orf1;TRINITY_DN124950_c0_g2_i1_orf1;TRINITY_DN2738_c1_g1_i3_orf1;TRINITY_DN33146_c0_g1_i1_orf1;TRINITY_DN30300_c0_g2_i1_orf1;TRINITY_DN1216_c0_g1_i4_orf1;TRINITY_DN142442_c0_g1_i1_orf1;TRINITY_DN24723_c2_g1_i1_orf1;TRINITY_DN31163_c1_g1_i4_orf1;TRINITY_DN8964_c0_g1_i4_orf1;TRINITY_DN29448_c0_g1_i1_orf1;TRINITY_DN11013_c0_g1_i3_orf1;TRINITY_DN6587_c0_g1_i3_orf1;TRINITY_DN21570_c0_g1_i1_orf1;TRINITY_DN1393_c0_g1_i2_orf1;TRINITY_DN95056_c0_g2_i2_orf1;TRINITY_DN74889_c0_g1_i1_orf1;TRINITY_DN3800_c0_g1_i7_orf1;TRINITY_DN135_c0_g1_i1_orf1;TRINITY_DN37165_c0_g1_i4_orf1;TRINITY_DN26195_c0_g1_i6_orf1;TRINITY_DN2401_c0_g2_i1_orf1;TRINITY_DN5952_c0_g1_i6_orf1;TRINITY_DN107261_c0_g1_i1_orf1;TRINITY_DN7808_c0_g1_i1_orf1;TRINITY_DN115210_c0_g4_i1_orf1;TRINITY_DN23360_c0_g1_i3_orf1;TRINITY_DN14398_c0_g1_i4_orf1;TRINITY_DN37532_c0_g1_i1_orf1;TRINITY_DN26947_c0_g1_i1_orf1;TRINITY_DN21567_c0_g1_i7_orf1;TRINITY_DN5211_c0_g1_i1_orf1;TRINITY_DN7512_c0_g1_i1_orf1;TRINITY_DN1578_c0_g3_i1_orf1;TRINITY_DN7583_c0_g1_i1_orf1;TRINITY_DN50787_c0_g2_i2_orf1;TRINITY_DN42759_c0_g3_i1_orf1;TRINITY_DN10430_c0_g1_i4_orf1;TRINITY_DN36144_c0_g1_i3_orf1;TRINITY_DN38644_c0_g1_i1_orf1;TRINITY_DN117844_c0_g1_i1_orf1;TRINITY_DN1999_c0_g1_i9_orf1;TRINITY_DN3073_c0_g1_i7_orf1;TRINITY_DN3733_c0_g1_i1_orf1;TRINITY_DN4408_c6_g1_i1_orf1;TRINITY_DN4707_c0_g1_i1_orf1;TRINITY_DN3472_c0_g1_i6_orf1;TRINITY_DN1718_c6_g1_i4_orf1;TRINITY_DN11948_c0_g1_i8_orf1;TRINITY_DN147458_c0_g1_i1_orf1;TRINITY_DN12397_c0_g1_i1_orf1;TRINITY_DN5064_c0_g1_i4_orf1;TRINITY_DN812_c2_g1_i1_orf1;TRINITY_DN48590_c0_g1_i1_orf1;TRINITY_DN5112_c0_g1_i1_orf1;TRINITY_DN1768_c0_g1_i2_orf1;TRINITY_DN1750_c1_g1_i5_orf1;TRINITY_DN6365_c0_g1_i4_orf1;TRINITY_DN127056_c0_g1_i1_orf1;TRINITY_DN39813_c0_g1_i1_orf1;TRINITY_DN6563_c0_g1_i1_orf1;TRINITY_DN140669_c0_g1_i1_orf1;TRINITY_DN4016_c0_g1_i1_orf1;TRINITY_DN934_c2_g1_i7_orf1;TRINITY_DN2803_c4_g1_i1_orf1;TRINITY_DN14477_c0_g1_i12_orf1;TRINITY_DN76283_c0_g2_i1_orf1;TRINITY_DN26293_c0_g1_i4_orf1;TRINITY_DN53311_c0_g2_i1_orf1;TRINITY_DN21367_c0_g1_i1_orf1;TRINITY_DN23042_c0_g1_i1_orf1;TRINITY_DN28299_c0_g1_i1_orf1;TRINITY_DN2618_c0_g1_i3_orf1;TRINITY_DN21971_c0_g1_i4_orf1;TRINITY_DN1957_c0_g1_i4_orf1;TRINITY_DN10548_c0_g2_i1_orf1;TRINITY_DN6813_c1_g1_i1_orf1;TRINITY_DN1084_c0_g2_i2_orf1;TRINITY_DN2265_c0_g2_i1_orf1;TRINITY_DN24970_c0_g1_i4_orf1;TRINITY_DN3482_c0_g2_i1_orf1;TRINITY_DN13651_c0_g1_i2_orf1;TRINITY_DN1045_c0_g1_i6_orf1;TRINITY_DN6027_c0_g1_i13_orf1;TRINITY_DN5200_c0_g1_i2_orf1;TRINITY_DN3991_c0_g1_i6_orf1;TRINITY_DN7134_c0_g1_i1_orf1;TRINITY_DN9028_c0_g1_i5_orf1;TRINITY_DN18782_c0_g1_i4_orf1;TRINITY_DN15222_c0_g1_i4_orf1;TRINITY_DN3826_c0_g1_i1_orf1;TRINITY_DN10070_c0_g1_i1_orf1;TRINITY_DN2338_c0_g1_i5_orf1;TRINITY_DN58125_c0_g1_i1_orf1;TRINITY_DN9591_c0_g1_i1_orf1;TRINITY_DN1084_c0_g1_i2_orf1;TRINITY_DN26824_c0_g1_i1_orf1;TRINITY_DN5697_c0_g1_i1_orf1
biological_process	cellular macromolecule metabolic process	GO:0044260	87	87/2360	TRINITY_DN5670_c0_g1_i2_orf1;TRINITY_DN13350_c0_g1_i4_orf1;TRINITY_DN181_c0_g1_i3_orf1;TRINITY_DN48983_c0_g1_i2_orf1;TRINITY_DN7122_c0_g1_i1_orf1;TRINITY_DN2054_c0_g1_i1_orf1;TRINITY_DN15040_c0_g4_i1_orf1;TRINITY_DN1344_c0_g1_i1_orf1;TRINITY_DN30300_c0_g2_i1_orf1;TRINITY_DN1768_c0_g1_i2_orf1;TRINITY_DN142442_c0_g1_i1_orf1;TRINITY_DN11817_c0_g1_i4_orf1;TRINITY_DN29448_c0_g1_i1_orf1;TRINITY_DN1393_c0_g1_i2_orf1;TRINITY_DN95056_c0_g2_i2_orf1;TRINITY_DN74889_c0_g1_i1_orf1;TRINITY_DN15256_c0_g1_i8_orf1;TRINITY_DN123184_c0_g1_i1_orf1;TRINITY_DN135_c0_g1_i1_orf1;TRINITY_DN41664_c0_g1_i4_orf1;TRINITY_DN10769_c0_g1_i1_orf1;TRINITY_DN20499_c0_g3_i1_orf1;TRINITY_DN14313_c0_g1_i1_orf1;TRINITY_DN3092_c0_g1_i2_orf1;TRINITY_DN17271_c0_g1_i1_orf1;TRINITY_DN23360_c0_g1_i3_orf1;TRINITY_DN45271_c0_g1_i1_orf1;TRINITY_DN37923_c0_g1_i1_orf1;TRINITY_DN1272_c1_g1_i4_orf1;TRINITY_DN26947_c0_g1_i1_orf1;TRINITY_DN81258_c0_g1_i2_orf1;TRINITY_DN57798_c0_g1_i1_orf1;TRINITY_DN7583_c0_g1_i1_orf1;TRINITY_DN50787_c0_g2_i2_orf1;TRINITY_DN12757_c0_g1_i1_orf1;TRINITY_DN1091_c0_g3_i1_orf1;TRINITY_DN3733_c0_g1_i1_orf1;TRINITY_DN9591_c0_g1_i1_orf1;TRINITY_DN6642_c0_g1_i2_orf1;TRINITY_DN4408_c6_g1_i1_orf1;TRINITY_DN9062_c0_g2_i3_orf1;TRINITY_DN139537_c0_g1_i1_orf1;TRINITY_DN147458_c0_g1_i1_orf1;TRINITY_DN4908_c1_g1_i5_orf1;TRINITY_DN28989_c0_g1_i7_orf1;TRINITY_DN5064_c0_g1_i4_orf1;TRINITY_DN291_c0_g1_i2_orf1;TRINITY_DN812_c2_g1_i1_orf1;TRINITY_DN5112_c0_g1_i1_orf1;TRINITY_DN1750_c1_g1_i5_orf1;TRINITY_DN6365_c0_g1_i4_orf1;TRINITY_DN9980_c0_g1_i1_orf1;TRINITY_DN4016_c0_g1_i1_orf1;TRINITY_DN934_c2_g1_i7_orf1;TRINITY_DN452_c1_g1_i3_orf1;TRINITY_DN34689_c0_g1_i4_orf1;TRINITY_DN4835_c0_g1_i2_orf1;TRINITY_DN7341_c0_g1_i8_orf1;TRINITY_DN19866_c0_g1_i4_orf1;TRINITY_DN5686_c0_g1_i4_orf1;TRINITY_DN21367_c0_g1_i1_orf1;TRINITY_DN38274_c0_g1_i1_orf1;TRINITY_DN44877_c0_g1_i2_orf1;TRINITY_DN14487_c0_g1_i4_orf1;TRINITY_DN96557_c0_g1_i1_orf1;TRINITY_DN21971_c0_g1_i4_orf1;TRINITY_DN2647_c0_g1_i3_orf1;TRINITY_DN6248_c0_g1_i1_orf1;TRINITY_DN46022_c0_g1_i1_orf1;TRINITY_DN2265_c0_g2_i1_orf1;TRINITY_DN2971_c0_g1_i1_orf1;TRINITY_DN67649_c0_g1_i1_orf1;TRINITY_DN13651_c0_g1_i2_orf1;TRINITY_DN104507_c0_g1_i2_orf1;TRINITY_DN143496_c0_g1_i1_orf1;TRINITY_DN5200_c0_g1_i2_orf1;TRINITY_DN1091_c0_g1_i1_orf1;TRINITY_DN3826_c0_g1_i1_orf1;TRINITY_DN12397_c0_g1_i1_orf1;TRINITY_DN5238_c0_g1_i2_orf1;TRINITY_DN5001_c0_g1_i4_orf1;TRINITY_DN110534_c0_g1_i3_orf1;TRINITY_DN5757_c0_g1_i1_orf1;TRINITY_DN10070_c0_g1_i1_orf1;TRINITY_DN346_c0_g1_i7_orf1;TRINITY_DN135188_c0_g1_i2_orf1;TRINITY_DN26824_c0_g1_i1_orf1
biological_process	cellular aromatic compound metabolic process	GO:0006725	163	163/2360	TRINITY_DN38230_c0_g1_i4_orf1;TRINITY_DN5670_c0_g1_i2_orf1;TRINITY_DN13350_c0_g1_i4_orf1;TRINITY_DN1827_c0_g1_i4_orf1;TRINITY_DN863_c0_g1_i6_orf1;TRINITY_DN7122_c0_g1_i1_orf1;TRINITY_DN35669_c0_g1_i1_orf1;TRINITY_DN2054_c0_g1_i1_orf1;TRINITY_DN124950_c0_g2_i1_orf1;TRINITY_DN2738_c1_g1_i3_orf1;TRINITY_DN15040_c0_g4_i1_orf1;TRINITY_DN34134_c0_g2_i1_orf1;TRINITY_DN33146_c0_g1_i1_orf1;TRINITY_DN1768_c0_g1_i2_orf1;TRINITY_DN44877_c0_g1_i2_orf1;TRINITY_DN31163_c1_g1_i4_orf1;TRINITY_DN11013_c0_g1_i3_orf1;TRINITY_DN15160_c0_g1_i1_orf1;TRINITY_DN6587_c0_g1_i3_orf1;TRINITY_DN4822_c0_g1_i6_orf1;TRINITY_DN2953_c1_g1_i2_orf1;TRINITY_DN1393_c0_g1_i2_orf1;TRINITY_DN5507_c0_g1_i1_orf1;TRINITY_DN3991_c0_g1_i6_orf1;TRINITY_DN29873_c0_g1_i1_orf1;TRINITY_DN3800_c0_g1_i7_orf1;TRINITY_DN47123_c0_g1_i1_orf1;TRINITY_DN15256_c0_g1_i8_orf1;TRINITY_DN123184_c0_g1_i1_orf1;TRINITY_DN27771_c0_g1_i1_orf1;TRINITY_DN2769_c0_g1_i1_orf1;TRINITY_DN37165_c0_g1_i4_orf1;TRINITY_DN26195_c0_g1_i6_orf1;TRINITY_DN2224_c0_g1_i1_orf1;TRINITY_DN2401_c0_g2_i1_orf1;TRINITY_DN20499_c0_g3_i1_orf1;TRINITY_DN5952_c0_g1_i6_orf1;TRINITY_DN4813_c0_g1_i5_orf1;TRINITY_DN107261_c0_g1_i1_orf1;TRINITY_DN15136_c0_g1_i2_orf1;TRINITY_DN107288_c0_g1_i2_orf1;TRINITY_DN7808_c0_g1_i1_orf1;TRINITY_DN56993_c0_g1_i4_orf1;TRINITY_DN14313_c0_g1_i1_orf1;TRINITY_DN1957_c0_g1_i4_orf1;TRINITY_DN3092_c0_g1_i2_orf1;TRINITY_DN1978_c0_g1_i4_orf1;TRINITY_DN17271_c0_g1_i1_orf1;TRINITY_DN115210_c0_g4_i1_orf1;TRINITY_DN45271_c0_g1_i1_orf1;TRINITY_DN18222_c0_g1_i5_orf1;TRINITY_DN1005_c0_g1_i5_orf1;TRINITY_DN37532_c0_g1_i1_orf1;TRINITY_DN51968_c0_g1_i1_orf1;TRINITY_DN15900_c0_g1_i6_orf1;TRINITY_DN81258_c0_g1_i2_orf1;TRINITY_DN19187_c0_g1_i1_orf1;TRINITY_DN21567_c0_g1_i7_orf1;TRINITY_DN30097_c0_g1_i2_orf1;TRINITY_DN1005_c0_g2_i1_orf1;TRINITY_DN8625_c0_g1_i1_orf1;TRINITY_DN779_c0_g1_i3_orf1;TRINITY_DN1607_c0_g1_i16_orf1;TRINITY_DN16978_c0_g1_i1_orf1;TRINITY_DN41664_c0_g1_i4_orf1;TRINITY_DN1091_c0_g3_i1_orf1;TRINITY_DN19034_c0_g1_i1_orf1;TRINITY_DN36144_c0_g1_i3_orf1;TRINITY_DN1616_c0_g1_i3_orf1;TRINITY_DN117844_c0_g1_i1_orf1;TRINITY_DN6642_c0_g1_i2_orf1;TRINITY_DN4408_c6_g1_i1_orf1;TRINITY_DN51568_c0_g1_i1_orf1;TRINITY_DN4707_c0_g1_i1_orf1;TRINITY_DN3472_c0_g1_i6_orf1;TRINITY_DN40945_c0_g1_i1_orf1;TRINITY_DN1718_c6_g1_i4_orf1;TRINITY_DN779_c0_g1_i12_orf1;TRINITY_DN31663_c0_g1_i2_orf1;TRINITY_DN139537_c0_g1_i1_orf1;TRINITY_DN1344_c0_g1_i1_orf1;TRINITY_DN107035_c0_g1_i1_orf1;TRINITY_DN4908_c1_g1_i5_orf1;TRINITY_DN291_c0_g1_i2_orf1;TRINITY_DN2749_c4_g1_i2_orf1;TRINITY_DN92153_c0_g2_i2_orf1;TRINITY_DN2749_c0_g1_i4_orf1;TRINITY_DN22951_c0_g1_i1_orf1;TRINITY_DN1750_c1_g1_i5_orf1;TRINITY_DN127056_c0_g1_i1_orf1;TRINITY_DN39813_c0_g1_i1_orf1;TRINITY_DN2718_c0_g1_i6_orf1;TRINITY_DN57918_c0_g1_i1_orf1;TRINITY_DN6563_c0_g1_i1_orf1;TRINITY_DN56270_c0_g1_i1_orf1;TRINITY_DN140669_c0_g1_i1_orf1;TRINITY_DN33346_c0_g1_i1_orf1;TRINITY_DN1554_c0_g1_i9_orf1;TRINITY_DN125_c0_g1_i2_orf1;TRINITY_DN49038_c0_g4_i1_orf1;TRINITY_DN38506_c0_g1_i4_orf1;TRINITY_DN2953_c1_g1_i10_orf1;TRINITY_DN2559_c0_g1_i4_orf1;TRINITY_DN98242_c0_g1_i1_orf1;TRINITY_DN14477_c0_g1_i12_orf1;TRINITY_DN452_c1_g1_i3_orf1;TRINITY_DN34689_c0_g1_i4_orf1;TRINITY_DN4135_c0_g1_i5_orf1;TRINITY_DN26293_c0_g1_i4_orf1;TRINITY_DN3062_c0_g1_i1_orf1;TRINITY_DN7341_c0_g1_i8_orf1;TRINITY_DN19866_c0_g1_i4_orf1;TRINITY_DN24322_c0_g1_i4_orf1;TRINITY_DN84322_c0_g2_i1_orf1;TRINITY_DN5686_c0_g1_i4_orf1;TRINITY_DN53311_c0_g2_i1_orf1;TRINITY_DN3082_c1_g1_i7_orf1;TRINITY_DN21367_c0_g1_i1_orf1;TRINITY_DN60787_c0_g1_i5_orf1;TRINITY_DN38274_c0_g1_i1_orf1;TRINITY_DN111_c0_g2_i2_orf1;TRINITY_DN14487_c0_g1_i4_orf1;TRINITY_DN23042_c0_g1_i1_orf1;TRINITY_DN28299_c0_g1_i1_orf1;TRINITY_DN2647_c0_g1_i3_orf1;TRINITY_DN120144_c0_g1_i1_orf1;TRINITY_DN6248_c0_g1_i1_orf1;TRINITY_DN10548_c0_g2_i1_orf1;TRINITY_DN6813_c1_g1_i1_orf1;TRINITY_DN46022_c0_g1_i1_orf1;TRINITY_DN1084_c0_g2_i2_orf1;TRINITY_DN1216_c0_g1_i4_orf1;TRINITY_DN2971_c0_g1_i1_orf1;TRINITY_DN67649_c0_g1_i1_orf1;TRINITY_DN4835_c0_g1_i2_orf1;TRINITY_DN38562_c0_g1_i3_orf1;TRINITY_DN3482_c0_g2_i1_orf1;TRINITY_DN13760_c1_g1_i1_orf1;TRINITY_DN15706_c0_g2_i5_orf1;TRINITY_DN4822_c0_g1_i9_orf1;TRINITY_DN104507_c0_g1_i2_orf1;TRINITY_DN87170_c0_g1_i3_orf1;TRINITY_DN5200_c0_g1_i2_orf1;TRINITY_DN34676_c1_g1_i3_orf1;TRINITY_DN2201_c0_g1_i1_orf1;TRINITY_DN2367_c1_g1_i20_orf1;TRINITY_DN1091_c0_g1_i1_orf1;TRINITY_DN18782_c0_g1_i4_orf1;TRINITY_DN15222_c0_g1_i4_orf1;TRINITY_DN23343_c0_g1_i9_orf1;TRINITY_DN5238_c0_g1_i2_orf1;TRINITY_DN19251_c0_g1_i8_orf1;TRINITY_DN13055_c0_g1_i5_orf1;TRINITY_DN34432_c0_g1_i1_orf1;TRINITY_DN110534_c0_g1_i3_orf1;TRINITY_DN5757_c0_g1_i1_orf1;TRINITY_DN2338_c0_g1_i5_orf1;TRINITY_DN6325_c0_g1_i8_orf1;TRINITY_DN58125_c0_g1_i1_orf1;TRINITY_DN21539_c0_g1_i1_orf1;TRINITY_DN346_c0_g1_i7_orf1;TRINITY_DN1084_c0_g1_i2_orf1;TRINITY_DN30950_c0_g1_i13_orf1
biological_process	cellular metabolic compound salvage	GO:0043094	5	5/2360	TRINITY_DN35763_c0_g1_i2_orf1;TRINITY_DN2738_c1_g1_i3_orf1;TRINITY_DN140669_c0_g1_i1_orf1;TRINITY_DN3073_c0_g1_i7_orf1;TRINITY_DN1718_c6_g1_i4_orf1
biological_process	neurotransmitter metabolic process	GO:0042133	2	2/2360	TRINITY_DN17693_c0_g1_i10_orf1;TRINITY_DN14565_c0_g1_i11_orf1
biological_process	cellular modified amino acid metabolic process	GO:0006575	4	4/2360	TRINITY_DN14398_c0_g1_i4_orf1;TRINITY_DN38506_c0_g1_i4_orf1;TRINITY_DN38562_c0_g1_i3_orf1;TRINITY_DN92153_c0_g2_i2_orf1
biological_process	translational initiation	GO:0006413	1	1/2360	TRINITY_DN48097_c0_g1_i1_orf1
biological_process	prenylation	GO:0097354	1	1/2360	TRINITY_DN5182_c0_g1_i5_orf1
biological_process	secondary metabolite biosynthetic process	GO:0044550	3	3/2360	TRINITY_DN31163_c1_g1_i4_orf1;TRINITY_DN58125_c0_g1_i1_orf1;TRINITY_DN2338_c0_g1_i5_orf1
biological_process	small molecule biosynthetic process	GO:0044283	27	27/2360	TRINITY_DN48590_c0_g1_i1_orf1;TRINITY_DN10722_c0_g3_i1_orf1;TRINITY_DN24970_c0_g1_i4_orf1;TRINITY_DN5564_c0_g1_i5_orf1;TRINITY_DN35763_c0_g1_i2_orf1;TRINITY_DN27885_c0_g1_i3_orf1;TRINITY_DN140669_c0_g1_i1_orf1;TRINITY_DN11948_c0_g1_i8_orf1;TRINITY_DN5211_c0_g1_i1_orf1;TRINITY_DN6027_c0_g1_i13_orf1;TRINITY_DN1718_c6_g1_i4_orf1;TRINITY_DN24723_c2_g1_i1_orf1;TRINITY_DN2803_c4_g1_i1_orf1;TRINITY_DN8964_c0_g1_i4_orf1;TRINITY_DN42759_c0_g3_i1_orf1;TRINITY_DN76283_c0_g2_i1_orf1;TRINITY_DN26293_c0_g1_i4_orf1;TRINITY_DN1999_c0_g1_i9_orf1;TRINITY_DN3991_c0_g1_i6_orf1;TRINITY_DN3073_c0_g1_i7_orf1;TRINITY_DN38644_c0_g1_i1_orf1;TRINITY_DN10430_c0_g1_i4_orf1;TRINITY_DN3472_c0_g1_i6_orf1;TRINITY_DN511_c0_g2_i1_orf1;TRINITY_DN37165_c0_g1_i4_orf1;TRINITY_DN18782_c0_g1_i4_orf1;TRINITY_DN1161_c0_g1_i2_orf1

biological_process	organic substance biosynthetic process	GO:1901576	107	107/2360	TRINITY_DN10722_c0_g3_i1_orf1;TRINITY_DN5564_c0_g1_i5_orf1;TRINITY_DN1578_c0_g3_i1_orf1;TRINITY_DN35763_c0_g1_i2_orf1;TRINITY_DN27885_c0_g1_i3_orf1;TRINITY_DN95850_c0_g1_i1_orf1;TRINITY_DN124950_c0_g2_i1_orf1;TRINITY_DN2738_c1_g1_i3_orf1;TRINITY_DN33146_c0_g1_i1_orf1;TRINITY_DN30300_c0_g2_i1_orf1;TRINITY_DN1216_c0_g1_i4_orf1;TRINITY_DN142442_c0_g1_i1_orf1;TRINITY_DN24723_c2_g1_i1_orf1;TRINITY_DN31163_c1_g1_i4_orf1;TRINITY_DN8964_c0_g1_i4_orf1;TRINITY_DN29448_c0_g1_i1_orf1;TRINITY_DN11013_c0_g1_i3_orf1;TRINITY_DN6587_c0_g1_i3_orf1;TRINITY_DN21570_c0_g1_i1_orf1;TRINITY_DN1393_c0_g1_i2_orf1;TRINITY_DN95056_c0_g2_i2_orf1;TRINITY_DN74889_c0_g1_i1_orf1;TRINITY_DN3800_c0_g1_i7_orf1;TRINITY_DN135_c0_g1_i1_orf1;TRINITY_DN37165_c0_g1_i4_orf1;TRINITY_DN26195_c0_g1_i6_orf1;TRINITY_DN2401_c0_g2_i1_orf1;TRINITY_DN5952_c0_g1_i6_orf1;TRINITY_DN107261_c0_g1_i1_orf1;TRINITY_DN7808_c0_g1_i1_orf1;TRINITY_DN1161_c0_g1_i2_orf1;TRINITY_DN115210_c0_g4_i1_orf1;TRINITY_DN23360_c0_g1_i3_orf1;TRINITY_DN14398_c0_g1_i4_orf1;TRINITY_DN37532_c0_g1_i1_orf1;TRINITY_DN26947_c0_g1_i1_orf1;TRINITY_DN21567_c0_g1_i7_orf1;TRINITY_DN5211_c0_g1_i1_orf1;TRINITY_DN7512_c0_g1_i1_orf1;TRINITY_DN8625_c0_g1_i1_orf1;TRINITY_DN7583_c0_g1_i1_orf1;TRINITY_DN50787_c0_g2_i2_orf1;TRINITY_DN42759_c0_g3_i1_orf1;TRINITY_DN10430_c0_g1_i4_orf1;TRINITY_DN36144_c0_g1_i3_orf1;TRINITY_DN38644_c0_g1_i1_orf1;TRINITY_DN117844_c0_g1_i1_orf1;TRINITY_DN1999_c0_g1_i9_orf1;TRINITY_DN3073_c0_g1_i7_orf1;TRINITY_DN3733_c0_g1_i1_orf1;TRINITY_DN4408_c6_g1_i1_orf1;TRINITY_DN4707_c0_g1_i1_orf1;TRINITY_DN3472_c0_g1_i6_orf1;TRINITY_DN511_c0_g2_i1_orf1;TRINITY_DN1718_c6_g1_i4_orf1;TRINITY_DN11948_c0_g1_i8_orf1;TRINITY_DN147458_c0_g1_i1_orf1;TRINITY_DN12397_c0_g1_i1_orf1;TRINITY_DN5064_c0_g1_i4_orf1;TRINITY_DN812_c2_g1_i1_orf1;TRINITY_DN48590_c0_g1_i1_orf1;TRINITY_DN5112_c0_g1_i1_orf1;TRINITY_DN1768_c0_g1_i2_orf1;TRINITY_DN1750_c1_g1_i5_orf1;TRINITY_DN6365_c0_g1_i4_orf1;TRINITY_DN127056_c0_g1_i1_orf1;TRINITY_DN39813_c0_g1_i1_orf1;TRINITY_DN6563_c0_g1_i1_orf1;TRINITY_DN140669_c0_g1_i1_orf1;TRINITY_DN4016_c0_g1_i1_orf1;TRINITY_DN934_c2_g1_i7_orf1;TRINITY_DN2803_c4_g1_i1_orf1;TRINITY_DN14477_c0_g1_i12_orf1;TRINITY_DN76283_c0_g2_i1_orf1;TRINITY_DN26293_c0_g1_i4_orf1;TRINITY_DN53311_c0_g2_i1_orf1;TRINITY_DN21367_c0_g1_i1_orf1;TRINITY_DN23042_c0_g1_i1_orf1;TRINITY_DN28299_c0_g1_i1_orf1;TRINITY_DN2618_c0_g1_i3_orf1;TRINITY_DN21971_c0_g1_i4_orf1;TRINITY_DN1957_c0_g1_i4_orf1;TRINITY_DN10548_c0_g2_i1_orf1;TRINITY_DN6813_c1_g1_i1_orf1;TRINITY_DN1084_c0_g2_i2_orf1;TRINITY_DN2265_c0_g2_i1_orf1;TRINITY_DN24970_c0_g1_i4_orf1;TRINITY_DN2425_c0_g1_i3_orf1;TRINITY_DN3482_c0_g2_i1_orf1;TRINITY_DN13651_c0_g1_i2_orf1;TRINITY_DN1045_c0_g1_i6_orf1;TRINITY_DN6027_c0_g1_i13_orf1;TRINITY_DN5200_c0_g1_i2_orf1;TRINITY_DN3991_c0_g1_i6_orf1;TRINITY_DN7134_c0_g1_i1_orf1;TRINITY_DN40197_c0_g1_i1_orf1;TRINITY_DN9028_c0_g1_i5_orf1;TRINITY_DN18782_c0_g1_i4_orf1;TRINITY_DN15222_c0_g1_i4_orf1;TRINITY_DN3826_c0_g1_i1_orf1;TRINITY_DN10070_c0_g1_i1_orf1;TRINITY_DN2338_c0_g1_i5_orf1;TRINITY_DN58125_c0_g1_i1_orf1;TRINITY_DN9591_c0_g1_i1_orf1;TRINITY_DN1084_c0_g1_i2_orf1;TRINITY_DN26824_c0_g1_i1_orf1;TRINITY_DN5697_c0_g1_i1_orf1
biological_process	small molecule catabolic process	GO:0044282	24	24/2360	TRINITY_DN2065_c1_g2_i1_orf1;TRINITY_DN5055_c0_g1_i12_orf1;TRINITY_DN863_c0_g1_i6_orf1;TRINITY_DN4822_c0_g1_i9_orf1;TRINITY_DN89483_c0_g1_i1_orf1;TRINITY_DN72017_c0_g1_i1_orf1;TRINITY_DN4451_c0_g2_i4_orf1;TRINITY_DN19187_c0_g1_i1_orf1;TRINITY_DN2890_c0_g1_i2_orf1;TRINITY_DN45220_c0_g1_i1_orf1;TRINITY_DN1034_c0_g1_i4_orf1;TRINITY_DN779_c0_g1_i3_orf1;TRINITY_DN87170_c0_g1_i3_orf1;TRINITY_DN2559_c0_g1_i4_orf1;TRINITY_DN98242_c0_g1_i1_orf1;TRINITY_DN4822_c0_g1_i6_orf1;TRINITY_DN43431_c0_g1_i1_orf1;TRINITY_DN38644_c0_g1_i1_orf1;TRINITY_DN3758_c0_g1_i2_orf1;TRINITY_DN1707_c0_g1_i1_orf1;TRINITY_DN779_c0_g1_i12_orf1;TRINITY_DN1262_c0_g1_i2_orf1;TRINITY_DN15136_c0_g1_i2_orf1;TRINITY_DN25997_c1_g2_i4_orf1
biological_process	organic substance catabolic process	GO:1901575	60	60/2360	TRINITY_DN2065_c1_g2_i1_orf1;TRINITY_DN38230_c0_g1_i4_orf1;TRINITY_DN6325_c0_g1_i8_orf1;TRINITY_DN5055_c0_g1_i12_orf1;TRINITY_DN98242_c0_g1_i1_orf1;TRINITY_DN10824_c0_g1_i3_orf1;TRINITY_DN18222_c0_g1_i5_orf1;TRINITY_DN1827_c0_g1_i4_orf1;TRINITY_DN863_c0_g1_i6_orf1;TRINITY_DN181_c0_g1_i3_orf1;TRINITY_DN48983_c0_g1_i2_orf1;TRINITY_DN4817_c0_g1_i4_orf1;TRINITY_DN4822_c0_g1_i9_orf1;TRINITY_DN37923_c0_g1_i1_orf1;TRINITY_DN1272_c1_g1_i4_orf1;TRINITY_DN12757_c0_g1_i1_orf1;TRINITY_DN72017_c0_g1_i1_orf1;TRINITY_DN4451_c0_g2_i4_orf1;TRINITY_DN19187_c0_g1_i1_orf1;TRINITY_DN3758_c0_g1_i2_orf1;TRINITY_DN45220_c0_g1_i1_orf1;TRINITY_DN1034_c0_g1_i4_orf1;TRINITY_DN2559_c0_g1_i4_orf1;TRINITY_DN87170_c0_g1_i3_orf1;TRINITY_DN34479_c0_g1_i2_orf1;TRINITY_DN21555_c0_g1_i4_orf1;TRINITY_DN779_c0_g1_i3_orf1;TRINITY_DN17003_c1_g1_i1_orf1;TRINITY_DN49047_c0_g1_i2_orf1;TRINITY_DN89483_c0_g1_i1_orf1;TRINITY_DN542_c0_g1_i4_orf1;TRINITY_DN34689_c0_g1_i4_orf1;TRINITY_DN4145_c0_g1_i1_orf1;TRINITY_DN96557_c0_g1_i1_orf1;TRINITY_DN4822_c0_g1_i6_orf1;TRINITY_DN1534_c0_g1_i3_orf1;TRINITY_DN43431_c0_g1_i1_orf1;TRINITY_DN29873_c0_g1_i1_orf1;TRINITY_DN38644_c0_g1_i1_orf1;TRINITY_DN57798_c0_g1_i1_orf1;TRINITY_DN46022_c0_g1_i1_orf1;TRINITY_DN2515_c0_g1_i6_orf1;TRINITY_DN5001_c0_g1_i4_orf1;TRINITY_DN1707_c0_g1_i1_orf1;TRINITY_DN9980_c0_g1_i1_orf1;TRINITY_DN44877_c0_g1_i2_orf1;TRINITY_DN779_c0_g1_i12_orf1;TRINITY_DN9062_c0_g2_i3_orf1;TRINITY_DN10769_c0_g1_i1_orf1;TRINITY_DN20499_c0_g3_i1_orf1;TRINITY_DN143496_c0_g1_i1_orf1;TRINITY_DN1262_c0_g1_i2_orf1;TRINITY_DN135188_c0_g1_i2_orf1;TRINITY_DN15136_c0_g1_i2_orf1;TRINITY_DN41_c0_g1_i5_orf1;TRINITY_DN28989_c0_g1_i7_orf1;TRINITY_DN5235_c0_g1_i7_orf1;TRINITY_DN25997_c1_g2_i4_orf1;TRINITY_DN2890_c0_g1_i2_orf1;TRINITY_DN650_c0_g1_i3_orf1
biological_process	formaldehyde metabolic process	GO:0046292	1	1/2360	TRINITY_DN3758_c0_g1_i2_orf1
biological_process	vitamin metabolic process	GO:0006766	2	2/2360	TRINITY_DN18782_c0_g1_i4_orf1;TRINITY_DN37165_c0_g1_i4_orf1
biological_process	urea metabolic process	GO:0019627	1	1/2360	TRINITY_DN24723_c2_g1_i1_orf1
biological_process	monosaccharide metabolic process	GO:0005996	12	12/2360	TRINITY_DN15222_c0_g1_i4_orf1;TRINITY_DN120089_c0_g1_i1_orf1;TRINITY_DN12545_c0_g1_i7_orf1;TRINITY_DN9109_c0_g1_i1_orf1;TRINITY_DN29873_c0_g1_i1_orf1;TRINITY_DN18650_c0_g1_i1_orf1;TRINITY_DN30713_c0_g1_i3_orf1;TRINITY_DN25997_c1_g2_i4_orf1;TRINITY_DN1161_c0_g1_i2_orf1;TRINITY_DN511_c0_g2_i1_orf1;TRINITY_DN31967_c0_g1_i5_orf1;TRINITY_DN1353_c0_g1_i1_orf1
biological_process	alcohol metabolic process	GO:0006066	9	9/2360	TRINITY_DN618_c0_g1_i3_orf1;TRINITY_DN1034_c0_g1_i4_orf1;TRINITY_DN10722_c0_g3_i1_orf1;TRINITY_DN36788_c0_g1_i2_orf1;TRINITY_DN38644_c0_g1_i1_orf1;TRINITY_DN9286_c0_g1_i2_orf1;TRINITY_DN3472_c0_g1_i6_orf1;TRINITY_DN1707_c0_g1_i1_orf1;TRINITY_DN11942_c0_g1_i1_orf1
biological_process	nucleobase-containing small molecule metaboli	GO:0055086	43	43/2360	TRINITY_DN38230_c0_g1_i4_orf1;TRINITY_DN6325_c0_g1_i8_orf1;TRINITY_DN15222_c0_g1_i4_orf1;TRINITY_DN115210_c0_g4_i1_orf1;TRINITY_DN18222_c0_g1_i5_orf1;TRINITY_DN1827_c0_g1_i4_orf1;TRINITY_DN60787_c0_g1_i5_orf1;TRINITY_DN39813_c0_g1_i1_orf1;TRINITY_DN140669_c0_g1_i1_orf1;TRINITY_DN2738_c1_g1_i3_orf1;TRINITY_DN125_c0_g1_i2_orf1;TRINITY_DN49038_c0_g4_i1_orf1;TRINITY_DN33146_c0_g1_i1_orf1;TRINITY_DN779_c0_g1_i3_orf1;TRINITY_DN1216_c0_g1_i4_orf1;TRINITY_DN8625_c0_g1_i1_orf1;TRINITY_DN2559_c0_g1_i4_orf1;TRINITY_DN98242_c0_g1_i1_orf1;TRINITY_DN14477_c0_g1_i12_orf1;TRINITY_DN11013_c0_g1_i3_orf1;TRINITY_DN26293_c0_g1_i4_orf1;TRINITY_DN779_c0_g1_i12_orf1;TRINITY_DN18782_c0_g1_i4_orf1;TRINITY_DN36144_c0_g1_i3_orf1;TRINITY_DN6587_c0_g1_i3_orf1;TRINITY_DN117844_c0_g1_i1_orf1;TRINITY_DN3991_c0_g1_i6_orf1;TRINITY_DN29873_c0_g1_i1_orf1;TRINITY_DN3800_c0_g1_i7_orf1;TRINITY_DN1718_c6_g1_i4_orf1;TRINITY_DN26195_c0_g1_i6_orf1;TRINITY_DN127056_c0_g1_i1_orf1;TRINITY_DN28299_c0_g1_i1_orf1;TRINITY_DN5952_c0_g1_i6_orf1;TRINITY_DN1957_c0_g1_i4_orf1;TRINITY_DN107261_c0_g1_i1_orf1;TRINITY_DN10548_c0_g2_i1_orf1;TRINITY_DN6813_c1_g1_i1_orf1;TRINITY_DN15136_c0_g1_i2_orf1;TRINITY_DN1084_c0_g1_i2_orf1;TRINITY_DN19251_c0_g1_i8_orf1;TRINITY_DN7808_c0_g1_i1_orf1;TRINITY_DN1084_c0_g2_i2_orf1
biological_process	urate metabolic process	GO:0046415	3	3/2360	TRINITY_DN779_c0_g1_i12_orf1;TRINITY_DN2559_c0_g1_i4_orf1;TRINITY_DN779_c0_g1_i3_orf1
biological_process	macromolecule glycosylation	GO:0043413	3	3/2360	TRINITY_DN103118_c0_g1_i4_orf1;TRINITY_DN1789_c0_g1_i5_orf1;TRINITY_DN53760_c0_g1_i1_orf1
biological_process	macromolecule methylation	GO:0043414	5	5/2360	TRINITY_DN15256_c0_g1_i8_orf1;TRINITY_DN41664_c0_g1_i4_orf1;TRINITY_DN14313_c0_g1_i1_orf1;TRINITY_DN13350_c0_g1_i4_orf1;TRINITY_DN1344_c0_g1_i1_orf1
biological_process	lipid metabolic process	GO:0006629	42	42/2360	TRINITY_DN48590_c0_g1_i1_orf1;TRINITY_DN10722_c0_g3_i1_orf1;TRINITY_DN5055_c0_g1_i12_orf1;TRINITY_DN12526_c0_g1_i5_orf1;TRINITY_DN2668_c0_g1_i7_orf1;TRINITY_DN1293_c0_g1_i4_orf1;TRINITY_DN482_c0_g1_i1_orf1;TRINITY_DN5841_c0_g1_i2_orf1;TRINITY_DN72017_c0_g1_i1_orf1;TRINITY_DN45220_c0_g1_i1_orf1;TRINITY_DN5211_c0_g1_i1_orf1;TRINITY_DN44658_c0_g1_i2_orf1;TRINITY_DN86833_c0_g3_i1_orf1;TRINITY_DN7134_c0_g1_i1_orf1;TRINITY_DN8964_c0_g1_i4_orf1;TRINITY_DN117_c0_g1_i6_orf1;TRINITY_DN117_c0_g1_i4_orf1;TRINITY_DN41_c0_g1_i3_orf1;TRINITY_DN42759_c0_g3_i1_orf1;TRINITY_DN10430_c0_g1_i4_orf1;TRINITY_DN9028_c0_g1_i5_orf1;TRINITY_DN2808_c0_g1_i8_orf1;TRINITY_DN21570_c0_g1_i1_orf1;TRINITY_DN3175_c0_g1_i7_orf1;TRINITY_DN117844_c0_g1_i1_orf1;TRINITY_DN1999_c0_g1_i9_orf1;TRINITY_DN3991_c0_g1_i6_orf1;TRINITY_DN38644_c0_g1_i1_orf1;TRINITY_DN84478_c0_g1_i8_orf1;TRINITY_DN4070_c0_g1_i4_orf1;TRINITY_DN76283_c0_g2_i1_orf1;TRINITY_DN40197_c0_g1_i1_orf1;TRINITY_DN7861_c0_g1_i5_orf1;TRINITY_DN52788_c0_g1_i1_orf1;TRINITY_DN2618_c0_g1_i3_orf1;TRINITY_DN6586_c0_g1_i1_orf1;TRINITY_DN1293_c1_g1_i4_orf1;TRINITY_DN41_c0_g1_i5_orf1;TRINITY_DN1084_c0_g1_i2_orf1;TRINITY_DN5697_c0_g1_i1_orf1;TRINITY_DN3545_c0_g1_i6_orf1;TRINITY_DN1084_c0_g2_i2_orf1

biological_process	nucleobase-containing compound metabolic pr	GO:0006139	148	148/2360	TRINITY_DN38230_c0_g1_i4_orf1;TRINITY_DN5670_c0_g1_i2_orf1;TRINITY_DN13350_c0_g1_i4_orf1;TRINITY_DN1827_c0_g1_i4_orf1;TRINITY_DN60787_c0_g1_i5_orf1;TRINITY_DN7122_c0_g1_i1_orf1;TRINITY_DN35669_c0_g1_i1_orf1;TRINITY_DN2054_c0_g1_i1_orf1;TRINITY_DN124950_c0_g2_i1_orf1;TRINITY_DN2738_c1_g1_i3_orf1;TRINITY_DN15040_c0_g4_i1_orf1;TRINITY_DN34134_c0_g2_i1_orf1;TRINITY_DN33146_c0_g1_i1_orf1;TRINITY_DN1768_c0_g1_i2_orf1;TRINITY_DN44877_c0_g1_i2_orf1;TRINITY_DN2953_c1_g1_i10_orf1;TRINITY_DN11013_c0_g1_i3_orf1;TRINITY_DN15160_c0_g1_i1_orf1;TRINITY_DN6587_c0_g1_i3_orf1;TRINITY_DN2953_c1_g1_i2_orf1;TRINITY_DN1393_c0_g1_i2_orf1;TRINITY_DN3991_c0_g1_i6_orf1;TRINITY_DN29873_c0_g1_i1_orf1;TRINITY_DN3800_c0_g1_i7_orf1;TRINITY_DN47123_c0_g1_i1_orf1;TRINITY_DN15256_c0_g1_i8_orf1;TRINITY_DN123184_c0_g1_i1_orf1;TRINITY_DN27771_c0_g1_i1_orf1;TRINITY_DN2769_c0_g1_i1_orf1;TRINITY_DN5507_c0_g1_i1_orf1;TRINITY_DN26195_c0_g1_i6_orf1;TRINITY_DN2224_c0_g1_i1_orf1;TRINITY_DN2401_c0_g2_i1_orf1;TRINITY_DN20499_c0_g3_i1_orf1;TRINITY_DN5952_c0_g1_i6_orf1;TRINITY_DN4813_c0_g1_i5_orf1;TRINITY_DN107261_c0_g1_i1_orf1;TRINITY_DN15136_c0_g1_i2_orf1;TRINITY_DN107288_c0_g1_i2_orf1;TRINITY_DN7808_c0_g1_i1_orf1;TRINITY_DN56993_c0_g1_i4_orf1;TRINITY_DN14313_c0_g1_i1_orf1;TRINITY_DN1957_c0_g1_i4_orf1;TRINITY_DN3092_c0_g1_i2_orf1;TRINITY_DN1978_c0_g1_i4_orf1;TRINITY_DN17271_c0_g1_i1_orf1;TRINITY_DN115210_c0_g4_i1_orf1;TRINITY_DN45271_c0_g1_i1_orf1;TRINITY_DN18222_c0_g1_i5_orf1;TRINITY_DN1005_c0_g1_i5_orf1;TRINITY_DN37532_c0_g1_i1_orf1;TRINITY_DN51968_c0_g1_i1_orf1;TRINITY_DN15900_c0_g1_i6_orf1;TRINITY_DN81258_c0_g1_i2_orf1;TRINITY_DN21567_c0_g1_i7_orf1;TRINITY_DN30097_c0_g1_i2_orf1;TRINITY_DN1005_c0_g2_i1_orf1;TRINITY_DN8625_c0_g1_i1_orf1;TRINITY_DN779_c0_g1_i3_orf1;TRINITY_DN1607_c0_g1_i16_orf1;TRINITY_DN16978_c0_g1_i1_orf1;TRINITY_DN41664_c0_g1_i4_orf1;TRINITY_DN1091_c0_g3_i1_orf1;TRINITY_DN19034_c0_g1_i1_orf1;TRINITY_DN36144_c0_g1_i3_orf1;TRINITY_DN1616_c0_g1_i3_orf1;TRINITY_DN117844_c0_g1_i1_orf1;TRINITY_DN6642_c0_g1_i2_orf1;TRINITY_DN4408_c6_g1_i1_orf1;TRINITY_DN51568_c0_g1_i1_orf1;TRINITY_DN4707_c0_g1_i1_orf1;TRINITY_DN40945_c0_g1_i1_orf1;TRINITY_DN1718_c6_g1_i4_orf1;TRINITY_DN779_c0_g1_i12_orf1;TRINITY_DN31663_c0_g1_i2_orf1;TRINITY_DN139537_c0_g1_i1_orf1;TRINITY_DN1344_c0_g1_i1_orf1;TRINITY_DN107035_c0_g1_i1_orf1;TRINITY_DN4908_c1_g1_i5_orf1;TRINITY_DN291_c0_g1_i2_orf1;TRINITY_DN2749_c4_g1_i2_orf1;TRINITY_DN6325_c0_g1_i8_orf1;TRINITY_DN2749_c0_g1_i4_orf1;TRINITY_DN22951_c0_g1_i1_orf1;TRINITY_DN1750_c1_g1_i5_orf1;TRINITY_DN127056_c0_g1_i1_orf1;TRINITY_DN39813_c0_g1_i1_orf1;TRINITY_DN2718_c0_g1_i6_orf1;TRINITY_DN57918_c0_g1_i1_orf1;TRINITY_DN56270_c0_g1_i1_orf1;TRINITY_DN140669_c0_g1_i1_orf1;TRINITY_DN33346_c0_g1_i1_orf1;TRINITY_DN1554_c0_g1_i9_orf1;TRINITY_DN125_c0_g1_i2_orf1;TRINITY_DN49038_c0_g4_i1_orf1;TRINITY_DN2559_c0_g1_i4_orf1;TRINITY_DN98242_c0_g1_i1_orf1;TRINITY_DN14477_c0_g1_i12_orf1;TRINITY_DN452_c1_g1_i3_orf1;TRINITY_DN34689_c0_g1_i4_orf1;TRINITY_DN4135_c0_g1_i5_orf1;TRINITY_DN26293_c0_g1_i4_orf1;TRINITY_DN3062_c0_g1_i1_orf1;TRINITY_DN7341_c0_g1_i8_orf1;TRINITY_DN19866_c0_g1_i4_orf1;TRINITY_DN24322_c0_g1_i4_orf1;TRINITY_DN84322_c0_g2_i1_orf1;TRINITY_DN5686_c0_g1_i4_orf1;TRINITY_DN53311_c0_g2_i1_orf1;TRINITY_DN3082_c1_g1_i7_orf1;TRINITY_DN21367_c0_g1_i1_orf1;TRINITY_DN38274_c0_g1_i1_orf1;TRINITY_DN111_c0_g2_i2_orf1;TRINITY_DN14487_c0_g1_i4_orf1;TRINITY_DN28299_c0_g1_i1_orf1;TRINITY_DN2647_c0_g1_i3_orf1;TRINITY_DN120144_c0_g1_i1_orf1;TRINITY_DN6248_c0_g1_i1_orf1;TRINITY_DN10548_c0_g2_i1_orf1;TRINITY_DN6813_c1_g1_i1_orf1;TRINITY_DN46022_c0_g1_i1_orf1;TRINITY_DN1084_c0_g2_i2_orf1;TRINITY_DN1216_c0_g1_i4_orf1;TRINITY_DN2971_c0_g1_i1_orf1;TRINITY_DN67649_c0_g1_i1_orf1;TRINITY_DN4835_c0_g1_i2_orf1;TRINITY_DN38562_c0_g1_i3_orf1;TRINITY_DN3482_c0_g2_i1_orf1;TRINITY_DN13760_c1_g1_i1_orf1;TRINITY_DN104507_c0_g1_i2_orf1;TRINITY_DN5200_c0_g1_i2_orf1;TRINITY_DN34676_c1_g1_i3_orf1;TRINITY_DN2201_c0_g1_i1_orf1;TRINITY_DN2367_c1_g1_i20_orf1;TRINITY_DN1091_c0_g1_i1_orf1;TRINITY_DN18782_c0_g1_i4_orf1;TRINITY_DN15222_c0_g1_i4_orf1;TRINITY_DN23343_c0_g1_i9_orf1;TRINITY_DN5238_c0_g1_i2_orf1;TRINITY_DN19251_c0_g1_i8_orf1;TRINITY_DN13055_c0_g1_i5_orf1;TRINITY_DN34432_c0_g1_i1_orf1;TRINITY_DN110534_c0_g1_i3_orf1;TRINITY_DN5757_c0_g1_i1_orf1;TRINITY_DN21539_c0_g1_i1_orf1;TRINITY_DN346_c0_g1_i7_orf1;TRINITY_DN1084_c0_g1_i2_orf1;TRINITY_DN30950_c0_g1_i13_orf1
biological_process	tricarboxylic acid cycle	GO:0006099	5	5/2360	TRINITY_DN36788_c0_g1_i2_orf1;TRINITY_DN5266_c0_g1_i1_orf1;TRINITY_DN3464_c0_g1_i1_orf1;TRINITY_DN146126_c0_g1_i1_orf1;TRINITY_DN19251_c0_g1_i8_orf1
biological_process	carbohydrate metabolic process	GO:0005975	67	67/2360	TRINITY_DN17003_c1_g1_i1_orf1;TRINITY_DN120089_c0_g1_i1_orf1;TRINITY_DN10722_c0_g3_i1_orf1;TRINITY_DN2474_c0_g1_i5_orf1;TRINITY_DN18650_c0_g1_i1_orf1;TRINITY_DN10824_c0_g1_i3_orf1;TRINITY_DN2425_c0_g1_i3_orf1;TRINITY_DN18222_c0_g1_i5_orf1;TRINITY_DN1827_c0_g1_i4_orf1;TRINITY_DN60787_c0_g1_i5_orf1;TRINITY_DN812_c2_g1_i1_orf1;TRINITY_DN22577_c0_g1_i2_orf1;TRINITY_DN25492_c0_g1_i1_orf1;TRINITY_DN1196_c0_g1_i5_orf1;TRINITY_DN11942_c0_g1_i1_orf1;TRINITY_DN31967_c0_g1_i5_orf1;TRINITY_DN95850_c0_g1_i1_orf1;TRINITY_DN11657_c0_g1_i2_orf1;TRINITY_DN49038_c0_g4_i1_orf1;TRINITY_DN1034_c0_g1_i4_orf1;TRINITY_DN12545_c0_g1_i7_orf1;TRINITY_DN9109_c0_g1_i1_orf1;TRINITY_DN5852_c0_g1_i13_orf1;TRINITY_DN4070_c0_g1_i4_orf1;TRINITY_DN28741_c0_g1_i3_orf1;TRINITY_DN7183_c0_g1_i2_orf1;TRINITY_DN11817_c0_g1_i4_orf1;TRINITY_DN361_c0_g1_i5_orf1;TRINITY_DN48237_c0_g1_i5_orf1;TRINITY_DN30713_c0_g1_i3_orf1;TRINITY_DN89483_c0_g1_i1_orf1;TRINITY_DN2894_c0_g2_i3_orf1;TRINITY_DN542_c0_g1_i4_orf1;TRINITY_DN7228_c0_g1_i6_orf1;TRINITY_DN15222_c0_g1_i4_orf1;TRINITY_DN1785_c0_g1_i5_orf1;TRINITY_DN18918_c0_g1_i3_orf1;TRINITY_DN9000_c0_g2_i1_orf1;TRINITY_DN29873_c0_g1_i1_orf1;TRINITY_DN38644_c0_g1_i1_orf1;TRINITY_DN650_c0_g1_i3_orf1;TRINITY_DN2061_c0_g1_i3_orf1;TRINITY_DN2170_c0_g2_i1_orf1;TRINITY_DN2515_c0_g1_i6_orf1;TRINITY_DN26688_c0_g1_i2_orf1;TRINITY_DN7534_c0_g1_i15_orf1;TRINITY_DN1707_c0_g1_i1_orf1;TRINITY_DN511_c0_g2_i1_orf1;TRINITY_DN14458_c0_g1_i2_orf1;TRINITY_DN7828_c0_g1_i2_orf1;TRINITY_DN6108_c0_g1_i5_orf1;TRINITY_DN6325_c0_g1_i8_orf1;TRINITY_DN21555_c0_g1_i4_orf1;TRINITY_DN5952_c0_g1_i6_orf1;TRINITY_DN1732_c0_g1_i17_orf1;TRINITY_DN3476_c0_g1_i5_orf1;TRINITY_DN479_c6_g1_i2_orf1;TRINITY_DN618_c0_g1_i3_orf1;TRINITY_DN82801_c0_g1_i1_orf1;TRINITY_DN103118_c0_g1_i4_orf1;TRINITY_DN13088_c0_g1_i5_orf1;TRINITY_DN2170_c1_g1_i3_orf1;TRINITY_DN36788_c0_g1_i2_orf1;TRINITY_DN5852_c0_g1_i6_orf1;TRINITY_DN25997_c1_g2_i4_orf1;TRINITY_DN1161_c0_g1_i2_orf1;TRINITY_DN1353_c0_g1_i1_orf1
biological_process	protein metabolic process	GO:0019538	185	185/2360	TRINITY_DN24469_c0_g2_i2_orf1;TRINITY_DN10364_c0_g1_i5_orf1;TRINITY_DN39404_c0_g1_i7_orf1;TRINITY_DN3194_c0_g1_i6_orf1;TRINITY_DN181_c0_g1_i3_orf1;TRINITY_DN71610_c0_g1_i1_orf1;TRINITY_DN1533_c0_g2_i1_orf1;TRINITY_DN48983_c0_g1_i2_orf1;TRINITY_DN135_c0_g1_i1_orf1;TRINITY_DN344_c1_g1_i1_orf1;TRINITY_DN2069_c1_g1_i8_orf1;TRINITY_DN103118_c0_g1_i4_orf1;TRINITY_DN4217_c0_g1_i2_orf1;TRINITY_DN38431_c0_g1_i1_orf1;TRINITY_DN3499_c0_g1_i8_orf1;TRINITY_DN30300_c0_g2_i1_orf1;TRINITY_DN2442_c0_g1_i2_orf1;TRINITY_DN8659_c0_g1_i1_orf1;TRINITY_DN70485_c0_g1_i2_orf1;TRINITY_DN142442_c0_g1_i1_orf1;TRINITY_DN4798_c0_g1_i3_orf1;TRINITY_DN16258_c0_g1_i2_orf1;TRINITY_DN2885_c1_g1_i2_orf1;TRINITY_DN14754_c0_g1_i6_orf1;TRINITY_DN29448_c0_g1_i1_orf1;TRINITY_DN41761_c0_g1_i4_orf1;TRINITY_DN14774_c0_g1_i4_orf1;TRINITY_DN875_c0_g1_i3_orf1;TRINITY_DN95056_c0_g2_i2_orf1;TRINITY_DN74889_c0_g1_i1_orf1;TRINITY_DN2794_c1_g1_i8_orf1;TRINITY_DN4125_c0_g1_i14_orf1;TRINITY_DN6185_c0_g1_i12_orf1;TRINITY_DN6205_c0_g1_i8_orf1;TRINITY_DN6436_c0_g1_i1_orf1;TRINITY_DN140538_c0_g2_i1_orf1;TRINITY_DN391_c0_g1_i4_orf1;TRINITY_DN10769_c0_g1_i1_orf1;TRINITY_DN15591_c0_g1_i3_orf1;TRINITY_DN1380_c0_g1_i5_orf1;TRINITY_DN46715_c0_g1_i1_orf1;TRINITY_DN1749_c0_g2_i2_orf1;TRINITY_DN1706_c0_g1_i7_orf1;TRINITY_DN23360_c0_g1_i3_orf1;TRINITY_DN45633_c0_g1_i1_orf1;TRINITY_DN10090_c0_g1_i1_orf1;TRINITY_DN29034_c0_g1_i2_orf1;TRINITY_DN19537_c0_g1_i1_orf1;TRINITY_DN4189_c0_g2_i1_orf1;TRINITY_DN37923_c0_g1_i1_orf1;TRINITY_DN1272_c1_g1_i4_orf1;TRINITY_DN53760_c0_g1_i1_orf1;TRINITY_DN26947_c0_g1_i1_orf1;TRINITY_DN57798_c0_g1_i1_orf1;TRINITY_DN9871_c0_g1_i11_orf1;TRINITY_DN36434_c0_g2_i3_orf1;TRINITY_DN1789_c0_g1_i5_orf1;TRINITY_DN42461_c0_g1_i4_orf1;TRINITY_DN1274_c0_g1_i4_orf1;TRINITY_DN11125_c0_g1_i1_orf1;TRINITY_DN6813_c1_g1_i1_orf1;TRINITY_DN21719_c0_g1_i2_orf1;TRINITY_DN7583_c0_g1_i1_orf1;TRINITY_DN277_c1_g1_i1_orf1;TRINITY_DN50787_c0_g2_i2_orf1;TRINITY_DN45948_c1_g1_i1_orf1;TRINITY_DN12757_c0_g1_i1_orf1;TRINITY_DN23167_c0_g2_i1_orf1;TRINITY_DN6423_c0_g1_i5_orf1;TRINITY_DN71863_c0_g1_i2_orf1;TRINITY_DN3733_c0_g1_i1_orf1;TRINITY_DN32700_c0_g1_i2_orf1;TRINITY_DN4125_c1_g1_i5_orf1;TRINITY_DN9591_c0_g1_i1_orf1;TRINITY_DN5444_c0_g2_i1_orf1;TRINITY_DN4408_c6_g1_i1_orf1;TRINITY_DN6423_c0_g1_i6_orf1;TRINITY_DN140_c1_g1_i2_orf1;TRINITY_DN1266_c2_g1_i1_orf1;TRINITY_DN83295_c0_g1_i3_orf1;TRINITY_DN3773_c0_g1_i4_orf1;TRINITY_DN9062_c0_g2_i3_orf1;TRINITY_DN1173_c1_g1_i10_orf1;TRINITY_DN1528_c0_g1_i4_orf1;TRINITY_DN7776_c0_g1_i5_orf1;TRINITY_DN10403_c0_g1_i1_orf1;TRINITY_DN1459_c1_g1_i1_orf1;TRINITY_DN3702_c0_g1_i1_orf1;TRINITY_DN147458_c0_g1_i1_orf1;TRINITY_DN21218_c0_g1_i4_orf1;TRINITY_DN12397_c0_g1_i1_orf1;TRINITY_DN8692_c0_g1_i2_orf1;TRINITY_DN5064_c0_g1_i4_orf1;TRINITY_DN5112_c0_g1_i1_orf1;TRINITY_DN25534_c0_g1_i1_orf1;TRINITY_DN10766_c0_g1_i1_orf1;TRINITY_DN6365_c0_g1_i4_orf1;TRINITY_DN46202_c0_g1_i1_orf1;TRINITY_DN1552_c0_g1_i3_orf1;TRINITY_DN5182_c0_g1_i5_orf1;TRINITY_DN4817_c0_g1_i4_orf1;TRINITY_DN81803_c0_g2_i1_orf1;TRINITY_DN70409_c0_g1_i3_orf1;TRINITY_DN57111_c0_g1_i1_orf1;TRINITY_DN5310_c2_g1_i2_orf1;TRINITY_DN2040_c0_g1_i6_orf1;TRINITY_DN4571_c0_g1_i4_orf1;TRINITY_DN52553_c0_g2_i1_orf1;TRINITY_DN62729_c0_g1_i13_orf1;TRINITY_DN1404_c0_g1_i6_orf1;TRINITY_DN4016_c0_g1_i1_orf1;TRINITY_DN934_c2_g1_i7_orf1;TRINITY_DN34479_c0_g1_i2_orf1;TRINITY_DN1405_c0_g1_i1_orf1;TRINITY_DN21719_c0_g2_i4_orf1;TRINITY_DN130778_c0_g1_i1_orf1;TRINITY_DN3343_c0_g2_i1_orf1;TRINITY_DN18172_c0_g1_i6_orf1;TRINITY_DN19866_c0_g1_i4_orf1;TRINITY_DN2673_c2_g1_i2_orf1;TRINITY_DN1308_c0_g1_i4_orf1;TRINITY_DN23167_c0_g1_i4_orf1;TRINITY_DN28661_c0_g1_i1_orf1;TRINITY_DN4228_c0_g1_i5_orf1;TRINITY_DN113327_c0_g1_i2_orf1;TRINITY_DN111110_c0_g1_i1_orf1;TRINITY_DN14487_c0_g1_i4_orf1;TRINITY_DN96557_c0_g1_i1_orf1;TRINITY_DN8659_c0_g2_i1_orf1;TRINITY_DN6470_c0_g3_i2_orf1;TRINITY_DN21971_c0_g1_i4_orf1;TRINITY_DN29034_c0_g1_i1_orf1;TRINITY_DN69697_c0_g1_i1_orf1;TRINITY_DN19990_c0_g1_i1_orf1;TRINITY_DN49047_c0_g1_i2_orf1;TRINITY_DN52553_c0_g1_i1_orf1;TRINITY_DN4898_c0_g1_i7_orf1;TRINITY_DN10385_c0_g1_i5_orf1;TRINITY_DN120593_c0_g1_i1_orf1;TRINITY_DN7316_c0_g2_i1_orf1;TRINITY_DN801_c0_g1_i2_orf1;TRINITY_DN24539_c0_g1_i4_orf1;TRINITY_DN753_c0_g1_i4_orf1;TRINITY_DN13686_c0_g2_i1_orf1;TRINITY_DN13651_c0_g1_i2_orf1;TRINITY_DN334_c0_g1_i4_orf1;TRINITY_DN26853_c0_g1_i1_orf1;TRINITY_DN334_c0_g1_i3_orf1;TRINITY_DN3119_c0_g1_i7_orf1;TRINITY_DN142588_c0_g1_i1_orf1;TRINITY_DN143496_c0_g1_i1_orf1;TRINITY_DN1173_c0_g1_i11_orf1;TRINITY_DN2043_c0_g1_i3_orf1;TRINITY_DN111621_c0_g3_i1_orf1;TRINITY_DN18273_c0_g1_i4_orf1;TRINITY_DN7134_c0_g1_i1_orf1;TRINITY_DN2673_c0_g3_i1_orf1;TRINITY_DN10994_c0_g1_i4_orf1;TRINITY_DN17759_c0_g1_i5_orf1;TRINITY_DN4886_c0_g1_i6_orf1;TRINITY_DN4494_c0_g1_i1_orf1;TRINITY_DN152_c0_g1_i4_orf1;TRINITY_DN3826_c0_g1_i1_orf1;TRINITY_DN747_c0_g1_i1_orf1;TRINITY_DN28989_c0_g1_i7_orf1;TRINITY_DN30154_c0_g1_i1_orf1;TRINITY_DN96_c0_g1_i1_orf1;TRINITY_DN15478_c0_g1_i1_orf1;TRINITY_DN4125_c0_g1_i6_orf1;TRINITY_DN5001_c0_g1_i4_orf1;TRINITY_DN9980_c0_g1_i1_orf1;TRINITY_DN248_c0_g1_i1_orf1;TRINITY_DN10070_c0_g1_i1_orf1;TRINITY_DN30_c0_g1_i6_orf1;TRINITY_DN701_c0_g1_i1_orf1;TRINITY_DN2043_c0_g1_i11_orf1;TRINITY_DN147475_c0_g1_i1_orf1;TRINITY_DN2207_c0_g1_i6_orf1;TRINITY_DN135188_c0_g1_i2_orf1;TRINITY_DN24121_c1_g1_i6_orf1;TRINITY_DN22797_c0_g1_i5_orf1;TRINITY_DN26824_c0_g1_i1_orf1;TRINITY_DN5697_c0_g1_i1_orf1;TRINITY_DN8480_c0_g1_i1_orf1;TRINITY_DN21367_c0_g1_i1_orf1
biological_process	cellular amino acid metabolic process	GO:0006520	42	42/2360	TRINITY_DN2065_c1_g2_i1_orf1;TRINITY_DN57918_c0_g1_i1_orf1;TRINITY_DN92153_c0_g2_i2_orf1;TRINITY_DN14565_c0_g1_i11_orf1;TRINITY_DN115210_c0_g4_i1_orf1;TRINITY_DN24970_c0_g1_i4_orf1;TRINITY_DN27771_c0_g1_i1_orf1;TRINITY_DN5564_c0_g1_i5_orf1;TRINITY_DN863_c0_g1_i6_orf1;TRINITY_DN100821_c0_g1_i1_orf1;TRINITY_DN35763_c0_g1_i2_orf1;TRINITY_DN4822_c0_g1_i9_orf1;TRINITY_DN6587_c0_g1_i3_orf1;TRINITY_DN4451_c0_g2_i4_orf1;TRINITY_DN19187_c0_g1_i1_orf1;TRINITY_DN2890_c0_g1_i2_orf1;TRINITY_DN11948_c0_g1_i8_orf1;TRINITY_DN87170_c0_g1_i3_orf1;TRINITY_DN2803_c4_g1_i1_orf1;TRINITY_DN2953_c1_g1_i10_orf1;TRINITY_DN24723_c2_g1_i1_orf1;TRINITY_DN1607_c0_g1_i16_orf1;TRINITY_DN89483_c0_g1_i1_orf1;TRINITY_DN2953_c1_g1_i2_orf1;TRINITY_DN11013_c0_g1_i3_orf1;TRINITY_DN2338_c0_g1_i5_orf1;TRINITY_DN84322_c0_g2_i1_orf1;TRINITY_DN20796_c0_g1_i4_orf1;TRINITY_DN4822_c0_g1_i6_orf1;TRINITY_DN17326_c0_g1_i8_orf1;TRINITY_DN43431_c0_g1_i1_orf1;TRINITY_DN3859_c0_g1_i5_orf1;TRINITY_DN3073_c0_g1_i7_orf1;TRINITY_DN15160_c0_g1_i1_orf1;TRINITY_DN2224_c0_g1_i1_orf1;TRINITY_DN31163_c1_g1_i4_orf1;TRINITY_DN21539_c0_g1_i1_orf1;TRINITY_DN1262_c0_g1_i2_orf1;TRINITY_DN11383_c0_g2_i4_orf1;TRINITY_DN15136_c0_g1_i2_orf1;TRINITY_DN107288_c0_g1_i2_orf1;TRINITY_DN1068_c0_g1_i3_orf1

biological_process	organophosphate metabolic process	GO:0019637	43	43/2360	TRINITY_DN5697_c0_g1_i1_orf1;TRINITY_DN143509_c0_g1_i1_orf1;TRINITY_DN62557_c0_g1_i1_orf1;TRINITY_DN47151_c0_g1_i1_orf1;TRINITY_DN38230_c0_g1_i4_orf1;TRINITY_DN10722_c0_g3_i1_orf1;TRINITY_DN15222_c0_g1_i4_orf1;TRINITY_DN115210_c0_g4_i1_orf1;TRINITY_DN18222_c0_g1_i5_orf1;TRINITY_DN1827_c0_g1_i4_orf1;TRINITY_DN60787_c0_g1_i5_orf1;TRINITY_DN39813_c0_g1_i1_orf1;TRINITY_DN2738_c1_g1_i3_orf1;TRINITY_DN6325_c0_g1_i8_orf1;TRINITY_DN1034_c0_g1_i4_orf1;TRINITY_DN33146_c0_g1_i1_orf1;TRINITY_DN1216_c0_g1_i4_orf1;TRINITY_DN7134_c0_g1_i1_orf1;TRINITY_DN116972_c0_g1_i1_orf1;TRINITY_DN14477_c0_g1_i12_orf1;TRINITY_DN18782_c0_g1_i4_orf1;TRINITY_DN36144_c0_g1_i3_orf1;TRINITY_DN6587_c0_g1_i3_orf1;TRINITY_DN117844_c0_g1_i1_orf1;TRINITY_DN3991_c0_g1_i6_orf1;TRINITY_DN29873_c0_g1_i1_orf1;TRINITY_DN38644_c0_g1_i1_orf1;TRINITY_DN3800_c0_g1_i7_orf1;TRINITY_DN1718_c6_g1_i4_orf1;TRINITY_DN49038_c0_g4_i1_orf1;TRINITY_DN26195_c0_g1_i6_orf1;TRINITY_DN127056_c0_g1_i1_orf1;TRINITY_DN28299_c0_g1_i1_orf1;TRINITY_DN2618_c0_g1_i3_orf1;TRINITY_DN1957_c0_g1_i4_orf1;TRINITY_DN107261_c0_g1_i1_orf1;TRINITY_DN10548_c0_g2_i1_orf1;TRINITY_DN6813_c1_g1_i1_orf1;TRINITY_DN15136_c0_g1_i2_orf1;TRINITY_DN1084_c0_g1_i2_orf1;TRINITY_DN19251_c0_g1_i8_orf1;TRINITY_DN7808_c0_g1_i1_orf1;TRINITY_DN1084_c0_g2_i2_orf1
biological_process	carbohydrate derivative metabolic process	GO:1901135	53	53/2360	TRINITY_DN5697_c0_g1_i1_orf1;TRINITY_DN62557_c0_g1_i1_orf1;TRINITY_DN47151_c0_g1_i1_orf1;TRINITY_DN6325_c0_g1_i8_orf1;TRINITY_DN15222_c0_g1_i4_orf1;TRINITY_DN115210_c0_g4_i1_orf1;TRINITY_DN98242_c0_g1_i1_orf1;TRINITY_DN10824_c0_g1_i3_orf1;TRINITY_DN18222_c0_g1_i5_orf1;TRINITY_DN1827_c0_g1_i4_orf1;TRINITY_DN60787_c0_g1_i5_orf1;TRINITY_DN39813_c0_g1_i1_orf1;TRINITY_DN1196_c0_g1_i5_orf1;TRINITY_DN140669_c0_g1_i1_orf1;TRINITY_DN2738_c1_g1_i3_orf1;TRINITY_DN125_c0_g1_i2_orf1;TRINITY_DN117844_c0_g1_i1_orf1;TRINITY_DN1034_c0_g1_i4_orf1;TRINITY_DN33146_c0_g1_i1_orf1;TRINITY_DN1216_c0_g1_i4_orf1;TRINITY_DN8625_c0_g1_i1_orf1;TRINITY_DN17003_c1_g1_i1_orf1;TRINITY_DN143509_c0_g1_i1_orf1;TRINITY_DN40197_c0_g1_i1_orf1;TRINITY_DN542_c0_g1_i4_orf1;TRINITY_DN11013_c0_g1_i3_orf1;TRINITY_DN26293_c0_g1_i4_orf1;TRINITY_DN1084_c0_g1_i2_orf1;TRINITY_DN6587_c0_g1_i3_orf1;TRINITY_DN1534_c0_g1_i3_orf1;TRINITY_DN3991_c0_g1_i6_orf1;TRINITY_DN29873_c0_g1_i1_orf1;TRINITY_DN3800_c0_g1_i7_orf1;TRINITY_DN2515_c0_g1_i6_orf1;TRINITY_DN11798_c0_g2_i1_orf1;TRINITY_DN1718_c6_g1_i4_orf1;TRINITY_DN49038_c0_g4_i1_orf1;TRINITY_DN26195_c0_g1_i6_orf1;TRINITY_DN127056_c0_g1_i1_orf1;TRINITY_DN21555_c0_g1_i4_orf1;TRINITY_DN28299_c0_g1_i1_orf1;TRINITY_DN5952_c0_g1_i6_orf1;TRINITY_DN18782_c0_g1_i4_orf1;TRINITY_DN107261_c0_g1_i1_orf1;TRINITY_DN10548_c0_g2_i1_orf1;TRINITY_DN6813_c1_g1_i1_orf1;TRINITY_DN15136_c0_g1_i2_orf1;TRINITY_DN116972_c0_g1_i1_orf1;TRINITY_DN19251_c0_g1_i8_orf1;TRINITY_DN5235_c0_g1_i7_orf1;TRINITY_DN7808_c0_g1_i1_orf1;TRINITY_DN1084_c0_g2_i2_orf1;TRINITY_DN650_c0_g1_i3_orf1
biological_process	organic hydroxy compound metabolic process	GO:1901615	14	14/2360	TRINITY_DN11942_c0_g1_i1_orf1;TRINITY_DN58125_c0_g1_i1_orf1;TRINITY_DN10722_c0_g3_i1_orf1;TRINITY_DN36788_c0_g1_i2_orf1;TRINITY_DN38644_c0_g1_i1_orf1;TRINITY_DN9286_c0_g1_i2_orf1;TRINITY_DN1034_c0_g1_i4_orf1;TRINITY_DN1707_c0_g1_i1_orf1;TRINITY_DN31163_c1_g1_i4_orf1;TRINITY_DN15706_c0_g2_i5_orf1;TRINITY_DN3472_c0_g1_i6_orf1;TRINITY_DN618_c0_g1_i3_orf1;TRINITY_DN37165_c0_g1_i4_orf1;TRINITY_DN2338_c0_g1_i5_orf1
biological_process	organic cyclic compound metabolic process	GO:1901360	166	166/2360	TRINITY_DN38230_c0_g1_i4_orf1;TRINITY_DN5670_c0_g1_i2_orf1;TRINITY_DN13350_c0_g1_i4_orf1;TRINITY_DN1827_c0_g1_i4_orf1;TRINITY_DN863_c0_g1_i6_orf1;TRINITY_DN7122_c0_g1_i1_orf1;TRINITY_DN35669_c0_g1_i1_orf1;TRINITY_DN2054_c0_g1_i1_orf1;TRINITY_DN124950_c0_g2_i1_orf1;TRINITY_DN2738_c1_g1_i3_orf1;TRINITY_DN15040_c0_g4_i1_orf1;TRINITY_DN34134_c0_g2_i1_orf1;TRINITY_DN33146_c0_g1_i1_orf1;TRINITY_DN1768_c0_g1_i2_orf1;TRINITY_DN44877_c0_g1_i2_orf1;TRINITY_DN31163_c1_g1_i4_orf1;TRINITY_DN11013_c0_g1_i3_orf1;TRINITY_DN15160_c0_g1_i1_orf1;TRINITY_DN6587_c0_g1_i3_orf1;TRINITY_DN4822_c0_g1_i6_orf1;TRINITY_DN2953_c1_g1_i2_orf1;TRINITY_DN1393_c0_g1_i2_orf1;TRINITY_DN5507_c0_g1_i1_orf1;TRINITY_DN3991_c0_g1_i6_orf1;TRINITY_DN29873_c0_g1_i1_orf1;TRINITY_DN3800_c0_g1_i7_orf1;TRINITY_DN47123_c0_g1_i1_orf1;TRINITY_DN15256_c0_g1_i8_orf1;TRINITY_DN123184_c0_g1_i1_orf1;TRINITY_DN27771_c0_g1_i1_orf1;TRINITY_DN2769_c0_g1_i1_orf1;TRINITY_DN37165_c0_g1_i4_orf1;TRINITY_DN26195_c0_g1_i6_orf1;TRINITY_DN2224_c0_g1_i1_orf1;TRINITY_DN2401_c0_g2_i1_orf1;TRINITY_DN20499_c0_g3_i1_orf1;TRINITY_DN5952_c0_g1_i6_orf1;TRINITY_DN4813_c0_g1_i5_orf1;TRINITY_DN107261_c0_g1_i1_orf1;TRINITY_DN15136_c0_g1_i2_orf1;TRINITY_DN107288_c0_g1_i2_orf1;TRINITY_DN7808_c0_g1_i1_orf1;TRINITY_DN56993_c0_g1_i4_orf1;TRINITY_DN14313_c0_g1_i1_orf1;TRINITY_DN1957_c0_g1_i4_orf1;TRINITY_DN3092_c0_g1_i2_orf1;TRINITY_DN1978_c0_g1_i4_orf1;TRINITY_DN17271_c0_g1_i1_orf1;TRINITY_DN115210_c0_g4_i1_orf1;TRINITY_DN45271_c0_g1_i1_orf1;TRINITY_DN18222_c0_g1_i5_orf1;TRINITY_DN1005_c0_g1_i5_orf1;TRINITY_DN37532_c0_g1_i1_orf1;TRINITY_DN51968_c0_g1_i1_orf1;TRINITY_DN15900_c0_g1_i6_orf1;TRINITY_DN81258_c0_g1_i2_orf1;TRINITY_DN19187_c0_g1_i1_orf1;TRINITY_DN21567_c0_g1_i7_orf1;TRINITY_DN30097_c0_g1_i2_orf1;TRINITY_DN1005_c0_g2_i1_orf1;TRINITY_DN8625_c0_g1_i1_orf1;TRINITY_DN779_c0_g1_i3_orf1;TRINITY_DN1607_c0_g1_i16_orf1;TRINITY_DN16978_c0_g1_i1_orf1;TRINITY_DN41664_c0_g1_i4_orf1;TRINITY_DN1091_c0_g3_i1_orf1;TRINITY_DN19034_c0_g1_i1_orf1;TRINITY_DN4145_c0_g1_i1_orf1;TRINITY_DN36144_c0_g1_i3_orf1;TRINITY_DN1616_c0_g1_i3_orf1;TRINITY_DN117844_c0_g1_i1_orf1;TRINITY_DN6642_c0_g1_i2_orf1;TRINITY_DN4408_c6_g1_i1_orf1;TRINITY_DN51568_c0_g1_i1_orf1;TRINITY_DN4707_c0_g1_i1_orf1;TRINITY_DN3472_c0_g1_i6_orf1;TRINITY_DN40945_c0_g1_i1_orf1;TRINITY_DN1718_c6_g1_i4_orf1;TRINITY_DN779_c0_g1_i12_orf1;TRINITY_DN31663_c0_g1_i2_orf1;TRINITY_DN139537_c0_g1_i1_orf1;TRINITY_DN1344_c0_g1_i1_orf1;TRINITY_DN107035_c0_g1_i1_orf1;TRINITY_DN4908_c1_g1_i5_orf1;TRINITY_DN291_c0_g1_i2_orf1;TRINITY_DN2749_c4_g1_i2_orf1;TRINITY_DN92153_c0_g2_i2_orf1;TRINITY_DN2749_c0_g1_i4_orf1;TRINITY_DN22951_c0_g1_i1_orf1;TRINITY_DN1750_c1_g1_i5_orf1;TRINITY_DN127056_c0_g1_i1_orf1;TRINITY_DN39813_c0_g1_i1_orf1;TRINITY_DN2718_c0_g1_i6_orf1;TRINITY_DN57918_c0_g1_i1_orf1;TRINITY_DN6563_c0_g1_i1_orf1;TRINITY_DN56270_c0_g1_i1_orf1;TRINITY_DN140669_c0_g1_i1_orf1;TRINITY_DN33346_c0_g1_i1_orf1;TRINITY_DN1554_c0_g1_i9_orf1;TRINITY_DN125_c0_g1_i2_orf1;TRINITY_DN49038_c0_g4_i1_orf1;TRINITY_DN38506_c0_g1_i4_orf1;TRINITY_DN2953_c1_g1_i10_orf1;TRINITY_DN2559_c0_g1_i4_orf1;TRINITY_DN2803_c4_g1_i1_orf1;TRINITY_DN98242_c0_g1_i1_orf1;TRINITY_DN14477_c0_g1_i12_orf1;TRINITY_DN452_c1_g1_i3_orf1;TRINITY_DN34689_c0_g1_i4_orf1;TRINITY_DN4135_c0_g1_i5_orf1;TRINITY_DN26293_c0_g1_i4_orf1;TRINITY_DN3062_c0_g1_i1_orf1;TRINITY_DN7341_c0_g1_i8_orf1;TRINITY_DN19866_c0_g1_i4_orf1;TRINITY_DN24322_c0_g1_i4_orf1;TRINITY_DN84322_c0_g2_i1_orf1;TRINITY_DN5686_c0_g1_i4_orf1;TRINITY_DN53311_c0_g2_i1_orf1;TRINITY_DN3082_c1_g1_i7_orf1;TRINITY_DN21367_c0_g1_i1_orf1;TRINITY_DN60787_c0_g1_i5_orf1;TRINITY_DN38274_c0_g1_i1_orf1;TRINITY_DN111_c0_g2_i2_orf1;TRINITY_DN14487_c0_g1_i4_orf1;TRINITY_DN23042_c0_g1_i1_orf1;TRINITY_DN28299_c0_g1_i1_orf1;TRINITY_DN2647_c0_g1_i3_orf1;TRINITY_DN120144_c0_g1_i1_orf1;TRINITY_DN6248_c0_g1_i1_orf1;TRINITY_DN10548_c0_g2_i1_orf1;TRINITY_DN6813_c1_g1_i1_orf1;TRINITY_DN46022_c0_g1_i1_orf1;TRINITY_DN1084_c0_g2_i2_orf1;TRINITY_DN1216_c0_g1_i4_orf1;TRINITY_DN2971_c0_g1_i1_orf1;TRINITY_DN67649_c0_g1_i1_orf1;TRINITY_DN24970_c0_g1_i4_orf1;TRINITY_DN4835_c0_g1_i2_orf1;TRINITY_DN38562_c0_g1_i3_orf1;TRINITY_DN3482_c0_g2_i1_orf1;TRINITY_DN13760_c1_g1_i1_orf1;TRINITY_DN15706_c0_g2_i5_orf1;TRINITY_DN4822_c0_g1_i9_orf1;TRINITY_DN104507_c0_g1_i2_orf1;TRINITY_DN87170_c0_g1_i3_orf1;TRINITY_DN5200_c0_g1_i2_orf1;TRINITY_DN34676_c1_g1_i3_orf1;TRINITY_DN2201_c0_g1_i1_orf1;TRINITY_DN2367_c1_g1_i20_orf1;TRINITY_DN1091_c0_g1_i1_orf1;TRINITY_DN18782_c0_g1_i4_orf1;TRINITY_DN15222_c0_g1_i4_orf1;TRINITY_DN23343_c0_g1_i9_orf1;TRINITY_DN5238_c0_g1_i2_orf1;TRINITY_DN19251_c0_g1_i8_orf1;TRINITY_DN13055_c0_g1_i5_orf1;TRINITY_DN34432_c0_g1_i1_orf1;TRINITY_DN110534_c0_g1_i3_orf1;TRINITY_DN5757_c0_g1_i1_orf1;TRINITY_DN2338_c0_g1_i5_orf1;TRINITY_DN6325_c0_g1_i8_orf1;TRINITY_DN58125_c0_g1_i1_orf1;TRINITY_DN21539_c0_g1_i1_orf1;TRINITY_DN346_c0_g1_i7_orf1;TRINITY_DN1084_c0_g1_i2_orf1;TRINITY_DN30950_c0_g1_i13_orf1
biological_process	thioester metabolic process	GO:0035383	7	7/2360	TRINITY_DN117844_c0_g1_i1_orf1;TRINITY_DN3991_c0_g1_i6_orf1;TRINITY_DN15136_c0_g1_i2_orf1;TRINITY_DN1084_c0_g1_i2_orf1;TRINITY_DN19251_c0_g1_i8_orf1;TRINITY_DN7808_c0_g1_i1_orf1;TRINITY_DN1084_c0_g2_i2_orf1
biological_process	macromolecule metabolic process	GO:0043170	298	298/2360	[illegible] TRINITY_DN24469_c0_g2_i2_orf1;TRINITY_DN7341_c0_g1_i8_orf1;TRINITY_DN10364_c0_g1_i5_orf1;TRINITY_DN32700_c0_g1_i2_orf1;TRINITY_DN5670_c0_g1_i2_orf1;TRINITY_DN39404_c0_g1_i7_orf1;TRINITY_DN3194_c0_g1_i6_orf1;TRINITY_DN27771_c0_g1_i1_orf1;TRINITY_DN181_c0_g1_i3_orf1;TRINITY_DN71610_c0_g1_i1_orf1;TRINITY_DN1533_c0_g2_i1_orf1;TRINITY_DN48983_c0_g1_i2_orf1;TRINITY_DN28661_c0_g1_i1_orf1;TRINITY_DN7122_c0_g1_i1_orf1;TRINITY_DN35669_c0_g1_i1_orf1;TRINITY_DN344_c1_g1_i1_orf1;TRINITY_DN2069_c1_g1_i8_orf1;TRINITY_DN124950_c0_g2_i1_orf1;TRINITY_DN4217_c0_g1_i2_orf1;TRINITY_DN38431_c0_g1_i1_orf1;TRINITY_DN34134_c0_g2_i1_orf1;TRINITY_DN3499_c0_g1_i8_orf1;TRINITY_DN30300_c0_g2_i1_orf1;TRINITY_DN2442_c0_g1_i2_orf1;TRINITY_DN8659_c0_g1_i1_orf1;TRINITY_DN1768_c0_g1_i2_orf1;TRINITY_DN70485_c0_g1_i2_orf1;TRINITY_DN142442_c0_g1_i1_orf1;TRINITY_DN44877_c0_g1_i2_orf1;TRINITY_DN16258_c0_g1_i2_orf1;TRINITY_DN2953_c1_g1_i10_orf1;TRINITY_DN14754_c0_g1_i6_orf1;TRINITY_DN29448_c0_g1_i1_orf1;TRINITY_DN41761_c0_g1_i4_orf1;TRINITY_DN15160_c0_g1_i1_orf1;TRINITY_DN14774_c0_g1_i4_orf1;TRINITY_DN50787_c0_g2_i2_orf1;TRINITY_DN2201_c0_g1_i1_orf1;TRINITY_DN875_c0_g1_i3_orf1;TRINITY_DN95056_c0_g2_i2_orf1;TRINITY_DN74889_c0_g1_i1_orf1;TRINITY_DN2794_c1_g1_i8_orf1;TRINITY_DN17759_c0_g1_i5_orf1;TRINITY_DN4125_c0_g1_i14_orf1;TRINITY_DN4817_c0_g1_i4_orf1;TRINITY_DN13350_c0_g1_i4_orf1;TRINITY_DN15256_c0_g1_i8_orf1;TRINITY_DN542_c0_g1_i4_orf1;TRINITY_DN2401_c0_g2_i1_orf1;TRINITY_DN123184_c0_g1_i1_orf1;TRINITY_DN6185_c0_g1_i12_orf1;TRINITY_DN2769_c0_g1_i1_orf1;TRINITY_DN135_c0_g1_i1_orf1;TRINITY_DN6436_c0_g1_i1_orf1;TRINITY_DN140538_c0_g2_i1_orf1;TRINITY_DN391_c0_g1_i4_orf1;TRINITY_DN7134_c0_g1_i1_orf1;TRINITY_DN2224_c0_g1_i1_orf1;TRINITY_DN10769_c0_g1_i1_orf1;TRINITY_DN20499_c0_g3_i1_orf1;TRINITY_DN4813_c0_g1_i5_orf1;TRINITY_DN5200_c0_g1_i2_orf1;TRINITY_DN15591_c0_g1_i3_orf1;TRINITY_DN1380_c0_g1_i5_orf1;TRINITY_DN107288_c0_g1_i2_orf1;TRINITY_DN46715_c0_g1_i1_orf1;TRINITY_DN56993_c0_g1_i4_orf1;TRINITY_DN14313_c0_g1_i1_orf1;TRINITY_DN1749_c0_g2_i2_orf1;TRINITY_DN3092_c0_g1_i2_orf1;TRINITY_DN1978_c0_g1_i4_orf1;TRINITY_DN17271_c0_g1_i1_orf1;TRINITY_DN28989_c0_g1_i7_orf1;TRINITY_DN1706_c0_g1_i7_orf1;TRINITY_DN23360_c0_g1_i3_orf1;TRINITY_DN10824_c0_g1_i3_orf1;TRINITY_DN45633_c0_g1_i1_orf1;TRINITY_DN12757_c0_g1_i1_orf1;TRINITY_DN1005_c0_g1_i5_orf1;TRINITY_DN29034_c0_g1_i2_orf1;TRINITY_DN11125_c0_g1_i1_orf1;TRINITY_DN38562_c0_g1_i3_orf1;TRINITY_DN19537_c0_g1_i1_orf1;TRINITY_DN4189_c0_g2_i1_orf1;TRINITY_DN37923_c0_g1_i1_orf1;TRINITY_DN1272_c1_g1_i4_orf1;TRINITY_DN53760_c0_g1_i1_orf1;TRINITY_DN15900_c0_g1_i6_orf1;TRINITY_DN81258_c0_g1_i2_orf1;TRINITY_DN57798_c0_g1_i1_orf1;TRINITY_DN9871_c0_g1_i11_orf1;TRINITY_DN21567_c0_g1_i7_orf1;TRINITY_DN57918_c0_g1_i1_orf1;TRINITY_DN30097_c0_g1_i2_orf1;TRINITY_DN1274_c0_g1_i4_orf1;TRINITY_DN1005_c0_g2_i1_orf1;TRINITY_DN30154_c0_g1_i1_orf1;TRINITY_DN6813_c1_g1_i1_orf1;TRINITY_DN21719_c0_g1_i2_orf1;TRINITY_DN7583_c0_g1_i1_orf1;TRINITY_DN1607_c0_g1_i16_orf1;TRINITY_DN277_c1_g1_i1_orf1;TRINITY_DN2054_c0_g1_i1_orf1;TRINITY_DN2043_c0_g1_i11_orf1;TRINITY_DN45948_c1_g1_i1_orf1;TRINITY_DN36434_c0_g2_i3_orf1;TRINITY_DN16978_c0_g1_i1_orf1;TRINITY_DN41664_c0_g1_i4_orf1;TRINITY_DN1091_c0_g3_i1_orf1;TRINITY_DN19034_c0_g1_i1_orf1;TRINITY_DN23167_c0_g2_i1_orf1;TRINITY_DN6423_c0_g1_i5_orf1;TRINITY_DN71863_c0_g1_i2_orf1;TRINITY_DN9591_c0_g1_i1_orf1;TRINITY_DN1616_c0_g1_i3_orf1;TRINITY_DN4125_c1_g1_i5_orf1;TRINITY_DN4571_c0_g1_i4_orf1;TRINITY_DN96_c0_g1_i1_orf1;TRINITY_DN3733_c0_g1_i1_orf1;TRINITY_DN6642_c0_g1_i2_orf1;TRINITY_DN5444_c0_g2_i1_orf1;TRINITY_DN753_c0_g1_i4_orf1;TRINITY_DN4408_c6_g1_i1_orf1;TRINITY_DN110534_c0_g1_i3_orf1;TRINITY_DN51568_c0_g1_i1_orf1;TRINITY_DN6205_c0_g1_i8_orf1;TRINITY_DN4707_c0_g1_i1_orf1;TRINITY_DN6423_c0_g1_i6_orf1;TRINITY_DN40945_c0_g1_i1_orf1;TRINITY_DN140_c1_g1_i2_orf1;TRINITY_DN1266_c2_g1_i1_orf1;TRINITY_DN15040_c0_g4_i1_orf1;TRINITY_DN3773_c0_g1_i4_orf1;TRINITY_DN9062_c0_g2_i3_orf1;TRINITY_DN1173_c1_g1_i10_orf1;TRINITY_DN1528_c0_g1_i4_orf1;TRINITY_DN139537_c0_g1_i1_orf1;TRINITY_DN7776_c0_g1_i5_orf1;TRINITY_DN1344_c0_g1_i1_orf1;TRINITY_DN10403_c0_g1_i1_orf1;TRINITY_DN120593_c0_g1_i1_orf1;TRINITY_DN107035_c0_g1_i1_orf1;TRINITY_DN147458_c0_g1_i1_orf1;TRINITY_DN4908_c1_g1_i5_orf1;TRINITY_DN12397_c0_g1_i1_orf1;TRINITY_DN8692_c0_g1_i2_orf1;TRINITY_DN5235_c0_g1_i7_orf1;TRINITY_DN5064_c0_g1_i4_orf1;TRINITY_DN291_c0_g1_i2_orf1;TRINITY_DN2749_c4_g1_i2_orf1;TRINITY_DN812_c2_g1_i1_orf1;TRINITY_DN5112_c0_g1_i1_orf1;TRINITY_DN25534_c0_g1_i1_orf1;TRINITY_DN10766_c0_g1_i1_orf1;TRINITY_DN22951_c0_g1_i1_orf1;TRINITY_DN1750_c1_g1_i5_orf1;TRINITY_DN4798_c0_g1_i3_orf1;TRINITY_DN6365_c0_g1_i4_orf1;TRINITY_DN46202_c0_g1_i1_orf1;TRINITY_DN1552_c0_g1_i3_orf1;TRINITY_DN5182_c0_g1_i5_orf1;TRINITY_DN2718_c0_g1_i6_orf1;TRINITY_DN81803_c0_g2_i1_orf1;TRINITY_DN70409_c0_g1_i3_orf1;TRINITY_DN11817_c0_g1_i4_orf1;TRINITY_DN57111_c0_g1_i1_orf1;TRINITY_DN56270_c0_g1_i1_orf1;TRINITY_DN5310_c2_g1_i2_orf1;TRINITY_DN2040_c0_g1_i6_orf1;TRINITY_DN33346_c0_g1_i1_orf1;TRINITY_DN1554_c0_g1_i9_orf1;TRINITY_DN5757_c0_g1_i1_orf1;TRINITY_DN62729_c0_g1_i13_orf1;TRINITY_DN1404_c0_g1_i6_orf1;TRINITY_DN4016_c0_g1_i1_orf1;TRINITY_DN934_c2_g1_i7_orf1;TRINITY_DN7316_c0_g2_i1_orf1;TRINITY_DN34479_c0_g1_i2_orf1;TRINITY_DN2885_c1_g1_i2_orf1;TRINITY_DN1405_c0_g1_i1_orf1;TRINITY_DN21719_c0_g2_i4_orf1;TRINITY_DN130778_c0_g1_i1_orf1;TRINITY_DN3342_c0_g3_i1_orf1;TRINITY_DN452_c1_g1_i3_orf1;TRINITY_DN34689_c0_g1_i4_orf1;TRINITY_DN4135_c0_g1_i5_orf1;TRINITY_DN3062_c0_g1_i1_orf1;TRINITY_DN45271_c0_g1_i1_orf1;TRINITY_DN18172_c0_g1 [illegible]
biological_process	S-adenosylmethionine metabolic process	GO:0046500	2	2/2360	TRINITY_DN7512_c0_g1_i1_orf1;TRINITY_DN1578_c0_g3_i1_orf1

biological_process	melanin metabolic process	GO:0006582	4	4/2360	TRINITY_DN31163_c1_g1_i4_orf1;TRINITY_DN58125_c0_g1_i1_orf1;TRINITY_DN15706_c0_g2_i5_orf1;TRINITY_DN2338_c0_g1_i5_orf1
biological_process	pigment biosynthetic process	GO:0046148	4	4/2360	TRINITY_DN31163_c1_g1_i4_orf1;TRINITY_DN2338_c0_g1_i5_orf1;TRINITY_DN58125_c0_g1_i1_orf1;TRINITY_DN6563_c0_g1_i1_orf1
biological_process	heme metabolic process	GO:0042168	1	1/2360	TRINITY_DN6563_c0_g1_i1_orf1
biological_process	mating behavior	GO:0007617	1	1/2360	TRINITY_DN58125_c0_g1_i1_orf1
biological_process	gamete generation	GO:0007276	2	2/2360	TRINITY_DN2652_c0_g2_i1_orf1;TRINITY_DN4813_c0_g1_i5_orf1
biological_process	germ cell development	GO:0007281	1	1/2360	TRINITY_DN2652_c0_g2_i1_orf1
biological_process	ovarian follicle cell development	GO:0030707	1	1/2360	TRINITY_DN15706_c0_g2_i5_orf1
biological_process	spermatogenesis	GO:0007283	1	1/2360	TRINITY_DN4813_c0_g1_i5_orf1
biological_process	killing of cells of another organism	GO:0031640	1	1/2360	TRINITY_DN6098_c1_g1_i5_orf1
biological_process	programmed cell death	GO:0012501	4	4/2360	TRINITY_DN18912_c1_g1_i1_orf1;TRINITY_DN87603_c0_g2_i1_orf1;TRINITY_DN50074_c0_g1_i1_orf1;TRINITY_DN2655_c0_g2_i1_orf1
biological_process	autophagy	GO:0006914	1	1/2360	TRINITY_DN5531_c0_g3_i3_orf1
biological_process	secretion by cell	GO:0032940	4	4/2360	TRINITY_DN25686_c0_g1_i4_orf1;TRINITY_DN47219_c0_g1_i3_orf1;TRINITY_DN1895_c0_g1_i2_orf1;TRINITY_DN33452_c0_g1_i1_orf1
biological_process	cell-substrate adhesion	GO:0031589	2	2/2360	TRINITY_DN2186_c0_g1_i17_orf1;TRINITY_DN2919_c0_g1_i5_orf1
biological_process	cell-cell adhesion	GO:0098609	2	2/2360	TRINITY_DN14389_c0_g1_i4_orf1;TRINITY_DN10070_c0_g1_i1_orf1
biological_process	cellular response to extracellular stimulus	GO:0031668	3	3/2360	TRINITY_DN1091_c0_g1_i1_orf1;TRINITY_DN1091_c0_g3_i1_orf1;TRINITY_DN2054_c0_g1_i1_orf1
biological_process	intermediate filament cytoskeleton organization	GO:0045104	4	4/2360	TRINITY_DN17137_c0_g1_i2_orf1;TRINITY_DN97097_c0_g1_i4_orf1;TRINITY_DN69557_c0_g1_i1_orf1;TRINITY_DN101991_c0_g1_i5_orf1
biological_process	maintenance of protein location in cell	GO:0032507	1	1/2360	TRINITY_DN245_c0_g1_i4_orf1
biological_process	cellular chemical homeostasis	GO:0055082	8	8/2360	TRINITY_DN46625_c0_g1_i1_orf1;TRINITY_DN65681_c0_g1_i1_orf1;TRINITY_DN1423_c0_g1_i4_orf1;TRINITY_DN3461_c0_g1_i1_orf1;TRINITY_DN1423_c0_g1_i8_orf1;TRINITY_DN2433_c0_g1_i3_orf1;TRINITY_DN96739_c0_g1_i1_orf1;TRINITY_DN44256_c0_g1_i1_orf1
biological_process	transposition, DNA-mediated	GO:0006313	1	1/2360	TRINITY_DN139537_c0_g1_i1_orf1
biological_process	mitotic cell cycle process	GO:1903047	3	3/2360	TRINITY_DN96557_c0_g1_i1_orf1;TRINITY_DN235_c0_g3_i1_orf1;TRINITY_DN31119_c0_g1_i1_orf1
biological_process	cell cycle phase transition	GO:0044770	1	1/2360	TRINITY_DN96557_c0_g1_i1_orf1
biological_process	spindle organization	GO:0007051	1	1/2360	TRINITY_DN31119_c0_g1_i1_orf1
biological_process	cytokinesis	GO:0000910	1	1/2360	TRINITY_DN235_c0_g3_i1_orf1
biological_process	sister chromatid cohesion	GO:0007062	1	1/2360	TRINITY_DN2638_c0_g1_i7_orf1
biological_process	cytokinetic process	GO:0032506	1	1/2360	TRINITY_DN96557_c0_g1_i1_orf1
biological_process	cellular macromolecule localization	GO:0070727	44	44/2360	TRINITY_DN245_c0_g1_i4_orf1;TRINITY_DN4790_c0_g1_i6_orf1;TRINITY_DN3209_c0_g1_i1_orf1;TRINITY_DN5182_c0_g1_i5_orf1;TRINITY_DN25681_c0_g1_i5_orf1;TRINITY_DN3450_c0_g1_i3_orf1;TRINITY_DN6243_c0_g1_i5_orf1;TRINITY_DN146119_c0_g1_i1_orf1;TRINITY_DN14677_c0_g2_i3_orf1;TRINITY_DN13139_c0_g1_i1_orf1;TRINITY_DN5982_c0_g1_i3_orf1;TRINITY_DN12767_c0_g1_i2_orf1;TRINITY_DN1447_c0_g1_i5_orf1;TRINITY_DN72859_c0_g1_i1_orf1;TRINITY_DN5118_c0_g1_i1_orf1;TRINITY_DN2879_c0_g1_i4_orf1;TRINITY_DN445_c0_g1_i2_orf1;TRINITY_DN13118_c0_g1_i6_orf1;TRINITY_DN1437_c0_g1_i6_orf1;TRINITY_DN41108_c0_g1_i1_orf1;TRINITY_DN6680_c0_g1_i1_orf1;TRINITY_DN48460_c0_g1_i1_orf1;TRINITY_DN42120_c0_g1_i2_orf1;TRINITY_DN11772_c0_g1_i1_orf1;TRINITY_DN44219_c0_g1_i1_orf1;TRINITY_DN4689_c0_g1_i5_orf1;TRINITY_DN8405_c0_g1_i4_orf1;TRINITY_DN486_c0_g1_i5_orf1;TRINITY_DN27721_c1_g1_i2_orf1;TRINITY_DN65299_c0_g4_i1_orf1;TRINITY_DN9592_c0_g1_i2_orf1;TRINITY_DN96557_c0_g1_i1_orf1;TRINITY_DN9741_c0_g1_i3_orf1;TRINITY_DN2172_c0_g2_i8_orf1;TRINITY_DN47219_c0_g1_i3_orf1;TRINITY_DN12777_c0_g1_i5_orf1;TRINITY_DN4859_c0_g1_i5_orf1;TRINITY_DN25210_c0_g1_i1_orf1;TRINITY_DN50875_c0_g1_i3_orf1;TRINITY_DN19702_c0_g1_i4_orf1;TRINITY_DN12320_c0_g1_i1_orf1;TRINITY_DN9931_c0_g1_i1_orf1;TRINITY_DN59042_c1_g1_i1_orf1;TRINITY_DN11693_c0_g1_i6_orf1
biological_process	intracellular transport	GO:0046907	38	38/2360	TRINITY_DN3835_c0_g1_i3_orf1;TRINITY_DN54586_c1_g1_i1_orf1;TRINITY_DN3209_c0_g1_i1_orf1;TRINITY_DN4770_c0_g1_i4_orf1;TRINITY_DN3821_c1_g1_i7_orf1;TRINITY_DN6231_c0_g1_i6_orf1;TRINITY_DN13626_c0_g2_i1_orf1;TRINITY_DN3450_c0_g1_i3_orf1;TRINITY_DN578_c0_g1_i3_orf1;TRINITY_DN5182_c0_g1_i5_orf1;TRINITY_DN14677_c0_g2_i3_orf1;TRINITY_DN855_c0_g1_i5_orf1;TRINITY_DN13139_c0_g1_i1_orf1;TRINITY_DN5982_c0_g1_i3_orf1;TRINITY_DN12767_c0_g1_i2_orf1;TRINITY_DN1437_c0_g1_i6_orf1;TRINITY_DN72859_c0_g1_i1_orf1;TRINITY_DN5118_c0_g1_i1_orf1;TRINITY_DN45037_c0_g1_i1_orf1;TRINITY_DN23416_c1_g1_i2_orf1;TRINITY_DN445_c0_g1_i2_orf1;TRINITY_DN13118_c0_g1_i6_orf1;TRINITY_DN1447_c0_g1_i5_orf1;TRINITY_DN25681_c0_g1_i5_orf1;TRINITY_DN48460_c0_g1_i1_orf1;TRINITY_DN21367_c0_g1_i1_orf1;TRINITY_DN8405_c0_g1_i4_orf1;TRINITY_DN486_c0_g1_i5_orf1;TRINITY_DN27721_c1_g1_i2_orf1;TRINITY_DN96557_c0_g1_i1_orf1;TRINITY_DN12777_c0_g1_i5_orf1;TRINITY_DN4859_c0_g1_i5_orf1;TRINITY_DN25210_c0_g1_i1_orf1;TRINITY_DN19702_c0_g1_i4_orf1;TRINITY_DN1245_c0_g1_i4_orf1;TRINITY_DN942_c0_g1_i1_orf1;TRINITY_DN59042_c1_g1_i1_orf1;TRINITY_DN5028_c0_g1_i11_orf1
biological_process	localization within membrane	GO:0051668	2	2/2360	TRINITY_DN96557_c0_g1_i1_orf1;TRINITY_DN48460_c0_g1_i1_orf1
biological_process	cellular component biogenesis	GO:0044085	6	6/2360	TRINITY_DN4016_c0_g1_i1_orf1;TRINITY_DN146217_c0_g1_i1_orf1;TRINITY_DN31225_c0_g1_i1_orf1;TRINITY_DN21367_c0_g1_i1_orf1;TRINITY_DN14313_c0_g1_i1_orf1;TRINITY_DN6239_c0_g1_i1_orf1
biological_process	cellular component organization	GO:0016043	90	90/2360	TRINITY_DN14920_c0_g1_i1_orf1;TRINITY_DN39404_c0_g1_i7_orf1;TRINITY_DN841_c0_g1_i4_orf1;TRINITY_DN11194_c0_g1_i4_orf1;TRINITY_DN3450_c0_g1_i3_orf1;TRINITY_DN19980_c0_g1_i4_orf1;TRINITY_DN698_c0_g1_i5_orf1;TRINITY_DN3126_c0_g1_i4_orf1;TRINITY_DN35669_c0_g1_i1_orf1;TRINITY_DN4237_c1_g1_i5_orf1;TRINITY_DN4010_c0_g2_i1_orf1;TRINITY_DN4217_c0_g1_i2_orf1;TRINITY_DN11069_c0_g2_i1_orf1;TRINITY_DN2904_c0_g1_i4_orf1;TRINITY_DN70485_c0_g1_i2_orf1;TRINITY_DN142442_c0_g1_i1_orf1;TRINITY_DN140538_c0_g2_i1_orf1;TRINITY_DN2638_c0_g1_i7_orf1;TRINITY_DN1639_c0_g2_i2_orf1;TRINITY_DN69557_c0_g1_i1_orf1;TRINITY_DN235_c0_g3_i1_orf1;TRINITY_DN36987_c0_g1_i1_orf1;TRINITY_DN4798_c0_g1_i3_orf1;TRINITY_DN2345_c0_g1_i4_orf1;TRINITY_DN110231_c0_g1_i1_orf1;TRINITY_DN3461_c0_g1_i1_orf1;TRINITY_DN14389_c0_g1_i4_orf1;TRINITY_DN18009_c0_g1_i1_orf1;TRINITY_DN23502_c0_g1_i1_orf1;TRINITY_DN4950_c0_g1_i2_orf1;TRINITY_DN1749_c0_g2_i2_orf1;TRINITY_DN11464_c0_g1_i3_orf1;TRINITY_DN20442_c0_g2_i1_orf1;TRINITY_DN34703_c0_g1_i4_orf1;TRINITY_DN146119_c0_g1_i1_orf1;TRINITY_DN23790_c0_g1_i1_orf1;TRINITY_DN71832_c0_g1_i1_orf1;TRINITY_DN298_c0_g1_i4_orf1;TRINITY_DN90497_c0_g1_i1_orf1;TRINITY_DN28018_c0_g6_i1_orf1;TRINITY_DN4108_c0_g1_i6_orf1;TRINITY_DN28622_c0_g1_i1_orf1;TRINITY_DN50085_c0_g1_i1_orf1;TRINITY_DN6642_c0_g1_i2_orf1;TRINITY_DN11772_c0_g1_i1_orf1;TRINITY_DN31119_c0_g1_i1_orf1;TRINITY_DN4689_c0_g1_i5_orf1;TRINITY_DN4439_c0_g1_i2_orf1;TRINITY_DN23020_c0_g1_i1_orf1;TRINITY_DN3702_c0_g1_i1_orf1;TRINITY_DN4908_c1_g1_i5_orf1;TRINITY_DN101991_c0_g1_i5_orf1;TRINITY_DN12771_c0_g1_i1_orf1;TRINITY_DN6239_c0_g1_i1_orf1;TRINITY_DN714_c0_g1_i3_orf1;TRINITY_DN8915_c0_g1_i3_orf1;TRINITY_DN3366_c0_g1_i6_orf1;TRINITY_DN70409_c0_g1_i3_orf1;TRINITY_DN4016_c0_g1_i1_orf1;TRINITY_DN14987_c0_g1_i3_orf1;TRINITY_DN8390_c0_g1_i2_orf1;TRINITY_DN43412_c0_g1_i2_orf1;TRINITY_DN452_c1_g1_i3_orf1;TRINITY_DN86309_c0_g1_i4_orf1;TRINITY_DN27960_c0_g1_i1_orf1;TRINITY_DN21367_c0_g1_i1_orf1;TRINITY_DN25976_c0_g1_i4_orf1;TRINITY_DN96557_c0_g1_i1_orf1;TRINITY_DN6248_c0_g1_i1_orf1;TRINITY_DN10455_c0_g1_i2_orf1;TRINITY_DN10385_c0_g1_i5_orf1;TRINITY_DN4159_c1_g1_i1_orf1;TRINITY_DN48097_c0_g1_i1_orf1;TRINITY_DN38540_c0_g1_i1_orf1;TRINITY_DN1298_c0_g1_i3_orf1;TRINITY_DN3887_c0_g1_i1_orf1;TRINITY_DN49872_c0_g1_i2_orf1;TRINITY_DN152_c0_g1_i4_orf1;TRINITY_DN35635_c0_g1_i1_orf1;TRINITY_DN24266_c0_g2_i2_orf1;TRINITY_DN30273_c1_g1_i1_orf1;TRINITY_DN5757_c0_g1_i1_orf1;TRINITY_DN10070_c0_g1_i1_orf1;TRINITY_DN19584_c0_g1_i2_orf1;TRINITY_DN96739_c0_g1_i1_orf1;TRINITY_DN147475_c0_g1_i1_orf1;TRINITY_DN80424_c0_g1_i1_orf1;TRINITY_DN17137_c0_g1_i2_orf1;TRINITY_DN97097_c0_g1_i4_orf1;TRINITY_DN9765_c0_g1_i6_orf1
biological_process	cell migration	GO:0016477	3	3/2360	TRINITY_DN110231_c0_g1_i1_orf1;TRINITY_DN96739_c0_g1_i1_orf1;TRINITY_DN15706_c0_g2_i5_orf1
biological_process	microtubule-based movement	GO:0007018	4	4/2360	TRINITY_DN14298_c0_g3_i1_orf1;TRINITY_DN122423_c0_g5_i1_orf1;TRINITY_DN14298_c0_g1_i1_orf1;TRINITY_DN122423_c0_g1_i1_orf1
biological_process	microtubule cytoskeleton organization	GO:0000226	5	5/2360	TRINITY_DN4689_c0_g1_i5_orf1;TRINITY_DN8390_c0_g1_i2_orf1;TRINITY_DN28018_c0_g6_i1_orf1;TRINITY_DN31119_c0_g1_i1_orf1;TRINITY_DN34703_c0_g1_i4_orf1
biological_process	cellular response to chemical stimulus	GO:0070887	2	2/2360	TRINITY_DN87603_c0_g2_i1_orf1;TRINITY_DN4016_c0_g1_i1_orf1
biological_process	cellular response to stress	GO:0033554	25	25/2360	TRINITY_DN3092_c0_g1_i2_orf1;TRINITY_DN2971_c0_g1_i1_orf1;TRINITY_DN7341_c0_g1_i8_orf1;TRINITY_DN17271_c0_g1_i1_orf1;TRINITY_DN104507_c0_g1_i2_orf1;TRINITY_DN2054_c0_g1_i1_orf1;TRINITY_DN452_c1_g1_i3_orf1;TRINITY_DN1091_c0_g1_i1_orf1;TRINITY_DN1091_c0_g3_i1_orf1;TRINITY_DN45271_c0_g1_i1_orf1;TRINITY_DN19866_c0_g1_i4_orf1;TRINITY_DN5238_c0_g1_i2_orf1;TRINITY_DN5686_c0_g1_i4_orf1;TRINITY_DN6642_c0_g1_i2_orf1;TRINITY_DN123184_c0_g1_i1_orf1;TRINITY_DN6185_c0_g1_i12_orf1;TRINITY_DN38274_c0_g1_i1_orf1;TRINITY_DN5757_c0_g1_i1_orf1;TRINITY_DN14487_c0_g1_i4_orf1;TRINITY_DN9062_c0_g2_i3_orf1;TRINITY_DN87603_c0_g2_i1_orf1;TRINITY_DN2647_c0_g1_i3_orf1;TRINITY_DN346_c0_g1_i7_orf1;TRINITY_DN15591_c0_g1_i3_orf1;TRINITY_DN28989_c0_g1_i7_orf1
biological_process	cell surface receptor signaling pathway	GO:0007166	7	7/2360	TRINITY_DN38371_c0_g1_i7_orf1;TRINITY_DN147475_c0_g1_i1_orf1;TRINITY_DN2170_c0_g2_i1_orf1;TRINITY_DN33418_c0_g1_i1_orf1;TRINITY_DN2170_c1_g1_i3_orf1;TRINITY_DN13216_c0_g1_i5_orf1;TRINITY_DN15247_c0_g1_i2_orf1
biological_process	hormone-mediated signaling pathway	GO:0009755	1	1/2360	TRINITY_DN147475_c0_g1_i1_orf1
biological_process	immune response-regulating signaling pathway	GO:0002764	2	2/2360	TRINITY_DN2170_c0_g2_i1_orf1;TRINITY_DN2170_c1_g1_i3_orf1
biological_process	G protein-coupled receptor signaling pathway	GO:0007186	2	2/2360	TRINITY_DN11245_c0_g1_i2_orf1;TRINITY_DN33418_c0_g1_i1_orf1
biological_process	intracellular signal transduction	GO:0035556	8	8/2360	TRINITY_DN32700_c0_g1_i2_orf1;TRINITY_DN15478_c0_g1_i1_orf1;TRINITY_DN802_c0_g1_i2_orf1;TRINITY_DN2623_c0_g1_i3_orf1;TRINITY_DN15706_c0_g2_i5_orf1;TRINITY_DN5182_c0_g1_i5_orf1;TRINITY_DN804_c0_g1_i7_orf1;TRINITY_DN79000_c1_g1_i1_orf1
biological_process	apoptotic signaling pathway	GO:0097190	1	1/2360	TRINITY_DN96739_c0_g1_i1_orf1
biological_process	cellular detoxification of aldehyde	GO:0110095	1	1/2360	TRINITY_DN3758_c0_g1_i2_orf1
biological_process	meiotic cell cycle	GO:0051321	3	3/2360	TRINITY_DN45271_c0_g1_i1_orf1;TRINITY_DN123184_c0_g1_i1_orf1;TRINITY_DN4813_c0_g1_i5_orf1
biological_process	mitotic cell cycle	GO:0000278	1	1/2360	TRINITY_DN31314_c0_g1_i4_orf1

biological_process	cell differentiation	GO:0030154	18	18/2360	TRINITY_DN928_c0_g1_i3_orf1;TRINITY_DN4813_c0_g1_i5_orf1;TRINITY_DN2012_c0_g1_i3_orf1;TRINITY_DN52395_c0_g2_i2_orf1;TRINITY_DN5954_c0_g1_i2_orf1;TRINITY_DN42461_c0_g1_i4_orf1;TRINITY_DN1173_c0_g1_i11_orf1;TRINITY_DN14389_c0_g1_i4_orf1;TRINITY_DN1173_c1_g1_i10_orf1;TRINITY_DN96739_c0_g1_i1_orf1;TRINITY_DN549_c0_g1_i14_orf1;TRINITY_DN11388_c0_g1_i4_orf1;TRINITY_DN21367_c0_g1_i1_orf1;TRINITY_DN549_c0_g1_i7_orf1;TRINITY_DN928_c0_g2_i1_orf1;TRINITY_DN248_c0_g1_i1_orf1;TRINITY_DN140538_c0_g2_i1_orf1;TRINITY_DN741_c0_g1_i10_orf1
biological_process	cell development	GO:0048468	17	17/2360	TRINITY_DN1749_c0_g2_i2_orf1;TRINITY_DN4571_c0_g1_i4_orf1;TRINITY_DN1710_c0_g2_i2_orf1;TRINITY_DN15706_c0_g2_i5_orf1;TRINITY_DN4217_c0_g1_i2_orf1;TRINITY_DN288_c0_g1_i9_orf1;TRINITY_DN39404_c0_g1_i7_orf1;TRINITY_DN20710_c0_g1_i2_orf1;TRINITY_DN71832_c0_g1_i1_orf1;TRINITY_DN25976_c0_g1_i4_orf1;TRINITY_DN36856_c0_g1_i1_orf1;TRINITY_DN19980_c0_g1_i4_orf1;TRINITY_DN237_c1_g1_i1_orf1;TRINITY_DN36987_c0_g1_i1_orf1;TRINITY_DN70409_c0_g1_i3_orf1;TRINITY_DN152_c0_g1_i4_orf1;TRINITY_DN2652_c0_g2_i1_orf1
biological_process	cell maturation	GO:0048469	1	1/2360	TRINITY_DN1272_c1_g1_i4_orf1
biological_process	mitochondrial transmembrane transport	GO:1990542	1	1/2360	TRINITY_DN44256_c0_g1_i1_orf1
biological_process	ion transmembrane transport	GO:0034220	4	4/2360	TRINITY_DN1661_c0_g1_i1_orf1;TRINITY_DN96739_c0_g1_i1_orf1;TRINITY_DN8306_c0_g1_i4_orf1;TRINITY_DN44256_c0_g1_i1_orf1
biological_process	actin cytoskeleton organization	GO:0030036	8	8/2360	TRINITY_DN4010_c0_g2_i1_orf1;TRINITY_DN86309_c0_g1_i4_orf1;TRINITY_DN8915_c0_g1_i3_orf1;TRINITY_DN235_c0_g3_i1_orf1;TRINITY_DN3887_c0_g1_i1_orf1;TRINITY_DN4159_c1_g1_i1_orf1;TRINITY_DN3126_c0_g1_i4_orf1;TRINITY_DN23790_c0_g1_i1_orf1
biological_process	anatomical structure maturation	GO:0071695	1	1/2360	TRINITY_DN1272_c1_g1_i4_orf1
biological_process	cellular component assembly involved in morph	GO:0010927	1	1/2360	TRINITY_DN235_c0_g3_i1_orf1
biological_process	establishment of tissue polarity	GO:0007164	1	1/2360	TRINITY_DN14389_c0_g1_i4_orf1
biological_process	tube morphogenesis	GO:0035239	2	2/2360	TRINITY_DN1639_c0_g2_i2_orf1;TRINITY_DN147475_c0_g1_i1_orf1
biological_process	embryonic morphogenesis	GO:0048598	1	1/2360	TRINITY_DN142442_c0_g1_i1_orf1
biological_process	tissue morphogenesis	GO:0048729	3	3/2360	TRINITY_DN36856_c0_g1_i1_orf1;TRINITY_DN237_c1_g1_i1_orf1;TRINITY_DN147475_c0_g1_i1_orf1
biological_process	animal organ morphogenesis	GO:0009887	4	4/2360	TRINITY_DN1639_c0_g2_i2_orf1;TRINITY_DN14389_c0_g1_i4_orf1;TRINITY_DN741_c0_g1_i10_orf1;TRINITY_DN5954_c0_g1_i2_orf1
biological_process	system development	GO:0048731	4	4/2360	TRINITY_DN1710_c0_g2_i2_orf1;TRINITY_DN20710_c0_g1_i2_orf1;TRINITY_DN142442_c0_g1_i1_orf1;TRINITY_DN288_c0_g1_i9_orf1
biological_process	multicellular organism development	GO:0007275	1	1/2360	TRINITY_DN1639_c0_g2_i2_orf1
biological_process	animal organ development	GO:0048513	8	8/2360	TRINITY_DN1639_c0_g2_i2_orf1;TRINITY_DN19980_c0_g1_i4_orf1;TRINITY_DN8044_c0_g1_i2_orf1;TRINITY_DN36856_c0_g1_i1_orf1;TRINITY_DN25976_c0_g1_i4_orf1;TRINITY_DN237_c1_g1_i1_orf1;TRINITY_DN36987_c0_g1_i1_orf1;TRINITY_DN71832_c0_g1_i1_orf1
biological_process	muscle structure development	GO:0061061	1	1/2360	TRINITY_DN8044_c0_g1_i2_orf1
biological_process	tissue development	GO:0009888	1	1/2360	TRINITY_DN142442_c0_g1_i1_orf1
biological_process	embryo development	GO:0009790	1	1/2360	TRINITY_DN1639_c0_g2_i2_orf1
biological_process	nervous system process	GO:0050877	5	5/2360	TRINITY_DN12256_c0_g1_i1_orf1;TRINITY_DN19951_c0_g1_i5_orf1;TRINITY_DN75086_c0_g1_i5_orf1;TRINITY_DN26337_c0_g1_i3_orf1;TRINITY_DN142442_c0_g1_i1_orf1
biological_process	regionalization	GO:0003002	1	1/2360	TRINITY_DN1639_c0_g2_i2_orf1
biological_process	reproductive behavior	GO:0019098	1	1/2360	TRINITY_DN58125_c0_g1_i1_orf1
biological_process	envenomation resulting in modulation of proces	GO:0035738	1	1/2360	TRINITY_DN1215_c0_g1_i2_orf1
biological_process	response to bacterium	GO:0009617	10	10/2360	TRINITY_DN1444_c1_g1_i5_orf1;TRINITY_DN479_c6_g1_i2_orf1;TRINITY_DN8685_c0_g1_i5_orf1;TRINITY_DN14019_c0_g1_i5_orf1;TRINITY_DN16840_c1_g1_i1_orf1;TRINITY_DN195_c8_g1_i1_orf1;TRINITY_DN1091_c0_g2_i10_orf1;TRINITY_DN1666_c0_g1_i2_orf1;TRINITY_DN29190_c0_g1_i4_orf1;TRINITY_DN5880_c0_g2_i2_orf1
biological_process	response to fungus	GO:0009620	1	1/2360	TRINITY_DN6098_c1_g1_i5_orf1
biological_process	defense response to other organism	GO:0098542	16	16/2360	TRINITY_DN1444_c1_g1_i5_orf1;TRINITY_DN1534_c0_g1_i3_orf1;TRINITY_DN479_c6_g1_i2_orf1;TRINITY_DN8685_c0_g1_i5_orf1;TRINITY_DN14019_c0_g1_i5_orf1;TRINITY_DN16840_c1_g1_i1_orf1;TRINITY_DN195_c8_g1_i1_orf1;TRINITY_DN1091_c0_g2_i10_orf1;TRINITY_DN6098_c1_g1_i5_orf1;TRINITY_DN2170_c0_g2_i1_orf1;TRINITY_DN2170_c1_g1_i3_orf1;TRINITY_DN15706_c0_g2_i5_orf1;TRINITY_DN1666_c0_g1_i2_orf1;TRINITY_DN5235_c0_g1_i7_orf1;TRINITY_DN29190_c0_g1_i4_orf1;TRINITY_DN5880_c0_g2_i2_orf1
biological_process	biological process involved in interaction with ho	GO:0051701	1	1/2360	TRINITY_DN96557_c0_g1_i1_orf1
biological_process	ribosomal subunit export from nucleus	GO:0000054	1	1/2360	TRINITY_DN21367_c0_g1_i1_orf1
biological_process	establishment of organelle localization	GO:0051656	2	2/2360	TRINITY_DN96557_c0_g1_i1_orf1;TRINITY_DN21367_c0_g1_i1_orf1
biological_process	chromosome localization	GO:0050000	1	1/2360	TRINITY_DN96557_c0_g1_i1_orf1
biological_process	ribosome localization	GO:0033750	1	1/2360	TRINITY_DN21367_c0_g1_i1_orf1
biological_process	lipid storage	GO:0019915	1	1/2360	TRINITY_DN11069_c0_g2_i1_orf1
biological_process	maintenance of protein location	GO:0045185	1	1/2360	TRINITY_DN245_c0_g1_i4_orf1
biological_process	maintenance of location in cell	GO:0051651	1	1/2360	TRINITY_DN245_c0_g1_i4_orf1
biological_process	establishment of protein localization	GO:0045184	43	43/2360	TRINITY_DN245_c0_g1_i4_orf1;TRINITY_DN4790_c0_g1_i6_orf1;TRINITY_DN3209_c0_g1_i1_orf1;TRINITY_DN5182_c0_g1_i5_orf1;TRINITY_DN25681_c0_g1_i5_orf1;TRINITY_DN3450_c0_g1_i3_orf1;TRINITY_DN6243_c0_g1_i5_orf1;TRINITY_DN146119_c0_g1_i1_orf1;TRINITY_DN14677_c0_g2_i3_orf1;TRINITY_DN13139_c0_g1_i1_orf1;TRINITY_DN5982_c0_g1_i3_orf1;TRINITY_DN12767_c0_g1_i2_orf1;TRINITY_DN1447_c0_g1_i5_orf1;TRINITY_DN72859_c0_g1_i1_orf1;TRINITY_DN5118_c0_g1_i1_orf1;TRINITY_DN2879_c0_g1_i4_orf1;TRINITY_DN445_c0_g1_i2_orf1;TRINITY_DN13118_c0_g1_i6_orf1;TRINITY_DN1437_c0_g1_i6_orf1;TRINITY_DN41108_c0_g1_i1_orf1;TRINITY_DN6680_c0_g1_i1_orf1;TRINITY_DN48460_c0_g1_i1_orf1;TRINITY_DN42120_c0_g1_i2_orf1;TRINITY_DN11772_c0_g1_i1_orf1;TRINITY_DN44219_c0_g1_i1_orf1;TRINITY_DN8405_c0_g1_i4_orf1;TRINITY_DN486_c0_g1_i5_orf1;TRINITY_DN27721_c1_g1_i2_orf1;TRINITY_DN65299_c0_g4_i1_orf1;TRINITY_DN9592_c0_g1_i2_orf1;TRINITY_DN96557_c0_g1_i1_orf1;TRINITY_DN9741_c0_g1_i3_orf1;TRINITY_DN2172_c0_g2_i8_orf1;TRINITY_DN47219_c0_g1_i3_orf1;TRINITY_DN12777_c0_g1_i5_orf1;TRINITY_DN4859_c0_g1_i5_orf1;TRINITY_DN25210_c0_g1_i1_orf1;TRINITY_DN50875_c0_g1_i3_orf1;TRINITY_DN19702_c0_g1_i4_orf1;TRINITY_DN12320_c0_g1_i1_orf1;TRINITY_DN9931_c0_g1_i1_orf1;TRINITY_DN59042_c1_g1_i1_orf1;TRINITY_DN11693_c0_g1_i6_orf1
biological_process	establishment of localization in cell	GO:0051649	41	41/2360	TRINITY_DN3835_c0_g1_i3_orf1;TRINITY_DN4790_c0_g1_i6_orf1;TRINITY_DN54586_c1_g1_i1_orf1;TRINITY_DN3209_c0_g1_i1_orf1;TRINITY_DN4770_c0_g1_i4_orf1;TRINITY_DN3821_c1_g1_i7_orf1;TRINITY_DN6231_c0_g1_i6_orf1;TRINITY_DN13626_c0_g2_i1_orf1;TRINITY_DN3450_c0_g1_i3_orf1;TRINITY_DN578_c0_g1_i3_orf1;TRINITY_DN5182_c0_g1_i5_orf1;TRINITY_DN14677_c0_g2_i3_orf1;TRINITY_DN855_c0_g1_i5_orf1;TRINITY_DN13139_c0_g1_i1_orf1;TRINITY_DN5982_c0_g1_i3_orf1;TRINITY_DN12767_c0_g1_i2_orf1;TRINITY_DN1437_c0_g1_i6_orf1;TRINITY_DN72859_c0_g1_i1_orf1;TRINITY_DN5118_c0_g1_i1_orf1;TRINITY_DN12951_c1_g1_i5_orf1;TRINITY_DN45037_c0_g1_i1_orf1;TRINITY_DN29144_c0_g3_i1_orf1;TRINITY_DN23416_c1_g1_i2_orf1;TRINITY_DN445_c0_g1_i2_orf1;TRINITY_DN13118_c0_g1_i6_orf1;TRINITY_DN1447_c0_g1_i5_orf1;TRINITY_DN25681_c0_g1_i5_orf1;TRINITY_DN48460_c0_g1_i1_orf1;TRINITY_DN21367_c0_g1_i1_orf1;TRINITY_DN8405_c0_g1_i4_orf1;TRINITY_DN486_c0_g1_i5_orf1;TRINITY_DN27721_c1_g1_i2_orf1;TRINITY_DN96557_c0_g1_i1_orf1;TRINITY_DN12777_c0_g1_i5_orf1;TRINITY_DN4859_c0_g1_i5_orf1;TRINITY_DN25210_c0_g1_i1_orf1;TRINITY_DN19702_c0_g1_i4_orf1;TRINITY_DN1245_c0_g1_i4_orf1;TRINITY_DN942_c0_g1_i1_orf1;TRINITY_DN59042_c1_g1_i1_orf1;TRINITY_DN5028_c0_g1_i11_orf1
biological_process	establishment of RNA localization	GO:0051236	5	5/2360	TRINITY_DN1245_c0_g1_i4_orf1;TRINITY_DN146119_c0_g1_i1_orf1;TRINITY_DN59042_c1_g1_i1_orf1;TRINITY_DN2879_c0_g1_i4_orf1;TRINITY_DN6680_c0_g1_i1_orf1
biological_process	transport	GO:0006810	99	99/2360	TRINITY_DN25681_c0_g1_i5_orf1;TRINITY_DN21872_c0_g1_i2_orf1;TRINITY_DN3450_c0_g1_i3_orf1;TRINITY_DN578_c0_g1_i3_orf1;TRINITY_DN44256_c0_g1_i1_orf1;TRINITY_DN14677_c0_g2_i3_orf1;TRINITY_DN12767_c0_g1_i2_orf1;TRINITY_DN15812_c0_g1_i2_orf1;TRINITY_DN7590_c0_g1_i4_orf1;TRINITY_DN23416_c1_g1_i2_orf1;TRINITY_DN46625_c0_g1_i1_orf1;TRINITY_DN1437_c0_g1_i6_orf1;TRINITY_DN41108_c0_g1_i1_orf1;TRINITY_DN8766_c0_g1_i1_orf1;TRINITY_DN9239_c0_g1_i1_orf1;TRINITY_DN51766_c0_g1_i2_orf1;TRINITY_DN56430_c0_g1_i1_orf1;TRINITY_DN1245_c0_g1_i4_orf1;TRINITY_DN63561_c1_g1_i2_orf1;TRINITY_DN65681_c0_g1_i1_orf1;TRINITY_DN198_c2_g1_i2_orf1;TRINITY_DN6231_c0_g1_i6_orf1;TRINITY_DN13626_c0_g2_i1_orf1;TRINITY_DN252_c0_g1_i3_orf1;TRINITY_DN146119_c0_g1_i1_orf1;TRINITY_DN1895_c0_g1_i2_orf1;TRINITY_DN132857_c0_g1_i1_orf1;TRINITY_DN9354_c0_g1_i7_orf1;TRINITY_DN1423_c0_g1_i4_orf1;TRINITY_DN13139_c0_g1_i1_orf1;TRINITY_DN5982_c0_g1_i3_orf1;TRINITY_DN45037_c0_g1_i1_orf1;TRINITY_DN13118_c0_g1_i6_orf1;TRINITY_DN29934_c0_g1_i6_orf1;TRINITY_DN1423_c0_g1_i8_orf1;TRINITY_DN11772_c0_g1_i1_orf1;TRINITY_DN741_c0_g1_i10_orf1;TRINITY_DN8405_c0_g1_i4_orf1;TRINITY_DN1407_c0_g1_i5_orf1;TRINITY_DN2172_c0_g2_i8_orf1;TRINITY_DN12777_c0_g1_i5_orf1;TRINITY_DN19702_c0_g1_i4_orf1;TRINITY_DN12320_c0_g1_i1_orf1;TRINITY_DN5064_c0_g1_i4_orf1;TRINITY_DN11693_c0_g1_i6_orf1;TRINITY_DN3835_c0_g1_i3_orf1;TRINITY_DN81488_c0_g1_i1_orf1;TRINITY_DN3209_c0_g1_i1_orf1;TRINITY_DN3821_c1_g1_i7_orf1;TRINITY_DN6243_c0_g1_i5_orf1;TRINITY_DN5182_c0_g1_i5_orf1;TRINITY_DN4016_c0_g1_i1_orf1;TRINITY_DN5118_c0_g1_i1_orf1;TRINITY_DN3219_c0_g1_i6_orf1;TRINITY_DN25686_c0_g1_i4_orf1;TRINITY_DN5666_c0_g1_i2_orf1;TRINITY_DN86621_c0_g1_i2_orf1;TRINITY_DN48460_c0_g1_i1_orf1;TRINITY_DN29144_c0_g3_i1_orf1;TRINITY_DN96739_c0_g1_i1_orf1;TRINITY_DN21367_c0_g1_i1_orf1;TRINITY_DN486_c0_g1_i5_orf1;TRINITY_DN9931_c0_g1_i1_orf1;TRINITY_DN65299_c0_g4_i1_orf1;TRINITY_DN8306_c0_g1_i4_orf1;TRINITY_DN96557_c0_g1_i1_orf1;TRINITY_DN45446_c0_g1_i2_orf1;TRINITY_DN110402_c0_g2_i1_orf1;TRINITY_DN47219_c0_g1_i3_orf1;TRINITY_DN59042_c1_g1_i1_orf1;TRINITY_DN5028_c0_g1_i11_orf1;TRINITY_DN245_c0_g1_i4_orf1;TRINITY_DN28759_c0_g1_i1_orf1;TRINITY_DN4790_c0_g1_i6_orf1;TRINITY_DN54586_c1_g1_i1_orf1;TRINITY_DN4770_c0_g1_i4_orf1;TRINITY_DN33272_c0_g1_i5_orf1;TRINITY_DN855_c0_g1_i5_orf1;TRINITY_DN72859_c0_g1_i1_orf1;TRINITY_DN12951_c1_g1_i5_orf1;TRINITY_DN445_c0_g1_i2_orf1;TRINITY_DN26429_c0_g1_i4_orf1;TRINITY_DN1447_c0_g1_i5_orf1;TRINITY_DN1661_c0_g1_i1_orf1;TRINITY_DN6680_c0_g1_i1_orf1;TRINITY_DN42120_c0_g1_i2_orf1;TRINITY_DN12286_c1_g1_i2_orf1;TRINITY_DN44219_c0_g1_i1_orf1;TRINITY_DN1114_c0_g1_i4_orf1;TRINITY_DN27721_c1_g1_i2_orf1;TRINITY_DN18912_c1_g1_i1_orf1;TRINITY_DN2879_c0_g1_i4_orf1;TRINITY_DN9741_c0_g1_i3_orf1;TRINITY_DN9592_c0_g1_i2_orf1;TRINITY_DN33452_c0_g1_i1_orf1;TRINITY_DN4859_c0_g1_i5_orf1;TRINITY_DN25210_c0_g1_i1_orf1;TRINITY_DN50875_c0_g1_i3_orf1;TRINITY_DN942_c0_g1_i1_orf1

biological_process	non-lytic viral release	GO:0046753	1	1/2360	TRINITY_DN96557_c0_g1_i1_orf1
biological_process	viral RNA genome replication	GO:0039694	1	1/2360	TRINITY_DN4408_c6_g1_i1_orf1
biological_process	viral budding via host ESCRT complex	GO:0039702	1	1/2360	TRINITY_DN96557_c0_g1_i1_orf1
biological_process	viral budding from plasma membrane	GO:0046761	1	1/2360	TRINITY_DN96557_c0_g1_i1_orf1
biological_process	response to external biotic stimulus	GO:0043207	25	25/2360	TRINITY_DN195_c8_g1_i1_orf1;TRINITY_DN8685_c0_g1_i5_orf1;TRINITY_DN16840_c1_g1_i1_orf1;TRINITY_DN2407_c0_g1_i2_orf1;TRINITY_DN15706_c0_g2_i5_orf1;TRINITY_DN1666_c0_g1_i2_orf1;TRINITY_DN29190_c0_g1_i4_orf1;TRINITY_DN12534_c0_g1_i4_orf1;TRINITY_DN479_c6_g1_i2_orf1;TRINITY_DN109503_c0_g1_i4_orf1;TRINITY_DN14019_c0_g1_i5_orf1;TRINITY_DN3166_c1_g1_i6_orf1;TRINITY_DN2407_c0_g1_i6_orf1;TRINITY_DN86772_c0_g1_i3_orfp1;TRINITY_DN1534_c0_g1_i3_orf1;TRINITY_DN1091_c0_g2_i10_orf1;TRINITY_DN6098_c1_g1_i5_orf1;TRINITY_DN2170_c0_g2_i1_orf1;TRINITY_DN4748_c0_g1_i5_orf1;TRINITY_DN4802_c0_g1_i4_orf1;TRINITY_DN1444_c1_g1_i5_orf1;TRINITY_DN59429_c0_g1_i6_orf1;TRINITY_DN2170_c1_g1_i3_orf1;TRINITY_DN5235_c0_g1_i7_orf1;TRINITY_DN5880_c0_g2_i2_orf1
biological_process	detection of biotic stimulus	GO:0009595	3	3/2360	TRINITY_DN1091_c0_g2_i10_orf1;TRINITY_DN8685_c0_g1_i5_orf1;TRINITY_DN5880_c0_g2_i2_orf1
biological_process	response to extracellular stimulus	GO:0009991	3	3/2360	TRINITY_DN1091_c0_g1_i1_orf1;TRINITY_DN1091_c0_g3_i1_orf1;TRINITY_DN2054_c0_g1_i1_orf1
biological_process	cellular response to external stimulus	GO:0071496	3	3/2360	TRINITY_DN1091_c0_g1_i1_orf1;TRINITY_DN1091_c0_g3_i1_orf1;TRINITY_DN2054_c0_g1_i1_orf1
biological_process	cellular response to endogenous stimulus	GO:0071495	1	1/2360	TRINITY_DN4016_c0_g1_i1_orf1
biological_process	response to hypoxia	GO:0001666	1	1/2360	TRINITY_DN140538_c0_g2_i1_orf1
biological_process	response to heat	GO:0009408	3	3/2360	TRINITY_DN12964_c0_g1_i1_orf1;TRINITY_DN15959_c0_g1_i1_orf1;TRINITY_DN5648_c0_g1_i5_orf1
biological_process	defense response	GO:0006952	26	26/2360	TRINITY_DN195_c8_g1_i1_orf1;TRINITY_DN8685_c0_g1_i5_orf1;TRINITY_DN16840_c1_g1_i1_orf1;TRINITY_DN2407_c0_g1_i2_orf1;TRINITY_DN15706_c0_g2_i5_orf1;TRINITY_DN1666_c0_g1_i2_orf1;TRINITY_DN29190_c0_g1_i4_orf1;TRINITY_DN12534_c0_g1_i4_orf1;TRINITY_DN479_c6_g1_i2_orf1;TRINITY_DN14019_c0_g1_i5_orf1;TRINITY_DN3166_c1_g1_i6_orf1;TRINITY_DN2407_c0_g1_i6_orf1;TRINITY_DN31163_c1_g1_i4_orf1;TRINITY_DN86772_c0_g1_i3_orfp1;TRINITY_DN1534_c0_g1_i3_orf1;TRINITY_DN1091_c0_g2_i10_orf1;TRINITY_DN6098_c1_g1_i5_orf1;TRINITY_DN2170_c0_g2_i1_orf1;TRINITY_DN2338_c0_g1_i5_orf1;TRINITY_DN4748_c0_g1_i5_orf1;TRINITY_DN4802_c0_g1_i4_orf1;TRINITY_DN1444_c1_g1_i5_orf1;TRINITY_DN59429_c0_g1_i6_orf1;TRINITY_DN2170_c1_g1_i3_orf1;TRINITY_DN5235_c0_g1_i7_orf1;TRINITY_DN5880_c0_g2_i2_orf1
biological_process	response to oxidative stress	GO:0006979	9	9/2360	TRINITY_DN87603_c0_g2_i1_orf1;TRINITY_DN51252_c0_g2_i1_orf1;TRINITY_DN2207_c0_g1_i6_orf1;TRINITY_DN80660_c0_g1_i1_orf1;TRINITY_DN5933_c0_g1_i1_orf1;TRINITY_DN285_c0_g1_i4_orf1;TRINITY_DN3321_c0_g1_i3_orf1;TRINITY_DN21420_c0_g1_i2_orf1;TRINITY_DN2652_c0_g2_i1_orf1
biological_process	response to oxygen-containing compound	GO:1901700	2	2/2360	TRINITY_DN87603_c0_g2_i1_orf1;TRINITY_DN4016_c0_g1_i1_orf1
biological_process	response to nitrogen compound	GO:1901698	3	3/2360	TRINITY_DN9062_c0_g2_i3_orf1;TRINITY_DN28989_c0_g1_i7_orf1;TRINITY_DN4016_c0_g1_i1_orf1
biological_process	response to inorganic substance	GO:0010035	3	3/2360	TRINITY_DN2433_c0_g1_i3_orf1;TRINITY_DN87603_c0_g2_i1_orf1;TRINITY_DN4016_c0_g1_i1_orf1
biological_process	response to organic substance	GO:0010033	7	7/2360	TRINITY_DN9062_c0_g2_i3_orf1;TRINITY_DN4016_c0_g1_i1_orf1;TRINITY_DN8685_c0_g1_i5_orf1;TRINITY_DN1091_c0_g2_i10_orf1;TRINITY_DN28989_c0_g1_i7_orf1;TRINITY_DN2227_c0_g1_i5_orf1;TRINITY_DN5880_c0_g2_i2_orf1
biological_process	response to temperature stimulus	GO:0009266	3	3/2360	TRINITY_DN12964_c0_g1_i1_orf1;TRINITY_DN15959_c0_g1_i1_orf1;TRINITY_DN5648_c0_g1_i5_orf1
biological_process	response to oxygen levels	GO:0070482	1	1/2360	TRINITY_DN140538_c0_g2_i1_orf1
biological_process	detection of chemical stimulus	GO:0009593	3	3/2360	TRINITY_DN1091_c0_g2_i10_orf1;TRINITY_DN8685_c0_g1_i5_orf1;TRINITY_DN5880_c0_g2_i2_orf1
cellular_component	nucleosome	GO:0000786	1	1/2360	TRINITY_DN20442_c0_g2_i1_orf1
cellular_component	cohesin complex	GO:0008278	1	1/2360	TRINITY_DN2638_c0_g1_i7_orf1
cellular_component	Mre11 complex	GO:0030870	2	2/2360	TRINITY_DN45271_c0_g1_i1_orf1;TRINITY_DN123184_c0_g1_i1_orf1
cellular_component	mRNA cleavage factor complex	GO:0005849	3	3/2360	TRINITY_DN2718_c0_g1_i6_orf1;TRINITY_DN1005_c0_g1_i5_orf1;TRINITY_DN1005_c0_g2_i1_orf1
cellular_component	Ku70:Ku80 complex	GO:0043564	1	1/2360	TRINITY_DN5757_c0_g1_i1_orf1
cellular_component	SWI/SNF superfamily-type complex	GO:0070603	5	5/2360	TRINITY_DN452_c1_g1_i3_orf1;TRINITY_DN3649_c0_g1_i6_orf1;TRINITY_DN45449_c0_g1_i1_orf1;TRINITY_DN9765_c0_g1_i6_orf1;TRINITY_DN5569_c0_g1_i1_orf1
cellular_component	U2AF complex	GO:0089701	1	1/2360	TRINITY_DN51968_c0_g1_i1_orf1
cellular_component	transcription elongation factor complex	GO:0008023	2	2/2360	TRINITY_DN5686_c0_g1_i4_orf1;TRINITY_DN3482_c0_g2_i1_orf1
cellular_component	PcG protein complex	GO:0031519	1	1/2360	TRINITY_DN1639_c0_g2_i2_orf1
cellular_component	spliceosomal complex	GO:0005681	15	15/2360	TRINITY_DN33346_c0_g1_i1_orf1;TRINITY_DN1554_c0_g1_i9_orf1;TRINITY_DN23502_c0_g1_i1_orf1;TRINITY_DN51568_c0_g1_i1_orf1;TRINITY_DN30097_c0_g1_i2_orf1;TRINITY_DN13055_c0_g1_i5_orf1;TRINITY_DN43412_c0_g1_i2_orf1;TRINITY_DN31663_c0_g1_i2_orf1;TRINITY_DN142652_c0_g1_i1_orf1;TRINITY_DN107035_c0_g1_i1_orf1;TRINITY_DN20215_c0_g2_i1_orf1;TRINITY_DN698_c0_g1_i5_orf1;TRINITY_DN4135_c0_g1_i5_orf1;TRINITY_DN44877_c0_g1_i2_orf1;TRINITY_DN14487_c0_g1_i4_orf1
cellular_component	BRISC complex	GO:0070552	2	2/2360	TRINITY_DN17655_c0_g1_i1_orf1;TRINITY_DN19866_c0_g1_i4_orf1
cellular_component	nuclear DNA-directed RNA polymerase comple	GO:0055029	1	1/2360	TRINITY_DN4707_c0_g1_i1_orf1
cellular_component	histone acetyltransferase complex	GO:0000123	1	1/2360	TRINITY_DN452_c1_g1_i3_orf1
cellular_component	exon-exon junction complex	GO:0035145	1	1/2360	TRINITY_DN5507_c0_g1_i1_orf1
cellular_component	small nuclear ribonucleoprotein complex	GO:0030532	8	8/2360	TRINITY_DN38540_c0_g1_i1_orf1;TRINITY_DN33346_c0_g1_i1_orf1;TRINITY_DN298_c0_g1_i4_orf1;TRINITY_DN1616_c0_g1_i3_orf1;TRINITY_DN31663_c0_g1_i2_orf1;TRINITY_DN43412_c0_g1_i2_orf1;TRINITY_DN5834_c0_g1_i2_orf1;TRINITY_DN4135_c0_g1_i5_orf1
cellular_component	nuclear ubiquitin ligase complex	GO:0000152	1	1/2360	TRINITY_DN146493_c0_g1_i1_orf1
cellular_component	BRCA1-A complex	GO:0070531	2	2/2360	TRINITY_DN17655_c0_g1_i1_orf1;TRINITY_DN19866_c0_g1_i4_orf1
cellular_component	histone methyltransferase complex	GO:0035097	1	1/2360	TRINITY_DN5569_c0_g1_i1_orf1
cellular_component	RNA polymerase II transcription regulator compl	GO:0090575	2	2/2360	TRINITY_DN346_c0_g1_i7_orf1;TRINITY_DN21567_c0_g1_i7_orf1
cellular_component	carboxy-terminal domain protein kinase comple	GO:0032806	1	1/2360	TRINITY_DN346_c0_g1_i7_orf1
cellular_component	nuclear pore outer ring	GO:0031080	1	1/2360	TRINITY_DN6680_c0_g1_i1_orf1
cellular_component	nuclear pore	GO:0005643	6	6/2360	TRINITY_DN96557_c0_g1_i1_orf1;TRINITY_DN1437_c0_g1_i6_orf1;TRINITY_DN59042_c1_g1_i1_orf1;TRINITY_DN146119_c0_g1_i1_orf1;TRINITY_DN10415_c0_g1_i5_orf1;TRINITY_DN2879_c0_g1_i4_orf1
cellular_component	ESCRT III complex	GO:0000815	1	1/2360	TRINITY_DN96557_c0_g1_i1_orf1
cellular_component	ESCRT I complex	GO:0000813	1	1/2360	TRINITY_DN4013_c0_g1_i4_orf1
cellular_component	transmembrane transporter complex	GO:1902495	3	3/2360	TRINITY_DN44256_c0_g1_i1_orf1;TRINITY_DN162_c0_g1_i4_orf1;TRINITY_DN29934_c0_g1_i6_orf1
cellular_component	dynein complex	GO:0030286	3	3/2360	TRINITY_DN4257_c0_g1_i2_orf1;TRINITY_DN122423_c0_g5_i1_orf1;TRINITY_DN122423_c0_g1_i1_orf1
cellular_component	catalytic step 2 spliceosome	GO:0071013	1	1/2360	TRINITY_DN30097_c0_g1_i2_orf1
cellular_component	proteasome core complex	GO:0005839	2	2/2360	TRINITY_DN113327_c0_g1_i2_orf1;TRINITY_DN10769_c0_g1_i1_orf1
cellular_component	cytochrome complex	GO:0070069	2	2/2360	TRINITY_DN5111_c0_g1_i2_orf1;TRINITY_DN136028_c0_g2_i1_orf1
cellular_component	tRNA-splicing ligase complex	GO:0072669	1	1/2360	TRINITY_DN19034_c0_g1_i1_orf1
cellular_component	transferase complex	GO:1990234	14	14/2360	TRINITY_DN81258_c0_g1_i2_orf1;TRINITY_DN2401_c0_g2_i1_orf1;TRINITY_DN15040_c0_g4_i1_orf1;TRINITY_DN143496_c0_g1_i1_orf1;TRINITY_DN9062_c0_g2_i3_orf1;TRINITY_DN4707_c0_g1_i1_orf1;TRINITY_DN70485_c0_g1_i2_orf1;TRINITY_DN147475_c0_g1_i1_orf1;TRINITY_DN452_c1_g1_i3_orf1;TRINITY_DN5182_c0_g1_i5_orf1;TRINITY_DN146493_c0_g1_i1_orf1;TRINITY_DN110534_c0_g1_i3_orf1;TRINITY_DN5569_c0_g1_i1_orf1;TRINITY_DN346_c0_g1_i7_orf1
cellular_component	peptidase complex	GO:1905368	12	12/2360	TRINITY_DN5775_c0_g1_i1_orf1;TRINITY_DN19260_c0_g1_i5_orf1;TRINITY_DN13384_c0_g1_i1_orf1;TRINITY_DN2058_c0_g1_i2_orf1;TRINITY_DN34479_c0_g1_i2_orf1;TRINITY_DN71610_c0_g1_i1_orf1;TRINITY_DN2591_c0_g1_i4_orf1;TRINITY_DN125967_c0_g1_i1_orf1;TRINITY_DN321_c0_g1_i1_orf1;TRINITY_DN6684_c0_g1_i4_orf1;TRINITY_DN49047_c0_g1_i2_orf1;TRINITY_DN32359_c0_g2_i1_orf1
cellular_component	aminoacyl-tRNA synthetase multienzyme compl	GO:0017101	4	4/2360	TRINITY_DN22572_c0_g1_i1_orf1;TRINITY_DN2953_c1_g1_i10_orf1;TRINITY_DN107288_c0_g1_i2_orf1;TRINITY_DN2953_c1_g1_i2_orf1
cellular_component	elongator holoenzyme complex	GO:0033588	1	1/2360	TRINITY_DN56270_c0_g1_i1_orf1
cellular_component	ATPase complex	GO:1904949	5	5/2360	TRINITY_DN452_c1_g1_i3_orf1;TRINITY_DN3649_c0_g1_i6_orf1;TRINITY_DN45449_c0_g1_i1_orf1;TRINITY_DN9765_c0_g1_i6_orf1;TRINITY_DN5569_c0_g1_i1_orf1
cellular_component	dystrophin-associated glycoprotein complex	GO:0016010	1	1/2360	TRINITY_DN7128_c0_g1_i7_orf1
cellular_component	GPI-anchor transamidase complex	GO:0042765	1	1/2360	TRINITY_DN71610_c0_g1_i1_orf1
cellular_component	plasma membrane protein complex	GO:0098797	6	6/2360	TRINITY_DN29934_c0_g1_i6_orf1;TRINITY_DN12777_c0_g1_i5_orf1;TRINITY_DN162_c0_g1_i4_orf1;TRINITY_DN7128_c0_g1_i7_orf1;TRINITY_DN5118_c0_g1_i1_orf1;TRINITY_DN8405_c0_g1_i4_orf1
cellular_component	outer mitochondrial membrane protein complex	GO:0098799	2	2/2360	TRINITY_DN9741_c0_g1_i3_orf1;TRINITY_DN27721_c1_g1_i2_orf1
cellular_component	proton-transporting two-sector ATPase comple	GO:0033178	1	1/2360	TRINITY_DN96080_c0_g2_i1_orf1
cellular_component	EMC complex	GO:0072546	1	1/2360	TRINITY_DN3838_c0_g1_i8_orf1
cellular_component	clathrin complex	GO:0071439	1	1/2360	TRINITY_DN8405_c0_g1_i4_orf1
cellular_component	respiratory chain complex	GO:0098803	2	2/2360	TRINITY_DN5111_c0_g1_i2_orf1;TRINITY_DN136028_c0_g2_i1_orf1
cellular_component	inner mitochondrial membrane protein complex	GO:0098800	6	6/2360	TRINITY_DN15222_c0_g1_i4_orf1;TRINITY_DN44219_c0_g1_i1_orf1;TRINITY_DN136028_c0_g2_i1_orf1;TRINITY_DN107261_c0_g1_i1_orf1;TRINITY_DN5111_c0_g1_i2_orf1;TRINITY_DN44256_c0_g1_i1_orf1

cellular_component	membrane coat	GO:0030117	10	10/2360	TRINITY_DN96557_c0_g1_i1_orf1;TRINITY_DN9592_c0_g1_i2_orf1;TRINITY_DN1447_c0_g1_i5_orf1;TRINITY_DN5982_c0_g1_i3_orf1;TRINITY_DN12767_c0_g1_i2_orf1;TRINITY_DN146119_c0_g1_i1_orf1;TRINITY_DN12777_c0_g1_i5_orf1;TRINITY_DN3209_c0_g1_i1_orf1;TRINITY_DN8405_c0_g1_i4_orf1;TRINITY_DN14677_c0_g2_i3_orf1
cellular_component	AP-type membrane coat adaptor complex	GO:0030119	6	6/2360	TRINITY_DN13118_c0_g1_i6_orf1;TRINITY_DN13139_c0_g1_i1_orf1;TRINITY_DN19702_c0_g1_i4_orf1;TRINITY_DN72859_c0_g1_i1_orf1;TRINITY_DN5118_c0_g1_i1_orf1;TRINITY_DN486_c0_g1_i5_orf1
cellular_component	proton-transporting two-sector ATPase comple	GO:0033177	3	3/2360	TRINITY_DN10458_c0_g1_i1_orf1;TRINITY_DN15222_c0_g1_i4_orf1;TRINITY_DN107261_c0_g1_i1_orf1
cellular_component	mitochondrial large ribosomal subunit	GO:0005762	1	1/2360	TRINITY_DN1313_c0_g1_i2_orf1
cellular_component	Golgi transport complex	GO:0017119	1	1/2360	TRINITY_DN50875_c0_g1_i3_orf1
cellular_component	exocyst	GO:0000145	2	2/2360	TRINITY_DN25686_c0_g1_i4_orf1;TRINITY_DN1895_c0_g1_i2_orf1
cellular_component	TRAPP complex	GO:0030008	1	1/2360	TRINITY_DN45037_c0_g1_i1_orf1
cellular_component	dynactin complex	GO:0005869	1	1/2360	TRINITY_DN8561_c0_g4_i1_orf1
cellular_component	kinesin complex	GO:0005871	1	1/2360	TRINITY_DN4808_c0_g1_i3_orf1
cellular_component	translation preinitiation complex	GO:0070993	4	4/2360	TRINITY_DN3366_c0_g1_i6_orf1;TRINITY_DN48097_c0_g1_i1_orf1;TRINITY_DN4237_c1_g1_i5_orf1;TRINITY_DN50085_c0_g1_i1_orf1
cellular_component	translation initiation complex	GO:0070992	1	1/2360	TRINITY_DN142442_c0_g1_i1_orf1
cellular_component	signal recognition particle	GO:0048500	1	1/2360	TRINITY_DN48460_c0_g1_i1_orf1
cellular_component	preribosome	GO:0030684	1	1/2360	TRINITY_DN3082_c1_g1_i7_orf1
cellular_component	ribosomal subunit	GO:0044391	11	11/2360	TRINITY_DN441_c0_g2_i1_orf1;TRINITY_DN87603_c0_g2_i1_orf1;TRINITY_DN4016_c0_g1_i1_orf1;TRINITY_DN95056_c0_g2_i2_orf1;TRINITY_DN30300_c0_g2_i1_orf1;TRINITY_DN1313_c0_g1_i2_orf1;TRINITY_DN142442_c0_g1_i1_orf1;TRINITY_DN50787_c0_g2_i2_orf1;TRINITY_DN13651_c0_g1_i2_orf1;TRINITY_DN10070_c0_g1_i1_orf1;TRINITY_DN21367_c0_g1_i1_orf1
cellular_component	mRNA cap binding complex	GO:0005845	1	1/2360	TRINITY_DN41664_c0_g1_i4_orf1
cellular_component	proteasome complex	GO:0000502	11	11/2360	TRINITY_DN5775_c0_g1_i1_orf1;TRINITY_DN19260_c0_g1_i5_orf1;TRINITY_DN13384_c0_g1_i1_orf1;TRINITY_DN34479_c0_g1_i2_orf1;TRINITY_DN125967_c0_g1_i1_orf1;TRINITY_DN6684_c0_g1_i4_orf1;TRINITY_DN49047_c0_g1_i2_orf1;TRINITY_DN32359_c0_g2_i1_orf1;TRINITY_DN2058_c0_g1_i2_orf1;TRINITY_DN321_c0_g1_i1_orf1;TRINITY_DN2591_c0_g1_i4_orf1
cellular_component	DNA polymerase complex	GO:0042575	4	4/2360	TRINITY_DN81258_c0_g1_i2_orf1;TRINITY_DN110534_c0_g1_i3_orf1;TRINITY_DN15040_c0_g4_i1_orf1;TRINITY_DN70485_c0_g1_i2_orf1
cellular_component	chaperone complex	GO:0101031	2	2/2360	TRINITY_DN5262_c0_g1_i7_orf1;TRINITY_DN1725_c0_g1_i7_orf1
cellular_component	ubiquitin ligase complex	GO:0000151	3	3/2360	TRINITY_DN146493_c0_g1_i1_orf1;TRINITY_DN9062_c0_g2_i3_orf1;TRINITY_DN143496_c0_g1_i1_orf1
cellular_component	RNA polymerase complex	GO:0030880	2	2/2360	TRINITY_DN2401_c0_g2_i1_orf1;TRINITY_DN4707_c0_g1_i1_orf1
cellular_component	protein acetyltransferase complex	GO:0031248	1	1/2360	TRINITY_DN452_c1_g1_i3_orf1
cellular_component	protein kinase CK2 complex	GO:0005956	1	1/2360	TRINITY_DN147475_c0_g1_i1_orf1
cellular_component	CIA complex	GO:0097361	1	1/2360	TRINITY_DN49872_c0_g1_i2_orf1
cellular_component	organelle lumen	GO:0043233	10	10/2360	TRINITY_DN38482_c0_g1_i4_orf1;TRINITY_DN14920_c0_g1_i1_orf1;TRINITY_DN17271_c0_g1_i1_orf1;TRINITY_DN9135_c0_g1_i4_orf1;TRINITY_DN21539_c0_g1_i1_orf1;TRINITY_DN33146_c0_g1_i1_orf1;TRINITY_DN47219_c0_g1_i3_orf1;TRINITY_DN147475_c0_g1_i1_orf1;TRINITY_DN9242_c0_g1_i1_orf1;TRINITY_DN3037_c0_g1_i1_orf1
cellular_component	chromosome, telomeric region	GO:0000781	1	1/2360	TRINITY_DN12771_c0_g1_i1_orf1
cellular_component	chromosome, centromeric region	GO:0000775	1	1/2360	TRINITY_DN31314_c0_g1_i4_orf1
cellular_component	cell cortex	GO:0005938	1	1/2360	TRINITY_DN2186_c0_g1_i17_orf1
cellular_component	extrinsic component of organelle membrane	GO:0031312	1	1/2360	TRINITY_DN6027_c0_g1_i13_orf1
cellular_component	heterochromatin	GO:0000792	2	2/2360	TRINITY_DN24266_c0_g2_i2_orf1;TRINITY_DN2345_c0_g1_i4_orf1
cellular_component	intrinsic component of plasma membrane	GO:0031226	2	2/2360	TRINITY_DN3833_c0_g1_i4_orf1;TRINITY_DN4464_c0_g2_i1_orf1
cellular_component	anchored component of membrane	GO:0031225	7	7/2360	TRINITY_DN5406_c0_g2_i1_orf1;TRINITY_DN9475_c0_g1_i6_orf1;TRINITY_DN4464_c0_g2_i1_orf1;TRINITY_DN1352_c0_g1_i5_orf1;TRINITY_DN5553_c0_g1_i4_orf1;TRINITY_DN2175_c0_g1_i4_orf1;TRINITY_DN3833_c0_g1_i4_orf1
cellular_component	integral component of membrane	GO:0016021	273	273/2360	TRINITY_DN129226_c0_g1_i2_orf1;TRINITY_DN38435_c0_g1_i1_orf1;TRINITY_DN9608_c0_g1_i3_orf1;TRINITY_DN3310_c0_g1_i1_orf1;TRINITY_DN120500_c0_g1_i1_orf1;TRINITY_DN79868_c0_g1_i1_orf1;TRINITY_DN12227_c0_g2_i3_orf1;TRINITY_DN5597_c0_g1_i2_orf1;TRINITY_DN3194_c0_g1_i6_orf1;TRINITY_DN6991_c0_g1_i24_orf1;TRINITY_DN26355_c0_g1_i4_orf1;TRINITY_DN21872_c0_g1_i2_orf1;TRINITY_DN6586_c0_g1_i1_orf1;TRINITY_DN72816_c0_g1_i2_orf1;TRINITY_DN246_c1_g1_i5_orf1;TRINITY_DN585_c0_g1_i5_orf1;TRINITY_DN9457_c0_g1_i9_orf1;TRINITY_DN7735_c1_g1_i1_orf1;TRINITY_DN38431_c0_g1_i1_orf1;TRINITY_DN15755_c0_g1_i1_orf1;TRINITY_DN3499_c0_g1_i8_orf1;TRINITY_DN11069_c0_g2_i1_orf1;TRINITY_DN8030_c0_g1_i2_orf1;TRINITY_DN29633_c0_g1_i8_orf1;TRINITY_DN1232_c0_g1_i1_orf1;TRINITY_DN1661_c0_g1_i1_orf1;TRINITY_DN2312_c0_g1_i4_orf1;TRINITY_DN3835_c0_g1_i3_orf1;TRINITY_DN8964_c0_g1_i4_orf1;TRINITY_DN1099_c1_g1_i2_orf1;TRINITY_DN20680_c0_g1_i5_orf1;TRINITY_DN2855_c0_g1_i6_orf1;TRINITY_DN7570_c0_g1_i18_orf1;TRINITY_DN4977_c0_g1_i2_orf1;TRINITY_DN7590_c0_g1_i4_orf1;TRINITY_DN4144_c0_g1_i7_orf1;TRINITY_DN585_c0_g1_i12_orf1;TRINITY_DN3196_c0_g1_i1_orf1;TRINITY_DN10774_c0_g2_i3_orf1;TRINITY_DN54925_c0_g1_i1_orf1;TRINITY_DN41708_c0_g1_i1_orf1;TRINITY_DN21570_c0_g1_i1_orf1;TRINITY_DN18338_c0_g1_i6_orf1;TRINITY_DN27114_c0_g1_i1_orf1;TRINITY_DN5337_c0_g1_i6_orf1;TRINITY_DN39404_c0_g1_i7_orf1;TRINITY_DN40197_c0_g1_i1_orf1;TRINITY_DN4125_c0_g1_i14_orf1;TRINITY_DN3177_c0_g1_i1_orf1;TRINITY_DN4757_c0_g1_i3_orf1;TRINITY_DN62707_c0_g1_i1_orf1;TRINITY_DN4886_c0_g1_i6_orf1;TRINITY_DN3355_c0_g2_i4_orf1;TRINITY_DN22443_c0_g2_i3_orf1;TRINITY_DN30932_c0_g1_i2_orf1;TRINITY_DN2508_c0_g1_i2_orf1;TRINITY_DN919_c0_g1_i7_orf1;TRINITY_DN2343_c1_g1_i2_orf1;TRINITY_DN6621_c0_g1_i1_orf1;TRINITY_DN29879_c0_g1_i3_orf1;TRINITY_DN51766_c0_g1_i2_orf1;TRINITY_DN91533_c0_g1_i1_orf1;TRINITY_DN56430_c0_g1_i1_orf1;TRINITY_DN10231_c0_g2_i1_orf1;TRINITY_DN3461_c0_g1_i1_orf1;TRINITY_DN12769_c0_g1_i5_orf1;TRINITY_DN14389_c0_g1_i4_orf1;TRINITY_DN11670_c0_g1_i1_orf1;TRINITY_DN2109_c0_g1_i4_orf1;TRINITY_DN3005_c0_g1_i7_orf1;TRINITY_DN4116_c0_g1_i3_orf1;TRINITY_DN3616_c0_g2_i1_orf1;TRINITY_DN82104_c0_g1_i5_orf1;TRINITY_DN5852_c0_g1_i6_orf1;TRINITY_DN3472_c1_g1_i4_orf1;TRINITY_DN4324_c0_g1_i1_orf1;TRINITY_DN4596_c0_g1_i14_orf1;TRINITY_DN13216_c0_g1_i5_orf1;TRINITY_DN1749_c0_g2_i2_orf1;TRINITY_DN6974_c0_g2_i1_orf1;TRINITY_DN30704_c0_g1_i1_orf1;TRINITY_DN198_c2_g1_i2_orf1;TRINITY_DN482_c0_g1_i1_orf1;TRINITY_DN75086_c0_g1_i5_orf1;TRINITY_DN252_c0_g1_i3_orf1;TRINITY_DN121650_c0_g1_i1_orf1;TRINITY_DN4612_c0_g1_i1_orf1;TRINITY_DN1664_c0_g1_i4_orf1;TRINITY_DN5046_c0_g3_i1_orf1;TRINITY_DN2177_c0_g1_i1_orf1;TRINITY_DN132857_c0_g1_i1_orf1;TRINITY_DN501_c0_g1_i5_orf1;TRINITY_DN79803_c0_g1_i7_orf1;TRINITY_DN9354_c0_g1_i7_orf1;TRINITY_DN1789_c0_g1_i5_orf1;TRINITY_DN5852_c0_g1_i13_orf1;TRINITY_DN2326_c0_g1_i1_orf1;TRINITY_DN1999_c0_g1_i9_orf1;TRINITY_DN5211_c0_g1_i1_orf1;TRINITY_DN45446_c0_g1_i2_orf1;TRINITY_DN21719_c0_g1_i2_orf1;TRINITY_DN1402_c1_g1_i6_orf1;TRINITY_DN172_c8_g2_i1_orf1;TRINITY_DN4273_c1_g1_i5_orf1;TRINITY_DN3647_c2_g1_i3_orf1;TRINITY_DN14429_c0_g1_i2_orf1;TRINITY_DN2808_c0_g1_i8_orf1;TRINITY_DN7336_c0_g1_i13_orf1;TRINITY_DN43369_c0_g2_i1_orf1;TRINITY_DN154_c0_g1_i4_orf1;TRINITY_DN9000_c0_g2_i1_orf1;TRINITY_DN2618_c0_g1_i3_orf1;TRINITY_DN5408_c0_g1_i5_orf1;TRINITY_DN9243_c0_g1_i4_orf1;TRINITY_DN4782_c0_g1_i1_orf1;TRINITY_DN7134_c0_g1_i1_orf1;TRINITY_DN867_c0_g1_i1_orf1;TRINITY_DN19917_c0_g1_i1_orf1;TRINITY_DN40945_c0_g1_i1_orf1;TRINITY_DN140_c1_g1_i2_orf1;TRINITY_DN7861_c0_g1_i5_orf1;TRINITY_DN52788_c0_g1_i1_orf1;TRINITY_DN83295_c0_g1_i3_orf1;TRINITY_DN7828_c0_g1_i2_orf1;TRINITY_DN2172_c0_g2_i8_orf1;TRINITY_DN28592_c0_g1_i2_orf1;TRINITY_DN31118_c0_g1_i1_orf1;TRINITY_DN48590_c0_g1_i1_orf1;TRINITY_DN4134_c2_g1_i2_orf1;TRINITY_DN9090_c0_g1_i9_orf1;TRINITY_DN57348_c0_g1_i4_orf1;TRINITY_DN1114_c0_g1_i4_orf1;TRINITY_DN1630_c0_g1_i6_orf1;TRINITY_DN12873_c0_g1_i3_orf1;TRINITY_DN448_c0_g1_i20_orf1;TRINITY_DN1480_c0_g1_i5_orf1;TRINITY_DN38371_c0_g1_i7_orf1;TRINITY_DN9475_c0_g1_i6_orf1;TRINITY_DN1750_c1_g1_i5_orf1;TRINITY_DN3821_c1_g1_i7_orf1;TRINITY_DN5697_c0_g1_i1_orf1;TRINITY_DN10220_c1_g1_i7_orf1;TRINITY_DN1768_c0_g1_i2_orf1;TRINITY_DN754_c1_g1_i8_orf1;TRINITY_DN29_c0_g1_i4_orf1;TRINITY_DN3637_c0_g1_i2_orf1;TRINITY_DN14262_c0_g1_i5_orf1;TRINITY_DN928_c0_g2_i1_orf1;TRINITY_DN625_c9_g1_i7_orf1;TRINITY_DN19951_c0_g1_i5_orf1;TRINITY_DN6563_c0_g1_i1_orf1;TRINITY_DN3962_c0_g1_i6_orf1;TRINITY_DN3616_c0_g2_i2_orf1;TRINITY_DN928_c0_g1_i3_orf1;TRINITY_DN7868_c0_g1_i8_orf1;TRINITY_DN19135_c0_g1_i1_orf1;TRINITY_DN8133_c0_g1_i4_orf1;TRINITY_DN2874_c0_g1_i4_orf1;TRINITY_DN21719_c0_g2_i4_orf1;TRINITY_DN48237_c0_g1_i5_orf1;TRINITY_DN27500_c0_g1_i4_orf1;TRINITY_DN14046_c0_g1_i1_orf1;TRINITY_DN5553_c0_g1_i4_orf1;TRINITY_DN1982_c0_g1_i24_orf1;TRINITY_DN3343_c0_g2_i1_orf1;TRINITY_DN11566_c0_g1_i6_orf1;TRINITY_DN38225_c0_g2_i1_orf1;TRINITY_DN1882_c0_g1_i4_orf1;TRINITY_DN15597_c0_g1_i1_orf1;TRINITY_DN18164_c0_g1_i7_orf1;TRINITY_DN5191_c0_g2_i1_orf1;TRINITY_DN20710_c0_g1_i2_orf1;TRINITY_DN114834_c0_g1_i1_orf1;TRINITY_DN60792_c0_g1_i2_orf1;TRINITY_DN25976_c0_g1_i4_orf1;TRINITY_DN4497_c2_g1_i3_orf1;TRINITY_DN7335_c0_g1_i1_orf1;TRINITY_DN9931_c0_g1_i1_orf1;TRINITY_DN2652_c0_g2_i1_orf1;TRINITY_DN12256_c0_g1_i1_orf1;TRINITY_DN5153_c1_g1_i1_orf1;TRINITY_DN23229_c0_g1_i2_orf1;TRINITY_DN49143_c0_g1_i1_orf1;TRINITY_DN6685_c0_g1_i8_orf1;TRINITY_DN31327_c0_g2_i1_orf1;TRINITY_DN6612_c0_g1_i4_orf1;TRINITY_DN1293_c0_g1_i4_orf1;TRINITY_DN139537_c0_g1_i1_orf1;TRINITY_DN1293_c1_g1_i4_orf1;TRINITY_DN257
cellular_component	intrinsic component of organelle membrane	GO:0031300	3	3/2360	TRINITY_DN8964_c0_g1_i4_orf1;TRINITY_DN91_c0_g1_i9_orf1;TRINITY_DN2172_c0_g2_i8_orf1
cellular_component	external side of plasma membrane	GO:0009897	1	1/2360	TRINITY_DN20339_c0_g1_i3_orf1
cellular_component	cytoplasmic side of membrane	GO:0098562	1	1/2360	TRINITY_DN96557_c0_g1_i1_orf1

cellular_component intracellular organelle	GO:0043229	212	212/2360	TRINITY_DN47731_c0_g1_i2_orf1;TRINITY_DN4380_c0_g1_i9_orf1;TRINITY_DN5670_c0_g1_i2_orf1;TRINITY_DN13350_c0_g1_i4_orf1;TRINITY_DN6991_c0_g1_i24_orf1;TRINITY_DN11194_c0_g1_i4_orf1;TRINITY_DN7122_c0_g1_i1_orf1;TRINITY_DN40176_c0_g1_i1_orf1;TRINITY_DN1921_c1_g1_i5_orf1;TRINITY_DN27885_c0_g1_i3_orf1;TRINITY_DN35669_c0_g1_i1_orf1;TRINITY_DN30663_c0_g1_i1_orf1;TRINITY_DN18804_c0_g1_i5_orf1;TRINITY_DN142442_c0_g1_i1_orf1;TRINITY_DN3457_c0_g1_i4_orf1;TRINITY_DN10769_c0_g1_i1_orf1;TRINITY_DN2638_c0_g1_i7_orf1;TRINITY_DN65299_c0_g4_i1_orf1;TRINITY_DN50074_c0_g1_i1_orf1;TRINITY_DN29448_c0_g1_i1_orf1;TRINITY_DN4711_c0_g1_i2_orf1;TRINITY_DN3464_c0_g1_i1_orf1;TRINITY_DN26789_c0_g1_i2_orf1;TRINITY_DN50787_c0_g2_i2_orf1;TRINITY_DN1639_c0_g2_i2_orf1;TRINITY_DN62_c1_g1_i3_orf1;TRINITY_DN21570_c0_g1_i1_orf1;TRINITY_DN6621_c0_g1_i1_orf1;TRINITY_DN24266_c0_g2_i2_orf1;TRINITY_DN5442_c0_g1_i4_orf1;TRINITY_DN74889_c0_g1_i1_orf1;TRINITY_DN51737_c0_g1_i3_orf1;TRINITY_DN99673_c0_g1_i1_orf1;TRINITY_DN50571_c1_g1_i1_orf1;TRINITY_DN84478_c0_g1_i8_orf1;TRINITY_DN146217_c0_g1_i1_orf1;TRINITY_DN14347_c0_g1_i1_orf1;TRINITY_DN235_c0_g3_i1_orf1;TRINITY_DN31314_c0_g1_i4_orf1;TRINITY_DN2058_c0_g1_i2_orf1;TRINITY_DN31585_c0_g1_i1_orf1;TRINITY_DN135_c0_g1_i1_orf1;TRINITY_DN30932_c0_g1_i2_orf1;TRINITY_DN140538_c0_g2_i1_orf1;TRINITY_DN2345_c0_g1_i4_orf1;TRINITY_DN110231_c0_g1_i1_orf1;TRINITY_DN2401_c0_g2_i1_orf1;TRINITY_DN33893_c0_g1_i1_orf1;TRINITY_DN9146_c0_g1_i1_orf1;TRINITY_DN2802_c1_g1_i1_orf1;TRINITY_DN31225_c0_g1_i1_orf1;TRINITY_DN7808_c0_g1_i1_orf1;TRINITY_DN56993_c0_g1_i4_orf1;TRINITY_DN14313_c0_g1_i1_orf1;TRINITY_DN445_c0_g1_i2_orf1;TRINITY_DN3092_c0_g1_i2_orf1;TRINITY_DN1706_c0_g1_i7_orf1;TRINITY_DN12526_c0_g1_i5_orf1;TRINITY_DN23360_c0_g1_i3_orf1;TRINITY_DN31503_c0_g1_i4_orf1;TRINITY_DN17905_c0_g3_i1_orf1;TRINITY_DN45633_c0_g1_i1_orf1;TRINITY_DN18009_c0_g1_i1_orf1;TRINITY_DN25783_c0_g1_i2_orf1;TRINITY_DN18933_c0_g1_i3_orf1;TRINITY_DN146119_c0_g1_i1_orf1;TRINITY_DN23790_c0_g1_i1_orf1;TRINITY_DN5893_c0_g1_i7_orf1;TRINITY_DN26947_c0_g1_i1_orf1;TRINITY_DN9871_c0_g1_i11_orf1;TRINITY_DN90497_c0_g1_i1_orf1;TRINITY_DN7583_c0_g1_i1_orf1;TRINITY_DN5531_c0_g3_i3_orf1;TRINITY_DN41_c0_g1_i3_orf1;TRINITY_DN45037_c0_g1_i1_orf1;TRINITY_DN12757_c0_g1_i1_orf1;TRINITY_DN3251_c0_g1_i6_orf1;TRINITY_DN40704_c0_g1_i2_orf1;TRINITY_DN9591_c0_g1_i1_orf1;TRINITY_DN141_c0_g1_i1_orf1;TRINITY_DN43431_c0_g1_i1_orf1;TRINITY_DN364_c1_g1_i2_orf1;TRINITY_DN3073_c0_g1_i7_orf1;TRINITY_DN3733_c0_g1_i1_orf1;TRINITY_DN3430_c0_g1_i1_orf1;TRINITY_DN28989_c0_g1_i7_orf1;TRINITY_DN18922_c0_g1_i1_orf1;TRINITY_DN11772_c0_g1_i1_orf1;TRINITY_DN741_c0_g1_i10_orf1;TRINITY_DN5664_c0_g1_i1_orf1;TRINITY_DN52893_c0_g1_i1_orf1;TRINITY_DN147691_c0_g1_i1_orf1;TRINITY_DN1718_c6_g1_i4_orf1;TRINITY_DN3335_c0_g1_i1_orf1;TRINITY_DN23734_c0_g1_i1_orf1;TRINITY_DN110460_c0_g2_i1_orf1;TRINITY_DN23020_c0_g1_i1_orf1;TRINITY_DN3702_c0_g1_i1_orf1;TRINITY_DN147458_c0_g1_i1_orf1;TRINITY_DN12397_c0_g1_i1_orf1;TRINITY_DN3860_c0_g1_i5_orf1;TRINITY_DN25997_c1_g2_i4_orf1;TRINITY_DN101991_c0_g1_i5_orf1;TRINITY_DN235_c0_g1_i2_orf1;TRINITY_DN2709_c0_g1_i4_orf1;TRINITY_DN291_c0_g1_i2_orf1;TRINITY_DN31119_c0_g1_i1_orf1;TRINITY_DN5112_c0_g1_i1_orf1;TRINITY_DN714_c0_g1_i3_orf1;TRINITY_DN5055_c0_g1_i12_orf1;TRINITY_DN22951_c0_g1_i1_orf1;TRINITY_DN26251_c0_g1_i1_orf1;TRINITY_DN15373_c0_g1_i2_orf1;TRINITY_DN6365_c0_g1_i4_orf1;TRINITY_DN2186_c0_g1_i17_orf1;TRINITY_DN17312_c0_g1_i1_orf1;TRINITY_DN3057_c0_g2_i1_orf1;TRINITY_DN72017_c0_g1_i1_orf1;TRINITY_DN5081_c0_g1_i5_orf1;TRINITY_DN34830_c0_g1_i1_orf1;TRINITY_DN19135_c0_g1_i1_orf1;TRINITY_DN4016_c0_g1_i1_orf1;TRINITY_DN934_c2_g1_i7_orf1;TRINITY_DN12771_c0_g1_i1_orf1;TRINITY_DN34479_c0_g1_i2_orf1;TRINITY_DN12858_c0_g1_i5_orf1;TRINITY_DN2559_c0_g1_i4_orf1;TRINITY_DN4793_c0_g1_i7_orf1;TRINITY_DN3219_c0_g1_i6_orf1;TRINITY_DN3062_c0_g1_i1_orf1;TRINITY_DN12576_c0_g1_i2_orf1;TRINITY_DN24322_c0_g1_i4_orf1;TRINITY_DN41736_c0_g2_i1_orf1;TRINITY_DN53311_c0_g2_i1_orf1;TRINITY_DN44288_c0_g1_i2_orf1;TRINITY_DN27960_c0_g1_i1_orf1;TRINITY_DN21367_c0_g1_i1_orf1;TRINITY_DN18036_c0_g1_i7_orf1;TRINITY_DN113327_c0_g1_i2_orf1;TRINITY_DN38274_c0_g1_i1_orf1;TRINITY_DN12820_c0_g1_i1_orf1;TRINITY_DN111_c0_g2_i2_orf1;TRINITY_DN19810_c1_g1_i7_orf1;TRINITY_DN72_c0_g1_i16_orf1;TRINITY_DN96557_c0_g1_i1_orf1;TRINITY_DN111110_c0_g1_i1_orf1;TRINITY_DN87603_c0_g2_i1_orf1;TRINITY_DN2802_c0_g1_i1_orf1;TRINITY_DN5962_c0_g1_i1_orf1;TRINITY_DN21971_c0_g1_i4_orf1;TRINITY_DN2647_c0_g1_i3_orf1;TRINITY_DN120144_c0_g1_i1_orf1;TRINITY_DN6248_c0_g1_i1_orf1;TRINITY_DN972_c0_g2_i1_orf1;TRINITY_DN5578_c0_g1_i4_orf1;TRINITY_DN26168_c0_g1_i1_orf1;TRINITY_DN46022_c0_g1_i1_orf1;TRINITY_DN10385_c0_g1_i5_orf1;TRINITY_DN787_c0_g1_i7_orf1;TRINITY_DN9517_c0_g1_i7_orf1;TRINITY_DN30673_c0_g1_i5_orf1;TRINITY_DN2265_c0_g2_i1_orf1;TRINITY_DN2971_c0_g1_i1_orf1;TRINITY_DN37532_c0_g1_i1_orf1;TRINITY_DN67649_c0_g1_i1_orf1;TRINITY_DN38211_c0_g1_i1_orf1;TRINITY_DN8044_c0_g1_i2_orf1;TRINITY_DN7128_c0_g1_i7_orf1;TRINITY_DN13760_c1_g1_i1_orf1;TRINITY_DN9938_c0_g2_i1_orf1;TRINITY_DN59291_c0_g1_i1_orf1;TRINITY_DN9302_c0_g1_i1_orf1;TRINITY_DN4056_c0_g1_i8_orf1;TRINITY_DN3521_c0_g2_i1_orf1;TRINITY_DN13711_c0_g1_i1_orf1;TRINITY_DN2140_c0_g1_i1_orf1;TRINITY_DN95056_c0_g2_i2_orf1;TRINITY_DN9383_c0_g1_i3_orf1;TRINITY_DN104507_c0_g1_i2_orf1;TRINITY_DN11596_c0_g1_i1_orf1;TRINITY_DN41108_c0_g1_i1_orf1;TRINITY_DN1298_c0_g1_i3_orf1;TRINITY_DN44633_c0_g1_i4_orf1;TRINITY_DN10994_c0_g1_i4_orf1;TRINITY_DN279_c0_g1_i10_orf1;TRINITY_DN3614_c0_g2_i1_orf1;TRINITY_DN166_c0_g1_i4_orf1;TRINITY_DN10745_c0_g1_i14_orf1;TRINITY_D
cellular_component non-membrane-bounded organelle	GO:0043228	66	66/2360	TRINITY_DN47731_c0_g1_i2_orf1;TRINITY_DN5112_c0_g1_i1_orf1;TRINITY_DN714_c0_g1_i3_orf1;TRINITY_DN23360_c0_g1_i3_orf1;TRINITY_DN3826_c0_g1_i1_orf1;TRINITY_DN6365_c0_g1_i4_orf1;TRINITY_DN7128_c0_g1_i7_orf1;TRINITY_DN2186_c0_g1_i17_orf1;TRINITY_DN9383_c0_g1_i3_orf1;TRINITY_DN10745_c0_g1_i14_orf1;TRINITY_DN23790_c0_g1_i1_orf1;TRINITY_DN5893_c0_g1_i7_orf1;TRINITY_DN4056_c0_g1_i8_orf1;TRINITY_DN26947_c0_g1_i1_orf1;TRINITY_DN4016_c0_g1_i1_orf1;TRINITY_DN90497_c0_g1_i1_orf1;TRINITY_DN934_c2_g1_i7_orf1;TRINITY_DN142442_c0_g1_i1_orf1;TRINITY_DN7583_c0_g1_i1_orf1;TRINITY_DN24322_c0_g1_i4_orf1;TRINITY_DN1298_c0_g1_i3_orf1;TRINITY_DN50787_c0_g2_i2_orf1;TRINITY_DN10994_c0_g1_i4_orf1;TRINITY_DN364_c1_g1_i2_orf1;TRINITY_DN5531_c0_g3_i3_orf1;TRINITY_DN1639_c0_g2_i2_orf1;TRINITY_DN3062_c0_g1_i1_orf1;TRINITY_DN9591_c0_g1_i1_orf1;TRINITY_DN12576_c0_g1_i2_orf1;TRINITY_DN95056_c0_g2_i2_orf1;TRINITY_DN74889_c0_g1_i1_orf1;TRINITY_DN47114_c0_g1_i5_orf1;TRINITY_DN29448_c0_g1_i1_orf1;TRINITY_DN50571_c1_g1_i1_orf1;TRINITY_DN34432_c0_g1_i1_orf1;TRINITY_DN7047_c0_g1_i1_orf1;TRINITY_DN26824_c0_g1_i1_orf1;TRINITY_DN146217_c0_g1_i1_orf1;TRINITY_DN235_c0_g3_i1_orf1;TRINITY_DN31314_c0_g1_i4_orf1;TRINITY_DN3733_c0_g1_i1_orf1;TRINITY_DN21367_c0_g1_i1_orf1;TRINITY_DN135_c0_g1_i1_orf1;TRINITY_DN147691_c0_g1_i1_orf1;TRINITY_DN30233_c0_g1_i2_orf1;TRINITY_DN10070_c0_g1_i1_orf1;TRINITY_DN6239_c0_g1_i1_orf1;TRINITY_DN96557_c0_g1_i1_orf1;TRINITY_DN110231_c0_g1_i1_orf1;TRINITY_DN31119_c0_g1_i1_orf1;TRINITY_DN87603_c0_g2_i1_orf1;TRINITY_DN8044_c0_g1_i2_orf1;TRINITY_DN9146_c0_g1_i1_orf1;TRINITY_DN97097_c0_g1_i4_orf1;TRINITY_DN21971_c0_g1_i4_orf1;TRINITY_DN6248_c0_g1_i1_orf1;TRINITY_DN31225_c0_g1_i1_orf1;TRINITY_DN147458_c0_g1_i1_orf1;TRINITY_DN12397_c0_g1_i1_orf1;TRINITY_DN17137_c0_g1_i2_orf1;TRINITY_DN5064_c0_g1_i4_orf1;TRINITY_DN101991_c0_g1_i5_orf1;TRINITY_DN235_c0_g1_i2_orf1;TRINITY_DN14313_c0_g1_i1_orf1;TRINITY_DN9765_c0_g1_i6_orf1;TRINITY_DN741_c0_g1_i10_orf1
cellular_component membrane-bounded organelle	GO:0043227	152	152/2360	TRINITY_DN5670_c0_g1_i2_orf1;TRINITY_DN13350_c0_g1_i4_orf1;TRINITY_DN6991_c0_g1_i24_orf1;TRINITY_DN11194_c0_g1_i4_orf1;TRINITY_DN7122_c0_g1_i1_orf1;TRINITY_DN40176_c0_g1_i1_orf1;TRINITY_DN1921_c1_g1_i5_orf1;TRINITY_DN27885_c0_g1_i3_orf1;TRINITY_DN35669_c0_g1_i1_orf1;TRINITY_DN30663_c0_g1_i1_orf1;TRINITY_DN18804_c0_g1_i5_orf1;TRINITY_DN30300_c0_g2_i1_orf1;TRINITY_DN3457_c0_g1_i4_orf1;TRINITY_DN2638_c0_g1_i7_orf1;TRINITY_DN19810_c1_g1_i7_orf1;TRINITY_DN50074_c0_g1_i1_orf1;TRINITY_DN4711_c0_g1_i2_orf1;TRINITY_DN3464_c0_g1_i1_orf1;TRINITY_DN26789_c0_g1_i2_orf1;TRINITY_DN7047_c0_g1_i1_orf1;TRINITY_DN21570_c0_g1_i1_orf1;TRINITY_DN6621_c0_g1_i1_orf1;TRINITY_DN74889_c0_g1_i1_orf1;TRINITY_DN51737_c0_g1_i3_orf1;TRINITY_DN99673_c0_g1_i1_orf1;TRINITY_DN84478_c0_g1_i8_orf1;TRINITY_DN14347_c0_g1_i1_orf1;TRINITY_DN31314_c0_g1_i4_orf1;TRINITY_DN2058_c0_g1_i2_orf1;TRINITY_DN30932_c0_g1_i2_orf1;TRINITY_DN140538_c0_g2_i1_orf1;TRINITY_DN2345_c0_g1_i4_orf1;TRINITY_DN5442_c0_g1_i4_orf1;TRINITY_DN10769_c0_g1_i1_orf1;TRINITY_DN33893_c0_g1_i1_orf1;TRINITY_DN2802_c1_g1_i1_orf1;TRINITY_DN7808_c0_g1_i1_orf1;TRINITY_DN56993_c0_g1_i4_orf1;TRINITY_DN3092_c0_g1_i2_orf1;TRINITY_DN1706_c0_g1_i7_orf1;TRINITY_DN12526_c0_g1_i5_orf1;TRINITY_DN23360_c0_g1_i3_orf1;TRINITY_DN17905_c0_g3_i1_orf1;TRINITY_DN45633_c0_g1_i1_orf1;TRINITY_DN18009_c0_g1_i1_orf1;TRINITY_DN18933_c0_g1_i3_orf1;TRINITY_DN146119_c0_g1_i1_orf1;TRINITY_DN5893_c0_g1_i7_orf1;TRINITY_DN7022_c0_g1_i7_orf1;TRINITY_DN9871_c0_g1_i11_orf1;TRINITY_DN2401_c0_g2_i1_orf1;TRINITY_DN50787_c0_g2_i2_orf1;TRINITY_DN41_c0_g1_i3_orf1;TRINITY_DN45037_c0_g1_i1_orf1;TRINITY_DN12757_c0_g1_i1_orf1;TRINITY_DN3251_c0_g1_i6_orf1;TRINITY_DN40704_c0_g1_i2_orf1;TRINITY_DN141_c0_g1_i1_orf1;TRINITY_DN43431_c0_g1_i1_orf1;TRINITY_DN3073_c0_g1_i7_orf1;TRINITY_DN9591_c0_g1_i1_orf1;TRINITY_DN3430_c0_g1_i1_orf1;TRINITY_DN18922_c0_g1_i1_orf1;TRINITY_DN11772_c0_g1_i1_orf1;TRINITY_DN5664_c0_g1_i1_orf1;TRINITY_DN52893_c0_g1_i1_orf1;TRINITY_DN1718_c6_g1_i4_orf1;TRINITY_DN3335_c0_g1_i1_orf1;TRINITY_DN110460_c0_g2_i1_orf1;TRINITY_DN23020_c0_g1_i1_orf1;TRINITY_DN3702_c0_g1_i1_orf1;TRINITY_DN147458_c0_g1_i1_orf1;TRINITY_DN28989_c0_g1_i7_orf1;TRINITY_DN3860_c0_g1_i5_orf1;TRINITY_DN25997_c1_g2_i4_orf1;TRINITY_DN291_c0_g1_i2_orf1;TRINITY_DN714_c0_g1_i3_orf1;TRINITY_DN5055_c0_g1_i12_orf1;TRINITY_DN22951_c0_g1_i1_orf1;TRINITY_DN26251_c0_g1_i1_orf1;TRINITY_DN6365_c0_g1_i4_orf1;TRINITY_DN17312_c0_g1_i1_orf1;TRINITY_DN3057_c0_g2_i1_orf1;TRINITY_DN72017_c0_g1_i1_orf1;TRINITY_DN5081_c0_g1_i5_orf1;TRINITY_DN72_c0_g1_i16_orf1;TRINITY_DN34830_c0_g1_i1_orf1;TRINITY_DN19135_c0_g1_i1_orf1;TRINITY_DN4016_c0_g1_i1_orf1;TRINITY_DN934_c2_g1_i7_orf1;TRINITY_DN12771_c0_g1_i1_orf1;TRINITY_DN34479_c0_g1_i2_orf1;TRINITY_DN12858_c0_g1_i5_orf1;TRINITY_DN2559_c0_g1_i4_orf1;TRINITY_DN4793_c0_g1_i7_orf1;TRINITY_DN3219_c0_g1_i6_orf1;TRINITY_DN41736_c0_g2_i1_orf1;TRINITY_DN53311_c0_g2_i1_orf1;TRINITY_DN44288_c0_g1_i2_orf1;TRINITY_DN27960_c0_g1_i1_orf1;TRINITY_DN21367_c0_g1_i1_orf1;TRINITY_DN18036_c0_g1_i7_orf1;TRINITY_DN113327_c0_g1_i2_orf1;TRINITY_DN38274_c0_g1_i1_orf1;TRINITY_DN111_c0_g2_i2_orf1;TRINITY_DN65299_c0_g4_i1_orf1;TRINITY_DN3521_c0_g2_i1_orf1;TRINITY_DN62_c1_g1_i3_orf1;TRINITY_DN111110_c0_g1_i1_orf1;TRINITY_DN2802_c0_g1_i1_orf1;TRINITY_DN2647_c0_g1_i3_orf1;TRINITY_DN120144_c0_g1_i1_orf1;TRINITY_DN6248_c0_g1_i1_orf1;TRINITY_DN972_c0_g2_i1_orf1;TRINITY_DN5578_c0_g1_i4_orf1;TRINITY_DN10385_c0_g1_i5_orf1;TRINITY_DN787_c0_g1_i7_orf1;TRINITY_DN9517_c0_g1_i7_orf1;TRINITY_DN30673_c0_g1_i5_orf1;TRINITY_DN2265_c0_g2_i1_orf1;TRINITY_DN2971_c0_g1_i1_orf1;TRINITY_DN1952_c0_g1_i2_orf1;TRINITY_DN67649_c0_g1_i1_orf1;TRINITY_DN38211_c0_g1_i1_orf1;TRINITY_DN12820_c0_g1_i1_orf1;TRINITY_DN13760_c1_g1_i1_orf1;TRINITY_DN15706_c0_g2_i5_orf1;TRINITY_DN9938_c0_g2_i1_orf1;TRINITY_DN104507_c0_g1_i2_orf1;TRINITY_DN13711_c0_g1_i1_orf1;TRINITY_DN2140_c0_g1_i1_orf1;TRINITY_DN5962_c0_g1_i1_orf1;TRINITY_DN41108_c0_g1_i1_orf1;TRINITY_DN44633_c0_g1_i4_orf1;TRINITY_DN279_c0_g1_i10_orf1;TRINITY_DN3614_c0_g2_i1_orf1;TRINITY_DN166_c0_g1_i4_orf1;TRINITY_DN445_c0_g1_i2_orf1;TRINITY_DN31585_c0_g1_i1_orf1;TRINITY_DN25345_c0_g1_i1_orf1;TRINITY_DN26429_c0_g1_i4_orf1;TRINITY_DN34432_c0_g1_i1_orf1;TRINITY_DN4070_c0_g1_i4_orf1;TRINITY_DN9980_c0_g1_i1_orf1;TRINITY_DN10070_c0_g1_i1_orf1;TRINITY_DN15256_c0_g1_i8_orf1;TRINITY_DN96739_c0_g1_i1_orf1;TRINITY_DN147475_c0_g1_i1_orf1;TRINITY_DN51045_c0_g1_i1_orf1;TRINITY_DN19251_c0_g1_i8_orf1;TRINITY_DN24266_c0_g2_i2_orf1;TRINITY_DN37532_c0_g1_i1_orf1
cellular_component postsynaptic specialization	GO:0099572	3	3/2360	TRINITY_DN33418_c0_g1_i1_orf1;TRINITY_DN4016_c0_g1_i1_orf1;TRINITY_DN142442_c0_g1_i1_orf1
cellular_component extracellular organelle	GO:0043230	5	5/2360	TRINITY_DN30300_c0_g2_i1_orf1;TRINITY_DN96739_c0_g1_i1_orf1;TRINITY_DN10070_c0_g1_i1_orf1;TRINITY_DN4016_c0_g1_i1_orf1;TRINITY_DN147475_c0_g1_i1_orf1
cellular_component striated muscle thin filament	GO:0005865	1	1/2360	TRINITY_DN235_c0_g3_i1_orf1
cellular_component neuronal cell body	GO:0043025	1	1/2360	TRINITY_DN10070_c0_g1_i1_orf1

cellular_component	organelle membrane	GO:0031090	51	51/2360	TRINITY_DN245_c0_g1_i4_orf1;TRINITY_DN445_c0_g1_i2_orf1;TRINITY_DN9608_c0_g1_i3_orf1;TRINITY_DN4790_c0_g1_i6_orf1;TRINITY_DN2312_c0_g1_i4_orf1;TRINITY_DN3209_c0_g1_i1_orf1;TRINITY_DN36324_c0_g1_i12_orf1;TRINITY_DN3821_c1_g1_i7_orf1;TRINITY_DN3461_c0_g1_i1_orf1;TRINITY_DN3450_c0_g1_i3_orf1;TRINITY_DN6243_c0_g1_i5_orf1;TRINITY_DN14262_c0_g1_i5_orf1;TRINITY_DN5867_c0_g1_i1_orf1;TRINITY_DN53760_c0_g1_i1_orf1;TRINITY_DN35635_c0_g1_i1_orf1;TRINITY_DN1789_c0_g1_i5_orf1;TRINITY_DN11069_c0_g2_i1_orf1;TRINITY_DN5982_c0_g1_i3_orf1;TRINITY_DN12767_c0_g1_i2_orf1;TRINITY_DN1134_c0_g1_i4_orf1;TRINITY_DN1280_c0_g1_i1_orf1;TRINITY_DN5531_c0_g3_i3_orf1;TRINITY_DN13186_c0_g1_i1_orf1;TRINITY_DN1362_c0_g1_i4_orf1;TRINITY_DN7336_c0_g1_i13_orf1;TRINITY_DN21570_c0_g1_i1_orf1;TRINITY_DN448_c0_g1_i20_orf1;TRINITY_DN154_c0_g1_i4_orf1;TRINITY_DN1447_c0_g1_i5_orf1;TRINITY_DN41108_c0_g1_i1_orf1;TRINITY_DN25210_c0_g1_i1_orf1;TRINITY_DN40197_c0_g1_i1_orf1;TRINITY_DN19303_c0_g1_i5_orf1;TRINITY_DN1960_c5_g1_i3_orf1;TRINITY_DN6680_c0_g1_i1_orf1;TRINITY_DN5439_c0_g1_i2_orf1;TRINITY_DN96739_c0_g1_i1_orf1;TRINITY_DN12286_c1_g1_i2_orf1;TRINITY_DN10745_c0_g1_i14_orf1;TRINITY_DN52887_c0_g1_i1_orf1;TRINITY_DN9931_c0_g1_i1_orf1;TRINITY_DN2879_c0_g1_i4_orf1;TRINITY_DN87603_c0_g2_i1_orf1;TRINITY_DN96557_c0_g1_i1_orf1;TRINITY_DN23042_c0_g1_i1_orf1;TRINITY_DN9592_c0_g1_i2_orf1;TRINITY_DN42120_c0_g1_i2_orf1;TRINITY_DN103118_c0_g1_i4_orf1;TRINITY_DN72816_c0_g1_i2_orf1;TRINITY_DN5064_c0_g1_i4_orf1;TRINITY_DN5697_c0_g1_i1_orf1
cellular_component	outer membrane	GO:0019867	3	3/2360	TRINITY_DN6656_c0_g1_i1_orf1;TRINITY_DN142657_c0_g1_i1_orf1;TRINITY_DN25210_c0_g1_i1_orf1
cellular_component	plasma membrane	GO:0005886	27	27/2360	TRINITY_DN28759_c0_g1_i1_orf1;TRINITY_DN6974_c0_g2_i1_orf1;TRINITY_DN23926_c0_g1_i4_orf1;TRINITY_DN7570_c0_g1_i18_orf1;TRINITY_DN198_c2_g1_i2_orf1;TRINITY_DN7128_c0_g1_i7_orf1;TRINITY_DN75086_c0_g1_i5_orf1;TRINITY_DN15706_c0_g2_i5_orf1;TRINITY_DN19951_c0_g1_i5_orf1;TRINITY_DN3962_c0_g1_i6_orf1;TRINITY_DN2735_c0_g1_i4_orf1;TRINITY_DN5406_c0_g2_i1_orf1;TRINITY_DN1352_c0_g1_i5_orf1;TRINITY_DN12256_c0_g1_i1_orf1;TRINITY_DN5553_c0_g1_i4_orf1;TRINITY_DN9475_c0_g1_i6_orf1;TRINITY_DN7590_c0_g1_i4_orf1;TRINITY_DN1012_c0_g1_i2_orf1;TRINITY_DN20710_c0_g1_i2_orf1;TRINITY_DN26337_c0_g1_i3_orf1;TRINITY_DN10070_c0_g1_i1_orf1;TRINITY_DN38431_c0_g1_i1_orf1;TRINITY_DN14389_c0_g1_i4_orf1;TRINITY_DN11670_c0_g1_i1_orf1;TRINITY_DN57348_c0_g1_i4_orf1;TRINITY_DN2175_c0_g1_i4_orf1;TRINITY_DN5064_c0_g1_i4_orf1
cellular_component	plasma membrane region	GO:0098590	3	3/2360	TRINITY_DN486_c0_g1_i5_orf1;TRINITY_DN10745_c0_g1_i14_orf1;TRINITY_DN7128_c0_g1_i7_orf1
cellular_component	phagophore assembly site membrane	GO:0034045	1	1/2360	TRINITY_DN5531_c0_g3_i3_orf1
cellular_component	dendritic spine	GO:0043197	1	1/2360	TRINITY_DN802_c0_g1_i2_orf1
cellular_component	extracellular matrix	GO:0031012	5	5/2360	TRINITY_DN10070_c0_g1_i1_orf1;TRINITY_DN96739_c0_g1_i1_orf1;TRINITY_DN3833_c0_g1_i4_orf1;TRINITY_DN2919_c0_g1_i5_orf1;TRINITY_DN4464_c0_g2_i1_orf1
cellular_component	egg chorion	GO:0042600	2	2/2360	TRINITY_DN5933_c0_g1_i1_orf1;TRINITY_DN51252_c0_g2_i1_orf1
cellular_component	synapse	GO:0045202	5	5/2360	TRINITY_DN10070_c0_g1_i1_orf1;TRINITY_DN17693_c0_g1_i10_orf1;TRINITY_DN142442_c0_g1_i1_orf1;TRINITY_DN4016_c0_g1_i1_orf1;TRINITY_DN140538_c0_g2_i1_orf1
cellular_component	anchoring junction	GO:0070161	15	15/2360	TRINITY_DN28759_c0_g1_i1_orf1;TRINITY_DN17693_c0_g1_i10_orf1;TRINITY_DN21367_c0_g1_i1_orf1;TRINITY_DN4016_c0_g1_i1_orf1;TRINITY_DN802_c0_g1_i2_orf1;TRINITY_DN30300_c0_g2_i1_orf1;TRINITY_DN9383_c0_g1_i3_orf1;TRINITY_DN7128_c0_g1_i7_orf1;TRINITY_DN2186_c0_g1_i17_orf1;TRINITY_DN96739_c0_g1_i1_orf1;TRINITY_DN33418_c0_g1_i1_orf1;TRINITY_DN142442_c0_g1_i1_orf1;TRINITY_DN364_c1_g1_i2_orf1;TRINITY_DN7590_c0_g1_i4_orf1;TRINITY_DN741_c0_g1_i10_orf1
cellular_component	Golgi apparatus subcompartment	GO:0098791	3	3/2360	TRINITY_DN9243_c0_g1_i4_orf1;TRINITY_DN22213_c0_g1_i3_orf1;TRINITY_DN4401_c0_g2_i1_orf1
cellular_component	plasma membrane bounded cell projection	GO:0120025	6	6/2360	TRINITY_DN4016_c0_g1_i1_orf1;TRINITY_DN5954_c0_g1_i2_orf1;TRINITY_DN9383_c0_g1_i3_orf1;TRINITY_DN802_c0_g1_i2_orf1;TRINITY_DN741_c0_g1_i10_orf1;TRINITY_DN364_c1_g1_i2_orf1
cellular_component	Cajal body	GO:0015030	1	1/2360	TRINITY_DN8044_c0_g1_i2_orf1
cellular_component	nuclear speck	GO:0016607	2	2/2360	TRINITY_DN140538_c0_g2_i1_orf1;TRINITY_DN47123_c0_g1_i1_orf1
cellular_component	cytosolic region	GO:0099522	1	1/2360	TRINITY_DN140538_c0_g2_i1_orf1
cellular_component	ciliary basal body	GO:0036064	1	1/2360	TRINITY_DN140538_c0_g2_i1_orf1
cellular_component	centrosome	GO:0005813	1	1/2360	TRINITY_DN4689_c0_g1_i5_orf1
cellular_component	kinetochore	GO:0000776	2	2/2360	TRINITY_DN96557_c0_g1_i1_orf1;TRINITY_DN31314_c0_g1_i4_orf1
cellular_component	ribonucleoprotein granule	GO:0035770	3	3/2360	TRINITY_DN1298_c0_g1_i3_orf1;TRINITY_DN4016_c0_g1_i1_orf1;TRINITY_DN12576_c0_g1_i2_orf1
cellular_component	supramolecular polymer	GO:0099081	18	18/2360	TRINITY_DN96557_c0_g1_i1_orf1;TRINITY_DN280_c0_g1_i8_orf1;TRINITY_DN2942_c0_g1_i6_orf1;TRINITY_DN4808_c0_g1_i3_orf1;TRINITY_DN14298_c0_g1_i1_orf1;TRINITY_DN63561_c1_g1_i2_orf1;TRINITY_DN8390_c0_g1_i2_orf1;TRINITY_DN122423_c0_g1_i1_orf1;TRINITY_DN28018_c0_g6_i1_orf1;TRINITY_DN34703_c0_g1_i4_orf1;TRINITY_DN5508_c0_g1_i1_orf1;TRINITY_DN46202_c0_g1_i1_orf1;TRINITY_DN2745_c0_g1_i2_orf1;TRINITY_DN97138_c0_g1_i2_orf1;TRINITY_DN14298_c0_g3_i1_orf1;TRINITY_DN235_c0_g3_i1_orf1;TRINITY_DN5893_c0_g1_i7_orf1;TRINITY_DN350_c0_g1_i5_orf1
molecular_function	translation factor activity, RNA binding	GO:0008135	23	23/2360	TRINITY_DN2265_c0_g2_i1_orf1;TRINITY_DN31503_c0_g1_i4_orf1;TRINITY_DN9575_c0_g1_i1_orf1;TRINITY_DN1074_c0_g1_i7_orf1;TRINITY_DN3366_c0_g1_i6_orf1;TRINITY_DN2716_c0_g2_i1_orf1;TRINITY_DN48097_c0_g1_i1_orf1;TRINITY_DN4237_c1_g1_i5_orf1;TRINITY_DN359_c0_g1_i5_orf1;TRINITY_DN11823_c1_g1_i2_orf1;TRINITY_DN44407_c0_g4_i2_orf1;TRINITY_DN22572_c0_g1_i1_orf1;TRINITY_DN36817_c0_g1_i1_orf1;TRINITY_DN50085_c0_g1_i1_orf1;TRINITY_DN9164_c0_g1_i3_orf1;TRINITY_DN53311_c0_g2_i1_orf1;TRINITY_DN15420_c0_g3_i2_orf1;TRINITY_DN9498_c0_g1_i3_orf1;TRINITY_DN11612_c0_g2_i1_orf1;TRINITY_DN24317_c0_g1_i7_orf1;TRINITY_DN21609_c0_g2_i1_orf1;TRINITY_DN10871_c0_g1_i3_orf1;TRINITY_DN6239_c0_g1_i1_orf1
molecular_function	transcription corepressor activity	GO:0003714	2	2/2360	TRINITY_DN34726_c0_g2_i1_orf1;TRINITY_DN1921_c1_g1_i5_orf1
molecular_function	transcription coactivator activity	GO:0003713	1	1/2360	TRINITY_DN77572_c0_g1_i1_orf1
molecular_function	RNA helicase activity	GO:0003724	14	14/2360	TRINITY_DN2904_c0_g1_i4_orf1;TRINITY_DN11596_c0_g1_i1_orf1;TRINITY_DN20499_c0_g3_i1_orf1;TRINITY_DN4380_c0_g1_i9_orf1;TRINITY_DN4950_c0_g1_i2_orf1;TRINITY_DN31503_c0_g1_i4_orf1;TRINITY_DN4408_c6_g1_i1_orf1;TRINITY_DN5675_c0_g1_i6_orf1;TRINITY_DN26168_c0_g1_i1_orf1;TRINITY_DN9302_c0_g1_i1_orf1;TRINITY_DN59291_c0_g1_i1_orf1;TRINITY_DN7213_c0_g1_i2_orf1;TRINITY_DN2709_c0_g1_i4_orf1;TRINITY_DN44288_c0_g1_i2_orf1
molecular_function	minus-end-directed microtubule motor activity	GO:0008569	2	2/2360	TRINITY_DN122423_c0_g5_i1_orf1;TRINITY_DN122423_c0_g1_i1_orf1
molecular_function	DNA helicase activity	GO:0003678	8	8/2360	TRINITY_DN2971_c0_g1_i1_orf1;TRINITY_DN25345_c0_g1_i1_orf1;TRINITY_DN6642_c0_g1_i2_orf1;TRINITY_DN7122_c0_g1_i1_orf1;TRINITY_DN452_c1_g1_i3_orf1;TRINITY_DN3057_c0_g2_i1_orf1;TRINITY_DN5757_c0_g1_i1_orf1;TRINITY_DN291_c0_g1_i2_orf1
molecular_function	ATP-dependent chromatin remodeler activity	GO:0140658	4	4/2360	TRINITY_DN3057_c0_g2_i1_orf1;TRINITY_DN45449_c0_g1_i1_orf1;TRINITY_DN25345_c0_g1_i1_orf1;TRINITY_DN12820_c0_g1_i1_orf1
molecular_function	DNA topoisomerase type II (double strand cut, A	GO:0003918	1	1/2360	TRINITY_DN4908_c1_g1_i5_orf1
molecular_function	DNA clamp loader activity	GO:0003689	1	1/2360	TRINITY_DN3092_c0_g1_i2_orf1
molecular_function	long-chain fatty acid-CoA ligase activity	GO:0004467	1	1/2360	TRINITY_DN2193_c0_g1_i7_orf1
molecular_function	ABC-type transporter activity	GO:0140359	5	5/2360	TRINITY_DN3637_c0_g1_i2_orf1;TRINITY_DN60792_c0_g1_i2_orf1;TRINITY_DN31327_c0_g2_i1_orf1;TRINITY_DN162_c0_g1_i4_orf1;TRINITY_DN2874_c0_g1_i4_orf1
molecular_function	P-type transmembrane transporter activity	GO:0140358	4	4/2360	TRINITY_DN7336_c0_g1_i13_orf1;TRINITY_DN7570_c0_g1_i18_orf1;TRINITY_DN4977_c0_g1_i2_orf1;TRINITY_DN9243_c0_g1_i4_orf1
molecular_function	ATPase-coupled cation transmembrane transpo	GO:0019829	6	6/2360	TRINITY_DN7336_c0_g1_i13_orf1;TRINITY_DN25975_c0_g3_i2_orf1;TRINITY_DN7570_c0_g1_i18_orf1;TRINITY_DN4977_c0_g1_i2_orf1;TRINITY_DN9243_c0_g1_i4_orf1;TRINITY_DN10458_c0_g1_i1_orf1
molecular_function	ATPase-coupled ion transmembrane transporter	GO:0042625	2	2/2360	TRINITY_DN10458_c0_g1_i1_orf1;TRINITY_DN25975_c0_g3_i2_orf1
molecular_function	cytoskeletal anchor activity	GO:0008093	2	2/2360	TRINITY_DN21559_c0_g2_i1_orf1;TRINITY_DN21559_c0_g1_i2_orf1
molecular_function	cargo adaptor activity	GO:0140312	1	1/2360	TRINITY_DN5118_c0_g1_i1_orf1
molecular_function	SNAP receptor activity	GO:0005484	1	1/2360	TRINITY_DN132857_c0_g1_i1_orf1
molecular_function	very-low-density lipoprotein particle receptor ac	GO:0030229	2	2/2360	TRINITY_DN585_c0_g1_i5_orf1;TRINITY_DN585_c0_g1_i12_orf1
molecular_function	copper chaperone activity	GO:0016531	1	1/2360	TRINITY_DN3461_c0_g1_i1_orf1
molecular_function	lactoperoxidase activity	GO:0140825	1	1/2360	TRINITY_DN3321_c0_g1_i3_orf1
molecular_function	peroxiredoxin activity	GO:0051920	3	3/2360	TRINITY_DN791_c0_g1_i2_orf1;TRINITY_DN7778_c0_g1_i1_orf1;TRINITY_DN69236_c0_g1_i1_orf1
molecular_function	catalase activity	GO:0004096	1	1/2360	TRINITY_DN285_c0_g1_i4_orf1
molecular_function	glutathione peroxidase activity	GO:0004602	2	2/2360	TRINITY_DN80660_c0_g1_i1_orf1;TRINITY_DN21420_c0_g1_i2_orf1
molecular_function	phospholipid transporter activity	GO:0005548	1	1/2360	TRINITY_DN2160_c0_g1_i13_orf1
molecular_function	intramembrane lipid transporter activity	GO:0140303	1	1/2360	TRINITY_DN252_c0_g1_i3_orf1
molecular_function	sulfur compound transmembrane transporter ac	GO:1901682	1	1/2360	TRINITY_DN268_c1_g1_i7_orf1
molecular_function	carbohydrate transmembrane transporter activit	GO:0015144	2	2/2360	TRINITY_DN2735_c0_g1_i4_orf1;TRINITY_DN57348_c0_g1_i4_orf1
molecular_function	passive transmembrane transporter activity	GO:0022803	7	7/2360	TRINITY_DN11566_c0_g1_i6_orf1;TRINITY_DN6974_c0_g2_i1_orf1;TRINITY_DN18338_c0_g1_i6_orf1;TRINITY_DN34821_c0_g1_i4_orf1;TRINITY_DN96739_c0_g1_i1_orf1;TRINITY_DN96080_c0_g2_i1_orf1;TRINITY_DN7787_c0_g1_i1_orf1
molecular_function	active transmembrane transporter activity	GO:0022804	15	15/2360	TRINITY_DN7336_c0_g1_i13_orf1;TRINITY_DN25975_c0_g3_i2_orf1;TRINITY_DN268_c1_g1_i7_orf1;TRINITY_DN31327_c0_g2_i1_orf1;TRINITY_DN7570_c0_g1_i18_orf1;TRINITY_DN2874_c0_g1_i4_orf1;TRINITY_DN4977_c0_g1_i2_orf1;TRINITY_DN12666_c0_g1_i2_orf1;TRINITY_DN26186_c0_g1_i7_orf1;TRINITY_DN9243_c0_g1_i4_orf1;TRINITY_DN29_c0_g1_i4_orf1;TRINITY_DN60792_c0_g1_i2_orf1;TRINITY_DN162_c0_g1_i4_orf1;TRINITY_DN3637_c0_g1_i2_orf1;TRINITY_DN10458_c0_g1_i1_orf1
molecular_function	ion transmembrane transporter activity	GO:0015075	19	19/2360	TRINITY_DN7336_c0_g1_i13_orf1;TRINITY_DN15222_c0_g1_i4_orf1;TRINITY_DN6974_c0_g2_i1_orf1;TRINITY_DN268_c1_g1_i7_orf1;TRINITY_DN1882_c0_g1_i4_orf1;TRINITY_DN9354_c0_g1_i7_orf1;TRINITY_DN25975_c0_g3_i2_orf1;TRINITY_DN7570_c0_g1_i18_orf1;TRINITY_DN4977_c0_g1_i2_orf1;TRINITY_DN9243_c0_g1_i4_orf1;TRINITY_DN96080_c0_g2_i1_orf1;TRINITY_DN12286_c1_g1_i2_orf1;TRINITY_DN107261_c0_g1_i1_orf1;TRINITY_DN26429_c0_g1_i4_orf1;TRINITY_DN10458_c0_g1_i1_orf1;TRINITY_DN7787_c0_g1_i1_orf1;TRINITY_DN91946_c0_g1_i1_orf1;TRINITY_DN96739_c0_g1_i1_orf1;TRINITY_DN34821_c0_g1_i4_orf1

molecular_function	inorganic molecular entity transmembrane trans	GO:0015318	17	17/2360	TRINITY_DN7336_c0_g1_i13_orf1;TRINITY_DN15222_c0_g1_i4_orf1;TRINITY_DN6974_c0_g2_i1_orf1;TRINITY_DN268_c1_g1_i7_orf1;TRINITY_DN1882_c0_g1_i4_orf1;TRINITY_DN9354_c0_g1_i7_orf1;TRINITY_DN25975_c0_g3_i2_orf1;TRINITY_DN7570_c0_g1_i18_orf1;TRINITY_DN4977_c0_g1_i2_orf1;TRINITY_DN9243_c0_g1_i4_orf1;TRINITY_DN96080_c0_g2_i1_orf1;TRINITY_DN107261_c0_g1_i1_orf1;TRINITY_DN10458_c0_g1_i1_orf1;TRINITY_DN7787_c0_g1_i1_orf1;TRINITY_DN91946_c0_g1_i1_orf1;TRINITY_DN96739_c0_g1_i1_orf1;TRINITY_DN34821_c0_g1_i4_orf1
molecular_function	channel inhibitor activity	GO:0016248	2	2/2360	TRINITY_DN6098_c1_g1_i5_orf1;TRINITY_DN4748_c0_g1_i5_orf1
molecular_function	ATPase inhibitor activity	GO:0042030	1	1/2360	TRINITY_DN5442_c0_g1_i4_orf1
molecular_function	ion channel regulator activity	GO:0099106	3	3/2360	TRINITY_DN6098_c1_g1_i5_orf1;TRINITY_DN4748_c0_g1_i5_orf1;TRINITY_DN10994_c0_g1_i4_orf1
molecular_function	ubiquitin-protein transferase regulator activity	GO:0055106	1	1/2360	TRINITY_DN21367_c0_g1_i1_orf1
molecular_function	kinase regulator activity	GO:0019207	2	2/2360	TRINITY_DN346_c0_g1_i7_orf1;TRINITY_DN147475_c0_g1_i1_orf1
molecular_function	nucleoside-triphosphatase regulator activity	GO:0060589	17	17/2360	TRINITY_DN21609_c0_g2_i1_orf1;TRINITY_DN2596_c0_g1_i2_orf1;TRINITY_DN138086_c0_g1_i1_orf1;TRINITY_DN42461_c0_g1_i4_orf1;TRINITY_DN1173_c0_g1_i11_orf1;TRINITY_DN518_c0_g1_i1_orf1;TRINITY_DN493_c0_g1_i4_orf1;TRINITY_DN69170_c0_g2_i1_orf1;TRINITY_DN9248_c0_g1_i10_orf1;TRINITY_DN802_c0_g1_i2_orf1;TRINITY_DN2623_c0_g1_i3_orf1;TRINITY_DN12320_c0_g1_i1_orf1;TRINITY_DN5182_c0_g1_i5_orf1;TRINITY_DN2596_c0_g1_i6_orf1;TRINITY_DN804_c0_g1_i7_orf1;TRINITY_DN15753_c0_g1_i1_orf1;TRINITY_DN1173_c1_g1_i10_orf1
molecular_function	peptidase regulator activity	GO:0061134	30	30/2360	TRINITY_DN122321_c0_g1_i1_orf1;TRINITY_DN4314_c0_g1_i9_orf1;TRINITY_DN7341_c0_g1_i8_orf1;TRINITY_DN9872_c0_g1_i2_orf1;TRINITY_DN1986_c0_g1_i1_orf1;TRINITY_DN4235_c0_g1_i2_orf1;TRINITY_DN501_c0_g1_i5_orf1;TRINITY_DN9732_c0_g1_i7_orf1;TRINITY_DN16234_c0_g2_i3_orf1;TRINITY_DN69697_c0_g1_i1_orf1;TRINITY_DN1328_c0_g1_i6_orf1;TRINITY_DN10994_c0_g1_i4_orf1;TRINITY_DN8258_c0_g1_i6_orf1;TRINITY_DN77425_c0_g1_i2_orf1;TRINITY_DN3609_c0_g1_i6_orf1;TRINITY_DN45948_c1_g1_i1_orf1;TRINITY_DN8780_c0_g1_i3_orf1;TRINITY_DN2097_c1_g2_i2_orf1;TRINITY_DN2323_c0_g1_i4_orf1;TRINITY_DN590_c0_g1_i4_orf1;TRINITY_DN7776_c0_g1_i5_orf1;TRINITY_DN1444_c1_g1_i5_orf1;TRINITY_DN1215_c0_g1_i2_orf1;TRINITY_DN7539_c0_g1_i2_orf1;TRINITY_DN10057_c0_g2_i1_orf1;TRINITY_DN1540_c0_g1_i9_orf1;TRINITY_DN135188_c0_g1_i2_orf1;TRINITY_DN9455_c0_g1_i6_orf1;TRINITY_DN1540_c0_g1_i14_orf1;TRINITY_DN712_c0_g2_i1_orf1
molecular_function	enzyme activator activity	GO:0008047	11	11/2360	TRINITY_DN346_c0_g1_i7_orf1;TRINITY_DN67649_c0_g1_i1_orf1;TRINITY_DN138086_c0_g1_i1_orf1;TRINITY_DN518_c0_g1_i1_orf1;TRINITY_DN7341_c0_g1_i8_orf1;TRINITY_DN69170_c0_g2_i1_orf1;TRINITY_DN9248_c0_g1_i10_orf1;TRINITY_DN802_c0_g1_i2_orf1;TRINITY_DN493_c0_g1_i4_orf1;TRINITY_DN5182_c0_g1_i5_orf1;TRINITY_DN46022_c0_g1_i1_orf1
molecular_function	enzyme inhibitor activity	GO:0004857	30	30/2360	TRINITY_DN122321_c0_g1_i1_orf1;TRINITY_DN4314_c0_g1_i9_orf1;TRINITY_DN9872_c0_g1_i2_orf1;TRINITY_DN1986_c0_g1_i1_orf1;TRINITY_DN4235_c0_g1_i2_orf1;TRINITY_DN501_c0_g1_i5_orf1;TRINITY_DN9732_c0_g1_i7_orf1;TRINITY_DN16234_c0_g2_i3_orf1;TRINITY_DN69697_c0_g1_i1_orf1;TRINITY_DN1328_c0_g1_i6_orf1;TRINITY_DN10994_c0_g1_i4_orf1;TRINITY_DN8258_c0_g1_i6_orf1;TRINITY_DN77425_c0_g1_i2_orf1;TRINITY_DN3609_c0_g1_i6_orf1;TRINITY_DN45948_c1_g1_i1_orf1;TRINITY_DN8780_c0_g1_i3_orf1;TRINITY_DN2097_c1_g2_i2_orf1;TRINITY_DN21367_c0_g1_i1_orf1;TRINITY_DN2323_c0_g1_i4_orf1;TRINITY_DN590_c0_g1_i4_orf1;TRINITY_DN7776_c0_g1_i5_orf1;TRINITY_DN1444_c1_g1_i5_orf1;TRINITY_DN1215_c0_g1_i2_orf1;TRINITY_DN7539_c0_g1_i2_orf1;TRINITY_DN10057_c0_g2_i1_orf1;TRINITY_DN1540_c0_g1_i9_orf1;TRINITY_DN135188_c0_g1_i2_orf1;TRINITY_DN9455_c0_g1_i6_orf1;TRINITY_DN1540_c0_g1_i14_orf1;TRINITY_DN712_c0_g2_i1_orf1
molecular_function	signaling receptor activator activity	GO:0030546	3	3/2360	TRINITY_DN22443_c0_g2_i3_orf1;TRINITY_DN18650_c0_g1_i1_orf1;TRINITY_DN2227_c0_g1_i5_orf1
molecular_function	nucleic acid binding	GO:0003676	171	171/2360	TRINITY_DN129226_c0_g1_i2_orf1;TRINITY_DN6380_c0_g1_i1_orf1;TRINITY_DN1856_c0_g1_i3_orf1;TRINITY_DN4380_c0_g1_i9_orf1;TRINITY_DN5670_c0_g1_i2_orf1;TRINITY_DN12227_c0_g2_i3_orf1;TRINITY_DN13350_c0_g1_i4_orf1;TRINITY_DN4262_c0_g1_i16_orf1;TRINITY_DN7122_c0_g1_i1_orf1;TRINITY_DN7213_c0_g1_i2_orf1;TRINITY_DN35669_c0_g1_i1_orf1;TRINITY_DN19651_c0_g1_i1_orf1;TRINITY_DN44407_c0_g4_i2_orf1;TRINITY_DN18804_c0_g1_i5_orf1;TRINITY_DN16965_c0_g2_i1_orf1;TRINITY_DN15040_c0_g4_i1_orf1;TRINITY_DN34134_c0_g2_i1_orf1;TRINITY_DN1344_c0_g1_i1_orf1;TRINITY_DN2904_c0_g1_i4_orf1;TRINITY_DN70485_c0_g1_i2_orf1;TRINITY_DN142442_c0_g1_i1_orf1;TRINITY_DN3457_c0_g1_i4_orf1;TRINITY_DN18681_c0_g1_i7_orf1;TRINITY_DN3521_c0_g2_i1_orf1;TRINITY_DN36817_c0_g1_i1_orf1;TRINITY_DN1639_c0_g2_i2_orf1;TRINITY_DN1710_c0_g2_i2_orf1;TRINITY_DN21570_c0_g1_i1_orf1;TRINITY_DN11596_c0_g1_i1_orf1;TRINITY_DN95056_c0_g2_i2_orf1;TRINITY_DN19746_c0_g1_i5_orf1;TRINITY_DN99673_c0_g1_i1_orf1;TRINITY_DN9575_c0_g1_i1_orf1;TRINITY_DN5296_c0_g2_i1_orf1;TRINITY_DN2769_c0_g1_i1_orf1;TRINITY_DN24317_c0_g1_i7_orf1;TRINITY_DN35582_c0_g1_i1_orf1;TRINITY_DN5442_c0_g1_i4_orf1;TRINITY_DN2401_c0_g2_i1_orf1;TRINITY_DN20499_c0_g3_i1_orf1;TRINITY_DN4813_c0_g1_i5_orf1;TRINITY_DN33893_c0_g1_i1_orf1;TRINITY_DN2802_c1_g1_i1_orf1;TRINITY_DN33346_c0_g1_i1_orf1;TRINITY_DN1245_c0_g1_i4_orf1;TRINITY_DN18563_c2_g1_i1_orf1;TRINITY_DN10558_c0_g1_i4_orf1;TRINITY_DN56993_c0_g1_i4_orf1;TRINITY_DN14313_c0_g1_i1_orf1;TRINITY_DN2647_c0_g1_i3_orf1;TRINITY_DN3092_c0_g1_i2_orf1;TRINITY_DN1978_c0_g1_i4_orf1;TRINITY_DN17271_c0_g1_i1_orf1;TRINITY_DN20442_c0_g2_i1_orf1;TRINITY_DN23360_c0_g1_i3_orf1;TRINITY_DN31503_c0_g1_i4_orf1;TRINITY_DN47723_c0_g1_i1_orf1;TRINITY_DN1074_c0_g1_i7_orf1;TRINITY_DN18242_c0_g1_i3_orf1;TRINITY_DN37532_c0_g1_i1_orf1;TRINITY_DN12242_c0_g1_i5_orf1;TRINITY_DN29743_c0_g1_i9_orf1;TRINITY_DN26947_c0_g1_i1_orf1;TRINITY_DN81258_c0_g1_i2_orf1;TRINITY_DN298_c0_g1_i4_orf1;TRINITY_DN4237_c1_g1_i5_orf1;TRINITY_DN51968_c0_g1_i1_orf1;TRINITY_DN359_c0_g1_i5_orf1;TRINITY_DN7583_c0_g1_i1_orf1;TRINITY_DN1607_c0_g1_i16_orf1;TRINITY_DN50787_c0_g2_i2_orf1;TRINITY_DN41664_c0_g1_i4_orf1;TRINITY_DN16978_c0_g1_i1_orf1;TRINITY_DN48641_c0_g1_i4_orf1;TRINITY_DN5675_c0_g1_i6_orf1;TRINITY_DN50085_c0_g1_i1_orf1;TRINITY_DN124950_c0_g2_i1_orf1;TRINITY_DN3733_c0_g1_i1_orf1;TRINITY_DN4408_c6_g1_i1_orf1;TRINITY_DN51568_c0_g1_i1_orf1;TRINITY_DN104597_c0_g1_i2_orf1;TRINITY_DN4707_c0_g1_i1_orf1;TRINITY_DN3366_c0_g1_i6_orf1;TRINITY_DN40945_c0_g1_i1_orf1;TRINITY_DN23502_c0_g1_i1_orf1;TRINITY_DN73224_c0_g4_i2_orf1;TRINITY_DN37055_c0_g1_i1_orf1;TRINITY_DN4950_c0_g1_i2_orf1;TRINITY_DN107035_c0_g1_i1_orf1;TRINITY_DN147458_c0_g1_i1_orf1;TRINITY_DN4908_c1_g1_i5_orf1;TRINITY_DN2709_c0_g1_i4_orf1;TRINITY_DN291_c0_g1_i2_orf1;TRINITY_DN2749_c4_g1_i2_orf1;TRINITY_DN6239_c0_g1_i1_orf1;TRINITY_DN257_c0_g1_i7_orf1;TRINITY_DN8008_c0_g1_i6_orf1;TRINITY_DN2749_c0_g1_i4_orf1;TRINITY_DN22951_c0_g1_i1_orf1;TRINITY_DN26251_c0_g1_i1_orf1;TRINITY_DN2117_c0_g1_i1_orf1;TRINITY_DN17312_c0_g1_i1_orf1;TRINITY_DN2718_c0_g1_i6_orf1;TRINITY_DN3057_c0_g2_i1_orf1;TRINITY_DN43076_c0_g1_i6_orf1;TRINITY_DN11823_c1_g1_i2_orf1;TRINITY_DN3335_c0_g1_i1_orf1;TRINITY_DN4016_c0_g1_i1_orf1;TRINITY_DN43412_c0_g1_i2_orf1;TRINITY_DN12858_c0_g1_i5_orf1;TRINITY_DN142652_c0_g1_i1_orf1;TRINITY_DN10070_c0_g1_i1_orf1;TRINITY_DN1066_c0_g1_i8_orf1;TRINITY_DN37599_c0_g1_i1_orf1;TRINITY_DN7251_c0_g1_i3_orf1;TRINITY_DN24322_c0_g1_i4_orf1;TRINITY_DN84322_c0_g2_i1_orf1;TRINITY_DN53311_c0_g2_i1_orf1;TRINITY_DN15420_c0_g3_i2_orf1;TRINITY_DN48460_c0_g1_i1_orf1;TRINITY_DN44288_c0_g1_i2_orf1;TRINITY_DN21367_c0_g1_i1_orf1;TRINITY_DN111_c0_g2_i2_orf1;TRINITY_DN19810_c1_g1_i7_orf1;TRINITY_DN15160_c0_g1_i1_orf1;TRINITY_DN87603_c0_g2_i1_orf1;TRINITY_DN2802_c0_g1_i1_orf1;TRINITY_DN5962_c0_g1_i1_orf1;TRINITY_DN36496_c0_g1_i1_orf1;TRINITY_DN6248_c0_g1_i1_orf1;TRINITY_DN972_c0_g2_i1_orf1;TRINITY_DN26168_c0_g1_i1_orf1;TRINITY_DN139537_c0_g1_i1_orf1;TRINITY_DN2265_c0_g2_i1_orf1;TRINITY_DN67649_c0_g1_i1_orf1;TRINITY_DN29579_c0_g1_i1_orf1;TRINITY_DN9637_c0_g1_i14_orf1;TRINITY_DN42205_c0_g1_i4_orf1;TRINITY_DN2716_c0_g2_i1_orf1;TRINITY_DN288_c0_g1_i9_orf1;TRINITY_DN9938_c0_g2_i1_orf1;TRINITY_DN59291_c0_g1_i1_orf1;TRINITY_DN9302_c0_g1_i1_orf1;TRINITY_DN48097_c0_g1_i1_orf1;TRINITY_DN4056_c0_g1_i8_orf1;TRINITY_DN38540_c0_g1_i1_orf1;TRINITY_DN21150_c0_g1_i4_orf1;TRINITY_DN27_c0_g1_i1_orf1;TRINITY_DN104507_c0_g1_i2_orf1;TRINITY_DN22572_c0_g1_i1_orf1;TRINITY_DN1298_c0_g1_i3_orf1;TRINITY_DN34676_c1_g1_i3_orf1;TRINITY_DN3127_c0_g1_i9_orf1;TRINITY_DN9164_c0_g1_i3_orf1;TRINITY_DN2367_c1_g1_i20_orf1;TRINITY_DN7112_c0_g1_i1_orf1;TRINITY_DN5238_c0_g1_i2_orf1;TRINITY_DN34432_c0_g1_i1_orf1;TRINITY_DN9498_c0_g1_i3_orf1;TRINITY_DN5653_c0_g1_i4_orf1;TRINITY_DN11612_c0_g2_i1_orf1;TRINITY_DN110534_c0_g1_i3_orf1;TRINITY_DN5757_c0_g1_i1_orf1;TRINITY_DN2879_c0_g1_i4_orf1;TRINITY_DN430_c0_g1_i5_orf1;TRINITY_DN21539_c0_g1_i1_orf1;TRINITY_DN21609_c0_g2_i1_orf1;TRINITY_DN51045_c0_g1_i1_orf1;TRINITY_DN63662_c0_g4_i1_orf1;TRINITY_DN10871_c0_g1_i3_orf1;TRINITY_DN20339_c0_g1_i3_orf1

molecular_function	nucleoside phosphate binding	GO:1901265	203	203/2360	TRINITY_DN38482_c0_g1_i4_orf1;TRINITY_DN280_c0_g1_i8_orf1;TRINITY_DN38230_c0_g1_i4_orf1;TRINITY_DN47731_c0_g1_i2_orf1;TRINITY_DN4380_c0_g1_i9_orf1;TRINITY_DN41311_c0_g2_i3_orf1;TRINITY_DN27771_c0_g1_i1_orf1;TRINITY_DN11194_c0_g1_i4_orf1;TRINITY_DN15959_c0_g1_i1_orf1;TRINITY_DN7122_c0_g1_i1_orf1;TRINITY_DN7213_c0_g1_i2_orf1;TRINITY_DN30932_c0_g1_i2_orf1;TRINITY_DN31967_c0_g1_i5_orf1;TRINITY_DN25341_c0_g1_i1_orf1;TRINITY_DN2738_c1_g1_i3_orf1;TRINITY_DN5262_c0_g1_i7_orf1;TRINITY_DN33146_c0_g1_i1_orf1;TRINITY_DN2904_c0_g1_i4_orf1;TRINITY_DN70485_c0_g1_i2_orf1;TRINITY_DN4798_c0_g1_i3_orf1;TRINITY_DN24723_c2_g1_i1_orf1;TRINITY_DN2953_c1_g1_i10_orf1;TRINITY_DN7388_c0_g1_i7_orf1;TRINITY_DN2638_c0_g1_i7_orf1;TRINITY_DN4320_c0_g1_i1_orf1;TRINITY_DN4711_c0_g1_i2_orf1;TRINITY_DN26789_c0_g1_i2_orf1;TRINITY_DN10774_c0_g2_i3_orf1;TRINITY_DN6587_c0_g1_i3_orf1;TRINITY_DN11596_c0_g1_i1_orf1;TRINITY_DN2953_c1_g1_i2_orf1;TRINITY_DN14298_c0_g1_i1_orf1;TRINITY_DN3991_c0_g1_i6_orf1;TRINITY_DN29873_c0_g1_i1_orf1;TRINITY_DN3800_c0_g1_i7_orf1;TRINITY_DN5354_c0_g1_i4_orf1;TRINITY_DN235_c0_g3_i1_orf1;TRINITY_DN6185_c0_g1_i12_orf1;TRINITY_DN6436_c0_g1_i1_orf1;TRINITY_DN37165_c0_g1_i4_orf1;TRINITY_DN26195_c0_g1_i6_orf1;TRINITY_DN2224_c0_g1_i1_orf1;TRINITY_DN20499_c0_g3_i1_orf1;TRINITY_DN987_c0_g1_i3_orf1;TRINITY_DN15591_c0_g1_i3_orf1;TRINITY_DN7247_c0_g1_i7_orf1;TRINITY_DN4501_c0_g1_i3_orf1;TRINITY_DN3637_c0_g1_i2_orf1;TRINITY_DN107288_c0_g1_i2_orf1;TRINITY_DN46715_c0_g1_i1_orf1;TRINITY_DN452_c1_g1_i3_orf1;TRINITY_DN20007_c0_g1_i1_orf1;TRINITY_DN4950_c0_g1_i2_orf1;TRINITY_DN63561_c1_g1_i2_orf1;TRINITY_DN3092_c0_g1_i2_orf1;TRINITY_DN39813_c0_g1_i1_orf1;TRINITY_DN2772_c0_g1_i3_orf1;TRINITY_DN115210_c0_g4_i1_orf1;TRINITY_DN31503_c0_g1_i4_orf1;TRINITY_DN4977_c0_g1_i2_orf1;TRINITY_DN252_c0_g1_i3_orf1;TRINITY_DN1921_c1_g1_i5_orf1;TRINITY_DN8659_c0_g2_i1_orf1;TRINITY_DN17935_c0_g1_i1_orf1;TRINITY_DN4451_c0_g2_i4_orf1;TRINITY_DN1604_c0_g1_i4_orf1;TRINITY_DN1034_c0_g1_i4_orf1;TRINITY_DN90497_c0_g1_i1_orf1;TRINITY_DN42461_c0_g1_i4_orf1;TRINITY_DN11125_c0_g1_i1_orf1;TRINITY_DN9575_c0_g1_i1_orf1;TRINITY_DN6813_c1_g1_i1_orf1;TRINITY_DN1578_c0_g3_i1_orf1;TRINITY_DN359_c0_g1_i5_orf1;TRINITY_DN1607_c0_g1_i16_orf1;TRINITY_DN277_c1_g1_i1_orf1;TRINITY_DN1725_c0_g1_i7_orf1;TRINITY_DN19034_c0_g1_i1_orf1;TRINITY_DN4794_c1_g1_i9_orf1;TRINITY_DN5675_c0_g1_i6_orf1;TRINITY_DN32700_c0_g1_i2_orf1;TRINITY_DN117844_c0_g1_i1_orf1;TRINITY_DN122423_c0_g1_i1_orf1;TRINITY_DN31225_c0_g1_i1_orf1;TRINITY_DN6642_c0_g1_i2_orf1;TRINITY_DN9243_c0_g1_i4_orf1;TRINITY_DN5508_c0_g1_i1_orf1;TRINITY_DN122423_c0_g5_i1_orf1;TRINITY_DN511_c0_g2_i1_orf1;TRINITY_DN1718_c6_g1_i4_orf1;TRINITY_DN1173_c1_g1_i10_orf1;TRINITY_DN2146_c0_g2_i1_orf1;TRINITY_DN7336_c0_g1_i13_orf1;TRINITY_DN15420_c0_g3_i2_orf1;TRINITY_DN4908_c1_g1_i5_orf1;TRINITY_DN28989_c0_g1_i7_orf1;TRINITY_DN25997_c1_g2_i4_orf1;TRINITY_DN47151_c0_g1_i1_orf1;TRINITY_DN235_c0_g1_i2_orf1;TRINITY_DN2709_c0_g1_i4_orf1;TRINITY_DN291_c0_g1_i2_orf1;TRINITY_DN1209_c0_g1_i9_orf1;TRINITY_DN16011_c0_g1_i3_orf1;TRINITY_DN8659_c0_g1_i1_orf1;TRINITY_DN33801_c0_g1_i1_orf1;TRINITY_DN5055_c0_g1_i12_orf1;TRINITY_DN7570_c0_g1_i18_orf1;TRINITY_DN127056_c0_g1_i1_orf1;TRINITY_DN2265_c0_g2_i1_orf1;TRINITY_DN1552_c0_g1_i3_orf1;TRINITY_DN3057_c0_g2_i1_orf1;TRINITY_DN11942_c0_g1_i1_orf1;TRINITY_DN72017_c0_g1_i1_orf1;TRINITY_DN1475_c0_g1_i6_orf1;TRINITY_DN62729_c0_g1_i13_orf1;TRINITY_DN49038_c0_g4_i1_orf1;TRINITY_DN38506_c0_g1_i4_orf1;TRINITY_DN9109_c0_g1_i1_orf1;TRINITY_DN36788_c0_g1_i2_orf1;TRINITY_DN8390_c0_g1_i2_orf1;TRINITY_DN34479_c0_g1_i2_orf1;TRINITY_DN16673_c0_g1_i1_orf1;TRINITY_DN143509_c0_g1_i1_orf1;TRINITY_DN3343_c0_g2_i1_orf1;TRINITY_DN29144_c0_g3_i1_orf1;TRINITY_DN26293_c0_g1_i4_orf1;TRINITY_DN82311_c0_g1_i1_orf1;TRINITY_DN4762_c0_g1_i2_orf1;TRINITY_DN4835_c0_g1_i2_orf1;TRINITY_DN3175_c0_g1_i7_orf1;TRINITY_DN84322_c0_g2_i1_orf1;TRINITY_DN36899_c0_g1_i1_orf1;TRINITY_DN162_c0_g1_i4_orf1;TRINITY_DN64126_c0_g1_i1_orf1;TRINITY_DN48460_c0_g1_i1_orf1;TRINITY_DN44288_c0_g1_i2_orf1;TRINITY_DN96739_c0_g1_i1_orf1;TRINITY_DN60792_c0_g1_i2_orf1;TRINITY_DN2193_c0_g1_i7_orf1;TRINITY_DN97138_c0_g1_i2_orf1;TRINITY_DN5099_c0_g1_i3_orf1;TRINITY_DN111110_c0_g1_i1_orf1;TRINITY_DN15160_c0_g1_i1_orf1;TRINITY_DN62_c1_g1_i3_orf1;TRINITY_DN129869_c0_g4_i1_orf1;TRINITY_DN8979_c0_g1_i5_orf1;TRINITY_DN31327_c0_g2_i1_orf1;TRINITY_DN7512_c0_g1_i1_orf1;TRINITY_DN49047_c0_g1_i2_orf1;TRINITY_DN26168_c0_g1_i1_orf1;TRINITY_DN38424_c0_g1_i1_orf1;TRINITY_DN1084_c0_g2_i2_orf1;TRINITY_DN100821_c0_g1_i1_orf1;TRINITY_DN5422_c0_g1_i1_orf1;TRINITY_DN62557_c0_g1_i1_orf1;TRINITY_DN57918_c0_g1_i1_orf1;TRINITY_DN15706_c0_g2_i5_orf1;TRINITY_DN4790_c0_g1_i6_orf1;TRINITY_DN2874_c0_g1_i4_orf1;TRINITY_DN3428_c0_g1_i1_orf1;TRINITY_DN12964_c0_g1_i1_orf1;TRINITY_DN12820_c0_g1_i1_orf1;TRINITY_DN5648_c0_g1_i5_orf1;TRINITY_DN1405_c0_g1_i1_orf1;TRINITY_DN59291_c0_g1_i1_orf1;TRINITY_DN9302_c0_g1_i1_orf1;TRINITY_DN618_c0_g1_i3_orf1;TRINITY_DN46090_c0_g2_i1_orf1;TRINITY_DN4056_c0_g1_i8_orf1;TRINITY_DN14572_c0_g1_i1_orf1;TRINITY_DN5954_c0_g1_i2_orf1;TRINITY_DN1173_c0_g1_i11_orf1;TRINITY_DN1285_c0_g1_i6_orf1;TRINITY_DN3859_c0_g1_i5_orf1;TRINITY_DN9242_c0_g1_i1_orf1;TRINITY_DN12951_c1_g1_i5_orf1;TRINITY_DN23946_c0_g1_i1_orf1;TRINITY_DN26961_c0_g1_i1_orf1;TRINITY_DN1125_c0_g1_i4_orf1;TRINITY_DN18782_c0_g1_i4_orf1;TRINITY_DN2432_c0_g1_i1_orf1;TRINITY_DN1266_c2_g1_i1_orf1;TRINITY_DN25[illegible]
molecular_function	L-ascorbic acid binding	GO:0031418	1	1/2360	TRINITY_DN43293_c0_g1_i2_orf1
molecular_function	nucleoside binding	GO:0001882	1	1/2360	TRINITY_DN2401_c0_g2_i1_orf1
molecular_function	tetrapyrrole binding	GO:0046906	32	32/2360	TRINITY_DN43369_c0_g2_i1_orf1;TRINITY_DN120500_c0_g1_i1_orf1;TRINITY_DN9608_c0_g1_i3_orf1;TRINITY_DN30704_c0_g1_i1_orf1;TRINITY_DN7580_c0_g1_i1_orf1;TRINITY_DN2392_c0_g2_i1_orf1;TRINITY_DN5933_c0_g1_i1_orf1;TRINITY_DN14262_c0_g1_i5_orf1;TRINITY_DN625_c9_g1_i7_orf1;TRINITY_DN1664_c0_g1_i4_orf1;TRINITY_DN3949_c0_g1_i1_orf1;TRINITY_DN1134_c0_g1_i4_orf1;TRINITY_DN2652_c0_g2_i1_orf1;TRINITY_DN829_c0_g1_i8_orf1;TRINITY_DN1363_c0_g1_i11_orf1;TRINITY_DN50743_c0_g1_i1_orf1;TRINITY_DN3675_c0_g1_i1_orf1;TRINITY_DN15755_c0_g1_i1_orf1;TRINITY_DN448_c0_g1_i20_orf1;TRINITY_DN27045_c0_g1_i1_orf1;TRINITY_DN1960_c5_g1_i3_orf1;TRINITY_DN57856_c0_g2_i1_orf1;TRINITY_DN5439_c0_g1_i2_orf1;TRINITY_DN23783_c0_g2_i1_orf1;TRINITY_DN51252_c0_g2_i1_orf1;TRINITY_DN52887_c0_g1_i1_orf1;TRINITY_DN4497_c2_g1_i3_orf1;TRINITY_DN7861_c0_g1_i5_orf1;TRINITY_DN5126_c0_g1_i3_orf1;TRINITY_DN5661_c0_g1_i5_orf1;TRINITY_DN3321_c0_g1_i3_orf1;TRINITY_DN285_c0_g1_i4_orf1
molecular_function	vitamin B6 binding	GO:0070279	11	11/2360	TRINITY_DN2065_c1_g2_i1_orf1;TRINITY_DN2890_c0_g1_i2_orf1;TRINITY_DN2688_c0_g2_i1_orf1;TRINITY_DN11948_c0_g1_i8_orf1;TRINITY_DN14565_c0_g1_i11_orf1;TRINITY_DN1262_c0_g1_i2_orf1;TRINITY_DN2684_c0_g2_i3_orf1;TRINITY_DN11817_c0_g1_i4_orf1;TRINITY_DN2803_c4_g1_i1_orf1;TRINITY_DN2688_c0_g1_i3_orf1;TRINITY_DN1068_c0_g1_i3_orf1
molecular_function	laminin binding	GO:0043236	1	1/2360	TRINITY_DN10070_c0_g1_i1_orf1
molecular_function	histone binding	GO:0042393	3	3/2360	TRINITY_DN12771_c0_g1_i1_orf1;TRINITY_DN7341_c0_g1_i8_orf1;TRINITY_DN45449_c0_g1_i1_orf1
molecular_function	identical protein binding	GO:0042802	5	5/2360	TRINITY_DN96557_c0_g1_i1_orf1;TRINITY_DN1639_c0_g2_i2_orf1;TRINITY_DN96739_c0_g1_i1_orf1;TRINITY_DN7787_c0_g1_i1_orf1;TRINITY_DN147475_c0_g1_i1_orf1
molecular_function	enzyme binding	GO:0019899	8	8/2360	TRINITY_DN8473_c0_g1_i6_orf1;TRINITY_DN143496_c0_g1_i1_orf1;TRINITY_DN41736_c0_g2_i1_orf1;TRINITY_DN4859_c0_g1_i5_orf1;TRINITY_DN80424_c0_g1_i1_orf1;TRINITY_DN3887_c0_g1_i1_orf1;TRINITY_DN4439_c0_g1_i2_orf1;TRINITY_DN140538_c0_g2_i1_orf1
molecular_function	SNARE binding	GO:0000149	1	1/2360	TRINITY_DN38301_c0_g1_i2_orf1
molecular_function	unfolded protein binding	GO:0051082	11	11/2360	TRINITY_DN25341_c0_g1_i1_orf1;TRINITY_DN5262_c0_g1_i7_orf1;TRINITY_DN1725_c0_g1_i7_orf1;TRINITY_DN16128_c0_g1_i5_orf1;TRINITY_DN7464_c1_g1_i1_orf1;TRINITY_DN15959_c0_g1_i1_orf1;TRINITY_DN5648_c0_g1_i5_orf1;TRINITY_DN33801_c0_g1_i1_orf1;TRINITY_DN4779_c0_g1_i5_orf1;TRINITY_DN12964_c0_g1_i1_orf1;TRINITY_DN6671_c0_g1_i6_orf1
molecular_function	calmodulin binding	GO:0005516	4	4/2360	TRINITY_DN3126_c0_g1_i4_orf1;TRINITY_DN4010_c0_g2_i1_orf1;TRINITY_DN6642_c0_g1_i2_orf1;TRINITY_DN32022_c0_g1_i1_orf1
molecular_function	MDM2/MDM4 family protein binding	GO:0097371	1	1/2360	TRINITY_DN21367_c0_g1_i1_orf1
molecular_function	heat shock protein binding	GO:0031072	5	5/2360	TRINITY_DN79083_c0_g1_i2_orf1;TRINITY_DN12964_c0_g1_i1_orf1;TRINITY_DN15959_c0_g1_i1_orf1;TRINITY_DN10694_c1_g2_i1_orf1;TRINITY_DN5648_c0_g1_i5_orf1
molecular_function	transcription factor binding	GO:0008134	1	1/2360	TRINITY_DN147475_c0_g1_i1_orf1
molecular_function	beta-catenin binding	GO:0008013	1	1/2360	TRINITY_DN140538_c0_g2_i1_orf1
molecular_function	dynein intermediate chain binding	GO:0045505	1	1/2360	TRINITY_DN122423_c0_g1_i1_orf1
molecular_function	modification-dependent protein binding	GO:0140030	1	1/2360	TRINITY_DN7341_c0_g1_i8_orf1
molecular_function	translation initiation factor binding	GO:0031369	3	3/2360	TRINITY_DN48097_c0_g1_i1_orf1;TRINITY_DN21609_c0_g2_i1_orf1;TRINITY_DN50085_c0_g1_i1_orf1
molecular_function	signaling receptor binding	GO:0005102	5	5/2360	TRINITY_DN22443_c0_g2_i3_orf1;TRINITY_DN18650_c0_g1_i1_orf1;TRINITY_DN33418_c0_g1_i1_orf1;TRINITY_DN2227_c0_g1_i5_orf1;TRINITY_DN147475_c0_g1_i1_orf1
molecular_function	dynein light intermediate chain binding	GO:0051959	1	1/2360	TRINITY_DN122423_c0_g1_i1_orf1
molecular_function	cytoskeletal protein binding	GO:0008092	40	40/2360	TRINITY_DN2942_c0_g1_i6_orf1;TRINITY_DN11464_c0_g1_i3_orf1;TRINITY_DN714_c0_g1_i3_orf1;TRINITY_DN350_c0_g1_i4_orf1;TRINITY_DN8915_c0_g1_i3_orf1;TRINITY_DN350_c0_g1_i5_orf1;TRINITY_DN4159_c1_g1_i1_orf1;TRINITY_DN3126_c0_g1_i4_orf1;TRINITY_DN23790_c0_g1_i1_orf1;TRINITY_DN1718_c1_g1_i5_orf1;TRINITY_DN129869_c0_g4_i1_orf1;TRINITY_DN4010_c0_g2_i1_orf1;TRINITY_DN5954_c0_g1_i2_orf1;TRINITY_DN28018_c0_g6_i1_orf1;TRINITY_DN9383_c0_g1_i3_orf1;TRINITY_DN16673_c0_g1_i1_orf1;TRINITY_DN3887_c0_g1_i1_orf1;TRINITY_DN28622_c0_g1_i1_orf1;TRINITY_DN364_c1_g1_i2_orf1;TRINITY_DN26961_c0_g1_i1_orf1;TRINITY_DN14298_c0_g1_i1_orf1;TRINITY_DN86309_c0_g1_i4_orf1;TRINITY_DN41736_c0_g2_i1_orf1;TRINITY_DN14298_c0_g3_i1_orf1;TRINITY_DN96739_c0_g1_i1_orf1;TRINITY_DN841_c0_g1_i4_orf1;TRINITY_DN21451_c0_g1_i3_orf1;TRINITY_DN286_c0_g1_i2_orf1;TRINITY_DN140538_c0_g2_i1_orf1;TRINITY_DN110231_c0_g1_i1_orf1;TRINITY_DN9146_c0_g1_i1_orf1;TRINITY_DN69557_c0_g1_i1_orf1;TRINITY_DN23020_c0_g1_i1_orf1;TRINITY_DN10455_c0_g1_i2_orf1;TRINITY_DN139335_c0_g2_i1_orf1;TRINITY_DN17137_c0_g1_i2_orf1;TRINITY_DN97097_c0_g1_i4_orf1;TRINITY_DN101991_c0_g1_i5_orf1;TRINITY_DN34703_c0_g1_i4_orf1;TRINITY_DN741_c0_g1_i10_orf1
molecular_function	protein dimerization activity	GO:0046983	5	5/2360	TRINITY_DN1639_c0_g2_i2_orf1;TRINITY_DN3457_c0_g1_i4_orf1;TRINITY_DN50471_c0_g1_i4_orf1;TRINITY_DN21567_c0_g1_i7_orf1;TRINITY_DN96557_c0_g1_i1_orf1
molecular_function	phosphoprotein binding	GO:0051219	1	1/2360	TRINITY_DN140538_c0_g2_i1_orf1
molecular_function	protein domain specific binding	GO:0019904	3	3/2360	TRINITY_DN1639_c0_g2_i2_orf1;TRINITY_DN18912_c1_g1_i1_orf1;TRINITY_DN147475_c0_g1_i1_orf1
molecular_function	clathrin binding	GO:0030276	4	4/2360	TRINITY_DN8405_c0_g1_i4_orf1;TRINITY_DN5118_c0_g1_i1_orf1;TRINITY_DN741_c0_g1_i10_orf1;TRINITY_DN13118_c0_g1_i6_orf1
molecular_function	ubiquitin-like protein binding	GO:0032182	3	3/2360	TRINITY_DN45633_c0_g1_i1_orf1;TRINITY_DN65299_c0_g4_i1_orf1;TRINITY_DN18620_c0_g1_i5_orf1
molecular_function	cell adhesion molecule binding	GO:0050839	1	1/2360	TRINITY_DN10070_c0_g1_i1_orf1
molecular_function	S100 protein binding	GO:0044548	1	1/2360	TRINITY_DN41736_c0_g2_i1_orf1
molecular_function	calcium-dependent protein binding	GO:0048306	1	1/2360	TRINITY_DN96739_c0_g1_i1_orf1
molecular_function	GTPase activating protein binding	GO:0032794	1	1/2360	TRINITY_DN140538_c0_g2_i1_orf1
molecular_function	chitin binding	GO:0008061	18	18/2360	TRINITY_DN77642_c0_g1_i1_orf1;TRINITY_DN26301_c0_g1_i1_orf1;TRINITY_DN21555_c0_g1_i4_orf1;TRINITY_DN9000_c0_g2_i1_orf1;TRINITY_DN17003_c0_g1_i1_orf1;TRINITY_DN3913_c0_g1_i6_orf1;TRINITY_DN10824_c0_g1_i3_orf1;TRINITY_DN619_c0_g1_i1_orf1;TRINITY_DN82801_c0_g1_i1_orf1;TRINITY_DN2061_c0_g1_i3_orf1;TRINITY_DN17003_c1_g1_i1_orf1;TRINITY_DN73923_c0_g1_i1_orf1;TRINITY_DN72999_c0_g1_i1_orf1;TRINITY_DN36061_c0_g4_i2_orf1;TRINITY_DN650_c0_g1_i3_orf1;TRINITY_DN54366_c0_g1_i1_orf1;TRINITY_DN6418_c0_g1_i28_orf1;TRINITY_DN3759_c0_g1_i1_orf1
molecular_function	lipopolysaccharide binding	GO:0001530	1	1/2360	TRINITY_DN2170_c0_g2_i1_orf1
molecular_function	lipoteichoic acid binding	GO:0070891	1	1/2360	TRINITY_DN2170_c0_g2_i1_orf1

molecular_function	ribonucleotide binding	GO:0032553	176	176/2360	TRINITY_DN38482_c0_g1_i4_orf1;TRINITY_DN280_c0_g1_i8_orf1;TRINITY_DN47731_c0_g1_i2_orf1;TRINITY_DN4380_c0_g1_i9_orf1;TRINITY_DN41311_c0_g2_i3_orf1;TRINITY_DN27771_c0_g1_i1_orf1;TRINITY_DN11194_c0_g1_i4_orf1;TRINITY_DN15959_c0_g1_i1_orf1;TRINITY_DN7122_c0_g1_i1_orf1;TRINITY_DN7213_c0_g1_i2_orf1;TRINITY_DN359_c0_g1_i5_orf1;TRINITY_DN31967_c0_g1_i5_orf1;TRINITY_DN25341_c0_g1_i1_orf1;TRINITY_DN2738_c1_g1_i3_orf1;TRINITY_DN5262_c0_g1_i7_orf1;TRINITY_DN33146_c0_g1_i1_orf1;TRINITY_DN2904_c0_g1_i4_orf1;TRINITY_DN70485_c0_g1_i2_orf1;TRINITY_DN4798_c0_g1_i3_orf1;TRINITY_DN24723_c2_g1_i1_orf1;TRINITY_DN2953_c1_g1_i10_orf1;TRINITY_DN143509_c0_g1_i1_orf1;TRINITY_DN2638_c0_g1_i7_orf1;TRINITY_DN4320_c0_g1_i1_orf1;TRINITY_DN10774_c0_g2_i3_orf1;TRINITY_DN6587_c0_g1_i3_orf1;TRINITY_DN11596_c0_g1_i1_orf1;TRINITY_DN2953_c1_g1_i2_orf1;TRINITY_DN14298_c0_g1_i1_orf1;TRINITY_DN3991_c0_g1_i6_orf1;TRINITY_DN3800_c0_g1_i7_orf1;TRINITY_DN9575_c0_g1_i1_orf1;TRINITY_DN235_c0_g3_i1_orf1;TRINITY_DN6185_c0_g1_i12_orf1;TRINITY_DN6436_c0_g1_i1_orf1;TRINITY_DN37165_c0_g1_i4_orf1;TRINITY_DN26195_c0_g1_i6_orf1;TRINITY_DN2224_c0_g1_i1_orf1;TRINITY_DN20499_c0_g3_i1_orf1;TRINITY_DN987_c0_g1_i3_orf1;TRINITY_DN26961_c0_g1_i1_orf1;TRINITY_DN15591_c0_g1_i3_orf1;TRINITY_DN7247_c0_g1_i7_orf1;TRINITY_DN4501_c0_g1_i3_orf1;TRINITY_DN31225_c0_g1_i1_orf1;TRINITY_DN107288_c0_g1_i2_orf1;TRINITY_DN46715_c0_g1_i1_orf1;TRINITY_DN452_c1_g1_i3_orf1;TRINITY_DN20007_c0_g1_i1_orf1;TRINITY_DN4950_c0_g1_i2_orf1;TRINITY_DN63561_c1_g1_i2_orf1;TRINITY_DN3092_c0_g1_i2_orf1;TRINITY_DN39813_c0_g1_i1_orf1;TRINITY_DN2772_c0_g1_i3_orf1;TRINITY_DN115210_c0_g4_i1_orf1;TRINITY_DN31503_c0_g1_i4_orf1;TRINITY_DN4977_c0_g1_i2_orf1;TRINITY_DN252_c0_g1_i3_orf1;TRINITY_DN33801_c0_g1_i1_orf1;TRINITY_DN8659_c0_g2_i1_orf1;TRINITY_DN17935_c0_g1_i1_orf1;TRINITY_DN1604_c0_g1_i4_orf1;TRINITY_DN1034_c0_g1_i4_orf1;TRINITY_DN90497_c0_g1_i1_orf1;TRINITY_DN42461_c0_g1_i4_orf1;TRINITY_DN11125_c0_g1_i1_orf1;TRINITY_DN6813_c1_g1_i1_orf1;TRINITY_DN1578_c0_g3_i1_orf1;TRINITY_DN1607_c0_g1_i16_orf1;TRINITY_DN277_c1_g1_i1_orf1;TRINITY_DN1725_c0_g1_i7_orf1;TRINITY_DN19034_c0_g1_i1_orf1;TRINITY_DN7336_c0_g1_i13_orf1;TRINITY_DN5675_c0_g1_i6_orf1;TRINITY_DN32700_c0_g1_i2_orf1;TRINITY_DN117844_c0_g1_i1_orf1;TRINITY_DN122423_c0_g1_i1_orf1;TRINITY_DN6642_c0_g1_i2_orf1;TRINITY_DN9243_c0_g1_i4_orf1;TRINITY_DN5508_c0_g1_i1_orf1;TRINITY_DN122423_c0_g5_i1_orf1;TRINITY_DN511_c0_g2_i1_orf1;TRINITY_DN1718_c6_g1_i4_orf1;TRINITY_DN1173_c1_g1_i10_orf1;TRINITY_DN2146_c0_g2_i1_orf1;TRINITY_DN3428_c0_g1_i1_orf1;TRINITY_DN4908_c1_g1_i5_orf1;TRINITY_DN28989_c0_g1_i7_orf1;TRINITY_DN25997_c1_g2_i4_orf1;TRINITY_DN47151_c0_g1_i1_orf1;TRINITY_DN235_c0_g1_i2_orf1;TRINITY_DN2709_c0_g1_i4_orf1;TRINITY_DN291_c0_g1_i2_orf1;TRINITY_DN16011_c0_g1_i3_orf1;TRINITY_DN8659_c0_g1_i1_orf1;TRINITY_DN7570_c0_g1_i18_orf1;TRINITY_DN127056_c0_g1_i1_orf1;TRINITY_DN3637_c0_g1_i2_orf1;TRINITY_DN1552_c0_g1_i3_orf1;TRINITY_DN3057_c0_g2_i1_orf1;TRINITY_DN11942_c0_g1_i1_orf1;TRINITY_DN1475_c0_g1_i6_orf1;TRINITY_DN62729_c0_g1_i13_orf1;TRINITY_DN38506_c0_g1_i4_orf1;TRINITY_DN9109_c0_g1_i1_orf1;TRINITY_DN1266_c2_g1_i1_orf1;TRINITY_DN8390_c0_g1_i2_orf1;TRINITY_DN34479_c0_g1_i2_orf1;TRINITY_DN16673_c0_g1_i1_orf1;TRINITY_DN7388_c0_g1_i7_orf1;TRINITY_DN3343_c0_g2_i1_orf1;TRINITY_DN29144_c0_g3_i1_orf1;TRINITY_DN82311_c0_g1_i1_orf1;TRINITY_DN4762_c0_g1_i2_orf1;TRINITY_DN4835_c0_g1_i2_orf1;TRINITY_DN12964_c0_g1_i1_orf1;TRINITY_DN15420_c0_g3_i2_orf1;TRINITY_DN162_c0_g1_i4_orf1;TRINITY_DN48460_c0_g1_i1_orf1;TRINITY_DN44288_c0_g1_i2_orf1;TRINITY_DN96739_c0_g1_i1_orf1;TRINITY_DN60792_c0_g1_i2_orf1;TRINITY_DN2193_c0_g1_i7_orf1;TRINITY_DN97138_c0_g1_i2_orf1;TRINITY_DN15160_c0_g1_i1_orf1;TRINITY_DN129869_c0_g4_i1_orf1;TRINITY_DN8979_c0_g1_i5_orf1;TRINITY_DN31327_c0_g2_i1_orf1;TRINITY_DN7512_c0_g1_i1_orf1;TRINITY_DN49047_c0_g1_i2_orf1;TRINITY_DN26168_c0_g1_i1_orf1;TRINITY_DN1084_c0_g2_i2_orf1;TRINITY_DN100821_c0_g1_i1_orf1;TRINITY_DN2265_c0_g2_i1_orf1;TRINITY_DN57918_c0_g1_i1_orf1;TRINITY_DN15706_c0_g2_i5_orf1;TRINITY_DN4790_c0_g1_i6_orf1;TRINITY_DN2874_c0_g1_i4_orf1;TRINITY_DN12820_c0_g1_i1_orf1;TRINITY_DN5648_c0_g1_i5_orf1;TRINITY_DN1405_c0_g1_i1_orf1;TRINITY_DN59291_c0_g1_i1_orf1;TRINITY_DN9302_c0_g1_i1_orf1;TRINITY_DN618_c0_g1_i3_orf1;TRINITY_DN46090_c0_g2_i1_orf1;TRINITY_DN4056_c0_g1_i8_orf1;TRINITY_DN14572_c0_g1_i1_orf1;TRINITY_DN5954_c0_g1_i2_orf1;TRINITY_DN1173_c0_g1_i11_orf1;TRINITY_DN1285_c0_g1_i6_orf1;TRINITY_DN12951_c1_g1_i5_orf1;TRINITY_DN23946_c0_g1_i1_orf1;TRINITY_DN62557_c0_g1_i1_orf1;TRINITY_DN84322_c0_g2_i1_orf1;TRINITY_DN18782_c0_g1_i4_orf1;TRINITY_DN2432_c0_g1_i1_orf1;TRINITY_DN25345_c0_g1_i1_orf1;TRINITY_DN30154_c0_g1_i1_orf1;TRINITY_DN13055_c0_g1_i5_orf1;TRINITY_DN15478_c0_g1_i1_orf1;TRINITY_DN28622_c0_g1_i1_orf1;TRINITY_DN2745_c0_g1_i2_orf1;TRINITY_DN11612_c0_g2_i1_orf1;TRINITY_DN248_c0_g1_i1_orf1;TRINITY_DN5757_c0_g1_i1_orf1;TRINITY_DN66442_c0_g2_i3_orf1;TRINITY_DN45449_c0_g1_i1_orf1;TRINITY_DN21539_c0_g1_i1_orf1;TRINITY_DN30_c0_g1_i6_orf1;TRINITY_DN7464_c1_g1_i1_orf1;TRINITY_DN1084_c0_g1_i2_orf1;TRINITY_DN19251_c0_g1_i8_orf1;TRINITY_DN14298_c0_g3_i1_orf1;TRINITY_DN10745_c0_g1_i14_orf1;TRINITY_DN4779_c0_g1_i5_orf1;TRINITY_DN4929_c1_g2_i5_orf1
molecular_function	glycosaminoglycan binding	GO:0005539	3	3/2360	TRINITY_DN5235_c0_g1_i7_orf1;TRINITY_DN96739_c0_g1_i1_orf1;TRINITY_DN1534_c0_g1_i3_orf1
molecular_function	chondroitin sulfate binding	GO:0035374	1	1/2360	TRINITY_DN96739_c0_g1_i1_orf1
molecular_function	peptide binding	GO:0042277	2	2/2360	TRINITY_DN245_c0_g1_i4_orf1;TRINITY_DN4016_c0_g1_i1_orf1
molecular_function	phosphopantetheine binding	GO:0031177	1	1/2360	TRINITY_DN10430_c0_g1_i4_orf1
molecular_function	cation binding	GO:0043169	253	253/2360	TRINITY_DN129226_c0_g1_i2_orf1;TRINITY_DN14920_c0_g1_i1_orf1;TRINITY_DN9608_c0_g1_i3_orf1;TRINITY_DN38230_c0_g1_i4_orf1;TRINITY_DN276_c0_g1_i1_orf1;TRINITY_DN84322_c0_g2_i1_orf1;TRINITY_DN3194_c0_g1_i6_orf1;TRINITY_DN7580_c0_g1_i1_orf1;TRINITY_DN863_c0_g1_i6_orf1;TRINITY_DN3461_c0_g1_i1_orf1;TRINITY_DN15959_c0_g1_i1_orf1;TRINITY_DN43293_c0_g1_i2_orf1;TRINITY_DN40176_c0_g1_i1_orf1;TRINITY_DN35763_c0_g1_i2_orf1;TRINITY_DN51342_c0_g1_i7_orf1;TRINITY_DN19651_c0_g1_i1_orf1;TRINITY_DN18804_c0_g1_i5_orf1;TRINITY_DN1231_c0_g1_i4_orf1;TRINITY_DN5628_c0_g1_i5_orf1;TRINITY_DN15040_c0_g4_i1_orf1;TRINITY_DN12545_c0_g1_i7_orf1;TRINITY_DN8771_c0_g1_i5_orf1;TRINITY_DN1492_c0_g1_i4_orf1;TRINITY_DN1134_c0_g1_i4_orf1;TRINITY_DN70485_c0_g1_i2_orf1;TRINITY_DN22928_c0_g1_i6_orf1;TRINITY_DN47151_c0_g1_i1_orf1;TRINITY_DN31163_c1_g1_i4_orf1;TRINITY_DN143509_c0_g1_i1_orf1;TRINITY_DN3235_c0_g1_i1_orf1;TRINITY_DN4711_c0_g1_i2_orf1;TRINITY_DN3675_c0_g1_i1_orf1;TRINITY_DN585_c0_g1_i12_orf1;TRINITY_DN14774_c0_g1_i4_orf1;TRINITY_DN15755_c0_g1_i1_orf1;TRINITY_DN4822_c0_g1_i6_orf1;TRINITY_DN448_c0_g1_i20_orf1;TRINITY_DN1534_c0_g1_i3_orf1;TRINITY_DN3991_c0_g1_i6_orf1;TRINITY_DN2794_c1_g1_i8_orf1;TRINITY_DN3800_c0_g1_i7_orf1;TRINITY_DN5439_c0_g1_i2_orf1;TRINITY_DN69557_c0_g1_i1_orf1;TRINITY_DN123184_c0_g1_i1_orf1;TRINITY_DN16400_c0_g2_i1_orf1;TRINITY_DN21943_c1_g1_i1_orf1;TRINITY_DN89483_c0_g1_i1_orf1;TRINITY_DN2117_c0_g1_i1_orf1;TRINITY_DN14347_c0_g1_i1_orf1;TRINITY_DN2688_c0_g2_i1_orf1;TRINITY_DN5952_c0_g1_i6_orf1;TRINITY_DN4813_c0_g1_i5_orf1;TRINITY_DN987_c0_g1_i3_orf1;TRINITY_DN14389_c0_g1_i4_orf1;TRINITY_DN1444_c1_g1_i5_orf1;TRINITY_DN2919_c0_g1_i5_orf1;TRINITY_DN1024_c0_g4_i1_orf1;TRINITY_DN78686_c0_g1_i1_orf1;TRINITY_DN2433_c0_g1_i3_orf1;TRINITY_DN5190_c0_g3_i1_orf1;TRINITY_DN1084_c0_g2_i2_orf1;TRINITY_DN46625_c0_g1_i1_orf1;TRINITY_DN1161_c0_g1_i2_orf1;TRINITY_DN2647_c0_g1_i3_orf1;TRINITY_DN120_c0_g1_i2_orf1;TRINITY_DN14262_c0_g1_i5_orf1;TRINITY_DN65681_c0_g1_i1_orf1;TRINITY_DN120089_c0_g1_i1_orf1;TRINITY_DN30704_c0_g1_i1_orf1;TRINITY_DN115210_c0_g4_i1_orf1;TRINITY_DN20442_c0_g2_i1_orf1;TRINITY_DN45271_c0_g1_i1_orf1;TRINITY_DN47723_c0_g1_i1_orf1;TRINITY_DN1423_c0_g1_i8_orf1;TRINITY_DN4367_c0_g1_i1_orf1;TRINITY_DN252_c0_g1_i3_orf1;TRINITY_DN37532_c0_g1_i1_orf1;TRINITY_DN2688_c0_g1_i3_orf1;TRINITY_DN4189_c0_g2_i1_orf1;TRINITY_DN27592_c0_g1_i1_orf1;TRINITY_DN1664_c0_g1_i4_orf1;TRINITY_DN1272_c1_g1_i4_orf1;TRINITY_DN501_c0_g1_i5_orf1;TRINITY_DN81258_c0_g1_i2_orf1;TRINITY_DN19187_c0_g1_i1_orf1;TRINITY_DN4125_c1_g1_i5_orf1;TRINITY_DN1423_c0_g1_i4_orf1;TRINITY_DN4501_c0_g1_i3_orf1;TRINITY_DN1999_c0_g1_i9_orf1;TRINITY_DN51968_c0_g1_i1_orf1;TRINITY_DN19990_c0_g1_i1_orf1;TRINITY_DN1578_c0_g3_i1_orf1;TRINITY_DN116972_c0_g1_i1_orf1;TRINITY_DN30713_c0_g1_i3_orf1;TRINITY_DN50787_c0_g2_i2_orf1;TRINITY_DN2338_c0_g2_i1_orf1;TRINITY_DN6602_c0_g1_i4_orf1;TRINITY_DN19034_c0_g1_i1_orf1;TRINITY_DN4145_c0_g1_i1_orf1;TRINITY_DN15327_c2_g1_i2_orf1;TRINITY_DN3733_c0_g1_i1_orf1;TRINITY_DN32700_c0_g1_i2_orf1;TRINITY_DN117844_c0_g1_i1_orf1;TRINITY_DN47842_c0_g1_i1_orf1;TRINITY_DN1083_c0_g1_i4_orf1;TRINITY_DN87522_c0_g2_i1_orf1;TRINITY_DN3073_c0_g1_i7_orf1;TRINITY_DN38644_c0_g1_i1_orf1;TRINITY_DN6642_c0_g1_i2_orf1;TRINITY_DN9243_c0_g1_i4_orf1;TRINITY_DN18922_c0_g1_i1_orf1;TRINITY_DN51568_c0_g1_i1_orf1;TRINITY_DN104597_c0_g1_i2_orf1;TRINITY_DN5664_c0_g1_i1_orf1;TRINITY_DN511_c0_g2_i1_orf1;TRINITY_DN7861_c0_g1_i5_orf1;TRINITY_DN38431_c0_g1_i1_orf1;TRINITY_DN1206_c0_g1_i6_orf1;TRINITY_DN23734_c0_g1_i1_orf1;TRINITY_DN46778_c0_g1_i2_orf1;TRINITY_DN69170_c0_g2_i1_orf1;TRINITY_DN5004_c0_g1_i2_orf1;TRINITY_DN107035_c0_g1_i1_orf1;TRINITY_DN103107_c0_g1_i2_orf1;TRINITY_DN4908_c1_g1_i5_orf1;TRINITY_DN36788_c0_g1_i2_orf1;TRINITY_DN5235_c0_g1_i7_orf1;TRINITY_DN25997_c1_g2_i4_orf1;TRINITY_DN5495_c0_g1_i5_orf1;TRINITY_DN125427_c0_g1_i1_orf1;TRINITY_DN812_c2_g1_i1_orf1;TRINITY_DN257_c0_g1_i7_orf1;TRINITY_DN120500_c0_g1_i1_orf1;TRINITY_DN64222_c0_g1_i1_orf1;TRINITY_DN10672_c0_g1_i3_orf1;TRINITY_DN5001_c0_g1_i4_orf1;TRINITY_DN7570_c0_g1_i18_orf1;TRINITY_DN6415_c0_g1_i1_orf1;TRINITY_DN36538_c0_g1_i2_orf1;TRINITY_DN2186_c0_g1_i17_orf1;TRINITY_DN73900_c0_g1_i1_orf1;TRINITY_DN8621_c0_g1_i5_orf1;TRINITY_DN53311_c0_g2_i1_orf1;TRINITY_DN3057_c0_g2_i1_orf1;TRINITY_DN1952_c0_g1_i2_orf1;TRINITY_DN5081_c0_g1_i5_orf1;TRINITY_DN7212_c0_g1_i4_orf1;TRINITY_DN23194_c0_g1_i4_orf1;TRINITY_DN2140_c0_g1_i1_orf1;TRINITY_DN934_c2_g1_i7_orf1;TRINITY_DN1266_c2_g1_i1_orf1;TRINITY_DN2825_c0_g1_i3_orf1;TRINITY_DN1363_c0_g1_i11_orf1;TRINITY_DN43412_c0_g1_i2_orf1;TRINITY_DN829_c0_g1_i8_orf1;TRINITY_DN4793_c0_g1_i7_orf1;TRINITY_DN142652_c0_g1_i1_orf1;TRINITY_DN276_c0_g1_i2_orf1;TRINITY_DN3343_c0_g2_i1_orf1;TRINITY_DN14477_c0_g1_i12_orf1;TRINITY_DN15812_c0_g1_i2_orf1;TRINITY_DN50743_c0_g1_i1_orf1;TRINITY_DN6415_c0_g2_i1_orf1;TRINITY_DN3464_c0_g1_i1_orf1;TRINITY_DN1989_c0_g1_i1_orf1;TRINITY_DN8771_c0_g2_i1_orf1;TRINITY_DN19866_c0_g1_i4_orf1;TRINITY_DN18164_c0_g1_i7_orf1;TRINITY_DN27045_c0_g1_i1_orf1;TRINITY_DN2338_c0_g2_i2_orf1;TRINITY_DN2673_c2_g1_i2_orf1;TRINITY_DN2986_c1_g1_i1_orf1;TRINITY_DN23783_c0_g2_i1_orf1;TRINITY_DN4497_c2_g1_i3_orf1;TRINITY_DN1707_c0_g1_i1_orf1;TRINITY_DN31348_c0_g1_i1_orf1;TRINITY_DN111110_c0_g1_i1_orf1;TRINITY_DN5126_c0_g1_i3_orf1;TRINITY_DN31943_c0_g1_i1_orf1;TRINITY_DN14398_c0_g1_i4_orf1;TRINITY_DN35582_c0_g1_i1_orf1;TRINITY_DN5661_c0_g1_i5_orf1;TRINITY_DN1957_c0_g1_i4_orf1;TRINITY_DN2472_c0_g1_i6_orf1;TRINITY_DN7512_c0_g1_i1_orf1;TRINITY_DN892_c0_g1_i9_orf1;TRINITY_DN10385_c0_g1_i5_orf1;TRINITY_DN1194_c0_g1_i5_orf1;TRINITY_DN18242_c0_g1_i[illegible]

molecular_function	anion binding	GO:0043168	202	202/2360	TRINITY_DN38482_c0_g1_i4_orf1;TRINITY_DN280_c0_g1_i8_orf1;TRINITY_DN2065_c1_g2_i1_orf1;TRINITY_DN47731_c0_g1_i2_orf1;TRINITY_DN4380_c0_g1_i9_orf1;TRINITY_DN41311_c0_g2_i3_orf1;TRINITY_DN27771_c0_g1_i1_orf1;TRINITY_DN11194_c0_g1_i4_orf1;TRINITY_DN15959_c0_g1_i1_orf1;TRINITY_DN43293_c0_g1_i2_orf1;TRINITY_DN7122_c0_g1_i1_orf1;TRINITY_DN7213_c0_g1_i2_orf1;TRINITY_DN30932_c0_g1_i2_orf1;TRINITY_DN31967_c0_g1_i5_orf1;TRINITY_DN25341_c0_g1_i1_orf1;TRINITY_DN2738_c1_g1_i3_orf1;TRINITY_DN5262_c0_g1_i7_orf1;TRINITY_DN33146_c0_g1_i1_orf1;TRINITY_DN2904_c0_g1_i4_orf1;TRINITY_DN70485_c0_g1_i2_orf1;TRINITY_DN11817_c0_g1_i4_orf1;TRINITY_DN24723_c2_g1_i1_orf1;TRINITY_DN2953_c1_g1_i10_orf1;TRINITY_DN143509_c0_g1_i1_orf1;TRINITY_DN2638_c0_g1_i7_orf1;TRINITY_DN4320_c0_g1_i1_orf1;TRINITY_DN4711_c0_g1_i2_orf1;TRINITY_DN26789_c0_g1_i2_orf1;TRINITY_DN10774_c0_g2_i3_orf1;TRINITY_DN6587_c0_g1_i3_orf1;TRINITY_DN11596_c0_g1_i1_orf1;TRINITY_DN2953_c1_g1_i2_orf1;TRINITY_DN14298_c0_g1_i1_orf1;TRINITY_DN3991_c0_g1_i6_orf1;TRINITY_DN3800_c0_g1_i7_orf1;TRINITY_DN9575_c0_g1_i1_orf1;TRINITY_DN235_c0_g3_i1_orf1;TRINITY_DN1068_c0_g1_i3_orf1;TRINITY_DN6185_c0_g1_i12_orf1;TRINITY_DN20499_c0_g3_i1_orf1;TRINITY_DN6436_c0_g1_i1_orf1;TRINITY_DN37165_c0_g1_i4_orf1;TRINITY_DN26195_c0_g1_i6_orf1;TRINITY_DN2224_c0_g1_i1_orf1;TRINITY_DN2688_c0_g2_i1_orf1;TRINITY_DN987_c0_g1_i3_orf1;TRINITY_DN15591_c0_g1_i3_orf1;TRINITY_DN7247_c0_g1_i7_orf1;TRINITY_DN4501_c0_g1_i3_orf1;TRINITY_DN3637_c0_g1_i2_orf1;TRINITY_DN107288_c0_g1_i2_orf1;TRINITY_DN46715_c0_g1_i1_orf1;TRINITY_DN452_c1_g1_i3_orf1;TRINITY_DN20007_c0_g1_i1_orf1;TRINITY_DN4950_c0_g1_i2_orf1;TRINITY_DN63561_c1_g1_i2_orf1;TRINITY_DN3092_c0_g1_i2_orf1;TRINITY_DN39813_c0_g1_i1_orf1;TRINITY_DN2772_c0_g1_i3_orf1;TRINITY_DN115210_c0_g4_i1_orf1;TRINITY_DN31503_c0_g1_i4_orf1;TRINITY_DN4977_c0_g1_i2_orf1;TRINITY_DN252_c0_g1_i3_orf1;TRINITY_DN2688_c0_g1_i3_orf1;TRINITY_DN8659_c0_g2_i1_orf1;TRINITY_DN17935_c0_g1_i1_orf1;TRINITY_DN1604_c0_g1_i4_orf1;TRINITY_DN1034_c0_g1_i4_orf1;TRINITY_DN90497_c0_g1_i1_orf1;TRINITY_DN42461_c0_g1_i4_orf1;TRINITY_DN11125_c0_g1_i1_orf1;TRINITY_DN6813_c1_g1_i1_orf1;TRINITY_DN1578_c0_g3_i1_orf1;TRINITY_DN359_c0_g1_i5_orf1;TRINITY_DN1607_c0_g1_i16_orf1;TRINITY_DN277_c1_g1_i1_orf1;TRINITY_DN1725_c0_g1_i7_orf1;TRINITY_DN19034_c0_g1_i1_orf1;TRINITY_DN7336_c0_g1_i13_orf1;TRINITY_DN5675_c0_g1_i6_orf1;TRINITY_DN32700_c0_g1_i2_orf1;TRINITY_DN117844_c0_g1_i1_orf1;TRINITY_DN122423_c0_g1_i1_orf1;TRINITY_DN14565_c0_g1_i11_orf1;TRINITY_DN31225_c0_g1_i1_orf1;TRINITY_DN6642_c0_g1_i2_orf1;TRINITY_DN9243_c0_g1_i4_orf1;TRINITY_DN5508_c0_g1_i1_orf1;TRINITY_DN122423_c0_g5_i1_orf1;TRINITY_DN511_c0_g2_i1_orf1;TRINITY_DN1718_c6_g1_i4_orf1;TRINITY_DN1173_c1_g1_i10_orf1;TRINITY_DN2146_c0_g2_i1_orf1;TRINITY_DN3428_c0_g1_i1_orf1;TRINITY_DN2684_c0_g2_i3_orf1;TRINITY_DN15420_c0_g3_i2_orf1;TRINITY_DN4908_c1_g1_i5_orf1;TRINITY_DN28989_c0_g1_i7_orf1;TRINITY_DN25997_c1_g2_i4_orf1;TRINITY_DN47151_c0_g1_i1_orf1;TRINITY_DN235_c0_g1_i2_orf1;TRINITY_DN2709_c0_g1_i4_orf1;TRINITY_DN291_c0_g1_i2_orf1;TRINITY_DN16011_c0_g1_i3_orf1;TRINITY_DN8659_c0_g1_i1_orf1;TRINITY_DN33801_c0_g1_i1_orf1;TRINITY_DN5055_c0_g1_i12_orf1;TRINITY_DN7570_c0_g1_i18_orf1;TRINITY_DN127056_c0_g1_i1_orf1;TRINITY_DN2265_c0_g2_i1_orf1;TRINITY_DN1552_c0_g1_i3_orf1;TRINITY_DN3057_c0_g2_i1_orf1;TRINITY_DN11942_c0_g1_i1_orf1;TRINITY_DN72017_c0_g1_i1_orf1;TRINITY_DN129869_c0_g4_i1_orf1;TRINITY_DN62729_c0_g1_i13_orf1;TRINITY_DN11948_c0_g1_i8_orf1;TRINITY_DN38506_c0_g1_i4_orf1;TRINITY_DN9109_c0_g1_i1_orf1;TRINITY_DN1266_c2_g1_i1_orf1;TRINITY_DN8390_c0_g1_i2_orf1;TRINITY_DN34479_c0_g1_i2_orf1;TRINITY_DN16673_c0_g1_i1_orf1;TRINITY_DN2803_c4_g1_i1_orf1;TRINITY_DN7388_c0_g1_i7_orf1;TRINITY_DN3343_c0_g2_i1_orf1;TRINITY_DN29144_c0_g3_i1_orf1;TRINITY_DN82311_c0_g1_i1_orf1;TRINITY_DN4762_c0_g1_i2_orf1;TRINITY_DN4835_c0_g1_i2_orf1;TRINITY_DN3175_c0_g1_i7_orf1;TRINITY_DN84322_c0_g2_i1_orf1;TRINITY_DN36899_c0_g1_i1_orf1;TRINITY_DN162_c0_g1_i4_orf1;TRINITY_DN64126_c0_g1_i1_orf1;TRINITY_DN48460_c0_g1_i1_orf1;TRINITY_DN44288_c0_g1_i2_orf1;TRINITY_DN96739_c0_g1_i1_orf1;TRINITY_DN60792_c0_g1_i2_orf1;TRINITY_DN2193_c0_g1_i7_orf1;TRINITY_DN97138_c0_g1_i2_orf1;TRINITY_DN111110_c0_g1_i1_orf1;TRINITY_DN15160_c0_g1_i1_orf1;TRINITY_DN62_c1_g1_i3_orf1;TRINITY_DN8979_c0_g1_i5_orf1;TRINITY_DN31327_c0_g2_i1_orf1;TRINITY_DN1262_c0_g1_i2_orf1;TRINITY_DN7512_c0_g1_i1_orf1;TRINITY_DN49047_c0_g1_i2_orf1;TRINITY_DN26168_c0_g1_i1_orf1;TRINITY_DN38424_c0_g1_i1_orf1;TRINITY_DN1084_c0_g2_i2_orf1;TRINITY_DN100821_c0_g1_i1_orf1;TRINITY_DN62557_c0_g1_i1_orf1;TRINITY_DN57918_c0_g1_i1_orf1;TRINITY_DN15706_c0_g2_i5_orf1;TRINITY_DN4790_c0_g1_i6_orf1;TRINITY_DN2874_c0_g1_i4_orf1;TRINITY_DN12964_c0_g1_i1_orf1;TRINITY_DN12820_c0_g1_i1_orf1;TRINITY_DN5648_c0_g1_i5_orf1;TRINITY_DN1405_c0_g1_i1_orf1;TRINITY_DN59291_c0_g1_i1_orf1;TRINITY_DN9302_c0_g1_i1_orf1;TRINITY_DN618_c0_g1_i3_orf1;TRINITY_DN46090_c0_g2_i1_orf1;TRINITY_DN4056_c0_g1_i8_orf1;TRINITY_DN4798_c0_g1_i3_orf1;TRINITY_DN14572_c0_g1_i1_orf1;TRINITY_DN2890_c0_g1_i2_orf1;TRINITY_DN5954_c0_g1_i2_orf1;TRINITY_DN1173_c0_g1_i11_orf1;TRINITY_DN1285_c0_g1_i6_orf1;TRINITY_DN3859_c0_g1_i5_orf1;TRINITY_DN9242_c0_g1_i1_orf1;TRINITY_DN12951_c1_g1_i5_orf1;TRINITY_DN23946_c0_g1_i1_orf1;TRINITY_DN26961_c0_g1_i1_orf1;TRINITY_DN1125_c0_g1_i4_orf1;TRINITY_DN18782_c0_g1_i4_orf1;TRINITY_DN2432_c0_g1_i1_orf1;TRINITY_DN25345_c0_g1_i1_orf1;TRINITY_DN30154_c0_g1_i1_orf1;TRINITY_DN13055_c0_g1_i5_orf1;TRINITY_DN15478_c0_g1_i1_orf1;TRINITY_DN38622_c0_g1_i1_orf1;TRINITY_DN3745_c0_g1_i2_orf1;TRINITY_DN11612_c0_g2_i1_orf1;TRINITY_DN348_c [illegible]
molecular_function	phospholipid binding	GO:0005543	7	7/2360	TRINITY_DN445_c0_g1_i2_orf1;TRINITY_DN96739_c0_g1_i1_orf1;TRINITY_DN6243_c0_g1_i5_orf1;TRINITY_DN119265_c0_g2_i1_orf1;TRINITY_DN11693_c0_g1_i6_orf1;TRINITY_DN65299_c0_g4_i1_orf1;TRINITY_DN18620_c0_g1_i5_orf1
molecular_function	steroid binding	GO:0005496	1	1/2360	TRINITY_DN96739_c0_g1_i1_orf1
molecular_function	monosaccharide binding	GO:0048029	2	2/2360	TRINITY_DN3835_c0_g1_i3_orf1;TRINITY_DN43293_c0_g1_i2_orf1
molecular_function	organic acid binding	GO:0043177	2	2/2360	TRINITY_DN115210_c0_g4_i1_orf1;TRINITY_DN43293_c0_g1_i2_orf1
molecular_function	alcohol binding	GO:0043178	1	1/2360	TRINITY_DN96739_c0_g1_i1_orf1
molecular_function	vitamin binding	GO:0019842	13	13/2360	TRINITY_DN2065_c1_g2_i1_orf1;TRINITY_DN2890_c0_g1_i2_orf1;TRINITY_DN2688_c0_g2_i1_orf1;TRINITY_DN11948_c0_g1_i8_orf1;TRINITY_DN14565_c0_g1_i11_orf1;TRINITY_DN1262_c0_g1_i2_orf1;TRINITY_DN2684_c0_g2_i3_orf1;TRINITY_DN11817_c0_g1_i4_orf1;TRINITY_DN2803_c4_g1_i1_orf1;TRINITY_DN43293_c0_g1_i2_orf1;TRINITY_DN10430_c0_g1_i4_orf1;TRINITY_DN2688_c0_g1_i3_orf1;TRINITY_DN1068_c0_g1_i3_orf1
molecular_function	nucleotide binding	GO:0000166	203	203/2360	TRINITY_DN38482_c0_g1_i4_orf1;TRINITY_DN280_c0_g1_i8_orf1;TRINITY_DN38230_c0_g1_i4_orf1;TRINITY_DN47731_c0_g1_i2_orf1;TRINITY_DN4380_c0_g1_i9_orf1;TRINITY_DN41311_c0_g2_i3_orf1;TRINITY_DN27771_c0_g1_i1_orf1;TRINITY_DN11194_c0_g1_i4_orf1;TRINITY_DN15959_c0_g1_i1_orf1;TRINITY_DN7122_c0_g1_i1_orf1;TRINITY_DN7213_c0_g1_i2_orf1;TRINITY_DN30932_c0_g1_i2_orf1;TRINITY_DN31967_c0_g1_i5_orf1;TRINITY_DN25341_c0_g1_i1_orf1;TRINITY_DN2738_c1_g1_i3_orf1;TRINITY_DN5262_c0_g1_i7_orf1;TRINITY_DN33146_c0_g1_i1_orf1;TRINITY_DN2904_c0_g1_i4_orf1;TRINITY_DN70485_c0_g1_i2_orf1;TRINITY_DN4798_c0_g1_i3_orf1;TRINITY_DN24723_c2_g1_i1_orf1;TRINITY_DN2953_c1_g1_i10_orf1;TRINITY_DN7388_c0_g1_i7_orf1;TRINITY_DN2638_c0_g1_i7_orf1;TRINITY_DN4320_c0_g1_i1_orf1;TRINITY_DN4711_c0_g1_i2_orf1;TRINITY_DN26789_c0_g1_i2_orf1;TRINITY_DN10774_c0_g2_i3_orf1;TRINITY_DN6587_c0_g1_i3_orf1;TRINITY_DN11596_c0_g1_i1_orf1;TRINITY_DN2953_c1_g1_i2_orf1;TRINITY_DN14298_c0_g1_i1_orf1;TRINITY_DN3991_c0_g1_i6_orf1;TRINITY_DN29873_c0_g1_i1_orf1;TRINITY_DN3800_c0_g1_i7_orf1;TRINITY_DN5354_c0_g1_i4_orf1;TRINITY_DN235_c0_g3_i1_orf1;TRINITY_DN6185_c0_g1_i12_orf1;TRINITY_DN6436_c0_g1_i1_orf1;TRINITY_DN37165_c0_g1_i4_orf1;TRINITY_DN26195_c0_g1_i6_orf1;TRINITY_DN2224_c0_g1_i1_orf1;TRINITY_DN20499_c0_g3_i1_orf1;TRINITY_DN987_c0_g1_i3_orf1;TRINITY_DN15591_c0_g1_i3_orf1;TRINITY_DN7247_c0_g1_i7_orf1;TRINITY_DN4501_c0_g1_i3_orf1;TRINITY_DN3637_c0_g1_i2_orf1;TRINITY_DN107288_c0_g1_i2_orf1;TRINITY_DN46715_c0_g1_i1_orf1;TRINITY_DN452_c1_g1_i3_orf1;TRINITY_DN20007_c0_g1_i1_orf1;TRINITY_DN4950_c0_g1_i2_orf1;TRINITY_DN63561_c1_g1_i2_orf1;TRINITY_DN3092_c0_g1_i2_orf1;TRINITY_DN39813_c0_g1_i1_orf1;TRINITY_DN2772_c0_g1_i3_orf1;TRINITY_DN115210_c0_g4_i1_orf1;TRINITY_DN31503_c0_g1_i4_orf1;TRINITY_DN4977_c0_g1_i2_orf1;TRINITY_DN252_c0_g1_i3_orf1;TRINITY_DN1921_c1_g1_i5_orf1;TRINITY_DN8659_c0_g2_i1_orf1;TRINITY_DN17935_c0_g1_i1_orf1;TRINITY_DN4451_c0_g2_i4_orf1;TRINITY_DN1604_c0_g1_i4_orf1;TRINITY_DN1034_c0_g1_i4_orf1;TRINITY_DN90497_c0_g1_i1_orf1;TRINITY_DN42461_c0_g1_i4_orf1;TRINITY_DN11125_c0_g1_i1_orf1;TRINITY_DN9575_c0_g1_i1_orf1;TRINITY_DN6813_c1_g1_i1_orf1;TRINITY_DN1578_c0_g3_i1_orf1;TRINITY_DN359_c0_g1_i5_orf1;TRINITY_DN1607_c0_g1_i16_orf1;TRINITY_DN277_c1_g1_i1_orf1;TRINITY_DN1725_c0_g1_i7_orf1;TRINITY_DN19034_c0_g1_i1_orf1;TRINITY_DN4794_c1_g1_i9_orf1;TRINITY_DN5675_c0_g1_i6_orf1;TRINITY_DN32700_c0_g1_i2_orf1;TRINITY_DN117844_c0_g1_i1_orf1;TRINITY_DN122423_c0_g1_i1_orf1;TRINITY_DN31225_c0_g1_i1_orf1;TRINITY_DN6642_c0_g1_i2_orf1;TRINITY_DN9243_c0_g1_i4_orf1;TRINITY_DN5508_c0_g1_i1_orf1;TRINITY_DN122423_c0_g5_i1_orf1;TRINITY_DN511_c0_g2_i1_orf1;TRINITY_DN1718_c6_g1_i4_orf1;TRINITY_DN1173_c1_g1_i10_orf1;TRINITY_DN2146_c0_g2_i1_orf1;TRINITY_DN7336_c0_g1_i13_orf1;TRINITY_DN15420_c0_g3_i2_orf1;TRINITY_DN4908_c1_g1_i5_orf1;TRINITY_DN28989_c0_g1_i7_orf1;TRINITY_DN25997_c1_g2_i4_orf1;TRINITY_DN47151_c0_g1_i1_orf1;TRINITY_DN235_c0_g1_i2_orf1;TRINITY_DN2709_c0_g1_i4_orf1;TRINITY_DN291_c0_g1_i2_orf1;TRINITY_DN1209_c0_g1_i9_orf1;TRINITY_DN16011_c0_g1_i3_orf1;TRINITY_DN8659_c0_g1_i1_orf1;TRINITY_DN33801_c0_g1_i1_orf1;TRINITY_DN5055_c0_g1_i12_orf1;TRINITY_DN7570_c0_g1_i18_orf1;TRINITY_DN127056_c0_g1_i1_orf1;TRINITY_DN2265_c0_g2_i1_orf1;TRINITY_DN1552_c0_g1_i3_orf1;TRINITY_DN3057_c0_g2_i1_orf1;TRINITY_DN11942_c0_g1_i1_orf1;TRINITY_DN72017_c0_g1_i1_orf1;TRINITY_DN1475_c0_g1_i6_orf1;TRINITY_DN62729_c0_g1_i13_orf1;TRINITY_DN49038_c0_g4_i1_orf1;TRINITY_DN38506_c0_g1_i4_orf1;TRINITY_DN9109_c0_g1_i1_orf1;TRINITY_DN36788_c0_g1_i2_orf1;TRINITY_DN8390_c0_g1_i2_orf1;TRINITY_DN34479_c0_g1_i2_orf1;TRINITY_DN16673_c0_g1_i1_orf1;TRINITY_DN143509_c0_g1_i1_orf1;TRINITY_DN3343_c0_g2_i1_orf1;TRINITY_DN29144_c0_g3_i1_orf1;TRINITY_DN26293_c0_g1_i4_orf1;TRINITY_DN82311_c0_g1_i1_orf1;TRINITY_DN4762_c0_g1_i2_orf1;TRINITY_DN4835_c0_g1_i2_orf1;TRINITY_DN3175_c0_g1_i7_orf1;TRINITY_DN84322_c0_g2_i1_orf1;TRINITY_DN36899_c0_g1_i1_orf1;TRINITY_DN162_c0_g1_i4_orf1;TRINITY_DN64126_c0_g1_i1_orf1;TRINITY_DN48460_c0_g1_i1_orf1;TRINITY_DN44288_c0_g1_i2_orf1;TRINITY_DN96739_c0_g1_i1_orf1;TRINITY_DN60792_c0_g1_i2_orf1;TRINITY_DN2193_c0_g1_i7_orf1;TRINITY_DN97138_c0_g1_i2_orf1;TRINITY_DN5099_c0_g1_i3_orf1;TRINITY_DN111110_c0_g1_i1_orf1;TRINITY_DN15160_c0_g1_i1_orf1;TRINITY_DN62_c1_g1_i3_orf1;TRINITY_DN129869_c0_g4_i1_orf1;TRINITY_DN8979_c0_g1_i5_orf1;TRINITY_DN31327_c0_g2_i1_orf1;TRINITY_DN7512_c0_g1_i1_orf1;TRINITY_DN49047_c0_g1_i2_orf1;TRINITY_DN26168_c0_g1_i1_orf1;TRINITY_DN38424_c0_g1_i1_orf1;TRINITY_DN1084_c0_g2_i2_orf1;TRINITY_DN100821_c0_g1_i1_orf1;TRINITY_DN5422_c0_g1_i1_orf1;TRINITY_DN62557_c0_g1_i1_orf1;TRINITY_DN57918_c0_g1_i1_orf1;TRINITY_DN15706_c0_g2_i5_orf1;TRINITY_DN4790_c0_g1_i6_orf1;TRINITY_DN2874_c0_g1_i4_orf1;TRINITY_DN3428_c0_g1_i1_orf1;TRINITY_DN12964_c0_g1_i1_orf1;TRINITY_DN12820_c0_g1_i1_orf1;TRINITY_DN5648_c0_g1_i5_orf1;TRINITY_DN1405_c0_g1_i1_orf1;TRINITY_DN59291_c0_g1_i1_orf1;TRINITY_DN9302_c0_g1_i1_orf1;TRINITY_DN618_c0_g1_i3_orf1;TRINITY_DN46090_c0_g2_i1_orf1;TRINITY_DN4056_c0_g1_i8_orf1;TRINITY_DN14572_c0_g1_i1_orf1;TRINITY_DN5954_c0_g1_i2_orf1;TRINITY_DN1173_c0_g1_i11_orf1;TRINITY_DN1285_c0_g1_i6_orf1;TRINITY_DN3859_c0_g1_i5_orf1;TRINITY_DN9242_c0_g1_i1_orf1;TRINITY_DN12951_c1_g1_i5_orf1;TRINITY_DN23946_c0_g1_i1_orf1;TRINITY_DN26961_c0_g1_i1_orf1;TRINITY_DN1125_c0_g1_i4_orf1;TRINITY_DN18782_c0_g1_i4_orf1;TRINITY_DN2432_c0_g1_i1_orf1;TRINITY_DN1266_c2_g1_i1_orf1;TRINITY_DN25345_c0_g1_i1_orf1;TRINITY_DN30154_c0_g1_i1_orf1;TRINITY_DN13055_c0_g1_i5_orf1;TRINITY_DN15478_c0_g1_i1_orf1;TRINITY_DN38622_c0_g1_i1_orf1;TRINITY_DN3745_c0_g1_i2_orf1;TRINITY_DN11612_c0 [illegible]
molecular_function	polysaccharide binding	GO:0030247	1	1/2360	TRINITY_DN2170_c0_g2_i1_orf1
molecular_function	phosphatidylserine binding	GO:0001786	1	1/2360	TRINITY_DN96739_c0_g1_i1_orf1
molecular_function	ribonucleoprotein complex binding	GO:0043021	7	7/2360	TRINITY_DN23360_c0_g1_i3_orf1;TRINITY_DN441_c0_g2_i1_orf1;TRINITY_DN3366_c0_g1_i6_orf1;TRINITY_DN48097_c0_g1_i1_orf1;TRINITY_DN44407_c0_g4_i2_orf1;TRINITY_DN10070_c0_g1_i1_orf1;TRINITY_DN6239_c0_g1_i1_orf1
molecular_function	dynein complex binding	GO:0070840	2	2/2360	TRINITY_DN21559_c0_g2_i1_orf1;TRINITY_DN21559_c0_g1_i2_orf1
molecular_function	proteasome binding	GO:0070628	2	2/2360	TRINITY_DN135188_c0_g1_i2_orf1;TRINITY_DN7341_c0_g1_i8_orf1
molecular_function	actin filament binding	GO:0051015	10	10/2360	TRINITY_DN110231_c0_g1_i1_orf1;TRINITY_DN129869_c0_g4_i1_orf1;TRINITY_DN86309_c0_g1_i4_orf1;TRINITY_DN8915_c0_g1_i3_orf1;TRINITY_DN1718_c1_g1_i5_orf1;TRINITY_DN96739_c0_g1_i1_orf1;TRINITY_DN9383_c0_g1_i3_orf1;TRINITY_DN21451_c0_g1_i3_orf1;TRINITY_DN26961_c0_g1_i1_orf1;TRINITY_DN741_c0_g1_i10_orf1
molecular_function	iron-sulfur cluster binding	GO:0051536	6	6/2360	TRINITY_DN14920_c0_g1_i1_orf1;TRINITY_DN2433_c0_g1_i3_orf1;TRINITY_DN5664_c0_g1_i1_orf1;TRINITY_DN4711_c0_g1_i2_orf1;TRINITY_DN3464_c0_g1_i1_orf1;TRINITY_DN9536_c0_g1_i4_orf1
molecular_function	virus receptor activity	GO:0001618	1	1/2360	TRINITY_DN10070_c0_g1_i1_orf1
molecular_function	protein-hormone receptor activity	GO:0016500	1	1/2360	TRINITY_DN3962_c0_g1_i6_orf1
molecular_function	transmembrane signaling receptor activity	GO:0004888	7	7/2360	TRINITY_DN34821_c0_g1_i4_orf1;TRINITY_DN38371_c0_g1_i7_orf1;TRINITY_DN15247_c0_g1_i2_orf1;TRINITY_DN20710_c0_g1_i2_orf1;TRINITY_DN13216_c0_g1_i5_orf1;TRINITY_DN46090_c0_g2_i1_orf1;TRINITY_DN3962_c0_g1_i6_orf1
molecular_function	pattern recognition receptor activity	GO:0038187	2	2/2360	TRINITY_DN2170_c0_g2_i1_orf1;TRINITY_DN2170_c1_g1_i3_orf1
molecular_function	ligase activity, forming carbon-oxygen bonds	GO:0016875	10	10/2360	TRINITY_DN2224_c0_g1_i1_orf1;TRINITY_DN57918_c0_g1_i1_orf1;TRINITY_DN21539_c0_g1_i1_orf1;TRINITY_DN84322_c0_g2_i1_orf1;TRINITY_DN1607_c0_g1_i16_orf1;TRINITY_DN27771_c0_g1_i1_orf1;TRINITY_DN2953_c1_g1_i10_orf1;TRINITY_DN107288_c0_g1_i2_orf1;TRINITY_DN2953_c1_g1_i2_orf1;TRINITY_DN15160_c0_g1_i1_orf1

molecular_function	ligase activity, forming carbon-sulfur bonds	GO:0016877	6	6/2360	TRINITY_DN8659_c0_g1_i1_orf1;TRINITY_DN22928_c0_g1_i6_orf1;TRINITY_DN2193_c0_g1_i7_orf1;TRINITY_DN19251_c0_g1_i8_orf1;TRINITY_DN8659_c0_g2_i1_orf1;TRINITY_DN120593_c0_g1_i1_orf1
molecular_function	ligase activity, forming carbon-nitrogen bonds	GO:0016879	8	8/2360	TRINITY_DN36144_c0_g1_i3_orf1;TRINITY_DN6587_c0_g1_i3_orf1;TRINITY_DN115210_c0_g4_i1_orf1;TRINITY_DN3800_c0_g1_i7_orf1;TRINITY_DN24723_c2_g1_i1_orf1;TRINITY_DN38506_c0_g1_i4_orf1;TRINITY_DN100821_c0_g1_i1_orf1;TRINITY_DN987_c0_g1_i3_orf1
molecular_function	proton-transporting ATP synthase activity, rotati	GO:0046933	1	1/2360	TRINITY_DN96080_c0_g2_i1_orf1
molecular_function	ligase activity, forming carbon-carbon bonds	GO:0016885	2	2/2360	TRINITY_DN3991_c0_g1_i6_orf1;TRINITY_DN511_c0_g2_i1_orf1
molecular_function	ligase activity, forming phosphoric ester bonds	GO:0016886	1	1/2360	TRINITY_DN19034_c0_g1_i1_orf1
molecular_function	carbon-sulfur lyase activity	GO:0016846	1	1/2360	TRINITY_DN11948_c0_g1_i8_orf1
molecular_function	carbon-nitrogen lyase activity	GO:0016840	2	2/2360	TRINITY_DN1716_c0_g1_i14_orf1;TRINITY_DN28299_c0_g1_i1_orf1
molecular_function	phosphorus-oxygen lyase activity	GO:0016849	3	3/2360	TRINITY_DN10774_c0_g2_i3_orf1;TRINITY_DN618_c0_g1_i3_orf1;TRINITY_DN11942_c0_g1_i1_orf1
molecular_function	carbon-oxygen lyase activity	GO:0016835	9	9/2360	TRINITY_DN87603_c0_g2_i1_orf1;TRINITY_DN42759_c0_g3_i1_orf1;TRINITY_DN2433_c0_g1_i3_orf1;TRINITY_DN73900_c0_g1_i1_orf1;TRINITY_DN10430_c0_g1_i4_orf1;TRINITY_DN2825_c0_g1_i3_orf1;TRINITY_DN35763_c0_g1_i2_orf1;TRINITY_DN3464_c0_g1_i1_orf1;TRINITY_DN89483_c0_g1_i1_orf1
molecular_function	carbon-carbon lyase activity	GO:0016830	7	7/2360	TRINITY_DN779_c0_g1_i12_orf1;TRINITY_DN1045_c0_g1_i6_orf1;TRINITY_DN6325_c0_g1_i8_orf1;TRINITY_DN10548_c0_g2_i1_orf1;TRINITY_DN109931_c0_g1_i1_orf1;TRINITY_DN2684_c0_g2_i3_orf1;TRINITY_DN779_c0_g1_i3_orf1
molecular_function	protein-malonyllysine demalonylase activity	GO:0036054	1	1/2360	TRINITY_DN111110_c0_g1_i1_orf1
molecular_function	peptide-lysine-N-acetyltransferase activity	GO:0061733	4	4/2360	TRINITY_DN20442_c0_g2_i1_orf1;TRINITY_DN12771_c0_g1_i1_orf1;TRINITY_DN46202_c0_g1_i1_orf1;TRINITY_DN51737_c0_g1_i3_orf1
molecular_function	palmitoyl-(protein) hydrolase activity	GO:0008474	1	1/2360	TRINITY_DN4817_c0_g1_i4_orf1
molecular_function	protein-disulfide reductase activity	GO:0015035	2	2/2360	TRINITY_DN10118_c0_g1_i4_orf1;TRINITY_DN79673_c0_g1_i1_orf1
molecular_function	protein-glutaryllysine deglutarylase activity	GO:0061697	1	1/2360	TRINITY_DN111110_c0_g1_i1_orf1
molecular_function	phosphoprotein phosphatase activity	GO:0004721	11	11/2360	TRINITY_DN1749_c0_g2_i2_orf1;TRINITY_DN4571_c0_g1_i4_orf1;TRINITY_DN3119_c0_g1_i7_orf1;TRINITY_DN34830_c0_g1_i1_orf1;TRINITY_DN39404_c0_g1_i7_orf1;TRINITY_DN24539_c0_g1_i4_orf1;TRINITY_DN7134_c0_g1_i1_orf1;TRINITY_DN40562_c0_g2_i1_orf1;TRINITY_DN4217_c0_g1_i2_orf1;TRINITY_DN70409_c0_g1_i3_orf1;TRINITY_DN152_c0_g1_i4_orf1
molecular_function	protein-N-terminal asparagine amidohydrolase	GO:0008418	2	2/2360	TRINITY_DN90327_c0_g1_i1_orf1;TRINITY_DN895_c0_g2_i1_orf1
molecular_function	ubiquitin-like protein transferase activity	GO:0019787	9	9/2360	TRINITY_DN24323_c0_g1_i3_orf1;TRINITY_DN9062_c0_g2_i3_orf1;TRINITY_DN7316_c0_g2_i1_orf1;TRINITY_DN11820_c0_g1_i1_orf1;TRINITY_DN1380_c0_g1_i5_orf1;TRINITY_DN48983_c0_g1_i2_orf1;TRINITY_DN9132_c0_g1_i5_orf1;TRINITY_DN1272_c1_g1_i4_orf1;TRINITY_DN14487_c0_g1_i4_orf1
molecular_function	aminoacyltransferase activity	GO:0016755	3	3/2360	TRINITY_DN52553_c0_g2_i1_orf1;TRINITY_DN52553_c0_g1_i1_orf1;TRINITY_DN4898_c0_g1_i7_orf1
molecular_function	protein lysine deacetylase activity	GO:0033558	2	2/2360	TRINITY_DN10385_c0_g1_i5_orf1;TRINITY_DN111110_c0_g1_i1_orf1
molecular_function	protein kinase activity	GO:0004672	24	24/2360	TRINITY_DN38482_c0_g1_i4_orf1;TRINITY_DN1552_c0_g1_i3_orf1;TRINITY_DN1405_c0_g1_i1_orf1;TRINITY_DN46090_c0_g2_i1_orf1;TRINITY_DN6436_c0_g1_i1_orf1;TRINITY_DN62729_c0_g1_i13_orf1;TRINITY_DN42461_c0_g1_i4_orf1;TRINITY_DN1173_c0_g1_i11_orf1;TRINITY_DN11125_c0_g1_i1_orf1;TRINITY_DN70485_c0_g1_i2_orf1;TRINITY_DN277_c1_g1_i1_orf1;TRINITY_DN10774_c0_g2_i3_orf1;TRINITY_DN32700_c0_g1_i2_orf1;TRINITY_DN30154_c0_g1_i1_orf1;TRINITY_DN15478_c0_g1_i1_orf1;TRINITY_DN6185_c0_g1_i12_orf1;TRINITY_DN248_c0_g1_i1_orf1;TRINITY_DN1266_c2_g1_i1_orf1;TRINITY_DN4798_c0_g1_i3_orf1;TRINITY_DN1173_c1_g1_i10_orf1;TRINITY_DN30_c0_g1_i6_orf1;TRINITY_DN15591_c0_g1_i3_orf1;TRINITY_DN147475_c0_g1_i1_orf1;TRINITY_DN46715_c0_g1_i1_orf1
molecular_function	peptidase activity	GO:0008233	101	101/2360	TRINITY_DN3194_c0_g1_i6_orf1;TRINITY_DN181_c0_g1_i3_orf1;TRINITY_DN1533_c0_g2_i1_orf1;TRINITY_DN344_c1_g1_i1_orf1;TRINITY_DN2069_c1_g1_i8_orf1;TRINITY_DN83295_c0_g1_i3_orf1;TRINITY_DN3499_c0_g1_i8_orf1;TRINITY_DN8692_c0_g1_i2_orf1;TRINITY_DN2442_c0_g1_i2_orf1;TRINITY_DN36434_c0_g2_i3_orf1;TRINITY_DN16258_c0_g1_i2_orf1;TRINITY_DN2885_c1_g1_i2_orf1;TRINITY_DN14754_c0_g1_i6_orf1;TRINITY_DN41761_c0_g1_i4_orf1;TRINITY_DN14774_c0_g1_i4_orf1;TRINITY_DN875_c0_g1_i3_orf1;TRINITY_DN18273_c0_g1_i4_orf1;TRINITY_DN2794_c1_g1_i8_orf1;TRINITY_DN4125_c0_g1_i14_orf1;TRINITY_DN10364_c0_g1_i5_orf1;TRINITY_DN6205_c0_g1_i8_orf1;TRINITY_DN391_c0_g1_i4_orf1;TRINITY_DN10769_c0_g1_i1_orf1;TRINITY_DN28428_c0_g1_i2_orf1;TRINITY_DN19651_c0_g1_i1_orf1;TRINITY_DN45633_c0_g1_i1_orf1;TRINITY_DN10090_c0_g1_i1_orf1;TRINITY_DN19537_c0_g1_i1_orf1;TRINITY_DN4189_c0_g2_i1_orf1;TRINITY_DN57798_c0_g1_i1_orf1;TRINITY_DN72541_c0_g1_i2_orf1;TRINITY_DN1274_c0_g1_i4_orf1;TRINITY_DN19990_c0_g1_i1_orf1;TRINITY_DN21719_c0_g1_i2_orf1;TRINITY_DN45948_c1_g1_i1_orf1;TRINITY_DN2040_c0_g1_i6_orf1;TRINITY_DN71863_c0_g1_i2_orf1;TRINITY_DN29034_c0_g1_i2_orf1;TRINITY_DN4125_c1_g1_i5_orf1;TRINITY_DN1703_c0_g1_i6_orf1;TRINITY_DN5444_c0_g2_i1_orf1;TRINITY_DN4408_c6_g1_i1_orf1;TRINITY_DN6423_c0_g1_i6_orf1;TRINITY_DN140_c1_g1_i2_orf1;TRINITY_DN38431_c0_g1_i1_orf1;TRINITY_DN1528_c0_g1_i4_orf1;TRINITY_DN7776_c0_g1_i5_orf1;TRINITY_DN10403_c0_g1_i1_orf1;TRINITY_DN81803_c0_g2_i1_orf1;TRINITY_DN3702_c0_g1_i1_orf1;TRINITY_DN4494_c0_g1_i1_orf1;TRINITY_DN21218_c0_g1_i4_orf1;TRINITY_DN28989_c0_g1_i7_orf1;TRINITY_DN25534_c0_g1_i1_orf1;TRINITY_DN10766_c0_g1_i1_orf1;TRINITY_DN554_c0_g1_i1_orf1;TRINITY_DN36538_c0_g1_i2_orf1;TRINITY_DN8621_c0_g1_i5_orf1;TRINITY_DN57111_c0_g1_i1_orf1;TRINITY_DN5310_c2_g1_i2_orf1;TRINITY_DN6423_c0_g1_i5_orf1;TRINITY_DN1404_c0_g1_i6_orf1;TRINITY_DN34479_c0_g1_i2_orf1;TRINITY_DN21719_c0_g2_i4_orf1;TRINITY_DN3343_c0_g2_i1_orf1;TRINITY_DN18172_c0_g1_i6_orf1;TRINITY_DN19866_c0_g1_i4_orf1;TRINITY_DN2673_c2_g1_i2_orf1;TRINITY_DN1308_c0_g1_i4_orf1;TRINITY_DN701_c0_g1_i1_orf1;TRINITY_DN28661_c0_g1_i1_orf1;TRINITY_DN4228_c0_g1_i5_orf1;TRINITY_DN113327_c0_g1_i2_orf1;TRINITY_DN6470_c0_g3_i2_orf1;TRINITY_DN29034_c0_g1_i1_orf1;TRINITY_DN69697_c0_g1_i1_orf1;TRINITY_DN6813_c1_g1_i1_orf1;TRINITY_DN49047_c0_g1_i2_orf1;TRINITY_DN1459_c1_g1_i1_orf1;TRINITY_DN125967_c0_g1_i1_orf1;TRINITY_DN801_c0_g1_i2_orf1;TRINITY_DN753_c0_g1_i4_orf1;TRINITY_DN13686_c0_g2_i1_orf1;TRINITY_DN334_c0_g1_i4_orf1;TRINITY_DN26853_c0_g1_i1_orf1;TRINITY_DN334_c0_g1_i3_orf1;TRINITY_DN2043_c0_g1_i3_orf1;TRINITY_DN111621_c0_g3_i1_orf1;TRINITY_DN2673_c0_g3_i1_orf1;TRINITY_DN10994_c0_g1_i4_orf1;TRINITY_DN17759_c0_g1_i5_orf1;TRINITY_DN4886_c0_g1_i6_orf1;TRINITY_DN747_c0_g1_i1_orf1;TRINITY_DN96_c0_g1_i1_orf1;TRINITY_DN23167_c0_g2_i1_orf1;TRINITY_DN23167_c0_g1_i4_orf1;TRINITY_DN2043_c0_g1_i11_orf1;TRINITY_DN24121_c1_g1_i6_orf1;TRINITY_DN22797_c0_g1_i5_orf1;TRINITY_DN4125_c0_g1_i6_orf1;TRINITY_DN8480_c0_g1_i1_orf1
molecular_function	peptidyl-prolyl cis-trans isomerase activity	GO:0003755	5	5/2360	TRINITY_DN3773_c0_g1_i4_orf1;TRINITY_DN14372_c0_g2_i1_orf1;TRINITY_DN142588_c0_g1_i1_orf1;TRINITY_DN140538_c0_g2_i1_orf1;TRINITY_DN2807_c0_g1_i4_orf1
molecular_function	ubiquitin-like modifier activating enzyme activity	GO:0008641	4	4/2360	TRINITY_DN22928_c0_g1_i6_orf1;TRINITY_DN8659_c0_g1_i1_orf1;TRINITY_DN8659_c0_g2_i1_orf1;TRINITY_DN120593_c0_g1_i1_orf1
molecular_function	hydrolase activity, acting on glycosyl bonds	GO:0016798	35	35/2360	TRINITY_DN24631_c0_g2_i1_orf1;TRINITY_DN25492_c0_g1_i1_orf1;TRINITY_DN18918_c0_g1_i3_orf1;TRINITY_DN6108_c0_g1_i5_orf1;TRINITY_DN22577_c0_g1_i2_orf1;TRINITY_DN11657_c0_g1_i2_orf1;TRINITY_DN3476_c0_g1_i5_orf1;TRINITY_DN467_c3_g1_i5_orf1;TRINITY_DN13088_c0_g1_i5_orf1;TRINITY_DN479_c6_g1_i2_orf1;TRINITY_DN5852_c0_g1_i13_orf1;TRINITY_DN28741_c0_g1_i3_orf1;TRINITY_DN7183_c0_g1_i2_orf1;TRINITY_DN812_c2_g1_i1_orf1;TRINITY_DN361_c0_g1_i5_orf1;TRINITY_DN48237_c0_g1_i5_orf1;TRINITY_DN2894_c0_g2_i3_orf1;TRINITY_DN542_c0_g1_i4_orf1;TRINITY_DN7228_c0_g1_i6_orf1;TRINITY_DN15222_c0_g1_i4_orf1;TRINITY_DN1785_c0_g1_i5_orf1;TRINITY_DN10824_c0_g1_i3_orf1;TRINITY_DN2170_c0_g2_i1_orf1;TRINITY_DN2515_c0_g1_i6_orf1;TRINITY_DN4070_c0_g1_i4_orf1;TRINITY_DN17003_c1_g1_i1_orf1;TRINITY_DN26688_c0_g1_i2_orf1;TRINITY_DN21555_c0_g1_i4_orf1;TRINITY_DN7828_c0_g1_i2_orf1;TRINITY_DN1732_c0_g1_i17_orf1;TRINITY_DN103118_c0_g1_i4_orf1;TRINITY_DN2170_c1_g1_i3_orf1;TRINITY_DN5852_c0_g1_i6_orf1;TRINITY_DN54410_c0_g2_i1_orf1;TRINITY_DN650_c0_g1_i3_orf1
molecular_function	hydrolase activity, acting on acid carbon-carbon	GO:0016822	1	1/2360	TRINITY_DN19187_c0_g1_i1_orf1
molecular_function	hydrolase activity, acting on carbon-nitrogen (bu	GO:0016810	20	20/2360	TRINITY_DN5422_c0_g1_i1_orf1;TRINITY_DN895_c0_g2_i1_orf1;TRINITY_DN5235_c0_g1_i7_orf1;TRINITY_DN1534_c0_g1_i3_orf1;TRINITY_DN17326_c0_g1_i8_orf1;TRINITY_DN90327_c0_g1_i1_orf1;TRINITY_DN115210_c0_g4_i1_orf1;TRINITY_DN87170_c0_g1_i3_orf1;TRINITY_DN8674_c0_g2_i1_orf1;TRINITY_DN1216_c0_g1_i4_orf1;TRINITY_DN2835_c0_g1_i6_orf1;TRINITY_DN82801_c0_g1_i1_orf1;TRINITY_DN38506_c0_g1_i4_orf1;TRINITY_DN98242_c0_g1_i1_orf1;TRINITY_DN13660_c0_g1_i1_orf1;TRINITY_DN542_c0_g1_i4_orf1;TRINITY_DN10385_c0_g1_i5_orf1;TRINITY_DN11383_c0_g2_i4_orf1;TRINITY_DN111110_c0_g1_i1_orf1;TRINITY_DN4145_c0_g1_i1_orf1
molecular_function	hydrolase activity, acting on acid anhydrides	GO:0016817	50	50/2360	TRINITY_DN280_c0_g1_i8_orf1;TRINITY_DN3092_c0_g1_i2_orf1;TRINITY_DN2265_c0_g2_i1_orf1;TRINITY_DN4790_c0_g1_i6_orf1;TRINITY_DN63561_c1_g1_i2_orf1;TRINITY_DN7570_c0_g1_i18_orf1;TRINITY_DN9575_c0_g1_i1_orf1;TRINITY_DN11194_c0_g1_i4_orf1;TRINITY_DN252_c0_g1_i3_orf1;TRINITY_DN15706_c0_g2_i5_orf1;TRINITY_DN7122_c0_g1_i1_orf1;TRINITY_DN33801_c0_g1_i1_orf1;TRINITY_DN2054_c0_g1_i1_orf1;TRINITY_DN25341_c0_g1_i1_orf1;TRINITY_DN5262_c0_g1_i7_orf1;TRINITY_DN90497_c0_g1_i1_orf1;TRINITY_DN11069_c0_g2_i1_orf1;TRINITY_DN34479_c0_g1_i2_orf1;TRINITY_DN29144_c0_g3_i1_orf1;TRINITY_DN4977_c0_g1_i2_orf1;TRINITY_DN7388_c0_g1_i7_orf1;TRINITY_DN2638_c0_g1_i7_orf1;TRINITY_DN3343_c0_g2_i1_orf1;TRINITY_DN1725_c0_g1_i7_orf1;TRINITY_DN452_c1_g1_i3_orf1;TRINITY_DN1091_c0_g1_i1_orf1;TRINITY_DN1091_c0_g3_i1_orf1;TRINITY_DN12951_c1_g1_i5_orf1;TRINITY_DN5422_c0_g1_i1_orf1;TRINITY_DN4762_c0_g1_i2_orf1;TRINITY_DN14572_c0_g1_i1_orf1;TRINITY_DN13055_c0_g1_i5_orf1;TRINITY_DN6642_c0_g1_i2_orf1;TRINITY_DN15420_c0_g3_i2_orf1;TRINITY_DN9243_c0_g1_i4_orf1;TRINITY_DN41311_c0_g2_i3_orf1;TRINITY_DN48460_c0_g1_i1_orf1;TRINITY_DN5508_c0_g1_i1_orf1;TRINITY_DN97138_c0_g1_i2_orf1;TRINITY_DN11612_c0_g2_i1_orf1;TRINITY_DN7565_c0_g1_i3_orf1;TRINITY_DN45449_c0_g1_i1_orf1;TRINITY_DN8979_c0_g1_i5_orf1;TRINITY_DN4779_c0_g1_i5_orf1;TRINITY_DN7464_c1_g1_i1_orf1;TRINITY_DN7336_c0_g1_i13_orf1;TRINITY_DN49047_c0_g1_i2_orf1;TRINITY_DN28989_c0_g1_i7_orf1;TRINITY_DN16011_c0_g1_i3_orf1;TRINITY_DN100821_c0_g1_i1_orf1

molecular_function	serine hydrolase activity	GO:0017171	49	49/2360	TRINITY_DN36434_c0_g2_i3_orf1;TRINITY_DN10766_c0_g1_i1_orf1;TRINITY_DN2043_c0_g1_i11_orf1;TRINITY_DN10090_c0_g1_i1_orf1;TRINITY_DN1533_c0_g2_i1_orf1;TRINITY_DN13686_c0_g2_i1_orf1;TRINITY_DN6205_c0_g1_i8_orf1;TRINITY_DN334_c0_g1_i4_orf1;TRINITY_DN111621_c0_g3_i1_orf1;TRINITY_DN344_c1_g1_i1_orf1;TRINITY_DN57111_c0_g1_i1_orf1;TRINITY_DN5310_c2_g1_i2_orf1;TRINITY_DN6423_c0_g1_i5_orf1;TRINITY_DN1404_c0_g1_i6_orf1;TRINITY_DN3499_c0_g1_i8_orf1;TRINITY_DN1274_c0_g1_i4_orf1;TRINITY_DN25534_c0_g1_i1_orf1;TRINITY_DN2043_c0_g1_i3_orf1;TRINITY_DN21719_c0_g1_i2_orf1;TRINITY_DN16258_c0_g1_i2_orf1;TRINITY_DN21719_c0_g2_i4_orf1;TRINITY_DN4886_c0_g1_i6_orf1;TRINITY_DN24121_c1_g1_i6_orf1;TRINITY_DN41761_c0_g1_i4_orf1;TRINITY_DN2040_c0_g1_i6_orf1;TRINITY_DN71863_c0_g1_i2_orf1;TRINITY_DN29034_c0_g1_i2_orf1;TRINITY_DN747_c0_g1_i1_orf1;TRINITY_DN18273_c0_g1_i4_orf1;TRINITY_DN96_c0_g1_i1_orf1;TRINITY_DN5444_c0_g2_i1_orf1;TRINITY_DN1308_c0_g1_i4_orf1;TRINITY_DN701_c0_g1_i1_orf1;TRINITY_DN10364_c0_g1_i5_orf1;TRINITY_DN4228_c0_g1_i5_orf1;TRINITY_DN6423_c0_g1_i6_orf1;TRINITY_DN23167_c0_g2_i1_orf1;TRINITY_DN140_c1_g1_i2_orf1;TRINITY_DN391_c0_g1_i4_orf1;TRINITY_DN6470_c0_g3_i2_orf1;TRINITY_DN1528_c0_g1_i4_orf1;TRINITY_DN10403_c0_g1_i1_orf1;TRINITY_DN23167_c0_g1_i4_orf1;TRINITY_DN29034_c0_g1_i1_orf1;TRINITY_DN334_c0_g1_i3_orf1;TRINITY_DN4494_c0_g1_i1_orf1;TRINITY_DN22797_c0_g1_i5_orf1;TRINITY_DN753_c0_g1_i4_orf1;TRINITY_DN8480_c0_g1_i1_orf1
molecular_function	hydrolase activity, acting on ester bonds	GO:0016788	69	69/2360	TRINITY_DN1749_c0_g2_i2_orf1;TRINITY_DN2749_c4_g1_i2_orf1;TRINITY_DN1978_c0_g1_i4_orf1;TRINITY_DN38230_c0_g1_i4_orf1;TRINITY_DN2772_c0_g1_i3_orf1;TRINITY_DN2749_c0_g1_i4_orf1;TRINITY_DN40562_c0_g2_i1_orf1;TRINITY_DN12227_c0_g2_i3_orf1;TRINITY_DN39404_c0_g1_i7_orf1;TRINITY_DN38562_c0_g1_i3_orf1;TRINITY_DN5238_c0_g1_i2_orf1;TRINITY_DN8771_c0_g2_i1_orf1;TRINITY_DN121650_c0_g1_i1_orf1;TRINITY_DN4817_c0_g1_i4_orf1;TRINITY_DN10644_c0_g1_i2_orf1;TRINITY_DN70409_c0_g1_i3_orf1;TRINITY_DN1952_c0_g1_i2_orf1;TRINITY_DN38274_c0_g1_i1_orf1;TRINITY_DN95850_c0_g1_i1_orf1;TRINITY_DN4571_c0_g1_i4_orf1;TRINITY_DN3758_c0_g1_i2_orf1;TRINITY_DN4217_c0_g1_i2_orf1;TRINITY_DN70485_c0_g1_i2_orf1;TRINITY_DN34830_c0_g1_i1_orf1;TRINITY_DN69713_c0_g1_i1_orf1;TRINITY_DN7134_c0_g1_i1_orf1;TRINITY_DN2812_c0_g1_i5_orf1;TRINITY_DN1884_c0_g2_i2_orf1;TRINITY_DN15865_c0_g1_i1_orf1;TRINITY_DN2367_c1_g1_i20_orf1;TRINITY_DN10430_c0_g1_i4_orf1;TRINITY_DN17693_c0_g1_i10_orf1;TRINITY_DN152_c0_g1_i4_orf1;TRINITY_DN616_c1_g1_i6_orf1;TRINITY_DN2808_c0_g1_i8_orf1;TRINITY_DN1249_c0_g1_i10_orf1;TRINITY_DN45271_c0_g1_i1_orf1;TRINITY_DN17437_c0_g1_i1_orf1;TRINITY_DN117_c0_g1_i6_orf1;TRINITY_DN13330_c0_g1_i4_orf1;TRINITY_DN87522_c0_g2_i1_orf1;TRINITY_DN3073_c0_g1_i7_orf1;TRINITY_DN42759_c0_g3_i1_orf1;TRINITY_DN1073_c0_g1_i3_orf1;TRINITY_DN34432_c0_g1_i1_orf1;TRINITY_DN18922_c0_g1_i1_orf1;TRINITY_DN3119_c0_g1_i7_orf1;TRINITY_DN123184_c0_g1_i1_orf1;TRINITY_DN24539_c0_g1_i4_orf1;TRINITY_DN38644_c0_g1_i1_orf1;TRINITY_DN935_c0_g1_i3_orf1;TRINITY_DN1841_c0_g1_i2_orf1;TRINITY_DN40945_c0_g1_i1_orf1;TRINITY_DN72707_c0_g1_i1_orf1;TRINITY_DN117_c0_g1_i4_orf1;TRINITY_DN44658_c0_g1_i2_orf1;TRINITY_DN4813_c0_g1_i5_orf1;TRINITY_DN2668_c0_g1_i7_orf1;TRINITY_DN2647_c0_g1_i3_orf1;TRINITY_DN42120_c0_g1_i2_orf1;TRINITY_DN15865_c0_g2_i2_orf1;TRINITY_DN6163_c0_g1_i4_orf1;TRINITY_DN11798_c0_g2_i1_orf1;TRINITY_DN41_c0_g1_i5_orf1;TRINITY_DN26168_c0_g1_i1_orf1;TRINITY_DN48023_c1_g1_i1_orf1;TRINITY_DN9931_c0_g1_i1_orf1;TRINITY_DN1161_c0_g1_i2_orf1;TRINITY_DN10662_c0_g1_i4_orf1
molecular_function	deacetylase activity	GO:0019213	4	4/2360	TRINITY_DN542_c0_g1_i4_orf1;TRINITY_DN10385_c0_g1_i5_orf1;TRINITY_DN82801_c0_g1_i1_orf1;TRINITY_DN111110_c0_g1_i1_orf1
molecular_function	deaminase activity	GO:0019239	2	2/2360	TRINITY_DN1196_c0_g1_i5_orf1;TRINITY_DN98242_c0_g1_i1_orf1
molecular_function	palmitoyl hydrolase activity	GO:0098599	1	1/2360	TRINITY_DN4817_c0_g1_i4_orf1
molecular_function	hydrolase activity, acting on ether bonds	GO:0016801	1	1/2360	TRINITY_DN5768_c0_g1_i2_orf1
molecular_function	FAD-AMP lyase (cyclizing) activity	GO:0034012	2	2/2360	TRINITY_DN11942_c0_g1_i1_orf1;TRINITY_DN618_c0_g1_i3_orf1
molecular_function	guanylate cyclase activity	GO:0004383	1	1/2360	TRINITY_DN10774_c0_g2_i3_orf1
molecular_function	oxidoreductase activity, acting on CH-OH group	GO:0016614	22	22/2360	TRINITY_DN5266_c0_g1_i1_orf1;TRINITY_DN146126_c0_g1_i1_orf1;TRINITY_DN1921_c1_g1_i5_orf1;TRINITY_DN4451_c0_g2_i4_orf1;TRINITY_DN49038_c0_g4_i1_orf1;TRINITY_DN36788_c0_g1_i2_orf1;TRINITY_DN29018_c0_g1_i4_orf1;TRINITY_DN9286_c0_g1_i2_orf1;TRINITY_DN4793_c0_g1_i7_orf1;TRINITY_DN42759_c0_g3_i1_orf1;TRINITY_DN10430_c0_g1_i4_orf1;TRINITY_DN26293_c0_g1_i4_orf1;TRINITY_DN4794_c1_g1_i9_orf1;TRINITY_DN3175_c0_g1_i7_orf1;TRINITY_DN36899_c0_g1_i1_orf1;TRINITY_DN5354_c0_g1_i4_orf1;TRINITY_DN3472_c0_g1_i6_orf1;TRINITY_DN1264_c0_g1_i2_orf1;TRINITY_DN1206_c0_g1_i6_orf1;TRINITY_DN77830_c0_g2_i2_orf1;TRINITY_DN38424_c0_g1_i1_orf1;TRINITY_DN1209_c0_g1_i9_orf1
molecular_function	oxidoreductase activity, acting on the aldehyde (	GO:0016903	15	15/2360	TRINITY_DN7075_c0_g2_i1_orf1;TRINITY_DN14755_c0_g1_i4_orf1;TRINITY_DN482_c0_g1_i1_orf1;TRINITY_DN40126_c0_g1_i1_orf1;TRINITY_DN29873_c0_g1_i1_orf1;TRINITY_DN4596_c0_g1_i14_orf1;TRINITY_DN1293_c0_g1_i4_orf1;TRINITY_DN1103_c0_g1_i15_orf1;TRINITY_DN6586_c0_g1_i1_orf1;TRINITY_DN7335_c0_g1_i1_orf1;TRINITY_DN40126_c0_g2_i1_orf1;TRINITY_DN1293_c1_g1_i4_orf1;TRINITY_DN7808_c0_g1_i1_orf1;TRINITY_DN2100_c0_g1_i2_orf1;TRINITY_DN52788_c0_g1_i1_orf1
molecular_function	oxidoreductase activity, acting on diphenols and	GO:0016679	1	1/2360	TRINITY_DN2140_c0_g1_i1_orf1
molecular_function	oxidoreductase activity, acting on the CH-NH gr	GO:0016645	4	4/2360	TRINITY_DN38506_c0_g1_i4_orf1;TRINITY_DN24970_c0_g1_i4_orf1;TRINITY_DN92153_c0_g2_i2_orf1;TRINITY_DN15136_c0_g1_i2_orf1
molecular_function	dioxygenase activity	GO:0051213	7	7/2360	TRINITY_DN44083_c0_g1_i2_orf1;TRINITY_DN4822_c0_g1_i6_orf1;TRINITY_DN38562_c0_g1_i3_orf1;TRINITY_DN14398_c0_g1_i4_orf1;TRINITY_DN43293_c0_g1_i2_orf1;TRINITY_DN57900_c0_g1_i2_orf1;TRINITY_DN4822_c0_g1_i9_orf1
molecular_function	electron transfer activity	GO:0009055	3	3/2360	TRINITY_DN14920_c0_g1_i1_orf1;TRINITY_DN9242_c0_g1_i1_orf1;TRINITY_DN9135_c0_g1_i4_orf1
molecular_function	oxidoreductase activity, acting on paired donors	GO:0016705	38	38/2360	TRINITY_DN43369_c0_g2_i1_orf1;TRINITY_DN120500_c0_g1_i1_orf1;TRINITY_DN9608_c0_g1_i3_orf1;TRINITY_DN30704_c0_g1_i1_orf1;TRINITY_DN64126_c0_g1_i1_orf1;TRINITY_DN7580_c0_g1_i1_orf1;TRINITY_DN863_c0_g1_i6_orf1;TRINITY_DN43293_c0_g1_i2_orf1;TRINITY_DN14262_c0_g1_i5_orf1;TRINITY_DN4998_c0_g1_i21_orf1;TRINITY_DN625_c9_g1_i7_orf1;TRINITY_DN1664_c0_g1_i4_orf1;TRINITY_DN7212_c0_g1_i4_orf1;TRINITY_DN3949_c0_g1_i1_orf1;TRINITY_DN1134_c0_g1_i4_orf1;TRINITY_DN31163_c1_g1_i4_orf1;TRINITY_DN6027_c0_g1_i13_orf1;TRINITY_DN829_c0_g1_i8_orf1;TRINITY_DN1363_c0_g1_i11_orf1;TRINITY_DN50743_c0_g1_i1_orf1;TRINITY_DN3675_c0_g1_i1_orf1;TRINITY_DN15755_c0_g1_i1_orf1;TRINITY_DN2392_c0_g2_i1_orf1;TRINITY_DN448_c0_g1_i20_orf1;TRINITY_DN1999_c0_g1_i9_orf1;TRINITY_DN27045_c0_g1_i1_orf1;TRINITY_DN1960_c5_g1_i3_orf1;TRINITY_DN57856_c0_g2_i1_orf1;TRINITY_DN5439_c0_g1_i2_orf1;TRINITY_DN52887_c0_g1_i1_orf1;TRINITY_DN4497_c2_g1_i3_orf1;TRINITY_DN5126_c0_g1_i3_orf1;TRINITY_DN14398_c0_g1_i4_orf1;TRINITY_DN5661_c0_g1_i5_orf1;TRINITY_DN48590_c0_g1_i1_orf1;TRINITY_DN5004_c0_g1_i2_orf1;TRINITY_DN82944_c0_g1_i4_orf1;TRINITY_DN2338_c0_g1_i5_orf1
molecular_function	oxidoreductase activity, acting on single donors	GO:0016701	4	4/2360	TRINITY_DN1707_c0_g1_i1_orf1;TRINITY_DN4822_c0_g1_i6_orf1;TRINITY_DN4822_c0_g1_i9_orf1;TRINITY_DN38562_c0_g1_i3_orf1
molecular_function	oxidoreductase activity, acting on the CH-CH gr	GO:0016627	9	9/2360	TRINITY_DN23042_c0_g1_i1_orf1;TRINITY_DN5055_c0_g1_i12_orf1;TRINITY_DN29018_c0_g1_i4_orf1;TRINITY_DN42759_c0_g3_i1_orf1;TRINITY_DN10430_c0_g1_i4_orf1;TRINITY_DN30932_c0_g1_i2_orf1;TRINITY_DN77830_c0_g2_i2_orf1;TRINITY_DN1125_c0_g1_i4_orf1;TRINITY_DN72017_c0_g1_i1_orf1
molecular_function	oxidoreductase activity, acting on NAD(P)H	GO:0016651	5	5/2360	TRINITY_DN1661_c0_g1_i1_orf1;TRINITY_DN1134_c0_g1_i4_orf1;TRINITY_DN8306_c0_g1_i4_orf1;TRINITY_DN52887_c0_g1_i1_orf1;TRINITY_DN6563_c0_g1_i1_orf1
molecular_function	oxidoreductase activity, acting on superoxide rac	GO:0016721	3	3/2360	TRINITY_DN103107_c0_g1_i2_orf1;TRINITY_DN16400_c0_g2_i1_orf1;TRINITY_DN1024_c0_g4_i1_orf1
molecular_function	oxidoreductase activity, acting on metal ions	GO:0016722	4	4/2360	TRINITY_DN46625_c0_g1_i1_orf1;TRINITY_DN1423_c0_g1_i8_orf1;TRINITY_DN65681_c0_g1_i1_orf1;TRINITY_DN1423_c0_g1_i4_orf1
molecular_function	oxidoreductase activity, acting on CH or CH2 grc	GO:0016725	1	1/2360	TRINITY_DN4835_c0_g1_i2_orf1
molecular_function	oxidoreductase activity, acting on peroxide as ac	GO:0016684	10	10/2360	TRINITY_DN7778_c0_g1_i1_orf1;TRINITY_DN791_c0_g1_i2_orf1;TRINITY_DN51252_c0_g2_i1_orf1;TRINITY_DN80660_c0_g1_i1_orf1;TRINITY_DN5933_c0_g1_i1_orf1;TRINITY_DN285_c0_g1_i4_orf1;TRINITY_DN69236_c0_g1_i1_orf1;TRINITY_DN3321_c0_g1_i3_orf1;TRINITY_DN21420_c0_g1_i2_orf1;TRINITY_DN2652_c0_g2_i1_orf1
molecular_function	monooxygenase activity	GO:0004497	41	41/2360	TRINITY_DN43369_c0_g2_i1_orf1;TRINITY_DN120500_c0_g1_i1_orf1;TRINITY_DN9608_c0_g1_i3_orf1;TRINITY_DN30704_c0_g1_i1_orf1;TRINITY_DN84357_c0_g1_i1_orf1;TRINITY_DN64126_c0_g1_i1_orf1;TRINITY_DN7580_c0_g1_i1_orf1;TRINITY_DN2392_c0_g2_i1_orf1;TRINITY_DN14262_c0_g1_i5_orf1;TRINITY_DN4998_c0_g1_i21_orf1;TRINITY_DN625_c9_g1_i7_orf1;TRINITY_DN51342_c0_g1_i7_orf1;TRINITY_DN7212_c0_g1_i4_orf1;TRINITY_DN1664_c0_g1_i4_orf1;TRINITY_DN3949_c0_g1_i1_orf1;TRINITY_DN1134_c0_g1_i4_orf1;TRINITY_DN31163_c1_g1_i4_orf1;TRINITY_DN6027_c0_g1_i13_orf1;TRINITY_DN829_c0_g1_i8_orf1;TRINITY_DN1363_c0_g1_i11_orf1;TRINITY_DN50743_c0_g1_i1_orf1;TRINITY_DN3675_c0_g1_i1_orf1;TRINITY_DN15755_c0_g1_i1_orf1;TRINITY_DN448_c0_g1_i20_orf1;TRINITY_DN27045_c0_g1_i1_orf1;TRINITY_DN2338_c0_g2_i2_orf1;TRINITY_DN1960_c5_g1_i3_orf1;TRINITY_DN57856_c0_g2_i1_orf1;TRINITY_DN5439_c0_g1_i2_orf1;TRINITY_DN52887_c0_g1_i1_orf1;TRINITY_DN4497_c2_g1_i3_orf1;TRINITY_DN1707_c0_g1_i1_orf1;TRINITY_DN5126_c0_g1_i3_orf1;TRINITY_DN863_c0_g1_i6_orf1;TRINITY_DN5661_c0_g1_i5_orf1;TRINITY_DN5004_c0_g1_i2_orf1;TRINITY_DN82944_c0_g1_i4_orf1;TRINITY_DN2338_c0_g2_i1_orf1;TRINITY_DN9198_c0_g1_i4_orf1;TRINITY_DN2338_c0_g1_i5_orf1;TRINITY_DN2338_c0_g1_i3_orf1
molecular_function	oxidoreductase activity, acting on the CH-NH2 g	GO:0016638	3	3/2360	TRINITY_DN3859_c0_g1_i5_orf1;TRINITY_DN37165_c0_g1_i4_orf1;TRINITY_DN43431_c0_g1_i1_orf1
molecular_function	oxidoreductase activity, acting on other nitrogen	GO:0016661	1	1/2360	TRINITY_DN2559_c0_g1_i4_orf1
molecular_function	oxidoreductase activity, acting on a sulfur group	GO:0016667	9	9/2360	TRINITY_DN25987_c0_g1_i5_orf1;TRINITY_DN1491_c0_g1_i8_orf1;TRINITY_DN10118_c0_g1_i4_orf1;TRINITY_DN2430_c0_g1_i1_orf1;TRINITY_DN79673_c0_g1_i1_orf1;TRINITY_DN81715_c0_g1_i1_orf1;TRINITY_DN2207_c0_g1_i6_orf1;TRINITY_DN920_c0_g1_i6_orf1;TRINITY_DN1491_c0_g1_i4_orf1
molecular_function	lysozyme activity	GO:0003796	2	2/2360	TRINITY_DN54410_c0_g2_i1_orf1;TRINITY_DN467_c3_g1_i5_orf1
molecular_function	N-acetylmuramoyl-L-alanine amidase activity	GO:0008745	2	2/2360	TRINITY_DN5235_c0_g1_i7_orf1;TRINITY_DN1534_c0_g1_i3_orf1
molecular_function	intramolecular lyase activity	GO:0016872	1	1/2360	TRINITY_DN10722_c0_g3_i1_orf1
molecular_function	DNA topoisomerase activity	GO:0003916	2	2/2360	TRINITY_DN4908_c1_g1_i5_orf1;TRINITY_DN6248_c0_g1_i1_orf1
molecular_function	intramolecular transferase activity	GO:0016866	6	6/2360	TRINITY_DN5952_c0_g1_i6_orf1;TRINITY_DN12545_c0_g1_i7_orf1;TRINITY_DN1827_c0_g1_i4_orf1;TRINITY_DN30713_c0_g1_i3_orf1;TRINITY_DN2769_c0_g1_i1_orf1;TRINITY_DN120089_c0_g1_i1_orf1
molecular_function	intramolecular oxidoreductase activity	GO:0016860	3	3/2360	TRINITY_DN6014_c1_g1_i2_orf1;TRINITY_DN1196_c0_g1_i5_orf1;TRINITY_DN3073_c0_g1_i7_orf1
molecular_function	racemase and epimerase activity	GO:0016854	1	1/2360	TRINITY_DN1353_c0_g1_i1_orf1
molecular_function	cis-trans isomerase activity	GO:0016859	5	5/2360	TRINITY_DN3773_c0_g1_i4_orf1;TRINITY_DN2807_c0_g1_i4_orf1;TRINITY_DN14372_c0_g2_i1_orf1;TRINITY_DN142588_c0_g1_i1_orf1;TRINITY_DN140538_c0_g2_i1_orf1

molecular_function	catalytic activity, acting on RNA	GO:0140098	36	36/2360	TRINITY_DN57918_c0_g1_i1_orf1;TRINITY_DN4380_c0_g1_i9_orf1;TRINITY_DN31503_c0_g1_i4_orf1;TRINITY_DN13350_c0_g1_i4_orf1;TRINITY_DN27771_c0_g1_i1_orf1;TRINITY_DN17312_c0_g1_i1_orf1;TRINITY_DN9302_c0_g1_i1_orf1;TRINITY_DN59291_c0_g1_i1_orf1;TRINITY_DN7213_c0_g1_i2_orf1;TRINITY_DN5962_c0_g1_i1_orf1;TRINITY_DN1344_c0_g1_i1_orf1;TRINITY_DN2904_c0_g1_i4_orf1;TRINITY_DN70485_c0_g1_i2_orf1;TRINITY_DN2953_c1_g1_i10_orf1;TRINITY_DN34676_c1_g1_i3_orf1;TRINITY_DN2953_c1_g1_i2_orf1;TRINITY_DN19034_c0_g1_i1_orf1;TRINITY_DN5675_c0_g1_i6_orf1;TRINITY_DN11596_c0_g1_i1_orf1;TRINITY_DN84322_c0_g2_i1_orf1;TRINITY_DN34432_c0_g1_i1_orf1;TRINITY_DN4408_c6_g1_i1_orf1;TRINITY_DN18922_c0_g1_i1_orf1;TRINITY_DN4707_c0_g1_i1_orf1;TRINITY_DN44288_c0_g1_i2_orf1;TRINITY_DN15160_c0_g1_i1_orf1;TRINITY_DN2224_c0_g1_i1_orf1;TRINITY_DN2401_c0_g2_i1_orf1;TRINITY_DN20499_c0_g3_i1_orf1;TRINITY_DN21539_c0_g1_i1_orf1;TRINITY_DN4813_c0_g1_i5_orf1;TRINITY_DN2709_c0_g1_i4_orf1;TRINITY_DN107288_c0_g1_i2_orf1;TRINITY_DN26168_c0_g1_i1_orf1;TRINITY_DN4950_c0_g1_i2_orf1;TRINITY_DN1607_c0_g1_i16_orf1
molecular_function	catalytic activity, acting on DNA	GO:0140097	20	20/2360	TRINITY_DN81258_c0_g1_i2_orf1;TRINITY_DN3092_c0_g1_i2_orf1;TRINITY_DN291_c0_g1_i2_orf1;TRINITY_DN2971_c0_g1_i1_orf1;TRINITY_DN15040_c0_g4_i1_orf1;TRINITY_DN87603_c0_g2_i1_orf1;TRINITY_DN45271_c0_g1_i1_orf1;TRINITY_DN6642_c0_g1_i2_orf1;TRINITY_DN70485_c0_g1_i2_orf1;TRINITY_DN12820_c0_g1_i1_orf1;TRINITY_DN110534_c0_g1_i3_orf1;TRINITY_DN6248_c0_g1_i1_orf1;TRINITY_DN123184_c0_g1_i1_orf1;TRINITY_DN4908_c1_g1_i5_orf1;TRINITY_DN7122_c0_g1_i1_orf1;TRINITY_DN452_c1_g1_i3_orf1;TRINITY_DN3057_c0_g2_i1_orf1;TRINITY_DN5757_c0_g1_i1_orf1;TRINITY_DN45449_c0_g1_i1_orf1;TRINITY_DN25345_c0_g1_i1_orf1
molecular_function	helicase activity	GO:0004386	23	23/2360	TRINITY_DN2971_c0_g1_i1_orf1;TRINITY_DN4380_c0_g1_i9_orf1;TRINITY_DN31503_c0_g1_i4_orf1;TRINITY_DN3057_c0_g2_i1_orf1;TRINITY_DN9302_c0_g1_i1_orf1;TRINITY_DN7122_c0_g1_i1_orf1;TRINITY_DN59291_c0_g1_i1_orf1;TRINITY_DN7213_c0_g1_i2_orf1;TRINITY_DN2904_c0_g1_i4_orf1;TRINITY_DN452_c1_g1_i3_orf1;TRINITY_DN5675_c0_g1_i6_orf1;TRINITY_DN11596_c0_g1_i1_orf1;TRINITY_DN25345_c0_g1_i1_orf1;TRINITY_DN6642_c0_g1_i2_orf1;TRINITY_DN4408_c6_g1_i1_orf1;TRINITY_DN45449_c0_g1_i1_orf1;TRINITY_DN5757_c0_g1_i1_orf1;TRINITY_DN20499_c0_g3_i1_orf1;TRINITY_DN44288_c0_g1_i2_orf1;TRINITY_DN4950_c0_g1_i2_orf1;TRINITY_DN26168_c0_g1_i1_orf1;TRINITY_DN2709_c0_g1_i4_orf1;TRINITY_DN291_c0_g1_i2_orf1
molecular_function	transferase activity, transferring alkyl or aryl (othe	GO:0016765	20	20/2360	TRINITY_DN10222_c0_g1_i2_orf1;TRINITY_DN9506_c0_g1_i2_orf1;TRINITY_DN1305_c0_g1_i6_orf1;TRINITY_DN2255_c0_g1_i1_orf1;TRINITY_DN128231_c0_g1_i5_orf1;TRINITY_DN4695_c0_g1_i4_orf1;TRINITY_DN15597_c0_g1_i1_orf1;TRINITY_DN8651_c0_g1_i18_orf1;TRINITY_DN2430_c0_g1_i1_orf1;TRINITY_DN12134_c0_g1_i4_orf1;TRINITY_DN1578_c0_g3_i1_orf1;TRINITY_DN4695_c0_g1_i3_orf1;TRINITY_DN7512_c0_g1_i1_orf1;TRINITY_DN8964_c0_g1_i4_orf1;TRINITY_DN8651_c0_g1_i16_orf1;TRINITY_DN62707_c0_g1_i1_orf1;TRINITY_DN20682_c0_g2_i1_orf1;TRINITY_DN4279_c0_g1_i4_orf1;TRINITY_DN8854_c0_g1_i2_orf1;TRINITY_DN920_c0_g1_i6_orf1
molecular_function	transferase activity, transferring nitrogenous gro	GO:0016769	7	7/2360	TRINITY_DN2890_c0_g1_i2_orf1;TRINITY_DN14565_c0_g1_i11_orf1;TRINITY_DN1262_c0_g1_i2_orf1;TRINITY_DN5564_c0_g1_i5_orf1;TRINITY_DN2803_c4_g1_i1_orf1;TRINITY_DN11013_c0_g1_i3_orf1;TRINITY_DN1068_c0_g1_i3_orf1
molecular_function	glycosyltransferase activity	GO:0016757	15	15/2360	TRINITY_DN38435_c0_g1_i1_orf1;TRINITY_DN125_c0_g1_i2_orf1;TRINITY_DN10548_c0_g2_i1_orf1;TRINITY_DN79868_c0_g1_i1_orf1;TRINITY_DN28592_c0_g1_i2_orf1;TRINITY_DN3355_c0_g2_i4_orf1;TRINITY_DN11817_c0_g1_i4_orf1;TRINITY_DN14018_c0_g1_i4_orf1;TRINITY_DN9079_c0_g1_i5_orf1;TRINITY_DN812_c2_g1_i1_orf1;TRINITY_DN31390_c0_g1_i2_orf1;TRINITY_DN40197_c0_g1_i1_orf1;TRINITY_DN14597_c0_g1_i5_orf1;TRINITY_DN53760_c0_g1_i1_orf1;TRINITY_DN140669_c0_g1_i1_orf1
molecular_function	transketolase or transaldolase activity	GO:0016744	1	1/2360	TRINITY_DN60787_c0_g1_i5_orf1
molecular_function	transferase activity, transferring phosphorus-con	GO:0016772	63	63/2360	TRINITY_DN38482_c0_g1_i4_orf1;TRINITY_DN14477_c0_g1_i12_orf1;TRINITY_DN47151_c0_g1_i1_orf1;TRINITY_DN39813_c0_g1_i1_orf1;TRINITY_DN18222_c0_g1_i5_orf1;TRINITY_DN127056_c0_g1_i1_orf1;TRINITY_DN62557_c0_g1_i1_orf1;TRINITY_DN277_c1_g1_i1_orf1;TRINITY_DN1552_c0_g1_i3_orf1;TRINITY_DN1405_c0_g1_i1_orf1;TRINITY_DN11942_c0_g1_i1_orf1;TRINITY_DN37923_c0_g1_i1_orf1;TRINITY_DN8261_c0_g1_i1_orf1;TRINITY_DN31967_c0_g1_i5_orf1;TRINITY_DN81258_c0_g1_i2_orf1;TRINITY_DN2738_c1_g1_i3_orf1;TRINITY_DN618_c0_g1_i3_orf1;TRINITY_DN62729_c0_g1_i13_orf1;TRINITY_DN15040_c0_g4_i1_orf1;TRINITY_DN1034_c0_g1_i4_orf1;TRINITY_DN33146_c0_g1_i1_orf1;TRINITY_DN42461_c0_g1_i4_orf1;TRINITY_DN46090_c0_g2_i1_orf1;TRINITY_DN11125_c0_g1_i1_orf1;TRINITY_DN30154_c0_g1_i1_orf1;TRINITY_DN70485_c0_g1_i2_orf1;TRINITY_DN1718_c6_g1_i4_orf1;TRINITY_DN248_c0_g1_i1_orf1;TRINITY_DN143509_c0_g1_i1_orf1;TRINITY_DN4320_c0_g1_i1_orf1;TRINITY_DN40197_c0_g1_i1_orf1;TRINITY_DN4056_c0_g1_i8_orf1;TRINITY_DN7247_c0_g1_i7_orf1;TRINITY_DN46715_c0_g1_i1_orf1;TRINITY_DN10774_c0_g2_i3_orf1;TRINITY_DN32700_c0_g1_i2_orf1;TRINITY_DN1475_c0_g1_i6_orf1;TRINITY_DN15478_c0_g1_i1_orf1;TRINITY_DN4408_c6_g1_i1_orf1;TRINITY_DN6185_c0_g1_i12_orf1;TRINITY_DN4707_c0_g1_i1_orf1;TRINITY_DN110534_c0_g1_i3_orf1;TRINITY_DN6436_c0_g1_i1_orf1;TRINITY_DN4929_c1_g2_i5_orf1;TRINITY_DN1266_c2_g1_i1_orf1;TRINITY_DN4798_c0_g1_i3_orf1;TRINITY_DN26195_c0_g1_i6_orf1;TRINITY_DN2401_c0_g2_i1_orf1;TRINITY_DN2618_c0_g1_i3_orf1;TRINITY_DN1173_c1_g1_i10_orf1;TRINITY_DN30_c0_g1_i6_orf1;TRINITY_DN1957_c0_g1_i4_orf1;TRINITY_DN18782_c0_g1_i4_orf1;TRINITY_DN15591_c0_g1_i3_orf1;TRINITY_DN147475_c0_g1_i1_orf1;TRINITY_DN9109_c0_g1_i1_orf1;TRINITY_DN6813_c1_g1_i1_orf1;TRINITY_DN116972_c0_g1_i1_orf1;TRINITY_DN1173_c0_g1_i11_orf1;TRINITY_DN25997_c1_g2_i4_orf1;TRINITY_DN5697_c0_g1_i1_orf1;TRINITY_DN2082_c0_g1_i2_orf1;TRINITY_DN1285_c0_g1_i6_orf1
molecular_function	transferase activity, transferring one-carbon gro	GO:0016741	14	14/2360	TRINITY_DN31431_c0_g1_i1_orf1;TRINITY_DN1344_c0_g1_i1_orf1;TRINITY_DN115210_c0_g4_i1_orf1;TRINITY_DN3800_c0_g1_i7_orf1;TRINITY_DN5748_c0_g1_i6_orf1;TRINITY_DN1216_c0_g1_i4_orf1;TRINITY_DN13350_c0_g1_i4_orf1;TRINITY_DN2114_c0_g1_i5_orf1;TRINITY_DN17312_c0_g1_i1_orf1;TRINITY_DN34676_c1_g1_i3_orf1;TRINITY_DN5748_c0_g1_i5_orf1;TRINITY_DN2457_c0_g1_i8_orf1;TRINITY_DN14313_c0_g1_i1_orf1;TRINITY_DN5962_c0_g1_i1_orf1
molecular_function	acyltransferase activity	GO:0016746	28	28/2360	TRINITY_DN2065_c1_g2_i1_orf1;TRINITY_DN86833_c0_g3_i1_orf1;TRINITY_DN20442_c0_g2_i1_orf1;TRINITY_DN20710_c0_g1_i2_orf1;TRINITY_DN46202_c0_g1_i1_orf1;TRINITY_DN5841_c0_g1_i2_orf1;TRINITY_DN52553_c0_g2_i1_orf1;TRINITY_DN5153_c1_g1_i1_orf1;TRINITY_DN12771_c0_g1_i1_orf1;TRINITY_DN5211_c0_g1_i1_orf1;TRINITY_DN3219_c0_g1_i6_orf1;TRINITY_DN12497_c0_g1_i1_orf1;TRINITY_DN42759_c0_g3_i1_orf1;TRINITY_DN1362_c0_g1_i4_orf1;TRINITY_DN10430_c0_g1_i4_orf1;TRINITY_DN21570_c0_g1_i1_orf1;TRINITY_DN117844_c0_g1_i1_orf1;TRINITY_DN3179_c0_g1_i1_orf1;TRINITY_DN51737_c0_g1_i3_orf1;TRINITY_DN15411_c0_g1_i4_orf1;TRINITY_DN16125_c0_g1_i3_orf1;TRINITY_DN76283_c0_g2_i1_orf1;TRINITY_DN22956_c0_g1_i1_orf1;TRINITY_DN52553_c0_g1_i1_orf1;TRINITY_DN1084_c0_g1_i2_orf1;TRINITY_DN4898_c0_g1_i7_orf1;TRINITY_DN3545_c0_g1_i6_orf1;TRINITY_DN1084_c0_g2_i2_orf1
