# Supplementary material for: Comparative Proteomics and Metabonomics Analysis of Different Diapause Stages Revealed a New Regulation Mechanism of Diapause in Loxostege sticticalis (Lepidoptera: Pyralidae)
Source: Molecules. 2024 Jul 25;29(15):3472. doi: 10.3390/molecules29153472 (PMC11314584; doi:10.3390/molecules29153472)
Supplement: Supplementary file 1 [file molecules-29-03472-s001.zip › analysis process/proteomic/GO annotations analysis/RDvsD all.pdf]

Term Type	GO Term	GO ID	JCZY_vs_ZY_all num	JCZY_vs_ZY_all percent	JCZY_vs_ZY_all Accession ids
biological_process	immune response-activating signal transduction	GO:0002757	2	2/2406	TRINITY_DN2170_c0_g1_i2_orf1;TRINITY_DN2170_c1_g1_i3_orf1
biological_process	activation of innate immune response	GO:0002218	5	5/2406	TRINITY_DN8685_c0_g1_i5_orf1;TRINITY_DN1091_c0_g2_i10_orf1;TRINITY_DN2170_c0_g1_i2_orf1;TRINITY_DN2170_c1_g1_i3_orf1;TRINITY_DN5880_c0_g2_i2_orf1
biological_process	cell activation involved in immune response	GO:0002263	1	1/2406	TRINITY_DN46409_c0_g1_i1_orf1
biological_process	lymphocyte activation	GO:0046649	1	1/2406	TRINITY_DN46409_c0_g1_i1_orf1
biological_process	leukocyte activation involved in immune response	GO:0002366	1	1/2406	TRINITY_DN46409_c0_g1_i1_orf1
biological_process	innate immune response	GO:0045087	14	14/2406	TRINITY_DN21545_c0_g1_i2_orf1;TRINITY_DN1534_c0_g1_i3_orf1;TRINITY_DN479_c6_g1_i2_orf1;TRINITY_DN8685_c0_g1_i5_orf1;TRINITY_DN1444_c1_g1_i5_orf1;TRINITY_DN2170_c0_g1_i2_orf1;TRINITY_DN1091_c0_g2_i10_orf1;TRINITY_DN6098_c1_g1_i5_orf1;TRINITY_DN2170_c1_g1_i3_orf1;TRINITY_DN15706_c0_g2_i5_orf1;TRINITY_DN1666_c0_g1_i2_orf1;TRINITY_DN5235_c0_g1_i7_orf1;TRINITY_DN9044_c0_g1_i2_orf1;TRINITY_DN5880_c0_g2_i2_orf1
biological_process	humoral immune response	GO:0006959	1	1/2406	TRINITY_DN2848_c0_g1_i1_orf1
biological_process	somatic diversification of immune receptors via germline recombination within a single locus	GO:0002562	1	1/2406	TRINITY_DN46409_c0_g1_i1_orf1
biological_process	somatic diversification of immunoglobulins	GO:0016445	1	1/2406	TRINITY_DN46409_c0_g1_i1_orf1
biological_process	regulation of catalytic activity	GO:0050790	15	15/2406	TRINITY_DN1475_c0_g1_i6_orf1;TRINITY_DN13999_c0_g1_i4_orf1;TRINITY_DN8473_c0_g1_i6_orf1;TRINITY_DN518_c0_g1_i1_orf1;TRINITY_DN11986_c0_g1_i1_orf1;TRINITY_DN975_c0_g1_i1_orf1;TRINITY_DN802_c0_g1_i2_orf1;TRINITY_DN1328_c0_g1_i6_orf1;TRINITY_DN46409_c0_g1_i1_orf1;TRINITY_DN2848_c0_g1_i1_orf1;TRINITY_DN50074_c0_g1_i1_orf1;TRINITY_DN147475_c0_g1_i1_orf1;TRINITY_DN23354_c0_g1_i7_orf1;TRINITY_DN55148_c0_g1_i1_orf1;TRINITY_DN28661_c0_g1_i1_orf1
biological_process	positive regulation of molecular function	GO:0044093	11	11/2406	TRINITY_DN5406_c0_g2_i1_orf1;TRINITY_DN9475_c0_g1_i6_orf1;TRINITY_DN23354_c0_g1_i7_orf1;TRINITY_DN802_c0_g1_i2_orf1;TRINITY_DN46409_c0_g1_i1_orf1;TRINITY_DN1352_c0_g1_i5_orf1;TRINITY_DN5553_c0_g1_i4_orf1;TRINITY_DN2175_c0_g1_i4_orf1;TRINITY_DN48097_c0_g1_i1_orf1;TRINITY_DN50074_c0_g1_i1_orf1;TRINITY_DN55148_c0_g1_i1_orf1
biological_process	negative regulation of molecular function	GO:0044092	7	7/2406	TRINITY_DN5442_c0_g1_i4_orf1;TRINITY_DN13999_c0_g1_i4_orf1;TRINITY_DN11986_c0_g1_i1_orf1;TRINITY_DN1328_c0_g1_i6_orf1;TRINITY_DN2848_c0_g1_i1_orf1;TRINITY_DN55148_c0_g1_i1_orf1;TRINITY_DN31584_c0_g2_i2_orf1
biological_process	regulation of binding	GO:0051098	3	3/2406	TRINITY_DN48097_c0_g1_i1_orf1;TRINITY_DN55148_c0_g1_i1_orf1;TRINITY_DN147475_c0_g1_i1_orf1
biological_process	regulation of ATP-dependent activity	GO:0043462	2	2/2406	TRINITY_DN5442_c0_g1_i4_orf1;TRINITY_DN11986_c0_g1_i1_orf1
biological_process	regulation of transporter activity	GO:0032409	6	6/2406	TRINITY_DN5406_c0_g2_i1_orf1;TRINITY_DN9475_c0_g1_i6_orf1;TRINITY_DN1352_c0_g1_i5_orf1;TRINITY_DN31584_c0_g2_i2_orf1;TRINITY_DN5553_c0_g1_i4_orf1;TRINITY_DN2175_c0_g1_i4_orf1
biological_process	regulation of metabolic process	GO:0019222	49	49/2406	TRINITY_DN13999_c0_g1_i4_orf1;TRINITY_DN19260_c0_g1_i5_orf1;TRINITY_DN8702_c0_g1_i1_orf1;TRINITY_DN1706_c0_g1_i7_orf1;TRINITY_DN23360_c0_g1_i3_orf1;TRINITY_DN31585_c0_g1_i1_orf1;TRINITY_DN46409_c0_g1_i1_orf1;TRINITY_DN31584_c0_g2_i2_orf1;TRINITY_DN288_c0_g1_i9_orf1;TRINITY_DN9938_c0_g2_i1_orf1;TRINITY_DN48097_c0_g1_i1_orf1;TRINITY_DN31851_c0_g1_i2_orf1;TRINITY_DN1475_c0_g1_i6_orf1;TRINITY_DN8473_c0_g1_i6_orf1;TRINITY_DN67649_c0_g1_i1_orf1;TRINITY_DN21150_c0_g1_i4_orf1;TRINITY_DN3457_c0_g1_i4_orf1;TRINITY_DN12771_c0_g1_i1_orf1;TRINITY_DN975_c0_g1_i1_orf1;TRINITY_DN142442_c0_g1_i1_orf1;TRINITY_DN55148_c0_g1_i1_orf1;TRINITY_DN1328_c0_g1_i6_orf1;TRINITY_DN3673_c0_g1_i10_orf1;TRINITY_DN41602_c0_g3_i1_orf1;TRINITY_DN11050_c0_g1_i8_orf1;TRINITY_DN2848_c0_g1_i1_orf1;TRINITY_DN50074_c0_g1_i1_orf1;TRINITY_DN34689_c0_g1_i4_orf1;TRINITY_DN18681_c0_g1_i7_orf1;TRINITY_DN12_c0_g1_i5_orf1;TRINITY_DN17655_c0_g1_i1_orf1;TRINITY_DN5562_c0_g1_i3_orf1;TRINITY_DN50085_c0_g1_i1_orf1;TRINITY_DN15256_c0_g1_i8_orf1;TRINITY_DN44070_c0_g2_i2_orf1;TRINITY_DN44877_c0_g1_i2_orf1;TRINITY_DN72_c0_g1_i16_orf1;TRINITY_DN96557_c0_g1_i1_orf1;TRINITY_DN51836_c0_g3_i1_orf1;TRINITY_DN16451_c0_g1_i7_orf1;TRINITY_DN2802_c0_g1_i1_orf1;TRINITY_DN33893_c0_g1_i1_orf1;TRINITY_DN4820_c0_g2_i2_orf1;TRINITY_DN2802_c1_g1_i1_orf1;TRINITY_DN147475_c0_g1_i1_orf1;TRINITY_DN18563_c2_g1_i1_orf1;TRINITY_DN4950_c0_g1_i2_orf1;TRINITY_DN6462_c0_g1_i5_orf1;TRINITY_DN4246_c0_g2_i3_orf1
biological_process	regulation of response to stimulus	GO:0048583	20	20/2406	TRINITY_DN1637_c0_g1_i5_orf1;TRINITY_DN51938_c0_g3_i1_orf1;TRINITY_DN17655_c0_g1_i1_orf1;TRINITY_DN21545_c0_g1_i2_orf1;TRINITY_DN479_c6_g1_i2_orf1;TRINITY_DN8685_c0_g1_i5_orf1;TRINITY_DN2170_c0_g1_i2_orf1;TRINITY_DN558_c0_g1_i4_orf1;TRINITY_DN4464_c0_g2_i1_orf1;TRINITY_DN147475_c0_g1_i1_orf1;TRINITY_DN1091_c0_g2_i10_orf1;TRINITY_DN46409_c0_g1_i1_orf1;TRINITY_DN31980_c0_g1_i1_orf1;TRINITY_DN2170_c1_g1_i3_orf1;TRINITY_DN2848_c0_g1_i1_orf1;TRINITY_DN146119_c0_g1_i1_orf1;TRINITY_DN34745_c0_g2_i1_orf1;TRINITY_DN3833_c0_g1_i4_orf1;TRINITY_DN55148_c0_g1_i1_orf1;TRINITY_DN5880_c0_g2_i2_orf1
biological_process	regulation of developmental process	GO:0050793	4	4/2406	TRINITY_DN51836_c0_g3_i1_orf1;TRINITY_DN41602_c0_g3_i1_orf1;TRINITY_DN3887_c0_g1_i1_orf1;TRINITY_DN20133_c0_g1_i1_orf1
biological_process	regulation of cellular process	GO:0050794	91	91/2406	TRINITY_DN142442_c0_g1_i1_orf1;TRINITY_DN21545_c0_g1_i2_orf1;TRINITY_DN1008_c0_g1_i2_orf1;TRINITY_DN8692_c0_g1_i2_orf1;TRINITY_DN558_c0_g1_i4_orf1;TRINITY_DN2983_c0_g1_i6_orf1;TRINITY_DN3457_c0_g1_i4_orf1;TRINITY_DN11050_c0_g1_i8_orf1;TRINITY_DN2848_c0_g1_i1_orf1;TRINITY_DN18681_c0_g1_i7_orf1;TRINITY_DN17655_c0_g1_i1_orf1;TRINITY_DN15256_c0_g1_i8_orf1;TRINITY_DN4798_c0_g1_i3_orf1;TRINITY_DN33893_c0_g1_i1_orf1;TRINITY_DN7102_c0_g1_i5_orf1;TRINITY_DN2802_c0_g1_i1_orf1;TRINITY_DN2802_c1_g1_i1_orf1;TRINITY_DN2170_c1_g1_i3_orf1;TRINITY_DN18563_c2_g1_i1_orf1;TRINITY_DN3833_c0_g1_i4_orf1;TRINITY_DN31584_c0_g2_i2_orf1;TRINITY_DN51938_c0_g3_i1_orf1;TRINITY_DN8702_c0_g1_i1_orf1;TRINITY_DN1706_c0_g1_i7_orf1;TRINITY_DN23360_c0_g1_i3_orf1;TRINITY_DN52649_c0_g1_i6_orf1;TRINITY_DN2793_c0_g2_i1_orf1;TRINITY_DN2623_c0_g1_i3_orf1;TRINITY_DN10629_c0_g1_i1_orf1;TRINITY_DN146119_c0_g1_i1_orf1;TRINITY_DN34745_c0_g2_i1_orf1;TRINITY_DN5406_c0_g2_i1_orf1;TRINITY_DN288_c0_g1_i9_orf1;TRINITY_DN3673_c0_g1_i10_orf1;TRINITY_DN33418_c0_g1_i1_orf1;TRINITY_DN12_c0_g1_i5_orf1;TRINITY_DN1637_c0_g1_i5_orf1;TRINITY_DN32700_c0_g1_i2_orf1;TRINITY_DN50085_c0_g1_i1_orf1;TRINITY_DN4439_c0_g1_i2_orf1;TRINITY_DN51836_c0_g3_i1_orf1;TRINITY_DN16451_c0_g1_i7_orf1;TRINITY_DN2170_c0_g1_i2_orf1;TRINITY_DN804_c0_g1_i7_orf1;TRINITY_DN16011_c0_g1_i3_orf1;TRINITY_DN13999_c0_g1_i4_orf1;TRINITY_DN9475_c0_g1_i6_orf1;TRINITY_DN4676_c0_g1_i16_orf1;TRINITY_DN46409_c0_g1_i1_orf1;TRINITY_DN40211_c0_g1_i1_orf1;TRINITY_DN13216_c0_g1_i5_orf1;TRINITY_DN5182_c0_g1_i5_orf1;TRINITY_DN31851_c0_g1_i2_orf1;TRINITY_DN1475_c0_g1_i6_orf1;TRINITY_DN14987_c0_g1_i3_orf1;TRINITY_DN5553_c0_g1_i4_orf1;TRINITY_DN4142_c0_g1_i5_orf1;TRINITY_DN5562_c0_g1_i3_orf1;TRINITY_DN11986_c0_g1_i1_orf1;TRINITY_DN44070_c0_g2_i2_orf1;TRINITY_DN72_c0_g1_i16_orf1;TRINITY_DN96557_c0_g1_i1_orf1;TRINITY_DN9146_c0_g1_i1_orf1;TRINITY_DN802_c0_g1_i2_orf1;TRINITY_DN42854_c0_g3_i2_orf1;TRINITY_DN31980_c0_g1_i1_orf1;TRINITY_DN10455_c0_g1_i2_orf1;TRINITY_DN46633_c0_g1_i4_orf1;TRINITY_DN2942_c0_g1_i6_orf1;TRINITY_DN79000_c1_g1_i1_orf1;TRINITY_DN67649_c0_g1_i1_orf1;TRINITY_DN9938_c0_g2_i1_orf1;TRINITY_DN15706_c0_g2_i5_orf1;TRINITY_DN4159_c1_g1_i1_orf1;TRINITY_DN1405_c0_g1_i1_orf1;TRINITY_DN48097_c0_g1_i1_orf1;TRINITY_DN55148_c0_g1_i1_orf1;TRINITY_DN8473_c0_g1_i6_orf1;TRINITY_DN21150_c0_g1_i4_orf1;TRINITY_DN975_c0_g1_i1_orf1;TRINITY_DN1352_c0_g1_i5_orf1;TRINITY_DN41602_c0_g3_i1_orf1;TRINITY_DN31585_c0_g1_i1_orf1;TRINITY_DN2175_c0_g1_i4_orf1;TRINITY_DN4410_c0_g1_i1_orf1;TRINITY_DN11245_c0_g1_i2_orf1;TRINITY_DN4464_c0_g2_i1_orf1;TRINITY_DN147475_c0_g1_i1_orf1;TRINITY_DN14684_c0_g2_i1_orf1;TRINITY_DN20133_c0_g1_i1_orf1;TRINITY_DN6462_
biological_process	regulation of locomotion	GO:0040012	2	2/2406	TRINITY_DN147475_c0_g1_i1_orf1;TRINITY_DN20133_c0_g1_i1_orf1
biological_process	regulation of localization	GO:0032879	7	7/2406	TRINITY_DN5406_c0_g2_i1_orf1;TRINITY_DN14987_c0_g1_i3_orf1;TRINITY_DN9475_c0_g1_i6_orf1;TRINITY_DN1352_c0_g1_i5_orf1;TRINITY_DN31584_c0_g2_i2_orf1;TRINITY_DN5553_c0_g1_i4_orf1;TRINITY_DN2175_c0_g1_i4_orf1
biological_process	regulation of multicellular organismal process	GO:0051239	7	7/2406	TRINITY_DN51836_c0_g3_i1_orf1;TRINITY_DN1455_c0_g1_i4_orf1;TRINITY_DN1455_c0_g1_i8_orf1;TRINITY_DN147475_c0_g1_i1_orf1;TRINITY_DN46409_c0_g1_i1_orf1;TRINITY_DN41602_c0_g3_i1_orf1;TRINITY_DN2848_c0_g1_i1_orf1
biological_process	regulation of membrane repolarization	GO:0060306	1	1/2406	TRINITY_DN31584_c0_g2_i2_orf1
biological_process	regulation of immune system process	GO:0002682	8	8/2406	TRINITY_DN21545_c0_g1_i2_orf1;TRINITY_DN479_c6_g1_i2_orf1;TRINITY_DN8685_c0_g1_i5_orf1;TRINITY_DN2170_c0_g1_i2_orf1;TRINITY_DN1091_c0_g2_i10_orf1;TRINITY_DN46409_c0_g1_i1_orf1;TRINITY_DN2170_c1_g1_i3_orf1;TRINITY_DN5880_c0_g2_i2_orf1
biological_process	positive regulation of biological process	GO:0048518	27	27/2406	TRINITY_DN51938_c0_g3_i1_orf1;TRINITY_DN15706_c0_g2_i5_orf1;TRINITY_DN8685_c0_g1_i5_orf1;TRINITY_DN9475_c0_g1_i6_orf1;TRINITY_DN46409_c0_g1_i1_orf1;TRINITY_DN31584_c0_g2_i2_orf1;TRINITY_DN288_c0_g1_i9_orf1;TRINITY_DN146119_c0_g1_i1_orf1;TRINITY_DN48097_c0_g1_i1_orf1;TRINITY_DN55148_c0_g1_i1_orf1;TRINITY_DN5406_c0_g2_i1_orf1;TRINITY_DN558_c0_g1_i4_orf1;TRINITY_DN3457_c0_g1_i4_orf1;TRINITY_DN1352_c0_g1_i5_orf1;TRINITY_DN2848_c0_g1_i1_orf1;TRINITY_DN5553_c0_g1_i4_orf1;TRINITY_DN17655_c0_g1_i1_orf1;TRINITY_DN50074_c0_g1_i1_orf1;TRINITY_DN2175_c0_g1_i4_orf1;TRINITY_DN1091_c0_g2_i10_orf1;TRINITY_DN51836_c0_g3_i1_orf1;TRINITY_DN2170_c0_g1_i2_orf1;TRINITY_DN147475_c0_g1_i1_orf1;TRINITY_DN2170_c1_g1_i3_orf1;TRINITY_DN20133_c0_g1_i1_orf1;TRINITY_DN3833_c0_g1_i4_orf1;TRINITY_DN5880_c0_g2_i2_orf1
biological_process	negative regulation of biological process	GO:0048519	20	20/2406	TRINITY_DN96557_c0_g1_i1_orf1;TRINITY_DN4820_c0_g2_i2_orf1;TRINITY_DN13999_c0_g1_i4_orf1;TRINITY_DN20133_c0_g1_i1_orf1;TRINITY_DN21545_c0_g1_i2_orf1;TRINITY_DN8702_c0_g1_i1_orf1;TRINITY_DN3673_c0_g1_i10_orf1;TRINITY_DN11986_c0_g1_i1_orf1;TRINITY_DN52649_c0_g1_i6_orf1;TRINITY_DN44070_c0_g2_i2_orf1;TRINITY_DN44877_c0_g1_i2_orf1;TRINITY_DN1328_c0_g1_i6_orf1;TRINITY_DN46409_c0_g1_i1_orf1;TRINITY_DN2848_c0_g1_i1_orf1;TRINITY_DN4159_c1_g1_i1_orf1;TRINITY_DN34689_c0_g1_i4_orf1;TRINITY_DN147475_c0_g1_i1_orf1;TRINITY_DN55148_c0_g1_i1_orf1;TRINITY_DN12771_c0_g1_i1_orf1;TRINITY_DN31584_c0_g2_i2_o

biological_process	regulation of signaling	GO:0023051	17	17/2406	TRINITY_DN1637_c0_g1_i5_orf1;TRINITY_DN51938_c0_g3_i1_orf1;TRINITY_DN5406_c0_g2_i1_orf1;TRINITY_DN21545_c0_g1_i2_orf1;TRINITY_DN9475_c0_g1_i6_orf1;TRINITY_DN2175_c0_g1_i4_orf1;TRINITY_DN558_c0_g1_i4_orf1;TRINITY_DN4464_c0_g2_i1_orf1;TRINITY_DN147475_c0_g1_i1_orf1;TRINITY_DN1352_c0_g1_i5_orf1;TRINITY_DN31980_c0_g1_i1_orf1;TRINITY_DN2848_c0_g1_i1_orf1;TRINITY_DN5553_c0_g1_i4_orf1;TRINITY_DN146119_c0_g1_i1_orf1;TRINITY_DN34745_c0_g2_i1_orf1;TRINITY_DN3833_c0_g1_i4_orf1;TRINITY_DN55148_c0_g1_i1_orf1
biological_process	regulation of growth	GO:0040008	1	1/2406	TRINITY_DN51836_c0_g3_i1_orf1
biological_process	regulation of membrane potential	GO:0042391	1	1/2406	TRINITY_DN31584_c0_g2_i2_orf1
biological_process	regulation of body fluid levels	GO:0050878	1	1/2406	TRINITY_DN4016_c0_g1_i1_orf1
biological_process	homeostatic process	GO:0042592	12	12/2406	TRINITY_DN96557_c0_g1_i1_orf1;TRINITY_DN46625_c0_g1_i1_orf1;TRINITY_DN65681_c0_g1_i1_orf1;TRINITY_DN1423_c0_g1_i4_orf1;TRINITY_DN1423_c0_g1_i8_orf1;TRINITY_DN136031_c0_g1_i7_orf1;TRINITY_DN3461_c0_g1_i1_orf1;TRINITY_DN15812_c0_g1_i2_orf1;TRINITY_DN31584_c0_g2_i2_orf1;TRINITY_DN20133_c0_g1_i1_orf1;TRINITY_DN44256_c0_g1_i1_orf1;TRINITY_DN5753_c0_g1_i10_orf1
biological_process	regulation of anatomical structure size	GO:0090066	4	4/2406	TRINITY_DN4439_c0_g1_i2_orf1;TRINITY_DN52649_c0_g1_i6_orf1;TRINITY_DN10455_c0_g1_i2_orf1;TRINITY_DN4159_c1_g1_i1_orf1
biological_process	regulation of protein stability	GO:0031647	3	3/2406	TRINITY_DN46409_c0_g1_i1_orf1;TRINITY_DN2848_c0_g1_i1_orf1;TRINITY_DN55148_c0_g1_i1_orf1
biological_process	NADH regeneration	GO:0006735	2	2/2406	TRINITY_DN2848_c0_g1_i1_orf1;TRINITY_DN20133_c0_g1_i1_orf1
biological_process	organonitrogen compound metabolic process	GO:1901564	291	291/2406	TRINITY_DN24469_c0_g2_i2_orf1;TRINITY_DN2065_c1_g2_i1_orf1;TRINITY_DN10364_c0_g1_i5_orf1;TRINITY_DN11948_c0_g1_i8_orf1;TRINITY_DN142442_c0_g1_i1_orf1;TRINITY_DN5564_c0_g1_i5_orf1;TRINITY_DN863_c0_g1_i6_orf1;TRINITY_DN1533_c0_g2_i1_orf1;TRINITY_DN28661_c0_g1_i1_orf1;TRINITY_DN3978_c0_g2_i1_orf1;TRINITY_DN1757_c0_g1_i4_orf1;TRINITY_DN35763_c0_g1_i2_orf1;TRINITY_DN1274_c0_g1_i4_orf1;TRINITY_DN2069_c1_g1_i8_orf1;TRINITY_DN1153_c1_g1_i1_orf1;TRINITY_DN4217_c0_g1_i2_orf1;TRINITY_DN38431_c0_g1_i1_orf1;TRINITY_DN58207_c0_g1_i1_orf1;TRINITY_DN17772_c0_g2_i3_orf1;TRINITY_DN8692_c0_g1_i2_orf1;TRINITY_DN2442_c0_g1_i2_orf1;TRINITY_DN8659_c0_g1_i1_orf1;TRINITY_DN1216_c0_g1_i4_orf1;TRINITY_DN41952_c0_g1_i4_orf1;TRINITY_DN2983_c0_g1_i6_orf1;TRINITY_DN97680_c0_g1_i1_orf1;TRINITY_DN24723_c2_g1_i1_orf1;TRINITY_DN16258_c0_g1_i2_orf1;TRINITY_DN2953_c1_g1_i10_orf1;TRINITY_DN277_c1_g1_i1_orf1;TRINITY_DN2848_c0_g1_i1_orf1;TRINITY_DN14754_c0_g1_i6_orf1;TRINITY_DN29448_c0_g1_i1_orf1;TRINITY_DN11013_c0_g1_i3_orf1;TRINITY_DN49047_c0_g1_i2_orf1;TRINITY_DN14217_c0_g1_i1_orf1;TRINITY_DN14774_c0_g1_i4_orf1;TRINITY_DN48020_c0_g1_i1_orf1;TRINITY_DN42753_c0_g1_i2_orf1;TRINITY_DN21570_c0_g1_i1_orf1;TRINITY_DN2885_c1_g1_i2_orf1;TRINITY_DN2953_c1_g1_i2_orf1;TRINITY_DN17326_c0_g1_i8_orf1;TRINITY_DN2224_c0_g1_i1_orf1;TRINITY_DN3991_c0_g1_i6_orf1;TRINITY_DN2794_c1_g1_i8_orf1;TRINITY_DN20796_c0_g1_i4_orf1;TRINITY_DN17759_c0_g1_i5_orf1;TRINITY_DN3800_c0_g1_i7_orf1;TRINITY_DN1760_c0_g1_i4_orf1;TRINITY_DN84478_c0_g1_i8_orf1;TRINITY_DN4886_c0_g1_i6_orf1;TRINITY_DN6185_c0_g1_i12_orf1;TRINITY_DN20527_c0_g1_i1_orf1;TRINITY_DN6205_c0_g1_i8_orf1;TRINITY_DN6436_c0_g1_i1_orf1;TRINITY_DN41086_c0_g1_i4_orf1;TRINITY_DN4798_c0_g1_i3_orf1;TRINITY_DN391_c0_g1_i4_orf1;TRINITY_DN875_c0_g1_i3_orf1;TRINITY_DN6563_c0_g1_i1_orf1;TRINITY_DN40_c0_g2_i1_orf1;TRINITY_DN4822_c0_g1_i9_orf1;TRINITY_DN19990_c0_g1_i1_orf1;TRINITY_DN13732_c0_g2_i3_orf1;TRINITY_DN15136_c0_g1_i2_orf1;TRINITY_DN46715_c0_g1_i1_orf1;TRINITY_DN10403_c0_g1_i3_orf1;TRINITY_DN338_c1_g1_i9_orf1;TRINITY_DN7808_c0_g1_i1_orf1;TRINITY_DN6967_c0_g1_i3_orf1;TRINITY_DN1749_c0_g2_i2_orf1;TRINITY_DN230_c2_g1_i5_orf1;TRINITY_DN73945_c0_g5_i3_orf1;TRINITY_DN143895_c0_g1_i1_orf1;TRINITY_DN13856_c0_g1_i1_orf1;TRINITY_DN12526_c0_g1_i5_orf1;TRINITY_DN23360_c0_g1_i3_orf1;TRINITY_DN10824_c0_g1_i3_orf1;TRINITY_DN18222_c0_g1_i5_orf1;TRINITY_DN14398_c0_g1_i4_orf1;TRINITY_DN6813_c1_g1_i1_orf1;TRINITY_DN10629_c0_g1_i1_orf1;TRINITY_DN6205_c0_g1_i1_orf1;TRINITY_DN2719_c1_g1_i6_orf1;TRINITY_DN19537_c0_g1_i1_orf1;TRINITY_DN4189_c0_g2_i1_orf1;TRINITY_DN1791_c0_g1_i3_orf1;TRINITY_DN37923_c0_g1_i1_orf1;TRINITY_DN97589_c0_g1_i3_orf1;TRINITY_DN4451_c0_g2_i4_orf1;TRINITY_DN57798_c0_g1_i1_orf1;TRINITY_DN5012_c0_g1_i6_orf1;TRINITY_DN4125_c1_g1_i5_orf1;TRINITY_DN42461_c0_g1_i4_orf1;TRINITY_DN344_c1_g1_i1_orf1;TRINITY_DN30154_c0_g1_i1_orf1;TRINITY_DN3836_c0_g1_i4_orf1;TRINITY_DN21719_c0_g1_i2_orf1;TRINITY_DN7583_c0_g1_i1_orf1;TRINITY_DN779_c0_g1_i3_orf1;TRINITY_DN1607_c0_g1_i16_orf1;TRINITY_DN7464_c0_g1_i14_orf1;TRINITY_DN244_c1_g1_i5_orf1;TRINITY_DN41_c0_g1_i3_orf1;TRINITY_DN45948_c1_g1_i1_orf1;TRINITY_DN36434_c0_g2_i3_orf1;TRINITY_DN5696_c0_g1_i4_orf1;TRINITY_DN4064_c0_g2_i1_orf1;TRINITY_DN4145_c0_g1_i1_orf1;TRINITY_DN23167_c0_g2_i1_orf1;TRINITY_DN5070_c0_g1_i1_orf1;TRINITY_DN29034_c0_g1_i2_orf1;TRINITY_DN32700_c0_g1_i2_orf1;TRINITY_DN117844_c0_g1_i1_orf1;TRINITY_DN43431_c0_g1_i1_orf1;TRINITY_DN3073_c0_g1_i7_orf1;TRINITY_DN9591_c0_g1_i1_orf1;TRINITY_DN2738_c1_g1_i3_orf1;TRINITY_DN5444_c0_g2_i1_orf1;TRINITY_DN4408_c6_g1_i1_orf1;TRINITY_DN95414_c0_g1_i1_orf1;TRINITY_DN11620_c0_g1_i2_orf1;TRINITY_DN27033_c1_g1_i3_orfp1;TRINITY_DN5281_c0_g2_i3_orf1;TRINITY_DN376_c1_g1_i1_orf1;TRINITY_DN1718_c6_g1_i4_orf1;TRINITY_DN1404_c0_g1_i6_orf1;TRINITY_DN779_c0_g1_i12_orf1;TRINITY_DN9062_c0_g2_i3_orf1;TRINITY_DN16451_c0_g1_i7_orf1;TRINITY_DN4767_c0_g1_i4_orf1;TRINITY_DN1262_c0_g1_i2_orf1;TRINITY_DN45633_c0_g1_i1_orf1;TRINITY_DN3499_c0_g1_i8_orf1;TRINITY_DN10831_c1_g1_i1_orf1;TRINITY_DN1459_c1_g1_i1_orf1;TRINITY_DN147458_c0_g1_i1_orf1;TRINITY_DN21218_c0_g1_i4_orf1;TRINITY_DN28989_c0_g1_i7_orf1;TRINITY_DN5235_c0_g1_i7_orf1;TRINITY_DN5513_c0_g1_i1_orf1;TRINITY_DN1068_c0_g1_i3_orf1;TRINITY_DN4248_c0_g1_i4_orf1;TRINITY_DN650_c0_g1_i3_orf1;TRINITY_DN1173_c0_g1_i12_orf1;TRINITY_DN2584_c0_g1_i7_orf1;TRINITY_DN92153_c0_g2_i2_orf1;TRINITY_DN25534_c0_g1_i1_orf1;TRINITY_DN10766_c0_g1_i1_orf1;TRINITY_DN6365_c0_g1_i4_orf1;TRINITY_DN704
biological_process	cellular nitrogen compound metabolic process	GO:0034641	181	181/2406	TRINITY_DN38230_c0_g1_i4_orf1;TRINITY_DN40434_c0_g1_i2_orf1;TRINITY_DN13350_c0_g1_i4_orf1;TRINITY_DN60787_c0_g1_i5_orf1;TRINITY_DN1116_c0_g1_i6_orf1;TRINITY_DN51813_c0_g1_i1_orf1;TRINITY_DN35669_c0_g1_i1_orf1;TRINITY_DN2054_c0_g1_i1_orf1;TRINITY_DN124950_c0_g2_i1_orf1;TRINITY_DN1153_c1_g1_i1_orf1;TRINITY_DN97680_c0_g1_i1_orf1;TRINITY_DN15040_c0_g4_i1_orf1;TRINITY_DN34134_c0_g2_i1_orf1;TRINITY_DN1344_c0_g1_i1_orf1;TRINITY_DN1216_c0_g1_i4_orf1;TRINITY_DN1607_c0_g1_i16_orf1;TRINITY_DN8603_c0_g1_i1_orf1;TRINITY_DN24723_c2_g1_i1_orf1;TRINITY_DN23616_c0_g1_i4_orf1;TRINITY_DN2953_c1_g1_i10_orf1;TRINITY_DN2848_c0_g1_i1_orf1;TRINITY_DN29448_c0_g1_i1_orf1;TRINITY_DN11013_c0_g1_i3_orf1;TRINITY_DN19251_c0_g1_i8_orf1;TRINITY_DN21570_c0_g1_i1_orf1;TRINITY_DN74889_c0_g1_i1_orf1;TRINITY_DN2794_c1_g1_i8_orf1;TRINITY_DN97589_c0_g1_i3_orf1;TRINITY_DN3800_c0_g1_i7_orf1;TRINITY_DN47123_c0_g1_i1_orf1;TRINITY_DN15256_c0_g1_i8_orf1;TRINITY_DN123184_c0_g1_i1_orf1;TRINITY_DN20527_c0_g1_i1_orf1;TRINITY_DN135_c0_g1_i1_orf1;TRINITY_DN5507_c0_g1_i1_orf1;TRINITY_DN2224_c0_g1_i1_orf1;TRINITY_DN2401_c0_g2_i1_orf1;TRINITY_DN5952_c0_g1_i6_orf1;TRINITY_DN5200_c0_g1_i2_orf1;TRINITY_DN15136_c0_g1_i2_orf1;TRINITY_DN7808_c0_g1_i1_orf1;TRINITY_DN89613_c0_g1_i13_orf1;TRINITY_DN14313_c0_g1_i1_orf1;TRINITY_DN230_c2_g1_i5_orf1;TRINITY_DN17271_c0_g1_i1_orf1;TRINITY_DN23360_c0_g1_i3_orf1;TRINITY_DN45271_c0_g1_i1_orf1;TRINITY_DN18222_c0_g1_i5_orf1;TRINITY_DN14398_c0_g1_i4_orf1;TRINITY_DN1005_c0_g1_i5_orf1;TRINITY_DN37532_c0_g1_i1_orf1;TRINITY_DN51968_c0_g1_i1_orf1;TRINITY_DN15900_c0_g1_i6_orf1;TRINITY_DN81258_c0_g1_i2_orf1;TRINITY_DN115658_c0_g1_i1_orf1;TRINITY_DN30131_c0_g1_i1_orf1;TRINITY_DN6813_c1_g1_i1_orf1;TRINITY_DN8625_c0_g1_i1_orf1;TRINITY_DN7583_c0_g1_i1_orf1;TRINITY_DN779_c0_g1_i3_orf1;TRINITY_DN18391_c0_g2_i8_orf1;TRINITY_DN7464_c0_g1_i14_orf1;TRINITY_DN2769_c0_g1_i1_orf1;TRINITY_DN41664_c0_g1_i4_orf1;TRINITY_DN1091_c0_g3_i1_orf1;TRINITY_DN4145_c0_g1_i1_orf1;TRINITY_DN5070_c0_g1_i1_orf1;TRINITY_DN1616_c0_g1_i3_orf1;TRINITY_DN117844_c0_g1_i1_orf1;TRINITY_DN2953_c1_g1_i11_orf1;TRINITY_DN9591_c0_g1_i1_orf1;TRINITY_DN6642_c0_g1_i2_orf1;TRINITY_DN2738_c1_g1_i3_orf1;TRINITY_DN13732_c0_g2_i3_orf1;TRINITY_DN4408_c6_g1_i1_orf1;TRINITY_DN5525_c0_g1_i4_orf1;TRINITY_DN4707_c0_g1_i1_orf1;TRINITY_DN6325_c0_g1_i9_orf1;TRINITY_DN1718_c6_g1_i4_orf1;TRINITY_DN779_c0_g1_i12_orf1;TRINITY_DN56993_c0_g1_i4_orf1;TRINITY_DN58207_c0_g1_i1_orf1;TRINITY_DN74037_c0_g5_i1_orf1;TRINITY_DN31663_c0_g1_i2_orf1;TRINITY_DN139537_c0_g1_i1_orf1;TRINITY_DN10831_c1_g1_i1_orf1;TRINITY_DN107035_c0_g1_i1_orf1;TRINITY_DN147458_c0_g1_i1_orf1;TRINITY_DN4908_c1_g1_i5_orf1;TRINITY_DN2299_c0_g1_i3_orf1;TRINITY_DN5064_c0_g1_i4_orf1;TRINITY_DN291_c0_g1_i2_orf1;TRINITY_DN2749_c4_g1_i2_orf1;TRINITY_DN6325_c0_g1_i8_orf1;TRINITY_DN2749_c0_g1_i4_orf1;TRINITY_DN1750_c1_g1_i5_orf1;TRINITY_DN27852_c0_g1_i1_orf1;TRINITY_DN6365_c0_g1_i4_orf1;TRINITY_DN77318_c0_g2_i1_orf1;TRINITY_DN46409_c0_g1_i1_orf1;TRINITY_DN58636_c0_g1_i1_orf1;TRINITY_DN142442_c0_g1_i1_orf1;TRINITY_DN51934_c0_g2_i1_orf1;TRINITY_DN2718_c0_g1_i6_orf1;TRINITY_DN57918_c0_g1_i1_orf1;TRINITY_DN6563_c0_g1_i1_orf1;TRINITY_DN33346_c0_g1_i1_orf1;TRINITY_DN1554_c0_g1_i9_orf1;TRINITY_DN42646_c0_g2_i1_orf1;TRINITY_DN49038_c0_g4_i1_orf1;TRINITY_DN4016_c0_g1_i1_orf1;TRINITY_DN934_c2_g1_i7_orf1;TRINITY_DN24_c0_g1_i1_orf1;TRINITY_DN17031_c0_g1_i1_orf1;TRINITY_DN2559_c0_g1_i4_orf1;TRINITY_DN98242_c0_g1_i1_orf1;TRINITY_DN10070_c0_g1_i1_orf1;TRINITY_DN14477_c0_g1_i12_orf1;TRINITY_DN34689_c0_g1_i4_orf1;TRINITY_DN147676_c0_g1_i1_orf1;TRINITY_DN26293_c0_g1_i4_orf1;TRINITY_DN2749_c0_g2_i3_orf1;TRINITY_DN47591_c0_g1_i2_orf1;TRINITY_DN3062_c0_g1_i1_orf1;TRINITY_DN21545_c0_g1_i2_orf1;TRINITY_DN24322_c0_g1_i4_orf1;TRINITY_DN11986_c0_g1_i1_orf1;TRINITY_DN64_c0_g1_i4_orf1;TRINITY_DN38274_c0_g1_i1_orf1;TRINITY_DN44877_c0_g1_i2_orf1;TRINITY_DN14487_c0_g1_i4_orf1;TRINITY_DN28299_c0_g1_i1_orf1;TRINITY_DN4820_c0_g2_i2_orf1;TRINITY_DN34509_c0_g1_i1_orf1;TRINITY_DN2647_c0_g1_i3_orf1;TRINITY_DN120144_c0_g1_i1_orf1;TRINITY_DN6248_c0_g1_i1_orf1;TRINITY_DN4955_c0_g1_i2_orf1;TRINITY_DN141353_c0_g1_i1_orf1;TRINITY_DN31980_c0_g1_i1_orf1;TRINITY_DN879_c0_g1_i2_orf1;TRINITY_DN38180_c0_g1_i3_orf1;TRINITY_DN1084_c0_g2_i2_orf1;TRINITY_DN2971_c0_g1_i1_orf1;TRINITY_DN67649_c0_g1_i1_orf1;TRINITY_DN2283_c0_g2_i1_orf1;TRINITY_DN44792_c0_g1_i1_orf1;TRINITY_DN8716_c0_g1_i3_orf1;TRINITY_DN4835_c0_g1_i2_orf1;TRINITY_DN38562_c0_g1_i3_orf1;TRINITY_DN1
biological_process	nitrogen cycle metabolic process	GO:0071941	2	2/2406	TRINITY_DN24723_c2_g1_i1_orf1;TRINITY_DN17031_c0_g1_i1_orf1
biological_process	cellular lipid metabolic process	GO:0044255	26	26/2406	TRINITY_DN34751_c0_g1_i1_orf1;TRINITY_DN48590_c0_g1_i1_orf1;TRINITY_DN10722_c0_g3_i1_orf1;TRINITY_DN22046_c1_g1_i5_orf1;TRINITY_DN12526_c0_g1_i5_orf1;TRINITY_DN5841_c0_g1_i2_orf1;TRINITY_DN72017_c0_g1_i1_orf1;TRINITY_DN27903_c0_g1_i1_orf1;TRINITY_DN45220_c0_g1_i1_orf1;TRINITY_DN5211_c0_g1_i1_orf1;TRINITY_DN86833_c0_g3_i1_orf1;TRINITY_DN8964_c0_g1_i4_orf1;TRINITY_DN41_c0_g1_i3_orf1;TRINITY_DN10430_c0_g1_i4_orf1;TRINITY_DN9028_c0_g1_i5_orf1;TRINITY_DN10742_c0_g1_i4_orf1;TRINITY_DN21570_c0_g1_i1_orf1;TRINITY_DN3175_c0_g1_i7_orf1;TRINITY_DN1999_c0_g1_i9_orf1;TRINITY_DN3991_c0_g1_i6_orf1;TRINITY_DN84478_c0_g1_i8_orf1;TRINITY_DN76283_c0_g2_i1_orf1;TRINITY_DN4321_c0_g1_i1_orf1;TRINITY_DN2618_c0_g1_i3_orf1;TRINITY_DN5697_c0_g1_i1_orf1;TRINIT

biological_process	generation of precursor metabolites and energy	GO:0006091	15	15/2406	TRINITY_DN60787_c0_g1_i5_orf1;TRINITY_DN49038_c0_g4_i1_orf1;TRINITY_DN6325_c0_g1_i8_orf1;TRINITY_DN76036_c0_g1_i1_orf1;TRINITY_DN86149_c0_g1_i1_orf1;TRINITY_DN9286_c0_g1_i2_orf1;TRINITY_DN18222_c0_g1_i5_orf1;TRINITY_DN9536_c0_g1_i4_orf1;TRINITY_DN2848_c0_g1_i1_orf1;TRINITY_DN20133_c0_g1_i1_orf1;TRINITY_DN6325_c0_g1_i9_orf1;TRINITY_DN1791_c0_g1_i3_orf1;TRINITY_DN140613_c0_g1_i1_orf1;TRINITY_DN5867_c0_g1_i1_orf1;TRINITY_DN812_c2_g1_i1_orf1
biological_process	one-carbon metabolic process	GO:0006730	8	8/2406	TRINITY_DN92153_c0_g2_i2_orf1;TRINITY_DN63536_c0_g1_i1_orf1;TRINITY_DN3263_c0_g1_i2_orf1;TRINITY_DN38506_c0_g1_i4_orf1;TRINITY_DN7512_c0_g1_i1_orf1;TRINITY_DN20527_c0_g1_i1_orf1;TRINITY_DN244_c1_g1_i5_orf1;TRINITY_DN5768_c0_g1_i2_orf1
biological_process	cellular ketone metabolic process	GO:0042180	6	6/2406	TRINITY_DN87170_c0_g1_i3_orf1;TRINITY_DN51813_c0_g1_i1_orf1;TRINITY_DN6027_c0_g1_i13_orf1;TRINITY_DN135781_c0_g1_i1_orf1;TRINITY_DN8964_c0_g1_i4_orf1;TRINITY_DN20133_c0_g1_i1_orf1
biological_process	heterocycle metabolic process	GO:0046483	156	156/2406	TRINITY_DN38230_c0_g1_i4_orf1;TRINITY_DN40434_c0_g1_i2_orf1;TRINITY_DN13350_c0_g1_i4_orf1;TRINITY_DN60787_c0_g1_i5_orf1;TRINITY_DN51813_c0_g1_i1_orf1;TRINITY_DN35669_c0_g1_i1_orf1;TRINITY_DN2054_c0_g1_i1_orf1;TRINITY_DN124950_c0_g2_i1_orf1;TRINITY_DN2738_c1_g1_i3_orf1;TRINITY_DN15040_c0_g4_i1_orf1;TRINITY_DN34134_c0_g2_i1_orf1;TRINITY_DN17772_c0_g2_i3_orf1;TRINITY_DN1216_c0_g1_i4_orf1;TRINITY_DN1607_c0_g1_i16_orf1;TRINITY_DN8603_c0_g1_i1_orf1;TRINITY_DN23616_c0_g1_i4_orf1;TRINITY_DN2953_c1_g1_i10_orf1;TRINITY_DN2848_c0_g1_i1_orf1;TRINITY_DN11013_c0_g1_i3_orf1;TRINITY_DN3991_c0_g1_i6_orf1;TRINITY_DN19251_c0_g1_i8_orf1;TRINITY_DN3800_c0_g1_i7_orf1;TRINITY_DN1760_c0_g1_i4_orf1;TRINITY_DN47123_c0_g1_i1_orf1;TRINITY_DN15256_c0_g1_i8_orf1;TRINITY_DN123184_c0_g1_i1_orf1;TRINITY_DN20527_c0_g1_i1_orf1;TRINITY_DN1116_c0_g1_i6_orf1;TRINITY_DN5507_c0_g1_i1_orf1;TRINITY_DN2224_c0_g1_i1_orf1;TRINITY_DN2401_c0_g2_i1_orf1;TRINITY_DN5952_c0_g1_i6_orf1;TRINITY_DN5200_c0_g1_i2_orf1;TRINITY_DN15136_c0_g1_i2_orf1;TRINITY_DN7808_c0_g1_i1_orf1;TRINITY_DN89613_c0_g1_i13_orf1;TRINITY_DN14313_c0_g1_i1_orf1;TRINITY_DN230_c2_g1_i5_orf1;TRINITY_DN17271_c0_g1_i1_orf1;TRINITY_DN45271_c0_g1_i1_orf1;TRINITY_DN18222_c0_g1_i5_orf1;TRINITY_DN1005_c0_g1_i5_orf1;TRINITY_DN37532_c0_g1_i1_orf1;TRINITY_DN51968_c0_g1_i1_orf1;TRINITY_DN15900_c0_g1_i6_orf1;TRINITY_DN81258_c0_g1_i2_orf1;TRINITY_DN115658_c0_g1_i1_orf1;TRINITY_DN6813_c1_g1_i1_orf1;TRINITY_DN8625_c0_g1_i1_orf1;TRINITY_DN779_c0_g1_i3_orf1;TRINITY_DN18391_c0_g2_i8_orf1;TRINITY_DN244_c1_g1_i5_orf1;TRINITY_DN2769_c0_g1_i1_orf1;TRINITY_DN41664_c0_g1_i4_orf1;TRINITY_DN1091_c0_g3_i1_orf1;TRINITY_DN4145_c0_g1_i1_orf1;TRINITY_DN5070_c0_g1_i1_orf1;TRINITY_DN1616_c0_g1_i3_orf1;TRINITY_DN117844_c0_g1_i1_orf1;TRINITY_DN2953_c1_g1_i11_orf1;TRINITY_DN6642_c0_g1_i2_orf1;TRINITY_DN13732_c0_g2_i3_orf1;TRINITY_DN4408_c6_g1_i1_orf1;TRINITY_DN5525_c0_g1_i4_orf1;TRINITY_DN4707_c0_g1_i1_orf1;TRINITY_DN6325_c0_g1_i9_orf1;TRINITY_DN1718_c6_g1_i4_orf1;TRINITY_DN779_c0_g1_i12_orf1;TRINITY_DN56993_c0_g1_i4_orf1;TRINITY_DN21545_c0_g1_i2_orf1;TRINITY_DN74037_c0_g5_i1_orf1;TRINITY_DN31663_c0_g1_i2_orf1;TRINITY_DN139537_c0_g1_i1_orf1;TRINITY_DN1344_c0_g1_i1_orf1;TRINITY_DN107035_c0_g1_i1_orf1;TRINITY_DN4908_c1_g1_i5_orf1;TRINITY_DN2299_c0_g1_i3_orf1;TRINITY_DN291_c0_g1_i2_orf1;TRINITY_DN2749_c4_g1_i2_orf1;TRINITY_DN92153_c0_g2_i2_orf1;TRINITY_DN2749_c0_g1_i4_orf1;TRINITY_DN1750_c1_g1_i5_orf1;TRINITY_DN27852_c0_g1_i1_orf1;TRINITY_DN77318_c0_g2_i1_orf1;TRINITY_DN46409_c0_g1_i1_orf1;TRINITY_DN58636_c0_g1_i1_orf1;TRINITY_DN51934_c0_g2_i1_orf1;TRINITY_DN2718_c0_g1_i6_orf1;TRINITY_DN57918_c0_g1_i1_orf1;TRINITY_DN6563_c0_g1_i1_orf1;TRINITY_DN33346_c0_g1_i1_orf1;TRINITY_DN1554_c0_g1_i9_orf1;TRINITY_DN49038_c0_g4_i1_orf1;TRINITY_DN38506_c0_g1_i4_orf1;TRINITY_DN24_c0_g1_i1_orf1;TRINITY_DN2559_c0_g1_i4_orf1;TRINITY_DN2803_c4_g1_i1_orf1;TRINITY_DN98242_c0_g1_i1_orf1;TRINITY_DN14477_c0_g1_i12_orf1;TRINITY_DN34689_c0_g1_i4_orf1;TRINITY_DN26293_c0_g1_i4_orf1;TRINITY_DN2749_c0_g2_i3_orf1;TRINITY_DN3062_c0_g1_i1_orf1;TRINITY_DN8716_c0_g1_i3_orf1;TRINITY_DN24322_c0_g1_i4_orf1;TRINITY_DN11986_c0_g1_i1_orf1;TRINITY_DN64_c0_g1_i4_orf1;TRINITY_DN38274_c0_g1_i1_orf1;TRINITY_DN44877_c0_g1_i2_orf1;TRINITY_DN14487_c0_g1_i4_orf1;TRINITY_DN28299_c0_g1_i1_orf1;TRINITY_DN4820_c0_g2_i2_orf1;TRINITY_DN34509_c0_g1_i1_orf1;TRINITY_DN2647_c0_g1_i3_orf1;TRINITY_DN3263_c0_g1_i2_orf1;TRINITY_DN120144_c0_g1_i1_orf1;TRINITY_DN6248_c0_g1_i1_orf1;TRINITY_DN4955_c0_g1_i2_orf1;TRINITY_DN141353_c0_g1_i1_orf1;TRINITY_DN31980_c0_g1_i1_orf1;TRINITY_DN879_c0_g1_i2_orf1;TRINITY_DN38180_c0_g1_i3_orf1;TRINITY_DN1084_c0_g2_i2_orf1;TRINITY_DN2971_c0_g1_i1_orf1;TRINITY_DN2719_c1_g1_i6_orf1;TRINITY_DN67649_c0_g1_i1_orf1;TRINITY_DN2283_c0_g2_i1_orf1;TRINITY_DN44792_c0_g1_i1_orf1;TRINITY_DN24970_c0_g1_i4_orf1;TRINITY_DN4835_c0_g1_i2_orf1;TRINITY_DN38562_c0_g1_i3_orf1;TRINITY_DN13760_c1_g1_i1_orf1;TRINITY_DN55148_c0_g1_i1_orf1;TRINITY_DN87170_c0_g1_i3_orf1;TRINITY_DN104507_c0_g1_i2_orf1;TRINITY_DN2953_c1_g1_i2_orf1;TRINITY_DN19115_c0_g1_i1_orf1;TRINITY_DN2367_c1_g1_i20_orf1;TRINITY_DN1091_c0_g1_i1_orf1;TRINITY_DN18782_c0_g1_i4_orf1;TRINITY_DN15222_c0_g1_i4_orf1;TRINITY_DN53311_c0_g2_i1_orf1;TRINITY_DN5238_c0_g1_i2_orf1;TRINITY_DN20133_c0_g1_i1_orf1;TRINITY_DN13055_c0_g1_i5_orf1;TRINITY_DN34432_c0_g1_i1_orf1;TRINITY_DN110534_c0_g1_i3_orf1;TRINITY_DN5757_c0_g1_i1_orf1;TRINITY_DN48619_c0_g1_i1_orf1;TRINITY_DN6325_c0_g1_i8_orf1;TRINITY_DN87
biological_process	reactive oxygen species metabolic process	GO:0072593	3	3/2406	TRINITY_DN285_c0_g1_i4_orf1;TRINITY_DN16400_c0_g2_i1_orf1;TRINITY_DN6580_c0_g1_i4_orf1
biological_process	cellular carbohydrate metabolic process	GO:0044262	11	11/2406	TRINITY_DN618_c0_g1_i3_orf1;TRINITY_DN52244_c1_g1_i1_orf1;TRINITY_DN10722_c0_g3_i1_orf1;TRINITY_DN53238_c1_g1_i5_orf1;TRINITY_DN36788_c0_g1_i2_orf1;TRINITY_DN1034_c0_g1_i4_orf1;TRINITY_DN812_c2_g1_i1_orf1;TRINITY_DN3010_c0_g1_i4_orf1;TRINITY_DN11657_c0_g1_i2_orf1;TRINITY_DN140613_c0_g1_i1_orf1;TRINITY_DN49038_c0_g4_i1_orf1
biological_process	sulfur compound metabolic process	GO:0006790	19	19/2406	TRINITY_DN14920_c0_g1_i1_orf1;TRINITY_DN92153_c0_g2_i2_orf1;TRINITY_DN117844_c0_g1_i1_orf1;TRINITY_DN3991_c0_g1_i6_orf1;TRINITY_DN3073_c0_g1_i7_orf1;TRINITY_DN19251_c0_g1_i8_orf1;TRINITY_DN5497_c0_g1_i6_orf1;TRINITY_DN38562_c0_g1_i3_orf1;TRINITY_DN34399_c0_g1_i1_orf1;TRINITY_DN7512_c0_g1_i1_orf1;TRINITY_DN11948_c0_g1_i8_orf1;TRINITY_DN1084_c0_g1_i2_orf1;TRINITY_DN49872_c0_g1_i2_orf1;TRINITY_DN35763_c0_g1_i2_orf1;TRINITY_DN85476_c0_g1_i1_orf1;TRINITY_DN15136_c0_g1_i2_orf1;TRINITY_DN7808_c0_g1_i1_orf1;TRINITY_DN1084_c0_g2_i2_orf1;TRINITY_DN54134_c0_g1_i1_orf1
biological_process	phosphorus metabolic process	GO:0006793	85	85/2406	TRINITY_DN1749_c0_g2_i2_orf1;TRINITY_DN1173_c0_g1_i12_orf1;TRINITY_DN10680_c0_g1_i5_orf1;TRINITY_DN14477_c0_g1_i12_orf1;TRINITY_DN47151_c0_g1_i1_orf1;TRINITY_DN38230_c0_g1_i4_orf1;TRINITY_DN10722_c0_g3_i1_orf1;TRINITY_DN5070_c0_g1_i1_orf1;TRINITY_DN18222_c0_g1_i5_orf1;TRINITY_DN2719_c1_g1_i6_orf1;TRINITY_DN1034_c0_g1_i4_orf1;TRINITY_DN60787_c0_g1_i5_orf1;TRINITY_DN2983_c0_g1_i6_orf1;TRINITY_DN62557_c0_g1_i1_orf1;TRINITY_DN41166_c0_g1_i1_orf1;TRINITY_DN277_c1_g1_i1_orf1;TRINITY_DN1552_c0_g1_i3_orf1;TRINITY_DN1405_c0_g1_i1_orf1;TRINITY_DN28299_c0_g1_i1_orf1;TRINITY_DN618_c0_g1_i3_orf1;TRINITY_DN37923_c0_g1_i1_orf1;TRINITY_DN5281_c0_g2_i3_orf1;TRINITY_DN30154_c0_g1_i1_orf1;TRINITY_DN1475_c0_g1_i6_orf1;TRINITY_DN2738_c1_g1_i3_orf1;TRINITY_DN3119_c0_g1_i7_orf1;TRINITY_DN4217_c0_g1_i2_orf1;TRINITY_DN6325_c0_g1_i8_orf1;TRINITY_DN21545_c0_g1_i2_orf1;TRINITY_DN42461_c0_g1_i4_orf1;TRINITY_DN1173_c0_g1_i11_orf1;TRINITY_DN24_c0_g1_i1_orf1;TRINITY_DN1216_c0_g1_i4_orf1;TRINITY_DN70485_c0_g1_i2_orf1;TRINITY_DN8603_c0_g1_i1_orf1;TRINITY_DN1718_c6_g1_i4_orf1;TRINITY_DN248_c0_g1_i1_orf1;TRINITY_DN143509_c0_g1_i1_orf1;TRINITY_DN2812_c0_g1_i5_orf1;TRINITY_DN2848_c0_g1_i1_orf1;TRINITY_DN4320_c0_g1_i1_orf1;TRINITY_DN19115_c0_g1_i1_orf1;TRINITY_DN15222_c0_g1_i4_orf1;TRINITY_DN73945_c0_g5_i3_orf1;TRINITY_DN26293_c0_g1_i4_orf1;TRINITY_DN12_c0_g1_i5_orf1;TRINITY_DN1084_c0_g1_i2_orf1;TRINITY_DN10742_c0_g1_i4_orf1;TRINITY_DN32700_c0_g1_i2_orf1;TRINITY_DN117844_c0_g1_i1_orf1;TRINITY_DN4571_c0_g1_i4_orf1;TRINITY_DN3991_c0_g1_i6_orf1;TRINITY_DN51813_c0_g1_i1_orf1;TRINITY_DN19251_c0_g1_i8_orf1;TRINITY_DN3800_c0_g1_i7_orf1;TRINITY_DN116972_c0_g1_i1_orf1;TRINITY_DN1154_c0_g1_i1_orf1;TRINITY_DN11015_c0_g1_i8_orf1;TRINITY_DN11013_c0_g1_i3_orf1;TRINITY_DN5525_c0_g1_i4_orf1;TRINITY_DN6185_c0_g1_i12_orf1;TRINITY_DN6325_c0_g1_i9_orf1;TRINITY_DN6436_c0_g1_i1_orf1;TRINITY_DN143637_c0_g1_i1_orf1;TRINITY_DN4798_c0_g1_i3_orf1;TRINITY_DN49038_c0_g4_i1_orf1;TRINITY_DN52244_c1_g1_i1_orf1;TRINITY_DN5952_c0_g1_i6_orf1;TRINITY_DN2618_c0_g1_i3_orf1;TRINITY_DN1173_c1_g1_i10_orf1;TRINITY_DN30_c0_g1_i6_orf1;TRINITY_DN18782_c0_g1_i4_orf1;TRINITY_DN141353_c0_g1_i1_orf1;TRINITY_DN147475_c0_g1_i1_orf1;TRINITY_DN6813_c1_g1_i1_orf1;TRINITY_DN15136_c0_g1_i2_orf1;TRINITY_DN40562_c0_g2_i1_orf1;TRINITY_DN46715_c0_g1_i1_orf1;TRINITY_DN24539_c0_g1_i4_orf1;TRINITY_DN20133_c0_g1_i1_orf1;TRINITY_DN11620_c0_g1_i2_orf1;TRINITY_DN7808_c0_g1_i1_orf1;TRINITY_DN5697_c0_g1_i1_orf1;TRINITY_DN4929_c1_g2_i5_orf1;TRINITY_DN1084_c0_g2_i2_orf1
biological_process	cellular aldehyde metabolic process	GO:0006081	3	3/2406	TRINITY_DN36788_c0_g1_i2_orf1;TRINITY_DN53238_c1_g1_i5_orf1;TRINITY_DN20133_c0_g1_i1_orf1
biological_process	organic acid metabolic process	GO:0006082	75	75/2406	TRINITY_DN34751_c0_g1_i1_orf1;TRINITY_DN48590_c0_g1_i1_orf1;TRINITY_DN2065_c1_g2_i1_orf1;TRINITY_DN57918_c0_g1_i1_orf1;TRINITY_DN146126_c0_g1_i1_orf1;TRINITY_DN92153_c0_g2_i2_orf1;TRINITY_DN5266_c0_g1_i1_orf1;TRINITY_DN24970_c0_g1_i4_orf1;TRINITY_DN8716_c0_g1_i3_orf1;TRINITY_DN5497_c0_g1_i6_orf1;TRINITY_DN5564_c0_g1_i5_orf1;TRINITY_DN863_c0_g1_i6_orf1;TRINITY_DN117844_c0_g1_i1_orf1;TRINITY_DN2719_c1_g1_i6_orf1;TRINITY_DN1264_c0_g1_i2_orf1;TRINITY_DN35763_c0_g1_i2_orf1;TRINITY_DN4822_c0_g1_i9_orf1;TRINITY_DN89483_c0_g1_i1_orf1;TRINITY_DN72017_c0_g1_i1_orf1;TRINITY_DN4451_c0_g2_i4_orf1;TRINITY_DN27903_c0_g1_i1_orf1;TRINITY_DN45220_c0_g1_i1_orf1;TRINITY_DN53238_c1_g1_i5_orf1;TRINITY_DN36788_c0_g1_i2_orf1;TRINITY_DN2803_c4_g1_i1_orf1;TRINITY_DN5211_c0_g1_i1_orf1;TRINITY_DN1607_c0_g1_i16_orf1;TRINITY_DN24723_c2_g1_i1_orf1;TRINITY_DN3991_c0_g1_i6_orf1;TRINITY_DN2953_c1_g1_i10_orf1;TRINITY_DN2848_c0_g1_i1_orf1;TRINITY_DN2953_c1_g1_i2_orf1;TRINITY_DN10430_c0_g1_i4_orf1;TRINITY_DN11013_c0_g1_i3_orf1;TRINITY_DN26293_c0_g1_i4_orf1;TRINITY_DN2953_c1_g1_i11_orf1;TRINITY_DN20796_c0_g1_i4_orf1;TRINITY_DN12293_c0_g1_i1_orf1;TRINITY_DN3175_c0_g1_i7_orf1;TRINITY_DN17326_c0_g1_i8_orf1;TRINITY_DN43431_c0_g1_i1_orf1;TRINITY_DN3859_c0_g1_i5_orf1;TRINITY_DN3073_c0_g1_i7_orf1;TRINITY_DN20133_c0_g1_i1_orf1;TRINITY_DN51813_c0_g1_i1_orf1;TRINITY_DN1760_c0_g1_i4_orf1;TRINITY_DN18222_c0_g1_i5_orf1;TRINITY_DN34399_c0_g1_i1_orf1;TRINITY_DN2570_c0_g1_i1_orf1;TRINITY_DN1999_c0_g1_i9_orf1;TRINITY_DN17031_c0_g1_i1_orf1;TRINITY_DN76283_c0_g2_i1_orf1;TRINITY_DN20527_c0_g1_i1_orf1;TRINITY_DN6325_c0_g1_i9_orf1;TRINITY_DN511_c0_g2_i1_orf1;TRINITY_DN48619_c0_g1_i1_orf1;TRINITY_DN49038_c0_g4_i1_orf1;TRINITY_DN2224_c0_g1_i1_orf1;TRINITY_DN6325_c0_g1_i8_orf1;TRINITY_DN11948_c0_g1_i8_orf1;TRINITY_DN21539_c0_g1_i1_orf1;TRINITY_DN1262_c0_g1_i2_orf1;TRINITY_DN4321_c0_g1_i1_orf1;TRINITY_DN11383_c0_g2_i4_orf1;TRINITY_DN3263_c0_g1_i2_orf1;TRINITY_DN2684_c0_g2_i3_orf1;TRINITY_DN3836_c0_g1_i4_orf1;TRINITY_DN15136_c0_g1_i2_orf1;TRINITY_DN1084_c0_g1_i2_orf1;TRINITY_DN87170_c0_g1_i3_orf1;TRINITY_DN7808_c0_g1_i1_orf1;TRINITY_DN905_c0_g1_i4_orf1;TRINITY_DN1068_c0_g1_i3_orf1;TRINITY_DN2890_c0_g1_i2_orf1;TRINITY_DN1084_c0_g2_i2_orf1

biological_process	cellular catabolic process	GO:0044248	48 48/2406	TRINITY_DN34751_c0_g1_i1_orf1;TRINITY_DN2065_c1_g2_i1_orf1;TRINITY_DN38230_c0_g1_i4_orf1;TRINITY_DN863_c0_g1_i6_orf1;TRINITY_DN1757_c0_g1_i4_orf1;TRINITY_DN3010_c0_g1_i4_orf1;TRINITY_DN4822_c0_g1_i9_orf1;TRINITY_DN37923_c0_g1_i1_orf1;TRINITY_DN2120_c0_g1_i2_orf1;TRINITY_DN89483_c0_g1_i1_orf1;TRINITY_DN72017_c0_g1_i1_orf1;TRINITY_DN4451_c0_g2_i4_orf1;TRINITY_DN57798_c0_g1_i1_orf1;TRINITY_DN2890_c0_g1_i2_orf1;TRINITY_DN45220_c0_g1_i1_orf1;TRINITY_DN21545_c0_g1_i2_orf1;TRINITY_DN19821_c0_g2_i4_orf1;TRINITY_DN87170_c0_g1_i3_orf1;TRINITY_DN24_c0_g1_i1_orf1;TRINITY_DN135188_c0_g1_i2_orf1;TRINITY_DN44877_c0_g1_i2_orf1;TRINITY_DN779_c0_g1_i3_orf1;TRINITY_DN98242_c0_g1_i1_orf1;TRINITY_DN5531_c0_g3_i3_orf1;TRINITY_DN6580_c0_g1_i4_orf1;TRINITY_DN34689_c0_g1_i4_orf1;TRINITY_DN11172_c0_g1_i4_orf1;TRINITY_DN4145_c0_g1_i1_orf1;TRINITY_DN96557_c0_g1_i1_orf1;TRINITY_DN43431_c0_g1_i1_orf1;TRINITY_DN51813_c0_g1_i1_orf1;TRINITY_DN285_c0_g1_i4_orf1;TRINITY_DN5001_c0_g1_i4_orf1;TRINITY_DN12293_c0_g1_i1_orf1;TRINITY_DN2559_c0_g1_i4_orf1;TRINITY_DN22242_c0_g1_i1_orf1;TRINITY_DN779_c0_g1_i12_orf1;TRINITY_DN9062_c0_g2_i3_orf1;TRINITY_DN1034_c0_g1_i4_orf1;TRINITY_DN1262_c0_g1_i2_orf1;TRINITY_DN31980_c0_g1_i1_orf1;TRINITY_DN46132_c0_g2_i2_orf1;TRINITY_DN3836_c0_g1_i4_orf1;TRINITY_DN15136_c0_g1_i2_orf1;TRINITY_DN113353_c0_g1_i1_orf1;TRINITY_DN28989_c0_g1_i7_orf1;TRINITY_DN11172_c1_g1_i1_orf1;TRINITY_DN38180_c0_g1_i3_orf1
biological_process	cellular biosynthetic process	GO:0044249	111 111/2406	TRINITY_DN10722_c0_g3_i1_orf1;TRINITY_DN5564_c0_g1_i5_orf1;TRINITY_DN35763_c0_g1_i2_orf1;TRINITY_DN124950_c0_g2_i1_orf1;TRINITY_DN1153_c1_g1_i1_orf1;TRINITY_DN97680_c0_g1_i1_orf1;TRINITY_DN58207_c0_g1_i1_orf1;TRINITY_DN1216_c0_g1_i4_orf1;TRINITY_DN8603_c0_g1_i1_orf1;TRINITY_DN24723_c2_g1_i1_orf1;TRINITY_DN17031_c0_g1_i1_orf1;TRINITY_DN8964_c0_g1_i4_orf1;TRINITY_DN47591_c0_g1_i2_orf1;TRINITY_DN29448_c0_g1_i1_orf1;TRINITY_DN11013_c0_g1_i3_orf1;TRINITY_DN10742_c0_g1_i4_orf1;TRINITY_DN21570_c0_g1_i1_orf1;TRINITY_DN74889_c0_g1_i1_orf1;TRINITY_DN3800_c0_g1_i7_orf1;TRINITY_DN1760_c0_g1_i4_orf1;TRINITY_DN20527_c0_g1_i1_orf1;TRINITY_DN135_c0_g1_i1_orf1;TRINITY_DN2401_c0_g2_i1_orf1;TRINITY_DN5952_c0_g1_i6_orf1;TRINITY_DN2265_c0_g1_i5_orf1;TRINITY_DN7808_c0_g1_i1_orf1;TRINITY_DN230_c2_g1_i5_orf1;TRINITY_DN23360_c0_g1_i3_orf1;TRINITY_DN5497_c0_g1_i6_orf1;TRINITY_DN14398_c0_g1_i4_orf1;TRINITY_DN2719_c1_g1_i6_orf1;TRINITY_DN97589_c0_g1_i3_orf1;TRINITY_DN115658_c0_g1_i1_orf1;TRINITY_DN5211_c0_g1_i1_orf1;TRINITY_DN6813_c1_g1_i1_orf1;TRINITY_DN8625_c0_g1_i1_orf1;TRINITY_DN7583_c0_g1_i1_orf1;TRINITY_DN7464_c0_g1_i14_orf1;TRINITY_DN10430_c0_g1_i4_orf1;TRINITY_DN117844_c0_g1_i1_orf1;TRINITY_DN1999_c0_g1_i9_orf1;TRINITY_DN3073_c0_g1_i7_orf1;TRINITY_DN9591_c0_g1_i1_orf1;TRINITY_DN2738_c1_g1_i3_orf1;TRINITY_DN13732_c0_g2_i3_orf1;TRINITY_DN4408_c6_g1_i1_orf1;TRINITY_DN5525_c0_g1_i4_orf1;TRINITY_DN4707_c0_g1_i1_orf1;TRINITY_DN1718_c6_g1_i4_orf1;TRINITY_DN16451_c0_g1_i7_orf1;TRINITY_DN74037_c0_g5_i1_orf1;TRINITY_DN10831_c1_g1_i1_orf1;TRINITY_DN147458_c0_g1_i1_orf1;TRINITY_DN2299_c0_g1_i3_orf1;TRINITY_DN5064_c0_g1_i4_orf1;TRINITY_DN812_c2_g1_i1_orf1;TRINITY_DN48590_c0_g1_i1_orf1;TRINITY_DN22046_c1_g1_i5_orf1;TRINITY_DN30131_c0_g1_i1_orf1;TRINITY_DN1750_c1_g1_i5_orf1;TRINITY_DN27852_c0_g1_i1_orf1;TRINITY_DN6365_c0_g1_i4_orf1;TRINITY_DN142442_c0_g1_i1_orf1;TRINITY_DN41645_c0_g1_i1_orf1;TRINITY_DN6563_c0_g1_i1_orf1;TRINITY_DN27903_c0_g1_i1_orf1;TRINITY_DN4016_c0_g1_i1_orf1;TRINITY_DN934_c2_g1_i7_orf1;TRINITY_DN2803_c4_g1_i1_orf1;TRINITY_DN14477_c0_g1_i12_orf1;TRINITY_DN76283_c0_g2_i1_orf1;TRINITY_DN147676_c0_g1_i1_orf1;TRINITY_DN26293_c0_g1_i4_orf1;TRINITY_DN53311_c0_g2_i1_orf1;TRINITY_DN4321_c0_g1_i1_orf1;TRINITY_DN28299_c0_g1_i1_orf1;TRINITY_DN34509_c0_g1_i1_orf1;TRINITY_DN3263_c0_g1_i2_orf1;TRINITY_DN141353_c0_g1_i1_orf1;TRINITY_DN11948_c0_g1_i8_orf1;TRINITY_DN879_c0_g1_i2_orf1;TRINITY_DN905_c0_g1_i4_orf1;TRINITY_DN1084_c0_g2_i2_orf1;TRINITY_DN37532_c0_g1_i1_orf1;TRINITY_DN44792_c0_g1_i1_orf1;TRINITY_DN24970_c0_g1_i4_orf1;TRINITY_DN13651_c0_g1_i2_orf1;TRINITY_DN55148_c0_g1_i1_orf1;TRINITY_DN1045_c0_g1_i6_orf1;TRINITY_DN975_c0_g1_i1_orf1;TRINITY_DN6027_c0_g1_i13_orf1;TRINITY_DN5200_c0_g1_i2_orf1;TRINITY_DN3991_c0_g1_i6_orf1;TRINITY_DN19115_c0_g1_i1_orf1;TRINITY_DN9028_c0_g1_i5_orf1;TRINITY_DN7512_c0_g1_i1_orf1;TRINITY_DN18782_c0_g1_i4_orf1;TRINITY_DN15222_c0_g1_i4_orf1;TRINITY_DN3826_c0_g1_i1_orf1;TRINITY_DN51813_c0_g1_i1_orf1;TRINITY_DN34399_c0_g1_i1_orf1;TRINITY_DN42646_c0_g2_i1_orf1;TRINITY_DN10070_c0_g1_i1_orf1;TRINITY_DN58125_c0_g1_i1_orf1;TRINITY_DN2618_c0_g1_i3_orf1;TRINITY_DN51498_c0_g1_i1_orf1;TRINITY_DN1084_c0_g1_i2_orf1;TRINITY_DN20133_c0_g1_i1_orf1;TRINITY_DN26824_c0_g1_i1_orf1;TRINITY_DN5697_c0_g1_i1_orf1;TRINITY_DN14
biological_process	cellular macromolecule metabolic process	GO:0044260	90 90/2406	TRINITY_DN40434_c0_g1_i2_orf1;TRINITY_DN13350_c0_g1_i4_orf1;TRINITY_DN55148_c0_g1_i1_orf1;TRINITY_DN1757_c0_g1_i4_orf1;TRINITY_DN2054_c0_g1_i1_orf1;TRINITY_DN97680_c0_g1_i1_orf1;TRINITY_DN15040_c0_g4_i1_orf1;TRINITY_DN58207_c0_g1_i1_orf1;TRINITY_DN1344_c0_g1_i1_orf1;TRINITY_DN142442_c0_g1_i1_orf1;TRINITY_DN47591_c0_g1_i2_orf1;TRINITY_DN29448_c0_g1_i1_orf1;TRINITY_DN74889_c0_g1_i1_orf1;TRINITY_DN15256_c0_g1_i8_orf1;TRINITY_DN123184_c0_g1_i1_orf1;TRINITY_DN135_c0_g1_i1_orf1;TRINITY_DN2265_c0_g1_i5_orf1;TRINITY_DN89613_c0_g1_i13_orf1;TRINITY_DN14313_c0_g1_i1_orf1;TRINITY_DN17271_c0_g1_i1_orf1;TRINITY_DN23360_c0_g1_i3_orf1;TRINITY_DN45271_c0_g1_i1_orf1;TRINITY_DN37923_c0_g1_i1_orf1;TRINITY_DN97589_c0_g1_i3_orf1;TRINITY_DN81258_c0_g1_i2_orf1;TRINITY_DN57798_c0_g1_i1_orf1;TRINITY_DN115658_c0_g1_i1_orf1;TRINITY_DN7583_c0_g1_i1_orf1;TRINITY_DN7464_c0_g1_i14_orf1;TRINITY_DN41664_c0_g1_i4_orf1;TRINITY_DN1091_c0_g3_i1_orf1;TRINITY_DN9591_c0_g1_i1_orf1;TRINITY_DN6642_c0_g1_i2_orf1;TRINITY_DN13732_c0_g2_i3_orf1;TRINITY_DN4408_c6_g1_i1_orf1;TRINITY_DN95414_c0_g1_i1_orf1;TRINITY_DN9062_c0_g2_i3_orf1;TRINITY_DN74037_c0_g5_i1_orf1;TRINITY_DN139537_c0_g1_i1_orf1;TRINITY_DN10831_c1_g1_i1_orf1;TRINITY_DN147458_c0_g1_i1_orf1;TRINITY_DN4908_c1_g1_i5_orf1;TRINITY_DN28989_c0_g1_i7_orf1;TRINITY_DN5064_c0_g1_i4_orf1;TRINITY_DN291_c0_g1_i2_orf1;TRINITY_DN812_c2_g1_i1_orf1;TRINITY_DN30131_c0_g1_i1_orf1;TRINITY_DN1750_c1_g1_i5_orf1;TRINITY_DN27852_c0_g1_i1_orf1;TRINITY_DN6365_c0_g1_i4_orf1;TRINITY_DN77318_c0_g2_i1_orf1;TRINITY_DN46409_c0_g1_i1_orf1;TRINITY_DN14734_c0_g1_i2_orf1;TRINITY_DN51934_c0_g2_i1_orf1;TRINITY_DN41645_c0_g1_i1_orf1;TRINITY_DN42646_c0_g2_i1_orf1;TRINITY_DN4016_c0_g1_i1_orf1;TRINITY_DN934_c2_g1_i7_orf1;TRINITY_DN34689_c0_g1_i4_orf1;TRINITY_DN147676_c0_g1_i1_orf1;TRINITY_DN4835_c0_g1_i2_orf1;TRINITY_DN11986_c0_g1_i1_orf1;TRINITY_DN38274_c0_g1_i1_orf1;TRINITY_DN44877_c0_g1_i2_orf1;TRINITY_DN14487_c0_g1_i4_orf1;TRINITY_DN96557_c0_g1_i1_orf1;TRINITY_DN2647_c0_g1_i3_orf1;TRINITY_DN6248_c0_g1_i1_orf1;TRINITY_DN31980_c0_g1_i1_orf1;TRINITY_DN2971_c0_g1_i1_orf1;TRINITY_DN67649_c0_g1_i1_orf1;TRINITY_DN13651_c0_g1_i2_orf1;TRINITY_DN104507_c0_g1_i2_orf1;TRINITY_DN975_c0_g1_i1_orf1;TRINITY_DN5200_c0_g1_i2_orf1;TRINITY_DN2120_c0_g1_i2_orf1;TRINITY_DN1091_c0_g1_i1_orf1;TRINITY_DN140613_c0_g1_i1_orf1;TRINITY_DN3826_c0_g1_i1_orf1;TRINITY_DN5238_c0_g1_i2_orf1;TRINITY_DN46132_c0_g2_i2_orf1;TRINITY_DN5001_c0_g1_i4_orf1;TRINITY_DN110534_c0_g1_i3_orf1;TRINITY_DN5757_c0_g1_i1_orf1;TRINITY_DN10070_c0_g1_i1_orf1;TRINITY_DN346_c0_g1_i7_orf1;TRINITY_DN135188_c0_g1_i2_orf1;TRINITY_DN26824_c0_g1_i1_orf1;TRINITY_DN6462_c0_g1_i5_orf1;TRINITY_DN14996_c0_g1_i2_orf1
biological_process	cellular aromatic compound metabolic process	GO:0006725	159 159/2406	TRINITY_DN38230_c0_g1_i4_orf1;TRINITY_DN40434_c0_g1_i2_orf1;TRINITY_DN13350_c0_g1_i4_orf1;TRINITY_DN863_c0_g1_i6_orf1;TRINITY_DN51813_c0_g1_i1_orf1;TRINITY_DN35669_c0_g1_i1_orf1;TRINITY_DN2054_c0_g1_i1_orf1;TRINITY_DN124950_c0_g2_i1_orf1;TRINITY_DN2738_c1_g1_i3_orf1;TRINITY_DN15040_c0_g4_i1_orf1;TRINITY_DN34134_c0_g2_i1_orf1;TRINITY_DN17772_c0_g2_i3_orf1;TRINITY_DN1216_c0_g1_i4_orf1;TRINITY_DN1607_c0_g1_i16_orf1;TRINITY_DN8603_c0_g1_i1_orf1;TRINITY_DN23616_c0_g1_i4_orf1;TRINITY_DN2953_c1_g1_i10_orf1;TRINITY_DN2848_c0_g1_i1_orf1;TRINITY_DN11013_c0_g1_i3_orf1;TRINITY_DN3991_c0_g1_i6_orf1;TRINITY_DN19251_c0_g1_i8_orf1;TRINITY_DN3800_c0_g1_i7_orf1;TRINITY_DN47123_c0_g1_i1_orf1;TRINITY_DN15256_c0_g1_i8_orf1;TRINITY_DN123184_c0_g1_i1_orf1;TRINITY_DN20527_c0_g1_i1_orf1;TRINITY_DN1116_c0_g1_i6_orf1;TRINITY_DN5507_c0_g1_i1_orf1;TRINITY_DN2224_c0_g1_i1_orf1;TRINITY_DN2401_c0_g2_i1_orf1;TRINITY_DN5952_c0_g1_i6_orf1;TRINITY_DN5200_c0_g1_i2_orf1;TRINITY_DN15136_c0_g1_i2_orf1;TRINITY_DN11172_c1_g1_i1_orf1;TRINITY_DN7808_c0_g1_i1_orf1;TRINITY_DN89613_c0_g1_i13_orf1;TRINITY_DN14313_c0_g1_i1_orf1;TRINITY_DN230_c2_g1_i5_orf1;TRINITY_DN17271_c0_g1_i1_orf1;TRINITY_DN45271_c0_g1_i1_orf1;TRINITY_DN18222_c0_g1_i5_orf1;TRINITY_DN1005_c0_g1_i5_orf1;TRINITY_DN37532_c0_g1_i1_orf1;TRINITY_DN51968_c0_g1_i1_orf1;TRINITY_DN15900_c0_g1_i6_orf1;TRINITY_DN81258_c0_g1_i2_orf1;TRINITY_DN115658_c0_g1_i1_orf1;TRINITY_DN22242_c0_g1_i1_orf1;TRINITY_DN6813_c1_g1_i1_orf1;TRINITY_DN8625_c0_g1_i1_orf1;TRINITY_DN779_c0_g1_i3_orf1;TRINITY_DN18391_c0_g2_i8_orf1;TRINITY_DN244_c1_g1_i5_orf1;TRINITY_DN2769_c0_g1_i1_orf1;TRINITY_DN41664_c0_g1_i4_orf1;TRINITY_DN1091_c0_g3_i1_orf1;TRINITY_DN5070_c0_g1_i1_orf1;TRINITY_DN1616_c0_g1_i3_orf1;TRINITY_DN117844_c0_g1_i1_orf1;TRINITY_DN2953_c1_g1_i11_orf1;TRINITY_DN6642_c0_g1_i2_orf1;TRINITY_DN13732_c0_g2_i3_orf1;TRINITY_DN4408_c6_g1_i1_orf1;TRINITY_DN5525_c0_g1_i4_orf1;TRINITY_DN4707_c0_g1_i1_orf1;TRINITY_DN6325_c0_g1_i9_orf1;TRINITY_DN1718_c6_g1_i4_orf1;TRINITY_DN779_c0_g1_i12_orf1;TRINITY_DN56993_c0_g1_i4_orf1;TRINITY_DN21545_c0_g1_i2_orf1;TRINITY_DN74037_c0_g5_i1_orf1;TRINITY_DN31663_c0_g1_i2_orf1;TRINITY_DN139537_c0_g1_i1_orf1;TRINITY_DN1344_c0_g1_i1_orf1;TRINITY_DN107035_c0_g1_i1_orf1;TRINITY_DN4908_c1_g1_i5_orf1;TRINITY_DN2299_c0_g1_i3_orf1;TRINITY_DN291_c0_g1_i2_orf1;TRINITY_DN2749_c4_g1_i2_orf1;TRINITY_DN92153_c0_g2_i2_orf1;TRINITY_DN2749_c0_g1_i4_orf1;TRINITY_DN1750_c1_g1_i5_orf1;TRINITY_DN27852_c0_g1_i1_orf1;TRINITY_DN77318_c0_g2_i1_orf1;TRINITY_DN46409_c0_g1_i1_orf1;TRINITY_DN58636_c0_g1_i1_orf1;TRINITY_DN51934_c0_g2_i1_orf1;TRINITY_DN2718_c0_g1_i6_orf1;TRINITY_DN57918_c0_g1_i1_orf1;TRINITY_DN6563_c0_g1_i1_orf1;TRINITY_DN33346_c0_g1_i1_orf1;TRINITY_DN1554_c0_g1_i9_orf1;TRINITY_DN49038_c0_g4_i1_orf1;TRINITY_DN38506_c0_g1_i4_orf1;TRINITY_DN24_c0_g1_i1_orf1;TRINITY_DN2559_c0_g1_i4_orf1;TRINITY_DN98242_c0_g1_i1_orf1;TRINITY_DN14477_c0_g1_i12_orf1;TRINITY_DN34689_c0_g1_i4_orf1;TRINITY_DN26293_c0_g1_i4_orf1;TRINITY_DN2749_c0_g2_i3_orf1;TRINITY_DN3062_c0_g1_i1_orf1;TRINITY_DN24322_c0_g1_i4_orf1;TRINITY_DN11986_c0_g1_i1_orf1;TRINITY_DN64_c0_g1_i4_orf1;TRINITY_DN60787_c0_g1_i5_orf1;TRINITY_DN12293_c0_g1_i1_orf1;TRINITY_DN38274_c0_g1_i1_orf1;TRINITY_DN44877_c0_g1_i2_orf1;TRINITY_DN14487_c0_g1_i4_orf1;TRINITY_DN28299_c0_g1_i1_orf1;TRINITY_DN4820_c0_g2_i2_orf1;TRINITY_DN34509_c0_g1_i1_orf1;TRINITY_DN2647_c0_g1_i3_orf1;TRINITY_DN3263_c0_g1_i2_orf1;TRINITY_DN120144_c0_g1_i1_orf1;TRINITY_DN6248_c0_g1_i1_orf1;TRINITY_DN4955_c0_g1_i2_orf1;TRINITY_DN141353_c0_g1_i1_orf1;TRINITY_DN31980_c0_g1_i1_orf1;TRINITY_DN879_c0_g1_i2_orf1;TRINITY_DN38180_c0_g1_i3_orf1;TRINITY_DN1084_c0_g2_i2_orf1;TRINITY_DN2971_c0_g1_i1_orf1;TRINITY_DN67649_c0_g1_i1_orf1;TRINITY_DN2283_c0_g2_i1_orf1;TRINITY_DN44792_c0_g1_i1_orf1;TRINITY_DN8716_c0_g1_i3_orf1;TRINITY_DN4835_c0_g1_i2_orf1;TRINITY_DN38562_c0_g1_i3_orf1;TRINITY_DN13760_c1_g1_i1_orf1;TRINITY_DN15706_c0_g2_i5_orf1;TRINITY_DN4822_c0_g1_i9_orf1;TRINITY_DN55148_c0_g1_i1_orf1;TRINITY_DN87170_c0_g1_i3_orf1;TRINITY_DN104507_c0_g1_i2_orf1;TRINITY_DN2953_c1_g1_i2_orf1;TRINITY_DN19115_c0_g1_i1_orf1;TRINITY_DN2367_c1_g1_i20_orf1;TRINITY_DN1091_c0_g1_i1_orf1;TRINITY_DN11172_c0_g1_i4_orf1;TRINITY_DN18782_c0_g1_i4_orf1;TRINITY_DN15222_c0_g1_i4_orf1;TRINITY_DN53311_c0_g2_i1_orf1;TRINITY_DN5238_c0_g1_i2_orf1;TRINITY_DN20133_c0_g1_i1_orf1;TRINITY_DN13055_c0_g1_i5_orf1;TRINITY_DN34432_c0_g1_i1_orf1;TRINITY_DN110534_c0_g1_i3_orf1;TRINITY_DN5757_c0_g1_i1_orf1;TRINITY_D

biological_process	cellular metabolic compound salvage	GO:0043094	6	6/2406	TRINITY_DN2738_c1_g1_i3_orf1;TRINITY_DN3073_c0_g1_i7_orf1;TRINITY_DN5497_c0_g1_i6_orf1;TRINITY_DN8603_c0_g1_i1_orf1;TRINITY_DN35763_c0_g1_i2_orf1;TRINITY_DN1718_c6_g1_i4_orf1
biological_process	cellular modified amino acid metabolic process	GO:0006575	9	9/2406	TRINITY_DN1153_c1_g1_i1_orf1;TRINITY_DN92153_c0_g2_i2_orf1;TRINITY_DN3263_c0_g1_i2_orf1;TRINITY_DN38562_c0_g1_i3_orf1;TRINITY_DN14398_c0_g1_i4_orf1;TRINITY_DN38506_c0_g1_i4_orf1;TRINITY_DN41166_c0_g1_i1_orf1;TRINITY_DN244_c1_g1_i5_orf1;TRINITY_DN20527_c0_g1_i1_orf1
biological_process	translational initiation	GO:0006413	1	1/2406	TRINITY_DN48097_c0_g1_i1_orf1
biological_process	prenylation	GO:0097354	1	1/2406	TRINITY_DN5182_c0_g1_i5_orf1
biological_process	secondary metabolite biosynthetic process	GO:0044550	1	1/2406	TRINITY_DN58125_c0_g1_i1_orf1
biological_process	small molecule biosynthetic process	GO:0044283	35	35/2406	TRINITY_DN48590_c0_g1_i1_orf1;TRINITY_DN230_c2_g1_i5_orf1;TRINITY_DN10722_c0_g3_i1_orf1;TRINITY_DN24970_c0_g1_i4_orf1;TRINITY_DN5497_c0_g1_i6_orf1;TRINITY_DN5564_c0_g1_i5_orf1;TRINITY_DN8603_c0_g1_i1_orf1;TRINITY_DN2719_c1_g1_i6_orf1;TRINITY_DN35763_c0_g1_i2_orf1;TRINITY_DN27903_c0_g1_i1_orf1;TRINITY_DN11948_c0_g1_i8_orf1;TRINITY_DN5211_c0_g1_i1_orf1;TRINITY_DN6027_c0_g1_i13_orf1;TRINITY_DN24723_c2_g1_i1_orf1;TRINITY_DN2803_c4_g1_i1_orf1;TRINITY_DN8964_c0_g1_i4_orf1;TRINITY_DN2848_c0_g1_i1_orf1;TRINITY_DN10430_c0_g1_i4_orf1;TRINITY_DN26293_c0_g1_i4_orf1;TRINITY_DN1999_c0_g1_i9_orf1;TRINITY_DN3991_c0_g1_i6_orf1;TRINITY_DN3073_c0_g1_i7_orf1;TRINITY_DN51813_c0_g1_i1_orf1;TRINITY_DN1760_c0_g1_i4_orf1;TRINITY_DN34399_c0_g1_i1_orf1;TRINITY_DN2570_c0_g1_i1_orf1;TRINITY_DN76283_c0_g2_i1_orf1;TRINITY_DN20527_c0_g1_i1_orf1;TRINITY_DN511_c0_g2_i1_orf1;TRINITY_DN1718_c6_g1_i4_orf1;TRINITY_DN4321_c0_g1_i1_orf1;TRINITY_DN3263_c0_g1_i2_orf1;TRINITY_DN18782_c0_g1_i4_orf1;TRINITY_DN20133_c0_g1_i1_orf1;TRINITY_DN905_c0_g1_i4_orf1
biological_process	organic substance biosynthetic process	GO:1901576	115	115/2406	TRINITY_DN10722_c0_g3_i1_orf1;TRINITY_DN5564_c0_g1_i5_orf1;TRINITY_DN35763_c0_g1_i2_orf1;TRINITY_DN124950_c0_g2_i1_orf1;TRINITY_DN1153_c1_g1_i1_orf1;TRINITY_DN97680_c0_g1_i1_orf1;TRINITY_DN58207_c0_g1_i1_orf1;TRINITY_DN1216_c0_g1_i4_orf1;TRINITY_DN8603_c0_g1_i1_orf1;TRINITY_DN24723_c2_g1_i1_orf1;TRINITY_DN17031_c0_g1_i1_orf1;TRINITY_DN8964_c0_g1_i4_orf1;TRINITY_DN2848_c0_g1_i1_orf1;TRINITY_DN29448_c0_g1_i1_orf1;TRINITY_DN11013_c0_g1_i3_orf1;TRINITY_DN10742_c0_g1_i4_orf1;TRINITY_DN21570_c0_g1_i1_orf1;TRINITY_DN3991_c0_g1_i6_orf1;TRINITY_DN3800_c0_g1_i7_orf1;TRINITY_DN1760_c0_g1_i4_orf1;TRINITY_DN20527_c0_g1_i1_orf1;TRINITY_DN135_c0_g1_i1_orf1;TRINITY_DN2401_c0_g2_i1_orf1;TRINITY_DN5952_c0_g1_i6_orf1;TRINITY_DN2265_c0_g1_i5_orf1;TRINITY_DN7808_c0_g1_i1_orf1;TRINITY_DN230_c2_g1_i5_orf1;TRINITY_DN23360_c0_g1_i3_orf1;TRINITY_DN5497_c0_g1_i6_orf1;TRINITY_DN14398_c0_g1_i4_orf1;TRINITY_DN37532_c0_g1_i1_orf1;TRINITY_DN97589_c0_g1_i3_orf1;TRINITY_DN115658_c0_g1_i1_orf1;TRINITY_DN5211_c0_g1_i1_orf1;TRINITY_DN6813_c1_g1_i1_orf1;TRINITY_DN8625_c0_g1_i1_orf1;TRINITY_DN7583_c0_g1_i1_orf1;TRINITY_DN7464_c0_g1_i14_orf1;TRINITY_DN10430_c0_g1_i4_orf1;TRINITY_DN117844_c0_g1_i1_orf1;TRINITY_DN1999_c0_g1_i9_orf1;TRINITY_DN3073_c0_g1_i7_orf1;TRINITY_DN9591_c0_g1_i1_orf1;TRINITY_DN2738_c1_g1_i3_orf1;TRINITY_DN13732_c0_g2_i3_orf1;TRINITY_DN4408_c6_g1_i1_orf1;TRINITY_DN5525_c0_g1_i4_orf1;TRINITY_DN4707_c0_g1_i1_orf1;TRINITY_DN511_c0_g2_i1_orf1;TRINITY_DN1718_c6_g1_i4_orf1;TRINITY_DN11948_c0_g1_i8_orf1;TRINITY_DN74037_c0_g5_i1_orf1;TRINITY_DN10831_c1_g1_i1_orf1;TRINITY_DN147458_c0_g1_i1_orf1;TRINITY_DN2299_c0_g1_i3_orf1;TRINITY_DN5064_c0_g1_i4_orf1;TRINITY_DN812_c2_g1_i1_orf1;TRINITY_DN48590_c0_g1_i1_orf1;TRINITY_DN22046_c1_g1_i5_orf1;TRINITY_DN30131_c0_g1_i1_orf1;TRINITY_DN1750_c1_g1_i5_orf1;TRINITY_DN27852_c0_g1_i1_orf1;TRINITY_DN6365_c0_g1_i4_orf1;TRINITY_DN142442_c0_g1_i1_orf1;TRINITY_DN41645_c0_g1_i1_orf1;TRINITY_DN6563_c0_g1_i1_orf1;TRINITY_DN27903_c0_g1_i1_orf1;TRINITY_DN4016_c0_g1_i1_orf1;TRINITY_DN934_c2_g1_i7_orf1;TRINITY_DN2803_c4_g1_i1_orf1;TRINITY_DN14477_c0_g1_i12_orf1;TRINITY_DN76283_c0_g2_i1_orf1;TRINITY_DN147676_c0_g1_i1_orf1;TRINITY_DN26293_c0_g1_i4_orf1;TRINITY_DN47591_c0_g1_i2_orf1;TRINITY_DN53311_c0_g2_i1_orf1;TRINITY_DN4321_c0_g1_i1_orf1;TRINITY_DN28299_c0_g1_i1_orf1;TRINITY_DN34509_c0_g1_i1_orf1;TRINITY_DN3263_c0_g1_i2_orf1;TRINITY_DN141353_c0_g1_i1_orf1;TRINITY_DN879_c0_g1_i2_orf1;TRINITY_DN905_c0_g1_i4_orf1;TRINITY_DN1084_c0_g2_i2_orf1;TRINITY_DN2719_c1_g1_i6_orf1;TRINITY_DN44792_c0_g1_i1_orf1;TRINITY_DN24970_c0_g1_i4_orf1;TRINITY_DN2425_c0_g1_i3_orf1;TRINITY_DN41166_c0_g1_i1_orf1;TRINITY_DN13651_c0_g1_i2_orf1;TRINITY_DN55148_c0_g1_i1_orf1;TRINITY_DN1045_c0_g1_i6_orf1;TRINITY_DN975_c0_g1_i1_orf1;TRINITY_DN6027_c0_g1_i13_orf1;TRINITY_DN5200_c0_g1_i2_orf1;TRINITY_DN74889_c0_g1_i1_orf1;TRINITY_DN19115_c0_g1_i1_orf1;TRINITY_DN9028_c0_g1_i5_orf1;TRINITY_DN7512_c0_g1_i1_orf1;TRINITY_DN18782_c0_g1_i4_orf1;TRINITY_DN15222_c0_g1_i4_orf1;TRINITY_DN3826_c0_g1_i1_orf1;TRINITY_DN51813_c0_g1_i1_orf1;TRINITY_DN34399_c0_g1_i1_orf1;TRINITY_DN2570_c0_g1_i1_orf1;TRINITY_DN42646_c0_g2_i1_orf1;TRINITY_DN10070_c0_g1_i1_orf1;TRINITY_DN58125_c0_g1_i1_orf1;TRINITY_DN2618_c0_g1_i3_orf1;TRINITY_DN51498_c0_g1_i1_orf1;TRINITY_DN108
biological_process	small molecule catabolic process	GO:0044282	24	24/2406	TRINITY_DN34751_c0_g1_i1_orf1;TRINITY_DN2065_c1_g2_i1_orf1;TRINITY_DN863_c0_g1_i6_orf1;TRINITY_DN3010_c0_g1_i4_orf1;TRINITY_DN4822_c0_g1_i9_orf1;TRINITY_DN89483_c0_g1_i1_orf1;TRINITY_DN72017_c0_g1_i1_orf1;TRINITY_DN4451_c0_g2_i4_orf1;TRINITY_DN2890_c0_g1_i2_orf1;TRINITY_DN45220_c0_g1_i1_orf1;TRINITY_DN1034_c0_g1_i4_orf1;TRINITY_DN2559_c0_g1_i4_orf1;TRINITY_DN87170_c0_g1_i3_orf1;TRINITY_DN779_c0_g1_i3_orf1;TRINITY_DN98242_c0_g1_i1_orf1;TRINITY_DN2848_c0_g1_i1_orf1;TRINITY_DN43431_c0_g1_i1_orf1;TRINITY_DN51813_c0_g1_i1_orf1;TRINITY_DN12293_c0_g1_i1_orf1;TRINITY_DN779_c0_g1_i12_orf1;TRINITY_DN1262_c0_g1_i2_orf1;TRINITY_DN3836_c0_g1_i4_orf1;TRINITY_DN15136_c0_g1_i2_orf1;TRINITY_DN20133_c0_g1_i1_orf1
biological_process	organic substance catabolic process	GO:1901575	57	57/2406	TRINITY_DN34751_c0_g1_i1_orf1;TRINITY_DN2065_c1_g2_i1_orf1;TRINITY_DN38230_c0_g1_i4_orf1;TRINITY_DN6325_c0_g1_i8_orf1;TRINITY_DN98242_c0_g1_i1_orf1;TRINITY_DN10824_c0_g1_i3_orf1;TRINITY_DN18222_c0_g1_i5_orf1;TRINITY_DN863_c0_g1_i6_orf1;TRINITY_DN1757_c0_g1_i4_orf1;TRINITY_DN3010_c0_g1_i4_orf1;TRINITY_DN4822_c0_g1_i9_orf1;TRINITY_DN37923_c0_g1_i1_orf1;TRINITY_DN2120_c0_g1_i2_orf1;TRINITY_DN89483_c0_g1_i1_orf1;TRINITY_DN72017_c0_g1_i1_orf1;TRINITY_DN4451_c0_g2_i4_orf1;TRINITY_DN57798_c0_g1_i1_orf1;TRINITY_DN2890_c0_g1_i2_orf1;TRINITY_DN45220_c0_g1_i1_orf1;TRINITY_DN21545_c0_g1_i2_orf1;TRINITY_DN2559_c0_g1_i4_orf1;TRINITY_DN87170_c0_g1_i3_orf1;TRINITY_DN24_c0_g1_i1_orf1;TRINITY_DN34479_c0_g1_i2_orf1;TRINITY_DN135188_c0_g1_i2_orf1;TRINITY_DN779_c0_g1_i3_orf1;TRINITY_DN17003_c1_g1_i1_orf1;TRINITY_DN49047_c0_g1_i2_orf1;TRINITY_DN2848_c0_g1_i1_orf1;TRINITY_DN542_c0_g1_i4_orf1;TRINITY_DN34689_c0_g1_i4_orf1;TRINITY_DN4145_c0_g1_i1_orf1;TRINITY_DN96557_c0_g1_i1_orf1;TRINITY_DN42753_c0_g1_i2_orf1;TRINITY_DN1534_c0_g1_i3_orf1;TRINITY_DN43431_c0_g1_i1_orf1;TRINITY_DN51813_c0_g1_i1_orf1;TRINITY_DN2515_c0_g1_i6_orf1;TRINITY_DN46132_c0_g2_i2_orf1;TRINITY_DN5001_c0_g1_i4_orf1;TRINITY_DN12293_c0_g1_i1_orf1;TRINITY_DN6325_c0_g1_i9_orf1;TRINITY_DN44877_c0_g1_i2_orf1;TRINITY_DN779_c0_g1_i12_orf1;TRINITY_DN9062_c0_g2_i3_orf1;TRINITY_DN21555_c0_g1_i4_orf1;TRINITY_DN1034_c0_g1_i4_orf1;TRINITY_DN1262_c0_g1_i2_orf1;TRINITY_DN31980_c0_g1_i1_orf1;TRINITY_DN3836_c0_g1_i4_orf1;TRINITY_DN15136_c0_g1_i2_orf1;TRINITY_DN41_c0_g1_i5_orf1;TRINITY_DN28989_c0_g1_i7_orf1;TRINITY_DN20133_c0_g1_i1_orf1;TRINITY_DN5235_c0_g1_i7_orf1;TRINITY_DN38180_
biological_process	vitamin metabolic process	GO:0006766	2	2/2406	TRINITY_DN135781_c0_g1_i1_orf1;TRINITY_DN18782_c0_g1_i4_orf1
biological_process	urea metabolic process	GO:0019627	2	2/2406	TRINITY_DN24723_c2_g1_i1_orf1;TRINITY_DN17031_c0_g1_i1_orf1
biological_process	monosaccharide metabolic process	GO:0005996	12	12/2406	TRINITY_DN15222_c0_g1_i4_orf1;TRINITY_DN120089_c0_g1_i1_orf1;TRINITY_DN12545_c0_g1_i7_orf1;TRINITY_DN18650_c0_g1_i1_orf1;TRINITY_DN2570_c0_g1_i1_orf1;TRINITY_DN30713_c0_g1_i3_orf1;TRINITY_DN2848_c0_g1_i1_orf1;TRINITY_DN20133_c0_g1_i1_orf1;TRINITY_DN4142_c0_g1_i5_orf1;TRINITY_DN511_c0_g2_i1_orf1;TRINITY_DN31967_c0_g1_i5_orf1;TRINITY_DN1353_c0_g1_i1_orf1
biological_process	alcohol metabolic process	GO:0006066	9	9/2406	TRINITY_DN230_c2_g1_i5_orf1;TRINITY_DN52244_c1_g1_i1_orf1;TRINITY_DN53238_c1_g1_i5_orf1;TRINITY_DN10722_c0_g3_i1_orf1;TRINITY_DN36788_c0_g1_i2_orf1;TRINITY_DN9286_c0_g1_i2_orf1;TRINITY_DN1034_c0_g1_i4_orf1;TRINITY_DN3010_c0_g1_i4_orf1;TRINITY_DN618_c0_g1_i3_orf1
biological_process	nucleobase-containing small molecule metabolic process	GO:0055086	41	41/2406	TRINITY_DN38230_c0_g1_i4_orf1;TRINITY_DN6325_c0_g1_i8_orf1;TRINITY_DN15222_c0_g1_i4_orf1;TRINITY_DN18222_c0_g1_i5_orf1;TRINITY_DN60787_c0_g1_i5_orf1;TRINITY_DN8625_c0_g1_i1_orf1;TRINITY_DN2738_c1_g1_i3_orf1;TRINITY_DN49038_c0_g4_i1_orf1;TRINITY_DN21545_c0_g1_i2_orf1;TRINITY_DN779_c0_g1_i3_orf1;TRINITY_DN24_c0_g1_i1_orf1;TRINITY_DN1216_c0_g1_i4_orf1;TRINITY_DN6813_c1_g1_i1_orf1;TRINITY_DN8603_c0_g1_i1_orf1;TRINITY_DN2559_c0_g1_i4_orf1;TRINITY_DN98242_c0_g1_i1_orf1;TRINITY_DN2848_c0_g1_i1_orf1;TRINITY_DN14477_c0_g1_i12_orf1;TRINITY_DN19115_c0_g1_i1_orf1;TRINITY_DN11013_c0_g1_i3_orf1;TRINITY_DN26293_c0_g1_i4_orf1;TRINITY_DN18782_c0_g1_i4_orf1;TRINITY_DN5070_c0_g1_i1_orf1;TRINITY_DN117844_c0_g1_i1_orf1;TRINITY_DN3991_c0_g1_i6_orf1;TRINITY_DN51813_c0_g1_i1_orf1;TRINITY_DN19251_c0_g1_i8_orf1;TRINITY_DN3800_c0_g1_i7_orf1;TRINITY_DN5525_c0_g1_i4_orf1;TRINITY_DN6325_c0_g1_i9_orf1;TRINITY_DN1718_c6_g1_i4_orf1;TRINITY_DN779_c0_g1_i12_orf1;TRINITY_DN28299_c0_g1_i1_orf1;TRINITY_DN5952_c0_g1_i6_orf1;TRINITY_DN141353_c0_g1_i1_orf1;TRINITY_DN15136_c0_g1_i2_orf1;TRINITY_DN1084_c0_g1_i2_orf1;TRINITY_DN20133_c0_g1_i1_orf1;TRINITY_DN7808_c0_g1_i1_orf1;TRINITY_DN38180_c0_g1_i3_orf1;TRINITY_
biological_process	urate metabolic process	GO:0046415	3	3/2406	TRINITY_DN779_c0_g1_i12_orf1;TRINITY_DN2559_c0_g1_i4_orf1;TRINITY_DN779_c0_g1_i3_orf1
biological_process	macromolecule glycosylation	GO:0043413	2	2/2406	TRINITY_DN5513_c0_g1_i1_orf1;TRINITY_DN4248_c0_g1_i4_orf1
biological_process	macromolecule methylation	GO:0043414	8	8/2406	TRINITY_DN1344_c0_g1_i1_orf1;TRINITY_DN13350_c0_g1_i4_orf1;TRINITY_DN15256_c0_g1_i8_orf1;TRINITY_DN95414_c0_g1_i1_orf1;TRINITY_DN14734_c0_g1_i2_orf1;TRINITY_DN41664_c0_g1_i4_orf1;TRINITY_DN14313_c0_g1_i1_orf1;TRINITY_DN6462_c0_g1_i5_orf1

biological_process	lipid metabolic process	GO:0006629	51	51/2406	TRINITY_DN34751_c0_g1_i1_orf1;TRINITY_DN48590_c0_g1_i1_orf1;TRINITY_DN10722_c0_g3_i1_orf1;TRINITY_DN22046_c1_g1_i5_orf1;TRINITY_DN12526_c0_g1_i5_orf1;TRINITY_DN2668_c0_g1_i7_orf1;TRINITY_DN44777_c0_g1_i2_orf1;TRINITY_DN1293_c0_g1_i4_orf1;TRINITY_DN482_c0_g1_i1_orf1;TRINITY_DN5841_c0_g1_i2_orf1;TRINITY_DN72017_c0_g1_i1_orf1;TRINITY_DN3784_c0_g1_i1_orf1;TRINITY_DN27903_c0_g1_i1_orf1;TRINITY_DN45220_c0_g1_i1_orf1;TRINITY_DN5211_c0_g1_i1_orf1;TRINITY_DN44658_c0_g1_i2_orf1;TRINITY_DN86833_c0_g3_i1_orf1;TRINITY_DN1038_c0_g1_i4_orf1;TRINITY_DN8964_c0_g1_i4_orf1;TRINITY_DN117_c0_g1_i6_orf1;TRINITY_DN117_c0_g1_i4_orf1;TRINITY_DN41_c0_g1_i3_orf1;TRINITY_DN10430_c0_g1_i4_orf1;TRINITY_DN9028_c0_g1_i5_orf1;TRINITY_DN117_c0_g1_i5_orf1;TRINITY_DN49508_c0_g2_i8_orf1;TRINITY_DN2808_c0_g1_i8_orf1;TRINITY_DN1109_c0_g1_i6_orf1;TRINITY_DN10742_c0_g1_i4_orf1;TRINITY_DN21570_c0_g1_i1_orf1;TRINITY_DN3175_c0_g1_i7_orf1;TRINITY_DN117844_c0_g1_i1_orf1;TRINITY_DN1999_c0_g1_i9_orf1;TRINITY_DN3991_c0_g1_i6_orf1;TRINITY_DN84478_c0_g1_i8_orf1;TRINITY_DN4070_c0_g1_i4_orf1;TRINITY_DN76283_c0_g2_i1_orf1;TRINITY_DN3529_c0_g1_i7_orf1;TRINITY_DN7861_c0_g1_i5_orf1;TRINITY_DN52788_c0_g1_i1_orf1;TRINITY_DN4321_c0_g1_i1_orf1;TRINITY_DN12024_c0_g1_i4_orf1;TRINITY_DN3545_c0_g1_i6_orf1;TRINITY_DN2618_c0_g1_i3_orf1;TRINITY_DN6586_c0_g1_i1_orf1;TRINITY_DN1293_c1_g1_i4_orf1;TRINITY_DN41_c0_g1_i5_orf1;TRINITY_DN1084_c0_g1_i2_orf1;TRINITY_DN5697_c0_g1_i1_orf1;TRINITY_DN905_c0_g1_i4_orf1;TRINITY_DN1084_c0_g2_i2_
biological_process	nucleobase-containing compound metabolic process	GO:0006139	141	141/2406	TRINITY_DN38230_c0_g1_i4_orf1;TRINITY_DN40434_c0_g1_i2_orf1;TRINITY_DN13350_c0_g1_i4_orf1;TRINITY_DN60787_c0_g1_i5_orf1;TRINITY_DN51813_c0_g1_i1_orf1;TRINITY_DN35669_c0_g1_i1_orf1;TRINITY_DN2054_c0_g1_i1_orf1;TRINITY_DN124950_c0_g2_i1_orf1;TRINITY_DN2738_c1_g1_i3_orf1;TRINITY_DN15040_c0_g4_i1_orf1;TRINITY_DN34134_c0_g2_i1_orf1;TRINITY_DN1344_c0_g1_i1_orf1;TRINITY_DN1216_c0_g1_i4_orf1;TRINITY_DN1607_c0_g1_i16_orf1;TRINITY_DN8603_c0_g1_i1_orf1;TRINITY_DN23616_c0_g1_i4_orf1;TRINITY_DN2953_c1_g1_i10_orf1;TRINITY_DN2848_c0_g1_i1_orf1;TRINITY_DN11013_c0_g1_i3_orf1;TRINITY_DN3991_c0_g1_i6_orf1;TRINITY_DN19251_c0_g1_i8_orf1;TRINITY_DN3800_c0_g1_i7_orf1;TRINITY_DN47123_c0_g1_i1_orf1;TRINITY_DN15256_c0_g1_i8_orf1;TRINITY_DN123184_c0_g1_i1_orf1;TRINITY_DN1116_c0_g1_i6_orf1;TRINITY_DN5507_c0_g1_i1_orf1;TRINITY_DN2224_c0_g1_i1_orf1;TRINITY_DN2401_c0_g2_i1_orf1;TRINITY_DN5952_c0_g1_i6_orf1;TRINITY_DN5200_c0_g1_i2_orf1;TRINITY_DN15136_c0_g1_i2_orf1;TRINITY_DN7808_c0_g1_i1_orf1;TRINITY_DN89613_c0_g1_i13_orf1;TRINITY_DN14313_c0_g1_i1_orf1;TRINITY_DN17271_c0_g1_i1_orf1;TRINITY_DN45271_c0_g1_i1_orf1;TRINITY_DN18222_c0_g1_i5_orf1;TRINITY_DN1005_c0_g1_i5_orf1;TRINITY_DN37532_c0_g1_i1_orf1;TRINITY_DN51968_c0_g1_i1_orf1;TRINITY_DN15900_c0_g1_i6_orf1;TRINITY_DN81258_c0_g1_i2_orf1;TRINITY_DN115658_c0_g1_i1_orf1;TRINITY_DN6813_c1_g1_i1_orf1;TRINITY_DN8625_c0_g1_i1_orf1;TRINITY_DN779_c0_g1_i3_orf1;TRINITY_DN18391_c0_g2_i8_orf1;TRINITY_DN2769_c0_g1_i1_orf1;TRINITY_DN41664_c0_g1_i4_orf1;TRINITY_DN1091_c0_g3_i1_orf1;TRINITY_DN5070_c0_g1_i1_orf1;TRINITY_DN1616_c0_g1_i3_orf1;TRINITY_DN117844_c0_g1_i1_orf1;TRINITY_DN2953_c1_g1_i11_orf1;TRINITY_DN6642_c0_g1_i2_orf1;TRINITY_DN13732_c0_g2_i3_orf1;TRINITY_DN4408_c6_g1_i1_orf1;TRINITY_DN5525_c0_g1_i4_orf1;TRINITY_DN4707_c0_g1_i1_orf1;TRINITY_DN6325_c0_g1_i9_orf1;TRINITY_DN1718_c6_g1_i4_orf1;TRINITY_DN779_c0_g1_i12_orf1;TRINITY_DN56993_c0_g1_i4_orf1;TRINITY_DN21545_c0_g1_i2_orf1;TRINITY_DN74037_c0_g5_i1_orf1;TRINITY_DN31663_c0_g1_i2_orf1;TRINITY_DN139537_c0_g1_i1_orf1;TRINITY_DN107035_c0_g1_i1_orf1;TRINITY_DN4908_c1_g1_i5_orf1;TRINITY_DN2299_c0_g1_i3_orf1;TRINITY_DN291_c0_g1_i2_orf1;TRINITY_DN2749_c4_g1_i2_orf1;TRINITY_DN6325_c0_g1_i8_orf1;TRINITY_DN2749_c0_g1_i4_orf1;TRINITY_DN1750_c1_g1_i5_orf1;TRINITY_DN27852_c0_g1_i1_orf1;TRINITY_DN77318_c0_g2_i1_orf1;TRINITY_DN46409_c0_g1_i1_orf1;TRINITY_DN58636_c0_g1_i1_orf1;TRINITY_DN51934_c0_g2_i1_orf1;TRINITY_DN2718_c0_g1_i6_orf1;TRINITY_DN57918_c0_g1_i1_orf1;TRINITY_DN33346_c0_g1_i1_orf1;TRINITY_DN1554_c0_g1_i9_orf1;TRINITY_DN49038_c0_g4_i1_orf1;TRINITY_DN24_c0_g1_i1_orf1;TRINITY_DN2559_c0_g1_i4_orf1;TRINITY_DN98242_c0_g1_i1_orf1;TRINITY_DN14477_c0_g1_i12_orf1;TRINITY_DN34689_c0_g1_i4_orf1;TRINITY_DN26293_c0_g1_i4_orf1;TRINITY_DN2749_c0_g2_i3_orf1;TRINITY_DN3062_c0_g1_i1_orf1;TRINITY_DN24322_c0_g1_i4_orf1;TRINITY_DN11986_c0_g1_i1_orf1;TRINITY_DN64_c0_g1_i4_orf1;TRINITY_DN38274_c0_g1_i1_orf1;TRINITY_DN44877_c0_g1_i2_orf1;TRINITY_DN14487_c0_g1_i4_orf1;TRINITY_DN28299_c0_g1_i1_orf1;TRINITY_DN4820_c0_g2_i2_orf1;TRINITY_DN34509_c0_g1_i1_orf1;TRINITY_DN2647_c0_g1_i3_orf1;TRINITY_DN120144_c0_g1_i1_orf1;TRINITY_DN6248_c0_g1_i1_orf1;TRINITY_DN4955_c0_g1_i2_orf1;TRINITY_DN141353_c0_g1_i1_orf1;TRINITY_DN31980_c0_g1_i1_orf1;TRINITY_DN879_c0_g1_i2_orf1;TRINITY_DN38180_c0_g1_i3_orf1;TRINITY_DN1084_c0_g2_i2_orf1;TRINITY_DN2971_c0_g1_i1_orf1;TRINITY_DN67649_c0_g1_i1_orf1;TRINITY_DN2283_c0_g2_i1_orf1;TRINITY_DN44792_c0_g1_i1_orf1;TRINITY_DN8716_c0_g1_i3_orf1;TRINITY_DN4835_c0_g1_i2_orf1;TRINITY_DN38562_c0_g1_i3_orf1;TRINITY_DN13760_c1_g1_i1_orf1;TRINITY_DN55148_c0_g1_i1_orf1;TRINITY_DN104507_c0_g1_i2_orf1;TRINITY_DN2953_c1_g1_i2_orf1;TRINITY_DN19115_c0_g1_i1_orf1;TRINITY_DN2367_c1_g1_i20_orf1;TRINITY_DN1091_c0_g1_i1_orf1;TRINITY_DN18782_c0_g1_i4_orf1;TRINITY_DN15222_c0_g1_i4_orf1;TRINITY_DN53311_c0_g2_i1_orf1;TRINITY_DN5238_c0_g1_i2_orf1;TRINITY_DN20133_c0_g1_i1_orf1;TRINITY_DN13055_c0_g1_i5_orf1;TRINITY_DN34432_c0_g1_i1_orf1;TRINITY_DN110534_c0_g1_i3_orf1;TRINITY_DN5757_c0_g1_i1_orf1;TRINITY_
biological_process	tricarboxylic acid cycle	GO:0006099	7	7/2406	TRINITY_DN5266_c0_g1_i1_orf1;TRINITY_DN146126_c0_g1_i1_orf1;TRINITY_DN53238_c1_g1_i5_orf1;TRINITY_DN36788_c0_g1_i2_orf1;TRINITY_DN19251_c0_g1_i8_orf1;TRINITY_DN2594_c0_g2_i4_orf1;TRINITY_DN3464_c0_g1_i1_orf1
biological_process	carbohydrate metabolic process	GO:0005975	61	61/2406	TRINITY_DN6510_c1_g1_i1_orf1;TRINITY_DN8703_c0_g1_i2_orf1;TRINITY_DN120089_c0_g1_i1_orf1;TRINITY_DN10722_c0_g3_i1_orf1;TRINITY_DN2474_c0_g1_i5_orf1;TRINITY_DN18650_c0_g1_i1_orf1;TRINITY_DN10824_c0_g1_i3_orf1;TRINITY_DN2425_c0_g1_i3_orf1;TRINITY_DN18222_c0_g1_i5_orf1;TRINITY_DN60787_c0_g1_i5_orf1;TRINITY_DN22577_c0_g1_i2_orf1;TRINITY_DN25492_c0_g1_i1_orf1;TRINITY_DN6325_c0_g1_i9_orf1;TRINITY_DN1196_c0_g1_i5_orf1;TRINITY_DN3010_c0_g1_i4_orf1;TRINITY_DN11657_c0_g1_i2_orf1;TRINITY_DN31967_c0_g1_i5_orf1;TRINITY_DN3476_c0_g1_i5_orf1;TRINITY_DN9044_c0_g1_i1_orf1;TRINITY_DN618_c0_g1_i3_orf1;TRINITY_DN49038_c0_g4_i1_orf1;TRINITY_DN1034_c0_g1_i4_orf1;TRINITY_DN12545_c0_g1_i7_orf1;TRINITY_DN36788_c0_g1_i2_orf1;TRINITY_DN4070_c0_g1_i4_orf1;TRINITY_DN28741_c0_g1_i3_orf1;TRINITY_DN7183_c0_g1_i2_orf1;TRINITY_DN812_c2_g1_i1_orf1;TRINITY_DN361_c0_g1_i5_orf1;TRINITY_DN17003_c1_g1_i1_orf1;TRINITY_DN30713_c0_g1_i3_orf1;TRINITY_DN2848_c0_g1_i1_orf1;TRINITY_DN2894_c0_g2_i3_orf1;TRINITY_DN4142_c0_g1_i5_orf1;TRINITY_DN3322_c0_g1_i2_orf1;TRINITY_DN140613_c0_g1_i1_orf1;TRINITY_DN15222_c0_g1_i4_orf1;TRINITY_DN89483_c0_g1_i1_orf1;TRINITY_DN9000_c0_g2_i1_orf1;TRINITY_DN53238_c1_g1_i5_orf1;TRINITY_DN542_c0_g1_i4_orf1;TRINITY_DN2515_c0_g1_i6_orf1;TRINITY_DN26688_c0_g1_i2_orf1;TRINITY_DN9044_c0_g1_i2_orf1;TRINITY_DN143603_c0_g1_i1_orf1;TRINITY_DN511_c0_g2_i1_orf1;TRINITY_DN14458_c0_g1_i2_orf1;TRINITY_DN7828_c0_g1_i2_orf1;TRINITY_DN6325_c0_g1_i8_orf1;TRINITY_DN21555_c0_g1_i4_orf1;TRINITY_DN52244_c1_g1_i1_orf1;TRINITY_DN5952_c0_g1_i6_orf1;TRINITY_DN2570_c0_g1_i1_orf1;TRINITY_DN479_c6_g1_i2_orf1;TRINITY_DN2170_c0_g1_i2_orf1;TRINITY_DN82801_c0_g1_i1_orf1;TRINITY_DN13088_c0_g1_i5_orf1;TRINITY_DN2170_c1_g1_i3_orf1;TRINITY_DN20133_c0_g1_i1_orf1;TRINITY_DN650_c0_g1_i3_orf1;TRINITY_DN1353_c0_

biological_process	protein metabolic process	GO:0019538	195	195/2406	TRINITY_DN24469_c0_g2_i2_orf1;TRINITY_DN10364_c0_g1_i5_orf1;TRINITY_DN1533_c0_g2_i1_orf1;TRINITY_DN3978_c0_g2_i1_orf1;TRINITY_DN1757_c0_g1_i4_orf1;TRINITY_DN1274_c0_g1_i4_orf1;TRINITY_DN2069_c1_g1_i8_orf1;TRINITY_DN4217_c0_g1_i2_orf1;TRINITY_DN38431_c0_g1_i1_orf1;TRINITY_DN58207_c0_g1_i1_orf1;TRINITY_DN3499_c0_g1_i8_orf1;TRINITY_DN8692_c0_g1_i2_orf1;TRINITY_DN2442_c0_g1_i2_orf1;TRINITY_DN8659_c0_g1_i1_orf1;TRINITY_DN70485_c0_g1_i2_orf1;TRINITY_DN2983_c0_g1_i6_orf1;TRINITY_DN16258_c0_g1_i2_orf1;TRINITY_DN2885_c1_g1_i2_orf1;TRINITY_DN277_c1_g1_i1_orf1;TRINITY_DN2848_c0_g1_i1_orf1;TRINITY_DN14754_c0_g1_i6_orf1;TRINITY_DN29448_c0_g1_i1_orf1;TRINITY_DN27033_c1_g1_i3_orfp1;TRINITY_DN49047_c0_g1_i2_orf1;TRINITY_DN14217_c0_g1_i1_orf1;TRINITY_DN14774_c0_g1_i4_orf1;TRINITY_DN48020_c0_g1_i1_orf1;TRINITY_DN42753_c0_g1_i2_orf1;TRINITY_DN875_c0_g1_i3_orf1;TRINITY_DN74889_c0_g1_i1_orf1;TRINITY_DN2794_c1_g1_i8_orf1;TRINITY_DN4125_c0_g1_i14_orf1;TRINITY_DN6185_c0_g1_i12_orf1;TRINITY_DN6205_c0_g1_i8_orf1;TRINITY_DN6436_c0_g1_i1_orf1;TRINITY_DN41086_c0_g1_i4_orf1;TRINITY_DN4798_c0_g1_i3_orf1;TRINITY_DN391_c0_g1_i4_orf1;TRINITY_DN40_c0_g2_i1_orf1;TRINITY_DN13732_c0_g2_i3_orf1;TRINITY_DN46715_c0_g1_i1_orf1;TRINITY_DN10403_c0_g1_i3_orf1;TRINITY_DN338_c1_g1_i9_orf1;TRINITY_DN1749_c0_g2_i2_orf1;TRINITY_DN73945_c0_g5_i3_orf1;TRINITY_DN143895_c0_g1_i1_orf1;TRINITY_DN13856_c0_g1_i1_orf1;TRINITY_DN23360_c0_g1_i3_orf1;TRINITY_DN45633_c0_g1_i1_orf1;TRINITY_DN10090_c0_g1_i1_orf1;TRINITY_DN10629_c0_g1_i1_orf1;TRINITY_DN6205_c0_g1_i1_orf1;TRINITY_DN19537_c0_g1_i1_orf1;TRINITY_DN4189_c0_g2_i1_orf1;TRINITY_DN1791_c0_g1_i3_orf1;TRINITY_DN37923_c0_g1_i1_orf1;TRINITY_DN97589_c0_g1_i3_orf1;TRINITY_DN57798_c0_g1_i1_orf1;TRINITY_DN5012_c0_g1_i6_orf1;TRINITY_DN36434_c0_g2_i3_orf1;TRINITY_DN42461_c0_g1_i4_orf1;TRINITY_DN344_c1_g1_i1_orf1;TRINITY_DN30154_c0_g1_i1_orf1;TRINITY_DN6813_c1_g1_i1_orf1;TRINITY_DN21719_c0_g1_i2_orf1;TRINITY_DN7583_c0_g1_i1_orf1;TRINITY_DN7464_c0_g1_i14_orf1;TRINITY_DN45948_c1_g1_i1_orf1;TRINITY_DN5696_c0_g1_i4_orf1;TRINITY_DN23167_c0_g2_i1_orf1;TRINITY_DN6423_c0_g1_i5_orf1;TRINITY_DN29034_c0_g1_i2_orf1;TRINITY_DN32700_c0_g1_i2_orf1;TRINITY_DN4125_c1_g1_i5_orf1;TRINITY_DN4064_c0_g2_i1_orf1;TRINITY_DN9591_c0_g1_i1_orf1;TRINITY_DN5444_c0_g2_i1_orf1;TRINITY_DN4408_c6_g1_i1_orf1;TRINITY_DN95414_c0_g1_i1_orf1;TRINITY_DN11620_c0_g1_i2_orf1;TRINITY_DN97680_c0_g1_i1_orf1;TRINITY_DN5281_c0_g2_i3_orf1;TRINITY_DN376_c1_g1_i1_orf1;TRINITY_DN9062_c0_g2_i3_orf1;TRINITY_DN16451_c0_g1_i7_orf1;TRINITY_DN4767_c0_g1_i4_orf1;TRINITY_DN1173_c1_g1_i10_orf1;TRINITY_DN10831_c1_g1_i1_orf1;TRINITY_DN120593_c0_g1_i1_orf1;TRINITY_DN147458_c0_g1_i1_orf1;TRINITY_DN21218_c0_g1_i4_orf1;TRINITY_DN28989_c0_g1_i7_orf1;TRINITY_DN5513_c0_g1_i1_orf1;TRINITY_DN4248_c0_g1_i4_orf1;TRINITY_DN1173_c0_g1_i12_orf1;TRINITY_DN2584_c0_g1_i7_orf1;TRINITY_DN25534_c0_g1_i1_orf1;TRINITY_DN10766_c0_g1_i1_orf1;TRINITY_DN6365_c0_g1_i4_orf1;TRINITY_DN9991_c0_g1_i4_orf1;TRINITY_DN46409_c0_g1_i1_orf1;TRINITY_DN14734_c0_g1_i2_orf1;TRINITY_DN142442_c0_g1_i1_orf1;TRINITY_DN1552_c0_g1_i3_orf1;TRINITY_DN5182_c0_g1_i5_orf1;TRINITY_DN81803_c0_g2_i1_orf1;TRINITY_DN57111_c0_g1_i1_orf1;TRINITY_DN5310_c2_g1_i2_orf1;TRINITY_DN2040_c0_g1_i6_orf1;TRINITY_DN4571_c0_g1_i4_orf1;TRINITY_DN140_c1_g1_i2_orf1;TRINITY_DN1404_c0_g1_i6_orf1;TRINITY_DN4016_c0_g1_i1_orf1;TRINITY_DN934_c2_g1_i7_orf1;TRINITY_DN1706_c0_g1_i7_orf1;TRINITY_DN34479_c0_g1_i2_orf1;TRINITY_DN1405_c0_g1_i1_orf1;TRINITY_DN21719_c0_g2_i4_orf1;TRINITY_DN147676_c0_g1_i1_orf1;TRINITY_DN18388_c0_g1_i6_orf1;TRINITY_DN47591_c0_g1_i2_orf1;TRINITY_DN135_c0_g1_i1_orf1;TRINITY_DN1309_c0_g2_i1_orf1;TRINITY_DN1308_c0_g1_i4_orf1;TRINITY_DN23167_c0_g1_i4_orf1;TRINITY_DN28661_c0_g1_i1_orf1;TRINITY_DN4228_c0_g1_i5_orf1;TRINITY_DN52553_c0_g2_i1_orf1;TRINITY_DN143637_c0_g1_i1_orf1;TRINITY_DN41761_c0_g1_i4_orf1;TRINITY_DN111110_c0_g1_i1_orf1;TRINITY_DN14487_c0_g1_i4_orf1;TRINITY_DN96557_c0_g1_i1_orf1;TRINITY_DN8659_c0_g2_i1_orf1;TRINITY_DN6470_c0_g3_i2_orf1;TRINITY_DN24121_c1_g1_i6_orf1;TRINITY_DN29034_c0_g1_i1_orf1;TRINITY_DN19990_c0_g1_i1_orf1;TRINITY_DN31980_c0_g1_i1_orf1;TRINITY_DN52553_c0_g1_i1_orf1;TRINITY_DN4898_c0_g1_i7_orf1;TRINITY_DN10385_c0_g1_i5_orf1;TRINITY_DN1459_c1_g1_i1_orf1;TRINITY_DN44792_c0_g1_i1_orf1;TRINITY_DN10403_c0_g1_i1_orf1;TRINITY_DN801_c0_g1_i2_orf1;TRINITY_DN24539_c0_g1_i4_orf1;TRINITY_DN13686_c0_g2_i1_orf1;TRINITY_DN13651_c0_g1_i2_orf1;TRINITY_DN334_c0_g1_i4_orf1;TRINITY_DN6423_c0_g1[illegible]
biological_process	cellular amino acid metabolic process	GO:0006520	43	43/2406	TRINITY_DN2065_c1_g2_i1_orf1;TRINITY_DN57918_c0_g1_i1_orf1;TRINITY_DN92153_c0_g2_i2_orf1;TRINITY_DN24970_c0_g1_i4_orf1;TRINITY_DN5497_c0_g1_i6_orf1;TRINITY_DN5564_c0_g1_i5_orf1;TRINITY_DN863_c0_g1_i6_orf1;TRINITY_DN2719_c1_g1_i6_orf1;TRINITY_DN35763_c0_g1_i2_orf1;TRINITY_DN4822_c0_g1_i9_orf1;TRINITY_DN89483_c0_g1_i1_orf1;TRINITY_DN4451_c0_g2_i4_orf1;TRINITY_DN2890_c0_g1_i2_orf1;TRINITY_DN11948_c0_g1_i8_orf1;TRINITY_DN87170_c0_g1_i3_orf1;TRINITY_DN2803_c4_g1_i1_orf1;TRINITY_DN2953_c1_g1_i10_orf1;TRINITY_DN24723_c2_g1_i1_orf1;TRINITY_DN1607_c0_g1_i16_orf1;TRINITY_DN2953_c1_g1_i2_orf1;TRINITY_DN11013_c0_g1_i3_orf1;TRINITY_DN2953_c1_g1_i11_orf1;TRINITY_DN20796_c0_g1_i4_orf1;TRINITY_DN8716_c0_g1_i3_orf1;TRINITY_DN17326_c0_g1_i8_orf1;TRINITY_DN43431_c0_g1_i1_orf1;TRINITY_DN3859_c0_g1_i5_orf1;TRINITY_DN3073_c0_g1_i7_orf1;TRINITY_DN51813_c0_g1_i1_orf1;TRINITY_DN1760_c0_g1_i4_orf1;TRINITY_DN34399_c0_g1_i1_orf1;TRINITY_DN17031_c0_g1_i1_orf1;TRINITY_DN12293_c0_g1_i1_orf1;TRINITY_DN20527_c0_g1_i1_orf1;TRINITY_DN48619_c0_g1_i1_orf1;TRINITY_DN2224_c0_g1_i1_orf1;TRINITY_DN21539_c0_g1_i1_orf1;TRINITY_DN1262_c0_g1_i2_orf1;TRINITY_DN11383_c0_g2_i4_orf1;TRINITY_DN3263_c0_g1_i2_orf1;TRINITY_DN3836_c0_g1_i4_orf1;TRINITY_DN15136_c0_g1_i2_orf1;TRINITY_DN1068_c0_g1_i3_orf1
biological_process	organophosphate metabolic process	GO:0019637	41	41/2406	TRINITY_DN5697_c0_g1_i1_orf1;TRINITY_DN143509_c0_g1_i1_orf1;TRINITY_DN62557_c0_g1_i1_orf1;TRINITY_DN47151_c0_g1_i1_orf1;TRINITY_DN38230_c0_g1_i4_orf1;TRINITY_DN6325_c0_g1_i8_orf1;TRINITY_DN5070_c0_g1_i1_orf1;TRINITY_DN18222_c0_g1_i5_orf1;TRINITY_DN60787_c0_g1_i5_orf1;TRINITY_DN15222_c0_g1_i4_orf1;TRINITY_DN41166_c0_g1_i1_orf1;TRINITY_DN2738_c1_g1_i3_orf1;TRINITY_DN10722_c0_g3_i1_orf1;TRINITY_DN21545_c0_g1_i2_orf1;TRINITY_DN24_c0_g1_i1_orf1;TRINITY_DN1216_c0_g1_i4_orf1;TRINITY_DN6813_c1_g1_i1_orf1;TRINITY_DN8603_c0_g1_i1_orf1;TRINITY_DN116972_c0_g1_i1_orf1;TRINITY_DN2848_c0_g1_i1_orf1;TRINITY_DN14477_c0_g1_i12_orf1;TRINITY_DN19115_c0_g1_i1_orf1;TRINITY_DN18782_c0_g1_i4_orf1;TRINITY_DN10742_c0_g1_i4_orf1;TRINITY_DN117844_c0_g1_i1_orf1;TRINITY_DN3991_c0_g1_i6_orf1;TRINITY_DN51813_c0_g1_i1_orf1;TRINITY_DN19251_c0_g1_i8_orf1;TRINITY_DN3800_c0_g1_i7_orf1;TRINITY_DN6325_c0_g1_i9_orf1;TRINITY_DN1718_c6_g1_i4_orf1;TRINITY_DN49038_c0_g4_i1_orf1;TRINITY_DN28299_c0_g1_i1_orf1;TRINITY_DN1034_c0_g1_i4_orf1;TRINITY_DN2618_c0_g1_i3_orf1;TRINITY_DN141353_c0_g1_i1_orf1;TRINITY_DN15136_c0_g1_i2_orf1;TRINITY_DN1084_c0_g1_i2_orf1;TRINITY_DN20133_c0_g1_i1_orf1;TRINITY_DN7808_c0_g1_i1_orf1;
biological_process	carbohydrate derivative metabolic process	GO:1901135	46	46/2406	TRINITY_DN5697_c0_g1_i1_orf1;TRINITY_DN62557_c0_g1_i1_orf1;TRINITY_DN47151_c0_g1_i1_orf1;TRINITY_DN6325_c0_g1_i8_orf1;TRINITY_DN98242_c0_g1_i1_orf1;TRINITY_DN10824_c0_g1_i3_orf1;TRINITY_DN18222_c0_g1_i5_orf1;TRINITY_DN60787_c0_g1_i5_orf1;TRINITY_DN8625_c0_g1_i1_orf1;TRINITY_DN1196_c0_g1_i5_orf1;TRINITY_DN2738_c1_g1_i3_orf1;TRINITY_DN117844_c0_g1_i1_orf1;TRINITY_DN1034_c0_g1_i4_orf1;TRINITY_DN1216_c0_g1_i4_orf1;TRINITY_DN6813_c1_g1_i1_orf1;TRINITY_DN8603_c0_g1_i1_orf1;TRINITY_DN17003_c1_g1_i1_orf1;TRINITY_DN143509_c0_g1_i1_orf1;TRINITY_DN2848_c0_g1_i1_orf1;TRINITY_DN19115_c0_g1_i1_orf1;TRINITY_DN542_c0_g1_i4_orf1;TRINITY_DN11013_c0_g1_i3_orf1;TRINITY_DN26293_c0_g1_i4_orf1;TRINITY_DN1084_c0_g1_i2_orf1;TRINITY_DN15222_c0_g1_i4_orf1;TRINITY_DN1534_c0_g1_i3_orf1;TRINITY_DN3991_c0_g1_i6_orf1;TRINITY_DN19251_c0_g1_i8_orf1;TRINITY_DN3800_c0_g1_i7_orf1;TRINITY_DN2515_c0_g1_i6_orf1;TRINITY_DN5525_c0_g1_i4_orf1;TRINITY_DN6325_c0_g1_i9_orf1;TRINITY_DN1718_c6_g1_i4_orf1;TRINITY_DN49038_c0_g4_i1_orf1;TRINITY_DN21555_c0_g1_i4_orf1;TRINITY_DN28299_c0_g1_i1_orf1;TRINITY_DN5952_c0_g1_i6_orf1;TRINITY_DN18782_c0_g1_i4_orf1;TRINITY_DN141353_c0_g1_i1_orf1;TRINITY_DN15136_c0_g1_i2_orf1;TRINITY_DN116972_c0_g1_i1_orf1;TRINITY_DN20133_c0_g1_i1_orf1;TRINITY_DN5235_c0_g1_i7_orf1;TRINITY_DN7808_c0_g1_i1_orf1;TRINITY_DN1084_c0_g2_i2_orf1;TRINIT
biological_process	organic hydroxy compound metabolic process	GO:1901615	11	11/2406	TRINITY_DN230_c2_g1_i5_orf1;TRINITY_DN52244_c1_g1_i1_orf1;TRINITY_DN58125_c0_g1_i1_orf1;TRINITY_DN10722_c0_g3_i1_orf1;TRINITY_DN36788_c0_g1_i2_orf1;TRINITY_DN9286_c0_g1_i2_orf1;TRINITY_DN1034_c0_g1_i4_orf1;TRINITY_DN15706_c0_g2_i5_orf1;TRINITY_DN53238_c1_g1_i5_orf1;TRINITY_DN3010_c0_g1_i4_orf1;TRINITY_DN618_c0_g1_i3_orf1

biological_process	organic cyclic compound metabolic process	GO:1901360	161	161/2406	TRINITY_DN38230_c0_g1_i4_orf1;TRINITY_DN40434_c0_g1_i2_orf1;TRINITY_DN13350_c0_g1_i4_orf1;TRINITY_DN863_c0_g1_i6_orf1;TRINITY_DN51813_c0_g1_i1_orf1;TRINITY_DN35669_c0_g1_i1_orf1;TRINITY_DN2054_c0_g1_i1_orf1;TRINITY_DN124950_c0_g2_i1_orf1;TRINITY_DN2738_c1_g1_i3_orf1;TRINITY_DN15040_c0_g4_i1_orf1;TRINITY_DN34134_c0_g2_i1_orf1;TRINITY_DN17772_c0_g2_i3_orf1;TRINITY_DN1216_c0_g1_i4_orf1;TRINITY_DN1607_c0_g1_i16_orf1;TRINITY_DN8603_c0_g1_i1_orf1;TRINITY_DN23616_c0_g1_i4_orf1;TRINITY_DN2953_c1_g1_i10_orf1;TRINITY_DN2848_c0_g1_i1_orf1;TRINITY_DN11013_c0_g1_i3_orf1;TRINITY_DN3991_c0_g1_i6_orf1;TRINITY_DN19251_c0_g1_i8_orf1;TRINITY_DN3800_c0_g1_i7_orf1;TRINITY_DN1760_c0_g1_i4_orf1;TRINITY_DN47123_c0_g1_i1_orf1;TRINITY_DN15256_c0_g1_i8_orf1;TRINITY_DN123184_c0_g1_i1_orf1;TRINITY_DN20527_c0_g1_i1_orf1;TRINITY_DN1116_c0_g1_i6_orf1;TRINITY_DN5507_c0_g1_i1_orf1;TRINITY_DN2224_c0_g1_i1_orf1;TRINITY_DN2401_c0_g2_i1_orf1;TRINITY_DN5952_c0_g1_i6_orf1;TRINITY_DN5200_c0_g1_i2_orf1;TRINITY_DN15136_c0_g1_i2_orf1;TRINITY_DN7808_c0_g1_i1_orf1;TRINITY_DN89613_c0_g1_i13_orf1;TRINITY_DN14313_c0_g1_i1_orf1;TRINITY_DN230_c2_g1_i5_orf1;TRINITY_DN17271_c0_g1_i1_orf1;TRINITY_DN45271_c0_g1_i1_orf1;TRINITY_DN18222_c0_g1_i5_orf1;TRINITY_DN1005_c0_g1_i5_orf1;TRINITY_DN37532_c0_g1_i1_orf1;TRINITY_DN51968_c0_g1_i1_orf1;TRINITY_DN15900_c0_g1_i6_orf1;TRINITY_DN81258_c0_g1_i2_orf1;TRINITY_DN115658_c0_g1_i1_orf1;TRINITY_DN6813_c1_g1_i1_orf1;TRINITY_DN8625_c0_g1_i1_orf1;TRINITY_DN779_c0_g1_i3_orf1;TRINITY_DN18391_c0_g2_i8_orf1;TRINITY_DN244_c1_g1_i5_orf1;TRINITY_DN2769_c0_g1_i1_orf1;TRINITY_DN41664_c0_g1_i4_orf1;TRINITY_DN1091_c0_g3_i1_orf1;TRINITY_DN4145_c0_g1_i1_orf1;TRINITY_DN5070_c0_g1_i1_orf1;TRINITY_DN1616_c0_g1_i3_orf1;TRINITY_DN117844_c0_g1_i1_orf1;TRINITY_DN2953_c1_g1_i11_orf1;TRINITY_DN6642_c0_g1_i2_orf1;TRINITY_DN13732_c0_g2_i3_orf1;TRINITY_DN4408_c6_g1_i1_orf1;TRINITY_DN5525_c0_g1_i4_orf1;TRINITY_DN4707_c0_g1_i1_orf1;TRINITY_DN6325_c0_g1_i9_orf1;TRINITY_DN1718_c6_g1_i4_orf1;TRINITY_DN779_c0_g1_i12_orf1;TRINITY_DN56993_c0_g1_i4_orf1;TRINITY_DN21545_c0_g1_i2_orf1;TRINITY_DN74037_c0_g5_i1_orf1;TRINITY_DN31663_c0_g1_i2_orf1;TRINITY_DN139537_c0_g1_i1_orf1;TRINITY_DN1344_c0_g1_i1_orf1;TRINITY_DN107035_c0_g1_i1_orf1;TRINITY_DN4908_c1_g1_i5_orf1;TRINITY_DN2299_c0_g1_i3_orf1;TRINITY_DN291_c0_g1_i2_orf1;TRINITY_DN2749_c4_g1_i2_orf1;TRINITY_DN92153_c0_g2_i2_orf1;TRINITY_DN2749_c0_g1_i4_orf1;TRINITY_DN1750_c1_g1_i5_orf1;TRINITY_DN27852_c0_g1_i1_orf1;TRINITY_DN77318_c0_g2_i1_orf1;TRINITY_DN46409_c0_g1_i1_orf1;TRINITY_DN58636_c0_g1_i1_orf1;TRINITY_DN51934_c0_g2_i1_orf1;TRINITY_DN2718_c0_g1_i6_orf1;TRINITY_DN57918_c0_g1_i1_orf1;TRINITY_DN6563_c0_g1_i1_orf1;TRINITY_DN33346_c0_g1_i1_orf1;TRINITY_DN1554_c0_g1_i9_orf1;TRINITY_DN49038_c0_g4_i1_orf1;TRINITY_DN38506_c0_g1_i4_orf1;TRINITY_DN24_c0_g1_i1_orf1;TRINITY_DN2559_c0_g1_i4_orf1;TRINITY_DN2803_c4_g1_i1_orf1;TRINITY_DN98242_c0_g1_i1_orf1;TRINITY_DN14477_c0_g1_i12_orf1;TRINITY_DN34689_c0_g1_i4_orf1;TRINITY_DN26293_c0_g1_i4_orf1;TRINITY_DN2749_c0_g2_i3_orf1;TRINITY_DN3062_c0_g1_i1_orf1;TRINITY_DN8716_c0_g1_i3_orf1;TRINITY_DN24322_c0_g1_i4_orf1;TRINITY_DN11986_c0_g1_i1_orf1;TRINITY_DN64_c0_g1_i4_orf1;TRINITY_DN60787_c0_g1_i5_orf1;TRINITY_DN12293_c0_g1_i1_orf1;TRINITY_DN38274_c0_g1_i1_orf1;TRINITY_DN44877_c0_g1_i2_orf1;TRINITY_DN14487_c0_g1_i4_orf1;TRINITY_DN28299_c0_g1_i1_orf1;TRINITY_DN4820_c0_g2_i2_orf1;TRINITY_DN34509_c0_g1_i1_orf1;TRINITY_DN2647_c0_g1_i3_orf1;TRINITY_DN3263_c0_g1_i2_orf1;TRINITY_DN120144_c0_g1_i1_orf1;TRINITY_DN6248_c0_g1_i1_orf1;TRINITY_DN4955_c0_g1_i2_orf1;TRINITY_DN141353_c0_g1_i1_orf1;TRINITY_DN31980_c0_g1_i1_orf1;TRINITY_DN879_c0_g1_i2_orf1;TRINITY_DN38180_c0_g1_i3_orf1;TRINITY_DN1084_c0_g2_i2_orf1;TRINITY_DN2971_c0_g1_i1_orf1;TRINITY_DN2719_c1_g1_i6_orf1;TRINITY_DN67649_c0_g1_i1_orf1;TRINITY_DN2283_c0_g2_i1_orf1;TRINITY_DN44792_c0_g1_i1_orf1;TRINITY_DN24970_c0_g1_i4_orf1;TRINITY_DN4835_c0_g1_i2_orf1;TRINITY_DN38562_c0_g1_i3_orf1;TRINITY_DN13760_c1_g1_i1_orf1;TRINITY_DN15706_c0_g2_i5_orf1;TRINITY_DN4822_c0_g1_i9_orf1;TRINITY_DN55148_c0_g1_i1_orf1;TRINITY_DN87170_c0_g1_i3_orf1;TRINITY_DN104507_c0_g1_i2_orf1;TRINITY_DN2953_c1_g1_i2_orf1;TRINITY_DN19115_c0_g1_i1_orf1;TRINITY_DN2367_c1_g1_i20_orf1;TRINITY_DN1091_c0_g1_i1_orf1;TRINITY_DN18782_c0_g1_i4_orf1;TRINITY_DN15222_c0_g1_i4_orf1;TRINITY_DN53311_c0_g2_i1_orf1;TRINITY_DN5238_c0_g1_i2_orf1;TRINITY_DN20133_c0_g1_i1_orf1;TRINITY_DN13055_c0_g1_i5_orf1;TRINITY_DN34432_c0_g1_i1_orf1;TRINITY_DN110534_c0
biological_process	thioester metabolic process	GO:0035383	7	7/2406	TRINITY_DN117844_c0_g1_i1_orf1;TRINITY_DN3991_c0_g1_i6_orf1;TRINITY_DN15136_c0_g1_i2_orf1;TRINITY_DN1084_c0_g1_i2_orf1;TRINITY_DN19251_c0_g1_i8_orf1;TRINITY_DN7808_c0_g1_i1_orf1;TRINITY_DN1084_c0_g2_i2_orf1
biological_process	macromolecule metabolic process	GO:0043170	300	300/2406	TRINITY_DN24469_c0_g2_i2_orf1;TRINITY_DN10364_c0_g1_i5_orf1;TRINITY_DN40434_c0_g1_i2_orf1;TRINITY_DN13350_c0_g1_i4_orf1;TRINITY_DN1533_c0_g2_i1_orf1;TRINITY_DN28661_c0_g1_i1_orf1;TRINITY_DN135_c0_g1_i1_orf1;TRINITY_DN1757_c0_g1_i4_orf1;TRINITY_DN35669_c0_g1_i1_orf1;TRINITY_DN1274_c0_g1_i4_orf1;TRINITY_DN2069_c1_g1_i8_orf1;TRINITY_DN51934_c0_g2_i1_orf1;TRINITY_DN124950_c0_g2_i1_orf1;TRINITY_DN4217_c0_g1_i2_orf1;TRINITY_DN38431_c0_g1_i1_orf1;TRINITY_DN58207_c0_g1_i1_orf1;TRINITY_DN3499_c0_g1_i8_orf1;TRINITY_DN8692_c0_g1_i2_orf1;TRINITY_DN2442_c0_g1_i2_orf1;TRINITY_DN8659_c0_g1_i1_orf1;TRINITY_DN41952_c0_g1_i4_orf1;TRINITY_DN2983_c0_g1_i6_orf1;TRINITY_DN97680_c0_g1_i1_orf1;TRINITY_DN16258_c0_g1_i2_orf1;TRINITY_DN2953_c1_g1_i10_orf1;TRINITY_DN277_c1_g1_i1_orf1;TRINITY_DN2848_c0_g1_i1_orf1;TRINITY_DN14754_c0_g1_i6_orf1;TRINITY_DN29448_c0_g1_i1_orf1;TRINITY_DN41761_c0_g1_i4_orf1;TRINITY_DN14487_c0_g1_i4_orf1;TRINITY_DN14217_c0_g1_i1_orf1;TRINITY_DN14774_c0_g1_i4_orf1;TRINITY_DN48020_c0_g1_i1_orf1;TRINITY_DN42753_c0_g1_i2_orf1;TRINITY_DN2953_c1_g1_i2_orf1;TRINITY_DN875_c0_g1_i3_orf1;TRINITY_DN5507_c0_g1_i1_orf1;TRINITY_DN74889_c0_g1_i1_orf1;TRINITY_DN2794_c1_g1_i8_orf1;TRINITY_DN4125_c0_g1_i14_orf1;TRINITY_DN47123_c0_g1_i1_orf1;TRINITY_DN15256_c0_g1_i8_orf1;TRINITY_DN542_c0_g1_i4_orf1;TRINITY_DN123184_c0_g1_i1_orf1;TRINITY_DN6185_c0_g1_i12_orf1;TRINITY_DN3978_c0_g2_i1_orf1;TRINITY_DN6436_c0_g1_i1_orf1;TRINITY_DN41086_c0_g1_i4_orf1;TRINITY_DN4798_c0_g1_i3_orf1;TRINITY_DN391_c0_g1_i4_orf1;TRINITY_DN2224_c0_g1_i1_orf1;TRINITY_DN2401_c0_g2_i1_orf1;TRINITY_DN40_c0_g2_i1_orf1;TRINITY_DN2265_c0_g1_i5_orf1;TRINITY_DN1091_c0_g3_i1_orf1;TRINITY_DN6470_c0_g3_i2_orf1;TRINITY_DN13732_c0_g2_i3_orf1;TRINITY_DN46715_c0_g1_i1_orf1;TRINITY_DN10403_c0_g1_i3_orf1;TRINITY_DN18391_c0_g2_i8_orf1;TRINITY_DN338_c1_g1_i9_orf1;TRINITY_DN89613_c0_g1_i13_orf1;TRINITY_DN14313_c0_g1_i1_orf1;TRINITY_DN1749_c0_g2_i2_orf1;TRINITY_DN73945_c0_g5_i3_orf1;TRINITY_DN17271_c0_g1_i1_orf1;TRINITY_DN143895_c0_g1_i1_orf1;TRINITY_DN1116_c0_g1_i6_orf1;TRINITY_DN13856_c0_g1_i1_orf1;TRINITY_DN24121_c1_g1_i6_orf1;TRINITY_DN23360_c0_g1_i3_orf1;TRINITY_DN10824_c0_g1_i3_orf1;TRINITY_DN45633_c0_g1_i1_orf1;TRINITY_DN10090_c0_g1_i1_orf1;TRINITY_DN1005_c0_g1_i5_orf1;TRINITY_DN10629_c0_g1_i1_orf1;TRINITY_DN6205_c0_g1_i1_orf1;TRINITY_DN37532_c0_g1_i1_orf1;TRINITY_DN19537_c0_g1_i1_orf1;TRINITY_DN4189_c0_g2_i1_orf1;TRINITY_DN1791_c0_g1_i3_orf1;TRINITY_DN37923_c0_g1_i1_orf1;TRINITY_DN32700_c0_g1_i2_orf1;TRINITY_DN97589_c0_g1_i3_orf1;TRINITY_DN81258_c0_g1_i2_orf1;TRINITY_DN57798_c0_g1_i1_orf1;TRINITY_DN5012_c0_g1_i6_orf1;TRINITY_DN115658_c0_g1_i1_orf1;TRINITY_DN57918_c0_g1_i1_orf1;TRINITY_DN30131_c0_g1_i1_orf1;TRINITY_DN42461_c0_g1_i4_orf1;TRINITY_DN344_c1_g1_i1_orf1;TRINITY_DN30154_c0_g1_i1_orf1;TRINITY_DN51968_c0_g1_i1_orf1;TRINITY_DN6813_c1_g1_i1_orf1;TRINITY_DN21719_c0_g1_i2_orf1;TRINITY_DN7583_c0_g1_i1_orf1;TRINITY_DN1607_c0_g1_i16_orf1;TRINITY_DN7464_c0_g1_i14_orf1;TRINITY_DN2054_c0_g1_i1_orf1;TRINITY_DN45948_c1_g1_i1_orf1;TRINITY_DN36434_c0_g2_i3_orf1;TRINITY_DN2769_c0_g1_i1_orf1;TRINITY_DN41664_c0_g1_i4_orf1;TRINITY_DN5696_c0_g1_i4_orf1;TRINITY_DN23167_c0_g2_i1_orf1;TRINITY_DN6423_c0_g1_i5_orf1;TRINITY_DN29034_c0_g1_i2_orf1;TRINITY_DN1616_c0_g1_i3_orf1;TRINITY_DN4125_c1_g1_i5_orf1;TRINITY_DN2953_c1_g1_i11_orf1;TRINITY_DN13055_c0_g1_i5_orf1;TRINITY_DN4064_c0_g2_i1_orf1;TRINITY_DN9591_c0_g1_i1_orf1;TRINITY_DN6642_c0_g1_i2_orf1;TRINITY_DN5444_c0_g2_i1_orf1;TRINITY_DN4408_c6_g1_i1_orf1;TRINITY_DN95414_c0_g1_i1_orf1;TRINITY_DN6205_c0_g1_i8_orf1;TRINITY_DN11620_c0_g1_i2_orf1;TRINITY_DN27033_c1_g1_i3_orfp1;TRINITY_DN5281_c0_g2_i3_orf1;TRINITY_DN376_c1_g1_i1_orf1;TRINITY_DN650_c0_g1_i3_orf1;TRINITY_DN15040_c0_g4_i1_orf1;TRINITY_DN9062_c0_g2_i3_orf1;TRINITY_DN56993_c0_g1_i4_orf1;TRINITY_DN34134_c0_g2_i1_orf1;TRINITY_DN4767_c0_g1_i4_orf1;TRINITY_DN74037_c0_g5_i1_orf1;TRINITY_DN31663_c0_g1_i2_orf1;TRINITY_DN139537_c0_g1_i1_orf1;TRINITY_DN49047_c0_g1_i2_orf1;TRINITY_DN1344_c0_g1_i1_orf1;TRINITY_DN10831_c1_g1_i1_orf1;TRINITY_DN120593_c0_g1_i1_orf1;TRINITY_DN140_c1_g1_i2_orf1;TRINITY_DN107035_c0_g1_i1_orf1;TRINITY_DN147458_c0_g1_i1_orf1;TRINITY_DN4908_c1_g1_i5_orf1;TRINITY_DN28989_c0_g1_i7_orf1;TRINITY_DN2299_c0_g1_i3_orf1;TRINITY_DN5235_c0_g1_i7_orf1;TRINITY_DN5513_c0_g1_i1_orf1;TRINITY_DN291_c0_g1_i2_orf1;TRINITY_DN4248_c0_g1_i4_orf1;TRINITY_DN812_c2_g1_i1_orf1;TRINITY_DN1173_c0_g1_i12_orf1;TRINITY_DN2584_c0_g1_i7_orf1;TRINITY_DN25534_c0_g
biological_process	S-adenosylmethionine metabolic process	GO:0046500	1	1/2406	TRINITY_DN7512_c0_g1_i1_orf1
biological_process	melanin metabolic process	GO:0006582	2	2/2406	TRINITY_DN58125_c0_g1_i1_orf1;TRINITY_DN15706_c0_g2_i5_orf1
biological_process	pigment biosynthetic process	GO:0046148	2	2/2406	TRINITY_DN58125_c0_g1_i1_orf1;TRINITY_DN6563_c0_g1_i1_orf1
biological_process	heme metabolic process	GO:0042168	2	2/2406	TRINITY_DN6563_c0_g1_i1_orf1;TRINITY_DN17772_c0_g2_i3_orf1
biological_process	mating behavior	GO:0007617	1	1/2406	TRINITY_DN58125_c0_g1_i1_orf1
biological_process	gamete generation	GO:0007276	2	2/2406	TRINITY_DN2652_c0_g2_i1_orf1;TRINITY_DN4820_c0_g2_i2_orf1
biological_process	germ cell development	GO:0007281	1	1/2406	TRINITY_DN2652_c0_g2_i1_orf1
biological_process	ovarian follicle cell development	GO:0030707	1	1/2406	TRINITY_DN15706_c0_g2_i5_orf1
biological_process	spermatogenesis	GO:0007283	1	1/2406	TRINITY_DN4820_c0_g2_i2_orf1
biological_process	binding of sperm to zona pellucida	GO:0007339	1	1/2406	TRINITY_DN20133_c0_g1_i1_orf1
biological_process	female pregnancy	GO:0007565	1	1/2406	TRINITY_DN2848_c0_g1_i1_orf1
biological_process	killing of cells of another organism	GO:0031640	2	2/2406	TRINITY_DN6098_c1_g1_i5_orf1;TRINITY_DN2848_c0_g1_i1_orf1
biological_process	leukocyte activation	GO:0045321	1	1/2406	TRINITY_DN46409_c0_g1_i1_orf1
biological_process	neuron death	GO:0070997	1	1/2406	TRINITY_DN2848_c0_g1_i1_orf1
biological_process	programmed cell death	GO:0012501	2	2/2406	TRINITY_DN2848_c0_g1_i1_orf1;TRINITY_DN50074_c0_g1_i1_orf1
biological_process	autophagy	GO:0006914	3	3/2406	TRINITY_DN113353_c0_g1_i1_orf1;TRINITY_DN5531_c0_g3_i3_orf1;TRINITY_DN19821_c0_g2_i4_orf1
biological_process	secretion by cell	GO:0032940	5	5/2406	TRINITY_DN25686_c0_g1_i4_orf1;TRINITY_DN47219_c0_g1_i3_orf1;TRINITY_DN33452_c0_g1_i1_orf1;TRINITY_DN1895_c0_g1_i2_orf1;TRINITY_DN33452_c0_g1_i3_orf1
biological_process	cell-substrate adhesion	GO:0031589	3	3/2406	TRINITY_DN2186_c0_g1_i17_orf1;TRINITY_DN6698_c0_g2_i1_orf1;TRINITY_DN2919_c0_g1_i5_orf1
biological_process	cell-cell adhesion	GO:0098609	4	4/2406	TRINITY_DN1008_c0_g1_i2_orf1;TRINITY_DN3469_c0_g1_i4_orf1;TRINITY_DN14389_c0_g1_i4_orf1;TRINITY_DN10070_c0_g1_i1_orf1

biological_process	cellular response to extracellular stimulus	GO:0031668	4	4/2406	TRINITY_DN1091_c0_g1_i1_orf1;TRINITY_DN1091_c0_g3_i1_orf1;TRINITY_DN51938_c0_g3_i1_orf1;TRINITY_DN2054_c0_g1_i1_orf1
biological_process	intermediate filament cytoskeleton organization	GO:0045104	8	8/2406	TRINITY_DN51836_c0_g3_i1_orf1;TRINITY_DN107962_c0_g1_i1_orf1;TRINITY_DN59852_c0_g1_i1_orf1;TRINITY_DN69557_c0_g1_i1_orf1;TRINITY_DN77480_c0_g1_i2_orf1;TRINITY_DN17137_c0_g1_i2_orf1;TRINITY_DN97097_c0_g1_i4_orf1;TRINITY_DN101991_c0_g1_i5_orf1
biological_process	vesicle tethering involved in exocytosis	GO:0090522	1	1/2406	TRINITY_DN16316_c0_g1_i7_orf1
biological_process	maintenance of protein location in cell	GO:0032507	1	1/2406	TRINITY_DN245_c0_g1_i4_orf1
biological_process	muscle cell cellular homeostasis	GO:0046716	1	1/2406	TRINITY_DN20133_c0_g1_i1_orf1
biological_process	cellular chemical homeostasis	GO:0055082	9	9/2406	TRINITY_DN46625_c0_g1_i1_orf1;TRINITY_DN65681_c0_g1_i1_orf1;TRINITY_DN1423_c0_g1_i4_orf1;TRINITY_DN1423_c0_g1_i8_orf1;TRINITY_DN136031_c0_g1_i7_orf1;TRINITY_DN3461_c0_g1_i1_orf1;TRINITY_DN31584_c0_g2_i2_orf1;TRINITY_DN44256_c0_g1_i1_orf1;TRINITY_DN5753_c0_g1_i10_orf1
biological_process	transposition, DNA-mediated	GO:0006313	1	1/2406	TRINITY_DN139537_c0_g1_i1_orf1
biological_process	leukocyte proliferation	GO:0070661	1	1/2406	TRINITY_DN46409_c0_g1_i1_orf1
biological_process	mitotic cell cycle process	GO:1903047	2	2/2406	TRINITY_DN96557_c0_g1_i1_orf1;TRINITY_DN235_c0_g3_i1_orf1
biological_process	cell cycle phase transition	GO:0044770	1	1/2406	TRINITY_DN96557_c0_g1_i1_orf1
biological_process	cytokinesis	GO:0000910	1	1/2406	TRINITY_DN235_c0_g3_i1_orf1
biological_process	sister chromatid cohesion	GO:0007062	1	1/2406	TRINITY_DN2638_c0_g1_i7_orf1
biological_process	cytokinetic process	GO:0032506	1	1/2406	TRINITY_DN96557_c0_g1_i1_orf1
biological_process	'de novo' protein folding	GO:0006458	1	1/2406	TRINITY_DN46409_c0_g1_i1_orf1
biological_process	protein refolding	GO:0042026	2	2/2406	TRINITY_DN46409_c0_g1_i1_orf1;TRINITY_DN18031_c0_g1_i1_orf1
biological_process	post-chaperonin tubulin folding pathway	GO:0007023	1	1/2406	TRINITY_DN104297_c0_g1_i1_orf1
biological_process	cellular macromolecule localization	GO:0070727	35	35/2406	TRINITY_DN245_c0_g1_i4_orf1;TRINITY_DN5182_c0_g1_i5_orf1;TRINITY_DN46409_c0_g1_i1_orf1;TRINITY_DN6243_c0_g1_i5_orf1;TRINITY_DN31584_c0_g2_i2_orf1;TRINITY_DN146119_c0_g1_i1_orf1;TRINITY_DN383_c0_g1_i1_orf1;TRINITY_DN45400_c0_g1_i1_orf1;TRINITY_DN14677_c0_g2_i3_orf1;TRINITY_DN55148_c0_g1_i1_orf1;TRINITY_DN34159_c0_g2_i1_orf1;TRINITY_DN558_c0_g1_i4_orf1;TRINITY_DN95971_c0_g5_i1_orf1;TRINITY_DN5118_c0_g1_i1_orf1;TRINITY_DN16316_c0_g1_i7_orf1;TRINITY_DN3513_c0_g1_i5_orf1;TRINITY_DN445_c0_g1_i2_orf1;TRINITY_DN13118_c0_g1_i6_orf1;TRINITY_DN1437_c0_g1_i6_orf1;TRINITY_DN2286_c2_g1_i1_orf1;TRINITY_DN6680_c0_g1_i1_orf1;TRINITY_DN146236_c0_g1_i1_orf1;TRINITY_DN11772_c0_g1_i1_orf1;TRINITY_DN959_c0_g1_i7_orf1;TRINITY_DN4689_c0_g1_i5_orf1;TRINITY_DN486_c0_g1_i5_orf1;TRINITY_DN2879_c0_g1_i4_orf1;TRINITY_DN96557_c0_g1_i1_orf1;TRINITY_DN2172_c0_g2_i8_orf1;TRINITY_DN4410_c0_g1_i1_orf1;TRINITY_DN47219_c0_g1_i3_orf1;TRINITY_DN69871_c0_g1_i1_orf1;TRINITY_DN12777_c0_g1_i5_orf1;TRINITY_DN50875_c0_g1_i3_orf1;TRINITY_DN38835_c0_g3_i1_orf1
biological_process	intracellular transport	GO:0046907	30	30/2406	TRINITY_DN54586_c1_g1_i1_orf1;TRINITY_DN3821_c1_g1_i7_orf1;TRINITY_DN6231_c0_g1_i6_orf1;TRINITY_DN13626_c0_g2_i1_orf1;TRINITY_DN46409_c0_g1_i1_orf1;TRINITY_DN13157_c0_g1_i1_orf1;TRINITY_DN31584_c0_g2_i2_orf1;TRINITY_DN5182_c0_g1_i5_orf1;TRINITY_DN45400_c0_g1_i1_orf1;TRINITY_DN14677_c0_g2_i3_orf1;TRINITY_DN95971_c0_g5_i1_orf1;TRINITY_DN34159_c0_g2_i1_orf1;TRINITY_DN55148_c0_g1_i1_orf1;TRINITY_DN5118_c0_g1_i1_orf1;TRINITY_DN16316_c0_g1_i7_orf1;TRINITY_DN3513_c0_g1_i5_orf1;TRINITY_DN445_c0_g1_i2_orf1;TRINITY_DN13118_c0_g1_i6_orf1;TRINITY_DN1437_c0_g1_i6_orf1;TRINITY_DN64_c0_g1_i4_orf1;TRINITY_DN35377_c0_g1_i3_orf1;TRINITY_DN486_c0_g1_i5_orf1;TRINITY_DN578_c0_g1_i5_orf1;TRINITY_DN96557_c0_g1_i1_orf1;TRINITY_DN69871_c0_g1_i1_orf1;TRINITY_DN12777_c0_g1_i5_orf1;TRINITY_DN1245_c0_g1_i4_orf1;TRINITY_DN942_c0_g1_i1_orf1;TRINITY_DN5028_c0_g1_i11_orf1;TRINITY_DN2338_c3_g2_i3_orf1
biological_process	localization within membrane	GO:0051668	3	3/2406	TRINITY_DN96557_c0_g1_i1_orf1;TRINITY_DN959_c0_g1_i7_orf1;TRINITY_DN69871_c0_g1_i1_orf1
biological_process	cellular component biogenesis	GO:0044085	7	7/2406	TRINITY_DN4016_c0_g1_i1_orf1;TRINITY_DN14391_c1_g1_i2_orf1;TRINITY_DN146217_c0_g1_i1_orf1;TRINITY_DN31225_c0_g1_i1_orf1;TRINITY_DN14313_c0_g1_i1_orf1;TRINITY_DN55148_c0_g1_i1_orf1;TRINITY_DN6239_c0_g1_i1_orf1
biological_process	cellular component organization	GO:0016043	102	102/2406	TRINITY_DN14920_c0_g1_i1_orf1;TRINITY_DN2304_c0_g1_i4_orf1;TRINITY_DN841_c0_g1_i4_orf1;TRINITY_DN11194_c0_g1_i4_orf1;TRINITY_DN34426_c0_g1_i1_orf1;TRINITY_DN19980_c0_g1_i4_orf1;TRINITY_DN35669_c0_g1_i1_orf1;TRINITY_DN4010_c0_g2_i1_orf1;TRINITY_DN4217_c0_g1_i2_orf1;TRINITY_DN91877_c0_g1_i1_orf1;TRINITY_DN35245_c0_g1_i1_orf1;TRINITY_DN104297_c0_g1_i1_orf1;TRINITY_DN70485_c0_g1_i2_orf1;TRINITY_DN142442_c0_g1_i1_orf1;TRINITY_DN59852_c0_g1_i1_orf1;TRINITY_DN2638_c0_g1_i7_orf1;TRINITY_DN2848_c0_g1_i1_orf1;TRINITY_DN69557_c0_g1_i1_orf1;TRINITY_DN235_c0_g3_i1_orf1;TRINITY_DN77480_c0_g1_i2_orf1;TRINITY_DN16316_c0_g1_i7_orf1;TRINITY_DN698_c0_g1_i5_orf1;TRINITY_DN4798_c0_g1_i3_orf1;TRINITY_DN2345_c0_g1_i4_orf1;TRINITY_DN110231_c0_g1_i1_orf1;TRINITY_DN662_c0_g1_i1_orf1;TRINITY_DN27276_c0_g1_i5_orf1;TRINITY_DN3461_c0_g1_i1_orf1;TRINITY_DN14389_c0_g1_i4_orf1;TRINITY_DN23502_c0_g1_i1_orf1;TRINITY_DN4950_c0_g1_i2_orf1;TRINITY_DN1749_c0_g2_i2_orf1;TRINITY_DN51938_c0_g3_i1_orf1;TRINITY_DN11464_c0_g1_i3_orf1;TRINITY_DN52649_c0_g1_i6_orf1;TRINITY_DN34703_c0_g1_i4_orf1;TRINITY_DN146119_c0_g1_i1_orf1;TRINITY_DN23790_c0_g1_i1_orf1;TRINITY_DN298_c0_g1_i4_orf1;TRINITY_DN90497_c0_g1_i1_orf1;TRINITY_DN30150_c0_g1_i7_orf1;TRINITY_DN28018_c0_g6_i1_orf1;TRINITY_DN28622_c0_g1_i1_orf1;TRINITY_DN10455_c0_g1_i2_orf1;TRINITY_DN50085_c0_g1_i1_orf1;TRINITY_DN6642_c0_g1_i2_orf1;TRINITY_DN11772_c0_g1_i1_orf1;TRINITY_DN4689_c0_g1_i5_orf1;TRINITY_DN4439_c0_g1_i2_orf1;TRINITY_DN51836_c0_g3_i1_orf1;TRINITY_DN6358_c0_g1_i5_orf1;TRINITY_DN3459_c0_g1_i4_orf1;TRINITY_DN4842_c0_g1_i5_orf1;TRINITY_DN16145_c0_g1_i12_orf1;TRINITY_DN4908_c1_g1_i5_orf1;TRINITY_DN101991_c0_g1_i5_orf1;TRINITY_DN12771_c0_g1_i1_orf1;TRINITY_DN6239_c0_g1_i1_orf1;TRINITY_DN714_c0_g1_i3_orf1;TRINITY_DN8915_c0_g1_i3_orf1;TRINITY_DN46409_c0_g1_i1_orf1;TRINITY_DN4016_c0_g1_i1_orf1;TRINITY_DN14987_c0_g1_i3_orf1;TRINITY_DN8390_c0_g1_i2_orf1;TRINITY_DN43412_c0_g1_i2_orf1;TRINITY_DN7794_c0_g1_i1_orf1;TRINITY_DN54134_c0_g1_i1_orf1;TRINITY_DN86309_c0_g1_i4_orf1;TRINITY_DN11746_c0_g2_i1_orf1;TRINITY_DN11986_c0_g1_i1_orf1;TRINITY_DN146236_c0_g1_i1_orf1;TRINITY_DN27960_c0_g1_i1_orf1;TRINITY_DN18009_c0_g1_i1_orf1;TRINITY_DN25976_c0_g1_i4_orf1;TRINITY_DN85476_c0_g1_i1_orf1;TRINITY_DN96557_c0_g1_i1_orf1;TRINITY_DN6248_c0_g1_i1_orf1;TRINITY_DN42854_c0_g3_i2_orf1;TRINITY_DN116467_c0_g1_i1_orf1;TRINITY_DN10385_c0_g1_i5_orf1;TRINITY_DN6071_c0_g1_i1_orf1;TRINITY_DN4182_c0_g1_i6_orf1;TRINITY_DN8087_c0_g1_i9_orf1;TRINITY_DN4159_c1_g1_i1_orf1;TRINITY_DN48097_c0_g1_i1_orf1;TRINITY_DN55148_c0_g1_i1_orf1;TRINITY_DN38540_c0_g1_i1_orf1;TRINITY_DN107962_c0_g1_i1_orf1;TRINITY_DN3887_c0_g1_i1_orf1;TRINITY_DN49872_c0_g1_i2_orf1;TRINITY_DN3513_c0_g1_i5_orf1;TRINITY_DN35635_c0_g1_i1_orf1;TRINITY_DN24266_c0_g2_i2_orf1;TRINITY_DN30273_c1_g1_i1_orf1;TRINITY_DN5757_c0_g1_i1_orf1;TRINITY_DN10070_c0_g1_i1_orf1;TRINITY_DN147475_c0_g1_i1_orf1;TRINITY_DN14684_c0_g2_i1_orf1;TRINITY_DN20133_c0_g1_i1_orf1;TRINITY_DN17137_c0_g1_i2_orf1;TRINITY_DN110231_c0_g1_i1_orf1;TRINITY_DN31584_c0_g2_i2_orf1;TRINITY_DN15706_c0_g2_i5_orf1
biological_process	cell migration	GO:0016477	3	3/2406	TRINITY_DN17995_c0_g4_i1_orf1;TRINITY_DN14298_c0_g3_i1_orf1;TRINITY_DN14298_c0_g1_i1_orf1;TRINITY_DN122423_c0_g1_i1_orf1
biological_process	microtubule-based movement	GO:0007018	4	4/2406	
biological_process	microtubule cytoskeleton organization	GO:0000226	6	6/2406	TRINITY_DN11746_c0_g2_i1_orf1;TRINITY_DN28018_c0_g6_i1_orf1;TRINITY_DN34703_c0_g1_i4_orf1;TRINITY_DN2848_c0_g1_i1_orf1;TRINITY_DN4689_c0_g1_i5_orf1;TRINITY_DN8390_c0_g1_i2_orf1
biological_process	cellular response to chemical stimulus	GO:0070887	5	5/2406	TRINITY_DN46409_c0_g1_i1_orf1;TRINITY_DN51938_c0_g3_i1_orf1;TRINITY_DN975_c0_g1_i1_orf1;TRINITY_DN4016_c0_g1_i1_orf1;TRINITY_DN2848_c0_g1_i1_orf1
biological_process	cellular response to stress	GO:0033554	24	24/2406	TRINITY_DN51938_c0_g3_i1_orf1;TRINITY_DN2971_c0_g1_i1_orf1;TRINITY_DN40434_c0_g1_i2_orf1;TRINITY_DN45271_c0_g1_i1_orf1;TRINITY_DN77318_c0_g2_i1_orf1;TRINITY_DN46409_c0_g1_i1_orf1;TRINITY_DN31584_c0_g2_i2_orf1;TRINITY_DN17271_c0_g1_i1_orf1;TRINITY_DN104507_c0_g1_i2_orf1;TRINITY_DN2054_c0_g1_i1_orf1;TRINITY_DN1091_c0_g1_i1_orf1;TRINITY_DN1091_c0_g3_i1_orf1;TRINITY_DN5238_c0_g1_i2_orf1;TRINITY_DN6642_c0_g1_i2_orf1;TRINITY_DN123184_c0_g1_i1_orf1;TRINITY_DN6185_c0_g1_i12_orf1;TRINITY_DN38274_c0_g1_i1_orf1;TRINITY_DN5757_c0_g1_i1_orf1;TRINITY_DN14487_c0_g1_i4_orf1;TRINITY_DN9062_c0_g2_i3_orf1;TRINITY_DN2647_c0_g1_i3_orf1;TRINITY_DN346_c0_g1_i7_orf1;TRINITY_DN31980_c0_g1_i1_orf1;TRINITY_DN28989_c0_g1_i7_orf1
biological_process	cell surface receptor signaling pathway	GO:0007166	8	8/2406	TRINITY_DN51938_c0_g3_i1_orf1;TRINITY_DN2170_c0_g1_i2_orf1;TRINITY_DN147475_c0_g1_i1_orf1;TRINITY_DN1008_c0_g1_i2_orf1;TRINITY_DN33418_c0_g1_i1_orf1;TRINITY_DN2170_c1_g1_i3_orf1;TRINITY_DN13216_c0_g1_i5_orf1;TRINITY_DN4142_c0_g1_i5_orf1
biological_process	hormone-mediated signaling pathway	GO:0009755	1	1/2406	TRINITY_DN147475_c0_g1_i1_orf1
biological_process	immune response-regulating signaling pathway	GO:0002764	3	3/2406	TRINITY_DN46409_c0_g1_i1_orf1;TRINITY_DN2170_c0_g1_i2_orf1;TRINITY_DN2170_c1_g1_i3_orf1
biological_process	G protein-coupled receptor signaling pathway	GO:0007186	3	3/2406	TRINITY_DN42854_c0_g3_i2_orf1;TRINITY_DN11245_c0_g1_i2_orf1;TRINITY_DN33418_c0_g1_i1_orf1
biological_process	intracellular signal transduction	GO:0035556	12	12/2406	TRINITY_DN32700_c0_g1_i2_orf1;TRINITY_DN79000_c1_g1_i1_orf1;TRINITY_DN4410_c0_g1_i1_orf1;TRINITY_DN2793_c0_g2_i1_orf1;TRINITY_DN2983_c0_g1_i6_orf1;TRINITY_DN802_c0_g1_i2_orf1;TRINITY_DN2623_c0_g1_i3_orf1;TRINITY_DN31584_c0_g2_i2_orf1;TRINITY_DN15706_c0_g2_i5_orf1;TRINITY_DN5182_c0_g1_i5_orf1;TRINITY_DN804_c0_g1_i7_orf1;TRINITY_DN2848_c0_g1_i1_orf1
biological_process	apoptotic signaling pathway	GO:0097190	1	1/2406	TRINITY_DN51938_c0_g3_i1_orf1
biological_process	meiotic cell cycle	GO:0051321	2	2/2406	TRINITY_DN45271_c0_g1_i1_orf1;TRINITY_DN123184_c0_g1_i1_orf1
biological_process	mitotic cell cycle	GO:0000278	1	1/2406	TRINITY_DN31314_c0_g1_i4_orf1
biological_process	cell differentiation	GO:0030154	19	19/2406	TRINITY_DN1173_c0_g1_i12_orf1;TRINITY_DN928_c0_g1_i3_orf1;TRINITY_DN51836_c0_g3_i1_orf1;TRINITY_DN14389_c0_g1_i4_orf1;TRINITY_DN52395_c0_g2_i2_orf1;TRINITY_DN5954_c0_g1_i2_orf1;TRINITY_DN42461_c0_g1_i4_orf1;TRINITY_DN1173_c0_g1_i11_orf1;TRINITY_DN4550_c1_g1_i19_orf1;TRINITY_DN1173_c1_g1_i10_orf1;TRINITY_DN4820_c0_g2_i2_orf1;TRINITY_DN549_c0_g1_i14_orf1;TRINITY_DN11388_c0_g1_i4_orf1;TRINITY_DN741_c0_g1_i10_orf1;TRINITY_DN549_c0_g1_i7_orf1;TRINITY_DN928_c0_g2_i1_orf1;TRINITY_DN2012_c0_g1_i3_orf1;TRINITY_DN248_c0_g1_i1_orf1;TRINITY_DN23746_c0_g1_i2_orf1

biological_process	cellular component morphogenesis	GO:0032989	1	1/2406	TRINITY_DN42854_c0_g3_i2_orf1
biological_process	cell development	GO:0048468	13	13/2406	TRINITY_DN1749_c0_g2_i2_orf1;TRINITY_DN4571_c0_g1_i4_orf1;TRINITY_DN4217_c0_g1_i2_orf1;TRINITY_DN288_c0_g1_i9_orf1;TRINITY_DN8087_c0_g1_i9_orf1;TRINITY_DN20710_c0_g1_i2_orf1;TRINITY_DN25976_c0_g1_i4_orf1;TRINITY_DN36856_c0_g1_i1_orf1;TRINITY_DN19980_c0_g1_i4_orf1;TRINITY_DN237_c1_g1_i1_orf1;TRINITY_DN15706_c0_g2_i5_orf1;TRINITY_DN2652_c0_g2_i1_orf1;TRINITY_DN20710_c0_g2_i2_orf1
biological_process	protein transmembrane transport	GO:0071806	1	1/2406	TRINITY_DN46409_c0_g1_i1_orf1
biological_process	mitochondrial transmembrane transport	GO:1990542	2	2/2406	TRINITY_DN46409_c0_g1_i1_orf1;TRINITY_DN44256_c0_g1_i1_orf1
biological_process	ion transmembrane transport	GO:0034220	4	4/2406	TRINITY_DN1661_c0_g1_i1_orf1;TRINITY_DN44256_c0_g1_i1_orf1;TRINITY_DN8306_c0_g1_i4_orf1;TRINITY_DN19115_c0_g1_i1_orf1
biological_process	cell-cell recognition	GO:0009988	1	1/2406	TRINITY_DN20133_c0_g1_i1_orf1
biological_process	actin cytoskeleton organization	GO:0030036	8	8/2406	TRINITY_DN4010_c0_g2_i1_orf1;TRINITY_DN30150_c0_g1_i7_orf1;TRINITY_DN4159_c1_g1_i1_orf1;TRINITY_DN8915_c0_g1_i3_orf1;TRINITY_DN235_c0_g3_i1_orf1;TRINITY_DN3887_c0_g1_i1_orf1;TRINITY_DN86309_c0_g1_i4_orf1;TRINITY_DN23790_c0_g1_i1_orf1
biological_process	actin filament severing	GO:0051014	1	1/2406	TRINITY_DN30150_c0_g1_i7_orf1
biological_process	cellular component assembly involved in morphogenesis	GO:0010927	1	1/2406	TRINITY_DN235_c0_g3_i1_orf1
biological_process	establishment of tissue polarity	GO:0007164	1	1/2406	TRINITY_DN14389_c0_g1_i4_orf1
biological_process	tube morphogenesis	GO:0035239	1	1/2406	TRINITY_DN147475_c0_g1_i1_orf1
biological_process	hair follicle morphogenesis	GO:0031069	1	1/2406	TRINITY_DN51836_c0_g3_i1_orf1
biological_process	embryonic morphogenesis	GO:0048598	1	1/2406	TRINITY_DN142442_c0_g1_i1_orf1
biological_process	tissue morphogenesis	GO:0048729	4	4/2406	TRINITY_DN51836_c0_g3_i1_orf1;TRINITY_DN36856_c0_g1_i1_orf1;TRINITY_DN237_c1_g1_i1_orf1;TRINITY_DN147475_c0_g1_i1_orf1
biological_process	animal organ morphogenesis	GO:0009887	4	4/2406	TRINITY_DN741_c0_g1_i10_orf1;TRINITY_DN14389_c0_g1_i4_orf1;TRINITY_DN23746_c0_g1_i2_orf1;TRINITY_DN5954_c0_g1_i2_orf1
biological_process	cell morphogenesis	GO:0000902	2	2/2406	TRINITY_DN7794_c0_g1_i1_orf1;TRINITY_DN34426_c0_g1_i1_orf1
biological_process	system development	GO:0048731	5	5/2406	TRINITY_DN42854_c0_g3_i2_orf1;TRINITY_DN20710_c0_g2_i2_orf1;TRINITY_DN20710_c0_g1_i2_orf1;TRINITY_DN142442_c0_g1_i1_orf1;TRINITY_DN288_c0_g1_i9_orf1
biological_process	hippocampus development	GO:0021766	1	1/2406	TRINITY_DN31584_c0_g2_i2_orf1
biological_process	animal organ development	GO:0048513	7	7/2406	TRINITY_DN19980_c0_g1_i4_orf1;TRINITY_DN8087_c0_g1_i9_orf1;TRINITY_DN36856_c0_g1_i1_orf1;TRINITY_DN34426_c0_g1_i1_orf1;TRINITY_DN25976_c0_g1_i4_orf1;TRINITY_DN237_c1_g1_i1_orf1;TRINITY_DN7794_c0_g1_i1_orf1
biological_process	cerebral cortex development	GO:0021987	1	1/2406	TRINITY_DN31584_c0_g2_i2_orf1
biological_process	tissue development	GO:0009888	1	1/2406	TRINITY_DN142442_c0_g1_i1_orf1
biological_process	hair cycle process	GO:0022405	1	1/2406	TRINITY_DN51836_c0_g3_i1_orf1
biological_process	nervous system process	GO:0050877	3	3/2406	TRINITY_DN19951_c0_g1_i5_orf1;TRINITY_DN26337_c0_g1_i3_orf1;TRINITY_DN142442_c0_g1_i1_orf1
biological_process	muscle system process	GO:0003012	1	1/2406	TRINITY_DN20133_c0_g1_i1_orf1
biological_process	reproductive behavior	GO:0019098	1	1/2406	TRINITY_DN58125_c0_g1_i1_orf1
biological_process	envenomation resulting in modulation of process in another organism	GO:0035738	1	1/2406	TRINITY_DN1215_c0_g1_i2_orf1
biological_process	response to virus	GO:0009615	1	1/2406	TRINITY_DN2836_c0_g1_i4_orf1
biological_process	response to bacterium	GO:0009617	11	11/2406	TRINITY_DN1444_c1_g1_i5_orf1;TRINITY_DN14904_c0_g1_i1_orf1;TRINITY_DN479_c6_g1_i2_orf1;TRINITY_DN8685_c0_g1_i5_orf1;TRINITY_DN16840_c1_g1_i1_orf1;TRINITY_DN195_c8_g1_i1_orf1;TRINITY_DN1091_c0_g2_i10_orf1;TRINITY_DN2836_c0_g1_i4_orf1;TRINITY_DN1666_c0_g1_i2_orf1;TRINITY_DN29190_c0_g1_i4_orf1;TRINITY_DN5880_c0_g2_i2_orf1
biological_process	response to fungus	GO:0009620	2	2/2406	TRINITY_DN6098_c1_g1_i5_orf1;TRINITY_DN2848_c0_g1_i1_orf1
biological_process	defense response to other organism	GO:0098542	20	20/2406	TRINITY_DN14904_c0_g1_i1_orf1;TRINITY_DN6098_c1_g1_i5_orf1;TRINITY_DN1444_c1_g1_i5_orf1;TRINITY_DN1534_c0_g1_i3_orf1;TRINITY_DN21545_c0_g1_i2_orf1;TRINITY_DN479_c6_g1_i2_orf1;TRINITY_DN8685_c0_g1_i5_orf1;TRINITY_DN16840_c1_g1_i1_orf1;TRINITY_DN195_c8_g1_i1_orf1;TRINITY_DN2170_c0_g1_i2_orf1;TRINITY_DN1091_c0_g2_i10_orf1;TRINITY_DN2836_c0_g1_i4_orf1;TRINITY_DN2170_c1_g1_i3_orf1;TRINITY_DN2848_c0_g1_i1_orf1;TRINITY_DN15706_c0_g2_i5_orf1;TRINITY_DN1666_c0_g1_i2_orf1;TRINITY_DN5235_c0_g1_i7_orf1;TRINITY_DN9044_c0_g1_i2_orf1;TRINITY_DN29190_c0_g1_i4_orf1;TRINITY_DN5880_c0_g2_i2_orf1
biological_process	biological process involved in interaction with symbiont	GO:0051702	3	3/2406	TRINITY_DN46409_c0_g1_i1_orf1;TRINITY_DN2848_c0_g1_i1_orf1;TRINITY_DN975_c0_g1_i1_orf1
biological_process	biological process involved in interaction with host	GO:0051701	1	1/2406	TRINITY_DN96557_c0_g1_i1_orf1
biological_process	establishment of organelle localization	GO:0051656	1	1/2406	TRINITY_DN96557_c0_g1_i1_orf1
biological_process	chromosome localization	GO:0050000	1	1/2406	TRINITY_DN96557_c0_g1_i1_orf1
biological_process	maintenance of protein location	GO:0045185	1	1/2406	TRINITY_DN245_c0_g1_i4_orf1
biological_process	maintenance of location in cell	GO:0051651	1	1/2406	TRINITY_DN245_c0_g1_i4_orf1
biological_process	establishment of protein localization	GO:0045184	34	34/2406	TRINITY_DN245_c0_g1_i4_orf1;TRINITY_DN5182_c0_g1_i5_orf1;TRINITY_DN46409_c0_g1_i1_orf1;TRINITY_DN6243_c0_g1_i5_orf1;TRINITY_DN31584_c0_g2_i2_orf1;TRINITY_DN146119_c0_g1_i1_orf1;TRINITY_DN383_c0_g1_i1_orf1;TRINITY_DN45400_c0_g1_i1_orf1;TRINITY_DN14677_c0_g2_i3_orf1;TRINITY_DN55148_c0_g1_i1_orf1;TRINITY_DN34159_c0_g2_i1_orf1;TRINITY_DN558_c0_g1_i4_orf1;TRINITY_DN95971_c0_g5_i1_orf1;TRINITY_DN5118_c0_g1_i1_orf1;TRINITY_DN16316_c0_g1_i7_orf1;TRINITY_DN3513_c0_g1_i5_orf1;TRINITY_DN445_c0_g1_i2_orf1;TRINITY_DN13118_c0_g1_i6_orf1;TRINITY_DN1437_c0_g1_i6_orf1;TRINITY_DN2286_c2_g1_i1_orf1;TRINITY_DN6680_c0_g1_i1_orf1;TRINITY_DN146236_c0_g1_i1_orf1;TRINITY_DN11772_c0_g1_i1_orf1;TRINITY_DN959_c0_g1_i7_orf1;TRINITY_DN486_c0_g1_i5_orf1;TRINITY_DN2879_c0_g1_i4_orf1;TRINITY_DN96557_c0_g1_i1_orf1;TRINITY_DN2172_c0_g2_i8_orf1;TRINITY_DN4410_c0_g1_i1_orf1;TRINITY_DN47219_c0_g1_i3_orf1;TRINITY_DN69871_c0_g1_i1_orf1;TRINITY_DN12777_c0_g1_i5_orf1;TRINITY_DN50875_c0_g1_i3_orf1;TRINITY_DN38835_c0_g3_i1_orf1
biological_process	establishment of localization in cell	GO:0051649	30	30/2406	TRINITY_DN54586_c1_g1_i1_orf1;TRINITY_DN3821_c1_g1_i7_orf1;TRINITY_DN6231_c0_g1_i6_orf1;TRINITY_DN13626_c0_g2_i1_orf1;TRINITY_DN46409_c0_g1_i1_orf1;TRINITY_DN13157_c0_g1_i1_orf1;TRINITY_DN31584_c0_g2_i2_orf1;TRINITY_DN5182_c0_g1_i5_orf1;TRINITY_DN45400_c0_g1_i1_orf1;TRINITY_DN14677_c0_g2_i3_orf1;TRINITY_DN95971_c0_g5_i1_orf1;TRINITY_DN34159_c0_g2_i1_orf1;TRINITY_DN55148_c0_g1_i1_orf1;TRINITY_DN5118_c0_g1_i1_orf1;TRINITY_DN16316_c0_g1_i7_orf1;TRINITY_DN3513_c0_g1_i5_orf1;TRINITY_DN445_c0_g1_i2_orf1;TRINITY_DN13118_c0_g1_i6_orf1;TRINITY_DN1437_c0_g1_i6_orf1;TRINITY_DN64_c0_g1_i4_orf1;TRINITY_DN35377_c0_g1_i3_orf1;TRINITY_DN486_c0_g1_i5_orf1;TRINITY_DN578_c0_g1_i5_orf1;TRINITY_DN96557_c0_g1_i1_orf1;TRINITY_DN69871_c0_g1_i1_orf1;TRINITY_DN12777_c0_g1_i5_orf1;TRINITY_DN1245_c0_g1_i4_orf1;TRINITY_DN942_c0_g1_i1_orf1;TRINITY_DN5028_c0_g1_i11_orf1;TRINITY_DN2338_c3_g2_i3_orf1
biological_process	establishment of RNA localization	GO:0051236	6	6/2406	TRINITY_DN64_c0_g1_i4_orf1;TRINITY_DN6680_c0_g1_i1_orf1;TRINITY_DN146119_c0_g1_i1_orf1;TRINITY_DN1245_c0_g1_i4_orf1;TRINITY_DN45400_c0_g1_i1_orf1;TRINITY_DN2879_c0_g1_i4_orf1
biological_process	transport	GO:0006810	85	85/2406	TRINITY_DN245_c0_g1_i4_orf1;TRINITY_DN28759_c0_g1_i1_orf1;TRINITY_DN69871_c0_g1_i1_orf1;TRINITY_DN65681_c0_g1_i1_orf1;TRINITY_DN105574_c0_g1_i1_orf1;TRINITY_DN13118_c0_g1_i6_orf1;TRINITY_DN8306_c0_g1_i4_orf1;TRINITY_DN1407_c0_g1_i12_orf1;TRINITY_DN35377_c0_g1_i3_orf1;TRINITY_DN146119_c0_g1_i1_orf1;TRINITY_DN3821_c1_g1_i7_orf1;TRINITY_DN198_c2_g1_i2_orf1;TRINITY_DN6231_c0_g1_i6_orf1;TRINITY_DN13626_c0_g2_i1_orf1;TRINITY_DN2338_c3_g2_i3_orf1;TRINITY_DN21872_c0_g1_i2_orf1;TRINITY_DN13157_c0_g1_i1_orf1;TRINITY_DN6243_c0_g1_i5_orf1;TRINITY_DN252_c0_g1_i3_orf1;TRINITY_DN3513_c0_g1_i5_orf1;TRINITY_DN5182_c0_g1_i5_orf1;TRINITY_DN383_c0_g1_i1_orf1;TRINITY_DN44256_c0_g1_i1_orf1;TRINITY_DN14677_c0_g2_i3_orf1;TRINITY_DN1895_c0_g1_i2_orf1;TRINITY_DN486_c0_g1_i5_orf1;TRINITY_DN445_c0_g1_i2_orf1;TRINITY_DN34159_c0_g2_i1_orf1;TRINITY_DN47389_c0_g1_i2_orf1;TRINITY_DN9354_c0_g1_i7_orf1;TRINITY_DN1423_c0_g1_i4_orf1;TRINITY_DN50875_c0_g1_i3_orf1;TRINITY_DN23354_c0_g1_i7_orf1;TRINITY_DN46409_c0_g1_i1_orf1;TRINITY_DN558_c0_g1_i4_orf1;TRINITY_DN95971_c0_g5_i1_orf1;TRINITY_DN5118_c0_g1_i1_orf1;TRINITY_DN9239_c0_g2_i2_orf1;TRINITY_DN19115_c0_g1_i1_orf1;TRINITY_DN25686_c0_g1_i4_orf1;TRINITY_DN16316_c0_g1_i7_orf1;TRINITY_DN7590_c0_g1_i4_orf1;TRINITY_DN110402_c0_g2_i1_orf1;TRINITY_DN86621_c0_g1_i2_orf1;TRINITY_DN46625_c0_g1_i1_orf1;TRINITY_DN29934_c0_g1_i6_orf1;TRINITY_DN1437_c0_g1_i6_orf1;TRINITY_DN1661_c0_g1_i1_orf1;TRINITY_DN1423_c0_g1_i8_orf1;TRINITY_DN2286_c2_g1_i1_orf1;TRINITY_DN64_c0_g1_i4_orf1;TRINITY_DN136031_c0_g1_i7_orf1;TRINITY_DN33272_c0_g1_i5_orf1;TRINITY_DN146236_c0_g1_i1_orf1;TRINITY_DN12286_c1_g1_i2_orf1;TRINITY_DN11772_c0_g1_i1_orf1;TRINITY_DN741_c0_g1_i10_orf1;TRINITY_DN54586_c1_g1_i1_orf1;TRINITY_DN12885_c0_g1_i1_orf1;TRINITY_DN9239_c0_g1_i1_orf1;TRINITY_DN33452_c0_g1_i3_orf1;TRINITY_DN2879_c0_g1_i4_orf1;TRINITY_DN15812_c0_g1_i2_orf1;TRINITY_DN578_c0_g1_i5_orf1;TRINITY_DN96557_c0_g1_i1_orf1;TRINITY_DN51766_c0_g1_i2_orf1;TRINITY_DN1407_c0_g1_i5_orf1;TRINITY_DN56430_c0_g1_i1_orf1;TRINITY_DN4016_c0_g1_i1_orf1;TRINITY_DN6680_c0_g1_i1_orf1;TRINITY_DN2172_c0_g2_i8_orf1;TRINITY_DN45400_c0_g1_i1_orf1;TRINITY_DN33452_c0_g1_i1_orf1;TRINITY_DN55148_c0_g1_i1_orf1;TRINITY_DN12777_c0_g1_i5_orf1;TRINITY_DN942_c0_g1_i1_orf1;TRINITY_DN81488_c0_g1_i1_orf1;TRINITY_DN4410_c0_g1_i1_orf1;TRINITY_DN113353_c0_g1_i1_orf1;TRINITY_DN47219_c0_g1_i3_orf1;TRINITY_DN1245_c0_g1_i4_orf1;TRINITY_DN5064_c0_g1_i4_orf1;TRINITY_DN38835_c0_g3_i1_orf1;TRINITY_DN5028_c0_g1_i11_orf1;TRINITY_DN31584_c0_g2_i2_orf1
biological_process	non-lytic viral release	GO:0046753	1	1/2406	TRINITY_DN96557_c0_g1_i1_orf1

biological_process	viral RNA genome replication	GO:0039694	1	1/2406	TRINITY_DN4408_c6_g1_i1_orf1
biological_process	viral budding via host ESCRT complex	GO:0039702	1	1/2406	TRINITY_DN96557_c0_g1_i1_orf1
biological_process	viral budding from plasma membrane	GO:0046761	1	1/2406	TRINITY_DN96557_c0_g1_i1_orf1
biological_process	response to external biotic stimulus	GO:0043207	28	28/2406	TRINITY_DN1444_c1_g1_i5_orf1;TRINITY_DN8685_c0_g1_i5_orf1;TRINITY_DN16840_c1_g1_i1_orf1;TRINITY_DN15706_c0_g2_i5_orf1;TRINITY_DN1666_c0_g1_i2_orf1;TRINITY_DN29190_c0_g1_i4_orf1;TRINITY_DN21545_c0_g1_i2_orf1;TRINITY_DN479_c6_g1_i2_orf1;TRINITY_DN109503_c0_g1_i4_orf1;TRINITY_DN3166_c1_g1_i6_orf1;TRINITY_DN2407_c0_g1_i6_orf1;TRINITY_DN2848_c0_g1_i1_orf1;TRINITY_DN86772_c0_g1_i3_orfp1;TRINITY_DN1534_c0_g1_i3_orf1;TRINITY_DN1091_c0_g2_i10_orf1;TRINITY_DN6098_c1_g1_i5_orf1;TRINITY_DN9044_c0_g1_i2_orf1;TRINITY_DN4748_c0_g1_i5_orf1;TRINITY_DN12534_c0_g1_i4_orf1;TRINITY_DN14904_c0_g1_i1_orf1;TRINITY_DN2836_c0_g1_i4_orf1;TRINITY_DN4802_c0_g1_i4_orf1;TRINITY_DN195_c8_g1_i1_orf1;TRINITY_DN2170_c0_g1_i2_orf1;TRINITY_DN59429_c0_g1_i6_orf1;TRINITY_DN2170_c1_g1_i3_orf1;TRINITY_DN5235_c0_g1_i7_orf1;TRINITY_DN5880_c0_g2_i2_orf1
biological_process	detection of biotic stimulus	GO:0009595	3	3/2406	TRINITY_DN1091_c0_g2_i10_orf1;TRINITY_DN8685_c0_g1_i5_orf1;TRINITY_DN5880_c0_g2_i2_orf1
biological_process	response to extracellular stimulus	GO:0009991	5	5/2406	TRINITY_DN51938_c0_g3_i1_orf1;TRINITY_DN1091_c0_g3_i1_orf1;TRINITY_DN143603_c0_g1_i1_orf1;TRINITY_DN2054_c0_g1_i1_orf1;TRINITY_DN1091_c0_g1_i1_orf1
biological_process	cellular response to external stimulus	GO:0071496	4	4/2406	TRINITY_DN1091_c0_g1_i1_orf1;TRINITY_DN1091_c0_g3_i1_orf1;TRINITY_DN51938_c0_g3_i1_orf1;TRINITY_DN2054_c0_g1_i1_orf1
biological_process	cellular response to endogenous stimulus	GO:0071495	3	3/2406	TRINITY_DN51938_c0_g3_i1_orf1;TRINITY_DN975_c0_g1_i1_orf1;TRINITY_DN4016_c0_g1_i1_orf1
biological_process	response to epidermal growth factor	GO:0070849	1	1/2406	TRINITY_DN975_c0_g1_i1_orf1
biological_process	response to transforming growth factor beta	GO:0071559	1	1/2406	TRINITY_DN51938_c0_g3_i1_orf1
biological_process	response to hormone	GO:0009725	1	1/2406	TRINITY_DN51938_c0_g3_i1_orf1
biological_process	response to hypoxia	GO:0001666	1	1/2406	TRINITY_DN51938_c0_g3_i1_orf1
biological_process	response to wounding	GO:0009611	1	1/2406	TRINITY_DN21545_c0_g1_i2_orf1
biological_process	response to topologically incorrect protein	GO:0035966	1	1/2406	TRINITY_DN46409_c0_g1_i1_orf1
biological_process	response to ischemia	GO:0002931	1	1/2406	TRINITY_DN51938_c0_g3_i1_orf1
biological_process	response to cold	GO:0009409	1	1/2406	TRINITY_DN46409_c0_g1_i1_orf1
biological_process	response to heat	GO:0009408	1	1/2406	TRINITY_DN31584_c0_g2_i2_orf1
biological_process	defense response	GO:0006952	27	27/2406	TRINITY_DN1444_c1_g1_i5_orf1;TRINITY_DN8685_c0_g1_i5_orf1;TRINITY_DN16840_c1_g1_i1_orf1;TRINITY_DN15706_c0_g2_i5_orf1;TRINITY_DN1666_c0_g1_i2_orf1;TRINITY_DN29190_c0_g1_i4_orf1;TRINITY_DN21545_c0_g1_i2_orf1;TRINITY_DN479_c6_g1_i2_orf1;TRINITY_DN3166_c1_g1_i6_orf1;TRINITY_DN2407_c0_g1_i6_orf1;TRINITY_DN2848_c0_g1_i1_orf1;TRINITY_DN86772_c0_g1_i3_orfp1;TRINITY_DN1534_c0_g1_i3_orf1;TRINITY_DN1091_c0_g2_i10_orf1;TRINITY_DN6098_c1_g1_i5_orf1;TRINITY_DN9044_c0_g1_i2_orf1;TRINITY_DN4748_c0_g1_i5_orf1;TRINITY_DN12534_c0_g1_i4_orf1;TRINITY_DN14904_c0_g1_i1_orf1;TRINITY_DN2836_c0_g1_i4_orf1;TRINITY_DN4802_c0_g1_i4_orf1;TRINITY_DN195_c8_g1_i1_orf1;TRINITY_DN2170_c0_g1_i2_orf1;TRINITY_DN59429_c0_g1_i6_orf1;TRINITY_DN2170_c1_g1_i3_orf1;TRINITY_DN5235_c0_g1_i7_orf1;TRINITY_DN5880_c0_g2_i2_orf1
biological_process	response to hyperoxia	GO:0055093	1	1/2406	TRINITY_DN51938_c0_g3_i1_orf1
biological_process	response to oxidative stress	GO:0006979	7	7/2406	TRINITY_DN6580_c0_g1_i4_orf1;TRINITY_DN3984_c0_g1_i4_orf1;TRINITY_DN5933_c0_g1_i1_orf1;TRINITY_DN2207_c0_g1_i6_orf1;TRINITY_DN1622_c0_g1_i6_orf1;TRINITY_DN285_c0_g1_i4_orf1;TRINITY_DN2652_c0_g2_i1_orf1
biological_process	response to oxygen-containing compound	GO:1901700	2	2/2406	TRINITY_DN51938_c0_g3_i1_orf1;TRINITY_DN4016_c0_g1_i1_orf1
biological_process	response to nitrogen compound	GO:1901698	4	4/2406	TRINITY_DN9062_c0_g2_i3_orf1;TRINITY_DN28989_c0_g1_i7_orf1;TRINITY_DN2848_c0_g1_i1_orf1;TRINITY_DN4016_c0_g1_i1_orf1
biological_process	response to nutrient	GO:0007584	1	1/2406	TRINITY_DN51938_c0_g3_i1_orf1
biological_process	response to inorganic substance	GO:0010035	2	2/2406	TRINITY_DN2848_c0_g1_i1_orf1;TRINITY_DN4016_c0_g1_i1_orf1
biological_process	response to organic substance	GO:0010033	12	12/2406	TRINITY_DN9062_c0_g2_i3_orf1;TRINITY_DN51938_c0_g3_i1_orf1;TRINITY_DN4016_c0_g1_i1_orf1;TRINITY_DN8685_c0_g1_i5_orf1;TRINITY_DN18218_c0_g1_i7_orf1;TRINITY_DN975_c0_g1_i1_orf1;TRINITY_DN1091_c0_g2_i10_orf1;TRINITY_DN46409_c0_g1_i1_orf1;TRINITY_DN2848_c0_g1_i1_orf1;TRINITY_DN28989_c0_g1_i7_orf1;TRINITY_DN2227_c0_g1_i5_orf1;TRINITY_DN5880_c0_g2_i2_orf1
biological_process	response to temperature stimulus	GO:0009266	2	2/2406	TRINITY_DN46409_c0_g1_i1_orf1;TRINITY_DN31584_c0_g2_i2_orf1
biological_process	response to oxygen levels	GO:0070482	1	1/2406	TRINITY_DN51938_c0_g3_i1_orf1
biological_process	detection of chemical stimulus	GO:0009593	3	3/2406	TRINITY_DN1091_c0_g2_i10_orf1;TRINITY_DN8685_c0_g1_i5_orf1;TRINITY_DN5880_c0_g2_i2_orf1
cellular_component	nucleosome	GO:0000786	2	2/2406	TRINITY_DN96801_c0_g1_i1_orf1;TRINITY_DN6358_c0_g1_i5_orf1
cellular_component	cohesin complex	GO:0008278	1	1/2406	TRINITY_DN2638_c0_g1_i7_orf1
cellular_component	Mre11 complex	GO:0030870	2	2/2406	TRINITY_DN45271_c0_g1_i1_orf1;TRINITY_DN123184_c0_g1_i1_orf1
cellular_component	mRNA cleavage factor complex	GO:0005849	2	2/2406	TRINITY_DN2718_c0_g1_i6_orf1;TRINITY_DN1005_c0_g1_i5_orf1
cellular_component	Ku70:Ku80 complex	GO:0043564	1	1/2406	TRINITY_DN5757_c0_g1_i1_orf1
cellular_component	SWI/SNF superfamily-type complex	GO:0070603	1	1/2406	TRINITY_DN9765_c0_g1_i6_orf1
cellular_component	U2AF complex	GO:0089701	1	1/2406	TRINITY_DN51968_c0_g1_i1_orf1
cellular_component	transcription elongation factor complex	GO:0008023	1	1/2406	TRINITY_DN44792_c0_g1_i1_orf1
cellular_component	spliceosomal complex	GO:0005681	16	16/2406	TRINITY_DN33346_c0_g1_i1_orf1;TRINITY_DN1554_c0_g1_i9_orf1;TRINITY_DN8717_c0_g1_i5_orf1;TRINITY_DN3459_c0_g1_i4_orf1;TRINITY_DN31663_c0_g1_i2_orf1;TRINITY_DN11746_c0_g2_i1_orf1;TRINITY_DN13055_c0_g1_i5_orf1;TRINITY_DN43412_c0_g1_i2_orf1;TRINITY_DN107035_c0_g1_i1_orf1;TRINITY_DN116467_c0_g1_i1_orf1;TRINITY_DN20215_c0_g2_i1_orf1;TRINITY_DN698_c0_g1_i5_orf1;TRINITY_DN23502_c0_g1_i1_orf1;TRINITY_DN27276_c0_g1_i5_orf1;TRINITY_DN44877_c0_g1_i2_orf1;TRINITY_DN14487_c0_g1_i4_orf1
cellular_component	BRISC complex	GO:0070552	1	1/2406	TRINITY_DN17655_c0_g1_i1_orf1
cellular_component	nuclear DNA-directed RNA polymerase complex	GO:0055029	1	1/2406	TRINITY_DN4707_c0_g1_i1_orf1
cellular_component	exon-exon junction complex	GO:0035145	1	1/2406	TRINITY_DN5507_c0_g1_i1_orf1
cellular_component	small nuclear ribonucleoprotein complex	GO:0030532	7	7/2406	TRINITY_DN38540_c0_g1_i1_orf1;TRINITY_DN33346_c0_g1_i1_orf1;TRINITY_DN298_c0_g1_i4_orf1;TRINITY_DN1616_c0_g1_i3_orf1;TRINITY_DN31663_c0_g1_i2_orf1;TRINITY_DN43412_c0_g1_i2_orf1;TRINITY_DN116467_c0_g1_i1_orf1
cellular_component	BRCA1-A complex	GO:0070531	1	1/2406	TRINITY_DN17655_c0_g1_i1_orf1
cellular_component	RNA polymerase II transcription regulator complex	GO:0090575	2	2/2406	TRINITY_DN34509_c0_g1_i1_orf1;TRINITY_DN346_c0_g1_i7_orf1
cellular_component	carboxy-terminal domain protein kinase complex	GO:0032806	1	1/2406	TRINITY_DN346_c0_g1_i7_orf1
cellular_component	THO complex	GO:0000347	1	1/2406	TRINITY_DN64_c0_g1_i4_orf1
cellular_component	nuclear pore outer ring	GO:0031080	2	2/2406	TRINITY_DN45400_c0_g1_i1_orf1;TRINITY_DN6680_c0_g1_i1_orf1
cellular_component	nuclear pore	GO:0005643	4	4/2406	TRINITY_DN96557_c0_g1_i1_orf1;TRINITY_DN146119_c0_g1_i1_orf1;TRINITY_DN2879_c0_g1_i4_orf1;TRINITY_DN1437_c0_g1_i6_orf1
cellular_component	ESCRT III complex	GO:0000815	1	1/2406	TRINITY_DN96557_c0_g1_i1_orf1
cellular_component	ESCRT I complex	GO:0000813	1	1/2406	TRINITY_DN4013_c0_g1_i4_orf1
cellular_component	transmembrane transporter complex	GO:1902495	4	4/2406	TRINITY_DN44256_c0_g1_i1_orf1;TRINITY_DN162_c0_g1_i4_orf1;TRINITY_DN29934_c0_g1_i6_orf1;TRINITY_DN20558_c0_g1_i2_orf1
cellular_component	dynein complex	GO:0030286	2	2/2406	TRINITY_DN17995_c0_g4_i1_orf1;TRINITY_DN122423_c0_g1_i1_orf1
cellular_component	cytochrome complex	GO:0070069	1	1/2406	TRINITY_DN76036_c0_g1_i1_orf1
cellular_component	oxidoreductase complex	GO:1990204	1	1/2406	TRINITY_DN2594_c0_g2_i4_orf1
cellular_component	tricarboxylic acid cycle enzyme complex	GO:0045239	1	1/2406	TRINITY_DN2594_c0_g2_i4_orf1
cellular_component	transferase complex	GO:1990234	17	17/2406	TRINITY_DN346_c0_g1_i7_orf1;TRINITY_DN81258_c0_g1_i2_orf1;TRINITY_DN2401_c0_g2_i1_orf1;TRINITY_DN15040_c0_g4_i1_orf1;TRINITY_DN9062_c0_g2_i3_orf1;TRINITY_DN4707_c0_g1_i1_orf1;TRINITY_DN70485_c0_g1_i2_orf1;TRINITY_DN147475_c0_g1_i1_orf1;TRINITY_DN2120_c0_g1_i2_orf1;TRINITY_DN879_c0_g1_i2_orf1;TRINITY_DN5182_c0_g1_i5_orf1;TRINITY_DN1757_c0_g1_i4_orf1;TRINITY_DN110534_c0_g1_i3_orf1;TRINITY_DN2299_c0_g1_i3_orf1;TRINITY_DN89613_c0_g1_i13_orf1;TRINITY_DN12_c0_g1_i5_orf1;TRINITY_DN74037_c0_g5_i1_orf1
cellular_component	peptidase complex	GO:1905368	9	9/2406	TRINITY_DN42753_c0_g1_i2_orf1;TRINITY_DN19260_c0_g1_i5_orf1;TRINITY_DN17133_c0_g1_i1_orf1;TRINITY_DN34479_c0_g1_i2_orf1;TRINITY_DN49047_c0_g1_i2_orf1;TRINITY_DN2591_c0_g1_i4_orf1;TRINITY_DN2058_c0_g1_i2_orf1;TRINITY_DN321_c0_g1_i1_orf1;TRINITY_DN32359_c0_g2_i1_orf1
cellular_component	aminoacyl-tRNA synthetase multienzyme complex	GO:0017101	3	3/2406	TRINITY_DN2953_c1_g1_i11_orf1;TRINITY_DN2953_c1_g1_i10_orf1;TRINITY_DN2953_c1_g1_i2_orf1
cellular_component	ATPase complex	GO:1904949	1	1/2406	TRINITY_DN9765_c0_g1_i6_orf1

cellular_component	dystrophin-associated glycoprotein complex	GO:0016010	1	1/2406	TRINITY_DN7128_c0_g1_i7_orf1
cellular_component	HOPS complex	GO:0030897	1	1/2406	TRINITY_DN3513_c0_g1_i5_orf1
cellular_component	lipopolysaccharide receptor complex	GO:0046696	1	1/2406	TRINITY_DN46409_c0_g1_i1_orf1
cellular_component	plasma membrane protein complex	GO:0098797	5	5/2406	TRINITY_DN5118_c0_g1_i1_orf1;TRINITY_DN12777_c0_g1_i5_orf1;TRINITY_DN162_c0_g1_i4_orf1;TRINITY_DN29934_c0_g1_i6_orf1;TRINITY_DN7128_c0_g1_i7_orf1
cellular_component	Tapasin-ERp57 complex	GO:0061779	1	1/2406	TRINITY_DN51938_c0_g3_i1_orf1
cellular_component	EMC complex	GO:0072546	2	2/2406	TRINITY_DN3838_c0_g1_i8_orf1;TRINITY_DN17828_c0_g1_i1_orf1
cellular_component	MHC class I peptide loading complex	GO:0042824	1	1/2406	TRINITY_DN51938_c0_g3_i1_orf1
cellular_component	TAP complex	GO:0042825	1	1/2406	TRINITY_DN51938_c0_g3_i1_orf1
cellular_component	respiratory chain complex	GO:0098803	1	1/2406	TRINITY_DN76036_c0_g1_i1_orf1
cellular_component	inner mitochondrial membrane protein complex	GO:0098800	4	4/2406	TRINITY_DN76036_c0_g1_i1_orf1;TRINITY_DN44256_c0_g1_i1_orf1;TRINITY_DN141353_c0_g1_i1_orf1;TRINITY_DN15222_c0_g1_i4_orf1
cellular_component	membrane coat	GO:0030117	5	5/2406	TRINITY_DN96557_c0_g1_i1_orf1;TRINITY_DN14677_c0_g2_i3_orf1;TRINITY_DN2286_c2_g1_i1_orf1;TRINITY_DN12777_c0_g1_i5_orf1;TRINITY_DN146119_c0_g1_i1_orf
cellular_component	AP-type membrane coat adaptor complex	GO:0030119	3	3/2406	TRINITY_DN13118_c0_g1_i6_orf1;TRINITY_DN5118_c0_g1_i1_orf1;TRINITY_DN486_c0_g1_i5_orf1
cellular_component	proton-transporting two-sector ATPase complex, proton-transporting domain	GO:0033177	4	4/2406	TRINITY_DN10458_c0_g1_i1_orf1;TRINITY_DN15222_c0_g1_i4_orf1;TRINITY_DN141353_c0_g1_i1_orf1;TRINITY_DN19115_c0_g1_i1_orf1
cellular_component	mitochondrial tricarboxylic acid cycle enzyme complex	GO:0030062	1	1/2406	TRINITY_DN2594_c0_g2_i4_orf1
cellular_component	mitochondrial large ribosomal subunit	GO:0005762	2	2/2406	TRINITY_DN97680_c0_g1_i1_orf1;TRINITY_DN1313_c0_g1_i2_orf1
cellular_component	Golgi transport complex	GO:0017119	1	1/2406	TRINITY_DN50875_c0_g1_i3_orf1
cellular_component	exocyst	GO:0000145	3	3/2406	TRINITY_DN25686_c0_g1_i4_orf1;TRINITY_DN16316_c0_g1_i7_orf1;TRINITY_DN1895_c0_g1_i2_orf1
cellular_component	CORVET complex	GO:0033263	1	1/2406	TRINITY_DN3513_c0_g1_i5_orf1
cellular_component	kinesin complex	GO:0005871	1	1/2406	TRINITY_DN4808_c0_g1_i3_orf1
cellular_component	translation preinitiation complex	GO:0070993	2	2/2406	TRINITY_DN48097_c0_g1_i1_orf1;TRINITY_DN50085_c0_g1_i1_orf1
cellular_component	translation initiation complex	GO:0070992	1	1/2406	TRINITY_DN142442_c0_g1_i1_orf1
cellular_component	ribosomal subunit	GO:0044391	12	12/2406	TRINITY_DN97680_c0_g1_i1_orf1;TRINITY_DN4016_c0_g1_i1_orf1;TRINITY_DN1313_c0_g1_i2_orf1;TRINITY_DN142442_c0_g1_i1_orf1;TRINITY_DN13732_c0_g2_i3_orf1;TRINITY_DN47591_c0_g1_i2_orf1;TRINITY_DN13651_c0_g1_i2_orf1;TRINITY_DN41645_c0_g1_i1_orf1;TRINITY_DN42646_c0_g2_i1_orf1;TRINITY_DN55148_c0_g1_i1_orf1;TRINITY_DN10070_c0_g1_i1_orf1;TRINITY_DN15234_c0_g1_i3_orf1
cellular_component	mRNA cap binding complex	GO:0005845	1	1/2406	TRINITY_DN41664_c0_g1_i4_orf1
cellular_component	proteasome complex	GO:0000502	9	9/2406	TRINITY_DN42753_c0_g1_i2_orf1;TRINITY_DN19260_c0_g1_i5_orf1;TRINITY_DN17133_c0_g1_i1_orf1;TRINITY_DN34479_c0_g1_i2_orf1;TRINITY_DN49047_c0_g1_i2_orf1;TRINITY_DN32359_c0_g2_i1_orf1;TRINITY_DN2058_c0_g1_i2_orf1;TRINITY_DN321_c0_g1_i1_orf1;TRINITY_DN2591_c0_g1_i4_orf1
cellular_component	DNA polymerase complex	GO:0042575	6	6/2406	TRINITY_DN81258_c0_g1_i2_orf1;TRINITY_DN15040_c0_g4_i1_orf1;TRINITY_DN74037_c0_g5_i1_orf1;TRINITY_DN70485_c0_g1_i2_orf1;TRINITY_DN110534_c0_g1_i3_orf1;TRINITY_DN89613_c0_g1_i13_orf1
cellular_component	ubiquitin ligase complex	GO:0000151	3	3/2406	TRINITY_DN1757_c0_g1_i4_orf1;TRINITY_DN9062_c0_g2_i3_orf1;TRINITY_DN2120_c0_g1_i2_orf1
cellular_component	TOR complex	GO:0038201	1	1/2406	TRINITY_DN40191_c2_g1_i1_orf1
cellular_component	CCR4-NOT core complex	GO:0030015	1	1/2406	TRINITY_DN41602_c0_g3_i1_orf1
cellular_component	guanyl-nucleotide exchange factor complex	GO:0032045	1	1/2406	TRINITY_DN34159_c0_g2_i1_orf1
cellular_component	RNA polymerase complex	GO:0030880	4	4/2406	TRINITY_DN2401_c0_g2_i1_orf1;TRINITY_DN879_c0_g1_i2_orf1;TRINITY_DN4707_c0_g1_i1_orf1;TRINITY_DN2299_c0_g1_i3_orf1
cellular_component	cAMP-dependent protein kinase complex	GO:0005952	1	1/2406	TRINITY_DN12_c0_g1_i5_orf1
cellular_component	protein kinase CK2 complex	GO:0005956	1	1/2406	TRINITY_DN147475_c0_g1_i1_orf1
cellular_component	CIA complex	GO:0097361	1	1/2406	TRINITY_DN49872_c0_g1_i2_orf1
cellular_component	organelle lumen	GO:0043233	17	17/2406	TRINITY_DN14920_c0_g1_i1_orf1;TRINITY_DN51938_c0_g3_i1_orf1;TRINITY_DN47219_c0_g1_i3_orf1;TRINITY_DN17271_c0_g1_i1_orf1;TRINITY_DN9135_c0_g1_i4_orf1;TRINITY_DN21539_c0_g1_i1_orf1;TRINITY_DN4842_c0_g1_i5_orf1;TRINITY_DN2299_c0_g1_i3_orf1;TRINITY_DN975_c0_g1_i1_orf1;TRINITY_DN147475_c0_g1_i1_orf1;TRINITY_DN42854_c0_g3_i2_orf1;TRINITY_DN46409_c0_g1_i1_orf1;TRINITY_DN9242_c0_g1_i1_orf1;TRINITY_DN2848_c0_g1_i1_orf1;TRINITY_DN20133_c0_g1_i1_orf1;TRINITY_DN3037_c0_g1_i1_orf1;TRINITY_DN1791_c0_g1_i3_orf1
cellular_component	chromosome, telomeric region	GO:0000781	1	1/2406	TRINITY_DN12771_c0_g1_i1_orf1
cellular_component	chromosome, centromeric region	GO:0000775	1	1/2406	TRINITY_DN31314_c0_g1_i4_orf1
cellular_component	cell cortex	GO:0005938	1	1/2406	TRINITY_DN2186_c0_g1_i17_orf1
cellular_component	extrinsic component of organelle membrane	GO:0031312	1	1/2406	TRINITY_DN6027_c0_g1_i13_orf1
cellular_component	heterochromatin	GO:0000792	3	3/2406	TRINITY_DN24266_c0_g2_i2_orf1;TRINITY_DN2345_c0_g1_i4_orf1;TRINITY_DN20133_c0_g1_i1_orf1
cellular_component	intrinsic component of plasma membrane	GO:0031226	2	2/2406	TRINITY_DN3833_c0_g1_i4_orf1;TRINITY_DN4464_c0_g2_i1_orf1
cellular_component	anchored component of membrane	GO:0031225	8	8/2406	TRINITY_DN5406_c0_g2_i1_orf1;TRINITY_DN56690_c0_g1_i4_orf1;TRINITY_DN9475_c0_g1_i6_orf1;TRINITY_DN4464_c0_g2_i1_orf1;TRINITY_DN1352_c0_g1_i5_orf1;TRINITY_DN5553_c0_g1_i4_orf1;TRINITY_DN2175_c0_g1_i4_orf1;TRINITY_DN3833_c0_g1_i4_orf1
cellular_component	integral component of membrane	GO:0016021	298	298/2406	TRINITY_DN6510_c1_g1_i1_orf1;TRINITY_DN38435_c0_g1_i1_orf1;TRINITY_DN13563_c0_g1_i1_orf1;TRINITY_DN9608_c0_g1_i3_orf1;TRINITY_DN3949_c1_g1_i1_orf1;TRINITY_DN3310_c0_g1_i1_orf1;TRINITY_DN120500_c0_g1_i1_orf1;TRINITY_DN79868_c0_g1_i1_orf1;TRINITY_DN7828_c0_g1_i2_orf1;TRINITY_DN2343_c1_g1_i8_orf1;TRINITY_DN12227_c0_g2_i3_orf1;TRINITY_DN5597_c0_g1_i2_orf1;TRINITY_DN104297_c0_g1_i1_orf1;TRINITY_DN6991_c0_g1_i24_orf1;TRINITY_DN496_c0_g1_i7_orf1;TRINITY_DN26355_c0_g1_i4_orf1;TRINITY_DN9615_c0_g1_i1_orf1;TRINITY_DN6586_c0_g1_i1_orf1;TRINITY_DN72816_c0_g1_i2_orf1;TRINITY_DN4394_c0_g2_i1_orf1;TRINITY_DN34426_c0_g1_i1_orf1;TRINITY_DN246_c1_g1_i5_orf1;TRINITY_DN383_c0_g1_i1_orf1;TRINITY_DN12392_c0_g1_i3_orf1;TRINITY_DN7735_c1_g1_i1_orf1;TRINITY_DN38431_c0_g1_i1_orf1;TRINITY_DN33705_c0_g1_i1_orf1;TRINITY_DN17772_c0_g2_i3_orf1;TRINITY_DN4134_c2_g1_i2_orf1;TRINITY_DN1622_c0_g1_i6_orf1;TRINITY_DN8030_c0_g1_i2_orf1;TRINITY_DN29633_c0_g1_i8_orf1;TRINITY_DN1232_c0_g1_i1_orf1;TRINITY_DN32780_c0_g1_i2_orf1;TRINITY_DN2312_c0_g1_i4_orf1;TRINITY_DN8964_c0_g1_i4_orf1;TRINITY_DN7590_c0_g1_i4_orf1;TRINITY_DN4144_c0_g1_i7_orf1;TRINITY_DN3196_c0_g1_i1_orf1;TRINITY_DN10774_c0_g2_i3_orf1;TRINITY_DN15755_c0_g1_i1_orf1;TRINITY_DN41708_c0_g1_i1_orf1;TRINITY_DN21570_c0_g1_i1_orf1;TRINITY_DN18338_c0_g1_i6_orf1;TRINITY_DN27114_c0_g1_i1_orf1;TRINITY_DN5337_c0_g1_i6_orf1;TRINITY_DN15157_c0_g1_i1_orf1;TRINITY_DN17505_c0_g1_i15_orf1;TRINITY_DN57105_c0_g1_i2_orf1;TRINITY_DN32479_c0_g1_i8_orf1;TRINITY_DN3177_c0_g1_i1_orf1;TRINITY_DN4757_c0_g1_i3_orf1;TRINITY_DN4886_c0_g1_i6_orf1;TRINITY_DN80245_c0_g1_i1_orf1;TRINITY_DN2794_c1_g1_i8_orf1;TRINITY_DN3355_c0_g2_i4_orf1;TRINITY_DN22443_c0_g2_i3_orf1;TRINITY_DN3978_c0_g2_i1_orf1;TRINITY_DN3529_c0_g1_i7_orf1;TRINITY_DN2967_c0_g1_i7_orf1;TRINITY_DN919_c0_g1_i7_orf1;TRINITY_DN2343_c1_g1_i2_orf1;TRINITY_DN6621_c0_g1_i1_orf1;TRINITY_DN29879_c0_g1_i3_orf1;TRINITY_DN51766_c0_g1_i2_orf1;TRINITY_DN56430_c0_g1_i1_orf1;TRINITY_DN29_c0_g1_i4_orf1;TRINITY_DN172_c8_g2_i1_orf1;TRINITY_DN14389_c0_g1_i4_orf1;TRINITY_DN3753_c0_g1_i7_orf1;TRINITY_DN2109_c0_g1_i4_orf1;TRINITY_DN11172_c0_g1_i4_orf1;TRINITY_DN3005_c0_g1_i7_orf1;TRINITY_DN4116_c0_g1_i3_orf1;TRINITY_DN3616_c0_g2_i1_orf1;TRINITY_DN8700_c9_g1_i1_orf1;TRINITY_DN12769_c0_g1_i5_orf1;TRINITY_DN11172_c1_g1_i1_orf1;TRINITY_DN4324_c0_g1_i1_orf1;TRINITY_DN113353_c0_g1_i1_orf1;TRINITY_DN52761_c0_g2_i1_orf1;TRINITY_DN1749_c0_g2_i2_orf1;TRINITY_DN64759_c0_g1_i1_orf1;TRINITY_DN6974_c0_g2_i1_orf1;TRINITY_DN11670_c0_g1_i1_orf1;TRINITY_DN30704_c0_g1_i1_orf1;TRINITY_DN13856_c0_g1_i1_orf1;TRINITY_DN198_c2_g1_i2_orf1;TRINITY_DN94337_c0_g1_i1_orf1;TRINITY_DN9079_c1_g1_i1_orf1;TRINITY_DN482_c0_g1_i1_orf1;TRINITY_DN252_c0_g1_i3_orf1;TRINITY_DN121650_c0_g1_i1_orf1;TRINITY_DN4612_c0_g1_i1_orf1;TRINITY_DN1664_c0_g1_i4_orf1;TRINITY_DN5046_c0_g3_i1_orf1;TRINITY_DN34821_c0_g1_i4_orf1;TRINITY_DN501_c0_g1_i5_orf1;TRINITY_DN42854_c0_g3_i2_orf1;TRINITY_DN5174_c0_g3_i1_orf1;TRINITY_DN79803_c0_g1_i7_orf1;TRINITY_DN5012_c0_g1_i6_orf1;TRINITY_DN9354_c0_g1_i7_orf1;TRINITY_DN5153_c1_g1_i1_orf1;TRINITY_DN1999_c0_g1_i9_orf1;TRINITY_DN5211_c0_g1_i1_orf1;TRINITY_DN483_c0_g1_i6_orf1;TRINITY_DN21719_c0_g1_i2_orf1;TRINITY_DN135781_c0_g1_i1_orf1;TRINITY_DN1008_c0_g1_i2_orf1;TRINITY_DN22678_c0_g1_i4_orf1;TRINITY_DN7464_c0_g1_i14_orf1;TRINITY_DN4273_c1_g1_i5_orf1;TRINITY_DN3461_c0_g1_i1_orf1;TRINITY_DN3647_c2_g1_i3_orf1;TRINITY_DN14429_c0_g1_i2_orf1;TRINITY_DN20558_c0_g1_i2_orf1;TRINITY_DN4425_c0_g1_i4_orf1;TRINITY_DN49508_c0_g2_i8_orf1;TRINITY_DN2808_c0_g1_i8_orf1;TRINITY_DN7336_c0_g1_i13_orf1;TRINITY_DN3469_c0_g1_i4_orf1;TRINITY_DN154_c0_g1_i4_orf1;TRINITY_DN9000_c0_g2_i1_orf1;TRINITY_DN5408_c0_g1_i5_orf1;TRINITY_DN4064_c0_g2_i1_orf1;TRINITY_DN15318_c0_g1_i1_orf1;TRINITY_DN62707_c0_g1_i1_orf1;TRINITY_DN4782_c0_g1_i1_orf1;TRINITY_DN27903_c0_g1_i1_orf1;TRINITY_DN22044_c0_g2_i1_orf1;TRINITY_DN32896_c0_g3_i1_orf1;TRINITY_DN3472_c1_g1_i4_orf1;TRINITY_DN140_c1_g1_i2_orf1;TRINITY_DN7861_c0_g1_i5_orf1;TRINITY_DN52788_c0_g1_i1_orf1;TRINITY_DN4321_c0_g1_i1_orf1;TRINITY_DN22_c0_g1_i3_orf1;TRINITY_DN23978_c0_g1_i2_orf1;TRINITY_DN16451_c0_g1_i7_orf1;TRINITY_DN2172_c0_g2_i8_orf1;TRINITY_DN1752_c0_g1_i18_orf1;TRINITY_DN61_c0_g2_i3_orf1;TRINITY_DN31118_c0_g1_i1_orf1;TRINITY_DN48590_c0_g1_i1_orf1;TRINITY_DN2618_c0_g1_i3_orf1;TRINITY_DN10581_c0_g1_i5_orf1;TRINITY_DN146364_c0_g1_i1_orf1;TRINITY_DN5513_c0_g1_i1_orf1;TRINITY_DN49786_c0_g1_i1_orf1;TRINITY_DN1630_c0_g1_i6_orf1;TRINITY_DN867_c0_g1_i1

cellular_component	intrinsic component of organelle membrane	GO:0031300	3	3/2406	TRINITY_DN8964_c0_g1_i4_orf1;TRINITY_DN483_c0_g1_i6_orf1;TRINITY_DN2172_c0_g2_i8_orf1
cellular_component	external side of plasma membrane	GO:0009897	1	1/2406	TRINITY_DN20339_c0_g1_i3_orf1
cellular_component	cytoplasmic side of membrane	GO:0098562	2	2/2406	TRINITY_DN96557_c0_g1_i1_orf1;TRINITY_DN975_c0_g1_i1_orf1
cellular_component	intracellular organelle	GO:0043229	220	220/2406	TRINITY_DN47731_c0_g1_i2_orf1;TRINITY_DN4380_c0_g1_i9_orf1;TRINITY_DN13350_c0_g1_i4_orf1;TRINITY_DN6991_c0_g1_i24_orf1;TRINITY_DN11194_c0_g1_i4_orf1;TRINITY_DN383_c0_g1_i1_orf1;TRINITY_DN1921_c1_g1_i5_orf1;TRINITY_DN5893_c0_g1_i7_orf1;TRINITY_DN35669_c0_g1_i1_orf1;TRINITY_DN38274_c0_g1_i1_orf1;TRINITY_DN18804_c0_g1_i5_orf1;TRINITY_DN110460_c0_g2_i1_orf1;TRINITY_DN58207_c0_g1_i1_orf1;TRINITY_DN80547_c0_g1_i5_orf1;TRINITY_DN558_c0_g1_i4_orf1;TRINITY_DN8603_c0_g1_i1_orf1;TRINITY_DN3457_c0_g1_i4_orf1;TRINITY_DN24266_c0_g2_i2_orf1;TRINITY_DN2638_c0_g1_i7_orf1;TRINITY_DN2848_c0_g1_i1_orf1;TRINITY_DN50074_c0_g1_i1_orf1;TRINITY_DN29448_c0_g1_i1_orf1;TRINITY_DN4711_c0_g1_i2_orf1;TRINITY_DN3464_c0_g1_i1_orf1;TRINITY_DN62_c1_g1_i3_orf1;TRINITY_DN21570_c0_g1_i1_orf1;TRINITY_DN6621_c0_g1_i1_orf1;TRINITY_DN1831_c0_g1_i3_orf1;TRINITY_DN5442_c0_g1_i4_orf1;TRINITY_DN74889_c0_g1_i1_orf1;TRINITY_DN364_c1_g1_i2_orf1;TRINITY_DN99673_c0_g1_i1_orf1;TRINITY_DN50571_c1_g1_i1_orf1;TRINITY_DN84478_c0_g1_i8_orf1;TRINITY_DN146217_c0_g1_i1_orf1;TRINITY_DN235_c0_g3_i1_orf1;TRINITY_DN31314_c0_g1_i4_orf1;TRINITY_DN2058_c0_g1_i2_orf1;TRINITY_DN31585_c0_g1_i1_orf1;TRINITY_DN135_c0_g1_i1_orf1;TRINITY_DN30932_c0_g1_i2_orf1;TRINITY_DN23746_c0_g1_i2_orf1;TRINITY_DN2345_c0_g1_i4_orf1;TRINITY_DN110231_c0_g1_i1_orf1;TRINITY_DN2401_c0_g2_i1_orf1;TRINITY_DN37532_c0_g1_i1_orf1;TRINITY_DN33893_c0_g1_i1_orf1;TRINITY_DN9146_c0_g1_i1_orf1;TRINITY_DN2802_c1_g1_i1_orf1;TRINITY_DN14391_c1_g1_i2_orf1;TRINITY_DN31225_c0_g1_i1_orf1;TRINITY_DN17312_c0_g1_i1_orf1;TRINITY_DN7808_c0_g1_i1_orf1;TRINITY_DN56993_c0_g1_i4_orf1;TRINITY_DN14313_c0_g1_i1_orf1;TRINITY_DN31584_c0_g2_i2_orf1;TRINITY_DN34751_c0_g1_i1_orf1;TRINITY_DN445_c0_g1_i2_orf1;TRINITY_DN51938_c0_g3_i1_orf1;TRINITY_DN1706_c0_g1_i7_orf1;TRINITY_DN12526_c0_g1_i5_orf1;TRINITY_DN23360_c0_g1_i3_orf1;TRINITY_DN52649_c0_g1_i6_orf1;TRINITY_DN17905_c0_g3_i1_orf1;TRINITY_DN5497_c0_g1_i6_orf1;TRINITY_DN18009_c0_g1_i1_orf1;TRINITY_DN18933_c0_g1_i3_orf1;TRINITY_DN146119_c0_g1_i1_orf1;TRINITY_DN23790_c0_g1_i1_orf1;TRINITY_DN2719_c1_g1_i6_orf1;TRINITY_DN97589_c0_g1_i3_orf1;TRINITY_DN9871_c0_g1_i11_orf1;TRINITY_DN90497_c0_g1_i1_orf1;TRINITY_DN53810_c0_g1_i1_orf1;TRINITY_DN7583_c0_g1_i1_orf1;TRINITY_DN18391_c0_g2_i8_orf1;TRINITY_DN7464_c0_g1_i14_orf1;TRINITY_DN5531_c0_g3_i3_orf1;TRINITY_DN41_c0_g1_i3_orf1;TRINITY_DN51737_c0_g1_i3_orf1;TRINITY_DN3251_c0_g1_i6_orf1;TRINITY_DN141_c0_g1_i1_orf1;TRINITY_DN43431_c0_g1_i1_orf1;TRINITY_DN3073_c0_g1_i7_orf1;TRINITY_DN9591_c0_g1_i1_orf1;TRINITY_DN106534_c0_g1_i1_orf1;TRINITY_DN14274_c0_g1_i3_orf1;TRINITY_DN3430_c0_g1_i1_orf1;TRINITY_DN18922_c0_g1_i1_orf1;TRINITY_DN11772_c0_g1_i1_orf1;TRINITY_DN741_c0_g1_i10_orf1;TRINITY_DN5664_c0_g1_i1_orf1;TRINITY_DN2140_c0_g1_i1_orf1;TRINITY_DN147691_c0_g1_i1_orf1;TRINITY_DN1718_c6_g1_i4_orf1;TRINITY_DN51836_c0_g3_i1_orf1;TRINITY_DN6358_c0_g1_i5_orf1;TRINITY_DN86149_c0_g1_i1_orf1;TRINITY_DN147458_c0_g1_i1_orf1;TRINITY_DN28989_c0_g1_i7_orf1;TRINITY_DN3860_c0_g1_i5_orf1;TRINITY_DN5064_c0_g1_i4_orf1;TRINITY_DN101991_c0_g1_i5_orf1;TRINITY_DN235_c0_g1_i2_orf1;TRINITY_DN2709_c0_g1_i4_orf1;TRINITY_DN291_c0_g1_i2_orf1;TRINITY_DN6239_c0_g1_i1_orf1;TRINITY_DN8703_c0_g1_i2_orf1;TRINITY_DN714_c0_g1_i3_orf1;TRINITY_DN30131_c0_g1_i1_orf1;TRINITY_DN26251_c0_g1_i1_orf1;TRINITY_DN15373_c0_g1_i2_orf1;TRINITY_DN6365_c0_g1_i4_orf1;TRINITY_DN114982_c0_g1_i1_orf1;TRINITY_DN46409_c0_g1_i1_orf1;TRINITY_DN58636_c0_g1_i1_orf1;TRINITY_DN14734_c0_g1_i2_orf1;TRINITY_DN142442_c0_g1_i1_orf1;TRINITY_DN8980_c0_g1_i2_orf1;TRINITY_DN1322_c0_g1_i4_orf1;TRINITY_DN31851_c0_g1_i2_orf1;TRINITY_DN72017_c0_g1_i1_orf1;TRINITY_DN5081_c0_g1_i5_orf1;TRINITY_DN34830_c0_g1_i1_orf1;TRINITY_DN3335_c0_g1_i1_orf1;TRINITY_DN4016_c0_g1_i1_orf1;TRINITY_DN23616_c0_g1_i4_orf1;TRINITY_DN934_c2_g1_i7_orf1;TRINITY_DN12771_c0_g1_i1_orf1;TRINITY_DN34479_c0_g1_i2_orf1;TRINITY_DN12858_c0_g1_i5_orf1;TRINITY_DN2559_c0_g1_i4_orf1;TRINITY_DN4793_c0_g1_i7_orf1;TRINITY_DN2710_c0_g1_i4_orf1;TRINITY_DN38211_c0_g1_i1_orf1;TRINITY_DN4142_c0_g1_i5_orf1;TRINITY_DN147676_c0_g1_i1_orf1;TRINITY_DN54134_c0_g1_i1_orf1;TRINITY_DN3062_c0_g1_i1_orf1;TRINITY_DN31503_c0_g1_i4_orf1;TRINITY_DN12576_c0_g1_i2_orf1;TRINITY_DN24322_c0_g1_i4_orf1;TRINITY_DN41736_c0_g2_i1_orf1;TRINITY_DN11986_c0_g1_i1_orf1;TRINITY_DN53311_c0_g2_i1_orf1;TRINITY_DN13094_c0_g1_i1_orf1;TRINITY_DN45633_c0_g1_i1_orf1;TRINITY_DN44288_c0_g1_i2_orf1;TRINITY_DN27960_c0_g1_i1_orf1;TRINITY_DN104507_c0_g1_i2_orf1;TRINITY_DN
cellular_component	non-membrane-bounded organelle	GO:0043228	75	75/2406	TRINITY_DN47731_c0_g1_i2_orf1;TRINITY_DN147596_c0_g1_i1_orf1;TRINITY_DN714_c0_g1_i3_orf1;TRINITY_DN30131_c0_g1_i1_orf1;TRINITY_DN23360_c0_g1_i3_orf1;TRINITY_DN52649_c0_g1_i6_orf1;TRINITY_DN3826_c0_g1_i1_orf1;TRINITY_DN6365_c0_g1_i4_orf1;TRINITY_DN7128_c0_g1_i7_orf1;TRINITY_DN114982_c0_g1_i1_orf1;TRINITY_DN58636_c0_g1_i1_orf1;TRINITY_DN9383_c0_g1_i3_orf1;TRINITY_DN23790_c0_g1_i1_orf1;TRINITY_DN5893_c0_g1_i7_orf1;TRINITY_DN9765_c0_g1_i6_orf1;TRINITY_DN55148_c0_g1_i1_orf1;TRINITY_DN24322_c0_g1_i4_orf1;TRINITY_DN107962_c0_g1_i1_orf1;TRINITY_DN58207_c0_g1_i1_orf1;TRINITY_DN90497_c0_g1_i1_orf1;TRINITY_DN934_c2_g1_i7_orf1;TRINITY_DN53810_c0_g1_i1_orf1;TRINITY_DN142442_c0_g1_i1_orf1;TRINITY_DN7583_c0_g1_i1_orf1;TRINITY_DN23616_c0_g1_i4_orf1;TRINITY_DN7464_c0_g1_i14_orf1;TRINITY_DN2848_c0_g1_i1_orf1;TRINITY_DN10994_c0_g1_i4_orf1;TRINITY_DN29448_c0_g1_i1_orf1;TRINITY_DN147676_c0_g1_i1_orf1;TRINITY_DN5531_c0_g3_i3_orf1;TRINITY_DN10745_c0_g1_i14_orf1;TRINITY_DN97589_c0_g1_i3_orf1;TRINITY_DN3062_c0_g1_i1_orf1;TRINITY_DN9591_c0_g1_i1_orf1;TRINITY_DN12576_c0_g1_i2_orf1;TRINITY_DN51836_c0_g3_i1_orf1;TRINITY_DN74889_c0_g1_i1_orf1;TRINITY_DN364_c1_g1_i2_orf1;TRINITY_DN77480_c0_g1_i2_orf1;TRINITY_DN50571_c1_g1_i1_orf1;TRINITY_DN34432_c0_g1_i1_orf1;TRINITY_DN26824_c0_g1_i1_orf1;TRINITY_DN146217_c0_g1_i1_orf1;TRINITY_DN235_c0_g3_i1_orf1;TRINITY_DN31314_c0_g1_i4_orf1;TRINITY_DN97097_c0_g1_i4_orf1;TRINITY_DN135_c0_g1_i1_orf1;TRINITY_DN147691_c0_g1_i1_orf1;TRINITY_DN23746_c0_g1_i2_orf1;TRINITY_DN10070_c0_g1_i1_orf1;TRINITY_DN6239_c0_g1_i1_orf1;TRINITY_DN96557_c0_g1_i1_orf1;TRINITY_DN110231_c0_g1_i1_orf1;TRINITY_DN975_c0_g1_i1_orf1;TRINITY_DN4016_c0_g1_i1_orf1;TRINITY_DN9146_c0_g1_i1_orf1;TRINITY_DN14391_c1_g1_i2_orf1;TRINITY_DN60821_c0_g1_i1_orf1;TRINITY_DN10831_c1_g1_i1_orf1;TRINITY_DN2186_c0_g1_i17_orf1;TRINITY_DN6248_c0_g1_i1_orf1;TRINITY_DN14684_c0_g2_i1_orf1;TRINITY_DN31225_c0_g1_i1_orf1;TRINITY_DN147458_c0_g1_i1_orf1;TRINITY_DN879_c0_g1_i2_orf1;TRINITY_DN20133_c0_g1_i1_orf1;TRINITY_DN17137_c0_g1_i2_orf1;TRINITY_DN5064_c0_g1_i4_orf1;TRINITY_DN101991_c0_g1_i5_orf1;TRINITY_DN235_c0_g1_i2_orf1;TRINITY_DN14313_c0_g1_i1_orf1;TRINITY_DN6462_c0_g1_i5_orf1;TRINITY_DN14996_c0_g1_i2_orf1;TRINITY_DN741_c0_g1_i10_orf1
cellular_component	membrane-bounded organelle	GO:0043227	154	154/2406	TRINITY_DN13350_c0_g1_i4_orf1;TRINITY_DN6991_c0_g1_i24_orf1;TRINITY_DN11194_c0_g1_i4_orf1;TRINITY_DN383_c0_g1_i1_orf1;TRINITY_DN1921_c1_g1_i5_orf1;TRINITY_DN5893_c0_g1_i7_orf1;TRINITY_DN35669_c0_g1_i1_orf1;TRINITY_DN18804_c0_g1_i5_orf1;TRINITY_DN110460_c0_g2_i1_orf1;TRINITY_DN80547_c0_g1_i5_orf1;TRINITY_DN558_c0_g1_i4_orf1;TRINITY_DN8603_c0_g1_i1_orf1;TRINITY_DN3457_c0_g1_i4_orf1;TRINITY_DN2638_c0_g1_i7_orf1;TRINITY_DN2848_c0_g1_i1_orf1;TRINITY_DN50074_c0_g1_i1_orf1;TRINITY_DN4711_c0_g1_i2_orf1;TRINITY_DN3464_c0_g1_i1_orf1;TRINITY_DN21570_c0_g1_i1_orf1;TRINITY_DN6621_c0_g1_i1_orf1;TRINITY_DN74889_c0_g1_i1_orf1;TRINITY_DN99673_c0_g1_i1_orf1;TRINITY_DN84478_c0_g1_i8_orf1;TRINITY_DN31314_c0_g1_i4_orf1;TRINITY_DN2058_c0_g1_i2_orf1;TRINITY_DN30932_c0_g1_i2_orf1;TRINITY_DN2345_c0_g1_i4_orf1;TRINITY_DN5442_c0_g1_i4_orf1;TRINITY_DN2401_c0_g2_i1_orf1;TRINITY_DN33893_c0_g1_i1_orf1;TRINITY_DN2802_c1_g1_i1_orf1;TRINITY_DN2265_c0_g1_i5_orf1;TRINITY_DN7808_c0_g1_i1_orf1;TRINITY_DN56993_c0_g1_i4_orf1;TRINITY_DN34751_c0_g1_i1_orf1;TRINITY_DN51938_c0_g3_i1_orf1;TRINITY_DN1706_c0_g1_i7_orf1;TRINITY_DN12526_c0_g1_i5_orf1;TRINITY_DN23360_c0_g1_i3_orf1;TRINITY_DN17905_c0_g3_i1_orf1;TRINITY_DN5497_c0_g1_i6_orf1;TRINITY_DN18009_c0_g1_i1_orf1;TRINITY_DN18933_c0_g1_i3_orf1;TRINITY_DN146119_c0_g1_i1_orf1;TRINITY_DN2719_c1_g1_i6_orf1;TRINITY_DN9871_c0_g1_i11_orf1;TRINITY_DN53810_c0_g1_i1_orf1;TRINITY_DN18391_c0_g2_i8_orf1;TRINITY_DN41_c0_g1_i3_orf1;TRINITY_DN51737_c0_g1_i3_orf1;TRINITY_DN3251_c0_g1_i6_orf1;TRINITY_DN141_c0_g1_i1_orf1;TRINITY_DN43431_c0_g1_i1_orf1;TRINITY_DN3073_c0_g1_i7_orf1;TRINITY_DN9591_c0_g1_i1_orf1;TRINITY_DN106534_c0_g1_i1_orf1;TRINITY_DN3430_c0_g1_i1_orf1;TRINITY_DN18922_c0_g1_i1_orf1;TRINITY_DN11772_c0_g1_i1_orf1;TRINITY_DN5664_c0_g1_i1_orf1;TRINITY_DN2140_c0_g1_i1_orf1;TRINITY_DN1718_c6_g1_i4_orf1;TRINITY_DN6358_c0_g1_i5_orf1;TRINITY_DN86149_c0_g1_i1_orf1;TRINITY_DN147458_c0_g1_i1_orf1;TRINITY_DN28989_c0_g1_i7_orf1;TRINITY_DN3860_c0_g1_i5_orf1;TRINITY_DN291_c0_g1_i2_orf1;TRINITY_DN8703_c0_g1_i2_orf1;TRINITY_DN714_c0_g1_i3_orf1;TRINITY_DN26251_c0_g1_i1_orf1;TRINITY_DN6365_c0_g1_i4_orf1;TRINITY_DN114982_c0_g1_i1_orf1;TRINITY_DN46409_c0_g1_i1_orf1;TRINITY_DN17312_c0_g1_i1_orf1;TRINITY_DN31584_c0_g2_i2_orf1;TRINITY_DN1322_c0_g1_i4_orf1;TRINITY_DN31851_c0_g1_i2_orf1;TRINITY_DN72017_c0_g1_i1_orf1;TRINITY_DN5081_c0_g1_i5_orf1;TRINITY_DN34830_c0_g1_i1_orf1;TRINITY_DN3335_c0_g1_i1_orf1;TRINITY_DN4016_c0_g1_i1_orf1;TRINITY_DN934_c2_g1_i7_orf1;TRINITY_DN12771_c0_g1_i1_orf1;TRINITY_DN34479_c0_g1_i2_orf1;TRINITY_DN12858_c0_g1_i5_orf1;TRINITY_DN2559_c0_g1_i4_orf1;TRINITY_DN4793_c0_g1_i7_orf1;TRINITY_DN2710_c0_g1_i4_orf1;TRINITY_DN4142_c0_g1_i5_orf1;TRINITY_DN54134_c0_g1_i1_orf1;TRINITY_DN41736_c0_g2_i1_orf1;TRINITY_DN11986_c0_g1_i1_orf1;TRINITY_DN53311_c0_g2_i1_orf1;TRINITY_DN45633_c0_g1_i1_orf1;TRINITY_DN44288_c0_g1_i2_orf1;TRINITY_DN27960_c0_g1_i1_orf1;TRINITY_DN104507_c0_g1_i2_orf1;TRINITY_DN38274_c0_g1_i1_orf1;TRINITY_DN19810_c1_g1_i7_orf1;TRINITY_DN72_c0_g1_i16_orf1;TRINITY_DN62_c1_g1_i3_orf1;TRINITY_DN111110_c0_g1_i1_orf1;TRINITY_DN2802_c0_g1_i1_orf1;TRINITY_DN5962_c0_g1_i1_orf1;TRINITY_DN2647_c0_g1_i3_orf1;TRINITY_DN120144_c0_g1_i1_orf1;TRINITY_DN6248_c0_g1_i1_orf1;TRINITY_DN42854_c0_g3_i2_orf1;TRINITY_DN5578_c0_g1_i4_orf1;TRINITY_DN47389_c0_g1_i2_orf1;TRINITY_DN10385_c0_g1_i5_orf1;TRINITY_DN787_c0_g1_i7_orf1;TRINITY_DN30673_c0_g1_i5_orf1;TRINITY_DN2971_c0_g1_i1_orf1;TRINITY_DN1952_c0_g1_i2_orf1;TRINITY_DN67649_c0_g1_i1_orf1;TRINITY_DN38211_c0_g1_i1_orf1;TRINITY_DN13157_c0_g1_i1_orf1;TRINITY_DN13760_c1_g1_i1_orf1;TRINITY_DN15706_c0_g2_i5_orf1;TRINITY_DN9938_c0_g2_i1_orf1;TRINITY_DN96801_c0_g1_i1_orf1;TRINITY_DN95971_c0_g5_i1_orf1;TRINITY_DN13711_c0_g1_i1_orf1;TRINITY_DN975_c0_g1_i1_orf1;TRINITY_DN55148_c0_g1_i1_orf1;TRINITY_DN41602_c0_g3_i1_orf1;TRINITY_DN1831_c0_g1_i3_orf1;TRINITY_DN19115_c0_g1_i1_orf1;TRINITY_DN279_c0_g1_i10_orf1;TRINITY_DN3614_c0_g2_i1_orf1;TRINITY_DN166_c0_g1_i4_orf1;TRINITY_DN445_c0_g1_i2_orf1;TRINITY_DN31585_c0_g1_i1_orf1;TRINITY_DN18031_c0_g1_i1_orf1;TRINITY_DN11736_c0_g1_i1_orf1;TRINITY_DN25345_c0_g1_i1_orf1;TRINITY_DN19251_c0_g1_i8_orf1;TRINITY_DN34432_c0_g1_i1_orf1;TRINITY_DN4070_c0_g1_i4_orf1;TRINITY_DN40704_c0_g1_i2_orf1;TRINITY_DN10070_c0_g1_i1_orf1;TRINITY_DN15256_c0_g1_i8_orf1;TRINITY_DN17133_c0_g1_i1_orf1;TRINITY_DN4182_c0_g1_i6_orf1;TRINITY_DN147475_c0_g1_i1_orf1;TRINITY_DN51045_c0_g1_i1_orf1;TRINITY_DN20133_c0_g1_i1_orf1;TRINITY_DN24266_c0
cellular_component	postsynaptic specialization	GO:0099572	3	3/2406	TRINITY_DN33418_c0_g1_i1_orf1;TRINITY_DN4016_c0_g1_i1_orf1;TRINITY_DN142442_c0_g1_i1_orf1
cellular_component	extracellular organelle	GO:0043230	8	8/2406	TRINITY_DN4016_c0_g1_i1_orf1;TRINITY_DN975_c0_g1_i1_orf1;TRINITY_DN147475_c0_g1_i1_orf1;TRINITY_DN42854_c0_g3_i2_orf1;TRINITY_DN46409_c0_g1_i1_orf1;TRINITY_DN20133_c0_g1_i1_orf1;TRINITY_DN55148_c0_g1_i1_orf1;TRINITY_DN10070_c0_g1_i1_orf1

cellular_component	striated muscle thin filament	GO:0005865	1	1/2406	TRINITY_DN235_c0_g3_i1_orf1
cellular_component	neuronal cell body	GO:0043025	1	1/2406	TRINITY_DN10070_c0_g1_i1_orf1
cellular_component	organelle membrane	GO:0031090	50	50/2406	TRINITY_DN245_c0_g1_i4_orf1;TRINITY_DN445_c0_g1_i2_orf1;TRINITY_DN9608_c0_g1_i3_orf1;TRINITY_DN2312_c0_g1_i4_orf1;TRINITY_DN32896_c0_g3_i1_orf1;TRINITY_DN44777_c0_g1_i2_orf1;TRINITY_DN46409_c0_g1_i1_orf1;TRINITY_DN72816_c0_g1_i2_orf1;TRINITY_DN6243_c0_g1_i5_orf1;TRINITY_DN45400_c0_g1_i1_orf1;TRINITY_DN5064_c0_g1_i4_orf1;TRINITY_DN5867_c0_g1_i1_orf1;TRINITY_DN35635_c0_g1_i1_orf1;TRINITY_DN22242_c0_g1_i1_orf1;TRINITY_DN19303_c0_g1_i5_orf1;TRINITY_DN1134_c0_g1_i4_orf1;TRINITY_DN1280_c0_g1_i1_orf1;TRINITY_DN31851_c0_g1_i2_orf1;TRINITY_DN135781_c0_g1_i1_orf1;TRINITY_DN2848_c0_g1_i1_orf1;TRINITY_DN1362_c0_g1_i4_orf1;TRINITY_DN3513_c0_g1_i5_orf1;TRINITY_DN11172_c0_g1_i4_orf1;TRINITY_DN5753_c0_g1_i10_orf1;TRINITY_DN5531_c0_g3_i3_orf1;TRINITY_DN7336_c0_g1_i13_orf1;TRINITY_DN21570_c0_g1_i1_orf1;TRINITY_DN448_c0_g1_i20_orf1;TRINITY_DN154_c0_g1_i4_orf1;TRINITY_DN3821_c1_g1_i7_orf1;TRINITY_DN2286_c2_g1_i1_orf1;TRINITY_DN24873_c0_g1_i4_orf1;TRINITY_DN1960_c5_g1_i3_orf1;TRINITY_DN6680_c0_g1_i1_orf1;TRINITY_DN5439_c0_g1_i2_orf1;TRINITY_DN12286_c1_g1_i2_orf1;TRINITY_DN10745_c0_g1_i14_orf1;TRINITY_DN22044_c0_g2_i1_orf1;TRINITY_DN5513_c0_g1_i1_orf1;TRINITY_DN8454_c0_g1_i4_orf1;TRINITY_DN32780_c0_g1_i2_orf1;TRINITY_DN2879_c0_g1_i4_orf1;TRINITY_DN96557_c0_g1_i1_orf1;TRINITY_DN4820_c0_g2_i2_orf1;TRINITY_DN69871_c0_g1_i1_orf1;TRINITY_DN42854_c0_g3_i2_orf1;TRINITY_DN3461_c0_g1_i1_orf1;TRINITY_DN11172_c1_g1_i1_orf1;TRINITY_DN5697_c0_g1_i1_orf1;TRINITY_DN4248_c0_g1_i4_orf1
cellular_component	outer membrane	GO:0019867	2	2/2406	TRINITY_DN6656_c0_g1_i1_orf1;TRINITY_DN4820_c0_g2_i2_orf1
cellular_component	plasma membrane	GO:0005886	38	38/2406	TRINITY_DN28759_c0_g1_i1_orf1;TRINITY_DN6974_c0_g2_i1_orf1;TRINITY_DN23926_c0_g1_i4_orf1;TRINITY_DN9475_c0_g1_i6_orf1;TRINITY_DN51938_c0_g3_i1_orf1;TRINITY_DN7128_c0_g1_i7_orf1;TRINITY_DN46409_c0_g1_i1_orf1;TRINITY_DN5011_c0_g1_i1_orf1;TRINITY_DN15706_c0_g2_i5_orf1;TRINITY_DN19951_c0_g1_i5_orf1;TRINITY_DN20710_c0_g2_i2_orf1;TRINITY_DN2735_c0_g1_i4_orf1;TRINITY_DN5406_c0_g2_i1_orf1;TRINITY_DN975_c0_g1_i1_orf1;TRINITY_DN1352_c0_g1_i5_orf1;TRINITY_DN2848_c0_g1_i1_orf1;TRINITY_DN5553_c0_g1_i4_orf1;TRINITY_DN7590_c0_g1_i4_orf1;TRINITY_DN140613_c0_g1_i1_orf1;TRINITY_DN3469_c0_g1_i4_orf1;TRINITY_DN2175_c0_g1_i4_orf1;TRINITY_DN1012_c0_g1_i2_orf1;TRINITY_DN198_c2_g1_i2_orf1;TRINITY_DN20710_c0_g1_i2_orf1;TRINITY_DN26337_c0_g1_i3_orf1;TRINITY_DN10581_c0_g1_i5_orf1;TRINITY_DN32780_c0_g1_i2_orf1;TRINITY_DN10070_c0_g1_i1_orf1;TRINITY_DN38431_c0_g1_i1_orf1;TRINITY_DN51836_c0_g3_i1_orf1;TRINITY_DN14389_c0_g1_i4_orf1;TRINITY_DN11670_c0_g1_i1_orf1;TRINITY_DN16145_c0_g1_i12_orf1;TRINITY_DN42854_c0_g3_i2_orf1;TRINITY_DN57348_c0_g1_i4_orf1;TRINITY_DN20133_c0_g1_i1_orf1;TRINITY_DN5064_c0_g1_i4_orf1;TRINITY_DN31584_c0_g2_i2_orf1
cellular_component	plasma membrane region	GO:0098590	6	6/2406	TRINITY_DN51938_c0_g3_i1_orf1;TRINITY_DN975_c0_g1_i1_orf1;TRINITY_DN7128_c0_g1_i7_orf1;TRINITY_DN46409_c0_g1_i1_orf1;TRINITY_DN10745_c0_g1_i14_orf1;TRINITY_DN486_c0_g1_i5_orf1
cellular_component	phagophore assembly site membrane	GO:0034045	2	2/2406	TRINITY_DN113353_c0_g1_i1_orf1;TRINITY_DN5531_c0_g3_i3_orf1
cellular_component	dendritic spine	GO:0043197	1	1/2406	TRINITY_DN802_c0_g1_i2_orf1
cellular_component	extracellular matrix	GO:0031012	5	5/2406	TRINITY_DN10070_c0_g1_i1_orf1;TRINITY_DN3833_c0_g1_i4_orf1;TRINITY_DN376_c1_g1_i1_orf1;TRINITY_DN2919_c0_g1_i5_orf1;TRINITY_DN4464_c0_g2_i1_orf1
cellular_component	egg chorion	GO:0042600	1	1/2406	TRINITY_DN5933_c0_g1_i1_orf1
cellular_component	synapse	GO:0045202	4	4/2406	TRINITY_DN10070_c0_g1_i1_orf1;TRINITY_DN142442_c0_g1_i1_orf1;TRINITY_DN4016_c0_g1_i1_orf1;TRINITY_DN2848_c0_g1_i1_orf1
cellular_component	anchoring junction	GO:0070161	13	13/2406	TRINITY_DN28759_c0_g1_i1_orf1;TRINITY_DN802_c0_g1_i2_orf1;TRINITY_DN4016_c0_g1_i1_orf1;TRINITY_DN364_c2_g1_i2_orf1;TRINITY_DN142442_c0_g1_i1_orf1;TRINITY_DN7128_c0_g1_i7_orf1;TRINITY_DN2186_c0_g1_i17_orf1;TRINITY_DN33418_c0_g1_i1_orf1;TRINITY_DN9383_c0_g1_i3_orf1;TRINITY_DN364_c1_g1_i2_orf1;TRINITY_DN7590_c0_g1_i4_orf1;TRINITY_DN23746_c0_g1_i2_orf1;TRINITY_DN741_c0_g1_i10_orf1
cellular_component	ruffle membrane	GO:0032587	1	1/2406	TRINITY_DN975_c0_g1_i1_orf1
cellular_component	plasma membrane bounded cell projection	GO:0120025	7	7/2406	TRINITY_DN4016_c0_g1_i1_orf1;TRINITY_DN5954_c0_g1_i2_orf1;TRINITY_DN364_c2_g1_i2_orf1;TRINITY_DN9383_c0_g1_i3_orf1;TRINITY_DN802_c0_g1_i2_orf1;TRINITY_DN741_c0_g1_i10_orf1;TRINITY_DN364_c1_g1_i2_orf1
cellular_component	nuclear speck	GO:0016607	1	1/2406	TRINITY_DN47123_c0_g1_i1_orf1
cellular_component	centrosome	GO:0005813	1	1/2406	TRINITY_DN4689_c0_g1_i5_orf1
cellular_component	kinetochore	GO:0000776	2	2/2406	TRINITY_DN96557_c0_g1_i1_orf1;TRINITY_DN31314_c0_g1_i4_orf1
cellular_component	ribonucleoprotein granule	GO:0035770	2	2/2406	TRINITY_DN4016_c0_g1_i1_orf1;TRINITY_DN12576_c0_g1_i2_orf1
cellular_component	supramolecular polymer	GO:0099081	18	18/2406	TRINITY_DN17995_c0_g4_i1_orf1;TRINITY_DN280_c0_g1_i8_orf1;TRINITY_DN2942_c0_g1_i6_orf1;TRINITY_DN4808_c0_g1_i3_orf1;TRINITY_DN14298_c0_g1_i1_orf1;TRINITY_DN122423_c0_g1_i1_orf1;TRINITY_DN8390_c0_g1_i2_orf1;TRINITY_DN51836_c0_g3_i1_orf1;TRINITY_DN28018_c0_g6_i1_orf1;TRINITY_DN34703_c0_g1_i4_orf1;TRINITY_DN350_c0_g1_i10_orf1;TRINITY_DN235_c0_g3_i1_orf1;TRINITY_DN2745_c0_g1_i2_orf1;TRINITY_DN97138_c0_g1_i2_orf1;TRINITY_DN14298_c0_g3_i1_orf1;TRINITY_DN350_c0_g1_i5_orf1;TRINITY_DN5893_c0_g1_i7_orf1;TRINITY_DN96557_c0_g1_i1_orf1
molecular_function	mRNA regulatory element binding translation repressor activity	GO:0000900	1	1/2406	TRINITY_DN3673_c0_g1_i10_orf1
molecular_function	translation factor activity, RNA binding	GO:0008135	19	19/2406	TRINITY_DN136906_c0_g1_i1_orf1;TRINITY_DN126648_c0_g1_i1_orf1;TRINITY_DN11823_c1_g1_i2_orf1;TRINITY_DN5086_c0_g1_i1_orf1;TRINITY_DN50085_c0_g1_i1_orf1;TRINITY_DN2265_c0_g1_i5_orf1;TRINITY_DN9164_c0_g1_i3_orf1;TRINITY_DN31503_c0_g1_i4_orf1;TRINITY_DN53311_c0_g2_i1_orf1;TRINITY_DN9575_c0_g1_i1_orf1;TRINITY_DN15420_c0_g3_i2_orf1;TRINITY_DN38412_c0_g1_i1_orf1;TRINITY_DN34509_c0_g1_i1_orf1;TRINITY_DN975_c0_g1_i1_orf1;TRINITY_DN48097_c0_g1_i1_orf1;TRINITY_DN19659_c1_g1_i1_orf1;TRINITY_DN6239_c0_g1_i1_orf1;TRINITY_DN1074_c0_g1_i7_orf1;TRINITY_DN36817_c0_g1_i1_orf1
molecular_function	transcription corepressor activity	GO:0003714	1	1/2406	TRINITY_DN1921_c1_g1_i5_orf1
molecular_function	RNA helicase activity	GO:0003724	12	12/2406	TRINITY_DN44288_c0_g1_i2_orf1;TRINITY_DN4380_c0_g1_i9_orf1;TRINITY_DN4950_c0_g1_i2_orf1;TRINITY_DN31503_c0_g1_i4_orf1;TRINITY_DN13094_c0_g1_i1_orf1;TRINITY_DN14274_c0_g1_i3_orf1;TRINITY_DN4408_c6_g1_i1_orf1;TRINITY_DN26168_c0_g1_i1_orf1;TRINITY_DN8980_c0_g1_i2_orf1;TRINITY_DN59291_c0_g1_i1_orf1;TRINITY_DN9302_c0_g1_i1_orf1;TRINITY_DN2709_c0_g1_i4_orf1
molecular_function	minus-end-directed microtubule motor activity	GO:0008569	1	1/2406	TRINITY_DN122423_c0_g1_i1_orf1
molecular_function	DNA helicase activity	GO:0003678	6	6/2406	TRINITY_DN2971_c0_g1_i1_orf1;TRINITY_DN25345_c0_g1_i1_orf1;TRINITY_DN11986_c0_g1_i1_orf1;TRINITY_DN6642_c0_g1_i2_orf1;TRINITY_DN5757_c0_g1_i1_orf1;TRINITY_DN291_c0_g1_i2_orf1
molecular_function	ATP-dependent chromatin remodeler activity	GO:0140658	1	1/2406	TRINITY_DN25345_c0_g1_i1_orf1
molecular_function	DNA topoisomerase type II (double strand cut, ATP-hydrolyzing) activity	GO:0003918	1	1/2406	TRINITY_DN4908_c1_g1_i5_orf1
molecular_function	long-chain fatty acid-CoA ligase activity	GO:0004467	1	1/2406	TRINITY_DN2193_c0_g1_i7_orf1
molecular_function	ABC-type transporter activity	GO:0140359	4	4/2406	TRINITY_DN3637_c0_g1_i2_orf1;TRINITY_DN13563_c0_g1_i1_orf1;TRINITY_DN60792_c0_g1_i2_orf1;TRINITY_DN162_c0_g1_i4_orf1
molecular_function	P-type transmembrane transporter activity	GO:0140358	1	1/2406	TRINITY_DN7336_c0_g1_i13_orf1
molecular_function	ATPase-coupled cation transmembrane transporter activity	GO:0019829	2	2/2406	TRINITY_DN7336_c0_g1_i13_orf1;TRINITY_DN10458_c0_g1_i1_orf1
molecular_function	ATPase-coupled ion transmembrane transporter activity	GO:0042625	1	1/2406	TRINITY_DN10458_c0_g1_i1_orf1
molecular_function	signaling adaptor activity	GO:0035591	1	1/2406	TRINITY_DN21545_c0_g1_i2_orf1
molecular_function	cytoskeletal anchor activity	GO:0008093	1	1/2406	TRINITY_DN21559_c0_g2_i1_orf1
molecular_function	cargo adaptor activity	GO:0140312	1	1/2406	TRINITY_DN5118_c0_g1_i1_orf1
molecular_function	SNAP receptor activity	GO:0005484	1	1/2406	TRINITY_DN383_c0_g1_i1_orf1
molecular_function	very-low-density lipoprotein particle receptor activity	GO:0030229	1	1/2406	TRINITY_DN585_c0_g1_i5_orf1
molecular_function	nuclear export signal receptor activity	GO:0005049	1	1/2406	TRINITY_DN95971_c0_g5_i1_orf1
molecular_function	membrane insertase activity	GO:0032977	1	1/2406	TRINITY_DN64759_c0_g1_i1_orf1
molecular_function	copper chaperone activity	GO:0016531	1	1/2406	TRINITY_DN3461_c0_g1_i1_orf1
molecular_function	ATP-dependent FeS chaperone activity	GO:0140663	1	1/2406	TRINITY_DN85476_c0_g1_i1_orf1
molecular_function	peroxiredoxin activity	GO:0051920	2	2/2406	TRINITY_DN7778_c0_g1_i1_orf1;TRINITY_DN69236_c0_g1_i1_orf1
molecular_function	catalase activity	GO:0004096	2	2/2406	TRINITY_DN285_c0_g1_i4_orf1;TRINITY_DN6580_c0_g1_i4_orf1
molecular_function	glutathione peroxidase activity	GO:0004602	1	1/2406	TRINITY_DN1622_c0_g1_i6_orf1
molecular_function	phospholipid transporter activity	GO:0005548	2	2/2406	TRINITY_DN2544_c1_g1_i2_orf1;TRINITY_DN2160_c0_g1_i13_orf1
molecular_function	intramembrane lipid transporter activity	GO:0140303	2	2/2406	TRINITY_DN2544_c1_g1_i2_orf1;TRINITY_DN252_c0_g1_i3_orf1
molecular_function	sulfur compound transmembrane transporter activity	GO:1901682	1	1/2406	TRINITY_DN268_c1_g1_i7_orf1
molecular_function	organic acid transmembrane transporter activity	GO:0005342	1	1/2406	TRINITY_DN32896_c0_g3_i1_orf1
molecular_function	carbohydrate transmembrane transporter activity	GO:0015144	3	3/2406	TRINITY_DN2735_c0_g1_i4_orf1;TRINITY_DN57348_c0_g1_i4_orf1;TRINITY_DN10581_c0_g1_i5_orf1

molecular_function	passive transmembrane transporter activity	GO:0022803	6	6/2406	TRINITY_DN6974_c0_g2_i1_orf1;TRINITY_DN18338_c0_g1_i6_orf1;TRINITY_DN34821_c0_g1_i4_orf1;TRINITY_DN20558_c0_g1_i2_orf1;TRINITY_DN10290_c0_g1_i7_orf1;TRINITY_DN5753_c0_g1_i10_orf1
molecular_function	active transmembrane transporter activity	GO:0022804	9	9/2406	TRINITY_DN7336_c0_g1_i13_orf1;TRINITY_DN268_c1_g1_i7_orf1;TRINITY_DN13563_c0_g1_i1_orf1;TRINITY_DN76036_c0_g1_i1_orf1;TRINITY_DN162_c0_g1_i4_orf1;TRINITY_DN29_c0_g1_i4_orf1;TRINITY_DN60792_c0_g1_i2_orf1;TRINITY_DN3637_c0_g1_i2_orf1;TRINITY_DN10458_c0_g1_i1_orf1
molecular_function	ion transmembrane transporter activity	GO:0015075	13	13/2406	TRINITY_DN7336_c0_g1_i13_orf1;TRINITY_DN15222_c0_g1_i4_orf1;TRINITY_DN6974_c0_g2_i1_orf1;TRINITY_DN268_c1_g1_i7_orf1;TRINITY_DN34821_c0_g1_i4_orf1;TRINITY_DN9354_c0_g1_i7_orf1;TRINITY_DN20558_c0_g1_i2_orf1;TRINITY_DN76036_c0_g1_i1_orf1;TRINITY_DN141353_c0_g1_i1_orf1;TRINITY_DN12286_c1_g1_i2_orf1;TRINITY_DN10458_c0_g1_i1_orf1;TRINITY_DN5753_c0_g1_i10_orf1;TRINITY_DN91946_c0_g1_i1_orf1
molecular_function	inorganic molecular entity transmembrane transporter activity	GO:0015318	12	12/2406	TRINITY_DN7336_c0_g1_i13_orf1;TRINITY_DN15222_c0_g1_i4_orf1;TRINITY_DN6974_c0_g2_i1_orf1;TRINITY_DN268_c1_g1_i7_orf1;TRINITY_DN34821_c0_g1_i4_orf1;TRINITY_DN9354_c0_g1_i7_orf1;TRINITY_DN20558_c0_g1_i2_orf1;TRINITY_DN76036_c0_g1_i1_orf1;TRINITY_DN141353_c0_g1_i1_orf1;TRINITY_DN10458_c0_g1_i1_orf1;TRINITY_DN5753_c0_g1_i10_orf1;TRINITY_DN91946_c0_g1_i1_orf1
molecular_function	channel inhibitor activity	GO:0016248	2	2/2406	TRINITY_DN6098_c1_g1_i5_orf1;TRINITY_DN4748_c0_g1_i5_orf1
molecular_function	ATPase inhibitor activity	GO:0042030	1	1/2406	TRINITY_DN5442_c0_g1_i4_orf1
molecular_function	ion channel regulator activity	GO:0099106	4	4/2406	TRINITY_DN6098_c1_g1_i5_orf1;TRINITY_DN4748_c0_g1_i5_orf1;TRINITY_DN31584_c0_g2_i2_orf1;TRINITY_DN10994_c0_g1_i4_orf1
molecular_function	ubiquitin-protein transferase regulator activity	GO:0055106	1	1/2406	TRINITY_DN55148_c0_g1_i1_orf1
molecular_function	kinase regulator activity	GO:0019207	3	3/2406	TRINITY_DN346_c0_g1_i7_orf1;TRINITY_DN12_c0_g1_i5_orf1;TRINITY_DN147475_c0_g1_i1_orf1
molecular_function	phosphatase regulator activity	GO:0019208	1	1/2406	TRINITY_DN13999_c0_g1_i4_orf1
molecular_function	nucleoside-triphosphatase regulator activity	GO:0060589	18	18/2406	TRINITY_DN1173_c0_g1_i12_orf1;TRINITY_DN802_c0_g1_i2_orf1;TRINITY_DN138086_c0_g1_i1_orf1;TRINITY_DN42461_c0_g1_i4_orf1;TRINITY_DN104297_c0_g1_i1_orf1;TRINITY_DN518_c0_g1_i1_orf1;TRINITY_DN493_c0_g1_i4_orf1;TRINITY_DN69170_c0_g2_i1_orf1;TRINITY_DN9248_c0_g1_i10_orf1;TRINITY_DN23354_c0_g1_i7_orf1;TRINITY_DN2623_c0_g1_i3_orf1;TRINITY_DN4410_c0_g1_i1_orf1;TRINITY_DN27491_c0_g1_i1_orf1;TRINITY_DN5182_c0_g1_i5_orf1;TRINITY_DN804_c0_g1_i7_orf1;TRINITY_DN15753_c0_g1_i1_orf1;TRINITY_DN1173_c0_g1_i11_orf1;TRINITY_DN1173_c1_g1_i10_orf1
molecular_function	peptidase regulator activity	GO:0061134	30	30/2406	TRINITY_DN122321_c0_g1_i1_orf1;TRINITY_DN4314_c0_g1_i9_orf1;TRINITY_DN8258_c0_g1_i3_orf1;TRINITY_DN9872_c0_g1_i2_orf1;TRINITY_DN1986_c0_g1_i1_orf1;TRINITY_DN2271_c0_g1_i12_orf1;TRINITY_DN501_c0_g1_i5_orf1;TRINITY_DN42854_c0_g3_i2_orf1;TRINITY_DN9732_c0_g1_i7_orf1;TRINITY_DN16234_c0_g2_i3_orf1;TRINITY_DN18196_c0_g1_i4_orf1;TRINITY_DN7776_c0_g1_i1_orf1;TRINITY_DN1328_c0_g1_i6_orf1;TRINITY_DN2848_c0_g1_i1_orf1;TRINITY_DN10994_c0_g1_i4_orf1;TRINITY_DN8258_c0_g1_i6_orf1;TRINITY_DN77425_c0_g1_i2_orf1;TRINITY_DN45948_c1_g1_i1_orf1;TRINITY_DN2097_c1_g2_i2_orf1;TRINITY_DN71308_c0_g1_i4_orf1;TRINITY_DN2323_c0_g1_i4_orf1;TRINITY_DN1444_c1_g1_i5_orf1;TRINITY_DN1215_c0_g1_i2_orf1;TRINITY_DN7539_c0_g1_i2_orf1;TRINITY_DN10057_c0_g2_i1_orf1;TRINITY_DN1540_c0_g1_i9_orf1;TRINITY_DN135188_c0_g1_i2_orf1;TRINITY_DN9455_c0_g1_i6_orf1;TRINITY_DN1540_c0_g1_i14_orf1;TRINITY_DN712_c0_g2_i1_orf1
molecular_function	enzyme activator activity	GO:0008047	12	12/2406	TRINITY_DN346_c0_g1_i7_orf1;TRINITY_DN67649_c0_g1_i1_orf1;TRINITY_DN138086_c0_g1_i1_orf1;TRINITY_DN4410_c0_g1_i1_orf1;TRINITY_DN104297_c0_g1_i1_orf1;TRINITY_DN518_c0_g1_i1_orf1;TRINITY_DN493_c0_g1_i4_orf1;TRINITY_DN69170_c0_g2_i1_orf1;TRINITY_DN9248_c0_g1_i10_orf1;TRINITY_DN23354_c0_g1_i7_orf1;TRINITY_DN5182_c0_g1_i5_orf1;TRINITY_DN802_c0_g1_i2_orf1
molecular_function	enzyme inhibitor activity	GO:0004857	32	32/2406	TRINITY_DN13999_c0_g1_i4_orf1;TRINITY_DN122321_c0_g1_i1_orf1;TRINITY_DN4314_c0_g1_i9_orf1;TRINITY_DN8258_c0_g1_i3_orf1;TRINITY_DN9872_c0_g1_i2_orf1;TRINITY_DN1986_c0_g1_i1_orf1;TRINITY_DN2271_c0_g1_i12_orf1;TRINITY_DN55148_c0_g1_i1_orf1;TRINITY_DN501_c0_g1_i5_orf1;TRINITY_DN42854_c0_g3_i2_orf1;TRINITY_DN9732_c0_g1_i7_orf1;TRINITY_DN16234_c0_g2_i3_orf1;TRINITY_DN18196_c0_g1_i4_orf1;TRINITY_DN7776_c0_g1_i1_orf1;TRINITY_DN1328_c0_g1_i6_orf1;TRINITY_DN2848_c0_g1_i1_orf1;TRINITY_DN10994_c0_g1_i4_orf1;TRINITY_DN8258_c0_g1_i6_orf1;TRINITY_DN77425_c0_g1_i2_orf1;TRINITY_DN45948_c1_g1_i1_orf1;TRINITY_DN2097_c1_g2_i2_orf1;TRINITY_DN71308_c0_g1_i4_orf1;TRINITY_DN2323_c0_g1_i4_orf1;TRINITY_DN1444_c1_g1_i5_orf1;TRINITY_DN1215_c0_g1_i2_orf1;TRINITY_DN7539_c0_g1_i2_orf1;TRINITY_DN10057_c0_g2_i1_orf1;TRINITY_DN1540_c0_g1_i9_orf1;TRINITY_DN135188_c0_g1_i2_orf1;TRINITY_DN9455_c0_g1_i6_orf1;TRINITY_DN1540_c0_g1_i14_orf1;TRINITY_DN712_c0_g2_i1_orf1
molecular_function	signaling receptor activator activity	GO:0030546	5	5/2406	TRINITY_DN22443_c0_g2_i3_orf1;TRINITY_DN2836_c0_g1_i4_orf1;TRINITY_DN18650_c0_g1_i1_orf1;TRINITY_DN18218_c0_g1_i7_orf1;TRINITY_DN2227_c0_g1_i5_orf1
molecular_function	nucleic acid binding	GO:0003676	174	174/2406	TRINITY_DN129226_c0_g1_i2_orf1;TRINITY_DN6380_c0_g1_i1_orf1;TRINITY_DN4380_c0_g1_i9_orf1;TRINITY_DN3301_c0_g1_i2_orf1;TRINITY_DN40434_c0_g1_i2_orf1;TRINITY_DN12227_c0_g2_i3_orf1;TRINITY_DN13350_c0_g1_i4_orf1;TRINITY_DN38412_c0_g1_i1_orf1;TRINITY_DN4262_c0_g1_i16_orf1;TRINITY_DN35669_c0_g1_i1_orf1;TRINITY_DN19651_c0_g1_i1_orf1;TRINITY_DN18804_c0_g1_i5_orf1;TRINITY_DN51934_c0_g2_i1_orf1;TRINITY_DN16965_c0_g2_i1_orf1;TRINITY_DN15040_c0_g4_i1_orf1;TRINITY_DN34134_c0_g2_i1_orf1;TRINITY_DN1344_c0_g1_i1_orf1;TRINITY_DN35245_c0_g1_i1_orf1;TRINITY_DN70485_c0_g1_i2_orf1;TRINITY_DN142442_c0_g1_i1_orf1;TRINITY_DN3457_c0_g1_i4_orf1;TRINITY_DN2304_c0_g1_i4_orf1;TRINITY_DN18681_c0_g1_i7_orf1;TRINITY_DN36817_c0_g1_i1_orf1;TRINITY_DN21570_c0_g1_i1_orf1;TRINITY_DN10070_c0_g1_i1_orf1;TRINITY_DN9164_c0_g1_i3_orf1;TRINITY_DN99673_c0_g1_i1_orf1;TRINITY_DN9575_c0_g1_i1_orf1;TRINITY_DN5296_c0_g2_i1_orf1;TRINITY_DN2676_c0_g1_i2_orf1;TRINITY_DN1116_c0_g1_i6_orf1;TRINITY_DN19659_c1_g1_i1_orf1;TRINITY_DN41664_c0_g1_i4_orf1;TRINITY_DN5442_c0_g1_i4_orf1;TRINITY_DN2401_c0_g2_i1_orf1;TRINITY_DN227_c0_g1_i1_orf1;TRINITY_DN33893_c0_g1_i1_orf1;TRINITY_DN251_c0_g1_i2_orf1;TRINITY_DN2802_c0_g1_i1_orf1;TRINITY_DN2802_c1_g1_i1_orf1;TRINITY_DN33346_c0_g1_i1_orf1;TRINITY_DN5210_c0_g1_i3_orf1;TRINITY_DN2265_c0_g1_i5_orf1;TRINITY_DN1245_c0_g1_i4_orf1;TRINITY_DN18563_c2_g1_i1_orf1;TRINITY_DN10558_c0_g1_i4_orf1;TRINITY_DN89613_c0_g1_i13_orf1;TRINITY_DN14313_c0_g1_i1_orf1;TRINITY_DN17271_c0_g1_i1_orf1;TRINITY_DN23360_c0_g1_i3_orf1;TRINITY_DN31503_c0_g1_i4_orf1;TRINITY_DN47723_c0_g1_i1_orf1;TRINITY_DN1074_c0_g1_i7_orf1;TRINITY_DN18242_c0_g1_i3_orf1;TRINITY_DN37532_c0_g1_i1_orf1;TRINITY_DN9637_c0_g1_i14_orf1;TRINITY_DN29743_c0_g1_i9_orf1;TRINITY_DN1870_c0_g1_i6_orf1;TRINITY_DN81258_c0_g1_i2_orf1;TRINITY_DN298_c0_g1_i4_orf1;TRINITY_DN51968_c0_g1_i1_orf1;TRINITY_DN7583_c0_g1_i1_orf1;TRINITY_DN3673_c0_g1_i10_orf1;TRINITY_DN1607_c0_g1_i16_orf1;TRINITY_DN7464_c0_g1_i14_orf1;TRINITY_DN2769_c0_g1_i1_orf1;TRINITY_DN48641_c0_g1_i4_orf1;TRINITY_DN26168_c0_g1_i1_orf1;TRINITY_DN50085_c0_g1_i1_orf1;TRINITY_DN124950_c0_g2_i1_orf1;TRINITY_DN14274_c0_g1_i3_orf1;TRINITY_DN4408_c6_g1_i1_orf1;TRINITY_DN11823_c1_g1_i2_orf1;TRINITY_DN4707_c0_g1_i1_orf1;TRINITY_DN27033_c1_g1_i3_orfp1;TRINITY_DN23502_c0_g1_i1_orf1;TRINITY_DN73224_c0_g4_i2_orf1;TRINITY_DN37055_c0_g1_i1_orf1;TRINITY_DN56993_c0_g1_i4_orf1;TRINITY_DN6358_c0_g1_i5_orf1;TRINITY_DN74037_c0_g5_i1_orf1;TRINITY_DN4950_c0_g1_i2_orf1;TRINITY_DN107035_c0_g1_i1_orf1;TRINITY_DN147458_c0_g1_i1_orf1;TRINITY_DN4908_c1_g1_i5_orf1;TRINITY_DN2299_c0_g1_i3_orf1;TRINITY_DN2709_c0_g1_i4_orf1;TRINITY_DN291_c0_g1_i2_orf1;TRINITY_DN2749_c4_g1_i2_orf1;TRINITY_DN6239_c0_g1_i1_orf1;TRINITY_DN257_c0_g1_i7_orf1;TRINITY_DN8008_c0_g1_i6_orf1;TRINITY_DN2749_c0_g1_i4_orf1;TRINITY_DN26251_c0_g1_i1_orf1;TRINITY_DN2117_c0_g1_i1_orf1;TRINITY_DN46409_c0_g1_i1_orf1;TRINITY_DN17312_c0_g1_i1_orf1;TRINITY_DN8980_c0_g1_i2_orf1;TRINITY_DN2718_c0_g1_i6_orf1;TRINITY_DN12242_c0_g1_i5_orf1;TRINITY_DN136906_c0_g1_i1_orf1;TRINITY_DN43076_c0_g1_i6_orf1;TRINITY_DN5757_c0_g1_i1_orf1;TRINITY_DN36494_c0_g1_i1_orf1;TRINITY_DN3335_c0_g1_i1_orf1;TRINITY_DN4016_c0_g1_i1_orf1;TRINITY_DN43412_c0_g1_i2_orf1;TRINITY_DN12858_c0_g1_i5_orf1;TRINITY_DN2879_c0_g1_i4_orf1;TRINITY_DN2710_c0_g1_i4_orf1;TRINITY_DN1066_c0_g1_i8_orf1;TRINITY_DN2749_c0_g2_i3_orf1;TRINITY_DN37599_c0_g1_i1_orf1;TRINITY_DN7251_c0_g1_i3_orf1;TRINITY_DN24322_c0_g1_i4_orf1;TRINITY_DN11746_c0_g2_i1_orf1;TRINITY_DN11986_c0_g1_i1_orf1;TRINITY_DN53311_c0_g2_i1_orf1;TRINITY_DN15420_c0_g3_i2_orf1;TRINITY_DN44288_c0_g1_i2_orf1;TRINITY_DN20339_c0_g1_i3_orf1;TRINITY_DN19810_c1_g1_i7_orf1;TRINITY_DN35582_c0_g1_i1_orf1;TRINITY_DN4820_c0_g2_i2_orf1;TRINITY_DN5962_c0_g1_i1_orf1;TRINITY_DN34509_c0_g1_i1_orf1;TRINITY_DN2647_c0_g1_i3_orf1;TRINITY_DN3459_c0_g1_i4_orf1;TRINITY_DN6248_c0_g1_i1_orf1;TRINITY_DN42854_c0_g3_i2_orf1;TRINITY_DN4955_c0_g1_i2_orf1;TRINITY_DN116467_c0_g1_i1_orf1;TRINITY_DN879_c0_g1_i2_orf1;TRINITY_DN139537_c0_g1_i1_orf1;TRINITY_DN4246_c0_g2_i3_orf1;TRINITY_DN8224_c0_g1_i7_orf1;TRINITY_DN67649_c0_g1_i1_orf1;TRINITY_DN2283_c0_g2_i1_orf1;TRINITY_DN29579_c0_g1_i1_orf1;TRINITY_DN8716_c0_g1_i3_orf1;TRINITY_DN42205_c0_g1_i4_orf1;TRINITY_DN288_c0_g1_i9_orf1;TRINITY_DN9938_c0_g2_i1_orf1;TRINITY_DN59291_c0_g1_i1_orf1;TRINITY_DN9302_c0_g1_i1_orf1;TRINITY_DN48097_c0_g1_i1_orf1;TRINITY_DN96801_c0_g1_i1_orf1;TRINITY_DN55148_c0_g1_i1_orf1;TRINITY_DN38540_c0_g1_i1_orf1;TRINITY_DN2[illegible]

molecular_function	nucleoside phosphate binding	GO:1901265	193	193/2406	TRINITY_DN280_c0_g1_i8_orf1;TRINITY_DN13563_c0_g1_i1_orf1;TRINITY_DN38230_c0_g1_i4_orf1;TRINITY_DN47731_c0_g1_i2_orf1;TRINITY_DN4380_c0_g1_i9_orf1;TRINITY_DN41311_c0_g2_i3_orf1;TRINITY_DN11194_c0_g1_i4_orf1;TRINITY_DN1921_c1_g1_i5_orf1;TRINITY_DN30932_c0_g1_i2_orf1;TRINITY_DN4744_c0_g1_i7_orf1;TRINITY_DN25341_c0_g1_i1_orf1;TRINITY_DN2738_c1_g1_i3_orf1;TRINITY_DN33705_c0_g1_i1_orf1;TRINITY_DN8659_c0_g1_i1_orf1;TRINITY_DN70485_c0_g1_i2_orf1;TRINITY_DN8603_c0_g1_i1_orf1;TRINITY_DN24723_c2_g1_i1_orf1;TRINITY_DN2953_c1_g1_i10_orf1;TRINITY_DN143509_c0_g1_i1_orf1;TRINITY_DN2638_c0_g1_i7_orf1;TRINITY_DN2848_c0_g1_i1_orf1;TRINITY_DN4320_c0_g1_i1_orf1;TRINITY_DN73945_c0_g5_i3_orf1;TRINITY_DN4711_c0_g1_i2_orf1;TRINITY_DN244_c1_g1_i5_orf1;TRINITY_DN10774_c0_g2_i3_orf1;TRINITY_DN42753_c0_g1_i2_orf1;TRINITY_DN2953_c1_g1_i2_orf1;TRINITY_DN14298_c0_g1_i1_orf1;TRINITY_DN3991_c0_g1_i6_orf1;TRINITY_DN3800_c0_g1_i7_orf1;TRINITY_DN5354_c0_g1_i4_orf1;TRINITY_DN235_c0_g3_i1_orf1;TRINITY_DN6185_c0_g1_i12_orf1;TRINITY_DN20527_c0_g1_i1_orf1;TRINITY_DN6436_c0_g1_i1_orf1;TRINITY_DN4798_c0_g1_i3_orf1;TRINITY_DN2224_c0_g1_i1_orf1;TRINITY_DN662_c0_g1_i1_orf1;TRINITY_DN987_c0_g1_i3_orf1;TRINITY_DN3131_c0_g1_i5_orf1;TRINITY_DN7247_c0_g1_i7_orf1;TRINITY_DN2265_c0_g1_i5_orf1;TRINITY_DN31225_c0_g1_i1_orf1;TRINITY_DN46715_c0_g1_i1_orf1;TRINITY_DN11620_c0_g1_i2_orf1;TRINITY_DN20007_c0_g1_i1_orf1;TRINITY_DN4950_c0_g1_i2_orf1;TRINITY_DN1173_c0_g1_i12_orf1;TRINITY_DN34751_c0_g1_i1_orf1;TRINITY_DN4501_c0_g1_i3_orf1;TRINITY_DN2772_c0_g1_i3_orf1;TRINITY_DN31503_c0_g1_i4_orf1;TRINITY_DN64126_c0_g1_i1_orf1;TRINITY_DN18031_c0_g1_i1_orf1;TRINITY_DN252_c0_g1_i3_orf1;TRINITY_DN2719_c1_g1_i6_orf1;TRINITY_DN33801_c0_g1_i1_orf1;TRINITY_DN8659_c0_g2_i1_orf1;TRINITY_DN17935_c0_g1_i1_orf1;TRINITY_DN4451_c0_g2_i4_orf1;TRINITY_DN1604_c0_g1_i4_orf1;TRINITY_DN53238_c1_g1_i5_orf1;TRINITY_DN90497_c0_g1_i1_orf1;TRINITY_DN42461_c0_g1_i4_orf1;TRINITY_DN9575_c0_g1_i1_orf1;TRINITY_DN6813_c1_g1_i1_orf1;TRINITY_DN1607_c0_g1_i16_orf1;TRINITY_DN277_c1_g1_i1_orf1;TRINITY_DN268_c3_g1_i2_orf1;TRINITY_DN31967_c0_g1_i5_orf1;TRINITY_DN28622_c0_g1_i1_orf1;TRINITY_DN12_c0_g1_i5_orf1;TRINITY_DN7336_c0_g1_i13_orf1;TRINITY_DN5070_c0_g1_i1_orf1;TRINITY_DN32700_c0_g1_i2_orf1;TRINITY_DN117844_c0_g1_i1_orf1;TRINITY_DN122423_c0_g1_i1_orf1;TRINITY_DN38424_c0_g1_i1_orf1;TRINITY_DN6642_c0_g1_i2_orf1;TRINITY_DN14274_c0_g1_i3_orf1;TRINITY_DN11015_c0_g1_i8_orf1;TRINITY_DN5281_c0_g2_i3_orf1;TRINITY_DN511_c0_g2_i1_orf1;TRINITY_DN1718_c6_g1_i4_orf1;TRINITY_DN1173_c1_g1_i10_orf1;TRINITY_DN119893_c0_g2_i3_orf1;TRINITY_DN3428_c0_g1_i1_orf1;TRINITY_DN52761_c0_g1_i2_orf1;TRINITY_DN15420_c0_g3_i2_orf1;TRINITY_DN4908_c1_g1_i5_orf1;TRINITY_DN28989_c0_g1_i7_orf1;TRINITY_DN235_c0_g1_i2_orf1;TRINITY_DN2709_c0_g1_i4_orf1;TRINITY_DN291_c0_g1_i2_orf1;TRINITY_DN1209_c0_g1_i9_orf1;TRINITY_DN12064_c0_g2_i1_orf1;TRINITY_DN16011_c0_g1_i3_orf1;TRINITY_DN7131_c0_g1_i2_orf1;TRINITY_DN46409_c0_g1_i1_orf1;TRINITY_DN3637_c0_g1_i2_orf1;TRINITY_DN2983_c0_g1_i6_orf1;TRINITY_DN1552_c0_g1_i3_orf1;TRINITY_DN8980_c0_g1_i2_orf1;TRINITY_DN47151_c0_g1_i1_orf1;TRINITY_DN143603_c0_g1_i1_orf1;TRINITY_DN141381_c0_g1_i1_orf1;TRINITY_DN72017_c0_g1_i1_orf1;TRINITY_DN136906_c0_g1_i1_orf1;TRINITY_DN2953_c1_g1_i11_orf1;TRINITY_DN5757_c0_g1_i1_orf1;TRINITY_DN49038_c0_g4_i1_orf1;TRINITY_DN38506_c0_g1_i4_orf1;TRINITY_DN36788_c0_g1_i2_orf1;TRINITY_DN8390_c0_g1_i2_orf1;TRINITY_DN24_c0_g1_i1_orf1;TRINITY_DN34479_c0_g1_i2_orf1;TRINITY_DN16673_c0_g1_i1_orf1;TRINITY_DN2146_c0_g1_i1_orf1;TRINITY_DN32769_c1_g1_i5_orf1;TRINITY_DN26293_c0_g1_i4_orf1;TRINITY_DN82311_c0_g1_i1_orf1;TRINITY_DN4762_c0_g1_i2_orf1;TRINITY_DN4835_c0_g1_i2_orf1;TRINITY_DN3175_c0_g1_i7_orf1;TRINITY_DN11986_c0_g1_i1_orf1;TRINITY_DN36899_c0_g1_i1_orf1;TRINITY_DN162_c0_g1_i4_orf1;TRINITY_DN2793_c0_g2_i1_orf1;TRINITY_DN44288_c0_g1_i2_orf1;TRINITY_DN60792_c0_g1_i2_orf1;TRINITY_DN2193_c0_g1_i7_orf1;TRINITY_DN97138_c0_g1_i2_orf1;TRINITY_DN5099_c0_g1_i3_orf1;TRINITY_DN85476_c0_g1_i1_orf1;TRINITY_DN143637_c0_g1_i1_orf1;TRINITY_DN111110_c0_g1_i1_orf1;TRINITY_DN62_c1_g1_i3_orf1;TRINITY_DN52244_c1_g1_i1_orf1;TRINITY_DN14301_c0_g1_i1_orf1;TRINITY_DN7512_c0_g1_i1_orf1;TRINITY_DN49047_c0_g1_i2_orf1;TRINITY_DN26168_c0_g1_i1_orf1;TRINITY_DN2146_c0_g2_i1_orf1;TRINITY_DN52761_c0_g2_i1_orf1;TRINITY_DN1084_c0_g2_i2_orf1;TRINITY_DN62557_c0_g1_i1_orf1;TRINITY_DN57918_c0_g1_i1_orf1;TRINITY_DN8716_c0_g1_i3_orf1;TRINITY_DN76815_c0_g1_i3_orf1;TRINITY_DN41166_c0_g1_i1_orf1;TRIN
molecular_function	nucleoside binding	GO:0001882	1	1/2406	TRINITY_DN2401_c0_g2_i1_orf1
molecular_function	tetrapyrrole binding	GO:0046906	41	41/2406	TRINITY_DN120500_c0_g1_i1_orf1;TRINITY_DN9608_c0_g1_i3_orf1;TRINITY_DN3949_c1_g1_i1_orf1;TRINITY_DN30704_c0_g1_i1_orf1;TRINITY_DN23564_c0_g1_i7_orf1;TRINITY_DN4497_c2_g1_i3_orf1;TRINITY_DN4497_c0_g1_i4_orf1;TRINITY_DN7580_c0_g1_i1_orf1;TRINITY_DN2392_c0_g2_i1_orf1;TRINITY_DN24873_c0_g1_i4_orf1;TRINITY_DN5933_c0_g1_i1_orf1;TRINITY_DN625_c9_g1_i7_orf1;TRINITY_DN9647_c0_g1_i1_orf1;TRINITY_DN1664_c0_g1_i4_orf1;TRINITY_DN3949_c0_g1_i1_orf1;TRINITY_DN1134_c0_g1_i4_orf1;TRINITY_DN3984_c0_g1_i4_orf1;TRINITY_DN3732_c0_g1_i2_orf1;TRINITY_DN829_c0_g1_i8_orf1;TRINITY_DN3732_c1_g1_i5_orf1;TRINITY_DN1363_c0_g1_i11_orf1;TRINITY_DN50743_c0_g1_i1_orf1;TRINITY_DN3675_c0_g1_i1_orf1;TRINITY_DN15755_c0_g1_i1_orf1;TRINITY_DN448_c0_g1_i20_orf1;TRINITY_DN6351_c0_g1_i4_orf1;TRINITY_DN27045_c0_g1_i1_orf1;TRINITY_DN1960_c5_g1_i3_orf1;TRINITY_DN57856_c0_g2_i1_orf1;TRINITY_DN5439_c0_g1_i2_orf1;TRINITY_DN285_c0_g1_i4_orf1;TRINITY_DN2652_c0_g2_i1_orf1;TRINITY_DN30037_c0_g1_i5_orf1;TRINITY_DN12997_c0_g2_i1_orf1;TRINITY_DN7861_c0_g1_i5_orf1;TRINITY_DN5126_c0_g1_i3_orf1;TRINITY_DN5126_c0_g2_i1_orf1;TRINITY_DN6580_c0_g1_i4_orf1;TRINITY_DN5661_c0_g1_i5_orf1;TRINITY_DN2442_c0_g1_i6_orf1;TRINITY_DN2676_c0_g1_
molecular_function	vitamin B6 binding	GO:0070279	12	12/2406	TRINITY_DN2065_c1_g2_i1_orf1;TRINITY_DN2890_c0_g1_i2_orf1;TRINITY_DN2688_c0_g2_i1_orf1;TRINITY_DN11948_c0_g1_i8_orf1;TRINITY_DN1262_c0_g1_i2_orf1;TRINITY_DN3263_c0_g1_i2_orf1;TRINITY_DN14935_c0_g1_i1_orf1;TRINITY_DN51813_c0_g1_i1_orf1;TRINITY_DN21035_c0_g1_i14_orf1;TRINITY_DN2803_c4_g1_i1_orf1;TRINITY_DN2684_c0_g2_i3_orf1;TRINITY_DN1068_c0_g1_i3_orf1
molecular_function	laminin binding	GO:0043236	1	1/2406	TRINITY_DN10070_c0_g1_i1_orf1
molecular_function	histone binding	GO:0042393	1	1/2406	TRINITY_DN12771_c0_g1_i1_orf1
molecular_function	identical protein binding	GO:0042802	7	7/2406	TRINITY_DN96557_c0_g1_i1_orf1;TRINITY_DN51938_c0_g3_i1_orf1;TRINITY_DN147475_c0_g1_i1_orf1;TRINITY_DN42854_c0_g3_i2_orf1;TRINITY_DN2848_c0_g1_i1_orf1;TRINITY_DN20133_c0_g1_i1_orf1;TRINITY_DN31584_c0_g2_i2_orf1
molecular_function	p53 binding	GO:0002039	1	1/2406	TRINITY_DN46409_c0_g1_i1_orf1
molecular_function	apolipoprotein binding	GO:0034185	1	1/2406	TRINITY_DN46409_c0_g1_i1_orf1
molecular_function	enzyme binding	GO:0019899	12	12/2406	TRINITY_DN8473_c0_g1_i6_orf1;TRINITY_DN41736_c0_g2_i1_orf1;TRINITY_DN975_c0_g1_i1_orf1;TRINITY_DN55148_c0_g1_i1_orf1;TRINITY_DN46409_c0_g1_i1_orf1;TRINITY_DN2120_c0_g1_i2_orf1;TRINITY_DN2848_c0_g1_i1_orf1;TRINITY_DN31584_c0_g2_i2_orf1;TRINITY_DN20133_c0_g1_i1_orf1;TRINITY_DN4439_c0_g1_i2_orf1;TRINITY_DN95971_c0_g5_i1_orf1;TRINITY_DN3887_c0_g1_i1_orf1
molecular_function	SNARE binding	GO:0000149	1	1/2406	TRINITY_DN38301_c0_g1_i2_orf1
molecular_function	chaperone binding	GO:0051087	1	1/2406	TRINITY_DN46409_c0_g1_i1_orf1
molecular_function	unfolded protein binding	GO:0051082	3	3/2406	TRINITY_DN46409_c0_g1_i1_orf1;TRINITY_DN25341_c0_g1_i1_orf1;TRINITY_DN33801_c0_g1_i1_orf1
molecular_function	calmodulin binding	GO:0005516	4	4/2406	TRINITY_DN140613_c0_g1_i1_orf1;TRINITY_DN4010_c0_g2_i1_orf1;TRINITY_DN6642_c0_g1_i2_orf1;TRINITY_DN975_c0_g1_i1_orf1
molecular_function	heat shock protein binding	GO:0031072	2	2/2406	TRINITY_DN79083_c0_g1_i2_orf1;TRINITY_DN10694_c1_g2_i1_orf1
molecular_function	transcription factor binding	GO:0008134	2	2/2406	TRINITY_DN6071_c0_g1_i1_orf1;TRINITY_DN147475_c0_g1_i1_orf1
molecular_function	dynein intermediate chain binding	GO:0045505	2	2/2406	TRINITY_DN17995_c0_g4_i1_orf1;TRINITY_DN122423_c0_g1_i1_orf1
molecular_function	translation initiation factor binding	GO:0031369	2	2/2406	TRINITY_DN48097_c0_g1_i1_orf1;TRINITY_DN50085_c0_g1_i1_orf1
molecular_function	signaling receptor binding	GO:0005102	8	8/2406	TRINITY_DN51938_c0_g3_i1_orf1;TRINITY_DN18650_c0_g1_i1_orf1;TRINITY_DN18218_c0_g1_i7_orf1;TRINITY_DN147475_c0_g1_i1_orf1;TRINITY_DN2836_c0_g1_i4_orf1;TRINITY_DN33418_c0_g1_i1_orf1;TRINITY_DN22443_c0_g2_i3_orf1;TRINITY_DN2227_c0_g1_i5_orf1
molecular_function	dynein light intermediate chain binding	GO:0051959	1	1/2406	TRINITY_DN122423_c0_g1_i1_orf1
molecular_function	cytoskeletal protein binding	GO:0008092	50	50/2406	TRINITY_DN2942_c0_g1_i6_orf1;TRINITY_DN11464_c0_g1_i3_orf1;TRINITY_DN714_c0_g1_i3_orf1;TRINITY_DN350_c0_g1_i4_orf1;TRINITY_DN104297_c0_g1_i1_orf1;TRINITY_DN52649_c0_g1_i6_orf1;TRINITY_DN841_c0_g1_i4_orf1;TRINITY_DN350_c0_g1_i5_orf1;TRINITY_DN4159_c1_g1_i1_orf1;TRINITY_DN23790_c0_g1_i1_orf1;TRINITY_DN129869_c0_g4_i1_orf1;TRINITY_DN107962_c0_g1_i1_orf1;TRINITY_DN4010_c0_g2_i1_orf1;TRINITY_DN8406_c0_g1_i4_orf1;TRINITY_DN5954_c0_g1_i2_orf1;TRINITY_DN30150_c0_g1_i7_orf1;TRINITY_DN28018_c0_g6_i1_orf1;TRINITY_DN9383_c0_g1_i3_orf1;TRINITY_DN741_c0_g1_i10_orf1;TRINITY_DN59852_c0_g1_i1_orf1;TRINITY_DN2848_c0_g1_i1_orf1;TRINITY_DN28622_c0_g1_i1_orf1;TRINITY_DN364_c1_g1_i2_orf1;TRINITY_DN26961_c0_g1_i1_orf1;TRINITY_DN3887_c0_g1_i1_orf1;TRINITY_DN14298_c0_g1_i1_orf1;TRINITY_DN86309_c0_g1_i4_orf1;TRINITY_DN41736_c0_g2_i1_orf1;TRINITY_DN16673_c0_g1_i1_orf1;TRINITY_DN14298_c0_g3_i1_orf1;TRINITY_DN69557_c0_g1_i1_orf1;TRINITY_DN8915_c0_g1_i3_orf1;TRINITY_DN21451_c0_g1_i3_orf1;TRINITY_DN286_c0_g1_i2_orf1;TRINITY_DN23746_c0_g1_i2_orf1;TRINITY_DN110231_c0_g1_i1_orf1;TRINITY_DN662_c0_g1_i1_orf1;TRINITY_DN104663_c1_g1_i2_orf1;TRINITY_DN350_c0_g1_i10_orf1;TRINITY_DN364_c2_g1_i2_orf1;TRINITY_DN9146_c0_g1_i1_orf1;TRINITY_DN1718_c1_g1_i5_orf1;TRINITY_DN14684_c0_g2_i1_orf1;TRINITY_DN10455_c0_g1_i2_orf1;TRINITY_DN20133_c0_g1_i1_orf1;TRINITY_DN17137_c0_g1_i2_orf1;TRINITY_DN97097_c0_g1_i4_orf1;TRINITY_DN101991_c0_g1_i5_orf1;TRINITY_DN34703_c0_g1_i4_orf1;TRINITY_DN77480_c0_g1_i2_orf1
molecular_function	protein dimerization activity	GO:0046983	7	7/2406	TRINITY_DN96557_c0_g1_i1_orf1;TRINITY_DN14301_c0_g1_i1_orf1;TRINITY_DN3457_c0_g1_i4_orf1;TRINITY_DN50471_c0_g1_i4_orf1;TRINITY_DN31584_c0_g2_i2_orf1;TRINITY_DN96801_c0_g1_i1_orf1;TRINITY_DN14611_c0_g1_i5_orf1
molecular_function	phosphoprotein binding	GO:0051219	1	1/2406	TRINITY_DN31584_c0_g2_i2_orf1
molecular_function	protein domain specific binding	GO:0019904	3	3/2406	TRINITY_DN31584_c0_g2_i2_orf1;TRINITY_DN2848_c0_g1_i1_orf1;TRINITY_DN147475_c0_g1_i1_orf1
molecular_function	clathrin binding	GO:0030276	3	3/2406	TRINITY_DN13118_c0_g1_i6_orf1;TRINITY_DN5118_c0_g1_i1_orf1;TRINITY_DN741_c0_g1_i10_orf1
molecular_function	ubiquitin-like protein binding	GO:0032182	1	1/2406	TRINITY_DN45633_c0_g1_i1_orf1
molecular_function	cell adhesion molecule binding	GO:0050839	2	2/2406	TRINITY_DN10070_c0_g1_i1_orf1;TRINITY_DN20133_c0_g1_i1_orf1
molecular_function	S100 protein binding	GO:0044548	1	1/2406	TRINITY_DN41736_c0_g2_i1_orf1

molecular_function	transmembrane transporter binding	GO:0044325	1	1/2406	TRINITY_DN31584_c0_g2_i2_orf1
molecular_function	scaffold protein binding	GO:0097110	1	1/2406	TRINITY_DN31584_c0_g2_i2_orf1
molecular_function	chitin binding	GO:0008061	18	18/2406	TRINITY_DN77642_c0_g1_i1_orf1;TRINITY_DN26301_c0_g1_i1_orf1;TRINITY_DN21555_c0_g1_i4_orf1;TRINITY_DN9000_c0_g2_i1_orf1;TRINITY_DN17003_c0_g1_i1_orf1;TRINITY_DN3913_c0_g1_i6_orf1;TRINITY_DN10824_c0_g1_i3_orf1;TRINITY_DN619_c0_g1_i1_orf1;TRINITY_DN17003_c1_g1_i1_orf1;TRINITY_DN82801_c0_g1_i1_orf1;TRINITY_DN2958_c0_g1_i2_orf1;TRINITY_DN73923_c0_g1_i1_orf1;TRINITY_DN72999_c0_g1_i1_orf1;TRINITY_DN36061_c0_g4_i2_orf1;TRINITY_DN650_c0_g1_i3_orf1;TRINITY_DN54366_c0_g1_i1_orf1;TRINITY_DN6418_c0_g1_i28_orf1;TRINITY_DN3759_c0_g1_i1_orf1
molecular_function	lipopolysaccharide binding	GO:0001530	2	2/2406	TRINITY_DN46409_c0_g1_i1_orf1;TRINITY_DN2170_c0_g1_i2_orf1
molecular_function	lipoteichoic acid binding	GO:0070891	1	1/2406	TRINITY_DN2170_c0_g1_i2_orf1
molecular_function	ribonucleotide binding	GO:0032553	157	157/2406	TRINITY_DN280_c0_g1_i8_orf1;TRINITY_DN13563_c0_g1_i1_orf1;TRINITY_DN47731_c0_g1_i2_orf1;TRINITY_DN4380_c0_g1_i9_orf1;TRINITY_DN41311_c0_g2_i3_orf1;TRINITY_DN11194_c0_g1_i4_orf1;TRINITY_DN33801_c0_g1_i1_orf1;TRINITY_DN31967_c0_g1_i5_orf1;TRINITY_DN25341_c0_g1_i1_orf1;TRINITY_DN2738_c1_g1_i3_orf1;TRINITY_DN33705_c0_g1_i1_orf1;TRINITY_DN8659_c0_g1_i1_orf1;TRINITY_DN70485_c0_g1_i2_orf1;TRINITY_DN8603_c0_g1_i1_orf1;TRINITY_DN24723_c2_g1_i1_orf1;TRINITY_DN2953_c1_g1_i10_orf1;TRINITY_DN143509_c0_g1_i1_orf1;TRINITY_DN2638_c0_g1_i7_orf1;TRINITY_DN4320_c0_g1_i1_orf1;TRINITY_DN73945_c0_g5_i3_orf1;TRINITY_DN10774_c0_g2_i3_orf1;TRINITY_DN42753_c0_g1_i2_orf1;TRINITY_DN2953_c1_g1_i2_orf1;TRINITY_DN14298_c0_g1_i1_orf1;TRINITY_DN3991_c0_g1_i6_orf1;TRINITY_DN3800_c0_g1_i7_orf1;TRINITY_DN9575_c0_g1_i1_orf1;TRINITY_DN10745_c0_g1_i14_orf1;TRINITY_DN6185_c0_g1_i12_orf1;TRINITY_DN52761_c0_g1_i2_orf1;TRINITY_DN6436_c0_g1_i1_orf1;TRINITY_DN4798_c0_g1_i3_orf1;TRINITY_DN2224_c0_g1_i1_orf1;TRINITY_DN662_c0_g1_i1_orf1;TRINITY_DN987_c0_g1_i3_orf1;TRINITY_DN2265_c0_g1_i5_orf1;TRINITY_DN31225_c0_g1_i1_orf1;TRINITY_DN46715_c0_g1_i1_orf1;TRINITY_DN11620_c0_g1_i2_orf1;TRINITY_DN20007_c0_g1_i1_orf1;TRINITY_DN4950_c0_g1_i2_orf1;TRINITY_DN52761_c0_g2_i1_orf1;TRINITY_DN4501_c0_g1_i3_orf1;TRINITY_DN2772_c0_g1_i3_orf1;TRINITY_DN31503_c0_g1_i4_orf1;TRINITY_DN2793_c0_g2_i1_orf1;TRINITY_DN18031_c0_g1_i1_orf1;TRINITY_DN252_c0_g1_i3_orf1;TRINITY_DN2719_c1_g1_i6_orf1;TRINITY_DN8659_c0_g2_i1_orf1;TRINITY_DN17935_c0_g1_i1_orf1;TRINITY_DN1604_c0_g1_i4_orf1;TRINITY_DN1034_c0_g1_i4_orf1;TRINITY_DN90497_c0_g1_i1_orf1;TRINITY_DN42461_c0_g1_i4_orf1;TRINITY_DN6813_c1_g1_i1_orf1;TRINITY_DN1607_c0_g1_i16_orf1;TRINITY_DN277_c1_g1_i1_orf1;TRINITY_DN244_c1_g1_i5_orf1;TRINITY_DN28622_c0_g1_i1_orf1;TRINITY_DN12_c0_g1_i5_orf1;TRINITY_DN7336_c0_g1_i13_orf1;TRINITY_DN5070_c0_g1_i1_orf1;TRINITY_DN32700_c0_g1_i2_orf1;TRINITY_DN117844_c0_g1_i1_orf1;TRINITY_DN122423_c0_g1_i1_orf1;TRINITY_DN6642_c0_g1_i2_orf1;TRINITY_DN14274_c0_g1_i3_orf1;TRINITY_DN11015_c0_g1_i8_orf1;TRINITY_DN5281_c0_g2_i3_orf1;TRINITY_DN511_c0_g2_i1_orf1;TRINITY_DN1718_c6_g1_i4_orf1;TRINITY_DN1173_c1_g1_i10_orf1;TRINITY_DN119893_c0_g2_i3_orf1;TRINITY_DN3428_c0_g1_i1_orf1;TRINITY_DN4908_c1_g1_i5_orf1;TRINITY_DN28989_c0_g1_i7_orf1;TRINITY_DN235_c0_g1_i2_orf1;TRINITY_DN2709_c0_g1_i4_orf1;TRINITY_DN291_c0_g1_i2_orf1;TRINITY_DN12064_c0_g2_i1_orf1;TRINITY_DN16011_c0_g1_i3_orf1;TRINITY_DN46409_c0_g1_i1_orf1;TRINITY_DN3637_c0_g1_i2_orf1;TRINITY_DN2983_c0_g1_i6_orf1;TRINITY_DN1552_c0_g1_i3_orf1;TRINITY_DN8980_c0_g1_i2_orf1;TRINITY_DN47151_c0_g1_i1_orf1;TRINITY_DN136906_c0_g1_i1_orf1;TRINITY_DN2953_c1_g1_i11_orf1;TRINITY_DN38506_c0_g1_i4_orf1;TRINITY_DN8390_c0_g1_i2_orf1;TRINITY_DN34479_c0_g1_i2_orf1;TRINITY_DN16673_c0_g1_i1_orf1;TRINITY_DN2146_c0_g1_i1_orf1;TRINITY_DN32769_c1_g1_i5_orf1;TRINITY_DN82311_c0_g1_i1_orf1;TRINITY_DN4762_c0_g1_i2_orf1;TRINITY_DN4835_c0_g1_i2_orf1;TRINITY_DN11986_c0_g1_i1_orf1;TRINITY_DN15420_c0_g3_i2_orf1;TRINITY_DN162_c0_g1_i4_orf1;TRINITY_DN44288_c0_g1_i2_orf1;TRINITY_DN60792_c0_g1_i2_orf1;TRINITY_DN2193_c0_g1_i7_orf1;TRINITY_DN97138_c0_g1_i2_orf1;TRINITY_DN85476_c0_g1_i1_orf1;TRINITY_DN143637_c0_g1_i1_orf1;TRINITY_DN52244_c1_g1_i1_orf1;TRINITY_DN7512_c0_g1_i1_orf1;TRINITY_DN49047_c0_g1_i2_orf1;TRINITY_DN26168_c0_g1_i1_orf1;TRINITY_DN2146_c0_g2_i1_orf1;TRINITY_DN1084_c0_g2_i2_orf1;TRINITY_DN62557_c0_g1_i1_orf1;TRINITY_DN57918_c0_g1_i1_orf1;TRINITY_DN8716_c0_g1_i3_orf1;TRINITY_DN76815_c0_g1_i3_orf1;TRINITY_DN41166_c0_g1_i1_orf1;TRINITY_DN15706_c0_g2_i5_orf1;TRINITY_DN1405_c0_g1_i1_orf1;TRINITY_DN59291_c0_g1_i1_orf1;TRINITY_DN9302_c0_g1_i1_orf1;TRINITY_DN1173_c0_g1_i12_orf1;TRINITY_DN618_c0_g1_i3_orf1;TRINITY_DN46090_c0_g2_i1_orf1;TRINITY_DN10680_c0_g1_i5_orf1;TRINITY_DN5954_c0_g1_i2_orf1;TRINITY_DN1173_c0_g1_i11_orf1;TRINITY_DN975_c0_g1_i1_orf1;TRINITY_DN23946_c0_g1_i1_orf1;TRINITY_DN26961_c0_g1_i1_orf1;TRINITY_DN7247_c0_g1_i7_orf1;TRINITY_DN18782_c0_g1_i4_orf1;TRINITY_DN1475_c0_g1_i6_orf1;TRINITY_DN25345_c0_g1_i1_orf1;TRINITY_DN30154_c0_g1_i1_orf1;TRINITY_DN13055_c0_g1_i5_orf1;TRINITY_DN13094_c0_g1_i1_orf1;TRINITY_DN2570_c0_g1_i1_orf1;TRINITY_DN2745_c0_g1_i2_orf1;TRINITY_DN248_c0_g1_i1_orf1;TRINITY_DN5757_c0_g1_i1_orf1;TRINITY_DN129869_c0_g4_i1_orf1;TRINITY_DN48619_c0_g1_i1_orf1;TRINITY_DN41697_c0_g1_i1_orf1;TRINITY_DN21539_c0_g1_i1_orf1;TRINITY_DN146236_c0_g1_i1_orf1;TRINITY_DN30_c0_g1_i6_orf1;TRINITY_DN60821_c0_g1_i1_orf1;TRINITY_DN126648_c0_g1
molecular_function	glycosaminoglycan binding	GO:0005539	3	3/2406	TRINITY_DN42854_c0_g3_i2_orf1;TRINITY_DN5235_c0_g1_i7_orf1;TRINITY_DN1534_c0_g1_i3_orf1
molecular_function	heparin binding	GO:0008201	1	1/2406	TRINITY_DN42854_c0_g3_i2_orf1
molecular_function	acyl-CoA binding	GO:0120227	1	1/2406	TRINITY_DN13563_c0_g1_i1_orf1
molecular_function	peptide binding	GO:0042277	2	2/2406	TRINITY_DN245_c0_g1_i4_orf1;TRINITY_DN4016_c0_g1_i1_orf1
molecular_function	phosphopantetheine binding	GO:0031177	1	1/2406	TRINITY_DN10430_c0_g1_i4_orf1
molecular_function	cation binding	GO:0043169	245	245/2406	TRINITY_DN129226_c0_g1_i2_orf1;TRINITY_DN14920_c0_g1_i1_orf1;TRINITY_DN9608_c0_g1_i3_orf1;TRINITY_DN38230_c0_g1_i4_orf1;TRINITY_DN276_c0_g1_i1_orf1;TRINITY_DN3301_c0_g1_i2_orf1;TRINITY_DN2986_c1_g1_i1_orf1;TRINITY_DN7580_c0_g1_i1_orf1;TRINITY_DN863_c0_g1_i6_orf1;TRINITY_DN42333_c0_g1_i5_orf1;TRINITY_DN49204_c0_g1_i1_orf1;TRINITY_DN21035_c0_g1_i14_orf1;TRINITY_DN35763_c0_g1_i2_orf1;TRINITY_DN19651_c0_g1_i1_orf1;TRINITY_DN3275_c0_g2_i3_orf1;TRINITY_DN18804_c0_g1_i5_orf1;TRINITY_DN1231_c0_g1_i4_orf1;TRINITY_DN1153_c1_g1_i1_orf1;TRINITY_DN5628_c0_g1_i5_orf1;TRINITY_DN15040_c0_g4_i1_orf1;TRINITY_DN17772_c0_g2_i3_orf1;TRINITY_DN8771_c0_g1_i5_orf1;TRINITY_DN8621_c0_g1_i4_orf1;TRINITY_DN125427_c0_g1_i1_orf1;TRINITY_DN1134_c0_g1_i4_orf1;TRINITY_DN70485_c0_g1_i2_orf1;TRINITY_DN59852_c0_g1_i1_orf1;TRINITY_DN17031_c0_g1_i1_orf1;TRINITY_DN143509_c0_g1_i1_orf1;TRINITY_DN15812_c0_g1_i2_orf1;TRINITY_DN78492_c0_g1_i1_orf1;TRINITY_DN31609_c0_g1_i3_orf1;TRINITY_DN3675_c0_g1_i1_orf1;TRINITY_DN14774_c0_g1_i4_orf1;TRINITY_DN48020_c0_g1_i1_orf1;TRINITY_DN15755_c0_g1_i1_orf1;TRINITY_DN448_c0_g1_i20_orf1;TRINITY_DN1534_c0_g1_i3_orf1;TRINITY_DN3464_c0_g1_i1_orf1;TRINITY_DN3991_c0_g1_i6_orf1;TRINITY_DN2794_c1_g1_i8_orf1;TRINITY_DN3800_c0_g1_i7_orf1;TRINITY_DN5439_c0_g1_i2_orf1;TRINITY_DN69557_c0_g1_i1_orf1;TRINITY_DN123184_c0_g1_i1_orf1;TRINITY_DN16400_c0_g2_i1_orf1;TRINITY_DN21943_c1_g1_i1_orf1;TRINITY_DN2117_c0_g1_i1_orf1;TRINITY_DN2688_c0_g2_i1_orf1;TRINITY_DN5952_c0_g1_i6_orf1;TRINITY_DN227_c0_g1_i1_orf1;TRINITY_DN987_c0_g1_i3_orf1;TRINITY_DN14389_c0_g1_i4_orf1;TRINITY_DN1444_c1_g1_i5_orf1;TRINITY_DN4822_c0_g1_i9_orf1;TRINITY_DN2919_c0_g1_i5_orf1;TRINITY_DN7247_c0_g1_i7_orf1;TRINITY_DN78686_c0_g1_i1_orf1;TRINITY_DN4501_c0_g1_i3_orf1;TRINITY_DN1084_c0_g2_i2_orf1;TRINITY_DN1024_c0_g4_i1_orf1;TRINITY_DN3949_c1_g1_i1_orf1;TRINITY_DN2722_c0_g1_i1_orf1;TRINITY_DN120_c0_g1_i2_orf1;TRINITY_DN230_c2_g1_i5_orf1;TRINITY_DN65681_c0_g1_i1_orf1;TRINITY_DN120089_c0_g1_i1_orf1;TRINITY_DN30704_c0_g1_i1_orf1;TRINITY_DN45271_c0_g1_i1_orf1;TRINITY_DN47723_c0_g1_i1_orf1;TRINITY_DN27833_c0_g2_i1_orf1;TRINITY_DN14398_c0_g1_i4_orf1;TRINITY_DN4367_c0_g1_i1_orf1;TRINITY_DN252_c0_g1_i3_orf1;TRINITY_DN37532_c0_g1_i1_orf1;TRINITY_DN9637_c0_g1_i14_orf1;TRINITY_DN4189_c0_g2_i1_orf1;TRINITY_DN3010_c0_g1_i4_orf1;TRINITY_DN1664_c0_g1_i4_orf1;TRINITY_DN501_c0_g1_i5_orf1;TRINITY_DN42854_c0_g3_i2_orf1;TRINITY_DN4125_c1_g1_i5_orf1;TRINITY_DN1423_c0_g1_i4_orf1;TRINITY_DN56690_c0_g1_i4_orf1;TRINITY_DN19990_c0_g1_i1_orf1;TRINITY_DN8224_c0_g1_i7_orf1;TRINITY_DN116972_c0_g1_i1_orf1;TRINITY_DN30713_c0_g1_i3_orf1;TRINITY_DN6602_c0_g1_i4_orf1;TRINITY_DN1423_c0_g1_i8_orf1;TRINITY_DN4145_c0_g1_i1_orf1;TRINITY_DN15327_c2_g1_i2_orf1;TRINITY_DN3469_c0_g1_i4_orf1;TRINITY_DN32700_c0_g1_i2_orf1;TRINITY_DN117844_c0_g1_i1_orf1;TRINITY_DN1999_c0_g1_i9_orf1;TRINITY_DN3073_c0_g1_i7_orf1;TRINITY_DN6415_c0_g1_i1_orf1;TRINITY_DN6642_c0_g1_i2_orf1;TRINITY_DN136031_c0_g1_i7_orf1;TRINITY_DN12024_c0_g1_i4_orf1;TRINITY_DN18922_c0_g1_i1_orf1;TRINITY_DN32896_c0_g3_i1_orf1;TRINITY_DN5664_c0_g1_i1_orf1;TRINITY_DN27033_c1_g1_i3_orfp1;TRINITY_DN376_c1_g1_i1_orf1;TRINITY_DN511_c0_g2_i1_orf1;TRINITY_DN7861_c0_g1_i5_orf1;TRINITY_DN38431_c0_g1_i1_orf1;TRINITY_DN1206_c0_g1_i6_orf1;TRINITY_DN4321_c0_g1_i1_orf1;TRINITY_DN69049_c0_g2_i1_orf1;TRINITY_DN12545_c0_g1_i7_orf1;TRINITY_DN69170_c0_g2_i1_orf1;TRINITY_DN107035_c0_g1_i1_orf1;TRINITY_DN3461_c0_g1_i1_orf1;TRINITY_DN4908_c1_g1_i5_orf1;TRINITY_DN2676_c0_g1_i2_orf1;TRINITY_DN2299_c0_g1_i3_orf1;TRINITY_DN5235_c0_g1_i7_orf1;TRINITY_DN140_c0_g1_i5_orf1;TRINITY_DN47151_c0_g1_i1_orf1;TRINITY_DN812_c2_g1_i1_orf1;TRINITY_DN257_c0_g1_i7_orf1;TRINITY_DN120500_c0_g1_i1_orf1;TRINITY_DN64222_c0_g1_i1_orf1;TRINITY_DN1154_c0_g1_i1_orf1;TRINITY_DN4497_c0_g1_i4_orf1;TRINITY_DN36538_c0_g1_i2_orf1;TRINITY_DN2186_c0_g1_i17_orf1;TRINITY_DN24873_c0_g1_i4_orf1;TRINITY_DN62557_c0_g1_i1_orf1;TRINITY_DN14734_c0_g1_i2_orf1;TRINITY_DN73900_c0_g1_i1_orf1;TRINITY_DN8621_c0_g1_i5_orf1;TRINITY_DN53311_c0_g2_i1_orf1;TRINITY_DN625_c9_g1_i7_orf1;TRINITY_DN31851_c0_g1_i2_orf1;TRINITY_DN1952_c0_g1_i2_orf1;TRINITY_DN5081_c0_g1_i5_orf1;TRINITY_DN2594_c0_g2_i4_orf1;TRINITY_DN2140_c0_g1_i1_orf1;TRINITY_DN3732_c1_g1_i5_orf1;TRINITY_DN934_c2_g1_i7_orf1;TRINITY_DN36788_c0_g1_i2_orf1;TRINITY_DN24_c0_g1_i1_orf1;TRINITY_DN1363_c0_g1_i11_orf1;TRINITY_DN43412_c0_g1_i2_orf1;TRINITY_DN829_c0_g1_i8_orf1;TRINITY_DN23564_c0_g1_i7_orf1;TRINITY_DN4793_c0_g1_i7_orf1;TRINITY_DN21218_c

molecular_function	anion binding	GO:0043168	188	188/2406	TRINITY_DN280_c0_g1_i8_orf1;TRINITY_DN2065_c1_g2_i1_orf1;TRINITY_DN47731_c0_g1_i2_orf1;TRINITY_DN4380_c0_g1_i9_orf1;TRINITY_DN41311_c0_g2_i3_orf1;TRINITY_DN11194_c0_g1_i4_orf1;TRINITY_DN33801_c0_g1_i1_orf1;TRINITY_DN30932_c0_g1_i2_orf1;TRINITY_DN4744_c0_g1_i7_orf1;TRINITY_DN25341_c0_g1_i1_orf1;TRINITY_DN2738_c1_g1_i3_orf1;TRINITY_DN33705_c0_g1_i1_orf1;TRINITY_DN8659_c0_g1_i1_orf1;TRINITY_DN70485_c0_g1_i2_orf1;TRINITY_DN8603_c0_g1_i1_orf1;TRINITY_DN24723_c2_g1_i1_orf1;TRINITY_DN2953_c1_g1_i10_orf1;TRINITY_DN143509_c0_g1_i1_orf1;TRINITY_DN2638_c0_g1_i7_orf1;TRINITY_DN4320_c0_g1_i1_orf1;TRINITY_DN73945_c0_g5_i3_orf1;TRINITY_DN4711_c0_g1_i2_orf1;TRINITY_DN244_c1_g1_i5_orf1;TRINITY_DN10774_c0_g2_i3_orf1;TRINITY_DN42753_c0_g1_i2_orf1;TRINITY_DN2953_c1_g1_i2_orf1;TRINITY_DN14298_c0_g1_i1_orf1;TRINITY_DN3991_c0_g1_i6_orf1;TRINITY_DN3800_c0_g1_i7_orf1;TRINITY_DN9575_c0_g1_i1_orf1;TRINITY_DN13563_c0_g1_i1_orf1;TRINITY_DN235_c0_g3_i1_orf1;TRINITY_DN1068_c0_g1_i3_orf1;TRINITY_DN6185_c0_g1_i12_orf1;TRINITY_DN52761_c0_g1_i2_orf1;TRINITY_DN6436_c0_g1_i1_orf1;TRINITY_DN4798_c0_g1_i3_orf1;TRINITY_DN2224_c0_g1_i1_orf1;TRINITY_DN662_c0_g1_i1_orf1;TRINITY_DN2688_c0_g2_i1_orf1;TRINITY_DN987_c0_g1_i3_orf1;TRINITY_DN3131_c0_g1_i5_orf1;TRINITY_DN7247_c0_g1_i7_orf1;TRINITY_DN2265_c0_g1_i5_orf1;TRINITY_DN31225_c0_g1_i1_orf1;TRINITY_DN46715_c0_g1_i1_orf1;TRINITY_DN11620_c0_g1_i2_orf1;TRINITY_DN20007_c0_g1_i1_orf1;TRINITY_DN4950_c0_g1_i2_orf1;TRINITY_DN1173_c0_g1_i12_orf1;TRINITY_DN34751_c0_g1_i1_orf1;TRINITY_DN4501_c0_g1_i3_orf1;TRINITY_DN2772_c0_g1_i3_orf1;TRINITY_DN31503_c0_g1_i4_orf1;TRINITY_DN64126_c0_g1_i1_orf1;TRINITY_DN252_c0_g1_i3_orf1;TRINITY_DN2719_c1_g1_i6_orf1;TRINITY_DN8659_c0_g2_i1_orf1;TRINITY_DN17935_c0_g1_i1_orf1;TRINITY_DN1604_c0_g1_i4_orf1;TRINITY_DN1034_c0_g1_i4_orf1;TRINITY_DN90497_c0_g1_i1_orf1;TRINITY_DN42461_c0_g1_i4_orf1;TRINITY_DN6813_c1_g1_i1_orf1;TRINITY_DN1607_c0_g1_i16_orf1;TRINITY_DN277_c1_g1_i1_orf1;TRINITY_DN31967_c0_g1_i5_orf1;TRINITY_DN28622_c0_g1_i1_orf1;TRINITY_DN12_c0_g1_i5_orf1;TRINITY_DN7336_c0_g1_i13_orf1;TRINITY_DN5070_c0_g1_i1_orf1;TRINITY_DN32700_c0_g1_i2_orf1;TRINITY_DN117844_c0_g1_i1_orf1;TRINITY_DN122423_c0_g1_i1_orf1;TRINITY_DN14935_c0_g1_i1_orf1;TRINITY_DN38424_c0_g1_i1_orf1;TRINITY_DN6642_c0_g1_i2_orf1;TRINITY_DN14274_c0_g1_i3_orf1;TRINITY_DN11015_c0_g1_i8_orf1;TRINITY_DN5281_c0_g2_i3_orf1;TRINITY_DN511_c0_g2_i1_orf1;TRINITY_DN1718_c6_g1_i4_orf1;TRINITY_DN1173_c1_g1_i10_orf1;TRINITY_DN119893_c0_g2_i3_orf1;TRINITY_DN3428_c0_g1_i1_orf1;TRINITY_DN2684_c0_g2_i3_orf1;TRINITY_DN15420_c0_g3_i2_orf1;TRINITY_DN4908_c1_g1_i5_orf1;TRINITY_DN28989_c0_g1_i7_orf1;TRINITY_DN235_c0_g1_i2_orf1;TRINITY_DN2709_c0_g1_i4_orf1;TRINITY_DN291_c0_g1_i2_orf1;TRINITY_DN12064_c0_g2_i1_orf1;TRINITY_DN16011_c0_g1_i3_orf1;TRINITY_DN7131_c0_g1_i2_orf1;TRINITY_DN46409_c0_g1_i1_orf1;TRINITY_DN3637_c0_g1_i2_orf1;TRINITY_DN2983_c0_g1_i6_orf1;TRINITY_DN1552_c0_g1_i3_orf1;TRINITY_DN8980_c0_g1_i2_orf1;TRINITY_DN47151_c0_g1_i1_orf1;TRINITY_DN143603_c0_g1_i1_orf1;TRINITY_DN72017_c0_g1_i1_orf1;TRINITY_DN136906_c0_g1_i1_orf1;TRINITY_DN2953_c1_g1_i11_orf1;TRINITY_DN141381_c0_g1_i1_orf1;TRINITY_DN11948_c0_g1_i8_orf1;TRINITY_DN38506_c0_g1_i4_orf1;TRINITY_DN8390_c0_g1_i2_orf1;TRINITY_DN34479_c0_g1_i2_orf1;TRINITY_DN16673_c0_g1_i1_orf1;TRINITY_DN2803_c4_g1_i1_orf1;TRINITY_DN2146_c0_g1_i1_orf1;TRINITY_DN32769_c1_g1_i5_orf1;TRINITY_DN82311_c0_g1_i1_orf1;TRINITY_DN4762_c0_g1_i2_orf1;TRINITY_DN4835_c0_g1_i2_orf1;TRINITY_DN3175_c0_g1_i7_orf1;TRINITY_DN11986_c0_g1_i1_orf1;TRINITY_DN51813_c0_g1_i1_orf1;TRINITY_DN36899_c0_g1_i1_orf1;TRINITY_DN162_c0_g1_i4_orf1;TRINITY_DN2793_c0_g2_i1_orf1;TRINITY_DN44288_c0_g1_i2_orf1;TRINITY_DN60792_c0_g1_i2_orf1;TRINITY_DN2193_c0_g1_i7_orf1;TRINITY_DN97138_c0_g1_i2_orf1;TRINITY_DN85476_c0_g1_i1_orf1;TRINITY_DN143637_c0_g1_i1_orf1;TRINITY_DN111110_c0_g1_i1_orf1;TRINITY_DN62_c1_g1_i3_orf1;TRINITY_DN52244_c1_g1_i1_orf1;TRINITY_DN14301_c0_g1_i1_orf1;TRINITY_DN1262_c0_g1_i2_orf1;TRINITY_DN3263_c0_g1_i2_orf1;TRINITY_DN7512_c0_g1_i1_orf1;TRINITY_DN49047_c0_g1_i2_orf1;TRINITY_DN26168_c0_g1_i1_orf1;TRINITY_DN2146_c0_g2_i1_orf1;TRINITY_DN52761_c0_g2_i1_orf1;TRINITY_DN1084_c0_g2_i2_orf1;TRINITY_DN62557_c0_g1_i1_orf1;TRINITY_DN57918_c0_g1_i1_orf1;TRINITY_DN8716_c0_g1_i3_orf1;TRINITY_DN76815_c0_g1_i3_orf1;TRINITY_DN41166_c0_g1_i1_orf1;TRINITY_DN15706_c0_g2_i5_orf1;TRINITY_DN1405_c0_g1_i1_orf1;TRINITY_DN59291_c0_g1_i1_orf1;TRINITY_DN9302_c0_g1_i1_orf1;TRINITY_DN618_c0_g1_i3_orf1;TRINITY
molecular_function	phospholipid binding	GO:0005543	5	5/2406	TRINITY_DN445_c0_g1_i2_orf1;TRINITY_DN119265_c0_g2_i1_orf1;TRINITY_DN143637_c0_g1_i1_orf1;TRINITY_DN6243_c0_g1_i5_orf1;TRINITY_DN21533_c0_g1_i7_orf1
molecular_function	fatty acid derivative binding	GO:1901567	1	1/2406	TRINITY_DN13563_c0_g1_i1_orf1
molecular_function	oxygen binding	GO:0019825	1	1/2406	TRINITY_DN30037_c0_g1_i5_orf1
molecular_function	monosaccharide binding	GO:0048029	1	1/2406	TRINITY_DN20133_c0_g1_i1_orf1
molecular_function	vitamin binding	GO:0019842	13	13/2406	TRINITY_DN2065_c1_g2_i1_orf1;TRINITY_DN2890_c0_g1_i2_orf1;TRINITY_DN2688_c0_g2_i1_orf1;TRINITY_DN11948_c0_g1_i8_orf1;TRINITY_DN1262_c0_g1_i2_orf1;TRINITY_DN3263_c0_g1_i2_orf1;TRINITY_DN14935_c0_g1_i1_orf1;TRINITY_DN51813_c0_g1_i1_orf1;TRINITY_DN21035_c0_g1_i14_orf1;TRINITY_DN2803_c4_g1_i1_orf1;TRINITY_DN2684_c0_g2_i3_orf1;TRINITY_DN10430_c0_g1_i4_orf1;TRINITY_DN1068_c0_g1_i3_orf1
molecular_function	nucleotide binding	GO:0000166	193	193/2406	TRINITY_DN280_c0_g1_i8_orf1;TRINITY_DN13563_c0_g1_i1_orf1;TRINITY_DN38230_c0_g1_i4_orf1;TRINITY_DN47731_c0_g1_i2_orf1;TRINITY_DN4380_c0_g1_i9_orf1;TRINITY_DN41311_c0_g2_i3_orf1;TRINITY_DN11194_c0_g1_i4_orf1;TRINITY_DN1921_c1_g1_i5_orf1;TRINITY_DN30932_c0_g1_i2_orf1;TRINITY_DN4744_c0_g1_i7_orf1;TRINITY_DN25341_c0_g1_i1_orf1;TRINITY_DN2738_c1_g1_i3_orf1;TRINITY_DN33705_c0_g1_i1_orf1;TRINITY_DN8659_c0_g1_i1_orf1;TRINITY_DN70485_c0_g1_i2_orf1;TRINITY_DN8603_c0_g1_i1_orf1;TRINITY_DN24723_c2_g1_i1_orf1;TRINITY_DN2953_c1_g1_i10_orf1;TRINITY_DN143509_c0_g1_i1_orf1;TRINITY_DN2638_c0_g1_i7_orf1;TRINITY_DN2848_c0_g1_i1_orf1;TRINITY_DN4320_c0_g1_i1_orf1;TRINITY_DN73945_c0_g5_i3_orf1;TRINITY_DN4711_c0_g1_i2_orf1;TRINITY_DN244_c1_g1_i5_orf1;TRINITY_DN10774_c0_g2_i3_orf1;TRINITY_DN42753_c0_g1_i2_orf1;TRINITY_DN2953_c1_g1_i2_orf1;TRINITY_DN14298_c0_g1_i1_orf1;TRINITY_DN3991_c0_g1_i6_orf1;TRINITY_DN3800_c0_g1_i7_orf1;TRINITY_DN5354_c0_g1_i4_orf1;TRINITY_DN235_c0_g3_i1_orf1;TRINITY_DN6185_c0_g1_i12_orf1;TRINITY_DN20527_c0_g1_i1_orf1;TRINITY_DN6436_c0_g1_i1_orf1;TRINITY_DN4798_c0_g1_i3_orf1;TRINITY_DN2224_c0_g1_i1_orf1;TRINITY_DN662_c0_g1_i1_orf1;TRINITY_DN987_c0_g1_i3_orf1;TRINITY_DN3131_c0_g1_i5_orf1;TRINITY_DN7247_c0_g1_i7_orf1;TRINITY_DN2265_c0_g1_i5_orf1;TRINITY_DN31225_c0_g1_i1_orf1;TRINITY_DN46715_c0_g1_i1_orf1;TRINITY_DN11620_c0_g1_i2_orf1;TRINITY_DN20007_c0_g1_i1_orf1;TRINITY_DN4950_c0_g1_i2_orf1;TRINITY_DN1173_c0_g1_i12_orf1;TRINITY_DN34751_c0_g1_i1_orf1;TRINITY_DN4501_c0_g1_i3_orf1;TRINITY_DN2772_c0_g1_i3_orf1;TRINITY_DN31503_c0_g1_i4_orf1;TRINITY_DN64126_c0_g1_i1_orf1;TRINITY_DN18031_c0_g1_i1_orf1;TRINITY_DN252_c0_g1_i3_orf1;TRINITY_DN2719_c1_g1_i6_orf1;TRINITY_DN33801_c0_g1_i1_orf1;TRINITY_DN8659_c0_g2_i1_orf1;TRINITY_DN17935_c0_g1_i1_orf1;TRINITY_DN4451_c0_g2_i4_orf1;TRINITY_DN1604_c0_g1_i4_orf1;TRINITY_DN53238_c1_g1_i5_orf1;TRINITY_DN90497_c0_g1_i1_orf1;TRINITY_DN42461_c0_g1_i4_orf1;TRINITY_DN9575_c0_g1_i1_orf1;TRINITY_DN6813_c1_g1_i1_orf1;TRINITY_DN1607_c0_g1_i16_orf1;TRINITY_DN277_c1_g1_i1_orf1;TRINITY_DN268_c3_g1_i2_orf1;TRINITY_DN31967_c0_g1_i5_orf1;TRINITY_DN28622_c0_g1_i1_orf1;TRINITY_DN12_c0_g1_i5_orf1;TRINITY_DN7336_c0_g1_i13_orf1;TRINITY_DN5070_c0_g1_i1_orf1;TRINITY_DN32700_c0_g1_i2_orf1;TRINITY_DN117844_c0_g1_i1_orf1;TRINITY_DN122423_c0_g1_i1_orf1;TRINITY_DN38424_c0_g1_i1_orf1;TRINITY_DN6642_c0_g1_i2_orf1;TRINITY_DN14274_c0_g1_i3_orf1;TRINITY_DN11015_c0_g1_i8_orf1;TRINITY_DN5281_c0_g2_i3_orf1;TRINITY_DN511_c0_g2_i1_orf1;TRINITY_DN1718_c6_g1_i4_orf1;TRINITY_DN1173_c1_g1_i10_orf1;TRINITY_DN119893_c0_g2_i3_orf1;TRINITY_DN3428_c0_g1_i1_orf1;TRINITY_DN52761_c0_g1_i2_orf1;TRINITY_DN15420_c0_g3_i2_orf1;TRINITY_DN4908_c1_g1_i5_orf1;TRINITY_DN28989_c0_g1_i7_orf1;TRINITY_DN235_c0_g1_i2_orf1;TRINITY_DN2709_c0_g1_i4_orf1;TRINITY_DN291_c0_g1_i2_orf1;TRINITY_DN1209_c0_g1_i9_orf1;TRINITY_DN12064_c0_g2_i1_orf1;TRINITY_DN16011_c0_g1_i3_orf1;TRINITY_DN7131_c0_g1_i2_orf1;TRINITY_DN46409_c0_g1_i1_orf1;TRINITY_DN3637_c0_g1_i2_orf1;TRINITY_DN2983_c0_g1_i6_orf1;TRINITY_DN1552_c0_g1_i3_orf1;TRINITY_DN8980_c0_g1_i2_orf1;TRINITY_DN47151_c0_g1_i1_orf1;TRINITY_DN143603_c0_g1_i1_orf1;TRINITY_DN141381_c0_g1_i1_orf1;TRINITY_DN72017_c0_g1_i1_orf1;TRINITY_DN136906_c0_g1_i1_orf1;TRINITY_DN2953_c1_g1_i11_orf1;TRINITY_DN5757_c0_g1_i1_orf1;TRINITY_DN49038_c0_g4_i1_orf1;TRINITY_DN38506_c0_g1_i4_orf1;TRINITY_DN36788_c0_g1_i2_orf1;TRINITY_DN8390_c0_g1_i2_orf1;TRINITY_DN24_c0_g1_i1_orf1;TRINITY_DN34479_c0_g1_i2_orf1;TRINITY_DN16673_c0_g1_i1_orf1;TRINITY_DN2146_c0_g1_i1_orf1;TRINITY_DN32769_c1_g1_i5_orf1;TRINITY_DN26293_c0_g1_i4_orf1;TRINITY_DN82311_c0_g1_i1_orf1;TRINITY_DN4762_c0_g1_i2_orf1;TRINITY_DN4835_c0_g1_i2_orf1;TRINITY_DN3175_c0_g1_i7_orf1;TRINITY_DN11986_c0_g1_i1_orf1;TRINITY_DN36899_c0_g1_i1_orf1;TRINITY_DN162_c0_g1_i4_orf1;TRINITY_DN2793_c0_g2_i1_orf1;TRINITY_DN44288_c0_g1_i2_orf1;TRINITY_DN60792_c0_g1_i2_orf1;TRINITY_DN2193_c0_g1_i7_orf1;TRINITY_DN97138_c0_g1_i2_orf1;TRINITY_DN5099_c0_g1_i3_orf1;TRINITY_DN85476_c0_g1_i1_orf1;TRINITY_DN143637_c0_g1_i1_orf1;TRINITY_DN111110_c0_g1_i1_orf1;TRINITY_DN62_c1_g1_i3_orf1;TRINITY_DN52244_c1_g1_i1_orf1;TRINITY_DN14301_c0_g1_i1_orf1;TRINITY_DN7512_c0_g1_i1_orf1;TRINITY_DN49047_c0_g1_i2_orf1;TRINITY_DN26168_c0_g1_i1_orf1;TRINITY_DN2146_c0_g2_i1_orf1;TRINITY_DN52761_c0_g2_i1_orf1;TRINITY_DN1084_c0_g2_i2_orf1;TRINITY_DN62557_c0_g1_i1_orf1;TRINITY_DN57918_c0_g1_i1_orf1;TRINITY_DN8716_c0_g1_i3_orf1;TRINITY_DN76815_c0_g1_i3_orf1;TRINITY_DN41166_c0_g1_i1_orf1;TRIN
molecular_function	polysaccharide binding	GO:0030247	1	1/2406	TRINITY_DN2170_c0_g1_i2_orf1
molecular_function	ribonucleoprotein complex binding	GO:0043021	4	4/2406	TRINITY_DN23360_c0_g1_i3_orf1;TRINITY_DN48097_c0_g1_i1_orf1;TRINITY_DN10070_c0_g1_i1_orf1;TRINITY_DN6239_c0_g1_i1_orf1
molecular_function	protein-lipid complex binding	GO:0071814	1	1/2406	TRINITY_DN46409_c0_g1_i1_orf1
molecular_function	dynein complex binding	GO:0070840	1	1/2406	TRINITY_DN21559_c0_g2_i1_orf1
molecular_function	proteasome binding	GO:0070628	1	1/2406	TRINITY_DN135188_c0_g1_i2_orf1
molecular_function	actin filament binding	GO:0051015	12	12/2406	TRINITY_DN110231_c0_g1_i1_orf1;TRINITY_DN104663_c1_g1_i2_orf1;TRINITY_DN129869_c0_g4_i1_orf1;TRINITY_DN86309_c0_g1_i4_orf1;TRINITY_DN364_c2_g1_i2_orf1;TRINITY_DN21451_c0_g1_i3_orf1;TRINITY_DN8915_c0_g1_i3_orf1;TRINITY_DN1718_c1_g1_i5_orf1;TRINITY_DN9383_c0_g1_i3_orf1;TRINITY_DN30150_c0_g1_i7_orf1;TRINITY_DN26961_c0_g1_i1_orf1;TRINITY_DN741_c0_g1_i10_orf1
molecular_function	iron-sulfur cluster binding	GO:0051536	10	10/2406	TRINITY_DN14920_c0_g1_i1_orf1;TRINITY_DN49221_c0_g1_i1_orf1;TRINITY_DN96566_c0_g1_i1_orf1;TRINITY_DN9536_c0_g1_i4_orf1;TRINITY_DN5664_c0_g1_i1_orf1;TRINITY_DN85476_c0_g1_i1_orf1;TRINITY_DN4711_c0_g1_i2_orf1;TRINITY_DN3464_c0_g1_i1_orf1;TRINITY_DN31851_c0_g1_i2_orf1;TRINITY_DN54134_c0_g1_i1_orf1
molecular_function	virus receptor activity	GO:0001618	1	1/2406	TRINITY_DN10070_c0_g1_i1_orf1
molecular_function	transmembrane signaling receptor activity	GO:0004888	8	8/2406	TRINITY_DN8953_c0_g1_i4_orf1;TRINITY_DN20710_c0_g1_i2_orf1;TRINITY_DN5011_c0_g1_i1_orf1;TRINITY_DN13216_c0_g1_i5_orf1;TRINITY_DN19662_c4_g1_i1_orf1;TRINITY_DN46090_c0_g2_i1_orf1;TRINITY_DN20710_c0_g2_i2_orf1;TRINITY_DN34821_c0_g1_i4_orf1
molecular_function	pattern recognition receptor activity	GO:0038187	2	2/2406	TRINITY_DN2170_c0_g1_i2_orf1;TRINITY_DN2170_c1_g1_i3_orf1

molecular_function	ligase activity, forming carbon-oxygen bonds	GO:0016875	9	9/2406	TRINITY_DN2953_c1_g1_i11_orf1;TRINITY_DN57918_c0_g1_i1_orf1;TRINITY_DN21539_c0_g1_i1_orf1;TRINITY_DN2224_c0_g1_i1_orf1;TRINITY_DN8716_c0_g1_i3_orf1;TRINITY_DN1607_c0_g1_i16_orf1;TRINITY_DN2953_c1_g1_i10_orf1;TRINITY_DN2953_c1_g1_i2_orf1;TRINITY_DN48619_c0_g1_i1_orf1
molecular_function	ligase activity, forming carbon-sulfur bonds	GO:0016877	5	5/2406	TRINITY_DN8659_c0_g1_i1_orf1;TRINITY_DN8659_c0_g2_i1_orf1;TRINITY_DN120593_c0_g1_i1_orf1;TRINITY_DN2193_c0_g1_i7_orf1;TRINITY_DN19251_c0_g1_i8_orf1
molecular_function	ligase activity, forming carbon-nitrogen bonds	GO:0016879	8	8/2406	TRINITY_DN16451_c0_g1_i7_orf1;TRINITY_DN987_c0_g1_i3_orf1;TRINITY_DN3800_c0_g1_i7_orf1;TRINITY_DN24723_c2_g1_i1_orf1;TRINITY_DN38506_c0_g1_i4_orf1;TRINITY_DN76815_c0_g1_i3_orf1;TRINITY_DN244_c1_g1_i5_orf1;TRINITY_DN41697_c0_g1_i1_orf1
molecular_function	ligase activity, forming carbon-carbon bonds	GO:0016885	3	3/2406	TRINITY_DN3991_c0_g1_i6_orf1;TRINITY_DN2570_c0_g1_i1_orf1;TRINITY_DN511_c0_g2_i1_orf1
molecular_function	carbon-sulfur lyase activity	GO:0016846	1	1/2406	TRINITY_DN11948_c0_g1_i8_orf1
molecular_function	carbon-nitrogen lyase activity	GO:0016840	2	2/2406	TRINITY_DN1716_c0_g1_i14_orf1;TRINITY_DN28299_c0_g1_i1_orf1
molecular_function	phosphorus-oxygen lyase activity	GO:0016849	3	3/2406	TRINITY_DN10774_c0_g2_i3_orf1;TRINITY_DN618_c0_g1_i3_orf1;TRINITY_DN52244_c1_g1_i1_orf1
molecular_function	carbon-oxygen lyase activity	GO:0016835	9	9/2406	TRINITY_DN230_c2_g1_i5_orf1;TRINITY_DN5070_c0_g1_i1_orf1;TRINITY_DN51498_c0_g1_i1_orf1;TRINITY_DN73900_c0_g1_i1_orf1;TRINITY_DN35763_c0_g1_i2_orf1;TRINITY_DN10430_c0_g1_i4_orf1;TRINITY_DN143603_c0_g1_i1_orf1;TRINITY_DN3464_c0_g1_i1_orf1;TRINITY_DN89483_c0_g1_i1_orf1
molecular_function	carbon-carbon lyase activity	GO:0016830	9	9/2406	TRINITY_DN779_c0_g1_i12_orf1;TRINITY_DN1045_c0_g1_i6_orf1;TRINITY_DN6325_c0_g1_i8_orf1;TRINITY_DN40434_c0_g1_i2_orf1;TRINITY_DN109931_c0_g1_i1_orf1;TRINITY_DN2684_c0_g2_i3_orf1;TRINITY_DN779_c0_g1_i3_orf1;TRINITY_DN20133_c0_g1_i1_orf1;TRINITY_DN6325_c0_g1_i9_orf1
molecular_function	protein-malonyllysine demalonylase activity	GO:0036054	1	1/2406	TRINITY_DN111110_c0_g1_i1_orf1
molecular_function	peptide-lysine-N-acetyltransferase activity	GO:0061733	2	2/2406	TRINITY_DN12771_c0_g1_i1_orf1;TRINITY_DN51737_c0_g1_i3_orf1
molecular_function	protein-disulfide reductase activity	GO:0015035	2	2/2406	TRINITY_DN51938_c0_g3_i1_orf1;TRINITY_DN79673_c0_g1_i1_orf1
molecular_function	protein-glutaryllysine deglutarylase activity	GO:0061697	1	1/2406	TRINITY_DN111110_c0_g1_i1_orf1
molecular_function	protein methyltransferase activity	GO:0008276	3	3/2406	TRINITY_DN95414_c0_g1_i1_orf1;TRINITY_DN14734_c0_g1_i2_orf1;TRINITY_DN6462_c0_g1_i5_orf1
molecular_function	phosphoprotein phosphatase activity	GO:0004721	7	7/2406	TRINITY_DN1749_c0_g2_i2_orf1;TRINITY_DN4571_c0_g1_i4_orf1;TRINITY_DN3119_c0_g1_i7_orf1;TRINITY_DN34830_c0_g1_i1_orf1;TRINITY_DN24539_c0_g1_i4_orf1;TRINITY_DN40562_c0_g2_i1_orf1;TRINITY_DN4217_c0_g1_i2_orf1
molecular_function	protein-N-terminal asparagine amidohydrolase activity	GO:0008418	2	2/2406	TRINITY_DN90327_c0_g1_i1_orf1;TRINITY_DN895_c0_g2_i1_orf1
molecular_function	peptidyl-cysteine S-nitrosylase activity	GO:0035605	1	1/2406	TRINITY_DN2848_c0_g1_i1_orf1
molecular_function	ubiquitin-like protein transferase activity	GO:0019787	5	5/2406	TRINITY_DN9062_c0_g2_i3_orf1;TRINITY_DN11820_c0_g1_i1_orf1;TRINITY_DN31980_c0_g1_i1_orf1;TRINITY_DN9132_c0_g1_i5_orf1;TRINITY_DN14487_c0_g1_i4_orf1
molecular_function	aminoacyltransferase activity	GO:0016755	3	3/2406	TRINITY_DN52553_c0_g2_i1_orf1;TRINITY_DN52553_c0_g1_i1_orf1;TRINITY_DN4898_c0_g1_i7_orf1
molecular_function	protein lysine deacetylase activity	GO:0033558	2	2/2406	TRINITY_DN10385_c0_g1_i5_orf1;TRINITY_DN111110_c0_g1_i1_orf1
molecular_function	protein kinase activity	GO:0004672	26	26/2406	TRINITY_DN1173_c0_g1_i12_orf1;TRINITY_DN1552_c0_g1_i3_orf1;TRINITY_DN1405_c0_g1_i1_orf1;TRINITY_DN46090_c0_g2_i1_orf1;TRINITY_DN5281_c0_g2_i3_orf1;TRINITY_DN10680_c0_g1_i5_orf1;TRINITY_DN42461_c0_g1_i4_orf1;TRINITY_DN1173_c0_g1_i11_orf1;TRINITY_DN70485_c0_g1_i2_orf1;TRINITY_DN2983_c0_g1_i6_orf1;TRINITY_DN248_c0_g1_i1_orf1;TRINITY_DN277_c1_g1_i1_orf1;TRINITY_DN19662_c4_g1_i1_orf1;TRINITY_DN73945_c0_g5_i3_orf1;TRINITY_DN10774_c0_g2_i3_orf1;TRINITY_DN32700_c0_g1_i2_orf1;TRINITY_DN30154_c0_g1_i1_orf1;TRINITY_DN6185_c0_g1_i12_orf1;TRINITY_DN6436_c0_g1_i1_orf1;TRINITY_DN143637_c0_g1_i1_orf1;TRINITY_DN4798_c0_g1_i3_orf1;TRINITY_DN1173_c1_g1_i10_orf1;TRINITY_DN30_c0_g1_i6_orf1;TRINITY_DN147475_c0_g1_i1_orf1;TRINITY_DN46715_c0_g1_i1_orf1;TRI
molecular_function	peptidase activity	GO:0008233	113	113/2406	TRINITY_DN1533_c0_g2_i1_orf1;TRINITY_DN344_c1_g1_i1_orf1;TRINITY_DN2069_c1_g1_i8_orf1;TRINITY_DN38431_c0_g1_i1_orf1;TRINITY_DN3499_c0_g1_i8_orf1;TRINITY_DN8692_c0_g1_i2_orf1;TRINITY_DN2442_c0_g1_i2_orf1;TRINITY_DN36434_c0_g2_i3_orf1;TRINITY_DN16258_c0_g1_i2_orf1;TRINITY_DN2885_c1_g1_i2_orf1;TRINITY_DN14754_c0_g1_i6_orf1;TRINITY_DN41761_c0_g1_i4_orf1;TRINITY_DN14217_c0_g1_i1_orf1;TRINITY_DN14774_c0_g1_i4_orf1;TRINITY_DN48020_c0_g1_i1_orf1;TRINITY_DN42753_c0_g1_i2_orf1;TRINITY_DN875_c0_g1_i3_orf1;TRINITY_DN2794_c1_g1_i8_orf1;TRINITY_DN4125_c0_g1_i14_orf1;TRINITY_DN10364_c0_g1_i5_orf1;TRINITY_DN6205_c0_g1_i8_orf1;TRINITY_DN391_c0_g1_i4_orf1;TRINITY_DN40_c0_g2_i1_orf1;TRINITY_DN28428_c0_g1_i2_orf1;TRINITY_DN19651_c0_g1_i1_orf1;TRINITY_DN10403_c0_g1_i3_orf1;TRINITY_DN338_c1_g1_i9_orf1;TRINITY_DN51938_c0_g3_i1_orf1;TRINITY_DN143895_c0_g1_i1_orf1;TRINITY_DN13856_c0_g1_i1_orf1;TRINITY_DN45633_c0_g1_i1_orf1;TRINITY_DN10090_c0_g1_i1_orf1;TRINITY_DN10629_c0_g1_i1_orf1;TRINITY_DN29034_c0_g1_i1_orf1;TRINITY_DN19537_c0_g1_i1_orf1;TRINITY_DN4189_c0_g2_i1_orf1;TRINITY_DN57798_c0_g1_i1_orf1;TRINITY_DN5012_c0_g1_i6_orf1;TRINITY_DN72541_c0_g1_i2_orf1;TRINITY_DN41086_c0_g1_i4_orf1;TRINITY_DN1274_c0_g1_i4_orf1;TRINITY_DN19990_c0_g1_i1_orf1;TRINITY_DN21719_c0_g1_i2_orf1;TRINITY_DN45948_c1_g1_i1_orf1;TRINITY_DN5696_c0_g1_i4_orf1;TRINITY_DN2040_c0_g1_i6_orf1;TRINITY_DN29034_c0_g1_i2_orf1;TRINITY_DN4125_c1_g1_i5_orf1;TRINITY_DN1703_c0_g1_i6_orf1;TRINITY_DN4064_c0_g2_i1_orf1;TRINITY_DN5444_c0_g2_i1_orf1;TRINITY_DN4408_c6_g1_i1_orf1;TRINITY_DN6423_c0_g1_i6_orf1;TRINITY_DN376_c1_g1_i1_orf1;TRINITY_DN4767_c0_g1_i4_orf1;TRINITY_DN10403_c0_g1_i1_orf1;TRINITY_DN1459_c1_g1_i1_orf1;TRINITY_DN4494_c0_g1_i1_orf1;TRINITY_DN21218_c0_g1_i4_orf1;TRINITY_DN28989_c0_g1_i7_orf1;TRINITY_DN8621_c0_g1_i4_orf1;TRINITY_DN2584_c0_g1_i7_orf1;TRINITY_DN25534_c0_g1_i1_orf1;TRINITY_DN10766_c0_g1_i1_orf1;TRINITY_DN554_c0_g1_i1_orf1;TRINITY_DN36538_c0_g1_i2_orf1;TRINITY_DN9991_c0_g1_i4_orf1;TRINITY_DN8621_c0_g1_i5_orf1;TRINITY_DN27033_c1_g1_i3_orfp1;TRINITY_DN6122_c0_g1_i6_orf1;TRINITY_DN57111_c0_g1_i1_orf1;TRINITY_DN5310_c2_g1_i2_orf1;TRINITY_DN6423_c0_g1_i5_orf1;TRINITY_DN140_c1_g1_i2_orf1;TRINITY_DN1404_c0_g1_i6_orf1;TRINITY_DN34479_c0_g1_i2_orf1;TRINITY_DN21719_c0_g2_i4_orf1;TRINITY_DN18388_c0_g1_i6_orf1;TRINITY_DN1309_c0_g2_i1_orf1;TRINITY_DN1308_c0_g1_i4_orf1;TRINITY_DN701_c0_g1_i1_orf1;TRINITY_DN28661_c0_g1_i1_orf1;TRINITY_DN4228_c0_g1_i5_orf1;TRINITY_DN6470_c0_g3_i2_orf1;TRINITY_DN6205_c0_g1_i1_orf1;TRINITY_DN6813_c1_g1_i1_orf1;TRINITY_DN49047_c0_g1_i2_orf1;TRINITY_DN81803_c0_g2_i1_orf1;TRINITY_DN801_c0_g1_i2_orf1;TRINITY_DN13686_c0_g2_i1_orf1;TRINITY_DN334_c0_g1_i4_orf1;TRINITY_DN334_c0_g1_i3_orf1;TRINITY_DN17329_c0_g2_i3_orf1;TRINITY_DN2043_c0_g1_i3_orf1;TRINITY_DN7776_c0_g1_i1_orf1;TRINITY_DN111621_c0_g3_i1_orf1;TRINITY_DN41952_c0_g1_i4_orf1;TRINITY_DN10994_c0_g1_i4_orf1;TRINITY_DN17759_c0_g1_i5_orf1;TRINITY_DN4886_c0_g1_i6_orf1;TRINITY_DN1592_c0_g1_i1_orf1;TRINITY_DN6967_c0_g1_i3_orf1;TRINITY_DN56690_c0_g1_i4_orf1;TRINITY_DN69049_c0_g2_i1_orf1;TRINITY_DN46132_c0_g2_i2_orf1;TRINITY_DN23167_c0_g2_i1_orf1;TRINITY_DN23167_c0_g1_i4_orf1;TRINITY_DN2043_c0_g1_i11_orf1;TRINITY_DN24121_c1_g1_i6_orf1;TRINITY_DN22797_c0_g1_i5_orf1;TRINITY_DN26130_c0_g1_i1_orf1;TRI
molecular_function	protein disulfide isomerase activity	GO:0003756	2	2/2406	TRINITY_DN51938_c0_g3_i1_orf1;TRINITY_DN1831_c0_g1_i3_orf1
molecular_function	peptidyl-prolyl cis-trans isomerase activity	GO:0003755	3	3/2406	TRINITY_DN14372_c0_g2_i1_orf1;TRINITY_DN2807_c0_g1_i4_orf1;TRINITY_DN142588_c0_g1_i1_orf1
molecular_function	ubiquitin-like modifier activating enzyme activity	GO:0008641	3	3/2406	TRINITY_DN8659_c0_g1_i1_orf1;TRINITY_DN8659_c0_g2_i1_orf1;TRINITY_DN120593_c0_g1_i1_orf1
molecular_function	peptide-O-fucosyltransferase activity	GO:0046922	1	1/2406	TRINITY_DN4142_c0_g1_i5_orf1
molecular_function	hydrolase activity, acting on glycosyl bonds	GO:0016798	32	32/2406	TRINITY_DN6510_c1_g1_i1_orf1;TRINITY_DN8703_c0_g1_i2_orf1;TRINITY_DN24631_c0_g2_i1_orf1;TRINITY_DN25492_c0_g1_i1_orf1;TRINITY_DN28741_c0_g1_i3_orf1;TRINITY_DN10824_c0_g1_i3_orf1;TRINITY_DN22577_c0_g1_i2_orf1;TRINITY_DN11657_c0_g1_i2_orf1;TRINITY_DN3476_c0_g1_i5_orf1;TRINITY_DN9044_c0_g1_i1_orf1;TRINITY_DN21545_c0_g1_i2_orf1;TRINITY_DN479_c6_g1_i2_orf1;TRINITY_DN6074_c0_g1_i1_orf1;TRINITY_DN7183_c0_g1_i2_orf1;TRINITY_DN361_c0_g1_i5_orf1;TRINITY_DN17003_c1_g1_i1_orf1;TRINITY_DN3322_c0_g1_i2_orf1;TRINITY_DN2894_c0_g2_i3_orf1;TRINITY_DN542_c0_g1_i4_orf1;TRINITY_DN15222_c0_g1_i4_orf1;TRINITY_DN2515_c0_g1_i6_orf1;TRINITY_DN4070_c0_g1_i4_orf1;TRINITY_DN9044_c0_g1_i2_orf1;TRINITY_DN143603_c0_g1_i1_orf1;TRINITY_DN26688_c0_g1_i2_orf1;TRINITY_DN21555_c0_g1_i4_orf1;TRINITY_DN7828_c0_g1_i2_orf1;TRINITY_DN2170_c0_g1_i2_orf1;TRINITY_DN13088_c0_g1_i5_orf1;TRINITY_DN2170_c1_g1_i3_orf1;TRINITY_DN812_c2_g1_i1_orf1;TRINITY_DN650_c0_g1_i3_orf1
molecular_function	hydrolase activity, acting on acid carbon-carbon bonds	GO:0016822	1	1/2406	TRINITY_DN51813_c0_g1_i1_orf1
molecular_function	hydrolase activity, acting on carbon-nitrogen (but not peptide) bonds	GO:0016810	21	21/2406	TRINITY_DN895_c0_g2_i1_orf1;TRINITY_DN1534_c0_g1_i3_orf1;TRINITY_DN17326_c0_g1_i8_orf1;TRINITY_DN90327_c0_g1_i1_orf1;TRINITY_DN87170_c0_g1_i3_orf1;TRINITY_DN5235_c0_g1_i7_orf1;TRINITY_DN1216_c0_g1_i4_orf1;TRINITY_DN2835_c0_g1_i6_orf1;TRINITY_DN82801_c0_g1_i1_orf1;TRINITY_DN38506_c0_g1_i4_orf1;TRINITY_DN17031_c0_g1_i1_orf1;TRINITY_DN98242_c0_g1_i1_orf1;TRINITY_DN17172_c0_g1_i5_orf1;TRINITY_DN244_c1_g1_i5_orf1;TRINITY_DN13660_c0_g1_i1_orf1;TRINITY_DN542_c0_g1_i4_orf1;TRINITY_DN10385_c0_g1_i5_orf1;TRINITY_DN38180_c0_g1_i3_orf1;TRINITY_DN11383_c0_g2_i4_orf1;TRINITY_DN111110_c0_g1_i1_orf1;TR
molecular_function	hydrolase activity, acting on acid anhydrides	GO:0016817	37	37/2406	TRINITY_DN12064_c0_g2_i1_orf1;TRINITY_DN280_c0_g1_i8_orf1;TRINITY_DN16011_c0_g1_i3_orf1;TRINITY_DN9575_c0_g1_i1_orf1;TRINITY_DN2793_c0_g2_i1_orf1;TRINITY_DN11194_c0_g1_i4_orf1;TRINITY_DN252_c0_g1_i3_orf1;TRINITY_DN15706_c0_g2_i5_orf1;TRINITY_DN33801_c0_g1_i1_orf1;TRINITY_DN2054_c0_g1_i1_orf1;TRINITY_DN136906_c0_g1_i1_orf1;TRINITY_DN25341_c0_g1_i1_orf1;TRINITY_DN33705_c0_g1_i1_orf1;TRINITY_DN90497_c0_g1_i1_orf1;TRINITY_DN34479_c0_g1_i2_orf1;TRINITY_DN975_c0_g1_i1_orf1;TRINITY_DN2638_c0_g1_i7_orf1;TRINITY_DN32769_c1_g1_i5_orf1;TRINITY_DN1091_c0_g1_i1_orf1;TRINITY_DN1091_c0_g3_i1_orf1;TRINITY_DN7336_c0_g1_i13_orf1;TRINITY_DN4762_c0_g1_i2_orf1;TRINITY_DN42753_c0_g1_i2_orf1;TRINITY_DN13055_c0_g1_i5_orf1;TRINITY_DN11986_c0_g1_i1_orf1;TRINITY_DN6642_c0_g1_i2_orf1;TRINITY_DN15420_c0_g3_i2_orf1;TRINITY_DN41311_c0_g2_i3_orf1;TRINITY_DN146236_c0_g1_i1_orf1;TRINITY_DN97138_c0_g1_i2_orf1;TRINITY_DN52761_c0_g1_i2_orf1;TRINITY_DN7565_c0_g1_i3_orf1;TRINITY_DN46409_c0_g1_i1_orf1;TRINITY_DN2265_c0_g1_i5_orf1;TRINITY_DN49047_c0_g1_i2_orf1;TRINITY_DN28989_c0_g1_i7_orf1;TRINITY_DN52761_c0_g2_i1_orf1

molecular_function	serine hydrolase activity	GO:0017171	58	58/2406	TRINITY_DN2584_c0_g1_i7_orf1;TRINITY_DN36434_c0_g2_i3_orf1;TRINITY_DN10766_c0_g1_i1_orf1;TRINITY_DN2043_c0_g1_i11_orf1;TRINITY_DN10090_c0_g1_i1_orf1;TRINITY_DN1533_c0_g2_i1_orf1;TRINITY_DN29034_c0_g1_i1_orf1;TRINITY_DN6423_c0_g1_i6_orf1;TRINITY_DN334_c0_g1_i4_orf1;TRINITY_DN111621_c0_g3_i1_orf1;TRINITY_DN344_c1_g1_i1_orf1;TRINITY_DN57111_c0_g1_i1_orf1;TRINITY_DN5310_c2_g1_i2_orf1;TRINITY_DN6423_c0_g1_i5_orf1;TRINITY_DN5012_c0_g1_i6_orf1;TRINITY_DN1404_c0_g1_i6_orf1;TRINITY_DN3499_c0_g1_i8_orf1;TRINITY_DN41086_c0_g1_i4_orf1;TRINITY_DN1274_c0_g1_i4_orf1;TRINITY_DN25534_c0_g1_i1_orf1;TRINITY_DN2043_c0_g1_i3_orf1;TRINITY_DN21719_c0_g1_i2_orf1;TRINITY_DN16258_c0_g1_i2_orf1;TRINITY_DN21719_c0_g2_i4_orf1;TRINITY_DN4886_c0_g1_i6_orf1;TRINITY_DN1592_c0_g1_i1_orf1;TRINITY_DN41761_c0_g1_i4_orf1;TRINITY_DN5696_c0_g1_i4_orf1;TRINITY_DN18388_c0_g1_i6_orf1;TRINITY_DN6967_c0_g1_i3_orf1;TRINITY_DN2040_c0_g1_i6_orf1;TRINITY_DN29034_c0_g1_i2_orf1;TRINITY_DN1309_c0_g2_i1_orf1;TRINITY_DN5444_c0_g2_i1_orf1;TRINITY_DN1308_c0_g1_i4_orf1;TRINITY_DN701_c0_g1_i1_orf1;TRINITY_DN10364_c0_g1_i5_orf1;TRINITY_DN4228_c0_g1_i5_orf1;TRINITY_DN6205_c0_g1_i8_orf1;TRINITY_DN23167_c0_g2_i1_orf1;TRINITY_DN140_c1_g1_i2_orf1;TRINITY_DN391_c0_g1_i4_orf1;TRINITY_DN9991_c0_g1_i4_orf1;TRINITY_DN40_c0_g2_i1_orf1;TRINITY_DN6470_c0_g3_i2_orf1;TRINITY_DN10403_c0_g1_i1_orf1;TRINITY_DN23167_c0_g1_i4_orf1;TRINITY_DN6205_c0_g1_i1_orf1;TRINITY_DN14217_c0_g1_i1_orf1;TRINITY_DN24121_c1_g1_i6_orf1;TRINITY_DN334_c0_g1_i3_orf1;TRINITY_DN4494_c0_g1_i1_orf1;TRINITY_DN22797_c0_g1_i5_orf1;TRINITY_DN10403_c0_g1_i3_orf1;TRINITY_DN8480_c0_g1_i1_orf1;TRINITY_DN338_c1_g1_i9_
molecular_function	hydrolase activity, acting on ester bonds	GO:0016788	68	68/2406	TRINITY_DN1749_c0_g2_i2_orf1;TRINITY_DN117_c0_g1_i5_orf1;TRINITY_DN2749_c4_g1_i2_orf1;TRINITY_DN38230_c0_g1_i4_orf1;TRINITY_DN2772_c0_g1_i3_orf1;TRINITY_DN2749_c0_g1_i4_orf1;TRINITY_DN11117_c0_g1_i1_orf1;TRINITY_DN45271_c0_g1_i1_orf1;TRINITY_DN1073_c0_g1_i3_orf1;TRINITY_DN38562_c0_g1_i3_orf1;TRINITY_DN5238_c0_g1_i2_orf1;TRINITY_DN42333_c0_g1_i5_orf1;TRINITY_DN8771_c0_g2_i1_orf1;TRINITY_DN121650_c0_g1_i1_orf1;TRINITY_DN27033_c1_g1_i3_orfp1;TRINITY_DN10644_c0_g1_i2_orf1;TRINITY_DN1952_c0_g1_i2_orf1;TRINITY_DN18909_c0_g1_i8_orf1;TRINITY_DN3784_c0_g1_i1_orf1;TRINITY_DN3119_c0_g1_i7_orf1;TRINITY_DN4217_c0_g1_i2_orf1;TRINITY_DN34465_c0_g1_i1_orf1;TRINITY_DN44517_c0_g1_i4_orf1;TRINITY_DN24_c0_g1_i1_orf1;TRINITY_DN70485_c0_g1_i2_orf1;TRINITY_DN34830_c0_g1_i1_orf1;TRINITY_DN69713_c0_g1_i1_orf1;TRINITY_DN40562_c0_g2_i1_orf1;TRINITY_DN2812_c0_g1_i5_orf1;TRINITY_DN1884_c0_g2_i2_orf1;TRINITY_DN15865_c0_g1_i1_orf1;TRINITY_DN2367_c1_g1_i20_orf1;TRINITY_DN10430_c0_g1_i4_orf1;TRINITY_DN616_c1_g1_i6_orf1;TRINITY_DN2808_c0_g1_i8_orf1;TRINITY_DN26168_c0_g1_i1_orf1;TRINITY_DN1249_c0_g1_i10_orf1;TRINITY_DN12227_c0_g2_i3_orf1;TRINITY_DN17437_c0_g1_i1_orf1;TRINITY_DN117_c0_g1_i6_orf1;TRINITY_DN4571_c0_g1_i4_orf1;TRINITY_DN4394_c0_g2_i1_orf1;TRINITY_DN38783_c0_g1_i1_orf1;TRINITY_DN3073_c0_g1_i7_orf1;TRINITY_DN34432_c0_g1_i1_orf1;TRINITY_DN12024_c0_g1_i4_orf1;TRINITY_DN18922_c0_g1_i1_orf1;TRINITY_DN123184_c0_g1_i1_orf1;TRINITY_DN19293_c0_g1_i4_orf1;TRINITY_DN935_c0_g1_i3_orf1;TRINITY_DN1116_c0_g1_i6_orf1;TRINITY_DN38274_c0_g1_i1_orf1;TRINITY_DN171_c0_g1_i1_orf1;TRINITY_DN117_c0_g1_i4_orf1;TRINITY_DN74037_c0_g5_i1_orf1;TRINITY_DN2668_c0_g1_i7_orf1;TRINITY_DN2647_c0_g1_i3_orf1;TRINITY_DN227_c0_g1_i1_orf1;TRINITY_DN4276_c0_g1_i6_orf1;TRINITY_DN15865_c0_g2_i2_orf1;TRINITY_DN44658_c0_g1_i2_orf1;TRINITY_DN64403_c0_g2_i1_orf1;TRINITY_DN41_c0_g1_i5_orf1;TRINITY_DN2749_c0_g2_i3_orf1;TRINITY_DN24539_c0_g1_i4_orf1;TRINITY_DN2798_c0_g
molecular_function	deacetylase activity	GO:0019213	4	4/2406	TRINITY_DN542_c0_g1_i4_orf1;TRINITY_DN10385_c0_g1_i5_orf1;TRINITY_DN82801_c0_g1_i1_orf1;TRINITY_DN111110_c0_g1_i1_orf1
molecular_function	deaminase activity	GO:0019239	3	3/2406	TRINITY_DN1196_c0_g1_i5_orf1;TRINITY_DN38180_c0_g1_i3_orf1;TRINITY_DN98242_c0_g1_i1_orf1
molecular_function	hydrolase activity, acting on ether bonds	GO:0016801	5	5/2406	TRINITY_DN5768_c0_g1_i2_orf1;TRINITY_DN11172_c1_g1_i1_orf1;TRINITY_DN63536_c0_g1_i1_orf1;TRINITY_DN11172_c0_g1_i4_orf1;TRINITY_DN22242_c0_g1_i1_orf1
molecular_function	FAD-AMP lyase (cyclizing) activity	GO:0034012	2	2/2406	TRINITY_DN618_c0_g1_i3_orf1;TRINITY_DN52244_c1_g1_i1_orf1
molecular_function	guanylate cyclase activity	GO:0004383	1	1/2406	TRINITY_DN10774_c0_g2_i3_orf1
molecular_function	oxidoreductase activity, acting on CH-OH group of donors	GO:0016614	23	23/2406	TRINITY_DN5266_c0_g1_i1_orf1;TRINITY_DN146126_c0_g1_i1_orf1;TRINITY_DN20658_c0_g2_i3_orf1;TRINITY_DN1921_c1_g1_i5_orf1;TRINITY_DN4451_c0_g2_i4_orf1;TRINITY_DN49038_c0_g4_i1_orf1;TRINITY_DN5161_c0_g1_i5_orf1;TRINITY_DN36788_c0_g1_i2_orf1;TRINITY_DN9286_c0_g1_i2_orf1;TRINITY_DN135781_c0_g1_i1_orf1;TRINITY_DN4793_c0_g1_i7_orf1;TRINITY_DN10430_c0_g1_i4_orf1;TRINITY_DN26293_c0_g1_i4_orf1;TRINITY_DN3175_c0_g1_i7_orf1;TRINITY_DN36899_c0_g1_i1_orf1;TRINITY_DN5354_c0_g1_i4_orf1;TRINITY_DN1264_c0_g1_i2_orf1;TRINITY_DN2594_c0_g2_i4_orf1;TRINITY_DN31609_c0_g1_i3_orf1;TRINITY_DN1206_c0_g1_i6_orf1;TRINITY_DN53238_c1_g1_i5_orf1;TRINITY_DN38424_c0_g1_i1_orf1;TRINITY_DN1209_c0_g1_i9_orf1
molecular_function	oxidoreductase activity, acting on the aldehyde or oxo group of donors	GO:0016903	15	15/2406	TRINITY_DN7075_c0_g2_i1_orf1;TRINITY_DN482_c0_g1_i1_orf1;TRINITY_DN40126_c0_g1_i1_orf1;TRINITY_DN7808_c0_g1_i1_orf1;TRINITY_DN1293_c1_g1_i4_orf1;TRINITY_DN1293_c0_g1_i4_orf1;TRINITY_DN6586_c0_g1_i1_orf1;TRINITY_DN2848_c0_g1_i1_orf1;TRINITY_DN2719_c1_g1_i6_orf1;TRINITY_DN40126_c0_g2_i1_orf1;TRINITY_DN3529_c0_g1_i7_orf1;TRINITY_DN2100_c0_g1_i2_orf1;TRINITY_DN49508_c0_g2_i8_orf1;TRINITY_DN3836_c0_g1_i4_orf1;TRINITY_DN52788_c0_g1_i1_orf1
molecular_function	oxidoreductase activity, acting on a heme group of donors	GO:0016675	1	1/2406	TRINITY_DN76036_c0_g1_i1_orf1
molecular_function	oxidoreductase activity, acting on diphenols and related substances as donors	GO:0016679	1	1/2406	TRINITY_DN2140_c0_g1_i1_orf1
molecular_function	oxidoreductase activity, acting on the CH-NH group of donors	GO:0016645	7	7/2406	TRINITY_DN92153_c0_g2_i2_orf1;TRINITY_DN24970_c0_g1_i4_orf1;TRINITY_DN1760_c0_g1_i4_orf1;TRINITY_DN38506_c0_g1_i4_orf1;TRINITY_DN15136_c0_g1_i2_orf1;TRINITY_DN244_c1_g1_i5_orf1;TRINITY_DN20527_c0_g1_i1_orf1
molecular_function	dioxygenase activity	GO:0051213	7	7/2406	TRINITY_DN44083_c0_g1_i2_orf1;TRINITY_DN1153_c1_g1_i1_orf1;TRINITY_DN5497_c0_g1_i6_orf1;TRINITY_DN38562_c0_g1_i3_orf1;TRINITY_DN14398_c0_g1_i4_orf1;TRINITY_DN57900_c0_g1_i2_orf1;TRINITY_DN4822_c0_g1_i9_orf1
molecular_function	electron transfer activity	GO:0009055	4	4/2406	TRINITY_DN14920_c0_g1_i1_orf1;TRINITY_DN76036_c0_g1_i1_orf1;TRINITY_DN9242_c0_g1_i1_orf1;TRINITY_DN9135_c0_g1_i4_orf1
molecular_function	oxidoreductase activity, acting on paired donors, with incorporation or reduction of molecular oxygen	GO:0016705	46	46/2406	TRINITY_DN48590_c0_g1_i1_orf1;TRINITY_DN120500_c0_g1_i1_orf1;TRINITY_DN9608_c0_g1_i3_orf1;TRINITY_DN3949_c1_g1_i1_orf1;TRINITY_DN30704_c0_g1_i1_orf1;TRINITY_DN23564_c0_g1_i7_orf1;TRINITY_DN4497_c0_g1_i4_orf1;TRINITY_DN64126_c0_g1_i1_orf1;TRINITY_DN7580_c0_g1_i1_orf1;TRINITY_DN863_c0_g1_i6_orf1;TRINITY_DN24873_c0_g1_i4_orf1;TRINITY_DN2442_c0_g1_i6_orf1;TRINITY_DN625_c9_g1_i7_orf1;TRINITY_DN1664_c0_g1_i4_orf1;TRINITY_DN27903_c0_g1_i1_orf1;TRINITY_DN17772_c0_g2_i3_orf1;TRINITY_DN3949_c0_g1_i1_orf1;TRINITY_DN1134_c0_g1_i4_orf1;TRINITY_DN6027_c0_g1_i13_orf1;TRINITY_DN3732_c0_g1_i2_orf1;TRINITY_DN829_c0_g1_i8_orf1;TRINITY_DN3732_c1_g1_i5_orf1;TRINITY_DN1363_c0_g1_i11_orf1;TRINITY_DN50743_c0_g1_i1_orf1;TRINITY_DN3675_c0_g1_i1_orf1;TRINITY_DN15755_c0_g1_i1_orf1;TRINITY_DN2392_c0_g2_i1_orf1;TRINITY_DN448_c0_g1_i20_orf1;TRINITY_DN6351_c0_g1_i4_orf1;TRINITY_DN27045_c0_g1_i1_orf1;TRINITY_DN1960_c5_g1_i3_orf1;TRINITY_DN57856_c0_g2_i1_orf1;TRINITY_DN5439_c0_g1_i2_orf1;TRINITY_DN4497_c2_g1_i3_orf1;TRINITY_DN12997_c0_g2_i1_orf1;TRINITY_DN9647_c0_g1_i1_orf1;TRINITY_DN5126_c0_g1_i3_orf1;TRINITY_DN14398_c0_g1_i4_orf1;TRINITY_DN5126_c0_g2_i1_orf1;TRINITY_DN2676_c0_g1_i2_orf1;TRINITY_DN4321_c0_g1_i1_orf1;TRINITY_DN5661_c0_g1_i5_orf1;TRINITY_DN3131_c0_g1_i5_orf1;TRINITY_DN1999_c0_g1_i9_orf1;TRINITY_DN82944_c0_g1_i4_orf1;TRINITY_DN905_c0_g
molecular_function	oxidoreductase activity, acting on single donors with incorporation of molecular oxygen	GO:0016701	4	4/2406	TRINITY_DN3010_c0_g1_i4_orf1;TRINITY_DN4822_c0_g1_i9_orf1;TRINITY_DN5497_c0_g1_i6_orf1;TRINITY_DN38562_c0_g1_i3_orf1
molecular_function	oxidoreductase activity, acting on the CH-CH group of donors	GO:0016627	11	11/2406	TRINITY_DN34751_c0_g1_i1_orf1;TRINITY_DN7131_c0_g1_i2_orf1;TRINITY_DN44777_c0_g1_i2_orf1;TRINITY_DN20658_c0_g2_i3_orf1;TRINITY_DN30932_c0_g1_i2_orf1;TRINITY_DN10430_c0_g1_i4_orf1;TRINITY_DN143603_c0_g1_i1_orf1;TRINITY_DN141381_c0_g1_i1_orf1;TRINITY_DN1125_c0_g1_i4_orf1;TRINITY_DN4744_c0_g1_i7_orf1;TRINITY_DN72017_c0_g1_i1_orf1
molecular_function	oxidoreductase activity, acting on NAD(P)H	GO:0016651	8	8/2406	TRINITY_DN8306_c0_g1_i4_orf1;TRINITY_DN49221_c0_g1_i1_orf1;TRINITY_DN1661_c0_g1_i1_orf1;TRINITY_DN1134_c0_g1_i4_orf1;TRINITY_DN4497_c0_g1_i4_orf1;TRINITY_DN22678_c0_g1_i4_orf1;TRINITY_DN96566_c0_g1_i1_orf1;TRINITY_DN6563_c0_g1_i1_orf1
molecular_function	oxidoreductase activity, acting on superoxide radicals as	GO:0016721	2	2/2406	TRINITY_DN16400_c0_g2_i1_orf1;TRINITY_DN1024_c0_g4_i1_orf1
molecular_function	oxidoreductase activity, acting on metal ions	GO:0016722	5	5/2406	TRINITY_DN46625_c0_g1_i1_orf1;TRINITY_DN1423_c0_g1_i8_orf1;TRINITY_DN65681_c0_g1_i1_orf1;TRINITY_DN136031_c0_g1_i7_orf1;TRINITY_DN1423_c0_g1_i4_orf1
molecular_function	oxidoreductase activity, acting on CH or CH2 groups	GO:0016725	2	2/2406	TRINITY_DN135781_c0_g1_i1_orf1;TRINITY_DN4835_c0_g1_i2_orf1
molecular_function	oxidoreductase activity, acting on peroxide as acceptor	GO:0016684	8	8/2406	TRINITY_DN7778_c0_g1_i1_orf1;TRINITY_DN6580_c0_g1_i4_orf1;TRINITY_DN1622_c0_g1_i6_orf1;TRINITY_DN3984_c0_g1_i4_orf1;TRINITY_DN5933_c0_g1_i1_orf1;TRINITY_DN285_c0_g1_i4_orf1;TRINITY_DN69236_c0_g1_i1_orf1;TRINITY_DN2652_c0_g2_i1_orf1
molecular_function	monooxygenase activity	GO:0004497	43	43/2406	TRINITY_DN120500_c0_g1_i1_orf1;TRINITY_DN9608_c0_g1_i3_orf1;TRINITY_DN3949_c1_g1_i1_orf1;TRINITY_DN30704_c0_g1_i1_orf1;TRINITY_DN23564_c0_g1_i7_orf1;TRINITY_DN4497_c0_g1_i4_orf1;TRINITY_DN64126_c0_g1_i1_orf1;TRINITY_DN7580_c0_g1_i1_orf1;TRINITY_DN2392_c0_g2_i1_orf1;TRINITY_DN24873_c0_g1_i4_orf1;TRINITY_DN31584_c0_g2_i2_orf1;TRINITY_DN625_c9_g1_i7_orf1;TRINITY_DN3010_c0_g1_i4_orf1;TRINITY_DN1664_c0_g1_i4_orf1;TRINITY_DN17772_c0_g2_i3_orf1;TRINITY_DN3949_c0_g1_i1_orf1;TRINITY_DN1134_c0_g1_i4_orf1;TRINITY_DN6027_c0_g1_i13_orf1;TRINITY_DN3732_c0_g1_i2_orf1;TRINITY_DN829_c0_g1_i8_orf1;TRINITY_DN3732_c1_g1_i5_orf1;TRINITY_DN1363_c0_g1_i11_orf1;TRINITY_DN50743_c0_g1_i1_orf1;TRINITY_DN3675_c0_g1_i1_orf1;TRINITY_DN15755_c0_g1_i1_orf1;TRINITY_DN448_c0_g1_i20_orf1;TRINITY_DN6351_c0_g1_i4_orf1;TRINITY_DN27045_c0_g1_i1_orf1;TRINITY_DN1960_c5_g1_i3_orf1;TRINITY_DN57856_c0_g2_i1_orf1;TRINITY_DN5439_c0_g1_i2_orf1;TRINITY_DN4497_c2_g1_i3_orf1;TRINITY_DN12997_c0_g2_i1_orf1;TRINITY_DN9647_c0_g1_i1_orf1;TRINITY_DN5126_c0_g1_i3_orf1;TRINITY_DN863_c0_g1_i6_orf1;TRINITY_DN5126_c0_g2_i1_orf1;TRINITY_DN2676_c0_g1_i2_orf1;TRINITY_DN5661_c0_g1_i5_orf1;TRINITY_DN3131_c0_g1_i5_orf1;TRINITY_DN2442_c0_g1_i6_orf1;TRINITY_DN82944_c0_g1_i4_orf1;TRINITY_DN9198_c0_g1_i4_orf1
molecular_function	oxidoreductase activity, acting on the CH-NH2 group of	GO:0016638	2	2/2406	TRINITY_DN3859_c0_g1_i5_orf1;TRINITY_DN43431_c0_g1_i1_orf1
molecular_function	oxidoreductase activity, acting on other nitrogenous compounds as donors	GO:0016661	1	1/2406	TRINITY_DN2559_c0_g1_i4_orf1

molecular_function	oxidoreductase activity, acting on a sulfur group of donors	GO:0016667	9	9/2406	TRINITY_DN920_c0_g1_i4_orf1;TRINITY_DN51938_c0_g3_i1_orf1;TRINITY_DN25987_c0_g1_i5_orf1;TRINITY_DN1491_c0_g1_i8_orf1;TRINITY_DN79673_c0_g1_i1_orf1;TRINITY_DN81715_c0_g1_i1_orf1;TRINITY_DN2207_c0_g1_i6_orf1;TRINITY_DN920_c0_g1_i6_orf1;TRINITY_DN1491_c0_g1_i4_orf1
molecular_function	N-acetylmuramoyl-L-alanine amidase activity	GO:0008745	2	2/2406	TRINITY_DN5235_c0_g1_i7_orf1;TRINITY_DN1534_c0_g1_i3_orf1
molecular_function	intramolecular lyase activity	GO:0016872	1	1/2406	TRINITY_DN10722_c0_g3_i1_orf1
molecular_function	DNA topoisomerase activity	GO:0003916	2	2/2406	TRINITY_DN4908_c1_g1_i5_orf1;TRINITY_DN6248_c0_g1_i1_orf1
molecular_function	intramolecular transferase activity	GO:0016866	7	7/2406	TRINITY_DN120089_c0_g1_i1_orf1;TRINITY_DN2283_c0_g2_i1_orf1;TRINITY_DN12545_c0_g1_i7_orf1;TRINITY_DN4955_c0_g1_i2_orf1;TRINITY_DN30713_c0_g1_i3_orf1;TRINITY_DN2769_c0_g1_i1_orf1;TRINITY_DN5952_c0_g1_i6_orf1
molecular_function	intramolecular oxidoreductase activity	GO:0016860	5	5/2406	TRINITY_DN6014_c1_g1_i2_orf1;TRINITY_DN1196_c0_g1_i5_orf1;TRINITY_DN51938_c0_g3_i1_orf1;TRINITY_DN3073_c0_g1_i7_orf1;TRINITY_DN1831_c0_g1_i3_orf1
molecular_function	racemase and epimerase activity	GO:0016854	1	1/2406	TRINITY_DN1353_c0_g1_i1_orf1
molecular_function	cis-trans isomerase activity	GO:0016859	4	4/2406	TRINITY_DN14372_c0_g2_i1_orf1;TRINITY_DN2807_c0_g1_i4_orf1;TRINITY_DN142588_c0_g1_i1_orf1;TRINITY_DN12293_c0_g1_i1_orf1
molecular_function	catalytic activity, acting on RNA	GO:0140098	34	34/2406	TRINITY_DN57918_c0_g1_i1_orf1;TRINITY_DN4380_c0_g1_i9_orf1;TRINITY_DN31503_c0_g1_i4_orf1;TRINITY_DN13350_c0_g1_i4_orf1;TRINITY_DN17312_c0_g1_i1_orf1;TRINITY_DN8980_c0_g1_i2_orf1;TRINITY_DN59291_c0_g1_i1_orf1;TRINITY_DN9302_c0_g1_i1_orf1;TRINITY_DN5962_c0_g1_i1_orf1;TRINITY_DN2953_c1_g1_i11_orf1;TRINITY_DN34465_c0_g1_i1_orf1;TRINITY_DN1344_c0_g1_i1_orf1;TRINITY_DN70485_c0_g1_i2_orf1;TRINITY_DN2953_c1_g1_i10_orf1;TRINITY_DN2953_c1_g1_i2_orf1;TRINITY_DN8716_c0_g1_i3_orf1;TRINITY_DN13094_c0_g1_i1_orf1;TRINITY_DN14274_c0_g1_i3_orf1;TRINITY_DN4408_c6_g1_i1_orf1;TRINITY_DN18922_c0_g1_i1_orf1;TRINITY_DN34432_c0_g1_i1_orf1;TRINITY_DN4707_c0_g1_i1_orf1;TRINITY_DN27033_c1_g1_i3_orfp1;TRINITY_DN143603_c0_g1_i1_orf1;TRINITY_DN48619_c0_g1_i1_orf1;TRINITY_DN2224_c0_g1_i1_orf1;TRINITY_DN2401_c0_g2_i1_orf1;TRINITY_DN21539_c0_g1_i1_orf1;TRINITY_DN44288_c0_g1_i2_orf1;TRINITY_DN4950_c0_g1_i2_orf1;TRINITY_DN26168_c0_g1_i1_orf1;TRINITY_DN2299_c0_g1_i3_orf1;TRINITY_DN2709_c0_g1_i4_orf1;TRINITY_DN1607_c0_g1_i16_orf1
molecular_function	catalytic activity, acting on DNA	GO:0140097	17	17/2406	TRINITY_DN81258_c0_g1_i2_orf1;TRINITY_DN11986_c0_g1_i1_orf1;TRINITY_DN2971_c0_g1_i1_orf1;TRINITY_DN15040_c0_g4_i1_orf1;TRINITY_DN74037_c0_g5_i1_orf1;TRINITY_DN40434_c0_g1_i2_orf1;TRINITY_DN45271_c0_g1_i1_orf1;TRINITY_DN6642_c0_g1_i2_orf1;TRINITY_DN70485_c0_g1_i2_orf1;TRINITY_DN6248_c0_g1_i1_orf1;TRINITY_DN123184_c0_g1_i1_orf1;TRINITY_DN4908_c1_g1_i5_orf1;TRINITY_DN110534_c0_g1_i3_orf1;TRINITY_DN5757_c0_g1_i1_orf1;TRINITY_DN291_c0_g1_i2_orf1;TRINITY_DN89613_c0_g1_i13_orf1;TRINITY_DN25345_c0_g1_i1_orf1
molecular_function	helicase activity	GO:0004386	18	18/2406	TRINITY_DN11986_c0_g1_i1_orf1;TRINITY_DN2971_c0_g1_i1_orf1;TRINITY_DN25345_c0_g1_i1_orf1;TRINITY_DN44288_c0_g1_i2_orf1;TRINITY_DN4380_c0_g1_i9_orf1;TRINITY_DN2709_c0_g1_i4_orf1;TRINITY_DN31503_c0_g1_i4_orf1;TRINITY_DN6642_c0_g1_i2_orf1;TRINITY_DN13094_c0_g1_i1_orf1;TRINITY_DN14274_c0_g1_i3_orf1;TRINITY_DN4408_c6_g1_i1_orf1;TRINITY_DN26168_c0_g1_i1_orf1;TRINITY_DN8980_c0_g1_i2_orf1;TRINITY_DN59291_c0_g1_i1_orf1;TRINITY_DN9302_c0_g1_i1_orf1;TRINITY_DN5757_c0_g1_i1_orf1;TRINITY_DN4950_c0_g1_i2_orf1;TRINITY_DN291_c0_g1_i2_orf1
molecular_function	transferase activity, transferring alkyl or aryl (other than methyl) groups	GO:0016765	22	22/2406	TRINITY_DN9506_c0_g1_i2_orf1;TRINITY_DN22046_c1_g1_i5_orf1;TRINITY_DN4695_c0_g1_i4_orf1;TRINITY_DN12134_c0_g1_i4_orf1;TRINITY_DN128231_c0_g1_i5_orf1;TRINITY_DN8651_c0_g1_i18_orf1;TRINITY_DN4695_c0_g1_i3_orf1;TRINITY_DN8964_c0_g1_i4_orf1;TRINITY_DN10222_c0_g1_i2_orf1;TRINITY_DN1305_c0_g1_i6_orf1;TRINITY_DN3929_c0_g3_i3_orf1;TRINITY_DN23732_c0_g1_i1_orf1;TRINITY_DN62707_c0_g1_i1_orf1;TRINITY_DN3332_c0_g1_i11_orf1;TRINITY_DN8651_c0_g1_i16_orf1;TRINITY_DN4279_c0_g1_i4_orf1;TRINITY_DN920_c0_g1_i6_orf1;TRINITY_DN920_c0_g1_i4_orf1;TRINITY_DN22_c0_g1_i3_orf1;TRINITY_DN2255_c0_g1_i1_orf1;TRINITY_DN7512_c0_g1_i1_orf1;TRINITY_DN3332_c0_g1_i9_orf1
molecular_function	transferase activity, transferring nitrogenous groups	GO:0016769	8	8/2406	TRINITY_DN2890_c0_g1_i2_orf1;TRINITY_DN1262_c0_g1_i2_orf1;TRINITY_DN6908_c0_g1_i3_orf1;TRINITY_DN5564_c0_g1_i5_orf1;TRINITY_DN2803_c4_g1_i1_orf1;TRINITY_DN2848_c0_g1_i1_orf1;TRINITY_DN11013_c0_g1_i3_orf1;TRINITY_DN1068_c0_g1_i3_orf1
molecular_function	transferase activity, transferring sulphur-containing groups	GO:0016782	3	3/2406	TRINITY_DN6693_c0_g1_i1_orf1;TRINITY_DN16516_c0_g1_i1_orf1;TRINITY_DN874_c2_g1_i1_orf1
molecular_function	glycosyltransferase activity	GO:0016757	16	16/2406	TRINITY_DN49786_c0_g1_i1_orf1;TRINITY_DN38435_c0_g1_i1_orf1;TRINITY_DN31390_c0_g1_i2_orf1;TRINITY_DN79868_c0_g1_i1_orf1;TRINITY_DN15157_c0_g1_i1_orf1;TRINITY_DN9079_c1_g1_i1_orf1;TRINITY_DN14018_c0_g1_i4_orf1;TRINITY_DN9079_c0_g1_i5_orf1;TRINITY_DN12508_c0_g1_i1_orf1;TRINITY_DN3355_c0_g2_i4_orf1;TRINITY_DN4142_c0_g1_i5_orf1;TRINITY_DN5513_c0_g1_i1_orf1;TRINITY_DN14597_c0_g1_i5_orf1;TRINITY_DN2967_c0_g1_i7_orf1;TRINITY_DN4248_c0_g1_i4_orf1;TRINITY_DN812_c2_g1_i1_orf1
molecular_function	transketolase or transaldolase activity	GO:0016744	1	1/2406	TRINITY_DN60787_c0_g1_i5_orf1
molecular_function	transferase activity, transferring phosphorus-containing groups	GO:0016772	67	67/2406	TRINITY_DN1173_c0_g1_i12_orf1;TRINITY_DN56998_c0_g1_i2_orf1;TRINITY_DN14477_c0_g1_i12_orf1;TRINITY_DN47151_c0_g1_i1_orf1;TRINITY_DN1552_c0_g1_i3_orf1;TRINITY_DN18222_c0_g1_i5_orf1;TRINITY_DN2719_c1_g1_i6_orf1;TRINITY_DN1173_c0_g1_i11_orf1;TRINITY_DN62557_c0_g1_i1_orf1;TRINITY_DN41166_c0_g1_i1_orf1;TRINITY_DN277_c1_g1_i1_orf1;TRINITY_DN21278_c0_g2_i2_orf1;TRINITY_DN1405_c0_g1_i1_orf1;TRINITY_DN618_c0_g1_i3_orf1;TRINITY_DN37923_c0_g1_i1_orf1;TRINITY_DN5281_c0_g2_i3_orf1;TRINITY_DN31967_c0_g1_i5_orf1;TRINITY_DN32700_c0_g1_i2_orf1;TRINITY_DN36632_c0_g1_i1_orf1;TRINITY_DN81258_c0_g1_i2_orf1;TRINITY_DN2738_c1_g1_i3_orf1;TRINITY_DN15040_c0_g4_i1_orf1;TRINITY_DN1034_c0_g1_i4_orf1;TRINITY_DN42461_c0_g1_i4_orf1;TRINITY_DN46090_c0_g2_i1_orf1;TRINITY_DN2983_c0_g1_i6_orf1;TRINITY_DN70485_c0_g1_i2_orf1;TRINITY_DN8603_c0_g1_i1_orf1;TRINITY_DN1718_c6_g1_i4_orf1;TRINITY_DN248_c0_g1_i1_orf1;TRINITY_DN143509_c0_g1_i1_orf1;TRINITY_DN19662_c4_g1_i1_orf1;TRINITY_DN73945_c0_g5_i3_orf1;TRINITY_DN12_c0_g1_i5_orf1;TRINITY_DN7247_c0_g1_i7_orf1;TRINITY_DN46715_c0_g1_i1_orf1;TRINITY_DN10774_c0_g2_i3_orf1;TRINITY_DN10742_c0_g1_i4_orf1;TRINITY_DN4320_c0_g1_i1_orf1;TRINITY_DN1475_c0_g1_i6_orf1;TRINITY_DN1154_c0_g1_i1_orf1;TRINITY_DN11015_c0_g1_i8_orf1;TRINITY_DN6185_c0_g1_i12_orf1;TRINITY_DN4707_c0_g1_i1_orf1;TRINITY_DN110534_c0_g1_i3_orf1;TRINITY_DN6436_c0_g1_i1_orf1;TRINITY_DN143637_c0_g1_i1_orf1;TRINITY_DN4798_c0_g1_i3_orf1;TRINITY_DN74037_c0_g5_i1_orf1;TRINITY_DN2401_c0_g2_i1_orf1;TRINITY_DN52244_c1_g1_i1_orf1;TRINITY_DN2618_c0_g1_i3_orf1;TRINITY_DN4408_c6_g1_i1_orf1;TRINITY_DN8261_c0_g1_i1_orf1;TRINITY_DN30_c0_g1_i6_orf1;TRINITY_DN1173_c1_g1_i10_orf1;TRINITY_DN147475_c0_g1_i1_orf1;TRINITY_DN18782_c0_g1_i4_orf1;TRINITY_DN6813_c1_g1_i1_orf1;TRINITY_DN10680_c0_g1_i5_orf1;TRINITY_DN116972_c0_g1_i1_orf1;TRINITY_DN30154_c0_g1_i1_orf1;TRINITY_DN2299_c0_g1_i3_orf1;TRINITY_DN11620_c0_g1_i2_orf1;TRINITY_DN5697_c0_g1_i1_orf1;TRINITY_
molecular_function	transferase activity, transferring one-carbon groups	GO:0016741	17	17/2406	TRINITY_DN2114_c0_g1_i5_orf1;TRINITY_DN1344_c0_g1_i1_orf1;TRINITY_DN3800_c0_g1_i7_orf1;TRINITY_DN5748_c0_g1_i6_orf1;TRINITY_DN3263_c0_g1_i2_orf1;TRINITY_DN1216_c0_g1_i4_orf1;TRINITY_DN13350_c0_g1_i4_orf1;TRINITY_DN77318_c0_g2_i1_orf1;TRINITY_DN95414_c0_g1_i1_orf1;TRINITY_DN17312_c0_g1_i1_orf1;TRINITY_DN6462_c0_g1_i5_orf1;TRINITY_DN33953_c0_g1_i4_orf1;TRINITY_DN5748_c0_g1_i5_orf1;TRINITY_DN14734_c0_g1_i2_orf1;TRINITY_DN2457_c0_g1_i8_orf1;TRINITY_DN14313_c0_g1_i1_orf1;TRINITY_DN5962_c0_g1_i1_orf1
molecular_function	acyltransferase activity	GO:0016746	29	29/2406	TRINITY_DN2065_c1_g2_i1_orf1;TRINITY_DN86833_c0_g3_i1_orf1;TRINITY_DN20710_c0_g1_i2_orf1;TRINITY_DN6871_c0_g1_i3_orf1;TRINITY_DN5841_c0_g1_i2_orf1;TRINITY_DN20710_c0_g2_i2_orf1;TRINITY_DN52553_c0_g2_i1_orf1;TRINITY_DN47389_c0_g1_i2_orf1;TRINITY_DN5153_c1_g1_i1_orf1;TRINITY_DN12771_c0_g1_i1_orf1;TRINITY_DN5211_c0_g1_i1_orf1;TRINITY_DN51737_c0_g1_i3_orf1;TRINITY_DN1362_c0_g1_i4_orf1;TRINITY_DN10430_c0_g1_i4_orf1;TRINITY_DN101358_c0_g2_i1_orf1;TRINITY_DN21570_c0_g1_i1_orf1;TRINITY_DN117844_c0_g1_i1_orf1;TRINITY_DN3179_c0_g1_i1_orf1;TRINITY_DN15411_c0_g1_i4_orf1;TRINITY_DN5525_c0_g1_i4_orf1;TRINITY_DN76283_c0_g2_i1_orf1;TRINITY_DN1081_c0_g1_i7_orf1;TRINITY_DN22956_c0_g1_i1_orf1;TRINITY_DN52553_c0_g1_i1_orf1;TRINITY_DN1084_c0_g1_i2_orf1;TRINITY_DN4898_c0_g1_i7_orf1;TRINITY_DN3545_c0_g1_i6_orf1;TRINITY_DN1084_c0_g2_i2_orf1;TRINITY_DN16125_c0_g1_i3_orf1
