# Supplementary material for: Comparative Proteomics and Metabonomics Analysis of Different Diapause Stages Revealed a New Regulation Mechanism of Diapause in Loxostege sticticalis (Lepidoptera: Pyralidae)
Source: Molecules. 2024 Jul 25;29(15):3472. doi: 10.3390/molecules29153472 (PMC11314584; doi:10.3390/molecules29153472)
Supplement: Supplementary file 1 [file molecules-29-03472-s001.zip › analysis process/proteomic/GO annotations analysis/RDvsPreD all.pdf]

Term Type	GO Term	GO ID	JCZY_vs_ZYQ_all num	JCZY_vs_ZYQ_all percent	JCZY_vs_ZYQ_all Accession ids
biological_process	immune response-activating signal transduction	GO:0002757	1	1/2397	TRINITY_DN2170_c1_g1_i3_orf1
biological_process	activation of innate immune response	GO:0002218	3	3/2397	TRINITY_DN8685_c0_g1_i5_orf1;TRINITY_DN2170_c1_g1_i3_orf1;TRINITY_DN5880_c0_g2_i2_orf1
biological_process	cell activation involved in immune response	GO:0002263	1	1/2397	TRINITY_DN46409_c0_g1_i1_orf1
biological_process	lymphocyte activation	GO:0046649	1	1/2397	TRINITY_DN46409_c0_g1_i1_orf1
biological_process	leukocyte activation involved in immune response	GO:0002366	1	1/2397	TRINITY_DN46409_c0_g1_i1_orf1
biological_process	innate immune response	GO:0045087	11	11/2397	TRINITY_DN827_c1_g1_i1_orf1;TRINITY_DN479_c6_g1_i2_orf1;TRINITY_DN8685_c0_g1_i5_orf1;TRINITY_DN1444_c1_g1_i5_orf1;TRINITY_DN195_c4_g1_i1_orf1;TRINITY_DN6098_c1_g1_i5_orf1;TRINITY_DN2170_c1_g1_i3_orf1;TRINITY_DN15706_c0_g2_i5_orf1;TRINITY_DN5235_c0_g1_i7_orf1;TRINITY_DN9044_c0_g1_i2_orf1;TRINITY_DN5880_c
biological_process	somatic diversification of immune receptors via germline recombination within a single locus	GO:0002562	1	1/2397	TRINITY_DN46409_c0_g1_i1_orf1
biological_process	somatic diversification of immunoglobulins	GO:0016445	1	1/2397	TRINITY_DN46409_c0_g1_i1_orf1
biological_process	regulation of catalytic activity	GO:0050790	14	14/2397	TRINITY_DN1475_c0_g1_i6_orf1;TRINITY_DN13999_c0_g1_i4_orf1;TRINITY_DN8473_c0_g1_i6_orf1;TRINITY_DN14154_c0_g1_i1_orf1;TRINITY_DN518_c0_g1_i1_orf1;TRINITY_DN11986_c0_g1_i1_orf1;TRINITY_DN802_c0_g1_i2_orf1;TRINITY_DN1328_c0_g1_i6_orf1;TRINITY_DN46409_c0_g1_i1_orf1;TRINITY_DN27021_c0_g1_i1_orf1;TRINITY_DN50074_c0_g1_i1_orf1;TRINITY_DN147475_c0_g1_i1_orf1;TRINITY_DN46022_c0_g1_i1_orf1;TRINITY_DN23354_c0_g1_i7_orf1
biological_process	positive regulation of molecular function	GO:0044093	12	12/2397	TRINITY_DN14154_c0_g1_i1_orf1;TRINITY_DN50074_c0_g1_i1_orf1;TRINITY_DN9475_c0_g1_i6_orf1;TRINITY_DN27021_c0_g1_i1_orf1;TRINITY_DN802_c0_g1_i2_orf1;TRINITY_DN46409_c0_g1_i1_orf1;TRINITY_DN1352_c0_g1_i5_orf1;TRINITY_DN5553_c0_g1_i4_orf1;TRINITY_DN2175_c0_g1_i4_orf1;TRINITY_DN46022_c0_g1_i1_orf1;TRINITY_DN48097_c0_g1_i1_orf1;TRINITY_DN23354_c0_g1_i7_orf1
biological_process	negative regulation of molecular function	GO:0044092	5	5/2397	TRINITY_DN1328_c0_g1_i6_orf1;TRINITY_DN5442_c0_g1_i4_orf1;TRINITY_DN13999_c0_g1_i4_orf1;TRINITY_DN11986_c0_g1_i1_orf1;TRINITY_DN31584_c0_g2_i2_orf1
biological_process	regulation of binding	GO:0051098	2	2/2397	TRINITY_DN48097_c0_g1_i1_orf1;TRINITY_DN147475_c0_g1_i1_orf1
biological_process	regulation of ATP-dependent activity	GO:0043462	2	2/2397	TRINITY_DN5442_c0_g1_i4_orf1;TRINITY_DN11986_c0_g1_i1_orf1
biological_process	regulation of transporter activity	GO:0032409	5	5/2397	TRINITY_DN9475_c0_g1_i6_orf1;TRINITY_DN1352_c0_g1_i5_orf1;TRINITY_DN31584_c0_g2_i2_orf1;TRINITY_DN5553_c0_g1_i4_orf1;TRINITY_DN2175_c0_g1_i4_orf1
biological_process	regulation of metabolic process	GO:0019222	48	48/2397	TRINITY_DN59804_c0_g1_i1_orf1;TRINITY_DN13999_c0_g1_i4_orf1;TRINITY_DN288_c0_g1_i9_orf1;TRINITY_DN8702_c0_g1_i1_orf1;TRINITY_DN1706_c0_g1_i7_orf1;TRINITY_DN28981_c0_g1_i1_orf1;TRINITY_DN20442_c0_g2_i1_orf1;TRINITY_DN23360_c0_g1_i3_orf1;TRINITY_DN1710_c0_g2_i2_orf1;TRINITY_DN46409_c0_g1_i1_orf1;TRINITY_DN31584_c0_g2_i2_orf1;TRINITY_DN53233_c0_g1_i1_orf1;TRINITY_DN52_c0_g1_i4_orf1;TRINITY_DN14701_c0_g1_i2_orf1;TRINITY_DN48097_c0_g1_i1_orf1;TRINITY_DN1475_c0_g1_i6_orf1;TRINITY_DN8473_c0_g1_i6_orf1;TRINITY_DN67649_c0_g1_i1_orf1;TRINITY_DN12771_c0_g1_i1_orf1;TRINITY_DN110400_c0_g1_i1_orf1;TRINITY_DN1328_c0_g1_i6_orf1;TRINITY_DN3673_c0_g1_i10_orf1;TRINITY_DN810_c0_g1_i4_orf1;TRINITY_DN50074_c0_g1_i1_orf1;TRINITY_DN11050_c0_g1_i8_orf1;TRINITY_DN12_c0_g1_i5_orf1;TRINITY_DN9510_c0_g2_i1_orf1;TRINITY_DN31585_c0_g1_i1_orf1;TRINITY_DN17655_c0_g1_i1_orf1;TRINITY_DN5562_c0_g1_i3_orf1;TRINITY_DN41573_c0_g1_i1_orf1;TRINITY_DN15256_c0_g1_i8_orf1;TRINITY_DN44070_c0_g2_i2_orf1;TRINITY_DN20009_c0_g1_i1_orf1;TRINITY_DN34689_c0_g1_i4_orf1;TRINITY_DN72_c0_g1_i16_orf1;TRINITY_DN96557_c0_g1_i1_orf1;TRINITY_DN45449_c0_g1_i1_orf1;TRINITY_DN2802_c0_g1_i1_orf1;TRINITY_DN33893_c0_g1_i1_orf1;TRINITY_DN4820_c0_g2_i2_orf1;TRINITY_DN2802_c1_g1_i1_orf1;TRINITY_DN147475_c0_g1_i1_orf1;TRINITY_DN3649_c0_g1_i6_orf1;TRINITY_DN46022_c0_g1_i1_orf1;TRINITY_DN18563_c2_g1_i1_orf1;TRINITY_
biological_process	regulation of response to stimulus	GO:0048583	14	14/2397	TRINITY_DN14154_c0_g1_i1_orf1;TRINITY_DN51938_c0_g3_i1_orf1;TRINITY_DN41573_c0_g1_i1_orf1;TRINITY_DN479_c6_g1_i2_orf1;TRINITY_DN8685_c0_g1_i5_orf1;TRINITY_DN17655_c0_g1_i1_orf1;TRINITY_DN147475_c0_g1_i1_orf1;TRINITY_DN46409_c0_g1_i1_orf1;TRINITY_DN2170_c1_g1_i3_orf1;TRINITY_DN146119_c0_g1_i1_orf1;TRINITY_DN34745_c0_g2_i1_orf1;TRINITY_DN1612_c0_g1_i3_orf1;TRINITY_DN3833_c0_g1_i4_orf1;TRINITY_DN5880_c0_g2_i2_orf1
biological_process	regulation of developmental process	GO:0050793	3	3/2397	TRINITY_DN52_c0_g1_i4_orf1;TRINITY_DN3887_c0_g1_i1_orf1;TRINITY_DN20133_c0_g1_i1_orf1
biological_process	regulation of cellular process	GO:0050794	79	79/2397	TRINITY_DN59804_c0_g1_i1_orf1;TRINITY_DN16011_c0_g1_i3_orf1;TRINITY_DN51938_c0_g3_i1_orf1;TRINITY_DN288_c0_g1_i9_orf1;TRINITY_DN8702_c0_g1_i1_orf1;TRINITY_DN1706_c0_g1_i7_orf1;TRINITY_DN28981_c0_g1_i1_orf1;TRINITY_DN20442_c0_g2_i1_orf1;TRINITY_DN23360_c0_g1_i3_orf1;TRINITY_DN5182_c0_g1_i5_orf1;TRINITY_DN13999_c0_g1_i4_orf1;TRINITY_DN4676_c0_g1_i16_orf1;TRINITY_DN2623_c0_g1_i3_orf1;TRINITY_DN46409_c0_g1_i1_orf1;TRINITY_DN9965_c0_g1_i1_orf1;TRINITY_DN31584_c0_g2_i2_orf1;TRINITY_DN15706_c0_g2_i5_orf1;TRINITY_DN146119_c0_g1_i1_orf1;TRINITY_DN34745_c0_g2_i1_orf1;TRINITY_DN48097_c0_g1_i1_orf1;TRINITY_DN2770_c0_g2_i4_orf1;TRINITY_DN1475_c0_g1_i6_orf1;TRINITY_DN6462_c0_g1_i5_orf1;TRINITY_DN17655_c0_g1_i1_orf1;TRINITY_DN67649_c0_g1_i1_orf1;TRINITY_DN25360_c0_g1_i2_orf1;TRINITY_DN3673_c0_g1_i10_orf1;TRINITY_DN8692_c0_g1_i2_orf1;TRINITY_DN110400_c0_g1_i1_orf1;TRINITY_DN2983_c0_g1_i6_orf1;TRINITY_DN1008_c0_g1_i2_orf1;TRINITY_DN1352_c0_g1_i5_orf1;TRINITY_DN11050_c0_g1_i8_orf1;TRINITY_DN3649_c0_g1_i6_orf1;TRINITY_DN5553_c0_g1_i4_orf1;TRINITY_DN9475_c0_g1_i6_orf1;TRINITY_DN44261_c0_g1_i1_orf1;TRINITY_DN1612_c0_g1_i3_orf1;TRINITY_DN12_c0_g1_i5_orf1;TRINITY_DN9510_c0_g2_i1_orf1;TRINITY_DN31585_c0_g1_i1_orf1;TRINITY_DN14154_c0_g1_i1_orf1;TRINITY_DN1710_c0_g2_i2_orf1;TRINITY_DN5562_c0_g1_i3_orf1;TRINITY_DN32700_c0_g1_i2_orf1;TRINITY_DN41573_c0_g1_i1_orf1;TRINITY_DN2175_c0_g1_i4_orf1;TRINITY_DN11986_c0_g1_i1_orf1;TRINITY_DN2793_c0_g2_i1_orf1;TRINITY_DN15256_c0_g1_i8_orf1;TRINITY_DN44070_c0_g2_i2_orf1;TRINITY_DN46633_c0_g1_i4_orf1;TRINITY_DN52_c0_g1_i4_orf1;TRINITY_DN20009_c0_g1_i1_orf1;TRINITY_DN8473_c0_g1_i6_orf1;TRINITY_DN4439_c0_g1_i2_orf1;TRINITY_DN147475_c0_g1_i1_orf1;TRINITY_DN72_c0_g1_i16_orf1;TRINITY_DN96557_c0_g1_i1_orf1;TRINITY_DN376_c0_g1_i1_orf1;TRINITY_DN45449_c0_g1_i1_orf1;TRINITY_DN7391_c0_g1_i2_orf1;TRINITY_DN2802_c0_g1_i1_orf1;TRINITY_DN33893_c0_g1_i1_orf1;TRINITY_DN7102_c0_g1_i5_orf1;TRINITY_DN11245_c0_g1_i2_orf1;TRINITY_DN9146_c0_g1_i1_orf1;TRINITY_DN2802_c1_g1_i1_orf1;TRINITY_DN802_c0_g1_i2_orf1;TRINITY_DN42854_c0_g3_i2_orf1;TRINITY_DN4410_c0_g1_i1_orf1;TRINITY_DN2170_c1_g1_i3_orf1;TRINITY_DN20133_c0_g1_i1_orf1;TRINITY_DN18563_c2_g1_i1_orf1;TRINITY_DN804_c0_g1_i7_orf1;TRINITY_DN3833_c0_g1_i4_orf1;TRINITY_DN15247_c0_g1_i2_orf1;TRINITY_DN8
biological_process	regulation of locomotion	GO:0040012	2	2/2397	TRINITY_DN147475_c0_g1_i1_orf1;TRINITY_DN20133_c0_g1_i1_orf1
biological_process	regulation of localization	GO:0032879	5	5/2397	TRINITY_DN9475_c0_g1_i6_orf1;TRINITY_DN1352_c0_g1_i5_orf1;TRINITY_DN31584_c0_g2_i2_orf1;TRINITY_DN5553_c0_g1_i4_orf1;TRINITY_DN2175_c0_g1_i4_orf1
biological_process	regulation of multicellular organismal process	GO:0051239	5	5/2397	TRINITY_DN1455_c0_g1_i8_orf1;TRINITY_DN52_c0_g1_i4_orf1;TRINITY_DN46409_c0_g1_i1_orf1;TRINITY_DN1455_c0_g1_i4_orf1;TRINITY_DN147475_c0_g1_i1_orf1
biological_process	regulation of membrane repolarization	GO:0060306	1	1/2397	TRINITY_DN31584_c0_g2_i2_orf1
biological_process	regulation of immune system process	GO:0002682	5	5/2397	TRINITY_DN8685_c0_g1_i5_orf1;TRINITY_DN46409_c0_g1_i1_orf1;TRINITY_DN2170_c1_g1_i3_orf1;TRINITY_DN5880_c0_g2_i2_orf1;TRINITY_DN479_c6_g1_i2_orf1
biological_process	positive regulation of biological process	GO:0048518	24	24/2397	TRINITY_DN51938_c0_g3_i1_orf1;TRINITY_DN15706_c0_g2_i5_orf1;TRINITY_DN44261_c0_g1_i1_orf1;TRINITY_DN8685_c0_g1_i5_orf1;TRINITY_DN9475_c0_g1_i6_orf1;TRINITY_DN1710_c0_g2_i2_orf1;TRINITY_DN46409_c0_g1_i1_orf1;TRINITY_DN31584_c0_g2_i2_orf1;TRINITY_DN288_c0_g1_i9_orf1;TRINITY_DN146119_c0_g1_i1_orf1;TRINITY_DN48097_c0_g1_i1_orf1;TRINITY_DN1352_c0_g1_i5_orf1;TRINITY_DN5553_c0_g1_i4_orf1;TRINITY_DN20009_c0_g1_i1_orf1;TRINITY_DN9510_c0_g2_i1_orf1;TRINITY_DN17655_c0_g1_i1_orf1;TRINITY_DN50074_c0_g1_i1_orf1;TRINITY_DN41573_c0_g1_i1_orf1;TRINITY_DN20133_c0_g1_i1_orf1;TRINITY_DN147475_c0_g1_i1_orf1;TRINITY_DN2170_c1_g1_i3_orf1;TRINITY_DN2175_c0_g1_i4_orf1;TRINITY_DN3833_c0_g1_i4_orf1;TRINITY_DN5880_c0_g2_i2_orf1
biological_process	negative regulation of biological process	GO:0048519	22	22/2397	TRINITY_DN59804_c0_g1_i1_orf1;TRINITY_DN13999_c0_g1_i4_orf1;TRINITY_DN8702_c0_g1_i1_orf1;TRINITY_DN46409_c0_g1_i1_orf1;TRINITY_DN31584_c0_g2_i2_orf1;TRINITY_DN53233_c0_g1_i1_orf1;TRINITY_DN14701_c0_g1_i2_orf1;TRINITY_DN12771_c0_g1_i1_orf1;TRINITY_DN1328_c0_g1_i6_orf1;TRINITY_DN3673_c0_g1_i10_orf1;TRINITY_DN810_c0_g1_i4_orf1;TRINITY_DN20009_c0_g1_i1_orf1;TRINITY_DN14154_c0_g1_i1_orf1;TRINITY_DN41573_c0_g1_i1_orf1;TRINITY_DN11986_c0_g1_i1_orf1;TRINITY_DN44070_c0_g2_i2_orf1;TRINITY_DN34689_c0_g1_i4_orf1;TRINITY_DN96557_c0_g1_i1_orf1;TRINITY_DN4820_c0_g2_i2_orf1;TRINITY_DN147475_c0_g1_i1_orf1;TRINITY_DN20133_c0_g1_i1_orf1;TRINITY_DN46022_c0_g1_i1_orf1
biological_process	regulation of signaling	GO:0023051	11	11/2397	TRINITY_DN14154_c0_g1_i1_orf1;TRINITY_DN51938_c0_g3_i1_orf1;TRINITY_DN9475_c0_g1_i6_orf1;TRINITY_DN2175_c0_g1_i4_orf1;TRINITY_DN147475_c0_g1_i1_orf1;TRINITY_DN1352_c0_g1_i5_orf1;TRINITY_DN5553_c0_g1_i4_orf1;TRINITY_DN146119_c0_g1_i1_orf1;TRINITY_DN34745_c0_g2_i1_orf1;TRINITY_DN1612_c0_g1_i3_orf1;TRINITY_DN3833_c0_g1_i4_orf1
biological_process	regulation of membrane potential	GO:0042391	1	1/2397	TRINITY_DN31584_c0_g2_i2_orf1
biological_process	regulation of neurotransmitter levels	GO:0001505	3	3/2397	TRINITY_DN17693_c0_g1_i10_orf1;TRINITY_DN82017_c0_g1_i5_orf1;TRINITY_DN14565_c0_g1_i11_orf1
biological_process	regulation of body fluid levels	GO:0050878	1	1/2397	TRINITY_DN4016_c0_g1_i1_orf1
biological_process	homeostatic process	GO:0042592	15	15/2397	TRINITY_DN96557_c0_g1_i1_orf1;TRINITY_DN46625_c0_g1_i1_orf1;TRINITY_DN65681_c0_g1_i1_orf1;TRINITY_DN1423_c0_g1_i4_orf1;TRINITY_DN1423_c0_g1_i8_orf1;TRINITY_DN4469_c0_g1_i2_orf1;TRINITY_DN136031_c0_g1_i7_orf1;TRINITY_DN376_c0_g1_i1_orf1;TRINITY_DN3461_c0_g1_i1_orf1;TRINITY_DN9965_c0_g1_i1_orf1;TRINITY_DN15812_c0_g1_i2_orf1;TRINITY_DN31584_c0_g2_i2_orf1;TRINITY_DN20133_c0_g1_i1_orf1;TRINITY_DN44256_c0_g1_i1_orf1;TRINITY_DN5753_c0_g1_i10_orf1
biological_process	regulation of anatomical structure size	GO:0090066	2	2/2397	TRINITY_DN4439_c0_g1_i2_orf1;TRINITY_DN80424_c0_g1_i1_orf1
biological_process	regulation of RNA stability	GO:0043487	1	1/2397	TRINITY_DN20009_c0_g1_i1_orf1
biological_process	regulation of protein stability	GO:0031647	1	1/2397	TRINITY_DN46409_c0_g1_i1_orf1
biological_process	NADH regeneration	GO:0006735	1	1/2397	TRINITY_DN20133_c0_g1_i1_orf1

biological_process	organonitrogen compound metabolic process	GO:1901564	311	311/2397	TRINITY_DN24469_c0_g2_i2_orf1;TRINITY_DN2065_c1_g2_i1_orf1;TRINITY_DN10364_c0_g1_i5_orf1;TRINITY_DN827_c1_g1_i1_orf1;TRINITY_DN14565_c0_g1_i11_orf1;TRINITY_DN4360_c0_g1_i4_orf1;TRINITY_DN3194_c0_g1_i6_orf1;TRINITY_DN137_c0_g1_i1_orf1;TRINITY_DN60787_c0_g1_i5_orf1;TRINITY_DN1533_c0_g2_i1_orf1;TRINITY_DN140_c1_g1_i2_orf1;TRINITY_DN3978_c0_g2_i1_orf1;TRINITY_DN23167_c0_g2_i1_orf1;TRINITY_DN35763_c0_g1_i2_orf1;TRINITY_DN56164_c0_g1_i1_orf1;TRINITY_DN5873_c0_g4_i1_orf1;TRINITY_DN1274_c0_g1_i4_orf1;TRINITY_DN6436_c0_g1_i1_orf1;TRINITY_DN2069_c1_g1_i8_orf1;TRINITY_DN15160_c0_g1_i1_orf1;TRINITY_DN4217_c0_g1_i2_orf1;TRINITY_DN38431_c0_g1_i1_orf1;TRINITY_DN2682_c0_g1_i4_orf1;TRINITY_DN17772_c0_g2_i3_orf1;TRINITY_DN8692_c0_g1_i2_orf1;TRINITY_DN2442_c0_g1_i2_orf1;TRINITY_DN8659_c0_g1_i1_orf1;TRINITY_DN41952_c0_g1_i4_orf1;TRINITY_DN2983_c0_g1_i6_orf1;TRINITY_DN11065_c0_g2_i1_orf1;TRINITY_DN24723_c2_g1_i1_orf1;TRINITY_DN16258_c0_g1_i2_orf1;TRINITY_DN2953_c1_g1_i10_orf1;TRINITY_DN7957_c0_g1_i5_orf1;TRINITY_DN14754_c0_g1_i6_orf1;TRINITY_DN29448_c0_g1_i1_orf1;TRINITY_DN11013_c0_g1_i3_orf1;TRINITY_DN2954_c0_g1_i1_orf1;TRINITY_DN14217_c0_g1_i1_orf1;TRINITY_DN14774_c0_g1_i4_orf1;TRINITY_DN4822_c0_g1_i6_orf1;TRINITY_DN21570_c0_g1_i1_orf1;TRINITY_DN2885_c1_g1_i2_orf1;TRINITY_DN2953_c1_g1_i2_orf1;TRINITY_DN17326_c0_g1_i8_orf1;TRINITY_DN2224_c0_g1_i1_orf1;TRINITY_DN3991_c0_g1_i6_orf1;TRINITY_DN2794_c1_g1_i8_orf1;TRINITY_DN20796_c0_g1_i4_orf1;TRINITY_DN59885_c0_g1_i3_orf1;TRINITY_DN3800_c0_g1_i7_orf1;TRINITY_DN4886_c0_g1_i6_orf1;TRINITY_DN1509_c0_g1_i1_orf1;TRINITY_DN6185_c0_g1_i12_orf1;TRINITY_DN28299_c0_g1_i1_orf1;TRINITY_DN817_c0_g1_i3_orf1;TRINITY_DN3985_c0_g2_i1_orf1;TRINITY_DN391_c0_g1_i4_orf1;TRINITY_DN875_c0_g1_i3_orf1;TRINITY_DN6563_c0_g1_i1_orf1;TRINITY_DN3483_c0_g1_i5_orf1;TRINITY_DN22674_c0_g1_i2_orf1;TRINITY_DN18593_c0_g1_i1_orf1;TRINITY_DN8908_c0_g1_i1_orf1;TRINITY_DN6470_c0_g3_i2_orf1;TRINITY_DN3826_c0_g1_i1_orf1;TRINITY_DN13732_c0_g2_i3_orf1;TRINITY_DN1380_c0_g1_i5_orf1;TRINITY_DN107288_c0_g1_i2_orf1;TRINITY_DN10403_c0_g1_i3_orf1;TRINITY_DN11620_c0_g1_i2_orf1;TRINITY_DN7808_c0_g1_i1_orf1;TRINITY_DN6967_c0_g1_i3_orf1;TRINITY_DN4767_c0_g1_i6_orf1;TRINITY_DN230_c2_g1_i5_orf1;TRINITY_DN6199_c2_g1_i3_orf1;TRINITY_DN18249_c0_g1_i1_orf1;TRINITY_DN143895_c0_g1_i1_orf1;TRINITY_DN13856_c0_g1_i1_orf1;TRINITY_DN12526_c0_g1_i5_orf1;TRINITY_DN23360_c0_g1_i3_orf1;TRINITY_DN10824_c0_g1_i3_orf1;TRINITY_DN19990_c0_g1_i1_orf1;TRINITY_DN14398_c0_g1_i4_orf1;TRINITY_DN6813_c1_g1_i1_orf1;TRINITY_DN18869_c0_g1_i1_orf1;TRINITY_DN2719_c1_g1_i6_orf1;TRINITY_DN19537_c0_g1_i1_orf1;TRINITY_DN4189_c0_g2_i1_orf1;TRINITY_DN1791_c0_g1_i3_orf1;TRINITY_DN37923_c0_g1_i1_orf1;TRINITY_DN97589_c0_g1_i3_orf1;TRINITY_DN4451_c0_g2_i4_orf1;TRINITY_DN57798_c0_g1_i1_orf1;TRINITY_DN1310_c0_g1_i4_orf1;TRINITY_DN5012_c0_g1_i6_orf1;TRINITY_DN4125_c1_g1_i5_orf1;TRINITY_DN57918_c0_g1_i1_orf1;TRINITY_DN42461_c0_g1_i4_orf1;TRINITY_DN30154_c0_g1_i1_orf1;TRINITY_DN46409_c0_g1_i1_orf1;TRINITY_DN3836_c0_g1_i4_orf1;TRINITY_DN21719_c0_g1_i2_orf1;TRINITY_DN7583_c0_g1_i1_orf1;TRINITY_DN779_c0_g1_i3_orf1;TRINITY_DN1607_c0_g1_i16_orf1;TRINITY_DN7464_c0_g1_i14_orf1;TRINITY_DN50787_c0_g2_i2_orf1;TRINITY_DN41_c0_g1_i3_orf1;TRINITY_DN45948_c1_g1_i1_orf1;TRINITY_DN36434_c0_g2_i3_orf1;TRINITY_DN5696_c0_g1_i4_orf1;TRINITY_DN4145_c0_g1_i1_orf1;TRINITY_DN36144_c0_g1_i3_orf1;TRINITY_DN6423_c0_g1_i5_orf1;TRINITY_DN71863_c0_g1_i2_orf1;TRINITY_DN29034_c0_g1_i2_orf1;TRINITY_DN32700_c0_g1_i2_orf1;TRINITY_DN117844_c0_g1_i1_orf1;TRINITY_DN9794_c0_g2_i8_orf1;TRINITY_DN3073_c0_g1_i7_orf1;TRINITY_DN9591_c0_g1_i1_orf1;TRINITY_DN49936_c0_g2_i1_orf1;TRINITY_DN5444_c0_g2_i1_orf1;TRINITY_DN4408_c6_g1_i1_orf1;TRINITY_DN95414_c0_g1_i1_orf1;TRINITY_DN6325_c0_g1_i9_orf1;TRINITY_DN5281_c0_g2_i3_orf1;TRINITY_DN376_c1_g1_i1_orf1;TRINITY_DN21357_c0_g1_i5_orf1;TRINITY_DN1954_c0_g1_i4_orf1;TRINITY_DN83295_c0_g1_i3_orf1;TRINITY_DN779_c0_g1_i12_orf1;TRINITY_DN9062_c0_g2_i3_orf1;TRINITY_DN58207_c0_g1_i1_orf1;TRINITY_DN4767_c0_g1_i4_orf1;TRINITY_DN1262_c0_g1_i2_orf1;TRINITY_DN1528_c0_g1_i4_orf1;TRINITY_DN3499_c0_g1_i8_orf1;TRINITY_DN10831_c1_g1_i1_orf1;TRINITY_DN9979_c0_g1_i1_orf1;TRINITY_DN17326_c0_g1_i5_orf1;TRINITY_DN15136_c0_g1_i2_orf1;TRINITY_DN147458_c0_g1_i1_orf1;TRINITY_DN21218_c0_g1_i4_orf1;TRINITY_DN10070_c0_g1_i1_orf1;TRINITY_DN52553_c0_g2_i1_orf1;TRINITY_DN5235_c0_g1_i7_orf1;TRINITY_DN5513_c0_g1_i1_orf1;TRINITY_DN143853_c0_g1_i1_orf1;TRINITY_DN1068_c0_g1_i3_orf1;TRINITY_DN4248_c0_g1_i4_orf1;TRINITY_DN650_c0_g1_i2_orf1;TRINITY_DN1172_c0_g1_i12_orf1;TRINITY_DN1749_c0
biological_process	cellular nitrogen compound metabolic process	GO:0034641	207	207/2397	TRINITY_DN38230_c0_g1_i4_orf1;TRINITY_DN4360_c0_g1_i4_orf1;TRINITY_DN40434_c0_g1_i2_orf1;TRINITY_DN13350_c0_g1_i4_orf1;TRINITY_DN137_c0_g1_i1_orf1;TRINITY_DN60787_c0_g1_i5_orf1;TRINITY_DN7122_c0_g1_i1_orf1;TRINITY_DN51813_c0_g1_i1_orf1;TRINITY_DN53311_c0_g2_i1_orf1;TRINITY_DN35669_c0_g1_i1_orf1;TRINITY_DN2054_c0_g1_i1_orf1;TRINITY_DN15160_c0_g1_i1_orf1;TRINITY_DN11065_c0_g2_i1_orf1;TRINITY_DN34134_c0_g2_i1_orf1;TRINITY_DN1344_c0_g1_i1_orf1;TRINITY_DN1768_c0_g1_i2_orf1;TRINITY_DN1607_c0_g1_i16_orf1;TRINITY_DN24723_c2_g1_i1_orf1;TRINITY_DN23616_c0_g1_i4_orf1;TRINITY_DN2953_c1_g1_i10_orf1;TRINITY_DN7957_c0_g1_i5_orf1;TRINITY_DN29448_c0_g1_i1_orf1;TRINITY_DN11013_c0_g1_i3_orf1;TRINITY_DN18404_c0_g1_i5_orf1;TRINITY_DN21570_c0_g1_i1_orf1;TRINITY_DN2953_c1_g1_i2_orf1;TRINITY_DN1393_c0_g1_i2_orf1;TRINITY_DN3991_c0_g1_i6_orf1;TRINITY_DN2794_c1_g1_i8_orf1;TRINITY_DN10070_c0_g1_i1_orf1;TRINITY_DN2682_c0_g1_i4_orf1;TRINITY_DN3800_c0_g1_i7_orf1;TRINITY_DN15370_c0_g1_i4_orf1;TRINITY_DN15256_c0_g1_i8_orf1;TRINITY_DN123184_c0_g1_i1_orf1;TRINITY_DN135_c0_g1_i1_orf1;TRINITY_DN817_c0_g1_i3_orf1;TRINITY_DN3985_c0_g2_i1_orf1;TRINITY_DN2224_c0_g1_i1_orf1;TRINITY_DN5952_c0_g1_i6_orf1;TRINITY_DN46409_c0_g1_i1_orf1;TRINITY_DN18593_c0_g1_i1_orf1;TRINITY_DN8908_c0_g1_i1_orf1;TRINITY_DN33883_c0_g1_i1_orf1;TRINITY_DN15136_c0_g1_i2_orf1;TRINITY_DN107288_c0_g1_i2_orf1;TRINITY_DN18869_c0_g1_i1_orf1;TRINITY_DN7808_c0_g1_i1_orf1;TRINITY_DN89613_c0_g1_i13_orf1;TRINITY_DN14313_c0_g1_i1_orf1;TRINITY_DN1957_c0_g1_i4_orf1;TRINITY_DN230_c2_g1_i5_orf1;TRINITY_DN17271_c0_g1_i1_orf1;TRINITY_DN115210_c0_g4_i1_orf1;TRINITY_DN140212_c0_g1_i1_orf1;TRINITY_DN23360_c0_g1_i3_orf1;TRINITY_DN45271_c0_g1_i1_orf1;TRINITY_DN3082_c1_g1_i7_orf1;TRINITY_DN14398_c0_g1_i4_orf1;TRINITY_DN1005_c0_g1_i5_orf1;TRINITY_DN37532_c0_g1_i1_orf1;TRINITY_DN19687_c0_g1_i1_orf1;TRINITY_DN15900_c0_g1_i6_orf1;TRINITY_DN81258_c0_g1_i2_orf1;TRINITY_DN18249_c0_g1_i1_orf1;TRINITY_DN5873_c0_g4_i1_orf1;TRINITY_DN115658_c0_g1_i1_orf1;TRINITY_DN141353_c0_g1_i1_orf1;TRINITY_DN8625_c0_g1_i1_orf1;TRINITY_DN7583_c0_g1_i1_orf1;TRINITY_DN779_c0_g1_i3_orf1;TRINITY_DN18391_c0_g2_i8_orf1;TRINITY_DN7464_c0_g1_i14_orf1;TRINITY_DN810_c0_g1_i4_orf1;TRINITY_DN50787_c0_g2_i2_orf1;TRINITY_DN16978_c0_g1_i1_orf1;TRINITY_DN4145_c0_g1_i1_orf1;TRINITY_DN36144_c0_g1_i3_orf1;TRINITY_DN12527_c0_g1_i4_orf1;TRINITY_DN1616_c0_g1_i3_orf1;TRINITY_DN41573_c0_g1_i1_orf1;TRINITY_DN12301_c0_g1_i1_orf1;TRINITY_DN9794_c0_g2_i8_orf1;TRINITY_DN6642_c0_g1_i2_orf1;TRINITY_DN9591_c0_g1_i1_orf1;TRINITY_DN49936_c0_g2_i1_orf1;TRINITY_DN13732_c0_g2_i3_orf1;TRINITY_DN4408_c6_g1_i1_orf1;TRINITY_DN6325_c0_g1_i9_orf1;TRINITY_DN21357_c0_g1_i5_orf1;TRINITY_DN1718_c6_g1_i4_orf1;TRINITY_DN779_c0_g1_i12_orf1;TRINITY_DN7991_c0_g1_i9_orf1;TRINITY_DN56993_c0_g1_i4_orf1;TRINITY_DN58207_c0_g1_i1_orf1;TRINITY_DN74037_c0_g5_i1_orf1;TRINITY_DN31663_c0_g1_i2_orf1;TRINITY_DN97589_c0_g1_i3_orf1;TRINITY_DN10831_c1_g1_i1_orf1;TRINITY_DN1091_c0_g1_i1_orf1;TRINITY_DN107035_c0_g1_i1_orf1;TRINITY_DN147458_c0_g1_i1_orf1;TRINITY_DN4908_c1_g1_i5_orf1;TRINITY_DN2299_c0_g1_i3_orf1;TRINITY_DN5064_c0_g1_i4_orf1;TRINITY_DN143852_c0_g1_i1_orf1;TRINITY_DN5029_c0_g1_i1_orf1;TRINITY_DN291_c0_g1_i2_orf1;TRINITY_DN6325_c0_g1_i8_orf1;TRINITY_DN30131_c0_g1_i1_orf1;TRINITY_DN1750_c1_g1_i5_orf1;TRINITY_DN27852_c0_g1_i1_orf1;TRINITY_DN6365_c0_g1_i4_orf1;TRINITY_DN77318_c0_g2_i1_orf1;TRINITY_DN27035_c0_g1_i1_orf1;TRINITY_DN1575_c0_g1_i7_orf1;TRINITY_DN24318_c0_g1_i1_orf1;TRINITY_DN53233_c0_g1_i1_orf1;TRINITY_DN51934_c0_g2_i1_orf1;TRINITY_DN2718_c0_g1_i6_orf1;TRINITY_DN2971_c0_g1_i1_orf1;TRINITY_DN6563_c0_g1_i1_orf1;TRINITY_DN56270_c0_g1_i1_orf1;TRINITY_DN1554_c0_g1_i9_orf1;TRINITY_DN42646_c0_g2_i1_orf1;TRINITY_DN49038_c0_g4_i1_orf1;TRINITY_DN4016_c0_g1_i1_orf1;TRINITY_DN24_c0_g1_i1_orf1;TRINITY_DN28221_c0_g2_i1_orf1;TRINITY_DN11297_c0_g1_i1_orf1;TRINITY_DN2559_c0_g1_i4_orf1;TRINITY_DN98242_c0_g1_i1_orf1;TRINITY_DN48619_c0_g1_i1_orf1;TRINITY_DN14477_c0_g1_i12_orf1;TRINITY_DN34689_c0_g1_i4_orf1;TRINITY_DN147676_c0_g1_i1_orf1;TRINITY_DN26293_c0_g1_i4_orf1;TRINITY_DN41179_c0_g1_i1_orf1;TRINITY_DN2749_c0_g2_i3_orf1;TRINITY_DN4835_c0_g1_i2_orf1;TRINITY_DN7341_c0_g1_i8_orf1;TRINITY_DN24322_c0_g1_i4_orf1;TRINITY_DN825_c23_g1_i5_orf1;TRINITY_DN11986_c0_g1_i1_orf1;TRINITY_DN64_c0_g1_i4_orf1;TRINITY_DN8012_c0_g1_i3_orf1;TRINITY_DN83150_c0_g1_i1_orf1;TRINITY_DN38274_c0_g1_i1_orf1;TRINITY_DN14487_c0_g1_i4_orf1;TRINITY_DN28299_c0_g1_i1_orf1;TRINITY_DN4820_c0_g2_i2_orf1;TRINITY_DN2647_c0_g1_i3_orf1;TRINITY_DN120144_c0_g1_i1_orf1;TRINITY_DN6248_c0_g1
biological_process	nitrogen cycle metabolic process	GO:0071941	2	2/2397	TRINITY_DN24723_c2_g1_i1_orf1;TRINITY_DN28221_c0_g2_i1_orf1
biological_process	cellular lipid metabolic process	GO:0044255	20	20/2397	TRINITY_DN34751_c0_g1_i1_orf1;TRINITY_DN48590_c0_g1_i1_orf1;TRINITY_DN38644_c0_g1_i1_orf1;TRINITY_DN45220_c0_g1_i1_orf1;TRINITY_DN10722_c0_g3_i1_orf1;TRINITY_DN1999_c0_g1_i9_orf1;TRINITY_DN12526_c0_g1_i5_orf1;TRINITY_DN44110_c0_g1_i4_orf1;TRINITY_DN5211_c0_g1_i1_orf1;TRINITY_DN86833_c0_g3_i1_orf1;TRINITY_DN21570_c0_g1_i1_orf1;TRINITY_DN8964_c0_g1_i4_orf1;TRINITY_DN41_c0_g1_i3_orf1;TRINITY_DN3991_c0_g1_i6_orf1;TRINITY_DN5841_c0_g1_i2_orf1;TRINITY_DN10430_c0_g1_i4_orf1;TRINITY_DN9028_c0_g1_i5_orf1;TRINITY_DN5697_c0_g1_i1_orf1;TRINITY_DN14306_c0_g1_i1_orf1;TRINITY_DN3175_c0_g1_i7_orf1
biological_process	generation of precursor metabolites and energy	GO:0006091	19	19/2397	TRINITY_DN1201_c0_g1_i4_orf1;TRINITY_DN49038_c0_g4_i1_orf1;TRINITY_DN6325_c0_g1_i8_orf1;TRINITY_DN136028_c0_g2_i1_orf1;TRINITY_DN4360_c0_g1_i4_orf1;TRINITY_DN20133_c0_g1_i1_orf1;TRINITY_DN9286_c0_g1_i2_orf1;TRINITY_DN5867_c0_g1_i1_orf1;TRINITY_DN60787_c0_g1_i5_orf1;TRINITY_DN27035_c0_g1_i1_orf1;TRINITY_DN9979_c0_g1_i1_orf1;TRINITY_DN4270_c0_g1_i1_orf1;TRINITY_DN83150_c0_g1_i1_orf1;TRINITY_DN6325_c0_g1_i9_orf1;TRINITY_DN11817_c0_g1_i4_orf1;TRINITY_DN1791_c0_g1_i3_orf1;TRINITY_DN140613_c0_g1_i1_orf1;TRINITY_DN9536_c0_g1_i4_orf1;TRINITY_DN812_c2_g1_i1_orf1
biological_process	one-carbon metabolic process	GO:0006730	6	6/2397	TRINITY_DN98313_c0_g1_i1_orf1;TRINITY_DN63536_c0_g1_i1_orf1;TRINITY_DN3263_c0_g1_i2_orf1;TRINITY_DN38506_c0_g1_i4_orf1;TRINITY_DN5768_c0_g1_i2_orf1;TRINITY_DN14107_c0_g1_i4_orf1
biological_process	cellular ketone metabolic process	GO:0042180	7	7/2397	TRINITY_DN87170_c0_g1_i3_orf1;TRINITY_DN51813_c0_g1_i1_orf1;TRINITY_DN6027_c0_g1_i13_orf1;TRINITY_DN135781_c0_g1_i1_orf1;TRINITY_DN6638_c0_g1_i1_orf1;TRINITY_DN8964_c0_g1_i4_orf1;TRINITY_DN20133_c0_g1_i1_orf1

biological_process	heterocycle metabolic process	GO:0046483	159	159/2397	TRINITY_DN38230_c0_g1_i4_orf1;TRINITY_DN4360_c0_g1_i4_orf1;TRINITY_DN40434_c0_g1_i2_orf1;TRINITY_DN13350_c0_g1_i4_orf1;TRINITY_DN60787_c0_g1_i5_orf1;TRINITY_DN7122_c0_g1_i1_orf1;TRINITY_DN51813_c0_g1_i1_orf1;TRINITY_DN35669_c0_g1_i1_orf1;TRINITY_DN2054_c0_g1_i1_orf1;TRINITY_DN15160_c0_g1_i1_orf1;TRINITY_DN34134_c0_g2_i1_orf1;TRINITY_DN17772_c0_g2_i3_orf1;TRINITY_DN1768_c0_g1_i2_orf1;TRINITY_DN1607_c0_g1_i16_orf1;TRINITY_DN23616_c0_g1_i4_orf1;TRINITY_DN2953_c1_g1_i10_orf1;TRINITY_DN11013_c0_g1_i3_orf1;TRINITY_DN18404_c0_g1_i5_orf1;TRINITY_DN2953_c1_g1_i2_orf1;TRINITY_DN1393_c0_g1_i2_orf1;TRINITY_DN3991_c0_g1_i6_orf1;TRINITY_DN3800_c0_g1_i7_orf1;TRINITY_DN15370_c0_g1_i4_orf1;TRINITY_DN15256_c0_g1_i8_orf1;TRINITY_DN123184_c0_g1_i1_orf1;TRINITY_DN817_c0_g1_i3_orf1;TRINITY_DN2224_c0_g1_i1_orf1;TRINITY_DN5952_c0_g1_i6_orf1;TRINITY_DN46409_c0_g1_i1_orf1;TRINITY_DN8908_c0_g1_i1_orf1;TRINITY_DN15136_c0_g1_i2_orf1;TRINITY_DN107288_c0_g1_i2_orf1;TRINITY_DN7808_c0_g1_i1_orf1;TRINITY_DN89613_c0_g1_i13_orf1;TRINITY_DN14313_c0_g1_i1_orf1;TRINITY_DN2647_c0_g1_i3_orf1;TRINITY_DN230_c2_g1_i5_orf1;TRINITY_DN17271_c0_g1_i1_orf1;TRINITY_DN115210_c0_g4_i1_orf1;TRINITY_DN140212_c0_g1_i1_orf1;TRINITY_DN45271_c0_g1_i1_orf1;TRINITY_DN3082_c1_g1_i7_orf1;TRINITY_DN1005_c0_g1_i5_orf1;TRINITY_DN37532_c0_g1_i1_orf1;TRINITY_DN19687_c0_g1_i1_orf1;TRINITY_DN15900_c0_g1_i6_orf1;TRINITY_DN81258_c0_g1_i2_orf1;TRINITY_DN115658_c0_g1_i1_orf1;TRINITY_DN6813_c1_g1_i1_orf1;TRINITY_DN8625_c0_g1_i1_orf1;TRINITY_DN779_c0_g1_i3_orf1;TRINITY_DN18391_c0_g2_i8_orf1;TRINITY_DN810_c0_g1_i4_orf1;TRINITY_DN16978_c0_g1_i1_orf1;TRINITY_DN4145_c0_g1_i1_orf1;TRINITY_DN36144_c0_g1_i3_orf1;TRINITY_DN12527_c0_g1_i4_orf1;TRINITY_DN1616_c0_g1_i3_orf1;TRINITY_DN41573_c0_g1_i1_orf1;TRINITY_DN12301_c0_g1_i1_orf1;TRINITY_DN9794_c0_g2_i8_orf1;TRINITY_DN6642_c0_g1_i2_orf1;TRINITY_DN13732_c0_g2_i3_orf1;TRINITY_DN4408_c6_g1_i1_orf1;TRINITY_DN6325_c0_g1_i9_orf1;TRINITY_DN1718_c6_g1_i4_orf1;TRINITY_DN779_c0_g1_i12_orf1;TRINITY_DN56993_c0_g1_i4_orf1;TRINITY_DN5811_c0_g1_i4_orf1;TRINITY_DN74037_c0_g5_i1_orf1;TRINITY_DN31663_c0_g1_i2_orf1;TRINITY_DN139537_c0_g1_i1_orf1;TRINITY_DN1344_c0_g1_i1_orf1;TRINITY_DN107035_c0_g1_i1_orf1;TRINITY_DN4908_c1_g1_i5_orf1;TRINITY_DN2299_c0_g1_i3_orf1;TRINITY_DN5029_c0_g1_i1_orf1;TRINITY_DN291_c0_g1_i2_orf1;TRINITY_DN6325_c0_g1_i8_orf1;TRINITY_DN1750_c1_g1_i5_orf1;TRINITY_DN27852_c0_g1_i1_orf1;TRINITY_DN77318_c0_g2_i1_orf1;TRINITY_DN27035_c0_g1_i1_orf1;TRINITY_DN1575_c0_g1_i7_orf1;TRINITY_DN53233_c0_g1_i1_orf1;TRINITY_DN51934_c0_g2_i1_orf1;TRINITY_DN2718_c0_g1_i6_orf1;TRINITY_DN57918_c0_g1_i1_orf1;TRINITY_DN6563_c0_g1_i1_orf1;TRINITY_DN56270_c0_g1_i1_orf1;TRINITY_DN1554_c0_g1_i9_orf1;TRINITY_DN49038_c0_g4_i1_orf1;TRINITY_DN38506_c0_g1_i4_orf1;TRINITY_DN24_c0_g1_i1_orf1;TRINITY_DN2559_c0_g1_i4_orf1;TRINITY_DN2803_c4_g1_i1_orf1;TRINITY_DN98242_c0_g1_i1_orf1;TRINITY_DN14477_c0_g1_i12_orf1;TRINITY_DN34689_c0_g1_i4_orf1;TRINITY_DN26293_c0_g1_i4_orf1;TRINITY_DN41179_c0_g1_i1_orf1;TRINITY_DN2749_c0_g2_i3_orf1;TRINITY_DN4835_c0_g1_i2_orf1;TRINITY_DN7341_c0_g1_i8_orf1;TRINITY_DN24322_c0_g1_i4_orf1;TRINITY_DN825_c23_g1_i5_orf1;TRINITY_DN11986_c0_g1_i1_orf1;TRINITY_DN64_c0_g1_i4_orf1;TRINITY_DN8012_c0_g1_i3_orf1;TRINITY_DN83150_c0_g1_i1_orf1;TRINITY_DN38274_c0_g1_i1_orf1;TRINITY_DN14487_c0_g1_i4_orf1;TRINITY_DN28299_c0_g1_i1_orf1;TRINITY_DN4820_c0_g2_i2_orf1;TRINITY_DN1957_c0_g1_i4_orf1;TRINITY_DN3263_c0_g1_i2_orf1;TRINITY_DN120144_c0_g1_i1_orf1;TRINITY_DN6248_c0_g1_i1_orf1;TRINITY_DN141353_c0_g1_i1_orf1;TRINITY_DN4429_c0_g1_i5_orf1;TRINITY_DN879_c0_g1_i2_orf1;TRINITY_DN46022_c0_g1_i1_orf1;TRINITY_DN9979_c0_g1_i1_orf1;TRINITY_DN1084_c0_g2_i2_orf1;TRINITY_DN59804_c0_g1_i1_orf1;TRINITY_DN2971_c0_g1_i1_orf1;TRINITY_DN2719_c1_g1_i6_orf1;TRINITY_DN67649_c0_g1_i1_orf1;TRINITY_DN44792_c0_g1_i1_orf1;TRINITY_DN24970_c0_g1_i4_orf1;TRINITY_DN38562_c0_g1_i3_orf1;TRINITY_DN13760_c1_g1_i1_orf1;TRINITY_DN26879_c0_g1_i1_orf1;TRINITY_DN25423_c0_g1_i1_orf1;TRINITY_DN1741_c0_g1_i5_orf1;TRINITY_DN104507_c0_g1_i2_orf1;TRINITY_DN87170_c0_g1_i3_orf1;TRINITY_DN5200_c0_g1_i2_orf1;TRINITY_DN117844_c0_g1_i1_orf1;TRINITY_DN34676_c1_g1_i3_orf1;TRINITY_DN2201_c0_g1_i1_orf1;TRINITY_DN19251_c0_g1_i8_orf1;TRINITY_DN1091_c0_g1_i1_orf1;TRINITY_DN1201_c0_g1_i4_orf1;TRINITY_DN15222_c0_g1_i4_orf1;TRINITY_DN323_c0_g2_i5_orf1;TRINITY_DN53311_c0_g2_i1_orf1;TRINITY_DN5238_c0_g1_i2_orf1;TRINITY_DN21596_c0_g1_i1_orf1;TRINITY_DN133760_c0_g1_i1_orf1;TRINITY_DN34432_c0_g1_i1_orf1;TRINITY_DN110534_c0_g1_i3_orf1;TRINITY_DN5757_c0_g1_i1_orf1;TRINITY_DN48619_c0_g1_i1
biological_process	reactive oxygen species metabolic process	GO:0072593	3	3/2397	TRINITY_DN15417_c0_g1_i6_orf1;TRINITY_DN285_c0_g1_i4_orf1;TRINITY_DN16400_c0_g2_i1_orf1
biological_process	cellular carbohydrate metabolic process	GO:0044262	11	11/2397	TRINITY_DN618_c0_g1_i3_orf1;TRINITY_DN52244_c1_g1_i1_orf1;TRINITY_DN49038_c0_g4_i1_orf1;TRINITY_DN10722_c0_g3_i1_orf1;TRINITY_DN1034_c0_g1_i4_orf1;TRINITY_DN38644_c0_g1_i1_orf1;TRINITY_DN11817_c0_g1_i4_orf1;TRINITY_DN3010_c0_g1_i4_orf1;TRINITY_DN11657_c0_g1_i2_orf1;TRINITY_DN140613_c0_g1_i1_orf1;TRINITY_DN812_c2_g1_i1_orf1
biological_process	sulfur compound metabolic process	GO:0006790	17	17/2397	TRINITY_DN14920_c0_g1_i1_orf1;TRINITY_DN117844_c0_g1_i1_orf1;TRINITY_DN11948_c0_g1_i8_orf1;TRINITY_DN9794_c0_g2_i8_orf1;TRINITY_DN3073_c0_g1_i7_orf1;TRINITY_DN19251_c0_g1_i8_orf1;TRINITY_DN38562_c0_g1_i3_orf1;TRINITY_DN28660_c0_g1_i4_orf1;TRINITY_DN34399_c0_g1_i1_orf1;TRINITY_DN3991_c0_g1_i6_orf1;TRINITY_DN35002_c0_g2_i2_orf1;TRINITY_DN15136_c0_g1_i2_orf1;TRINITY_DN49872_c0_g1_i2_orf1;TRINITY_DN35763_c0_g1_i2_orf1;TRINITY_DN85476_c0_g1_i1_orf1;TRINITY_DN7808_c0_g1_i1_orf1;TRINITY_DN1084_c0_g2_i2_orf1
biological_process	phosphorus metabolic process	GO:0006793	88	88/2397	TRINITY_DN38230_c0_g1_i4_orf1;TRINITY_DN10722_c0_g3_i1_orf1;TRINITY_DN4360_c0_g1_i4_orf1;TRINITY_DN60787_c0_g1_i5_orf1;TRINITY_DN4217_c0_g1_i2_orf1;TRINITY_DN70485_c0_g1_i2_orf1;TRINITY_DN2983_c0_g1_i6_orf1;TRINITY_DN143509_c0_g1_i1_orf1;TRINITY_DN4320_c0_g1_i1_orf1;TRINITY_DN11013_c0_g1_i3_orf1;TRINITY_DN3991_c0_g1_i6_orf1;TRINITY_DN3800_c0_g1_i7_orf1;TRINITY_DN6185_c0_g1_i12_orf1;TRINITY_DN52244_c1_g1_i1_orf1;TRINITY_DN6436_c0_g1_i1_orf1;TRINITY_DN5952_c0_g1_i6_orf1;TRINITY_DN8908_c0_g1_i1_orf1;TRINITY_DN15136_c0_g1_i2_orf1;TRINITY_DN11620_c0_g1_i2_orf1;TRINITY_DN7808_c0_g1_i1_orf1;TRINITY_DN1749_c0_g2_i2_orf1;TRINITY_DN115210_c0_g4_i1_orf1;TRINITY_DN2719_c1_g1_i6_orf1;TRINITY_DN37923_c0_g1_i1_orf1;TRINITY_DN1201_c0_g1_i4_orf1;TRINITY_DN1034_c0_g1_i4_orf1;TRINITY_DN42461_c0_g1_i4_orf1;TRINITY_DN6813_c1_g1_i1_orf1;TRINITY_DN277_c1_g1_i1_orf1;TRINITY_DN59885_c0_g1_i3_orf1;TRINITY_DN12_c0_g1_i5_orf1;TRINITY_DN36144_c0_g1_i3_orf1;TRINITY_DN32700_c0_g1_i2_orf1;TRINITY_DN117844_c0_g1_i1_orf1;TRINITY_DN12301_c0_g1_i1_orf1;TRINITY_DN9794_c0_g2_i8_orf1;TRINITY_DN38644_c0_g1_i1_orf1;TRINITY_DN6325_c0_g1_i9_orf1;TRINITY_DN5281_c0_g2_i3_orf1;TRINITY_DN1266_c2_g1_i1_orf1;TRINITY_DN1954_c0_g1_i4_orf1;TRINITY_DN5811_c0_g1_i4_orf1;TRINITY_DN1173_c1_g1_i10_orf1;TRINITY_DN5029_c0_g1_i1_orf1;TRINITY_DN1173_c0_g1_i12_orf1;TRINITY_DN6325_c0_g1_i8_orf1;TRINITY_DN27035_c0_g1_i1_orf1;TRINITY_DN1575_c0_g1_i7_orf1;TRINITY_DN2770_c0_g2_i4_orf1;TRINITY_DN1475_c0_g1_i6_orf1;TRINITY_DN28729_c0_g1_i9_orf1;TRINITY_DN49038_c0_g4_i1_orf1;TRINITY_DN24_c0_g1_i1_orf1;TRINITY_DN1718_c6_g1_i4_orf1;TRINITY_DN14477_c0_g1_i12_orf1;TRINITY_DN26293_c0_g1_i4_orf1;TRINITY_DN51813_c0_g1_i1_orf1;TRINITY_DN8012_c0_g1_i3_orf1;TRINITY_DN83150_c0_g1_i1_orf1;TRINITY_DN143637_c0_g1_i1_orf1;TRINITY_DN28299_c0_g1_i1_orf1;TRINITY_DN1957_c0_g1_i4_orf1;TRINITY_DN141353_c0_g1_i1_orf1;TRINITY_DN40562_c0_g2_i1_orf1;TRINITY_DN116972_c0_g1_i1_orf1;TRINITY_DN9979_c0_g1_i1_orf1;TRINITY_DN1084_c0_g2_i2_orf1;TRINITY_DN62557_c0_g1_i1_orf1;TRINITY_DN47151_c0_g1_i1_orf1;TRINITY_DN24539_c0_g1_i4_orf1;TRINITY_DN41166_c0_g1_i1_orf1;TRINITY_DN1741_c0_g1_i5_orf1;TRINITY_DN618_c0_g1_i3_orf1;TRINITY_DN4571_c0_g1_i4_orf1;TRINITY_DN10680_c0_g1_i5_orf1;TRINITY_DN3119_c0_g1_i7_orf1;TRINITY_DN1173_c0_g1_i11_orf1;TRINITY_DN44110_c0_g1_i4_orf1;TRINITY_DN15222_c0_g1_i4_orf1;TRINITY_DN30154_c0_g1_i1_orf1;TRINITY_DN20133_c0_g1_i1_orf1;TRINITY_DN248_c0_g1_i1_orf1;TRINITY_DN30_c0_g1_i6_orf1;TRINITY_DN147475_c0_g1_i1_orf1;TRINITY_DN18782_c0_g1_i4_orf1;TRINITY_DN19251_c0_g1_i8_orf1;TRINITY_DN5697_c0_g1_i1_orf1;TRINITY_DN49
biological_process	cellular aldehyde metabolic process	GO:0006081	2	2/2397	TRINITY_DN125150_c0_g1_i1_orf1;TRINITY_DN20133_c0_g1_i1_orf1
biological_process	organic acid metabolic process	GO:0006082	72	72/2397	TRINITY_DN34751_c0_g1_i1_orf1;TRINITY_DN48590_c0_g1_i1_orf1;TRINITY_DN2065_c1_g2_i1_orf1;TRINITY_DN57918_c0_g1_i1_orf1;TRINITY_DN146126_c0_g1_i1_orf1;TRINITY_DN6325_c0_g1_i8_orf1;TRINITY_DN14565_c0_g1_i11_orf1;TRINITY_DN115210_c0_g4_i1_orf1;TRINITY_DN4360_c0_g1_i4_orf1;TRINITY_DN24970_c0_g1_i4_orf1;TRINITY_DN6199_c2_g1_i3_orf1;TRINITY_DN27035_c0_g1_i1_orf1;TRINITY_DN11159_c0_g1_i5_orf1;TRINITY_DN117844_c0_g1_i1_orf1;TRINITY_DN5218_c0_g1_i4_orf1;TRINITY_DN2719_c1_g1_i6_orf1;TRINITY_DN5266_c0_g1_i1_orf1;TRINITY_DN35763_c0_g1_i2_orf1;TRINITY_DN4822_c0_g1_i9_orf1;TRINITY_DN89483_c0_g1_i1_orf1;TRINITY_DN3175_c0_g1_i7_orf1;TRINITY_DN1201_c0_g1_i4_orf1;TRINITY_DN4451_c0_g2_i4_orf1;TRINITY_DN2890_c0_g1_i2_orf1;TRINITY_DN45220_c0_g1_i1_orf1;TRINITY_DN11948_c0_g1_i8_orf1;TRINITY_DN87170_c0_g1_i3_orf1;TRINITY_DN2803_c4_g1_i1_orf1;TRINITY_DN5211_c0_g1_i1_orf1;TRINITY_DN1607_c0_g1_i16_orf1;TRINITY_DN24723_c2_g1_i1_orf1;TRINITY_DN9794_c0_g2_i8_orf1;TRINITY_DN2953_c1_g1_i10_orf1;TRINITY_DN28221_c0_g2_i1_orf1;TRINITY_DN14306_c0_g1_i1_orf1;TRINITY_DN2953_c1_g1_i2_orf1;TRINITY_DN3991_c0_g1_i6_orf1;TRINITY_DN10430_c0_g1_i4_orf1;TRINITY_DN11013_c0_g1_i3_orf1;TRINITY_DN26293_c0_g1_i4_orf1;TRINITY_DN20796_c0_g1_i4_orf1;TRINITY_DN4822_c0_g1_i6_orf1;TRINITY_DN49038_c0_g4_i1_orf1;TRINITY_DN17326_c0_g1_i8_orf1;TRINITY_DN1999_c0_g1_i9_orf1;TRINITY_DN3859_c0_g1_i5_orf1;TRINITY_DN825_c23_g1_i5_orf1;TRINITY_DN3073_c0_g1_i7_orf1;TRINITY_DN51813_c0_g1_i1_orf1;TRINITY_DN34399_c0_g1_i1_orf1;TRINITY_DN2570_c0_g1_i1_orf1;TRINITY_DN83150_c0_g1_i1_orf1;TRINITY_DN6325_c0_g1_i9_orf1;TRINITY_DN817_c0_g1_i3_orf1;TRINITY_DN511_c0_g2_i1_orf1;TRINITY_DN48619_c0_g1_i1_orf1;TRINITY_DN15160_c0_g1_i1_orf1;TRINITY_DN2224_c0_g1_i1_orf1;TRINITY_DN21539_c0_g1_i1_orf1;TRINITY_DN1262_c0_g1_i2_orf1;TRINITY_DN11383_c0_g2_i4_orf1;TRINITY_DN3263_c0_g1_i2_orf1;TRINITY_DN2684_c0_g2_i3_orf1;TRINITY_DN17326_c0_g1_i5_orf1;TRINITY_DN3836_c0_g1_i4_orf1;TRINITY_DN15136_c0_g1_i2_orf1;TRINITY_DN107288_c0_g1_i2_orf1;TRINITY_DN20133_c0_g1_i1_orf1;TRINITY_DN7808_c0_g1_i1_orf1;TRINITY_DN1068_c0_g1_i3_orf1;TRINITY_DN9979_c0_g1_i1_orf1;TRINITY_DN1084_c0_g2_i2_orf
biological_process	cellular catabolic process	GO:0044248	47	47/2397	TRINITY_DN34751_c0_g1_i1_orf1;TRINITY_DN2065_c1_g2_i1_orf1;TRINITY_DN38230_c0_g1_i4_orf1;TRINITY_DN14565_c0_g1_i11_orf1;TRINITY_DN15930_c0_g1_i5_orf1;TRINITY_DN6199_c2_g1_i3_orf1;TRINITY_DN53233_c0_g1_i1_orf1;TRINITY_DN3010_c0_g1_i4_orf1;TRINITY_DN4822_c0_g1_i9_orf1;TRINITY_DN37923_c0_g1_i1_orf1;TRINITY_DN89483_c0_g1_i1_orf1;TRINITY_DN4451_c0_g2_i4_orf1;TRINITY_DN57798_c0_g1_i1_orf1;TRINITY_DN2890_c0_g1_i2_orf1;TRINITY_DN45220_c0_g1_i1_orf1;TRINITY_DN1034_c0_g1_i4_orf1;TRINITY_DN19821_c0_g2_i4_orf1;TRINITY_DN87170_c0_g1_i3_orf1;TRINITY_DN24_c0_g1_i1_orf1;TRINITY_DN135188_c0_g1_i2_orf1;TRINITY_DN11375_c0_g1_i4_orf1;TRINITY_DN779_c0_g1_i3_orf1;TRINITY_DN98242_c0_g1_i1_orf1;TRINITY_DN82017_c0_g1_i5_orf1;TRINITY_DN5531_c0_g3_i3_orf1;TRINITY_DN34689_c0_g1_i4_orf1;TRINITY_DN11172_c0_g1_i4_orf1;TRINITY_DN4145_c0_g1_i1_orf1;TRINITY_DN96557_c0_g1_i1_orf1;TRINITY_DN4822_c0_g1_i6_orf1;TRINITY_DN17693_c0_g1_i10_orf1;TRINITY_DN51813_c0_g1_i1_orf1;TRINITY_DN8012_c0_g1_i3_orf1;TRINITY_DN285_c0_g1_i4_orf1;TRINITY_DN5001_c0_g1_i4_orf1;TRINITY_DN4121_c0_g1_i1_orf1;TRINITY_DN2559_c0_g1_i4_orf1;TRINITY_DN779_c0_g1_i12_orf1;TRINITY_DN9062_c0_g2_i3_orf1;TRINITY_DN1262_c0_g1_i2_orf1;TRINITY_DN8908_c0_g1_i1_orf1;TRINITY_DN3836_c0_g1_i4_orf1;TRINITY_DN15136_c0_g1_i2_orf1;TRINITY_DN113353_c0_g1_i1_orf1;TRINITY_DN25423_c0_g1_i1_orf1;TRINITY_DN46022_c0_g1_i1_orf1;TRINITY_

biological_process	cellular biosynthetic process	GO:0044249	124	124/2397	TRINITY_DN10722_c0_g3_i1_orf1;TRINITY_DN137_c0_g1_i1_orf1;TRINITY_DN51813_c0_g1_i1_orf1;TRINITY_DN35763_c0_g1_i2_orf1;TRINITY_DN5873_c0_g4_i1_orf1;TRINITY_DN11065_c0_g2_i1_orf1;TRINITY_DN2682_c0_g1_i4_orf1;TRINITY_DN1768_c0_g1_i2_orf1;TRINITY_DN24723_c2_g1_i1_orf1;TRINITY_DN28221_c0_g2_i1_orf1;TRINITY_DN8964_c0_g1_i4_orf1;TRINITY_DN29448_c0_g1_i1_orf1;TRINITY_DN11013_c0_g1_i3_orf1;TRINITY_DN21570_c0_g1_i1_orf1;TRINITY_DN1393_c0_g1_i2_orf1;TRINITY_DN3991_c0_g1_i6_orf1;TRINITY_DN3800_c0_g1_i7_orf1;TRINITY_DN9028_c0_g1_i5_orf1;TRINITY_DN28299_c0_g1_i1_orf1;TRINITY_DN1509_c0_g1_i1_orf1;TRINITY_DN135_c0_g1_i1_orf1;TRINITY_DN58207_c0_g1_i1_orf1;TRINITY_DN3985_c0_g2_i1_orf1;TRINITY_DN5952_c0_g1_i6_orf1;TRINITY_DN7808_c0_g1_i1_orf1;TRINITY_DN230_c2_g1_i5_orf1;TRINITY_DN18249_c0_g1_i1_orf1;TRINITY_DN115210_c0_g4_i1_orf1;TRINITY_DN23360_c0_g1_i3_orf1;TRINITY_DN14398_c0_g1_i4_orf1;TRINITY_DN2719_c1_g1_i6_orf1;TRINITY_DN14306_c0_g1_i1_orf1;TRINITY_DN97589_c0_g1_i3_orf1;TRINITY_DN115658_c0_g1_i1_orf1;TRINITY_DN1999_c0_g1_i9_orf1;TRINITY_DN5211_c0_g1_i1_orf1;TRINITY_DN141353_c0_g1_i1_orf1;TRINITY_DN8625_c0_g1_i1_orf1;TRINITY_DN7583_c0_g1_i1_orf1;TRINITY_DN7464_c0_g1_i14_orf1;TRINITY_DN50787_c0_g2_i2_orf1;TRINITY_DN10430_c0_g1_i4_orf1;TRINITY_DN36144_c0_g1_i3_orf1;TRINITY_DN12527_c0_g1_i4_orf1;TRINITY_DN38644_c0_g1_i1_orf1;TRINITY_DN117844_c0_g1_i1_orf1;TRINITY_DN12301_c0_g1_i1_orf1;TRINITY_DN9794_c0_g2_i8_orf1;TRINITY_DN3073_c0_g1_i7_orf1;TRINITY_DN9591_c0_g1_i1_orf1;TRINITY_DN49936_c0_g2_i1_orf1;TRINITY_DN13732_c0_g2_i3_orf1;TRINITY_DN4408_c6_g1_i1_orf1;TRINITY_DN21357_c0_g1_i5_orf1;TRINITY_DN1718_c6_g1_i4_orf1;TRINITY_DN7991_c0_g1_i9_orf1;TRINITY_DN5811_c0_g1_i4_orf1;TRINITY_DN74037_c0_g5_i1_orf1;TRINITY_DN13233_c0_g1_i3_orf1;TRINITY_DN10831_c1_g1_i1_orf1;TRINITY_DN147458_c0_g1_i1_orf1;TRINITY_DN2299_c0_g1_i3_orf1;TRINITY_DN5064_c0_g1_i4_orf1;TRINITY_DN143852_c0_g1_i1_orf1;TRINITY_DN5029_c0_g1_i1_orf1;TRINITY_DN812_c2_g1_i1_orf1;TRINITY_DN48590_c0_g1_i1_orf1;TRINITY_DN30131_c0_g1_i1_orf1;TRINITY_DN1750_c1_g1_i5_orf1;TRINITY_DN27852_c0_g1_i1_orf1;TRINITY_DN6365_c0_g1_i4_orf1;TRINITY_DN1575_c0_g1_i7_orf1;TRINITY_DN24318_c0_g1_i1_orf1;TRINITY_DN5009_c0_g1_i2_orf1;TRINITY_DN41645_c0_g1_i1_orf1;TRINITY_DN6563_c0_g1_i1_orf1;TRINITY_DN4016_c0_g1_i1_orf1;TRINITY_DN11297_c0_g1_i1_orf1;TRINITY_DN2803_c4_g1_i1_orf1;TRINITY_DN7957_c0_g1_i5_orf1;TRINITY_DN10070_c0_g1_i1_orf1;TRINITY_DN14477_c0_g1_i12_orf1;TRINITY_DN147676_c0_g1_i1_orf1;TRINITY_DN26293_c0_g1_i4_orf1;TRINITY_DN24970_c0_g1_i4_orf1;TRINITY_DN53311_c0_g2_i1_orf1;TRINITY_DN42506_c0_g1_i1_orf1;TRINITY_DN18869_c0_g1_i1_orf1;TRINITY_DN33883_c0_g1_i1_orf1;TRINITY_DN1957_c0_g1_i4_orf1;TRINITY_DN3263_c0_g1_i2_orf1;TRINITY_DN6813_c1_g1_i1_orf1;TRINITY_DN11948_c0_g1_i8_orf1;TRINITY_DN879_c0_g1_i2_orf1;TRINITY_DN1084_c0_g2_i2_orf1;TRINITY_DN40650_c0_g1_i1_orf1;TRINITY_DN5031_c0_g1_i1_orf1;TRINITY_DN37532_c0_g1_i1_orf1;TRINITY_DN44792_c0_g1_i1_orf1;TRINITY_DN36893_c0_g1_i1_orf1;TRINITY_DN18593_c0_g1_i1_orf1;TRINITY_DN1741_c0_g1_i5_orf1;TRINITY_DN8949_c0_g1_i2_orf1;TRINITY_DN2265_c0_g2_i1_orf1;TRINITY_DN1045_c0_g1_i6_orf1;TRINITY_DN6027_c0_g1_i13_orf1;TRINITY_DN5200_c0_g1_i2_orf1;TRINITY_DN6638_c0_g1_i1_orf1;TRINITY_DN8824_c0_g2_i1_orf1;TRINITY_DN15222_c0_g1_i4_orf1;TRINITY_DN3826_c0_g1_i1_orf1;TRINITY_DN323_c0_g2_i5_orf1;TRINITY_DN10234_c0_g1_i1_orf1;TRINITY_DN34399_c0_g1_i1_orf1;TRINITY_DN42646_c0_g2_i1_orf1;TRINITY_DN82324_c0_g1_i4_orf1;TRINITY_DN58125_c0_g1_i1_orf1;TRINITY_DN31253_c0_g1_i2_orf1;TRINITY_DN26879_c0_g1_i1_orf1;TRINITY_DN9862_c0_g2_i1_orf1;TRINITY_DN18782_c0_g1_i4_orf1;TRINITY_DN20133_c0_g1_i1_orf1;TRINITY_DN5697_c0_g1_i1_orf1;TRINI
biological_process	cellular macromolecule metabolic process	GO:0044260	115	115/2397	TRINITY_DN40434_c0_g1_i2_orf1;TRINITY_DN13350_c0_g1_i4_orf1;TRINITY_DN137_c0_g1_i1_orf1;TRINITY_DN7122_c0_g1_i1_orf1;TRINITY_DN5873_c0_g4_i1_orf1;TRINITY_DN2054_c0_g1_i1_orf1;TRINITY_DN11065_c0_g2_i1_orf1;TRINITY_DN2682_c0_g1_i4_orf1;TRINITY_DN1344_c0_g1_i1_orf1;TRINITY_DN1768_c0_g1_i2_orf1;TRINITY_DN11817_c0_g1_i4_orf1;TRINITY_DN29448_c0_g1_i1_orf1;TRINITY_DN42506_c0_g1_i1_orf1;TRINITY_DN1393_c0_g1_i2_orf1;TRINITY_DN15370_c0_g1_i4_orf1;TRINITY_DN15256_c0_g1_i8_orf1;TRINITY_DN1509_c0_g1_i1_orf1;TRINITY_DN135_c0_g1_i1_orf1;TRINITY_DN3985_c0_g2_i1_orf1;TRINITY_DN22674_c0_g1_i2_orf1;TRINITY_DN18869_c0_g1_i1_orf1;TRINITY_DN89613_c0_g1_i13_orf1;TRINITY_DN14313_c0_g1_i1_orf1;TRINITY_DN17271_c0_g1_i1_orf1;TRINITY_DN140212_c0_g1_i1_orf1;TRINITY_DN23360_c0_g1_i3_orf1;TRINITY_DN45271_c0_g1_i1_orf1;TRINITY_DN2971_c0_g1_i1_orf1;TRINITY_DN37923_c0_g1_i1_orf1;TRINITY_DN97589_c0_g1_i3_orf1;TRINITY_DN81258_c0_g1_i2_orf1;TRINITY_DN57798_c0_g1_i1_orf1;TRINITY_DN115658_c0_g1_i1_orf1;TRINITY_DN7583_c0_g1_i1_orf1;TRINITY_DN7464_c0_g1_i14_orf1;TRINITY_DN50787_c0_g2_i2_orf1;TRINITY_DN41573_c0_g1_i1_orf1;TRINITY_DN6642_c0_g1_i2_orf1;TRINITY_DN9591_c0_g1_i1_orf1;TRINITY_DN49936_c0_g2_i1_orf1;TRINITY_DN13732_c0_g2_i3_orf1;TRINITY_DN4408_c6_g1_i1_orf1;TRINITY_DN95414_c0_g1_i1_orf1;TRINITY_DN21357_c0_g1_i5_orf1;TRINITY_DN9062_c0_g2_i3_orf1;TRINITY_DN58207_c0_g1_i1_orf1;TRINITY_DN74037_c0_g5_i1_orf1;TRINITY_DN13233_c0_g1_i3_orf1;TRINITY_DN139537_c0_g1_i1_orf1;TRINITY_DN10831_c1_g1_i1_orf1;TRINITY_DN1091_c0_g1_i1_orf1;TRINITY_DN147458_c0_g1_i1_orf1;TRINITY_DN4908_c1_g1_i5_orf1;TRINITY_DN5064_c0_g1_i4_orf1;TRINITY_DN143852_c0_g1_i1_orf1;TRINITY_DN291_c0_g1_i2_orf1;TRINITY_DN812_c2_g1_i1_orf1;TRINITY_DN30131_c0_g1_i1_orf1;TRINITY_DN1750_c1_g1_i5_orf1;TRINITY_DN27852_c0_g1_i1_orf1;TRINITY_DN6365_c0_g1_i4_orf1;TRINITY_DN77318_c0_g2_i1_orf1;TRINITY_DN46409_c0_g1_i1_orf1;TRINITY_DN40650_c0_g1_i1_orf1;TRINITY_DN24318_c0_g1_i1_orf1;TRINITY_DN53233_c0_g1_i1_orf1;TRINITY_DN51934_c0_g2_i1_orf1;TRINITY_DN18249_c0_g1_i1_orf1;TRINITY_DN41645_c0_g1_i1_orf1;TRINITY_DN5757_c0_g1_i1_orf1;TRINITY_DN4016_c0_g1_i1_orf1;TRINITY_DN11297_c0_g1_i1_orf1;TRINITY_DN10070_c0_g1_i1_orf1;TRINITY_DN34689_c0_g1_i4_orf1;TRINITY_DN147676_c0_g1_i1_orf1;TRINITY_DN4835_c0_g1_i2_orf1;TRINITY_DN7341_c0_g1_i8_orf1;TRINITY_DN7991_c0_g1_i9_orf1;TRINITY_DN11986_c0_g1_i1_orf1;TRINITY_DN4121_c0_g1_i1_orf1;TRINITY_DN38274_c0_g1_i1_orf1;TRINITY_DN14487_c0_g1_i4_orf1;TRINITY_DN96557_c0_g1_i1_orf1;TRINITY_DN10234_c0_g1_i1_orf1;TRINITY_DN33883_c0_g1_i1_orf1;TRINITY_DN2647_c0_g1_i3_orf1;TRINITY_DN6248_c0_g1_i1_orf1;TRINITY_DN4429_c0_g1_i5_orf1;TRINITY_DN46022_c0_g1_i1_orf1;TRINITY_DN59804_c0_g1_i1_orf1;TRINITY_DN2265_c0_g2_i1_orf1;TRINITY_DN5031_c0_g1_i1_orf1;TRINITY_DN67649_c0_g1_i1_orf1;TRINITY_DN36893_c0_g1_i1_orf1;TRINITY_DN18593_c0_g1_i1_orf1;TRINITY_DN8949_c0_g1_i2_orf1;TRINITY_DN104507_c0_g1_i2_orf1;TRINITY_DN5009_c0_g1_i2_orf1;TRINITY_DN5200_c0_g1_i2_orf1;TRINITY_DN8824_c0_g2_i1_orf1;TRINITY_DN140613_c0_g1_i1_orf1;TRINITY_DN3826_c0_g1_i1_orf1;TRINITY_DN5238_c0_g1_i2_orf1;TRINITY_DN46132_c0_g2_i2_orf1;TRINITY_DN5001_c0_g1_i4_orf1;TRINITY_DN110534_c0_g1_i3_orf1;TRINITY_DN42646_c0_g2_i1_orf1;TRINITY_DN82324_c0_g1_i4_orf1;TRINITY_DN31253_c0_g1_i2_orf1;TRINITY_DN26879_c0_g1_i1_orf1;TRINITY_DN135188_c0_g1_i2_orf1;TRINITY_DN9862_c0_g2_i1_orf1;TRINITY_DN12
biological_process	cellular aromatic compound metabolic process	GO:0006725	158	158/2397	TRINITY_DN38230_c0_g1_i4_orf1;TRINITY_DN4360_c0_g1_i4_orf1;TRINITY_DN40434_c0_g1_i2_orf1;TRINITY_DN13350_c0_g1_i4_orf1;TRINITY_DN60787_c0_g1_i5_orf1;TRINITY_DN7122_c0_g1_i1_orf1;TRINITY_DN51813_c0_g1_i1_orf1;TRINITY_DN35669_c0_g1_i1_orf1;TRINITY_DN2054_c0_g1_i1_orf1;TRINITY_DN15160_c0_g1_i1_orf1;TRINITY_DN34134_c0_g2_i1_orf1;TRINITY_DN17772_c0_g2_i3_orf1;TRINITY_DN1768_c0_g1_i2_orf1;TRINITY_DN1607_c0_g1_i16_orf1;TRINITY_DN23616_c0_g1_i4_orf1;TRINITY_DN2953_c1_g1_i10_orf1;TRINITY_DN11013_c0_g1_i3_orf1;TRINITY_DN18404_c0_g1_i5_orf1;TRINITY_DN4822_c0_g1_i6_orf1;TRINITY_DN2953_c1_g1_i2_orf1;TRINITY_DN1393_c0_g1_i2_orf1;TRINITY_DN3991_c0_g1_i6_orf1;TRINITY_DN3800_c0_g1_i7_orf1;TRINITY_DN15370_c0_g1_i4_orf1;TRINITY_DN15256_c0_g1_i8_orf1;TRINITY_DN123184_c0_g1_i1_orf1;TRINITY_DN817_c0_g1_i3_orf1;TRINITY_DN2224_c0_g1_i1_orf1;TRINITY_DN5952_c0_g1_i6_orf1;TRINITY_DN46409_c0_g1_i1_orf1;TRINITY_DN8908_c0_g1_i1_orf1;TRINITY_DN15136_c0_g1_i2_orf1;TRINITY_DN107288_c0_g1_i2_orf1;TRINITY_DN7808_c0_g1_i1_orf1;TRINITY_DN89613_c0_g1_i13_orf1;TRINITY_DN14313_c0_g1_i1_orf1;TRINITY_DN2647_c0_g1_i3_orf1;TRINITY_DN230_c2_g1_i5_orf1;TRINITY_DN17271_c0_g1_i1_orf1;TRINITY_DN115210_c0_g4_i1_orf1;TRINITY_DN140212_c0_g1_i1_orf1;TRINITY_DN45271_c0_g1_i1_orf1;TRINITY_DN3082_c1_g1_i7_orf1;TRINITY_DN1005_c0_g1_i5_orf1;TRINITY_DN37532_c0_g1_i1_orf1;TRINITY_DN19687_c0_g1_i1_orf1;TRINITY_DN15900_c0_g1_i6_orf1;TRINITY_DN81258_c0_g1_i2_orf1;TRINITY_DN115658_c0_g1_i1_orf1;TRINITY_DN6813_c1_g1_i1_orf1;TRINITY_DN8625_c0_g1_i1_orf1;TRINITY_DN779_c0_g1_i3_orf1;TRINITY_DN18391_c0_g2_i8_orf1;TRINITY_DN810_c0_g1_i4_orf1;TRINITY_DN16978_c0_g1_i1_orf1;TRINITY_DN36144_c0_g1_i3_orf1;TRINITY_DN12527_c0_g1_i4_orf1;TRINITY_DN1616_c0_g1_i3_orf1;TRINITY_DN41573_c0_g1_i1_orf1;TRINITY_DN12301_c0_g1_i1_orf1;TRINITY_DN9794_c0_g2_i8_orf1;TRINITY_DN6642_c0_g1_i2_orf1;TRINITY_DN13732_c0_g2_i3_orf1;TRINITY_DN4408_c6_g1_i1_orf1;TRINITY_DN6325_c0_g1_i9_orf1;TRINITY_DN1718_c6_g1_i4_orf1;TRINITY_DN779_c0_g1_i12_orf1;TRINITY_DN56993_c0_g1_i4_orf1;TRINITY_DN5811_c0_g1_i4_orf1;TRINITY_DN74037_c0_g5_i1_orf1;TRINITY_DN31663_c0_g1_i2_orf1;TRINITY_DN139537_c0_g1_i1_orf1;TRINITY_DN1344_c0_g1_i1_orf1;TRINITY_DN107035_c0_g1_i1_orf1;TRINITY_DN4908_c1_g1_i5_orf1;TRINITY_DN2299_c0_g1_i3_orf1;TRINITY_DN5029_c0_g1_i1_orf1;TRINITY_DN291_c0_g1_i2_orf1;TRINITY_DN6325_c0_g1_i8_orf1;TRINITY_DN1750_c1_g1_i5_orf1;TRINITY_DN27852_c0_g1_i1_orf1;TRINITY_DN77318_c0_g2_i1_orf1;TRINITY_DN27035_c0_g1_i1_orf1;TRINITY_DN1575_c0_g1_i7_orf1;TRINITY_DN53233_c0_g1_i1_orf1;TRINITY_DN51934_c0_g2_i1_orf1;TRINITY_DN2718_c0_g1_i6_orf1;TRINITY_DN57918_c0_g1_i1_orf1;TRINITY_DN6563_c0_g1_i1_orf1;TRINITY_DN56270_c0_g1_i1_orf1;TRINITY_DN1554_c0_g1_i9_orf1;TRINITY_DN49038_c0_g4_i1_orf1;TRINITY_DN38506_c0_g1_i4_orf1;TRINITY_DN24_c0_g1_i1_orf1;TRINITY_DN2559_c0_g1_i4_orf1;TRINITY_DN98242_c0_g1_i1_orf1;TRINITY_DN14477_c0_g1_i12_orf1;TRINITY_DN34689_c0_g1_i4_orf1;TRINITY_DN26293_c0_g1_i4_orf1;TRINITY_DN41179_c0_g1_i1_orf1;TRINITY_DN2749_c0_g2_i3_orf1;TRINITY_DN4835_c0_g1_i2_orf1;TRINITY_DN7341_c0_g1_i8_orf1;TRINITY_DN24322_c0_g1_i4_orf1;TRINITY_DN825_c23_g1_i5_orf1;TRINITY_DN11986_c0_g1_i1_orf1;TRINITY_DN64_c0_g1_i4_orf1;TRINITY_DN8012_c0_g1_i3_orf1;TRINITY_DN83150_c0_g1_i1_orf1;TRINITY_DN38274_c0_g1_i1_orf1;TRINITY_DN14487_c0_g1_i4_orf1;TRINITY_DN28299_c0_g1_i1_orf1;TRINITY_DN4820_c0_g2_i2_orf1;TRINITY_DN1957_c0_g1_i4_orf1;TRINITY_DN3263_c0_g1_i2_orf1;TRINITY_DN120144_c0_g1_i1_orf1;TRINITY_DN6248_c0_g1_i1_orf1;TRINITY_DN141353_c0_g1_i1_orf1;TRINITY_DN4429_c0_g1_i5_orf1;TRINITY_DN879_c0_g1_i2_orf1;TRINITY_DN46022_c0_g1_i1_orf1;TRINITY_DN9979_c0_g1_i1_orf1;TRINITY_DN1084_c0_g2_i2_orf1;TRINITY_DN59804_c0_g1_i1_orf1;TRINITY_DN2971_c0_g1_i1_orf1;TRINITY_DN67649_c0_g1_i1_orf1;TRINITY_DN44792_c0_g1_i1_orf1;TRINITY_DN38562_c0_g1_i3_orf1;TRINITY_DN13760_c1_g1_i1_orf1;TRINITY_DN26879_c0_g1_i1_orf1;TRINITY_DN15706_c0_g2_i5_orf1;TRINITY_DN4822_c0_g1_i9_orf1;TRINITY_DN104507_c0_g1_i2_orf1;TRINITY_DN87170_c0_g1_i3_orf1;TRINITY_DN5200_c0_g1_i2_orf1;TRINITY_DN117844_c0_g1_i1_orf1;TRINITY_DN34676_c1_g1_i3_orf1;TRINITY_DN2201_c0_g1_i1_orf1;TRINITY_DN19251_c0_g1_i8_orf1;TRINITY_DN1091_c0_g1_i1_orf1;TRINITY_DN1201_c0_g1_i4_orf1;TRINITY_DN11172_c0_g1_i4_orf1;TRINITY_DN15222_c0_g1_i4_orf1;TRINITY_DN323_c0_g2_i5_orf1;TRINITY_DN53311_c0_g2_i1_orf1;TRINITY_DN5238_c0_g1_i2_orf1;TRINITY_DN21596_c0_g1_i1_orf1;TRINITY_DN133760_c0_g1_i1_orf1;TRINITY_DN34432_c0_g1_i1_orf1;TRINITY_DN110534_c0_g1_i3_orf1;TRINITY_DN5757_c0_g1_i1_orf1;TRINITY_DN48619_c0_g1_i1_orf1;TRINITY_DN5998_c0_g2_i1_orf1;TRINITY_DN58125_c0_g1
biological_process	cellular metabolic compound salvage	GO:0043094	5	5/2397	TRINITY_DN35763_c0_g1_i2_orf1;TRINITY_DN3073_c0_g1_i7_orf1;TRINITY_DN1575_c0_g1_i7_orf1;TRINITY_DN5811_c0_g1_i4_orf1;TRINITY_DN1718_c6_g1_i4_orf1
biological_process	neurotransmitter metabolic process	GO:0042133	3	3/2397	TRINITY_DN17693_c0_g1_i10_orf1;TRINITY_DN82017_c0_g1_i5_orf1;TRINITY_DN14565_c0_g1_i11_orf1
biological_process	cellular modified amino acid metabolic process	GO:0006575	5	5/2397	TRINITY_DN14398_c0_g1_i4_orf1;TRINITY_DN38506_c0_g1_i4_orf1;TRINITY_DN41166_c0_g1_i1_orf1;TRINITY_DN3263_c0_g1_i2_orf1;TRINITY_DN38562_c0_g1_i3_orf1
biological_process	translational initiation	GO:0006413	1	1/2397	TRINITY_DN48097_c0_g1_i1_orf1
biological_process	prenylation	GO:0097354	1	1/2397	TRINITY_DN5182_c0_g1_i5_orf1
biological_process	secondary metabolite biosynthetic process	GO:0044550	1	1/2397	TRINITY_DN58125_c0_g1_i1_orf1

biological_process	small molecule biosynthetic process	GO:0044283	33	33/2397	TRINITY_DN48590_c0_g1_i1_orf1;TRINITY_DN230_c2_g1_i5_orf1;TRINITY_DN10722_c0_g3_i1_orf1;TRINITY_DN4360_c0_g1_i4_orf1;TRINITY_DN24970_c0_g1_i4_orf1;TRINITY_DN27035_c0_g1_i1_orf1;TRINITY_DN1575_c0_g1_i7_orf1;TRINITY_DN2719_c1_g1_i6_orf1;TRINITY_DN35763_c0_g1_i2_orf1;TRINITY_DN14306_c0_g1_i1_orf1;TRINITY_DN1201_c0_g1_i4_orf1;TRINITY_DN11948_c0_g1_i8_orf1;TRINITY_DN5211_c0_g1_i1_orf1;TRINITY_DN28221_c0_g2_i1_orf1;TRINITY_DN6027_c0_g1_i13_orf1;TRINITY_DN24723_c2_g1_i1_orf1;TRINITY_DN2803_c4_g1_i1_orf1;TRINITY_DN8964_c0_g1_i4_orf1;TRINITY_DN10430_c0_g1_i4_orf1;TRINITY_DN26293_c0_g1_i4_orf1;TRINITY_DN1999_c0_g1_i9_orf1;TRINITY_DN3991_c0_g1_i6_orf1;TRINITY_DN3073_c0_g1_i7_orf1;TRINITY_DN38644_c0_g1_i1_orf1;TRINITY_DN51813_c0_g1_i1_orf1;TRINITY_DN6638_c0_g1_i1_orf1;TRINITY_DN34399_c0_g1_i1_orf1;TRINITY_DN2570_c0_g1_i1_orf1;TRINITY_DN511_c0_g2_i1_orf1;TRINITY_DN1718_c6_g1_i4_orf1;TRINITY_DN3263_c0_g1_i2_orf1;TRINITY_DN18782_c0_g1_i4_orf1;TRINITY_DN20133_c0_g1_i1_orf1
biological_process	organic substance biosynthetic process	GO:1901576	132	132/2397	TRINITY_DN10722_c0_g3_i1_orf1;TRINITY_DN4360_c0_g1_i4_orf1;TRINITY_DN137_c0_g1_i1_orf1;TRINITY_DN51813_c0_g1_i1_orf1;TRINITY_DN35763_c0_g1_i2_orf1;TRINITY_DN5873_c0_g4_i1_orf1;TRINITY_DN11065_c0_g2_i1_orf1;TRINITY_DN2682_c0_g1_i4_orf1;TRINITY_DN1768_c0_g1_i2_orf1;TRINITY_DN24723_c2_g1_i1_orf1;TRINITY_DN28221_c0_g2_i1_orf1;TRINITY_DN7957_c0_g1_i5_orf1;TRINITY_DN29448_c0_g1_i1_orf1;TRINITY_DN11013_c0_g1_i3_orf1;TRINITY_DN21570_c0_g1_i1_orf1;TRINITY_DN1393_c0_g1_i2_orf1;TRINITY_DN3991_c0_g1_i6_orf1;TRINITY_DN3800_c0_g1_i7_orf1;TRINITY_DN9028_c0_g1_i5_orf1;TRINITY_DN1509_c0_g1_i1_orf1;TRINITY_DN135_c0_g1_i1_orf1;TRINITY_DN3985_c0_g2_i1_orf1;TRINITY_DN10785_c0_g1_i4_orf1;TRINITY_DN5952_c0_g1_i6_orf1;TRINITY_DN18869_c0_g1_i1_orf1;TRINITY_DN7808_c0_g1_i1_orf1;TRINITY_DN230_c2_g1_i5_orf1;TRINITY_DN18249_c0_g1_i1_orf1;TRINITY_DN15222_c0_g1_i4_orf1;TRINITY_DN115210_c0_g4_i1_orf1;TRINITY_DN23360_c0_g1_i3_orf1;TRINITY_DN14398_c0_g1_i4_orf1;TRINITY_DN2719_c1_g1_i6_orf1;TRINITY_DN14306_c0_g1_i1_orf1;TRINITY_DN97589_c0_g1_i3_orf1;TRINITY_DN115658_c0_g1_i1_orf1;TRINITY_DN1999_c0_g1_i9_orf1;TRINITY_DN5211_c0_g1_i1_orf1;TRINITY_DN141353_c0_g1_i1_orf1;TRINITY_DN8625_c0_g1_i1_orf1;TRINITY_DN7583_c0_g1_i1_orf1;TRINITY_DN7464_c0_g1_i14_orf1;TRINITY_DN50787_c0_g2_i2_orf1;TRINITY_DN10430_c0_g1_i4_orf1;TRINITY_DN36144_c0_g1_i3_orf1;TRINITY_DN12527_c0_g1_i4_orf1;TRINITY_DN38644_c0_g1_i1_orf1;TRINITY_DN117844_c0_g1_i1_orf1;TRINITY_DN12301_c0_g1_i1_orf1;TRINITY_DN9794_c0_g2_i8_orf1;TRINITY_DN3073_c0_g1_i7_orf1;TRINITY_DN9591_c0_g1_i1_orf1;TRINITY_DN49936_c0_g2_i1_orf1;TRINITY_DN13732_c0_g2_i3_orf1;TRINITY_DN4408_c6_g1_i1_orf1;TRINITY_DN21357_c0_g1_i5_orf1;TRINITY_DN1718_c6_g1_i4_orf1;TRINITY_DN7991_c0_g1_i9_orf1;TRINITY_DN58207_c0_g1_i1_orf1;TRINITY_DN74037_c0_g5_i1_orf1;TRINITY_DN13233_c0_g1_i3_orf1;TRINITY_DN10831_c1_g1_i1_orf1;TRINITY_DN147458_c0_g1_i1_orf1;TRINITY_DN2299_c0_g1_i3_orf1;TRINITY_DN5064_c0_g1_i4_orf1;TRINITY_DN143852_c0_g1_i1_orf1;TRINITY_DN5029_c0_g1_i1_orf1;TRINITY_DN812_c2_g1_i1_orf1;TRINITY_DN48590_c0_g1_i1_orf1;TRINITY_DN30131_c0_g1_i1_orf1;TRINITY_DN1750_c1_g1_i5_orf1;TRINITY_DN27852_c0_g1_i1_orf1;TRINITY_DN6365_c0_g1_i4_orf1;TRINITY_DN27035_c0_g1_i1_orf1;TRINITY_DN1575_c0_g1_i7_orf1;TRINITY_DN24318_c0_g1_i1_orf1;TRINITY_DN5009_c0_g1_i2_orf1;TRINITY_DN41645_c0_g1_i1_orf1;TRINITY_DN6563_c0_g1_i1_orf1;TRINITY_DN4016_c0_g1_i1_orf1;TRINITY_DN511_c0_g2_i1_orf1;TRINITY_DN11297_c0_g1_i1_orf1;TRINITY_DN2803_c4_g1_i1_orf1;TRINITY_DN8964_c0_g1_i4_orf1;TRINITY_DN10070_c0_g1_i1_orf1;TRINITY_DN14477_c0_g1_i12_orf1;TRINITY_DN147676_c0_g1_i1_orf1;TRINITY_DN26293_c0_g1_i4_orf1;TRINITY_DN24970_c0_g1_i4_orf1;TRINITY_DN53311_c0_g2_i1_orf1;TRINITY_DN42506_c0_g1_i1_orf1;TRINITY_DN28299_c0_g1_i1_orf1;TRINITY_DN33883_c0_g1_i1_orf1;TRINITY_DN1957_c0_g1_i4_orf1;TRINITY_DN3263_c0_g1_i2_orf1;TRINITY_DN6813_c1_g1_i1_orf1;TRINITY_DN11948_c0_g1_i8_orf1;TRINITY_DN879_c0_g1_i2_orf1;TRINITY_DN1084_c0_g2_i2_orf1;TRINITY_DN40650_c0_g1_i1_orf1;TRINITY_DN5031_c0_g1_i1_orf1;TRINITY_DN37532_c0_g1_i1_orf1;TRINITY_DN44792_c0_g1_i1_orf1;TRINITY_DN36893_c0_g1_i1_orf1;TRINITY_DN2425_c0_g1_i3_orf1;TRINITY_DN41166_c0_g1_i1_orf1;TRINITY_DN18593_c0_g1_i1_orf1;TRINITY_DN1741_c0_g1_i5_orf1;TRINITY_DN8949_c0_g1_i2_orf1;TRINITY_DN2265_c0_g2_i1_orf1;TRINITY_DN1045_c0_g1_i6_orf1;TRINITY_DN6027_c0_g1_i13_orf1;TRINITY_DN5200_c0_g1_i2_orf1;TRINITY_DN6638_c0_g1_i1_orf1;TRINITY_DN8824_c0_g2_i1_orf1;TRINITY_DN1201_c0_g1_i4_orf1;TRINITY_DN3826_c0_g1_i1_orf1;TRINITY_DN5811_c0_g1_i4_orf1;TRINITY_DN323_c0_g2_i5_orf1;TRINITY_DN10234_c0_g1_i1_orf1;TRINITY_DN34399_c0_g1_i1_orf1;TRINITY_DN2570_c0_g1_i1_orf1;TRINITY_DN42646_c0_g2_i1_orf1;TRINITY_DN82324_c0_g1_i4_orf1;TRINITY_DN58125_c0_g1_i1_orf1;TRINITY_DN31253_c0_g1_i2_orf1;TRINITY_DN26879_c0_g1_i1_orf1;TRINITY_DN9862_c0_g2_i1_orf1;TRINITY_DN1
biological_process	small molecule catabolic process	GO:0044282	23	23/2397	TRINITY_DN34751_c0_g1_i1_orf1;TRINITY_DN6199_c2_g1_i3_orf1;TRINITY_DN2065_c1_g2_i1_orf1;TRINITY_DN3010_c0_g1_i4_orf1;TRINITY_DN4822_c0_g1_i9_orf1;TRINITY_DN89483_c0_g1_i1_orf1;TRINITY_DN4451_c0_g2_i4_orf1;TRINITY_DN2890_c0_g1_i2_orf1;TRINITY_DN45220_c0_g1_i1_orf1;TRINITY_DN1034_c0_g1_i4_orf1;TRINITY_DN2559_c0_g1_i4_orf1;TRINITY_DN87170_c0_g1_i3_orf1;TRINITY_DN779_c0_g1_i3_orf1;TRINITY_DN98242_c0_g1_i1_orf1;TRINITY_DN4822_c0_g1_i6_orf1;TRINITY_DN38644_c0_g1_i1_orf1;TRINITY_DN51813_c0_g1_i1_orf1;TRINITY_DN779_c0_g1_i12_orf1;TRINITY_DN1262_c0_g1_i2_orf1;TRINITY_DN3836_c0_g1_i4_orf1;TRINITY_DN15136_c0_g1_i2_orf1;TRINITY_DN20133_c0_g1_i1_orf1;TRINITY_DN25997_c1_g2_i4_orf1
biological_process	organic substance catabolic process	GO:1901575	58	58/2397	TRINITY_DN34751_c0_g1_i1_orf1;TRINITY_DN2065_c1_g2_i1_orf1;TRINITY_DN38230_c0_g1_i4_orf1;TRINITY_DN827_c1_g1_i1_orf1;TRINITY_DN4360_c0_g1_i4_orf1;TRINITY_DN10824_c0_g1_i3_orf1;TRINITY_DN6199_c2_g1_i3_orf1;TRINITY_DN27035_c0_g1_i1_orf1;TRINITY_DN53233_c0_g1_i1_orf1;TRINITY_DN4121_c0_g1_i1_orf1;TRINITY_DN3010_c0_g1_i4_orf1;TRINITY_DN4822_c0_g1_i9_orf1;TRINITY_DN37923_c0_g1_i1_orf1;TRINITY_DN89483_c0_g1_i1_orf1;TRINITY_DN1201_c0_g1_i4_orf1;TRINITY_DN4451_c0_g2_i4_orf1;TRINITY_DN57798_c0_g1_i1_orf1;TRINITY_DN2890_c0_g1_i2_orf1;TRINITY_DN45220_c0_g1_i1_orf1;TRINITY_DN1034_c0_g1_i4_orf1;TRINITY_DN2559_c0_g1_i4_orf1;TRINITY_DN542_c0_g2_i1_orf1;TRINITY_DN87170_c0_g1_i3_orf1;TRINITY_DN24_c0_g1_i1_orf1;TRINITY_DN135188_c0_g1_i2_orf1;TRINITY_DN779_c0_g1_i3_orf1;TRINITY_DN17003_c1_g1_i1_orf1;TRINITY_DN98242_c0_g1_i1_orf1;TRINITY_DN542_c0_g1_i4_orf1;TRINITY_DN34689_c0_g1_i4_orf1;TRINITY_DN4145_c0_g1_i1_orf1;TRINITY_DN96557_c0_g1_i1_orf1;TRINITY_DN4822_c0_g1_i6_orf1;TRINITY_DN38644_c0_g1_i1_orf1;TRINITY_DN51813_c0_g1_i1_orf1;TRINITY_DN8012_c0_g1_i3_orf1;TRINITY_DN46022_c0_g1_i1_orf1;TRINITY_DN2515_c0_g1_i6_orf1;TRINITY_DN46132_c0_g2_i2_orf1;TRINITY_DN83150_c0_g1_i1_orf1;TRINITY_DN5001_c0_g1_i4_orf1;TRINITY_DN6325_c0_g1_i9_orf1;TRINITY_DN779_c0_g1_i12_orf1;TRINITY_DN9062_c0_g2_i3_orf1;TRINITY_DN6325_c0_g1_i8_orf1;TRINITY_DN21555_c0_g1_i4_orf1;TRINITY_DN1262_c0_g1_i2_orf1;TRINITY_DN1287_c0_g1_i5_orf1;TRINITY_DN8908_c0_g1_i1_orf1;TRINITY_DN3836_c0_g1_i4_orf1;TRINITY_DN15136_c0_g1_i2_orf1;TRINITY_DN41_c0_g1_i5_orf1;TRINITY_DN25423_c0_g1_i1_orf1;TRINITY_DN20133_c0_g1_i1_orf1;TRINITY_DN5235_c0_g1_i7_orf1;TRINITY_DN25997_c1_g2_i4_orf1;TRINITY_DN9979_c0_g1_i1_orf
biological_process	vitamin metabolic process	GO:0006766	2	2/2397	TRINITY_DN135781_c0_g1_i1_orf1;TRINITY_DN18782_c0_g1_i4_orf1
biological_process	urea metabolic process	GO:0019627	2	2/2397	TRINITY_DN24723_c2_g1_i1_orf1;TRINITY_DN28221_c0_g2_i1_orf1
biological_process	monosaccharide metabolic process	GO:0005996	15	15/2397	TRINITY_DN1201_c0_g1_i4_orf1;TRINITY_DN15222_c0_g1_i4_orf1;TRINITY_DN120089_c0_g1_i1_orf1;TRINITY_DN12545_c0_g1_i7_orf1;TRINITY_DN9109_c0_g1_i1_orf1;TRINITY_DN4360_c0_g1_i4_orf1;TRINITY_DN18650_c0_g1_i1_orf1;TRINITY_DN27035_c0_g1_i1_orf1;TRINITY_DN2570_c0_g1_i1_orf1;TRINITY_DN30713_c0_g1_i3_orf1;TRINITY_DN20133_c0_g1_i1_orf1;TRINITY_DN25997_c1_g2_i4_orf1;TRINITY_DN511_c0_g2_i1_orf1;TRINITY_DN31967_c0_g1_i5_orf1;TRINITY_DN1353_c0_g1_i1_orf1
biological_process	alcohol metabolic process	GO:0006066	8	8/2397	TRINITY_DN230_c2_g1_i5_orf1;TRINITY_DN52244_c1_g1_i1_orf1;TRINITY_DN1034_c0_g1_i4_orf1;TRINITY_DN10722_c0_g3_i1_orf1;TRINITY_DN38644_c0_g1_i1_orf1;TRINITY_DN9286_c0_g1_i2_orf1;TRINITY_DN3010_c0_g1_i4_orf1;TRINITY_DN618_c0_g1_i3_orf1
biological_process	nucleobase-containing small molecule metabolic process	GO:0055086	45	45/2397	TRINITY_DN38230_c0_g1_i4_orf1;TRINITY_DN6325_c0_g1_i8_orf1;TRINITY_DN115210_c0_g4_i1_orf1;TRINITY_DN4360_c0_g1_i4_orf1;TRINITY_DN60787_c0_g1_i5_orf1;TRINITY_DN27035_c0_g1_i1_orf1;TRINITY_DN1575_c0_g1_i7_orf1;TRINITY_DN1201_c0_g1_i4_orf1;TRINITY_DN49038_c0_g4_i1_orf1;TRINITY_DN2559_c0_g1_i4_orf1;TRINITY_DN779_c0_g1_i3_orf1;TRINITY_DN24_c0_g1_i1_orf1;TRINITY_DN6813_c1_g1_i1_orf1;TRINITY_DN8625_c0_g1_i1_orf1;TRINITY_DN9794_c0_g2_i8_orf1;TRINITY_DN98242_c0_g1_i1_orf1;TRINITY_DN14477_c0_g1_i12_orf1;TRINITY_DN11013_c0_g1_i3_orf1;TRINITY_DN26293_c0_g1_i4_orf1;TRINITY_DN36144_c0_g1_i3_orf1;TRINITY_DN15222_c0_g1_i4_orf1;TRINITY_DN117844_c0_g1_i1_orf1;TRINITY_DN12301_c0_g1_i1_orf1;TRINITY_DN3991_c0_g1_i6_orf1;TRINITY_DN51813_c0_g1_i1_orf1;TRINITY_DN19251_c0_g1_i8_orf1;TRINITY_DN3800_c0_g1_i7_orf1;TRINITY_DN8012_c0_g1_i3_orf1;TRINITY_DN83150_c0_g1_i1_orf1;TRINITY_DN6325_c0_g1_i9_orf1;TRINITY_DN1718_c6_g1_i4_orf1;TRINITY_DN5952_c0_g1_i6_orf1;TRINITY_DN779_c0_g1_i12_orf1;TRINITY_DN28299_c0_g1_i1_orf1;TRINITY_DN5811_c0_g1_i4_orf1;TRINITY_DN8908_c0_g1_i1_orf1;TRINITY_DN9979_c0_g1_i1_orf1;TRINITY_DN141353_c0_g1_i1_orf1;TRINITY_DN15136_c0_g1_i2_orf1;TRINITY_DN18782_c0_g1_i4_orf1;TRINITY_DN20133_c0_g1_i1_orf1;TRINITY_DN7808_c0_g1_i1_orf1;TRINITY_DN5029_c0_g1_i1_orf1;TRINITY_DN1957_c0_g1_i4_orf1;TRINITY_DN1084_c0_g2_i2_orf1
biological_process	urate metabolic process	GO:0046415	3	3/2397	TRINITY_DN779_c0_g1_i12_orf1;TRINITY_DN2559_c0_g1_i4_orf1;TRINITY_DN779_c0_g1_i3_orf1
biological_process	macromolecule glycosylation	GO:0043413	5	5/2397	TRINITY_DN5513_c0_g1_i1_orf1;TRINITY_DN56164_c0_g1_i1_orf1;TRINITY_DN31676_c0_g1_i4_orf1;TRINITY_DN4248_c0_g1_i4_orf1;TRINITY_DN1789_c0_g1_i5_orf1
biological_process	macromolecule methylation	GO:0043414	7	7/2397	TRINITY_DN1344_c0_g1_i1_orf1;TRINITY_DN22674_c0_g1_i2_orf1;TRINITY_DN13350_c0_g1_i4_orf1;TRINITY_DN15256_c0_g1_i8_orf1;TRINITY_DN95414_c0_g1_i1_orf1;TRINITY_DN14313_c0_g1_i1_orf1;TRINITY_DN6462_c0_g1_i5_orf1
biological_process	molybdopterin cofactor metabolic process	GO:0043545	1	1/2397	TRINITY_DN1741_c0_g1_i5_orf1
biological_process	lipid metabolic process	GO:0006629	41	41/2397	TRINITY_DN34751_c0_g1_i1_orf1;TRINITY_DN48590_c0_g1_i1_orf1;TRINITY_DN10722_c0_g3_i1_orf1;TRINITY_DN12526_c0_g1_i5_orf1;TRINITY_DN2668_c0_g1_i7_orf1;TRINITY_DN1293_c0_g1_i4_orf1;TRINITY_DN482_c0_g1_i1_orf1;TRINITY_DN5841_c0_g1_i2_orf1;TRINITY_DN14306_c0_g1_i1_orf1;TRINITY_DN3784_c0_g1_i1_orf1;TRINITY_DN45220_c0_g1_i1_orf1;TRINITY_DN44110_c0_g1_i4_orf1;TRINITY_DN5211_c0_g1_i1_orf1;TRINITY_DN44658_c0_g1_i2_orf1;TRINITY_DN86833_c0_g3_i1_orf1;TRINITY_DN8964_c0_g1_i4_orf1;TRINITY_DN117_c0_g1_i6_orf1;TRINITY_DN52788_c0_g1_i1_orf1;TRINITY_DN41_c0_g1_i3_orf1;TRINITY_DN10430_c0_g1_i4_orf1;TRINITY_DN9028_c0_g1_i5_orf1;TRINITY_DN12024_c0_g2_i2_orf1;TRINITY_DN21570_c0_g1_i1_orf1;TRINITY_DN3175_c0_g1_i7_orf1;TRINITY_DN117844_c0_g1_i1_orf1;TRINITY_DN1999_c0_g1_i9_orf1;TRINITY_DN3991_c0_g1_i6_orf1;TRINITY_DN38644_c0_g1_i1_orf1;TRINITY_DN12024_c0_g1_i4_orf1;TRINITY_DN4070_c0_g1_i4_orf1;TRINITY_DN3529_c0_g1_i7_orf1;TRINITY_DN12806_c0_g2_i1_orf1;TRINITY_DN10785_c0_g1_i4_orf1;TRINITY_DN6586_c0_g1_i1_orf1;TRINITY_DN1293_c1_g1_i4_orf1;TRINITY_DN41_c0_g1_i5_orf1;TRINITY_DN1109_c0_g1_i6_orf1;TRINITY_DN117_c0_g1_i4_orf1;TRINITY_DN5697_c0_g1_i1_orf1;TRINITY_DN3545_c0_g1_i6_orf1;TRINITY_DN1084_c0_g2_i2_orf1

biological_process	nucleobase-containing compound metabolic process	GO:0006139	146	146/2397	TRINITY_DN38230_c0_g1_i4_orf1;TRINITY_DN4360_c0_g1_i4_orf1;TRINITY_DN40434_c0_g1_i2_orf1;TRINITY_DN13350_c0_g1_i4_orf1;TRINITY_DN60787_c0_g1_i5_orf1;TRINITY_DN7122_c0_g1_i1_orf1;TRINITY_DN51813_c0_g1_i1_orf1;TRINITY_DN35669_c0_g1_i1_orf1;TRINITY_DN2054_c0_g1_i1_orf1;TRINITY_DN15160_c0_g1_i1_orf1;TRINITY_DN34134_c0_g2_i1_orf1;TRINITY_DN1344_c0_g1_i1_orf1;TRINITY_DN1768_c0_g1_i2_orf1;TRINITY_DN1607_c0_g1_i16_orf1;TRINITY_DN23616_c0_g1_i4_orf1;TRINITY_DN2953_c1_g1_i10_orf1;TRINITY_DN11013_c0_g1_i3_orf1;TRINITY_DN18404_c0_g1_i5_orf1;TRINITY_DN2953_c1_g1_i2_orf1;TRINITY_DN1393_c0_g1_i2_orf1;TRINITY_DN3991_c0_g1_i6_orf1;TRINITY_DN3800_c0_g1_i7_orf1;TRINITY_DN15370_c0_g1_i4_orf1;TRINITY_DN15256_c0_g1_i8_orf1;TRINITY_DN123184_c0_g1_i1_orf1;TRINITY_DN817_c0_g1_i3_orf1;TRINITY_DN2224_c0_g1_i1_orf1;TRINITY_DN5952_c0_g1_i6_orf1;TRINITY_DN46409_c0_g1_i1_orf1;TRINITY_DN8908_c0_g1_i1_orf1;TRINITY_DN15136_c0_g1_i2_orf1;TRINITY_DN107288_c0_g1_i2_orf1;TRINITY_DN7808_c0_g1_i1_orf1;TRINITY_DN89613_c0_g1_i13_orf1;TRINITY_DN14313_c0_g1_i1_orf1;TRINITY_DN2647_c0_g1_i3_orf1;TRINITY_DN17271_c0_g1_i1_orf1;TRINITY_DN115210_c0_g4_i1_orf1;TRINITY_DN140212_c0_g1_i1_orf1;TRINITY_DN45271_c0_g1_i1_orf1;TRINITY_DN3082_c1_g1_i7_orf1;TRINITY_DN1005_c0_g1_i5_orf1;TRINITY_DN37532_c0_g1_i1_orf1;TRINITY_DN19687_c0_g1_i1_orf1;TRINITY_DN15900_c0_g1_i6_orf1;TRINITY_DN81258_c0_g1_i2_orf1;TRINITY_DN115658_c0_g1_i1_orf1;TRINITY_DN6813_c1_g1_i1_orf1;TRINITY_DN8625_c0_g1_i1_orf1;TRINITY_DN779_c0_g1_i3_orf1;TRINITY_DN18391_c0_g2_i8_orf1;TRINITY_DN810_c0_g1_i4_orf1;TRINITY_DN16978_c0_g1_i1_orf1;TRINITY_DN36144_c0_g1_i3_orf1;TRINITY_DN12527_c0_g1_i4_orf1;TRINITY_DN1616_c0_g1_i3_orf1;TRINITY_DN41573_c0_g1_i1_orf1;TRINITY_DN12301_c0_g1_i1_orf1;TRINITY_DN9794_c0_g2_i8_orf1;TRINITY_DN6642_c0_g1_i2_orf1;TRINITY_DN13732_c0_g2_i3_orf1;TRINITY_DN4408_c6_g1_i1_orf1;TRINITY_DN6325_c0_g1_i9_orf1;TRINITY_DN1718_c6_g1_i4_orf1;TRINITY_DN779_c0_g1_i12_orf1;TRINITY_DN56993_c0_g1_i4_orf1;TRINITY_DN5811_c0_g1_i4_orf1;TRINITY_DN74037_c0_g5_i1_orf1;TRINITY_DN31663_c0_g1_i2_orf1;TRINITY_DN139537_c0_g1_i1_orf1;TRINITY_DN107035_c0_g1_i1_orf1;TRINITY_DN4908_c1_g1_i5_orf1;TRINITY_DN2299_c0_g1_i3_orf1;TRINITY_DN5029_c0_g1_i1_orf1;TRINITY_DN291_c0_g1_i2_orf1;TRINITY_DN6325_c0_g1_i8_orf1;TRINITY_DN1750_c1_g1_i5_orf1;TRINITY_DN27852_c0_g1_i1_orf1;TRINITY_DN77318_c0_g2_i1_orf1;TRINITY_DN27035_c0_g1_i1_orf1;TRINITY_DN1575_c0_g1_i7_orf1;TRINITY_DN53233_c0_g1_i1_orf1;TRINITY_DN51934_c0_g2_i1_orf1;TRINITY_DN2718_c0_g1_i6_orf1;TRINITY_DN57918_c0_g1_i1_orf1;TRINITY_DN56270_c0_g1_i1_orf1;TRINITY_DN1554_c0_g1_i9_orf1;TRINITY_DN49038_c0_g4_i1_orf1;TRINITY_DN24_c0_g1_i1_orf1;TRINITY_DN2559_c0_g1_i4_orf1;TRINITY_DN98242_c0_g1_i1_orf1;TRINITY_DN14477_c0_g1_i12_orf1;TRINITY_DN34689_c0_g1_i4_orf1;TRINITY_DN26293_c0_g1_i4_orf1;TRINITY_DN41179_c0_g1_i1_orf1;TRINITY_DN2749_c0_g2_i3_orf1;TRINITY_DN4835_c0_g1_i2_orf1;TRINITY_DN7341_c0_g1_i8_orf1;TRINITY_DN24322_c0_g1_i4_orf1;TRINITY_DN825_c23_g1_i5_orf1;TRINITY_DN11986_c0_g1_i1_orf1;TRINITY_DN64_c0_g1_i4_orf1;TRINITY_DN8012_c0_g1_i3_orf1;TRINITY_DN83150_c0_g1_i1_orf1;TRINITY_DN38274_c0_g1_i1_orf1;TRINITY_DN14487_c0_g1_i4_orf1;TRINITY_DN28299_c0_g1_i1_orf1;TRINITY_DN4820_c0_g2_i2_orf1;TRINITY_DN1957_c0_g1_i4_orf1;TRINITY_DN120144_c0_g1_i1_orf1;TRINITY_DN6248_c0_g1_i1_orf1;TRINITY_DN141353_c0_g1_i1_orf1;TRINITY_DN4429_c0_g1_i5_orf1;TRINITY_DN879_c0_g1_i2_orf1;TRINITY_DN46022_c0_g1_i1_orf1;TRINITY_DN9979_c0_g1_i1_orf1;TRINITY_DN1084_c0_g2_i2_orf1;TRINITY_DN59804_c0_g1_i1_orf1;TRINITY_DN2971_c0_g1_i1_orf1;TRINITY_DN67649_c0_g1_i1_orf1;TRINITY_DN44792_c0_g1_i1_orf1;TRINITY_DN38562_c0_g1_i3_orf1;TRINITY_DN13760_c1_g1_i1_orf1;TRINITY_DN26879_c0_g1_i1_orf1;TRINITY_DN104507_c0_g1_i2_orf1;TRINITY_DN5200_c0_g1_i2_orf1;TRINITY_DN117844_c0_g1_i1_orf1;TRINITY_DN34676_c1_g1_i3_orf1;TRINITY_DN2201_c0_g1_i1_orf1;TRINITY_DN19251_c0_g1_i8_orf1;TRINITY_DN1091_c0_g1_i1_orf1;TRINITY_DN1201_c0_g1_i4_orf1;TRINITY_DN15222_c0_g1_i4_orf1;TRINITY_DN53311_c0_g2_i1_orf1;TRINITY_DN5238_c0_g1_i2_orf1;TRINITY_DN21596_c0_g1_i1_orf1;TRINITY_DN133760_c0_g1_i1_orf1;TRINITY_DN34432_c0_g1_i1_orf1;TRINITY_DN110534_c0_g1_i3_orf1;TRINITY_DN5757_c0_g1_i1_orf1;TRINITY_DN48619_c0_g1_i1_orf1;TRINITY_DN5998_c0_g2_i1_orf1;TRINITY_DN21539_c0_g1_i1_orf1;TRINITY_DN52
biological_process	tricarboxylic acid cycle	GO:0006099	4	4/2397	TRINITY_DN5266_c0_g1_i1_orf1;TRINITY_DN3464_c0_g1_i1_orf1;TRINITY_DN146126_c0_g1_i1_orf1;TRINITY_DN19251_c0_g1_i8_orf1
biological_process	carbohydrate metabolic process	GO:0005975	77	77/2397	TRINITY_DN17003_c1_g1_i1_orf1;TRINITY_DN14235_c0_g1_i1_orf1;TRINITY_DN8703_c0_g1_i2_orf1;TRINITY_DN120089_c0_g1_i1_orf1;TRINITY_DN10722_c0_g3_i1_orf1;TRINITY_DN2474_c0_g1_i5_orf1;TRINITY_DN4360_c0_g1_i4_orf1;TRINITY_DN18650_c0_g1_i1_orf1;TRINITY_DN10824_c0_g1_i3_orf1;TRINITY_DN2425_c0_g1_i3_orf1;TRINITY_DN28741_c0_g1_i3_orf1;TRINITY_DN60787_c0_g1_i5_orf1;TRINITY_DN27035_c0_g1_i1_orf1;TRINITY_DN664_c0_g1_i18_orf1;TRINITY_DN22577_c0_g1_i2_orf1;TRINITY_DN25492_c0_g1_i1_orf1;TRINITY_DN6325_c0_g1_i9_orf1;TRINITY_DN1196_c0_g1_i5_orf1;TRINITY_DN3010_c0_g1_i4_orf1;TRINITY_DN11657_c0_g1_i2_orf1;TRINITY_DN31967_c0_g1_i5_orf1;TRINITY_DN1201_c0_g1_i4_orf1;TRINITY_DN618_c0_g1_i3_orf1;TRINITY_DN2205_c0_g1_i3_orf1;TRINITY_DN49038_c0_g4_i1_orf1;TRINITY_DN1034_c0_g1_i4_orf1;TRINITY_DN12545_c0_g1_i7_orf1;TRINITY_DN9109_c0_g1_i1_orf1;TRINITY_DN5852_c0_g1_i13_orf1;TRINITY_DN4070_c0_g1_i4_orf1;TRINITY_DN53167_c0_g1_i3_orf1;TRINITY_DN7183_c0_g1_i2_orf1;TRINITY_DN11817_c0_g1_i4_orf1;TRINITY_DN361_c0_g1_i5_orf1;TRINITY_DN48237_c0_g1_i5_orf1;TRINITY_DN3322_c0_g1_i2_orf1;TRINITY_DN89483_c0_g1_i1_orf1;TRINITY_DN83150_c0_g1_i1_orf1;TRINITY_DN2894_c0_g2_i3_orf1;TRINITY_DN542_c0_g1_i4_orf1;TRINITY_DN30713_c0_g1_i3_orf1;TRINITY_DN7228_c0_g1_i6_orf1;TRINITY_DN140613_c0_g1_i1_orf1;TRINITY_DN15222_c0_g1_i4_orf1;TRINITY_DN9000_c0_g2_i1_orf1;TRINITY_DN38644_c0_g1_i1_orf1;TRINITY_DN812_c2_g1_i1_orf1;TRINITY_DN195_c4_g1_i1_orf1;TRINITY_DN2061_c0_g1_i3_orf1;TRINITY_DN2570_c0_g1_i1_orf1;TRINITY_DN2515_c0_g1_i6_orf1;TRINITY_DN26688_c0_g1_i2_orf1;TRINITY_DN9044_c0_g1_i2_orf1;TRINITY_DN511_c0_g2_i1_orf1;TRINITY_DN14458_c0_g1_i2_orf1;TRINITY_DN1732_c0_g1_i15_orf1;TRINITY_DN7828_c0_g1_i2_orf1;TRINITY_DN670_c0_g1_i3_orf1;TRINITY_DN6325_c0_g1_i8_orf1;TRINITY_DN21555_c0_g1_i4_orf1;TRINITY_DN52244_c1_g1_i1_orf1;TRINITY_DN5952_c0_g1_i6_orf1;TRINITY_DN1732_c0_g1_i17_orf1;TRINITY_DN1287_c0_g1_i5_orf1;TRINITY_DN479_c6_g1_i2_orf1;TRINITY_DN82801_c0_g1_i1_orf1;TRINITY_DN14409_c0_g1_i1_orf1;TRINITY_DN542_c0_g2_i1_orf1;TRINITY_DN13088_c0_g1_i5_orf1;TRINITY_DN2170_c1_g1_i3_orf1;TRINITY_DN20133_c0_g1_i1_orf1;TRINITY_DN5852_c0_g1_i6_orf1;TRINITY_DN25997_c1_g2_i4_orf1;TRINITY_DN650_c0_g1_i3_orf1;TRINITY_DN9979_c0_g1_i1_orf1;TRINITY_DN27332_c0_g2_i1_orf1;TRINITY_DN1353_c0_g1_i1_orf1
biological_process	protein metabolic process	GO:0019538	210	210/2397	TRINITY_DN24469_c0_g2_i2_orf1;TRINITY_DN10364_c0_g1_i5_orf1;TRINITY_DN3194_c0_g1_i6_orf1;TRINITY_DN137_c0_g1_i1_orf1;TRINITY_DN1533_c0_g2_i1_orf1;TRINITY_DN135_c0_g1_i1_orf1;TRINITY_DN11928_c0_g1_i3_orf1;TRINITY_DN56164_c0_g1_i1_orf1;TRINITY_DN5873_c0_g4_i1_orf1;TRINITY_DN1274_c0_g1_i4_orf1;TRINITY_DN2069_c1_g1_i8_orf1;TRINITY_DN4217_c0_g1_i2_orf1;TRINITY_DN38431_c0_g1_i1_orf1;TRINITY_DN2682_c0_g1_i4_orf1;TRINITY_DN3499_c0_g1_i8_orf1;TRINITY_DN8692_c0_g1_i2_orf1;TRINITY_DN2442_c0_g1_i2_orf1;TRINITY_DN8659_c0_g1_i1_orf1;TRINITY_DN70485_c0_g1_i2_orf1;TRINITY_DN2983_c0_g1_i6_orf1;TRINITY_DN11065_c0_g2_i1_orf1;TRINITY_DN16258_c0_g1_i2_orf1;TRINITY_DN2885_c1_g1_i2_orf1;TRINITY_DN277_c1_g1_i1_orf1;TRINITY_DN14754_c0_g1_i6_orf1;TRINITY_DN29448_c0_g1_i1_orf1;TRINITY_DN41761_c0_g1_i4_orf1;TRINITY_DN2954_c0_g1_i1_orf1;TRINITY_DN14217_c0_g1_i1_orf1;TRINITY_DN14774_c0_g1_i4_orf1;TRINITY_DN6813_c1_g1_i1_orf1;TRINITY_DN875_c0_g1_i3_orf1;TRINITY_DN10070_c0_g1_i1_orf1;TRINITY_DN2794_c1_g1_i8_orf1;TRINITY_DN59885_c0_g1_i3_orf1;TRINITY_DN4125_c0_g1_i14_orf1;TRINITY_DN1509_c0_g1_i1_orf1;TRINITY_DN6185_c0_g1_i12_orf1;TRINITY_DN3978_c0_g2_i1_orf1;TRINITY_DN6436_c0_g1_i1_orf1;TRINITY_DN3985_c0_g2_i1_orf1;TRINITY_DN391_c0_g1_i4_orf1;TRINITY_DN46409_c0_g1_i1_orf1;TRINITY_DN22674_c0_g1_i2_orf1;TRINITY_DN6470_c0_g3_i2_orf1;TRINITY_DN3826_c0_g1_i1_orf1;TRINITY_DN13732_c0_g2_i3_orf1;TRINITY_DN1380_c0_g1_i5_orf1;TRINITY_DN10403_c0_g1_i3_orf1;TRINITY_DN11620_c0_g1_i2_orf1;TRINITY_DN6967_c0_g1_i3_orf1;TRINITY_DN4767_c0_g1_i6_orf1;TRINITY_DN18249_c0_g1_i1_orf1;TRINITY_DN143895_c0_g1_i1_orf1;TRINITY_DN13856_c0_g1_i1_orf1;TRINITY_DN23360_c0_g1_i3_orf1;TRINITY_DN45633_c0_g1_i1_orf1;TRINITY_DN10090_c0_g1_i1_orf1;TRINITY_DN18869_c0_g1_i1_orf1;TRINITY_DN19537_c0_g1_i1_orf1;TRINITY_DN4189_c0_g2_i1_orf1;TRINITY_DN1791_c0_g1_i3_orf1;TRINITY_DN37923_c0_g1_i1_orf1;TRINITY_DN97589_c0_g1_i3_orf1;TRINITY_DN57798_c0_g1_i1_orf1;TRINITY_DN1310_c0_g1_i4_orf1;TRINITY_DN5012_c0_g1_i6_orf1;TRINITY_DN36434_c0_g2_i3_orf1;TRINITY_DN1789_c0_g1_i5_orf1;TRINITY_DN42461_c0_g1_i4_orf1;TRINITY_DN30154_c0_g1_i1_orf1;TRINITY_DN13233_c0_g1_i3_orf1;TRINITY_DN21719_c0_g1_i2_orf1;TRINITY_DN7583_c0_g1_i1_orf1;TRINITY_DN7464_c0_g1_i14_orf1;TRINITY_DN50787_c0_g2_i2_orf1;TRINITY_DN45948_c1_g1_i1_orf1;TRINITY_DN5696_c0_g1_i4_orf1;TRINITY_DN23167_c0_g2_i1_orf1;TRINITY_DN6423_c0_g1_i5_orf1;TRINITY_DN71863_c0_g1_i2_orf1;TRINITY_DN29034_c0_g1_i2_orf1;TRINITY_DN32700_c0_g1_i2_orf1;TRINITY_DN4125_c1_g1_i5_orf1;TRINITY_DN9591_c0_g1_i1_orf1;TRINITY_DN49936_c0_g2_i1_orf1;TRINITY_DN5444_c0_g2_i1_orf1;TRINITY_DN4408_c6_g1_i1_orf1;TRINITY_DN95414_c0_g1_i1_orf1;TRINITY_DN6423_c0_g1_i6_orf1;TRINITY_DN5281_c0_g2_i3_orf1;TRINITY_DN376_c1_g1_i1_orf1;TRINITY_DN21357_c0_g1_i5_orf1;TRINITY_DN1954_c0_g1_i4_orf1;TRINITY_DN83295_c0_g1_i3_orf1;TRINITY_DN7991_c0_g1_i9_orf1;TRINITY_DN58207_c0_g1_i1_orf1;TRINITY_DN4767_c0_g1_i4_orf1;TRINITY_DN1173_c1_g1_i10_orf1;TRINITY_DN1528_c0_g1_i4_orf1;TRINITY_DN10831_c1_g1_i1_orf1;TRINITY_DN81803_c0_g2_i1_orf1;TRINITY_DN147458_c0_g1_i1_orf1;TRINITY_DN21218_c0_g1_i4_orf1;TRINITY_DN52553_c0_g2_i1_orf1;TRINITY_DN5513_c0_g1_i1_orf1;TRINITY_DN143852_c0_g1_i1_orf1;TRINITY_DN4248_c0_g1_i4_orf1;TRINITY_DN1173_c0_g1_i12_orf1;TRINITY_DN1749_c0_g2_i2_orf1;TRINITY_DN2584_c0_g1_i7_orf1;TRINITY_DN25534_c0_g1_i1_orf1;TRINITY_DN10766_c0_g1_i1_orf1;TRINITY_DN6365_c0_g1_i4_orf1;TRINITY_DN3483_c0_g1_i5_orf1;TRINITY_DN24318_c0_g1_i1_orf1;TRINITY_DN5182_c0_g1_i5_orf1;TRINITY_DN5009_c0_g1_i2_orf1;TRINITY_DN57111_c0_g1_i1_orf1;TRINITY_DN2040_c0_g1_i6_orf1;TRINITY_DN4571_c0_g1_i4_orf1;TRINITY_DN140_c1_g1_i2_orf1;TRINITY_DN28729_c0_g1_i9_orf1;TRINITY_DN1404_c0_g1_i6_orf1;TRINITY_DN4016_c0_g1_i1_orf1;TRINITY_DN74116_c0_g1_i2_orf1;TRINITY_DN1706_c0_g1_i7_orf1;TRINITY_DN11297_c0_g1_i1_orf1;TRINITY_DN21719_c0_g2_i4_orf1;TRINITY_DN3343_c0_g2_i1_orf1;TRINITY_DN147676_c0_g1_i1_orf1;TRINITY_DN18388_c0_g1_i6_orf1;TRINITY_DN18172_c0_g1_i6_orf1;TRINITY_DN9062_c0_g2_i3_orf1;TRINITY_DN1309_c0_g2_i1_orf1;TRINITY_DN1308_c0_g1_i4_orf1;TRINITY_DN23167_c0_g1_i4_orf1;TRINITY_DN4228_c0_g1_i5_orf1;TRINITY_DN4121_c0_g1_i1_orf1;TRINITY_DN143637_c0_g1_i1_orf1;TRINITY_DN111110_c0_g1_i1_orf1;TRINITY_DN42506_c0_g1_i1_orf1;TRINITY_DN96557_c0_g1_i1_orf1;TRINITY_DN8659_c0_g2_i1_orf1;TRINITY_DN10234_c0_g1_i1_orf1;TRINITY_DN33883_c0_g1_i1_orf1;TRINITY_DN24121_c1_g1_i6_orf1;TRINITY_DN29034_c0_g1_i1_orf1;TRINITY_DN3975_c0_g1_i7_orf1;T[illegible]
biological_process	cellular amino acid metabolic process	GO:0006520	43	43/2397	TRINITY_DN2065_c1_g2_i1_orf1;TRINITY_DN57918_c0_g1_i1_orf1;TRINITY_DN14565_c0_g1_i11_orf1;TRINITY_DN115210_c0_g4_i1_orf1;TRINITY_DN24970_c0_g1_i4_orf1;TRINITY_DN6199_c2_g1_i3_orf1;TRINITY_DN2719_c1_g1_i6_orf1;TRINITY_DN35763_c0_g1_i2_orf1;TRINITY_DN4822_c0_g1_i9_orf1;TRINITY_DN89483_c0_g1_i1_orf1;TRINITY_DN4451_c0_g2_i4_orf1;TRINITY_DN2890_c0_g1_i2_orf1;TRINITY_DN11948_c0_g1_i8_orf1;TRINITY_DN1607_c0_g1_i16_orf1;TRINITY_DN87170_c0_g1_i3_orf1;TRINITY_DN2803_c4_g1_i1_orf1;TRINITY_DN2953_c1_g1_i10_orf1;TRINITY_DN24723_c2_g1_i1_orf1;TRINITY_DN28221_c0_g2_i1_orf1;TRINITY_DN2953_c1_g1_i2_orf1;TRINITY_DN11013_c0_g1_i3_orf1;TRINITY_DN20796_c0_g1_i4_orf1;TRINITY_DN4822_c0_g1_i6_orf1;TRINITY_DN17326_c0_g1_i8_orf1;TRINITY_DN3859_c0_g1_i5_orf1;TRINITY_DN825_c23_g1_i5_orf1;TRINITY_DN3073_c0_g1_i7_orf1;TRINITY_DN51813_c0_g1_i1_orf1;TRINITY_DN34399_c0_g1_i1_orf1;TRINITY_DN817_c0_g1_i3_orf1;TRINITY_DN48619_c0_g1_i1_orf1;TRINITY_DN15160_c0_g1_i1_orf1;TRINITY_DN2224_c0_g1_i1_orf1;TRINITY_DN21539_c0_g1_i1_orf1;TRINITY_DN1262_c0_g1_i2_orf1;TRINITY_DN11383_c0_g2_i4_orf1;TRINITY_DN3263_c0_g1_i2_orf1;TRINITY_DN5218_c0_g1_i4_orf1;TRINITY_DN17326_c0_g1_i5_orf1;TRINITY_DN3836_c0_g1_i4_orf1;TRINITY_DN15136_c0_g1_i2_orf1;TRINITY_DN107288_c0_g1_i2_orf1;TRINITY_DN1068_c0_g1_i3_orf1

biological_process	organophosphate metabolic process	GO:0019637	48	48/2397	TRINITY_DN143509_c0_g1_i1_orf1;TRINITY_DN62557_c0_g1_i1_orf1;TRINITY_DN47151_c0_g1_i1_orf1;TRINITY_DN38230_c0_g1_i4_orf1;TRINITY_DN10722_c0_g3_i1_orf1;TRINITY_DN115210_c0_g4_i1_orf1;TRINITY_DN4360_c0_g1_i4_orf1;TRINITY_DN5697_c0_g1_i1_orf1;TRINITY_DN60787_c0_g1_i5_orf1;TRINITY_DN27035_c0_g1_i1_orf1;TRINITY_DN1575_c0_g1_i7_orf1;TRINITY_DN41166_c0_g1_i1_orf1;TRINITY_DN1741_c0_g1_i5_orf1;TRINITY_DN1201_c0_g1_i4_orf1;TRINITY_DN6325_c0_g1_i8_orf1;TRINITY_DN1034_c0_g1_i4_orf1;TRINITY_DN24_c0_g1_i1_orf1;TRINITY_DN6813_c1_g1_i1_orf1;TRINITY_DN9794_c0_g2_i8_orf1;TRINITY_DN116972_c0_g1_i1_orf1;TRINITY_DN14477_c0_g1_i12_orf1;TRINITY_DN19251_c0_g1_i8_orf1;TRINITY_DN36144_c0_g1_i3_orf1;TRINITY_DN15222_c0_g1_i4_orf1;TRINITY_DN117844_c0_g1_i1_orf1;TRINITY_DN12301_c0_g1_i1_orf1;TRINITY_DN3991_c0_g1_i6_orf1;TRINITY_DN51813_c0_g1_i1_orf1;TRINITY_DN38644_c0_g1_i1_orf1;TRINITY_DN3800_c0_g1_i7_orf1;TRINITY_DN44110_c0_g1_i4_orf1;TRINITY_DN8012_c0_g1_i3_orf1;TRINITY_DN83150_c0_g1_i1_orf1;TRINITY_DN6325_c0_g1_i9_orf1;TRINITY_DN1718_c6_g1_i4_orf1;TRINITY_DN49038_c0_g4_i1_orf1;TRINITY_DN28299_c0_g1_i1_orf1;TRINITY_DN5811_c0_g1_i4_orf1;TRINITY_DN8908_c0_g1_i1_orf1;TRINITY_DN9979_c0_g1_i1_orf1;TRINITY_DN141353_c0_g1_i1_orf1;TRINITY_DN15136_c0_g1_i2_orf1;TRINITY_DN18782_c0_g1_i4_orf1;TRINITY_DN20133_c0_g1_i1_orf1;TRINITY_DN7808_c0_g1_i1_orf1;TRINITY_DN5029_c0_g1_i1_orf1;T
biological_process	carbohydrate derivative metabolic process	GO:1901135	53	53/2397	TRINITY_DN62557_c0_g1_i1_orf1;TRINITY_DN47151_c0_g1_i1_orf1;TRINITY_DN827_c1_g1_i1_orf1;TRINITY_DN115210_c0_g4_i1_orf1;TRINITY_DN4360_c0_g1_i4_orf1;TRINITY_DN10824_c0_g1_i3_orf1;TRINITY_DN5697_c0_g1_i1_orf1;TRINITY_DN60787_c0_g1_i5_orf1;TRINITY_DN27035_c0_g1_i1_orf1;TRINITY_DN1575_c0_g1_i7_orf1;TRINITY_DN1196_c0_g1_i5_orf1;TRINITY_DN1201_c0_g1_i4_orf1;TRINITY_DN6325_c0_g1_i8_orf1;TRINITY_DN1034_c0_g1_i4_orf1;TRINITY_DN542_c0_g2_i1_orf1;TRINITY_DN6813_c1_g1_i1_orf1;TRINITY_DN8625_c0_g1_i1_orf1;TRINITY_DN9794_c0_g2_i8_orf1;TRINITY_DN17003_c1_g1_i1_orf1;TRINITY_DN143509_c0_g1_i1_orf1;TRINITY_DN542_c0_g1_i4_orf1;TRINITY_DN11013_c0_g1_i3_orf1;TRINITY_DN26293_c0_g1_i4_orf1;TRINITY_DN18782_c0_g1_i4_orf1;TRINITY_DN15222_c0_g1_i4_orf1;TRINITY_DN49038_c0_g4_i1_orf1;TRINITY_DN117844_c0_g1_i1_orf1;TRINITY_DN12301_c0_g1_i1_orf1;TRINITY_DN3991_c0_g1_i6_orf1;TRINITY_DN19251_c0_g1_i8_orf1;TRINITY_DN3800_c0_g1_i7_orf1;TRINITY_DN98242_c0_g1_i1_orf1;TRINITY_DN2515_c0_g1_i6_orf1;TRINITY_DN83150_c0_g1_i1_orf1;TRINITY_DN6325_c0_g1_i9_orf1;TRINITY_DN11798_c0_g2_i1_orf1;TRINITY_DN1718_c6_g1_i4_orf1;TRINITY_DN5811_c0_g1_i4_orf1;TRINITY_DN21555_c0_g1_i4_orf1;TRINITY_DN28299_c0_g1_i1_orf1;TRINITY_DN5952_c0_g1_i6_orf1;TRINITY_DN1287_c0_g1_i5_orf1;TRINITY_DN8908_c0_g1_i1_orf1;TRINITY_DN141353_c0_g1_i1_orf1;TRINITY_DN15136_c0_g1_i2_orf1;TRINITY_DN116972_c0_g1_i1_orf1;TRINITY_DN20133_c0_g1_i1_orf1;TRINITY_DN5235_c0_g1_i7_orf1;TRINITY_DN7808_c0_g1_i1_orf1;TRINITY_DN5029_c0_g1_i1_orf1;TRINITY_DN9979_c0_g1_i1_orf1;TRINITY_D
biological_process	organic hydroxy compound metabolic process	GO:1901615	10	10/2397	TRINITY_DN230_c2_g1_i5_orf1;TRINITY_DN52244_c1_g1_i1_orf1;TRINITY_DN58125_c0_g1_i1_orf1;TRINITY_DN10722_c0_g3_i1_orf1;TRINITY_DN38644_c0_g1_i1_orf1;TRINITY_DN9286_c0_g1_i2_orf1;TRINITY_DN1034_c0_g1_i4_orf1;TRINITY_DN15706_c0_g2_i5_orf1;TRINITY_DN3010_c0_g1_i4_orf1;TRINITY_DN618_c0_g1_i3_orf1
biological_process	organic cyclic compound metabolic process	GO:1901360	163	163/2397	TRINITY_DN38230_c0_g1_i4_orf1;TRINITY_DN4360_c0_g1_i4_orf1;TRINITY_DN40434_c0_g1_i2_orf1;TRINITY_DN13350_c0_g1_i4_orf1;TRINITY_DN60787_c0_g1_i5_orf1;TRINITY_DN7122_c0_g1_i1_orf1;TRINITY_DN51813_c0_g1_i1_orf1;TRINITY_DN35669_c0_g1_i1_orf1;TRINITY_DN2054_c0_g1_i1_orf1;TRINITY_DN15160_c0_g1_i1_orf1;TRINITY_DN34134_c0_g2_i1_orf1;TRINITY_DN17772_c0_g2_i3_orf1;TRINITY_DN1768_c0_g1_i2_orf1;TRINITY_DN1607_c0_g1_i16_orf1;TRINITY_DN23616_c0_g1_i4_orf1;TRINITY_DN2953_c1_g1_i10_orf1;TRINITY_DN11013_c0_g1_i3_orf1;TRINITY_DN18404_c0_g1_i5_orf1;TRINITY_DN4822_c0_g1_i6_orf1;TRINITY_DN2953_c1_g1_i2_orf1;TRINITY_DN1393_c0_g1_i2_orf1;TRINITY_DN3991_c0_g1_i6_orf1;TRINITY_DN3800_c0_g1_i7_orf1;TRINITY_DN15370_c0_g1_i4_orf1;TRINITY_DN15256_c0_g1_i8_orf1;TRINITY_DN123184_c0_g1_i1_orf1;TRINITY_DN15706_c0_g2_i5_orf1;TRINITY_DN817_c0_g1_i3_orf1;TRINITY_DN2224_c0_g1_i1_orf1;TRINITY_DN5952_c0_g1_i6_orf1;TRINITY_DN46409_c0_g1_i1_orf1;TRINITY_DN8908_c0_g1_i1_orf1;TRINITY_DN15136_c0_g1_i2_orf1;TRINITY_DN107288_c0_g1_i2_orf1;TRINITY_DN7808_c0_g1_i1_orf1;TRINITY_DN89613_c0_g1_i13_orf1;TRINITY_DN14313_c0_g1_i1_orf1;TRINITY_DN2647_c0_g1_i3_orf1;TRINITY_DN230_c2_g1_i5_orf1;TRINITY_DN17271_c0_g1_i1_orf1;TRINITY_DN115210_c0_g4_i1_orf1;TRINITY_DN140212_c0_g1_i1_orf1;TRINITY_DN45271_c0_g1_i1_orf1;TRINITY_DN3082_c1_g1_i7_orf1;TRINITY_DN1005_c0_g1_i5_orf1;TRINITY_DN37532_c0_g1_i1_orf1;TRINITY_DN19687_c0_g1_i1_orf1;TRINITY_DN15900_c0_g1_i6_orf1;TRINITY_DN81258_c0_g1_i2_orf1;TRINITY_DN115658_c0_g1_i1_orf1;TRINITY_DN6813_c1_g1_i1_orf1;TRINITY_DN8625_c0_g1_i1_orf1;TRINITY_DN779_c0_g1_i3_orf1;TRINITY_DN18391_c0_g2_i8_orf1;TRINITY_DN810_c0_g1_i4_orf1;TRINITY_DN16978_c0_g1_i1_orf1;TRINITY_DN4145_c0_g1_i1_orf1;TRINITY_DN36144_c0_g1_i3_orf1;TRINITY_DN12527_c0_g1_i4_orf1;TRINITY_DN1616_c0_g1_i3_orf1;TRINITY_DN41573_c0_g1_i1_orf1;TRINITY_DN12301_c0_g1_i1_orf1;TRINITY_DN9794_c0_g2_i8_orf1;TRINITY_DN6642_c0_g1_i2_orf1;TRINITY_DN13732_c0_g2_i3_orf1;TRINITY_DN4408_c6_g1_i1_orf1;TRINITY_DN6325_c0_g1_i9_orf1;TRINITY_DN1718_c6_g1_i4_orf1;TRINITY_DN779_c0_g1_i12_orf1;TRINITY_DN56993_c0_g1_i4_orf1;TRINITY_DN5811_c0_g1_i4_orf1;TRINITY_DN74037_c0_g5_i1_orf1;TRINITY_DN31663_c0_g1_i2_orf1;TRINITY_DN139537_c0_g1_i1_orf1;TRINITY_DN1344_c0_g1_i1_orf1;TRINITY_DN107035_c0_g1_i1_orf1;TRINITY_DN4908_c1_g1_i5_orf1;TRINITY_DN2299_c0_g1_i3_orf1;TRINITY_DN5029_c0_g1_i1_orf1;TRINITY_DN291_c0_g1_i2_orf1;TRINITY_DN6325_c0_g1_i8_orf1;TRINITY_DN1750_c1_g1_i5_orf1;TRINITY_DN27852_c0_g1_i1_orf1;TRINITY_DN77318_c0_g2_i1_orf1;TRINITY_DN27035_c0_g1_i1_orf1;TRINITY_DN1575_c0_g1_i7_orf1;TRINITY_DN53233_c0_g1_i1_orf1;TRINITY_DN51934_c0_g2_i1_orf1;TRINITY_DN2718_c0_g1_i6_orf1;TRINITY_DN57918_c0_g1_i1_orf1;TRINITY_DN6563_c0_g1_i1_orf1;TRINITY_DN56270_c0_g1_i1_orf1;TRINITY_DN1554_c0_g1_i9_orf1;TRINITY_DN49038_c0_g4_i1_orf1;TRINITY_DN38506_c0_g1_i4_orf1;TRINITY_DN24_c0_g1_i1_orf1;TRINITY_DN2559_c0_g1_i4_orf1;TRINITY_DN2803_c4_g1_i1_orf1;TRINITY_DN98242_c0_g1_i1_orf1;TRINITY_DN14477_c0_g1_i12_orf1;TRINITY_DN34689_c0_g1_i4_orf1;TRINITY_DN26293_c0_g1_i4_orf1;TRINITY_DN41179_c0_g1_i1_orf1;TRINITY_DN2749_c0_g2_i3_orf1;TRINITY_DN4835_c0_g1_i2_orf1;TRINITY_DN7341_c0_g1_i8_orf1;TRINITY_DN24322_c0_g1_i4_orf1;TRINITY_DN825_c23_g1_i5_orf1;TRINITY_DN11986_c0_g1_i1_orf1;TRINITY_DN64_c0_g1_i4_orf1;TRINITY_DN8012_c0_g1_i3_orf1;TRINITY_DN83150_c0_g1_i1_orf1;TRINITY_DN38274_c0_g1_i1_orf1;TRINITY_DN14487_c0_g1_i4_orf1;TRINITY_DN28299_c0_g1_i1_orf1;TRINITY_DN4820_c0_g2_i2_orf1;TRINITY_DN1957_c0_g1_i4_orf1;TRINITY_DN3263_c0_g1_i2_orf1;TRINITY_DN120144_c0_g1_i1_orf1;TRINITY_DN6248_c0_g1_i1_orf1;TRINITY_DN141353_c0_g1_i1_orf1;TRINITY_DN4429_c0_g1_i5_orf1;TRINITY_DN879_c0_g1_i2_orf1;TRINITY_DN46022_c0_g1_i1_orf1;TRINITY_DN9979_c0_g1_i1_orf1;TRINITY_DN1084_c0_g2_i2_orf1;TRINITY_DN59804_c0_g1_i1_orf1;TRINITY_DN2971_c0_g1_i1_orf1;TRINITY_DN2719_c1_g1_i6_orf1;TRINITY_DN67649_c0_g1_i1_orf1;TRINITY_DN44792_c0_g1_i1_orf1;TRINITY_DN24970_c0_g1_i4_orf1;TRINITY_DN38562_c0_g1_i3_orf1;TRINITY_DN13760_c1_g1_i1_orf1;TRINITY_DN26879_c0_g1_i1_orf1;TRINITY_DN25423_c0_g1_i1_orf1;TRINITY_DN1741_c0_g1_i5_orf1;TRINITY_DN4822_c0_g1_i9_orf1;TRINITY_DN104507_c0_g1_i2_orf1;TRINITY_DN87170_c0_g1_i3_orf1;TRINITY_DN5200_c0_g1_i2_orf1;TRINITY_DN117844_c0_g1_i1_orf1;TRINITY_DN34676_c1_g1_i3_orf1;TRINITY_DN2201_c0_g1_i1_orf1;TRINITY_DN19251_c0_g1_i8_orf1;TRINITY_DN1091_c0_g1_i1_orf1;TRINITY_DN1201_c0_g1_i4_orf1;TRINITY_DN15222_c0_g1_i4_orf1;TRINITY_DN323_c0_g2_i5_orf1;TRINITY_DN53311_c0_g2_i1_orf1;TRINITY_DN5238_c0_g1_i2_orf1;TRINITY_DN21596_c0_g1_i1_orf1;TRINITY_DN133760_c0_g1_i1_orf1;TRINITY_DN34432_c0_g1_i1_or
biological_process	thioester metabolic process	GO:0035383	7	7/2397	TRINITY_DN117844_c0_g1_i1_orf1;TRINITY_DN9794_c0_g2_i8_orf1;TRINITY_DN3991_c0_g1_i6_orf1;TRINITY_DN15136_c0_g1_i2_orf1;TRINITY_DN19251_c0_g1_i8_orf1;TRINITY_DN7808_c0_g1_i1_orf1;TRINITY_DN1084_c0_g2_i2_orf1
biological_process	macromolecule metabolic process	GO:0043170	321	321/2397	TRINITY_DN24469_c0_g2_i2_orf1;TRINITY_DN7341_c0_g1_i8_orf1;TRINITY_DN10364_c0_g1_i5_orf1;TRINITY_DN827_c1_g1_i1_orf1;TRINITY_DN8824_c0_g2_i1_orf1;TRINITY_DN40434_c0_g1_i2_orf1;TRINITY_DN3194_c0_g1_i6_orf1;TRINITY_DN5757_c0_g1_i1_orf1;TRINITY_DN137_c0_g1_i1_orf1;TRINITY_DN107288_c0_g1_i2_orf1;TRINITY_DN1533_c0_g2_i1_orf1;TRINITY_DN140_c1_g1_i2_orf1;TRINITY_DN3978_c0_g2_i1_orf1;TRINITY_DN11928_c0_g1_i3_orf1;TRINITY_DN56164_c0_g1_i1_orf1;TRINITY_DN35669_c0_g1_i1_orf1;TRINITY_DN1274_c0_g1_i4_orf1;TRINITY_DN6436_c0_g1_i1_orf1;TRINITY_DN2054_c0_g1_i1_orf1;TRINITY_DN51934_c0_g2_i1_orf1;TRINITY_DN15160_c0_g1_i1_orf1;TRINITY_DN56993_c0_g1_i4_orf1;TRINITY_DN4217_c0_g1_i2_orf1;TRINITY_DN38431_c0_g1_i1_orf1;TRINITY_DN58207_c0_g1_i1_orf1;TRINITY_DN3499_c0_g1_i8_orf1;TRINITY_DN8692_c0_g1_i2_orf1;TRINITY_DN2442_c0_g1_i2_orf1;TRINITY_DN8659_c0_g1_i1_orf1;TRINITY_DN1768_c0_g1_i2_orf1;TRINITY_DN41952_c0_g1_i4_orf1;TRINITY_DN2983_c0_g1_i6_orf1;TRINITY_DN11065_c0_g2_i1_orf1;TRINITY_DN16258_c0_g1_i2_orf1;TRINITY_DN2953_c1_g1_i10_orf1;TRINITY_DN277_c1_g1_i1_orf1;TRINITY_DN14754_c0_g1_i6_orf1;TRINITY_DN29448_c0_g1_i1_orf1;TRINITY_DN11817_c0_g1_i4_orf1;TRINITY_DN41761_c0_g1_i4_orf1;TRINITY_DN18404_c0_g1_i5_orf1;TRINITY_DN14217_c0_g1_i1_orf1;TRINITY_DN14774_c0_g1_i4_orf1;TRINITY_DN50787_c0_g2_i2_orf1;TRINITY_DN33883_c0_g1_i1_orf1;TRINITY_DN2201_c0_g1_i1_orf1;TRINITY_DN875_c0_g1_i3_orf1;TRINITY_DN10070_c0_g1_i1_orf1;TRINITY_DN2794_c1_g1_i8_orf1;TRINITY_DN45948_c1_g1_i1_orf1;TRINITY_DN4125_c0_g1_i14_orf1;TRINITY_DN15370_c0_g1_i4_orf1;TRINITY_DN13350_c0_g1_i4_orf1;TRINITY_DN15256_c0_g1_i8_orf1;TRINITY_DN4886_c0_g1_i6_orf1;TRINITY_DN123184_c0_g1_i1_orf1;TRINITY_DN6185_c0_g1_i12_orf1;TRINITY_DN135_c0_g1_i1_orf1;TRINITY_DN817_c0_g1_i3_orf1;TRINITY_DN77318_c0_g2_i1_orf1;TRINITY_DN3985_c0_g2_i1_orf1;TRINITY_DN391_c0_g1_i4_orf1;TRINITY_DN2224_c0_g1_i1_orf1;TRINITY_DN1393_c0_g1_i2_orf1;TRINITY_DN2682_c0_g1_i4_orf1;TRINITY_DN3483_c0_g1_i5_orf1;TRINITY_DN22674_c0_g1_i2_orf1;TRINITY_DN18593_c0_g1_i1_orf1;TRINITY_DN4820_c0_g2_i2_orf1;TRINITY_DN3826_c0_g1_i1_orf1;TRINITY_DN13732_c0_g2_i3_orf1;TRINITY_DN1380_c0_g1_i5_orf1;TRINITY_DN52553_c0_g1_i1_orf1;TRINITY_DN10403_c0_g1_i3_orf1;TRINITY_DN18391_c0_g2_i8_orf1;TRINITY_DN11620_c0_g1_i2_orf1;TRINITY_DN6967_c0_g1_i3_orf1;TRINITY_DN14313_c0_g1_i1_orf1;TRINITY_DN4767_c0_g1_i6_orf1;TRINITY_DN17271_c0_g1_i1_orf1;TRINITY_DN143895_c0_g1_i1_orf1;TRINITY_DN13856_c0_g1_i1_orf1;TRINITY_DN24121_c1_g1_i6_orf1;TRINITY_DN140212_c0_g1_i1_orf1;TRINITY_DN23360_c0_g1_i3_orf1;TRINITY_DN10824_c0_g1_i3_orf1;TRINITY_DN45633_c0_g1_i1_orf1;TRINITY_DN10090_c0_g1_i1_orf1;TRINITY_DN1005_c0_g1_i5_orf1;TRINITY_DN18869_c0_g1_i1_orf1;TRINITY_DN37532_c0_g1_i1_orf1;TRINITY_DN19537_c0_g1_i1_orf1;TRINITY_DN4189_c0_g2_i1_orf1;TRINITY_DN19687_c0_g1_i1_orf1;TRINITY_DN1791_c0_g1_i3_orf1;TRINITY_DN37923_c0_g1_i1_orf1;TRINITY_DN32700_c0_g1_i2_orf1;TRINITY_DN97589_c0_g1_i3_orf1;TRINITY_DN81258_c0_g1_i2_orf1;TRINITY_DN18249_c0_g1_i1_orf1;TRINITY_DN5873_c0_g4_i1_orf1;TRINITY_DN5012_c0_g1_i6_orf1;TRINITY_DN115658_c0_g1_i1_orf1;TRINITY_DN57918_c0_g1_i1_orf1;TRINITY_DN57798_c0_g1_i1_orf1;TRINITY_DN42461_c0_g1_i4_orf1;TRINITY_DN30154_c0_g1_i1_orf1;TRINITY_DN13233_c0_g1_i3_orf1;TRINITY_DN21719_c0_g1_i2_orf1;TRINITY_DN7583_c0_g1_i1_orf1;TRINITY_DN1607_c0_g1_i16_orf1;TRINITY_DN7464_c0_g1_i14_orf1;TRINITY_DN810_c0_g1_i4_orf1;TRINITY_DN2069_c1_g1_i8_orf1;TRINITY_DN59885_c0_g1_i3_orf1;TRINITY_DN36434_c0_g2_i3_orf1;TRINITY_DN16978_c0_g1_i1_orf1;TRINITY_DN5696_c0_g1_i4_orf1;TRINITY_DN6813_c1_g1_i1_orf1;TRINITY_DN23167_c0_g2_i1_orf1;TRINITY_DN6423_c0_g1_i5_orf1;TRINITY_DN12527_c0_g1_i4_orf1;TRINITY_DN29034_c0_g1_i2_orf1;TRINITY_DN1616_c0_g1_i3_orf1;TRINITY_DN41573_c0_g1_i1_orf1;TRINITY_DN4571_c0_g1_i4_orf1;TRINITY_DN6642_c0_g1_i2_orf1;TRINITY_DN9591_c0_g1_i1_orf1;TRINITY_DN49936_c0_g2_i1_orf1;TRINITY_DN17759_c0_g1_i5_orf1;TRINITY_DN5444_c0_g2_i1_orf1;TRINITY_DN4408_c6_g1_i1_orf1;TRINITY_DN95414_c0_g1_i1_orf1;TRINITY_DN6423_c0_g1_i6_orf1;TRINITY_DN5281_c0_g2_i3_orf1;TRINITY_DN376_c1_g1_i1_orf1;TRINITY_DN21357_c0_g1_i5_orf1;TRINITY_DN1954_c0_g1_i4_orf1;TRINITY_DN83295_c0_g1_i3_orf1;TRINITY_DN9062_c0_g2_i3_orf1;TRINITY_DN89613_c0_g1_i13_orf1;TRINITY_DN34134_c0_g2_i1_orf1;TRINITY_DN4767_c0_g1_i4_orf1;TRINITY_DN74037_c0_g5_i1_orf1;TRINITY_DN1528_c0_g1_i4_orf1;TRINITY_DN139537_c0_g1_i1_orf1;TRINITY_DN1344_c0_g1_i1_orf1;TRINITY_DN10831_c1_g1_i1_orf1;TRINITY_DN81803_c0_g2_i1_orf1;TRINITY_DN107035_c0_g1_i1_orf1;TRINITY_DN147458_c0_g1_i1_orf1;TRINITY_DN4908_c1_g1_i5_orf1;TRINITY_DN2299_c0_g1_i3_orf1;TRINITY_DN71863_c0_g1_i2
biological_process	melanin metabolic process	GO:0006582	2	2/2397	TRINITY_DN58125_c0_g1_i1_orf1;TRINITY_DN15706_c0_g2_i5_orf1

biological_process	pigment biosynthetic process	GO:0046148	2	2/2397	TRINITY_DN58125_c0_g1_i1_orf1;TRINITY_DN6563_c0_g1_i1_orf1
biological_process	heme metabolic process	GO:0042168	2	2/2397	TRINITY_DN6563_c0_g1_i1_orf1;TRINITY_DN17772_c0_g2_i3_orf1
biological_process	mating behavior	GO:0007617	1	1/2397	TRINITY_DN58125_c0_g1_i1_orf1
biological_process	gamete generation	GO:0007276	2	2/2397	TRINITY_DN2652_c0_g2_i1_orf1;TRINITY_DN4820_c0_g2_i2_orf1
biological_process	germ cell development	GO:0007281	1	1/2397	TRINITY_DN2652_c0_g2_i1_orf1
biological_process	ovarian follicle cell development	GO:0030707	1	1/2397	TRINITY_DN15706_c0_g2_i5_orf1
biological_process	spermatogenesis	GO:0007283	1	1/2397	TRINITY_DN4820_c0_g2_i2_orf1
biological_process	binding of sperm to zona pellucida	GO:0007339	1	1/2397	TRINITY_DN20133_c0_g1_i1_orf1
biological_process	killing of cells of another organism	GO:0031640	1	1/2397	TRINITY_DN6098_c1_g1_i5_orf1
biological_process	leukocyte activation	GO:0045321	1	1/2397	TRINITY_DN46409_c0_g1_i1_orf1
biological_process	programmed cell death	GO:0012501	4	4/2397	TRINITY_DN50074_c0_g1_i1_orf1;TRINITY_DN18912_c1_g1_i1_orf1;TRINITY_DN87603_c0_g2_i1_orf1;TRINITY_DN41573_c0_g1_i1_orf1
biological_process	autophagy	GO:0006914	5	5/2397	TRINITY_DN15930_c0_g1_i5_orf1;TRINITY_DN11375_c0_g1_i4_orf1;TRINITY_DN113353_c0_g1_i1_orf1;TRINITY_DN5531_c0_g3_i3_orf1;TRINITY_DN19821_c0_g2_i4_orf1
biological_process	secretion by cell	GO:0032940	4	4/2397	TRINITY_DN25686_c0_g1_i4_orf1;TRINITY_DN47219_c0_g1_i3_orf1;TRINITY_DN1895_c0_g1_i2_orf1;TRINITY_DN33452_c0_g1_i3_orf1
biological_process	cell-substrate adhesion	GO:0031589	3	3/2397	TRINITY_DN2186_c0_g1_i17_orf1;TRINITY_DN6698_c0_g2_i1_orf1;TRINITY_DN2186_c0_g1_i13_orf1
biological_process	cell-cell adhesion	GO:0098609	2	2/2397	TRINITY_DN1008_c0_g1_i2_orf1;TRINITY_DN10070_c0_g1_i1_orf1
biological_process	cellular response to extracellular stimulus	GO:0031668	4	4/2397	TRINITY_DN140212_c0_g1_i1_orf1;TRINITY_DN1091_c0_g1_i1_orf1;TRINITY_DN51938_c0_g3_i1_orf1;TRINITY_DN2054_c0_g1_i1_orf1
biological_process	intermediate filament cytoskeleton organization	GO:0045104	8	8/2397	TRINITY_DN107962_c0_g1_i1_orf1;TRINITY_DN59852_c0_g1_i1_orf1;TRINITY_DN69557_c0_g1_i1_orf1;TRINITY_DN77480_c0_g1_i2_orf1;TRINITY_DN97097_c0_g1_i4_orf1;TRINITY_DN17137_c0_g1_i2_orf1;TRINITY_DN20009_c0_g1_i1_orf1;TRINITY_DN101991_c0_g1_i5_orf1
biological_process	maintenance of protein location in cell	GO:0032507	1	1/2397	TRINITY_DN245_c0_g1_i4_orf1
biological_process	muscle cell cellular homeostasis	GO:0046716	1	1/2397	TRINITY_DN20133_c0_g1_i1_orf1
biological_process	cell redox homeostasis	GO:0045454	2	2/2397	TRINITY_DN376_c0_g1_i1_orf1;TRINITY_DN9965_c0_g1_i1_orf1
biological_process	cellular chemical homeostasis	GO:0055082	9	9/2397	TRINITY_DN46625_c0_g1_i1_orf1;TRINITY_DN65681_c0_g1_i1_orf1;TRINITY_DN1423_c0_g1_i4_orf1;TRINITY_DN1423_c0_g1_i8_orf1;TRINITY_DN136031_c0_g1_i7_orf1;TRINITY_DN3461_c0_g1_i1_orf1;TRINITY_DN31584_c0_g2_i2_orf1;TRINITY_DN44256_c0_g1_i1_orf1;TRINITY_DN5753_c0_g1_i10_orf1
biological_process	transposition, DNA-mediated	GO:0006313	1	1/2397	TRINITY_DN139537_c0_g1_i1_orf1
biological_process	leukocyte proliferation	GO:0070661	1	1/2397	TRINITY_DN46409_c0_g1_i1_orf1
biological_process	mitotic cell cycle process	GO:1903047	3	3/2397	TRINITY_DN96557_c0_g1_i1_orf1;TRINITY_DN235_c0_g3_i1_orf1;TRINITY_DN41573_c0_g1_i1_orf1
biological_process	cell cycle phase transition	GO:0044770	1	1/2397	TRINITY_DN96557_c0_g1_i1_orf1
biological_process	spindle organization	GO:0007051	1	1/2397	TRINITY_DN25960_c0_g1_i1_orf1
biological_process	DNA replication preinitiation complex assembly	GO:0071163	1	1/2397	TRINITY_DN110400_c0_g1_i1_orf1
biological_process	cytokinesis	GO:0000910	1	1/2397	TRINITY_DN235_c0_g3_i1_orf1
biological_process	sister chromatid cohesion	GO:0007062	1	1/2397	TRINITY_DN2638_c0_g1_i7_orf1
biological_process	cytokinetic process	GO:0032506	1	1/2397	TRINITY_DN96557_c0_g1_i1_orf1
biological_process	'de novo' protein folding	GO:0006458	1	1/2397	TRINITY_DN46409_c0_g1_i1_orf1
biological_process	protein refolding	GO:0042026	1	1/2397	TRINITY_DN46409_c0_g1_i1_orf1
biological_process	cellular macromolecule localization	GO:0070727	36	36/2397	TRINITY_DN245_c0_g1_i4_orf1;TRINITY_DN5910_c1_g1_i6_orf1;TRINITY_DN5182_c0_g1_i5_orf1;TRINITY_DN15811_c0_g1_i7_orf1;TRINITY_DN46409_c0_g1_i1_orf1;TRINITY_DN3450_c0_g1_i3_orf1;TRINITY_DN31584_c0_g2_i2_orf1;TRINITY_DN146119_c0_g1_i1_orf1;TRINITY_DN15930_c0_g1_i5_orf1;TRINITY_DN95971_c0_g5_i1_orf1;TRINITY_DN34159_c0_g2_i1_orf1;TRINITY_DN13139_c0_g1_i1_orf1;TRINITY_DN3299_c0_g1_i2_orf1;TRINITY_DN11375_c0_g1_i4_orf1;TRINITY_DN41108_c0_g1_i1_orf1;TRINITY_DN959_c0_g1_i7_orf1;TRINITY_DN445_c0_g1_i2_orf1;TRINITY_DN9741_c0_g1_i3_orf1;TRINITY_DN1437_c0_g1_i6_orf1;TRINITY_DN3698_c0_g1_i4_orf1;TRINITY_DN3747_c1_g2_i1_orf1;TRINITY_DN146236_c0_g1_i1_orf1;TRINITY_DN42120_c0_g1_i2_orf1;TRINITY_DN11772_c0_g1_i1_orf1;TRINITY_DN7233_c0_g2_i1_orf1;TRINITY_DN44219_c0_g1_i1_orf1;TRINITY_DN4689_c0_g1_i5_orf1;TRINITY_DN486_c0_g1_i5_orf1;TRINITY_DN2879_c0_g1_i4_orf1;TRINITY_DN96557_c0_g1_i1_orf1;TRINITY_DN4410_c0_g1_i1_orf1;TRINITY_DN47219_c0_g1_i3_orf1;TRINITY_DN69871_c0_g1_i1_orf1;TRINITY_DN50875_c0_g1_i3_orf1;TRINITY_DN4394_c0_g1_i4_orf1;TRINITY_DN4207_c0_g1_i1_orf1
biological_process	intracellular transport	GO:0046907	28	28/2397	TRINITY_DN5910_c1_g1_i6_orf1;TRINITY_DN3821_c1_g1_i7_orf1;TRINITY_DN46409_c0_g1_i1_orf1;TRINITY_DN3450_c0_g1_i3_orf1;TRINITY_DN31584_c0_g2_i2_orf1;TRINITY_DN5182_c0_g1_i5_orf1;TRINITY_DN95971_c0_g5_i1_orf1;TRINITY_DN34159_c0_g2_i1_orf1;TRINITY_DN13139_c0_g1_i1_orf1;TRINITY_DN45037_c0_g1_i1_orf1;TRINITY_DN445_c0_g1_i2_orf1;TRINITY_DN96557_c0_g1_i1_orf1;TRINITY_DN1437_c0_g1_i6_orf1;TRINITY_DN15811_c0_g1_i7_orf1;TRINITY_DN64_c0_g1_i4_orf1;TRINITY_DN3747_c1_g2_i1_orf1;TRINITY_DN4394_c0_g1_i4_orf1;TRINITY_DN7233_c0_g2_i1_orf1;TRINITY_DN35377_c0_g1_i3_orf1;TRINITY_DN486_c0_g1_i5_orf1;TRINITY_DN578_c0_g1_i5_orf1;TRINITY_DN69871_c0_g1_i1_orf1;TRINITY_DN4814_c0_g1_i6_orf1;TRINITY_DN5558_c0_g1_i4_orf1;TRINITY_DN1245_c0_g1_i4_orf1;TRINITY_DN4207_c0_g1_i1_orf1;TRINITY_DN942_c0_g1_i1_orf1;TRINITY_DN5028_c0_g1_i11_orf1
biological_process	localization within membrane	GO:0051668	4	4/2397	TRINITY_DN96557_c0_g1_i1_orf1;TRINITY_DN959_c0_g1_i7_orf1;TRINITY_DN69871_c0_g1_i1_orf1;TRINITY_DN15811_c0_g1_i7_orf1
biological_process	cellular component biogenesis	GO:0044085	10	10/2397	TRINITY_DN3618_c0_g1_i4_orf1;TRINITY_DN4016_c0_g1_i1_orf1;TRINITY_DN14391_c1_g1_i2_orf1;TRINITY_DN14313_c0_g1_i1_orf1;TRINITY_DN13496_c0_g1_i7_orf1;TRINITY_DN146217_c0_g1_i1_orf1;TRINITY_DN31225_c0_g1_i1_orf1;TRINITY_DN102260_c0_g1_i1_orf1;TRINITY_DN41179_c0_g1_i1_orf1;TRINITY_DN6239_c0_g1_i1_orf1
biological_process	cellular component organization	GO:0016043	96	96/2397	TRINITY_DN14920_c0_g1_i1_orf1;TRINITY_DN25960_c0_g1_i1_orf1;TRINITY_DN44261_c0_g1_i1_orf1;TRINITY_DN15811_c0_g1_i7_orf1;TRINITY_DN11194_c0_g1_i4_orf1;TRINITY_DN3450_c0_g1_i3_orf1;TRINITY_DN34426_c0_g1_i1_orf1;TRINITY_DN3126_c0_g1_i4_orf1;TRINITY_DN35669_c0_g1_i1_orf1;TRINITY_DN4010_c0_g2_i1_orf1;TRINITY_DN4217_c0_g1_i2_orf1;TRINITY_DN91877_c0_g1_i1_orf1;TRINITY_DN110400_c0_g1_i1_orf1;TRINITY_DN70485_c0_g1_i2_orf1;TRINITY_DN59852_c0_g1_i1_orf1;TRINITY_DN2638_c0_g1_i7_orf1;TRINITY_DN69557_c0_g1_i1_orf1;TRINITY_DN235_c0_g3_i1_orf1;TRINITY_DN77480_c0_g1_i2_orf1;TRINITY_DN36987_c0_g1_i1_orf1;TRINITY_DN2345_c0_g1_i4_orf1;TRINITY_DN110231_c0_g1_i1_orf1;TRINITY_DN27276_c0_g1_i5_orf1;TRINITY_DN3461_c0_g1_i1_orf1;TRINITY_DN28660_c0_g1_i4_orf1;TRINITY_DN4950_c0_g1_i2_orf1;TRINITY_DN1749_c0_g2_i2_orf1;TRINITY_DN51938_c0_g3_i1_orf1;TRINITY_DN11464_c0_g1_i3_orf1;TRINITY_DN20442_c0_g2_i1_orf1;TRINITY_DN34703_c0_g1_i4_orf1;TRINITY_DN146119_c0_g1_i1_orf1;TRINITY_DN23790_c0_g1_i1_orf1;TRINITY_DN6248_c0_g1_i1_orf1;TRINITY_DN71832_c0_g1_i1_orf1;TRINITY_DN298_c0_g1_i4_orf1;TRINITY_DN90497_c0_g1_i1_orf1;TRINITY_DN28018_c0_g6_i1_orf1;TRINITY_DN11375_c0_g1_i4_orf1;TRINITY_DN28622_c0_g1_i1_orf1;TRINITY_DN14209_c0_g1_i1_orf1;TRINITY_DN41573_c0_g1_i1_orf1;TRINITY_DN6642_c0_g1_i2_orf1;TRINITY_DN37986_c0_g1_i2_orf1;TRINITY_DN11772_c0_g1_i1_orf1;TRINITY_DN40911_c0_g1_i1_orf1;TRINITY_DN4689_c0_g1_i5_orf1;TRINITY_DN4439_c0_g1_i2_orf1;TRINITY_DN6358_c0_g1_i5_orf1;TRINITY_DN3459_c0_g1_i4_orf1;TRINITY_DN16145_c0_g1_i12_orf1;TRINITY_DN4908_c1_g1_i5_orf1;TRINITY_DN101991_c0_g1_i5_orf1;TRINITY_DN12771_c0_g1_i1_orf1;TRINITY_DN13371_c0_g1_i4_orf1;TRINITY_DN6239_c0_g1_i1_orf1;TRINITY_DN2904_c0_g1_i4_orf1;TRINITY_DN714_c0_g1_i3_orf1;TRINITY_DN8915_c0_g1_i3_orf1;TRINITY_DN46409_c0_g1_i1_orf1;TRINITY_DN89083_c0_g1_i1_orf1;TRINITY_DN4016_c0_g1_i1_orf1;TRINITY_DN8390_c0_g1_i2_orf1;TRINITY_DN37418_c0_g1_i4_orf1;TRINITY_DN86309_c0_g1_i4_orf1;TRINITY_DN11986_c0_g1_i1_orf1;TRINITY_DN146236_c0_g1_i1_orf1;TRINITY_DN27960_c0_g1_i1_orf1;TRINITY_DN18009_c0_g1_i1_orf1;TRINITY_DN25976_c0_g1_i4_orf1;TRINITY_DN20009_c0_g1_i1_orf1;TRINITY_DN85476_c0_g1_i1_orf1;TRINITY_DN96557_c0_g1_i1_orf1;TRINITY_DN18869_c0_g1_i1_orf1;TRINITY_DN14904_c1_g2_i2_orf1;TRINITY_DN42854_c0_g3_i2_orf1;TRINITY_DN116467_c0_g1_i1_orf1;TRINITY_DN10385_c0_g1_i5_orf1;TRINITY_DN59804_c0_g1_i1_orf1;TRINITY_DN8087_c0_g1_i9_orf1;TRINITY_DN48097_c0_g1_i1_orf1;TRINITY_DN38540_c0_g1_i1_orf1;TRINITY_DN107962_c0_g1_i1_orf1;TRINITY_DN99900_c0_g1_i3_orf1;TRINITY_DN3887_c0_g1_i1_orf1;TRINITY_DN49872_c0_g1_i2_orf1;TRINITY_DN24266_c0_g2_i2_orf1;TRINITY_DN30273_c1_g1_i1_orf1;TRINITY_DN5757_c0_g1_i1_orf1;TRINITY_DN10070_c0_g1_i1_orf1;TRINITY_DN19584_c0_g1_i2_orf1;TRINITY_DN147475_c0_g1_i1_orf1;TRINITY_DN80424_c0_g1_i1_orf1;TRINITY_DN20133_c0_g1_i1_orf1;TRINITY_DN17137_c0_g1_i2_orf1;TRINITY_DN97097_c0_g1_i4_orf1
biological_process	cell migration	GO:0016477	3	3/2397	TRINITY_DN110231_c0_g1_i1_orf1;TRINITY_DN31584_c0_g2_i2_orf1;TRINITY_DN15706_c0_g2_i5_orf1
biological_process	microtubule-based movement	GO:0007018	4	4/2397	TRINITY_DN17995_c0_g4_i1_orf1;TRINITY_DN14298_c0_g3_i1_orf1;TRINITY_DN14298_c0_g1_i3_orf1;TRINITY_DN14298_c0_g1_i1_orf1
biological_process	microtubule cytoskeleton organization	GO:0000226	5	5/2397	TRINITY_DN4689_c0_g1_i5_orf1;TRINITY_DN28018_c0_g6_i1_orf1;TRINITY_DN8390_c0_g1_i2_orf1;TRINITY_DN25960_c0_g1_i1_orf1;TRINITY_DN34703_c0_g1_i4_orf1
biological_process	cellular response to chemical stimulus	GO:0070887	5	5/2397	TRINITY_DN46409_c0_g1_i1_orf1;TRINITY_DN51938_c0_g3_i1_orf1;TRINITY_DN87603_c0_g2_i1_orf1;TRINITY_DN4016_c0_g1_i1_orf1;TRINITY_DN20009_c0_g1_i1_orf1
biological_process	cellular response to stress	GO:0033554	26	26/2397	TRINITY_DN59804_c0_g1_i1_orf1;TRINITY_DN51938_c0_g3_i1_orf1;TRINITY_DN2971_c0_g1_i1_orf1;TRINITY_DN140212_c0_g1_i1_orf1;TRINITY_DN40434_c0_g1_i2_orf1;TRINITY_DN7341_c0_g1_i8_orf1;TRINITY_DN77318_c0_g2_i1_orf1;TRINITY_DN46409_c0_g1_i1_orf1;TRINITY_DN31584_c0_g2_i2_orf1;TRINITY_DN17271_c0_g1_i1_orf1;TRINITY_DN104507_c0_g1_i2_orf1;TRINITY_DN2054_c0_g1_i1_orf1;TRINITY_DN1091_c0_g1_i1_orf1;TRINITY_DN45271_c0_g1_i1_orf1;TRINITY_DN41573_c0_g1_i1_orf1;TRINITY_DN5238_c0_g1_i2_orf1;TRINITY_DN6642_c0_g1_i2_orf1;TRINITY_DN123184_c0_g1_i1_orf1;TRINITY_DN6185_c0_g1_i12_orf1;TRINITY_DN38274_c0_g1_i1_orf1;TRINITY_DN5757_c0_g1_i1_orf1;TRINITY_DN14487_c0_g1_i4_orf1;TRINITY_DN9062_c0_g2_i3_orf1;TRINITY_DN87603_c0_g2_i1_orf1;TRINITY_DN2647_c0_g1_i3_orf1;TRINITY_DN44
biological_process	cellular response to biotic stimulus	GO:0071216	1	1/2397	TRINITY_DN20009_c0_g1_i1_orf1
biological_process	establishment or maintenance of cytoskeleton polarity	GO:0030952	1	1/2397	TRINITY_DN25960_c0_g1_i1_orf1
biological_process	cell surface receptor signaling pathway	GO:0007166	7	7/2397	TRINITY_DN51938_c0_g3_i1_orf1;TRINITY_DN147475_c0_g1_i1_orf1;TRINITY_DN1008_c0_g1_i2_orf1;TRINITY_DN2170_c1_g1_i3_orf1;TRINITY_DN13216_c0_g1_i5_orf1;TRINITY_DN25360_c0_g1_i2_orf1;TRINITY_DN15247_c0_g1_i2_orf1

biological_process	hormone-mediated signaling pathway	GO:0009755	1	1/2397	TRINITY_DN147475_c0_g1_i1_orf1
biological_process	immune response-regulating signaling pathway	GO:0002764	2	2/2397	TRINITY_DN46409_c0_g1_i1_orf1;TRINITY_DN2170_c1_g1_i3_orf1
biological_process	SMAD protein signal transduction	GO:0060395	1	1/2397	TRINITY_DN20009_c0_g1_i1_orf1
biological_process	G protein-coupled receptor signaling pathway	GO:0007186	2	2/2397	TRINITY_DN42854_c0_g3_i2_orf1;TRINITY_DN11245_c0_g1_i2_orf1
biological_process	intracellular signal transduction	GO:0035556	14	14/2397	TRINITY_DN32700_c0_g1_i2_orf1;TRINITY_DN41573_c0_g1_i1_orf1;TRINITY_DN4410_c0_g1_i1_orf1;TRINITY_DN7391_c0_g1_i2_orf1;TRINITY_DN25360_c0_g1_i2_orf1;TRINITY_DN2793_c0_g2_i1_orf1;TRINITY_DN2983_c0_g1_i6_orf1;TRINITY_DN802_c0_g1_i2_orf1;TRINITY_DN2623_c0_g1_i3_orf1;TRINITY_DN31584_c0_g2_i2_orf1;TRINITY_DN15706_c0_g2_i5_orf1;TRINITY_DN5182_c0_g1_i5_orf1;TRINITY_DN804_c0_g1_i7_orf1;TRINITY_DN2770_c0_g2_i4_orf1
biological_process	apoptotic signaling pathway	GO:0097190	1	1/2397	TRINITY_DN51938_c0_g3_i1_orf1
biological_process	meiotic cell cycle	GO:0051321	2	2/2397	TRINITY_DN45271_c0_g1_i1_orf1;TRINITY_DN123184_c0_g1_i1_orf1
biological_process	mitotic cell cycle	GO:0000278	1	1/2397	TRINITY_DN31314_c0_g1_i4_orf1
biological_process	cell differentiation	GO:0030154	20	20/2397	TRINITY_DN1173_c0_g1_i12_orf1;TRINITY_DN928_c0_g1_i3_orf1;TRINITY_DN741_c0_g1_i10_orf1;TRINITY_DN4820_c0_g2_i2_orf1;TRINITY_DN52395_c0_g2_i2_orf1;TRINITY_DN50725_c0_g1_i6_orf1;TRINITY_DN5954_c0_g1_i2_orf1;TRINITY_DN42461_c0_g1_i4_orf1;TRINITY_DN1173_c0_g1_i11_orf1;TRINITY_DN4550_c1_g1_i19_orf1;TRINITY_DN1173_c1_g1_i10_orf1;TRINITY_DN549_c0_g1_i14_orf1;TRINITY_DN11388_c0_g1_i4_orf1;TRINITY_DN2468_c0_g1_i7_orf1;TRINITY_DN549_c0_g1_i7_orf1;TRINITY_DN20009_c0_g1_i1_orf1;TRINITY_DN248_c0_g1_i1_orf1;TRINITY_DN26790_c0_g1_i3_orf1;TRINITY_DN23746_c0_g1_i2_orf1;TRINITY_DN2012_c0_g1_i3_orf1
biological_process	cellular component morphogenesis	GO:0032989	1	1/2397	TRINITY_DN42854_c0_g3_i2_orf1
biological_process	cell development	GO:0048468	17	17/2397	TRINITY_DN1749_c0_g2_i2_orf1;TRINITY_DN4571_c0_g1_i4_orf1;TRINITY_DN1710_c0_g2_i2_orf1;TRINITY_DN4217_c0_g1_i2_orf1;TRINITY_DN25976_c0_g1_i4_orf1;TRINITY_DN8087_c0_g1_i9_orf1;TRINITY_DN237_c1_g1_i1_orf1;TRINITY_DN20710_c0_g1_i2_orf1;TRINITY_DN2652_c0_g2_i1_orf1;TRINITY_DN36856_c0_g1_i1_orf1;TRINITY_DN288_c0_g1_i9_orf1;TRINITY_DN25360_c0_g1_i2_orf1;TRINITY_DN20009_c0_g1_i1_orf1;TRINITY_DN15706_c0_g2_i5_orf1;TRINITY_DN99900_c0_g1_i3_orf1;TRINITY_DN36987_c0_g1_i1_orf1;TRINITY_DN71832_c0_g1_i1_orf1
biological_process	protein transmembrane transport	GO:0071806	2	2/2397	TRINITY_DN46409_c0_g1_i1_orf1;TRINITY_DN4207_c0_g1_i1_orf1
biological_process	mitochondrial transmembrane transport	GO:1990542	3	3/2397	TRINITY_DN46409_c0_g1_i1_orf1;TRINITY_DN4207_c0_g1_i1_orf1;TRINITY_DN44256_c0_g1_i1_orf1
biological_process	ion transmembrane transport	GO:0034220	3	3/2397	TRINITY_DN1661_c0_g1_i1_orf1;TRINITY_DN8306_c0_g1_i4_orf1;TRINITY_DN44256_c0_g1_i1_orf1
biological_process	cell-cell recognition	GO:0009988	1	1/2397	TRINITY_DN20133_c0_g1_i1_orf1
biological_process	actin cytoskeleton organization	GO:0030036	7	7/2397	TRINITY_DN4010_c0_g2_i1_orf1;TRINITY_DN86309_c0_g1_i4_orf1;TRINITY_DN8915_c0_g1_i3_orf1;TRINITY_DN235_c0_g3_i1_orf1;TRINITY_DN3887_c0_g1_i1_orf1;TRINITY_DN3126_c0_g1_i4_orf1;TRINITY_DN23790_c0_g1_i1_orf1
biological_process	cellular component assembly involved in morphogenesis	GO:0010927	1	1/2397	TRINITY_DN235_c0_g3_i1_orf1
biological_process	tube morphogenesis	GO:0035239	1	1/2397	TRINITY_DN147475_c0_g1_i1_orf1
biological_process	tissue morphogenesis	GO:0048729	3	3/2397	TRINITY_DN36856_c0_g1_i1_orf1;TRINITY_DN237_c1_g1_i1_orf1;TRINITY_DN147475_c0_g1_i1_orf1
biological_process	animal organ morphogenesis	GO:0009887	4	4/2397	TRINITY_DN741_c0_g1_i10_orf1;TRINITY_DN2468_c0_g1_i7_orf1;TRINITY_DN23746_c0_g1_i2_orf1;TRINITY_DN5954_c0_g1_i2_orf1
biological_process	cell morphogenesis	GO:0000902	3	3/2397	TRINITY_DN14209_c0_g1_i1_orf1;TRINITY_DN34426_c0_g1_i1_orf1;TRINITY_DN99900_c0_g1_i3_orf1
biological_process	system development	GO:0048731	5	5/2397	TRINITY_DN42854_c0_g3_i2_orf1;TRINITY_DN1710_c0_g2_i2_orf1;TRINITY_DN20710_c0_g1_i2_orf1;TRINITY_DN288_c0_g1_i9_orf1;TRINITY_DN99900_c0_g1_i3_orf1
biological_process	hippocampus development	GO:0021766	1	1/2397	TRINITY_DN31584_c0_g2_i2_orf1
biological_process	animal organ development	GO:0048513	10	10/2397	TRINITY_DN8087_c0_g1_i9_orf1;TRINITY_DN99900_c0_g1_i3_orf1;TRINITY_DN36856_c0_g1_i1_orf1;TRINITY_DN26790_c0_g1_i3_orf1;TRINITY_DN34426_c0_g1_i1_orf1;TRINITY_DN25976_c0_g1_i4_orf1;TRINITY_DN237_c1_g1_i1_orf1;TRINITY_DN36987_c0_g1_i1_orf1;TRINITY_DN14209_c0_g1_i1_orf1;TRINITY_DN71832_c0_g1_i1_orf1
biological_process	muscle structure development	GO:0061061	1	1/2397	TRINITY_DN26790_c0_g1_i3_orf1
biological_process	cerebral cortex development	GO:0021987	1	1/2397	TRINITY_DN31584_c0_g2_i2_orf1
biological_process	nervous system process	GO:0050877	2	2/2397	TRINITY_DN19951_c0_g1_i5_orf1;TRINITY_DN26337_c0_g1_i3_orf1
biological_process	muscle system process	GO:0003012	1	1/2397	TRINITY_DN20133_c0_g1_i1_orf1
biological_process	reproductive behavior	GO:0019098	1	1/2397	TRINITY_DN58125_c0_g1_i1_orf1
biological_process	envenomation resulting in modulation of process in another organism	GO:0035738	1	1/2397	TRINITY_DN1215_c0_g1_i2_orf1
biological_process	response to bacterium	GO:0009617	8	8/2397	TRINITY_DN1444_c1_g1_i5_orf1;TRINITY_DN14904_c0_g1_i1_orf1;TRINITY_DN479_c6_g1_i2_orf1;TRINITY_DN8685_c0_g1_i5_orf1;TRINITY_DN14019_c0_g1_i5_orf1;TRINITY_DN195_c8_g1_i1_orf1;TRINITY_DN29190_c0_g1_i4_orf1;TRINITY_DN5880_c0_g2_i2_orf1
biological_process	response to host	GO:0075136	1	1/2397	TRINITY_DN3159_c0_g1_i4_orf1
biological_process	response to defenses of other organism	GO:0052173	1	1/2397	TRINITY_DN3159_c0_g1_i4_orf1
biological_process	response to fungus	GO:0009620	1	1/2397	TRINITY_DN6098_c1_g1_i5_orf1
biological_process	defense response to other organism	GO:0098542	15	15/2397	TRINITY_DN1444_c1_g1_i5_orf1;TRINITY_DN14904_c0_g1_i1_orf1;TRINITY_DN827_c1_g1_i1_orf1;TRINITY_DN479_c6_g1_i2_orf1;TRINITY_DN8685_c0_g1_i5_orf1;TRINITY_DN14019_c0_g1_i5_orf1;TRINITY_DN195_c8_g1_i1_orf1;TRINITY_DN195_c4_g1_i1_orf1;TRINITY_DN6098_c1_g1_i5_orf1;TRINITY_DN2170_c1_g1_i3_orf1;TRINITY_DN15706_c0_g2_i5_orf1;TRINITY_DN5235_c0_g1_i7_orf1;TRINITY_DN9044_c0_g1_i2_orf1;TRINITY_DN29190_c0_g1_i4_orf1;TRINITY_DN5880_c0_g2_i2_orf1
biological_process	biological process involved in interaction with symbiont	GO:0051702	1	1/2397	TRINITY_DN46409_c0_g1_i1_orf1
biological_process	biological process involved in interaction with host	GO:0051701	2	2/2397	TRINITY_DN96557_c0_g1_i1_orf1;TRINITY_DN3159_c0_g1_i4_orf1
biological_process	establishment of organelle localization	GO:0051656	1	1/2397	TRINITY_DN96557_c0_g1_i1_orf1
biological_process	chromosome localization	GO:0050000	1	1/2397	TRINITY_DN96557_c0_g1_i1_orf1
biological_process	maintenance of protein location	GO:0045185	1	1/2397	TRINITY_DN245_c0_g1_i4_orf1
biological_process	maintenance of location in cell	GO:0051651	1	1/2397	TRINITY_DN245_c0_g1_i4_orf1
biological_process	establishment of protein localization	GO:0045184	35	35/2397	TRINITY_DN245_c0_g1_i4_orf1;TRINITY_DN5910_c1_g1_i6_orf1;TRINITY_DN5182_c0_g1_i5_orf1;TRINITY_DN15811_c0_g1_i7_orf1;TRINITY_DN46409_c0_g1_i1_orf1;TRINITY_DN3450_c0_g1_i3_orf1;TRINITY_DN31584_c0_g2_i2_orf1;TRINITY_DN146119_c0_g1_i1_orf1;TRINITY_DN95971_c0_g5_i1_orf1;TRINITY_DN34159_c0_g2_i1_orf1;TRINITY_DN50875_c0_g1_i3_orf1;TRINITY_DN13139_c0_g1_i1_orf1;TRINITY_DN3299_c0_g1_i2_orf1;TRINITY_DN11375_c0_g1_i4_orf1;TRINITY_DN15930_c0_g1_i5_orf1;TRINITY_DN959_c0_g1_i7_orf1;TRINITY_DN445_c0_g1_i2_orf1;TRINITY_DN9741_c0_g1_i3_orf1;TRINITY_DN1437_c0_g1_i6_orf1;TRINITY_DN41108_c0_g1_i1_orf1;TRINITY_DN3747_c1_g2_i1_orf1;TRINITY_DN146236_c0_g1_i1_orf1;TRINITY_DN42120_c0_g1_i2_orf1;TRINITY_DN11772_c0_g1_i1_orf1;TRINITY_DN7233_c0_g2_i1_orf1;TRINITY_DN44219_c0_g1_i1_orf1;TRINITY_DN486_c0_g1_i5_orf1;TRINITY_DN2879_c0_g1_i4_orf1;TRINITY_DN96557_c0_g1_i1_orf1;TRINITY_DN3698_c0_g1_i4_orf1;TRINITY_DN47219_c0_g1_i3_orf1;TRINITY_DN69871_c0_g1_i1_orf1;TRINITY_DN4410_c0_g1_i1_orf1;TRINITY_DN4394_c0_g1_i4_orf1;TRINITY_DN4207_c0_g1_i1_orf1
biological_process	establishment of localization in cell	GO:0051649	29	29/2397	TRINITY_DN5910_c1_g1_i6_orf1;TRINITY_DN3821_c1_g1_i7_orf1;TRINITY_DN46409_c0_g1_i1_orf1;TRINITY_DN3450_c0_g1_i3_orf1;TRINITY_DN31584_c0_g2_i2_orf1;TRINITY_DN5182_c0_g1_i5_orf1;TRINITY_DN95971_c0_g5_i1_orf1;TRINITY_DN34159_c0_g2_i1_orf1;TRINITY_DN13139_c0_g1_i1_orf1;TRINITY_DN45037_c0_g1_i1_orf1;TRINITY_DN445_c0_g1_i2_orf1;TRINITY_DN96557_c0_g1_i1_orf1;TRINITY_DN1437_c0_g1_i6_orf1;TRINITY_DN15811_c0_g1_i7_orf1;TRINITY_DN64_c0_g1_i4_orf1;TRINITY_DN3747_c1_g2_i1_orf1;TRINITY_DN4394_c0_g1_i4_orf1;TRINITY_DN7233_c0_g2_i1_orf1;TRINITY_DN35377_c0_g1_i3_orf1;TRINITY_DN42185_c0_g1_i7_orf1;TRINITY_DN486_c0_g1_i5_orf1;TRINITY_DN578_c0_g1_i5_orf1;TRINITY_DN69871_c0_g1_i1_orf1;TRINITY_DN4814_c0_g1_i6_orf1;TRINITY_DN5558_c0_g1_i4_orf1;TRINITY_DN1245_c0_g1_i4_orf1;TRINITY_DN4207_c0_g1_i1_orf1;TRINITY_DN942_c0_g1_i1_orf1;TRINITY_DN5028_c0_g1_i11_orf1
biological_process	establishment of RNA localization	GO:0051236	4	4/2397	TRINITY_DN1245_c0_g1_i4_orf1;TRINITY_DN64_c0_g1_i4_orf1;TRINITY_DN2879_c0_g1_i4_orf1;TRINITY_DN146119_c0_g1_i1_orf1

biological_process	transport	GO:0006810	85	85/2397	TRINITY_DN245_c0_g1_i4_orf1;TRINITY_DN28759_c0_g1_i1_orf1;TRINITY_DN65681_c0_g1_i1_orf1;TRINITY_DN44219_c0_g1_i1_orf1;TRINITY_DN81488_c0_g1_i1_orf1;TRINITY_DN5910_c1_g1_i6_orf1;TRINITY_DN146119_c0_g1_i1_orf1;TRINITY_DN15811_c0_g1_i7_orf1;TRINITY_DN198_c2_g1_i2_orf1;TRINITY_DN146236_c0_g1_i1_orf1;TRINITY_DN33452_c0_g1_i3_orf1;TRINITY_DN21872_c0_g1_i2_orf1;TRINITY_DN3450_c0_g1_i3_orf1;TRINITY_DN252_c0_g1_i3_orf1;TRINITY_DN5182_c0_g1_i5_orf1;TRINITY_DN44256_c0_g1_i1_orf1;TRINITY_DN15930_c0_g1_i5_orf1;TRINITY_DN1895_c0_g1_i2_orf1;TRINITY_DN42185_c0_g1_i7_orf1;TRINITY_DN445_c0_g1_i2_orf1;TRINITY_DN132857_c0_g1_i1_orf1;TRINITY_DN34159_c0_g2_i1_orf1;TRINITY_DN19521_c0_g1_i1_orf1;TRINITY_DN50875_c0_g1_i3_orf1;TRINITY_DN4410_c0_g1_i1_orf1;TRINITY_DN1423_c0_g1_i4_orf1;TRINITY_DN13139_c0_g1_i1_orf1;TRINITY_DN18912_c1_g1_i1_orf1;TRINITY_DN3299_c0_g1_i2_orf1;TRINITY_DN1407_c0_g1_i12_orf1;TRINITY_DN95971_c0_g5_i1_orf1;TRINITY_DN11375_c0_g1_i4_orf1;TRINITY_DN41108_c0_g1_i1_orf1;TRINITY_DN9554_c0_g1_i1_orf1;TRINITY_DN9239_c0_g2_i2_orf1;TRINITY_DN45037_c0_g1_i1_orf1;TRINITY_DN25686_c0_g1_i4_orf1;TRINITY_DN15812_c0_g1_i2_orf1;TRINITY_DN7590_c0_g1_i4_orf1;TRINITY_DN110402_c0_g2_i1_orf1;TRINITY_DN113353_c0_g1_i1_orf1;TRINITY_DN86621_c0_g1_i2_orf1;TRINITY_DN46625_c0_g1_i1_orf1;TRINITY_DN9354_c0_g1_i7_orf1;TRINITY_DN1437_c0_g1_i6_orf1;TRINITY_DN3698_c0_g1_i4_orf1;TRINITY_DN3821_c1_g1_i7_orf1;TRINITY_DN1423_c0_g1_i8_orf1;TRINITY_DN64_c0_g1_i4_orf1;TRINITY_DN136031_c0_g1_i7_orf1;TRINITY_DN33272_c0_g1_i5_orf1;TRINITY_DN3747_c1_g2_i1_orf1;TRINITY_DN4394_c0_g1_i4_orf1;TRINITY_DN42120_c0_g1_i2_orf1;TRINITY_DN12286_c1_g1_i2_orf1;TRINITY_DN11772_c0_g1_i1_orf1;TRINITY_DN741_c0_g1_i10_orf1;TRINITY_DN9741_c0_g1_i3_orf1;TRINITY_DN35377_c0_g1_i3_orf1;TRINITY_DN9239_c0_g1_i1_orf1;TRINITY_DN23354_c0_g1_i7_orf1;TRINITY_DN486_c0_g1_i5_orf1;TRINITY_DN2879_c0_g1_i4_orf1;TRINITY_DN8306_c0_g1_i4_orf1;TRINITY_DN578_c0_g1_i5_orf1;TRINITY_DN96557_c0_g1_i1_orf1;TRINITY_DN51766_c0_g1_i2_orf1;TRINITY_DN1407_c0_g1_i5_orf1;TRINITY_DN56430_c0_g1_i1_orf1;TRINITY_DN4016_c0_g1_i1_orf1;TRINITY_DN46409_c0_g1_i1_orf1;TRINITY_DN1661_c0_g1_i1_orf1;TRINITY_DN37654_c0_g1_i5_orf1;TRINITY_DN7233_c0_g2_i1_orf1;TRINITY_DN69871_c0_g1_i1_orf1;TRINITY_DN4814_c0_g1_i6_orf1;TRINITY_DN5558_c0_g1_i4_orf1;TRINITY_DN5028_c0_g1_i11_orf1;TRINITY_DN47219_c0_g1_i3_orf1;TRINITY_DN1245_c0_g1_i4_orf1;TRINITY_DN5064_c0_g1_i4_orf1;TRINITY_DN4207_c0_g1_i1_orf1;TRINITY
biological_process	non-lytic viral release	GO:0046753	1	1/2397	TRINITY_DN96557_c0_g1_i1_orf1
biological_process	viral RNA genome replication	GO:0039694	1	1/2397	TRINITY_DN4408_c6_g1_i1_orf1
biological_process	viral budding via host ESCRT complex	GO:0039702	1	1/2397	TRINITY_DN96557_c0_g1_i1_orf1
biological_process	viral budding from plasma membrane	GO:0046761	1	1/2397	TRINITY_DN96557_c0_g1_i1_orf1
biological_process	response to external biotic stimulus	GO:0043207	27	27/2397	TRINITY_DN1444_c1_g1_i5_orf1;TRINITY_DN827_c1_g1_i1_orf1;TRINITY_DN8685_c0_g1_i5_orf1;TRINITY_DN3159_c0_g1_i4_orf1;TRINITY_DN2407_c0_g1_i2_orf1;TRINITY_DN15706_c0_g2_i5_orf1;TRINITY_DN29190_c0_g1_i4_orf1;TRINITY_DN66287_c0_g1_i1_orfp1;TRINITY_DN12534_c0_g1_i4_orf1;TRINITY_DN479_c6_g1_i2_orf1;TRINITY_DN14019_c0_g1_i5_orf1;TRINITY_DN3166_c1_g1_i6_orf1;TRINITY_DN2407_c0_g1_i6_orf1;TRINITY_DN20009_c0_g1_i1_orf1;TRINITY_DN195_c4_g1_i1_orf1;TRINITY_DN6098_c1_g1_i5_orf1;TRINITY_DN86772_c0_g1_i3_orfp1;TRINITY_DN9044_c0_g1_i2_orf1;TRINITY_DN85161_c0_g1_i2_orf1;TRINITY_DN4748_c0_g1_i5_orf1;TRINITY_DN14904_c0_g1_i1_orf1;TRINITY_DN4802_c0_g1_i4_orf1;TRINITY_DN195_c8_g1_i1_orf1;TRINITY_DN59429_c0_g1_i6_orf1;TRINITY_DN2170_c1_g1_i3_orf1;TRINITY_DN5235_c0_g1_i7_o
biological_process	detection of biotic stimulus	GO:0009595	2	2/2397	TRINITY_DN8685_c0_g1_i5_orf1;TRINITY_DN5880_c0_g2_i2_orf1
biological_process	response to extracellular stimulus	GO:0009991	4	4/2397	TRINITY_DN140212_c0_g1_i1_orf1;TRINITY_DN51938_c0_g3_i1_orf1;TRINITY_DN1091_c0_g1_i1_orf1;TRINITY_DN2054_c0_g1_i1_orf1
biological_process	cellular response to external stimulus	GO:0071496	4	4/2397	TRINITY_DN140212_c0_g1_i1_orf1;TRINITY_DN1091_c0_g1_i1_orf1;TRINITY_DN51938_c0_g3_i1_orf1;TRINITY_DN2054_c0_g1_i1_orf1
biological_process	cellular response to endogenous stimulus	GO:0071495	3	3/2397	TRINITY_DN20009_c0_g1_i1_orf1;TRINITY_DN51938_c0_g3_i1_orf1;TRINITY_DN4016_c0_g1_i1_orf1
biological_process	response to transforming growth factor beta	GO:0071559	1	1/2397	TRINITY_DN51938_c0_g3_i1_orf1
biological_process	response to hormone	GO:0009725	1	1/2397	TRINITY_DN51938_c0_g3_i1_orf1
biological_process	response to hypoxia	GO:0001666	1	1/2397	TRINITY_DN51938_c0_g3_i1_orf1
biological_process	response to topologically incorrect protein	GO:0035966	1	1/2397	TRINITY_DN46409_c0_g1_i1_orf1
biological_process	response to ischemia	GO:0002931	1	1/2397	TRINITY_DN51938_c0_g3_i1_orf1
biological_process	response to cold	GO:0009409	1	1/2397	TRINITY_DN46409_c0_g1_i1_orf1
biological_process	response to heat	GO:0009408	2	2/2397	TRINITY_DN31584_c0_g2_i2_orf1;TRINITY_DN5648_c0_g1_i5_orf1
biological_process	defense response	GO:0006952	25	25/2397	TRINITY_DN195_c8_g1_i1_orf1;TRINITY_DN827_c1_g1_i1_orf1;TRINITY_DN8685_c0_g1_i5_orf1;TRINITY_DN2407_c0_g1_i2_orf1;TRINITY_DN15706_c0_g2_i5_orf1;TRINITY_DN29190_c0_g1_i4_orf1;TRINITY_DN66287_c0_g1_i1_orfp1;TRINITY_DN12534_c0_g1_i4_orf1;TRINITY_DN479_c6_g1_i2_orf1;TRINITY_DN14019_c0_g1_i5_orf1;TRINITY_DN3166_c1_g1_i6_orf1;TRINITY_DN2407_c0_g1_i6_orf1;TRINITY_DN86772_c0_g1_i3_orfp1;TRINITY_DN195_c4_g1_i1_orf1;TRINITY_DN6098_c1_g1_i5_orf1;TRINITY_DN9044_c0_g1_i2_orf1;TRINITY_DN85161_c0_g1_i2_orf1;TRINITY_DN4748_c0_g1_i5_orf1;TRINITY_DN14904_c0_g1_i1_orf1;TRINITY_DN4802_c0_g1_i4_orf1;TRINITY_DN1444_c1_g1_i5_orf1;TRINITY_DN59429_c0_g1_i6_orf1;TRINITY_DN2170_c1_g1_i3_orf1;TRINITY_DN5235_c0_g1_i7_orf1;TRINITY_DN5880_c0_g2_i2_orf1
biological_process	response to hyperoxia	GO:0055093	1	1/2397	TRINITY_DN51938_c0_g3_i1_orf1
biological_process	response to oxidative stress	GO:0006979	9	9/2397	TRINITY_DN87603_c0_g2_i1_orf1;TRINITY_DN1622_c0_g1_i6_orf1;TRINITY_DN2207_c0_g1_i6_orf1;TRINITY_DN5933_c0_g1_i1_orf1;TRINITY_DN285_c0_g1_i4_orf1;TRINITY_DN54387_c0_g1_i1_orf1;TRINITY_DN51252_c0_g2_i1_orf1;TRINITY_DN21420_c0_g1_i2_orf1;TRINITY_DN2652_c0_g2_i1_orf1
biological_process	response to oxygen-containing compound	GO:1901700	4	4/2397	TRINITY_DN20009_c0_g1_i1_orf1;TRINITY_DN51938_c0_g3_i1_orf1;TRINITY_DN87603_c0_g2_i1_orf1;TRINITY_DN4016_c0_g1_i1_orf1
biological_process	response to nitrogen compound	GO:1901698	3	3/2397	TRINITY_DN9062_c0_g2_i3_orf1;TRINITY_DN4016_c0_g1_i1_orf1;TRINITY_DN20009_c0_g1_i1_orf1
biological_process	response to nutrient	GO:0007584	1	1/2397	TRINITY_DN51938_c0_g3_i1_orf1
biological_process	response to inorganic substance	GO:0010035	2	2/2397	TRINITY_DN87603_c0_g2_i1_orf1;TRINITY_DN4016_c0_g1_i1_orf1
biological_process	response to organic substance	GO:0010033	9	9/2397	TRINITY_DN9062_c0_g2_i3_orf1;TRINITY_DN51938_c0_g3_i1_orf1;TRINITY_DN4016_c0_g1_i1_orf1;TRINITY_DN8685_c0_g1_i5_orf1;TRINITY_DN18218_c0_g1_i7_orf1;TRINITY_DN46409_c0_g1_i1_orf1;TRINITY_DN20009_c0_g1_i1_orf1;TRINITY_DN2227_c0_g1_i5_orf1;TRINITY_DN5880_c0_g2_i2_orf1
biological_process	response to temperature stimulus	GO:0009266	3	3/2397	TRINITY_DN46409_c0_g1_i1_orf1;TRINITY_DN31584_c0_g2_i2_orf1;TRINITY_DN5648_c0_g1_i5_orf1
biological_process	response to radiation	GO:0009314	1	1/2397	TRINITY_DN41573_c0_g1_i1_orf1
biological_process	response to oxygen levels	GO:0070482	1	1/2397	TRINITY_DN51938_c0_g3_i1_orf1
biological_process	detection of chemical stimulus	GO:0009593	2	2/2397	TRINITY_DN8685_c0_g1_i5_orf1;TRINITY_DN5880_c0_g2_i2_orf1
cellular_componen	nucleosome	GO:0000786	3	3/2397	TRINITY_DN20442_c0_g2_i1_orf1;TRINITY_DN24917_c0_g2_i1_orf1;TRINITY_DN6358_c0_g1_i5_orf1
cellular_componen	cohesin complex	GO:0008278	1	1/2397	TRINITY_DN2638_c0_g1_i7_orf1
cellular_componen	Mre11 complex	GO:0030870	2	2/2397	TRINITY_DN45271_c0_g1_i1_orf1;TRINITY_DN123184_c0_g1_i1_orf1
cellular_componen	mRNA cleavage factor complex	GO:0005849	2	2/2397	TRINITY_DN2718_c0_g1_i6_orf1;TRINITY_DN1005_c0_g1_i5_orf1
cellular_componen	Ku70:Ku80 complex	GO:0043564	1	1/2397	TRINITY_DN5757_c0_g1_i1_orf1
cellular_componen	SWI/SNF superfamily-type complex	GO:0070603	2	2/2397	TRINITY_DN3649_c0_g1_i6_orf1;TRINITY_DN45449_c0_g1_i1_orf1
cellular_componen	transcription elongation factor complex	GO:0008023	1	1/2397	TRINITY_DN44792_c0_g1_i1_orf1
cellular_componen	spliceosomal complex	GO:0005681	9	9/2397	TRINITY_DN1554_c0_g1_i9_orf1;TRINITY_DN27276_c0_g1_i5_orf1;TRINITY_DN3459_c0_g1_i4_orf1;TRINITY_DN31663_c0_g1_i2_orf1;TRINITY_DN107035_c0_g1_i1_orf1;TRINITY_DN116467_c0_g1_i1_orf1;TRINITY_DN53233_c0_g1_i1_orf1;TRINITY_DN20215_c0_g2_i1_orf1;TRINITY_DN14487_c0_g1_i4_orf1
cellular_componen	BRISC complex	GO:0070552	2	2/2397	TRINITY_DN17655_c0_g1_i1_orf1;TRINITY_DN41573_c0_g1_i1_orf1
cellular_componen	nuclear DNA-directed RNA polymerase complex	GO:0055029	1	1/2397	TRINITY_DN12527_c0_g1_i4_orf1
cellular_componen	histone acetyltransferase complex	GO:0000123	1	1/2397	TRINITY_DN59804_c0_g1_i1_orf1
cellular_componen	small nuclear ribonucleoprotein complex	GO:0030532	6	6/2397	TRINITY_DN38540_c0_g1_i1_orf1;TRINITY_DN298_c0_g1_i4_orf1;TRINITY_DN1616_c0_g1_i3_orf1;TRINITY_DN31663_c0_g1_i2_orf1;TRINITY_DN116467_c0_g1_i1_orf1;TRINITY_DN5834_c0_g1_i2_orf1
cellular_componen	BRCA1-A complex	GO:0070531	2	2/2397	TRINITY_DN17655_c0_g1_i1_orf1;TRINITY_DN41573_c0_g1_i1_orf1
cellular_componen	THO complex	GO:0000347	2	2/2397	TRINITY_DN64_c0_g1_i4_orf1;TRINITY_DN133760_c0_g1_i1_orf1
cellular_componen	nuclear pore	GO:0005643	5	5/2397	TRINITY_DN96557_c0_g1_i1_orf1;TRINITY_DN146119_c0_g1_i1_orf1;TRINITY_DN10415_c0_g1_i5_orf1;TRINITY_DN2879_c0_g1_i4_orf1;TRINITY_DN1437_c0_g1_i6_orf1
cellular_componen	ESCRT III complex	GO:0000815	1	1/2397	TRINITY_DN96557_c0_g1_i1_orf1
cellular_componen	ESCRT I complex	GO:0000813	1	1/2397	TRINITY_DN4013_c0_g1_i4_orf1
cellular_componen	mitochondrial intermembrane space protein transporter complex	GO:0042719	1	1/2397	TRINITY_DN15811_c0_g1_i7_orf1
cellular_componen	transmembrane transporter complex	GO:1902495	6	6/2397	TRINITY_DN20346_c0_g1_i1_orf1;TRINITY_DN19521_c0_g1_i1_orf1;TRINITY_DN20558_c0_g1_i2_orf1;TRINITY_DN162_c0_g1_i4_orf1;TRINITY_DN4270_c0_g1_i1_orf1;TRINITY_DN44256_c0_g1_i1_orf1
cellular_componen	dynein complex	GO:0030286	1	1/2397	TRINITY_DN17995_c0_g4_i1_orf1
cellular_componen	cytochrome complex	GO:0070069	2	2/2397	TRINITY_DN4270_c0_g1_i1_orf1;TRINITY_DN136028_c0_g2_i1_orf1
cellular_componen	phosphatase complex	GO:1903293	1	1/2397	TRINITY_DN2257_c0_g1_i4_orf1
cellular_componen	oxidoreductase complex	GO:1990204	3	3/2397	TRINITY_DN20346_c0_g1_i1_orf1;TRINITY_DN6199_c2_g1_i3_orf1;TRINITY_DN4270_c0_g1_i1_orf1

cellular_componen	transferase complex	GO:1990234	13	13/2397	TRINITY_DN59804_c0_g1_i1_orf1;TRINITY_DN81258_c0_g1_i2_orf1;TRINITY_DN12527_c0_g1_i4_orf1;TRINITY_DN9062_c0_g2_i3_orf1;TRINITY_DN74037_c0_g5_i1_orf1;TRINITY_DN70485_c0_g1_i2_orf1;TRINITY_DN147475_c0_g1_i1_orf1;TRINITY_DN879_c0_g1_i2_orf1;TRINITY_DN5182_c0_g1_i5_orf1;TRINITY_DN110534_c0_g1_i3_orf1;TRINITY_DN2299_c0_g1_i3_orf1;TRINITY_DN89613_c0_g1_i13_orf1;TRINITY_DN12_c0_g1_i5_orf1
cellular_componen	peptidase complex	GO:1905368	3	3/2397	TRINITY_DN321_c0_g1_i1_orf1;TRINITY_DN2591_c0_g1_i4_orf1;TRINITY_DN2058_c0_g1_i2_orf1
cellular_componen	aminoacyl-tRNA synthetase multienzyme complex	GO:0017101	5	5/2397	TRINITY_DN825_c23_g1_i5_orf1;TRINITY_DN5857_c0_g1_i13_orf1;TRINITY_DN107288_c0_g1_i2_orf1;TRINITY_DN2953_c1_g1_i10_orf1;TRINITY_DN2953_c1_g1_i2_orf1
cellular_componen	elongator holoenzyme complex	GO:0033588	1	1/2397	TRINITY_DN56270_c0_g1_i1_orf1
cellular_componen	ATPase complex	GO:1904949	2	2/2397	TRINITY_DN3649_c0_g1_i6_orf1;TRINITY_DN45449_c0_g1_i1_orf1
cellular_componen	dystrophin-associated glycoprotein complex	GO:0016010	1	1/2397	TRINITY_DN7128_c0_g1_i7_orf1
cellular_componen	lipopolysaccharide receptor complex	GO:0046696	1	1/2397	TRINITY_DN46409_c0_g1_i1_orf1
cellular_componen	plasma membrane protein complex	GO:0098797	3	3/2397	TRINITY_DN19521_c0_g1_i1_orf1;TRINITY_DN162_c0_g1_i4_orf1;TRINITY_DN7128_c0_g1_i7_orf1
cellular_componen	outer mitochondrial membrane protein complex	GO:0098799	2	2/2397	TRINITY_DN9741_c0_g1_i3_orf1;TRINITY_DN3299_c0_g1_i2_orf1
cellular_componen	Tapasin-ERp57 complex	GO:0061779	1	1/2397	TRINITY_DN51938_c0_g3_i1_orf1
cellular_componen	translocon complex	GO:0071256	1	1/2397	TRINITY_DN7233_c0_g2_i1_orf1
cellular_componen	proton-transporting two-sector ATPase complex, catalytic domain	GO:0033178	1	1/2397	TRINITY_DN96080_c0_g2_i1_orf1
cellular_componen	EMC complex	GO:0072546	1	1/2397	TRINITY_DN9002_c0_g1_i1_orf1
cellular_componen	MHC class I peptide loading complex	GO:0042824	1	1/2397	TRINITY_DN51938_c0_g3_i1_orf1
cellular_componen	TAP complex	GO:0042825	1	1/2397	TRINITY_DN51938_c0_g3_i1_orf1
cellular_componen	NADH dehydrogenase complex	GO:0030964	1	1/2397	TRINITY_DN20346_c0_g1_i1_orf1
cellular_componen	respiratory chain complex	GO:0098803	3	3/2397	TRINITY_DN20346_c0_g1_i1_orf1;TRINITY_DN4270_c0_g1_i1_orf1;TRINITY_DN136028_c0_g2_i1_orf1
cellular_componen	inner mitochondrial membrane protein complex	GO:0098800	8	8/2397	TRINITY_DN20346_c0_g1_i1_orf1;TRINITY_DN136028_c0_g2_i1_orf1;TRINITY_DN15222_c0_g1_i4_orf1;TRINITY_DN141353_c0_g1_i1_orf1;TRINITY_DN4270_c0_g1_i1_orf1;TRINITY_DN44219_c0_g1_i1_orf1;TRINITY_DN44256_c0_g1_i1_orf1;TRINITY_DN4207_c0_g1_i1_orf1
cellular_componen	membrane coat	GO:0030117	2	2/2397	TRINITY_DN96557_c0_g1_i1_orf1;TRINITY_DN146119_c0_g1_i1_orf1
cellular_componen	AP-type membrane coat adaptor complex	GO:0030119	2	2/2397	TRINITY_DN486_c0_g1_i5_orf1;TRINITY_DN13139_c0_g1_i1_orf1
cellular_componen	proton-transporting two-sector ATPase complex, proton-transporting domain	GO:0033177	2	2/2397	TRINITY_DN15222_c0_g1_i4_orf1;TRINITY_DN141353_c0_g1_i1_orf1
cellular_componen	mitochondrial large ribosomal subunit	GO:0005762	1	1/2397	TRINITY_DN1313_c0_g1_i2_orf1
cellular_componen	Golgi transport complex	GO:0017119	1	1/2397	TRINITY_DN50875_c0_g1_i3_orf1
cellular_componen	exocyst	GO:0000145	2	2/2397	TRINITY_DN25686_c0_g1_i4_orf1;TRINITY_DN1895_c0_g1_i2_orf1
cellular_componen	TRAPP complex	GO:0030008	1	1/2397	TRINITY_DN45037_c0_g1_i1_orf1
cellular_componen	kinesin complex	GO:0005871	1	1/2397	TRINITY_DN4808_c0_g1_i3_orf1
cellular_componen	sno(s)RNA-containing ribonucleoprotein complex	GO:0005732	1	1/2397	TRINITY_DN13496_c0_g1_i7_orf1
cellular_componen	polysome	GO:0005844	1	1/2397	TRINITY_DN20009_c0_g1_i1_orf1
cellular_componen	translation preinitiation complex	GO:0070993	1	1/2397	TRINITY_DN48097_c0_g1_i1_orf1
cellular_componen	RNAi effector complex	GO:0031332	1	1/2397	TRINITY_DN14701_c0_g1_i2_orf1
cellular_componen	preribosome	GO:0030684	2	2/2397	TRINITY_DN3082_c1_g1_i7_orf1;TRINITY_DN13496_c0_g1_i7_orf1
cellular_componen	ribosomal subunit	GO:0044391	16	16/2397	TRINITY_DN18869_c0_g1_i1_orf1;TRINITY_DN2682_c0_g1_i4_orf1;TRINITY_DN4016_c0_g1_i1_orf1;TRINITY_DN87603_c0_g2_i1_orf1;TRINITY_DN10070_c0_g1_i1_orf1;TRINITY_DN36893_c0_g1_i1_orf1;TRINITY_DN1313_c0_g1_i2_orf1;TRINITY_DN11297_c0_g1_i1_orf1;TRINITY_DN137_c0_g1_i1_orf1;TRINITY_DN13732_c0_g2_i3_orf1;TRINITY_DN50787_c0_g2_i2_orf1;TRINITY_DN41645_c0_g1_i1_orf1;TRINITY_DN42646_c0_g2_i1_orf1;TRINITY_DN8949_c0_g1_i2_orf1;TRINITY_DN82324_c0_g1_i4_orf1;TRINITY_DN
cellular_componen	proteasome complex	GO:0000502	3	3/2397	TRINITY_DN321_c0_g1_i1_orf1;TRINITY_DN2591_c0_g1_i4_orf1;TRINITY_DN2058_c0_g1_i2_orf1
cellular_componen	DNA polymerase complex	GO:0042575	5	5/2397	TRINITY_DN81258_c0_g1_i2_orf1;TRINITY_DN110534_c0_g1_i3_orf1;TRINITY_DN89613_c0_g1_i13_orf1;TRINITY_DN70485_c0_g1_i2_orf1;TRINITY_DN74037_c0_g5_i1_orf1
cellular_componen	ubiquitin ligase complex	GO:0000151	1	1/2397	TRINITY_DN9062_c0_g2_i3_orf1
cellular_componen	TOR complex	GO:0038201	1	1/2397	TRINITY_DN40191_c2_g1_i1_orf1
cellular_componen	guanyl-nucleotide exchange factor complex	GO:0032045	1	1/2397	TRINITY_DN34159_c0_g2_i1_orf1
cellular_componen	RNA polymerase complex	GO:0030880	3	3/2397	TRINITY_DN12527_c0_g1_i4_orf1;TRINITY_DN879_c0_g1_i2_orf1;TRINITY_DN2299_c0_g1_i3_orf1
cellular_componen	protein acetyltransferase complex	GO:0031248	1	1/2397	TRINITY_DN59804_c0_g1_i1_orf1
cellular_componen	cAMP-dependent protein kinase complex	GO:0005952	1	1/2397	TRINITY_DN12_c0_g1_i5_orf1
cellular_componen	protein kinase CK2 complex	GO:0005956	1	1/2397	TRINITY_DN147475_c0_g1_i1_orf1
cellular_componen	CIA complex	GO:0097361	1	1/2397	TRINITY_DN49872_c0_g1_i2_orf1
cellular_componen	organelle lumen	GO:0043233	14	14/2397	TRINITY_DN14920_c0_g1_i1_orf1;TRINITY_DN51938_c0_g3_i1_orf1;TRINITY_DN17271_c0_g1_i1_orf1;TRINITY_DN21539_c0_g1_i1_orf1;TRINITY_DN47219_c0_g1_i3_orf1;TRINITY_DN5122_c0_g1_i3_orf1;TRINITY_DN68401_c1_g1_i1_orf1;TRINITY_DN4424_c0_g1_i1_orf1;TRINITY_DN42854_c0_g3_i2_orf1;TRINITY_DN46409_c0_g1_i1_orf1;TRINITY_DN20133_c0_g1_i1_orf1;TRINITY_DN1791_c0_g1_i3_orf1;TRINITY_DN2299_c0_g1_i3_orf1;TRINITY_DN147475_c0_g1_i1_orf1
cellular_componen	chromosome, telomeric region	GO:0000781	1	1/2397	TRINITY_DN12771_c0_g1_i1_orf1
cellular_componen	chromosome, centromeric region	GO:0000775	1	1/2397	TRINITY_DN31314_c0_g1_i4_orf1
cellular_componen	cell cortex	GO:0005938	2	2/2397	TRINITY_DN2186_c0_g1_i17_orf1;TRINITY_DN2186_c0_g1_i13_orf1
cellular_componen	extrinsic component of organelle membrane	GO:0031312	2	2/2397	TRINITY_DN6638_c0_g1_i1_orf1;TRINITY_DN6027_c0_g1_i13_orf1
cellular_componen	heterochromatin	GO:0000792	3	3/2397	TRINITY_DN24266_c0_g2_i2_orf1;TRINITY_DN2345_c0_g1_i4_orf1;TRINITY_DN20133_c0_g1_i1_orf1
cellular_componen	intrinsic component of plasma membrane	GO:0031226	1	1/2397	TRINITY_DN3833_c0_g1_i4_orf1
cellular_componen	anchored component of membrane	GO:0031225	6	6/2397	TRINITY_DN56690_c0_g1_i4_orf1;TRINITY_DN9475_c0_g1_i6_orf1;TRINITY_DN1352_c0_g1_i5_orf1;TRINITY_DN5553_c0_g1_i4_orf1;TRINITY_DN2175_c0_g1_i4_orf1;TRINITY_DN3833_c0_g1_i4_orf1

cellular_componen	integral component of membrane	GO:0016021	286	286/2397	TRINITY_DN129226_c0_g1_i2_orf1;TRINITY_DN38435_c0_g1_i1_orf1;TRINITY_DN13563_c0_g1_i1_orf1;TRINITY_DN4289_c0_g1_i5_orf1;TRINITY_DN9608_c0_g1_i3_orf1;TRINITY_DN827_c1_g1_i1_orf1;TRINITY_DN120500_c0_g1_i1_orf1;TRINITY_DN2312_c0_g1_i4_orf1;TRINITY_DN139537_c0_g1_i1_orf1;TRINITY_DN3194_c0_g1_i6_orf1;TRINITY_DN6991_c0_g1_i24_orf1;TRINITY_DN496_c0_g1_i7_orf1;TRINITY_DN26355_c0_g1_i4_orf1;TRINITY_DN9615_c0_g1_i1_orf1;TRINITY_DN6586_c0_g1_i1_orf1;TRINITY_DN72816_c0_g1_i2_orf1;TRINITY_DN4394_c0_g2_i1_orf1;TRINITY_DN34426_c0_g1_i1_orf1;TRINITY_DN140_c1_g1_i2_orf1;TRINITY_DN246_c1_g1_i5_orf1;TRINITY_DN11928_c0_g1_i3_orf1;TRINITY_DN3529_c0_g1_i7_orf1;TRINITY_DN7735_c1_g1_i1_orf1;TRINITY_DN38431_c0_g1_i1_orf1;TRINITY_DN17772_c0_g2_i3_orf1;TRINITY_DN4134_c2_g1_i2_orf1;TRINITY_DN1622_c0_g1_i6_orf1;TRINITY_DN8030_c0_g1_i2_orf1;TRINITY_DN29633_c0_g1_i8_orf1;TRINITY_DN1232_c0_g1_i1_orf1;TRINITY_DN32780_c0_g1_i2_orf1;TRINITY_DN3105_c0_g1_i4_orf1;TRINITY_DN9554_c0_g1_i1_orf1;TRINITY_DN8964_c0_g1_i4_orf1;TRINITY_DN7570_c0_g1_i18_orf1;TRINITY_DN4273_c1_g1_i5_orf1;TRINITY_DN3066_c0_g1_i5_orf1;TRINITY_DN7590_c0_g1_i4_orf1;TRINITY_DN4144_c0_g1_i7_orf1;TRINITY_DN585_c0_g1_i12_orf1;TRINITY_DN5753_c0_g1_i10_orf1;TRINITY_DN10774_c0_g2_i3_orf1;TRINITY_DN54925_c0_g1_i1_orf1;TRINITY_DN41708_c0_g1_i1_orf1;TRINITY_DN21570_c0_g1_i1_orf1;TRINITY_DN18338_c0_g1_i6_orf1;TRINITY_DN3014_c0_g1_i4_orf1;TRINITY_DN27114_c0_g1_i1_orf1;TRINITY_DN3821_c1_g1_i7_orf1;TRINITY_DN57105_c0_g1_i2_orf1;TRINITY_DN4125_c0_g1_i14_orf1;TRINITY_DN3177_c0_g1_i1_orf1;TRINITY_DN4757_c0_g1_i3_orf1;TRINITY_DN4886_c0_g1_i6_orf1;TRINITY_DN22443_c0_g2_i3_orf1;TRINITY_DN3978_c0_g2_i1_orf1;TRINITY_DN30932_c0_g1_i2_orf1;TRINITY_DN2508_c0_g1_i2_orf1;TRINITY_DN919_c0_g1_i7_orf1;TRINITY_DN10785_c0_g1_i4_orf1;TRINITY_DN2343_c1_g1_i2_orf1;TRINITY_DN6621_c0_g1_i1_orf1;TRINITY_DN29879_c0_g1_i3_orf1;TRINITY_DN51766_c0_g1_i2_orf1;TRINITY_DN91533_c0_g1_i1_orf1;TRINITY_DN56430_c0_g1_i1_orf1;TRINITY_DN10231_c0_g2_i1_orf1;TRINITY_DN29_c0_g1_i4_orf1;TRINITY_DN172_c8_g2_i1_orf1;TRINITY_DN11670_c0_g1_i1_orf1;TRINITY_DN4814_c0_g1_i6_orf1;TRINITY_DN11172_c0_g1_i4_orf1;TRINITY_DN3005_c0_g1_i7_orf1;TRINITY_DN3616_c0_g2_i1_orf1;TRINITY_DN8700_c9_g1_i1_orf1;TRINITY_DN82104_c0_g1_i5_orf1;TRINITY_DN12769_c0_g1_i5_orf1;TRINITY_DN5852_c0_g1_i6_orf1;TRINITY_DN3472_c1_g1_i4_orf1;TRINITY_DN4324_c0_g1_i1_orf1;TRINITY_DN113353_c0_g1_i1_orf1;TRINITY_DN14597_c0_g1_i5_orf1;TRINITY_DN7633_c0_g1_i1_orf1;TRINITY_DN13216_c0_g1_i5_orf1;TRINITY_DN1749_c0_g2_i2_orf1;TRINITY_DN6974_c0_g2_i1_orf1;TRINITY_DN13856_c0_g1_i1_orf1;TRINITY_DN198_c2_g1_i2_orf1;TRINITY_DN482_c0_g1_i1_orf1;TRINITY_DN252_c0_g1_i3_orf1;TRINITY_DN121650_c0_g1_i1_orf1;TRINITY_DN957_c0_g1_i18_orf1;TRINITY_DN5046_c0_g3_i1_orf1;TRINITY_DN14306_c0_g1_i1_orf1;TRINITY_DN132857_c0_g1_i1_orf1;TRINITY_DN501_c0_g1_i5_orf1;TRINITY_DN79803_c0_g1_i7_orf1;TRINITY_DN5012_c0_g1_i6_orf1;TRINITY_DN1789_c0_g1_i5_orf1;TRINITY_DN5852_c0_g1_i13_orf1;TRINITY_DN1999_c0_g1_i9_orf1;TRINITY_DN5211_c0_g1_i1_orf1;TRINITY_DN21719_c0_g1_i2_orf1;TRINITY_DN135781_c0_g1_i1_orf1;TRINITY_DN1008_c0_g1_i2_orf1;TRINITY_DN2109_c0_g1_i4_orf1;TRINITY_DN7464_c0_g1_i14_orf1;TRINITY_DN810_c0_g1_i4_orf1;TRINITY_DN3647_c2_g1_i3_orf1;TRINITY_DN14429_c0_g1_i2_orf1;TRINITY_DN20558_c0_g1_i2_orf1;TRINITY_DN1237_c0_g1_i4_orf1;TRINITY_DN7336_c0_g1_i13_orf1;TRINITY_DN3227_c0_g1_i5_orf1;TRINITY_DN43369_c0_g2_i1_orf1;TRINITY_DN34821_c0_g1_i4_orf1;TRINITY_DN9000_c0_g2_i1_orf1;TRINITY_DN5408_c0_g1_i5_orf1;TRINITY_DN1720_c0_g1_i3_orf1;TRINITY_DN15318_c0_g1_i1_orf1;TRINITY_DN585_c0_g1_i5_orf1;TRINITY_DN62707_c0_g1_i1_orf1;TRINITY_DN10581_c0_g1_i5_orf1;TRINITY_DN4394_c0_g1_i4_orf1;TRINITY_DN2706_c0_g1_i3_orf1;TRINITY_DN22044_c0_g2_i1_orf1;TRINITY_DN2826_c0_g1_i7_orf1;TRINITY_DN1768_c0_g1_i2_orf1;TRINITY_DN30509_c0_g1_i9_orf1;TRINITY_DN52788_c0_g1_i1_orf1;TRINITY_DN83295_c0_g1_i3_orf1;TRINITY_DN23978_c0_g1_i2_orf1;TRINITY_DN7828_c0_g1_i2_orf1;TRINITY_DN1752_c0_g1_i18_orf1;TRINITY_DN3499_c0_g1_i8_orf1;TRINITY_DN31118_c0_g1_i1_orf1;TRINITY_DN42120_c0_g1_i2_orf1;TRINITY_DN48590_c0_g1_i1_orf1;TRINITY_DN3244_c0_g1_i4_orf1;TRINITY_DN3461_c0_g1_i1_orf1;TRINITY_DN52761_c0_g2_i1_orf1;TRINITY_DN57348_c0_g1_i4_orf1;TRINITY_DN1318_c0_g1_i5_orf1;TRINITY_DN5513_c0_g1_i1_orf1;TRINITY_DN1630_c0_g1_i6_orf1;TRINITY_DN867_c0_g1_i1_orf1;TRINITY_DN448_c0_g1_i20_orf1;TRINITY_DN245_c0_g1_i4_orf1;TRINITY_DN9475_c0_g1_i6_orf1;TRINITY_DN1750_c1_g1_i5_or[illegible]
cellular_componen	intrinsic component of organelle membrane	GO:0031300	1	1/2397	TRINITY_DN8964_c0_g1_i4_orf1
cellular_componen	cytoplasmic side of membrane	GO:0098562	1	1/2397	TRINITY_DN96557_c0_g1_i1_orf1
cellular_componen	intracellular organelle	GO:0043229	232	232/2397	TRINITY_DN47731_c0_g1_i2_orf1;TRINITY_DN25960_c0_g1_i1_orf1;TRINITY_DN44261_c0_g1_i1_orf1;TRINITY_DN12526_c0_g1_i5_orf1;TRINITY_DN13350_c0_g1_i4_orf1;TRINITY_DN6991_c0_g1_i24_orf1;TRINITY_DN137_c0_g1_i1_orf1;TRINITY_DN11194_c0_g1_i4_orf1;TRINITY_DN11263_c0_g1_i5_orf1;TRINITY_DN7122_c0_g1_i1_orf1;TRINITY_DN1921_c1_g1_i5_orf1;TRINITY_DN5893_c0_g1_i7_orf1;TRINITY_DN35669_c0_g1_i1_orf1;TRINITY_DN18804_c0_g1_i5_orf1;TRINITY_DN110460_c0_g2_i1_orf1;TRINITY_DN11065_c0_g2_i1_orf1;TRINITY_DN58207_c0_g1_i1_orf1;TRINITY_DN7277_c0_g1_i1_orf1;TRINITY_DN110400_c0_g1_i1_orf1;TRINITY_DN23746_c0_g1_i2_orf1;TRINITY_DN23616_c0_g1_i4_orf1;TRINITY_DN2638_c0_g1_i7_orf1;TRINITY_DN111110_c0_g1_i1_orf1;TRINITY_DN50074_c0_g1_i1_orf1;TRINITY_DN29448_c0_g1_i1_orf1;TRINITY_DN2954_c0_g1_i1_orf1;TRINITY_DN3464_c0_g1_i1_orf1;TRINITY_DN5531_c0_g3_i3_orf1;TRINITY_DN62_c1_g1_i3_orf1;TRINITY_DN21570_c0_g1_i1_orf1;TRINITY_DN6621_c0_g1_i1_orf1;TRINITY_DN1831_c0_g1_i3_orf1;TRINITY_DN5442_c0_g1_i4_orf1;TRINITY_DN41108_c0_g1_i1_orf1;TRINITY_DN51737_c0_g1_i3_orf1;TRINITY_DN99673_c0_g1_i1_orf1;TRINITY_DN50571_c1_g1_i1_orf1;TRINITY_DN15256_c0_g1_i8_orf1;TRINITY_DN146217_c0_g1_i1_orf1;TRINITY_DN9146_c0_g1_i1_orf1;TRINITY_DN235_c0_g3_i1_orf1;TRINITY_DN31314_c0_g1_i4_orf1;TRINITY_DN2058_c0_g1_i2_orf1;TRINITY_DN31585_c0_g1_i1_orf1;TRINITY_DN4929_c0_g1_i1_orf1;TRINITY_DN30932_c0_g1_i2_orf1;TRINITY_DN3985_c0_g2_i1_orf1;TRINITY_DN6239_c0_g1_i1_orf1;TRINITY_DN2345_c0_g1_i4_orf1;TRINITY_DN110231_c0_g1_i1_orf1;TRINITY_DN7909_c0_g2_i1_orf1;TRINITY_DN7976_c0_g1_i4_orf1;TRINITY_DN33893_c0_g1_i1_orf1;TRINITY_DN3618_c0_g1_i4_orf1;TRINITY_DN33883_c0_g1_i1_orf1;TRINITY_DN2802_c1_g1_i1_orf1;TRINITY_DN14391_c1_g1_i2_orf1;TRINITY_DN31225_c0_g1_i1_orf1;TRINITY_DN135_c0_g1_i1_orf1;TRINITY_DN7808_c0_g1_i1_orf1;TRINITY_DN56993_c0_g1_i4_orf1;TRINITY_DN14313_c0_g1_i1_orf1;TRINITY_DN34751_c0_g1_i1_orf1;TRINITY_DN445_c0_g1_i2_orf1;TRINITY_DN6199_c2_g1_i3_orf1;TRINITY_DN18249_c0_g1_i1_orf1;TRINITY_DN1706_c0_g1_i7_orf1;TRINITY_DN4991_c0_g1_i1_orf1;TRINITY_DN23360_c0_g1_i3_orf1;TRINITY_DN31503_c0_g1_i4_orf1;TRINITY_DN17905_c0_g3_i1_orf1;TRINITY_DN45633_c0_g1_i1_orf1;TRINITY_DN18009_c0_g1_i1_orf1;TRINITY_DN108433_c0_g1_i1_orf1;TRINITY_DN18933_c0_g1_i3_orf1;TRINITY_DN146119_c0_g1_i1_orf1;TRINITY_DN23790_c0_g1_i1_orf1;TRINITY_DN2971_c0_g1_i1_orf1;TRINITY_DN2719_c1_g1_i6_orf1;TRINITY_DN97589_c0_g1_i3_orf1;TRINITY_DN5873_c0_g4_i1_orf1;TRINITY_DN9871_c0_g1_i11_orf1;TRINITY_DN90497_c0_g1_i1_orf1;TRINITY_DN7583_c0_g1_i1_orf1;TRINITY_DN18391_c0_g2_i8_orf1;TRINITY_DN7464_c0_g1_i14_orf1;TRINITY_DN50787_c0_g2_i2_orf1;TRINITY_DN41_c0_g1_i3_orf1;TRINITY_DN45037_c0_g1_i1_orf1;TRINITY_DN937_c0_g1_i2_orf1;TRINITY_DN2621_c0_g1_i1_orf1;TRINITY_DN23534_c0_g2_i2_orf1;TRINITY_DN106534_c0_g1_i1_orf1;TRINITY_DN3073_c0_g1_i7_orf1;TRINITY_DN9591_c0_g1_i1_orf1;TRINITY_DN49936_c0_g2_i1_orf1;TRINITY_DN14274_c0_g1_i3_orf1;TRINITY_DN3430_c0_g1_i1_orf1;TRINITY_DN11772_c0_g1_i1_orf1;TRINITY_DN40911_c0_g1_i1_orf1;TRINITY_DN22272_c0_g1_i1_orf1;TRINITY_DN5664_c0_g1_i1_orf1;TRINITY_DN2140_c0_g1_i1_orf1;TRINITY_DN4938_c0_g1_i13_orf1;TRINITY_DN21357_c0_g1_i5_orf1;TRINITY_DN1718_c6_g1_i4_orf1;TRINITY_DN7991_c0_g1_i9_orf1;TRINITY_DN6358_c0_g1_i5_orf1;TRINITY_DN13233_c0_g1_i3_orf1;TRINITY_DN24917_c0_g2_i1_orf1;TRINITY_DN10831_c1_g1_i1_orf1;TRINITY_DN147458_c0_g1_i1_orf1;TRINITY_DN2468_c0_g1_i7_orf1;TRINITY_DN25997_c1_g2_i4_orf1;TRINITY_DN101991_c0_g1_i5_orf1;TRINITY_DN235_c0_g1_i2_orf1;TRINITY_DN2709_c0_g1_i4_orf1;TRINITY_DN291_c0_g1_i2_orf1;TRINITY_DN13371_c0_g1_i4_orf1;TRINITY_DN741_c0_g1_i10_orf1;TRINITY_DN8703_c0_g1_i2_orf1;TRINITY_DN714_c0_g1_i3_orf1;TRINITY_DN30131_c0_g1_i1_orf1;TRINITY_DN15373_c0_g1_i2_orf1;TRINITY_DN51938_c0_g3_i1_orf1;TRINITY_DN6365_c0_g1_i4_orf1;TRINITY_DN37418_c0_g1_i4_orf1;TRINITY_DN114982_c0_g1_i1_orf1;TRINITY_DN46409_c0_g1_i1_orf1;TRINITY_DN89083_c0_g1_i1_orf1;TRINITY_DN40650_c0_g1_i1_orf1;TRINITY_DN24318_c0_g1_i1_orf1;TRINITY_DN31584_c0_g2_i2_orf1;TRINITY_DN102260_c0_g1_i1_orf1;TRINITY_DN3057_c0_g2_i1_orf1;TRINITY_DN19186_c0_g1_i1_orf1;TRINITY_DN1322_c0_g1_i4_orf1;TRINITY_DN1952_c0_g1_i2_orf1;TRINITY_DN72_c0_g1_i16_orf1;TRINITY_DN147691_c0_g1_i1_orf1;TRINITY_DN34830_c0_g1_i1_orf1;TRINITY_DN4016_c0_g1_i1_orf1;TRINITY_DN2186_c0_g1_i13_orf1;TRINITY_DN12771_c0_g1_i1_orf1;TRINITY_DN166_c0_g1_i4_orf1;TRINITY_DN12858_c0_g1_i5_orf1;TRINITY_DN2559_c0_g1_i4_orf1;TRINITY_DN4793_c0_g1_i7_orf1;TRINITY_DN20009_c0_g[illegible]
cellular_componen	non-membrane-bounded organelle	GO:0043228	95	95/2397	TRINITY_DN47731_c0_g1_i2_orf1;TRINITY_DN25960_c0_g1_i1_orf1;TRINITY_DN137_c0_g1_i1_orf1;TRINITY_DN5893_c0_g1_i7_orf1;TRINITY_DN5873_c0_g4_i1_orf1;TRINITY_DN11065_c0_g2_i1_orf1;TRINITY_DN58207_c0_g1_i1_orf1;TRINITY_DN7277_c0_g1_i1_orf1;TRINITY_DN23746_c0_g1_i2_orf1;TRINITY_DN23616_c0_g1_i4_orf1;TRINITY_DN29448_c0_g1_i1_orf1;TRINITY_DN50787_c0_g2_i2_orf1;TRINITY_DN50571_c1_g1_i1_orf1;TRINITY_DN146217_c0_g1_i1_orf1;TRINITY_DN235_c0_g3_i1_orf1;TRINITY_DN31314_c0_g1_i4_orf1;TRINITY_DN135_c0_g1_i1_orf1;TRINITY_DN3985_c0_g2_i1_orf1;TRINITY_DN3618_c0_g1_i4_orf1;TRINITY_DN110231_c0_g1_i1_orf1;TRINITY_DN14391_c1_g1_i2_orf1;TRINITY_DN33883_c0_g1_i1_orf1;TRINITY_DN31225_c0_g1_i1_orf1;TRINITY_DN14313_c0_g1_i1_orf1;TRINITY_DN18249_c0_g1_i1_orf1;TRINITY_DN23360_c0_g1_i3_orf1;TRINITY_DN23790_c0_g1_i1_orf1;TRINITY_DN97589_c0_g1_i3_orf1;TRINITY_DN90497_c0_g1_i1_orf1;TRINITY_DN7583_c0_g1_i1_orf1;TRINITY_DN7464_c0_g1_i14_orf1;TRINITY_DN5531_c0_g3_i3_orf1;TRINITY_DN9591_c0_g1_i1_orf1;TRINITY_DN49936_c0_g2_i1_orf1;TRINITY_DN741_c0_g1_i10_orf1;TRINITY_DN22272_c0_g1_i1_orf1;TRINITY_DN147691_c0_g1_i1_orf1;TRINITY_DN2186_c0_g1_i13_orf1;TRINITY_DN7991_c0_g1_i9_orf1;TRINITY_DN13233_c0_g1_i3_orf1;TRINITY_DN10831_c1_g1_i1_orf1;TRINITY_DN147458_c0_g1_i1_orf1;TRINITY_DN2468_c0_g1_i7_orf1;TRINITY_DN5064_c0_g1_i4_orf1;TRINITY_DN143852_c0_g1_i1_orf1;TRINITY_DN235_c0_g1_i2_orf1;TRINITY_DN6239_c0_g1_i1_orf1;TRINITY_DN714_c0_g1_i3_orf1;TRINITY_DN30131_c0_g1_i1_orf1;TRINITY_DN6365_c0_g1_i4_orf1;TRINITY_DN114982_c0_g1_i1_orf1;TRINITY_DN24318_c0_g1_i1_orf1;TRINITY_DN102260_c0_g1_i1_orf1;TRINITY_DN5031_c0_g1_i1_orf1;TRINITY_DN19186_c0_g1_i1_orf1;TRINITY_DN4016_c0_g1_i1_orf1;TRINITY_DN21357_c0_g1_i5_orf1;TRINITY_DN20009_c0_g1_i1_orf1;TRINITY_DN147676_c0_g1_i1_orf1;TRINITY_DN1509_c0_g1_i1_orf1;TRINITY_DN12576_c0_g1_i2_orf1;TRINITY_DN24322_c0_g1_i4_orf1;TRINITY_DN42506_c0_g1_i1_orf1;TRINITY_DN96557_c0_g1_i1_orf1;TRINITY_DN10234_c0_g1_i1_orf1;TRINITY_DN87603_c0_g2_i1_orf1;TRINITY_DN9146_c0_g1_i1_orf1;TRINITY_DN2186_c0_g1_i17_orf1;TRINITY_DN6248_c0_g1_i1_orf1;TRINITY_DN4429_c0_g1_i5_orf1;TRINITY_DN879_c0_g1_i2_orf1;TRINITY_DN40650_c0_g1_i1_orf1;TRINITY_DN5009_c0_g1_i2_orf1;TRINITY_DN7128_c0_g1_i7_orf1;TRINITY_DN107962_c0_g1_i1_orf1;TRINITY_DN50725_c0_g1_i6_orf1;TRINITY_DN13496_c0_g1_i7_orf1;TRINITY_DN10994_c0_g1_i4_orf1;TRINITY_DN8824_c0_g2_i1_orf1;TRINITY_DN3826_c0_g1_i1_orf1;TRINITY_DN34432_c0_g1_i1_orf1;TRINITY_DN97097_c0_g1_i4_orf1;TRINITY_DN101991_c0_g1_i5_orf1;TRINITY_DN10070_c0_g1_i1_orf1;TRINITY_DN31253_c0_g1_i2_orf1;TRINITY_DN60821_c0_g1_i1_orf1;TRINITY_DN18593_c0_g1_i1_orf1;TRINITY_DN9862_c0_g2_i1_orf1;TRINITY_DN20133_c0_g1_i1_orf1;TRINITY_DN17137_c0_g1_i2_orf1;TRINITY_DN10745_c0_g1_i14_orf1;TRINITY_DN7241_c0_g2_i2_

cellular_componen	membrane-bounded organelle	GO:0043227	150	150/2397	TRINITY_DN44261_c0_g1_i1_orf1;TRINITY_DN12526_c0_g1_i5_orf1;TRINITY_DN13350_c0_g1_i4_orf1;TRINITY_DN6991_c0_g1_i24_orf1;TRINITY_DN11194_c0_g1_i4_orf1;TRINITY_DN7122_c0_g1_i1_orf1;TRINITY_DN1921_c1_g1_i5_orf1;TRINITY_DN5893_c0_g1_i7_orf1;TRINITY_DN35669_c0_g1_i1_orf1;TRINITY_DN18804_c0_g1_i5_orf1;TRINITY_DN110460_c0_g2_i1_orf1;TRINITY_DN110400_c0_g1_i1_orf1;TRINITY_DN2638_c0_g1_i7_orf1;TRINITY_DN111110_c0_g1_i1_orf1;TRINITY_DN50074_c0_g1_i1_orf1;TRINITY_DN2954_c0_g1_i1_orf1;TRINITY_DN3464_c0_g1_i1_orf1;TRINITY_DN21570_c0_g1_i1_orf1;TRINITY_DN6621_c0_g1_i1_orf1;TRINITY_DN41108_c0_g1_i1_orf1;TRINITY_DN51737_c0_g1_i3_orf1;TRINITY_DN99673_c0_g1_i1_orf1;TRINITY_DN15370_c0_g1_i4_orf1;TRINITY_DN15256_c0_g1_i8_orf1;TRINITY_DN31314_c0_g1_i4_orf1;TRINITY_DN2058_c0_g1_i2_orf1;TRINITY_DN4929_c0_g1_i1_orf1;TRINITY_DN30932_c0_g1_i2_orf1;TRINITY_DN3985_c0_g2_i1_orf1;TRINITY_DN2345_c0_g1_i4_orf1;TRINITY_DN5442_c0_g1_i4_orf1;TRINITY_DN7909_c0_g2_i1_orf1;TRINITY_DN7976_c0_g1_i4_orf1;TRINITY_DN33893_c0_g1_i1_orf1;TRINITY_DN2802_c1_g1_i1_orf1;TRINITY_DN7808_c0_g1_i1_orf1;TRINITY_DN56993_c0_g1_i4_orf1;TRINITY_DN34751_c0_g1_i1_orf1;TRINITY_DN6199_c2_g1_i3_orf1;TRINITY_DN1706_c0_g1_i7_orf1;TRINITY_DN4991_c0_g1_i1_orf1;TRINITY_DN23360_c0_g1_i3_orf1;TRINITY_DN17905_c0_g3_i1_orf1;TRINITY_DN45633_c0_g1_i1_orf1;TRINITY_DN18009_c0_g1_i1_orf1;TRINITY_DN108433_c0_g1_i1_orf1;TRINITY_DN18933_c0_g1_i3_orf1;TRINITY_DN146119_c0_g1_i1_orf1;TRINITY_DN2719_c1_g1_i6_orf1;TRINITY_DN7022_c0_g1_i7_orf1;TRINITY_DN9871_c0_g1_i11_orf1;TRINITY_DN18391_c0_g2_i8_orf1;TRINITY_DN50787_c0_g2_i2_orf1;TRINITY_DN41_c0_g1_i3_orf1;TRINITY_DN45037_c0_g1_i1_orf1;TRINITY_DN937_c0_g1_i2_orf1;TRINITY_DN2621_c0_g1_i1_orf1;TRINITY_DN23534_c0_g2_i2_orf1;TRINITY_DN3073_c0_g1_i7_orf1;TRINITY_DN9591_c0_g1_i1_orf1;TRINITY_DN106534_c0_g1_i1_orf1;TRINITY_DN4938_c0_g1_i13_orf1;TRINITY_DN11772_c0_g1_i1_orf1;TRINITY_DN40911_c0_g1_i1_orf1;TRINITY_DN22272_c0_g1_i1_orf1;TRINITY_DN5664_c0_g1_i1_orf1;TRINITY_DN2140_c0_g1_i1_orf1;TRINITY_DN3430_c0_g1_i1_orf1;TRINITY_DN1718_c6_g1_i4_orf1;TRINITY_DN6358_c0_g1_i5_orf1;TRINITY_DN24917_c0_g2_i1_orf1;TRINITY_DN147458_c0_g1_i1_orf1;TRINITY_DN25997_c1_g2_i4_orf1;TRINITY_DN291_c0_g1_i2_orf1;TRINITY_DN13371_c0_g1_i4_orf1;TRINITY_DN8703_c0_g1_i2_orf1;TRINITY_DN714_c0_g1_i3_orf1;TRINITY_DN51938_c0_g3_i1_orf1;TRINITY_DN6365_c0_g1_i4_orf1;TRINITY_DN37418_c0_g1_i4_orf1;TRINITY_DN114982_c0_g1_i1_orf1;TRINITY_DN46409_c0_g1_i1_orf1;TRINITY_DN89083_c0_g1_i1_orf1;TRINITY_DN31584_c0_g2_i2_orf1;TRINITY_DN3057_c0_g2_i1_orf1;TRINITY_DN1322_c0_g1_i4_orf1;TRINITY_DN72_c0_g1_i16_orf1;TRINITY_DN34830_c0_g1_i1_orf1;TRINITY_DN4016_c0_g1_i1_orf1;TRINITY_DN12771_c0_g1_i1_orf1;TRINITY_DN166_c0_g1_i4_orf1;TRINITY_DN12858_c0_g1_i5_orf1;TRINITY_DN2559_c0_g1_i4_orf1;TRINITY_DN4793_c0_g1_i7_orf1;TRINITY_DN20009_c0_g1_i1_orf1;TRINITY_DN41179_c0_g1_i1_orf1;TRINITY_DN14154_c0_g1_i1_orf1;TRINITY_DN41736_c0_g2_i1_orf1;TRINITY_DN5122_c0_g1_i3_orf1;TRINITY_DN53311_c0_g2_i1_orf1;TRINITY_DN63568_c0_g1_i1_orf1;TRINITY_DN44288_c0_g1_i2_orf1;TRINITY_DN27960_c0_g1_i1_orf1;TRINITY_DN38274_c0_g1_i1_orf1;TRINITY_DN19810_c1_g1_i7_orf1;TRINITY_DN3521_c0_g2_i1_orf1;TRINITY_DN62_c1_g1_i3_orf1;TRINITY_DN2802_c0_g1_i1_orf1;TRINITY_DN2647_c0_g1_i3_orf1;TRINITY_DN120144_c0_g1_i1_orf1;TRINITY_DN6248_c0_g1_i1_orf1;TRINITY_DN42854_c0_g3_i2_orf1;TRINITY_DN972_c0_g2_i1_orf1;TRINITY_DN4429_c0_g1_i5_orf1;TRINITY_DN10385_c0_g1_i5_orf1;TRINITY_DN787_c0_g1_i7_orf1;TRINITY_DN2265_c0_g2_i1_orf1;TRINITY_DN2971_c0_g1_i1_orf1;TRINITY_DN1952_c0_g1_i2_orf1;TRINITY_DN67649_c0_g1_i1_orf1;TRINITY_DN28981_c0_g1_i1_orf1;TRINITY_DN38211_c0_g1_i1_orf1;TRINITY_DN12820_c0_g1_i1_orf1;TRINITY_DN13760_c1_g1_i1_orf1;TRINITY_DN15706_c0_g2_i5_orf1;TRINITY_DN95971_c0_g5_i1_orf1;TRINITY_DN13711_c0_g1_i1_orf1;TRINITY_DN104507_c0_g1_i2_orf1;TRINITY_DN3698_c0_g1_i4_orf1;TRINITY_DN44633_c0_g1_i4_orf1;TRINITY_DN1831_c0_g1_i3_orf1;TRINITY_DN279_c0_g1_i10_orf1;TRINITY_DN3614_c0_g2_i1_orf1;TRINITY_DN9510_c0_g2_i1_orf1;TRINITY_DN445_c0_g1_i2_orf1;TRINITY_DN31585_c0_g1_i1_orf1;TRINITY_DN11986_c0_g1_i1_orf1;TRINITY_DN25345_c0_g1_i1_orf1;TRINITY_DN19251_c0_g1_i8_orf1;TRINITY_DN34432_c0_g1_i1_orf1;TRINITY_DN4070_c0_g1_i4_orf1;TRINITY_DN40704_c0_g1_i2_orf1;TRINITY_DN10070_c0_g1_i1_orf1;TRINITY_DN147475_c
cellular_componen	postsynaptic specialization	GO:0099572	1	1/2397	TRINITY_DN4016_c0_g1_i1_orf1
cellular_componen	extracellular organelle	GO:0043230	7	7/2397	TRINITY_DN4016_c0_g1_i1_orf1;TRINITY_DN147475_c0_g1_i1_orf1;TRINITY_DN42854_c0_g3_i2_orf1;TRINITY_DN46409_c0_g1_i1_orf1;TRINITY_DN20133_c0_g1_i1_orf1;TRINITY_DN20009_c0_g1_i1_orf1;TRINITY_DN10070_c0_g1_i1_orf1
cellular_componen	striated muscle thin filament	GO:0005865	1	1/2397	TRINITY_DN235_c0_g3_i1_orf1
cellular_componen	neuronal cell body	GO:0043025	1	1/2397	TRINITY_DN10070_c0_g1_i1_orf1
cellular_componen	organelle membrane	GO:0031090	56	56/2397	TRINITY_DN245_c0_g1_i4_orf1;TRINITY_DN445_c0_g1_i2_orf1;TRINITY_DN9608_c0_g1_i3_orf1;TRINITY_DN2312_c0_g1_i4_orf1;TRINITY_DN3821_c1_g1_i7_orf1;TRINITY_DN46409_c0_g1_i1_orf1;TRINITY_DN72816_c0_g1_i2_orf1;TRINITY_DN14262_c0_g1_i5_orf1;TRINITY_DN31676_c0_g1_i4_orf1;TRINITY_DN56164_c0_g1_i1_orf1;TRINITY_DN5867_c0_g1_i1_orf1;TRINITY_DN135781_c0_g1_i1_orf1;TRINITY_DN1789_c0_g1_i5_orf1;TRINITY_DN1134_c0_g1_i4_orf1;TRINITY_DN1280_c0_g1_i1_orf1;TRINITY_DN11375_c0_g1_i4_orf1;TRINITY_DN37418_c0_g1_i4_orf1;TRINITY_DN5531_c0_g3_i3_orf1;TRINITY_DN1362_c0_g1_i4_orf1;TRINITY_DN5432_c1_g1_i3_orf1;TRINITY_DN4289_c0_g1_i5_orf1;TRINITY_DN11172_c0_g1_i4_orf1;TRINITY_DN5753_c0_g1_i10_orf1;TRINITY_DN642_c0_g1_i6_orf1;TRINITY_DN7336_c0_g1_i13_orf1;TRINITY_DN5697_c0_g1_i1_orf1;TRINITY_DN21570_c0_g1_i1_orf1;TRINITY_DN448_c0_g1_i20_orf1;TRINITY_DN41108_c0_g1_i1_orf1;TRINITY_DN5122_c0_g1_i3_orf1;TRINITY_DN24873_c0_g1_i4_orf1;TRINITY_DN1960_c5_g1_i3_orf1;TRINITY_DN5439_c0_g1_i2_orf1;TRINITY_DN42120_c0_g1_i2_orf1;TRINITY_DN12286_c1_g1_i2_orf1;TRINITY_DN10745_c0_g1_i14_orf1;TRINITY_DN40911_c0_g1_i1_orf1;TRINITY_DN5064_c0_g1_i4_orf1;TRINITY_DN8454_c0_g1_i4_orf1;TRINITY_DN32780_c0_g1_i2_orf1;TRINITY_DN2879_c0_g1_i4_orf1;TRINITY_DN96557_c0_g1_i1_orf1;TRINITY_DN87603_c0_g2_i1_orf1;TRINITY_DN4820_c0_g2_i2_orf1;TRINITY_DN69871_c0_g1_i1_orf1;TRINITY_DN4814_c0_g1_i6_orf1;TRINITY_DN42854_c0_g3_i2_orf1;TRINITY_DN5558_c0_g1_i4_orf1;TRINITY_DN3450_c0_g1_i3_orf1;TRINITY_DN3461_c0_g1_i1_orf1;TRINITY_DN4248_c0_g1_i4_orf1;TRINITY_DN4394_c0_g1_i4_orf1;TRINITY_DN5513_c0_g1_i1_orf1;TRINITY_DN8747_c0_g1_i2_orf1;TRINITY_DN9002_c0_g1_i1_orf1;TRINITY_DN22044_c0_g2_i1_orf1
cellular_componen	outer membrane	GO:0019867	2	2/2397	TRINITY_DN6656_c0_g1_i1_orf1;TRINITY_DN4820_c0_g2_i2_orf1
cellular_componen	plasma membrane	GO:0005886	32	32/2397	TRINITY_DN28759_c0_g1_i1_orf1;TRINITY_DN6974_c0_g2_i1_orf1;TRINITY_DN23926_c0_g1_i4_orf1;TRINITY_DN7570_c0_g1_i18_orf1;TRINITY_DN20710_c0_g1_i2_orf1;TRINITY_DN7128_c0_g1_i7_orf1;TRINITY_DN46409_c0_g1_i1_orf1;TRINITY_DN31584_c0_g2_i2_orf1;TRINITY_DN15706_c0_g2_i5_orf1;TRINITY_DN19951_c0_g1_i5_orf1;TRINITY_DN3962_c0_g1_i6_orf1;TRINITY_DN38431_c0_g1_i1_orf1;TRINITY_DN51938_c0_g3_i1_orf1;TRINITY_DN1352_c0_g1_i5_orf1;TRINITY_DN5553_c0_g1_i4_orf1;TRINITY_DN9475_c0_g1_i6_orf1;TRINITY_DN20009_c0_g1_i1_orf1;TRINITY_DN7590_c0_g1_i4_orf1;TRINITY_DN140613_c0_g1_i1_orf1;TRINITY_DN2175_c0_g1_i4_orf1;TRINITY_DN1012_c0_g1_i2_orf1;TRINITY_DN198_c2_g1_i2_orf1;TRINITY_DN26337_c0_g1_i3_orf1;TRINITY_DN10581_c0_g1_i5_orf1;TRINITY_DN32780_c0_g1_i2_orf1;TRINITY_DN10070_c0_g1_i1_orf1;TRINITY_DN11670_c0_g1_i1_orf1;TRINITY_DN16145_c0_g1_i12_orf1;TRINITY_DN42854_c0_g3_i2_orf1;TRINITY_DN57348_c0_g1_i4_orf1;TRINITY_DN20133_c0
cellular_componen	plasma membrane region	GO:0098590	6	6/2397	TRINITY_DN51938_c0_g3_i1_orf1;TRINITY_DN2257_c0_g1_i4_orf1;TRINITY_DN7128_c0_g1_i7_orf1;TRINITY_DN46409_c0_g1_i1_orf1;TRINITY_DN10745_c0_g1_i14_orf1;TRINITY_DN486_c0_g1_i5_orf1
cellular_componen	phagophore assembly site membrane	GO:0034045	2	2/2397	TRINITY_DN113353_c0_g1_i1_orf1;TRINITY_DN5531_c0_g3_i3_orf1
cellular_componen	dendritic spine	GO:0043197	1	1/2397	TRINITY_DN802_c0_g1_i2_orf1
cellular_componen	extracellular matrix	GO:0031012	3	3/2397	TRINITY_DN3833_c0_g1_i4_orf1;TRINITY_DN10070_c0_g1_i1_orf1;TRINITY_DN376_c1_g1_i1_orf1
cellular_componen	egg chorion	GO:0042600	2	2/2397	TRINITY_DN5933_c0_g1_i1_orf1;TRINITY_DN51252_c0_g2_i1_orf1
cellular_componen	synapse	GO:0045202	4	4/2397	TRINITY_DN17693_c0_g1_i10_orf1;TRINITY_DN82017_c0_g1_i5_orf1;TRINITY_DN10070_c0_g1_i1_orf1;TRINITY_DN4016_c0_g1_i1_orf1
cellular_componen	anchoring junction	GO:0070161	11	11/2397	TRINITY_DN28759_c0_g1_i1_orf1;TRINITY_DN17693_c0_g1_i10_orf1;TRINITY_DN4016_c0_g1_i1_orf1;TRINITY_DN7128_c0_g1_i7_orf1;TRINITY_DN2186_c0_g1_i17_orf1;TRINITY_DN802_c0_g1_i2_orf1;TRINITY_DN741_c0_g1_i10_orf1;TRINITY_DN20009_c0_g1_i1_orf1;TRINITY_DN7590_c0_g1_i4_orf1;TRINITY_DN2186_c0_g1_i13_orf1;TRINITY_DN23746_c0_g1_i2_orf1
cellular_componen	Golgi apparatus subcompartment	GO:0098791	2	2/2397	TRINITY_DN4401_c0_g2_i1_orf1;TRINITY_DN7976_c0_g1_i4_orf1
cellular_componen	plasma membrane bounded cell projection	GO:0120025	5	5/2397	TRINITY_DN20009_c0_g1_i1_orf1;TRINITY_DN802_c0_g1_i2_orf1;TRINITY_DN741_c0_g1_i10_orf1;TRINITY_DN4016_c0_g1_i1_orf1;TRINITY_DN5954_c0_g1_i2_orf1
cellular_componen	centrosome	GO:0005813	1	1/2397	TRINITY_DN4689_c0_g1_i5_orf1
cellular_componen	kinetochore	GO:0000776	2	2/2397	TRINITY_DN96557_c0_g1_i1_orf1;TRINITY_DN31314_c0_g1_i4_orf1
cellular_componen	ribonucleoprotein granule	GO:0035770	3	3/2397	TRINITY_DN4016_c0_g1_i1_orf1;TRINITY_DN50725_c0_g1_i6_orf1;TRINITY_DN12576_c0_g1_i2_orf1
cellular_componen	supramolecular polymer	GO:0099081	17	17/2397	TRINITY_DN17995_c0_g4_i1_orf1;TRINITY_DN280_c0_g1_i8_orf1;TRINITY_DN4808_c0_g1_i3_orf1;TRINITY_DN14298_c0_g1_i1_orf1;TRINITY_DN350_c0_g1_i10_orf1;TRINITY_DN96557_c0_g1_i1_orf1;TRINITY_DN8390_c0_g1_i2_orf1;TRINITY_DN28018_c0_g6_i1_orf1;TRINITY_DN34703_c0_g1_i4_orf1;TRINITY_DN235_c0_g3_i1_orf1;TRINITY_DN2745_c0_g1_i2_orf1;TRINITY_DN97138_c0_g1_i2_orf1;TRINITY_DN14298_c0_g3_i1_orf1;TRINITY_DN20009_c0_g1_i1_orf1;TRINITY_DN5893_c0_g1_i7_orf1;TRINITY_DN14298_c0_g1_i3_orf1;TRINITY_DN350_c0_g1_i5_orf1
molecular_functior	mRNA regulatory element binding translation repressor activity	GO:0000900	1	1/2397	TRINITY_DN3673_c0_g1_i10_orf1
molecular_functior	translation factor activity, RNA binding	GO:0008135	21	21/2397	TRINITY_DN136906_c0_g1_i1_orf1;TRINITY_DN14498_c0_g1_i1_orf1;TRINITY_DN19659_c1_g1_i1_orf1;TRINITY_DN2265_c0_g2_i1_orf1;TRINITY_DN1771_c0_g2_i1_orf1;TRINITY_DN126648_c0_g1_i1_orf1;TRINITY_DN28039_c0_g1_i1_orf1;TRINITY_DN6239_c0_g1_i1_orf1;TRINITY_DN31503_c0_g1_i4_orf1;TRINITY_DN53311_c0_g2_i1_orf1;TRINITY_DN15420_c0_g3_i2_orf1;TRINITY_DN1074_c0_g1_i7_orf1;TRINITY_DN38412_c0_g1_i1_orf1;TRINITY_DN11823_c1_g1_i2_orf1;TRINITY_DN29521_c0_g1_i1_orf1;TRINITY_DN11612_c0_g2_i1_orf1;TRINITY_DN107708_c0_g1_i1_orf1;TRINITY_DN48097_c0_g1_i1_orf1;TRINITY_DN36817_c0_g1_i1_orf1;TRINITY_DN21000_c0_g1_i1_orf1;TRINITY_
molecular_functior	transcription corepressor activity	GO:0003714	2	2/2397	TRINITY_DN34726_c0_g2_i1_orf1;TRINITY_DN1921_c1_g1_i5_orf1
molecular_functior	transcription coactivator activity	GO:0003713	1	1/2397	TRINITY_DN9510_c0_g2_i1_orf1
molecular_functior	DNA-binding transcription factor activity, RNA polymerase II-specific	GO:0000981	1	1/2397	TRINITY_DN1926_c0_g1_i5_orf1
molecular_functior	DNA-binding transcription repressor activity	GO:0001217	1	1/2397	TRINITY_DN1926_c0_g1_i5_orf1
molecular_functior	RNA helicase activity	GO:0003724	7	7/2397	TRINITY_DN31503_c0_g1_i4_orf1;TRINITY_DN4408_c6_g1_i1_orf1;TRINITY_DN4950_c0_g1_i2_orf1;TRINITY_DN2904_c0_g1_i4_orf1;TRINITY_DN14274_c0_g1_i3_orf1;TRINITY_DN44288_c0_g1_i2_orf1;TRINITY_DN2709_c0_g1_i4_orf1

molecular_functior	DNA helicase activity	GO:0003678	9	9/2397	TRINITY_DN2971_c0_g1_i1_orf1;TRINITY_DN25345_c0_g1_i1_orf1;TRINITY_DN11986_c0_g1_i1_orf1;TRINITY_DN6642_c0_g1_i2_orf1;TRINITY_DN15370_c0_g1_i4_orf1;TRINITY_DN7122_c0_g1_i1_orf1;TRINITY_DN3057_c0_g2_i1_orf1;TRINITY_DN5757_c0_g1_i1_orf1;TRINITY_DN291_c0_g1_i2_orf1
molecular_functior	ATP-dependent chromatin remodeler activity	GO:0140658	5	5/2397	TRINITY_DN3057_c0_g2_i1_orf1;TRINITY_DN45449_c0_g1_i1_orf1;TRINITY_DN25345_c0_g1_i1_orf1;TRINITY_DN63568_c0_g1_i1_orf1;TRINITY_DN12820_c0_g1_i1_orf1
molecular_functior	DNA topoisomerase type II (double strand cut, ATP-hydrolyzing)	GO:0003918	1	1/2397	TRINITY_DN4908_c1_g1_i5_orf1
molecular_functior	long-chain fatty acid-CoA ligase activity	GO:0004467	1	1/2397	TRINITY_DN2193_c0_g1_i7_orf1
molecular_functior	ABC-type transporter activity	GO:0140359	6	6/2397	TRINITY_DN13563_c0_g1_i1_orf1;TRINITY_DN2874_c0_g1_i4_orf1;TRINITY_DN162_c0_g1_i4_orf1;TRINITY_DN60792_c0_g1_i2_orf1;TRINITY_DN2826_c0_g1_i7_orf1;TRINITY_DN2706_c0_g1_i3_orf1
molecular_functior	P-type transmembrane transporter activity	GO:0140358	2	2/2397	TRINITY_DN7336_c0_g1_i13_orf1;TRINITY_DN7570_c0_g1_i18_orf1
molecular_functior	ATPase-coupled cation transmembrane transporter activity	GO:0019829	3	3/2397	TRINITY_DN7336_c0_g1_i13_orf1;TRINITY_DN25975_c0_g3_i2_orf1;TRINITY_DN7570_c0_g1_i18_orf1
molecular_functior	ATPase-coupled ion transmembrane transporter activity	GO:0042625	1	1/2397	TRINITY_DN25975_c0_g3_i2_orf1
molecular_functior	cytoskeletal anchor activity	GO:0008093	1	1/2397	TRINITY_DN21559_c0_g2_i1_orf1
molecular_functior	SNAP receptor activity	GO:0005484	2	2/2397	TRINITY_DN5558_c0_g1_i4_orf1;TRINITY_DN132857_c0_g1_i1_orf1
molecular_functior	very-low-density lipoprotein particle receptor activity	GO:0030229	2	2/2397	TRINITY_DN585_c0_g1_i5_orf1;TRINITY_DN585_c0_g1_i12_orf1
molecular_functior	nuclear export signal receptor activity	GO:0005049	2	2/2397	TRINITY_DN3747_c1_g2_i1_orf1;TRINITY_DN95971_c0_g5_i1_orf1
molecular_functior	copper chaperone activity	GO:0016531	1	1/2397	TRINITY_DN3461_c0_g1_i1_orf1
molecular_functior	ATP-dependent FeS chaperone activity	GO:0140663	1	1/2397	TRINITY_DN85476_c0_g1_i1_orf1
molecular_functior	peroxiredoxin activity	GO:0051920	3	3/2397	TRINITY_DN2542_c0_g2_i1_orf1;TRINITY_DN7778_c0_g1_i1_orf1;TRINITY_DN69236_c0_g1_i1_orf1
molecular_functior	catalase activity	GO:0004096	2	2/2397	TRINITY_DN54387_c0_g1_i1_orf1;TRINITY_DN285_c0_g1_i4_orf1
molecular_functior	glutathione peroxidase activity	GO:0004602	2	2/2397	TRINITY_DN1622_c0_g1_i6_orf1;TRINITY_DN21420_c0_g1_i2_orf1
molecular_functior	phospholipid transporter activity	GO:0005548	1	1/2397	TRINITY_DN2160_c0_g1_i13_orf1
molecular_functior	intramembrane lipid transporter activity	GO:0140303	1	1/2397	TRINITY_DN252_c0_g1_i3_orf1
molecular_functior	sulfur compound transmembrane transporter activity	GO:1901682	1	1/2397	TRINITY_DN268_c1_g1_i7_orf1
molecular_functior	carbohydrate transmembrane transporter activity	GO:0015144	2	2/2397	TRINITY_DN57348_c0_g1_i4_orf1;TRINITY_DN10581_c0_g1_i5_orf1
molecular_functior	passive transmembrane transporter activity	GO:0022803	10	10/2397	TRINITY_DN11566_c0_g1_i6_orf1;TRINITY_DN6974_c0_g2_i1_orf1;TRINITY_DN18338_c0_g1_i6_orf1;TRINITY_DN34821_c0_g1_i4_orf1;TRINITY_DN20558_c0_g1_i2_orf1;TRINITY_DN96080_c0_g2_i1_orf1;TRINITY_DN10290_c0_g1_i7_orf1;TRINITY_DN957_c0_g1_i18_orf1;TRINITY_DN7787_c0_g1_i1_orf1;TRINITY_DN5753_c0_g1_i10_orf1
molecular_functior	active transmembrane transporter activity	GO:0022804	13	13/2397	TRINITY_DN7336_c0_g1_i13_orf1;TRINITY_DN25975_c0_g3_i2_orf1;TRINITY_DN13563_c0_g1_i1_orf1;TRINITY_DN268_c1_g1_i7_orf1;TRINITY_DN2874_c0_g1_i4_orf1;TRINITY_DN7570_c0_g1_i18_orf1;TRINITY_DN12666_c0_g1_i2_orf1;TRINITY_DN26186_c0_g1_i7_orf1;TRINITY_DN29_c0_g1_i4_orf1;TRINITY_DN60792_c0_g1_i2_orf1;TRINITY_DN162_c0_g1_i4_orf1;TRINITY_DN2826_c0_g1_i7_orf1;TRINITY_DN2706_c0_g1_i3_orf1
molecular_functior	ion transmembrane transporter activity	GO:0015075	17	17/2397	TRINITY_DN7336_c0_g1_i13_orf1;TRINITY_DN15222_c0_g1_i4_orf1;TRINITY_DN6974_c0_g2_i1_orf1;TRINITY_DN268_c1_g1_i7_orf1;TRINITY_DN34821_c0_g1_i4_orf1;TRINITY_DN9354_c0_g1_i7_orf1;TRINITY_DN20558_c0_g1_i2_orf1;TRINITY_DN7570_c0_g1_i18_orf1;TRINITY_DN96080_c0_g2_i1_orf1;TRINITY_DN25975_c0_g3_i2_orf1;TRINITY_DN9554_c0_g1_i1_orf1;TRINITY_DN12286_c1_g1_i2_orf1;TRINITY_DN957_c0_g1_i18_orf1;TRINITY_DN7787_c0_g1_i1_orf1;TRINITY_DN141353_c0_g1_i1_orf1;TRINITY_DN5753_c0_g1_i10_orf1;TRINITY_DN91946_c0_g1_i1_orf1
molecular_functior	inorganic molecular entity transmembrane transporter activity	GO:0015318	15	15/2397	TRINITY_DN7336_c0_g1_i13_orf1;TRINITY_DN15222_c0_g1_i4_orf1;TRINITY_DN6974_c0_g2_i1_orf1;TRINITY_DN268_c1_g1_i7_orf1;TRINITY_DN34821_c0_g1_i4_orf1;TRINITY_DN9354_c0_g1_i7_orf1;TRINITY_DN20558_c0_g1_i2_orf1;TRINITY_DN7570_c0_g1_i18_orf1;TRINITY_DN141353_c0_g1_i1_orf1;TRINITY_DN25975_c0_g3_i2_orf1;TRINITY_DN96080_c0_g2_i1_orf1;TRINITY_DN957_c0_g1_i18_orf1;TRINITY_DN7787_c0_g1_i1_orf1;TRINITY_DN5753_c0_g1_i10_orf1;TRINITY_DN91946_c0_g1_i1_orf1
molecular_functior	channel inhibitor activity	GO:0016248	2	2/2397	TRINITY_DN6098_c1_g1_i5_orf1;TRINITY_DN4748_c0_g1_i5_orf1
molecular_functior	ATPase inhibitor activity	GO:0042030	1	1/2397	TRINITY_DN5442_c0_g1_i4_orf1
molecular_functior	ion channel regulator activity	GO:0099106	4	4/2397	TRINITY_DN6098_c1_g1_i5_orf1;TRINITY_DN4748_c0_g1_i5_orf1;TRINITY_DN31584_c0_g2_i2_orf1;TRINITY_DN10994_c0_g1_i4_orf1
molecular_functior	kinase regulator activity	GO:0019207	2	2/2397	TRINITY_DN12_c0_g1_i5_orf1;TRINITY_DN147475_c0_g1_i1_orf1
molecular_functior	phosphatase regulator activity	GO:0019208	1	1/2397	TRINITY_DN13999_c0_g1_i4_orf1
molecular_functior	nucleoside-triphosphatase regulator activity	GO:0060589	18	18/2397	TRINITY_DN1173_c0_g1_i12_orf1;TRINITY_DN14154_c0_g1_i1_orf1;TRINITY_DN138086_c0_g1_i1_orf1;TRINITY_DN42461_c0_g1_i4_orf1;TRINITY_DN1173_c0_g1_i11_orf1;TRINITY_DN518_c0_g1_i1_orf1;TRINITY_DN1173_c1_g1_i10_orf1;TRINITY_DN9248_c0_g1_i10_orf1;TRINITY_DN23354_c0_g1_i7_orf1;TRINITY_DN2623_c0_g1_i3_orf1;TRINITY_DN4410_c0_g1_i1_orf1;TRINITY_DN27021_c0_g1_i1_orf1;TRINITY_DN27491_c0_g1_i1_orf1;TRINITY_DN5182_c0_g1_i5_orf1;TRINITY_DN2596_c0_g1_i6_orf1;TRINITY_DN804_c0_g1_i7_orf1;TRINITY_DN802_c0_g1_i2_orf1;TRINITY_DN20614_c0_g1_i1_orf1
molecular_functior	peptidase regulator activity	GO:0061134	30	30/2397	TRINITY_DN4314_c0_g1_i9_orf1;TRINITY_DN8258_c0_g1_i3_orf1;TRINITY_DN7341_c0_g1_i8_orf1;TRINITY_DN9872_c0_g1_i2_orf1;TRINITY_DN3055_c0_g1_i9_orf1;TRINITY_DN1986_c0_g1_i1_orf1;TRINITY_DN501_c0_g1_i5_orf1;TRINITY_DN42854_c0_g3_i2_orf1;TRINITY_DN9732_c0_g1_i7_orf1;TRINITY_DN18196_c0_g1_i4_orf1;TRINITY_DN1328_c0_g1_i6_orf1;TRINITY_DN10994_c0_g1_i4_orf1;TRINITY_DN8258_c0_g1_i6_orf1;TRINITY_DN77425_c0_g1_i2_orf1;TRINITY_DN3609_c0_g1_i6_orf1;TRINITY_DN45948_c1_g1_i1_orf1;TRINITY_DN399_c3_g2_i6_orf1;TRINITY_DN2097_c1_g2_i2_orf1;TRINITY_DN71308_c0_g1_i4_orf1;TRINITY_DN1540_c0_g1_i7_orf1;TRINITY_DN2323_c0_g1_i4_orf1;TRINITY_DN590_c0_g1_i4_orf1;TRINITY_DN1444_c1_g1_i5_orf1;TRINITY_DN1215_c0_g1_i2_orf1;TRINITY_DN7539_c0_g1_i2_orf1;TRINITY_DN10057_c0_g2_i1_orf1;TRINITY_DN69697_c0_g1_i1_orf1;TRINITY_DN135188_c0_g1_i2_orf1;TRINITY_DN9455_c0_g1_i6_orf1;TRINITY_DN712_c0_g2_i1_orf1
molecular_functior	enzyme activator activity	GO:0008047	12	12/2397	TRINITY_DN14154_c0_g1_i1_orf1;TRINITY_DN67649_c0_g1_i1_orf1;TRINITY_DN138086_c0_g1_i1_orf1;TRINITY_DN4410_c0_g1_i1_orf1;TRINITY_DN518_c0_g1_i1_orf1;TRINITY_DN7341_c0_g1_i8_orf1;TRINITY_DN9248_c0_g1_i10_orf1;TRINITY_DN23354_c0_g1_i7_orf1;TRINITY_DN27021_c0_g1_i1_orf1;TRINITY_DN5182_c0_g1_i5_orf1;TRINITY_DN46022_c0_g1_i1_orf1;TRINITY_DN802_c0_g1_i2_orf1
molecular_functior	enzyme inhibitor activity	GO:0004857	30	30/2397	TRINITY_DN13999_c0_g1_i4_orf1;TRINITY_DN4314_c0_g1_i9_orf1;TRINITY_DN8258_c0_g1_i3_orf1;TRINITY_DN9872_c0_g1_i2_orf1;TRINITY_DN3055_c0_g1_i9_orf1;TRINITY_DN1986_c0_g1_i1_orf1;TRINITY_DN501_c0_g1_i5_orf1;TRINITY_DN42854_c0_g3_i2_orf1;TRINITY_DN9732_c0_g1_i7_orf1;TRINITY_DN18196_c0_g1_i4_orf1;TRINITY_DN1328_c0_g1_i6_orf1;TRINITY_DN10994_c0_g1_i4_orf1;TRINITY_DN8258_c0_g1_i6_orf1;TRINITY_DN77425_c0_g1_i2_orf1;TRINITY_DN3609_c0_g1_i6_orf1;TRINITY_DN45948_c1_g1_i1_orf1;TRINITY_DN399_c3_g2_i6_orf1;TRINITY_DN2097_c1_g2_i2_orf1;TRINITY_DN71308_c0_g1_i4_orf1;TRINITY_DN1540_c0_g1_i7_orf1;TRINITY_DN2323_c0_g1_i4_orf1;TRINITY_DN590_c0_g1_i4_orf1;TRINITY_DN1444_c1_g1_i5_orf1;TRINITY_DN1215_c0_g1_i2_orf1;TRINITY_DN7539_c0_g1_i2_orf1;TRINITY_DN10057_c0_g2_i1_orf1;TRINITY_DN69697_c0_g1_i1_orf1;TRINITY_DN135188_c0_g1_i2_orf1;TRINITY_DN9455_c0_g1_i6_orf1;TRINITY_DN712_c0_g2_i1_orf1
molecular_functior	signaling receptor activator activity	GO:0030546	4	4/2397	TRINITY_DN22443_c0_g2_i3_orf1;TRINITY_DN18650_c0_g1_i1_orf1;TRINITY_DN18218_c0_g1_i7_orf1;TRINITY_DN2227_c0_g1_i5_orf1
molecular_functior	signaling receptor inhibitor activity	GO:0030547	1	1/2397	TRINITY_DN108433_c0_g1_i1_orf1

molecular_functior	nucleic acid binding	GO:0003676	165	165/2397	TRINITY_DN129226_c0_g1_i2_orf1;TRINITY_DN17271_c0_g1_i1_orf1;TRINITY_DN6380_c0_g1_i1_orf1;TRINITY_DN110400_c0_g1_i1_orf1;TRINITY_DN3301_c0_g1_i2_orf1;TRINITY_DN40434_c0_g1_i2_orf1;TRINITY_DN13350_c0_g1_i4_orf1;TRINITY_DN137_c0_g1_i1_orf1;TRINITY_DN38412_c0_g1_i1_orf1;TRINITY_DN4262_c0_g1_i16_orf1;TRINITY_DN7122_c0_g1_i1_orf1;TRINITY_DN35669_c0_g1_i1_orf1;TRINITY_DN19651_c0_g1_i1_orf1;TRINITY_DN18804_c0_g1_i5_orf1;TRINITY_DN7583_c0_g1_i1_orf1;TRINITY_DN34134_c0_g2_i1_orf1;TRINITY_DN1344_c0_g1_i1_orf1;TRINITY_DN2904_c0_g1_i4_orf1;TRINITY_DN70485_c0_g1_i2_orf1;TRINITY_DN24689_c0_g1_i1_orf1;TRINITY_DN29521_c0_g1_i1_orf1;TRINITY_DN15160_c0_g1_i1_orf1;TRINITY_DN36817_c0_g1_i1_orf1;TRINITY_DN1710_c0_g2_i2_orf1;TRINITY_DN21570_c0_g1_i1_orf1;TRINITY_DN10070_c0_g1_i1_orf1;TRINITY_DN19746_c0_g1_i5_orf1;TRINITY_DN2682_c0_g1_i4_orf1;TRINITY_DN99673_c0_g1_i1_orf1;TRINITY_DN15370_c0_g1_i4_orf1;TRINITY_DN2676_c0_g1_i2_orf1;TRINITY_DN4929_c0_g1_i1_orf1;TRINITY_DN817_c0_g1_i3_orf1;TRINITY_DN19659_c1_g1_i1_orf1;TRINITY_DN3985_c0_g2_i1_orf1;TRINITY_DN35582_c0_g1_i1_orf1;TRINITY_DN5442_c0_g1_i4_orf1;TRINITY_DN227_c0_g1_i1_orf1;TRINITY_DN33893_c0_g1_i1_orf1;TRINITY_DN251_c0_g1_i2_orf1;TRINITY_DN4820_c0_g2_i2_orf1;TRINITY_DN2802_c1_g1_i1_orf1;TRINITY_DN1245_c0_g1_i4_orf1;TRINITY_DN18563_c2_g1_i1_orf1;TRINITY_DN89613_c0_g1_i13_orf1;TRINITY_DN14313_c0_g1_i1_orf1;TRINITY_DN2647_c0_g1_i3_orf1;TRINITY_DN1771_c0_g2_i1_orf1;TRINITY_DN20442_c0_g2_i1_orf1;TRINITY_DN23360_c0_g1_i3_orf1;TRINITY_DN31503_c0_g1_i4_orf1;TRINITY_DN47723_c0_g1_i1_orf1;TRINITY_DN1074_c0_g1_i7_orf1;TRINITY_DN3712_c0_g1_i1_orf1;TRINITY_DN37532_c0_g1_i1_orf1;TRINITY_DN12242_c0_g1_i5_orf1;TRINITY_DN19687_c0_g1_i1_orf1;TRINITY_DN6248_c0_g1_i1_orf1;TRINITY_DN1870_c0_g1_i6_orf1;TRINITY_DN81258_c0_g1_i2_orf1;TRINITY_DN298_c0_g1_i4_orf1;TRINITY_DN16965_c0_g2_i1_orf1;TRINITY_DN3673_c0_g1_i10_orf1;TRINITY_DN1607_c0_g1_i16_orf1;TRINITY_DN7464_c0_g1_i14_orf1;TRINITY_DN810_c0_g1_i4_orf1;TRINITY_DN50787_c0_g2_i2_orf1;TRINITY_DN16978_c0_g1_i1_orf1;TRINITY_DN48641_c0_g1_i4_orf1;TRINITY_DN14498_c0_g1_i1_orf1;TRINITY_DN12527_c0_g1_i4_orf1;TRINITY_DN49936_c0_g2_i1_orf1;TRINITY_DN14274_c0_g1_i3_orf1;TRINITY_DN28039_c0_g1_i1_orf1;TRINITY_DN4408_c6_g1_i1_orf1;TRINITY_DN11823_c1_g1_i2_orf1;TRINITY_DN36494_c0_g1_i1_orf1;TRINITY_DN73224_c0_g4_i2_orf1;TRINITY_DN56993_c0_g1_i4_orf1;TRINITY_DN6358_c0_g1_i5_orf1;TRINITY_DN8458_c0_g2_i1_orf1;TRINITY_DN4950_c0_g1_i2_orf1;TRINITY_DN24917_c0_g2_i1_orf1;TRINITY_DN3459_c0_g1_i4_orf1;TRINITY_DN107035_c0_g1_i1_orf1;TRINITY_DN147458_c0_g1_i1_orf1;TRINITY_DN4908_c1_g1_i5_orf1;TRINITY_DN2299_c0_g1_i3_orf1;TRINITY_DN4429_c0_g1_i5_orf1;TRINITY_DN2709_c0_g1_i4_orf1;TRINITY_DN291_c0_g1_i2_orf1;TRINITY_DN21000_c0_g1_i1_orf1;TRINITY_DN8008_c0_g1_i6_orf1;TRINITY_DN46409_c0_g1_i1_orf1;TRINITY_DN7251_c0_g1_i3_orf1;TRINITY_DN53233_c0_g1_i1_orf1;TRINITY_DN51934_c0_g2_i1_orf1;TRINITY_DN2718_c0_g1_i6_orf1;TRINITY_DN3057_c0_g2_i1_orf1;TRINITY_DN15362_c0_g1_i1_orf1;TRINITY_DN136906_c0_g1_i1_orf1;TRINITY_DN5757_c0_g1_i1_orf1;TRINITY_DN107708_c0_g1_i1_orf1;TRINITY_DN4016_c0_g1_i1_orf1;TRINITY_DN12858_c0_g1_i5_orf1;TRINITY_DN2879_c0_g1_i4_orf1;TRINITY_DN20009_c0_g1_i1_orf1;TRINITY_DN2749_c0_g2_i3_orf1;TRINITY_DN24322_c0_g1_i4_orf1;TRINITY_DN11986_c0_g1_i1_orf1;TRINITY_DN53311_c0_g2_i1_orf1;TRINITY_DN15420_c0_g3_i2_orf1;TRINITY_DN63568_c0_g1_i1_orf1;TRINITY_DN44288_c0_g1_i2_orf1;TRINITY_DN19810_c1_g1_i7_orf1;TRINITY_DN42506_c0_g1_i1_orf1;TRINITY_DN10234_c0_g1_i1_orf1;TRINITY_DN87603_c0_g2_i1_orf1;TRINITY_DN2802_c0_g1_i1_orf1;TRINITY_DN36496_c0_g1_i1_orf1;TRINITY_DN74037_c0_g5_i1_orf1;TRINITY_DN14904_c1_g2_i2_orf1;TRINITY_DN42854_c0_g3_i2_orf1;TRINITY_DN972_c0_g2_i1_orf1;TRINITY_DN103_c0_g1_i1_orf1;TRINITY_DN116467_c0_g1_i1_orf1;TRINITY_DN879_c0_g1_i2_orf1;TRINITY_DN139537_c0_g1_i1_orf1;TRINITY_DN2265_c0_g2_i1_orf1;TRINITY_DN8224_c0_g1_i7_orf1;TRINITY_DN67649_c0_g1_i1_orf1;TRINITY_DN28981_c0_g1_i1_orf1;TRINITY_DN36893_c0_g1_i1_orf1;TRINITY_DN42205_c0_g1_i4_orf1;TRINITY_DN9637_c0_g1_i14_orf1;TRINITY_DN288_c0_g1_i9_orf1;TRINITY_DN48097_c0_g1_i1_orf1;TRINITY_DN104507_c0_g1_i2_orf1;TRINITY_DN38540_c0_g1_i1_orf1;TRINITY_DN3521_c0_g2_i1_orf1;TRINITY_DN271_c0_g2_i6_orf1;TRINITY_DN13496_c0_g1_i7_orf1;TRINITY_DN15234_c0_g1_i3_orf1;TRINITY_DN9510_c0_g2_i1_orf1;TRINITY_DN23004_c0_g1_i1_orf1;TRINITY_DN129226_c0_g4_i1_orf1;TRINITY_DN7112_c0_g1_i1_orf1;TRINITY_DN5238_c0_g1_i2_orf1;TRINITY_DN21596_c0_g1_i1_orf1;TRINITY_DN34432_c0_g1_i1_orf1;TRINITY_DN5653_c0_g1_i4_orf1;TRINITY_DN11612_c0_g2_i1_orf1;TRINITY_DN110534_c0_g1_i3_orf1;TRINITY_DN42646_c
molecular_functior	nucleoside phosphate binding	GO:1901265	191	191/2397	TRINITY_DN98313_c0_g1_i1_orf1;TRINITY_DN280_c0_g1_i8_orf1;TRINITY_DN13563_c0_g1_i1_orf1;TRINITY_DN38230_c0_g1_i4_orf1;TRINITY_DN47731_c0_g1_i2_orf1;TRINITY_DN97138_c0_g1_i2_orf1;TRINITY_DN41311_c0_g2_i3_orf1;TRINITY_DN11194_c0_g1_i4_orf1;TRINITY_DN7122_c0_g1_i1_orf1;TRINITY_DN1921_c1_g1_i5_orf1;TRINITY_DN817_c0_g1_i3_orf1;TRINITY_DN4744_c0_g1_i7_orf1;TRINITY_DN25341_c0_g1_i1_orf1;TRINITY_DN2904_c0_g1_i4_orf1;TRINITY_DN70485_c0_g1_i2_orf1;TRINITY_DN2983_c0_g1_i6_orf1;TRINITY_DN24723_c2_g1_i1_orf1;TRINITY_DN2953_c1_g1_i10_orf1;TRINITY_DN143509_c0_g1_i1_orf1;TRINITY_DN2638_c0_g1_i7_orf1;TRINITY_DN4320_c0_g1_i1_orf1;TRINITY_DN810_c0_g1_i4_orf1;TRINITY_DN10774_c0_g2_i3_orf1;TRINITY_DN2953_c1_g1_i2_orf1;TRINITY_DN14298_c0_g1_i1_orf1;TRINITY_DN3991_c0_g1_i6_orf1;TRINITY_DN3800_c0_g1_i7_orf1;TRINITY_DN15370_c0_g1_i4_orf1;TRINITY_DN235_c0_g3_i1_orf1;TRINITY_DN6185_c0_g1_i12_orf1;TRINITY_DN52761_c0_g1_i2_orf1;TRINITY_DN52244_c1_g1_i1_orf1;TRINITY_DN6436_c0_g1_i1_orf1;TRINITY_DN2224_c0_g1_i1_orf1;TRINITY_DN7909_c0_g2_i1_orf1;TRINITY_DN987_c0_g1_i3_orf1;TRINITY_DN26961_c0_g1_i1_orf1;TRINITY_DN3131_c0_g1_i5_orf1;TRINITY_DN7247_c0_g1_i7_orf1;TRINITY_DN4501_c0_g1_i3_orf1;TRINITY_DN31225_c0_g1_i1_orf1;TRINITY_DN107288_c0_g1_i2_orf1;TRINITY_DN11620_c0_g1_i2_orf1;TRINITY_DN20007_c0_g1_i1_orf1;TRINITY_DN4950_c0_g1_i2_orf1;TRINITY_DN52761_c0_g2_i1_orf1;TRINITY_DN34751_c0_g1_i1_orf1;TRINITY_DN7336_c0_g1_i13_orf1;TRINITY_DN2772_c0_g1_i3_orf1;TRINITY_DN115210_c0_g4_i1_orf1;TRINITY_DN31503_c0_g1_i4_orf1;TRINITY_DN64126_c0_g1_i1_orf1;TRINITY_DN252_c0_g1_i3_orf1;TRINITY_DN2719_c1_g1_i6_orf1;TRINITY_DN8659_c0_g2_i1_orf1;TRINITY_DN17935_c0_g1_i1_orf1;TRINITY_DN4451_c0_g2_i4_orf1;TRINITY_DN1604_c0_g1_i4_orf1;TRINITY_DN1034_c0_g1_i4_orf1;TRINITY_DN90497_c0_g1_i1_orf1;TRINITY_DN42461_c0_g1_i4_orf1;TRINITY_DN1607_c0_g1_i16_orf1;TRINITY_DN277_c1_g1_i1_orf1;TRINITY_DN268_c3_g1_i2_orf1;TRINITY_DN31967_c0_g1_i5_orf1;TRINITY_DN28622_c0_g1_i1_orf1;TRINITY_DN12_c0_g1_i5_orf1;TRINITY_DN2621_c0_g1_i1_orf1;TRINITY_DN32700_c0_g1_i2_orf1;TRINITY_DN117844_c0_g1_i1_orf1;TRINITY_DN12301_c0_g1_i1_orf1;TRINITY_DN9794_c0_g2_i8_orf1;TRINITY_DN6642_c0_g1_i2_orf1;TRINITY_DN14274_c0_g1_i3_orf1;TRINITY_DN28039_c0_g1_i1_orf1;TRINITY_DN2826_c0_g1_i7_orf1;TRINITY_DN42185_c0_g1_i7_orf1;TRINITY_DN511_c0_g2_i1_orf1;TRINITY_DN1954_c0_g1_i4_orf1;TRINITY_DN2706_c0_g1_i3_orf1;TRINITY_DN5811_c0_g1_i4_orf1;TRINITY_DN1173_c1_g1_i10_orf1;TRINITY_DN119893_c0_g2_i3_orf1;TRINITY_DN4908_c1_g1_i5_orf1;TRINITY_DN25997_c1_g2_i4_orf1;TRINITY_DN47151_c0_g1_i1_orf1;TRINITY_DN5029_c0_g1_i1_orf1;TRINITY_DN4501_c0_g2_i1_orf1;TRINITY_DN291_c0_g1_i2_orf1;TRINITY_DN21000_c0_g1_i1_orf1;TRINITY_DN1173_c0_g1_i12_orf1;TRINITY_DN16011_c0_g1_i3_orf1;TRINITY_DN8659_c0_g1_i1_orf1;TRINITY_DN28221_c0_g2_i1_orf1;TRINITY_DN7570_c0_g1_i18_orf1;TRINITY_DN376_c0_g1_i1_orf1;TRINITY_DN46409_c0_g1_i1_orf1;TRINITY_DN2265_c0_g2_i1_orf1;TRINITY_DN3057_c0_g2_i1_orf1;TRINITY_DN2770_c0_g2_i4_orf1;TRINITY_DN5281_c0_g2_i3_orf1;TRINITY_DN136906_c0_g1_i1_orf1;TRINITY_DN1475_c0_g1_i6_orf1;TRINITY_DN141381_c0_g1_i1_orf1;TRINITY_DN28729_c0_g1_i9_orf1;TRINITY_DN49038_c0_g4_i1_orf1;TRINITY_DN38506_c0_g1_i4_orf1;TRINITY_DN9109_c0_g1_i1_orf1;TRINITY_DN1266_c2_g1_i1_orf1;TRINITY_DN8390_c0_g1_i2_orf1;TRINITY_DN24_c0_g1_i1_orf1;TRINITY_DN46090_c0_g3_i1_orf1;TRINITY_DN1718_c6_g1_i4_orf1;TRINITY_DN16673_c0_g1_i1_orf1;TRINITY_DN3343_c0_g2_i1_orf1;TRINITY_DN26293_c0_g1_i4_orf1;TRINITY_DN4762_c0_g1_i2_orf1;TRINITY_DN4835_c0_g1_i2_orf1;TRINITY_DN3175_c0_g1_i7_orf1;TRINITY_DN825_c23_g1_i5_orf1;TRINITY_DN11986_c0_g1_i1_orf1;TRINITY_DN36899_c0_g1_i1_orf1;TRINITY_DN162_c0_g1_i4_orf1;TRINITY_DN2793_c0_g2_i1_orf1;TRINITY_DN44288_c0_g1_i2_orf1;TRINITY_DN60792_c0_g1_i2_orf1;TRINITY_DN2193_c0_g1_i7_orf1;TRINITY_DN659_c0_g2_i1_orf1;TRINITY_DN5099_c0_g1_i3_orf1;TRINITY_DN85476_c0_g1_i1_orf1;TRINITY_DN143637_c0_g1_i1_orf1;TRINITY_DN111110_c0_g1_i1_orf1;TRINITY_DN15160_c0_g1_i1_orf1;TRINITY_DN62_c1_g1_i3_orf1;TRINITY_DN63568_c0_g1_i1_orf1;TRINITY_DN6813_c1_g1_i1_orf1;TRINITY_DN70236_c0_g1_i1_orf1;TRINITY_DN2146_c0_g2_i1_orf1;TRINITY_DN1084_c0_g2_i2_orf1;TRINITY_DN62557_c0_g1_i1_orf1;TRINITY_DN57918_c0_g1_i1_orf1;TRINITY_DN15706_c0_g2_i5_orf1;TRINITY_DN2874_c0_g1_i4_orf1;TRINITY_DN12820_c0_g1_i1_orf1;TRINITY_DN33430_c0_g1_i5_orf1;TRINITY_DN9965_c0_g1_i1_orf1;TRINITY_DN76815_c0_g1_i3_orf1;TRINITY_DN41166_c0_g1_i1_orf1;TRINITY_DN5648_c0_g1_i5_orf1;TRINITY_DN1741_c0_g1_i5_orf1;TRINITY_DN618_c0_g1_i3_orf1;TRINITY_DN46090_c0_g2_i1_orf1;TRINITY_DN10680_c0_g1_i5_orf1;TRINITY_DN5954_c0_g1_i2_orf1;TRINITY_DN1173_c0_g
molecular_functior	L-ascorbic acid binding	GO:0031418	1	1/2397	TRINITY_DN43293_c0_g1_i2_orf1
molecular_functior	tetrapyrrole binding	GO:0046906	36	36/2397	TRINITY_DN43369_c0_g2_i1_orf1;TRINITY_DN8985_c0_g1_i4_orf1;TRINITY_DN9608_c0_g1_i3_orf1;TRINITY_DN120500_c0_g1_i1_orf1;TRINITY_DN23564_c0_g1_i7_orf1;TRINITY_DN4497_c2_g1_i3_orf1;TRINITY_DN7580_c0_g1_i1_orf1;TRINITY_DN24873_c0_g1_i4_orf1;TRINITY_DN35002_c0_g2_i2_orf1;TRINITY_DN5933_c0_g1_i1_orf1;TRINITY_DN14262_c0_g1_i5_orf1;TRINITY_DN625_c9_g1_i7_orf1;TRINITY_DN3949_c0_g1_i1_orf1;TRINITY_DN1134_c0_g1_i4_orf1;TRINITY_DN2652_c0_g2_i1_orf1;TRINITY_DN829_c0_g1_i8_orf1;TRINITY_DN3732_c1_g1_i5_orf1;TRINITY_DN1363_c0_g1_i11_orf1;TRINITY_DN54387_c0_g1_i1_orf1;TRINITY_DN3675_c0_g1_i1_orf1;TRINITY_DN15755_c0_g1_i1_orf1;TRINITY_DN448_c0_g1_i20_orf1;TRINITY_DN6351_c0_g1_i4_orf1;TRINITY_DN27045_c0_g1_i1_orf1;TRINITY_DN1960_c5_g1_i3_orf1;TRINITY_DN57856_c0_g2_i1_orf1;TRINITY_DN5439_c0_g1_i2_orf1;TRINITY_DN23783_c0_g2_i1_orf1;TRINITY_DN51252_c0_g2_i1_orf1;TRINITY_DN30037_c0_g1_i5_orf1;TRINITY_DN9647_c0_g1_i1_orf1;TRINITY_DN2676_c0_g1_i2_orf1;TRINITY_DN5661_c0_g1_i5_orf1;TRINITY_DN3732_c0_g1_i6_orf1;TRINITY_DN23398_c0_g1_i1_orf1;TRINITY_DN285_c0_g1_i4_orf1
molecular_functior	vitamin B6 binding	GO:0070279	15	15/2397	TRINITY_DN2065_c1_g2_i1_orf1;TRINITY_DN2890_c0_g1_i2_orf1;TRINITY_DN2688_c0_g2_i1_orf1;TRINITY_DN11948_c0_g1_i8_orf1;TRINITY_DN14565_c0_g1_i11_orf1;TRINITY_DN3263_c0_g1_i2_orf1;TRINITY_DN1262_c0_g1_i2_orf1;TRINITY_DN51813_c0_g1_i1_orf1;TRINITY_DN21035_c0_g1_i14_orf1;TRINITY_DN11817_c0_g1_i4_orf1;TRINITY_DN2803_c4_g1_i1_orf1;TRINITY_DN11159_c0_g1_i5_orf1;TRINITY_DN2684_c0_g2_i3_orf1;TRINITY_DN2688_c0_g1_i3_orf1;TRINITY_DN1068_c0_g1_i3_orf1
molecular_functior	laminin binding	GO:0043236	1	1/2397	TRINITY_DN10070_c0_g1_i1_orf1
molecular_functior	histone binding	GO:0042393	3	3/2397	TRINITY_DN12771_c0_g1_i1_orf1;TRINITY_DN7341_c0_g1_i8_orf1;TRINITY_DN45449_c0_g1_i1_orf1
molecular_functior	identical protein binding	GO:0042802	8	8/2397	TRINITY_DN96557_c0_g1_i1_orf1;TRINITY_DN51938_c0_g3_i1_orf1;TRINITY_DN147475_c0_g1_i1_orf1;TRINITY_DN42854_c0_g3_i2_orf1;TRINITY_DN31584_c0_g2_i2_orf1;TRINITY_DN20133_c0_g1_i1_orf1;TRINITY_DN20009_c0_g1_i1_orf1;TRINITY_DN7787_c0_g1_i1_orf1
molecular_functior	p53 binding	GO:0002039	1	1/2397	TRINITY_DN46409_c0_g1_i1_orf1
molecular_functior	apolipoprotein binding	GO:0034185	1	1/2397	TRINITY_DN46409_c0_g1_i1_orf1
molecular_functior	enzyme binding	GO:0019899	11	11/2397	TRINITY_DN8473_c0_g1_i6_orf1;TRINITY_DN41736_c0_g2_i1_orf1;TRINITY_DN46409_c0_g1_i1_orf1;TRINITY_DN39975_c0_g1_i4_orf1;TRINITY_DN31584_c0_g2_i2_orf1;TRINITY_DN20133_c0_g1_i1_orf1;TRINITY_DN4439_c0_g1_i2_orf1;TRINITY_DN80424_c0_g1_i1_orf1;TRINITY_DN3747_c1_g2_i1_orf1;TRINITY_DN95971_c0_g5_i1_orf1;TRINITY_DN3887_c0_g1_i1_orf1
molecular_functior	SNARE binding	GO:0000149	1	1/2397	TRINITY_DN38301_c0_g1_i2_orf1
molecular_functior	chaperone binding	GO:0051087	2	2/2397	TRINITY_DN46409_c0_g1_i1_orf1;TRINITY_DN5563_c1_g2_i2_orf1
molecular_functior	unfolded protein binding	GO:0051082	4	4/2397	TRINITY_DN46409_c0_g1_i1_orf1;TRINITY_DN4424_c0_g1_i1_orf1;TRINITY_DN5648_c0_g1_i5_orf1;TRINITY_DN25341_c0_g1_i1_orf1
molecular_functior	calmodulin binding	GO:0005516	4	4/2397	TRINITY_DN3126_c0_g1_i4_orf1;TRINITY_DN140613_c0_g1_i1_orf1;TRINITY_DN4010_c0_g2_i1_orf1;TRINITY_DN6642_c0_g1_i2_orf1

molecular_functior	heat shock protein binding	GO:0031072	2	2/2397	TRINITY_DN10694_c1_g2_i1_orf1;TRINITY_DN5648_c0_g1_i5_orf1
molecular_functior	transcription factor binding	GO:0008134	1	1/2397	TRINITY_DN147475_c0_g1_i1_orf1
molecular_functior	dynein intermediate chain binding	GO:0045505	1	1/2397	TRINITY_DN17995_c0_g4_i1_orf1
molecular_functior	modification-dependent protein binding	GO:0140030	2	2/2397	TRINITY_DN7341_c0_g1_i8_orf1;TRINITY_DN41573_c0_g1_i1_orf1
molecular_functior	translation initiation factor binding	GO:0031369	1	1/2397	TRINITY_DN48097_c0_g1_i1_orf1
molecular_functior	signaling receptor binding	GO:0005102	7	7/2397	TRINITY_DN51938_c0_g3_i1_orf1;TRINITY_DN18650_c0_g1_i1_orf1;TRINITY_DN18218_c0_g1_i7_orf1;TRINITY_DN147475_c0_g1_i1_orf1;TRINITY_DN108433_c0_g1_i1_orf1;TRINITY_DN22443_c0_g2_i3_orf1;TRINITY_DN2227_c0_g1_i5_orf1
molecular_functior	cytoskeletal protein binding	GO:0008092	42	42/2397	TRINITY_DN11464_c0_g1_i3_orf1;TRINITY_DN714_c0_g1_i3_orf1;TRINITY_DN350_c0_g1_i4_orf1;TRINITY_DN28018_c0_g6_i1_orf1;TRINITY_DN8915_c0_g1_i3_orf1;TRINITY_DN350_c0_g1_i5_orf1;TRINITY_DN3126_c0_g1_i4_orf1;TRINITY_DN23790_c0_g1_i1_orf1;TRINITY_DN129869_c0_g4_i1_orf1;TRINITY_DN107962_c0_g1_i1_orf1;TRINITY_DN4010_c0_g2_i1_orf1;TRINITY_DN8406_c0_g1_i4_orf1;TRINITY_DN5954_c0_g1_i2_orf1;TRINITY_DN44261_c0_g1_i1_orf1;TRINITY_DN8406_c0_g1_i2_orf1;TRINITY_DN59852_c0_g1_i1_orf1;TRINITY_DN3887_c0_g1_i1_orf1;TRINITY_DN28622_c0_g1_i1_orf1;TRINITY_DN26961_c0_g1_i1_orf1;TRINITY_DN14298_c0_g1_i1_orf1;TRINITY_DN22824_c0_g1_i4_orf1;TRINITY_DN86309_c0_g1_i4_orf1;TRINITY_DN41736_c0_g2_i1_orf1;TRINITY_DN16673_c0_g1_i1_orf1;TRINITY_DN25960_c0_g1_i1_orf1;TRINITY_DN14298_c0_g3_i1_orf1;TRINITY_DN69557_c0_g1_i1_orf1;TRINITY_DN77480_c0_g1_i2_orf1;TRINITY_DN286_c0_g1_i2_orf1;TRINITY_DN23746_c0_g1_i2_orf1;TRINITY_DN110231_c0_g1_i1_orf1;TRINITY_DN104663_c1_g1_i2_orf1;TRINITY_DN350_c0_g1_i10_orf1;TRINITY_DN14298_c0_g1_i3_orf1;TRINITY_DN9146_c0_g1_i1_orf1;TRINITY_DN1718_c1_g1_i5_orf1;TRINITY_DN20133_c0_g1_i1_orf1;TRINITY_DN17137_c0_g1_i2_orf1;TRINITY_DN97097_c0_g1_i4_orf1;TRINITY_DN101991_c0_g1_i5_orf1;TRINITY_DN34703_c0_g1_i4
molecular_functior	protein dimerization activity	GO:0046983	5	5/2397	TRINITY_DN96557_c0_g1_i1_orf1;TRINITY_DN24917_c0_g2_i1_orf1;TRINITY_DN50471_c0_g1_i4_orf1;TRINITY_DN31584_c0_g2_i2_orf1;TRINITY_DN14611_c0_g1_i5_orf1
molecular_functior	phosphoprotein binding	GO:0051219	1	1/2397	TRINITY_DN31584_c0_g2_i2_orf1
molecular_functior	protein domain specific binding	GO:0019904	4	4/2397	TRINITY_DN20009_c0_g1_i1_orf1;TRINITY_DN18912_c1_g1_i1_orf1;TRINITY_DN31584_c0_g2_i2_orf1;TRINITY_DN147475_c0_g1_i1_orf1
molecular_functior	clathrin binding	GO:0030276	1	1/2397	TRINITY_DN741_c0_g1_i10_orf1
molecular_functior	ubiquitin-like protein binding	GO:0032182	2	2/2397	TRINITY_DN45633_c0_g1_i1_orf1;TRINITY_DN18620_c0_g1_i5_orf1
molecular_functior	cell adhesion molecule binding	GO:0050839	2	2/2397	TRINITY_DN10070_c0_g1_i1_orf1;TRINITY_DN20133_c0_g1_i1_orf1
molecular_functior	S100 protein binding	GO:0044548	1	1/2397	TRINITY_DN41736_c0_g2_i1_orf1
molecular_functior	transmembrane transporter binding	GO:0044325	1	1/2397	TRINITY_DN31584_c0_g2_i2_orf1
molecular_functior	scaffold protein binding	GO:0097110	2	2/2397	TRINITY_DN20009_c0_g1_i1_orf1;TRINITY_DN31584_c0_g2_i2_orf1
molecular_functior	chitin binding	GO:0008061	20	20/2397	TRINITY_DN26301_c0_g1_i1_orf1;TRINITY_DN664_c0_g1_i18_orf1;TRINITY_DN21555_c0_g1_i4_orf1;TRINITY_DN2205_c0_g1_i3_orf1;TRINITY_DN9000_c0_g2_i1_orf1;TRINITY_DN17003_c0_g1_i1_orf1;TRINITY_DN3913_c0_g1_i6_orf1;TRINITY_DN10824_c0_g1_i3_orf1;TRINITY_DN619_c0_g1_i1_orf1;TRINITY_DN17003_c1_g1_i1_orf1;TRINITY_DN82801_c0_g1_i1_orf1;TRINITY_DN2061_c0_g1_i3_orf1;TRINITY_DN2958_c0_g1_i2_orf1;TRINITY_DN73923_c0_g1_i1_orf1;TRINITY_DN1287_c0_g1_i5_orf1;TRINITY_DN72999_c0_g1_i1_orf1;TRINITY_DN36061_c0_g4_i2_orf1;TRINITY_DN650_c0_g1_i3_orf1;TRINITY_DN54366_c0_g1_i1_orf1;TRINITY_DN3759_c0_g1_i1_orf1
molecular_functior	lipopolysaccharide binding	GO:0001530	1	1/2397	TRINITY_DN46409_c0_g1_i1_orf1
molecular_functior	ribonucleotide binding	GO:0032553	163	163/2397	TRINITY_DN98313_c0_g1_i1_orf1;TRINITY_DN280_c0_g1_i8_orf1;TRINITY_DN13563_c0_g1_i1_orf1;TRINITY_DN47731_c0_g1_i2_orf1;TRINITY_DN41311_c0_g2_i3_orf1;TRINITY_DN11194_c0_g1_i4_orf1;TRINITY_DN7122_c0_g1_i1_orf1;TRINITY_DN6436_c0_g1_i1_orf1;TRINITY_DN31967_c0_g1_i5_orf1;TRINITY_DN25341_c0_g1_i1_orf1;TRINITY_DN2904_c0_g1_i4_orf1;TRINITY_DN70485_c0_g1_i2_orf1;TRINITY_DN2983_c0_g1_i6_orf1;TRINITY_DN24723_c2_g1_i1_orf1;TRINITY_DN2953_c1_g1_i10_orf1;TRINITY_DN143509_c0_g1_i1_orf1;TRINITY_DN2638_c0_g1_i7_orf1;TRINITY_DN4320_c0_g1_i1_orf1;TRINITY_DN10774_c0_g2_i3_orf1;TRINITY_DN2953_c1_g1_i2_orf1;TRINITY_DN14298_c0_g1_i1_orf1;TRINITY_DN3991_c0_g1_i6_orf1;TRINITY_DN3800_c0_g1_i7_orf1;TRINITY_DN15370_c0_g1_i4_orf1;TRINITY_DN235_c0_g3_i1_orf1;TRINITY_DN6185_c0_g1_i12_orf1;TRINITY_DN52761_c0_g1_i2_orf1;TRINITY_DN52244_c1_g1_i1_orf1;TRINITY_DN817_c0_g1_i3_orf1;TRINITY_DN2224_c0_g1_i1_orf1;TRINITY_DN987_c0_g1_i3_orf1;TRINITY_DN26961_c0_g1_i1_orf1;TRINITY_DN4501_c0_g1_i3_orf1;TRINITY_DN31225_c0_g1_i1_orf1;TRINITY_DN107288_c0_g1_i2_orf1;TRINITY_DN11620_c0_g1_i2_orf1;TRINITY_DN20007_c0_g1_i1_orf1;TRINITY_DN4950_c0_g1_i2_orf1;TRINITY_DN52761_c0_g2_i1_orf1;TRINITY_DN7336_c0_g1_i13_orf1;TRINITY_DN2772_c0_g1_i3_orf1;TRINITY_DN115210_c0_g4_i1_orf1;TRINITY_DN31503_c0_g1_i4_orf1;TRINITY_DN2793_c0_g2_i1_orf1;TRINITY_DN252_c0_g1_i3_orf1;TRINITY_DN2719_c1_g1_i6_orf1;TRINITY_DN8659_c0_g2_i1_orf1;TRINITY_DN17935_c0_g1_i1_orf1;TRINITY_DN1604_c0_g1_i4_orf1;TRINITY_DN1034_c0_g1_i4_orf1;TRINITY_DN90497_c0_g1_i1_orf1;TRINITY_DN42461_c0_g1_i4_orf1;TRINITY_DN1607_c0_g1_i16_orf1;TRINITY_DN277_c1_g1_i1_orf1;TRINITY_DN810_c0_g1_i4_orf1;TRINITY_DN28622_c0_g1_i1_orf1;TRINITY_DN12_c0_g1_i5_orf1;TRINITY_DN2621_c0_g1_i1_orf1;TRINITY_DN32700_c0_g1_i2_orf1;TRINITY_DN117844_c0_g1_i1_orf1;TRINITY_DN12301_c0_g1_i1_orf1;TRINITY_DN9794_c0_g2_i8_orf1;TRINITY_DN6642_c0_g1_i2_orf1;TRINITY_DN14274_c0_g1_i3_orf1;TRINITY_DN28039_c0_g1_i1_orf1;TRINITY_DN2826_c0_g1_i7_orf1;TRINITY_DN42185_c0_g1_i7_orf1;TRINITY_DN511_c0_g2_i1_orf1;TRINITY_DN1954_c0_g1_i4_orf1;TRINITY_DN2706_c0_g1_i3_orf1;TRINITY_DN5811_c0_g1_i4_orf1;TRINITY_DN1173_c1_g1_i10_orf1;TRINITY_DN119893_c0_g2_i3_orf1;TRINITY_DN4908_c1_g1_i5_orf1;TRINITY_DN25997_c1_g2_i4_orf1;TRINITY_DN47151_c0_g1_i1_orf1;TRINITY_DN5029_c0_g1_i1_orf1;TRINITY_DN4501_c0_g2_i1_orf1;TRINITY_DN291_c0_g1_i2_orf1;TRINITY_DN21000_c0_g1_i1_orf1;TRINITY_DN1173_c0_g1_i12_orf1;TRINITY_DN16011_c0_g1_i3_orf1;TRINITY_DN8659_c0_g1_i1_orf1;TRINITY_DN28221_c0_g2_i1_orf1;TRINITY_DN7570_c0_g1_i18_orf1;TRINITY_DN46409_c0_g1_i1_orf1;TRINITY_DN62557_c0_g1_i1_orf1;TRINITY_DN3057_c0_g2_i1_orf1;TRINITY_DN2770_c0_g2_i4_orf1;TRINITY_DN5281_c0_g2_i3_orf1;TRINITY_DN136906_c0_g1_i1_orf1;TRINITY_DN1475_c0_g1_i6_orf1;TRINITY_DN28729_c0_g1_i9_orf1;TRINITY_DN38506_c0_g1_i4_orf1;TRINITY_DN9109_c0_g1_i1_orf1;TRINITY_DN1266_c2_g1_i1_orf1;TRINITY_DN8390_c0_g1_i2_orf1;TRINITY_DN46090_c0_g3_i1_orf1;TRINITY_DN1718_c6_g1_i4_orf1;TRINITY_DN16673_c0_g1_i1_orf1;TRINITY_DN3343_c0_g2_i1_orf1;TRINITY_DN4762_c0_g1_i2_orf1;TRINITY_DN4835_c0_g1_i2_orf1;TRINITY_DN825_c23_g1_i5_orf1;TRINITY_DN11986_c0_g1_i1_orf1;TRINITY_DN15420_c0_g3_i2_orf1;TRINITY_DN162_c0_g1_i4_orf1;TRINITY_DN44288_c0_g1_i2_orf1;TRINITY_DN60792_c0_g1_i2_orf1;TRINITY_DN2193_c0_g1_i7_orf1;TRINITY_DN97138_c0_g1_i2_orf1;TRINITY_DN85476_c0_g1_i1_orf1;TRINITY_DN143637_c0_g1_i1_orf1;TRINITY_DN15160_c0_g1_i1_orf1;TRINITY_DN63568_c0_g1_i1_orf1;TRINITY_DN6813_c1_g1_i1_orf1;TRINITY_DN2146_c0_g2_i1_orf1;TRINITY_DN1084_c0_g2_i2_orf1;TRINITY_DN2265_c0_g2_i1_orf1;TRINITY_DN57918_c0_g1_i1_orf1;TRINITY_DN15706_c0_g2_i5_orf1;TRINITY_DN2874_c0_g1_i4_orf1;TRINITY_DN12820_c0_g1_i1_orf1;TRINITY_DN76815_c0_g1_i3_orf1;TRINITY_DN41166_c0_g1_i1_orf1;TRINITY_DN5648_c0_g1_i5_orf1;TRINITY_DN1741_c0_g1_i5_orf1;TRINITY_DN618_c0_g1_i3_orf1;TRINITY_DN46090_c0_g2_i1_orf1;TRINITY_DN10680_c0_g1_i5_orf1;TRINITY_DN5954_c0_g1_i2_orf1;TRINITY_DN1173_c0_g1_i11_orf1;TRINITY_DN23946_c0_g1_i1_orf1;TRINITY_DN3916_c0_g1_i6_orf1;TRINITY_DN14298_c0_g1_i3_orf1;TRINITY_DN7247_c0_g1_i7_orf1;TRINITY_DN6653_c0_g1_i1_orf1;TRINITY_DN25345_c0_g1_i1_orf1;TRINITY_DN30154_c0_g1_i1_orf1;TRINITY_DN2570_c0_g1_i1_orf1;TRINITY_DN2709_c0_g1_i4_orf1;TRINITY_DN2745_c0_g1_i2_orf1;TRINITY_DN11612_c0_g2_i1_orf1;TRINITY_DN248_c0_g1_i1_orf1;TRINITY_DN5757_c0_g1_i1_orf1;TRINITY_DN129869_c0_g4_i1_orf1;TRINITY_DN48619_c0_g1_i1_orf1;TRINITY_DN41697_c0_g1_i1_orf1;TRINITY_DN68401_c1_g1_i1_orf1;TRINITY_DN235_c0_g1_i2_orf1;TRINITY_DN45449_c0_g1_i1_orf1;TRINITY_DN21539_c0_g1_i1_orf1;TRINITY_DN146236_c0_g1_i1_orf1;TRINITY_DN30_c0_g1_i6_orf1;TRINITY_DN6
molecular_functior	glycosaminoglycan binding	GO:0005539	3	3/2397	TRINITY_DN108433_c0_g1_i1_orf1;TRINITY_DN42854_c0_g3_i2_orf1;TRINITY_DN5235_c0_g1_i7_orf1
molecular_functior	heparin binding	GO:0008201	2	2/2397	TRINITY_DN42854_c0_g3_i2_orf1;TRINITY_DN108433_c0_g1_i1_orf1
molecular_functior	acyl-CoA binding	GO:0120227	1	1/2397	TRINITY_DN13563_c0_g1_i1_orf1
molecular_functior	peptide binding	GO:0042277	2	2/2397	TRINITY_DN245_c0_g1_i4_orf1;TRINITY_DN4016_c0_g1_i1_orf1
molecular_functior	phosphopantetheine binding	GO:0031177	1	1/2397	TRINITY_DN10430_c0_g1_i4_orf1

molecular_functior	cation binding	GO:0043169	259	259/2397	TRINITY_DN129226_c0_g1_i2_orf1;TRINITY_DN14920_c0_g1_i1_orf1;TRINITY_DN9608_c0_g1_i3_orf1;TRINITY_DN38230_c0_g1_i4_orf1;TRINITY_DN827_c1_g1_i1_orf1;TRINITY_DN276_c0_g1_i1_orf1;TRINITY_DN3301_c0_g1_i2_orf1;TRINITY_DN3194_c0_g1_i6_orf1;TRINITY_DN7580_c0_g1_i1_orf1;TRINITY_DN42333_c0_g1_i5_orf1;TRINITY_DN35002_c0_g2_i2_orf1;TRINITY_DN3732_c0_g1_i6_orf1;TRINITY_DN43293_c0_g1_i2_orf1;TRINITY_DN21035_c0_g1_i14_orf1;TRINITY_DN35763_c0_g1_i2_orf1;TRINITY_DN56164_c0_g1_i1_orf1;TRINITY_DN19651_c0_g1_i1_orf1;TRINITY_DN2694_c0_g1_i3_orf1;TRINITY_DN18804_c0_g1_i5_orf1;TRINITY_DN8241_c0_g1_i3_orf1;TRINITY_DN1231_c0_g1_i4_orf1;TRINITY_DN5628_c0_g1_i5_orf1;TRINITY_DN38431_c0_g1_i1_orf1;TRINITY_DN17772_c0_g2_i3_orf1;TRINITY_DN8771_c0_g1_i5_orf1;TRINITY_DN8621_c0_g1_i4_orf1;TRINITY_DN125427_c0_g1_i1_orf1;TRINITY_DN1134_c0_g1_i4_orf1;TRINITY_DN99673_c0_g1_i1_orf1;TRINITY_DN70485_c0_g1_i2_orf1;TRINITY_DN24689_c0_g1_i1_orf1;TRINITY_DN59852_c0_g1_i1_orf1;TRINITY_DN143509_c0_g1_i1_orf1;TRINITY_DN3235_c0_g1_i1_orf1;TRINITY_DN3433_c0_g1_i6_orf1;TRINITY_DN810_c0_g1_i4_orf1;TRINITY_DN3675_c0_g1_i1_orf1;TRINITY_DN585_c0_g1_i12_orf1;TRINITY_DN14774_c0_g1_i4_orf1;TRINITY_DN15755_c0_g1_i1_orf1;TRINITY_DN4822_c0_g1_i6_orf1;TRINITY_DN448_c0_g1_i20_orf1;TRINITY_DN625_c9_g1_i7_orf1;TRINITY_DN41179_c0_g1_i1_orf1;TRINITY_DN3991_c0_g1_i6_orf1;TRINITY_DN2794_c1_g1_i8_orf1;TRINITY_DN4125_c0_g1_i14_orf1;TRINITY_DN1999_c0_g1_i9_orf1;TRINITY_DN5439_c0_g1_i2_orf1;TRINITY_DN69557_c0_g1_i1_orf1;TRINITY_DN123184_c0_g1_i1_orf1;TRINITY_DN42159_c0_g1_i6_orf1;TRINITY_DN21943_c1_g1_i1_orf1;TRINITY_DN817_c0_g1_i3_orf1;TRINITY_DN10785_c0_g1_i4_orf1;TRINITY_DN5952_c0_g1_i6_orf1;TRINITY_DN2688_c0_g2_i1_orf1;TRINITY_DN7909_c0_g2_i1_orf1;TRINITY_DN227_c0_g1_i1_orf1;TRINITY_DN987_c0_g1_i3_orf1;TRINITY_DN1444_c1_g1_i5_orf1;TRINITY_DN2627_c0_g2_i1_orf1;TRINITY_DN7247_c0_g1_i7_orf1;TRINITY_DN4501_c0_g1_i3_orf1;TRINITY_DN46625_c0_g1_i1_orf1;TRINITY_DN2647_c0_g1_i3_orf1;TRINITY_DN7633_c0_g1_i1_orf1;TRINITY_DN14262_c0_g1_i5_orf1;TRINITY_DN230_c2_g1_i5_orf1;TRINITY_DN65681_c0_g1_i1_orf1;TRINITY_DN120089_c0_g1_i1_orf1;TRINITY_DN115210_c0_g4_i1_orf1;TRINITY_DN20442_c0_g2_i1_orf1;TRINITY_DN45271_c0_g1_i1_orf1;TRINITY_DN47723_c0_g1_i1_orf1;TRINITY_DN14398_c0_g1_i4_orf1;TRINITY_DN4367_c0_g1_i1_orf1;TRINITY_DN82810_c0_g1_i1_orf1;TRINITY_DN252_c0_g1_i3_orf1;TRINITY_DN37532_c0_g1_i1_orf1;TRINITY_DN22018_c0_g1_i3_orf1;TRINITY_DN4189_c0_g2_i1_orf1;TRINITY_DN27592_c0_g1_i1_orf1;TRINITY_DN3010_c0_g1_i4_orf1;TRINITY_DN1024_c0_g4_i1_orf1;TRINITY_DN120_c0_g1_i2_orf1;TRINITY_DN501_c0_g1_i5_orf1;TRINITY_DN81258_c0_g1_i2_orf1;TRINITY_DN5998_c0_g2_i1_orf1;TRINITY_DN1423_c0_g1_i4_orf1;TRINITY_DN56690_c0_g1_i4_orf1;TRINITY_DN3275_c0_g2_i3_orf1;TRINITY_DN116972_c0_g1_i1_orf1;TRINITY_DN30713_c0_g1_i3_orf1;TRINITY_DN50787_c0_g2_i2_orf1;TRINITY_DN1423_c0_g1_i8_orf1;TRINITY_DN4145_c0_g1_i1_orf1;TRINITY_DN15327_c2_g1_i2_orf1;TRINITY_DN6415_c0_g1_i1_orf1;TRINITY_DN32700_c0_g1_i2_orf1;TRINITY_DN117844_c0_g1_i1_orf1;TRINITY_DN12301_c0_g1_i1_orf1;TRINITY_DN9794_c0_g2_i8_orf1;TRINITY_DN6642_c0_g1_i2_orf1;TRINITY_DN3073_c0_g1_i7_orf1;TRINITY_DN38644_c0_g1_i1_orf1;TRINITY_DN2825_c0_g1_i3_orf1;TRINITY_DN136031_c0_g1_i7_orf1;TRINITY_DN47842_c0_g1_i1_orf1;TRINITY_DN12024_c0_g1_i4_orf1;TRINITY_DN48638_c0_g1_i5_orf1;TRINITY_DN5664_c0_g1_i1_orf1;TRINITY_DN376_c1_g1_i1_orf1;TRINITY_DN511_c0_g2_i1_orf1;TRINITY_DN23398_c0_g1_i1_orf1;TRINITY_DN83295_c0_g1_i3_orf1;TRINITY_DN1206_c0_g1_i6_orf1;TRINITY_DN8458_c0_g2_i1_orf1;TRINITY_DN12545_c0_g1_i7_orf1;TRINITY_DN4424_c0_g1_i1_orf1;TRINITY_DN5004_c0_g1_i2_orf1;TRINITY_DN107035_c0_g1_i1_orf1;TRINITY_DN103107_c0_g1_i2_orf1;TRINITY_DN4908_c1_g1_i5_orf1;TRINITY_DN2299_c0_g1_i3_orf1;TRINITY_DN5235_c0_g1_i7_orf1;TRINITY_DN25997_c1_g2_i4_orf1;TRINITY_DN47151_c0_g1_i1_orf1;TRINITY_DN5029_c0_g1_i1_orf1;TRINITY_DN4501_c0_g2_i1_orf1;TRINITY_DN812_c2_g1_i1_orf1;TRINITY_DN8985_c0_g1_i4_orf1;TRINITY_DN120500_c0_g1_i1_orf1;TRINITY_DN64222_c0_g1_i1_orf1;TRINITY_DN46778_c0_g1_i2_orf1;TRINITY_DN7570_c0_g1_i18_orf1;TRINITY_DN30037_c0_g1_i5_orf1;TRINITY_DN16400_c0_g2_i1_orf1;TRINITY_DN2186_c0_g1_i17_orf1;TRINITY_DN24873_c0_g1_i4_orf1;TRINITY_DN89083_c0_g1_i1_orf1;TRINITY_DN62557_c0_g1_i1_orf1;TRINITY_DN73900_c0_g1_i1_orf1;TRINITY_DN8621_c0_g1_i5_orf1;TRINITY_DN53311_c0_g2_i1_orf1;TRINITY_DN3057_c0_g2_i1_orf1;TRINITY_DN8224_c0_g1_i7_orf1;TRINITY_DN2140_c0_g1_i1_orf1;TRINITY_DN3732_c1_g1_i5_orf1;TRINITY_DN542_c0_g2_i1_orf1;TRINITY_DN2186_c0_g1_i12_orf1;TRINITY_DN5122_c0_g1_i2_orf1;TRINITY_DN24_c0_g1_i1_orf1;TRINITY_DN1363_c0_g1_i11_orf1;TRINITY_DN839_c
molecular_functior	anion binding	GO:0043168	196	196/2397	TRINITY_DN98315_c0_g1_i1_orf1;TRINITY_DN280_c0_g1_i8_orf1;TRINITY_DN2065_c1_g2_i1_orf1;TRINITY_DN47731_c0_g1_i2_orf1;TRINITY_DN97138_c0_g1_i2_orf1;TRINITY_DN41311_c0_g2_i3_orf1;TRINITY_DN11194_c0_g1_i4_orf1;TRINITY_DN11159_c0_g1_i5_orf1;TRINITY_DN43293_c0_g1_i2_orf1;TRINITY_DN7122_c0_g1_i1_orf1;TRINITY_DN2688_c0_g1_i3_orf1;TRINITY_DN6436_c0_g1_i1_orf1;TRINITY_DN4744_c0_g1_i7_orf1;TRINITY_DN25341_c0_g1_i1_orf1;TRINITY_DN13563_c0_g1_i1_orf1;TRINITY_DN2904_c0_g1_i4_orf1;TRINITY_DN70485_c0_g1_i2_orf1;TRINITY_DN2983_c0_g1_i6_orf1;TRINITY_DN11817_c0_g1_i4_orf1;TRINITY_DN24723_c2_g1_i1_orf1;TRINITY_DN2953_c1_g1_i10_orf1;TRINITY_DN143509_c0_g1_i1_orf1;TRINITY_DN2638_c0_g1_i7_orf1;TRINITY_DN4320_c0_g1_i1_orf1;TRINITY_DN10774_c0_g2_i3_orf1;TRINITY_DN2953_c1_g1_i2_orf1;TRINITY_DN14298_c0_g1_i1_orf1;TRINITY_DN3991_c0_g1_i6_orf1;TRINITY_DN3800_c0_g1_i7_orf1;TRINITY_DN15370_c0_g1_i4_orf1;TRINITY_DN235_c0_g3_i1_orf1;TRINITY_DN1068_c0_g1_i3_orf1;TRINITY_DN6185_c0_g1_i12_orf1;TRINITY_DN52761_c0_g1_i2_orf1;TRINITY_DN52244_c1_g1_i1_orf1;TRINITY_DN817_c0_g1_i3_orf1;TRINITY_DN2224_c0_g1_i1_orf1;TRINITY_DN2688_c0_g2_i1_orf1;TRINITY_DN7909_c0_g2_i1_orf1;TRINITY_DN987_c0_g1_i3_orf1;TRINITY_DN26961_c0_g1_i1_orf1;TRINITY_DN3131_c0_g1_i5_orf1;TRINITY_DN7247_c0_g1_i7_orf1;TRINITY_DN4501_c0_g1_i3_orf1;TRINITY_DN31225_c0_g1_i1_orf1;TRINITY_DN107288_c0_g1_i2_orf1;TRINITY_DN11620_c0_g1_i2_orf1;TRINITY_DN20007_c0_g1_i1_orf1;TRINITY_DN4950_c0_g1_i2_orf1;TRINITY_DN52761_c0_g2_i1_orf1;TRINITY_DN34751_c0_g1_i1_orf1;TRINITY_DN7336_c0_g1_i13_orf1;TRINITY_DN2772_c0_g1_i3_orf1;TRINITY_DN115210_c0_g4_i1_orf1;TRINITY_DN31503_c0_g1_i4_orf1;TRINITY_DN64126_c0_g1_i1_orf1;TRINITY_DN252_c0_g1_i3_orf1;TRINITY_DN2719_c1_g1_i6_orf1;TRINITY_DN8659_c0_g2_i1_orf1;TRINITY_DN17935_c0_g1_i1_orf1;TRINITY_DN1604_c0_g1_i4_orf1;TRINITY_DN1034_c0_g1_i4_orf1;TRINITY_DN90497_c0_g1_i1_orf1;TRINITY_DN42461_c0_g1_i4_orf1;TRINITY_DN9794_c0_g2_i8_orf1;TRINITY_DN1607_c0_g1_i16_orf1;TRINITY_DN277_c1_g1_i1_orf1;TRINITY_DN810_c0_g1_i4_orf1;TRINITY_DN31967_c0_g1_i5_orf1;TRINITY_DN28622_c0_g1_i1_orf1;TRINITY_DN12_c0_g1_i5_orf1;TRINITY_DN2621_c0_g1_i1_orf1;TRINITY_DN32700_c0_g1_i2_orf1;TRINITY_DN117844_c0_g1_i1_orf1;TRINITY_DN12301_c0_g1_i1_orf1;TRINITY_DN14565_c0_g1_i11_orf1;TRINITY_DN6642_c0_g1_i2_orf1;TRINITY_DN14274_c0_g1_i3_orf1;TRINITY_DN28039_c0_g1_i1_orf1;TRINITY_DN2826_c0_g1_i7_orf1;TRINITY_DN42185_c0_g1_i7_orf1;TRINITY_DN511_c0_g2_i1_orf1;TRINITY_DN1954_c0_g1_i4_orf1;TRINITY_DN2706_c0_g1_i3_orf1;TRINITY_DN5811_c0_g1_i4_orf1;TRINITY_DN1173_c1_g1_i10_orf1;TRINITY_DN2146_c0_g2_i1_orf1;TRINITY_DN2684_c0_g2_i3_orf1;TRINITY_DN4908_c1_g1_i5_orf1;TRINITY_DN25997_c1_g2_i4_orf1;TRINITY_DN47151_c0_g1_i1_orf1;TRINITY_DN5029_c0_g1_i1_orf1;TRINITY_DN4501_c0_g2_i1_orf1;TRINITY_DN291_c0_g1_i2_orf1;TRINITY_DN21000_c0_g1_i1_orf1;TRINITY_DN1173_c0_g1_i12_orf1;TRINITY_DN16011_c0_g1_i3_orf1;TRINITY_DN8659_c0_g1_i1_orf1;TRINITY_DN28221_c0_g2_i1_orf1;TRINITY_DN7570_c0_g1_i18_orf1;TRINITY_DN376_c0_g1_i1_orf1;TRINITY_DN46409_c0_g1_i1_orf1;TRINITY_DN2265_c0_g2_i1_orf1;TRINITY_DN3057_c0_g2_i1_orf1;TRINITY_DN2770_c0_g2_i4_orf1;TRINITY_DN5281_c0_g2_i3_orf1;TRINITY_DN136906_c0_g1_i1_orf1;TRINITY_DN129869_c0_g4_i1_orf1;TRINITY_DN141381_c0_g1_i1_orf1;TRINITY_DN28729_c0_g1_i9_orf1;TRINITY_DN11948_c0_g1_i8_orf1;TRINITY_DN38506_c0_g1_i4_orf1;TRINITY_DN9109_c0_g1_i1_orf1;TRINITY_DN1266_c2_g1_i1_orf1;TRINITY_DN8390_c0_g1_i2_orf1;TRINITY_DN46090_c0_g3_i1_orf1;TRINITY_DN1718_c6_g1_i4_orf1;TRINITY_DN16673_c0_g1_i1_orf1;TRINITY_DN2803_c4_g1_i1_orf1;TRINITY_DN3343_c0_g2_i1_orf1;TRINITY_DN4762_c0_g1_i2_orf1;TRINITY_DN4835_c0_g1_i2_orf1;TRINITY_DN3175_c0_g1_i7_orf1;TRINITY_DN825_c23_g1_i5_orf1;TRINITY_DN11986_c0_g1_i1_orf1;TRINITY_DN51813_c0_g1_i1_orf1;TRINITY_DN36899_c0_g1_i1_orf1;TRINITY_DN162_c0_g1_i4_orf1;TRINITY_DN2793_c0_g2_i1_orf1;TRINITY_DN44288_c0_g1_i2_orf1;TRINITY_DN60792_c0_g1_i2_orf1;TRINITY_DN2193_c0_g1_i7_orf1;TRINITY_DN659_c0_g2_i1_orf1;TRINITY_DN85476_c0_g1_i1_orf1;TRINITY_DN143637_c0_g1_i1_orf1;TRINITY_DN111110_c0_g1_i1_orf1;TRINITY_DN15160_c0_g1_i1_orf1;TRINITY_DN62_c1_g1_i3_orf1;TRINITY_DN63568_c0_g1_i1_orf1;TRINITY_DN1262_c0_g1_i2_orf1;TRINITY_DN3263_c0_g1_i2_orf1;TRINITY_DN6813_c1_g1_i1_orf1;TRINITY_DN70236_c0_g1_i1_orf1;TRINITY_DN119893_c0_g2_i3_orf1;TRINITY_DN1084_c0_g2_i2_orf1;TRINITY_DN62557_c0_g1_i1_orf1;TRINITY_DN57918_c0_g1_i1_orf1;TRINITY_DN15706_c0_g2_i5_orf1;TRINITY_DN2874_c0_g1_i4_orf1;TRINITY_DN12820_c0_g1_i1_orf1;TRINITY_DN9965_c0_g1_i1_orf1;TRINITY_DN76815_c0_g1_i3_orf1;TRINITY_DN41166_c0_g1_i1_orf1;TRINITY_DN5648_c0_g1_i5_orf1;TRINITY_DN1741_c
molecular_functior	phospholipid binding	GO:0005543	4	4/2397	TRINITY_DN445_c0_g1_i2_orf1;TRINITY_DN143637_c0_g1_i1_orf1;TRINITY_DN2770_c0_g2_i4_orf1;TRINITY_DN18620_c0_g1_i5_orf1
molecular_functior	fatty acid derivative binding	GO:1901567	1	1/2397	TRINITY_DN13563_c0_g1_i1_orf1
molecular_functior	oxygen binding	GO:0019825	1	1/2397	TRINITY_DN30037_c0_g1_i5_orf1
molecular_functior	monosaccharide binding	GO:0048029	2	2/2397	TRINITY_DN43293_c0_g1_i2_orf1;TRINITY_DN20133_c0_g1_i1_orf1
molecular_functior	organic acid binding	GO:0043177	2	2/2397	TRINITY_DN115210_c0_g4_i1_orf1;TRINITY_DN43293_c0_g1_i2_orf1
molecular_functior	vitamin binding	GO:0019842	17	17/2397	TRINITY_DN2065_c1_g2_i1_orf1;TRINITY_DN2890_c0_g1_i2_orf1;TRINITY_DN2688_c0_g2_i1_orf1;TRINITY_DN11948_c0_g1_i8_orf1;TRINITY_DN14565_c0_g1_i11_orf1;TRINITY_DN3263_c0_g1_i2_orf1;TRINITY_DN1262_c0_g1_i2_orf1;TRINITY_DN51813_c0_g1_i1_orf1;TRINITY_DN21035_c0_g1_i14_orf1;TRINITY_DN11817_c0_g1_i4_orf1;TRINITY_DN2803_c4_g1_i1_orf1;TRINITY_DN11159_c0_g1_i5_orf1;TRINITY_DN43293_c0_g1_i2_orf1;TRINITY_DN2684_c0_g2_i3_orf1;TRINITY_DN10430_c0_g1_i4_orf1;TRINITY_DN2688_c0_g1_i3_orf1;TRINITY_DN1068_c0_g1_i3_orf1

molecular_function	nucleotide binding	GO:0000166	191	191/2397	TRINITY_DN98313_c0_g1_i1_orf1;TRINITY_DN280_c0_g1_i8_orf1;TRINITY_DN13563_c0_g1_i1_orf1;TRINITY_DN38230_c0_g1_i4_orf1;TRINITY_DN47731_c0_g1_i2_orf1;TRINITY_DN97138_c0_g1_i2_orf1;TRINITY_DN41311_c0_g2_i3_orf1;TRINITY_DN11194_c0_g1_i4_orf1;TRINITY_DN7122_c0_g1_i1_orf1;TRINITY_DN1921_c1_g1_i5_orf1;TRINITY_DN817_c0_g1_i3_orf1;TRINITY_DN4744_c0_g1_i7_orf1;TRINITY_DN25341_c0_g1_i1_orf1;TRINITY_DN2904_c0_g1_i4_orf1;TRINITY_DN70485_c0_g1_i2_orf1;TRINITY_DN2983_c0_g1_i6_orf1;TRINITY_DN24723_c2_g1_i1_orf1;TRINITY_DN2953_c1_g1_i10_orf1;TRINITY_DN143509_c0_g1_i1_orf1;TRINITY_DN2638_c0_g1_i7_orf1;TRINITY_DN4320_c0_g1_i1_orf1;TRINITY_DN810_c0_g1_i4_orf1;TRINITY_DN10774_c0_g2_i3_orf1;TRINITY_DN2953_c1_g1_i2_orf1;TRINITY_DN14298_c0_g1_i1_orf1;TRINITY_DN3991_c0_g1_i6_orf1;TRINITY_DN3800_c0_g1_i7_orf1;TRINITY_DN15370_c0_g1_i4_orf1;TRINITY_DN235_c0_g3_i1_orf1;TRINITY_DN6185_c0_g1_i12_orf1;TRINITY_DN52761_c0_g1_i2_orf1;TRINITY_DN52244_c1_g1_i1_orf1;TRINITY_DN6436_c0_g1_i1_orf1;TRINITY_DN2224_c0_g1_i1_orf1;TRINITY_DN7909_c0_g2_i1_orf1;TRINITY_DN987_c0_g1_i3_orf1;TRINITY_DN26961_c0_g1_i1_orf1;TRINITY_DN3131_c0_g1_i5_orf1;TRINITY_DN7247_c0_g1_i7_orf1;TRINITY_DN4501_c0_g1_i3_orf1;TRINITY_DN31225_c0_g1_i1_orf1;TRINITY_DN107288_c0_g1_i2_orf1;TRINITY_DN11620_c0_g1_i2_orf1;TRINITY_DN20007_c0_g1_i1_orf1;TRINITY_DN4950_c0_g1_i2_orf1;TRINITY_DN52761_c0_g2_i1_orf1;TRINITY_DN34751_c0_g1_i1_orf1;TRINITY_DN7336_c0_g1_i13_orf1;TRINITY_DN2772_c0_g1_i3_orf1;TRINITY_DN115210_c0_g4_i1_orf1;TRINITY_DN31503_c0_g1_i4_orf1;TRINITY_DN64126_c0_g1_i1_orf1;TRINITY_DN252_c0_g1_i3_orf1;TRINITY_DN2719_c1_g1_i6_orf1;TRINITY_DN8659_c0_g2_i1_orf1;TRINITY_DN17935_c0_g1_i1_orf1;TRINITY_DN4451_c0_g2_i4_orf1;TRINITY_DN1604_c0_g1_i4_orf1;TRINITY_DN1034_c0_g1_i4_orf1;TRINITY_DN90497_c0_g1_i1_orf1;TRINITY_DN42461_c0_g1_i4_orf1;TRINITY_DN1607_c0_g1_i16_orf1;TRINITY_DN277_c1_g1_i1_orf1;TRINITY_DN268_c3_g1_i2_orf1;TRINITY_DN31967_c0_g1_i5_orf1;TRINITY_DN28622_c0_g1_i1_orf1;TRINITY_DN12_c0_g1_i5_orf1;TRINITY_DN2621_c0_g1_i1_orf1;TRINITY_DN32700_c0_g1_i2_orf1;TRINITY_DN117844_c0_g1_i1_orf1;TRINITY_DN12301_c0_g1_i1_orf1;TRINITY_DN9794_c0_g2_i8_orf1;TRINITY_DN6642_c0_g1_i2_orf1;TRINITY_DN14274_c0_g1_i3_orf1;TRINITY_DN28039_c0_g1_i1_orf1;TRINITY_DN2826_c0_g1_i7_orf1;TRINITY_DN42185_c0_g1_i7_orf1;TRINITY_DN511_c0_g2_i1_orf1;TRINITY_DN1954_c0_g1_i4_orf1;TRINITY_DN2706_c0_g1_i3_orf1;TRINITY_DN5811_c0_g1_i4_orf1;TRINITY_DN1173_c1_g1_i10_orf1;TRINITY_DN119893_c0_g2_i3_orf1;TRINITY_DN4908_c1_g1_i5_orf1;TRINITY_DN25997_c1_g2_i4_orf1;TRINITY_DN47151_c0_g1_i1_orf1;TRINITY_DN5029_c0_g1_i1_orf1;TRINITY_DN4501_c0_g2_i1_orf1;TRINITY_DN291_c0_g1_i2_orf1;TRINITY_DN21000_c0_g1_i1_orf1;TRINITY_DN1173_c0_g1_i12_orf1;TRINITY_DN16011_c0_g1_i3_orf1;TRINITY_DN8659_c0_g1_i1_orf1;TRINITY_DN28221_c0_g2_i1_orf1;TRINITY_DN7570_c0_g1_i18_orf1;TRINITY_DN376_c0_g1_i1_orf1;TRINITY_DN46409_c0_g1_i1_orf1;TRINITY_DN2265_c0_g2_i1_orf1;TRINITY_DN3057_c0_g2_i1_orf1;TRINITY_DN2770_c0_g2_i4_orf1;TRINITY_DN5281_c0_g2_i3_orf1;TRINITY_DN136906_c0_g1_i1_orf1;TRINITY_DN1475_c0_g1_i6_orf1;TRINITY_DN141381_c0_g1_i1_orf1;TRINITY_DN28729_c0_g1_i9_orf1;TRINITY_DN49038_c0_g4_i1_orf1;TRINITY_DN38506_c0_g1_i4_orf1;TRINITY_DN9109_c0_g1_i1_orf1;TRINITY_DN1266_c2_g1_i1_orf1;TRINITY_DN8390_c0_g1_i2_orf1;TRINITY_DN24_c0_g1_i1_orf1;TRINITY_DN46090_c0_g3_i1_orf1;TRINITY_DN1718_c6_g1_i4_orf1;TRINITY_DN16673_c0_g1_i1_orf1;TRINITY_DN3343_c0_g2_i1_orf1;TRINITY_DN26293_c0_g1_i4_orf1;TRINITY_DN4762_c0_g1_i2_orf1;TRINITY_DN4835_c0_g1_i2_orf1;TRINITY_DN3175_c0_g1_i7_orf1;TRINITY_DN825_c23_g1_i5_orf1;TRINITY_DN11986_c0_g1_i1_orf1;TRINITY_DN36899_c0_g1_i1_orf1;TRINITY_DN162_c0_g1_i4_orf1;TRINITY_DN2793_c0_g2_i1_orf1;TRINITY_DN44288_c0_g1_i2_orf1;TRINITY_DN60792_c0_g1_i2_orf1;TRINITY_DN2193_c0_g1_i7_orf1;TRINITY_DN659_c0_g2_i1_orf1;TRINITY_DN5099_c0_g1_i3_orf1;TRINITY_DN85476_c0_g1_i1_orf1;TRINITY_DN143637_c0_g1_i1_orf1;TRINITY_DN111110_c0_g1_i1_orf1;TRINITY_DN15160_c0_g1_i1_orf1;TRINITY_DN62_c1_g1_i3_orf1;TRINITY_DN63568_c0_g1_i1_orf1;TRINITY_DN6813_c1_g1_i1_orf1;TRINITY_DN70236_c0_g1_i1_orf1;TRINITY_DN2146_c0_g2_i1_orf1;TRINITY_DN1084_c0_g2_i2_orf1;TRINITY_DN62557_c0_g1_i1_orf1;TRINITY_DN57918_c0_g1_i1_orf1;TRINITY_DN15706_c0_g2_i5_orf1;TRINITY_DN2874_c0_g1_i4_orf1;TRINITY_DN12820_c0_g1_i1_orf1;TRINITY_DN33430_c0_g1_i5_orf1;TRINITY_DN9965_c0_g1_i1_orf1;TRINITY_DN76815_c0_g1_i3_orf1;TRINITY_DN41166_c0_g1_i1_orf1;TRINITY_DN5648_c0_g1_i5_orf1;TRINITY_DN1741_c0_g1_i5_orf1;TRINITY_DN618_c0_g1_i3_orf1;TRINITY_DN46090_c0_g2_i1_orf1;TRINITY_DN10680_c0_g1_i5_orf1;TRINITY_DN5954_c0_g1_i2_orf1;TRINITY_DN1173_c0_g
molecular_function	nucleosome binding	GO:0031491	1	1/2397	TRINITY_DN63568_c0_g1_i1_orf1
molecular_function	ribonucleoprotein complex binding	GO:0043021	4	4/2397	TRINITY_DN23360_c0_g1_i3_orf1;TRINITY_DN48097_c0_g1_i1_orf1;TRINITY_DN10070_c0_g1_i1_orf1;TRINITY_DN6239_c0_g1_i1_orf1
molecular_function	protein-lipid complex binding	GO:0071814	1	1/2397	TRINITY_DN46409_c0_g1_i1_orf1
molecular_function	intermediate filament binding	GO:0019215	1	1/2397	TRINITY_DN20009_c0_g1_i1_orf1
molecular_function	dynein complex binding	GO:0070840	1	1/2397	TRINITY_DN21559_c0_g2_i1_orf1
molecular_function	proteasome binding	GO:0070628	2	2/2397	TRINITY_DN135188_c0_g1_i2_orf1;TRINITY_DN7341_c0_g1_i8_orf1
molecular_function	actin filament binding	GO:0051015	9	9/2397	TRINITY_DN110231_c0_g1_i1_orf1;TRINITY_DN104663_c1_g1_i2_orf1;TRINITY_DN129869_c0_g4_i1_orf1;TRINITY_DN86309_c0_g1_i4_orf1;TRINITY_DN22824_c0_g1_i4_orf1;TRINITY_DN8915_c0_g1_i3_orf1;TRINITY_DN1718_c1_g1_i5_orf1;TRINITY_DN741_c0_g1_i10_orf1;TRINITY_DN26961_c0_g1_i1_orf1
molecular_function	iron-sulfur cluster binding	GO:0051536	9	9/2397	TRINITY_DN14920_c0_g1_i1_orf1;TRINITY_DN7909_c0_g2_i1_orf1;TRINITY_DN28660_c0_g1_i4_orf1;TRINITY_DN48638_c0_g1_i5_orf1;TRINITY_DN5664_c0_g1_i1_orf1;TRINITY_DN85476_c0_g1_i1_orf1;TRINITY_DN5432_c1_g1_i3_orf1;TRINITY_DN3464_c0_g1_i1_orf1;TRINITY_DN9536_c0_g1_i4_orf1
molecular_function	virus receptor activity	GO:0001618	1	1/2397	TRINITY_DN10070_c0_g1_i1_orf1
molecular_function	protein-hormone receptor activity	GO:0016500	1	1/2397	TRINITY_DN3962_c0_g1_i6_orf1
molecular_function	transmembrane signaling receptor activity	GO:0004888	9	9/2397	TRINITY_DN8953_c0_g1_i4_orf1;TRINITY_DN46090_c0_g2_i1_orf1;TRINITY_DN20710_c0_g1_i2_orf1;TRINITY_DN46090_c0_g3_i1_orf1;TRINITY_DN13216_c0_g1_i5_orf1;TRINITY_DN19662_c4_g1_i1_orf1;TRINITY_DN15247_c0_g1_i2_orf1;TRINITY_DN3962_c0_g1_i6_orf1;TRINITY_DN34821_c0_g1_i4_orf1
molecular_function	pattern recognition receptor activity	GO:0038187	1	1/2397	TRINITY_DN2170_c1_g1_i3_orf1
molecular_function	ligase activity, forming carbon-oxygen bonds	GO:0016875	12	12/2397	TRINITY_DN2224_c0_g1_i1_orf1;TRINITY_DN57918_c0_g1_i1_orf1;TRINITY_DN21539_c0_g1_i1_orf1;TRINITY_DN825_c23_g1_i5_orf1;TRINITY_DN1607_c0_g1_i16_orf1;TRINITY_DN5218_c0_g1_i4_orf1;TRINITY_DN2953_c1_g1_i10_orf1;TRINITY_DN107288_c0_g1_i2_orf1;TRINITY_DN2953_c1_g1_i2_orf1;TRINITY_DN817_c0_g1_i3_orf1;TRINITY_DN48619_c0_g1_i1_orf1;TRINITY_DN15160_c0_g1_i1_orf1
molecular_function	ligase activity, forming carbon-sulfur bonds	GO:0016877	8	8/2397	TRINITY_DN9794_c0_g2_i8_orf1;TRINITY_DN8659_c0_g1_i1_orf1;TRINITY_DN15930_c0_g1_i5_orf1;TRINITY_DN2193_c0_g1_i7_orf1;TRINITY_DN19251_c0_g1_i8_orf1;TRINITY_DN8659_c0_g2_i1_orf1;TRINITY_DN120593_c0_g1_i1_orf1;TRINITY_DN6653_c0_g1_i1_orf1
molecular_function	ligase activity, forming carbon-nitrogen bonds	GO:0016879	10	10/2397	TRINITY_DN98313_c0_g1_i1_orf1;TRINITY_DN36144_c0_g1_i3_orf1;TRINITY_DN115210_c0_g4_i1_orf1;TRINITY_DN3800_c0_g1_i7_orf1;TRINITY_DN24723_c2_g1_i1_orf1;TRINITY_DN38506_c0_g1_i4_orf1;TRINITY_DN28221_c0_g2_i1_orf1;TRINITY_DN76815_c0_g1_i3_orf1;TRINITY_DN987_c0_g1_i3_orf1;TRINITY_DN41697_c0_g1_i1_orf1
molecular_function	proton-transporting ATP synthase activity, rotational mechanism	GO:0046933	1	1/2397	TRINITY_DN96080_c0_g2_i1_orf1
molecular_function	ligase activity, forming carbon-carbon bonds	GO:0016885	3	3/2397	TRINITY_DN3991_c0_g1_i6_orf1;TRINITY_DN2570_c0_g1_i1_orf1;TRINITY_DN511_c0_g2_i1_orf1
molecular_function	carbon-sulfur lyase activity	GO:0016846	1	1/2397	TRINITY_DN11948_c0_g1_i8_orf1
molecular_function	carbon-nitrogen lyase activity	GO:0016840	3	3/2397	TRINITY_DN16868_c0_g2_i1_orf1;TRINITY_DN1716_c0_g1_i14_orf1;TRINITY_DN28299_c0_g1_i1_orf1
molecular_function	phosphorus-oxygen lyase activity	GO:0016849	4	4/2397	TRINITY_DN10774_c0_g2_i3_orf1;TRINITY_DN3712_c0_g1_i1_orf1;TRINITY_DN618_c0_g1_i3_orf1;TRINITY_DN52244_c1_g1_i1_orf1
molecular_function	carbon-oxygen lyase activity	GO:0016835	9	9/2397	TRINITY_DN230_c2_g1_i5_orf1;TRINITY_DN82810_c0_g1_i1_orf1;TRINITY_DN87603_c0_g2_i1_orf1;TRINITY_DN73900_c0_g1_i1_orf1;TRINITY_DN35763_c0_g1_i2_orf1;TRINITY_DN2825_c0_g1_i3_orf1;TRINITY_DN10430_c0_g1_i4_orf1;TRINITY_DN3464_c0_g1_i1_orf1;TRINITY_DN89483_c0_g1_i1_orf1
molecular_function	carbon-carbon lyase activity	GO:0016830	12	12/2397	TRINITY_DN779_c0_g1_i12_orf1;TRINITY_DN1045_c0_g1_i6_orf1;TRINITY_DN6325_c0_g1_i8_orf1;TRINITY_DN40434_c0_g1_i2_orf1;TRINITY_DN109931_c0_g1_i1_orf1;TRINITY_DN2684_c0_g2_i3_orf1;TRINITY_DN779_c0_g1_i3_orf1;TRINITY_DN11159_c0_g1_i5_orf1;TRINITY_DN83150_c0_g1_i1_orf1;TRINITY_DN20133_c0_g1_i1_orf1;TRINITY_DN6325_c0_g1_i9_orf1;TRINITY_DN9998_c0_g1_i2_orf1
molecular_function	protein-malonyllysine demalonylase activity	GO:0036054	1	1/2397	TRINITY_DN111110_c0_g1_i1_orf1
molecular_function	peptide-lysine-N-acetyltransferase activity	GO:0061733	3	3/2397	TRINITY_DN20442_c0_g2_i1_orf1;TRINITY_DN12771_c0_g1_i1_orf1;TRINITY_DN51737_c0_g1_i3_orf1
molecular_function	protein-disulfide reductase activity	GO:0015035	6	6/2397	TRINITY_DN51938_c0_g3_i1_orf1;TRINITY_DN10118_c0_g1_i4_orf1;TRINITY_DN79673_c0_g1_i1_orf1;TRINITY_DN24689_c0_g1_i1_orf1;TRINITY_DN376_c0_g1_i1_orf1;TRINITY_DN14306_c0_g1_i1_orf1
molecular_function	protein-glutaryllysine deglutarylase activity	GO:0061697	1	1/2397	TRINITY_DN111110_c0_g1_i1_orf1
molecular_function	protein methyltransferase activity	GO:0008276	3	3/2397	TRINITY_DN22674_c0_g1_i2_orf1;TRINITY_DN95414_c0_g1_i1_orf1;TRINITY_DN6462_c0_g1_i5_orf1
molecular_function	phosphoprotein phosphatase activity	GO:0004721	9	9/2397	TRINITY_DN1749_c0_g2_i2_orf1;TRINITY_DN4571_c0_g1_i4_orf1;TRINITY_DN3119_c0_g1_i7_orf1;TRINITY_DN34830_c0_g1_i1_orf1;TRINITY_DN24539_c0_g1_i4_orf1;TRINITY_DN40562_c0_g2_i1_orf1;TRINITY_DN59885_c0_g1_i3_orf1;TRINITY_DN4217_c0_g1_i2_orf1;TRINITY_DN2257_c0_g1_i4_orf1
molecular_function	protein-N-terminal asparagine amidohydrolase activity	GO:0008418	1	1/2397	TRINITY_DN895_c0_g2_i1_orf1
molecular_function	ubiquitin-like protein transferase activity	GO:0019787	4	4/2397	TRINITY_DN9062_c0_g2_i3_orf1;TRINITY_DN11820_c0_g1_i1_orf1;TRINITY_DN1380_c0_g1_i5_orf1;TRINITY_DN14487_c0_g1_i4_orf1
molecular_function	aminoacyltransferase activity	GO:0016755	3	3/2397	TRINITY_DN52553_c0_g2_i1_orf1;TRINITY_DN52553_c0_g1_i1_orf1;TRINITY_DN4898_c0_g1_i7_orf1
molecular_function	protein lysine deacetylase activity	GO:0033558	3	3/2397	TRINITY_DN10385_c0_g1_i5_orf1;TRINITY_DN70236_c0_g1_i1_orf1;TRINITY_DN111110_c0_g1_i1_orf1
molecular_function	protein kinase activity	GO:0004672	25	25/2397	TRINITY_DN1173_c0_g1_i12_orf1;TRINITY_DN5281_c0_g2_i3_orf1;TRINITY_DN70485_c0_g1_i2_orf1;TRINITY_DN46090_c0_g2_i1_orf1;TRINITY_DN248_c0_g1_i1_orf1;TRINITY_DN10680_c0_g1_i5_orf1;TRINITY_DN28729_c0_g1_i9_orf1;TRINITY_DN42461_c0_g1_i4_orf1;TRINITY_DN1173_c0_g1_i11_orf1;TRINITY_DN46090_c0_g3_i1_orf1;TRINITY_DN2983_c0_g1_i6_orf1;TRINITY_DN277_c1_g1_i1_orf1;TRINITY_DN19662_c4_g1_i1_orf1;TRINITY_DN10774_c0_g2_i3_orf1;TRINITY_DN32700_c0_g1_i2_orf1;TRINITY_DN30154_c0_g1_i1_orf1;TRINITY_DN6185_c0_g1_i12_orf1;TRINITY_DN6436_c0_g1_i1_orf1;TRINITY_DN143637_c0_g1_i1_orf1;TRINITY_DN1266_c2_g1_i1_orf1;TRINITY_DN1954_c0_g1_i4_orf1;TRINITY_DN1173_c1_g1_i10_orf1;TRINITY_DN30_c0_g1_i6_orf1;TRINITY_DN147475_c0_g1_i1_orf1;TRINITY_DN11620_c0_g1_i2_orf1

molecular_functior	peptidase activity	GO:0008233	107	107/2397	TRINITY_DN3194_c0_g1_i6_orf1;TRINITY_DN1533_c0_g2_i1_orf1;TRINITY_DN11928_c0_g1_i3_orf1;TRINITY_DN1274_c0_g1_i4_orf1;TRINITY_DN2069_c1_g1_i8_orf1;TRINITY_DN83295_c0_g1_i3_orf1;TRINITY_DN3499_c0_g1_i8_orf1;TRINITY_DN8692_c0_g1_i2_orf1;TRINITY_DN2442_c0_g1_i2_orf1;TRINITY_DN36434_c0_g2_i3_orf1;TRINITY_DN16258_c0_g1_i2_orf1;TRINITY_DN2885_c1_g1_i2_orf1;TRINITY_DN14754_c0_g1_i6_orf1;TRINITY_DN41761_c0_g1_i4_orf1;TRINITY_DN3975_c0_g1_i7_orf1;TRINITY_DN14774_c0_g1_i4_orf1;TRINITY_DN875_c0_g1_i3_orf1;TRINITY_DN2794_c1_g1_i8_orf1;TRINITY_DN4125_c0_g1_i14_orf1;TRINITY_DN10364_c0_g1_i5_orf1;TRINITY_DN391_c0_g1_i4_orf1;TRINITY_DN28428_c0_g1_i2_orf1;TRINITY_DN10403_c0_g1_i3_orf1;TRINITY_DN4767_c0_g1_i6_orf1;TRINITY_DN51938_c0_g3_i1_orf1;TRINITY_DN143895_c0_g1_i1_orf1;TRINITY_DN13856_c0_g1_i1_orf1;TRINITY_DN45633_c0_g1_i1_orf1;TRINITY_DN10090_c0_g1_i1_orf1;TRINITY_DN19537_c0_g1_i1_orf1;TRINITY_DN4189_c0_g2_i1_orf1;TRINITY_DN57798_c0_g1_i1_orf1;TRINITY_DN1310_c0_g1_i4_orf1;TRINITY_DN5012_c0_g1_i6_orf1;TRINITY_DN72541_c0_g1_i2_orf1;TRINITY_DN19651_c0_g1_i1_orf1;TRINITY_DN19990_c0_g1_i1_orf1;TRINITY_DN21719_c0_g1_i2_orf1;TRINITY_DN45948_c1_g1_i1_orf1;TRINITY_DN5696_c0_g1_i4_orf1;TRINITY_DN2040_c0_g1_i6_orf1;TRINITY_DN71863_c0_g1_i2_orf1;TRINITY_DN29034_c0_g1_i2_orf1;TRINITY_DN4125_c1_g1_i5_orf1;TRINITY_DN1703_c0_g1_i6_orf1;TRINITY_DN5444_c0_g2_i1_orf1;TRINITY_DN4408_c6_g1_i1_orf1;TRINITY_DN6423_c0_g1_i6_orf1;TRINITY_DN140_c1_g1_i2_orf1;TRINITY_DN74116_c0_g1_i2_orf1;TRINITY_DN38431_c0_g1_i1_orf1;TRINITY_DN4767_c0_g1_i4_orf1;TRINITY_DN1528_c0_g1_i4_orf1;TRINITY_DN10403_c0_g1_i1_orf1;TRINITY_DN4494_c0_g1_i1_orf1;TRINITY_DN21218_c0_g1_i4_orf1;TRINITY_DN8621_c0_g1_i4_orf1;TRINITY_DN2584_c0_g1_i7_orf1;TRINITY_DN25534_c0_g1_i1_orf1;TRINITY_DN10766_c0_g1_i1_orf1;TRINITY_DN554_c0_g1_i1_orf1;TRINITY_DN3483_c0_g1_i5_orf1;TRINITY_DN8621_c0_g1_i5_orf1;TRINITY_DN6122_c0_g1_i6_orf1;TRINITY_DN57111_c0_g1_i1_orf1;TRINITY_DN6423_c0_g1_i5_orf1;TRINITY_DN376_c1_g1_i1_orf1;TRINITY_DN1404_c0_g1_i6_orf1;TRINITY_DN21719_c0_g2_i4_orf1;TRINITY_DN3343_c0_g2_i1_orf1;TRINITY_DN18388_c0_g1_i6_orf1;TRINITY_DN18172_c0_g1_i6_orf1;TRINITY_DN1309_c0_g2_i1_orf1;TRINITY_DN1308_c0_g1_i4_orf1;TRINITY_DN701_c0_g1_i1_orf1;TRINITY_DN4228_c0_g1_i5_orf1;TRINITY_DN4121_c0_g1_i1_orf1;TRINITY_DN6470_c0_g3_i2_orf1;TRINITY_DN29034_c0_g1_i1_orf1;TRINITY_DN14217_c0_g1_i1_orf1;TRINITY_DN69697_c0_g1_i1_orf1;TRINITY_DN6813_c1_g1_i1_orf1;TRINITY_DN81803_c0_g2_i1_orf1;TRINITY_DN801_c0_g1_i2_orf1;TRINITY_DN13686_c0_g2_i1_orf1;TRINITY_DN14874_c0_g1_i6_orf1;TRINITY_DN334_c0_g1_i3_orf1;TRINITY_DN4030_c0_g2_i1_orf1;TRINITY_DN17329_c0_g2_i3_orf1;TRINITY_DN2043_c0_g1_i3_orf1;TRINITY_DN111621_c0_g3_i1_orf1;TRINITY_DN41952_c0_g1_i4_orf1;TRINITY_DN10994_c0_g1_i4_orf1;TRINITY_DN17759_c0_g1_i5_orf1;TRINITY_DN4886_c0_g1_i6_orf1;TRINITY_DN1592_c0_g1_i1_orf1;TRINITY_DN6967_c0_g1_i3_orf1;TRINITY_DN56690_c0_g1_i4_orf1;TRINITY_DN4189_c0_g1_i4_orf1;TRINITY_DN46132_c0_g2_i2_orf1;TRINITY_DN23167_c0_g2_i1_orf1;TRINITY_DN23167_c0_g1_i4_orf1;TRINITY_DN24121_c1_g1_i6_orf1;TRI
molecular_functior	protein disulfide isomerase activity	GO:0003756	2	2/2397	TRINITY_DN51938_c0_g3_i1_orf1;TRINITY_DN1831_c0_g1_i3_orf1
molecular_functior	peptidyl-prolyl cis-trans isomerase activity	GO:0003755	2	2/2397	TRINITY_DN21596_c0_g1_i1_orf1;TRINITY_DN142588_c0_g1_i1_orf1
molecular_functior	protein demethylase activity	GO:0140457	1	1/2397	TRINITY_DN89083_c0_g1_i1_orf1
molecular_functior	ubiquitin-like modifier activating enzyme activity	GO:0008641	5	5/2397	TRINITY_DN15930_c0_g1_i5_orf1;TRINITY_DN8659_c0_g1_i1_orf1;TRINITY_DN8659_c0_g2_i1_orf1;TRINITY_DN120593_c0_g1_i1_orf1;TRINITY_DN6653_c0_g1_i1_orf1
molecular_functior	microtubule plus end polymerase	GO:0061863	1	1/2397	TRINITY_DN25960_c0_g1_i1_orf1
molecular_functior	hydrolase activity, acting on glycosyl bonds	GO:0016798	41	41/2397	TRINITY_DN14235_c0_g1_i1_orf1;TRINITY_DN8703_c0_g1_i2_orf1;TRINITY_DN24631_c0_g2_i1_orf1;TRINITY_DN25492_c0_g1_i1_orf1;TRINITY_DN53167_c0_g1_i3_orf1;TRINITY_DN10824_c0_g1_i3_orf1;TRINITY_DN467_c3_g1_i5_orf1;TRINITY_DN22577_c0_g1_i2_orf1;TRINITY_DN11657_c0_g1_i2_orf1;TRINITY_DN2205_c0_g1_i3_orf1;TRINITY_DN13088_c0_g1_i5_orf1;TRINITY_DN479_c6_g1_i2_orf1;TRINITY_DN5852_c0_g1_i13_orf1;TRINITY_DN28741_c0_g1_i3_orf1;TRINITY_DN7183_c0_g1_i2_orf1;TRINITY_DN6074_c0_g1_i1_orf1;TRINITY_DN361_c0_g1_i5_orf1;TRINITY_DN48237_c0_g1_i5_orf1;TRINITY_DN3322_c0_g1_i2_orf1;TRINITY_DN2894_c0_g2_i3_orf1;TRINITY_DN542_c0_g1_i4_orf1;TRINITY_DN7228_c0_g1_i6_orf1;TRINITY_DN15222_c0_g1_i4_orf1;TRINITY_DN195_c4_g1_i1_orf1;TRINITY_DN2515_c0_g1_i6_orf1;TRINITY_DN4070_c0_g1_i4_orf1;TRINITY_DN9044_c0_g1_i2_orf1;TRINITY_DN1732_c0_g1_i15_orf1;TRINITY_DN17003_c1_g1_i1_orf1;TRINITY_DN1098_c1_g1_i4_orf1;TRINITY_DN670_c0_g1_i3_orf1;TRINITY_DN26688_c0_g1_i2_orf1;TRINITY_DN21555_c0_g1_i4_orf1;TRINITY_DN7828_c0_g1_i2_orf1;TRINITY_DN1732_c0_g1_i17_orf1;TRINITY_DN1287_c0_g1_i5_orf1;TRINITY_DN14409_c0_g1_i1_orf1;TRINITY_DN2170_c1_g1_i3_orf1;TRINITY_DN5852_c0_g1_i6_orf1;TRINITY_DN812_c2_g1_i1_orf1;TRINITY_DN650_c0_g1_i3_orf1
molecular_functior	hydrolase activity, acting on acid carbon-carbon bonds	GO:0016822	1	1/2397	TRINITY_DN51813_c0_g1_i1_orf1
molecular_functior	hydrolase activity, acting on carbon-nitrogen (but not peptide) bonds	GO:0016810	22	22/2397	TRINITY_DN895_c0_g2_i1_orf1;TRINITY_DN827_c1_g1_i1_orf1;TRINITY_DN115210_c0_g4_i1_orf1;TRINITY_DN25423_c0_g1_i1_orf1;TRINITY_DN542_c0_g2_i1_orf1;TRINITY_DN87170_c0_g1_i3_orf1;TRINITY_DN38506_c0_g1_i4_orf1;TRINITY_DN98242_c0_g1_i1_orf1;TRINITY_DN13660_c0_g1_i1_orf1;TRINITY_DN542_c0_g1_i4_orf1;TRINITY_DN14107_c0_g1_i4_orf1;TRINITY_DN4145_c0_g1_i1_orf1;TRINITY_DN17326_c0_g1_i8_orf1;TRINITY_DN2835_c0_g1_i6_orf1;TRINITY_DN111110_c0_g1_i1_orf1;TRINITY_DN8674_c0_g2_i1_orf1;TRINITY_DN82801_c0_g1_i1_orf1;TRINITY_DN17326_c0_g1_i5_orf1;TRINITY_DN70236_c0_g1_i1_orf1;TRINITY_DN5235_c0_g1_i7_orf1;TRINITY_DN10385_c0_g1_i5_orf1;TRINITY_DN11383_c0_g2_i4_orf1
molecular_functior	hydrolase activity, acting on acid anhydrides	GO:0016817	37	37/2397	TRINITY_DN280_c0_g1_i8_orf1;TRINITY_DN2265_c0_g2_i1_orf1;TRINITY_DN16011_c0_g1_i3_orf1;TRINITY_DN140212_c0_g1_i1_orf1;TRINITY_DN41311_c0_g2_i3_orf1;TRINITY_DN2793_c0_g2_i1_orf1;TRINITY_DN11194_c0_g1_i4_orf1;TRINITY_DN252_c0_g1_i3_orf1;TRINITY_DN15706_c0_g2_i5_orf1;TRINITY_DN7122_c0_g1_i1_orf1;TRINITY_DN2054_c0_g1_i1_orf1;TRINITY_DN136906_c0_g1_i1_orf1;TRINITY_DN25341_c0_g1_i1_orf1;TRINITY_DN11612_c0_g2_i1_orf1;TRINITY_DN90497_c0_g1_i1_orf1;TRINITY_DN2638_c0_g1_i7_orf1;TRINITY_DN3343_c0_g2_i1_orf1;TRINITY_DN7570_c0_g1_i18_orf1;TRINITY_DN1091_c0_g1_i1_orf1;TRINITY_DN7336_c0_g1_i13_orf1;TRINITY_DN4762_c0_g1_i2_orf1;TRINITY_DN11986_c0_g1_i1_orf1;TRINITY_DN6642_c0_g1_i2_orf1;TRINITY_DN15370_c0_g1_i4_orf1;TRINITY_DN15420_c0_g3_i2_orf1;TRINITY_DN28039_c0_g1_i1_orf1;TRINITY_DN146236_c0_g1_i1_orf1;TRINITY_DN97138_c0_g1_i2_orf1;TRINITY_DN52761_c0_g1_i2_orf1;TRINITY_DN42185_c0_g1_i7_orf1;TRINITY_DN7565_c0_g1_i3_orf1;TRINITY_DN45449_c0_g1_i1_orf1;TRINITY_DN46409_c0_g1_i1_orf1;TRINITY_DN1665_c1_g1_i2_orf1;TRINITY_DN2621_c0_g1_i1_orf1;TRINITY_DN52761_c0_g2_i
molecular_functior	serine hydrolase activity	GO:0017171	54	54/2397	TRINITY_DN2584_c0_g1_i7_orf1;TRINITY_DN36434_c0_g2_i3_orf1;TRINITY_DN10766_c0_g1_i1_orf1;TRINITY_DN10090_c0_g1_i1_orf1;TRINITY_DN3483_c0_g1_i5_orf1;TRINITY_DN1533_c0_g2_i1_orf1;TRINITY_DN13686_c0_g2_i1_orf1;TRINITY_DN111621_c0_g3_i1_orf1;TRINITY_DN1274_c0_g1_i4_orf1;TRINITY_DN57111_c0_g1_i1_orf1;TRINITY_DN6423_c0_g1_i5_orf1;TRINITY_DN1310_c0_g1_i4_orf1;TRINITY_DN5012_c0_g1_i6_orf1;TRINITY_DN1404_c0_g1_i6_orf1;TRINITY_DN3499_c0_g1_i8_orf1;TRINITY_DN25534_c0_g1_i1_orf1;TRINITY_DN2043_c0_g1_i3_orf1;TRINITY_DN21719_c0_g1_i2_orf1;TRINITY_DN16258_c0_g1_i2_orf1;TRINITY_DN21719_c0_g2_i4_orf1;TRINITY_DN4886_c0_g1_i6_orf1;TRINITY_DN1592_c0_g1_i1_orf1;TRINITY_DN41761_c0_g1_i4_orf1;TRINITY_DN5696_c0_g1_i4_orf1;TRINITY_DN3975_c0_g1_i7_orf1;TRINITY_DN6967_c0_g1_i3_orf1;TRINITY_DN2040_c0_g1_i6_orf1;TRINITY_DN71863_c0_g1_i2_orf1;TRINITY_DN29034_c0_g1_i2_orf1;TRINITY_DN1309_c0_g2_i1_orf1;TRINITY_DN5444_c0_g2_i1_orf1;TRINITY_DN1308_c0_g1_i4_orf1;TRINITY_DN701_c0_g1_i1_orf1;TRINITY_DN10364_c0_g1_i5_orf1;TRINITY_DN4228_c0_g1_i5_orf1;TRINITY_DN6423_c0_g1_i6_orf1;TRINITY_DN23167_c0_g2_i1_orf1;TRINITY_DN140_c1_g1_i2_orf1;TRINITY_DN74116_c0_g1_i2_orf1;TRINITY_DN391_c0_g1_i4_orf1;TRINITY_DN4030_c0_g2_i1_orf1;TRINITY_DN6470_c0_g3_i2_orf1;TRINITY_DN1528_c0_g1_i4_orf1;TRINITY_DN10403_c0_g1_i1_orf1;TRINITY_DN14217_c0_g1_i1_orf1;TRINITY_DN29034_c0_g1_i1_orf1;TRINITY_DN18388_c0_g1_i6_orf1;TRINITY_DN24121_c1_g1_i6_orf1;TRINITY_DN334_c0_g1_i3_orf1;TRINITY_DN4494_c0_g1_i1_orf1;TRINITY_DN22797_c0_g1_i5_orf1;TRINITY_DN10403_c0_g1
molecular_functior	hydrolase activity, acting on ester bonds	GO:0016788	73	73/2397	TRINITY_DN1749_c0_g2_i2_orf1;TRINITY_DN38230_c0_g1_i4_orf1;TRINITY_DN2772_c0_g1_i3_orf1;TRINITY_DN40562_c0_g2_i1_orf1;TRINITY_DN45271_c0_g1_i1_orf1;TRINITY_DN10644_c0_g1_i2_orf1;TRINITY_DN38562_c0_g1_i3_orf1;TRINITY_DN5238_c0_g1_i2_orf1;TRINITY_DN42333_c0_g1_i5_orf1;TRINITY_DN17693_c0_g1_i10_orf1;TRINITY_DN3712_c0_g1_i1_orf1;TRINITY_DN121650_c0_g1_i1_orf1;TRINITY_DN14701_c0_g1_i2_orf1;TRINITY_DN2257_c0_g1_i4_orf1;TRINITY_DN1330_c0_g1_i1_orf1;TRINITY_DN1952_c0_g1_i2_orf1;TRINITY_DN3784_c0_g1_i1_orf1;TRINITY_DN53294_c0_g1_i1_orf1;TRINITY_DN4217_c0_g1_i2_orf1;TRINITY_DN34465_c0_g1_i1_orf1;TRINITY_DN44517_c0_g1_i4_orf1;TRINITY_DN24_c0_g1_i1_orf1;TRINITY_DN70485_c0_g1_i2_orf1;TRINITY_DN69713_c0_g1_i1_orf1;TRINITY_DN13330_c0_g1_i4_orf1;TRINITY_DN82017_c0_g1_i5_orf1;TRINITY_DN12024_c0_g2_i2_orf1;TRINITY_DN59885_c0_g1_i3_orf1;TRINITY_DN10430_c0_g1_i4_orf1;TRINITY_DN227_c0_g1_i1_orf1;TRINITY_DN41179_c0_g1_i1_orf1;TRINITY_DN616_c1_g1_i6_orf1;TRINITY_DN2749_c0_g2_i3_orf1;TRINITY_DN1109_c0_g1_i6_orf1;TRINITY_DN38644_c0_g1_i1_orf1;TRINITY_DN17437_c0_g1_i1_orf1;TRINITY_DN117_c0_g1_i6_orf1;TRINITY_DN4571_c0_g1_i4_orf1;TRINITY_DN4394_c0_g2_i1_orf1;TRINITY_DN38783_c0_g1_i1_orf1;TRINITY_DN3073_c0_g1_i7_orf1;TRINITY_DN15865_c0_g1_i1_orf1;TRINITY_DN1073_c0_g1_i3_orf1;TRINITY_DN34432_c0_g1_i1_orf1;TRINITY_DN12024_c0_g1_i4_orf1;TRINITY_DN42120_c0_g1_i2_orf1;TRINITY_DN3119_c0_g1_i7_orf1;TRINITY_DN123184_c0_g1_i1_orf1;TRINITY_DN24539_c0_g1_i4_orf1;TRINITY_DN34830_c0_g1_i1_orf1;TRINITY_DN38274_c0_g1_i1_orf1;TRINITY_DN53847_c0_g1_i7_orf1;TRINITY_DN12806_c0_g2_i1_orf1;TRINITY_DN30509_c0_g1_i9_orf1;TRINITY_DN117_c0_g1_i4_orf1;TRINITY_DN74037_c0_g5_i1_orf1;TRINITY_DN810_c0_g1_i4_orf1;TRINITY_DN1557_c0_g1_i9_orf1;TRINITY_DN7976_c0_g1_i4_orf1;TRINITY_DN2668_c0_g1_i7_orf1;TRINITY_DN2647_c0_g1_i3_orf1;TRINITY_DN8458_c0_g2_i1_orf1;TRINITY_DN2627_c0_g2_i1_orf1;TRINITY_DN4276_c0_g1_i6_orf1;TRINITY_DN15865_c0_g2_i2_orf1;TRINITY_DN44658_c0_g1_i2_orf1;TRINITY_DN11798_c0_g2_i1_orf1;TRINITY_DN41_c0_g1_i5_orf1;TRINITY_DN23004_c0_g1_i1_orf1;TRINITY_DN42159_c0_g1_i6_orf1;TRINITY_DN48023_c1_g1_i1_orf1;TRINITY_DN10662_c0_g1_i4_orf1;T
molecular_functior	deacetylase activity	GO:0019213	6	6/2397	TRINITY_DN542_c0_g2_i1_orf1;TRINITY_DN82801_c0_g1_i1_orf1;TRINITY_DN70236_c0_g1_i1_orf1;TRINITY_DN542_c0_g1_i4_orf1;TRINITY_DN10385_c0_g1_i5_orf1;TRINITY_DN111110_c0_g1_i1_orf1
molecular_functior	deaminase activity	GO:0019239	2	2/2397	TRINITY_DN1196_c0_g1_i5_orf1;TRINITY_DN98242_c0_g1_i1_orf1
molecular_functior	hydrolase activity, acting on ether bonds	GO:0016801	3	3/2397	TRINITY_DN5768_c0_g1_i2_orf1;TRINITY_DN63536_c0_g1_i1_orf1;TRINITY_DN11172_c0_g1_i4_orf1
molecular_functior	FAD-AMP lyase (cyclizing) activity	GO:0034012	2	2/2397	TRINITY_DN618_c0_g1_i3_orf1;TRINITY_DN52244_c1_g1_i1_orf1
molecular_functior	guanylate cyclase activity	GO:0004383	1	1/2397	TRINITY_DN10774_c0_g2_i3_orf1
molecular_functior	oxidoreductase activity, acting on CH-OH group of donors	GO:0016614	18	18/2397	TRINITY_DN4451_c0_g2_i4_orf1;TRINITY_DN5266_c0_g1_i1_orf1;TRINITY_DN77830_c0_g2_i2_orf1;TRINITY_DN146126_c0_g1_i1_orf1;TRINITY_DN3175_c0_g1_i7_orf1;TRINITY_DN1206_c0_g1_i6_orf1;TRINITY_DN3053_c0_g1_i2_orf1;TRINITY_DN9286_c0_g1_i2_orf1;TRINITY_DN36899_c0_g1_i1_orf1;TRINITY_DN20658_c0_g2_i3_orf1;TRINITY_DN4793_c0_g1_i7_orf1;TRINITY_DN1209_c0_g1_i9_orf1;TRINITY_DN12193_c0_g1_i6_orf1;TRINITY_DN26293_c0_g1_i4_orf1;TRINITY_DN10430_c0_g1_i4_orf1;TRINITY_DN1921_c1_g1_i5_orf1;TRINITY_DN135781_c0_g1_i1_orf1;TRINITY_DN49038_c0_g4_i1_orf1

molecular_functior	oxidoreductase activity, acting on the aldehyde or oxo group of donors	GO:0016903	19	19/2397	TRINITY_DN2100_c0_g1_i2_orf1;TRINITY_DN482_c0_g1_i1_orf1;TRINITY_DN40126_c0_g1_i1_orf1;TRINITY_DN3836_c0_g1_i4_orf1;TRINITY_DN7808_c0_g1_i1_orf1;TRINITY_DN1293_c0_g1_i4_orf1;TRINITY_DN1293_c1_g1_i4_orf1;TRINITY_DN81031_c0_g1_i1_orf1;TRINITY_DN6586_c0_g1_i1_orf1;TRINITY_DN11826_c0_g1_i4_orf1;TRINITY_DN64772_c0_g1_i1_orf1;TRINITY_DN1103_c0_g1_i15_orf1;TRINITY_DN2719_c1_g1_i6_orf1;TRINITY_DN7335_c0_g1_i1_orf1;TRINITY_DN40126_c0_g2_i1_orf1;TRINITY_DN64892_c0_g1_i1_orf1;TRINITY_DN3529_c0_g1_i7_orf1;TRINITY_DN1103_c0_g1_i19_orf1;TRINITY_DN52788_c0_g1_i1_orf1
molecular_functior	oxidoreductase activity, acting on diphenols and related substances as donors	GO:0016679	1	1/2397	TRINITY_DN2140_c0_g1_i1_orf1
molecular_functior	oxidoreductase activity, acting on the CH-NH group of donors	GO:0016645	5	5/2397	TRINITY_DN38506_c0_g1_i4_orf1;TRINITY_DN5432_c1_g1_i3_orf1;TRINITY_DN24970_c0_g1_i4_orf1;TRINITY_DN14107_c0_g1_i4_orf1;TRINITY_DN15136_c0_g1_i2_orf1
molecular_functior	dioxygenase activity	GO:0051213	8	8/2397	TRINITY_DN44083_c0_g1_i2_orf1;TRINITY_DN4822_c0_g1_i6_orf1;TRINITY_DN38562_c0_g1_i3_orf1;TRINITY_DN14398_c0_g1_i4_orf1;TRINITY_DN89083_c0_g1_i1_orf1;TRINITY_DN43293_c0_g1_i2_orf1;TRINITY_DN57900_c0_g1_i2_orf1;TRINITY_DN4822_c0_g1_i9_orf1
molecular_functior	electron transfer activity	GO:0009055	2	2/2397	TRINITY_DN14920_c0_g1_i1_orf1;TRINITY_DN5432_c1_g1_i3_orf1
molecular_functior	oxidoreductase activity, acting on paired donors, with incorporation or reduction of molecular oxygen	GO:0016705	40	40/2397	TRINITY_DN43369_c0_g2_i1_orf1;TRINITY_DN8985_c0_g1_i4_orf1;TRINITY_DN9608_c0_g1_i3_orf1;TRINITY_DN120500_c0_g1_i1_orf1;TRINITY_DN23564_c0_g1_i7_orf1;TRINITY_DN64126_c0_g1_i1_orf1;TRINITY_DN7580_c0_g1_i1_orf1;TRINITY_DN14398_c0_g1_i4_orf1;TRINITY_DN24873_c0_g1_i4_orf1;TRINITY_DN89083_c0_g1_i1_orf1;TRINITY_DN3732_c0_g1_i6_orf1;TRINITY_DN43293_c0_g1_i2_orf1;TRINITY_DN14262_c0_g1_i5_orf1;TRINITY_DN4998_c0_g1_i21_orf1;TRINITY_DN625_c9_g1_i7_orf1;TRINITY_DN48590_c0_g1_i1_orf1;TRINITY_DN17772_c0_g2_i3_orf1;TRINITY_DN3949_c0_g1_i1_orf1;TRINITY_DN1134_c0_g1_i4_orf1;TRINITY_DN6027_c0_g1_i13_orf1;TRINITY_DN23398_c0_g1_i1_orf1;TRINITY_DN829_c0_g1_i8_orf1;TRINITY_DN3732_c1_g1_i5_orf1;TRINITY_DN1363_c0_g1_i11_orf1;TRINITY_DN3675_c0_g1_i1_orf1;TRINITY_DN15755_c0_g1_i1_orf1;TRINITY_DN448_c0_g1_i20_orf1;TRINITY_DN6351_c0_g1_i4_orf1;TRINITY_DN27045_c0_g1_i1_orf1;TRINITY_DN1960_c5_g1_i3_orf1;TRINITY_DN57856_c0_g2_i1_orf1;TRINITY_DN5439_c0_g1_i2_orf1;TRINITY_DN4497_c2_g1_i3_orf1;TRINITY_DN9647_c0_g1_i1_orf1;TRINITY_DN2676_c0_g1_i2_orf1;TRINITY_DN5661_c0_g1_i5_orf1;TRINITY_DN3131_c0_g1_i5_orf1;TRINITY_DN1999_c0_g1_i9_orf1;TRINITY_DN5004_c0_g1_i2_orf1;TRINITY_DN82944_c0_g1_i4_orf1
molecular_functior	oxidoreductase activity, acting on single donors with incorporation of molecular oxygen	GO:0016701	4	4/2397	TRINITY_DN4822_c0_g1_i6_orf1;TRINITY_DN4822_c0_g1_i9_orf1;TRINITY_DN3010_c0_g1_i4_orf1;TRINITY_DN38562_c0_g1_i3_orf1
molecular_functior	oxidoreductase activity, acting on the CH-CH group of donors	GO:0016627	12	12/2397	TRINITY_DN34751_c0_g1_i1_orf1;TRINITY_DN141381_c0_g1_i1_orf1;TRINITY_DN323_c0_g2_i5_orf1;TRINITY_DN659_c0_g2_i1_orf1;TRINITY_DN20658_c0_g2_i3_orf1;TRINITY_DN3053_c0_g1_i2_orf1;TRINITY_DN10430_c0_g1_i4_orf1;TRINITY_DN30932_c0_g1_i2_orf1;TRINITY_DN77830_c0_g2_i2_orf1;TRINITY_DN1125_c0_g1_i4_orf1;TRINITY_DN4744_c0_g1_i7_orf1;TRINITY_DN3478_c0_g1_i10_orf1
molecular_functior	oxidoreductase activity, acting on NAD(P)H	GO:0016651	5	5/2397	TRINITY_DN1661_c0_g1_i1_orf1;TRINITY_DN1134_c0_g1_i4_orf1;TRINITY_DN6563_c0_g1_i1_orf1;TRINITY_DN8306_c0_g1_i4_orf1;TRINITY_DN33430_c0_g1_i5_orf1
molecular_functior	oxidoreductase activity, acting on superoxide radicals as acceptor	GO:0016721	5	5/2397	TRINITY_DN15417_c0_g1_i6_orf1;TRINITY_DN37307_c0_g1_i4_orf1;TRINITY_DN103107_c0_g1_i2_orf1;TRINITY_DN16400_c0_g2_i1_orf1;TRINITY_DN1024_c0_g4_i1_orf1
molecular_functior	oxidoreductase activity, acting on metal ions	GO:0016722	5	5/2397	TRINITY_DN46625_c0_g1_i1_orf1;TRINITY_DN1423_c0_g1_i8_orf1;TRINITY_DN65681_c0_g1_i1_orf1;TRINITY_DN136031_c0_g1_i7_orf1;TRINITY_DN1423_c0_g1_i4_orf1
molecular_functior	oxidoreductase activity, acting on CH or CH2 groups	GO:0016725	2	2/2397	TRINITY_DN135781_c0_g1_i1_orf1;TRINITY_DN4835_c0_g1_i2_orf1
molecular_functior	oxidoreductase activity, acting on peroxide as acceptor	GO:0016684	10	10/2397	TRINITY_DN7778_c0_g1_i1_orf1;TRINITY_DN1622_c0_g1_i6_orf1;TRINITY_DN51252_c0_g2_i1_orf1;TRINITY_DN5933_c0_g1_i1_orf1;TRINITY_DN285_c0_g1_i4_orf1;TRINITY_DN69236_c0_g1_i1_orf1;TRINITY_DN54387_c0_g1_i1_orf1;TRINITY_DN2542_c0_g2_i1_orf1;TRINITY_DN21420_c0_g1_i2_orf1;TRINITY_DN2652_c0_g2_i1_orf1
molecular_functior	monooxygenase activity	GO:0004497	39	39/2397	TRINITY_DN43369_c0_g2_i1_orf1;TRINITY_DN8985_c0_g1_i4_orf1;TRINITY_DN9608_c0_g1_i3_orf1;TRINITY_DN120500_c0_g1_i1_orf1;TRINITY_DN23564_c0_g1_i7_orf1;TRINITY_DN64126_c0_g1_i1_orf1;TRINITY_DN7580_c0_g1_i1_orf1;TRINITY_DN24873_c0_g1_i4_orf1;TRINITY_DN3732_c0_g1_i6_orf1;TRINITY_DN14262_c0_g1_i5_orf1;TRINITY_DN4998_c0_g1_i21_orf1;TRINITY_DN625_c9_g1_i7_orf1;TRINITY_DN3010_c0_g1_i4_orf1;TRINITY_DN17772_c0_g2_i3_orf1;TRINITY_DN3949_c0_g1_i1_orf1;TRINITY_DN1134_c0_g1_i4_orf1;TRINITY_DN6027_c0_g1_i13_orf1;TRINITY_DN23398_c0_g1_i1_orf1;TRINITY_DN829_c0_g1_i8_orf1;TRINITY_DN3732_c1_g1_i5_orf1;TRINITY_DN1363_c0_g1_i11_orf1;TRINITY_DN3675_c0_g1_i1_orf1;TRINITY_DN15755_c0_g1_i1_orf1;TRINITY_DN448_c0_g1_i20_orf1;TRINITY_DN6351_c0_g1_i4_orf1;TRINITY_DN27045_c0_g1_i1_orf1;TRINITY_DN1960_c5_g1_i3_orf1;TRINITY_DN57856_c0_g2_i1_orf1;TRINITY_DN5439_c0_g1_i2_orf1;TRINITY_DN4497_c2_g1_i3_orf1;TRINITY_DN9647_c0_g1_i1_orf1;TRINITY_DN84357_c0_g1_i1_orf1;TRINITY_DN2676_c0_g1_i2_orf1;TRINITY_DN5661_c0_g1_i5_orf1;TRINITY_DN3131_c0_g1_i5_orf1;TRINITY_DN5004_c0_g1_i2_orf1;TRINITY_DN82944_c0_g1_i4_orf1;TRINITY_DN9198_c0_g1_i4_orf1;TRINITY_DN31584_c0_g2_i2_orf1
molecular_functior	oxidoreductase activity, acting on the CH-NH2 group of donors	GO:0016638	1	1/2397	TRINITY_DN3859_c0_g1_i5_orf1
molecular_functior	oxidoreductase activity, acting on other nitrogenous compounds as	GO:0016661	1	1/2397	TRINITY_DN2559_c0_g1_i4_orf1
molecular_functior	oxidoreductase activity, acting on a sulfur group of donors	GO:0016667	13	13/2397	TRINITY_DN920_c0_g1_i4_orf1;TRINITY_DN81715_c0_g1_i1_orf1;TRINITY_DN51938_c0_g3_i1_orf1;TRINITY_DN25987_c0_g1_i5_orf1;TRINITY_DN1491_c0_g1_i8_orf1;TRINITY_DN10118_c0_g1_i4_orf1;TRINITY_DN79673_c0_g1_i1_orf1;TRINITY_DN24689_c0_g1_i1_orf1;TRINITY_DN376_c0_g1_i1_orf1;TRINITY_DN2207_c0_g1_i6_orf1;TRINITY_DN9965_c0_g1_i1_orf1;TRINITY_DN1491_c0_g1_i4_orf1;TRINITY_DN14306_c0_g1_i1_orf1
molecular_functior	lysozyme activity	GO:0003796	2	2/2397	TRINITY_DN467_c3_g1_i5_orf1;TRINITY_DN1098_c1_g1_i4_orf1
molecular_functior	N-acetylmuramoyl-L-alanine amidase activity	GO:0008745	2	2/2397	TRINITY_DN5235_c0_g1_i7_orf1;TRINITY_DN827_c1_g1_i1_orf1
molecular_functior	intramolecular lyase activity	GO:0016872	1	1/2397	TRINITY_DN10722_c0_g3_i1_orf1
molecular_functior	DNA topoisomerase activity	GO:0003916	2	2/2397	TRINITY_DN4908_c1_g1_i5_orf1;TRINITY_DN6248_c0_g1_i1_orf1
molecular_functior	intramolecular transferase activity	GO:0016866	5	5/2397	TRINITY_DN5952_c0_g1_i6_orf1;TRINITY_DN30713_c0_g1_i3_orf1;TRINITY_DN120089_c0_g1_i1_orf1;TRINITY_DN27332_c0_g2_i1_orf1;TRINITY_DN12545_c0_g1_i7_orf1
molecular_functior	intramolecular oxidoreductase activity	GO:0016860	9	9/2397	TRINITY_DN6014_c1_g1_i2_orf1;TRINITY_DN1201_c0_g1_i4_orf1;TRINITY_DN51938_c0_g3_i1_orf1;TRINITY_DN4360_c0_g1_i4_orf1;TRINITY_DN3073_c0_g1_i7_orf1;TRINITY_DN27035_c0_g1_i1_orf1;TRINITY_DN1831_c0_g1_i3_orf1;TRINITY_DN1196_c0_g1_i5_orf1;TRINITY_DN14306_c0_g1_i1_orf1
molecular_functior	racemase and epimerase activity	GO:0016854	1	1/2397	TRINITY_DN1353_c0_g1_i1_orf1
molecular_functior	cis-trans isomerase activity	GO:0016859	2	2/2397	TRINITY_DN21596_c0_g1_i1_orf1;TRINITY_DN142588_c0_g1_i1_orf1
molecular_functior	catalytic activity, acting on RNA	GO:0140098	32	32/2397	TRINITY_DN57918_c0_g1_i1_orf1;TRINITY_DN31503_c0_g1_i4_orf1;TRINITY_DN13350_c0_g1_i4_orf1;TRINITY_DN3712_c0_g1_i1_orf1;TRINITY_DN14701_c0_g1_i2_orf1;TRINITY_DN34465_c0_g1_i1_orf1;TRINITY_DN1344_c0_g1_i1_orf1;TRINITY_DN2904_c0_g1_i4_orf1;TRINITY_DN70485_c0_g1_i2_orf1;TRINITY_DN2953_c1_g1_i10_orf1;TRINITY_DN810_c0_g1_i4_orf1;TRINITY_DN34676_c1_g1_i3_orf1;TRINITY_DN2953_c1_g1_i2_orf1;TRINITY_DN41179_c0_g1_i1_orf1;TRINITY_DN48619_c0_g1_i1_orf1;TRINITY_DN825_c23_g1_i5_orf1;TRINITY_DN14274_c0_g1_i3_orf1;TRINITY_DN4408_c6_g1_i1_orf1;TRINITY_DN34432_c0_g1_i1_orf1;TRINITY_DN817_c0_g1_i3_orf1;TRINITY_DN44288_c0_g1_i2_orf1;TRINITY_DN15160_c0_g1_i1_orf1;TRINITY_DN2224_c0_g1_i1_orf1;TRINITY_DN21539_c0_g1_i1_orf1;TRINITY_DN8458_c0_g2_i1_orf1;TRINITY_DN4950_c0_g1_i2_orf1;TRINITY_DN5218_c0_g1_i4_orf1;TRINITY_DN107288_c0_g1_i2_orf1;TRINITY_DN23004_c0_g1_i1_orf1;TRINITY_DN2299_c0_g1_i3_orf1;TRINITY_DN2709_c0_g1_i4_
molecular_functior	catalytic activity, acting on DNA	GO:0140097	23	23/2397	TRINITY_DN2971_c0_g1_i1_orf1;TRINITY_DN40434_c0_g1_i2_orf1;TRINITY_DN45271_c0_g1_i1_orf1;TRINITY_DN12820_c0_g1_i1_orf1;TRINITY_DN7122_c0_g1_i1_orf1;TRINITY_DN3057_c0_g2_i1_orf1;TRINITY_DN81258_c0_g1_i2_orf1;TRINITY_DN70485_c0_g1_i2_orf1;TRINITY_DN25345_c0_g1_i1_orf1;TRINITY_DN11986_c0_g1_i1_orf1;TRINITY_DN6642_c0_g1_i2_orf1;TRINITY_DN15370_c0_g1_i4_orf1;TRINITY_DN63568_c0_g1_i1_orf1;TRINITY_DN123184_c0_g1_i1_orf1;TRINITY_DN110534_c0_g1_i3_orf1;TRINITY_DN5757_c0_g1_i1_orf1;TRINITY_DN45449_c0_g1_i1_orf1;TRINITY_DN87603_c0_g2_i1_orf1;TRINITY_DN74037_c0_g5_i1_orf1;TRINITY_DN6248_c0_g1_i1_orf1;TRINITY_DN4908_c1_g1_i5_orf1;TRINITY_DN89613_c0_g1_i13_orf1;TRINITY_DN291_c0_g1_i2_orf1
molecular_functior	helicase activity	GO:0004386	18	18/2397	TRINITY_DN2904_c0_g1_i4_orf1;TRINITY_DN2971_c0_g1_i1_orf1;TRINITY_DN25345_c0_g1_i1_orf1;TRINITY_DN44288_c0_g1_i2_orf1;TRINITY_DN2709_c0_g1_i4_orf1;TRINITY_DN11986_c0_g1_i1_orf1;TRINITY_DN31503_c0_g1_i4_orf1;TRINITY_DN6642_c0_g1_i2_orf1;TRINITY_DN15370_c0_g1_i4_orf1;TRINITY_DN14274_c0_g1_i3_orf1;TRINITY_DN291_c0_g1_i2_orf1;TRINITY_DN4408_c6_g1_i1_orf1;TRINITY_DN810_c0_g1_i4_orf1;TRINITY_DN7122_c0_g1_i1_orf1;TRINITY_DN3057_c0_g2_i1_orf1;TRINITY_DN5757_c0_g1_i1_orf1;TRINITY_DN4950_c0_g1_i2_orf1;TRINITY_DN45449_c0_g1_i1_orf1
molecular_functior	transferase activity, transferring alkyl or aryl (other than methyl) groups	GO:0016765	17	17/2397	TRINITY_DN920_c0_g1_i4_orf1;TRINITY_DN10222_c0_g1_i2_orf1;TRINITY_DN9506_c0_g1_i2_orf1;TRINITY_DN20682_c0_g2_i1_orf1;TRINITY_DN3929_c0_g3_i3_orf1;TRINITY_DN4695_c0_g1_i4_orf1;TRINITY_DN23732_c0_g1_i1_orf1;TRINITY_DN8651_c0_g1_i18_orf1;TRINITY_DN82320_c0_g1_i2_orf1;TRINITY_DN12134_c0_g1_i4_orf1;TRINITY_DN57462_c0_g1_i1_orf1;TRINITY_DN4695_c0_g1_i3_orf1;TRINITY_DN3332_c0_g1_i11_orf1;TRINITY_DN8964_c0_g1_i4_orf1;TRINITY_DN62707_c0_g1_i1_orf1;TRINITY_DN3332_c0_g1_i9_orf1;TRINITY_DN225_c0_g1_i6_orf1
molecular_functior	transferase activity, transferring nitrogenous groups	GO:0016769	6	6/2397	TRINITY_DN2890_c0_g1_i2_orf1;TRINITY_DN1262_c0_g1_i2_orf1;TRINITY_DN14565_c0_g1_i11_orf1;TRINITY_DN2803_c4_g1_i1_orf1;TRINITY_DN11013_c0_g1_i3_orf1;TRINITY_DN1068_c0_g1_i3_orf1
molecular_functior	transferase activity, transferring sulphur-containing groups	GO:0016782	2	2/2397	TRINITY_DN6693_c0_g1_i1_orf1;TRINITY_DN16516_c0_g1_i1_orf1
molecular_functior	glycosyltransferase activity	GO:0016757	15	15/2397	TRINITY_DN38435_c0_g1_i1_orf1;TRINITY_DN31390_c0_g1_i2_orf1;TRINITY_DN31676_c0_g1_i4_orf1;TRINITY_DN9079_c0_g1_i5_orf1;TRINITY_DN8908_c0_g1_i1_orf1;TRINITY_DN6992_c0_g1_i6_orf1;TRINITY_DN11817_c0_g1_i4_orf1;TRINITY_DN1575_c0_g1_i7_orf1;TRINITY_DN12508_c0_g1_i1_orf1;TRINITY_DN5513_c0_g1_i1_orf1;TRINITY_DN56164_c0_g1_i1_orf1;TRINITY_DN812_c2_g1_i1_orf1;TRINITY_DN14597_c0_g1_i5_orf1;TRINITY_DN4248_c0_g1_i4_orf1;TRINITY_DN98091_c0_g1_i3_orf1
molecular_functior	transketolase or transaldolase activity	GO:0016744	1	1/2397	TRINITY_DN60787_c0_g1_i5_orf1
molecular_functior	molybdopterin molybdotransferase activity	GO:0061599	1	1/2397	TRINITY_DN1741_c0_g1_i5_orf1

molecular_functior	transferase activity, transferring phosphorus-containing groups	GO:0016772	64	64/2397	TRINITY_DN1173_c0_g1_i12_orf1;TRINITY_DN14477_c0_g1_i12_orf1;TRINITY_DN47151_c0_g1_i1_orf1;TRINITY_DN6185_c0_g1_i12_orf1;TRINITY_DN1475_c0_g1_i6_orf1;TRINITY_DN46090_c0_g3_i1_orf1;TRINITY_DN1173_c0_g1_i11_orf1;TRINITY_DN62557_c0_g1_i1_orf1;TRINITY_DN41166_c0_g1_i1_orf1;TRINITY_DN277_c1_g1_i1_orf1;TRINITY_DN2719_c1_g1_i6_orf1;TRINITY_DN1741_c0_g1_i5_orf1;TRINITY_DN2770_c0_g2_i4_orf1;TRINITY_DN37923_c0_g1_i1_orf1;TRINITY_DN5281_c0_g2_i3_orf1;TRINITY_DN31967_c0_g1_i5_orf1;TRINITY_DN32700_c0_g1_i2_orf1;TRINITY_DN81258_c0_g1_i2_orf1;TRINITY_DN618_c0_g1_i3_orf1;TRINITY_DN28729_c0_g1_i9_orf1;TRINITY_DN1034_c0_g1_i4_orf1;TRINITY_DN42461_c0_g1_i4_orf1;TRINITY_DN46090_c0_g2_i1_orf1;TRINITY_DN2082_c0_g1_i2_orf1;TRINITY_DN30154_c0_g1_i1_orf1;TRINITY_DN70485_c0_g1_i2_orf1;TRINITY_DN2983_c0_g1_i6_orf1;TRINITY_DN1718_c6_g1_i4_orf1;TRINITY_DN248_c0_g1_i1_orf1;TRINITY_DN143509_c0_g1_i1_orf1;TRINITY_DN19662_c4_g1_i1_orf1;TRINITY_DN12_c0_g1_i5_orf1;TRINITY_DN7247_c0_g1_i7_orf1;TRINITY_DN18782_c0_g1_i4_orf1;TRINITY_DN10774_c0_g2_i3_orf1;TRINITY_DN4320_c0_g1_i1_orf1;TRINITY_DN12301_c0_g1_i1_orf1;TRINITY_DN4408_c6_g1_i1_orf1;TRINITY_DN36632_c0_g1_i1_orf1;TRINITY_DN25997_c1_g2_i4_orf1;TRINITY_DN110534_c0_g1_i3_orf1;TRINITY_DN6436_c0_g1_i1_orf1;TRINITY_DN143637_c0_g1_i1_orf1;TRINITY_DN1266_c2_g1_i1_orf1;TRINITY_DN1954_c0_g1_i4_orf1;TRINITY_DN4929_c1_g2_i5_orf1;TRINITY_DN5029_c0_g1_i1_orf1;TRINITY_DN52244_c1_g1_i1_orf1;TRINITY_DN5811_c0_g1_i4_orf1;TRINITY_DN1173_c1_g1_i10_orf1;TRINITY_DN8261_c0_g1_i1_orf1;TRINITY_DN30_c0_g1_i6_orf1;TRINITY_DN1957_c0_g1_i4_orf1;TRINITY_DN74037_c0_g5_i1_orf1;TRINITY_DN147475_c0_g1_i1_orf1;TRINITY_DN9109_c0_g1_i1_orf1;TRINITY_DN6813_c1_g1_i1_orf1;TRINITY_DN10680_c0_g1_i5_orf1;TRINITY_DN116972_c0_g1_i1_orf1;TRINITY_DN2299_c0_g1_i3_orf1;TRINITY_DN11620_c0_g1_i2_orf1;TRINITY_DN5697_c0
molecular_functior	transferase activity, transferring one-carbon groups	GO:0016741	14	14/2397	TRINITY_DN1344_c0_g1_i1_orf1;TRINITY_DN115210_c0_g4_i1_orf1;TRINITY_DN3263_c0_g1_i2_orf1;TRINITY_DN5748_c0_g1_i6_orf1;TRINITY_DN3800_c0_g1_i7_orf1;TRINITY_DN13350_c0_g1_i4_orf1;TRINITY_DN77318_c0_g2_i1_orf1;TRINITY_DN22674_c0_g1_i2_orf1;TRINITY_DN95414_c0_g1_i1_orf1;TRINITY_DN34676_c1_g1_i3_orf1;TRINITY_DN5748_c0_g1_i5_orf1;TRINITY_DN2457_c0_g1_i8_orf1;TRINITY_DN14313_c0_g1_i1_orf1;TRINITY_DN6462_c0_g1_i5_orf1
molecular_functior	acyltransferase activity	GO:0016746	23	23/2397	TRINITY_DN2065_c1_g2_i1_orf1;TRINITY_DN86833_c0_g3_i1_orf1;TRINITY_DN20442_c0_g2_i1_orf1;TRINITY_DN20710_c0_g1_i2_orf1;TRINITY_DN5841_c0_g1_i2_orf1;TRINITY_DN52553_c0_g2_i1_orf1;TRINITY_DN5153_c1_g1_i1_orf1;TRINITY_DN12771_c0_g1_i1_orf1;TRINITY_DN5211_c0_g1_i1_orf1;TRINITY_DN3105_c0_g1_i4_orf1;TRINITY_DN2365_c0_g1_i6_orf1;TRINITY_DN51737_c0_g1_i3_orf1;TRINITY_DN1362_c0_g1_i4_orf1;TRINITY_DN10430_c0_g1_i4_orf1;TRINITY_DN21570_c0_g1_i1_orf1;TRINITY_DN117844_c0_g1_i1_orf1;TRINITY_DN15411_c0_g1_i4_orf1;TRINITY_DN16125_c0_g1_i3_orf1;TRINITY_DN22956_c0_g1_i1_orf1;TRINITY_DN52553_c0_g1_i1_orf1;TRINITY_DN4898_c0_g1_i7_orf1;TRINITY_DN3545_c0_g1_i6_orf1;TRINITY_DN1084_c0_g2_i2_orf1
