# Supplementary material for: Comparative Proteomics and Metabonomics Analysis of Different Diapause Stages Revealed a New Regulation Mechanism of Diapause in Loxostege sticticalis (Lepidoptera: Pyralidae)
Source: Molecules. 2024 Jul 25;29(15):3472. doi: 10.3390/molecules29153472 (PMC11314584; doi:10.3390/molecules29153472)
Supplement: Supplementary file 1 [file molecules-29-03472-s001.zip › analysis process/proteomic/GO enrichment analysis/turquoise.pdf]

Protein Number	GO ID	GO Description	GO Term(Level1)	Ratio_in_study	Ratio_in_pop	Pvalue_uncorrected	Pvalue_corrected
106	GO:0034645	cellular macromolecule biosynthetic process	BP	106/1493	124/3664	1.50E-06	6.20E-05
104	GO:0006518	peptide metabolic process	BP	104/1493	126/3664	1.82E-06	5.64E-05
107	GO:0043604	amide biosynthetic process	BP	107/1493	132/3664	2.00E-06	4.38E-05
102	GO:0006412	translation	BP	102/1493	117/3664	2.01E-06	4.16E-05
111	GO:0043603	cellular amide metabolic process	BP	111/1493	149/3664	2.23E-06	3.78E-05
103	GO:0043043	peptide biosynthetic process	BP	103/1493	119/3664	2.40E-06	3.72E-05
146	GO:1901566	organonitrogen compound biosynthetic process	BP	146/1493	221/3664	2.67E-06	3.82E-05
120	GO:0016070	RNA metabolic process	BP	120/1493	194/3664	2.68E-06	3.69E-05
194	GO:0044249	cellular biosynthetic process	BP	194/1493	325/3664	2.69E-06	3.58E-05
78	GO:0006396	RNA processing	BP	78/1493	121/3664	2.95E-06	3.78E-05
119	GO:0009059	macromolecule biosynthetic process	BP	119/1493	159/3664	3.05E-06	3.43E-05
196	GO:1901576	organic substance biosynthetic process	BP	196/1493	339/3664	3.14E-06	3.24E-05
162	GO:0044260	cellular macromolecule metabolic process	BP	162/1493	272/3664	3.45E-06	3.46E-05
154	GO:0044271	cellular nitrogen compound biosynthetic process	BP	154/1493	241/3664	3.48E-06	3.41E-05
297	GO:0034641	cellular nitrogen compound metabolic process	BP	297/1493	525/3664	3.89E-06	3.62E-05
206	GO:0009058	biosynthetic process	BP	206/1493	362/3664	4.62E-06	3.82E-05
462	GO:0044237	cellular metabolic process	BP	462/1493	935/3664	5.26E-06	4.07E-05
392	GO:0043170	macromolecule metabolic process	BP	392/1493	794/3664	5.76E-06	4.20E-05
142	GO:0090304	nucleic acid metabolic process	BP	142/1493	262/3664	8.29E-06	5.41E-05
539	GO:0044238	primary metabolic process	BP	539/1493	1165/3664	8.85E-06	5.68E-05
615	GO:0008152	metabolic process	BP	615/1493	1346/3664	1.10E-05	6.83E-05
17	GO:0022900	electron transport chain	BP	17/1493	19/3664	1.46E-05	8.60E-05
467	GO:0006807	nitrogen compound metabolic process	BP	467/1493	1004/3664	2.03E-05	0.000116216
56	GO:0034660	ncRNA metabolic process	BP	56/1493	89/3664	2.53E-05	0.000142729
657	GO:0009987	cellular process	BP	657/1493	1463/3664	3.78E-05	0.000207032
13	GO:0022904	respiratory electron transport chain	BP	13/1493	14/3664	7.23E-05	0.000363242
557	GO:0071704	organic substance metabolic process	BP	557/1493	1229/3664	7.68E-05	0.000380691
42	GO:0016071	mRNA metabolic process	BP	42/1493	65/3664	0.000106131	0.00049351
12	GO:0009060	aerobic respiration	BP	12/1493	13/3664	0.000166004	0.000693861
12	GO:0045333	cellular respiration	BP	12/1493	13/3664	0.000166004	0.000693861
27	GO:0006364	rRNA processing	BP	27/1493	38/3664	0.00018366	0.000742624
27	GO:0016072	rRNA metabolic process	BP	27/1493	38/3664	0.00018366	0.000742624
25	GO:0071826	ribonucleoprotein complex subunit organization	BP	25/1493	35/3664	0.000367897	0.001315939
25	GO:0022618	ribonucleoprotein complex assembly	BP	25/1493	35/3664	0.000367897	0.001315939
11	GO:0019646	aerobic electron transport chain	BP	11/1493	12/3664	0.00037919	0.001294115
249	GO:0019538	protein metabolic process	BP	249/1493	522/3664	0.000530132	0.001776657
34	GO:0034470	ncRNA processing	BP	34/1493	54/3664	0.00114875	0.003621484
35	GO:0006397	mRNA processing	BP	35/1493	56/3664	0.001388467	0.004304247
7	GO:0072655	establishment of protein localization to mitochondrion	BP	7/1493	7/3664	0.001849709	0.005504735
7	GO:0070585	protein localization to mitochondrion	BP	7/1493	7/3664	0.001849709	0.005504735
179	GO:0006139	nucleobase-containing compound metabolic process	BP	179/1493	372/3664	0.002618627	0.007008125
11	GO:0001732	formation of cytoplasmic translation initiation complex	BP	11/1493	13/3664	0.002645108	0.007028431
30	GO:0006399	tRNA metabolic process	BP	30/1493	48/3664	0.002769517	0.007105243
21	GO:0006418	tRNA aminoacylation for protein translation	BP	21/1493	31/3664	0.002851516	0.007265506
197	GO:0006725	cellular aromatic compound metabolic process	BP	197/1493	414/3664	0.002923337	0.007298533

347	GO:1901564	organonitrogen compound metabolic process	BP	347/1493	764/3664	0.003303584	0.008138631
198	GO:1901360	organic cyclic compound metabolic process	BP	198/1493	417/3664	0.00355713	0.008648708
191	GO:0046483	heterocycle metabolic process	BP	191/1493	402/3664	0.003671026	0.008867672
8	GO:0009220	pyrimidine ribonucleotide biosynthetic process	BP	8/1493	9/3664	0.004322216	0.009986736
8	GO:0006221	pyrimidine nucleotide biosynthetic process	BP	8/1493	9/3664	0.004322216	0.009986736
8	GO:0009218	pyrimidine ribonucleotide metabolic process	BP	8/1493	9/3664	0.004322216	0.009986736
8	GO:0006220	pyrimidine nucleotide metabolic process	BP	8/1493	9/3664	0.004322216	0.009986736
8	GO:1990542	mitochondrial transmembrane transport	BP	8/1493	9/3664	0.004322216	0.009986736
8	GO:0072528	pyrimidine-containing compound biosynthetic process	BP	8/1493	9/3664	0.004322216	0.009986736
8	GO:0072527	pyrimidine-containing compound metabolic process	BP	8/1493	9/3664	0.004322216	0.009986736
6	GO:0044743	protein transmembrane import into intracellular organelle	BP	6/1493	6/3664	0.00455026	0.009898811
51	GO:0022607	cellular component assembly	BP	51/1493	92/3664	0.00495124	0.010232563
21	GO:0043038	amino acid activation	BP	21/1493	32/3664	0.00588198	0.011701051
21	GO:0043039	tRNA aminoacylation	BP	21/1493	32/3664	0.00588198	0.011701051
26	GO:0008380	RNA splicing	BP	26/1493	42/3664	0.006685502	0.012886045
42	GO:0065003	protein-containing complex assembly	BP	42/1493	75/3664	0.008611015	0.015474867
7	GO:0006839	mitochondrial transport	BP	7/1493	8/3664	0.009536347	0.016973785
54	GO:0010468	regulation of gene expression	BP	54/1493	101/3664	0.010017431	0.017495232
10	GO:0009142	nucleoside triphosphate biosynthetic process	BP	10/1493	13/3664	0.010076331	0.017515866
10	GO:0009201	ribonucleoside triphosphate biosynthetic process	BP	10/1493	13/3664	0.010076331	0.017515866
14	GO:0006099	tricarboxylic acid cycle	BP	14/1493	20/3664	0.01062065	0.018291119
16	GO:0009451	RNA modification	BP	16/1493	24/3664	0.011810312	0.019701507
17	GO:0000375	RNA splicing, via transesterification reactions	BP	17/1493	26/3664	0.014775496	0.023691744
17	GO:0000377	RNA splicing, via transesterification reactions with bulged ade	BP	17/1493	26/3664	0.014775496	0.023691744
17	GO:0000398	mRNA splicing, via spliceosome	BP	17/1493	26/3664	0.014775496	0.023691744
37	GO:0009889	regulation of biosynthetic process	BP	37/1493	67/3664	0.016974693	0.026531873
37	GO:0010556	regulation of macromolecule biosynthetic process	BP	37/1493	67/3664	0.016974693	0.026531873
37	GO:0031326	regulation of cellular biosynthetic process	BP	37/1493	67/3664	0.016974693	0.026531873
6	GO:0009130	pyrimidine nucleoside monophosphate biosynthetic process	BP	6/1493	7/3664	0.020753565	0.030758271
6	GO:0071806	protein transmembrane transport	BP	6/1493	7/3664	0.020753565	0.030758271
6	GO:0046049	UMP metabolic process	BP	6/1493	7/3664	0.020753565	0.030758271
6	GO:0065002	intracellular protein transmembrane transport	BP	6/1493	7/3664	0.020753565	0.030758271
6	GO:0006222	UMP biosynthetic process	BP	6/1493	7/3664	0.020753565	0.030758271
6	GO:0009174	pyrimidine ribonucleoside monophosphate biosynthetic proce	BP	6/1493	7/3664	0.020753565	0.030758271
6	GO:0009173	pyrimidine ribonucleoside monophosphate metabolic process	BP	6/1493	7/3664	0.020753565	0.030758271
6	GO:0009129	pyrimidine nucleoside monophosphate metabolic process	BP	6/1493	7/3664	0.020753565	0.030758271
43	GO:0043933	protein-containing complex organization	BP	43/1493	80/3664	0.020910922	0.029918704
19	GO:0032259	methylation	BP	19/1493	31/3664	0.026281032	0.035422261
4	GO:0006241	CTP biosynthetic process	BP	4/1493	4/3664	0.027503128	0.036409835
4	GO:0009148	pyrimidine nucleoside triphosphate biosynthetic process	BP	4/1493	4/3664	0.027503128	0.036409835
4	GO:0009147	pyrimidine nucleoside triphosphate metabolic process	BP	4/1493	4/3664	0.027503128	0.036409835
4	GO:0006120	mitochondrial electron transport, NADH to ubiquinone	BP	4/1493	4/3664	0.027503128	0.036409835
4	GO:0006446	regulation of translational initiation	BP	4/1493	4/3664	0.027503128	0.036409835
4	GO:0009209	pyrimidine ribonucleoside triphosphate biosynthetic process	BP	4/1493	4/3664	0.027503128	0.036409835
4	GO:0046036	CTP metabolic process	BP	4/1493	4/3664	0.027503128	0.036409835
4	GO:0009081	branched-chain amino acid metabolic process	BP	4/1493	4/3664	0.027503128	0.036409835

4	GO:0030150	protein import into mitochondrial matrix	BP	4/1493	4/3664	0.027503128	0.036409835
4	GO:0032543	mitochondrial translation	BP	4/1493	4/3664	0.027503128	0.036409835
4	GO:0009208	pyrimidine ribonucleoside triphosphate metabolic process	BP	4/1493	4/3664	0.027503128	0.036409835
4	GO:0006123	mitochondrial electron transport, cytochrome c to oxygen	BP	4/1493	4/3664	0.027503128	0.036409835
12	GO:0015980	energy derivation by oxidation of organic compounds	BP	12/1493	18/3664	0.030328159	0.037112089
29	GO:0051252	regulation of RNA metabolic process	BP	29/1493	52/3664	0.032363923	0.039088894
7	GO:0006400	tRNA modification	BP	7/1493	9/3664	0.036727061	0.041653862
11	GO:0043414	macromolecule methylation	BP	11/1493	16/3664	0.037721605	0.042522537
48	GO:0031323	regulation of cellular metabolic process	BP	48/1493	94/3664	0.043303791	0.047659793
5	GO:0006448	regulation of translational elongation	BP	5/1493	6/3664	0.044383384	0.04870389
14	GO:0044085	cellular component biogenesis	BP	14/1493	22/3664	0.047456556	0.047713078
14	GO:0022613	ribonucleoprotein complex biogenesis	BP	14/1493	22/3664	0.047456556	0.047713078
23	GO:0015935	small ribosomal subunit	CC	23/1493	25/3664	1.10E-07	4.09E-05
42	GO:0044391	ribosomal subunit	CC	42/1493	50/3664	5.84E-07	3.62E-05
35	GO:0098798	mitochondrial protein-containing complex	CC	35/1493	40/3664	6.93E-07	3.68E-05
38	GO:0005730	nucleolus	CC	38/1493	51/3664	1.82E-06	5.22E-05
27	GO:0019866	organelle inner membrane	CC	27/1493	33/3664	2.19E-06	4.28E-05
27	GO:0005743	mitochondrial inner membrane	CC	27/1493	33/3664	2.19E-06	4.28E-05
92	GO:0005840	ribosome	CC	92/1493	109/3664	2.27E-06	3.67E-05
174	GO:1990904	ribonucleoprotein complex	CC	174/1493	219/3664	2.45E-06	3.65E-05
142	GO:0043232	intracellular non-membrane-bounded organelle	CC	142/1493	223/3664	2.95E-06	3.66E-05
142	GO:0043228	non-membrane-bounded organelle	CC	142/1493	223/3664	2.95E-06	3.66E-05
23	GO:0098800	inner mitochondrial membrane protein complex	CC	23/1493	27/3664	3.11E-06	3.30E-05
69	GO:0098796	membrane protein complex	CC	69/1493	110/3664	4.22E-06	3.65E-05
340	GO:0032991	protein-containing complex	CC	340/1493	573/3664	4.39E-06	3.71E-05
303	GO:0043229	intracellular organelle	CC	303/1493	606/3664	4.70E-06	3.80E-05
306	GO:0043226	organelle	CC	306/1493	610/3664	4.95E-06	3.92E-05
33	GO:0031966	mitochondrial membrane	CC	33/1493	44/3664	6.85E-06	4.55E-05
15	GO:0098803	respiratory chain complex	CC	15/1493	16/3664	1.35E-05	8.10E-05
10	GO:0033178	proton-transporting two-sector ATPase complex, catalytic do	CC	10/1493	10/3664	0.000123955	0.000569275
928	GO:0005575	cellular_component	CC	928/1493	2139/3664	0.000132853	0.000602698
25	GO:0005681	spliceosomal complex	CC	25/1493	34/3664	0.000141381	0.000618752
14	GO:1990204	oxidoreductase complex	CC	14/1493	16/3664	0.000155903	0.000659046
13	GO:0033177	proton-transporting two-sector ATPase complex, proton-trar	CC	13/1493	15/3664	0.000338524	0.001234616
19	GO:0015934	large ribosomal subunit	CC	19/1493	25/3664	0.00067797	0.002212324
70	GO:1902494	catalytic complex	CC	70/1493	129/3664	0.001839756	0.005519269
7	GO:0045271	respiratory chain complex I	CC	7/1493	7/3664	0.001849709	0.005504735
7	GO:0030964	NADH dehydrogenase complex	CC	7/1493	7/3664	0.001849709	0.005504735
7	GO:0033179	proton-transporting V-type ATPase, V0 domain	CC	7/1493	7/3664	0.001849709	0.005504735
7	GO:0070469	respirasome	CC	7/1493	7/3664	0.001849709	0.005504735
11	GO:0016282	eukaryotic 43S preinitiation complex	CC	11/1493	13/3664	0.002645108	0.007028431
11	GO:0033290	eukaryotic 48S preinitiation complex	CC	11/1493	13/3664	0.002645108	0.007028431
11	GO:0070993	translation preinitiation complex	CC	11/1493	13/3664	0.002645108	0.007028431
11	GO:0005852	eukaryotic translation initiation factor 3 complex	CC	11/1493	13/3664	0.002645108	0.007028431
8	GO:0070069	cytochrome complex	CC	8/1493	9/3664	0.004322216	0.009986736
6	GO:0045277	respiratory chain complex IV	CC	6/1493	6/3664	0.00455026	0.009898811

6	GO:0030684	preribosome	CC	6/1493	6/3664	0.00455026	0.009898811
6	GO:0005747	mitochondrial respiratory chain complex I	CC	6/1493	6/3664	0.00455026	0.009898811
5	GO:0016272	prefoldin complex	CC	5/1493	5/3664	0.011189114	0.019181338
5	GO:0005751	mitochondrial respiratory chain complex IV	CC	5/1493	5/3664	0.011189114	0.019181338
5	GO:0033180	proton-transporting V-type ATPase, V1 domain	CC	5/1493	5/3664	0.011189114	0.019181338
8	GO:0022627	cytosolic small ribosomal subunit	CC	8/1493	10/3664	0.01915064	0.028959505
6	GO:0031461	cullin-RING ubiquitin ligase complex	CC	6/1493	7/3664	0.020753565	0.030758271
54	GO:0140513	nuclear protein-containing complex	CC	54/1493	105/3664	0.02652566	0.035494769
4	GO:0045261	proton-transporting ATP synthase complex, catalytic core F(1)	CC	4/1493	4/3664	0.027503128	0.036409835
39	GO:0005739	mitochondrion	CC	39/1493	73/3664	0.030053731	0.03689765
46	GO:0140535	intracellular protein-containing complex	CC	46/1493	89/3664	0.037823005	0.042379993
5	GO:1902493	acetyltransferase complex	CC	5/1493	6/3664	0.044383384	0.04870389
5	GO:0055029	nuclear DNA-directed RNA polymerase complex	CC	5/1493	6/3664	0.044383384	0.04870389
5	GO:0031248	protein acetyltransferase complex	CC	5/1493	6/3664	0.044383384	0.04870389
9	GO:0120114	Sm-like protein family complex	CC	9/1493	13/3664	0.04704678	0.047687744
9	GO:0005759	mitochondrial matrix	CC	9/1493	13/3664	0.04704678	0.047687744
16	GO:0046961	proton-transporting ATPase activity, rotational mechanism	MF	16/1493	16/3664	5.51E-07	0.000102423
16	GO:0044769	ATPase activity, coupled to transmembrane movement of ions	MF	16/1493	16/3664	5.51E-07	0.000102423
16	GO:0042625	ATPase-coupled ion transmembrane transporter activity	MF	16/1493	16/3664	5.51E-07	0.000102423
16	GO:0009678	pyrophosphate hydrolysis-driven proton transmembrane tran	MF	16/1493	16/3664	5.51E-07	0.000102423
25	GO:0051082	unfolded protein binding	MF	25/1493	29/3664	7.68E-07	3.57E-05
25	GO:0015078	proton transmembrane transporter activity	MF	25/1493	28/3664	1.54E-06	5.73E-05
47	GO:0008135	translation factor activity, RNA binding	MF	47/1493	63/3664	1.63E-06	5.50E-05
48	GO:0090079	translation regulator activity, nucleic acid binding	MF	48/1493	64/3664	2.00E-06	5.30E-05
48	GO:0045182	translation regulator activity	MF	48/1493	64/3664	2.00E-06	5.30E-05
106	GO:0003735	structural constituent of ribosome	MF	106/1493	123/3664	2.23E-06	3.95E-05
84	GO:0140657	ATP-dependent activity	MF	84/1493	137/3664	3.10E-06	3.39E-05
145	GO:0003723	RNA binding	MF	145/1493	228/3664	3.75E-06	3.58E-05
249	GO:0003676	nucleic acid binding	MF	249/1493	440/3664	3.93E-06	3.56E-05
32	GO:0003743	translation initiation factor activity	MF	32/1493	42/3664	3.96E-06	3.51E-05
476	GO:0097159	organic cyclic compound binding	MF	476/1493	989/3664	5.41E-06	4.11E-05
476	GO:1901363	heterocyclic compound binding	MF	476/1493	989/3664	5.41E-06	4.11E-05
22	GO:0044183	protein folding chaperone	MF	22/1493	26/3664	6.29E-06	4.50E-05
22	GO:0140662	ATP-dependent protein folding chaperone	MF	22/1493	26/3664	6.29E-06	4.50E-05
18	GO:0019829	ATPase-coupled cation transmembrane transporter activity	MF	18/1493	20/3664	6.53E-06	4.41E-05
23	GO:0022853	active ion transmembrane transporter activity	MF	23/1493	28/3664	1.66E-05	9.64E-05
32	GO:0022890	inorganic cation transmembrane transporter activity	MF	32/1493	45/3664	4.51E-05	0.000242983
33	GO:0008324	cation transmembrane transporter activity	MF	33/1493	47/3664	6.44E-05	0.000328251
17	GO:0019843	rRNA binding	MF	17/1493	20/3664	8.76E-05	0.000428589
38	GO:0022804	active transmembrane transporter activity	MF	38/1493	58/3664	0.000136568	0.000612086
74	GO:0022857	transmembrane transporter activity	MF	74/1493	130/3664	0.000175944	0.000719244
34	GO:0015318	inorganic molecular entity transmembrane transporter activity	MF	34/1493	51/3664	0.000248755	0.000953988
36	GO:0015075	ion transmembrane transporter activity	MF	36/1493	55/3664	0.000253905	0.000963804
31	GO:0015399	primary active transmembrane transporter activity	MF	31/1493	46/3664	0.000383685	0.001297554
58	GO:0140098	catalytic activity, acting on RNA	MF	58/1493	100/3664	0.000590855	0.001962483
33	GO:0016887	ATP hydrolysis activity	MF	33/1493	51/3664	0.000782028	0.002529691

26	GO:0140101	catalytic activity, acting on a tRNA	MF	26/1493	39/3664	0.001495381	0.004559688
7	GO:0046933	proton-transporting ATP synthase activity, rotational mechani	MF	7/1493	7/3664	0.001849709	0.005504735
21	GO:0051540	metal cluster binding	MF	21/1493	31/3664	0.002851516	0.007265506
21	GO:0051536	iron-sulfur cluster binding	MF	21/1493	31/3664	0.002851516	0.007265506
26	GO:0042626	ATPase-coupled transmembrane transporter activity	MF	26/1493	41/3664	0.003714931	0.008915835
18	GO:0008186	ATP-dependent activity, acting on RNA	MF	18/1493	26/3664	0.004252576	0.009949423
18	GO:0003724	RNA helicase activity	MF	18/1493	26/3664	0.004252576	0.009949423
8	GO:0031072	heat shock protein binding	MF	8/1493	9/3664	0.004322216	0.009986736
8	GO:0019205	nucleobase-containing compound kinase activity	MF	8/1493	9/3664	0.004322216	0.009986736
21	GO:0016875	ligase activity, forming carbon-oxygen bonds	MF	21/1493	32/3664	0.00588198	0.011701051
21	GO:0004812	aminoacyl-tRNA ligase activity	MF	21/1493	32/3664	0.00588198	0.011701051
81	GO:0005215	transporter activity	MF	81/1493	157/3664	0.00599273	0.011671704
15	GO:0003746	translation elongation factor activity	MF	15/1493	21/3664	0.006183122	0.011979799
7	GO:0015252	proton channel activity	MF	7/1493	8/3664	0.009536347	0.016973785
7	GO:0003712	transcription coregulator activity	MF	7/1493	8/3664	0.009536347	0.016973785
5	GO:0016776	phosphotransferase activity, phosphate group as acceptor	MF	5/1493	5/3664	0.011189114	0.019181338
5	GO:0015453	oxidoreduction-driven active transmembrane transporter acti	MF	5/1493	5/3664	0.011189114	0.019181338
72	GO:0016817	hydrolase activity, acting on acid anhydrides	MF	72/1493	142/3664	0.014756156	0.02376316
11	GO:0043021	ribonucleoprotein complex binding	MF	11/1493	15/3664	0.015192578	0.024049527
69	GO:0140640	catalytic activity, acting on a nucleic acid	MF	69/1493	135/3664	0.015681108	0.024717679
22	GO:0008168	methyltransferase activity	MF	22/1493	36/3664	0.016169402	0.025379821
13	GO:0016298	lipase activity	MF	13/1493	19/3664	0.018062626	0.02776569
18	GO:0004177	aminopeptidase activity	MF	18/1493	28/3664	0.018756421	0.028595855
8	GO:0003729	mRNA binding	MF	8/1493	10/3664	0.01915064	0.028959505
9	GO:0009055	electron transfer activity	MF	9/1493	12/3664	0.019183517	0.028775276
71	GO:0016818	hydrolase activity, acting on acid anhydrides, in phosphorus-c	MF	71/1493	141/3664	0.02273874	0.032040951
239	GO:0000166	nucleotide binding	MF	239/1493	528/3664	0.024415895	0.034274389
239	GO:1901265	nucleoside phosphate binding	MF	239/1493	528/3664	0.024415895	0.034274389
63	GO:0017111	ribonucleoside triphosphate phosphatase activity	MF	63/1493	124/3664	0.025288567	0.034208534
4	GO:0016624	oxidoreductase activity, acting on the aldehyde or oxo group	MF	4/1493	4/3664	0.027503128	0.036409835
4	GO:0050136	NADH dehydrogenase (quinone) activity	MF	4/1493	4/3664	0.027503128	0.036409835
4	GO:0003954	NADH dehydrogenase activity	MF	4/1493	4/3664	0.027503128	0.036409835
4	GO:0050145	nucleoside monophosphate kinase activity	MF	4/1493	4/3664	0.027503128	0.036409835
4	GO:0003955	NAD(P)H dehydrogenase (quinone) activity	MF	4/1493	4/3664	0.027503128	0.036409835
4	GO:0016655	oxidoreductase activity, acting on NAD(P)H, quinone or simila	MF	4/1493	4/3664	0.027503128	0.036409835
4	GO:0008137	NADH dehydrogenase (ubiquinone) activity	MF	4/1493	4/3664	0.027503128	0.036409835
4	GO:0004176	ATP-dependent peptidase activity	MF	4/1493	4/3664	0.027503128	0.036409835
70	GO:0016462	pyrophosphatase activity	MF	70/1493	140/3664	0.028024225	0.034519906
26	GO:0004386	helicase activity	MF	26/1493	46/3664	0.034024007	0.039927226
127	GO:0005198	structural molecule activity	MF	127/1493	271/3664	0.034171705	0.039974447
23	GO:0016741	transferase activity, transferring one-carbon groups	MF	23/1493	40/3664	0.035200993	0.040415955
254	GO:0036094	small molecule binding	MF	254/1493	567/3664	0.036399003	0.04140804
7	GO:0008276	protein methyltransferase activity	MF	7/1493	9/3664	0.036727061	0.041653862
11	GO:0016651	oxidoreductase activity, acting on NAD(P)H	MF	11/1493	16/3664	0.037721605	0.042522537
36	GO:0008237	metallopeptidase activity	MF	36/1493	68/3664	0.045815256	0.049688849
230	GO:0043168	anion binding	MF	230/1493	514/3664	0.047318439	0.047703142

14	GO:0008757	S-adenosylmethionine-dependent methyltransferase activity	MF	14/1493	22/3664	0.047456556	0.047713078
