# Supplementary material for: Comparative Proteomics and Metabonomics Analysis of Different Diapause Stages Revealed a New Regulation Mechanism of Diapause in Loxostege sticticalis (Lepidoptera: Pyralidae)
Source: Molecules. 2024 Jul 25;29(15):3472. doi: 10.3390/molecules29153472 (PMC11314584; doi:10.3390/molecules29153472)
Supplement: Supplementary file 1 [file molecules-29-03472-s001.zip › analysis process/proteomic/KEGG enrichment analysis/turquoise.pdf]

Protein Number	Pathway_ID	KEGG Description	Database	Ratio_in_study	Ratio_in_pop	P_value_uncorre	P_value_corrected
111	map03010	Ribosome	KEGG PATHWAY	111/1610	126/3750	0	0
76	map00190	Oxidative phosphorylation	KEGG PATHWAY	76/1610	79/3750	0	0
87	map05171	Coronavirus disease - COVID-19	KEGG PATHWAY	87/1610	108/3750	1.00E-15	5.70E-14
77	map04714	Thermogenesis	KEGG PATHWAY	77/1610	99/3750	8.67E-13	7.20E-11
49	map04932	Non-alcoholic fatty liver disease	KEGG PATHWAY	49/1610	64/3750	3.58E-08	2.38E-06
67	map03040	Spliceosome	KEGG PATHWAY	67/1610	96/3750	6.66E-08	3.68E-06
29	map04723	Retrograde endocannabinoid signaling	KEGG PATHWAY	29/1610	34/3750	3.85E-07	1.60E-05
65	map05415	Diabetic cardiomyopathy	KEGG PATHWAY	65/1610	95/3750	3.41E-07	1.62E-05
13	map04966	Collecting duct acid secretion	KEGG PATHWAY	13/1610	13/3750	1.64E-05	0.00060419
17	map05323	Rheumatoid arthritis	KEGG PATHWAY	17/1610	20/3750	0.000132709	0.004405932
26	map00970	Aminoacyl-tRNA biosynthesis	KEGG PATHWAY	26/1610	36/3750	0.000338982	0.010231096
23	map04721	Synaptic vesicle cycle	KEGG PATHWAY	23/1610	32/3750	0.000842024	0.021504009
66	map05208	Chemical carcinogenesis - reactive oxygen species	KEGG PATHWAY	66/1610	114/3750	0.000781256	0.02161474
76	map05020	Prion disease	KEGG PATHWAY	76/1610	135/3750	0.001006115	0.02226867
20	map05110	Vibrio cholerae infection	KEGG PATHWAY	20/1610	27/3750	0.001001457	0.023748843
21	map05120	Epithelial cell signaling in Helicobacter pylori infection	KEGG PATHWAY	21/1610	30/3750	0.002429499	0.050412101
30	map03008	Ribosome biogenesis in eukaryotes	KEGG PATHWAY	30/1610	47/3750	0.002971294	0.058027618
78	map05012	Parkinson disease	KEGG PATHWAY	78/1610	145/3750	0.004721441	0.087084352
25	map01232	Nucleotide metabolism	KEGG PATHWAY	25/1610	39/3750	0.00602916	0.105351632
79	map05016	Huntington disease	KEGG PATHWAY	79/1610	149/3750	0.007301111	0.121198443
13	map04640	Hematopoietic cell lineage	KEGG PATHWAY	13/1610	18/3750	0.011456317	0.172886242
38	map00983	Drug metabolism - other enzymes	KEGG PATHWAY	38/1610	66/3750	0.011099297	0.175474598
40	map03013	Nucleocytoplasmic transport	KEGG PATHWAY	40/1610	72/3750	0.019962	0.276141
20	map00020	Citrate cycle (TCA cycle)	KEGG PATHWAY	20/1610	32/3750	0.019861813	0.286700957
15	map04080	Neuroactive ligand-receptor interaction	KEGG PATHWAY	15/1610	23/3750	0.025805144	0.329511839
97	map05014	Amyotrophic lateral sclerosis	KEGG PATHWAY	97/1610	194/3750	0.024951457	0.331355344
32	map04972	Pancreatic secretion	KEGG PATHWAY	32/1610	57/3750	0.029631911	0.364362756
99	map05022	Pathways of neurodegeneration - multiple diseases	KEGG PATHWAY	99/1610	200/3750	0.032221026	0.382049311
42	map05165	Human papillomavirus infection	KEGG PATHWAY	42/1610	81/3750	0.064100785	0.686498726
20	map00860	Porphyrin metabolism	KEGG PATHWAY	20/1610	35/3750	0.063137288	0.698719315
22	map05152	Tuberculosis	KEGG PATHWAY	22/1610	39/3750	0.061683398	0.706168557
6	map00910	Nitrogen metabolism	KEGG PATHWAY	6/1610	8/3750	0.070324592	0.729617645
15	map00640	Propanoate metabolism	KEGG PATHWAY	15/1610	26/3750	0.092903807	0.934668608
15	map05162	Measles	KEGG PATHWAY	15/1610	26/3750	0.092903807	0.934668608
24	map05164	Influenza A	KEGG PATHWAY	24/1610	45/3750	0.103161571	0.978561192
2	map00030	Pentose phosphate pathway	KEGG PATHWAY	2/1610	42/3750	0.999999998	0.999999998
22	map03015	mRNA surveillance pathway	KEGG PATHWAY	22/1610	41/3750	0.108611428	1
5	map00730	Thiamine metabolism	KEGG PATHWAY	5/1610	7/3750	0.127246008	1
12	map00130	Ubiquinone and other terpenoid-quinone biosynthesis	KEGG PATHWAY	12/1610	21/3750	0.136332567	1
19	map03018	RNA degradation	KEGG PATHWAY	19/1610	36/3750	0.151591516	1
79	map05010	Alzheimer disease	KEGG PATHWAY	79/1610	168/3750	0.154782083	1
25	map04974	Protein digestion and absorption	KEGG PATHWAY	25/1610	49/3750	0.157220117	1
13	map04614	Renin-angiotensin system	KEGG PATHWAY	13/1610	24/3750	0.18149352	1
13	map00140	Steroid hormone biosynthesis	KEGG PATHWAY	13/1610	24/3750	0.18149352	1

2	map00261	Monobactam biosynthesis	KEGG PATHWAY	2/1610	2/3750	0.184261759	1
2	map04122	Sulfur relay system	KEGG PATHWAY	2/1610	2/3750	0.184261759	1
8	map00450	Selenocompound metabolism	KEGG PATHWAY	8/1610	14/3750	0.209434118	1
15	map00240	Pyrimidine metabolism	KEGG PATHWAY	15/1610	29/3750	0.219315958	1
4	map05216	Thyroid cancer	KEGG PATHWAY	4/1610	6/3750	0.222005329	1
5	map00563	Glycosylphosphatidylinositol (GPI)-anchor biosynthesis	KEGG PATHWAY	5/1610	8/3750	0.222115033	1
17	map04260	Cardiac muscle contraction	KEGG PATHWAY	17/1610	34/3750	0.252736032	1
17	map00830	Retinol metabolism	KEGG PATHWAY	17/1610	34/3750	0.252736032	1
16	map05134	Legionellosis	KEGG PATHWAY	16/1610	32/3750	0.26245144	1
9	map05226	Gastric cancer	KEGG PATHWAY	9/1610	17/3750	0.275706099	1
8	map00062	Fatty acid elongation	KEGG PATHWAY	8/1610	15/3750	0.287583733	1
8	map03020	RNA polymerase	KEGG PATHWAY	8/1610	15/3750	0.287583733	1
12	map05161	Hepatitis B	KEGG PATHWAY	12/1610	24/3750	0.308236542	1
5	map05412	Arrhythmogenic right ventricular cardiomyopathy	KEGG PATHWAY	5/1610	9/3750	0.330471584	1
4	map04215	Apoptosis - multiple species	KEGG PATHWAY	4/1610	7/3750	0.34835109	1
9	map05416	Viral myocarditis	KEGG PATHWAY	9/1610	18/3750	0.353166935	1
9	map05211	Renal cell carcinoma	KEGG PATHWAY	9/1610	18/3750	0.353166935	1
8	map04657	IL-17 signaling pathway	KEGG PATHWAY	8/1610	16/3750	0.371265022	1
8	map04350	TGF-beta signaling pathway	KEGG PATHWAY	8/1610	16/3750	0.371265022	1
2	map00470	D-Amino acid metabolism	KEGG PATHWAY	2/1610	3/3750	0.394678068	1
2	map00440	Phosphonate and phosphinate metabolism	KEGG PATHWAY	2/1610	3/3750	0.394678068	1
2	map00120	Primary bile acid biosynthesis	KEGG PATHWAY	2/1610	3/3750	0.394678068	1
29	map05203	Viral carcinogenesis	KEGG PATHWAY	29/1610	64/3750	0.395267715	1
14	map04666	Fc gamma R-mediated phagocytosis	KEGG PATHWAY	14/1610	30/3750	0.406238961	1
14	map04390	Hippo signaling pathway	KEGG PATHWAY	14/1610	30/3750	0.406238961	1
23	map03320	PPAR signaling pathway	KEGG PATHWAY	23/1610	51/3750	0.429300442	1
1	map00300	Lysine biosynthesis	KEGG PATHWAY	1/1610	1/3750	0.429333333	1
1	map00780	Biotin metabolism	KEGG PATHWAY	1/1610	1/3750	0.429333333	1
1	map00311	Penicillin and cephalosporin biosynthesis	KEGG PATHWAY	1/1610	1/3750	0.429333333	1
1	map05033	Nicotine addiction	KEGG PATHWAY	1/1610	1/3750	0.429333333	1
1	map00290	Valine, leucine and isoleucine biosynthesis	KEGG PATHWAY	1/1610	1/3750	0.429333333	1
1	map05340	Primary immunodeficiency	KEGG PATHWAY	1/1610	1/3750	0.429333333	1
5	map04115	p53 signaling pathway	KEGG PATHWAY	5/1610	10/3750	0.441834661	1
18	map05225	Hepatocellular carcinoma	KEGG PATHWAY	18/1610	40/3750	0.455375733	1
8	map05210	Colorectal cancer	KEGG PATHWAY	8/1610	17/3750	0.456206287	1
8	map00061	Fatty acid biosynthesis	KEGG PATHWAY	8/1610	17/3750	0.456206287	1
24	map00280	Valine, leucine and isoleucine degradation	KEGG PATHWAY	24/1610	54/3750	0.462668999	1
11	map04020	Calcium signaling pathway	KEGG PATHWAY	11/1610	24/3750	0.463935288	1
7	map00650	Butanoate metabolism	KEGG PATHWAY	7/1610	15/3750	0.482561659	1
7	map05140	Leishmaniasis	KEGG PATHWAY	7/1610	15/3750	0.482561659	1
22	map04120	Ubiquitin mediated proteolysis	KEGG PATHWAY	22/1610	50/3750	0.493527125	1
28	map05207	Chemical carcinogenesis - receptor activation	KEGG PATHWAY	28/1610	64/3750	0.495371317	1
9	map01040	Biosynthesis of unsaturated fatty acids	KEGG PATHWAY	9/1610	20/3750	0.51128984	1
6	map04650	Natural killer cell mediated cytotoxicity	KEGG PATHWAY	6/1610	13/3750	0.512675456	1
6	map05224	Breast cancer	KEGG PATHWAY	6/1610	13/3750	0.512675456	1

6	map04964	Proximal tubule bicarbonate reclamation	KEGG PATHWAY	6/1610	13/3750	0.512675456	1
6	map01521	EGFR tyrosine kinase inhibitor resistance	KEGG PATHWAY	6/1610	13/3750	0.512675456	1
3	map04711	Circadian rhythm - fly	KEGG PATHWAY	3/1610	6/3750	0.51636658	1
23	map05205	Proteoglycans in cancer	KEGG PATHWAY	23/1610	53/3750	0.525770296	1
11	map04214	Apoptosis - fly	KEGG PATHWAY	11/1610	25/3750	0.533770491	1
5	map00920	Sulfur metabolism	KEGG PATHWAY	5/1610	11/3750	0.54778839	1
2	map00232	Caffeine metabolism	KEGG PATHWAY	2/1610	4/3750	0.574854403	1
2	map05217	Basal cell carcinoma	KEGG PATHWAY	2/1610	4/3750	0.574854403	1
2	map00512	Mucin type O-glycan biosynthesis	KEGG PATHWAY	2/1610	4/3750	0.574854403	1
22	map05417	Lipid and atherosclerosis	KEGG PATHWAY	22/1610	52/3750	0.589521621	1
4	map00360	Phenylalanine metabolism	KEGG PATHWAY	4/1610	9/3750	0.589879597	1
4	map04923	Regulation of lipolysis in adipocytes	KEGG PATHWAY	4/1610	9/3750	0.589879597	1
4	map00100	Steroid biosynthesis	KEGG PATHWAY	4/1610	9/3750	0.589879597	1
11	map03250	Viral life cycle - HIV-1	KEGG PATHWAY	11/1610	26/3750	0.600199861	1
11	map04915	Estrogen signaling pathway	KEGG PATHWAY	11/1610	26/3750	0.600199861	1
6	map05031	Amphetamine addiction	KEGG PATHWAY	6/1610	14/3750	0.603503079	1
21	map04150	mTOR signaling pathway	KEGG PATHWAY	21/1610	50/3750	0.606980652	1
8	map04933	AGE-RAGE signaling pathway in diabetic complications	KEGG PATHWAY	8/1610	19/3750	0.615623615	1
10	map04391	Hippo signaling pathway - fly	KEGG PATHWAY	10/1610	24/3750	0.626564017	1
12	map01524	Platinum drug resistance	KEGG PATHWAY	12/1610	29/3750	0.636574782	1
3	map03022	Basal transcription factors	KEGG PATHWAY	3/1610	7/3750	0.642378197	1
3	map00514	Other types of O-glycan biosynthesis	KEGG PATHWAY	3/1610	7/3750	0.642378197	1
5	map03030	DNA replication	KEGG PATHWAY	5/1610	12/3750	0.642816007	1
5	map04623	Cytosolic DNA-sensing pathway	KEGG PATHWAY	5/1610	12/3750	0.642816007	1
5	map05322	Systemic lupus erythematosus	KEGG PATHWAY	5/1610	12/3750	0.642816007	1
5	map03420	Nucleotide excision repair	KEGG PATHWAY	5/1610	12/3750	0.642816007	1
24	map00982	Drug metabolism - cytochrome P450	KEGG PATHWAY	24/1610	58/3750	0.64389058	1
9	map00562	Inositol phosphate metabolism	KEGG PATHWAY	9/1610	22/3750	0.654820407	1
28	map05130	Pathogenic Escherichia coli infection	KEGG PATHWAY	28/1610	68/3750	0.660528015	1
11	map04371	Apelin signaling pathway	KEGG PATHWAY	11/1610	27/3750	0.661797315	1
25	map05204	Chemical carcinogenesis - DNA adducts	KEGG PATHWAY	25/1610	61/3750	0.668308292	1
1	map00524	Neomycin, kanamycin and gentamicin biosynthesis	KEGG PATHWAY	1/1610	2/3750	0.674404908	1
1	map00400	Phenylalanine, tyrosine and tryptophan biosynthesis	KEGG PATHWAY	1/1610	2/3750	0.674404908	1
55	map01240	Biosynthesis of cofactors	KEGG PATHWAY	55/1610	133/3750	0.677495863	1
4	map04914	Progesterone-mediated oocyte maturation	KEGG PATHWAY	4/1610	10/3750	0.688576222	1
18	map05168	Herpes simplex virus 1 infection	KEGG PATHWAY	18/1610	45/3750	0.70739075	1
2	map05218	Melanoma	KEGG PATHWAY	2/1610	5/3750	0.711966703	1
2	map00750	Vitamin B6 metabolism	KEGG PATHWAY	2/1610	5/3750	0.711966703	1
2	map05030	Cocaine addiction	KEGG PATHWAY	2/1610	5/3750	0.711966703	1
2	map00565	Ether lipid metabolism	KEGG PATHWAY	2/1610	5/3750	0.711966703	1
7	map02010	ABC transporters	KEGG PATHWAY	7/1610	18/3750	0.717871193	1
5	map04130	SNARE interactions in vesicular transport	KEGG PATHWAY	5/1610	13/3750	0.724153852	1
5	map04917	Prolactin signaling pathway	KEGG PATHWAY	5/1610	13/3750	0.724153852	1
5	map04341	Hedgehog signaling pathway - fly	KEGG PATHWAY	5/1610	13/3750	0.724153852	1
21	map00230	Purine metabolism	KEGG PATHWAY	21/1610	53/3750	0.734219988	1

3	map04745	Phototransduction - fly	KEGG PATHWAY	3/1610	8/3750	0.743052828	1
3	map05213	Endometrial cancer	KEGG PATHWAY	3/1610	8/3750	0.743052828	1
3	map04664	Fc epsilon RI signaling pathway	KEGG PATHWAY	3/1610	8/3750	0.743052828	1
10	map00564	Glycerophospholipid metabolism	KEGG PATHWAY	10/1610	26/3750	0.743567935	1
10	map00510	N-Glycan biosynthesis	KEGG PATHWAY	10/1610	26/3750	0.743567935	1
10	map05145	Toxoplasmosis	KEGG PATHWAY	10/1610	26/3750	0.743567935	1
26	map00980	Metabolism of xenobiotics by cytochrome P450	KEGG PATHWAY	26/1610	66/3750	0.760603002	1
15	map05135	Yersinia infection	KEGG PATHWAY	15/1610	39/3750	0.765892446	1
4	map04726	Serotonergic synapse	KEGG PATHWAY	4/1610	11/3750	0.769026411	1
25	map04145	Phagosome	KEGG PATHWAY	25/1610	64/3750	0.775012833	1
5	map04916	Melanogenesis	KEGG PATHWAY	5/1610	14/3750	0.791182059	1
5	map04340	Hedgehog signaling pathway	KEGG PATHWAY	5/1610	14/3750	0.791182059	1
30	map00480	Glutathione metabolism	KEGG PATHWAY	30/1610	77/3750	0.795572173	1
8	map04612	Antigen processing and presentation	KEGG PATHWAY	8/1610	22/3750	0.798462643	1
8	map04110	Cell cycle	KEGG PATHWAY	8/1610	22/3750	0.798462643	1
23	map04910	Insulin signaling pathway	KEGG PATHWAY	23/1610	60/3750	0.803781957	1
2	map04744	Phototransduction	KEGG PATHWAY	2/1610	6/3750	0.809766764	1
2	map00740	Riboflavin metabolism	KEGG PATHWAY	2/1610	6/3750	0.809766764	1
6	map00350	Tyrosine metabolism	KEGG PATHWAY	6/1610	17/3750	0.810386978	1
6	map05231	Choline metabolism in cancer	KEGG PATHWAY	6/1610	17/3750	0.810386978	1
6	map05133	Pertussis	KEGG PATHWAY	6/1610	17/3750	0.810386978	1
1	map00532	Glycosaminoglycan biosynthesis - chondroitin sulfate / de	KEGG PATHWAY	1/1610	3/3750	0.814268328	1
9	map04068	FoxO signaling pathway	KEGG PATHWAY	9/1610	25/3750	0.816722644	1
3	map00592	alpha-Linolenic acid metabolism	KEGG PATHWAY	3/1610	9/3750	0.819639443	1
3	map04928	Parathyroid hormone synthesis, secretion and action	KEGG PATHWAY	3/1610	9/3750	0.819639443	1
3	map00900	Terpenoid backbone biosynthesis	KEGG PATHWAY	3/1610	9/3750	0.819639443	1
16	map05206	MicroRNAs in cancer	KEGG PATHWAY	16/1610	43/3750	0.820210019	1
12	map00270	Cysteine and methionine metabolism	KEGG PATHWAY	12/1610	33/3750	0.826691342	1
12	map05100	Bacterial invasion of epithelial cells	KEGG PATHWAY	12/1610	33/3750	0.826691342	1
4	map04973	Carbohydrate digestion and absorption	KEGG PATHWAY	4/1610	12/3750	0.832131613	1
10	map04921	Oxytocin signaling pathway	KEGG PATHWAY	10/1610	28/3750	0.83281136	1
19	map04530	Tight junction	KEGG PATHWAY	19/1610	51/3750	0.833170712	1
37	map05200	Pathways in cancer	KEGG PATHWAY	37/1610	96/3750	0.837674535	1
13	map00310	Lysine degradation	KEGG PATHWAY	13/1610	36/3750	0.841366472	1
5	map00670	One carbon pool by folate	KEGG PATHWAY	5/1610	15/3750	0.844707743	1
5	map04012	ErbB signaling pathway	KEGG PATHWAY	5/1610	15/3750	0.844707743	1
12	map05160	Hepatitis C	KEGG PATHWAY	12/1610	34/3750	0.859880439	1
19	map00071	Fatty acid degradation	KEGG PATHWAY	19/1610	52/3750	0.860061822	1
17	map04152	AMPK signaling pathway	KEGG PATHWAY	17/1610	47/3750	0.862717196	1
7	map04072	Phospholipase D signaling pathway	KEGG PATHWAY	7/1610	21/3750	0.867738067	1
13	map04210	Apoptosis	KEGG PATHWAY	13/1610	37/3750	0.871363051	1
18	map00590	Arachidonic acid metabolism	KEGG PATHWAY	18/1610	50/3750	0.873599782	1
3	map04550	Signaling pathways regulating pluripotency of stem cells	KEGG PATHWAY	3/1610	10/3750	0.875809395	1
2	map00073	Cutin, suberine and wax biosynthesis	KEGG PATHWAY	2/1610	7/3750	0.87672219	1
2	map04320	Dorso-ventral axis formation	KEGG PATHWAY	2/1610	7/3750	0.87672219	1

2	map04940	Type I diabetes mellitus	KEGG PATHWAY	2/1610	7/3750	0.87672219	1
16	map04976	Bile secretion	KEGG PATHWAY	16/1610	45/3750	0.877094812	1
16	map00040	Pentose and glucuronate interconversions	KEGG PATHWAY	16/1610	45/3750	0.877094812	1
11	map05167	Kaposi sarcoma-associated herpesvirus infection	KEGG PATHWAY	11/1610	32/3750	0.878125102	1
4	map04659	Th17 cell differentiation	KEGG PATHWAY	4/1610	13/3750	0.880121729	1
4	map05214	Glioma	KEGG PATHWAY	4/1610	13/3750	0.880121729	1
4	map04713	Circadian entrainment	KEGG PATHWAY	4/1610	13/3750	0.880121729	1
21	map04212	Longevity regulating pathway - worm	KEGG PATHWAY	21/1610	58/3750	0.880954446	1
14	map00380	Tryptophan metabolism	KEGG PATHWAY	14/1610	40/3750	0.881726149	1
5	map04971	Gastric acid secretion	KEGG PATHWAY	5/1610	16/3750	0.886332316	1
5	map05215	Prostate cancer	KEGG PATHWAY	5/1610	16/3750	0.886332316	1
20	map04151	PI3K-Akt signaling pathway	KEGG PATHWAY	20/1610	56/3750	0.892513207	1
6	map04660	T cell receptor signaling pathway	KEGG PATHWAY	6/1610	19/3750	0.893166066	1
1	map03410	Base excision repair	KEGG PATHWAY	1/1610	4/3750	0.89407297	1
1	map05219	Bladder cancer	KEGG PATHWAY	1/1610	4/3750	0.89407297	1
1	map00430	Taurine and hypotaurine metabolism	KEGG PATHWAY	1/1610	4/3750	0.89407297	1
10	map04114	Oocyte meiosis	KEGG PATHWAY	10/1610	30/3750	0.895949602	1
10	map04218	Cellular senescence	KEGG PATHWAY	10/1610	30/3750	0.895949602	1
7	map00513	Various types of N-glycan biosynthesis	KEGG PATHWAY	7/1610	22/3750	0.900066599	1
19	map05170	Human immunodeficiency virus 1 infection	KEGG PATHWAY	19/1610	54/3750	0.903708756	1
4	map04620	Toll-like receptor signaling pathway	KEGG PATHWAY	4/1610	14/3750	0.915697597	1
4	map05212	Pancreatic cancer	KEGG PATHWAY	4/1610	14/3750	0.915697597	1
4	map04960	Aldosterone-regulated sodium reabsorption	KEGG PATHWAY	4/1610	14/3750	0.915697597	1
3	map04724	Glutamatergic synapse	KEGG PATHWAY	3/1610	11/3750	0.915853013	1
2	map05223	Non-small cell lung cancer	KEGG PATHWAY	2/1610	8/3750	0.921278645	1
2	map05032	Morphine addiction	KEGG PATHWAY	2/1610	8/3750	0.921278645	1
2	map04710	Circadian rhythm	KEGG PATHWAY	2/1610	8/3750	0.921278645	1
11	map00790	Folate biosynthesis	KEGG PATHWAY	11/1610	34/3750	0.924867067	1
7	map04934	Cushing syndrome	KEGG PATHWAY	7/1610	23/3750	0.925404563	1
23	map04936	Alcoholic liver disease	KEGG PATHWAY	23/1610	66/3750	0.929640946	1
12	map04261	Adrenergic signaling in cardiomyocytes	KEGG PATHWAY	12/1610	37/3750	0.930148839	1
12	map04066	HIF-1 signaling pathway	KEGG PATHWAY	12/1610	37/3750	0.930148839	1
9	map04975	Fat digestion and absorption	KEGG PATHWAY	9/1610	29/3750	0.933781972	1
1	map04929	GnRH secretion	KEGG PATHWAY	1/1610	5/3750	0.939599536	1
1	map04392	Hippo signaling pathway - multiple species	KEGG PATHWAY	1/1610	5/3750	0.939599536	1
1	map03450	Non-homologous end-joining	KEGG PATHWAY	1/1610	5/3750	0.939599536	1
4	map04662	B cell receptor signaling pathway	KEGG PATHWAY	4/1610	15/3750	0.94151209	1
4	map04720	Long-term potentiation	KEGG PATHWAY	4/1610	15/3750	0.94151209	1
4	map04935	Growth hormone synthesis, secretion and action	KEGG PATHWAY	4/1610	15/3750	0.94151209	1
5	map03060	Protein export	KEGG PATHWAY	5/1610	18/3750	0.941565182	1
6	map05230	Central carbon metabolism in cancer	KEGG PATHWAY	6/1610	21/3750	0.942895857	1
3	map04727	GABAergic synapse	KEGG PATHWAY	3/1610	12/3750	0.943760147	1
3	map04370	VEGF signaling pathway	KEGG PATHWAY	3/1610	12/3750	0.943760147	1
7	map04728	Dopaminergic synapse	KEGG PATHWAY	7/1610	24/3750	0.944941017	1
8	map04520	Adherens junction	KEGG PATHWAY	8/1610	27/3750	0.947373499	1

8	map04970	Salivary secretion	KEGG PATHWAY	8/1610	27/3750	0.947373499	1
9	map04062	Chemokine signaling pathway	KEGG PATHWAY	9/1610	30/3750	0.949995846	1
9	map04919	Thyroid hormone signaling pathway	KEGG PATHWAY	9/1610	30/3750	0.949995846	1
2	map04740	Olfactory transduction	KEGG PATHWAY	2/1610	9/3750	0.950318417	1
2	map04725	Cholinergic synapse	KEGG PATHWAY	2/1610	9/3750	0.950318417	1
42	map04141	Protein processing in endoplasmic reticulum	KEGG PATHWAY	42/1610	117/3750	0.952193367	1
10	map00630	Glyoxylate and dicarboxylate metabolism	KEGG PATHWAY	10/1610	33/3750	0.952685884	1
11	map04022	cGMP-PKG signaling pathway	KEGG PATHWAY	11/1610	36/3750	0.955367021	1
16	map05418	Fluid shear stress and atherosclerosis	KEGG PATHWAY	16/1610	50/3750	0.95867164	1
16	map00410	beta-Alanine metabolism	KEGG PATHWAY	16/1610	50/3750	0.95867164	1
16	map04024	cAMP signaling pathway	KEGG PATHWAY	16/1610	50/3750	0.95867164	1
6	map04270	Vascular smooth muscle contraction	KEGG PATHWAY	6/1610	22/3750	0.958956829	1
6	map05142	Chagas disease	KEGG PATHWAY	6/1610	22/3750	0.958956829	1
7	map04071	Sphingolipid signaling pathway	KEGG PATHWAY	7/1610	25/3750	0.959782989	1
4	map04911	Insulin secretion	KEGG PATHWAY	4/1610	16/3750	0.959905347	1
4	map04961	Endocrine and other factor-regulated calcium reabsorptic	KEGG PATHWAY	4/1610	16/3750	0.959905347	1
4	map04912	GnRH signaling pathway	KEGG PATHWAY	4/1610	16/3750	0.959905347	1
20	map04810	Regulation of actin cytoskeleton	KEGG PATHWAY	20/1610	61/3750	0.96098824	1
20	map04146	Peroxisome	KEGG PATHWAY	20/1610	61/3750	0.96098824	1
8	map05034	Alcoholism	KEGG PATHWAY	8/1610	28/3750	0.961053234	1
3	map04514	Cell adhesion molecules	KEGG PATHWAY	3/1610	13/3750	0.962851672	1
14	map05169	Epstein-Barr virus infection	KEGG PATHWAY	14/1610	45/3750	0.962961558	1
1	map00604	Glycosphingolipid biosynthesis - ganglio series	KEGG PATHWAY	1/1610	6/3750	0.965566091	1
1	map04060	Cytokine-cytokine receptor interaction	KEGG PATHWAY	1/1610	6/3750	0.965566091	1
1	map04630	JAK-STAT signaling pathway	KEGG PATHWAY	1/1610	6/3750	0.965566091	1
2	map04658	Th1 and Th2 cell differentiation	KEGG PATHWAY	2/1610	10/3750	0.968945672	1
2	map04730	Long-term depression	KEGG PATHWAY	2/1610	10/3750	0.968945672	1
6	map04211	Longevity regulating pathway	KEGG PATHWAY	6/1610	23/3750	0.970798805	1
5	map05222	Small cell lung cancer	KEGG PATHWAY	5/1610	20/3750	0.971326707	1
5	map04070	Phosphatidylinositol signaling system	KEGG PATHWAY	5/1610	20/3750	0.971326707	1
4	map05220	Chronic myeloid leukemia	KEGG PATHWAY	4/1610	17/3750	0.972806621	1
4	map04668	TNF signaling pathway	KEGG PATHWAY	4/1610	17/3750	0.972806621	1
19	map05017	Spinocerebellar ataxia	KEGG PATHWAY	19/1610	60/3750	0.973368254	1
16	map00053	Ascorbate and aldarate metabolism	KEGG PATHWAY	16/1610	52/3750	0.974581106	1
3	map04750	Inflammatory mediator regulation of TRP channels	KEGG PATHWAY	3/1610	14/3750	0.975711875	1
3	map04924	Renin secretion	KEGG PATHWAY	3/1610	14/3750	0.975711875	1
3	map01523	Antifolate resistance	KEGG PATHWAY	3/1610	14/3750	0.975711875	1
14	map05163	Human cytomegalovirus infection	KEGG PATHWAY	14/1610	47/3750	0.977922561	1
7	map04310	Wnt signaling pathway	KEGG PATHWAY	7/1610	27/3750	0.979144961	1
6	map04670	Leukocyte transendothelial migration	KEGG PATHWAY	6/1610	24/3750	0.979418068	1
10	map05202	Transcriptional misregulation in cancer	KEGG PATHWAY	10/1610	36/3750	0.980132973	1
5	map04361	Axon regeneration	KEGG PATHWAY	5/1610	21/3750	0.980211348	1
1	map03430	Mismatch repair	KEGG PATHWAY	1/1610	7/3750	0.980373408	1
2	map05221	Acute myeloid leukemia	KEGG PATHWAY	2/1610	11/3750	0.980744041	1
2	map01522	Endocrine resistance	KEGG PATHWAY	2/1610	11/3750	0.980744041	1

2	map04930	Type II diabetes mellitus	KEGG PATHWAY	2/1610	11/3750	0.980744041	1
12	map04015	Rap1 signaling pathway	KEGG PATHWAY	12/1610	42/3750	0.981529706	1
4	map04136	Autophagy - other	KEGG PATHWAY	4/1610	18/3750	0.981732747	1
4	map04962	Vasopressin-regulated water reabsorption	KEGG PATHWAY	4/1610	18/3750	0.981732747	1
3	map04622	RIG-I-like receptor signaling pathway	KEGG PATHWAY	3/1610	15/3750	0.984261821	1
8	map00250	Alanine, aspartate and glutamate metabolism	KEGG PATHWAY	8/1610	31/3750	0.985032597	1
9	map04722	Neurotrophin signaling pathway	KEGG PATHWAY	9/1610	34/3750	0.98511192	1
9	map04922	Glucagon signaling pathway	KEGG PATHWAY	9/1610	34/3750	0.98511192	1
12	map05166	Human T-cell leukemia virus 1 infection	KEGG PATHWAY	12/1610	43/3750	0.986155688	1
5	map04625	C-type lectin receptor signaling pathway	KEGG PATHWAY	5/1610	22/3750	0.986462677	1
13	map00620	Pyruvate metabolism	KEGG PATHWAY	13/1610	46/3750	0.98665445	1
15	map04010	MAPK signaling pathway	KEGG PATHWAY	15/1610	52/3750	0.987745934	1
4	map04512	ECM-receptor interaction	KEGG PATHWAY	4/1610	19/3750	0.987834658	1
4	map04920	Adipocytokine signaling pathway	KEGG PATHWAY	4/1610	19/3750	0.987834658	1
2	map04064	NF-kappa B signaling pathway	KEGG PATHWAY	2/1610	12/3750	0.98814082	1
29	map04144	Endocytosis	KEGG PATHWAY	29/1610	91/3750	0.989150179	1
9	map00260	Glycine, serine and threonine metabolism	KEGG PATHWAY	9/1610	35/3750	0.989216744	1
8	map05410	Hypertrophic cardiomyopathy	KEGG PATHWAY	8/1610	32/3750	0.989290974	1
11	map00330	Arginine and proline metabolism	KEGG PATHWAY	11/1610	41/3750	0.989447562	1
3	map04540	Gap junction	KEGG PATHWAY	3/1610	16/3750	0.989882545	1
6	map05414	Dilated cardiomyopathy	KEGG PATHWAY	6/1610	26/3750	0.99003183	1
5	map04613	Neutrophil extracellular trap formation	KEGG PATHWAY	5/1610	23/3750	0.990813753	1
7	map04611	Platelet activation	KEGG PATHWAY	7/1610	30/3750	0.992681273	1
2	map00220	Arginine biosynthesis	KEGG PATHWAY	2/1610	13/3750	0.992738846	1
3	map04978	Mineral absorption	KEGG PATHWAY	3/1610	17/3750	0.993541672	1
3	map04380	Osteoclast differentiation	KEGG PATHWAY	3/1610	17/3750	0.993541672	1
3	map04918	Thyroid hormone synthesis	KEGG PATHWAY	3/1610	17/3750	0.993541672	1
3	map04216	Ferroptosis	KEGG PATHWAY	3/1610	17/3750	0.993541672	1
4	map04926	Relaxin signaling pathway	KEGG PATHWAY	4/1610	21/3750	0.994726421	1
7	map00500	Starch and sucrose metabolism	KEGG PATHWAY	7/1610	31/3750	0.994912137	1
7	map04360	Axon guidance	KEGG PATHWAY	7/1610	31/3750	0.994912137	1
1	map03440	Homologous recombination	KEGG PATHWAY	1/1610	10/3750	0.996370096	1
4	map04137	Mitophagy - animal	KEGG PATHWAY	4/1610	22/3750	0.996562808	1
6	map04621	NOD-like receptor signaling pathway	KEGG PATHWAY	6/1610	29/3750	0.996820333	1
12	map00561	Glycerolipid metabolism	KEGG PATHWAY	12/1610	48/3750	0.997021302	1
32	map05132	Salmonella infection	KEGG PATHWAY	32/1610	105/3750	0.99710605	1
7	map04013	MAPK signaling pathway - fly	KEGG PATHWAY	7/1610	33/3750	0.997588013	1
14	map04140	Autophagy - animal	KEGG PATHWAY	14/1610	55/3750	0.997829989	1
6	map00770	Pantothenate and CoA biosynthesis	KEGG PATHWAY	6/1610	30/3750	0.997855098	1
8	map03050	Proteasome	KEGG PATHWAY	8/1610	37/3750	0.99818501	1
7	map01250	Biosynthesis of nucleotide sugars	KEGG PATHWAY	7/1610	34/3750	0.998354006	1
7	map04217	Necroptosis	KEGG PATHWAY	7/1610	34/3750	0.998354006	1
2	map00600	Sphingolipid metabolism	KEGG PATHWAY	2/1610	16/3750	0.998379547	1
6	map00052	Galactose metabolism	KEGG PATHWAY	6/1610	31/3750	0.998561408	1
9	map04931	Insulin resistance	KEGG PATHWAY	9/1610	41/3750	0.998641564	1

8	map04213	Longevity regulating pathway - multiple species	KEGG PATHWAY	8/1610	38/3750	0.998749835	1
8	map04014	Ras signaling pathway	KEGG PATHWAY	8/1610	38/3750	0.998749835	1
5	map04925	Aldosterone synthesis and secretion	KEGG PATHWAY	5/1610	28/3750	0.998805694	1
1	map00760	Nicotinate and nicotinamide metabolism	KEGG PATHWAY	1/1610	12/3750	0.998822873	1
2	map04977	Vitamin digestion and absorption	KEGG PATHWAY	2/1610	17/3750	0.999024597	1
3	map00340	Histidine metabolism	KEGG PATHWAY	3/1610	22/3750	0.999370587	1
5	map00981	Insect hormone biosynthesis	KEGG PATHWAY	5/1610	30/3750	0.999492154	1
1	map05235	PD-L1 expression and PD-1 checkpoint pathway in cance	KEGG PATHWAY	1/1610	15/3750	0.999782952	1
1	map00511	Other glycan degradation	KEGG PATHWAY	1/1610	15/3750	0.999782952	1
5	map05146	Amoebiasis	KEGG PATHWAY	5/1610	32/3750	0.99978796	1
14	map04510	Focal adhesion	KEGG PATHWAY	14/1610	63/3750	0.999845728	1
21	map05131	Shigellosis	KEGG PATHWAY	21/1610	89/3750	0.9999635	1
4	map00051	Fructose and mannose metabolism	KEGG PATHWAY	4/1610	32/3750	0.99996361	1
20	map04142	Lysosome	KEGG PATHWAY	20/1610	86/3750	0.999964289	1
5	map04624	Toll and Imd signaling pathway	KEGG PATHWAY	5/1610	40/3750	0.999994432	1
7	map00520	Amino sugar and nucleotide sugar metabolism	KEGG PATHWAY	7/1610	49/3750	0.99999686	1
4	map04979	Cholesterol metabolism	KEGG PATHWAY	4/1610	38/3750	0.999997979	1
8	map00010	Glycolysis / Gluconeogenesis	KEGG PATHWAY	8/1610	64/3750	0.99999998	1
